# Environmental induced transgenerational inheritance impacts systems epigenetics in disease etiology

**DOI:** 10.1038/s41598-022-09336-0

**Published:** 2022-04-19

**Authors:** Daniel Beck, Eric E. Nilsson, Millissia Ben Maamar, Michael K. Skinner

**Affiliations:** Center for Reproductive Biology, School of Biological Sciences, Washington State University, Pullman, WA 99164-4236 USA

**Keywords:** Molecular biology, Systems biology, Molecular medicine

## Abstract

Environmental toxicants have been shown to promote the epigenetic transgenerational inheritance of disease through exposure specific epigenetic alterations in the germline. The current study examines the actions of hydrocarbon jet fuel, dioxin, pesticides (permethrin and methoxychlor), plastics, and herbicides (glyphosate and atrazine) in the promotion of transgenerational disease in the great grand-offspring rats that correlates with specific disease associated differential DNA methylation regions (DMRs). The transgenerational disease observed was similar for all exposures and includes pathologies of the kidney, prostate, and testis, pubertal abnormalities, and obesity. The disease specific DMRs in sperm were exposure specific for each pathology with negligible overlap. Therefore, for each disease the DMRs and associated genes were distinct for each exposure generational lineage. Observations suggest a large number of DMRs and associated genes are involved in a specific pathology, and various environmental exposures influence unique subsets of DMRs and genes to promote the transgenerational developmental origins of disease susceptibility later in life. A novel multiscale systems biology basis of disease etiology is proposed involving an integration of environmental epigenetics, genetics and generational toxicology.

## Introduction

Chronic disease has been shown to impact over 75% of the world-wide human population^1^. The frequency of disease has increased dramatically in the past decades at all ages, suggesting a major environmental impact on disease etiology^1,2^. Each generation continues to have an extended life span due to our more efficient medical capacity, but the percentage of disease continues to increase at all ages within the population^1,2^. Although currently we have a high incidence of infectious disease^3^, this is far less than the level of chronic disease in the human population^1,2^. Environmental factors such as diet, lifestyle and pollutants are considered to be the risk factors for this increased generational occurrence of disease within the population. Since the vast majority of environmental factors cannot act as mutagens and directly change DNA sequence^4^, our understanding of disease etiology needs to be expanded.

The classic current paradigm for the molecular basis of disease etiology involves genetics^5^. The development of genetic mutations has been, and is still, thought to be the causal factor for phenotypic variation and disease development. Genome-wide association studies (GWAS) have demonstrated in large population-based studies that most diseases have specific associated gene mutations. The issue is that generally less than 1% of the specific diseased population have the associated genetic mutations^5^, so these specific mutations have negligible impact on the specific disease population. Environmental factors such as toxicant exposures have been shown to promote disease development, but these exposures generally do not have the capacity to directly induce genetic mutations^6,7^. There is a growing appreciation that a combination of environmental factors and epigenetics are integrated in disease etiology^8,9^. Advancing age has been shown to correlate in the etiology of nearly all pathologies. The concept that early life exposures promote molecular alterations to induce the developmental origins of health and disease (DOHAD) has been established^10^. Studies to examine the impacts of race, ethnic background and regional alterations of disease etiology have suggested that environmental factors such as diet, exercise, toxicants, and lifestyle are the primary elements inducing the disease frequency differences, rather than genetics^6,11^.

Epigenetics is defined as “molecular factors and processes around DNA that regulate genome activity independent of DNA sequence, and are mitotically stable”^12^. The epigenetic factors such as DNA methylation, non-coding RNA (ncRNA), histone modifications, chromatin structure alterations, and RNA methylation have the ability to integrate with genetics to impact all areas of biology^6,13^. When environmental factors influence the epigenetics of the germ cells (sperm and egg), the environmental factors have the potential to promote the epigenetic transgenerational inheritance of phenotypic variation and disease^6,14^. Environmental epigenetics provides a molecular mechanism to explain the developmental origins of health and disease (DOHAD) theory, phenotypic variation and adaptation associated with evolutionary biology, and the etiology of disease^6,13^. The integration of epigenetic and genetic molecular mechanisms is required for most biological processes and associated disease. However, the current paradigms in science generally focus only on genetics.

A large number of environmental factors have been shown to induce the epigenetic transgenerational inheritance of pathologies and disease^6,15^. The first observation involved the use of the agricultural fungicide vinclozolin^14^, followed by a stress-induced behavior alteration transgenerationally^16^. Since then, a large variety of environmental toxicants from dioxin^17^ to glyphosate^18^, or nutritional abnormalities^19^, or more recently infectious disease^20^ have been shown to promote the epigenetic transgenerational inheritance of disease^6^. This non-genetic form of environmentally induced disease in subsequent generations needs to be considered as a component in disease etiology. The rapid increase in specific disease frequency within the population^1,2^ will likely involve this environmentally induced epigenetic transgenerational inheritance of pathology phenomenon.

Recently, a number of epigenome-wide association studies (EWAS) have been shown to identify epigenetic alterations (i.e., epimutations) associated with diseases. Sperm epimutations involved in the transgenerational inheritance of specific pathologies have been identified^21–27^. Transgenerational sperm epimutations associated with kidney, prostate, puberty, testis, obesity, and multiple pathologies have been identified for a variety of environmental toxicants including dioxin^21^, plastics^22^, pesticides^23^, glyphosate^24^, methoxychlor^25^, atrazine^26^, and jet fuel^27^ in animal studies. The transgenerational sperm epimutations for exposure and disease-specific epimutations have been identified in these EWAS studies^21–27^ and in EWAS human studies^28,29^.

The current study used the sperm samples and histological sections from these previous toxicant-induced epigenetic transgenerational inheritance EWAS rat studies to identify with more advanced protocols epigenetic alterations for specific diseases and associate with the genetics of the specific diseases. The common and distinct differential DNA methylation regions (DMRs) for the different exposure lineage diseases were correlated with known disease associated genes. Observations provide new insights into the integration of epigenetics and genetics in disease etiology. A novel systems epigenetics and multiscale framework is suggested to explain the apparent stochastic genetic events and variation within a disease population.

## Results

Previously, a variety of distinct environmental toxicant exposures have been used to promote the epigenetic transgenerational inheritance of a number of pathologies and phenotypic variation. This initially involved an outbred rat model, but has now been found in all organisms examined from plants to humans^6^. Gestating female F0 generation rats were exposed transiently to a specific toxicant during the period of fetal gonadal sex determination (i.e., embryonic days 8–14 in the rat). The F1 generation offspring were obtained and aged to 3 months to be bred within the exposure lineage and avoid any inbreeding to obtain the F2 generation grand offspring. The F2 generation was then bred at 3 months of age to generate the F3 generation great-grand offspring. Interbreeding unrelated males and females within the exposure lineages was used to avoid any inbreeding and optimize the pathology observed by obtaining both maternal and paternal lineage contributions to the F3 generation, as previously described^30^. All animals were aged to 1 year in order to assess pathology and disease phenotypes, and the sperm collected to assess epigenetic (DNA methylation) alterations. This was previously accomplished with jet fuel (JP8)^31^, pesticides (permethrin and diethyltoluamide (DEET))^32^, plastics (bisphenol A (BPA) and phthalates)^33^, dioxin^17^, methoxychlor^34^, glyphosate^18^, and atrazine^26^. Control populations of animals were also prepared for comparison to identify the exposure-specific disease and epigenetic alterations of differential DNA methylation regions (DMRs) in the sperm, Supplemental Fig. S1. For the current study, the previous studies archived histology tissue slides and archived frozen (− 80 °C) sperm were reanalyzed to identify exposure specific pathologies (Supplemental Tables S1–S8) exposure specific DMRs (Supplemental Tables S9–S15) and control population disease specific DMR sets in the sperm for each of the F3 generation males with a single specific disease or multiple disease (Supplemental Tables S16–S20). The reanalysis was performed with more advanced histology and pathology analysis of the slides and with more advanced technology for the sperm DMR analysis, as described in the Methods. This includes the use of updated methylated DNA immunoprecipitation (MeDIP) procedure and bioinformatics^35^, in comparison to tiling arrays and the earlier MeDIP procedures used in the past. More advanced reagents were used to improve reproducibility and accuracy of the MeDIP and generation of sequencing libraries, as described in the Methods (i.e., MeDIP-Seq Analysis). A 1 kb DMR size was used instead of 100 bp to improve the bioinformatics, as described in the Methods. The updated histopathology procedure used three histologists blinded to the slide identity and the counting of larger tissue section regions than used previously^17,18,26,27,31–33^. The analysis of larger regions allowed more efficient detection of various abnormal histology and pathology. Only animals with a specific disease were utilized for that specific disease. The disease specific DMRs of transgenerational animals for jet fuel^27^, pesticides^23^, plastics^22^, dioxin^21^, methoxychlor^25^, glyphosate^24^, and atrazine^36^ were previously reported, so are not included in the Supplemental Tables. Those males that only had an individual specific disease (i.e., no other disease present) were used to identify the pathology specific sperm epigenetic biomarkers for that disease. Animals with multiple diseases were used and referred to as “multiple disease” groups. The current study was designed to compare the different toxicant exposures and assess the transgenerational disease specific DMRs to provide insights into the role of epigenetics in disease etiology and generational toxicology.

As previously observed^17,18,31–34,36^, the control lineage F3 generation generally does not have appreciable pathology. The pathologies investigated were testis disease, prostate disease, kidney disease, obesity, pubertal onset abnormalities, and tumors. The specific histology analysis and tissue-specific pathology analysis are described in the Methods section. Three different individuals blinded to the slide identity separately assessed the pathology as described^21–27^ in the Methods. Due to the reduced number of pathologies in the control lineages, all the different previous study controls were combined for the current study to identify potential control lineage epigenetic biomarkers for disease between individuals without and with specific pathologies, Supplemental Table S1. The pathologies observed were testis disease, prostate disease, kidney disease, obesity and multiple disease, with no tumors observed and negligible pubertal abnormalities, Supplemental Table S1. This allowed for sufficient numbers of individuals and the ability to identify a control lineage epigenetic biomarker for each of the pathologies, Supplemental Fig. S1. The reanalysis of the F3 generation exposure specific pathologies is presented in Supplemental Tables S2–S8. As can be seen in Supplemental Tables S1–S8, here are generally sufficient numbers of animals with a single disease, such that individual disease epigenetic biomarkers can be identified. Only animals with a specific disease were used to identify DMR for the specific disease. Those animals with multiple disease were grouped and designated “multiple disease” and the associated epigenetic biomarkers identified. The DMRs in sperm were assessed for each of the individual pathologies at *p* < 1e−04 (Supplemental Fig. S1A), and the negligible overlap was observed for the various disease specific DMRs (Supplemental Fig. S1B). The disease control lineage DMR lists, and gene associations are presented in Supplemental Tables S16–S20. The principal component analysis (PCA) of the different control disease specific and non-disease specific and non-disease DMR comparison are presented in Supplemental Fig. S1, showing generally good separation of the DMRs. This combined F3 generation control disease specific information is used to compare with the various toxicant exposure disease DMR biomarkers previously identified^21–27^.

**Figure 1:**
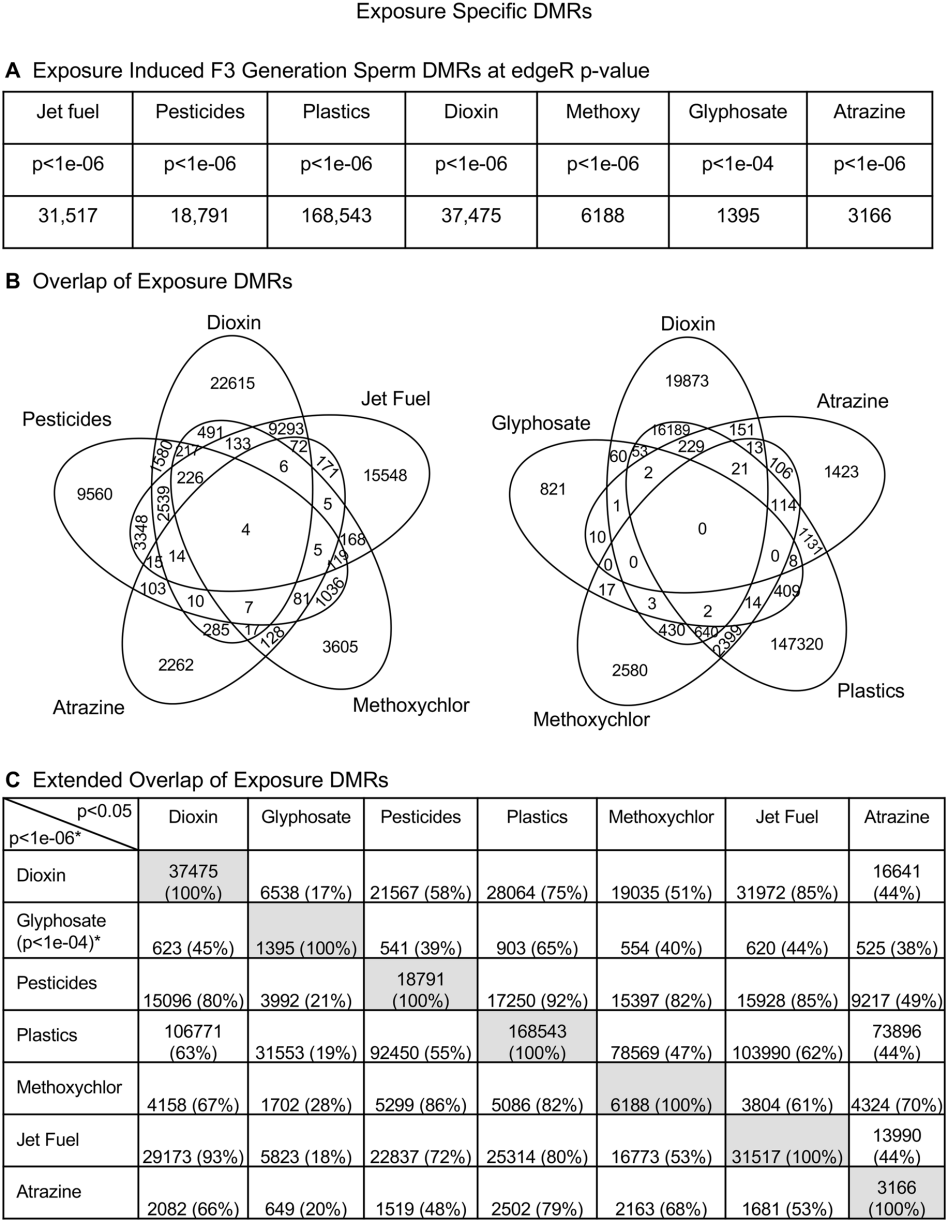
Exposure specific DMRs. (**A**) Exposure DMRs at using a *p* < 1e−6 edgeR *p* value threshold for everything except glyphosate at *p* < 1e−4. (**B**) Overlap of Exposure DMRs (*p* < 1e−06), *except glyphosate at *p* < 1e−04. (**C**) Extended Overlap of Exposure DMRs. The overlapping DMRs and percent overlap are indicated.

The reanalysis of the archived sperm from the F3 generation males of all the different exposures used a combined set of control samples, except for glyphosate and atrazine. Due to the lower number of transgenerational DMRs with glyphosate and atrazine, only the original control sets were used. A more advanced technology of methylated DNA immunoprecipitation (MeDIP) followed by DNA sequencing for an MeDIP-Seq procedure was used, which examines over 95% of the genome DNA methylation sites^35^. This is in contrast to the tiling array technology used previously for several of the exposures. The specific exposure DMRs at an edgeR *p* value of *p* < 1e−06, in all but glyphosate, is shown in Fig. 1A. Due to the low DMR numbers with glyphosate exposure, the imbalance was addressed by using *p* < 1e−04 for glyphosate. The exposure-specific DMR lists that provide chromosomal position, CpG density, log-fold change for the increase or decrease in DNA methylation, statistical *p* value and gene associations for only the DMR with associated genes are presented (Supplemental Tables S9–S20). An overlap of the different exposure DMRs at *p* < 1e−06, except for glyphosate at *p* < 1e−04, demonstrated minimal overlap among all exposure lineages, Fig. 1B, but some overlap was observed between specific exposures. An extended overlap with a comparison of the *p* < 1e−06, or *p* < 1e−04 for glyphosate, with others at *p* < 0.05 demonstrated higher (17–85%) overlaps, such as plastics and pesticides and lower overlaps with the dioxin and glyphosate, Fig. 1C. This is likely in part due to common or distinct signal transduction of the various exposures. For example, dioxin and jet fuel at 85% overlap both use the aryl hydrocarbon receptor (AHR) system, or methoxychlor and plastics at 82% overlap both use the estrogen receptor system. Therefore, the different environmental toxicants promoted the epigenetic transgenerational inheritance of common and exposure-specific DMRs in sperm from the F3 generation males.

**Figure 2:**
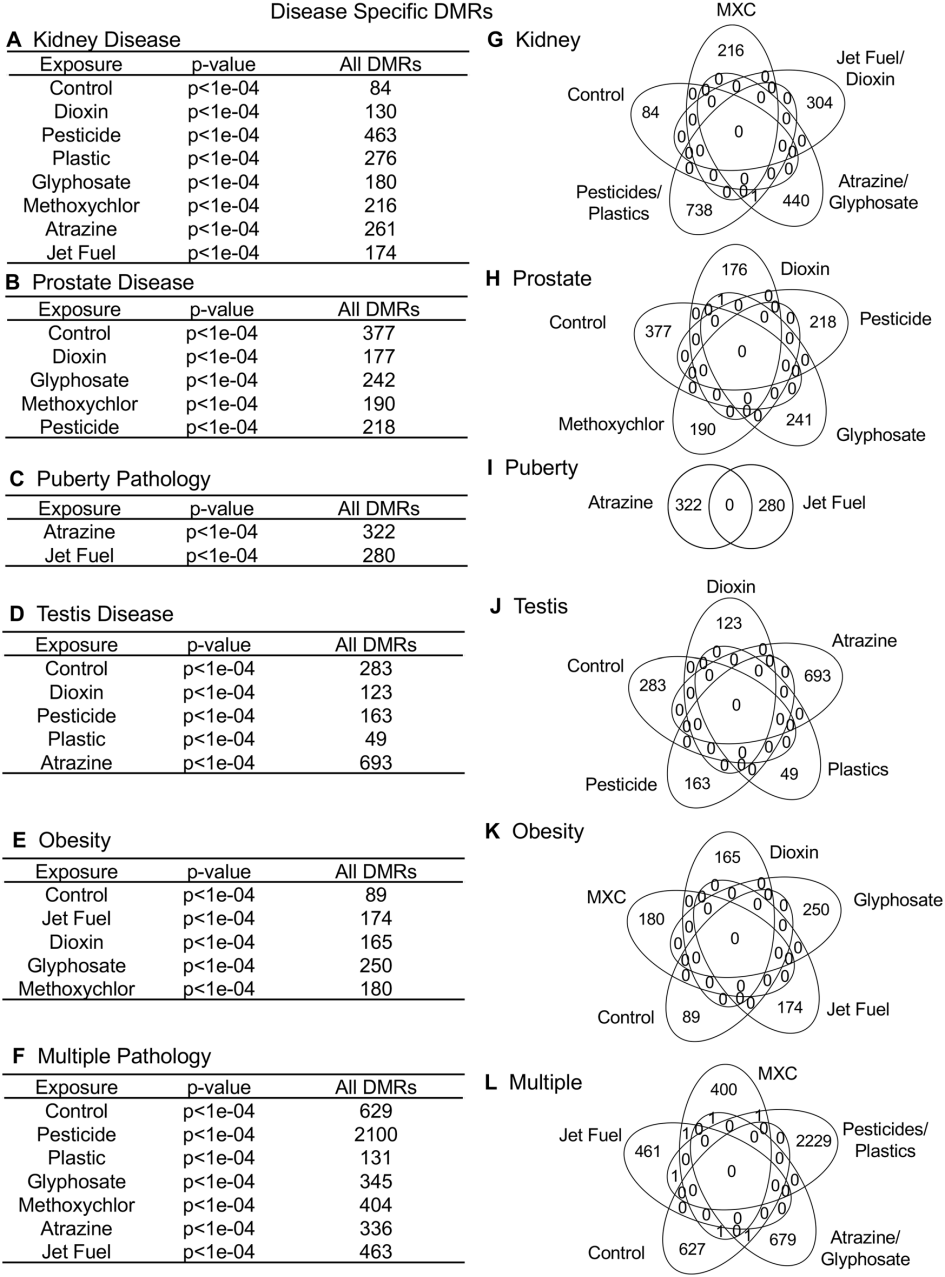
Specific disease DMRs. (**A**) Kidney disease; (**B**) Prostate disease; (**C**) Puberty pathology; (**D**) Testis disease; (**E**) Obesity; (**F**) Multiple pathology. The exposure, *p* value and number of DMR are presented. Venn diagram overlaps for each exposure DMR set are shown for (**G**) Kidney; (**H**) Prostate; (**I**) Puberty; (**J**) Testis; (**K**) Obesity; (**L**) Multiple.

**Figure 3:**
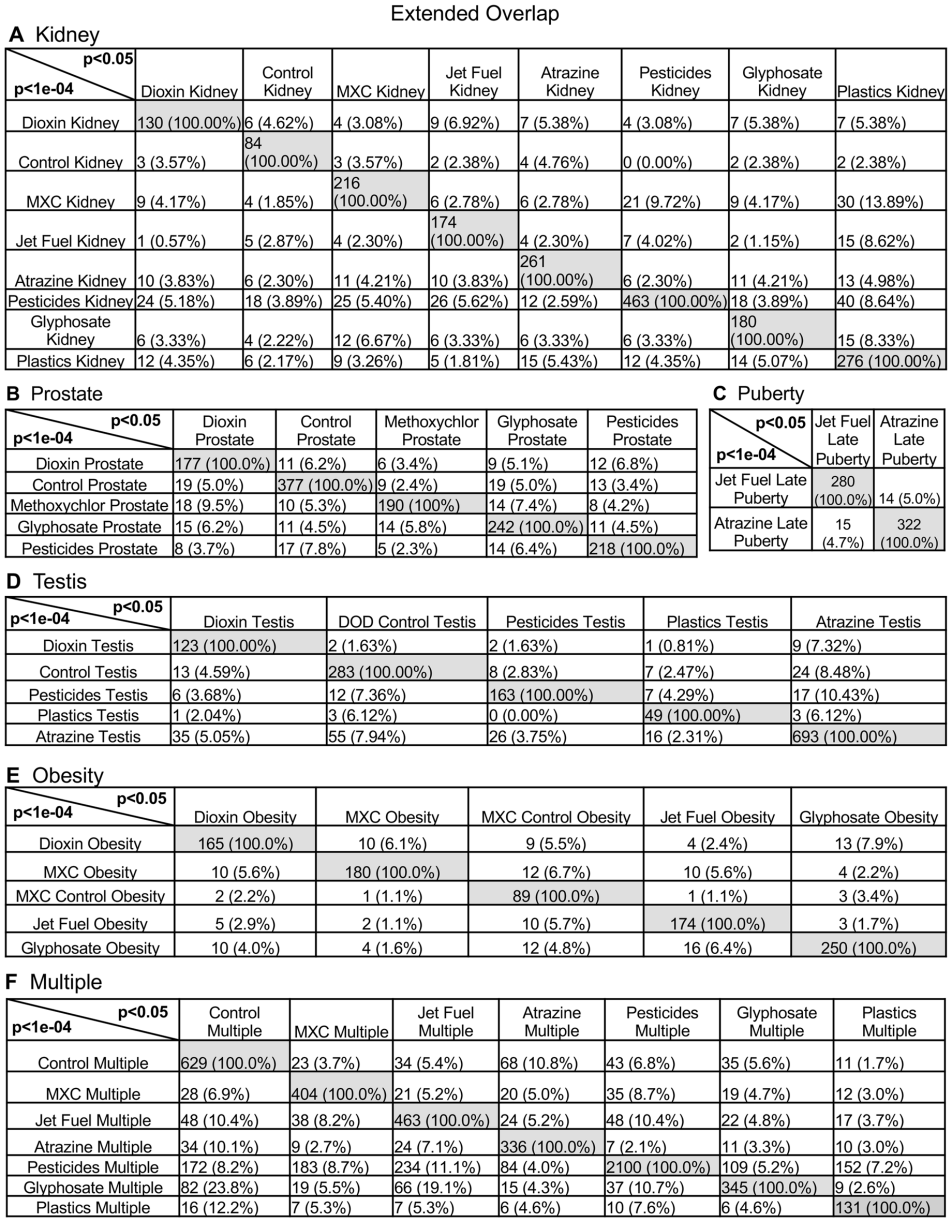
Extended disease specific DMR overlap at *p* < 1e−04 versus *p* < 0.05. (**A**) Kidney; (**B**) Prostate; (**C**) Puberty; (**D**) Testis; (**E**) Obesity; (**F**) Multiple. The DMR number and percentage overlap presented. The horizontal row overlap identifies DMR number and percentage for each exposure.

Each of the environmental toxicant exposures promoted transgenerational disease and pathology, Supplemental Tables S2–S8, that was common between the different exposures, Fig. 2. As previously described, the individuals with only a single specific disease were used to identify potential sperm epigenetic biomarkers for disease^21–27^, Supplemental Tables S2–S8. A summary of the different exposures for each disease is presented in Fig. 2. The sperm DMRs at *p* < 1e−04 are presented for all 1 kb windows throughout the genome for each kidney disease (Fig. 2A), prostate disease (Fig. 2B), puberty abnormalities (Fig. 2C), testis disease (Fig. 2D), obesity (Fig. 2E), and the presence of multiple pathology (Fig. 2F). The overlap of the various disease-specific DMRs for each exposure were found to be distinct with negligible overlap (Fig. 2G–L), for all the different pathologies. An expanded overlap of the exposure-specific DMRs at *p* < 1e−04 with a less stringent threshold of *p* < 0.05 also demonstrated negligible overlap with less than 10% overlap observed, Fig. 3. This was similar for all the different pathologies and diseases. Therefore, the different toxicant exposures promoted similar pathology, but the disease-specific epigenetic biomarkers were distinct. A further analysis examined the chromosomal locations of the sperm DMRs in the rat genome for each of the different exposures for each of the different pathologies, Supplemental Fig. S2. A genome-wide distribution of the exposure’s disease-specific DMRs were observed for the kidney disease (Supplemental Fig. S2A), prostate disease (Supplemental Fig. S2B), puberty pathology (Supplemental Fig. S2C), testis disease (Supplemental Fig. S2D), obesity (Supplemental Fig. S2E), and multiple pathologies (Supplemental Fig. S2F). Clusters of DMRs on the chromosomes were also observed for each, but were distinct between the exposures and disease.

**Figure 4:**
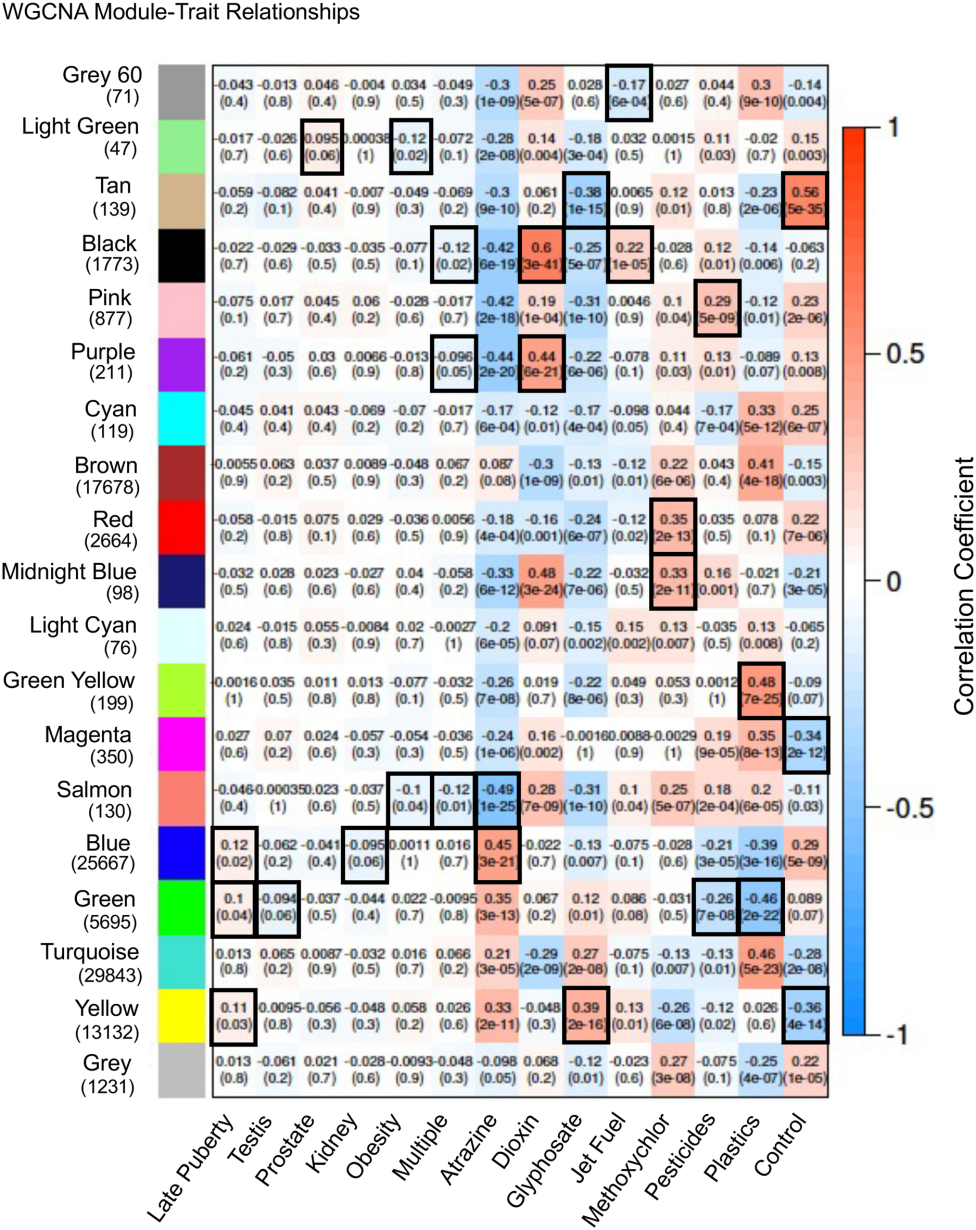
Weighted co-expression network analysis WGCNA module-trait relationships. Module colors and genomic window numbers listed correlate to specific diseases and exposures. The Correlation Coefficient and *p* value (brackets) for each presented. Black outline correlations used for subsequent analysis.

Observations with differential DNA methylated regions (DMRs) identified exposure-specific DMR sets that correlated with similar transgenerational disease, but each disease had unique DMR biomarkers and associated genes for the specific pathology, Supplemental Tables S21–S26. Therefore, similar transgenerational pathologies and diseases were induced by the various toxicant exposures with exposure and pathology distinct DNA methylation alterations. An additional analysis that was used to help elucidate this phenomenon was weighted genome coexpression network analysis (WGCNA)^37,38^. This bioinformatics procedure takes all the genome-wide sequencing data from the MeDIP-Seq analysis for each transgenerational exposure and pathology to assess DNA methylation patterns in the genome that correlate with the exposures and disease. Although this WGCNA has not been used extensively with DNA methylation, it has been extensively used to assess transcriptomes for gene correlations^39^. Previously, we have used this approach for gene predictions for developmental systems^40^, so the current study extends this to epigenetic alterations correlated with associated genes. In the current study, all MeDIP-Seq data was used in the WGCNA (https://doi.org/10.1186/1471-2105-9-559) to initially establish dendrograms of correlated DNA methylation site information to identify coexpression clusters, Supplemental Fig. S3. The exposure and control data generated large clusters of correlation specific DNA methylation data, but the pathology specific DNA methylation data generated smaller clusters throughout the genome, which often associated with the exposure clusters, Supplemental Fig. S3. Due to computational limitations (i.e., extended > 7 day periods), the 100,000 1 kb genomic windows with the highest total read depth were selected for inclusion in the WGCNA analysis. Genomic windows were clustered into modules based on methylation levels in all samples. These modules were then correlated with the disease and exposure characteristics of the samples. A summary with correlation coefficient and *p* value statistics is presented in Fig. 4. The various pathologies and exposures are correlated to a number of modules of DNA methylation site data. The modules are identified with different colors listed and the number of DNA methylation sites for each listed next to the color module, Fig. 4. Black outlined boxes identify significant correlations for the pathologies and exposures that were selected for further analysis. For the exposures, two of the top statistically significant modules were identified. Generally, the exposures had much higher levels of correlations, and in general each exposure had correlations with different modules. Some overlap is observed, such as between the control and glyphosate modules, Fig. 4. The pathology correlations had lower statistical significance, but correlations with one or two modules were observed, with multiple pathology having three correlated modules, Fig. 4. The DNA methylation sites within each of the WGCNA modules were associated with genes within 10 kb of the site(s) to incorporate distal and proximal promoter regions, Supplemental Tables S27–S32, similar to exposure specific DMR associated genes within 10 kb, Supplemental Tables S21–S26. Only the DMR sites or DNA methylation sites with associated genes are presented in Supplemental Tables S9–S20. This is an underestimate of potential regulatory sites due to not considering distal ncRNA regulation. The WGCNA provided DNA methylation correlations with exposure and pathology that had gene associations for further analysis, Supplemental Tables S27–S32.

**Figure 5:**
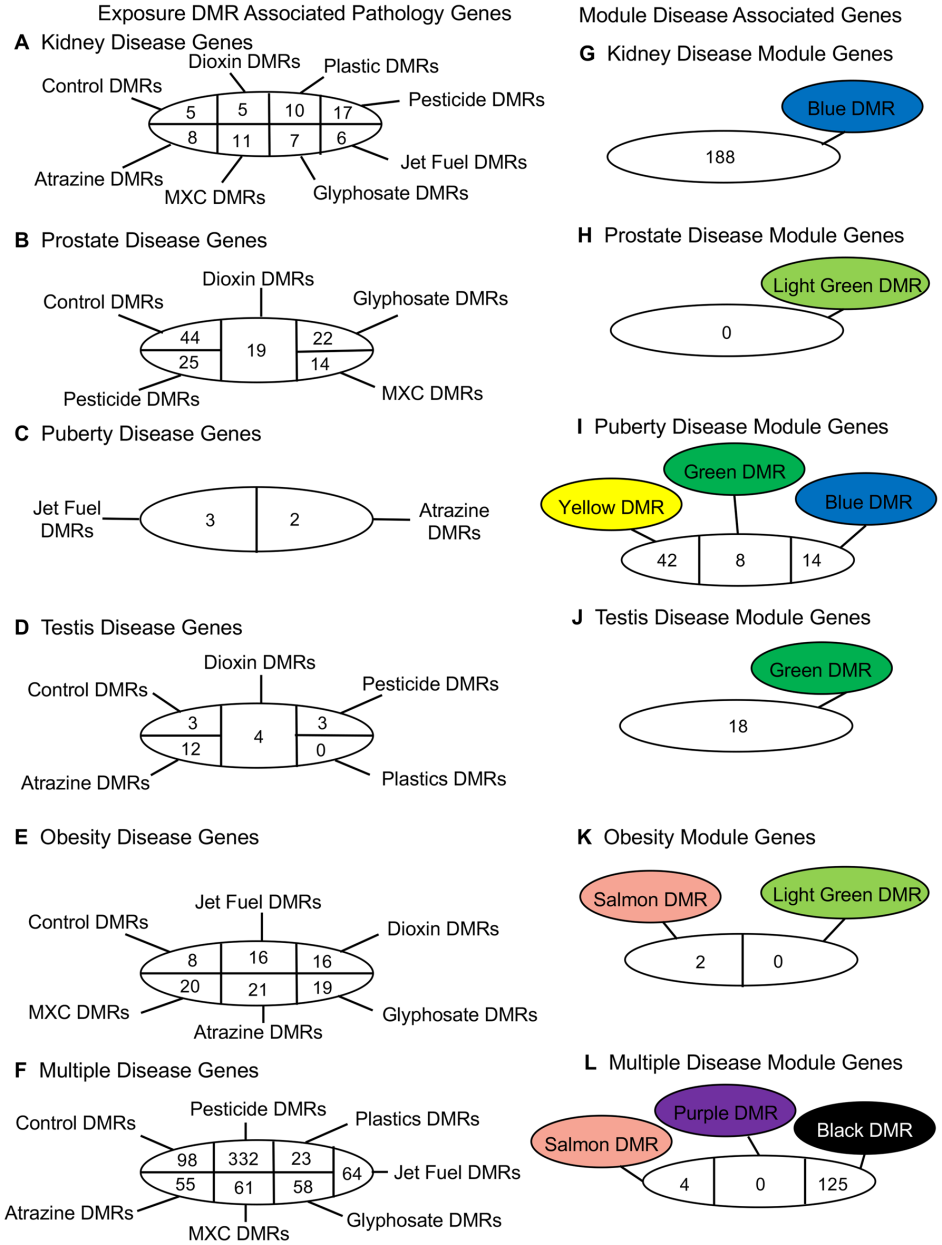
Exposure DMR associated pathology genes. Pathologies and specific number of DMR per exposure: (**A**) Kidney; (**B**) Prostate; (**C**) Puberty; (**D**) Testis; (**E**) Obesity; (**F**) Multiple. Module disease associated genes; (**G**) Kidney disease module associated genes; (**H**) Prostate disease module associated genes; (**I**) Puberty disease module associated genes; (**J**) Testis disease module associated genes; (**K**) Obesity disease module associated genes. (**L**) Multiple disease module associated genes.

**Figure 6:**
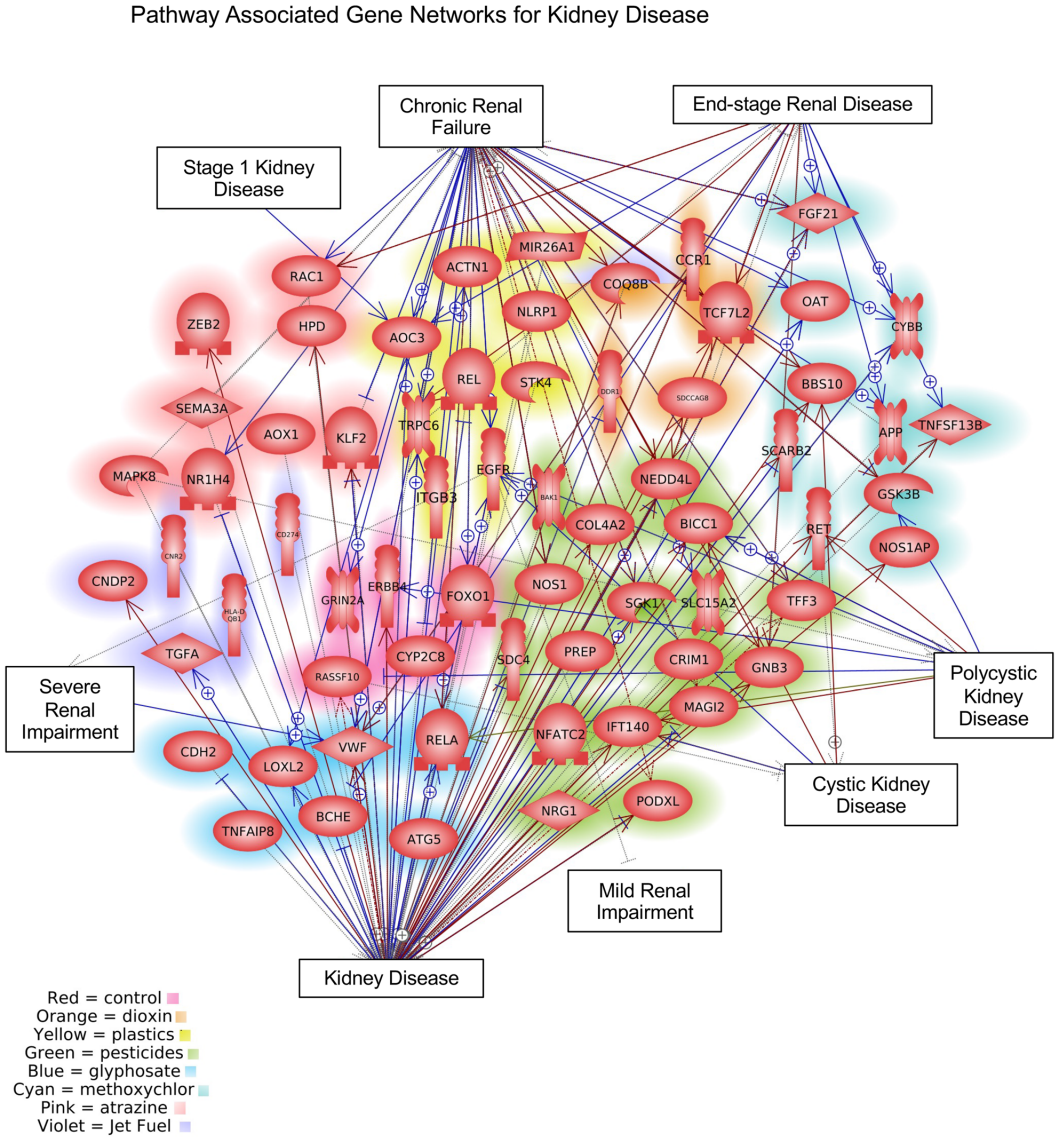
Kidney disease DMR associated genes and network. The index presents the color and exposure group for DMR associated genes. The disease pathways and processes with gene links identified.

A summary of the exposure DMR associated pathology genes is presented in Fig. 5A–F. The number of genes associated with the exposure DMRs that have been shown to correlate to specific disease are provided. A list of the pathology associated genes in the various exposure DMRs is presented in Supplemental Table S21–S26. Figure 5 presents the gene numbers for kidney disease (A), prostate disease (B), puberty abnormalities (C), testis disease (D), obesity (E), and multiple pathologies (F). Each of the exposure toxicants that promote the pathology has associated pathology genes, as indicated. Although the exposure DMRs are distinct, all the exposures have known pathology associated genes. A summary of the WGCNA module DNA methylation associated genes is also presented in Fig. 5. The DNA methylation site modules that correlate with specific pathologies are presented and the number of associated genes within 10 kb of the DMR are listed for each, Fig. 5G–L. The WGCNA module DNA methylation site associated genes for each pathology are listed in Supplemental Table S27–S32. Although different exposures were found to have different correlated modules, all had specific disease or pathology associated genes. Further network analysis of the DMR associated disease specific processes are presented for kidney disease in Fig. 6. Each of the different exposure kidney disease associated DMR associated genes are identified with a different color, Fig. 6. Unique subsets of genes specific for the different exposures are identified. Similar analyses of the other disease network analyses are presented in Supplemental Fig. S4 for prostate disease (Fig. S4A), puberty abnormality (Fig. S4B), testis disease (Fig. S4C), and obesity (Fig. S4D). The multiple disease associated genes were not analyzed for a network due to presence of multiple pathologies. In each of the disease DMR associated gene networks, there were exposure specific genes identified that were distinct. Therefore, the exposure specific epigenetic alterations were associated with unique subsets of disease specific genes.

## Discussion

**Figure 7:**
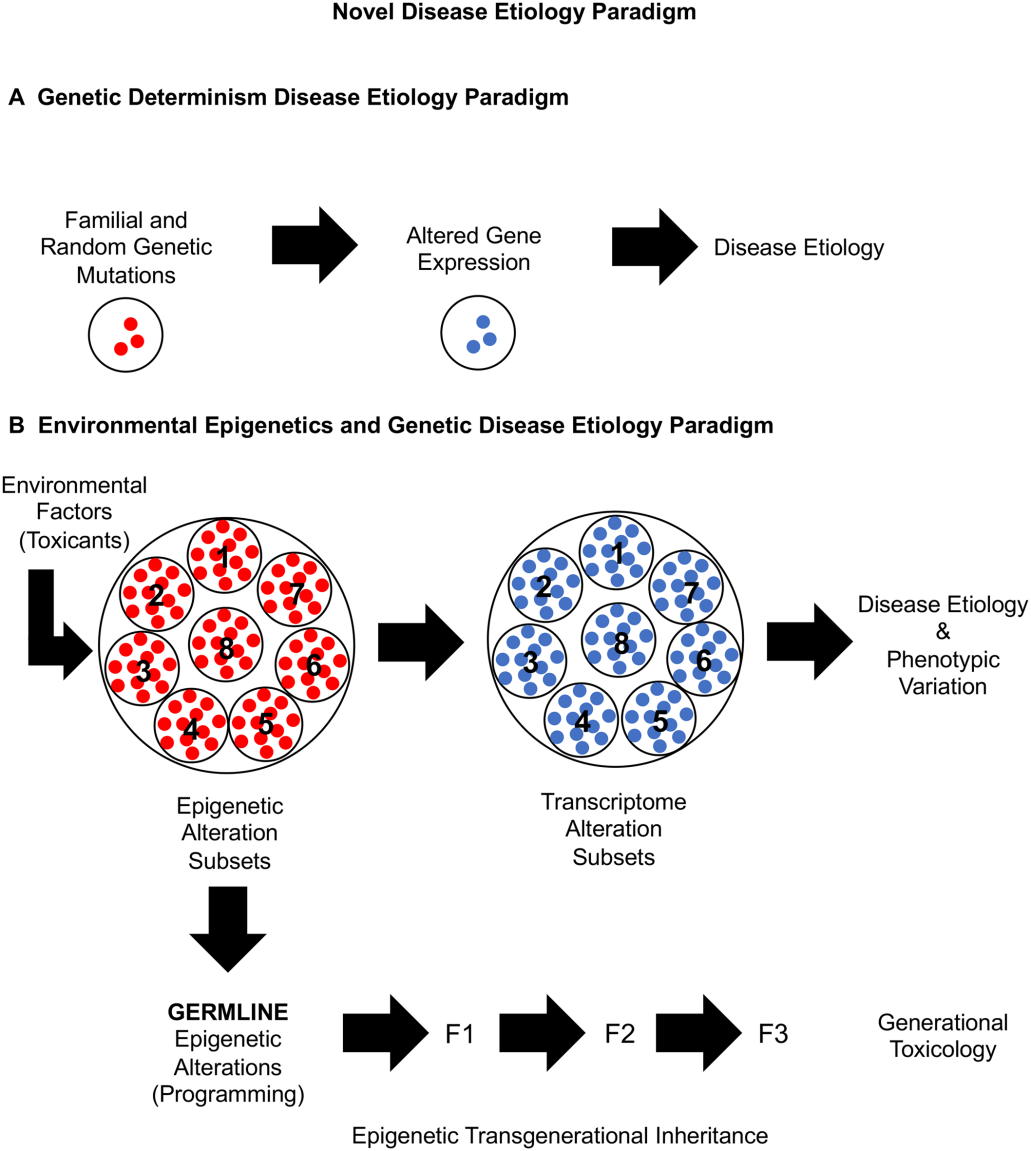
Novel disease etiology paradigm. (**A**) Genetic determination disease etiology paradigm. (**B**) Environmental epigenetics and genetic disease etiology paradigm. Environmental exposures promote subsets of distinct subsets of DMR associated sites that are associated with subsets of DMR linked genes that when altered promote disease etiology and phenotypic variation. The subsets of altered epimutations are transmitted to subsequent generations through the germline to promote generational toxicology.

Over the past fifty years, there have been many observations that suggest the environment has significant impacts on disease etiology, but the vast majority of the environmental impacts cannot directly alter DNA sequence or promote genetic mutations. This includes regional impacts on disease frequencies^1,41^, the low frequency of associated genetic mutations within a disease population^5^, the dramatic increase in disease frequency over the last several decades^1,2^, discordant monozygotic twin disease as the twins age^42^, and direct impacts of environmental factors such as diet and toxicants on disease etiology^9,43^. The current paradigm primarily considered in disease etiology involves genetic determinism, where familial inheritance or random genetic mutations promote altered gene expression to promote abnormal cell or tissue biology to induce disease, Fig. 7A. Although this has developed over the past century, the sequencing of the human genome in the early 2000s significantly expanded support for this paradigm. The inability of this genetic determinism paradigm to incorporate the growing literature observations on environmental impacts on disease etiology suggests an additional molecular component needs to be incorporated into a new scientific paradigm for disease etiology.

Epigenetics provides an additional molecular mechanism that is now known to be essential for gene expression and is environmentally responsive. The incorporation of environmental epigenetics into a new paradigm for disease etiology will address the limitations of the more classic genetic determinism disease etiology paradigm. The observations that environmentally induced epigenetic alterations in the germline can promote the epigenetic transgenerational inheritance of disease allows a more generational component of disease etiology to be considered. This non-genetic form of inheritance is known to be influenced by a variety of environmental factors, from nutrition to toxicants, in all species investigated from plants to humans^6^. The current study used a number of previously published toxicant induced epigenetic transgenerational inheritance studies^17,18,31–34,36^ to compare and develop greater insights into the molecular mechanisms involved in disease etiology.

The various environmental toxicant exposures of a gestating female rat around the time of gonadal sex determination during fetal development all promoted the epigenetic transgenerational inheritance of disease to the F3 generation great-grand offspring. The male sperm transmitting these transgenerational disease phenotypes all had exposure specific epigenetic DNA methylation alterations called epimutations (Fig. 1). Although the different toxicants promoted distinct subsets of epigenetic alterations, the disease and pathology phenotypes observed were similar for the different exposures, Fig. 2. Therefore, the alterations of various exposure subsets of unique differential DNA methylation regions (DMRs) were observed and associated with unique subsets of genes to promote altered transcriptomes associated with similar disease phenotypes, Fig. 7B.

The exposure-induced epigenetic transgenerational inheritance of disease etiology was further investigated with an examination of pathology specific epigenetic biomarkers for disease, Fig. 2. The various toxicants were found to promote similar transgenerational disease and pathologies^21–27^. Comparison of the exposure-specific disease DMR biomarkers demonstrated negligible overlap, Fig. 2. Therefore, the different exposure disease versus non-disease DMR sets were found to be primarily distinct and associated with unique genes, but promote similar disease phenotypes. When the associated genes were identified for specific diseases and pathologies, unique subsets of genes previously associated with the specific pathologies were identified, Fig. 6, Supplemental Fig. S4, and Fig. 7B. Observations suggest large numbers of genes are associated with specific pathologies and small subsets induced through environmental epigenetics can promote disease susceptibility and etiology. These observations are in contrast to the classic genetic determinism concept where a limited number of specific genetic mutations and associated genes are the primary regulators of disease etiology.

Analysis of control animals in the absence of any environmental toxicant exposures also had similar pathologies when sufficient numbers of animals were combined, due to the low frequency of pathology in the control populations, Supplemental Table S1 and Fig. S1. The epigenetic DMR biomarkers for the specific pathologies were compared with the toxicant exposure diseased vs non-diseased transgenerational DMR sets. The control populations also had a unique subset of epimutations that were associated with genes previously shown to be involved in those pathologies, Supplemental Table S1. This observation suggests any exposure or natural environment impacts that alters specific subsets of epimutations will then alter small subsets of genes associated with specific pathologies to increase disease susceptibility and etiology, Fig. 7B.

The alternate approach used for the analysis examined the correlations of DNA methylation sites throughout the genome, in contrast to DMR analysis. The weighted genome coexpression network analysis (WGCNA) is useful to assess genome-wide trends and patterns in the genome to correlate with genomic characteristics^37,38,44^. The current study extends this to the epigenome (i.e., DNA methylation) and compared this with the gene associations. The WGCNA identified strong correlations with the exposures and different modules of DNA methylation site information, Fig. 4. Although the pathology data from all the exposures was also found to correlate, this was at a reduced significance compared to the exposures. The modules with significant correlations were identified, and the associated DNA methylation site genes identified. As was observed with DMRs, the WGCNA module data had exposure specific patterns, as well as pathology specific module patterns. When the module associated genes for specific pathologies were examined, subsets of DNA methylation sites were identified. Therefore, as was observed with the DMR analysis data in Figs. 1 and 3, the WGCNA data also support the disease etiology paradigm of environmental epigenetics and genetics, Fig. 7B. Environmentally induced epigenetic alterations promote subsets of epimutations that impact expression of subsets of genes that promotes disease susceptibility and pathologies. Examples are provided for specific disease in Fig. 6 and Supplemental Fig. S4.

The current study focused on transgenerational disease observed in the F3 generation following the exposure of gestating females in the F0 generation^17,18,31–34,36^. The epigenetic transgenerational inheritance of disease supports this novel disease etiology paradigm of the inclusion of epigenetics and genetics, Fig. 7. The direct exposure of toxicants to promote epigenetic alterations and later life disease etiology involves the same disease etiology paradigm as the transgenerational model. The inclusion of environmental epigenetics in disease etiology is required to integrate with the classic genetic determinism paradigm. Observations demonstrate that the toxicants not only affect the first generation exposed, but also transmit this through the germline to subsequent generations. This is referred to as “generational toxicology”^18^. Previously, this has not been considered in disease etiology nor in toxicology. The ability of an environmental toxicant to promote pathologies in subsequent generations dramatically impacts the hazards of toxicants^6^. Exposures such as glyphosate, atrazine, or pesticides had negligible effects on the F1 generation, so negligible direct exposure toxicity, but had dramatic effects at later generations, such as the great grand-offspring F3 generation, that does not have any direct exposure^18,32,36^. The proposed environmental epigenetics disease etiology paradigm, Fig. 7B, helps explain this generational toxicology phenomenon, and further supports the integration of environmental epigenetics in disease etiology.

The current study used seven different environmental toxicants to induce the epigenetic transgenerational inheritance of various diseases and pathologies. Comparison of the DMRs demonstrated subsets of distinct epimutations in sperm that had subsets of distal gene associations that promoted similar pathologies. This was also assessed in a WGCNA and demonstrated DNA methylation site modules that supported the same inclusion of environmental epigenetics and genetics in disease etiology, Fig. 7B. Therefore, environmental epigenetics impacts subsets of epimutations, that impact subsets of gene expression alterations, that promote disease susceptibility and etiology. This novel paradigm for disease etiology incorporates epigenetics and genetics to address the limitations of the classic disease etiology paradigm of genetic determinism. In addition, this helps clarify the molecular mechanisms of generational toxicology.

## Methods

### Animal studies and breeding

As previously described^17,18,31–34,36^, female and male rats of an outbred strain Hsd:Sprague Dawley SD (Harlan) at 70–100 days of age were fed ad lib with a standard rat diet and ad lib tap water. All animal cages were housed in the same room and environment with gestating females and females with litters being housed individually within cages. Conditions were designed to minimize differences that would cause maternal effects. The breeding of unrelated males and females within specific exposure lineages (interbreeding) was used to optimize the maternal and paternal lineage contributions to the phenotypes observed^30^. No inbreeding within the colonies was performed. Generally, six unrelated breeding pairs at the F0 generation were used to generate the subsequent generations. Timed-pregnant females were mated and on embryonic days 8 through 14 (E8–E14) of gestation were administered daily intraperitoneal injections of the treatment compounds (Dioxin TCDD 100 ng/kg BW/day; glyphosate (25 mg/kg BW/day; JP-8 hydrocarbon jet fuel 500 mg/kg BW/day; permethrin 150 mg/kg BW/day and insect repellent DEET 40 mg/kg/BW/day; BPA 50 mg/kg BW/day, phthalate DEHP 750 mg/kg BW/day and phthalate DBP 66 mg/kg/BW/day; methoxychlor 200 mg/kg BW/day; atrazine 25 mg/kg BW/day) or vehicle control dimethyl sulfoxide (DMSO) or in the case of glyphosate, phosphate buffered saline (PBS), as previously described^17,18,31–34,36^.

The gestating female rats exposed were designated as the F0 generation. F1–F3 generation control and exposure lineages were housed in the same room and racks with lighting, food and water. Non-littermate females and males aged 70–100 days from the F1 generation of exposure or control lineages were bred within their treatment group to obtain F2 generation offspring. Unrelated F2 generation rats were bred to obtain F3 generation offspring. No sibling or cousin breeding was used to avoid any inbreeding artifacts. Only the F0 generation received exposure treatments. All animals were aged to 1 year for pathology and epigenetic analysis. All experimental protocols for the procedures with rats were pre-approved by the Washington State University Animal Care and Use Committee (IACUC approval # 2568 & 6931). All methods were performed in accordance with the relevant guidelines and regulations. The excess sperm samples and paraffin tissue sections stored (i.e., archived) from the previous studies^17,18,31–34,36^ were used and reanalyzed for the current study.

### Tissue harvest and histology processing

As previously described^17,18,31–34,36^, at 12 months of age, rats were euthanized by CO_2_ inhalation and cervical dislocation for tissue harvest. Testis, prostate, and kidney were fixed in Bouin’s solution (Sigma) followed by 70% ethanol, then processed for paraffin embedding, and hematoxylin and eosin (H & E) staining by standard procedures for histopathological examination. Paraffin five micron sections were processed and stained by Nationwide Histology, Spokane WA, USA. Archived glass slides with hematoxylin and eosin stained tissue sections were stored at room temperature for use and reanalysis for the current study.

### Histopathology examination and disease classification

Archived histology slides from previous studies^17,18,31–34,36^, were reanalyzed and used for a new histology analysis for the current study. Stained testis, prostate, and kidney slides were imaged through a microscope using 4 × objective lenses (testis and prostate) or 10 × objective lenses (kidney). Tiled images were captured using a digital camera. Tiled images for each tissue were photo-merged into a single image using Adobe Photoshop (ver. 21.1.2, Adobe, Inc.). Images were evaluated and pathology features digitally marked using Photoshop software. The Washington Animal Disease Diagnostic Laboratory (WADDL) at the Washington State University College of Veterinary Medicine has board certified veterinary pathologists and assisted in initially establishing the criteria for the pathology analyses and identifying parameters to assess. The tissue pathology evaluated used previously described histological criteria described in transgenerational models with representative images^17,31–34^. Histopathology readers were trained to recognize the specific abnormalities evaluated for this study in rat testis, ventral prostate and kidney. Two individuals blinded to the exposure evaluated each tissue image for abnormalities. In the event of a disagreement about the disease status, a third individual blinded to the exposure evaluated the tissue. Sets of quality control (QC) slides were generated for each tissue and were read by each reader prior to evaluating any set of experimental slides. These QC slide results were monitored for reader accuracy and concordance. The more advanced pathology analysis used involved larger areas of the tissue sections to be analyzed to optimize pathology detection and more consistent multiple readers blinded to the section identity used for assessment of pathology.

Specific descriptions of histopathological analysis and example images were previously reported^17,18,31–34,36^. Testis histopathology criteria included the presence of vacuoles in the seminiferous tubules, azoospermic atretic seminiferous tubules, and ‘other’ abnormalities including sloughed spermatogenic cells in center of the tubule and a lack of a tubule lumen, Supplemental Fig. S5. Prostate histopathology criteria included the presence of vacuoles in the glandular epithelium, atrophic glandular epithelium and hyperplasia of prostatic gland epithelium, Supplemental Fig. S6. Kidney histopathology criteria included reduced size of glomerulus, thickened Bowman’s capsule, and the presence of proteinaceous fluid-filled cysts > 50 μm in diameter, Supplemental Fig. S7. A cutoff was established to declare a tissue ‘diseased’ based on the mean number of histopathological abnormalities plus two standard deviations from the mean of control group tissues, as assessed by each of the individual observers blinded to the treatment groups. This number (i.e., greater than two standard deviations) was used to classify rats into those with and without testis, prostate, or kidney disease in each lineage. A rat tissue section was finally declared ‘diseased’ only when at least two of the three observers marked the same tissue section ‘diseased’. Onset of puberty was assessed in males starting at 35 days of age by the presence of balano-preputial separation. Obesity was assessed with an increase in body mass and a qualitative evaluation of abdominal adiposity. The statistical analyses for pathology results were expressed as the proportion of affected animals that exceeded a pre-determined threshold (testis, prostate or kidney disease frequency, tumor frequency, obesity frequency). Groups were analyzed using Fisher’s exact test.

### Epididymal sperm collection and DNA isolation

The protocol used is as previously described^45^. Briefly, the epididymis was dissected free of fat and connective tissue, then, after cutting open the cauda, placed into 6 ml of phosphate buffer saline (PBS) for 20 min at room temperature. Further incubation at 4 °C will immobilize the sperm. The tissue was then minced, the released sperm pelleted at 4 °C 3000×*g* for 10 min, then resuspended in 250 µL NIM buffer and stored at − 80 °C for further processing. An appropriate amount of rat sperm suspension (approximately 50 µl) was used for DNA extraction. Previous studies have shown mammalian sperm heads are resistant to sonication unlike somatic cells^46,47^. Somatic cell contamination and debris were removed by brief sonication (Fisher Sonic Dismembrator, model 300, power level 25), which destroys the somatic cells, then centrifugation and washing 1–2 times in 1× PBS. The resulting purified sperm pellet was resuspended in 820 µl DNA extraction buffer and 80 µl 0.1 M DTT added, then incubated at 65 °C for 15 min. Proteinase K (80 µl of 20 mg/ml) was added and the sample was incubated at 55 °C for 2–3 h under constant rotation. Protein was removed by addition of protein precipitation solution (300 µl, Promega A795A), incubation for 15 min on ice, then centrifugation at 13,500×*g* for 30 min at 4 °C. One ml of the supernatant was precipitated with 2 µl of GlycoBlue (Invitrogen, AM9516) and 1 ml of cold 100% isopropanol. After incubation, the sample was spun at 13,500×*g* for 30 min at 4 °C, then washed with 70% cold ethanol. The pellet was air-dried for about 5 min then resuspended in 100 µl of nuclease free water.

### Methylated DNA immunoprecipitation (MeDIP)

The archived sperm samples were prepared as previously described^45^. Genomic DNA was sonicated and run on 1.5% agarose gel for fragment size verification. The sonicated DNA was then diluted with 1× TE buffer to 400 μl, then heat-denatured for 10 min at 95 °C, and immediately cooled on ice for 10 min to create single-stranded DNA fragments. Then 100 μl of 5× IP buffer and 5 μg of antibody (monoclonal mouse anti 5-methyl cytidine; Diagenode #C15200006) were added, and the mixture was incubated overnight on a rotator at 4 °C. The following day magnetic beads (Dynabeads M280 Sheep anti-Mouse IgG; Life Technologies 11201D) were pre-washed per manufacturer’s instructions, and 50 μl of beads were added to the 500 μl of DNA-antibody mixture from the overnight incubation, then incubated for 2 h on a rotator at 4 °C. After this incubation, the samples were washed three times with 1× IP buffer using a magnetic rack. The washed samples were then resuspended in 250 μl digestion buffer (5 mM Tris PH 8, 10.mM EDTA, 0.5% SDS) with 3.5 μl Proteinase K (20 mg/ml), and incubated for 2–3 h on a rotator at 55 °C. DNA clean-up was performed using a Phenol–Chloroform–Isoamyl–Alcohol extraction, and the supernatant precipitated with 2 μl of GlycoBlue (20 mg/ml), 20 μl of 5 M NaCl and 500 μl ethanol in − 20 °C freezer for one to several hours. The DNA precipitate was pelleted, washed with 70% ethanol, then dried and resuspended in 20 μl H_2_O or 1× TE. DNA concentration was measured in a Qubit apparatus (Life Technologies) with the ssDNA analysis kit (Molecular Probes Q10212). The more advanced protocol used involved the new reagent kits and optimal procedures for the MeDIP^30^.

### MeDIP-Seq analysis

MeDIP DNA was used to create libraries for next generation sequencing (NGS) using the NEBNext Ultra RNA Library Prep Kit for Illumina (San Diego, CA) starting at step 1.4 of the manufacturer’s protocol to generate double stranded DNA from the single-stranded DNA resulting from MeDIP^30^. After this step, the manufacturer’s protocol was followed indexing each sample individually with NEBNext Multiplex Oligos for Illumina^30^. The WSU Spokane Genomics Core sequenced the samples on the Illumina HiSeq 2500 at PE50, with a read size of approximately 50 bp and approximately 10–20 million reads per pool. Twelve libraries were run in one lane.

### Statistics and bioinformatics

The DMR identification and annotation methods follow those presented in previous published papers^36,45^. Data quality was assessed using the FastQC program (https://www.bioinformatics.babraham.ac.uk/projects/fastqc/). The data was cleaned and filtered to remove adapters and low-quality bases using Trimmomatic^48^. The reads for each MeDIP sample were mapped to the Rnor 6.0 rat genome using Bowtie2^49^ with default parameter options. The mapped read files were then converted to sorted BAM files using SAMtools^50^. The MEDIPS R package^51^ was used to calculate differential coverage between disease and non-disease sample groups. The edgeR *p* value^52^ was used to determine the relative difference between the two groups for each genomic window. Windows with an edgeR *p* value less than the selected *p* < 1e−06 threshold for all exposures, except glyphosate at *p* < 1e−04, were considered DMR. The site edges were extended until no genomic window with an edgeR *p* value less than 0.1 remained within 1000 bp of the DMR. The edgeR *p* value was used to assess the significance of the DMR identified. A false discovery rate (FDR) analysis for each comparison was performed and provided a *p* < 0.1 for all the exposure comparisons, Supplemental Tables S9–S15. Due to the relatively low number of individuals with one specific disease type, the previous published FDR analysis of the specific disease DMR biomarkers^53–58^ were generally between 0.1 and 0.3, depending on the specific exposure diseases^53–58^, (Supplemental Tables S16–S20). The toxicant exposure associated disease DMRs were annotated using the biomaRt R package^59^ to access the Ensembl database^60^, as described in the original publications. The DMR associated genes were then automatically sorted into functional groups using Panther^61^ (www.skinner.wsu.edu under genomic data). The exposure DMRs, WGCNA methylation sites, and the control disease DMRs were annotated using a modified method. These sites were annotated using NCBI provided gene information. Genes were sorted into categories by converting Panther (25) protein classifications into more general groups. A Pathway Studio, Elsevier, database and network tool was used to assess physiological and disease process gene correlations. All molecular data has been deposited into the public database at NCBI [GEO # GSE98683 (atrazine), GSE155922 (jet fuel), GSE157539 (dioxin), GSE158254 (pesticides), GSE158086 (methoxychlor), GSE163412 (plastics), GSE152678 (glyphosate), and GSE198452 (control)] and R code computational tools available at GitHub (https://github.com/skinnerlab/MeDIP-seq) and www.skinner.wsu.edu.

### Weighted gene coexpression network analysis (WGCNA)

The weighted correlation network analysis (WGCNA)^62^ was performed using the WGCNA R package^63^. All MeDIP-Seq genomic windows were ranked by the mean RPKM read depth across all samples. The top 100,000 sites were chosen for inclusion in the analysis. The size of this subset was chosen to allow for a reasonable read depth to be considered and to limit computational time (< 1 week) requirements. WGCNA is a parameter rich analysis and only limited exploration of parameter variations was performed. Modules were calculated using the *blockwiseModules* function with the following parameters: maxBlockSize = 15,000, power = 4, TOMType = "unsigned", minModuleSize = 30, reassignThreshold = 0, and mergeCutHeight = 0.25. The Pearson correlation was calculated for each development stage and module. The *p* value for each correlation was calculated using the *corPvalueStudent* function. Sites within each module were annotated using the same methods as the DMRs.

### Ethics

All experimental protocols for the procedures with rats were pre-approved by the Washington State University Animal Care and Use Committee (IACUC approval # 2568 & 6931). All methods were performed in accordance with the relevant guidelines and regulations. This study was carried out in compliance with the ARRIVE guidelines.

## Supplementary Information

Supplementary Legends.

Supplementary Figure S1.

Supplementary Figure S2.

Supplementary Figure S3.

Supplementary Figure S4.

Supplementary Figure S5.

Supplementary Figure S6.

Supplementary Figure S7.

Supplementary Table S1.

Supplementary Table S2.

Supplementary Table S3.

Supplementary Table S4.

Supplementary Table S5.

Supplementary Table S6.

Supplementary Table S7.

Supplementary Table S8.

Supplementary Table S9.

Supplementary Table S10.

Supplementary Table S11.

Supplementary Table S12.

Supplementary Table S13.

Supplementary Table S14.

Supplementary Table S15.

Supplementary Table S16.

Supplementary Table S17.

Supplementary Table S18.

Supplementary Table S19.

Supplementary Table S20.

Supplementary Table S21.

Supplementary Table S22.

Supplementary Table S23.

Supplementary Table S24.

Supplementary Table S25.

Supplementary Table S26.

Supplementary Table S27.

Supplementary Table S28.

Supplementary Table S29.

Supplementary Table S30.

Supplementary Table S31.

Supplementary Table S32.

## Data Availability

All molecular data has been deposited into the public database at NCBI [GEO # GSE98683 (atrazine), GSE155922 (jet fuel), GSE157539 (dioxin), GSE158254 (pesticides), GSE158086 (methoxychlor), GSE163412 (plastics), GSE152678 (glyphosate), and GSE198452 (control)] and R code computational tools available at GitHub (https://github.com/skinnerlab/MeDIP-seq) and www.skinner.wsu.edu.

## Abbreviations

DMRs: DNA methylation regions
WGCNA: Whole genome coexpression network analysis
GWAS: Genome-wide association studies
DOHAD: Developmental origins of health and disease
ncRNA: Non-coding RNA
EWAS: Epigenome-wide association studies
MeDIP: Methylated DNA immunoprecipitation
MeDIP-Seq: Methylated DNA immunoprecipitation followed by next generation sequencing
JP8: Jet fuel
DEET: Diethyltoluamide
BPA: Bisphenol A
PCA: Principal component analysis
AHR: Aryl hydrocarbon receptor
