# Supplementary material for: Environmental induced transgenerational inheritance impacts systems epigenetics in disease etiology
Source: Sci Rep. 2022 Apr 19;12:5452. doi: 10.1038/s41598-022-09336-0 (PMC9018793; doi:10.1038/s41598-022-09336-0)
Supplement: Supplementary file 1 — Supplementary Legends. [file 41598_2022_9336_MOESM1_ESM.pdf]

Supplemental Figure and Table Legends

Supplemental Figure S1. Control pathology DMRs and Principal Component Analysis (PCA). **(A)** Control pathology DMR identification. **(B)** Venn diagram overlap disease DMRs. **(C)** Kidney pathology, **(D)** Prostate pathology, **(E)** Obesity, **(F)** Testis pathology and **(G)** Multiple pathologies DMR PCA. The samples plotted by the first two principal components. The underlying data is the RPKM read depth for all genomic windows with an edgeR p-value < 1e-04.

Supplemental Figure S2. DMR chromosomal locations. **(A)** Kidney pathology and exposure DMRs; **(B)** Prostate pathology and exposure DMRs; **(C)** Puberty pathology and exposure DMRs; **(D)** Testis pathology and exposure DMRs; **(E)** Obesity and exposure DMRs; and **(F)** Multiple pathologies and exposure DMRs.

Supplemental Figure S3. WGCNA sample dendrogram and trait heatmap. The dendrogram and height correlation. The different pathology and exposure DMR associated clusters (red) are presented.

Supplemental Figure S4. DMR associated gene networks for specific pathology. **(A)** Prostate disease; **(B)** Puberty abnormalities; **(C)** Testis disease; and **(D)** Obesity. The index with exposure and color for gene associations.

Supplemental Figure S5. Testis histopathology. **(A)** Atrophied testes seminiferous tubules marked with black numbers 1, 2, and 3 generally appear smaller than normal tubules and lack basal cells; these tubules may or may not have lumens. **(B)** Vacuoles in seminiferous tubules marked by green numbers 2 and 3 appear as round, crisp-edged holes and must occur in the basal cell layer of the tubule. **(C)** Maturation arrest of seminiferous tubules marked with black numbers 1 through 9 and appear as clumps of basal cells in the tubule lumen; these cells are generally larger and darker than spermatid heads which are normally found in tubule lumens.

Supplemental Figure S6. Prostate histopathology. **(A)** Vacuoles in the lining of a prostatic gland marked by green numbers 1 and 2. Similar to testes, prostate vacuoles appear as round holes and must exist within the epithelial lining of a prostate gland. **(B)** Atrophied prostatic gland lining marked with yellow arrows are thinned epithelial layers and unevenly spaced cell nuclei over at least one-third of the gland compared to normal prostatic glands (black arrows). **(C)** Prostatic hyperplasia marked by black numbers 4 and 5 characterized by clumping epithelial cells in the gland lining; black arrow points to an infolding of the prostate gland that is not hyperplasia.

Supplemental Figure S7. Kidney histopathology. The same criteria apply for both male and female kidney. **(A)** Reduced glomerulus marked by black number 1 is half the size or less than the other three glomeruli on the right side of the image. **(B)** Kidney cysts marked by black numbers 3, 4, and 5; can be pink or white fluid-filled spaces that are surrounded by flattened or irregular epithelium compared to normal kidney tubules. Orange arrow

points to a blood vessel, not a cyst. Black number 2 is a thickened Bowman's capsule. **(C)** Thickened Bowman's capsules marked in black numbers 6 and 7 are thick pink bands surrounding at least half of the glomerulus. Yellow number 1 is a reduced glomerulus also with a thickened Bowman's capsule.

Supplemental Table S1. Control transgenerational pathology from all studies. F3 generation control lineage male rat pathology. Vehicle dimethyl sulfoxide (DMSO) exposure used in the F0 generation gestating female control. The individual animals for the control lineage males are listed with a (+) to indicate the presence of the pathology or (-) for absence of the pathology. The number of disease/total animals is presented and percentage disease. The shaded boxes are used to identify the single disease or multiple disease animals.

Supplemental Table S2. F3 generation jet fuel lineage male pathology. The individual animals for the exposure lineage males are listed and a (+) indicates presence of disease and (-) absence of disease. The animals with shaded (+) were used for the epigenetic analysis due to the presence of only one disease, except the multiple (≥2) disease. The no disease animals used were identified with a shaded (0). n/a indicates not applicable due to absent pathology. The ratio of number disease / total is presented and percentage with disease.

Supplemental Table S3. F3 generation dioxin lineage pathology. The individual animals for the exposure lineage males are listed and a (+) indicates presence of disease and (-)

absence of disease. The animals with shaded (+) were used for the epigenetic analysis due to the presence of only one disease, except the multiple (≥2) disease. The no disease animals used were identified with a shaded (0). n/a indicates not applicable due to absent pathology. The ratio of number disease / total is presented and percentage with disease.

Supplemental Table S4. F3 generation pesticides lineage males pathology. The individual animals for the exposure lineage males are listed and a (+) indicates presence of disease and (-) absence of disease. The animals with shaded (+) were used for the epigenetic analysis due to the presence of only one disease, except the multiple (≥2) disease. The no disease animals used were identified with a shaded (0). n/a indicates not applicable due to absent pathology. The ratio of number disease / total is presented and percentage with disease.

Supplemental Table S5. F3 generation plastics lineage males pathology. The individual animals for the exposure lineage males are listed and a (+) indicates presence of disease and (-) absence of disease. The animals with shaded (+) were used for the epigenetic analysis due to the presence of only one disease, except the multiple (≥2) disease. The no disease animals used were identified with a shaded (0). n/a indicates not applicable due to absent pathology. The ratio of number disease / total is presented and percentage with disease.

Supplemental Table S6. F3 generation methoxychlor lineage male pathology. The individual animals for the exposure lineage males are listed and a (+) indicates presence

of disease and (-) absence of disease. The animals with shaded (+) were used for the epigenetic analysis due to the presence of only one disease, except the multiple (≥2) disease. The no disease animals used were identified with a shaded (0). n/a indicates not applicable due to absent pathology. The ratio of number disease / total is presented and percentage with disease.

Supplemental Table S7. F3 generation atrazine lineage males pathology. The individual animals for the exposure lineage males are listed and a (+) indicates presence of disease and (-) absence of disease. The animals with shaded (+) were used for the epigenetic analysis due to the presence of only one disease, except the multiple (≥2) disease. The no disease animals used were identified with a shaded (0). n/a indicates not applicable due to absent pathology. The ratio of number disease / total is presented and percentage with disease.

Supplemental Table S8. F3 generation glyphosate lineage male pathology. The individual animals for the exposure lineage males are listed and a (+) indicates presence of disease and (-) absence of disease. The animals with shaded (+) were used for the epigenetic analysis due to the presence of only one disease, except the multiple (≥2) disease. The no disease animals used were identified with a shaded (0). n/a indicates not applicable due to absent pathology. The ratio of number disease / total is presented and percentage with disease.

Supplemental Table S9. Jet fuel DMR with gene associations. List, chromosomal site, start, stop, length (bp), p-value, maximum log fold change (LFC), CpG number and density, gene annotation, and gene category.

Supplemental Table S10. Pesticides DMR with gene associations. List, chromosomal site, start, stop, length (bp), p-value, maximum log fold change (LFC), CpG number and density, gene annotation, and gene category.

Supplemental Table S11. Plastics DMR with gene associations. List, chromosomal site, start, stop, length (bp), p-value, maximum log fold change (LFC), CpG number and density, gene annotation, and gene category.

Supplemental Table S12. Dioxin DMR with gene associations. List, chromosomal site, start, stop, length (bp), p-value, maximum log fold change (LFC), CpG number and density, gene annotation, and gene category.

Supplemental Table S13. Methoxychlor DMR with gene associations. List, chromosomal site, start, stop, length (bp), p-value, maximum log fold change (LFC), CpG number and density, gene annotation, and gene category.

Supplemental Table S14. Atrazine DMR with gene associations. List, chromosomal site, start, stop, length (bp), p-value, maximum log fold change (LFC), CpG number and density, gene annotation, and gene category.

Supplemental Table S15. Glyphosate DMR with gene associations. List, chromosomal site, start, stop, length (bp), p-value, maximum log fold change (LFC), CpG number and density, gene annotation, and gene category.

Supplemental Table S16. Control kidney disease specific DMR with gene associations. List, chromosomal site, start, stop, length (bp), p-value, maximum log fold change (LFC), CpG number and density, gene annotation, and gene category

Supplemental Table S17. Control prostate disease specific DMR with gene associations. List, chromosomal site, start, stop, length (bp), p-value, maximum log fold change (LFC), CpG number and density, gene annotation, and gene category.

Supplemental Table S18. Control testis disease specific DMR with gene associations. List, chromosomal site, start, stop, length (bp), p-value, maximum log fold change (LFC), CpG number and density, gene annotation, and gene category.

Supplemental Table S19. Control obesity specific DMR with gene associations. List, chromosomal site, start, stop, length (bp), p-value, maximum log fold change (LFC), CpG number and density, gene annotation, and gene category.

Supplemental Table S20. Control multiple disease specific DMR with gene associations. List, chromosomal site, start, stop, length (bp), p-value, maximum log fold change (LFC), CpG number and density, gene annotation, and gene category.

Supplemental Table S21. Kidney disease correlated DMR associated genes. Disease DMR associated genes for specific exposures.

Supplemental Table S22. Prostate disease correlated DMR associated genes. Disease DMR associated genes for specific exposures.

Supplemental Table S23. Puberty abnormalities correlated DMR associated genes. Disease DMR associated genes for specific exposures.

Supplemental Table S24. Testis disease correlated DMR associated genes. Disease DMR associated genes for specific exposures.

Supplemental Table S25. Obesity correlated DMR associated genes. Disease DMR associated genes for specific exposures.

Supplemental Table S26. Multiple diseases correlated DMR associated genes. Disease DMR associated genes for specific exposures.

Supplemental Table S27. Kidney disease associated module genes. Disease DMR associated genes for modules.

Supplemental Table S28. Prostate disease associated module genes. Disease DMR associated genes for modules.

Supplemental Table S29. Puberty abnormalities associated module genes. Disease DMR associated genes for modules.

Supplemental Table S30. Testis disease associated module genes. Disease DMR associated genes for modules.

Supplemental Table S31. Obesity associated module genes. Disease DMR associated genes for modules.

Supplemental Table S32. Multiple disease associated module genes. Disease DMR associated genes for modules.
