# Supplementary material for: Environmental induced transgenerational inheritance impacts systems epigenetics in disease etiology
Source: Sci Rep. 2022 Apr 19;12:5452. doi: 10.1038/s41598-022-09336-0 (PMC9018793; doi:10.1038/s41598-022-09336-0)

Supplemental Figure S1

A Control Pathology DMR Identification

Pathology	p-value	All DMRs (Disease Specific)
Kidney	p<1e-04	84
Prostate	p<1e-04	377
Testis	p<1e-04	283
Obesity	p<1e-04	89
Multiple	p<1e-04	629

B Venn diagram overlap disease DMRs

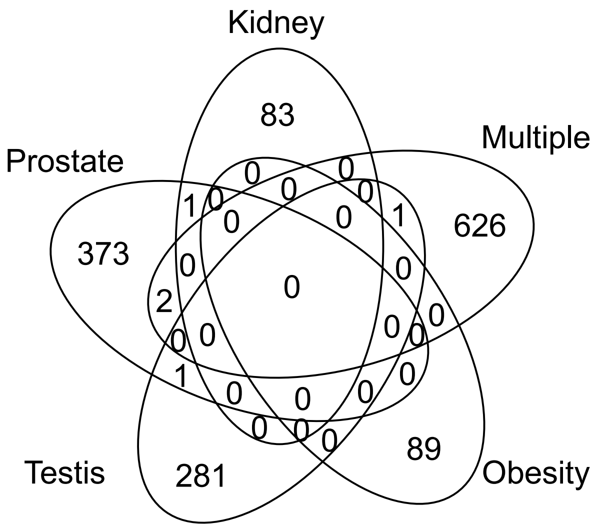

C Kidney Pathology DMR PCA

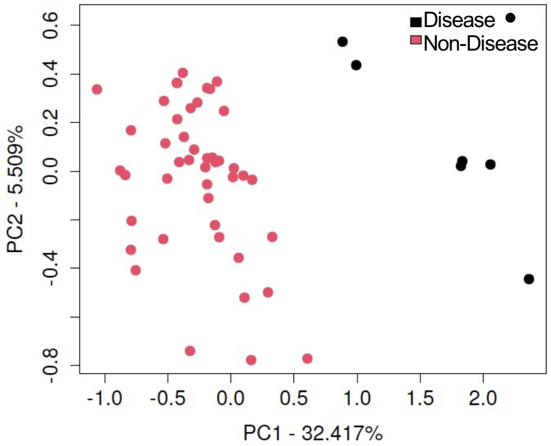

D Prostate Pathology DMR PCA

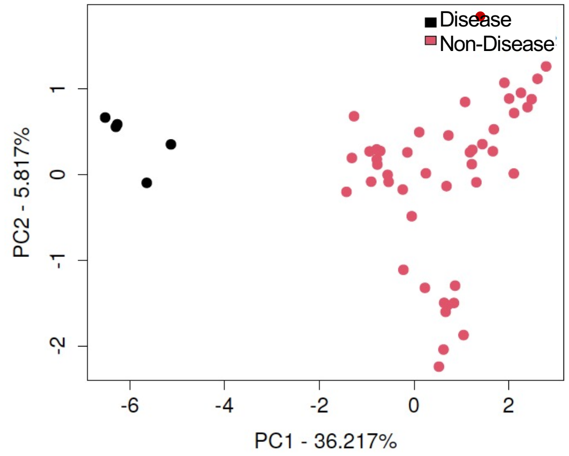

E Obesity DMR PCA

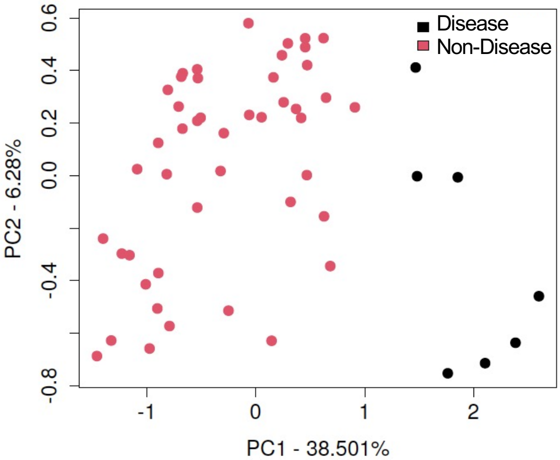

F Testis Pathology DMR PCA

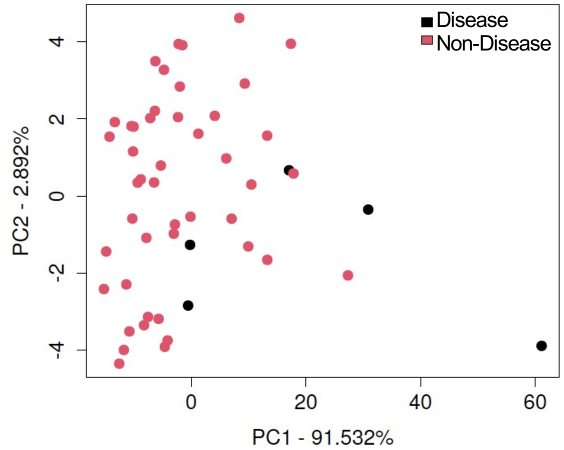

G Multiple Pathologies DMR PCA

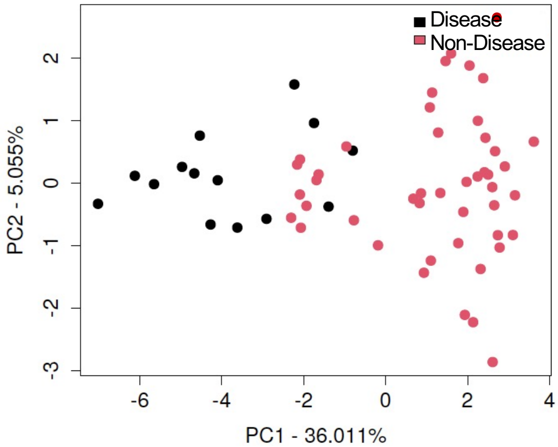

Supplement: Supplementary file 2 — Supplementary Figure S1. [file 41598_2022_9336_MOESM2_ESM.pdf]
