# Supplementary material for: Environmental induced transgenerational inheritance impacts systems epigenetics in disease etiology
Source: Sci Rep. 2022 Apr 19;12:5452. doi: 10.1038/s41598-022-09336-0 (PMC9018793; doi:10.1038/s41598-022-09336-0)

Supplemental Figure S2 DMR Chromosomal Locations

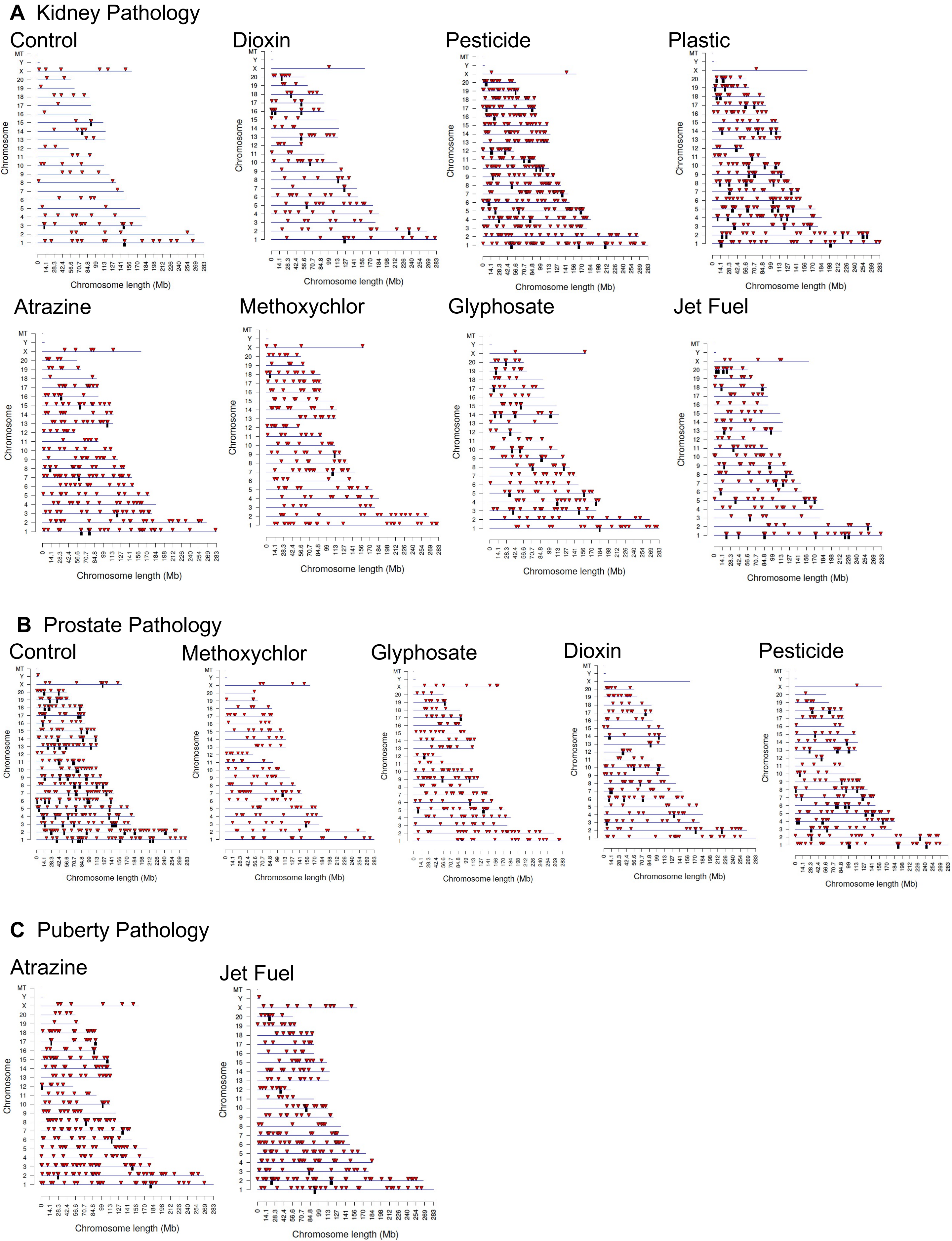

Supplemental Figure S2 (continued) DMR Chromosomal Locations

D Testis Pathology

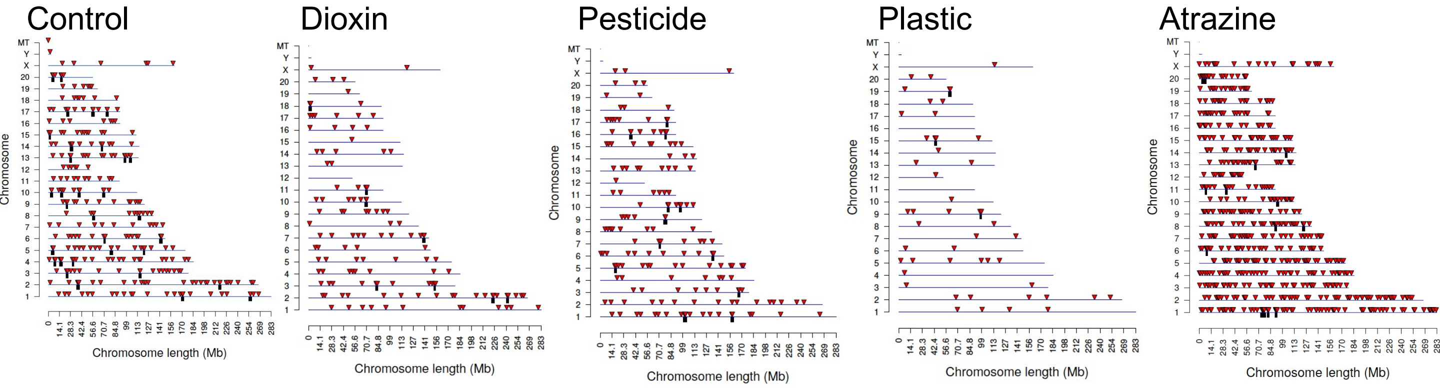

E Obesity

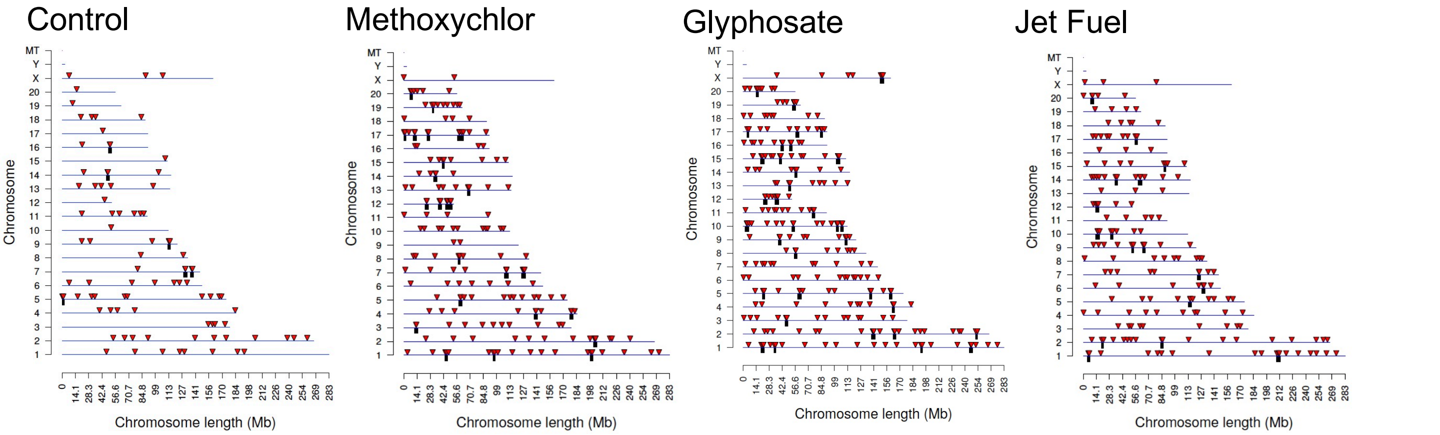

F Multiple Pathologies

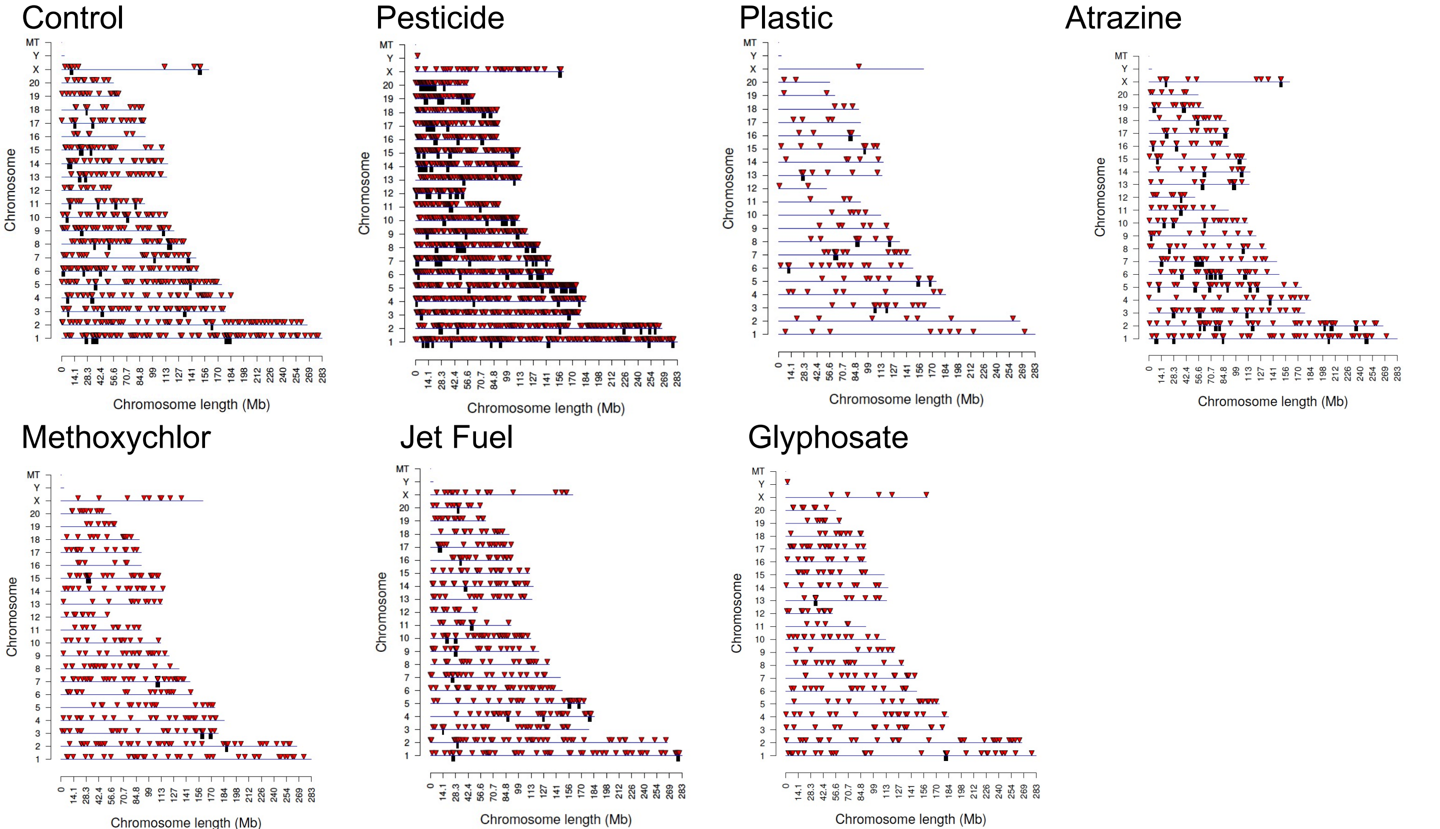

Supplement: Supplementary file 3 — Supplementary Figure S2. [file 41598_2022_9336_MOESM3_ESM.pdf]
