# Supplementary figures and images for: Environmental induced transgenerational inheritance impacts systems epigenetics in disease etiology
Source: Sci Rep. 2022 Apr 19;12:5452. doi: 10.1038/s41598-022-09336-0 (PMC9018793; doi:10.1038/s41598-022-09336-0)

## Sample Dendrogram and Trait Heatmap

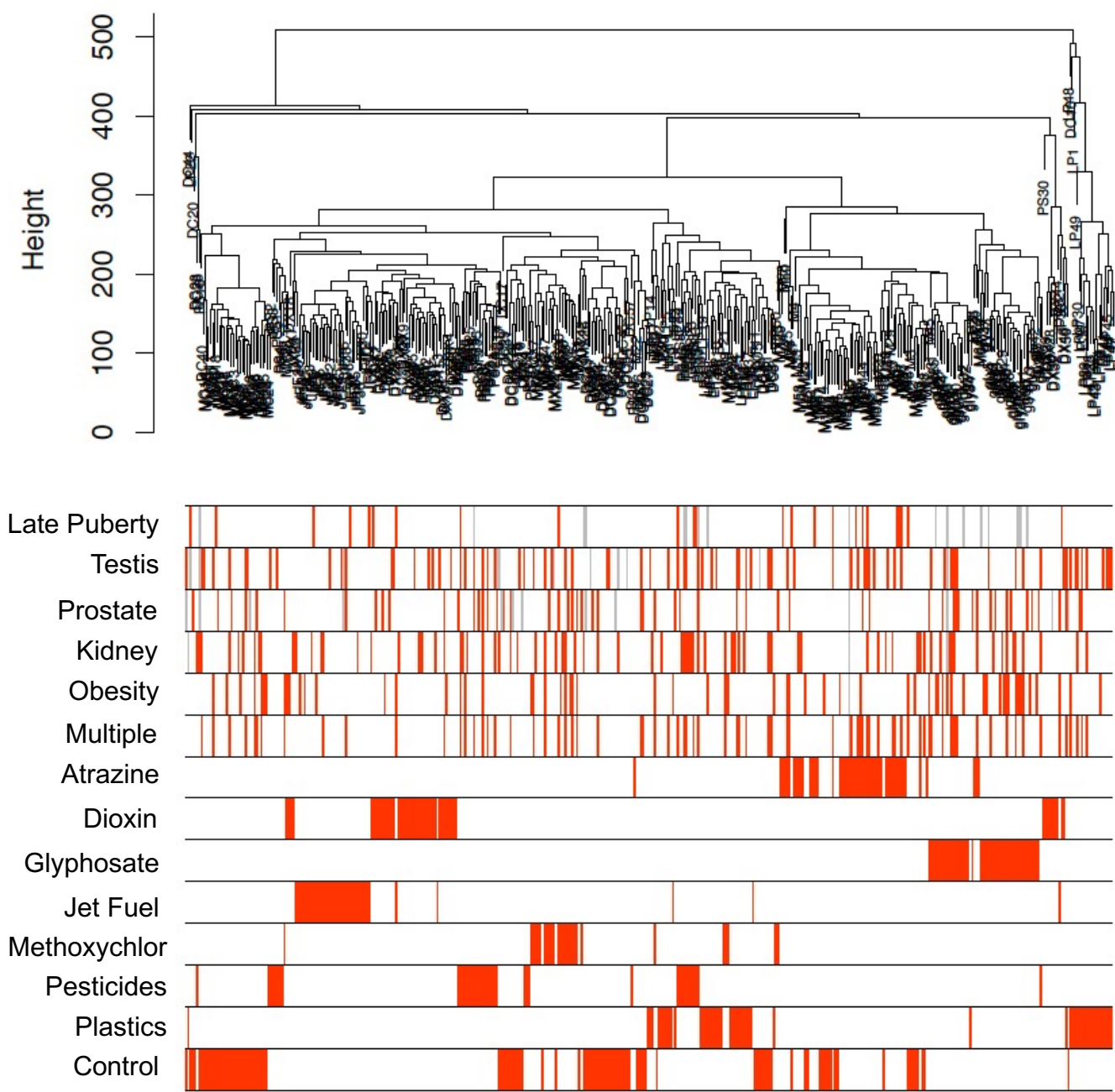

Supplement: Supplementary file 4 — Supplementary Figure S3. [file 41598_2022_9336_MOESM4_ESM.pdf]

F3 generation plastics lineage males pathology

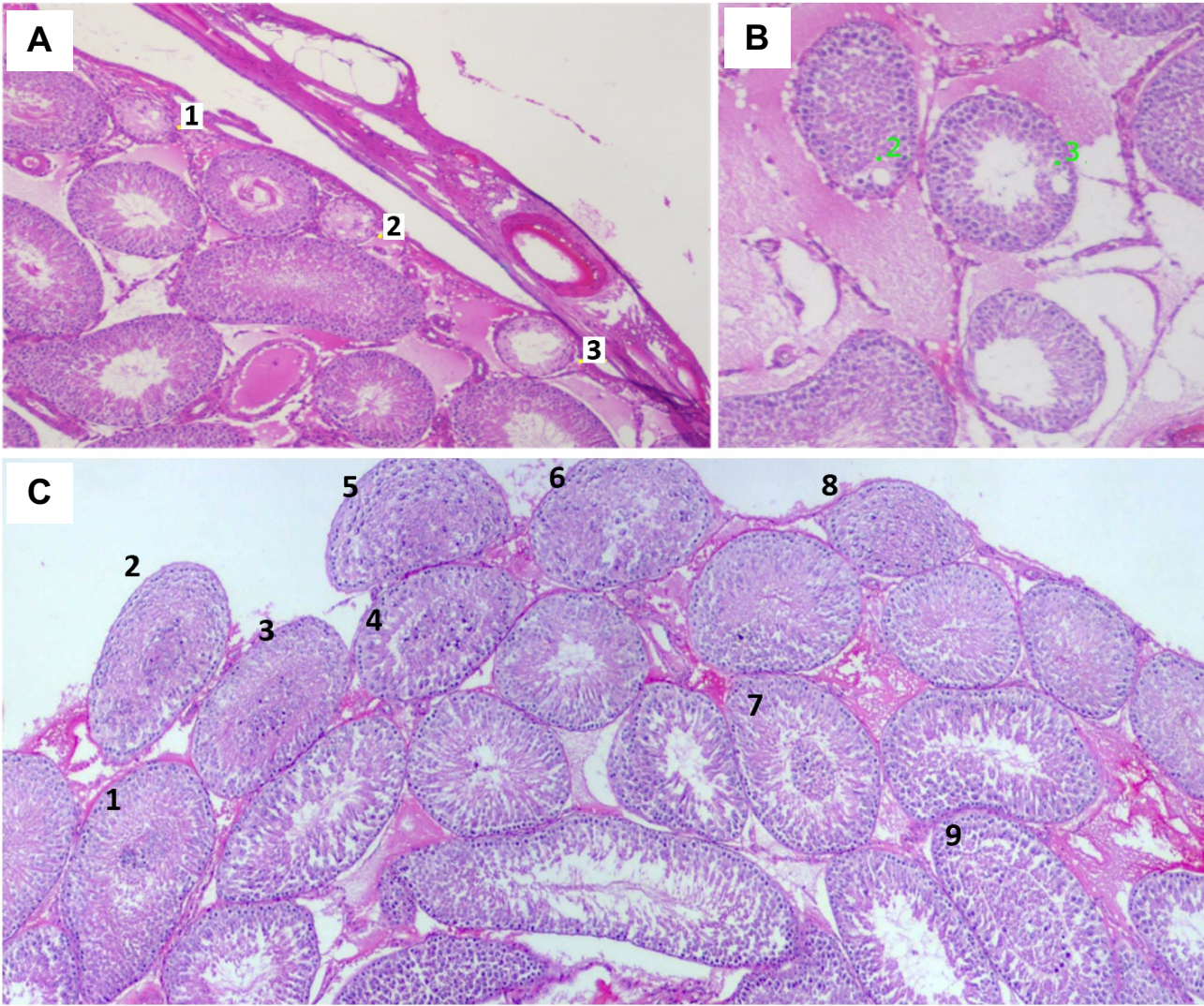

Supplement: Supplementary file 6 — Supplementary Figure S5. [file 41598_2022_9336_MOESM6_ESM.pdf]

F3 generation methoxychlor lineage male pathology

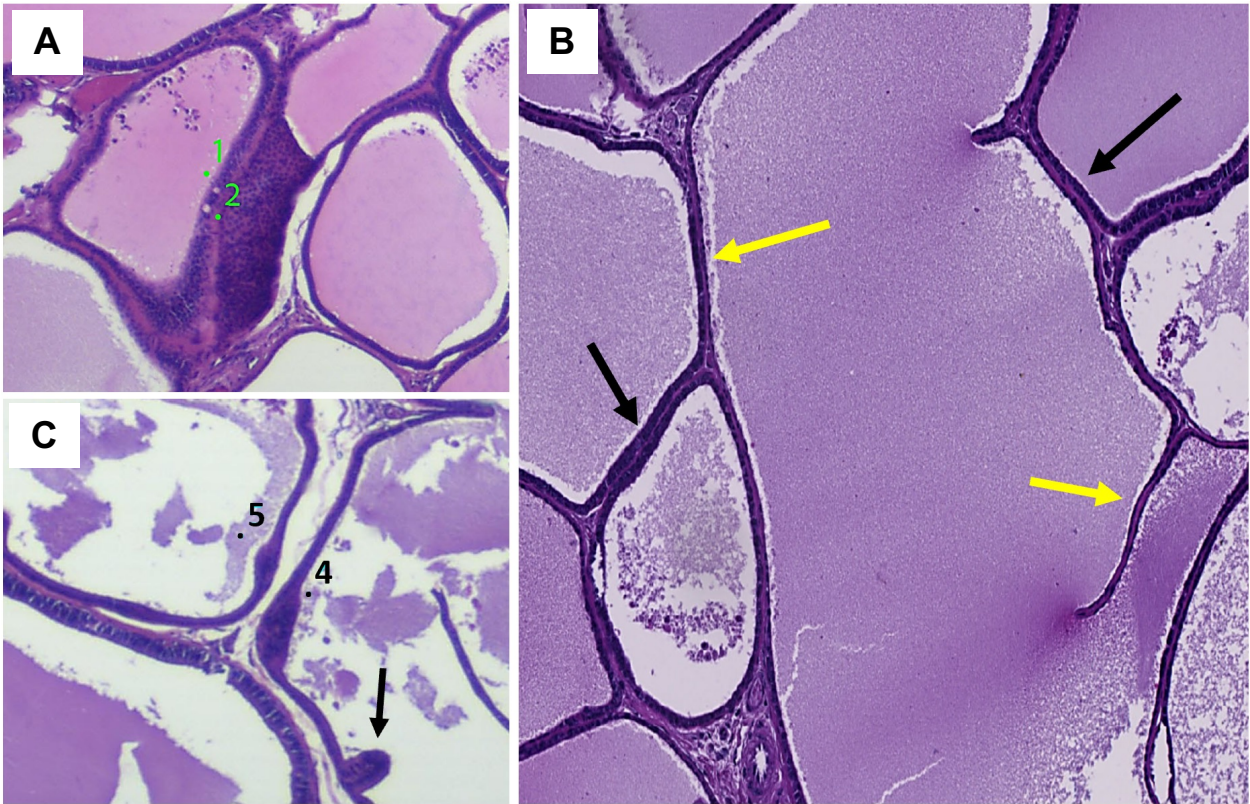

Supplement: Supplementary file 7 — Supplementary Figure S6. [file 41598_2022_9336_MOESM7_ESM.pdf]

F3 generation atrazine lineage males pathology

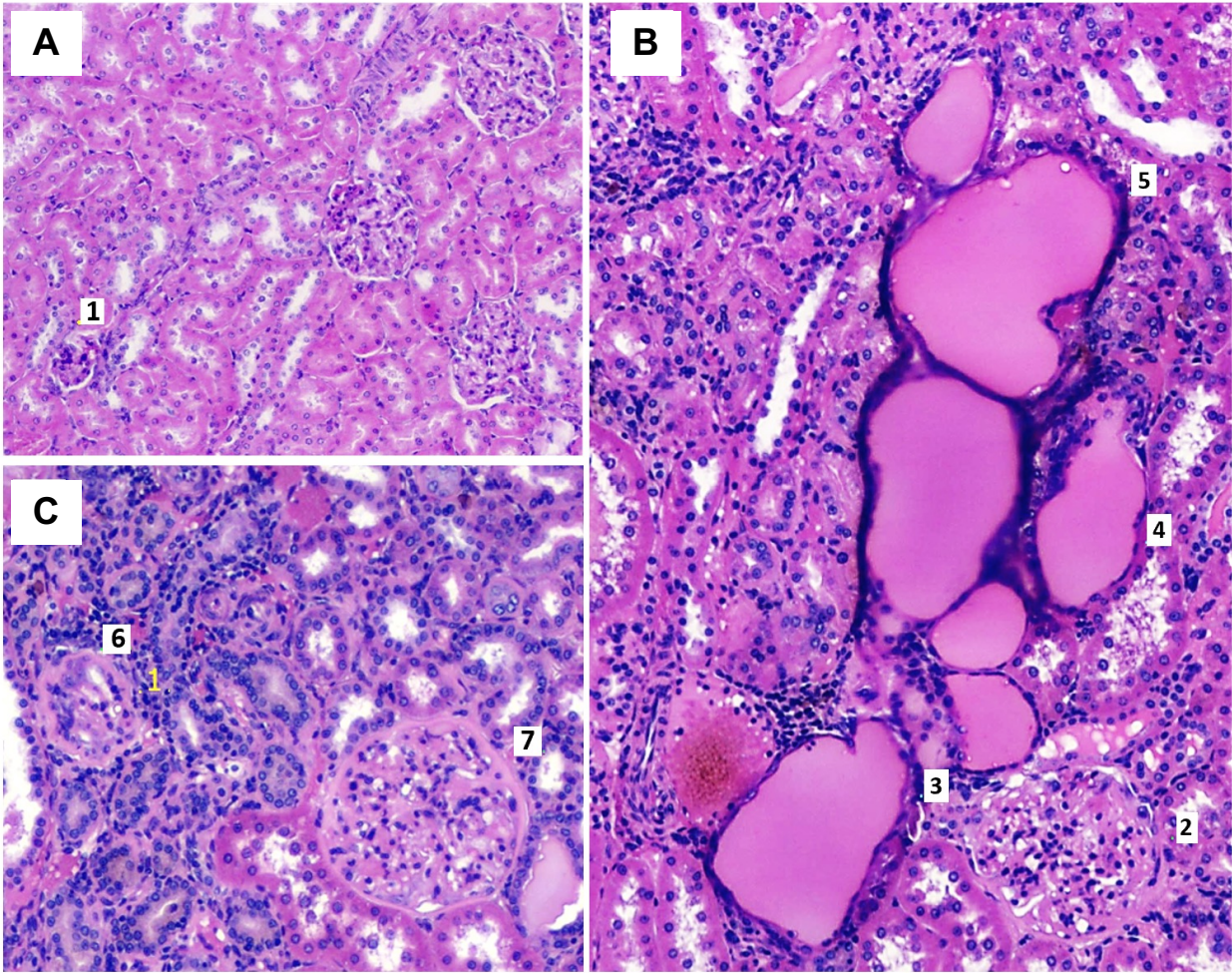

Supplement: Supplementary file 8 — Supplementary Figure S7. [file 41598_2022_9336_MOESM8_ESM.pdf]
