# Supplementary material for: Environmental induced transgenerational inheritance impacts systems epigenetics in disease etiology
Source: Sci Rep. 2022 Apr 19;12:5452. doi: 10.1038/s41598-022-09336-0 (PMC9018793; doi:10.1038/s41598-022-09336-0)

Supplemental Figure S4

A. Prostate Disease DMR Associated Gene Network

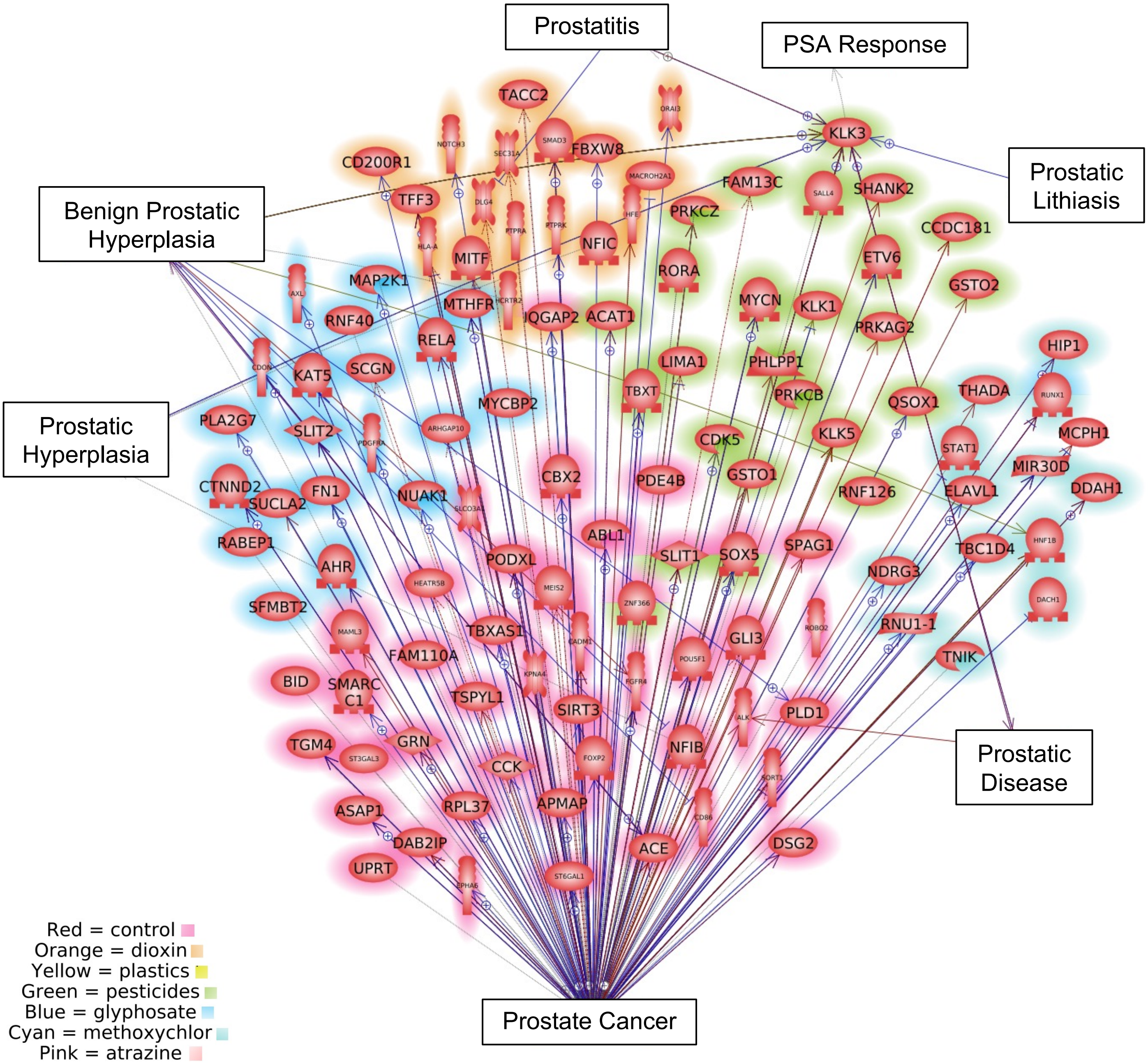

Red = control
Orange = dioxin
Yellow = plastics
Green = pesticides
Blue = glyphosate
Cyan = methoxychlor
Pink = atrazine

Supplemental Figure S4 (Continued)

B. Puberty Disease DMR Associated Gene Network

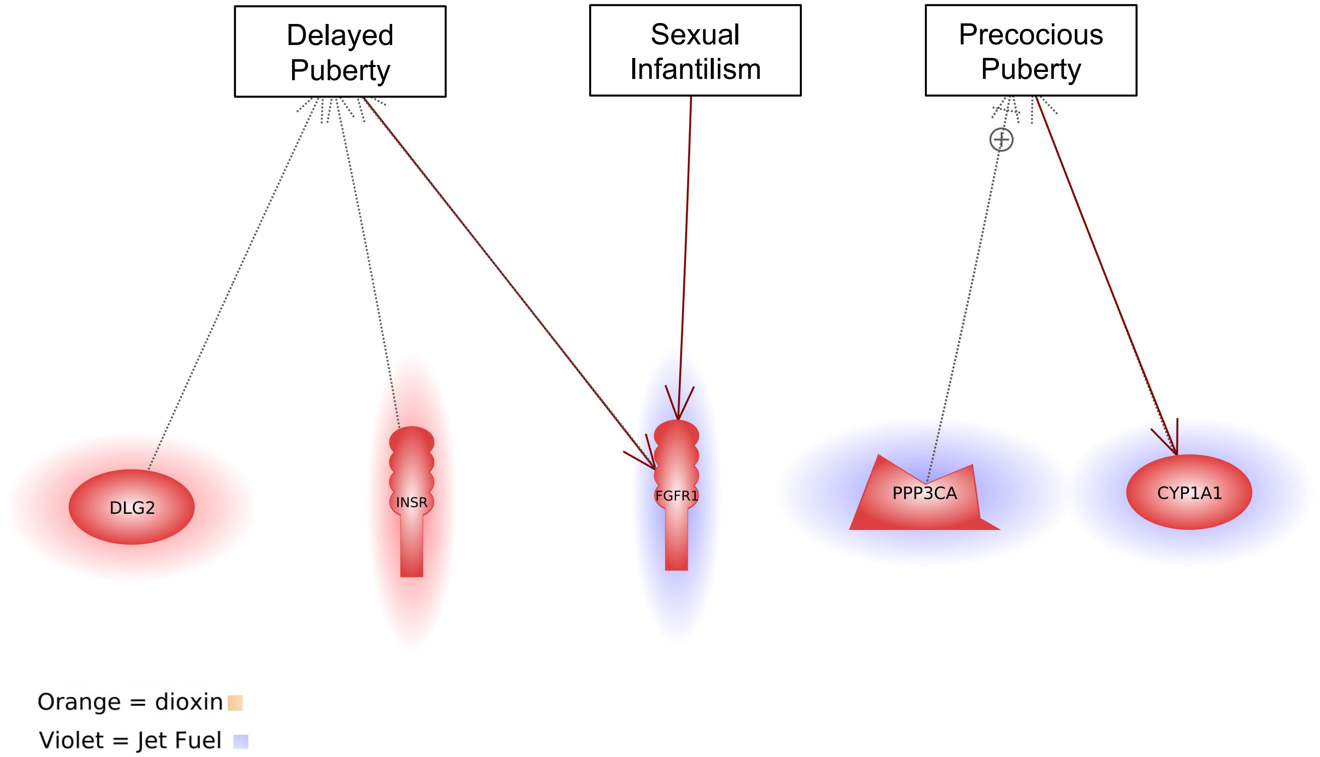

Supplemental Figure S4 (Continued)

C. Testis Disease DMR Associated Gene Network

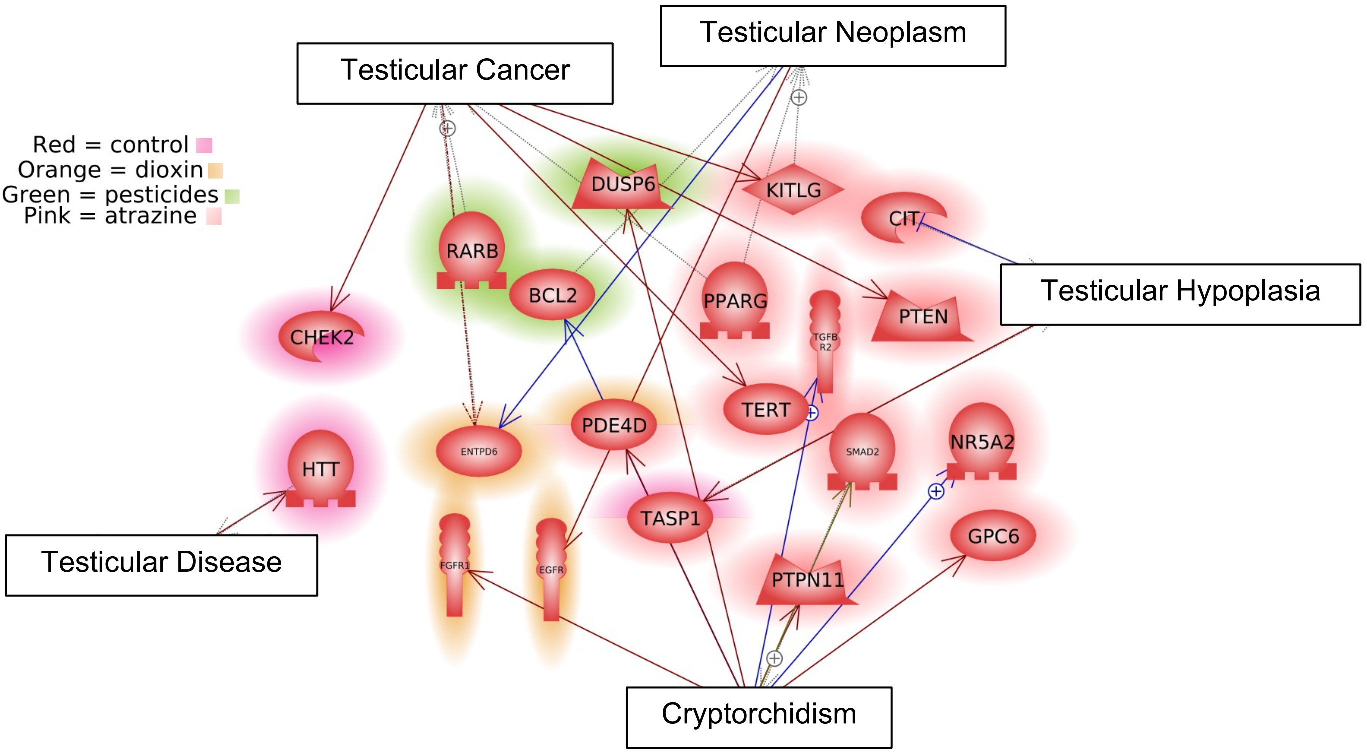

Supplemental Figure S4 (Continued)

D. Obesity Disease DMR Associated Gene Network

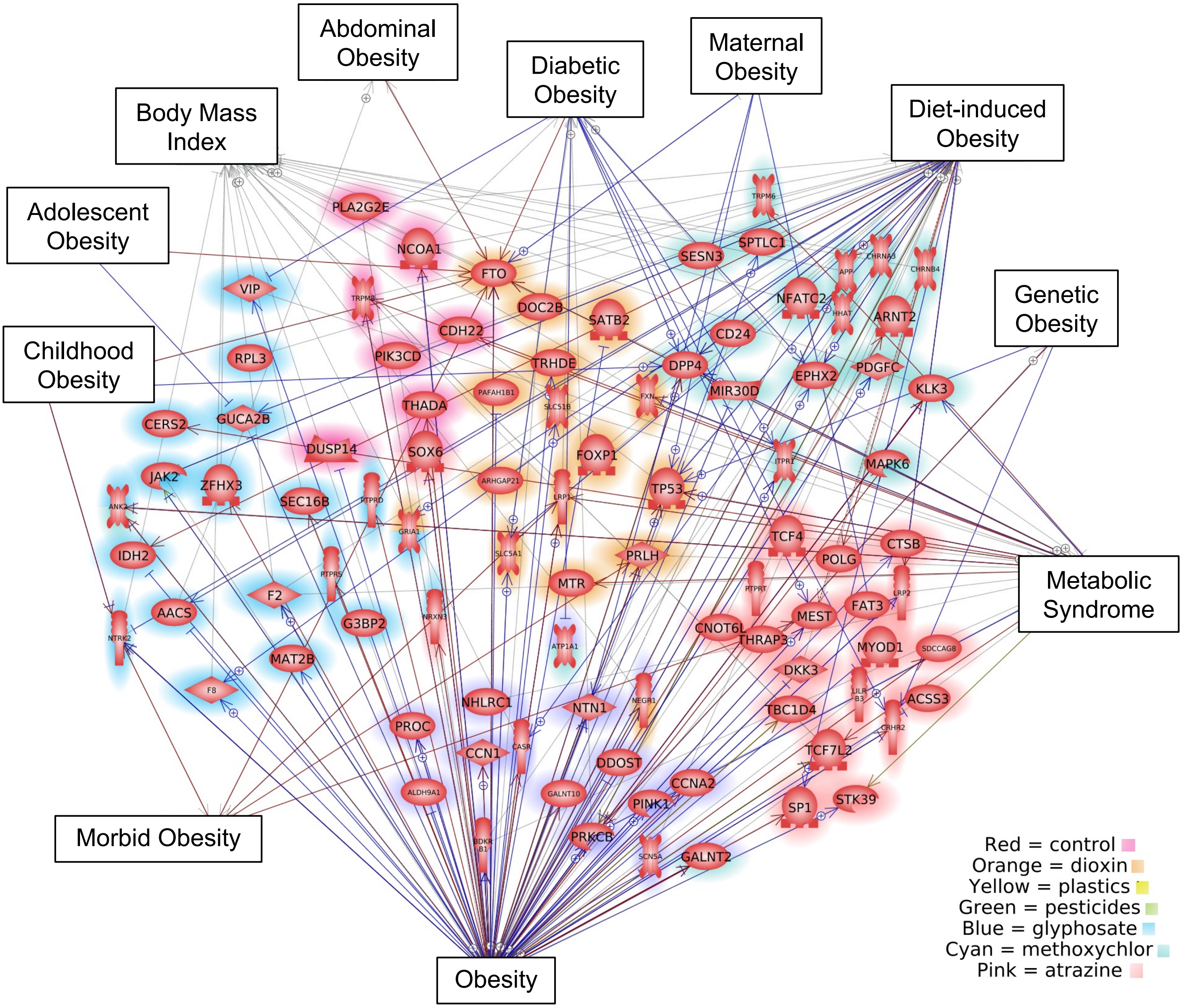

Supplement: Supplementary file 5 — Supplementary Figure S4. [file 41598_2022_9336_MOESM5_ESM.pdf]
