# Supplementary material for: Environmental induced transgenerational inheritance impacts systems epigenetics in disease etiology
Source: Sci Rep. 2022 Apr 19;12:5452. doi: 10.1038/s41598-022-09336-0 (PMC9018793; doi:10.1038/s41598-022-09336-0)
Supplement: Supplementary file 9 — Supplementary Table S1. [file 41598_2022_9336_MOESM9_ESM.pdf]

Supplemental Table S1
Control Lineage F3 Generation Male Transgenerational Pathology

Molecular ID	Early Puberty	Late Puberty	Testis Disease	Prostate Disease	Kidney Disease	Obesity	Tumor	Multiple Disease	Total Disease
DC51	-	-	+	-	-	-	-	-	1
DC1	-	-	-	-	-	-	-	-	0
DC2	-	-	+	-	+	-	-	+	2
DC3	-	-	+	-	+	-	-	+	2
DC52	-	-	+	-	-	-	-	-	1
DC4	-	-	n/a	-	-	-	-	-	n/a
DC5	-	-	-	-	-	-	-	-	0
DC6	-	-	-	-	-	-	-	-	0
DC7	-	-	-	-	-	-	-	-	0
DC8	-	-	-	-	-	-	-	-	0
DC9	-	-	-	-	-	-	-	-	0
DC10	-	-	-	-	-	-	-	-	0
DC11	-	-	-	+	-	-	-	-	1
DC12	-	-	-	-	-	-	-	-	0
DC13	-	-	+	-	-	-	-	-	1
DC14	-	-	-	n/a	-	-	-	-	n/a
DC15	-	-	-	n/a	-	-	-	-	n/a
DC16	-	-	-	n/a	-	-	-	-	n/a
DC17	-	-	n/a	-	+	-	-	-	1
DC53	-	-	-	-	-	-	-	-	0
DC18	-	-	-	-	-	-	-	-	0
DC19	-	-	-	-	+	-	-	-	1
DC20	-	+	n/a	-	-	-	-	-	1
DC54	-	-	-	-	-	-	-	-	0
DC55	-	-	-	-	-	-	-	-	0
DC21	-	-	-	-	-	-	-	-	0
DC22	-	-	-	-	-	-	-	-	0
DC56	-	-	-	n/a	-	-	-	-	n/a
DC57	-	-	n/a	-	-	-	-	-	n/a
DC23	-	-	-	+	-	-	-	-	1
DC24	-	-	-	+	+	-	-	+	2
DC25	+	-	-	+	-	-	-	+	2
DC26	+	-	-	+	-	-	-	+	2
DC27	+	-	-	-	-	-	-	-	1
DC28	-	-	-	-	-	-	-	-	0
DC29	-	-	-	n/a	-	-	-	-	n/a
DC30	-	-	-	-	-	-	-	-	0
DC31	-	-	-	+	-	-	-	-	1
DC32	-	-	-	+	-	-	-	-	1
DC33	-	-	-	-	-	-	-	-	0
DC34	-	-	-	+	+	-	-	+	2
DC35	-	-	-	-	-	-	-	-	0
DC36	-	-	-	-	-	-	-	-	0
DC60	-	-	n/a	-	+	-	-	-	1
DC37	-	-	n/a	-	-	-	-	-	n/a
DC38	-	-	+	+	-	-	-	+	2
DC39	n/a	n/a	-	-	+	-	-	-	1
DC40	n/a	n/a	n/a	n/a	+	-	-	-	1
DC41	n/a	n/a	-	n/a	-	-	-	-	n/a
DC42	-	-	-	-	-	-	-	-	0
DC43	-	-	-	-	+	-	-	-	1
DC44	-	-	+	n/a	-	-	-	-	1

DC45	-	-	-	-	-	-	-	-	0
DC46	-	-	-	-	-	-	-	-	0
DC47	-	-	-	-	-	-	-	-	0
DC48	-	-	-	-	-	-	-	-	0
DC49	-	-	-	-	-	-	-	-	0
DC50	-	-	-	-	-	-	-	-	0
MC2	-		-	-	-	+	-	-	1
MC3	-		-	+	-	-	-	-	1
MC4	-		-	-	-	-	-	-	0
MC5	-		-	-	-	-	-	-	0
MC6	-		-	-	+	-	-	-	1
MC7	-		-	+	-	-	-	-	1
MC8	-		-	-	+	-	-	-	1
MC9	-		+	+	-	-	-	+	2
MC10	+		-	-	-	-	-	-	1
MC11	-		-	-	-	-	-	-	0
MC12	-		-	-	-	-	-	-	0
MC13	-		-	-	-	-	-	-	0
MC14	-		+	-	+	-	-	+	2
MC15	-		+	-	-	-	-	-	1
MC16	-		-	-	-	-	-	-	0
MC17	-		-	-	-	+	-	-	1
MC18	-		+	-	-	+	-	+	2
MC19	-		+	-	+	-	-	+	2
MC20	-		-	-	-	-	-	-	0
MC21	-		-	-	-	-	-	-	0
MC22	-		-	-	-	-	-	-	0
MC23	-		-	-	-	+	-	-	1
MC24	-		-	-	-	+	-	-	1
MC25	-		-	-	+	+	-	+	2
MC26	-		+	-	-	-	-	-	1
MC27	-		-	n/a	-	-	-	-	0
MC28	-		-	-	-	-	-	-	0
MC29	-		-	-	+	+	-	+	2
MC30	-		-	-	+	-	-	-	1
CM1	-	-	-	-	-	+	-	-	1
CM2	-	-	-	-	-	-	-	-	0
CM3	-	-	-	-	-	-	-	-	0
CM4	-	-		-	-	-	-	-	0
CM5	-	-	-	-	-	-	-	-	0
CM6	-	-		-	+	+	-	+	2
CM7	-	-	-	-	-	+	-	-	n/a
CM8	-	-	-	-	-	-	-	-	0
CM9	-	-	-	-	-	+	-	-	n/a
CM10	-	-	-	-	-	+	-	-	n/a
CM11	-	-	-	-	-		-	-	0
CM12	-	-	+	+	-	-	-	+	2
CM13	-	-	-	-	-	-	-	-	0
CM14	-	-	-	-	-	-	-	-	0
CM16	-	-	-	-	-	-	-	-	0
GCM17	-	-				-	-	-	-
CM17	-	+	-	-	-	+	-	+	2
CM18	-	+	-	-	-	-	-	-	1
CM19	-	-	-	-	-	+	-	-	1
CM20	-	-	-	-	-	-	-	-	0
CM21	-	-	-	-	-	-	-	-	0
CM22	-	-	-	-	+	-	-	-	1

CM23	**-**	**-**	**-**	**-**	**-**	**-**	**-**	**-**	0
CM24	**-**	**-**	**-**	**-**	**-**	**-**	**-**	**-**	0
CM25	**-**	**-**	**-**	**-**	**-**	**-**	**-**	**-**	0
CM26	**-**	**-**	**-**	**-**	**-**	**+**	**-**	**-**	**1**
CM27	**-**	**-**	**-**	**-**	**-**		**-**	**-**	**0**
**Totals**	**4/111 =3.6%**	**3/82 =3.7%**	**14/104 =13.5%**	**13/104 =12.5%**	**19/113 =16.8%**	**15/112 =13.4%**	**0/114 =0%**	**16/114 =14%**	
