# Supplementary material for: Environmental induced transgenerational inheritance impacts systems epigenetics in disease etiology
Source: Sci Rep. 2022 Apr 19;12:5452. doi: 10.1038/s41598-022-09336-0 (PMC9018793; doi:10.1038/s41598-022-09336-0)
Supplement: Supplementary file 10 — Supplementary Table S2. [file 41598_2022_9336_MOESM10_ESM.pdf]

Supplemental Table S2
Jet Fuel Lineage F3 Generation Male Transgenerational Pathology

Molecular ID	Early Puberty	Late Puberty	Testis Disease	Prostate Disease	Kidney Disease	Obesity	Tumor	Multiple Disease	Total Disease
JF6	-	+	-	-	-	-	-	-	1
JF7	-	-	-	-	-	-	-	-	0
JF8	-	+	-	-	-	+	-	+	2
JF9	-	+	-	-	-	-	-	-	1
JF10	-	-	-	-	-	-	-	-	0
JF5	-	-	-	-	+	-	-	-	1
JF1	-	-	-	-	-	+	-	-	1
JF2	-	-	-	-	-	-	-	-	0
JF3	-	-	-	-	-	-	-	-	0
JF4	-	-	-	-	-	-	-	-	0
JF13	-	-	-	-	-	+	-	-	1
JF14	-	-	-	-	-	-	-	-	0
JF11	-	-	-	-	-	-	-	-	0
JF12	-	-	-	-	+	-	-	-	1
JF18	+	-	-	-	+	-	-	+	2
JF19	-	-	-	-	-	-	-	-	0
JF20	-	-	-	-	-	+	-	-	1
JF15	-	-	-	-	-	-	-	-	0
JF16	-	-	+	-	-	-	-	-	1
JF24	-	-	-	-	-	-	-	-	0
JF21	-	-	+	-	-	-	-	-	1
JF22	-	-	-	-	-	-	-	-	0
JF23	-	-	-	-	-	+	-	-	1
JF25	-	-	-	-	-	-	-	-	0
JF26	-	-	-	-	-	-	-	-	0
JF27	-	-	-	-	-	-	-	-	0
JF37	-	-	+	+	-	-	-	+	2
JF38	-	-	-	+	-	+	-	+	2
JF39	-	-	-	-	-	-	-	-	0
JF28	-	-	-	-	+	-	-	-	1
JF29	-	-	-	-	-	-	-	-	0
JF30	-	-	-	-	-	-	-	-	0
JF31	-	-	-	-	-	-	-	-	0
JF32	-	+	-	-	-	-	-	-	1
JF33	-	-	n/a	n/a	-	-	-	-	n/a
JF34	-	-	-	-	-	-	-	-	0
JF35	-	-	-	-	-	-	-	-	0
JF36	-	-	-	-	+	-	-	-	1
**Totals**	1/38 = 3%	4/38 = 11%	3/37 = 8%	2/37 = 5%	5/38 = 13%	6/38 = 16%	0/38 = 0%	4/38 = 11%	
