# Supplementary material for: Environmental induced transgenerational inheritance impacts systems epigenetics in disease etiology
Source: Sci Rep. 2022 Apr 19;12:5452. doi: 10.1038/s41598-022-09336-0 (PMC9018793; doi:10.1038/s41598-022-09336-0)
Supplement: Supplementary file 11 — Supplementary Table S3. [file 41598_2022_9336_MOESM11_ESM.pdf]

Supplemental Table S3
Dioxin Lineage F3 Generation Male Transgenerational Pathology

Molecular ID	Late Puberty	Testis Disease	Prostate Disease	Kidney Disease	Obesity	Tumor	Multiple Disease	Total Disease
DX14	-	-	+	+	-	-	+	2
DX9	-	-	-	-	-	-	-	0
DX10	-	-	-	-	-	-	-	0
DX11	-	+	-	-	-	-	-	1
DX12	-	-	-	-	-	-	-	0
DX13	-	-	-	+	-	-	-	1
DX1	-	-	-	+	-	-	-	1
DX2	+	-	-	-	-	-	-	1
DX4	-	-	-	-	+	-	-	1
DX5	-	-	-	-	-	-	-	0
DX6	-	-	-	-	-	-	-	0
DX7	-	-	-	-	+	-	-	1
DX8	-	-	-	-	-	-	-	0
DX18	-	-	-	-	+	-	-	1
DX15	-	+	-	-	-	-	-	1
DX16	-	+	-	-	-	-	-	1
DX25	-	-	-	-	+	-	-	1
DX26	-	-	-	-	-	-	-	0
DX27	-	-	-	-	-	-	-	0
DX28	-	-	-	-	-	-	-	0
DX29	-	-	-	-	-	-	-	0
DX34	-	-	-	+	-	-	-	1
DX35	-	-	-	-	-	-	-	0
DX30	-	-	-	-	-	-	-	0
DX31	-	-	-	-	-	-	-	0
DX32	-	-	n/a	-	-	-	-	n/a
DX33	-	-	-	+	-	-	-	1
DX21	-	-	+	-	-	-	-	1
DX19	-	-	+	-	-	-	-	1
DX20	-	-	-	-	-	-	-	0
DX22	-	+	-	-	-	-	-	1
DX23	-	-	+	-	-	-	-	1
DX24	-	+	-	-	-	-	-	1
DX41	-	+	-	-	-	-	-	1
DX42	-	+	-	-	-	-	-	1
DX43	-	-	-	-	-	-	-	0
DX40	-	-	-	+	-	-	-	1
DX36	-	-	-	-	-	-	-	0
DX37	-	-	-	-	-	-	-	0
DX38	-	-	-	-	-	-	-	0
DX39	-	-	-	-	-	-	-	0
DX53	-	-	-	+	-	-	-	1
DX50	-	+	-	-	-	-	-	1
DX51	-	-	-	+	-	-	-	1
DX52	-	-	-	-	-	-	-	0
DX44	-	-	-	-	-	-	-	0
DX45	-	-	-	-	-	-	-	0
DX46	+	-	-	-	-	-	-	1
DX47	-	-	-	-	-	-	-	0
DX48	-	-	-	-	-	-	-	0
DX49	-	-	+	-	-	-	-	1
**Totals**	2/51 = 4%	8/51 = 16%	5/50 = 10%	8/51 = 16%	4/51 = 8%	0/51 = 0%	1/51 = 2%	
