# Supplementary material for: Environmental induced transgenerational inheritance impacts systems epigenetics in disease etiology
Source: Sci Rep. 2022 Apr 19;12:5452. doi: 10.1038/s41598-022-09336-0 (PMC9018793; doi:10.1038/s41598-022-09336-0)
Supplement: Supplementary file 12 — Supplementary Table S4. [file 41598_2022_9336_MOESM12_ESM.pdf]

Supplemental Table S4
Pesticides Lineage F3 Generation Male Transgenerational Pathologies

Molecular ID	Late Puberty	Testis Disease	Prostate Disease	Kidney Disease	Obesity	Tumor	Multiple Disease	Total Disease
PS1	-	-	-	-	-	-	-	0
PS2	-	-	-	-	-	-	-	0
PS3	-	+	-	-	-	-	-	1
PS4	-	+	-	-	-	-	-	1
PS5	-	-	-	-	-	-	-	0
PS6	-	-	-	-	-	-	-	0
PS7	-	-	-	-	-	-	-	0
PS8	-	-	-	-	-	-	-	0
PS9	-	+	-	-	-	-	-	1
PS10	-	-	-	-	-	-	-	0
PS11	-	-	-	-	-	-	-	0
PS12	-	-	-	-	-	-	-	0
PS13	-	-	-	-	-	-	-	0
PS14	-	+	-	+	-	-	+	2
PS15	-	-	-	-	-	-	-	0
PS16	-	-	-	+	-	-	-	1
PS17	-	n/a	+	-	-	-	-	1
PS18	-	-	-	-	-	-	-	0
PS19	-	-	+	-	-	-	-	1
PS20	-	+	+	+	+	-	+	4
PS21	-	-	-	-	-	-	-	0
PS22	-	+	+	-	-	-	+	2
PS23	-	-	-	+	-	-	-	1
PS24	-	-	-	-	-	-	-	0
PS25	-	-	-	+	-	-	-	1
PS26	-	-	+	-	-	-	-	1
PS27	+	+	-	+	+	-	+	4
PS28	-	-	-	+	-	-	-	1
PS29	-	+	-	-	+	-	+	2
PS30	-	+	-	+	-	-	+	2
PS31	-	-	-	+	-	-	-	1
PS32	+	+	-	-	-	-	+	2
PS33	+	-	-	-	-	-	-	1
PS34	+	-	-	+	-	-	+	2
PS35	n/a	+	+	-	-	-	+	2
PS36	n/a	+	-	+	-	-	+	2
PS37	n/a	+	+	+	-	-	+	3
PS38	n/a	+	-	+	-	-	+	2
PS39	-	-	-	-	-	+	-	1
PS40	-	-	+	-	-	-	-	1
PS41	-	-	-	+	-	-	-	1
**Totals**	4/37 = 11%	14/40 = 35%	8/41 = 20%	14/41 = 34%	3/41 = 7%	1/41 = 2%	12/41 = 29%	
