# Supplementary material for: Environmental induced transgenerational inheritance impacts systems epigenetics in disease etiology
Source: Sci Rep. 2022 Apr 19;12:5452. doi: 10.1038/s41598-022-09336-0 (PMC9018793; doi:10.1038/s41598-022-09336-0)
Supplement: Supplementary file 13 — Supplementary Table S5. [file 41598_2022_9336_MOESM13_ESM.pdf]

Supplemental Table S5
Plastics Lineage F3 Generation Male Transgenerational Pathology

Molecular ID	Puberty	Testis Disease	Prostate Disease	Kidney Disease	Obesity	Tumor	Multiple Disease	Total Disease
LP1	-	+	-	-	+	-	+	2
LP2	-	+	+	-	-	-	+	2
LP3	-	+	-	-	-	+	+	2
LP4	-	+	-	-	-	-	-	1
LP5	-	-	-	-	-	-	-	0
LP6	-	+	-	-	-	-	-	1
LP7	-	-	-	+	-	-	-	1
LP8	-	-	-	-	-	-	-	0
LP9	-	-	-	-	-	-	-	0
LP10	-	-	-	-	-	-	-	0
LP11	-	-	-	-	-	-	-	0
LP12	-	+	-	+	-	-	+	2
LP13	-	-	-	-	-	-	-	0
LP14	-	-	-	-	-	-	-	0
LP15	-	-	-	-	-	-	-	0
LP16	-	-	-	+	-	-	-	1
LP19	-	-	-	-	-	-	-	0
LP20	-	-	-	+	-	-	-	1
LP21	-	-	-	-	-	-	-	0
LP17	-	+	-	-	-	-	-	1
LP18	n/a	-	-	-	+	-	-	1
LP22	-	-	-	n/a	-	-	-	n/a
LP23	-	-	-	-	-	-	-	0
LP24	-	-	-	+	-	-	-	1
LP25	-	-	-	+	-	-	-	1
LP26	-	+	-	-	-	-	-	1
LP27	-	+	-	+	-	-	+	2
LP28	-	+	-	+	-	+	+	3
LP29	-	+	+	-	-	-	+	2
LP30	-	+	-	-	-	-	-	1
LP31	-	-	-	-	-	-	-	0
LP32	-	-	-	-	-	-	-	0
LP33	-	-	-	-	-	-	-	0
LP34	-	-	+	-	-	-	-	1
LP35	-	-	+	-	-	-	-	1
LP36	-	-	-	-	-	-	-	0
LP37	-	+	+	-	-	-	+	2
LP38	-	-	-	-	-	-	-	0
LP40	-	-	-	+	-	-	-	1
LP39	-	+	-	-	-	-	-	1
LP41	-	-	-	-	+	-	-	1
LP42	-	-	-	-	-	-	-	0
LP43	-	-	-	-	-	-	-	0
LP44	-	+	-	-	-	-	-	1
LP45	-	-	-	-	-	-	-	0
LP46	-	+	-	-	-	-	-	1
LP47	-	+	-	-	-	-	-	1
LP48	-	+	+	-	-	-	+	2
LP49	-	-	-	-	-	-	-	0
LP50	-	-	-	-	+	-	-	1
LP51	-	-	-	-	-	-	-	0
LP52	-	-	-	-	-	-	-	0
**Totals**	0/48 = 0%	18/52 = 35%	6/52 = 12%	9/51 = 18%	4/52 = 7%	2/52 = 4%	9/52 = 17%	
