# Supplementary material for: Environmental induced transgenerational inheritance impacts systems epigenetics in disease etiology
Source: Sci Rep. 2022 Apr 19;12:5452. doi: 10.1038/s41598-022-09336-0 (PMC9018793; doi:10.1038/s41598-022-09336-0)
Supplement: Supplementary file 14 — Supplementary Table S6. [file 41598_2022_9336_MOESM14_ESM.pdf]

Supplemental Table S6
Methoxychlor Lineage F3 Generation Male Transgenerational Pathology

Molecular ID	Puberty	Testis Disease	Prostate Disease	Kidney Disease	Obesity	Tumor	Multiple Disease	Total Disease
MX1	-	-	+	-	-	-	-	1
MX2	-	+	-	+	+	-	+	3
MX3	-	-	+	-	+	-	+	2
MX4	+	-	+	-	-	-	+	2
MX5	-	+	-	-	+	-	+	2
MX6	-	-	-	+	-	-	-	1
MX7	-	-	-	-	+	-	-	1
MX8	-	-	-	-	+	-	-	1
MX9	-	+	+	-	+	-	+	2
MX10	-	-	-	-	+	-	-	1
MX11	-	-	-	+	-	-	-	1
MX12	-	-	-	-	+	-	-	1
MX13	-	-	+	-	+	-	+	2
MX14	-	+	-	+	+	-	+	3
MX15	-	-	-	+	+	-	+	2
MX16	-	-	+	+	-	-	+	2
MX17	-	-	-	-	-	-	-	0
MX18	-	-	-	-	-	-	-	0
MX19	-	-	-	+	-	-	-	1
MX20	-	-	-	-	-	-	-	0
MX21	-	-	-	-	-	-	-	0
MX22	-	+	-	-	-	-	-	1
MX23	-	-	-	-	-	-	-	0
MX24	-	n/a	-	-	-	-	-	n/a
MX25	-	-	+	-	-	-	-	1
MX26	-	-	+	-	-	-	-	1
MX27	-	-	-	-	+	-	-	1
MX28	-	-	+	-	+	-	+	2
MX29	-	-	+	-	-	-	-	1
**Totals**	1/29 = 3%	5/28 = 18%	10/29 = 34%	7/29 = 24%	13/29 = 45%	0/29 = 0%	10/29 = 34%	
