# Supplementary material for: Environmental induced transgenerational inheritance impacts systems epigenetics in disease etiology
Source: Sci Rep. 2022 Apr 19;12:5452. doi: 10.1038/s41598-022-09336-0 (PMC9018793; doi:10.1038/s41598-022-09336-0)
Supplement: Supplementary file 15 — Supplementary Table S7. [file 41598_2022_9336_MOESM15_ESM.pdf]

Supplemental Table S7
Atrazine Lineage F3 Generation Male Transgenerational Pathology

Animal ID	Early Puberty	Late Puberty	Testis Disease	Prostate Disease	Kidney Disease	Lean	Obese	Tumor	Multiple Disease	Total Disease
AM1	-	-	-	-	+	-	-	-	-	1
AM2	-	-	-	+	+	-	-	-	+	2
AM3	-	-	-	-	-	-	-	-	-	0
AM4	-	-	-	-	+	+	-	-	+	2
AM5	-	-	-	-	+	-	-	-	-	1
AM6	-	-	+	-	-	+	-	-	+	2
AM7	-	-	-	-	-	-	-	-	-	0
AM8	-	-	-	-	-	-	-	-	-	0
AM9	-	-	-	-	-	-	-	-	-	0
AM10	-	-	+	-	-	-	-	-	-	1
AM11	-	-	-	-	-	+	-	-	-	1
AM12	-	-	-	-	-	-	-	-	-	0
AM13	-	-	-	-	-	-	-	-	-	0
AM14	-	-	+	-	-	+	-	-	+	2
AM15	-	-	-	-	-	-	-	-	-	0
AM16	-	-	-	-	+	-	-	-	-	1
AM17	-	-	-	-	-	-	-	-	-	0
AM18	-	-	-	-	-	-	-	-	-	0
AM19	-	-	+	-	-	-	+	-	+	2
AM20	-	-	-	+	-	-	+	-	+	2
AM21	-	-	+	+	-	+	-	-	+	3
AM22	-	-	-	-	-	-	-	-	-	0
AM23	-	-	-	-	-	+	-	-	-	1
AM24	-	+	+	-	-	-	-	-	+	2
AM25	-	+	-	-	-	-	-	-	-	1
AM26	-	+	-	-	-	-	-	-	-	1
AM27	-	+	+	-	-	+	-	-	+	3
AM28	-	+	-	-	-	-	-	-	-	1
AM29	-	+	-	-	-	-	-	-	-	1
AM30	-	-	-	-	-	-	+	+	+	2
AM31	-	-	-	-	-	-	-	-	-	0
AM32	-	-	-	-	-	-	-	-	-	0
AM33	-	-	-	-	-	-	-	-	-	0
AM34	-	-	-	-	-	-	-	-	-	0
AM35	-	-	-	-	-	-	-	-	-	0
AM36	-	-	+	-	-	-	-	-	-	1
AM37	-	-	+	-	-	+	-	-	+	2
AM38	-	-	-	-	-	-	-	-	-	0
AM39	-	-	+	-	-	-	-	-	-	1
AM40	-	-	+	-	-	-	-	-	-	1
AM41	-	-	-	-	-	-	-	-	-	0
AM42	-	-	-	-	-	+	-	-	-	1
AM43	-	-	-	-	-	-	-	-	-	0
AM44	-	-	-	-	-	-	+	-	-	1
AM45	-	-	-	-	-	-	-	-	-	0
AM46	-	+	+	-	+	+	-	-	+	4
AM47	-	+	-	+	-	+	-	-	+	3
AM48	-	+	-	-	-	-	-	-	-	1
AM49	-	+	-	-	-	-	-	-	-	1
AM50	-	-	-	-	-	+	-	-	-	1
AM51	-	-	-	-	-	-	-	-	-	0
AM52	-	-	-	-	-	+	-	-	-	1
AM53	-	-	-	-	+	+	-	-	+	2
AM54	-	-	-	-	+	+	-	-	+	2
**# affected**	**0**	**10**	**12**	**4**	**8**	**15**	**4**	**1**		
**# evaluated**	**55**	**55**	**55**	**55**	**55**	**55**	**55**	**55**		
