# Supplementary material for: Environmental induced transgenerational inheritance impacts systems epigenetics in disease etiology
Source: Sci Rep. 2022 Apr 19;12:5452. doi: 10.1038/s41598-022-09336-0 (PMC9018793; doi:10.1038/s41598-022-09336-0)
Supplement: Supplementary file 16 — Supplementary Table S8. [file 41598_2022_9336_MOESM16_ESM.pdf]

Supplemental Table S8
Glyphosate Lineage F3 Generation Male Pathology

ID	Puberty	Testis Disease	Prostate Disease	Kidney Disease	Tumor	Lean	Obese	Multiple Disease	Total Disease
Gly-1		-	-	-	-	-	+	-	**1**
Gly-2		-	-	-	-	-	+	-	**1**
Gly-3		-	+	-	-	-	-	-	**1**
Gly-4		+	-	-	-	-	-	-	**1**
Gly-5	-	-	-	-	-	-	+	-	**1**
Gly-6	-	+	-	+	-	-	+	+	**3**
Gly-7	-	-	-	-	-	-	+	-	**1**
Gly-8	-	-	-	+	-	-	-	-	**1**
Gly-9	-	-	+	-	-	-	-	-	**1**
Gly-10	-	-	-	-	-	-	-	-	**0**
Gly-11	-	-	-	-	-	-	-	-	**0**
Gly-12	-	-	-	-	-	-	+	-	**1**
Gly-13	-	+	+	-	-	-	-	+	**2**
Gly-14	-	-	-	+	-	-	-	-	**1**
Gly-15	-	-	+	-	-	-	-	-	**1**
Gly-16	-	-	-	-	-	-	+	-	**1**
Gly-17	-	-	-	+	-	-	-	-	**1**
Gly-18	-	-	-	-	-	-	-	-	**0**
Gly-19	-	+	-	+	-	-	+	+	**3**
Gly-20	-	+	+	+	-	-	-	+	**3**
Gly-21	-	+	-	+	-	-	+	+	**3**
Gly-22						-	+	-	**1**
Gly-23		-	-	-	-	-	+	-	**1**
Gly-24	-	-	-	+	-	-	-	-	**1**
Gly-25	-	+	-	+	-	-	-	+	**2**
Gly-26		-	-	-	-	-	+	-	**1**
Gly-27	-	-	-	-	-	-	-	-	**0**
Gly-28	-	-	+	+	-	-	+	+	**3**
Gly-29	-	-	-	-	-	-	+	-	**1**
Gly-30	-	-	+	-	-	-	+	+	**2**
Gly-31	-	-	+	-	-	-	+	+	**2**
Gly-32	-	-	-	-	-	-	-	-	**0**
Gly-33	-	+	-	-	-	-	-	-	**1**
Gly-34	-	-	-	-	-	-	-	-	**0**
Gly-35	-	-	-	-	-	-	+	-	**1**
Gly-36	-	-	+	-	-	-	-	-	**1**
Gly-37		-	-	-	-	-	-	-	**0**
Gly-38	-	-	-	-	-	-	-	-	**0**
Gly-39	-	-	-	-	-	-	+	-	**1**
Gly-40	-	-	+	-	+	-	-	+	**2**
Gly-41	-	-	+	-	-	-	-	-	**1**
Gly-42	-	-	+	-	-	-	-	-	**1**
Gly-43	-	-	-	-	-	-	+	-	**1**
Gly-44	-	-	-	+	-	-	-	-	**1**
Gly-45	-	-	+	-	-	-	-	-	**1**
**Affected**	**0**	**8**	**13**	**11**	**1**	**0**	**19**	**10**	
**Population**	**37**	**44**	**44**	**44**	**44**	**45**	**45**	**45**	
