# Supplementary material for: Environmental induced transgenerational inheritance impacts systems epigenetics in disease etiology
Source: Sci Rep. 2022 Apr 19;12:5452. doi: 10.1038/s41598-022-09336-0 (PMC9018793; doi:10.1038/s41598-022-09336-0)
Supplement: Supplementary file 17 — Supplementary Table S9. [file 41598_2022_9336_MOESM17_ESM.pdf]

Supplemental Table S9
Jet Fuel DMR p<1e-06

DMR Name	Chr	Start	Stop	Length	# Sig Win	minP	maxLFC	CpG #	CpG Density	Gene Annotation	Gene Category
DMR1:567001	1	567001	570000	3000	1	1.20E-07	-0.36	23	0.77	Vom2r-ps5	
DMR1:649001	1	649001	650000	1000	1	2.40E-07	-0.44	19	1.9	Vom2r-ps5	
DMR1:656001	1	656001	660000	4000	1	4.70E-08	-0.47	150	3.75	Vom2r-ps5	
DMR1:721001	1	721001	727000	6000	1	3.60E-14	-0.43	48	0.8	Vom2r4	Signaling
DMR1:1682001	1	1682001	1685000	3000	1	5.80E-07	-0.53	22	0.73	LOC108348623;RGD1561843	
DMR1:1746001	1	1746001	1747000	1000	1	2.40E-07	-0.38	16	1.6	Pcmt1	Epigenetic
DMR1:1795001	1	1795001	1796000	1000	1	3.60E-07	-0.53	15	1.5	Lats1	Signaling
DMR1:1947001	1	1947001	1949000	2000	1	7.30E-09	0.36	29	1.45	Zc3h12d	Translation
DMR1:1976001	1	1976001	1977000	1000	1	5.20E-10	-0.44	18	1.8	Zc3h12d	Translation
DMR1:2335001	1	2335001	2337000	2000	1	9.10E-11	0.54	18	0.9	Ust;LOC108348767	Transport
DMR1:2442001	1	2442001	2447000	5000	1	1.40E-14	0.41	47	0.94	Ust	Transport
DMR1:2471001	1	2471001	2474000	3000	1	1.40E-07	-0.56	51	1.7	Ust	Transport
DMR1:2598001	1	2598001	2599000	1000	1	5.50E-08	0.66	16	1.6	Ust	Transport
DMR1:2856001	1	2856001	2859000	3000	3	1.50E-11	-0.59	60	2	Sash1	
DMR1:2927001	1	2927001	2929000	2000	1	1.60E-07	0.36	48	2.4	Sash1	
DMR1:2953001	1	2953001	2955000	2000	1	2.30E-07	-0.46	27	1.35	Sash1	
DMR1:3736001	1	3736001	3737000	1000	1	1.00E-09	0.43	4	0.4	Samd5	
DMR1:3853001	1	3853001	3856000	3000	1	2.80E-09	0.6	53	1.77	RGD1560633;Stxbp5	Translation;Transport
DMR1:4001001	1	4001001	4002000	1000	1	1.20E-08	0.29	3	0.3	Stxbp5	Transport
DMR1:4469001	1	4469001	4472000	3000	2	1.90E-10	-0.4	19	0.63	Adgb	
DMR1:4524001	1	4524001	4529000	5000	1	5.10E-07	-0.41	43	0.86	Adgb	
DMR1:4559001	1	4559001	4566000	7000	1	4.20E-07	-0.34	82	1.17	Adgb	
DMR1:4754001	1	4754001	4756000	2000	1	1.40E-08	0.64	35	1.75	Grm1	Signaling
DMR1:4786001	1	4786001	4787000	1000	1	6.90E-12	0.98	32	3.2	Grm1	Signaling
DMR1:5027001	1	5027001	5028000	1000	1	1.50E-14	0.77	22	2.2	Grm1	Signaling
DMR1:5127001	1	5127001	5128000	1000	1	1.20E-12	0.72	10	1	Grm1	Signaling
DMR1:5149001	1	5149001	5150000	1000	1	5.20E-07	-0.53	19	1.9	Grm1	Signaling
DMR1:5568001	1	5568001	5570000	2000	1	5.40E-08	0.39	4	0.2	Epm2a	
DMR1:5574001	1	5574001	5577000	3000	1	3.20E-07	-0.47	8	0.27	Epm2a	
DMR1:6469001	1	6469001	6470000	1000	1	6.00E-08	-0.46	17	1.7	Utrn	
DMR1:6565001	1	6565001	6567000	2000	1	1.20E-08	-0.42	13	0.65	Utrn	
DMR1:6596001	1	6596001	6603000	7000	2	1.90E-13	-0.53	85	1.21	Utrn	
DMR1:6642001	1	6642001	6643000	1000	1	5.80E-07	-0.41	23	2.3	Utrn	
DMR1:6685001	1	6685001	6686000	1000	1	6.90E-19	0.42	12	1.2	Utrn	
DMR1:6783001	1	6783001	6784000	1000	1	1.10E-07	-0.51	13	1.3	Utrn	
DMR1:6828001	1	6828001	6830000	2000	1	3.40E-07	0.39	27	1.35	Utrn	
DMR1:6863001	1	6863001	6864000	1000	1	2.00E-09	0.57	1	0.1	Utrn	
DMR1:6872001	1	6872001	6877000	5000	2	1.30E-08	-0.54	67	1.34	Utrn	
DMR1:6879001	1	6879001	6880000	1000	1	1.50E-09	-0.48	11	1.1	Utrn	
DMR1:6971001	1	6971001	6973000	2000	1	4.10E-10	0.43	21	1.05	Utrn	
DMR1:7031001	1	7031001	7034000	3000	1	7.60E-07	0.58	69	2.3	Stx11	Transcription
DMR1:7048001	1	7048001	7049000	1000	1	2.10E-13	0.87	30	3	Stx11	Transcription
DMR1:7283001	1	7283001	7285000	2000	1	1.60E-07	-0.43	27	1.35	Zc2hc1b	Transcription
DMR1:7358001	1	7358001	7362000	4000	1	7.60E-08	-0.51	50	1.25	Phactr2	Signaling
DMR1:7511001	1	7511001	7513000	2000	1	4.40E-07	-0.41	20	1	Phactr2	Signaling
DMR1:7581001	1	7581001	7587000	6000	2	7.00E-15	0.46	98	1.63	Phactr2	Signaling
DMR1:7595001	1	7595001	7601000	6000	1	2.10E-07	-0.67	98	1.63	Phactr2	Signaling
DMR1:7604001	1	7604001	7605000	1000	1	2.40E-08	0.36	10	1	Phactr2	Signaling
DMR1:7635001	1	7635001	7640000	5000	1	2.00E-07	0.35	88	1.76	Phactr2;LOC103690919	Signaling
DMR1:7702001	1	7702001	7704000	2000	2	6.70E-08	-0.46	10	0.5	Pex3	
DMR1:7746001	1	7746001	7747000	1000	1	2.50E-13	0.72	29	2.9	Adat2	Metabolism
DMR1:7914001	1	7914001	7916000	2000	1	5.20E-07	-0.41	47	2.35	Aig1	
DMR1:7963001	1	7963001	7967000	4000	1	4.60E-09	-0.38	58	1.45	Aig1	
DMR1:7999001	1	7999001	8001000	2000	1	6.00E-08	0.49	42	2.1	Aig1	
DMR1:8011001	1	8011001	8016000	5000	1	2.60E-07	-0.53	118	2.36	Aig1	
DMR1:8020001	1	8020001	8022000	2000	1	2.30E-08	-0.4	33	1.65	Aig1	
DMR1:8026001	1	8026001	8028000	2000	1	9.80E-08	-0.44	62	3.1	Aig1	
DMR1:8036001	1	8036001	8038000	2000	1	8.70E-09	-0.55	43	2.15	Aig1	
DMR1:8149001	1	8149001	8152000	3000	2	5.40E-07	-0.59	42	1.4	Hivep2	

DMR1:8177001	1	8177001	8181000	4000	1	5.90E-07	0.32	64	1.6	Hivep2	
DMR1:8189001	1	8189001	8191000	2000	1	4.50E-07	0.36	24	1.2	Hivep2	
DMR1:8197001	1	8197001	8201000	4000	1	6.80E-08	-0.45	69	1.73	Hivep2;LOC102551141;LOC108348466	
DMR1:8219001	1	8219001	8220000	1000	1	4.40E-07	-0.39	17	1.7	Hivep2;LOC108348466	
DMR1:8233001	1	8233001	8235000	2000	1	2.70E-07	-0.52	24	1.2	Hivep2	
DMR1:8239001	1	8239001	8242000	3000	1	9.80E-07	-0.4	64	2.13	Hivep2	
DMR1:8300001	1	8300001	8302000	2000	1	4.40E-09	-0.53	34	1.7	Hivep2	
DMR1:8338001	1	8338001	8341000	3000	1	6.40E-09	-0.56	41	1.37	Hivep2	
DMR1:11799001	1	11799001	11800000	1000	1	8.60E-08	0.44	10	1	Ect2l	
DMR1:12103001	1	12103001	12105000	2000	1	1.10E-09	-0.45	14	0.7	Mcc	
DMR1:12919001	1	12919001	12922000	3000	1	8.50E-08	0.46	51	1.7	LOC102554594;Txlnb	Transport
DMR1:12945001	1	12945001	12947000	2000	1	8.10E-10	0.46	44	2.2	Txlnb;LOC100360362	Transport
DMR1:12955001	1	12955001	12957000	2000	1	7.30E-07	0.38	10	0.5	Txlnb;LOC100360362	Transport
DMR1:12965001	1	12965001	12970000	5000	1	6.70E-08	-0.42	48	0.96	Txlnb	Transport
DMR1:13166001	1	13166001	13167000	1000	1	2.70E-07	-0.49	17	1.7	LOC108349444;Abracl	
DMR1:13286001	1	13286001	13287000	1000	1	3.90E-07	-0.43	14	1.4	Reps1	Transport
DMR1:13340001	1	13340001	13342000	2000	1	1.20E-11	0.43	28	1.4	NEWGENE_2319083	
DMR1:13391001	1	13391001	13392000	1000	1	2.00E-09	0.5	8	0.8	Ccdc28a;LOC102549233	Receptor
DMR1:13463001	1	13463001	13465000	2000	2	1.80E-07	0.53	42	2.1	LOC102549339;Nhsl1	
DMR1:13513001	1	13513001	13516000	3000	1	5.20E-07	-0.38	68	2.27	Nhsl1	
DMR1:13549001	1	13549001	13550000	1000	1	4.90E-07	-0.31	20	2	Nhsl1	
DMR1:13573001	1	13573001	13577000	4000	1	1.70E-08	-0.44	80	2	Nhsl1	
DMR1:13630001	1	13630001	13631000	1000	1	1.60E-12	-0.63	38	3.8	Nhsl1	
DMR1:13640001	1	13640001	13643000	3000	1	3.40E-08	-0.42	83	2.77	Nhsl1	
DMR1:13662001	1	13662001	13663000	1000	1	4.10E-08	-0.35	21	2.1	Nhsl1	
DMR1:13664001	1	13664001	13665000	1000	1	1.50E-07	-0.34	22	2.2	Nhsl1	
DMR1:13700001	1	13700001	13705000	5000	1	1.10E-10	0.59	137	2.74	Nhsl1;Hebp2	
DMR1:13854001	1	13854001	13855000	1000	1	9.40E-07	0.47	20	2	Arfgef3	Transcription
DMR1:13881001	1	13881001	13883000	2000	1	9.30E-07	0.33	29	1.45	Arfgef3	Transcription
DMR1:13886001	1	13886001	13887000	1000	1	1.40E-08	0.64	22	2.2	Arfgef3	Transcription
DMR1:14116001	1	14116001	14122000	6000	1	1.70E-07	0.35	92	1.53	RGD1560303	Signaling
DMR1:15060001	1	15060001	15061000	1000	1	1.70E-07	0.37	12	1.2	Ifngr1	Receptor
DMR1:15072001	1	15072001	15074000	2000	1	5.40E-07	0.43	41	2.05	Ifngr1	Receptor
DMR1:15083001	1	15083001	15087000	4000	1	1.10E-08	0.42	70	1.75	Ifngr1;Il22ra2	Receptor
DMR1:15100001	1	15100001	15103000	3000	1	6.10E-14	0.86	51	1.7	Il22ra2	Receptor
DMR1:15106001	1	15106001	15108000	2000	1	7.00E-07	-0.38	24	1.2	Il22ra2	Receptor
DMR1:15112001	1	15112001	15115000	3000	1	3.90E-07	-0.4	49	1.63	Il22ra2	Receptor
DMR1:15116001	1	15116001	15117000	1000	1	8.50E-09	-0.5	9	0.9	Il22ra2	Receptor
DMR1:15188001	1	15188001	15189000	1000	1	1.00E-07	-0.41	10	1	Il20ra	Receptor
DMR1:15200001	1	15200001	15201000	1000	1	5.00E-07	0.3	13	1.3	Il20ra	Receptor
DMR1:15205001	1	15205001	15206000	1000	1	2.40E-07	-0.41	11	1.1	Il20ra	Receptor
DMR1:15229001	1	15229001	15230000	1000	1	2.70E-07	0.34	6	0.6	Il20ra	Receptor
DMR1:15305001	1	15305001	15309000	4000	2	1.60E-08	-0.41	69	1.73	Slc35d3;Pex7	Transport
DMR1:15328001	1	15328001	15331000	3000	1	1.60E-09	0.5	25	0.83	Pex7	
DMR1:15369001	1	15369001	15373000	4000	1	1.00E-07	0.35	87	2.17	Pex7	
DMR1:15381001	1	15381001	15383000	2000	1	6.30E-07	0.48	26	1.3	Pex7;LOC102547604	
DMR1:15405001	1	15405001	15409000	4000	2	7.30E-11	-0.6	48	1.2	LOC102547411;Map3k5	Signaling
DMR1:15451001	1	15451001	15452000	1000	1	3.20E-08	-0.42	21	2.1	Map3k5	Signaling
DMR1:15523001	1	15523001	15525000	2000	1	8.10E-07	-0.49	55	2.75	Map3k5	Signaling
DMR1:15545001	1	15545001	15548000	3000	1	9.30E-07	-0.39	64	2.13	Map3k5;LOC102547350	Signaling
DMR1:15569001	1	15569001	15576000	7000	1	3.00E-07	0.36	47	0.67	Map3k5;LOC102547350	Signaling
DMR1:15629001	1	15629001	15633000	4000	2	9.70E-10	-0.66	87	2.17	Map7;LOC108349472	Cytoskeleton
DMR1:15643001	1	15643001	15645000	2000	1	9.40E-07	0.39	25	1.25	Map7;LOC108349472	Cytoskeleton
DMR1:15646001	1	15646001	15651000	5000	1	9.50E-12	-0.47	81	1.62	Map7	Cytoskeleton
DMR1:15668001	1	15668001	15669000	1000	1	3.50E-08	-0.51	16	1.6	Map7	Cytoskeleton
DMR1:15694001	1	15694001	15697000	3000	1	7.90E-08	-0.4	68	2.27	Map7	Cytoskeleton
DMR1:15793001	1	15793001	15795000	2000	1	2.80E-07	-0.46	38	1.9	Bclaf1	
DMR1:15840001	1	15840001	15844000	4000	2	1.60E-08	-0.36	80	2	Mtfr2	
DMR1:15893001	1	15893001	15894000	1000	1	4.30E-12	0.77	46	4.6	Pde7b	Signaling
DMR1:15897001	1	15897001	15899000	2000	1	6.00E-10	0.41	27	1.35	Pde7b	Signaling
DMR1:15926001	1	15926001	15928000	2000	1	7.20E-09	0.42	22	1.1	Pde7b	Signaling
DMR1:15988001	1	15988001	15993000	5000	1	4.00E-07	0.41	62	1.24	Pde7b	Signaling

DMR1:15998001	1	15998001	15999000	1000	1	3.50E-08	0.7	35	3.5	Pde7b	Signaling
DMR1:16033001	1	16033001	16035000	2000	1	4.30E-09	-0.49	38	1.9	Pde7b	Signaling
DMR1:16064001	1	16064001	16068000	4000	1	1.40E-09	0.51	50	1.25	Pde7b	Signaling
DMR1:16100001	1	16100001	16101000	1000	1	9.20E-09	0.54	19	1.9	Pde7b	Signaling
DMR1:16150001	1	16150001	16153000	3000	1	7.50E-07	0.41	33	1.1	Pde7b	Signaling
DMR1:16207001	1	16207001	16213000	6000	1	1.50E-08	-0.49	75	1.25	Pde7b	Signaling
DMR1:16687001	1	16687001	16689000	2000	1	9.60E-07	-0.43	17	0.85	RGD1560020_predicted	
DMR1:16921001	1	16921001	16922000	1000	1	1.50E-07	0.46	29	2.9	Aldh8a1	Metabolism
DMR1:17227001	1	17227001	17232000	5000	1	8.20E-07	0.46	42	0.84	Themis	
DMR1:17327001	1	17327001	17328000	1000	1	2.10E-07	-0.5	3	0.3	Themis	
DMR1:17339001	1	17339001	17340000	1000	1	4.60E-07	0.41	18	1.8	Themis	
DMR1:17662001	1	17662001	17663000	1000	1	2.80E-11	0.36	16	1.6	Ptprk	Signaling
DMR1:17787001	1	17787001	17790000	3000	1	9.10E-07	-0.4	64	2.13	Ptprk	Signaling
DMR1:17817001	1	17817001	17819000	2000	1	1.30E-09	-0.43	34	1.7	Ptprk	Signaling
DMR1:17870001	1	17870001	17873000	3000	1	5.10E-10	0.84	43	1.43	Ptprk	Signaling
DMR1:17930001	1	17930001	17931000	1000	1	3.60E-07	-0.44	24	2.4	Ptprk	Signaling
DMR1:18052001	1	18052001	18054000	2000	1	1.10E-15	0.4	18	0.9	Ptprk	Signaling
DMR1:18599001	1	18599001	18602000	3000	1	1.10E-09	-0.55	8	0.27	Lama2	Extracellular Matrix
DMR1:18736001	1	18736001	18740000	4000	1	3.30E-11	0.72	33	0.82	Lama2	Extracellular Matrix
DMR1:18886001	1	18886001	18887000	1000	1	1.10E-17	0.51	8	0.8	Lama2	Extracellular Matrix
DMR1:18959001	1	18959001	18965000	6000	1	7.10E-09	-0.47	77	1.28	Lama2	Extracellular Matrix
DMR1:19003001	1	19003001	19007000	4000	1	9.10E-08	-0.24	34	0.85	Lama2	Extracellular Matrix
DMR1:19031001	1	19031001	19032000	1000	1	2.10E-07	0.41	3	0.3	Lama2	Extracellular Matrix
DMR1:19063001	1	19063001	19064000	1000	1	3.70E-10	0.47	11	1.1	Lama2	Extracellular Matrix
DMR1:19068001	1	19068001	19069000	1000	1	1.90E-07	0.39	11	1.1	Lama2	Extracellular Matrix
DMR1:19138001	1	19138001	19139000	1000	1	2.90E-08	-0.54	9	0.9	Lama2	Extracellular Matrix
DMR1:19257001	1	19257001	19259000	2000	2	2.20E-08	0.39	86	4.3	Arhgap18	Signaling
DMR1:20026001	1	20026001	20031000	5000	1	3.00E-08	-0.45	54	1.08	L3mbtl3	Epigenetic
DMR1:20108001	1	20108001	20110000	2000	1	3.10E-08	0.37	20	1	Samd3;LOC100360791	
DMR1:20117001	1	20117001	20123000	6000	2	4.70E-12	0.44	55	0.92	Samd3;LOC100360791	
DMR1:20334001	1	20334001	20336000	2000	1	2.80E-07	-0.48	20	1	Tmem200a	
DMR1:20366001	1	20366001	20367000	1000	1	2.90E-09	0.57	10	1	Tmem200a	
DMR1:20409001	1	20409001	20411000	2000	1	2.60E-08	0.76	66	3.3	Tmem200a;RGD1559962	
DMR1:20929001	1	20929001	20930000	1000	1	8.40E-10	0.46	3	0.3	Epb41l2	
DMR1:21016001	1	21016001	21018000	2000	1	3.80E-07	-0.31	17	0.85	Epb41l2	
DMR1:21031001	1	21031001	21034000	3000	1	3.60E-12	-0.68	16	0.53	Epb41l2	
DMR1:21122001	1	21122001	21124000	2000	1	9.00E-08	0.66	41	2.05	LOC102552728;Akap7	Translation
DMR1:21157001	1	21157001	21158000	1000	1	5.10E-08	0.33	2	0.2	Akap7	Translation
DMR1:21630001	1	21630001	21632000	2000	1	3.60E-07	0.36	22	1.1	Enpp3	
DMR1:21670001	1	21670001	21672000	2000	1	1.90E-09	-0.56	39	1.95	Enpp3	
DMR1:21740001	1	21740001	21743000	3000	2	9.40E-11	-0.34	31	1.03	Enpp1	
DMR1:21770001	1	21770001	21772000	2000	1	6.90E-07	-0.44	31	1.55	Enpp1	
DMR1:21773001	1	21773001	21778000	5000	3	2.00E-18	-0.52	61	1.22	Enpp1	
DMR1:21793001	1	21793001	21794000	1000	1	2.10E-08	-0.57	14	1.4	Enpp1	
DMR1:22259001	1	22259001	22264000	5000	1	1.30E-07	-0.35	96	1.92	Stx7	Transcription
DMR1:22319001	1	22319001	22322000	3000	1	1.50E-08	0.69	72	2.4	Taar9	Signaling
DMR1:22335001	1	22335001	22340000	5000	1	2.00E-07	-0.29	38	0.76	Taar8c	Signaling
DMR1:22419001	1	22419001	22422000	3000	1	4.60E-07	-0.41	11	0.37	LOC108349728;Taar7i-ps	
DMR1:22436001	1	22436001	22438000	2000	1	6.80E-07	-0.33	5	0.25	Taar7i-ps	
DMR1:22494001	1	22494001	22496000	2000	1	5.30E-08	-0.25	23	1.15	Taar7d	Signaling
DMR1:22728001	1	22728001	22732000	4000	2	4.20E-07	-0.43	26	0.65	Slc18b1	Transport
DMR1:23351001	1	23351001	23353000	2000	1	2.80E-07	0.36	14	0.7	Eya4	
DMR1:23403001	1	23403001	23404000	1000	1	4.00E-07	0.44	8	0.8	Eya4	
DMR1:23490001	1	23490001	23491000	1000	1	7.10E-07	0.4	7	0.7	Eya4	
DMR1:23593001	1	23593001	23594000	1000	1	2.80E-08	-0.36	4	0.4	Eya4	
DMR1:23595001	1	23595001	23596000	1000	1	5.00E-07	-0.51	8	0.8	Eya4	
DMR1:23903001	1	23903001	23904000	1000	1	1.40E-07	-0.55	17	1.7	Tcf21	Transcription
DMR1:23916001	1	23916001	23918000	2000	1	1.70E-07	-0.37	13	0.65	Tcf21	Transcription
DMR1:24014001	1	24014001	24017000	3000	1	7.20E-10	0.43	24	0.8	Slc2a12	
DMR1:24177001	1	24177001	24179000	2000	1	1.70E-08	0.54	23	1.15	Sgk1	Signaling
DMR1:24188001	1	24188001	24189000	1000	1	3.60E-07	0.43	18	1.8	Sgk1	Signaling
DMR1:25165001	1	25165001	25168000	3000	1	3.00E-08	-0.65	19	0.63	Clvs2	Transport
DMR1:25174001	1	25174001	25175000	1000	1	7.60E-11	0.81	18	1.8	Clvs2	Transport

DMR1:25703001	1	25703001	25707000	4000	1	3.60E-07	-0.32	33	0.82	Trdn	
DMR1:25743001	1	25743001	25749000	6000	3	4.30E-14	-0.39	73	1.22	Trdn	
DMR1:28350001	1	28350001	28352000	2000	1	4.00E-10	-0.8	20	1	Rnf217	Proteolysis
DMR1:28387001	1	28387001	28389000	2000	1	1.10E-10	0.39	13	0.65	Rnf217	Proteolysis
DMR1:28459001	1	28459001	28461000	2000	2	2.30E-07	-0.4	10	0.5	Tpd52l1	
DMR1:28465001	1	28465001	28467000	2000	1	6.10E-07	-0.44	7	0.35	Tpd52l1	
DMR1:28569001	1	28569001	28571000	2000	1	2.40E-07	0.34	28	1.4	Tpd52l1;Hddc2	
DMR1:29199001	1	29199001	29200000	1000	1	2.00E-17	1.12	34	3.4	Hey2	Transcription
DMR1:29267001	1	29267001	29269000	2000	1	3.30E-09	0.49	15	0.75	Ncoa7	
DMR1:29358001	1	29358001	29361000	3000	1	8.10E-07	-0.24	31	1.03	Ncoa7;LOC108348877	
DMR1:30737001	1	30737001	30739000	2000	1	1.90E-09	-0.53	20	1	Rspo3	
DMR1:30863001	1	30863001	30864000	1000	1	1.90E-07	-0.38	5	0.5	Rnf146	
DMR1:31034001	1	31034001	31037000	3000	1	2.40E-08	-0.44	6	0.2	LOC102554862;Soga3	
DMR1:31118001	1	31118001	31120000	2000	1	1.80E-07	0.36	20	1	Soga3	
DMR1:31639001	1	31639001	31641000	2000	1	1.50E-17	0.48	27	1.35	Ahrr	Transcription
DMR1:31713001	1	31713001	31716000	3000	1	6.50E-07	-0.49	54	1.8	Exoc3	Transport
DMR1:31869001	1	31869001	31871000	2000	1	9.70E-07	0.38	36	1.8	Cep72;Tppp	Cytoskeleton
DMR1:31914001	1	31914001	31915000	1000	1	5.00E-08	-0.45	6	0.6	Zdhhc11	
DMR1:32046001	1	32046001	32049000	3000	1	1.20E-07	0.46	40	1.33	Nkd2	
DMR1:32279001	1	32279001	32280000	1000	1	7.40E-09	-0.52	15	1.5	Tert;Clptm1l	Transcription
DMR1:32374001	1	32374001	32375000	1000	1	2.90E-08	0.5	2	0.2	Lpcat1	Metabolism
DMR1:32411001	1	32411001	32413000	2000	1	6.90E-07	0.46	13	0.65	Lpcat1	Metabolism
DMR1:33281001	1	33281001	33282000	1000	1	1.20E-07	0.48	14	1.4	Irx2;LOC103690947	Development
DMR1:35083001	1	35083001	35084000	1000	1	9.50E-07	-0.37	8	0.8	Adamts16	Protease
DMR1:35150001	1	35150001	35157000	7000	1	3.20E-08	-0.26	77	1.1	Adamts16	Protease
DMR1:35283001	1	35283001	35284000	1000	1	5.90E-07	-0.49	6	0.6	Ice1	Golgi
DMR1:36122001	1	36122001	36125000	3000	1	8.70E-07	-0.37	29	0.97	Med10	Transcription
DMR1:37323001	1	37323001	37325000	2000	1	9.90E-08	-0.45	9	0.45	Adcy2	
DMR1:37400001	1	37400001	37401000	1000	1	5.60E-09	-0.46	5	0.5	Adcy2	
DMR1:37469001	1	37469001	37471000	2000	1	1.10E-07	0.76	46	2.3	Adcy2	
DMR1:37648001	1	37648001	37655000	7000	2	3.50E-08	-0.43	33	0.47	Adcy2	
DMR1:37672001	1	37672001	37682000	10000	1	1.20E-07	-0.33	69	0.69	Adcy2	
DMR1:37973001	1	37973001	37975000	2000	1	1.50E-07	-0.38	7	0.35	LOC102549842;Zfp748	Transcription
DMR1:38517001	1	38517001	38519000	2000	2	1.30E-15	1.13	90	4.5	RGD1564382;LOC102551340	Transcription
DMR1:39832001	1	39832001	39837000	5000	1	8.70E-07	-0.29	52	1.04	Ppp1r14c	Signaling
DMR1:39847001	1	39847001	39850000	3000	1	7.50E-09	-0.38	18	0.6	Ppp1r14c	Signaling
DMR1:39868001	1	39868001	39869000	1000	1	4.20E-10	0.43	2	0.2	Ppp1r14c	Signaling
DMR1:39873001	1	39873001	39874000	1000	1	3.20E-07	0.5	5	0.5	Ppp1r14c	Signaling
DMR1:40105001	1	40105001	40109000	4000	2	7.00E-09	-0.27	29	0.72	Iyd	Metabolism
DMR1:40290001	1	40290001	40296000	6000	1	3.70E-08	-0.55	39	0.65	LOC103690956;Plekhg1	
DMR1:40299001	1	40299001	40301000	2000	1	3.60E-07	0.37	20	1	Plekhg1	
DMR1:40326001	1	40326001	40328000	2000	1	5.40E-07	-0.45	25	1.25	Plekhg1	
DMR1:40647001	1	40647001	40648000	1000	1	6.90E-08	-0.44	18	1.8	Mthfd1l;LOC102546677	
DMR1:41258001	1	41258001	41262000	4000	1	3.70E-08	-0.38	37	0.92	Esr1	
DMR1:41264001	1	41264001	41266000	2000	1	1.40E-08	0.49	27	1.35	Esr1	
DMR1:41373001	1	41373001	41375000	2000	1	1.60E-09	-0.77	11	0.55	Esr1	
DMR1:41413001	1	41413001	41414000	1000	1	1.10E-08	0.4	3	0.3	Esr1	
DMR1:41469001	1	41469001	41470000	1000	1	5.50E-10	-0.74	9	0.9	Esr1	
DMR1:41880001	1	41880001	41883000	3000	1	5.20E-08	-0.62	25	0.83	Syne1	
DMR1:41928001	1	41928001	41929000	1000	1	4.30E-07	-0.45	23	2.3	Syne1	
DMR1:41961001	1	41961001	41963000	2000	1	3.20E-07	0.33	11	0.55	Syne1	
DMR1:42020001	1	42020001	42022000	2000	1	1.50E-07	-0.45	23	1.15	Syne1	
DMR1:42036001	1	42036001	42037000	1000	1	8.20E-07	0.38	10	1	Syne1	
DMR1:42082001	1	42082001	42084000	2000	1	1.20E-10	0.36	18	0.9	Syne1;LOC102546605	
DMR1:43702001	1	43702001	43703000	1000	1	8.90E-07	0.35	11	1.1	Oprm1;Ipcef1	Signaling
DMR1:43731001	1	43731001	43733000	2000	1	5.90E-07	-0.33	10	0.5	Ipcef1;LOC680118	
DMR1:43864001	1	43864001	43866000	2000	1	7.50E-12	0.44	18	0.9	Cnksr3	
DMR1:44351001	1	44351001	44354000	3000	1	1.20E-07	0.29	38	1.27	Tiam2	
DMR1:44379001	1	44379001	44382000	3000	2	4.80E-09	-0.57	29	0.97	Tiam2	
DMR1:44439001	1	44439001	44441000	2000	1	3.30E-07	-0.33	19	0.95	Tiam2;Tfb1m;Cldn20;LOC102547288	Epigenetic;Cell Junction
DMR1:44537001	1	44537001	44544000	7000	1	7.10E-08	-0.34	88	1.26	LOC103690965;Nox3	Metabolism
DMR1:44616001	1	44616001	44617000	1000	1	8.90E-09	0.43	9	0.9	Nox3	Metabolism

DMR1:45987001	1	45987001	45989000	2000	1	2.40E-11	-0.5	19	0.95	Arid1b	
DMR1:46012001	1	46012001	46015000	3000	1	8.50E-07	-0.59	38	1.27	Arid1b	
DMR1:46028001	1	46028001	46029000	1000	1	3.40E-08	-0.58	21	2.1	Arid1b	
DMR1:46101001	1	46101001	46103000	2000	1	3.50E-10	-0.47	36	1.8	Arid1b	
DMR1:46232001	1	46232001	46233000	1000	1	3.80E-07	-0.44	23	2.3	Arid1b	
DMR1:46298001	1	46298001	46300000	2000	1	9.80E-07	-0.41	41	2.05	Tmem242;Ldhal6b	
DMR1:46324001	1	46324001	46327000	3000	1	7.00E-07	0.45	35	1.17	Tmem242	
DMR1:46442001	1	46442001	46445000	3000	1	2.60E-11	0.43	27	0.9	Zdhhc14	
DMR1:46449001	1	46449001	46454000	5000	1	4.80E-08	0.44	64	1.28	Zdhhc14	
DMR1:46461001	1	46461001	46463000	2000	1	3.90E-09	0.53	27	1.35	Zdhhc14	
DMR1:46542001	1	46542001	46544000	2000	1	1.50E-08	0.65	50	2.5	Zdhhc14	
DMR1:46788001	1	46788001	46792000	4000	1	3.70E-07	-0.4	51	1.27	Snx9	Cytoskeleton
DMR1:46827001	1	46827001	46828000	1000	1	6.60E-07	-0.43	15	1.5	Snx9;LOC100365065;Synj2	Cytoskeleton;Signaling
DMR1:47126001	1	47126001	47127000	1000	1	3.30E-07	-0.38	23	2.3	Tulp4	
DMR1:47157001	1	47157001	47160000	3000	1	3.00E-07	0.44	35	1.17	Tmem181	
DMR1:47167001	1	47167001	47171000	4000	1	4.90E-11	0.38	64	1.6	Tmem181	
DMR1:47269001	1	47269001	47273000	4000	1	8.30E-07	-0.27	69	1.73	Sytl3	
DMR1:47276001	1	47276001	47277000	1000	1	4.80E-07	0.36	12	1.2	Sytl3	
DMR1:47314001	1	47314001	47316000	2000	1	1.60E-08	-0.38	37	1.85	Ezr	Cytoskeleton
DMR1:47420001	1	47420001	47422000	2000	1	2.00E-08	0.34	20	1	Rsph3	Development
DMR1:47423001	1	47423001	47425000	2000	1	2.60E-08	0.52	27	1.35	Rsph3	Development
DMR1:47490001	1	47490001	47492000	2000	1	8.50E-07	-0.39	32	1.6	Tagap	Signaling
DMR1:47586001	1	47586001	47588000	2000	1	8.30E-09	0.74	39	1.95	RGD1560015	
DMR1:47655001	1	47655001	47656000	1000	1	2.90E-20	1.13	39	3.9	Fndc1	
DMR1:47668001	1	47668001	47673000	5000	1	5.70E-09	0.51	97	1.94	Fndc1	
DMR1:47927001	1	47927001	47931000	4000	1	1.70E-08	-0.42	57	1.43	Sod2	Metabolism
DMR1:47945001	1	47945001	47946000	1000	1	4.80E-09	-0.54	10	1	Wtap	
DMR1:48011001	1	48011001	48016000	5000	1	1.20E-08	0.37	86	1.72	Acat2l1;LOC108349718;Tcp1	Metabolism;Translation
DMR1:48162001	1	48162001	48164000	2000	1	3.10E-07	-0.44	24	1.2	Mas1l	
DMR1:48204001	1	48204001	48206000	2000	1	9.10E-14	0.97	99	4.95	Mas1l;Igf2r	Transport
DMR1:48219001	1	48219001	48220000	1000	1	6.00E-08	0.45	12	1.2	Igf2r	Transport
DMR1:48254001	1	48254001	48256000	2000	1	3.50E-07	-0.36	44	2.2	Igf2r	Transport
DMR1:48346001	1	48346001	48347000	1000	1	1.80E-07	0.36	9	0.9	Slc22a2;LOC103690972	Transport
DMR1:48665001	1	48665001	48666000	1000	1	4.70E-08	-0.45	11	1.1	Map3k4	Signaling
DMR1:48680001	1	48680001	48681000	1000	1	1.40E-07	0.37	11	1.1	Map3k4	Signaling
DMR1:48832001	1	48832001	48835000	3000	1	3.40E-08	0.52	53	1.77	Agpat4	Metabolism
DMR1:48906001	1	48906001	48907000	1000	1	1.90E-07	0.34	15	1.5	Park2;LOC103690973	
DMR1:48919001	1	48919001	48920000	1000	1	2.10E-11	0.35	10	1	Park2	
DMR1:48942001	1	48942001	48943000	1000	1	4.60E-09	0.55	36	3.6	Park2	
DMR1:48952001	1	48952001	48953000	1000	1	7.90E-12	0.71	22	2.2	Park2	
DMR1:49058001	1	49058001	49059000	1000	1	2.80E-07	0.29	5	0.5	Park2	
DMR1:49082001	1	49082001	49083000	1000	1	2.90E-10	0.58	4	0.4	Park2	
DMR1:49422001	1	49422001	49423000	1000	1	2.50E-10	0.35	2	0.2	Park2	
DMR1:49448001	1	49448001	49450000	2000	1	1.20E-10	0.7	42	2.1	Park2	
DMR1:49478001	1	49478001	49480000	2000	1	4.80E-07	0.42	21	1.05	Park2	
DMR1:49493001	1	49493001	49494000	1000	1	3.70E-09	0.44	14	1.4	Park2	
DMR1:49699001	1	49699001	49701000	2000	1	5.60E-08	-0.4	26	1.3	Park2	
DMR1:49795001	1	49795001	49797000	2000	2	3.20E-09	-0.53	24	1.2	Park2	
DMR1:49916001	1	49916001	49922000	6000	2	6.90E-08	-0.4	73	1.22	Park2	
DMR1:49955001	1	49955001	49959000	4000	1	1.20E-09	0.58	58	1.45	Park2;LOC108349802	
DMR1:49995001	1	49995001	49996000	1000	1	2.70E-08	0.36	4	0.4	Park2	
DMR1:50040001	1	50040001	50042000	2000	1	2.00E-09	-0.45	11	0.55	Park2	
DMR1:50284001	1	50284001	50285000	1000	1	4.90E-07	0.41	5	0.5	Pacrg	
DMR1:50349001	1	50349001	50350000	1000	1	2.50E-07	0.38	12	1.2	Pacrg	
DMR1:50358001	1	50358001	50361000	3000	1	1.40E-08	0.43	36	1.2	Pacrg	
DMR1:50377001	1	50377001	50378000	1000	1	4.70E-07	0.33	13	1.3	Pacrg	
DMR1:51619001	1	51619001	51623000	4000	1	6.50E-08	0.63	103	2.58	LOC108348950;Pabpc6	
DMR1:52323001	1	52323001	52327000	4000	1	1.30E-08	0.41	46	1.15	MGC94891	
DMR1:52407001	1	52407001	52409000	2000	1	9.90E-07	-0.49	49	2.45	Pde10a	Signaling
DMR1:52478001	1	52478001	52480000	2000	1	2.10E-07	0.3	23	1.15	Pde10a	Signaling
DMR1:52504001	1	52504001	52505000	1000	1	2.70E-07	-0.55	20	2	Pde10a	Signaling
DMR1:52902001	1	52902001	52905000	3000	2	1.10E-08	0.42	30	1	T;T2	
DMR1:52909001	1	52909001	52911000	2000	1	7.80E-07	0.53	8	0.4	T;T2	

DMR1:52923001	1	52923001	52924000	1000	1	2.90E-11	0.37	8	0.8	T2	
DMR1:53077001	1	53077001	53078000	1000	1	1.60E-08	0.4	8	0.8	Ccr6	
DMR1:53505001	1	53505001	53508000	3000	1	4.70E-08	-0.65	74	2.47	Rps6ka2	Golgi
DMR1:53563001	1	53563001	53567000	4000	2	2.90E-08	0.48	34	0.85	Tcp10b;Ttll2;Unc93a	Cytoskeleton;Signaling
DMR1:53853001	1	53853001	53854000	1000	1	1.70E-16	-0.66	12	1.2	Afdn	Cytoskeleton
DMR1:54038001	1	54038001	54042000	4000	1	4.30E-10	0.3	27	0.68	RGD1560718	Signaling
DMR1:54043001	1	54043001	54044000	1000	1	6.20E-08	0.2	5	0.5	RGD1560718	Signaling
DMR1:54046001	1	54046001	54048000	2000	1	4.30E-09	0.44	14	0.7	RGD1560718;LOC102552087;LOC108349470	Signaling
DMR1:54846001	1	54846001	54847000	1000	1	4.10E-09	0.33	20	2	Smok2a	Signaling
DMR1:55232001	1	55232001	55233000	1000	1	2.50E-10	0.22	14	1.4	RGD1561667	Signaling
DMR1:55898001	1	55898001	55900000	2000	1	4.90E-08	0.47	18	0.9	Vom2r9	Signaling
DMR1:56173001	1	56173001	56174000	1000	1	1.10E-07	0.42	10	1	Dact2	
DMR1:56796001	1	56796001	56799000	3000	1	5.00E-08	0.42	24	0.8	Wdr27	
DMR1:56811001	1	56811001	56813000	2000	1	1.20E-07	0.36	10	0.5	Wdr27	
DMR1:56842001	1	56842001	56846000	4000	1	2.30E-07	-0.37	27	0.68	Wdr27	
DMR1:56896001	1	56896001	56898000	2000	1	8.60E-07	-0.49	9	0.45	Wdr27	
DMR1:56993001	1	56993001	56994000	1000	1	9.10E-08	-0.59	12	1.2	Ermard	
DMR1:57012001	1	57012001	57013000	1000	1	1.80E-07	0.4	7	0.7	Ermard	
DMR1:57348001	1	57348001	57351000	3000	1	4.20E-09	-0.47	54	1.8	Fam120b	
DMR1:57377001	1	57377001	57380000	3000	1	3.90E-07	-0.35	41	1.37	Fam120b	
DMR1:57475001	1	57475001	57478000	3000	1	1.10E-07	0.44	24	0.8	Psmb1	Protease
DMR1:57704001	1	57704001	57706000	2000	1	1.10E-08	-0.44	24	1.2	Chd1	
DMR1:57714001	1	57714001	57715000	1000	1	1.70E-12	-0.58	4	0.4	Chd1	
DMR1:57716001	1	57716001	57719000	3000	2	3.40E-09	-0.49	46	1.53	Chd1	
DMR1:57796001	1	57796001	57802000	6000	1	1.20E-07	-0.5	83	1.38	Rgmb	
DMR1:59200001	1	59200001	59202000	2000	1	2.60E-11	0.62	42	2.1	Lix1	
DMR1:59736001	1	59736001	59738000	2000	1	2.80E-08	0.45	10	0.5	Has1	Golgi
DMR1:60040001	1	60040001	60042000	2000	1	2.80E-10	-0.52	13	0.65	LOC108349105;Vom1r6	Receptor
DMR1:60091001	1	60091001	60092000	1000	1	1.30E-08	-0.42	5	0.5	Vom1r7	Receptor
DMR1:60625001	1	60625001	60627000	2000	1	1.90E-09	-0.53	5	0.25	Vom1r15	Receptor
DMR1:60677001	1	60677001	60680000	3000	1	2.40E-09	-0.39	22	0.73	Vom1r-ps14	
DMR1:61123001	1	61123001	61128000	5000	1	9.30E-09	-0.41	37	0.74	Vom1r-ps24;Vom1r20	Receptor
DMR1:61301001	1	61301001	61304000	3000	1	3.10E-07	-0.4	22	0.73	Zfp51	Transcription
DMR1:61410001	1	61410001	61415000	5000	2	7.10E-10	-0.45	30	0.6	Zfp52	Transcription
DMR1:61511001	1	61511001	61517000	6000	1	3.90E-09	-0.32	68	1.13	LOC108348215;Znf761	Transcription
DMR1:61688001	1	61688001	61689000	1000	1	1.10E-11	0.53	12	1.2	Vom1r23	Receptor
DMR1:61691001	1	61691001	61694000	3000	1	6.70E-07	-0.33	16	0.53	Vom1r23;LOC102553001	Receptor
DMR1:62286001	1	62286001	62290000	4000	1	2.10E-07	-0.38	21	0.52	RGD1566248	
DMR1:62491001	1	62491001	62495000	4000	1	4.30E-09	-0.49	71	1.77	Vom2r80	Signaling
DMR1:62886001	1	62886001	62889000	3000	1	9.20E-09	-0.5	47	1.57	Vom2r-ps32	
DMR1:63437001	1	63437001	63442000	5000	3	9.20E-10	-0.36	43	0.86	Vom2r-ps41	
DMR1:63552001	1	63552001	63555000	3000	1	4.60E-07	-0.28	25	0.83	Vom2r26	Signaling
DMR1:63696001	1	63696001	63702000	6000	2	1.50E-11	-0.44	60	1	RGD1562625	
DMR1:63704001	1	63704001	63708000	4000	1	1.00E-12	-0.75	26	0.65	RGD1562625	
DMR1:63725001	1	63725001	63732000	7000	1	4.20E-11	-0.37	70	1	RGD1562625;Pirb	
DMR1:63768001	1	63768001	63774000	6000	2	4.70E-07	-0.51	35	0.58	Lilrb3l	Immune
DMR1:63855001	1	63855001	63861000	6000	2	1.50E-09	-0.38	79	1.32	Lilrb3l	Immune
DMR1:63892001	1	63892001	63898000	6000	1	5.20E-07	-0.41	66	1.1	Lilrb1	
DMR1:63936001	1	63936001	63941000	5000	2	1.40E-11	-0.33	46	0.92	Lilra3	
DMR1:64176001	1	64176001	64183000	7000	1	2.20E-08	-0.35	58	0.83	Tfpt;Ndufa3;Oscar	Metabolism;Immune
DMR1:64188001	1	64188001	64192000	4000	1	1.40E-08	-0.41	36	0.9	Oscar	Immune
DMR1:64397001	1	64397001	64398000	1000	1	5.30E-07	0.46	11	1.1	LOC103691003;Cacng7;Prkcg	Transport;Signaling
DMR1:64490001	1	64490001	64491000	1000	1	9.70E-07	0.31	10	1	Olr1l;LOC103689958;Olr386	Receptor
DMR1:64689001	1	64689001	64692000	3000	1	6.70E-08	-0.37	14	0.47	Vom2r27	Signaling
DMR1:64863001	1	64863001	64866000	3000	1	1.20E-09	-0.37	20	0.67	Vom2r12	
DMR1:64875001	1	64875001	64881000	6000	1	5.60E-07	-0.58	114	1.9	Vom2r12	
DMR1:65657001	1	65657001	65658000	1000	1	3.40E-09	0.57	12	1.2	LOC102552901;Rnf225;Rps5	Translation
DMR1:65771001	1	65771001	65774000	3000	1	2.90E-09	-0.38	63	2.1	LOC108348438;Zfp329	Transcription
DMR1:65927001	1	65927001	65932000	5000	1	2.70E-07	-0.32	45	0.9	LOC108349536;Vom2r36;Vom2r33	Signaling
DMR1:65986001	1	65986001	65991000	5000	3	4.00E-11	-0.34	44	0.88	Vom2r36	Signaling
DMR1:66005001	1	66005001	66014000	9000	2	1.80E-11	-0.57	76	0.84	Vom2r36;Vom2r35	Signaling
DMR1:66027001	1	66027001	66030000	3000	1	1.10E-10	-0.55	30	1	Vom2r36;Vom2r35;LOC108348434	Signaling

DMR1:66170001	1	66170001	66175000	5000	1	5.20E-08	-0.53	37	0.74	Vom2r36;LOC691722	Signaling
DMR1:66698001	1	66698001	66707000	9000	1	1.70E-08	-0.35	90	1	Vom1r57;Vom1r-ps65	Receptor
DMR1:66859001	1	66859001	66864000	5000	2	2.90E-08	-0.38	45	0.9	Vom1r51	Receptor
DMR1:67022001	1	67022001	67025000	3000	1	9.80E-07	-0.43	20	0.67	Vom1r47	Receptor
DMR1:67194001	1	67194001	67200000	6000	1	1.60E-10	-0.47	44	0.73	Vom1r43	Receptor
DMR1:67216001	1	67216001	67217000	1000	1	8.90E-07	-0.43	6	0.6	Vom1r43	Receptor
DMR1:67263001	1	67263001	67271000	8000	1	2.10E-08	-0.57	48	0.6	LOC103691020;Vom1r42	
DMR1:67344001	1	67344001	67349000	5000	1	6.90E-09	-0.39	31	0.62	LOC100362054;Vom1r-ps108	Transcription
DMR1:68099001	1	68099001	68103000	4000	1	2.80E-08	-0.31	31	0.78	LOC683242;Vom2r-ps57	
DMR1:68205001	1	68205001	68209000	4000	1	1.00E-10	-0.34	37	0.92	Vom1r-ps38	
DMR1:69382001	1	69382001	69385000	3000	2	6.80E-09	-0.41	23	0.77	Nlrp4a	
DMR1:69392001	1	69392001	69397000	5000	2	1.00E-08	-0.34	46	0.92	Nlrp4a	
DMR1:69438001	1	69438001	69442000	4000	1	1.90E-07	-0.32	31	0.78	Nlrp4a	
DMR1:69509001	1	69509001	69514000	5000	1	3.50E-07	-0.34	49	0.98	Vom2r-ps45;LOC100362711	
DMR1:69783001	1	69783001	69785000	2000	1	8.50E-16	-0.53	12	0.6	Mfsd14a;Hiat1-ps1	
DMR1:69787001	1	69787001	69789000	2000	1	2.40E-07	-0.31	60	3	Mfsd14a;Hiat1-ps1	
DMR1:70037001	1	70037001	70040000	3000	1	6.80E-07	0.48	38	1.27	Usp29;LOC102547903	Protease
DMR1:70050001	1	70050001	70053000	3000	1	1.30E-10	0.41	41	1.37	Usp29;LOC102547903	Protease
DMR1:70230001	1	70230001	70231000	1000	1	1.60E-08	0.77	44	4.4	Peg3;Apeg3	Transcription
DMR1:70250001	1	70250001	70254000	4000	1	3.60E-07	0.31	49	1.23	Zim1	
DMR1:70489001	1	70489001	70490000	1000	1	1.20E-07	0.34	17	1.7	Olr6;Olr7;Olr8	Signaling
DMR1:71145001	1	71145001	71146000	1000	1	7.40E-07	-0.44	0	0	Smim17	
DMR1:71289001	1	71289001	71291000	2000	1	1.80E-08	0.51	76	3.8	Zfp667;LOC108349542;LOC102548473;LOC103691035	
DMR1:71347001	1	71347001	71348000	1000	1	3.10E-08	0.47	19	1.9	Zscan5b;Galp	Transcription
DMR1:71350001	1	71350001	71353000	3000	2	2.20E-19	0.64	62	2.07	Zscan5b;Galp	Transcription
DMR1:71500001	1	71500001	71503000	3000	1	9.60E-11	-1.11	73	2.43	Nlrp5;LOC108349747	
DMR1:72122001	1	72122001	72125000	3000	1	4.60E-08	-0.44	7	0.23	Vom1r35	Receptor
DMR1:72188001	1	72188001	72192000	4000	1	2.60E-08	-0.36	24	0.6	Vom1r36;LOC108349748	Receptor
DMR1:72236001	1	72236001	72239000	3000	1	1.70E-07	-0.27	24	0.8	Vom1r38;Vom2r-ps47	Receptor
DMR1:72365001	1	72365001	72367000	2000	1	5.20E-08	0.59	81	4.05	Zfp865;LOC100911196;Zfp524	Transcription
DMR1:72815001	1	72815001	72816000	1000	1	7.70E-07	-0.55	17	1.7	Tmem86b;Ptprh	Signaling
DMR1:72826001	1	72826001	72831000	5000	1	2.30E-09	-0.74	51	1.02	Ptprh	Signaling
DMR1:72936001	1	72936001	72937000	1000	1	3.60E-11	0.96	50	5	Ppp1r12c;Eps8l1	Signaling;Cytoskeleton
DMR1:72966001	1	72966001	72969000	3000	2	1.60E-09	-0.45	27	0.9	Rdh13	Golgi
DMR1:73250001	1	73250001	73251000	1000	1	7.00E-10	-0.74	8	0.8	Fcar;LOC108349720	Immune
DMR1:73406001	1	73406001	73408000	2000	1	3.10E-07	0.51	11	0.55	Lilrb4	Immune
DMR1:73846001	1	73846001	73849000	3000	1	1.80E-07	-0.45	24	0.8	Lair1	Immune
DMR1:74773001	1	74773001	74776000	3000	1	5.20E-09	-0.39	20	0.67	Vom2r30	
DMR1:74987001	1	74987001	74989000	2000	2	6.20E-13	0.58	7	0.35	Vom2r31	Signaling
DMR1:74994001	1	74994001	74996000	2000	1	9.90E-13	0.64	16	0.8	Vom2r31	Signaling
DMR1:75002001	1	75002001	75003000	1000	1	8.10E-10	0.86	6	0.6	Vom2r31	Signaling
DMR1:75138001	1	75138001	75141000	3000	1	7.20E-08	-0.36	24	0.8	Vom1r58;Vom1r-ps66	Receptor
DMR1:75204001	1	75204001	75205000	1000	1	5.10E-07	0.31	11	1.1	Vom1r60	Receptor
DMR1:75300001	1	75300001	75301000	1000	1	3.90E-07	0.4	5	0.5	Vom1r61	Receptor
DMR1:75308001	1	75308001	75309000	1000	1	2.20E-07	-0.41	6	0.6	Vom1r61	Receptor
DMR1:75336001	1	75336001	75338000	2000	1	6.70E-08	0.53	59	2.95	Vom1r62;RGD1310257	Receptor
DMR1:75435001	1	75435001	75439000	4000	1	8.70E-07	-0.26	29	0.72	Pla2g4c	Metabolism
DMR1:75457001	1	75457001	75463000	6000	2	4.40E-12	-0.32	74	1.23	Pla2g4c	Metabolism
DMR1:75490001	1	75490001	75491000	1000	1	6.80E-08	-0.63	6	0.6	Pla2g4c;LOC108348107	Metabolism
DMR1:75647001	1	75647001	75653000	6000	1	5.80E-07	-0.29	57	0.95	Peli1-ps1	
DMR1:75763001	1	75763001	75764000	1000	1	8.70E-07	-0.23	5	0.5	Bsph1	
DMR1:76309001	1	76309001	76312000	3000	1	4.50E-07	-0.49	12	0.4	Sult2a2	
DMR1:76412001	1	76412001	76413000	1000	1	6.90E-08	-0.52	4	0.4	Sult2a2	
DMR1:76653001	1	76653001	76656000	3000	1	4.30E-07	-0.58	22	0.73	Sult2a6;Srp72-ps1	
DMR1:77539001	1	77539001	77541000	2000	1	4.50E-07	-0.39	16	0.8	Sepw1;LOC103690015	Translation
DMR1:77623001	1	77623001	77627000	4000	2	6.00E-12	-0.62	18	0.45	Obox1;LOC102554917	
DMR1:77869001	1	77869001	77871000	2000	2	2.40E-21	1	68	3.4	Ehd2;Gltscr1;LOC103691043	Transport
DMR1:78184001	1	78184001	78188000	4000	1	2.60E-07	0.54	104	2.6	C5ar2;LOC684925;C5ar1	Signaling
DMR1:78382001	1	78382001	78383000	1000	1	1.40E-07	0.45	12	1.2	Zc3h4	Transcription
DMR1:78673001	1	78673001	78675000	2000	1	2.80E-07	-0.4	36	1.8	Ceacam9;Ap2s1	Transport
DMR1:78676001	1	78676001	78679000	3000	1	2.40E-07	0.43	48	1.6	Ap2s1;LOC679748	Transport
DMR1:78816001	1	78816001	78817000	1000	1	1.90E-07	0.44	9	0.9	Dact3;Gng8	Signaling

DMR1:78869001	1	78869001	78871000	2000	1	2.00E-08	0.4	14	0.7	Pnmal2	
DMR1:78877001	1	78877001	78878000	1000	1	1.90E-08	0.85	47	4.7	Pnmal2;LOC103691045;Pnmal1	
DMR1:79115001	1	79115001	79121000	6000	2	1.40E-08	-0.36	77	1.28	Ceacam3	Immune
DMR1:79170001	1	79170001	79175000	5000	2	3.60E-09	-0.37	54	1.08	Psgb1	
DMR1:79178001	1	79178001	79180000	2000	1	8.60E-08	-0.67	4	0.2	Psgb1	
DMR1:79196001	1	79196001	79203000	7000	1	1.20E-07	-0.48	51	0.73	Psgb1	
DMR1:79756001	1	79756001	79758000	2000	1	1.20E-10	-0.39	31	1.55	LOC100362871;Micb	Immune
DMR1:79770001	1	79770001	79773000	3000	1	4.40E-10	0.74	64	2.13	Micb	Immune
DMR1:79793001	1	79793001	79794000	1000	1	1.10E-08	0.52	10	1	Pglyrp1;Ccdc61	Epigenetic
DMR1:79887001	1	79887001	79890000	3000	1	1.80E-13	0.64	43	1.43	Nanos2;Mypop	Metabolism;Transcription
DMR1:79977001	1	79977001	79979000	2000	1	5.40E-07	0.38	39	1.95	Rsph6a;LOC108349062;Dmwd;Dmpk	Cytoskeleton;Signaling
DMR1:80026001	1	80026001	80027000	1000	1	1.10E-07	0.65	67	6.7	LOC102553424;Fbxo46	
DMR1:80071001	1	80071001	80073000	2000	1	3.70E-08	0.66	57	2.85	Gipr	Receptor
DMR1:80107001	1	80107001	80110000	3000	1	1.20E-07	0.33	63	2.1	Eml2	
DMR1:80129001	1	80129001	80130000	1000	1	1.90E-07	0.39	5	0.5	Gpr4	Signaling
DMR1:80287001	1	80287001	80290000	3000	1	2.70E-07	0.5	73	2.43	Ppp1r13l;Ercc2;Mir343	Epigenetic
DMR1:80362001	1	80362001	80364000	2000	1	4.60E-07	0.38	30	1.5	Mark4;Exoc3l2	Signaling;Transport
DMR1:80376001	1	80376001	80378000	2000	1	6.70E-08	0.31	24	1.2	Mark4;Exoc3l2	Signaling;Transport
DMR1:80395001	1	80395001	80397000	2000	1	2.90E-07	0.51	35	1.75	Exoc3l2	Transport
DMR1:80494001	1	80494001	80495000	1000	1	6.10E-07	0.42	22	2.2	Gemin7;Zfp296;Clasrp	Transcription;Translation
DMR1:80655001	1	80655001	80656000	1000	1	2.10E-07	0.52	35	3.5	Nectin2	
DMR1:80717001	1	80717001	80718000	1000	1	1.90E-09	0.48	8	0.8	Cblc	Metabolism
DMR1:80742001	1	80742001	80743000	1000	1	3.90E-07	0.68	21	2.1	Bcl3	Transport
DMR1:80851001	1	80851001	80854000	3000	2	2.50E-10	0.75	63	2.1	Igsf23;LOC108349553	
DMR1:80856001	1	80856001	80860000	4000	1	9.90E-07	-0.36	74	1.85	Igsf23;LOC108349553	
DMR1:80865001	1	80865001	80868000	3000	1	7.70E-08	0.38	51	1.7	Igsf23	
DMR1:80892001	1	80892001	80893000	1000	1	7.30E-07	-0.38	23	2.3	Ceacam20;LOC102548132	
DMR1:80954001	1	80954001	80956000	2000	1	1.10E-08	0.39	28	1.4	Zfp112;Znf235	Transcription
DMR1:80962001	1	80962001	80964000	2000	1	9.20E-07	0.28	20	1	Zfp112;Znf235	Transcription
DMR1:81115001	1	81115001	81116000	1000	1	7.60E-10	0.71	26	2.6	Zfp61	Transcription
DMR1:81127001	1	81127001	81130000	3000	1	5.10E-07	-0.37	59	1.97	Zfp61;Zfp94	Transcription
DMR1:81181001	1	81181001	81187000	6000	1	4.00E-08	0.43	120	2	LOC102548695;Tescl	
DMR1:81200001	1	81200001	81203000	3000	1	2.50E-08	0.64	77	2.57	Tescl;Lypd5	Receptor
DMR1:81222001	1	81222001	81224000	2000	1	1.20E-07	0.31	19	0.95	Lypd5;Kcnn4	Receptor;Transport
DMR1:81226001	1	81226001	81230000	4000	1	1.40E-07	0.52	55	1.38	Kcnn4	Transport
DMR1:81290001	1	81290001	81292000	2000	1	4.20E-07	0.36	39	1.95	Smg9;Irgc	
DMR1:81397001	1	81397001	81399000	2000	1	6.50E-10	0.74	68	3.4	Irgq;Pinlyp	Cytoskeleton
DMR1:81428001	1	81428001	81430000	2000	1	4.10E-07	0.6	49	2.45	Xrcc1	Transcription
DMR1:81448001	1	81448001	81449000	1000	1	9.60E-08	0.81	53	5.3	Xrcc1;Zfp575;Ethe1	Transcription;Transcription;Metabolism
DMR1:81752001	1	81752001	81754000	2000	1	6.70E-07	-0.45	24	1.2	Rps19;Cd79a	Translation;Immune
DMR1:81811001	1	81811001	81812000	1000	1	6.70E-08	0.44	16	1.6	RGD1563034	
DMR1:81875001	1	81875001	81877000	2000	1	7.80E-08	0.55	51	2.55	Atp1a3;Grik5	Transport;Receptor
DMR1:81912001	1	81912001	81914000	2000	1	3.70E-09	0.44	5	0.25	Grik5	Receptor
DMR1:81923001	1	81923001	81925000	2000	1	3.50E-07	-0.39	41	2.05	Grik5	Receptor
DMR1:82008001	1	82008001	82009000	1000	1	1.30E-07	0.33	8	0.8	Pou2f2;LOC103691054	
DMR1:82196001	1	82196001	82198000	2000	1	7.60E-07	0.33	37	1.85	Megf8	Extracellular Matrix
DMR1:82241001	1	82241001	82244000	3000	1	6.90E-07	0.35	46	1.53	Megf8;Cnfn;LOC102549342;Lipe	Extracellular Matrix;Metabolism
DMR1:82316001	1	82316001	82321000	5000	1	9.20E-10	-0.44	78	1.56	LOC102549342;Ceacam1	
DMR1:82328001	1	82328001	82331000	3000	2	2.70E-07	-0.41	70	2.33	LOC102549342;Ceacam1	
DMR1:82593001	1	82593001	82596000	3000	2	1.10E-08	0.47	58	1.93	Cyp2s1	Metabolism
DMR1:83207001	1	83207001	83210000	3000	1	3.40E-07	-0.52	34	1.13	Cyp2b3	Metabolism
DMR1:83224001	1	83224001	83229000	5000	1	5.30E-08	-0.39	41	0.82	Cyp2b3	Metabolism
DMR1:83816001	1	83816001	83818000	2000	1	2.70E-10	-0.46	26	1.3	LOC108349422;Sdccag1-ps1;LOC365218	
DMR1:84116001	1	84116001	84123000	7000	3	5.30E-11	0.77	144	2.06	Ltbp4	Extracellular Matrix
DMR1:84138001	1	84138001	84141000	3000	2	4.50E-07	0.7	100	3.33	Ltbp4	Extracellular Matrix
DMR1:84151001	1	84151001	84152000	1000	1	8.80E-08	0.66	25	2.5	Ltbp4;Shkbp1	Extracellular Matrix;Cytoskeleton
DMR1:84170001	1	84170001	84173000	3000	1	1.10E-08	0.39	44	1.47	Shkbp1;Sptbn4	Cytoskeleton

DMR1:84188001	1	84188001	84191000	3000	2	2.80E-10	0.48	27	0.9	Sptbn4	
DMR1:84454001	1	84454001	84456000	2000	1	1.70E-08	-0.4	58	2.9	Akt2	Signaling
DMR1:84932001	1	84932001	84933000	1000	1	6.20E-07	0.52	4	0.4	Zfp59	
DMR1:84961001	1	84961001	84962000	1000	1	1.30E-07	-0.48	11	1.1	Zfp59	
DMR1:85022001	1	85022001	85023000	1000	1	5.10E-08	0.4	14	1.4	Fcgbp	Extracellular Matrix
DMR1:85047001	1	85047001	85050000	3000	1	7.30E-07	-0.47	49	1.63	Fcgbp;Fcgbpl1	Extracellular Matrix
DMR1:85051001	1	85051001	85052000	1000	1	3.30E-07	-0.36	12	1.2	Fcgbp;Fcgbpl1	Extracellular Matrix
DMR1:85208001	1	85208001	85211000	3000	1	1.80E-08	0.53	14	0.47	Pak4;Nccrp1;Sycn	Signaling
DMR1:85243001	1	85243001	85244000	1000	1	2.40E-09	0.4	18	1.8	Ifnl3;Ifnl1	Cytokine
DMR1:85463001	1	85463001	85464000	1000	1	1.60E-07	0.33	22	2.2	Supt5h;Timm50	
DMR1:86015001	1	86015001	86018000	3000	2	1.00E-10	-0.68	17	0.57	LOC690191;Vom1r-ps4	
DMR1:86047001	1	86047001	86054000	7000	3	4.40E-08	-0.41	82	1.17	Vom1r-ps5	
DMR1:86095001	1	86095001	86097000	2000	1	2.90E-08	-0.4	9	0.45	Vom2r10	Signaling
DMR1:86985001	1	86985001	86986000	1000	1	1.40E-07	0.47	10	1	Rinl;Hnrnpl	Transcription
DMR1:86999001	1	86999001	87003000	4000	1	1.80E-11	-0.53	79	1.98	Hnrnpl;Ech1	Metabolism
DMR1:87021001	1	87021001	87023000	2000	1	1.10E-07	0.36	26	1.3	Ech1;Lgals4	Metabolism;Extracellular Matrix
DMR1:87047001	1	87047001	87048000	1000	1	7.80E-09	0.64	30	3	Lgals7	Extracellular Matrix
DMR1:87059001	1	87059001	87063000	4000	1	8.40E-07	0.4	42	1.05	Capn12;LOC102550499	Protease
DMR1:87069001	1	87069001	87071000	2000	1	2.90E-07	0.34	37	1.85	Capn12;LOC102550499;Actn4	Protease
DMR1:87076001	1	87076001	87077000	1000	1	1.20E-07	0.45	16	1.6	Capn12;LOC102550499;Actn4	Protease
DMR1:87210001	1	87210001	87211000	1000	1	4.70E-07	-0.43	5	0.5	Spint2	Protease; Proteolysis
DMR1:87464001	1	87464001	87466000	2000	2	6.90E-10	-0.51	34	1.7	Sipa1l3	Signaling
DMR1:87654001	1	87654001	87658000	4000	1	9.40E-08	-0.41	88	2.2	Zfp84;LOC102547413	Transcription
DMR1:87965001	1	87965001	87966000	1000	1	1.80E-08	0.57	21	2.1	Map4k1;Ryr1	Ion Channel
DMR1:88047001	1	88047001	88051000	4000	1	2.40E-07	0.47	84	2.1	Ryr1	Ion Channel
DMR1:88073001	1	88073001	88075000	2000	2	1.20E-07	0.53	18	0.9	Ryr1;Rasgrp4	Ion Channel;Transcription
DMR1:88115001	1	88115001	88116000	1000	1	2.50E-09	0.84	43	4.3	Spred3;Ggn;Psmd8	Cytoskeleton;Protease
DMR1:88461001	1	88461001	88463000	2000	1	9.10E-08	-0.57	45	2.25	Zfp260	
DMR1:88600001	1	88600001	88601000	1000	1	1.70E-07	0.75	49	4.9	Zfp382;LOC100912964	
DMR1:88871001	1	88871001	88872000	1000	1	1.20E-11	-0.54	19	1.9	LOC688666;Tyrobp;Hcst	Cytoskeleton
DMR1:88913001	1	88913001	88916000	3000	1	5.30E-07	0.41	40	1.33	Aplp1;Kirrel2;Nphs1	Protease; Proteolysis
DMR1:88926001	1	88926001	88928000	2000	1	4.00E-08	0.37	18	0.9	Kirrel2;Nphs1	
DMR1:88969001	1	88969001	88971000	2000	1	1.10E-07	0.59	44	2.2	Prodh2;RGD1560986;Arhgap33	Metabolism;Signaling
DMR1:88996001	1	88996001	88998000	2000	1	2.80E-08	0.62	36	1.8	Arhgap33;Proser3	Signaling
DMR1:89126001	1	89126001	89127000	1000	1	1.20E-08	0.44	0	0	Haus5;LOC100912333;LOC688924	
DMR1:89165001	1	89165001	89166000	1000	1	8.50E-09	0.58	43	4.3	Atp4a	Transport
DMR1:89192001	1	89192001	89193000	1000	1	3.40E-07	0.4	16	1.6	Gapdhs;Sbsn	Metabolism
DMR1:89235001	1	89235001	89236000	1000	1	4.90E-09	0.77	26	2.6	Dmkn;Krtdap	
DMR1:89267001	1	89267001	89269000	2000	1	6.10E-07	0.43	23	1.15	LOC100361079;Ffar2	Translation
DMR1:89280001	1	89280001	89281000	1000	1	5.50E-08	0.49	13	1.3	Ffar2;LOC102554169	
DMR1:89313001	1	89313001	89322000	9000	1	2.30E-07	0.57	174	1.93	Ffar3;Ffar1;Cd22	
DMR1:89405001	1	89405001	89407000	2000	1	9.90E-07	0.5	20	1	Lsr;LOC100909893;Fam187b	Immune;Immune
DMR1:89491001	1	89491001	89496000	5000	1	5.20E-07	0.36	78	1.56	Fxyd7;Fxyd1;Lgi4;Fxyd3	Transport
DMR1:89533001	1	89533001	89535000	2000	1	1.50E-08	0.71	44	2.2	Hpn	Protease
DMR1:89639001	1	89639001	89641000	2000	1	6.70E-07	-0.55	22	1.1	LOC108349560;RGD1561430;RGD1563307	Epigenetic
DMR1:89644001	1	89644001	89646000	2000	1	2.60E-07	0.63	28	1.4	RGD1561430;RGD1563307	Epigenetic
DMR1:89793001	1	89793001	89796000	3000	1	7.00E-07	-0.65	25	0.83	Apbh	
DMR1:90234001	1	90234001	90236000	2000	1	4.90E-07	-0.57	29	1.45	Lsm14a	Metabolism
DMR1:90615001	1	90615001	90617000	2000	1	6.20E-11	0.59	37	1.85	Chst8	Transport
DMR1:90641001	1	90641001	90642000	1000	1	1.90E-08	-0.48	30	3	Chst8	Transport
DMR1:90657001	1	90657001	90661000	4000	2	7.30E-12	0.85	88	2.2	Chst8	Transport
DMR1:90727001	1	90727001	90731000	4000	2	3.80E-11	-0.57	79	1.98	RGD1560088	
DMR1:90836001	1	90836001	90841000	5000	2	3.20E-09	-0.39	41	0.82	Pepd	Protease
DMR1:91427001	1	91427001	91428000	1000	1	9.40E-10	-0.44	20	2	Slc7a10	Transport
DMR1:91472001	1	91472001	91474000	2000	1	4.60E-09	-0.33	40	2	Lrp3	Binding Proteins
DMR1:91492001	1	91492001	91493000	1000	1	1.00E-11	0.34	13	1.3	Trnat-agu;Wdr88	
DMR1:91499001	1	91499001	91505000	6000	1	4.50E-10	-0.45	99	1.65	Wdr88	
DMR1:91526001	1	91526001	91528000	2000	1	5.80E-08	0.69	50	2.5	Wdr88;Gpatch1	
DMR1:91539001	1	91539001	91541000	2000	1	5.60E-09	-0.49	30	1.5	Gpatch1	
DMR1:91619001	1	91619001	91621000	2000	1	3.60E-22	0.53	19	0.95	Rhpn2	Cytoskeleton

DMR1:91865001	1	91865001	91867000	2000	1	6.40E-09	-0.5	32	1.6	Rgs9bp;Ankrd27	
DMR1:91991001	1	91991001	91994000	3000	1	6.70E-09	0.74	52	1.73	Dpy19l3	
DMR1:91998001	1	91998001	91999000	1000	1	8.90E-09	0.65	21	2.1	Dpy19l3	
DMR1:92764001	1	92764001	92766000	2000	1	1.60E-09	-0.44	47	2.35	Tshz3;LOC108349564	Transcription
DMR1:93715001	1	93715001	93716000	1000	1	2.90E-09	0.73	33	3.3	Zfp536	Transcription
DMR1:93730001	1	93730001	93732000	2000	1	1.30E-08	0.59	48	2.4	Zfp536	Transcription
DMR1:93753001	1	93753001	93757000	4000	1	6.00E-07	0.59	92	2.3	Zfp536	Transcription
DMR1:93789001	1	93789001	93791000	2000	1	1.10E-08	0.49	8	0.4	Zfp536;LOC108349103	Transcription
DMR1:93855001	1	93855001	93856000	1000	1	5.80E-08	0.44	13	1.3	Zfp536	Transcription
DMR1:93904001	1	93904001	93905000	1000	1	2.30E-08	-0.59	8	0.8	Zfp536	Transcription
DMR1:93972001	1	93972001	93975000	3000	1	9.40E-09	0.77	60	2	Zfp536	Transcription
DMR1:93981001	1	93981001	93982000	1000	1	4.00E-07	-0.33	20	2	Zfp536	Transcription
DMR1:94018001	1	94018001	94020000	2000	1	2.80E-08	-0.49	36	1.8	Zfp536	Transcription
DMR1:94030001	1	94030001	94033000	3000	1	6.60E-08	-0.48	37	1.23	Zfp536	Transcription
DMR1:94037001	1	94037001	94042000	5000	1	3.80E-08	-0.43	87	1.74	Zfp536	Transcription
DMR1:94155001	1	94155001	94156000	1000	1	1.70E-08	0.48	30	3	Zfp536	Transcription
DMR1:94164001	1	94164001	94166000	2000	1	1.70E-08	0.32	17	0.85	Zfp536	Transcription
DMR1:94336001	1	94336001	94338000	2000	1	1.60E-07	-0.35	26	1.3	LOC108349449;Uri1	Epigenetic
DMR1:94349001	1	94349001	94351000	2000	2	6.70E-07	-0.45	35	1.75	Uri1	Epigenetic
DMR1:94360001	1	94360001	94362000	2000	1	1.10E-08	-0.45	17	0.85	Uri1	Epigenetic
DMR1:94486001	1	94486001	94488000	2000	1	1.20E-07	-0.41	30	1.5	Ccne1	Signaling
DMR1:94612001	1	94612001	94615000	3000	1	4.70E-11	-0.45	40	1.33	Plekhf1;LOC108349450	
DMR1:95080001	1	95080001	95082000	2000	1	9.30E-07	0.41	19	0.95	Rps27a-ps30	
DMR1:98419001	1	98419001	98426000	7000	1	1.50E-09	0.48	77	1.1	Siglecl1;LOC690483	Immune
DMR1:98450001	1	98450001	98453000	3000	1	1.40E-10	0.49	34	1.13	Iglon5;Vsig10l	Immune
DMR1:98643001	1	98643001	98644000	1000	1	8.80E-08	0.51	40	4	RGD1565346	
DMR1:99019001	1	99019001	99020000	1000	1	4.70E-09	-0.47	6	0.6	Vom2r38;Vom2r37	Signaling
DMR1:99035001	1	99035001	99038000	3000	2	5.40E-15	-0.29	20	0.67	Vom2r38;Vom2r37	Signaling
DMR1:99056001	1	99056001	99063000	7000	1	9.20E-08	-0.18	61	0.87	Vom2r38;Vom2r37;LOC100911353;LOC108349760	Signaling
DMR1:99079001	1	99079001	99080000	1000	1	8.80E-08	0.23	19	1.9	Vom2r38;Vom2r37;LOC108349760;LOC103691104	Signaling
DMR1:99083001	1	99083001	99089000	6000	1	5.20E-10	0.24	49	0.82	Vom2r38;Vom2r37;LOC108349760;LOC103691104	Signaling
DMR1:99115001	1	99115001	99120000	5000	2	1.20E-11	-0.5	79	1.58	Vom2r38;Vom2r37;LOC100912942;LOC108349568	Signaling
DMR1:99124001	1	99124001	99125000	1000	1	4.80E-09	-0.42	21	2.1	Vom2r38;Vom2r37;LOC108349568	Signaling
DMR1:99542001	1	99542001	99545000	3000	1	5.80E-07	-0.42	48	1.6	Zfp819	Transcription
DMR1:99602001	1	99602001	99603000	1000	1	3.40E-07	0.68	51	5.1	Ctu1	
DMR1:99611001	1	99611001	99614000	3000	1	4.30E-08	0.33	41	1.37	Ctu1;Klk14	Protease
DMR1:99662001	1	99662001	99663000	1000	1	1.40E-08	0.44	4	0.4	Klk13	Protease
DMR1:99668001	1	99668001	99672000	4000	1	4.50E-07	0.41	36	0.9	Klk13	Protease
DMR1:99736001	1	99736001	99737000	1000	1	2.00E-07	0.62	20	2	Klk9;Klk8	Protease
DMR1:100307001	1	100307001	100308000	1000	1	7.00E-07	0.46	11	1.1	Shank1	
DMR1:100454001	1	100454001	100455000	1000	1	2.90E-07	0.57	5	0.5	Lrrc4b	
DMR1:100576001	1	100576001	100580000	4000	1	6.80E-11	0.45	56	1.4	Napsa	
DMR1:100584001	1	100584001	100586000	2000	2	9.70E-14	0.69	42	2.1	Napsa;Kcnc3	Transport
DMR1:100637001	1	100637001	100640000	3000	2	1.60E-10	0.46	72	2.4	Myh14	
DMR1:100949001	1	100949001	100951000	2000	1	4.80E-09	0.72	51	2.55	Tsks;Cpt1c	Metabolism
DMR1:100959001	1	100959001	100961000	2000	1	7.60E-07	0.4	36	1.8	Tsks;Cpt1c	Metabolism
DMR1:101174001	1	101174001	101177000	3000	2	9.60E-09	0.43	44	1.47	Slc17a7;Gfy;Pth2;Ccdc155	Transport
DMR1:101195001	1	101195001	101196000	1000	1	8.40E-11	0.47	3	0.3	Ccdc155;Dkkl1	
DMR1:101227001	1	101227001	101231000	4000	1	8.20E-08	0.52	88	2.2	Tead2;Cd37;Slc6a16	Transcription;Transport
DMR1:101633001	1	101633001	101634000	1000	1	4.20E-12	0.87	43	4.3	Mamstr;Fut2	Golgi
DMR1:101692001	1	101692001	101693000	1000	1	6.30E-07	-0.44	18	1.8	Car11;Dbp;Sphk2;Rpl18	Transcription;Signaling;Translation
DMR1:101742001	1	101742001	101745000	3000	1	3.00E-09	0.55	23	0.77	Sult2b1	Transport
DMR1:101777001	1	101777001	101783000	6000	1	3.90E-16	1.13	145	2.42	Sult2b1;Lmtk3	Transport
DMR1:101784001	1	101784001	101785000	1000	1	1.40E-09	0.89	53	5.3	Sult2b1;Lmtk3	Transport
DMR1:101789001	1	101789001	101790000	1000	1	7.00E-09	0.81	37	3.7	Lmtk3;Cyth2	Transcription
DMR1:101911001	1	101911001	101912000	1000	1	2.80E-10	0.54	35	3.5	Emp3;Ccdc114	Cytoskeleton
DMR1:101925001	1	101925001	101927000	2000	2	2.10E-08	0.6	43	2.15	Ccdc114	
DMR1:102006001	1	102006001	102008000	2000	1	7.80E-08	-0.35	30	1.5	Abcc6;Nomo1	Transport

DMR1:102053001	1	102053001	102055000	2000	1	6.20E-07	-0.57	25	1.25	Nomo1;LOC103691113	
DMR1:102066001	1	102066001	102076000	10000	1	1.30E-07	-0.4	152	1.52	Nomo1;LOC103691113;Ncr3lg1	
DMR1:102091001	1	102091001	102092000	1000	1	3.40E-09	0.39	6	0.6	Ncr3lg1;LOC102550828	
DMR1:102104001	1	102104001	102106000	2000	1	2.20E-10	0.71	80	4	LOC102550828;Kcnj11;Abcc8	Transport;Transport
DMR1:102161001	1	102161001	102166000	5000	3	1.80E-09	0.36	67	1.34	Abcc8	Transport
DMR1:102221001	1	102221001	102222000	1000	1	1.90E-07	0.4	19	1.9	Ush1c	Cytoskeleton
DMR1:102276001	1	102276001	102278000	2000	1	6.80E-08	0.34	31	1.55	Otog	Extracellular Matrix
DMR1:102289001	1	102289001	102290000	1000	1	4.30E-08	0.46	16	1.6	Otog;LOC103691114	Extracellular Matrix
DMR1:102295001	1	102295001	102296000	1000	1	1.20E-08	-0.4	15	1.5	Otog;LOC103691114	Extracellular Matrix
DMR1:102479001	1	102479001	102481000	2000	1	1.20E-07	0.38	35	1.75	Sergef	Proteolysis
DMR1:102517001	1	102517001	102518000	1000	1	1.70E-07	-0.53	13	1.3	Sergef	Proteolysis
DMR1:102526001	1	102526001	102527000	1000	1	1.90E-07	0.31	12	1.2	Sergef	Proteolysis
DMR1:102584001	1	102584001	102587000	3000	1	2.10E-09	-0.57	45	1.5	Sergef	Proteolysis
DMR1:102648001	1	102648001	102652000	4000	1	6.40E-08	0.42	50	1.25	Sergef	Proteolysis
DMR1:102767001	1	102767001	102769000	2000	1	3.80E-07	0.34	21	1.05	LOC108349573;Saa4	
DMR1:103163001	1	103163001	103165000	2000	1	5.60E-10	0.5	27	1.35	LOC108349285;Tmem86a	
DMR1:103235001	1	103235001	103237000	2000	1	1.40E-07	-0.46	28	1.4	Ptpn5	
DMR1:103298001	1	103298001	103303000	5000	2	8.30E-08	0.6	56	1.12	Mrgprx3	Signaling
DMR1:103328001	1	103328001	103329000	1000	1	6.80E-13	0.5	12	1.2	Mrgprx3	Signaling
DMR1:103420001	1	103420001	103423000	3000	1	2.40E-09	-0.29	30	1	Mrgprb4	Signaling
DMR1:103733001	1	103733001	103736000	3000	1	7.60E-08	-0.44	27	0.9	Mrgprx2	Signaling
DMR1:104004001	1	104004001	104006000	2000	1	9.30E-07	-0.47	12	0.6	RGD1560730	Signaling
DMR1:104056001	1	104056001	104059000	3000	1	6.50E-07	-0.42	11	0.37	Mrgprb2	
DMR1:104640001	1	104640001	104644000	4000	1	3.00E-07	-0.43	77	1.93	Nav2	
DMR1:104772001	1	104772001	104777000	5000	1	8.30E-07	-0.39	84	1.68	Nav2	
DMR1:104843001	1	104843001	104844000	1000	1	2.40E-08	-0.41	19	1.9	Nav2	
DMR1:105175001	1	105175001	105177000	2000	1	8.20E-07	0.44	23	1.15	Prmt3	Golgi
DMR1:105405001	1	105405001	105407000	2000	1	2.90E-07	-0.51	23	1.15	Nell1	Signaling
DMR1:105477001	1	105477001	105480000	3000	1	7.20E-08	-0.42	30	1	Nell1	Signaling
DMR1:105794001	1	105794001	105796000	2000	1	3.00E-08	0.43	45	2.25	Nell1	Signaling
DMR1:105808001	1	105808001	105810000	2000	1	3.60E-07	0.38	13	0.65	Nell1	Signaling
DMR1:105873001	1	105873001	105879000	6000	1	1.20E-08	-0.41	50	0.83	Nell1	Signaling
DMR1:106006001	1	106006001	106007000	1000	1	4.00E-08	0.33	15	1.5	Nell1	Signaling
DMR1:111991001	1	111991001	111992000	1000	1	5.90E-08	0.37	8	0.8	Luzp2	
DMR1:112393001	1	112393001	112394000	1000	1	8.00E-08	0.51	5	0.5	Gabrg3	Ion Channel
DMR1:112758001	1	112758001	112760000	2000	1	7.40E-08	-0.61	5	0.25	Gabrg3	Ion Channel
DMR1:112824001	1	112824001	112827000	3000	1	1.90E-07	-0.47	19	0.63	Gabra5	Ion Channel
DMR1:113449001	1	113449001	113451000	2000	1	6.20E-09	-0.41	14	0.7	Luzp2	
DMR1:113515001	1	113515001	113516000	1000	1	5.60E-12	0.64	14	1.4	Luzp2;LOC103691130	
DMR1:114132001	1	114132001	114134000	2000	1	1.80E-07	-0.5	8	0.4	Siglech	
DMR1:114135001	1	114135001	114141000	6000	1	8.20E-18	-0.6	71	1.18	Siglech;LOC108349722	
DMR1:114267001	1	114267001	114268000	1000	1	6.10E-09	0.58	22	2.2	Cyfip1	Cytoskeleton
DMR1:114324001	1	114324001	114326000	2000	1	2.80E-07	-0.39	26	1.3	Cyfip1;LOC102549565	Cytoskeleton
DMR1:114608001	1	114608001	114614000	6000	1	7.10E-08	-0.44	61	1.02	Herc2	Transcription
DMR1:116008001	1	116008001	116009000	1000	1	1.30E-11	-0.45	8	0.8	Atp10a	Transport
DMR1:116632001	1	116632001	116634000	2000	1	1.70E-07	0.5	19	0.95	Ube3a	Proteolysis
DMR1:116670001	1	116670001	116673000	3000	1	6.80E-09	-0.44	23	0.77	Ube3a;LOC103691125;LOC103691124	Proteolysis
DMR1:123105001	1	123105001	123109000	4000	1	1.40E-07	-0.24	56	1.4	Peg12;LOC108349584;LOC679961	
DMR1:124475001	1	124475001	124477000	2000	2	1.70E-07	-0.48	11	0.55	Otud7a;Hmgn5b	Protease
DMR1:124632001	1	124632001	124634000	2000	1	3.70E-08	0.42	27	1.35	Otud7a	Protease
DMR1:125010001	1	125010001	125011000	1000	1	1.20E-08	0.39	17	1.7	Trpm1	Transport
DMR1:125213001	1	125213001	125214000	1000	1	9.70E-09	-0.45	12	1.2	Fan1;Mphosph10	Metabolism
DMR1:125409001	1	125409001	125410000	1000	1	6.30E-08	0.27	5	0.5	Apba2	Transport
DMR1:125429001	1	125429001	125430000	1000	1	1.30E-09	0.43	14	1.4	Apba2	Transport
DMR1:125624001	1	125624001	125625000	1000	1	9.40E-07	-0.54	13	1.3	Fam189a1	
DMR1:125637001	1	125637001	125640000	3000	1	2.10E-11	-0.48	62	2.07	Fam189a1	
DMR1:125732001	1	125732001	125733000	1000	1	1.30E-07	-0.42	25	2.5	Fam189a1	
DMR1:125754001	1	125754001	125756000	2000	1	8.30E-08	-0.41	44	2.2	Fam189a1	
DMR1:125782001	1	125782001	125787000	5000	1	6.80E-10	-0.62	51	1.02	Fam189a1	
DMR1:125834001	1	125834001	125835000	1000	1	4.70E-08	0.36	16	1.6	Fam189a1	
DMR1:125855001	1	125855001	125857000	2000	1	1.90E-07	-0.35	27	1.35	Fam189a1	
DMR1:125882001	1	125882001	125884000	2000	1	4.00E-08	0.47	14	0.7	Fam189a1;Matr3-ps1	

DMR1:125948001	1	125948001	125950000	2000	1	6.10E-07	-0.39	18	0.9	Fam189a1	
DMR1:126172001	1	126172001	126177000	5000	1	1.10E-08	-0.57	56	1.12	Tjp1	Cell Junction
DMR1:126194001	1	126194001	126196000	2000	1	1.30E-07	-0.4	29	1.45	Tjp1	Cell Junction
DMR1:126343001	1	126343001	126345000	2000	1	2.40E-08	-0.45	38	1.9	Tjp1	Cell Junction
DMR1:126359001	1	126359001	126364000	5000	1	9.40E-07	-0.26	54	1.08	Tjp1	Cell Junction
DMR1:126372001	1	126372001	126375000	3000	1	1.20E-13	0.75	53	1.77	Tjp1	Cell Junction
DMR1:126385001	1	126385001	126388000	3000	1	7.70E-07	-0.34	72	2.4	Tjp1	Cell Junction
DMR1:126482001	1	126482001	126486000	4000	1	3.70E-08	0.4	58	1.45	Tjp1;LOC102546680	Cell Junction
DMR1:126538001	1	126538001	126539000	1000	1	4.90E-07	0.33	14	1.4	Tarsl2	Translation
DMR1:126755001	1	126755001	126756000	1000	1	2.50E-07	-0.46	21	2.1	Pcsk6	Protease
DMR1:126773001	1	126773001	126774000	1000	1	8.80E-07	-0.4	17	1.7	Pcsk6;LOC108349587	Protease
DMR1:126777001	1	126777001	126780000	3000	1	7.60E-07	-0.42	37	1.23	Pcsk6;LOC108349587	Protease
DMR1:126802001	1	126802001	126805000	3000	1	4.70E-09	0.46	42	1.4	Pcsk6	Protease
DMR1:126863001	1	126863001	126864000	1000	1	6.30E-07	0.51	12	1.2	Pcsk6	Protease
DMR1:126888001	1	126888001	126896000	8000	1	8.20E-07	-0.23	83	1.04	Pcsk6	Protease
DMR1:126905001	1	126905001	126907000	2000	1	9.20E-07	-0.33	38	1.9	Pcsk6	Protease
DMR1:127018001	1	127018001	127020000	2000	1	6.90E-07	0.5	17	0.85	LOC108349588;Chsy1	Golgi
DMR1:127205001	1	127205001	127206000	1000	1	4.60E-07	-0.45	21	2.1	Lrrk1	Signaling
DMR1:127260001	1	127260001	127262000	2000	1	2.30E-08	-0.47	42	2.1	Lrrk1	Signaling
DMR1:127560001	1	127560001	127562000	2000	1	2.50E-07	-0.5	36	1.8	Asb7	Transport
DMR1:127585001	1	127585001	127591000	6000	1	2.90E-14	-0.67	87	1.45	Asb7;Lins1	Transport
DMR1:127722001	1	127722001	127724000	2000	1	7.70E-07	-0.61	17	0.85	Cers3	
DMR1:127821001	1	127821001	127823000	2000	1	3.40E-07	0.31	19	0.95	Adamts17	Protease
DMR1:127916001	1	127916001	127921000	5000	1	1.80E-07	-0.35	37	0.74	Adamts17	Protease
DMR1:128119001	1	128119001	128121000	2000	1	8.80E-07	-0.36	5	0.25	Adamts17	Protease
DMR1:128196001	1	128196001	128197000	1000	1	9.10E-08	0.48	20	2	Lysmd4	
DMR1:128255001	1	128255001	128256000	1000	1	2.40E-09	-0.42	9	0.9	Mef2a	Transcription
DMR1:128296001	1	128296001	128297000	1000	1	4.80E-10	-0.61	5	0.5	Mef2a	Transcription
DMR1:128503001	1	128503001	128504000	1000	1	1.20E-07	-0.43	18	1.8	Lrrc28	
DMR1:128570001	1	128570001	128571000	1000	1	5.40E-07	-0.42	17	1.7	Lrrc28	
DMR1:128607001	1	128607001	128609000	2000	2	2.40E-11	-0.61	28	1.4	Lrrc28;Ttc23;LOC108349234	
DMR1:128652001	1	128652001	128653000	1000	1	4.20E-07	-0.46	22	2.2	Ttc23	
DMR1:128928001	1	128928001	128930000	2000	1	3.10E-11	-0.47	30	1.5	Igf1r	Receptor
DMR1:128941001	1	128941001	128944000	3000	2	6.30E-14	-0.59	88	2.93	Igf1r	Receptor
DMR1:128949001	1	128949001	128952000	3000	2	1.20E-12	-0.66	54	1.8	Igf1r	Receptor
DMR1:128966001	1	128966001	128970000	4000	1	2.00E-08	-0.49	49	1.23	Igf1r	Receptor
DMR1:129021001	1	129021001	129022000	1000	1	8.00E-09	-0.53	16	1.6	Igf1r	Receptor
DMR1:129047001	1	129047001	129051000	4000	1	9.50E-07	-0.36	81	2.02	Igf1r	Receptor
DMR1:129150001	1	129150001	129151000	1000	1	1.90E-07	0.35	13	1.3	Igf1r	Receptor
DMR1:129163001	1	129163001	129165000	2000	1	3.50E-14	-0.79	55	2.75	Igf1r	Receptor
DMR1:129252001	1	129252001	129255000	3000	2	3.70E-11	0.78	64	2.13	Pgpep1l;Fam169b	Protease
DMR1:129261001	1	129261001	129264000	3000	1	1.70E-07	-0.35	66	2.2	Fam169b	
DMR1:129279001	1	129279001	129280000	1000	1	1.70E-07	-0.31	16	1.6	Fam169b	
DMR1:129283001	1	129283001	129284000	1000	1	1.10E-07	0.41	7	0.7	Fam169b	
DMR1:132188001	1	132188001	132189000	1000	1	1.20E-09	0.52	13	1.3	RGD1562781	
DMR1:132196001	1	132196001	132197000	1000	1	4.90E-07	-0.53	15	1.5	RGD1562781	
DMR1:133435001	1	133435001	133436000	1000	1	4.20E-07	0.3	11	1.1	Mctp2	
DMR1:133469001	1	133469001	133473000	4000	1	3.00E-08	0.55	64	1.6	Mctp2	
DMR1:133480001	1	133480001	133482000	2000	1	2.80E-12	-0.58	19	0.95	Mctp2	
DMR1:133513001	1	133513001	133514000	1000	1	3.30E-09	0.49	4	0.4	Mctp2	
DMR1:133529001	1	133529001	133535000	6000	1	1.90E-07	-0.26	46	0.77	Mctp2	
DMR1:134831001	1	134831001	134833000	2000	1	1.20E-13	-0.52	17	0.85	Chd2	
DMR1:135103001	1	135103001	135109000	6000	1	2.10E-08	-0.58	75	1.25	Fam174b	
DMR1:135876001	1	135876001	135878000	2000	1	7.70E-08	0.42	11	0.55	Slco3a1	Transport
DMR1:135897001	1	135897001	135899000	2000	1	5.80E-07	0.34	11	0.55	Slco3a1	Transport
DMR1:136007001	1	136007001	136008000	1000	1	6.80E-07	0.43	20	2	Slco3a1	Transport
DMR1:136024001	1	136024001	136027000	3000	1	9.90E-07	-0.44	29	0.97	Slco3a1	Transport
DMR1:136657001	1	136657001	136658000	1000	1	5.70E-08	0.43	8	0.8	Sv2b	
DMR1:136804001	1	136804001	136806000	2000	2	7.20E-09	-0.45	24	1.2	Sv2b	
DMR1:136830001	1	136830001	136832000	2000	1	2.00E-09	0.84	35	1.75	Sv2b;LOC108349288	
DMR1:137224001	1	137224001	137225000	1000	1	4.80E-07	-0.51	10	1	Akap13	
DMR1:137316001	1	137316001	137318000	2000	1	9.30E-07	-0.45	10	0.5	Akap13	
DMR1:137913001	1	137913001	137915000	2000	1	9.70E-07	0.43	39	1.95	Agbl1;LOC108349814	Protease

DMR1:138164001	1	138164001	138167000	3000	1	8.60E-10	-0.42	23	0.77	Agbl1	Protease
DMR1:138265001	1	138265001	138268000	3000	1	4.10E-12	-0.34	21	0.7	Agbl1	Protease
DMR1:138320001	1	138320001	138326000	6000	2	1.80E-10	-0.41	66	1.1	Agbl1	Protease
DMR1:138558001	1	138558001	138560000	2000	1	4.10E-07	0.35	18	0.9	Agbl1	Protease
DMR1:138632001	1	138632001	138633000	1000	1	3.30E-09	-0.57	4	0.4	Agbl1	Protease
DMR1:138826001	1	138826001	138827000	1000	1	2.10E-07	0.41	11	1.1	Vom1r-ps67	
DMR1:138841001	1	138841001	138845000	4000	2	5.20E-09	-0.32	28	0.7	Vom1r-ps67	
DMR1:140494001	1	140494001	140497000	3000	1	2.30E-07	0.45	33	1.1	Mrps11	Translation
DMR1:141383001	1	141383001	141384000	1000	1	9.00E-08	0.36	9	0.9	LOC103691171;Ticrr	
DMR1:141589001	1	141589001	141592000	3000	2	7.50E-10	0.83	55	1.83	Anpep	Protease
DMR1:141872001	1	141872001	141874000	2000	1	9.70E-18	-0.68	39	1.95	Zfp710;Idh2	Transcription;Metabolism
DMR1:142152001	1	142152001	142154000	2000	1	1.60E-07	0.44	41	2.05	Man2a2	
DMR1:142509001	1	142509001	142514000	5000	1	2.80E-07	0.36	75	1.5	Crtc3;LOC103691178	Transcription
DMR1:142542001	1	142542001	142543000	1000	1	4.30E-08	-0.5	20	2	Iqgap1	Signaling
DMR1:142588001	1	142588001	142593000	5000	1	2.50E-08	-0.46	68	1.36	Iqgap1	Signaling
DMR1:142612001	1	142612001	142613000	1000	1	2.50E-10	-0.41	17	1.7	Iqgap1	Signaling
DMR1:142677001	1	142677001	142678000	1000	1	2.40E-07	0.45	20	2	Zscan2	Transcription
DMR1:142890001	1	142890001	142891000	1000	1	3.70E-07	0.35	9	0.9	Alpk3;LOC102555867	Signaling
DMR1:142902001	1	142902001	142907000	5000	2	1.50E-07	0.49	82	1.64	Alpk3;LOC102555867	Signaling
DMR1:142915001	1	142915001	142916000	1000	1	8.20E-08	-0.36	17	1.7	Alpk3;LOC102555867	Signaling
DMR1:142946001	1	142946001	142949000	3000	1	1.10E-07	0.38	44	1.47	Slc28a1	Transport
DMR1:142956001	1	142956001	142959000	3000	1	9.40E-11	0.39	36	1.2	Slc28a1	Transport
DMR1:143157001	1	143157001	143158000	1000	1	1.70E-07	0.49	12	1.2	Pde8a;Rps17	Signaling;Translation
DMR1:143200001	1	143200001	143201000	1000	1	4.50E-07	-0.89	4	0.4	Cpeb1;LOC103691174	Translation
DMR1:143323001	1	143323001	143325000	2000	1	1.90E-09	-0.5	15	0.75	Ap3b2;LOC102555928;LOC100360933;LOC102556224	Transport
DMR1:143359001	1	143359001	143360000	1000	1	6.20E-08	-0.42	21	2.1	LOC102556224;Scarna15;Fsd2	Proteolysis
DMR1:143457001	1	143457001	143460000	3000	1	3.20E-07	-0.4	59	1.97	Homer2	
DMR1:143500001	1	143500001	143502000	2000	2	7.10E-08	0.41	19	0.95	Homer2	
DMR1:143663001	1	143663001	143664000	1000	1	8.30E-07	-0.38	7	0.7	Tm6sf1	
DMR1:143669001	1	143669001	143670000	1000	1	1.20E-11	0.48	7	0.7	Tm6sf1;LOC102556283	
DMR1:143697001	1	143697001	143699000	2000	1	2.30E-07	-0.58	28	1.4	Tm6sf1;Hdgfrp3	
DMR1:144173001	1	144173001	144176000	3000	1	2.70E-07	-0.43	42	1.4	Sh3gl3	
DMR1:144243001	1	144243001	144246000	3000	1	3.70E-07	-0.4	38	1.27	Adamtsl3	Protease
DMR1:144414001	1	144414001	144419000	5000	1	6.10E-08	-0.31	39	0.78	Adamtsl3	Protease
DMR1:144420001	1	144420001	144421000	1000	1	3.20E-08	0.4	9	0.9	Adamtsl3	Protease
DMR1:144557001	1	144557001	144558000	1000	1	1.50E-08	0.35	3	0.3	Adamtsl3	Protease
DMR1:145795001	1	145795001	145798000	3000	1	1.60E-07	-0.33	25	0.83	Il16;LOC103691182	Cytokine
DMR1:145827001	1	145827001	145829000	2000	1	5.40E-07	0.43	21	1.05	Il16	Cytokine
DMR1:145858001	1	145858001	145863000	5000	1	6.70E-08	-0.3	57	1.14	Il16	Cytokine
DMR1:145940001	1	145940001	145945000	5000	1	1.30E-11	-0.53	44	0.88	Cfap161	Development
DMR1:146294001	1	146294001	146299000	5000	1	3.80E-11	-0.35	49	0.98	Abhd17c;LOC108349171	Protease
DMR1:147048001	1	147048001	147049000	1000	1	1.40E-09	0.6	20	2	Xlr3a	
DMR1:147058001	1	147058001	147061000	3000	1	3.10E-07	0.45	55	1.83	Xlr3a;LOC100366231	
DMR1:147440001	1	147440001	147443000	3000	1	9.60E-08	-0.32	21	0.7	Cyp2c7	Metabolism
DMR1:147552001	1	147552001	147556000	4000	2	1.20E-08	-0.3	44	1.1	Cyp2c7;LOC100361434	Metabolism
DMR1:147561001	1	147561001	147563000	2000	1	2.10E-07	-0.35	6	0.3	Cyp2c7;LOC100361434	Metabolism
DMR1:147570001	1	147570001	147572000	2000	1	3.10E-10	-0.39	8	0.4	Cyp2c7;LOC100361434	Metabolism
DMR1:147716001	1	147716001	147718000	2000	2	4.50E-09	-0.38	17	0.85	Cyp2c7;Cyp2c6v1	Metabolism
DMR1:147783001	1	147783001	147788000	5000	1	2.60E-10	-0.36	37	0.74	Cyp2c7;Cyp2c6v1	Metabolism
DMR1:147792001	1	147792001	147796000	4000	1	7.00E-07	-0.29	30	0.75	Cyp2c7;Cyp2c6v1	Metabolism
DMR1:147828001	1	147828001	147832000	4000	1	1.30E-10	0.52	30	0.75	Cyp2c7	Metabolism
DMR1:147847001	1	147847001	147851000	4000	1	1.70E-09	-0.37	25	0.62	Cyp2c7	Metabolism
DMR1:147870001	1	147870001	147871000	1000	1	1.00E-08	-0.36	6	0.6	Cyp2c7	Metabolism
DMR1:147892001	1	147892001	147899000	7000	1	2.00E-08	-0.36	41	0.59	Cyp2c7;LOC100911718	Metabolism
DMR1:148061001	1	148061001	148066000	5000	1	3.30E-09	-0.46	44	0.88	Cyp2c7	Metabolism
DMR1:148091001	1	148091001	148095000	4000	1	5.80E-07	-0.4	16	0.4	Cyp2c7	Metabolism
DMR1:148415001	1	148415001	148418000	3000	1	1.40E-09	0.59	131	4.37	Vbp1	Transcription
DMR1:148428001	1	148428001	148431000	3000	3	3.80E-18	1.04	224	7.47	Vbp1	Transcription
DMR1:148446001	1	148446001	148448000	2000	2	1.30E-15	0.79	65	3.25	Vbp1;LOC102546990;Mpp1	Transcription
DMR1:148835001	1	148835001	148837000	2000	1	2.50E-07	-0.41	12	0.6	LOC100912014;Olr225-ps;Olr226	Receptor
DMR1:148912001	1	148912001	148918000	6000	1	6.80E-08	-0.35	34	0.57	Vom2r40	

DMR1:148919001	1	148919001	148922000	3000	1	1.10E-08	-0.51	17	0.57	Vom2r40	
DMR1:149826001	1	149826001	149829000	3000	1	1.50E-07	-0.28	21	0.7	Olr14;Olr15-ps	Signaling
DMR1:150014001	1	150014001	150017000	3000	2	1.70E-07	-0.52	13	0.43	Olr21-ps	
DMR1:150149001	1	150149001	150150000	1000	1	6.50E-08	-0.49	5	0.5	Olr26-ps	
DMR1:150278001	1	150278001	150280000	2000	1	1.70E-11	-0.44	14	0.7	Olr33-ps	
DMR1:150789001	1	150789001	150792000	3000	1	5.10E-08	-0.57	6	0.2	LOC100360510;LOC100910339;Nox4	Metabolism
DMR1:150869001	1	150869001	150870000	1000	1	5.10E-09	0.42	3	0.3	Nox4	Metabolism
DMR1:151293001	1	151293001	151295000	2000	1	7.00E-10	-0.41	10	0.5	Grm5	Signaling
DMR1:151349001	1	151349001	151351000	2000	1	6.00E-07	-0.45	12	0.6	Grm5;LOC102555017	Signaling
DMR1:151604001	1	151604001	151605000	1000	1	8.00E-09	-0.41	7	0.7	Grm5	Signaling
DMR1:151697001	1	151697001	151699000	2000	1	5.90E-07	-0.3	16	0.8	Grm5	Signaling
DMR1:152063001	1	152063001	152065000	2000	1	9.40E-07	-0.41	6	0.3	Rab38	
DMR1:152100001	1	152100001	152104000	4000	1	2.60E-08	-0.35	37	0.92	Rab38	
DMR1:152892001	1	152892001	152896000	4000	1	8.40E-08	-0.43	46	1.15	Tmem135	
DMR1:152927001	1	152927001	152933000	6000	1	2.30E-07	-0.33	59	0.98	Tmem135	
DMR1:153020001	1	153020001	153021000	1000	1	8.60E-07	-0.46	9	0.9	Tmem135	
DMR1:153035001	1	153035001	153037000	2000	1	6.60E-07	-0.42	3	0.15	Tmem135	
DMR1:153058001	1	153058001	153064000	6000	3	5.50E-09	-0.36	53	0.88	Tmem135	
DMR1:153953001	1	153953001	153955000	2000	1	3.60E-07	0.41	17	0.85	Me3	Metabolism
DMR1:154092001	1	154092001	154099000	7000	1	1.70E-08	-0.35	78	1.11	Ccdc81	
DMR1:154409001	1	154409001	154410000	1000	1	5.70E-07	-0.52	8	0.8	Picalm	Transport
DMR1:154482001	1	154482001	154484000	2000	1	1.10E-08	0.67	32	1.6	Ccdc83	
DMR1:154488001	1	154488001	154493000	5000	1	1.80E-07	-0.39	41	0.82	Ccdc83	
DMR1:155101001	1	155101001	155102000	1000	1	1.60E-08	0.66	16	1.6	Dlg2	Cytoskeleton
DMR1:155340001	1	155340001	155343000	3000	1	7.10E-07	-0.36	25	0.83	Dlg2	Cytoskeleton
DMR1:155566001	1	155566001	155572000	6000	1	3.90E-07	-0.32	57	0.95	Dlg2	Cytoskeleton
DMR1:155711001	1	155711001	155716000	5000	1	1.10E-09	-0.35	40	0.8	Dlg2	Cytoskeleton
DMR1:155980001	1	155980001	155981000	1000	1	9.70E-07	0.39	2	0.2	Dlg2;LOC108349816	Cytoskeleton
DMR1:156112001	1	156112001	156117000	5000	1	7.70E-08	-0.31	35	0.7	Dlg2;LOC108349816	Cytoskeleton
DMR1:156240001	1	156240001	156243000	3000	1	2.30E-07	-0.43	9	0.3	Dlg2;LOC108349816	Cytoskeleton
DMR1:156447001	1	156447001	156448000	1000	1	8.70E-07	-0.52	5	0.5	Dlg2;Sytl2	Cytoskeleton
DMR1:156474001	1	156474001	156481000	7000	1	1.70E-07	-0.28	64	0.91	Dlg2;Ccdc83	Cytoskeleton
DMR1:156748001	1	156748001	156749000	1000	1	5.20E-08	-0.47	8	0.8	Dlg2	Cytoskeleton
DMR1:156763001	1	156763001	156764000	1000	1	9.30E-07	-0.53	6	0.6	Dlg2	Cytoskeleton
DMR1:156858001	1	156858001	156862000	4000	1	5.50E-07	-0.25	28	0.7	Dlg2	Cytoskeleton
DMR1:156971001	1	156971001	156975000	4000	1	1.20E-08	-0.34	39	0.98	Dlg2	Cytoskeleton
DMR1:157037001	1	157037001	157039000	2000	1	2.70E-10	-0.72	13	0.65	Dlg2	Cytoskeleton
DMR1:157060001	1	157060001	157061000	1000	1	8.20E-07	0.32	8	0.8	Dlg2	Cytoskeleton
DMR1:157102001	1	157102001	157103000	1000	1	4.10E-10	0.65	5	0.5	Dlg2	Cytoskeleton
DMR1:157395001	1	157395001	157397000	2000	1	5.10E-07	-0.46	19	0.95	LOC100361001;Ccdc90b	
DMR1:157601001	1	157601001	157608000	7000	3	1.40E-07	-0.28	79	1.13	Rab30	
DMR1:161563001	1	161563001	161565000	2000	1	4.30E-07	0.44	20	1	Tenm4	
DMR1:161637001	1	161637001	161641000	4000	1	2.80E-07	-0.39	60	1.5	Tenm4	
DMR1:161739001	1	161739001	161741000	2000	1	3.10E-08	0.34	20	1	Tenm4	
DMR1:161877001	1	161877001	161878000	1000	1	1.30E-07	0.28	6	0.6	Tenm4	
DMR1:161886001	1	161886001	161887000	1000	1	2.80E-07	0.29	11	1.1	Tenm4	
DMR1:161938001	1	161938001	161944000	6000	1	6.00E-07	-0.33	38	0.63	Nars2	Translation
DMR1:162002001	1	162002001	162004000	2000	1	2.20E-07	-0.53	16	0.8	Nars2;LOC108349194	Translation
DMR1:162017001	1	162017001	162024000	7000	1	1.40E-12	-0.38	82	1.17	Nars2	Translation
DMR1:162387001	1	162387001	162389000	2000	1	8.80E-09	0.38	25	1.25	Thrsp;RGD1562118	
DMR1:162410001	1	162410001	162414000	4000	1	8.30E-07	0.37	43	1.07	LOC102557251;Kctd14	Cytoskeleton
DMR1:162756001	1	162756001	162760000	4000	1	1.70E-09	-0.28	31	0.78	LOC102550562;Pak1	Signaling
DMR1:162944001	1	162944001	162945000	1000	1	1.00E-06	-0.49	10	1	Gdpd4;LOC293133	Signaling
DMR1:162955001	1	162955001	162956000	1000	1	7.70E-08	-0.48	11	1.1	Gdpd4	Signaling
DMR1:162967001	1	162967001	162972000	5000	2	8.90E-12	0.58	61	1.22	Gdpd4	Signaling
DMR1:162973001	1	162973001	162976000	3000	1	2.40E-08	0.34	19	0.63	Gdpd4	Signaling
DMR1:163075001	1	163075001	163077000	2000	1	8.50E-07	0.52	33	1.65	Myo7a;Capn5	Cytoskeleton;Protease
DMR1:163133001	1	163133001	163136000	3000	1	5.30E-09	0.48	39	1.3	Capn5;B3gnt6	Protease;Golgi
DMR1:163229001	1	163229001	163234000	5000	2	3.90E-10	-0.44	24	0.48	Acer3	
DMR1:163239001	1	163239001	163244000	5000	1	5.50E-10	-0.31	39	0.78	Acer3	
DMR1:163802001	1	163802001	163806000	4000	1	9.40E-07	0.45	78	1.95	Wnt11;RGD1561870	Signaling;Translation
DMR1:164012001	1	164012001	164014000	2000	1	4.80E-07	-0.33	14	0.7	Uvrag	

DMR1:164053001	1	164053001	164055000	2000	2	1.40E-08	0.45	16	0.8	Uvrag	
DMR1:164196001	1	164196001	164197000	1000	1	9.00E-09	0.55	36	3.6	Mogat2	Metabolism
DMR1:164295001	1	164295001	164298000	3000	1	7.30E-08	0.47	24	0.8	Map6;Serpinh1	Protease; Proteolysis
DMR1:164378001	1	164378001	164379000	1000	1	3.10E-07	-0.52	14	1.4	Gdpd5	Signaling
DMR1:164412001	1	164412001	164414000	2000	1	6.40E-09	0.35	15	0.75	Gdpd5;Klhl35	Signaling;Cytoskeleton
DMR1:164559001	1	164559001	164560000	1000	1	1.80E-08	0.45	10	1	Arrb1	Cytoskeleton
DMR1:164620001	1	164620001	164622000	2000	1	9.20E-08	-0.34	21	1.05	LOC108349134;Slco2b1	Transport
DMR1:164661001	1	164661001	164668000	7000	2	6.10E-09	0.44	82	1.17	Slco2b1	Transport
DMR1:164793001	1	164793001	164797000	4000	2	1.00E-07	-0.37	34	0.85	Neu3	Metabolism
DMR1:165006001	1	165006001	165008000	2000	1	5.60E-08	0.46	22	1.1	Chrdl2	
DMR1:165040001	1	165040001	165042000	2000	1	3.20E-07	-0.47	33	1.65	Chrdl2	
DMR1:165195001	1	165195001	165202000	7000	1	5.80E-08	-0.42	97	1.39	LOC100912071;Kcne3	Transport
DMR1:165293001	1	165293001	165294000	1000	1	5.50E-07	-0.34	15	1.5	Pgm2l1;P4ha3	Metabolism;Golgi
DMR1:165329001	1	165329001	165330000	1000	1	8.20E-10	0.41	4	0.4	P4ha3;Ppme1	Golgi
DMR1:165544001	1	165544001	165547000	3000	1	1.10E-12	0.97	42	1.4	LOC103691200;Coa4	Transcription
DMR1:165594001	1	165594001	165595000	1000	1	7.10E-08	-0.64	13	1.3	Mrpl48;LOC102548233	Translation
DMR1:165917001	1	165917001	165919000	2000	1	1.40E-08	0.37	54	2.7	Mir3102	
DMR1:165974001	1	165974001	165978000	4000	2	4.80E-13	0.87	81	2.02	P2ry6	Signaling
DMR1:165982001	1	165982001	165984000	2000	1	2.30E-09	0.47	16	0.8	P2ry6	Signaling
DMR1:166188001	1	166188001	166190000	2000	1	1.20E-09	-0.55	12	0.6	Fchsd2	
DMR1:166204001	1	166204001	166206000	2000	1	1.30E-07	-0.27	36	1.8	Fchsd2	
DMR1:166229001	1	166229001	166232000	3000	1	5.60E-09	-0.59	18	0.6	Fchsd2	
DMR1:166241001	1	166241001	166247000	6000	2	2.50E-09	-0.43	59	0.98	Fchsd2	
DMR1:166354001	1	166354001	166360000	6000	1	9.70E-07	-0.3	72	1.2	Fchsd2	
DMR1:166506001	1	166506001	166509000	3000	1	5.90E-08	0.31	50	1.67	Arap1	Signaling
DMR1:166535001	1	166535001	166538000	3000	1	1.60E-07	0.34	36	1.2	Arap1;Pde2a	Signaling;Signaling
DMR1:166542001	1	166542001	166544000	2000	1	1.80E-07	0.5	17	0.85	Pde2a	Signaling
DMR1:166579001	1	166579001	166580000	1000	1	8.10E-08	0.27	13	1.3	Pde2a;Mir139	Signaling
DMR1:166669001	1	166669001	166670000	1000	1	1.20E-07	-0.51	20	2	Art2b	
DMR1:166890001	1	166890001	166896000	6000	1	9.80E-08	0.39	144	2.4	LOC102547177;Phox2a;Inppl1	Development
DMR1:166899001	1	166899001	166900000	1000	1	3.80E-08	0.45	29	2.9	Phox2a;Inppl1	Development
DMR1:167103001	1	167103001	167104000	1000	1	3.30E-18	0.8	13	1.3	Il18bp;Rnf121	Proteolysis
DMR1:167147001	1	167147001	167149000	2000	1	6.40E-07	-0.43	19	0.95	Rnf121;LOC102549471	Proteolysis
DMR1:167153001	1	167153001	167154000	1000	1	2.10E-08	-0.53	7	0.7	Rnf121;LOC102549471;LOC108348605	Proteolysis
DMR1:167163001	1	167163001	167166000	3000	1	6.40E-10	-0.47	41	1.37	Rnf121;LOC102549471;LOC108348605	Proteolysis
DMR1:167196001	1	167196001	167197000	1000	1	1.90E-07	0.47	23	2.3	Trpc2;Art5;Art1;Chrna10	Transport;Transport;Ion Channel
DMR1:167228001	1	167228001	167231000	3000	1	9.40E-07	-0.25	31	1.03	Nup98	Transport
DMR1:167600001	1	167600001	167606000	6000	3	3.80E-14	0.58	80	1.33	Olr40	Receptor
DMR1:167691001	1	167691001	167696000	5000	1	3.10E-08	-0.28	52	1.04	Olr42-ps;Trim21	Proteolysis
DMR1:167720001	1	167720001	167721000	1000	1	4.60E-07	0.61	20	2	Olr43	Receptor
DMR1:167727001	1	167727001	167729000	2000	1	1.90E-07	-0.42	8	0.4	Olr43;Olr44	Receptor
DMR1:167765001	1	167765001	167766000	1000	1	4.30E-09	0.68	34	3.4	Olr46;Bcl2l1-ps1;Olr47	Receptor
DMR1:168103001	1	168103001	168108000	5000	2	1.10E-11	-0.41	39	0.78	Or51t1;Olr66-ps;Olr67	Receptor
DMR1:168186001	1	168186001	168191000	5000	2	4.90E-08	-0.53	29	0.58	Olr72;Olr73-ps;Olr74	Receptor
DMR1:168274001	1	168274001	168279000	5000	1	1.80E-07	-0.37	39	0.78	Olr80	Receptor
DMR1:168371001	1	168371001	168373000	2000	1	3.70E-07	0.33	20	1	Olr86	Receptor
DMR1:168429001	1	168429001	168437000	8000	2	1.40E-10	-0.55	75	0.94	Olr90-ps	
DMR1:168586001	1	168586001	168587000	1000	1	1.60E-10	0.6	18	1.8	Olr103;Olr104	Receptor
DMR1:168627001	1	168627001	168632000	5000	4	5.60E-10	-0.42	46	0.92	Olr107;Olr108	Receptor
DMR1:168645001	1	168645001	168646000	1000	1	6.70E-07	0.47	4	0.4	Olr108;Olr109;Olr110	Receptor
DMR1:168731001	1	168731001	168738000	7000	3	5.10E-10	-0.31	61	0.87	Olr115;LOC499215;Olr116-ps	Receptor
DMR1:168894001	1	168894001	168898000	4000	1	1.80E-08	-0.41	36	0.9	Olr127	Receptor
DMR1:168947001	1	168947001	168953000	6000	2	6.70E-10	-0.36	55	0.92	Hbb-b1;LOC100134871	
DMR1:168973001	1	168973001	168974000	1000	1	1.40E-07	0.37	6	0.6	LOC689064;Hbb	
DMR1:169013001	1	169013001	169014000	1000	1	3.30E-08	0.58	7	0.7	Hbe1	
DMR1:169058001	1	169058001	169063000	5000	2	1.80E-10	-0.35	39	0.78	Olr131	Receptor
DMR1:169064001	1	169064001	169068000	4000	1	1.70E-08	-0.34	35	0.88	Olr132	Receptor
DMR1:169131001	1	169131001	169133000	2000	1	3.80E-07	-0.33	11	0.55	Olr136	Receptor
DMR1:169190001	1	169190001	169196000	6000	1	9.70E-07	-0.28	85	1.42	LOC102555557;Olr139;LOC689243	Receptor
DMR1:169215001	1	169215001	169221000	6000	1	6.00E-07	-0.27	66	1.1	Olr140;Olr141;Olr142	Receptor

DMR1:169264001	1	169264001	169268000	4000	1	9.90E-07	-0.2	34	0.85	Olr145	Receptor
DMR1:169355001	1	169355001	169357000	2000	1	9.60E-07	0.37	12	0.6	RGD1310717	Development
DMR1:169377001	1	169377001	169384000	7000	1	9.50E-07	-0.25	91	1.3	Olr149;Olr150;LOC108348458	Receptor
DMR1:169579001	1	169579001	169583000	4000	1	5.90E-07	-0.31	37	0.92	Olr155;Olr154	Receptor
DMR1:169831001	1	169831001	169836000	5000	1	8.60E-09	-0.26	50	1	Olr179	Receptor
DMR1:169929001	1	169929001	169931000	2000	1	1.40E-12	-0.47	21	1.05	Olr186;Olr187-ps	Receptor
DMR1:170077001	1	170077001	170081000	4000	1	2.10E-07	-0.33	36	0.9	Olr196	Receptor
DMR1:170129001	1	170129001	170136000	7000	1	5.90E-08	-0.28	51	0.73	Olr199;Olr200	Receptor
DMR1:170255001	1	170255001	170256000	1000	1	5.90E-08	-0.36	11	1.1	Cnga4;LOC108349007;Cckbr	Ion Channel;Signaling
DMR1:170263001	1	170263001	170265000	2000	1	6.60E-07	-0.54	16	0.8	LOC108349007;Cckbr	Signaling
DMR1:170271001	1	170271001	170272000	1000	1	7.20E-14	0.64	30	3	LOC108349007;Cckbr	Signaling
DMR1:170564001	1	170564001	170565000	1000	1	2.80E-14	0.78	21	2.1	Dnhd1;Rrp8	Cytoskeleton
DMR1:170642001	1	170642001	170644000	2000	1	1.10E-08	-0.39	16	0.8	Mrpl17	Translation
DMR1:170718001	1	170718001	170721000	3000	1	4.20E-07	-0.57	21	0.7	Olr207-ps	
DMR1:170971001	1	170971001	170973000	2000	1	6.70E-07	-0.39	16	0.8	Olr220	
DMR1:171188001	1	171188001	171189000	1000	1	1.20E-09	0.68	13	1.3	Olr230;Olr231;Olr232	Receptor
DMR1:171621001	1	171621001	171624000	3000	1	5.30E-12	-0.46	25	0.83	Syt9	Transport
DMR1:171727001	1	171727001	171729000	2000	1	1.30E-09	-0.4	12	0.6	Syt9	Transport
DMR1:171789001	1	171789001	171791000	2000	1	3.40E-07	0.42	17	0.85	Olfml1	Development
DMR1:171925001	1	171925001	171927000	2000	1	9.90E-07	0.41	24	1.2	Ppfibp2	
DMR1:172038001	1	172038001	172040000	2000	1	1.30E-07	-0.44	5	0.25	Ovch2	Protease
DMR1:172076001	1	172076001	172086000	10000	2	8.40E-08	-0.37	86	0.86	Olr238-ps;Olr239;Olr240	Signaling
DMR1:172270001	1	172270001	172274000	4000	1	3.10E-07	-0.46	36	0.9	Olr246;Olr247	Signaling
DMR1:172280001	1	172280001	172281000	1000	1	7.30E-07	-0.63	5	0.5	Olr247;Olr248-ps	Signaling
DMR1:172560001	1	172560001	172562000	2000	1	7.50E-07	0.33	28	1.4	Olr257;Olr258-ps;Olr259	Signaling
DMR1:172675001	1	172675001	172682000	7000	1	1.20E-09	-0.29	58	0.83	Olr264;Olr265-ps	Signaling
DMR1:172745001	1	172745001	172747000	2000	1	3.50E-09	-0.44	9	0.45	Olr268	Signaling
DMR1:173891001	1	173891001	173897000	6000	2	2.40E-07	-0.42	37	0.62	Stk33	Signaling
DMR1:173935001	1	173935001	173937000	2000	1	9.50E-09	0.51	13	0.65	Stk33	Signaling
DMR1:173990001	1	173990001	173993000	3000	2	2.90E-07	0.42	25	0.83	Stk33	Signaling
DMR1:174108001	1	174108001	174109000	1000	1	3.80E-08	-0.42	6	0.6	Trim66	Epigenetic
DMR1:174222001	1	174222001	174225000	3000	1	3.00E-07	0.33	16	0.53	St5	
DMR1:174266001	1	174266001	174269000	3000	1	2.60E-08	0.42	33	1.1	St5;LOC108349575	
DMR1:174295001	1	174295001	174296000	1000	1	6.00E-07	-0.26	14	1.4	St5	
DMR1:174452001	1	174452001	174453000	1000	1	2.70E-08	0.35	18	1.8	Scube2	Extracellular Matrix
DMR1:174470001	1	174470001	174473000	3000	1	7.00E-07	-0.3	44	1.47	Scube2	Extracellular Matrix
DMR1:174601001	1	174601001	174602000	1000	1	5.40E-10	0.41	35	3.5	Tmem41b	
DMR1:174872001	1	174872001	174874000	2000	2	6.30E-11	-0.7	19	0.95	Swap70	Cytoskeleton
DMR1:174877001	1	174877001	174880000	3000	1	7.30E-08	-0.39	45	1.5	Swap70	Cytoskeleton
DMR1:175108001	1	175108001	175113000	5000	1	3.30E-08	-0.28	43	0.86	Sbf2	Signaling
DMR1:175129001	1	175129001	175134000	5000	2	1.60E-10	-0.42	57	1.14	Sbf2	Signaling
DMR1:175269001	1	175269001	175275000	6000	1	5.40E-08	-0.35	41	0.68	Sbf2	Signaling
DMR1:175453001	1	175453001	175455000	2000	1	1.70E-07	0.35	28	1.4	Adm	Hormone
DMR1:175616001	1	175616001	175621000	5000	1	3.70E-07	0.54	95	1.9	Ampd3	Metabolism
DMR1:175723001	1	175723001	175725000	2000	2	2.80E-09	-0.47	18	0.9	Mrvi1;LOC103691205	Cytoskeleton
DMR1:175891001	1	175891001	175894000	3000	1	1.70E-14	-0.7	25	0.83	Eif4g2	Translation
DMR1:176305001	1	176305001	176310000	5000	2	6.10E-18	0.41	52	1.04	Galnt18	Golgi
DMR1:176430001	1	176430001	176436000	6000	2	2.80E-08	-0.38	61	1.02	Galnt18	Golgi
DMR1:176451001	1	176451001	176453000	2000	1	5.20E-07	0.38	16	0.8	Galnt18	Golgi
DMR1:176456001	1	176456001	176457000	1000	1	7.50E-07	0.48	8	0.8	Galnt18	Golgi
DMR1:176498001	1	176498001	176500000	2000	1	1.30E-07	-0.39	27	1.35	Galnt18	Golgi
DMR1:176537001	1	176537001	176539000	2000	1	6.90E-10	0.47	20	1	Galnt18	Golgi
DMR1:177131001	1	177131001	177132000	1000	1	3.00E-08	0.35	5	0.5	Mical2	
DMR1:177194001	1	177194001	177195000	1000	1	1.10E-09	0.44	15	1.5	Micalcl	
DMR1:177296001	1	177296001	177299000	3000	1	5.00E-10	0.57	32	1.07	Parva	Cytoskeleton
DMR1:177317001	1	177317001	177318000	1000	1	7.40E-07	-0.52	17	1.7	Parva	Cytoskeleton
DMR1:177413001	1	177413001	177414000	1000	1	6.50E-07	0.41	12	1.2	Parva	Cytoskeleton
DMR1:177489001	1	177489001	177490000	1000	1	2.60E-08	0.56	8	0.8	Tead1	Transcription
DMR1:177553001	1	177553001	177554000	1000	1	5.30E-08	-0.42	16	1.6	Tead1	Transcription
DMR1:177698001	1	177698001	177700000	2000	1	3.30E-07	-0.46	27	1.35	Tead1	Transcription
DMR1:178100001	1	178100001	178101000	1000	1	3.90E-10	0.37	7	0.7	Arntl	Transcription
DMR1:178122001	1	178122001	178123000	1000	1	5.80E-07	0.43	7	0.7	Arntl	Transcription
DMR1:178674001	1	178674001	178676000	2000	1	3.50E-11	0.41	7	0.35	Spon1	Cytoskeleton

DMR1:178680001	1	178680001	178688000	8000	2	1.40E-08	-0.38	94	1.18	Spon1	Cytoskeleton
DMR1:178777001	1	178777001	178780000	3000	2	6.00E-09	-0.4	37	1.23	Spon1	Cytoskeleton
DMR1:178887001	1	178887001	178892000	5000	2	9.60E-12	-0.48	39	0.78	Spon1	Cytoskeleton
DMR1:178907001	1	178907001	178911000	4000	1	2.60E-07	0.35	52	1.3	Spon1	Cytoskeleton
DMR1:178915001	1	178915001	178917000	2000	1	2.60E-07	0.32	23	1.15	Spon1	Cytoskeleton
DMR1:182062001	1	182062001	182064000	2000	2	3.50E-12	0.59	34	1.7	Ythdc2	Transcription
DMR1:182067001	1	182067001	182071000	4000	2	3.70E-13	0.68	69	1.73	Ythdc2	Transcription
DMR1:182102001	1	182102001	182103000	1000	1	4.90E-07	-0.44	4	0.4	Ythdc2	Transcription
DMR1:183688001	1	183688001	183690000	2000	1	3.70E-08	0.49	16	0.8	RGD1565301;Copb1	Transport
DMR1:183935001	1	183935001	183938000	3000	1	5.20E-07	-0.27	29	0.97	Pde3b	Signaling
DMR1:184025001	1	184025001	184030000	5000	1	3.50E-08	-0.32	41	0.82	Pde3b	Signaling
DMR1:184279001	1	184279001	184281000	2000	1	1.10E-07	-0.47	18	0.9	Calcb	Hormone
DMR1:185118001	1	185118001	185122000	4000	1	2.80E-07	-0.41	47	1.18	Nucb2	Signaling
DMR1:185691001	1	185691001	185692000	1000	1	7.80E-10	0.34	9	0.9	Sox6	
DMR1:185755001	1	185755001	185759000	4000	1	5.30E-09	-0.27	33	0.82	Sox6	
DMR1:185794001	1	185794001	185799000	5000	2	4.60E-08	-0.37	36	0.72	Sox6	
DMR1:186065001	1	186065001	186066000	1000	1	3.30E-08	0.59	12	1.2	Sox6;LOC103691214	
DMR1:187034001	1	187034001	187035000	1000	1	6.90E-08	0.6	10	1	Xylt1	Transport
DMR1:187050001	1	187050001	187051000	1000	1	1.80E-07	0.57	16	1.6	Xylt1	Transport
DMR1:187243001	1	187243001	187245000	2000	1	9.10E-07	0.38	32	1.6	Xylt1	Transport
DMR1:187751001	1	187751001	187752000	1000	1	1.20E-07	0.44	7	0.7	Rps15a	Translation
DMR1:187836001	1	187836001	187837000	1000	1	1.20E-08	0.47	4	0.4	Smg1	
DMR1:188185001	1	188185001	188189000	4000	1	1.10E-08	-0.34	34	0.85	Coq7;Tmc7	
DMR1:188199001	1	188199001	188203000	4000	1	1.20E-07	0.34	53	1.32	Coq7;Tmc7;LOC102550534	
DMR1:188233001	1	188233001	188236000	3000	1	9.00E-07	-0.45	43	1.43	Tmc7	
DMR1:188281001	1	188281001	188283000	2000	1	5.90E-09	-0.44	21	1.05	Tmc5	
DMR1:188332001	1	188332001	188335000	3000	1	4.10E-08	-0.42	33	1.1	Tmc5	
DMR1:188434001	1	188434001	188436000	2000	1	3.90E-07	-0.45	14	0.7	Ccp110	
DMR1:188558001	1	188558001	188563000	5000	2	2.40E-07	-0.28	32	0.64	LOC361635;Knop1;Iqck	
DMR1:188615001	1	188615001	188616000	1000	1	2.10E-11	0.49	2	0.2	Iqck	
DMR1:188694001	1	188694001	188696000	2000	1	5.90E-08	0.34	21	1.05	Iqck;Gprc5b;LOC102550985	Signaling
DMR1:189158001	1	189158001	189159000	1000	1	2.50E-18	0.66	9	0.9	Gp2	Receptor
DMR1:189266001	1	189266001	189267000	1000	1	6.70E-15	1	15	1.5	Acsm5	Metabolism
DMR1:189490001	1	189490001	189494000	4000	1	7.00E-10	-0.51	115	2.88	Thumpd1;LOC102551569	
DMR1:189821001	1	189821001	189822000	1000	1	1.20E-09	-0.67	9	0.9	Thumpd1;Dnah3	
DMR1:190028001	1	190028001	190029000	1000	1	9.40E-07	0.37	12	1.2	Thumpd1;Abca14	Transport
DMR1:190107001	1	190107001	190108000	1000	1	6.40E-07	0.44	13	1.3	Thumpd1;Abca15	Transport
DMR1:190125001	1	190125001	190132000	7000	1	1.10E-08	-0.31	76	1.09	Thumpd1;Abca15;Hspd1-ps5	Transport
DMR1:190146001	1	190146001	190148000	2000	1	7.80E-07	-0.35	24	1.2	Thumpd1;Abca15;Hspd1-ps5	Transport
DMR1:190157001	1	190157001	190159000	2000	1	3.80E-07	-0.86	11	0.55	Thumpd1;Abca15	Transport
DMR1:190684001	1	190684001	190685000	1000	1	3.50E-12	-0.64	7	0.7	LOC102555900;Vwa3a	
DMR1:190813001	1	190813001	190815000	2000	1	4.70E-08	0.31	29	1.45	Eef2k	
DMR1:191021001	1	191021001	191024000	3000	1	8.20E-07	-0.48	22	0.73	Otoa	Cytoskeleton
DMR1:191025001	1	191025001	191026000	1000	1	9.00E-08	0.68	27	2.7	Otoa	Cytoskeleton
DMR1:191032001	1	191032001	191035000	3000	1	9.00E-07	0.34	46	1.53	Otoa	Cytoskeleton
DMR1:191072001	1	191072001	191074000	2000	1	4.60E-08	0.51	20	1	Otoa	Cytoskeleton
DMR1:191459001	1	191459001	191466000	7000	1	1.00E-07	-0.24	80	1.14	Hs3st2	Transport
DMR1:191697001	1	191697001	191699000	2000	1	9.00E-11	-0.6	21	1.05	Scnn1g	Transport
DMR1:191840001	1	191840001	191841000	1000	1	2.70E-07	0.38	9	0.9	Scnn1b	Transport
DMR1:191999001	1	191999001	1.92E+08	1000	1	1.30E-09	-0.5	9	0.9	Ears2	Translation
DMR1:192055001	1	192055001	192056000	1000	1	8.30E-07	-0.45	10	1	Ndufab1;Palb2	Transport
DMR1:192291001	1	192291001	192293000	2000	1	2.60E-09	0.45	16	0.8	Prkcb	Signaling
DMR1:192377001	1	192377001	192378000	1000	1	4.40E-08	0.38	4	0.4	Prkcb	Signaling
DMR1:192389001	1	192389001	192392000	3000	1	2.10E-13	0.31	48	1.6	Prkcb	Signaling
DMR1:192532001	1	192532001	192534000	2000	1	1.10E-08	-0.54	46	2.3	Prkcb	Signaling
DMR1:192962001	1	192962001	192963000	1000	1	2.40E-07	-0.35	12	1.2	Rbbp6	Proteolysis
DMR1:192964001	1	192964001	192966000	2000	1	4.70E-17	-0.54	29	1.45	Rbbp6	Proteolysis
DMR1:193112001	1	193112001	193115000	3000	1	7.30E-08	-0.44	44	1.47	Tnrc6a	Metabolism
DMR1:193230001	1	193230001	193233000	3000	1	3.70E-07	-0.47	9	0.3	Slc5a11;Arhgap17	Transport
DMR1:194769001	1	194769001	194772000	3000	1	3.70E-07	0.3	16	0.53	Nupr1	
DMR1:197301001	1	197301001	197303000	2000	1	9.90E-07	-0.41	27	1.35	Gsg1l	Cytoskeleton
DMR1:197405001	1	197405001	197408000	3000	1	9.10E-07	0.41	47	1.57	Gsg1l	Cytoskeleton
DMR1:197426001	1	197426001	197431000	5000	1	9.10E-07	-0.33	108	2.16	Gsg1l	Cytoskeleton

DMR1:197478001	1	197478001	197480000	2000	1	4.20E-07	0.43	21	1.05	Gsg1l	Cytoskeleton
DMR1:197631001	1	197631001	197632000	1000	1	1.40E-08	0.43	14	1.4	LOC108349640;Sbk1	Signaling
DMR1:197804001	1	197804001	197805000	1000	1	5.70E-07	0.46	22	2.2	Nfatc2ip;LOC100361060	
DMR1:197868001	1	197868001	197869000	1000	1	2.50E-07	-0.47	17	1.7	Atp2a1;Sh2b1	Transport;Cytoskeleton
DMR1:198104001	1	198104001	198105000	1000	1	1.70E-07	-0.54	8	0.8	Sgf29;Sult1a1;Slx1b	Transport;Transcription
DMR1:198135001	1	198135001	198136000	1000	1	2.80E-09	0.42	6	0.6	Coro1a;LOC108349642	Cytoskeleton
DMR1:198264001	1	198264001	198267000	3000	1	3.00E-08	0.45	55	1.83	Fam57b;RGD1563217	
DMR1:198375001	1	198375001	198376000	1000	1	2.40E-07	0.34	23	2.3	Kctd13;Asphd1;Sez6l2	Metabolism
DMR1:198546001	1	198546001	198547000	1000	1	1.60E-07	0.39	8	0.8	Qprt;LOC108349646	
DMR1:198656001	1	198656001	198657000	1000	1	3.40E-08	0.42	9	0.9	Tbc1d10b;Mylpf;Sept1	Signaling;Cytoskeleton
DMR1:199038001	1	199038001	199042000	4000	1	3.90E-08	-0.46	88	2.2	Phkg2;Ccdc189;Rnf40	Signaling
DMR1:199072001	1	199072001	199074000	2000	1	1.10E-10	-0.5	30	1.5	Zfp629	
DMR1:199203001	1	199203001	199210000	7000	1	1.80E-11	-0.62	96	1.37	LOC102554987;Fbxl19;Orai3	Transport
DMR1:199713001	1	199713001	199715000	2000	1	1.10E-07	-0.64	20	1	RGD1310127;LOC102556439;Ahsp	Transcription
DMR1:199727001	1	199727001	199729000	2000	1	3.00E-09	0.38	18	0.9	Ahsp	Transcription
DMR1:199875001	1	199875001	199876000	1000	1	5.90E-07	0.39	19	1.9	Tial1;LOC687874;LOC108349783	Translation
DMR1:201120001	1	201120001	201121000	1000	1	9.80E-08	-0.44	26	2.6	Nsmce4a	
DMR1:201220001	1	201220001	201223000	3000	1	3.00E-07	-0.42	45	1.5	Tacc2	
DMR1:201320001	1	201320001	201322000	2000	1	3.70E-08	-0.42	37	1.85	Tacc2	
DMR1:201338001	1	201338001	201340000	2000	1	6.10E-08	0.38	17	0.85	Tacc2;Btbd16	
DMR1:201367001	1	201367001	201370000	3000	1	9.10E-12	0.44	17	0.57	Btbd16	
DMR1:201496001	1	201496001	201498000	2000	1	2.90E-10	0.54	33	1.65	Htra1	Protease
DMR1:202448001	1	202448001	202451000	3000	1	6.90E-10	-0.53	32	1.07	Plpp4	Signaling
DMR1:202550001	1	202550001	202553000	3000	1	9.00E-08	0.42	26	0.87	Plpp4	Signaling
DMR1:202755001	1	202755001	202761000	6000	1	1.40E-07	-0.25	67	1.12	Wdr11	
DMR1:203191001	1	203191001	203192000	1000	1	6.40E-07	0.39	18	1.8	Dmbt1	Protease
DMR1:204060001	1	204060001	204062000	2000	1	7.00E-07	0.4	29	1.45	Cpxm2	Protease
DMR1:204255001	1	204255001	204258000	3000	1	2.50E-07	0.31	51	1.7	Chst15	Transport
DMR1:204270001	1	204270001	204271000	1000	1	5.60E-09	0.37	16	1.6	Chst15	Transport
DMR1:204600001	1	204600001	204601000	1000	1	9.70E-07	0.37	16	1.6	Nkx1-2	Development
DMR1:204627001	1	204627001	204630000	3000	1	1.70E-07	0.37	38	1.27	Lhpp	Signaling
DMR1:204663001	1	204663001	204665000	2000	1	2.40E-07	0.37	32	1.6	Lhpp	Signaling
DMR1:204763001	1	204763001	204764000	1000	1	6.70E-07	0.52	10	1	Fam53b	
DMR1:204885001	1	204885001	204886000	1000	1	5.20E-08	-0.54	15	1.5	Fam175b	
DMR1:205001001	1	205001001	205002000	1000	1	2.20E-07	-0.55	14	1.4	Zranb1;Ctbp2	Protease;Transcription
DMR1:205627001	1	205627001	205628000	1000	1	4.00E-07	0.35	9	0.9	Tex36;LOC102552830	
DMR1:205718001	1	205718001	205720000	2000	1	2.00E-07	-0.51	10	0.5	Edrf1	
DMR1:205794001	1	205794001	205795000	1000	1	4.90E-07	-0.44	18	1.8	Bccip;Dhx32	Transcription
DMR1:205799001	1	205799001	205800000	1000	1	1.10E-08	0.41	10	1	Bccip;Dhx32	Transcription
DMR1:205874001	1	205874001	205876000	2000	1	8.70E-08	0.39	17	0.85	Fank1;LOC108349724	
DMR1:205943001	1	205943001	205948000	5000	1	5.30E-07	-0.35	40	0.8	Fank1;Adam12	Protease
DMR1:206052001	1	206052001	206054000	2000	1	9.00E-07	0.38	28	1.4	Adam12	Protease
DMR1:206107001	1	206107001	206110000	3000	2	1.10E-09	0.47	32	1.07	Adam12;LOC108349655	Protease
DMR1:206194001	1	206194001	206197000	3000	1	3.40E-08	-0.5	13	0.43	Adam12	Protease
DMR1:206216001	1	206216001	206217000	1000	1	9.20E-07	0.43	11	1.1	Adam12	Protease
DMR1:206236001	1	206236001	206240000	4000	2	7.70E-08	-0.28	36	0.9	Adam12	Protease
DMR1:206273001	1	206273001	206275000	2000	1	5.70E-07	-0.36	21	1.05	Adam12	Protease
DMR1:207248001	1	207248001	207249000	1000	1	8.90E-07	0.42	10	1	Dock1	Transcription
DMR1:207252001	1	207252001	207253000	1000	1	5.30E-14	0.53	0	0	Dock1	Transcription
DMR1:207304001	1	207304001	207305000	1000	1	7.40E-09	0.48	6	0.6	Dock1	Transcription
DMR1:207378001	1	207378001	207380000	2000	1	1.50E-08	0.42	21	1.05	Dock1;LOC103691254	Transcription
DMR1:207400001	1	207400001	207403000	3000	1	1.10E-10	0.5	25	0.83	Dock1	Transcription
DMR1:207651001	1	207651001	207654000	3000	1	8.20E-08	0.35	26	0.87	Foxi2	Transcription
DMR1:207749001	1	207749001	207750000	1000	1	1.50E-08	0.37	11	1.1	Clrn3	
DMR1:207940001	1	207940001	207943000	3000	1	3.00E-21	0.97	38	1.27	Ptpre;LOC108349658;LOC102551963	Signaling
DMR1:207971001	1	207971001	207974000	3000	1	9.80E-13	0.47	45	1.5	Ptpre	Signaling
DMR1:209257001	1	209257001	209258000	1000	1	5.00E-07	-0.45	12	1.2	Mgmt	
DMR1:209583001	1	209583001	209586000	3000	1	1.10E-07	-0.5	47	1.57	Ebf3	Transcription
DMR1:209772001	1	209772001	209773000	1000	1	2.00E-08	-0.63	4	0.4	Glrx3	Metabolism
DMR1:209799001	1	209799001	209800000	1000	1	1.10E-08	-0.53	15	1.5	Glrx3;LOC103691257	Metabolism
DMR1:210597001	1	210597001	210598000	1000	1	1.80E-07	0.36	1	0.1	Tcerg1l	Transcription
DMR1:210618001	1	210618001	210622000	4000	2	1.40E-09	0.51	31	0.78	Tcerg1l	Transcription

DMR1:210639001	1	210639001	210640000	1000	1	2.20E-08	0.42	5	0.5	Tcerg1l	Transcription
DMR1:210729001	1	210729001	210731000	2000	1	9.00E-09	-0.48	25	1.25	Tcerg1l	Transcription
DMR1:211262001	1	211262001	211264000	2000	1	1.30E-07	-0.65	26	1.3	Bnip3;LOC102553541	
DMR1:211272001	1	211272001	211274000	2000	1	9.90E-10	0.46	32	1.6	Bnip3;LOC102553541	
DMR1:211328001	1	211328001	211329000	1000	1	1.20E-07	0.37	4	0.4	Jakmip3	
DMR1:211411001	1	211411001	211414000	3000	1	4.80E-07	0.49	21	0.7	Jakmip3;Dpysl4	Metabolism
DMR1:211506001	1	211506001	211508000	2000	1	1.00E-07	-0.45	18	0.9	Stk32c	Signaling
DMR1:211515001	1	211515001	211517000	2000	2	4.10E-08	0.49	12	0.6	Stk32c	Signaling
DMR1:211524001	1	211524001	211525000	1000	1	4.00E-08	0.36	7	0.7	Stk32c	Signaling
DMR1:211533001	1	211533001	211538000	5000	1	2.80E-09	-0.4	44	0.88	Lrrc27	
DMR1:211606001	1	211606001	211608000	2000	2	4.50E-10	0.47	12	0.6	Pwwp2b	Epigenetic
DMR1:211800001	1	211800001	211801000	1000	1	1.20E-07	0.44	14	1.4	Inpp5a	Signaling
DMR1:212208001	1	212208001	212209000	1000	1	2.40E-07	-0.57	5	0.5	Adgra1	Signaling
DMR1:212249001	1	212249001	212251000	2000	1	8.30E-08	0.65	45	2.25	Kndc1	Transcription
DMR1:212298001	1	212298001	212300000	2000	1	1.20E-08	0.41	22	1.1	LOC102553731;Mir202;LOC108349785;LOC103691261	
DMR1:212302001	1	212302001	212305000	3000	1	1.80E-07	0.41	30	1	Mir202;LOC108349785;LOC103691261	
DMR1:212587001	1	212587001	212589000	2000	1	4.80E-07	-0.62	22	1.1	Echs1;Paox	Metabolism;Metabolism
DMR1:212655001	1	212655001	212656000	1000	1	3.80E-09	0.42	12	1.2	Olr287	Receptor
DMR1:212668001	1	212668001	212669000	1000	1	5.60E-08	-0.35	14	1.4	Olr288	Receptor
DMR1:212851001	1	212851001	212857000	6000	1	5.90E-11	-0.34	53	0.88	Olr292;Olr290-ps	
DMR1:212895001	1	212895001	212904000	9000	1	1.20E-09	-0.39	92	1.02	Olr292;Olr293-ps;Olr294-ps;Olr295	Signaling
DMR1:212911001	1	212911001	212912000	1000	1	1.10E-07	0.51	11	1.1	Olr294-ps;Olr295	Signaling
DMR1:213034001	1	213034001	213035000	1000	1	6.40E-08	-0.55	2	0.2	Olr300;Olr301-ps	
DMR1:213198001	1	213198001	213199000	1000	1	4.00E-08	-0.49	5	0.5	Olr306	Signaling
DMR1:213202001	1	213202001	213205000	3000	1	2.30E-08	0.32	27	0.9	Olr306	Signaling
DMR1:213365001	1	213365001	213370000	5000	2	1.10E-07	-0.35	50	1	Olr311	Signaling
DMR1:213384001	1	213384001	213385000	1000	1	4.90E-07	-0.43	2	0.2	Olr311	Signaling
DMR1:213411001	1	213411001	213414000	3000	1	3.30E-07	-0.36	25	0.83	Olr312	Signaling
DMR1:213432001	1	213432001	213436000	4000	1	8.70E-08	-0.28	32	0.8	Olr313;Olr314-ps	Signaling
DMR1:213528001	1	213528001	213529000	1000	1	1.40E-12	0.45	3	0.3	Cyp2e1;Syce1;LOC103691262	Metabolism
DMR1:213585001	1	213585001	213592000	7000	2	6.90E-10	-0.69	73	1.04	RGD1309350;LOC108349668;Scgb1c1;Odf3	Metabolism;Growth Factors;Development
DMR1:213601001	1	213601001	213603000	2000	1	1.50E-07	-0.42	30	1.5	Scgb1c1;Odf3;Bet1l;Ric8a	Growth Factors;Development;Transcription
DMR1:213834001	1	213834001	213835000	1000	1	5.30E-07	0.49	16	1.6	Ifitm6	
DMR1:214170001	1	214170001	214171000	1000	1	5.40E-09	0.44	3	0.3	Rnh1;Hras	Signaling
DMR1:214686001	1	214686001	214687000	1000	1	6.80E-08	0.55	5	0.5	Muc2	Extracellular Matrix
DMR1:214771001	1	214771001	214773000	2000	1	3.40E-07	0.43	28	1.4	Muc5b	Extracellular Matrix
DMR1:214779001	1	214779001	214780000	1000	1	9.80E-07	0.45	12	1.2	Muc5b	Extracellular Matrix
DMR1:215126001	1	215126001	215128000	2000	1	5.10E-11	0.49	12	0.6	LOC108349786;Krtap5-1;LOC103691269	
DMR1:215691001	1	215691001	215692000	1000	1	2.00E-07	-0.41	19	1.9	Tnnt3;Mrpl23	Cytoskeleton;Translation
DMR1:215714001	1	215714001	215716000	2000	2	3.30E-10	0.46	18	0.9	Mrpl23	Translation
DMR1:215719001	1	215719001	215721000	2000	1	7.90E-11	0.65	30	1.5	Mrpl23	Translation
DMR1:215743001	1	215743001	215747000	4000	2	2.60E-07	0.41	80	2	H19;Mir675;LOC102547221;LOC102548505	
DMR1:215823001	1	215823001	215827000	4000	1	1.10E-09	0.46	29	0.72	Igf2;Mir483	Growth Factors
DMR1:216158001	1	216158001	216160000	2000	1	1.10E-07	0.41	22	1.1	Ascl2	Transcription
DMR1:216206001	1	216206001	216207000	1000	1	2.20E-09	0.43	9	0.9	Tspan32	
DMR1:216319001	1	216319001	216322000	3000	1	3.60E-09	-0.44	45	1.5	Kcnq1	Transport
DMR1:216363001	1	216363001	216366000	3000	2	2.40E-07	-0.5	42	1.4	Kcnq1	Transport
DMR1:216471001	1	216471001	216472000	1000	1	7.00E-08	0.49	10	1	Kcnq1	Transport
DMR1:216570001	1	216570001	216572000	2000	1	2.90E-09	-0.39	32	1.6	Kcnq1	Transport
DMR1:216693001	1	216693001	216695000	2000	1	2.70E-08	0.48	24	1.2	Slc22a18;Phlda2	Transport
DMR1:216877001	1	216877001	216879000	2000	1	6.70E-08	0.51	24	1.2	Osbpl5	
DMR1:216946001	1	216946001	216949000	3000	1	1.40E-07	0.48	26	0.87	LOC103691270;RGD1562011;Mrgprg	Signaling
DMR1:217206001	1	217206001	217209000	3000	1	7.10E-07	0.41	23	0.77	Shank2	
DMR1:217273001	1	217273001	217274000	1000	1	9.60E-10	0.4	11	1.1	Shank2	
DMR1:217309001	1	217309001	217311000	2000	1	4.00E-07	0.47	33	1.65	Shank2	

DMR1:217321001	1	217321001	217326000	5000	1	7.50E-07	0.41	50	1	Shank2	
DMR1:217376001	1	217376001	217377000	1000	1	1.50E-08	0.49	5	0.5	Shank2	
DMR1:217396001	1	217396001	217397000	1000	1	3.20E-07	0.45	9	0.9	Shank2	
DMR1:217445001	1	217445001	217449000	4000	1	2.40E-10	0.45	53	1.32	Shank2	
DMR1:217493001	1	217493001	217495000	2000	1	6.00E-08	0.46	10	0.5	Shank2	
DMR1:217743001	1	217743001	217746000	3000	1	7.40E-07	-0.5	25	0.83	LOC100364769;Fadd;Ano1	
DMR1:217775001	1	217775001	217777000	2000	2	6.20E-10	0.59	32	1.6	Ano1	
DMR1:218456001	1	218456001	218457000	1000	1	1.10E-08	-0.42	10	1	Tpcn2;Mrgprf	Transport;Signaling
DMR1:218460001	1	218460001	218462000	2000	2	1.40E-13	0.51	22	1.1	Mrgprf	Signaling
DMR1:218473001	1	218473001	218474000	1000	1	4.20E-07	0.41	6	0.6	Mrgprf;Mrgprd	Signaling
DMR1:218564001	1	218564001	218566000	2000	1	6.00E-07	0.38	23	1.15	Cpt1a	Metabolism
DMR1:218871001	1	218871001	218874000	3000	1	5.70E-07	0.43	41	1.37	Lrp5	Receptor
DMR1:219175001	1	219175001	219180000	5000	2	5.10E-15	0.64	95	1.9	Aldh3b1;Unc93b1	Metabolism
DMR1:219208001	1	219208001	219210000	2000	1	1.00E-06	0.31	20	1	LOC688778;Aldh3b2	Metabolism
DMR1:219212001	1	219212001	219216000	4000	2	2.20E-10	0.33	66	1.65	LOC688778;Aldh3b2	Metabolism
DMR1:219226001	1	219226001	219230000	4000	1	1.70E-08	-0.43	79	1.98	Aldh3b2;Acy3;Tbx10	Transcription
DMR1:219242001	1	219242001	219245000	3000	1	1.20E-07	0.39	64	2.13	Acy3;Tbx10;LOC103691273;Nudt8;Doc2g;Ndufv1	Transcription;Signaling;Metabolism
DMR1:219324001	1	219324001	219325000	1000	1	4.00E-09	-0.46	12	1.2	Cabp2	
DMR1:219608001	1	219608001	219610000	2000	1	9.60E-08	-0.57	16	0.8	Kdm2a	
DMR1:220109001	1	220109001	220110000	1000	1	4.00E-11	-0.49	11	1.1	Ccdc87;Ctsf	Protease
DMR1:220519001	1	220519001	220520000	1000	1	4.50E-07	0.34	9	0.9	Pacs1	
DMR1:220531001	1	220531001	220532000	1000	1	5.70E-07	0.46	9	0.9	Pacs1	
DMR1:220782001	1	220782001	220783000	1000	1	6.00E-10	0.46	3	0.3	LOC102555676;Tsga10ip	
DMR1:220787001	1	220787001	220788000	1000	1	9.20E-08	-0.37	15	1.5	Tsga10ip	
DMR1:220891001	1	220891001	220893000	2000	1	5.10E-08	-0.38	52	2.6	Snx32;LOC102551929;LOC102556290	Cytoskeleton
DMR1:220958001	1	220958001	220959000	1000	1	1.90E-07	0.39	5	0.5	Ap5b1;Rnaseh2c;Kat5	Epigenetic
DMR1:221076001	1	221076001	221077000	1000	1	6.70E-09	0.4	8	0.8	Ehbp1l1;Fam89b	Cytoskeleton
DMR1:221338001	1	221338001	221339000	1000	1	6.10E-07	0.24	19	1.9	Slc22a20;Capn1	Transport;Protease
DMR1:221508001	1	221508001	221509000	1000	1	1.30E-07	-0.36	30	3	Arl2	
DMR1:221536001	1	221536001	221537000	1000	1	3.90E-09	0.48	12	1.2	Batf2;Majin	Transcription
DMR1:221560001	1	221560001	221562000	2000	1	7.40E-07	-0.45	21	1.05	Majin	
DMR1:221588001	1	221588001	221590000	2000	1	8.00E-09	0.4	21	1.05	Majin;Gpha2;Ppp2r5b	Hormone;Signaling
DMR1:221684001	1	221684001	221685000	1000	1	6.50E-07	0.42	21	2.1	Cdc42bpg	Signaling
DMR1:221697001	1	221697001	221699000	2000	1	4.50E-07	-0.65	14	0.7	Cdc42bpg;Men1	Signaling
DMR1:221804001	1	221804001	221807000	3000	1	1.50E-08	0.5	17	0.57	Nrxn2	
DMR1:221842001	1	221842001	221843000	1000	1	3.70E-07	0.4	7	0.7	Nrxn2	
DMR1:221921001	1	221921001	221930000	9000	1	5.00E-07	0.41	131	1.46	Slc22a12	Transport
DMR1:222100001	1	222100001	222101000	1000	1	1.40E-07	-0.41	14	1.4	Rps6ka4;Ccdc88b	Golgi;Transport
DMR1:222115001	1	222115001	222116000	1000	1	1.50E-11	0.78	46	4.6	Ccdc88b	Transport
DMR1:222373001	1	222373001	222375000	2000	1	7.70E-08	0.37	24	1.2	Macrod1;Flrt1	
DMR1:222484001	1	222484001	222487000	3000	1	3.00E-08	-0.44	57	1.9	Naa40;LOC102551606	
DMR1:222589001	1	222589001	222590000	1000	1	7.20E-07	-0.47	33	3.3	Mark2	Signaling
DMR1:222814001	1	222814001	222818000	4000	1	3.40E-07	-0.23	35	0.88	LOC686151;RGD1563402	
DMR1:222819001	1	222819001	222821000	2000	1	4.00E-07	-0.28	18	0.9	LOC686151;RGD1563402	
DMR1:222822001	1	222822001	222823000	1000	1	7.50E-08	0.58	16	1.6	RGD1563402	
DMR1:222882001	1	222882001	222886000	4000	1	7.90E-09	-0.46	30	0.75	Pla2g16;Lgals12	Extracellular Matrix
DMR1:223364001	1	223364001	223365000	1000	1	2.90E-07	-0.34	6	0.6	Slc22a24	
DMR1:223393001	1	223393001	223395000	2000	1	1.00E-08	-0.28	16	0.8	Slc22a24	
DMR1:224313001	1	224313001	224319000	6000	1	7.00E-09	-0.33	54	0.9	UST4r	Transport
DMR1:224334001	1	224334001	224340000	6000	2	8.40E-08	-0.43	44	0.73	UST4r	Transport
DMR1:224348001	1	224348001	224350000	2000	1	7.80E-07	-0.41	14	0.7	UST4r	Transport
DMR1:224399001	1	224399001	224404000	5000	2	4.70E-12	-0.35	43	0.86	UST4r	Transport
DMR1:224466001	1	224466001	224473000	7000	2	2.10E-08	-0.34	67	0.96	Ust5r	Transport
DMR1:224493001	1	224493001	224500000	7000	1	1.20E-07	-0.28	77	1.1	Ust5r	Transport
DMR1:224516001	1	224516001	224521000	5000	1	9.20E-07	-0.27	61	1.22	Ust5r	Transport
DMR1:224669001	1	224669001	224672000	3000	1	9.90E-07	-0.33	25	0.83	Slc22a25	Transport
DMR1:224895001	1	224895001	224898000	3000	1	7.00E-08	0.44	26	0.87	Chrm1;Slc3a2	Signaling
DMR1:225142001	1	225142001	225146000	4000	1	2.40E-07	-0.38	70	1.75	Eml3;Mta2;Tut1	Development;Metabolism
DMR1:225280001	1	225280001	225282000	2000	1	2.20E-07	0.39	13	0.65	Ahnak;Scgb1a1	Growth Factors
DMR1:225349001	1	225349001	225355000	6000	1	2.20E-07	-0.3	61	1.02	Ahnak	

DMR1:225364001	1	225364001	225371000	7000	1	2.10E-08	-0.4	141	2.01	Ahnak	
DMR1:225575001	1	225575001	225580000	5000	1	3.90E-07	-0.29	35	0.7	RGD1566289;LOC690454	
DMR1:225597001	1	225597001	225598000	1000	1	2.00E-08	-0.47	5	0.5	Scgb1d2	
DMR1:225603001	1	225603001	225605000	2000	1	1.00E-08	-0.42	12	0.6	Scgb1d2	
DMR1:226016001	1	226016001	226018000	2000	1	4.60E-07	0.47	46	2.3	Fads2;LOC102552566;LOC102552394	
DMR1:226133001	1	226133001	226135000	2000	1	2.10E-08	-0.4	28	1.4	Fads2	
DMR1:226275001	1	226275001	226281000	6000	1	2.00E-08	0.47	93	1.55	Myrf	
DMR1:226324001	1	226324001	226325000	1000	1	3.60E-08	0.52	9	0.9	Dagla	Metabolism
DMR1:226445001	1	226445001	226447000	2000	1	5.60E-07	0.3	27	1.35	Syt7	Transport
DMR1:226659001	1	226659001	226660000	1000	1	9.70E-08	-0.53	8	0.8	Tkfc;Ddb1	Metabolism;Translation
DMR1:226871001	1	226871001	226875000	4000	1	5.00E-08	-0.35	50	1.25	Cd6	Protease
DMR1:226889001	1	226889001	226890000	1000	1	5.20E-09	0.5	13	1.3	Cd6;Slc15a3	Protease;Transport
DMR1:227014001	1	227014001	227016000	2000	1	2.70E-07	0.33	12	0.6	Ms4a10	Transport
DMR1:227512001	1	227512001	227513000	1000	1	6.60E-08	-0.43	14	1.4	Ms4a7	Transport
DMR1:227516001	1	227516001	227517000	1000	1	1.60E-07	-0.52	9	0.9	Ms4a7	Transport
DMR1:227661001	1	227661001	227662000	1000	1	2.60E-07	0.51	7	0.7	Ms4a4c;Ms4a6c	Transport
DMR1:227681001	1	227681001	227684000	3000	1	4.80E-07	0.36	18	0.6	Ms4a6c;LOC690870	
DMR1:228109001	1	228109001	228115000	6000	1	2.90E-07	-0.33	63	1.05	Gif	
DMR1:228457001	1	228457001	228458000	1000	1	3.70E-07	0.48	8	0.8	LOC100363176;Olr320-ps	
DMR1:228496001	1	228496001	228502000	6000	1	3.20E-07	-0.27	55	0.92	Olr322	Receptor
DMR1:228616001	1	228616001	228621000	5000	2	8.80E-10	-0.36	46	0.92	Olr330	Signaling
DMR1:229525001	1	229525001	229527000	2000	1	2.50E-07	-0.41	3	0.15	Glyatl2	
DMR1:229675001	1	229675001	229677000	2000	1	6.40E-07	-0.38	15	0.75	Lpxn	Cytoskeleton
DMR1:229715001	1	229715001	229716000	1000	1	7.10E-11	-0.41	6	0.6	Olr339	Receptor
DMR1:229738001	1	229738001	229747000	9000	1	2.00E-07	0.19	108	1.2	Olr341;Olr340	Receptor
DMR1:229791001	1	229791001	229792000	1000	1	3.80E-08	-0.49	9	0.9	Olr342-ps	
DMR1:229965001	1	229965001	229970000	5000	1	1.40E-09	-0.3	45	0.9	Olr350-ps	
DMR1:229982001	1	229982001	229988000	6000	1	6.10E-09	-0.25	70	1.17	Olr351-ps	
DMR1:230046001	1	230046001	230047000	1000	1	5.20E-09	0.44	15	1.5	Olr355-ps;Olr356-ps;Olr357	Receptor
DMR1:230089001	1	230089001	230092000	3000	1	7.00E-10	-0.5	26	0.87	Olr358;Olr359-ps	Receptor
DMR1:230113001	1	230113001	230114000	1000	1	6.90E-09	0.55	10	1	Olr360;RGD1559854	Receptor
DMR1:230152001	1	230152001	230158000	6000	2	2.70E-10	-0.36	58	0.97	Olr361	Receptor
DMR1:230387001	1	230387001	230391000	4000	1	2.10E-07	-0.37	26	0.65	Olr367;Olr368-ps	Receptor
DMR1:230556001	1	230556001	230561000	5000	1	2.70E-07	-0.38	48	0.96	Olr373	Signaling
DMR1:230723001	1	230723001	230728000	5000	2	1.10E-07	-0.32	46	0.92	Olr382	Receptor
DMR1:231528001	1	231528001	231529000	1000	1	1.70E-07	-0.61	8	0.8	Tle4	Transcription
DMR1:231534001	1	231534001	231536000	2000	1	8.40E-09	-0.51	17	0.85	Tle4	Transcription
DMR1:233177001	1	233177001	233179000	2000	1	9.10E-07	-0.4	28	1.4	Cep78	
DMR1:233402001	1	233402001	233403000	1000	1	4.70E-12	0.44	9	0.9	Gnaq	Signaling
DMR1:233415001	1	233415001	233416000	1000	1	3.50E-07	-0.48	12	1.2	Gnaq	Signaling
DMR1:234317001	1	234317001	234318000	1000	1	7.30E-09	-0.67	10	1	Rorb;LOC102554891	Transcription
DMR1:234413001	1	234413001	234414000	1000	1	1.80E-10	0.59	20	2	Rorb	Transcription
DMR1:234434001	1	234434001	234436000	2000	1	1.50E-07	0.4	28	1.4	Rorb	Transcription
DMR1:234743001	1	234743001	234745000	2000	1	4.40E-08	-0.53	15	0.75	Nmrk1;LOC108349697;Ostf1	Signaling
DMR1:235368001	1	235368001	235369000	1000	1	5.90E-07	0.54	7	0.7	Vps13a	
DMR1:235456001	1	235456001	235459000	3000	1	5.30E-09	-0.59	12	0.4	Vps13a	
DMR1:235493001	1	235493001	235495000	2000	1	8.50E-07	-0.27	13	0.65	Vps13a	
DMR1:235585001	1	235585001	235586000	1000	1	5.10E-07	-0.51	16	1.6	Vps13a	
DMR1:235622001	1	235622001	235623000	1000	1	2.20E-08	0.42	18	1.8	Vps13a	
DMR1:235696001	1	235696001	235699000	3000	1	8.40E-07	-0.25	16	0.53	Vps13a	
DMR1:235768001	1	235768001	235771000	3000	1	1.60E-08	-0.45	22	0.73	Vps13a	
DMR1:235782001	1	235782001	235789000	7000	1	1.10E-07	-0.36	73	1.04	Vps13a	
DMR1:235805001	1	235805001	235807000	2000	1	1.10E-08	-0.41	22	1.1	Vps13a	
DMR1:236165001	1	236165001	236166000	1000	1	7.00E-07	-0.33	10	1	Pcsk5	Protease
DMR1:236744001	1	236744001	236747000	3000	1	8.70E-07	0.43	29	0.97	Prune2	
DMR1:236883001	1	236883001	236884000	1000	1	4.80E-07	0.47	8	0.8	Prune2	
DMR1:238259001	1	238259001	238260000	1000	1	1.40E-08	-0.43	14	1.4	Aldh1a1	Metabolism
DMR1:238440001	1	238440001	238442000	2000	1	2.30E-07	0.45	14	0.7	Tmc1	
DMR1:238506001	1	238506001	238512000	6000	1	5.10E-12	-0.36	54	0.9	Tmc1	
DMR1:239237001	1	239237001	239238000	1000	1	2.80E-09	-0.59	6	0.6	RGD1359158	
DMR1:239427001	1	239427001	239429000	2000	1	2.60E-07	0.36	46	2.3	Tmem2	
DMR1:239442001	1	239442001	239445000	3000	1	2.40E-07	-0.44	44	1.47	Tmem2	

DMR1:239777001	1	239777001	239778000	1000	1	2.40E-08	-0.63	10	1	Trpm3	Transport
DMR1:240103001	1	240103001	240104000	1000	1	4.20E-07	-0.5	6	0.6	Trpm3	Transport
DMR1:240324001	1	240324001	240325000	1000	1	4.10E-07	-0.35	10	1	Trpm3	Transport
DMR1:240397001	1	240397001	240398000	1000	1	1.30E-07	0.34	8	0.8	Trpm3;Mir204	Transport
DMR1:240932001	1	240932001	240933000	1000	1	7.20E-10	-0.52	19	1.9	Klf9	Transcription
DMR1:241026001	1	241026001	241028000	2000	1	6.20E-07	-0.38	13	0.65	Smc5	
DMR1:241523001	1	241523001	241525000	2000	1	7.90E-07	-0.37	19	0.95	Ptar1	Metabolism
DMR1:241601001	1	241601001	241603000	2000	1	1.90E-08	0.37	17	0.85	Apba1	Transport
DMR1:241622001	1	241622001	241623000	1000	1	8.40E-07	0.33	10	1	Apba1	Transport
DMR1:241658001	1	241658001	241660000	2000	1	7.80E-09	-0.49	20	1	Apba1	Transport
DMR1:241744001	1	241744001	241746000	2000	1	4.00E-08	0.39	17	0.85	Apba1	Transport
DMR1:241782001	1	241782001	241783000	1000	1	5.30E-11	-0.47	27	2.7	Apba1	Transport
DMR1:241790001	1	241790001	241792000	2000	1	9.70E-09	-0.43	43	2.15	Apba1	Transport
DMR1:241854001	1	241854001	241858000	4000	2	2.20E-14	-0.52	46	1.15	Fam189a2	
DMR1:241983001	1	241983001	241985000	2000	1	5.80E-07	-0.45	32	1.6	Tjp2	Cell Junction
DMR1:242026001	1	242026001	242029000	3000	1	7.80E-08	-0.44	36	1.2	Tjp2	Cell Junction
DMR1:242085001	1	242085001	242087000	2000	1	2.20E-08	-0.54	13	0.65	Tjp2	Cell Junction
DMR1:242171001	1	242171001	242175000	4000	2	2.50E-11	0.72	57	1.43	Pip5k1b	Signaling
DMR1:242210001	1	242210001	242213000	3000	1	3.80E-12	0.82	115	3.83	Pip5k1b	Signaling
DMR1:242336001	1	242336001	242342000	6000	3	2.30E-10	-0.36	39	0.65	Pip5k1b	Signaling
DMR1:242344001	1	242344001	242346000	2000	1	9.20E-07	-0.44	23	1.15	Pip5k1b	Signaling
DMR1:242349001	1	242349001	242352000	3000	1	1.70E-08	-0.49	64	2.13	Pip5k1b	Signaling
DMR1:242408001	1	242408001	242411000	3000	1	4.60E-08	-0.45	59	1.97	Pip5k1b	Signaling
DMR1:242412001	1	242412001	242418000	6000	1	4.00E-07	-0.42	119	1.98	Pip5k1b	Signaling
DMR1:242436001	1	242436001	242439000	3000	1	1.80E-09	-0.47	52	1.73	Pip5k1b	Signaling
DMR1:242563001	1	242563001	242565000	2000	1	5.30E-07	-0.54	29	1.45	Tmem252	
DMR1:242658001	1	242658001	242660000	2000	1	3.70E-07	0.4	24	1.2	Pgm5	Metabolism
DMR1:242675001	1	242675001	242681000	6000	3	3.80E-15	0.48	47	0.78	Pgm5	Metabolism
DMR1:242682001	1	242682001	242683000	1000	1	5.30E-07	-0.48	17	1.7	Pgm5	Metabolism
DMR1:242687001	1	242687001	242689000	2000	1	2.30E-08	0.39	18	0.9	Pgm5	Metabolism
DMR1:242747001	1	242747001	242749000	2000	1	7.00E-10	0.4	16	0.8	Pgm5	Metabolism
DMR1:242939001	1	242939001	242945000	6000	1	5.80E-11	0.43	78	1.3	Dock8	Transcription
DMR1:243139001	1	243139001	243140000	1000	1	3.40E-08	0.59	15	1.5	Dock8	Transcription
DMR1:243283001	1	243283001	243284000	1000	1	4.10E-10	0.65	7	0.7	Kank1	Cytoskeleton
DMR1:243314001	1	243314001	243317000	3000	2	8.90E-09	-0.38	52	1.73	Kank1;LOC102554752	Cytoskeleton
DMR1:243333001	1	243333001	243336000	3000	1	4.40E-09	0.39	40	1.33	Kank1	Cytoskeleton
DMR1:243338001	1	243338001	243339000	1000	1	3.10E-08	0.37	15	1.5	Kank1	Cytoskeleton
DMR1:243348001	1	243348001	243349000	1000	1	5.20E-07	-0.6	13	1.3	Kank1	Cytoskeleton
DMR1:243502001	1	243502001	243504000	2000	1	9.80E-07	0.39	26	1.3	Dmrt1	Transcription
DMR1:243537001	1	243537001	243539000	2000	1	1.60E-07	-0.41	38	1.9	Dmrt1	Transcription
DMR1:243556001	1	243556001	243561000	5000	2	5.40E-09	0.44	68	1.36	Dmrt1	Transcription
DMR1:243656001	1	243656001	243657000	1000	1	6.10E-07	0.55	31	3.1	Dmrt2	Transcription
DMR1:244628001	1	244628001	244629000	1000	1	4.30E-07	-0.32	20	2	Smarca2	Epigenetic
DMR1:244683001	1	244683001	244684000	1000	1	9.30E-09	0.4	3	0.3	Smarca2	Epigenetic
DMR1:244691001	1	244691001	244692000	1000	1	5.80E-08	0.49	16	1.6	Smarca2	Epigenetic
DMR1:244757001	1	244757001	244758000	1000	1	9.80E-14	-0.42	27	2.7	Smarca2	Epigenetic
DMR1:245493001	1	245493001	245501000	8000	1	4.20E-07	-0.33	67	0.84	Pum3	
DMR1:245946001	1	245946001	245947000	1000	1	4.00E-07	0.43	15	1.5	Rfx3	Transcription
DMR1:246568001	1	246568001	246573000	5000	2	1.80E-08	-0.48	75	1.5	Glis3;LOC102556263	Transcription
DMR1:246692001	1	246692001	246694000	2000	1	5.20E-07	-0.3	40	2	NEWGENE_1565505;LOC102550766	
DMR1:246697001	1	246697001	246698000	1000	1	2.70E-08	-0.41	18	1.8	NEWGENE_1565505;LOC102550766	
DMR1:246728001	1	246728001	246730000	2000	1	6.50E-08	-0.51	27	1.35	NEWGENE_1565505;LOC103691328	
DMR1:246739001	1	246739001	246742000	3000	2	4.90E-08	-0.49	55	1.83	NEWGENE_1565505;LOC103691328	
DMR1:246746001	1	246746001	246750000	4000	1	1.40E-13	-0.59	44	1.1	NEWGENE_1565505;LOC103691328	
DMR1:246809001	1	246809001	246811000	2000	1	7.90E-07	0.28	13	0.65	NEWGENE_1565505	
DMR1:246983001	1	246983001	246989000	6000	1	8.40E-07	-0.45	108	1.8	Slc1a1	Transport
DMR1:247002001	1	247002001	247005000	3000	2	7.80E-11	0.43	16	0.53	Slc1a1	Transport
DMR1:247099001	1	247099001	247103000	4000	1	1.80E-08	0.29	35	0.88	LOC680727;RGD1565057;Cdc37l1	Transcription

DMR1:247118001	1	247118001	247120000	2000	1	7.20E-07	-0.43	17	0.85	RGD1565057;Cdc37l1	Transcription
DMR1:247127001	1	247127001	247131000	4000	1	3.40E-07	-0.36	34	0.85	Cdc37l1	Transcription
DMR1:247231001	1	247231001	247232000	1000	1	3.20E-10	-0.51	6	0.6	Rcl1	Metabolism
DMR1:247417001	1	247417001	247420000	3000	1	1.10E-08	-0.46	32	1.07	Jak2	
DMR1:247467001	1	247467001	247469000	2000	1	8.60E-10	0.64	28	1.4	Jak2;Insl6;LOC102546806	Hormone
DMR1:247478001	1	247478001	247480000	2000	1	7.80E-08	0.48	24	1.2	Insl6;LOC102546806;Rln1	Hormone
DMR1:247483001	1	247483001	247485000	2000	1	2.60E-08	0.7	45	2.25	Insl6;LOC102546806;Rln1	Hormone
DMR1:247487001	1	247487001	247489000	2000	2	3.70E-10	-0.54	20	1	LOC102546806;Rln1	Hormone
DMR1:247528001	1	247528001	247530000	2000	1	9.90E-09	0.38	13	0.65	Cd274	Immune
DMR1:247577001	1	247577001	247578000	1000	1	2.10E-14	-0.68	2	0.2	Pdcd1lg2;LOC102547032	Immune
DMR1:247629001	1	247629001	247631000	2000	2	1.00E-09	0.45	16	0.8	Pdcd1lg2	Immune
DMR1:247865001	1	247865001	247868000	3000	1	8.30E-08	0.45	21	0.7	Mlana	
DMR1:247902001	1	247902001	247904000	2000	1	8.00E-07	-0.33	30	1.5	RGD1311595	
DMR1:247973001	1	247973001	247975000	2000	1	3.80E-07	-0.43	20	1	RGD1311595;Ranbp6	Transport
DMR1:248112001	1	248112001	248114000	2000	1	6.70E-12	-0.49	31	1.55	Il33	
DMR1:248124001	1	248124001	248127000	3000	1	2.10E-08	0.7	51	1.7	Il33	
DMR1:248182001	1	248182001	248187000	5000	1	3.10E-07	-0.27	40	0.8	Tpd52l3	
DMR1:248188001	1	248188001	248189000	1000	1	1.20E-09	0.72	30	3	Tpd52l3	
DMR1:248245001	1	248245001	248246000	1000	1	6.00E-08	-0.52	9	0.9	Uhrf2	Proteolysis
DMR1:248890001	1	248890001	248891000	1000	1	1.50E-08	0.56	7	0.7	Dkk1	
DMR1:249024001	1	249024001	249025000	1000	1	1.20E-09	0.42	17	1.7	Prkg1	
DMR1:249030001	1	249030001	249031000	1000	1	7.30E-11	0.43	7	0.7	Prkg1	
DMR1:249304001	1	249304001	249309000	5000	3	4.80E-09	-0.38	43	0.86	Prkg1	
DMR1:249402001	1	249402001	249403000	1000	1	1.80E-07	-0.37	13	1.3	Prkg1	
DMR1:249406001	1	249406001	249408000	2000	1	1.30E-08	0.38	9	0.45	Prkg1	
DMR1:249457001	1	249457001	249459000	2000	1	4.10E-07	0.38	14	0.7	Prkg1	
DMR1:249476001	1	249476001	249481000	5000	1	2.20E-10	-0.49	46	0.92	Prkg1	
DMR1:249527001	1	249527001	249529000	2000	1	1.60E-07	0.37	31	1.55	Prkg1	
DMR1:249596001	1	249596001	249600000	4000	2	3.00E-12	0.63	66	1.65	Prkg1	
DMR1:249628001	1	249628001	249630000	2000	1	6.30E-07	0.29	28	1.4	Prkg1	
DMR1:249637001	1	249637001	249639000	2000	1	1.10E-07	0.47	26	1.3	Prkg1	
DMR1:249640001	1	249640001	249642000	2000	1	3.90E-10	-0.5	14	0.7	Prkg1	
DMR1:249644001	1	249644001	249645000	1000	1	5.50E-08	0.49	4	0.4	Prkg1	
DMR1:249684001	1	249684001	249686000	2000	1	7.70E-08	0.34	19	0.95	Prkg1	
DMR1:249708001	1	249708001	249715000	7000	1	2.00E-07	-0.32	85	1.21	Prkg1	
DMR1:249733001	1	249733001	249735000	2000	1	5.80E-07	-0.24	24	1.2	Prkg1	
DMR1:249812001	1	249812001	249815000	3000	1	2.40E-07	-0.41	49	1.63	Prkg1	
DMR1:250139001	1	250139001	250140000	1000	1	7.80E-07	-0.39	6	0.6	Prkg1	
DMR1:250166001	1	250166001	250168000	2000	1	4.20E-10	-0.43	25	1.25	Prkg1	
DMR1:250228001	1	250228001	250230000	2000	1	1.80E-07	-0.51	25	1.25	Prkg1	
DMR1:250488001	1	250488001	250489000	1000	1	1.70E-07	0.47	8	0.8	A1cf	Metabolism
DMR1:250493001	1	250493001	250494000	1000	1	2.60E-08	-0.49	42	4.2	A1cf	Metabolism
DMR1:250612001	1	250612001	250614000	2000	1	3.20E-07	-0.37	34	1.7	Asah2	
DMR1:250824001	1	250824001	250826000	2000	1	4.20E-10	0.48	32	1.6	Sgms1	
DMR1:250831001	1	250831001	250832000	1000	1	2.60E-07	-0.34	14	1.4	Sgms1	
DMR1:251160001	1	251160001	251163000	3000	1	1.60E-07	0.4	35	1.17	Papss2	
DMR1:251877001	1	251877001	251882000	5000	1	7.50E-12	-0.49	37	0.74	Rnls	
DMR1:251954001	1	251954001	251960000	6000	1	1.40E-07	-0.36	55	0.92	Rnls	
DMR1:252058001	1	252058001	252062000	4000	2	2.20E-07	-0.34	33	0.82	Rnls	
DMR1:252078001	1	252078001	252083000	5000	2	9.70E-08	-0.36	39	0.78	Rnls	
DMR1:252282001	1	252282001	252284000	2000	1	9.00E-08	0.45	7	0.35	Lipf	Metabolism
DMR1:252292001	1	252292001	252293000	1000	1	5.40E-09	-0.46	12	1.2	Lipf	Metabolism
DMR1:252301001	1	252301001	252302000	1000	1	2.00E-10	-0.64	14	1.4	Lipf	Metabolism
DMR1:252314001	1	252314001	252316000	2000	1	3.70E-12	0.57	24	1.2	Lipk	Metabolism
DMR1:252357001	1	252357001	252359000	2000	1	3.20E-11	-0.65	18	0.9	Lipk	Metabolism
DMR1:252449001	1	252449001	252450000	1000	1	6.30E-08	0.37	2	0.2	Ankrd22	
DMR1:252487001	1	252487001	252488000	1000	1	6.10E-07	-0.53	11	1.1	Stambpl1	Protease
DMR1:252556001	1	252556001	252557000	1000	1	3.20E-10	0.59	31	3.1	Acta2;LOC687722	Cytoskeleton
DMR1:252846001	1	252846001	252848000	2000	1	7.20E-07	-0.47	43	2.15	Lipa	Metabolism
DMR1:252856001	1	252856001	252859000	3000	1	5.30E-08	0.42	33	1.1	Lipa	Metabolism
DMR1:252934001	1	252934001	252938000	4000	1	1.80E-07	0.34	62	1.55	Lipa;Ifit1bl;Ifit1	Metabolism
DMR1:253105001	1	253105001	253107000	2000	2	2.90E-13	0.46	17	0.85	Pank1	Signaling
DMR1:253160001	1	253160001	253161000	1000	1	6.10E-08	-0.6	13	1.3	Pank1	Signaling

DMR1:253267001	1	253267001	253269000	2000	1	3.00E-08	-0.7	22	1.1	Kif20b	Cytoskeleton
DMR1:253286001	1	253286001	253293000	7000	1	5.10E-10	-0.42	82	1.17	Ears2l1	
DMR1:254574001	1	254574001	254578000	4000	1	5.10E-07	-0.58	30	0.75	Htr7	Signaling
DMR1:254605001	1	254605001	254609000	4000	1	9.20E-07	0.38	52	1.3	Htr7	Signaling
DMR1:254612001	1	254612001	254615000	3000	2	4.60E-10	-0.4	18	0.6	Htr7;LOC108349452	Signaling
DMR1:254732001	1	254732001	254733000	1000	1	6.90E-07	-0.42	19	1.9	Ankrd1	
DMR1:255060001	1	255060001	255061000	1000	1	2.80E-07	-0.44	19	1.9	Pcgf5	Epigenetic
DMR1:255063001	1	255063001	255064000	1000	1	1.60E-09	-0.52	14	1.4	Pcgf5	Epigenetic
DMR1:255208001	1	255208001	255210000	2000	1	8.50E-08	-0.48	13	0.65	Hectd2	Proteolysis
DMR1:255371001	1	255371001	255373000	2000	1	1.30E-09	-0.62	34	1.7	Ppp1r3c	Signaling
DMR1:255382001	1	255382001	255387000	5000	1	2.40E-07	-0.33	27	0.54	Ppp1r3c	Signaling
DMR1:255573001	1	255573001	255574000	1000	1	1.80E-07	-0.45	13	1.3	Btaf1;Rps27a-ps28	Epigenetic
DMR1:255660001	1	255660001	255666000	6000	2	1.50E-11	-0.58	140	2.33	Btaf1;Cpeb3	Epigenetic;Translation
DMR1:255902001	1	255902001	255906000	4000	1	4.90E-09	0.4	46	1.15	LOC102549247;Ide	Protease
DMR1:255969001	1	255969001	255971000	2000	1	1.60E-08	-0.37	22	1.1	Ide	Protease
DMR1:255979001	1	255979001	255986000	7000	2	2.20E-08	-0.37	74	1.06	Ide	Protease
DMR1:256017001	1	256017001	256020000	3000	1	8.20E-07	0.47	33	1.1	Ide	Protease
DMR1:256044001	1	256044001	256045000	1000	1	1.20E-09	-0.72	15	1.5	Kif11	Cytoskeleton
DMR1:256052001	1	256052001	256057000	5000	1	1.20E-10	-0.31	46	0.92	Kif11	Cytoskeleton
DMR1:256077001	1	256077001	256079000	2000	1	1.80E-09	0.49	24	1.2	Kif11	Cytoskeleton
DMR1:256221001	1	256221001	256222000	1000	1	2.50E-10	0.38	10	1	Exoc6	Transport
DMR1:256252001	1	256252001	256255000	3000	1	4.10E-07	-0.38	54	1.8	Exoc6	Transport
DMR1:256289001	1	256289001	256292000	3000	2	9.30E-11	-0.42	22	0.73	Exoc6	Transport
DMR1:256347001	1	256347001	256349000	2000	1	9.60E-07	0.43	22	1.1	Exoc6	Transport
DMR1:256377001	1	256377001	256378000	1000	1	4.20E-08	0.64	23	2.3	Exoc6;Cyp26c1;LOC102549441;Cyp26a1	Transport;Metabolism
DMR1:256612001	1	256612001	256613000	1000	1	4.20E-13	0.51	9	0.9	Myof	Transport
DMR1:256638001	1	256638001	256640000	2000	1	1.10E-07	0.42	23	1.15	Myof	Transport
DMR1:256738001	1	256738001	256739000	1000	1	3.80E-08	0.39	13	1.3	Myof;LOC108349463;Cep55	Transport
DMR1:256832001	1	256832001	256833000	1000	1	1.30E-07	0.49	15	1.5	Pde6c	Signaling
DMR1:256852001	1	256852001	256854000	2000	1	5.60E-13	0.63	54	2.7	Pde6c	Signaling
DMR1:256953001	1	256953001	256955000	2000	1	3.60E-08	0.38	38	1.9	RGD1561251;Lgi1;LOC103691354	
DMR1:257161001	1	257161001	257164000	3000	1	1.50E-08	-0.55	37	1.23	Plce1	Metabolism
DMR1:257215001	1	257215001	257218000	3000	1	2.60E-08	-0.56	27	0.9	Plce1;LOC102550070	Metabolism
DMR1:257297001	1	257297001	257300000	3000	2	1.10E-07	0.37	40	1.33	Plce1	Metabolism
DMR1:257382001	1	257382001	257384000	2000	1	8.00E-07	-0.34	12	0.6	Plce1	Metabolism
DMR1:257486001	1	257486001	257487000	1000	1	3.10E-07	0.29	25	2.5	Noc3l	
DMR1:258103001	1	258103001	258105000	2000	1	1.90E-11	-0.6	6	0.3	Cyp2c24	Metabolism
DMR1:258148001	1	258148001	258152000	4000	1	8.60E-08	-0.29	26	0.65	Cyp2c24	Metabolism
DMR1:258846001	1	258846001	258853000	7000	1	8.10E-10	-0.54	57	0.81	Cyp2c13	Metabolism
DMR1:258884001	1	258884001	258887000	3000	1	6.90E-07	-0.37	38	1.27	Cyp2c13	Metabolism
DMR1:259317001	1	259317001	259318000	1000	1	8.30E-07	-0.47	4	0.4	Pdlim1	Cytoskeleton
DMR1:259412001	1	259412001	259413000	1000	1	9.40E-08	0.59	24	2.4	Sorbs1	
DMR1:259414001	1	259414001	259415000	1000	1	1.20E-07	0.41	15	1.5	Sorbs1	
DMR1:259631001	1	259631001	259636000	5000	1	6.80E-10	-0.34	47	0.94	Aldh18a1	Metabolism
DMR1:259813001	1	259813001	259814000	1000	1	1.60E-08	-0.39	12	1.2	Entpd1	Signaling
DMR1:259995001	1	259995001	259996000	1000	1	3.50E-07	-0.57	9	0.9	LOC103689954;Cc2d2b	Signaling
DMR1:260113001	1	260113001	260115000	2000	1	1.50E-07	0.42	25	1.25	Ccnj	Signaling
DMR1:260158001	1	260158001	260161000	3000	1	9.50E-07	-0.36	21	0.7	Zfp518a	
DMR1:260201001	1	260201001	260203000	2000	2	2.80E-11	0.65	37	1.85	Blnk	Cytoskeleton
DMR1:260286001	1	260286001	260288000	2000	2	5.40E-11	0.4	12	0.6	Dntt	Transcription
DMR1:260479001	1	260479001	260481000	2000	1	6.40E-07	-0.58	23	1.15	Tm9sf3	Transport
DMR1:260596001	1	260596001	260599000	3000	1	1.30E-10	-0.56	44	1.47	Pik3ap1	
DMR1:260602001	1	260602001	260603000	1000	1	7.30E-09	-0.42	29	2.9	Pik3ap1	
DMR1:260616001	1	260616001	260620000	4000	1	2.00E-12	-0.56	65	1.62	Pik3ap1	
DMR1:260772001	1	260772001	260773000	1000	1	6.70E-09	-0.51	12	1.2	Lcor	
DMR1:260819001	1	260819001	260820000	1000	1	9.80E-08	-0.4	19	1.9	Lcor	
DMR1:260851001	1	260851001	260854000	3000	1	6.50E-07	-0.44	39	1.3	Slit1	
DMR1:260879001	1	260879001	260881000	2000	1	1.30E-07	-0.35	37	1.85	Slit1	
DMR1:260933001	1	260933001	260935000	2000	1	2.10E-07	0.38	27	1.35	Slit1	
DMR1:260947001	1	260947001	260948000	1000	1	1.20E-07	0.41	12	1.2	Slit1;LOC102553433	
DMR1:260962001	1	260962001	260963000	1000	1	9.80E-07	-0.45	27	2.7	Slit1	
DMR1:260998001	1	260998001	261001000	3000	1	9.60E-08	0.39	50	1.67	Slit1	

DMR1:261127001	1	261127001	261128000	1000	1	8.00E-07	-0.42	17	1.7	Rrp12	
DMR1:261141001	1	261141001	261143000	2000	1	1.20E-07	0.29	25	1.25	Rrp12	
DMR1:261395001	1	261395001	261397000	2000	1	2.40E-09	0.56	36	1.8	Marveld1	
DMR1:261579001	1	261579001	261582000	3000	1	6.90E-09	0.41	68	2.27	Crtac1	
DMR1:261589001	1	261589001	261593000	4000	1	9.40E-08	-0.42	63	1.57	Crtac1	
DMR1:261785001	1	261785001	261788000	3000	1	2.50E-11	0.51	19	0.63	R3hcc1l	
DMR1:261962001	1	261962001	261963000	1000	1	4.40E-11	0.43	16	1.6	Pyroxd2	Metabolism
DMR1:262336001	1	262336001	262342000	6000	1	2.30E-07	-0.33	68	1.13	Hpse2	
DMR1:262389001	1	262389001	262392000	3000	1	6.40E-08	-0.33	29	0.97	Hpse2	
DMR1:262512001	1	262512001	262519000	7000	1	5.50E-09	0.33	78	1.11	Hpse2	
DMR1:262587001	1	262587001	262591000	4000	2	7.10E-10	-0.34	37	0.92	Hpse2	
DMR1:262632001	1	262632001	262633000	1000	1	1.90E-12	0.42	7	0.7	Hpse2	
DMR1:262723001	1	262723001	262729000	6000	2	1.50E-09	-0.39	52	0.87	Hpse2	
DMR1:262908001	1	262908001	262909000	1000	1	2.80E-17	0.38	1	0.1	Hpse2;LOC100911951	
DMR1:263059001	1	263059001	263062000	3000	1	1.60E-07	-0.3	23	0.77	Hpse2;LOC100911855	
DMR1:263068001	1	263068001	263073000	5000	1	7.00E-07	-0.37	61	1.22	Hpse2	
DMR1:263083001	1	263083001	263084000	1000	1	2.00E-07	-0.45	10	1	Hpse2	
DMR1:263105001	1	263105001	263111000	6000	2	9.60E-08	0.38	47	0.78	Hpse2	
DMR1:263194001	1	263194001	263198000	4000	1	2.40E-08	-0.39	47	1.18	Cnnm1	
DMR1:263246001	1	263246001	263250000	4000	1	1.90E-07	-0.37	77	1.93	Cnnm1;Got1	Metabolism
DMR1:263278001	1	263278001	263279000	1000	1	6.60E-08	-0.65	22	2.2	Got1;LOC102546432	Metabolism
DMR1:263445001	1	263445001	263447000	2000	1	1.80E-07	0.3	12	0.6	Entpd7	Signaling
DMR1:263562001	1	263562001	263564000	2000	1	5.50E-08	0.47	58	2.9	LOC102546509;Abcc2	Transport
DMR1:263571001	1	263571001	263574000	3000	1	8.50E-07	-0.46	41	1.37	Abcc2	Transport
DMR1:263576001	1	263576001	263577000	1000	1	4.20E-08	-0.51	24	2.4	Abcc2	Transport
DMR1:263596001	1	263596001	263597000	1000	1	9.00E-18	0.92	29	2.9	Abcc2	Transport
DMR1:263619001	1	263619001	263621000	2000	1	4.50E-07	0.32	21	1.05	Abcc2;Dnmbp	Transport
DMR1:263636001	1	263636001	263640000	4000	2	4.80E-09	-0.45	57	1.43	Dnmbp	
DMR1:263665001	1	263665001	263668000	3000	1	1.30E-08	-0.48	68	2.27	Dnmbp	
DMR1:263689001	1	263689001	263691000	2000	1	2.30E-10	-0.45	36	1.8	Dnmbp	
DMR1:263734001	1	263734001	263735000	1000	1	3.60E-07	0.49	9	0.9	Cpn1	Protease
DMR1:263761001	1	263761001	263762000	1000	1	1.10E-09	0.48	26	2.6	Cpn1;LOC102554493	Protease
DMR1:263798001	1	263798001	263800000	2000	1	1.10E-09	0.53	35	1.75	Cyp2c23	Metabolism
DMR1:263816001	1	263816001	263820000	4000	1	4.50E-08	-0.55	28	0.7	Erlin1	
DMR1:263932001	1	263932001	263933000	1000	1	5.30E-11	0.55	20	2	Pkd2l1	Transport
DMR1:264071001	1	264071001	264073000	2000	1	3.20E-08	-0.44	43	2.15	Scd2	
DMR1:264163001	1	264163001	264167000	4000	1	1.60E-09	-0.49	78	1.95	Scd	
DMR1:264244001	1	264244001	264247000	3000	1	2.00E-12	-0.55	64	2.13	Wnt8b	Signaling
DMR1:264255001	1	264255001	264260000	5000	1	5.70E-07	0.3	58	1.16	Wnt8b;Sec31b	Signaling;Transport
DMR1:264513001	1	264513001	264515000	2000	1	6.60E-07	-0.4	37	1.85	Pax2	
DMR1:264583001	1	264583001	264586000	3000	2	1.50E-08	0.83	150	5	Pax2	
DMR1:264773001	1	264773001	264776000	3000	1	2.60E-07	0.49	82	2.73	Lzts2;Pdzd7	Cytoskeleton
DMR1:264783001	1	264783001	264786000	3000	1	1.10E-07	0.44	72	2.4	Lzts2;Pdzd7	Cytoskeleton
DMR1:264793001	1	264793001	264795000	2000	1	4.60E-07	0.63	37	1.85	Pdzd7;Sfxn3	Cytoskeleton;Transport
DMR1:264906001	1	264906001	264907000	1000	1	2.70E-10	0.37	6	0.6	Tlx1	
DMR1:265155001	1	265155001	265157000	2000	1	5.70E-08	0.44	15	0.75	Btrc	Cytoskeleton
DMR1:265500001	1	265500001	265503000	3000	1	1.20E-07	0.67	68	2.27	Fgf8;Npm3;Mgea5	Growth Factors;Transcription
DMR1:265557001	1	265557001	265558000	1000	1	9.30E-13	0.33	3	0.3	Kcnip2	
DMR1:265679001	1	265679001	265682000	3000	1	3.90E-07	-0.34	27	0.9	C1H10orf76	
DMR1:265749001	1	265749001	265751000	2000	1	7.70E-07	-0.39	25	1.25	C1H10orf76	
DMR1:265936001	1	265936001	265938000	2000	1	9.10E-09	0.46	13	0.65	Gbf1	Transcription
DMR1:266057001	1	266057001	266063000	6000	2	1.40E-07	0.5	147	2.45	LOC102551485;Nfkb2;Psd	Transcription;Transcription
DMR1:266281001	1	266281001	266282000	1000	1	7.20E-09	0.38	5	0.5	Arl3;LOC108349707	
DMR1:266304001	1	266304001	266305000	1000	1	6.70E-08	-0.53	10	1	Arl3;LOC108349707	
DMR1:266311001	1	266311001	266314000	3000	1	1.10E-07	0.35	16	0.53	Arl3	
DMR1:266362001	1	266362001	266366000	4000	2	1.00E-07	-0.45	76	1.9	Wbp1l	
DMR1:266371001	1	266371001	266374000	3000	1	8.00E-09	-0.39	70	2.33	Wbp1l	
DMR1:266393001	1	266393001	266394000	1000	1	1.90E-12	-0.43	24	2.4	Wbp1l	
DMR1:266523001	1	266523001	266524000	1000	1	7.60E-07	0.33	7	0.7	As3mt;LOC103691369;Cnnm2	Epigenetic
DMR1:266534001	1	266534001	266536000	2000	1	1.00E-09	0.29	15	0.75	LOC103691369;Cnnm2	
DMR1:266540001	1	266540001	266542000	2000	1	9.30E-07	-0.32	29	1.45	Cnnm2	

DMR1:266627001	1	266627001	266631000	4000	1	6.80E-07	-0.37	61	1.52	Cnnm2	
DMR1:266633001	1	266633001	266634000	1000	1	7.30E-07	-0.47	14	1.4	Cnnm2	
DMR1:266675001	1	266675001	266676000	1000	1	4.60E-07	-0.42	12	1.2	Nt5c2	Signaling
DMR1:266864001	1	266864001	266866000	2000	1	8.80E-11	-0.62	17	0.85	Taf5;Usmg5;Pdcd11	Metabolism
DMR1:267001001	1	267001001	267003000	2000	1	5.10E-07	0.4	5	0.25	Neurl1;LOC108349530	Proteolysis
DMR1:267010001	1	267010001	267016000	6000	1	8.30E-09	0.34	97	1.62	Neurl1;LOC108349530	Proteolysis
DMR1:267348001	1	267348001	267351000	3000	1	6.00E-07	-0.46	38	1.27	LOC108349794;Slk	
DMR1:267355001	1	267355001	267356000	1000	1	3.80E-07	0.46	22	2.2	LOC108349794;Slk	
DMR1:267367001	1	267367001	267372000	5000	1	5.30E-07	-0.38	53	1.06	Slk	
DMR1:267423001	1	267423001	267427000	4000	1	2.90E-07	0.38	76	1.9	Slk;Col17a1	Extracellular Matrix
DMR1:267431001	1	267431001	267432000	1000	1	3.50E-07	0.47	12	1.2	Col17a1;LOC103691371	Extracellular Matrix
DMR1:267564001	1	267564001	267567000	3000	1	1.00E-11	0.6	62	2.07	Cfap43	
DMR1:267574001	1	267574001	267575000	1000	1	1.20E-09	0.41	23	2.3	Cfap43	
DMR1:268032001	1	268032001	268033000	1000	1	5.80E-09	0.3	0	0	Sorcs3	Transport
DMR1:268078001	1	268078001	268080000	2000	1	1.90E-07	0.35	26	1.3	Sorcs3	Transport
DMR1:268129001	1	268129001	268131000	2000	1	1.90E-07	0.25	17	0.85	Sorcs3	Transport
DMR1:268307001	1	268307001	268308000	1000	1	3.50E-08	-0.51	12	1.2	Sorcs3	Transport
DMR1:268323001	1	268323001	268327000	4000	1	1.30E-10	0.53	59	1.48	Sorcs3	Transport
DMR1:268487001	1	268487001	268488000	1000	1	7.30E-08	0.49	7	0.7	Sorcs3	Transport
DMR1:268566001	1	268566001	268568000	2000	1	7.10E-07	0.4	24	1.2	Sorcs3	Transport
DMR1:270059001	1	270059001	270064000	5000	1	6.60E-11	-0.43	38	0.76	Sorcs1	Transport
DMR1:270153001	1	270153001	270155000	2000	1	7.90E-07	-0.56	6	0.3	Sorcs1	Transport
DMR1:270180001	1	270180001	270183000	3000	1	4.00E-08	-0.55	11	0.37	Sorcs1	Transport
DMR1:270336001	1	270336001	270339000	3000	1	3.20E-07	-0.57	36	1.2	Sorcs1	Transport
DMR1:271288001	1	271288001	271290000	2000	1	1.10E-07	-0.6	38	1.9	Ccdc147	
DMR1:271325001	1	271325001	271328000	3000	1	4.30E-07	-0.44	67	2.23	Ccdc147	
DMR1:271405001	1	271405001	271410000	5000	1	4.10E-08	-0.39	61	1.22	Ccdc147	
DMR1:273549001	1	273549001	273555000	6000	1	2.70E-07	-0.26	67	1.12	RGD1561333	
DMR1:273708001	1	273708001	273710000	2000	1	3.10E-07	-0.39	32	1.6	Xpnpep1	Protease
DMR1:273892001	1	273892001	273900000	8000	1	2.10E-10	-0.72	102	1.27	Add3	Cytoskeleton
DMR1:273955001	1	273955001	273958000	3000	2	1.20E-08	0.32	42	1.4	Add3	Cytoskeleton
DMR1:273965001	1	273965001	273966000	1000	1	5.20E-08	-0.36	14	1.4	Add3	Cytoskeleton
DMR1:274064001	1	274064001	274069000	5000	1	3.40E-07	-0.45	100	2	Mxi1;LOC108349609	Transcription
DMR1:274070001	1	274070001	274072000	2000	1	7.60E-09	-0.41	37	1.85	Mxi1;LOC108349609	Transcription
DMR1:274078001	1	274078001	274085000	7000	1	4.20E-07	-0.47	129	1.84	Mxi1;LOC108349609	Transcription
DMR1:274305001	1	274305001	274307000	2000	1	8.50E-08	0.42	33	1.65	Rps12l2;Smc3	
DMR1:274311001	1	274311001	274313000	2000	1	7.90E-08	-0.41	27	1.35	Rps12l2;Smc3	
DMR1:274328001	1	274328001	274331000	3000	1	2.80E-07	-0.42	36	1.2	Smc3	
DMR1:274392001	1	274392001	274395000	3000	1	8.50E-07	0.62	88	2.93	Rbm20	
DMR1:274397001	1	274397001	274400000	3000	1	1.20E-07	0.44	56	1.87	Rbm20	
DMR1:274493001	1	274493001	274496000	3000	2	2.10E-09	-0.52	58	1.93	Rbm20	
DMR1:274500001	1	274500001	274501000	1000	1	2.20E-07	-0.27	9	0.9	Rbm20	
DMR1:274521001	1	274521001	274524000	3000	1	8.50E-07	-0.44	54	1.8	Rbm20	
DMR1:274586001	1	274586001	274587000	1000	1	1.60E-07	0.4	10	1	Rbm20	
DMR1:274590001	1	274590001	274591000	1000	1	1.10E-07	0.42	1	0.1	Rbm20	
DMR1:274697001	1	274697001	274702000	5000	1	2.90E-07	-0.56	47	0.94	Shoc2	Cytoskeleton
DMR1:274717001	1	274717001	274722000	5000	1	1.60E-08	-0.62	56	1.12	Shoc2	Cytoskeleton
DMR1:274737001	1	274737001	274740000	3000	1	3.40E-09	-0.3	24	0.8	Shoc2	Cytoskeleton
DMR1:275831001	1	275831001	275837000	6000	1	3.80E-07	0.65	107	1.78	LOC108349650;Gpam	Metabolism
DMR1:275868001	1	275868001	275869000	1000	1	9.00E-07	-0.57	12	1.2	Gpam	Metabolism
DMR1:275880001	1	275880001	275882000	2000	1	2.50E-09	-0.32	23	1.15	Gpam	Metabolism
DMR1:275985001	1	275985001	275986000	1000	1	2.40E-07	-0.45	23	2.3	Tectb;LOC108348158	Receptor
DMR1:276180001	1	276180001	276184000	4000	1	7.40E-11	-0.46	71	1.77	Gucy2g	Signaling
DMR1:276223001	1	276223001	276225000	2000	1	2.10E-07	0.39	16	0.8	Gucy2g	Signaling
DMR1:276320001	1	276320001	276324000	4000	1	3.80E-08	0.38	59	1.48	Vti1a	Transcription
DMR1:276350001	1	276350001	276351000	1000	1	6.10E-10	-0.46	7	0.7	Vti1a	Transcription
DMR1:276378001	1	276378001	276380000	2000	1	5.10E-07	-0.47	29	1.45	Vti1a	Transcription
DMR1:276462001	1	276462001	276463000	1000	1	3.30E-08	0.46	12	1.2	Vti1a;LOC103691376	Transcription
DMR1:276728001	1	276728001	276729000	1000	1	1.10E-11	0.78	27	2.7	Tcf7l2	Transcription
DMR1:277071001	1	277071001	277072000	1000	1	6.50E-07	0.37	8	0.8	LOC102546765;Habp2	Protease
DMR1:277149001	1	277149001	277150000	1000	1	1.10E-07	0.48	10	1	Nrap	
DMR1:277176001	1	277176001	277178000	2000	1	2.30E-08	0.49	52	2.6	Nrap	
DMR1:277180001	1	277180001	277186000	6000	2	8.90E-09	-0.57	91	1.52	Nrap;Casp7	Protease

DMR1:277218001	1	277218001	277220000	2000	1	2.10E-07	-0.41	21	1.05	Casp7	Protease
DMR1:277240001	1	277240001	277242000	2000	1	3.20E-08	0.46	29	1.45	Casp7	Protease
DMR1:277382001	1	277382001	277383000	1000	1	1.20E-08	-0.46	9	0.9	Nhlrc2	
DMR1:277605001	1	277605001	277607000	2000	1	4.80E-08	-0.82	28	1.4	Ccdc186	
DMR1:277615001	1	277615001	277621000	6000	1	3.20E-09	-0.43	96	1.6	Ccdc186	
DMR1:277631001	1	277631001	277633000	2000	1	2.20E-09	-0.55	16	0.8	Ccdc186;Tdrd1	Cytoskeleton
DMR1:277641001	1	277641001	277642000	1000	1	6.50E-11	0.87	55	5.5	Ccdc186;Tdrd1	Cytoskeleton
DMR1:277647001	1	277647001	277649000	2000	1	8.80E-07	-0.34	25	1.25	Tdrd1	Cytoskeleton
DMR1:277660001	1	277660001	277663000	3000	1	4.20E-09	-0.5	36	1.2	Tdrd1	Cytoskeleton
DMR1:277694001	1	277694001	277695000	1000	1	4.90E-11	-0.58	22	2.2	Tdrd1;Vwa2	Cytoskeleton;Extracellular Matrix
DMR1:277703001	1	277703001	277705000	2000	1	2.50E-08	0.43	15	0.75	Vwa2	Extracellular Matrix
DMR1:277706001	1	277706001	277708000	2000	1	6.20E-11	-0.59	27	1.35	Vwa2	Extracellular Matrix
DMR1:277717001	1	277717001	277718000	1000	1	9.80E-09	0.49	9	0.9	Vwa2	Extracellular Matrix
DMR1:277776001	1	277776001	277779000	3000	1	1.30E-08	0.39	35	1.17	Afap1l2	
DMR1:277841001	1	277841001	277844000	3000	1	8.50E-07	0.35	55	1.83	Ablim1	
DMR1:277856001	1	277856001	277858000	2000	1	3.60E-08	-0.57	49	2.45	Ablim1	
DMR1:277954001	1	277954001	277956000	2000	1	7.20E-13	0.55	40	2	Ablim1	
DMR1:278390001	1	278390001	278402000	12000	2	3.90E-13	0.39	71	0.59	Trub1	Metabolism
DMR1:278665001	1	278665001	278668000	3000	1	8.50E-10	-0.53	25	0.83	Atrnl1	Extracellular Matrix
DMR1:278815001	1	278815001	278821000	6000	2	4.30E-13	-0.56	109	1.82	Atrnl1;LOC108349827	Extracellular Matrix
DMR1:278937001	1	278937001	278938000	1000	1	9.50E-08	0.6	31	3.1	Atrnl1	Extracellular Matrix
DMR1:278944001	1	278944001	278945000	1000	1	9.50E-14	0.49	11	1.1	Atrnl1	Extracellular Matrix
DMR1:278988001	1	278988001	278990000	2000	1	2.60E-07	-0.45	28	1.4	Atrnl1	Extracellular Matrix
DMR1:279046001	1	279046001	279047000	1000	1	9.40E-08	0.33	10	1	Atrnl1	Extracellular Matrix
DMR1:279237001	1	279237001	279243000	6000	2	5.20E-09	-0.29	59	0.98	Gfra1;LOC102556644	Receptor
DMR1:279662001	1	279662001	279665000	3000	1	8.90E-07	-0.49	22	0.73	Ccdc172;LOC108349704	
DMR1:279942001	1	279942001	279945000	3000	2	1.90E-11	0.56	30	1	LOC681006;Hspa12a	
DMR1:279973001	1	279973001	279976000	3000	1	2.10E-07	0.5	53	1.77	Hspa12a	
DMR1:279984001	1	279984001	279986000	2000	1	8.20E-09	0.37	16	0.8	Hspa12a	
DMR1:280012001	1	280012001	280013000	1000	1	2.50E-07	0.39	10	1	Hspa12a;LOC103691386	
DMR1:280046001	1	280046001	280050000	4000	1	1.80E-12	0.63	32	0.8	Hspa12a;LOC100363557	
DMR1:280137001	1	280137001	280138000	1000	1	1.90E-08	0.38	11	1.1	Eno4;Shtn1	Metabolism
DMR1:280171001	1	280171001	280175000	4000	1	4.70E-07	0.31	48	1.2	Shtn1	
DMR1:280213001	1	280213001	280215000	2000	1	5.60E-07	0.38	22	1.1	Shtn1	
DMR1:280235001	1	280235001	280236000	1000	1	2.00E-08	0.45	14	1.4	Shtn1	
DMR1:280387001	1	280387001	280390000	3000	2	4.70E-08	0.39	30	1	Kcnk18;Slc18a2	Transport;Transport
DMR1:280424001	1	280424001	280427000	3000	1	5.70E-08	0.47	29	0.97	Slc18a2	Transport
DMR1:281383001	1	281383001	281385000	2000	1	4.80E-11	0.44	14	0.7	Fam204a	
DMR1:281395001	1	281395001	281397000	2000	1	4.40E-07	-0.32	26	1.3	Fam204a;LOC108349713	
DMR1:282152001	1	282152001	282153000	1000	1	1.20E-08	-0.45	15	1.5	Eif3a	Translation
DMR2:1397001	2	1397001	1402000	5000	2	4.70E-07	0.46	98	1.96	LOC102548635;Erap1	Protease
DMR2:1432001	2	1432001	1438000	6000	1	3.70E-08	-0.37	63	1.05	Erap1	Protease
DMR2:1523001	2	1523001	1527000	4000	1	8.40E-08	-0.52	70	1.75	Cast	Protease; Proteolysis
DMR2:1546001	2	1546001	1548000	2000	1	8.20E-08	0.38	7	0.35	Cast	Protease; Proteolysis
DMR2:2131001	2	2131001	2134000	3000	1	4.40E-07	-0.52	30	1	Rpl17-ps1	
DMR2:2467001	2	2467001	2468000	1000	1	1.40E-09	0.64	3	0.3	Ell2;LOC108349911	Transcription
DMR2:2729001	2	2729001	2732000	3000	1	1.10E-09	0.52	20	0.67	Spata9	
DMR2:2772001	2	2772001	2777000	5000	1	4.40E-08	-0.5	32	0.64	Spata9;Rfesd;LOC108349912	Metabolism
DMR2:2792001	2	2792001	2793000	1000	1	1.40E-07	0.38	8	0.8	Rfesd;LOC108349912	Metabolism
DMR2:2827001	2	2827001	2829000	2000	1	9.60E-08	-0.44	38	1.9	Arsk	
DMR2:2899001	2	2899001	2904000	5000	1	2.30E-11	-0.49	55	1.1	Arsk;Ttc37	
DMR2:2928001	2	2928001	2929000	1000	1	7.70E-08	-0.48	2	0.2	Ttc37	
DMR2:2938001	2	2938001	2940000	2000	1	1.30E-07	-0.56	16	0.8	Ttc37	
DMR2:2979001	2	2979001	2980000	1000	1	1.30E-11	0.4	9	0.9	Ttc37	
DMR2:2981001	2	2981001	2985000	4000	3	3.00E-09	-0.29	45	1.12	Ttc37	
DMR2:3085001	2	3085001	3087000	2000	1	7.30E-08	-0.45	4	0.2	Fam81b	
DMR2:3101001	2	3101001	3102000	1000	1	3.60E-08	-0.48	4	0.4	Fam81b	
DMR2:3417001	2	3417001	3420000	3000	1	5.10E-07	-0.46	9	0.3	Mctp1	
DMR2:3465001	2	3465001	3466000	1000	1	5.70E-07	0.51	13	1.3	Mctp1	
DMR2:3572001	2	3572001	3579000	7000	2	7.00E-08	-0.37	81	1.16	Mctp1	
DMR2:3600001	2	3600001	3606000	6000	1	1.60E-08	-0.37	86	1.43	Mctp1	
DMR2:3631001	2	3631001	3633000	2000	1	9.00E-07	0.49	17	0.85	Mctp1	

DMR2:3713001	2	3713001	3719000	6000	1	2.00E-07	-0.37	52	0.87	Mctp1	
DMR2:3720001	2	3720001	3723000	3000	1	8.30E-07	-0.38	19	0.63	Mctp1	
DMR2:4131001	2	4131001	4133000	2000	1	3.80E-07	-0.36	18	0.9	Slf1	
DMR2:4167001	2	4167001	4170000	3000	1	8.30E-11	-0.52	16	0.53	Slf1	
DMR2:4385001	2	4385001	4392000	7000	1	6.90E-09	-0.36	51	0.73	RGD1560883	
DMR2:4398001	2	4398001	4401000	3000	1	5.20E-10	-0.6	22	0.73	RGD1560883	
DMR2:4422001	2	4422001	4424000	2000	1	3.60E-13	-0.59	43	2.15	RGD1560883	
DMR2:4450001	2	4450001	4457000	7000	2	1.10E-10	-0.3	59	0.84	RGD1560883	
DMR2:4559001	2	4559001	4562000	3000	2	5.90E-10	-0.29	31	1.03	RGD1560883	
DMR2:4576001	2	4576001	4577000	1000	1	2.50E-08	0.48	3	0.3	RGD1560883	
DMR2:4618001	2	4618001	4625000	7000	3	1.20E-08	-0.38	79	1.13	RGD1560883	
DMR2:4673001	2	4673001	4680000	7000	1	2.30E-09	-0.41	54	0.77	RGD1560883	
DMR2:4724001	2	4724001	4728000	4000	1	1.30E-07	-0.51	34	0.85	RGD1560883	
DMR2:4759001	2	4759001	4764000	5000	1	3.60E-07	-0.27	52	1.04	RGD1560883	
DMR2:5018001	2	5018001	5020000	2000	1	8.30E-09	-0.41	20	1	Fam172a	
DMR2:5040001	2	5040001	5047000	7000	1	1.40E-07	-0.39	80	1.14	Fam172a	
DMR2:5320001	2	5320001	5324000	4000	1	8.60E-11	-0.36	36	0.9	Fam172a	
DMR2:5345001	2	5345001	5355000	10000	2	1.20E-08	-0.4	75	0.75	Fam172a	
DMR2:5396001	2	5396001	5402000	6000	1	5.70E-11	-0.34	47	0.78	Fam172a	
DMR2:5413001	2	5413001	5416000	3000	1	6.20E-07	0.36	22	0.73	Fam172a	
DMR2:5452001	2	5452001	5460000	8000	1	5.80E-08	-0.28	91	1.14	Fam172a	
DMR2:5469001	2	5469001	5474000	5000	1	1.70E-10	-0.7	54	1.08	Fam172a	
DMR2:5489001	2	5489001	5490000	1000	1	2.90E-09	0.37	11	1.1	Fam172a	
DMR2:5514001	2	5514001	5516000	2000	1	6.00E-07	-0.35	19	0.95	Fam172a	
DMR2:8568001	2	8568001	8572000	4000	1	3.70E-07	-0.39	30	0.75	LOC100360354;Rps8-ps1	
DMR2:8641001	2	8641001	8646000	5000	1	6.20E-07	-0.28	39	0.78	Tdg-ps1	
DMR2:8974001	2	8974001	8976000	2000	1	2.20E-07	-0.58	27	1.35	Adgrv1	Signaling
DMR2:9010001	2	9010001	9012000	2000	2	9.30E-11	0.41	18	0.9	Adgrv1	Signaling
DMR2:9028001	2	9028001	9033000	5000	2	1.50E-10	-0.4	33	0.66	Adgrv1	Signaling
DMR2:9167001	2	9167001	9169000	2000	1	1.10E-07	-0.56	37	1.85	Adgrv1	Signaling
DMR2:9300001	2	9300001	9302000	2000	1	2.10E-10	0.6	28	1.4	Adgrv1	Signaling
DMR2:9380001	2	9380001	9384000	4000	3	2.10E-09	-0.5	59	1.48	Adgrv1	Signaling
DMR2:9399001	2	9399001	9403000	4000	1	2.60E-09	-0.55	69	1.73	Adgrv1	Signaling
DMR2:9449001	2	9449001	9450000	1000	1	7.70E-08	-0.35	17	1.7	Adgrv1	Signaling
DMR2:9461001	2	9461001	9464000	3000	1	2.30E-07	-0.43	60	2	Adgrv1	Signaling
DMR2:9472001	2	9472001	9474000	2000	1	2.40E-08	-0.44	25	1.25	Adgrv1	Signaling
DMR2:9499001	2	9499001	9501000	2000	1	4.60E-09	-0.49	33	1.65	Adgrv1;LOC499494	Signaling
DMR2:9511001	2	9511001	9513000	2000	1	8.90E-07	-0.42	48	2.4	Adgrv1;LOC685441	Signaling
DMR2:9535001	2	9535001	9539000	4000	1	4.30E-09	0.4	38	0.95	Lysmd3;Polr3g	Transcription
DMR2:9590001	2	9590001	9593000	3000	1	6.00E-07	-0.41	15	0.5	Mblac2	Metabolism
DMR2:11064001	2	11064001	11065000	1000	1	1.30E-24	1.04	10	1	Rps27a-ps29	
DMR2:11805001	2	11805001	11808000	3000	1	1.20E-08	0.32	29	0.97	Mef2c;LOC103691412	Transcription
DMR2:12302001	2	12302001	12304000	2000	1	6.20E-07	-0.62	6	0.3	Tmem161b	
DMR2:12406001	2	12406001	12408000	2000	1	3.90E-09	-0.52	11	0.55	Tmem161b	
DMR2:12417001	2	12417001	12418000	1000	1	2.80E-07	0.4	6	0.6	Tmem161b	
DMR2:12489001	2	12489001	12491000	2000	1	4.40E-08	0.34	14	0.7	Tmem161b;LOC102554243	
DMR2:12524001	2	12524001	12526000	2000	1	3.20E-07	-0.52	11	0.55	Tmem161b;LOC103691414	
DMR2:13660001	2	13660001	13662000	2000	1	2.40E-08	-0.49	13	0.65	Rasa1	Signaling
DMR2:14272001	2	14272001	14275000	3000	1	6.40E-07	-0.48	11	0.37	RGD1559647	
DMR2:14711001	2	14711001	14714000	3000	2	2.70E-11	-0.41	25	0.83	Cox7c	Metabolism
DMR2:17827001	2	17827001	17828000	1000	1	2.10E-07	0.41	3	0.3	Edil3	Metabolism
DMR2:17830001	2	17830001	17832000	2000	1	4.50E-09	0.45	16	0.8	Edil3	Metabolism
DMR2:18093001	2	18093001	18094000	1000	1	3.20E-07	-0.59	9	0.9	Edil3	Metabolism
DMR2:18130001	2	18130001	18131000	1000	1	4.30E-10	0.66	12	1.2	Edil3	Metabolism
DMR2:18412001	2	18412001	18415000	3000	1	2.70E-12	0.37	31	1.03	Hapln1	Extracellular Matrix
DMR2:18505001	2	18505001	18506000	1000	1	4.40E-11	0.4	8	0.8	Vcan	Extracellular Matrix
DMR2:18574001	2	18574001	18576000	2000	1	1.90E-09	-0.5	25	1.25	Vcan	Extracellular Matrix
DMR2:18748001	2	18748001	18753000	5000	1	1.50E-07	-0.36	53	1.06	Xrcc4	Transcription
DMR2:18862001	2	18862001	18868000	6000	1	3.80E-08	-0.25	65	1.08	Xrcc4;LOC108350153	Transcription
DMR2:18891001	2	18891001	18895000	4000	1	2.40E-09	-0.38	31	0.78	Xrcc4;LOC108350153	Transcription
DMR2:19814001	2	19814001	19816000	2000	1	1.20E-07	-0.41	31	1.55	Atp6ap1l;Rps23	Metabolism;Translation
DMR2:19824001	2	19824001	19825000	1000	1	1.10E-07	-0.43	12	1.2	Rps23	Translation
DMR2:19844001	2	19844001	19846000	2000	1	5.40E-07	-0.44	45	2.25	Atg10	Proteolysis

DMR2:19894001	2	19894001	19896000	2000	1	1.70E-10	0.46	20	1	Atg10	Proteolysis
DMR2:19983001	2	19983001	19985000	2000	1	1.80E-08	-0.45	35	1.75	Atg10	Proteolysis
DMR2:20012001	2	20012001	20015000	3000	1	8.20E-07	-0.25	23	0.77	Atg10	Proteolysis
DMR2:20086001	2	20086001	20088000	2000	1	3.40E-08	-0.44	34	1.7	Atg10	Proteolysis
DMR2:20093001	2	20093001	20094000	1000	1	3.50E-08	0.54	17	1.7	Atg10	Proteolysis
DMR2:20409001	2	20409001	20411000	2000	1	6.40E-07	-0.55	26	1.3	Ssbp2	Transcription
DMR2:20489001	2	20489001	20490000	1000	1	5.50E-07	-0.42	8	0.8	Ssbp2	Transcription
DMR2:20508001	2	20508001	20512000	4000	1	5.40E-07	-0.5	40	1	Ssbp2	Transcription
DMR2:20553001	2	20553001	20556000	3000	2	1.40E-08	-0.51	36	1.2	Ssbp2	Transcription
DMR2:20631001	2	20631001	20632000	1000	1	6.60E-07	0.45	11	1.1	Ssbp2	Transcription
DMR2:20715001	2	20715001	20722000	7000	1	4.40E-08	-0.25	72	1.03	Ssbp2	Transcription
DMR2:20845001	2	20845001	20851000	6000	2	4.30E-11	0.45	87	1.45	Acot12	Metabolism
DMR2:20853001	2	20853001	20856000	3000	1	4.90E-09	0.49	41	1.37	Acot12	Metabolism
DMR2:20872001	2	20872001	20877000	5000	1	1.00E-07	-0.37	90	1.8	Acot12	Metabolism
DMR2:21427001	2	21427001	21428000	1000	1	1.10E-07	0.56	33	3.3	Ckmt2	Signaling
DMR2:22052001	2	22052001	22058000	6000	1	2.40E-08	-0.44	60	1	Fam151b;Zfyve16	
DMR2:22101001	2	22101001	22103000	2000	1	2.20E-09	-0.76	21	1.05	Zfyve16	
DMR2:22155001	2	22155001	22157000	2000	1	1.50E-07	0.35	24	1.2	Spz1	Transcription
DMR2:22306001	2	22306001	22307000	1000	1	2.00E-08	0.51	11	1.1	Serinc5;LOC108350165	Signaling
DMR2:22696001	2	22696001	22697000	1000	1	2.60E-23	0.81	2	0.2	Cmya5	Proteolysis
DMR2:22741001	2	22741001	22742000	1000	1	6.10E-09	0.33	8	0.8	Cmya5;Papd4	Proteolysis
DMR2:22766001	2	22766001	22768000	2000	1	5.80E-09	-0.31	21	1.05	Papd4	
DMR2:22769001	2	22769001	22772000	3000	1	6.10E-07	-0.38	31	1.03	Papd4	
DMR2:22901001	2	22901001	22903000	2000	1	4.30E-07	0.39	21	1.05	Homer1	
DMR2:22922001	2	22922001	22924000	2000	1	2.50E-08	-0.36	32	1.6	Homer1	
DMR2:23005001	2	23005001	23007000	2000	1	3.10E-08	-0.34	32	1.6	Homer1	
DMR2:23226001	2	23226001	23228000	2000	1	4.40E-08	0.53	30	1.5	Bhmt	Epigenetic
DMR2:23251001	2	23251001	23254000	3000	2	6.50E-10	0.7	65	2.17	Bhmt;LOC103691423	Epigenetic
DMR2:23273001	2	23273001	23274000	1000	1	4.40E-21	0.73	16	1.6	Bhmt2	Epigenetic
DMR2:23285001	2	23285001	23287000	2000	1	4.00E-09	0.65	37	1.85	Bhmt2;Dmgdh	Epigenetic;Metabolism
DMR2:23418001	2	23418001	23419000	1000	1	2.70E-09	-0.46	19	1.9	Arsb	
DMR2:23426001	2	23426001	23430000	4000	1	4.60E-08	-0.38	65	1.62	Arsb	
DMR2:23459001	2	23459001	23462000	3000	2	8.80E-12	0.44	28	0.93	Arsb	
DMR2:23471001	2	23471001	23473000	2000	1	1.80E-09	-0.41	47	2.35	Arsb	
DMR2:23488001	2	23488001	23490000	2000	1	7.00E-09	-0.46	15	0.75	Arsb	
DMR2:23532001	2	23532001	23534000	2000	1	2.90E-07	-0.45	25	1.25	Arsb	
DMR2:23551001	2	23551001	23553000	2000	1	3.20E-07	0.32	14	0.7	Arsb	
DMR2:23730001	2	23730001	23732000	2000	1	2.10E-09	0.52	26	1.3	Lhfpl2	
DMR2:23734001	2	23734001	23741000	7000	1	4.80E-07	-0.51	136	1.94	Lhfpl2	
DMR2:23758001	2	23758001	23759000	1000	1	2.90E-08	0.48	4	0.4	Lhfpl2	
DMR2:23782001	2	23782001	23785000	3000	1	1.70E-16	-0.54	59	1.97	Lhfpl2	
DMR2:23812001	2	23812001	23814000	2000	1	4.90E-07	0.37	29	1.45	Lhfpl2	
DMR2:23859001	2	23859001	23860000	1000	1	1.10E-07	-0.32	11	1.1	Lhfpl2;Scamp1	Transport
DMR2:23873001	2	23873001	23875000	2000	1	1.90E-07	-0.39	44	2.2	Scamp1	Transport
DMR2:24029001	2	24029001	24031000	2000	1	4.20E-13	-0.56	23	1.15	Ap3b1	Transport
DMR2:24148001	2	24148001	24149000	1000	1	3.90E-08	0.45	13	1.3	Ap3b1	Transport
DMR2:24155001	2	24155001	24157000	2000	1	6.70E-07	0.4	28	1.4	Ap3b1	Transport
DMR2:24205001	2	24205001	24209000	4000	2	2.20E-08	-0.55	87	2.17	Ap3b1	Transport
DMR2:24656001	2	24656001	24659000	3000	1	4.30E-08	-0.44	56	1.87	Wdr41	
DMR2:24753001	2	24753001	24756000	3000	1	2.60E-08	0.37	25	0.83	Pde8b	Signaling
DMR2:24825001	2	24825001	24827000	2000	1	2.40E-09	0.37	10	0.5	Pde8b	Signaling
DMR2:24872001	2	24872001	24878000	6000	1	2.00E-10	0.43	89	1.48	Pde8b	Signaling
DMR2:24894001	2	24894001	24899000	5000	1	4.20E-07	0.58	84	1.68	Pde8b	Signaling
DMR2:25146001	2	25146001	25149000	3000	1	7.20E-07	-0.47	45	1.5	Crhbp	
DMR2:25187001	2	25187001	25189000	2000	1	3.20E-11	0.83	51	2.55	S100z	Signaling
DMR2:25244001	2	25244001	25246000	2000	1	8.60E-09	0.58	30	1.5	F2rl1;LOC102550768	Signaling
DMR2:25746001	2	25746001	25747000	1000	1	1.90E-07	-0.41	22	2.2	Arhgef28	
DMR2:25768001	2	25768001	25771000	3000	1	4.00E-07	-0.43	55	1.83	Arhgef28	
DMR2:25837001	2	25837001	25843000	6000	1	2.00E-10	0.42	107	1.78	Arhgef28	
DMR2:25863001	2	25863001	25864000	1000	1	1.30E-09	0.4	3	0.3	Arhgef28	
DMR2:25865001	2	25865001	25867000	2000	1	7.40E-07	-0.33	30	1.5	Arhgef28	
DMR2:25956001	2	25956001	25957000	1000	1	2.20E-07	-0.39	26	2.6	Arhgef28	
DMR2:25987001	2	25987001	25989000	2000	2	1.10E-16	0.93	44	2.2	Arhgef28	

DMR2:26006001	2	26006001	26008000	2000	1	9.50E-07	0.44	32	1.6	Arhgef28;RGD1566212	
DMR2:26011001	2	26011001	26012000	1000	1	2.70E-08	-0.36	26	2.6	Arhgef28;RGD1566212	
DMR2:26050001	2	26050001	26053000	3000	1	2.50E-08	-0.46	10	0.33	RGD1566212	
DMR2:26121001	2	26121001	26123000	2000	1	7.40E-12	0.91	77	3.85	F2r	Signaling
DMR2:26124001	2	26124001	26126000	2000	1	6.90E-07	0.44	22	1.1	F2r	Signaling
DMR2:26235001	2	26235001	26238000	3000	1	1.10E-09	0.4	45	1.5	Iqgap2;F2rl2	Signaling;Signaling
DMR2:26299001	2	26299001	26300000	1000	1	7.40E-10	-0.46	26	2.6	Iqgap2	Signaling
DMR2:26313001	2	26313001	26318000	5000	1	8.60E-07	-0.34	82	1.64	Iqgap2	Signaling
DMR2:26423001	2	26423001	26424000	1000	1	2.00E-08	-0.48	4	0.4	Iqgap2	Signaling
DMR2:26440001	2	26440001	26441000	1000	1	4.10E-09	0.51	27	2.7	Iqgap2	Signaling
DMR2:26521001	2	26521001	26523000	2000	1	1.10E-07	-0.34	31	1.55	Sv2c	
DMR2:26545001	2	26545001	26550000	5000	1	1.70E-07	-0.39	43	0.86	Sv2c	
DMR2:26649001	2	26649001	26652000	3000	2	1.50E-11	0.38	29	0.97	Sv2c	
DMR2:26670001	2	26670001	26672000	2000	1	1.60E-10	-0.57	26	1.3	Sv2c	
DMR2:26687001	2	26687001	26688000	1000	1	1.50E-13	0.59	22	2.2	Sv2c	
DMR2:26701001	2	26701001	26703000	2000	1	4.50E-08	-0.4	25	1.25	Sv2c	
DMR2:27280001	2	27280001	27281000	1000	1	1.70E-08	0.45	16	1.6	Ankdd1b	
DMR2:27335001	2	27335001	27339000	4000	2	7.10E-10	-0.54	55	1.38	Polk	Transcription
DMR2:27387001	2	27387001	27391000	4000	1	7.10E-11	-0.62	22	0.55	Col4a3bp	
DMR2:27677001	2	27677001	27678000	1000	1	8.00E-07	-0.38	13	1.3	Ankrd31;LOC102554085	
DMR2:27701001	2	27701001	27702000	1000	1	9.50E-08	-0.41	0	0	Ankrd31	
DMR2:27938001	2	27938001	27939000	1000	1	9.10E-07	-0.47	9	0.9	Fam169a;Nsa2	
DMR2:28011001	2	28011001	28012000	1000	1	8.00E-08	0.38	13	1.3	Hexb	Metabolism
DMR2:28054001	2	28054001	28060000	6000	1	2.00E-09	-0.55	126	2.1	Enc1	
DMR2:28346001	2	28346001	28347000	1000	1	4.30E-08	0.58	25	2.5	LOC108349925;Utp15	Translation
DMR2:28758001	2	28758001	28759000	1000	1	8.40E-08	0.68	16	1.6	Tmem174	
DMR2:29080001	2	29080001	29085000	5000	1	5.30E-09	-0.48	70	1.4	Tnpo1	Transport
DMR2:29475001	2	29475001	29476000	1000	1	8.70E-09	-0.58	13	1.3	Zfp366;LOC108349928	Transcription
DMR2:29624001	2	29624001	29627000	3000	1	5.10E-11	-0.59	50	1.67	Mrps27	Translation
DMR2:29686001	2	29686001	29691000	5000	1	1.20E-07	-0.35	143	2.86	Map1b	Cytoskeleton
DMR2:29742001	2	29742001	29744000	2000	2	7.30E-08	0.37	24	1.2	Map1b	Cytoskeleton
DMR2:29755001	2	29755001	29757000	2000	1	3.30E-11	-0.51	35	1.75	Map1b	Cytoskeleton
DMR2:30114001	2	30114001	30116000	2000	1	2.00E-07	0.6	51	2.55	LOC680951;Cartpt	
DMR2:30188001	2	30188001	30189000	1000	1	9.80E-22	0.39	4	0.4	Mccc2	Metabolism
DMR2:30242001	2	30242001	30243000	1000	1	6.60E-11	-0.42	14	1.4	Mccc2;Bdp1	Metabolism;Transcription
DMR2:30270001	2	30270001	30271000	1000	1	2.30E-07	-0.61	17	1.7	Bdp1	Transcription
DMR2:30515001	2	30515001	30519000	4000	2	2.30E-09	-0.51	28	0.7	Gtf2h2;LOC365655;Ocln	Transcription
DMR2:30574001	2	30574001	30576000	2000	1	3.60E-07	-0.43	39	1.95	Ocln	Transcription
DMR2:30666001	2	30666001	30669000	3000	1	1.60E-09	-0.46	33	1.1	Rad17;Ak6;Taf9	Epigenetic
DMR2:30685001	2	30685001	30688000	3000	1	3.40E-08	0.56	75	2.5	Ak6;Ccdc125	Epigenetic
DMR2:30743001	2	30743001	30746000	3000	1	1.50E-08	-0.53	36	1.2	Cdk7;Mrps36	Signaling;Translation
DMR2:31744001	2	31744001	31754000	10000	2	8.90E-15	-0.6	190	1.9	Pik3r1	Signaling
DMR2:31768001	2	31768001	31770000	2000	1	4.70E-08	-0.37	32	1.6	Pik3r1	Signaling
DMR2:31771001	2	31771001	31776000	5000	1	1.60E-07	-0.4	98	1.96	Pik3r1	Signaling
DMR2:31831001	2	31831001	31834000	3000	1	1.40E-10	0.38	43	1.43	Pik3r1;LOC103691440;LOC103691441	Signaling
DMR2:32489001	2	32489001	32493000	4000	1	6.20E-08	-0.47	91	2.28	Mast4;LOC108349933	Signaling
DMR2:32918001	2	32918001	32922000	4000	1	1.60E-07	-0.49	106	2.65	NEWGENE_1310139	
DMR2:32973001	2	32973001	32975000	2000	1	1.30E-07	0.38	20	1	NEWGENE_1310139	
DMR2:33042001	2	33042001	33043000	1000	1	7.20E-08	-0.48	11	1.1	NEWGENE_1310139	
DMR2:33066001	2	33066001	33067000	1000	1	3.00E-07	0.37	17	1.7	NEWGENE_1310139	
DMR2:33068001	2	33068001	33070000	2000	1	1.00E-07	-0.61	25	1.25	NEWGENE_1310139	
DMR2:33143001	2	33143001	33145000	2000	1	5.50E-08	-0.52	21	1.05	NEWGENE_1310139	
DMR2:33161001	2	33161001	33162000	1000	1	1.90E-08	-0.37	15	1.5	NEWGENE_1310139	
DMR2:33163001	2	33163001	33170000	7000	1	1.30E-08	0.35	77	1.1	NEWGENE_1310139	
DMR2:33182001	2	33182001	33184000	2000	1	4.20E-07	0.52	32	1.6	NEWGENE_1310139	
DMR2:33204001	2	33204001	33205000	1000	1	9.80E-09	0.3	7	0.7	NEWGENE_1310139	
DMR2:33257001	2	33257001	33262000	5000	2	4.70E-08	-0.51	71	1.42	NEWGENE_1310139	
DMR2:33301001	2	33301001	33304000	3000	1	7.10E-07	0.53	65	2.17	NEWGENE_1310139	
DMR2:33321001	2	33321001	33323000	2000	1	1.00E-08	-0.42	14	0.7	NEWGENE_1310139	
DMR2:33802001	2	33802001	33809000	7000	1	4.00E-07	-0.4	93	1.33	Srek1	Translation
DMR2:33848001	2	33848001	33854000	6000	1	6.70E-07	0.39	74	1.23	Srek1	Translation

DMR2:34218001	2	34218001	34221000	3000	1	3.00E-08	0.42	54	1.8	Sgtb;Trappc13	
DMR2:34409001	2	34409001	34413000	4000	1	9.20E-08	-0.28	35	0.88	Adamts6;LOC108349936	Protease
DMR2:34444001	2	34444001	34449000	5000	1	4.50E-07	-0.24	44	0.88	Adamts6	Protease
DMR2:34470001	2	34470001	34473000	3000	1	9.40E-08	-0.44	27	0.9	Adamts6	Protease
DMR2:34481001	2	34481001	34484000	3000	1	3.60E-09	-0.45	37	1.23	Adamts6	Protease
DMR2:34594001	2	34594001	34595000	1000	1	1.50E-08	0.64	18	1.8	Adamts6	Protease
DMR2:34752001	2	34752001	34757000	5000	1	7.90E-11	-0.48	41	0.82	Cwc27	Transcription
DMR2:34805001	2	34805001	34806000	1000	1	1.40E-07	-0.35	8	0.8	Cwc27	Transcription
DMR2:35344001	2	35344001	35345000	1000	1	9.40E-07	-0.34	8	0.8	Rgs7bp;LOC100910253	
DMR2:35602001	2	35602001	35603000	1000	1	2.40E-12	0.79	32	3.2	Rgs7bp	
DMR2:35628001	2	35628001	35632000	4000	2	3.60E-11	0.78	52	1.3	Rgs7bp	
DMR2:35771001	2	35771001	35774000	3000	1	7.40E-07	-0.42	10	0.33	Rnf180	Proteolysis
DMR2:35836001	2	35836001	35838000	2000	1	8.20E-07	-0.61	16	0.8	Rnf180	Proteolysis
DMR2:35876001	2	35876001	35879000	3000	1	9.80E-08	-0.58	43	1.43	Rnf180	Proteolysis
DMR2:36237001	2	36237001	36238000	1000	1	1.50E-08	0.47	24	2.4	Htr1a	Signaling
DMR2:38991001	2	38991001	38994000	3000	1	9.00E-09	-0.58	37	1.23	Zswim6	
DMR2:39048001	2	39048001	39049000	1000	1	5.80E-07	-0.31	16	1.6	Zswim6	
DMR2:39327001	2	39327001	39328000	1000	1	1.90E-07	-0.45	19	1.9	Smim15;Ndufaf2	Transcription
DMR2:39442001	2	39442001	39443000	1000	1	4.10E-07	-0.65	7	0.7	Ndufaf2;Ercc8	Transcription;Transcription
DMR2:40020001	2	40020001	40024000	4000	1	4.50E-07	0.36	77	1.93	Depdc1b	Cytoskeleton
DMR2:40045001	2	40045001	40046000	1000	1	2.60E-07	0.43	20	2	Depdc1b	Cytoskeleton
DMR2:40695001	2	40695001	40696000	1000	1	1.40E-09	0.5	5	0.5	Pde4d	Signaling
DMR2:40709001	2	40709001	40711000	2000	1	4.00E-08	0.34	16	0.8	Pde4d	Signaling
DMR2:40891001	2	40891001	40893000	2000	1	7.70E-07	-0.32	29	1.45	Pde4d;LOC108350210	Signaling
DMR2:41006001	2	41006001	41007000	1000	1	6.00E-07	0.41	6	0.6	Pde4d	Signaling
DMR2:41030001	2	41030001	41032000	2000	1	1.70E-07	-0.49	29	1.45	Pde4d	Signaling
DMR2:41078001	2	41078001	41079000	1000	1	1.00E-09	-0.58	16	1.6	Pde4d	Signaling
DMR2:41478001	2	41478001	41481000	3000	2	1.70E-14	0.43	13	0.43	Pde4d	Signaling
DMR2:41575001	2	41575001	41577000	2000	1	4.10E-07	0.65	33	1.65	Rab3c	
DMR2:41579001	2	41579001	41580000	1000	1	3.20E-10	0.47	3	0.3	Rab3c	
DMR2:41657001	2	41657001	41658000	1000	1	7.60E-08	0.43	2	0.2	Rab3c	
DMR2:42839001	2	42839001	42840000	1000	1	5.80E-07	-0.44	12	1.2	LOC103691452;Actbl2	Cytoskeleton
DMR2:43359001	2	43359001	43361000	2000	1	4.30E-09	-0.68	36	1.8	Map3k1	
DMR2:43872001	2	43872001	43874000	2000	1	1.50E-13	0.42	25	1.25	Ankrd55	Cytoskeleton
DMR2:43966001	2	43966001	43967000	1000	1	3.60E-08	-0.66	20	2	Ankrd55;LOC100912399	Cytoskeleton;Signaling
DMR2:44142001	2	44142001	44144000	2000	1	1.30E-07	0.31	22	1.1	Ankrd55;LOC103691456	Cytoskeleton
DMR2:44327001	2	44327001	44328000	1000	1	8.30E-08	0.3	7	0.7	Il6st;Il31ra	Receptor
DMR2:44407001	2	44407001	44408000	1000	1	2.00E-07	-0.41	12	1.2	Il31ra	Receptor
DMR2:44417001	2	44417001	44419000	2000	1	1.70E-09	-0.49	24	1.2	Il31ra	Receptor
DMR2:44591001	2	44591001	44593000	2000	1	2.00E-08	-0.39	47	2.35	Slc38a9	Transport
DMR2:44671001	2	44671001	44672000	1000	1	6.10E-09	-0.52	12	1.2	Plpp1	Signaling
DMR2:44708001	2	44708001	44714000	6000	1	1.40E-07	-0.45	64	1.07	Plpp1	Signaling
DMR2:44848001	2	44848001	44850000	2000	1	3.30E-09	0.67	5	0.25	Ccno	Signaling
DMR2:44940001	2	44940001	44941000	1000	1	6.60E-07	-0.37	11	1.1	Cdc20b	
DMR2:44990001	2	44990001	44994000	4000	2	3.30E-13	0.72	44	1.1	Gzma	Protease
DMR2:45094001	2	45094001	45095000	1000	1	6.80E-07	0.41	10	1	Esm1	
DMR2:45193001	2	45193001	45194000	1000	1	8.90E-07	-0.35	15	1.5	RGD1561161	
DMR2:45446001	2	45446001	45447000	1000	1	4.20E-09	0.6	30	3	Snx18	Cytoskeleton
DMR2:45461001	2	45461001	45463000	2000	1	5.00E-07	-0.39	38	1.9	Snx18	Cytoskeleton
DMR2:45486001	2	45486001	45487000	1000	1	9.70E-09	-0.52	14	1.4	Snx18;LOC108349945	Cytoskeleton
DMR2:45670001	2	45670001	45678000	8000	1	1.40E-10	-0.48	132	1.65	Arl15	
DMR2:45727001	2	45727001	45733000	6000	1	1.70E-07	-0.37	95	1.58	Arl15	
DMR2:45736001	2	45736001	45739000	3000	1	1.00E-08	-0.64	46	1.53	Arl15	
DMR2:45756001	2	45756001	45763000	7000	1	6.00E-07	-0.4	96	1.37	Arl15;LOC100362624	
DMR2:45827001	2	45827001	45829000	2000	1	4.00E-08	-0.5	34	1.7	Arl15	
DMR2:45856001	2	45856001	45858000	2000	1	4.00E-07	-0.42	33	1.65	Arl15	
DMR2:45859001	2	45859001	45862000	3000	1	1.00E-08	-0.5	64	2.13	Arl15	
DMR2:45917001	2	45917001	45922000	5000	1	3.90E-09	0.6	57	1.14	Arl15	
DMR2:46048001	2	46048001	46049000	1000	1	5.40E-11	0.5	9	0.9	Arl15	
DMR2:46448001	2	46448001	46451000	3000	1	4.90E-07	0.37	25	0.83	Ndufs4	Metabolism
DMR2:46539001	2	46539001	46543000	4000	1	1.60E-09	0.45	76	1.9	Fst	Protease; Proteolysis
DMR2:47159001	2	47159001	47160000	1000	1	1.10E-15	0.64	16	1.6	Itga1	Extracellular Matrix

DMR2:49197001	2	49197001	49198000	1000	1	1.40E-08	0.45	10	1	Parp8	
DMR2:49332001	2	49332001	49334000	2000	1	1.90E-08	0.41	31	1.55	Parp8;LOC100910954	
DMR2:49336001	2	49336001	49338000	2000	1	8.30E-08	0.5	36	1.8	Parp8;LOC100910954	
DMR2:49352001	2	49352001	49353000	1000	1	3.40E-07	0.33	27	2.7	Parp8;LOC100910954	
DMR2:49370001	2	49370001	49372000	2000	2	9.30E-19	0.68	42	2.1	Parp8;LOC100910954	
DMR2:49460001	2	49460001	49463000	3000	1	3.90E-19	-0.68	49	1.63	Parp8	
DMR2:49694001	2	49694001	49695000	1000	1	4.60E-08	-0.43	12	1.2	Emb	Cytoskeleton
DMR2:50179001	2	50179001	50180000	1000	1	2.80E-09	0.5	8	0.8	Hcn1	Transport
DMR2:50202001	2	50202001	50204000	2000	1	1.50E-09	0.49	15	0.75	Hcn1	Transport
DMR2:50227001	2	50227001	50232000	5000	3	1.40E-07	-0.43	43	0.86	Hcn1	Transport
DMR2:50332001	2	50332001	50339000	7000	1	4.50E-10	-0.45	74	1.06	Hcn1	Transport
DMR2:50430001	2	50430001	50432000	2000	1	2.60E-13	-0.56	18	0.9	Hcn1	Transport
DMR2:51035001	2	51035001	51036000	1000	1	1.10E-08	-0.75	10	1	Mrps30	Translation
DMR2:51687001	2	51687001	51688000	1000	1	8.40E-07	-0.47	8	0.8	Fgf10	Growth Factors
DMR2:52172001	2	52172001	52175000	3000	1	2.80E-07	-0.37	41	1.37	RGD1561520	
DMR2:52304001	2	52304001	52306000	2000	1	3.80E-09	-0.65	11	0.55	Paip1	Metabolism
DMR2:53171001	2	53171001	53175000	4000	1	1.50E-13	-0.3	33	0.82	Ghr	Receptor
DMR2:53223001	2	53223001	53228000	5000	1	4.30E-07	-0.34	43	0.86	Ghr	Receptor
DMR2:53268001	2	53268001	53270000	2000	1	1.40E-10	-0.54	9	0.45	Ghr	Receptor
DMR2:53273001	2	53273001	53275000	2000	1	2.90E-07	-0.41	18	0.9	Ghr	Receptor
DMR2:53288001	2	53288001	53289000	1000	1	3.30E-08	0.37	9	0.9	Ghr	Receptor
DMR2:53795001	2	53795001	53797000	2000	1	4.10E-08	0.48	32	1.6	Fbxo4	
DMR2:53852001	2	53852001	53853000	1000	1	1.10E-10	-0.58	15	1.5	Oxct1	Transport
DMR2:53864001	2	53864001	53866000	2000	1	4.70E-07	-0.42	27	1.35	Oxct1	Transport
DMR2:53888001	2	53888001	53889000	1000	1	6.60E-08	-0.65	15	1.5	Oxct1	Transport
DMR2:54145001	2	54145001	54150000	5000	1	1.30E-08	-0.3	32	0.64	RGD1566344	
DMR2:54288001	2	54288001	54293000	5000	1	8.30E-11	-0.27	56	1.12	Plcxd3	
DMR2:54348001	2	54348001	54351000	3000	1	5.00E-07	-0.46	12	0.4	Plcxd3	
DMR2:54535001	2	54535001	54537000	2000	1	9.30E-09	-0.62	23	1.15	C6	
DMR2:54657001	2	54657001	54658000	1000	1	1.60E-07	0.38	11	1.1	Mroh2b	
DMR2:54665001	2	54665001	54668000	3000	1	4.90E-07	0.31	20	0.67	Mroh2b;LOC102553573	
DMR2:54712001	2	54712001	54713000	1000	1	1.40E-09	0.37	4	0.4	C7	
DMR2:54747001	2	54747001	54751000	4000	1	8.80E-11	-0.45	38	0.95	C7	
DMR2:54760001	2	54760001	54765000	5000	1	5.80E-08	-0.41	53	1.06	C7	
DMR2:54818001	2	54818001	54820000	2000	1	1.70E-07	0.61	33	1.65	Card6	
DMR2:54883001	2	54883001	54886000	3000	2	6.70E-10	0.64	23	0.77	Prkaa1	Signaling
DMR2:54902001	2	54902001	54904000	2000	1	8.90E-08	-0.58	23	1.15	Prkaa1;Ttc33	Signaling
DMR2:54947001	2	54947001	54949000	2000	1	4.80E-11	0.52	8	0.4	Ttc33;Ptger4	Signaling
DMR2:54957001	2	54957001	54962000	5000	2	8.00E-12	-0.68	70	1.4	Ptger4	Signaling
DMR2:55061001	2	55061001	55062000	1000	1	1.40E-07	0.48	5	0.5	Rps15al4	
DMR2:55801001	2	55801001	55802000	1000	1	8.00E-07	-0.42	10	1	C9	
DMR2:55967001	2	55967001	55971000	4000	1	2.70E-08	-0.29	42	1.05	Fyb	
DMR2:56145001	2	56145001	56147000	2000	1	6.80E-07	-0.36	37	1.85	Osmr	Receptor
DMR2:56463001	2	56463001	56469000	6000	1	1.60E-07	-0.35	94	1.57	Lifr	Receptor
DMR2:56538001	2	56538001	56542000	4000	1	2.70E-07	0.33	44	1.1	Egflam;LOC108349957	Extracellular Matrix
DMR2:56686001	2	56686001	56687000	1000	1	8.80E-11	-0.47	10	1	Egflam;LOC108349956	Extracellular Matrix
DMR2:56913001	2	56913001	56915000	2000	1	2.30E-09	0.39	20	1	Gdnf	Growth Factors
DMR2:56973001	2	56973001	56980000	7000	3	1.30E-10	-0.42	76	1.09	Wdr70	
DMR2:57008001	2	57008001	57009000	1000	1	8.00E-08	0.37	12	1.2	Wdr70	
DMR2:57097001	2	57097001	57099000	2000	1	9.90E-08	-0.43	29	1.45	Wdr70	
DMR2:57141001	2	57141001	57147000	6000	1	5.60E-09	-0.35	48	0.8	Wdr70	
DMR2:57583001	2	57583001	57584000	1000	1	3.80E-08	-0.48	13	1.3	Nipbl	Epigenetic
DMR2:57615001	2	57615001	57617000	2000	1	9.00E-08	-0.61	17	0.85	Nipbl	Epigenetic
DMR2:57624001	2	57624001	57625000	1000	1	4.90E-10	-0.67	5	0.5	Nipbl	Epigenetic
DMR2:57869001	2	57869001	57871000	2000	1	2.60E-08	0.61	41	2.05	Slc1a3	Transport
DMR2:57942001	2	57942001	57946000	4000	2	1.60E-07	-0.46	55	1.38	Slc1a3;LOC108349962	Transport
DMR2:58226001	2	58226001	58227000	1000	1	3.80E-15	0.98	34	3.4	Ranbp3l;LOC679871	Cytoskeleton
DMR2:58248001	2	58248001	58250000	2000	1	7.80E-07	0.5	15	0.75	Ranbp3l	Cytoskeleton
DMR2:58276001	2	58276001	58280000	4000	1	7.50E-11	-0.28	29	0.72	Ranbp3l;LOC365693	Cytoskeleton
DMR2:58313001	2	58313001	58314000	1000	1	1.30E-07	0.38	5	0.5	Ranbp3l	Cytoskeleton
DMR2:58319001	2	58319001	58320000	1000	1	3.30E-10	-0.56	15	1.5	Ranbp3l	Cytoskeleton
DMR2:58351001	2	58351001	58358000	7000	1	1.10E-08	-0.39	78	1.11	Ranbp3l;LOC108350155	Cytoskeleton
DMR2:58489001	2	58489001	58490000	1000	1	5.70E-07	0.29	15	1.5	Nadk2	

DMR2:58491001	2	58491001	58495000	4000	1	6.50E-09	-0.47	61	1.52	Nadk2	
DMR2:58500001	2	58500001	58503000	3000	1	2.20E-08	-0.46	41	1.37	Nadk2;Skp2	
DMR2:58519001	2	58519001	58522000	3000	1	1.40E-09	0.42	50	1.67	Skp2	
DMR2:58545001	2	58545001	58547000	2000	1	3.40E-08	0.36	38	1.9	Lmbrd2	
DMR2:59048001	2	59048001	59049000	1000	1	3.80E-17	0.65	23	2.3	Spef2	
DMR2:60377001	2	60377001	60379000	2000	1	9.50E-07	0.48	37	1.85	Agxt2	
DMR2:60386001	2	60386001	60394000	8000	1	3.10E-09	-0.44	86	1.07	Agxt2	
DMR2:60422001	2	60422001	60427000	5000	4	3.80E-08	-0.37	50	1	Dnajc21	Transcription
DMR2:60502001	2	60502001	60505000	3000	2	1.10E-07	-0.47	9	0.3	Ttc23l	
DMR2:60549001	2	60549001	60553000	4000	1	9.50E-08	-0.44	97	2.42	Rai14	
DMR2:60562001	2	60562001	60563000	1000	1	3.00E-07	0.38	23	2.3	Rai14	
DMR2:60924001	2	60924001	60928000	4000	1	1.90E-08	-0.53	55	1.38	C1qtnf3	
DMR2:60977001	2	60977001	60982000	5000	1	5.30E-08	-0.37	48	0.96	Slc45a2	Transport
DMR2:61036001	2	61036001	61037000	1000	1	8.20E-08	-0.5	9	0.9	Adamts12	Protease
DMR2:61419001	2	61419001	61424000	5000	1	1.10E-07	-0.3	46	0.92	Tars	
DMR2:61959001	2	61959001	61962000	3000	1	7.70E-07	-0.37	42	1.4	Npr3	Receptor
DMR2:62188001	2	62188001	62189000	1000	1	3.10E-10	-0.59	8	0.8	Zfr	Metabolism
DMR2:62230001	2	62230001	62231000	1000	1	1.50E-08	0.39	11	1.1	Mtmr12	Signaling
DMR2:62288001	2	62288001	62289000	1000	1	2.60E-08	0.43	15	1.5	Mtmr12	Signaling
DMR2:62386001	2	62386001	62388000	2000	1	9.30E-08	-0.49	28	1.4	Golph3	
DMR2:62391001	2	62391001	62393000	2000	1	1.10E-08	-0.54	33	1.65	Golph3;Pdzd2	Cytokine
DMR2:62449001	2	62449001	62451000	2000	1	5.40E-08	0.35	26	1.3	Pdzd2	Cytokine
DMR2:62489001	2	62489001	62494000	5000	1	6.20E-08	0.48	80	1.6	Pdzd2	Cytokine
DMR2:63006001	2	63006001	63009000	3000	1	9.30E-10	-0.5	48	1.6	Drosha	Translation
DMR2:63149001	2	63149001	63154000	5000	3	2.10E-07	-0.41	51	1.02	Cdh6	Cytoskeleton
DMR2:63178001	2	63178001	63182000	4000	1	4.40E-10	-0.36	28	0.7	Cdh6	Cytoskeleton
DMR2:66593001	2	66593001	66594000	1000	1	6.00E-07	-0.35	8	0.8	RGD1564125	
DMR2:67036001	2	67036001	67039000	3000	2	3.50E-08	-0.45	19	0.63	Cdh9	Cytoskeleton
DMR2:69522001	2	69522001	69523000	1000	1	1.70E-08	-0.66	7	0.7	Cdh10	Cytoskeleton
DMR2:69560001	2	69560001	69561000	1000	1	4.10E-07	-0.44	11	1.1	Cdh10	Cytoskeleton
DMR2:69585001	2	69585001	69586000	1000	1	1.70E-08	-0.55	9	0.9	Cdh10	Cytoskeleton
DMR2:71344001	2	71344001	71345000	1000	1	5.90E-08	0.49	6	0.6	Cdh12	Cytoskeleton
DMR2:71455001	2	71455001	71456000	1000	1	1.40E-07	0.55	9	0.9	Cdh12	Cytoskeleton
DMR2:71622001	2	71622001	71623000	1000	1	7.10E-07	0.43	10	1	Cdh12	Cytoskeleton
DMR2:71784001	2	71784001	71785000	1000	1	1.60E-07	0.4	3	0.3	Cdh12	Cytoskeleton
DMR2:71966001	2	71966001	71969000	3000	1	1.80E-08	-0.41	6	0.2	Cdh12	Cytoskeleton
DMR2:72065001	2	72065001	72066000	1000	1	1.00E-06	-0.34	7	0.7	Cdh12	Cytoskeleton
DMR2:72296001	2	72296001	72302000	6000	2	1.60E-13	-0.42	55	0.92	Cdh12	Cytoskeleton
DMR2:72519001	2	72519001	72521000	2000	1	9.80E-08	-0.33	18	0.9	Cdh12	Cytoskeleton
DMR2:73760001	2	73760001	73761000	1000	1	2.40E-08	-0.49	6	0.6	Cdh18	Cytoskeleton
DMR2:73983001	2	73983001	73987000	4000	1	2.90E-10	-0.37	37	0.92	Cdh18	Cytoskeleton
DMR2:74057001	2	74057001	74058000	1000	1	5.00E-09	-0.69	8	0.8	Cdh18	Cytoskeleton
DMR2:74180001	2	74180001	74181000	1000	1	2.50E-07	0.49	9	0.9	Cdh18	Cytoskeleton
DMR2:74315001	2	74315001	74321000	6000	1	5.00E-10	-0.56	57	0.95	Cdh18	Cytoskeleton
DMR2:74425001	2	74425001	74427000	2000	1	1.70E-08	0.48	5	0.25	Cdh18	Cytoskeleton
DMR2:74429001	2	74429001	74430000	1000	1	2.20E-07	0.53	11	1.1	Cdh18	Cytoskeleton
DMR2:77929001	2	77929001	77930000	1000	1	1.80E-07	-0.37	12	1.2	Myo10	
DMR2:77986001	2	77986001	77990000	4000	1	7.00E-08	-0.43	67	1.68	Myo10;LOC103691488	
DMR2:79055001	2	79055001	79058000	3000	1	2.40E-07	-0.38	18	0.6	Fbxl7	
DMR2:79127001	2	79127001	79128000	1000	1	3.30E-07	0.35	13	1.3	Fbxl7	
DMR2:79206001	2	79206001	79209000	3000	1	9.50E-15	0.56	24	0.8	Fbxl7	
DMR2:79384001	2	79384001	79388000	4000	1	2.90E-07	-0.29	27	0.68	Fbxl7	
DMR2:80179001	2	80179001	80183000	4000	1	3.90E-10	-0.5	63	1.57	Ankh	
DMR2:80385001	2	80385001	80388000	3000	1	2.80E-07	-0.39	69	2.3	Fam105a	
DMR2:80463001	2	80463001	80464000	1000	1	5.90E-07	0.28	9	0.9	Trio	Transcription
DMR2:80511001	2	80511001	80515000	4000	1	4.80E-08	-0.36	103	2.58	Trio	Transcription
DMR2:80526001	2	80526001	80528000	2000	1	4.40E-07	-0.57	24	1.2	Trio	Transcription
DMR2:80570001	2	80570001	80574000	4000	2	1.50E-09	-0.43	61	1.52	Trio	Transcription
DMR2:80592001	2	80592001	80596000	4000	1	5.90E-11	0.48	62	1.55	Trio	Transcription
DMR2:80618001	2	80618001	80623000	5000	1	2.70E-08	-0.6	90	1.8	Trio	Transcription
DMR2:80747001	2	80747001	80749000	2000	1	1.60E-07	-0.39	24	1.2	Trio	Transcription
DMR2:80960001	2	80960001	80961000	1000	1	6.10E-08	0.62	24	2.4	Dnah5	Cytoskeleton
DMR2:81305001	2	81305001	81307000	2000	1	1.30E-08	0.41	23	1.15	Dnah5	Cytoskeleton

DMR2:81339001	2	81339001	81343000	4000	2	4.80E-10	-0.3	27	0.68	Dnah5	Cytoskeleton
DMR2:83459001	2	83459001	83464000	5000	1	1.40E-08	-0.29	47	0.94	Ctnnd2	Cytoskeleton
DMR2:83619001	2	83619001	83621000	2000	2	5.60E-08	0.5	24	1.2	Ctnnd2;LOC102553378	Cytoskeleton
DMR2:83708001	2	83708001	83710000	2000	1	1.90E-09	0.42	8	0.4	Ctnnd2	Cytoskeleton
DMR2:83775001	2	83775001	83776000	1000	1	6.40E-07	0.34	11	1.1	Ctnnd2	Cytoskeleton
DMR2:83957001	2	83957001	83958000	1000	1	2.00E-07	-0.44	17	1.7	Ctnnd2	Cytoskeleton
DMR2:84362001	2	84362001	84363000	1000	1	1.60E-07	0.46	25	2.5	Ankrd33b	
DMR2:84553001	2	84553001	84559000	6000	1	8.70E-08	-0.24	79	1.32	6-Mar	Proteolysis
DMR2:85511001	2	85511001	85515000	4000	1	4.00E-09	0.47	24	0.6	Sema5a	Signaling
DMR2:85533001	2	85533001	85536000	3000	1	1.80E-07	-0.43	16	0.53	Sema5a	Signaling
DMR2:85560001	2	85560001	85562000	2000	1	7.80E-07	0.36	15	0.75	Sema5a	Signaling
DMR2:85768001	2	85768001	85770000	2000	1	4.80E-08	0.54	39	1.95	Sema5a	Signaling
DMR2:88126001	2	88126001	88127000	1000	1	4.20E-07	-0.59	7	0.7	Car3	
DMR2:89312001	2	89312001	89313000	1000	1	1.30E-09	0.48	9	0.9	Ralyl	
DMR2:89341001	2	89341001	89349000	8000	1	5.00E-08	-0.29	61	0.76	Ralyl;LOC108349976	
DMR2:89586001	2	89586001	89587000	1000	1	1.50E-07	0.44	7	0.7	Ralyl	
DMR2:89605001	2	89605001	89611000	6000	1	2.20E-07	-0.5	55	0.92	Ralyl	
DMR2:89662001	2	89662001	89665000	3000	1	2.60E-07	0.47	39	1.3	Ralyl;LOC103695106	
DMR2:89770001	2	89770001	89772000	2000	1	1.70E-10	0.52	20	1	Ralyl	
DMR2:89836001	2	89836001	89837000	1000	1	2.10E-09	0.37	8	0.8	Ralyl	
DMR2:91462001	2	91462001	91463000	1000	1	2.40E-10	0.53	6	0.6	Pcsk1	Protease
DMR2:91486001	2	91486001	91487000	1000	1	9.60E-09	0.34	10	1	Pcsk1;LOC102548697	Protease
DMR2:92564001	2	92564001	92566000	2000	1	7.20E-10	0.53	8	0.4	tGap1;LOC102553273;LOC100363165	Signaling
DMR2:94372001	2	94372001	94375000	3000	1	1.20E-07	0.41	32	1.07	LOC102556687;Zfp704	Transcription
DMR2:94520001	2	94520001	94521000	1000	1	4.10E-08	0.36	16	1.6	Zfp704	Transcription
DMR2:94527001	2	94527001	94530000	3000	1	9.30E-09	-0.58	77	2.57	Zfp704	Transcription
DMR2:94536001	2	94536001	94537000	1000	1	1.10E-08	0.37	9	0.9	Zfp704	Transcription
DMR2:95034001	2	95034001	95036000	2000	1	7.80E-07	-0.36	25	1.25	Tpd52	
DMR2:95075001	2	95075001	95076000	1000	1	8.20E-09	0.45	8	0.8	Mrps28;LOC499573	Translation
DMR2:95317001	2	95317001	95319000	2000	1	7.40E-09	0.69	23	1.15	Hey1;LOC108349980	Transcription
DMR2:95459001	2	95459001	95462000	3000	2	9.20E-09	0.52	46	1.53	Stmn2	
DMR2:96482001	2	96482001	96485000	3000	1	1.20E-08	-0.5	26	0.87	Il7;Zc2hc1a	Transcription
DMR2:96623001	2	96623001	96626000	3000	1	1.70E-07	-0.4	19	0.63	Pkia	Signaling
DMR2:98426001	2	98426001	98427000	1000	1	4.50E-07	0.43	15	1.5	Zfhx4	Transcription
DMR2:100665001	2	100665001	100668000	3000	1	1.40E-07	-0.28	27	0.9	Ythdf3	
DMR2:103945001	2	103945001	103949000	4000	2	9.40E-09	-0.3	47	1.18	Armc1	
DMR2:103963001	2	103963001	103965000	2000	1	1.30E-10	-0.56	13	0.65	Armc1	
DMR2:104429001	2	104429001	104430000	1000	1	6.20E-08	-0.7	11	1.1	Trim55	Proteolysis
DMR2:104764001	2	104764001	104771000	7000	1	5.90E-07	-0.39	79	1.13	Cp	Metabolism
DMR2:104879001	2	104879001	104881000	2000	1	1.20E-07	0.43	34	1.7	Hltf	
DMR2:104888001	2	104888001	104892000	4000	1	6.70E-09	0.44	30	0.75	Hltf	
DMR2:105022001	2	105022001	105026000	4000	1	1.90E-09	-0.4	33	0.82	Cpa3	Protease
DMR2:105052001	2	105052001	105058000	6000	4	1.60E-08	-0.29	74	1.23	Cpa3;Cpb1	Protease
DMR2:105062001	2	105062001	105063000	1000	1	1.80E-09	-0.51	7	0.7	Cpb1	Protease
DMR2:105064001	2	105064001	105066000	2000	1	6.00E-09	-0.62	11	0.55	Cpb1	Protease
DMR2:105161001	2	105161001	105162000	1000	1	3.80E-08	0.32	22	2.2	Agtr1b	Signaling
DMR2:109320001	2	109320001	109321000	1000	1	1.60E-08	-0.52	19	1.9	Naaladl2;LOC682571	
DMR2:109485001	2	109485001	109486000	1000	1	5.80E-08	0.35	12	1.2	Naaladl2	
DMR2:109551001	2	109551001	109556000	5000	1	6.80E-07	-0.29	46	0.92	Naaladl2	
DMR2:110028001	2	110028001	110029000	1000	1	1.70E-08	0.46	19	1.9	Naaladl2	
DMR2:110305001	2	110305001	110306000	1000	1	2.50E-11	0.97	10	1	Naaladl2;LOC499584	
DMR2:110364001	2	110364001	110367000	3000	1	8.00E-07	0.47	16	0.53	Naaladl2	
DMR2:111099001	2	111099001	111105000	6000	3	6.60E-10	-0.38	55	0.92	Nlgn1	Cytoskeleton
DMR2:111356001	2	111356001	111361000	5000	1	5.00E-09	-0.33	46	0.92	Nlgn1	Cytoskeleton
DMR2:111685001	2	111685001	111688000	3000	1	2.30E-07	-0.25	29	0.97	Nlgn1	Cytoskeleton
DMR2:111898001	2	111898001	111900000	2000	1	3.30E-07	0.4	19	0.95	Nlgn1	Cytoskeleton
DMR2:111917001	2	111917001	111918000	1000	1	6.80E-07	-0.46	7	0.7	Nlgn1	Cytoskeleton
DMR2:112323001	2	112323001	112327000	4000	2	6.50E-07	0.38	52	1.3	Spata16	
DMR2:112328001	2	112328001	112330000	2000	1	3.90E-09	-0.31	13	0.65	Spata16	
DMR2:112351001	2	112351001	112352000	1000	1	2.30E-10	0.51	15	1.5	Spata16	
DMR2:112354001	2	112354001	112361000	7000	2	3.60E-07	-0.4	75	1.07	Spata16	
DMR2:112367001	2	112367001	112373000	6000	1	7.60E-08	-0.23	73	1.22	Spata16	

DMR2:112389001	2	112389001	112390000	1000	1	1.90E-08	0.39	12	1.2	Spata16	
DMR2:112424001	2	112424001	112430000	6000	1	4.00E-08	-0.31	55	0.92	Spata16	
DMR2:112476001	2	112476001	112479000	3000	1	2.40E-11	0.4	17	0.57	Spata16	
DMR2:112601001	2	112601001	112602000	1000	1	9.40E-07	-0.48	27	2.7	Spata16	
DMR2:112758001	2	112758001	112761000	3000	1	2.80E-07	0.38	27	0.9	LOC102555205;Ect2	Transcription
DMR2:112779001	2	112779001	112781000	2000	1	5.00E-10	-0.43	16	0.8	Ect2	Transcription
DMR2:113057001	2	113057001	113058000	1000	1	4.40E-07	-0.51	5	0.5	LOC103691520;Ghsr	Signaling
DMR2:113080001	2	113080001	113082000	2000	1	4.40E-08	0.39	15	0.75	Ghsr	Signaling
DMR2:113367001	2	113367001	113370000	3000	1	2.80E-07	-0.43	36	1.2	Fndc3b	Proteolysis
DMR2:113604001	2	113604001	113610000	6000	1	1.60E-08	0.41	81	1.35	Tmem212;LOC102555566	Transport
DMR2:113817001	2	113817001	113819000	2000	1	8.60E-08	0.42	25	1.25	Pld1	Metabolism
DMR2:114003001	2	114003001	114004000	1000	1	1.40E-08	0.42	5	0.5	Tnik	Signaling
DMR2:114200001	2	114200001	114202000	2000	1	1.10E-08	-0.38	16	0.8	Tnik	Signaling
DMR2:114425001	2	114425001	114427000	2000	1	1.60E-08	-0.6	21	1.05	Slc2a2	
DMR2:115364001	2	115364001	115367000	3000	2	7.00E-10	-0.48	30	1	Eif5a2;Rpl22l1	Translation;Translation
DMR2:115822001	2	115822001	115825000	3000	1	2.00E-08	0.81	57	1.9	Cldn11	Cell Junction
DMR2:115829001	2	115829001	115831000	2000	1	2.40E-07	0.74	56	2.8	Cldn11	Cell Junction
DMR2:115842001	2	115842001	115843000	1000	1	4.80E-11	-0.56	8	0.8	Cldn11	Cell Junction
DMR2:115948001	2	115948001	115952000	4000	1	6.20E-07	-0.37	78	1.95	Prkci	Signaling
DMR2:115959001	2	115959001	115961000	2000	1	3.60E-07	-0.33	14	0.7	Prkci	Signaling
DMR2:115981001	2	115981001	115984000	3000	1	2.00E-07	0.47	28	0.93	Prkci	Signaling
DMR2:116085001	2	116085001	116086000	1000	1	4.80E-07	-0.45	9	0.9	Phc3	Epigenetic
DMR2:116092001	2	116092001	116095000	3000	1	5.10E-07	-0.46	43	1.43	Phc3	Epigenetic
DMR2:116124001	2	116124001	116127000	3000	1	1.20E-07	-0.37	18	0.6	Gpr160	Signaling
DMR2:116134001	2	116134001	116137000	3000	1	4.30E-07	-0.35	24	0.8	Gpr160	Signaling
DMR2:116297001	2	116297001	116299000	2000	1	4.30E-09	-0.39	46	2.3	LOC685065;Lrrc31	
DMR2:116359001	2	116359001	116362000	3000	1	2.10E-08	-0.28	27	0.9	Lrriq4;Lrrc34	Cytoskeleton
DMR2:116514001	2	116514001	116516000	2000	1	1.20E-09	-0.45	11	0.55	Egfem1	
DMR2:116594001	2	116594001	116595000	1000	1	7.20E-07	-0.56	9	0.9	Egfem1	
DMR2:116600001	2	116600001	116604000	4000	1	2.10E-14	-0.7	33	0.82	Egfem1	
DMR2:116638001	2	116638001	116644000	6000	1	5.90E-07	-0.35	54	0.9	Egfem1	
DMR2:116649001	2	116649001	116650000	1000	1	1.10E-08	-0.44	10	1	Egfem1	
DMR2:116764001	2	116764001	116766000	2000	1	2.00E-07	0.39	9	0.45	Egfem1	
DMR2:116775001	2	116775001	116782000	7000	1	6.50E-07	0.35	54	0.77	Egfem1	
DMR2:116817001	2	116817001	116818000	1000	1	5.80E-09	0.39	11	1.1	Egfem1	
DMR2:116892001	2	116892001	116893000	1000	1	4.10E-09	0.68	15	1.5	Egfem1	
DMR2:116912001	2	116912001	116918000	6000	1	1.40E-07	-0.28	52	0.87	Egfem1	
DMR2:116951001	2	116951001	116956000	5000	2	5.00E-10	-0.33	39	0.78	Egfem1;LOC103691523	
DMR2:116979001	2	116979001	116984000	5000	1	4.60E-08	-0.3	47	0.94	Egfem1	
DMR2:117044001	2	117044001	117049000	5000	1	3.80E-08	-0.29	36	0.72	Egfem1	
DMR2:117102001	2	117102001	117105000	3000	1	1.30E-07	0.5	31	1.03	Egfem1	
DMR2:117414001	2	117414001	117416000	2000	1	6.90E-07	0.36	19	0.95	Mecom	Transcription
DMR2:117779001	2	117779001	117780000	1000	1	2.90E-08	-0.47	17	1.7	Mecom;LOC103691544	Transcription
DMR2:117804001	2	117804001	117808000	4000	1	3.70E-10	0.45	42	1.05	Mecom;LOC103691544	Transcription
DMR2:117858001	2	117858001	117860000	2000	1	1.50E-07	-0.45	24	1.2	Mecom;LOC103691544	Transcription
DMR2:118382001	2	118382001	118383000	1000	1	6.10E-09	-0.43	7	0.7	Kcnmb2;LOC102548736	Transport
DMR2:118487001	2	118487001	118494000	7000	1	1.00E-08	-0.42	81	1.16	Kcnmb2	Transport
DMR2:118721001	2	118721001	118723000	2000	1	2.80E-10	0.71	54	2.7	Zmat3	
DMR2:119208001	2	119208001	119209000	1000	1	3.80E-08	0.43	6	0.6	Usp13	Protease
DMR2:119413001	2	119413001	119414000	1000	1	5.30E-09	0.37	6	0.6	Pex5l	Transport
DMR2:120258001	2	120258001	120259000	1000	1	5.60E-10	-0.53	7	0.7	Ttc14	
DMR2:120580001	2	120580001	120581000	1000	1	3.50E-08	0.41	13	1.3	Fxr1	Translation
DMR2:122466001	2	122466001	122469000	3000	1	2.90E-09	0.38	42	1.4	Dcun1d1	
DMR2:122486001	2	122486001	122489000	3000	1	1.20E-08	-0.54	41	1.37	Dcun1d1	
DMR2:122552001	2	122552001	122553000	1000	1	6.60E-07	0.36	13	1.3	Mccc1	Metabolism
DMR2:122707001	2	122707001	122709000	2000	1	1.50E-07	-0.45	24	1.2	Ccdc144b	
DMR2:122713001	2	122713001	122714000	1000	1	3.40E-07	-0.39	5	0.5	Ccdc144b	
DMR2:122798001	2	122798001	122799000	1000	1	1.20E-09	-0.39	12	1.2	Acad9	Metabolism
DMR2:122806001	2	122806001	122807000	1000	1	4.80E-08	-0.4	9	0.9	Acad9	Metabolism
DMR2:122863001	2	122863001	122864000	1000	1	5.20E-08	0.68	31	3.1	Rslcan18;LOC102556166;LOC108349996	
DMR2:122878001	2	122878001	122880000	2000	2	1.90E-10	0.72	56	2.8	Rslcan18;LOC108349996;Arse	Metabolism
DMR2:122881001	2	122881001	122884000	3000	1	2.60E-13	0.97	94	3.13	Rslcan18;Arse;Qrfpr	Metabolism;Signaling

DMR2:123420001	2	123420001	123421000	1000	1	6.10E-09	0.33	18	1.8	Trpc3;LOC365768	Transport
DMR2:123435001	2	123435001	123441000	6000	1	3.00E-08	-0.3	72	1.2	Trpc3;LOC365768	Transport
DMR2:123465001	2	123465001	123467000	2000	2	1.20E-09	0.42	11	0.55	Trpc3	Transport
DMR2:123624001	2	123624001	123627000	3000	1	4.20E-08	-0.24	30	1	RGD1307100	
DMR2:123666001	2	123666001	123674000	8000	1	2.10E-08	-0.42	89	1.11	RGD1307100	
DMR2:123725001	2	123725001	123729000	4000	1	7.90E-09	-0.42	43	1.07	RGD1307100	
DMR2:123765001	2	123765001	123766000	1000	1	3.80E-10	0.54	6	0.6	RGD1307100	
DMR2:123790001	2	123790001	123792000	2000	1	5.10E-14	1.05	69	3.45	Adad1	Metabolism
DMR2:123978001	2	123978001	123979000	1000	1	5.40E-10	0.63	9	0.9	Il21	Cytokine
DMR2:124031001	2	124031001	124038000	7000	1	1.80E-07	-0.24	73	1.04	Cetn4	Signaling
DMR2:124071001	2	124071001	124073000	2000	1	6.50E-10	-0.49	19	0.95	Fgf2	Growth Factors
DMR2:124132001	2	124132001	124133000	1000	1	4.50E-10	0.4	3	0.3	Fgf2;Nudt6	Growth Factors;Signaling
DMR2:124196001	2	124196001	124198000	2000	1	1.40E-09	-0.37	18	0.9	Spata5;LOC108349998	
DMR2:124414001	2	124414001	124416000	2000	1	1.40E-08	-0.42	43	2.15	Spry1	Cytoskeleton
DMR2:125777001	2	125777001	125778000	1000	1	1.90E-10	-0.51	16	1.6	Fat4	Cytoskeleton
DMR2:125802001	2	125802001	125803000	1000	1	4.20E-08	0.32	4	0.4	Fat4	Cytoskeleton
DMR2:127505001	2	127505001	127506000	1000	1	9.10E-07	-0.36	4	0.4	Intu	
DMR2:127602001	2	127602001	127604000	2000	1	3.40E-08	0.47	13	0.65	Intu	
DMR2:127778001	2	127778001	127779000	1000	1	1.50E-08	0.87	25	2.5	Mfsd8;Abhd18;LOC365778	
DMR2:128011001	2	128011001	128015000	4000	1	2.10E-10	-0.47	49	1.23	Pgrmc2;LOC108350245	Receptor
DMR2:128454001	2	128454001	128455000	1000	1	1.50E-13	1.04	29	2.9	Jade1	Transcription
DMR2:128504001	2	128504001	128506000	2000	1	1.70E-07	-0.6	16	0.8	Jade1	Transcription
DMR2:128657001	2	128657001	128662000	5000	1	9.90E-07	-0.27	46	0.92	Sclt1	
DMR2:128686001	2	128686001	128690000	4000	2	7.20E-07	-0.29	48	1.2	RGD1359508	
DMR2:140362001	2	140362001	140367000	5000	1	4.40E-09	-0.49	77	1.54	Elf2	Transcription
DMR2:140530001	2	140530001	140532000	2000	1	1.70E-09	-0.55	20	1	Naa15;Rab33b	Metabolism
DMR2:140561001	2	140561001	140565000	4000	1	4.80E-07	-0.41	60	1.5	Rab33b	
DMR2:140888001	2	140888001	140889000	1000	1	8.30E-08	0.46	10	1	Maml3	
DMR2:140996001	2	140996001	1.41E+08	4000	1	3.00E-08	-0.48	85	2.12	Maml3	
DMR2:141024001	2	141024001	141025000	1000	1	3.90E-08	0.57	32	3.2	Maml3	
DMR2:141181001	2	141181001	141183000	2000	1	1.00E-08	0.43	18	0.9	Maml3	
DMR2:141224001	2	141224001	141226000	2000	1	2.20E-08	-0.47	28	1.4	Maml3	
DMR2:141270001	2	141270001	141272000	2000	1	5.70E-08	-0.4	41	2.05	Maml3	
DMR2:141459001	2	141459001	141462000	3000	1	1.40E-07	-0.59	22	0.73	LOC103691555;Foxo1	
DMR2:142325001	2	142325001	142326000	1000	1	9.50E-07	-0.39	26	2.6	Lhfp	
DMR2:142340001	2	142340001	142341000	1000	1	1.20E-08	-0.44	26	2.6	Lhfp	
DMR2:142424001	2	142424001	142426000	2000	1	2.70E-08	0.6	43	2.15	Lhfp	
DMR2:142702001	2	142702001	142704000	2000	1	6.10E-24	0.99	67	3.35	Proser1	
DMR2:142738001	2	142738001	142739000	1000	1	1.80E-08	0.65	26	2.6	Frem2	
DMR2:142789001	2	142789001	142794000	5000	1	1.40E-09	0.52	75	1.5	Frem2	
DMR2:142859001	2	142859001	142860000	1000	1	3.40E-07	-0.35	19	1.9	Frem2	
DMR2:142887001	2	142887001	142889000	2000	1	2.10E-07	0.35	12	0.6	Frem2	
DMR2:143482001	2	143482001	143486000	4000	1	3.80E-09	-0.33	33	0.82	Trpc4	Transport
DMR2:143566001	2	143566001	143567000	1000	1	6.80E-10	0.51	10	1	Trpc4	Transport
DMR2:143695001	2	143695001	143696000	1000	1	5.30E-08	0.48	6	0.6	Postn	Cytoskeleton
DMR2:143959001	2	143959001	143960000	1000	1	7.10E-07	0.43	3	0.3	Smad9	Transcription
DMR2:143975001	2	143975001	143976000	1000	1	2.10E-14	0.35	3	0.3	Smad9	Transcription
DMR2:144221001	2	144221001	144222000	1000	1	2.70E-09	0.53	3	0.3	LOC103691556;Rfxapl1	
DMR2:144293001	2	144293001	144295000	2000	1	5.40E-07	0.34	17	0.85	Sertm1	
DMR2:144525001	2	144525001	144527000	2000	1	2.90E-10	-0.6	15	0.75	Spg20	
DMR2:144566001	2	144566001	144567000	1000	1	1.30E-07	0.43	12	1.2	Ccdc169	
DMR2:144823001	2	144823001	144824000	1000	1	8.90E-10	0.57	21	2.1	Dclk1	Signaling
DMR2:144938001	2	144938001	144939000	1000	1	1.50E-11	-0.68	17	1.7	Dclk1	Signaling
DMR2:145098001	2	145098001	145106000	8000	1	1.90E-11	-0.32	91	1.14	Nbea	
DMR2:145310001	2	145310001	145316000	6000	1	2.20E-07	-0.28	50	0.83	Nbea	
DMR2:147472001	2	147472001	147473000	1000	1	1.60E-10	-0.57	3	0.3	RGD1563943	
DMR2:147642001	2	147642001	147643000	1000	1	1.90E-07	-0.48	11	1.1	Wwtr1	Transcription
DMR2:147793001	2	147793001	147797000	4000	1	7.30E-07	-0.44	39	0.98	LOC108350015;Ankub1	
DMR2:147928001	2	147928001	147929000	1000	1	9.40E-07	0.39	4	0.4	Rnf13	
DMR2:147966001	2	147966001	147973000	7000	1	6.80E-07	-0.22	60	0.86	Pfn2	Cytoskeleton
DMR2:148843001	2	148843001	148846000	3000	2	2.90E-08	-0.45	46	1.53	Erich6	
DMR2:148847001	2	148847001	148849000	2000	1	1.40E-10	0.4	14	0.7	Erich6	

DMR2:149203001	2	149203001	149204000	1000	1	8.00E-12	-0.43	9	0.9	Med12l	Transcription
DMR2:149221001	2	149221001	149222000	1000	1	3.80E-08	0.48	8	0.8	Med12l	Transcription
DMR2:149239001	2	149239001	149241000	2000	1	4.70E-07	-0.37	40	2	Med12l	Transcription
DMR2:149256001	2	149256001	149257000	1000	1	1.80E-09	0.43	12	1.2	Med12l	Transcription
DMR2:149347001	2	149347001	149349000	2000	2	1.60E-08	-0.53	33	1.65	P2ry14	Signaling
DMR2:149386001	2	149386001	149391000	5000	3	8.90E-11	0.49	23	0.46	P2ry14;Gpr87	Signaling
DMR2:149405001	2	149405001	149407000	2000	1	1.50E-07	-0.47	29	1.45	Gpr87	Signaling
DMR2:149745001	2	149745001	149749000	4000	1	1.30E-10	-0.52	21	0.52	RGD1559622;LOC103691561	Metabolism
DMR2:150715001	2	150715001	150721000	6000	1	9.00E-07	-0.31	66	1.1	Mbnl1	Translation
DMR2:153904001	2	153904001	153907000	3000	1	1.80E-08	0.46	42	1.4	LOC108350023;RGD1560703	
DMR2:154386001	2	154386001	154393000	7000	1	4.10E-12	-0.39	71	1.01	Plch1	Metabolism
DMR2:154417001	2	154417001	154420000	3000	1	2.40E-08	-0.75	31	1.03	Plch1	Metabolism
DMR2:154438001	2	154438001	154440000	2000	1	5.00E-07	-0.62	22	1.1	Plch1	Metabolism
DMR2:154516001	2	154516001	154517000	1000	1	9.00E-08	-0.57	7	0.7	RGD1565059;Slc33a1	Transport
DMR2:155358001	2	155358001	155360000	2000	1	5.30E-10	-0.51	5	0.25	Vom2r47	Signaling
DMR2:155567001	2	155567001	155569000	2000	2	1.10E-07	0.32	4	0.2	Kcnab1	
DMR2:155669001	2	155669001	155673000	4000	1	4.70E-07	-0.24	46	1.15	Kcnab1	
DMR2:155784001	2	155784001	155787000	3000	1	1.60E-11	0.67	12	0.4	Kcnab1	
DMR2:155947001	2	155947001	155954000	7000	1	1.30E-07	-0.41	74	1.06	Kcnab1	
DMR2:156028001	2	156028001	156034000	6000	2	1.00E-09	-0.42	42	0.7	Ssr3	
DMR2:157335001	2	157335001	157336000	1000	1	2.00E-07	-0.47	9	0.9	Tiparp;LOC108350031	
DMR2:157471001	2	157471001	157473000	2000	1	3.90E-07	-0.59	23	1.15	Lekr1	
DMR2:157544001	2	157544001	157548000	4000	1	3.80E-07	0.42	50	1.25	Lekr1	
DMR2:157561001	2	157561001	157563000	2000	1	2.70E-08	-0.54	18	0.9	Lekr1	
DMR2:157898001	2	157898001	157901000	3000	1	2.40E-09	0.59	34	1.13	Veph1	
DMR2:158154001	2	158154001	158157000	3000	1	3.60E-08	-0.57	32	1.07	Veph1	
DMR2:162411001	2	162411001	162420000	9000	5	1.20E-36	0.82	152	1.69	Olr1818-ps	
DMR2:162429001	2	162429001	162432000	3000	1	9.00E-07	0.4	19	0.63	Olr1818-ps	
DMR2:164170001	2	164170001	164172000	2000	1	1.50E-07	-0.39	5	0.25	Rsrc1	
DMR2:164266001	2	164266001	164267000	1000	1	1.10E-09	-0.48	6	0.6	Rsrc1	
DMR2:164276001	2	164276001	164277000	1000	1	1.10E-07	-0.53	5	0.5	Rsrc1	
DMR2:164283001	2	164283001	164285000	2000	2	2.70E-13	-0.54	18	0.9	Rsrc1	
DMR2:164349001	2	164349001	164350000	1000	1	4.70E-07	-0.39	4	0.4	Rsrc1	
DMR2:164378001	2	164378001	164382000	4000	1	5.00E-07	-0.25	33	0.82	Rsrc1	
DMR2:164459001	2	164459001	164461000	2000	1	6.20E-09	0.58	35	1.75	Rsrc1	
DMR2:164490001	2	164490001	164492000	2000	1	4.90E-08	-0.46	15	0.75	Rsrc1	
DMR2:164653001	2	164653001	164658000	5000	1	5.40E-07	-0.36	59	1.18	Gfm1;Rarres1	Protease; Proteolysis
DMR2:164679001	2	164679001	164682000	3000	1	2.90E-10	-0.63	65	2.17	Rarres1	Protease; Proteolysis
DMR2:164743001	2	164743001	164746000	3000	1	8.40E-09	-0.45	22	0.73	Mfsd1	
DMR2:165067001	2	165067001	165068000	1000	1	2.30E-10	0.45	22	2.2	Il12a	
DMR2:165085001	2	165085001	165087000	2000	1	3.30E-07	0.25	27	1.35	Il12a	
DMR2:165753001	2	165753001	165754000	1000	1	7.40E-09	0.44	22	2.2	Arl14	Signaling
DMR2:165803001	2	165803001	165805000	2000	1	3.10E-07	-0.54	15	0.75	RGD1563962	
DMR2:165806001	2	165806001	165807000	1000	1	7.10E-09	0.29	4	0.4	RGD1563962	
DMR2:165988001	2	165988001	165990000	2000	1	9.30E-12	-0.71	37	1.85	Ppm1l	Signaling
DMR2:165991001	2	165991001	165993000	2000	1	6.80E-07	-0.37	24	1.2	Ppm1l	Signaling
DMR2:166127001	2	166127001	166129000	2000	1	1.60E-09	-0.46	24	1.2	Ppm1l	Signaling
DMR2:166142001	2	166142001	166143000	1000	1	8.80E-08	0.41	29	2.9	Ppm1l	Signaling
DMR2:166399001	2	166399001	166401000	2000	1	9.60E-07	-0.34	34	1.7	Nmd3	
DMR2:170436001	2	170436001	170437000	1000	1	5.90E-09	0.51	7	0.7	Slitrk3	
DMR2:172378001	2	172378001	172379000	1000	1	8.00E-07	-0.39	13	1.3	Schip1	
DMR2:172537001	2	172537001	172539000	2000	1	1.80E-07	0.52	21	1.05	Schip1	
DMR2:172626001	2	172626001	172628000	2000	1	5.90E-12	-0.57	33	1.65	Schip1	
DMR2:172695001	2	172695001	172699000	4000	1	4.60E-07	0.51	17	0.42	Schip1	
DMR2:172839001	2	172839001	172842000	3000	1	1.70E-08	0.38	15	0.5	Schip1	
DMR2:172892001	2	172892001	172896000	4000	1	9.10E-07	-0.37	33	0.82	Schip1	
DMR2:172953001	2	172953001	172954000	1000	1	2.40E-10	0.39	4	0.4	Schip1	
DMR2:173059001	2	173059001	173063000	4000	1	9.20E-08	-0.51	28	0.7	Schip1	
DMR2:173443001	2	173443001	173447000	4000	1	1.10E-08	-0.49	22	0.55	LOC365821;Zbbx	
DMR2:173477001	2	173477001	173481000	4000	2	1.20E-10	-0.55	15	0.38	Zbbx	
DMR2:173717001	2	173717001	173719000	2000	1	7.50E-07	-0.5	7	0.35	Wdr49	
DMR2:173807001	2	173807001	173812000	5000	1	1.60E-07	-0.35	32	0.64	Wdr49	
DMR2:174373001	2	174373001	174379000	6000	1	2.20E-08	-0.28	66	1.1	Golim4	

DMR2:174544001	2	174544001	174545000	1000	1	5.30E-08	-0.6	7	0.7	Fstl5	Protease; Proteolysis
DMR2:174636001	2	174636001	174644000	8000	1	6.60E-07	-0.21	76	0.95	Fstl5	Protease; Proteolysis
DMR2:178342001	2	178342001	178345000	3000	1	2.70E-08	0.41	24	0.8	Fnip2;Ppid	Transcription
DMR2:178393001	2	178393001	178394000	1000	1	4.50E-08	-0.52	7	0.7	Etfdh;RGD1560010;Rxfp1	Metabolism;Signaling
DMR2:178406001	2	178406001	178411000	5000	1	2.10E-07	-0.37	76	1.52	Rxfp1	Signaling
DMR2:178474001	2	178474001	178478000	4000	1	2.40E-08	-0.43	23	0.58	Rxfp1	Signaling
DMR2:178683001	2	178683001	178684000	1000	1	7.20E-07	-0.5	10	1	Fam198b	
DMR2:178723001	2	178723001	178726000	3000	1	7.20E-07	-0.29	51	1.7	Fam198b	
DMR2:179635001	2	179635001	179637000	2000	1	1.20E-07	-0.37	12	0.6	Gria2	Receptor
DMR2:179711001	2	179711001	179712000	1000	1	7.00E-07	-0.67	2	0.2	Gria2	Receptor
DMR2:179714001	2	179714001	179720000	6000	1	2.80E-07	-0.28	60	1	Gria2	Receptor
DMR2:180954001	2	180954001	180955000	1000	1	1.90E-20	0.68	3	0.3	Asic5	Transport
DMR2:181345001	2	181345001	181346000	1000	1	1.20E-07	-0.39	19	1.9	Map9	
DMR2:182097001	2	182097001	182104000	7000	3	4.90E-11	-0.34	89	1.27	Dchs2	
DMR2:182135001	2	182135001	182137000	2000	1	3.50E-07	0.43	5	0.25	Dchs2	
DMR2:182154001	2	182154001	182158000	4000	1	1.30E-07	-0.4	52	1.3	Dchs2	
DMR2:182176001	2	182176001	182177000	1000	1	4.20E-10	0.44	6	0.6	Dchs2	
DMR2:182215001	2	182215001	182217000	2000	1	2.20E-12	0.42	29	1.45	Dchs2	
DMR2:182262001	2	182262001	182268000	6000	1	1.50E-08	-0.42	84	1.4	Dchs2	
DMR2:182738001	2	182738001	182740000	2000	1	7.80E-07	0.36	32	1.6	LOC108350198;Sfrp2	Receptor
DMR2:182831001	2	182831001	182836000	5000	1	8.10E-07	-0.25	44	0.88	LOC102551394;Tlr2	
DMR2:183064001	2	183064001	183065000	1000	1	6.00E-07	-0.37	12	1.2	LOC103691596;Mnd1	
DMR2:183133001	2	183133001	183134000	1000	1	1.50E-09	0.58	20	2	Mnd1	
DMR2:183439001	2	183439001	183444000	5000	1	8.40E-10	-0.3	41	0.82	Fhdc1	
DMR2:183461001	2	183461001	183465000	4000	1	7.60E-07	-0.32	80	2	Fhdc1	
DMR2:183531001	2	183531001	183533000	2000	1	1.40E-08	-0.37	22	1.1	Arfip1	Transport
DMR2:183589001	2	183589001	183595000	6000	2	2.00E-10	-0.41	67	1.12	Arfip1;Tigd4	Transport;Epigenetic
DMR2:183677001	2	183677001	183678000	1000	1	1.00E-07	-0.37	9	0.9	Tmem154;LOC108350047	
DMR2:183687001	2	183687001	183689000	2000	1	6.50E-08	-0.51	27	1.35	Tmem154;LOC108350047	
DMR2:184275001	2	184275001	184279000	4000	2	2.00E-10	-0.82	45	1.12	Fbxw7	Proteolysis
DMR2:184281001	2	184281001	184283000	2000	1	2.10E-07	-0.44	28	1.4	Fbxw7	Proteolysis
DMR2:184602001	2	184602001	184604000	2000	1	6.00E-07	-0.5	24	1.2	LOC108350050;Gatb	Metabolism
DMR2:184963001	2	184963001	184965000	2000	1	1.80E-07	-0.41	31	1.55	Fam160a1	
DMR2:185291001	2	185291001	185292000	1000	1	4.50E-08	-0.48	15	1.5	Sh3d19;Prss48	Protease
DMR2:185320001	2	185320001	185322000	2000	1	2.60E-08	-0.37	24	1.2	Sh3d19	
DMR2:185453001	2	185453001	185458000	5000	2	4.50E-09	-0.43	87	1.74	Rps3a	Translation
DMR2:185741001	2	185741001	185745000	4000	1	3.50E-10	-0.43	30	0.75	Lrba	
DMR2:185759001	2	185759001	185763000	4000	1	6.60E-10	-0.43	39	0.98	Lrba	
DMR2:185852001	2	185852001	185853000	1000	1	6.20E-10	0.64	27	2.7	Lrba;Mab21l2	
DMR2:185854001	2	185854001	185855000	1000	1	4.00E-07	0.63	19	1.9	Lrba;Mab21l2	
DMR2:186154001	2	186154001	186157000	3000	1	1.70E-07	0.33	49	1.63	Dclk2	Signaling
DMR2:186339001	2	186339001	186344000	5000	1	1.20E-08	-0.39	39	0.78	Cd1d1	
DMR2:186448001	2	186448001	186450000	2000	1	2.00E-07	0.61	34	1.7	Kirrel	
DMR2:186701001	2	186701001	186702000	1000	1	1.30E-08	0.48	7	0.7	Cd5l;Fcrl1	Immune
DMR2:187126001	2	187126001	187127000	1000	1	1.80E-09	0.44	16	1.6	Pear1	Signaling
DMR2:187207001	2	187207001	187210000	3000	1	3.30E-09	-0.4	22	0.73	Sh2d2a	Immune
DMR2:187531001	2	187531001	187533000	2000	1	3.00E-07	-0.39	21	1.05	Mef2d	Transcription
DMR2:187894001	2	187894001	187898000	4000	1	8.50E-09	-0.39	113	2.83	Mex3a;Rab25	Metabolism
DMR2:188245001	2	188245001	188246000	1000	1	1.30E-07	-0.4	10	1	Dap3;Ash1l	Translation;Epigenetic
DMR2:188284001	2	188284001	188285000	1000	1	8.50E-08	-0.42	17	1.7	Ash1l	Epigenetic
DMR2:188308001	2	188308001	188310000	2000	1	1.20E-12	-0.64	23	1.15	Ash1l	Epigenetic
DMR2:188451001	2	188451001	188452000	1000	1	8.50E-08	0.42	7	0.7	Pklr;Hcn3	Signaling;Transport
DMR2:188464001	2	188464001	188466000	2000	1	1.90E-07	0.45	49	2.45	Pklr;Hcn3	Signaling;Transport
DMR2:188493001	2	188493001	188494000	1000	1	1.00E-08	0.49	7	0.7	Clk2;Mir3541;Scamp3;Fam189b	Signaling
DMR2:188555001	2	188555001	188558000	3000	1	6.80E-08	0.35	37	1.23	Muc1;Trim46;Krtcap2	Cytoskeleton;Proteolysis
DMR2:188690001	2	188690001	188692000	2000	1	1.60E-08	0.4	32	1.6	Dcst1;Dcst2	
DMR2:188705001	2	188705001	188707000	2000	1	4.30E-11	0.95	64	3.2	Dcst2;Zbtb7b	
DMR2:188954001	2	188954001	188957000	3000	1	3.50E-07	0.4	37	1.23	Kcnn3	Transport
DMR2:189093001	2	189093001	189094000	1000	1	3.10E-07	0.5	40	4	Adar;Chrnb2	Metabolism;Ion Channel
DMR2:189194001	2	189194001	189197000	3000	1	7.90E-13	0.41	29	0.97	She;Il6r	Receptor
DMR2:189370001	2	189370001	189371000	1000	1	4.70E-08	-0.36	7	0.7	Atp8b2	Transport
DMR2:189434001	2	189434001	189439000	5000	1	2.60E-07	-0.58	70	1.4	Tpm3	Cytoskeleton
DMR2:189448001	2	189448001	189450000	2000	1	1.20E-13	-0.56	25	1.25	Tpm3;Nup210l	Cytoskeleton;Transport

DMR2:189485001	2	189485001	189486000	1000	1	3.40E-08	0.49	13	1.3	Nup210l	Transport
DMR2:189501001	2	189501001	189505000	4000	1	1.60E-07	-0.43	24	0.6	Nup210l	Transport
DMR2:189516001	2	189516001	189517000	1000	1	8.70E-07	-0.36	37	3.7	Nup210l	Transport
DMR2:189594001	2	189594001	189595000	1000	1	5.00E-10	0.44	2	0.2	Rab13;Jtb;Creb3l4	
DMR2:189705001	2	189705001	189706000	1000	1	1.70E-11	0.57	6	0.6	Gatad2b	Transcription
DMR2:189999001	2	189999001	1.90E+08	1000	1	7.70E-07	0.55	10	1	S100a3;S100a4;S100a5;S100a6	Signaling
DMR2:190181001	2	190181001	190182000	1000	1	3.00E-07	0.48	20	2	Pglyrp4	
DMR2:191930001	2	191930001	191931000	1000	1	8.60E-07	0.54	8	0.8	Pglyrp3;LOC365846	
DMR2:191943001	2	191943001	191946000	3000	1	7.80E-08	-0.43	18	0.6	Pglyrp3;LOC365846;LOC100911387	
DMR2:191989001	2	191989001	191991000	2000	1	2.70E-08	-0.52	15	0.75	Pglyrp3;Lor	
DMR2:192090001	2	192090001	192091000	1000	1	2.40E-07	0.45	11	1.1	Pglyrp3	
DMR2:193133001	2	193133001	193134000	1000	1	1.90E-17	1.04	42	4.2	Kprp	
DMR2:193572001	2	193572001	193574000	2000	2	5.20E-22	1.07	92	4.6	LOC108350062;Flg	
DMR2:194606001	2	194606001	194610000	4000	1	2.30E-09	-0.28	35	0.88	LOC689026;Tdpoz1	
DMR2:194873001	2	194873001	194875000	2000	1	6.80E-08	-0.43	18	0.9	RGD1566337	Proteolysis
DMR2:194923001	2	194923001	194930000	7000	1	1.60E-09	-0.5	45	0.64	RGD1562104;RGD1564386	
DMR2:195529001	2	195529001	195531000	2000	1	3.30E-09	-0.41	8	0.4	RGD1563667;RGD1560554	Proteolysis
DMR2:195563001	2	195563001	195564000	1000	1	9.00E-07	-0.38	13	1.3	RGD1563667;RGD1560554;Them4	Proteolysis;Metabolism
DMR2:195623001	2	195623001	195624000	1000	1	1.00E-07	0.48	11	1.1	Rorc;Lingo4	Transcription;Receptor
DMR2:195803001	2	195803001	195805000	2000	1	3.70E-10	-0.44	52	2.6	Snx27	Cytoskeleton
DMR2:195842001	2	195842001	195848000	6000	2	1.20E-09	-0.52	84	1.4	RGD1560263	
DMR2:195914001	2	195914001	195916000	2000	1	8.40E-09	0.47	36	1.8	Tuft1;Cgn	
DMR2:196047001	2	196047001	196048000	1000	1	3.90E-07	0.41	7	0.7	Pogz;Psmb4;LOC108350065	Transcription;Protease
DMR2:196293001	2	196293001	196294000	1000	1	8.00E-09	0.74	33	3.3	Vps72;Tmod4;Scnm1	Transcription;Cytoskeleton;Transport
DMR2:196319001	2	196319001	196322000	3000	1	2.60E-09	0.34	18	0.6	Lysmd1;Tnfaip8l2;Sema6c	Signaling
DMR2:196488001	2	196488001	196490000	2000	1	2.50E-07	-0.38	61	3.05	Anxa9;Cers2;Setdb1	Signaling;Epigenetic
DMR2:196959001	2	196959001	196960000	1000	1	6.60E-07	0.33	13	1.3	RGD1566258	
DMR2:197658001	2	197658001	197662000	4000	1	4.50E-07	-0.47	62	1.55	Ctss	Protease
DMR2:197665001	2	197665001	197669000	4000	1	5.00E-08	0.45	58	1.45	Ctss	Protease
DMR2:197701001	2	197701001	197704000	3000	1	5.40E-07	-0.32	18	0.6	Hormad1;LOC102547423	
DMR2:197856001	2	197856001	197859000	3000	1	8.80E-08	0.5	86	2.87	Ecm1;Tars2	Translation
DMR2:198564001	2	198564001	198565000	1000	1	3.20E-07	0.58	25	2.5	Trnag-ccc;Trnak-cuu	
DMR2:198590001	2	198590001	198592000	2000	1	1.20E-25	1.6	35	1.75	LOC103691644;LOC100360229;Trnah-gug;Trnan-guu;LOC108350070	
DMR2:198660001	2	198660001	198663000	3000	1	6.20E-08	0.35	32	1.07	Hfe2	
DMR2:198688001	2	198688001	198689000	1000	1	7.20E-07	-0.47	22	2.2	LOC103691645;Txnip;Polr3gl	Transcription
DMR2:198924001	2	198924001	198928000	4000	1	3.10E-10	-0.36	30	0.75	Rnf115;Cd160	Proteolysis
DMR2:198940001	2	198940001	198941000	1000	1	4.30E-11	-0.59	22	2.2	Cd160	
DMR2:198947001	2	198947001	198951000	4000	1	1.50E-09	0.53	69	1.73	Cd160	
DMR2:198998001	2	198998001	1.99E+08	2000	1	2.30E-08	-0.37	44	2.2	Pdzk1;Gpr89b	Signaling
DMR2:199193001	2	199193001	199195000	2000	1	8.90E-07	0.33	27	1.35	Gja5;LOC100909441	Cytoskeleton;Cytoskeleton
DMR2:199362001	2	199362001	199366000	4000	1	8.30E-07	-0.36	82	2.05	Bcl9	
DMR2:199413001	2	199413001	199414000	1000	1	2.10E-11	0.87	30	3	Bcl9;LOC102549654	
DMR2:199429001	2	199429001	199430000	1000	1	4.20E-09	0.42	3	0.3	Bcl9;LOC102549654;LOC102550046	
DMR2:199705001	2	199705001	199711000	6000	1	7.60E-08	-0.26	53	0.88	Chd1l	
DMR2:199781001	2	199781001	199786000	5000	2	3.40E-08	-0.36	36	0.72	Chd1l	
DMR2:199802001	2	199802001	199803000	1000	1	1.40E-10	0.5	13	1.3	Chd1l;Fmo5	Metabolism
DMR2:199820001	2	199820001	199823000	3000	1	1.10E-09	0.66	35	1.17	Fmo5;Prkab2	Metabolism;Signaling
DMR2:199953001	2	199953001	199954000	1000	1	1.80E-07	-0.55	16	1.6	Pde4dip	
DMR2:200184001	2	200184001	200186000	2000	1	1.30E-07	-0.43	20	1	Notch2	Extracellular Matrix
DMR2:200196001	2	200196001	200197000	1000	1	4.00E-07	-0.57	17	1.7	Notch2	Extracellular Matrix
DMR2:200324001	2	200324001	200325000	1000	1	3.90E-08	0.38	10	1	Notch2;Adam30;LOC100361630	Extracellular Matrix;Protease
DMR2:200541001	2	200541001	200543000	2000	1	6.60E-07	-0.41	18	0.9	Zfp697	Transcription
DMR2:200747001	2	200747001	200749000	2000	1	4.40E-08	-0.48	5	0.25	Hsd3b3	
DMR2:200784001	2	200784001	200787000	3000	1	1.10E-07	-0.5	23	0.77	Hao2	Metabolism
DMR2:201186001	2	201186001	201188000	2000	1	8.90E-07	0.41	17	0.85	Wars2	Translation
DMR2:202178001	2	202178001	202181000	3000	1	5.50E-07	-0.26	32	1.07	Spag17	
DMR2:202281001	2	202281001	202282000	1000	1	2.00E-12	0.86	30	3	Spag17	
DMR2:202534001	2	202534001	202535000	1000	1	1.10E-08	-0.61	1	0.1	Gdap2	

DMR2:203030001	2	203030001	203031000	1000	1	5.70E-08	-0.58	9	0.9	Man1a2;LOC683192	Golgi
DMR2:203040001	2	203040001	203042000	2000	1	1.30E-07	-0.5	13	0.65	Man1a2;LOC683192	Golgi
DMR2:203231001	2	203231001	203233000	2000	1	4.30E-09	0.53	44	2.2	Vtcn1	Immune
DMR2:203242001	2	203242001	203243000	1000	1	6.60E-10	0.49	11	1.1	Vtcn1	Immune
DMR2:203398001	2	203398001	203399000	1000	1	7.00E-07	-0.43	14	1.4	Cd101	Immune
DMR2:203447001	2	203447001	203449000	2000	1	3.30E-08	-0.43	33	1.65	Ptgfrn;LOC108350075	Immune
DMR2:203450001	2	203450001	203453000	3000	1	4.80E-08	0.35	28	0.93	Ptgfrn;LOC108350075	Immune
DMR2:203471001	2	203471001	203473000	2000	1	8.30E-07	0.47	43	2.15	Ptgfrn;LOC108350075	Immune
DMR2:203687001	2	203687001	203688000	1000	1	1.20E-07	0.44	16	1.6	Cd2	Immune
DMR2:203999001	2	203999001	204003000	4000	1	1.50E-08	-0.59	71	1.77	Atp1a1	Transport
DMR2:204008001	2	204008001	204011000	3000	1	8.40E-07	-0.34	46	1.53	Atp1a1	Transport
DMR2:204239001	2	204239001	204242000	3000	1	1.30E-09	0.74	26	0.87	Mab21l3	
DMR2:204272001	2	204272001	204273000	1000	1	1.10E-09	0.56	9	0.9	Mab21l3;Slc22a15	Transport
DMR2:205174001	2	205174001	205176000	2000	1	1.80E-07	0.31	15	0.75	LOC108350076;Tspan2	
DMR2:205196001	2	205196001	205200000	4000	1	3.80E-09	-0.66	34	0.85	Tspan2;Tshb	Hormone
DMR2:205296001	2	205296001	205298000	2000	1	3.30E-08	-0.38	13	0.65	Sycp1	
DMR2:205342001	2	205342001	205347000	5000	1	8.70E-07	-0.4	42	0.84	Sycp1	
DMR2:205348001	2	205348001	205359000	11000	2	1.70E-09	-0.41	110	1	Sycp1	
DMR2:205382001	2	205382001	205383000	1000	1	9.50E-09	-0.65	6	0.6	Sycp1	
DMR2:205384001	2	205384001	205387000	3000	1	1.60E-07	-0.42	20	0.67	Sycp1	
DMR2:205394001	2	205394001	205398000	4000	1	3.30E-08	-0.41	31	0.78	Sycp1	
DMR2:205482001	2	205482001	205483000	1000	1	4.80E-08	0.35	8	0.8	LOC108350203;Sike1	
DMR2:205582001	2	205582001	205584000	2000	1	3.50E-07	0.38	39	1.95	Ampd1;Dennd2c	Metabolism
DMR2:205670001	2	205670001	205671000	1000	1	3.60E-07	-0.37	20	2	Dennd2c;Bcas2	Translation
DMR2:206113001	2	206113001	206115000	2000	1	1.70E-07	-0.35	31	1.55	Syt6	Transport
DMR2:206367001	2	206367001	206368000	1000	1	4.20E-08	0.51	5	0.5	Ptpn22	
DMR2:206477001	2	206477001	206480000	3000	1	8.30E-07	-0.35	37	1.23	Phtf1	Development
DMR2:206505001	2	206505001	206506000	1000	1	6.20E-08	-0.44	10	1	Phtf1;Magi3	Development
DMR2:206581001	2	206581001	206582000	1000	1	1.00E-07	-0.6	3	0.3	Magi3	
DMR2:206626001	2	206626001	206629000	3000	2	5.70E-08	-0.49	45	1.5	Magi3	
DMR2:206900001	2	206900001	206903000	3000	1	3.70E-08	0.58	26	0.87	LOC103691658;RGD1564469	
DMR2:207297001	2	207297001	207298000	1000	1	7.60E-07	0.47	0	0	Mov10;Capza1	Cytoskeleton
DMR2:207361001	2	207361001	207362000	1000	1	4.70E-07	0.42	2	0.2	St7l	
DMR2:207388001	2	207388001	207393000	5000	1	1.20E-08	-0.28	43	0.86	St7l	
DMR2:207489001	2	207489001	207491000	2000	1	1.70E-09	-0.58	26	1.3	Cttnbp2nl	
DMR2:207933001	2	207933001	207934000	1000	1	8.60E-07	0.41	5	0.5	Kcnd3	Transport
DMR2:208183001	2	208183001	208184000	1000	1	3.80E-07	0.45	21	2.1	Fam212b;Rap1a	Signaling
DMR2:208754001	2	208754001	208758000	4000	1	1.40E-07	-0.35	36	0.9	Chia	Metabolism
DMR2:208760001	2	208760001	208765000	5000	1	1.50E-08	-0.3	43	0.86	Chia	Metabolism
DMR2:208788001	2	208788001	208796000	8000	1	5.20E-10	-0.38	103	1.29	Chia	Metabolism
DMR2:209761001	2	209761001	209764000	3000	1	6.00E-08	-0.37	23	0.77	LOC108350087;Kcna3;LOC103691668	Transport
DMR2:209930001	2	209930001	209932000	2000	1	8.40E-07	0.49	27	1.35	Kcna10;LOC102557194	Transport
DMR2:209950001	2	209950001	209955000	5000	1	2.80E-08	-0.25	43	0.86	LOC102557194;Cym	
DMR2:209959001	2	209959001	209960000	1000	1	2.70E-07	0.31	6	0.6	LOC102557194;Cym	
DMR2:210004001	2	210004001	210006000	2000	1	1.30E-09	0.43	13	0.65	Prok1;LOC103691669	Hormone
DMR2:210665001	2	210665001	210666000	1000	1	2.30E-07	0.33	8	0.8	Eps8l3	Cytoskeleton
DMR2:210790001	2	210790001	210792000	2000	1	2.10E-07	-0.5	22	1.1	Gstm2	Transport
DMR2:210917001	2	210917001	210918000	1000	1	5.00E-10	-0.71	3	0.3	Gnai3	Signaling
DMR2:210945001	2	210945001	210946000	1000	1	1.60E-07	-0.55	10	1	LOC108350090;Gpr61	Signaling
DMR2:211053001	2	211053001	211059000	6000	1	3.40E-07	-0.39	56	0.93	Psma5	Protease
DMR2:211297001	2	211297001	211301000	4000	1	1.90E-09	-0.5	60	1.5	RGD1310209	
DMR2:211313001	2	211313001	211315000	2000	1	7.10E-08	-0.42	29	1.45	RGD1310209;RGD1309139	
DMR2:211326001	2	211326001	211327000	1000	1	6.30E-07	-0.4	19	1.9	RGD1310209;RGD1309139	
DMR2:211401001	2	211401001	211403000	2000	1	1.10E-07	-0.29	14	0.7	Wdr47	
DMR2:211483001	2	211483001	211493000	10000	3	4.50E-10	-0.43	133	1.33	Clcc1;Gpsm2	Transport
DMR2:211498001	2	211498001	211501000	3000	1	2.30E-10	-0.73	31	1.03	Gpsm2	
DMR2:211505001	2	211505001	211508000	3000	1	1.00E-07	-0.55	51	1.7	Gpsm2	
DMR2:211635001	2	211635001	211637000	2000	1	5.70E-08	-0.34	23	1.15	Stxbp3	Transport
DMR2:211708001	2	211708001	211709000	1000	1	4.20E-08	-0.4	22	2.2	Prpf38b	
DMR2:211787001	2	211787001	211788000	1000	1	3.90E-07	0.4	14	1.4	Fam102b	
DMR2:211939001	2	211939001	211940000	1000	1	2.50E-10	0.37	12	1.2	Slc25a24	Transport
DMR2:211944001	2	211944001	211946000	2000	1	8.90E-09	0.32	16	0.8	Slc25a24	Transport

DMR2:211960001	2	211960001	211963000	3000	1	8.40E-10	-0.52	34	1.13	Slc25a24	Transport
DMR2:211973001	2	211973001	211975000	2000	1	1.70E-08	0.48	29	1.45	Slc25a24	Transport
DMR2:212286001	2	212286001	212288000	2000	1	9.60E-09	-0.48	32	1.6	Vav3	
DMR2:212305001	2	212305001	212307000	2000	1	8.10E-07	-0.52	27	1.35	Vav3	
DMR2:212359001	2	212359001	212365000	6000	3	9.10E-09	-0.38	66	1.1	Vav3	
DMR2:212439001	2	212439001	212443000	4000	1	1.10E-08	-0.39	33	0.82	Vav3;Mrps17-ps1	
DMR2:212450001	2	212450001	212452000	2000	1	1.40E-07	-0.43	19	0.95	Vav3	
DMR2:212481001	2	212481001	212486000	5000	1	5.50E-08	-0.52	58	1.16	Vav3	
DMR2:212547001	2	212547001	212548000	1000	1	5.30E-09	-0.57	11	1.1	Vav3	
DMR2:213035001	2	213035001	213036000	1000	1	6.90E-08	0.49	12	1.2	Ntng1	Extracellular Matrix
DMR2:213165001	2	213165001	213166000	1000	1	1.10E-10	0.73	19	1.9	Prmt6	Golgi
DMR2:216447001	2	216447001	216449000	2000	1	9.10E-11	0.55	10	0.5	Amy1a	Metabolism
DMR2:217910001	2	217910001	217916000	6000	1	7.90E-07	0.3	47	0.78	Olfm3	Development
DMR2:218668001	2	218668001	218671000	3000	1	4.20E-07	-0.43	23	0.77	S1pr1	Signaling
DMR2:218901001	2	218901001	218903000	2000	1	7.30E-07	-0.51	38	1.9	Slc30a7	
DMR2:219332001	2	219332001	219335000	3000	1	7.90E-07	-0.38	41	1.37	Cdc14a	Signaling
DMR2:219407001	2	219407001	219409000	2000	1	2.60E-08	-0.57	26	1.3	Cdc14a	Signaling
DMR2:219883001	2	219883001	219889000	6000	2	4.00E-07	-0.32	45	0.75	Plppr5	Signaling
DMR2:219970001	2	219970001	219972000	2000	1	2.70E-07	-0.4	18	0.9	Plppr5	Signaling
DMR2:220031001	2	220031001	220034000	3000	1	7.20E-07	-0.33	27	0.9	Plppr5;LOC108348210	Signaling
DMR2:220316001	2	220316001	220317000	1000	1	1.30E-08	-0.64	12	1.2	Plppr4	Signaling
DMR2:220430001	2	220430001	220431000	1000	1	2.00E-09	0.49	16	1.6	Frrs1	Metabolism
DMR2:220437001	2	220437001	220438000	1000	1	4.10E-07	0.48	16	1.6	Frrs1	Metabolism
DMR2:221083001	2	221083001	221084000	1000	1	6.30E-08	0.57	23	2.3	Snx7	Cytoskeleton
DMR2:221869001	2	221869001	221874000	5000	2	5.30E-11	-0.38	66	1.32	Dpyd	Metabolism
DMR2:222226001	2	222226001	222228000	2000	1	1.80E-10	0.49	19	0.95	Dpyd	Metabolism
DMR2:222385001	2	222385001	222391000	6000	1	1.40E-08	-0.26	74	1.23	Dpyd	Metabolism
DMR2:222535001	2	222535001	222537000	2000	1	3.10E-12	0.6	23	1.15	Dpyd	Metabolism
DMR2:222667001	2	222667001	222673000	6000	2	2.20E-10	-0.38	64	1.07	Dpyd	Metabolism
DMR2:222699001	2	222699001	222701000	2000	1	1.00E-08	0.53	12	0.6	Dpyd	Metabolism
DMR2:223253001	2	223253001	223256000	3000	1	3.20E-07	-0.49	24	0.8	LOC103691683;Ptbp2	
DMR2:223328001	2	223328001	223332000	4000	2	3.00E-13	-0.57	39	0.98	Ptbp2	
DMR2:224847001	2	224847001	224848000	1000	1	6.90E-09	0.85	32	3.2	Tmem56;Alg14	Transport
DMR2:224897001	2	224897001	224898000	1000	1	2.80E-07	-0.5	22	2.2	Alg14	Transport
DMR2:225317001	2	225317001	225319000	2000	1	6.90E-10	-0.61	35	1.75	LOC102555482;F3	Receptor
DMR2:225385001	2	225385001	225387000	2000	1	3.30E-08	-0.38	26	1.3	Abcd3;LOC108350099	Transport
DMR2:225590001	2	225590001	225591000	1000	1	5.40E-07	-0.42	16	1.6	Arhgap29	
DMR2:225640001	2	225640001	225642000	2000	2	5.80E-09	0.54	15	0.75	Abca4	Transport
DMR2:225712001	2	225712001	225714000	2000	1	5.70E-08	0.42	37	1.85	Abca4	Transport
DMR2:225715001	2	225715001	225716000	1000	1	4.50E-09	0.55	21	2.1	Abca4	Transport
DMR2:225778001	2	225778001	225780000	2000	1	7.20E-09	0.47	22	1.1	Abca4	Transport
DMR2:225783001	2	225783001	225788000	5000	1	7.30E-11	0.62	61	1.22	Abca4	Transport
DMR2:226643001	2	226643001	226645000	2000	1	5.60E-07	-0.34	36	1.8	Bcar3;LOC108350102	
DMR2:226704001	2	226704001	226706000	2000	1	5.90E-08	-0.61	15	0.75	Fnbp1l	
DMR2:226934001	2	226934001	226935000	1000	1	8.40E-09	-0.41	9	0.9	Pde5a	Signaling
DMR2:227013001	2	227013001	227014000	1000	1	3.50E-08	-0.63	19	1.9	Pde5a	Signaling
DMR2:227021001	2	227021001	227024000	3000	1	3.40E-09	-0.44	37	1.23	Pde5a	Signaling
DMR2:227041001	2	227041001	227044000	3000	1	2.20E-08	-0.48	64	2.13	Pde5a	Signaling
DMR2:227112001	2	227112001	227114000	2000	1	2.90E-09	-0.47	17	0.85	Usp53	Protease
DMR2:227119001	2	227119001	227122000	3000	1	4.40E-10	-0.41	25	0.83	Usp53	Protease
DMR2:227248001	2	227248001	227251000	3000	2	4.90E-08	0.54	42	1.4	Synpo2	Cytoskeleton
DMR2:227268001	2	227268001	227271000	3000	1	2.40E-09	0.64	50	1.67	Synpo2	Cytoskeleton
DMR2:227294001	2	227294001	227296000	2000	2	3.00E-12	0.91	55	2.75	Synpo2	Cytoskeleton
DMR2:227312001	2	227312001	227316000	4000	1	1.30E-07	-0.63	50	1.25	Synpo2	Cytoskeleton
DMR2:227355001	2	227355001	227356000	1000	1	1.40E-07	0.31	10	1	Synpo2	Cytoskeleton
DMR2:227370001	2	227370001	227374000	4000	1	8.70E-09	-0.39	49	1.23	Synpo2	Cytoskeleton
DMR2:227463001	2	227463001	227464000	1000	1	3.80E-07	0.38	5	0.5	Sec24d	Transport
DMR2:227533001	2	227533001	227534000	1000	1	2.30E-08	0.43	4	0.4	Sec24d	Transport
DMR2:227798001	2	227798001	227801000	3000	1	2.30E-07	-0.49	26	0.87	Ndst3	Transport
DMR2:227842001	2	227842001	227845000	3000	1	8.30E-07	-0.51	28	0.93	Ndst3	Transport
DMR2:227847001	2	227847001	227849000	2000	1	8.30E-11	-0.45	40	2	Ndst3	Transport
DMR2:229306001	2	229306001	229308000	2000	1	3.20E-07	-0.43	14	0.7	Ndst4	Transport
DMR2:229362001	2	229362001	229367000	5000	1	3.20E-07	-0.37	53	1.06	Ndst4	Transport

DMR2:229474001	2	229474001	229475000	1000	1	1.00E-10	0.79	16	1.6	Ndst4	Transport
DMR2:230204001	2	230204001	230206000	2000	1	4.60E-07	-0.45	22	1.1	Arsj;LOC103689977;Mcub	
DMR2:230398001	2	230398001	230400000	2000	2	1.40E-12	0.75	38	1.9	Arsj	
DMR2:230423001	2	230423001	230424000	1000	1	1.50E-07	-0.5	34	3.4	Arsj	
DMR2:230442001	2	230442001	230445000	3000	1	1.20E-08	0.52	18	0.6	Arsj	
DMR2:230482001	2	230482001	230484000	2000	1	4.80E-10	-0.56	40	2	Arsj;LOC102553088	
DMR2:230648001	2	230648001	230651000	3000	1	1.70E-10	0.49	34	1.13	Arsj	
DMR2:231232001	2	231232001	231234000	2000	1	1.40E-07	-0.47	36	1.8	Ank2	
DMR2:231249001	2	231249001	231251000	2000	1	9.70E-14	-0.51	51	2.55	Ank2	
DMR2:231300001	2	231300001	231301000	1000	1	2.30E-07	-0.47	27	2.7	Ank2	
DMR2:231388001	2	231388001	231390000	2000	1	6.90E-07	0.43	14	0.7	Ank2	
DMR2:231411001	2	231411001	231412000	1000	1	1.40E-10	-0.51	21	2.1	Ank2	
DMR2:231416001	2	231416001	231419000	3000	1	6.90E-08	-0.51	51	1.7	Ank2	
DMR2:232008001	2	232008001	232010000	2000	1	8.70E-08	0.42	38	1.9	Alpk1	Signaling
DMR2:232017001	2	232017001	232018000	1000	1	5.80E-08	0.38	9	0.9	Alpk1	Signaling
DMR2:232056001	2	232056001	232062000	6000	2	8.30E-08	0.55	138	2.3	Alpk1	Signaling
DMR2:232111001	2	232111001	232114000	3000	2	1.10E-08	0.4	33	1.1	Alpk1;Tifa	Signaling
DMR2:234202001	2	234202001	234204000	2000	1	4.80E-08	-0.58	35	1.75	Elovl6	Metabolism
DMR2:234205001	2	234205001	234207000	2000	1	2.90E-07	-0.41	38	1.9	Elovl6	Metabolism
DMR2:234240001	2	234240001	234242000	2000	1	1.50E-08	-0.54	35	1.75	Elovl6	Metabolism
DMR2:235245001	2	235245001	235249000	4000	1	9.10E-07	-0.34	45	1.12	Rrh;Gar1	Signaling;Metabolism
DMR2:235332001	2	235332001	235337000	5000	1	1.40E-07	-0.36	91	1.82	Pla2g12a;Casp6	Metabolism;Protease
DMR2:235582001	2	235582001	235584000	2000	1	4.50E-07	-0.61	28	1.4	Col25a1	Extracellular Matrix
DMR2:235638001	2	235638001	235641000	3000	1	5.30E-09	-0.54	72	2.4	Col25a1	Extracellular Matrix
DMR2:235692001	2	235692001	235694000	2000	1	8.10E-10	0.43	31	1.55	Col25a1	Extracellular Matrix
DMR2:236253001	2	236253001	236256000	3000	1	7.40E-09	0.68	59	1.97	Lef1;LOC103691708	Transcription
DMR2:236523001	2	236523001	236529000	6000	1	9.50E-07	-0.51	111	1.85	Sgms2;LOC108350109;LOC108350110	
DMR2:237230001	2	237230001	237232000	2000	1	2.60E-10	0.54	14	0.7	Dkk2	
DMR2:237696001	2	237696001	237697000	1000	1	9.90E-07	0.44	6	0.6	Gimd1	Signaling
DMR2:237818001	2	237818001	237824000	6000	2	1.20E-08	0.3	59	0.98	Tbck	Signaling
DMR2:237858001	2	237858001	237864000	6000	1	5.90E-07	-0.3	67	1.12	Tbck	Signaling
DMR2:237964001	2	237964001	237967000	3000	1	7.90E-08	-0.31	40	1.33	Tbck;LOC680335;LOC102556143	Signaling
DMR2:238029001	2	238029001	238031000	2000	1	2.10E-09	0.39	39	1.95	Npnt	
DMR2:238047001	2	238047001	238048000	1000	1	3.30E-07	-0.38	23	2.3	Npnt	
DMR2:238107001	2	238107001	238108000	1000	1	8.20E-08	0.54	24	2.4	LOC102548236;Gstcd	
DMR2:238205001	2	238205001	238208000	3000	1	3.60E-09	-0.48	28	0.93	Gstcd	
DMR2:238232001	2	238232001	238234000	2000	1	6.10E-08	-0.43	29	1.45	Gstcd	
DMR2:238240001	2	238240001	238242000	2000	2	2.50E-10	-0.59	33	1.65	Gstcd	
DMR2:238274001	2	238274001	238277000	3000	1	6.20E-08	-0.43	51	1.7	Ints12;Arhgef38	
DMR2:238537001	2	238537001	238541000	4000	1	8.70E-10	0.32	80	2	LOC102546359;Ppa2	Signaling
DMR2:238542001	2	238542001	238544000	2000	1	8.60E-07	-0.45	45	2.25	Ppa2	Signaling
DMR2:238570001	2	238570001	238572000	2000	1	8.20E-09	0.75	33	1.65	Ppa2	Signaling
DMR2:238584001	2	238584001	238585000	1000	1	7.80E-13	0.7	20	2	Ppa2	Signaling
DMR2:238587001	2	238587001	238591000	4000	1	6.80E-16	-0.68	66	1.65	Ppa2	Signaling
DMR2:238711001	2	238711001	238712000	1000	1	6.30E-13	0.83	31	3.1	Tet2	
DMR2:238776001	2	238776001	238777000	1000	1	1.90E-07	0.4	9	0.9	Tet2	
DMR2:238804001	2	238804001	238805000	1000	1	4.20E-07	0.51	21	2.1	Tet2;LOC108350114	
DMR2:240041001	2	240041001	240043000	2000	1	6.20E-07	-0.49	17	0.85	Tacr3	Signaling
DMR2:240077001	2	240077001	240080000	3000	1	1.20E-14	0.53	18	0.6	Tacr3	Signaling
DMR2:240121001	2	240121001	240122000	1000	1	5.60E-09	-0.43	17	1.7	Tacr3	Signaling
DMR2:240517001	2	240517001	240519000	2000	1	3.80E-09	0.31	31	1.55	Slc9b2;Slc9b1	
DMR2:240538001	2	240538001	240540000	2000	1	1.60E-14	-0.56	24	1.2	Slc9b1	
DMR2:240592001	2	240592001	240596000	4000	3	6.80E-10	-0.41	36	0.9	Cisd2	
DMR2:240695001	2	240695001	240696000	1000	1	8.20E-07	0.44	25	2.5	Manba	Golgi
DMR2:240712001	2	240712001	240718000	6000	1	1.20E-08	-0.34	59	0.98	Manba	Golgi
DMR2:240824001	2	240824001	240830000	6000	1	2.80E-10	-0.6	131	2.18	Nfkb1	Transcription
DMR2:240882001	2	240882001	240884000	2000	1	8.30E-09	-0.46	34	1.7	Nfkb1	Transcription
DMR2:240886001	2	240886001	240888000	2000	1	4.80E-08	-0.42	30	1.5	Nfkb1	Transcription
DMR2:240891001	2	240891001	240893000	2000	1	1.20E-07	0.38	17	0.85	Nfkb1	Transcription
DMR2:241047001	2	241047001	241052000	5000	1	2.90E-08	0.34	85	1.7	Slc39a8	Transport
DMR2:241393001	2	241393001	241394000	1000	1	3.40E-07	0.29	9	0.9	Bank1	
DMR2:241914001	2	241914001	241917000	3000	1	2.90E-07	-0.41	48	1.6	Ppp3ca	Signaling

DMR2:242006001	2	242006001	242008000	2000	1	4.10E-09	-0.42	14	0.7	Ppp3ca	Signaling
DMR2:242170001	2	242170001	242175000	5000	1	6.80E-07	0.38	66	1.32	Ppp3ca	Signaling
DMR2:243133001	2	243133001	243135000	2000	1	6.00E-07	-0.54	17	0.85	Dnajb14	
DMR2:243364001	2	243364001	243368000	4000	1	6.10E-08	-0.54	57	1.43	Mttp	Transport
DMR2:243385001	2	243385001	243386000	1000	1	2.10E-08	-0.4	15	1.5	Mttp	Transport
DMR2:243405001	2	243405001	243407000	2000	1	3.50E-07	0.35	22	1.1	Mttp	Transport
DMR2:243490001	2	243490001	243491000	1000	1	8.30E-07	0.33	12	1.2	LOC681022;Adh7	Metabolism
DMR2:243582001	2	243582001	243587000	5000	2	6.20E-07	-0.28	42	0.84	LOC102556144;Adh6a	Metabolism
DMR2:243589001	2	243589001	243590000	1000	1	2.90E-07	0.41	10	1	Adh6a	Metabolism
DMR2:243600001	2	243600001	243601000	1000	1	2.20E-07	0.42	10	1	Adh6a	Metabolism
DMR2:243809001	2	243809001	243810000	1000	1	1.10E-11	-0.48	19	1.9	LOC102556448;Eif4e;Mir1956	Translation
DMR2:244083001	2	244083001	244085000	2000	1	4.10E-07	-0.35	49	2.45	Tspan5	
DMR2:244119001	2	244119001	244120000	1000	1	1.40E-08	-0.45	15	1.5	Tspan5	
DMR2:244125001	2	244125001	244128000	3000	1	6.90E-08	-0.44	48	1.6	Tspan5	
DMR2:244160001	2	244160001	244164000	4000	1	2.60E-08	-0.62	59	1.48	Tspan5	
DMR2:244315001	2	244315001	244319000	4000	1	6.00E-09	-0.46	44	1.1	Rap1gds1	
DMR2:244354001	2	244354001	244356000	2000	1	6.90E-07	-0.36	24	1.2	Rap1gds1	
DMR2:244546001	2	244546001	244550000	4000	1	5.50E-07	-0.28	35	0.88	Stpg2	Development
DMR2:244551001	2	244551001	244554000	3000	1	1.10E-08	-0.48	27	0.9	Stpg2	Development
DMR2:244567001	2	244567001	244570000	3000	1	9.00E-09	-0.44	16	0.53	Stpg2	Development
DMR2:244671001	2	244671001	244673000	2000	1	5.60E-09	-0.46	18	0.9	Stpg2	Development
DMR2:244737001	2	244737001	244739000	2000	1	5.70E-08	-0.49	15	0.75	Stpg2	Development
DMR2:244794001	2	244794001	244796000	2000	1	3.30E-08	0.36	12	0.6	Stpg2	Development
DMR2:244829001	2	244829001	244831000	2000	1	2.70E-09	0.34	22	1.1	Stpg2;LOC100910816	Development
DMR2:244849001	2	244849001	244854000	5000	2	1.40E-08	-0.36	47	0.94	Stpg2	Development
DMR2:244855001	2	244855001	244860000	5000	1	5.60E-07	-0.45	51	1.02	Stpg2	Development
DMR2:244890001	2	244890001	244891000	1000	1	9.20E-10	0.52	16	1.6	Stpg2	Development
DMR2:244936001	2	244936001	244937000	1000	1	5.30E-07	0.37	14	1.4	Stpg2	Development
DMR2:247097001	2	247097001	247098000	1000	1	2.10E-08	-0.6	13	1.3	Unc5c	Receptor
DMR2:247205001	2	247205001	247206000	1000	1	7.00E-07	0.38	2	0.2	Unc5c	Receptor
DMR2:247272001	2	247272001	247276000	4000	1	7.20E-08	-0.43	36	0.9	Unc5c	Receptor
DMR2:247330001	2	247330001	247332000	2000	1	7.00E-08	0.43	22	1.1	Unc5c	Receptor
DMR2:247655001	2	247655001	247657000	2000	1	4.90E-07	-0.39	35	1.75	Bmpr1b	Signaling
DMR2:247821001	2	247821001	247824000	3000	1	2.10E-07	-0.41	52	1.73	Pdlim5	Cytoskeleton
DMR2:248201001	2	248201001	248204000	3000	1	1.20E-08	-0.35	10	0.33	Gbp5	Signaling
DMR2:248429001	2	248429001	248432000	3000	1	2.50E-07	-0.85	14	0.47	Gbp1	Signaling
DMR2:248555001	2	248555001	248559000	4000	1	9.20E-07	-0.35	28	0.7	RGD1560801	
DMR2:248690001	2	248690001	248693000	3000	3	1.70E-17	0.83	59	1.97	Kyat3	Metabolism
DMR2:250213001	2	250213001	250220000	7000	1	6.40E-09	0.61	133	1.9	Lmo4	
DMR2:250488001	2	250488001	250489000	1000	1	2.40E-07	-0.56	24	2.4	Hs2st1	Transport
DMR2:250700001	2	250700001	250702000	2000	1	3.40E-08	0.34	48	2.4	Sh3glb1	
DMR2:250741001	2	250741001	250742000	1000	1	3.90E-07	-0.46	10	1	Sh3glb1	
DMR2:250781001	2	250781001	250783000	2000	1	5.00E-08	0.5	23	1.15	Clca2;Clca4l;LOC108350129	Transport
DMR2:250790001	2	250790001	250793000	3000	1	1.60E-09	-0.41	57	1.9	Clca4l;LOC108350129;LOC102549543	Transport
DMR2:250905001	2	250905001	250908000	3000	1	4.30E-07	0.35	41	1.37	Clca1	Transport
DMR2:250953001	2	250953001	250954000	1000	1	1.10E-08	0.76	22	2.2	Clca5	Transport
DMR2:251014001	2	251014001	251017000	3000	1	7.50E-08	-0.38	25	0.83	Odf2l	
DMR2:251232001	2	251232001	251233000	1000	1	2.20E-09	0.62	9	0.9	Col24a1	Extracellular Matrix
DMR2:251252001	2	251252001	251254000	2000	1	6.30E-12	0.46	14	0.7	Col24a1	Extracellular Matrix
DMR2:251317001	2	251317001	251320000	3000	1	3.90E-07	-0.36	32	1.07	Col24a1	Extracellular Matrix
DMR2:251401001	2	251401001	251404000	3000	1	9.40E-07	-0.42	54	1.8	Col24a1	Extracellular Matrix
DMR2:251642001	2	251642001	251647000	5000	1	1.60E-07	-0.39	100	2	Ddah1;LOC108350132	Metabolism
DMR2:251685001	2	251685001	251686000	1000	1	4.20E-10	0.47	9	0.9	Ddah1	Metabolism
DMR2:251700001	2	251700001	251703000	3000	2	8.30E-09	-0.55	57	1.9	Ddah1	Metabolism
DMR2:251738001	2	251738001	251746000	8000	1	2.20E-08	0.37	122	1.52	Ddah1;LOC103691727	Metabolism
DMR2:251757001	2	251757001	251759000	2000	2	4.80E-09	0.36	19	0.95	Ddah1	Metabolism
DMR2:251864001	2	251864001	251867000	3000	1	2.10E-07	-0.35	52	1.73	Syde2	Signaling
DMR2:251887001	2	251887001	251888000	1000	1	5.10E-07	-0.34	24	2.4	Syde2	Signaling
DMR2:251900001	2	251900001	251903000	3000	1	3.50E-12	-0.67	61	2.03	Syde2;Wdr63	Signaling;Cytoskeleton
DMR2:251914001	2	251914001	251919000	5000	1	1.40E-07	-0.47	87	1.74	Wdr63	Cytoskeleton
DMR2:251927001	2	251927001	251928000	1000	1	2.00E-13	0.84	36	3.6	Wdr63;Mcoln3	Cytoskeleton;Transport
DMR2:251944001	2	251944001	251945000	1000	1	6.10E-07	-0.36	27	2.7	Wdr63;Mcoln3	Cytoskeleton;Transport

DMR2:251947001	2	251947001	251949000	2000	1	2.40E-07	0.37	18	0.9	Wdr63;Mcoln3	Cytoskeleton;Transport
DMR2:251964001	2	251964001	251965000	1000	1	5.50E-13	0.6	3	0.3	Wdr63;Mcoln3	Cytoskeleton;Transport
DMR2:252006001	2	252006001	252007000	1000	1	1.10E-10	0.45	15	1.5	Mcoln3	Transport
DMR2:252008001	2	252008001	252012000	4000	1	1.00E-07	0.61	91	2.28	Mcoln3;Mcoln2	Transport
DMR2:252016001	2	252016001	252017000	1000	1	1.10E-07	0.45	15	1.5	Mcoln3;Mcoln2	Transport
DMR2:252100001	2	252100001	252101000	1000	1	1.50E-07	-0.39	17	1.7	Lpar3	Signaling
DMR2:252167001	2	252167001	252169000	2000	1	7.60E-08	0.63	46	2.3	Lpar3	Signaling
DMR2:252278001	2	252278001	252280000	2000	1	9.00E-14	-0.63	33	1.65	Ssx2ip	
DMR2:252343001	2	252343001	252349000	6000	2	3.40E-07	-0.39	51	0.85	Spata1	
DMR2:252373001	2	252373001	252380000	7000	1	2.70E-07	-0.39	105	1.5	Gng5;Rpf1	Signaling;Translation
DMR2:252792001	2	252792001	252797000	5000	1	6.00E-08	0.27	78	1.56	Ttll7	Cytoskeleton
DMR2:252805001	2	252805001	252809000	4000	1	2.20E-14	0.39	74	1.85	Ttll7	Cytoskeleton
DMR2:252830001	2	252830001	252837000	7000	3	1.10E-07	0.45	56	0.8	Ttll7	Cytoskeleton
DMR2:252851001	2	252851001	252852000	1000	1	2.70E-07	-0.45	10	1	Ttll7	Cytoskeleton
DMR2:255909001	2	255909001	255910000	1000	1	8.20E-07	-0.45	13	1.3	LOC102548237;RGD1566247	
DMR2:256614001	2	256614001	256615000	1000	1	1.40E-07	-0.42	14	1.4	Adgrl4	Signaling
DMR2:256652001	2	256652001	256653000	1000	1	2.20E-07	0.48	12	1.2	Adgrl4	Signaling
DMR2:256692001	2	256692001	256695000	3000	2	9.00E-08	0.32	28	0.93	Adgrl4	Signaling
DMR2:256982001	2	256982001	256983000	1000	1	4.50E-10	0.59	27	2.7	Ifi44l	
DMR2:257293001	2	257293001	257294000	1000	1	1.20E-07	0.68	34	3.4	Gipc2	Cytoskeleton
DMR2:257346001	2	257346001	257347000	1000	1	4.80E-10	0.38	2	0.2	Gipc2	Cytoskeleton
DMR2:257362001	2	257362001	257365000	3000	1	7.50E-09	0.41	35	1.17	Gipc2	Cytoskeleton
DMR2:257380001	2	257380001	257382000	2000	1	7.50E-21	1.18	37	1.85	Gipc2	Cytoskeleton
DMR2:257427001	2	257427001	257428000	1000	1	2.80E-07	-0.49	10	1	Dnajb4;Fubp1	Transcription;Metabolism
DMR2:257464001	2	257464001	257469000	5000	1	1.30E-07	-0.54	56	1.12	Nexn;LOC102548862	Cytoskeleton
DMR2:257482001	2	257482001	257484000	2000	1	1.50E-08	0.61	34	1.7	Nexn;Miga1	Cytoskeleton
DMR2:257942001	2	257942001	257944000	2000	1	1.00E-07	-0.53	50	2.5	Pigk	
DMR2:258007001	2	258007001	258009000	2000	1	1.00E-07	-0.4	35	1.75	Pigk	
DMR2:258026001	2	258026001	258027000	1000	1	3.40E-07	0.54	17	1.7	St6galnac5	
DMR2:258121001	2	258121001	258125000	4000	2	1.30E-12	-0.37	38	0.95	St6galnac5	
DMR2:258412001	2	258412001	258413000	1000	1	1.10E-11	0.78	20	2	St6galnac5	
DMR2:258434001	2	258434001	258436000	2000	1	3.90E-10	0.82	36	1.8	St6galnac5	
DMR2:258439001	2	258439001	258440000	1000	1	1.30E-09	0.5	7	0.7	St6galnac5	
DMR2:258441001	2	258441001	258443000	2000	1	2.30E-07	0.45	24	1.2	St6galnac5	
DMR2:258494001	2	258494001	258495000	1000	1	9.30E-09	-0.42	13	1.3	St6galnac5	
DMR2:258534001	2	258534001	258535000	1000	1	8.20E-11	0.5	2	0.2	St6galnac5	
DMR2:258556001	2	258556001	258557000	1000	1	1.90E-08	0.49	12	1.2	St6galnac5	
DMR2:258566001	2	258566001	258569000	3000	1	4.00E-07	-0.37	44	1.47	St6galnac5	
DMR2:258619001	2	258619001	258620000	1000	1	9.90E-08	-0.56	10	1	St6galnac5	
DMR2:258632001	2	258632001	258636000	4000	2	6.40E-08	-0.39	30	0.75	St6galnac5	
DMR2:258734001	2	258734001	258738000	4000	1	3.10E-10	0.68	54	1.35	St6galnac5	
DMR2:258803001	2	258803001	258809000	6000	2	2.30E-07	-0.57	120	2	St6galnac5;Adgrl2	Signaling
DMR2:258826001	2	258826001	258829000	3000	1	2.20E-07	-0.46	61	2.03	St6galnac5;Adgrl2	Signaling
DMR2:258830001	2	258830001	258833000	3000	1	4.40E-10	-0.37	59	1.97	St6galnac5;Adgrl2	Signaling
DMR2:258906001	2	258906001	258908000	2000	1	6.60E-09	0.52	35	1.75	St6galnac5;Adgrl2	Signaling
DMR2:259030001	2	259030001	259033000	3000	1	3.40E-09	0.56	42	1.4	St6galnac5	
DMR2:259118001	2	259118001	259120000	2000	1	2.50E-07	0.37	24	1.2	St6galnac5	
DMR2:259368001	2	259368001	259374000	6000	1	7.50E-07	0.37	95	1.58	St6galnac3	
DMR2:259389001	2	259389001	259394000	5000	1	5.40E-07	-0.39	35	0.7	St6galnac3	
DMR2:259420001	2	259420001	259422000	2000	1	5.20E-07	0.52	53	2.65	St6galnac3	
DMR2:259428001	2	259428001	259431000	3000	1	5.70E-07	0.34	49	1.63	St6galnac3	
DMR2:259600001	2	259600001	259602000	2000	1	6.80E-08	-0.48	47	2.35	St6galnac3	
DMR2:259768001	2	259768001	259769000	1000	1	7.80E-07	-0.48	23	2.3	St6galnac3;LOC108350219	
DMR2:259910001	2	259910001	259916000	6000	2	4.30E-14	0.93	134	2.23	St6galnac3;LOC103691740	
DMR2:260113001	2	260113001	260116000	3000	2	3.20E-10	-0.56	59	1.97	Msh4;Rabggtb;Acadm	Transcription;Metabolism;Metabolism
DMR2:260258001	2	260258001	260264000	6000	1	4.70E-07	0.56	106	1.77	Slc44a5	Transport
DMR2:260292001	2	260292001	260293000	1000	1	7.90E-11	0.68	23	2.3	Slc44a5	Transport
DMR2:260300001	2	260300001	260302000	2000	2	7.80E-12	0.82	63	3.15	Slc44a5	Transport
DMR2:260331001	2	260331001	260332000	1000	1	1.50E-20	0.95	12	1.2	Slc44a5;LOC102553583	Transport
DMR2:260377001	2	260377001	260379000	2000	1	7.40E-09	0.42	25	1.25	Slc44a5	Transport
DMR2:260380001	2	260380001	260384000	4000	1	3.00E-07	-0.62	72	1.8	Slc44a5	Transport

DMR2:260386001	2	260386001	260388000	2000	1	1.80E-10	0.86	34	1.7	Slc44a5	Transport
DMR2:260421001	2	260421001	260425000	4000	1	1.80E-07	0.38	72	1.8	Slc44a5	Transport
DMR2:260434001	2	260434001	260437000	3000	1	2.40E-10	-0.61	38	1.27	Slc44a5	Transport
DMR2:260476001	2	260476001	260479000	3000	1	2.10E-10	0.79	68	2.27	Slc44a5	Transport
DMR2:260493001	2	260493001	260495000	2000	1	2.90E-10	-0.41	27	1.35	Slc44a5	Transport
DMR2:260535001	2	260535001	260536000	1000	1	6.80E-07	0.41	14	1.4	Slc44a5	Transport
DMR2:260855001	2	260855001	260857000	2000	1	3.90E-18	0.58	16	0.8	Tyw3	Epigenetic
DMR2:260868001	2	260868001	260872000	4000	1	2.20E-08	-0.49	26	0.65	Tyw3	Epigenetic
DMR2:260897001	2	260897001	260899000	2000	1	9.90E-08	-0.42	40	2	Cryz;LOC108350160	Metabolism
DMR2:260916001	2	260916001	260917000	1000	1	9.60E-09	-0.54	4	0.4	Cryz	Metabolism
DMR2:261045001	2	261045001	261048000	3000	1	8.30E-11	0.59	54	1.8	Erich3	
DMR2:261116001	2	261116001	261119000	3000	1	9.70E-11	-0.56	35	1.17	LOC102555624;Tnni3k	
DMR2:261135001	2	261135001	261138000	3000	1	7.10E-08	-0.48	53	1.77	LOC102555624;Tnni3k	
DMR2:261342001	2	261342001	261343000	1000	1	1.80E-09	0.37	4	0.4	Tnni3k	
DMR2:261369001	2	261369001	261381000	12000	12	6.70E-17	1.33	368	3.07	Fpgt	Transport
DMR2:261408001	2	261408001	261410000	2000	1	6.60E-07	0.37	12	0.6	Fpgt;Lrriq3	Transport
DMR2:261434001	2	261434001	261435000	1000	1	1.50E-07	0.36	10	1	Lrriq3	
DMR2:261474001	2	261474001	261476000	2000	1	4.90E-07	-0.6	11	0.55	Lrriq3	
DMR2:262909001	2	262909001	262911000	2000	1	5.70E-08	0.37	9	0.45	Negr1	Immune
DMR2:262947001	2	262947001	262949000	2000	1	3.50E-09	0.41	24	1.2	Negr1	Immune
DMR2:263005001	2	263005001	263006000	1000	1	2.20E-07	0.57	23	2.3	Negr1	Immune
DMR2:263038001	2	263038001	263039000	1000	1	1.70E-07	0.56	8	0.8	Negr1	Immune
DMR2:263104001	2	263104001	263106000	2000	1	8.50E-07	0.38	23	1.15	Negr1	Immune
DMR2:265047001	2	265047001	265048000	1000	1	3.00E-07	0.31	5	0.5	Lrrc7	Cytoskeleton
DMR2:265184001	2	265184001	265185000	1000	1	1.60E-08	0.46	9	0.9	Lrrc7	Cytoskeleton
DMR2:266178001	2	266178001	266179000	1000	1	2.00E-10	0.74	6	0.6	Rpe65	Metabolism
DMR2:266306001	2	266306001	266308000	2000	1	5.80E-10	0.46	15	0.75	Trnai-aau;Wls;LOC108350151	
DMR2:266364001	2	266364001	266365000	1000	1	1.30E-07	-0.39	10	1	Wls	
DMR2:266383001	2	266383001	266384000	1000	1	1.60E-08	0.49	7	0.7	Wls	
DMR2:266406001	2	266406001	266410000	4000	1	3.40E-08	-0.34	32	0.8	Wls	
DMR3:260001	3	260001	261000	1000	1	5.30E-11	0.72	24	2.4	Nxph2;LOC679914	Signaling
DMR3:475001	3	475001	477000	2000	1	3.00E-08	-0.36	16	0.8	Spopl	Proteolysis
DMR3:567001	3	567001	572000	5000	1	9.30E-07	-0.39	26	0.52	Psx1	
DMR3:895001	3	895001	896000	1000	1	1.00E-10	0.53	7	0.7	Hnmt	
DMR3:1299001	3	1299001	1300000	1000	1	8.60E-09	-0.34	8	0.8	Il36g	Cytokine
DMR3:1394001	3	1394001	1397000	3000	1	8.30E-09	-0.49	18	0.6	Il36b;Il36rn	Cytokine
DMR3:1398001	3	1398001	1399000	1000	1	7.20E-08	-0.43	8	0.8	Il36b;Il36rn;Il1f10	Cytokine
DMR3:1491001	3	1491001	1493000	2000	1	4.60E-07	0.38	29	1.45	Psd4	Transcription
DMR3:1537001	3	1537001	1539000	2000	1	3.80E-11	-0.6	16	0.8	Pax8;LOC102549321	
DMR3:1743001	3	1743001	1744000	1000	1	3.00E-18	0.8	31	3.1	Cacna1b	Transport
DMR3:1746001	3	1746001	1750000	4000	1	5.20E-09	0.37	39	0.98	Cacna1b	Transport
DMR3:1812001	3	1812001	1814000	2000	2	6.30E-08	0.45	26	1.3	Cacna1b	Transport
DMR3:1829001	3	1829001	1832000	3000	1	1.50E-08	-0.49	36	1.2	Cacna1b	Transport
DMR3:1908001	3	1908001	1911000	3000	2	7.70E-09	0.4	36	1.2	Cacna1b	Transport
DMR3:1930001	3	1930001	1931000	1000	1	7.70E-07	0.35	5	0.5	Cacna1b	Transport
DMR3:2315001	3	2315001	2317000	2000	1	2.00E-08	0.33	19	0.95	Noxa1	Signaling
DMR3:2446001	3	2446001	2448000	2000	1	6.10E-12	0.45	14	0.7	Fam166a;Tubb4b;Slc34a3;Rnf224	Cytoskeleton;Transport
DMR3:2469001	3	2469001	2470000	1000	1	1.50E-07	0.5	15	1.5	Cysrt1;Rnf208;Ndor1;Tmem203	Metabolism
DMR3:2605001	3	2605001	2607000	2000	1	1.50E-07	-0.4	31	1.55	Sapcd2	
DMR3:2629001	3	2629001	2630000	1000	1	7.10E-09	0.44	21	2.1	Entpd2;Npdc1;LOC366006;Fut7	Signaling;Golgi
DMR3:2652001	3	2652001	2656000	4000	1	2.00E-09	0.51	69	1.73	Fut7;Abca2	Golgi;Transport
DMR3:2665001	3	2665001	2667000	2000	1	3.70E-09	0.35	73	3.65	Abca2;Clic3	Transport;Transport
DMR3:2740001	3	2740001	2743000	3000	1	2.30E-09	-0.46	58	1.93	Fbxw5;Traf2	Cytoskeleton
DMR3:2800001	3	2800001	2801000	1000	1	1.20E-09	0.86	46	4.6	Mamdc4;Phpt1;RGD1560470;Rabl6	Signaling
DMR3:3252001	3	3252001	3255000	3000	1	4.30E-11	-0.55	57	1.9	LOC100359901;LOC108350339;Glt6d1	Golgi
DMR3:3345001	3	3345001	3347000	2000	1	1.80E-07	0.41	29	1.45	Kcnt1	Transport
DMR3:3348001	3	3348001	3351000	3000	1	1.70E-12	0.61	56	1.87	Kcnt1	Transport
DMR3:3424001	3	3424001	3427000	3000	1	4.40E-08	-0.39	31	1.03	Camsap1;LOC102550543	
DMR3:3502001	3	3502001	3503000	1000	1	1.70E-07	0.37	7	0.7	Nacc2	
DMR3:3528001	3	3528001	3535000	7000	1	7.20E-07	0.32	135	1.93	Nacc2	
DMR3:3781001	3	3781001	3785000	4000	1	1.30E-07	0.44	55	1.38	Gpsm1	
DMR3:3956001	3	3956001	3958000	2000	1	3.30E-07	0.4	30	1.5	Notch1;LOC103691754	Extracellular Matrix

DMR3:4061001	3	4061001	4062000	1000	1	5.80E-07	0.41	6	0.6	Agpat2	Metabolism
DMR3:4384001	3	4384001	4386000	2000	1	5.40E-07	-0.32	20	1	LOC100911704;Abo	Golgi
DMR3:5383001	3	5383001	5385000	2000	1	1.60E-14	0.73	48	2.4	Abo2	Golgi
DMR3:5538001	3	5538001	5539000	1000	1	7.20E-10	-0.42	26	2.6	Adamts13	Protease
DMR3:5576001	3	5576001	5577000	1000	1	5.10E-07	0.41	3	0.3	Cacfd1;Slc2a6	
DMR3:5637001	3	5637001	5638000	1000	1	5.90E-10	0.62	14	1.4	Adamtsl2	Protease
DMR3:5683001	3	5683001	5684000	1000	1	3.00E-10	0.53	9	0.9	Fam163b	
DMR3:5769001	3	5769001	5771000	2000	1	8.00E-08	0.42	34	1.7	Sardh;LOC102548789	Metabolism
DMR3:5831001	3	5831001	5833000	2000	1	7.60E-09	0.54	25	1.25	Vav2	
DMR3:5923001	3	5923001	5926000	3000	1	1.50E-07	-0.42	36	1.2	Vav2	
DMR3:5985001	3	5985001	5988000	3000	1	2.70E-09	0.44	24	0.8	Vav2;LOC100911432;Brd3	
DMR3:6020001	3	6020001	6022000	2000	1	3.80E-08	-0.75	27	1.35	Brd3	
DMR3:6436001	3	6436001	6437000	1000	1	3.00E-08	0.34	7	0.7	Col5a1	Extracellular Matrix
DMR3:6478001	3	6478001	6481000	3000	1	9.00E-09	0.43	48	1.6	Col5a1	Extracellular Matrix
DMR3:6511001	3	6511001	6514000	3000	1	2.70E-11	0.55	40	1.33	Col5a1	Extracellular Matrix
DMR3:6789001	3	6789001	6791000	2000	1	2.20E-07	0.44	30	1.5	Olfm1	Development
DMR3:6969001	3	6969001	6972000	3000	2	2.60E-10	-0.79	27	0.9	RGD1564492	
DMR3:7170001	3	7170001	7172000	2000	1	3.60E-07	0.38	28	1.4	Gtf3c5	Transcription
DMR3:7291001	3	7291001	7294000	3000	1	2.40E-10	0.5	38	1.27	Ak8	Signaling
DMR3:7299001	3	7299001	7302000	3000	1	6.60E-08	0.37	62	2.07	Ak8	Signaling
DMR3:7395001	3	7395001	7396000	1000	1	1.20E-09	0.37	6	0.6	Ak8;Gtf3c4	Signaling;Transcription
DMR3:7401001	3	7401001	7402000	1000	1	1.20E-09	0.58	4	0.4	Ak8;Gtf3c4	Signaling;Transcription
DMR3:7504001	3	7504001	7505000	1000	1	8.10E-07	0.39	16	1.6	Barhl1;Cfap77	Development;Development
DMR3:7595001	3	7595001	7598000	3000	1	5.90E-08	0.41	36	1.2	Cfap77	Development
DMR3:7774001	3	7774001	7776000	2000	1	6.30E-07	0.61	47	2.35	Ntng2	Extracellular Matrix
DMR3:8052001	3	8052001	8058000	6000	1	1.80E-07	0.41	75	1.25	Med27	Transcription
DMR3:8402001	3	8402001	8406000	4000	1	1.90E-08	0.49	46	1.15	Urm1;Mir2964;Mir219-2	
DMR3:8446001	3	8446001	8448000	2000	1	1.50E-07	0.27	21	1.05	Cercam;LOC108350590;Odf2	Golgi
DMR3:8551001	3	8551001	8553000	2000	1	4.30E-07	-0.37	21	1.05	Sptan1	
DMR3:8734001	3	8734001	8736000	2000	1	7.10E-07	-0.28	10	0.5	Endog;LOC499770	
DMR3:9274001	3	9274001	9275000	1000	1	5.00E-07	0.35	8	0.8	Lamc3	Extracellular Matrix
DMR3:9292001	3	9292001	9295000	3000	1	5.00E-07	0.45	56	1.87	Lamc3	Extracellular Matrix
DMR3:9305001	3	9305001	9307000	2000	2	4.50E-09	0.55	25	1.25	Lamc3	Extracellular Matrix
DMR3:9650001	3	9650001	9652000	2000	1	4.50E-07	-0.35	47	2.35	Ntmt1;Asb6	Epigenetic;Cytoskeleton
DMR3:9668001	3	9668001	9669000	1000	1	3.90E-09	0.38	9	0.9	Ntmt1;Asb6	Epigenetic;Cytoskeleton
DMR3:9804001	3	9804001	9806000	2000	1	4.60E-07	-0.39	36	1.8	Tor1b;Tor1a;RGD1305178	Transcription
DMR3:9836001	3	9836001	9838000	2000	1	3.00E-08	0.42	35	1.75	Usp20	Protease
DMR3:9851001	3	9851001	9853000	2000	1	5.40E-07	0.47	29	1.45	Usp20	Protease
DMR3:9938001	3	9938001	9940000	2000	1	2.40E-10	0.61	25	1.25	Fnbp1;LOC102554659	
DMR3:10412001	3	10412001	10413000	1000	1	9.90E-07	0.47	13	1.3	Hmcn2	
DMR3:10464001	3	10464001	10466000	2000	1	3.30E-09	0.49	39	1.95	Hmcn2;LOC103695151	
DMR3:10545001	3	10545001	10546000	1000	1	6.10E-08	0.58	21	2.1	Hmcn2;LOC108350350	
DMR3:10563001	3	10563001	10567000	4000	1	1.80E-08	0.38	74	1.85	Ncs1	
DMR3:11094001	3	11094001	11098000	4000	1	4.30E-07	0.43	63	1.57	Fam78a	
DMR3:11128001	3	11128001	11129000	1000	1	2.20E-07	0.53	20	2	Plpp7	Signaling
DMR3:11169001	3	11169001	11175000	6000	2	2.10E-13	-0.62	147	2.45	Prrc2b	Metabolism
DMR3:11179001	3	11179001	11181000	2000	1	9.70E-07	-0.38	48	2.4	Prrc2b	Metabolism
DMR3:11194001	3	11194001	11196000	2000	1	1.70E-09	-0.49	46	2.3	Prrc2b	Metabolism
DMR3:11402001	3	11402001	11404000	2000	1	4.20E-07	0.45	34	1.7	Ciz1;RGD1561113	
DMR3:11606001	3	11606001	11607000	1000	1	1.30E-07	-0.4	18	1.8	Pip5kl1;St6galnac4	Signaling
DMR3:11712001	3	11712001	11713000	1000	1	8.20E-07	0.41	18	1.8	Eng;Fpgs	Receptor;Metabolism
DMR3:11719001	3	11719001	11721000	2000	1	1.40E-07	0.44	36	1.8	Eng;Fpgs	Receptor;Metabolism
DMR3:11737001	3	11737001	11739000	2000	1	1.50E-07	-0.5	32	1.6	Fpgs;LOC103691767;Cdk9	Metabolism;Signaling
DMR3:11801001	3	11801001	11803000	2000	1	4.20E-08	0.39	20	1	Sh2d3c;Tor2a;Ttc16;Ptrh1	Transcription;Metabolism
DMR3:11843001	3	11843001	11845000	2000	1	8.10E-07	-0.53	26	1.3	Stxbp1	Transport
DMR3:12022001	3	12022001	12023000	1000	1	3.20E-07	0.5	12	1.2	Slc2a8	
DMR3:12103001	3	12103001	12104000	1000	1	2.80E-09	0.65	20	2	Garnl3;LOC102557449	Signaling
DMR3:12220001	3	12220001	12221000	1000	1	2.50E-09	-0.65	9	0.9	Ralgps1	Transcription
DMR3:12371001	3	12371001	12373000	2000	1	2.50E-11	0.59	22	1.1	Ralgps1	Transcription
DMR3:12515001	3	12515001	12517000	2000	1	3.50E-07	0.41	19	0.95	Zbtb43;LOC102550017	Cytoskeleton
DMR3:12641001	3	12641001	12643000	2000	1	3.00E-09	0.45	19	0.95	Lmx1b	Development

DMR3:12794001	3	12794001	12798000	4000	1	2.80E-09	0.32	75	1.88	Mvb12b	
DMR3:13355001	3	13355001	13356000	1000	1	2.20E-07	-0.4	8	0.8	Pbx3	Development
DMR3:13386001	3	13386001	13387000	1000	1	4.10E-07	0.59	9	0.9	Pbx3;LOC103691768	Development
DMR3:13766001	3	13766001	13767000	1000	1	4.50E-07	-0.42	12	1.2	Gapvd1	Transcription
DMR3:13793001	3	13793001	13794000	1000	1	6.20E-09	0.4	9	0.9	Gapvd1	Transcription
DMR3:13841001	3	13841001	13843000	2000	1	1.00E-08	-0.42	22	1.1	Hspa5;Rabepk	
DMR3:13922001	3	13922001	13926000	4000	1	8.00E-07	-0.41	62	1.55	Psmd5	Protease
DMR3:14006001	3	14006001	14007000	1000	1	1.20E-13	0.45	12	1.2	Traf1	Cytoskeleton
DMR3:14067001	3	14067001	14068000	1000	1	3.30E-08	0.5	10	1	C5	
DMR3:14097001	3	14097001	14098000	1000	1	7.00E-07	-0.46	5	0.5	C5	
DMR3:14115001	3	14115001	14118000	3000	1	2.10E-07	-0.37	24	0.8	C5	
DMR3:14214001	3	14214001	14217000	3000	1	2.00E-07	0.41	53	1.77	C5	
DMR3:14231001	3	14231001	14234000	3000	2	5.30E-11	-0.38	20	0.67	C5	
DMR3:14347001	3	14347001	14348000	1000	1	2.60E-10	-0.44	14	1.4	Cntrl	
DMR3:14479001	3	14479001	14480000	1000	1	7.10E-09	0.37	10	1	Gsn	Cytoskeleton
DMR3:14488001	3	14488001	14490000	2000	1	1.20E-09	0.41	20	1	Gsn	Cytoskeleton
DMR3:14711001	3	14711001	14712000	1000	1	3.60E-08	0.4	13	1.3	Ggta1	Golgi
DMR3:15105001	3	15105001	15107000	2000	1	3.40E-07	0.32	21	1.05	Ttll11	Cytoskeleton
DMR3:15120001	3	15120001	15127000	7000	1	2.40E-11	-0.52	154	2.2	Ttll11	Cytoskeleton
DMR3:15200001	3	15200001	15201000	1000	1	2.90E-07	0.42	14	1.4	Ttll11;LOC108350357	Cytoskeleton
DMR3:15213001	3	15213001	15217000	4000	1	3.40E-10	-0.49	69	1.73	Ttll11	Cytoskeleton
DMR3:15304001	3	15304001	15306000	2000	1	2.50E-08	-0.43	44	2.2	Ttll11	Cytoskeleton
DMR3:15376001	3	15376001	15378000	2000	1	4.90E-07	-0.37	37	1.85	Ndufa8;Morn5	Metabolism
DMR3:15476001	3	15476001	15477000	1000	1	2.10E-07	-0.35	13	1.3	Mrrf	Translation
DMR3:15628001	3	15628001	15634000	6000	1	3.30E-07	-0.36	68	1.13	Olr393-ps;Olr394-ps	
DMR3:15754001	3	15754001	15757000	3000	1	1.10E-07	-0.31	24	0.8	Olr396	Receptor
DMR3:15772001	3	15772001	15773000	1000	1	1.20E-07	0.45	63	6.3	Klf5-ps1	
DMR3:15928001	3	15928001	15930000	2000	1	5.60E-07	-0.62	7	0.35	Olr401	Receptor
DMR3:16232001	3	16232001	16238000	6000	2	3.10E-09	-0.41	71	1.18	Olr407	Receptor
DMR3:18308001	3	18308001	18310000	2000	1	1.20E-07	-0.5	9	0.45	RGD1565546	
DMR3:21216001	3	21216001	21221000	5000	1	3.90E-07	-0.35	41	0.82	Olr427	Receptor
DMR3:21564001	3	21564001	21566000	2000	2	5.70E-20	1.07	59	2.95	LOC100911251;Klf5-ps2	
DMR3:21570001	3	21570001	21575000	5000	1	2.20E-07	-0.48	32	0.64	LOC100911251;Klf5-ps2;LOC690273;LOC108350364	
DMR3:21594001	3	21594001	21595000	1000	1	9.80E-07	0.37	9	0.9	LOC108350364;Pdcl	
DMR3:21663001	3	21663001	21666000	3000	1	7.90E-08	0.38	19	0.63	Rc3h2	
DMR3:21687001	3	21687001	21690000	3000	1	3.90E-08	-0.67	20	0.67	Zbtb6;LOC108350365;Zbtb26;Rabgap1	Transcription;Signaling
DMR3:22038001	3	22038001	22039000	1000	1	1.70E-07	0.63	35	3.5	Crb2	Cytoskeleton
DMR3:22328001	3	22328001	22329000	1000	1	6.00E-07	0.38	8	0.8	Dennd1a	
DMR3:22343001	3	22343001	22344000	1000	1	7.80E-08	-0.61	7	0.7	Dennd1a	
DMR3:25589001	3	25589001	25595000	6000	1	2.70E-07	-0.27	70	1.17	Lrp1b	
DMR3:25882001	3	25882001	25886000	4000	2	2.40E-07	-0.44	16	0.4	Lrp1b	
DMR3:25910001	3	25910001	25913000	3000	1	3.20E-10	0.52	29	0.97	Lrp1b;LOC108350579	
DMR3:26069001	3	26069001	26070000	1000	1	5.00E-07	0.36	8	0.8	Lrp1b	
DMR3:26431001	3	26431001	26432000	1000	1	7.80E-10	0.45	6	0.6	Lrp1b	
DMR3:26494001	3	26494001	26497000	3000	2	1.20E-11	0.83	12	0.4	Lrp1b;LOC103691806	
DMR3:26565001	3	26565001	26570000	5000	1	2.60E-07	-0.38	55	1.1	Lrp1b	
DMR3:26596001	3	26596001	26598000	2000	1	2.60E-07	0.48	9	0.45	Lrp1b	
DMR3:26935001	3	26935001	26936000	1000	1	3.20E-08	0.54	5	0.5	Lrp1b	
DMR3:27161001	3	27161001	27162000	1000	1	7.30E-07	0.57	11	1.1	Lrp1b	
DMR3:27301001	3	27301001	27302000	1000	1	2.30E-07	-0.57	5	0.5	Lrp1b	
DMR3:27356001	3	27356001	27357000	1000	1	2.00E-07	0.52	18	1.8	Lrp1b	
DMR3:28438001	3	28438001	28439000	1000	1	8.50E-07	-0.37	2	0.2	Kynu;LOC102547849	Metabolism
DMR3:28486001	3	28486001	28487000	1000	1	9.80E-08	0.48	16	1.6	Kynu	Metabolism
DMR3:28690001	3	28690001	28692000	2000	1	6.90E-10	0.71	30	1.5	Arhgap15	Signaling
DMR3:28788001	3	28788001	28789000	1000	1	1.40E-07	-0.54	10	1	Arhgap15	Signaling
DMR3:28875001	3	28875001	28876000	1000	1	7.50E-14	-0.55	11	1.1	Arhgap15	Signaling
DMR3:28916001	3	28916001	28918000	2000	1	5.50E-11	0.52	38	1.9	Arhgap15	Signaling
DMR3:28971001	3	28971001	28972000	1000	1	5.30E-09	0.44	11	1.1	Arhgap15	Signaling
DMR3:29014001	3	29014001	29015000	1000	1	2.70E-09	-0.42	5	0.5	Arhgap15	Signaling
DMR3:29123001	3	29123001	29124000	1000	1	2.00E-07	-0.49	10	1	Arhgap15	Signaling
DMR3:29154001	3	29154001	29156000	2000	1	7.40E-08	0.45	31	1.55	Arhgap15	Signaling

DMR3:29646001	3	29646001	29647000	1000	1	5.00E-07	0.35	8	0.8	Gtdc1	Golgi
DMR3:29711001	3	29711001	29718000	7000	3	8.00E-09	-0.67	68	0.97	Gtdc1	Golgi
DMR3:29845001	3	29845001	29848000	3000	1	3.60E-08	0.7	47	1.57	Zeb2	Transcription
DMR3:29862001	3	29862001	29863000	1000	1	1.60E-09	0.8	39	3.9	Zeb2	Transcription
DMR3:29957001	3	29957001	29959000	2000	1	2.60E-10	-0.51	38	1.9	Zeb2	Transcription
DMR3:33395001	3	33395001	33399000	4000	1	6.40E-09	-0.6	45	1.12	Mbd5	
DMR3:33464001	3	33464001	33465000	1000	1	2.20E-07	0.51	5	0.5	Mbd5	
DMR3:33707001	3	33707001	33709000	2000	1	3.00E-07	-0.41	15	0.75	Epc2	Epigenetic
DMR3:35007001	3	35007001	35010000	3000	1	3.90E-10	0.47	48	1.6	Kif5c	
DMR3:35113001	3	35113001	35114000	1000	1	3.30E-07	0.58	10	1	Kif5c	
DMR3:35149001	3	35149001	35153000	4000	1	4.40E-07	-0.46	69	1.73	Kif5c	
DMR3:35192001	3	35192001	35194000	2000	1	6.90E-07	-0.34	25	1.25	Kif5c	
DMR3:35531001	3	35531001	35533000	2000	2	4.00E-08	-0.48	20	1	Ns5atp4	
DMR3:35668001	3	35668001	35670000	2000	1	2.60E-08	-0.5	23	1.15	Lypd6;LOC102555146	
DMR3:35680001	3	35680001	35681000	1000	1	7.90E-07	0.39	15	1.5	Lypd6	
DMR3:37149001	3	37149001	37150000	1000	1	6.40E-08	0.61	25	2.5	Tas2r134	Receptor
DMR3:37475001	3	37475001	37478000	3000	1	1.90E-09	-0.39	41	1.37	Nmi	Transcription
DMR3:37569001	3	37569001	37572000	3000	1	3.20E-07	-0.76	33	1.1	Tnfaip6	
DMR3:37756001	3	37756001	37757000	1000	1	7.00E-08	0.4	6	0.6	Neb	
DMR3:37773001	3	37773001	37777000	4000	1	1.60E-07	-0.25	36	0.9	Neb	
DMR3:37828001	3	37828001	37830000	2000	1	3.80E-08	-0.53	33	1.65	Neb	
DMR3:37832001	3	37832001	37835000	3000	1	1.60E-07	0.41	38	1.27	Neb	
DMR3:37856001	3	37856001	37857000	1000	1	5.70E-07	-0.62	10	1	Neb	
DMR3:37965001	3	37965001	37968000	3000	1	2.50E-07	-0.4	37	1.23	Cacnb4	Transport
DMR3:38014001	3	38014001	38022000	8000	2	2.30E-09	-0.28	119	1.49	Cacnb4	Transport
DMR3:38170001	3	38170001	38171000	1000	1	4.00E-07	-0.4	19	1.9	Cacnb4	Transport
DMR3:38411001	3	38411001	38412000	1000	1	2.30E-09	-0.45	19	1.9	RGD1560248	
DMR3:38596001	3	38596001	38599000	3000	1	9.50E-11	-0.66	44	1.47	RGD1560248	
DMR3:40040001	3	40040001	40042000	2000	1	7.60E-07	-0.35	16	0.8	Galnt13	Golgi
DMR3:40234001	3	40234001	40235000	1000	1	7.20E-10	0.58	8	0.8	Galnt13	Golgi
DMR3:40258001	3	40258001	40260000	2000	1	2.10E-09	0.6	19	0.95	Galnt13	Golgi
DMR3:40344001	3	40344001	40347000	3000	2	1.20E-07	-0.44	27	0.9	Galnt13	Golgi
DMR3:40512001	3	40512001	40514000	2000	1	3.80E-07	-0.52	7	0.35	Galnt13	Golgi
DMR3:40515001	3	40515001	40517000	2000	1	1.20E-07	0.43	29	1.45	Galnt13	Golgi
DMR3:40630001	3	40630001	40632000	2000	1	3.60E-08	0.54	18	0.9	Galnt13	Golgi
DMR3:41017001	3	41017001	41018000	1000	1	1.10E-13	-0.75	11	1.1	LOC108350374;Kcnj3	Transport
DMR3:41035001	3	41035001	41037000	2000	1	9.60E-09	-0.45	20	1	Kcnj3	Transport
DMR3:43133001	3	43133001	43135000	2000	1	8.80E-08	0.42	17	0.85	Nr4a2	Transcription
DMR3:43280001	3	43280001	43286000	6000	1	2.00E-09	-0.43	61	1.02	Gpd2	Metabolism
DMR3:44018001	3	44018001	44019000	1000	1	1.00E-07	0.45	12	1.2	Galnt5	Golgi
DMR3:44075001	3	44075001	44076000	1000	1	5.30E-09	-0.55	13	1.3	Galnt5;Ermn	Golgi
DMR3:44077001	3	44077001	44078000	1000	1	5.80E-07	0.29	2	0.2	Galnt5;Ermn	Golgi
DMR3:44178001	3	44178001	44179000	1000	1	4.10E-10	-0.42	23	2.3	Cytip	
DMR3:44502001	3	44502001	44505000	3000	1	4.80E-09	-0.56	35	1.17	Acvr1	Signaling
DMR3:44747001	3	44747001	44749000	2000	1	2.10E-08	0.33	39	1.95	Gapdh-ps1	
DMR3:45123001	3	45123001	45129000	6000	2	2.10E-08	-0.3	52	0.87	Ccdc148	
DMR3:45144001	3	45144001	45146000	2000	1	6.90E-07	0.33	23	1.15	Ccdc148	
DMR3:45172001	3	45172001	45173000	1000	1	1.00E-06	-0.42	7	0.7	Ccdc148	
DMR3:45175001	3	45175001	45178000	3000	1	7.40E-07	-0.6	27	0.9	Ccdc148	
DMR3:45421001	3	45421001	45422000	1000	1	4.40E-08	0.35	3	0.3	Pkp4	Cytoskeleton
DMR3:45564001	3	45564001	45565000	1000	1	6.70E-07	-0.37	14	1.4	LOC108350378;Dapl1	
DMR3:45688001	3	45688001	45690000	2000	1	5.10E-13	-0.63	39	1.95	Tanc1	
DMR3:45694001	3	45694001	45695000	1000	1	1.10E-10	-0.6	13	1.3	Tanc1	
DMR3:45774001	3	45774001	45775000	1000	1	1.30E-08	-0.68	16	1.6	Tanc1	
DMR3:45827001	3	45827001	45833000	6000	1	3.80E-07	-0.38	115	1.92	Tanc1	
DMR3:45975001	3	45975001	45984000	9000	1	3.90E-07	-0.42	157	1.74	Baz2b	Epigenetic
DMR3:46338001	3	46338001	46340000	2000	1	6.20E-08	-0.46	18	0.9	Cd302	
DMR3:46364001	3	46364001	46365000	1000	1	9.10E-13	0.71	30	3	Cd302;Mir6216;Ly75	
DMR3:46414001	3	46414001	46415000	1000	1	6.10E-09	0.34	7	0.7	Ly75	
DMR3:46511001	3	46511001	46515000	4000	1	4.10E-07	0.65	74	1.85	LOC108350581;Pla2r1	
DMR3:46726001	3	46726001	46730000	4000	1	7.10E-07	-0.39	67	1.68	Itgb6	Extracellular Matrix
DMR3:46979001	3	46979001	46985000	6000	1	1.80E-13	-0.38	67	1.12	Rbms1	
DMR3:47499001	3	47499001	47500000	1000	1	1.60E-07	0.48	4	0.4	Tank	

DMR3:48183001	3	48183001	48189000	6000	3	5.00E-09	-0.39	53	0.88	Slc4a10	Transport
DMR3:48218001	3	48218001	48219000	1000	1	9.20E-07	-0.43	11	1.1	Slc4a10	Transport
DMR3:48320001	3	48320001	48323000	3000	1	3.60E-11	-0.55	59	1.97	Dpp4	Protease
DMR3:48441001	3	48441001	48442000	1000	1	6.70E-10	0.44	8	0.8	Gcg	
DMR3:48487001	3	48487001	48489000	2000	1	1.40E-09	-0.52	12	0.6	Fap	Protease
DMR3:48496001	3	48496001	48499000	3000	1	1.00E-06	-0.29	28	0.93	Fap	Protease
DMR3:49051001	3	49051001	49059000	8000	2	3.90E-10	0.49	81	1.01	Kcnh7	Transport
DMR3:49134001	3	49134001	49135000	1000	1	3.50E-07	-0.48	9	0.9	Kcnh7	Transport
DMR3:49994001	3	49994001	49995000	1000	1	6.00E-09	0.66	31	3.1	Fign	Cytoskeleton
DMR3:50060001	3	50060001	50064000	4000	1	3.50E-07	0.42	41	1.02	Fign	Cytoskeleton
DMR3:50955001	3	50955001	50957000	2000	1	1.70E-08	0.38	12	0.6	Grb14	Cytoskeleton
DMR3:51001001	3	51001001	51002000	1000	1	4.10E-07	0.39	24	2.4	Grb14	Cytoskeleton
DMR3:51150001	3	51150001	51156000	6000	1	9.00E-07	-0.23	68	1.13	Cobll1	
DMR3:51227001	3	51227001	51228000	1000	1	1.00E-09	0.5	11	1.1	Cobll1	
DMR3:51248001	3	51248001	51249000	1000	1	1.40E-07	0.5	10	1	Cobll1	
DMR3:51360001	3	51360001	51361000	1000	1	1.80E-13	-3.05	10	1	Slc38a11	Transport
DMR3:51593001	3	51593001	51595000	2000	1	2.00E-09	-0.44	21	1.05	Scn3a	Transport
DMR3:51683001	3	51683001	51684000	1000	1	1.80E-09	0.63	23	2.3	Scn2a	Transport
DMR3:51886001	3	51886001	51889000	3000	1	5.00E-07	-0.5	49	1.63	Csrnp3	
DMR3:52014001	3	52014001	52018000	4000	1	1.80E-07	-0.58	39	0.98	Csrnp3	
DMR3:52055001	3	52055001	52064000	9000	4	1.40E-19	0.61	84	0.93	Csrnp3	
DMR3:52065001	3	52065001	52071000	6000	4	2.40E-14	0.63	48	0.8	Csrnp3	
DMR3:52166001	3	52166001	52168000	2000	1	1.90E-07	0.34	35	1.75	Galnt3	Golgi
DMR3:52185001	3	52185001	52187000	2000	1	2.10E-07	-0.44	35	1.75	Galnt3	Golgi
DMR3:52222001	3	52222001	52223000	1000	1	1.00E-07	-0.34	13	1.3	Galnt3	Golgi
DMR3:52442001	3	52442001	52449000	7000	1	3.50E-07	0.46	106	1.51	Scn1a	Transport
DMR3:52571001	3	52571001	52574000	3000	2	5.10E-08	-0.43	20	0.67	LOC108350387;Scn9a	Transport
DMR3:52596001	3	52596001	52599000	3000	1	2.70E-07	-0.49	34	1.13	LOC108350387;Scn9a	Transport
DMR3:52791001	3	52791001	52792000	1000	1	1.30E-07	0.41	12	1.2	Scn7a	Transport
DMR3:53661001	3	53661001	53662000	1000	1	6.40E-08	0.43	13	1.3	Xirp2	Cytoskeleton
DMR3:53710001	3	53710001	53711000	1000	1	3.10E-08	-0.49	14	1.4	B3galt1	Golgi
DMR3:53760001	3	53760001	53762000	2000	1	2.60E-12	0.78	29	1.45	B3galt1	Golgi
DMR3:53911001	3	53911001	53915000	4000	1	2.10E-07	-0.27	30	0.75	B3galt1	Golgi
DMR3:54055001	3	54055001	54059000	4000	1	1.20E-07	-0.36	29	0.72	B3galt1	Golgi
DMR3:54063001	3	54063001	54065000	2000	1	9.10E-08	0.44	12	0.6	B3galt1	Golgi
DMR3:54085001	3	54085001	54086000	1000	1	1.20E-08	-0.44	6	0.6	B3galt1	Golgi
DMR3:54251001	3	54251001	54253000	2000	1	1.20E-08	0.45	27	1.35	B3galt1	Golgi
DMR3:54255001	3	54255001	54257000	2000	1	4.30E-08	-0.35	10	0.5	B3galt1	Golgi
DMR3:54259001	3	54259001	54260000	1000	1	3.50E-08	0.66	14	1.4	B3galt1	Golgi
DMR3:54269001	3	54269001	54270000	1000	1	3.20E-07	-0.39	22	2.2	B3galt1	Golgi
DMR3:54529001	3	54529001	54534000	5000	1	1.20E-08	-0.32	60	1.2	Stk39	
DMR3:54535001	3	54535001	54537000	2000	1	5.90E-07	0.32	34	1.7	Stk39	
DMR3:54544001	3	54544001	54546000	2000	1	3.10E-08	-0.57	37	1.85	Stk39	
DMR3:54557001	3	54557001	54558000	1000	1	6.80E-09	-0.54	17	1.7	Stk39	
DMR3:54560001	3	54560001	54563000	3000	1	5.40E-11	-0.46	51	1.7	Stk39	
DMR3:54567001	3	54567001	54570000	3000	1	6.10E-07	-0.46	69	2.3	Stk39	
DMR3:54573001	3	54573001	54574000	1000	1	1.70E-07	-0.4	16	1.6	Stk39	
DMR3:54619001	3	54619001	54622000	3000	1	2.80E-07	-0.6	38	1.27	Stk39	
DMR3:55225001	3	55225001	55226000	1000	1	7.60E-07	0.47	13	1.3	Cers6	
DMR3:55230001	3	55230001	55232000	2000	1	2.90E-08	0.45	6	0.3	Cers6	
DMR3:55240001	3	55240001	55242000	2000	1	8.30E-08	0.51	34	1.7	Cers6	
DMR3:55316001	3	55316001	55318000	2000	1	2.00E-07	0.53	26	1.3	Cers6	
DMR3:55429001	3	55429001	55430000	1000	1	3.60E-07	0.31	10	1	Nostrin;Spc25	
DMR3:55450001	3	55450001	55451000	1000	1	1.20E-07	-0.57	17	1.7	Spc25;LOC108350389	
DMR3:55479001	3	55479001	55480000	1000	1	1.80E-07	0.34	4	0.4	LOC108350389;Abcb11	Transport
DMR3:55595001	3	55595001	55596000	1000	1	5.20E-07	-0.36	8	0.8	Abcb11	Transport
DMR3:55654001	3	55654001	55657000	3000	1	3.20E-07	0.43	28	0.93	Dhrs9;Lrp2	Metabolism
DMR3:55690001	3	55690001	55691000	1000	1	1.10E-07	-0.35	26	2.6	Lrp2	
DMR3:55703001	3	55703001	55705000	2000	1	2.10E-08	-0.45	23	1.15	Lrp2	
DMR3:55716001	3	55716001	55718000	2000	1	4.20E-08	-0.47	55	2.75	Lrp2	
DMR3:55727001	3	55727001	55729000	2000	1	2.50E-08	0.42	18	0.9	Lrp2	
DMR3:56005001	3	56005001	56007000	2000	1	1.90E-08	-0.48	14	0.7	Ccdc173	
DMR3:56013001	3	56013001	56014000	1000	1	4.40E-07	0.42	7	0.7	Ccdc173	

DMR3:56069001	3	56069001	56070000	1000	1	2.80E-07	-0.48	22	2.2	Klhl23	Cytoskeleton
DMR3:56387001	3	56387001	56390000	3000	1	6.80E-07	-0.4	55	1.83	Myo3b	
DMR3:56439001	3	56439001	56441000	2000	1	1.60E-07	-0.43	11	0.55	Myo3b	
DMR3:56452001	3	56452001	56453000	1000	1	4.10E-07	0.42	8	0.8	Myo3b	
DMR3:56513001	3	56513001	56514000	1000	1	1.40E-10	0.58	26	2.6	Myo3b	
DMR3:56638001	3	56638001	56640000	2000	1	3.60E-12	0.49	18	0.9	Myo3b	
DMR3:56713001	3	56713001	56715000	2000	1	2.40E-08	-0.4	27	1.35	Myo3b	
DMR3:56827001	3	56827001	56829000	2000	1	4.10E-08	-0.36	18	0.9	Erich2	
DMR3:57017001	3	57017001	57019000	2000	1	2.70E-07	-0.78	26	1.3	Tlk1	Signaling
DMR3:57187001	3	57187001	57189000	2000	1	4.60E-09	-0.5	37	1.85	Tlk1;RGD1565767	Signaling;Translation
DMR3:57601001	3	57601001	57603000	2000	1	3.60E-07	0.35	17	0.85	Mettl8;LOC102548511	
DMR3:57607001	3	57607001	57610000	3000	1	7.10E-09	-0.47	40	1.33	Mettl8;LOC102548511	
DMR3:57747001	3	57747001	57750000	3000	1	5.10E-09	-0.46	42	1.4	Cybrd1	Metabolism
DMR3:58664001	3	58664001	58665000	1000	1	2.00E-07	0.61	17	1.7	Rapgef4	Transcription
DMR3:58760001	3	58760001	58761000	1000	1	4.10E-09	0.48	13	1.3	Rapgef4	Transcription
DMR3:58884001	3	58884001	58885000	1000	1	1.40E-08	-0.67	9	0.9	Rapgef4;LOC103695171	Transcription
DMR3:58961001	3	58961001	58966000	5000	1	9.80E-07	-0.39	90	1.8	Zak	
DMR3:58990001	3	58990001	58996000	6000	2	8.50E-20	-0.84	101	1.68	Zak	
DMR3:59022001	3	59022001	59025000	3000	1	8.30E-08	-0.4	33	1.1	Zak	
DMR3:59597001	3	59597001	59599000	2000	1	1.40E-09	-0.43	57	2.85	LOC108350397;RGD1563716	
DMR3:59868001	3	59868001	59870000	2000	1	5.60E-10	0.44	9	0.45	Ola1	Signaling
DMR3:60015001	3	60015001	60016000	1000	1	3.60E-07	-0.37	15	1.5	Cir1;LOC108350582;Scrn3	
DMR3:60067001	3	60067001	60076000	9000	1	1.20E-07	-0.26	103	1.14	Gpr155	Transcription
DMR3:60112001	3	60112001	60113000	1000	1	7.00E-12	-0.49	7	0.7	Gpr155	Transcription
DMR3:60154001	3	60154001	60159000	5000	1	1.00E-17	0.96	119	2.38	Wipf1	Cytoskeleton
DMR3:60163001	3	60163001	60166000	3000	1	9.50E-07	-0.36	47	1.57	Wipf1	Cytoskeleton
DMR3:60206001	3	60206001	60207000	1000	1	1.20E-07	0.68	26	2.6	Wipf1	Cytoskeleton
DMR3:60608001	3	60608001	60610000	2000	1	4.30E-08	-0.37	16	0.8	Chn1	
DMR3:60642001	3	60642001	60645000	3000	1	3.60E-07	-0.5	24	0.8	Chn1	
DMR3:61652001	3	61652001	61653000	1000	1	2.30E-09	-0.64	10	1	Hoxd4;Mir10b;Hoxd3	
DMR3:61679001	3	61679001	61681000	2000	1	1.90E-07	0.39	22	1.1	Hoxd3;Hoxd1	Development
DMR3:61792001	3	61792001	61797000	5000	1	2.30E-08	-0.52	26	0.52	Mtx2	
DMR3:61802001	3	61802001	61806000	4000	2	3.50E-07	-0.42	17	0.42	Mtx2	
DMR3:62713001	3	62713001	62714000	1000	1	5.30E-07	-0.47	12	1.2	Agps	
DMR3:62895001	3	62895001	62897000	2000	1	1.60E-07	-0.49	44	2.2	Pde11a	Signaling
DMR3:62990001	3	62990001	62993000	3000	1	2.70E-08	0.38	32	1.07	Pde11a	Signaling
DMR3:63046001	3	63046001	63048000	2000	1	2.70E-07	-0.46	27	1.35	Pde11a	Signaling
DMR3:63110001	3	63110001	63113000	3000	2	3.30E-08	-0.25	26	0.87	Pde11a	Signaling
DMR3:63115001	3	63115001	63121000	6000	1	3.90E-07	-0.37	57	0.95	Pde11a	Signaling
DMR3:63138001	3	63138001	63143000	5000	1	1.80E-08	0.75	76	1.52	Pde11a	Signaling
DMR3:63144001	3	63144001	63147000	3000	2	5.60E-08	0.4	25	0.83	Pde11a	Signaling
DMR3:63150001	3	63150001	63151000	1000	1	6.10E-09	0.34	3	0.3	Pde11a	Signaling
DMR3:63166001	3	63166001	63171000	5000	1	6.10E-07	0.48	97	1.94	Pde11a	Signaling
DMR3:63172001	3	63172001	63177000	5000	1	4.80E-09	-0.41	43	0.86	Pde11a	Signaling
DMR3:63194001	3	63194001	63196000	2000	1	1.00E-08	0.71	37	1.85	Pde11a;Cyct	Signaling
DMR3:63230001	3	63230001	63233000	3000	2	3.10E-11	0.75	68	2.27	Rbm45	
DMR3:63271001	3	63271001	63274000	3000	1	9.60E-08	0.38	49	1.63	Osbpl6	
DMR3:63279001	3	63279001	63280000	1000	1	2.30E-07	-0.53	12	1.2	Osbpl6	
DMR3:63335001	3	63335001	63342000	7000	1	7.40E-07	-0.24	79	1.13	Osbpl6	
DMR3:63344001	3	63344001	63351000	7000	1	2.20E-12	-0.49	119	1.7	Osbpl6	
DMR3:63359001	3	63359001	63361000	2000	1	4.30E-09	-0.45	34	1.7	Osbpl6	
DMR3:63540001	3	63540001	63541000	1000	1	8.90E-08	-0.51	10	1	Fkbp7;Plekha3	Transcription
DMR3:63546001	3	63546001	63552000	6000	1	4.60E-11	-0.67	83	1.38	Plekha3	
DMR3:63565001	3	63565001	63569000	4000	1	5.10E-11	-0.57	78	1.95	Plekha3;Ttn	
DMR3:63571001	3	63571001	63574000	3000	1	1.40E-08	-0.44	140	4.67	Ttn	
DMR3:63596001	3	63596001	63605000	9000	1	9.50E-09	-0.49	266	2.96	Ttn	
DMR3:63618001	3	63618001	63625000	7000	2	7.10E-07	-0.48	140	2	Ttn	
DMR3:63644001	3	63644001	63649000	5000	1	1.40E-07	-0.47	130	2.6	Ttn	
DMR3:63685001	3	63685001	63688000	3000	1	3.30E-08	-0.42	50	1.67	Ttn	
DMR3:63729001	3	63729001	63735000	6000	1	1.20E-08	-0.49	105	1.75	Ttn	
DMR3:63919001	3	63919001	63920000	1000	1	3.30E-07	0.38	7	0.7	Ccdc141	
DMR3:64128001	3	64128001	64133000	5000	1	1.60E-12	-0.76	68	1.36	Sestd1	
DMR3:64312001	3	64312001	64316000	4000	1	2.40E-07	0.29	86	2.15	Zfp385b	

DMR3:64341001	3	64341001	64342000	1000	1	3.20E-08	0.5	22	2.2	Zfp385b	
DMR3:64411001	3	64411001	64414000	3000	1	4.70E-07	-0.45	34	1.13	Zfp385b	
DMR3:64430001	3	64430001	64432000	2000	1	1.20E-09	-0.43	18	0.9	Zfp385b	
DMR3:64454001	3	64454001	64459000	5000	1	3.40E-07	-0.35	75	1.5	Zfp385b	
DMR3:64788001	3	64788001	64793000	5000	1	1.60E-07	-0.4	35	0.7	Cwc22;LOC295681	Translation
DMR3:66230001	3	66230001	66233000	3000	1	1.10E-07	0.46	19	0.63	Itga4	Extracellular Matrix
DMR3:66299001	3	66299001	66300000	1000	1	1.10E-07	-0.54	14	1.4	Cerkl	Signaling
DMR3:66306001	3	66306001	66308000	2000	1	3.20E-15	0.73	31	1.55	Cerkl	Signaling
DMR3:66316001	3	66316001	66324000	8000	1	4.00E-10	-0.33	83	1.04	Cerkl	Signaling
DMR3:66616001	3	66616001	66617000	1000	1	2.50E-08	-0.49	9	0.9	Ssfa2	
DMR3:66669001	3	66669001	66672000	3000	1	1.20E-08	0.47	15	0.5	Ppp1r1c	Signaling
DMR3:66686001	3	66686001	66688000	2000	1	4.70E-13	0.49	26	1.3	Ppp1r1c	Signaling
DMR3:66745001	3	66745001	66746000	1000	1	3.40E-07	0.35	18	1.8	Ppp1r1c	Signaling
DMR3:66912001	3	66912001	66916000	4000	1	1.60E-09	-0.36	47	1.18	Pde1a	Signaling
DMR3:67070001	3	67070001	67076000	6000	1	6.00E-08	-0.32	64	1.07	Pde1a	Signaling
DMR3:67212001	3	67212001	67216000	4000	1	6.30E-07	-0.31	29	0.72	Pde1a	Signaling
DMR3:67526001	3	67526001	67529000	3000	1	9.80E-10	0.56	17	0.57	Dnajc10	Transcription
DMR3:67530001	3	67530001	67531000	1000	1	3.00E-08	0.44	14	1.4	Dnajc10	Transcription
DMR3:67557001	3	67557001	67560000	3000	1	4.10E-07	-0.4	26	0.87	Dnajc10	Transcription
DMR3:67812001	3	67812001	67816000	4000	1	7.90E-10	-0.51	56	1.4	Nckap1	
DMR3:67876001	3	67876001	67878000	2000	1	4.40E-07	-0.51	9	0.45	Nup35	
DMR3:69369001	3	69369001	69371000	2000	2	2.60E-08	0.59	26	1.3	Zfp804a	
DMR3:70276001	3	70276001	70279000	3000	1	2.80E-08	-0.69	13	0.43	Fsip2	
DMR3:70328001	3	70328001	70331000	3000	1	9.60E-10	-0.51	53	1.77	Fsip2	
DMR3:71304001	3	71304001	71307000	3000	1	2.00E-11	-0.7	26	0.87	Zswim2;Trnap-agg	Proteolysis
DMR3:71766001	3	71766001	71768000	2000	1	8.50E-08	0.45	12	0.6	Calcrl	Receptor
DMR3:71770001	3	71770001	71773000	3000	1	3.20E-10	0.43	39	1.3	Calcrl	Receptor
DMR3:71781001	3	71781001	71784000	3000	1	3.60E-08	-0.62	31	1.03	Calcrl	Receptor
DMR3:71822001	3	71822001	71826000	4000	1	2.50E-09	0.43	38	0.95	Calcrl;LOC108350411	Receptor
DMR3:71865001	3	71865001	71867000	2000	1	2.60E-07	0.46	12	0.6	Tfpi	Protease; Proteolysis
DMR3:71898001	3	71898001	71901000	3000	1	4.90E-07	-0.46	35	1.17	Tfpi	Protease; Proteolysis
DMR3:72086001	3	72086001	72087000	1000	1	3.90E-14	0.58	6	0.6	Tmx2;Med19;Zdhhc5	Metabolism
DMR3:72113001	3	72113001	72114000	1000	1	4.70E-10	-0.52	11	1.1	Zdhhc5	
DMR3:72251001	3	72251001	72252000	1000	1	4.10E-07	0.5	15	1.5	Slc43a1	
DMR3:72400001	3	72400001	72401000	1000	1	1.20E-08	-0.51	15	1.5	Prg3;P2rx3	Extracellular Matrix;Ion Channel
DMR3:72429001	3	72429001	72431000	2000	1	1.20E-07	0.54	26	1.3	P2rx3	Ion Channel
DMR3:72530001	3	72530001	72531000	1000	1	4.40E-07	-0.49	17	1.7	Aplnr	Signaling
DMR3:72610001	3	72610001	72611000	1000	1	1.90E-11	-0.45	16	1.6	Lrrc55	
DMR3:72797001	3	72797001	72801000	4000	1	7.00E-07	-0.47	28	0.7	Olr440;Olr441	Receptor
DMR3:72973001	3	72973001	72974000	1000	1	2.10E-07	0.47	1	0.1	Olr443	Signaling
DMR3:73018001	3	73018001	73019000	1000	1	2.60E-08	0.39	9	0.9	Olr447	Signaling
DMR3:73336001	3	73336001	73337000	1000	1	2.00E-08	-0.75	9	0.9	LOC684683;Olr470;Olr471	Receptor
DMR3:73340001	3	73340001	73341000	1000	1	1.30E-08	0.56	13	1.3	Olr470;Olr471	Receptor
DMR3:73380001	3	73380001	73381000	1000	1	4.50E-08	0.41	10	1	LOC103691839;Olr475	Receptor
DMR3:73440001	3	73440001	73446000	6000	2	3.10E-09	-0.46	47	0.78	Olr478-ps;Olr479	Receptor
DMR3:73460001	3	73460001	73461000	1000	1	1.20E-07	0.54	9	0.9	Olr479	Receptor
DMR3:73536001	3	73536001	73537000	1000	1	9.50E-07	-0.45	9	0.9	Olr485	Receptor
DMR3:73547001	3	73547001	73554000	7000	1	8.10E-08	-0.29	78	1.11	Olr485;Olr486	Receptor
DMR3:73555001	3	73555001	73556000	1000	1	3.40E-15	0.78	5	0.5	Olr486	Receptor
DMR3:73685001	3	73685001	73688000	3000	1	3.00E-08	-0.58	11	0.37	LOC100912505;Olr497-ps	Receptor
DMR3:73691001	3	73691001	73698000	7000	2	1.30E-08	-0.43	67	0.96	Olr497-ps;Olr498-ps	
DMR3:73764001	3	73764001	73765000	1000	1	2.90E-07	0.53	14	1.4	Olr500;Olr502;Olr503-ps	Receptor
DMR3:73805001	3	73805001	73806000	1000	1	2.10E-09	0.38	3	0.3	Olr507	Receptor
DMR3:73954001	3	73954001	73960000	6000	2	1.40E-10	-0.44	71	1.18	Olr514	Receptor
DMR3:74411001	3	74411001	74412000	1000	1	1.80E-07	0.5	7	0.7	Olr525-ps;Olr526	Receptor
DMR3:74467001	3	74467001	74468000	1000	1	4.20E-09	0.55	19	1.9	Olr528	Receptor
DMR3:74494001	3	74494001	74496000	2000	1	3.50E-07	-0.45	10	0.5	Olr529	Receptor
DMR3:74505001	3	74505001	74508000	3000	1	4.30E-07	-0.27	19	0.63	Olr529	Receptor
DMR3:74706001	3	74706001	74708000	2000	1	5.00E-08	-0.33	16	0.8	Olr541	Signaling
DMR3:74939001	3	74939001	74945000	6000	1	2.00E-09	-0.65	81	1.35	Olr549-ps	
DMR3:75030001	3	75030001	75031000	1000	1	2.20E-07	-0.42	7	0.7	Olr547-ps	
DMR3:75079001	3	75079001	75085000	6000	1	8.30E-09	-0.35	53	0.88	Olr542	Signaling

DMR3:75304001	3	75304001	75309000	5000	1	6.60E-11	-0.35	48	0.96	Olr555	Receptor
DMR3:75310001	3	75310001	75311000	1000	1	1.40E-07	0.26	18	1.8	Olr555	Receptor
DMR3:75360001	3	75360001	75361000	1000	1	9.10E-07	-0.3	4	0.4	Olr557	Receptor
DMR3:75386001	3	75386001	75388000	2000	1	1.10E-08	-0.41	19	0.95	Olr558;Olr559	Receptor
DMR3:75392001	3	75392001	75399000	7000	1	1.80E-09	-0.33	66	0.94	Olr559;LOC685743	Receptor
DMR3:75414001	3	75414001	75416000	2000	1	1.90E-08	0.54	41	2.05	LOC685743;Olr560;LOC685772	Receptor
DMR3:75422001	3	75422001	75428000	6000	2	2.90E-10	-0.47	55	0.92	Olr560;LOC685772;Olr561	Receptor
DMR3:75446001	3	75446001	75452000	6000	1	1.70E-07	-0.45	54	0.9	Olr561	Receptor
DMR3:75646001	3	75646001	75648000	2000	1	3.20E-08	-0.49	22	1.1	Olr572-ps;LOC100361457;LOC100360176	
DMR3:75727001	3	75727001	75728000	1000	1	2.10E-09	-0.53	9	0.9	Olr576;Olr577	Signaling;Receptor
DMR3:75729001	3	75729001	75730000	1000	1	3.70E-07	0.36	12	1.2	Olr576;Olr577	Signaling;Receptor
DMR3:75753001	3	75753001	75754000	1000	1	6.70E-09	0.54	9	0.9	Olr578	Signaling
DMR3:75828001	3	75828001	75834000	6000	2	2.00E-08	-0.43	52	0.87	Olr582	Signaling
DMR3:75898001	3	75898001	75904000	6000	2	1.20E-08	-0.37	52	0.87	Olr585-ps;Olr586	Signaling
DMR3:76122001	3	76122001	76126000	4000	1	4.30E-09	-0.28	33	0.82	LOC102555599;Olr603-ps;Olr604	Receptor
DMR3:76149001	3	76149001	76151000	2000	1	6.40E-07	0.5	20	1	Olr605-ps	
DMR3:76306001	3	76306001	76310000	4000	1	9.90E-07	-0.29	35	0.88	Olr610	
DMR3:76668001	3	76668001	76673000	5000	1	6.60E-07	-0.33	41	0.82	Olr627-ps	
DMR3:76686001	3	76686001	76688000	2000	1	2.20E-10	-0.48	15	0.75	Olr628-ps;Olr629	Receptor
DMR3:76689001	3	76689001	76690000	1000	1	1.70E-07	0.33	9	0.9	Olr628-ps;Olr629	Receptor
DMR3:77084001	3	77084001	77090000	6000	2	2.50E-09	-0.33	66	1.1	Olr650	Receptor
DMR3:77441001	3	77441001	77450000	9000	2	4.30E-08	-0.26	72	0.8	Olr660	Receptor
DMR3:77528001	3	77528001	77531000	3000	1	2.30E-08	-0.4	14	0.47	Olr663	Receptor
DMR3:77649001	3	77649001	77650000	1000	1	9.40E-07	0.46	5	0.5	Olr667-ps	
DMR3:77718001	3	77718001	77722000	4000	1	2.50E-07	-0.38	18	0.45	Olr671;Olr672	Receptor
DMR3:78417001	3	78417001	78419000	2000	1	1.20E-07	-0.34	18	0.9	Olr704;Olr705	Receptor
DMR3:78549001	3	78549001	78550000	1000	1	2.80E-07	-0.39	6	0.6	Olr710;Olr711	Receptor
DMR3:78560001	3	78560001	78561000	1000	1	2.00E-07	0.44	5	0.5	Olr711;Olr712	Receptor
DMR3:78704001	3	78704001	78706000	2000	1	1.10E-08	-0.37	15	0.75	Olr719-ps	
DMR3:78732001	3	78732001	78734000	2000	1	1.80E-08	-0.52	13	0.65	Olr721	Receptor
DMR3:78739001	3	78739001	78740000	1000	1	3.10E-08	0.43	8	0.8	Olr722	Receptor
DMR3:78867001	3	78867001	78869000	2000	1	3.40E-07	0.34	18	0.9	Olr727;Olr728	Receptor
DMR3:78926001	3	78926001	78932000	6000	2	5.90E-07	-0.27	60	1	Olr730-ps;Olr731	Receptor
DMR3:78962001	3	78962001	78965000	3000	1	6.20E-09	-0.43	24	0.8	Olr733;Olr732-ps	Receptor
DMR3:78999001	3	78999001	79001000	2000	1	5.40E-07	-0.38	10	0.5	Olr735	Receptor
DMR3:79002001	3	79002001	79007000	5000	2	1.20E-09	-0.3	45	0.9	Olr735	Receptor
DMR3:79024001	3	79024001	79027000	3000	1	6.60E-07	-0.4	12	0.4	Olr736	Receptor
DMR3:79039001	3	79039001	79041000	2000	1	9.80E-08	-0.43	14	0.7	Olr737	Receptor
DMR3:79174001	3	79174001	79182000	8000	2	2.90E-08	-0.4	86	1.07	Olr744;Olr745	Receptor
DMR3:79278001	3	79278001	79282000	4000	1	1.90E-07	0.32	69	1.73	Ptprj	Signaling
DMR3:79356001	3	79356001	79361000	5000	1	6.40E-07	-0.45	80	1.6	Ptprj	Signaling
DMR3:79487001	3	79487001	79490000	3000	2	1.70E-10	-0.42	26	0.87	LOC102555600;LOC103691844;Nup160	Transport
DMR3:79547001	3	79547001	79548000	1000	1	9.30E-08	-0.5	13	1.3	Nup160	Transport
DMR3:79554001	3	79554001	79557000	3000	1	4.90E-07	0.47	24	0.8	Nup160	Transport
DMR3:79939001	3	79939001	79941000	2000	1	4.70E-11	0.49	30	1.5	Spi1;Mybpc3	Transcription
DMR3:80121001	3	80121001	80123000	2000	1	1.30E-07	0.51	18	0.9	RGD1309540	
DMR3:80887001	3	80887001	80888000	1000	1	9.50E-07	0.41	21	2.1	Dgkz;LOC103691847;Creb3l1	Signaling
DMR3:80908001	3	80908001	80913000	5000	1	6.30E-08	0.38	63	1.26	LOC103691847;Creb3l1	
DMR3:80938001	3	80938001	80941000	3000	1	3.90E-10	0.41	33	1.1	LOC103691847;Creb3l1	
DMR3:81357001	3	81357001	81358000	1000	1	3.30E-07	-0.35	17	1.7	Slc35c1	Transport
DMR3:81867001	3	81867001	81868000	1000	1	7.10E-08	0.41	11	1.1	Prdm11	Transcription
DMR3:81887001	3	81887001	81891000	4000	1	1.50E-08	0.36	45	1.12	Prdm11;LOC102547078	Transcription
DMR3:82105001	3	82105001	82106000	1000	1	1.60E-07	0.6	9	0.9	Tp53i11;Tspan18	
DMR3:82149001	3	82149001	82150000	1000	1	3.10E-07	-0.45	17	1.7	Tspan18	
DMR3:82167001	3	82167001	82171000	4000	1	4.10E-07	-0.44	43	1.07	Tspan18	
DMR3:82187001	3	82187001	82190000	3000	1	4.10E-10	0.33	45	1.5	Tspan18	
DMR3:82219001	3	82219001	82223000	4000	1	1.90E-08	0.4	42	1.05	Tspan18	
DMR3:82338001	3	82338001	82340000	2000	1	2.80E-08	0.38	11	0.55	Cd82	
DMR3:82563001	3	82563001	82564000	1000	1	7.20E-07	0.5	11	1.1	Alx4	Development
DMR3:82720001	3	82720001	82724000	4000	1	7.50E-07	-0.33	70	1.75	Ext2	Golgi
DMR3:82728001	3	82728001	82731000	3000	1	7.10E-07	-0.43	35	1.17	Ext2	Golgi

DMR3:82775001	3	82775001	82776000	1000	1	5.50E-08	0.44	11	1.1	Accsl	Metabolism
DMR3:82857001	3	82857001	82858000	1000	1	7.80E-07	0.4	17	1.7	RGD1564664	
DMR3:83009001	3	83009001	83012000	3000	1	7.70E-08	-0.52	22	0.73	Hsd17b12	
DMR3:83254001	3	83254001	83255000	1000	1	4.70E-07	0.38	5	0.5	Ttc17	
DMR3:85475001	3	85475001	85476000	1000	1	4.10E-07	0.48	11	1.1	Lrrc4c	
DMR3:85582001	3	85582001	85584000	2000	1	8.50E-08	-0.28	19	0.95	Lrrc4c	
DMR3:85856001	3	85856001	85857000	1000	1	4.00E-10	-0.59	8	0.8	Lrrc4c;LOC366130	
DMR3:85952001	3	85952001	85954000	2000	1	9.90E-07	-0.4	10	0.5	Lrrc4c	
DMR3:86787001	3	86787001	86788000	1000	1	3.30E-07	0.45	7	0.7	Lrrc4c	
DMR3:86790001	3	86790001	86791000	1000	1	9.60E-07	0.46	4	0.4	Lrrc4c	
DMR3:86793001	3	86793001	86794000	1000	1	4.20E-09	0.43	5	0.5	Lrrc4c	
DMR3:91185001	3	91185001	91188000	3000	1	2.50E-08	-0.47	36	1.2	RGD1309730;Rag2	
DMR3:91190001	3	91190001	91192000	2000	1	1.40E-08	-0.43	19	0.95	RGD1309730;Rag2	
DMR3:91439001	3	91439001	91441000	2000	1	4.80E-07	0.63	42	2.1	Prr5l	
DMR3:91467001	3	91467001	91469000	2000	1	4.00E-12	-0.74	23	1.15	Prr5l;Commd9	
DMR3:91488001	3	91488001	91489000	1000	1	9.20E-09	-0.59	11	1.1	Commd9	
DMR3:91660001	3	91660001	91661000	1000	1	1.70E-07	0.43	1	0.1	Ldlrad3	Binding Proteins
DMR3:91744001	3	91744001	91745000	1000	1	5.90E-11	0.4	8	0.8	Ldlrad3	Binding Proteins
DMR3:92137001	3	92137001	92138000	1000	1	7.80E-08	0.34	3	0.3	Trim44;LOC102551100	Proteolysis
DMR3:92960001	3	92960001	92962000	2000	1	4.10E-07	-0.52	17	0.85	Apip	Metabolism
DMR3:93343001	3	93343001	93344000	1000	1	3.50E-09	-0.47	21	2.1	Elf5;LOC102548892	Transcription
DMR3:93397001	3	93397001	93399000	2000	1	1.80E-07	0.37	23	1.15	Cat;LOC108350544	Metabolism
DMR3:93693001	3	93693001	93694000	1000	1	7.00E-07	-0.66	15	1.5	Nat10;Caprin1	Translation;Metabolism
DMR3:93695001	3	93695001	93696000	1000	1	4.10E-07	-0.71	13	1.3	Nat10;Caprin1	Translation;Metabolism
DMR3:93769001	3	93769001	93772000	3000	1	4.50E-08	0.64	34	1.13	Caprin1;LOC108350425	Metabolism
DMR3:94374001	3	94374001	94376000	2000	1	1.40E-07	-0.34	11	0.55	Hipk3	
DMR3:94645001	3	94645001	94649000	4000	1	2.30E-07	-0.35	38	0.95	Tcp11l1	Cytoskeleton
DMR3:94880001	3	94880001	94887000	7000	2	4.20E-08	-0.32	89	1.27	Ccdc73	
DMR3:94921001	3	94921001	94928000	7000	3	1.60E-07	-0.32	71	1.01	Ccdc73	
DMR3:95170001	3	95170001	95172000	2000	1	1.40E-08	0.38	20	1	Wt1	Transcription
DMR3:95688001	3	95688001	95691000	3000	1	8.10E-10	0.37	38	1.27	Pax6	
DMR3:95907001	3	95907001	95913000	6000	1	1.00E-09	-0.38	48	0.8	Elp4	
DMR3:97283001	3	97283001	97284000	1000	1	3.40E-09	0.5	16	1.6	Dcdc5	
DMR3:97365001	3	97365001	97369000	4000	1	3.40E-08	-0.29	38	0.95	Dcdc5	
DMR3:97370001	3	97370001	97371000	1000	1	4.60E-13	0.5	15	1.5	Dcdc5	
DMR3:97711001	3	97711001	97713000	2000	1	6.70E-09	-0.61	24	1.2	LOC103691853;Mpped2	Metabolism
DMR3:97803001	3	97803001	97806000	3000	1	6.60E-07	-0.41	76	2.53	Mpped2	Metabolism
DMR3:97809001	3	97809001	97811000	2000	1	1.70E-07	-0.38	44	2.2	Mpped2	Metabolism
DMR3:97859001	3	97859001	97860000	1000	1	7.70E-08	0.35	20	2	Mpped2	Metabolism
DMR3:97882001	3	97882001	97884000	2000	1	1.60E-09	-0.45	38	1.9	Mpped2	Metabolism
DMR3:98298001	3	98298001	98300000	2000	1	3.90E-10	0.91	46	2.3	Kcna4	Transport
DMR3:100197001	3	100197001	100198000	1000	1	1.60E-14	0.39	4	0.4	Mettl15	Epigenetic
DMR3:100359001	3	100359001	100361000	2000	1	7.80E-08	-0.42	11	0.55	Mettl15;Kif18a	Epigenetic;Cytoskeleton
DMR3:101167001	3	101167001	101171000	4000	1	8.70E-09	-0.42	21	0.52	Ccdc34	
DMR3:101196001	3	101196001	101201000	5000	2	2.00E-11	-0.43	34	0.68	Ccdc34	
DMR3:101484001	3	101484001	101489000	5000	2	1.30E-07	-0.39	24	0.48	Bbox1	Metabolism
DMR3:101857001	3	101857001	101858000	1000	1	5.30E-08	-0.48	9	0.9	Ano3	
DMR3:101975001	3	101975001	101981000	6000	1	3.60E-07	-0.32	51	0.85	Ano3	
DMR3:102058001	3	102058001	102063000	5000	1	3.30E-07	-0.39	38	0.76	Ano3	
DMR3:102081001	3	102081001	102082000	1000	1	1.80E-09	0.55	15	1.5	Ano3	
DMR3:102183001	3	102183001	102185000	2000	2	9.70E-10	0.47	8	0.4	Ano3	
DMR3:102387001	3	102387001	102389000	2000	1	4.60E-08	0.27	41	2.05	Ankrd30a	
DMR3:102390001	3	102390001	102395000	5000	3	3.90E-08	-0.36	45	0.9	Ankrd30a	
DMR3:102494001	3	102494001	102496000	2000	1	4.90E-07	0.37	8	0.4	Olr750	Receptor
DMR3:102539001	3	102539001	102544000	5000	2	1.30E-08	-0.3	47	0.94	Olr752;Olr753	Receptor
DMR3:102568001	3	102568001	102570000	2000	2	6.00E-08	0.53	10	0.5	Olr754	Receptor
DMR3:102662001	3	102662001	102665000	3000	1	2.30E-07	0.43	30	1	Olr758;RGD1561276;Olr760	Receptor
DMR3:102765001	3	102765001	102766000	1000	1	1.90E-07	-0.37	3	0.3	Olr767	Receptor
DMR3:103010001	3	103010001	103015000	5000	1	2.00E-08	-0.41	25	0.5	Olr774	Receptor
DMR3:103160001	3	103160001	103163000	3000	1	8.70E-10	-0.48	19	0.63	Olr781;Olr782	Receptor
DMR3:103274001	3	103274001	103276000	2000	1	5.80E-10	-0.38	21	1.05	Olr787-ps	
DMR3:103302001	3	103302001	103303000	1000	1	4.00E-09	-0.47	8	0.8	Olr788	Receptor
DMR3:103389001	3	103389001	103390000	1000	1	4.60E-09	-0.43	9	0.9	Olr790	Receptor

DMR3:103391001	3	103391001	103394000	3000	1	7.30E-07	-0.34	21	0.7	Olr790	Receptor
DMR3:103552001	3	103552001	103553000	1000	1	1.10E-07	0.45	4	0.4	Olr792	Receptor
DMR3:103644001	3	103644001	103650000	6000	2	1.70E-07	-0.35	68	1.13	Olr795;Olr796	Receptor
DMR3:103848001	3	103848001	103850000	2000	2	2.50E-07	0.4	24	1.2	Slc12a6;Emc4	Transport
DMR3:104031001	3	104031001	104032000	1000	1	3.60E-08	0.4	7	0.7	Aven	
DMR3:104060001	3	104060001	104062000	2000	1	2.80E-08	-0.52	25	1.25	Aven;LOC108350586;LOC102551301	
DMR3:104141001	3	104141001	104143000	2000	1	2.20E-07	-0.45	28	1.4	Ryr3	Ion Channel
DMR3:104213001	3	104213001	104215000	2000	1	2.10E-13	0.71	30	1.5	Ryr3;LOC103691859	Ion Channel
DMR3:104309001	3	104309001	104310000	1000	1	1.10E-07	0.8	27	2.7	Ryr3	Ion Channel
DMR3:104314001	3	104314001	104315000	1000	1	9.40E-08	-0.53	8	0.8	Ryr3	Ion Channel
DMR3:104660001	3	104660001	104662000	2000	1	1.60E-09	-0.52	24	1.2	Ryr3	Ion Channel
DMR3:104677001	3	104677001	104684000	7000	1	6.80E-11	-0.43	81	1.16	Hmgn4	
DMR3:104741001	3	104741001	104749000	8000	2	6.50E-09	-0.38	56	0.7	Tmco5b;LOC102553315	
DMR3:105013001	3	105013001	105015000	2000	1	3.50E-09	-0.45	21	1.05	Fmn1	
DMR3:105026001	3	105026001	105029000	3000	1	8.10E-15	-0.62	34	1.13	Fmn1	
DMR3:105147001	3	105147001	105148000	1000	1	8.90E-08	0.4	15	1.5	Fmn1;LOC102551877	
DMR3:105220001	3	105220001	105221000	1000	1	4.10E-07	-0.37	10	1	Grem1	
DMR3:107213001	3	107213001	107215000	2000	1	8.20E-08	-0.44	17	0.85	RGD1563680;LOC103691863	
DMR3:108538001	3	108538001	108539000	1000	1	5.10E-09	-0.32	7	0.7	Tmco5a	
DMR3:108951001	3	108951001	108957000	6000	1	1.40E-08	-0.36	61	1.02	Fam98b	
DMR3:110094001	3	110094001	110099000	5000	1	2.10E-10	-0.58	57	1.14	Gpr176	Signaling
DMR3:110174001	3	110174001	110176000	2000	1	7.70E-08	0.52	20	1	Eif2ak4	Signaling
DMR3:110298001	3	110298001	110300000	2000	1	6.90E-09	0.45	15	0.75	Bmf	
DMR3:110624001	3	110624001	110626000	2000	1	3.60E-07	-0.34	16	0.8	Knstrn	
DMR3:110694001	3	110694001	110698000	4000	1	3.40E-09	0.37	62	1.55	Ivd	Metabolism
DMR3:110971001	3	110971001	110972000	1000	1	3.70E-07	-0.45	18	1.8	Rmdn3;LOC108350564;Gchfr;Dnajc17;LOC691418	Signaling;Transcription
DMR3:111008001	3	111008001	111009000	1000	1	6.70E-07	-0.48	15	1.5	Dnajc17;Zfyve19	Transcription
DMR3:111052001	3	111052001	111054000	2000	1	1.30E-08	-0.45	18	0.9	Spint1	Protease; Proteolysis
DMR3:111095001	3	111095001	111097000	2000	1	2.80E-08	0.53	21	1.05	Rhov;Vps18	Signaling;Transport
DMR3:111142001	3	111142001	111143000	1000	1	1.60E-09	0.43	18	1.8	LOC103691871;Dll4	
DMR3:111843001	3	111843001	111844000	1000	1	3.60E-07	0.39	16	1.6	Pla2g4b;Sptbn5	Metabolism
DMR3:112045001	3	112045001	112048000	3000	1	3.90E-11	-0.52	34	1.13	Pla2g4d	Metabolism
DMR3:112200001	3	112200001	112202000	2000	1	2.10E-07	-0.51	16	0.8	Ganc;LOC108350445	Metabolism
DMR3:112240001	3	112240001	112241000	1000	1	5.90E-07	0.52	10	1	Capn3	Protease
DMR3:112287001	3	112287001	112288000	1000	1	1.60E-08	-0.48	15	1.5	Capn3;Zfp106	Protease
DMR3:112348001	3	112348001	112350000	2000	1	2.90E-08	-0.43	18	0.9	Snap23	Transcription
DMR3:112648001	3	112648001	112653000	5000	1	4.40E-07	0.53	65	1.3	Stard9;Cdan1	Cytoskeleton
DMR3:112829001	3	112829001	112831000	2000	1	5.50E-08	-0.47	21	1.05	Ubr1	Proteolysis
DMR3:112871001	3	112871001	112872000	1000	1	7.70E-09	-0.73	13	1.3	Ubr1	Proteolysis
DMR3:113014001	3	113014001	113015000	1000	1	2.20E-07	0.39	13	1.3	Tgm7l1	
DMR3:113144001	3	113144001	113146000	2000	1	1.40E-09	0.44	4	0.2	Tubgcp4	Cytoskeleton
DMR3:113176001	3	113176001	113178000	2000	1	6.00E-07	0.27	12	0.6	Tp53bp1	Transcription
DMR3:113214001	3	113214001	113221000	7000	1	3.70E-09	-0.72	54	0.77	Tp53bp1	Transcription
DMR3:113432001	3	113432001	113433000	1000	1	1.20E-10	0.56	3	0.3	Serinc4;Hypk;Mfap1a	Signaling
DMR3:113460001	3	113460001	113461000	1000	1	7.20E-07	0.48	1	0.1	Wdr76	
DMR3:113489001	3	113489001	113490000	1000	1	4.60E-09	0.5	20	2	Wdr76;Frmd5	
DMR3:113815001	3	113815001	113816000	1000	1	2.50E-07	-0.43	16	1.6	Casc4	
DMR3:113852001	3	113852001	113853000	1000	1	2.80E-09	0.6	16	1.6	Casc4	
DMR3:114059001	3	114059001	114060000	1000	1	5.80E-07	-0.42	22	2.2	Spg11;Patl2	Translation
DMR3:114115001	3	114115001	114118000	3000	1	7.90E-10	0.53	28	0.93	Trim69	Proteolysis
DMR3:114289001	3	114289001	114290000	1000	1	2.30E-09	0.47	13	1.3	Duox1;Shf	Metabolism
DMR3:114352001	3	114352001	114355000	3000	2	3.90E-08	-0.37	23	0.77	LOC102557338;Slc28a2	Transport
DMR3:114430001	3	114430001	114431000	1000	1	2.00E-07	-0.38	6	0.6	Slc28a2	Transport
DMR3:114532001	3	114532001	114534000	2000	1	2.10E-07	-0.33	12	0.6	Slc28a2	Transport
DMR3:114626001	3	114626001	114627000	1000	1	6.80E-09	0.46	5	0.5	Slc28a2	Transport
DMR3:114716001	3	114716001	114717000	1000	1	8.10E-11	0.34	12	1.2	Gatm	Transport
DMR3:114767001	3	114767001	114771000	4000	1	4.70E-09	-0.4	77	1.93	Spata5l1;MGC105649;Mir147	
DMR3:114837001	3	114837001	114840000	3000	1	5.30E-07	0.45	22	0.73	Slc30a4;LOC108350451	Transport
DMR3:114904001	3	114904001	114910000	6000	1	8.00E-07	-0.47	84	1.4	Sqrdl	
DMR3:116931001	3	116931001	116933000	2000	1	3.10E-09	-0.63	30	1.5	Sema6d	Signaling
DMR3:117441001	3	117441001	117443000	2000	1	3.60E-09	-0.51	19	0.95	Slc12a1	Transport

DMR3:117450001	3	117450001	117452000	2000	1	4.10E-07	0.59	32	1.6	Slc12a1	Transport
DMR3:117492001	3	117492001	117496000	4000	1	1.40E-10	0.48	24	0.6	Slc12a1;LOC102555270	Transport
DMR3:117619001	3	117619001	117620000	1000	1	4.70E-07	-0.46	27	2.7	Fbn1	Extracellular Matrix
DMR3:117843001	3	117843001	117844000	1000	1	1.20E-07	-0.5	18	1.8	Cep152;LOC102547425	
DMR3:117845001	3	117845001	117848000	3000	2	1.80E-08	-0.49	80	2.67	Cep152;LOC102547425	
DMR3:117886001	3	117886001	117888000	2000	1	1.10E-12	-0.61	20	1	Cep152;Shc4	Cytoskeleton
DMR3:117905001	3	117905001	117909000	4000	1	2.80E-12	-0.48	64	1.6	Shc4;LOC108350457	Cytoskeleton
DMR3:118013001	3	118013001	118014000	1000	1	5.50E-09	-0.64	12	1.2	Secisbp2l	
DMR3:118040001	3	118040001	118042000	2000	1	2.50E-08	-0.52	32	1.6	Secisbp2l;LOC102548101	
DMR3:118108001	3	118108001	118110000	2000	1	1.10E-08	-0.67	27	1.35	Cops2	
DMR3:118238001	3	118238001	118245000	7000	1	7.50E-07	-0.67	38	0.54	Galk2	Metabolism
DMR3:118288001	3	118288001	118293000	5000	1	1.60E-08	-0.31	44	0.88	Fam227b	
DMR3:118376001	3	118376001	118381000	5000	1	1.10E-10	-0.45	19	0.38	Fam227b;Fgf7	Growth Factors
DMR3:118409001	3	118409001	118416000	7000	2	4.60E-09	-0.47	70	1	Fam227b	
DMR3:118598001	3	118598001	118600000	2000	1	3.70E-08	-0.46	5	0.25	Atp8b4	Transport
DMR3:118675001	3	118675001	118676000	1000	1	2.10E-09	1.02	8	0.8	Atp8b4	Transport
DMR3:119080001	3	119080001	119082000	2000	1	5.20E-08	0.64	33	1.65	Hdc;Gabpb1	Metabolism
DMR3:119095001	3	119095001	119099000	4000	1	6.00E-13	0.48	43	1.07	Gabpb1	
DMR3:119318001	3	119318001	119319000	1000	1	8.60E-10	-0.37	4	0.4	Trpm7	Transport
DMR3:119539001	3	119539001	119544000	5000	1	4.10E-08	-0.65	39	0.78	Ap4e1;Blvra	Transport;Metabolism
DMR3:119788001	3	119788001	119789000	1000	1	1.00E-08	0.31	9	0.9	Dusp2;Astl	Signaling;Protease
DMR3:120022001	3	120022001	120024000	2000	1	7.00E-08	0.4	41	2.05	RGD1561323;Kcnip3	
DMR3:120182001	3	120182001	120186000	4000	1	3.90E-07	-0.4	64	1.6	Mrps5;LOC108350459	Translation
DMR3:120301001	3	120301001	120302000	1000	1	2.20E-09	0.4	11	1.1	Mall	Transport
DMR3:120313001	3	120313001	120315000	2000	1	1.30E-07	0.32	11	0.55	Mall;Nphp1	Transport;Cytoskeleton
DMR3:120414001	3	120414001	120415000	1000	1	5.20E-07	0.52	16	1.6	Bub1;Acoxl	Signaling;Metabolism
DMR3:120626001	3	120626001	120627000	1000	1	4.80E-10	0.59	10	1	Acoxl	Metabolism
DMR3:120648001	3	120648001	120649000	1000	1	1.00E-11	0.38	22	2.2	Acoxl	Metabolism
DMR3:120704001	3	120704001	120710000	6000	1	1.20E-07	-0.37	57	0.95	Acoxl	Metabolism
DMR3:121296001	3	121296001	121298000	2000	1	4.90E-08	0.33	16	0.8	Mertk	Receptor
DMR3:121533001	3	121533001	121535000	2000	1	4.90E-07	-0.41	23	1.15	Zc3h6	Transcription
DMR3:121627001	3	121627001	121629000	2000	1	2.50E-08	0.51	15	0.75	Ttl;Polr1b	Transcription
DMR3:122538001	3	122538001	122540000	2000	1	7.70E-07	-0.38	27	1.35	LOC108350463;Tgm3	Transport
DMR3:122753001	3	122753001	122754000	1000	1	1.80E-07	0.4	4	0.4	Tmc2	
DMR3:122812001	3	122812001	122813000	1000	1	4.00E-07	-0.42	12	1.2	Nop56;Idh3B;RGD1561317;Rps12l3	Metabolism;Metabolism
DMR3:122881001	3	122881001	122883000	2000	1	1.60E-07	0.5	40	2	Ebf4	Transcription
DMR3:122885001	3	122885001	122887000	2000	1	5.60E-09	0.65	39	1.95	Ebf4	Transcription
DMR3:123110001	3	123110001	123115000	5000	1	2.70E-07	-0.26	52	1.04	Oxt;Avp	Signaling
DMR3:123358001	3	123358001	123359000	1000	1	8.70E-09	-0.64	6	0.6	RGD1565616	
DMR3:123571001	3	123571001	123572000	1000	1	2.60E-09	-0.4	6	0.6	Atrn;Gfra4	Extracellular Matrix;Receptor
DMR3:123631001	3	123631001	123632000	1000	1	9.90E-08	-0.52	12	1.2	Siglec1	
DMR3:125009001	3	125009001	125011000	2000	1	4.90E-08	0.42	49	2.45	Prokr2	Signaling
DMR3:125031001	3	125031001	125034000	3000	1	9.80E-09	0.29	30	1	Prokr2	Signaling
DMR3:125159001	3	125159001	125160000	1000	1	2.30E-08	0.59	27	2.7	Gpcpd1	Signaling
DMR3:125439001	3	125439001	125440000	1000	1	8.00E-13	0.75	26	2.6	Chgb	
DMR3:127473001	3	127473001	127477000	4000	1	1.20E-07	-0.27	39	0.98	Hao1;LOC108350470	Metabolism
DMR3:127826001	3	127826001	127829000	3000	1	8.10E-11	0.51	20	0.67	Plcb1;LOC102549293	Metabolism
DMR3:127838001	3	127838001	127840000	2000	1	2.30E-07	0.33	14	0.7	Plcb1	Metabolism
DMR3:128012001	3	128012001	128018000	6000	1	8.00E-11	-0.29	83	1.38	Plcb1	Metabolism
DMR3:128389001	3	128389001	128390000	1000	1	1.60E-08	0.45	7	0.7	Plcb1	Metabolism
DMR3:128415001	3	128415001	128417000	2000	1	9.60E-07	0.43	28	1.4	Plcb1	Metabolism
DMR3:128662001	3	128662001	128666000	4000	1	6.70E-08	-0.38	70	1.75	Plcb4	Metabolism
DMR3:128675001	3	128675001	128677000	2000	1	1.60E-08	0.41	6	0.3	Plcb4	Metabolism
DMR3:128697001	3	128697001	128701000	4000	1	4.30E-08	-0.43	73	1.82	Plcb4;LOC102549547	Metabolism
DMR3:128727001	3	128727001	128730000	3000	1	7.30E-12	-0.65	28	0.93	Plcb4	Metabolism
DMR3:128762001	3	128762001	128763000	1000	1	8.60E-07	-0.5	10	1	Plcb4	Metabolism
DMR3:129120001	3	129120001	129121000	1000	1	7.90E-08	-0.5	13	1.3	Pak7	
DMR3:129283001	3	129283001	129286000	3000	1	2.40E-07	0.37	48	1.6	Pak7	
DMR3:129306001	3	129306001	129307000	1000	1	2.70E-07	-0.48	10	1	Pak7	
DMR3:129469001	3	129469001	129470000	1000	1	3.10E-15	0.76	7	0.7	Ankef1	
DMR3:129971001	3	129971001	129979000	8000	1	8.00E-08	-0.63	51	0.64	Slx4ip	
DMR3:132586001	3	132586001	132591000	5000	1	1.10E-07	-0.33	42	0.84	Sptlc3	Metabolism

DMR3:132614001	3	132614001	132617000	3000	1	9.50E-09	0.46	16	0.53	Sptlc3	Metabolism
DMR3:132647001	3	132647001	132651000	4000	2	1.70E-07	-0.27	30	0.75	Sptlc3	Metabolism
DMR3:132685001	3	132685001	132686000	1000	1	4.90E-07	-0.42	7	0.7	Sptlc3	Metabolism
DMR3:132791001	3	132791001	132794000	3000	1	6.20E-07	-0.46	27	0.9	Ism1	
DMR3:132992001	3	132992001	132994000	2000	1	5.00E-08	0.45	13	0.65	Tasp1	Protease
DMR3:134302001	3	134302001	134303000	1000	1	8.80E-07	0.37	16	1.6	Sel1l2	
DMR3:134698001	3	134698001	134702000	4000	1	8.50E-11	0.58	53	1.32	Flrt3	
DMR3:135725001	3	135725001	135727000	2000	1	2.80E-08	0.36	11	0.55	Macrod2	
DMR3:135920001	3	135920001	135922000	2000	1	8.30E-10	0.52	9	0.45	Macrod2	
DMR3:136130001	3	136130001	136131000	1000	1	5.00E-10	0.36	10	1	Macrod2	
DMR3:136393001	3	136393001	136397000	4000	1	2.10E-07	0.42	32	0.8	Macrod2	
DMR3:136738001	3	136738001	136740000	2000	1	1.70E-07	-0.63	14	0.7	Kif16b	Cytoskeleton
DMR3:137663001	3	137663001	137670000	7000	1	8.10E-09	-0.31	57	0.81	Pcsk2;LOC108350478	Protease
DMR3:137732001	3	137732001	137739000	7000	1	3.80E-08	0.4	68	0.97	Pcsk2	Protease
DMR3:137745001	3	137745001	137747000	2000	1	1.20E-07	0.38	26	1.3	Pcsk2	Protease
DMR3:137784001	3	137784001	137785000	1000	1	5.00E-10	-0.61	7	0.7	Pcsk2	Protease
DMR3:137956001	3	137956001	137960000	4000	1	5.60E-08	-0.6	57	1.43	Bfsp1	
DMR3:138442001	3	138442001	138445000	3000	1	3.80E-08	-0.42	55	1.83	Ovol2	
DMR3:138587001	3	138587001	138588000	1000	1	8.20E-07	-0.36	21	2.1	Zfp133	Transcription
DMR3:138624001	3	138624001	138625000	1000	1	8.30E-08	0.35	4	0.4	Zfp133;Dzank1	Transcription
DMR3:138862001	3	138862001	138864000	2000	1	9.20E-08	-0.58	13	0.65	Dtd1	Metabolism
DMR3:139321001	3	139321001	139325000	4000	1	8.50E-08	-0.49	51	1.27	Slc24a3	Transport
DMR3:139389001	3	139389001	139391000	2000	1	8.90E-07	0.43	28	1.4	Slc24a3	Transport
DMR3:139705001	3	139705001	139707000	2000	1	1.10E-11	0.6	12	0.6	Slc24a3	Transport
DMR3:139766001	3	139766001	139770000	4000	1	5.00E-12	-0.4	51	1.27	Slc24a3	Transport
DMR3:139913001	3	139913001	139915000	2000	1	1.70E-07	-0.46	13	0.65	Rin2	Transcription
DMR3:139990001	3	139990001	139993000	3000	1	8.30E-10	0.44	26	0.87	Rin2	Transcription
DMR3:140176001	3	140176001	140178000	2000	1	1.30E-09	-0.3	17	0.85	Cfap61	
DMR3:140449001	3	140449001	140453000	4000	1	5.10E-09	-0.54	42	1.05	Ralgapa2	Signaling
DMR3:140536001	3	140536001	140540000	4000	1	5.10E-10	-0.4	51	1.27	Ralgapa2	Signaling
DMR3:140546001	3	140546001	140552000	6000	2	1.10E-09	-0.41	64	1.07	Ralgapa2	Signaling
DMR3:140634001	3	140634001	140635000	1000	1	8.30E-08	-0.54	14	1.4	Ralgapa2	Signaling
DMR3:141121001	3	141121001	141126000	5000	1	3.50E-08	-0.32	38	0.76	Kiz	
DMR3:141183001	3	141183001	141189000	6000	3	7.20E-10	-0.37	52	0.87	Kiz;LOC688692	
DMR3:141244001	3	141244001	141247000	3000	1	6.30E-08	-0.44	25	0.83	Xrn2	Transcription
DMR3:142996001	3	142996001	142997000	1000	1	4.70E-07	-0.46	13	1.3	Nxt1;Sert1;Gzf1	Transcription
DMR3:143053001	3	143053001	143057000	4000	2	4.20E-07	-0.33	38	0.95	Napb	Transport
DMR3:143120001	3	143120001	143121000	1000	1	6.00E-07	0.37	13	1.3	LOC257643;Cst12;Cst8	
DMR3:143162001	3	143162001	143166000	4000	1	5.40E-07	-0.33	34	0.85	Cst13;Cst9l	
DMR3:143598001	3	143598001	143601000	3000	1	1.50E-07	-0.53	12	0.4	RGD1563136	
DMR3:143625001	3	143625001	143627000	2000	1	4.40E-07	-0.44	8	0.4	RGD1563136	
DMR3:143899001	3	143899001	143903000	4000	1	7.30E-09	-0.55	22	0.55	Andpro	
DMR3:145086001	3	145086001	145089000	3000	1	2.70E-08	0.48	40	1.33	Syndig1;LOC108350492	
DMR3:145158001	3	145158001	145159000	1000	1	3.80E-07	-0.42	7	0.7	Syndig1	
DMR3:146357001	3	146357001	146362000	5000	1	2.00E-08	-0.4	23	0.46	RGD1565143;LOC689686;Cst7	
DMR3:146611001	3	146611001	146612000	1000	1	2.40E-08	0.43	17	1.7	Pygb	Golgi
DMR3:146729001	3	146729001	146736000	7000	1	4.30E-07	0.47	86	1.23	Ninl	
DMR3:146998001	3	146998001	147001000	3000	1	6.90E-11	-0.46	13	0.43	Nsfl1c	Signaling
DMR3:147083001	3	147083001	147085000	2000	1	4.40E-08	-0.58	19	0.95	Sdcbp2	Transport
DMR3:147086001	3	147086001	147088000	2000	1	2.40E-07	-0.28	37	1.85	Sdcbp2	Transport
DMR3:147268001	3	147268001	147270000	2000	1	8.90E-08	0.35	27	1.35	Psmf1	Protease; Proteolysis
DMR3:147489001	3	147489001	147491000	2000	1	7.30E-13	0.57	43	2.15	Fam110a;LOC108350501	
DMR3:147709001	3	147709001	147710000	1000	1	2.70E-07	-0.46	14	1.4	Csnk2a1	
DMR3:147770001	3	147770001	147772000	2000	1	8.90E-07	0.52	50	2.5	Csnk2a1;Tbc1d20	Signaling
DMR3:147929001	3	147929001	147931000	2000	1	5.50E-07	-0.37	21	1.05	Defb23	
DMR3:148559001	3	148559001	148564000	5000	3	2.80E-08	0.69	81	1.62	Ccm2l	
DMR3:148565001	3	148565001	148568000	3000	1	2.20E-08	0.53	36	1.2	Ccm2l	
DMR3:148698001	3	148698001	148699000	1000	1	5.20E-07	0.37	20	2	Tm9sf4;Tspy26	Transport;Epigenetic
DMR3:148753001	3	148753001	148756000	3000	1	4.50E-08	0.36	41	1.37	Pofut1;LOC690246	Golgi
DMR3:148784001	3	148784001	148785000	1000	1	6.20E-07	-0.33	17	1.7	Kif3b	Cytoskeleton
DMR3:149160001	3	149160001	149163000	3000	1	4.60E-08	-0.52	46	1.53	Dnmt3b;Dnmt3b-ps1	Epigenetic
DMR3:149258001	3	149258001	149259000	1000	1	6.00E-09	0.6	17	1.7	Mapre1;Efcab8	Cytoskeleton
DMR3:149287001	3	149287001	149292000	5000	2	2.70E-07	-0.48	50	1	Efcab8	

DMR3:149514001	3	149514001	149516000	2000	1	4.10E-07	-0.52	12	0.6	Bpifa2f;RGD1564277	
DMR3:149537001	3	149537001	149538000	1000	1	1.60E-07	-0.47	9	0.9	Bpifa2f	
DMR3:149626001	3	149626001	149631000	5000	1	4.90E-08	0.39	48	0.96	Bpifa3;Bpifa1	
DMR3:149699001	3	149699001	149701000	2000	1	8.00E-08	0.31	23	1.15	Bpifb1;Bpifb5	
DMR3:150060001	3	150060001	150061000	1000	1	1.00E-11	0.45	20	2	Necab3;RGD1561517;Actl10;E2f1	Cytoskeleton;Transcription
DMR3:150141001	3	150141001	150145000	4000	1	3.70E-08	-0.39	52	1.3	Zfp341;LOC103691953;Trnaa-agc;LOC103691939	
DMR3:150169001	3	150169001	150170000	1000	1	5.30E-08	0.53	15	1.5	Zfp341	
DMR3:150177001	3	150177001	150178000	1000	1	5.50E-07	-0.33	17	1.7	Zfp341	
DMR3:150180001	3	150180001	150181000	1000	1	4.60E-11	0.54	8	0.8	Zfp341;Chmp4b	Transport
DMR3:150390001	3	150390001	150393000	3000	1	3.10E-07	-0.41	39	1.3	Raly;Eif2s2	Translation
DMR3:150406001	3	150406001	150407000	1000	1	7.30E-09	-0.43	8	0.8	Eif2s2	Translation
DMR3:150498001	3	150498001	150505000	7000	2	6.60E-09	0.53	124	1.77	Asip	Signaling
DMR3:150571001	3	150571001	150572000	1000	1	1.60E-12	0.81	25	2.5	Asip	Signaling
DMR3:150606001	3	150606001	150608000	2000	2	2.00E-10	0.41	29	1.45	Ahcy	Metabolism
DMR3:150817001	3	150817001	150819000	2000	1	3.80E-08	-0.6	28	1.4	Pigu	
DMR3:150894001	3	150894001	150896000	2000	1	1.50E-07	-0.42	21	1.05	Pigu;LOC108350506	
DMR3:151116001	3	151116001	151118000	2000	1	2.60E-07	0.48	24	1.2	Gss;Myh7b	Metabolism
DMR3:151129001	3	151129001	151131000	2000	1	1.90E-08	0.63	51	2.55	Myh7b;Mir499	
DMR3:151177001	3	151177001	151180000	3000	1	9.80E-07	0.35	29	0.97	Trpc4ap	
DMR3:151295001	3	151295001	151296000	1000	1	1.00E-12	-0.51	24	2.4	Procr	Signaling
DMR3:151320001	3	151320001	151327000	7000	1	1.40E-14	0.72	124	1.77	Mmp24	Protease
DMR3:151339001	3	151339001	151341000	2000	1	2.40E-12	0.55	28	1.4	Mmp24	Protease
DMR3:151369001	3	151369001	151372000	3000	1	3.30E-09	0.63	60	2	Eif6;Fam83c	Translation
DMR3:151556001	3	151556001	151558000	2000	1	3.20E-07	-0.41	43	2.15	LOC102550306;Ergic3;Fer1l4	Transport
DMR3:151571001	3	151571001	151575000	4000	2	9.40E-12	0.91	41	1.02	Ergic3;Fer1l4	Transport
DMR3:151604001	3	151604001	151606000	2000	1	1.20E-07	-0.5	30	1.5	Fer1l4;Spag4	Transport;Cytoskeleton
DMR3:152307001	3	152307001	152310000	3000	1	1.10E-08	-0.56	47	1.57	Phf20	
DMR3:152338001	3	152338001	152344000	6000	1	6.60E-10	-0.51	70	1.17	Phf20	
DMR3:152406001	3	152406001	152408000	2000	1	4.30E-09	0.69	40	2	Cnbd2	
DMR3:152427001	3	152427001	152429000	2000	1	3.10E-08	0.38	15	0.75	Cnbd2	
DMR3:152767001	3	152767001	152768000	1000	1	3.00E-07	-0.32	22	2.2	Dlgap4	Cytoskeleton
DMR3:152774001	3	152774001	152776000	2000	1	4.50E-07	0.32	24	1.2	Dlgap4	Cytoskeleton
DMR3:152837001	3	152837001	152840000	3000	1	3.90E-07	0.37	57	1.9	Dlgap4;LOC103691944	Cytoskeleton
DMR3:152892001	3	152892001	152897000	5000	1	3.30E-09	0.42	70	1.4	LOC102551821;Tgif2	Development
DMR3:152949001	3	152949001	152950000	1000	1	2.10E-10	0.43	21	2.1	RGD1307752;Sla2	Signaling
DMR3:153602001	3	153602001	153605000	3000	1	1.80E-07	0.42	22	0.73	Src	
DMR3:154217001	3	154217001	154219000	2000	1	5.80E-12	-0.57	32	1.6	Ctnnbl1	
DMR3:154237001	3	154237001	154239000	2000	1	7.10E-07	-0.33	26	1.3	Ctnnbl1;LOC102554875	
DMR3:154326001	3	154326001	154328000	2000	1	3.00E-07	0.37	31	1.55	Ctnnbl1	
DMR3:154561001	3	154561001	154563000	2000	1	2.10E-09	0.48	28	1.4	Rprd1b;LOC108350509	Signaling
DMR3:154697001	3	154697001	154699000	2000	1	1.10E-08	0.49	21	1.05	RGD1563354	Transcription
DMR3:154762001	3	154762001	154763000	1000	1	6.60E-09	-0.49	16	1.6	Bpi	
DMR3:154808001	3	154808001	154811000	3000	1	4.00E-08	0.36	41	1.37	Lbp	
DMR3:155245001	3	155245001	155250000	5000	1	5.20E-07	0.24	88	1.76	Ppp1r16b	Signaling
DMR3:156770001	3	156770001	156772000	2000	1	7.50E-09	0.35	12	0.6	Zhx3	Development
DMR3:156824001	3	156824001	156831000	7000	1	1.20E-13	-0.37	88	1.26	Zhx3	Development
DMR3:156927001	3	156927001	156928000	1000	1	6.10E-08	-0.34	19	1.9	Chd6	
DMR3:157067001	3	157067001	157070000	3000	1	5.50E-07	-0.4	48	1.6	Chd6	
DMR3:157564001	3	157564001	157566000	2000	1	2.70E-09	0.74	35	1.75	Ptprt	Signaling
DMR3:157618001	3	157618001	157623000	5000	1	7.30E-07	-0.3	58	1.16	Ptprt;LOC108350516	Signaling
DMR3:157727001	3	157727001	157729000	2000	1	2.00E-07	0.43	21	1.05	Ptprt	Signaling
DMR3:157841001	3	157841001	157845000	4000	1	4.80E-07	-0.33	36	0.9	Ptprt	Signaling
DMR3:158013001	3	158013001	158015000	2000	1	6.80E-10	0.41	53	2.65	Ptprt	Signaling
DMR3:158191001	3	158191001	158193000	2000	1	2.10E-07	0.38	12	0.6	Ptprt	Signaling
DMR3:158236001	3	158236001	158239000	3000	1	4.40E-10	0.36	23	0.77	Ptprt	Signaling
DMR3:159303001	3	159303001	159304000	1000	1	9.70E-07	0.33	18	1.8	LOC100362466;Srsf6	Translation
DMR3:159306001	3	159306001	159309000	3000	1	1.80E-07	-0.43	65	2.17	LOC100362466;Srsf6;L3mbtl1	Translation;Epigenetic
DMR3:159333001	3	159333001	159335000	2000	1	4.40E-07	0.39	32	1.6	L3mbtl1	Epigenetic
DMR3:159393001	3	159393001	159394000	1000	1	1.70E-09	-0.33	13	1.3	Sgk2;Ift52	Signaling
DMR3:159582001	3	159582001	159583000	1000	1	3.20E-07	0.41	6	0.6	Tox2	
DMR3:159591001	3	159591001	159592000	1000	1	1.60E-08	0.62	35	3.5	Tox2	

DMR3:159653001	3	159653001	159656000	3000	1	5.00E-09	0.64	54	1.8	Tox2	
DMR3:159671001	3	159671001	159673000	2000	1	3.10E-11	0.66	35	1.75	Tox2	
DMR3:159680001	3	159680001	159682000	2000	1	5.40E-07	-0.37	45	2.25	Tox2	
DMR3:159683001	3	159683001	159685000	2000	1	9.30E-08	0.52	28	1.4	Tox2	
DMR3:159698001	3	159698001	159701000	3000	2	4.40E-08	0.33	27	0.9	Tox2;Jph2	
DMR3:159704001	3	159704001	159707000	3000	1	2.60E-07	0.33	21	0.7	Tox2;Jph2	
DMR3:159709001	3	159709001	159713000	4000	2	8.90E-07	0.42	76	1.9	Jph2	
DMR3:159768001	3	159768001	159770000	2000	1	7.10E-07	-0.32	23	1.15	Jph2	
DMR3:159774001	3	159774001	159776000	2000	1	2.60E-16	0.97	47	2.35	Jph2;Oser1	
DMR3:159807001	3	159807001	159809000	2000	1	5.80E-07	-0.42	26	1.3	Oser1	
DMR3:159907001	3	159907001	159914000	7000	3	6.40E-15	0.79	150	2.14	LOC102548241;R3hdml;Hnf4a	Immune;Transcription
DMR3:159931001	3	159931001	159938000	7000	1	1.10E-09	0.42	101	1.44	Hnf4a	Transcription
DMR3:159949001	3	159949001	159950000	1000	1	4.30E-07	0.35	11	1.1	Hnf4a	Transcription
DMR3:160137001	3	160137001	160138000	1000	1	5.70E-08	0.46	10	1	Ada	Metabolism
DMR3:160214001	3	160214001	160215000	1000	1	7.80E-08	0.43	3	0.3	Wisp2	
DMR3:160216001	3	160216001	160217000	1000	1	2.00E-08	0.37	22	2.2	Wisp2	
DMR3:160404001	3	160404001	160406000	2000	1	2.20E-10	-0.48	58	2.9	Ywhab;Pabpc1l	Cytoskeleton
DMR3:160476001	3	160476001	160481000	5000	1	6.00E-08	-0.5	102	2.04	Stk4	Signaling
DMR3:160491001	3	160491001	160496000	5000	1	7.50E-07	0.48	90	1.8	Stk4	Signaling
DMR3:160500001	3	160500001	160503000	3000	1	8.00E-08	-0.36	59	1.97	Stk4	Signaling
DMR3:160521001	3	160521001	160523000	2000	1	1.50E-08	-0.57	37	1.85	Stk4	Signaling
DMR3:160539001	3	160539001	160540000	1000	1	7.60E-07	0.36	13	1.3	Stk4	Signaling
DMR3:160613001	3	160613001	160618000	5000	1	5.50E-11	-0.63	67	1.34	Wfdc15b;Semg1	Protease; Proteolysis
DMR3:160748001	3	160748001	160750000	2000	1	7.50E-08	-0.45	17	0.85	Slpil2	Protease; Proteolysis
DMR3:160766001	3	160766001	160770000	4000	1	6.40E-07	-0.27	33	0.82	Slpil3	Protease; Proteolysis
DMR3:160782001	3	160782001	160785000	3000	1	1.70E-10	-0.91	42	1.4	Slpil3	Protease; Proteolysis
DMR3:160871001	3	160871001	160872000	1000	1	6.70E-07	0.54	35	3.5	Rbpjl;Sdc4	Transcription;Receptor
DMR3:161064001	3	161064001	161065000	1000	1	8.90E-07	-0.44	24	2.4	Wfdc8;Wfdc6b	
DMR3:161073001	3	161073001	161078000	5000	1	2.30E-10	0.69	45	0.9	Wfdc8;Wfdc6b;LOC100362743	
DMR3:161112001	3	161112001	161114000	2000	1	1.40E-10	-0.71	30	1.5	Wfdc9;Wfdc10	Protease; Proteolysis
DMR3:161185001	3	161185001	161186000	1000	1	9.80E-09	-0.42	16	1.6	Spint5p;LOC108350622;Wfdc3	Protease; Proteolysis
DMR3:161192001	3	161192001	161193000	1000	1	3.90E-08	0.36	29	2.9	LOC108350622;Wfdc3	Protease; Proteolysis
DMR3:161326001	3	161326001	161327000	1000	1	3.00E-08	0.38	13	1.3	Pltp	
DMR3:161334001	3	161334001	161338000	4000	1	2.10E-11	0.41	46	1.15	Pcif1;LOC103691999	
DMR3:161386001	3	161386001	161387000	1000	1	2.00E-07	0.43	22	2.2	Zfp335	
DMR3:161422001	3	161422001	161425000	3000	1	1.70E-08	0.42	38	1.27	Mmp9;LOC108350521;Slc12a5	Protease;Transport
DMR3:161503001	3	161503001	161504000	1000	1	9.60E-07	0.45	8	0.8	Ncoa5	Transcription
DMR3:161543001	3	161543001	161545000	2000	1	7.10E-08	0.38	28	1.4	Cd40	Receptor
DMR3:161566001	3	161566001	161569000	3000	1	2.10E-08	0.36	34	1.13	Cdh22	Cytoskeleton
DMR3:161585001	3	161585001	161587000	2000	1	1.10E-08	0.38	40	2	Cdh22	Cytoskeleton
DMR3:161792001	3	161792001	161796000	4000	1	1.80E-07	0.44	59	1.48	Cdh22;Slc35c2	Cytoskeleton;Transport
DMR3:161912001	3	161912001	161914000	2000	1	1.10E-11	-0.52	33	1.65	Cdh22	Cytoskeleton
DMR3:162028001	3	162028001	162033000	5000	2	4.00E-07	-0.47	99	1.98	Zfp663	Transcription
DMR3:162046001	3	162046001	162050000	4000	1	1.50E-09	0.61	85	2.12	Zfp663;LOC685574	Transcription
DMR3:162075001	3	162075001	162078000	3000	1	1.40E-07	0.37	93	3.1	LOC685574;Zfp334;Slc13a3	Transport
DMR3:162144001	3	162144001	162146000	2000	1	2.40E-07	0.48	35	1.75	Slc13a3	Transport
DMR3:162325001	3	162325001	162326000	1000	1	1.20E-07	0.4	34	3.4	Eya2	
DMR3:162361001	3	162361001	162362000	1000	1	4.60E-08	-0.36	29	2.9	Eya2	
DMR3:162383001	3	162383001	162384000	1000	1	3.70E-08	0.39	7	0.7	Eya2	
DMR3:162385001	3	162385001	162388000	3000	1	6.30E-08	0.29	35	1.17	Eya2	
DMR3:162412001	3	162412001	162416000	4000	1	2.10E-07	0.4	50	1.25	Eya2	
DMR3:162452001	3	162452001	162454000	2000	1	2.00E-07	0.3	10	0.5	Eya2	
DMR3:162542001	3	162542001	162544000	2000	1	3.90E-07	-0.44	36	1.8	Zmynd8	
DMR3:162557001	3	162557001	162559000	2000	1	2.90E-08	0.53	22	1.1	Zmynd8	
DMR3:162602001	3	162602001	162604000	2000	1	3.10E-07	0.36	26	1.3	Zmynd8	
DMR3:162719001	3	162719001	162721000	2000	1	3.10E-15	-0.75	20	1	Ncoa3	Epigenetic
DMR3:162810001	3	162810001	162812000	2000	1	5.40E-07	0.53	29	1.45	Sulf2	Metabolism
DMR3:162840001	3	162840001	162841000	1000	1	2.10E-09	0.35	15	1.5	Sulf2;LOC108350522	Metabolism
DMR3:163337001	3	163337001	163339000	2000	1	5.00E-07	-0.38	24	1.2	Prex1	Transcription
DMR3:163681001	3	163681001	163683000	2000	1	1.80E-08	-0.49	21	1.05	Cse1l	Transport
DMR3:163698001	3	163698001	163700000	2000	1	6.30E-07	-0.48	23	1.15	Cse1l;Stau1	Transport
DMR3:164038001	3	164038001	164039000	1000	1	1.80E-07	0.39	12	1.2	B4galt5	Golgi
DMR3:164666001	3	164666001	164669000	3000	1	1.70E-10	-0.67	42	1.4	Ptpn1	Signaling

DMR3:164740001	3	164740001	164744000	4000	1	5.50E-08	0.43	71	1.77	Fam65c	
DMR3:164755001	3	164755001	164757000	2000	1	2.40E-08	0.37	28	1.4	Fam65c	
DMR3:164760001	3	164760001	164762000	2000	1	2.20E-10	0.38	22	1.1	Fam65c	
DMR3:164789001	3	164789001	164791000	2000	2	4.70E-11	0.72	60	3	Fam65c	
DMR3:165020001	3	165020001	165028000	8000	1	5.90E-11	0.7	157	1.96	Kcng1	Transport
DMR3:165239001	3	165239001	165240000	1000	1	1.80E-07	0.74	22	2.2	LOC102554533;Nfatc2	Transcription
DMR3:165283001	3	165283001	165284000	1000	1	6.40E-07	-0.42	32	3.2	Nfatc2	Transcription
DMR3:165338001	3	165338001	165341000	3000	2	3.10E-08	0.91	93	3.1	Nfatc2	Transcription
DMR3:165347001	3	165347001	165350000	3000	1	8.40E-13	0.71	53	1.77	Nfatc2	Transcription
DMR3:165540001	3	165540001	165541000	1000	1	3.60E-07	0.5	13	1.3	Sall4	Transcription
DMR3:165708001	3	165708001	165710000	2000	1	3.50E-10	0.59	58	2.9	Zfp93;Zfp64	Transcription
DMR3:165725001	3	165725001	165727000	2000	1	8.60E-08	0.33	43	2.15	Zfp64	Transcription
DMR3:166601001	3	166601001	166602000	1000	1	1.30E-08	0.35	10	1	Tshz2	Transcription
DMR3:166604001	3	166604001	166607000	3000	3	2.10E-20	0.86	66	2.2	Tshz2	Transcription
DMR3:166684001	3	166684001	166688000	4000	2	2.70E-09	0.53	77	1.93	Tshz2	Transcription
DMR3:166714001	3	166714001	166715000	1000	1	1.20E-07	0.4	10	1	Tshz2	Transcription
DMR3:167021001	3	167021001	167023000	2000	1	1.10E-07	-0.44	42	2.1	Zfp217	Transcription
DMR3:167970001	3	167970001	167973000	3000	1	5.70E-16	0.86	82	2.73	Bcas1	
DMR3:168023001	3	168023001	168027000	4000	1	1.80E-10	0.38	52	1.3	Bcas1	
DMR3:168106001	3	168106001	168109000	3000	1	2.50E-07	0.31	36	1.2	Cyp24a1	Metabolism
DMR3:168361001	3	168361001	168364000	3000	1	6.50E-09	0.36	69	2.3	Dok5	
DMR3:168400001	3	168400001	168402000	2000	1	3.90E-15	0.84	45	2.25	Dok5	
DMR3:168470001	3	168470001	168473000	3000	1	8.00E-07	0.45	65	2.17	Dok5	
DMR3:170042001	3	170042001	170043000	1000	1	7.80E-08	0.43	8	0.8	Cbln4	
DMR3:170359001	3	170359001	170363000	4000	1	6.30E-08	0.42	89	2.22	Fam210b;Aurka	Signaling
DMR3:170391001	3	170391001	170393000	2000	1	6.80E-07	-0.39	32	1.6	Cstf1;Cass4	
DMR3:170395001	3	170395001	170396000	1000	1	1.90E-08	0.6	12	1.2	Cstf1;Cass4	
DMR3:170986001	3	170986001	170990000	4000	1	1.00E-07	-0.35	64	1.6	Spo11	Transcription
DMR3:171142001	3	171142001	171147000	5000	1	1.10E-10	0.43	98	1.96	LOC102550488;Uchl3-ps1;Ctcfl	Transcription
DMR3:171167001	3	171167001	171168000	1000	1	5.70E-11	0.68	27	2.7	Ctcfl;LOC103691985	Transcription
DMR3:171169001	3	171169001	171175000	6000	1	1.60E-08	0.44	63	1.05	Ctcfl;LOC103691985	Transcription
DMR3:171272001	3	171272001	171273000	1000	1	5.60E-09	0.61	19	1.9	Zbp1	Transcription
DMR3:171274001	3	171274001	171276000	2000	1	1.40E-09	0.62	38	1.9	Zbp1	Transcription
DMR3:171303001	3	171303001	171309000	6000	1	7.20E-08	0.46	110	1.83	Pmepa1;LOC102547304	
DMR3:171925001	3	171925001	171927000	2000	1	1.60E-10	-0.43	29	1.45	Apcdd1l	
DMR3:172170001	3	172170001	172173000	3000	1	1.60E-10	-0.41	24	0.8	Stx16	Transcription
DMR3:172367001	3	172367001	172369000	2000	1	5.50E-09	-0.35	35	1.75	Mir296;Mir298;Gnas;LOC102548488	Signaling
DMR3:172438001	3	172438001	172442000	4000	1	3.10E-07	0.64	45	1.12	Gnas	Signaling
DMR3:172552001	3	172552001	172557000	5000	2	1.70E-08	0.76	94	1.88	Tubb1;Atp5e	Cytoskeleton
DMR3:172568001	3	172568001	172570000	2000	1	3.70E-09	-0.48	16	0.8	Tubb1;Atp5e;Prelid3b	Cytoskeleton
DMR3:172681001	3	172681001	172684000	3000	1	2.70E-10	-0.44	40	1.33	Zfp831	
DMR3:172737001	3	172737001	172739000	2000	1	6.20E-09	0.43	13	0.65	Zfp831;LOC108350546	
DMR3:172871001	3	172871001	172873000	2000	1	3.60E-07	0.35	23	1.15	Edn3	Hormone
DMR3:173961001	3	173961001	173962000	1000	1	4.00E-07	-0.32	17	1.7	Ppp1r3d;Fam217b;Cdh26	Signaling;Cytoskeleton
DMR3:174010001	3	174010001	174014000	4000	2	6.60E-09	0.55	51	1.27	Cdh26	Cytoskeleton
DMR3:175419001	3	175419001	175423000	4000	2	1.40E-09	-0.46	62	1.55	Lsm14b;Psma7;Ss18l1	Metabolism;Protease;Transcription
DMR3:175445001	3	175445001	175447000	2000	1	3.40E-09	-0.48	49	2.45	Ss18l1;Mtg2	Transcription
DMR3:175758001	3	175758001	175760000	2000	1	5.00E-08	0.53	25	1.25	LOC102552888;Mir1b	
DMR3:175775001	3	175775001	175776000	1000	1	3.80E-11	0.42	6	0.6	LOC102552888;Mir1b	
DMR3:175990001	3	175990001	175993000	3000	2	5.00E-10	0.46	43	1.43	Ntsr1	Signaling
DMR3:176015001	3	176015001	176016000	1000	1	2.60E-07	-0.61	24	2.4	Ntsr1	Signaling
DMR3:176042001	3	176042001	176044000	2000	1	1.60E-08	0.6	57	2.85	Ntsr1	Signaling
DMR3:176164001	3	176164001	176165000	1000	1	2.00E-11	0.81	40	4	LOC108350539;Dido1	Transcription
DMR3:176466001	3	176466001	176467000	1000	1	3.70E-08	0.55	24	2.4	Nkain4;Arfgap1	Signaling
DMR3:176527001	3	176527001	176529000	2000	1	2.50E-10	-0.49	25	1.25	Col20a1;LOC102546950;Chrna4	Extracellular Matrix;Ion Channel
DMR3:176921001	3	176921001	176922000	1000	1	6.40E-07	0.42	9	0.9	Zbtb46	Cytoskeleton
DMR3:176938001	3	176938001	176939000	1000	1	6.80E-18	0.52	1	0.1	Zbtb46	Cytoskeleton
DMR3:177057001	3	177057001	177058000	1000	1	1.60E-10	-0.8	4	0.4	Dnajc5;Uckl1	Transcription;Signaling
DMR3:177102001	3	177102001	177106000	4000	1	6.60E-09	-0.44	33	0.82	Samd10;Prpf6	Translation
DMR3:177158001	3	177158001	177159000	1000	1	9.80E-09	0.41	10	1	Prpf6;RGD1561282	Translation

DMR3:177271001	3	177271001	177272000	1000	1	5.00E-12	0.47	6	0.6	Myt1	Transcription
DMR4:355001	4	355001	357000	2000	1	6.50E-08	0.42	15	0.75	Insig1	
DMR4:718001	4	718001	719000	1000	1	7.00E-08	0.75	63	6.3	Shh;LOC108350640;LOC102546975	
DMR4:1649001	4	1649001	1655000	6000	1	5.10E-07	-0.23	47	0.78	Olr1249;Olr1250	Receptor
DMR4:1836001	4	1836001	1840000	4000	1	4.60E-08	-0.24	38	0.95	Olr1096	Receptor
DMR4:2044001	4	2044001	2046000	2000	1	6.20E-07	0.42	16	0.8	LOC108350641;Rnf32	
DMR4:2067001	4	2067001	2071000	4000	1	6.40E-08	-0.54	18	0.45	Rnf32	
DMR4:2179001	4	2179001	2184000	5000	2	2.70E-07	-0.3	46	0.92	Lmbr1	Receptor
DMR4:2259001	4	2259001	2261000	2000	1	8.00E-09	-0.71	10	0.5	Lmbr1	Receptor
DMR4:2330001	4	2330001	2332000	2000	1	7.00E-08	-0.36	12	0.6	Nom1	Translation
DMR4:4008001	4	4008001	4012000	4000	1	2.70E-07	-0.4	56	1.4	Paxip1;LOC108350643;Dpp6	Protease
DMR4:4028001	4	4028001	4030000	2000	1	2.30E-08	-0.57	15	0.75	Dpp6	Protease
DMR4:4113001	4	4113001	4119000	6000	2	1.80E-10	-0.43	70	1.17	Dpp6	Protease
DMR4:4123001	4	4123001	4125000	2000	1	7.90E-07	0.35	27	1.35	Dpp6	Protease
DMR4:4198001	4	4198001	4199000	1000	1	8.40E-07	0.41	2	0.2	Dpp6	Protease
DMR4:4217001	4	4217001	4218000	1000	1	5.60E-09	0.38	12	1.2	Dpp6	Protease
DMR4:4294001	4	4294001	4296000	2000	1	3.80E-07	-0.39	19	0.95	Dpp6;LOC108350828	Protease
DMR4:4441001	4	4441001	4442000	1000	1	3.10E-07	0.38	9	0.9	Dpp6	Protease
DMR4:4741001	4	4741001	4743000	2000	1	1.50E-07	0.45	28	1.4	Dpp6	Protease
DMR4:4758001	4	4758001	4760000	2000	1	1.20E-09	0.53	11	0.55	Dpp6	Protease
DMR4:4827001	4	4827001	4830000	3000	1	1.50E-07	0.39	19	0.63	Dpp6	Protease
DMR4:4879001	4	4879001	4880000	1000	1	9.70E-08	0.39	2	0.2	Dpp6	Protease
DMR4:4887001	4	4887001	4893000	6000	2	1.20E-07	-0.44	49	0.82	Dpp6	Protease
DMR4:4913001	4	4913001	4916000	3000	1	9.30E-07	-0.37	21	0.7	Dpp6	Protease
DMR4:5637001	4	5637001	5638000	1000	1	1.30E-07	0.38	14	1.4	LOC102548175;Actr3b	Cytoskeleton
DMR4:5643001	4	5643001	5646000	3000	1	1.20E-07	0.39	40	1.33	Actr3b	Cytoskeleton
DMR4:5650001	4	5650001	5653000	3000	1	3.10E-07	0.35	49	1.63	Actr3b	Cytoskeleton
DMR4:5668001	4	5668001	5674000	6000	1	9.20E-08	0.46	100	1.67	Actr3b	Cytoskeleton
DMR4:5853001	4	5853001	5855000	2000	1	3.90E-07	-0.35	30	1.5	Xrcc2	Transcription
DMR4:6303001	4	6303001	6306000	3000	1	3.30E-07	-0.35	33	1.1	Kmt2c;Galnt11;LOC108350646	Golgi
DMR4:6624001	4	6624001	6628000	4000	2	9.60E-08	0.43	65	1.62	Prkag2	Signaling
DMR4:6660001	4	6660001	6662000	2000	1	5.70E-07	0.49	21	1.05	Prkag2;LOC102554253	Signaling
DMR4:6689001	4	6689001	6692000	3000	1	4.30E-07	0.33	53	1.77	Prkag2;LOC103692022	Signaling
DMR4:6719001	4	6719001	6721000	2000	1	3.00E-10	0.46	28	1.4	Prkag2;LOC102554193	Signaling
DMR4:6754001	4	6754001	6756000	2000	1	3.80E-08	-0.44	56	2.8	Prkag2	Signaling
DMR4:6895001	4	6895001	6898000	3000	1	2.00E-08	0.48	57	1.9	Crygn	
DMR4:7292001	4	7292001	7294000	2000	1	9.50E-11	0.69	50	2.5	Slc4a2;Cdk5;Asic3;Abcb8	Transport;Signaling;Transport;Transport
DMR4:7347001	4	7347001	7348000	1000	1	9.40E-10	0.41	10	1	Nos3;Kcnh2	Metabolism;Transport
DMR4:7517001	4	7517001	7519000	2000	1	1.70E-08	0.38	22	1.1	Nupl2	
DMR4:7574001	4	7574001	7577000	3000	1	2.50E-08	-0.37	52	1.73	Klhl7;LOC100364673;LOC108350648	Cytoskeleton
DMR4:7697001	4	7697001	7699000	2000	1	2.70E-08	-0.39	27	1.35	Fam126a;LOC108350649	
DMR4:7719001	4	7719001	7722000	3000	1	1.60E-07	-0.44	54	1.8	Fam126a	
DMR4:7762001	4	7762001	7767000	5000	1	2.80E-13	0.61	65	1.3	Fam126a;LOC103692024	
DMR4:7877001	4	7877001	7881000	4000	1	2.60E-08	-0.45	63	1.57	Rint1;Pus7	
DMR4:7989001	4	7989001	7990000	1000	1	1.40E-09	0.46	22	2.2	Srpk2	Signaling
DMR4:8104001	4	8104001	8105000	1000	1	7.70E-08	-0.55	7	0.7	Srpk2	Signaling
DMR4:8110001	4	8110001	8112000	2000	1	8.30E-07	-0.38	30	1.5	Srpk2	Signaling
DMR4:8252001	4	8252001	8253000	1000	1	2.80E-08	-0.44	11	1.1	Kmt2e	
DMR4:9401001	4	9401001	9403000	2000	1	1.20E-09	0.4	17	0.85	Reln	Extracellular Matrix
DMR4:9494001	4	9494001	9496000	2000	1	7.70E-08	-0.46	31	1.55	Reln	Extracellular Matrix
DMR4:9701001	4	9701001	9705000	4000	1	5.60E-10	-0.58	101	2.52	Reln	Extracellular Matrix
DMR4:9738001	4	9738001	9740000	2000	1	1.60E-07	0.47	23	1.15	Reln;LOC103692036	Extracellular Matrix
DMR4:9783001	4	9783001	9791000	8000	1	3.90E-07	-0.36	92	1.15	Reln;Slc26a5	Extracellular Matrix;Transport
DMR4:9800001	4	9800001	9802000	2000	1	4.00E-07	0.59	31	1.55	Slc26a5;LOC102555727	Transport
DMR4:9806001	4	9806001	9807000	1000	1	8.10E-09	0.61	17	1.7	Slc26a5;LOC102555727	Transport
DMR4:9831001	4	9831001	9834000	3000	1	1.00E-12	0.79	59	1.97	Slc26a5	Transport
DMR4:9835001	4	9835001	9838000	3000	1	1.40E-07	0.3	42	1.4	Slc26a5	Transport
DMR4:9844001	4	9844001	9847000	3000	1	1.80E-11	0.71	42	1.4	Slc26a5	Transport
DMR4:9871001	4	9871001	9874000	3000	1	2.10E-07	-0.59	46	1.53	Psmc2;Dnajc2	Protease;Transcription
DMR4:9896001	4	9896001	9899000	3000	1	6.80E-07	-0.31	46	1.53	Dnajc2;Pmpcb	Transcription;Protease

DMR4:10063001	4	10063001	10066000	3000	1	2.60E-07	-0.32	23	0.77	Fbxl13	
DMR4:10108001	4	10108001	10109000	1000	1	2.50E-07	-0.43	25	2.5	Fbxl13;Lrrc17	Receptor
DMR4:10114001	4	10114001	10115000	1000	1	7.80E-07	0.49	22	2.2	Fbxl13;Lrrc17	Receptor
DMR4:10132001	4	10132001	10134000	2000	1	9.70E-07	0.51	42	2.1	Fbxl13;Lrrc17	Receptor
DMR4:10140001	4	10140001	10141000	1000	1	2.80E-08	0.36	12	1.2	Fbxl13;Lrrc17	Receptor
DMR4:10217001	4	10217001	10218000	1000	1	9.50E-09	-0.54	10	1	Fam185a	
DMR4:10274001	4	10274001	10276000	2000	1	3.40E-07	-0.37	27	1.35	Fam185a;Ccdc146	Development
DMR4:10326001	4	10326001	10327000	1000	1	2.20E-07	-0.48	26	2.6	Ccdc146;Fgl2	Development;Signaling
DMR4:10410001	4	10410001	10412000	2000	1	2.70E-10	0.53	34	1.7	Ccdc146	Development
DMR4:10475001	4	10475001	10476000	1000	1	7.30E-08	-0.55	17	1.7	Gsap	Signaling
DMR4:10493001	4	10493001	10498000	5000	1	1.90E-08	0.5	61	1.22	Gsap	Signaling
DMR4:10621001	4	10621001	10623000	2000	1	9.80E-09	0.42	26	1.3	LOC102556034;Ptpn12	
DMR4:10625001	4	10625001	10626000	1000	1	3.30E-07	-0.66	14	1.4	LOC102556034;Ptpn12	
DMR4:10776001	4	10776001	10778000	2000	1	2.70E-08	-0.43	31	1.55	Rsbn1l	
DMR4:10800001	4	10800001	10804000	4000	1	8.40E-07	-0.57	34	0.85	Rsbn1l	
DMR4:10885001	4	10885001	10886000	1000	1	2.90E-08	0.48	9	0.9	Phtf2	Development
DMR4:10889001	4	10889001	10891000	2000	1	5.20E-09	-0.42	32	1.6	Phtf2	Development
DMR4:10989001	4	10989001	10990000	1000	1	1.50E-08	0.54	26	2.6	LOC102556088;Magi2	
DMR4:10999001	4	10999001	1.10E+07	1000	1	2.20E-08	0.71	21	2.1	Magi2	
DMR4:11038001	4	11038001	11041000	3000	1	3.90E-09	0.47	34	1.13	Magi2	
DMR4:11063001	4	11063001	11064000	1000	1	9.20E-11	-0.48	22	2.2	Magi2	
DMR4:11087001	4	11087001	11090000	3000	2	6.80E-11	-0.52	15	0.5	Magi2	
DMR4:11101001	4	11101001	11102000	1000	1	1.40E-08	-0.54	21	2.1	Magi2	
DMR4:11116001	4	11116001	11117000	1000	1	1.30E-09	0.43	29	2.9	Magi2	
DMR4:11118001	4	11118001	11120000	2000	1	4.30E-08	0.53	35	1.75	Magi2	
DMR4:11182001	4	11182001	11186000	4000	1	2.70E-12	0.85	84	2.1	Magi2	
DMR4:11239001	4	11239001	11241000	2000	1	1.00E-08	0.62	38	1.9	Magi2	
DMR4:11264001	4	11264001	11267000	3000	1	2.10E-08	-0.46	15	0.5	Magi2	
DMR4:11280001	4	11280001	11282000	2000	1	1.20E-08	-0.5	21	1.05	Magi2	
DMR4:11295001	4	11295001	11296000	1000	1	4.00E-07	0.28	17	1.7	Magi2	
DMR4:11395001	4	11395001	11397000	2000	1	4.20E-10	-0.57	34	1.7	Magi2;LOC100909926	
DMR4:11405001	4	11405001	11407000	2000	1	5.10E-07	-0.46	31	1.55	Magi2;LOC100909926	
DMR4:11458001	4	11458001	11461000	3000	1	8.90E-07	-0.52	51	1.7	Magi2	
DMR4:11510001	4	11510001	11512000	2000	1	1.90E-07	0.47	16	0.8	Magi2	
DMR4:11780001	4	11780001	11781000	1000	1	1.80E-07	0.48	15	1.5	Magi2	
DMR4:11980001	4	11980001	11982000	2000	1	2.80E-12	-0.59	19	0.95	Magi2	
DMR4:12039001	4	12039001	12043000	4000	1	1.40E-11	-0.62	39	0.98	Magi2	
DMR4:12187001	4	12187001	12189000	2000	1	5.60E-11	0.64	36	1.8	Magi2;LOC103692037	
DMR4:12283001	4	12283001	12289000	6000	2	2.10E-08	0.45	81	1.35	Magi2	
DMR4:12323001	4	12323001	12324000	1000	1	2.90E-09	0.52	11	1.1	Magi2	
DMR4:12401001	4	12401001	12404000	3000	2	1.50E-08	-0.33	33	1.1	Magi2	
DMR4:14122001	4	14122001	14123000	1000	1	1.50E-08	0.64	33	3.3	RGD1565355;LOC108350652	
DMR4:14333001	4	14333001	14341000	8000	2	9.30E-13	-0.32	81	1.01	Sema3c	Signaling
DMR4:14459001	4	14459001	14461000	2000	1	2.50E-08	0.46	20	1	Sema3c	Signaling
DMR4:15692001	4	15692001	15698000	6000	2	7.20E-09	-0.38	59	0.98	Cacna2d1	Transport
DMR4:15722001	4	15722001	15724000	2000	1	1.50E-09	0.37	18	0.9	Cacna2d1	Transport
DMR4:15815001	4	15815001	15816000	1000	1	3.40E-08	0.43	13	1.3	Cacna2d1	Transport
DMR4:16051001	4	16051001	16052000	1000	1	4.50E-13	0.41	4	0.4	Cacna2d1	Transport
DMR4:16102001	4	16102001	16104000	2000	1	2.20E-09	0.44	18	0.9	Cacna2d1	Transport
DMR4:16558001	4	16558001	16559000	1000	1	1.10E-09	-0.34	10	1	Pclo	
DMR4:16650001	4	16650001	16655000	5000	1	9.10E-11	0.67	115	2.3	Pclo	
DMR4:16720001	4	16720001	16722000	2000	1	5.20E-09	0.41	10	0.5	Pclo	
DMR4:17030001	4	17030001	17032000	2000	1	2.10E-07	0.38	17	0.85	Pclo	
DMR4:18229001	4	18229001	18230000	1000	1	6.20E-07	0.42	17	1.7	Sema3a;LOC108350657	Signaling
DMR4:18275001	4	18275001	18276000	1000	1	7.20E-08	-0.4	7	0.7	Sema3a	Signaling
DMR4:18449001	4	18449001	18452000	3000	1	5.70E-09	-0.52	24	0.8	Sema3a	Signaling
DMR4:18466001	4	18466001	18467000	1000	1	4.30E-07	-0.44	7	0.7	Sema3a	Signaling
DMR4:18469001	4	18469001	18470000	1000	1	9.40E-08	-0.53	10	1	Sema3a	Signaling
DMR4:20789001	4	20789001	20791000	2000	1	5.50E-07	0.36	10	0.5	RGD1564798	
DMR4:21419001	4	21419001	21428000	9000	2	4.00E-09	-0.44	87	0.97	Grm3	Signaling
DMR4:21690001	4	21690001	21692000	2000	1	7.30E-08	-0.57	28	1.4	RGD1563349	
DMR4:21728001	4	21728001	21730000	2000	1	3.40E-07	0.39	24	1.2	RGD1563349;LOC108350658	
DMR4:21738001	4	21738001	21739000	1000	1	3.50E-07	-0.45	14	1.4	RGD1563349;LOC108350658	

DMR4:22106001	4	22106001	22110000	4000	1	2.50E-07	-0.59	55	1.38	Crot	Metabolism
DMR4:22157001	4	22157001	22160000	3000	1	9.20E-07	-0.42	34	1.13	Abcb4;LOC108350661	Transport
DMR4:22180001	4	22180001	22181000	1000	1	3.60E-07	-0.61	9	0.9	Abcb4	Transport
DMR4:22186001	4	22186001	22188000	2000	1	7.30E-07	-0.31	19	0.95	Abcb4	Transport
DMR4:22189001	4	22189001	22191000	2000	1	6.50E-07	-0.48	27	1.35	Abcb4	Transport
DMR4:22255001	4	22255001	22263000	8000	1	1.50E-08	-0.3	90	1.12	Abcb1b	
DMR4:22283001	4	22283001	22286000	3000	1	2.80E-08	-0.3	38	1.27	Abcb1b	
DMR4:22290001	4	22290001	22291000	1000	1	6.70E-09	-0.51	1	0.1	Abcb1b	
DMR4:22333001	4	22333001	22334000	1000	1	5.30E-14	0.76	27	2.7	Abcb1a	
DMR4:22348001	4	22348001	22350000	2000	1	2.90E-08	0.34	14	0.7	Abcb1a	
DMR4:22534001	4	22534001	22537000	3000	1	6.10E-07	0.4	28	0.93	Rundc3b	
DMR4:22641001	4	22641001	22644000	3000	2	6.80E-10	-0.52	18	0.6	Slc25a40	
DMR4:22660001	4	22660001	22661000	1000	1	6.00E-07	-0.38	8	0.8	Slc25a40;LOC103690019	
DMR4:22876001	4	22876001	22877000	1000	1	1.30E-07	0.4	1	0.1	Adam22	Protease
DMR4:22879001	4	22879001	22881000	2000	1	3.10E-07	-0.43	35	1.75	Adam22	Protease
DMR4:22985001	4	22985001	22987000	2000	1	5.30E-08	-0.58	30	1.5	Adam22	Protease
DMR4:23127001	4	23127001	23135000	8000	1	4.30E-07	0.34	87	1.09	Steap4	
DMR4:23785001	4	23785001	23787000	2000	2	6.40E-12	0.48	17	0.85	Zfp804b	
DMR4:23926001	4	23926001	23929000	3000	1	1.00E-08	0.34	20	0.67	Zfp804b	
DMR4:23964001	4	23964001	23965000	1000	1	9.50E-09	0.36	7	0.7	Zfp804b	
DMR4:23972001	4	23972001	23977000	5000	1	3.10E-07	-0.41	44	0.88	Zfp804b	
DMR4:24047001	4	24047001	24050000	3000	1	4.70E-07	-0.43	24	0.8	Zfp804b	
DMR4:24148001	4	24148001	24150000	2000	1	4.30E-08	0.65	25	1.25	Zfp804b	
DMR4:25448001	4	25448001	25454000	6000	1	7.80E-11	-0.47	55	0.92	Steap1	
DMR4:25528001	4	25528001	25531000	3000	1	2.70E-07	-0.37	29	0.97	Steap2;Cfap69	Development
DMR4:25827001	4	25827001	25830000	3000	1	5.40E-09	-0.53	45	1.5	Cdk14	Signaling
DMR4:25886001	4	25886001	25891000	5000	3	1.80E-07	-0.38	47	0.94	Cdk14	Signaling
DMR4:25903001	4	25903001	25904000	1000	1	1.20E-07	0.53	14	1.4	Cdk14	Signaling
DMR4:25942001	4	25942001	25944000	2000	1	2.70E-07	-0.34	36	1.8	Cdk14	Signaling
DMR4:25945001	4	25945001	25946000	1000	1	3.80E-07	-0.47	22	2.2	Cdk14	Signaling
DMR4:25994001	4	25994001	26001000	7000	2	1.00E-11	-0.46	70	1	Cdk14	Signaling
DMR4:26024001	4	26024001	26027000	3000	1	1.20E-07	-0.44	38	1.27	Cdk14	Signaling
DMR4:26033001	4	26033001	26037000	4000	1	4.80E-09	-0.32	39	0.98	Cdk14	Signaling
DMR4:26083001	4	26083001	26087000	4000	1	2.10E-08	-0.6	50	1.25	Cdk14	Signaling
DMR4:26184001	4	26184001	26186000	2000	1	6.00E-09	0.42	13	0.65	Cdk14	Signaling
DMR4:26189001	4	26189001	26192000	3000	1	7.40E-07	0.39	49	1.63	Cdk14	Signaling
DMR4:26204001	4	26204001	26209000	5000	1	3.60E-10	-0.48	46	0.92	Cdk14	Signaling
DMR4:26289001	4	26289001	26299000	10000	1	2.10E-10	-0.32	103	1.03	Cdk14	Signaling
DMR4:26304001	4	26304001	26305000	1000	1	7.00E-07	0.43	14	1.4	Cdk14	Signaling
DMR4:26330001	4	26330001	26333000	3000	1	3.00E-09	-0.61	32	1.07	Cdk14	Signaling
DMR4:26423001	4	26423001	26425000	2000	1	9.20E-07	-0.45	34	1.7	Cdk14	Signaling
DMR4:27227001	4	27227001	27230000	3000	1	7.40E-09	-0.52	47	1.57	Akap9	
DMR4:27241001	4	27241001	27243000	2000	1	6.00E-08	0.35	11	0.55	Akap9	
DMR4:27262001	4	27262001	27264000	2000	1	2.40E-08	-0.51	23	1.15	Akap9	
DMR4:27280001	4	27280001	27282000	2000	1	3.60E-10	-0.51	28	1.4	Akap9	
DMR4:27299001	4	27299001	27302000	3000	2	1.40E-08	-0.59	61	2.03	Akap9	
DMR4:27305001	4	27305001	27307000	2000	1	1.40E-11	-0.48	26	1.3	Akap9	
DMR4:27413001	4	27413001	27421000	8000	1	4.00E-09	-0.45	63	0.79	Lrrd1	Cytoskeleton
DMR4:27756001	4	27756001	27765000	9000	1	9.10E-08	-0.39	118	1.31	Fam133b	Metabolism
DMR4:27867001	4	27867001	27870000	3000	1	2.40E-07	-0.38	54	1.8	Cdk6	Signaling
DMR4:27898001	4	27898001	27901000	3000	1	2.20E-08	-0.44	67	2.23	Cdk6;LOC102550744	Signaling
DMR4:27902001	4	27902001	27905000	3000	1	2.20E-10	-0.55	49	1.63	Cdk6;LOC102550744	Signaling
DMR4:27963001	4	27963001	27965000	2000	1	8.90E-07	-0.38	28	1.4	Cdk6	Signaling
DMR4:28224001	4	28224001	28227000	3000	1	4.30E-07	0.28	32	1.07	RGD1565361;LOC100911141	
DMR4:28295001	4	28295001	28297000	2000	1	2.50E-07	-0.4	48	2.4	LOC103690231;LOC100359932;RGD1563091	
DMR4:28402001	4	28402001	28403000	1000	1	3.70E-07	0.44	18	1.8	Hepacam2	
DMR4:28433001	4	28433001	28434000	1000	1	5.30E-07	-0.4	17	1.7	Hepacam2;Vps50	
DMR4:28652001	4	28652001	28654000	2000	1	1.10E-07	0.5	12	0.6	Calcr;Mir653;Mir489	Receptor
DMR4:29097001	4	29097001	29098000	1000	1	4.30E-11	0.8	17	1.7	Bet1	Transcription
DMR4:29729001	4	29729001	29732000	3000	1	4.70E-07	0.52	26	0.87	Sgce	Cytoskeleton
DMR4:29750001	4	29750001	29752000	2000	1	4.70E-08	-0.43	36	1.8	Sgce	Cytoskeleton
DMR4:29792001	4	29792001	29793000	1000	1	1.50E-07	-0.47	17	1.7	Peg10	

DMR4:30100001	4	30100001	30101000	1000	1	4.00E-10	-0.56	17	1.7	Ppp1r9a	
DMR4:30122001	4	30122001	30123000	1000	1	2.20E-08	-0.45	11	1.1	Ppp1r9a	
DMR4:30236001	4	30236001	30240000	4000	1	1.90E-10	-0.41	56	1.4	Ppp1r9a;Pon1	
DMR4:30270001	4	30270001	30274000	4000	1	6.40E-09	-0.4	71	1.77	Pon1;LOC102552373	
DMR4:30467001	4	30467001	30472000	5000	1	6.50E-07	0.43	85	1.7	Asb4	Cytoskeleton
DMR4:30500001	4	30500001	30501000	1000	1	4.30E-07	-0.46	9	0.9	Asb4	Cytoskeleton
DMR4:30845001	4	30845001	30846000	1000	1	1.80E-07	0.56	12	1.2	Dync1i1	Cytoskeleton
DMR4:30907001	4	30907001	30915000	8000	2	1.90E-10	0.42	98	1.23	Dync1i1	Cytoskeleton
DMR4:31034001	4	31034001	31035000	1000	1	3.30E-10	0.46	8	0.8	Dync1i1	Cytoskeleton
DMR4:31118001	4	31118001	31119000	1000	1	2.40E-11	-0.66	12	1.2	Dync1i1	Cytoskeleton
DMR4:31192001	4	31192001	31194000	2000	1	6.10E-08	-0.48	56	2.8	Slc25a13	Transport
DMR4:31221001	4	31221001	31222000	1000	1	1.60E-07	0.49	8	0.8	Slc25a13;LOC103692066	Transport
DMR4:31452001	4	31452001	31453000	1000	1	1.90E-07	0.53	1	0.1	Slc25a13	Transport
DMR4:31700001	4	31700001	31705000	5000	1	9.60E-09	-0.58	74	1.48	Slc25a13;LOC103692067;LOC108350671	Transport
DMR4:31725001	4	31725001	31727000	2000	1	7.40E-07	0.36	20	1	Slc25a13	Transport
DMR4:32529001	4	32529001	32533000	4000	1	6.80E-13	-0.32	35	0.88	Sdhaf3	
DMR4:33910001	4	33910001	33911000	1000	1	7.70E-07	-0.31	10	1	C1galt1	Transport
DMR4:34425001	4	34425001	34426000	1000	1	1.20E-07	0.43	8	0.8	Glcci1	
DMR4:34539001	4	34539001	34541000	2000	1	3.70E-08	0.52	21	1.05	Glcci1	
DMR4:34633001	4	34633001	34634000	1000	1	1.80E-15	1.06	20	2	Ica1	
DMR4:34669001	4	34669001	34670000	1000	1	9.60E-10	-0.65	15	1.5	Ica1	
DMR4:35004001	4	35004001	35005000	1000	1	7.10E-07	0.47	11	1.1	Nxph1	Signaling
DMR4:35065001	4	35065001	35071000	6000	3	7.60E-11	-0.36	53	0.88	Nxph1	Signaling
DMR4:35101001	4	35101001	35102000	1000	1	4.40E-10	0.79	24	2.4	Nxph1	Signaling
DMR4:37554001	4	37554001	37556000	2000	1	1.70E-08	-0.29	19	0.95	RGD1562387	
DMR4:38218001	4	38218001	38224000	6000	3	1.40E-07	-0.37	57	0.95	Ndufa4	Metabolism
DMR4:38275001	4	38275001	38277000	2000	1	2.90E-07	-0.45	15	0.75	Phf14	Transcription
DMR4:38888001	4	38888001	38889000	1000	1	2.20E-07	0.44	5	0.5	Thsd7a;LOC102548177	Cytoskeleton
DMR4:39010001	4	39010001	39013000	3000	1	7.90E-07	-0.37	24	0.8	Thsd7a	Cytoskeleton
DMR4:39543001	4	39543001	39546000	3000	2	4.20E-10	-0.52	14	0.47	Tmem106b	
DMR4:39681001	4	39681001	39683000	2000	1	3.50E-07	-0.43	9	0.45	Vwde	
DMR4:39685001	4	39685001	39689000	4000	1	7.00E-08	-0.4	21	0.52	Vwde	
DMR4:40037001	4	40037001	40039000	2000	1	6.10E-07	-0.39	14	0.7	Tmem168	
DMR4:40103001	4	40103001	40105000	2000	1	5.10E-07	-0.38	36	1.8	Bmt2	
DMR4:40116001	4	40116001	40120000	4000	1	1.40E-09	-0.45	26	0.65	Bmt2	
DMR4:41220001	4	41220001	41224000	4000	1	5.00E-08	-0.37	40	1	Ppp1r3a	Signaling
DMR4:41460001	4	41460001	41461000	1000	1	8.30E-09	0.53	5	0.5	Foxp2	
DMR4:41480001	4	41480001	41483000	3000	1	3.70E-08	-0.36	28	0.93	Foxp2	
DMR4:41529001	4	41529001	41533000	4000	1	5.40E-12	-0.33	36	0.9	Foxp2	
DMR4:41559001	4	41559001	41562000	3000	1	1.90E-07	-0.46	10	0.33	Foxp2	
DMR4:41605001	4	41605001	41606000	1000	1	3.20E-07	0.33	15	1.5	Foxp2	
DMR4:41622001	4	41622001	41624000	2000	1	1.30E-10	0.6	28	1.4	Foxp2	
DMR4:41628001	4	41628001	41629000	1000	1	5.10E-08	-0.59	10	1	Foxp2	
DMR4:41668001	4	41668001	41670000	2000	1	1.20E-08	0.41	15	0.75	Foxp2	
DMR4:41681001	4	41681001	41683000	2000	1	1.30E-08	-0.48	33	1.65	Foxp2	
DMR4:41685001	4	41685001	41686000	1000	1	1.10E-14	0.6	15	1.5	Foxp2	
DMR4:41704001	4	41704001	41706000	2000	1	2.50E-09	0.43	24	1.2	Foxp2	
DMR4:42197001	4	42197001	42200000	3000	1	9.00E-08	0.43	36	1.2	Rbmxl1;Mdfic	
DMR4:42211001	4	42211001	42215000	4000	1	8.10E-12	-0.62	44	1.1	Mdfic	
DMR4:42849001	4	42849001	42854000	5000	1	4.20E-08	-0.41	45	0.9	Cftr	Transport
DMR4:44144001	4	44144001	44150000	6000	1	8.70E-07	-0.27	55	0.92	Tfec	
DMR4:44782001	4	44782001	44785000	3000	1	1.10E-08	-0.6	65	2.17	Met	Receptor
DMR4:44805001	4	44805001	44807000	2000	1	7.10E-08	-0.55	23	1.15	Met	Receptor
DMR4:45006001	4	45006001	45008000	2000	1	4.30E-07	0.59	10	0.5	ST7	
DMR4:45037001	4	45037001	45040000	3000	1	3.60E-07	-0.36	22	0.73	ST7	
DMR4:45069001	4	45069001	45074000	5000	1	2.10E-07	-0.49	46	0.92	ST7	
DMR4:45123001	4	45123001	45124000	1000	1	6.40E-09	0.43	6	0.6	ST7;LOC108350800;LOC103692064	
DMR4:45322001	4	45322001	45323000	1000	1	2.20E-11	-0.73	11	1.1	Wnt2	Signaling
DMR4:45340001	4	45340001	45342000	2000	1	1.80E-08	0.41	21	1.05	Wnt2	Signaling
DMR4:45394001	4	45394001	45396000	2000	1	3.50E-07	-0.3	20	1	Asz1	
DMR4:45412001	4	45412001	45413000	1000	1	1.90E-07	-0.51	3	0.3	Asz1	
DMR4:48387001	4	48387001	48388000	1000	1	1.40E-10	0.41	10	1	Kcnd2	Transport

DMR4:48396001	4	48396001	48397000	1000	1	2.60E-07	-0.59	8	0.8	Kcnd2	Transport
DMR4:48406001	4	48406001	48407000	1000	1	1.60E-07	-0.4	13	1.3	Kcnd2	Transport
DMR4:48440001	4	48440001	48442000	2000	1	1.30E-14	0.45	29	1.45	Kcnd2	Transport
DMR4:48581001	4	48581001	48585000	4000	2	2.70E-08	-0.33	35	0.88	Kcnd2	Transport
DMR4:48610001	4	48610001	48611000	1000	1	2.10E-08	-0.65	11	1.1	Kcnd2	Transport
DMR4:48870001	4	48870001	48872000	2000	1	1.20E-07	0.34	28	1.4	Tspan12	
DMR4:48882001	4	48882001	48886000	4000	2	7.50E-12	-0.46	39	0.98	Tspan12	
DMR4:48887001	4	48887001	48889000	2000	1	9.70E-08	-0.44	49	2.45	Tspan12	
DMR4:48897001	4	48897001	48898000	1000	1	4.70E-07	-0.44	22	2.2	Tspan12	
DMR4:49025001	4	49025001	49027000	2000	1	1.50E-07	-0.42	16	0.8	Ing3	Epigenetic
DMR4:49060001	4	49060001	49061000	1000	1	1.30E-11	-0.55	15	1.5	Cped1	
DMR4:49183001	4	49183001	49186000	3000	1	6.20E-07	0.46	58	1.93	Cped1	
DMR4:49190001	4	49190001	49192000	2000	1	2.70E-09	-0.39	29	1.45	Cped1	
DMR4:49280001	4	49280001	49282000	2000	1	3.20E-09	0.46	18	0.9	Cped1	
DMR4:49320001	4	49320001	49322000	2000	1	2.40E-09	-0.66	37	1.85	Cped1	
DMR4:49359001	4	49359001	49361000	2000	1	9.80E-10	0.32	16	0.8	Wnt16	Signaling
DMR4:49389001	4	49389001	49392000	3000	1	2.40E-07	-0.46	74	2.47	Wnt16;Fam3c;LOC103692083	Signaling;Signaling
DMR4:49405001	4	49405001	49409000	4000	1	4.40E-08	-0.48	112	2.8	Fam3c;LOC103692083	Signaling
DMR4:50186001	4	50186001	50187000	1000	1	6.80E-08	0.47	8	0.8	Aass	Metabolism
DMR4:50211001	4	50211001	50213000	2000	1	8.10E-07	0.43	34	1.7	Aass	Metabolism
DMR4:50331001	4	50331001	50334000	3000	1	2.60E-10	-0.63	43	1.43	Cadps2	Transport
DMR4:50362001	4	50362001	50365000	3000	2	1.20E-11	-0.58	18	0.6	Cadps2	Transport
DMR4:50373001	4	50373001	50374000	1000	1	7.90E-07	0.46	13	1.3	Cadps2	Transport
DMR4:50419001	4	50419001	50422000	3000	1	1.80E-07	-0.55	22	0.73	Cadps2	Transport
DMR4:50449001	4	50449001	50452000	3000	1	1.20E-07	-0.45	80	2.67	Cadps2	Transport
DMR4:50471001	4	50471001	50473000	2000	1	6.10E-07	-0.58	31	1.55	Cadps2	Transport
DMR4:50543001	4	50543001	50547000	4000	1	8.40E-07	0.32	37	0.92	Cadps2	Transport
DMR4:50575001	4	50575001	50580000	5000	3	2.60E-11	-0.59	66	1.32	Cadps2	Transport
DMR4:50778001	4	50778001	50780000	2000	1	6.60E-07	-0.38	13	0.65	Cadps2	Transport
DMR4:50801001	4	50801001	50802000	1000	1	1.70E-07	-0.45	19	1.9	Cadps2	Transport
DMR4:51009001	4	51009001	51011000	2000	1	6.40E-08	0.36	16	0.8	Tas2r118	
DMR4:51137001	4	51137001	51138000	1000	1	2.60E-12	0.9	33	3.3	Slc13a1	Transport
DMR4:51511001	4	51511001	51514000	3000	1	1.90E-07	0.36	34	1.13	Iqub	
DMR4:51628001	4	51628001	51629000	1000	1	7.70E-07	0.41	24	2.4	Asb15;LOC108350676	
DMR4:51631001	4	51631001	51632000	1000	1	2.40E-11	0.66	13	1.3	Asb15;LOC108350676	
DMR4:51722001	4	51722001	51724000	2000	1	3.50E-08	-0.4	14	0.7	Wasl	
DMR4:51823001	4	51823001	51824000	1000	1	3.90E-10	0.72	34	3.4	Gpr37	Signaling
DMR4:51906001	4	51906001	51909000	3000	1	7.60E-08	0.49	40	1.33	Pot1	Transcription
DMR4:52118001	4	52118001	52122000	4000	2	9.80E-12	-0.56	23	0.58	Hyal6	Metabolism
DMR4:52239001	4	52239001	52240000	1000	1	7.30E-09	0.43	16	1.6	Hyal5	Metabolism
DMR4:52303001	4	52303001	52304000	1000	1	3.30E-08	-0.6	14	1.4	Hyal5	Metabolism
DMR4:54486001	4	54486001	54491000	5000	2	1.80E-08	-0.29	43	0.86	Grm8	Signaling
DMR4:54517001	4	54517001	54520000	3000	1	7.30E-11	-0.48	37	1.23	Grm8	Signaling
DMR4:54522001	4	54522001	54528000	6000	1	9.60E-08	-0.28	59	0.98	Grm8	Signaling
DMR4:54620001	4	54620001	54623000	3000	2	8.50E-11	-0.35	28	0.93	Grm8	Signaling
DMR4:54630001	4	54630001	54631000	1000	1	1.10E-08	-0.43	4	0.4	Grm8	Signaling
DMR4:54635001	4	54635001	54636000	1000	1	7.90E-08	0.6	18	1.8	Grm8	Signaling
DMR4:54677001	4	54677001	54684000	7000	1	6.10E-09	-0.3	79	1.13	Grm8	Signaling
DMR4:54765001	4	54765001	54771000	6000	1	9.50E-10	-0.26	66	1.1	Grm8	Signaling
DMR4:54838001	4	54838001	54842000	4000	2	3.30E-08	-0.31	38	0.95	Grm8	Signaling
DMR4:54848001	4	54848001	54853000	5000	1	9.80E-07	-0.36	44	0.88	Grm8	Signaling
DMR4:54943001	4	54943001	54945000	2000	1	3.40E-08	-0.59	5	0.25	Grm8	Signaling
DMR4:54993001	4	54993001	54994000	1000	1	4.20E-07	0.38	9	0.9	Grm8	Signaling
DMR4:55013001	4	55013001	55015000	2000	1	1.10E-07	-0.41	7	0.35	Grm8	Signaling
DMR4:55017001	4	55017001	55021000	4000	2	1.20E-09	-0.53	22	0.55	Grm8	Signaling
DMR4:55138001	4	55138001	55140000	2000	1	1.60E-10	0.64	14	0.7	Grm8	Signaling
DMR4:55252001	4	55252001	55258000	6000	1	1.50E-08	-0.38	44	0.73	Grm8	Signaling
DMR4:55387001	4	55387001	55394000	7000	1	3.50E-09	0.43	71	1.01	Grm8	Signaling
DMR4:55419001	4	55419001	55421000	2000	1	2.50E-12	0.36	26	1.3	Grm8	Signaling
DMR4:55752001	4	55752001	55753000	1000	1	2.30E-07	0.36	11	1.1	Pax4	
DMR4:55788001	4	55788001	55790000	2000	1	2.90E-07	-0.39	17	0.85	Snd1	
DMR4:55810001	4	55810001	55812000	2000	1	3.60E-07	-0.48	21	1.05	Snd1	
DMR4:55842001	4	55842001	55844000	2000	1	1.00E-15	-0.67	25	1.25	Snd1	

DMR4:55909001	4	55909001	55912000	3000	2	5.60E-09	-0.6	25	0.83	Snd1	
DMR4:55940001	4	55940001	55943000	3000	1	9.80E-07	0.43	16	0.53	Snd1	
DMR4:56055001	4	56055001	56056000	1000	1	3.70E-09	-0.64	8	0.8	Snd1	
DMR4:56096001	4	56096001	56097000	1000	1	4.60E-07	-0.31	14	1.4	Snd1	
DMR4:56391001	4	56391001	56394000	3000	1	1.10E-07	0.56	39	1.3	Rbm28	
DMR4:56614001	4	56614001	56617000	3000	1	3.80E-07	0.72	63	2.1	Fam71f1;Calu	Signaling
DMR4:56717001	4	56717001	56720000	3000	1	6.00E-07	0.35	69	2.3	Flnc	
DMR4:56773001	4	56773001	56775000	2000	1	4.00E-22	0.92	45	2.25	Kcp	
DMR4:56835001	4	56835001	56839000	4000	1	8.50E-08	-0.35	23	0.58	Tnpo3	Transport
DMR4:56901001	4	56901001	56902000	1000	1	6.40E-11	0.41	7	0.7	Tnpo3	Transport
DMR4:57022001	4	57022001	57028000	6000	1	1.10E-07	0.36	93	1.55	Smo	Receptor
DMR4:57279001	4	57279001	57280000	1000	1	2.40E-07	0.42	16	1.6	Smkr1	
DMR4:57329001	4	57329001	57331000	2000	1	1.30E-07	0.37	14	0.7	Nrf1	
DMR4:57453001	4	57453001	57455000	2000	2	4.00E-12	0.59	35	1.75	Nrf1;LOC108350684;Mir182;Mir96;Mir183;Mir3553	
DMR4:57520001	4	57520001	57522000	2000	1	8.20E-07	0.39	25	1.25	Ube2h	Proteolysis
DMR4:57763001	4	57763001	57765000	2000	1	2.80E-07	0.41	21	1.05	Klhdc10	
DMR4:57770001	4	57770001	57771000	1000	1	6.50E-10	-0.5	17	1.7	Klhdc10;LOC108350685	
DMR4:57781001	4	57781001	57783000	2000	1	4.30E-08	-0.57	50	2.5	Klhdc10;LOC108350685	
DMR4:57880001	4	57880001	57882000	2000	1	3.20E-10	0.48	14	0.7	Cpa2;Cpa4	Protease
DMR4:57944001	4	57944001	57947000	3000	2	1.90E-08	0.45	43	1.43	Cpa5;Cpa1	Protease
DMR4:57952001	4	57952001	57957000	5000	1	8.80E-07	0.45	93	1.86	Cpa5;Cpa1;Cep41	Protease
DMR4:58045001	4	58045001	58049000	4000	2	1.50E-08	0.82	101	2.52	Mest	Protease
DMR4:58220001	4	58220001	58223000	3000	3	1.20E-16	-0.53	57	1.9	Tsga13	
DMR4:58236001	4	58236001	58239000	3000	2	4.60E-08	-0.38	38	1.27	LOC103692110;Klf14	Transcription
DMR4:58336001	4	58336001	58338000	2000	1	1.20E-08	-0.52	37	1.85	Mir3556a;Mir29a;Mir3587;Mir29b1	
DMR4:58876001	4	58876001	58878000	2000	1	6.60E-07	-0.47	16	0.8	Podxl;LOC103692091	Cytoskeleton
DMR4:58895001	4	58895001	58899000	4000	1	1.30E-08	0.48	44	1.1	Podxl	Cytoskeleton
DMR4:60056001	4	60056001	60058000	2000	1	7.40E-09	0.41	12	0.6	RGD1563055	
DMR4:60118001	4	60118001	60119000	1000	1	1.30E-16	-0.67	1	0.1	Chchd3	
DMR4:60157001	4	60157001	60158000	1000	1	3.00E-07	0.38	19	1.9	Chchd3	
DMR4:60274001	4	60274001	60279000	5000	1	3.70E-11	-0.35	49	0.98	Chchd3	
DMR4:60540001	4	60540001	60548000	8000	2	9.00E-09	-0.31	92	1.15	RGD1565435;Exoc4	Transport
DMR4:60711001	4	60711001	60713000	2000	1	4.60E-08	0.35	9	0.45	Exoc4	Transport
DMR4:60814001	4	60814001	60818000	4000	1	3.60E-07	-0.24	50	1.25	Exoc4	Transport
DMR4:60917001	4	60917001	60918000	1000	1	3.20E-07	0.47	5	0.5	Exoc4;LOC108350837	Transport
DMR4:61320001	4	61320001	61324000	4000	1	1.50E-09	-0.4	38	0.95	Exoc4	Transport
DMR4:61360001	4	61360001	61362000	2000	1	1.40E-08	-0.38	28	1.4	Exoc4;LOC108350836	Transport
DMR4:61423001	4	61423001	61425000	2000	1	4.20E-09	0.44	51	2.55	Lrguk	Signaling
DMR4:61518001	4	61518001	61520000	2000	1	5.10E-10	0.29	25	1.25	Lrguk	Signaling
DMR4:61920001	4	61920001	61921000	1000	1	7.30E-07	-0.34	21	2.1	Bpgm	Metabolism
DMR4:61927001	4	61927001	61929000	2000	1	2.90E-08	-0.49	20	1	Bpgm	Metabolism
DMR4:62099001	4	62099001	62100000	1000	1	2.00E-08	-0.46	6	0.6	Cald1	Cytoskeleton
DMR4:62187001	4	62187001	62189000	2000	1	5.80E-08	-0.38	38	1.9	Cald1	Cytoskeleton
DMR4:62417001	4	62417001	62422000	5000	2	5.20E-09	-0.36	57	1.14	Wdr91	
DMR4:62451001	4	62451001	62452000	1000	1	1.70E-12	0.87	34	3.4	Stra8	
DMR4:62496001	4	62496001	62499000	3000	1	2.90E-07	0.38	52	1.73	RGD1565367;LOC103692099	Transport
DMR4:62550001	4	62550001	62552000	2000	1	8.40E-07	-0.36	33	1.65	Cnot4	Proteolysis
DMR4:62665001	4	62665001	62669000	4000	1	8.70E-09	-0.39	44	1.1	Cnot4;LOC102553126	Proteolysis
DMR4:62699001	4	62699001	62700000	1000	1	1.90E-07	0.52	19	1.9	Nup205	
DMR4:63338001	4	63338001	63339000	1000	1	7.50E-07	0.48	2	0.2	Mir490	
DMR4:64365001	4	64365001	64367000	2000	1	2.60E-08	0.74	36	1.8	Dgki	Signaling
DMR4:64446001	4	64446001	64449000	3000	1	3.80E-07	-0.52	48	1.6	Dgki	Signaling
DMR4:64566001	4	64566001	64569000	3000	1	1.90E-07	-0.4	72	2.4	Dgki	Signaling
DMR4:64584001	4	64584001	64588000	4000	1	1.30E-08	-0.45	59	1.48	Dgki	Signaling
DMR4:64663001	4	64663001	64665000	2000	1	3.50E-08	-0.41	18	0.9	Dgki	Signaling
DMR4:64813001	4	64813001	64817000	4000	1	1.90E-07	-0.27	45	1.12	Dgki	Signaling
DMR4:64909001	4	64909001	64917000	8000	2	1.10E-08	-0.47	139	1.74	Creb3l2	
DMR4:64969001	4	64969001	64970000	1000	1	8.60E-07	-0.44	25	2.5	Creb3l2	
DMR4:65627001	4	65627001	65632000	5000	1	3.30E-09	-0.39	47	0.94	Trim24	Epigenetic
DMR4:65644001	4	65644001	65645000	1000	1	8.90E-09	0.57	12	1.2	Trim24	Epigenetic
DMR4:65710001	4	65710001	65712000	2000	1	8.70E-08	0.6	28	1.4	Svopl	Transport

DMR4:65748001	4	65748001	65751000	3000	1	2.60E-07	0.27	33	1.1	Atp6v0a4	Metabolism
DMR4:65909001	4	65909001	65911000	2000	1	9.90E-07	0.38	26	1.3	RGD1306271	
DMR4:66092001	4	66092001	66093000	1000	1	1.60E-07	-0.51	8	0.8	Ttc26;LOC108350693	
DMR4:66331001	4	66331001	66332000	1000	1	2.70E-07	-0.43	6	0.6	Luc7l2;LOC103692107;LOC108350694	
DMR4:66514001	4	66514001	66516000	2000	1	2.20E-07	0.44	10	0.5	Hipk2	
DMR4:66670001	4	66670001	66675000	5000	2	2.00E-10	-0.57	71	1.42	Tbxas1	Metabolism
DMR4:66701001	4	66701001	66702000	1000	1	1.90E-10	0.5	10	1	Tbxas1	Metabolism
DMR4:66755001	4	66755001	66757000	2000	1	7.50E-07	0.41	19	0.95	Tbxas1	Metabolism
DMR4:66814001	4	66814001	66818000	4000	2	1.20E-09	0.47	64	1.6	Tbxas1	Metabolism
DMR4:66833001	4	66833001	66834000	1000	1	3.30E-07	0.43	19	1.9	Tbxas1;Parp12	Metabolism
DMR4:67314001	4	67314001	67315000	1000	1	3.30E-08	0.44	21	2.1	Dennd2a	
DMR4:67464001	4	67464001	67468000	4000	1	1.70E-09	-0.34	31	0.78	Braf	Signaling
DMR4:67594001	4	67594001	67599000	5000	1	3.10E-07	-0.28	47	0.94	Mrps33	Translation
DMR4:67714001	4	67714001	67715000	1000	1	4.20E-07	0.38	11	1.1	Tmem178b	
DMR4:67863001	4	67863001	67864000	1000	1	5.20E-08	0.61	19	1.9	Tmem178b	
DMR4:68157001	4	68157001	68163000	6000	1	4.30E-07	-0.39	48	0.8	Vom2r-ps15	
DMR4:68519001	4	68519001	68524000	5000	1	3.90E-07	-0.26	43	0.86	Agk	Signaling
DMR4:68582001	4	68582001	68584000	2000	1	7.50E-07	-0.43	39	1.95	RGD1563986;LOC108350697	
DMR4:68615001	4	68615001	68616000	1000	1	1.60E-08	-0.65	6	0.6	Wee2	Signaling
DMR4:69082001	4	69082001	69084000	2000	1	9.90E-07	0.5	8	0.4	Mgam	Metabolism
DMR4:69107001	4	69107001	69108000	1000	1	1.80E-08	0.32	2	0.2	Mgam;Moxd2	Metabolism;Metabolism
DMR4:70702001	4	70702001	70710000	8000	2	2.20E-08	-0.36	84	1.05	LOC108348060;Prss2	Protease
DMR4:70921001	4	70921001	70922000	1000	1	6.10E-09	-0.45	8	0.8	Ephb6;Trpv6	Receptor;Transport
DMR4:71378001	4	71378001	71380000	2000	1	2.70E-11	-0.32	15	0.75	Sval1	
DMR4:71611001	4	71611001	71615000	4000	1	2.20E-07	-0.24	34	0.85	Gstk1	Transport
DMR4:71633001	4	71633001	71639000	6000	3	7.80E-11	-0.38	56	0.93	Gstk1;LOC100360758;Tmem139	Transport
DMR4:71673001	4	71673001	71676000	3000	1	2.00E-08	0.47	39	1.3	Casp2;Clcn1	Protease;Transport
DMR4:71684001	4	71684001	71685000	1000	1	1.20E-11	-0.76	5	0.5	Clcn1	Transport
DMR4:71745001	4	71745001	71747000	2000	1	7.60E-09	0.36	25	1.25	Zyx;Epha1	Cytoskeleton;Receptor
DMR4:71843001	4	71843001	71844000	1000	1	9.20E-07	-0.4	4	0.4	Tas2r126	
DMR4:71976001	4	71976001	71981000	5000	2	5.10E-08	-0.33	54	1.08	Olr804	Receptor
DMR4:72155001	4	72155001	72160000	5000	1	6.00E-09	-0.28	50	1	Tcaf1	
DMR4:72197001	4	72197001	72202000	5000	2	8.10E-08	-0.38	34	0.68	Olr805-ps;LOC108350808	
DMR4:72246001	4	72246001	72252000	6000	1	5.70E-07	-0.32	43	0.72	Olr807	Receptor
DMR4:72535001	4	72535001	72540000	5000	1	6.50E-07	-0.46	51	1.02	Olr815-ps	
DMR4:72552001	4	72552001	72559000	7000	1	9.20E-07	-0.28	78	1.11	Olr816	Receptor
DMR4:72573001	4	72573001	72578000	5000	1	6.70E-07	-0.27	41	0.82	Olr816	Receptor
DMR4:72628001	4	72628001	72629000	1000	1	9.00E-08	-0.52	3	0.3	Olr819;LOC108350809	Receptor
DMR4:72674001	4	72674001	72675000	1000	1	3.20E-07	-0.28	8	0.8	Olr821	Receptor
DMR4:72676001	4	72676001	72679000	3000	1	3.10E-07	-0.31	24	0.8	Olr821	Receptor
DMR4:72779001	4	72779001	72785000	6000	1	9.10E-07	-0.26	53	0.88	Tpk1	Signaling
DMR4:72952001	4	72952001	72954000	2000	1	2.20E-09	-0.38	11	0.55	Tpk1	Signaling
DMR4:72975001	4	72975001	72976000	1000	1	7.50E-07	0.41	10	1	Tpk1	Signaling
DMR4:73156001	4	73156001	73159000	3000	1	1.50E-07	0.54	25	0.83	Tpk1	Signaling
DMR4:74828001	4	74828001	74829000	1000	1	2.00E-07	0.55	8	0.8	Cntnap2	
DMR4:75200001	4	75200001	75202000	2000	1	3.90E-08	-0.49	12	0.6	Cntnap2	
DMR4:75463001	4	75463001	75468000	5000	1	4.70E-08	-0.3	37	0.74	Cntnap2	
DMR4:75516001	4	75516001	75517000	1000	1	7.00E-07	0.45	6	0.6	Cntnap2	
DMR4:76205001	4	76205001	76207000	2000	1	1.90E-09	-0.39	12	0.6	Cntnap2	
DMR4:76223001	4	76223001	76224000	1000	1	8.60E-07	-0.43	1	0.1	Cntnap2	
DMR4:76517001	4	76517001	76519000	2000	1	2.40E-07	-0.27	24	1.2	Cntnap2	
DMR4:76836001	4	76836001	76837000	1000	1	6.70E-07	0.39	4	0.4	Cntnap2	
DMR4:76948001	4	76948001	76954000	6000	2	2.00E-11	-0.33	67	1.12	Cntnap2	
DMR4:77575001	4	77575001	77576000	1000	1	4.80E-09	-0.39	12	1.2	Zfp282	Transcription
DMR4:77896001	4	77896001	77900000	4000	1	9.10E-07	-0.33	14	0.35	LOC108350703;Trnac-gca	
DMR4:78070001	4	78070001	78073000	3000	1	5.40E-09	1	123	4.1	Zfp467;Sspo	Extracellular Matrix
DMR4:78085001	4	78085001	78086000	1000	1	1.20E-10	0.42	7	0.7	Zfp467;Sspo	Extracellular Matrix
DMR4:78165001	4	78165001	78166000	1000	1	4.00E-07	0.36	19	1.9	Zfp862;Atp6v0e2	Metabolism
DMR4:78313001	4	78313001	78317000	4000	1	5.80E-10	0.39	32	0.8	LOC681294;Gimap9;Gimap4	Signaling
DMR4:78439001	4	78439001	78441000	2000	1	1.10E-07	-0.47	3	0.15	Tmem176b	
DMR4:78650001	4	78650001	78655000	5000	2	5.50E-08	-0.26	43	0.86	Svs1	Metabolism
DMR4:78704001	4	78704001	78705000	1000	1	2.20E-07	0.34	9	0.9	Gpnmb	Signaling

DMR4:78798001	4	78798001	78800000	2000	1	1.60E-07	0.49	57	2.85	Igf2bp3	Metabolism
DMR4:78818001	4	78818001	78821000	3000	1	1.80E-08	-0.61	37	1.23	Igf2bp3	Metabolism
DMR4:79163001	4	79163001	79167000	4000	1	6.90E-07	-0.41	16	0.4	Stk31	
DMR4:79835001	4	79835001	79838000	3000	1	3.80E-07	-0.5	36	1.2	Mpp6	Cytoskeleton
DMR4:80154001	4	80154001	80155000	1000	1	1.70E-08	-0.52	18	1.8	Osbpl3	
DMR4:80208001	4	80208001	80210000	2000	1	4.50E-07	-0.37	23	1.15	Osbpl3	
DMR4:81240001	4	81240001	81241000	1000	1	2.10E-08	-0.49	7	0.7	Nfe2l3;Hnrnpa2b1;Cbx3	Transcription
DMR4:81294001	4	81294001	81297000	3000	1	6.50E-07	-0.32	64	2.13	RGD1561341	
DMR4:81379001	4	81379001	81381000	2000	1	5.40E-08	-0.41	48	2.4	Snx10	
DMR4:82138001	4	82138001	82139000	1000	1	9.60E-07	0.62	27	2.7	Hotairm1;Hoxa2;Hoxa3;LOC102554823	
DMR4:82161001	4	82161001	82165000	4000	1	1.00E-10	-0.64	79	1.98	Hoxa3;Hoxa4;LOC102547399;Hoxaas3;LOC103689922;Hoxa6	Development
DMR4:82878001	4	82878001	82879000	1000	1	1.00E-10	0.54	8	0.8	Jazf1	
DMR4:82889001	4	82889001	82892000	3000	1	1.20E-08	0.43	33	1.1	Jazf1;LOC108350712	
DMR4:82931001	4	82931001	82934000	3000	1	5.90E-08	-0.47	38	1.27	Jazf1	
DMR4:82974001	4	82974001	82975000	1000	1	9.50E-07	-0.35	11	1.1	Jazf1	
DMR4:82990001	4	82990001	82991000	1000	1	2.50E-13	-0.68	11	1.1	Jazf1	
DMR4:83838001	4	83838001	83839000	1000	1	1.30E-07	0.55	22	2.2	Creb5	Transcription
DMR4:84015001	4	84015001	84021000	6000	2	4.90E-09	-0.34	53	0.88	Cpvl	
DMR4:84064001	4	84064001	84065000	1000	1	1.30E-10	0.34	6	0.6	Cpvl;LOC108350713	
DMR4:84490001	4	84490001	84493000	3000	2	2.30E-08	-0.42	36	1.2	Prr15	
DMR4:84621001	4	84621001	84624000	3000	1	5.30E-08	0.37	32	1.07	Wipf3	Cytoskeleton
DMR4:84646001	4	84646001	84649000	3000	1	4.60E-07	-0.41	35	1.17	Wipf3;LOC103692134	Cytoskeleton
DMR4:84747001	4	84747001	84750000	3000	1	1.30E-10	0.46	22	0.73	Scrn1;LOC102553885;Fkbp14	Transcription
DMR4:84764001	4	84764001	84765000	1000	1	2.70E-07	-0.64	8	0.8	LOC102553885;Fkbp14;Plekha8	Transcription;Transport
DMR4:84851001	4	84851001	84853000	2000	1	9.10E-07	0.45	28	1.4	Mturn	
DMR4:85287001	4	85287001	85291000	4000	1	5.70E-07	0.55	59	1.48	Crhr2	Receptor
DMR4:85419001	4	85419001	85425000	6000	1	6.00E-08	-0.42	36	0.6	Fam188b	
DMR4:85526001	4	85526001	85527000	1000	1	2.50E-08	0.47	10	1	Fam188b;LOC108350810	
DMR4:86159001	4	86159001	86162000	3000	1	1.70E-07	-0.39	9	0.3	Ccdc129	
DMR4:86711001	4	86711001	86716000	5000	1	9.90E-07	-0.26	33	0.66	Pde1c	Signaling
DMR4:86786001	4	86786001	86787000	1000	1	5.80E-07	0.44	2	0.2	Pde1c	Signaling
DMR4:86939001	4	86939001	86945000	6000	1	8.10E-07	-0.27	66	1.1	RGD1565107	
DMR4:87163001	4	87163001	87165000	2000	1	3.40E-10	-0.4	20	1	Fkbp9	
DMR4:87178001	4	87178001	87180000	2000	1	9.10E-07	-0.46	33	1.65	Fkbp9	
DMR4:87260001	4	87260001	87262000	2000	1	4.90E-09	-0.41	30	1.5	Nt5c3a;LOC103692137	Metabolism
DMR4:87284001	4	87284001	87290000	6000	1	1.00E-09	-0.4	57	0.95	Nt5c3a;LOC103692137;LOC688782	Metabolism
DMR4:87306001	4	87306001	87310000	4000	1	2.60E-08	-0.39	25	0.62	Vom1r65	Receptor
DMR4:87760001	4	87760001	87762000	2000	1	1.20E-12	-0.49	18	0.9	Vom1r73	Receptor
DMR4:87849001	4	87849001	87850000	1000	1	1.30E-09	0.44	8	0.8	Vom1r76	Receptor
DMR4:87859001	4	87859001	87860000	1000	1	4.70E-09	0.6	17	1.7	Vom1r76;LOC689033	Receptor
DMR4:88130001	4	88130001	88135000	5000	2	6.30E-09	-0.41	49	0.98	Vom1r82;Vom1r-ps73	Receptor
DMR4:88192001	4	88192001	88193000	1000	1	6.20E-10	-0.54	3	0.3	Vom1r83	Receptor
DMR4:88241001	4	88241001	88244000	3000	1	3.40E-07	-0.2	33	1.1	Vom1r85	Receptor
DMR4:88323001	4	88323001	88325000	2000	1	8.30E-08	0.41	16	0.8	Vom1r87	Receptor
DMR4:88416001	4	88416001	88417000	1000	1	6.80E-09	0.74	5	0.5	Vom1r88	Receptor
DMR4:88657001	4	88657001	88661000	4000	1	2.30E-08	-0.29	44	1.1	Herc6	Proteolysis
DMR4:88688001	4	88688001	88689000	1000	1	2.50E-08	0.47	2	0.2	LOC500148;Ppm1k	
DMR4:89118001	4	89118001	89122000	4000	1	1.80E-09	-0.56	53	1.32	Herc3	Proteolysis
DMR4:89249001	4	89249001	89253000	4000	1	8.60E-08	-0.25	38	0.95	Fam13a	
DMR4:89273001	4	89273001	89274000	1000	1	4.90E-10	0.42	13	1.3	Fam13a	
DMR4:89718001	4	89718001	89725000	7000	2	1.80E-09	-0.39	59	0.84	Gprin3	
DMR4:89788001	4	89788001	89789000	1000	1	5.00E-08	0.49	7	0.7	Gprin3	
DMR4:90845001	4	90845001	90848000	3000	1	8.40E-07	-0.39	25	0.83	Snca	Transport
DMR4:91272001	4	91272001	91274000	2000	1	4.40E-09	-0.41	19	0.95	Ccser1	
DMR4:91298001	4	91298001	91301000	3000	1	6.20E-11	0.49	21	0.7	Ccser1	
DMR4:91449001	4	91449001	91450000	1000	1	1.20E-08	0.54	7	0.7	Ccser1	
DMR4:91502001	4	91502001	91506000	4000	1	3.40E-07	-0.45	29	0.72	Ccser1	
DMR4:91655001	4	91655001	91658000	3000	1	8.20E-07	0.46	22	0.73	Ccser1;LOC103692146	
DMR4:91722001	4	91722001	91724000	2000	1	2.10E-07	-0.44	11	0.55	Ccser1	
DMR4:91782001	4	91782001	91783000	1000	1	8.40E-11	0.47	11	1.1	Ccser1	
DMR4:91856001	4	91856001	91857000	1000	1	4.70E-07	-0.51	10	1	Ccser1	

DMR4:91876001	4	91876001	91877000	1000	1	2.40E-07	-0.59	11	1.1	Ccser1	
DMR4:92027001	4	92027001	92028000	1000	1	9.40E-15	0.57	12	1.2	Ccser1;LOC108350840	
DMR4:92046001	4	92046001	92048000	2000	1	4.60E-07	-0.46	16	0.8	Ccser1	
DMR4:92320001	4	92320001	92321000	1000	1	2.50E-08	0.53	10	1	Ccser1	
DMR4:92389001	4	92389001	92393000	4000	1	6.70E-08	-0.24	49	1.23	Ccser1	
DMR4:92458001	4	92458001	92459000	1000	1	2.30E-08	0.54	13	1.3	Ccser1	
DMR4:92460001	4	92460001	92463000	3000	1	1.70E-07	-0.25	25	0.83	Ccser1	
DMR4:92516001	4	92516001	92520000	4000	1	1.10E-13	0.58	39	0.98	Ccser1	
DMR4:94056001	4	94056001	94061000	5000	1	1.30E-07	-0.28	36	0.72	Grid2	Receptor
DMR4:94197001	4	94197001	94198000	1000	1	6.50E-08	0.45	4	0.4	Grid2	Receptor
DMR4:94199001	4	94199001	94201000	2000	1	5.40E-10	-0.64	12	0.6	Grid2	Receptor
DMR4:94204001	4	94204001	94206000	2000	1	6.80E-07	-0.38	9	0.45	Grid2	Receptor
DMR4:94248001	4	94248001	94250000	2000	1	9.00E-07	0.4	10	0.5	Grid2	Receptor
DMR4:94274001	4	94274001	94277000	3000	1	3.90E-07	-0.23	27	0.9	Grid2	Receptor
DMR4:94736001	4	94736001	94737000	1000	1	2.20E-10	0.51	4	0.4	Grid2	Receptor
DMR4:95236001	4	95236001	95239000	3000	1	1.10E-07	-0.38	30	1	Grid2	Receptor
DMR4:95280001	4	95280001	95285000	5000	1	8.60E-08	-0.39	57	1.14	Grid2	Receptor
DMR4:95324001	4	95324001	95326000	2000	1	1.80E-07	0.4	8	0.4	Grid2	Receptor
DMR4:95463001	4	95463001	95465000	2000	1	9.20E-11	0.42	13	0.65	Grid2	Receptor
DMR4:95978001	4	95978001	95980000	2000	1	1.00E-07	-0.31	24	1.2	Hpgds	Transport
DMR4:96262001	4	96262001	96263000	1000	1	1.40E-08	0.44	9	0.9	RGD1560028	Signaling
DMR4:96400001	4	96400001	96401000	1000	1	3.50E-08	-0.53	14	1.4	Tnip3	
DMR4:96667001	4	96667001	96669000	2000	1	1.20E-09	-0.53	18	0.9	Prdm5	Transcription
DMR4:96821001	4	96821001	96826000	5000	2	1.80E-08	0.4	50	1	RSA-14-44	Signaling
DMR4:98037001	4	98037001	98039000	2000	2	6.50E-12	-0.78	11	0.55	Serbp1	Metabolism
DMR4:98229001	4	98229001	98231000	2000	1	7.50E-07	-0.41	24	1.2	Il23r	Receptor
DMR4:98287001	4	98287001	98292000	5000	1	3.20E-09	-0.52	36	0.72	Il23r	Receptor
DMR4:98314001	4	98314001	98319000	5000	1	3.10E-07	-0.35	41	0.82	Il23r	Receptor
DMR4:98655001	4	98655001	98657000	2000	1	1.30E-08	-0.41	21	1.05	LOC102549525;Eif2ak3	Signaling
DMR4:99101001	4	99101001	99105000	4000	1	1.30E-07	0.47	42	1.05	Smyd1	Epigenetic
DMR4:99146001	4	99146001	99147000	1000	1	2.10E-07	-0.41	6	0.6	Krcc1	
DMR4:99388001	4	99388001	99394000	6000	2	1.30E-07	-0.39	49	0.82	Rnf103	Proteolysis
DMR4:99447001	4	99447001	99448000	1000	1	3.30E-11	-0.58	13	1.3	Chmp3	Transport
DMR4:99655001	4	99655001	99656000	1000	1	7.90E-08	0.34	14	1.4	Reep1	Transport
DMR4:99686001	4	99686001	99690000	4000	1	5.00E-10	-0.68	40	1	Reep1	Transport
DMR4:99910001	4	99910001	99915000	5000	1	1.20E-07	-0.37	35	0.7	Polr1a;LOC100361613	Transcription
DMR4:99949001	4	99949001	99950000	1000	1	3.00E-08	-0.52	11	1.1	St3gal5	Transport
DMR4:99952001	4	99952001	99954000	2000	1	4.70E-07	0.33	9	0.45	St3gal5	Transport
DMR4:100670001	4	100670001	100672000	2000	1	4.10E-07	0.58	36	1.8	Tcf7l1	Transcription
DMR4:100773001	4	100773001	100777000	4000	1	3.50E-09	-0.53	46	1.15	Kcmf1	Proteolysis
DMR4:100866001	4	100866001	100874000	8000	1	8.60E-10	0.43	71	0.89	Tmsb10	
DMR4:101003001	4	101003001	101006000	3000	1	4.10E-09	-0.45	33	1.1	Dnah6	Cytoskeleton
DMR4:101008001	4	101008001	101011000	3000	1	2.60E-07	0.51	22	0.73	Dnah6	Cytoskeleton
DMR4:101020001	4	101020001	101021000	1000	1	4.20E-07	0.39	13	1.3	Dnah6	Cytoskeleton
DMR4:101358001	4	101358001	101361000	3000	1	8.40E-11	0.66	23	0.77	Olr822-ps	
DMR4:102881001	4	102881001	102882000	1000	1	2.80E-08	0.46	8	0.8	RGD1565490	
DMR4:107944001	4	107944001	107946000	2000	1	5.70E-08	-0.34	15	0.75	Ctnna2	Cytoskeleton
DMR4:107947001	4	107947001	107950000	3000	1	2.30E-08	0.48	37	1.23	Ctnna2	Cytoskeleton
DMR4:108223001	4	108223001	108228000	5000	1	4.40E-10	0.49	43	0.86	Ctnna2	Cytoskeleton
DMR4:108245001	4	108245001	108248000	3000	1	3.10E-08	-0.35	27	0.9	Ctnna2	Cytoskeleton
DMR4:108805001	4	108805001	108809000	4000	1	5.00E-07	-0.4	36	0.9	Ctnna2	Cytoskeleton
DMR4:108857001	4	108857001	108858000	1000	1	6.30E-08	-0.4	10	1	Ctnna2	Cytoskeleton
DMR4:108897001	4	108897001	108899000	2000	1	3.20E-08	-0.45	15	0.75	Ctnna2	Cytoskeleton
DMR4:108903001	4	108903001	108906000	3000	1	7.30E-07	-0.44	22	0.73	Ctnna2	Cytoskeleton
DMR4:109508001	4	109508001	109511000	3000	1	3.70E-10	-0.45	13	0.43	Reg1a	
DMR4:110731001	4	110731001	110733000	2000	1	3.50E-07	0.53	51	2.55	Lrrtm4	Receptor
DMR4:110889001	4	110889001	110890000	1000	1	5.80E-07	0.41	6	0.6	Lrrtm4	Receptor
DMR4:111134001	4	111134001	111135000	1000	1	7.70E-08	-0.41	7	0.7	Lrrtm4	Receptor
DMR4:111150001	4	111150001	111151000	1000	1	8.10E-07	0.38	7	0.7	Lrrtm4	Receptor
DMR4:111170001	4	111170001	111171000	1000	1	2.50E-07	0.5	6	0.6	Lrrtm4	Receptor
DMR4:111258001	4	111258001	111260000	2000	1	7.80E-07	-0.4	10	0.5	Lrrtm4;LOC108350842	Receptor
DMR4:112713001	4	112713001	112714000	1000	1	1.20E-07	-0.46	19	1.9	Mrpl19;Eva1a	Translation
DMR4:112727001	4	112727001	112731000	4000	1	2.90E-08	-0.43	59	1.48	Eva1a;LOC102547556	

DMR4:112740001	4	112740001	112742000	2000	1	8.70E-11	0.52	14	0.7	Eva1a;LOC102547556	
DMR4:113283001	4	113283001	113287000	4000	1	8.40E-08	-0.42	35	0.88	Tacr1	Signaling
DMR4:113375001	4	113375001	113377000	2000	1	5.90E-07	-0.51	19	0.95	Tacr1	Signaling
DMR4:113560001	4	113560001	113562000	2000	1	8.00E-07	-0.31	41	2.05	Hk2	Signaling
DMR4:113563001	4	113563001	113565000	2000	1	1.90E-09	-0.5	48	2.4	Hk2	Signaling
DMR4:113771001	4	113771001	113773000	2000	1	4.20E-08	0.83	32	1.6	Sema4f;M1ap	Signaling
DMR4:113841001	4	113841001	113842000	1000	1	9.70E-16	-0.62	14	1.4	M1ap	
DMR4:113848001	4	113848001	113849000	1000	1	4.20E-09	-0.61	4	0.4	M1ap	
DMR4:113861001	4	113861001	113862000	1000	1	8.20E-08	0.39	9	0.9	M1ap;Dok1;Loxl3	Metabolism
DMR4:114912001	4	114912001	114913000	1000	1	1.60E-07	-0.48	8	0.8	Slc4a5	Transport
DMR4:114938001	4	114938001	114942000	4000	1	3.80E-08	0.37	48	1.2	Slc4a5	Transport
DMR4:115269001	4	115269001	115273000	4000	1	3.40E-08	-0.37	78	1.95	Stambp	Protease
DMR4:115572001	4	115572001	115575000	3000	1	2.80E-09	0.52	41	1.37	Zfp638;LOC502853	
DMR4:115632001	4	115632001	115635000	3000	1	7.20E-10	-0.53	16	0.53	Zfp638	
DMR4:115745001	4	115745001	115746000	1000	1	4.50E-08	0.34	10	1	Dysf	Transport
DMR4:115802001	4	115802001	115804000	2000	1	1.30E-07	-0.36	35	1.75	Dysf	Transport
DMR4:115864001	4	115864001	115866000	2000	1	3.40E-07	-0.52	24	1.2	Dysf	Transport
DMR4:116407001	4	116407001	116408000	1000	1	2.10E-08	-0.65	10	1	Exoc6b	Transport
DMR4:117028001	4	117028001	117030000	2000	1	3.90E-09	0.41	20	1	Sfxn5	Transport
DMR4:117480001	4	117480001	117485000	5000	1	3.20E-08	-0.41	39	0.78	Alms1;Nat8f3	Metabolism
DMR4:117530001	4	117530001	117535000	5000	2	7.40E-08	-0.57	72	1.44	Nat8f5;Nat8	Metabolism
DMR4:117879001	4	117879001	117883000	4000	1	3.70E-11	0.41	54	1.35	Add2	Cytoskeleton
DMR4:117892001	4	117892001	117893000	1000	1	8.80E-09	-0.48	24	2.4	Add2	Cytoskeleton
DMR4:117975001	4	117975001	117980000	5000	3	3.00E-09	-0.51	86	1.72	Tgfa	Growth Factors
DMR4:117985001	4	117985001	117986000	1000	1	3.70E-09	0.56	5	0.5	Tgfa	Growth Factors
DMR4:118195001	4	118195001	118196000	1000	1	1.70E-07	-0.46	15	1.5	Pcyox1	Metabolism
DMR4:118200001	4	118200001	118201000	1000	1	2.80E-07	0.35	6	0.6	Pcyox1;Trnag-ccc;Tia1	Metabolism;Translation
DMR4:118262001	4	118262001	118265000	3000	2	2.30E-09	-0.49	31	1.03	RGD1306746	
DMR4:118281001	4	118281001	118283000	2000	1	2.00E-08	-0.47	37	1.85	RGD1306746	
DMR4:118424001	4	118424001	118426000	2000	1	2.00E-10	0.5	15	0.75	Asprv1	
DMR4:118550001	4	118550001	118551000	1000	1	4.20E-07	0.36	9	0.9	Anxa4	Signaling
DMR4:118570001	4	118570001	118571000	1000	1	1.40E-07	0.35	2	0.2	Anxa4	Signaling
DMR4:118584001	4	118584001	118587000	3000	1	1.30E-07	0.36	36	1.2	Anxa4	Signaling
DMR4:118593001	4	118593001	118595000	2000	1	3.20E-07	0.42	21	1.05	Anxa4;LOC103692178	Signaling
DMR4:118955001	4	118955001	118958000	3000	1	1.30E-18	0.4	36	1.2	Antxr1	Cytoskeleton
DMR4:118969001	4	118969001	118970000	1000	1	7.90E-09	0.41	9	0.9	Antxr1	Cytoskeleton
DMR4:118985001	4	118985001	118987000	2000	1	3.00E-09	0.41	23	1.15	Antxr1;LOC108350726	Cytoskeleton
DMR4:119071001	4	119071001	119073000	2000	1	1.30E-09	0.44	30	1.5	Antxr1	Cytoskeleton
DMR4:119142001	4	119142001	119144000	2000	1	4.20E-08	-0.47	25	1.25	Gkn2;Gkn1	
DMR4:119250001	4	119250001	119252000	2000	2	7.40E-11	-0.54	21	1.05	Arhgap25	
DMR4:119664001	4	119664001	119666000	2000	1	1.50E-07	-0.44	20	1	Efcc1	Translation
DMR4:120126001	4	120126001	120127000	1000	1	2.90E-16	0.66	31	3.1	Gata2	Transcription
DMR4:120131001	4	120131001	120132000	1000	1	7.10E-07	0.59	27	2.7	Gata2	Transcription
DMR4:120177001	4	120177001	120182000	5000	1	7.60E-09	0.45	85	1.7	Tcp1-ps1	
DMR4:120199001	4	120199001	120200000	1000	1	2.60E-09	0.52	9	0.9	Eefsec	Translation
DMR4:120542001	4	120542001	120548000	6000	1	1.80E-09	0.49	77	1.28	Kbtbd12	Cytoskeleton
DMR4:120742001	4	120742001	120744000	2000	1	5.80E-09	-0.54	36	1.8	Mgll;LOC102554878	Metabolism
DMR4:120747001	4	120747001	120750000	3000	1	1.70E-08	-0.44	52	1.73	Mgll;LOC102554878	Metabolism
DMR4:120818001	4	120818001	120823000	5000	1	6.60E-07	0.31	67	1.34	Podxl2;Mcm2	Transcription
DMR4:121031001	4	121031001	121036000	5000	1	9.80E-13	0.37	70	1.4	Prr20e	
DMR4:121571001	4	121571001	121577000	6000	1	1.30E-07	-0.39	41	0.68	Chchd6	
DMR4:121781001	4	121781001	121787000	6000	1	1.10E-07	-0.27	60	1	Vom1r93	Receptor
DMR4:121831001	4	121831001	121832000	1000	1	1.10E-07	-0.37	7	0.7	Vom1r95	Receptor
DMR4:121836001	4	121836001	121840000	4000	1	7.30E-07	-0.23	26	0.65	Vom1r-ps77	
DMR4:121854001	4	121854001	121858000	4000	1	1.80E-07	-0.24	31	0.78	Vom1r-ps77	
DMR4:121900001	4	121900001	121901000	1000	1	1.50E-10	0.61	2	0.2	Vom1r97;Vom1r-ps78	Receptor
DMR4:122636001	4	122636001	122637000	1000	1	1.50E-07	-0.36	29	2.9	LOC108350819;Nup210	Transport
DMR4:122697001	4	122697001	122699000	2000	1	1.20E-09	0.53	14	0.7	Nup210	Transport
DMR4:122872001	4	122872001	122875000	3000	2	5.60E-17	0.38	27	0.9	Fbln2	Extracellular Matrix
DMR4:123137001	4	123137001	123139000	2000	1	1.80E-08	0.45	40	2	Tmem43;Xpc	DNA Repair
DMR4:123501001	4	123501001	123502000	1000	1	1.00E-08	0.43	6	0.6	Slc41a3	Transport
DMR4:123514001	4	123514001	123516000	2000	1	2.00E-08	0.37	16	0.8	Slc41a3;Aldh1l1	Transport;Metabolism
DMR4:123563001	4	123563001	123565000	2000	1	1.60E-08	0.36	26	1.3	Aldh1l1;LOC108350870	Metabolism

DMR4:123627001	4	123627001	123630000	3000	1	6.80E-10	0.46	24	0.8	Grip2;Slc6a6	Transport
DMR4:123753001	4	123753001	123755000	2000	1	4.20E-08	0.54	22	1.1	Ccdc174;LOC108350801	
DMR4:123768001	4	123768001	123772000	4000	1	2.80E-10	-0.34	35	0.88	Ccdc174;LOC108350801	
DMR4:124101001	4	124101001	124102000	1000	1	6.70E-08	0.5	5	0.5	Trh	Hormone
DMR4:124335001	4	124335001	124336000	1000	1	1.60E-07	-0.41	21	2.1	Prickle2	Cytoskeleton
DMR4:124412001	4	124412001	124413000	1000	1	7.40E-13	0.5	8	0.8	Prickle2	Cytoskeleton
DMR4:124458001	4	124458001	124460000	2000	1	9.70E-08	0.55	12	0.6	Prickle2	Cytoskeleton
DMR4:125912001	4	125912001	125913000	1000	1	4.20E-11	-0.52	13	1.3	Magi1	
DMR4:126002001	4	126002001	126008000	6000	1	8.50E-08	0.37	77	1.28	Magi1;LOC102556397	
DMR4:126151001	4	126151001	126153000	2000	1	3.60E-09	-0.45	38	1.9	Magi1	
DMR4:126623001	4	126623001	126625000	2000	1	1.50E-07	-0.45	29	1.45	Slc25a26;Lrig1	Transport;Receptor
DMR4:126666001	4	126666001	126671000	5000	1	1.70E-08	-0.49	65	1.3	Lrig1	Receptor
DMR4:127555001	4	127555001	127558000	3000	1	2.00E-09	-0.27	26	0.87	Suclg2;LOC103692194	Metabolism
DMR4:127559001	4	127559001	127564000	5000	1	7.80E-07	0.3	60	1.2	Suclg2;LOC103692194	Metabolism
DMR4:127667001	4	127667001	127674000	7000	2	1.50E-08	0.32	94	1.34	Suclg2	Metabolism
DMR4:127728001	4	127728001	127729000	1000	1	4.90E-10	-0.78	9	0.9	Suclg2	Metabolism
DMR4:127812001	4	127812001	127814000	2000	1	2.60E-07	-0.4	20	1	Suclg2	Metabolism
DMR4:128864001	4	128864001	128865000	1000	1	1.10E-09	0.5	11	1.1	Fam19a1	
DMR4:128891001	4	128891001	128892000	1000	1	2.60E-14	0.8	30	3	Fam19a1	
DMR4:129294001	4	129294001	129296000	2000	1	2.20E-09	0.35	17	0.85	Fam19a4	
DMR4:129307001	4	129307001	129308000	1000	1	2.10E-09	0.49	8	0.8	Fam19a4	
DMR4:129392001	4	129392001	129393000	1000	1	3.00E-08	-0.5	7	0.7	Fam19a4	
DMR4:129468001	4	129468001	129469000	1000	1	3.70E-11	-0.55	17	1.7	Eogt	Golgi
DMR4:129681001	4	129681001	129684000	3000	1	5.10E-11	-0.56	76	2.53	Frmd4b;LOC108350736	
DMR4:129692001	4	129692001	129694000	2000	1	4.80E-08	-0.4	36	1.8	Frmd4b;LOC108350736	
DMR4:129863001	4	129863001	129864000	1000	1	1.30E-07	-0.33	17	1.7	Frmd4b;LOC108350735	
DMR4:129885001	4	129885001	129887000	2000	1	1.30E-07	0.37	37	1.85	Frmd4b;LOC108350735	
DMR4:129891001	4	129891001	129892000	1000	1	8.40E-07	0.31	20	2	Frmd4b	
DMR4:129894001	4	129894001	129898000	4000	1	5.90E-10	0.42	57	1.43	Frmd4b	
DMR4:129944001	4	129944001	129946000	2000	1	2.60E-08	0.37	19	0.95	Frmd4b	
DMR4:130388001	4	130388001	130389000	1000	1	3.70E-08	0.35	7	0.7	Mitf	
DMR4:130423001	4	130423001	130424000	1000	1	1.80E-07	-0.64	16	1.6	Mitf	
DMR4:131365001	4	131365001	131368000	3000	1	8.00E-07	-0.38	92	3.07	Foxp1	
DMR4:131419001	4	131419001	131420000	1000	1	2.80E-07	-0.48	18	1.8	Foxp1	
DMR4:131493001	4	131493001	131495000	2000	1	5.00E-09	-0.74	25	1.25	Foxp1	
DMR4:131502001	4	131502001	131506000	4000	1	3.90E-08	0.53	44	1.1	Foxp1	
DMR4:131530001	4	131530001	131533000	3000	1	2.40E-08	-0.43	59	1.97	Foxp1	
DMR4:131534001	4	131534001	131538000	4000	1	1.20E-08	-0.48	64	1.6	Foxp1	
DMR4:131709001	4	131709001	131712000	3000	1	5.20E-10	-0.48	55	1.83	Foxp1	
DMR4:131861001	4	131861001	131864000	3000	1	1.40E-07	0.35	50	1.67	Foxp1	
DMR4:131868001	4	131868001	131869000	1000	1	9.30E-07	-0.42	14	1.4	Foxp1	
DMR4:132091001	4	132091001	132093000	2000	1	4.40E-09	0.36	27	1.35	Eif4e3	Translation
DMR4:133067001	4	133067001	133068000	1000	1	1.70E-09	-0.51	15	1.5	Shq1	
DMR4:133170001	4	133170001	133172000	2000	1	8.90E-07	-0.49	37	1.85	Gxylt2	
DMR4:133190001	4	133190001	133193000	3000	1	2.70E-09	0.58	33	1.1	Gxylt2	
DMR4:134800001	4	134800001	134801000	1000	1	4.10E-07	0.43	5	0.5	Cntn3	
DMR4:135005001	4	135005001	135011000	6000	1	6.50E-07	-0.37	48	0.8	Cntn3	
DMR4:135036001	4	135036001	135038000	2000	1	1.60E-07	-0.41	20	1	Cntn3	
DMR4:135129001	4	135129001	135130000	1000	1	6.50E-09	0.49	12	1.2	Cntn3	
DMR4:136864001	4	136864001	136865000	1000	1	2.10E-07	0.35	12	1.2	Cntn6	
DMR4:136883001	4	136883001	136888000	5000	1	6.00E-09	-0.23	54	1.08	Cntn6	
DMR4:137769001	4	137769001	137776000	7000	2	1.50E-12	-0.42	76	1.09	Cntn4	Cytoskeleton
DMR4:138043001	4	138043001	138046000	3000	1	8.70E-08	-0.31	27	0.9	Cntn4	Cytoskeleton
DMR4:138525001	4	138525001	138527000	2000	1	8.70E-07	0.39	13	0.65	Cntn4	Cytoskeleton
DMR4:138594001	4	138594001	138595000	1000	1	3.20E-08	0.37	8	0.8	Cntn4	Cytoskeleton
DMR4:138645001	4	138645001	138648000	3000	1	6.70E-10	-0.42	28	0.93	Cntn4	Cytoskeleton
DMR4:138880001	4	138880001	138882000	2000	1	8.30E-10	-0.5	26	1.3	Crbn	Proteolysis
DMR4:139666001	4	139666001	139667000	1000	1	9.60E-08	-0.6	10	1	Lrrn1	Receptor
DMR4:140148001	4	140148001	140149000	1000	1	4.10E-08	-0.42	7	0.7	Sumf1	
DMR4:140212001	4	140212001	140217000	5000	1	2.10E-14	-0.42	31	0.62	Sumf1	
DMR4:140242001	4	140242001	140244000	2000	1	8.20E-07	-0.65	20	1	Itpr1	Ion Channel
DMR4:140330001	4	140330001	140331000	1000	1	1.50E-15	-0.67	17	1.7	Itpr1	Ion Channel
DMR4:140343001	4	140343001	140344000	1000	1	3.20E-07	-0.41	13	1.3	Itpr1	Ion Channel

DMR4:140353001	4	140353001	140354000	1000	1	5.60E-07	0.35	10	1	Itpr1	Ion Channel
DMR4:140542001	4	140542001	140544000	2000	1	7.10E-08	-0.45	28	1.4	Itpr1	Ion Channel
DMR4:140906001	4	140906001	140908000	2000	1	1.30E-07	0.55	24	1.2	Edem1	
DMR4:142717001	4	142717001	142721000	4000	1	3.30E-08	-0.29	38	0.95	Grm7	Signaling
DMR4:142815001	4	142815001	142816000	1000	1	7.70E-11	0.8	16	1.6	Grm7	Signaling
DMR4:143102001	4	143102001	143106000	4000	1	1.90E-08	-0.35	34	0.85	Grm7	Signaling
DMR4:144375001	4	144375001	144377000	2000	1	7.90E-07	-0.41	20	1	Cav3	Cytoskeleton
DMR4:144567001	4	144567001	144573000	6000	1	7.20E-08	-0.44	64	1.07	Rad18	Proteolysis
DMR4:144592001	4	144592001	144594000	2000	1	4.10E-07	-0.5	19	0.95	Rad18	Proteolysis
DMR4:144693001	4	144693001	144694000	1000	1	6.40E-10	0.51	12	1.2	LOC102548080;Srgap3	Signaling
DMR4:144713001	4	144713001	144714000	1000	1	5.60E-12	-0.51	9	0.9	Srgap3	Signaling
DMR4:144977001	4	144977001	144979000	2000	1	7.70E-10	-0.48	30	1.5	LOC102548337;Thumpd3	Epigenetic
DMR4:145243001	4	145243001	145245000	2000	1	9.00E-07	0.33	20	1	Mtmr14;Cpne9	Signaling
DMR4:145382001	4	145382001	145384000	2000	1	8.10E-07	0.34	26	1.3	LOC102548450;Cidec	
DMR4:145400001	4	145400001	145402000	2000	1	9.10E-11	0.56	41	2.05	Cidec;LOC100361844;Jagn1	
DMR4:145407001	4	145407001	145408000	1000	1	9.40E-07	-0.42	8	0.8	LOC100361844;Jagn1;Il17re	Receptor
DMR4:145502001	4	145502001	145504000	2000	1	7.60E-08	0.33	4	0.2	Fancd2	
DMR4:145596001	4	145596001	145597000	1000	1	7.90E-07	-0.46	21	2.1	Vhl;Irak2	Proteolysis
DMR4:145610001	4	145610001	145612000	2000	1	2.20E-07	-0.36	28	1.4	Irak2	
DMR4:145823001	4	145823001	145825000	2000	1	2.70E-07	0.38	12	0.6	Atp2b2	Transport
DMR4:145892001	4	145892001	145893000	1000	1	6.80E-07	0.35	8	0.8	Atp2b2	Transport
DMR4:146140001	4	146140001	146141000	1000	1	8.00E-10	0.47	3	0.3	Slc6a11	Transport
DMR4:146302001	4	146302001	146304000	2000	1	8.20E-10	-0.4	30	1.5	Slc6a1;LOC102548867	Transport
DMR4:146420001	4	146420001	146423000	3000	1	7.30E-08	0.45	32	1.07	Hrh1	Signaling
DMR4:146561001	4	146561001	146564000	3000	3	2.00E-09	0.59	71	2.37	LOC108350747;Atg7	Proteolysis
DMR4:146627001	4	146627001	146631000	4000	1	3.90E-09	0.41	47	1.18	Atg7	Proteolysis
DMR4:146651001	4	146651001	146654000	3000	1	3.80E-07	-0.42	35	1.17	Atg7	Proteolysis
DMR4:146678001	4	146678001	146679000	1000	1	7.00E-08	-0.58	15	1.5	Atg7	Proteolysis
DMR4:146856001	4	146856001	146862000	6000	2	1.80E-07	0.48	53	0.88	Vgll4	Transcription
DMR4:146885001	4	146885001	146886000	1000	1	1.70E-07	-0.42	11	1.1	Vgll4	Transcription
DMR4:147111001	4	147111001	147116000	5000	1	2.60E-08	-0.36	51	1.02	Syn2;LOC100365210;LOC100911724	Transport
DMR4:147706001	4	147706001	147707000	1000	1	4.30E-08	0.38	14	1.4	Cand2;Rpl32	Proteolysis;Translation
DMR4:147911001	4	147911001	147913000	2000	1	1.30E-07	0.41	35	1.75	Tmcc1	
DMR4:148047001	4	148047001	148048000	1000	1	2.80E-07	0.31	1	0.1	Tmcc1	
DMR4:148278001	4	148278001	148279000	1000	1	1.20E-07	0.48	10	1	Zfand4;March8	
DMR4:148280001	4	148280001	148285000	5000	1	1.50E-12	-0.38	56	1.12	Zfand4;March8	
DMR4:148512001	4	148512001	148513000	1000	1	1.80E-07	-0.55	4	0.4	Olr824	Receptor
DMR4:148524001	4	148524001	148526000	2000	1	7.80E-09	-0.49	4	0.2	Olr824	Receptor
DMR4:148554001	4	148554001	148559000	5000	1	6.30E-08	-0.32	40	0.8	Olr825;Olr826	Receptor
DMR4:148598001	4	148598001	148605000	7000	1	3.90E-07	-0.29	66	0.94	Olr827	Receptor
DMR4:148665001	4	148665001	148666000	1000	1	8.30E-09	0.76	27	2.7	Olr830	Receptor
DMR4:148710001	4	148710001	148712000	2000	1	4.70E-11	0.74	43	2.15	Olr832;Trnac-gca	Receptor
DMR4:149972001	4	149972001	149974000	2000	2	1.20E-12	-0.5	10	0.5	Hnrnpf	Translation
DMR4:150071001	4	150071001	150074000	3000	1	3.50E-14	0.47	54	1.8	Rasgef1a	Transcription
DMR4:150120001	4	150120001	150122000	2000	1	7.10E-09	0.39	34	1.7	Rasgef1a	Transcription
DMR4:150167001	4	150167001	150169000	2000	1	3.40E-07	-0.34	29	1.45	Csgalnact2	Golgi
DMR4:150188001	4	150188001	150191000	3000	1	1.40E-08	-0.54	34	1.13	Csgalnact2;LOC685129	Golgi
DMR4:150442001	4	150442001	150446000	4000	1	1.20E-07	0.41	57	1.43	Bms1;LOC108350752	
DMR4:150453001	4	150453001	150455000	2000	1	3.50E-14	-0.55	23	1.15	Bms1;LOC108350752	
DMR4:150493001	4	150493001	150495000	2000	2	6.50E-09	-0.53	22	1.1	Zfp9	
DMR4:150500001	4	150500001	150501000	1000	1	4.50E-07	-0.44	12	1.2	Zfp9	
DMR4:150522001	4	150522001	150523000	1000	1	1.10E-09	0.48	13	1.3	Zfp9	
DMR4:150587001	4	150587001	150589000	2000	1	4.50E-11	0.38	21	1.05	Ankrd26	
DMR4:150890001	4	150890001	150891000	1000	1	2.60E-08	0.46	6	0.6	Cacna1c	Transport
DMR4:151178001	4	151178001	151179000	1000	1	6.20E-07	0.45	25	2.5	Cacna1c;LOC108350846	Transport
DMR4:151220001	4	151220001	151221000	1000	1	5.50E-08	-0.54	8	0.8	Cacna1c	Transport
DMR4:151306001	4	151306001	151307000	1000	1	6.00E-09	0.36	11	1.1	Cacna2d4	Transport
DMR4:151436001	4	151436001	151437000	1000	1	2.40E-08	0.3	1	0.1	Adipor2	Signaling
DMR4:151524001	4	151524001	151525000	1000	1	2.30E-07	0.39	8	0.8	Wnt5b	Signaling
DMR4:152094001	4	152094001	152095000	1000	1	7.50E-07	0.46	7	0.7	Erc1	Transport
DMR4:152103001	4	152103001	152104000	1000	1	9.40E-09	-0.58	11	1.1	Erc1	Transport
DMR4:152116001	4	152116001	152118000	2000	1	1.50E-07	-0.47	35	1.75	Erc1	Transport

DMR4:152162001	4	152162001	152165000	3000	1	1.40E-09	-0.56	30	1	Erc1	Transport
DMR4:152184001	4	152184001	152187000	3000	1	1.90E-08	-0.36	24	0.8	Erc1	Transport
DMR4:152249001	4	152249001	152250000	1000	1	1.10E-09	0.43	9	0.9	Erc1	Transport
DMR4:152338001	4	152338001	152340000	2000	2	2.00E-10	-0.41	16	0.8	Erc1;LOC102552689	Transport
DMR4:152478001	4	152478001	152481000	3000	1	1.80E-07	-0.43	32	1.07	Wnk1	Signaling
DMR4:152651001	4	152651001	152654000	3000	1	4.60E-09	0.33	32	1.07	Ninj2	Cytoskeleton
DMR4:152774001	4	152774001	152776000	2000	1	2.80E-08	0.43	15	0.75	B4galnt3	Golgi
DMR4:152938001	4	152938001	152939000	1000	1	1.30E-11	0.57	10	1	Kdm5a	Epigenetic
DMR4:153003001	4	153003001	153006000	3000	1	7.10E-07	0.32	32	1.07	Il17ra	Receptor
DMR4:153031001	4	153031001	153032000	1000	1	3.40E-18	0.45	9	0.9	Cecr6	
DMR4:153486001	4	153486001	153487000	1000	1	3.90E-08	0.49	26	2.6	Mical3	
DMR4:153593001	4	153593001	153597000	4000	1	9.90E-08	-0.65	75	1.88	Mical3	
DMR4:153607001	4	153607001	153612000	5000	1	2.00E-07	-0.31	91	1.82	Mical3	
DMR4:153739001	4	153739001	153741000	2000	1	1.10E-07	0.63	44	2.2	Pex26	Transcription
DMR4:153753001	4	153753001	153754000	1000	1	1.70E-07	-0.38	14	1.4	Pex26	Transcription
DMR4:153793001	4	153793001	153797000	4000	2	2.30E-10	0.41	45	1.12	Tuba8	Cytoskeleton
DMR4:153813001	4	153813001	153815000	2000	1	2.80E-07	-0.37	34	1.7	Usp18	Protease
DMR4:153882001	4	153882001	153887000	5000	1	5.50E-10	-0.45	62	1.24	LOC102553128;Slc6a13	Transport
DMR4:153895001	4	153895001	153896000	1000	1	7.50E-07	0.33	9	0.9	Slc6a13	Transport
DMR4:154113001	4	154113001	154114000	1000	1	1.30E-08	0.5	17	1.7	RGD1564159	
DMR4:154230001	4	154230001	154237000	7000	1	4.10E-07	-0.35	71	1.01	Mug2	Protease; Proteolysis
DMR4:154697001	4	154697001	154701000	4000	1	6.60E-08	-0.47	35	0.88	Mug1;LOC103692217	Protease; Proteolysis
DMR4:154874001	4	154874001	154879000	5000	1	1.30E-09	-0.31	58	1.16	Cpamd8	
DMR4:154890001	4	154890001	154892000	2000	1	1.00E-08	0.38	35	1.75	Cpamd8	
DMR4:154943001	4	154943001	154950000	7000	2	1.20E-07	-0.33	78	1.11	Cpamd8;LOC102554538	
DMR4:154955001	4	154955001	154960000	5000	1	5.80E-08	-0.32	36	0.72	Cpamd8	
DMR4:155052001	4	155052001	155058000	6000	3	1.30E-10	0.41	69	1.15	Klrg1	Receptor
DMR4:155185001	4	155185001	155187000	2000	1	1.90E-07	0.46	25	1.25	LOC103692219;Trnaa-agc	
DMR4:155328001	4	155328001	155330000	2000	1	5.00E-07	0.56	45	2.25	Mfap5	
DMR4:155354001	4	155354001	155356000	2000	1	7.20E-07	0.35	11	0.55	Aicda	Translation
DMR4:155361001	4	155361001	155363000	2000	1	2.90E-08	0.68	43	2.15	Aicda	Translation
DMR4:155368001	4	155368001	155369000	1000	1	2.10E-10	0.59	17	1.7	Aicda	Translation
DMR4:155413001	4	155413001	155415000	2000	1	3.30E-10	-0.53	29	1.45	Apobec1;Gdf3	Translation;Growth Factors
DMR4:155444001	4	155444001	155446000	2000	1	3.60E-08	0.51	49	2.45	Dppa3	
DMR4:155586001	4	155586001	155587000	1000	1	1.40E-08	0.53	30	3	Slc2a3	
DMR4:155612001	4	155612001	155616000	4000	1	1.20E-07	0.38	44	1.1	Slc2a3	
DMR4:155780001	4	155780001	155785000	5000	1	1.50E-07	-0.47	49	0.98	Clec4a2	Transport
DMR4:155799001	4	155799001	155801000	2000	2	2.40E-08	-0.37	18	0.9	Clec4a2	Transport
DMR4:155815001	4	155815001	155817000	2000	1	4.80E-07	-0.43	13	0.65	Clec4a2	Transport
DMR4:155932001	4	155932001	155934000	2000	1	3.60E-09	-0.51	18	0.9	Clec4a3	Transport
DMR4:155966001	4	155966001	155971000	5000	1	1.70E-07	-0.51	34	0.68	Clec4a1;LOC686015	Transport
DMR4:156133001	4	156133001	156139000	6000	2	3.20E-09	-0.38	67	1.12	Clec4b2	Transport
DMR4:156140001	4	156140001	156146000	6000	1	7.60E-08	-0.44	42	0.7	Clec4b2	Transport
DMR4:156322001	4	156322001	156325000	3000	1	1.10E-08	0.47	28	0.93	LOC108350823;Vom2r48	Signaling
DMR4:156341001	4	156341001	156345000	4000	1	4.10E-07	-0.45	23	0.58	Vom2r48	Signaling
DMR4:156478001	4	156478001	156479000	1000	1	1.80E-10	-0.57	2	0.2	Vom2r50	Signaling
DMR4:156535001	4	156535001	156544000	9000	2	3.00E-08	-0.41	91	1.01	Vom2r51	Signaling
DMR4:156601001	4	156601001	156603000	2000	1	1.60E-08	0.35	32	1.6	Vom2r52	Signaling
DMR4:156901001	4	156901001	156903000	2000	1	7.10E-11	-0.49	12	0.6	RGD1307916	
DMR4:156916001	4	156916001	156919000	3000	2	1.10E-08	-0.44	10	0.33	RGD1307916	
DMR4:157018001	4	157018001	157021000	3000	1	2.50E-10	-0.62	13	0.43	Pex5;LOC103692221	Transport
DMR4:157104001	4	157104001	157109000	5000	2	3.60E-09	0.68	68	1.36	LOC102553636;C1rl	Protease
DMR4:157140001	4	157140001	157142000	2000	1	6.00E-07	0.44	19	0.95	C1r;LOC108350760;C1s	Protease
DMR4:157244001	4	157244001	157247000	3000	1	7.60E-11	0.66	47	1.57	Phb2;Mir141;Mir3575;Mir200c;Ptpn6	Signaling
DMR4:157370001	4	157370001	157376000	6000	1	7.20E-07	0.39	197	3.28	P3h3;Gpr162;Cd4	Extracellular Matrix;Signaling
DMR4:157428001	4	157428001	157432000	4000	2	1.40E-11	0.51	76	1.9	Lag3;Ptms;LOC102553981	Receptor
DMR4:157447001	4	157447001	157449000	2000	1	5.00E-07	-0.35	46	2.3	Ptms;LOC102553981;Mlf2	Signaling
DMR4:157602001	4	157602001	157603000	1000	1	8.40E-07	0.37	11	1.1	Lpar5;Chd4	Signaling
DMR4:157617001	4	157617001	157619000	2000	1	7.80E-13	-0.49	55	2.75	Chd4	
DMR4:157731001	4	157731001	157732000	1000	1	3.90E-09	-0.5	11	1.1	Vamp1;Tapbpl	Immune

DMR4:157743001	4	157743001	157745000	2000	2	3.40E-11	0.47	22	1.1	Vamp1;Tapbpl;Cd27;LOC102555485	Immune
DMR4:157876001	4	157876001	157877000	1000	1	1.10E-13	0.7	32	3.2	Tnfrsf1a;Plekhg6	Receptor
DMR4:158212001	4	158212001	158213000	1000	1	1.00E-08	0.47	12	1.2	Vwf;Ano2	
DMR4:158218001	4	158218001	158221000	3000	1	1.30E-07	-0.39	34	1.13	Vwf;Ano2	
DMR4:158528001	4	158528001	158532000	4000	1	3.40E-07	0.32	43	1.07	Ano2	
DMR4:158548001	4	158548001	158549000	1000	1	1.60E-07	-0.48	6	0.6	Ano2	
DMR4:158551001	4	158551001	158552000	1000	1	3.10E-07	0.67	12	1.2	Ano2	
DMR4:158704001	4	158704001	158705000	1000	1	4.60E-07	0.56	35	3.5	Ntf3	Growth Factors
DMR4:158713001	4	158713001	158715000	2000	2	3.00E-08	-0.49	27	1.35	Ntf3	Growth Factors
DMR4:159068001	4	159068001	159069000	1000	1	4.20E-07	0.5	9	0.9	Kcna5	Transport
DMR4:159263001	4	159263001	159266000	3000	1	2.40E-08	0.39	53	1.77	Kcna6	Transport
DMR4:159278001	4	159278001	159280000	2000	1	1.30E-07	0.38	17	0.85	Kcna6	Transport
DMR4:159379001	4	159379001	159382000	3000	1	5.30E-08	-0.41	46	1.53	Ndufa9	Metabolism
DMR4:159437001	4	159437001	159439000	2000	1	8.00E-07	0.37	27	1.35	Dyrk4	
DMR4:159538001	4	159538001	159541000	3000	1	4.30E-08	0.35	47	1.57	LOC689087;RGD1559795	
DMR4:159546001	4	159546001	159548000	2000	1	3.20E-07	-0.57	16	0.8	LOC689087;RGD1559795	
DMR4:159564001	4	159564001	159567000	3000	1	2.60E-11	0.47	53	1.77	Fgf6	Growth Factors
DMR4:159620001	4	159620001	159621000	1000	1	2.50E-09	0.36	11	1.1	Fgf23	Growth Factors
DMR4:159650001	4	159650001	159652000	2000	1	4.70E-07	-0.4	19	0.95	Tigar	Signaling
DMR4:161561001	4	161561001	161563000	2000	1	3.90E-07	0.39	38	1.9	Tead4	Transcription
DMR4:161621001	4	161621001	161624000	3000	1	1.40E-14	0.64	34	1.13	Tead4;LOC100362138	Transcription
DMR4:161913001	4	161913001	161915000	2000	1	2.10E-08	-0.43	48	2.4	A2ml1	Protease; Proteolysis
DMR4:162018001	4	162018001	162021000	3000	1	1.90E-08	0.37	41	1.37	Klrb1b	Receptor
DMR4:162302001	4	162302001	162304000	2000	1	1.70E-07	-0.5	25	1.25	Clec2d;LOC102547086	
DMR4:162305001	4	162305001	162312000	7000	1	2.40E-10	-0.45	61	0.87	Clec2d;LOC102547086	
DMR4:162851001	4	162851001	162852000	1000	1	1.10E-08	-0.51	9	0.9	Clec2h;Clec2e	
DMR4:163076001	4	163076001	163083000	7000	1	9.50E-09	-0.41	48	0.69	RGD1564770	
DMR4:163118001	4	163118001	163119000	1000	1	9.30E-08	0.56	25	2.5	Clec12a;Chtop	Metabolism
DMR4:163159001	4	163159001	163161000	2000	1	1.70E-08	0.54	14	0.7	Clec12b;Clec1b	
DMR4:163185001	4	163185001	163186000	1000	1	4.20E-08	0.46	3	0.3	Clec9a;Clec1a	
DMR4:163372001	4	163372001	163381000	9000	2	9.10E-09	-0.39	63	0.7	Klrd1	
DMR4:163509001	4	163509001	163514000	5000	1	1.60E-07	-0.35	37	0.74	Klri1	
DMR4:164381001	4	164381001	164383000	2000	1	1.80E-08	-0.36	16	0.8	Klra22	
DMR4:164472001	4	164472001	164474000	2000	1	3.50E-07	-0.41	13	0.65	Ly49s4	
DMR4:165429001	4	165429001	165433000	4000	1	3.90E-07	-0.55	31	0.78	Klra1	
DMR4:165462001	4	165462001	165466000	4000	1	3.00E-07	-0.25	41	1.02	Klra1	
DMR4:165525001	4	165525001	165526000	1000	1	2.90E-07	-0.48	11	1.1	Magohb;Styk1	
DMR4:165776001	4	165776001	165783000	7000	1	2.70E-07	-0.25	62	0.89	Tas2r107;Tas2r106	Receptor
DMR4:166050001	4	166050001	166051000	1000	1	9.00E-07	0.37	8	0.8	Prr21	
DMR4:166052001	4	166052001	166057000	5000	1	2.30E-07	-0.22	55	1.1	Prr21	
DMR4:166913001	4	166913001	166914000	1000	1	5.30E-09	0.44	13	1.3	Tas2r124;Tas2r102	Receptor
DMR4:167073001	4	167073001	167078000	5000	2	1.80E-09	0.3	64	1.28	Tas2r125	Receptor
DMR4:167430001	4	167430001	167432000	2000	1	2.20E-07	-0.34	12	0.6	Grpcb	Signaling
DMR4:167435001	4	167435001	167439000	4000	1	2.30E-08	-0.32	56	1.4	Grpcb	Signaling
DMR4:167800001	4	167800001	167801000	1000	1	1.10E-07	-0.43	10	1	Etv6	Transcription
DMR4:167821001	4	167821001	167823000	2000	1	1.50E-08	-0.46	34	1.7	Etv6	Transcription
DMR4:167824001	4	167824001	167828000	4000	1	5.10E-07	-0.37	96	2.4	Etv6	Transcription
DMR4:167941001	4	167941001	167943000	2000	1	1.80E-10	-0.53	34	1.7	Etv6	Transcription
DMR4:167994001	4	167994001	167997000	3000	1	1.80E-07	-0.32	69	2.3	Etv6	Transcription
DMR4:168319001	4	168319001	168321000	2000	1	2.90E-08	-0.5	27	1.35	Lrp6;LOC102551409	Binding Proteins
DMR4:168405001	4	168405001	168408000	3000	1	1.70E-07	-0.5	37	1.23	Borcs5	
DMR4:168417001	4	168417001	168419000	2000	1	2.20E-08	-0.63	23	1.15	Borcs5	
DMR4:168670001	4	168670001	168671000	1000	1	5.90E-08	-0.72	5	0.5	Gpr19	Signaling
DMR4:168837001	4	168837001	168840000	3000	1	9.60E-08	0.25	47	1.57	Gprc5a	Signaling
DMR4:168841001	4	168841001	168846000	5000	1	1.90E-08	0.42	71	1.42	Gprc5a	Signaling
DMR4:168897001	4	168897001	168898000	1000	1	6.10E-07	0.42	23	2.3	LOC108350826;Hebp1	
DMR4:168906001	4	168906001	168909000	3000	1	2.90E-07	0.29	58	1.93	Hebp1	
DMR4:168914001	4	168914001	168916000	2000	1	1.80E-07	0.44	53	2.65	Hebp1	
DMR4:168974001	4	168974001	168975000	1000	1	9.70E-08	-0.45	19	1.9	Fam234b	
DMR4:168978001	4	168978001	168979000	1000	1	2.50E-07	-0.53	13	1.3	Fam234b	
DMR4:168992001	4	168992001	168994000	2000	1	2.80E-07	0.28	24	1.2	Fam234b	
DMR4:169036001	4	169036001	169037000	1000	1	2.80E-07	0.57	24	2.4	Fam234b;Gsg1	Cytoskeleton

DMR4:169552001	4	169552001	169555000	3000	2	7.50E-11	-0.53	49	1.63	Grin2b	Receptor
DMR4:169561001	4	169561001	169563000	2000	1	4.40E-12	0.89	72	3.6	Grin2b	Receptor
DMR4:169794001	4	169794001	169796000	2000	1	5.00E-08	0.73	42	2.1	Grin2b	Receptor
DMR4:169808001	4	169808001	169812000	4000	1	3.60E-11	0.58	59	1.48	Grin2b	Receptor
DMR4:169879001	4	169879001	169880000	1000	1	5.40E-07	-0.33	20	2	Grin2b	Receptor
DMR4:170502001	4	170502001	170504000	2000	1	4.80E-08	-0.44	13	0.65	Atf7ip	Transcription
DMR4:170521001	4	170521001	170523000	2000	1	1.10E-07	0.42	9	0.45	Atf7ip	Transcription
DMR4:170575001	4	170575001	170580000	5000	1	3.80E-08	-0.4	77	1.54	Plbd1	Metabolism
DMR4:170583001	4	170583001	170584000	1000	1	1.90E-09	0.49	10	1	Plbd1	Metabolism
DMR4:170596001	4	170596001	170597000	1000	1	2.90E-07	0.41	7	0.7	Plbd1	Metabolism
DMR4:170623001	4	170623001	170624000	1000	1	1.40E-07	0.43	2	0.2	Plbd1	Metabolism
DMR4:170648001	4	170648001	170656000	8000	1	1.60E-07	-0.31	79	0.99	Gucy2c	Signaling
DMR4:170690001	4	170690001	170691000	1000	1	8.20E-11	0.51	10	1	Gucy2c	Signaling
DMR4:170698001	4	170698001	170700000	2000	1	5.90E-07	-0.45	22	1.1	Gucy2c	Signaling
DMR4:170855001	4	170855001	170858000	3000	1	3.10E-09	0.46	63	2.1	Mgp	
DMR4:170888001	4	170888001	170889000	1000	1	7.30E-07	0.41	21	2.1	Erp27	Transcription
DMR4:171070001	4	171070001	171072000	2000	1	2.10E-07	0.79	63	3.15	LOC102551070;Rerg	Signaling
DMR4:171076001	4	171076001	171084000	8000	1	3.40E-12	0.6	179	2.24	Rerg	Signaling
DMR4:171162001	4	171162001	171164000	2000	1	6.20E-10	0.8	29	1.45	Rerg	Signaling
DMR4:171249001	4	171249001	171250000	1000	1	1.50E-14	0.45	32	3.2	Ptpro	Receptor
DMR4:171273001	4	171273001	171275000	2000	1	3.00E-12	0.39	16	0.8	Ptpro	Receptor
DMR4:171359001	4	171359001	171360000	1000	1	2.20E-07	0.34	9	0.9	Ptpro	Receptor
DMR4:171366001	4	171366001	171369000	3000	1	2.00E-08	-0.42	35	1.17	Ptpro	Receptor
DMR4:171432001	4	171432001	171434000	2000	1	4.00E-08	0.35	17	0.85	Ptpro	Receptor
DMR4:171455001	4	171455001	171461000	6000	1	2.80E-08	-0.41	123	2.05	Ptpro	Receptor
DMR4:171477001	4	171477001	171483000	6000	1	3.30E-14	-0.49	120	2	Eps8	Cytoskeleton
DMR4:171519001	4	171519001	171527000	8000	1	5.70E-07	0.31	130	1.62	Eps8	Cytoskeleton
DMR4:171537001	4	171537001	171540000	3000	1	3.60E-07	-0.39	52	1.73	Eps8	Cytoskeleton
DMR4:171541001	4	171541001	171543000	2000	1	2.70E-08	-0.43	37	1.85	Eps8	Cytoskeleton
DMR4:171551001	4	171551001	171553000	2000	2	8.00E-08	-0.48	40	2	Eps8	Cytoskeleton
DMR4:171568001	4	171568001	171572000	4000	1	1.00E-08	0.38	52	1.3	Eps8	Cytoskeleton
DMR4:171582001	4	171582001	171585000	3000	1	8.50E-07	-0.43	58	1.93	Eps8	Cytoskeleton
DMR4:171588001	4	171588001	171590000	2000	1	4.10E-08	-0.4	32	1.6	Eps8	Cytoskeleton
DMR4:171632001	4	171632001	171634000	2000	1	2.60E-07	-0.46	33	1.65	Eps8	Cytoskeleton
DMR4:171640001	4	171640001	171642000	2000	1	2.00E-12	-0.51	39	1.95	Eps8	Cytoskeleton
DMR4:171649001	4	171649001	171651000	2000	1	4.70E-08	-0.6	25	1.25	Eps8	Cytoskeleton
DMR4:171750001	4	171750001	171751000	1000	1	6.30E-07	0.32	13	1.3	Strap;Dera;LOC103692250;LOC103692249	Translation;Metabolism
DMR4:171985001	4	171985001	171988000	3000	1	4.20E-07	-0.44	39	1.3	Slc15a5	Transport
DMR4:172012001	4	172012001	172017000	5000	1	9.20E-15	0.9	68	1.36	Slc15a5	Transport
DMR4:172027001	4	172027001	172029000	2000	1	3.40E-07	0.33	28	1.4	Slc15a5	Transport
DMR4:172072001	4	172072001	172073000	1000	1	3.70E-15	0.88	26	2.6	Slc15a5	Transport
DMR4:172135001	4	172135001	172137000	2000	1	7.90E-07	-0.46	20	1	Mgst1	Transport
DMR4:172143001	4	172143001	172146000	3000	1	1.40E-07	0.39	11	0.37	Mgst1	Transport
DMR4:172967001	4	172967001	172969000	2000	1	3.00E-09	0.54	44	2.2	Lmo3	
DMR4:172978001	4	172978001	172979000	1000	1	1.20E-15	0.86	27	2.7	Lmo3	
DMR4:173764001	4	173764001	173768000	4000	1	1.50E-07	-0.42	84	2.1	Pik3c2g	Signaling
DMR4:173799001	4	173799001	173804000	5000	1	3.00E-07	0.53	89	1.78	Pik3c2g	Signaling
DMR4:173860001	4	173860001	173862000	2000	1	6.20E-13	0.79	26	1.3	Pik3c2g	Signaling
DMR4:173870001	4	173870001	173872000	2000	1	1.30E-08	-0.47	27	1.35	Pik3c2g	Signaling
DMR4:173903001	4	173903001	173910000	7000	3	4.90E-09	-0.35	86	1.23	Pik3c2g	Signaling
DMR4:174012001	4	174012001	174013000	1000	1	4.30E-07	-0.42	19	1.9	Pik3c2g	Signaling
DMR4:174069001	4	174069001	174071000	2000	1	4.90E-10	0.47	21	1.05	Pik3c2g	Signaling
DMR4:174080001	4	174080001	174083000	3000	2	2.30E-12	0.36	36	1.2	Pik3c2g	Signaling
DMR4:174121001	4	174121001	174123000	2000	2	1.70E-09	0.43	21	1.05	Plcz1	Metabolism
DMR4:174161001	4	174161001	174162000	1000	1	1.00E-15	0.43	4	0.4	Plcz1	Metabolism
DMR4:174638001	4	174638001	174640000	2000	1	2.10E-10	-0.63	11	0.55	Plekha5	
DMR4:174837001	4	174837001	174839000	2000	1	4.70E-07	-0.37	27	1.35	Aebp2	
DMR4:174866001	4	174866001	174867000	1000	1	2.70E-08	0.43	9	0.9	Aebp2	
DMR4:175451001	4	175451001	175453000	2000	1	7.00E-10	0.63	41	2.05	Pde3a	Signaling
DMR4:175491001	4	175491001	175492000	1000	1	2.10E-07	-0.46	16	1.6	Pde3a	Signaling
DMR4:175542001	4	175542001	175544000	2000	1	4.60E-07	0.44	28	1.4	Pde3a	Signaling
DMR4:175581001	4	175581001	175583000	2000	1	2.90E-12	0.41	24	1.2	Pde3a	Signaling

DMR4:175586001	4	175586001	175587000	1000	1	2.50E-09	0.37	9	0.9	Pde3a	Signaling
DMR4:175615001	4	175615001	175616000	1000	1	3.20E-07	0.53	3	0.3	Pde3a	Signaling
DMR4:175629001	4	175629001	175632000	3000	2	8.50E-14	0.41	21	0.7	Pde3a	Signaling
DMR4:175652001	4	175652001	175653000	1000	1	3.90E-08	0.79	26	2.6	Pde3a	Signaling
DMR4:175655001	4	175655001	175656000	1000	1	4.60E-10	0.54	5	0.5	Pde3a	Signaling
DMR4:175738001	4	175738001	175739000	1000	1	1.20E-07	-0.47	12	1.2	Slco1c1	Transport
DMR4:175773001	4	175773001	175777000	4000	1	3.20E-10	0.39	93	2.33	Slco1c1;LOC103692253	Transport
DMR4:175822001	4	175822001	175829000	7000	4	1.30E-07	-0.35	73	1.04	LOC103692254;Slco1b2	Transport
DMR4:175975001	4	175975001	175978000	3000	2	9.30E-10	-0.28	22	0.73	Slco1a2	
DMR4:176150001	4	176150001	176152000	2000	2	7.60E-10	-0.54	16	0.8	Slco1a1	Transport
DMR4:176266001	4	176266001	176268000	2000	1	2.00E-09	0.67	23	1.15	Slc21a4	
DMR4:176290001	4	176290001	176291000	1000	1	1.00E-08	0.59	5	0.5	Slc21a4	
DMR4:176516001	4	176516001	176518000	2000	1	8.20E-08	-0.49	43	2.15	Slco1a5;Iapp	Transport;Hormone
DMR4:176666001	4	176666001	176668000	2000	1	1.80E-09	0.43	22	1.1	Gys2	
DMR4:176710001	4	176710001	176712000	2000	1	3.40E-07	0.41	63	3.15	Ldhb	Metabolism
DMR4:176808001	4	176808001	176810000	2000	1	1.30E-10	0.44	15	0.75	Abcc9	Transport
DMR4:176890001	4	176890001	176891000	1000	1	2.50E-08	0.5	25	2.5	Abcc9	Transport
DMR4:177023001	4	177023001	177024000	1000	1	5.00E-07	0.65	22	2.2	RGD1561551	Transport
DMR4:177081001	4	177081001	177086000	5000	1	2.90E-07	0.34	78	1.56	St8sia1	Transport
DMR4:177161001	4	177161001	177165000	4000	1	2.30E-10	0.8	100	2.5	St8sia1	Transport
DMR4:177173001	4	177173001	177176000	3000	1	4.00E-07	0.32	47	1.57	St8sia1	Transport
DMR4:177238001	4	177238001	177240000	2000	2	1.10E-08	0.49	26	1.3	C2cd5	
DMR4:177285001	4	177285001	177286000	1000	1	8.10E-07	-0.6	11	1.1	C2cd5	
DMR4:177302001	4	177302001	177303000	1000	1	1.90E-07	-0.35	8	0.8	C2cd5	
DMR4:177337001	4	177337001	177339000	2000	1	4.70E-07	0.51	46	2.3	C2cd5	
DMR4:178068001	4	178068001	178070000	2000	1	6.60E-08	0.62	50	2.5	Sox5	
DMR4:178090001	4	178090001	178092000	2000	1	3.00E-07	0.34	27	1.35	Sox5	
DMR4:178127001	4	178127001	178130000	3000	1	3.60E-10	-0.52	58	1.93	Sox5	
DMR4:178166001	4	178166001	178168000	2000	1	1.10E-12	-0.61	44	2.2	Sox5	
DMR4:178192001	4	178192001	178195000	3000	1	6.80E-11	0.84	66	2.2	Sox5	
DMR4:178206001	4	178206001	178210000	4000	3	2.60E-09	0.64	91	2.28	Sox5	
DMR4:178238001	4	178238001	178244000	6000	1	1.70E-07	0.33	91	1.52	Sox5	
DMR4:178290001	4	178290001	178292000	2000	1	1.50E-07	0.41	31	1.55	Sox5	
DMR4:178335001	4	178335001	178337000	2000	1	9.50E-07	-0.36	32	1.6	Sox5;LOC108350847	
DMR4:178342001	4	178342001	178344000	2000	1	1.10E-07	0.39	7	0.35	Sox5;LOC108350847	
DMR4:178358001	4	178358001	178359000	1000	1	4.10E-10	0.42	9	0.9	Sox5	
DMR4:178373001	4	178373001	178374000	1000	1	1.10E-08	0.36	10	1	Sox5	
DMR4:178446001	4	178446001	178447000	1000	1	6.70E-07	0.45	8	0.8	Sox5	
DMR4:178490001	4	178490001	178492000	2000	1	6.20E-08	0.34	10	0.5	Sox5	
DMR4:178497001	4	178497001	178502000	5000	1	9.80E-07	-0.34	109	2.18	Sox5	
DMR4:178561001	4	178561001	178564000	3000	1	1.50E-13	0.76	56	1.87	Sox5	
DMR4:178569001	4	178569001	178573000	4000	1	7.30E-09	0.4	77	1.93	Sox5	
DMR4:178677001	4	178677001	178678000	1000	1	9.10E-07	0.35	8	0.8	Sox5	
DMR4:178682001	4	178682001	178683000	1000	1	3.00E-07	0.3	4	0.4	Sox5	
DMR4:178695001	4	178695001	178697000	2000	1	2.00E-09	-0.49	37	1.85	Sox5	
DMR4:178763001	4	178763001	178765000	2000	1	8.90E-09	0.79	50	2.5	Sox5	
DMR4:178771001	4	178771001	178772000	1000	1	3.50E-11	0.66	26	2.6	Sox5	
DMR4:178793001	4	178793001	178796000	3000	2	9.70E-12	0.84	74	2.47	Sox5	
DMR4:178893001	4	178893001	178894000	1000	1	8.30E-07	0.35	16	1.6	Sox5	
DMR4:178917001	4	178917001	178922000	5000	1	5.40E-11	0.52	80	1.6	Sox5	
DMR4:178924001	4	178924001	178929000	5000	1	1.40E-08	0.36	72	1.44	Sox5	
DMR4:178963001	4	178963001	178965000	2000	1	3.10E-08	0.62	42	2.1	Sox5	
DMR4:178967001	4	178967001	178976000	9000	1	1.30E-16	0.87	157	1.74	Sox5	
DMR4:178980001	4	178980001	178983000	3000	1	7.30E-18	0.75	50	1.67	Sox5	
DMR4:179001001	4	179001001	179002000	1000	1	3.80E-14	0.73	28	2.8	Sox5	
DMR4:179028001	4	179028001	179030000	2000	1	6.40E-08	-0.34	31	1.55	Sox5	
DMR4:179340001	4	179340001	179343000	3000	2	2.70E-08	0.68	39	1.3	Bcat1	Metabolism
DMR4:179433001	4	179433001	179434000	1000	1	4.20E-08	0.51	38	3.8	Lrmp;Casc1	
DMR4:179462001	4	179462001	179467000	5000	1	1.80E-07	0.35	79	1.58	Casc1;Lyrm5	
DMR4:179520001	4	179520001	179523000	3000	1	3.60E-07	0.66	59	1.97	Kras	Signaling
DMR4:179673001	4	179673001	179674000	1000	1	3.30E-10	0.64	12	1.2	Lmntd1	
DMR4:179675001	4	179675001	179676000	1000	1	1.20E-07	0.5	9	0.9	Lmntd1	
DMR4:179677001	4	179677001	179681000	4000	3	9.20E-09	0.64	22	0.55	Lmntd1	

DMR4:179682001	4	179682001	179685000	3000	3	1.90E-18	0.68	57	1.9	Lmntd1	
DMR4:179745001	4	179745001	179748000	3000	1	3.70E-07	-0.48	72	2.4	Lmntd1;LOC108350788	
DMR4:179757001	4	179757001	179760000	3000	1	4.00E-09	-0.55	68	2.27	Lmntd1;LOC108350788;LOC108350787	
DMR4:179796001	4	179796001	179801000	5000	2	9.00E-11	-0.4	42	0.84	Lmntd1;LOC108350787	
DMR4:179863001	4	179863001	179864000	1000	1	1.10E-09	-0.65	16	1.6	Lmntd1	
DMR4:180082001	4	180082001	180089000	7000	2	3.20E-10	-0.64	112	1.6	Rassf8	Cytoskeleton
DMR4:180138001	4	180138001	180142000	4000	3	2.40E-10	0.51	70	1.75	Rassf8;LOC100912509	Cytoskeleton
DMR4:180506001	4	180506001	180508000	2000	1	3.10E-12	0.72	31	1.55	Itpr2	Ion Channel
DMR4:180680001	4	180680001	180681000	1000	1	2.30E-17	0.73	32	3.2	Itpr2	Ion Channel
DMR4:180695001	4	180695001	180699000	4000	1	7.50E-10	0.39	83	2.08	Itpr2	Ion Channel
DMR4:180707001	4	180707001	180710000	3000	1	3.10E-07	0.37	28	0.93	Itpr2	Ion Channel
DMR4:180723001	4	180723001	180724000	1000	1	3.40E-08	0.35	10	1	Itpr2	Ion Channel
DMR4:180846001	4	180846001	180847000	1000	1	5.40E-08	0.59	46	4.6	Asun	
DMR4:180977001	4	180977001	180978000	1000	1	1.60E-07	-0.48	13	1.3	Med21	Transcription
DMR4:181098001	4	181098001	181099000	1000	1	7.00E-10	0.47	7	0.7	Stk38l;Arntl2	Signaling;Transcription
DMR4:181273001	4	181273001	181275000	2000	1	8.70E-08	0.67	36	1.8	Smco2	
DMR4:181393001	4	181393001	181396000	3000	1	1.70E-11	-0.45	48	1.6	Ppfibp1;LOC108350794	
DMR4:181404001	4	181404001	181406000	2000	1	1.70E-07	-0.38	31	1.55	Ppfibp1;LOC108350794	
DMR4:181448001	4	181448001	181451000	3000	1	2.10E-15	-0.69	52	1.73	Mrps35	Translation
DMR4:181464001	4	181464001	181466000	2000	1	3.30E-07	0.32	28	1.4	Mrps35;Mansc4	Translation
DMR4:181683001	4	181683001	181684000	1000	1	1.70E-08	0.75	25	2.5	Pthlh;LOC108350879	Hormone
DMR4:181864001	4	181864001	181866000	2000	1	9.00E-19	0.78	47	2.35	Ccdc91	
DMR4:181881001	4	181881001	181884000	3000	1	9.10E-07	-0.39	40	1.33	Ccdc91	
DMR4:182022001	4	182022001	182024000	2000	1	3.30E-09	-0.48	29	1.45	Ccdc91	
DMR4:182475001	4	182475001	182477000	2000	1	9.70E-11	0.44	13	0.65	Far2	
DMR4:182478001	4	182478001	182480000	2000	1	3.20E-09	-0.5	31	1.55	Far2	
DMR4:182546001	4	182546001	182547000	1000	1	1.70E-07	0.34	6	0.6	Far2	
DMR4:183419001	4	183419001	183420000	1000	1	9.80E-07	-0.38	8	0.8	Fam60a	
DMR4:183647001	4	183647001	183653000	6000	3	2.30E-08	-0.29	60	1	Etfbkmt	Epigenetic
DMR4:184034001	4	184034001	184035000	1000	1	1.30E-08	-0.69	15	1.5	Bicd1	
DMR4:184098001	4	184098001	184103000	5000	1	8.70E-07	-0.36	45	0.9	Bicd1;LOC100362344	Translation
DMR5:563001	5	563001	564000	1000	1	5.70E-07	-0.41	7	0.7	Cct6a-ps3	
DMR5:653001	5	653001	658000	5000	1	1.20E-07	-0.25	54	1.08	Crispld1	Immune
DMR5:1351001	5	1351001	1352000	1000	1	5.00E-07	-0.4	7	0.7	Gdap1	
DMR5:1733001	5	1733001	1734000	1000	1	2.80E-07	-0.6	0	0	LOC100360433;Rps4x-ps8;LOC679619	
DMR5:1738001	5	1738001	1746000	8000	2	7.50E-10	-0.41	90	1.12	Rps4x-ps8;LOC679619	
DMR5:2347001	5	2347001	2349000	2000	1	6.80E-16	-0.58	38	1.9	Stau2	
DMR5:2465001	5	2465001	2467000	2000	1	3.20E-07	0.47	17	0.85	Stau2	
DMR5:2634001	5	2634001	2635000	1000	1	5.20E-07	-0.36	22	2.2	Rdh10;Rpl7;LOC102546601;LOC100912291	Translation
DMR5:2636001	5	2636001	2637000	1000	1	1.40E-07	-0.41	15	1.5	Rdh10;Rpl7;LOC102546601;LOC100912291	Translation
DMR5:2821001	5	2821001	2822000	1000	1	5.20E-07	-0.39	11	1.1	Sbspon	
DMR5:2841001	5	2841001	2842000	1000	1	7.10E-07	-0.42	9	0.9	Sbspon	
DMR5:2878001	5	2878001	2879000	1000	1	2.00E-07	-0.65	13	1.3	Terf1;LOC100361352	
DMR5:3032001	5	3032001	3034000	2000	1	7.80E-07	0.42	18	0.9	Kcnb2	Transport
DMR5:3168001	5	3168001	3175000	7000	2	2.90E-07	-0.27	78	1.11	Kcnb2	Transport
DMR5:3189001	5	3189001	3191000	2000	1	4.90E-09	0.37	14	0.7	Kcnb2	Transport
DMR5:3269001	5	3269001	3270000	1000	1	5.40E-07	-0.27	7	0.7	Kcnb2	Transport
DMR5:3302001	5	3302001	3306000	4000	1	2.40E-07	-0.43	110	2.75	Kcnb2	Transport
DMR5:3324001	5	3324001	3326000	2000	1	1.50E-07	0.39	7	0.35	Kcnb2	Transport
DMR5:4285001	5	4285001	4287000	2000	1	6.30E-10	-0.65	11	0.55	Eya1	
DMR5:4289001	5	4289001	4294000	5000	1	8.40E-08	-0.31	46	0.92	Eya1	
DMR5:4300001	5	4300001	4302000	2000	1	1.40E-07	0.61	11	0.55	Eya1	
DMR5:4359001	5	4359001	4361000	2000	1	1.10E-08	0.51	21	1.05	Eya1	
DMR5:5000001	5	5000001	5002000	2000	1	7.00E-07	-0.55	35	1.75	Lactb2;LOC102547823	Translation
DMR5:5469001	5	5469001	5470000	1000	1	9.40E-07	-0.65	10	1	LOC108350921;Ncoa2;LOC108351077	Epigenetic
DMR5:5548001	5	5548001	5551000	3000	1	2.70E-07	-0.37	35	1.17	Ncoa2	Epigenetic
DMR5:5565001	5	5565001	5572000	7000	1	2.10E-08	-0.29	93	1.33	Ncoa2;Mir378b	Epigenetic
DMR5:5647001	5	5647001	5648000	1000	1	1.90E-08	-0.32	17	1.7	Ncoa2	Epigenetic

DMR5:5894001	5	5894001	5896000	2000	1	3.40E-09	-0.49	25	1.25	Slco5a1	Transport
DMR5:5984001	5	5984001	5988000	4000	1	9.30E-08	-0.39	56	1.4	Slco5a1	Transport
DMR5:6954001	5	6954001	6962000	8000	2	1.30E-08	-0.28	63	0.79	RGD1564053	
DMR5:6968001	5	6968001	6969000	1000	1	1.00E-07	-0.45	2	0.2	RGD1564053	
DMR5:6988001	5	6988001	6996000	8000	1	9.10E-07	-0.36	73	0.91	RGD1564053	
DMR5:7318001	5	7318001	7319000	1000	1	7.20E-08	-0.48	9	0.9	RGD1564053	
DMR5:7832001	5	7832001	7834000	2000	1	8.60E-07	-0.41	15	0.75	Prex2	Transcription
DMR5:8276001	5	8276001	8278000	2000	1	8.50E-09	-0.28	24	1.2	Cpa6	Protease
DMR5:8397001	5	8397001	8400000	3000	1	3.20E-09	-0.52	13	0.43	Cpa6	Protease
DMR5:8417001	5	8417001	8419000	2000	1	3.30E-07	0.37	15	0.75	Cpa6	Protease
DMR5:8461001	5	8461001	8463000	2000	1	1.50E-07	0.36	11	0.55	Cpa6	Protease
DMR5:8476001	5	8476001	8478000	2000	1	5.90E-07	-0.54	16	0.8	Cpa6	Protease
DMR5:8493001	5	8493001	8497000	4000	1	5.70E-07	-0.35	26	0.65	Cpa6	Protease
DMR5:8506001	5	8506001	8507000	1000	1	3.50E-07	0.4	13	1.3	Cpa6	Protease
DMR5:8562001	5	8562001	8565000	3000	1	4.70E-09	0.78	41	1.37	Cpa6	Protease
DMR5:8572001	5	8572001	8574000	2000	1	2.40E-08	0.54	34	1.7	Cpa6	Protease
DMR5:8774001	5	8774001	8775000	1000	1	9.70E-07	-0.44	29	2.9	Cspp1	Cell Cycle
DMR5:8785001	5	8785001	8786000	1000	1	1.00E-08	-0.58	16	1.6	Cspp1	Cell Cycle
DMR5:8796001	5	8796001	8802000	6000	2	2.20E-08	-0.5	47	0.78	Cspp1	Cell Cycle
DMR5:8830001	5	8830001	8834000	4000	1	2.60E-08	-0.53	45	1.12	Cspp1	Cell Cycle
DMR5:10138001	5	10138001	10140000	2000	1	5.60E-07	0.36	26	1.3	Sntg1	
DMR5:10314001	5	10314001	10316000	2000	1	5.20E-07	0.32	14	0.7	Sntg1	
DMR5:10420001	5	10420001	10423000	3000	1	8.20E-09	-0.23	28	0.93	Sntg1	
DMR5:12737001	5	12737001	12745000	8000	3	1.00E-10	-0.47	47	0.59	St18	Transcription
DMR5:12907001	5	12907001	12908000	1000	1	9.00E-07	-0.25	5	0.5	Fam150a	
DMR5:13382001	5	13382001	13383000	1000	1	7.10E-07	-0.45	10	1	Npbwr1	Signaling
DMR5:13763001	5	13763001	13769000	6000	2	6.20E-10	-0.29	58	0.97	Oprk1	Signaling
DMR5:14254001	5	14254001	14257000	3000	1	1.40E-07	-0.52	9	0.3	Atp6v1h	Metabolism
DMR5:14336001	5	14336001	14338000	2000	1	1.20E-07	-0.46	7	0.35	Atp6v1h	Metabolism
DMR5:14355001	5	14355001	14356000	1000	1	7.30E-09	-0.63	12	1.2	Atp6v1h	Metabolism
DMR5:14402001	5	14402001	14408000	6000	2	1.30E-13	-0.41	45	0.75	Rgs20	Signaling
DMR5:14975001	5	14975001	14976000	1000	1	1.10E-07	0.47	26	2.6	Sumo4	
DMR5:15786001	5	15786001	15787000	1000	1	9.90E-07	0.44	3	0.3	Xkr4	
DMR5:15820001	5	15820001	15821000	1000	1	3.10E-07	0.42	10	1	Xkr4	
DMR5:15828001	5	15828001	15829000	1000	1	1.30E-08	-0.45	3	0.3	Xkr4	
DMR5:15890001	5	15890001	15892000	2000	1	3.80E-07	0.43	13	0.65	Xkr4	
DMR5:16055001	5	16055001	16056000	1000	1	4.90E-07	0.52	9	0.9	Xkr4	
DMR5:16084001	5	16084001	16088000	4000	1	4.40E-08	-0.41	29	0.72	Xkr4	
DMR5:16090001	5	16090001	16093000	3000	1	9.30E-07	-0.38	25	0.83	Xkr4	
DMR5:16144001	5	16144001	16145000	1000	1	7.20E-09	0.35	13	1.3	Xkr4	
DMR5:16146001	5	16146001	16150000	4000	1	1.70E-07	-0.35	34	0.85	Xkr4	
DMR5:16504001	5	16504001	16506000	2000	1	8.20E-11	-0.69	17	0.85	RGD1564981	
DMR5:16592001	5	16592001	16593000	1000	1	3.80E-07	-0.7	16	1.6	Lyn	
DMR5:16755001	5	16755001	16761000	6000	1	1.40E-08	-0.42	53	0.88	Mos;LOC685119;LOC100364265	Signaling;Epigenetic
DMR5:16810001	5	16810001	16819000	9000	2	3.10E-10	-0.54	90	1	Plag1	Transcription
DMR5:16853001	5	16853001	16857000	4000	1	7.40E-07	-0.68	41	1.02	Chchd7	
DMR5:17400001	5	17400001	17401000	1000	1	2.90E-09	0.9	22	2.2	RGD1563405	
DMR5:18777001	5	18777001	18778000	1000	1	3.50E-09	0.41	12	1.2	Fam110b	
DMR5:18980001	5	18980001	18984000	4000	2	4.70E-07	-0.29	29	0.72	RGD1565372	
DMR5:19293001	5	19293001	19295000	2000	1	1.80E-07	-0.46	27	1.35	Ubxn2b	Signaling
DMR5:19904001	5	19904001	19905000	1000	1	1.80E-08	0.42	3	0.3	Tox;LOC108350926	
DMR5:19992001	5	19992001	19993000	1000	1	3.90E-07	-0.55	8	0.8	Tox	
DMR5:20011001	5	20011001	20015000	4000	1	1.60E-07	-0.5	42	1.05	Tox	
DMR5:20036001	5	20036001	20038000	2000	1	1.50E-07	-0.47	17	0.85	Tox	
DMR5:20067001	5	20067001	20068000	1000	1	6.50E-07	0.58	21	2.1	Tox	
DMR5:21787001	5	21787001	21790000	3000	1	9.10E-07	-0.38	67	2.23	Chd7	
DMR5:21867001	5	21867001	21869000	2000	1	2.70E-08	-0.37	37	1.85	Chd7;LOC108350929	
DMR5:21886001	5	21886001	21888000	2000	1	5.60E-07	-0.44	66	3.3	Chd7;LOC108350929	
DMR5:21904001	5	21904001	21906000	2000	1	2.70E-07	0.31	23	1.15	Chd7	
DMR5:22121001	5	22121001	22123000	2000	1	3.90E-10	0.39	12	0.6	Lnc056	
DMR5:22386001	5	22386001	22388000	2000	1	5.50E-07	0.37	14	0.7	Clvs1	Transport
DMR5:22431001	5	22431001	22433000	2000	1	8.50E-09	-0.62	26	1.3	Clvs1	Transport
DMR5:22480001	5	22480001	22481000	1000	1	8.70E-09	-0.4	16	1.6	Clvs1	Transport

DMR5:22565001	5	22565001	22566000	1000	1	5.00E-08	-0.41	7	0.7	Clvs1	Transport
DMR5:22567001	5	22567001	22570000	3000	1	9.30E-07	0.37	24	0.8	Clvs1;Asph	Transport;Golgi
DMR5:22680001	5	22680001	22681000	1000	1	5.30E-11	-0.37	14	1.4	Asph	Golgi
DMR5:23046001	5	23046001	23048000	2000	1	2.10E-07	-0.54	26	1.3	Gdf6	Growth Factors
DMR5:24475001	5	24475001	24477000	2000	1	5.60E-07	-0.49	40	2	Ints8	
DMR5:24609001	5	24609001	24612000	3000	1	3.00E-07	-0.42	63	2.1	Esrp1	Translation
DMR5:24885001	5	24885001	24886000	1000	1	1.20E-07	0.37	17	1.7	RGD1559441	
DMR5:24905001	5	24905001	24908000	3000	2	8.00E-08	0.51	64	2.13	RGD1559441	
DMR5:25073001	5	25073001	25076000	3000	1	4.90E-08	-0.26	29	0.97	RGD1559904	
DMR5:25432001	5	25432001	25433000	1000	1	3.70E-16	0.39	3	0.3	Cdh17	Cytoskeleton
DMR5:25675001	5	25675001	25677000	2000	1	4.60E-07	-0.44	38	1.9	Tmem67	
DMR5:25685001	5	25685001	25688000	3000	1	6.40E-07	-0.46	12	0.4	Tmem67	
DMR5:27389001	5	27389001	27390000	1000	1	4.50E-12	0.44	8	0.8	Runx1t1	Transcription
DMR5:27990001	5	27990001	27993000	3000	1	2.70E-09	-0.61	25	0.83	Slc26a7	Transport
DMR5:27999001	5	27999001	28001000	2000	1	3.20E-08	-0.3	17	0.85	Slc26a7	Transport
DMR5:28002001	5	28002001	28003000	1000	1	1.40E-08	0.41	10	1	Slc26a7	Transport
DMR5:28066001	5	28066001	28070000	4000	2	2.20E-08	-0.3	31	0.78	Slc26a7	Transport
DMR5:28274001	5	28274001	28275000	1000	1	1.30E-07	0.54	9	0.9	Lrrc69	Cytoskeleton
DMR5:28286001	5	28286001	28289000	3000	1	2.90E-11	0.87	52	1.73	Lrrc69	Cytoskeleton
DMR5:28356001	5	28356001	28359000	3000	2	5.30E-09	0.69	33	1.1	Otud6b	Protease
DMR5:28529001	5	28529001	28534000	5000	3	1.10E-08	-0.45	46	0.92	Necab1	
DMR5:28612001	5	28612001	28617000	5000	1	7.40E-07	-0.23	44	0.88	Necab1	
DMR5:28659001	5	28659001	28666000	7000	1	2.00E-08	-0.33	62	0.89	Necab1	
DMR5:28728001	5	28728001	28729000	1000	1	1.40E-07	0.41	21	2.1	Necab1	
DMR5:28841001	5	28841001	28843000	2000	1	4.40E-07	-0.43	15	0.75	Tmem64;LOC108350938	
DMR5:28852001	5	28852001	28855000	3000	1	7.50E-07	-0.54	49	1.63	Tmem64;LOC108350938	
DMR5:28881001	5	28881001	28883000	2000	1	6.20E-07	0.54	32	1.6	Tmem64	
DMR5:29573001	5	29573001	29575000	2000	1	3.00E-09	-0.41	32	1.6	Decr1	Metabolism
DMR5:29581001	5	29581001	29582000	1000	1	6.70E-07	-0.47	11	1.1	Decr1	Metabolism
DMR5:32744001	5	32744001	32745000	1000	1	9.10E-09	-0.54	5	0.5	Cnbd1	
DMR5:32922001	5	32922001	32926000	4000	1	3.20E-08	-0.35	40	1	Cnbd1	
DMR5:33280001	5	33280001	33281000	1000	1	7.90E-07	0.44	13	1.3	Cngb3	Ion Channel
DMR5:33344001	5	33344001	33345000	1000	1	3.80E-07	0.32	11	1.1	Cngb3	Ion Channel
DMR5:33395001	5	33395001	33396000	1000	1	3.40E-09	0.57	5	0.5	Cngb3;LOC100909929;LOC102549738	Ion Channel
DMR5:33436001	5	33436001	33437000	1000	1	5.40E-07	0.39	14	1.4	Cngb3	Ion Channel
DMR5:33472001	5	33472001	33476000	4000	2	6.50E-11	-0.34	39	0.98	Cngb3	Ion Channel
DMR5:33499001	5	33499001	33502000	3000	1	7.50E-08	-0.55	22	0.73	Cngb3	Ion Channel
DMR5:33676001	5	33676001	33680000	4000	1	1.60E-07	-0.63	31	0.78	Wwp1	Proteolysis
DMR5:33787001	5	33787001	33788000	1000	1	6.60E-11	0.58	5	0.5	Slc7a13	Transport
DMR5:34052001	5	34052001	34054000	2000	1	4.40E-07	-0.49	23	1.15	Ggh	Protease
DMR5:34068001	5	34068001	34070000	2000	1	1.80E-07	0.58	27	1.35	Ggh	Protease
DMR5:34137001	5	34137001	34138000	1000	1	2.10E-11	0.45	6	0.6	Nkain3	
DMR5:34454001	5	34454001	34459000	5000	2	1.40E-11	-0.37	44	0.88	Nkain3	
DMR5:34616001	5	34616001	34620000	4000	1	2.20E-08	-0.29	44	1.1	Nkain3	
DMR5:35905001	5	35905001	35913000	8000	2	5.20E-07	-0.36	89	1.11	Tstd3;Usp45	Transport;Protease
DMR5:36005001	5	36005001	36007000	2000	1	2.20E-10	-0.41	25	1.25	Pnisr	
DMR5:36068001	5	36068001	36069000	1000	1	3.90E-07	0.56	16	1.6	Faxc	
DMR5:36133001	5	36133001	36134000	1000	1	6.80E-12	-0.57	13	1.3	Faxc	
DMR5:36624001	5	36624001	36625000	1000	1	5.00E-10	-0.68	12	1.2	Fbxl4	
DMR5:38833001	5	38833001	38835000	2000	1	6.10E-07	-0.42	9	0.45	Mms22l	
DMR5:39055001	5	39055001	39056000	1000	1	8.10E-08	-0.52	5	0.5	Klhl32	
DMR5:39076001	5	39076001	39078000	2000	1	4.00E-08	-0.37	18	0.9	Klhl32	
DMR5:39103001	5	39103001	39109000	6000	2	1.20E-07	0.4	54	0.9	Klhl32	
DMR5:39181001	5	39181001	39183000	2000	1	9.20E-07	0.36	12	0.6	Klhl32	
DMR5:39293001	5	39293001	39296000	3000	1	3.60E-07	-0.31	20	0.67	Gpr63;LOC103695246	Signaling
DMR5:39602001	5	39602001	39608000	6000	1	1.80E-07	-0.28	73	1.22	Fhl5;Ufl1	Transcription;Proteolysis
DMR5:39626001	5	39626001	39628000	2000	1	7.20E-07	-0.5	11	0.55	Ufl1	Proteolysis
DMR5:40229001	5	40229001	40230000	1000	1	2.50E-09	0.5	8	0.8	Fut9	Golgi
DMR5:40835001	5	40835001	40836000	1000	1	2.90E-08	-0.4	10	1	Manea	
DMR5:40877001	5	40877001	40882000	5000	1	2.30E-09	-0.45	47	0.94	Manea	
DMR5:43617001	5	43617001	43618000	1000	1	3.40E-13	0.87	19	1.9	Epha7	Receptor
DMR5:43688001	5	43688001	43691000	3000	1	2.70E-07	-0.6	36	1.2	Epha7	Receptor

DMR5:47449001	5	47449001	47451000	2000	1	3.10E-12	0.82	47	2.35	LOC108350945;LOC102551110;Bach2	
DMR5:47555001	5	47555001	47557000	2000	1	1.80E-08	-0.46	47	2.35	Bach2	
DMR5:47645001	5	47645001	47646000	1000	1	4.80E-07	-0.39	30	3	Bach2	
DMR5:47678001	5	47678001	47681000	3000	1	2.40E-07	-0.36	44	1.47	Bach2	
DMR5:47755001	5	47755001	47758000	3000	1	6.70E-08	-0.41	52	1.73	Bach2	
DMR5:47876001	5	47876001	47879000	3000	1	3.10E-09	-0.43	27	0.9	Casp8ap2	Signaling
DMR5:47928001	5	47928001	47929000	1000	1	2.30E-08	-0.45	9	0.9	Mdn1	
DMR5:48163001	5	48163001	48166000	3000	1	6.00E-09	-0.52	52	1.73	Ankrd6	
DMR5:48550001	5	48550001	48552000	2000	1	9.40E-11	0.53	35	1.75	LOC108350948;Rngtt	Translation
DMR5:48581001	5	48581001	48582000	1000	1	5.10E-07	0.38	9	0.9	Rngtt	Translation
DMR5:48671001	5	48671001	48672000	1000	1	5.40E-07	0.37	8	0.8	Rngtt	Translation
DMR5:48759001	5	48759001	48764000	5000	1	2.70E-08	-0.31	46	0.92	Rngtt	Translation
DMR5:49445001	5	49445001	49447000	2000	1	4.60E-07	-0.43	5	0.25	Spaca1	
DMR5:50147001	5	50147001	50149000	2000	1	8.30E-08	0.3	21	1.05	Slc35a1;Cfap206	Transport
DMR5:50167001	5	50167001	50168000	1000	1	4.10E-07	-0.6	4	0.4	Cfap206	
DMR5:50187001	5	50187001	50188000	1000	1	3.70E-09	0.54	7	0.7	Cfap206	
DMR5:50201001	5	50201001	50205000	4000	2	1.00E-10	-0.31	33	0.82	Cfap206;RGD1563056	
DMR5:50213001	5	50213001	50215000	2000	1	2.30E-07	0.49	13	0.65	RGD1563056	
DMR5:50284001	5	50284001	50286000	2000	1	6.50E-07	-0.49	18	0.9	Zfp292	Transcription
DMR5:50307001	5	50307001	50311000	4000	1	4.10E-07	-0.44	48	1.2	Zfp292	Transcription
DMR5:50355001	5	50355001	50357000	2000	1	3.60E-07	-0.53	17	0.85	Zfp292;Cga	Transcription;Hormone
DMR5:50429001	5	50429001	50431000	2000	1	1.50E-08	-0.39	9	0.45	Mob3b	Signaling
DMR5:50512001	5	50512001	50514000	2000	1	4.50E-10	0.7	51	2.55	Mob3b	Signaling
DMR5:50586001	5	50586001	50588000	2000	2	5.90E-16	0.91	38	1.9	Mob3b	Signaling
DMR5:56439001	5	56439001	56440000	1000	1	5.70E-07	0.34	15	1.5	Aco1	Metabolism
DMR5:56464001	5	56464001	56467000	3000	1	7.60E-11	-0.57	64	2.13	Aco1	Metabolism
DMR5:56479001	5	56479001	56480000	1000	1	6.40E-08	-0.48	33	3.3	Aco1;Ddx58	Metabolism
DMR5:56485001	5	56485001	56490000	5000	1	6.40E-07	-0.38	89	1.78	Aco1;Ddx58	Metabolism
DMR5:56491001	5	56491001	56494000	3000	2	1.80E-13	0.36	33	1.1	Aco1;Ddx58	Metabolism
DMR5:56540001	5	56540001	56545000	5000	1	8.80E-08	-0.5	56	1.12	Ddx58;Topors	Proteolysis
DMR5:56555001	5	56555001	56559000	4000	1	1.10E-07	-0.4	93	2.33	Topors;LOC102552818;Ndufb6	Proteolysis;Metabolism
DMR5:56563001	5	56563001	56564000	1000	1	5.30E-08	0.49	7	0.7	Topors;LOC102552818;Ndufb6	Proteolysis;Metabolism
DMR5:56758001	5	56758001	56759000	1000	1	2.00E-07	0.52	9	0.9	Sec61gl	
DMR5:57234001	5	57234001	57235000	1000	1	4.50E-07	-0.44	14	1.4	Spink4	Protease; Proteolysis
DMR5:57241001	5	57241001	57243000	2000	1	5.80E-07	0.26	28	1.4	Spink4	Protease; Proteolysis
DMR5:57301001	5	57301001	57302000	1000	1	2.80E-09	-0.56	12	1.2	Nfx1	Transcription
DMR5:57303001	5	57303001	57305000	2000	1	1.20E-07	0.4	18	0.9	Nfx1	Transcription
DMR5:57370001	5	57370001	57372000	2000	1	1.10E-09	0.47	30	1.5	Aqp7	Transport
DMR5:57373001	5	57373001	57375000	2000	1	2.00E-07	0.43	24	1.2	Aqp7	Transport
DMR5:57439001	5	57439001	57441000	2000	1	2.50E-08	0.61	43	2.15	Aqp3;Nol6	Transport;Metabolism
DMR5:57527001	5	57527001	57528000	1000	1	8.50E-07	-0.36	16	1.6	Ube2r2;Ubap2	
DMR5:57728001	5	57728001	57730000	2000	1	3.40E-07	0.57	30	1.5	LOC690990;Ubap1	
DMR5:57902001	5	57902001	57905000	3000	1	1.90E-08	0.31	42	1.4	RGD1561916;LOC102553821;Fam219a	
DMR5:58010001	5	58010001	58011000	1000	1	1.10E-08	0.42	10	1	Dnai1;Enho	Cytoskeleton
DMR5:58041001	5	58041001	58042000	1000	1	6.80E-07	0.4	24	2.4	LOC108350957;LOC103692336;Cntfr	Receptor
DMR5:58141001	5	58141001	58142000	1000	1	6.30E-07	-0.36	40	4	Galt;Il11ra1	Metabolism;Receptor
DMR5:58175001	5	58175001	58177000	2000	1	1.70E-12	-0.6	21	1.05	Ccl27;LOC102547621;Ccl19;LOC689481	Growth Factors;Growth Factors
DMR5:58180001	5	58180001	58183000	3000	1	1.30E-07	0.53	77	2.57	LOC102547621;Ccl19;LOC689481	Growth Factors
DMR5:58192001	5	58192001	58193000	1000	1	5.20E-09	0.46	21	2.1	Ccl19;LOC689481;Ccl21	Growth Factors
DMR5:58201001	5	58201001	58204000	3000	1	2.10E-07	0.62	90	3	Ccl21	Growth Factors
DMR5:58277001	5	58277001	58282000	5000	1	9.50E-07	0.38	43	0.86	Fam205a	
DMR5:58348001	5	58348001	58350000	2000	2	2.20E-08	-0.45	25	1.25	Phf24	Signaling
DMR5:58859001	5	58859001	58860000	1000	1	1.30E-07	0.5	9	0.9	Rusc2	
DMR5:58928001	5	58928001	58932000	4000	1	7.00E-11	-0.6	55	1.38	LOC103692345;Tesk1	
DMR5:58968001	5	58968001	58970000	2000	1	3.50E-07	0.62	33	1.65	RGD1560723	
DMR5:59114001	5	59114001	59115000	1000	1	5.50E-07	0.33	14	1.4	RGD1562259	
DMR5:59238001	5	59238001	59240000	2000	1	4.40E-08	0.4	32	1.6	Hrct1	
DMR5:59301001	5	59301001	59304000	3000	1	4.70E-10	-0.45	16	0.53	Olr838;Olr839	Receptor
DMR5:59316001	5	59316001	59317000	1000	1	6.50E-08	0.51	6	0.6	Olr839;Olr840	Receptor

DMR5:59409001	5	59409001	59410000	1000	1	2.10E-08	0.47	16	1.6	Reck;Glipr2	Protease; Proteolysis;Immune
DMR5:59549001	5	59549001	59557000	8000	3	4.30E-09	-0.52	144	1.8	Gne	Transcription
DMR5:59558001	5	59558001	59559000	1000	1	9.60E-17	0.4	8	0.8	Gne	Transcription
DMR5:59659001	5	59659001	59662000	3000	1	4.50E-07	-0.43	34	1.13	Rnf38	
DMR5:59703001	5	59703001	59704000	1000	1	1.90E-10	0.39	7	0.7	Rnf38	
DMR5:59718001	5	59718001	59720000	2000	1	3.40E-09	0.31	8	0.4	Rnf38	
DMR5:59794001	5	59794001	59800000	6000	1	6.10E-09	-0.76	76	1.27	Melk	Signaling
DMR5:60088001	5	60088001	60089000	1000	1	8.60E-07	0.44	19	1.9	Pax5	
DMR5:60105001	5	60105001	60106000	1000	1	1.40E-07	0.41	13	1.3	Pax5	
DMR5:60111001	5	60111001	60113000	2000	1	3.30E-08	-0.47	52	2.6	Pax5	
DMR5:60151001	5	60151001	60154000	3000	1	4.00E-09	0.44	39	1.3	Pax5	
DMR5:60156001	5	60156001	60159000	3000	1	7.00E-08	0.41	40	1.33	Pax5	
DMR5:60366001	5	60366001	60367000	1000	1	7.60E-07	-0.44	6	0.6	Zcchc7;LOC108350960	
DMR5:60781001	5	60781001	60783000	2000	1	2.40E-07	0.47	14	0.7	Frmpd1	
DMR5:60926001	5	60926001	60927000	1000	1	7.40E-09	0.56	5	0.5	Dcaf10;LOC108350961	
DMR5:61019001	5	61019001	61022000	3000	1	5.60E-11	-0.48	59	1.97	Shb	
DMR5:61528001	5	61528001	61532000	4000	1	1.60E-11	-0.48	32	0.8	Ccdc180	
DMR5:61663001	5	61663001	61667000	4000	1	1.00E-16	0.38	61	1.52	Tmod1	Cytoskeleton
DMR5:61738001	5	61738001	61739000	1000	1	1.30E-08	-0.76	11	1.1	Tstd2;Ncbp1	Transport;Translation
DMR5:61751001	5	61751001	61754000	3000	1	9.70E-08	0.41	31	1.03	Ncbp1;Xpa	Translation;DNA Repair
DMR5:61784001	5	61784001	61788000	4000	1	2.90E-08	-0.29	38	0.95	Xpa;LOC102550553	DNA Repair
DMR5:61966001	5	61966001	61967000	1000	1	3.90E-09	0.43	7	0.7	Foxe1	Transcription
DMR5:62102001	5	62102001	62104000	2000	1	1.30E-07	-0.48	30	1.5	Anp32b;Nans	Epigenetic;Metabolism
DMR5:62159001	5	62159001	62160000	1000	1	1.10E-07	0.31	19	1.9	Trim14;Coro2a	Proteolysis;Cytoskeleton
DMR5:62364001	5	62364001	62365000	1000	1	8.30E-13	0.58	17	1.7	Gabbr2	Signaling
DMR5:62466001	5	62466001	62467000	1000	1	3.00E-07	0.44	8	0.8	Gabbr2	Signaling
DMR5:62469001	5	62469001	62472000	3000	1	3.50E-18	0.8	65	2.17	Gabbr2	Signaling
DMR5:62476001	5	62476001	62479000	3000	1	2.60E-07	0.47	31	1.03	Gabbr2	Signaling
DMR5:62512001	5	62512001	62514000	2000	1	8.50E-08	-0.49	41	2.05	Gabbr2	Signaling
DMR5:62547001	5	62547001	62548000	1000	1	9.50E-07	0.38	30	3	Gabbr2;LOC102550819	Signaling
DMR5:62580001	5	62580001	62582000	2000	1	1.10E-07	0.44	13	0.65	Gabbr2	Signaling
DMR5:62909001	5	62909001	62911000	2000	1	2.90E-07	-0.49	29	1.45	Col15a1	Extracellular Matrix
DMR5:63810001	5	63810001	63815000	5000	1	7.50E-07	-0.41	80	1.6	Nr4a3	Transcription
DMR5:63869001	5	63869001	63871000	2000	1	1.50E-09	-0.52	8	0.4	Stx17	Transcription
DMR5:63922001	5	63922001	63924000	2000	1	1.30E-07	-0.55	25	1.25	Stx17;LOC102551558	Transcription
DMR5:63953001	5	63953001	63955000	2000	1	1.30E-07	-0.51	29	1.45	Erp44	
DMR5:63957001	5	63957001	63962000	5000	1	4.80E-07	-0.28	46	0.92	Erp44	
DMR5:63971001	5	63971001	63974000	3000	1	1.90E-07	-0.62	26	0.87	Erp44	
DMR5:64034001	5	64034001	64035000	1000	1	8.50E-07	0.38	10	1	Erp44;Invs	
DMR5:64045001	5	64045001	64046000	1000	1	8.50E-07	-0.58	7	0.7	Invs	
DMR5:64051001	5	64051001	64052000	1000	1	1.20E-08	0.37	14	1.4	Invs;LOC108350967	
DMR5:64072001	5	64072001	64074000	2000	1	4.30E-08	-0.45	23	1.15	Invs;LOC108350967	
DMR5:64106001	5	64106001	64108000	2000	1	9.00E-10	-0.51	21	1.05	Invs;LOC102551711	
DMR5:64216001	5	64216001	64218000	2000	2	2.90E-11	-0.56	17	0.85	Tex10;LOC108350966	
DMR5:64271001	5	64271001	64275000	4000	2	1.60E-15	0.74	72	1.8	Msantd3	
DMR5:64316001	5	64316001	64317000	1000	1	1.20E-10	-0.44	15	1.5	Msantd3;LOC108350968;Tmeff1	
DMR5:64714001	5	64714001	64715000	1000	1	1.00E-08	0.52	16	1.6	Acnat1	Metabolism
DMR5:67864001	5	67864001	67867000	3000	1	1.20E-08	0.54	33	1.1	Plppr1	Signaling
DMR5:68713001	5	68713001	68715000	2000	2	5.30E-10	-0.52	12	0.6	Olr848;Smc2	Receptor
DMR5:69027001	5	69027001	69029000	2000	2	2.50E-08	0.49	23	1.15	Olr848	Receptor
DMR5:69045001	5	69045001	69046000	1000	1	7.30E-07	0.51	8	0.8	Olr848	Receptor
DMR5:69049001	5	69049001	69051000	2000	1	1.80E-07	0.54	25	1.25	Olr848	Receptor
DMR5:69782001	5	69782001	69783000	1000	1	9.90E-07	-0.38	12	1.2	Olr853	Receptor
DMR5:69856001	5	69856001	69860000	4000	1	1.30E-08	-0.59	50	1.25	Nipsnap3b;Abca1	Transport
DMR5:69863001	5	69863001	69866000	3000	1	7.30E-07	-0.48	52	1.73	Abca1	Transport
DMR5:69921001	5	69921001	69927000	6000	1	1.00E-08	-0.55	100	1.67	Abca1	Transport
DMR5:70260001	5	70260001	70262000	2000	1	1.90E-08	-0.44	42	2.1	Slc44a1	Transport
DMR5:70348001	5	70348001	70350000	2000	1	9.30E-07	-0.37	24	1.2	Slc44a1	Transport
DMR5:72108001	5	72108001	72110000	2000	1	6.90E-08	-0.36	14	0.7	Rad23b	DNA Repair
DMR5:72136001	5	72136001	72137000	1000	1	1.40E-07	-0.64	7	0.7	Rad23b	DNA Repair
DMR5:73519001	5	73519001	73520000	1000	1	9.00E-07	-0.4	10	1	Ikbkap	
DMR5:73591001	5	73591001	73592000	1000	1	3.50E-12	-0.53	11	1.1	Ctnnal1	

DMR5:74036001	5	74036001	74040000	4000	2	1.60E-09	0.35	156	3.9	Frrs1l;Epb41l4b	
DMR5:74099001	5	74099001	74101000	2000	1	9.00E-08	-0.48	34	1.7	Epb41l4b	
DMR5:74675001	5	74675001	74677000	2000	1	9.10E-07	0.39	15	0.75	Palm2	
DMR5:74773001	5	74773001	74777000	4000	1	3.30E-24	0.71	83	2.08	Palm2	
DMR5:74790001	5	74790001	74791000	1000	1	1.10E-07	-0.52	13	1.3	Palm2	
DMR5:75046001	5	75046001	75048000	2000	1	1.10E-07	0.47	35	1.75	Txn1	
DMR5:75066001	5	75066001	75067000	1000	1	1.90E-07	0.41	5	0.5	Txn1;LOC102556461	
DMR5:75295001	5	75295001	75298000	3000	1	1.60E-11	0.62	46	1.53	Svep1	
DMR5:75403001	5	75403001	75405000	2000	1	8.30E-09	0.5	19	0.95	Musk	Receptor
DMR5:75457001	5	75457001	75459000	2000	2	2.80E-09	-0.32	20	1	Musk	Receptor
DMR5:75492001	5	75492001	75494000	2000	1	3.90E-07	0.61	29	1.45	Musk	Receptor
DMR5:75580001	5	75580001	75582000	2000	1	9.10E-07	-0.48	20	1	Lpar1	Signaling
DMR5:75987001	5	75987001	75988000	1000	1	3.10E-08	-0.48	8	0.8	RGD1306148;LOC103692363	
DMR5:76033001	5	76033001	76035000	2000	1	7.70E-09	-0.52	7	0.35	RGD1306148	
DMR5:76153001	5	76153001	76155000	2000	1	1.30E-09	-0.46	33	1.65	Dnajc25	Transcription
DMR5:76418001	5	76418001	76420000	2000	1	2.60E-12	-0.56	30	1.5	Ugcg	Golgi
DMR5:76426001	5	76426001	76428000	2000	1	7.00E-07	0.32	22	1.1	Ugcg;LOC102551074	Golgi
DMR5:76557001	5	76557001	76559000	2000	1	1.00E-06	-0.49	25	1.25	Susd1	Extracellular Matrix
DMR5:76581001	5	76581001	76585000	4000	1	4.40E-07	-0.46	59	1.48	Susd1	Extracellular Matrix
DMR5:76908001	5	76908001	76909000	1000	1	1.50E-07	-0.42	9	0.9	RGD1310951	
DMR5:76950001	5	76950001	76958000	8000	2	1.50E-09	-0.32	99	1.24	RGD1310951	
DMR5:76986001	5	76986001	76989000	3000	1	2.90E-08	-0.33	26	0.87	RGD1310951	
DMR5:77097001	5	77097001	77098000	1000	1	7.10E-10	-0.64	18	1.8	Snx30	Cytoskeleton
DMR5:77123001	5	77123001	77130000	7000	3	8.40E-09	-0.36	98	1.4	Snx30	Cytoskeleton
DMR5:77776001	5	77776001	77778000	2000	1	2.80E-08	-0.34	18	0.9	Mup5	Transport
DMR5:77795001	5	77795001	77797000	2000	1	5.30E-09	0.26	13	0.65	Mup5	Transport
DMR5:77859001	5	77859001	77860000	1000	1	4.30E-08	-0.44	7	0.7	Mup5;Zfp37	Transport;Transcription
DMR5:77864001	5	77864001	77869000	5000	4	3.50E-08	-0.36	49	0.98	Mup5;Zfp37	Transport;Transcription
DMR5:77870001	5	77870001	77876000	6000	1	4.90E-08	-0.54	88	1.47	Mup5;Zfp37	Transport;Transcription
DMR5:78458001	5	78458001	78460000	2000	1	1.20E-08	-0.47	36	1.8	Rgs3	
DMR5:78501001	5	78501001	78502000	1000	1	2.70E-07	-0.54	14	1.4	Rgs3	
DMR5:78857001	5	78857001	78858000	1000	1	6.20E-08	-0.39	9	0.9	Zfp618	Transcription
DMR5:78869001	5	78869001	78871000	2000	1	5.60E-07	-0.52	16	0.8	Zfp618	Transcription
DMR5:79565001	5	79565001	79566000	1000	1	1.40E-08	0.38	19	1.9	Tnfsf15;LOC108350984	
DMR5:81396001	5	81396001	81398000	2000	1	1.60E-08	-0.46	36	1.8	Astn2	
DMR5:81540001	5	81540001	81541000	1000	1	8.80E-10	-0.43	8	0.8	Astn2	
DMR5:81829001	5	81829001	81836000	7000	1	1.10E-09	-0.3	67	0.96	Astn2	
DMR5:82014001	5	82014001	82018000	4000	2	2.30E-09	-0.44	32	0.8	Astn2	
DMR5:85070001	5	85070001	85073000	3000	1	2.20E-09	-0.39	23	0.77	Brinp1	
DMR5:85089001	5	85089001	85091000	2000	1	3.30E-08	-0.45	13	0.65	Brinp1	
DMR5:86651001	5	86651001	86654000	3000	1	4.10E-07	-0.28	24	0.8	Megf9	Extracellular Matrix
DMR5:87347001	5	87347001	87348000	1000	1	3.90E-08	0.29	10	1	RGD1560539	Immune
DMR5:90067001	5	90067001	90069000	2000	1	9.10E-09	-0.34	14	0.7	Frmd3	
DMR5:90141001	5	90141001	90142000	1000	1	4.20E-08	-0.44	8	0.8	Frmd3	
DMR5:90189001	5	90189001	90195000	6000	1	1.80E-09	-0.27	56	0.93	Frmd3;LOC688541	
DMR5:90216001	5	90216001	90222000	6000	1	1.00E-08	-0.39	53	0.88	Frmd3	
DMR5:90224001	5	90224001	90231000	7000	1	3.60E-07	-0.39	83	1.19	Frmd3	
DMR5:90283001	5	90283001	90284000	1000	1	4.10E-08	0.51	8	0.8	Frmd3	
DMR5:90797001	5	90797001	90798000	1000	1	2.90E-09	0.59	18	1.8	Dppa3-ps2;Kdm4c	
DMR5:90944001	5	90944001	90952000	8000	2	3.20E-07	-0.45	87	1.09	Kdm4c	
DMR5:91505001	5	91505001	91509000	4000	1	2.60E-08	-0.38	30	0.75	Vom2r-ps76	
DMR5:91540001	5	91540001	91542000	2000	1	5.20E-09	-0.49	7	0.35	Vom2r-ps76	
DMR5:93207001	5	93207001	93208000	1000	1	1.60E-07	0.41	6	0.6	Ptprd	Signaling
DMR5:93230001	5	93230001	93231000	1000	1	1.90E-11	0.39	5	0.5	Ptprd	Signaling
DMR5:98409001	5	98409001	98410000	1000	1	1.10E-09	-0.65	8	0.8	Tyrp1;LOC102556039	Metabolism
DMR5:98566001	5	98566001	98567000	1000	1	3.80E-07	0.36	16	1.6	RGD1565987	
DMR5:100461001	5	100461001	100464000	3000	1	5.90E-07	-0.47	44	1.47	Nfib	Transcription
DMR5:100507001	5	100507001	100509000	2000	1	8.00E-07	-0.41	37	1.85	Nfib	Transcription
DMR5:100563001	5	100563001	100566000	3000	1	5.90E-07	0.47	29	0.97	Nfib	Transcription
DMR5:100638001	5	100638001	100642000	4000	1	1.60E-08	-0.55	73	1.82	Nfib;LOC100910558	Transcription
DMR5:100762001	5	100762001	100763000	1000	1	4.80E-07	-0.41	14	1.4	LOC102551858;Trnah-gug	
DMR5:101009001	5	101009001	101010000	1000	1	2.80E-07	0.49	20	2	Cer1;Frem1	
DMR5:101124001	5	101124001	101126000	2000	1	2.60E-07	0.31	14	0.7	Frem1;LOC102552173	

DMR5:101773001	5	101773001	101775000	2000	1	1.10E-07	0.49	14	0.7	Ccdc171	
DMR5:101808001	5	101808001	101817000	9000	1	1.20E-07	-0.21	100	1.11	Ccdc171	
DMR5:101884001	5	101884001	101886000	2000	1	9.90E-07	-0.31	14	0.7	Ccdc171	
DMR5:101936001	5	101936001	101937000	1000	1	5.20E-09	0.73	20	2	Ccdc171	
DMR5:102455001	5	102455001	102457000	2000	1	4.90E-08	-0.63	28	1.4	Bnc2	Transcription
DMR5:102583001	5	102583001	102586000	3000	1	8.10E-07	-0.46	33	1.1	Bnc2	Transcription
DMR5:102673001	5	102673001	102674000	1000	1	1.90E-07	-0.53	15	1.5	Bnc2	Transcription
DMR5:102691001	5	102691001	102696000	5000	1	2.60E-07	0.38	60	1.2	Bnc2	Transcription
DMR5:103799001	5	103799001	103804000	5000	1	9.40E-08	-0.33	38	0.76	Adamtsl1	
DMR5:103951001	5	103951001	103957000	6000	2	1.80E-09	-0.26	62	1.03	Adamtsl1	
DMR5:104030001	5	104030001	104032000	2000	1	3.00E-07	-0.34	19	0.95	Adamtsl1	
DMR5:104301001	5	104301001	104307000	6000	4	4.10E-09	-0.4	61	1.02	Adamtsl1	
DMR5:104546001	5	104546001	104547000	1000	1	3.20E-08	-0.51	11	1.1	Adamtsl1	
DMR5:104764001	5	104764001	104766000	2000	2	4.20E-08	-0.39	50	2.5	Fam154a	
DMR5:104896001	5	104896001	104898000	2000	1	4.50E-08	0.64	33	1.65	Fam154a	
DMR5:104919001	5	104919001	104921000	2000	1	1.30E-07	0.5	39	1.95	Fam154a	
DMR5:104926001	5	104926001	104928000	2000	1	1.20E-08	-0.39	19	0.95	Fam154a	
DMR5:105219001	5	105219001	105221000	2000	1	5.40E-08	0.54	8	0.4	LOC100911372;Acer2	
DMR5:105266001	5	105266001	105268000	2000	1	6.60E-08	-0.36	57	2.85	Acer2	
DMR5:105328001	5	105328001	105329000	1000	1	6.90E-07	0.39	16	1.6	Slc24a2	Transport
DMR5:105496001	5	105496001	105497000	1000	1	2.20E-08	0.38	6	0.6	Slc24a2	Transport
DMR5:106429001	5	106429001	106432000	3000	1	2.60E-08	-0.29	28	0.93	Focad	
DMR5:106450001	5	106450001	106452000	2000	1	6.60E-08	0.4	14	0.7	Focad	
DMR5:106534001	5	106534001	106536000	2000	1	8.10E-07	-0.44	27	1.35	Focad	
DMR5:106593001	5	106593001	106598000	5000	2	4.30E-10	-0.34	45	0.9	Focad	
DMR5:106642001	5	106642001	106643000	1000	1	3.40E-09	-0.5	8	0.8	Focad;LOC102549336	
DMR5:106655001	5	106655001	106659000	4000	1	7.80E-07	-0.34	38	0.95	Focad	
DMR5:107324001	5	107324001	107325000	1000	1	1.20E-10	-0.76	22	2.2	LOC680462;Klhl9;LOC108350997	
DMR5:107423001	5	107423001	107424000	1000	1	2.70E-08	0.35	22	2.2	Ifna2;RGD1564637	Immune
DMR5:107435001	5	107435001	107440000	5000	2	1.70E-09	0.51	42	0.84	RGD1564637;Ifna16l1	Immune
DMR5:108612001	5	108612001	108618000	6000	1	8.00E-07	-0.27	46	0.77	RGD1560729	
DMR5:108757001	5	108757001	108758000	1000	1	7.20E-08	0.59	18	1.8	Zfp353;LOC500497	Transcription
DMR5:109498001	5	109498001	109500000	2000	1	7.30E-07	-0.51	24	1.2	Elavl2	Translation
DMR5:113638001	5	113638001	113644000	6000	1	3.90E-07	-0.26	72	1.2	Ift74	
DMR5:113648001	5	113648001	113651000	3000	1	4.90E-08	-0.5	25	0.83	Ift74;Mir872	
DMR5:113652001	5	113652001	113654000	2000	2	6.50E-08	-0.41	18	0.9	Ift74;Mir872	
DMR5:113814001	5	113814001	113819000	5000	1	3.60E-10	-0.35	47	0.94	Tek	Receptor
DMR5:114569001	5	114569001	114574000	5000	1	1.90E-09	-0.32	43	0.86	Fggy	Metabolism
DMR5:115006001	5	115006001	115009000	3000	1	1.50E-07	-0.41	14	0.47	Hook1	Transport
DMR5:116419001	5	116419001	116424000	5000	1	8.10E-07	-0.43	88	1.76	Nfia	Transcription
DMR5:116443001	5	116443001	116448000	5000	1	2.10E-08	0.32	61	1.22	Nfia	Transcription
DMR5:116498001	5	116498001	116500000	2000	1	1.10E-12	-0.48	25	1.25	Nfia	Transcription
DMR5:116562001	5	116562001	116563000	1000	1	8.50E-07	-0.47	22	2.2	Nfia	Transcription
DMR5:117008001	5	117008001	117009000	1000	1	4.30E-07	-0.4	15	1.5	Tm2d1	
DMR5:117011001	5	117011001	117015000	4000	1	3.90E-07	-0.45	40	1	Tm2d1	
DMR5:117135001	5	117135001	117137000	2000	1	1.20E-07	-0.39	12	0.6	Patj	
DMR5:117198001	5	117198001	117201000	3000	1	2.20E-07	-0.37	25	0.83	Patj	
DMR5:117263001	5	117263001	117265000	2000	1	1.90E-09	-0.42	14	0.7	Patj	
DMR5:117352001	5	117352001	117357000	5000	1	7.30E-09	0.5	91	1.82	L1td1	Epigenetic
DMR5:117390001	5	117390001	117392000	2000	2	6.20E-13	0.49	20	1	Kank4	Cytoskeleton
DMR5:117636001	5	117636001	117637000	1000	1	4.70E-07	-0.49	17	1.7	Dock7	Transcription
DMR5:117644001	5	117644001	117650000	6000	1	2.30E-14	0.38	36	0.6	Dock7	Transcription
DMR5:117684001	5	117684001	117686000	2000	1	2.10E-07	-0.7	18	0.9	Dock7	Transcription
DMR5:117787001	5	117787001	117790000	3000	1	1.20E-07	-0.51	59	1.97	Dock7;LOC100366073	Transcription
DMR5:118413001	5	118413001	118414000	1000	1	2.00E-07	0.33	6	0.6	Alg6	Golgi
DMR5:118426001	5	118426001	118431000	5000	1	5.60E-10	-0.4	47	0.94	Alg6	Golgi
DMR5:118584001	5	118584001	118588000	4000	1	1.30E-07	-0.6	39	0.98	Ube2u	
DMR5:118730001	5	118730001	118733000	3000	2	2.70E-08	0.47	12	0.4	Efcab7	Signaling
DMR5:118958001	5	118958001	118961000	3000	1	5.70E-07	-0.49	51	1.7	Ror1	Receptor
DMR5:119734001	5	119734001	119737000	3000	1	1.40E-09	-0.54	38	1.27	Cachd1	Transport
DMR5:119825001	5	119825001	119829000	4000	1	4.20E-10	0.48	53	1.32	Cachd1	Transport
DMR5:119973001	5	119973001	119977000	4000	1	8.50E-07	-0.42	67	1.68	Raver2;Jak1	Metabolism
DMR5:120004001	5	120004001	120006000	2000	1	6.40E-07	0.4	33	1.65	Jak1	

DMR5:120255001	5	120255001	120257000	2000	1	5.00E-07	-0.38	22	1.1	Ak4	Signaling
DMR5:120259001	5	120259001	120264000	5000	1	9.70E-07	-0.43	58	1.16	Ak4	Signaling
DMR5:120302001	5	120302001	120305000	3000	1	8.70E-07	0.31	39	1.3	Ak4	Signaling
DMR5:120308001	5	120308001	120310000	2000	1	2.10E-09	-0.7	29	1.45	Ak4	Signaling
DMR5:120400001	5	120400001	120401000	1000	1	2.00E-09	0.68	6	0.6	Dnajc6	Transport
DMR5:120472001	5	120472001	120480000	8000	2	1.50E-11	-0.32	80	1	Dnajc6	Transport
DMR5:120504001	5	120504001	120505000	1000	1	4.50E-07	-0.4	13	1.3	Leprot;Lepr	Receptor
DMR5:121898001	5	121898001	121904000	6000	2	7.10E-08	-0.32	61	1.02	Pde4b	Signaling
DMR5:122027001	5	122027001	122028000	1000	1	2.40E-07	-0.35	10	1	Pde4b	Signaling
DMR5:122459001	5	122459001	122462000	3000	1	1.40E-08	0.36	55	1.83	Sgip1;LOC102548308	Cytoskeleton
DMR5:122522001	5	122522001	122529000	7000	2	8.80E-11	0.65	99	1.41	Tctex1d1;LOC103692428;Insl5	Cytoskeleton
DMR5:122571001	5	122571001	122574000	3000	1	1.30E-08	-0.28	28	0.93	Wdr78	Cytoskeleton
DMR5:122575001	5	122575001	122576000	1000	1	2.80E-07	-0.34	12	1.2	Wdr78	Cytoskeleton
DMR5:122592001	5	122592001	122595000	3000	1	7.60E-07	-0.42	48	1.6	Wdr78	Cytoskeleton
DMR5:122643001	5	122643001	122645000	2000	1	1.90E-10	-0.81	22	1.1	Wdr78;Mier1	Cytoskeleton;Development
DMR5:122701001	5	122701001	122704000	3000	1	4.00E-07	0.45	39	1.3	Mier1;Slc35d1	Development;Transport
DMR5:122775001	5	122775001	122778000	3000	1	2.50E-08	-0.29	27	0.9	RGD1562532	
DMR5:122821001	5	122821001	122824000	3000	1	4.60E-07	-0.52	37	1.23	RGD1562532	
DMR5:122885001	5	122885001	122890000	5000	1	7.00E-08	-0.34	34	0.68	Oma1	Protease
DMR5:123443001	5	123443001	123445000	2000	1	1.40E-08	0.38	14	0.7	Dab1	Cytoskeleton
DMR5:123665001	5	123665001	123666000	1000	1	4.50E-07	0.51	4	0.4	Dab1	Cytoskeleton
DMR5:123711001	5	123711001	123713000	2000	1	1.30E-08	0.5	16	0.8	Dab1	Cytoskeleton
DMR5:123800001	5	123800001	123803000	3000	2	2.20E-08	-0.38	30	1	Dab1	Cytoskeleton
DMR5:123862001	5	123862001	123869000	7000	1	9.10E-08	-0.27	72	1.03	Dab1	Cytoskeleton
DMR5:123923001	5	123923001	123926000	3000	1	1.80E-07	-0.6	24	0.8	Dab1	Cytoskeleton
DMR5:124186001	5	124186001	124187000	1000	1	3.90E-08	-0.51	8	0.8	Dab1	Cytoskeleton
DMR5:124404001	5	124404001	124406000	2000	1	2.20E-07	0.33	18	0.9	C8a	
DMR5:124480001	5	124480001	124482000	2000	1	7.00E-07	0.36	29	1.45	RGD1564074	
DMR5:126077001	5	126077001	126079000	2000	1	2.20E-07	0.37	22	1.1	Bsnd;LOC100909776	
DMR5:126333001	5	126333001	126336000	3000	1	4.40E-07	0.4	32	1.07	Mroh7;Fam151a	
DMR5:126384001	5	126384001	126389000	5000	2	5.00E-11	0.41	80	1.6	Acot11	Metabolism
DMR5:126404001	5	126404001	126405000	1000	1	2.40E-07	0.45	3	0.3	Acot11	Metabolism
DMR5:126568001	5	126568001	126569000	1000	1	6.40E-08	0.37	13	1.3	Ssbp3	Transcription
DMR5:126791001	5	126791001	126792000	1000	1	1.30E-08	-0.4	17	1.7	Tceanc2;Tmem59	Transcription
DMR5:126826001	5	126826001	126828000	2000	1	7.60E-09	-0.43	29	1.45	Ldlrad1;Lrrc42	
DMR5:126837001	5	126837001	126838000	1000	1	1.20E-08	-0.42	18	1.8	Lrrc42	
DMR5:126907001	5	126907001	126909000	2000	1	1.10E-08	0.35	19	0.95	Dio1	
DMR5:126964001	5	126964001	126965000	1000	1	1.60E-07	0.39	14	1.4	Yipf1	
DMR5:126995001	5	126995001	126996000	1000	1	3.60E-08	-0.41	15	1.5	Ndc1	
DMR5:127051001	5	127051001	127053000	2000	1	6.40E-07	-0.52	62	3.1	Glis1	Transcription
DMR5:127096001	5	127096001	127098000	2000	1	6.80E-07	0.41	26	1.3	Glis1	Transcription
DMR5:127195001	5	127195001	127197000	2000	1	9.60E-07	0.29	22	1.1	Glis1	Transcription
DMR5:127274001	5	127274001	127275000	1000	1	2.00E-09	0.58	27	2.7	Dmrtb1	Transcription
DMR5:127508001	5	127508001	127511000	3000	1	4.80E-08	-0.52	45	1.5	RGD1559786;Cpt2	Metabolism
DMR5:127596001	5	127596001	127600000	4000	2	6.30E-13	0.6	75	1.88	Slc1a7	Transport
DMR5:127662001	5	127662001	127667000	5000	3	1.00E-08	-0.57	50	1	Scp2	Transport
DMR5:127870001	5	127870001	127872000	2000	1	7.40E-08	-0.4	20	1	Zyg11b	
DMR5:127938001	5	127938001	127939000	1000	1	5.00E-07	-0.41	11	1.1	Coa7	
DMR5:127962001	5	127962001	127965000	3000	1	1.10E-08	0.36	30	1	Fam159a	
DMR5:127982001	5	127982001	127984000	2000	1	2.70E-08	0.52	87	4.35	Fam159a	
DMR5:127995001	5	127995001	127998000	3000	2	3.60E-08	0.39	25	0.83	Gpx7	Metabolism
DMR5:128289001	5	128289001	128293000	4000	1	1.50E-07	-0.47	34	0.85	Zfyve9	
DMR5:128482001	5	128482001	128483000	1000	1	2.80E-09	-0.67	18	1.8	Txndc12	Metabolism
DMR5:128558001	5	128558001	128560000	2000	1	2.60E-08	-0.48	28	1.4	Rab3b	
DMR5:128694001	5	128694001	128697000	3000	1	5.40E-10	-0.51	44	1.47	Nrdc;Osbpl9	Protease
DMR5:128795001	5	128795001	128797000	2000	1	7.30E-07	-0.45	39	1.95	Osbpl9	
DMR5:128958001	5	128958001	128960000	2000	1	8.90E-09	-0.58	19	0.95	Eps15	Transport
DMR5:128997001	5	128997001	128998000	1000	1	1.80E-07	-0.58	8	0.8	Eps15	Transport
DMR5:129033001	5	129033001	129035000	2000	1	8.50E-09	0.45	10	0.5	Ttc39a	
DMR5:129416001	5	129416001	129423000	7000	2	5.80E-10	-0.33	81	1.16	Faf1	
DMR5:129503001	5	129503001	129506000	3000	1	3.20E-07	-0.32	18	0.6	Faf1	
DMR5:129512001	5	129512001	129513000	1000	1	4.00E-08	-0.45	5	0.5	Faf1	

DMR5:129593001	5	129593001	129597000	4000	1	3.20E-08	-0.29	44	1.1	Faf1	
DMR5:129650001	5	129650001	129656000	6000	1	3.00E-07	-0.26	52	0.87	Faf1	
DMR5:130031001	5	130031001	130034000	3000	1	3.80E-07	-0.45	33	1.1	Elavl4	Translation
DMR5:130305001	5	130305001	130306000	1000	1	2.10E-07	-0.38	7	0.7	Agbl4	Protease
DMR5:131297001	5	131297001	131298000	1000	1	8.60E-07	-0.46	19	1.9	LOC100911395;Bend5	
DMR5:131755001	5	131755001	131756000	1000	1	5.50E-07	-0.46	11	1.1	Spata6	
DMR5:133382001	5	133382001	133384000	2000	1	3.70E-13	0.47	19	0.95	Trabd2b	Protease
DMR5:133966001	5	133966001	133971000	5000	1	6.40E-08	-0.43	40	0.8	Cyp4x1;Cyp4a8	Metabolism
DMR5:134011001	5	134011001	134013000	2000	1	5.00E-11	-0.55	10	0.5	Cyp4a8	
DMR5:134210001	5	134210001	134214000	4000	1	1.20E-08	-0.3	34	0.85	Cyp4a2;LOC108351019	Metabolism
DMR5:134506001	5	134506001	134508000	2000	1	6.00E-07	0.31	18	0.9	Cyp4a1;Cyp4b1	Metabolism
DMR5:134658001	5	134658001	134662000	4000	2	1.20E-07	0.42	66	1.65	Atpaf1;LOC103692454	Transcription
DMR5:134704001	5	134704001	134706000	2000	1	3.10E-07	0.34	25	1.25	Mknk1	Signaling
DMR5:134734001	5	134734001	134735000	1000	1	7.40E-07	0.44	11	1.1	Mknk1;Kncn	Signaling
DMR5:135028001	5	135028001	135030000	2000	1	9.00E-08	-0.41	28	1.4	Tspan1;LOC100911669	
DMR5:135206001	5	135206001	135209000	3000	1	2.10E-09	0.4	43	1.43	Mast2	Signaling
DMR5:135449001	5	135449001	135450000	1000	1	1.80E-07	-0.5	15	1.5	Gpbp1l1;Ccdc17;Nasp	Transcription
DMR5:135495001	5	135495001	135497000	2000	1	2.30E-10	-0.49	17	0.85	Akr1a1	Metabolism
DMR5:135549001	5	135549001	135551000	2000	1	2.20E-07	-0.45	13	0.65	Prdx1;Mmachc	Metabolism
DMR5:135753001	5	135753001	135756000	3000	1	5.00E-07	-0.31	25	0.83	Zswim5	
DMR5:135862001	5	135862001	135863000	1000	1	9.00E-07	0.35	19	1.9	Zswim5;Urod;Hectd3	Epigenetic;Proteolysis
DMR5:136001001	5	136001001	136002000	1000	1	3.50E-07	0.36	13	1.3	Btbd19;Tctex1d4;Plk3	Cytoskeleton;Signaling
DMR5:136230001	5	136230001	136233000	3000	2	1.10E-21	0.7	64	2.13	Rnf220	
DMR5:136294001	5	136294001	136298000	4000	1	1.00E-09	0.39	53	1.32	Rnf220	
DMR5:136497001	5	136497001	136498000	1000	1	1.10E-08	-0.4	27	2.7	Eri3	Transcription
DMR5:136594001	5	136594001	136598000	4000	1	8.80E-08	-0.34	20	0.5	Klf17	Transcription
DMR5:136610001	5	136610001	136613000	3000	1	5.10E-07	0.33	47	1.57	Klf17	Transcription
DMR5:136620001	5	136620001	136623000	3000	1	7.70E-07	0.31	29	0.97	Klf17	Transcription
DMR5:136857001	5	136857001	136862000	5000	1	1.20E-07	0.37	71	1.42	St3gal3	Transport
DMR5:136940001	5	136940001	136942000	2000	1	2.10E-09	-0.47	37	1.85	St3gal3	Transport
DMR5:136955001	5	136955001	136957000	2000	1	6.30E-08	0.34	14	0.7	St3gal3	Transport
DMR5:137270001	5	137270001	137271000	1000	1	8.00E-07	0.37	12	1.2	Elovl1;Cdc20;LOC103692460;Mpl;LOC102554074	Metabolism;Proteolysis;Receptor
DMR5:137823001	5	137823001	137829000	6000	1	3.90E-09	-0.37	53	0.88	Olr865	Receptor
DMR5:137853001	5	137853001	137858000	5000	1	3.00E-07	0.57	57	1.14	Olr866	Receptor
DMR5:137949001	5	137949001	137954000	5000	1	5.50E-07	-0.4	25	0.5	Olr870-ps;Olr871-ps	
DMR5:137971001	5	137971001	137976000	5000	2	7.40E-08	-0.29	44	0.88	Olr872-ps	
DMR5:138014001	5	138014001	138019000	5000	1	6.80E-07	-0.35	68	1.36	Lao1	Metabolism
DMR5:138302001	5	138302001	138303000	1000	1	7.70E-11	0.45	14	1.4	P3h1;Cldn19	Extracellular Matrix;Cell Junction
DMR5:138352001	5	138352001	138355000	3000	1	4.40E-07	0.36	35	1.17	Ppih;LOC103692462;Ccdc30	Transcription
DMR5:138386001	5	138386001	138389000	3000	1	2.70E-08	0.39	50	1.67	Ccdc30	
DMR5:138456001	5	138456001	138458000	2000	1	2.00E-09	-0.46	29	1.45	Ccdc30;Ppcs	Transport
DMR5:138473001	5	138473001	138475000	2000	1	9.90E-07	-0.47	22	1.1	Ppcs;Zmynd12	Transport
DMR5:138704001	5	138704001	138710000	6000	1	3.00E-07	0.39	66	1.1	Guca2b	Signaling
DMR5:138894001	5	138894001	138895000	1000	1	2.70E-09	-0.7	6	0.6	Hivep3;LOC103689960	
DMR5:138941001	5	138941001	138945000	4000	1	3.40E-07	0.36	47	1.18	Hivep3	
DMR5:139024001	5	139024001	139025000	1000	1	3.80E-09	-0.47	12	1.2	Hivep3	
DMR5:139207001	5	139207001	139208000	1000	1	2.20E-08	0.68	31	3.1	Foxo6	
DMR5:139218001	5	139218001	139222000	4000	1	8.20E-08	0.39	46	1.15	Foxo6	
DMR5:139230001	5	139230001	139233000	3000	1	1.70E-08	0.41	42	1.4	Foxo6	
DMR5:139409001	5	139409001	139414000	5000	4	1.60E-09	-0.38	51	1.02	Scmh1	Epigenetic
DMR5:139501001	5	139501001	139503000	2000	1	6.70E-07	-0.55	19	0.95	Ctps1	Metabolism
DMR5:139626001	5	139626001	139627000	1000	1	2.60E-09	0.74	25	2.5	Kcnq4	Transport
DMR5:139658001	5	139658001	139659000	1000	1	7.10E-08	0.43	13	1.3	Kcnq4	Transport
DMR5:139807001	5	139807001	139810000	3000	1	6.90E-08	0.42	49	1.63	Rims3	Transport
DMR5:139812001	5	139812001	139814000	2000	1	1.40E-07	0.4	29	1.45	Rims3	Transport
DMR5:140583001	5	140583001	140585000	2000	1	1.70E-11	-0.39	21	1.05	Cap1	Cytoskeleton
DMR5:140667001	5	140667001	140668000	1000	1	1.00E-11	0.55	15	1.5	Mfsd2a	
DMR5:140761001	5	140761001	140767000	6000	1	1.30E-08	0.4	64	1.07	Trit1	Translation
DMR5:140799001	5	140799001	140803000	4000	1	4.40E-07	0.39	64	1.6	Bmp8b	Growth Factors
DMR5:140828001	5	140828001	140831000	3000	1	2.80E-07	0.46	52	1.73	Bmp8b;Ppie	Growth Factors;Transcription

DMR5:140946001	5	140946001	140951000	5000	1	5.70E-07	0.4	43	0.86	Heyl;LOC108351027	Transcription
DMR5:141007001	5	141007001	141010000	3000	1	2.10E-11	0.5	47	1.57	Bmp8a;LOC100361036	Growth Factors
DMR5:141014001	5	141014001	141015000	1000	1	5.80E-07	-0.41	23	2.3	Bmp8a;LOC100361036	Growth Factors
DMR5:141368001	5	141368001	141370000	2000	1	2.40E-07	0.36	23	1.15	Macf1;LOC108351106	Cytoskeleton
DMR5:141429001	5	141429001	141430000	1000	1	3.60E-09	-0.57	17	1.7	Akirin1	
DMR5:141585001	5	141585001	141587000	2000	1	5.30E-07	-0.37	29	1.45	Rragc	Signaling
DMR5:142682001	5	142682001	142684000	2000	1	2.40E-07	-0.32	28	1.4	Utp11;Fhl3	Metabolism;Transcription
DMR5:142716001	5	142716001	142717000	1000	1	4.60E-08	-0.43	23	2.3	Sf3a3;LOC103692473	Translation
DMR5:142725001	5	142725001	142726000	1000	1	7.20E-07	0.34	13	1.3	Sf3a3;Inpp5b	Translation;Signaling
DMR5:142879001	5	142879001	142882000	3000	1	4.30E-09	0.72	71	2.37	Epha10;LOC108351028	Receptor
DMR5:142885001	5	142885001	142886000	1000	1	8.80E-07	-0.38	16	1.6	Epha10;LOC108351028	Receptor
DMR5:142918001	5	142918001	142922000	4000	2	5.30E-08	-0.4	45	1.12	Epha10;Cdca8	Receptor
DMR5:143050001	5	143050001	143051000	1000	1	4.70E-07	-0.46	17	1.7	Gnl2;Dnali1	Cytoskeleton
DMR5:143091001	5	143091001	143095000	4000	1	8.90E-08	-0.41	57	1.43	Meaf6	
DMR5:143110001	5	143110001	143113000	3000	1	1.20E-07	0.47	51	1.7	Meaf6;Zc3h12a	Translation
DMR5:143608001	5	143608001	143609000	1000	1	6.30E-09	0.47	9	0.9	Grik3	Receptor
DMR5:143628001	5	143628001	143630000	2000	1	1.70E-07	-0.43	39	1.95	Grik3	Receptor
DMR5:143636001	5	143636001	143639000	3000	1	6.60E-07	0.3	45	1.5	Grik3	Receptor
DMR5:144190001	5	144190001	144196000	6000	1	1.50E-11	-0.53	83	1.38	Thrap3	Metabolism
DMR5:144276001	5	144276001	144277000	1000	1	6.50E-13	0.68	23	2.3	Map7d1;Trappc3	Cytoskeleton
DMR5:144343001	5	144343001	144345000	2000	1	1.30E-08	0.6	44	2.2	Col8a2;Adprhl2;Tekt2	Extracellular Matrix;Metabolism;Cytoskeleton
DMR5:144361001	5	144361001	144362000	1000	1	5.20E-07	-0.34	14	1.4	Ago3	Translation
DMR5:144415001	5	144415001	144416000	1000	1	6.90E-09	-0.52	8	0.8	Ago3	Translation
DMR5:144506001	5	144506001	144508000	2000	1	4.50E-07	-0.4	20	1	Ago4	Translation
DMR5:144611001	5	144611001	144612000	1000	1	3.50E-09	-0.51	18	1.8	Clspn	
DMR5:145407001	5	145407001	145409000	2000	1	1.60E-10	0.61	44	2.2	Gjb3;Gjb4	Cytoskeleton
DMR5:146269001	5	146269001	146271000	2000	1	7.50E-10	0.57	23	1.15	Csmd2;LOC108351071	
DMR5:146300001	5	146300001	146302000	2000	1	4.90E-07	-0.6	40	2	Csmd2	
DMR5:146358001	5	146358001	146360000	2000	1	3.60E-07	0.41	22	1.1	Csmd2	
DMR5:146429001	5	146429001	146430000	1000	1	8.70E-07	0.29	11	1.1	Csmd2	
DMR5:146495001	5	146495001	146496000	1000	1	7.70E-13	0.63	21	2.1	Csmd2	
DMR5:146497001	5	146497001	146504000	7000	1	3.70E-08	-0.35	82	1.17	Csmd2	
DMR5:146547001	5	146547001	146548000	1000	1	1.50E-11	-0.51	9	0.9	Csmd2	
DMR5:146567001	5	146567001	146568000	1000	1	7.70E-09	0.41	10	1	Csmd2	
DMR5:146575001	5	146575001	146576000	1000	1	3.90E-07	0.5	22	2.2	Csmd2	
DMR5:146596001	5	146596001	146597000	1000	1	3.20E-10	0.48	12	1.2	Csmd2	
DMR5:146598001	5	146598001	146600000	2000	1	1.20E-07	-0.41	45	2.25	Csmd2	
DMR5:146610001	5	146610001	146612000	2000	1	5.40E-09	-0.58	49	2.45	Csmd2	
DMR5:146645001	5	146645001	146647000	2000	1	9.50E-07	-0.37	48	2.4	Csmd2	
DMR5:146665001	5	146665001	146668000	3000	1	1.00E-07	0.43	45	1.5	Csmd2	
DMR5:146726001	5	146726001	146727000	1000	1	2.80E-07	0.46	13	1.3	Csmd2	
DMR5:146823001	5	146823001	146825000	2000	1	4.20E-10	0.46	30	1.5	Phc2	Epigenetic
DMR5:146909001	5	146909001	146910000	1000	1	3.00E-07	-0.38	22	2.2	Phc2	Epigenetic
DMR5:146980001	5	146980001	146981000	1000	1	1.50E-08	0.45	19	1.9	Zfp362;LOC102551009	Transcription
DMR5:147157001	5	147157001	147158000	1000	1	5.10E-07	0.32	21	2.1	Azin2	Metabolism
DMR5:147279001	5	147279001	147282000	3000	1	4.80E-07	0.5	48	1.6	Rnf19b;Tmem54	Proteolysis
DMR5:147436001	5	147436001	147437000	1000	1	9.10E-07	0.52	18	1.8	RGD1561149	
DMR5:147506001	5	147506001	147507000	1000	1	1.60E-07	-0.55	10	1	Sync;Rbbp4	
DMR5:148026001	5	148026001	148030000	4000	1	3.80E-08	0.45	22	0.55	Ptp4a2	Signaling
DMR5:148151001	5	148151001	148152000	1000	1	6.00E-08	0.47	22	2.2	Spocd1	
DMR5:148155001	5	148155001	148159000	4000	1	1.70E-07	0.43	66	1.65	Spocd1;Adgrb2	Signaling
DMR5:148307001	5	148307001	148311000	4000	1	2.40E-07	0.38	57	1.43	Col16a1;Pef1	Extracellular Matrix
DMR5:148349001	5	148349001	148350000	1000	1	1.60E-08	-0.48	21	2.1	Pef1;Hcrtr1	Signaling
DMR5:148360001	5	148360001	148361000	1000	1	6.70E-07	0.35	10	1	Hcrtr1	Signaling
DMR5:148371001	5	148371001	148373000	2000	1	3.40E-07	-0.43	35	1.75	Tinagl1	Protease
DMR5:148382001	5	148382001	148384000	2000	1	2.70E-07	0.35	27	1.35	Tinagl1	Protease
DMR5:148475001	5	148475001	148478000	3000	1	6.80E-09	0.49	49	1.63	LOC102552786;Serinc2	Signaling
DMR5:148480001	5	148480001	148482000	2000	1	8.00E-07	0.65	49	2.45	Serinc2	Signaling
DMR5:148498001	5	148498001	148503000	5000	1	4.60E-07	-0.3	49	0.98	Serinc2	Signaling
DMR5:148563001	5	148563001	148567000	4000	1	8.40E-09	-0.54	81	2.02	Zcchc17	

DMR5:148663001	5	148663001	148664000	1000	1	1.10E-12	-0.55	20	2	Nkain1;LOC103692481	
DMR5:148670001	5	148670001	148671000	1000	1	1.40E-07	0.48	9	0.9	Nkain1;LOC103692481	
DMR5:148914001	5	148914001	148918000	4000	1	9.30E-08	0.41	61	1.52	Pum1;Sdc3	Metabolism;Receptor
DMR5:149082001	5	149082001	149083000	1000	1	8.60E-08	0.39	21	2.1	Matn1	Extracellular Matrix
DMR5:149986001	5	149986001	149987000	1000	1	3.60E-08	0.41	13	1.3	Ptpru	Signaling
DMR5:150118001	5	150118001	150119000	1000	1	5.30E-08	-0.4	13	1.3	Epb41	
DMR5:150130001	5	150130001	150132000	2000	1	9.30E-07	-0.39	26	1.3	Epb41	
DMR5:150180001	5	150180001	150185000	5000	1	9.70E-09	-0.67	60	1.2	Epb41	
DMR5:150229001	5	150229001	150230000	1000	1	2.50E-07	0.35	9	0.9	Epb41	
DMR5:150312001	5	150312001	150315000	3000	1	7.80E-07	0.43	53	1.77	Oprd1	Signaling
DMR5:150582001	5	150582001	150584000	2000	1	5.00E-07	-0.43	40	2	Phactr4	Signaling
DMR5:150751001	5	150751001	150755000	4000	2	1.10E-07	0.29	50	1.25	Dnajc8;Ptafr	Transcription;Signaling
DMR5:150772001	5	150772001	150773000	1000	1	2.20E-07	0.44	29	2.9	Ptafr	Signaling
DMR5:150781001	5	150781001	150782000	1000	1	9.10E-07	0.47	13	1.3	Ptafr	Signaling
DMR5:150908001	5	150908001	150910000	2000	1	5.00E-08	-0.3	29	1.45	Eya3;Xkr8	
DMR5:150962001	5	150962001	150970000	8000	2	6.00E-08	-0.48	116	1.45	Rpa2;Themis2	
DMR5:151038001	5	151038001	151040000	2000	1	5.40E-11	-0.46	19	0.95	Ppp1r8;Stx12	Translation;Transcription
DMR5:151202001	5	151202001	151203000	1000	1	9.60E-07	0.74	33	3.3	Fgr;Ahdc1	
DMR5:151321001	5	151321001	151322000	1000	1	9.60E-08	-0.43	17	1.7	Wasf2	Cytoskeleton
DMR5:151395001	5	151395001	151396000	1000	1	1.40E-08	0.66	29	2.9	Wasf2;Gpr3;Cd164l2	Cytoskeleton;Signaling
DMR5:151589001	5	151589001	151592000	3000	1	8.50E-09	0.32	27	0.9	Slc9a1	Transport
DMR5:151634001	5	151634001	151636000	2000	2	1.00E-11	0.4	28	1.4	Slc9a1	Transport
DMR5:151810001	5	151810001	151812000	2000	1	3.70E-08	-0.58	23	1.15	Gpatch3;Gpn2	Signaling
DMR5:151858001	5	151858001	151860000	2000	1	2.20E-07	-0.36	34	1.7	Zdhhc18;LOC366479	
DMR5:151951001	5	151951001	151953000	2000	1	1.10E-08	-0.63	22	1.1	Arid1a	
DMR5:152076001	5	152076001	152077000	1000	1	1.20E-08	0.51	12	1.2	RGD1562725;Rps6ka1	Golgi
DMR5:152085001	5	152085001	152087000	2000	1	1.70E-08	-0.54	29	1.45	Rps6ka1	Golgi
DMR5:152257001	5	152257001	152258000	1000	1	1.30E-08	0.34	6	0.6	Lin28a	Metabolism
DMR5:152325001	5	152325001	152327000	2000	1	4.00E-07	0.48	58	2.9	Aim1l;Cd52;LOC100360087;Ubxn11	Signaling
DMR5:152428001	5	152428001	152429000	1000	1	5.00E-09	0.53	14	1.4	Umodl;Catsper4	Receptor
DMR5:152549001	5	152549001	152551000	2000	1	6.70E-07	-0.39	13	0.65	Trim63;Slc30a2	Proteolysis;Transport
DMR5:152640001	5	152640001	152643000	3000	1	5.50E-09	0.54	55	1.83	Pafah2;LOC102546373	Golgi
DMR5:152669001	5	152669001	152673000	4000	1	5.50E-08	0.47	61	1.52	LOC102546373;Stmn1	
DMR5:152717001	5	152717001	152721000	4000	1	1.20E-07	0.37	100	2.5	LOC102546455;Paqr7;Aunip	Signaling
DMR5:152764001	5	152764001	152771000	7000	1	3.10E-07	0.37	93	1.33	Sepn1;Man1c1	Golgi
DMR5:152776001	5	152776001	152779000	3000	1	5.80E-07	0.38	59	1.97	Man1c1	Golgi
DMR5:152889001	5	152889001	152892000	3000	1	6.30E-11	0.4	34	1.13	Man1c1	Golgi
DMR5:153154001	5	153154001	153155000	1000	1	2.30E-07	-0.46	18	1.8	Tmem57	
DMR5:153500001	5	153500001	153501000	1000	1	2.60E-07	0.55	16	1.6	Runx3	Transcription
DMR5:153691001	5	153691001	153695000	4000	1	2.80E-07	-0.36	60	1.5	Srrm1	Translation
DMR5:153699001	5	153699001	153701000	2000	1	5.70E-07	-0.41	29	1.45	Srrm1	Translation
DMR5:153780001	5	153780001	153786000	6000	1	3.40E-09	-0.53	113	1.88	Rcan3	Signaling
DMR5:153862001	5	153862001	153864000	2000	1	1.50E-08	0.41	30	1.5	Stpg1	Development
DMR5:153903001	5	153903001	153904000	1000	1	1.90E-07	0.64	32	3.2	Grhl3	Transcription
DMR5:153906001	5	153906001	153908000	2000	1	9.40E-07	0.35	46	2.3	Grhl3	Transcription
DMR5:153921001	5	153921001	153924000	3000	1	4.50E-08	-0.43	90	3	Grhl3	Transcription
DMR5:154031001	5	154031001	154034000	3000	1	6.70E-08	-0.57	61	2.03	Ifnlr1	Receptor
DMR5:154051001	5	154051001	154053000	2000	2	2.30E-14	0.42	23	1.15	Ifnlr1	Receptor
DMR5:154129001	5	154129001	154131000	2000	1	4.30E-10	0.49	33	1.65	Myom3	
DMR5:154169001	5	154169001	154170000	1000	1	3.60E-08	-0.37	7	0.7	Myom3	
DMR5:154232001	5	154232001	154238000	6000	2	1.40E-07	-0.43	81	1.35	Pnrc2;Cnr2	Signaling
DMR5:154287001	5	154287001	154288000	1000	1	4.00E-07	0.41	16	1.6	Fuca1;Hmgcl	Metabolism;Metabolism
DMR5:154387001	5	154387001	154390000	3000	1	2.60E-07	-0.3	83	2.77	Rpl11;LOC102551799	Translation
DMR5:154499001	5	154499001	154500000	1000	1	2.40E-08	0.55	10	1	Id3	Transcription
DMR5:154501001	5	154501001	154504000	3000	1	3.10E-08	0.32	26	0.87	Id3	Transcription
DMR5:154610001	5	154610001	154611000	1000	1	4.00E-07	0.39	17	1.7	Tcea3	Transcription
DMR5:154647001	5	154647001	154651000	4000	1	4.40E-08	-0.47	72	1.8	RGD1564482;Zfp46	
DMR5:154769001	5	154769001	154771000	2000	2	3.90E-11	0.63	29	1.45	Htr1d	Signaling
DMR5:154800001	5	154800001	154802000	2000	1	7.80E-13	0.82	53	2.65	Htr1d	Signaling
DMR5:154899001	5	154899001	154901000	2000	1	1.50E-11	-0.5	39	1.95	Luzp1;LOC103692495;Kdm1a	Metabolism
DMR5:154952001	5	154952001	154954000	2000	1	4.20E-08	-0.38	19	0.95	Kdm1a	Metabolism
DMR5:155038001	5	155038001	155040000	2000	1	1.10E-14	0.85	56	2.8	Ephb2	Receptor
DMR5:155096001	5	155096001	155097000	1000	1	4.90E-07	0.38	4	0.4	Ephb2	Receptor

DMR5:155130001	5	155130001	155133000	3000	1	1.90E-07	0.33	39	1.3	Ephb2	Receptor
DMR5:155362001	5	155362001	155364000	2000	1	3.00E-07	0.41	23	1.15	Zbtb40	Transcription
DMR5:155377001	5	155377001	155379000	2000	1	4.90E-07	-0.38	32	1.6	Zbtb40	Transcription
DMR5:155634001	5	155634001	155641000	7000	2	7.90E-08	-0.46	120	1.71	Wnt4	Signaling
DMR5:155767001	5	155767001	155771000	4000	1	2.70E-08	0.39	51	1.27	Cela3b	Protease
DMR5:155816001	5	155816001	155817000	1000	1	3.40E-08	0.46	8	0.8	Hspg2	
DMR5:155898001	5	155898001	155900000	2000	1	8.20E-07	0.41	22	1.1	Hspg2	
DMR5:155904001	5	155904001	155907000	3000	1	1.10E-07	0.41	36	1.2	Hspg2;Ldlrad2	
DMR5:155952001	5	155952001	155953000	1000	1	4.30E-07	-0.35	19	1.9	Usp48	Protease
DMR5:156106001	5	156106001	156108000	2000	1	4.40E-08	-0.37	39	1.95	Alpl	Signaling
DMR5:156225001	5	156225001	156228000	3000	1	6.20E-10	0.44	45	1.5	Ece1	Protease
DMR5:156275001	5	156275001	156276000	1000	1	7.80E-07	0.35	12	1.2	Ece1	Protease
DMR5:156345001	5	156345001	156352000	7000	3	3.30E-10	-0.53	120	1.71	Eif4g3	Translation
DMR5:156493001	5	156493001	156496000	3000	1	8.40E-07	-0.44	33	1.1	Eif4g3	Translation
DMR5:156579001	5	156579001	156581000	2000	1	7.90E-15	0.64	30	1.5	LOC102553854;Hp1bp3	Cytoskeleton
DMR5:156677001	5	156677001	156681000	4000	1	1.90E-07	0.44	78	1.95	Ddost;Pink1	Golgi;Signaling
DMR5:157336001	5	157336001	157339000	3000	1	5.80E-10	0.49	30	1	Pla2g2e;Otud3	Protease
DMR5:157494001	5	157494001	157497000	3000	1	8.10E-08	0.53	45	1.5	Tmco4;Htr6	Signaling
DMR5:157540001	5	157540001	157541000	1000	1	5.60E-07	0.39	8	0.8	Nbl1;Minos1	
DMR5:157797001	5	157797001	157798000	1000	1	4.10E-07	0.38	9	0.9	LOC108351050;Akr7a3	Metabolism
DMR5:157892001	5	157892001	157894000	2000	1	2.80E-07	-0.52	35	1.75	Ubr4;Trnav-aac	Proteolysis
DMR5:157920001	5	157920001	157924000	4000	2	4.20E-10	0.48	70	1.75	Ubr4	Proteolysis
DMR5:158511001	5	158511001	158513000	2000	1	3.20E-09	0.4	48	2.4	LOC108351052;Igsf21	
DMR5:158558001	5	158558001	158560000	2000	1	5.70E-07	0.31	41	2.05	Igsf21;LOC102547437	
DMR5:158592001	5	158592001	158593000	1000	1	5.60E-09	0.56	13	1.3	Igsf21	
DMR5:158615001	5	158615001	158617000	2000	1	6.00E-10	0.49	25	1.25	Igsf21	
DMR5:159009001	5	159009001	159010000	1000	1	2.80E-07	0.55	32	3.2	Arhgef10l	Transcription
DMR5:159017001	5	159017001	159019000	2000	1	1.50E-09	-0.42	45	2.25	Arhgef10l	Transcription
DMR5:159027001	5	159027001	159029000	2000	1	2.10E-07	0.36	29	1.45	Arhgef10l	Transcription
DMR5:159056001	5	159056001	159059000	3000	1	4.90E-07	0.35	74	2.47	Arhgef10l	Transcription
DMR5:159060001	5	159060001	159066000	6000	1	3.20E-08	0.56	103	1.72	Arhgef10l	Transcription
DMR5:159078001	5	159078001	159079000	1000	1	3.50E-07	0.43	13	1.3	Arhgef10l	Transcription
DMR5:159279001	5	159279001	159285000	6000	1	1.70E-11	-0.44	102	1.7	Padi4	
DMR5:159300001	5	159300001	159302000	2000	1	3.50E-07	0.27	20	1	Padi4;Padi3	
DMR5:159341001	5	159341001	159342000	1000	1	1.20E-12	0.7	12	1.2	Padi3;Padi1	
DMR5:159460001	5	159460001	159462000	2000	2	5.00E-08	0.42	42	2.1	Padi2	
DMR5:159621001	5	159621001	159625000	4000	1	5.60E-07	0.33	62	1.55	Spata21	Signaling
DMR5:159702001	5	159702001	159704000	2000	1	5.20E-09	-0.53	32	1.6	Fbxo42	
DMR5:159747001	5	159747001	159752000	5000	2	3.30E-07	0.38	83	1.66	Trnai-aau;LOC108351054;Arhgef19	Transcription
DMR5:159764001	5	159764001	159765000	1000	1	3.20E-15	0.75	55	5.5	Trnai-aau;LOC108351054;Arhgef19	Transcription
DMR5:159891001	5	159891001	159894000	3000	2	1.10E-07	0.36	49	1.63	Rps27a-ps2	
DMR5:159933001	5	159933001	159936000	3000	1	4.20E-08	0.41	39	1.3	Fam131c;Clcnka	Transport
DMR5:159955001	5	159955001	159958000	3000	1	8.00E-07	0.62	69	2.3	Clcnka;Clcnkb	Transport
DMR5:159967001	5	159967001	159971000	4000	1	5.90E-07	0.38	73	1.82	Clcnkb;Hspb7	Transport
DMR5:160024001	5	160024001	160027000	3000	1	6.20E-09	-0.39	118	3.93	Zbtb17;Spen	Transcription;Metabolism
DMR5:160036001	5	160036001	160039000	3000	1	7.90E-07	-0.49	48	1.6	Spen	Metabolism
DMR5:160065001	5	160065001	160069000	4000	1	5.50E-07	-0.66	88	2.2	Spen	Metabolism
DMR5:160184001	5	160184001	160187000	3000	1	1.50E-07	0.49	82	2.73	Tmem82;Slc25a34;Plekhm2	
DMR5:160277001	5	160277001	160279000	2000	1	3.20E-09	-0.56	28	1.4	Ddi2	Proteolysis
DMR5:160306001	5	160306001	160307000	1000	1	7.10E-14	0.53	9	0.9	Agmat	Metabolism
DMR5:160494001	5	160494001	160495000	1000	1	7.50E-07	-0.35	15	1.5	Fhad1	
DMR5:160520001	5	160520001	160521000	1000	1	1.50E-08	0.32	13	1.3	Fhad1	
DMR5:160762001	5	160762001	160767000	5000	2	2.50E-10	-0.49	91	1.82	Kazn	
DMR5:160780001	5	160780001	160781000	1000	1	2.20E-10	0.7	32	3.2	Kazn	
DMR5:160794001	5	160794001	160796000	2000	1	4.10E-11	0.74	44	2.2	Kazn	
DMR5:160808001	5	160808001	160810000	2000	1	7.30E-09	0.38	25	1.25	Kazn	
DMR5:160872001	5	160872001	160874000	2000	1	1.60E-07	0.34	26	1.3	Kazn	
DMR5:160926001	5	160926001	160929000	3000	1	1.50E-10	0.38	44	1.47	Kazn	
DMR5:161071001	5	161071001	161074000	3000	2	1.40E-10	0.43	35	1.17	Kazn	
DMR5:161082001	5	161082001	161086000	4000	1	2.30E-08	0.45	98	2.45	Kazn	
DMR5:161182001	5	161182001	161183000	1000	1	7.30E-07	0.32	4	0.4	Kazn	
DMR5:161195001	5	161195001	161197000	2000	2	2.60E-09	0.43	14	0.7	Kazn	

DMR5:161335001	5	161335001	161340000	5000	1	3.50E-08	0.44	90	1.8	Kazn	
DMR5:161560001	5	161560001	161562000	2000	1	2.40E-07	-0.48	24	1.2	Kazn	
DMR5:161598001	5	161598001	161601000	3000	1	5.80E-07	-0.27	29	0.97	Kazn	
DMR5:161615001	5	161615001	161617000	2000	1	8.40E-09	0.48	51	2.55	Kazn	
DMR5:161637001	5	161637001	161638000	1000	1	8.30E-07	0.4	8	0.8	Kazn	
DMR5:161658001	5	161658001	161661000	3000	1	1.10E-09	0.52	43	1.43	Kazn	
DMR5:161673001	5	161673001	161674000	1000	1	2.30E-09	0.5	24	2.4	Kazn	
DMR5:161792001	5	161792001	161793000	1000	1	5.50E-12	0.59	12	1.2	Prdm2;LOC103692503	Transcription
DMR5:161848001	5	161848001	161850000	2000	1	1.90E-09	0.45	13	0.65	Prdm2	Transcription
DMR5:161886001	5	161886001	161888000	2000	1	2.30E-07	-0.36	43	2.15	Prdm2;LOC100362684	Transcription
DMR5:161889001	5	161889001	161890000	1000	1	7.90E-07	0.51	25	2.5	Prdm2;LOC100362684	Transcription
DMR5:161974001	5	161974001	161976000	2000	1	1.20E-08	0.62	30	1.5	Pdpn	
DMR5:162157001	5	162157001	162159000	2000	1	1.70E-17	0.99	69	3.45	Pramef27	
DMR5:162378001	5	162378001	162379000	1000	1	1.10E-14	1	29	2.9	LOC691162;Pramef12	
DMR5:162584001	5	162584001	162587000	3000	1	2.50E-08	-0.49	14	0.47	RGD1563334	Metabolism
DMR5:162958001	5	162958001	162960000	2000	1	8.10E-07	-0.5	20	1	Vps13d	Transport
DMR5:163128001	5	163128001	163129000	1000	1	3.20E-09	0.6	23	2.3	Vps13d;LOC102556804;Tnfrsf1b	Transport;Receptor
DMR5:163208001	5	163208001	163211000	3000	1	5.30E-08	0.32	29	0.97	Tnfrsf8	Receptor
DMR5:163217001	5	163217001	163220000	3000	1	2.30E-11	0.53	35	1.17	Tnfrsf8	Receptor
DMR5:163227001	5	163227001	163230000	3000	1	3.70E-08	0.35	42	1.4	Tnfrsf8	Receptor
DMR5:164504001	5	164504001	164508000	4000	1	1.20E-07	-0.55	23	0.58	RGD1561413;LOC103692508	
DMR5:164512001	5	164512001	164518000	6000	1	1.80E-09	-0.53	62	1.03	RGD1561413;LOC103692508	
DMR5:164744001	5	164744001	164746000	2000	1	3.50E-07	0.39	37	1.85	Plod1;RGD1305350	Golgi
DMR5:164873001	5	164873001	164875000	2000	1	6.70E-08	0.48	22	1.1	Mthfr;LOC103692509;LOC102551309	Metabolism
DMR5:164930001	5	164930001	164933000	3000	1	1.20E-15	0.75	60	2	Draxin;LOC108351058	
DMR5:165613001	5	165613001	165618000	5000	1	1.30E-07	-0.39	111	2.22	Casz1	Transcription
DMR5:165816001	5	165816001	165818000	2000	1	6.20E-10	0.45	14	0.7	Pex14	Transport
DMR5:165897001	5	165897001	165898000	1000	1	5.00E-07	-0.3	19	1.9	Pex14	Transport
DMR5:165957001	5	165957001	165959000	2000	1	8.40E-10	0.68	52	2.6	Apitd1;Pgd	Metabolism
DMR5:165991001	5	165991001	165993000	2000	1	2.80E-07	-0.47	33	1.65	Pgd;Kif1b	Metabolism;Cytoskeleton
DMR5:166285001	5	166285001	166287000	2000	1	7.10E-07	0.34	30	1.5	LOC103692512;Rbp7;LOC691196	Metabolism
DMR5:166411001	5	166411001	166414000	3000	1	3.20E-08	0.35	41	1.37	Nmnat1	Metabolism
DMR5:166521001	5	166521001	166522000	1000	1	1.40E-08	0.48	16	1.6	Ctnnbip1	
DMR5:166982001	5	166982001	166985000	3000	1	2.70E-07	0.64	63	2.1	Spsb1;LOC108351061;H6pd	Metabolism
DMR5:167231001	5	167231001	167233000	2000	1	4.40E-07	-0.34	41	2.05	Car6	
DMR5:167365001	5	167365001	167367000	2000	1	9.50E-09	-0.51	19	0.95	Rere;LOC108351064	
DMR5:167370001	5	167370001	167371000	1000	1	1.40E-07	-0.38	20	2	Rere;LOC108351064	
DMR5:167391001	5	167391001	167395000	4000	1	5.50E-07	-0.38	64	1.6	Rere;Mir6332	
DMR5:167429001	5	167429001	167430000	1000	1	9.20E-07	-0.4	21	2.1	Rere	
DMR5:167525001	5	167525001	167528000	3000	1	1.20E-07	-0.37	57	1.9	Rere	
DMR5:167697001	5	167697001	167698000	1000	1	2.40E-09	0.43	22	2.2	Slc45a1	Transport
DMR5:167989001	5	167989001	167991000	2000	1	7.70E-08	-0.42	35	1.75	Park7	
DMR5:167996001	5	167996001	167998000	2000	1	2.10E-08	-0.55	34	1.7	Park7	
DMR5:168033001	5	168033001	168039000	6000	1	7.90E-10	-0.48	111	1.85	Tnfrsf9	Receptor
DMR5:168102001	5	168102001	168103000	1000	1	9.20E-07	-0.43	39	3.9	Per3	Transcription
DMR5:168116001	5	168116001	168122000	6000	1	8.60E-09	-0.48	145	2.42	Per3;Vamp3	Transcription
DMR5:168153001	5	168153001	168154000	1000	1	5.40E-08	-0.56	28	2.8	Camta1	Transcription
DMR5:168178001	5	168178001	168181000	3000	1	7.80E-09	-0.37	85	2.83	Camta1	Transcription
DMR5:168254001	5	168254001	168255000	1000	1	2.70E-07	-0.45	25	2.5	Camta1	Transcription
DMR5:168307001	5	168307001	168309000	2000	1	1.70E-08	0.38	41	2.05	Camta1	Transcription
DMR5:168411001	5	168411001	168412000	1000	1	2.90E-09	0.78	28	2.8	Camta1	Transcription
DMR5:168431001	5	168431001	168432000	1000	1	1.30E-07	0.64	26	2.6	Camta1	Transcription
DMR5:168474001	5	168474001	168475000	1000	1	2.70E-08	-0.42	56	5.6	Camta1;LOC102546456	Transcription
DMR5:168496001	5	168496001	168497000	1000	1	5.20E-07	0.44	16	1.6	Camta1	Transcription
DMR5:168542001	5	168542001	168545000	3000	3	3.40E-15	0.9	93	3.1	Camta1;LOC102546564	Transcription
DMR5:168567001	5	168567001	168570000	3000	1	6.20E-10	0.43	62	2.07	Camta1	Transcription
DMR5:168613001	5	168613001	168616000	3000	1	1.10E-10	0.51	58	1.93	Camta1	Transcription
DMR5:168658001	5	168658001	168660000	2000	1	1.30E-07	0.39	35	1.75	Camta1	Transcription
DMR5:168666001	5	168666001	168668000	2000	2	4.90E-09	0.55	55	2.75	Camta1	Transcription
DMR5:168670001	5	168670001	168671000	1000	1	1.10E-17	0.93	36	3.6	Camta1	Transcription
DMR5:168693001	5	168693001	168694000	1000	1	1.50E-08	0.39	11	1.1	Camta1	Transcription

DMR5:168738001	5	168738001	168740000	2000	1	3.30E-14	0.46	29	1.45	Camta1	Transcription
DMR5:168786001	5	168786001	168790000	4000	2	1.30E-10	0.51	76	1.9	Camta1	Transcription
DMR5:168793001	5	168793001	168795000	2000	2	3.40E-13	0.56	39	1.95	Camta1	Transcription
DMR5:168837001	5	168837001	168839000	2000	1	2.10E-08	0.6	49	2.45	Camta1;LOC102546752	Transcription
DMR5:168880001	5	168880001	168881000	1000	1	8.30E-08	0.52	23	2.3	Camta1	Transcription
DMR5:168895001	5	168895001	168896000	1000	1	4.30E-15	0.94	29	2.9	Camta1	Transcription
DMR5:169010001	5	169010001	169012000	2000	1	1.60E-08	-0.57	29	1.45	Camta1;LOC100362830	Transcription
DMR5:169014001	5	169014001	169016000	2000	1	7.40E-07	-0.32	34	1.7	Camta1;LOC100362830	Transcription
DMR5:169022001	5	169022001	169023000	1000	1	7.10E-08	0.43	21	2.1	Camta1;LOC100362830	Transcription
DMR5:169129001	5	169129001	169131000	2000	1	1.90E-09	-0.62	33	1.65	Dnajc11	Transcription
DMR5:169261001	5	169261001	169262000	1000	1	1.40E-10	-0.53	21	2.1	Plekhg5	
DMR5:169449001	5	169449001	169451000	2000	1	3.50E-07	0.38	37	1.85	Acot7;Gpr153	Metabolism;Signaling
DMR5:169456001	5	169456001	169459000	3000	1	1.70E-07	0.37	75	2.5	Acot7;Gpr153;Hes3	Metabolism;Signaling;Transcription
DMR5:169498001	5	169498001	169499000	1000	1	2.90E-07	0.57	20	2	Rnf207;Rpl22	Translation
DMR5:169552001	5	169552001	169554000	2000	1	3.20E-07	0.36	37	1.85	Chd5	
DMR5:169697001	5	169697001	169699000	2000	1	6.60E-07	0.32	45	2.25	Nphp4	
DMR5:171364001	5	171364001	171365000	1000	1	1.20E-07	0.31	10	1	LOC102551562;Tp73	Transcription
DMR5:171397001	5	171397001	171399000	2000	1	4.60E-07	0.52	46	2.3	Tp73	Transcription
DMR5:171598001	5	171598001	171599000	1000	1	7.30E-07	0.44	11	1.1	Megf6	Extracellular Matrix
DMR5:171607001	5	171607001	171608000	1000	1	2.10E-07	0.45	12	1.2	Megf6	Extracellular Matrix
DMR5:171673001	5	171673001	171674000	1000	1	2.80E-09	0.63	38	3.8	Prdm16	Transcription
DMR5:172358001	5	172358001	172360000	2000	1	1.20E-09	0.48	24	1.2	Hes5;Pank4	Transcription;Signaling
DMR5:172403001	5	172403001	172406000	3000	1	7.00E-09	0.39	82	2.73	Plch2	Metabolism
DMR5:172419001	5	172419001	172420000	1000	1	1.60E-07	0.5	26	2.6	Plch2	Metabolism
DMR5:172445001	5	172445001	172448000	3000	2	2.60E-08	0.42	35	1.17	Plch2	Metabolism
DMR5:172456001	5	172456001	172459000	3000	1	2.10E-08	0.52	41	1.37	Plch2	Metabolism
DMR5:172529001	5	172529001	172538000	9000	1	2.50E-07	0.48	154	1.71	Morn1;LOC108351067	Signaling
DMR5:172594001	5	172594001	172598000	4000	2	5.80E-07	-0.5	79	1.98	Ski	
DMR5:172605001	5	172605001	172609000	4000	1	6.00E-07	-0.39	56	1.4	Ski	
DMR5:172659001	5	172659001	172663000	4000	1	5.00E-07	-0.44	86	2.15	Faap20;Prkcz	Signaling
DMR5:172696001	5	172696001	172697000	1000	1	2.20E-07	-0.41	22	2.2	Prkcz	Signaling
DMR5:172898001	5	172898001	172899000	1000	1	6.70E-07	0.54	31	3.1	Tmem52	
DMR5:173588001	5	173588001	173590000	2000	1	7.40E-08	-0.4	16	0.8	LOC100362942;Agrn	Extracellular Matrix
DMR5:173591001	5	173591001	173594000	3000	1	8.00E-08	0.35	39	1.3	LOC100362942;Agrn	Extracellular Matrix
DMR6:800001	6	800001	801000	1000	1	4.60E-08	-0.45	21	2.1	Crim1	
DMR6:802001	6	802001	804000	2000	1	1.90E-07	-0.47	19	0.95	Crim1	
DMR6:818001	6	818001	822000	4000	1	2.00E-08	-0.52	72	1.8	Crim1	
DMR6:914001	6	914001	916000	2000	1	4.70E-08	-0.42	36	1.8	Crim1	
DMR6:947001	6	947001	951000	4000	1	2.30E-07	-0.47	102	2.55	Crim1	
DMR6:1103001	6	1103001	1105000	2000	1	7.30E-07	0.4	45	2.25	Vit	Extracellular Matrix
DMR6:1132001	6	1132001	1135000	3000	1	4.80E-09	-0.34	21	0.7	Vit	Extracellular Matrix
DMR6:1161001	6	1161001	1166000	5000	1	1.20E-07	-0.44	82	1.64	Vit	Extracellular Matrix
DMR6:1232001	6	1232001	1233000	1000	1	5.10E-07	-0.48	14	1.4	Strn	
DMR6:1328001	6	1328001	1333000	5000	1	3.80E-08	-0.5	87	1.74	Strn;Heatr5b	
DMR6:1396001	6	1396001	1398000	2000	1	4.30E-09	-0.45	29	1.45	Heatr5b	
DMR6:1565001	6	1565001	1566000	1000	1	9.90E-07	-0.53	23	2.3	Prkd3	Signaling
DMR6:1664001	6	1664001	1665000	1000	1	1.50E-12	0.86	19	1.9	Qpct	Transport
DMR6:2231001	6	2231001	2234000	3000	1	8.20E-07	-0.42	62	2.07	Rmdn2	
DMR6:2266001	6	2266001	2267000	1000	1	7.40E-08	0.44	3	0.3	Rmdn2	
DMR6:2721001	6	2721001	2723000	2000	1	6.40E-08	-0.42	12	0.6	Hnrnpll	
DMR6:2916001	6	2916001	2917000	1000	1	2.30E-09	0.68	41	4.1	Gemin6;Dhx57	Transcription
DMR6:2976001	6	2976001	2978000	2000	1	2.90E-07	0.38	22	1.1	Morn2;LOC500607;Arhgef33	Transcription
DMR6:3281001	6	3281001	3282000	1000	1	6.10E-09	-0.48	22	2.2	Cdkl4;Map4k3	Signaling
DMR6:3363001	6	3363001	3367000	4000	1	2.50E-09	-0.45	39	0.98	Map4k3;LOC102551744	
DMR6:3409001	6	3409001	3410000	1000	1	3.40E-09	-0.48	16	1.6	Map4k3	
DMR6:3414001	6	3414001	3417000	3000	2	2.20E-10	-0.33	25	0.83	Map4k3	
DMR6:3446001	6	3446001	3448000	2000	1	8.00E-07	-0.58	20	1	Map4k3;LOC108351160	
DMR6:3661001	6	3661001	3662000	1000	1	2.80E-13	0.77	38	3.8	Tmem178a	
DMR6:3713001	6	3713001	3716000	3000	1	3.10E-14	-0.55	42	1.4	Tmem178a	
DMR6:3759001	6	3759001	3761000	2000	1	4.00E-10	-0.33	30	1.5	Thumpd2	Epigenetic
DMR6:3762001	6	3762001	3765000	3000	1	2.70E-08	-0.35	28	0.93	Thumpd2	Epigenetic
DMR6:4296001	6	4296001	4297000	1000	1	4.20E-10	0.35	5	0.5	Slc8a1	Transport

DMR6:4298001	6	4298001	4301000	3000	1	9.90E-07	-0.57	52	1.73	Slc8a1	Transport
DMR6:4340001	6	4340001	4341000	1000	1	2.80E-09	0.39	8	0.8	Slc8a1	Transport
DMR6:4480001	6	4480001	4481000	1000	1	2.50E-10	0.57	8	0.8	Slc8a1	Transport
DMR6:6830001	6	6830001	6831000	1000	1	8.50E-08	0.51	12	1.2	Kcng3	Transport
DMR6:6832001	6	6832001	6834000	2000	1	1.30E-07	-0.35	33	1.65	Kcng3	Transport
DMR6:6909001	6	6909001	6912000	3000	1	9.00E-07	-0.55	49	1.63	Mta3	Development
DMR6:6968001	6	6968001	6969000	1000	1	1.70E-08	0.37	24	2.4	Mta3	Development
DMR6:7005001	6	7005001	7007000	2000	1	4.40E-14	1.02	65	3.25	Mta3	Development
DMR6:7015001	6	7015001	7017000	2000	1	2.40E-07	-0.49	35	1.75	Mta3	Development
DMR6:7047001	6	7047001	7049000	2000	1	2.10E-07	0.51	29	1.45	Haao	Metabolism
DMR6:7052001	6	7052001	7053000	1000	1	4.40E-10	0.39	9	0.9	Haao	Metabolism
DMR6:7056001	6	7056001	7058000	2000	1	5.90E-09	0.59	51	2.55	Haao	Metabolism
DMR6:7071001	6	7071001	7072000	1000	1	1.50E-07	-0.39	20	2	LOC102548459;Trnai-uau	
DMR6:7406001	6	7406001	7408000	2000	1	1.90E-08	0.62	40	2	LOC102548121;Zfp36l2	Metabolism
DMR6:7612001	6	7612001	7614000	2000	1	3.20E-10	-0.49	26	1.3	Thada	Cytoskeleton
DMR6:7639001	6	7639001	7644000	5000	1	5.40E-07	-0.26	40	0.8	Thada	Cytoskeleton
DMR6:7681001	6	7681001	7685000	4000	1	4.70E-08	-0.41	28	0.7	Thada	Cytoskeleton
DMR6:7740001	6	7740001	7741000	1000	1	1.20E-11	-0.51	19	1.9	Thada	Cytoskeleton
DMR6:7749001	6	7749001	7750000	1000	1	3.60E-07	0.39	5	0.5	Thada	Cytoskeleton
DMR6:7797001	6	7797001	7801000	4000	1	2.50E-07	-0.38	47	1.18	Plekhh2	
DMR6:7821001	6	7821001	7827000	6000	1	3.20E-07	-0.43	80	1.33	Plekhh2	
DMR6:7845001	6	7845001	7852000	7000	1	5.90E-14	0.47	127	1.81	Plekhh2	
DMR6:7919001	6	7919001	7927000	8000	1	1.20E-11	0.82	124	1.55	Dync2li1;Abcg5	Cytoskeleton;Transport
DMR6:7970001	6	7970001	7972000	2000	1	1.40E-07	0.43	59	2.95	Abcg5;Abcg8	Transport
DMR6:8019001	6	8019001	8020000	1000	1	1.60E-09	-0.48	20	2	Lrpprc	
DMR6:8039001	6	8039001	8041000	2000	1	6.30E-08	-0.36	26	1.3	Lrpprc	
DMR6:8396001	6	8396001	8397000	1000	1	5.50E-07	-0.64	8	0.8	Camkmt	Golgi
DMR6:8414001	6	8414001	8415000	1000	1	3.40E-10	-0.79	7	0.7	Camkmt	Golgi
DMR6:8429001	6	8429001	8430000	1000	1	4.50E-07	0.42	7	0.7	Camkmt	Golgi
DMR6:8471001	6	8471001	8473000	2000	1	5.60E-07	-0.4	25	1.25	Camkmt	Golgi
DMR6:8555001	6	8555001	8556000	1000	1	2.50E-08	0.31	5	0.5	Camkmt	Golgi
DMR6:8581001	6	8581001	8584000	3000	1	3.90E-07	0.3	34	1.13	Camkmt	Golgi
DMR6:8596001	6	8596001	8597000	1000	1	7.90E-07	-0.48	13	1.3	Camkmt	Golgi
DMR6:8628001	6	8628001	8630000	2000	1	2.80E-07	0.35	33	1.65	Camkmt	Golgi
DMR6:8737001	6	8737001	8738000	1000	1	2.00E-08	0.51	13	1.3	Camkmt	Golgi
DMR6:8739001	6	8739001	8741000	2000	1	2.50E-07	0.56	26	1.3	Camkmt	Golgi
DMR6:8876001	6	8876001	8878000	2000	1	9.80E-08	-0.47	26	1.3	LOC108351167;Six3	Development
DMR6:9408001	6	9408001	9411000	3000	1	2.10E-07	-0.59	9	0.3	Srbd1	Translation
DMR6:9533001	6	9533001	9537000	4000	1	2.40E-07	0.33	43	1.07	Prkce	Signaling
DMR6:9560001	6	9560001	9561000	1000	1	6.40E-09	0.36	6	0.6	Prkce	Signaling
DMR6:9591001	6	9591001	9592000	1000	1	7.20E-07	0.46	12	1.2	Prkce	Signaling
DMR6:9596001	6	9596001	9597000	1000	1	4.20E-07	-0.39	21	2.1	Prkce	Signaling
DMR6:9610001	6	9610001	9616000	6000	1	5.70E-10	-0.57	96	1.6	Prkce;LOC102551687	Signaling
DMR6:9672001	6	9672001	9673000	1000	1	5.30E-07	-0.38	26	2.6	Prkce	Signaling
DMR6:9930001	6	9930001	9932000	2000	1	1.10E-07	0.44	16	0.8	Prkce;LOC102557388	Signaling
DMR6:9968001	6	9968001	9969000	1000	1	4.90E-07	0.57	12	1.2	Prkce	Signaling
DMR6:10390001	6	10390001	10393000	3000	1	3.70E-07	0.35	43	1.43	Epas1	Transcription
DMR6:10483001	6	10483001	10485000	2000	1	5.30E-11	0.77	46	2.3	Tmem247	
DMR6:10543001	6	10543001	10545000	2000	1	1.10E-09	-0.51	51	2.55	Rhoq	Signaling
DMR6:10883001	6	10883001	10884000	1000	1	2.80E-08	0.49	10	1	Mcfd2;LOC103692553	Transport
DMR6:10996001	6	10996001	10997000	1000	1	9.40E-08	0.35	22	2.2	Ttc7a;Mir3558	
DMR6:11069001	6	11069001	11072000	3000	1	3.10E-07	-0.45	32	1.07	LOC681766;Calm2	Signaling
DMR6:11242001	6	11242001	11244000	2000	1	1.20E-07	0.36	29	1.45	Msh2	Transcription
DMR6:11269001	6	11269001	11271000	2000	1	8.20E-08	-0.46	23	1.15	Msh2	Transcription
DMR6:11361001	6	11361001	11365000	4000	1	5.30E-16	0.99	143	3.58	Kcnk12	Transport
DMR6:11376001	6	11376001	11377000	1000	1	3.00E-15	0.95	23	2.3	Kcnk12	Transport
DMR6:11406001	6	11406001	11408000	2000	1	5.00E-07	0.32	24	1.2	Kcnk12	Transport
DMR6:11419001	6	11419001	11422000	3000	1	1.90E-07	0.38	62	2.07	Kcnk12;LOC103689957	Transport
DMR6:11485001	6	11485001	11486000	1000	1	2.40E-07	-0.35	15	1.5	Kcnk12	Transport
DMR6:11724001	6	11724001	11726000	2000	1	4.30E-08	-0.47	37	1.85	Fbxo11	Proteolysis
DMR6:12251001	6	12251001	12253000	2000	1	6.90E-08	0.59	19	0.95	Ppp1r21	
DMR6:12289001	6	12289001	12290000	1000	1	9.10E-07	-0.52	15	1.5	Ppp1r21	
DMR6:12302001	6	12302001	12303000	1000	1	1.70E-07	0.41	2	0.2	Ppp1r21	

DMR6:12333001	6	12333001	12336000	3000	1	5.10E-10	0.38	41	1.37	Ppp1r21;LOC103692558;Ston1	Transport
DMR6:12365001	6	12365001	12366000	1000	1	1.50E-09	0.46	24	2.4	Ston1	Transport
DMR6:12389001	6	12389001	12390000	1000	1	6.80E-07	-0.38	3	0.3	Ston1	Transport
DMR6:14237001	6	14237001	14238000	1000	1	5.30E-09	-0.43	5	0.5	Nrxn1	
DMR6:14260001	6	14260001	14262000	2000	1	1.20E-07	-0.45	18	0.9	Nrxn1	
DMR6:14389001	6	14389001	14390000	1000	1	1.50E-11	0.78	15	1.5	Nrxn1	
DMR6:14487001	6	14487001	14489000	2000	2	1.70E-10	0.58	38	1.9	Nrxn1;LOC102550556	
DMR6:14756001	6	14756001	14757000	1000	1	9.40E-09	0.5	13	1.3	Nrxn1;LOC103692561	
DMR6:14853001	6	14853001	14858000	5000	1	6.10E-08	-0.38	66	1.32	Nrxn1	
DMR6:14891001	6	14891001	14897000	6000	1	2.70E-08	-0.34	58	0.97	Nrxn1	
DMR6:14958001	6	14958001	14959000	1000	1	6.00E-07	0.34	7	0.7	Nrxn1	
DMR6:15016001	6	15016001	15020000	4000	1	9.20E-12	-0.33	32	0.8	Nrxn1	
DMR6:18998001	6	18998001	19003000	5000	1	9.30E-08	-0.31	36	0.72	Cwf19l2	
DMR6:21041001	6	21041001	21044000	3000	1	5.50E-09	-0.45	49	1.63	Fam98a	Translation
DMR6:21140001	6	21140001	21141000	1000	1	4.00E-09	-0.44	11	1.1	Rasgrp3;LOC102549280	Transcription
DMR6:21163001	6	21163001	21164000	1000	1	6.50E-08	0.47	11	1.1	Rasgrp3	Transcription
DMR6:21254001	6	21254001	21256000	2000	2	5.30E-08	-0.43	15	0.75	Ltbp1	Extracellular Matrix
DMR6:21392001	6	21392001	21394000	2000	1	5.00E-07	-0.72	16	0.8	Ltbp1	Extracellular Matrix
DMR6:21551001	6	21551001	21552000	1000	1	1.70E-10	0.56	29	2.9	Ltbp1	Extracellular Matrix
DMR6:21767001	6	21767001	21769000	2000	1	8.50E-07	0.39	23	1.15	Ttc27	
DMR6:22025001	6	22025001	22031000	6000	1	2.50E-07	-0.34	52	0.87	Birc6	
DMR6:22156001	6	22156001	22165000	9000	1	2.70E-08	-0.41	116	1.29	Nlrc4	
DMR6:22738001	6	22738001	22740000	2000	1	9.40E-11	-0.45	15	0.75	Alk	Receptor
DMR6:22817001	6	22817001	22818000	1000	1	3.00E-09	0.44	8	0.8	Alk	Receptor
DMR6:22891001	6	22891001	22893000	2000	1	3.60E-08	0.39	14	0.7	Alk	Receptor
DMR6:22902001	6	22902001	22903000	1000	1	4.10E-08	0.4	9	0.9	Alk	Receptor
DMR6:23045001	6	23045001	23050000	5000	2	1.30E-23	1.11	71	1.42	Alk	Receptor
DMR6:23194001	6	23194001	23196000	2000	1	3.60E-08	-0.47	23	1.15	Alk;LOC108351181	Receptor
DMR6:23199001	6	23199001	23202000	3000	2	6.10E-12	0.76	65	2.17	Alk;LOC108351181	Receptor
DMR6:23209001	6	23209001	23210000	1000	1	9.40E-10	0.69	21	2.1	Alk;LOC108351181	Receptor
DMR6:23223001	6	23223001	23226000	3000	1	1.10E-09	0.43	31	1.03	LOC108351181;Clip4	Transcription
DMR6:23316001	6	23316001	23317000	1000	1	4.10E-08	0.55	30	3	Clip4	Transcription
DMR6:23329001	6	23329001	23331000	2000	1	1.90E-07	-0.52	14	0.7	RGD1304963	
DMR6:23364001	6	23364001	23365000	1000	1	1.30E-07	0.32	17	1.7	Fam179a	
DMR6:23462001	6	23462001	23463000	1000	1	4.10E-07	0.45	27	2.7	Wdr43	
DMR6:23492001	6	23492001	23493000	1000	1	2.80E-07	-0.55	11	1.1	Trnac-gca;Spdya	
DMR6:23563001	6	23563001	23564000	1000	1	2.60E-08	-0.55	8	0.8	Ppp1cb	Signaling
DMR6:24144001	6	24144001	24147000	3000	1	2.80E-08	-0.47	71	2.37	Lbh	
DMR6:24436001	6	24436001	24437000	1000	1	4.00E-07	-0.57	10	1	Lclat1	Metabolism
DMR6:24485001	6	24485001	24489000	4000	1	2.20E-07	-0.3	32	0.8	Lclat1	Metabolism
DMR6:24570001	6	24570001	24571000	1000	1	4.80E-08	-0.66	18	1.8	LOC685881;Capn13	Protease
DMR6:24577001	6	24577001	24579000	2000	2	8.40E-11	0.82	39	1.95	Capn13	Protease
DMR6:24640001	6	24640001	24641000	1000	1	8.00E-07	0.52	19	1.9	Capn13;LOC102554046	Protease
DMR6:25100001	6	25100001	25101000	1000	1	7.60E-07	0.42	15	1.5	Ehd3	Transport
DMR6:25211001	6	25211001	25212000	1000	1	1.60E-09	-0.44	20	2	Xdh	Metabolism
DMR6:25430001	6	25430001	25432000	2000	1	5.00E-07	0.36	16	0.8	Plb1	Metabolism
DMR6:25700001	6	25700001	25703000	3000	1	1.40E-10	-0.4	20	0.67	Bre	
DMR6:25791001	6	25791001	25792000	1000	1	1.90E-07	0.41	15	1.5	Bre	
DMR6:25898001	6	25898001	25900000	2000	1	1.50E-08	-0.53	10	0.5	Bre	
DMR6:26384001	6	26384001	26385000	1000	1	1.80E-07	-0.39	11	1.1	Gckr;LOC100910821;Ift172	Signaling;Development
DMR6:26626001	6	26626001	26628000	2000	1	4.70E-08	-0.36	25	1.25	Trim54;Dnajc5g;Slc30a3	Proteolysis;Transcription;Transport
DMR6:26827001	6	26827001	26832000	5000	1	2.10E-08	0.49	56	1.12	Khk;Emilin1;LOC103692571;Ost4;Agbl5	Metabolism;Golgi;Protease
DMR6:26917001	6	26917001	26919000	2000	1	7.00E-09	0.42	15	0.75	Mapre3;LOC108351189	Cytoskeleton
DMR6:26922001	6	26922001	26923000	1000	1	8.60E-07	0.57	27	2.7	Mapre3;LOC108351189;LOC108351188	Cytoskeleton
DMR6:27195001	6	27195001	27198000	3000	1	6.50E-07	0.44	39	1.3	Kcnk3	Transport
DMR6:27235001	6	27235001	27237000	2000	1	8.60E-07	0.37	29	1.45	Cib4	
DMR6:27275001	6	27275001	27277000	2000	1	1.30E-08	0.61	43	2.15	Cib4	
DMR6:27279001	6	27279001	27282000	3000	1	1.40E-09	0.52	66	2.2	Cib4	
DMR6:27350001	6	27350001	27352000	2000	1	1.30E-07	-0.43	19	0.95	Otof	Transport
DMR6:27371001	6	27371001	27372000	1000	1	3.70E-07	0.4	13	1.3	Otof	Transport

DMR6:27408001	6	27408001	27409000	1000	1	5.40E-07	0.42	17	1.7	Otof	Transport
DMR6:27418001	6	27418001	27419000	1000	1	3.60E-08	0.37	11	1.1	Otof;Drc1	Transport
DMR6:27521001	6	27521001	27522000	1000	1	1.10E-09	-0.5	16	1.6	Seli	
DMR6:27690001	6	27690001	27691000	1000	1	7.00E-08	-0.62	9	0.9	Rab10	
DMR6:27731001	6	27731001	27735000	4000	1	1.10E-07	0.29	43	1.07	Rab10	
DMR6:27984001	6	27984001	27987000	3000	1	1.30E-08	-0.45	33	1.1	Dtnb	Proteolysis
DMR6:28043001	6	28043001	28044000	1000	1	1.30E-07	-0.42	15	1.5	Dtnb;LOC103692574	Proteolysis
DMR6:28086001	6	28086001	28089000	3000	1	3.90E-10	-0.39	42	1.4	Dtnb	Proteolysis
DMR6:28106001	6	28106001	28107000	1000	1	5.90E-07	-0.56	14	1.4	Dtnb	Proteolysis
DMR6:28148001	6	28148001	28149000	1000	1	4.60E-07	0.39	15	1.5	Dtnb	Proteolysis
DMR6:28408001	6	28408001	28409000	1000	1	4.40E-09	0.5	24	2.4	Efr3b	
DMR6:28453001	6	28453001	28455000	2000	1	4.60E-07	-0.55	27	1.35	Efr3b	
DMR6:28572001	6	28572001	28573000	1000	1	8.40E-13	0.77	42	4.2	Adcy3	
DMR6:28780001	6	28780001	28784000	4000	1	6.00E-07	-0.31	25	0.62	Ncoa1	Epigenetic
DMR6:28805001	6	28805001	28810000	5000	1	1.80E-09	-0.36	46	0.92	Ncoa1;LOC103692576	Epigenetic
DMR6:28825001	6	28825001	28828000	3000	3	1.20E-10	-0.42	24	0.8	Ncoa1;LOC103692576	Epigenetic
DMR6:28845001	6	28845001	28847000	2000	1	2.00E-07	-0.43	14	0.7	Ncoa1	Epigenetic
DMR6:28899001	6	28899001	28900000	1000	1	4.30E-08	-0.57	9	0.9	Ncoa1	Epigenetic
DMR6:29200001	6	29200001	29203000	3000	1	4.10E-07	0.39	38	1.27	Klhl29	Cytoskeleton
DMR6:29259001	6	29259001	29260000	1000	1	2.10E-07	0.4	6	0.6	Klhl29	Cytoskeleton
DMR6:29311001	6	29311001	29312000	1000	1	3.10E-07	0.37	7	0.7	Klhl29	Cytoskeleton
DMR6:29318001	6	29318001	29320000	2000	1	1.00E-06	-0.43	36	1.8	Klhl29	Cytoskeleton
DMR6:29386001	6	29386001	29387000	1000	1	7.00E-13	0.4	12	1.2	Klhl29;LOC102554916	Cytoskeleton
DMR6:29456001	6	29456001	29457000	1000	1	2.50E-07	0.45	7	0.7	Klhl29	Cytoskeleton
DMR6:30067001	6	30067001	30068000	1000	1	1.50E-07	-0.52	10	1	Fam228b;Fam228a	
DMR6:30113001	6	30113001	30114000	1000	1	3.30E-08	0.36	1	0.1	Itsn2	Transport
DMR6:33487001	6	33487001	33490000	3000	1	6.90E-07	0.3	37	1.23	Ldah;Gdf7	Metabolism;Growth Factors
DMR6:33814001	6	33814001	33815000	1000	1	2.90E-10	-0.57	10	1	Pum2	Metabolism
DMR6:33914001	6	33914001	33916000	2000	2	2.20E-11	0.47	22	1.1	Sdc1	Receptor
DMR6:34054001	6	34054001	34057000	3000	1	2.90E-07	-0.78	25	0.83	Laptm4a	Transport
DMR6:35316001	6	35316001	35317000	1000	1	4.60E-11	0.67	27	2.7	Osr1	Transcription
DMR6:35320001	6	35320001	35322000	2000	1	7.80E-07	0.55	72	3.6	Osr1	Transcription
DMR6:36958001	6	36958001	36960000	2000	1	2.10E-08	-0.45	18	0.9	Smc6	Transcription
DMR6:36969001	6	36969001	36971000	2000	1	5.40E-07	-0.64	16	0.8	Smc6	Transcription
DMR6:36974001	6	36974001	36976000	2000	1	2.60E-09	-0.61	20	1	Smc6	Transcription
DMR6:38013001	6	38013001	38016000	3000	1	2.70E-07	0.3	29	0.97	RGD1565679	
DMR6:38214001	6	38214001	38215000	1000	1	6.40E-12	0.48	16	1.6	Mycn	Transcription
DMR6:38223001	6	38223001	38225000	2000	1	6.70E-07	-0.41	68	3.4	Mycn	Transcription
DMR6:38461001	6	38461001	38466000	5000	1	1.20E-07	-0.44	34	0.68	Ddx1;Nbas	
DMR6:38533001	6	38533001	38536000	3000	1	2.40E-07	-0.43	24	0.8	Nbas	
DMR6:38573001	6	38573001	38577000	4000	2	8.60E-08	-0.38	45	1.12	Nbas;LOC108351202	
DMR6:38727001	6	38727001	38729000	2000	1	6.50E-10	-0.48	31	1.55	Nbas	
DMR6:38772001	6	38772001	38775000	3000	2	2.50E-07	-0.33	20	0.67	Nbas	
DMR6:41899001	6	41899001	41900000	1000	1	1.50E-07	-0.46	18	1.8	Lpin1	
DMR6:42303001	6	42303001	42305000	2000	1	4.10E-10	0.57	25	1.25	Pqlc3;LOC690276	
DMR6:42464001	6	42464001	42466000	2000	1	8.40E-08	0.47	18	0.9	Kcnf1	Transport
DMR6:42700001	6	42700001	42701000	1000	1	2.10E-07	-0.46	11	1.1	Nol10	
DMR6:42708001	6	42708001	42709000	1000	1	3.70E-10	-0.54	20	2	Nol10	
DMR6:42950001	6	42950001	42952000	2000	1	4.20E-07	0.34	27	1.35	LOC100909414;RGD1563157	
DMR6:43045001	6	43045001	43050000	5000	1	2.50E-08	0.38	66	1.32	Hpcal1	
DMR6:43080001	6	43080001	43082000	2000	1	4.60E-10	0.4	29	1.45	Hpcal1	
DMR6:43444001	6	43444001	43446000	2000	1	2.80E-07	-0.42	27	1.35	Adam17	Protease
DMR6:43639001	6	43639001	43640000	1000	1	7.70E-08	0.42	13	1.3	Taf1b	
DMR6:44126001	6	44126001	44127000	1000	1	1.60E-07	-0.45	24	2.4	Mboat2	Metabolism
DMR6:44145001	6	44145001	44149000	4000	2	1.50E-07	-0.34	39	0.98	Mboat2;LOC103692604	Metabolism
DMR6:45543001	6	45543001	45544000	1000	1	6.20E-07	0.53	11	1.1	Rnf144a	Proteolysis
DMR6:45549001	6	45549001	45554000	5000	1	1.40E-07	-0.36	90	1.8	Rnf144a	Proteolysis
DMR6:45575001	6	45575001	45577000	2000	1	3.40E-08	0.39	8	0.4	Rnf144a	Proteolysis
DMR6:45586001	6	45586001	45589000	3000	1	2.30E-09	-0.51	39	1.3	Rnf144a	Proteolysis
DMR6:47817001	6	47817001	47821000	4000	2	3.40E-08	-0.41	35	0.88	Dcdc2c;Allc	
DMR6:48069001	6	48069001	48070000	1000	1	1.80E-07	0.36	11	1.1	Tssc1	
DMR6:49029001	6	49029001	49036000	7000	1	9.10E-07	-0.3	63	0.9	Tpo	Metabolism

DMR6:49052001	6	49052001	49057000	5000	2	2.70E-10	0.41	37	0.74	Tpo	Metabolism
DMR6:49090001	6	49090001	49092000	2000	1	4.10E-10	0.55	11	0.55	Tpo	Metabolism
DMR6:49265001	6	49265001	49266000	1000	1	2.10E-10	-0.45	7	0.7	Sntg2	
DMR6:49885001	6	49885001	49887000	2000	1	5.20E-07	-0.44	12	0.6	Sh3yl1	Cytoskeleton
DMR6:50731001	6	50731001	50732000	1000	1	5.10E-09	0.4	10	1	Slc26a3	Transport
DMR6:50758001	6	50758001	50760000	2000	1	1.00E-07	0.36	17	0.85	Slc26a3	Transport
DMR6:50802001	6	50802001	50804000	2000	1	2.20E-09	-0.47	25	1.25	LOC102555378;Slc26a4	Transport
DMR6:50832001	6	50832001	50834000	2000	1	1.70E-08	0.38	16	0.8	Slc26a4	Transport
DMR6:50856001	6	50856001	50857000	1000	1	5.90E-09	-0.41	3	0.3	Slc26a4;LOC108351219	Transport
DMR6:50900001	6	50900001	50906000	6000	1	1.30E-09	-0.41	57	0.95	Bcap29	Transport
DMR6:51074001	6	51074001	51075000	1000	1	6.70E-07	-0.45	12	1.2	Cog5	
DMR6:51242001	6	51242001	51243000	1000	1	6.90E-07	-0.47	9	0.9	Hbp1;LOC102555645	
DMR6:51280001	6	51280001	51285000	5000	1	1.80E-09	-0.46	66	1.32	Prkar2b	Signaling
DMR6:51480001	6	51480001	51482000	2000	1	2.50E-10	0.69	27	1.35	Pik3cg	Signaling
DMR6:52160001	6	52160001	52161000	1000	1	3.70E-07	-0.34	18	1.8	Nampt	Transport
DMR6:52480001	6	52480001	52481000	1000	1	6.70E-08	-0.47	18	1.8	Atxn7l1	
DMR6:52504001	6	52504001	52505000	1000	1	8.90E-09	-0.41	7	0.7	Atxn7l1	
DMR6:52591001	6	52591001	52592000	1000	1	2.90E-07	0.34	9	0.9	Atxn7l1;LOC102549100	
DMR6:52604001	6	52604001	52610000	6000	2	1.80E-10	-0.49	97	1.62	Atxn7l1;LOC102549100	
DMR6:52628001	6	52628001	52630000	2000	1	1.60E-07	-0.47	25	1.25	Atxn7l1	
DMR6:53396001	6	53396001	53398000	2000	1	7.10E-08	-0.45	20	1	Twist1	Transcription
DMR6:53550001	6	53550001	53552000	2000	1	2.10E-08	-0.5	18	0.9	Hdac9	
DMR6:53704001	6	53704001	53705000	1000	1	8.30E-08	0.47	4	0.4	Hdac9	
DMR6:53758001	6	53758001	53759000	1000	1	2.30E-07	0.52	7	0.7	Hdac9	
DMR6:53899001	6	53899001	53901000	2000	2	2.60E-08	-0.47	38	1.9	Hdac9	
DMR6:54065001	6	54065001	54067000	2000	1	2.20E-08	-0.51	22	1.1	Hdac9	
DMR6:54111001	6	54111001	54112000	1000	1	1.10E-07	-0.44	9	0.9	Hdac9	
DMR6:54406001	6	54406001	54414000	8000	1	5.00E-08	-0.42	67	0.84	Prps1l1	Signaling
DMR6:54502001	6	54502001	54503000	1000	1	8.00E-08	-0.35	11	1.1	Snx13	Cytoskeleton
DMR6:55599001	6	55599001	55600000	1000	1	8.60E-08	-0.53	34	3.4	Bzw2	Transcription
DMR6:55611001	6	55611001	55614000	3000	1	8.30E-08	0.52	25	0.83	Bzw2	Transcription
DMR6:55649001	6	55649001	55651000	2000	1	1.00E-10	-0.6	29	1.45	Bzw2;Ankmy2;LOC102556505	Transcription
DMR6:55826001	6	55826001	55828000	2000	1	7.40E-08	0.39	18	0.9	Sostdc1	Signaling
DMR6:56095001	6	56095001	56102000	7000	1	1.20E-11	-0.44	80	1.14	Ispd	
DMR6:56105001	6	56105001	56110000	5000	1	3.80E-07	0.51	30	0.6	Ispd	
DMR6:56614001	6	56614001	56617000	3000	1	9.60E-09	-0.33	27	0.9	Meox2	Development
DMR6:56723001	6	56723001	56727000	4000	1	8.50E-09	-0.35	33	0.82	Vom2r49	Signaling
DMR6:57039001	6	57039001	57040000	1000	1	3.20E-12	-0.46	10	1	Agmo	
DMR6:57167001	6	57167001	57169000	2000	1	1.90E-07	0.34	10	0.5	Agmo	
DMR6:57548001	6	57548001	57552000	4000	1	1.90E-09	-0.62	32	0.8	Dgkb	Signaling
DMR6:57567001	6	57567001	57573000	6000	1	2.00E-08	-0.35	54	0.9	Dgkb	Signaling
DMR6:57598001	6	57598001	57600000	2000	1	1.80E-07	-0.46	18	0.9	Dgkb;LOC102549779	Signaling
DMR6:57620001	6	57620001	57622000	2000	1	1.10E-07	0.4	10	0.5	Dgkb	Signaling
DMR6:57710001	6	57710001	57711000	1000	1	1.30E-08	-0.53	7	0.7	Dgkb;LOC103692620	Signaling
DMR6:57740001	6	57740001	57742000	2000	1	8.60E-08	-0.5	27	1.35	Dgkb	Signaling
DMR6:57760001	6	57760001	57761000	1000	1	8.90E-07	0.39	12	1.2	Dgkb	Signaling
DMR6:57881001	6	57881001	57883000	2000	1	7.30E-07	-0.46	5	0.25	Dgkb	Signaling
DMR6:57916001	6	57916001	57919000	3000	1	5.90E-07	0.43	36	1.2	Dgkb	Signaling
DMR6:57972001	6	57972001	57978000	6000	1	7.30E-07	-0.21	62	1.03	Dgkb	Signaling
DMR6:57987001	6	57987001	57992000	5000	3	2.40E-10	-0.34	40	0.8	Dgkb	Signaling
DMR6:58193001	6	58193001	58200000	7000	1	2.00E-09	-0.37	61	0.87	Dgkb	Signaling
DMR6:58264001	6	58264001	58270000	6000	1	1.60E-09	-0.27	56	0.93	Dgkb	Signaling
DMR6:60245001	6	60245001	60246000	1000	1	6.20E-07	0.41	7	0.7	Zfp277	Transcription
DMR6:60285001	6	60285001	60287000	2000	1	5.50E-08	0.34	25	1.25	Zfp277	Transcription
DMR6:60296001	6	60296001	60297000	1000	1	2.90E-12	0.63	10	1	Zfp277	Transcription
DMR6:60537001	6	60537001	60539000	2000	1	1.40E-07	-0.69	21	1.05	Dock4	Transcription
DMR6:61033001	6	61033001	61035000	2000	1	6.70E-07	0.36	20	1	Immp2l;LOC102552766	
DMR6:61206001	6	61206001	61209000	3000	1	4.00E-09	0.36	31	1.03	Immp2l	
DMR6:61299001	6	61299001	61302000	3000	1	9.10E-07	0.33	37	1.23	Immp2l	
DMR6:61534001	6	61534001	61536000	2000	1	5.80E-11	-0.57	39	1.95	Immp2l	
DMR6:61549001	6	61549001	61550000	1000	1	6.00E-07	-0.34	15	1.5	Immp2l	
DMR6:61568001	6	61568001	61570000	2000	1	1.10E-12	0.54	15	0.75	Immp2l	
DMR6:61609001	6	61609001	61610000	1000	1	5.70E-11	0.79	20	2	Immp2l	

DMR6:64244001	6	64244001	64246000	2000	1	1.50E-08	-0.38	20	1	Pnpla8	Metabolism
DMR6:64471001	6	64471001	64477000	6000	1	1.90E-07	-0.28	75	1.25	Nrcam	Cytoskeleton
DMR6:64803001	6	64803001	64807000	4000	1	1.90E-09	-0.4	16	0.4	Nrcam	Cytoskeleton
DMR6:65198001	6	65198001	65199000	1000	1	4.00E-07	-0.52	8	0.8	Stxbp6	Transcription
DMR6:66980001	6	66980001	66981000	1000	1	5.70E-10	0.61	7	0.7	Nova1	Metabolism
DMR6:67089001	6	67089001	67091000	2000	1	1.60E-07	0.53	22	1.1	Nova1	Metabolism
DMR6:71211001	6	71211001	71216000	5000	2	7.90E-19	0.39	43	0.86	Prkd1	Signaling
DMR6:71273001	6	71273001	71276000	3000	1	4.30E-09	-0.62	27	0.9	Prkd1	Signaling
DMR6:71327001	6	71327001	71330000	3000	1	2.30E-07	0.48	31	1.03	Prkd1	Signaling
DMR6:72441001	6	72441001	72442000	1000	1	2.20E-10	-0.5	12	1.2	Strn3	
DMR6:72500001	6	72500001	72501000	1000	1	5.40E-07	-0.55	12	1.2	Ap4s1;Hectd1	Transport;Proteolysis
DMR6:72517001	6	72517001	72518000	1000	1	1.20E-09	-0.53	19	1.9	Hectd1	Proteolysis
DMR6:72604001	6	72604001	72607000	3000	1	7.80E-09	0.41	43	1.43	Hectd1	Proteolysis
DMR6:72689001	6	72689001	72695000	6000	3	8.60E-11	-0.45	56	0.93	Heatr5a	
DMR6:72801001	6	72801001	72803000	2000	1	1.00E-07	-0.31	27	1.35	Gpr33	Signaling
DMR6:72896001	6	72896001	72897000	1000	1	7.40E-07	0.34	27	2.7	Nubpl	
DMR6:73046001	6	73046001	73049000	3000	2	2.00E-07	0.49	30	1	Nubpl	
DMR6:73337001	6	73337001	73341000	4000	1	7.00E-08	0.44	69	1.73	Arhgap5	Signaling
DMR6:73639001	6	73639001	73641000	2000	1	1.00E-07	-0.39	26	1.3	Akap6	
DMR6:73652001	6	73652001	73653000	1000	1	1.50E-08	0.82	27	2.7	Akap6	
DMR6:73695001	6	73695001	73697000	2000	1	7.90E-09	-0.49	36	1.8	Akap6	
DMR6:73734001	6	73734001	73735000	1000	1	4.30E-08	0.47	31	3.1	Akap6	
DMR6:73753001	6	73753001	73754000	1000	1	3.00E-08	-0.55	10	1	Akap6	
DMR6:73878001	6	73878001	73880000	2000	1	4.00E-07	-0.38	21	1.05	Akap6	
DMR6:73930001	6	73930001	73936000	6000	1	4.10E-10	-0.34	53	0.88	Akap6	
DMR6:74238001	6	74238001	74239000	1000	1	1.40E-08	-0.45	8	0.8	Npas3;LOC102547735	
DMR6:74415001	6	74415001	74417000	2000	1	2.00E-07	-0.42	35	1.75	Npas3	
DMR6:74433001	6	74433001	74436000	3000	1	3.20E-10	-0.51	46	1.53	Npas3	
DMR6:74670001	6	74670001	74671000	1000	1	9.30E-10	0.42	5	0.5	Npas3	
DMR6:75050001	6	75050001	75052000	2000	1	4.60E-11	-0.54	47	2.35	Egln3	
DMR6:75434001	6	75434001	75435000	1000	1	3.10E-09	0.55	14	1.4	Rps10l1	Translation
DMR6:75529001	6	75529001	75531000	2000	2	2.10E-11	-0.46	37	1.85	Sptssa	Golgi
DMR6:75624001	6	75624001	75626000	2000	1	3.10E-09	0.72	78	3.9	LOC102548522;Snx6	Cytoskeleton
DMR6:75772001	6	75772001	75775000	3000	1	8.30E-08	-0.36	49	1.63	Cfl2	Cytoskeleton
DMR6:76129001	6	76129001	76130000	1000	1	8.30E-09	-0.6	14	1.4	RGD1305089	
DMR6:76131001	6	76131001	76132000	1000	1	3.10E-07	0.37	12	1.2	RGD1305089	
DMR6:76359001	6	76359001	76360000	1000	1	1.80E-09	0.62	14	1.4	Aldoart2	Metabolism
DMR6:76593001	6	76593001	76595000	2000	1	3.50E-08	-0.39	18	0.9	Ralgapa1	Signaling
DMR6:77417001	6	77417001	77418000	1000	1	4.50E-12	-0.5	11	1.1	LOC100911923;Nkx2-1;LOC102552092	Development
DMR6:77626001	6	77626001	77628000	2000	1	7.20E-07	-0.34	29	1.45	Pax9;Slc25a21	Transport
DMR6:77689001	6	77689001	77694000	5000	1	3.30E-07	-0.35	47	0.94	Slc25a21;LOC102554460	Transport
DMR6:77744001	6	77744001	77745000	1000	1	2.40E-08	0.75	19	1.9	Slc25a21;LOC108351240	Transport
DMR6:77809001	6	77809001	77812000	3000	1	6.00E-07	0.28	29	0.97	Slc25a21	Transport
DMR6:77836001	6	77836001	77841000	5000	2	1.50E-08	-0.27	47	0.94	Slc25a21	Transport
DMR6:77897001	6	77897001	77899000	2000	1	3.60E-09	0.71	36	1.8	Slc25a21;LOC103692639	Transport
DMR6:77923001	6	77923001	77925000	2000	1	3.00E-07	0.39	26	1.3	Slc25a21	Transport
DMR6:77964001	6	77964001	77966000	2000	1	1.20E-11	-0.61	11	0.55	Slc25a21	Transport
DMR6:77976001	6	77976001	77979000	3000	1	2.30E-07	-0.38	26	0.87	Slc25a21	Transport
DMR6:77998001	6	77998001	77999000	1000	1	4.30E-10	0.51	10	1	Slc25a21	Transport
DMR6:78100001	6	78100001	78103000	3000	1	1.20E-13	0.43	28	0.93	Slc25a21;LOC102552151	Transport
DMR6:78205001	6	78205001	78211000	6000	2	5.20E-10	-0.4	58	0.97	Mipol1	
DMR6:78359001	6	78359001	78366000	7000	1	1.70E-08	-0.55	82	1.17	Mipol1	
DMR6:78454001	6	78454001	78456000	2000	1	9.40E-07	-0.44	26	1.3	Mipol1	
DMR6:78544001	6	78544001	78546000	2000	1	4.60E-07	-0.65	32	1.6	Foxa1	Transcription
DMR6:78750001	6	78750001	78754000	4000	1	7.20E-08	-0.47	29	0.72	RGD1560556	
DMR6:78782001	6	78782001	78787000	5000	2	8.00E-09	-0.42	37	0.74	RGD1560556	
DMR6:79251001	6	79251001	79253000	2000	1	2.60E-07	0.41	12	0.6	Sstr1	Signaling
DMR6:80067001	6	80067001	80069000	2000	1	1.30E-09	0.91	95	4.75	Sec23a;LOC102553646	Transport
DMR6:80087001	6	80087001	80090000	3000	1	3.30E-09	-0.48	40	1.33	Sec23a	Transport
DMR6:80161001	6	80161001	80162000	1000	1	2.10E-09	-0.47	19	1.9	Trappc6b;Pnn	
DMR6:80292001	6	80292001	80293000	1000	1	6.40E-08	-0.47	2	0.2	LOC100912115;Fbxo33	
DMR6:81052001	6	81052001	81056000	4000	1	5.10E-07	0.62	45	1.12	RGD1563850	

DMR6:83426001	6	83426001	83427000	1000	1	5.40E-07	0.48	14	1.4	Lrfn5	
DMR6:86690001	6	86690001	86691000	1000	1	6.30E-10	-0.45	5	0.5	Klhl28	Cytoskeleton
DMR6:86752001	6	86752001	86753000	1000	1	7.90E-07	-0.46	8	0.8	Fam179b	
DMR6:86805001	6	86805001	86806000	1000	1	6.70E-08	-0.68	11	1.1	Prpf39;Fkbp3	Translation;Transcription
DMR6:88511001	6	88511001	88515000	4000	1	1.60E-10	-0.31	32	0.8	Mdga2	
DMR6:88567001	6	88567001	88574000	7000	2	1.30E-09	-0.38	78	1.11	Mdga2;LOC108351351	
DMR6:88605001	6	88605001	88606000	1000	1	2.60E-08	0.55	10	1	Mdga2	
DMR6:88612001	6	88612001	88613000	1000	1	3.20E-16	0.65	27	2.7	Mdga2	
DMR6:88720001	6	88720001	88721000	1000	1	7.30E-07	-0.57	6	0.6	Mdga2	
DMR6:88725001	6	88725001	88727000	2000	1	2.00E-07	-0.54	18	0.9	Mdga2	
DMR6:88750001	6	88750001	88751000	1000	1	8.60E-08	0.4	13	1.3	Mdga2	
DMR6:88990001	6	88990001	88992000	2000	1	2.60E-07	-0.58	17	0.85	Mdga2	
DMR6:89021001	6	89021001	89022000	1000	1	1.40E-07	-0.59	4	0.4	Mdga2	
DMR6:91457001	6	91457001	91460000	3000	1	3.80E-08	-1.52	49	1.63	Rps29;Rn7sl1;Lrr1	Translation;Cytoskeleton
DMR6:91556001	6	91556001	91557000	1000	1	1.40E-09	0.37	2	0.2	Klhdc1	
DMR6:91680001	6	91680001	91681000	1000	1	3.40E-09	-2.24	53	5.3	Nemf	
DMR6:91688001	6	91688001	91690000	2000	1	9.40E-07	-0.47	29	1.45	Arf6	Signaling
DMR6:91893001	6	91893001	91895000	2000	1	1.30E-08	0.43	27	1.35	Vcpkmt;Sos2	Transcription
DMR6:91990001	6	91990001	91992000	2000	1	6.60E-11	-0.54	25	1.25	Sos2;LOC103692654	Transcription
DMR6:92021001	6	92021001	92028000	7000	1	9.60E-07	-0.4	55	0.79	LOC103692650;L2hgdh	Metabolism
DMR6:92101001	6	92101001	92102000	1000	1	3.30E-08	0.37	12	1.2	Cdkl1;LOC102548655	Signaling
DMR6:92339001	6	92339001	92340000	1000	1	8.80E-08	-0.39	10	1	Atl1	Signaling
DMR6:92631001	6	92631001	92634000	3000	1	1.20E-08	-0.43	93	3.1	Pygl	Golgi
DMR6:92644001	6	92644001	92647000	3000	1	5.30E-07	0.64	51	1.7	Pygl	Golgi
DMR6:93321001	6	93321001	93326000	5000	1	3.00E-08	-0.49	75	1.5	Frmd6	
DMR6:93513001	6	93513001	93523000	10000	1	3.40E-09	-0.48	188	1.88	Arid4a	Transcription
DMR6:94493001	6	94493001	94496000	3000	1	3.50E-12	-0.61	34	1.13	Ppm1a;LOC108351251	
DMR6:94640001	6	94640001	94642000	2000	1	1.40E-10	-0.51	25	1.25	Daam1	
DMR6:94735001	6	94735001	94738000	3000	1	1.70E-08	0.72	65	2.17	Daam1;LOC108351252	
DMR6:94853001	6	94853001	94854000	1000	1	5.70E-07	0.33	12	1.2	Jkamp;Ccdc175	Cytoskeleton
DMR6:94870001	6	94870001	94871000	1000	1	3.80E-07	-0.36	17	1.7	Ccdc175	
DMR6:94964001	6	94964001	94967000	3000	1	4.60E-08	0.67	55	1.83	Rtn1	
DMR6:95020001	6	95020001	95022000	2000	1	1.70E-10	0.51	13	0.65	Rtn1	
DMR6:95041001	6	95041001	95042000	1000	1	9.70E-07	-0.47	13	1.3	Rtn1	
DMR6:95120001	6	95120001	95121000	1000	1	2.90E-08	-0.52	9	0.9	Rtn1	
DMR6:95225001	6	95225001	95228000	3000	1	3.40E-07	0.35	14	0.47	Lrrc9	
DMR6:95311001	6	95311001	95313000	2000	1	6.50E-09	-0.47	28	1.4	LOC102549502;Pcnx4	
DMR6:95365001	6	95365001	95366000	1000	1	8.50E-07	0.29	14	1.4	LOC102549502;Pcnx4	
DMR6:95411001	6	95411001	95415000	4000	2	1.10E-09	-0.58	48	1.2	Pcnx4;Dhrs7	Metabolism
DMR6:95428001	6	95428001	95431000	3000	1	7.60E-15	-0.59	61	2.03	Pcnx4;Dhrs7	Metabolism
DMR6:95983001	6	95983001	95984000	1000	1	5.80E-08	0.4	12	1.2	LOC690390;Six4	Development
DMR6:96038001	6	96038001	96041000	3000	1	6.70E-07	-0.5	32	1.07	Mnat1	Transcription
DMR6:96138001	6	96138001	96140000	2000	1	1.10E-12	-0.69	18	0.9	Mnat1	Transcription
DMR6:96144001	6	96144001	96146000	2000	1	1.80E-10	-0.45	17	0.85	Mnat1	Transcription
DMR6:96201001	6	96201001	96203000	2000	1	1.20E-10	-0.49	33	1.65	Slc38a6;LOC688981	Transport;Translation
DMR6:96435001	6	96435001	96438000	3000	1	1.70E-10	0.35	30	1	Tmem30b	
DMR6:96446001	6	96446001	96447000	1000	1	5.40E-07	-0.48	12	1.2	Tmem30b	
DMR6:96507001	6	96507001	96509000	2000	1	1.10E-07	-0.43	34	1.7	Prkch	Signaling
DMR6:96652001	6	96652001	96653000	1000	1	9.70E-08	0.4	14	1.4	Prkch;LOC108351320	Signaling
DMR6:96860001	6	96860001	96862000	2000	1	2.70E-09	0.63	35	1.75	Hif1a;Snapc1	Transcription
DMR6:96869001	6	96869001	96870000	1000	1	9.10E-08	0.41	2	0.2	Snapc1;LOC102550623	
DMR6:97122001	6	97122001	97123000	1000	1	1.50E-08	0.52	8	0.8	Syt16;LOC102550910	
DMR6:97131001	6	97131001	97133000	2000	1	9.80E-09	0.72	43	2.15	Syt16;LOC102550910	
DMR6:97935001	6	97935001	97937000	2000	1	9.40E-07	-0.35	24	1.2	Kcnh5	Transport
DMR6:97945001	6	97945001	97947000	2000	1	6.30E-07	0.47	17	0.85	Kcnh5	Transport
DMR6:98001001	6	98001001	98002000	1000	1	5.70E-07	0.42	9	0.9	Kcnh5	Transport
DMR6:98357001	6	98357001	98359000	2000	1	2.50E-07	-0.75	39	1.95	Rhoj	Signaling
DMR6:98374001	6	98374001	98375000	1000	1	1.90E-09	0.44	8	0.8	Rhoj;Gphb5	Signaling;Hormone
DMR6:98391001	6	98391001	98392000	1000	1	2.00E-07	-0.45	8	0.8	Gphb5	Hormone
DMR6:98421001	6	98421001	98423000	2000	1	4.70E-08	-0.58	40	2	Ppp2r5e	Signaling
DMR6:98450001	6	98450001	98453000	3000	1	3.70E-11	-0.53	40	1.33	Ppp2r5e;LOC108351261	Signaling
DMR6:98577001	6	98577001	98579000	2000	1	5.50E-09	0.49	37	1.85	Ppp2r5e	Signaling
DMR6:99013001	6	99013001	99015000	2000	1	6.90E-08	-0.46	49	2.45	Syne2	

DMR6:99054001	6	99054001	99058000	4000	1	3.40E-09	-0.45	42	1.05	Syne2	
DMR6:99093001	6	99093001	99097000	4000	1	9.30E-07	-0.43	81	2.02	Syne2	
DMR6:99205001	6	99205001	99207000	2000	1	3.90E-09	0.45	25	1.25	Esr2	
DMR6:99411001	6	99411001	99412000	1000	1	6.30E-07	-0.38	19	1.9	Zbtb1;LOC108351262	Transcription
DMR6:99414001	6	99414001	99417000	3000	1	1.70E-07	-0.44	89	2.97	Zbtb1;LOC108351262	Transcription
DMR6:99625001	6	99625001	99627000	2000	1	2.00E-07	-0.44	34	1.7	Plekhg3	
DMR6:99777001	6	99777001	99782000	5000	1	6.80E-07	-0.47	55	1.1	Sptb	
DMR6:99784001	6	99784001	99785000	1000	1	9.20E-10	0.71	20	2	Sptb	
DMR6:99912001	6	99912001	99913000	1000	1	9.70E-08	-0.41	18	1.8	Fntb	Metabolism
DMR6:99920001	6	99920001	99922000	2000	1	6.10E-07	-0.52	15	0.75	Fntb	Metabolism
DMR6:100375001	6	100375001	100381000	6000	1	1.90E-07	-0.31	49	0.82	Fut8	Golgi
DMR6:100476001	6	100476001	100481000	5000	1	5.30E-07	-0.26	51	1.02	Fut8	Golgi
DMR6:101281001	6	101281001	101284000	3000	1	6.90E-07	0.46	86	2.87	RGD1562540	
DMR6:101294001	6	101294001	101299000	5000	3	2.70E-08	-0.3	52	1.04	RGD1562540	
DMR6:101303001	6	101303001	101304000	1000	1	3.70E-07	0.54	15	1.5	RGD1562540	
DMR6:101344001	6	101344001	101345000	1000	1	2.40E-07	-0.67	5	0.5	Gphn	
DMR6:101354001	6	101354001	101355000	1000	1	4.30E-07	-0.37	5	0.5	Gphn	
DMR6:101401001	6	101401001	101402000	1000	1	1.60E-09	-0.74	7	0.7	Gphn;LOC108351264;LOC102547341	
DMR6:101404001	6	101404001	101408000	4000	1	1.40E-09	-0.54	29	0.72	Gphn;LOC108351264;LOC102547341	
DMR6:101704001	6	101704001	101707000	3000	1	1.40E-08	-0.38	26	0.87	Gphn	
DMR6:101729001	6	101729001	101730000	1000	1	3.20E-07	0.48	7	0.7	Gphn	
DMR6:101799001	6	101799001	101800000	1000	1	3.40E-07	0.42	7	0.7	Gphn	
DMR6:101815001	6	101815001	101820000	5000	1	2.70E-08	-0.3	37	0.74	Gphn	
DMR6:101821001	6	101821001	101827000	6000	1	1.50E-08	-0.48	59	0.98	Gphn	
DMR6:101904001	6	101904001	101905000	1000	1	2.80E-07	-0.37	15	1.5	Fam71d	
DMR6:101973001	6	101973001	101974000	1000	1	3.00E-11	-0.57	13	1.3	Mpp5	Cytoskeleton
DMR6:102002001	6	102002001	102006000	4000	2	1.70E-07	-0.72	30	0.75	Mpp5	Cytoskeleton
DMR6:102032001	6	102032001	102034000	2000	1	6.90E-08	-0.58	21	1.05	Mpp5;Atp6v1d	Cytoskeleton;Metabolism
DMR6:102247001	6	102247001	102248000	1000	1	8.70E-07	0.5	16	1.6	Plekhh1;LOC102547892	
DMR6:102494001	6	102494001	102498000	4000	1	7.60E-07	-0.28	26	0.65	Rad51b	Transcription
DMR6:102522001	6	102522001	102524000	2000	1	7.20E-07	0.42	13	0.65	Rad51b	Transcription
DMR6:102627001	6	102627001	102628000	1000	1	7.20E-07	-0.41	9	0.9	Rad51b	Transcription
DMR6:102696001	6	102696001	102699000	3000	1	8.80E-07	0.39	32	1.07	Rad51b;LOC102546422	Transcription
DMR6:102733001	6	102733001	102736000	3000	1	3.30E-08	-0.36	25	0.83	Rad51b	Transcription
DMR6:102742001	6	102742001	102743000	1000	1	2.60E-08	-0.44	15	1.5	Rad51b	Transcription
DMR6:102839001	6	102839001	102840000	1000	1	2.40E-10	0.58	7	0.7	Rad51b	Transcription
DMR6:103007001	6	103007001	103009000	2000	1	5.50E-09	0.48	21	1.05	Rad51b	Transcription
DMR6:103022001	6	103022001	103023000	1000	1	7.30E-08	0.47	15	1.5	Rad51b	Transcription
DMR6:103457001	6	103457001	103459000	2000	1	1.90E-07	0.55	7	0.35	Actn1;LOC100359669	
DMR6:103553001	6	103553001	103554000	1000	1	8.60E-09	-0.43	17	1.7	Dcaf5	Proteolysis
DMR6:103568001	6	103568001	103569000	1000	1	4.90E-07	-0.55	13	1.3	Dcaf5;Scarna3	Proteolysis
DMR6:103640001	6	103640001	103641000	1000	1	4.50E-09	0.85	42	4.2	RGD1562299;RGD1561024	
DMR6:103820001	6	103820001	103821000	1000	1	1.50E-08	0.6	17	1.7	LOC100911289;RGD1565752;Mphosph6-ps1;LOC103692709;LOC685142	
DMR6:103824001	6	103824001	103826000	2000	1	1.10E-08	0.62	30	1.5	LOC100911289;RGD1565752;Mphosph6-ps1;LOC103692709;LOC685142	
DMR6:104053001	6	104053001	104055000	2000	1	5.10E-08	-0.5	19	0.95	Exd2	
DMR6:104234001	6	104234001	104235000	1000	1	7.20E-09	-0.44	10	1	Galnt16	Golgi
DMR6:104315001	6	104315001	104316000	1000	1	5.80E-07	-0.53	8	0.8	Slc39a9	Transport
DMR6:104347001	6	104347001	104350000	3000	2	2.60E-07	0.53	26	0.87	Plekhd1	Cytoskeleton
DMR6:104526001	6	104526001	104527000	1000	1	1.40E-07	0.44	17	1.7	Susd6	
DMR6:104612001	6	104612001	104616000	4000	1	5.70E-09	-0.45	78	1.95	Srsf5;Slc10a1	Translation;Transport
DMR6:104735001	6	104735001	104737000	2000	1	2.10E-08	0.41	23	1.15	Smoc1	Signaling
DMR6:105058001	6	105058001	105059000	1000	1	3.60E-11	-0.51	19	1.9	Slc8a3	Transport
DMR6:105162001	6	105162001	105165000	3000	1	2.20E-08	-0.48	15	0.5	Adam4	Protease
DMR6:105324001	6	105324001	105327000	3000	1	2.50E-07	-0.59	23	0.77	Med6	Transcription
DMR6:105388001	6	105388001	105390000	2000	1	2.60E-07	0.46	25	1.25	Ttc9	Transcription
DMR6:105522001	6	105522001	105523000	1000	1	2.80E-07	0.42	12	1.2	Map3k9;LOC102552601	Signaling

DMR6:105647001	6	105647001	105650000	3000	1	1.20E-09	-0.63	29	0.97	Pcnx1	
DMR6:105698001	6	105698001	105700000	2000	1	3.50E-08	-0.44	27	1.35	Pcnx1	
DMR6:106316001	6	106316001	106319000	3000	1	9.90E-10	0.35	28	0.93	Rgs6	
DMR6:106408001	6	106408001	106411000	3000	1	4.00E-08	-0.45	14	0.47	Rgs6	
DMR6:106491001	6	106491001	106492000	1000	1	1.60E-15	0.63	9	0.9	Rgs6	
DMR6:106683001	6	106683001	106686000	3000	1	2.70E-08	0.66	65	2.17	Dpf3	Epigenetic
DMR6:106708001	6	106708001	106709000	1000	1	8.60E-08	0.34	7	0.7	Dpf3	Epigenetic
DMR6:106872001	6	106872001	106873000	1000	1	7.70E-07	0.38	14	1.4	Dpf3	Epigenetic
DMR6:106897001	6	106897001	106898000	1000	1	2.00E-09	0.4	18	1.8	Dpf3	Epigenetic
DMR6:106977001	6	106977001	106978000	1000	1	3.70E-13	0.71	25	2.5	Dpf3	Epigenetic
DMR6:106992001	6	106992001	106993000	1000	1	1.30E-07	-0.46	17	1.7	Dcaf4	
DMR6:107142001	6	107142001	107144000	2000	1	1.20E-08	-0.78	14	0.7	Rbm25	
DMR6:107258001	6	107258001	107260000	2000	1	7.60E-13	0.85	66	3.3	Papln	Protease
DMR6:107295001	6	107295001	107296000	1000	1	2.00E-10	-0.63	7	0.7	Numb	Cytoskeleton
DMR6:107593001	6	107593001	107594000	1000	1	5.20E-07	-0.32	11	1.1	Acot6;LOC108351272;Dnal1	Metabolism;Cytoskeleton
DMR6:108268001	6	108268001	108270000	2000	1	3.00E-08	0.72	49	2.45	Lin52	
DMR6:108298001	6	108298001	108301000	3000	1	4.00E-07	0.28	40	1.33	Vsx2	Development
DMR6:108306001	6	108306001	108307000	1000	1	4.30E-08	0.5	27	2.7	Vsx2;Abcd4	Development;Transport
DMR6:108383001	6	108383001	108385000	2000	1	6.00E-07	0.65	31	1.55	LOC108351273;Syndig1l	
DMR6:108396001	6	108396001	108397000	1000	1	1.60E-08	0.61	24	2.4	LOC108351273;Syndig1l	
DMR6:108460001	6	108460001	108462000	2000	1	4.00E-10	0.43	29	1.45	Npc2	
DMR6:109040001	6	109040001	109042000	2000	1	9.00E-08	0.34	24	1.2	Eif2b2	Translation
DMR6:109201001	6	109201001	109203000	2000	1	2.10E-09	-0.5	26	1.3	Tmed10	Transport
DMR6:109308001	6	109308001	109311000	3000	1	2.00E-07	-0.55	42	1.4	Fos;LOC102548751	Transcription
DMR6:109483001	6	109483001	109484000	1000	1	3.60E-08	0.41	5	0.5	Jdp2	Transcription
DMR6:109508001	6	109508001	109510000	2000	1	4.20E-09	0.41	22	1.1	Jdp2	Transcription
DMR6:109811001	6	109811001	109813000	2000	1	2.90E-07	-0.35	30	1.5	Ttll5	Cytoskeleton
DMR6:109881001	6	109881001	109882000	1000	1	1.10E-09	0.4	14	1.4	Ttll5	Cytoskeleton
DMR6:109891001	6	109891001	109893000	2000	1	5.60E-07	-0.37	16	0.8	Ttll5	Cytoskeleton
DMR6:109995001	6	109995001	109998000	3000	1	5.70E-09	0.33	46	1.53	Ift43	
DMR6:110420001	6	110420001	110424000	4000	1	6.40E-07	0.34	65	1.62	Esrrb	
DMR6:110441001	6	110441001	110442000	1000	1	1.90E-15	0.74	32	3.2	Esrrb	
DMR6:110719001	6	110719001	110720000	1000	1	3.40E-08	0.51	14	1.4	Lrrc74a	
DMR6:110774001	6	110774001	110776000	2000	1	4.00E-07	0.47	35	1.75	Lrrc74a	
DMR6:110993001	6	110993001	110994000	1000	1	1.00E-07	-0.45	19	1.9	Cipc	
DMR6:111129001	6	111129001	111131000	2000	1	6.70E-07	0.4	29	1.45	Tmem63c;Ngb;Pomt2	Transport;Transport
DMR6:111319001	6	111319001	111321000	2000	1	1.30E-08	0.48	23	1.15	Ism2	
DMR6:111415001	6	111415001	111416000	1000	1	2.80E-07	-0.37	12	1.2	Sptlc2	Metabolism
DMR6:111485001	6	111485001	111488000	3000	1	6.40E-07	-0.59	33	1.1	Alkbh1;Slirp;Snw1	Transcription;Translation
DMR6:111512001	6	111512001	111514000	2000	1	4.90E-07	0.55	44	2.2	Snw1;Gle1-ps1	Translation
DMR6:112423001	6	112423001	112429000	6000	1	1.40E-07	-0.26	51	0.85	Nrxn3	
DMR6:112639001	6	112639001	112640000	1000	1	2.70E-09	-0.58	7	0.7	Nrxn3	
DMR6:112641001	6	112641001	112644000	3000	1	3.50E-09	-0.56	19	0.63	Nrxn3	
DMR6:112974001	6	112974001	112975000	1000	1	1.60E-08	-0.51	4	0.4	Nrxn3	
DMR6:113102001	6	113102001	113107000	5000	1	4.50E-07	-0.26	45	0.9	Nrxn3	
DMR6:113387001	6	113387001	113388000	1000	1	2.00E-07	0.46	13	1.3	Nrxn3	
DMR6:113439001	6	113439001	113440000	1000	1	3.20E-08	0.33	6	0.6	Nrxn3	
DMR6:113511001	6	113511001	113513000	2000	1	1.60E-08	0.52	18	0.9	Nrxn3	
DMR6:113626001	6	113626001	113628000	2000	1	6.30E-12	-0.6	15	0.75	Nrxn3;LOC100363383	
DMR6:113671001	6	113671001	113673000	2000	1	8.30E-07	-0.38	10	0.5	Nrxn3	
DMR6:113707001	6	113707001	113709000	2000	1	1.60E-07	0.44	14	0.7	Nrxn3	
DMR6:113718001	6	113718001	113723000	5000	2	5.70E-10	-0.41	48	0.96	Nrxn3	
DMR6:114496001	6	114496001	114500000	4000	1	1.30E-10	-0.43	20	0.5	Dio2;LOC103692683	
DMR6:114765001	6	114765001	114767000	2000	1	3.90E-10	0.47	10	0.5	Cep128	
DMR6:114844001	6	114844001	114845000	1000	1	3.20E-08	-0.52	7	0.7	Cep128	
DMR6:114858001	6	114858001	114862000	4000	1	1.70E-07	-0.31	57	1.43	Cep128	
DMR6:114866001	6	114866001	114867000	1000	1	7.30E-07	0.46	16	1.6	Cep128	
DMR6:115023001	6	115023001	115026000	3000	1	4.60E-07	-0.48	45	1.5	Cep128	
DMR6:115160001	6	115160001	115163000	3000	1	1.90E-09	-0.48	21	0.7	Cep128;Tshr	Signaling
DMR6:115354001	6	115354001	115358000	4000	1	6.50E-07	-0.39	50	1.25	Gtf2a1	Transcription
DMR6:115406001	6	115406001	115408000	2000	2	1.10E-09	0.46	13	0.65	Ston2	Transport
DMR6:115438001	6	115438001	115440000	2000	1	3.30E-08	-0.53	38	1.9	Ston2;LOC102551017	Transport

DMR6:115513001	6	115513001	115515000	2000	1	7.10E-08	-0.48	34	1.7	Ston2	Transport
DMR6:119607001	6	119607001	119608000	1000	1	4.70E-11	0.81	27	2.7	Flrt2	
DMR6:122248001	6	122248001	122250000	2000	1	2.70E-08	-0.5	27	1.35	Galc;Gpr65	Metabolism;Signaling
DMR6:122442001	6	122442001	122445000	3000	1	1.40E-14	0.43	45	1.5	Kcnk10	Transport
DMR6:122484001	6	122484001	122485000	1000	1	9.60E-07	-0.45	9	0.9	Kcnk10	Transport
DMR6:122516001	6	122516001	122522000	6000	1	9.60E-11	-0.38	67	1.12	Kcnk10	Transport
DMR6:122647001	6	122647001	122649000	2000	1	1.60E-07	-0.5	26	1.3	Spata7;Ptpn21	Signaling
DMR6:122774001	6	122774001	122775000	1000	1	2.90E-12	-0.48	12	1.2	Zc3h14;Eml5	
DMR6:122842001	6	122842001	122847000	5000	1	4.00E-08	-0.46	60	1.2	Eml5	
DMR6:122884001	6	122884001	122888000	4000	2	6.40E-10	-0.34	31	0.78	Eml5	
DMR6:123200001	6	123200001	123205000	5000	2	5.70E-07	-0.47	35	0.7	Foxn3	
DMR6:123269001	6	123269001	123272000	3000	1	3.60E-12	-0.57	60	2	Foxn3	
DMR6:123331001	6	123331001	123332000	1000	1	5.80E-09	-0.49	13	1.3	Foxn3;LOC103692689	
DMR6:123397001	6	123397001	123404000	7000	1	8.00E-07	-0.46	100	1.43	Foxn3	
DMR6:123409001	6	123409001	123410000	1000	1	1.50E-07	-0.66	31	3.1	Foxn3	
DMR6:123436001	6	123436001	123438000	2000	1	7.90E-07	0.35	25	1.25	Foxn3	
DMR6:123457001	6	123457001	123459000	2000	1	2.70E-07	-0.43	41	2.05	Foxn3	
DMR6:123468001	6	123468001	123469000	1000	1	1.80E-08	0.35	22	2.2	Foxn3	
DMR6:123487001	6	123487001	123489000	2000	1	5.70E-09	0.4	32	1.6	Foxn3;LOC103692688	
DMR6:123558001	6	123558001	123561000	3000	1	8.80E-08	0.33	29	0.97	Foxn3	
DMR6:123579001	6	123579001	123580000	1000	1	3.20E-07	0.33	12	1.2	Foxn3;LOC102550560	
DMR6:123871001	6	123871001	123873000	2000	1	5.70E-07	-0.36	40	2	Efcab11	Signaling
DMR6:123994001	6	123994001	123999000	5000	1	5.20E-10	-0.38	31	0.62	Kcnk13	Transport
DMR6:124047001	6	124047001	124050000	3000	1	1.10E-07	-0.33	31	1.03	Kcnk13	Transport
DMR6:124079001	6	124079001	124081000	2000	1	7.50E-09	0.63	44	2.2	Kcnk13	Transport
DMR6:124116001	6	124116001	124117000	1000	1	9.30E-08	0.35	9	0.9	Psmc1	Protease
DMR6:124187001	6	124187001	124188000	1000	1	3.40E-07	0.55	23	2.3	Nrde2	
DMR6:124476001	6	124476001	124479000	3000	1	6.50E-08	-0.42	46	1.53	Ttc7b	
DMR6:124490001	6	124490001	124493000	3000	1	8.90E-08	-0.44	32	1.07	Ttc7b	
DMR6:124643001	6	124643001	124645000	2000	1	3.40E-09	0.3	16	0.8	Rps6ka5;LOC688724	Golgi
DMR6:124717001	6	124717001	124719000	2000	1	6.00E-08	-0.38	32	1.6	Rps6ka5	Golgi
DMR6:124741001	6	124741001	124743000	2000	1	5.90E-07	-0.45	32	1.6	Rps6ka5	Golgi
DMR6:124780001	6	124780001	124785000	5000	1	9.30E-09	-0.42	63	1.26	RGD1311756	
DMR6:124921001	6	124921001	124925000	4000	1	1.20E-07	-0.48	80	2	Ccdc88c	Transport
DMR6:124941001	6	124941001	124942000	1000	1	7.40E-13	0.39	17	1.7	Ccdc88c	Transport
DMR6:125013001	6	125013001	125014000	1000	1	7.70E-07	-0.5	15	1.5	Ccdc88c	Transport
DMR6:125361001	6	125361001	125364000	3000	1	8.60E-12	0.4	46	1.53	Catsperb	
DMR6:125379001	6	125379001	125380000	1000	1	6.30E-09	-0.49	8	0.8	Catsperb	
DMR6:125487001	6	125487001	125488000	1000	1	3.80E-07	-0.36	18	1.8	Tc2n	
DMR6:125504001	6	125504001	125506000	2000	1	1.20E-09	0.35	16	0.8	Tc2n	
DMR6:125516001	6	125516001	125522000	6000	2	1.10E-07	-0.36	39	0.65	Tc2n	
DMR6:125604001	6	125604001	125609000	5000	1	2.50E-07	-0.29	44	0.88	Tc2n	
DMR6:125670001	6	125670001	125673000	3000	2	1.20E-09	0.41	19	0.63	Fbln5	Extracellular Matrix
DMR6:125691001	6	125691001	125692000	1000	1	7.00E-08	0.37	6	0.6	Fbln5	Extracellular Matrix
DMR6:125725001	6	125725001	125728000	3000	1	2.20E-11	-0.53	31	1.03	Fbln5	Extracellular Matrix
DMR6:125729001	6	125729001	125732000	3000	1	8.80E-09	0.46	29	0.97	Fbln5;Trip11	Extracellular Matrix;Cytoskeleton
DMR6:126045001	6	126045001	126046000	1000	1	3.20E-07	-0.34	19	1.9	Slc24a4	Transport
DMR6:126097001	6	126097001	126100000	3000	1	2.70E-08	0.47	32	1.07	Slc24a4	Transport
DMR6:126118001	6	126118001	126120000	2000	1	7.10E-07	0.56	40	2	Slc24a4	Transport
DMR6:126233001	6	126233001	126235000	2000	1	8.50E-07	0.29	16	0.8	Rin3	Transcription
DMR6:126257001	6	126257001	126259000	2000	2	1.90E-07	0.54	55	2.75	Rin3	Transcription
DMR6:126304001	6	126304001	126309000	5000	1	1.90E-07	0.38	64	1.28	Lgmn	Protease
DMR6:126360001	6	126360001	126364000	4000	1	7.60E-08	-0.4	42	1.05	Golga5	
DMR6:126432001	6	126432001	126434000	2000	1	8.50E-07	0.39	29	1.45	Chga	
DMR6:126730001	6	126730001	126733000	3000	1	9.10E-07	-0.44	25	0.83	Btbd7	
DMR6:126766001	6	126766001	126768000	2000	1	7.90E-07	0.71	49	2.45	Cox8c	Metabolism
DMR6:126862001	6	126862001	126863000	1000	1	9.00E-08	-0.41	26	2.6	Unc79	
DMR6:126875001	6	126875001	126879000	4000	1	6.60E-08	-0.46	77	1.93	Unc79	
DMR6:126897001	6	126897001	126900000	3000	1	5.80E-07	-0.47	51	1.7	Unc79;LOC108351291	
DMR6:126916001	6	126916001	126921000	5000	1	4.00E-07	0.54	104	2.08	Unc79;LOC108351291	
DMR6:126945001	6	126945001	126946000	1000	1	1.00E-07	-0.44	25	2.5	Unc79	
DMR6:126979001	6	126979001	126981000	2000	1	1.80E-08	-0.51	34	1.7	Unc79	

DMR6:126989001	6	126989001	126998000	9000	1	4.30E-07	-0.42	148	1.64	Unc79	
DMR6:127003001	6	127003001	127005000	2000	1	3.60E-08	-0.46	53	2.65	Unc79	
DMR6:127022001	6	127022001	127024000	2000	2	1.00E-08	-0.45	48	2.4	Unc79	
DMR6:127031001	6	127031001	127032000	1000	1	1.40E-08	0.39	11	1.1	Unc79	
DMR6:127120001	6	127120001	127122000	2000	1	1.50E-07	-0.48	43	2.15	Prima1	
DMR6:127271001	6	127271001	127273000	2000	1	6.90E-13	0.6	40	2	LOC102549032;Otub2	Protease
DMR6:127426001	6	127426001	127427000	1000	1	1.30E-07	-0.49	5	0.5	Ppp4r4	Signaling
DMR6:127681001	6	127681001	127684000	3000	1	3.90E-08	-0.52	28	0.93	Serpina9	Protease; Proteolysis
DMR6:127821001	6	127821001	127826000	5000	1	4.20E-07	-0.33	51	1.02	Serpina3m	Protease; Proteolysis
DMR6:128086001	6	128086001	128088000	2000	2	1.90E-08	0.65	46	2.3	Serpina3n;LOC690435	Protease; Proteolysis
DMR6:128680001	6	128680001	128684000	4000	1	6.40E-07	0.35	68	1.7	Syne3	
DMR6:128691001	6	128691001	128693000	2000	1	6.60E-08	0.37	30	1.5	Syne3	
DMR6:128710001	6	128710001	128711000	1000	1	3.60E-08	-0.51	27	2.7	Syne3	
DMR6:128734001	6	128734001	128736000	2000	1	4.30E-07	0.39	24	1.2	Rpl6-ps1	Translation
DMR6:128902001	6	128902001	128904000	2000	1	2.10E-10	-0.42	46	2.3	Tcl1a;LOC108351373;LOC108351374	
DMR6:129590001	6	129590001	129595000	5000	1	3.00E-07	0.34	92	1.84	Ak7	Signaling
DMR6:129598001	6	129598001	129602000	4000	1	6.00E-16	0.98	82	2.05	Ak7;Papola	Signaling;Translation
DMR6:131833001	6	131833001	131834000	1000	1	1.00E-11	0.71	28	2.8	Bcl11b	Transcription
DMR6:131836001	6	131836001	131837000	1000	1	1.20E-09	0.73	34	3.4	Bcl11b	Transcription
DMR6:131841001	6	131841001	131842000	1000	1	1.50E-08	0.41	7	0.7	Bcl11b	Transcription
DMR6:131849001	6	131849001	131851000	2000	1	1.50E-07	-0.36	65	3.25	Bcl11b	Transcription
DMR6:131882001	6	131882001	131885000	3000	1	7.50E-10	-0.48	78	2.6	Bcl11b	Transcription
DMR6:131896001	6	131896001	131899000	3000	1	1.30E-07	-0.39	71	2.37	Bcl11b;LOC103692702	Transcription
DMR6:132142001	6	132142001	132146000	4000	1	1.40E-10	0.55	78	1.95	Ccdc85c	
DMR6:132385001	6	132385001	132388000	3000	1	8.80E-07	0.29	62	2.07	Eml1	
DMR6:132410001	6	132410001	132411000	1000	1	1.40E-07	-0.52	17	1.7	Eml1	
DMR6:132428001	6	132428001	132430000	2000	1	3.10E-07	-0.55	39	1.95	Eml1	
DMR6:132494001	6	132494001	132498000	4000	1	4.00E-07	-0.47	43	1.07	Evl	Cytoskeleton
DMR6:132538001	6	132538001	132540000	2000	1	8.20E-10	-0.71	15	0.75	Evl;LOC102555052;LOC108351298	Cytoskeleton
DMR6:132592001	6	132592001	132593000	1000	1	1.20E-07	0.36	14	1.4	Evl;Degs2	Cytoskeleton;Metabolism
DMR6:132919001	6	132919001	132922000	3000	1	4.80E-07	-0.38	62	2.07	Wdr25	Cytoskeleton
DMR6:132930001	6	132930001	132933000	3000	2	2.20E-07	0.58	48	1.6	Wdr25;Begain	Cytoskeleton;Cell Junction
DMR6:132940001	6	132940001	132941000	1000	1	1.20E-07	0.59	28	2.8	Wdr25;Begain	Cytoskeleton;Cell Junction
DMR6:133694001	6	133694001	133702000	8000	1	1.70E-07	0.4	101	1.26	RGD1566401;Mir673;Mir493;Mir337;Mir3544;Mir540;Mir665;Rtl1;Mir431	
DMR6:133743001	6	133743001	133749000	6000	1	8.80E-07	0.33	67	1.12	Mir341;Mir1188;Mir370	
DMR6:133873001	6	133873001	133874000	1000	1	1.80E-17	0.51	22	2.2	Mir494;Mir1193;Mir666;Mir543;Mir495;Mir667;Mir376c;Mir376b;Mir3595;Mir376a;Mir300;Mir381;Mir487b;Mir3576;Mir539;Mir6331;Mir544	
DMR6:134830001	6	134830001	134833000	3000	1	1.60E-07	-0.4	57	1.9	Ppp2r5c;LOC102547242;LOC102547175	Signaling
DMR6:135151001	6	135151001	135153000	2000	1	6.10E-08	0.32	22	1.1	Wdr20	
DMR6:135503001	6	135503001	135506000	3000	1	4.20E-11	-0.5	45	1.5	Rcor1	
DMR6:135720001	6	135720001	135721000	1000	1	5.70E-11	-0.69	9	0.9	Traf3;Amn	Cytoskeleton
DMR6:135770001	6	135770001	135772000	2000	1	2.00E-10	-0.47	49	2.45	Cdc42bpb	Signaling
DMR6:135852001	6	135852001	135856000	4000	2	5.60E-12	0.81	66	1.65	RGD1560608	
DMR6:136275001	6	136275001	136278000	3000	1	3.80E-07	-0.54	50	1.67	NEWGENE_1310847;Apopt1	
DMR6:136306001	6	136306001	136309000	3000	1	2.40E-11	0.51	19	0.63	Apopt1	
DMR6:136462001	6	136462001	136465000	3000	1	3.00E-14	-0.62	39	1.3	Ppp1r13b;LOC100363116	Signaling
DMR6:136541001	6	136541001	136547000	6000	1	4.20E-07	0.44	71	1.18	LOC100912557;Tdrd9;LOC691437;Rd3l	Transcription
DMR6:136548001	6	136548001	136552000	4000	1	6.20E-10	-0.59	68	1.7	Tdrd9;LOC691437;Rd3l	Transcription
DMR6:136554001	6	136554001	136556000	2000	1	7.40E-07	-0.34	18	0.9	Tdrd9;Rd3l	Transcription
DMR6:136592001	6	136592001	136593000	1000	1	4.60E-07	0.37	11	1.1	Tdrd9	Transcription
DMR6:136651001	6	136651001	136654000	3000	1	8.00E-10	-0.53	62	2.07	Tdrd9	Transcription
DMR6:137172001	6	137172001	137174000	2000	1	9.10E-09	0.39	43	2.15	Inf2	

DMR6:137180001	6	137180001	137182000	2000	1	8.20E-10	0.42	40	2	Inf2;Adssl1	Metabolism
DMR6:137430001	6	137430001	137431000	1000	1	6.60E-07	-0.45	36	3.6	Cdca4	Transcription
DMR6:137704001	6	137704001	137705000	1000	1	1.90E-07	0.35	12	1.2	Jag2	
DMR6:137721001	6	137721001	137724000	3000	1	2.20E-07	0.36	59	1.97	Jag2	
DMR6:139260001	6	139260001	139266000	6000	1	3.10E-07	-0.52	34	0.57	Ighg	
DMR6:139291001	6	139291001	139293000	2000	1	1.20E-11	-0.78	11	0.55	Ighg;LOC100360344	
DMR6:139485001	6	139485001	139487000	2000	1	5.80E-09	-0.57	12	0.6	Ighg;LOC100361945	
DMR6:139768001	6	139768001	139770000	2000	1	1.60E-07	-0.63	2	0.1	Ighg	
DMR6:139785001	6	139785001	139786000	1000	1	6.10E-15	-0.69	7	0.7	Ighg	
DMR6:140921001	6	140921001	140928000	7000	1	3.40E-07	-0.28	68	0.97	RGD1560842;LOC100360610	
DMR6:142890001	6	142890001	142896000	6000	1	1.20E-10	-0.45	46	0.77	RGD1561247;LOC299458	
DMR6:144112001	6	144112001	144113000	1000	1	7.70E-12	0.84	19	1.9	Wdr60	Cytoskeleton
DMR6:144557001	6	144557001	144560000	3000	1	1.10E-08	-0.41	29	0.97	Ptprn2	Signaling
DMR6:144740001	6	144740001	144741000	1000	1	9.40E-07	0.5	13	1.3	Ptprn2	Signaling
DMR6:145070001	6	145070001	145071000	1000	1	4.60E-07	0.4	13	1.3	Ptprn2;LOC108351314	Signaling
DMR6:145420001	6	145420001	145422000	2000	1	9.30E-08	0.37	17	0.85	Rapgef5	Transcription
DMR6:145531001	6	145531001	145533000	2000	1	3.50E-10	-0.46	21	1.05	Rapgef5;LOC108351316;LOC102556588	Transcription
DMR6:145754001	6	145754001	145759000	5000	1	8.10E-07	-0.32	47	0.94	Cdca7l	
DMR6:145868001	6	145868001	145869000	1000	1	5.20E-07	0.45	6	0.6	Dnah11	Cytoskeleton
DMR6:145894001	6	145894001	145896000	2000	1	1.20E-08	-0.42	20	1	Dnah11	Cytoskeleton
DMR6:145955001	6	145955001	145956000	1000	1	2.70E-07	0.55	11	1.1	Dnah11	Cytoskeleton
DMR6:146022001	6	146022001	146026000	4000	1	1.70E-07	-0.35	61	1.52	Dnah11	Cytoskeleton
DMR6:146031001	6	146031001	146034000	3000	1	2.90E-07	-0.27	43	1.43	Dnah11	Cytoskeleton
DMR6:146167001	6	146167001	146168000	1000	1	4.70E-09	0.45	9	0.9	Sp4	Transcription
DMR6:146205001	6	146205001	146206000	1000	1	3.80E-07	0.48	4	0.4	Sp4	Transcription
DMR6:146782001	6	146782001	146784000	2000	1	2.60E-07	-0.54	24	1.2	Sp8	Transcription
DMR6:146830001	6	146830001	146832000	2000	1	6.40E-10	0.43	16	0.8	Abcb5	Transport
DMR6:147325001	6	147325001	147327000	2000	1	2.00E-10	-0.41	14	0.7	Macc1	
DMR7:1225001	7	1225001	1227000	2000	1	3.60E-08	-0.37	10	0.5	Pros1	
DMR7:2506001	7	2506001	2508000	2000	1	7.50E-09	-0.57	22	1.1	Ptges3;Atp5b;LOC103692761	Transcription
DMR7:2574001	7	2574001	2576000	2000	2	6.70E-09	0.38	10	0.5	Rbms2	
DMR7:2687001	7	2687001	2689000	2000	1	8.60E-07	0.35	15	0.75	Apon;LOC102551925;Apof;Stat2	Binding Proteins;Transcription
DMR7:3059001	7	3059001	3063000	4000	1	3.80E-10	0.5	82	2.05	Rps26;Ikzf4	Translation;Transcription
DMR7:3079001	7	3079001	3081000	2000	2	6.50E-14	0.75	10	0.5	Ikzf4;LOC103692757	Transcription
DMR7:3085001	7	3085001	3087000	2000	1	2.70E-08	0.3	14	0.7	Ikzf4;LOC103692757	Transcription
DMR7:3154001	7	3154001	3157000	3000	1	9.70E-08	-0.37	45	1.5	Dgka;Robld3-ps1	Signaling
DMR7:3192001	7	3192001	3193000	1000	1	2.60E-07	-0.41	22	2.2	Pym1	
DMR7:3274001	7	3274001	3276000	2000	1	1.20E-07	-0.4	16	0.8	Sarnp	
DMR7:3324001	7	3324001	3327000	3000	1	8.00E-14	0.4	32	1.07	Gdf11;LOC102553109;Cd63;Rdh5	Growth Factors;Metabolism
DMR7:3379001	7	3379001	3381000	2000	1	4.60E-10	0.39	16	0.8	Itga7;Mettl7b	Extracellular Matrix;Epigenetic
DMR7:3418001	7	3418001	3419000	1000	1	2.70E-08	-0.36	6	0.6	Olr875	Signaling
DMR7:3452001	7	3452001	3453000	1000	1	4.10E-20	0.93	23	2.3	Olr877	Receptor
DMR7:3735001	7	3735001	3744000	9000	2	2.20E-07	-0.35	68	0.76	Olr880	Receptor
DMR7:3798001	7	3798001	3802000	4000	1	5.00E-07	0.35	26	0.65	Olr881	Receptor
DMR7:3812001	7	3812001	3817000	5000	1	8.60E-07	-0.26	37	0.74	Olr881;Olr882-ps	Receptor
DMR7:4330001	7	4330001	4339000	9000	2	3.00E-11	0.66	151	1.68	Olr986-ps	
DMR7:4341001	7	4341001	4342000	1000	1	4.00E-10	0.65	20	2	Olr986-ps	
DMR7:5164001	7	5164001	5166000	2000	1	2.80E-07	0.32	26	1.3	Olr894;Olr895-ps	Receptor
DMR7:5180001	7	5180001	5181000	1000	1	4.80E-07	-0.48	10	1	Olr895-ps	
DMR7:5209001	7	5209001	5223000	14000	4	2.60E-10	0.71	252	1.8	Olr897-ps	
DMR7:5408001	7	5408001	5410000	2000	1	2.20E-07	-0.44	14	0.7	Olr903	Receptor
DMR7:6019001	7	6019001	6023000	4000	1	8.40E-07	-0.3	31	0.78	Olr1008-ps	
DMR7:6573001	7	6573001	6579000	6000	1	8.60E-07	-0.21	77	1.28	Olr966-ps	
DMR7:7086001	7	7086001	7088000	2000	1	2.50E-10	0.49	10	0.5	Olr1013-ps	
DMR7:7322001	7	7322001	7324000	2000	1	9.40E-07	0.48	13	0.65	LOC108351564;Olr953-ps	
DMR7:7764001	7	7764001	7765000	1000	1	2.90E-07	-0.35	11	1.1	Olr1030-ps	
DMR7:9417001	7	9417001	9418000	1000	1	1.20E-07	-0.4	8	0.8	Olr1068	Signaling
DMR7:9579001	7	9579001	9583000	4000	1	2.50E-07	-0.56	18	0.45	Olr1071	Signaling
DMR7:9614001	7	9614001	9623000	9000	1	2.20E-07	-0.34	70	0.78	Olr1072	Signaling

DMR7:9638001	7	9638001	9640000	2000	1	8.00E-09	-0.44	12	0.6	Olr1072	Signaling
DMR7:10947001	7	10947001	10952000	5000	1	2.70E-08	0.63	75	1.5	Ankrd24;Sirt6;LOC102551996	
DMR7:11012001	7	11012001	11014000	2000	1	7.00E-07	-0.48	41	2.05	Tle2;Aes	Transcription
DMR7:11039001	7	11039001	11041000	2000	1	3.40E-08	0.57	20	1	Gna11	Signaling
DMR7:11065001	7	11065001	11067000	2000	1	4.80E-07	0.43	41	2.05	Gna15	Signaling
DMR7:11181001	7	11181001	11185000	4000	3	2.20E-08	0.57	52	1.3	Nfic;Smim24;LOC102552675;Dohh	Transcription;Metabolism
DMR7:11226001	7	11226001	11228000	2000	1	5.50E-07	-0.39	48	2.4	Mfsd12;LOC690617	
DMR7:11252001	7	11252001	11254000	2000	1	1.80E-12	0.64	54	2.7	Hmg20b;LOC102552827;Gipc3;Tbxa2r;Cactin	Cytoskeleton;Signaling
DMR7:11358001	7	11358001	11364000	6000	1	4.50E-10	0.5	77	1.28	Zfr2;Atcay	Metabolism
DMR7:11377001	7	11377001	11379000	2000	1	3.30E-08	0.58	28	1.4	Atcay;Nmrk2	Signaling
DMR7:11384001	7	11384001	11388000	4000	1	8.30E-08	0.37	104	2.6	Atcay;Nmrk2;Dapk3	Signaling;Signaling
DMR7:11599001	7	11599001	11601000	2000	1	1.80E-07	0.34	19	0.95	Gng7;LOC102553301	Signaling
DMR7:11619001	7	11619001	11624000	5000	1	8.00E-07	0.5	58	1.16	Gng7	Signaling
DMR7:11655001	7	11655001	11656000	1000	1	6.80E-13	0.6	18	1.8	Gadd45b;Lmnb2	
DMR7:11704001	7	11704001	11705000	1000	1	8.20E-07	0.39	20	2	Tmprss9;Sppl2b	Protease;Proteolysis
DMR7:11710001	7	11710001	11712000	2000	1	8.20E-09	0.44	29	1.45	Tmprss9;Sppl2b	Protease;Proteolysis
DMR7:11733001	7	11733001	11734000	1000	1	4.80E-08	0.4	19	1.9	Sppl2b;Lsm7;Lingo3	Proteolysis;Translation;Receptor
DMR7:11990001	7	11990001	11991000	1000	1	3.80E-07	0.41	7	0.7	Scamp4;Adat3;LOC102554028;Abhd17a	Transport;Metabolism;Protease
DMR7:12051001	7	12051001	12053000	2000	2	5.90E-10	0.55	24	1.2	Rexo1;Atp8b3	Transcription;Transport
DMR7:12062001	7	12062001	12064000	2000	1	1.00E-14	0.68	58	2.9	Atp8b3	Transport
DMR7:12065001	7	12065001	12068000	3000	1	9.70E-09	0.36	36	1.2	Atp8b3	Transport
DMR7:12072001	7	12072001	12074000	2000	1	3.70E-09	0.67	15	0.75	Atp8b3	Transport
DMR7:12425001	7	12425001	12426000	1000	1	1.40E-09	0.72	15	1.5	Midn;Atp5d;Cbarp	Transport
DMR7:12459001	7	12459001	12460000	1000	1	1.50E-07	-0.41	21	2.1	Stk11	
DMR7:12587001	7	12587001	12588000	1000	1	6.60E-12	0.77	34	3.4	Arhgap45;Arid3a;LOC103692777	Transcription
DMR7:12666001	7	12666001	12669000	3000	1	1.90E-07	-0.37	95	3.17	Arhgap45;Plppr3;Ptbp1	Signaling
DMR7:12715001	7	12715001	12717000	2000	1	2.10E-14	0.34	36	1.8	Arhgap45;Misp;Palm	
DMR7:12727001	7	12727001	12733000	6000	1	6.80E-10	0.62	158	2.63	Arhgap45;Palm;Abca7	Transport
DMR7:12848001	7	12848001	12851000	3000	2	7.60E-12	0.59	95	3.17	Polrmt;Hcn2	Transcription;Transport
DMR7:12852001	7	12852001	12853000	1000	1	7.10E-07	0.46	46	4.6	Polrmt;Hcn2	Transcription;Transport
DMR7:12945001	7	12945001	12947000	2000	1	1.30E-09	0.57	43	2.15	Odf3l2;Shc2	Development;Cytoskeleton
DMR7:12958001	7	12958001	12961000	3000	1	2.70E-08	0.7	57	1.9	Shc2	Cytoskeleton
DMR7:12979001	7	12979001	12981000	2000	1	4.00E-09	0.44	25	1.25	Shc2;C2cd4c	Cytoskeleton
DMR7:13016001	7	13016001	13019000	3000	2	9.70E-11	0.74	69	2.3	Theg	
DMR7:13026001	7	13026001	13029000	3000	1	1.60E-10	0.48	46	1.53	Theg	
DMR7:13134001	7	13134001	13137000	3000	1	3.50E-07	-0.23	26	0.87	LOC100910608;Vom2r53	Signaling
DMR7:13372001	7	13372001	13374000	2000	1	3.20E-07	-0.5	7	0.35	Olr1073	Receptor
DMR7:13448001	7	13448001	13454000	6000	1	2.70E-11	-0.38	50	0.83	Olr1077	Receptor
DMR7:13469001	7	13469001	13475000	6000	1	6.60E-08	-0.37	51	0.85	Olr1077;Olr1078	Receptor
DMR7:13563001	7	13563001	13569000	6000	1	2.80E-09	-0.36	53	0.88	Olr1082	Receptor
DMR7:13633001	7	13633001	13639000	6000	2	1.40E-07	-0.34	57	0.95	Olr1084	Receptor
DMR7:13641001	7	13641001	13647000	6000	1	6.00E-12	-0.49	78	1.3	Olr1084	Receptor
DMR7:13706001	7	13706001	13709000	3000	1	3.20E-08	-0.4	18	0.6	Olr1086	Receptor
DMR7:13756001	7	13756001	13758000	2000	1	1.20E-07	0.41	35	1.75	Slc1a6;LOC102549402	Transport
DMR7:13908001	7	13908001	13911000	3000	1	6.50E-11	-0.37	32	1.07	Ccdc105	
DMR7:13918001	7	13918001	13920000	2000	2	7.80E-18	0.89	56	2.8	Ccdc105	
DMR7:13939001	7	13939001	13945000	6000	2	6.30E-09	0.33	86	1.43	Casp14	Protease
DMR7:14152001	7	14152001	14153000	1000	1	3.00E-08	0.37	12	1.2	Notch3	
DMR7:14176001	7	14176001	14177000	1000	1	3.00E-10	0.62	35	3.5	Notch3	
DMR7:14195001	7	14195001	14196000	1000	1	2.40E-08	0.39	14	1.4	Notch3	
DMR7:14234001	7	14234001	14235000	1000	1	3.10E-08	-0.65	10	1	Brd4	
DMR7:14388001	7	14388001	14389000	1000	1	3.20E-08	0.43	15	1.5	Wiz	Transcription
DMR7:14412001	7	14412001	14417000	5000	1	5.10E-13	0.52	126	2.52	Wiz;Rasal3;Pglyrp2	Transcription;Signaling
DMR7:14445001	7	14445001	14450000	5000	1	2.80E-13	0.6	113	2.26	Pglyrp2;Cyp4f39	Metabolism
DMR7:14485001	7	14485001	14487000	2000	2	1.20E-10	0.48	35	1.75	Cyp4f39;LOC102555438	Metabolism
DMR7:14515001	7	14515001	14516000	1000	1	1.30E-10	0.69	22	2.2	Cyp4f39	Metabolism
DMR7:14524001	7	14524001	14527000	3000	1	4.90E-07	0.45	66	2.2	Cyp4f39;Cyp4f17	Metabolism
DMR7:15399001	7	15399001	15405000	6000	1	2.70E-07	-0.45	47	0.78	Zfp563	

DMR7:15491001	7	15491001	15498000	7000	1	5.20E-07	-0.36	57	0.81	Morc2b	
DMR7:15615001	7	15615001	15618000	3000	1	9.50E-09	-0.39	22	0.73	Olr1095	Signaling
DMR7:16489001	7	16489001	16491000	2000	1	3.20E-07	-0.42	15	0.75	Olr934-ps	
DMR7:16816001	7	16816001	16819000	3000	1	2.70E-07	-0.38	24	0.8	Vom2r-ps84	
DMR7:16951001	7	16951001	16957000	6000	1	2.10E-08	-0.34	65	1.08	Vom2r54	Signaling
DMR7:16974001	7	16974001	16979000	5000	1	1.90E-07	-0.26	42	0.84	Vom2r54	Signaling
DMR7:17056001	7	17056001	17061000	5000	1	2.40E-09	-0.32	46	0.92	Vom2r55	
DMR7:17079001	7	17079001	17083000	4000	2	9.90E-09	-0.33	29	0.72	Vom2r55	
DMR7:17343001	7	17343001	17348000	5000	2	1.50E-08	-0.41	40	0.8	Vom2r-ps82;Vom2r-ps83	
DMR7:17484001	7	17484001	17486000	2000	1	2.70E-08	-0.4	8	0.4	Vom1r-ps93	
DMR7:17770001	7	17770001	17774000	4000	1	5.20E-07	-0.31	24	0.6	Vom2r-ps85	
DMR7:17862001	7	17862001	17867000	5000	1	3.70E-07	-0.24	56	1.12	Zscan4f	Transcription
DMR7:18117001	7	18117001	18120000	3000	1	7.20E-09	-0.42	14	0.47	Vom1r108	Receptor
DMR7:18125001	7	18125001	18128000	3000	1	6.20E-09	-0.5	11	0.37	Vom1r108	Receptor
DMR7:18252001	7	18252001	18253000	1000	1	5.90E-07	0.3	25	2.5	Vom1r-ps102;LOC108351411	
DMR7:18265001	7	18265001	18271000	6000	2	2.30E-14	-0.36	52	0.87	LOC108351411;RGD1565010	
DMR7:18274001	7	18274001	18278000	4000	1	5.20E-07	-0.41	18	0.45	RGD1565010	
DMR7:18539001	7	18539001	18541000	2000	1	6.40E-09	-0.51	16	0.8	Hnrnpm	Translation
DMR7:18557001	7	18557001	18558000	1000	1	5.10E-07	0.48	5	0.5	Hnrnpm;March2	Translation
DMR7:18911001	7	18911001	18913000	2000	1	2.60E-07	-0.37	4	0.2	Vom2r56	
DMR7:19080001	7	19080001	19082000	2000	1	1.40E-07	-0.5	6	0.3	Vom2r57	Signaling
DMR7:20094001	7	20094001	20096000	2000	1	2.90E-07	0.35	16	0.8	RGD1565071	Signaling
DMR7:20361001	7	20361001	20363000	2000	1	9.20E-07	0.3	17	0.85	RGD1564409;LOC100912403	
DMR7:20376001	7	20376001	20379000	3000	2	6.20E-09	-0.48	27	0.9	RGD1564409;LOC100912403;LOC102552467;LOC103692792	
DMR7:21603001	7	21603001	21604000	1000	1	4.40E-10	0.77	31	3.1	Olr1829-ps	
DMR7:23408001	7	23408001	23413000	5000	1	1.80E-07	0.37	87	1.74	Syn3	Transport
DMR7:23497001	7	23497001	23499000	2000	1	2.70E-08	0.41	10	0.5	Syn3	Transport
DMR7:23525001	7	23525001	23528000	3000	1	2.20E-09	-0.46	37	1.23	Syn3	Transport
DMR7:23748001	7	23748001	23751000	3000	1	1.10E-07	0.5	30	1	Syn3	Transport
DMR7:23795001	7	23795001	23798000	3000	2	8.50E-10	0.43	46	1.53	Syn3;LOC102551348	Transport
DMR7:23831001	7	23831001	23834000	3000	1	2.20E-07	-0.47	30	1	Fbxo7	
DMR7:23887001	7	23887001	23888000	1000	1	1.60E-10	-0.44	17	1.7	Bpifc	
DMR7:23900001	7	23900001	23903000	3000	1	3.00E-08	0.36	49	1.63	Bpifc	
DMR7:23936001	7	23936001	23941000	5000	1	5.40E-09	-0.37	48	0.96	Rtcb;Ascl4	Transcription
DMR7:24032001	7	24032001	24035000	3000	1	1.00E-09	-0.57	59	1.97	Btbd11	Cytoskeleton
DMR7:24057001	7	24057001	24063000	6000	1	1.40E-13	0.36	128	2.13	Btbd11	Cytoskeleton
DMR7:24074001	7	24074001	24075000	1000	1	3.70E-08	0.4	33	3.3	Btbd11	Cytoskeleton
DMR7:24180001	7	24180001	24181000	1000	1	4.50E-08	0.63	21	2.1	Btbd11	Cytoskeleton
DMR7:24202001	7	24202001	24203000	1000	1	6.90E-08	0.37	13	1.3	Btbd11	Cytoskeleton
DMR7:24248001	7	24248001	24252000	4000	1	3.60E-08	-0.49	72	1.8	Btbd11	Cytoskeleton
DMR7:24272001	7	24272001	24273000	1000	1	3.00E-07	0.4	9	0.9	Btbd11	Cytoskeleton
DMR7:24301001	7	24301001	24304000	3000	1	4.80E-07	0.27	36	1.2	Btbd11	Cytoskeleton
DMR7:24610001	7	24610001	24612000	2000	1	1.10E-07	0.34	10	0.5	Cry1	DNA Repair
DMR7:24619001	7	24619001	24621000	2000	1	2.90E-09	0.32	35	1.75	Cry1	DNA Repair
DMR7:24828001	7	24828001	24829000	1000	1	4.00E-07	-0.68	15	1.5	Polr3b	Transcription
DMR7:25051001	7	25051001	25054000	3000	1	9.00E-10	0.4	49	1.63	Nuak1;LOC103692812	Signaling
DMR7:25101001	7	25101001	25102000	1000	1	1.20E-07	-0.38	19	1.9	Nuak1	Signaling
DMR7:25112001	7	25112001	25115000	3000	1	2.80E-07	0.36	36	1.2	Nuak1	Signaling
DMR7:25831001	7	25831001	25834000	3000	1	8.20E-07	0.29	56	1.87	LOC100910996;Rfx4	Transcription
DMR7:25846001	7	25846001	25849000	3000	1	9.70E-07	-0.37	41	1.37	LOC100910996;Rfx4	Transcription
DMR7:25870001	7	25870001	25873000	3000	1	1.50E-08	0.38	39	1.3	LOC100910996;Rfx4	Transcription
DMR7:26273001	7	26273001	26274000	1000	1	3.30E-07	0.44	15	1.5	Appl2	Cytoskeleton
DMR7:26340001	7	26340001	26342000	2000	1	8.50E-12	-0.49	19	0.95	RGD1309995	
DMR7:26345001	7	26345001	26348000	3000	1	4.00E-08	-0.5	43	1.43	RGD1309995	
DMR7:26414001	7	26414001	26417000	3000	2	2.50E-07	0.42	30	1	Aldh1l2	Metabolism
DMR7:26560001	7	26560001	26562000	2000	1	2.20E-08	-0.39	34	1.7	Slc41a2;LOC103692827	Transport
DMR7:26583001	7	26583001	26589000	6000	1	8.60E-07	-0.42	135	2.25	Slc41a2	Transport
DMR7:26598001	7	26598001	26603000	5000	1	1.00E-07	-0.5	87	1.74	Slc41a2	Transport
DMR7:26634001	7	26634001	26636000	2000	1	2.60E-07	0.53	28	1.4	Chst11	Transport
DMR7:26694001	7	26694001	26695000	1000	1	5.90E-07	0.29	23	2.3	Chst11;LOC103692813	Transport
DMR7:26801001	7	26801001	26802000	1000	1	2.60E-07	0.45	14	1.4	Chst11	Transport
DMR7:26973001	7	26973001	26976000	3000	1	2.70E-07	-0.35	50	1.67	Txnrd1;Eid3;LOC108351425	Metabolism

DMR7:27162001	7	27162001	27164000	2000	1	4.00E-15	0.77	60	3	Glt8d2	Golgi
DMR7:27297001	7	27297001	27301000	4000	1	4.80E-07	-0.44	50	1.25	LOC362863;LOC102550216;Nt5dc3	Signaling
DMR7:27403001	7	27403001	27404000	1000	1	3.50E-09	0.64	29	2.9	Stab2	Transport
DMR7:27437001	7	27437001	27439000	2000	2	3.40E-19	0.85	46	2.3	Stab2;LOC102550284	Transport
DMR7:27619001	7	27619001	27621000	2000	1	2.70E-08	0.43	23	1.15	RGD1560034	
DMR7:28070001	7	28070001	28072000	2000	1	1.50E-10	-0.58	18	0.9	Pah	
DMR7:28096001	7	28096001	28101000	5000	2	7.70E-10	0.59	73	1.46	Pah	
DMR7:28133001	7	28133001	28137000	4000	1	3.80E-08	-0.48	42	1.05	Pah;LOC100359965	
DMR7:28409001	7	28409001	28414000	5000	1	1.00E-09	-0.67	101	2.02	Igf1	Growth Factors
DMR7:28753001	7	28753001	28754000	1000	1	2.20E-08	0.65	28	2.8	Nup37;Ccdc53	Transport
DMR7:28917001	7	28917001	28920000	3000	1	6.00E-11	0.82	71	2.37	Dram1	
DMR7:29030001	7	29030001	29032000	2000	1	7.80E-07	0.54	35	1.75	Gnptab;Chpt1;Sycp3	Transport
DMR7:29054001	7	29054001	29055000	1000	1	9.20E-09	0.37	15	1.5	Chpt1;Sycp3	Transport
DMR7:29057001	7	29057001	29061000	4000	1	2.70E-07	-0.37	40	1	Chpt1;LOC102554140	Transport
DMR7:29172001	7	29172001	29173000	1000	1	4.00E-07	0.37	8	0.8	Mybpc1	
DMR7:29436001	7	29436001	29438000	2000	1	3.70E-07	0.47	43	2.15	Slc5a8	Transport
DMR7:29496001	7	29496001	29497000	1000	1	1.70E-09	0.53	9	0.9	Ano4	
DMR7:29544001	7	29544001	29545000	1000	1	3.00E-07	-0.47	8	0.8	Ano4	
DMR7:29612001	7	29612001	29614000	2000	1	4.80E-07	0.4	27	1.35	Ano4	
DMR7:29695001	7	29695001	29697000	2000	1	1.40E-10	-0.64	9	0.45	Ano4	
DMR7:29739001	7	29739001	29741000	2000	1	4.70E-07	-0.46	10	0.5	Ano4	
DMR7:29748001	7	29748001	29750000	2000	1	3.60E-09	-0.43	25	1.25	Ano4	
DMR7:29769001	7	29769001	29770000	1000	1	9.00E-09	0.46	10	1	Ano4	
DMR7:29895001	7	29895001	29896000	1000	1	3.10E-11	0.78	24	2.4	Ano4;LOC102554867	
DMR7:29900001	7	29900001	29901000	1000	1	7.70E-12	0.75	27	2.7	Ano4;LOC102554867;LOC100909955	
DMR7:30067001	7	30067001	30069000	2000	1	3.20E-09	0.37	14	0.7	Nr1h4	Transcription
DMR7:30077001	7	30077001	30078000	1000	1	3.50E-08	-0.52	34	3.4	Nr1h4	Transcription
DMR7:30123001	7	30123001	30124000	1000	1	2.40E-07	-0.52	23	2.3	Nr1h4;LOC100910056	Transcription
DMR7:30206001	7	30206001	30209000	3000	1	1.90E-10	-0.46	18	0.6	Slc17a8	Transport
DMR7:30276001	7	30276001	30279000	3000	1	4.80E-11	0.48	49	1.63	Slc17a8	Transport
DMR7:30349001	7	30349001	30350000	1000	1	2.50E-09	0.44	9	0.9	Scyl2	Signaling
DMR7:30458001	7	30458001	30459000	1000	1	2.40E-08	-0.38	13	1.3	Uhrf1bp1l	
DMR7:30465001	7	30465001	30468000	3000	1	1.40E-09	-0.29	48	1.6	Uhrf1bp1l	
DMR7:30563001	7	30563001	30569000	6000	1	1.70E-07	-0.3	77	1.28	Anks1b	Cytoskeleton
DMR7:30572001	7	30572001	30574000	2000	1	5.80E-08	0.39	21	1.05	Anks1b	Cytoskeleton
DMR7:30579001	7	30579001	30582000	3000	2	1.30E-11	-0.38	26	0.87	Anks1b	Cytoskeleton
DMR7:30583001	7	30583001	30584000	1000	1	3.50E-09	-0.3	9	0.9	Anks1b	Cytoskeleton
DMR7:30686001	7	30686001	30689000	3000	2	4.20E-09	-0.64	42	1.4	Anks1b	Cytoskeleton
DMR7:30690001	7	30690001	30691000	1000	1	9.50E-10	0.63	13	1.3	Anks1b	Cytoskeleton
DMR7:31020001	7	31020001	31021000	1000	1	1.40E-07	0.33	9	0.9	Anks1b	Cytoskeleton
DMR7:31022001	7	31022001	31025000	3000	1	2.40E-08	0.39	46	1.53	Anks1b	Cytoskeleton
DMR7:31078001	7	31078001	31080000	2000	2	1.90E-08	0.4	21	1.05	Anks1b	Cytoskeleton
DMR7:31157001	7	31157001	31158000	1000	1	1.10E-08	0.54	27	2.7	Anks1b	Cytoskeleton
DMR7:31218001	7	31218001	31219000	1000	1	6.70E-09	-0.65	11	1.1	Anks1b	Cytoskeleton
DMR7:31228001	7	31228001	31229000	1000	1	4.30E-07	0.36	5	0.5	Anks1b	Cytoskeleton
DMR7:31254001	7	31254001	31255000	1000	1	2.80E-10	0.69	24	2.4	Anks1b	Cytoskeleton
DMR7:31262001	7	31262001	31268000	6000	1	4.00E-09	0.49	117	1.95	Anks1b	Cytoskeleton
DMR7:31306001	7	31306001	31307000	1000	1	9.50E-10	-0.47	8	0.8	Anks1b	Cytoskeleton
DMR7:31335001	7	31335001	31336000	1000	1	4.10E-09	-0.52	19	1.9	Anks1b	Cytoskeleton
DMR7:31425001	7	31425001	31427000	2000	1	1.70E-07	0.71	56	2.8	Anks1b	Cytoskeleton
DMR7:31498001	7	31498001	31501000	3000	1	3.20E-07	0.51	37	1.23	Anks1b	Cytoskeleton
DMR7:31576001	7	31576001	31577000	1000	1	2.40E-08	0.33	17	1.7	Anks1b	Cytoskeleton
DMR7:31583001	7	31583001	31585000	2000	1	6.60E-07	0.37	40	2	Anks1b;LOC102547065	Cytoskeleton
DMR7:31627001	7	31627001	31630000	3000	1	4.30E-09	-0.5	66	2.2	Anks1b	Cytoskeleton
DMR7:31683001	7	31683001	31684000	1000	1	2.40E-07	0.45	20	2	Anks1b	Cytoskeleton
DMR7:31726001	7	31726001	31728000	2000	1	4.80E-07	0.34	8	0.4	Apaf1	
DMR7:31751001	7	31751001	31753000	2000	1	4.50E-07	0.34	26	1.3	Apaf1	
DMR7:31795001	7	31795001	31796000	1000	1	3.00E-07	-0.45	24	2.4	Ikbip	
DMR7:31825001	7	31825001	31826000	1000	1	2.90E-08	0.64	17	1.7	Slc25a3	Transport
DMR7:31848001	7	31848001	31851000	3000	1	8.60E-09	-0.58	62	2.07	Tmpo	Hormone
DMR7:32885001	7	32885001	32886000	1000	1	4.10E-09	-0.55	2	0.2	Mir135a	
DMR7:33625001	7	33625001	33626000	1000	1	3.00E-09	-0.45	6	0.6	RGD1565866	

DMR7:33839001	7	33839001	33842000	3000	1	2.60E-07	-0.32	56	1.87	RGD1565866	
DMR7:33872001	7	33872001	33874000	2000	1	1.70E-07	0.48	57	2.85	RGD1565866	
DMR7:34050001	7	34050001	34055000	5000	1	3.40E-07	-0.76	86	1.72	Cdk17	Signaling
DMR7:34075001	7	34075001	34076000	1000	1	2.00E-07	-0.52	23	2.3	Cdk17	Signaling
DMR7:34077001	7	34077001	34085000	8000	2	2.30E-07	-0.53	131	1.64	Cdk17;Elk3	Signaling;Transcription
DMR7:34087001	7	34087001	34091000	4000	1	1.70E-07	-0.55	75	1.88	Cdk17;Elk3	Signaling;Transcription
DMR7:34309001	7	34309001	34316000	7000	1	4.50E-08	-0.45	134	1.91	Lta4h;LOC102553601;LOC102553539	
DMR7:34470001	7	34470001	34471000	1000	1	7.00E-07	-0.46	32	3.2	Ccdc38;Snrpf	Translation
DMR7:34495001	7	34495001	34498000	3000	1	8.80E-11	0.42	42	1.4	Snrpf	Translation
DMR7:34628001	7	34628001	34629000	1000	1	2.60E-12	0.73	21	2.1	Ntn4	Extracellular Matrix
DMR7:34774001	7	34774001	34776000	2000	1	8.30E-07	-0.38	29	1.45	Metap2	Protease
DMR7:34793001	7	34793001	34795000	2000	1	2.10E-09	-0.48	35	1.75	Metap2	Protease
DMR7:34998001	7	34998001	35003000	5000	1	1.40E-09	0.29	65	1.3	Fgd6	Transcription
DMR7:35007001	7	35007001	35009000	2000	1	1.70E-07	-0.5	40	2	Fgd6	Transcription
DMR7:35041001	7	35041001	35044000	3000	1	3.70E-09	-0.41	46	1.53	Fgd6	Transcription
DMR7:35304001	7	35304001	35305000	1000	1	2.10E-07	0.42	4	0.4	Tmcc3	
DMR7:35313001	7	35313001	35322000	9000	1	2.40E-07	-0.42	152	1.69	Tmcc3	
DMR7:35355001	7	35355001	35358000	3000	1	6.60E-08	-0.54	9	0.3	Tmcc3	
DMR7:35362001	7	35362001	35364000	2000	1	4.20E-09	0.36	29	1.45	Tmcc3	
DMR7:35410001	7	35410001	35411000	1000	1	6.10E-07	0.4	7	0.7	Tmcc3;LOC102554269	
DMR7:35455001	7	35455001	35457000	2000	1	5.10E-09	0.53	50	2.5	Tmcc3	
DMR7:35515001	7	35515001	35516000	1000	1	2.70E-11	0.45	6	0.6	Tmcc3	
DMR7:36008001	7	36008001	36009000	1000	1	8.20E-08	0.41	17	1.7	Plxnc1	
DMR7:36486001	7	36486001	36488000	2000	1	9.70E-09	0.39	45	2.25	Socs2	Signaling
DMR7:36491001	7	36491001	36492000	1000	1	1.90E-09	-0.54	30	3	Socs2	Signaling
DMR7:36589001	7	36589001	36590000	1000	1	3.90E-07	-0.47	10	1	Mrpl42	Translation
DMR7:36835001	7	36835001	36837000	2000	1	2.30E-10	0.55	31	1.55	Ndufa13;LOC108351434	Metabolism
DMR7:37105001	7	37105001	37106000	1000	1	6.90E-07	-0.62	7	0.7	Eea1	Transport
DMR7:37177001	7	37177001	37179000	2000	1	4.00E-07	-0.38	20	1	Eea1	Transport
DMR7:38957001	7	38957001	38958000	1000	1	6.60E-07	-0.41	6	0.6	Ccer1	
DMR7:40218001	7	40218001	40223000	5000	1	1.00E-07	-0.48	69	1.38	Tmtc3;Cep290	
DMR7:41121001	7	41121001	41123000	2000	1	2.00E-07	-0.48	27	1.35	Atp2b1	Transport
DMR7:41126001	7	41126001	41129000	3000	1	5.10E-09	-0.62	38	1.27	Atp2b1	Transport
DMR7:41195001	7	41195001	41205000	10000	1	4.20E-07	-0.46	204	2.04	Atp2b1	Transport
DMR7:41210001	7	41210001	41216000	6000	1	1.30E-09	-0.59	147	2.45	Atp2b1;LOC102553961	Transport
DMR7:41219001	7	41219001	41220000	1000	1	1.10E-07	-0.55	8	0.8	Atp2b1;LOC102553961	Transport
DMR7:41226001	7	41226001	41228000	2000	1	1.70E-08	-0.41	29	1.45	Atp2b1;LOC102553961	Transport
DMR7:41310001	7	41310001	41313000	3000	1	7.70E-07	-0.39	33	1.1	Poc1b;Galnt4	
DMR7:41356001	7	41356001	41359000	3000	1	1.90E-07	0.47	37	1.23	Poc1b	
DMR7:41411001	7	41411001	41412000	1000	1	3.10E-12	0.82	28	2.8	Poc1b;LOC108351444	
DMR7:42071001	7	42071001	42072000	1000	1	2.90E-12	0.42	8	0.8	RGD1565753	
DMR7:42355001	7	42355001	42358000	3000	1	1.90E-10	0.94	66	2.2	Kitlg;LOC108351446	
DMR7:43276001	7	43276001	43281000	5000	1	9.70E-07	-0.38	52	1.04	Mgat4c	Transport
DMR7:43386001	7	43386001	43387000	1000	1	7.10E-07	0.42	10	1	Mgat4c	Transport
DMR7:43637001	7	43637001	43640000	3000	1	6.70E-08	-0.35	21	0.7	Mgat4c	Transport
DMR7:43697001	7	43697001	43698000	1000	1	9.50E-07	-0.5	8	0.8	Mgat4c	Transport
DMR7:43805001	7	43805001	43806000	1000	1	2.20E-07	0.47	10	1	Mgat4c	Transport
DMR7:43833001	7	43833001	43835000	2000	1	6.70E-10	-0.68	16	0.8	Mgat4c	Transport
DMR7:43854001	7	43854001	43856000	2000	1	5.60E-08	0.67	24	1.2	Mgat4c	Transport
DMR7:43891001	7	43891001	43894000	3000	1	3.90E-09	0.53	21	0.7	Mgat4c	Transport
DMR7:43956001	7	43956001	43957000	1000	1	7.10E-08	0.58	6	0.6	Mgat4c	Transport
DMR7:44768001	7	44768001	44769000	1000	1	4.50E-08	0.8	11	1.1	Alx1;LOC102555472;LOC102555532	Development
DMR7:45079001	7	45079001	45080000	1000	1	9.60E-09	0.48	4	0.4	Lrriq1	
DMR7:45178001	7	45178001	45180000	2000	1	4.80E-08	0.4	10	0.5	Lrriq1	
DMR7:45380001	7	45380001	45381000	1000	1	6.20E-13	0.72	31	3.1	Slc6a15	Transport
DMR7:47314001	7	47314001	47315000	1000	1	1.10E-07	-0.73	12	1.2	Tmtc2	Golgi
DMR7:47351001	7	47351001	47357000	6000	1	1.70E-10	-0.4	56	0.93	Tmtc2	Golgi
DMR7:47364001	7	47364001	47368000	4000	1	1.40E-08	-0.55	65	1.62	Tmtc2	Golgi
DMR7:47476001	7	47476001	47477000	1000	1	4.00E-14	1.04	25	2.5	Tmtc2	Golgi
DMR7:47875001	7	47875001	47877000	2000	1	2.30E-09	-0.41	9	0.45	Mettl25	Epigenetic
DMR7:48619001	7	48619001	48621000	2000	1	2.40E-07	-0.44	2	0.1	Ppfia2	

DMR7:48636001	7	48636001	48637000	1000	1	4.20E-07	-0.46	13	1.3	Ppfia2	
DMR7:48681001	7	48681001	48682000	1000	1	3.20E-07	0.58	26	2.6	Ppfia2	
DMR7:48744001	7	48744001	48745000	1000	1	2.00E-08	0.64	19	1.9	Ppfia2	
DMR7:48828001	7	48828001	48832000	4000	2	1.50E-08	0.54	33	0.82	Ppfia2	
DMR7:49087001	7	49087001	49092000	5000	1	9.40E-11	-0.32	45	0.9	Acss3	Metabolism
DMR7:49161001	7	49161001	49166000	5000	2	2.40E-09	-0.32	46	0.92	Acss3	Metabolism
DMR7:49211001	7	49211001	49212000	1000	1	3.50E-07	0.35	6	0.6	Acss3	Metabolism
DMR7:49255001	7	49255001	49257000	2000	1	1.40E-10	-0.58	18	0.9	Acss3	Metabolism
DMR7:49490001	7	49490001	49497000	7000	1	2.60E-10	-0.31	96	1.37	Lin7a	Cytoskeleton
DMR7:49562001	7	49562001	49563000	1000	1	1.70E-08	0.55	8	0.8	Lin7a	Cytoskeleton
DMR7:49721001	7	49721001	49722000	1000	1	1.20E-07	0.44	12	1.2	Myf5	Transcription
DMR7:49838001	7	49838001	49841000	3000	1	5.10E-09	0.59	24	0.8	Ptprq	Receptor
DMR7:49873001	7	49873001	49877000	4000	1	2.60E-09	-0.4	31	0.78	Ptprq;LOC103690159	Receptor
DMR7:50106001	7	50106001	50107000	1000	1	3.10E-08	0.35	11	1.1	Syt1	Transport
DMR7:50232001	7	50232001	50236000	4000	3	2.70E-09	-0.58	53	1.32	Syt1;LOC102547595	Transport
DMR7:50283001	7	50283001	50284000	1000	1	8.10E-16	0.65	19	1.9	Syt1;LOC103692848	Transport
DMR7:50310001	7	50310001	50318000	8000	1	9.10E-08	-0.26	106	1.32	Syt1	Transport
DMR7:50423001	7	50423001	50428000	5000	1	2.10E-08	-0.32	42	0.84	Syt1	Transport
DMR7:50471001	7	50471001	50474000	3000	1	3.10E-08	-0.37	21	0.7	Syt1	Transport
DMR7:50519001	7	50519001	50521000	2000	1	2.50E-09	-0.48	37	1.85	Syt1	Transport
DMR7:50524001	7	50524001	50527000	3000	1	3.00E-08	0.46	48	1.6	Syt1	Transport
DMR7:51265001	7	51265001	51266000	1000	1	6.10E-08	-0.58	19	1.9	Pawr	
DMR7:51274001	7	51274001	51275000	1000	1	8.00E-07	-0.41	22	2.2	Pawr	
DMR7:51461001	7	51461001	51462000	1000	1	8.30E-08	0.5	5	0.5	Ppp1r12a	Signaling
DMR7:51463001	7	51463001	51464000	1000	1	3.40E-08	-0.43	10	1	Ppp1r12a	Signaling
DMR7:51830001	7	51830001	51833000	3000	1	3.90E-07	-0.41	24	0.8	Otogl	Extracellular Matrix
DMR7:51850001	7	51850001	51856000	6000	1	2.40E-09	-0.4	65	1.08	Otogl	Extracellular Matrix
DMR7:52162001	7	52162001	52165000	3000	1	2.40E-09	0.4	36	1.2	Nav3	
DMR7:52182001	7	52182001	52185000	3000	1	9.50E-09	-0.5	17	0.57	Nav3	
DMR7:52264001	7	52264001	52266000	2000	1	8.00E-10	-0.55	18	0.9	Nav3	
DMR7:52410001	7	52410001	52412000	2000	1	1.10E-07	-0.37	17	0.85	Nav3	
DMR7:53265001	7	53265001	53267000	2000	1	5.90E-08	-0.39	32	1.6	E2f7	Transcription
DMR7:53325001	7	53325001	53328000	3000	1	3.20E-11	0.44	29	0.97	E2f7	Transcription
DMR7:53665001	7	53665001	53667000	2000	1	1.40E-07	-0.57	24	1.2	Zdhhc17	
DMR7:53869001	7	53869001	53872000	3000	2	4.50E-07	-0.6	31	1.03	LOC100361991;Osbpl8	
DMR7:53969001	7	53969001	53971000	2000	1	2.00E-07	-0.66	19	0.95	Osbpl8	
DMR7:54017001	7	54017001	54021000	4000	1	4.10E-10	-0.44	26	0.65	Osbpl8;Bbs10	
DMR7:54762001	7	54762001	54764000	2000	1	1.20E-07	-0.49	33	1.65	Krr1;Glipr1	Metabolism;Immune
DMR7:54826001	7	54826001	54827000	1000	1	1.70E-11	0.84	26	2.6	Glipr1l2;Glipr1l1	Immune
DMR7:54835001	7	54835001	54837000	2000	1	3.40E-08	0.61	39	1.95	Glipr1l1	Immune
DMR7:54841001	7	54841001	54846000	5000	1	1.10E-07	-0.21	51	1.02	Glipr1l1	Immune
DMR7:54847001	7	54847001	54849000	2000	1	1.30E-07	-0.41	10	0.5	Glipr1l1;Caps2	Immune;Signaling
DMR7:54867001	7	54867001	54868000	1000	1	4.40E-07	-0.35	6	0.6	Caps2	Signaling
DMR7:54920001	7	54920001	54921000	1000	1	1.90E-07	-0.58	18	1.8	Caps2	Signaling
DMR7:54923001	7	54923001	54924000	1000	1	3.40E-07	0.31	4	0.4	Caps2	Signaling
DMR7:55129001	7	55129001	55132000	3000	1	5.90E-07	-0.37	87	2.9	Kcnc2;LOC103692854	Transport
DMR7:57302001	7	57302001	57308000	6000	1	2.80E-07	-0.22	49	0.82	Trhde	Protease
DMR7:57618001	7	57618001	57619000	1000	1	3.00E-08	0.73	20	2	Trhde	Protease
DMR7:57680001	7	57680001	57682000	2000	2	1.20E-11	0.57	42	2.1	Trhde	Protease
DMR7:58049001	7	58049001	58051000	2000	1	1.40E-07	0.32	25	1.25	Tph2	
DMR7:58072001	7	58072001	58073000	1000	1	1.50E-07	0.3	8	0.8	Tph2	
DMR7:58077001	7	58077001	58078000	1000	1	2.40E-07	0.34	9	0.9	Tph2	
DMR7:58107001	7	58107001	58108000	1000	1	8.80E-08	0.53	6	0.6	Tph2;LOC103692856	
DMR7:58119001	7	58119001	58121000	2000	1	3.30E-10	-0.53	27	1.35	Tph2	
DMR7:58168001	7	58168001	58172000	4000	1	5.00E-07	-0.59	31	0.78	Tbc1d15	Signaling
DMR7:58256001	7	58256001	58259000	3000	1	1.80E-08	-0.49	39	1.3	Rab21	
DMR7:58326001	7	58326001	58327000	1000	1	1.40E-08	0.32	5	0.5	Tmem19	
DMR7:58503001	7	58503001	58505000	2000	1	2.80E-09	0.43	28	1.4	Lgr5	Signaling
DMR7:58514001	7	58514001	58519000	5000	1	2.50E-12	0.53	50	1	Lgr5	Signaling
DMR7:58580001	7	58580001	58581000	1000	1	4.60E-11	0.46	7	0.7	Lgr5;LOC685207	Signaling
DMR7:59077001	7	59077001	59078000	1000	1	3.80E-11	-0.64	15	1.5	Ptprr	
DMR7:59139001	7	59139001	59141000	2000	1	2.00E-17	1.14	62	3.1	Ptprr;Taf7l-ps1	
DMR7:59222001	7	59222001	59223000	1000	1	2.20E-07	0.43	10	1	Ptprr	

DMR7:59241001	7	59241001	59246000	5000	1	3.40E-07	-0.26	42	0.84	Ptprr	
DMR7:59261001	7	59261001	59265000	4000	1	1.20E-08	0.38	65	1.62	Ptprr	
DMR7:59293001	7	59293001	59294000	1000	1	1.00E-08	0.36	7	0.7	Ptprr	
DMR7:59513001	7	59513001	59514000	1000	1	4.50E-08	0.43	21	2.1	Kcnmb4	Transport
DMR7:59531001	7	59531001	59533000	2000	1	5.00E-09	-0.54	23	1.15	Cnot2	Transcription
DMR7:59836001	7	59836001	59837000	1000	1	2.90E-07	0.49	30	3	Myrfl	
DMR7:59928001	7	59928001	59931000	3000	1	2.00E-07	-0.36	51	1.7	LOC688961;Rab3ip	Transcription
DMR7:59952001	7	59952001	59955000	3000	1	5.30E-10	-0.49	57	1.9	Rab3ip	Transcription
DMR7:59976001	7	59976001	59978000	2000	1	1.40E-07	0.45	29	1.45	Rab3ip;Best3	Transcription;Transport
DMR7:59998001	7	59998001	60001000	3000	1	4.60E-07	0.38	70	2.33	Best3	Transport
DMR7:60047001	7	60047001	60048000	1000	1	3.10E-08	-0.58	19	1.9	Best3	Transport
DMR7:60090001	7	60090001	60094000	4000	1	4.40E-08	-0.29	44	1.1	Lrrc10;LOC100911101	
DMR7:60147001	7	60147001	60150000	3000	1	2.90E-07	-0.47	62	2.07	Frs2	
DMR7:60165001	7	60165001	60166000	1000	1	4.20E-09	-0.52	4	0.4	Frs2	
DMR7:60323001	7	60323001	60327000	4000	1	6.70E-08	0.39	35	0.88	Lyc2;Lyz2	
DMR7:60773001	7	60773001	60776000	3000	1	1.00E-08	-0.39	47	1.57	LOC108351461;Slc35e3;Nup107	Transport;Transport
DMR7:60826001	7	60826001	60828000	2000	1	2.10E-08	0.52	20	1	Nup107	Transport
DMR7:60986001	7	60986001	60987000	1000	1	8.60E-07	-0.41	14	1.4	LOC100911247;RGD1306282	
DMR7:61169001	7	61169001	61172000	3000	1	4.80E-07	-0.49	54	1.8	LOC102551023;Mdm1	
DMR7:61347001	7	61347001	61350000	3000	1	4.30E-08	-0.51	29	0.97	Ifng	Immune
DMR7:61782001	7	61782001	61786000	4000	1	5.00E-08	-0.38	82	2.05	Dyrk2	
DMR7:61794001	7	61794001	61798000	4000	1	2.60E-09	-0.62	54	1.35	Dyrk2	
DMR7:61801001	7	61801001	61803000	2000	1	3.20E-08	0.39	21	1.05	Dyrk2	
DMR7:62880001	7	62880001	62883000	3000	1	1.40E-08	-0.79	47	1.57	Msrb3	Metabolism
DMR7:62893001	7	62893001	62897000	4000	1	7.50E-07	-0.34	76	1.9	Msrb3	Metabolism
DMR7:62930001	7	62930001	62931000	1000	1	3.30E-09	-0.53	33	3.3	Msrb3	Metabolism
DMR7:62947001	7	62947001	62948000	1000	1	1.90E-14	0.95	39	3.9	Msrb3	Metabolism
DMR7:62966001	7	62966001	62967000	1000	1	4.30E-08	0.67	33	3.3	Msrb3;Lemd3	Metabolism
DMR7:63113001	7	63113001	63114000	1000	1	3.20E-08	0.67	16	1.6	Wif1	Signaling
DMR7:63133001	7	63133001	63134000	1000	1	3.70E-10	-0.45	15	1.5	Wif1	Signaling
DMR7:63173001	7	63173001	63175000	2000	1	2.70E-07	0.3	18	0.9	Wif1	Signaling
DMR7:63397001	7	63397001	63399000	2000	1	3.20E-07	0.48	30	1.5	Tbc1d30	Signaling
DMR7:63419001	7	63419001	63422000	3000	1	1.80E-07	-0.41	49	1.63	Tbc1d30;LOC102548194	Signaling
DMR7:63542001	7	63542001	63545000	3000	1	7.50E-07	-0.37	73	2.43	Rassf3	Cytoskeleton
DMR7:63920001	7	63920001	63921000	1000	1	8.60E-07	-0.47	17	1.7	RGD1565498	
DMR7:63927001	7	63927001	63933000	6000	1	6.20E-08	0.48	82	1.37	RGD1565498	
DMR7:63958001	7	63958001	63961000	3000	1	6.10E-09	0.42	32	1.07	Srgap1	Signaling
DMR7:64101001	7	64101001	64103000	2000	1	4.70E-08	-0.49	31	1.55	Srgap1	Signaling
DMR7:64158001	7	64158001	64160000	2000	1	5.70E-08	0.51	22	1.1	Srgap1	Signaling
DMR7:64348001	7	64348001	64349000	1000	1	2.20E-13	0.81	28	2.8	Tmem5	
DMR7:64402001	7	64402001	64403000	1000	1	1.40E-07	0.35	14	1.4	Vps54-ps1	
DMR7:64856001	7	64856001	64858000	2000	1	5.40E-08	-0.41	34	1.7	Grip1;Helb	Transcription
DMR7:64914001	7	64914001	64918000	4000	1	8.00E-12	0.51	82	2.05	Irak3	
DMR7:64926001	7	64926001	64929000	3000	1	1.30E-07	-0.46	67	2.23	Irak3	
DMR7:65177001	7	65177001	65182000	5000	1	5.00E-08	-0.51	76	1.52	Hmga2;LOC102549373	Transcription
DMR7:66083001	7	66083001	66085000	2000	1	7.50E-13	0.59	33	1.65	Fam19a2	
DMR7:66219001	7	66219001	66221000	2000	1	7.80E-07	-0.54	27	1.35	Fam19a2	
DMR7:66229001	7	66229001	66231000	2000	1	3.30E-08	0.41	20	1	Fam19a2	
DMR7:66354001	7	66354001	66357000	3000	2	4.00E-15	0.53	34	1.13	Fam19a2	
DMR7:66398001	7	66398001	66400000	2000	1	7.70E-07	0.52	12	0.6	Fam19a2	
DMR7:66735001	7	66735001	66737000	2000	1	6.40E-07	-0.52	17	0.85	Mon2;LOC108351468;LOC680056	Transcription
DMR7:66834001	7	66834001	66839000	5000	1	2.50E-08	-0.36	68	1.36	Ppm1h	Signaling
DMR7:66850001	7	66850001	66853000	3000	1	6.80E-07	0.48	46	1.53	Ppm1h	Signaling
DMR7:66865001	7	66865001	66866000	1000	1	3.80E-07	-0.41	17	1.7	Ppm1h	Signaling
DMR7:66884001	7	66884001	66887000	3000	1	2.70E-07	-0.57	51	1.7	Ppm1h	Signaling
DMR7:66890001	7	66890001	66899000	9000	1	3.70E-09	-0.34	141	1.57	Ppm1h	Signaling
DMR7:66908001	7	66908001	66911000	3000	1	4.40E-07	-0.36	64	2.13	Ppm1h	Signaling
DMR7:67054001	7	67054001	67055000	1000	1	3.20E-08	-0.65	21	2.1	Ppm1h	Signaling
DMR7:68446001	7	68446001	68448000	2000	1	2.80E-07	-0.5	21	1.05	Slc16a7	Transport
DMR7:68614001	7	68614001	68619000	5000	1	9.10E-07	0.32	54	1.08	Slc16a7;LOC103692869	Transport
DMR7:69228001	7	69228001	69230000	2000	1	2.20E-09	-0.49	32	1.6	Lrig3	
DMR7:69979001	7	69979001	69980000	1000	1	6.60E-12	0.55	7	0.7	RGD1559995	
DMR7:70440001	7	70440001	70442000	2000	1	1.00E-10	0.41	18	0.9	B4galnt1;RGD1565117	Translation

DMR7:70457001	7	70457001	70461000	4000	1	7.70E-15	0.86	113	2.83	B4galnt1;Slc26a10;Arhgef25	Transport;Transcription
DMR7:70477001	7	70477001	70478000	1000	1	1.30E-11	0.71	18	1.8	Slc26a10;Arhgef25;Dtx3;Pip4k2c	Transport;Transcription;Proteolysis;Signaling
DMR7:70495001	7	70495001	70498000	3000	1	2.70E-07	0.44	27	0.9	Pip4k2c	Signaling
DMR7:70607001	7	70607001	70610000	3000	1	5.00E-08	0.43	31	1.03	Mars;LOC108351474;Arhgap9	Translation;Signaling
DMR7:70621001	7	70621001	70623000	2000	1	7.80E-07	0.63	54	2.7	Arhgap9;Gli1	Signaling;Transcription
DMR7:70708001	7	70708001	70710000	2000	1	7.10E-07	-0.41	30	1.5	R3hdm2	
DMR7:70838001	7	70838001	70841000	3000	1	5.30E-09	-0.43	58	1.93	Shmt2;Nxph4;Lrp1	Epigenetic;Signaling;Binding Proteins
DMR7:70867001	7	70867001	70869000	2000	1	6.10E-10	0.44	60	3	Lrp1	Binding Proteins
DMR7:70890001	7	70890001	70891000	1000	1	1.10E-11	0.89	42	4.2	Lrp1	Binding Proteins
DMR7:70893001	7	70893001	70897000	4000	1	1.50E-09	0.55	77	1.93	Lrp1	Binding Proteins
DMR7:70906001	7	70906001	70908000	2000	1	1.40E-21	-0.96	43	2.15	Lrp1	Binding Proteins
DMR7:70913001	7	70913001	70915000	2000	1	4.30E-10	0.45	20	1	Lrp1	Binding Proteins
DMR7:71019001	7	71019001	71024000	5000	1	7.80E-08	0.43	73	1.46	Myo1a;Tac3	Cytoskeleton
DMR7:71256001	7	71256001	71258000	2000	1	8.90E-07	-0.51	7	0.35	Uqcrb	Metabolism
DMR7:71306001	7	71306001	71309000	3000	1	1.40E-09	-0.36	37	1.23	Ptdss1	Transport
DMR7:71391001	7	71391001	71393000	2000	1	7.40E-09	-0.44	12	0.6	Esco2-ps1	
DMR7:71621001	7	71621001	71622000	1000	1	2.60E-09	-0.54	9	0.9	Sdc2	Receptor
DMR7:71625001	7	71625001	71629000	4000	1	4.10E-09	0.42	30	0.75	Sdc2	Receptor
DMR7:71696001	7	71696001	71699000	3000	1	1.30E-07	0.3	18	0.6	Sdc2	Receptor
DMR7:71722001	7	71722001	71723000	1000	1	9.40E-09	-0.5	12	1.2	Cpq	Protease
DMR7:71787001	7	71787001	71790000	3000	1	5.30E-08	-0.47	32	1.07	Cpq	Protease
DMR7:71796001	7	71796001	71797000	1000	1	2.30E-07	0.27	20	2	Cpq	Protease
DMR7:71806001	7	71806001	71811000	5000	1	1.80E-07	-0.29	44	0.88	Cpq;LOC103692875	Protease
DMR7:71856001	7	71856001	71857000	1000	1	3.60E-13	-0.66	8	0.8	Cpq	Protease
DMR7:72074001	7	72074001	72075000	1000	1	2.60E-09	0.48	7	0.7	Cpq	Protease
DMR7:72147001	7	72147001	72152000	5000	1	9.30E-10	-0.32	57	1.14	Cpq	Protease
DMR7:73012001	7	73012001	73017000	5000	1	2.30E-07	-0.29	52	1.04	Matn2	Extracellular Matrix
DMR7:73169001	7	73169001	73170000	1000	1	2.70E-10	-0.43	3	0.3	Matn2	Extracellular Matrix
DMR7:73305001	7	73305001	73306000	1000	1	5.60E-07	0.38	4	0.4	Pop1	
DMR7:73401001	7	73401001	73403000	2000	1	8.40E-07	0.32	16	0.8	Nipal2	
DMR7:73557001	7	73557001	73558000	1000	1	2.90E-07	-0.46	11	1.1	Kcns2	Transport
DMR7:73657001	7	73657001	73658000	1000	1	9.60E-08	-0.24	5	0.5	Stk3	Signaling
DMR7:73659001	7	73659001	73662000	3000	1	4.10E-10	-0.43	28	0.93	Stk3	Signaling
DMR7:73710001	7	73710001	73712000	2000	1	5.60E-08	-0.46	12	0.6	Stk3	Signaling
DMR7:73804001	7	73804001	73805000	1000	1	3.80E-07	-0.55	10	1	Stk3	Signaling
DMR7:73850001	7	73850001	73858000	8000	1	7.60E-08	-0.24	83	1.04	Stk3	Signaling
DMR7:74136001	7	74136001	74138000	2000	1	5.90E-07	-0.44	14	0.7	Vps13b	
DMR7:74192001	7	74192001	74197000	5000	1	1.50E-08	-0.37	38	0.76	Vps13b;LOC103692884	
DMR7:74557001	7	74557001	74558000	1000	1	1.00E-06	0.48	7	0.7	Vps13b	
DMR7:74693001	7	74693001	74694000	1000	1	1.80E-07	-0.57	7	0.7	Vps13b	
DMR7:74700001	7	74700001	74703000	3000	1	3.20E-08	0.4	26	0.87	Vps13b	
DMR7:74838001	7	74838001	74841000	3000	1	1.70E-07	-0.44	30	1	Rgs22	
DMR7:74842001	7	74842001	74845000	3000	1	3.10E-07	-0.34	27	0.9	Rgs22	
DMR7:75035001	7	75035001	75037000	2000	1	1.10E-10	0.4	59	2.95	Spag1	
DMR7:75314001	7	75314001	75316000	2000	1	6.80E-07	-0.38	12	0.6	Snx31	Cytoskeleton
DMR7:75411001	7	75411001	75414000	3000	1	9.30E-08	-0.52	35	1.17	Pabpc1;LOC102555439	
DMR7:75419001	7	75419001	75420000	1000	1	9.50E-08	-0.48	14	1.4	Pabpc1;LOC102555439	
DMR7:75582001	7	75582001	75583000	1000	1	2.10E-07	-0.48	12	1.2	Ywhaz	Cytoskeleton
DMR7:75589001	7	75589001	75590000	1000	1	5.20E-09	-0.69	9	0.9	Ywhaz	Cytoskeleton
DMR7:76066001	7	76066001	76067000	1000	1	1.90E-08	-0.42	19	1.9	Grhl2	Transcription
DMR7:76117001	7	76117001	76118000	1000	1	2.50E-08	0.53	11	1.1	Grhl2	Transcription
DMR7:76153001	7	76153001	76154000	1000	1	5.70E-07	-0.37	13	1.3	Grhl2	Transcription
DMR7:76470001	7	76470001	76472000	2000	1	8.60E-08	0.53	20	1	Ncald	
DMR7:76511001	7	76511001	76519000	8000	1	5.00E-09	-0.38	63	0.79	Ncald	
DMR7:76537001	7	76537001	76539000	2000	1	5.50E-07	0.5	15	0.75	Ncald	
DMR7:76571001	7	76571001	76573000	2000	1	5.90E-09	0.41	20	1	Ncald;LOC102549311	
DMR7:76611001	7	76611001	76613000	2000	1	1.60E-13	0.61	35	1.75	Ncald;LOC102549311	
DMR7:76751001	7	76751001	76753000	2000	1	6.60E-07	-0.42	18	0.9	Rrm2b	Metabolism
DMR7:76814001	7	76814001	76817000	3000	1	1.30E-08	-0.49	38	1.27	Ubr5	Proteolysis
DMR7:76895001	7	76895001	76897000	2000	1	8.00E-07	-0.42	36	1.8	Ubr5	Proteolysis
DMR7:77669001	7	77669001	77673000	4000	1	8.50E-09	0.45	69	1.73	Atp6v1c1	Metabolism

DMR7:77756001	7	77756001	77758000	2000	1	3.60E-07	0.36	20	1	Baalc	
DMR7:77802001	7	77802001	77804000	2000	1	4.60E-09	0.78	41	2.05	Baalc	
DMR7:77816001	7	77816001	77818000	2000	1	4.50E-07	-0.67	18	0.9	Baalc	
DMR7:77841001	7	77841001	77842000	1000	1	2.90E-08	0.37	8	0.8	Baalc	
DMR7:77891001	7	77891001	77898000	7000	3	7.80E-10	-0.37	72	1.03	Fzd6	Receptor
DMR7:78025001	7	78025001	78031000	6000	1	7.00E-07	-0.76	38	0.63	Dcaf13	Metabolism
DMR7:78153001	7	78153001	78158000	5000	1	5.70E-09	-0.38	27	0.54	Rims2	Transport
DMR7:78181001	7	78181001	78185000	4000	2	3.00E-07	-0.51	54	1.35	Rims2	Transport
DMR7:78222001	7	78222001	78225000	3000	1	9.50E-09	-0.34	23	0.77	Rims2	Transport
DMR7:78231001	7	78231001	78238000	7000	1	5.60E-07	-0.44	54	0.77	Rims2	Transport
DMR7:78241001	7	78241001	78243000	2000	2	1.40E-11	0.71	54	2.7	Rims2	Transport
DMR7:78246001	7	78246001	78248000	2000	1	1.90E-08	-0.61	22	1.1	Rims2	Transport
DMR7:78260001	7	78260001	78265000	5000	3	3.30E-11	-0.38	46	0.92	Rims2	Transport
DMR7:78783001	7	78783001	78784000	1000	1	3.50E-09	-0.38	10	1	Dpys	Metabolism
DMR7:78884001	7	78884001	78886000	2000	2	5.20E-13	-0.72	26	1.3	Lrp12	Binding Proteins
DMR7:78917001	7	78917001	78920000	3000	1	6.10E-07	-0.4	33	1.1	Lrp12	Binding Proteins
DMR7:79497001	7	79497001	79498000	1000	1	3.90E-07	0.38	17	1.7	Zfpm2	Transcription
DMR7:79621001	7	79621001	79624000	3000	1	1.40E-07	-0.4	29	0.97	Zfpm2	Transcription
DMR7:79625001	7	79625001	79627000	2000	1	1.10E-08	-0.61	22	1.1	Zfpm2	Transcription
DMR7:79890001	7	79890001	79892000	2000	1	6.90E-08	-0.47	27	1.35	Zfpm2	Transcription
DMR7:79928001	7	79928001	79930000	2000	1	8.00E-09	0.38	24	1.2	Zfpm2	Transcription
DMR7:80710001	7	80710001	80711000	1000	1	1.20E-08	-0.56	6	0.6	Oxr1	
DMR7:80714001	7	80714001	80716000	2000	2	1.60E-09	-0.62	23	1.15	Oxr1	
DMR7:81414001	7	81414001	81416000	2000	1	3.20E-07	-0.48	15	0.75	Angpt1	Signaling
DMR7:81422001	7	81422001	81424000	2000	1	3.50E-07	-0.42	18	0.9	Angpt1	Signaling
DMR7:81473001	7	81473001	81480000	7000	2	1.10E-08	-0.31	68	0.97	Angpt1	Signaling
DMR7:81571001	7	81571001	81572000	1000	1	9.30E-08	0.46	13	1.3	Angpt1	Signaling
DMR7:81912001	7	81912001	81914000	2000	1	4.80E-08	0.41	15	0.75	Rspo2	
DMR7:83405001	7	83405001	83411000	6000	3	1.10E-10	-0.33	54	0.9	Pkhd1l1	
DMR7:84031001	7	84031001	84034000	3000	1	2.80E-08	-0.6	12	0.4	Kcnv1	Transport
DMR7:87050001	7	87050001	87052000	2000	1	8.80E-08	-0.4	41	2.05	Csmd3	
DMR7:87064001	7	87064001	87065000	1000	1	6.10E-08	0.44	14	1.4	Csmd3	
DMR7:87103001	7	87103001	87104000	1000	1	6.00E-09	0.42	6	0.6	Csmd3	
DMR7:87201001	7	87201001	87203000	2000	1	8.00E-11	0.66	19	0.95	Csmd3	
DMR7:87226001	7	87226001	87230000	4000	1	1.00E-08	-0.38	30	0.75	Csmd3	
DMR7:87363001	7	87363001	87364000	1000	1	2.00E-08	0.48	3	0.3	Csmd3	
DMR7:87387001	7	87387001	87388000	1000	1	6.50E-07	-0.46	5	0.5	Csmd3	
DMR7:87533001	7	87533001	87536000	3000	3	7.60E-10	-0.42	28	0.93	Csmd3	
DMR7:87608001	7	87608001	87611000	3000	1	2.10E-07	-0.31	20	0.67	Csmd3	
DMR7:87641001	7	87641001	87643000	2000	1	2.40E-07	-0.38	12	0.6	Csmd3	
DMR7:87678001	7	87678001	87679000	1000	1	1.40E-08	-0.56	4	0.4	Csmd3	
DMR7:87712001	7	87712001	87720000	8000	1	2.00E-08	-0.26	87	1.09	Csmd3	
DMR7:87752001	7	87752001	87755000	3000	1	4.10E-07	-0.35	29	0.97	Csmd3	
DMR7:87823001	7	87823001	87828000	5000	1	4.90E-07	-0.26	59	1.18	Csmd3	
DMR7:87917001	7	87917001	87921000	4000	1	6.10E-08	0.44	51	1.27	Csmd3	
DMR7:87936001	7	87936001	87940000	4000	1	1.50E-09	0.58	53	1.32	Csmd3	
DMR7:87990001	7	87990001	87994000	4000	1	1.80E-08	0.66	73	1.82	Csmd3	
DMR7:90322001	7	90322001	90324000	2000	1	5.00E-07	-0.43	33	1.65	Trps1	Transcription
DMR7:91515001	7	91515001	91516000	1000	1	8.00E-07	-0.65	8	0.8	Rad21	
DMR7:92666001	7	92666001	92667000	1000	1	2.80E-09	0.43	7	0.7	Ext1	Golgi
DMR7:92677001	7	92677001	92678000	1000	1	4.80E-09	0.42	7	0.7	Ext1	Golgi
DMR7:92740001	7	92740001	92742000	2000	1	1.30E-08	-0.4	27	1.35	Ext1	Golgi
DMR7:92996001	7	92996001	92997000	1000	1	4.10E-07	0.47	11	1.1	Samd12	
DMR7:93023001	7	93023001	93029000	6000	1	3.50E-07	-0.22	56	0.93	Samd12	
DMR7:93034001	7	93034001	93039000	5000	1	7.50E-12	-0.37	41	0.82	Samd12	
DMR7:93267001	7	93267001	93271000	4000	1	1.40E-08	-0.26	32	0.8	Samd12	
DMR7:93967001	7	93967001	93971000	4000	1	6.50E-09	-0.4	16	0.4	Colec10	Transport
DMR7:94014001	7	94014001	94019000	5000	1	1.10E-07	-0.37	50	1	Colec10	Transport
DMR7:94716001	7	94716001	94719000	3000	1	3.40E-14	-0.67	30	1	Taf2	Transcription
DMR7:94752001	7	94752001	94754000	2000	1	3.60E-07	-0.68	13	0.65	Taf2;Dscc1	Transcription;Cell Cycle
DMR7:95125001	7	95125001	95126000	1000	1	7.60E-07	0.25	16	1.6	Col14a1	Extracellular Matrix
DMR7:95167001	7	95167001	95170000	3000	1	9.70E-12	-0.37	18	0.6	Col14a1	Extracellular Matrix
DMR7:95328001	7	95328001	95331000	3000	1	1.90E-08	-0.37	28	0.93	Mtbp	

DMR7:95441001	7	95441001	95445000	4000	2	1.10E-08	-0.41	33	0.82	Sntb1	
DMR7:95527001	7	95527001	95529000	2000	1	5.00E-07	-0.54	13	0.65	Sntb1	
DMR7:96473001	7	96473001	96474000	1000	1	3.30E-07	0.35	5	0.5	Has2;LOC108351495	Golgi
DMR7:97668001	7	97668001	97670000	2000	1	7.90E-10	0.59	30	1.5	Zhx2	Development
DMR7:97695001	7	97695001	97697000	2000	1	2.10E-07	-0.41	52	2.6	Zhx2;LOC102553429	Development
DMR7:97813001	7	97813001	97817000	4000	1	1.50E-07	-0.34	68	1.7	Tbc1d31;LOC102553732	
DMR7:97827001	7	97827001	97828000	1000	1	2.80E-07	-0.34	18	1.8	Tbc1d31;LOC102553732	
DMR7:97837001	7	97837001	97839000	2000	1	3.70E-07	-0.44	34	1.7	Tbc1d31;LOC102553732;Trnam-cau	
DMR7:97856001	7	97856001	97858000	2000	1	9.40E-07	0.41	28	1.4	Fam83a	
DMR7:97894001	7	97894001	97897000	3000	1	2.30E-07	-0.56	44	1.47	RGD1310852	
DMR7:98200001	7	98200001	98201000	1000	1	1.80E-07	0.52	10	1	Klhl38	Cytoskeleton
DMR7:98287001	7	98287001	98293000	6000	1	4.70E-08	-0.35	55	0.92	Fam91a1	
DMR7:98294001	7	98294001	98295000	1000	1	2.20E-07	-0.38	18	1.8	Fam91a1	
DMR7:98401001	7	98401001	98404000	3000	1	2.80E-11	0.51	25	0.83	Fer1l6	Transport
DMR7:98473001	7	98473001	98479000	6000	2	4.80E-10	-0.46	65	1.08	Fer1l6	Transport
DMR7:98492001	7	98492001	98495000	3000	1	1.20E-10	0.45	23	0.77	Fer1l6	Transport
DMR7:98542001	7	98542001	98544000	2000	1	5.90E-07	-0.35	19	0.95	Fer1l6	Transport
DMR7:98674001	7	98674001	98675000	1000	1	1.00E-07	-0.66	9	0.9	Tmem65	
DMR7:98761001	7	98761001	98763000	2000	1	1.00E-08	-0.45	4	0.2	Rnf139	
DMR7:98818001	7	98818001	98820000	2000	1	2.00E-09	-0.69	23	1.15	Tatdn1;Ndufb9;Mtss1	Metabolism;Cytoskeleton
DMR7:98847001	7	98847001	98854000	7000	1	3.00E-07	-0.36	128	1.83	Mtss1	Cytoskeleton
DMR7:98894001	7	98894001	98899000	5000	1	3.10E-07	-0.52	97	1.94	Mtss1	Cytoskeleton
DMR7:99716001	7	99716001	99719000	3000	1	6.80E-10	-0.54	27	0.9	Nsmce2	
DMR7:99731001	7	99731001	99733000	2000	1	7.90E-13	0.57	27	1.35	Nsmce2	
DMR7:99755001	7	99755001	99758000	3000	1	5.40E-08	-0.54	30	1	Nsmce2	
DMR7:99771001	7	99771001	99773000	2000	1	1.20E-07	-0.35	26	1.3	Nsmce2	
DMR7:99807001	7	99807001	99813000	6000	1	6.10E-09	-0.28	74	1.23	Nsmce2	
DMR7:99840001	7	99840001	99841000	1000	1	7.00E-08	-0.45	17	1.7	Nsmce2	
DMR7:99863001	7	99863001	99864000	1000	1	1.60E-09	0.43	9	0.9	Nsmce2	
DMR7:99943001	7	99943001	99945000	2000	1	1.30E-08	0.43	33	1.65	LOC108351497;LOC690120;Trib1	Signaling
DMR7:102647001	7	102647001	102648000	1000	1	1.70E-11	0.81	26	2.6	Pvt1;LOC108351503	
DMR7:102676001	7	102676001	102681000	5000	1	4.60E-08	-0.26	46	0.92	Pvt1	
DMR7:102772001	7	102772001	102774000	2000	1	3.10E-08	-0.35	21	1.05	Pvt1	
DMR7:104500001	7	104500001	104501000	1000	1	4.20E-09	0.46	20	2	Gsdmc;RGD1359449	
DMR7:104789001	7	104789001	104794000	5000	1	5.60E-08	-0.35	44	0.88	Asap1	
DMR7:105367001	7	105367001	105372000	5000	1	6.00E-07	-0.29	48	0.96	Adcy8	
DMR7:105447001	7	105447001	105450000	3000	1	4.70E-07	-0.57	18	0.6	Adcy8	
DMR7:106643001	7	106643001	106644000	1000	1	3.70E-08	0.62	2	0.2	Oc90	Metabolism
DMR7:107133001	7	107133001	107136000	3000	1	7.40E-07	0.38	24	0.8	Lrrc6	
DMR7:107574001	7	107574001	107578000	4000	1	2.70E-08	-0.33	34	0.85	Tg;Sla	Cytoskeleton
DMR7:107646001	7	107646001	107647000	1000	1	4.90E-07	0.36	3	0.3	Tg	Cytoskeleton
DMR7:107887001	7	107887001	107889000	2000	1	2.80E-07	-0.36	25	1.25	St3gal1	Transport
DMR7:107892001	7	107892001	107894000	2000	1	4.20E-08	0.54	18	0.9	St3gal1	Transport
DMR7:108763001	7	108763001	108764000	1000	1	8.90E-11	0.61	21	2.1	Phf20l1	
DMR7:109041001	7	109041001	109042000	1000	1	1.70E-07	0.37	14	1.4	Zfat	Transcription
DMR7:109054001	7	109054001	109057000	3000	1	1.30E-08	0.67	30	1	Zfat	Transcription
DMR7:109076001	7	109076001	109077000	1000	1	4.20E-08	0.54	7	0.7	Zfat	Transcription
DMR7:109078001	7	109078001	109079000	1000	1	4.60E-07	0.49	11	1.1	Zfat	Transcription
DMR7:109092001	7	109092001	109094000	2000	1	3.40E-07	0.67	14	0.7	Zfat	Transcription
DMR7:109096001	7	109096001	109099000	3000	1	5.90E-07	0.55	28	0.93	Zfat	Transcription
DMR7:109101001	7	109101001	109102000	1000	1	1.60E-18	0.68	7	0.7	Zfat	Transcription
DMR7:109107001	7	109107001	109108000	1000	1	9.10E-10	0.45	20	2	Zfat	Transcription
DMR7:109123001	7	109123001	109125000	2000	2	2.90E-14	0.67	15	0.75	Zfat	Transcription
DMR7:109134001	7	109134001	109137000	3000	1	1.20E-07	0.53	18	0.6	Zfat	Transcription
DMR7:109145001	7	109145001	109146000	1000	1	1.70E-17	0.73	8	0.8	Zfat	Transcription
DMR7:109147001	7	109147001	109150000	3000	1	2.50E-09	0.52	24	0.8	Zfat	Transcription
DMR7:109156001	7	109156001	109161000	5000	1	1.10E-07	0.5	66	1.32	Zfat	Transcription
DMR7:109165001	7	109165001	109169000	4000	4	1.60E-14	0.67	35	0.88	Zfat	Transcription
DMR7:109172001	7	109172001	109176000	4000	1	1.70E-10	0.6	38	0.95	Zfat	Transcription
DMR7:109181001	7	109181001	109182000	1000	1	4.50E-08	0.44	4	0.4	Zfat	Transcription
DMR7:109194001	7	109194001	109204000	10000	1	3.60E-10	0.57	102	1.02	Zfat	Transcription

DMR7:109207001	7	109207001	109208000	1000	1	7.50E-11	0.63	5	0.5	Zfat	Transcription
DMR7:109211001	7	109211001	109213000	2000	1	6.00E-07	0.51	12	0.6	Zfat	Transcription
DMR7:109281001	7	109281001	109284000	3000	1	8.90E-10	0.61	19	0.63	Mir30b;Mir30d	
DMR7:109291001	7	109291001	109292000	1000	1	1.60E-09	0.63	7	0.7	Mir30b;Mir30d	
DMR7:110148001	7	110148001	110151000	3000	1	3.40E-08	-0.52	18	0.6	Khdrbs3	Translation
DMR7:110156001	7	110156001	110157000	1000	1	5.70E-08	0.39	10	1	Khdrbs3	Translation
DMR7:111727001	7	111727001	111729000	2000	1	9.70E-08	-0.4	14	0.7	Rps19l1	Translation
DMR7:113248001	7	113248001	113249000	1000	1	2.30E-09	-0.5	9	0.9	Col22a1	Extracellular Matrix
DMR7:113988001	7	113988001	113989000	1000	1	2.30E-07	0.41	12	1.2	Trappc9	
DMR7:114097001	7	114097001	114098000	1000	1	3.50E-08	0.43	11	1.1	Trappc9	
DMR7:114184001	7	114184001	114185000	1000	1	1.60E-07	0.37	25	2.5	Trappc9	
DMR7:114293001	7	114293001	114296000	3000	1	2.00E-10	0.38	46	1.53	Trappc9	
DMR7:115007001	7	115007001	115008000	1000	1	1.40E-12	0.6	10	1	Ptp4a3;Mroh5	Signaling
DMR7:115838001	7	115838001	115842000	4000	1	1.50E-08	-0.46	47	1.18	Adgrb1;LOC102550505;LOC102550355;Mroh4	Signaling
DMR7:115865001	7	115865001	115867000	2000	1	6.00E-08	0.39	20	1	LOC102550355;Mroh4	
DMR7:115934001	7	115934001	115935000	1000	1	2.60E-07	-0.38	7	0.7	LOC103692938;Jrk	Epigenetic
DMR7:116250001	7	116250001	116255000	5000	1	3.80E-07	0.39	63	1.26	Cyp11b2	
DMR7:116351001	7	116351001	116352000	1000	1	1.40E-07	0.48	9	0.9	Ly6e	
DMR7:116436001	7	116436001	116439000	3000	1	1.60E-08	0.5	48	1.6	LOC108351510;Ly6l	
DMR7:116508001	7	116508001	116510000	2000	1	1.20E-08	-0.51	10	0.5	RGD1565410	
DMR7:116650001	7	116650001	116651000	1000	1	2.60E-08	0.42	12	1.2	Zfp41	Transcription
DMR7:116705001	7	116705001	116706000	1000	1	2.20E-08	-0.42	26	2.6	Top1mt	Transcription
DMR7:116718001	7	116718001	116720000	2000	2	1.00E-12	0.44	11	0.55	Top1mt	Transcription
DMR7:116721001	7	116721001	116722000	1000	1	2.50E-07	0.5	0	0	Top1mt	Transcription
DMR7:116723001	7	116723001	116725000	2000	1	1.20E-07	0.42	25	1.25	Top1mt	Transcription
DMR7:116848001	7	116848001	116849000	1000	1	4.50E-07	0.46	1	0.1	Zc3h3	
DMR7:116898001	7	116898001	116899000	1000	1	2.60E-08	0.4	9	0.9	Gsdmd	
DMR7:116909001	7	116909001	116910000	1000	1	1.40E-07	-0.49	11	1.1	Mroh6	
DMR7:117370001	7	117370001	117372000	2000	1	1.50E-07	0.43	19	0.95	Oplah	Metabolism
DMR7:117380001	7	117380001	117382000	2000	1	8.20E-10	0.4	31	1.55	Oplah;Exosc4	Metabolism;Transcription
DMR7:117387001	7	117387001	117389000	2000	1	2.20E-07	-0.38	30	1.5	Oplah;Exosc4;Gpaa1	Metabolism;Transcription
DMR7:117406001	7	117406001	117408000	2000	1	6.40E-07	0.36	12	0.6	Gpaa1;Cyc1;Sharpin;Maf1	Proteolysis;Transcription
DMR7:117464001	7	117464001	117466000	2000	1	1.00E-07	0.37	36	1.8	Mroh1	
DMR7:117505001	7	117505001	117506000	1000	1	4.10E-07	0.36	14	1.4	Bop1	Translation
DMR7:117535001	7	117535001	117538000	3000	1	2.30E-13	-0.73	43	1.43	Bop1;Hsf1	Translation;Transcription
DMR7:117560001	7	117560001	117561000	1000	1	1.80E-07	0.53	8	0.8	Hsf1;Dgat1	Transcription;Metabolism
DMR7:117790001	7	117790001	117791000	1000	1	1.30E-08	0.55	18	1.8	Lrrc24;MGC94207;Arhgap39	Immune
DMR7:117833001	7	117833001	117836000	3000	1	5.20E-07	-0.34	47	1.57	Arhgap39	
DMR7:119181001	7	119181001	119182000	1000	1	3.30E-07	0.41	19	1.9	Foxred2;Eif3d	Metabolism;Translation
DMR7:119257001	7	119257001	119261000	4000	1	3.70E-07	0.5	48	1.2	Cacng2	Transport
DMR7:119275001	7	119275001	119276000	1000	1	1.10E-08	0.38	7	0.7	Cacng2	Transport
DMR7:119405001	7	119405001	119407000	2000	1	3.40E-08	0.39	24	1.2	Ift27	
DMR7:119559001	7	119559001	119560000	1000	1	5.90E-08	0.6	6	0.6	Csf2rb	Receptor
DMR7:119652001	7	119652001	119653000	1000	1	7.20E-07	0.47	15	1.5	Kctd17;Tmprss6	Protease
DMR7:119663001	7	119663001	119666000	3000	1	7.40E-10	0.5	53	1.77	Kctd17;Tmprss6	Protease
DMR7:119801001	7	119801001	119803000	2000	1	2.00E-08	0.46	16	0.8	Rac2	Signaling
DMR7:119814001	7	119814001	119818000	4000	1	3.50E-08	0.73	97	2.42	Cyth4	Transcription
DMR7:119902001	7	119902001	119903000	1000	1	6.60E-07	0.35	11	1.1	Elfn2;LOC102549375	Receptor
DMR7:119970001	7	119970001	119973000	3000	1	4.00E-07	0.42	25	0.83	Mfng	Golgi
DMR7:119993001	7	119993001	119994000	1000	1	2.00E-07	0.39	11	1.1	Mfng;Card10	Golgi
DMR7:120054001	7	120054001	120057000	3000	1	3.20E-08	0.52	35	1.17	LOC108351511;Cdc42ep1	
DMR7:120059001	7	120059001	120061000	2000	1	2.60E-07	0.3	20	1	Cdc42ep1	
DMR7:120096001	7	120096001	120097000	1000	1	1.00E-08	-0.45	24	2.4	Gga1	
DMR7:120175001	7	120175001	120176000	1000	1	2.10E-07	0.35	11	1.1	Nol12;Triobp	Cytoskeleton
DMR7:120659001	7	120659001	120662000	3000	1	2.20E-07	-0.45	79	2.63	Csnk1e	Signaling
DMR7:120813001	7	120813001	120814000	1000	1	1.80E-12	0.51	16	1.6	Dmc1	Transcription
DMR7:120838001	7	120838001	120845000	7000	1	2.70E-07	0.64	103	1.47	Dmc1;Fam227a	Transcription
DMR7:121116001	7	121116001	121121000	5000	1	8.20E-10	0.38	73	1.46	LOC102547383;Apobec3b	
DMR7:121127001	7	121127001	121128000	1000	1	3.00E-18	0.95	26	2.6	Apobec3b;Cbx7	Proteolysis

DMR7:121208001	7	121208001	121211000	3000	1	1.80E-08	0.42	73	2.43	Pdgfb	Growth Factors
DMR7:121291001	7	121291001	121293000	2000	1	2.10E-11	0.52	29	1.45	LOC108351514;LOC102547448;Rpl3	Translation
DMR7:121432001	7	121432001	121433000	1000	1	4.60E-10	0.59	31	3.1	Mgat3	Golgi
DMR7:121482001	7	121482001	121483000	1000	1	1.50E-07	-0.46	9	0.9	Mief1;Atf4;Rps19bp1	Transcription
DMR7:121766001	7	121766001	121767000	1000	1	1.30E-08	-0.39	13	1.3	Enthd1;LOC102550152	
DMR7:121987001	7	121987001	121989000	2000	1	3.10E-09	-0.43	28	1.4	Tnrc6b	Metabolism
DMR7:122076001	7	122076001	122077000	1000	1	5.60E-07	-0.39	14	1.4	Tnrc6b	Metabolism
DMR7:122109001	7	122109001	122113000	4000	1	4.60E-11	-0.52	40	1	Tnrc6b	Metabolism
DMR7:122353001	7	122353001	122355000	2000	1	3.20E-13	-0.58	45	2.25	Mkl1	
DMR7:122557001	7	122557001	122560000	3000	1	9.20E-08	-0.39	48	1.6	Slc25a17	
DMR7:122643001	7	122643001	122644000	1000	1	3.70E-07	-0.46	21	2.1	St13;Xpnpep3;Dnajb7	Protease
DMR7:123176001	7	123176001	123179000	3000	1	5.70E-07	0.5	52	1.73	LOC102549144;Csdc2;Pmm1	Metabolism;Metabolism
DMR7:123282001	7	123282001	123286000	4000	1	1.30E-07	-0.52	48	1.2	Xrcc6;Nhp2l1;LOC100362109	Epigenetic
DMR7:123294001	7	123294001	123295000	1000	1	1.10E-09	0.88	27	2.7	Nhp2l1;LOC100362109	
DMR7:123383001	7	123383001	123389000	6000	2	3.80E-09	-0.37	89	1.48	Ccdc134;Srebf2	
DMR7:123450001	7	123450001	123451000	1000	1	1.60E-07	0.62	26	2.6	Shisa8;Tnfrsf13c	Receptor
DMR7:123519001	7	123519001	123522000	3000	1	1.90E-07	0.28	38	1.27	Sept3;Wbp2nl	
DMR7:123544001	7	123544001	123548000	4000	1	4.00E-07	-0.45	77	1.93	Wbp2nl	
DMR7:123637001	7	123637001	123638000	1000	1	5.30E-07	0.37	16	1.6	Cyp2d1;Cyp2d3	Metabolism
DMR7:123852001	7	123852001	123853000	1000	1	9.20E-07	0.33	20	2	Nfam1	
DMR7:124006001	7	124006001	124009000	3000	1	2.50E-07	-0.45	30	1	Poldip3	Metabolism
DMR7:124046001	7	124046001	124049000	3000	1	9.10E-07	0.36	31	1.03	Cyb5r3	Metabolism
DMR7:124370001	7	124370001	124373000	3000	1	6.20E-12	0.46	21	0.7	Ttll1	Cytoskeleton
DMR7:124377001	7	124377001	124378000	1000	1	2.40E-07	-0.36	30	3	Ttll1	Cytoskeleton
DMR7:124508001	7	124508001	124509000	1000	1	3.00E-08	0.41	11	1.1	Scube1	Extracellular Matrix
DMR7:124552001	7	124552001	124555000	3000	1	1.50E-10	0.49	35	1.17	Scube1	Extracellular Matrix
DMR7:124583001	7	124583001	124588000	5000	1	1.40E-08	0.39	71	1.42	Scube1	Extracellular Matrix
DMR7:124705001	7	124705001	124706000	1000	1	5.90E-07	-0.41	18	1.8	Mpped1	Metabolism
DMR7:124751001	7	124751001	124754000	3000	1	3.90E-08	0.58	22	0.73	Mpped1;Efcab6	Metabolism;Signaling
DMR7:124895001	7	124895001	124896000	1000	1	3.00E-07	0.56	12	1.2	Efcab6	Signaling
DMR7:125230001	7	125230001	125231000	1000	1	1.30E-07	0.39	16	1.6	Parvb	Cytoskeleton
DMR7:125236001	7	125236001	125237000	1000	1	3.10E-08	-0.46	26	2.6	Parvb	Cytoskeleton
DMR7:125242001	7	125242001	125247000	5000	3	7.50E-19	1	60	1.2	Parvb	Cytoskeleton
DMR7:125308001	7	125308001	125309000	1000	1	4.80E-15	0.46	11	1.1	Parvg;LOC108351521	Cytoskeleton
DMR7:125358001	7	125358001	125360000	2000	1	5.30E-09	0.41	19	0.95	RGD1566029	
DMR7:125461001	7	125461001	125462000	1000	1	5.50E-07	0.46	15	1.5	Ldoc1l	
DMR7:125497001	7	125497001	125498000	1000	1	4.50E-08	-0.54	20	2	RGD1560568	
DMR7:125597001	7	125597001	125599000	2000	1	1.80E-07	-0.42	31	1.55	Prr5	
DMR7:125917001	7	125917001	125918000	1000	1	1.60E-10	0.58	20	2	Upk3a	
DMR7:126044001	7	126044001	126046000	2000	1	3.20E-07	0.39	16	0.8	Ribc2	Cytoskeleton
DMR7:126166001	7	126166001	126167000	1000	1	6.40E-07	0.3	19	1.9	Fbln1	Extracellular Matrix
DMR7:126251001	7	126251001	126254000	3000	1	3.30E-07	-0.51	35	1.17	Atxn10	
DMR7:126300001	7	126300001	126302000	2000	1	6.00E-07	-0.49	22	1.1	Atxn10	
DMR7:126328001	7	126328001	126330000	2000	1	6.90E-10	-0.38	27	1.35	Atxn10	
DMR7:126416001	7	126416001	126419000	3000	1	1.30E-07	0.36	32	1.07	Wnt7b	Signaling
DMR7:126435001	7	126435001	126440000	5000	2	9.40E-13	0.53	64	1.28	Wnt7b	Signaling
DMR7:126649001	7	126649001	126656000	7000	1	2.00E-08	-0.49	129	1.84	Ppara	Transcription
DMR7:126671001	7	126671001	126674000	3000	1	6.20E-09	0.5	23	0.77	Ppara	Transcription
DMR7:126805001	7	126805001	126807000	2000	1	1.20E-09	0.47	36	1.8	Celsr1	Cytoskeleton
DMR7:126821001	7	126821001	126825000	4000	1	2.70E-08	0.33	60	1.5	Celsr1	Cytoskeleton
DMR7:126841001	7	126841001	126846000	5000	1	1.10E-09	0.41	69	1.38	Celsr1	Cytoskeleton
DMR7:126863001	7	126863001	126866000	3000	1	2.80E-09	-0.53	34	1.13	Celsr1	Cytoskeleton
DMR7:128529001	7	128529001	128531000	2000	1	3.50E-08	0.39	14	0.7	Fam19a5	
DMR7:128627001	7	128627001	128629000	2000	1	2.20E-08	0.51	30	1.5	Fam19a5	
DMR7:129589001	7	129589001	129591000	2000	1	7.50E-10	-0.42	40	2	Zdhhc25	
DMR7:129719001	7	129719001	129721000	2000	1	8.50E-12	-0.52	51	2.55	Brd1	Transcription
DMR7:129988001	7	129988001	129993000	5000	3	1.10E-08	0.47	79	1.58	Mov10l1	
DMR7:130031001	7	130031001	130032000	1000	1	4.70E-07	-0.44	20	2	Mov10l1	
DMR7:130165001	7	130165001	130168000	3000	1	1.00E-08	0.51	58	1.93	Plxnb2;LOC102549176;Dennd6b	
DMR7:130333001	7	130333001	130334000	1000	1	2.20E-10	0.32	10	1	Lmf2;Ncaph2;Tymp	Golgi
DMR7:130362001	7	130362001	130366000	4000	1	1.90E-07	0.52	37	0.92	Klhdc7b;Syce3	
DMR7:130480001	7	130480001	130481000	1000	1	6.40E-07	0.34	8	0.8	Shank3	

DMR7:130483001	7	130483001	130487000	4000	1	8.70E-07	-0.43	69	1.73	Shank3	
DMR7:131682001	7	131682001	131684000	2000	1	7.90E-10	0.43	18	0.9	Cpne8	
DMR7:131690001	7	131690001	131691000	1000	1	4.20E-08	-0.54	13	1.3	Cpne8	
DMR7:131782001	7	131782001	131784000	2000	1	1.30E-13	0.81	47	2.35	Cpne8	
DMR7:131789001	7	131789001	131791000	2000	1	8.10E-12	0.48	17	0.85	Cpne8	
DMR7:132105001	7	132105001	132114000	9000	1	3.50E-08	-0.43	208	2.31	Kif21a	Cytoskeleton
DMR7:132155001	7	132155001	132156000	1000	1	7.60E-10	-0.56	15	1.5	Kif21a	Cytoskeleton
DMR7:132164001	7	132164001	132166000	2000	1	3.20E-07	-0.47	45	2.25	Kif21a	Cytoskeleton
DMR7:132344001	7	132344001	132345000	1000	1	2.90E-11	0.71	15	1.5	Abcd2;LOC690142	Transport
DMR7:132428001	7	132428001	132429000	1000	1	8.60E-07	-0.41	10	1	Slc2a13	Transport
DMR7:132608001	7	132608001	132611000	3000	1	1.10E-08	-0.49	10	0.33	Slc2a13	Transport
DMR7:132619001	7	132619001	132623000	4000	1	2.00E-08	-0.35	38	0.95	Slc2a13	Transport
DMR7:132654001	7	132654001	132657000	3000	2	3.60E-08	-0.32	30	1	Slc2a13	Transport
DMR7:132708001	7	132708001	132709000	1000	1	1.90E-08	0.32	8	0.8	Slc2a13	Transport
DMR7:132877001	7	132877001	132879000	2000	1	9.20E-07	-0.37	20	1	Lrrk2	Cytoskeleton
DMR7:133062001	7	133062001	133068000	6000	1	8.30E-07	-0.26	66	1.1	Muc19;Smgc	
DMR7:133306001	7	133306001	133309000	3000	1	2.50E-08	0.45	24	0.8	Cntn1	
DMR7:133473001	7	133473001	133474000	1000	1	1.10E-08	0.27	15	1.5	Cntn1	
DMR7:133885001	7	133885001	133887000	2000	1	2.30E-07	0.4	19	0.95	Pdzrn4	
DMR7:134445001	7	134445001	134446000	1000	1	1.60E-08	-0.51	23	2.3	LOC102552792;LOC103692960;Gxylt1	
DMR7:134448001	7	134448001	134454000	6000	1	5.70E-07	-0.38	80	1.33	Gxylt1	
DMR7:134581001	7	134581001	134584000	3000	1	2.90E-07	0.64	49	1.63	Zcrb1	
DMR7:134614001	7	134614001	134616000	2000	1	1.50E-12	-0.56	27	1.35	Pphln1;LOC108351532	
DMR7:134746001	7	134746001	134748000	2000	1	1.50E-07	0.33	20	1	Prickle1	Cytoskeleton
DMR7:134756001	7	134756001	134757000	1000	1	1.20E-07	0.38	7	0.7	Prickle1	Cytoskeleton
DMR7:135469001	7	135469001	135471000	2000	1	9.90E-07	0.3	23	1.15	Adamts20	Protease
DMR7:135492001	7	135492001	135493000	1000	1	2.10E-10	0.71	23	2.3	Adamts20	Protease
DMR7:135514001	7	135514001	135515000	1000	1	6.00E-07	0.36	35	3.5	Adamts20	Protease
DMR7:135526001	7	135526001	135528000	2000	1	1.60E-12	0.86	65	3.25	Adamts20	Protease
DMR7:135530001	7	135530001	135535000	5000	1	5.90E-08	-0.47	121	2.42	Adamts20	Protease
DMR7:135540001	7	135540001	135541000	1000	1	2.50E-10	-0.79	21	2.1	Adamts20	Protease
DMR7:135563001	7	135563001	135564000	1000	1	6.80E-09	0.51	4	0.4	Adamts20	Protease
DMR7:135902001	7	135902001	135905000	3000	1	1.60E-15	0.32	47	1.57	Tmem117	
DMR7:135930001	7	135930001	135935000	5000	1	9.80E-16	-0.69	89	1.78	Tmem117	
DMR7:135992001	7	135992001	135993000	1000	1	3.20E-07	0.42	12	1.2	Tmem117	
DMR7:136005001	7	136005001	136006000	1000	1	9.30E-07	-0.41	27	2.7	Tmem117	
DMR7:136187001	7	136187001	136189000	2000	1	1.30E-08	0.36	21	1.05	Tmem117	
DMR7:136191001	7	136191001	136192000	1000	1	2.00E-08	0.39	8	0.8	Tmem117	
DMR7:136260001	7	136260001	136261000	1000	1	4.10E-07	0.65	17	1.7	Tmem117;LOC103690215;LOC102546578	
DMR7:136317001	7	136317001	136318000	1000	1	2.50E-10	0.71	24	2.4	Tmem117	
DMR7:136337001	7	136337001	136340000	3000	1	4.40E-08	0.57	60	2	Tmem117	
DMR7:136530001	7	136530001	136531000	1000	1	3.10E-07	-0.42	16	1.6	Nell2	Signaling
DMR7:136755001	7	136755001	136758000	3000	2	5.50E-15	0.92	99	3.3	Nell2	Signaling
DMR7:136800001	7	136800001	136801000	1000	1	5.10E-07	-0.45	15	1.5	Nell2	Signaling
DMR7:136820001	7	136820001	136824000	4000	2	4.50E-09	-0.49	38	0.95	Nell2	Signaling
DMR7:136990001	7	136990001	136994000	4000	1	5.00E-14	-0.58	79	1.98	Dbx2	
DMR7:137152001	7	137152001	137158000	6000	1	9.50E-09	-0.32	108	1.8	Ano6	
DMR7:137166001	7	137166001	137171000	5000	1	2.80E-07	-0.44	103	2.06	Ano6;LOC102547300	
DMR7:137339001	7	137339001	137344000	5000	2	1.10E-14	0.61	100	2	Ano6	
DMR7:137773001	7	137773001	137776000	3000	1	1.20E-07	-0.47	46	1.53	Arid2	
DMR7:137782001	7	137782001	137784000	2000	1	3.60E-08	0.33	39	1.95	Arid2	
DMR7:137822001	7	137822001	137823000	1000	1	1.30E-09	-0.56	10	1	Scaf11	
DMR7:138015001	7	138015001	138018000	3000	1	6.00E-07	-0.35	53	1.77	Slc38a1	Transport
DMR7:138019001	7	138019001	138023000	4000	1	2.40E-08	0.66	124	3.1	Slc38a1	Transport
DMR7:138032001	7	138032001	138036000	4000	1	2.20E-07	0.36	90	2.25	Slc38a1;LOC108351543	Transport
DMR7:138093001	7	138093001	138100000	7000	4	2.40E-11	-0.85	199	2.84	Slc38a2	Transport
DMR7:138105001	7	138105001	138108000	3000	1	3.80E-08	-0.49	63	2.1	Slc38a2;LOC108351544	Transport
DMR7:138467001	7	138467001	138468000	1000	1	4.10E-09	0.52	22	2.2	Slc38a4;LOC108351546	Transport
DMR7:138705001	7	138705001	138707000	2000	1	1.80E-07	-0.6	83	4.15	Amigo2;LOC102551484;Pced1b	
DMR7:138726001	7	138726001	138731000	5000	3	4.00E-10	0.71	61	1.22	LOC102551484;Pced1b	
DMR7:138742001	7	138742001	138744000	2000	1	1.00E-06	0.48	57	2.85	LOC102551484;Pced1b	

DMR7:138786001	7	138786001	138787000	1000	1	1.60E-12	0.86	37	3.7	LOC102551484;Pced1b	
DMR7:138795001	7	138795001	138800000	5000	1	9.00E-07	-0.33	86	1.72	LOC102551484;Pced1b	
DMR7:138838001	7	138838001	138839000	1000	1	7.30E-08	0.71	41	4.1	Pced1b	
DMR7:138905001	7	138905001	138909000	4000	1	1.00E-08	0.35	54	1.35	RGD1565798	
DMR7:139190001	7	139190001	139192000	2000	1	1.90E-08	0.39	27	1.35	Rpap3	
DMR7:139214001	7	139214001	139220000	6000	1	1.30E-07	0.37	83	1.38	Endou	
DMR7:139230001	7	139230001	139231000	1000	1	6.60E-08	0.28	16	1.6	Endou;Rapgef3	Transcription
DMR7:139256001	7	139256001	139262000	6000	2	5.70E-08	0.36	89	1.48	Rapgef3;LOC102548155;Slc48a1	Transcription;Transport
DMR7:139268001	7	139268001	139270000	2000	1	4.10E-09	0.52	40	2	LOC102548155;Slc48a1	Transport
DMR7:139281001	7	139281001	139282000	1000	1	4.70E-07	0.56	13	1.3	Slc48a1;Hdac7	Transport;Epigenetic
DMR7:139302001	7	139302001	139304000	2000	1	5.40E-08	0.42	36	1.8	Hdac7	Epigenetic
DMR7:139324001	7	139324001	139325000	1000	1	3.50E-07	0.36	8	0.8	Hdac7;LOC108351548;LOC103692974	Epigenetic
DMR7:139399001	7	139399001	139400000	1000	1	3.10E-07	-0.36	24	2.4	Vdr	Transcription
DMR7:139489001	7	139489001	139491000	2000	1	7.90E-07	0.46	43	2.15	Col2a1	Extracellular Matrix
DMR7:139662001	7	139662001	139663000	1000	1	1.90E-07	-0.5	13	1.3	Senp1	Protease
DMR7:139720001	7	139720001	139722000	2000	2	3.10E-10	0.52	15	0.75	Pfkm;Asb8	Metabolism
DMR7:139726001	7	139726001	139729000	3000	1	2.00E-09	-0.41	43	1.43	Pfkm;Asb8	Metabolism
DMR7:139736001	7	139736001	139741000	5000	2	8.80E-12	0.7	95	1.9	Asb8	
DMR7:139744001	7	139744001	139745000	1000	1	7.90E-08	0.53	21	2.1	Asb8	
DMR7:139779001	7	139779001	139782000	3000	2	3.80E-13	0.66	63	2.1	Or10ad1	
DMR7:139852001	7	139852001	139854000	2000	1	5.40E-07	0.33	22	1.1	Olr1877;LOC100911698	
DMR7:139929001	7	139929001	139930000	1000	1	1.30E-15	0.99	35	3.5	Olr1104	Receptor
DMR7:140038001	7	140038001	140040000	2000	1	7.10E-07	-0.52	9	0.45	Olr1107;LOC680635	Receptor
DMR7:140050001	7	140050001	140053000	3000	1	1.00E-09	0.61	70	2.33	LOC680635;Olr1108	Receptor
DMR7:140054001	7	140054001	140056000	2000	1	1.60E-07	0.42	55	2.75	LOC680635;Olr1108	Receptor
DMR7:140058001	7	140058001	140059000	1000	1	5.60E-07	-0.47	14	1.4	LOC680635;Olr1108	Receptor
DMR7:140063001	7	140063001	140065000	2000	1	6.20E-10	0.39	20	1	Olr1108	Receptor
DMR7:140078001	7	140078001	140082000	4000	2	1.20E-10	0.53	70	1.75	Olr1109-ps;Olr1110-ps;Lalba	
DMR7:140116001	7	140116001	140119000	3000	2	1.10E-11	0.64	48	1.6	Olr1111;LOC103692975	Receptor
DMR7:140373001	7	140373001	140375000	2000	1	2.70E-12	0.52	29	1.45	LOC103692978;Ccdc65	
DMR7:140396001	7	140396001	140398000	2000	1	5.60E-08	-0.44	48	2.4	Ccdc65;Fkbp11	
DMR7:140412001	7	140412001	140414000	2000	1	1.40E-09	-0.4	24	1.2	Fkbp11;Arf3	Signaling
DMR7:140474001	7	140474001	140475000	1000	1	1.20E-08	0.48	22	2.2	Wnt10b;Wnt1;Ddn;LOC102555574	Signaling
DMR7:140476001	7	140476001	140477000	1000	1	1.30E-08	0.56	7	0.7	Wnt10b;Wnt1;Ddn;LOC102555574	Signaling
DMR7:140606001	7	140606001	140608000	2000	1	2.30E-09	0.55	24	1.2	Lmbr1l;LOC102556368;LOC680861;Tuba1b	Receptor;Cytoskeleton
DMR7:140708001	7	140708001	140710000	2000	2	3.40E-15	0.92	50	2.5	Tuba1c	Cytoskeleton
DMR7:140935001	7	140935001	140938000	3000	1	1.50E-10	0.43	60	2	LOC103692979;Fam186b	
DMR7:140954001	7	140954001	140955000	1000	1	1.40E-09	0.41	12	1.2	Fam186b;Prpf40b	
DMR7:141038001	7	141038001	141039000	1000	1	3.00E-07	0.34	17	1.7	Fmnl3;Tmbim6;LOC103692980	
DMR7:141375001	7	141375001	141376000	1000	1	3.00E-08	0.58	25	2.5	Smarcd1;Gpd1;Cox14	Epigenetic;Metabolism;Transcription
DMR7:141428001	7	141428001	141431000	3000	1	3.40E-09	0.87	101	3.37	Cers5;Lima1	Cytoskeleton
DMR7:141756001	7	141756001	141759000	3000	1	4.40E-10	-0.5	35	1.17	Dip2b	
DMR7:141795001	7	141795001	141797000	2000	1	5.60E-14	-0.87	28	1.4	Dip2b;LOC102550811	
DMR7:141841001	7	141841001	141842000	1000	1	1.30E-07	0.36	18	1.8	Dip2b	
DMR7:141934001	7	141934001	141935000	1000	1	3.80E-08	0.38	7	0.7	Atf1	
DMR7:141977001	7	141977001	141980000	3000	1	4.80E-12	0.42	35	1.17	Mettl7a	Epigenetic
DMR7:141999001	7	141999001	142003000	4000	1	1.00E-09	0.83	103	2.58	Mettl7a;Higd1c	Epigenetic
DMR7:142055001	7	142055001	142057000	2000	1	1.10E-07	-0.52	30	1.5	Slc11a2	Transport
DMR7:142193001	7	142193001	142194000	1000	1	9.00E-12	0.65	4	0.4	Pou6f1	
DMR7:142355001	7	142355001	142358000	3000	1	2.60E-08	0.39	26	0.87	Galnt6	Golgi
DMR7:142368001	7	142368001	142369000	1000	1	3.40E-12	0.75	28	2.8	Galnt6	Golgi
DMR7:142370001	7	142370001	142376000	6000	3	4.10E-11	0.55	62	1.03	Galnt6	Golgi
DMR7:142595001	7	142595001	142597000	2000	1	3.20E-08	0.3	34	1.7	Scn8a	Transport
DMR7:142639001	7	142639001	142644000	5000	1	9.10E-07	-0.34	57	1.14	Scn8a	Transport
DMR7:142646001	7	142646001	142647000	1000	1	1.20E-07	0.34	7	0.7	Scn8a	Transport
DMR7:142651001	7	142651001	142653000	2000	1	4.00E-17	0.85	32	1.6	Scn8a	Transport
DMR7:142716001	7	142716001	142718000	2000	1	6.20E-08	0.41	28	1.4	Fignl2	
DMR7:142776001	7	142776001	142779000	3000	1	5.30E-08	0.44	58	1.93	Acvrl1	Signaling
DMR7:142807001	7	142807001	142810000	3000	1	3.50E-13	-0.55	35	1.17	Acvr1b	Signaling
DMR7:142919001	7	142919001	142920000	1000	1	5.50E-10	0.43	30	3	Nr4a1;Atg101	Transcription

DMR7:143058001	7	143058001	143060000	2000	1	4.80E-09	0.34	13	0.65	LOC103692999;Krt7	
DMR7:143065001	7	143065001	143068000	3000	1	1.20E-09	0.37	35	1.17	Krt7	
DMR7:143165001	7	143165001	143168000	3000	1	4.40E-08	0.59	43	1.43	Krt85	
DMR7:143196001	7	143196001	143200000	4000	1	8.60E-07	0.46	63	1.57	Krt84;Krt82;Kb15	
DMR7:143343001	7	143343001	143346000	3000	1	2.00E-07	0.42	22	0.73	Krt71	
DMR7:143407001	7	143407001	143408000	1000	1	3.40E-08	0.44	17	1.7	Krt73;Krt2	
DMR7:143419001	7	143419001	143420000	1000	1	4.80E-10	0.8	37	3.7	Krt2	
DMR7:143429001	7	143429001	143430000	1000	1	3.40E-07	0.31	7	0.7	Krt2	
DMR7:143445001	7	143445001	143447000	2000	2	4.70E-10	0.45	13	0.65	LOC300249;Krt1	
DMR7:143506001	7	143506001	143507000	1000	1	8.90E-07	0.35	12	1.2	Krt76	
DMR7:143539001	7	143539001	143543000	4000	1	1.50E-10	0.41	52	1.3	Krt79;Krt78	
DMR7:143607001	7	143607001	143608000	1000	1	8.30E-07	-0.46	29	2.9	Krt8	
DMR7:143719001	7	143719001	143720000	1000	1	1.30E-08	0.5	3	0.3	Tns2;Spryd3	Cytoskeleton;Cytoskeleton
DMR7:143755001	7	143755001	143756000	1000	1	1.70E-09	0.6	25	2.5	Igfbp6;LOC102551965;Soat2	Protease; Proteolysis;Metabolism
DMR7:143757001	7	143757001	143759000	2000	1	2.40E-07	0.37	22	1.1	Igfbp6;LOC102551965;Soat2	Protease; Proteolysis;Metabolism
DMR7:143787001	7	143787001	143792000	5000	2	2.10E-10	-0.69	57	1.14	Csad	Metabolism
DMR7:143797001	7	143797001	143798000	1000	1	7.40E-08	-0.44	15	1.5	Csad	Metabolism
DMR7:143827001	7	143827001	143828000	1000	1	7.80E-09	0.48	33	3.3	Znf740;Itgb7	Transcription;Extracellular Matrix
DMR7:143842001	7	143842001	143843000	1000	1	1.00E-07	0.36	12	1.2	Itgb7;Rarg	Extracellular Matrix;Transcription
DMR7:143874001	7	143874001	143876000	2000	1	8.90E-09	0.46	42	2.1	LOC102552775;Mfsd5	Transport
DMR7:143937001	7	143937001	143938000	1000	1	2.00E-07	0.43	19	1.9	Pfdn5;Myg1;Aaas	Transcription;Metabolism
DMR7:144153001	7	144153001	144156000	3000	1	2.40E-07	0.43	24	0.8	Atf7	Transcription
DMR7:144572001	7	144572001	144574000	2000	1	2.00E-09	-0.38	29	1.45	Hoxc11;Hoxc10	Development
DMR7:144598001	7	144598001	144601000	3000	1	1.60E-09	-0.37	46	1.53	LOC103692987;Hoxc9;Hoxc8	
DMR7:144621001	7	144621001	144622000	1000	1	1.90E-08	-0.36	11	1.1	Hoxc6;Hoxc5;Mir615	Development
DMR7:144940001	7	144940001	144941000	1000	1	4.70E-10	0.87	42	4.2	Gpr84;Zfp385a	Signaling
DMR7:144998001	7	144998001	144999000	1000	1	2.50E-11	0.53	5	0.5	Itga5	Extracellular Matrix
DMR7:145074001	7	145074001	145077000	3000	1	3.60E-09	-0.52	22	0.73	LOC102547836;RGD1563200;Nckap1l	
DMR7:145136001	7	145136001	145139000	3000	1	3.50E-10	0.48	57	1.9	Pde1b;Ppp1r1a	Signaling;Signaling
DMR7:145322001	7	145322001	145323000	1000	1	7.40E-09	0.51	6	0.6	LOC300274;Spt1	
DMR7:145586001	7	145586001	145588000	2000	1	7.00E-07	0.44	17	0.85	LOC102555445;Mucl1	
DMR8:462001	8	462001	464000	2000	1	5.40E-09	-0.35	12	0.6	Gucy1a2	Signaling
DMR8:675001	8	675001	678000	3000	1	6.20E-07	-0.37	33	1.1	Gucy1a2	Signaling
DMR8:700001	8	700001	702000	2000	1	5.20E-07	-0.47	12	0.6	Gucy1a2	Signaling
DMR8:724001	8	724001	730000	6000	1	6.30E-07	-0.34	73	1.22	Gucy1a2	Signaling
DMR8:801001	8	801001	806000	5000	1	1.90E-07	-0.27	46	0.92	Gucy1a2	Signaling
DMR8:861001	8	861001	866000	5000	1	2.70E-09	-0.44	49	0.98	Gucy1a2	Signaling
DMR8:1492001	8	1492001	1497000	5000	3	7.30E-10	-0.38	44	0.88	LOC102549625;Msantd4	
DMR8:1510001	8	1510001	1513000	3000	1	1.10E-08	-0.5	49	1.63	LOC102549625;Msantd4	
DMR8:1774001	8	1774001	1775000	1000	1	3.70E-07	-0.6	5	0.5	Gria4	Receptor
DMR8:1832001	8	1832001	1833000	1000	1	1.40E-07	-0.39	3	0.3	Gria4	Receptor
DMR8:1952001	8	1952001	1960000	8000	1	5.00E-08	-0.33	43	0.54	Gria4	Receptor
DMR8:4060001	8	4060001	4062000	2000	1	6.00E-08	0.43	8	0.4	Vom2r23	Signaling
DMR8:4077001	8	4077001	4082000	5000	1	8.40E-08	-0.36	39	0.78	Actl9b	
DMR8:4564001	8	4564001	4565000	1000	1	8.20E-07	0.47	13	1.3	Pdgfd;LOC108351647	Growth Factors
DMR8:4643001	8	4643001	4644000	1000	1	3.80E-07	0.62	26	2.6	Pdgfd	Growth Factors
DMR8:5225001	8	5225001	5230000	5000	2	7.60E-09	-0.42	26	0.52	Dync2h1	Cytoskeleton
DMR8:5232001	8	5232001	5233000	1000	1	7.20E-14	0.53	18	1.8	Dync2h1	Cytoskeleton
DMR8:5334001	8	5334001	5337000	3000	1	3.80E-07	0.38	30	1	Dync2h1	Cytoskeleton
DMR8:5419001	8	5419001	5420000	1000	1	3.70E-07	-0.56	17	1.7	Dync2h1	Cytoskeleton
DMR8:5541001	8	5541001	5546000	5000	1	3.20E-07	-0.4	43	0.86	Mmp13;LOC689310	Protease
DMR8:5702001	8	5702001	5704000	2000	1	1.10E-10	0.42	21	1.05	Mmp3;Mmp1	Protease
DMR8:5726001	8	5726001	5729000	3000	1	9.50E-08	-0.34	22	0.73	Mmp1;Mmp10	Protease
DMR8:5815001	8	5815001	5817000	2000	1	3.80E-09	0.48	28	1.4	Mmp20	Protease
DMR8:5843001	8	5843001	5846000	3000	1	1.40E-09	0.42	41	1.37	Mmp20	Protease
DMR8:5968001	8	5968001	5970000	2000	1	1.70E-07	-0.53	27	1.35	LOC102556477;Tmem123	

DMR8:6005001	8	6005001	6008000	3000	1	2.20E-07	-0.25	28	0.93	Tmem123;Birc2	Protease; Proteolysis
DMR8:6289001	8	6289001	6292000	3000	1	1.50E-07	0.47	20	0.67	Cep126	Transcription
DMR8:6849001	8	6849001	6850000	1000	1	9.20E-10	0.61	8	0.8	Trpc6	Transport
DMR8:6867001	8	6867001	6868000	1000	1	1.30E-08	-0.49	14	1.4	Trpc6	Transport
DMR8:6887001	8	6887001	6889000	2000	1	7.80E-07	0.33	22	1.1	Trpc6	Transport
DMR8:6899001	8	6899001	6900000	1000	1	3.50E-08	0.52	6	0.6	Trpc6	Transport
DMR8:7269001	8	7269001	7271000	2000	1	1.00E-09	0.71	31	1.55	Arhgap42	Signaling
DMR8:7334001	8	7334001	7336000	2000	1	1.60E-07	-0.69	27	1.35	Arhgap42	Signaling
DMR8:7848001	8	7848001	7851000	3000	1	5.20E-07	0.4	33	1.1	Cntn5	
DMR8:8159001	8	8159001	8163000	4000	1	1.60E-07	-0.35	28	0.7	Cntn5	
DMR8:8477001	8	8477001	8478000	1000	1	1.00E-07	0.49	3	0.3	Cntn5	
DMR8:8566001	8	8566001	8568000	2000	1	4.90E-07	-0.36	43	2.15	Cntn5	
DMR8:8672001	8	8672001	8678000	6000	1	5.50E-07	-0.33	39	0.65	Cntn5	
DMR8:8767001	8	8767001	8768000	1000	1	7.40E-07	0.42	12	1.2	Cntn5;LOC102551865	
DMR8:8781001	8	8781001	8784000	3000	1	4.30E-08	0.48	22	0.73	Cntn5	
DMR8:8798001	8	8798001	8804000	6000	1	8.30E-08	-0.34	53	0.88	Cntn5	
DMR8:8930001	8	8930001	8936000	6000	1	8.00E-08	0.39	58	0.97	Cntn5	
DMR8:11893001	8	11893001	11894000	1000	1	1.70E-07	-0.44	14	1.4	Jrkl;Ccdc82	Epigenetic
DMR8:11981001	8	11981001	11986000	5000	2	1.20E-12	0.53	43	0.86	Maml2	Transcription
DMR8:12024001	8	12024001	12026000	2000	1	9.60E-08	0.4	19	0.95	Maml2	Transcription
DMR8:12060001	8	12060001	12062000	2000	1	4.30E-11	-0.61	43	2.15	Maml2	Transcription
DMR8:12099001	8	12099001	12100000	1000	1	3.60E-10	0.48	11	1.1	Maml2	Transcription
DMR8:12112001	8	12112001	12114000	2000	1	7.60E-08	0.44	21	1.05	Maml2	Transcription
DMR8:12115001	8	12115001	12116000	1000	1	7.20E-07	0.32	18	1.8	Maml2	Transcription
DMR8:12125001	8	12125001	12128000	3000	1	3.80E-10	-0.59	38	1.27	Maml2	Transcription
DMR8:12170001	8	12170001	12174000	4000	1	7.60E-08	0.28	60	1.5	Maml2	Transcription
DMR8:12213001	8	12213001	12215000	2000	1	9.00E-08	0.52	25	1.25	Maml2	Transcription
DMR8:13055001	8	13055001	13059000	4000	1	2.30E-07	-0.46	78	1.95	Amotl1	
DMR8:13077001	8	13077001	13078000	1000	1	2.20E-07	0.38	19	1.9	Amotl1	
DMR8:13118001	8	13118001	13120000	2000	1	1.60E-11	0.51	43	2.15	Amotl1	
DMR8:13251001	8	13251001	13253000	2000	1	1.30E-07	0.53	37	1.85	Piwil4;LOC102549248;Fut4	Translation;Golgi
DMR8:13302001	8	13302001	13303000	1000	1	1.40E-08	-0.49	11	1.1	RGD1561795;Ankrd49;Mre11a	
DMR8:13349001	8	13349001	13350000	1000	1	4.50E-09	-0.65	17	1.7	Mre11a	
DMR8:13565001	8	13565001	13568000	3000	1	1.00E-09	-0.65	69	2.3	Med17;Panx1	Transcription
DMR8:13573001	8	13573001	13574000	1000	1	7.60E-09	0.37	13	1.3	Med17;Panx1	Transcription
DMR8:13620001	8	13620001	13623000	3000	1	4.40E-09	-0.51	51	1.7	Med17;Hephl1	Transcription;Metabolism
DMR8:13633001	8	13633001	13636000	3000	1	8.20E-08	0.47	60	2	Med17;Hephl1	Transcription;Metabolism
DMR8:13663001	8	13663001	13666000	3000	1	3.20E-12	0.63	39	1.3	Med17;Hephl1;RGD1562937	Transcription;Metabolism
DMR8:13691001	8	13691001	13692000	1000	1	2.60E-07	-0.44	6	0.6	Med17	Transcription
DMR8:13698001	8	13698001	13704000	6000	1	3.90E-09	-0.64	80	1.33	Med17;LOC108351808	Transcription
DMR8:13791001	8	13791001	13792000	1000	1	2.80E-08	0.36	14	1.4	Med17;LOC102551966;Vstm5	Transcription;Immune
DMR8:14023001	8	14023001	14026000	3000	1	3.00E-12	0.45	32	1.07	Smco4	
DMR8:14053001	8	14053001	14056000	3000	1	2.40E-12	-0.71	57	1.9	Smco4	
DMR8:14125001	8	14125001	14128000	3000	1	7.90E-10	0.63	45	1.5	Deup1;LOC108351654	Cytoskeleton
DMR8:14136001	8	14136001	14139000	3000	1	7.10E-09	-0.43	41	1.37	Deup1	Cytoskeleton
DMR8:14383001	8	14383001	14385000	2000	2	6.40E-10	-0.6	31	1.55	Mtnr1b	Signaling
DMR8:14408001	8	14408001	14410000	2000	1	3.80E-07	0.51	10	0.5	Fat3	Cytoskeleton
DMR8:14446001	8	14446001	14448000	2000	1	8.90E-07	0.36	23	1.15	Fat3	Cytoskeleton
DMR8:14454001	8	14454001	14457000	3000	1	2.70E-08	-0.45	31	1.03	Fat3	Cytoskeleton
DMR8:14683001	8	14683001	14686000	3000	1	5.90E-07	-0.44	26	0.87	Fat3	Cytoskeleton
DMR8:14691001	8	14691001	14695000	4000	1	4.40E-11	0.52	32	0.8	Fat3	Cytoskeleton
DMR8:14700001	8	14700001	14705000	5000	1	2.30E-10	0.49	45	0.9	Fat3	Cytoskeleton
DMR8:14845001	8	14845001	14848000	3000	1	8.30E-10	0.36	57	1.9	Fat3	Cytoskeleton
DMR8:15937001	8	15937001	15938000	1000	1	4.40E-07	-0.25	9	0.9	LOC108351816;Nek2l1	Signaling
DMR8:18521001	8	18521001	18522000	1000	1	4.60E-08	0.45	5	0.5	Muc16	
DMR8:18544001	8	18544001	18549000	5000	2	8.80E-09	-0.4	52	1.04	Muc16	
DMR8:18755001	8	18755001	18757000	2000	1	6.90E-07	-0.28	17	0.85	Olr1122	Receptor
DMR8:18947001	8	18947001	18954000	7000	2	5.00E-10	-0.47	69	0.99	Olr1131-ps	
DMR8:19090001	8	19090001	19096000	6000	1	3.60E-09	-0.31	48	0.8	Olr1135;Olr1136-ps	Receptor
DMR8:19335001	8	19335001	19338000	3000	1	8.00E-07	-0.56	8	0.27	Olr1144	Receptor

DMR8:19720001	8	19720001	19723000	3000	1	1.30E-10	-0.54	14	0.47	Olr1153-ps	
DMR8:19821001	8	19821001	19825000	4000	1	2.00E-08	-0.66	52	1.3	Olr1156	Receptor
DMR8:19839001	8	19839001	19845000	6000	1	4.40E-10	-0.29	71	1.18	Olr1157-ps	
DMR8:20179001	8	20179001	20181000	2000	1	7.20E-07	-0.36	6	0.3	Olr1163;RGD1563757	Receptor
DMR8:20233001	8	20233001	20237000	4000	1	2.20E-07	-0.38	103	2.58	Olr1165;LOC108351836	Receptor
DMR8:20713001	8	20713001	20720000	7000	1	3.20E-07	-0.37	41	0.59	Olr1179	Receptor
DMR8:21002001	8	21002001	21006000	4000	1	7.90E-07	-0.29	51	1.27	Olr1187-ps;Olfr873	Receptor
DMR8:21166001	8	21166001	21167000	1000	1	3.50E-08	0.47	5	0.5	Olr1185	Receptor
DMR8:21550001	8	21550001	21552000	2000	1	1.10E-09	0.46	15	0.75	Zfp266;LOC367035	
DMR8:21721001	8	21721001	21723000	2000	1	2.30E-07	0.37	18	0.9	Olfm2	Development
DMR8:21745001	8	21745001	21747000	2000	1	9.80E-07	0.42	31	1.55	Olfm2;LOC102552496	Development
DMR8:21791001	8	21791001	21792000	1000	1	2.40E-07	0.36	12	1.2	Col5a3	Extracellular Matrix
DMR8:21937001	8	21937001	21939000	2000	1	1.70E-07	-0.4	44	2.2	Dnmt1	Epigenetic
DMR8:21974001	8	21974001	21978000	4000	2	2.10E-15	0.78	86	2.15	Dnmt1;LOC367036;S1pr2	Epigenetic;Signaling
DMR8:21990001	8	21990001	21993000	3000	1	3.10E-08	0.44	45	1.5	S1pr2	Signaling
DMR8:22267001	8	22267001	22270000	3000	1	5.50E-08	0.5	89	2.97	Keap1;S1pr5	Cytoskeleton;Signaling
DMR8:22568001	8	22568001	22570000	2000	1	5.90E-10	0.48	22	1.1	LOC691141;Carm1	Golgi
DMR8:22708001	8	22708001	22709000	1000	1	7.00E-08	-0.54	6	0.6	Smarca4	Epigenetic
DMR8:22745001	8	22745001	22750000	5000	1	2.30E-08	0.45	62	1.24	Smarca4;Ldlr	Epigenetic;Binding Proteins
DMR8:22764001	8	22764001	22767000	3000	1	1.30E-10	0.43	57	1.9	Ldlr	Binding Proteins
DMR8:22816001	8	22816001	22820000	4000	1	2.20E-10	0.56	118	2.95	Kank2;Dock6	Cytoskeleton;Transcription
DMR8:22846001	8	22846001	22847000	1000	1	1.10E-07	0.41	28	2.8	Dock6;Angptl8	Transcription
DMR8:22864001	8	22864001	22865000	1000	1	5.10E-07	0.38	14	1.4	Dock6;Angptl8	Transcription
DMR8:22943001	8	22943001	22945000	2000	1	3.30E-08	0.67	53	2.65	Tmem205;Ccdc159;Plppr2	Signaling
DMR8:22957001	8	22957001	22959000	2000	1	5.30E-07	0.72	76	3.8	Plppr2;Swsap1	Signaling
DMR8:22964001	8	22964001	22966000	2000	1	1.50E-08	0.41	32	1.6	Plppr2;Swsap1;Epor	Signaling;Receptor
DMR8:22985001	8	22985001	22988000	3000	1	9.90E-08	0.36	53	1.77	Rgl3	Transcription
DMR8:23392001	8	23392001	23395000	3000	1	3.90E-11	-0.53	25	0.83	Anln;RGD1561444	Cytoskeleton
DMR8:23405001	8	23405001	23407000	2000	1	1.40E-07	0.44	22	1.1	Anln;RGD1561444	Cytoskeleton
DMR8:23490001	8	23490001	23491000	1000	1	4.20E-08	0.75	28	2.8	Rp9;Bbs9	
DMR8:23666001	8	23666001	23667000	1000	1	2.90E-08	0.65	17	1.7	Bbs9;LOC103693028	
DMR8:23938001	8	23938001	23939000	1000	1	6.00E-07	-0.41	20	2	Bbs9	
DMR8:23948001	8	23948001	23950000	2000	1	8.90E-07	-0.42	25	1.25	Bbs9	
DMR8:24400001	8	24400001	24403000	3000	1	1.90E-09	-0.51	68	2.27	Bmper	Extracellular Matrix
DMR8:24410001	8	24410001	24413000	3000	1	7.40E-07	-0.44	42	1.4	Bmper	Extracellular Matrix
DMR8:25372001	8	25372001	25374000	2000	1	2.10E-10	-0.46	12	0.6	Npsr1	Signaling
DMR8:25380001	8	25380001	25383000	3000	1	6.70E-08	-0.39	27	0.9	Npsr1	Signaling
DMR8:25462001	8	25462001	25463000	1000	1	5.60E-09	0.35	10	1	Npsr1	Signaling
DMR8:25636001	8	25636001	25639000	3000	2	1.10E-07	0.4	86	2.87	Dpy19l1	
DMR8:25733001	8	25733001	25734000	1000	1	7.50E-09	0.52	13	1.3	Dpy19l2	
DMR8:25755001	8	25755001	25757000	2000	1	2.80E-07	-0.53	1	0.05	Dpy19l2	
DMR8:25763001	8	25763001	25766000	3000	1	2.20E-07	-0.37	27	0.9	Dpy19l2	
DMR8:25767001	8	25767001	25768000	1000	1	5.70E-07	-0.25	5	0.5	Dpy19l2	
DMR8:25847001	8	25847001	25848000	1000	1	1.70E-07	0.33	10	1	Tbx20	Transcription
DMR8:26481001	8	26481001	26483000	2000	1	4.40E-07	-0.38	12	0.6	7-Sep	
DMR8:26658001	8	26658001	26665000	7000	2	6.20E-10	-0.43	82	1.17	Eepd1	
DMR8:26724001	8	26724001	26725000	1000	1	3.00E-07	-0.47	11	1.1	Eepd1	
DMR8:27796001	8	27796001	27797000	1000	1	2.80E-07	-0.34	14	1.4	B3gat1;Glb1l2	Golgi;Metabolism
DMR8:27825001	8	27825001	27827000	2000	1	5.60E-07	-0.45	42	2.1	Glb1l2	Metabolism
DMR8:27890001	8	27890001	27893000	3000	1	1.60E-07	-0.4	12	0.4	Glb1l3	Metabolism
DMR8:28098001	8	28098001	28100000	2000	1	1.70E-11	-0.45	27	1.35	Ncapd3	Epigenetic
DMR8:28386001	8	28386001	28387000	1000	1	9.90E-08	0.71	36	3.6	Igsf9b	
DMR8:28445001	8	28445001	28446000	1000	1	6.90E-08	-0.41	10	1	Spata19	
DMR8:28460001	8	28460001	28462000	2000	1	1.60E-07	0.37	25	1.25	Spata19	
DMR8:28989001	8	28989001	28992000	3000	1	1.70E-07	0.43	50	1.67	Opcml	Immune
DMR8:29176001	8	29176001	29177000	1000	1	2.40E-09	0.52	5	0.5	Opcml	Immune
DMR8:29374001	8	29374001	29377000	3000	2	4.90E-08	-0.37	23	0.77	Opcml	Immune
DMR8:29540001	8	29540001	29541000	1000	1	5.30E-08	0.32	7	0.7	Opcml	Immune
DMR8:29619001	8	29619001	29621000	2000	2	3.60E-11	0.47	11	0.55	Opcml	Immune
DMR8:29630001	8	29630001	29631000	1000	1	3.20E-07	0.36	6	0.6	Opcml	Immune
DMR8:29762001	8	29762001	29765000	3000	1	1.40E-08	-0.51	15	0.5	Opcml	Immune

DMR8:29932001	8	29932001	29934000	2000	1	1.90E-07	0.44	12	0.6	Opcml	Immune
DMR8:29966001	8	29966001	29967000	1000	1	1.30E-08	-0.48	17	1.7	Opcml	Immune
DMR8:29977001	8	29977001	29979000	2000	1	8.30E-08	-0.67	22	1.1	Opcml	Immune
DMR8:30096001	8	30096001	30097000	1000	1	1.70E-09	-0.43	15	1.5	Ntm	Immune
DMR8:30350001	8	30350001	30351000	1000	1	9.30E-08	0.39	15	1.5	Ntm	Immune
DMR8:30395001	8	30395001	30397000	2000	2	9.40E-09	-0.37	18	0.9	Ntm	Immune
DMR8:30431001	8	30431001	30432000	1000	1	5.30E-07	-0.55	2	0.2	Ntm	Immune
DMR8:30489001	8	30489001	30491000	2000	1	9.40E-07	0.44	18	0.9	Ntm	Immune
DMR8:30547001	8	30547001	30549000	2000	1	2.50E-08	0.43	30	1.5	Ntm	Immune
DMR8:30619001	8	30619001	30620000	1000	1	1.20E-07	0.56	12	1.2	Ntm	Immune
DMR8:30727001	8	30727001	30730000	3000	1	1.10E-07	0.32	37	1.23	Ntm	Immune
DMR8:32001001	8	32001001	32002000	1000	1	4.50E-08	0.39	15	1.5	Adamts15	Protease
DMR8:32192001	8	32192001	32199000	7000	2	4.20E-10	-0.4	68	0.97	Zbtb44	Transcription
DMR8:32300001	8	32300001	32303000	3000	1	1.60E-08	-0.42	41	1.37	Aplp2	Protease; Proteolysis
DMR8:32583001	8	32583001	32584000	1000	1	2.70E-10	0.58	22	2.2	Tmem45b	
DMR8:33135001	8	33135001	33138000	3000	1	3.80E-07	0.38	39	1.3	LOC102551288;Arhgap32	Signaling
DMR8:33176001	8	33176001	33180000	4000	1	1.30E-10	-0.59	55	1.38	Arhgap32	Signaling
DMR8:33261001	8	33261001	33262000	1000	1	7.10E-07	0.29	13	1.3	Arhgap32	Signaling
DMR8:33278001	8	33278001	33284000	6000	1	1.60E-07	-0.45	72	1.2	Arhgap32	Signaling
DMR8:33290001	8	33290001	33292000	2000	1	1.40E-07	-0.46	28	1.4	Arhgap32	Signaling
DMR8:33347001	8	33347001	33348000	1000	1	1.60E-07	0.37	8	0.8	Arhgap32;LOC367050	Signaling
DMR8:33368001	8	33368001	33372000	4000	1	2.00E-07	-0.5	76	1.9	Arhgap32	Signaling
DMR8:33373001	8	33373001	33374000	1000	1	3.90E-07	-0.34	19	1.9	Arhgap32	Signaling
DMR8:33390001	8	33390001	33392000	2000	1	3.50E-11	-0.62	44	2.2	Arhgap32;LOC103693036	Signaling
DMR8:33428001	8	33428001	33430000	2000	1	9.20E-07	0.37	23	1.15	Kcnj5	Transport
DMR8:33571001	8	33571001	33576000	5000	1	5.40E-08	0.31	50	1	Fli1	Transcription
DMR8:33763001	8	33763001	33764000	1000	1	2.20E-08	0.45	14	1.4	Ets1	Transcription
DMR8:33766001	8	33766001	33767000	1000	1	6.00E-14	0.81	41	4.1	Ets1	Transcription
DMR8:33813001	8	33813001	33815000	2000	1	3.20E-11	-0.5	36	1.8	Ets1	Transcription
DMR8:33817001	8	33817001	33818000	1000	1	1.70E-07	-0.44	11	1.1	Ets1	Transcription
DMR8:35722001	8	35722001	35725000	3000	2	3.30E-09	0.59	93	3.1	Kirrel3;LOC102555390	
DMR8:35773001	8	35773001	35774000	1000	1	8.20E-07	0.36	7	0.7	Kirrel3	
DMR8:35777001	8	35777001	35778000	1000	1	8.30E-07	0.34	4	0.4	Kirrel3	
DMR8:35835001	8	35835001	35838000	3000	1	2.80E-08	-0.41	48	1.6	Kirrel3	
DMR8:35947001	8	35947001	35950000	3000	1	2.20E-07	-0.44	39	1.3	Kirrel3	
DMR8:36040001	8	36040001	36041000	1000	1	7.10E-07	-0.34	13	1.3	Kirrel3	
DMR8:36187001	8	36187001	36188000	1000	1	8.40E-07	0.45	22	2.2	Kirrel3	
DMR8:36648001	8	36648001	36649000	1000	1	3.80E-12	0.39	12	1.2	Cdon	
DMR8:36785001	8	36785001	36788000	3000	1	1.30E-08	-0.31	22	0.73	Pate2	
DMR8:37100001	8	37100001	37106000	6000	1	1.10E-07	-0.32	72	1.2	Pate-f	
DMR8:37894001	8	37894001	37896000	2000	1	4.80E-07	-0.36	30	1.5	RGD1560348	
DMR8:38057001	8	38057001	38062000	5000	1	2.20E-09	-0.3	65	1.3	Trnaa-ugc	
DMR8:39533001	8	39533001	39534000	1000	1	7.20E-08	-0.59	9	0.9	Pknox2;LOC108351675	Development
DMR8:39558001	8	39558001	39560000	2000	1	5.70E-07	0.35	21	1.05	Pknox2	Development
DMR8:39701001	8	39701001	39703000	2000	1	2.50E-11	-0.47	33	1.65	Tmem218;Slc37a2	Transport
DMR8:39729001	8	39729001	39733000	4000	1	5.90E-09	0.45	54	1.35	Slc37a2	Transport
DMR8:39923001	8	39923001	39926000	3000	1	8.20E-07	-0.44	34	1.13	Robo3	
DMR8:39991001	8	39991001	39992000	1000	1	2.20E-11	0.58	16	1.6	Msantd2;LOC102549473;Esam	
DMR8:40147001	8	40147001	40149000	2000	1	1.80E-08	0.38	12	0.6	Panx3;LOC103693044	
DMR8:40396001	8	40396001	40402000	6000	2	3.10E-08	-0.4	49	0.82	Olr1202	Receptor
DMR8:40404001	8	40404001	40405000	1000	1	8.90E-07	0.4	12	1.2	Olr1202	Receptor
DMR8:40433001	8	40433001	40439000	6000	1	4.70E-09	-0.29	77	1.28	Olr1203	Receptor
DMR8:42129001	8	42129001	42131000	2000	1	1.60E-09	-0.5	10	0.5	RGD1563738;LOC108351681;LOC102555114;LOC108351680	
DMR8:42155001	8	42155001	42159000	4000	1	3.20E-07	-0.41	26	0.65	RGD1563738	
DMR8:43123001	8	43123001	43125000	2000	1	2.90E-07	-0.38	79	3.95	Olr1298-ps	
DMR8:43216001	8	43216001	43218000	2000	1	6.20E-07	0.42	28	1.4	Olr1303	
DMR8:43219001	8	43219001	43223000	4000	2	1.70E-07	-0.3	63	1.57	Olr1303	
DMR8:43427001	8	43427001	43433000	6000	1	5.20E-08	-0.37	72	1.2	Olr1314	Receptor
DMR8:43544001	8	43544001	43558000	14000	3	1.50E-10	-0.4	125	0.89	LOC103690283;Olr1316	Receptor
DMR8:43569001	8	43569001	43574000	5000	2	4.30E-09	-0.35	48	0.96	Olr1316;Olr1317-ps	Receptor
DMR8:43725001	8	43725001	43727000	2000	1	2.20E-07	-0.41	13	0.65	Olr1325;Olr1326	Receptor
DMR8:43877001	8	43877001	43882000	5000	2	3.20E-08	-0.31	50	1	Olr1337	Receptor

DMR8:44211001	8	44211001	44216000	5000	1	6.20E-07	0.35	80	1.6	Gramd1b	
DMR8:44391001	8	44391001	44392000	1000	1	5.90E-08	-0.4	17	1.7	Gramd1b;LOC102553969	
DMR8:44882001	8	44882001	44883000	1000	1	5.20E-10	0.69	23	2.3	Clmp	
DMR8:44916001	8	44916001	44918000	2000	1	2.70E-07	0.49	30	1.5	Clmp	
DMR8:45052001	8	45052001	45054000	2000	1	1.70E-10	0.46	27	1.35	LOC300647;LOC688977;Bsx	Development
DMR8:45121001	8	45121001	45123000	2000	1	5.50E-13	0.75	64	3.2	RGD1309108	
DMR8:45358001	8	45358001	45365000	7000	1	9.20E-11	-0.61	95	1.36	Ubash3b	
DMR8:45741001	8	45741001	45742000	1000	1	3.50E-09	-0.42	25	2.5	Lnc215;Mir100	
DMR8:46286001	8	46286001	46288000	2000	1	2.30E-07	-0.41	49	2.45	Sorl1	Transport
DMR8:46637001	8	46637001	46638000	1000	1	7.90E-07	0.33	6	0.6	Tecta	
DMR8:46673001	8	46673001	46678000	5000	2	2.60E-10	0.39	59	1.18	Tecta	
DMR8:46853001	8	46853001	46864000	11000	1	3.00E-10	0.38	153	1.39	Grik4	Receptor
DMR8:46899001	8	46899001	46902000	3000	1	4.90E-08	0.45	60	2	Grik4	Receptor
DMR8:46967001	8	46967001	46970000	3000	1	2.40E-08	0.46	35	1.17	Grik4	Receptor
DMR8:46980001	8	46980001	46982000	2000	1	1.40E-10	0.35	30	1.5	Grik4	Receptor
DMR8:47047001	8	47047001	47049000	2000	1	8.40E-07	0.31	23	1.15	Grik4;LOC102549858	Receptor
DMR8:47074001	8	47074001	47079000	5000	1	7.20E-13	-0.56	61	1.22	Grik4	Receptor
DMR8:47129001	8	47129001	47132000	3000	1	4.10E-08	0.4	44	1.47	Grik4	Receptor
DMR8:47243001	8	47243001	47244000	1000	1	3.60E-07	0.42	9	0.9	Grik4;LOC102549942	Receptor
DMR8:47245001	8	47245001	47247000	2000	1	4.80E-07	-0.4	28	1.4	Grik4;LOC102549942	Receptor
DMR8:47673001	8	47673001	47674000	1000	1	5.70E-07	0.6	15	1.5	Trim29	
DMR8:47706001	8	47706001	47709000	3000	1	7.30E-07	0.31	51	1.7	Trim29	
DMR8:48530001	8	48530001	48532000	2000	2	2.20E-17	-0.75	21	1.05	Cbl	Metabolism
DMR8:48737001	8	48737001	48739000	2000	1	2.80E-10	0.59	28	1.4	Trappc4;Rps25;Ccdc84;Mettl9-ps1	Transport;Translation
DMR8:48776001	8	48776001	48778000	2000	1	1.60E-09	0.42	19	0.95	Upk2	
DMR8:48783001	8	48783001	48787000	4000	1	1.80E-07	0.33	40	1	Upk2	
DMR8:48927001	8	48927001	48929000	2000	1	1.70E-09	-0.54	22	1.1	Ddx6	
DMR8:49002001	8	49002001	49003000	1000	1	1.80E-07	-0.49	19	1.9	Treh;Phldb1	
DMR8:49042001	8	49042001	49044000	2000	1	2.00E-15	0.9	60	3	Phldb1;Arcn1	Transport
DMR8:49066001	8	49066001	49067000	1000	1	1.00E-08	-0.68	10	1	Arcn1;Ift46	Transport
DMR8:49336001	8	49336001	49338000	2000	1	7.40E-07	0.36	20	1	Mpzl2	Cytoskeleton
DMR8:49468001	8	49468001	49471000	3000	1	1.40E-07	-0.37	33	1.1	Tmprss4	Protease
DMR8:49635001	8	49635001	49639000	4000	1	2.60E-10	0.42	51	1.27	Tmprss13	Protease
DMR8:49700001	8	49700001	49701000	1000	1	7.70E-08	0.38	4	0.4	Fxyd6;Fxyd2	Transport
DMR8:49957001	8	49957001	49958000	1000	1	2.10E-08	0.4	7	0.7	Dscaml1	Cytoskeleton
DMR8:49959001	8	49959001	49962000	3000	1	9.10E-09	0.51	28	0.93	Dscaml1	Cytoskeleton
DMR8:49965001	8	49965001	49966000	1000	1	3.50E-08	0.35	9	0.9	Dscaml1	Cytoskeleton
DMR8:49968001	8	49968001	49971000	3000	1	8.80E-08	0.35	57	1.9	Dscaml1	Cytoskeleton
DMR8:50069001	8	50069001	50070000	1000	1	1.50E-08	-0.42	18	1.8	Cep164	Cytoskeleton
DMR8:51920001	8	51920001	51921000	1000	1	2.90E-07	-0.51	15	1.5	Cadm1;LOC102550632	
DMR8:51951001	8	51951001	51953000	2000	1	3.10E-08	-0.46	34	1.7	Cadm1;LOC102550632	
DMR8:52019001	8	52019001	52021000	2000	1	6.10E-08	-0.34	57	2.85	Cadm1	
DMR8:52717001	8	52717001	52721000	4000	1	1.70E-10	-0.54	83	2.08	Nxpe4	
DMR8:52761001	8	52761001	52763000	2000	1	3.60E-07	-0.37	20	1	Nxpe1	
DMR8:52796001	8	52796001	52797000	1000	1	2.60E-07	0.59	25	2.5	Rexo2	
DMR8:52975001	8	52975001	52976000	1000	1	1.50E-07	0.31	4	0.4	Zbtb16	Transcription
DMR8:52981001	8	52981001	52987000	6000	1	8.80E-09	0.53	89	1.48	Zbtb16	Transcription
DMR8:53085001	8	53085001	53088000	3000	1	8.50E-07	-0.39	65	2.17	Zbtb16	Transcription
DMR8:53156001	8	53156001	53158000	2000	1	2.00E-09	-0.5	19	0.95	Zbtb16	Transcription
DMR8:53250001	8	53250001	53253000	3000	2	7.30E-10	0.51	35	1.17	Htr3b	Ion Channel
DMR8:53363001	8	53363001	53365000	2000	1	3.50E-08	0.44	19	0.95	Usp28;LOC100360390;Zw10	Protease;Cytoskeleton
DMR8:53436001	8	53436001	53437000	1000	1	1.90E-09	-0.41	21	2.1	Tmprss5	Protease
DMR8:53742001	8	53742001	53743000	1000	1	4.00E-08	0.5	14	1.4	Drd2;Ankk1	Signaling;Signaling
DMR8:53832001	8	53832001	53833000	1000	1	4.80E-09	0.55	5	0.5	LOC102556026;Ncam1	
DMR8:53961001	8	53961001	53962000	1000	1	1.90E-07	0.46	14	1.4	Ncam1	
DMR8:53967001	8	53967001	53968000	1000	1	1.20E-07	0.48	10	1	Ncam1	
DMR8:54929001	8	54929001	54931000	2000	1	7.10E-07	0.3	31	1.55	Plet1	
DMR8:54973001	8	54973001	54975000	2000	1	1.90E-09	0.37	36	1.8	Bco2	Metabolism
DMR8:54996001	8	54996001	54997000	1000	1	1.60E-18	0.98	43	4.3	Bco2;Tex12	Metabolism
DMR8:55070001	8	55070001	55071000	1000	1	3.50E-08	-0.5	6	0.6	Pih1d2;Dlat	Metabolism
DMR8:55146001	8	55146001	55148000	2000	1	1.40E-10	-0.72	20	1	Dixdc1	Cytoskeleton
DMR8:55211001	8	55211001	55213000	2000	1	6.20E-07	-0.49	36	1.8	Fdxacb1;Alg9	Translation;Golgi
DMR8:55232001	8	55232001	55234000	2000	1	4.90E-23	0.6	20	1	Alg9	Golgi

DMR8:55267001	8	55267001	55273000	6000	1	7.80E-08	-0.43	99	1.65	Alg9;LOC108351703;Ppp2r1b	Golgi;Translation;Signaling
DMR8:55510001	8	55510001	55512000	2000	1	5.40E-08	-0.55	22	1.1	Btg4;LOC102553270	
DMR8:55593001	8	55593001	55596000	3000	1	1.50E-07	-0.42	34	1.13	Pou2af1	
DMR8:56268001	8	56268001	56274000	6000	1	1.20E-09	-0.49	67	1.12	Arhgap20	Signaling
DMR8:56390001	8	56390001	56391000	1000	1	9.40E-07	0.32	9	0.9	Fdx1	Metabolism
DMR8:58350001	8	58350001	58355000	5000	2	4.50E-08	-0.53	66	1.32	Slc35f2	
DMR8:58420001	8	58420001	58423000	3000	1	4.50E-10	0.58	54	1.8	Sln	
DMR8:58516001	8	58516001	58517000	1000	1	7.20E-07	0.35	4	0.4	Elmod1	Cytoskeleton
DMR8:58620001	8	58620001	58621000	1000	1	6.70E-10	0.62	24	2.4	Tnfaip8l3	
DMR8:58892001	8	58892001	58895000	3000	1	3.80E-08	-0.4	66	2.2	Gldn	Development
DMR8:58913001	8	58913001	58915000	2000	1	4.50E-07	-0.41	36	1.8	Gldn	Development
DMR8:59195001	8	59195001	59202000	7000	1	2.30E-09	0.39	82	1.17	Acsbg1	Metabolism
DMR8:59206001	8	59206001	59207000	1000	1	5.70E-07	0.39	9	0.9	Acsbg1	Metabolism
DMR8:59518001	8	59518001	59521000	3000	1	1.60E-10	0.43	32	1.07	Hykk	
DMR8:59574001	8	59574001	59575000	1000	1	8.80E-11	0.63	23	2.3	Chrna5;LOC690408	Ion Channel
DMR8:59577001	8	59577001	59581000	4000	1	9.80E-07	-0.36	64	1.6	Chrna5;LOC690408	Ion Channel
DMR8:60096001	8	60096001	60097000	1000	1	8.40E-11	0.6	23	2.3	Etfa;Bola2	Metabolism
DMR8:60399001	8	60399001	60402000	3000	1	8.30E-07	-0.35	34	1.13	Scaper;LOC102554336;LOC108348227	
DMR8:60487001	8	60487001	60489000	2000	1	3.50E-07	-0.45	3	0.15	Scaper	
DMR8:60503001	8	60503001	60508000	5000	1	4.10E-09	-0.34	41	0.82	Scaper	
DMR8:60572001	8	60572001	60573000	1000	1	6.20E-09	-0.41	6	0.6	Scaper	
DMR8:60600001	8	60600001	60603000	3000	2	4.40E-07	-0.3	30	1	Scaper;RGD1563578	
DMR8:60623001	8	60623001	60625000	2000	1	1.10E-09	-0.36	11	0.55	RGD1563578;LOC691004	
DMR8:60679001	8	60679001	60680000	1000	1	1.10E-08	0.41	6	0.6	RGD1563578;Dppa3l1	
DMR8:60701001	8	60701001	60703000	2000	1	1.10E-07	-0.35	75	3.75	LOC103690189;Rcn2	Signaling
DMR8:60779001	8	60779001	60781000	2000	2	2.10E-19	0.38	16	0.8	Pstpip1	Cytoskeleton
DMR8:60918001	8	60918001	60921000	3000	1	3.10E-09	-0.44	73	2.43	Peak1	Signaling
DMR8:60931001	8	60931001	60933000	2000	1	3.80E-10	-0.44	21	1.05	Peak1	Signaling
DMR8:61042001	8	61042001	61045000	3000	1	5.70E-08	-0.26	24	0.8	Peak1	Signaling
DMR8:61273001	8	61273001	61276000	3000	1	3.60E-09	0.82	114	3.8	Lingo1	Receptor
DMR8:61321001	8	61321001	61325000	4000	1	3.20E-07	0.51	48	1.2	Lingo1	Receptor
DMR8:61790001	8	61790001	61791000	1000	1	8.20E-08	-0.59	13	1.3	Sin3a	Epigenetic
DMR8:62178001	8	62178001	62185000	7000	2	2.10E-08	-0.38	82	1.17	Ppcdc	Metabolism
DMR8:62407001	8	62407001	62408000	1000	1	6.30E-07	0.41	18	1.8	Lman1l;Csk	Transport
DMR8:62451001	8	62451001	62455000	4000	1	1.30E-08	-0.46	48	1.2	Cyp1a2	Metabolism
DMR8:62602001	8	62602001	62604000	2000	1	5.70E-08	-0.39	27	1.35	Arid3b	Transcription
DMR8:62836001	8	62836001	62837000	1000	1	1.80E-16	0.61	2	0.2	Ccdc33	
DMR8:62918001	8	62918001	62920000	2000	1	9.00E-07	0.43	32	1.6	Ccdc33;Stra6	
DMR8:63140001	8	63140001	63142000	2000	1	9.20E-07	0.36	35	1.75	Tbc1d21	Signaling
DMR8:63537001	8	63537001	63539000	2000	1	6.60E-11	0.47	16	0.8	Rec114	
DMR8:63626001	8	63626001	63628000	2000	1	6.50E-10	0.64	37	1.85	Hcn4	Transport
DMR8:63636001	8	63636001	63637000	1000	1	1.50E-09	0.75	33	3.3	Hcn4	Transport
DMR8:64110001	8	64110001	64111000	1000	1	3.90E-23	1.34	49	4.9	Adpgk;Bbs4;LOC108351713	Signaling
DMR8:64394001	8	64394001	64397000	3000	1	1.70E-07	0.32	40	1.33	Celf6	
DMR8:64407001	8	64407001	64411000	4000	1	3.90E-07	-0.32	36	0.9	Celf6	
DMR8:64448001	8	64448001	64453000	5000	2	6.00E-08	-0.54	49	0.98	Parp6	
DMR8:64505001	8	64505001	64508000	3000	1	6.20E-09	0.54	34	1.13	Pkm;Gramd2	Signaling
DMR8:64509001	8	64509001	64512000	3000	1	6.30E-07	0.4	30	1	Pkm;Gramd2	Signaling
DMR8:64650001	8	64650001	64651000	1000	1	2.50E-08	0.46	10	1	Myo9a	
DMR8:64684001	8	64684001	64685000	1000	1	7.20E-07	0.4	5	0.5	Myo9a;LOC102546766	
DMR8:64709001	8	64709001	64711000	2000	1	6.00E-07	-0.59	18	0.9	Myo9a;LOC102546688	
DMR8:65119001	8	65119001	65120000	1000	1	1.20E-14	0.84	31	3.1	Thsd4	
DMR8:65140001	8	65140001	65145000	5000	2	1.60E-12	-0.47	47	0.94	Thsd4	
DMR8:65264001	8	65264001	65270000	6000	2	2.00E-09	-0.28	67	1.12	Thsd4	
DMR8:65323001	8	65323001	65326000	3000	1	1.50E-07	-0.47	45	1.5	Thsd4	
DMR8:65387001	8	65387001	65389000	2000	1	5.90E-09	0.49	23	1.15	Thsd4	
DMR8:65395001	8	65395001	65402000	7000	2	5.10E-09	-0.56	133	1.9	Thsd4	
DMR8:65490001	8	65490001	65492000	2000	1	9.30E-07	0.35	10	0.5	Lrrc49	Signaling
DMR8:65539001	8	65539001	65540000	1000	1	7.90E-09	0.48	7	0.7	Lrrc49	Signaling
DMR8:65752001	8	65752001	65754000	2000	1	4.90E-09	-0.49	30	1.5	Uaca	
DMR8:65779001	8	65779001	65783000	4000	1	1.90E-08	-0.45	72	1.8	Uaca	

DMR8:66963001	8	66963001	66965000	2000	2	4.10E-09	0.31	42	2.1	Paqr5	Signaling
DMR8:67059001	8	67059001	67060000	1000	1	1.60E-08	0.5	6	0.6	Glce	Metabolism
DMR8:67226001	8	67226001	67233000	7000	1	1.40E-07	0.82	134	1.91	Spesp1	
DMR8:67389001	8	67389001	67390000	1000	1	4.60E-08	0.36	9	0.9	Coro2b	Cytoskeleton
DMR8:67408001	8	67408001	67411000	3000	1	1.80E-09	-0.53	34	1.13	Coro2b	Cytoskeleton
DMR8:67443001	8	67443001	67445000	2000	1	3.60E-08	-0.41	29	1.45	Coro2b	Cytoskeleton
DMR8:67494001	8	67494001	67496000	2000	1	5.70E-10	0.63	45	2.25	Coro2b;LOC103693114	Cytoskeleton
DMR8:67502001	8	67502001	67504000	2000	2	2.90E-08	0.45	33	1.65	Coro2b	Cytoskeleton
DMR8:67564001	8	67564001	67567000	3000	1	3.60E-07	0.45	38	1.27	LOC102556363;Itga11	Extracellular Matrix
DMR8:67624001	8	67624001	67627000	3000	1	4.50E-09	0.36	28	0.93	Itga11;LOC102546312;LOC103693115	Extracellular Matrix
DMR8:67671001	8	67671001	67675000	4000	1	2.50E-08	0.43	66	1.65	Itga11	Extracellular Matrix
DMR8:67755001	8	67755001	67757000	2000	1	1.50E-07	0.39	33	1.65	Cln6;LOC102546618;Calml4	Signaling
DMR8:68056001	8	68056001	68058000	2000	1	8.90E-08	0.39	27	1.35	LOC103693116;Map2k5	Signaling
DMR8:68128001	8	68128001	68133000	5000	1	4.00E-07	-0.41	69	1.38	Map2k5	Signaling
DMR8:68280001	8	68280001	68282000	2000	2	1.60E-09	0.36	19	0.95	Map2k5	Signaling
DMR8:68350001	8	68350001	68353000	3000	1	9.90E-08	-0.41	45	1.5	LOC103693118;Iqch	
DMR8:68494001	8	68494001	68495000	1000	1	1.60E-13	0.5	12	1.2	Iqch	
DMR8:68496001	8	68496001	68498000	2000	1	7.40E-07	-0.57	20	1	Iqch	
DMR8:68673001	8	68673001	68675000	2000	1	3.00E-10	-0.4	31	1.55	Smad3	Transcription
DMR8:68898001	8	68898001	68900000	2000	1	3.60E-08	0.57	68	3.4	Smad6	Transcription
DMR8:69473001	8	69473001	69474000	1000	1	1.50E-07	0.37	11	1.1	Map2k1;Ids;LOC102547109	Signaling
DMR8:69525001	8	69525001	69526000	1000	1	4.30E-07	-0.44	6	0.6	Map2k1	Signaling
DMR8:69986001	8	69986001	69988000	2000	1	5.60E-07	0.3	21	1.05	Megf11	Extracellular Matrix
DMR8:70034001	8	70034001	70035000	1000	1	1.80E-07	0.34	18	1.8	Megf11	Extracellular Matrix
DMR8:70119001	8	70119001	70121000	2000	1	5.00E-07	0.49	19	0.95	Megf11	Extracellular Matrix
DMR8:70221001	8	70221001	70224000	3000	1	1.90E-08	0.27	36	1.2	Rab11a;Trnaq-cug;LOC367101	
DMR8:70313001	8	70313001	70314000	1000	1	3.90E-07	-0.42	10	1	Dennd4a	
DMR8:70472001	8	70472001	70474000	2000	1	9.20E-09	-0.44	24	1.2	Vwa9;Hacd3	
DMR8:70533001	8	70533001	70534000	1000	1	3.60E-08	0.36	29	2.9	Dpp8	Protease
DMR8:70614001	8	70614001	70615000	1000	1	9.50E-07	0.44	16	1.6	Igdcc4	
DMR8:70901001	8	70901001	70903000	2000	1	3.70E-08	0.49	20	1	Ubap1l;Kbtbd13	
DMR8:70981001	8	70981001	70982000	1000	1	9.00E-07	0.36	9	0.9	Mtfmt	Translation
DMR8:71092001	8	71092001	71093000	1000	1	1.80E-08	0.34	10	1	Plekho2	
DMR8:71112001	8	71112001	71114000	2000	1	1.20E-07	-0.47	34	1.7	Plekho2	
DMR8:71129001	8	71129001	71130000	1000	1	5.70E-07	0.4	12	1.2	Pif1	Epigenetic
DMR8:71265001	8	71265001	71266000	1000	1	4.90E-10	0.77	5	0.5	Zfp609	
DMR8:71430001	8	71430001	71431000	1000	1	2.10E-09	0.72	43	4.3	Trip4;LOC108351729	Transcription
DMR8:71480001	8	71480001	71487000	7000	1	3.70E-10	-0.34	53	0.76	Trip4	Transcription
DMR8:71589001	8	71589001	71590000	1000	1	7.60E-07	-0.42	7	0.7	Csnk1g1	Signaling
DMR8:71694001	8	71694001	71697000	3000	1	9.10E-10	-0.41	33	1.1	Csnk1g1	Signaling
DMR8:72142001	8	72142001	72148000	6000	2	7.30E-09	-0.37	46	0.77	Herc1	Transcription
DMR8:72414001	8	72414001	72415000	1000	1	8.80E-12	0.43	9	0.9	Car12	
DMR8:72738001	8	72738001	72740000	2000	1	2.00E-09	0.43	24	1.2	LOC102554897;Rps27l	Translation
DMR8:72807001	8	72807001	72809000	2000	1	1.50E-09	-0.59	25	1.25	Tpm1	Cytoskeleton
DMR8:73007001	8	73007001	73009000	2000	1	3.40E-10	0.48	24	1.2	Tln2	
DMR8:73067001	8	73067001	73070000	3000	1	2.10E-08	-0.61	42	1.4	Tln2	
DMR8:73253001	8	73253001	73254000	1000	1	2.40E-09	0.48	7	0.7	Tln2	
DMR8:73345001	8	73345001	73347000	2000	1	4.70E-09	0.41	26	1.3	Tln2	
DMR8:73402001	8	73402001	73409000	7000	1	5.80E-07	-0.27	97	1.39	Tln2	
DMR8:73580001	8	73580001	73584000	4000	1	1.90E-15	0.39	48	1.2	LOC108351734;C2cd4b	
DMR8:73684001	8	73684001	73686000	2000	1	6.50E-09	-0.58	28	1.4	C2cd4a;Vps13c	Transport
DMR8:73721001	8	73721001	73726000	5000	2	1.10E-14	-0.58	69	1.38	Vps13c	Transport
DMR8:73750001	8	73750001	73752000	2000	1	7.70E-07	-0.46	27	1.35	Vps13c	Transport
DMR8:75646001	8	75646001	75647000	1000	1	2.90E-08	0.38	12	1.2	Ice2	
DMR8:76454001	8	76454001	76456000	2000	1	8.10E-07	-0.43	26	1.3	LOC108351740;Gcnt3	Golgi
DMR8:76527001	8	76527001	76529000	2000	1	7.80E-07	0.37	29	1.45	Fam81a	
DMR8:76539001	8	76539001	76540000	1000	1	7.40E-11	0.39	14	1.4	Fam81a	
DMR8:76586001	8	76586001	76587000	1000	1	3.90E-08	0.58	63	6.3	Fam81a	
DMR8:76991001	8	76991001	76994000	3000	1	6.80E-07	-0.36	22	0.73	Sltm	
DMR8:77004001	8	77004001	77006000	2000	1	1.10E-11	-0.5	26	1.3	Sltm	
DMR8:77009001	8	77009001	77012000	3000	1	4.60E-08	-0.4	32	1.07	Sltm	
DMR8:77074001	8	77074001	77076000	2000	1	1.70E-08	0.48	17	0.85	Fam63b;LOC102551907	

DMR8:77091001	8	77091001	77092000	1000	1	1.40E-08	-0.48	4	0.4	Fam63b;LOC102551907	
DMR8:77097001	8	77097001	77099000	2000	1	1.10E-08	0.46	28	1.4	Fam63b;LOC102551907;Adam10	Protease
DMR8:77232001	8	77232001	77233000	1000	1	2.50E-07	0.32	9	0.9	Adam10	Protease
DMR8:77295001	8	77295001	77296000	1000	1	1.10E-07	0.38	11	1.1	Lipc	Metabolism
DMR8:77377001	8	77377001	77379000	2000	1	3.00E-07	0.46	14	0.7	Lipc	Metabolism
DMR8:77580001	8	77580001	77584000	4000	1	1.10E-08	-0.49	48	1.2	Aqp9	Transport
DMR8:77638001	8	77638001	77639000	1000	1	4.80E-08	0.7	28	2.8	Aldh1a2	Metabolism
DMR8:77667001	8	77667001	77669000	2000	1	6.90E-07	-0.34	20	1	Aldh1a2	Metabolism
DMR8:77977001	8	77977001	77981000	4000	1	2.00E-07	-0.58	45	1.12	Polr2m	
DMR8:77989001	8	77989001	77991000	2000	1	9.90E-11	-0.54	19	0.95	Polr2m	
DMR8:78120001	8	78120001	78122000	2000	1	1.10E-07	-0.57	15	0.75	Cgnl1	
DMR8:78227001	8	78227001	78230000	3000	1	1.50E-07	-0.4	41	1.37	Cgnl1	
DMR8:78244001	8	78244001	78247000	3000	1	2.40E-07	-0.41	36	1.2	Cgnl1	
DMR8:78258001	8	78258001	78260000	2000	1	4.00E-11	0.45	16	0.8	Cgnl1	
DMR8:78342001	8	78342001	78343000	1000	1	6.70E-07	0.39	15	1.5	Tcf12	Transcription
DMR8:78406001	8	78406001	78408000	2000	2	1.60E-09	0.46	7	0.35	Tcf12	Transcription
DMR8:78917001	8	78917001	78920000	3000	2	2.80E-07	-0.46	17	0.57	Zfp280d	Transcription
DMR8:79053001	8	79053001	79057000	4000	3	7.30E-09	-0.69	88	2.2	LOC103693138;Mns1	Development
DMR8:79064001	8	79064001	79066000	2000	1	5.20E-11	-0.51	37	1.85	Mns1	Development
DMR8:79081001	8	79081001	79082000	1000	1	5.60E-07	-0.48	9	0.9	Mns1;Tex9	Development;Cytoskeleto n
DMR8:79218001	8	79218001	79220000	2000	1	9.20E-09	-0.73	11	0.55	Rfx7	Transcription
DMR8:79355001	8	79355001	79356000	1000	1	3.40E-09	-0.39	25	2.5	Nedd4	Proteolysis
DMR8:79393001	8	79393001	79394000	1000	1	7.90E-08	-0.48	8	0.8	Nedd4	Proteolysis
DMR8:79497001	8	79497001	79498000	1000	1	2.20E-07	0.36	10	1	Prtg	
DMR8:79515001	8	79515001	79518000	3000	1	4.70E-07	0.29	23	0.77	Prtg	
DMR8:79580001	8	79580001	79581000	1000	1	1.40E-07	-0.61	8	0.8	Prtg	
DMR8:79702001	8	79702001	79704000	2000	1	6.20E-07	0.35	35	1.75	Ccpg1;Pigb	Golgi
DMR8:79934001	8	79934001	79940000	6000	2	3.90E-10	-0.47	30	0.5	LOC102554551;RGD1564166	
DMR8:79950001	8	79950001	79951000	1000	1	8.80E-09	0.53	4	0.4	LOC102554551;RGD1564166	
DMR8:80193001	8	80193001	80196000	3000	1	2.10E-11	0.65	36	1.2	LOC108351745;Unc13c	
DMR8:80321001	8	80321001	80323000	2000	1	3.70E-07	0.39	20	1	Unc13c	
DMR8:80419001	8	80419001	80420000	1000	1	2.80E-07	0.46	6	0.6	Unc13c	
DMR8:80461001	8	80461001	80462000	1000	1	1.00E-08	0.46	3	0.3	Unc13c	
DMR8:80482001	8	80482001	80486000	4000	1	7.00E-08	-0.54	56	1.4	Unc13c	
DMR8:81014001	8	81014001	81015000	1000	1	1.60E-08	0.29	11	1.1	Wdr72	
DMR8:81029001	8	81029001	81030000	1000	1	8.20E-07	-0.49	9	0.9	Wdr72	
DMR8:81855001	8	81855001	81856000	1000	1	2.80E-08	-0.41	13	1.3	Fam214a	
DMR8:81899001	8	81899001	81901000	2000	1	1.00E-07	-0.43	19	0.95	Fam214a	
DMR8:81943001	8	81943001	81944000	1000	1	3.00E-07	-0.43	15	1.5	Fam214a;Arpp19	
DMR8:82209001	8	82209001	82211000	2000	1	4.80E-11	0.42	24	1.2	Myo5c	Cytoskeleton
DMR8:82268001	8	82268001	82270000	2000	1	9.00E-12	0.42	29	1.45	Gnb5	Signaling
DMR8:82290001	8	82290001	82293000	3000	1	2.00E-07	-0.28	23	0.77	Gnb5;Bcl2l10	Signaling
DMR8:82318001	8	82318001	82320000	2000	1	9.20E-10	-0.48	17	0.85	Mapk6	Signaling
DMR8:82477001	8	82477001	82480000	3000	1	1.30E-07	-0.42	40	1.33	Tmod3	Cytoskeleton
DMR8:82523001	8	82523001	82524000	1000	1	5.20E-09	0.5	4	0.4	Tmod2	Cytoskeleton
DMR8:82585001	8	82585001	82588000	3000	1	1.00E-07	0.38	29	0.97	Lysmd2;Scg3	
DMR8:82620001	8	82620001	82622000	2000	1	7.90E-09	0.54	32	1.6	Scg3	
DMR8:82639001	8	82639001	82640000	1000	1	8.10E-08	-0.52	24	2.4	Scg3	
DMR8:82691001	8	82691001	82693000	2000	1	8.50E-07	-0.51	21	1.05	Bmp5	Growth Factors
DMR8:82814001	8	82814001	82815000	1000	1	4.70E-07	0.79	41	4.1	Bmp5	Growth Factors
DMR8:83092001	8	83092001	83093000	1000	1	5.60E-10	-0.57	13	1.3	Hmgcll1	Metabolism
DMR8:83170001	8	83170001	83173000	3000	1	1.10E-07	-0.36	65	2.17	Hmgcll1	Metabolism
DMR8:84507001	8	84507001	84511000	4000	2	6.30E-09	-0.31	33	0.82	Mlip	
DMR8:84565001	8	84565001	84567000	2000	1	2.70E-17	0.79	34	1.7	Mlip	
DMR8:85382001	8	85382001	85387000	5000	2	6.20E-09	0.45	46	0.92	LOC691940;Fbxo9	
DMR8:85464001	8	85464001	85467000	3000	1	5.80E-10	0.34	32	1.07	Ick	
DMR8:85524001	8	85524001	85526000	2000	2	4.30E-07	0.41	11	0.55	Gsta4;LOC100909835	Transport
DMR8:85545001	8	85545001	85546000	1000	1	2.80E-09	0.68	34	3.4	Gsta5	Transport
DMR8:85701001	8	85701001	85703000	2000	2	7.50E-09	0.64	46	2.3	Dppa5	
DMR8:85756001	8	85756001	85757000	1000	1	1.00E-07	0.44	10	1	Ddx43	
DMR8:85839001	8	85839001	85841000	2000	2	3.80E-12	-0.61	43	2.15	Mto1;Eef1a1	Translation
DMR8:85911001	8	85911001	85920000	9000	1	4.30E-07	0.43	119	1.32	Slc17a5	Transport

DMR8:85959001	8	85959001	85970000	11000	2	1.20E-07	-0.44	137	1.25	Cd109	Protease; Proteolysis
DMR8:86020001	8	86020001	86023000	3000	1	5.10E-10	-0.61	20	0.67	Cd109	Protease; Proteolysis
DMR8:86049001	8	86049001	86050000	1000	1	1.20E-16	0.4	4	0.4	Cd109	Protease; Proteolysis
DMR8:86070001	8	86070001	86071000	1000	1	2.20E-10	-0.5	11	1.1	Cd109	Protease; Proteolysis
DMR8:87271001	8	87271001	87273000	2000	1	5.60E-14	0.86	67	3.35	Filip1	
DMR8:87299001	8	87299001	87303000	4000	2	8.00E-12	-0.4	36	0.9	Filip1	
DMR8:87330001	8	87330001	87332000	2000	1	2.90E-07	0.26	29	1.45	Filip1	
DMR8:87360001	8	87360001	87361000	1000	1	4.10E-07	-0.34	17	1.7	Filip1	
DMR8:87366001	8	87366001	87368000	2000	1	6.10E-08	0.33	24	1.2	Filip1	
DMR8:87479001	8	87479001	87480000	1000	1	4.50E-07	0.31	14	1.4	Senp6	Protease
DMR8:87509001	8	87509001	87513000	4000	1	5.80E-07	-0.54	37	0.92	Senp6	Protease
DMR8:87555001	8	87555001	87556000	1000	1	7.20E-07	-0.51	14	1.4	Senp6	Protease
DMR8:87596001	8	87596001	87597000	1000	1	2.60E-07	-0.38	18	1.8	Myo6	Cytoskeleton
DMR8:87705001	8	87705001	87706000	1000	1	5.10E-07	-0.43	18	1.8	Myo6	Cytoskeleton
DMR8:87793001	8	87793001	87800000	7000	2	4.10E-09	-0.4	75	1.07	Impg1	Extracellular Matrix
DMR8:87820001	8	87820001	87822000	2000	1	1.10E-08	0.55	52	2.6	Impg1	Extracellular Matrix
DMR8:89467001	8	89467001	89470000	3000	1	8.30E-10	-0.42	33	1.1	Mei4;LOC102553441	
DMR8:89481001	8	89481001	89482000	1000	1	3.40E-12	-0.49	7	0.7	Mei4;LOC102553441	
DMR8:89516001	8	89516001	89518000	2000	1	9.60E-08	-0.51	20	1	Mei4;LOC102553441	
DMR8:90301001	8	90301001	90302000	1000	1	2.30E-09	0.53	8	0.8	RGD1562068	
DMR8:90345001	8	90345001	90347000	2000	1	8.60E-08	0.44	19	0.95	Irak1bp1	
DMR8:90392001	8	90392001	90393000	1000	1	6.50E-07	-0.42	12	1.2	Phip	
DMR8:90638001	8	90638001	90639000	1000	1	3.90E-08	-0.59	22	2.2	Hmgn3	Epigenetic
DMR8:90671001	8	90671001	90676000	5000	1	1.40E-07	-0.25	51	1.02	Hmgn3	Epigenetic
DMR8:90920001	8	90920001	90922000	2000	1	6.50E-09	0.37	16	0.8	Lca5	
DMR8:90968001	8	90968001	90973000	5000	2	1.70E-09	-0.56	56	1.12	Lca5;LOC103690486	
DMR8:91108001	8	91108001	91110000	2000	1	3.90E-07	-0.41	38	1.9	LOC102554622;Sh3bgrl2;LOC102554552	
DMR8:91111001	8	91111001	91115000	4000	1	8.80E-07	-0.49	37	0.92	LOC102554622;Sh3bgrl2	
DMR8:91310001	8	91310001	91312000	2000	1	4.50E-08	-0.49	28	1.4	RGD1561944;Elovl4	Metabolism
DMR8:91403001	8	91403001	91404000	1000	1	2.00E-07	-0.47	10	1	Ttk	Signaling
DMR8:91412001	8	91412001	91415000	3000	1	8.50E-07	-0.42	50	1.67	Ttk;LOC108351756	Signaling
DMR8:91610001	8	91610001	91613000	3000	1	7.50E-08	0.29	27	0.9	Bckdhb	Metabolism
DMR8:91615001	8	91615001	91621000	6000	2	2.80E-07	-0.38	78	1.3	Bckdhb	Metabolism
DMR8:91653001	8	91653001	91657000	4000	1	3.90E-08	-0.6	61	1.52	Bckdhb	Metabolism
DMR8:91661001	8	91661001	91662000	1000	1	5.10E-09	-0.55	14	1.4	Bckdhb	Metabolism
DMR8:93471001	8	93471001	93474000	3000	1	8.60E-08	-0.52	28	0.93	RGD1564645	
DMR8:94034001	8	94034001	94041000	7000	1	2.60E-07	-0.37	53	0.76	Ube3d;LOC102551813	Proteolysis
DMR8:94435001	8	94435001	94438000	3000	1	5.30E-07	0.34	49	1.63	Prss35;Snap91	Protease;Transport
DMR8:94516001	8	94516001	94522000	6000	1	2.00E-08	-0.38	64	1.07	Snap91	Transport
DMR8:94527001	8	94527001	94529000	2000	1	7.90E-07	0.3	40	2	Snap91	Transport
DMR8:94552001	8	94552001	94553000	1000	1	9.80E-07	-0.44	12	1.2	Snap91	Transport
DMR8:94682001	8	94682001	94684000	2000	1	2.50E-09	0.38	18	0.9	Ripply2;Cyb5r4	Metabolism
DMR8:94700001	8	94700001	94701000	1000	1	3.30E-07	-0.49	44	4.4	Ripply2;Cyb5r4	Metabolism
DMR8:94711001	8	94711001	94714000	3000	1	4.70E-09	-0.42	31	1.03	Cyb5r4	Metabolism
DMR8:94766001	8	94766001	94770000	4000	1	8.30E-07	-0.56	46	1.15	Cyb5r4	Metabolism
DMR8:94910001	8	94910001	94911000	1000	1	8.20E-08	-0.44	10	1	Cep162	
DMR8:95354001	8	95354001	95356000	2000	1	1.00E-07	-0.36	31	1.55	Tbx18	Transcription
DMR8:95393001	8	95393001	95394000	1000	1	1.00E-07	-0.58	16	1.6	Tbx18;LOC100909636	Transcription
DMR8:95896001	8	95896001	95898000	2000	1	3.30E-07	0.62	36	1.8	RGD1561192	
DMR8:96037001	8	96037001	96038000	1000	1	1.00E-09	-0.55	11	1.1	Snx14;LOC102554498	Cytoskeleton
DMR8:96366001	8	96366001	96367000	1000	1	3.40E-07	0.37	2	0.2	Zfp949;LOC108351760	Transcription
DMR8:96447001	8	96447001	96453000	6000	1	2.50E-07	-0.29	57	0.95	RGD1560775	
DMR8:96590001	8	96590001	96593000	3000	1	6.10E-07	-0.36	20	0.67	Mthfs	Metabolism
DMR8:97362001	8	97362001	97368000	6000	1	3.70E-08	-0.42	57	0.95	Rasgrf1	Transcription
DMR8:97403001	8	97403001	97405000	2000	1	5.40E-08	0.44	28	1.4	Rasgrf1	Transcription
DMR8:97546001	8	97546001	97547000	1000	1	4.60E-07	0.47	33	3.3	Adamts7	Protease
DMR8:99663001	8	99663001	99664000	1000	1	3.60E-07	0.46	8	0.8	Plscr2	
DMR8:99896001	8	99896001	99898000	2000	1	4.60E-07	0.36	20	1	Plscr4	Transport
DMR8:100069001	8	100069001	100071000	2000	1	2.20E-08	-0.51	29	1.45	Plod2	Golgi
DMR8:102302001	8	102302001	102304000	2000	1	3.70E-07	-0.46	27	1.35	LOC102552777;Slc9a9	Transport
DMR8:102379001	8	102379001	102380000	1000	1	3.90E-07	0.41	8	0.8	Slc9a9	Transport
DMR8:102592001	8	102592001	102593000	1000	1	5.10E-07	-0.46	16	1.6	Slc9a9;LOC103693164	Transport

DMR8:102614001	8	102614001	102616000	2000	2	4.20E-19	1.03	70	3.5	Slc9a9	Transport
DMR8:102617001	8	102617001	102618000	1000	1	8.20E-07	-0.48	15	1.5	Slc9a9	Transport
DMR8:102749001	8	102749001	102751000	2000	1	2.20E-07	0.31	24	1.2	Slc9a9	Transport
DMR8:102790001	8	102790001	102792000	2000	1	6.10E-10	0.62	19	0.95	Slc9a9	Transport
DMR8:103008001	8	103008001	103012000	4000	1	1.40E-09	-0.42	49	1.23	Slc9a9	Transport
DMR8:103241001	8	103241001	103242000	1000	1	2.50E-07	-0.38	20	2	U2surp	Translation
DMR8:103292001	8	103292001	103293000	1000	1	3.40E-07	-0.34	10	1	U2surp	Translation
DMR8:103512001	8	103512001	103518000	6000	1	1.60E-07	-0.26	60	1	Trpc1	Transport
DMR8:103949001	8	103949001	103950000	1000	1	4.40E-07	-0.38	14	1.4	Gk5	Metabolism
DMR8:104341001	8	104341001	104343000	2000	1	6.30E-08	0.61	34	1.7	Rnf7	Proteolysis
DMR8:104463001	8	104463001	104465000	2000	1	6.00E-07	-0.42	19	0.95	Rasa2	Signaling
DMR8:104557001	8	104557001	104560000	3000	1	7.90E-08	0.53	35	1.17	Zbtb38	Transcription
DMR8:104568001	8	104568001	104569000	1000	1	2.40E-09	0.71	24	2.4	Zbtb38	Transcription
DMR8:104583001	8	104583001	104585000	2000	1	3.40E-07	-0.47	38	1.9	Zbtb38	Transcription
DMR8:104594001	8	104594001	104597000	3000	1	5.70E-07	0.32	22	0.73	Zbtb38;LOC100909637	Transcription
DMR8:104618001	8	104618001	104622000	4000	1	2.70E-08	-0.56	84	2.1	Zbtb38	Transcription
DMR8:104631001	8	104631001	104633000	2000	1	7.20E-09	-0.4	38	1.9	Zbtb38	Transcription
DMR8:104658001	8	104658001	104662000	4000	1	2.20E-08	0.44	43	1.07	Zbtb38	Transcription
DMR8:104686001	8	104686001	104689000	3000	2	9.80E-12	0.38	39	1.3	Zbtb38	Transcription
DMR8:104753001	8	104753001	104759000	6000	1	4.30E-08	-0.39	110	1.83	Pxylp1	Signaling
DMR8:104767001	8	104767001	104772000	5000	1	8.20E-08	-0.65	78	1.56	Pxylp1	Signaling
DMR8:104786001	8	104786001	104788000	2000	1	4.80E-07	-0.45	21	1.05	Pxylp1	Signaling
DMR8:104800001	8	104800001	104801000	1000	1	9.60E-10	-0.5	14	1.4	Pxylp1	Signaling
DMR8:104829001	8	104829001	104831000	2000	1	7.20E-07	-0.35	35	1.75	Spsb4	
DMR8:104840001	8	104840001	104843000	3000	1	3.10E-10	0.47	51	1.7	Spsb4	
DMR8:105212001	8	105212001	105214000	2000	1	1.10E-11	-0.68	24	1.2	Trim42	
DMR8:105445001	8	105445001	105446000	1000	1	7.40E-07	0.41	16	1.6	Clstn2	Transport
DMR8:106489001	8	106489001	106490000	1000	1	7.00E-10	0.63	24	2.4	Rbp2	
DMR8:107185001	8	107185001	107186000	1000	1	1.40E-07	0.35	11	1.1	LOC102547261;Foxl2	Transcription
DMR8:107377001	8	107377001	107380000	3000	1	3.20E-10	-0.42	50	1.67	Pik3cb	Signaling
DMR8:107388001	8	107388001	107390000	2000	1	2.30E-07	0.35	19	0.95	Pik3cb	Signaling
DMR8:107552001	8	107552001	107553000	1000	1	1.20E-07	-0.57	11	1.1	Cep70;Esyt3	
DMR8:107718001	8	107718001	107719000	1000	1	7.50E-07	-0.32	15	1.5	Nme9;Armc8	
DMR8:107809001	8	107809001	107811000	2000	1	5.00E-12	-0.46	20	1	Armc8	
DMR8:107920001	8	107920001	107922000	2000	1	5.00E-07	0.56	40	2	Dzip1l	
DMR8:107927001	8	107927001	107928000	1000	1	2.00E-10	0.58	25	2.5	Cldn18	Cell Junction
DMR8:108780001	8	108780001	108781000	1000	1	2.30E-08	-0.61	14	1.4	Il20rb;Nck1	Receptor;Cytoskeleton
DMR8:108813001	8	108813001	108815000	2000	1	2.00E-07	-0.35	51	2.55	Nck1	Cytoskeleton
DMR8:108816001	8	108816001	108820000	4000	1	6.10E-08	-0.43	31	0.78	Nck1	Cytoskeleton
DMR8:109016001	8	109016001	109017000	1000	1	6.80E-18	0.46	14	1.4	Stag1	Epigenetic
DMR8:109029001	8	109029001	109033000	4000	1	1.30E-08	-0.37	32	0.8	Stag1	Epigenetic
DMR8:109061001	8	109061001	109064000	3000	2	2.40E-08	-0.43	20	0.67	Stag1	Epigenetic
DMR8:109133001	8	109133001	109135000	2000	1	1.50E-07	0.47	8	0.4	Stag1	Epigenetic
DMR8:109141001	8	109141001	109144000	3000	1	2.80E-09	-0.51	20	0.67	Stag1	Epigenetic
DMR8:109195001	8	109195001	109199000	4000	2	8.10E-09	-0.46	34	0.85	Stag1	Epigenetic
DMR8:109259001	8	109259001	109260000	1000	1	3.00E-09	-0.46	13	1.3	Stag1	Epigenetic
DMR8:109362001	8	109362001	109363000	1000	1	8.70E-08	-0.56	16	1.6	LOC686039;Pccb	Metabolism
DMR8:109387001	8	109387001	109388000	1000	1	6.70E-07	0.37	8	0.8	Pccb	Metabolism
DMR8:109446001	8	109446001	109449000	3000	1	2.90E-08	-0.38	40	1.33	Msl2	
DMR8:109579001	8	109579001	109582000	3000	1	2.10E-07	-0.36	20	0.67	Ppp2r3a	Signaling
DMR8:110430001	8	110430001	110435000	5000	3	2.60E-10	-0.46	39	0.78	Ephb1	Receptor
DMR8:110523001	8	110523001	110525000	2000	2	1.70E-10	0.46	10	0.5	Ephb1	Receptor
DMR8:110800001	8	110800001	110802000	2000	1	2.20E-08	0.37	12	0.6	Ephb1	Receptor
DMR8:110818001	8	110818001	110821000	3000	1	3.60E-08	0.62	44	1.47	Ephb1	Receptor
DMR8:111027001	8	111027001	111028000	1000	1	2.70E-11	0.38	4	0.4	Ky	
DMR8:111225001	8	111225001	111227000	2000	2	4.50E-09	-0.55	33	1.65	Amotl2;LOC102549628	
DMR8:111320001	8	111320001	111323000	3000	1	2.60E-07	-0.48	43	1.43	Ryk	Receptor
DMR8:111621001	8	111621001	111625000	4000	1	7.30E-07	0.55	57	1.43	Rab6b	
DMR8:111663001	8	111663001	111664000	1000	1	9.10E-08	0.34	21	2.1	Rab6b;Srprb	Transport
DMR8:111904001	8	111904001	111908000	4000	1	1.30E-08	0.44	74	1.85	Bfsp2	
DMR8:112058001	8	112058001	112060000	2000	1	9.10E-07	-0.53	32	1.6	Tmem108	
DMR8:112120001	8	112120001	112123000	3000	1	2.60E-07	0.36	27	0.9	Tmem108	
DMR8:112126001	8	112126001	112128000	2000	1	2.50E-07	-0.42	25	1.25	Tmem108	

DMR8:112244001	8	112244001	112245000	1000	1	1.70E-07	-0.37	17	1.7	Tmem108	
DMR8:112275001	8	112275001	112277000	2000	1	4.40E-08	0.41	22	1.1	Tmem108	
DMR8:112394001	8	112394001	112395000	1000	1	5.50E-12	0.72	28	2.8	Rpl27-ps1	
DMR8:112529001	8	112529001	112531000	2000	1	5.90E-08	0.55	47	2.35	Nphp3	
DMR8:112672001	8	112672001	112673000	1000	1	1.90E-07	0.37	13	1.3	Acad11	Metabolism
DMR8:112691001	8	112691001	112695000	4000	1	6.60E-07	0.35	28	0.7	Dnajc13	Transcription
DMR8:112789001	8	112789001	112790000	1000	1	8.60E-07	-0.45	12	1.2	Dnajc13;LOC108351774	Transcription
DMR8:112888001	8	112888001	112896000	8000	1	6.20E-10	-0.47	135	1.69	Acpp	
DMR8:112951001	8	112951001	112956000	5000	2	3.90E-08	0.57	72	1.44	Trnac-gca;Trnae-uuc	
DMR8:113323001	8	113323001	113325000	2000	1	3.90E-07	0.36	13	0.65	Cpne4	
DMR8:113329001	8	113329001	113330000	1000	1	3.60E-07	-0.59	9	0.9	Cpne4	
DMR8:113549001	8	113549001	113551000	2000	1	2.70E-09	-0.46	16	0.8	Cpne4	
DMR8:113621001	8	113621001	113623000	2000	1	1.10E-09	-0.51	26	1.3	Mrpl3	Translation
DMR8:113760001	8	113760001	113761000	1000	1	2.90E-09	0.34	15	1.5	Nek11;LOC102551579	Signaling
DMR8:113818001	8	113818001	113820000	2000	1	3.70E-07	0.31	23	1.15	Nek11;LOC108351775	Signaling
DMR8:113841001	8	113841001	113843000	2000	1	3.30E-07	-0.38	34	1.7	Nek11	Signaling
DMR8:114005001	8	114005001	114008000	3000	1	4.50E-09	-0.42	49	1.63	Atp2c1	Transport
DMR8:114037001	8	114037001	114039000	2000	1	9.60E-07	-0.45	28	1.4	Atp2c1	Transport
DMR8:114043001	8	114043001	114045000	2000	1	3.60E-09	0.43	18	0.9	Atp2c1	Transport
DMR8:114301001	8	114301001	114302000	1000	1	3.20E-11	-0.7	23	2.3	Pik3r4	Signaling
DMR8:114327001	8	114327001	114328000	1000	1	1.80E-07	-0.47	19	1.9	Pik3r4	Signaling
DMR8:114334001	8	114334001	114336000	2000	1	1.70E-08	-0.5	21	1.05	Pik3r4	Signaling
DMR8:114625001	8	114625001	114627000	2000	1	1.20E-07	0.53	29	1.45	Col6a5	
DMR8:114932001	8	114932001	114937000	5000	1	4.50E-07	-0.44	87	1.74	Alas1;LOC102549759	Metabolism
DMR8:115157001	8	115157001	115158000	1000	1	7.80E-08	0.46	11	1.1	Abhd14a;Abhd14b;Pcbp4;Gpr62	Metabolism;Signaling
DMR8:115184001	8	115184001	115186000	2000	1	4.10E-07	0.31	25	1.25	Parp3;Rrp9;Iqcf1	Transcription
DMR8:115710001	8	115710001	115716000	6000	1	1.20E-08	-0.27	65	1.08	Dock3	Transcription
DMR8:115717001	8	115717001	115719000	2000	1	1.40E-08	-0.33	17	0.85	Dock3	Transcription
DMR8:115762001	8	115762001	115764000	2000	1	6.10E-11	-0.4	15	0.75	Dock3	Transcription
DMR8:115777001	8	115777001	115780000	3000	1	3.60E-10	0.45	24	0.8	Dock3	Transcription
DMR8:115845001	8	115845001	115848000	3000	1	9.20E-08	-0.28	26	0.87	Dock3	Transcription
DMR8:115855001	8	115855001	115858000	3000	1	3.40E-08	-0.4	17	0.57	Dock3	Transcription
DMR8:115915001	8	115915001	115921000	6000	1	1.60E-08	-0.54	54	0.9	Dock3;LOC102550698	Transcription
DMR8:116031001	8	116031001	116037000	6000	1	4.20E-12	0.55	70	1.17	Mapkapk3	Signaling
DMR8:116292001	8	116292001	116294000	2000	1	6.70E-07	-0.42	31	1.55	Cacna2d2;Tmem115;Cyb561d2;Nprl2;Zmynd10	Transport;Transport;Signaling
DMR8:116316001	8	116316001	116317000	1000	1	6.40E-08	-0.46	9	0.9	Zmynd10;Rassf1;Tusc2;Hyal2	Cytoskeleton;Metabolism
DMR8:116354001	8	116354001	116356000	2000	1	7.10E-15	0.63	62	3.1	Ifrd2;Lsmem2;Sema3b	Signaling
DMR8:116670001	8	116670001	116671000	1000	1	8.00E-07	-0.49	11	1.1	Mon1a	
DMR8:116802001	8	116802001	116806000	4000	1	8.70E-10	-0.47	51	1.27	Ip6k1	Signaling
DMR8:116944001	8	116944001	116946000	2000	1	3.60E-08	-0.52	31	1.55	Bsn	
DMR8:117215001	8	117215001	117216000	1000	1	1.30E-08	0.72	33	3.3	Ccdc36;LOC680045	
DMR8:117479001	8	117479001	117481000	2000	1	1.80E-09	0.38	22	1.1	Slc25a20;LOC679586;Prkar2a	Transport;Signaling
DMR8:117502001	8	117502001	117503000	1000	1	3.50E-08	-0.48	14	1.4	Prkar2a	Signaling
DMR8:117674001	8	117674001	117679000	5000	1	2.40E-07	-0.34	62	1.24	LOC108351778;Uqcrc1	Protease
DMR8:117720001	8	117720001	117723000	3000	1	2.30E-07	0.47	47	1.57	Col7a1;Mir711;Ucn2	Extracellular Matrix
DMR8:117729001	8	117729001	117732000	3000	2	1.40E-07	0.46	2	0.07	Col7a1;Ucn2;Pfkfb4	Extracellular Matrix;Metabolism
DMR8:117961001	8	117961001	117963000	2000	1	3.30E-08	-0.41	23	1.15	Cdc25a	Signaling
DMR8:118049001	8	118049001	118052000	3000	1	2.80E-09	-0.45	25	0.83	Map4	
DMR8:118141001	8	118141001	118144000	3000	1	4.00E-07	-0.56	23	0.77	Map4	
DMR8:118269001	8	118269001	118270000	1000	1	1.10E-10	0.43	6	0.6	Smarcc1	Epigenetic
DMR8:118272001	8	118272001	118275000	3000	1	4.90E-07	-0.41	36	1.2	Smarcc1	Epigenetic
DMR8:118291001	8	118291001	118293000	2000	1	1.90E-08	-0.42	24	1.2	Smarcc1	Epigenetic
DMR8:118593001	8	118593001	118594000	1000	1	3.60E-14	-0.51	14	1.4	Scap	
DMR8:118678001	8	118678001	118680000	2000	1	5.00E-09	-0.46	42	2.1	Klhl18	Cytoskeleton
DMR8:118741001	8	118741001	118742000	1000	1	4.00E-07	0.42	7	0.7	Klhl18;Kif9	Cytoskeleton;Cytoskeleton
DMR8:118821001	8	118821001	118826000	5000	1	1.40E-09	-0.45	74	1.48	Setd2	Epigenetic
DMR8:118895001	8	118895001	118897000	2000	1	3.60E-08	0.49	48	2.4	Setd2;LOC108351781;Nradd;Nbeal2	Epigenetic;Receptor
DMR8:118899001	8	118899001	118900000	1000	1	3.80E-07	0.46	38	3.8	Nradd;Nbeal2	Receptor

DMR8:118946001	8	118946001	118949000	3000	1	5.30E-07	-0.41	37	1.23	Ccdc12	Translation
DMR8:119019001	8	119019001	119020000	1000	1	7.60E-09	0.32	10	1	Pth1r	Receptor
DMR8:119099001	8	119099001	119103000	4000	2	9.20E-09	0.48	78	1.95	Prss44;LOC103690462;LOC100360828;Prss45	Protease
DMR8:119115001	8	119115001	119117000	2000	1	4.40E-07	-0.37	25	1.25	LOC100360828;Prss45;Prss46	Protease
DMR8:119332001	8	119332001	119336000	4000	1	9.40E-07	-0.39	76	1.9	Ccrl2	
DMR8:119407001	8	119407001	119408000	1000	1	4.40E-07	0.31	12	1.2	Lrrfip2	Transcription
DMR8:119570001	8	119570001	119571000	1000	1	1.40E-09	-0.55	17	1.7	Trank1	
DMR8:119582001	8	119582001	119586000	4000	1	2.70E-13	0.39	42	1.05	Trank1	
DMR8:119679001	8	119679001	119682000	3000	1	4.60E-08	-0.47	35	1.17	LOC102552712;Dclk3	Signaling
DMR8:119711001	8	119711001	119712000	1000	1	5.70E-08	0.39	4	0.4	Dclk3	Signaling
DMR8:119718001	8	119718001	119719000	1000	1	1.10E-07	-0.46	20	2	Dclk3	Signaling
DMR8:119736001	8	119736001	119740000	4000	1	7.30E-07	0.38	62	1.55	Dclk3	Signaling
DMR8:120311001	8	120311001	120314000	3000	1	9.40E-08	0.45	43	1.43	Arpp21	
DMR8:120352001	8	120352001	120355000	3000	1	9.60E-07	0.37	49	1.63	Arpp21	
DMR8:120374001	8	120374001	120376000	2000	1	1.70E-08	-0.36	27	1.35	Arpp21;Mir128-2	
DMR8:120384001	8	120384001	120385000	1000	1	1.10E-08	0.53	22	2.2	Arpp21;Mir128-2	
DMR8:120432001	8	120432001	120436000	4000	1	3.60E-08	-0.56	70	1.75	Arpp21;LOC108351783	
DMR8:120459001	8	120459001	120461000	2000	1	5.10E-08	0.48	31	1.55	Arpp21	
DMR8:120480001	8	120480001	120481000	1000	1	2.30E-10	0.77	24	2.4	Arpp21	
DMR8:122029001	8	122029001	122030000	1000	1	4.40E-07	0.35	4	0.4	Clasp2	Cytoskeleton
DMR8:122099001	8	122099001	122102000	3000	1	3.30E-07	0.35	40	1.33	Clasp2	Cytoskeleton
DMR8:122156001	8	122156001	122157000	1000	1	3.30E-10	0.39	14	1.4	Clasp2	Cytoskeleton
DMR8:122220001	8	122220001	122222000	2000	1	8.90E-07	-0.4	21	1.05	Ubp1	Transcription
DMR8:122252001	8	122252001	122259000	7000	2	1.80E-07	-0.59	122	1.74	Fbxl2	
DMR8:122277001	8	122277001	122280000	3000	1	1.70E-08	0.36	61	2.03	Fbxl2;LOC102554690	
DMR8:122327001	8	122327001	122331000	4000	2	6.80E-10	0.45	62	1.55	LOC108351786;Susd5	
DMR8:122355001	8	122355001	122359000	4000	1	1.50E-07	0.4	45	1.12	Susd5	
DMR8:122366001	8	122366001	122368000	2000	1	5.70E-08	0.49	44	2.2	Susd5	
DMR8:122371001	8	122371001	122372000	1000	1	5.30E-10	0.55	11	1.1	Susd5;LOC108351787	
DMR8:122380001	8	122380001	122382000	2000	1	1.10E-07	0.5	45	2.25	Susd5;LOC108351787	
DMR8:122392001	8	122392001	122395000	3000	1	4.30E-08	-0.42	44	1.47	Crtap	
DMR8:122487001	8	122487001	122488000	1000	1	6.00E-08	0.59	29	2.9	Glb1	Metabolism
DMR8:122529001	8	122529001	122530000	1000	1	1.30E-07	0.83	9	0.9	Ccr4	
DMR8:122545001	8	122545001	122547000	2000	1	3.80E-07	0.38	39	1.95	Ccr4;Trim71	Proteolysis
DMR8:122827001	8	122827001	122831000	4000	1	3.20E-07	0.41	86	2.15	Cmtm7	Transport
DMR8:122874001	8	122874001	122876000	2000	1	1.00E-08	0.51	16	0.8	Cmtm8	Transport
DMR8:122967001	8	122967001	122968000	1000	1	5.40E-12	-0.47	16	1.6	Gpd1l	Metabolism
DMR8:123022001	8	123022001	123025000	3000	1	1.30E-07	0.41	63	2.1	LOC108351788;Osbpl10	
DMR8:123033001	8	123033001	123035000	2000	1	1.60E-11	0.63	44	2.2	LOC108351788;Osbpl10	
DMR8:123096001	8	123096001	123097000	1000	1	2.20E-09	0.39	18	1.8	Osbpl10	
DMR8:123112001	8	123112001	123113000	1000	1	1.80E-18	1.11	32	3.2	Osbpl10	
DMR8:123143001	8	123143001	123146000	3000	1	1.40E-07	0.4	46	1.53	Osbpl10	
DMR8:123351001	8	123351001	123353000	2000	1	4.60E-07	-0.42	34	1.7	Stt3b	Golgi
DMR8:124240001	8	124240001	124244000	4000	1	2.30E-13	0.52	25	0.62	Gadl1	
DMR8:124293001	8	124293001	124298000	5000	1	3.60E-11	0.64	114	2.28	Gadl1	
DMR8:124834001	8	124834001	124835000	1000	1	5.80E-08	0.52	27	2.7	Rbms3	
DMR8:124873001	8	124873001	124874000	1000	1	4.50E-08	0.33	9	0.9	Rbms3	
DMR8:124893001	8	124893001	124896000	3000	1	7.00E-07	-0.43	35	1.17	Rbms3	
DMR8:124921001	8	124921001	124924000	3000	1	1.20E-09	-0.41	42	1.4	Rbms3	
DMR8:124945001	8	124945001	124946000	1000	1	5.50E-10	0.63	17	1.7	Rbms3	
DMR8:124992001	8	124992001	124993000	1000	1	6.30E-08	0.37	5	0.5	Rbms3	
DMR8:125010001	8	125010001	125012000	2000	1	3.70E-08	0.36	19	0.95	Rbms3	
DMR8:125020001	8	125020001	125026000	6000	1	1.10E-07	0.61	75	1.25	Rbms3	
DMR8:125118001	8	125118001	125121000	3000	1	1.20E-07	-0.49	36	1.2	Rbms3	
DMR8:125187001	8	125187001	125188000	1000	1	5.60E-09	0.37	16	1.6	Rbms3	
DMR8:125223001	8	125223001	125226000	3000	1	3.60E-09	0.48	58	1.93	Rbms3;LOC103693177	
DMR8:125256001	8	125256001	125257000	1000	1	2.60E-07	0.45	13	1.3	Rbms3	
DMR8:125268001	8	125268001	125271000	3000	1	1.70E-10	-0.53	49	1.63	Rbms3	
DMR8:125294001	8	125294001	125295000	1000	1	4.90E-07	0.4	12	1.2	Rbms3	
DMR8:125383001	8	125383001	125388000	5000	1	1.30E-12	0.64	106	2.12	Rbms3	
DMR8:125437001	8	125437001	125440000	3000	2	1.20E-07	0.61	74	2.47	Rbms3	
DMR8:125445001	8	125445001	125447000	2000	2	6.30E-18	0.61	11	0.55	Rbms3	

DMR8:125495001	8	125495001	125496000	1000	1	3.70E-17	1	25	2.5	Rbms3	
DMR8:125512001	8	125512001	125515000	3000	1	1.10E-10	0.41	28	0.93	Rbms3	
DMR8:125596001	8	125596001	125597000	1000	1	1.30E-09	0.4	7	0.7	Rbms3	
DMR8:126129001	8	126129001	126132000	3000	1	7.10E-07	0.34	59	1.97	Zcwpw2;RGD1559743	
DMR8:126149001	8	126149001	126153000	4000	1	4.80E-07	0.47	65	1.62	Zcwpw2	
DMR8:126161001	8	126161001	126164000	3000	1	2.00E-08	0.4	49	1.63	Zcwpw2	
DMR8:126167001	8	126167001	126171000	4000	1	7.80E-15	0.57	71	1.77	Zcwpw2	
DMR8:126182001	8	126182001	126184000	2000	1	1.00E-07	0.4	20	1	Zcwpw2	
DMR8:126188001	8	126188001	126192000	4000	2	6.10E-08	0.56	51	1.27	Zcwpw2	
DMR8:126226001	8	126226001	126227000	1000	1	2.10E-07	0.7	18	1.8	Zcwpw2	
DMR8:126228001	8	126228001	126229000	1000	1	9.20E-07	-0.38	6	0.6	Zcwpw2	
DMR8:127073001	8	127073001	127076000	3000	1	1.60E-09	-0.51	54	1.8	Ctdspl	
DMR8:127089001	8	127089001	127091000	2000	1	1.40E-07	0.33	33	1.65	Ctdspl	
DMR8:127121001	8	127121001	127123000	2000	1	4.40E-07	0.55	39	1.95	Ctdspl	
DMR8:127192001	8	127192001	127194000	2000	2	8.10E-08	-0.51	27	1.35	Ctdspl;Golga4	Transport
DMR8:127221001	8	127221001	127227000	6000	3	2.20E-09	-0.57	130	2.17	Ctdspl;Golga4	Transport
DMR8:127252001	8	127252001	127255000	3000	1	3.60E-08	-0.44	56	1.87	Ctdspl;Golga4	Transport
DMR8:127282001	8	127282001	127284000	2000	1	2.70E-08	0.44	28	1.4	Ctdspl;Itga9	Extracellular Matrix
DMR8:127329001	8	127329001	127334000	5000	1	2.80E-08	-0.47	110	2.2	Ctdspl;Itga9	Extracellular Matrix
DMR8:127369001	8	127369001	127371000	2000	1	4.20E-07	0.3	37	1.85	Ctdspl;Itga9	Extracellular Matrix
DMR8:127405001	8	127405001	127406000	1000	1	4.40E-07	0.37	18	1.8	Ctdspl;Itga9	Extracellular Matrix
DMR8:127474001	8	127474001	127477000	3000	1	5.90E-09	0.49	37	1.23	Ctdspl;Itga9	Extracellular Matrix
DMR8:127490001	8	127490001	127493000	3000	1	3.80E-07	0.33	36	1.2	Ctdspl;Itga9	Extracellular Matrix
DMR8:127522001	8	127522001	127524000	2000	2	2.20E-10	0.44	19	0.95	Ctdspl;Itga9	Extracellular Matrix
DMR8:127547001	8	127547001	127548000	1000	1	2.20E-15	0.64	11	1.1	Ctdspl;Itga9	Extracellular Matrix
DMR8:127552001	8	127552001	127553000	1000	1	3.60E-09	0.47	6	0.6	Ctdspl;Itga9	Extracellular Matrix
DMR8:127630001	8	127630001	127631000	1000	1	5.10E-13	-0.59	32	3.2	Ctdspl	
DMR8:127665001	8	127665001	127671000	6000	1	1.80E-07	-0.37	115	1.92	Ctdspl;LOC108351797	
DMR8:127699001	8	127699001	127702000	3000	1	3.30E-12	0.45	47	1.57	Ctdspl;LOC108351798	
DMR8:127745001	8	127745001	127747000	2000	1	3.70E-08	0.48	50	2.5	Vill;Plcd1	Cytoskeleton;Metabolism
DMR8:127782001	8	127782001	127785000	3000	2	2.70E-07	0.49	70	2.33	Plcd1;LOC102548352;Dlec1	Metabolism
DMR8:127810001	8	127810001	127815000	5000	1	5.10E-07	0.35	95	1.9	Dlec1	
DMR8:127865001	8	127865001	127866000	1000	1	6.50E-14	0.48	10	1	Slc22a14	
DMR8:127891001	8	127891001	127894000	3000	1	6.30E-07	0.47	53	1.77	Slc22a13	Transport
DMR8:128023001	8	128023001	128026000	3000	1	2.10E-07	0.41	54	1.8	Myd88;Acaa1	Cytoskeleton
DMR8:128112001	8	128112001	128115000	3000	1	2.50E-13	0.69	84	2.8	Acvr2b	Signaling
DMR8:128154001	8	128154001	128157000	3000	1	7.90E-07	0.35	55	1.83	Exog	
DMR8:128224001	8	128224001	128225000	1000	1	4.90E-11	0.46	8	0.8	Scn5a	Transport
DMR8:128251001	8	128251001	128252000	1000	1	8.00E-09	0.39	10	1	Scn5a	Transport
DMR8:128254001	8	128254001	128262000	8000	1	9.00E-08	0.35	144	1.8	Scn5a	Transport
DMR8:128270001	8	128270001	128271000	1000	1	2.70E-08	-0.42	21	2.1	Scn5a	Transport
DMR8:128295001	8	128295001	128297000	2000	1	2.30E-12	0.42	19	0.95	Scn10a	Transport
DMR8:128303001	8	128303001	128309000	6000	2	3.20E-08	-0.5	98	1.63	Scn10a	Transport
DMR8:128466001	8	128466001	128467000	1000	1	1.50E-10	0.85	24	2.4	Scn11a	Transport
DMR8:128507001	8	128507001	128511000	4000	1	4.70E-09	0.57	73	1.82	Scn11a	Transport
DMR8:128646001	8	128646001	128650000	4000	2	2.80E-09	0.51	78	1.95	Ttc21a;Csrnp1	
DMR8:128858001	8	128858001	128860000	2000	1	3.20E-11	0.74	54	2.7	Mobp;LOC102549288	Cytoskeleton
DMR8:129007001	8	129007001	129010000	3000	1	1.30E-07	0.47	24	0.8	Myrip;LOC108351802	Cytoskeleton
DMR8:129093001	8	129093001	129096000	3000	2	1.40E-15	0.5	34	1.13	Myrip	Cytoskeleton
DMR8:129192001	8	129192001	129193000	1000	1	1.50E-16	0.84	15	1.5	LOC367190;Entpd3	Signaling
DMR8:129194001	8	129194001	129195000	1000	1	7.20E-07	0.5	27	2.7	LOC367190;Entpd3	Signaling
DMR8:129225001	8	129225001	129230000	5000	1	1.60E-07	0.39	56	1.12	Entpd3	Signaling
DMR8:129233001	8	129233001	129234000	1000	1	6.60E-09	0.43	7	0.7	Entpd3;Rpl14	Signaling;Translation
DMR8:129642001	8	129642001	129644000	2000	1	1.10E-07	0.41	17	0.85	Ulk4	Signaling
DMR8:129677001	8	129677001	129679000	2000	1	1.20E-09	0.53	18	0.9	Ulk4	Signaling
DMR8:129810001	8	129810001	129811000	1000	1	2.70E-07	0.36	4	0.4	Ulk4	Signaling
DMR8:129871001	8	129871001	129876000	5000	2	1.60E-08	0.46	118	2.36	Ulk4	Signaling
DMR8:129902001	8	129902001	129904000	2000	1	7.30E-07	0.24	33	1.65	Ulk4	Signaling
DMR8:129937001	8	129937001	129940000	3000	1	1.50E-13	0.72	73	2.43	Trak1	Transport
DMR8:130137001	8	130137001	130138000	1000	1	4.50E-07	0.38	15	1.5	Cck	
DMR8:130221001	8	130221001	130223000	2000	1	1.70E-07	0.46	28	1.4	Lyzl4	
DMR8:130233001	8	130233001	130235000	2000	1	3.00E-08	0.62	35	1.75	Lyzl4	

DMR8:130542001	8	130542001	130545000	3000	1	9.10E-07	0.28	46	1.53	Ackr2;Cyp8b1;LOC102551973	
DMR8:130770001	8	130770001	130771000	1000	1	1.30E-08	-0.49	11	1.1	Snrk	Signaling
DMR8:130888001	8	130888001	130889000	1000	1	5.50E-07	-0.44	11	1.1	Ano10	
DMR8:131730001	8	131730001	131732000	2000	1	3.50E-10	0.47	22	1.1	Mir138-1	
DMR8:131801001	8	131801001	131804000	3000	1	2.20E-08	-0.41	35	1.17	Topaz1;LOC102550701	
DMR8:131895001	8	131895001	131896000	1000	1	2.10E-09	-0.5	5	0.5	Zfp445;LOC102552507	Transcription
DMR8:132028001	8	132028001	132031000	3000	1	3.60E-08	-0.52	23	0.77	LOC367195;RGD1311745;Kif15	Translation;Cytoskeleton
DMR8:132115001	8	132115001	132117000	2000	1	3.40E-07	0.37	13	0.65	Tmem42;Tgm4	Transport
DMR8:132473001	8	132473001	132476000	3000	1	7.00E-09	0.33	24	0.8	Lars2	Translation
DMR8:132676001	8	132676001	132681000	5000	1	6.30E-08	-0.37	27	0.54	RGD1566368	
DMR8:133017001	8	133017001	133018000	1000	1	5.00E-09	-0.53	5	0.5	Ccr3	
DMR9:39001	9	39001	41000	2000	1	5.90E-07	-0.37	33	1.65	Efhb	Signaling
DMR9:1167001	9	1167001	1168000	1000	1	7.50E-08	-0.59	1	0.1	Tbc1d5	Signaling
DMR9:1403001	9	1403001	1409000	6000	1	5.50E-08	-0.51	32	0.53	Tbc1d5;LOC108348193	Signaling
DMR9:1411001	9	1411001	1413000	2000	1	1.20E-11	0.3	16	0.8	Tbc1d5;LOC108348193	Signaling
DMR9:1420001	9	1420001	1426000	6000	1	2.80E-10	0.35	45	0.75	Tbc1d5;LOC108348193	Signaling
DMR9:1460001	9	1460001	1469000	9000	1	7.50E-07	-0.27	106	1.18	Tbc1d5	Signaling
DMR9:1471001	9	1471001	1473000	2000	1	2.00E-07	-0.57	19	0.95	Tbc1d5	Signaling
DMR9:1498001	9	1498001	1501000	3000	1	1.80E-07	-0.23	27	0.9	Tbc1d5	Signaling
DMR9:1538001	9	1538001	1539000	1000	1	2.70E-14	0.59	20	2	Tbc1d5	Signaling
DMR9:1556001	9	1556001	1561000	5000	3	1.90E-07	-0.33	47	0.94	Tbc1d5	Signaling
DMR9:1609001	9	1609001	1611000	2000	1	6.60E-09	0.42	15	0.75	Tbc1d5;LOC102555421	Signaling
DMR9:1730001	9	1730001	1733000	3000	2	3.10E-08	0.34	21	0.7	Tbc1d5	Signaling
DMR9:1735001	9	1735001	1737000	2000	1	2.10E-08	-0.48	16	0.8	Tbc1d5	Signaling
DMR9:1840001	9	1840001	1842000	2000	1	2.30E-09	-0.43	69	3.45	Plcl2	Metabolism
DMR9:1892001	9	1892001	1897000	5000	1	1.90E-07	-0.63	39	0.78	Plcl2	Metabolism
DMR9:1990001	9	1990001	1992000	2000	1	3.10E-07	0.32	20	1	Plcl2;LOC103693197	Metabolism
DMR9:2209001	9	2209001	2216000	7000	1	4.00E-07	-0.46	115	1.64	Satb1	Epigenetic
DMR9:2272001	9	2272001	2274000	2000	1	2.40E-09	0.41	47	2.35	Satb1	Epigenetic
DMR9:3140001	9	3140001	3145000	5000	1	2.50E-08	-0.32	38	0.76	Kcnh8;LOC103690491	Transport
DMR9:3152001	9	3152001	3153000	1000	1	6.00E-08	-0.52	8	0.8	Kcnh8	Transport
DMR9:3215001	9	3215001	3218000	3000	1	5.20E-07	-0.27	27	0.9	Kcnh8;LOC102551325	Transport
DMR9:3245001	9	3245001	3250000	5000	1	1.40E-09	-0.58	23	0.46	Kcnh8;RGD1559808	Transport;Translation
DMR9:3286001	9	3286001	3287000	1000	1	9.80E-07	0.46	6	0.6	Kcnh8	Transport
DMR9:3340001	9	3340001	3341000	1000	1	2.60E-08	0.4	17	1.7	Kcnh8	Transport
DMR9:3345001	9	3345001	3347000	2000	1	6.60E-09	0.45	12	0.6	Kcnh8	Transport
DMR9:3537001	9	3537001	3540000	3000	1	2.10E-08	-0.38	14	0.47	Kcnh8	Transport
DMR9:3543001	9	3543001	3546000	3000	1	5.70E-09	-0.43	31	1.03	Kcnh8	Transport
DMR9:3655001	9	3655001	3662000	7000	1	2.00E-07	-0.26	60	0.86	Kcnh8	Transport
DMR9:4158001	9	4158001	4164000	6000	1	2.70E-07	-0.21	58	0.97	Sult1c2a	Transport
DMR9:4165001	9	4165001	4166000	1000	1	1.30E-07	0.31	4	0.4	Sult1c2a	Transport
DMR9:4334001	9	4334001	4335000	1000	1	2.60E-07	0.31	8	0.8	RGD1562392;LOC100910235	Transport
DMR9:4363001	9	4363001	4365000	2000	1	1.50E-08	0.36	19	0.95	RGD1562392	Transport
DMR9:4374001	9	4374001	4376000	2000	2	8.30E-11	0.47	8	0.4	RGD1562392;LOC100359586	Transport
DMR9:4400001	9	4400001	4402000	2000	1	1.10E-09	0.29	10	0.5	RGD1562392;LOC100359586	Transport
DMR9:4407001	9	4407001	4408000	1000	1	2.40E-08	-0.33	12	1.2	RGD1562392	Transport
DMR9:4431001	9	4431001	4432000	1000	1	4.60E-07	-0.41	20	2	RGD1562392;Sgo1;Kat2b	Transport;Cell Cycle
DMR9:4667001	9	4667001	4668000	1000	1	4.50E-08	0.3	5	0.5	Sult1c2	Transport
DMR9:5303001	9	5303001	5305000	2000	1	6.60E-07	0.45	13	0.65	Slc5a7	Transport
DMR9:7740001	9	7740001	7741000	1000	1	6.90E-07	0.51	26	2.6	Vom2r77	Signaling
DMR9:7859001	9	7859001	7861000	2000	1	6.20E-07	-0.35	16	0.8	Pot1b	Transcription
DMR9:7895001	9	7895001	7896000	1000	1	5.60E-07	0.51	5	0.5	Pot1b;LOC102548597	Transcription
DMR9:8047001	9	8047001	8048000	1000	1	6.40E-08	-0.55	1	0.1	LOC100911278;Adgre4	Signaling
DMR9:8097001	9	8097001	8099000	2000	1	1.40E-08	-0.43	7	0.35	Adgre4	Signaling
DMR9:8154001	9	8154001	8157000	3000	1	9.70E-10	-0.39	14	0.47	Adgre4	Signaling
DMR9:8172001	9	8172001	8176000	4000	1	9.00E-07	-0.36	25	0.62	Adgre4	Signaling
DMR9:9153001	9	9153001	9155000	2000	1	6.40E-08	0.36	21	1.05	MGC116197;Vom2r-ps141	
DMR9:9169001	9	9169001	9170000	1000	1	2.50E-07	-0.51	5	0.5	Vom2r-ps141	
DMR9:9172001	9	9172001	9173000	1000	1	1.70E-07	-0.31	6	0.6	Vom2r-ps141	
DMR9:9270001	9	9270001	9273000	3000	1	5.70E-07	-0.34	19	0.63	Vom2r79	Signaling
DMR9:9460001	9	9460001	9465000	5000	1	3.60E-08	-0.42	38	0.76	Adgre1;LOC103690497	Signaling
DMR9:9532001	9	9532001	9534000	2000	1	3.10E-08	-0.4	13	0.65	Adgre1;LOC102549477	Signaling
DMR9:9707001	9	9707001	9708000	1000	1	6.60E-07	0.43	11	1.1	Trip10;Gpr108	Signaling

DMR9:9846001	9	9846001	9847000	1000	1	4.20E-12	0.42	4	0.4	Cd70	
DMR9:9967001	9	9967001	9969000	2000	1	4.10E-07	0.49	51	2.55	LOC108351881;Tubb4a;Dennd1c	Cytoskeleton
DMR9:10165001	9	10165001	10168000	3000	1	3.40E-07	0.45	33	1.1	Mllt1;LOC316124;Acsbg2	Transcription;Metabolism
DMR9:10192001	9	10192001	10196000	4000	1	4.00E-08	0.37	42	1.05	LOC316124;Acsbg2	Metabolism
DMR9:10208001	9	10208001	10209000	1000	1	6.40E-08	0.62	35	3.5	Acsbg2;LOC501280;Rfx2	Metabolism;Transcription
DMR9:10607001	9	10607001	10609000	2000	1	5.50E-08	0.49	31	1.55	Ptprs	Signaling
DMR9:10663001	9	10663001	10665000	2000	1	1.30E-08	0.43	26	1.3	Kdm4b	Epigenetic
DMR9:10741001	9	10741001	10743000	2000	1	1.70E-12	0.57	37	1.85	Kdm4b;LOC100362987;Uhrf1	Epigenetic;Translation;Proteolysis
DMR9:10877001	9	10877001	10882000	5000	1	4.30E-07	0.38	79	1.58	Dpp9;Mydgf	Protease;Growth Factors
DMR9:10950001	9	10950001	10951000	1000	1	7.20E-07	0.62	70	7	Sema6b;Lrg1;Plin5	Signaling;Cytoskeleton
DMR9:11095001	9	11095001	11097000	2000	1	9.20E-07	0.57	59	2.95	Shd;Ccdc94	
DMR9:11103001	9	11103001	11104000	1000	1	4.40E-08	0.49	19	1.9	Shd;Ccdc94;Ebi3	Receptor
DMR9:11153001	9	11153001	11156000	3000	2	3.00E-12	0.58	60	2	LOC102555289;Uxs1	Metabolism
DMR9:12748001	9	12748001	12751000	3000	1	3.60E-07	-0.54	51	1.7	Rftn1	
DMR9:12875001	9	12875001	12876000	1000	1	4.40E-08	0.52	20	2	Rftn1	
DMR9:13068001	9	13068001	13069000	1000	1	8.20E-08	0.32	13	1.3	Kif6	Cytoskeleton
DMR9:13070001	9	13070001	13071000	1000	1	1.10E-17	1	32	3.2	Kif6	Cytoskeleton
DMR9:13112001	9	13112001	13116000	4000	1	4.90E-09	0.38	62	1.55	Kif6	Cytoskeleton
DMR9:13226001	9	13226001	13227000	1000	1	2.00E-07	0.37	3	0.3	Kif6	Cytoskeleton
DMR9:13229001	9	13229001	13230000	1000	1	3.50E-07	-0.46	21	2.1	Kif6	Cytoskeleton
DMR9:13241001	9	13241001	13243000	2000	1	8.20E-08	-0.37	25	1.25	Kif6	Cytoskeleton
DMR9:13317001	9	13317001	13320000	3000	1	1.20E-07	0.36	33	1.1	Kif6	Cytoskeleton
DMR9:13858001	9	13858001	13861000	3000	1	2.70E-15	0.44	52	1.73	Lrfn2	
DMR9:13872001	9	13872001	13875000	3000	1	5.50E-08	-0.44	41	1.37	Lrfn2	
DMR9:13943001	9	13943001	13945000	2000	1	1.10E-12	0.59	36	1.8	Lrfn2	
DMR9:13984001	9	13984001	13985000	1000	1	4.90E-11	0.45	16	1.6	Lrfn2	
DMR9:14570001	9	14570001	14574000	4000	1	3.80E-08	-0.42	42	1.05	Nfya	Transcription
DMR9:14579001	9	14579001	14580000	1000	1	1.80E-08	-0.46	12	1.2	Nfya	Transcription
DMR9:14641001	9	14641001	14645000	4000	2	1.40E-08	0.39	61	1.52	LOC102548435;RGD1307182	
DMR9:14686001	9	14686001	14688000	2000	1	3.70E-07	-0.48	16	0.8	Treml4	
DMR9:14789001	9	14789001	14794000	5000	2	2.50E-07	0.27	54	1.08	Ncr2	
DMR9:14797001	9	14797001	14803000	6000	3	5.40E-16	0.4	93	1.55	Ncr2	
DMR9:14901001	9	14901001	14903000	2000	1	1.30E-09	0.44	29	1.45	RGD1565959;LOC680955	
DMR9:15077001	9	15077001	15079000	2000	1	1.20E-07	-0.41	34	1.7	Foxp4	
DMR9:15122001	9	15122001	15126000	4000	2	1.90E-16	0.53	71	1.77	Foxp4	
DMR9:15131001	9	15131001	15140000	9000	3	1.80E-11	0.63	136	1.51	Foxp4	
DMR9:15157001	9	15157001	15160000	3000	1	5.40E-12	-0.44	16	0.53	Mdfi	
DMR9:15274001	9	15274001	15276000	2000	1	9.10E-13	0.51	48	2.4	Tfeb;Pgc;LOC102554309	Proteolysis
DMR9:15342001	9	15342001	15344000	2000	1	3.80E-07	-0.47	23	1.15	Usp49;LOC103690499	Protease
DMR9:15505001	9	15505001	15510000	5000	1	1.60E-08	-0.49	74	1.48	LOC103690500;Taf8	
DMR9:15573001	9	15573001	15574000	1000	1	1.20E-07	-0.3	26	2.6	RGD1561662;LOC100912849	
DMR9:15651001	9	15651001	15654000	3000	1	1.30E-08	-0.55	63	2.1	Mrps10;Trerf1	Translation
DMR9:15690001	9	15690001	15691000	1000	1	2.20E-09	0.8	36	3.6	Trerf1	
DMR9:15704001	9	15704001	15706000	2000	1	1.40E-10	0.3	45	2.25	Trerf1	
DMR9:15744001	9	15744001	15745000	1000	1	3.60E-10	-0.53	9	0.9	Trerf1	
DMR9:15853001	9	15853001	15855000	2000	1	2.00E-09	-0.4	51	2.55	Trerf1	
DMR9:15866001	9	15866001	15868000	2000	1	5.70E-09	0.71	51	2.55	Trerf1	
DMR9:15883001	9	15883001	15884000	1000	1	5.00E-11	0.47	14	1.4	Trerf1	
DMR9:16080001	9	16080001	16082000	2000	1	4.80E-07	-0.53	45	2.25	Ubr2;Prph2	Proteolysis
DMR9:16211001	9	16211001	16212000	1000	1	8.80E-08	0.42	14	1.4	Prph2	
DMR9:16531001	9	16531001	16533000	2000	1	9.70E-08	0.31	29	1.45	Ptcra	
DMR9:16537001	9	16537001	16539000	2000	1	4.10E-07	0.43	28	1.4	Ptcra;Cnpy3	
DMR9:16552001	9	16552001	16553000	1000	1	6.10E-10	0.35	15	1.5	Cnpy3	
DMR9:16624001	9	16624001	16626000	2000	1	8.90E-11	0.47	49	2.45	Mea1;Klhdc3;Rrp36;LOC680835	
DMR9:16669001	9	16669001	16672000	3000	1	1.80E-07	-0.45	62	2.07	Klc4;Ptk7	Cytoskeleton;Receptor
DMR9:16698001	9	16698001	16700000	2000	1	7.80E-08	0.33	30	1.5	Ptk7	Receptor
DMR9:16869001	9	16869001	16870000	1000	1	4.80E-07	0.47	14	1.4	Ttbk1	Signaling
DMR9:16905001	9	16905001	16907000	2000	1	1.10E-08	0.4	30	1.5	Ttbk1	Signaling
DMR9:16940001	9	16940001	16941000	1000	1	9.90E-07	0.37	9	0.9	Slc22a7;Crip3;Zfp318	Transport;Cytoskeleton
DMR9:17035001	9	17035001	17036000	1000	1	3.60E-07	-0.42	12	1.2	Abcc10	Transport

DMR9:17343001	9	17343001	17344000	1000	1	4.30E-07	0.54	8	0.8	Vegfa	Growth Factors
DMR9:17719001	9	17719001	17723000	4000	2	1.60E-08	0.53	87	2.17	Tmem63b;Capn11	Protease
DMR9:17756001	9	17756001	17758000	2000	1	3.10E-11	0.71	39	1.95	Capn11	Protease
DMR9:17850001	9	17850001	17851000	1000	1	1.80E-07	0.65	30	3	Tmem151b;Tcte1	Cytoskeleton
DMR9:17857001	9	17857001	17858000	1000	1	8.50E-11	0.79	38	3.8	Tcte1	Cytoskeleton
DMR9:18240001	9	18240001	18241000	1000	1	3.50E-10	0.44	12	1.2	Supt3h	Transcription
DMR9:18289001	9	18289001	18291000	2000	1	2.00E-10	0.42	31	1.55	Supt3h	Transcription
DMR9:18407001	9	18407001	18413000	6000	2	8.00E-08	-0.51	92	1.53	Supt3h	Transcription
DMR9:18463001	9	18463001	18466000	3000	1	9.10E-07	-0.35	17	0.57	Supt3h	Transcription
DMR9:18483001	9	18483001	18485000	2000	1	1.50E-09	0.5	15	0.75	Supt3h;Znrd1-as1	Transcription
DMR9:18596001	9	18596001	18598000	2000	1	1.60E-07	-0.51	21	1.05	Supt3h;Runx2	Transcription;Transcriptio n
DMR9:18636001	9	18636001	18637000	1000	1	8.90E-07	0.64	34	3.4	Runx2	Transcription
DMR9:18646001	9	18646001	18647000	1000	1	3.60E-09	0.52	15	1.5	Runx2	Transcription
DMR9:18653001	9	18653001	18656000	3000	1	2.90E-08	-0.37	47	1.57	Runx2	Transcription
DMR9:18703001	9	18703001	18704000	1000	1	8.80E-07	-0.35	32	3.2	Runx2	Transcription
DMR9:19240001	9	19240001	19242000	2000	1	1.70E-07	0.38	19	0.95	Clic5	Transport
DMR9:19262001	9	19262001	19266000	4000	1	9.30E-09	0.7	57	1.43	Clic5	Transport
DMR9:19379001	9	19379001	19381000	2000	1	5.90E-07	0.31	19	0.95	Clic5;LOC108351895	Transport
DMR9:19709001	9	19709001	19710000	1000	1	9.00E-07	-0.48	16	1.6	Rcan2	Signaling
DMR9:19896001	9	19896001	19897000	1000	1	7.20E-07	0.51	20	2	Slc25a27	Transport
DMR9:19979001	9	19979001	19981000	2000	1	8.90E-08	0.47	11	0.55	Pla2g7;Ankrd66	Golgi;Signaling
DMR9:20062001	9	20062001	20065000	3000	1	5.70E-08	0.36	31	1.03	Mep1a	Protease
DMR9:20120001	9	20120001	20123000	3000	1	4.90E-11	0.44	40	1.33	Adgrf5	
DMR9:20141001	9	20141001	20144000	3000	1	1.10E-08	-0.31	23	0.77	Adgrf5	
DMR9:20331001	9	20331001	20334000	3000	1	2.90E-07	0.32	26	0.87	Adgrf1	
DMR9:20354001	9	20354001	20356000	2000	1	3.30E-11	0.56	17	0.85	Adgrf1	
DMR9:20375001	9	20375001	20377000	2000	1	8.60E-08	0.32	23	1.15	Adgrf1	
DMR9:20823001	9	20823001	20825000	2000	1	1.00E-10	-0.36	22	1.1	Cd2ap	
DMR9:20835001	9	20835001	20842000	7000	1	7.40E-08	-0.41	55	0.79	Cd2ap	
DMR9:21057001	9	21057001	21059000	2000	1	8.10E-09	0.31	28	1.4	Opn5	Signaling
DMR9:21061001	9	21061001	21064000	3000	1	8.50E-14	0.8	71	2.37	Opn5	Signaling
DMR9:21144001	9	21144001	21151000	7000	2	1.40E-07	-0.42	81	1.16	Ptchd4	Signaling
DMR9:21223001	9	21223001	21224000	1000	1	1.60E-07	-0.42	7	0.7	Ptchd4	Signaling
DMR9:21236001	9	21236001	21237000	1000	1	2.20E-08	-0.38	9	0.9	Ptchd4	Signaling
DMR9:21904001	9	21904001	21906000	2000	1	1.20E-13	-0.47	18	0.9	Olr1828-ps	
DMR9:23445001	9	23445001	23451000	6000	1	8.70E-07	-0.32	53	0.88	LOC108351992;Cyp2ac1	
DMR9:23479001	9	23479001	23481000	2000	1	6.70E-07	0.38	14	0.7	Rhag	Transport
DMR9:23487001	9	23487001	23490000	3000	1	6.30E-08	0.58	32	1.07	Rhag	Transport
DMR9:25326001	9	25326001	25327000	1000	1	7.20E-09	0.41	14	1.4	Tfap2d	Transcription
DMR9:25374001	9	25374001	25383000	9000	1	2.20E-08	-0.95	91	1.01	Tfap2d	Transcription
DMR9:26558001	9	26558001	26559000	1000	1	4.00E-07	0.52	8	0.8	Pkhd1	
DMR9:26560001	9	26560001	26566000	6000	1	4.80E-07	-0.55	36	0.6	Pkhd1	
DMR9:26702001	9	26702001	26703000	1000	1	1.20E-07	0.42	7	0.7	Pkhd1	
DMR9:26739001	9	26739001	26741000	2000	1	4.90E-07	-0.38	21	1.05	Pkhd1	
DMR9:26801001	9	26801001	26803000	2000	1	2.60E-09	0.52	13	0.65	Mir206;LOC103690506;Mir133b	
DMR9:26896001	9	26896001	26900000	4000	1	6.80E-07	0.26	46	1.15	Il17f	
DMR9:27007001	9	27007001	27008000	1000	1	4.30E-07	-0.38	18	1.8	Paqr8	Signaling
DMR9:27056001	9	27056001	27060000	4000	1	3.90E-10	0.53	105	2.62	Paqr8;LOC102549795;Efhc1	Signaling;Signaling
DMR9:27072001	9	27072001	27073000	1000	1	1.90E-07	-0.47	15	1.5	LOC102549795;Efhc1	Signaling
DMR9:27168001	9	27168001	27170000	2000	1	1.40E-07	0.34	28	1.4	Tram2	
DMR9:27337001	9	27337001	27339000	2000	1	2.70E-07	0.53	32	1.6	LOC102550123;Tmem14a;LOC1036 93210	
DMR9:27566001	9	27566001	27568000	2000	1	8.80E-16	0.97	54	2.7	Khdc1b;Kcnq5	Transport
DMR9:27582001	9	27582001	27584000	2000	1	1.60E-08	0.42	13	0.65	Kcnq5	Transport
DMR9:27744001	9	27744001	27747000	3000	1	9.90E-07	0.36	11	0.37	Kcnq5	Transport
DMR9:27766001	9	27766001	27768000	2000	1	8.00E-11	-0.41	32	1.6	Kcnq5	Transport
DMR9:28108001	9	28108001	28110000	2000	1	9.70E-07	-0.4	35	1.75	Kcnq5	Transport
DMR9:28130001	9	28130001	28132000	2000	1	8.80E-11	0.89	42	2.1	Kcnq5	Transport
DMR9:28437001	9	28437001	28438000	1000	1	3.90E-08	-0.43	9	0.9	Rims1	Transport
DMR9:28440001	9	28440001	28441000	1000	1	1.90E-08	-0.43	26	2.6	Rims1	Transport
DMR9:28521001	9	28521001	28523000	2000	1	1.30E-09	-0.51	32	1.6	Rims1	Transport
DMR9:28569001	9	28569001	28570000	1000	1	6.40E-07	-0.41	23	2.3	Rims1	Transport

DMR9:28643001	9	28643001	28645000	2000	1	5.30E-07	-0.37	29	1.45	Rims1	Transport
DMR9:28770001	9	28770001	28771000	1000	1	3.10E-08	0.35	8	0.8	Rims1	Transport
DMR9:28812001	9	28812001	28813000	1000	1	9.20E-09	0.42	12	1.2	Rims1	Transport
DMR9:30259001	9	30259001	30261000	2000	1	1.40E-08	-0.54	19	0.95	Sdhaf4;Fam135a	Transcription
DMR9:30413001	9	30413001	30420000	7000	1	8.70E-08	-0.27	69	0.99	Col9a1	Extracellular Matrix
DMR9:30626001	9	30626001	30627000	1000	1	4.70E-08	0.47	12	1.2	Col19a1	Extracellular Matrix
DMR9:30782001	9	30782001	30783000	1000	1	2.00E-07	0.41	9	0.9	Col19a1	Extracellular Matrix
DMR9:30861001	9	30861001	30864000	3000	2	1.60E-09	0.71	43	1.43	Col19a1	Extracellular Matrix
DMR9:30963001	9	30963001	30964000	1000	1	4.50E-07	-0.55	1	0.1	Lmbrd1;LOC103690508	Transport
DMR9:31399001	9	31399001	31406000	7000	1	2.20E-09	0.54	86	1.23	Adgrb3	Signaling
DMR9:31560001	9	31560001	31562000	2000	1	2.30E-07	-0.62	20	1	Adgrb3	Signaling
DMR9:31822001	9	31822001	31823000	1000	1	3.70E-09	0.46	34	3.4	Adgrb3	Signaling
DMR9:31930001	9	31930001	31932000	2000	1	3.40E-08	-0.41	28	1.4	Adgrb3	Signaling
DMR9:37090001	9	37090001	37092000	2000	1	2.90E-08	-0.47	12	0.6	Phf3	Transcription
DMR9:37093001	9	37093001	37098000	5000	2	1.80E-10	-0.57	42	0.84	Phf3	Transcription
DMR9:37784001	9	37784001	37785000	1000	1	1.50E-07	-0.46	27	2.7	Imp4;Ccdc115;Trnae-uuc;LOC102551518	Translation
DMR9:37787001	9	37787001	37788000	1000	1	3.80E-08	0.42	15	1.5	Ccdc115;Trnae-uuc;LOC102551518	
DMR9:37912001	9	37912001	37913000	1000	1	2.40E-08	-0.46	27	2.7	Dst	Cytoskeleton
DMR9:37942001	9	37942001	37945000	3000	1	2.50E-08	-0.42	69	2.3	Dst	Cytoskeleton
DMR9:37967001	9	37967001	37974000	7000	1	2.20E-09	-0.64	195	2.79	Dst	Cytoskeleton
DMR9:38052001	9	38052001	38054000	2000	2	1.70E-07	-0.49	55	2.75	Dst	Cytoskeleton
DMR9:38055001	9	38055001	38058000	3000	1	3.60E-07	-0.37	67	2.23	Dst	Cytoskeleton
DMR9:38069001	9	38069001	38070000	1000	1	1.70E-07	-0.29	27	2.7	Dst	Cytoskeleton
DMR9:38088001	9	38088001	38092000	4000	2	2.00E-11	-0.52	87	2.17	Dst	Cytoskeleton
DMR9:38213001	9	38213001	38214000	1000	1	5.70E-09	-0.46	13	1.3	Dst	Cytoskeleton
DMR9:38253001	9	38253001	38255000	2000	1	2.70E-07	-0.28	37	1.85	Dst	Cytoskeleton
DMR9:38272001	9	38272001	38277000	5000	1	1.50E-09	0.4	65	1.3	Dst	Cytoskeleton
DMR9:38301001	9	38301001	38302000	1000	1	2.80E-07	-0.38	18	1.8	Dst;Bend6	Cytoskeleton
DMR9:38587001	9	38587001	38589000	2000	1	6.60E-07	-0.36	32	1.6	Prim2	Cell Cycle
DMR9:38594001	9	38594001	38595000	1000	1	1.10E-08	-0.46	15	1.5	Prim2	Cell Cycle
DMR9:38639001	9	38639001	38642000	3000	1	1.60E-11	-0.65	35	1.17	Prim2	Cell Cycle
DMR9:38649001	9	38649001	38650000	1000	1	1.30E-07	0.41	11	1.1	Prim2	Cell Cycle
DMR9:38693001	9	38693001	38695000	2000	1	2.60E-09	0.44	19	0.95	Prim2	Cell Cycle
DMR9:39589001	9	39589001	39590000	1000	1	1.30E-11	0.77	23	2.3	Khdrbs2	Translation
DMR9:39596001	9	39596001	39597000	1000	1	7.00E-09	0.53	18	1.8	Khdrbs2	Translation
DMR9:39808001	9	39808001	39809000	1000	1	2.80E-07	-0.69	5	0.5	Khdrbs2	Translation
DMR9:39908001	9	39908001	39909000	1000	1	9.90E-09	0.49	8	0.8	Khdrbs2	Translation
DMR9:40976001	9	40976001	40977000	1000	1	8.10E-07	-0.29	22	2.2	Ptpn18	
DMR9:41006001	9	41006001	41007000	1000	1	1.20E-09	0.52	25	2.5	Prss39;LOC102554718	
DMR9:41058001	9	41058001	41060000	2000	2	9.50E-10	0.46	11	0.55	Cfc1;Prss40	Signaling;Protease
DMR9:41066001	9	41066001	41069000	3000	1	8.00E-08	0.35	28	0.93	Prss40	Protease
DMR9:41099001	9	41099001	41100000	1000	1	1.00E-09	0.43	12	1.2	Amer3	
DMR9:41150001	9	41150001	41152000	2000	1	6.90E-17	0.79	40	2	LOC108351913;RGD1562461;Arhgef4	Transcription
DMR9:41171001	9	41171001	41172000	1000	1	1.50E-09	-0.59	13	1.3	Arhgef4	Transcription
DMR9:41176001	9	41176001	41178000	2000	1	1.20E-09	-0.42	27	1.35	Arhgef4	Transcription
DMR9:41281001	9	41281001	41283000	2000	1	7.00E-07	-0.4	44	2.2	Arhgef4	Transcription
DMR9:43347001	9	43347001	43351000	4000	2	2.40E-16	0.84	79	1.98	Zap70;Tmem131	
DMR9:43352001	9	43352001	43354000	2000	1	7.70E-07	0.75	48	2.4	Zap70;Tmem131	
DMR9:43358001	9	43358001	43359000	1000	1	8.20E-10	-0.4	26	2.6	Zap70;Tmem131	
DMR9:43693001	9	43693001	43694000	1000	1	1.20E-07	-0.51	6	0.6	Vwa3b;LOC102554996	
DMR9:43786001	9	43786001	43789000	3000	1	6.10E-10	0.64	68	2.27	Vwa3b	
DMR9:43954001	9	43954001	43963000	9000	1	4.00E-07	0.34	101	1.12	Inpp4a	
DMR9:44003001	9	44003001	44008000	5000	2	4.00E-08	-0.47	82	1.64	Inpp4a;Coa5	Transcription
DMR9:44022001	9	44022001	44024000	2000	1	3.30E-07	-0.58	27	1.35	Coa5;Unc50;Mgat4a	Transcription;Transport
DMR9:44037001	9	44037001	44042000	5000	1	1.30E-08	-0.52	69	1.38	Unc50;Mgat4a	Transport
DMR9:44247001	9	44247001	44251000	4000	1	6.40E-16	-0.52	70	1.75	RGD1310819	
DMR9:44270001	9	44270001	44272000	2000	1	4.90E-09	-0.55	19	0.95	RGD1310819	
DMR9:44351001	9	44351001	44354000	3000	1	3.10E-10	-0.58	34	1.13	Tsga10	Epigenetic
DMR9:44387001	9	44387001	44388000	1000	1	6.80E-09	-0.51	17	1.7	Tsga10	Epigenetic
DMR9:44681001	9	44681001	44683000	2000	2	7.00E-09	-0.36	25	1.25	Eif5b	Translation
DMR9:44792001	9	44792001	44793000	1000	1	6.20E-12	-0.51	16	1.6	Rev1	Transcription

DMR9:44845001	9	44845001	44846000	1000	1	1.30E-07	0.34	5	0.5	Aff3	Transcription
DMR9:44870001	9	44870001	44875000	5000	1	3.50E-09	0.45	101	2.02	Aff3	Transcription
DMR9:44883001	9	44883001	44885000	2000	1	8.80E-08	0.35	19	0.95	Aff3	Transcription
DMR9:45133001	9	45133001	45134000	1000	1	3.50E-07	0.36	8	0.8	Aff3	Transcription
DMR9:45151001	9	45151001	45152000	1000	1	2.50E-07	0.56	11	1.1	Aff3	Transcription
DMR9:45491001	9	45491001	45494000	3000	1	4.90E-08	-0.7	23	0.77	Lonrf2	Proteolysis
DMR9:45503001	9	45503001	45504000	1000	1	1.40E-07	0.4	2	0.2	Lonrf2	Proteolysis
DMR9:45533001	9	45533001	45534000	1000	1	5.30E-07	0.46	14	1.4	Chst10	Transport
DMR9:45595001	9	45595001	45596000	1000	1	2.60E-07	0.62	17	1.7	NMS	
DMR9:45677001	9	45677001	45679000	2000	1	1.20E-07	-0.48	53	2.65	Pdcl3	Epigenetic
DMR9:45953001	9	45953001	45955000	2000	1	3.30E-07	0.34	22	1.1	Npas2	
DMR9:45956001	9	45956001	45958000	2000	1	1.30E-07	0.4	14	0.7	Npas2	
DMR9:45971001	9	45971001	45974000	3000	1	8.90E-08	0.46	26	0.87	Npas2	
DMR9:45982001	9	45982001	45984000	2000	1	8.60E-07	0.42	27	1.35	Npas2	
DMR9:46001001	9	46001001	46003000	2000	1	6.00E-07	-0.51	49	2.45	Npas2	
DMR9:46033001	9	46033001	46034000	1000	1	7.00E-08	0.68	16	1.6	Npas2	
DMR9:46204001	9	46204001	46206000	2000	1	1.50E-07	-0.42	27	1.35	Tbc1d8	Signaling
DMR9:46331001	9	46331001	46333000	2000	1	5.60E-10	-0.47	21	1.05	Creg2	
DMR9:46382001	9	46382001	46383000	1000	1	8.20E-10	0.6	19	1.9	Creg2	
DMR9:46425001	9	46425001	46426000	1000	1	7.80E-07	0.44	6	0.6	Rfx8	Transcription
DMR9:46450001	9	46450001	46453000	3000	1	1.80E-07	0.5	36	1.2	Rfx8	Transcription
DMR9:46688001	9	46688001	46690000	2000	1	2.30E-07	-0.4	41	2.05	Map4k4	Signaling
DMR9:46774001	9	46774001	46777000	3000	1	1.90E-07	-0.6	41	1.37	Map4k4	Signaling
DMR9:46863001	9	46863001	46864000	1000	1	3.90E-13	-0.73	17	1.7	Il1r2	Receptor
DMR9:46867001	9	46867001	46868000	1000	1	2.60E-16	0.42	4	0.4	Il1r2	Receptor
DMR9:47055001	9	47055001	47059000	4000	1	6.50E-07	-0.43	38	0.95	Il1rl2	Receptor
DMR9:47134001	9	47134001	47136000	2000	1	7.20E-08	0.61	30	1.5	Il1rl1	Receptor
DMR9:47244001	9	47244001	47247000	3000	1	8.20E-07	0.37	34	1.13	Il18rap	Receptor
DMR9:47305001	9	47305001	47308000	3000	1	1.50E-08	0.62	42	1.4	Slc9a4	Transport
DMR9:47378001	9	47378001	47380000	2000	1	4.40E-13	-0.46	19	0.95	LOC103690524;Slc9a2	Transport
DMR9:47393001	9	47393001	47399000	6000	1	8.30E-23	0.53	49	0.82	Slc9a2;LOC103690525	Transport
DMR9:47457001	9	47457001	47458000	1000	1	1.30E-07	-0.53	12	1.2	Slc9a2	Transport
DMR9:49405001	9	49405001	49410000	5000	1	8.20E-07	0.39	80	1.6	RGD1562818	
DMR9:49433001	9	49433001	49435000	2000	1	6.60E-07	-0.53	23	1.15	Linc01158	
DMR9:49456001	9	49456001	49457000	1000	1	1.40E-10	0.46	5	0.5	Linc01158;LOC103690527	
DMR9:49886001	9	49886001	49888000	2000	1	1.60E-15	-0.79	7	0.35	Tgfbrap1	
DMR9:49933001	9	49933001	49934000	1000	1	2.10E-07	-0.37	14	1.4	Fhl2	Transcription
DMR9:50336001	9	50336001	50337000	1000	1	2.00E-08	0.4	18	1.8	Nck2	Cytoskeleton
DMR9:50546001	9	50546001	50548000	2000	1	1.30E-07	-0.42	33	1.65	RGD1305645	
DMR9:50963001	9	50963001	50964000	1000	1	3.80E-08	-0.5	6	0.6	Ercc5	Transcription
DMR9:50976001	9	50976001	50980000	4000	1	3.70E-08	-0.46	22	0.55	Ercc5	Transcription
DMR9:51118001	9	51118001	51124000	6000	2	7.20E-10	-0.42	64	1.07	RGD1562067	
DMR9:51362001	9	51362001	51367000	5000	2	1.90E-09	-0.41	47	0.94	Gulp1	Cytoskeleton
DMR9:51442001	9	51442001	51446000	4000	1	5.20E-07	-0.5	24	0.6	Gulp1	Cytoskeleton
DMR9:51488001	9	51488001	51489000	1000	1	6.90E-09	-0.78	2	0.2	Gulp1	Cytoskeleton
DMR9:52179001	9	52179001	52182000	3000	2	2.00E-08	-0.29	27	0.9	Col5a2	Extracellular Matrix
DMR9:52428001	9	52428001	52429000	1000	1	3.80E-08	0.5	18	1.8	Vom1r-ps54	
DMR9:52486001	9	52486001	52487000	1000	1	3.90E-09	-0.48	5	0.5	RGD1562143	
DMR9:53061001	9	53061001	53064000	3000	1	4.50E-07	-0.43	13	0.43	Ankar	
DMR9:53111001	9	53111001	53112000	1000	1	6.00E-07	-0.44	23	2.3	LOC100911809;Ormdl1;Pms1	Transcription
DMR9:53148001	9	53148001	53149000	1000	1	2.80E-07	0.41	13	1.3	Pms1;LOC108351924	Transcription
DMR9:53169001	9	53169001	53171000	2000	1	1.70E-07	0.41	12	0.6	Pms1	Transcription
DMR9:53173001	9	53173001	53175000	2000	1	5.70E-11	-0.42	13	0.65	Pms1	Transcription
DMR9:53495001	9	53495001	53499000	4000	1	5.50E-08	-0.4	28	0.7	Hibch	Metabolism
DMR9:53666001	9	53666001	53668000	2000	1	7.00E-10	-0.54	14	0.7	Mfsd6	
DMR9:53729001	9	53729001	53731000	2000	1	7.10E-07	-0.48	33	1.65	Nemp2;LOC100912307	
DMR9:53737001	9	53737001	53738000	1000	1	8.20E-08	-0.37	15	1.5	Nemp2;LOC100912307	
DMR9:54294001	9	54294001	54296000	2000	1	1.90E-08	0.36	18	0.9	Gls;Stat1	Metabolism;Transcription
DMR9:54323001	9	54323001	54325000	2000	2	1.20E-10	-0.54	41	2.05	Stat1	Transcription
DMR9:54336001	9	54336001	54338000	2000	1	9.70E-07	-0.4	30	1.5	Stat1;Stat4	Transcription
DMR9:54369001	9	54369001	54370000	1000	1	7.40E-11	0.57	26	2.6	Stat4	
DMR9:54416001	9	54416001	54417000	1000	1	4.90E-07	0.47	8	0.8	Stat4	

DMR9:55527001	9	55527001	55533000	6000	1	1.50E-07	-0.34	59	0.98	Tmeff2	
DMR9:55608001	9	55608001	55611000	3000	1	6.00E-09	0.41	32	1.07	Tmeff2	
DMR9:59561001	9	59561001	59562000	1000	1	1.10E-15	0.6	34	3.4	Dnah7	Cytoskeleton
DMR9:59991001	9	59991001	59992000	1000	1	1.40E-07	-0.5	6	0.6	Slc39a10	Transport
DMR9:60014001	9	60014001	60016000	2000	1	4.40E-08	-0.35	30	1.5	Slc39a10	Transport
DMR9:60047001	9	60047001	60051000	4000	1	3.90E-08	-0.48	63	1.57	Slc39a10	Transport
DMR9:60108001	9	60108001	60112000	4000	1	3.40E-09	0.35	46	1.15	Dnah7	Cytoskeleton
DMR9:60146001	9	60146001	60147000	1000	1	4.90E-08	-0.3	10	1	Dnah7	Cytoskeleton
DMR9:60185001	9	60185001	60193000	8000	2	2.60E-07	-0.36	70	0.88	Dnah7	Cytoskeleton
DMR9:60224001	9	60224001	60232000	8000	1	6.90E-07	-0.39	94	1.18	Dnah7	Cytoskeleton
DMR9:60315001	9	60315001	60316000	1000	1	8.00E-08	-0.55	12	1.2	Dnah7	Cytoskeleton
DMR9:60442001	9	60442001	60445000	3000	2	3.20E-13	0.42	24	0.8	Hecw2	Proteolysis
DMR9:60596001	9	60596001	60601000	5000	2	8.20E-07	0.38	43	0.86	Hecw2	Proteolysis
DMR9:60607001	9	60607001	60609000	2000	1	8.60E-07	-0.44	4	0.2	Hecw2	Proteolysis
DMR9:60646001	9	60646001	60647000	1000	1	7.50E-08	-0.44	15	1.5	Hecw2	Proteolysis
DMR9:60923001	9	60923001	60926000	3000	1	5.40E-07	-0.35	27	0.9	Ccdc150;LOC108351931	
DMR9:60927001	9	60927001	60931000	4000	1	2.80E-09	-0.4	27	0.68	Ccdc150;LOC108351931	
DMR9:60949001	9	60949001	60950000	1000	1	2.20E-08	-0.36	2	0.2	Ccdc150	
DMR9:61059001	9	61059001	61061000	2000	1	5.10E-08	-0.49	38	1.9	Pgap1	Transport
DMR9:61229001	9	61229001	61230000	1000	1	9.80E-07	-0.42	17	1.7	Ankrd44	Cytoskeleton
DMR9:61284001	9	61284001	61286000	2000	1	6.40E-08	-0.58	10	0.5	Ankrd44	Cytoskeleton
DMR9:61368001	9	61368001	61370000	2000	2	5.90E-09	-0.52	8	0.4	Ankrd44	Cytoskeleton
DMR9:61494001	9	61494001	61500000	6000	1	6.90E-07	-0.35	66	1.1	Ankrd44	Cytoskeleton
DMR9:61552001	9	61552001	61558000	6000	1	1.10E-14	-0.34	63	1.05	Tmem258b	
DMR9:61902001	9	61902001	61906000	4000	1	3.40E-08	-0.45	31	0.78	Boll	Metabolism
DMR9:62077001	9	62077001	62083000	6000	1	4.30E-08	-0.38	26	0.43	Plcl1	Metabolism
DMR9:62129001	9	62129001	62131000	2000	1	2.80E-08	-0.41	36	1.8	Plcl1	Metabolism
DMR9:62175001	9	62175001	62176000	1000	1	4.20E-08	-0.46	17	1.7	Plcl1	Metabolism
DMR9:62188001	9	62188001	62190000	2000	2	2.40E-09	-0.42	26	1.3	Plcl1	Metabolism
DMR9:62232001	9	62232001	62237000	5000	1	8.80E-10	-0.44	23	0.46	Plcl1;LOC108351933	Metabolism
DMR9:62315001	9	62315001	62316000	1000	1	1.20E-07	-0.53	4	0.4	Plcl1;LOC103690535	Metabolism
DMR9:63471001	9	63471001	63476000	5000	1	5.20E-08	-0.51	41	0.82	Satb2	Epigenetic
DMR9:63594001	9	63594001	63595000	1000	1	6.30E-07	0.33	14	1.4	Satb2	Epigenetic
DMR9:63646001	9	63646001	63648000	2000	1	3.00E-07	-0.35	25	1.25	Satb2;LOC102549320;LOC108351934	Epigenetic
DMR9:64852001	9	64852001	64855000	3000	1	3.70E-09	-0.47	42	1.4	Spats2l;Kctd18	Cytoskeleton
DMR9:64895001	9	64895001	64897000	2000	1	1.80E-09	0.41	25	1.25	Kctd18;LOC102550420;Sgo2	Cytoskeleton;Cell Cycle
DMR9:64913001	9	64913001	64915000	2000	1	5.00E-08	-0.5	19	0.95	Kctd18;Sgo2	Cytoskeleton;Cell Cycle
DMR9:65200001	9	65200001	65201000	1000	1	1.50E-11	0.46	8	0.8	Aox2;LOC108351937	Metabolism
DMR9:65665001	9	65665001	65666000	1000	1	1.60E-12	-0.57	16	1.6	Casp8;Als2cr12	Protease
DMR9:65812001	9	65812001	65814000	2000	1	7.30E-11	-0.57	14	0.7	LOC102552195;Als2cr11	
DMR9:66037001	9	66037001	66038000	1000	1	7.10E-08	0.33	30	3	Als2;RGD1562399	
DMR9:66354001	9	66354001	66355000	1000	1	9.20E-11	0.48	1	0.1	RGD1562029	
DMR9:66398001	9	66398001	66399000	1000	1	1.20E-08	0.54	7	0.7	RGD1562029	
DMR9:66426001	9	66426001	66427000	1000	1	9.10E-08	-0.51	20	2	RGD1562029	
DMR9:66434001	9	66434001	66435000	1000	1	4.10E-10	0.72	19	1.9	RGD1562029	
DMR9:66487001	9	66487001	66488000	1000	1	2.50E-07	-0.35	14	1.4	Sumo1;LOC689131;Nop58	Metabolism
DMR9:66811001	9	66811001	66812000	1000	1	1.20E-09	0.41	10	1	Ica1l	
DMR9:66869001	9	66869001	66875000	6000	2	1.30E-08	-0.39	55	0.92	Wdr12;Carf	Transcription
DMR9:66978001	9	66978001	66982000	4000	1	6.50E-08	-0.49	19	0.48	Nbeal1	
DMR9:67138001	9	67138001	67139000	1000	1	3.00E-07	-0.37	26	2.6	Cyp20a1	Metabolism
DMR9:67179001	9	67179001	67185000	6000	1	2.90E-08	-0.26	84	1.4	Cyp20a1	Metabolism
DMR9:67310001	9	67310001	67312000	2000	2	3.80E-11	-0.86	15	0.75	Raph1	Cytoskeleton
DMR9:67743001	9	67743001	67744000	1000	1	4.00E-08	-0.4	22	2.2	Icos	
DMR9:68455001	9	68455001	68459000	4000	1	1.40E-08	-0.29	37	0.92	Pard3b;LOC103690539	
DMR9:68571001	9	68571001	68572000	1000	1	5.00E-11	-0.53	4	0.4	Pard3b	
DMR9:68680001	9	68680001	68681000	1000	1	9.00E-07	0.44	6	0.6	Pard3b	
DMR9:68828001	9	68828001	68833000	5000	2	1.10E-08	-0.29	61	1.22	Pard3b	
DMR9:68878001	9	68878001	68880000	2000	1	9.00E-11	0.51	19	0.95	Pard3b	
DMR9:69197001	9	69197001	69198000	1000	1	2.30E-07	0.32	15	1.5	Pard3b;LOC108352006	
DMR9:69418001	9	69418001	69419000	1000	1	5.00E-07	0.52	11	1.1	Pard3b	
DMR9:69422001	9	69422001	69427000	5000	1	3.70E-08	0.47	48	0.96	Pard3b	
DMR9:69541001	9	69541001	69542000	1000	1	2.10E-07	-0.53	14	1.4	Nrp2	

DMR9:69547001	9	69547001	69548000	1000	1	9.60E-08	0.32	13	1.3	Nrp2	
DMR9:69587001	9	69587001	69592000	5000	1	3.30E-10	0.63	79	1.58	Nrp2	
DMR9:69607001	9	69607001	69609000	2000	1	2.70E-08	0.52	18	0.9	Nrp2	
DMR9:69925001	9	69925001	69926000	1000	1	4.00E-09	0.44	9	0.9	LOC102548599;Ndufs1	Metabolism
DMR9:69976001	9	69976001	69977000	1000	1	7.50E-16	0.92	32	3.2	Gpr1	Signaling
DMR9:69982001	9	69982001	69984000	2000	1	7.70E-08	0.32	15	0.75	Gpr1	Signaling
DMR9:70041001	9	70041001	70048000	7000	1	4.10E-07	-0.38	171	2.44	Zdbf2	
DMR9:70292001	9	70292001	70294000	2000	1	1.80E-08	-0.49	34	1.7	Adam23	Protease
DMR9:70296001	9	70296001	70297000	1000	1	1.10E-09	0.4	6	0.6	Adam23	Protease
DMR9:70328001	9	70328001	70329000	1000	1	3.90E-07	0.42	14	1.4	LOC103690541;Dytn	Proteolysis
DMR9:70346001	9	70346001	70347000	1000	1	3.80E-07	-0.38	2	0.2	Dytn	Proteolysis
DMR9:70351001	9	70351001	70355000	4000	1	4.90E-08	0.56	64	1.6	Dytn	Proteolysis
DMR9:70391001	9	70391001	70397000	6000	3	3.00E-09	-0.39	66	1.1	Dytn;Mdh1b	Proteolysis;Metabolism
DMR9:70411001	9	70411001	70415000	4000	2	2.30E-11	-0.47	24	0.6	Mdh1b	Metabolism
DMR9:71265001	9	71265001	71266000	1000	1	1.40E-07	0.5	8	0.8	Creb1	
DMR9:71280001	9	71280001	71281000	1000	1	3.90E-11	-0.57	11	1.1	Creb1	
DMR9:71614001	9	71614001	71616000	2000	2	1.90E-10	-0.53	28	1.4	Plekhm3	
DMR9:71909001	9	71909001	71910000	1000	1	2.30E-09	-0.37	14	1.4	Idh1;Pikfyve	Metabolism;Signaling
DMR9:71984001	9	71984001	71990000	6000	1	1.80E-08	-0.38	67	1.12	Pikfyve	Signaling
DMR9:72052001	9	72052001	72053000	1000	1	8.00E-10	0.66	22	2.2	LOC108351946;Pth2r	Receptor
DMR9:73276001	9	73276001	73277000	1000	1	6.70E-07	0.29	8	0.8	Map2	
DMR9:73736001	9	73736001	73737000	1000	1	1.60E-07	-0.56	9	0.9	Kansl1l	
DMR9:73805001	9	73805001	73806000	1000	1	4.70E-07	-0.46	9	0.9	Kansl1l	
DMR9:73829001	9	73829001	73833000	4000	1	8.30E-08	-0.25	53	1.32	Kansl1l;Acadl	Metabolism
DMR9:73860001	9	73860001	73862000	2000	1	1.20E-07	0.35	25	1.25	Acadl	Metabolism
DMR9:74108001	9	74108001	74110000	2000	1	6.00E-10	0.43	18	0.9	LOC103690552;Cps1	Metabolism
DMR9:74120001	9	74120001	74121000	1000	1	1.70E-08	-0.54	13	1.3	Cps1	Metabolism
DMR9:74215001	9	74215001	74217000	2000	1	2.70E-07	-0.4	17	0.85	Cps1	Metabolism
DMR9:75080001	9	75080001	75082000	2000	1	1.40E-11	-0.42	11	0.55	Erbb4	Receptor
DMR9:75239001	9	75239001	75240000	1000	1	1.20E-08	-0.59	8	0.8	Erbb4	Receptor
DMR9:75398001	9	75398001	75403000	5000	3	2.70E-11	-0.5	46	0.92	Erbb4	Receptor
DMR9:75433001	9	75433001	75438000	5000	1	1.30E-10	-0.37	48	0.96	Erbb4	Receptor
DMR9:75537001	9	75537001	75543000	6000	2	3.40E-08	-0.33	61	1.02	Erbb4	Receptor
DMR9:75768001	9	75768001	75769000	1000	1	5.30E-07	0.46	17	1.7	Erbb4	Receptor
DMR9:76073001	9	76073001	76076000	3000	1	2.80E-19	1.14	57	1.9	Erbb4	Receptor
DMR9:76081001	9	76081001	76083000	2000	1	1.10E-07	0.5	12	0.6	Erbb4	Receptor
DMR9:76123001	9	76123001	76124000	1000	1	7.40E-20	0.75	17	1.7	Erbb4	Receptor
DMR9:76439001	9	76439001	76440000	1000	1	1.80E-10	0.67	23	2.3	RGD1562431	
DMR9:76441001	9	76441001	76446000	5000	2	6.80E-17	0.67	66	1.32	RGD1562431	
DMR9:76633001	9	76633001	76637000	4000	1	1.00E-06	-0.55	32	0.8	Ikzf2	Transcription
DMR9:77015001	9	77015001	77018000	3000	1	5.10E-07	-0.51	9	0.3	Spag16	Cytoskeleton
DMR9:77099001	9	77099001	77102000	3000	1	4.50E-11	-0.43	25	0.83	Spag16	Cytoskeleton
DMR9:77249001	9	77249001	77250000	1000	1	6.20E-07	-0.58	4	0.4	Spag16	Cytoskeleton
DMR9:77345001	9	77345001	77347000	2000	1	5.50E-07	0.4	3	0.15	Spag16	Cytoskeleton
DMR9:77354001	9	77354001	77355000	1000	1	4.60E-07	0.4	7	0.7	Spag16;LOC103690554	Cytoskeleton
DMR9:77391001	9	77391001	77394000	3000	1	2.70E-09	-0.48	24	0.8	Spag16	Cytoskeleton
DMR9:77418001	9	77418001	77419000	1000	1	6.30E-08	-0.44	5	0.5	Spag16	Cytoskeleton
DMR9:77495001	9	77495001	77496000	1000	1	3.70E-07	0.36	7	0.7	Spag16	Cytoskeleton
DMR9:77542001	9	77542001	77546000	4000	2	1.20E-07	-0.44	35	0.88	Spag16	Cytoskeleton
DMR9:77679001	9	77679001	77686000	7000	1	7.70E-08	-0.32	72	1.03	Spag16	Cytoskeleton
DMR9:77881001	9	77881001	77885000	4000	1	4.10E-08	0.55	33	0.82	Vwc2l	
DMR9:77901001	9	77901001	77906000	5000	2	1.60E-07	0.34	47	0.94	Vwc2l	
DMR9:77916001	9	77916001	77917000	1000	1	1.30E-15	0.66	7	0.7	Vwc2l	
DMR9:78319001	9	78319001	78322000	3000	1	5.20E-08	0.44	30	1	Bard1	
DMR9:78327001	9	78327001	78329000	2000	1	1.30E-10	-0.74	15	0.75	Bard1	
DMR9:78342001	9	78342001	78343000	1000	1	3.20E-07	-0.42	17	1.7	Bard1	
DMR9:78574001	9	78574001	78577000	3000	1	6.00E-08	-0.54	41	1.37	Abca12	Transport
DMR9:78632001	9	78632001	78633000	1000	1	2.90E-10	0.38	5	0.5	Abca12	Transport
DMR9:78948001	9	78948001	78950000	2000	1	2.90E-08	-0.46	16	0.8	Fn1	Signaling
DMR9:79479001	9	79479001	79484000	5000	1	2.30E-08	-0.4	37	0.74	Mreg	
DMR9:79511001	9	79511001	79513000	2000	1	7.00E-10	0.35	35	1.75	Mreg	
DMR9:79523001	9	79523001	79524000	1000	1	2.90E-07	0.36	20	2	Mreg	
DMR9:79551001	9	79551001	79553000	2000	1	8.50E-07	-0.46	32	1.6	Mreg	

DMR9:79568001	9	79568001	79573000	5000	2	2.50E-08	-0.38	46	0.92	Mreg;LOC108351947	
DMR9:79796001	9	79796001	79797000	1000	1	5.60E-08	0.38	5	0.5	LOC108351949;March4;LOC102555358	Proteolysis
DMR9:79818001	9	79818001	79820000	2000	1	1.10E-10	0.43	23	1.15	March4;LOC102555358	Proteolysis
DMR9:79853001	9	79853001	79858000	5000	2	1.40E-07	-0.35	56	1.12	4-Mar	Proteolysis
DMR9:79872001	9	79872001	79874000	2000	1	3.40E-07	0.23	22	1.1	4-Mar	Proteolysis
DMR9:79941001	9	79941001	79943000	2000	1	3.80E-08	0.41	44	2.2	Smarcal1	Transcription
DMR9:79945001	9	79945001	79950000	5000	1	2.00E-09	-0.5	84	1.68	Smarcal1	Transcription
DMR9:79969001	9	79969001	79970000	1000	1	2.50E-07	0.32	23	2.3	Smarcal1	Transcription
DMR9:80136001	9	80136001	80137000	1000	1	1.30E-08	0.37	25	2.5	Igfbp2	Protease; Proteolysis
DMR9:80156001	9	80156001	80158000	2000	1	2.00E-08	0.57	32	1.6	Igfbp5	Protease; Proteolysis
DMR9:81225001	9	81225001	81226000	1000	1	1.30E-07	0.45	8	0.8	Tns1	Cytoskeleton
DMR9:81280001	9	81280001	81282000	2000	1	3.40E-08	0.46	15	0.75	Tns1	Cytoskeleton
DMR9:81283001	9	81283001	81285000	2000	1	3.30E-07	0.28	8	0.4	Tns1	Cytoskeleton
DMR9:81349001	9	81349001	81350000	1000	1	8.70E-09	0.44	11	1.1	Tns1;LOC102548628	Cytoskeleton
DMR9:81396001	9	81396001	81397000	1000	1	2.80E-07	0.41	12	1.2	Tns1;Rufy4	Cytoskeleton
DMR9:81601001	9	81601001	81602000	1000	1	3.50E-07	0.29	17	1.7	Pnkd	
DMR9:81651001	9	81651001	81652000	1000	1	2.60E-08	0.64	43	4.3	Catip;Slc11a1	Transport
DMR9:81825001	9	81825001	81827000	2000	1	1.70E-07	-0.44	18	0.9	Usp37;Plcd4	Protease;Metabolism
DMR9:82047001	9	82047001	82048000	1000	1	4.90E-07	0.46	9	0.9	Wnt6;Wnt10a	Signaling
DMR9:82254001	9	82254001	82260000	6000	1	1.50E-07	-0.28	95	1.58	Nhej1;LOC100361833	
DMR9:82308001	9	82308001	82310000	2000	1	2.90E-07	0.35	6	0.3	Nhej1	
DMR9:82439001	9	82439001	82440000	1000	1	6.70E-07	-0.3	21	2.1	LOC102554903;Dnajb2;Ptprn	Transcription;Signaling
DMR9:82483001	9	82483001	82485000	2000	1	2.90E-07	-0.37	40	2	Resp18	
DMR9:82518001	9	82518001	82519000	1000	1	1.20E-07	-0.53	9	0.9	Dnpep	Protease
DMR9:82558001	9	82558001	82561000	3000	1	8.00E-09	0.35	70	2.33	Des;LOC102550477	
DMR9:82579001	9	82579001	82583000	4000	1	2.60E-07	0.46	83	2.08	LOC102550477;Speg	
DMR9:83103001	9	83103001	83109000	6000	1	1.10E-07	-0.32	95	1.58	Epha4	Receptor
DMR9:83188001	9	83188001	83189000	1000	1	3.60E-09	0.75	23	2.3	Epha4	Receptor
DMR9:83227001	9	83227001	83228000	1000	1	4.40E-07	0.53	6	0.6	Epha4	Receptor
DMR9:83245001	9	83245001	83246000	1000	1	3.00E-08	0.8	31	3.1	Epha4	Receptor
DMR9:84006001	9	84006001	84009000	3000	1	6.80E-07	-0.35	43	1.43	Pax3	
DMR9:84300001	9	84300001	84303000	3000	1	4.50E-07	0.36	29	0.97	Sgpp2	Signaling
DMR9:84692001	9	84692001	84694000	2000	1	3.60E-09	-0.52	36	1.8	LOC108351957;Kcne4	Transport
DMR9:84887001	9	84887001	84889000	2000	1	2.50E-07	0.37	30	1.5	Hdac1l	
DMR9:85402001	9	85402001	85406000	4000	1	9.70E-07	-0.44	62	1.55	Ap1s3	Transport
DMR9:85452001	9	85452001	85454000	2000	1	5.20E-08	0.52	17	0.85	Ap1s3	Transport
DMR9:85502001	9	85502001	85503000	1000	1	1.70E-10	0.57	23	2.3	Wdfy1	
DMR9:85526001	9	85526001	85527000	1000	1	4.20E-07	-0.45	11	1.1	Wdfy1;LOC103690560	
DMR9:85598001	9	85598001	85599000	1000	1	6.60E-12	0.54	10	1	Serpine2	Protease; Proteolysis
DMR9:86257001	9	86257001	86258000	1000	1	8.50E-07	-0.39	19	1.9	RGD1563713	
DMR9:86491001	9	86491001	86499000	8000	1	5.10E-07	-0.27	88	1.1	Dock10	
DMR9:86530001	9	86530001	86531000	1000	1	7.70E-09	-0.43	10	1	Dock10	
DMR9:87021001	9	87021001	87029000	8000	2	2.00E-15	1.11	115	1.44	NEWGENE_1305560	
DMR9:87066001	9	87066001	87068000	2000	1	1.80E-07	0.43	21	1.05	NEWGENE_1305560	
DMR9:87109001	9	87109001	87110000	1000	1	6.20E-08	0.49	9	0.9	NEWGENE_1305560	
DMR9:88173001	9	88173001	88175000	2000	1	1.30E-08	0.59	14	0.7	Rhbdd1	Protease
DMR9:88600001	9	88600001	88602000	2000	1	1.50E-10	-0.59	30	1.5	Agfg1	
DMR9:88648001	9	88648001	88651000	3000	1	4.10E-09	0.4	11	0.37	Agfg1	
DMR9:88668001	9	88668001	88670000	2000	1	8.30E-07	-0.52	15	0.75	Agfg1	
DMR9:88928001	9	88928001	88930000	2000	1	6.10E-07	-0.38	24	1.2	Ccl20	Growth Factors
DMR9:88980001	9	88980001	88983000	3000	1	5.00E-12	0.51	40	1.33	Daw1	
DMR9:91657001	9	91657001	91662000	5000	2	3.00E-07	-0.29	45	0.9	Pid1	
DMR9:92197001	9	92197001	92199000	2000	1	3.10E-07	0.53	24	1.2	Dner	
DMR9:92215001	9	92215001	92217000	2000	1	2.80E-07	0.47	32	1.6	Dner	
DMR9:92267001	9	92267001	92269000	2000	1	6.50E-07	0.39	14	0.7	Dner	
DMR9:92289001	9	92289001	92290000	1000	1	1.00E-09	0.77	37	3.7	Dner	
DMR9:92475001	9	92475001	92477000	2000	1	2.30E-07	-0.46	21	1.05	Fbxo36	
DMR9:92701001	9	92701001	92703000	2000	1	9.10E-07	-0.52	27	1.35	Sp100	
DMR9:92830001	9	92830001	92831000	1000	1	4.20E-08	0.7	15	1.5	Cab39	
DMR9:93153001	9	93153001	93156000	3000	1	9.00E-07	-0.41	43	1.43	Psmd1	Protease
DMR9:93246001	9	93246001	93248000	2000	1	2.10E-09	0.5	10	0.5	Armc9	Cytoskeleton
DMR9:93907001	9	93907001	93908000	1000	1	1.20E-08	0.5	17	1.7	Dis3l2	Transcription

DMR9:93909001	9	93909001	93914000	5000	1	2.30E-07	-0.34	43	0.86	Dis3l2	Transcription
DMR9:93967001	9	93967001	93973000	6000	1	4.20E-09	-0.43	68	1.13	Dis3l2	Transcription
DMR9:94124001	9	94124001	94125000	1000	1	7.20E-07	0.42	4	0.4	Dis3l2	Transcription
DMR9:94231001	9	94231001	94236000	5000	2	6.30E-10	-0.49	82	1.64	Akp3;Ecel1	Signaling;Protease
DMR9:94507001	9	94507001	94510000	3000	1	9.10E-07	-0.42	42	1.4	Gigyf2	
DMR9:94520001	9	94520001	94522000	2000	1	6.00E-07	-0.38	27	1.35	Gigyf2	
DMR9:94573001	9	94573001	94576000	3000	1	3.10E-11	0.47	48	1.6	RGD1311447;Ngef	Transcription
DMR9:94734001	9	94734001	94735000	1000	1	2.50E-14	0.86	23	2.3	Neu2	Metabolism
DMR9:94892001	9	94892001	94893000	1000	1	9.80E-07	-0.39	11	1.1	Atg16l1	
DMR9:95427001	9	95427001	95428000	1000	1	6.10E-07	-0.46	7	0.7	Trpm8	Transport
DMR9:95439001	9	95439001	95443000	4000	1	1.50E-07	0.47	48	1.2	Trpm8	Transport
DMR9:96786001	9	96786001	96789000	3000	1	1.30E-07	-0.54	60	2	Agap1	
DMR9:96808001	9	96808001	96813000	5000	1	1.50E-10	-0.55	133	2.66	Agap1	
DMR9:96912001	9	96912001	96916000	4000	1	2.60E-09	0.58	60	1.5	Agap1	
DMR9:96954001	9	96954001	96955000	1000	1	2.80E-07	0.38	15	1.5	Agap1	
DMR9:96983001	9	96983001	96986000	3000	1	5.30E-07	-0.47	87	2.9	Agap1	
DMR9:97126001	9	97126001	97129000	3000	1	2.80E-09	0.44	49	1.63	Asb18	Transport
DMR9:97145001	9	97145001	97147000	2000	1	6.80E-07	0.31	21	1.05	Asb18	Transport
DMR9:97238001	9	97238001	97241000	3000	1	3.90E-08	0.36	23	0.77	Iqca1	
DMR9:97285001	9	97285001	97289000	4000	2	3.70E-08	-0.59	58	1.45	Iqca1	
DMR9:97959001	9	97959001	97961000	2000	1	6.60E-08	0.41	28	1.4	Col6a3	
DMR9:98135001	9	98135001	98136000	1000	1	9.10E-12	0.46	9	0.9	Rab17;Lrrfip1	Transcription
DMR9:98251001	9	98251001	98253000	2000	1	2.10E-08	-0.42	45	2.25	Lrrfip1	Transcription
DMR9:98254001	9	98254001	98258000	4000	1	7.10E-07	-0.66	58	1.45	Lrrfip1	Transcription
DMR9:98413001	9	98413001	98416000	3000	1	3.90E-07	-0.45	31	1.03	Ube2f	Proteolysis
DMR9:98445001	9	98445001	98447000	2000	1	2.60E-10	-0.48	43	2.15	LOC102547942;Scly	Metabolism
DMR9:98522001	9	98522001	98523000	1000	1	7.70E-07	-0.5	13	1.3	Erfe;Ilkap	Hormone;Signaling
DMR9:98617001	9	98617001	98618000	1000	1	4.40E-08	0.37	0	0	Traf3ip1	
DMR9:98967001	9	98967001	98968000	1000	1	9.50E-09	0.39	16	1.6	Twist2;Rps27a-ps9	Transcription
DMR9:99246001	9	99246001	99247000	1000	1	7.50E-08	-0.42	10	1	Hdac4	
DMR9:99358001	9	99358001	99359000	1000	1	5.30E-08	0.39	15	1.5	RGD1564730;LOC108351974	
DMR9:99559001	9	99559001	99561000	2000	1	1.20E-07	-0.43	28	1.4	Ppp1r7	Signaling
DMR9:99587001	9	99587001	99588000	1000	1	1.10E-12	0.59	13	1.3	Ppp1r7	Signaling
DMR9:99877001	9	99877001	99879000	2000	1	4.30E-07	0.29	18	0.9	Ppp1r7	Signaling
DMR9:99922001	9	99922001	99923000	1000	1	7.90E-09	0.37	8	0.8	Ppp1r7	Signaling
DMR9:100014001	9	100014001	100015000	1000	1	1.90E-09	0.7	45	4.5	Ppp1r7;Gpc1;Mir149	Signaling
DMR9:100133001	9	100133001	100136000	3000	1	3.40E-07	0.36	47	1.57	Ppp1r7;Gpr35	Signaling;Signaling
DMR9:100192001	9	100192001	100193000	1000	1	1.60E-07	0.49	16	1.6	Ppp1r7;Kif1a	Signaling;Cytoskeleton
DMR9:100196001	9	100196001	100199000	3000	1	2.20E-07	0.4	42	1.4	Ppp1r7;Kif1a	Signaling;Cytoskeleton
DMR9:100238001	9	100238001	100241000	3000	1	8.40E-07	-0.46	39	1.3	Ppp1r7;Kif1a	Signaling;Cytoskeleton
DMR9:100327001	9	100327001	100330000	3000	1	5.00E-07	0.5	41	1.37	Ppp1r7;Crocc2	Signaling
DMR9:100399001	9	100399001	100404000	5000	1	3.50E-07	0.5	87	1.74	Ppp1r7;Sned1;LOC108352012	Signaling
DMR9:100737001	9	100737001	100738000	1000	1	1.80E-14	-0.53	6	0.6	Farp2	
DMR9:100934001	9	100934001	100936000	2000	1	1.30E-12	0.36	18	0.9	Dtymk;Ing5	Signaling;Epigenetic
DMR9:100976001	9	100976001	100977000	1000	1	4.40E-08	0.4	13	1.3	D2hgdh;Gal3st2	Metabolism;Transport
DMR9:101228001	9	101228001	101232000	4000	1	5.30E-08	-0.64	50	1.25	Vom1r-ps44;Vom1r64;Cct6a-ps6;LOC292581	Receptor
DMR9:104328001	9	104328001	104331000	3000	1	8.00E-08	0.43	41	1.37	Slco4c1	Transport
DMR9:104471001	9	104471001	104476000	5000	1	2.00E-07	-0.43	30	0.6	Slco6b1	Transport
DMR9:104722001	9	104722001	104729000	7000	1	7.90E-08	-0.36	79	1.13	Slco6d1;LOC102554041	Transport
DMR9:104852001	9	104852001	104853000	1000	1	2.50E-12	-0.77	9	0.9	Slco6d1	Transport
DMR9:105611001	9	105611001	105612000	1000	1	3.20E-07	0.49	24	2.4	RGD1560925	
DMR9:105688001	9	105688001	105690000	2000	1	1.20E-12	0.54	42	2.1	Nudt12	Metabolism
DMR9:110047001	9	110047001	110049000	2000	1	1.20E-11	0.4	25	1.25	Efna5	Signaling
DMR9:110077001	9	110077001	110078000	1000	1	2.50E-09	-0.4	12	1.2	Efna5	Signaling
DMR9:110235001	9	110235001	110236000	1000	1	3.40E-10	-0.55	29	2.9	Efna5	Signaling
DMR9:110598001	9	110598001	110605000	7000	2	2.20E-09	-0.59	118	1.69	Fbxl17;LOC103690581	Metabolism
DMR9:110673001	9	110673001	110675000	2000	1	5.40E-07	-0.6	33	1.65	Fbxl17	Metabolism
DMR9:110730001	9	110730001	110732000	2000	1	3.20E-08	-0.68	29	1.45	Fbxl17	Metabolism
DMR9:110752001	9	110752001	110753000	1000	1	2.20E-09	0.39	8	0.8	Fbxl17	Metabolism
DMR9:110812001	9	110812001	110817000	5000	1	1.90E-08	-0.45	84	1.68	Fbxl17	Metabolism
DMR9:110880001	9	110880001	110884000	4000	1	7.50E-12	0.32	63	1.57	Fbxl17	Metabolism
DMR9:110913001	9	110913001	110918000	5000	1	1.60E-17	-0.66	93	1.86	Fbxl17	Metabolism

DMR9:110928001	9	110928001	110930000	2000	1	4.10E-08	-0.46	33	1.65	Fbxl17	Metabolism
DMR9:111247001	9	111247001	111248000	1000	1	1.50E-08	0.4	13	1.3	Ppip5k2	Signaling
DMR9:111553001	9	111553001	111556000	3000	1	6.90E-09	0.34	29	0.97	Fer	
DMR9:111625001	9	111625001	111630000	5000	2	2.60E-13	0.63	111	2.22	Fer	
DMR9:111638001	9	111638001	111646000	8000	1	6.30E-07	-0.55	100	1.25	Fer	
DMR9:111699001	9	111699001	111700000	1000	1	2.20E-21	0.89	2	0.2	Fer	
DMR9:111811001	9	111811001	111813000	2000	1	1.90E-07	0.52	26	1.3	Fer	
DMR9:112298001	9	112298001	112299000	1000	1	4.80E-09	-0.41	16	1.6	Man2a1	
DMR9:112302001	9	112302001	112304000	2000	1	2.10E-07	-0.38	39	1.95	Man2a1	
DMR9:112322001	9	112322001	112327000	5000	2	6.40E-10	-0.55	123	2.46	Man2a1	
DMR9:113003001	9	113003001	113005000	2000	1	5.70E-10	-0.47	35	1.75	Tmem232	
DMR9:113028001	9	113028001	113031000	3000	1	1.60E-11	0.93	63	2.1	Tmem232	
DMR9:113038001	9	113038001	113041000	3000	2	1.30E-13	0.88	49	1.63	Tmem232	
DMR9:113056001	9	113056001	113060000	4000	1	5.00E-08	0.37	63	1.57	Tmem232	
DMR9:113065001	9	113065001	113069000	4000	1	6.10E-09	0.38	49	1.23	Tmem232	
DMR9:113093001	9	113093001	113094000	1000	1	3.40E-07	-0.37	18	1.8	Tmem232	
DMR9:113153001	9	113153001	113156000	3000	1	4.40E-08	-0.58	30	1	Tmem232;LOC102552718	
DMR9:113182001	9	113182001	113183000	1000	1	2.30E-09	0.68	21	2.1	Tmem232	
DMR9:113190001	9	113190001	113193000	3000	1	1.70E-07	0.35	28	0.93	Tmem232	
DMR9:113269001	9	113269001	113271000	2000	1	3.50E-12	-0.87	26	1.3	Tmem232;LOC103690585	
DMR9:113662001	9	113662001	113665000	3000	1	4.50E-09	-0.4	59	1.97	Ankrd12	
DMR9:113898001	9	113898001	113899000	1000	1	8.30E-07	-0.32	9	0.9	Ndufv2	Metabolism
DMR9:113912001	9	113912001	113916000	4000	1	1.30E-09	-0.31	49	1.23	Wash1;LOC102553152	
DMR9:113918001	9	113918001	113920000	2000	1	6.00E-09	0.42	31	1.55	Wash1;LOC102553152	
DMR9:113937001	9	113937001	113938000	1000	1	4.70E-07	0.31	4	0.4	Wash1;LOC102553152;LOC102553382	
DMR9:114115001	9	114115001	114118000	3000	1	9.80E-09	-0.34	59	1.97	LOC103693248;Ddx11	Epigenetic
DMR9:114128001	9	114128001	114131000	3000	1	5.90E-10	-0.38	51	1.7	Ddx11	Epigenetic
DMR9:114541001	9	114541001	114546000	5000	2	1.20E-15	0.96	140	2.8	Mtcl1	
DMR9:114584001	9	114584001	114587000	3000	1	1.50E-07	-0.41	70	2.33	Mtcl1	
DMR9:114603001	9	114603001	114608000	5000	1	3.80E-07	-0.43	105	2.1	Mtcl1	
DMR9:114707001	9	114707001	114708000	1000	1	3.30E-20	-0.63	21	2.1	Rab12;LOC108352037	
DMR9:114838001	9	114838001	114844000	6000	2	9.10E-09	-0.57	88	1.47	Ptprm	Signaling
DMR9:114876001	9	114876001	114880000	4000	1	6.50E-07	-0.44	86	2.15	Ptprm	Signaling
DMR9:114970001	9	114970001	114973000	3000	1	1.80E-07	-0.37	22	0.73	Ptprm	Signaling
DMR9:115044001	9	115044001	115045000	1000	1	2.10E-07	-0.66	17	1.7	Ptprm	Signaling
DMR9:115126001	9	115126001	115127000	1000	1	2.20E-08	-0.39	10	1	Ptprm	Signaling
DMR9:115252001	9	115252001	115253000	1000	1	7.10E-07	-0.61	6	0.6	Ptprm	Signaling
DMR9:115335001	9	115335001	115339000	4000	1	2.10E-07	-0.48	68	1.7	Ptprm	Signaling
DMR9:115423001	9	115423001	115425000	2000	1	1.90E-07	0.33	14	0.7	Ptprm	Signaling
DMR9:115494001	9	115494001	115495000	1000	1	2.20E-07	0.36	3	0.3	Ptprm	Signaling
DMR9:115855001	9	115855001	115860000	5000	1	3.30E-11	0.71	84	1.68	Lrrc30	Cytoskeleton
DMR9:115933001	9	115933001	115935000	2000	1	1.10E-07	0.36	14	0.7	Lama1;LOC103690592	Extracellular Matrix
DMR9:115973001	9	115973001	115974000	1000	1	6.80E-12	0.54	5	0.5	Lama1	Extracellular Matrix
DMR9:116059001	9	116059001	116061000	2000	1	9.80E-07	0.49	34	1.7	Arhgap28	Signaling
DMR9:116062001	9	116062001	116065000	3000	2	6.80E-09	0.63	67	2.23	Arhgap28	Signaling
DMR9:116077001	9	116077001	116081000	4000	1	1.00E-08	0.33	36	0.9	Arhgap28	Signaling
DMR9:116171001	9	116171001	116173000	2000	1	1.30E-08	0.34	14	0.7	Arhgap28	Signaling
DMR9:116190001	9	116190001	116191000	1000	1	2.50E-07	-0.42	18	1.8	Arhgap28	Signaling
DMR9:116557001	9	116557001	116559000	2000	1	1.00E-06	-0.62	17	0.85	L3mbtl4	
DMR9:116595001	9	116595001	116597000	2000	1	9.10E-09	-0.55	30	1.5	L3mbtl4	
DMR9:116689001	9	116689001	116690000	1000	1	4.10E-08	0.38	11	1.1	L3mbtl4	
DMR9:117051001	9	117051001	117052000	1000	1	1.00E-09	0.56	7	0.7	Tmem200c	
DMR9:117385001	9	117385001	117387000	2000	1	3.00E-11	0.76	59	2.95	Epb41l3	
DMR9:117409001	9	117409001	117411000	2000	1	5.10E-07	-0.37	14	0.7	Epb41l3;LOC100361186;LOC689002	
DMR9:117532001	9	117532001	117533000	1000	1	8.70E-07	0.41	5	0.5	Epb41l3	
DMR9:117564001	9	117564001	117567000	3000	1	3.50E-09	0.69	51	1.7	Epb41l3	
DMR9:117602001	9	117602001	117607000	5000	1	7.20E-09	-0.53	87	1.74	Epb41l3	
DMR9:117610001	9	117610001	117612000	2000	1	3.20E-08	0.37	35	1.75	Epb41l3	
DMR9:117741001	9	117741001	117746000	5000	1	7.40E-08	-0.38	145	2.9	LOC102552880;Zfp161	Transcription
DMR9:117750001	9	117750001	117752000	2000	1	6.80E-07	0.59	61	3.05	LOC102552880;Zfp161	Transcription
DMR9:117817001	9	117817001	117818000	1000	1	8.90E-08	0.73	32	3.2	Akain1;LOC108351986	

DMR9:118272001	9	118272001	118273000	1000	1	6.80E-08	-0.37	7	0.7	Dlgap1	Cytoskeleton
DMR9:118330001	9	118330001	118331000	1000	1	4.60E-07	0.43	6	0.6	Dlgap1;Tas2r143	Cytoskeleton;Receptor
DMR9:118704001	9	118704001	118707000	3000	1	1.20E-07	-0.56	19	0.63	Dlgap1;LOC108352015	Cytoskeleton
DMR9:118749001	9	118749001	118750000	1000	1	1.90E-09	0.52	20	2	Dlgap1	Cytoskeleton
DMR9:118768001	9	118768001	118770000	2000	1	1.40E-07	0.3	18	0.9	Dlgap1	Cytoskeleton
DMR9:118783001	9	118783001	118789000	6000	2	1.90E-07	-0.3	66	1.1	Dlgap1	Cytoskeleton
DMR9:118837001	9	118837001	118838000	1000	1	8.00E-07	-0.53	16	1.6	Dlgap1	Cytoskeleton
DMR9:119010001	9	119010001	119013000	3000	1	3.70E-10	0.49	33	1.1	Dlgap1	Cytoskeleton
DMR9:119063001	9	119063001	119064000	1000	1	4.40E-12	0.35	8	0.8	Dlgap1	Cytoskeleton
DMR9:119172001	9	119172001	119176000	4000	1	2.50E-09	-0.43	76	1.9	LOC108351987;Tgif1;LOC102555570	Development
DMR9:119327001	9	119327001	119330000	3000	1	6.80E-08	-0.42	55	1.83	Myl12b;Myl12a;LOC102555687	Cytoskeleton
DMR9:119388001	9	119388001	119392000	4000	1	8.30E-10	0.4	40	1	Myom1	
DMR9:119428001	9	119428001	119430000	2000	1	3.50E-07	0.61	45	2.25	Myom1	
DMR9:119447001	9	119447001	119448000	1000	1	3.10E-08	-0.46	19	1.9	Myom1	
DMR9:119756001	9	119756001	119759000	3000	2	4.20E-10	-0.49	24	0.8	Smchd1	
DMR9:121449001	9	121449001	121451000	2000	1	5.50E-10	0.51	15	0.75	LOC678715;LOC103690596;Ppidl1	Transcription
DMR9:121455001	9	121455001	121458000	3000	1	2.00E-10	0.66	37	1.23	LOC103690596;Ppidl1	Transcription
DMR9:121849001	9	121849001	121852000	3000	1	1.00E-06	-0.31	26	0.87	Yes1	
DMR9:121938001	9	121938001	121941000	3000	1	3.80E-08	0.53	28	0.93	Tyms;Clul1	Epigenetic
DMR10:825001	10	825001	831000	6000	2	3.30E-08	-0.29	76	1.27	LOC103693254;Myh11	
DMR10:835001	10	835001	836000	1000	1	1.10E-09	0.39	7	0.7	LOC103693254;Myh11	
DMR10:1296001	10	1296001	1302000	6000	1	2.30E-07	-0.32	46	0.77	Parn	Translation
DMR10:1373001	10	1373001	1374000	1000	1	8.50E-07	-0.51	6	0.6	Parn	Translation
DMR10:1447001	10	1447001	1450000	3000	1	3.70E-07	-0.57	24	0.8	Parn	Translation
DMR10:2815001	10	2815001	2817000	2000	1	2.20E-07	-0.6	18	0.9	Shisa9	
DMR10:2855001	10	2855001	2859000	4000	1	1.30E-07	-0.44	29	0.72	Shisa9	
DMR10:2988001	10	2988001	2994000	6000	1	5.90E-07	-0.34	58	0.97	Shisa9	
DMR10:3028001	10	3028001	3029000	1000	1	8.50E-09	0.39	8	0.8	Shisa9	
DMR10:3075001	10	3075001	3077000	2000	2	2.80E-07	0.35	18	0.9	Shisa9	
DMR10:3742001	10	3742001	3743000	1000	1	1.20E-07	-0.45	28	2.8	Cpped1	
DMR10:3745001	10	3745001	3750000	5000	2	6.50E-07	-0.31	37	0.74	Cpped1	
DMR10:3759001	10	3759001	3760000	1000	1	3.00E-07	0.45	6	0.6	Cpped1	
DMR10:4012001	10	4012001	4014000	2000	1	8.00E-07	-0.33	17	0.85	Snx29	Cytoskeleton
DMR10:4129001	10	4129001	4130000	1000	1	4.10E-07	0.44	9	0.9	Snx29	Cytoskeleton
DMR10:4163001	10	4163001	4164000	1000	1	5.80E-09	0.46	9	0.9	Snx29	Cytoskeleton
DMR10:4744001	10	4744001	4749000	5000	2	7.40E-09	-0.45	89	1.78	LOC108352049;LOC108352050;Litaf	Cytoskeleton
DMR10:4772001	10	4772001	4775000	3000	1	1.00E-10	0.63	49	1.63	Litaf;LOC103693267;LOC102552269	Cytoskeleton
DMR10:4951001	10	4951001	4952000	1000	1	3.60E-09	0.4	25	2.5	Prm1;Prm2;Prm3;Tnp2;Socs1	Epigenetic;Epigenetic;Signaling
DMR10:4965001	10	4965001	4967000	2000	1	2.30E-10	0.38	22	1.1	Socs1	Signaling
DMR10:5238001	10	5238001	5239000	1000	1	3.90E-08	0.33	9	0.9	Ciita	
DMR10:5263001	10	5263001	5267000	4000	1	2.20E-09	0.44	53	1.32	Ciita	
DMR10:5299001	10	5299001	5301000	2000	1	1.10E-10	0.61	39	1.95	LOC100361397;Tvp23a	
DMR10:5304001	10	5304001	5306000	2000	1	5.70E-07	0.37	14	0.7	Tvp23a	
DMR10:5311001	10	5311001	5312000	1000	1	8.00E-07	0.3	15	1.5	Tvp23a	
DMR10:5313001	10	5313001	5316000	3000	1	5.90E-08	0.29	38	1.27	Tvp23a	
DMR10:5317001	10	5317001	5322000	5000	3	3.50E-11	0.47	50	1	Tvp23a	
DMR10:5734001	10	5734001	5735000	1000	1	2.00E-15	0.7	24	2.4	Grin2a	Receptor
DMR10:5740001	10	5740001	5742000	2000	1	2.20E-08	-0.34	35	1.75	Grin2a	Receptor
DMR10:5745001	10	5745001	5748000	3000	1	2.30E-08	-0.53	30	1	Grin2a	Receptor
DMR10:5835001	10	5835001	5837000	2000	1	1.50E-08	0.35	12	0.6	Grin2a	Receptor
DMR10:5950001	10	5950001	5952000	2000	1	2.10E-07	-0.41	17	0.85	Grin2a	Receptor
DMR10:6926001	10	6926001	6932000	6000	2	9.60E-12	0.81	85	1.42	Usp7	Protease
DMR10:7002001	10	7002001	7004000	2000	1	3.00E-07	-0.56	24	1.2	Usp7	Protease
DMR10:7018001	10	7018001	7020000	2000	1	4.50E-09	-0.64	35	1.75	Usp7;RGD1564086	Protease
DMR10:7130001	10	7130001	7132000	2000	1	2.40E-09	0.49	9	0.45	Abat	Metabolism
DMR10:7139001	10	7139001	7140000	1000	1	6.40E-07	-0.51	15	1.5	Abat	Metabolism
DMR10:7208001	10	7208001	7210000	2000	1	7.50E-10	0.57	26	1.3	Abat	Metabolism
DMR10:7302001	10	7302001	7304000	2000	1	1.80E-08	0.35	26	1.3	Tmem114;LOC102546970	
DMR10:8409001	10	8409001	8411000	2000	1	3.20E-07	0.47	28	1.4	Rbfox1	Translation

DMR10:8425001	10	8425001	8429000	4000	2	5.40E-11	0.8	92	2.3	Rbfox1	Translation
DMR10:8515001	10	8515001	8516000	1000	1	4.20E-11	-0.54	11	1.1	Rbfox1	Translation
DMR10:8692001	10	8692001	8693000	1000	1	1.50E-07	0.37	12	1.2	Rbfox1	Translation
DMR10:8704001	10	8704001	8706000	2000	1	6.90E-08	0.35	26	1.3	Rbfox1	Translation
DMR10:8815001	10	8815001	8817000	2000	1	9.20E-07	0.34	28	1.4	Rbfox1	Translation
DMR10:8903001	10	8903001	8904000	1000	1	6.30E-07	-0.32	12	1.2	Rbfox1	Translation
DMR10:9318001	10	9318001	9320000	2000	1	9.60E-14	0.51	15	0.75	Rbfox1	Translation
DMR10:9358001	10	9358001	9361000	3000	1	7.40E-07	0.42	21	0.7	Rbfox1	Translation
DMR10:9422001	10	9422001	9425000	3000	2	4.10E-08	0.38	35	1.17	Rbfox1	Translation
DMR10:9626001	10	9626001	9628000	2000	1	3.00E-09	0.45	16	0.8	Rbfox1	Translation
DMR10:9641001	10	9641001	9643000	2000	1	5.40E-08	0.48	22	1.1	Rbfox1	Translation
DMR10:9820001	10	9820001	9821000	1000	1	9.60E-07	-0.7	9	0.9	Rbfox1	Translation
DMR10:9847001	10	9847001	9848000	1000	1	7.00E-07	-0.4	6	0.6	Rbfox1	Translation
DMR10:10027001	10	10027001	10029000	2000	1	2.00E-08	0.47	24	1.2	Rbfox1	Translation
DMR10:10170001	10	10170001	10172000	2000	1	5.40E-11	-0.54	48	2.4	Rbfox1	Translation
DMR10:10177001	10	10177001	10179000	2000	2	2.50E-13	0.37	23	1.15	Rbfox1	Translation
DMR10:10225001	10	10225001	10227000	2000	1	7.10E-07	0.37	20	1	Rbfox1	Translation
DMR10:10229001	10	10229001	10231000	2000	1	2.60E-09	-0.44	29	1.45	Rbfox1	Translation
DMR10:10232001	10	10232001	10233000	1000	1	1.80E-07	0.32	11	1.1	Rbfox1	Translation
DMR10:10268001	10	10268001	10271000	3000	1	7.50E-10	0.34	23	0.77	Rbfox1	Translation
DMR10:10423001	10	10423001	10424000	1000	1	1.70E-07	-0.38	28	2.8	Rbfox1	Translation
DMR10:10627001	10	10627001	10630000	3000	1	2.70E-08	0.33	63	2.1	Sec14l5	
DMR10:10764001	10	10764001	10766000	2000	1	3.40E-07	0.4	36	1.8	Glyr1;Rogdi;Smim22;Sept12	Metabolism;Transcription
DMR10:10778001	10	10778001	10779000	1000	1	2.90E-12	0.34	4	0.4	Smim22;Sept12	
DMR10:11031001	10	11031001	11032000	1000	1	6.60E-07	-0.38	9	0.9	Hmox2	Metabolism
DMR10:11239001	10	11239001	11241000	2000	1	2.90E-11	0.82	45	2.25	Srl	Transport
DMR10:11244001	10	11244001	11246000	2000	1	2.50E-09	-0.38	33	1.65	Srl	Transport
DMR10:11266001	10	11266001	11267000	1000	1	3.30E-07	0.51	28	2.8	Srl	Transport
DMR10:11280001	10	11280001	11282000	2000	1	3.70E-07	0.61	51	2.55	Srl;LOC103693274	Transport
DMR10:11425001	10	11425001	11428000	3000	1	3.40E-09	-0.49	48	1.6	Adcy9	
DMR10:11518001	10	11518001	11520000	2000	1	6.30E-08	0.65	36	1.8	Adcy9;LOC102551062	
DMR10:11585001	10	11585001	11586000	1000	1	4.90E-08	0.4	9	0.9	LOC103693277;Crebbp	Epigenetic
DMR10:11675001	10	11675001	11678000	3000	1	3.50E-12	-0.73	27	0.9	Crebbp	Epigenetic
DMR10:11872001	10	11872001	11877000	5000	1	7.40E-08	0.46	72	1.44	Cluap1;RGD1561796;Naa60;LOC102551430	Metabolism
DMR10:11931001	10	11931001	11933000	2000	1	6.30E-07	0.34	16	0.8	Zfp174;Olr1356	Transcription;Receptor
DMR10:11940001	10	11940001	11942000	2000	1	9.10E-08	-0.43	30	1.5	Zfp174;Olr1356	Transcription;Receptor
DMR10:12053001	10	12053001	12056000	3000	2	5.50E-07	-0.4	56	1.87	Mefv	Proteolysis
DMR10:12065001	10	12065001	12066000	1000	1	2.00E-07	-0.49	2	0.2	Mefv	Proteolysis
DMR10:12342001	10	12342001	12345000	3000	1	1.10E-07	-0.35	10	0.33	Olr1361	Receptor
DMR10:12512001	10	12512001	12515000	3000	1	1.70E-07	-0.53	36	1.2	Olr1369;Olr1370	Receptor
DMR10:12579001	10	12579001	12586000	7000	2	2.30E-08	0.44	68	0.97	Olr1372	Receptor
DMR10:12652001	10	12652001	12660000	8000	2	9.20E-08	-0.33	103	1.29	Olr1375	Receptor
DMR10:12718001	10	12718001	12723000	5000	2	2.00E-09	-0.39	44	0.88	Olr1377-ps;Olr1378	Receptor
DMR10:12727001	10	12727001	12729000	2000	1	2.20E-07	0.3	14	0.7	Olr1378;LOC100910926	Receptor
DMR10:12784001	10	12784001	12789000	5000	1	1.50E-10	-0.42	48	0.96	Olr1380;Rps4x-ps5	Receptor
DMR10:12828001	10	12828001	12835000	7000	1	2.80E-07	-0.38	98	1.4	Olr1382;LOC102553477	Receptor
DMR10:12866001	10	12866001	12868000	2000	2	7.30E-09	0.49	32	1.6	Trnap-agg;Trnap-ugg;Trnap-cgg;Trnak-cuu;RGD1563656;LOC100910623;Trnar-ccu	
DMR10:12909001	10	12909001	12910000	1000	1	7.00E-08	0.76	57	5.7	Zfp13	Transcription
DMR10:12958001	10	12958001	12961000	3000	1	2.30E-07	0.33	53	1.77	Mmp25;LOC102557038	
DMR10:13004001	10	13004001	13007000	3000	1	2.70E-07	0.4	46	1.53	Thoc6;Hcfc1r1;Tnfrsf12a;Cldn6;Cldn9	Receptor;Cell Junction
DMR10:13144001	10	13144001	13149000	5000	3	1.10E-07	-0.48	62	1.24	Srrm2	
DMR10:13174001	10	13174001	13175000	1000	1	1.00E-09	0.68	27	2.7	Tceb2;Prss33;Prss41	Protease
DMR10:13335001	10	13335001	13338000	3000	1	1.00E-07	0.47	25	0.83	Sbpl	
DMR10:13346001	10	13346001	13347000	1000	1	3.10E-07	0.34	17	1.7	Prss30	Protease
DMR10:13366001	10	13366001	13367000	1000	1	6.10E-12	0.77	26	2.6	Prss22	Protease
DMR10:13436001	10	13436001	13437000	1000	1	3.60E-10	-0.71	7	0.7	Kctd5;Pdpk1	Signaling
DMR10:13556001	10	13556001	13558000	2000	1	5.30E-07	-0.38	45	2.25	Tbc1d24	Signaling

DMR10:13634001	10	13634001	13637000	3000	1	3.70E-11	-0.54	40	1.33	Abca17	Transport
DMR10:13660001	10	13660001	13661000	1000	1	2.20E-08	-0.46	20	2	Abca17	Transport
DMR10:13757001	10	13757001	13759000	2000	1	1.60E-07	0.49	34	1.7	Abca3	Transport
DMR10:14027001	10	14027001	14029000	2000	1	5.90E-07	0.51	11	0.55	Npw;Zfp598	Proteolysis
DMR10:14166001	10	14166001	14168000	2000	1	5.40E-07	-0.3	47	2.35	Meiob	
DMR10:14224001	10	14224001	14227000	3000	1	1.70E-09	0.37	45	1.5	Fahd1;Hagh	Metabolism
DMR10:14385001	10	14385001	14388000	3000	1	1.40E-09	-0.34	94	3.13	Ift140	Development
DMR10:14586001	10	14586001	14588000	2000	1	9.10E-07	-0.5	30	1.5	Unkl;Gnptg	Signaling
DMR10:14650001	10	14650001	14653000	3000	1	1.50E-08	0.61	36	1.2	Prss34	Protease
DMR10:14660001	10	14660001	14662000	2000	1	5.30E-07	0.3	18	0.9	Prss34;LOC685321	Protease
DMR10:14663001	10	14663001	14667000	4000	1	1.40E-07	-0.34	76	1.9	Prss34;LOC685321	Protease
DMR10:14701001	10	14701001	14704000	3000	1	3.40E-07	0.39	58	1.93	RGD1559662;Tpsab1	Protease
DMR10:15127001	10	15127001	15129000	2000	1	9.80E-07	0.42	28	1.4	Mslnl;Msln	Cytoskeleton
DMR10:15208001	10	15208001	15209000	1000	1	8.50E-07	0.34	25	2.5	Jmjd8;Stub1;Rhbdl1;Rhot2;Wdr90	Golgi;Proteolysis;Signaling
DMR10:15277001	10	15277001	15280000	3000	1	9.00E-09	-0.49	43	1.43	Rab40c;Pigq	Golgi
DMR10:15321001	10	15321001	15322000	1000	1	2.10E-11	0.52	31	3.1	Prr35;LOC108352065;Capn15	Protease
DMR10:15330001	10	15330001	15333000	3000	1	5.30E-09	0.42	23	0.77	Capn15	Protease
DMR10:15360001	10	15360001	15362000	2000	1	7.70E-07	-0.34	25	1.25	Rab11fip3	
DMR10:15674001	10	15674001	15676000	2000	1	5.20E-09	-0.46	45	2.25	Mpg;Rhbdf1;Snrnp25	Epigenetic;Protease
DMR10:15720001	10	15720001	15722000	2000	1	3.50E-07	0.37	33	1.65	RGD1560789	
DMR10:15868001	10	15868001	15870000	2000	1	1.20E-16	1.06	56	2.8	Nsg2	
DMR10:15879001	10	15879001	15880000	1000	1	6.70E-07	0.39	22	2.2	Nsg2	
DMR10:15909001	10	15909001	15911000	2000	1	2.80E-12	-0.4	52	2.6	Nsg2	
DMR10:15960001	10	15960001	15962000	2000	1	5.90E-14	0.9	64	3.2	RGD1311343	
DMR10:16008001	10	16008001	16009000	1000	1	1.70E-08	-0.49	8	0.8	Cpeb4	Translation
DMR10:16553001	10	16553001	16556000	3000	1	4.30E-14	0.64	74	2.47	Stc2	Hormone
DMR10:16770001	10	16770001	16777000	7000	1	6.00E-07	-0.47	114	1.63	Atp6v0e1	Metabolism
DMR10:17438001	10	17438001	17439000	1000	1	2.50E-08	0.52	18	1.8	Stk10	
DMR10:17460001	10	17460001	17464000	4000	2	7.50E-10	0.62	54	1.35	Stk10	
DMR10:17522001	10	17522001	17525000	3000	1	1.80E-09	-0.68	51	1.7	Stk10	
DMR10:17530001	10	17530001	17533000	3000	1	3.80E-07	-0.66	34	1.13	Stk10;Fbxw11	
DMR10:17555001	10	17555001	17557000	2000	1	2.70E-08	-0.44	25	1.25	Fbxw11	
DMR10:17562001	10	17562001	17565000	3000	1	5.10E-08	-0.47	38	1.27	Fbxw11	
DMR10:17600001	10	17600001	17602000	2000	1	6.20E-08	-0.49	23	1.15	Fbxw11	
DMR10:17610001	10	17610001	17611000	1000	1	3.90E-10	0.37	6	0.6	Fbxw11	
DMR10:17724001	10	17724001	17729000	5000	1	2.60E-07	-0.32	60	1.2	Smim23	
DMR10:18100001	10	18100001	18101000	1000	1	1.80E-15	0.59	28	2.8	Npm1;LOC102546482	Transcription
DMR10:18354001	10	18354001	18356000	2000	1	8.80E-07	-0.48	19	0.95	Ranbp17	Transport
DMR10:18418001	10	18418001	18422000	4000	1	9.50E-08	-0.3	38	0.95	Ranbp17;LOC108352172	Transport
DMR10:18477001	10	18477001	18483000	6000	1	9.30E-08	-0.34	64	1.07	Gabrp	Ion Channel
DMR10:18571001	10	18571001	18572000	1000	1	1.80E-07	0.42	20	2	LOC108352198;Kcnip1	
DMR10:18643001	10	18643001	18646000	3000	1	4.40E-11	0.46	87	2.9	Kcnip1;LOC102547076	
DMR10:18681001	10	18681001	18683000	2000	1	3.80E-07	0.49	25	1.25	Kcnip1	
DMR10:18810001	10	18810001	18811000	1000	1	2.00E-07	-0.32	17	1.7	Kcnip1	
DMR10:18854001	10	18854001	18855000	1000	1	1.70E-09	0.42	12	1.2	Kcnip1	
DMR10:18932001	10	18932001	18935000	3000	1	1.30E-10	0.39	32	1.07	Kcnip1;Kcnmb1	Transport
DMR10:19152001	10	19152001	19154000	2000	1	1.10E-07	0.66	38	1.9	Foxi1	Transcription
DMR10:19251001	10	19251001	19252000	1000	1	3.80E-08	0.49	12	1.2	Dock2	
DMR10:19394001	10	19394001	19395000	1000	1	1.30E-08	0.49	14	1.4	Dock2;Fam196b	
DMR10:19600001	10	19600001	19604000	4000	1	1.70E-07	0.39	46	1.15	Dock2	
DMR10:19634001	10	19634001	19635000	1000	1	4.50E-07	-0.41	4	0.4	Spdl1	Transport
DMR10:19951001	10	19951001	19953000	2000	1	5.70E-08	-0.42	49	2.45	Slit3	
DMR10:20024001	10	20024001	20026000	2000	1	8.50E-07	0.3	18	0.9	Slit3	
DMR10:20135001	10	20135001	20136000	1000	1	1.90E-09	0.42	3	0.3	Slit3	
DMR10:20194001	10	20194001	20196000	2000	1	3.40E-07	0.36	20	1	Slit3	
DMR10:20256001	10	20256001	20259000	3000	1	3.80E-07	-0.51	47	1.57	Slit3	
DMR10:20261001	10	20261001	20262000	1000	1	6.30E-07	-0.36	23	2.3	Slit3	
DMR10:20285001	10	20285001	20288000	3000	1	6.00E-08	0.31	61	2.03	Slit3	
DMR10:20502001	10	20502001	20505000	3000	1	5.00E-07	0.43	26	0.87	Slit3	
DMR10:20582001	10	20582001	20583000	1000	1	1.60E-11	-0.61	10	1	Pank3	Signaling
DMR10:20666001	10	20666001	20667000	1000	1	9.00E-09	0.57	40	4	Rars;Wwc1	
DMR10:20730001	10	20730001	20733000	3000	1	5.10E-08	-0.55	43	1.43	Wwc1	

DMR10:20824001	10	20824001	20828000	4000	1	1.50E-08	-0.4	69	1.73	Wwc1	
DMR10:20842001	10	20842001	20844000	2000	1	9.80E-07	-0.48	35	1.75	Tenm2	
DMR10:20845001	10	20845001	20848000	3000	1	1.60E-10	0.55	97	3.23	Tenm2	
DMR10:20875001	10	20875001	20876000	1000	1	1.30E-10	0.55	33	3.3	Tenm2	
DMR10:20947001	10	20947001	20949000	2000	1	1.60E-07	0.34	28	1.4	Tenm2;LOC108352203	
DMR10:21023001	10	21023001	21026000	3000	1	5.90E-09	-0.53	27	0.9	Tenm2	
DMR10:21138001	10	21138001	21140000	2000	1	1.70E-08	-0.36	34	1.7	Tenm2	
DMR10:21244001	10	21244001	21245000	1000	1	4.80E-07	0.57	33	3.3	Tenm2	
DMR10:21319001	10	21319001	21322000	3000	1	1.50E-08	0.33	45	1.5	Tenm2	
DMR10:21419001	10	21419001	21424000	5000	2	2.40E-08	0.45	80	1.6	Tenm2	
DMR10:21432001	10	21432001	21435000	3000	1	1.20E-07	-0.45	55	1.83	Tenm2	
DMR10:21526001	10	21526001	21529000	3000	1	8.40E-08	-0.48	54	1.8	Tenm2	
DMR10:21677001	10	21677001	21679000	2000	1	1.30E-08	-0.41	50	2.5	Tenm2	
DMR10:21776001	10	21776001	21777000	1000	1	2.60E-07	0.5	21	2.1	Tenm2	
DMR10:21796001	10	21796001	21798000	2000	1	6.00E-16	0.36	14	0.7	Tenm2	
DMR10:23693001	10	23693001	23695000	2000	1	1.90E-11	0.52	20	1	Ebf1	Transcription
DMR10:23729001	10	23729001	23730000	1000	1	5.20E-10	-0.38	17	1.7	Ebf1	Transcription
DMR10:23816001	10	23816001	23817000	1000	1	1.80E-09	-0.62	19	1.9	Ebf1	Transcription
DMR10:23916001	10	23916001	23917000	1000	1	1.90E-07	0.32	7	0.7	Ebf1	Transcription
DMR10:27372001	10	27372001	27373000	1000	1	1.10E-08	0.4	8	0.8	Gabra1	Ion Channel
DMR10:28196001	10	28196001	28200000	4000	1	7.80E-08	-0.53	18	0.45	Gabrb2	Ion Channel
DMR10:28201001	10	28201001	28202000	1000	1	1.20E-08	0.52	6	0.6	Gabrb2	Ion Channel
DMR10:28542001	10	28542001	28546000	4000	1	1.40E-08	-0.33	32	0.8	Atp10b	Transport
DMR10:28573001	10	28573001	28576000	3000	1	2.00E-15	-0.93	34	1.13	Atp10b	Transport
DMR10:28614001	10	28614001	28615000	1000	1	1.00E-18	1.23	16	1.6	Atp10b	Transport
DMR10:28818001	10	28818001	28823000	5000	1	3.00E-08	-0.28	50	1	Atp10b	Transport
DMR10:29030001	10	29030001	29035000	5000	1	1.70E-09	-0.5	64	1.28	Pttg1;Slu7	Translation
DMR10:29171001	10	29171001	29173000	2000	1	7.10E-07	0.46	28	1.4	Ccnjl	Signaling
DMR10:29412001	10	29412001	29413000	1000	1	1.00E-09	0.53	10	1	Adra1b	Signaling
DMR10:29459001	10	29459001	29461000	2000	1	3.10E-07	-0.49	12	0.6	Adra1b;LOC108352076	Signaling
DMR10:29808001	10	29808001	29809000	1000	1	3.40E-09	0.42	5	0.5	LOC102549660;LOC108352079;RGD1562355	
DMR10:30057001	10	30057001	30060000	3000	1	1.40E-10	-0.49	66	2.2	Il12b	Receptor
DMR10:30848001	10	30848001	30849000	1000	1	7.00E-08	0.62	20	2	LOC102550742;Clint1	
DMR10:30881001	10	30881001	30886000	5000	1	3.80E-08	-0.4	84	1.68	LOC102550742;Clint1	
DMR10:30896001	10	30896001	30903000	7000	1	1.40E-07	-0.42	76	1.09	LOC102550742;Clint1	
DMR10:30996001	10	30996001	30999000	3000	1	6.30E-07	-0.38	43	1.43	Clint1	
DMR10:31245001	10	31245001	31247000	2000	1	1.60E-07	-0.48	24	1.2	Adam19;Nipal4	Protease
DMR10:31406001	10	31406001	31407000	1000	1	6.60E-08	-0.59	16	1.6	Cyfip2;LOC108352178	Cytoskeleton
DMR10:31520001	10	31520001	31524000	4000	2	2.20E-07	-0.43	81	2.02	Fam71b;Med7	Immune
DMR10:31569001	10	31569001	31570000	1000	1	9.30E-08	0.48	12	1.2	Havcr2	Immune
DMR10:31827001	10	31827001	31829000	2000	1	9.00E-09	0.43	13	0.65	Havcr1	
DMR10:32140001	10	32140001	32142000	2000	1	1.70E-08	0.42	18	0.9	Sgcd	Cytoskeleton
DMR10:32183001	10	32183001	32184000	1000	1	2.20E-07	-0.37	17	1.7	Sgcd	Cytoskeleton
DMR10:32295001	10	32295001	32297000	2000	1	8.30E-08	-0.46	30	1.5	Sgcd	Cytoskeleton
DMR10:34226001	10	34226001	34228000	2000	1	1.90E-11	0.5	41	2.05	Irgm;LOC108352081;RGD1559575	
DMR10:34309001	10	34309001	34312000	3000	1	4.70E-08	-0.36	23	0.77	Olr1383;LOC690160;RGD1560464	Receptor
DMR10:34337001	10	34337001	34338000	1000	1	1.10E-09	-0.52	11	1.1	Olr1384;LOC100360198	Receptor
DMR10:34582001	10	34582001	34583000	1000	1	3.00E-08	-0.36	8	0.8	Olr1389;Olr1390-ps	Receptor
DMR10:34651001	10	34651001	34655000	4000	1	8.50E-08	-0.27	38	0.95	Olr1392;Olr1393	Receptor
DMR10:34738001	10	34738001	34739000	1000	1	9.50E-08	0.47	10	1	Olr1398	Receptor
DMR10:34774001	10	34774001	34776000	2000	1	2.30E-07	-0.44	5	0.25	Olr1400	Receptor
DMR10:34808001	10	34808001	34811000	3000	2	4.10E-09	-0.34	41	1.37	Olr1401	Receptor
DMR10:35027001	10	35027001	35029000	2000	1	9.80E-08	0.48	8	0.4	Trappc2b	
DMR10:35081001	10	35081001	35085000	4000	2	5.60E-14	0.64	55	1.38	Flt4	Receptor
DMR10:35106001	10	35106001	35108000	2000	1	1.90E-09	0.54	43	2.15	Flt4	Receptor
DMR10:35121001	10	35121001	35122000	1000	1	1.60E-07	0.37	21	2.1	Flt4	Receptor
DMR10:35200001	10	35200001	35202000	2000	2	2.40E-08	0.45	16	0.8	Cnot6;LOC108352082	Translation
DMR10:35276001	10	35276001	35279000	3000	1	1.50E-07	0.52	52	1.73	Gfpt2	Metabolism
DMR10:35293001	10	35293001	35294000	1000	1	6.20E-08	-0.47	21	2.1	Gfpt2	Metabolism
DMR10:35416001	10	35416001	35418000	2000	1	1.80E-08	0.46	33	1.65	Rasgef1c	Transcription
DMR10:35855001	10	35855001	35856000	1000	1	1.10E-07	-0.39	20	2	Cby3	
DMR10:35929001	10	35929001	35931000	2000	1	3.40E-14	0.46	21	1.05	Rufy1	

DMR10:35937001	10	35937001	35940000	3000	1	1.70E-10	0.67	56	1.87	Rufy1	
DMR10:36160001	10	36160001	36162000	2000	2	2.90E-11	0.33	21	1.05	Adamts2	Protease
DMR10:36169001	10	36169001	36171000	2000	1	3.10E-08	0.46	49	2.45	Adamts2	Protease
DMR10:36337001	10	36337001	36339000	2000	1	2.20E-10	-0.5	23	1.15	Zfp879;Grm6	Signaling
DMR10:36340001	10	36340001	36342000	2000	1	7.90E-07	0.32	29	1.45	Zfp879;Grm6	Signaling
DMR10:36430001	10	36430001	36436000	6000	1	2.40E-09	-0.41	50	0.83	Znf354b	
DMR10:36449001	10	36449001	36451000	2000	1	4.00E-10	0.41	43	2.15	Prop1;Olr1403-ps	Development
DMR10:37648001	10	37648001	37653000	5000	1	3.90E-07	0.38	59	1.18	Tcf7	Transcription
DMR10:38100001	10	38100001	38102000	2000	1	4.50E-09	0.39	21	1.05	Fstl4	Protease; Proteolysis
DMR10:38112001	10	38112001	38116000	4000	1	4.60E-10	0.44	58	1.45	Fstl4	Protease; Proteolysis
DMR10:38136001	10	38136001	38137000	1000	1	4.90E-09	-0.4	21	2.1	Fstl4	Protease; Proteolysis
DMR10:38152001	10	38152001	38155000	3000	2	9.70E-08	0.41	58	1.93	Fstl4	Protease; Proteolysis
DMR10:38198001	10	38198001	38200000	2000	1	9.40E-07	0.45	35	1.75	Fstl4	Protease; Proteolysis
DMR10:38228001	10	38228001	38231000	3000	1	4.80E-07	-0.39	23	0.77	Fstl4	Protease; Proteolysis
DMR10:38289001	10	38289001	38290000	1000	1	1.10E-08	0.4	13	1.3	Fstl4	Protease; Proteolysis
DMR10:38297001	10	38297001	38299000	2000	1	4.30E-07	0.41	21	1.05	Fstl4	Protease; Proteolysis
DMR10:38363001	10	38363001	38366000	3000	1	4.30E-07	-0.38	48	1.6	Fstl4	Protease; Proteolysis
DMR10:38429001	10	38429001	38430000	1000	1	3.10E-07	0.35	15	1.5	Fstl4	Protease; Proteolysis
DMR10:38537001	10	38537001	38539000	2000	1	8.70E-07	0.31	24	1.2	Fstl4;LOC108352083	Protease; Proteolysis
DMR10:38760001	10	38760001	38761000	1000	1	1.30E-08	-0.39	10	1	Aff4	Transcription
DMR10:38907001	10	38907001	38908000	1000	1	2.90E-10	0.4	10	1	8-Sep	
DMR10:39022001	10	39022001	39025000	3000	1	1.70E-07	0.41	34	1.13	Rad50	
DMR10:39236001	10	39236001	39238000	2000	1	9.60E-09	-0.48	21	1.05	Slc22a5	Transport
DMR10:39703001	10	39703001	39708000	5000	1	9.20E-18	0.74	94	1.88	Acsl6	Metabolism
DMR10:39899001	10	39899001	39903000	4000	1	9.50E-13	-0.33	37	0.92	Rapgef6;LOC108352084	Transcription
DMR10:40112001	10	40112001	40114000	2000	1	6.50E-07	-0.37	20	1	Cdc42se2	
DMR10:40189001	10	40189001	40192000	3000	1	1.30E-08	-0.53	39	1.3	Lyrm7	Transcription
DMR10:40285001	10	40285001	40286000	1000	1	4.20E-08	-0.66	5	0.5	Tnip1	
DMR10:40534001	10	40534001	40536000	2000	1	9.60E-07	0.52	39	1.95	Slc36a2;Slc36a1	Transport
DMR10:40772001	10	40772001	40774000	2000	1	9.90E-09	0.39	41	2.05	Sparc;LOC108352218	Extracellular Matrix
DMR10:40852001	10	40852001	40856000	4000	1	1.90E-12	0.65	74	1.85	G3bp1;Glra1	Metabolism;Ion Channel
DMR10:40900001	10	40900001	40908000	8000	1	6.00E-10	-0.33	74	0.92	Glra1;LOC103693333	Ion Channel
DMR10:42437001	10	42437001	42439000	2000	1	2.50E-07	0.41	16	0.8	Gria1	Receptor
DMR10:42453001	10	42453001	42455000	2000	1	3.30E-08	0.38	6	0.3	Gria1	Receptor
DMR10:42757001	10	42757001	42758000	1000	1	4.80E-07	-0.48	7	0.7	Gria1	Receptor
DMR10:43202001	10	43202001	43203000	1000	1	7.70E-08	0.47	18	1.8	Galnt10	Golgi
DMR10:43209001	10	43209001	43211000	2000	1	9.30E-07	0.39	35	1.75	Galnt10	Golgi
DMR10:43247001	10	43247001	43248000	1000	1	9.80E-07	-0.43	9	0.9	Hand1	Transcription
DMR10:43515001	10	43515001	43517000	2000	1	1.80E-07	0.33	23	1.15	Faxdc2	Metabolism
DMR10:43948001	10	43948001	43953000	5000	1	7.60E-07	-0.54	36	0.72	LOC100361716;Olr1416	Receptor
DMR10:43999001	10	43999001	44003000	4000	1	9.70E-08	-0.55	23	0.58	Olr1417	Receptor
DMR10:44011001	10	44011001	44022000	11000	1	6.40E-08	-0.38	100	0.91	Olr1418	Receptor
DMR10:44128001	10	44128001	44137000	9000	3	1.70E-08	-0.36	72	0.8	Olr1423;Olr1424	Receptor
DMR10:44258001	10	44258001	44259000	1000	1	4.00E-08	0.33	8	0.8	Olr1432	Signaling
DMR10:44267001	10	44267001	44269000	2000	1	4.30E-08	0.45	47	2.35	Trim58	Proteolysis
DMR10:44282001	10	44282001	44284000	2000	1	3.00E-20	0.87	35	1.75	Trim58;Olr1433	Proteolysis;Receptor
DMR10:44338001	10	44338001	44344000	6000	1	2.70E-07	-0.24	55	0.92	Olr1434;Olr1435	Receptor
DMR10:44661001	10	44661001	44664000	3000	1	2.30E-08	-0.42	17	0.57	Olr1449;RGD1559534	Receptor
DMR10:44667001	10	44667001	44668000	1000	1	3.00E-08	-0.42	4	0.4	RGD1559534	
DMR10:44669001	10	44669001	44673000	4000	2	1.10E-09	-0.49	32	0.8	RGD1559534;Olr1450	Receptor
DMR10:44723001	10	44723001	44726000	3000	1	1.70E-07	-0.4	40	1.33	Olr1454;Olr1456	Receptor
DMR10:44783001	10	44783001	44785000	2000	1	8.90E-08	-0.44	8	0.4	Olr1458;Olr1459	Signaling
DMR10:45146001	10	45146001	45149000	3000	2	1.50E-09	0.46	34	1.13	LOC691460;RGD1560444	
DMR10:45227001	10	45227001	45230000	3000	1	2.60E-08	-0.46	52	1.73	Zfp39	Transcription
DMR10:45248001	10	45248001	45251000	3000	2	1.40E-09	-0.39	19	0.63	Zfp39;Btnl10	Transcription;Immune
DMR10:45428001	10	45428001	45436000	8000	2	7.40E-10	0.51	112	1.4	Obscn	
DMR10:45457001	10	45457001	45459000	2000	1	4.30E-09	0.33	39	1.95	Obscn	
DMR10:45468001	10	45468001	45470000	2000	1	2.40E-10	0.38	27	1.35	Obscn	
DMR10:45483001	10	45483001	45484000	1000	1	1.80E-08	0.54	17	1.7	Obscn	
DMR10:45733001	10	45733001	45734000	1000	1	1.50E-07	-0.5	6	0.6	Prss38;LOC102546363;LOC103693339	Protease
DMR10:45809001	10	45809001	45810000	1000	1	4.60E-07	-0.5	9	0.9	Jmjd4;Zfp867	Golgi

DMR10:45822001	10	45822001	45825000	3000	2	6.00E-09	0.65	54	1.8	Zfp867;LOC108352088;LOC100912120;Zfp496	Transcription
DMR10:46310001	10	46310001	46312000	2000	1	1.00E-07	0.48	37	1.85	Med9	Transcription
DMR10:46320001	10	46320001	46322000	2000	1	1.30E-07	-0.51	24	1.2	Med9;Rasd1	Transcription
DMR10:46376001	10	46376001	46377000	1000	1	5.80E-07	-0.45	16	1.6	Pemt	Epigenetic
DMR10:46512001	10	46512001	46514000	2000	1	3.60E-07	0.37	37	1.85	Rai1	Transcription
DMR10:46841001	10	46841001	46844000	3000	2	2.90E-18	1.03	79	2.63	Drg2;Myo15a	Cytoskeleton
DMR10:46863001	10	46863001	46864000	1000	1	1.50E-09	0.54	19	1.9	Myo15a	Cytoskeleton
DMR10:46964001	10	46964001	46965000	1000	1	8.20E-07	0.47	18	1.8	Llgl1;Flii;Mief2	Transport;Cytoskeleton
DMR10:47101001	10	47101001	47102000	1000	1	8.90E-08	-0.35	8	0.8	Dhrs7b	Metabolism
DMR10:47308001	10	47308001	47316000	8000	2	1.70E-10	-0.54	117	1.46	Kcnj12	Transport
DMR10:47485001	10	47485001	47490000	5000	1	6.20E-11	0.5	61	1.22	Aldh3a1	Metabolism
DMR10:47519001	10	47519001	47521000	2000	1	4.90E-08	0.45	19	0.95	RGD1566257;Aldh3a2	Metabolism
DMR10:47717001	10	47717001	47721000	4000	1	5.30E-07	0.54	79	1.98	LOC108352219;Rnf112	Signaling
DMR10:47848001	10	47848001	47851000	3000	1	2.60E-08	-0.46	24	0.8	Epn2	
DMR10:48000001	10	48000001	48003000	3000	1	7.10E-07	-0.38	31	1.03	Slc5a10;Prpsap2	Transport;Signaling
DMR10:48263001	10	48263001	48264000	1000	1	6.60E-07	-0.34	10	1	Specc1	
DMR10:48290001	10	48290001	48292000	2000	1	4.40E-08	0.4	19	0.95	Specc1	
DMR10:48419001	10	48419001	48421000	2000	1	6.10E-16	-0.53	27	1.35	Specc1	
DMR10:48600001	10	48600001	48603000	3000	1	9.20E-11	-0.52	51	1.7	Zswim7;Ttc19	
DMR10:48649001	10	48649001	48650000	1000	1	3.40E-08	-0.43	25	2.5	Ncor1	Epigenetic
DMR10:48901001	10	48901001	48903000	2000	1	9.50E-07	0.3	26	1.3	LOC103693343;Trpv2	Transport
DMR10:48916001	10	48916001	48921000	5000	2	3.90E-11	0.46	67	1.34	Trpv2;LOC102552369;Lrrc75a	Transport
DMR10:49376001	10	49376001	49379000	3000	1	3.30E-07	-0.37	41	1.37	LOC102552952;Cdrt4	
DMR10:49568001	10	49568001	49570000	2000	2	1.50E-10	0.68	45	2.25	Pmp22	Cytoskeleton
DMR10:50437001	10	50437001	50438000	1000	1	3.00E-09	0.53	16	1.6	Cox10	Metabolism
DMR10:51567001	10	51567001	51569000	2000	1	9.60E-08	0.61	56	2.8	Arhgap44	
DMR10:51660001	10	51660001	51664000	4000	1	1.40E-07	-0.41	59	1.48	Arhgap44	
DMR10:51670001	10	51670001	51673000	3000	1	5.80E-07	0.45	37	1.23	Arhgap44;Myocd	Transcription
DMR10:51740001	10	51740001	51744000	4000	1	2.80E-08	0.48	33	0.82	Myocd	Transcription
DMR10:52204001	10	52204001	52209000	5000	1	8.70E-09	-0.64	47	0.94	Map2k4	Signaling
DMR10:52474001	10	52474001	52484000	10000	1	3.20E-10	-0.26	104	1.04	Dnah9	Cytoskeleton
DMR10:52638001	10	52638001	52644000	6000	1	3.10E-09	-0.6	33	0.55	Dnah9	Cytoskeleton
DMR10:52814001	10	52814001	52815000	1000	1	5.20E-07	0.43	6	0.6	Shisa6	
DMR10:52925001	10	52925001	52926000	1000	1	3.20E-08	0.47	10	1	Shisa6	
DMR10:52977001	10	52977001	52982000	5000	1	1.10E-07	0.34	37	0.74	Shisa6	
DMR10:53564001	10	53564001	53568000	4000	2	1.90E-07	-0.39	54	1.35	Tmem220	
DMR10:53623001	10	53623001	53625000	2000	1	2.10E-08	0.58	47	2.35	Myh3	
DMR10:53636001	10	53636001	53638000	2000	1	1.00E-13	0.93	71	3.55	Myh3	
DMR10:54194001	10	54194001	54198000	4000	1	4.70E-07	0.35	30	0.75	Gas7	Cytoskeleton
DMR10:54215001	10	54215001	54218000	3000	1	1.00E-07	0.49	29	0.97	Gas7	Cytoskeleton
DMR10:54247001	10	54247001	54249000	2000	1	8.10E-07	-0.33	20	1	Gas7;Rcvrn	Cytoskeleton
DMR10:54399001	10	54399001	54401000	2000	1	1.50E-08	0.42	18	0.9	Usp43	Protease
DMR10:54461001	10	54461001	54464000	3000	1	4.30E-07	-0.34	28	0.93	Usp43;LOC100911946;Cfap52	Protease
DMR10:54465001	10	54465001	54466000	1000	1	2.30E-07	-0.32	16	1.6	Usp43;LOC100911946;Cfap52	Protease
DMR10:54490001	10	54490001	54491000	1000	1	2.50E-08	0.34	3	0.3	Cfap52	
DMR10:54850001	10	54850001	54853000	3000	1	3.30E-07	0.51	38	1.27	Ntn1	Extracellular Matrix
DMR10:54970001	10	54970001	54972000	2000	2	4.00E-08	-0.58	31	1.55	Ntn1	Extracellular Matrix
DMR10:55076001	10	55076001	55078000	2000	1	3.60E-07	0.41	47	2.35	Pik3r5;Pik3r6	Signaling
DMR10:55222001	10	55222001	55224000	2000	1	2.90E-07	0.47	64	3.2	RGD1564148	
DMR10:55269001	10	55269001	55271000	2000	1	2.70E-07	-0.46	35	1.75	Myh10	
DMR10:55543001	10	55543001	55544000	1000	1	1.00E-07	0.41	21	2.1	Arhgef15	Transcription
DMR10:55725001	10	55725001	55727000	2000	1	1.20E-08	0.39	25	1.25	Aloxe3;LOC103693355	Metabolism
DMR10:55728001	10	55728001	55735000	7000	1	1.50E-07	0.61	113	1.61	Aloxe3;LOC103693355;LOC100359495;LOC102555853;Alox12b	Metabolism
DMR10:55763001	10	55763001	55766000	3000	1	3.00E-09	-0.62	42	1.4	Alox12b;Alox15b	Metabolism
DMR10:55841001	10	55841001	55843000	2000	1	1.30E-09	0.74	45	2.25	Gucy2e	Signaling
DMR10:56014001	10	56014001	56015000	1000	1	9.60E-08	-0.57	12	1.2	Kdm6b;LOC102555947	Epigenetic
DMR10:56128001	10	56128001	56132000	4000	1	1.90E-10	0.46	56	1.4	Dnah2	Cytoskeleton
DMR10:56141001	10	56141001	56146000	5000	2	5.80E-17	0.47	52	1.04	Dnah2	Cytoskeleton
DMR10:56196001	10	56196001	56201000	5000	1	2.70E-08	0.4	75	1.5	Tp53;Atp1b2	Transcription;Transport
DMR10:56213001	10	56213001	56219000	6000	1	6.50E-08	0.44	74	1.23	Atp1b2;Shbg;Sat2	Transport;Metabolism

DMR10:56427001	10	56427001	56436000	9000	1	1.20E-07	-0.62	181	2.01	LOC102546484;LOC497938;Spem1;Nlgn2	Cytoskeleton
DMR10:56502001	10	56502001	56503000	1000	1	6.20E-08	0.37	17	1.7	Acap1;LOC497940;Neurl4	Signaling;Proteolysis
DMR10:56719001	10	56719001	56721000	2000	1	3.60E-07	0.53	36	1.8	Asgr2	Transport
DMR10:56766001	10	56766001	56769000	3000	1	1.90E-07	0.65	52	1.73	Clec10a	Transport
DMR10:57070001	10	57070001	57071000	1000	1	7.10E-07	0.63	41	4.1	Cxcl16;Zmynd15	Growth Factors
DMR10:57104001	10	57104001	57105000	1000	1	4.90E-07	0.38	21	2.1	Tm4sf5	
DMR10:57333001	10	57333001	57335000	2000	1	2.20E-07	0.46	30	1.5	Kif1c	Cytoskeleton
DMR10:57348001	10	57348001	57352000	4000	1	1.10E-09	0.7	88	2.2	Kif1c	Cytoskeleton
DMR10:57717001	10	57717001	57719000	2000	1	4.00E-09	-0.41	10	0.5	Nlrp1a	
DMR10:58717001	10	58717001	58719000	2000	1	4.50E-07	0.35	37	1.85	Pitpnm3;RGD1304728	Metabolism
DMR10:58809001	10	58809001	58810000	1000	1	2.70E-07	0.43	15	1.5	Slc13a5	Transport
DMR10:58902001	10	58902001	58905000	3000	1	1.20E-07	0.49	34	1.13	Tekt1;LOC103693361	Cytoskeleton
DMR10:58931001	10	58931001	58933000	2000	1	8.90E-08	-0.6	30	1.5	Tekt1	Cytoskeleton
DMR10:59027001	10	59027001	59029000	2000	1	2.10E-07	0.33	22	1.1	Spns2	Transport
DMR10:59059001	10	59059001	59062000	3000	1	5.00E-07	0.46	40	1.33	Spns2;Spns3	Transport
DMR10:59217001	10	59217001	59219000	2000	1	6.20E-07	-0.45	12	0.6	Ube2g1	
DMR10:59376001	10	59376001	59381000	5000	1	6.80E-09	-0.32	40	0.8	Zzef1;LOC102548894	
DMR10:59526001	10	59526001	59528000	2000	1	1.20E-07	0.38	13	0.65	Atp2a3	Transport
DMR10:59539001	10	59539001	59540000	1000	1	1.80E-07	0.73	19	1.9	Atp2a3	Transport
DMR10:59634001	10	59634001	59635000	1000	1	5.30E-07	-0.45	19	1.9	Ncbp3	
DMR10:59707001	10	59707001	59708000	1000	1	2.60E-08	-0.43	13	1.3	Itgae;Gsg2	Extracellular Matrix
DMR10:59724001	10	59724001	59725000	1000	1	3.10E-08	0.42	15	1.5	LOC108352105;P2rx5	Ion Channel
DMR10:59816001	10	59816001	59818000	2000	1	4.60E-07	0.5	24	1.2	Trpv1	Transport
DMR10:59921001	10	59921001	59923000	2000	1	6.70E-09	0.4	11	0.55	Olr1466	
DMR10:59945001	10	59945001	59947000	2000	1	4.10E-07	0.46	26	1.3	Olr1467;LOC103693362;Olr1468	Receptor
DMR10:60148001	10	60148001	60151000	3000	1	9.20E-07	0.49	5	0.17	Olr1475	
DMR10:60172001	10	60172001	60174000	2000	1	5.40E-07	0.41	28	1.4	Olr1476-ps	
DMR10:60284001	10	60284001	60285000	1000	1	2.60E-07	-0.38	4	0.4	Olr1485	
DMR10:60399001	10	60399001	60401000	2000	1	3.10E-07	-0.57	8	0.4	Olr1490;Olr1491	Receptor
DMR10:60926001	10	60926001	60929000	3000	1	1.00E-07	-0.27	39	1.3	Olr1508-ps	
DMR10:60950001	10	60950001	60956000	6000	2	8.70E-10	-0.47	57	0.95	Olr1509;Olr1510-ps;LOC497952	Receptor
DMR10:61184001	10	61184001	61185000	1000	1	6.10E-07	0.38	6	0.6	Rap1gap2	Signaling
DMR10:61205001	10	61205001	61207000	2000	2	4.10E-08	0.41	12	0.6	Rap1gap2	Signaling
DMR10:61319001	10	61319001	61323000	4000	2	2.90E-11	-0.49	65	1.62	Rap1gap2	Signaling
DMR10:61359001	10	61359001	61362000	3000	1	2.60E-08	0.34	37	1.23	Rap1gap2	Signaling
DMR10:61466001	10	61466001	61468000	2000	1	4.20E-09	-0.62	17	0.85	Pafah1b1	
DMR10:61530001	10	61530001	61531000	1000	1	7.10E-07	-0.44	11	1.1	Pafah1b1;Smg6	Metabolism
DMR10:62022001	10	62022001	62023000	1000	1	9.30E-07	0.45	10	1	Mir212;Mir132;Ovca2;Dph1	
DMR10:62250001	10	62250001	62252000	2000	2	1.00E-15	0.51	13	0.65	Serpinf1	Protease; Proteolysis
DMR10:62595001	10	62595001	62596000	1000	1	7.60E-16	0.66	7	0.7	Taok1	Signaling
DMR10:62689001	10	62689001	62690000	1000	1	1.20E-09	0.51	32	3.2	Ankrd13b;Coro6	Cytoskeleton
DMR10:62812001	10	62812001	62813000	1000	1	4.40E-07	-0.44	8	0.8	Ssh2	Signaling
DMR10:63076001	10	63076001	63082000	6000	1	1.90E-07	-0.41	77	1.28	Efcab5	Signaling
DMR10:63733001	10	63733001	63736000	3000	1	7.50E-09	-0.5	35	1.17	Pitpna	Transport
DMR10:63862001	10	63862001	63863000	1000	1	3.50E-09	0.69	21	2.1	Crk	Cytoskeleton
DMR10:63908001	10	63908001	63909000	1000	1	8.60E-07	-0.55	6	0.6	Ywhae	Cytoskeleton
DMR10:63935001	10	63935001	63937000	2000	1	5.60E-07	0.3	20	1	Doc2b	
DMR10:64006001	10	64006001	64009000	3000	1	3.50E-11	-0.64	35	1.17	Rph3al;LOC102549294	
DMR10:64021001	10	64021001	64025000	4000	1	1.60E-07	0.36	57	1.43	Rph3al;LOC102549294	
DMR10:64040001	10	64040001	64041000	1000	1	2.30E-13	0.45	5	0.5	Rph3al;LOC102549294	
DMR10:64045001	10	64045001	64048000	3000	1	3.70E-08	0.44	49	1.63	Rph3al;LOC102549294	
DMR10:64056001	10	64056001	64057000	1000	1	3.80E-07	0.45	8	0.8	Rph3al;LOC102549419	
DMR10:64063001	10	64063001	64064000	1000	1	1.80E-07	-0.54	21	2.1	Rph3al;LOC102549419	
DMR10:64145001	10	64145001	64146000	1000	1	9.90E-07	0.36	13	1.3	Rph3al	
DMR10:64419001	10	64419001	64423000	4000	1	5.40E-07	-0.32	65	1.62	Nxn	Metabolism
DMR10:64507001	10	64507001	64509000	2000	1	1.60E-07	-0.44	26	1.3	Nxn;LOC108352108	Metabolism
DMR10:64546001	10	64546001	64547000	1000	1	3.20E-08	-0.52	8	0.8	Nxn;Timm22	Metabolism;Transport
DMR10:64850001	10	64850001	64851000	1000	1	4.70E-08	-0.5	18	1.8	Phf12	Epigenetic
DMR10:64867001	10	64867001	64869000	2000	1	7.20E-07	0.36	32	1.6	Phf12;Sez6	Epigenetic
DMR10:64903001	10	64903001	64904000	1000	1	4.70E-07	0.31	9	0.9	Sez6	
DMR10:64968001	10	64968001	64970000	2000	2	6.60E-15	0.81	48	2.4	Pipox;LOC103693392;Myo18a	Metabolism

DMR10:64984001	10	64984001	64987000	3000	1	3.00E-12	0.68	78	2.6	LOC103693392;Myo18a;LOC102557169	
DMR10:65043001	10	65043001	65044000	1000	1	1.90E-08	0.42	13	1.3	Myo18a;LOC103693393	
DMR10:65665001	10	65665001	65667000	2000	1	3.90E-07	0.47	35	1.75	Slc13a2	Transport
DMR10:65833001	10	65833001	65837000	4000	1	7.70E-07	0.4	54	1.35	LOC100361067;Nlk	Signaling
DMR10:65850001	10	65850001	65851000	1000	1	7.30E-07	-0.49	10	1	Nlk	Signaling
DMR10:66345001	10	66345001	66346000	1000	1	9.40E-09	-0.46	13	1.3	Ksr1	Signaling
DMR10:66413001	10	66413001	66414000	1000	1	1.50E-07	0.38	9	0.9	Ksr1	Signaling
DMR10:66873001	10	66873001	66876000	3000	1	9.00E-07	-0.4	23	0.77	Nf1;Evi2a	Signaling
DMR10:67247001	10	67247001	67250000	3000	3	7.60E-15	0.64	20	0.67	LOC103693400;LOC363652;Utp6	Metabolism
DMR10:67422001	10	67422001	67423000	1000	1	1.30E-07	0.39	8	0.8	Atad5	Transcription
DMR10:67518001	10	67518001	67521000	3000	1	2.50E-07	0.3	36	1.2	Adap2	
DMR10:67668001	10	67668001	67671000	3000	2	6.00E-08	-0.53	46	1.53	Rhbdl3	
DMR10:67834001	10	67834001	67835000	1000	1	8.70E-09	0.45	8	0.8	Psmd11	
DMR10:67980001	10	67980001	67986000	6000	1	1.70E-07	-0.5	89	1.48	Myo1d	Cytoskeleton
DMR10:68144001	10	68144001	68147000	3000	1	8.60E-14	-0.54	40	1.33	Myo1d	Cytoskeleton
DMR10:68224001	10	68224001	68226000	2000	1	1.80E-09	0.41	32	1.6	Spaca3	
DMR10:68233001	10	68233001	68234000	1000	1	1.10E-07	0.43	26	2.6	Spaca3	
DMR10:68288001	10	68288001	68292000	4000	1	2.30E-08	0.41	68	1.7	Asic2	Transport
DMR10:68351001	10	68351001	68352000	1000	1	3.50E-07	0.36	5	0.5	Asic2	Transport
DMR10:68411001	10	68411001	68415000	4000	1	2.40E-08	-0.42	44	1.1	Asic2	Transport
DMR10:68470001	10	68470001	68471000	1000	1	7.90E-07	0.4	4	0.4	Asic2	Transport
DMR10:68565001	10	68565001	68567000	2000	1	9.00E-07	0.23	11	0.55	Asic2;LOC688465	Transport
DMR10:68594001	10	68594001	68596000	2000	1	3.70E-08	0.69	44	2.2	Asic2;LOC108352207	Transport
DMR10:68925001	10	68925001	68929000	4000	1	7.40E-08	0.56	58	1.45	Asic2;Phb-ps1	Transport
DMR10:68964001	10	68964001	68965000	1000	1	4.90E-09	-0.58	4	0.4	Asic2;LOC103693401	Transport
DMR10:68991001	10	68991001	68993000	2000	1	7.60E-07	-0.37	43	2.15	Asic2	Transport
DMR10:70109001	10	70109001	70111000	2000	1	1.00E-08	0.61	34	1.7	Cct6b;Zfp830	Translation
DMR10:70189001	10	70189001	70195000	6000	1	1.30E-08	-0.43	67	1.12	Rffl	Proteolysis
DMR10:70234001	10	70234001	70235000	1000	1	9.20E-13	0.54	9	0.9	Rad51d;Fndc8	Transcription
DMR10:70385001	10	70385001	70390000	5000	1	9.90E-08	-0.3	29	0.58	Slfn2;Slfn1	
DMR10:70454001	10	70454001	70456000	2000	1	2.10E-07	-0.41	20	1	Slfn3	
DMR10:70458001	10	70458001	70464000	6000	2	1.80E-10	-0.39	51	0.85	Slfn3	
DMR10:70494001	10	70494001	70497000	3000	1	2.00E-07	-0.48	48	1.6	Slfn14;LOC100909755	
DMR10:70570001	10	70570001	70572000	2000	1	2.90E-10	-0.51	25	1.25	Ap2b1	Transport
DMR10:70656001	10	70656001	70658000	2000	1	9.70E-08	0.49	21	1.05	Gas2l2;LOC689039;Mmp28	Protease
DMR10:70879001	10	70879001	70881000	2000	1	2.10E-07	0.4	18	0.9	Ccl3;Ccl4	Growth Factors
DMR10:71154001	10	71154001	71155000	1000	1	4.30E-10	0.48	7	0.7	Hnf1b	Transcription
DMR10:71241001	10	71241001	71244000	3000	1	7.90E-08	0.42	31	1.03	Ddx52	
DMR10:71325001	10	71325001	71327000	2000	1	1.60E-07	-0.39	38	1.9	Synrg	
DMR10:71615001	10	71615001	71616000	1000	1	3.40E-07	0.48	8	0.8	Acaca	
DMR10:71700001	10	71700001	71701000	1000	1	1.20E-07	-0.56	15	1.5	Acaca	
DMR10:71834001	10	71834001	71836000	2000	1	7.60E-09	-0.5	22	1.1	Aatf;Lhx1	Epigenetic;Development
DMR10:72146001	10	72146001	72148000	2000	1	9.90E-08	0.54	53	2.65	Mrm1;Dhrs11;LOC102553386;Ggnbp2	Epigenetic;Metabolism
DMR10:72162001	10	72162001	72163000	1000	1	1.10E-08	-0.51	18	1.8	Dhrs11;Ggnbp2	Metabolism
DMR10:72245001	10	72245001	72246000	1000	1	5.60E-08	0.49	6	0.6	Znhit3;LOC100362647	
DMR10:72381001	10	72381001	72383000	2000	1	2.80E-07	-0.58	18	0.9	Usp32	Protease
DMR10:73217001	10	73217001	73220000	3000	1	6.40E-09	0.48	28	0.93	Bcas3	
DMR10:73740001	10	73740001	73742000	2000	1	2.10E-07	-0.49	18	0.9	Med13	
DMR10:73847001	10	73847001	73849000	2000	1	1.10E-07	0.36	18	0.9	Rps6kb1	Golgi
DMR10:73902001	10	73902001	73903000	1000	1	1.30E-08	-0.46	20	2	Vmp1;Mir21	Receptor
DMR10:74233001	10	74233001	74235000	2000	1	4.20E-09	0.29	25	1.25	Ypel2	
DMR10:74244001	10	74244001	74245000	1000	1	1.70E-09	-0.46	15	1.5	Ypel2	
DMR10:74258001	10	74258001	74260000	2000	1	8.20E-08	-0.51	31	1.55	Ypel2	
DMR10:74590001	10	74590001	74592000	2000	1	1.90E-10	-0.43	26	1.3	Ppm1e	Signaling
DMR10:74596001	10	74596001	74598000	2000	2	2.50E-08	0.42	12	0.6	Ppm1e	Signaling
DMR10:74613001	10	74613001	74617000	4000	3	4.00E-08	0.41	20	0.5	Ppm1e;LOC102557246	Signaling
DMR10:74636001	10	74636001	74637000	1000	1	4.20E-08	-0.46	11	1.1	Ppm1e	Signaling
DMR10:74669001	10	74669001	74672000	3000	1	9.10E-07	-0.43	44	1.47	Ppm1e	Signaling
DMR10:74749001	10	74749001	74750000	1000	1	7.10E-07	0.33	4	0.4	Tex14	
DMR10:74986001	10	74986001	74988000	2000	1	1.10E-10	-0.51	33	1.65	Rnf43	
DMR10:75054001	10	75054001	75057000	3000	1	2.30E-13	0.55	53	1.77	Mir142;Tspoap1	

DMR10:75093001	10	75093001	75094000	1000	1	6.80E-07	0.34	4	0.4	Mpo;Lpo	Metabolism
DMR10:75210001	10	75210001	75213000	3000	1	3.00E-07	-0.38	31	1.03	Olr1523	Receptor
DMR10:75278001	10	75278001	75281000	3000	1	1.60E-08	0.43	24	0.8	Dynll2	Cytoskeleton
DMR10:75282001	10	75282001	75284000	2000	1	3.00E-10	0.49	20	1	Dynll2	Cytoskeleton
DMR10:75525001	10	75525001	75528000	3000	1	8.50E-07	0.46	35	1.17	Cuedc1;Mrps23	Translation
DMR10:75609001	10	75609001	75613000	4000	1	4.30E-14	0.35	36	0.9	Ccdc182	
DMR10:75759001	10	75759001	75762000	3000	1	1.30E-07	-0.43	52	1.73	Msi2	
DMR10:75939001	10	75939001	75941000	2000	1	2.80E-07	-0.39	41	2.05	Msi2	
DMR10:75999001	10	75999001	7.60E+07	1000	1	6.50E-07	-0.44	17	1.7	Msi2	
DMR10:76016001	10	76016001	76018000	2000	2	9.00E-08	-0.48	32	1.6	Msi2	
DMR10:76030001	10	76030001	76032000	2000	1	1.20E-10	0.36	40	2	Msi2;LOC102549226	
DMR10:76325001	10	76325001	76327000	2000	1	3.70E-07	0.33	24	1.2	Coil	Transcription
DMR10:76347001	10	76347001	76350000	3000	1	5.10E-09	-0.69	51	1.7	Trim25	
DMR10:76394001	10	76394001	76396000	2000	1	3.30E-09	-0.72	18	0.9	Dgke;LOC103693414	Signaling
DMR10:76410001	10	76410001	76411000	1000	1	6.50E-09	0.33	1	0.1	Dgke	Signaling
DMR10:77487001	10	77487001	77488000	1000	1	1.60E-08	-0.46	22	2.2	Pctp	
DMR10:77530001	10	77530001	77534000	4000	1	2.60E-08	0.32	40	1	Pctp;Tmem100	
DMR10:77540001	10	77540001	77542000	2000	1	2.50E-10	0.74	34	1.7	Pctp;Tmem100;LOC103693415	
DMR10:77757001	10	77757001	77760000	3000	1	7.20E-08	-0.47	48	1.6	Mmd	Signaling
DMR10:77762001	10	77762001	77765000	3000	1	3.60E-08	-0.43	78	2.6	Mmd;LOC108352118	Signaling
DMR10:77780001	10	77780001	77784000	4000	1	3.00E-13	-0.55	94	2.35	Mmd;LOC108352118	Signaling
DMR10:77787001	10	77787001	77788000	1000	1	2.60E-07	0.4	14	1.4	Mmd;LOC108352118	Signaling
DMR10:77838001	10	77838001	77841000	3000	1	4.80E-08	0.5	54	1.8	LOC103693417;Hlf;LOC103693416	Transcription
DMR10:78064001	10	78064001	78066000	2000	1	5.70E-08	0.57	22	1.1	Stxbp4	
DMR10:78072001	10	78072001	78074000	2000	1	1.90E-07	-0.35	28	1.4	Stxbp4	
DMR10:78139001	10	78139001	78141000	2000	2	5.70E-07	0.3	18	0.9	Stxbp4;Cox11	Transcription
DMR10:78148001	10	78148001	78150000	2000	1	7.30E-08	0.31	32	1.6	Stxbp4;Cox11	Transcription
DMR10:78182001	10	78182001	78185000	3000	1	5.70E-09	-0.37	44	1.47	Cox11;LOC103693419;Tom1l1	Transcription
DMR10:78202001	10	78202001	78204000	2000	1	4.50E-07	-0.34	44	2.2	Tom1l1;LOC100363423	
DMR10:80944001	10	80944001	80946000	2000	1	9.60E-07	-0.55	19	0.95	Car10;LOC100911361	Translation
DMR10:81029001	10	81029001	81033000	4000	1	8.80E-07	-0.45	27	0.68	Car10	
DMR10:81258001	10	81258001	81261000	3000	1	3.90E-08	0.39	36	1.2	Car10	
DMR10:81554001	10	81554001	81558000	4000	1	1.00E-07	-0.61	58	1.45	Utp18	
DMR10:81564001	10	81564001	81568000	4000	1	8.30E-07	-0.6	38	0.95	Utp18	
DMR10:81585001	10	81585001	81587000	2000	2	4.20E-11	-0.43	31	1.55	Utp18;Mbtd1	Epigenetic
DMR10:81815001	10	81815001	81816000	1000	1	7.80E-07	-0.4	12	1.2	Spag9	Cytoskeleton
DMR10:81820001	10	81820001	81821000	1000	1	5.30E-10	-0.43	16	1.6	Spag9	Cytoskeleton
DMR10:81834001	10	81834001	81835000	1000	1	8.10E-07	-0.36	14	1.4	Spag9;LOC103693421	Cytoskeleton
DMR10:81992001	10	81992001	81994000	2000	1	8.00E-08	-0.36	29	1.45	Luc7l3	
DMR10:82006001	10	82006001	82007000	1000	1	1.60E-07	-0.41	45	4.5	Luc7l3	
DMR10:82035001	10	82035001	82038000	3000	1	7.10E-09	-0.47	23	0.77	Luc7l3;Ankrd40;Abcc3	Transport
DMR10:82107001	10	82107001	82108000	1000	1	2.60E-07	-0.44	15	1.5	Abcc3	Transport
DMR10:82115001	10	82115001	82116000	1000	1	2.10E-07	0.5	30	3	Abcc3	Transport
DMR10:82294001	10	82294001	82296000	2000	1	1.80E-07	0.41	33	1.65	Acsf2;Chad	Metabolism;Receptor
DMR10:82433001	10	82433001	82437000	4000	1	1.70E-10	0.71	73	1.82	Trnar-ccu	
DMR10:82665001	10	82665001	82668000	3000	1	4.80E-10	0.44	28	0.93	LOC108352184;RGD1563563	
DMR10:82849001	10	82849001	82851000	2000	1	3.30E-07	-0.51	34	1.7	Pdk2;Itga3	Signaling;Extracellular Matrix
DMR10:82940001	10	82940001	82941000	1000	1	2.10E-07	0.42	21	2.1	Dlx3	Development
DMR10:83394001	10	83394001	83395000	1000	1	2.50E-07	0.33	15	1.5	Ngfr	Receptor
DMR10:83819001	10	83819001	83822000	3000	1	8.30E-07	-0.45	55	1.83	Igf2bp1;LOC102550169	Metabolism
DMR10:83962001	10	83962001	83964000	2000	1	5.10E-12	0.43	19	0.95	Calcoco2;Ttll6	Cytoskeleton
DMR10:84113001	10	84113001	84116000	3000	1	2.30E-08	0.41	32	1.07	LOC100911410;Mir196c;Hoxb9	
DMR10:84165001	10	84165001	84166000	1000	1	3.90E-07	-0.41	23	2.3	Hoxb5os;Mir10a;Hoxb4;Hoxb3	
DMR10:84170001	10	84170001	84175000	5000	1	6.50E-08	-0.42	61	1.22	Mir10a;Hoxb4;Hoxb3	
DMR10:84216001	10	84216001	84218000	2000	1	1.40E-08	0.58	32	1.6	Hoxb1	Development
DMR10:84305001	10	84305001	84306000	1000	1	3.10E-07	0.43	9	0.9	Skap1	Cytoskeleton
DMR10:84358001	10	84358001	84359000	1000	1	5.70E-16	0.98	27	2.7	Skap1	Cytoskeleton
DMR10:84361001	10	84361001	84368000	7000	1	8.90E-07	-0.3	70	1	Skap1	Cytoskeleton
DMR10:84386001	10	84386001	84388000	2000	1	1.50E-07	-0.41	50	2.5	Skap1	Cytoskeleton
DMR10:84390001	10	84390001	84395000	5000	2	1.50E-10	-0.44	121	2.42	Skap1	Cytoskeleton
DMR10:84399001	10	84399001	84404000	5000	2	5.20E-09	-0.59	121	2.42	Skap1	Cytoskeleton
DMR10:84450001	10	84450001	84454000	4000	1	2.30E-07	-0.36	88	2.2	Skap1	Cytoskeleton

DMR10:84471001	10	84471001	84473000	2000	1	6.70E-07	-0.39	35	1.75	Skap1	Cytoskeleton
DMR10:84502001	10	84502001	84503000	1000	1	8.40E-07	-0.43	23	2.3	Skap1	Cytoskeleton
DMR10:84560001	10	84560001	84562000	2000	1	7.60E-08	0.5	30	1.5	Skap1	Cytoskeleton
DMR10:84576001	10	84576001	84577000	1000	1	1.10E-08	0.62	28	2.8	Skap1	Cytoskeleton
DMR10:84600001	10	84600001	84605000	5000	1	8.90E-07	-0.37	101	2.02	Skap1	Cytoskeleton
DMR10:84608001	10	84608001	84610000	2000	1	1.90E-07	-0.42	33	1.65	Skap1	Cytoskeleton
DMR10:84641001	10	84641001	84644000	3000	1	2.00E-08	-0.38	59	1.97	Snx11	
DMR10:84772001	10	84772001	84773000	1000	1	8.40E-09	0.52	12	1.2	Cdk5rap3	
DMR10:84861001	10	84861001	84864000	3000	1	2.50E-07	-0.34	26	0.87	LOC102552748;Prr15l	
DMR10:84912001	10	84912001	84913000	1000	1	3.50E-07	-0.36	12	1.2	LOC102547955;Sp2;LOC102547278	Transcription
DMR10:84951001	10	84951001	84952000	1000	1	2.00E-07	0.43	9	0.9	Sp6	Transcription
DMR10:85057001	10	85057001	85061000	4000	1	1.60E-11	0.47	54	1.35	Tbx21	Transcription
DMR10:85288001	10	85288001	85290000	2000	1	1.10E-09	0.4	42	2.1	Gpr179;LOC108352126	Signaling
DMR10:85293001	10	85293001	85296000	3000	1	3.10E-07	0.59	66	2.2	Gpr179;LOC108352126;Socs7	Signaling;Signaling
DMR10:85376001	10	85376001	85378000	2000	1	3.90E-07	0.4	33	1.65	LOC102557607;Arhgap23	
DMR10:85451001	10	85451001	85456000	5000	1	1.20E-07	0.47	88	1.76	Arhgap23;Srcin1	
DMR10:85461001	10	85461001	85463000	2000	1	8.10E-09	0.47	36	1.8	Srcin1	
DMR10:85720001	10	85720001	85722000	2000	1	3.50E-07	0.49	21	1.05	Trnac-gca;LOC691189;Rpl23	Translation
DMR10:86299001	10	86299001	86304000	5000	1	1.70E-07	0.37	130	2.6	Ppp1r1b;Stard3	Signaling
DMR10:86307001	10	86307001	86309000	2000	1	7.80E-07	-0.32	26	1.3	Ppp1r1b;Stard3	Signaling
DMR10:86419001	10	86419001	86425000	6000	2	2.00E-09	0.53	124	2.07	Ikzf3	Transcription
DMR10:86465001	10	86465001	86470000	5000	1	2.10E-09	0.65	54	1.08	Ikzf3;LOC108352128	Transcription
DMR10:86502001	10	86502001	86503000	1000	1	1.20E-08	0.36	6	0.6	Ikzf3	Transcription
DMR10:86564001	10	86564001	86567000	3000	1	4.40E-07	0.42	77	2.57	Ormdl3;Lrrc3c;Gsdma;LOC108352129	
DMR10:86568001	10	86568001	86570000	2000	2	4.00E-18	0.97	61	3.05	Lrrc3c;Gsdma;LOC108352129	
DMR10:86714001	10	86714001	86715000	1000	1	1.40E-13	-0.59	14	1.4	LOC108352130;Msl1;Casc3	Epigenetic
DMR10:86780001	10	86780001	86784000	4000	1	5.10E-08	-0.41	69	1.73	Wipf2	Cytoskeleton
DMR10:86796001	10	86796001	86800000	4000	1	6.80E-08	-0.44	39	0.98	Wipf2	Cytoskeleton
DMR10:86886001	10	86886001	86888000	2000	1	3.80E-07	-0.43	46	2.3	Rara;Gjd3	Transcription;Cytoskeleton
DMR10:86968001	10	86968001	86971000	3000	2	6.90E-08	0.58	67	2.23	Igfbp4;Tns4	Protease; Proteolysis;Cytoskeleton
DMR10:87000001	10	87000001	87007000	7000	2	2.60E-09	0.56	123	1.76	Tns4	Cytoskeleton
DMR10:87228001	10	87228001	87229000	1000	1	4.70E-07	0.5	20	2	Krt25	
DMR10:87239001	10	87239001	87240000	1000	1	1.40E-09	-0.38	21	2.1	Krt25;Krt26	
DMR10:87248001	10	87248001	87251000	3000	1	1.40E-09	0.6	64	2.13	Krt25;Krt26;Krt27	
DMR10:87335001	10	87335001	87336000	1000	1	2.20E-10	0.79	30	3	Krt12;Krt20;LOC103695157	
DMR10:87382001	10	87382001	87384000	2000	1	3.50E-07	0.42	43	2.15	Krt23	
DMR10:87417001	10	87417001	87418000	1000	1	8.80E-07	0.38	12	1.2	Krt23;LOC108352133	
DMR10:87489001	10	87489001	87491000	2000	2	3.10E-08	0.4	27	1.35	Krtap3-1;Krtap1-5	
DMR10:87496001	10	87496001	87497000	1000	1	5.00E-10	-0.6	9	0.9	Krtap1-5;Krtap1-1	
DMR10:87546001	10	87546001	87548000	2000	1	5.80E-08	-0.69	28	1.4	RGD1561684;LOC680160;LOC100361969	
DMR10:87551001	10	87551001	87552000	1000	1	6.80E-07	0.41	10	1	RGD1561684;LOC680160;LOC100361969	
DMR10:87656001	10	87656001	87657000	1000	1	3.20E-07	-0.51	7	0.7	Cct6a-ps2	
DMR10:87949001	10	87949001	87950000	1000	1	1.50E-07	-0.45	9	0.9	Krt34;Krt31	
DMR10:88011001	10	88011001	88014000	3000	3	4.80E-12	0.54	19	0.63	Krt35;Krt36	
DMR10:88021001	10	88021001	88023000	2000	1	5.90E-07	0.32	16	0.8	Krt36;Krt13	
DMR10:88200001	10	88200001	88204000	4000	1	5.30E-09	0.41	54	1.35	Krt42	
DMR10:88255001	10	88255001	88258000	3000	1	8.00E-08	0.31	35	1.17	Gast;Hap1	Hormone;Transport
DMR10:88281001	10	88281001	88282000	1000	1	9.00E-09	0.52	16	1.6	Jup	
DMR10:88290001	10	88290001	88291000	1000	1	6.60E-08	0.37	26	2.6	Jup	
DMR10:88292001	10	88292001	88294000	2000	2	2.40E-08	0.6	61	3.05	Jup	
DMR10:88308001	10	88308001	88312000	4000	1	1.00E-08	0.45	74	1.85	Jup;LOC108352135;P3h4	Golgi
DMR10:88317001	10	88317001	88324000	7000	1	2.40E-08	0.42	120	1.71	Jup;LOC108352135;P3h4;Fkbp10	Golgi
DMR10:88504001	10	88504001	88508000	4000	1	9.90E-07	-0.4	46	1.15	Cnp;Dnajc7	Signaling;Transcription
DMR10:89046001	10	89046001	89048000	2000	1	9.40E-07	-0.48	22	1.1	Tubg1	Cytoskeleton
DMR10:89078001	10	89078001	89080000	2000	1	9.70E-08	0.78	82	4.1	Tubg2;Plekhh3;Ccr10;Cntnap1	Cytoskeleton
DMR10:89189001	10	89189001	89191000	2000	1	1.50E-12	0.55	52	2.6	Vps25;Wnk4;LOC103693430;Coa3;Cntd1	Transport;Signaling;Transcription
DMR10:89415001	10	89415001	89416000	1000	1	1.70E-07	-0.45	14	1.4	Brca1	Proteolysis

DMR10:89574001	10	89574001	89575000	1000	1	9.40E-10	0.72	12	1.2	Arl4d	Signaling
DMR10:89807001	10	89807001	89808000	1000	1	2.20E-10	0.42	11	1.1	Meox1	Development
DMR10:89908001	10	89908001	89914000	6000	1	6.30E-08	0.33	102	1.7	Sost;Dusp3;RGD1565533	Signaling
DMR10:90312001	10	90312001	90313000	1000	1	2.30E-07	0.32	20	2	Slc4a1	Transport
DMR10:90404001	10	90404001	90407000	3000	1	3.20E-07	0.39	46	1.53	Itga2b	Extracellular Matrix
DMR10:90422001	10	90422001	90427000	5000	1	4.90E-07	0.33	112	2.24	Itga2b;Gpatch8	Extracellular Matrix
DMR10:90623001	10	90623001	90624000	1000	1	3.30E-10	1.02	69	6.9	Meioc	
DMR10:90646001	10	90646001	90648000	2000	1	4.20E-08	-0.6	28	1.4	Meioc;Ccdc43	
DMR10:90665001	10	90665001	90666000	1000	1	2.90E-07	-0.56	19	1.9	Ccdc43	
DMR10:90702001	10	90702001	90703000	1000	1	5.20E-07	0.27	17	1.7	LOC102547686;LOC103693436;Dbf4b	
DMR10:91307001	10	91307001	91309000	2000	1	1.80E-07	0.37	35	1.75	Spata32;Map3k14	Signaling
DMR10:91322001	10	91322001	91327000	5000	1	6.30E-07	-0.46	99	1.98	Map3k14	Signaling
DMR10:91440001	10	91440001	91443000	3000	1	7.30E-09	-0.36	62	2.07	Arhgap27;Plekhm1	Signaling
DMR10:91634001	10	91634001	91638000	4000	1	9.90E-08	-0.36	38	0.95	Prp2l1	Receptor
DMR10:91719001	10	91719001	91721000	2000	1	1.20E-09	-0.8	26	1.3	Rprml	
DMR10:91811001	10	91811001	91812000	1000	1	2.70E-07	0.34	13	1.3	Wnt9b	Signaling
DMR10:91878001	10	91878001	91880000	2000	1	5.10E-09	-0.56	37	1.85	Wnt3;Nsf	Signaling;Transport
DMR10:91887001	10	91887001	91889000	2000	1	1.20E-07	-0.43	42	2.1	Nsf	Transport
DMR10:91938001	10	91938001	91939000	1000	1	1.90E-08	0.39	10	1	Nsf	Transport
DMR10:91994001	10	91994001	91995000	1000	1	6.30E-10	-0.36	20	2	Nsf	Transport
DMR10:91996001	10	91996001	91998000	2000	1	8.10E-07	-0.53	23	1.15	Nsf	Transport
DMR10:92208001	10	92208001	92209000	1000	1	3.50E-10	0.44	2	0.2	Crhr1	Receptor
DMR10:92278001	10	92278001	92280000	2000	1	1.70E-12	0.48	31	1.55	LOC108352187;Mapt	
DMR10:92299001	10	92299001	92300000	1000	1	3.50E-07	0.36	7	0.7	LOC108352187;Mapt;LOC100912629	
DMR10:92308001	10	92308001	92315000	7000	1	2.70E-07	0.43	99	1.41	Mapt;LOC100912629	
DMR10:92496001	10	92496001	92498000	2000	1	7.50E-10	0.4	16	0.8	Kansl1	
DMR10:92587001	10	92587001	92589000	2000	1	2.10E-07	-0.39	22	1.1	Cdc27	Proteolysis
DMR10:92635001	10	92635001	92636000	1000	1	7.00E-09	0.49	34	3.4	Myl4	Cytoskeleton
DMR10:92671001	10	92671001	92672000	1000	1	6.00E-10	0.4	9	0.9	Itgb3	Extracellular Matrix
DMR10:92692001	10	92692001	92693000	1000	1	1.00E-07	0.49	14	1.4	Itgb3	Extracellular Matrix
DMR10:92739001	10	92739001	92742000	3000	1	6.00E-08	-0.27	27	0.9	Itgb3	Extracellular Matrix
DMR10:92813001	10	92813001	92815000	2000	1	1.00E-08	-0.48	22	1.1	LOC102552446;Efcab13	
DMR10:92845001	10	92845001	92849000	4000	1	2.70E-07	-0.49	70	1.75	Efcab13	
DMR10:92868001	10	92868001	92871000	3000	1	7.80E-12	-0.54	39	1.3	Efcab13	
DMR10:92881001	10	92881001	92886000	5000	2	9.40E-08	-0.42	37	0.74	Efcab13	
DMR10:93183001	10	93183001	93184000	1000	1	1.80E-07	-0.59	20	2	Efcab13;LOC686084	
DMR10:93348001	10	93348001	93350000	2000	1	1.80E-09	-0.58	7	0.35	Mettl2b	Epigenetic
DMR10:93567001	10	93567001	93571000	4000	1	6.40E-08	0.61	77	1.93	Mrc2	
DMR10:93601001	10	93601001	93604000	3000	1	1.10E-08	0.43	51	1.7	10-Mar	
DMR10:93680001	10	93680001	93681000	1000	1	7.50E-10	0.49	18	1.8	March10;LOC108352144	
DMR10:93894001	10	93894001	93898000	4000	1	5.60E-08	0.39	37	0.92	Tanc2	
DMR10:93900001	10	93900001	93902000	2000	2	5.80E-08	0.49	17	0.85	Tanc2	
DMR10:93985001	10	93985001	93988000	3000	2	1.20E-08	-0.46	27	0.9	Tanc2	
DMR10:94261001	10	94261001	94262000	1000	1	2.30E-09	-0.57	9	0.9	Dcaf7;Taco1	
DMR10:94403001	10	94403001	94404000	1000	1	1.20E-08	-0.41	15	1.5	Ccdc47;Ddx42	
DMR10:94437001	10	94437001	94441000	4000	1	3.10E-16	-0.68	64	1.6	Ddx42;Ftsj3;Psmc5	Epigenetic;Protease
DMR10:94442001	10	94442001	94444000	2000	1	2.50E-07	-0.41	36	1.8	Ddx42;Ftsj3;Psmc5;Smarcd2	Epigenetic;Protease;Epigenetic
DMR10:94492001	10	94492001	94493000	1000	1	2.20E-11	0.38	8	0.8	Gh1;Cd79b	Hormone;Immune
DMR10:94509001	10	94509001	94510000	1000	1	9.60E-09	0.4	7	0.7	Cd79b;Scn4a	Immune;Transport
DMR10:94514001	10	94514001	94516000	2000	1	4.20E-07	0.31	51	2.55	Scn4a	Transport
DMR10:94679001	10	94679001	94680000	1000	1	1.50E-07	-0.43	13	1.3	Ern1	Translation
DMR10:94702001	10	94702001	94704000	2000	1	6.30E-07	-0.42	22	1.1	LOC102546698;Tex2	
DMR10:94767001	10	94767001	94769000	2000	1	2.50E-07	-0.41	25	1.25	Tex2	
DMR10:94777001	10	94777001	94779000	2000	1	6.20E-08	0.34	18	0.9	Tex2	
DMR10:94998001	10	94998001	94999000	1000	1	3.90E-07	-0.55	7	0.7	Ddx5;Cep95	
DMR10:95010001	10	95010001	95012000	2000	1	6.50E-12	-0.49	29	1.45	Cep95;Smurf2	Proteolysis
DMR10:95040001	10	95040001	95041000	1000	1	4.90E-07	0.37	10	1	Smurf2	Proteolysis
DMR10:95259001	10	95259001	95260000	1000	1	1.00E-07	-0.49	11	1.1	Bptf	
DMR10:95414001	10	95414001	95416000	2000	1	7.60E-08	0.46	36	1.8	Nol11;RGD1562577	
DMR10:95462001	10	95462001	95464000	2000	1	1.50E-07	-0.34	20	1	Pitpnc1	

DMR10:95522001	10	95522001	95525000	3000	1	1.50E-08	0.37	42	1.4	Pitpnc1	
DMR10:96112001	10	96112001	96113000	1000	1	3.90E-09	0.7	20	2	Cacng5	Transport
DMR10:96146001	10	96146001	96149000	3000	1	3.20E-08	0.31	34	1.13	Cacng5	Transport
DMR10:96181001	10	96181001	96183000	2000	1	3.80E-08	0.45	15	0.75	Prkca	Signaling
DMR10:96225001	10	96225001	96226000	1000	1	7.20E-09	0.36	16	1.6	Prkca	Signaling
DMR10:96236001	10	96236001	96237000	1000	1	9.00E-07	0.38	12	1.2	Prkca	Signaling
DMR10:96327001	10	96327001	96328000	1000	1	2.50E-07	0.32	6	0.6	Prkca;Trnaa-agc	Signaling
DMR10:96338001	10	96338001	96341000	3000	1	3.10E-07	0.46	33	1.1	Prkca	Signaling
DMR10:96345001	10	96345001	96346000	1000	1	3.10E-10	0.44	18	1.8	Prkca	Signaling
DMR10:96375001	10	96375001	96376000	1000	1	1.20E-07	0.42	13	1.3	Prkca	Signaling
DMR10:96471001	10	96471001	96475000	4000	1	6.80E-09	0.43	56	1.4	Prkca	Signaling
DMR10:96476001	10	96476001	96478000	2000	1	4.80E-09	-0.46	40	2	Prkca	Signaling
DMR10:96685001	10	96685001	96689000	4000	1	3.50E-08	-0.37	24	0.6	Cep112	
DMR10:96747001	10	96747001	96752000	5000	3	2.30E-08	-0.35	49	0.98	Cep112	
DMR10:96767001	10	96767001	96770000	3000	1	1.50E-08	-0.44	69	2.3	Cep112	
DMR10:96820001	10	96820001	96821000	1000	1	7.40E-07	0.34	8	0.8	Cep112	
DMR10:96868001	10	96868001	96870000	2000	1	4.40E-07	-0.46	35	1.75	Cep112	
DMR10:96900001	10	96900001	96903000	3000	1	1.60E-07	0.48	14	0.47	Cep112	
DMR10:96945001	10	96945001	96948000	3000	1	1.10E-07	-0.43	41	1.37	Cep112;LOC108352152	
DMR10:96959001	10	96959001	96961000	2000	1	6.90E-07	-0.48	18	0.9	Cep112;LOC108352152	
DMR10:97518001	10	97518001	97520000	2000	1	3.50E-07	-0.33	30	1.5	Rgs9	
DMR10:97527001	10	97527001	97528000	1000	1	8.60E-08	0.39	9	0.9	Rgs9	
DMR10:97660001	10	97660001	97662000	2000	1	4.10E-10	-0.48	27	1.35	Gna13	Signaling
DMR10:97700001	10	97700001	97702000	2000	1	5.10E-10	0.45	29	1.45	Amz2	
DMR10:97751001	10	97751001	97753000	2000	1	9.30E-12	0.39	22	1.1	Arsg;Slc16a6	Metabolism;Transport
DMR10:97765001	10	97765001	97766000	1000	1	7.90E-07	0.36	3	0.3	Arsg;Slc16a6	Metabolism;Transport
DMR10:97833001	10	97833001	97835000	2000	1	7.60E-13	0.4	34	1.7	Arsg	Metabolism
DMR10:97836001	10	97836001	97838000	2000	1	3.40E-08	-0.47	37	1.85	Arsg	Metabolism
DMR10:97852001	10	97852001	97854000	2000	2	9.40E-09	0.38	21	1.05	Arsg;Wipi1	Metabolism
DMR10:97856001	10	97856001	97858000	2000	1	2.20E-07	-0.46	44	2.2	Arsg;Wipi1	Metabolism
DMR10:97880001	10	97880001	97881000	1000	1	7.20E-09	-0.54	21	2.1	Wipi1	
DMR10:97965001	10	97965001	97966000	1000	1	4.60E-11	0.71	31	3.1	Prkar1a;Fam20a	Signaling
DMR10:97993001	10	97993001	97996000	3000	1	5.50E-07	-0.23	34	1.13	Fam20a	
DMR10:98085001	10	98085001	98088000	3000	2	3.90E-10	0.63	17	0.57	RGD1559578	
DMR10:98254001	10	98254001	98259000	5000	3	2.10E-08	0.7	63	1.26	Abca8	Transport
DMR10:98293001	10	98293001	98294000	1000	1	5.20E-07	-0.61	11	1.1	Abca8	Transport
DMR10:98309001	10	98309001	98310000	1000	1	1.20E-07	0.46	9	0.9	Abca8;Abca8a	Transport
DMR10:98347001	10	98347001	98350000	3000	1	1.50E-08	-0.38	19	0.63	Abca8a	Transport
DMR10:98373001	10	98373001	98374000	1000	1	1.20E-07	-0.49	17	1.7	Abca8a	Transport
DMR10:98533001	10	98533001	98536000	3000	1	1.70E-07	0.31	47	1.57	Abca6	Transport
DMR10:98589001	10	98589001	98592000	3000	2	6.30E-08	-0.4	20	0.67	Abca5	Transport
DMR10:98638001	10	98638001	98640000	2000	1	1.40E-09	-0.44	19	0.95	Abca5	Transport
DMR10:98696001	10	98696001	98699000	3000	1	3.10E-07	0.41	36	1.2	Map2k6	Signaling
DMR10:98702001	10	98702001	98703000	1000	1	1.00E-07	0.32	22	2.2	Map2k6	Signaling
DMR10:98716001	10	98716001	98718000	2000	1	1.90E-07	0.65	54	2.7	Map2k6	Signaling
DMR10:98805001	10	98805001	98806000	1000	1	8.80E-13	0.65	26	2.6	Map2k6	Signaling
DMR10:101804001	10	101804001	101806000	2000	1	8.80E-07	0.44	32	1.6	Slc39a11	Transport
DMR10:101844001	10	101844001	101850000	6000	1	2.30E-07	-0.37	116	1.93	Slc39a11	Transport
DMR10:101929001	10	101929001	101931000	2000	1	8.40E-08	-0.43	48	2.4	Slc39a11	Transport
DMR10:101997001	10	101997001	102005000	8000	1	4.60E-09	-0.46	118	1.48	Slc39a11	Transport
DMR10:102011001	10	102011001	102012000	1000	1	1.20E-08	0.5	14	1.4	Slc39a11	Transport
DMR10:102061001	10	102061001	102063000	2000	1	9.70E-08	0.47	40	2	Slc39a11	Transport
DMR10:102134001	10	102134001	102136000	2000	1	1.20E-07	0.57	64	3.2	Slc39a11;Sstr2	Transport;Signaling
DMR10:102141001	10	102141001	102143000	2000	1	1.50E-09	0.25	63	3.15	Slc39a11;Sstr2	Transport;Signaling
DMR10:102145001	10	102145001	102147000	2000	1	2.60E-08	0.4	43	2.15	Slc39a11;Sstr2	Transport;Signaling
DMR10:102224001	10	102224001	102230000	6000	3	2.60E-10	0.65	87	1.45	Cpsf4l	
DMR10:102303001	10	102303001	102306000	3000	1	9.70E-12	0.59	50	1.67	Sdk2	
DMR10:102381001	10	102381001	102384000	3000	1	4.00E-10	0.47	48	1.6	Sdk2	
DMR10:102393001	10	102393001	102397000	4000	1	1.30E-11	0.94	97	2.42	Sdk2	
DMR10:102400001	10	102400001	102401000	1000	1	4.40E-07	0.35	9	0.9	Sdk2	
DMR10:102431001	10	102431001	102432000	1000	1	4.00E-07	0.36	24	2.4	Sdk2	
DMR10:102434001	10	102434001	102438000	4000	1	5.40E-08	0.54	60	1.5	Sdk2	
DMR10:102451001	10	102451001	102452000	1000	1	9.40E-07	0.38	5	0.5	Sdk2	

DMR10:102486001	10	102486001	102488000	2000	1	5.30E-09	0.55	21	1.05	Sdk2	
DMR10:102490001	10	102490001	102494000	4000	2	3.30E-14	0.58	47	1.18	Sdk2	
DMR10:102496001	10	102496001	102497000	1000	1	1.10E-08	-0.49	24	2.4	Sdk2	
DMR10:102498001	10	102498001	102501000	3000	1	1.80E-07	0.44	35	1.17	Sdk2	
DMR10:102519001	10	102519001	102520000	1000	1	7.30E-10	-0.37	28	2.8	Sdk2	
DMR10:103191001	10	103191001	103192000	1000	1	7.50E-10	0.58	28	2.8	Rpl38	Translation
DMR10:103225001	10	103225001	103229000	4000	1	9.10E-08	0.32	87	2.17	Ttyh2	Transport
DMR10:103243001	10	103243001	103245000	2000	1	5.60E-08	0.29	38	1.9	Ttyh2	Transport
DMR10:103283001	10	103283001	103287000	4000	1	5.10E-09	0.4	74	1.85	Dnai2	Cytoskeleton
DMR10:103461001	10	103461001	103464000	3000	1	5.10E-14	0.74	64	2.13	Cd300a;Cd300lb	Immune
DMR10:103575001	10	103575001	103577000	2000	1	8.80E-07	0.61	28	1.4	Cd300c;Cd300le	Immune
DMR10:103598001	10	103598001	103600000	2000	1	3.20E-07	-0.4	78	3.9	Cd300le;Cd300e	Immune
DMR10:103648001	10	103648001	103649000	1000	1	7.00E-07	0.38	10	1	Rab37	
DMR10:103786001	10	103786001	103787000	1000	1	1.20E-07	0.49	16	1.6	Tmem104;LOC102554289	
DMR10:103817001	10	103817001	103820000	3000	1	4.80E-08	0.51	74	2.47	Grin2c;Fdxr	Receptor;Metabolism
DMR10:104010001	10	104010001	104012000	2000	1	5.20E-07	0.6	34	1.7	Trim80;Slc16a5	Transport
DMR10:104133001	10	104133001	104134000	1000	1	5.40E-08	0.42	11	1.1	Nup85;Gga3	Development
DMR10:104350001	10	104350001	104351000	1000	1	5.10E-09	0.42	9	0.9	Mir3577;Caskin2;Tsen54	Cytoskeleton;Translation
DMR10:104370001	10	104370001	104372000	2000	1	4.60E-07	-0.58	37	1.85	Tsen54;Llgl2	Translation;Transport
DMR10:104408001	10	104408001	104410000	2000	1	3.30E-07	0.46	21	1.05	Llgl2;LOC690323	Transport
DMR10:104434001	10	104434001	104436000	2000	2	8.60E-08	0.61	49	2.45	LOC690323;Recql5	Epigenetic
DMR10:104538001	10	104538001	104540000	2000	2	1.30E-13	0.65	54	2.7	Itgb4	Extracellular Matrix
DMR10:104565001	10	104565001	104566000	1000	1	4.20E-09	0.55	11	1.1	Itgb4;Galk1;H3f3b	Extracellular Matrix;Metabolism
DMR10:104601001	10	104601001	104602000	1000	1	4.80E-07	0.45	10	1	Unk	
DMR10:104639001	10	104639001	104640000	1000	1	3.60E-10	0.43	13	1.3	Wbp2;LOC102552044	
DMR10:104861001	10	104861001	104862000	1000	1	4.70E-12	0.38	7	0.7	Cd300ld	
DMR10:104878001	10	104878001	104881000	3000	1	4.20E-07	-0.3	16	0.53	Cd300ld	
DMR10:105051001	10	105051001	105052000	1000	1	3.20E-07	0.46	12	1.2	RGD1565046	
DMR10:105109001	10	105109001	105111000	2000	1	2.30E-07	0.52	31	1.55	Evpl	Cytoskeleton
DMR10:105319001	10	105319001	105321000	2000	1	1.10E-07	-0.4	33	1.65	Rnf157	Proteolysis
DMR10:105497001	10	105497001	105499000	2000	1	4.60E-07	0.32	24	1.2	Prpsap1;Khps1a;Sphk1;Ube2o	Signaling;Signaling
DMR10:105613001	10	105613001	105614000	1000	1	2.80E-17	0.56	17	1.7	Cygb	
DMR10:105637001	10	105637001	105639000	2000	1	1.50E-08	0.35	25	1.25	Cygb;Prcd;LOC100912247;LOC108352161	
DMR10:105669001	10	105669001	105671000	2000	1	1.40E-11	0.63	57	2.85	St6galnac2	
DMR10:105733001	10	105733001	105734000	1000	1	6.00E-07	0.32	17	1.7	St6galnac1;Mxra7	
DMR10:105775001	10	105775001	105780000	5000	2	3.10E-09	0.8	76	1.52	Mxra7;Jmjd6;Mettl23	Golgi
DMR10:105876001	10	105876001	105878000	2000	2	1.70E-09	0.59	60	3	Mgat5b;LOC108352162	Golgi
DMR10:105886001	10	105886001	105887000	1000	1	2.60E-08	0.37	4	0.4	Mgat5b;LOC108352162	Golgi
DMR10:105917001	10	105917001	105920000	3000	1	9.40E-07	0.3	31	1.03	Mgat5b	Golgi
DMR10:106067001	10	106067001	106070000	3000	1	7.80E-09	-0.54	51	1.7	Sec14l1	
DMR10:106075001	10	106075001	106076000	1000	1	6.90E-12	0.57	13	1.3	Sec14l1	
DMR10:106088001	10	106088001	106092000	4000	1	6.50E-07	-0.39	53	1.32	Sec14l1	
DMR10:106120001	10	106120001	106121000	1000	1	4.30E-08	0.39	18	1.8	Sec14l1;LOC102547461	
DMR10:106255001	10	106255001	106258000	3000	1	3.20E-09	0.37	50	1.67	9-Sep	
DMR10:106821001	10	106821001	106823000	2000	1	4.60E-11	0.42	39	1.95	Syngr2;Tk1;Afmid	Transport;Signaling;Metabolism
DMR10:106824001	10	106824001	106825000	1000	1	1.60E-09	0.42	17	1.7	Syngr2;Tk1;Afmid	Transport;Signaling;Metabolism
DMR10:106838001	10	106838001	106839000	1000	1	8.90E-08	-0.42	25	2.5	Tk1;Afmid;LOC102548046	Signaling;Metabolism
DMR10:107038001	10	107038001	107041000	3000	1	6.40E-08	0.4	102	3.4	Pgs1;Dnah17	Transport;Cytoskeleton
DMR10:107054001	10	107054001	107055000	1000	1	4.10E-08	0.29	11	1.1	Dnah17	Cytoskeleton
DMR10:107337001	10	107337001	107342000	5000	1	3.30E-13	0.57	95	1.9	Timp2	Protease; Proteolysis
DMR10:107484001	10	107484001	107488000	4000	2	3.70E-10	0.49	49	1.23	C1qtnf1	
DMR10:107501001	10	107501001	107502000	1000	1	9.40E-10	0.57	14	1.4	Engase	Metabolism
DMR10:107524001	10	107524001	107526000	2000	1	5.00E-07	0.37	26	1.3	Engase;Rbfox3	Metabolism;Translation
DMR10:107589001	10	107589001	107591000	2000	1	2.10E-07	0.28	20	1	Rbfox3	Translation
DMR10:107643001	10	107643001	107644000	1000	1	1.20E-09	0.76	19	1.9	Rbfox3	Translation
DMR10:107653001	10	107653001	107655000	2000	1	3.20E-09	0.51	18	0.9	Rbfox3	Translation
DMR10:107665001	10	107665001	107668000	3000	1	5.10E-07	0.39	40	1.33	Rbfox3	Translation
DMR10:107740001	10	107740001	107742000	2000	1	4.60E-09	0.38	19	0.95	Rbfox3	Translation
DMR10:107793001	10	107793001	107795000	2000	1	7.60E-09	0.45	35	1.75	Rbfox3	Translation

DMR10:107819001	10	107819001	107821000	2000	1	8.70E-07	0.36	23	1.15	Rbfox3	Translation
DMR10:108060001	10	108060001	108064000	4000	1	2.90E-07	0.35	63	1.57	LOC108348054;Ccdc40	
DMR10:108086001	10	108086001	108087000	1000	1	8.60E-07	0.3	12	1.2	Ccdc40;Enpp7	
DMR10:108098001	10	108098001	108100000	2000	1	9.00E-07	0.35	32	1.6	Ccdc40;Enpp7	
DMR10:108342001	10	108342001	108343000	1000	1	6.40E-07	-0.52	24	2.4	Ccdc40	
DMR10:108435001	10	108435001	108437000	2000	1	9.70E-07	0.33	16	0.8	Eif4a3;LOC108352165;Card14	
DMR10:108480001	10	108480001	108482000	2000	1	2.00E-08	0.48	28	1.4	Sgsh;Slc26a11	Transport
DMR10:108554001	10	108554001	108556000	2000	1	6.50E-09	0.41	27	1.35	Rnf213	
DMR10:108567001	10	108567001	108571000	4000	1	9.90E-09	0.48	38	0.95	Rnf213	
DMR10:108780001	10	108780001	108783000	3000	1	4.40E-08	-0.43	43	1.43	Rptor	
DMR10:109054001	10	109054001	109056000	2000	1	5.50E-08	0.51	28	1.4	Rptor;LOC108352229	
DMR10:109227001	10	109227001	109230000	3000	2	2.70E-12	0.44	29	0.97	Aatk	Signaling
DMR10:109232001	10	109232001	109233000	1000	1	3.20E-08	-0.43	25	2.5	Aatk	Signaling
DMR10:109303001	10	109303001	109305000	2000	1	5.70E-08	0.39	30	1.5	Slc38a10	Transport
DMR10:109331001	10	109331001	109332000	1000	1	9.30E-08	0.3	14	1.4	Slc38a10	Transport
DMR10:109428001	10	109428001	109431000	3000	1	4.10E-10	-0.47	75	2.5	Bahcc1	Transcription
DMR10:109444001	10	109444001	109447000	3000	1	2.10E-07	0.59	50	1.67	Bahcc1	Transcription
DMR10:110131001	10	110131001	110134000	3000	1	2.20E-14	0.47	47	1.57	LOC102550455;LOC100364110;Slc16a3	Transport
DMR10:110276001	10	110276001	110277000	1000	1	8.20E-07	-0.44	23	2.3	Sectm1a	
DMR10:110391001	10	110391001	110392000	1000	1	4.10E-11	-0.59	22	2.2	Ogfod3	Golgi
DMR10:110493001	10	110493001	110494000	1000	1	5.00E-08	-0.58	9	0.9	Foxk2	
DMR10:110683001	10	110683001	110685000	2000	1	3.30E-07	-0.47	17	0.85	Tbcd	Transcription
DMR10:110736001	10	110736001	110737000	1000	1	2.90E-07	-0.53	20	2	Tbcd	Transcription
DMR10:110792001	10	110792001	110794000	2000	1	1.80E-08	-0.38	18	0.9	Tbcd;B3gntl1	Transcription;Golgi
DMR10:110913001	10	110913001	110914000	1000	1	4.40E-07	-0.36	16	1.6	Metrnl	
DMR11:604001	11	604001	609000	5000	1	6.50E-07	-0.32	34	0.68	Epha3	Receptor
DMR11:723001	11	723001	725000	2000	1	3.30E-07	-0.53	19	0.95	Epha3	Receptor
DMR11:1421001	11	1421001	1426000	5000	1	4.70E-09	-0.6	29	0.58	Csnka2ip	
DMR11:1432001	11	1432001	1434000	2000	1	1.30E-08	0.41	16	0.8	Csnka2ip	
DMR11:1437001	11	1437001	1439000	2000	2	1.10E-12	0.89	42	2.1	Csnka2ip	
DMR11:1453001	11	1453001	1457000	4000	1	8.30E-14	-0.53	24	0.6	Csnka2ip	
DMR11:1519001	11	1519001	1521000	2000	1	7.40E-09	-0.77	10	0.5	Csnka2ip	
DMR11:1577001	11	1577001	1580000	3000	2	9.40E-08	-0.46	22	0.73	Csnka2ip	
DMR11:4268001	11	4268001	4273000	5000	2	7.10E-10	-0.45	48	0.96	Cadm2	
DMR11:7213001	11	7213001	7216000	3000	1	4.20E-07	0.32	30	1	Gbe1	Metabolism
DMR11:7380001	11	7380001	7381000	1000	1	1.40E-08	0.6	2	0.2	Gbe1;LOC102549549	Metabolism
DMR11:7456001	11	7456001	7457000	1000	1	6.00E-07	0.43	10	1	Gbe1	Metabolism
DMR11:9085001	11	9085001	9087000	2000	1	1.70E-08	-0.51	10	0.5	Robo1	
DMR11:9186001	11	9186001	9188000	2000	1	6.60E-08	-0.35	13	0.65	Robo1	
DMR11:9274001	11	9274001	9276000	2000	1	1.40E-11	0.41	16	0.8	Robo1	
DMR11:9294001	11	9294001	9295000	1000	1	8.50E-08	0.42	13	1.3	Robo1	
DMR11:9316001	11	9316001	9317000	1000	1	4.30E-08	-0.29	5	0.5	Robo1	
DMR11:9332001	11	9332001	9333000	1000	1	7.30E-13	0.38	4	0.4	Robo1	
DMR11:9411001	11	9411001	9412000	1000	1	5.00E-07	0.46	11	1.1	Robo1	
DMR11:9497001	11	9497001	9499000	2000	1	3.80E-14	0.7	26	1.3	Robo1	
DMR11:9604001	11	9604001	9606000	2000	1	2.00E-08	0.29	24	1.2	Robo1;LOC102551469	
DMR11:9812001	11	9812001	9813000	1000	1	6.80E-09	0.57	22	2.2	Robo1	
DMR11:9823001	11	9823001	9825000	2000	2	2.60E-07	0.3	12	0.6	Robo1	
DMR11:9878001	11	9878001	9885000	7000	2	7.80E-09	-0.29	83	1.19	Robo1	
DMR11:9929001	11	9929001	9930000	1000	1	1.50E-08	0.44	8	0.8	Robo1	
DMR11:9936001	11	9936001	9938000	2000	1	9.70E-08	0.27	31	1.55	Robo1	
DMR11:10052001	11	10052001	10053000	1000	1	1.10E-07	-0.59	14	1.4	Robo1	
DMR11:10054001	11	10054001	10057000	3000	1	1.30E-09	-0.81	52	1.73	Robo1	
DMR11:10087001	11	10087001	10094000	7000	1	2.30E-09	-0.73	117	1.67	Robo1	
DMR11:10124001	11	10124001	10126000	2000	1	2.10E-08	-0.45	49	2.45	Robo1	
DMR11:11124001	11	11124001	11126000	2000	1	6.70E-09	0.42	18	0.9	Robo2	
DMR11:11576001	11	11576001	11577000	1000	1	6.10E-11	0.63	15	1.5	Robo2	
DMR11:11666001	11	11666001	11669000	3000	1	3.40E-08	0.35	41	1.37	Robo2	
DMR11:11784001	11	11784001	11785000	1000	1	4.10E-09	0.57	5	0.5	Robo2	
DMR11:11801001	11	11801001	11802000	1000	1	5.90E-07	0.58	15	1.5	Robo2	
DMR11:11900001	11	11900001	11901000	1000	1	2.80E-07	-0.47	13	1.3	Robo2	
DMR11:11902001	11	11902001	11905000	3000	1	3.70E-08	-0.59	44	1.47	Robo2	

DMR11:11913001	11	11913001	11915000	2000	1	1.90E-09	0.47	9	0.45	Robo2	
DMR11:12002001	11	12002001	12004000	2000	1	4.00E-07	-0.42	14	0.7	Robo2	
DMR11:12130001	11	12130001	12132000	2000	1	2.30E-08	-0.52	3	0.15	Robo2	
DMR11:12172001	11	12172001	12174000	2000	1	3.80E-07	0.41	17	0.85	Robo2	
DMR11:12272001	11	12272001	12276000	4000	1	8.20E-07	-0.49	39	0.98	Robo2;LOC680624	
DMR11:12410001	11	12410001	12411000	1000	1	5.00E-07	-0.5	9	0.9	Robo2	
DMR11:12518001	11	12518001	12523000	5000	1	1.30E-08	-0.45	23	0.46	Robo2	
DMR11:12596001	11	12596001	12597000	1000	1	1.10E-07	0.53	4	0.4	Robo2	
DMR11:14238001	11	14238001	14243000	5000	1	4.30E-07	-0.33	43	0.86	Samsn1	
DMR11:14251001	11	14251001	14253000	2000	1	9.60E-07	0.33	7	0.35	Samsn1	
DMR11:14382001	11	14382001	14384000	2000	1	3.60E-07	0.38	19	0.95	Samsn1	
DMR11:14410001	11	14410001	14411000	1000	1	2.40E-07	-0.72	8	0.8	Samsn1	
DMR11:14415001	11	14415001	14418000	3000	1	3.00E-10	-0.36	29	0.97	Samsn1	
DMR11:14699001	11	14699001	14701000	2000	1	1.30E-07	-0.42	37	1.85	Nrip1	
DMR11:14720001	11	14720001	14729000	9000	1	6.60E-08	-0.5	124	1.38	Nrip1	
DMR11:14732001	11	14732001	14738000	6000	2	7.10E-11	-0.7	104	1.73	Nrip1	
DMR11:14739001	11	14739001	14740000	1000	1	1.10E-07	-0.46	16	1.6	Nrip1	
DMR11:15474001	11	15474001	15478000	4000	1	3.30E-08	-0.29	46	1.15	Usp25	Protease
DMR11:16097001	11	16097001	16098000	1000	1	2.00E-07	-0.58	12	1.2	Mir3588;Mir125b-2	
DMR11:16886001	11	16886001	16888000	2000	1	1.30E-08	-0.62	12	0.6	Btg3	
DMR11:17585001	11	17585001	17590000	5000	1	3.60E-07	-0.24	66	1.32	Tmprss15	Protease
DMR11:17598001	11	17598001	17599000	1000	1	1.90E-10	0.54	21	2.1	Tmprss15	Protease
DMR11:19713001	11	19713001	19714000	1000	1	5.70E-07	-0.32	11	1.1	Ncam2	
DMR11:19758001	11	19758001	19760000	2000	1	3.30E-07	-0.51	6	0.3	Ncam2;LOC681289	
DMR11:19770001	11	19770001	19772000	2000	1	1.90E-10	0.39	14	0.7	Ncam2;LOC681289	
DMR11:19828001	11	19828001	19829000	1000	1	7.70E-07	0.42	5	0.5	Ncam2	
DMR11:19854001	11	19854001	19856000	2000	1	1.50E-07	0.39	20	1	Ncam2	
DMR11:19942001	11	19942001	19944000	2000	1	7.70E-08	0.44	12	0.6	Ncam2	
DMR11:19971001	11	19971001	19972000	1000	1	5.90E-08	0.55	11	1.1	Ncam2	
DMR11:20082001	11	20082001	20084000	2000	2	1.20E-07	-0.38	19	0.95	Ncam2	
DMR11:20188001	11	20188001	20189000	1000	1	4.70E-07	0.5	13	1.3	Ncam2	
DMR11:20447001	11	20447001	20452000	5000	1	9.80E-07	-0.2	46	0.92	Ncam2	
DMR11:20486001	11	20486001	20487000	1000	1	3.70E-08	0.44	10	1	Ncam2	
DMR11:20574001	11	20574001	20576000	2000	1	2.40E-07	0.47	23	1.15	Ncam2	
DMR11:20579001	11	20579001	20582000	3000	1	2.90E-07	0.39	29	0.97	Ncam2	
DMR11:24327001	11	24327001	24333000	6000	1	3.90E-07	-0.42	80	1.33	Gabpa	Transcription
DMR11:24412001	11	24412001	24416000	4000	1	4.90E-11	0.52	46	1.15	App	Protease; Proteolysis
DMR11:24418001	11	24418001	24420000	2000	1	6.70E-08	0.34	13	0.65	App	Protease; Proteolysis
DMR11:24479001	11	24479001	24481000	2000	1	7.00E-18	0.88	54	2.7	App	Protease; Proteolysis
DMR11:24625001	11	24625001	24627000	2000	1	8.00E-08	-0.42	35	1.75	App	Protease; Proteolysis
DMR11:24984001	11	24984001	24985000	1000	1	3.70E-07	0.38	13	1.3	Cyyr1	
DMR11:25087001	11	25087001	25088000	1000	1	2.00E-09	0.42	5	0.5	Cyyr1	
DMR11:25401001	11	25401001	25402000	1000	1	6.00E-09	0.38	7	0.7	Adamts5	Protease
DMR11:25460001	11	25460001	25462000	2000	1	5.70E-07	0.31	23	1.15	Adamts5;LOC102552725	Protease
DMR11:27219001	11	27219001	27220000	1000	1	9.40E-07	-0.56	8	0.8	Map3k7cl	
DMR11:27819001	11	27819001	27823000	4000	1	1.90E-10	0.42	49	1.23	Grik1;LOC108352302	Receptor
DMR11:28009001	11	28009001	28012000	3000	1	5.00E-08	0.56	37	1.23	Grik1	Receptor
DMR11:28127001	11	28127001	28128000	1000	1	1.40E-07	-0.52	16	1.6	Grik1;LOC108352301	Receptor
DMR11:28699001	11	28699001	28701000	2000	1	6.10E-13	0.82	26	1.3	Krtap13-1	Development
DMR11:28731001	11	28731001	28733000	2000	1	6.50E-09	0.57	30	1.5	LOC100363287;Krtap14l;Krtap13-2	Development
DMR11:29608001	11	29608001	29614000	6000	1	7.00E-08	-0.27	64	1.07	Krtap8-1	
DMR11:29963001	11	29963001	29965000	2000	1	3.40E-08	-0.34	60	3	Tiam1	
DMR11:30438001	11	30438001	30444000	6000	1	2.10E-07	0.49	87	1.45	Scaf4;LOC108352372	Translation
DMR11:30552001	11	30552001	30556000	4000	1	1.00E-07	-0.52	67	1.68	Hunk	
DMR11:30608001	11	30608001	30610000	2000	1	5.20E-07	-0.43	31	1.55	Hunk	
DMR11:30669001	11	30669001	30670000	1000	1	9.80E-18	0.36	8	0.8	Hunk	
DMR11:30982001	11	30982001	30989000	7000	1	2.70E-07	-0.53	94	1.34	Urb1	
DMR11:31041001	11	31041001	31042000	1000	1	2.60E-09	0.35	6	0.6	Eva1c	
DMR11:31109001	11	31109001	31111000	2000	1	1.40E-07	-0.42	28	1.4	RGD1306954;Synj1	Signaling
DMR11:31161001	11	31161001	31162000	1000	1	1.20E-08	-0.6	10	1	Synj1	Signaling
DMR11:31517001	11	31517001	31519000	2000	1	1.50E-07	0.35	32	1.6	Ifnar2	Receptor
DMR11:31769001	11	31769001	31772000	3000	1	6.00E-10	-0.53	56	1.87	Dnajc28;Gart	Transport
DMR11:31817001	11	31817001	31819000	2000	1	8.40E-08	-0.63	42	2.1	Son	Translation

DMR11:31822001	11	31822001	31824000	2000	1	9.60E-08	-0.4	24	1.2	Son	Translation
DMR11:31861001	11	31861001	31865000	4000	1	6.20E-10	0.43	26	0.65	Cryzl1	Metabolism
DMR11:31884001	11	31884001	31886000	2000	1	2.80E-08	0.45	27	1.35	Cryzl1	Metabolism
DMR11:31938001	11	31938001	31942000	4000	1	9.00E-07	-0.36	43	1.07	Itsn1	Transport
DMR11:31943001	11	31943001	31947000	4000	1	4.90E-10	-0.56	54	1.35	Itsn1	Transport
DMR11:31994001	11	31994001	31995000	1000	1	5.70E-09	-0.51	25	2.5	Itsn1	Transport
DMR11:32204001	11	32204001	32205000	1000	1	7.00E-07	0.39	15	1.5	Mrps6	Translation
DMR11:32269001	11	32269001	32271000	2000	1	6.30E-08	-0.41	30	1.5	Mrps6	Translation
DMR11:32434001	11	32434001	32437000	3000	1	1.70E-12	-0.52	46	1.53	LOC102548387;Kcne2	Transport
DMR11:32452001	11	32452001	32456000	4000	1	2.20E-12	0.49	68	1.7	Kcne2;Smim11	Transport
DMR11:32495001	11	32495001	32496000	1000	1	5.00E-08	0.45	4	0.4	Kcne1;LOC108352307	Transport
DMR11:32592001	11	32592001	32593000	1000	1	9.70E-09	0.38	18	1.8	Rcan1	Signaling
DMR11:32686001	11	32686001	32687000	1000	1	2.10E-08	0.46	11	1.1	Clic6	Transport
DMR11:32755001	11	32755001	32756000	1000	1	1.30E-08	0.35	7	0.7	Runx1	Transcription
DMR11:32769001	11	32769001	32772000	3000	1	2.10E-09	0.83	90	3	Runx1	Transcription
DMR11:32847001	11	32847001	32855000	8000	2	2.10E-08	-0.48	176	2.2	Runx1	Transcription
DMR11:32878001	11	32878001	32879000	1000	1	4.00E-07	0.39	3	0.3	Runx1	Transcription
DMR11:32886001	11	32886001	32889000	3000	1	1.20E-07	0.47	38	1.27	Runx1	Transcription
DMR11:32939001	11	32939001	32941000	2000	1	1.10E-07	-0.52	25	1.25	Runx1	Transcription
DMR11:32976001	11	32976001	32983000	7000	3	7.60E-08	-0.53	86	1.23	Runx1	Transcription
DMR11:34031001	11	34031001	34035000	4000	1	1.00E-06	-0.35	62	1.55	Dopey2;Ifitm7	
DMR11:34129001	11	34129001	34130000	1000	1	4.60E-09	-0.46	7	0.7	Chaf1b;Cldn14	Epigenetic;Cell Junction
DMR11:34326001	11	34326001	34329000	3000	2	3.50E-09	-0.6	67	2.23	Sim2	Transcription
DMR11:34566001	11	34566001	34567000	1000	1	1.70E-07	-0.4	7	0.7	Ripply3	
DMR11:34624001	11	34624001	34625000	1000	1	6.50E-07	-0.52	16	1.6	Ttc3	Proteolysis
DMR11:34636001	11	34636001	34637000	1000	1	8.90E-10	-0.74	6	0.6	Ttc3	Proteolysis
DMR11:34733001	11	34733001	34734000	1000	1	7.50E-08	-0.55	12	1.2	Ttc3;LOC108352310	Proteolysis
DMR11:34901001	11	34901001	34903000	2000	1	5.40E-09	-0.43	21	1.05	LOC103693522;Dyrk1a	
DMR11:34945001	11	34945001	34947000	2000	1	2.50E-08	-0.4	27	1.35	Dyrk1a	
DMR11:35017001	11	35017001	35020000	3000	2	7.40E-18	0.67	62	2.07	Kcnj6	Transport
DMR11:35039001	11	35039001	35040000	1000	1	3.40E-09	0.46	7	0.7	Kcnj6	Transport
DMR11:35083001	11	35083001	35089000	6000	1	3.40E-07	0.47	89	1.48	Kcnj6	Transport
DMR11:35147001	11	35147001	35149000	2000	1	2.50E-07	0.44	36	1.8	Kcnj6	Transport
DMR11:35197001	11	35197001	35201000	4000	1	2.90E-08	-0.36	72	1.8	Kcnj6	Transport
DMR11:35229001	11	35229001	35230000	1000	1	6.60E-10	0.44	14	1.4	Kcnj6	Transport
DMR11:35535001	11	35535001	35536000	1000	1	4.10E-07	0.34	4	0.4	LOC102550135;Kcnj15	Transport
DMR11:35641001	11	35641001	35644000	3000	1	3.20E-07	0.32	43	1.43	Erg	Transcription
DMR11:36329001	11	36329001	36332000	3000	1	3.20E-07	0.37	48	1.6	Psmg1;Brwd1	Transcription
DMR11:36378001	11	36378001	36380000	2000	1	1.70E-07	-0.48	24	1.2	Brwd1;Mir6324	
DMR11:36588001	11	36588001	36589000	1000	1	9.00E-08	0.48	38	3.8	Sh3bgr	
DMR11:36647001	11	36647001	36648000	1000	1	1.20E-07	-0.41	19	1.9	B3galt5	Golgi
DMR11:36785001	11	36785001	36787000	2000	1	2.90E-07	0.44	19	0.95	Igsf5;LOC689402	Immune
DMR11:36903001	11	36903001	36904000	1000	1	2.00E-07	-0.57	15	1.5	Pcp4	
DMR11:37133001	11	37133001	37136000	3000	1	1.10E-09	0.42	14	0.47	Dscam	Cytoskeleton
DMR11:37207001	11	37207001	37208000	1000	1	8.90E-10	0.4	2	0.2	Dscam	Cytoskeleton
DMR11:37345001	11	37345001	37346000	1000	1	7.00E-08	0.46	8	0.8	Dscam	Cytoskeleton
DMR11:37803001	11	37803001	37804000	1000	1	2.00E-07	-0.41	9	0.9	Bace2	Protease
DMR11:38210001	11	38210001	38211000	1000	1	1.60E-08	0.4	12	1.2	Oaz1-ps	
DMR11:38373001	11	38373001	38375000	2000	1	5.60E-11	-0.53	19	0.95	LOC108352316;C2cd2	
DMR11:38531001	11	38531001	38533000	2000	1	1.70E-07	-0.33	19	0.95	LOC108352317;Nsun3	Epigenetic
DMR11:38548001	11	38548001	38550000	2000	1	9.40E-23	0.64	34	1.7	LOC108352317;Nsun3;LOC103693528	Epigenetic
DMR11:38747001	11	38747001	38754000	7000	2	2.80E-08	-0.31	64	0.91	Zfp758	
DMR11:39763001	11	39763001	39764000	1000	1	4.80E-08	0.45	10	1	LOC108352320;RGD1565472	
DMR11:41888001	11	41888001	41889000	1000	1	3.80E-09	-0.58	10	1	Epha6;LOC102556000	Receptor
DMR11:41906001	11	41906001	41907000	1000	1	1.60E-07	-0.41	12	1.2	Epha6	Receptor
DMR11:42190001	11	42190001	42192000	2000	1	3.80E-13	0.61	19	0.95	Epha6	Receptor
DMR11:42554001	11	42554001	42556000	2000	1	1.90E-07	0.38	18	0.9	Epha6	Receptor
DMR11:42564001	11	42564001	42568000	4000	3	4.90E-09	-0.55	40	1	Epha6	Receptor
DMR11:42602001	11	42602001	42604000	2000	1	1.80E-07	-0.41	19	0.95	Epha6	Receptor
DMR11:42615001	11	42615001	42617000	2000	2	2.00E-11	0.65	43	2.15	Epha6	Receptor
DMR11:42647001	11	42647001	42648000	1000	1	6.60E-07	0.41	12	1.2	Epha6	Receptor
DMR11:43091001	11	43091001	43092000	1000	1	4.70E-07	0.28	11	1.1	Gabrr3	Ion Channel

DMR11:43105001	11	43105001	43108000	3000	1	1.90E-07	-0.28	31	1.03	Gabrr3	Ion Channel
DMR11:43155001	11	43155001	43158000	3000	1	8.20E-08	0.4	28	0.93	Olr1529;Olr1530	Signaling
DMR11:43289001	11	43289001	43291000	2000	1	2.20E-07	-0.26	16	0.8	Olr1538	Signaling
DMR11:43299001	11	43299001	43305000	6000	2	3.90E-07	-0.38	51	0.85	Olr1537	
DMR11:43513001	11	43513001	43516000	3000	1	7.00E-08	-0.48	9	0.3	Olr1550-ps;Olr1551	Signaling
DMR11:43534001	11	43534001	43536000	2000	1	3.30E-08	-0.4	16	0.8	Olr1552-ps	
DMR11:43553001	11	43553001	43559000	6000	1	2.40E-07	-0.28	49	0.82	Olr1553	Signaling
DMR11:43623001	11	43623001	43625000	2000	1	2.00E-08	0.39	18	0.9	Olr1557	Signaling
DMR11:43770001	11	43770001	43773000	3000	1	5.80E-08	-0.5	17	0.57	Olr1562	
DMR11:43872001	11	43872001	43877000	5000	2	6.40E-07	-0.37	50	1	Olr1561	Signaling
DMR11:44050001	11	44050001	44052000	2000	1	7.80E-09	0.47	24	1.2	Cpox	Metabolism
DMR11:44179001	11	44179001	44182000	3000	1	3.50E-07	-0.39	51	1.7	St3gal6	Transport
DMR11:44206001	11	44206001	44208000	2000	1	2.80E-07	0.33	24	1.2	St3gal6	Transport
DMR11:44972001	11	44972001	44975000	3000	1	8.20E-07	-0.48	34	1.13	Col8a1	Extracellular Matrix
DMR11:45006001	11	45006001	45008000	2000	1	3.40E-13	0.67	48	2.4	Col8a1	Extracellular Matrix
DMR11:45041001	11	45041001	45042000	1000	1	9.00E-08	0.34	20	2	Cmss1;Filip1l	Metabolism
DMR11:45044001	11	45044001	45047000	3000	1	3.00E-07	-0.43	34	1.13	Cmss1;Filip1l	Metabolism
DMR11:45058001	11	45058001	45060000	2000	1	8.90E-07	-0.4	33	1.65	Cmss1;Filip1l;LOC103693552	Metabolism
DMR11:45247001	11	45247001	45249000	2000	1	3.20E-09	0.52	18	0.9	Cmss1;Filip1l	Metabolism
DMR11:45325001	11	45325001	45326000	1000	1	7.20E-10	-0.63	16	1.6	Cmss1	Metabolism
DMR11:45348001	11	45348001	45351000	3000	1	2.30E-07	0.41	40	1.33	Tmem30c;LOC108352325	
DMR11:45473001	11	45473001	45474000	1000	1	4.40E-10	-0.56	8	0.8	Nit2;Tomm70	Metabolism;Transport
DMR11:45654001	11	45654001	45661000	7000	1	4.40E-08	-0.26	74	1.06	Olr1534-ps;Olr1535	Signaling
DMR11:45909001	11	45909001	45910000	1000	1	5.40E-07	-0.37	47	4.7	Lnp1	
DMR11:46138001	11	46138001	46143000	5000	1	1.70E-09	-0.53	36	0.72	Adgrg7	Signaling
DMR11:46641001	11	46641001	46643000	2000	1	6.70E-10	0.4	31	1.55	Impg2	Extracellular Matrix
DMR11:46938001	11	46938001	46940000	2000	1	2.80E-07	-0.38	20	1	Senp7	Protease
DMR11:46978001	11	46978001	46980000	2000	2	3.60E-07	-0.32	17	0.85	Senp7	Protease
DMR11:47124001	11	47124001	47125000	1000	1	1.40E-08	0.46	14	1.4	Zbtb11os1;Rpl24	Translation
DMR11:47192001	11	47192001	47193000	1000	1	6.50E-08	-0.57	14	1.4	Nxpe3	
DMR11:47236001	11	47236001	47237000	1000	1	6.80E-08	-0.45	23	2.3	Nxpe3;Nfkbiz	Signaling
DMR11:47266001	11	47266001	47268000	2000	1	7.50E-07	-0.4	38	1.9	Nfkbiz	Signaling
DMR11:47933001	11	47933001	47934000	1000	1	2.50E-08	0.54	7	0.7	Zpld1	Receptor
DMR11:50811001	11	50811001	50816000	5000	1	1.60E-09	-0.73	40	0.8	Alcam	Immune
DMR11:50868001	11	50868001	50869000	1000	1	3.40E-08	-0.63	13	1.3	Alcam	Immune
DMR11:50883001	11	50883001	50885000	2000	1	3.80E-08	0.41	18	0.9	Alcam	Immune
DMR11:50962001	11	50962001	50963000	1000	1	8.90E-07	-0.41	7	0.7	Alcam	Immune
DMR11:51157001	11	51157001	51161000	4000	2	6.00E-08	-0.32	53	1.32	Cblb	Metabolism
DMR11:51195001	11	51195001	51197000	2000	1	6.10E-11	-0.46	17	0.85	Cblb	Metabolism
DMR11:53042001	11	53042001	53046000	4000	1	2.80E-09	0.42	48	1.2	Bbx	Transcription
DMR11:53503001	11	53503001	53504000	1000	1	6.80E-13	0.35	10	1	Cd47	Cytoskeleton
DMR11:53559001	11	53559001	53562000	3000	1	4.60E-07	-0.34	44	1.47	Cd47	Cytoskeleton
DMR11:54279001	11	54279001	54280000	1000	1	1.60E-07	0.43	4	0.4	Myh15	
DMR11:54652001	11	54652001	54656000	4000	1	1.30E-07	-0.25	32	0.8	Trat1	Immune
DMR11:54859001	11	54859001	54860000	1000	1	1.80E-10	-0.81	7	0.7	Morc1	
DMR11:54879001	11	54879001	54881000	2000	1	3.50E-07	-0.24	20	1	Morc1	
DMR11:55021001	11	55021001	55025000	4000	1	8.00E-07	-0.38	37	0.92	Morc1	
DMR11:55039001	11	55039001	55040000	1000	1	9.50E-08	-0.38	9	0.9	Morc1;LOC108352393	
DMR11:55044001	11	55044001	55045000	1000	1	3.90E-10	0.91	26	2.6	Morc1;LOC108352393	
DMR11:55485001	11	55485001	55487000	2000	1	6.60E-07	-0.45	8	0.4	Olr1453	Receptor
DMR11:57111001	11	57111001	57112000	1000	1	7.60E-07	-0.57	6	0.6	Cd96	Immune
DMR11:57237001	11	57237001	57238000	1000	1	1.20E-08	-0.54	13	1.3	Plcxd2	
DMR11:57361001	11	57361001	57363000	2000	1	3.30E-07	0.47	31	1.55	Phldb2	
DMR11:58692001	11	58692001	58694000	2000	1	7.00E-12	0.49	27	1.35	Gap43	
DMR11:58796001	11	58796001	58798000	2000	1	5.40E-07	0.5	36	1.8	Lsamp	Immune
DMR11:58866001	11	58866001	58867000	1000	1	2.30E-07	0.44	8	0.8	Lsamp	Immune
DMR11:59115001	11	59115001	59116000	1000	1	6.50E-09	0.62	19	1.9	Lsamp	Immune
DMR11:59333001	11	59333001	59334000	1000	1	2.90E-12	0.71	21	2.1	Lsamp	Immune
DMR11:59336001	11	59336001	59337000	1000	1	2.90E-09	-0.4	12	1.2	Lsamp	Immune
DMR11:59450001	11	59450001	59451000	1000	1	4.10E-07	-0.42	11	1.1	Lsamp	Immune
DMR11:60109001	11	60109001	60111000	2000	1	1.60E-07	-0.44	27	1.35	Tmprss7	Protease
DMR11:60549001	11	60549001	60550000	1000	1	1.50E-07	-0.66	6	0.6	Btla	
DMR11:60824001	11	60824001	60826000	2000	1	6.30E-09	0.45	27	1.35	Cd200r1l	Receptor

DMR11:60900001	11	60900001	60902000	2000	1	6.20E-07	0.39	21	1.05	Gtpbp8	
DMR11:61206001	11	61206001	61209000	3000	1	7.00E-08	-0.36	34	1.13	Cfap44	
DMR11:61213001	11	61213001	61215000	2000	1	1.70E-08	0.52	33	1.65	Cfap44	
DMR11:61216001	11	61216001	61217000	1000	1	3.10E-15	0.74	25	2.5	Cfap44	
DMR11:61328001	11	61328001	61329000	1000	1	3.60E-07	0.38	7	0.7	Sidt1	
DMR11:61474001	11	61474001	61475000	1000	1	1.60E-07	-0.49	12	1.2	Usf3;LOC102553099	Metabolism
DMR11:61682001	11	61682001	61684000	2000	1	2.90E-08	0.36	3	0.15	Zdhhc23;Ccdc191	
DMR11:61831001	11	61831001	61833000	2000	1	1.80E-07	0.35	12	0.6	Drd3	Signaling
DMR11:61840001	11	61840001	61841000	1000	1	2.30E-15	0.75	24	2.4	Drd3	Signaling
DMR11:61842001	11	61842001	61845000	3000	2	4.90E-08	0.6	50	1.67	Drd3	Signaling
DMR11:61960001	11	61960001	61961000	1000	1	6.50E-08	0.38	12	1.2	Tigit	
DMR11:61994001	11	61994001	61997000	3000	1	8.70E-07	-0.36	51	1.7	Tigit;Mir568	
DMR11:62033001	11	62033001	62036000	3000	1	2.00E-09	-0.65	28	0.93	Zbtb20	Transcription
DMR11:62103001	11	62103001	62105000	2000	1	5.00E-07	-0.43	17	0.85	Zbtb20	Transcription
DMR11:62106001	11	62106001	62108000	2000	1	6.50E-10	0.36	14	0.7	Zbtb20	Transcription
DMR11:62118001	11	62118001	62122000	4000	1	8.80E-07	-0.48	43	1.07	Zbtb20	Transcription
DMR11:62167001	11	62167001	62170000	3000	1	7.50E-07	-0.42	39	1.3	Zbtb20	Transcription
DMR11:62195001	11	62195001	62198000	3000	1	6.90E-08	-0.44	35	1.17	Zbtb20	Transcription
DMR11:62220001	11	62220001	62221000	1000	1	2.30E-07	-0.44	9	0.9	Zbtb20	Transcription
DMR11:62222001	11	62222001	62224000	2000	1	4.00E-07	-0.58	23	1.15	Zbtb20	Transcription
DMR11:62358001	11	62358001	62360000	2000	1	6.10E-07	-0.48	6	0.3	Zbtb20	Transcription
DMR11:62381001	11	62381001	62385000	4000	1	9.60E-07	-0.48	66	1.65	Zbtb20	Transcription
DMR11:62401001	11	62401001	62407000	6000	1	6.80E-07	-0.34	48	0.8	Zbtb20	Transcription
DMR11:62415001	11	62415001	62416000	1000	1	7.90E-08	-0.62	9	0.9	Zbtb20	Transcription
DMR11:62481001	11	62481001	62484000	3000	1	4.80E-12	-0.48	34	1.13	Zbtb20	Transcription
DMR11:62596001	11	62596001	62598000	2000	1	1.60E-08	0.34	21	1.05	Gramd1c	
DMR11:64288001	11	64288001	64289000	1000	1	1.00E-16	0.77	66	6.6	Igsf11	Immune
DMR11:64357001	11	64357001	64358000	1000	1	3.30E-07	-0.47	8	0.8	Igsf11	Immune
DMR11:64426001	11	64426001	64427000	1000	1	4.90E-07	0.39	1	0.1	Igsf11	Immune
DMR11:64516001	11	64516001	64520000	4000	1	2.00E-11	0.5	42	1.05	RGD1306995;Upk1b	
DMR11:64531001	11	64531001	64534000	3000	1	1.60E-09	0.39	32	1.07	Upk1b	
DMR11:64556001	11	64556001	64562000	6000	1	7.60E-07	-0.34	108	1.8	Upk1b;B4galt4;LOC103693580	Golgi
DMR11:64628001	11	64628001	64629000	1000	1	4.10E-10	0.45	2	0.2	Arhgap31	Signaling
DMR11:64830001	11	64830001	64832000	2000	1	3.30E-12	-0.61	36	1.8	Cd80	Immune
DMR11:64846001	11	64846001	64851000	5000	2	1.70E-08	0.35	67	1.34	Cd80;LOC108352341	Immune
DMR11:64857001	11	64857001	64860000	3000	2	1.30E-08	0.5	46	1.53	Cd80;LOC108352341;Adprh	Immune;Metabolism
DMR11:64888001	11	64888001	64889000	1000	1	1.50E-07	0.31	4	0.4	Pla1a	Metabolism
DMR11:64892001	11	64892001	64895000	3000	1	7.80E-08	-0.49	54	1.8	Pla1a	Metabolism
DMR11:64961001	11	64961001	64963000	2000	1	1.10E-08	1.05	15	0.75	Popdc2;Cox17	Transcription
DMR11:64981001	11	64981001	64982000	1000	1	1.60E-07	0.3	22	2.2	Maats1	
DMR11:64989001	11	64989001	64991000	2000	1	9.20E-07	0.41	27	1.35	Maats1	
DMR11:65022001	11	65022001	65023000	1000	1	3.20E-07	0.44	17	1.7	Maats1;Nr1i2	Transcription
DMR11:65786001	11	65786001	65787000	1000	1	4.30E-07	0.42	18	1.8	LOC102552404;Fstl1	Protease; Proteolysis
DMR11:65881001	11	65881001	65882000	1000	1	1.40E-11	-0.47	16	1.6	RGD1565413	
DMR11:65964001	11	65964001	65965000	1000	1	2.80E-09	-0.43	10	1	LOC102552623;Ndufb4;RGD1561232	Metabolism
DMR11:65986001	11	65986001	65987000	1000	1	1.90E-10	0.45	12	1.2	Hgd	Metabolism
DMR11:65997001	11	65997001	65998000	1000	1	4.40E-07	0.36	15	1.5	Hgd	Metabolism
DMR11:66067001	11	66067001	66072000	5000	1	9.20E-10	-0.69	83	1.66	Rabl3;Gtf2e1	Transcription
DMR11:66330001	11	66330001	66332000	2000	1	3.60E-08	-0.49	7	0.35	Stxbp5l	Transport
DMR11:66407001	11	66407001	66408000	1000	1	2.10E-07	0.53	4	0.4	Stxbp5l	Transport
DMR11:66669001	11	66669001	66671000	2000	1	2.20E-09	-0.47	11	0.55	Polq;LOC103693585	Transcription
DMR11:66750001	11	66750001	66751000	1000	1	4.80E-07	-0.63	7	0.7	Hcls1	Cytoskeleton
DMR11:66777001	11	66777001	66783000	6000	2	1.40E-08	-0.44	110	1.83	Golgb1	
DMR11:66784001	11	66784001	66785000	1000	1	2.60E-10	-0.56	7	0.7	Golgb1	
DMR11:66830001	11	66830001	66836000	6000	1	1.10E-08	-0.32	54	0.9	LOC108352342;Iqcb1	
DMR11:66911001	11	66911001	66912000	1000	1	6.90E-08	-0.83	24	2.4	Eaf2	
DMR11:67018001	11	67018001	67022000	4000	1	2.50E-07	0.64	87	2.17	Ildr1	Immune
DMR11:67023001	11	67023001	67026000	3000	1	5.20E-07	-0.33	38	1.27	Ildr1	Immune
DMR11:67057001	11	67057001	67066000	9000	1	5.20E-07	-0.37	132	1.47	Cd86	Immune
DMR11:67127001	11	67127001	67130000	3000	1	1.10E-07	0.4	8	0.27	Cd86	Immune
DMR11:67239001	11	67239001	67240000	1000	1	2.60E-09	0.73	34	3.4	Casr	Signaling
DMR11:67268001	11	67268001	67271000	3000	1	2.70E-09	0.48	44	1.47	Casr	Signaling

DMR11:67299001	11	67299001	67300000	1000	1	7.20E-10	0.57	8	0.8	Stfa3	
DMR11:67394001	11	67394001	67395000	1000	1	7.00E-08	0.46	2	0.2	Stfa2l1	Protease; Proteolysis
DMR11:68189001	11	68189001	68192000	3000	1	6.00E-08	-0.51	45	1.5	Hspbap1;Dirc2	
DMR11:68207001	11	68207001	68209000	2000	1	3.80E-11	-0.44	28	1.4	Hspbap1;Dirc2;LOC108352343	
DMR11:68220001	11	68220001	68223000	3000	1	4.30E-08	-0.54	29	0.97	Dirc2;LOC108352343	
DMR11:68321001	11	68321001	68324000	3000	1	2.00E-16	0.53	14	0.47	Sema5b	Signaling
DMR11:68401001	11	68401001	68402000	1000	1	1.80E-09	-0.59	12	1.2	Sema5b	Signaling
DMR11:68406001	11	68406001	68407000	1000	1	3.70E-09	0.47	4	0.4	Sema5b	Signaling
DMR11:68408001	11	68408001	68409000	1000	1	1.00E-08	0.47	17	1.7	Sema5b	Signaling
DMR11:68432001	11	68432001	68434000	2000	2	1.10E-09	0.64	55	2.75	Sema5b	Signaling
DMR11:68440001	11	68440001	68441000	1000	1	4.50E-07	-0.35	16	1.6	Sema5b	Signaling
DMR11:68639001	11	68639001	68641000	2000	1	7.30E-08	-0.41	22	1.1	Sec22a	
DMR11:68921001	11	68921001	68924000	3000	1	2.00E-07	-0.49	34	1.13	Hacd2	
DMR11:69038001	11	69038001	69039000	1000	1	3.80E-09	0.52	15	1.5	Mylk	
DMR11:69053001	11	69053001	69054000	1000	1	5.20E-07	0.3	10	1	Mylk	
DMR11:69169001	11	69169001	69172000	3000	1	6.60E-07	0.31	50	1.67	Mylk;LOC102548830	
DMR11:69185001	11	69185001	69186000	1000	1	4.40E-08	0.42	10	1	Mylk	
DMR11:69343001	11	69343001	69345000	2000	1	7.10E-09	-0.48	45	2.25	Ropn1	
DMR11:69498001	11	69498001	69499000	1000	1	2.30E-08	0.51	1	0.1	Kalrn	Transcription
DMR11:69701001	11	69701001	69703000	2000	1	9.90E-07	-0.5	20	1	Kalrn	Transcription
DMR11:69732001	11	69732001	69738000	6000	2	1.90E-15	-0.58	82	1.37	Kalrn	Transcription
DMR11:69766001	11	69766001	69767000	1000	1	1.90E-07	0.41	18	1.8	Kalrn	Transcription
DMR11:69943001	11	69943001	69944000	1000	1	5.20E-10	0.47	3	0.3	Kalrn	Transcription
DMR11:70001001	11	70001001	70003000	2000	1	9.40E-08	0.34	15	0.75	Kalrn;LOC103693587	Transcription
DMR11:70010001	11	70010001	70012000	2000	1	8.20E-09	0.36	31	1.55	Kalrn;LOC103693587	Transcription
DMR11:70272001	11	70272001	70276000	4000	1	3.70E-08	-0.56	72	1.8	Heg1	
DMR11:70299001	11	70299001	70300000	1000	1	2.40E-09	0.55	30	3	Heg1	
DMR11:70416001	11	70416001	70418000	2000	1	3.90E-07	0.39	32	1.6	Slc12a8	Transport
DMR11:70455001	11	70455001	70456000	1000	1	1.60E-08	0.52	29	2.9	Slc12a8	Transport
DMR11:70470001	11	70470001	70475000	5000	2	2.30E-10	0.52	58	1.16	Slc12a8	Transport
DMR11:70492001	11	70492001	70495000	3000	2	2.40E-09	0.66	55	1.83	Slc12a8	Transport
DMR11:70888001	11	70888001	70891000	3000	1	1.70E-09	-0.71	47	1.57	Lmln	Protease
DMR11:70920001	11	70920001	70925000	5000	1	3.30E-08	-0.35	47	0.94	Lmln	Protease
DMR11:71002001	11	71002001	71005000	3000	1	3.70E-07	-0.4	42	1.4	Iqcg;Lrch3	Cytoskeleton
DMR11:71062001	11	71062001	71065000	3000	1	2.20E-07	-0.32	14	0.47	Lrch3	
DMR11:71082001	11	71082001	71084000	2000	1	1.20E-08	0.52	11	0.55	Lrch3	
DMR11:71237001	11	71237001	71239000	2000	1	3.90E-07	0.46	51	2.55	Muc20;Muc4	
DMR11:71380001	11	71380001	71382000	2000	1	7.00E-07	-0.55	20	1	Tnk2	
DMR11:71386001	11	71386001	71387000	1000	1	9.70E-07	-0.49	21	2.1	Tnk2	
DMR11:71404001	11	71404001	71406000	2000	1	1.50E-10	-0.65	22	1.1	Tfrc	Protease
DMR11:71552001	11	71552001	71553000	1000	1	7.40E-08	-0.39	11	1.1	Slc51a;Pcyt1a	Transport;Transport
DMR11:71572001	11	71572001	71573000	1000	1	2.10E-08	-0.45	14	1.4	Pcyt1a	Transport
DMR11:71885001	11	71885001	71886000	1000	1	4.10E-07	-0.52	14	1.4	Nrros	
DMR11:72104001	11	72104001	72105000	1000	1	5.10E-10	0.41	16	1.6	Ncbp2;LOC108352349;LOC108352348	Metabolism
DMR11:72216001	11	72216001	72217000	1000	1	7.60E-08	0.48	17	1.7	Dlg1	Cytoskeleton
DMR11:72229001	11	72229001	72230000	1000	1	8.70E-07	-0.39	10	1	Dlg1	Cytoskeleton
DMR11:72979001	11	72979001	72982000	3000	1	2.80E-08	-0.53	50	1.67	Ppp1r2	Signaling
DMR11:73043001	11	73043001	73048000	5000	1	1.70E-11	0.5	82	1.64	Ppp1r2;LOC108352350	Signaling
DMR11:73070001	11	73070001	73071000	1000	1	6.20E-08	0.44	4	0.4	LOC108352350;Acap2	
DMR11:73085001	11	73085001	73088000	3000	1	6.20E-07	-0.38	57	1.9	Acap2	
DMR11:73115001	11	73115001	73116000	1000	1	6.20E-07	-0.57	12	1.2	Acap2	
DMR11:73126001	11	73126001	73127000	1000	1	2.30E-07	-0.43	17	1.7	Acap2	
DMR11:73261001	11	73261001	73263000	2000	1	7.00E-09	-0.55	41	2.05	Xxylt1	Transport
DMR11:73727001	11	73727001	73729000	2000	1	9.00E-08	-0.4	41	2.05	Lsg1;Tmem44	
DMR11:73748001	11	73748001	73751000	3000	1	3.00E-10	0.55	66	2.2	Tmem44;LOC102548792	
DMR11:73757001	11	73757001	73758000	1000	1	5.00E-07	0.37	13	1.3	Tmem44;LOC102548792	
DMR11:73932001	11	73932001	73934000	2000	1	1.00E-06	0.39	17	0.85	RGD1562415;Atp13a3	Translation
DMR11:74032001	11	74032001	74034000	2000	1	2.30E-09	0.38	26	1.3	Lrrc15	
DMR11:74318001	11	74318001	74321000	3000	1	5.80E-07	-0.44	34	1.13	Hes1	Transcription
DMR11:74708001	11	74708001	74713000	5000	2	1.20E-08	-0.48	118	2.36	Opa1	Transport
DMR11:74905001	11	74905001	74906000	1000	1	1.60E-07	-0.39	9	0.9	Atp13a4	
DMR11:74921001	11	74921001	74922000	1000	1	2.00E-10	0.37	14	1.4	Atp13a4	

DMR11:75034001	11	75034001	75035000	1000	1	1.60E-08	-0.39	13	1.3	Atp13a5	
DMR11:75081001	11	75081001	75082000	1000	1	1.90E-13	0.4	14	1.4	Atp13a5	
DMR11:75441001	11	75441001	75444000	3000	1	3.70E-10	-0.64	54	1.8	Mb21d2	
DMR11:75640001	11	75640001	75642000	2000	1	1.50E-10	-0.47	18	0.9	Fgf12	Growth Factors
DMR11:75704001	11	75704001	75707000	3000	1	6.40E-07	0.47	42	1.4	Fgf12	Growth Factors
DMR11:75939001	11	75939001	75940000	1000	1	8.80E-11	-0.48	15	1.5	Fgf12	Growth Factors
DMR11:76012001	11	76012001	76013000	1000	1	4.80E-08	-0.38	11	1.1	Fgf12	Growth Factors
DMR11:76139001	11	76139001	76140000	1000	1	2.10E-07	0.36	12	1.2	Fgf12	Growth Factors
DMR11:76740001	11	76740001	76741000	1000	1	6.80E-15	0.84	39	3.9	Ccdc50	
DMR11:76888001	11	76888001	76892000	4000	1	3.90E-07	0.48	49	1.23	Ostn	
DMR11:77446001	11	77446001	77447000	1000	1	9.80E-09	0.35	19	1.9	Il1rap	Receptor
DMR11:77705001	11	77705001	77707000	2000	1	2.90E-09	0.33	8	0.4	Cldn16	Cell Junction
DMR11:77710001	11	77710001	77711000	1000	1	1.40E-08	0.63	29	2.9	Cldn16	Cell Junction
DMR11:78042001	11	78042001	78044000	2000	1	2.90E-08	-0.46	15	0.75	P3h2;LOC103690757	Extracellular Matrix
DMR11:78091001	11	78091001	78092000	1000	1	7.60E-07	-0.43	23	2.3	P3h2	Extracellular Matrix
DMR11:78281001	11	78281001	78282000	1000	1	1.30E-07	-0.4	14	1.4	Tp63	Transcription
DMR11:78376001	11	78376001	78377000	1000	1	9.80E-07	-0.45	10	1	Tp63	Transcription
DMR11:78392001	11	78392001	78394000	2000	1	1.00E-07	0.34	17	0.85	Tp63	Transcription
DMR11:78740001	11	78740001	78741000	1000	1	1.70E-09	-0.77	13	1.3	Tprg1;LOC102554536	
DMR11:78863001	11	78863001	78864000	1000	1	1.20E-09	0.43	14	1.4	Tprg1	
DMR11:79261001	11	79261001	79262000	1000	1	5.10E-07	-0.47	6	0.6	Lpp	Signaling
DMR11:79560001	11	79560001	79562000	2000	1	3.20E-09	-0.51	24	1.2	Lpp	Signaling
DMR11:79673001	11	79673001	79674000	1000	1	7.40E-07	-0.5	11	1.1	Lpp	Signaling
DMR11:79798001	11	79798001	79799000	1000	1	9.30E-08	0.31	8	0.8	Lpp	Signaling
DMR11:80323001	11	80323001	80327000	4000	1	3.50E-08	-0.32	73	1.82	Rtp2	
DMR11:80528001	11	80528001	80529000	1000	1	8.50E-08	-0.51	11	1.1	RGD1564585	
DMR11:80986001	11	80986001	80988000	2000	1	8.90E-13	-0.45	17	0.85	St6gal1;LOC108352356	Transport
DMR11:81332001	11	81332001	81333000	1000	1	2.10E-08	0.42	22	2.2	Adipoq	
DMR11:81338001	11	81338001	81339000	1000	1	3.80E-10	-0.52	18	1.8	Adipoq	
DMR11:81373001	11	81373001	81378000	5000	1	7.60E-16	-0.68	46	0.92	Rfc4;Eif4a2	Transcription
DMR11:81454001	11	81454001	81455000	1000	1	7.70E-08	-0.29	10	1	Kng1l1	
DMR11:81517001	11	81517001	81519000	2000	1	7.80E-07	0.41	13	0.65	Kng1	Protease; Proteolysis
DMR11:81667001	11	81667001	81668000	1000	1	5.60E-09	0.37	3	0.3	Fetub	Protease; Proteolysis
DMR11:81714001	11	81714001	81717000	3000	1	1.60E-10	0.66	66	2.2	Ahsg;LOC103690621	Protease; Proteolysis
DMR11:81790001	11	81790001	81793000	3000	2	5.20E-10	-0.73	59	1.97	Tbccd1;Crygs	
DMR11:81975001	11	81975001	81978000	3000	2	4.10E-10	-0.44	50	1.67	LOC102552590;Dgkg	Signaling
DMR11:81979001	11	81979001	81984000	5000	2	1.70E-10	-0.54	81	1.62	LOC102552590;Dgkg	Signaling
DMR11:82003001	11	82003001	82006000	3000	2	5.70E-08	0.45	38	1.27	Dgkg	Signaling
DMR11:82074001	11	82074001	82076000	2000	1	4.80E-08	0.39	30	1.5	Dgkg	Signaling
DMR11:82111001	11	82111001	82113000	2000	1	8.30E-07	0.39	26	1.3	Dgkg	Signaling
DMR11:82125001	11	82125001	82126000	1000	1	8.30E-07	0.41	7	0.7	Dgkg	Signaling
DMR11:82160001	11	82160001	82161000	1000	1	4.60E-08	0.64	32	3.2	Dgkg	Signaling
DMR11:82172001	11	82172001	82174000	2000	1	9.10E-14	0.76	41	2.05	Dgkg	Signaling
DMR11:82364001	11	82364001	82367000	3000	1	2.40E-15	0.75	75	2.5	LOC691006;RGD1559972;Tra2b	
DMR11:82380001	11	82380001	82381000	1000	1	1.80E-08	-0.58	6	0.6	Tra2b	
DMR11:82485001	11	82485001	82486000	1000	1	2.80E-12	0.52	4	0.4	Igf2bp2	Metabolism
DMR11:82640001	11	82640001	82643000	3000	1	3.20E-08	-0.38	38	1.27	Senp2	Protease
DMR11:82743001	11	82743001	82745000	2000	1	7.40E-07	-0.41	33	1.65	Tmem41a;Map3k13	Signaling
DMR11:82793001	11	82793001	82795000	2000	1	1.10E-07	-0.53	32	1.6	Map3k13	Signaling
DMR11:82957001	11	82957001	82959000	2000	1	3.00E-08	0.57	35	1.75	Ehhadh	Metabolism
DMR11:82984001	11	82984001	82987000	3000	1	8.20E-08	0.42	44	1.47	Ehhadh	Metabolism
DMR11:83083001	11	83083001	83085000	2000	1	3.10E-08	0.44	22	1.1	RGD1562339	
DMR11:83126001	11	83126001	83127000	1000	1	8.50E-07	0.43	10	1	Vps8;LOC108352385	Cytoskeleton
DMR11:83188001	11	83188001	83193000	5000	1	2.70E-07	-0.44	70	1.4	Vps8	Cytoskeleton
DMR11:83205001	11	83205001	83207000	2000	1	4.30E-07	-0.35	32	1.6	Vps8	Cytoskeleton
DMR11:83265001	11	83265001	83272000	7000	1	4.40E-07	-0.48	163	2.33	Vps8	Cytoskeleton
DMR11:83312001	11	83312001	83314000	2000	1	7.70E-07	-0.55	43	2.15	Vps8	Cytoskeleton
DMR11:83520001	11	83520001	83521000	1000	1	6.60E-07	0.39	7	0.7	Ephb3	Receptor
DMR11:83850001	11	83850001	83851000	1000	1	7.50E-07	-0.43	24	2.4	RGD1563956;Chrd	
DMR11:83967001	11	83967001	83968000	1000	1	1.10E-08	0.48	31	3.1	Ece2;Camk2n2;LOC102551435	Protease;Signaling
DMR11:84019001	11	84019001	84021000	2000	1	1.40E-07	0.41	24	1.2	LOC102551647;Abcf3	Translation
DMR11:84315001	11	84315001	84319000	4000	1	6.10E-10	-0.56	39	0.98	LOC108352401;Map6d1	
DMR11:84433001	11	84433001	84435000	2000	1	8.40E-13	0.41	52	2.6	Yeats2;Abcc5	Transcription;Transport

DMR11:84465001	11	84465001	84466000	1000	1	1.50E-09	0.43	10	1	Yeats2;Abcc5	Transcription;Transport
DMR11:84523001	11	84523001	84525000	2000	1	2.90E-08	0.39	15	0.75	Yeats2;Cyp2ab1;Parl	Transcription;Metabolism;Protease
DMR11:84562001	11	84562001	84563000	1000	1	3.90E-09	-0.47	7	0.7	Yeats2	Transcription
DMR11:85144001	11	85144001	85149000	5000	1	3.50E-09	-0.32	48	0.96	Olr1565	Receptor
DMR11:85296001	11	85296001	85297000	1000	1	3.50E-07	-0.39	3	0.3	Olr1568	
DMR11:85306001	11	85306001	85311000	5000	2	1.40E-10	-0.37	42	0.84	Olr1569	Receptor
DMR11:85322001	11	85322001	85325000	3000	1	1.70E-07	-0.25	21	0.7	Olr1569	Receptor
DMR11:86165001	11	86165001	86166000	1000	1	7.20E-08	-0.68	3	0.3	Hira	Epigenetic
DMR11:86606001	11	86606001	86608000	2000	1	5.60E-07	0.4	20	1	Gnb1l	Cytoskeleton
DMR11:86617001	11	86617001	86621000	4000	1	3.70E-08	-0.32	34	0.85	Gnb1l	Cytoskeleton
DMR11:86637001	11	86637001	86638000	1000	1	1.40E-08	-0.43	15	1.5	Gnb1l	Cytoskeleton
DMR11:87084001	11	87084001	87085000	1000	1	6.10E-08	0.42	3	0.3	Prodh1;Dgcr6;LOC108352363	Metabolism
DMR11:87157001	11	87157001	87158000	1000	1	4.20E-08	0.42	19	1.9	LOC103690707;Vpreb2	Immune
DMR11:87160001	11	87160001	87163000	3000	1	7.00E-08	-0.54	30	1	LOC103690707;Vpreb2	Immune
DMR11:87979001	11	87979001	87980000	1000	1	5.60E-07	0.45	17	1.7	Pi4ka;Tmem191c;Hic2	Signaling;Transcription
DMR11:88133001	11	88133001	88136000	3000	1	2.00E-07	0.33	41	1.37	Sdf2l1;RGD1308065;Mir301b;Mir130b	Transport
DMR11:88433001	11	88433001	88440000	7000	1	3.80E-08	-0.46	68	0.97	Spag6	Cytoskeleton
DMR11:88498001	11	88498001	88504000	6000	1	5.30E-09	-0.32	78	1.3	Spag6	Cytoskeleton
DMR11:88736001	11	88736001	88739000	3000	1	7.40E-07	-0.6	3	0.1	Fgd4	Transcription
DMR11:88784001	11	88784001	88787000	3000	1	4.20E-09	-0.29	28	0.93	Fgd4	Transcription
DMR11:88956001	11	88956001	88957000	1000	1	4.00E-07	0.41	6	0.6	Pkp2;LOC683148	
DMR11:89242001	11	89242001	89243000	1000	1	1.40E-08	-0.48	2	0.2	Spidr	Transcription
DMR11:89265001	11	89265001	89268000	3000	1	3.90E-08	-0.58	26	0.87	Spidr;Mzt2b	Transcription
DMR11:89362001	11	89362001	89364000	2000	1	1.10E-08	-0.39	14	0.7	Prkdc	Signaling
DMR11:89491001	11	89491001	89496000	5000	2	4.00E-09	-0.34	52	1.04	Prkdc	Signaling
DMR11:89552001	11	89552001	89555000	3000	1	8.60E-07	-0.37	10	0.33	Ube2v2	Proteolysis
DMR12:581001	12	581001	583000	2000	1	1.70E-14	0.55	16	0.8	N4bp2l1;LOC100909860	
DMR12:761001	12	761001	763000	2000	1	1.90E-07	-0.4	20	1	Pds5b	Epigenetic
DMR12:967001	12	967001	968000	1000	1	1.90E-07	0.33	20	2	Kl	Metabolism
DMR12:981001	12	981001	983000	2000	1	6.90E-15	1	50	2.5	Kl	Metabolism
DMR12:985001	12	985001	987000	2000	2	5.40E-19	0.83	66	3.3	Kl;LOC102548964	Metabolism
DMR12:1103001	12	1103001	1107000	4000	2	1.50E-10	0.73	65	1.62	Stard13	Signaling
DMR12:1114001	12	1114001	1115000	1000	1	3.70E-09	0.43	12	1.2	Stard13	Signaling
DMR12:1126001	12	1126001	1130000	4000	1	6.00E-08	-0.37	83	2.08	Stard13	Signaling
DMR12:1148001	12	1148001	1151000	3000	1	2.00E-07	-0.46	68	2.27	Stard13	Signaling
DMR12:1158001	12	1158001	1159000	1000	1	2.30E-08	0.35	14	1.4	Stard13	Signaling
DMR12:1210001	12	1210001	1212000	2000	1	1.50E-07	-0.51	36	1.8	Stard13	Signaling
DMR12:1218001	12	1218001	1220000	2000	1	5.50E-07	-0.41	34	1.7	Stard13	Signaling
DMR12:1233001	12	1233001	1234000	1000	1	6.50E-09	-0.47	16	1.6	Stard13	Signaling
DMR12:1246001	12	1246001	1250000	4000	1	3.20E-08	-0.44	76	1.9	Stard13;LOC108352405	Signaling
DMR12:1259001	12	1259001	1262000	3000	1	1.40E-10	0.49	35	1.17	Stard13	Signaling
DMR12:1265001	12	1265001	1266000	1000	1	6.70E-10	0.37	3	0.3	Stard13	Signaling
DMR12:1294001	12	1294001	1296000	2000	2	2.20E-09	0.66	55	2.75	Stard13	Signaling
DMR12:1483001	12	1483001	1484000	1000	1	6.20E-08	-0.45	1	0.1	Vom2r-ps97;LOC103693612;LOC102554571	
DMR12:1672001	12	1672001	1673000	1000	1	3.70E-07	-0.33	25	2.5	Insr	Receptor
DMR12:1751001	12	1751001	1753000	2000	1	6.00E-12	0.46	16	0.8	Insr	Receptor
DMR12:1773001	12	1773001	1782000	9000	1	4.40E-07	-0.41	108	1.2	Insr	Receptor
DMR12:1792001	12	1792001	1793000	1000	1	6.80E-10	0.51	0	0	Insr	Receptor
DMR12:1880001	12	1880001	1881000	1000	1	1.00E-07	0.4	4	0.4	Arhgef18	
DMR12:1958001	12	1958001	1960000	2000	1	8.80E-10	-0.45	43	2.15	Arhgef18	
DMR12:2010001	12	2010001	2013000	3000	1	1.20E-07	0.43	35	1.17	Pex11g;RGD1563425	
DMR12:2034001	12	2034001	2038000	4000	1	1.10E-07	-0.41	21	0.52	RGD1563425;Zfp358	Transcription
DMR12:2105001	12	2105001	2112000	7000	1	3.70E-07	-0.35	87	1.24	Pnpla6	Metabolism
DMR12:2217001	12	2217001	2220000	3000	1	4.30E-10	0.45	24	0.8	Mcemp1;Trappc5	
DMR12:2468001	12	2468001	2474000	6000	1	4.50E-09	-0.54	54	0.9	LOC102552452;Elavl1	Translation
DMR12:2545001	12	2545001	2550000	5000	1	2.10E-08	0.46	82	1.64	Ctxn1;Snapc2;Tgfbr3l;Map2k7;Lrrc8e	Receptor;Signaling;Cytoskeleton
DMR12:2654001	12	2654001	2656000	2000	1	3.40E-07	0.52	23	1.15	LOC103690865;Cd209f	Transport
DMR12:2665001	12	2665001	2666000	1000	1	4.30E-08	-0.53	12	1.2	LOC103690865;Cd209f;LOC688763	Transport

DMR12:2855001	12	2855001	2856000	1000	1	6.90E-07	-0.49	6	0.6	Cd209c	Transport
DMR12:3923001	12	3923001	3924000	1000	1	1.80E-10	0.8	39	3.9	Vom2r-ps94;RGD1562451;Vom2r-ps95	
DMR12:4104001	12	4104001	4107000	3000	2	5.40E-10	-0.63	38	1.27	Vom2r60;LOC688812	Signaling
DMR12:4109001	12	4109001	4110000	1000	1	1.60E-07	-0.37	12	1.2	Vom2r60;LOC688812	Signaling
DMR12:4112001	12	4112001	4113000	1000	1	1.40E-12	-0.57	12	1.2	Vom2r60;LOC688812	Signaling
DMR12:4138001	12	4138001	4142000	4000	1	5.90E-08	-0.34	37	0.92	Vom2r60;LOC102555699	Signaling
DMR12:4232001	12	4232001	4236000	4000	1	8.10E-07	-0.38	30	0.75	Vom2r60	Signaling
DMR12:4330001	12	4330001	4335000	5000	2	4.50E-07	-0.37	50	1	Vom2r60	Signaling
DMR12:4421001	12	4421001	4426000	5000	1	8.40E-08	-0.28	50	1	Vom2r60	Signaling
DMR12:4451001	12	4451001	4452000	1000	1	1.40E-07	-0.54	10	1	Cers4	
DMR12:5494001	12	5494001	5499000	5000	1	1.40E-09	-0.42	50	1	Zfp958	Transcription
DMR12:5642001	12	5642001	5645000	3000	1	7.40E-18	0.56	58	1.93	Fry	Cytoskeleton
DMR12:5694001	12	5694001	5696000	2000	1	7.70E-07	0.33	38	1.9	Fry	Cytoskeleton
DMR12:5725001	12	5725001	5727000	2000	2	5.80E-12	0.88	56	2.8	Fry	Cytoskeleton
DMR12:5780001	12	5780001	5783000	3000	1	1.90E-07	-0.47	67	2.23	Fry	Cytoskeleton
DMR12:5796001	12	5796001	5799000	3000	1	3.70E-07	0.48	54	1.8	Fry	Cytoskeleton
DMR12:5992001	12	5992001	5994000	2000	1	1.80E-08	-0.38	34	1.7	Lnc001;LOC100361192	
DMR12:6027001	12	6027001	6030000	3000	1	3.20E-07	0.41	58	1.93	Rxfp2;LOC102549667	Signaling
DMR12:6035001	12	6035001	6036000	1000	1	6.30E-14	0.83	25	2.5	Rxfp2	Signaling
DMR12:6042001	12	6042001	6044000	2000	1	6.00E-09	0.57	36	1.8	Rxfp2	Signaling
DMR12:6060001	12	6060001	6063000	3000	1	6.90E-08	-0.47	51	1.7	Rxfp2	Signaling
DMR12:6070001	12	6070001	6071000	1000	1	3.50E-12	0.98	25	2.5	Rxfp2	Signaling
DMR12:6074001	12	6074001	6075000	1000	1	2.90E-12	1.06	33	3.3	Rxfp2	Signaling
DMR12:6086001	12	6086001	6093000	7000	1	4.40E-07	0.44	167	2.39	Rxfp2;LOC102549494	Signaling
DMR12:6273001	12	6273001	6275000	2000	1	1.60E-08	0.38	26	1.3	Wdr95	
DMR12:6396001	12	6396001	6402000	6000	1	5.70E-07	0.4	117	1.95	B3glct	Golgi
DMR12:6413001	12	6413001	6414000	1000	1	1.90E-09	0.66	32	3.2	B3glct	Golgi
DMR12:6442001	12	6442001	6446000	4000	2	2.10E-08	0.64	82	2.05	B3glct;LOC103690977	Golgi
DMR12:6450001	12	6450001	6451000	1000	1	1.90E-08	0.36	6	0.6	B3glct;LOC103690977	Golgi
DMR12:6453001	12	6453001	6455000	2000	1	9.60E-08	0.65	38	1.9	B3glct;LOC103690977	Golgi
DMR12:6462001	12	6462001	6464000	2000	1	1.10E-07	0.46	41	2.05	B3glct	Golgi
DMR12:6470001	12	6470001	6473000	3000	1	4.40E-07	0.58	51	1.7	B3glct	Golgi
DMR12:6705001	12	6705001	6708000	3000	1	3.80E-07	-0.43	67	2.23	Tex26;Medag	
DMR12:6850001	12	6850001	6854000	4000	1	2.20E-11	0.73	48	1.2	Alox5ap	Transport
DMR12:6855001	12	6855001	6858000	3000	2	4.20E-12	0.68	94	3.13	Alox5ap	Transport
DMR12:6864001	12	6864001	6868000	4000	1	1.30E-07	-0.38	79	1.98	Alox5ap	Transport
DMR12:6882001	12	6882001	6884000	2000	1	4.70E-09	0.4	44	2.2	Alox5ap	Transport
DMR12:6886001	12	6886001	6888000	2000	1	4.60E-07	0.64	63	3.15	Alox5ap	Transport
DMR12:7075001	12	7075001	7077000	2000	1	2.70E-07	0.4	26	1.3	Hmgb1	
DMR12:7544001	12	7544001	7545000	1000	1	2.90E-07	0.31	17	1.7	Katnal1	Cytoskeleton
DMR12:8068001	12	8068001	8069000	1000	1	2.90E-08	-0.4	34	3.4	Slc7a1	Transport
DMR12:8071001	12	8071001	8073000	2000	1	2.40E-08	-0.43	41	2.05	Slc7a1	Transport
DMR12:8118001	12	8118001	8119000	1000	1	1.70E-14	0.65	15	1.5	Slc7a1;Mtus2	Transport
DMR12:8171001	12	8171001	8173000	2000	1	4.20E-08	-0.48	50	2.5	Mtus2;LOC102547400	
DMR12:8274001	12	8274001	8275000	1000	1	2.00E-09	-0.5	19	1.9	Mtus2	
DMR12:8278001	12	8278001	8282000	4000	3	9.80E-08	-0.59	112	2.8	Mtus2	
DMR12:8294001	12	8294001	8295000	1000	1	6.30E-11	0.74	18	1.8	Mtus2	
DMR12:8311001	12	8311001	8313000	2000	1	8.20E-09	-0.48	17	0.85	Mtus2	
DMR12:8340001	12	8340001	8341000	1000	1	6.30E-13	0.51	25	2.5	Mtus2	
DMR12:8349001	12	8349001	8351000	2000	1	3.20E-08	0.7	33	1.65	Mtus2	
DMR12:8356001	12	8356001	8358000	2000	1	1.80E-09	0.53	37	1.85	Mtus2	
DMR12:8359001	12	8359001	8362000	3000	1	2.00E-07	0.44	44	1.47	Mtus2	
DMR12:8411001	12	8411001	8416000	5000	3	2.90E-14	0.86	109	2.18	Mtus2	
DMR12:8418001	12	8418001	8421000	3000	1	3.70E-10	0.7	71	2.37	Mtus2	
DMR12:8449001	12	8449001	8450000	1000	1	5.40E-07	0.45	29	2.9	Mtus2	
DMR12:8471001	12	8471001	8474000	3000	1	4.20E-07	-0.39	67	2.23	Mtus2	
DMR12:8744001	12	8744001	8745000	1000	1	8.50E-08	0.52	9	0.9	Slc46a3;Pomp	Transcription
DMR12:9045001	12	9045001	9047000	2000	1	1.90E-08	0.46	13	0.65	Flt1	Receptor
DMR12:9123001	12	9123001	9124000	1000	1	4.50E-07	-0.42	28	2.8	Flt1	Receptor
DMR12:9145001	12	9145001	9148000	3000	1	4.60E-13	0.67	68	2.27	Flt1	Receptor
DMR12:9165001	12	9165001	9167000	2000	1	5.80E-08	0.44	32	1.6	Flt1	Receptor
DMR12:9255001	12	9255001	9257000	2000	1	4.60E-09	-0.49	25	1.25	Pan3	Transcription

DMR12:9281001	12	9281001	9282000	1000	1	4.50E-09	-0.39	12	1.2	Pan3	Transcription
DMR12:9451001	12	9451001	9452000	1000	1	1.00E-07	0.61	28	2.8	Urad;LOC103691014	Metabolism
DMR12:9478001	12	9478001	9480000	2000	1	7.70E-08	0.77	37	1.85	Cdx2	Development
DMR12:9686001	12	9686001	9687000	1000	1	6.70E-08	0.46	12	1.2	Polr1d	
DMR12:10009001	12	10009001	10010000	1000	1	3.70E-09	0.61	30	3	Rpl21;LOC102549133	Translation
DMR12:10050001	12	10050001	10054000	4000	1	5.40E-07	-0.46	56	1.4	Usp12;LOC108352420	Protease
DMR12:10256001	12	10256001	10257000	1000	1	8.80E-12	0.53	33	3.3	Gpr12	Signaling
DMR12:10261001	12	10261001	10262000	1000	1	5.30E-07	0.3	14	1.4	Gpr12	Signaling
DMR12:10324001	12	10324001	10326000	2000	1	5.20E-07	0.5	39	1.95	Wasf3	Cytoskeleton
DMR12:10370001	12	10370001	10371000	1000	1	2.30E-08	-0.38	20	2	Wasf3	Cytoskeleton
DMR12:10389001	12	10389001	10390000	1000	1	1.50E-07	0.45	9	0.9	Wasf3;LOC684427	Cytoskeleton
DMR12:10501001	12	10501001	10503000	2000	1	1.90E-07	-0.59	31	1.55	Cdk8	
DMR12:10506001	12	10506001	10507000	1000	1	1.30E-09	-0.46	20	2	Cdk8	
DMR12:10583001	12	10583001	10584000	1000	1	8.40E-07	-0.52	20	2	Rnf6	
DMR12:10641001	12	10641001	10645000	4000	2	7.20E-15	0.73	70	1.75	Cyp3a18	Metabolism
DMR12:10892001	12	10892001	10895000	3000	1	8.20E-07	-0.41	18	0.6	Cyp3a71-ps	
DMR12:11183001	12	11183001	11187000	4000	1	8.70E-07	0.32	76	1.9	Zkscan5;Zfp394;Atp5j2	
DMR12:11193001	12	11193001	11196000	3000	1	3.10E-08	-0.54	50	1.67	Zfp394;Atp5j2;Cpsf4	Translation
DMR12:11302001	12	11302001	11307000	5000	2	1.60E-11	0.57	85	1.7	Arpc1a;Rps15al1	Cytoskeleton
DMR12:11310001	12	11310001	11313000	3000	1	2.50E-12	0.56	25	0.83	Rps15al1	
DMR12:11337001	12	11337001	11338000	1000	1	4.70E-07	0.43	9	0.9	LOC108352477;Kpna7	Transport
DMR12:11339001	12	11339001	11340000	1000	1	6.60E-07	0.54	17	1.7	LOC108352477;Kpna7	Transport
DMR12:11348001	12	11348001	11357000	9000	2	7.20E-08	0.6	104	1.16	LOC108352477;Kpna7	Transport
DMR12:11483001	12	11483001	11484000	1000	1	2.30E-08	-0.43	25	2.5	Smurf1	Proteolysis
DMR12:11766001	12	11766001	11768000	2000	1	6.00E-07	-0.32	19	0.95	Zfp498	
DMR12:11812001	12	11812001	11813000	1000	1	8.70E-07	0.35	13	1.3	LOC103690102;Tmem130	Signaling
DMR12:12011001	12	12011001	12014000	3000	2	4.00E-11	1.01	21	0.7	Nptx2	
DMR12:12600001	12	12600001	12604000	4000	2	1.80E-11	0.65	70	1.75	Ocm2	
DMR12:12606001	12	12606001	12609000	3000	1	7.00E-07	0.5	78	2.6	Ocm2	
DMR12:12646001	12	12646001	12650000	4000	1	2.10E-07	0.32	62	1.55	Ccz1b	
DMR12:12672001	12	12672001	12673000	1000	1	1.60E-09	0.49	5	0.5	Ccz1b;Rsph10b	
DMR12:12720001	12	12720001	12721000	1000	1	7.70E-07	-0.45	14	1.4	Rsph10b;Pms2	Transcription
DMR12:12777001	12	12777001	12781000	4000	1	2.80E-07	-0.34	60	1.5	Eif2ak1	Signaling
DMR12:12885001	12	12885001	12887000	2000	1	1.70E-09	0.44	45	2.25	Usp42;Cyth3	Protease;Transcription
DMR12:13074001	12	13074001	13078000	4000	2	8.70E-09	0.7	76	1.9	Fam220a	
DMR12:13101001	12	13101001	13103000	2000	1	3.00E-07	-0.47	32	1.6	Rac1	Signaling
DMR12:13164001	12	13164001	13166000	2000	1	8.30E-07	-0.31	28	1.4	Daglb	Metabolism
DMR12:13240001	12	13240001	13242000	2000	2	4.40E-10	0.44	32	1.6	Grid2ip	
DMR12:13506001	12	13506001	13508000	2000	1	1.30E-07	0.47	25	1.25	Rnf216	Proteolysis
DMR12:13660001	12	13660001	13661000	1000	1	5.80E-07	0.39	20	2	Fscn1	Cytoskeleton
DMR12:14055001	12	14055001	14057000	2000	1	4.20E-07	0.32	30	1.5	Mmd2	Signaling
DMR12:14434001	12	14434001	14437000	3000	1	2.20E-08	-0.58	39	1.3	Sdk1	
DMR12:14503001	12	14503001	14505000	2000	1	7.40E-07	0.41	25	1.25	Sdk1	
DMR12:14527001	12	14527001	14528000	1000	1	8.70E-09	0.49	17	1.7	Sdk1	
DMR12:14581001	12	14581001	14583000	2000	1	3.60E-08	0.43	28	1.4	Sdk1	
DMR12:14618001	12	14618001	14619000	1000	1	6.60E-10	0.56	6	0.6	Sdk1	
DMR12:14663001	12	14663001	14664000	1000	1	5.70E-07	0.47	17	1.7	Sdk1	
DMR12:14696001	12	14696001	14700000	4000	1	8.40E-10	0.46	42	1.05	Sdk1	
DMR12:14735001	12	14735001	14737000	2000	1	2.90E-08	0.41	21	1.05	Sdk1;RGD1564193	
DMR12:14804001	12	14804001	14805000	1000	1	2.20E-11	0.4	10	1	Sdk1	
DMR12:14843001	12	14843001	14844000	1000	1	2.20E-14	0.85	24	2.4	Sdk1	
DMR12:14845001	12	14845001	14849000	4000	1	8.80E-07	0.53	54	1.35	Sdk1	
DMR12:14856001	12	14856001	14857000	1000	1	6.10E-07	0.47	16	1.6	Sdk1	
DMR12:14870001	12	14870001	14874000	4000	1	2.90E-07	0.33	46	1.15	Sdk1	
DMR12:14884001	12	14884001	14885000	1000	1	6.50E-07	0.36	12	1.2	Sdk1	
DMR12:14897001	12	14897001	14898000	1000	1	4.00E-07	-0.66	18	1.8	Sdk1	
DMR12:14903001	12	14903001	14906000	3000	1	2.20E-07	0.38	29	0.97	Sdk1	
DMR12:14918001	12	14918001	14920000	2000	1	3.40E-13	0.48	20	1	Sdk1	
DMR12:14937001	12	14937001	14941000	4000	2	6.30E-10	0.51	45	1.12	Sdk1	
DMR12:15089001	12	15089001	15091000	2000	1	7.00E-08	0.59	40	2	Sdk1	
DMR12:15095001	12	15095001	15099000	4000	1	8.10E-07	0.3	68	1.7	Sdk1	
DMR12:15107001	12	15107001	15111000	4000	1	5.00E-13	0.64	66	1.65	Sdk1	
DMR12:15203001	12	15203001	15207000	4000	1	1.70E-07	-0.36	47	1.18	Sdk1;LOC108352492	

DMR12:15244001	12	15244001	15246000	2000	1	2.50E-07	-0.36	40	2	Sdk1	
DMR12:15271001	12	15271001	15273000	2000	1	9.40E-10	-0.51	30	1.5	Sdk1	
DMR12:15379001	12	15379001	15382000	3000	1	1.90E-08	-0.56	34	1.13	Sdk1	
DMR12:15410001	12	15410001	15411000	1000	1	4.70E-07	-0.45	19	1.9	Sdk1	
DMR12:15743001	12	15743001	15745000	2000	1	2.20E-07	0.42	33	1.65	Card11	
DMR12:15803001	12	15803001	15808000	5000	1	4.50E-12	0.74	65	1.3	Card11	
DMR12:15818001	12	15818001	15819000	1000	1	7.60E-07	0.28	4	0.4	Card11	
DMR12:15910001	12	15910001	15912000	2000	1	7.60E-08	0.46	13	0.65	Gna12	Signaling
DMR12:15957001	12	15957001	15959000	2000	2	3.70E-08	0.49	10	0.5	Gna12	Signaling
DMR12:16113001	12	16113001	16114000	1000	1	5.40E-07	-0.59	12	1.2	Ttyh3;Lfng	Transport;Golgi
DMR12:16164001	12	16164001	16167000	3000	1	2.40E-08	0.66	67	2.23	Grifin	Extracellular Matrix
DMR12:16225001	12	16225001	16227000	2000	1	1.10E-07	0.41	15	0.75	Chst12;LOC679924	Transport
DMR12:16314001	12	16314001	16317000	3000	1	5.70E-11	0.74	50	1.67	Eif3b;LOC108352425	Translation
DMR12:16334001	12	16334001	16336000	2000	1	4.50E-07	0.41	12	0.6	LOC108352425;Snx8	Cytoskeleton
DMR12:16399001	12	16399001	16402000	3000	1	4.80E-07	0.32	64	2.13	Snx8;Nudt1;Mrm2;Mad1l1	Cytoskeleton;Signaling;Epigenetic
DMR12:16489001	12	16489001	16491000	2000	1	3.80E-07	0.41	20	1	Mad1l1	
DMR12:16649001	12	16649001	16654000	5000	1	4.80E-08	0.42	73	1.46	Mad1l1	
DMR12:16917001	12	16917001	16919000	2000	1	3.30E-08	0.6	60	3	Tmem184a;Mafk	Transport;Transcription
DMR12:17318001	12	17318001	17319000	1000	1	5.30E-07	0.5	13	1.3	LOC498154;Gper1;LOC102546864	Signaling
DMR12:17351001	12	17351001	17354000	3000	1	7.40E-07	0.34	30	1	LOC498154;Gpr146	Signaling
DMR12:17413001	12	17413001	17415000	2000	1	3.40E-07	0.31	21	1.05	Cox19;Adap1	Transcription
DMR12:17913001	12	17913001	17921000	8000	1	3.80E-07	0.28	117	1.46	LOC680273;Fam20c	Transcription
DMR12:17960001	12	17960001	17962000	2000	1	3.60E-08	0.44	18	0.9	Fam20c	
DMR12:18518001	12	18518001	18520000	2000	2	1.60E-08	0.76	67	3.35	Dhrsx;Asmt;Akap17a	Metabolism;Epigenetic
DMR12:18522001	12	18522001	18523000	1000	1	5.00E-13	0.95	26	2.6	Dhrsx;Asmt;Akap17a;Asmtl	Metabolism;Epigenetic
DMR12:18525001	12	18525001	18530000	5000	5	9.50E-15	0.91	211	4.22	Dhrsx;Asmt;Akap17a;Asmtl;Il3ra	Metabolism;Epigenetic
DMR12:18936001	12	18936001	18940000	4000	4	8.60E-19	1.22	138	3.45	LOC102554070;Spry3	Cytoskeleton
DMR12:18945001	12	18945001	18947000	2000	1	9.20E-08	-0.41	14	0.7	Spry3	Cytoskeleton
DMR12:19005001	12	19005001	19007000	2000	1	8.60E-08	-0.39	27	1.35	Vamp7	Transcription
DMR12:19023001	12	19023001	19026000	3000	2	7.80E-10	-0.43	29	0.97	Vamp7	Transcription
DMR12:19081001	12	19081001	19086000	5000	1	6.00E-11	-0.44	46	0.92	Cyp3a9	Metabolism
DMR12:19098001	12	19098001	19100000	2000	1	2.50E-11	0.47	8	0.4	Cyp3a9	Metabolism
DMR12:19151001	12	19151001	19153000	2000	1	4.50E-07	-0.39	14	0.7	Rpl31l4	Translation
DMR12:19418001	12	19418001	19421000	3000	1	3.20E-11	0.4	41	1.37	Nxpe5	
DMR12:19429001	12	19429001	19431000	2000	1	6.70E-18	0.62	23	1.15	Nxpe5	
DMR12:19554001	12	19554001	19555000	1000	1	1.90E-07	0.61	16	1.6	RGD1562319	
DMR12:19567001	12	19567001	19569000	2000	1	4.10E-08	0.61	38	1.9	RGD1562319;Lamtor4;RGD1305455	
DMR12:19638001	12	19638001	19644000	6000	1	2.50E-07	-0.36	70	1.17	Stag3	Epigenetic
DMR12:20827001	12	20827001	20829000	2000	1	4.00E-08	-0.54	47	2.35	Vom2r-ps100	
DMR12:20981001	12	20981001	20987000	6000	2	2.70E-13	-0.39	57	0.95	Vom2r64	Signaling
DMR12:22156001	12	22156001	22159000	3000	1	6.20E-07	0.48	68	2.27	Fbxo24;LOC108352432;Pcolce;Mospd3	Protease;Transport
DMR12:22216001	12	22216001	22220000	4000	1	2.10E-12	0.81	95	2.38	Actl6b;LOC108352483;Gnb2	Cytoskeleton;Signaling
DMR12:22318001	12	22318001	22322000	4000	1	7.20E-12	-0.43	92	2.3	Zan	
DMR12:22347001	12	22347001	22348000	1000	1	9.60E-11	0.49	17	1.7	Zan	
DMR12:22551001	12	22551001	22553000	2000	1	3.10E-08	-0.36	23	1.15	Muc3	
DMR12:22732001	12	22732001	22735000	3000	1	2.30E-07	0.38	45	1.5	Plod3;Znhit1;Cldn15	Golgi;Epigenetic;Cell Junction
DMR12:22793001	12	22793001	22795000	2000	1	8.30E-08	0.48	23	1.15	LOC100910802;Ift22	
DMR12:22797001	12	22797001	22799000	2000	1	1.80E-07	0.38	28	1.4	LOC100910802;Ift22	
DMR12:22857001	12	22857001	22860000	3000	1	2.70E-07	0.36	41	1.37	Col26a1	
DMR12:22874001	12	22874001	22879000	5000	1	1.50E-08	0.35	67	1.34	Col26a1	
DMR12:22900001	12	22900001	22903000	3000	1	9.20E-17	0.83	78	2.6	Col26a1	
DMR12:22928001	12	22928001	22930000	2000	1	1.40E-07	0.37	26	1.3	Col26a1	
DMR12:22951001	12	22951001	22953000	2000	1	7.70E-09	0.44	25	1.25	Col26a1	
DMR12:22963001	12	22963001	22965000	2000	1	3.80E-07	0.41	48	2.4	Col26a1	
DMR12:23003001	12	23003001	23005000	2000	1	2.20E-09	0.44	30	1.5	Myl10	Cytoskeleton
DMR12:23067001	12	23067001	23070000	3000	1	3.00E-07	0.37	33	1.1	Myl10	Cytoskeleton
DMR12:23082001	12	23082001	23085000	3000	1	3.30E-09	0.47	57	1.9	Myl10	Cytoskeleton
DMR12:23242001	12	23242001	23243000	1000	1	2.20E-09	-0.64	18	1.8	Cux1	Development
DMR12:23553001	12	23553001	23556000	3000	1	7.90E-07	0.36	52	1.73	Orai2	Transport

DMR12:23577001	12	23577001	23580000	3000	1	3.20E-07	0.41	70	2.33	Alkbh4;Lrwd1;Polr2j	Metabolism;Extracellular Matrix;Transcription
DMR12:23602001	12	23602001	23603000	1000	1	3.10E-07	0.41	13	1.3	Polr2j;Rasa4	Transcription;Signaling
DMR12:23657001	12	23657001	23661000	4000	1	1.00E-09	0.4	67	1.68	Upk3bl	
DMR12:23680001	12	23680001	23682000	2000	1	1.40E-13	0.65	39	1.95	Upk3b;Dtx2	Proteolysis
DMR12:23772001	12	23772001	23776000	4000	1	4.40E-10	0.44	53	1.32	Ssc4d	Protease
DMR12:23854001	12	23854001	23855000	1000	1	3.20E-08	0.45	12	1.2	Srrm3	
DMR12:23883001	12	23883001	23887000	4000	1	3.80E-07	0.57	60	1.5	Srrm3	
DMR12:23919001	12	23919001	23920000	1000	1	9.20E-07	0.44	22	2.2	Srrm3;LOC108352435	
DMR12:24026001	12	24026001	24027000	1000	1	8.30E-07	-0.52	7	0.7	Por	Metabolism
DMR12:24121001	12	24121001	24126000	5000	1	1.90E-09	0.37	64	1.28	Ccl24	Growth Factors
DMR12:24172001	12	24172001	24175000	3000	1	7.00E-08	0.62	45	1.5	LOC108352436;LOC363903;Hip1	Cytoskeleton
DMR12:24210001	12	24210001	24211000	1000	1	5.80E-14	0.54	6	0.6	Hip1	Cytoskeleton
DMR12:24223001	12	24223001	24226000	3000	1	5.20E-10	0.45	50	1.67	Hip1	Cytoskeleton
DMR12:24283001	12	24283001	24284000	1000	1	1.70E-07	0.38	17	1.7	Hip1	Cytoskeleton
DMR12:24289001	12	24289001	24295000	6000	1	6.70E-10	0.72	142	2.37	Hip1	Cytoskeleton
DMR12:24424001	12	24424001	24425000	1000	1	3.80E-07	0.48	6	0.6	Fkbp6	Transcription
DMR12:24595001	12	24595001	24597000	2000	1	7.20E-13	0.62	22	1.1	Mlxipl	Transcription
DMR12:24731001	12	24731001	24734000	3000	1	1.20E-07	-0.38	82	2.73	Abhd11os;Abhd11	
DMR12:25065001	12	25065001	25066000	1000	1	3.90E-10	0.49	16	1.6	Limk1	
DMR12:25165001	12	25165001	25168000	3000	3	6.50E-12	0.73	92	3.07	LOC363906;Syna;Clip2	Transcription
DMR12:25233001	12	25233001	25234000	1000	1	5.10E-09	0.62	24	2.4	Clip2	Transcription
DMR12:25312001	12	25312001	25314000	2000	1	1.80E-12	0.76	47	2.35	Gtf2ird1	Transcription
DMR12:25408001	12	25408001	25410000	2000	1	2.40E-07	-0.65	29	1.45	Gtf2i	Transcription
DMR12:28411001	12	28411001	28414000	3000	2	7.60E-07	-0.38	74	2.47	Wbscr17	
DMR12:28577001	12	28577001	28579000	2000	1	1.40E-08	-0.46	12	0.6	Wbscr17	
DMR12:28584001	12	28584001	28586000	2000	1	4.60E-13	0.69	51	2.55	Wbscr17	
DMR12:28610001	12	28610001	28611000	1000	1	1.40E-07	0.53	9	0.9	Wbscr17	
DMR12:28660001	12	28660001	28663000	3000	1	4.40E-07	-0.42	38	1.27	Wbscr17	
DMR12:28725001	12	28725001	28726000	1000	1	1.20E-12	0.85	22	2.2	Wbscr17	
DMR12:28736001	12	28736001	28741000	5000	1	6.90E-09	0.4	65	1.3	Wbscr17	
DMR12:28800001	12	28800001	28804000	4000	1	2.00E-08	0.4	54	1.35	Wbscr17	
DMR12:28810001	12	28810001	28813000	3000	1	3.80E-11	0.48	54	1.8	Wbscr17	
DMR12:28843001	12	28843001	28848000	5000	3	5.30E-11	0.73	97	1.94	Wbscr17	
DMR12:28937001	12	28937001	28940000	3000	1	2.10E-07	0.48	51	1.7	Wbscr17	
DMR12:29019001	12	29019001	29021000	2000	1	2.10E-08	0.55	49	2.45	Wbscr17;LOC100912262	
DMR12:29215001	12	29215001	29217000	2000	1	4.30E-14	-0.6	49	2.45	Wbscr17;LOC103690026	
DMR12:29254001	12	29254001	29257000	3000	1	1.30E-10	0.69	70	2.33	Wbscr17	
DMR12:29302001	12	29302001	29303000	1000	1	2.80E-07	0.35	6	0.6	LOC102550645;Caln1	
DMR12:29306001	12	29306001	29307000	1000	1	2.20E-07	0.76	25	2.5	Caln1	
DMR12:29341001	12	29341001	29345000	4000	1	3.20E-08	0.54	88	2.2	Caln1	
DMR12:29381001	12	29381001	29382000	1000	1	1.50E-13	0.68	24	2.4	Caln1	
DMR12:29455001	12	29455001	29457000	2000	1	2.30E-07	0.43	17	0.85	Caln1	
DMR12:29739001	12	29739001	29741000	2000	1	4.40E-08	0.35	24	1.2	Caln1	
DMR12:29743001	12	29743001	29745000	2000	1	6.80E-08	0.52	36	1.8	Caln1	
DMR12:29856001	12	29856001	29859000	3000	1	7.60E-12	-0.63	68	2.27	Tyw1	Metabolism
DMR12:30036001	12	30036001	30039000	3000	1	1.30E-07	0.28	39	1.3	Kctd7;Tpst1	Cytoskeleton;Transport
DMR12:30092001	12	30092001	30093000	1000	1	3.40E-07	0.32	13	1.3	Tpst1	Transport
DMR12:30113001	12	30113001	30114000	1000	1	4.20E-10	0.38	5	0.5	Tpst1	Transport
DMR12:30129001	12	30129001	30132000	3000	1	9.80E-07	-0.45	60	2	Crcp	Signaling
DMR12:30187001	12	30187001	30188000	1000	1	5.00E-10	0.7	31	3.1	Asl;LOC501835	Metabolism
DMR12:30444001	12	30444001	30446000	2000	1	8.40E-07	-0.46	29	1.45	LOC100910788;Phkg1	Signaling
DMR12:30496001	12	30496001	30499000	3000	1	1.60E-11	-0.61	36	1.2	Sumf2;Cct6a	Translation
DMR12:30720001	12	30720001	30721000	1000	1	3.40E-07	0.42	11	1.1	Mmp17	Protease
DMR12:31182001	12	31182001	31185000	3000	1	7.00E-08	0.41	39	1.3	Adgrd1	Signaling
DMR12:31195001	12	31195001	31198000	3000	1	1.10E-08	-0.47	78	2.6	Adgrd1	Signaling
DMR12:31219001	12	31219001	31223000	4000	1	9.60E-07	0.33	75	1.88	Adgrd1	Signaling
DMR12:31238001	12	31238001	31241000	3000	1	8.30E-12	0.74	61	2.03	Adgrd1	Signaling
DMR12:31334001	12	31334001	31335000	1000	1	4.00E-10	0.77	21	2.1	Ran;Stx2	Signaling;Transcription
DMR12:31343001	12	31343001	31345000	2000	1	4.10E-07	0.35	20	1	Stx2	Transcription
DMR12:31366001	12	31366001	31368000	2000	1	4.50E-08	0.42	20	1	Stx2	Transcription
DMR12:31410001	12	31410001	31413000	3000	1	6.70E-08	0.53	61	2.03	Rimbp2	
DMR12:31529001	12	31529001	31532000	3000	1	9.20E-11	0.45	47	1.57	Rimbp2	

DMR12:31580001	12	31580001	31581000	1000	1	3.00E-11	0.77	24	2.4	Rimbp2	
DMR12:31588001	12	31588001	31590000	2000	1	3.40E-07	0.66	55	2.75	Rimbp2	
DMR12:31591001	12	31591001	31592000	1000	1	3.10E-07	0.48	14	1.4	Rimbp2	
DMR12:32132001	12	32132001	32133000	1000	1	2.00E-12	0.98	28	2.8	Tmem132d	
DMR12:32177001	12	32177001	32180000	3000	1	3.40E-07	0.38	39	1.3	Tmem132d	
DMR12:32210001	12	32210001	32212000	2000	1	4.00E-10	0.38	24	1.2	Tmem132d	
DMR12:32374001	12	32374001	32376000	2000	1	4.10E-07	0.36	19	0.95	Tmem132d	
DMR12:32383001	12	32383001	32387000	4000	1	3.00E-07	-0.3	30	0.75	Tmem132d	
DMR12:32392001	12	32392001	32394000	2000	1	8.30E-07	0.33	37	1.85	Tmem132d	
DMR12:32458001	12	32458001	32460000	2000	1	2.90E-17	0.96	37	1.85	Tmem132d	
DMR12:32466001	12	32466001	32470000	4000	1	1.30E-10	0.55	62	1.55	Tmem132d	
DMR12:32504001	12	32504001	32505000	1000	1	2.60E-07	-0.47	23	2.3	Tmem132d	
DMR12:32523001	12	32523001	32524000	1000	1	4.60E-07	0.49	20	2	Tmem132d	
DMR12:32563001	12	32563001	32567000	4000	1	2.60E-11	0.67	78	1.95	Tmem132d	
DMR12:32884001	12	32884001	32885000	1000	1	1.40E-07	0.33	13	1.3	Tmem132c	
DMR12:32907001	12	32907001	32911000	4000	1	6.70E-08	0.52	51	1.27	Tmem132c;LOC108352442	
DMR12:32938001	12	32938001	32939000	1000	1	3.20E-08	0.57	6	0.6	Tmem132c	
DMR12:32974001	12	32974001	32975000	1000	1	6.80E-11	0.53	29	2.9	Tmem132c	
DMR12:33178001	12	33178001	33179000	1000	1	1.20E-09	0.68	28	2.8	RGD1559821	
DMR12:33186001	12	33186001	33188000	2000	1	8.00E-08	0.43	40	2	RGD1559821	
DMR12:34001001	12	34001001	34005000	4000	1	2.30E-08	-0.42	56	1.4	Frg1l1	
DMR12:35963001	12	35963001	35964000	1000	1	3.40E-07	0.45	14	1.4	Tmem132b	
DMR12:36031001	12	36031001	36036000	5000	1	1.10E-10	0.49	78	1.56	Tmem132b	
DMR12:36046001	12	36046001	36050000	4000	2	1.00E-09	0.67	66	1.65	Tmem132b	
DMR12:36051001	12	36051001	36057000	6000	2	3.10E-09	0.61	122	2.03	Tmem132b	
DMR12:36082001	12	36082001	36084000	2000	1	3.70E-15	0.65	25	1.25	Tmem132b	
DMR12:36197001	12	36197001	36198000	1000	1	1.40E-07	0.5	17	1.7	Tmem132b;LOC100911699	
DMR12:36207001	12	36207001	36209000	2000	1	1.80E-10	0.36	22	1.1	Tmem132b;LOC100911699	
DMR12:36227001	12	36227001	36228000	1000	1	1.90E-08	0.42	3	0.3	Tmem132b	
DMR12:36251001	12	36251001	36256000	5000	1	9.90E-08	0.59	112	2.24	Tmem132b	
DMR12:36265001	12	36265001	36269000	4000	1	1.90E-07	0.25	66	1.65	Tmem132b	
DMR12:36289001	12	36289001	36293000	4000	2	6.80E-13	0.82	70	1.75	Tmem132b	
DMR12:36295001	12	36295001	36297000	2000	1	1.90E-07	0.35	25	1.25	Tmem132b	
DMR12:36394001	12	36394001	36399000	5000	1	4.90E-08	0.6	110	2.2	Tmem132b	
DMR12:36652001	12	36652001	36654000	2000	1	3.40E-07	-0.43	86	4.3	Ubc	
DMR12:36761001	12	36761001	36766000	5000	1	1.80E-09	0.55	71	1.42	Scarb1	Transport
DMR12:36972001	12	36972001	36973000	1000	1	1.60E-10	0.41	17	1.7	Ncor2	Epigenetic
DMR12:37203001	12	37203001	37204000	1000	1	8.10E-07	-0.4	9	0.9	LOC102556092;RGD1561886;Ccdc92	Transcription
DMR12:37502001	12	37502001	37505000	3000	1	3.00E-07	-0.46	42	1.4	Rilpl1	
DMR12:37543001	12	37543001	37544000	1000	1	5.40E-07	0.42	17	1.7	Snrnp35;Rilpl2	Translation
DMR12:37572001	12	37572001	37574000	2000	1	5.40E-07	-0.41	40	2	Kmt5a	Golgi
DMR12:37651001	12	37651001	37655000	4000	2	5.20E-09	-0.46	68	1.7	Sbno1	
DMR12:37882001	12	37882001	37886000	4000	1	3.60E-07	-0.32	91	2.28	Pitpnm2	Transport
DMR12:37922001	12	37922001	37923000	1000	1	9.00E-15	-0.58	22	2.2	LOC103691351;Abcb9	Transport
DMR12:37932001	12	37932001	37933000	1000	1	4.10E-08	0.41	24	2.4	Abcb9	Transport
DMR12:38031001	12	38031001	38033000	2000	1	3.40E-08	0.45	44	2.2	Vps37b;Hip1r	Cytoskeleton
DMR12:38297001	12	38297001	38298000	1000	1	2.50E-07	-0.37	13	1.3	Rsrc2;Zcchc8	Metabolism
DMR12:38340001	12	38340001	38341000	1000	1	2.40E-11	0.63	26	2.6	LOC100359550;Clip1	Transcription
DMR12:38421001	12	38421001	38425000	4000	1	5.20E-12	0.44	63	1.57	Clip1	Transcription
DMR12:38605001	12	38605001	38607000	2000	1	1.30E-11	0.59	9	0.45	Mlxip	Transcription
DMR12:38612001	12	38612001	38613000	1000	1	1.60E-11	0.59	2	0.2	Mlxip	Transcription
DMR12:38657001	12	38657001	38664000	7000	1	2.60E-08	0.65	192	2.74	Bcl7a	
DMR12:38728001	12	38728001	38731000	3000	1	3.80E-07	-0.36	59	1.97	Wdr66	
DMR12:38745001	12	38745001	38748000	3000	1	5.10E-07	-0.85	80	2.67	Wdr66	
DMR12:38821001	12	38821001	38824000	3000	1	5.40E-09	0.55	44	1.47	LOC102547495;Hpd	Metabolism
DMR12:38843001	12	38843001	38847000	4000	1	4.60E-09	0.46	82	2.05	Hpd;LOC100359816	Metabolism
DMR12:38901001	12	38901001	38902000	1000	1	7.00E-08	-0.41	28	2.8	Rhof;Tmem120b	Signaling
DMR12:38943001	12	38943001	38944000	1000	1	3.90E-13	0.57	17	1.7	LOC687612;Morn3	
DMR12:39032001	12	39032001	39034000	2000	1	1.80E-07	0.35	23	1.15	Kdm2b	
DMR12:39173001	12	39173001	39174000	1000	1	7.70E-07	-0.45	11	1.1	Rnf34	Proteolysis
DMR12:39197001	12	39197001	39200000	3000	1	2.10E-07	0.69	44	1.47	Anapc5	
DMR12:39247001	12	39247001	39249000	2000	1	6.30E-07	-0.41	38	1.9	Anapc5;Camkk2	

DMR12:39302001	12	39302001	39305000	3000	1	4.60E-07	-0.41	78	2.6	Camkk2;P2rx4	Ion Channel
DMR12:39493001	12	39493001	39495000	2000	1	4.30E-09	-0.37	33	1.65	Ift81;LOC108352446	
DMR12:39589001	12	39589001	39592000	3000	1	8.60E-08	-0.41	46	1.53	Atp2a2	Transport
DMR12:39691001	12	39691001	39692000	1000	1	6.40E-07	-0.37	15	1.5	Fam216a;Vps29;Rad9b	Transport;DNA Repair
DMR12:39778001	12	39778001	39782000	4000	1	8.60E-09	0.45	68	1.7	Pptc7;Tctn1	Signaling
DMR12:39831001	12	39831001	39836000	5000	2	1.50E-11	0.56	69	1.38	Tctn1;Hvcn1	
DMR12:39837001	12	39837001	39838000	1000	1	1.10E-07	0.55	29	2.9	Tctn1;Hvcn1	
DMR12:40040001	12	40040001	40041000	1000	1	1.10E-10	0.53	23	2.3	Cux2	Development
DMR12:40048001	12	40048001	40052000	4000	2	3.10E-09	0.5	77	1.93	Cux2	Development
DMR12:40095001	12	40095001	40096000	1000	1	8.90E-07	0.53	43	4.3	Cux2	Development
DMR12:40112001	12	40112001	40114000	2000	1	1.70E-07	0.43	29	1.45	Cux2	Development
DMR12:40126001	12	40126001	40128000	2000	1	2.90E-07	0.42	27	1.35	Cux2	Development
DMR12:40134001	12	40134001	40137000	3000	1	2.40E-08	0.34	52	1.73	Cux2	Development
DMR12:40321001	12	40321001	40322000	1000	1	4.90E-07	0.37	28	2.8	Atxn2;LOC102556457	Metabolism
DMR12:40467001	12	40467001	40468000	1000	1	1.40E-12	-0.68	16	1.6	Acad10;Aldh2	Metabolism
DMR12:40684001	12	40684001	40686000	2000	1	5.70E-07	0.35	24	1.2	Naa25;LOC100360290;Trafd1	Metabolism
DMR12:40782001	12	40782001	40785000	3000	1	1.20E-09	0.41	49	1.63	Hectd4	Proteolysis
DMR12:40815001	12	40815001	40819000	4000	1	3.40E-10	-0.52	54	1.35	Hectd4	Proteolysis
DMR12:40881001	12	40881001	40882000	1000	1	9.00E-09	-0.75	56	5.6	Rpl6	Translation
DMR12:41096001	12	41096001	41098000	2000	1	7.40E-10	0.62	39	1.95	Rph3a	
DMR12:41145001	12	41145001	41146000	1000	1	2.10E-08	0.39	16	1.6	Rph3a;Oas1i	Metabolism
DMR12:41194001	12	41194001	41195000	1000	1	1.70E-08	-0.44	22	2.2	Oas1d;Oas1a	Metabolism
DMR12:41245001	12	41245001	41248000	3000	1	9.10E-12	0.45	12	0.4	Oas1f;Oas1b	Metabolism
DMR12:41314001	12	41314001	41317000	3000	1	2.70E-07	0.38	41	1.37	Oas1k;Oas3	Metabolism
DMR12:41325001	12	41325001	41326000	1000	1	3.20E-14	0.61	25	2.5	Oas3	Metabolism
DMR12:41351001	12	41351001	41355000	4000	1	9.50E-09	0.36	70	1.75	Oas3;Oas2	Metabolism
DMR12:41391001	12	41391001	41392000	1000	1	8.70E-07	0.49	12	1.2	Dtx1	Proteolysis
DMR12:41404001	12	41404001	41406000	2000	1	5.20E-08	0.38	24	1.2	Dtx1	Proteolysis
DMR12:41434001	12	41434001	41435000	1000	1	1.60E-07	0.41	13	1.3	Rasal1	Signaling
DMR12:41917001	12	41917001	41919000	2000	1	2.40E-08	0.36	35	1.75	Rbm19	Translation
DMR12:42051001	12	42051001	42052000	1000	1	3.90E-12	0.97	36	3.6	Tbx5	Transcription
DMR12:42082001	12	42082001	42087000	5000	2	6.40E-08	0.49	62	1.24	Tbx5;LOC102549962	Transcription
DMR12:42129001	12	42129001	42130000	1000	1	7.60E-08	0.76	32	3.2	Tbx5	Transcription
DMR12:42146001	12	42146001	42147000	1000	1	1.60E-12	0.7	30	3	Tbx5	Transcription
DMR12:42486001	12	42486001	42487000	1000	1	2.90E-13	-0.58	13	1.3	Tbx3	Transcription
DMR12:42489001	12	42489001	42491000	2000	1	7.00E-10	-0.53	50	2.5	Tbx3	Transcription
DMR12:43446001	12	43446001	43448000	2000	1	8.60E-07	-0.32	55	2.75	Med13l	
DMR12:43512001	12	43512001	43516000	4000	1	8.50E-10	-0.48	62	1.55	Med13l	
DMR12:43933001	12	43933001	43935000	2000	1	1.50E-09	0.55	16	0.8	RGD1562310;Rnft2	
DMR12:43957001	12	43957001	43960000	3000	1	3.70E-07	0.42	46	1.53	Rnft2	
DMR12:43996001	12	43996001	43997000	1000	1	4.70E-09	0.7	5	0.5	Rnft2;LOC102553921	
DMR12:44003001	12	44003001	44004000	1000	1	5.30E-07	0.46	11	1.1	Rnft2;Hrk	
DMR12:44052001	12	44052001	44054000	2000	1	1.30E-09	0.86	18	0.9	Fbxw8	
DMR12:44148001	12	44148001	44149000	1000	1	6.50E-07	0.33	18	1.8	Fbxw8;Tesc	
DMR12:44163001	12	44163001	44164000	1000	1	9.20E-07	0.36	13	1.3	Tesc	
DMR12:44284001	12	44284001	44285000	1000	1	3.80E-09	-0.42	12	1.2	Nos1;Fbxo21	Metabolism
DMR12:44624001	12	44624001	44625000	1000	1	1.80E-07	0.41	1	0.1	Ksr2	Signaling
DMR12:44659001	12	44659001	44661000	2000	1	1.40E-07	0.33	20	1	Ksr2	Signaling
DMR12:44718001	12	44718001	44719000	1000	1	2.60E-09	0.5	9	0.9	Ksr2	Signaling
DMR12:44761001	12	44761001	44763000	2000	1	1.60E-08	0.53	48	2.4	Ksr2	Signaling
DMR12:44767001	12	44767001	44770000	3000	2	1.00E-09	0.68	55	1.83	Ksr2	Signaling
DMR12:44822001	12	44822001	44826000	4000	1	3.40E-07	0.43	61	1.52	Ksr2	Signaling
DMR12:44852001	12	44852001	44853000	1000	1	9.10E-08	0.68	26	2.6	Ksr2	Signaling
DMR12:44857001	12	44857001	44859000	2000	1	3.00E-08	0.37	22	1.1	Ksr2	Signaling
DMR12:44860001	12	44860001	44864000	4000	1	2.50E-07	-0.43	67	1.68	Ksr2	Signaling
DMR12:45002001	12	45002001	45003000	1000	1	1.20E-08	-0.45	10	1	Vsig10	
DMR12:45078001	12	45078001	45080000	2000	1	8.80E-20	0.46	19	0.95	Taok3	Signaling
DMR12:45189001	12	45189001	45190000	1000	1	4.10E-08	0.6	23	2.3	Suds3	
DMR12:45801001	12	45801001	45802000	1000	1	9.30E-11	0.42	9	0.9	Srrm4	
DMR12:45818001	12	45818001	45820000	2000	1	1.60E-09	0.84	59	2.95	Srrm4	
DMR12:45893001	12	45893001	45894000	1000	1	1.60E-08	0.37	23	2.3	Srrm4	
DMR12:46077001	12	46077001	46081000	4000	1	1.10E-08	0.47	106	2.65	Ccdc60	
DMR12:46129001	12	46129001	46132000	3000	2	6.20E-10	0.37	51	1.7	Ccdc60	

DMR12:46159001	12	46159001	46160000	1000	1	2.90E-08	0.34	10	1	Ccdc60	
DMR12:46296001	12	46296001	46298000	2000	1	3.20E-09	0.73	41	2.05	Tmem233	
DMR12:46307001	12	46307001	46311000	4000	1	1.80E-09	0.66	78	1.95	Tmem233;Prkab1	Signaling
DMR12:46328001	12	46328001	46333000	5000	1	2.90E-07	-0.38	117	2.34	Prkab1;LOC108352463;Cit	Signaling;Signaling
DMR12:46353001	12	46353001	46357000	4000	1	2.60E-07	-0.46	94	2.35	Cit	Signaling
DMR12:46376001	12	46376001	46380000	4000	1	7.10E-09	0.46	98	2.45	Cit	Signaling
DMR12:46459001	12	46459001	46462000	3000	1	8.70E-10	0.37	58	1.93	Cit	Signaling
DMR12:46478001	12	46478001	46479000	1000	1	3.50E-07	0.45	12	1.2	Cit	Signaling
DMR12:46631001	12	46631001	46632000	1000	1	6.80E-13	-0.54	26	2.6	Bicdl1	Transport
DMR12:46647001	12	46647001	46651000	4000	1	2.80E-07	-0.38	70	1.75	Bicdl1	Transport
DMR12:46670001	12	46670001	46671000	1000	1	3.00E-08	-0.4	11	1.1	Bicdl1	Transport
DMR12:46723001	12	46723001	46727000	4000	1	8.40E-07	-0.33	54	1.35	Rab35;Gcn1l1	
DMR12:46788001	12	46788001	46789000	1000	1	4.10E-07	-0.47	14	1.4	Gcn1l1;Rplp0;Pxn	Translation;Cytoskeleton
DMR12:46835001	12	46835001	46838000	3000	1	1.40E-07	-0.5	84	2.8	Pxn	Cytoskeleton
DMR12:46871001	12	46871001	46875000	4000	1	4.80E-07	0.48	104	2.6	Sirt4;Pla2g1b	Metabolism
DMR12:46942001	12	46942001	46947000	5000	1	2.30E-09	0.42	106	2.12	Msi1;LOC102547958	
DMR12:47259001	12	47259001	47261000	2000	1	2.90E-12	0.55	47	2.35	Unc119b;Acads	Metabolism
DMR12:47315001	12	47315001	47317000	2000	1	4.30E-07	-0.63	22	1.1	Sppl3	Proteolysis
DMR12:47402001	12	47402001	47403000	1000	1	2.20E-08	0.42	6	0.6	LOC102546369;Hnf1a	Transcription
DMR12:47451001	12	47451001	47452000	1000	1	1.70E-10	0.68	8	0.8	Oasl	Metabolism
DMR12:47648001	12	47648001	47649000	1000	1	9.00E-09	0.31	12	1.2	Tchp;LOC102547088	
DMR12:47733001	12	47733001	47734000	1000	1	8.20E-08	0.63	23	2.3	Trpv4;Fam222a	Transport
DMR12:48074001	12	48074001	48075000	1000	1	3.30E-09	0.43	11	1.1	Myo1h	
DMR12:48115001	12	48115001	48116000	1000	1	1.30E-07	0.37	21	2.1	Foxn4	
DMR12:48169001	12	48169001	48174000	5000	1	4.40E-07	0.37	127	2.54	Acacb	
DMR12:48192001	12	48192001	48195000	3000	1	1.20E-08	0.38	46	1.53	Acacb	
DMR12:48268001	12	48268001	48269000	1000	1	2.30E-08	-0.43	24	2.4	Alkbh2;Usp30;LOC103691493	Metabolism;Protease
DMR12:48315001	12	48315001	48318000	3000	1	5.10E-07	0.42	64	2.13	Usp30;Svop	Protease;Transport
DMR12:48355001	12	48355001	48356000	1000	1	4.50E-10	0.4	11	1.1	Svop;Dao	Transport;Metabolism
DMR12:48405001	12	48405001	48407000	2000	1	1.70E-07	-0.45	33	1.65	Ssh1	Signaling
DMR12:48485001	12	48485001	48487000	2000	1	7.40E-10	-0.46	39	1.95	Coro1c	Cytoskeleton
DMR12:48545001	12	48545001	48547000	2000	1	1.30E-07	0.44	55	2.75	Coro1c	Cytoskeleton
DMR12:48622001	12	48622001	48625000	3000	1	4.60E-07	0.31	45	1.5	Iscu;Sart3	Transcription;Translation
DMR12:48655001	12	48655001	48657000	2000	1	4.00E-07	0.42	40	2	Sart3;Ficd	Translation
DMR12:48744001	12	48744001	48748000	4000	2	2.30E-08	0.77	85	2.12	Cmklr1	Signaling
DMR12:48771001	12	48771001	48775000	4000	1	1.80E-09	0.73	87	2.17	Cmklr1	Signaling
DMR12:48797001	12	48797001	48801000	4000	2	3.60E-08	-0.46	70	1.75	Cmklr1	Signaling
DMR12:48820001	12	48820001	48821000	1000	1	8.80E-07	0.43	19	1.9	Wscd2	
DMR12:48825001	12	48825001	48830000	5000	1	3.30E-07	0.5	112	2.24	Wscd2	
DMR12:49335001	12	49335001	49337000	2000	1	3.90E-07	0.4	33	1.65	Sgsm1	Signaling
DMR12:49352001	12	49352001	49355000	3000	2	6.20E-11	0.52	42	1.4	Sgsm1	Signaling
DMR12:49395001	12	49395001	49398000	3000	1	4.60E-07	0.35	25	0.83	Sgsm1;Tmem211	Signaling
DMR12:49402001	12	49402001	49405000	3000	1	9.50E-07	0.33	27	0.9	Sgsm1;Tmem211;LOC108352470	Signaling
DMR12:49441001	12	49441001	49442000	1000	1	6.00E-07	0.42	47	4.7	RGD1306556	
DMR12:49503001	12	49503001	49504000	1000	1	2.40E-07	0.52	11	1.1	RGD1306556;LOC102552141	
DMR12:49596001	12	49596001	49598000	2000	2	8.20E-09	0.53	12	0.6	Crybb2	
DMR12:49623001	12	49623001	49625000	2000	1	2.80E-07	0.4	23	1.15	Grk3	Signaling
DMR12:49697001	12	49697001	49698000	1000	1	6.80E-08	0.36	9	0.9	Grk3	Signaling
DMR12:49779001	12	49779001	49783000	4000	1	5.10E-08	0.61	90	2.25	Myo18b	
DMR12:49797001	12	49797001	49798000	1000	1	2.30E-14	0.75	36	3.6	Myo18b	
DMR12:49914001	12	49914001	49921000	7000	2	6.60E-11	0.49	119	1.7	Myo18b	
DMR12:49971001	12	49971001	49974000	3000	1	5.20E-07	0.37	34	1.13	Myo18b	
DMR12:50176001	12	50176001	50178000	2000	1	7.40E-07	0.42	29	1.45	Sez6l	
DMR12:50246001	12	50246001	50249000	3000	1	4.20E-08	0.42	24	0.8	Sez6l	
DMR12:50267001	12	50267001	50268000	1000	1	2.60E-07	0.37	16	1.6	Asphd2	Metabolism
DMR12:51228001	12	51228001	51230000	2000	1	3.70E-07	0.41	50	2.5	Mn1	
DMR12:51257001	12	51257001	51259000	2000	1	2.10E-08	0.52	15	0.75	Mn1	
DMR12:51275001	12	51275001	51276000	1000	1	8.70E-08	0.43	6	0.6	Pitpnb	Transport
DMR12:51375001	12	51375001	51379000	4000	1	7.30E-08	0.44	48	1.2	LOC102554233;Ttc28	Cytoskeleton
DMR12:51400001	12	51400001	51401000	1000	1	1.20E-13	0.54	22	2.2	Ttc28	Cytoskeleton
DMR12:51430001	12	51430001	51434000	4000	1	1.20E-11	0.39	44	1.1	Ttc28	Cytoskeleton
DMR12:51547001	12	51547001	51548000	1000	1	3.50E-08	0.37	8	0.8	Ttc28;LOC102553486;LOC108352474	Cytoskeleton

DMR12:51642001	12	51642001	51645000	3000	1	2.20E-07	0.49	38	1.27	Ttc28	Cytoskeleton
DMR12:51749001	12	51749001	51752000	3000	1	1.50E-08	0.41	39	1.3	Ttc28	Cytoskeleton
DMR12:51766001	12	51766001	51768000	2000	1	6.70E-07	0.41	16	0.8	Ttc28	Cytoskeleton
DMR12:51846001	12	51846001	51847000	1000	1	1.90E-07	-0.43	22	2.2	Ttc28;Chek2	Cytoskeleton;Signaling
DMR12:51863001	12	51863001	51864000	1000	1	6.30E-07	0.37	9	0.9	Chek2	Signaling
DMR12:52309001	12	52309001	52313000	4000	1	1.50E-08	-0.5	51	1.27	Fbrsl1	
DMR13:1956001	13	1956001	1958000	2000	1	1.80E-07	-0.38	20	1	Dsel	
DMR13:6420001	13	6420001	6423000	3000	1	3.40E-07	-0.38	20	0.67	Cntnap5c	
DMR13:6424001	13	6424001	6426000	2000	1	4.90E-08	-0.54	15	0.75	Cntnap5c	
DMR13:6441001	13	6441001	6446000	5000	1	1.90E-07	-0.4	39	0.78	Cntnap5c	
DMR13:6739001	13	6739001	6745000	6000	2	1.40E-11	0.48	44	0.73	Cntnap5c	
DMR13:7063001	13	7063001	7068000	5000	3	6.40E-10	-0.4	40	0.8	Cntnap5c	
DMR13:7070001	13	7070001	7071000	1000	1	5.80E-09	0.55	9	0.9	Cntnap5c	
DMR13:7086001	13	7086001	7087000	1000	1	3.90E-07	0.49	3	0.3	Cntnap5c	
DMR13:21758001	13	21758001	21760000	2000	1	3.60E-07	-0.34	80	4	Cntnap5b	
DMR13:21799001	13	21799001	21806000	7000	1	3.40E-11	-0.41	63	0.9	Cntnap5b	
DMR13:21867001	13	21867001	21868000	1000	1	4.00E-07	0.52	4	0.4	Cntnap5b	
DMR13:22098001	13	22098001	22104000	6000	1	6.70E-08	-0.34	64	1.07	Cntnap5b	
DMR13:22108001	13	22108001	22113000	5000	1	8.70E-07	-0.64	48	0.96	Cntnap5b	
DMR13:22137001	13	22137001	22140000	3000	1	4.00E-08	-0.37	18	0.6	Cntnap5b	
DMR13:22213001	13	22213001	22216000	3000	1	6.00E-07	-0.64	66	2.2	Cntnap5b	
DMR13:22301001	13	22301001	22303000	2000	1	4.80E-08	-0.4	23	1.15	Cntnap5b	
DMR13:22313001	13	22313001	22314000	1000	1	8.20E-09	0.4	5	0.5	Cntnap5b	
DMR13:22447001	13	22447001	22450000	3000	1	7.20E-07	-0.49	10	0.33	Cntnap5b	
DMR13:24750001	13	24750001	24754000	4000	1	3.70E-07	-0.38	26	0.65	Cdh20	Cytoskeleton
DMR13:24856001	13	24856001	24859000	3000	1	2.90E-07	-0.35	27	0.9	Cdh20	Cytoskeleton
DMR13:25517001	13	25517001	25518000	1000	1	3.60E-07	0.39	8	0.8	Pign	Extracellular Matrix
DMR13:25557001	13	25557001	25561000	4000	1	6.10E-08	0.33	38	0.95	Pign	Extracellular Matrix
DMR13:25665001	13	25665001	25669000	4000	1	6.80E-09	-0.42	28	0.7	Pign;RGD1307235	Extracellular Matrix
DMR13:25770001	13	25770001	25771000	1000	1	1.30E-07	0.33	14	1.4	Tnfrsf11a	Receptor
DMR13:25786001	13	25786001	25787000	1000	1	5.70E-13	0.35	8	0.8	Tnfrsf11a	Receptor
DMR13:25818001	13	25818001	25820000	2000	1	3.20E-08	0.37	27	1.35	Tnfrsf11a	Receptor
DMR13:25834001	13	25834001	25837000	3000	2	5.30E-10	0.59	52	1.73	Tnfrsf11a	Receptor
DMR13:26836001	13	26836001	26837000	1000	1	4.60E-07	-0.64	10	1	Vps4b	Cytoskeleton
DMR13:27039001	13	27039001	27040000	1000	1	7.60E-08	-0.42	9	0.9	Serpinb13	Protease; Proteolysis
DMR13:27178001	13	27178001	27179000	1000	1	6.40E-08	0.47	1	0.1	Serpinb3	Protease; Proteolysis
DMR13:27266001	13	27266001	27268000	2000	1	1.50E-08	-0.44	11	0.55	Serpinb11;LOC680414	Protease; Proteolysis
DMR13:27864001	13	27864001	27869000	5000	2	2.30E-09	-0.46	35	0.7	Serpinb8	Protease; Proteolysis
DMR13:27915001	13	27915001	27917000	2000	1	2.30E-07	-0.35	58	2.9	Serpinb8	Protease; Proteolysis
DMR13:30504001	13	30504001	30510000	6000	1	3.40E-07	-0.33	55	0.92	RGD1560523	
DMR13:30543001	13	30543001	30549000	6000	1	9.20E-07	-0.36	87	1.45	RGD1565627	
DMR13:34244001	13	34244001	34245000	1000	1	3.80E-07	-0.45	5	0.5	Tsn	
DMR13:34257001	13	34257001	34264000	7000	1	1.00E-06	-0.43	62	0.89	Tsn;Nifk	
DMR13:34271001	13	34271001	34273000	2000	2	1.70E-14	-0.52	20	1	Nifk	
DMR13:34554001	13	34554001	34556000	2000	1	9.50E-13	-0.69	23	1.15	Clasp1	Cytoskeleton
DMR13:34643001	13	34643001	34645000	2000	1	5.20E-20	0.7	27	1.35	Tfcp2l1	Transcription
DMR13:34675001	13	34675001	34678000	3000	2	7.50E-07	0.38	21	0.7	Tfcp2l1	Transcription
DMR13:35014001	13	35014001	35015000	1000	1	1.30E-10	0.37	9	0.9	Gli2	Transcription
DMR13:35019001	13	35019001	35023000	4000	1	2.30E-07	0.38	46	1.15	Gli2	Transcription
DMR13:35663001	13	35663001	35665000	2000	1	1.20E-09	0.52	8	0.4	Epb4.1l5	
DMR13:35798001	13	35798001	35805000	7000	3	1.00E-09	-0.43	51	0.73	Ptpn4	Signaling
DMR13:36021001	13	36021001	36023000	2000	1	2.30E-08	-0.54	23	1.15	Cfap221	Development
DMR13:36035001	13	36035001	36036000	1000	1	2.80E-10	0.47	9	0.9	Sctr	Receptor
DMR13:36048001	13	36048001	36054000	6000	1	5.10E-10	0.47	93	1.55	Sctr	Receptor
DMR13:36096001	13	36096001	36099000	3000	1	6.10E-09	0.37	33	1.1	Sctr;Tmem37	Receptor;Transport
DMR13:36261001	13	36261001	36265000	4000	1	4.80E-09	0.48	64	1.6	Steap3	
DMR13:36528001	13	36528001	36529000	1000	1	5.80E-07	-0.39	21	2.1	En1	Development
DMR13:37253001	13	37253001	37256000	3000	1	2.20E-09	0.42	31	1.03	Insig2	
DMR13:37393001	13	37393001	37394000	1000	1	1.20E-07	-0.48	20	2	Ccdc93	
DMR13:37507001	13	37507001	37513000	6000	2	1.10E-08	-0.31	65	1.08	Ddx18	
DMR13:39769001	13	39769001	39770000	1000	1	3.10E-07	0.46	5	0.5	Dpp10	Protease
DMR13:40031001	13	40031001	40033000	2000	1	4.20E-07	0.48	17	0.85	Dpp10	Protease
DMR13:40071001	13	40071001	40072000	1000	1	4.60E-07	-0.45	3	0.3	Dpp10	Protease

DMR13:40208001	13	40208001	40212000	4000	1	7.30E-08	-0.32	36	0.9	Dpp10	Protease
DMR13:40322001	13	40322001	40327000	5000	1	3.80E-09	-0.3	68	1.36	Dpp10	Protease
DMR13:40349001	13	40349001	40355000	6000	2	6.20E-09	-0.37	52	0.87	Dpp10	Protease
DMR13:40459001	13	40459001	40461000	2000	1	1.90E-07	0.42	11	0.55	Dpp10	Protease
DMR13:40470001	13	40470001	40476000	6000	2	2.00E-13	-0.4	71	1.18	Dpp10	Protease
DMR13:40839001	13	40839001	40840000	1000	1	5.60E-07	0.6	19	1.9	Dpp10	Protease
DMR13:40950001	13	40950001	40951000	1000	1	9.40E-08	0.47	4	0.4	Dpp10	Protease
DMR13:41093001	13	41093001	41094000	1000	1	3.00E-09	-0.33	6	0.6	Dpp10	Protease
DMR13:41119001	13	41119001	41125000	6000	1	2.80E-14	-0.57	53	0.88	Dpp10	Protease
DMR13:41138001	13	41138001	41139000	1000	1	8.90E-14	0.75	22	2.2	Dpp10	Protease
DMR13:41896001	13	41896001	41897000	1000	1	1.80E-20	0.89	8	0.8	Slc35f5	Transport
DMR13:42045001	13	42045001	42046000	1000	1	3.50E-07	0.44	7	0.7	Gpr39	Signaling
DMR13:42097001	13	42097001	42101000	4000	1	1.10E-11	0.48	22	0.55	Gpr39	Signaling
DMR13:42127001	13	42127001	42129000	2000	1	5.30E-08	0.57	30	1.5	Gpr39	Signaling
DMR13:42132001	13	42132001	42135000	3000	1	1.00E-08	-0.29	27	0.9	Gpr39	Signaling
DMR13:42136001	13	42136001	42137000	1000	1	7.20E-09	-0.26	10	1	Gpr39	Signaling
DMR13:42426001	13	42426001	42431000	5000	2	3.50E-09	-0.39	47	0.94	Nckap5	
DMR13:42622001	13	42622001	42628000	6000	2	5.60E-08	-0.29	76	1.27	Nckap5	
DMR13:42640001	13	42640001	42641000	1000	1	5.70E-07	0.38	4	0.4	Nckap5	
DMR13:42760001	13	42760001	42764000	4000	1	6.00E-07	-0.38	61	1.52	Nckap5	
DMR13:42835001	13	42835001	42836000	1000	1	1.40E-09	0.43	6	0.6	Nckap5	
DMR13:42862001	13	42862001	42863000	1000	1	3.30E-08	0.46	9	0.9	Nckap5	
DMR13:42955001	13	42955001	42957000	2000	1	7.80E-07	0.44	13	0.65	Nckap5	
DMR13:42966001	13	42966001	42967000	1000	1	1.50E-08	0.49	4	0.4	Nckap5	
DMR13:43047001	13	43047001	43049000	2000	1	3.10E-07	-0.39	24	1.2	Nckap5	
DMR13:43990001	13	43990001	43992000	2000	1	1.30E-07	0.33	13	0.65	Mgat5	Golgi
DMR13:44048001	13	44048001	44050000	2000	1	8.10E-07	0.25	40	2	Mgat5	Golgi
DMR13:44183001	13	44183001	44188000	5000	3	2.00E-09	0.35	62	1.24	Tmem163	
DMR13:44291001	13	44291001	44294000	3000	1	2.60E-07	0.35	50	1.67	Tmem163	
DMR13:44309001	13	44309001	44313000	4000	1	6.00E-08	0.33	39	0.98	Tmem163	
DMR13:44323001	13	44323001	44327000	4000	1	2.30E-07	0.36	35	0.88	Tmem163	
DMR13:44435001	13	44435001	44440000	5000	1	3.40E-07	-0.51	15	0.3	Acmsd	Metabolism
DMR13:44482001	13	44482001	44483000	1000	1	7.80E-08	-0.48	12	1.2	LOC108352526;Ccnt2	Signaling
DMR13:44520001	13	44520001	44524000	4000	1	2.50E-07	0.35	49	1.23	Ccnt2;Map3k19	Signaling
DMR13:44645001	13	44645001	44646000	1000	1	1.90E-10	0.41	12	1.2	Rab3gap1	Signaling
DMR13:44823001	13	44823001	44825000	2000	1	4.30E-07	-0.55	7	0.35	R3hdm1;LOC102548453	
DMR13:46077001	13	46077001	46079000	2000	1	4.20E-09	0.61	28	1.4	Thsd7b	Cytoskeleton
DMR13:46299001	13	46299001	46303000	4000	2	1.00E-08	-0.82	31	0.78	Thsd7b	Cytoskeleton
DMR13:46399001	13	46399001	46404000	5000	1	6.60E-08	-0.35	42	0.84	Thsd7b	Cytoskeleton
DMR13:46566001	13	46566001	46567000	1000	1	3.90E-11	0.48	9	0.9	Thsd7b	Cytoskeleton
DMR13:46705001	13	46705001	46706000	1000	1	4.60E-08	0.46	10	1	Thsd7b	Cytoskeleton
DMR13:46740001	13	46740001	46745000	5000	1	2.00E-08	-0.25	55	1.1	Thsd7b	Cytoskeleton
DMR13:46879001	13	46879001	46880000	1000	1	2.40E-10	-0.49	8	0.8	Thsd7b	Cytoskeleton
DMR13:47271001	13	47271001	47277000	6000	2	1.00E-08	-0.36	60	1	Zp3r	
DMR13:47306001	13	47306001	47309000	3000	1	1.20E-08	-0.36	25	0.83	Zp3r	
DMR13:47671001	13	47671001	47673000	2000	2	4.00E-09	0.73	49	2.45	Il19	
DMR13:47777001	13	47777001	47778000	1000	1	7.20E-10	0.62	23	2.3	Mapkapk2	Signaling
DMR13:47896001	13	47896001	47899000	3000	1	1.90E-10	0.74	79	2.63	Dyrk3	
DMR13:47933001	13	47933001	47934000	1000	1	3.90E-09	0.47	4	0.4	Eif2d	Translation
DMR13:48482001	13	48482001	48484000	2000	1	1.70E-08	0.58	40	2	Rab7b	
DMR13:48599001	13	48599001	48601000	2000	1	4.60E-07	-0.42	36	1.8	Pm20d1;Slc41a1	Metabolism;Transport
DMR13:48737001	13	48737001	48738000	1000	1	7.50E-07	-0.35	21	2.1	Slc45a3	Transport
DMR13:48777001	13	48777001	48779000	2000	2	1.00E-09	0.37	7	0.35	Elk4	Transcription
DMR13:48812001	13	48812001	48813000	1000	1	2.90E-08	0.54	21	2.1	Elk4;Mfsd4	Transcription
DMR13:49063001	13	49063001	49065000	2000	1	9.00E-07	-0.55	23	1.15	Klhdc8a	
DMR13:49171001	13	49171001	49173000	2000	1	5.80E-10	0.56	47	2.35	Tmcc2	
DMR13:49321001	13	49321001	49327000	6000	1	1.00E-07	0.36	66	1.1	Cntn2;Nfasc	
DMR13:49384001	13	49384001	49385000	1000	1	8.60E-07	-0.46	19	1.9	Nfasc	
DMR13:49416001	13	49416001	49419000	3000	1	2.70E-07	-0.48	59	1.97	Nfasc	
DMR13:49529001	13	49529001	49530000	1000	1	4.40E-07	0.4	10	1	Nfasc;LOC100360239	
DMR13:49686001	13	49686001	49687000	1000	1	6.40E-07	0.62	28	2.8	Lrrn2	Receptor
DMR13:49728001	13	49728001	49729000	1000	1	2.10E-07	0.55	34	3.4	Lrrn2	Receptor
DMR13:49837001	13	49837001	49839000	2000	1	6.60E-08	-0.38	23	1.15	Mdm4;Pik3c2b	Epigenetic;Signaling

DMR13:50138001	13	50138001	50141000	3000	1	7.00E-08	0.42	55	1.83	Atp2b4	Transport
DMR13:50153001	13	50153001	50155000	2000	1	2.70E-10	0.46	23	1.15	Atp2b4;Lax1	Transport;Immune
DMR13:50471001	13	50471001	50472000	1000	1	2.20E-07	0.42	16	1.6	Sox13;Etnk2	Signaling
DMR13:50774001	13	50774001	50776000	2000	1	7.70E-07	0.37	20	1	Prelp;LOC102557118	
DMR13:50912001	13	50912001	50915000	3000	1	7.60E-10	-0.41	57	1.9	LOC103691904;Btg2	
DMR13:50947001	13	50947001	50948000	1000	1	4.10E-08	-0.39	17	1.7	Chit1	Metabolism
DMR13:50988001	13	50988001	50989000	1000	1	1.30E-10	0.45	9	0.9	Chit1	Metabolism
DMR13:51012001	13	51012001	51015000	3000	1	8.50E-08	-0.57	46	1.53	LOC102552406;Chi3l1	Metabolism
DMR13:51172001	13	51172001	51174000	2000	2	7.10E-15	0.87	51	2.55	Ppfia4	
DMR13:51178001	13	51178001	51180000	2000	1	7.50E-08	0.47	30	1.5	Ppfia4;Tmem183a	
DMR13:51365001	13	51365001	51366000	1000	1	4.30E-08	-0.41	24	2.4	LOC108352535;Mgat4e;LOC103691910	Transport
DMR13:51570001	13	51570001	51572000	2000	1	4.70E-07	0.42	24	1.2	Syt2	Transport
DMR13:51650001	13	51650001	51654000	4000	1	1.00E-07	0.35	45	1.12	Ppp1r12b	Signaling
DMR13:51912001	13	51912001	51915000	3000	1	2.60E-07	-0.35	33	1.1	Lgr6	
DMR13:52205001	13	52205001	52206000	1000	1	3.50E-07	-0.43	13	1.3	Shisa4;Ipo9	Cytoskeleton;Transport
DMR13:52282001	13	52282001	52285000	3000	1	9.70E-09	-0.42	51	1.7	Nav1	
DMR13:52377001	13	52377001	52379000	2000	1	6.40E-08	0.59	25	1.25	Nav1	
DMR13:52381001	13	52381001	52382000	1000	1	5.10E-07	0.32	9	0.9	Nav1	
DMR13:52395001	13	52395001	52397000	2000	1	1.80E-07	0.51	38	1.9	Nav1	
DMR13:52667001	13	52667001	52670000	3000	2	6.20E-08	0.52	61	2.03	Lad1;Tnnt2	Development;Cytoskeleton
DMR13:52698001	13	52698001	52702000	4000	1	5.90E-10	0.38	37	0.92	Pkp1	Cytoskeleton
DMR13:52712001	13	52712001	52714000	2000	1	6.20E-07	-0.35	33	1.65	Pkp1	Cytoskeleton
DMR13:52876001	13	52876001	52878000	2000	1	1.20E-07	0.39	30	1.5	Tmem9;Ascl5	Transcription
DMR13:53147001	13	53147001	53148000	1000	1	1.80E-07	-0.47	15	1.5	Gpr25;Camsap2	Signaling
DMR13:53404001	13	53404001	53406000	2000	1	3.50E-07	-0.49	31	1.55	Kif14	Cytoskeleton
DMR13:53927001	13	53927001	53929000	2000	1	2.30E-09	0.71	31	1.55	RGD1562134	
DMR13:53936001	13	53936001	53938000	2000	1	3.90E-07	0.35	13	0.65	RGD1562134	
DMR13:55172001	13	55172001	55176000	4000	2	2.20E-08	-0.68	47	1.18	Ptprc	Signaling
DMR13:55484001	13	55484001	55485000	1000	1	2.80E-07	-0.44	10	1	Nek7	Signaling
DMR13:55513001	13	55513001	55517000	4000	1	2.40E-08	-0.48	99	2.48	Nek7	Signaling
DMR13:55557001	13	55557001	55560000	3000	1	2.50E-12	-0.43	36	1.2	Nek7	Signaling
DMR13:56084001	13	56084001	56085000	1000	1	4.70E-09	-0.43	7	0.7	Dennd1b	
DMR13:56110001	13	56110001	56114000	4000	1	2.20E-12	-0.62	21	0.52	Dennd1b	
DMR13:56226001	13	56226001	56228000	2000	1	6.70E-12	0.48	18	0.9	Dennd1b	
DMR13:56564001	13	56564001	56565000	1000	1	4.40E-07	-0.43	28	2.8	Aspm	
DMR13:56567001	13	56567001	56572000	5000	1	9.60E-09	-0.45	57	1.14	Aspm	
DMR13:56893001	13	56893001	56894000	1000	1	2.60E-10	0.58	16	1.6	RGD1564614;LOC108352543	
DMR13:56910001	13	56910001	56912000	2000	1	5.80E-07	-0.39	9	0.45	RGD1564614	
DMR13:56995001	13	56995001	56998000	3000	1	4.80E-08	0.43	26	0.87	Cfh	
DMR13:57038001	13	57038001	57043000	5000	2	2.30E-07	-0.4	42	0.84	Cfh	
DMR13:57050001	13	57050001	57056000	6000	1	1.50E-09	-0.42	68	1.13	Cfh	
DMR13:57325001	13	57325001	57326000	1000	1	9.20E-11	-0.41	8	0.8	Kcnt2	Transport
DMR13:57527001	13	57527001	57528000	1000	1	1.40E-13	0.68	16	1.6	Kcnt2	Transport
DMR13:60846001	13	60846001	60849000	3000	1	1.40E-09	-0.59	51	1.7	Rgs2	Signaling
DMR13:62653001	13	62653001	62658000	5000	2	2.20E-07	-0.37	44	0.88	RGD1565548	
DMR13:63543001	13	63543001	63545000	2000	1	5.20E-10	0.78	23	1.15	Brinp3	
DMR13:63683001	13	63683001	63684000	1000	1	2.80E-08	0.51	8	0.8	Brinp3	
DMR13:63748001	13	63748001	63752000	4000	2	7.70E-09	-0.41	40	1	Brinp3	
DMR13:67166001	13	67166001	67169000	3000	1	7.70E-13	-0.66	23	0.77	Pla2g4a	Metabolism
DMR13:67197001	13	67197001	67199000	2000	1	8.70E-16	-0.96	15	0.75	Pla2g4a	Metabolism
DMR13:67557001	13	67557001	67558000	1000	1	1.10E-07	0.43	20	2	Pdc	
DMR13:67641001	13	67641001	67644000	3000	3	2.30E-10	-0.46	32	1.07	Tpr	Transport
DMR13:68139001	13	68139001	68141000	2000	1	3.30E-08	-0.47	32	1.6	Hmcn1	
DMR13:68146001	13	68146001	68150000	4000	2	8.30E-10	-0.35	36	0.9	Hmcn1	
DMR13:68224001	13	68224001	68226000	2000	2	1.60E-12	0.44	12	0.6	Hmcn1	
DMR13:68235001	13	68235001	68238000	3000	1	2.20E-11	-0.39	11	0.37	Hmcn1	
DMR13:68303001	13	68303001	68304000	1000	1	1.20E-09	-0.53	12	1.2	Hmcn1	
DMR13:68317001	13	68317001	68319000	2000	1	1.10E-07	-0.44	29	1.45	Hmcn1	
DMR13:68698001	13	68698001	68701000	3000	1	8.30E-10	0.46	24	0.8	Ivns1abp	Cytoskeleton
DMR13:68715001	13	68715001	68717000	2000	1	2.70E-09	-0.51	25	1.25	Ivns1abp	Cytoskeleton
DMR13:69007001	13	69007001	69009000	2000	1	2.80E-07	-0.31	36	1.8	Fam129a	

DMR13:69017001	13	69017001	69021000	4000	1	8.30E-07	0.44	16	0.4	Fam129a	
DMR13:69028001	13	69028001	69029000	1000	1	8.00E-09	-0.45	16	1.6	Fam129a	
DMR13:69065001	13	69065001	69066000	1000	1	6.30E-09	-0.56	18	1.8	Fam129a	
DMR13:69421001	13	69421001	69422000	1000	1	1.40E-11	-0.51	13	1.3	RGD1309104	
DMR13:69440001	13	69440001	69443000	3000	1	4.20E-07	-0.39	61	2.03	RGD1309104;LOC108352552	
DMR13:69786001	13	69786001	69789000	3000	1	1.20E-08	-0.34	37	1.23	Tsen15;Colgalt2	Golgi
DMR13:69967001	13	69967001	69971000	4000	1	1.30E-09	0.4	40	1	Rgl1;LOC102551528	Transcription
DMR13:69975001	13	69975001	69976000	1000	1	2.50E-07	0.28	6	0.6	Rgl1	Transcription
DMR13:70031001	13	70031001	70032000	1000	1	3.90E-08	-0.61	11	1.1	Rgl1	Transcription
DMR13:70079001	13	70079001	70084000	5000	1	4.80E-07	-0.4	74	1.48	Rgl1	Transcription
DMR13:70122001	13	70122001	70123000	1000	1	2.20E-08	-0.39	28	2.8	Rgl1	Transcription
DMR13:70222001	13	70222001	70223000	1000	1	3.30E-12	0.68	28	2.8	LOC689881;Ncf2	Signaling
DMR13:70247001	13	70247001	70250000	3000	1	7.70E-17	1.01	74	2.47	Ncf2;Smg7	Signaling;Metabolism
DMR13:70327001	13	70327001	70328000	1000	1	9.80E-07	-0.42	13	1.3	Smg7	Metabolism
DMR13:70376001	13	70376001	70378000	2000	1	4.00E-10	0.34	29	1.45	Nmnat2	Metabolism
DMR13:70394001	13	70394001	70395000	1000	1	1.90E-09	0.68	32	3.2	Nmnat2	Metabolism
DMR13:70426001	13	70426001	70432000	6000	1	2.40E-07	-0.47	59	0.98	Nmnat2	Metabolism
DMR13:70490001	13	70490001	70492000	2000	1	8.60E-10	-0.36	41	2.05	Nmnat2;LOC102551684	Metabolism
DMR13:70493001	13	70493001	70498000	5000	1	6.80E-07	-0.46	14	0.28	Nmnat2;LOC102551684	Metabolism
DMR13:70528001	13	70528001	70529000	1000	1	6.80E-09	0.43	14	1.4	Nmnat2	Metabolism
DMR13:70549001	13	70549001	70550000	1000	1	2.10E-07	0.43	15	1.5	Nmnat2	Metabolism
DMR13:70583001	13	70583001	70585000	2000	1	9.70E-09	0.34	25	1.25	Lamc2;LOC102552142	Extracellular Matrix
DMR13:70688001	13	70688001	70689000	1000	1	9.50E-07	-0.45	6	0.6	Lamc1	Extracellular Matrix
DMR13:70748001	13	70748001	70749000	1000	1	3.70E-08	-0.44	8	0.8	Lamc1;LOC102551797	Extracellular Matrix
DMR13:70753001	13	70753001	70754000	1000	1	4.80E-08	-0.42	13	1.3	Lamc1;LOC102551797	Extracellular Matrix
DMR13:70770001	13	70770001	70772000	2000	1	1.20E-09	-0.55	29	1.45	Lamc1	Extracellular Matrix
DMR13:70790001	13	70790001	70794000	4000	1	9.60E-10	-0.3	37	0.92	Lamc1	Extracellular Matrix
DMR13:70851001	13	70851001	70852000	1000	1	4.10E-11	0.69	33	3.3	Shcbp1l	
DMR13:70951001	13	70951001	70953000	2000	1	6.70E-07	0.41	26	1.3	Npl	Metabolism
DMR13:71171001	13	71171001	71173000	2000	1	2.80E-07	0.36	25	1.25	LOC102552085;Rgs16	Signaling
DMR13:71187001	13	71187001	71189000	2000	1	2.30E-08	-0.66	42	2.1	Rgs16;Rnasel	Signaling
DMR13:71324001	13	71324001	71326000	2000	1	4.30E-15	-0.5	31	1.55	Teddm1;Glul	Metabolism
DMR13:71327001	13	71327001	71330000	3000	1	9.70E-11	-0.55	57	1.9	Teddm1;Glul	Metabolism
DMR13:71666001	13	71666001	71667000	1000	1	2.90E-08	-0.71	12	1.2	Zfp648	Transcription
DMR13:71669001	13	71669001	71670000	1000	1	1.20E-07	0.57	26	2.6	Zfp648	Transcription
DMR13:71907001	13	71907001	71908000	1000	1	2.30E-07	0.69	24	2.4	Cacna1e	Transport
DMR13:71942001	13	71942001	71943000	1000	1	1.30E-08	0.46	8	0.8	Cacna1e;LOC108352605	Transport
DMR13:72017001	13	72017001	72021000	4000	1	1.70E-08	-0.42	34	0.85	Cacna1e	Transport
DMR13:72424001	13	72424001	72426000	2000	1	4.00E-07	0.83	45	2.25	Cacna1e	Transport
DMR13:72437001	13	72437001	72439000	2000	1	7.70E-07	0.44	26	1.3	Cacna1e	Transport
DMR13:72504001	13	72504001	72507000	3000	1	3.20E-07	-0.37	10	0.33	Cacna1e	Transport
DMR13:72540001	13	72540001	72541000	1000	1	8.30E-07	0.31	0	0	Cacna1e	Transport
DMR13:72793001	13	72793001	72794000	1000	1	1.20E-10	0.39	11	1.1	Mr1;LOC102552997	Immune
DMR13:73052001	13	73052001	73054000	2000	1	3.30E-11	0.45	11	0.55	Xpr1	Transport
DMR13:73495001	13	73495001	73496000	1000	1	2.10E-18	1.13	24	2.4	Cep350	Cytoskeleton
DMR13:73906001	13	73906001	73908000	2000	1	3.40E-13	-0.6	46	2.3	Tdrd5	Cytoskeleton
DMR13:73942001	13	73942001	73947000	5000	1	5.50E-07	-0.41	49	0.98	Nphs2;Axdnd1	Cytoskeleton
DMR13:73971001	13	73971001	73973000	2000	1	9.90E-07	-0.44	24	1.2	Axdnd1	
DMR13:74032001	13	74032001	74033000	1000	1	6.90E-10	-0.46	12	1.2	Axdnd1;Soat1	Metabolism
DMR13:74259001	13	74259001	74260000	1000	1	5.20E-10	-0.38	12	1.2	Abl2;Tor3a	Transcription
DMR13:74501001	13	74501001	74505000	4000	2	1.90E-08	-0.39	35	0.88	Ralgps2	Transcription
DMR13:74899001	13	74899001	74904000	5000	2	2.00E-08	-0.34	42	0.84	Rasal2	Signaling
DMR13:74913001	13	74913001	74914000	1000	1	2.00E-07	-0.53	4	0.4	Rasal2	Signaling
DMR13:75120001	13	75120001	75123000	3000	1	6.60E-07	-0.42	41	1.37	Tp53i3	
DMR13:75175001	13	75175001	75177000	2000	1	2.90E-07	0.39	19	0.95	Sec16b	
DMR13:75203001	13	75203001	75204000	1000	1	5.50E-08	0.61	23	2.3	Sec16b	
DMR13:75986001	13	75986001	75988000	2000	1	1.40E-07	0.45	16	0.8	Brinp2	
DMR13:76235001	13	76235001	76239000	4000	1	5.40E-09	-0.49	36	0.9	Astn1	
DMR13:76642001	13	76642001	76644000	2000	1	4.50E-07	-0.28	15	0.75	Pappa2	
DMR13:78005001	13	78005001	78007000	2000	1	1.60E-09	-0.57	27	1.35	Rabgap1l	Signaling
DMR13:78118001	13	78118001	78121000	3000	1	2.40E-07	-0.36	12	0.4	Rabgap1l	Signaling
DMR13:78254001	13	78254001	78256000	2000	1	1.20E-07	0.42	15	0.75	Rabgap1l	Signaling
DMR13:78455001	13	78455001	78459000	4000	1	3.80E-08	-0.44	35	0.88	Rabgap1l	Signaling

DMR13:78511001	13	78511001	78514000	3000	1	7.60E-07	-0.37	25	0.83	Rabgap1l	Signaling
DMR13:78557001	13	78557001	78562000	5000	2	1.00E-11	-0.38	45	0.9	Rabgap1l	Signaling
DMR13:78741001	13	78741001	78743000	2000	1	1.00E-08	-0.48	11	0.55	Rc3h1;LOC108352560	
DMR13:79053001	13	79053001	79059000	6000	1	3.10E-11	-0.32	68	1.13	Slc9c2	
DMR13:79371001	13	79371001	79372000	1000	1	2.30E-07	0.36	8	0.8	LOC103692000;Tnfsf18	
DMR13:79773001	13	79773001	79776000	3000	1	3.00E-07	-0.39	17	0.57	Suco	Development
DMR13:79876001	13	79876001	79878000	2000	1	1.60E-07	0.33	27	1.35	RGD1309106;Pigc	Golgi
DMR13:79940001	13	79940001	79942000	2000	1	5.10E-07	0.4	30	1.5	Dnm3;LOC102554129	Transport
DMR13:80093001	13	80093001	80095000	2000	1	6.10E-07	-0.42	24	1.2	Dnm3	Transport
DMR13:80172001	13	80172001	80173000	1000	1	6.70E-07	-0.37	6	0.6	Dnm3	Transport
DMR13:80226001	13	80226001	80230000	4000	1	1.40E-10	-0.64	55	1.38	Dnm3	Transport
DMR13:80384001	13	80384001	80386000	2000	2	1.60E-08	-0.46	11	0.55	Dnm3;LOC102554073;LOC102555077	Transport
DMR13:80544001	13	80544001	80546000	2000	1	4.00E-07	-0.52	19	0.95	Prrc2c	
DMR13:80728001	13	80728001	80730000	2000	1	3.40E-07	0.49	13	0.65	Fmo1	Metabolism
DMR13:80968001	13	80968001	80969000	1000	1	4.50E-07	-0.55	14	1.4	Mroh9	
DMR13:81179001	13	81179001	81181000	2000	1	3.90E-08	0.57	33	1.65	Prrx1	
DMR13:81190001	13	81190001	81192000	2000	1	2.80E-09	0.43	19	0.95	Prrx1	
DMR13:81989001	13	81989001	81991000	2000	1	1.80E-08	0.38	48	2.4	Mettl11b	Epigenetic
DMR13:82026001	13	82026001	82027000	1000	1	2.90E-10	0.58	1	0.1	Mettl11b	Epigenetic
DMR13:82124001	13	82124001	82132000	8000	2	1.10E-09	-0.49	73	0.91	Kifap3	Cytoskeleton
DMR13:82137001	13	82137001	82139000	2000	2	3.30E-10	-0.48	23	1.15	Kifap3	Cytoskeleton
DMR13:82481001	13	82481001	82483000	2000	1	4.50E-09	0.33	17	0.85	F5	Metabolism
DMR13:82565001	13	82565001	82568000	3000	1	1.80E-10	-0.7	39	1.3	Slc19a2;Ccdc181	Transport
DMR13:82578001	13	82578001	82580000	2000	1	2.10E-08	-0.39	41	2.05	Ccdc181	
DMR13:82686001	13	82686001	82689000	3000	1	7.20E-08	-0.59	33	1.1	Nme7	Signaling
DMR13:83092001	13	83092001	83095000	3000	1	8.40E-07	0.33	35	1.17	Dpt;LOC102546371	Cytoskeleton
DMR13:83195001	13	83195001	83197000	2000	1	5.30E-11	0.48	33	1.65	Xcl1	Growth Factors
DMR13:83432001	13	83432001	83435000	3000	1	8.40E-07	-0.49	44	1.47	Tbx19;Sft2d2	Transcription
DMR13:83615001	13	83615001	83616000	1000	1	2.10E-11	0.55	5	0.5	Dcaf6	Proteolysis
DMR13:83630001	13	83630001	83632000	2000	1	1.40E-07	-0.42	22	1.1	Dcaf6	Proteolysis
DMR13:83878001	13	83878001	83879000	1000	1	9.30E-08	0.42	12	1.2	Rcsd1	Cytoskeleton
DMR13:83992001	13	83992001	83996000	4000	2	7.70E-08	0.8	68	1.7	Creg1;Cd247	Immune
DMR13:84000001	13	84000001	84001000	1000	1	1.40E-07	0.38	22	2.2	Cd247	Immune
DMR13:84011001	13	84011001	84013000	2000	1	4.00E-08	0.71	41	2.05	Cd247	Immune
DMR13:84018001	13	84018001	84019000	1000	1	3.50E-07	0.51	22	2.2	Cd247	Immune
DMR13:84020001	13	84020001	84021000	1000	1	3.00E-07	-0.53	14	1.4	Cd247	Immune
DMR13:84115001	13	84115001	84116000	1000	1	4.70E-08	0.52	6	0.6	Pou2f1	
DMR13:84134001	13	84134001	84135000	1000	1	4.50E-07	-0.42	6	0.6	Pou2f1	
DMR13:84305001	13	84305001	84307000	2000	1	3.90E-08	0.58	51	2.55	Dusp27	
DMR13:84364001	13	84364001	84369000	5000	1	1.40E-09	0.85	76	1.52	Gpa33	
DMR13:84452001	13	84452001	84456000	4000	1	5.20E-07	-0.42	82	2.05	Mael	
DMR13:84462001	13	84462001	84467000	5000	1	2.40E-07	-0.31	33	0.66	Mael;Ildr2	Immune
DMR13:84470001	13	84470001	84473000	3000	1	1.70E-07	0.45	39	1.3	Ildr2	Immune
DMR13:84487001	13	84487001	84490000	3000	1	3.30E-07	-0.35	57	1.9	Ildr2	Immune
DMR13:85215001	13	85215001	85218000	3000	2	7.70E-07	0.34	35	1.17	Fam78b	
DMR13:85373001	13	85373001	85374000	1000	1	3.20E-09	0.4	18	1.8	Uck2	Signaling
DMR13:85488001	13	85488001	85489000	1000	1	3.50E-08	0.62	11	1.1	Tmco1;LOC100910278	
DMR13:85826001	13	85826001	85827000	1000	1	8.40E-13	0.73	24	2.4	LOC103692054;Rxrg	Transcription
DMR13:85859001	13	85859001	85862000	3000	1	1.40E-07	0.42	64	2.13	Rxrg;LOC102553986	Transcription
DMR13:86478001	13	86478001	86479000	1000	1	1.90E-07	0.4	10	1	Pbx1	Development
DMR13:87816001	13	87816001	87817000	1000	1	4.10E-07	-0.5	25	2.5	Nuf2	Cytoskeleton
DMR13:87818001	13	87818001	87821000	3000	1	2.60E-07	-0.44	41	1.37	Nuf2	Cytoskeleton
DMR13:87832001	13	87832001	87833000	1000	1	8.90E-08	0.43	42	4.2	Nuf2	Cytoskeleton
DMR13:88005001	13	88005001	88008000	3000	1	1.20E-09	0.68	34	1.13	Rgs5	Signaling
DMR13:88369001	13	88369001	88370000	1000	1	1.70E-07	0.48	1	0.1	Ddr2	Receptor
DMR13:88371001	13	88371001	88372000	1000	1	2.00E-07	0.45	10	1	Ddr2	Receptor
DMR13:88427001	13	88427001	88430000	3000	1	6.40E-08	0.36	36	1.2	Ddr2	Receptor
DMR13:88438001	13	88438001	88440000	2000	1	1.10E-07	-0.41	34	1.7	Ddr2	Receptor
DMR13:88459001	13	88459001	88461000	2000	1	2.00E-07	-0.53	19	0.95	LOC100911901;Uap1	
DMR13:88486001	13	88486001	88487000	1000	1	8.50E-07	0.37	5	0.5	Uap1	
DMR13:88528001	13	88528001	88531000	3000	1	2.30E-07	-0.34	26	0.87	Uhmk1;LOC102556681	Signaling
DMR13:88858001	13	88858001	88861000	3000	1	6.80E-07	0.44	38	1.27	Nos1ap	Cytoskeleton

DMR13:88866001	13	88866001	88869000	3000	1	1.80E-08	0.66	72	2.4	Nos1ap	Cytoskeleton
DMR13:88872001	13	88872001	88874000	2000	1	2.00E-07	-0.38	36	1.8	Nos1ap	Cytoskeleton
DMR13:88875001	13	88875001	88876000	1000	1	2.00E-07	0.39	22	2.2	Nos1ap	Cytoskeleton
DMR13:89081001	13	89081001	89087000	6000	2	6.00E-09	-0.3	55	0.92	Atf6	
DMR13:89110001	13	89110001	89115000	5000	2	4.20E-10	-0.31	46	0.92	Atf6	
DMR13:89163001	13	89163001	89165000	2000	2	1.30E-10	-0.61	31	1.55	Atf6	
DMR13:89252001	13	89252001	89253000	1000	1	6.10E-07	-0.36	39	3.9	Atf6;LOC100362384;Dnmt3b-ps2;Dusp12	Transport;Signaling
DMR13:89275001	13	89275001	89277000	2000	1	7.10E-07	-0.38	36	1.8	Dusp12;Fcrlb	Signaling;Immune
DMR13:89298001	13	89298001	89301000	3000	1	5.30E-10	0.64	70	2.33	Fcrlb;Fcrla	Immune
DMR13:89344001	13	89344001	89345000	1000	1	7.30E-11	0.5	10	1	Fcgr2b	
DMR13:89437001	13	89437001	89440000	3000	3	7.50E-13	0.65	121	4.03	LOC108348047;Trnae-cuc;Trnag-ucc;Trnad-guc;Trnal-cag;Trnag-gcc;LOC102557067	
DMR13:89441001	13	89441001	89449000	8000	5	1.00E-12	0.8	301	3.76	LOC108348047;Trnae-cuc;Trnag-ucc;Trnad-guc;Trnal-cag;Trnag-gcc;LOC102557067	
DMR13:89569001	13	89569001	89572000	3000	1	1.50E-07	-0.61	21	0.7	Pcp4l1	
DMR13:89626001	13	89626001	89630000	4000	1	4.30E-07	0.44	54	1.35	Ndufs2;Adamts4	Metabolism;Protease
DMR13:89781001	13	89781001	89785000	4000	1	1.00E-11	0.48	43	1.07	Nectin4;Arhgap30	Signaling
DMR13:89838001	13	89838001	89840000	2000	1	3.40E-07	-0.42	28	1.4	F11r	
DMR13:89936001	13	89936001	89938000	2000	1	8.70E-08	-0.37	26	1.3	Itln1;LOC688577	
DMR13:89955001	13	89955001	89956000	1000	1	1.20E-07	0.39	14	1.4	Cd244	Immune
DMR13:89982001	13	89982001	89987000	5000	2	3.20E-11	0.38	62	1.24	Cd244;LOC108352572	Immune
DMR13:90048001	13	90048001	90049000	1000	1	2.60E-14	1	18	1.8	LOC102549964;Slamf7	Immune
DMR13:90142001	13	90142001	90151000	9000	3	3.50E-11	-0.58	96	1.07	Cd48	Immune
DMR13:90240001	13	90240001	90241000	1000	1	1.80E-07	0.47	5	0.5	LOC103692288;Cd84	Immune
DMR13:90267001	13	90267001	90271000	4000	1	1.10E-08	-0.37	33	0.82	LOC103692288;Cd84;LOC103692287	Immune
DMR13:90385001	13	90385001	90388000	3000	1	9.50E-07	0.51	93	3.1	Vangl2;LOC108352574	
DMR13:90603001	13	90603001	90605000	2000	1	1.10E-07	0.34	30	1.5	Casq1;Atp1a4	Signaling;Transport
DMR13:90645001	13	90645001	90647000	2000	1	7.60E-07	-0.34	45	2.25	Atp1a4;Atp1a2	Transport
DMR13:90695001	13	90695001	90696000	1000	1	4.90E-09	0.44	13	1.3	Igsf8;Kcnj9	Immune;Transport
DMR13:90698001	13	90698001	90700000	2000	1	5.00E-11	0.81	72	3.6	Igsf8;Kcnj9	Immune;Transport
DMR13:90708001	13	90708001	90710000	2000	1	3.10E-11	0.53	36	1.8	Igsf8;Kcnj9	Immune;Transport
DMR13:90763001	13	90763001	90764000	1000	1	8.80E-08	-0.48	14	1.4	Kcnj10;Pigm	Transport;Golgi
DMR13:90772001	13	90772001	90774000	2000	1	1.90E-07	0.32	32	1.6	Pigm	Golgi
DMR13:90818001	13	90818001	90820000	2000	1	1.20E-09	0.45	45	2.25	Tagln2;Igsf9	Cytoskeleton
DMR13:90903001	13	90903001	90905000	2000	1	6.10E-08	-0.43	43	2.15	LOC102555374;Cfap45	
DMR13:90919001	13	90919001	90921000	2000	1	5.30E-07	-0.36	43	2.15	Cfap45	
DMR13:90940001	13	90940001	90942000	2000	1	4.40E-07	-0.33	21	1.05	Cfap45;Vsig8	Immune
DMR13:90945001	13	90945001	90949000	4000	1	3.90E-10	0.4	46	1.15	Vsig8	Immune
DMR13:90970001	13	90970001	90971000	1000	1	8.20E-07	0.35	12	1.2	Slamf8	Immune
DMR13:91076001	13	91076001	91080000	4000	1	2.60E-08	-0.45	30	0.75	Crp	
DMR13:91159001	13	91159001	91162000	3000	3	2.50E-17	0.67	130	4.33	Fcgr2a;Trnad-guc;Trnal-cag;LOC498276	
DMR13:91205001	13	91205001	91207000	2000	1	5.50E-16	0.83	59	2.95	LOC103693683;Trnag-ucc;LOC108352576;Trnag-gcc	
DMR13:91214001	13	91214001	91217000	3000	1	3.70E-12	0.73	78	2.6	Trnag-ucc;LOC108352576;Trnag-gcc;LOC100911825	
DMR13:91645001	13	91645001	91647000	2000	1	5.30E-09	-0.46	22	1.1	Olr1579;Olr1580-ps	
DMR13:91797001	13	91797001	91799000	2000	1	9.10E-07	-0.41	14	0.7	Mptx1	
DMR13:91920001	13	91920001	91921000	1000	1	9.40E-07	-0.47	16	1.6	Aim2	Transcription
DMR13:91987001	13	91987001	91989000	2000	1	7.60E-07	-0.53	11	0.55	LOC102556096;RGD1562462	
DMR13:92064001	13	92064001	92066000	2000	1	2.30E-07	-0.79	7	0.35	Olr1584;Mnda	Transcription
DMR13:92157001	13	92157001	92162000	5000	1	3.20E-07	-0.37	45	0.9	Olr1588;Olr1589	Receptor
DMR13:92230001	13	92230001	92232000	2000	1	1.30E-12	-0.65	14	0.7	Olr1593;Olr1594-ps;Olr1595	
DMR13:92242001	13	92242001	92243000	1000	1	7.30E-08	-0.57	5	0.5	Olr1594-ps;Olr1595;Olr1596	
DMR13:92313001	13	92313001	92321000	8000	1	6.20E-09	-0.43	86	1.07	Spta1	
DMR13:92382001	13	92382001	92387000	5000	2	6.70E-08	-0.35	43	0.86	Olr1600	Signaling
DMR13:92507001	13	92507001	92508000	1000	1	2.10E-07	0.45	4	0.4	Olr1602	Receptor
DMR13:92513001	13	92513001	92517000	4000	1	1.10E-07	0.3	16	0.4	Olr1602	Receptor
DMR13:92747001	13	92747001	92748000	1000	1	8.00E-09	-0.71	9	0.9	Fmn2	

DMR13:92763001	13	92763001	92765000	2000	1	1.80E-08	0.42	17	0.85	Fmn2	
DMR13:93385001	13	93385001	93386000	1000	1	2.20E-09	0.47	10	1	Rgs7	
DMR13:93685001	13	93685001	93686000	1000	1	1.70E-08	0.45	3	0.3	Fh;Kmo	Metabolism;Metabolism
DMR13:93694001	13	93694001	93695000	1000	1	3.40E-07	0.44	15	1.5	Kmo	Metabolism
DMR13:93696001	13	93696001	93697000	1000	1	6.10E-07	0.63	10	1	Kmo	Metabolism
DMR13:93788001	13	93788001	93789000	1000	1	2.50E-10	-0.49	8	0.8	LOC103692323;Wdr64	
DMR13:93811001	13	93811001	93812000	1000	1	3.80E-08	0.3	11	1.1	Wdr64	
DMR13:93834001	13	93834001	93835000	1000	1	6.50E-08	0.54	18	1.8	Wdr64	
DMR13:93878001	13	93878001	93879000	1000	1	4.80E-07	-0.51	13	1.3	Wdr64;LOC103692322;Uba2-ps1	
DMR13:93974001	13	93974001	93975000	1000	1	8.40E-07	0.38	15	1.5	Becn2	Protease; Proteolysis
DMR13:94118001	13	94118001	94122000	4000	1	3.00E-09	0.32	45	1.12	Pld5	Metabolism
DMR13:94139001	13	94139001	94143000	4000	1	4.30E-10	-0.31	19	0.48	Pld5	Metabolism
DMR13:94291001	13	94291001	94292000	1000	1	3.00E-10	0.86	23	2.3	Pld5	Metabolism
DMR13:94350001	13	94350001	94352000	2000	1	2.30E-10	-0.61	21	1.05	Pld5	Metabolism
DMR13:94833001	13	94833001	94834000	1000	1	9.60E-09	-0.69	13	1.3	Cep170	Cytoskeleton
DMR13:94896001	13	94896001	94897000	1000	1	3.60E-07	-0.59	9	0.9	Cep170;Sdccag8	Cytoskeleton
DMR13:95032001	13	95032001	95034000	2000	1	1.90E-09	0.42	19	0.95	Sdccag8;LOC108352628	
DMR13:95102001	13	95102001	95103000	1000	1	5.90E-08	0.39	4	0.4	Sdccag8;Akt3	Signaling
DMR13:95176001	13	95176001	95182000	6000	1	9.30E-10	-0.31	64	1.07	Akt3	Signaling
DMR13:95200001	13	95200001	95204000	4000	2	1.00E-07	-0.39	18	0.45	Akt3	Signaling
DMR13:95409001	13	95409001	95414000	5000	1	4.70E-07	-0.36	50	1	Rpl30l1	Translation
DMR13:95969001	13	95969001	95971000	2000	1	2.40E-07	0.34	9	0.45	RGD1565309	
DMR13:95977001	13	95977001	95978000	1000	1	4.40E-09	-0.42	4	0.4	RGD1565309	
DMR13:96174001	13	96174001	96176000	2000	1	7.50E-07	0.38	21	1.05	RGD1566217	
DMR13:96417001	13	96417001	96420000	3000	1	3.70E-08	-0.43	34	1.13	Efcab2;LOC102550369	Cytoskeleton
DMR13:96791001	13	96791001	96792000	1000	1	4.40E-09	0.64	39	3.9	Kif26b	Cytoskeleton
DMR13:96998001	13	96998001	97001000	3000	1	1.50E-07	-0.41	44	1.47	Kif26b	Cytoskeleton
DMR13:97094001	13	97094001	97096000	2000	2	3.90E-12	0.44	26	1.3	Kif26b	Cytoskeleton
DMR13:97121001	13	97121001	97123000	2000	1	5.50E-09	0.38	23	1.15	Kif26b;LOC102553199	Cytoskeleton
DMR13:97141001	13	97141001	97142000	1000	1	8.60E-07	0.43	7	0.7	Kif26b	Cytoskeleton
DMR13:97326001	13	97326001	97330000	4000	2	1.10E-13	-0.53	93	2.33	Smyd3	
DMR13:97331001	13	97331001	97334000	3000	1	1.10E-07	0.37	40	1.33	Smyd3	
DMR13:97335001	13	97335001	97337000	2000	1	5.00E-07	-0.4	42	2.1	Smyd3	
DMR13:97361001	13	97361001	97363000	2000	1	5.30E-08	-0.43	39	1.95	Smyd3	
DMR13:97447001	13	97447001	97449000	2000	1	9.70E-09	0.38	13	0.65	Smyd3;LOC108352579	
DMR13:97473001	13	97473001	97476000	3000	1	1.20E-08	-0.62	34	1.13	Smyd3	
DMR13:97516001	13	97516001	97518000	2000	1	3.30E-07	-0.52	42	2.1	Smyd3	
DMR13:97606001	13	97606001	97607000	1000	1	8.60E-07	-0.45	31	3.1	Smyd3;LOC108348143	
DMR13:97749001	13	97749001	97752000	3000	1	1.40E-07	0.46	31	1.03	Smyd3	
DMR13:97784001	13	97784001	97787000	3000	1	5.80E-08	-0.54	63	2.1	Smyd3	
DMR13:97791001	13	97791001	97795000	4000	1	2.30E-07	0.4	55	1.38	Smyd3;LOC102552208	
DMR13:97873001	13	97873001	97876000	3000	1	3.70E-08	-0.46	48	1.6	Cnst	
DMR13:97986001	13	97986001	97987000	1000	1	1.20E-08	0.47	11	1.1	Kif28p	Cytoskeleton
DMR13:98006001	13	98006001	98008000	2000	1	4.20E-07	0.49	42	2.1	Kif28p	Cytoskeleton
DMR13:98030001	13	98030001	98033000	3000	1	4.00E-07	-0.38	49	1.63	Kif28p;Ahctf1	Cytoskeleton;Cytoskeleton
DMR13:98053001	13	98053001	98055000	2000	1	6.40E-11	-0.47	17	0.85	Ahctf1	Cytoskeleton
DMR13:98302001	13	98302001	98304000	2000	1	4.80E-08	-0.43	23	1.15	Cdc42bpa	Signaling
DMR13:98325001	13	98325001	98327000	2000	1	4.20E-07	-0.53	6	0.3	Cdc42bpa	Signaling
DMR13:98385001	13	98385001	98387000	2000	1	1.00E-07	-0.49	20	1	Cdc42bpa	Signaling
DMR13:98406001	13	98406001	98409000	3000	1	2.90E-09	-0.57	36	1.2	Cdc42bpa	Signaling
DMR13:98424001	13	98424001	98426000	2000	1	8.10E-07	0.47	17	0.85	Cdc42bpa	Signaling
DMR13:98624001	13	98624001	98626000	2000	1	4.50E-09	0.41	17	0.85	Itpkb	Signaling
DMR13:98678001	13	98678001	98681000	3000	1	1.30E-08	0.37	48	1.6	Itpkb	Signaling
DMR13:99016001	13	99016001	99020000	4000	1	6.70E-07	-0.37	61	1.52	Acbd3	
DMR13:99183001	13	99183001	99184000	1000	1	2.20E-08	0.52	28	2.8	Lefty2;Pycr2	Growth Factors;Metabolism
DMR13:99254001	13	99254001	99256000	2000	1	8.50E-09	0.37	23	1.15	Tmem63a	
DMR13:99530001	13	99530001	99533000	3000	1	1.50E-07	-0.55	30	1	Wdr26;LOC103693699	
DMR13:99736001	13	99736001	99738000	2000	1	3.40E-12	0.48	29	1.45	Cnih3	Transport
DMR13:99811001	13	99811001	99812000	1000	1	7.60E-08	-0.62	8	0.8	Ccdc121	
DMR13:99827001	13	99827001	99828000	1000	1	6.20E-10	0.33	9	0.9	Ccdc121	
DMR13:100317001	13	100317001	100318000	1000	1	2.30E-13	0.66	32	3.2	Enah	Cytoskeleton

DMR13:100393001	13	100393001	100396000	3000	1	8.10E-08	-0.43	33	1.1	Enah	Cytoskeleton
DMR13:100704001	13	100704001	100706000	2000	1	3.90E-08	-0.4	14	0.7	Fbxo28	
DMR13:100750001	13	100750001	100751000	1000	1	1.90E-11	0.49	17	1.7	Fbxo28	
DMR13:100818001	13	100818001	100822000	4000	1	3.50E-10	-0.65	60	1.5	Tp53bp2	Signaling
DMR13:100849001	13	100849001	100853000	4000	1	3.50E-12	0.33	75	1.88	Tp53bp2	Signaling
DMR13:100881001	13	100881001	100887000	6000	1	2.30E-08	0.38	121	2.02	Tp53bp2;Capn2	Signaling;Protease
DMR13:100910001	13	100910001	100913000	3000	1	4.50E-08	0.42	36	1.2	Capn2;LOC102555308	Protease
DMR13:101159001	13	101159001	101162000	3000	1	1.60E-17	1.04	50	1.67	Ccdc185	
DMR13:101190001	13	101190001	101194000	4000	1	6.40E-07	0.33	73	1.82	Susd4	
DMR13:101230001	13	101230001	101232000	2000	1	6.10E-07	0.43	26	1.3	Susd4	
DMR13:101504001	13	101504001	101507000	3000	1	5.90E-10	0.38	39	1.3	Disp1	
DMR13:101558001	13	101558001	101559000	1000	1	7.30E-11	0.47	2	0.2	Disp1	
DMR13:101674001	13	101674001	101677000	3000	1	7.50E-09	-0.49	44	1.47	Fam177b;LOC108352623;Brox	
DMR13:101778001	13	101778001	101780000	2000	1	1.70E-07	-0.45	29	1.45	Mia3;Taf1a	Transport
DMR13:101794001	13	101794001	101798000	4000	1	2.40E-07	-0.37	44	1.1	Taf1a	
DMR13:101818001	13	101818001	101819000	1000	1	7.80E-11	0.84	29	2.9	Hhipl2	Signaling
DMR13:101825001	13	101825001	101828000	3000	1	2.90E-09	0.61	50	1.67	Hhipl2	Signaling
DMR13:101843001	13	101843001	101846000	3000	1	9.00E-07	0.38	45	1.5	Hhipl2	Signaling
DMR13:102648001	13	102648001	102650000	2000	1	1.60E-09	-0.42	38	1.9	Hlx	Development
DMR13:102651001	13	102651001	102654000	3000	1	3.40E-08	0.53	49	1.63	Hlx	Development
DMR13:102807001	13	102807001	102810000	3000	1	1.50E-07	-0.49	78	2.6	Mark1	Signaling
DMR13:102932001	13	102932001	102934000	2000	1	9.10E-10	-0.5	19	0.95	Mark1;LOC108352588	Signaling
DMR13:103219001	13	103219001	103223000	4000	1	5.80E-07	-0.49	64	1.6	Rab3gap2;Iars2	Signaling;Translation
DMR13:103297001	13	103297001	103298000	1000	1	2.90E-08	-0.51	18	1.8	Bpnt1;Eprs	Signaling;Translation
DMR13:103338001	13	103338001	103340000	2000	1	8.30E-07	-0.51	26	1.3	Eprs	Translation
DMR13:103391001	13	103391001	103392000	1000	1	2.80E-08	0.36	11	1.1	LOC100911719;Slc30a10	
DMR13:103414001	13	103414001	103415000	1000	1	8.50E-07	0.39	7	0.7	Slc30a10	
DMR13:104156001	13	104156001	104158000	2000	1	4.20E-09	0.59	31	1.55	LOC103693693;RGD1561704	
DMR13:104312001	13	104312001	104315000	3000	1	1.10E-09	-0.6	38	1.27	Dusp10	Signaling
DMR13:105094001	13	105094001	105101000	7000	2	8.00E-09	-0.4	69	0.99	Tgfb2	Growth Factors
DMR13:105104001	13	105104001	105110000	6000	1	5.90E-08	0.39	75	1.25	Tgfb2	Growth Factors
DMR13:105180001	13	105180001	105181000	1000	1	1.90E-09	0.54	13	1.3	Rrp15	
DMR13:105406001	13	105406001	105409000	3000	2	5.90E-12	1	94	3.13	Ddx3y	
DMR13:105480001	13	105480001	105486000	6000	1	1.40E-09	-0.42	79	1.32	Spata17	
DMR13:105622001	13	105622001	105623000	1000	1	1.60E-08	0.54	9	0.9	Spata17	
DMR13:105801001	13	105801001	105803000	2000	1	3.10E-08	-0.46	29	1.45	Gpatch2	Metabolism
DMR13:106052001	13	106052001	106055000	3000	1	1.30E-07	0.36	46	1.53	Esrrg	
DMR13:106060001	13	106060001	106062000	2000	2	2.80E-08	0.54	69	3.45	Esrrg	
DMR13:106136001	13	106136001	106138000	2000	1	8.70E-07	-0.42	32	1.6	Esrrg	
DMR13:106213001	13	106213001	106215000	2000	1	3.10E-07	0.35	24	1.2	Esrrg	
DMR13:106228001	13	106228001	106231000	3000	1	2.00E-09	0.41	36	1.2	Esrrg	
DMR13:106255001	13	106255001	106256000	1000	1	2.50E-07	0.53	11	1.1	Esrrg	
DMR13:106299001	13	106299001	106304000	5000	1	5.30E-11	0.69	84	1.68	Esrrg	
DMR13:106314001	13	106314001	106317000	3000	1	8.10E-08	0.32	46	1.53	Esrrg	
DMR13:106318001	13	106318001	106320000	2000	1	5.30E-10	0.68	50	2.5	Esrrg	
DMR13:106378001	13	106378001	106379000	1000	1	9.50E-10	0.62	23	2.3	Esrrg	
DMR13:106425001	13	106425001	106427000	2000	1	7.40E-11	0.64	46	2.3	Esrrg	
DMR13:106565001	13	106565001	106566000	1000	1	2.80E-08	-0.45	16	1.6	Esrrg	
DMR13:106575001	13	106575001	106576000	1000	1	1.30E-09	0.28	9	0.9	Esrrg	
DMR13:106650001	13	106650001	106651000	1000	1	1.60E-07	-0.43	8	0.8	Esrrg	
DMR13:106657001	13	106657001	106660000	3000	1	3.30E-10	0.55	38	1.27	Esrrg	
DMR13:106753001	13	106753001	106754000	1000	1	1.60E-10	0.82	33	3.3	Ush2a	Extracellular Matrix
DMR13:106793001	13	106793001	106795000	2000	1	1.50E-08	0.33	20	1	Ush2a	Extracellular Matrix
DMR13:106820001	13	106820001	106823000	3000	1	2.80E-09	0.41	52	1.73	Ush2a	Extracellular Matrix
DMR13:106885001	13	106885001	106886000	1000	1	1.00E-07	-0.44	11	1.1	Ush2a	Extracellular Matrix
DMR13:106987001	13	106987001	106989000	2000	1	1.30E-07	0.34	22	1.1	Ush2a	Extracellular Matrix
DMR13:107007001	13	107007001	107008000	1000	1	2.80E-07	-0.38	15	1.5	Ush2a	Extracellular Matrix
DMR13:107018001	13	107018001	107022000	4000	1	1.50E-08	-0.46	75	1.88	Ush2a	Extracellular Matrix
DMR13:107151001	13	107151001	107158000	7000	4	5.60E-12	0.48	96	1.37	Ush2a	Extracellular Matrix
DMR13:107305001	13	107305001	107306000	1000	1	4.40E-11	0.4	13	1.3	Ush2a	Extracellular Matrix
DMR13:107374001	13	107374001	107375000	1000	1	8.80E-08	-0.47	29	2.9	Ush2a;LOC103692406	Extracellular Matrix
DMR13:107384001	13	107384001	107388000	4000	1	2.20E-12	0.51	63	1.57	Ush2a;LOC103692406	Extracellular Matrix
DMR13:107717001	13	107717001	107720000	3000	2	5.60E-08	0.72	77	2.57	Kcnk2	Transport

DMR13:107784001	13	107784001	107788000	4000	1	1.90E-11	0.41	53	1.32	Kcnk2	Transport
DMR13:107886001	13	107886001	107887000	1000	1	1.70E-07	-0.46	30	3	Kcnk2	Transport
DMR13:108374001	13	108374001	108375000	1000	1	2.40E-07	0.34	8	0.8	LOC108352595;Prox1	Development
DMR13:108674001	13	108674001	108678000	4000	1	3.70E-09	-0.68	112	2.8	Smyd2	Epigenetic
DMR13:108738001	13	108738001	108740000	2000	1	4.60E-07	-0.43	28	1.4	Ptpn14	Signaling
DMR13:108768001	13	108768001	108769000	1000	1	1.30E-09	-0.41	36	3.6	Ptpn14	Signaling
DMR13:108817001	13	108817001	108820000	3000	1	4.50E-07	0.27	45	1.5	Ptpn14	Signaling
DMR13:109413001	13	109413001	109415000	2000	1	2.50E-08	0.35	20	1	Rps6kc1	Signaling
DMR13:109534001	13	109534001	109535000	1000	1	1.70E-08	0.39	10	1	Rps6kc1;Vash2	Signaling
DMR13:109538001	13	109538001	109540000	2000	1	7.70E-08	-0.43	40	2	Rps6kc1;Vash2	Signaling
DMR13:109573001	13	109573001	109577000	4000	2	6.80E-15	-0.61	44	1.1	Rps6kc1;LOC108348225	Signaling
DMR13:109780001	13	109780001	109781000	1000	1	3.10E-07	-0.57	14	1.4	Fam71a;LOC100911428	
DMR13:109979001	13	109979001	109983000	4000	2	4.00E-11	0.58	53	1.32	Nenf	Receptor
DMR13:110054001	13	110054001	110055000	1000	1	4.20E-07	0.33	14	1.4	Ppp2r5a	Signaling
DMR13:110275001	13	110275001	110276000	1000	1	5.50E-12	0.44	15	1.5	Ints7;LOC108352606	
DMR13:110853001	13	110853001	110854000	1000	1	1.30E-07	0.37	19	1.9	Rcor3	
DMR13:110994001	13	110994001	110996000	2000	1	1.60E-10	0.85	50	2.5	Kcnh1	Transport
DMR13:111006001	13	111006001	111008000	2000	1	6.60E-07	-0.38	37	1.85	Kcnh1	Transport
DMR13:111050001	13	111050001	111053000	3000	1	1.00E-13	0.67	39	1.3	Kcnh1	Transport
DMR13:111057001	13	111057001	111058000	1000	1	1.70E-07	0.37	17	1.7	Kcnh1	Transport
DMR13:111079001	13	111079001	111082000	3000	1	9.10E-07	0.5	27	0.9	Kcnh1	Transport
DMR13:111165001	13	111165001	111168000	3000	1	2.70E-10	0.55	30	1	Kcnh1	Transport
DMR13:111337001	13	111337001	111342000	5000	1	5.20E-09	-0.41	42	0.84	Hhat	Metabolism
DMR13:111362001	13	111362001	111364000	2000	1	1.40E-10	0.29	25	1.25	Hhat	Metabolism
DMR13:111376001	13	111376001	111378000	2000	1	3.50E-08	0.38	34	1.7	Hhat	Metabolism
DMR13:111420001	13	111420001	111422000	2000	1	1.50E-08	0.57	30	1.5	Hhat	Metabolism
DMR13:111429001	13	111429001	111432000	3000	1	5.60E-10	0.56	42	1.4	Hhat	Metabolism
DMR13:111451001	13	111451001	111454000	3000	1	2.50E-09	0.42	35	1.17	Hhat	Metabolism
DMR13:111656001	13	111656001	111657000	1000	1	9.30E-10	-0.59	8	0.8	Syt14	
DMR13:111740001	13	111740001	111744000	4000	1	6.20E-07	-0.41	26	0.65	Syt14	
DMR13:111852001	13	111852001	111853000	1000	1	3.80E-07	0.35	11	1.1	Diexf	Metabolism
DMR13:111923001	13	111923001	111924000	1000	1	1.50E-08	0.48	2	0.2	Traf3ip3	Cytoskeleton
DMR13:111975001	13	111975001	111976000	1000	1	6.40E-07	0.34	14	1.4	Hsd11b1	Metabolism
DMR13:111988001	13	111988001	111991000	3000	1	3.80E-10	0.46	18	0.6	Hsd11b1	Metabolism
DMR13:112103001	13	112103001	112106000	3000	1	1.80E-12	0.46	15	0.5	Camk1g	Signaling
DMR13:113382001	13	113382001	113383000	1000	1	9.40E-07	0.45	10	1	Plxna2	
DMR13:113455001	13	113455001	113457000	2000	1	1.20E-07	0.49	9	0.45	Plxna2	
DMR13:113577001	13	113577001	113584000	7000	3	1.20E-07	-0.33	80	1.14	Plxna2	
DMR14:651001	14	651001	654000	3000	1	1.80E-10	-0.59	18	0.6	RGD1562423	
DMR14:685001	14	685001	687000	2000	1	7.80E-07	-0.37	12	0.6	RGD1561977	
DMR14:957001	14	957001	963000	6000	1	3.20E-09	-0.41	59	0.98	Vom2r-ps116	
DMR14:1023001	14	1023001	1024000	1000	1	9.30E-09	0.63	12	1.2	Vom2r69	Signaling
DMR14:1156001	14	1156001	1159000	3000	1	9.10E-08	-0.55	36	1.2	Vom2r-ps115	
DMR14:1392001	14	1392001	1394000	2000	1	9.90E-07	-0.38	15	0.75	Vom2r66	
DMR14:1456001	14	1456001	1460000	4000	2	3.90E-12	0.96	122	3.05	Crlf2;Csf2ra;LOC100365289;Ppp2r3b	Receptor;Signaling
DMR14:1465001	14	1465001	1466000	1000	1	4.30E-10	0.7	31	3.1	Crlf2;Csf2ra;LOC100365289;Ppp2r3b	Receptor;Signaling
DMR14:1472001	14	1472001	1476000	4000	2	7.30E-10	0.91	140	3.5	Csf2ra;LOC100365289;Ppp2r3b	Receptor;Signaling
DMR14:1692001	14	1692001	1698000	6000	1	7.10E-07	-0.24	52	0.87	Ccdc18	Cytoskeleton
DMR14:2058001	14	2058001	2062000	4000	1	2.40E-11	0.58	70	1.75	Idua;Slc26a1;Dgkq	Metabolism;Transport;Signaling
DMR14:2151001	14	2151001	2153000	2000	1	6.60E-07	-0.46	15	0.75	Gak	Transport
DMR14:2264001	14	2264001	2266000	2000	1	1.10E-15	-0.62	9	0.45	Pcgf3	Epigenetic
DMR14:2862001	14	2862001	2863000	1000	1	1.10E-11	-0.53	10	1	Fam69a;Rpl5;LOC689892	Translation
DMR14:3122001	14	3122001	3126000	4000	1	1.20E-07	-0.4	19	0.48	Rpap2	
DMR14:3157001	14	3157001	3158000	1000	1	9.70E-07	-0.43	16	1.6	Rpap2;LOC103692490	
DMR14:3193001	14	3193001	3195000	2000	1	3.90E-07	-0.46	30	1.5	Rpap2;Glmn	Proteolysis
DMR14:3196001	14	3196001	3198000	2000	1	1.90E-08	-0.51	28	1.4	Rpap2;Glmn	Proteolysis
DMR14:3378001	14	3378001	3379000	1000	1	3.00E-07	0.45	17	1.7	LOC108352691;Ephx4	Metabolism
DMR14:3392001	14	3392001	3395000	3000	1	2.00E-09	0.43	29	0.97	Ephx4;LOC108352690	Metabolism
DMR14:3434001	14	3434001	3436000	2000	1	6.20E-08	-0.32	23	1.15	Brdt	
DMR14:3642001	14	3642001	3644000	2000	1	2.10E-08	-0.61	47	2.35	Tgfbr3	Receptor

DMR14:3654001	14	3654001	3663000	9000	1	3.80E-07	-0.41	146	1.62	Tgfbr3	Receptor
DMR14:3682001	14	3682001	3686000	4000	1	2.80E-07	-0.39	50	1.25	Tgfbr3	Receptor
DMR14:4176001	14	4176001	4184000	8000	1	1.50E-09	-0.58	175	2.19	Zfp644	Transcription
DMR14:4185001	14	4185001	4186000	1000	1	7.80E-08	-0.62	10	1	Zfp644	Transcription
DMR14:4192001	14	4192001	4195000	3000	1	2.50E-10	-0.55	42	1.4	Zfp644	Transcription
DMR14:4992001	14	4992001	4993000	1000	1	1.50E-07	-0.41	24	2.4	Zfp326	Cytoskeleton
DMR14:5086001	14	5086001	5089000	3000	1	5.10E-08	-0.48	114	3.8	Lrrc8d	Cytoskeleton
DMR14:5101001	14	5101001	5102000	1000	1	3.30E-08	-0.52	31	3.1	Lrrc8d	Cytoskeleton
DMR14:5183001	14	5183001	5185000	2000	1	1.60E-07	-0.5	29	1.45	Lrrc8d	Cytoskeleton
DMR14:5188001	14	5188001	5191000	3000	1	2.40E-07	0.4	53	1.77	Lrrc8d	Cytoskeleton
DMR14:5315001	14	5315001	5316000	1000	1	1.50E-09	0.71	38	3.8	Lrrc8c	Cytoskeleton
DMR14:5605001	14	5605001	5606000	1000	1	1.40E-08	0.3	12	1.2	Abcg3l4	
DMR14:5608001	14	5608001	5610000	2000	1	3.00E-09	0.31	20	1	Abcg3l4	
DMR14:5623001	14	5623001	5626000	3000	1	4.70E-09	-0.4	47	1.57	Abcg3l4	
DMR14:5860001	14	5860001	5867000	7000	1	5.40E-10	-0.41	44	0.63	Abcg3l3;LOC100359633;LOC100912618;LOC102554096	Transport;Proteolysis
DMR14:6512001	14	6512001	6513000	1000	1	3.30E-07	-0.43	20	2	Abcg3l1	Transport
DMR14:6777001	14	6777001	6780000	3000	1	8.00E-13	0.64	61	2.03	LOC100910198;Mepe	
DMR14:6790001	14	6790001	6793000	3000	1	3.10E-12	0.56	49	1.63	LOC100910198;Mepe;Ibsp	Extracellular Matrix
DMR14:6800001	14	6800001	6801000	1000	1	2.50E-07	0.35	7	0.7	LOC100910198;Mepe;Ibsp	Extracellular Matrix
DMR14:6802001	14	6802001	6803000	1000	1	5.20E-12	0.93	41	4.1	LOC100910198;Mepe;Ibsp	Extracellular Matrix
DMR14:6805001	14	6805001	6807000	2000	1	2.10E-08	0.77	53	2.65	Ibsp	Extracellular Matrix
DMR14:6814001	14	6814001	6817000	3000	1	6.60E-07	-0.42	52	1.73	Ibsp	Extracellular Matrix
DMR14:6880001	14	6880001	6882000	2000	1	2.40E-10	0.57	43	2.15	Dmp1	
DMR14:6891001	14	6891001	6892000	1000	1	4.70E-11	0.73	34	3.4	Dmp1	
DMR14:6899001	14	6899001	6900000	1000	1	7.90E-10	0.46	6	0.6	Dmp1;LOC108352697	
DMR14:6930001	14	6930001	6932000	2000	1	2.10E-16	0.88	52	2.6	Dmp1;LOC108352697;Dspp	
DMR14:7017001	14	7017001	7021000	4000	1	3.20E-07	0.53	68	1.7	Sparcl1;LOC100359907	Extracellular Matrix
DMR14:7079001	14	7079001	7083000	4000	2	8.60E-14	-0.52	48	1.2	Hsd17b11;LOC108352698	
DMR14:7085001	14	7085001	7086000	1000	1	1.50E-09	-0.86	13	1.3	Hsd17b11;LOC108352698	
DMR14:7087001	14	7087001	7089000	2000	1	1.30E-07	-0.47	25	1.25	Hsd17b11;LOC108352698	
DMR14:7218001	14	7218001	7222000	4000	1	2.80E-08	-0.43	77	1.93	Klhl8;Aff1	Cytoskeleton;Transcription
DMR14:7246001	14	7246001	7248000	2000	1	3.30E-07	-0.3	64	3.2	Aff1	Transcription
DMR14:7250001	14	7250001	7252000	2000	1	4.80E-07	-0.4	36	1.8	Aff1	Transcription
DMR14:7264001	14	7264001	7266000	2000	1	1.80E-08	-0.54	35	1.75	Aff1	Transcription
DMR14:7268001	14	7268001	7270000	2000	1	3.80E-11	0.48	41	2.05	Aff1	Transcription
DMR14:7276001	14	7276001	7277000	1000	1	1.20E-07	-0.35	20	2	Aff1	Transcription
DMR14:7303001	14	7303001	7307000	4000	1	5.20E-07	-0.5	68	1.7	Aff1	Transcription
DMR14:7660001	14	7660001	7661000	1000	1	1.40E-10	0.49	6	0.6	Slc10a6;LOC102546534	Transport
DMR14:7683001	14	7683001	7684000	1000	1	5.20E-10	-0.61	19	1.9	Slc10a6;Ptpn13	Transport;Signaling
DMR14:7758001	14	7758001	7760000	2000	2	1.20E-07	-0.54	36	1.8	Ptpn13	Signaling
DMR14:7767001	14	7767001	7769000	2000	1	1.80E-07	-0.37	42	2.1	Ptpn13	Signaling
DMR14:7783001	14	7783001	7786000	3000	1	7.60E-07	-0.37	41	1.37	Ptpn13	Signaling
DMR14:7807001	14	7807001	7813000	6000	1	6.20E-07	-0.42	125	2.08	Ptpn13	Signaling
DMR14:7819001	14	7819001	7824000	5000	1	1.30E-09	-0.59	122	2.44	Ptpn13	Signaling
DMR14:7835001	14	7835001	7841000	6000	1	4.90E-07	-0.38	121	2.02	Ptpn13	Signaling
DMR14:7843001	14	7843001	7850000	7000	1	9.60E-08	-0.56	121	1.73	Ptpn13	Signaling
DMR14:7858001	14	7858001	7859000	1000	1	4.10E-07	-0.35	17	1.7	Ptpn13	Signaling
DMR14:8084001	14	8084001	8088000	4000	1	9.00E-08	0.5	93	2.33	Mapk10;LOC108352700	Signaling
DMR14:8104001	14	8104001	8105000	1000	1	5.40E-09	-0.48	4	0.4	Mapk10	Signaling
DMR14:8144001	14	8144001	8147000	3000	2	8.40E-11	-0.48	49	1.63	Mapk10;LOC103692565	Signaling
DMR14:8156001	14	8156001	8157000	1000	1	1.70E-10	0.9	46	4.6	Mapk10;LOC103692565	Signaling
DMR14:8181001	14	8181001	8183000	2000	1	1.90E-08	0.71	31	1.55	Mapk10	Signaling
DMR14:8259001	14	8259001	8264000	5000	1	2.80E-13	-0.59	96	1.92	Mapk10	Signaling
DMR14:8300001	14	8300001	8303000	3000	1	2.70E-08	0.33	27	0.9	Mapk10	Signaling
DMR14:8322001	14	8322001	8326000	4000	1	3.90E-07	-0.35	38	0.95	Mapk10	Signaling
DMR14:8372001	14	8372001	8376000	4000	1	3.60E-07	-0.48	52	1.3	Mapk10;Arhgap24	Signaling
DMR14:8434001	14	8434001	8436000	2000	1	1.70E-08	0.49	29	1.45	Arhgap24;LOC108352702	
DMR14:8591001	14	8591001	8592000	1000	1	2.40E-08	0.35	19	1.9	Arhgap24	
DMR14:8610001	14	8610001	8611000	1000	1	3.20E-08	-0.43	24	2.4	Arhgap24	
DMR14:9022001	14	9022001	9025000	3000	1	9.70E-11	0.96	80	2.67	RGD1560931	
DMR14:9035001	14	9035001	9037000	2000	1	8.30E-09	0.63	25	1.25	RGD1560931	

DMR14:9047001	14	9047001	9048000	1000	1	1.30E-07	0.39	7	0.7	RGD1560931	
DMR14:9161001	14	9161001	9165000	4000	1	1.80E-13	-0.57	85	2.12	Wdfy3	
DMR14:9199001	14	9199001	9202000	3000	1	1.20E-09	-0.39	59	1.97	Wdfy3	
DMR14:9295001	14	9295001	9300000	5000	1	3.70E-08	0.4	125	2.5	Wdfy3	
DMR14:9338001	14	9338001	9340000	2000	1	8.90E-07	-0.39	61	3.05	Wdfy3	
DMR14:9348001	14	9348001	9350000	2000	2	1.30E-10	0.52	22	1.1	Wdfy3	
DMR14:9399001	14	9399001	9401000	2000	1	8.10E-07	-0.4	51	2.55	Cds1	Transport
DMR14:9463001	14	9463001	9464000	1000	1	7.10E-08	0.4	21	2.1	Cds1;LOC108352705	Transport
DMR14:10390001	14	10390001	10393000	3000	1	6.20E-07	0.3	36	1.2	Gpat3	Metabolism
DMR14:10438001	14	10438001	10442000	4000	1	1.60E-07	-0.48	60	1.5	Fam175a;Mrps18c;Helq	Translation
DMR14:10554001	14	10554001	10559000	5000	1	9.90E-07	-0.31	65	1.3	Hpse	Metabolism
DMR14:10567001	14	10567001	10574000	7000	1	6.10E-11	0.74	143	2.04	Hpse;Coq2	Metabolism;Metabolism
DMR14:10587001	14	10587001	10589000	2000	1	2.90E-09	-0.45	28	1.4	Coq2	Metabolism
DMR14:10597001	14	10597001	10599000	2000	1	2.20E-07	0.45	27	1.35	Coq2	Metabolism
DMR14:10688001	14	10688001	10689000	1000	1	6.90E-08	0.3	10	1	LOC103693725;Plac8	
DMR14:10747001	14	10747001	10748000	1000	1	5.70E-08	-0.42	19	1.9	Cops4;LOC102550950	Protease
DMR14:11082001	14	11082001	11086000	4000	1	1.60E-09	0.6	68	1.7	LOC108352712;Tmem150c	
DMR14:11093001	14	11093001	11094000	1000	1	5.40E-08	0.4	15	1.5	LOC108352712;Tmem150c	
DMR14:11130001	14	11130001	11131000	1000	1	7.40E-10	0.36	14	1.4	Tmem150c	
DMR14:11275001	14	11275001	11277000	2000	1	7.70E-07	-0.42	20	1	Hnrnpd;LOC102557303	
DMR14:11280001	14	11280001	11282000	2000	2	1.30E-08	-0.47	31	1.55	Hnrnpd;LOC102557303;LOC681250	
DMR14:11491001	14	11491001	11492000	1000	1	5.60E-08	0.66	21	2.1	Rasgef1b	Transcription
DMR14:11549001	14	11549001	11550000	1000	1	2.50E-07	0.4	2	0.2	Rasgef1b	Transcription
DMR14:11576001	14	11576001	11581000	5000	1	1.00E-17	0.85	91	1.82	Rasgef1b	Transcription
DMR14:11888001	14	11888001	11890000	2000	1	1.50E-07	-0.46	46	2.3	Rasgef1b	Transcription
DMR14:11999001	14	11999001	1.20E+07	1000	1	5.80E-08	-0.55	28	2.8	Rasgef1b	Transcription
DMR14:12001001	14	12001001	12010000	9000	1	6.70E-11	-0.43	213	2.37	Rasgef1b	Transcription
DMR14:12012001	14	12012001	12015000	3000	1	6.90E-08	-0.43	67	2.23	Rasgef1b	Transcription
DMR14:12253001	14	12253001	12255000	2000	1	2.30E-07	-0.44	79	3.95	Prkg2	Signaling
DMR14:12256001	14	12256001	12259000	3000	1	1.50E-07	-0.46	43	1.43	Prkg2	Signaling
DMR14:12270001	14	12270001	12274000	4000	1	5.00E-07	-0.37	58	1.45	Prkg2	Signaling
DMR14:12353001	14	12353001	12356000	3000	1	5.40E-07	-0.44	50	1.67	Bmp3	Growth Factors
DMR14:12367001	14	12367001	12369000	2000	1	5.90E-08	-0.46	33	1.65	Bmp3	Growth Factors
DMR14:13201001	14	13201001	13205000	4000	1	1.30E-08	-0.44	51	1.27	Antxr2	Cytoskeleton
DMR14:13268001	14	13268001	13271000	3000	1	3.80E-07	-0.35	8	0.27	Antxr2	Cytoskeleton
DMR14:13296001	14	13296001	13298000	2000	1	1.30E-07	0.52	31	1.55	Antxr2	Cytoskeleton
DMR14:13755001	14	13755001	13758000	3000	1	2.00E-07	-0.25	21	0.7	Gk2	Metabolism
DMR14:13827001	14	13827001	13828000	1000	1	6.50E-08	0.37	41	4.1	LOC102554702;Naa11	Metabolism
DMR14:14106001	14	14106001	14108000	2000	1	3.50E-07	0.32	69	3.45	Paqr3	Signaling
DMR14:14202001	14	14202001	14204000	2000	1	1.10E-07	-0.52	37	1.85	Bmp2k	Signaling
DMR14:14421001	14	14421001	14424000	3000	1	2.50E-09	-0.6	46	1.53	Anxa3	Signaling
DMR14:14437001	14	14437001	14442000	5000	1	2.00E-07	0.48	96	1.92	Fras1	
DMR14:14447001	14	14447001	14448000	1000	1	3.90E-08	-0.56	12	1.2	Fras1	
DMR14:14475001	14	14475001	14476000	1000	1	1.90E-07	-0.48	10	1	Fras1	
DMR14:14481001	14	14481001	14483000	2000	1	1.10E-12	0.46	18	0.9	Fras1;LOC103693728	
DMR14:14806001	14	14806001	14809000	3000	1	6.10E-08	-0.43	55	1.83	Fras1	
DMR14:14844001	14	14844001	14845000	1000	1	8.30E-08	0.39	2	0.2	Fras1;LOC103693729	
DMR14:14943001	14	14943001	14948000	5000	1	7.50E-08	-0.35	71	1.42	Mrpl1	Translation
DMR14:15428001	14	15428001	15430000	2000	1	3.10E-08	0.41	18	0.9	LOC100909497;RGD1561699;LOC108348100	
DMR14:15667001	14	15667001	15671000	4000	1	1.70E-07	-0.42	35	0.88	pramef20l	
DMR14:15734001	14	15734001	15740000	6000	2	2.10E-07	-0.31	50	0.83	RGD1559999;RGD1561226	
DMR14:16277001	14	16277001	16279000	2000	1	7.10E-12	-0.84	29	1.45	Ccng2	Signaling
DMR14:16653001	14	16653001	16657000	4000	1	4.30E-10	-0.56	96	2.4	Shroom3	Cytoskeleton
DMR14:16670001	14	16670001	16673000	3000	2	4.50E-07	-0.48	69	2.3	Shroom3	Cytoskeleton
DMR14:16718001	14	16718001	16719000	1000	1	4.30E-08	0.37	14	1.4	Shroom3	Cytoskeleton
DMR14:16762001	14	16762001	16765000	3000	1	2.10E-07	0.4	32	1.07	Shroom3	Cytoskeleton
DMR14:16786001	14	16786001	16788000	2000	1	1.50E-09	-0.54	37	1.85	Shroom3;LOC108352720	Cytoskeleton
DMR14:16800001	14	16800001	16801000	1000	1	2.40E-07	0.36	15	1.5	Shroom3	Cytoskeleton
DMR14:16813001	14	16813001	16814000	1000	1	4.40E-13	0.78	25	2.5	Shroom3	Cytoskeleton
DMR14:16819001	14	16819001	16820000	1000	1	3.10E-08	-0.48	30	3	Shroom3	Cytoskeleton
DMR14:16846001	14	16846001	16847000	1000	1	5.70E-08	0.65	33	3.3	Shroom3	Cytoskeleton
DMR14:17102001	14	17102001	17104000	2000	1	9.90E-07	-0.39	23	1.15	Scarb2	Transport

DMR14:17209001	14	17209001	17211000	2000	1	1.80E-08	-0.5	22	1.1	Art3;Cxcl10	Transport;Growth Factors
DMR14:17232001	14	17232001	17235000	3000	1	7.60E-08	-0.39	51	1.7	Art3;Cxcl9	Transport;Growth Factors
DMR14:17238001	14	17238001	17240000	2000	1	8.90E-08	0.33	37	1.85	Cxcl9;Sdad1	Growth Factors
DMR14:17269001	14	17269001	17271000	2000	1	8.90E-08	-0.56	49	2.45	Sdad1	
DMR14:17315001	14	17315001	17316000	1000	1	1.90E-07	0.54	13	1.3	Ppef2	Signaling
DMR14:17455001	14	17455001	17456000	1000	1	2.10E-07	-0.47	29	2.9	G3bp2	Metabolism
DMR14:17550001	14	17550001	17551000	1000	1	8.40E-08	0.64	8	0.8	Cdkl2;LOC108352722	Signaling
DMR14:17983001	14	17983001	17985000	2000	1	3.20E-09	0.44	28	1.4	Parm1	
DMR14:18539001	14	18539001	18540000	1000	1	3.70E-13	0.87	9	0.9	Areg	Growth Factors
DMR14:18643001	14	18643001	18645000	2000	1	2.20E-07	0.32	17	0.85	Epgn;Mthfd2l	Growth Factors
DMR14:18706001	14	18706001	18707000	1000	1	5.40E-07	0.53	14	1.4	Mthfd2l	
DMR14:18708001	14	18708001	18709000	1000	1	9.80E-07	-0.34	17	1.7	Mthfd2l	
DMR14:18816001	14	18816001	18819000	3000	1	1.20E-08	0.27	24	0.8	Cxcl3	Growth Factors
DMR14:18895001	14	18895001	18898000	3000	1	1.20E-07	0.35	57	1.9	Cxcl6	
DMR14:18919001	14	18919001	18922000	3000	1	8.90E-08	0.66	65	2.17	Cxcl6	
DMR14:19000001	14	19000001	19002000	2000	1	5.40E-07	0.65	44	2.2	Rassf6	Cytoskeleton
DMR14:19147001	14	19147001	19149000	2000	1	1.70E-07	0.48	40	2	Afp	Transport
DMR14:19404001	14	19404001	19408000	4000	1	9.90E-07	-0.45	48	1.2	Ankrd17	Immune
DMR14:19448001	14	19448001	19451000	3000	1	4.90E-07	-0.45	42	1.4	Cox18	Transport
DMR14:19863001	14	19863001	19865000	2000	1	1.30E-07	0.36	17	0.85	Adamts3	Protease
DMR14:19907001	14	19907001	19908000	1000	1	1.00E-08	-0.64	10	1	Adamts3	Protease
DMR14:20194001	14	20194001	20195000	1000	1	3.20E-16	0.53	14	1.4	Npffr2	Signaling
DMR14:20484001	14	20484001	20486000	2000	1	2.70E-10	0.45	9	0.45	LOC498339;Slc4a4	Transport
DMR14:20499001	14	20499001	20501000	2000	1	3.50E-10	-0.55	42	2.1	Slc4a4	Transport
DMR14:20509001	14	20509001	20512000	3000	1	1.50E-07	-0.51	41	1.37	Slc4a4	Transport
DMR14:20595001	14	20595001	20596000	1000	1	1.50E-10	-0.39	12	1.2	Slc4a4;LOC103692660	Transport
DMR14:20645001	14	20645001	20646000	1000	1	5.40E-07	-0.56	10	1	Slc4a4	Transport
DMR14:20655001	14	20655001	20656000	1000	1	1.20E-07	-0.42	25	2.5	Slc4a4	Transport
DMR14:20657001	14	20657001	20660000	3000	1	3.30E-08	-0.52	47	1.57	Slc4a4	Transport
DMR14:20687001	14	20687001	20689000	2000	1	2.70E-08	-0.37	30	1.5	Slc4a4	Transport
DMR14:20733001	14	20733001	20739000	6000	1	3.00E-10	-0.41	70	1.17	Slc4a4	Transport
DMR14:20772001	14	20772001	20777000	5000	1	3.40E-07	-0.45	111	2.22	Slc4a4	Transport
DMR14:20923001	14	20923001	20926000	3000	1	2.80E-08	-0.47	41	1.37	LOC108352724;Dck	Signaling
DMR14:21032001	14	21032001	21033000	1000	1	3.80E-07	-0.35	18	1.8	Grsf1	Translation
DMR14:21153001	14	21153001	21155000	2000	2	2.10E-07	-0.56	31	1.55	Rufy3;Utp3;RGD1561381	Metabolism
DMR14:21170001	14	21170001	21172000	2000	2	7.20E-08	-0.56	15	0.75	RGD1561381;Jchain	Immune
DMR14:21204001	14	21204001	21205000	1000	1	2.30E-08	-0.46	2	0.2	LOC102554948;Enam	Development
DMR14:21279001	14	21279001	21282000	3000	1	1.40E-07	0.47	25	0.83	LOC102554948;Amtn	
DMR14:21349001	14	21349001	21350000	1000	1	1.30E-08	0.67	20	2	Prol1	
DMR14:21571001	14	21571001	21575000	4000	1	3.40E-09	-0.42	37	0.92	Smr3b;Vcsa2	Protease; Proteolysis
DMR14:21598001	14	21598001	21602000	4000	1	8.30E-09	-0.46	31	0.78	Smr3b;LOC108352726;LOC108352824	
DMR14:21842001	14	21842001	21843000	1000	1	1.20E-07	0.4	9	0.9	Odam;LOC108352727	
DMR14:21844001	14	21844001	21847000	3000	1	5.20E-08	0.63	33	1.1	Odam;LOC108352727	
DMR14:21897001	14	21897001	21899000	2000	1	2.70E-10	0.66	36	1.8	Prr27;Csn1s2b	
DMR14:21946001	14	21946001	21948000	2000	1	3.30E-07	0.42	16	0.8	Csn1s2a;Stath	
DMR14:22006001	14	22006001	22007000	1000	1	1.50E-07	0.53	8	0.8	Csn1s1	Transport
DMR14:22028001	14	22028001	22035000	7000	2	2.10E-13	0.51	28	0.4	RGD1562394	
DMR14:22218001	14	22218001	22226000	8000	1	4.30E-11	-0.3	87	1.09	Ugt2a1	
DMR14:22456001	14	22456001	22459000	3000	1	3.10E-08	0.5	23	0.77	Ugt2b17	
DMR14:22612001	14	22612001	22617000	5000	2	1.60E-07	-0.3	44	0.88	Ugt2b7	
DMR14:22762001	14	22762001	22767000	5000	1	6.00E-08	-0.35	36	0.72	RGD1565664	
DMR14:22991001	14	22991001	22992000	1000	1	9.90E-08	-0.61	8	0.8	Ythdc1	
DMR14:23156001	14	23156001	23157000	1000	1	1.50E-07	0.51	15	1.5	Tmprss11g	Protease
DMR14:23268001	14	23268001	23270000	2000	1	1.80E-10	0.34	7	0.35	Tmprss11a	Protease
DMR14:23338001	14	23338001	23343000	5000	1	3.80E-07	-0.29	49	0.98	Tmprss11d	Protease
DMR14:23402001	14	23402001	23406000	4000	1	3.80E-13	0.73	58	1.45	Tmprss11d;Tmprss11c	Protease
DMR14:23484001	14	23484001	23485000	1000	1	1.10E-08	0.35	6	0.6	LOC103692776;Gnrhr	Signaling
DMR14:23562001	14	23562001	23567000	5000	1	4.00E-09	-0.32	47	0.94	Uba6;Stap1	Proteolysis;Cytoskeleton
DMR14:24057001	14	24057001	24062000	5000	1	4.00E-07	-0.28	50	1	Tmprss11f;LOC100364381	Protease
DMR14:25636001	14	25636001	25640000	4000	1	9.90E-09	-0.38	31	0.78	Epha5	Receptor

DMR14:25776001	14	25776001	25777000	1000	1	7.70E-09	0.55	17	1.7	Epha5	Receptor
DMR14:25961001	14	25961001	25963000	2000	1	5.70E-07	-0.46	22	1.1	Epha5	Receptor
DMR14:26675001	14	26675001	26676000	1000	1	9.50E-08	-0.4	7	0.7	Tecrl	Metabolism
DMR14:28394001	14	28394001	28397000	3000	1	5.90E-10	0.71	50	1.67	Adgrl3	Signaling
DMR14:28564001	14	28564001	28565000	1000	1	7.20E-07	-0.49	20	2	Adgrl3	Signaling
DMR14:28590001	14	28590001	28592000	2000	2	4.40E-11	0.55	5	0.25	Adgrl3	Signaling
DMR14:29186001	14	29186001	29188000	2000	1	2.50E-08	0.37	24	1.2	Adgrl3	Signaling
DMR14:33057001	14	33057001	33059000	2000	1	1.50E-09	-0.42	33	1.65	Igfbp7	
DMR14:33150001	14	33150001	33151000	1000	1	6.70E-08	-0.47	20	2	Rest	Transcription
DMR14:33342001	14	33342001	33347000	5000	2	1.60E-08	-0.5	82	1.64	Hopx	
DMR14:33454001	14	33454001	33456000	2000	1	5.60E-07	-0.6	10	0.5	Arl9;LOC108352736;Srp72	
DMR14:33657001	14	33657001	33658000	1000	1	1.70E-08	-0.46	22	2.2	Aasdh;LOC108352737;RGD1311575	Metabolism
DMR14:33663001	14	33663001	33664000	1000	1	3.30E-13	0.61	44	4.4	RGD1311575	
DMR14:33680001	14	33680001	33683000	3000	1	4.20E-07	-0.38	52	1.73	RGD1311575	
DMR14:33688001	14	33688001	33690000	2000	1	5.00E-08	-0.36	39	1.95	RGD1311575	
DMR14:34083001	14	34083001	34084000	1000	1	6.00E-09	-0.52	16	1.6	Cep135	Epigenetic
DMR14:34157001	14	34157001	34160000	3000	1	7.00E-07	0.37	33	1.1	Exoc1	Transcription
DMR14:34415001	14	34415001	34417000	2000	1	5.00E-08	-0.42	26	1.3	Clock	
DMR14:34457001	14	34457001	34458000	1000	1	7.30E-07	-0.39	13	1.3	Clock	
DMR14:34466001	14	34466001	34467000	1000	1	2.80E-09	-0.57	10	1	Clock	
DMR14:34534001	14	34534001	34539000	5000	1	4.10E-07	-0.38	77	1.54	Tmem165	
DMR14:34564001	14	34564001	34565000	1000	1	5.90E-07	-0.38	30	3	Srd5a3	
DMR14:34577001	14	34577001	34583000	6000	3	2.00E-07	-0.3	75	1.25	Srd5a3	
DMR14:35554001	14	35554001	35555000	1000	1	2.20E-08	0.4	4	0.4	Pdgfra;LOC102554024	Receptor
DMR14:35565001	14	35565001	35567000	2000	1	1.90E-07	-0.38	43	2.15	Pdgfra;LOC102554024	Receptor
DMR14:35680001	14	35680001	35683000	3000	1	1.70E-07	-0.37	36	1.2	LOC102556465;Chic2	
DMR14:36295001	14	36295001	36296000	1000	1	7.00E-07	-0.51	16	1.6	Scfd2	Transport
DMR14:36312001	14	36312001	36322000	10000	2	3.50E-08	-0.33	115	1.15	Scfd2	Transport
DMR14:36332001	14	36332001	36333000	1000	1	2.80E-07	0.44	10	1	Scfd2	Transport
DMR14:36334001	14	36334001	36336000	2000	1	3.20E-08	-0.4	16	0.8	Scfd2	Transport
DMR14:36349001	14	36349001	36350000	1000	1	8.80E-07	0.39	2	0.2	Scfd2	Transport
DMR14:36397001	14	36397001	36398000	1000	1	9.50E-07	0.49	6	0.6	Scfd2	Transport
DMR14:36433001	14	36433001	36435000	2000	1	2.90E-08	0.39	22	1.1	Scfd2	Transport
DMR14:36454001	14	36454001	36456000	2000	1	1.70E-07	0.39	13	0.65	Scfd2	Transport
DMR14:36462001	14	36462001	36463000	1000	1	2.30E-07	-0.62	7	0.7	Scfd2	Transport
DMR14:36507001	14	36507001	36513000	6000	1	7.50E-09	-0.31	48	0.8	Scfd2	Transport
DMR14:36526001	14	36526001	36528000	2000	1	6.90E-08	-0.33	13	0.65	Scfd2	Transport
DMR14:37249001	14	37249001	37252000	3000	1	2.10E-08	0.43	31	1.03	Dcun1d4	
DMR14:37255001	14	37255001	37260000	5000	1	3.80E-08	0.43	50	1	Dcun1d4	
DMR14:37644001	14	37644001	37647000	3000	1	4.40E-08	-0.58	52	1.73	Fryl	Cytoskeleton
DMR14:37663001	14	37663001	37665000	2000	1	4.60E-07	-0.36	33	1.65	Fryl	Cytoskeleton
DMR14:37671001	14	37671001	37673000	2000	1	8.80E-08	-0.51	34	1.7	Fryl	Cytoskeleton
DMR14:37847001	14	37847001	37853000	6000	1	3.70E-07	-0.35	66	1.1	Slain2	
DMR14:37950001	14	37950001	37952000	2000	1	5.90E-08	-0.4	22	1.1	Tec	
DMR14:38060001	14	38060001	38061000	1000	1	1.40E-09	0.43	6	0.6	Txk	
DMR14:38174001	14	38174001	38177000	3000	2	9.60E-10	0.39	10	0.33	Cnga1	Ion Channel
DMR14:38227001	14	38227001	38234000	7000	3	3.20E-08	-0.74	88	1.26	Nfxl1	Transcription
DMR14:38360001	14	38360001	38361000	1000	1	2.10E-08	-0.5	24	2.4	Corin;LOC680377;LOC102550273	Binding Proteins
DMR14:38553001	14	38553001	38554000	1000	1	8.60E-10	0.41	16	1.6	Atp10d	Transport
DMR14:38567001	14	38567001	38569000	2000	1	4.40E-07	0.37	15	0.75	Atp10d	Transport
DMR14:38816001	14	38816001	38817000	1000	1	6.70E-11	0.57	18	1.8	Gabrb1	Ion Channel
DMR14:38852001	14	38852001	38857000	5000	1	5.80E-07	-0.25	48	0.96	Gabrb1	Ion Channel
DMR14:38982001	14	38982001	38984000	2000	1	4.40E-11	0.47	18	0.9	Gabrb1	Ion Channel
DMR14:39122001	14	39122001	39129000	7000	1	4.80E-07	-0.33	70	1	Gabrb1	Ion Channel
DMR14:39297001	14	39297001	39299000	2000	1	1.80E-08	0.5	13	0.65	Cox7b2	Metabolism
DMR14:41710001	14	41710001	41714000	4000	1	4.40E-07	-0.44	22	0.55	Grxcr1	Metabolism
DMR14:41718001	14	41718001	41721000	3000	1	1.30E-07	-0.57	40	1.33	Grxcr1	Metabolism
DMR14:42049001	14	42049001	42050000	1000	1	1.00E-07	0.36	16	1.6	Atp8a1	Transport
DMR14:42052001	14	42052001	42055000	3000	1	2.10E-07	-0.5	34	1.13	Atp8a1	Transport
DMR14:42161001	14	42161001	42163000	2000	1	7.40E-11	0.64	40	2	Atp8a1	Transport
DMR14:42452001	14	42452001	42456000	4000	1	2.00E-09	0.48	74	1.85	LOC108352852;Slc30a9	Transport
DMR14:42494001	14	42494001	42497000	3000	1	2.60E-07	-0.35	25	0.83	Slc30a9	Transport

DMR14:42702001	14	42702001	42703000	1000	1	8.10E-08	0.34	5	0.5	Phox2b	Development
DMR14:42765001	14	42765001	42766000	1000	1	1.70E-07	-0.42	20	2	Limch1	Cytoskeleton
DMR14:42802001	14	42802001	42806000	4000	1	9.90E-07	-0.46	58	1.45	Limch1	Cytoskeleton
DMR14:42928001	14	42928001	42930000	2000	1	5.50E-07	-0.49	16	0.8	Limch1	Cytoskeleton
DMR14:43250001	14	43250001	43252000	2000	1	1.50E-07	-0.41	31	1.55	Apbb2	
DMR14:43318001	14	43318001	43319000	1000	1	5.50E-09	-0.46	18	1.8	Apbb2	
DMR14:43558001	14	43558001	43562000	4000	1	6.40E-07	-0.46	40	1	Nsun7	
DMR14:43756001	14	43756001	43759000	3000	3	2.70E-11	-0.46	31	1.03	Rbm47	Metabolism
DMR14:43849001	14	43849001	43858000	9000	1	8.70E-09	-0.43	91	1.01	Rbm47	Metabolism
DMR14:43989001	14	43989001	43992000	3000	1	1.40E-07	-0.42	45	1.5	Rhoh;N4bp2;LOC108352747	Signaling
DMR14:44192001	14	44192001	44193000	1000	1	7.40E-11	-0.59	4	0.4	Pds5a	Epigenetic
DMR14:44225001	14	44225001	44227000	2000	1	1.30E-09	-0.61	20	1	Pds5a;LOC680579	Epigenetic
DMR14:44843001	14	44843001	44846000	3000	1	1.40E-07	-0.34	49	1.63	Klhl5;LOC102556650	Cytoskeleton
DMR14:44892001	14	44892001	44893000	1000	1	6.20E-08	0.43	3	0.3	Tmem156	
DMR14:44933001	14	44933001	44934000	1000	1	2.90E-07	-0.37	19	1.9	Fam114a1	
DMR14:44936001	14	44936001	44938000	2000	1	2.30E-07	-0.35	31	1.55	Fam114a1	
DMR14:46157001	14	46157001	46161000	4000	1	1.10E-08	-0.33	38	0.95	Nwd2	
DMR14:46628001	14	46628001	46640000	12000	3	1.20E-09	0.45	849	7.08	LOC102554740;Rn45s;Rn18s;Rn5-8s;Rn28s	
DMR14:46656001	14	46656001	46662000	6000	1	9.10E-10	0.4	144	2.4	Rn45s;Rn5-8s;Rn28s	
DMR14:46665001	14	46665001	46672000	7000	1	1.70E-07	-0.37	76	1.09	Rn45s;Rn28s;LOC108352829	
DMR14:47203001	14	47203001	47204000	1000	1	8.30E-12	0.57	16	1.6	LOC108352753;Slc25a52;LOC108352830	
DMR14:48616001	14	48616001	48622000	6000	2	1.20E-07	-0.38	60	1	LOC685935;Dthd1	
DMR14:48799001	14	48799001	48802000	3000	2	7.70E-14	-0.38	22	0.73	Arap2	Signaling
DMR14:48912001	14	48912001	48913000	1000	1	6.10E-07	0.43	9	0.9	Arap2	Signaling
DMR14:51473001	14	51473001	51479000	6000	1	2.30E-08	-0.76	57	0.95	Amd1-ps2	
DMR14:54671001	14	54671001	54672000	1000	1	5.50E-07	0.57	12	1.2	Pcdh7	Cytoskeleton
DMR14:54760001	14	54760001	54761000	1000	1	2.00E-11	0.47	14	1.4	Pcdh7	Cytoskeleton
DMR14:54814001	14	54814001	54815000	1000	1	6.50E-09	0.42	13	1.3	Pcdh7	Cytoskeleton
DMR14:54849001	14	54849001	54850000	1000	1	2.90E-07	-0.35	12	1.2	Pcdh7	Cytoskeleton
DMR14:54851001	14	54851001	54852000	1000	1	5.50E-08	0.53	14	1.4	Pcdh7	Cytoskeleton
DMR14:54924001	14	54924001	54930000	6000	1	2.30E-08	-0.32	79	1.32	Pcdh7;LOC103693027	Cytoskeleton
DMR14:55025001	14	55025001	55026000	1000	1	1.20E-09	-0.59	6	0.6	Pcdh7	Cytoskeleton
DMR14:59419001	14	59419001	59420000	1000	1	1.50E-09	0.46	2	0.2	Tbc1d19	
DMR14:59484001	14	59484001	59485000	1000	1	1.30E-07	0.58	25	2.5	Tbc1d19	
DMR14:59789001	14	59789001	59790000	1000	1	7.00E-07	0.4	8	0.8	Rbpj;LOC108352754	Transcription
DMR14:60076001	14	60076001	60079000	3000	1	7.80E-13	0.79	94	3.13	Smim20	
DMR14:60131001	14	60131001	60134000	3000	1	8.40E-08	-0.35	58	1.93	LOC108352755;Sel1l3	
DMR14:60686001	14	60686001	60688000	2000	1	8.30E-09	-0.53	17	0.85	Sepsecs	
DMR14:60867001	14	60867001	60869000	2000	1	6.10E-07	-0.46	15	0.75	Ccdc149	
DMR14:60906001	14	60906001	60908000	2000	1	1.60E-22	0.39	16	0.8	Ccdc149	
DMR14:60958001	14	60958001	60964000	6000	1	2.30E-23	1.11	113	1.88	Ccdc149;Sod3	Metabolism
DMR14:61927001	14	61927001	61933000	6000	1	3.00E-10	0.49	47	0.78	Kctd8	
DMR14:61939001	14	61939001	61940000	1000	1	2.90E-09	0.53	12	1.2	Kctd8	
DMR14:62082001	14	62082001	62085000	3000	1	5.80E-07	-0.47	24	0.8	Kctd8	
DMR14:62297001	14	62297001	62298000	1000	1	3.80E-10	0.68	22	2.2	Vom1r-ps31	
DMR14:62651001	14	62651001	62657000	6000	1	1.30E-09	-0.38	53	0.88	Gnpda2	Metabolism
DMR14:64696001	14	64696001	64697000	1000	1	1.60E-07	-0.52	12	1.2	Adgra3	Signaling
DMR14:64698001	14	64698001	64699000	1000	1	2.60E-08	-0.45	17	1.7	Adgra3	Signaling
DMR14:64711001	14	64711001	64713000	2000	1	4.80E-08	-0.58	14	0.7	Adgra3	Signaling
DMR14:65637001	14	65637001	65638000	1000	1	1.10E-10	0.39	10	1	Kcnip4	
DMR14:65658001	14	65658001	65660000	2000	1	1.20E-09	0.53	25	1.25	Kcnip4	
DMR14:66257001	14	66257001	66259000	2000	1	6.70E-07	-0.43	12	0.6	Kcnip4	
DMR14:66333001	14	66333001	66335000	2000	1	2.00E-08	0.38	9	0.45	Kcnip4;LOC103693056	
DMR14:66538001	14	66538001	66541000	3000	1	3.10E-08	0.58	23	0.77	Kcnip4;LOC364172	
DMR14:66937001	14	66937001	66938000	1000	1	2.20E-08	0.53	11	1.1	Slit2	
DMR14:67019001	14	67019001	67021000	2000	1	7.70E-07	-0.45	37	1.85	Slit2	
DMR14:67050001	14	67050001	67051000	1000	1	4.20E-07	0.46	11	1.1	Slit2	
DMR14:67098001	14	67098001	67100000	2000	1	4.80E-08	0.53	33	1.65	Slit2	
DMR14:69757001	14	69757001	69762000	5000	2	2.00E-08	-0.35	49	0.98	Lcorl	Transcription
DMR14:69787001	14	69787001	69789000	2000	1	1.30E-08	-0.81	23	1.15	Lcorl	Transcription
DMR14:70067001	14	70067001	70068000	1000	1	2.60E-08	0.52	10	1	Fam184b	

DMR14:70922001	14	70922001	70924000	2000	1	5.50E-08	-0.38	30	1.5	Ldb2	Transcription
DMR14:70988001	14	70988001	70990000	2000	1	6.40E-08	0.79	42	2.1	Ldb2	Transcription
DMR14:71052001	14	71052001	71055000	3000	1	5.80E-12	0.59	11	0.37	Ldb2	Transcription
DMR14:71075001	14	71075001	71076000	1000	1	3.10E-07	-0.57	6	0.6	Ldb2	Transcription
DMR14:71538001	14	71538001	71541000	3000	1	3.40E-08	-0.47	23	0.77	Prom1	
DMR14:71611001	14	71611001	71612000	1000	1	2.00E-09	-0.45	9	0.9	Prom1	
DMR14:71973001	14	71973001	71978000	5000	1	5.40E-09	-0.44	70	1.4	Cc2d2a	
DMR14:72385001	14	72385001	72386000	1000	1	7.10E-13	-0.43	9	0.9	Cpeb2	Translation
DMR14:73760001	14	73760001	73764000	4000	2	1.90E-09	-0.45	53	1.32	Bod1l1	
DMR14:73800001	14	73800001	73802000	2000	1	6.70E-09	-0.42	27	1.35	Bod1l1	
DMR14:73891001	14	73891001	73892000	1000	1	9.40E-07	-0.47	8	0.8	LOC100361803;Rab28	
DMR14:73912001	14	73912001	73915000	3000	2	4.60E-07	-0.43	20	0.67	Rab28	
DMR14:73918001	14	73918001	73919000	1000	1	4.60E-17	-0.93	7	0.7	Rab28	
DMR14:73941001	14	73941001	73947000	6000	2	2.30E-07	-0.29	70	1.17	Rab28	
DMR14:76695001	14	76695001	76697000	2000	1	9.30E-08	0.42	28	1.4	Clnk	Cytoskeleton
DMR14:76731001	14	76731001	76734000	3000	1	3.50E-08	0.37	33	1.1	Clnk;LOC108352770	Cytoskeleton
DMR14:76810001	14	76810001	76814000	4000	1	5.60E-07	-0.5	16	0.4	Clnk	Cytoskeleton
DMR14:76878001	14	76878001	76880000	2000	1	2.40E-15	0.85	32	1.6	Zfp518b;LOC498391	Transcription
DMR14:76914001	14	76914001	76915000	1000	1	4.40E-07	0.71	29	2.9	Rrm1-ps1;LOC108352833	
DMR14:77283001	14	77283001	77286000	3000	1	8.70E-08	-0.56	39	1.3	Tmem128	
DMR14:77795001	14	77795001	77797000	2000	1	7.80E-08	0.43	12	0.6	Cytl1	Growth Factors
DMR14:77812001	14	77812001	77814000	2000	1	3.70E-08	0.48	17	0.85	Cytl1	Growth Factors
DMR14:78148001	14	78148001	78150000	2000	1	1.40E-09	-0.46	27	1.35	Evc2	
DMR14:78172001	14	78172001	78173000	1000	1	1.00E-08	0.49	6	0.6	Evc2	
DMR14:78216001	14	78216001	78219000	3000	2	2.80E-07	0.54	40	1.33	Evc2;Evc	
DMR14:78232001	14	78232001	78237000	5000	1	1.40E-11	0.65	47	0.94	Evc	
DMR14:78453001	14	78453001	78455000	2000	1	7.30E-07	0.44	23	1.15	Jakmip1	
DMR14:78950001	14	78950001	78953000	3000	1	1.70E-07	0.61	45	1.5	Man2b2	
DMR14:79428001	14	79428001	79431000	3000	1	1.10E-07	-0.33	35	1.17	Tbc1d14;LOC108352776	Signaling
DMR14:79611001	14	79611001	79613000	2000	1	1.20E-07	0.36	33	1.65	Sorcs2	Transport
DMR14:79632001	14	79632001	79634000	2000	1	1.80E-07	0.42	18	0.9	Sorcs2	Transport
DMR14:79766001	14	79766001	79767000	1000	1	9.20E-09	0.42	3	0.3	Sorcs2	Transport
DMR14:79769001	14	79769001	79773000	4000	1	1.70E-10	0.4	78	1.95	Sorcs2	Transport
DMR14:79805001	14	79805001	79808000	3000	1	1.70E-10	0.51	51	1.7	Sorcs2	Transport
DMR14:79825001	14	79825001	79826000	1000	1	1.70E-09	0.46	11	1.1	Sorcs2	Transport
DMR14:79898001	14	79898001	79899000	1000	1	8.90E-08	0.36	13	1.3	Sorcs2	Transport
DMR14:79906001	14	79906001	79907000	1000	1	3.30E-11	0.65	12	1.2	Sorcs2	Transport
DMR14:79940001	14	79940001	79942000	2000	1	6.10E-10	-0.56	40	2	Afap1	
DMR14:80137001	14	80137001	80138000	1000	1	2.80E-07	0.35	13	1.3	Ablim2	Cytoskeleton
DMR14:80211001	14	80211001	80213000	2000	1	4.50E-07	0.46	19	0.95	Sh3tc1	
DMR14:80216001	14	80216001	80218000	2000	1	1.40E-12	0.4	12	0.6	Sh3tc1	
DMR14:80256001	14	80256001	80260000	4000	1	2.90E-08	0.43	62	1.55	Htra3	Protease
DMR14:80275001	14	80275001	80278000	3000	1	1.20E-11	0.52	40	1.33	Htra3	Protease
DMR14:80344001	14	80344001	80351000	7000	2	2.80E-10	-0.4	88	1.26	Acox3	Metabolism
DMR14:80386001	14	80386001	80387000	1000	1	6.30E-07	0.39	19	1.9	Trmt44	
DMR14:80405001	14	80405001	80406000	1000	1	1.90E-10	0.57	16	1.6	Cpz	Protease
DMR14:80905001	14	80905001	80907000	2000	1	1.40E-09	0.45	15	0.75	LOC108352779;Lrpap1	Transcription
DMR14:80926001	14	80926001	80929000	3000	1	1.00E-08	0.42	66	2.2	Lrpap1;Dok7	Transcription
DMR14:80940001	14	80940001	80944000	4000	1	7.70E-07	0.43	51	1.27	Dok7	
DMR14:80992001	14	80992001	80997000	5000	1	1.00E-09	0.4	62	1.24	Rgs12	
DMR14:80999001	14	80999001	81001000	2000	1	6.90E-08	0.52	22	1.1	Rgs12	
DMR14:81204001	14	81204001	81210000	6000	2	5.40E-07	-0.45	50	0.83	Htt	
DMR14:81222001	14	81222001	81223000	1000	1	8.50E-07	-0.47	3	0.3	Htt	
DMR14:81311001	14	81311001	81313000	2000	1	6.20E-10	0.46	9	0.45	Grk4	Signaling
DMR14:81423001	14	81423001	81426000	3000	1	8.10E-09	-0.61	37	1.23	Add1;Sh3bp2	Cytoskeleton
DMR14:81439001	14	81439001	81440000	1000	1	1.20E-08	0.38	19	1.9	Sh3bp2	
DMR14:81733001	14	81733001	81735000	2000	1	4.30E-07	0.32	27	1.35	Cfap99;Zfyve28	Development
DMR14:81865001	14	81865001	81869000	4000	1	7.90E-08	-0.33	40	1	Poln;Haus3	Transcription
DMR14:82021001	14	82021001	82022000	1000	1	8.00E-09	-0.47	8	0.8	Poln	Transcription
DMR14:82102001	14	82102001	82103000	1000	1	3.80E-07	-0.42	16	1.6	Nelfa	
DMR14:82173001	14	82173001	82174000	1000	1	5.20E-07	-0.5	4	0.4	Whsc1	
DMR14:82393001	14	82393001	82395000	2000	1	2.20E-09	-0.4	22	1.1	Fam53a	
DMR14:82620001	14	82620001	82621000	1000	1	5.50E-08	-0.54	11	1.1	Uvssa	

DMR14:83106001	14	83106001	83107000	1000	1	2.10E-07	0.4	6	0.6	Depdc5	Signaling
DMR14:83280001	14	83280001	83281000	1000	1	1.20E-11	-0.42	15	1.5	Prr14l	
DMR14:83458001	14	83458001	83459000	1000	1	2.50E-07	-0.4	13	1.3	Eif4enif1;Drg1	Transport
DMR14:83494001	14	83494001	83500000	6000	1	1.10E-11	0.41	49	0.82	Patz1	Transcription
DMR14:83525001	14	83525001	83527000	2000	1	9.40E-10	0.42	35	1.75	Patz1	Transcription
DMR14:83721001	14	83721001	83723000	2000	1	8.70E-07	-0.45	29	1.45	Pla2g3;Inpp5j	Signaling
DMR14:83739001	14	83739001	83741000	2000	1	1.70E-17	0.93	52	2.6	Pla2g3;Inpp5j	Signaling
DMR14:83760001	14	83760001	83765000	5000	2	9.50E-11	0.49	130	2.6	Selm;Smtn	
DMR14:83778001	14	83778001	83780000	2000	1	5.30E-12	0.9	37	1.85	Smtn	
DMR14:83927001	14	83927001	83928000	1000	1	1.90E-09	0.44	18	1.8	Morc2	
DMR14:83979001	14	83979001	83984000	5000	1	6.90E-09	-0.44	73	1.46	Osbp2	
DMR14:84327001	14	84327001	84332000	5000	1	3.50E-07	-0.41	50	1	Sec14l3;Mtfp1;LOC102549281;Sec14l2	
DMR14:84615001	14	84615001	84616000	1000	1	2.90E-08	-0.46	13	1.3	Hormad2;LOC103693146	
DMR14:84670001	14	84670001	84671000	1000	1	1.30E-12	0.85	27	2.7	Hormad2	
DMR14:84733001	14	84733001	84734000	1000	1	6.20E-09	-0.63	8	0.8	Mtmr3	Signaling
DMR14:84789001	14	84789001	84794000	5000	1	5.40E-08	-0.3	80	1.6	Mtmr3	Signaling
DMR14:84909001	14	84909001	84910000	1000	1	3.90E-08	0.62	8	0.8	Ascc2	
DMR14:84960001	14	84960001	84961000	1000	1	1.70E-07	-0.4	19	1.9	Zmat5;Cabp7	
DMR14:85071001	14	85071001	85073000	2000	1	2.50E-07	-0.43	19	0.95	Nf2	Cytoskeleton
DMR14:85128001	14	85128001	85129000	1000	1	4.10E-08	0.53	21	2.1	Nipsnap1;Thoc5	
DMR14:85141001	14	85141001	85146000	5000	1	3.20E-09	-0.56	75	1.5	Nipsnap1;Thoc5	
DMR14:85181001	14	85181001	85184000	3000	1	8.80E-07	-0.37	59	1.97	Thoc5;Nefh	
DMR14:85483001	14	85483001	85484000	1000	1	9.20E-07	-0.57	16	1.6	Kremen1	Receptor
DMR14:85570001	14	85570001	85573000	3000	1	5.70E-12	-0.42	33	1.1	Znrf3	
DMR14:85749001	14	85749001	85753000	4000	1	5.10E-08	0.34	68	1.7	LOC102549967;Xbp1	
DMR14:85761001	14	85761001	85764000	3000	1	3.70E-07	-0.4	40	1.33	Xbp1;Ccdc117	
DMR14:85767001	14	85767001	85768000	1000	1	4.20E-07	-0.41	10	1	Xbp1;Ccdc117	
DMR14:85842001	14	85842001	85846000	4000	1	1.00E-09	0.46	73	1.82	Ankrd36	
DMR14:85866001	14	85866001	85868000	2000	1	2.40E-07	0.33	32	1.6	Ankrd36	
DMR14:85891001	14	85891001	85893000	2000	1	3.10E-07	-0.43	36	1.8	Ankrd36;RGD1560623;LOC364191	
DMR14:85909001	14	85909001	85911000	2000	1	2.30E-10	0.45	8	0.4	Ankrd36;LOC364191	
DMR14:85917001	14	85917001	85922000	5000	2	1.70E-08	-0.55	46	0.92	Ankrd36;LOC102550119	
DMR14:86054001	14	86054001	86057000	3000	1	2.50E-07	-0.34	48	1.6	Dbnl;Pgam2	Cytoskeleton;Metabolism
DMR14:86127001	14	86127001	86137000	10000	1	9.70E-07	-0.26	115	1.15	Pold2;LOC108352788;Myl7	Transcription;Cytoskeleton
DMR14:86218001	14	86218001	86219000	1000	1	1.10E-07	0.54	24	2.4	Camk2b;LOC108352789	Signaling
DMR14:86462001	14	86462001	86466000	4000	1	1.70E-07	-0.27	33	0.82	Npc1l1;Ogdh	Metabolism
DMR14:86559001	14	86559001	86561000	2000	1	9.50E-11	0.69	54	2.7	Npc1l1	
DMR14:86782001	14	86782001	86784000	2000	1	2.30E-10	0.6	37	1.85	LOC103693157;Myo1g	Cytoskeleton
DMR14:86818001	14	86818001	86820000	2000	1	9.00E-09	-0.68	26	1.3	Ccm2;LOC108352791	
DMR14:87348001	14	87348001	87349000	1000	1	1.90E-07	0.57	19	1.9	Adcy1	
DMR14:88696001	14	88696001	88697000	1000	1	7.40E-10	-0.4	13	1.3	Tns3	Cytoskeleton
DMR14:88752001	14	88752001	88754000	2000	1	2.50E-08	-0.61	33	1.65	Tns3	Cytoskeleton
DMR14:88783001	14	88783001	88786000	3000	1	3.50E-13	-0.53	68	2.27	Tns3	Cytoskeleton
DMR14:89047001	14	89047001	89050000	3000	1	1.20E-13	-0.48	44	1.47	Pkd1l1	Transport
DMR14:89191001	14	89191001	89193000	2000	1	1.60E-07	0.52	15	0.75	Pkd1l1;Hus1	Transport;Transcription
DMR14:89244001	14	89244001	89251000	7000	1	7.30E-07	-0.32	73	1.04	Sun3;LOC688553	Cytoskeleton
DMR14:89258001	14	89258001	89259000	1000	1	3.90E-08	0.42	15	1.5	Sun3;LOC688553	Cytoskeleton
DMR14:89417001	14	89417001	89423000	6000	1	3.70E-09	0.37	23	0.38	Abca13	Transport
DMR14:89465001	14	89465001	89468000	3000	1	8.90E-07	-0.44	21	0.7	Abca13	Transport
DMR14:89527001	14	89527001	89530000	3000	1	5.80E-07	-0.48	24	0.8	Abca13	Transport
DMR14:89543001	14	89543001	89544000	1000	1	4.10E-07	0.41	9	0.9	Abca13	Transport
DMR14:89549001	14	89549001	89553000	4000	2	1.10E-07	-0.29	37	0.92	Abca13	Transport
DMR14:89716001	14	89716001	89719000	3000	2	1.70E-08	-0.35	30	1	Abca13	Transport
DMR14:89764001	14	89764001	89766000	2000	1	1.00E-07	0.42	14	0.7	Abca13	Transport
DMR14:89880001	14	89880001	89882000	2000	1	2.90E-09	-0.63	13	0.65	Abca13	Transport
DMR14:91395001	14	91395001	91398000	3000	1	7.90E-10	-0.49	17	0.57	Zpbp	
DMR14:91461001	14	91461001	91463000	2000	1	2.00E-07	-0.43	16	0.8	Zpbp	
DMR14:91546001	14	91546001	91550000	4000	2	6.40E-08	-0.28	33	0.82	Zpbp;RGD1309870	
DMR14:91591001	14	91591001	91592000	1000	1	3.30E-08	0.38	7	0.7	RGD1309870	
DMR14:91607001	14	91607001	91611000	4000	2	5.40E-15	0.85	63	1.57	RGD1309870;LOC364198	

DMR14:92006001	14	92006001	92007000	1000	1	4.10E-09	-0.42	18	1.8	Ddc;LOC103693175	Metabolism
DMR14:92110001	14	92110001	92112000	2000	1	4.50E-08	0.38	21	1.05	Grb10	Cytoskeleton
DMR14:92391001	14	92391001	92392000	1000	1	7.60E-08	0.36	16	1.6	Cobl	
DMR14:92399001	14	92399001	92401000	2000	1	1.70E-09	0.36	30	1.5	Cobl	
DMR14:92665001	14	92665001	92668000	3000	1	3.10E-07	-0.3	24	0.8	Trnar-ucu	
DMR14:93500001	14	93500001	93501000	1000	1	3.10E-07	0.42	14	1.4	RGD1561286	
DMR14:99552001	14	99552001	99555000	3000	1	1.50E-08	-0.51	39	1.3	Vstm2a	Immune
DMR14:100086001	14	100086001	100089000	3000	1	2.80E-08	-0.36	27	0.9	Egfr	Receptor
DMR14:101758001	14	101758001	101761000	3000	1	2.10E-08	-0.29	23	0.77	Olr1827-ps	
DMR14:102108001	14	102108001	102111000	3000	1	2.10E-11	0.49	53	1.77	Olr1806-ps	
DMR14:103199001	14	103199001	103200000	1000	1	8.60E-09	-0.44	25	2.5	Meis1	Development
DMR14:103204001	14	103204001	103206000	2000	1	1.50E-08	-0.5	38	1.9	Meis1	Development
DMR14:103209001	14	103209001	103211000	2000	1	2.30E-07	0.41	18	0.9	Meis1	Development
DMR14:104258001	14	104258001	104260000	2000	1	8.10E-07	-0.44	35	1.75	Spred2	Cytoskeleton
DMR14:104288001	14	104288001	104289000	1000	1	3.70E-08	0.53	24	2.4	Spred2	Cytoskeleton
DMR14:104290001	14	104290001	104291000	1000	1	1.30E-11	0.99	23	2.3	Spred2	Cytoskeleton
DMR14:104299001	14	104299001	104300000	1000	1	1.50E-08	0.37	11	1.1	Spred2	Cytoskeleton
DMR14:104344001	14	104344001	104346000	2000	1	9.60E-08	-0.43	33	1.65	Actr2	Cytoskeleton
DMR14:104587001	14	104587001	104588000	1000	1	3.80E-10	0.83	36	3.6	Slc1a4	Transport
DMR14:104798001	14	104798001	104803000	5000	1	1.50E-07	-0.44	96	1.92	Sertad2	Transcription
DMR14:104832001	14	104832001	104833000	1000	1	3.00E-10	-0.62	12	1.2	Sertad2	Transcription
DMR14:104856001	14	104856001	104858000	2000	1	4.30E-09	-0.62	29	1.45	Sertad2	Transcription
DMR14:104884001	14	104884001	104886000	2000	1	1.20E-08	-0.53	45	2.25	Sertad2	Transcription
DMR14:104945001	14	104945001	104947000	2000	1	5.80E-07	-0.41	29	1.45	Aftph	
DMR14:105045001	14	105045001	105046000	1000	1	5.20E-08	-0.59	26	2.6	Lgalsl;LOC108352836	Extracellular Matrix
DMR14:106071001	14	106071001	106074000	3000	1	1.30E-16	0.9	58	1.93	Peli1	Proteolysis
DMR14:106162001	14	106162001	106163000	1000	1	1.20E-08	-0.45	15	1.5	Vps54	Transport
DMR14:106378001	14	106378001	106380000	2000	1	1.50E-07	-0.48	42	2.1	Mdh1	Metabolism
DMR14:106402001	14	106402001	106403000	1000	1	1.00E-07	0.34	13	1.3	Mdh1;Wdpcp	Metabolism
DMR14:106618001	14	106618001	106620000	2000	1	2.60E-08	-0.37	13	0.65	Wdpcp	
DMR14:106769001	14	106769001	106773000	4000	1	7.70E-08	-0.62	38	0.95	Wdpcp	
DMR14:106878001	14	106878001	106883000	5000	1	4.60E-09	-0.58	87	1.74	Ehbp1	
DMR14:106885001	14	106885001	106886000	1000	1	8.50E-08	-0.49	16	1.6	Ehbp1	
DMR14:106927001	14	106927001	106928000	1000	1	9.00E-08	0.45	6	0.6	Ehbp1	
DMR14:107043001	14	107043001	107045000	2000	1	1.60E-10	-0.59	36	1.8	Ehbp1;LOC102550584	
DMR14:107064001	14	107064001	107067000	3000	3	6.20E-13	0.79	168	5.6	Ehbp1;LOC102550584;LOC108352821	
DMR14:107099001	14	107099001	107100000	1000	1	7.80E-07	0.3	14	1.4	Ehbp1	
DMR14:107274001	14	107274001	107278000	4000	1	1.80E-10	-0.42	66	1.65	Tmem17	
DMR14:107583001	14	107583001	107585000	2000	1	9.70E-08	-0.39	33	1.65	LOC103693217;B3gnt2	Golgi
DMR14:107708001	14	107708001	107711000	3000	1	5.90E-10	0.51	23	0.77	Commd1	
DMR14:107733001	14	107733001	107734000	1000	1	8.20E-11	0.55	10	1	Commd1	
DMR14:107790001	14	107790001	107792000	2000	1	8.70E-09	0.38	30	1.5	Cct4;Fam161a	Translation
DMR14:107805001	14	107805001	107808000	3000	1	1.90E-08	0.31	50	1.67	Fam161a	
DMR14:108076001	14	108076001	108078000	2000	1	1.70E-08	0.42	23	1.15	Usp34;LOC108352814	Protease
DMR14:108122001	14	108122001	108124000	2000	1	9.00E-09	-0.47	21	1.05	Usp34	Protease
DMR14:108192001	14	108192001	108193000	1000	1	6.10E-08	0.27	9	0.9	Usp34	Protease
DMR14:108197001	14	108197001	108199000	2000	1	3.70E-14	-0.66	21	1.05	Usp34	Protease
DMR14:108250001	14	108250001	108252000	2000	1	1.30E-07	-0.42	37	1.85	Usp34	Protease
DMR14:108383001	14	108383001	108385000	2000	1	3.00E-10	-0.58	18	0.9	RGD1305110;Pex13	Transport
DMR14:108831001	14	108831001	108832000	1000	1	1.40E-08	0.73	22	2.2	Bcl11a	Transcription
DMR14:108859001	14	108859001	108863000	4000	1	2.00E-07	0.33	76	1.9	Bcl11a	Transcription
DMR14:108911001	14	108911001	108915000	4000	1	7.10E-09	0.85	130	3.25	Bcl11a	Transcription
DMR14:110742001	14	110742001	110744000	2000	2	2.70E-08	-0.51	26	1.3	Fancl;Vrk2	Proteolysis;Signaling
DMR14:110816001	14	110816001	110819000	3000	1	5.50E-08	-0.43	24	0.8	Vrk2	Signaling
DMR14:110834001	14	110834001	110842000	8000	2	1.30E-07	-0.36	87	1.09	Vrk2	Signaling
DMR14:112744001	14	112744001	112747000	3000	1	2.20E-07	-0.54	7	0.23	Ccdc85a	
DMR14:113122001	14	113122001	113123000	1000	1	7.70E-10	-0.43	3	0.3	LOC102550342;Mir216a;Mir217	
DMR14:113129001	14	113129001	113130000	1000	1	7.70E-07	-0.6	3	0.3	LOC102550342;Mir217	
DMR14:113288001	14	113288001	113290000	2000	1	9.10E-07	-0.46	4	0.2	Efemp1	Extracellular Matrix
DMR14:113804001	14	113804001	113808000	4000	1	3.40E-08	-0.44	15	0.38	Ccdc88a	Transport
DMR14:113881001	14	113881001	113885000	4000	1	1.70E-07	-0.32	36	0.9	Ccdc88a	Transport
DMR14:113887001	14	113887001	113888000	1000	1	1.20E-09	-0.43	15	1.5	Ccdc88a	Transport

DMR14:113998001	14	113998001	114004000	6000	1	5.70E-10	-0.35	64	1.07	Clhc1	Transport
DMR14:114254001	14	114254001	114261000	7000	1	8.30E-07	-0.34	57	0.81	Eml6	
DMR14:114285001	14	114285001	114286000	1000	1	2.80E-07	-0.5	10	1	Eml6	
DMR14:114544001	14	114544001	114549000	5000	1	3.50E-07	-0.38	41	0.82	Sptbn1	
DMR14:114630001	14	114630001	114633000	3000	1	4.90E-07	-0.52	8	0.27	Sptbn1	
DMR14:114635001	14	114635001	114636000	1000	1	5.40E-07	0.4	11	1.1	Sptbn1	
DMR14:114902001	14	114902001	114905000	3000	2	2.20E-09	-0.31	26	0.87	Acyp2	Signaling
DMR14:114911001	14	114911001	114914000	3000	1	5.00E-07	-0.53	5	0.17	Acyp2	Signaling
DMR14:115056001	14	115056001	115057000	1000	1	5.80E-13	1.02	20	2	Acyp2	Signaling
DMR14:115306001	14	115306001	115312000	6000	2	5.50E-09	-0.3	48	0.8	Erlec1	
DMR14:115452001	14	115452001	115454000	2000	1	7.20E-07	-0.35	60	3	Asb3	
DMR15:462001	15	462001	463000	1000	1	5.50E-07	0.41	4	0.4	Kcnma1	Transport
DMR15:633001	15	633001	634000	1000	1	1.90E-07	0.53	8	0.8	Kcnma1	Transport
DMR15:832001	15	832001	833000	1000	1	9.00E-08	0.53	6	0.6	Kcnma1	Transport
DMR15:2627001	15	2627001	2629000	2000	1	1.20E-07	0.53	41	2.05	Comtd1;Vdac2	Epigenetic;Transport
DMR15:2775001	15	2775001	2776000	1000	1	2.60E-08	0.47	23	2.3	Dupd1;LOC108352871	
DMR15:2798001	15	2798001	2799000	1000	1	2.80E-08	0.71	39	3.9	Dupd1;LOC108352871	
DMR15:2856001	15	2856001	2858000	2000	1	1.50E-07	-0.45	41	2.05	Kat6b	Epigenetic
DMR15:2876001	15	2876001	2878000	2000	2	2.10E-07	-0.49	37	1.85	Kat6b	Epigenetic
DMR15:2959001	15	2959001	2962000	3000	1	7.90E-07	-0.38	44	1.47	Kat6b;LOC103693811	Epigenetic
DMR15:3043001	15	3043001	3046000	3000	1	3.40E-08	0.42	43	1.43	Adk	
DMR15:3102001	15	3102001	3103000	1000	1	9.50E-10	-0.54	28	2.8	Adk	
DMR15:3142001	15	3142001	3147000	5000	1	4.90E-08	-0.54	97	1.94	Adk	
DMR15:3153001	15	3153001	3156000	3000	1	1.50E-10	-0.52	21	0.7	Adk	
DMR15:3216001	15	3216001	3217000	1000	1	3.20E-07	-0.48	17	1.7	Adk	
DMR15:3243001	15	3243001	3246000	3000	1	1.40E-07	-0.44	84	2.8	Adk	
DMR15:3261001	15	3261001	3262000	1000	1	6.90E-07	-0.52	8	0.8	Adk	
DMR15:3307001	15	3307001	3312000	5000	2	1.70E-16	-0.6	109	2.18	Adk	
DMR15:3323001	15	3323001	3326000	3000	1	2.40E-09	0.66	68	2.27	Adk	
DMR15:3408001	15	3408001	3409000	1000	1	1.20E-07	-0.5	14	1.4	Adk	
DMR15:3420001	15	3420001	3424000	4000	1	7.20E-07	-0.36	55	1.38	Adk	
DMR15:3432001	15	3432001	3433000	1000	1	1.10E-07	-0.43	12	1.2	Adk;Ap3m1	Transport
DMR15:3463001	15	3463001	3466000	3000	1	2.70E-07	-0.47	38	1.27	Ap3m1;Vcl	Transport
DMR15:3470001	15	3470001	3473000	3000	1	1.00E-07	0.49	40	1.33	Vcl	
DMR15:3497001	15	3497001	3500000	3000	1	2.40E-07	-0.56	42	1.4	Vcl	
DMR15:3541001	15	3541001	3542000	1000	1	4.10E-07	-0.68	8	0.8	Vcl	
DMR15:3740001	15	3740001	3741000	1000	1	4.50E-11	0.62	7	0.7	NEWGENE_621802;NEWGENE_1304700	
DMR15:4184001	15	4184001	4186000	2000	1	5.20E-07	-0.48	25	1.25	Usp54;LOC103693236	Protease
DMR15:4198001	15	4198001	4199000	1000	1	6.60E-13	-0.91	5	0.5	Usp54;LOC103693236	Protease
DMR15:4324001	15	4324001	4325000	1000	1	7.40E-07	-0.4	25	2.5	Cfap70	Development
DMR15:4441001	15	4441001	4442000	1000	1	2.20E-09	0.7	30	3	Ecd;Nudt13	
DMR15:4459001	15	4459001	4460000	1000	1	7.30E-08	0.3	12	1.2	Nudt13	
DMR15:4478001	15	4478001	4482000	4000	1	6.30E-09	0.62	84	2.1	Kcnk16;LOC108352873	Transport
DMR15:4487001	15	4487001	4489000	2000	1	3.20E-08	0.77	51	2.55	Kcnk16;LOC108352873	Transport
DMR15:4576001	15	4576001	4578000	2000	1	2.00E-07	0.38	28	1.4	Kcnk5	Transport
DMR15:4738001	15	4738001	4740000	2000	1	2.80E-07	-0.34	35	1.75	Gng2	Signaling
DMR15:4762001	15	4762001	4765000	3000	1	3.90E-08	-0.43	43	1.43	Gng2	Signaling
DMR15:4816001	15	4816001	4817000	1000	1	4.00E-08	-0.43	21	2.1	Gng2	Signaling
DMR15:5295001	15	5295001	5298000	3000	1	1.80E-07	0.45	43	1.43	LOC102546376;Cd99l2	
DMR15:5482001	15	5482001	5491000	9000	1	2.10E-08	-0.45	68	0.76	Spetex-2F;LOC102547093;LOC108352879	
DMR15:5501001	15	5501001	5503000	2000	1	3.70E-07	0.26	16	0.8	Spetex-2F;LOC108352879;Spetex-2C	
DMR15:5551001	15	5551001	5554000	3000	1	4.50E-10	-0.41	28	0.93	Spetex-2C;LOC102549465	
DMR15:5645001	15	5645001	5646000	1000	1	5.00E-08	0.29	6	0.6	Spetex-2C;LOC108349412	
DMR15:6213001	15	6213001	6214000	1000	1	1.30E-07	-0.47	5	0.5	Zfp385d	
DMR15:6241001	15	6241001	6242000	1000	1	4.00E-07	0.44	10	1	Zfp385d	
DMR15:6253001	15	6253001	6254000	1000	1	1.50E-11	0.6	9	0.9	Zfp385d	
DMR15:6315001	15	6315001	6317000	2000	1	3.10E-10	-0.68	25	1.25	Zfp385d	
DMR15:6332001	15	6332001	6334000	2000	1	4.80E-08	-0.54	22	1.1	Zfp385d	
DMR15:6462001	15	6462001	6463000	1000	1	7.40E-11	-0.65	15	1.5	Zfp385d	
DMR15:6639001	15	6639001	6644000	5000	2	3.40E-08	-0.33	47	0.94	Zfp385d	

DMR15:6850001	15	6850001	6853000	3000	1	5.80E-11	-0.44	23	0.77	Zfp385d	
DMR15:6920001	15	6920001	6921000	1000	1	9.80E-08	0.45	17	1.7	Zfp385d	
DMR15:6967001	15	6967001	6969000	2000	1	8.30E-10	-0.56	11	0.55	Zfp385d	
DMR15:7511001	15	7511001	7516000	5000	2	4.30E-09	-0.41	44	0.88	Ube2e2	Proteolysis
DMR15:7578001	15	7578001	7579000	1000	1	1.40E-07	-0.41	5	0.5	Ube2e2	Proteolysis
DMR15:7690001	15	7690001	7691000	1000	1	3.50E-10	-0.69	22	2.2	Ube2e2;LOC103693837	Proteolysis
DMR15:7730001	15	7730001	7731000	1000	1	7.40E-08	0.51	19	1.9	Ube2e2	Proteolysis
DMR15:7893001	15	7893001	7894000	1000	1	2.10E-07	0.44	10	1	Ube2e2	Proteolysis
DMR15:7900001	15	7900001	7902000	2000	1	2.80E-07	0.39	26	1.3	Ube2e2	Proteolysis
DMR15:7913001	15	7913001	7917000	4000	1	8.90E-09	-0.39	17	0.42	Ube2e2	Proteolysis
DMR15:8116001	15	8116001	8117000	1000	1	3.70E-08	0.38	23	2.3	Ube2e1	Proteolysis
DMR15:8144001	15	8144001	8145000	1000	1	8.90E-07	0.27	15	1.5	Ube2e1;LOC102552559	Proteolysis
DMR15:8152001	15	8152001	8156000	4000	1	3.30E-18	-0.64	51	1.27	Ube2e1;LOC102552559	Proteolysis
DMR15:8159001	15	8159001	8163000	4000	2	2.80E-09	-0.57	46	1.15	Ube2e1;LOC102552559	Proteolysis
DMR15:8938001	15	8938001	8940000	2000	1	1.80E-07	0.31	38	1.9	Thrb;LOC103693275	Transcription
DMR15:8982001	15	8982001	8983000	1000	1	1.70E-07	0.29	23	2.3	Thrb	Transcription
DMR15:8987001	15	8987001	8989000	2000	1	2.10E-08	0.32	35	1.75	Thrb	Transcription
DMR15:9007001	15	9007001	9013000	6000	2	1.70E-07	-0.26	57	0.95	Thrb	Transcription
DMR15:9074001	15	9074001	9080000	6000	1	5.20E-09	0.76	104	1.73	Thrb	Transcription
DMR15:9108001	15	9108001	9110000	2000	1	7.30E-09	0.35	30	1.5	Thrb	Transcription
DMR15:9148001	15	9148001	9149000	1000	1	6.30E-08	0.33	12	1.2	Thrb	Transcription
DMR15:9153001	15	9153001	9156000	3000	1	5.60E-09	0.33	30	1	Thrb	Transcription
DMR15:9170001	15	9170001	9172000	2000	1	1.60E-12	0.51	22	1.1	Thrb	Transcription
DMR15:9232001	15	9232001	9234000	2000	1	2.10E-07	0.47	50	2.5	Thrb	Transcription
DMR15:10054001	15	10054001	10055000	1000	1	1.70E-09	0.42	9	0.9	Rarb	Transcription
DMR15:10092001	15	10092001	10094000	2000	1	6.10E-14	0.63	10	0.5	Rarb	Transcription
DMR15:10228001	15	10228001	10231000	3000	2	9.60E-12	0.39	21	0.7	Rarb	Transcription
DMR15:11661001	15	11661001	11662000	1000	1	1.70E-11	0.52	16	1.6	Nek10;LOC498453	Signaling
DMR15:11721001	15	11721001	11724000	3000	1	2.80E-07	-0.39	10	0.33	Nek10	Signaling
DMR15:11915001	15	11915001	11921000	6000	1	3.40E-08	-0.45	68	1.13	Slc4a7	Transport
DMR15:12423001	15	12423001	12426000	3000	1	6.00E-09	-0.72	39	1.3	Psmd6;Atxn7;LOC108352888	
DMR15:12467001	15	12467001	12469000	2000	1	2.00E-13	-0.68	19	0.95	Atxn7	
DMR15:12524001	15	12524001	12525000	1000	1	1.30E-08	0.4	9	0.9	Atxn7	
DMR15:12574001	15	12574001	12577000	3000	1	9.40E-07	0.37	36	1.2	Atxn7;Thoc7;LOC102550375	
DMR15:12586001	15	12586001	12588000	2000	1	2.70E-07	0.44	16	0.8	Thoc7;LOC102550375	
DMR15:12604001	15	12604001	12607000	3000	3	4.90E-08	-0.52	31	1.03	LOC102550375;RGD1565725	
DMR15:12619001	15	12619001	12626000	7000	1	8.90E-08	-0.39	70	1	RGD1565725	
DMR15:12939001	15	12939001	12944000	5000	1	7.10E-08	-0.24	46	0.92	Ptprg	Signaling
DMR15:13053001	15	13053001	13055000	2000	1	1.90E-08	0.4	22	1.1	Ptprg	Signaling
DMR15:13075001	15	13075001	13080000	5000	1	3.90E-08	0.35	66	1.32	Ptprg	Signaling
DMR15:13279001	15	13279001	13281000	2000	1	5.20E-08	-0.43	45	2.25	Ptprg	Signaling
DMR15:13285001	15	13285001	13286000	1000	1	7.40E-07	0.32	10	1	Ptprg	Signaling
DMR15:13415001	15	13415001	13417000	2000	1	5.50E-07	0.63	34	1.7	Ptprg	Signaling
DMR15:13475001	15	13475001	13477000	2000	1	9.50E-07	0.39	25	1.25	Ptprg	Signaling
DMR15:13512001	15	13512001	13513000	1000	1	5.30E-11	-0.55	12	1.2	Ptprg	Signaling
DMR15:13556001	15	13556001	13558000	2000	1	5.20E-07	0.36	40	2	Ptprg	Signaling
DMR15:13560001	15	13560001	13565000	5000	1	5.00E-07	-0.33	66	1.32	Ptprg	Signaling
DMR15:13873001	15	13873001	13876000	3000	1	2.40E-07	-0.44	19	0.63	RGD1563739	
DMR15:14461001	15	14461001	14467000	6000	1	5.90E-09	-0.4	60	1	RGD1559573	
DMR15:14516001	15	14516001	14521000	5000	1	2.10E-07	-0.47	20	0.4	Sntn	Signaling
DMR15:14797001	15	14797001	14798000	1000	1	7.50E-07	0.44	7	0.7	Synpr;LOC108352889	Transport
DMR15:14813001	15	14813001	14818000	5000	2	1.30E-08	0.42	38	0.76	Synpr;LOC108352889	Transport
DMR15:15564001	15	15564001	15565000	1000	1	6.20E-07	0.37	4	0.4	Cadps	Transport
DMR15:15589001	15	15589001	15592000	3000	1	3.90E-07	-0.44	47	1.57	Cadps	Transport
DMR15:15684001	15	15684001	15686000	2000	1	7.70E-08	-0.47	19	0.95	Cadps	Transport
DMR15:15896001	15	15896001	15898000	2000	1	7.20E-07	-0.41	16	0.8	Fhit	Signaling
DMR15:15955001	15	15955001	15956000	1000	1	1.10E-08	-0.62	6	0.6	Fhit	Signaling
DMR15:15971001	15	15971001	15972000	1000	1	1.30E-09	-0.61	19	1.9	Fhit	Signaling
DMR15:16434001	15	16434001	16435000	1000	1	2.60E-11	0.76	34	3.4	Fhit	Signaling
DMR15:16478001	15	16478001	16480000	2000	1	7.80E-07	-0.43	19	0.95	Fhit	Signaling
DMR15:16509001	15	16509001	16510000	1000	1	2.30E-08	0.49	5	0.5	Fhit	Signaling
DMR15:16626001	15	16626001	16629000	3000	1	1.40E-07	-0.39	36	1.2	Fhit	Signaling
DMR15:16813001	15	16813001	16819000	6000	3	1.20E-09	-0.45	53	0.88	Fhit	Signaling

DMR15:18327001	15	18327001	18328000	1000	1	2.70E-10	0.38	11	1.1	Fam3d	Signaling
DMR15:18402001	15	18402001	18403000	1000	1	2.00E-07	0.43	13	1.3	Fam107a	
DMR15:18483001	15	18483001	18490000	7000	1	2.80E-07	-0.45	150	2.14	Acox2;Kctd6	Metabolism;Cytoskeleton
DMR15:18502001	15	18502001	18505000	3000	1	3.70E-11	-0.51	41	1.37	Kctd6;LOC103693296	Cytoskeleton
DMR15:18589001	15	18589001	18592000	3000	1	1.40E-18	0.99	87	2.9	Pxk	
DMR15:18611001	15	18611001	18615000	4000	1	2.60E-09	0.62	73	1.82	Pxk	
DMR15:18696001	15	18696001	18701000	5000	1	3.40E-07	0.63	82	1.64	Abhd6;Dnase1l3	Metabolism
DMR15:18791001	15	18791001	18794000	3000	1	3.80E-07	0.46	53	1.77	Flnb	
DMR15:18809001	15	18809001	18815000	6000	1	1.10E-07	-0.53	101	1.68	Flnb	
DMR15:18821001	15	18821001	18828000	7000	2	1.70E-07	-0.51	110	1.57	Flnb	
DMR15:18844001	15	18844001	18847000	3000	2	1.60E-08	-0.49	52	1.73	Flnb;LOC102548801	
DMR15:19187001	15	19187001	19192000	5000	2	2.10E-10	-0.4	52	1.04	Ptgdrl	Signaling
DMR15:19353001	15	19353001	19356000	3000	1	1.00E-08	0.63	66	2.2	Ptger2	Signaling
DMR15:19493001	15	19493001	19496000	3000	1	4.00E-09	-0.5	19	0.63	Txndc16	Metabolism
DMR15:19578001	15	19578001	19581000	3000	1	2.00E-07	0.5	43	1.43	Gpr137c	
DMR15:19618001	15	19618001	19619000	1000	1	7.90E-08	-0.43	14	1.4	Gpr137c;LOC102552640;Ero1a	Metabolism
DMR15:19633001	15	19633001	19636000	3000	2	6.40E-11	0.4	55	1.83	Ero1a	Metabolism
DMR15:19742001	15	19742001	19745000	3000	1	9.70E-07	0.3	28	0.93	Gnpnat1	Metabolism
DMR15:19825001	15	19825001	19830000	5000	1	2.10E-10	-0.43	86	1.72	Fermt2	
DMR15:19842001	15	19842001	19844000	2000	1	8.60E-10	-0.44	24	1.2	Fermt2	
DMR15:20040001	15	20040001	20048000	8000	1	7.50E-12	-0.46	143	1.79	Ddhd1;LOC108352895	Metabolism
DMR15:20764001	15	20764001	20768000	4000	1	6.20E-07	0.29	67	1.68	LOC108352897;Bmp4	Growth Factors
DMR15:23022001	15	23022001	23026000	4000	2	4.50E-34	0.95	77	1.93	Olr1809-ps	
DMR15:23562001	15	23562001	23564000	2000	1	2.20E-07	-0.42	30	1.5	Cdkn3;Cnih1	Signaling;Transport
DMR15:23582001	15	23582001	23583000	1000	1	8.10E-08	-0.42	16	1.6	Cnih1	Transport
DMR15:23602001	15	23602001	23607000	5000	1	4.90E-09	-0.48	82	1.64	Gmfb	Signaling
DMR15:23689001	15	23689001	23690000	1000	1	1.80E-09	0.73	31	3.1	Samd4a	
DMR15:23720001	15	23720001	23721000	1000	1	2.50E-10	0.48	10	1	Samd4a	
DMR15:23729001	15	23729001	23731000	2000	1	2.10E-07	0.44	41	2.05	Samd4a	
DMR15:23785001	15	23785001	23786000	1000	1	2.70E-07	-0.47	32	3.2	Samd4a	
DMR15:23842001	15	23842001	23843000	1000	1	3.50E-12	0.43	19	1.9	Samd4a	
DMR15:23851001	15	23851001	23852000	1000	1	1.60E-07	0.38	4	0.4	Samd4a	
DMR15:24054001	15	24054001	24055000	1000	1	5.80E-07	-0.37	14	1.4	Wdhd1;Socs4	Epigenetic;Signaling
DMR15:24134001	15	24134001	24138000	4000	1	3.90E-13	0.37	47	1.18	RGD1564783	
DMR15:24159001	15	24159001	24162000	3000	1	4.00E-07	-0.46	57	1.9	Lgals3;Dlgap5	Extracellular Matrix;Cytoskeleton
DMR15:24190001	15	24190001	24191000	1000	1	5.80E-08	0.37	12	1.2	Dlgap5	Cytoskeleton
DMR15:24194001	15	24194001	24197000	3000	2	5.00E-08	-0.49	76	2.53	Dlgap5	Cytoskeleton
DMR15:24201001	15	24201001	24203000	2000	1	4.70E-07	-0.61	25	1.25	Dlgap5	Cytoskeleton
DMR15:24493001	15	24493001	24497000	4000	1	6.50E-08	-0.4	57	1.43	Ktn1	
DMR15:24504001	15	24504001	24505000	1000	1	9.60E-07	0.37	15	1.5	Ktn1	
DMR15:24937001	15	24937001	24940000	3000	1	2.10E-07	-0.4	50	1.67	Peli2	Proteolysis
DMR15:24973001	15	24973001	24980000	7000	1	3.80E-07	-0.51	140	2	Peli2	Proteolysis
DMR15:25000001	15	25000001	25003000	3000	1	4.60E-08	0.37	61	2.03	Peli2	Proteolysis
DMR15:25315001	15	25315001	25316000	1000	1	2.40E-08	0.39	6	0.6	Tmem260	
DMR15:26174001	15	26174001	26179000	5000	1	4.80E-10	0.64	90	1.8	Slc35f4	
DMR15:26216001	15	26216001	26220000	4000	1	3.70E-08	-0.31	30	0.75	Slc35f4	
DMR15:26369001	15	26369001	26370000	1000	1	2.70E-10	0.47	10	1	Slc35f4	
DMR15:26931001	15	26931001	26933000	2000	1	8.20E-08	-0.48	36	1.8	Olr1629;Olr1630	Receptor
DMR15:27212001	15	27212001	27218000	6000	1	6.70E-07	-0.24	56	0.93	Olr1614	Signaling
DMR15:27388001	15	27388001	27394000	6000	2	6.40E-09	-0.44	71	1.18	Olr1620;LOC690055	Signaling
DMR15:27411001	15	27411001	27416000	5000	1	4.10E-07	-0.41	23	0.46	Olr1620;LOC690055;RGD1562558;Olr1621	Signaling
DMR15:27508001	15	27508001	27515000	7000	2	7.90E-09	-0.4	50	0.71	Olr1632	Signaling
DMR15:27607001	15	27607001	27614000	7000	1	4.10E-08	0.3	69	0.99	Olr1634-ps	
DMR15:27748001	15	27748001	27749000	1000	1	6.00E-07	-0.39	10	1	Rmrp;Parp2;Tep1	Transcription
DMR15:27782001	15	27782001	27787000	5000	1	2.50E-12	-0.47	81	1.62	Tep1	Transcription
DMR15:27887001	15	27887001	27888000	1000	1	6.50E-07	-0.37	12	1.2	Pnp	Signaling
DMR15:27977001	15	27977001	27979000	2000	1	9.20E-09	-0.4	31	1.55	Rnase9;Rnase11;Rnase12	
DMR15:28104001	15	28104001	28110000	6000	1	2.50E-08	0.61	42	0.7	Eddm3b;LOC103693840;Ang2	
DMR15:28163001	15	28163001	28166000	3000	1	3.40E-07	-0.47	74	2.47	Rnase2	
DMR15:28219001	15	28219001	28222000	3000	1	1.40E-08	-0.31	44	1.47	Ear11	

DMR15:28384001	15	28384001	28385000	1000	1	5.40E-08	0.5	15	1.5	Arhgef40	Transcription
DMR15:28576001	15	28576001	28577000	1000	1	1.30E-07	-0.42	16	1.6	Rpgrip1;Supt16h	Epigenetic
DMR15:28586001	15	28586001	28587000	1000	1	4.20E-09	0.44	14	1.4	Supt16h	Epigenetic
DMR15:28659001	15	28659001	28661000	2000	1	6.50E-10	-0.48	20	1	Chd8	
DMR15:28730001	15	28730001	28731000	1000	1	3.70E-13	0.86	28	2.8	Mettl3;Trnas-aga;Sall2	Epigenetic;Transcription
DMR15:28809001	15	28809001	28810000	1000	1	2.30E-09	0.48	2	0.2	LOC690444;Olr1640	Receptor
DMR15:28827001	15	28827001	28832000	5000	1	1.60E-07	-0.24	49	0.98	Olr1641	Receptor
DMR15:28833001	15	28833001	28836000	3000	1	3.80E-09	-0.37	26	0.87	Olr1641	Receptor
DMR15:28846001	15	28846001	28847000	1000	1	8.50E-10	0.52	19	1.9	Olr1641;Olr1642	Receptor
DMR15:28862001	15	28862001	28864000	2000	1	4.50E-09	0.43	27	1.35	Olr1642;LOC100363123	Receptor
DMR15:30074001	15	30074001	30077000	3000	1	7.70E-09	-0.65	13	0.43	RGD1561152;LOC103690223	
DMR15:30542001	15	30542001	30548000	6000	1	1.40E-07	-0.48	89	1.48	LOC102552674;Tcrva8;Trav14s1;LOC100360242	
DMR15:32418001	15	32418001	32425000	7000	1	3.80E-12	0.67	111	1.59	LOC685078;LOC103693549;RGD1560771;LOC102547213	
DMR15:33406001	15	33406001	33409000	3000	1	4.30E-08	0.34	39	1.3	Slc7a8	Transport
DMR15:33626001	15	33626001	33629000	3000	1	3.20E-07	0.37	52	1.73	Myh6;Myh7	
DMR15:33659001	15	33659001	33660000	1000	1	1.30E-09	0.59	31	3.1	Myh7	
DMR15:33668001	15	33668001	33669000	1000	1	5.20E-14	0.47	4	0.4	Ngdn	Metabolism
DMR15:33721001	15	33721001	33723000	2000	1	4.40E-08	0.54	49	2.45	Zfhx2	Transcription
DMR15:33725001	15	33725001	33727000	2000	1	2.90E-08	0.4	45	2.25	Zfhx2	Transcription
DMR15:33730001	15	33730001	33734000	4000	2	1.20E-08	0.45	109	2.72	Zfhx2	Transcription
DMR15:33814001	15	33814001	33816000	2000	1	1.60E-19	0.67	40	2	RGD1564324	Metabolism
DMR15:33846001	15	33846001	33849000	3000	1	1.70E-07	0.43	35	1.17	RGD1564324	Metabolism
DMR15:33869001	15	33869001	33871000	2000	1	8.30E-08	-0.7	10	0.5	Dhrs2	Metabolism
DMR15:33872001	15	33872001	33880000	8000	2	6.00E-13	0.83	123	1.54	Dhrs2	Metabolism
DMR15:34043001	15	34043001	34052000	9000	1	1.40E-08	0.71	123	1.37	LOC102551831;RGD1565258	
DMR15:34179001	15	34179001	34183000	4000	1	1.50E-07	0.43	65	1.62	Carmil3;LOC102552093;Cpne6	
DMR15:34235001	15	34235001	34236000	1000	1	3.10E-07	-0.54	16	1.6	Dcaf11	Proteolysis
DMR15:34366001	15	34366001	34372000	6000	1	8.80E-09	0.49	63	1.05	Gmpr2;Tinf2;Tgm1	Metabolism;Transport
DMR15:34387001	15	34387001	34388000	1000	1	6.80E-09	0.6	28	2.8	Tgm1;Rabggta	Transport;Metabolism
DMR15:34514001	15	34514001	34516000	2000	2	4.30E-10	0.36	16	0.8	LOC102552484;Nynrin	Translation
DMR15:34522001	15	34522001	34528000	6000	1	1.10E-09	-0.4	103	1.72	LOC102552484;Nynrin	Translation
DMR15:34560001	15	34560001	34561000	1000	1	3.10E-07	0.46	10	1	Cbln3;Khnyn;Sdr39u1	Translation;Metabolism
DMR15:34658001	15	34658001	34661000	3000	1	5.20E-09	-0.34	26	0.87	Mcpt8l3	
DMR15:34683001	15	34683001	34692000	9000	1	5.10E-08	-0.33	69	0.77	LOC498518;Mcpt8	Protease
DMR15:34760001	15	34760001	34762000	2000	1	1.90E-07	-0.37	16	0.8	Mcpt4;RGD1562290	Protease
DMR15:34930001	15	34930001	34931000	1000	1	9.00E-08	-0.58	8	0.8	Mcpt1l3	Protease
DMR15:35117001	15	35117001	35118000	1000	1	9.20E-07	0.33	10	1	Ctsg	
DMR15:36591001	15	36591001	36592000	1000	1	2.80E-09	0.6	7	0.7	Atp12a	Transport
DMR15:36628001	15	36628001	36634000	6000	1	1.80E-07	-0.34	53	0.88	Rnf17	
DMR15:36996001	15	36996001	36998000	2000	2	1.80E-08	-0.51	15	0.75	Pspc1	Metabolism
DMR15:37129001	15	37129001	37134000	5000	1	1.10E-08	0.65	52	1.04	RGD1563527	
DMR15:37143001	15	37143001	37149000	6000	1	1.20E-08	-0.34	44	0.73	RGD1563527;Zmym2	Transcription
DMR15:37388001	15	37388001	37389000	1000	1	3.10E-07	0.42	21	2.1	Gjb2	Cytoskeleton
DMR15:37553001	15	37553001	37556000	3000	1	1.50E-08	0.37	66	2.2	Cryl1	Metabolism
DMR15:37651001	15	37651001	37652000	1000	1	9.70E-07	-0.39	12	1.2	Cryl1	Metabolism
DMR15:37836001	15	37836001	37839000	3000	1	1.20E-11	-0.5	33	1.1	Eef1akmt1;Xpo4	Transport
DMR15:37910001	15	37910001	37915000	5000	1	1.70E-11	-0.46	41	0.82	Xpo4	Transport
DMR15:38061001	15	38061001	38062000	1000	1	2.30E-07	0.5	19	1.9	LOC103693597;Sap18	
DMR15:38128001	15	38128001	38129000	1000	1	4.00E-07	0.36	5	0.5	Zdhhc20	
DMR15:38169001	15	38169001	38171000	2000	1	7.70E-09	0.59	55	2.75	Zdhhc20	
DMR15:38238001	15	38238001	38242000	4000	1	2.20E-08	-0.38	44	1.1	Micu2	Signaling
DMR15:38854001	15	38854001	38855000	1000	1	4.40E-07	-0.36	16	1.6	Phf11;Setdb2	Transcription;Epigenetic
DMR15:39009001	15	39009001	39010000	1000	1	1.50E-07	0.4	2	0.2	Phf11;Setdb2;LOC102551719	Transcription;Epigenetic
DMR15:39221001	15	39221001	39227000	6000	2	3.30E-07	-0.37	71	1.18	Phf11;Setdb2	Transcription;Epigenetic
DMR15:39545001	15	39545001	39548000	3000	1	6.80E-09	-0.46	71	2.37	Phf11;Setdb2	Transcription;Epigenetic
DMR15:39609001	15	39609001	39612000	3000	1	8.90E-09	0.39	1	0.03	Phf11;Setdb2;RGD1563302;LOC102552417	Transcription;Epigenetic
DMR15:39801001	15	39801001	39802000	1000	1	9.00E-07	-0.37	5	0.5	Cab39l	
DMR15:39938001	15	39938001	39941000	3000	2	1.10E-10	0.45	40	1.33	Shisa2	Cytoskeleton
DMR15:39947001	15	39947001	39953000	6000	2	1.00E-08	0.51	131	2.18	Shisa2;Atp8a2;LOC102552603	Cytoskeleton;Transport
DMR15:40043001	15	40043001	40046000	3000	1	7.70E-08	-0.5	38	1.27	Atp8a2	Transport

DMR15:40165001	15	40165001	40169000	4000	2	5.00E-07	-0.43	56	1.4	Atp8a2	Transport
DMR15:40338001	15	40338001	40340000	2000	1	7.90E-07	-0.63	12	0.6	Atp8a2	Transport
DMR15:40410001	15	40410001	40412000	2000	1	8.20E-07	-0.42	28	1.4	Atp8a2	Transport
DMR15:40483001	15	40483001	40487000	4000	2	1.30E-09	-0.48	71	1.77	Atp8a2;LOC103693635;Nup58	Transport
DMR15:40604001	15	40604001	40607000	3000	1	4.80E-07	0.43	29	0.97	Mtmr6	Signaling
DMR15:41107001	15	41107001	41108000	1000	1	9.40E-07	-0.41	14	1.4	Mipep	Protease
DMR15:41278001	15	41278001	41281000	3000	1	1.70E-07	-0.4	55	1.83	Tnfrsf19	
DMR15:41534001	15	41534001	41536000	2000	1	7.50E-09	-0.38	49	2.45	Sacs	
DMR15:41860001	15	41860001	41862000	2000	1	2.90E-08	-0.36	18	0.9	Spryd7	
DMR15:41905001	15	41905001	41906000	1000	1	2.30E-08	0.6	61	6.1	Trim13	Proteolysis
DMR15:42634001	15	42634001	42635000	1000	1	2.30E-07	0.33	12	1.2	Scara3;Clu	Extracellular Matrix
DMR15:42804001	15	42804001	42805000	1000	1	1.80E-07	0.45	10	1	Ephx2;Chrna2	Metabolism;Ion Channel
DMR15:42978001	15	42978001	42980000	2000	1	9.80E-08	-0.41	14	0.7	Trim35	Proteolysis
DMR15:43021001	15	43021001	43023000	2000	1	1.30E-07	-0.35	37	1.85	Stmn4	
DMR15:43288001	15	43288001	43289000	1000	1	6.70E-07	0.33	11	1.1	Adra1a	Signaling
DMR15:43330001	15	43330001	43332000	2000	1	5.00E-07	-0.53	12	0.6	Adra1a	Signaling
DMR15:43502001	15	43502001	43504000	2000	1	8.70E-08	0.41	8	0.4	Dpysl2	Metabolism
DMR15:43583001	15	43583001	43584000	1000	1	2.10E-09	0.51	17	1.7	Dpysl2;Pnma2	Metabolism
DMR15:43894001	15	43894001	43896000	2000	1	3.80E-07	0.37	18	0.9	Ebf2	Transcription
DMR15:44043001	15	44043001	44045000	2000	1	3.20E-08	0.43	22	1.1	Ebf2	Transcription
DMR15:44531001	15	44531001	44532000	1000	1	6.70E-07	0.43	7	0.7	Dock5	Transcription
DMR15:44557001	15	44557001	44558000	1000	1	3.80E-07	-0.43	11	1.1	Dock5	Transcription
DMR15:44809001	15	44809001	44811000	2000	1	3.60E-12	-0.48	14	0.7	LOC108352935;Nefl	
DMR15:44868001	15	44868001	44870000	2000	1	7.40E-09	0.65	8	0.4	Nefm	
DMR15:45368001	15	45368001	45369000	1000	1	4.30E-08	-0.43	17	1.7	Dleu7	
DMR15:45536001	15	45536001	45538000	2000	2	3.30E-09	0.47	9	0.45	Gucy1b2	Signaling
DMR15:45732001	15	45732001	45734000	2000	1	8.20E-09	0.52	46	2.3	Fam124a	
DMR15:45735001	15	45735001	45738000	3000	1	6.30E-07	0.42	42	1.4	Fam124a	
DMR15:45828001	15	45828001	45831000	3000	1	1.70E-08	-0.49	21	0.7	Serpine3	Protease; Proteolysis
DMR15:46049001	15	46049001	46050000	1000	1	4.30E-07	-0.44	17	1.7	Wdfy2	
DMR15:46083001	15	46083001	46086000	3000	1	1.60E-08	0.34	31	1.03	Wdfy2	
DMR15:46092001	15	46092001	46094000	2000	1	8.30E-07	-0.35	31	1.55	Wdfy2	
DMR15:46152001	15	46152001	46154000	2000	1	3.10E-08	-0.38	10	0.5	Defb42;LOC364402	Signaling
DMR15:46406001	15	46406001	46409000	3000	1	1.90E-07	0.39	30	1	Gata4	Transcription
DMR15:46591001	15	46591001	46592000	1000	1	2.90E-07	0.43	14	1.4	Blk	
DMR15:46864001	15	46864001	46865000	1000	1	7.50E-07	0.49	15	1.5	Xkr6	
DMR15:47143001	15	47143001	47145000	2000	1	1.40E-08	0.47	27	1.35	Xkr6	
DMR15:47172001	15	47172001	47173000	1000	1	2.00E-10	0.64	25	2.5	Xkr6	
DMR15:47288001	15	47288001	47289000	1000	1	5.60E-08	-0.37	10	1	Sox7	Development
DMR15:47661001	15	47661001	47664000	3000	1	4.70E-10	-0.51	47	1.57	Msra	Metabolism
DMR15:48800001	15	48800001	48801000	1000	1	1.60E-07	0.45	19	1.9	Zfp395;Pnoc	Transcription;Signaling
DMR15:48949001	15	48949001	48951000	2000	1	2.30E-07	0.37	41	2.05	Elp3	Epigenetic
DMR15:48977001	15	48977001	48978000	1000	1	1.80E-13	-0.53	16	1.6	Elp3;Anxa2-ps1	Epigenetic
DMR15:48991001	15	48991001	48992000	1000	1	9.80E-07	-0.4	18	1.8	Elp3	Epigenetic
DMR15:49125001	15	49125001	49126000	1000	1	1.40E-08	-0.51	13	1.3	Scara5	Protease
DMR15:49459001	15	49459001	49462000	3000	1	7.10E-08	-0.36	17	0.57	Adam7	Protease
DMR15:51149001	15	51149001	51150000	1000	1	3.10E-11	0.48	15	1.5	Slc25a37	
DMR15:51670001	15	51670001	51671000	1000	1	2.00E-07	0.36	11	1.1	Pebp4	
DMR15:51704001	15	51704001	51706000	2000	1	5.40E-10	0.4	22	1.1	Pebp4	
DMR15:52122001	15	52122001	52123000	1000	1	7.90E-08	-0.43	7	0.7	Piwil2;Polr3d	Translation;Transcription
DMR15:52177001	15	52177001	52179000	2000	1	4.30E-09	0.41	36	1.8	Bmp1	Protease
DMR15:52332001	15	52332001	52334000	2000	1	4.70E-11	0.88	47	2.35	Fgf17;Npm2	Growth Factors;Transcription
DMR15:52433001	15	52433001	52435000	2000	1	8.70E-09	-0.54	16	0.8	Xpo7	Transport
DMR15:52561001	15	52561001	52565000	4000	1	2.00E-07	0.42	32	0.8	Gfra2	Receptor
DMR15:52618001	15	52618001	52620000	2000	1	1.60E-09	0.47	11	0.55	Gfra2	Receptor
DMR15:52847001	15	52847001	52853000	6000	1	2.30E-09	-0.26	69	1.15	Dpm3-ps1	
DMR15:54371001	15	54371001	54377000	6000	2	1.50E-17	-0.52	63	1.05	Fndc3a	Proteolysis
DMR15:54420001	15	54420001	54421000	1000	1	5.80E-07	-0.37	8	0.8	Fndc3a	Proteolysis
DMR15:54523001	15	54523001	54527000	4000	1	5.00E-07	-0.26	33	0.82	Fndc3a	Proteolysis
DMR15:54939001	15	54939001	54941000	2000	1	5.20E-09	-0.52	21	1.05	Cysltr2	Signaling
DMR15:55127001	15	55127001	55129000	2000	1	7.90E-08	-0.63	41	2.05	Rb1;Lpar6	Epigenetic;Signaling
DMR15:55398001	15	55398001	55400000	2000	1	6.50E-08	0.41	31	1.55	LOC103693716;Med4	Transcription

DMR15:56682001	15	56682001	56685000	3000	1	5.20E-37	1.13	36	1.2	Htr2a	Signaling
DMR15:56729001	15	56729001	56732000	3000	2	4.30E-07	-0.62	45	1.5	Htr2a	Signaling
DMR15:57200001	15	57200001	57201000	1000	1	2.90E-08	-0.57	14	1.4	Lcp1	Cytoskeleton
DMR15:57202001	15	57202001	57205000	3000	2	1.10E-08	-0.53	28	0.93	Lcp1	Cytoskeleton
DMR15:57238001	15	57238001	57240000	2000	1	2.90E-07	-0.38	25	1.25	Lcp1	Cytoskeleton
DMR15:57281001	15	57281001	57282000	1000	1	5.50E-13	-0.55	5	0.5	Lcp1;Cpb2	Cytoskeleton;Protease
DMR15:57309001	15	57309001	57311000	2000	1	2.80E-07	-0.54	37	1.85	Cpb2;LOC691812	Protease
DMR15:57376001	15	57376001	57378000	2000	1	9.80E-08	-0.46	24	1.2	Zc3h13	
DMR15:57388001	15	57388001	57390000	2000	1	4.50E-07	0.54	47	2.35	Zc3h13	
DMR15:57503001	15	57503001	57504000	1000	1	9.50E-07	-0.48	12	1.2	Siah3	Proteolysis
DMR15:57567001	15	57567001	57571000	4000	2	7.70E-12	0.88	78	1.95	Siah3	Proteolysis
DMR15:57695001	15	57695001	57696000	1000	1	2.20E-09	0.57	6	0.6	Spert;Erich6b	
DMR15:57738001	15	57738001	57740000	2000	1	4.80E-19	1.08	79	3.95	Spert;Erich6b	
DMR15:57883001	15	57883001	57884000	1000	1	1.00E-08	0.67	25	2.5	LOC102552389;Tpt1	Cytoskeleton
DMR15:58045001	15	58045001	58047000	2000	1	1.30E-08	-0.65	15	0.75	Gtf2f2	Transcription
DMR15:58073001	15	58073001	58074000	1000	1	2.90E-07	-0.66	10	1	Gtf2f2	Transcription
DMR15:58182001	15	58182001	58186000	4000	1	2.50E-10	-0.55	40	1	Nufip1	Metabolism
DMR15:58596001	15	58596001	58599000	3000	1	1.40E-10	-0.66	42	1.4	Tsc22d1	
DMR15:58616001	15	58616001	58621000	5000	2	6.20E-09	-0.56	80	1.6	Tsc22d1	
DMR15:58657001	15	58657001	58661000	4000	1	1.30E-08	-0.47	68	1.7	Tsc22d1;LOC108352945	
DMR15:58697001	15	58697001	58698000	1000	1	2.80E-10	0.45	13	1.3	Serp2	Transcription
DMR15:59155001	15	59155001	59156000	1000	1	6.10E-07	-0.44	16	1.6	LOC108353005;Ccdc122	
DMR15:59164001	15	59164001	59166000	2000	1	3.10E-11	0.51	38	1.9	LOC108353005;Ccdc122	
DMR15:59290001	15	59290001	59291000	1000	1	2.50E-10	-0.65	8	0.8	Ccdc122	
DMR15:59354001	15	59354001	59358000	4000	1	5.60E-07	-0.42	79	1.98	Enox1	Metabolism
DMR15:59394001	15	59394001	59397000	3000	1	2.80E-09	-0.49	44	1.47	Enox1	Metabolism
DMR15:59402001	15	59402001	59405000	3000	1	5.00E-09	0.53	20	0.67	Enox1	Metabolism
DMR15:59436001	15	59436001	59440000	4000	1	7.80E-08	-0.4	73	1.82	Enox1	Metabolism
DMR15:59495001	15	59495001	59497000	2000	1	3.80E-10	-0.57	9	0.45	Enox1	Metabolism
DMR15:59517001	15	59517001	59519000	2000	1	1.70E-07	0.46	22	1.1	Enox1	Metabolism
DMR15:59560001	15	59560001	59562000	2000	1	8.40E-07	0.33	26	1.3	Enox1	Metabolism
DMR15:59638001	15	59638001	59639000	1000	1	1.70E-07	0.38	10	1	Enox1	Metabolism
DMR15:59681001	15	59681001	59682000	1000	1	1.90E-10	-0.51	14	1.4	Enox1	Metabolism
DMR15:59741001	15	59741001	59743000	2000	1	9.80E-11	0.37	23	1.15	Enox1	Metabolism
DMR15:60121001	15	60121001	60122000	1000	1	1.60E-09	0.41	6	0.6	Epsti1	
DMR15:60475001	15	60475001	60477000	2000	1	8.10E-07	0.34	34	1.7	Tnfsf11	
DMR15:60810001	15	60810001	60811000	1000	1	8.90E-09	-0.56	17	1.7	Dgkh	Signaling
DMR15:60827001	15	60827001	60828000	1000	1	2.20E-10	-0.73	14	1.4	Dgkh	Signaling
DMR15:60891001	15	60891001	60894000	3000	1	2.10E-07	0.37	49	1.63	Dgkh	Signaling
DMR15:61073001	15	61073001	61075000	2000	1	1.00E-11	0.38	13	0.65	Vwa8	
DMR15:61105001	15	61105001	61107000	2000	1	9.60E-07	-0.67	19	0.95	Vwa8	
DMR15:61438001	15	61438001	61440000	2000	1	3.50E-10	0.39	21	1.05	Vwa8	
DMR15:61594001	15	61594001	61595000	1000	1	1.70E-07	0.66	25	2.5	LOC100909790;Naa16	Metabolism
DMR15:61734001	15	61734001	61740000	6000	1	8.10E-08	-0.26	57	0.95	Kbtbd6;Wbp4	Cytoskeleton
DMR15:61879001	15	61879001	61881000	2000	1	9.10E-09	-0.51	22	1.1	LOC100360244;Sugt1	
DMR15:61913001	15	61913001	61916000	3000	1	1.90E-07	-0.59	34	1.13	Sugt1;Lect1	
DMR15:70155001	15	70155001	70159000	4000	1	8.70E-09	-0.27	34	0.85	Diaph3	
DMR15:70211001	15	70211001	70213000	2000	2	1.20E-10	-0.66	18	0.9	Diaph3	
DMR15:70276001	15	70276001	70279000	3000	1	1.70E-07	-0.47	32	1.07	Diaph3	
DMR15:70286001	15	70286001	70290000	4000	1	4.40E-09	-0.64	33	0.82	Diaph3	
DMR15:70292001	15	70292001	70297000	5000	1	1.30E-08	-0.35	43	0.86	Diaph3	
DMR15:70298001	15	70298001	70299000	1000	1	3.40E-10	0.47	13	1.3	Diaph3	
DMR15:70387001	15	70387001	70388000	1000	1	9.10E-08	0.44	12	1.2	Diaph3	
DMR15:70405001	15	70405001	70406000	1000	1	1.50E-07	-0.42	14	1.4	Diaph3	
DMR15:76923001	15	76923001	76928000	5000	2	6.30E-10	-0.36	39	0.78	Pcdh9	Cytoskeleton
DMR15:76934001	15	76934001	76935000	1000	1	3.80E-07	-0.55	13	1.3	Pcdh9	Cytoskeleton
DMR15:77073001	15	77073001	77075000	2000	1	1.90E-11	-0.42	18	0.9	Pcdh9	Cytoskeleton
DMR15:77136001	15	77136001	77138000	2000	1	1.60E-07	0.48	14	0.7	Pcdh9	Cytoskeleton
DMR15:77213001	15	77213001	77216000	3000	1	9.80E-07	0.42	30	1	Pcdh9	Cytoskeleton
DMR15:77464001	15	77464001	77465000	1000	1	8.30E-07	0.4	12	1.2	Pcdh9	Cytoskeleton
DMR15:77604001	15	77604001	77607000	3000	1	9.50E-09	0.55	34	1.13	Pcdh9	Cytoskeleton
DMR15:77623001	15	77623001	77625000	2000	1	4.20E-09	0.45	19	0.95	Pcdh9	Cytoskeleton
DMR15:77666001	15	77666001	77667000	1000	1	2.70E-12	0.54	6	0.6	Pcdh9	Cytoskeleton

DMR15:77684001	15	77684001	77686000	2000	1	2.00E-07	-0.37	26	1.3	Pcdh9	Cytoskeleton
DMR15:80382001	15	80382001	80384000	2000	1	9.80E-09	0.45	16	0.8	Klhl1	Cytoskeleton
DMR15:80515001	15	80515001	80518000	3000	1	9.20E-07	-0.31	24	0.8	Klhl1	Cytoskeleton
DMR15:80536001	15	80536001	80539000	3000	1	8.80E-12	0.54	25	0.83	Klhl1	Cytoskeleton
DMR15:80578001	15	80578001	80582000	4000	1	4.10E-09	-0.25	39	0.98	Klhl1	Cytoskeleton
DMR15:81438001	15	81438001	81439000	1000	1	3.10E-07	0.34	10	1	Matr3-ps2	
DMR15:82079001	15	82079001	82082000	3000	2	2.10E-07	0.62	53	1.77	Setsip	
DMR15:82178001	15	82178001	82179000	1000	1	8.70E-10	-0.63	22	2.2	Dach1	Transcription
DMR15:82375001	15	82375001	82378000	3000	1	6.20E-07	-0.52	34	1.13	Dach1	Transcription
DMR15:82406001	15	82406001	82407000	1000	1	6.50E-09	0.64	21	2.1	Dach1	Transcription
DMR15:83542001	15	83542001	83544000	2000	1	9.90E-08	-0.46	19	0.95	Pibf1	
DMR15:83562001	15	83562001	83563000	1000	1	4.90E-07	0.42	17	1.7	Pibf1	
DMR15:83601001	15	83601001	83605000	4000	1	2.90E-08	-0.36	32	0.8	Pibf1	
DMR15:83713001	15	83713001	83715000	2000	1	9.70E-09	-0.55	26	1.3	Klf5	Transcription
DMR15:84416001	15	84416001	84417000	1000	1	1.80E-07	0.42	3	0.3	Klf12	Transcription
DMR15:84457001	15	84457001	84462000	5000	1	1.10E-09	-0.6	69	1.38	Klf12	Transcription
DMR15:84609001	15	84609001	84610000	1000	1	7.70E-11	0.47	4	0.4	Klf12	Transcription
DMR15:84662001	15	84662001	84663000	1000	1	5.80E-15	-0.55	10	1	Klf12	Transcription
DMR15:84690001	15	84690001	84693000	3000	1	4.50E-08	-0.56	20	0.67	Klf12	Transcription
DMR15:84694001	15	84694001	84695000	1000	1	3.20E-07	0.35	10	1	Klf12	Transcription
DMR15:85122001	15	85122001	85124000	2000	1	1.40E-07	-0.37	15	0.75	RGD1560797;LOC108352953;LOC108352954	
DMR15:85917001	15	85917001	85919000	2000	1	3.80E-08	0.56	10	0.5	Tbc1d4	Signaling
DMR15:86018001	15	86018001	86022000	4000	1	2.10E-08	0.45	60	1.5	Tbc1d4	Signaling
DMR15:86036001	15	86036001	86037000	1000	1	2.10E-08	-0.42	15	1.5	Tbc1d4	Signaling
DMR15:86133001	15	86133001	86136000	3000	1	5.60E-07	0.46	48	1.6	RGD2320734;Commd6	
DMR15:86235001	15	86235001	86239000	4000	1	1.50E-08	-0.44	45	1.12	Lmo7	
DMR15:86262001	15	86262001	86264000	2000	1	1.50E-07	0.42	17	0.85	Lmo7	
DMR15:86341001	15	86341001	86342000	1000	1	4.20E-07	0.49	12	1.2	Lmo7	
DMR15:86348001	15	86348001	86349000	1000	1	1.00E-07	-0.52	9	0.9	Lmo7	
DMR15:86416001	15	86416001	86418000	2000	1	1.20E-09	-0.37	35	1.75	Lmo7	
DMR15:86468001	15	86468001	86470000	2000	1	2.30E-09	-0.54	41	2.05	Lmo7	
DMR15:86609001	15	86609001	86610000	1000	1	5.30E-08	0.49	15	1.5	LOC290444;RGD1562819	
DMR15:86611001	15	86611001	86612000	1000	1	8.20E-08	-0.39	16	1.6	LOC290444;RGD1562819	
DMR15:87733001	15	87733001	87737000	4000	1	1.00E-07	0.41	52	1.3	Scel	
DMR15:87740001	15	87740001	87741000	1000	1	1.60E-10	0.79	27	2.7	Scel	
DMR15:87788001	15	87788001	87790000	2000	1	2.30E-08	-0.36	15	0.75	Scel	
DMR15:87808001	15	87808001	87809000	1000	1	6.10E-09	0.53	7	0.7	Scel	
DMR15:87888001	15	87888001	87890000	2000	1	1.90E-07	0.38	35	1.75	Slain1	
DMR15:88007001	15	88007001	88009000	2000	1	4.70E-07	0.36	33	1.65	Ednrb	
DMR15:88020001	15	88020001	88022000	2000	1	4.10E-07	0.42	5	0.25	Ednrb	
DMR15:89331001	15	89331001	89332000	1000	1	2.70E-14	-0.51	4	0.4	Rbm26	Metabolism
DMR15:89445001	15	89445001	89447000	2000	1	6.50E-11	0.63	51	2.55	Ndfip2	
DMR15:90171001	15	90171001	90175000	4000	1	5.40E-07	-0.36	79	1.98	Spry2	Cytoskeleton
DMR15:90361001	15	90361001	90363000	2000	1	1.40E-12	0.68	27	1.35	LOC103693771;Trim52	Proteolysis
DMR15:90384001	15	90384001	90385000	1000	1	1.00E-07	0.43	7	0.7	Trim52	Proteolysis
DMR15:93669001	15	93669001	93674000	5000	2	5.90E-08	-0.57	65	1.3	Fbxl3;Mycbp2	Proteolysis
DMR15:93689001	15	93689001	93690000	1000	1	1.80E-07	-0.37	13	1.3	Mycbp2	Proteolysis
DMR15:93775001	15	93775001	93776000	1000	1	3.90E-08	-0.53	15	1.5	Mycbp2	Proteolysis
DMR15:93804001	15	93804001	93809000	5000	1	6.60E-11	-0.52	31	0.62	Mycbp2	Proteolysis
DMR15:95502001	15	95502001	95505000	3000	1	8.00E-08	-0.32	37	1.23	Slitrk6	
DMR15:96663001	15	96663001	96666000	3000	1	1.20E-07	0.28	16	0.53	RGD1559833	
DMR15:98647001	15	98647001	98648000	1000	1	6.00E-07	-0.35	8	0.8	Vom1r-ps104	
DMR15:100287001	15	100287001	100289000	2000	1	2.10E-07	-0.48	25	1.25	LOC102548462;Gpc5	
DMR15:100347001	15	100347001	100349000	2000	1	1.40E-07	0.35	12	0.6	Gpc5	
DMR15:100394001	15	100394001	100397000	3000	1	3.40E-07	-0.27	28	0.93	Gpc5	
DMR15:100402001	15	100402001	100404000	2000	1	6.00E-07	-0.46	14	0.7	Gpc5	
DMR15:100473001	15	100473001	100479000	6000	2	3.90E-10	0.37	57	0.95	Gpc5	
DMR15:100504001	15	100504001	100506000	2000	1	1.80E-08	-0.4	16	0.8	Gpc5	
DMR15:100730001	15	100730001	100734000	4000	1	6.60E-08	-0.41	18	0.45	Gpc5	
DMR15:100898001	15	100898001	100903000	5000	1	1.90E-07	0.39	50	1	Gpc5	
DMR15:101086001	15	101086001	101087000	1000	1	2.50E-08	0.5	9	0.9	Gpc5	
DMR15:101228001	15	101228001	101229000	1000	1	9.70E-07	0.4	7	0.7	Gpc5	

DMR15:101474001	15	101474001	101476000	2000	1	2.90E-08	0.42	22	1.1	Gpc5	
DMR15:101482001	15	101482001	101483000	1000	1	9.60E-07	0.43	11	1.1	Gpc5	
DMR15:102157001	15	102157001	102158000	1000	1	4.90E-08	0.46	16	1.6	Gpc6	
DMR15:102218001	15	102218001	102222000	4000	1	1.70E-12	0.48	49	1.23	Gpc6	
DMR15:102267001	15	102267001	102269000	2000	1	1.50E-07	0.48	21	1.05	Gpc6	
DMR15:102335001	15	102335001	102337000	2000	1	6.00E-09	-0.58	21	1.05	Gpc6	
DMR15:102448001	15	102448001	102450000	2000	1	4.90E-10	0.66	29	1.45	Gpc6	
DMR15:102457001	15	102457001	102458000	1000	1	2.70E-08	0.58	8	0.8	Gpc6	
DMR15:102506001	15	102506001	102509000	3000	1	6.30E-16	0.78	51	1.7	Gpc6;LOC103693784	
DMR15:102523001	15	102523001	102525000	2000	1	1.40E-14	0.35	32	1.6	Gpc6	
DMR15:102630001	15	102630001	102631000	1000	1	1.10E-08	-0.52	9	0.9	Gpc6;LOC108353029	
DMR15:102792001	15	102792001	102793000	1000	1	1.30E-07	0.36	5	0.5	Gpc6	
DMR15:102801001	15	102801001	102803000	2000	1	5.20E-07	0.4	19	0.95	Gpc6	
DMR15:102874001	15	102874001	102877000	3000	1	1.50E-07	-0.41	48	1.6	Gpc6	
DMR15:102898001	15	102898001	102900000	2000	1	9.00E-07	-0.36	32	1.6	Gpc6	
DMR15:102923001	15	102923001	102924000	1000	1	8.10E-07	0.48	3	0.3	Gpc6	
DMR15:102950001	15	102950001	102952000	2000	1	1.80E-07	-0.57	10	0.5	Gpc6	
DMR15:102965001	15	102965001	102967000	2000	1	6.90E-07	-0.39	35	1.75	Gpc6	
DMR15:102987001	15	102987001	102988000	1000	1	4.90E-10	0.51	25	2.5	Gpc6	
DMR15:103092001	15	103092001	103093000	1000	1	1.30E-07	0.4	10	1	Gpc6	
DMR15:103117001	15	103117001	103118000	1000	1	1.20E-10	-0.42	18	1.8	Gpc6	
DMR15:103122001	15	103122001	103123000	1000	1	2.50E-14	0.87	45	4.5	Gpc6	
DMR15:103124001	15	103124001	103125000	1000	1	5.70E-10	0.47	21	2.1	Gpc6	
DMR15:103133001	15	103133001	103136000	3000	1	2.10E-07	-0.45	61	2.03	Gpc6	
DMR15:103236001	15	103236001	103241000	5000	1	6.90E-10	0.44	83	1.66	Dct	
DMR15:103363001	15	103363001	103364000	1000	1	3.00E-08	0.4	21	2.1	Gpr180	
DMR15:103694001	15	103694001	103697000	3000	1	5.80E-08	0.38	58	1.93	Abcc4	Transport
DMR15:103737001	15	103737001	103742000	5000	1	5.70E-07	-0.43	141	2.82	Abcc4	Transport
DMR15:103830001	15	103830001	103833000	3000	1	9.20E-07	-0.45	72	2.4	Abcc4	Transport
DMR15:103904001	15	103904001	103906000	2000	1	1.80E-07	0.4	32	1.6	Abcc4	Transport
DMR15:104051001	15	104051001	104053000	2000	1	7.80E-07	0.46	22	1.1	Cldn10	Cell Junction
DMR15:104074001	15	104074001	104075000	1000	1	5.70E-07	0.46	31	3.1	Cldn10	Cell Junction
DMR15:104077001	15	104077001	104079000	2000	1	2.50E-11	0.42	19	0.95	Cldn10	Cell Junction
DMR15:104141001	15	104141001	104144000	3000	1	8.60E-10	-0.55	69	2.3	Dzip1	
DMR15:104147001	15	104147001	104150000	3000	1	3.90E-11	-0.42	77	2.57	Dzip1	
DMR15:104203001	15	104203001	104208000	5000	1	1.10E-09	-0.33	53	1.06	Dnajc3	
DMR15:104388001	15	104388001	104390000	2000	1	8.20E-08	-0.44	15	0.75	Uggt2	
DMR15:104402001	15	104402001	104404000	2000	1	1.10E-07	-0.5	22	1.1	Uggt2	
DMR15:104413001	15	104413001	104414000	1000	1	7.60E-09	0.42	8	0.8	Uggt2	
DMR15:104440001	15	104440001	104446000	6000	2	3.30E-08	0.65	117	1.95	Hs6st3	
DMR15:104466001	15	104466001	104467000	1000	1	4.60E-07	-0.29	16	1.6	Hs6st3	
DMR15:104574001	15	104574001	104575000	1000	1	4.10E-08	-0.43	16	1.6	Hs6st3	
DMR15:104620001	15	104620001	104625000	5000	1	3.40E-07	-0.4	60	1.2	Hs6st3	
DMR15:104638001	15	104638001	104645000	7000	2	1.20E-09	-0.39	94	1.34	Hs6st3	
DMR15:104938001	15	104938001	104940000	2000	1	1.90E-12	-0.38	15	0.75	Hs6st3	
DMR15:105042001	15	105042001	105043000	1000	1	4.80E-08	-0.41	16	1.6	Hs6st3	
DMR15:105182001	15	105182001	105183000	1000	1	4.60E-11	0.89	31	3.1	Hs6st3	
DMR15:105192001	15	105192001	105193000	1000	1	8.40E-07	0.36	7	0.7	Hs6st3	
DMR15:105202001	15	105202001	105203000	1000	1	3.90E-10	-0.45	20	2	Hs6st3	
DMR15:105247001	15	105247001	105250000	3000	1	3.80E-11	0.44	30	1	Hs6st3	
DMR15:105395001	15	105395001	105397000	2000	1	4.10E-10	0.94	46	2.3	LOC290492;LOC680256;Oxgr1;LOC108352979	Signaling
DMR15:105426001	15	105426001	105431000	5000	1	2.50E-07	0.52	104	2.08	Oxgr1;LOC108353051	Signaling
DMR15:105635001	15	105635001	105637000	2000	1	6.80E-08	0.39	24	1.2	Mbnl2	Translation
DMR15:105662001	15	105662001	105663000	1000	1	4.20E-15	-0.71	23	2.3	Mbnl2	Translation
DMR15:105692001	15	105692001	105695000	3000	1	7.20E-08	0.37	45	1.5	Mbnl2	Translation
DMR15:106387001	15	106387001	106391000	4000	1	1.40E-07	0.37	39	0.98	Farp1	
DMR15:106398001	15	106398001	106401000	3000	1	2.70E-08	0.44	42	1.4	Farp1	
DMR15:106464001	15	106464001	106467000	3000	1	6.10E-08	-0.55	43	1.43	Farp1	
DMR15:106481001	15	106481001	106483000	2000	1	2.30E-10	-0.56	41	2.05	Farp1	
DMR15:106563001	15	106563001	106565000	2000	1	3.10E-08	0.39	23	1.15	Farp1	
DMR15:106639001	15	106639001	106641000	2000	1	9.40E-08	-0.49	29	1.45	Stk24	
DMR15:106676001	15	106676001	106677000	1000	1	8.30E-08	-0.5	18	1.8	Stk24	

DMR15:106678001	15	106678001	106679000	1000	1	1.00E-07	-0.45	27	2.7	Stk24	
DMR15:106836001	15	106836001	106840000	4000	2	6.10E-08	0.45	46	1.15	Slc15a1	Transport
DMR15:107170001	15	107170001	107172000	2000	1	6.10E-32	1.16	32	1.6	Olr1865-ps	
DMR15:107384001	15	107384001	107389000	5000	2	2.80E-25	0.74	90	1.8	Olr1775-ps	
DMR15:107939001	15	107939001	107943000	4000	1	2.10E-07	-0.52	30	0.75	Dock9	Transcription
DMR15:107951001	15	107951001	107952000	1000	1	6.10E-08	0.54	23	2.3	Dock9	Transcription
DMR15:108076001	15	108076001	108081000	5000	1	3.70E-08	0.45	72	1.44	Dock9	Transcription
DMR15:108355001	15	108355001	108357000	2000	1	4.00E-09	-0.53	28	1.4	Ubac2;Gpr183	Signaling
DMR15:108413001	15	108413001	108414000	1000	1	4.00E-07	-0.46	21	2.1	Ubac2	
DMR15:108794001	15	108794001	108796000	2000	1	9.40E-08	0.6	33	1.65	Clybl	Metabolism
DMR15:108807001	15	108807001	108809000	2000	1	4.30E-07	-0.37	43	2.15	Clybl	Metabolism
DMR15:109013001	15	109013001	109016000	3000	1	5.40E-08	-0.38	25	0.83	Pcca	Metabolism
DMR15:109043001	15	109043001	109044000	1000	1	7.00E-10	0.36	10	1	Pcca	Metabolism
DMR15:109060001	15	109060001	109061000	1000	1	5.00E-10	0.34	24	2.4	Pcca	Metabolism
DMR15:109219001	15	109219001	109220000	1000	1	2.20E-09	-0.48	14	1.4	Pcca	Metabolism
DMR15:109880001	15	109880001	109883000	3000	1	1.70E-07	-0.35	12	0.4	Nalcn	Transport
DMR15:109928001	15	109928001	109932000	4000	2	3.10E-09	-0.63	30	0.75	Nalcn	Transport
DMR15:110030001	15	110030001	110031000	1000	1	3.70E-07	0.55	18	1.8	Nalcn	Transport
DMR15:110110001	15	110110001	110111000	1000	1	8.10E-08	0.54	9	0.9	Itgbl1	Extracellular Matrix
DMR15:110134001	15	110134001	110137000	3000	1	8.50E-08	-0.71	22	0.73	Itgbl1	Extracellular Matrix
DMR15:110160001	15	110160001	110161000	1000	1	6.50E-07	0.4	12	1.2	Itgbl1	Extracellular Matrix
DMR15:110200001	15	110200001	110201000	1000	1	5.70E-09	0.53	20	2	Itgbl1	Extracellular Matrix
DMR15:110273001	15	110273001	110275000	2000	1	9.50E-07	-0.39	21	1.05	Itgbl1	Extracellular Matrix
DMR15:110473001	15	110473001	110475000	2000	2	4.60E-09	0.62	21	1.05	Fgf14	Growth Factors
DMR15:110507001	15	110507001	110513000	6000	1	3.80E-08	-0.28	62	1.03	Fgf14	Growth Factors
DMR15:110556001	15	110556001	110562000	6000	1	4.50E-07	-0.25	64	1.07	Fgf14	Growth Factors
DMR16:1919001	16	1919001	1920000	1000	1	9.50E-08	0.47	19	1.9	Zmiz1	
DMR16:1994001	16	1994001	1996000	2000	1	3.30E-08	0.39	22	1.1	Ppif;Zcchc24	Transcription
DMR16:2040001	16	2040001	2043000	3000	1	9.00E-08	0.33	38	1.27	Zcchc24	
DMR16:2215001	16	2215001	2217000	2000	1	3.00E-07	0.37	16	0.8	Slmap;LOC108353067	Cytoskeleton
DMR16:2340001	16	2340001	2341000	1000	1	1.80E-07	-0.43	8	0.8	Arf4	Signaling
DMR16:2399001	16	2399001	2404000	5000	2	4.70E-08	-0.38	53	1.06	Dnah12	Cytoskeleton
DMR16:2434001	16	2434001	2439000	5000	1	1.50E-07	0.4	61	1.22	Dnah12	Cytoskeleton
DMR16:2484001	16	2484001	2487000	3000	1	1.60E-09	-0.51	34	1.13	Dnah12	Cytoskeleton
DMR16:2620001	16	2620001	2621000	1000	1	3.60E-10	0.79	28	2.8	Hesx1	Development
DMR16:2628001	16	2628001	2629000	1000	1	1.00E-08	0.57	29	2.9	Hesx1	Development
DMR16:2733001	16	2733001	2741000	8000	1	5.00E-08	-0.44	141	1.76	Il17rd;LOC102548056;Arhgef3	Receptor;Transcription
DMR16:2802001	16	2802001	2804000	2000	1	1.20E-07	-0.37	32	1.6	Arhgef3	Transcription
DMR16:2856001	16	2856001	2861000	5000	1	5.10E-10	0.36	73	1.46	Arhgef3	Transcription
DMR16:2865001	16	2865001	2868000	3000	1	6.30E-08	0.36	26	0.87	Arhgef3	Transcription
DMR16:2890001	16	2890001	2891000	1000	1	6.40E-07	0.45	15	1.5	Arhgef3	Transcription
DMR16:2946001	16	2946001	2948000	2000	1	1.10E-07	-0.4	31	1.55	Arhgef3	Transcription
DMR16:3015001	16	3015001	3019000	4000	1	1.20E-11	0.37	63	1.57	Arhgef3	Transcription
DMR16:3036001	16	3036001	3039000	3000	1	1.50E-07	0.43	22	0.73	Arhgef3	Transcription
DMR16:3281001	16	3281001	3284000	3000	1	5.40E-07	-0.53	29	0.97	Erc2	Transport
DMR16:3382001	16	3382001	3383000	1000	1	3.70E-19	0.98	30	3	Erc2;LOC108348452	Transport
DMR16:3465001	16	3465001	3466000	1000	1	2.10E-09	0.55	11	1.1	Erc2	Transport
DMR16:3524001	16	3524001	3525000	1000	1	3.30E-07	0.43	13	1.3	Erc2	Transport
DMR16:3588001	16	3588001	3591000	3000	1	3.10E-11	0.56	40	1.33	Erc2;LOC108353109	Transport
DMR16:3630001	16	3630001	3631000	1000	1	6.60E-07	0.36	6	0.6	Erc2	Transport
DMR16:3828001	16	3828001	3831000	3000	1	4.10E-10	-0.5	97	3.23	Erc2;Fam213a	Transport
DMR16:3917001	16	3917001	3918000	1000	1	3.60E-07	0.33	6	0.6	Erc2;Anxa11;LOC108353089	Transport;Signaling
DMR16:3969001	16	3969001	3970000	1000	1	2.40E-07	0.36	10	1	Erc2	Transport
DMR16:4047001	16	4047001	4048000	1000	1	7.80E-08	-0.37	14	1.4	Erc2	Transport
DMR16:4272001	16	4272001	4273000	1000	1	1.80E-09	-0.64	16	1.6	Erc2	Transport
DMR16:4479001	16	4479001	4483000	4000	1	1.10E-07	0.53	61	1.52	Wnt5a	Signaling
DMR16:5239001	16	5239001	5243000	4000	1	3.40E-07	0.37	26	0.65	Cacna2d3	Transport
DMR16:5299001	16	5299001	5300000	1000	1	8.50E-07	-0.39	17	1.7	Cacna2d3	Transport
DMR16:5321001	16	5321001	5322000	1000	1	2.50E-07	0.43	8	0.8	Cacna2d3	Transport
DMR16:5523001	16	5523001	5524000	1000	1	8.50E-08	0.41	7	0.7	Cacna2d3	Transport
DMR16:5584001	16	5584001	5585000	1000	1	7.30E-07	0.34	12	1.2	Cacna2d3	Transport
DMR16:5676001	16	5676001	5677000	1000	1	8.90E-19	0.42	7	0.7	Cacna2d3	Transport
DMR16:5715001	16	5715001	5718000	3000	1	3.20E-08	0.68	53	1.77	Cacna2d3	Transport

DMR16:6054001	16	6054001	6059000	5000	1	2.90E-08	-0.48	131	2.62	Selk;Actr8;Il17rb	Cytoskeleton;Receptor
DMR16:6063001	16	6063001	6069000	6000	1	9.10E-07	0.34	79	1.32	Actr8;Il17rb;Chdh	Cytoskeleton;Receptor;Metabolism
DMR16:6289001	16	6289001	6292000	3000	1	4.60E-07	-0.41	40	1.33	Cacna1d;LOC103693892	Transport
DMR16:6338001	16	6338001	6339000	1000	1	8.00E-07	0.48	11	1.1	Cacna1d	Transport
DMR16:6413001	16	6413001	6415000	2000	1	4.40E-07	0.47	28	1.4	Cacna1d	Transport
DMR16:6565001	16	6565001	6566000	1000	1	8.00E-07	-0.44	21	2.1	Dcp1a	Translation
DMR16:6647001	16	6647001	6648000	1000	1	1.30E-08	0.37	12	1.2	LOC108348374;Prkcd	Signaling
DMR16:6812001	16	6812001	6813000	1000	1	1.60E-07	-0.47	13	1.3	Sfmbt1	Epigenetic
DMR16:6906001	16	6906001	6908000	2000	1	5.50E-07	-0.43	37	1.85	Sfmbt1;LOC103693895;Tmem110;LOC102555619	Epigenetic
DMR16:7168001	16	7168001	7169000	1000	1	3.90E-10	-0.43	10	1	Pbrm1	Epigenetic
DMR16:7310001	16	7310001	7312000	2000	1	7.50E-08	0.42	38	1.9	Sema3g	Signaling
DMR16:7394001	16	7394001	7396000	2000	1	9.90E-08	0.61	37	1.85	Dnah1	Cytoskeleton
DMR16:7408001	16	7408001	7410000	2000	1	3.90E-08	0.41	25	1.25	Dnah1;RGD1564325;LOC680729	Cytoskeleton
DMR16:7422001	16	7422001	7423000	1000	1	2.60E-07	0.37	10	1	RGD1564325;LOC680729	
DMR16:7519001	16	7519001	7521000	2000	1	8.80E-09	-0.52	35	1.75	Sh3bp5	Cytoskeleton
DMR16:7774001	16	7774001	7775000	1000	1	7.20E-08	0.65	37	3.7	Btd	Metabolism
DMR16:7875001	16	7875001	7876000	1000	1	2.40E-10	0.73	4	0.4	Ankrd28	Cytoskeleton
DMR16:8126001	16	8126001	8128000	2000	1	2.20E-17	0.97	46	2.3	Galnt15	Golgi
DMR16:8312001	16	8312001	8313000	1000	1	8.60E-08	-0.57	7	0.7	Ncoa4	Transcription
DMR16:8373001	16	8373001	8374000	1000	1	1.40E-07	-0.36	21	2.1	Parg;LOC108348371	Metabolism
DMR16:8649001	16	8649001	8650000	1000	1	1.60E-08	-0.52	10	1	Chat	Metabolism
DMR16:8664001	16	8664001	8665000	1000	1	4.20E-12	0.8	29	2.9	Chat	Metabolism
DMR16:8779001	16	8779001	8780000	1000	1	6.00E-08	-0.44	12	1.2	Ercc6	
DMR16:8803001	16	8803001	8806000	3000	1	3.70E-11	-0.7	52	1.73	Ercc6	
DMR16:8814001	16	8814001	8817000	3000	1	1.00E-07	-0.43	39	1.3	Ercc6;Drgx	Development
DMR16:8830001	16	8830001	8832000	2000	1	1.50E-07	-0.5	42	2.1	Drgx	Development
DMR16:8881001	16	8881001	8882000	1000	1	1.60E-07	0.45	18	1.8	RGD1564899	
DMR16:8888001	16	8888001	8891000	3000	1	1.20E-08	-0.5	40	1.33	RGD1564899	
DMR16:9050001	16	9050001	9054000	4000	1	3.30E-08	-0.44	77	1.93	RGD1561145	
DMR16:9059001	16	9059001	9060000	1000	1	8.90E-07	0.38	10	1	RGD1561145	
DMR16:9167001	16	9167001	9172000	5000	1	1.10E-08	-0.43	86	1.72	Vstm4	Immune
DMR16:9288001	16	9288001	9289000	1000	1	3.30E-07	0.47	5	0.5	Wdfy4	
DMR16:9341001	16	9341001	9343000	2000	1	1.90E-07	0.46	24	1.2	Wdfy4	
DMR16:9384001	16	9384001	9387000	3000	1	6.10E-07	-0.4	44	1.47	Wdfy4	
DMR16:9442001	16	9442001	9445000	3000	1	5.30E-08	-0.4	56	1.87	Arhgap22	
DMR16:9467001	16	9467001	9468000	1000	1	1.80E-08	0.42	10	1	Arhgap22	
DMR16:9489001	16	9489001	9492000	3000	1	7.00E-08	0.42	20	0.67	Arhgap22	
DMR16:9560001	16	9560001	9563000	3000	1	2.70E-07	-0.54	36	1.2	Arhgap22	
DMR16:9567001	16	9567001	9569000	2000	1	1.60E-08	0.56	39	1.95	Arhgap22	
DMR16:9605001	16	9605001	9606000	1000	1	9.40E-08	0.46	29	2.9	Arhgap22	
DMR16:9623001	16	9623001	9624000	1000	1	1.50E-08	-0.42	11	1.1	Arhgap22;Mapk8	Signaling
DMR16:9678001	16	9678001	9679000	1000	1	1.20E-07	-0.53	16	1.6	Mapk8	Signaling
DMR16:9700001	16	9700001	9701000	1000	1	3.70E-07	-0.63	5	0.5	Mapk8	Signaling
DMR16:9733001	16	9733001	9736000	3000	1	9.00E-08	-0.51	14	0.47	Frmpd2	
DMR16:9741001	16	9741001	9742000	1000	1	2.70E-08	0.56	21	2.1	Frmpd2	
DMR16:9906001	16	9906001	9909000	3000	1	1.00E-16	0.9	53	1.77	LOC108353074;Ptpn20	Signaling
DMR16:9931001	16	9931001	9932000	1000	1	4.60E-10	0.41	7	0.7	Ptpn20	Signaling
DMR16:10406001	16	10406001	10408000	2000	1	2.10E-10	0.4	24	1.2	Antxrl;Anxa8	Cytoskeleton;Signaling
DMR16:10572001	16	10572001	10574000	2000	1	2.70E-09	-0.59	34	1.7	Syt15;Fam35a	Transport
DMR16:10647001	16	10647001	10648000	1000	1	7.50E-08	-0.37	11	1.1	Fam35a	
DMR16:10725001	16	10725001	10728000	3000	1	4.30E-07	0.5	70	2.33	Sncg;Mmrn2	Transport
DMR16:10751001	16	10751001	10752000	1000	1	4.60E-08	0.65	8	0.8	Mmrn2;Bmpr1a	Signaling
DMR16:10780001	16	10780001	10781000	1000	1	7.10E-10	0.51	15	1.5	Bmpr1a;LOC108353075	Signaling
DMR16:10973001	16	10973001	10975000	2000	1	2.90E-09	0.55	46	2.3	Opn4	Signaling
DMR16:11021001	16	11021001	11023000	2000	1	8.50E-11	0.46	22	1.1	Wapl	
DMR16:11220001	16	11220001	11223000	3000	1	9.70E-11	0.42	53	1.77	Grid1;Rpl38-ps3	Receptor
DMR16:11243001	16	11243001	11246000	3000	1	4.90E-09	-0.45	34	1.13	Grid1;Mir346	Receptor
DMR16:11372001	16	11372001	11373000	1000	1	5.10E-20	0.36	9	0.9	Grid1	Receptor
DMR16:11539001	16	11539001	11542000	3000	1	4.80E-07	-0.38	22	0.73	Grid1	Receptor
DMR16:11573001	16	11573001	11575000	2000	1	3.30E-09	0.45	11	0.55	Grid1	Receptor
DMR16:11699001	16	11699001	11702000	3000	1	5.90E-07	-0.37	35	1.17	Grid1	Receptor

DMR16:12652001	16	12652001	12659000	7000	1	6.80E-12	0.4	89	1.27	LOC108353079;LOC102551632;RGD1559508;LOC108353078	
DMR16:12667001	16	12667001	12668000	1000	1	1.70E-07	-0.4	6	0.6	RGD1559508;LOC108353078	
DMR16:16132001	16	16132001	16134000	2000	1	1.20E-23	0.97	24	1.2	Nrg3	Growth Factors
DMR16:16178001	16	16178001	16180000	2000	1	3.00E-08	0.37	18	0.9	Nrg3	Growth Factors
DMR16:16399001	16	16399001	16401000	2000	1	9.10E-07	-0.32	19	0.95	Nrg3	Growth Factors
DMR16:16424001	16	16424001	16426000	2000	1	9.90E-07	0.33	8	0.4	Nrg3	Growth Factors
DMR16:16533001	16	16533001	16534000	1000	1	3.80E-14	0.65	11	1.1	Nrg3	Growth Factors
DMR16:16764001	16	16764001	16765000	1000	1	2.90E-08	0.43	14	1.4	Nrg3	Growth Factors
DMR16:16868001	16	16868001	16870000	2000	1	4.70E-08	-0.42	7	0.35	Nrg3	Growth Factors
DMR16:16950001	16	16950001	16951000	1000	1	1.60E-10	0.6	19	1.9	LOC108348383;RGD1564958	
DMR16:17516001	16	17516001	17517000	1000	1	2.80E-07	0.28	23	2.3	RGD1309676	
DMR16:17522001	16	17522001	17523000	1000	1	5.70E-07	0.34	16	1.6	RGD1309676	
DMR16:17573001	16	17573001	17574000	1000	1	6.90E-07	-0.52	11	1.1	Tspan14	
DMR16:17591001	16	17591001	17594000	3000	1	1.80E-09	-0.57	57	1.9	Tspan14	
DMR16:17643001	16	17643001	17645000	2000	1	4.50E-08	-0.38	30	1.5	Sh2d4b	Immune
DMR16:17704001	16	17704001	17706000	2000	2	9.10E-09	0.51	8	0.4	Sh2d4b	Immune
DMR16:18637001	16	18637001	18639000	2000	1	3.30E-09	-0.47	41	2.05	Dydc2;Dydc1	
DMR16:19222001	16	19222001	19223000	1000	1	1.70E-08	0.59	32	3.2	Klf2	Transcription
DMR16:19406001	16	19406001	19408000	2000	2	4.30E-08	0.41	8	0.4	Tpm4;LOC108348385	Cytoskeleton
DMR16:19431001	16	19431001	19432000	1000	1	2.80E-07	0.4	13	1.3	LOC108348385;Olr1648-ps	
DMR16:19515001	16	19515001	19516000	1000	1	7.20E-07	0.57	25	2.5	Cyp4f18	
DMR16:19646001	16	19646001	19650000	4000	1	6.70E-07	-0.34	24	0.6	Zfp709;Haus8	Transcription
DMR16:19759001	16	19759001	19760000	1000	1	3.10E-08	-0.49	9	0.9	Myo9b;Use1;Ocel1;Nr2f6	Transcription;Transcription
DMR16:19888001	16	19888001	19890000	2000	1	3.30E-12	0.94	85	4.25	Dda1;Ano8;Gtpbp3	Epigenetic
DMR16:19964001	16	19964001	19966000	2000	1	6.60E-07	-0.48	41	2.05	LOC108348437;Mvb12a	
DMR16:20072001	16	20072001	20075000	3000	1	1.30E-07	0.3	29	0.97	Unc13a	
DMR16:20389001	16	20389001	20390000	1000	1	4.80E-07	0.48	20	2	Il12rb1;Mast3	Receptor;Signaling
DMR16:20745001	16	20745001	20749000	4000	3	3.50E-11	0.55	77	1.93	Crtc1;LOC102549994	Transcription
DMR16:20834001	16	20834001	20836000	2000	1	8.70E-08	-0.52	39	1.95	Upf1;Cers1;Gdf1	Growth Factors
DMR16:21099001	16	21099001	21101000	2000	1	2.20E-07	0.58	43	2.15	Hapln4;Tm6sf2;Sugp1	Extracellular Matrix;Translation
DMR16:21112001	16	21112001	21113000	1000	1	1.10E-07	-0.28	10	1	Sugp1	Translation
DMR16:21164001	16	21164001	21170000	6000	1	2.10E-08	-0.53	69	1.15	Mau2;Gatad2a	Transcription
DMR16:21295001	16	21295001	21297000	2000	1	9.90E-10	0.47	59	2.95	Yjefn3;Cilp2;Pbx4	Metabolism;Development
DMR16:21298001	16	21298001	21299000	1000	1	2.00E-10	0.66	10	1	Yjefn3;Cilp2;Pbx4	Metabolism;Development
DMR16:21311001	16	21311001	21314000	3000	1	3.80E-10	0.56	49	1.63	Pbx4	Development
DMR16:21357001	16	21357001	21358000	1000	1	2.40E-16	0.89	47	4.7	Lpar2;Gmip;Atp13a1	Signaling
DMR16:21989001	16	21989001	21991000	2000	1	9.10E-07	-0.45	8	0.4	RGD1563748;RGD1564941	
DMR16:22005001	16	22005001	22020000	15000	6	6.20E-27	0.59	155	1.03	RGD1563748;RGD1564941	
DMR16:22030001	16	22030001	22037000	7000	1	8.30E-09	-0.27	69	0.99	RGD1563748;RGD1564941	
DMR16:22043001	16	22043001	22046000	3000	1	2.20E-07	0.24	20	0.67	RGD1563748;RGD1564941	
DMR16:22047001	16	22047001	22055000	8000	3	2.60E-15	0.51	73	0.91	RGD1563748;RGD1564941	
DMR16:22058001	16	22058001	22060000	2000	1	4.30E-09	0.23	26	1.3	RGD1563748;RGD1564941	
DMR16:22068001	16	22068001	22069000	1000	1	1.90E-16	0.34	11	1.1	RGD1563748;RGD1564941	
DMR16:22070001	16	22070001	22075000	5000	1	2.20E-07	-0.23	41	0.82	RGD1563748;RGD1564941	
DMR16:22119001	16	22119001	22120000	1000	1	6.10E-14	0.37	13	1.3	RGD1563748;RGD1564941;LOC108353083	
DMR16:22128001	16	22128001	22131000	3000	1	4.90E-08	0.29	15	0.5	RGD1563748;RGD1564941;LOC108353083;LOC108353082	
DMR16:22338001	16	22338001	22339000	1000	1	5.10E-07	0.33	6	0.6	Atp6v1b2	Metabolism
DMR16:22554001	16	22554001	22555000	1000	1	7.40E-10	-0.57	20	2	Lpl	Metabolism
DMR16:22627001	16	22627001	22629000	2000	1	1.30E-09	-0.57	20	1	Ints10	
DMR16:22838001	16	22838001	22840000	2000	1	1.00E-08	-0.69	16	0.8	Csgalnact1	Golgi
DMR16:22949001	16	22949001	22950000	1000	1	8.90E-08	0.44	3	0.3	Csgalnact1	Golgi
DMR16:23061001	16	23061001	23063000	2000	1	2.50E-10	0.49	26	1.3	Csgalnact1	Golgi
DMR16:23284001	16	23284001	23285000	1000	1	1.30E-07	0.45	6	0.6	Psd3	Transcription
DMR16:23314001	16	23314001	23317000	3000	1	3.30E-11	0.52	68	2.27	Psd3	Transcription
DMR16:23352001	16	23352001	23358000	6000	1	4.80E-08	-0.3	50	0.83	Psd3	Transcription
DMR16:23385001	16	23385001	23386000	1000	1	9.70E-07	-0.51	10	1	Psd3	Transcription

DMR16:23496001	16	23496001	23498000	2000	1	1.20E-10	-0.44	25	1.25	Psd3	Transcription
DMR16:23608001	16	23608001	23609000	1000	1	4.40E-08	0.3	4	0.4	Psd3	Transcription
DMR16:24950001	16	24950001	24951000	1000	1	1.80E-10	0.87	40	4	Tktl2	Metabolism
DMR16:25195001	16	25195001	25200000	5000	1	4.70E-07	-0.22	49	0.98	March1;LOC102552063	
DMR16:25207001	16	25207001	25214000	7000	1	1.70E-07	-0.29	80	1.14	1-Mar	
DMR16:25232001	16	25232001	25239000	7000	1	8.10E-10	-0.27	75	1.07	1-Mar	
DMR16:25468001	16	25468001	25469000	1000	1	3.00E-07	0.35	8	0.8	1-Mar	
DMR16:25761001	16	25761001	25765000	4000	1	4.60E-07	-0.29	38	0.95	March1;Gapdh-ps2	
DMR16:26636001	16	26636001	26637000	1000	1	1.40E-07	0.38	7	0.7	Tmem192	
DMR16:26744001	16	26744001	26747000	3000	2	1.20E-10	-0.64	37	1.23	Klhl2	Cytoskeleton
DMR16:26782001	16	26782001	26783000	1000	1	2.50E-10	-0.56	17	1.7	Klhl2	Cytoskeleton
DMR16:27024001	16	27024001	27025000	1000	1	9.00E-08	0.51	6	0.6	Cpe	Protease
DMR16:27233001	16	27233001	27235000	2000	1	9.80E-08	0.38	32	1.6	Grlf1-ps1	
DMR16:27404001	16	27404001	27406000	2000	1	9.20E-07	-0.53	14	0.7	Tll1;LOC108348393	Protease
DMR16:28425001	16	28425001	28426000	1000	1	1.10E-08	0.5	5	0.5	Spock3	Extracellular Matrix
DMR16:28496001	16	28496001	28498000	2000	1	1.10E-08	-0.34	19	0.95	Spock3	Extracellular Matrix
DMR16:28671001	16	28671001	28674000	3000	1	3.90E-08	-0.39	12	0.4	Spock3	Extracellular Matrix
DMR16:29748001	16	29748001	29752000	4000	1	3.90E-07	-0.37	58	1.45	Anxa10	Signaling
DMR16:29834001	16	29834001	29835000	1000	1	1.80E-07	0.57	10	1	Ddx60	
DMR16:29850001	16	29850001	29855000	5000	3	8.40E-11	-0.39	40	0.8	Ddx60	
DMR16:31391001	16	31391001	31394000	3000	1	1.30E-07	0.45	21	0.7	Palld	Cytoskeleton
DMR16:32064001	16	32064001	32065000	1000	1	2.50E-09	-0.54	19	1.9	Sh3rf1	
DMR16:32124001	16	32124001	32129000	5000	1	7.70E-09	-0.55	95	1.9	Sh3rf1	
DMR16:32180001	16	32180001	32183000	3000	1	1.90E-07	-0.34	45	1.5	Sh3rf1	
DMR16:32187001	16	32187001	32188000	1000	1	7.10E-07	0.41	4	0.4	Sh3rf1	
DMR16:32191001	16	32191001	32196000	5000	1	2.00E-11	-0.53	144	2.88	Sh3rf1	
DMR16:32223001	16	32223001	32225000	2000	1	4.00E-10	-0.44	26	1.3	Sh3rf1	
DMR16:32331001	16	32331001	32333000	2000	1	2.00E-07	-0.42	40	2	LOC102547442;Nek1	
DMR16:32389001	16	32389001	32391000	2000	2	8.90E-09	-0.62	8	0.4	Nek1	
DMR16:32509001	16	32509001	32511000	2000	1	3.40E-10	-0.49	23	1.15	Clcn3;LOC100360260	Transport
DMR16:32520001	16	32520001	32524000	4000	1	4.10E-09	-0.48	35	0.88	Clcn3;LOC100360260;Hpf1	Transport;Epigenetic
DMR16:34581001	16	34581001	34584000	3000	1	9.50E-10	-0.45	59	1.97	Galntl6	Golgi
DMR16:34614001	16	34614001	34622000	8000	1	3.90E-08	-0.29	88	1.1	Galntl6	Golgi
DMR16:34803001	16	34803001	34807000	4000	1	1.50E-07	-0.25	33	0.82	Galntl6;LOC100912321	Golgi
DMR16:34933001	16	34933001	34934000	1000	1	6.80E-08	0.46	8	0.8	Galntl6	Golgi
DMR16:35066001	16	35066001	35067000	1000	1	7.80E-07	-0.45	3	0.3	Galntl6	Golgi
DMR16:35142001	16	35142001	35144000	2000	1	8.00E-07	0.39	12	0.6	Galntl6	Golgi
DMR16:35171001	16	35171001	35176000	5000	1	2.10E-07	-0.4	49	0.98	Galntl6	Golgi
DMR16:35260001	16	35260001	35262000	2000	1	4.40E-07	0.42	14	0.7	Galntl6	Golgi
DMR16:35556001	16	35556001	35557000	1000	1	3.90E-07	0.39	1	0.1	Galntl6	Golgi
DMR16:35628001	16	35628001	35636000	8000	1	1.10E-07	-0.38	87	1.09	Galntl6	Golgi
DMR16:35717001	16	35717001	35721000	4000	2	4.10E-10	-0.42	36	0.9	Galntl6	Golgi
DMR16:35773001	16	35773001	35775000	2000	1	1.20E-09	0.95	41	2.05	Galntl6	Golgi
DMR16:35944001	16	35944001	35945000	1000	1	1.60E-11	-0.44	16	1.6	LOC102548526;Galnt7	Golgi
DMR16:35978001	16	35978001	35979000	1000	1	6.10E-07	-0.4	15	1.5	Galnt7	Golgi
DMR16:36021001	16	36021001	36022000	1000	1	3.30E-07	-0.41	27	2.7	Galnt7	Golgi
DMR16:36060001	16	36060001	36061000	1000	1	3.90E-07	-0.43	30	3	Galnt7	Golgi
DMR16:37129001	16	37129001	37130000	1000	1	8.80E-08	0.58	3	0.3	Fbxo8	Transcription
DMR16:37492001	16	37492001	37493000	1000	1	7.80E-07	0.31	13	1.3	Hpgd;LOC690117	Metabolism
DMR16:37713001	16	37713001	37714000	1000	1	5.20E-07	0.4	7	0.7	Glra3	Ion Channel
DMR16:37748001	16	37748001	37786000	38000	25	3.30E-12	0.34	371	0.98	Glra3	Ion Channel
DMR16:39889001	16	39889001	39891000	2000	1	3.80E-07	-0.32	12	0.6	Wdr17	
DMR16:40459001	16	40459001	40462000	3000	1	1.50E-08	-0.34	17	0.57	Vegfc	Growth Factors
DMR16:41067001	16	41067001	41073000	6000	1	9.70E-07	-0.52	24	0.4	Neil3	Epigenetic
DMR16:41095001	16	41095001	41101000	6000	2	4.00E-08	-0.34	55	0.92	Neil3	Epigenetic
DMR16:46409001	16	46409001	46413000	4000	1	8.70E-07	-0.38	48	1.2	Tenm3	
DMR16:46420001	16	46420001	46424000	4000	1	1.20E-14	0.9	100	2.5	Tenm3	
DMR16:46430001	16	46430001	46431000	1000	1	5.90E-07	0.37	6	0.6	Tenm3	
DMR16:46432001	16	46432001	46435000	3000	1	5.20E-07	-0.32	43	1.43	Tenm3	
DMR16:46471001	16	46471001	46473000	2000	1	1.60E-07	-0.52	22	1.1	Tenm3	
DMR16:46493001	16	46493001	46495000	2000	1	4.00E-07	-0.39	37	1.85	Tenm3;LOC102552351	
DMR16:46550001	16	46550001	46553000	3000	1	4.10E-07	-0.43	22	0.73	Tenm3;LOC108353111	
DMR16:46578001	16	46578001	46579000	1000	1	6.50E-07	-0.56	11	1.1	Tenm3	
DMR16:46601001	16	46601001	46602000	1000	1	1.20E-07	-0.35	21	2.1	Tenm3	

DMR16:46684001	16	46684001	46686000	2000	1	1.00E-07	-0.45	41	2.05	Tenm3	
DMR16:46713001	16	46713001	46717000	4000	1	4.30E-07	-0.36	73	1.82	Tenm3	
DMR16:46751001	16	46751001	46753000	2000	1	4.50E-08	0.32	32	1.6	Tenm3	
DMR16:46781001	16	46781001	46787000	6000	1	2.90E-07	-0.39	110	1.83	Tenm3	
DMR16:46804001	16	46804001	46810000	6000	1	7.60E-08	-0.41	137	2.28	Tenm3	
DMR16:46927001	16	46927001	46928000	1000	1	4.00E-09	0.45	19	1.9	Tenm3;LOC102552563	
DMR16:47206001	16	47206001	47208000	2000	1	5.00E-07	-0.44	26	1.3	Dctd	Metabolism
DMR16:47487001	16	47487001	47492000	5000	1	5.10E-07	-0.36	88	1.76	Wwc2	
DMR16:47543001	16	47543001	47545000	2000	1	2.10E-12	0.46	24	1.2	Wwc2;Cldn22;Cldn24	Cell Junction
DMR16:47659001	16	47659001	47660000	1000	1	6.70E-08	-0.46	8	0.8	Cdkn2aip	Transcription
DMR16:47992001	16	47992001	47994000	2000	1	2.00E-09	0.49	24	1.2	Stox2	
DMR16:48002001	16	48002001	48004000	2000	1	3.70E-08	-0.62	23	1.15	Stox2	
DMR16:48014001	16	48014001	48015000	1000	1	8.20E-07	-0.4	22	2.2	Stox2	
DMR16:48057001	16	48057001	48060000	3000	1	1.10E-10	-0.4	63	2.1	Stox2	
DMR16:48094001	16	48094001	48095000	1000	1	8.90E-09	0.42	10	1	Stox2	
DMR16:48130001	16	48130001	48132000	2000	1	4.10E-08	-0.39	29	1.45	Stox2	
DMR16:48175001	16	48175001	48178000	3000	1	2.60E-11	0.5	26	0.87	Stox2	
DMR16:48237001	16	48237001	48243000	6000	1	3.50E-07	0.34	90	1.5	Stox2	
DMR16:48304001	16	48304001	48306000	2000	1	4.90E-07	-0.29	13	0.65	LOC103693959;Enpp6	
DMR16:48331001	16	48331001	48337000	6000	1	6.70E-07	-0.25	63	1.05	LOC103693959;Enpp6	
DMR16:48397001	16	48397001	48398000	1000	1	1.40E-08	0.54	13	1.3	Enpp6	
DMR16:48431001	16	48431001	48432000	1000	1	1.80E-10	0.68	23	2.3	Enpp6	
DMR16:48614001	16	48614001	48618000	4000	1	9.70E-08	-0.38	52	1.3	Irf2;LOC102552290	Transcription
DMR16:48633001	16	48633001	48634000	1000	1	1.10E-10	0.41	9	0.9	Irf2	Transcription
DMR16:48929001	16	48929001	48932000	3000	1	1.80E-08	0.36	35	1.17	Cenpu;Acsl1	Metabolism
DMR16:48947001	16	48947001	48949000	2000	1	1.60E-09	0.4	23	1.15	Acsl1;LOC108348396	Metabolism
DMR16:48992001	16	48992001	48993000	1000	1	7.60E-07	0.43	9	0.9	Acsl1	Metabolism
DMR16:49175001	16	49175001	49177000	2000	1	2.00E-07	0.36	12	0.6	Helt	Transcription
DMR16:49291001	16	49291001	49296000	5000	1	6.40E-10	-0.39	47	0.94	Cfap97	
DMR16:49332001	16	49332001	49336000	4000	1	1.10E-07	-0.45	35	0.88	Cfap97;Snx25	Cytoskeleton
DMR16:49337001	16	49337001	49340000	3000	1	1.40E-09	-0.61	36	1.2	Cfap97;Snx25	Cytoskeleton
DMR16:49349001	16	49349001	49354000	5000	1	9.80E-07	-0.39	58	1.16	Snx25	Cytoskeleton
DMR16:49535001	16	49535001	49536000	1000	1	7.50E-09	0.42	9	0.9	Pdlim3	Cytoskeleton
DMR16:49617001	16	49617001	49619000	2000	1	3.70E-09	0.45	10	0.5	Sorbs2	
DMR16:49657001	16	49657001	49659000	2000	1	2.30E-11	0.8	65	3.25	Sorbs2	
DMR16:49696001	16	49696001	49699000	3000	1	2.40E-07	-0.47	34	1.13	Sorbs2	
DMR16:49732001	16	49732001	49733000	1000	1	9.90E-07	-0.39	23	2.3	Sorbs2	
DMR16:49736001	16	49736001	49737000	1000	1	8.40E-07	-0.39	9	0.9	Sorbs2	
DMR16:49827001	16	49827001	49828000	1000	1	1.00E-08	-0.3	7	0.7	Sorbs2	
DMR16:50046001	16	50046001	50047000	1000	1	5.90E-15	-0.58	11	1.1	Fam149a	
DMR16:50063001	16	50063001	50068000	5000	1	7.40E-08	-0.26	45	0.9	Fam149a	
DMR16:50126001	16	50126001	50127000	1000	1	2.20E-08	0.59	13	1.3	Cyp4v3	
DMR16:50143001	16	50143001	50145000	2000	1	7.70E-11	-0.49	60	3	Cyp4v3;LOC498634;Klkb1	Protease
DMR16:50366001	16	50366001	50367000	1000	1	1.10E-07	-0.55	17	1.7	Mtnr1a;Fat1	Signaling;Cytoskeleton
DMR16:50372001	16	50372001	50375000	3000	1	1.20E-08	-0.47	82	2.73	Fat1	Cytoskeleton
DMR16:50403001	16	50403001	50412000	9000	2	3.60E-10	-0.44	155	1.72	Fat1	Cytoskeleton
DMR16:50419001	16	50419001	50428000	9000	1	9.30E-09	-0.67	156	1.73	Fat1	Cytoskeleton
DMR16:50511001	16	50511001	50514000	3000	1	2.40E-08	0.39	15	0.5	Fat1	Cytoskeleton
DMR16:52022001	16	52022001	52023000	1000	1	1.50E-08	-0.52	7	0.7	Zfp42	
DMR16:52648001	16	52648001	52654000	6000	2	7.10E-09	-0.34	54	0.9	Rps27a-ps15	
DMR16:53118001	16	53118001	53119000	1000	1	4.80E-07	0.37	13	1.3	Rps27a-ps16	
DMR16:54000001	16	54000001	54002000	2000	1	5.50E-15	-0.5	21	1.05	LOC102547443;Asah1	Protease
DMR16:54007001	16	54007001	54010000	3000	2	3.30E-08	0.3	41	1.37	LOC102547443;Asah1	Protease
DMR16:54133001	16	54133001	54134000	1000	1	4.60E-10	-0.6	7	0.7	Pcm1	
DMR16:54198001	16	54198001	54201000	3000	1	7.60E-09	-0.46	37	1.23	Fgl1	Signaling
DMR16:54278001	16	54278001	54279000	1000	1	7.30E-07	-0.47	12	1.2	Mtus1;LOC103693968	
DMR16:54329001	16	54329001	54333000	4000	1	2.80E-08	-0.47	61	1.52	Mtus1	
DMR16:54461001	16	54461001	54464000	3000	1	1.30E-07	-0.54	56	1.87	Slc7a2	Transport
DMR16:54516001	16	54516001	54517000	1000	1	1.00E-11	0.69	19	1.9	Slc7a2	Transport
DMR16:54641001	16	54641001	54643000	2000	1	1.50E-08	-0.36	14	0.7	Adam24;LOC100911065	Protease
DMR16:54808001	16	54808001	54813000	5000	1	3.70E-09	-0.45	55	1.1	Mtmr7	Signaling
DMR16:54880001	16	54880001	54885000	5000	1	2.40E-08	-0.39	44	0.88	Vps37a	
DMR16:54954001	16	54954001	54955000	1000	1	5.70E-07	-0.38	8	0.8	Zdhhc2	

DMR16:55008001	16	55008001	55009000	1000	1	1.30E-07	0.3	9	0.9	Zdhhc2	
DMR16:56254001	16	56254001	56256000	2000	1	1.20E-13	0.45	22	1.1	Tusc3	Golgi
DMR16:56323001	16	56323001	56325000	2000	1	2.70E-07	-0.42	65	3.25	Tusc3	Golgi
DMR16:56820001	16	56820001	56821000	1000	1	2.30E-08	-0.57	4	0.4	Msr1;LOC100362275	Protease
DMR16:56865001	16	56865001	56866000	1000	1	8.70E-10	0.55	10	1	Msr1	Protease
DMR16:56888001	16	56888001	56892000	4000	1	2.80E-07	-0.3	34	0.85	Msr1	Protease
DMR16:56908001	16	56908001	56909000	1000	1	3.90E-07	0.43	19	1.9	Msr1	Protease
DMR16:57537001	16	57537001	57538000	1000	1	8.50E-07	0.56	4	0.4	Sgcz	
DMR16:57564001	16	57564001	57572000	8000	2	5.50E-15	-0.51	58	0.72	Sgcz	
DMR16:57578001	16	57578001	57579000	1000	1	5.30E-07	0.32	16	1.6	Sgcz	
DMR16:57688001	16	57688001	57689000	1000	1	1.20E-08	0.42	9	0.9	Sgcz	
DMR16:57768001	16	57768001	57770000	2000	1	7.60E-07	-0.38	22	1.1	Sgcz	
DMR16:57837001	16	57837001	57838000	1000	1	1.30E-07	-0.57	6	0.6	Sgcz	
DMR16:57918001	16	57918001	57922000	4000	1	4.70E-07	-0.3	35	0.88	Sgcz	
DMR16:58016001	16	58016001	58017000	1000	1	8.20E-07	0.52	5	0.5	Sgcz	
DMR16:58018001	16	58018001	58019000	1000	1	1.60E-07	0.49	7	0.7	Sgcz	
DMR16:58159001	16	58159001	58160000	1000	1	1.00E-07	0.39	17	1.7	Sgcz	
DMR16:58843001	16	58843001	58846000	3000	1	2.10E-07	-0.49	33	1.1	Dlc1	Signaling
DMR16:58903001	16	58903001	58904000	1000	1	2.00E-09	0.57	14	1.4	Dlc1	Signaling
DMR16:58983001	16	58983001	58985000	2000	1	8.60E-07	0.34	19	0.95	Dlc1	Signaling
DMR16:59029001	16	59029001	59032000	3000	2	1.60E-09	0.41	24	0.8	Dlc1;LOC102552115	Signaling
DMR16:59122001	16	59122001	59123000	1000	1	8.00E-07	0.5	11	1.1	Dlc1	Signaling
DMR16:59151001	16	59151001	59153000	2000	1	2.60E-07	-0.36	37	1.85	Dlc1	Signaling
DMR16:59201001	16	59201001	59204000	3000	1	8.00E-09	-0.77	42	1.4	Dlc1;LOC103693970	Signaling
DMR16:59235001	16	59235001	59237000	2000	1	6.40E-07	-0.34	55	2.75	Dlc1	Signaling
DMR16:59332001	16	59332001	59336000	4000	1	4.40E-11	0.88	85	2.12	RGD1304810;LOC108348403;LOC103693971	
DMR16:59688001	16	59688001	59693000	5000	1	2.40E-08	-0.32	98	1.96	Pragmin	
DMR16:59730001	16	59730001	59732000	2000	1	1.10E-07	0.37	30	1.5	Pragmin	
DMR16:60110001	16	60110001	60111000	1000	1	6.20E-07	-0.35	10	1	Mfhas1	Cytoskeleton
DMR16:60161001	16	60161001	60163000	2000	1	3.20E-08	-0.51	33	1.65	Mfhas1	Cytoskeleton
DMR16:60167001	16	60167001	60169000	2000	1	1.30E-07	-0.37	23	1.15	Mfhas1	Cytoskeleton
DMR16:60191001	16	60191001	60193000	2000	1	1.50E-08	-0.49	43	2.15	Mfhas1	Cytoskeleton
DMR16:60212001	16	60212001	60214000	2000	1	6.30E-11	-0.53	32	1.6	Mfhas1	Cytoskeleton
DMR16:60416001	16	60416001	60417000	1000	1	7.70E-07	-0.39	10	1	Ppp1r3b	Signaling
DMR16:61057001	16	61057001	61059000	2000	1	4.60E-07	-0.39	37	1.85	Tnks	Signaling
DMR16:61095001	16	61095001	61096000	1000	1	6.40E-09	0.55	22	2.2	Dusp4	Signaling
DMR16:61749001	16	61749001	61752000	3000	1	9.40E-08	-0.48	28	0.93	Saraf;Leprotl1	Receptor
DMR16:61770001	16	61770001	61773000	3000	1	1.20E-07	-0.45	51	1.7	Leprotl1;Mboat4	Receptor;Metabolism
DMR16:61822001	16	61822001	61823000	1000	1	1.90E-07	0.48	17	1.7	Dctn6	Cytoskeleton
DMR16:61824001	16	61824001	61826000	2000	1	8.20E-07	0.67	34	1.7	Dctn6	Cytoskeleton
DMR16:61980001	16	61980001	61981000	1000	1	2.80E-07	-0.55	21	2.1	Rbpms	Translation
DMR16:62002001	16	62002001	62003000	1000	1	2.70E-08	-0.52	26	2.6	Rbpms	Translation
DMR16:62023001	16	62023001	62024000	1000	1	6.20E-09	-0.49	22	2.2	Rbpms	Translation
DMR16:62034001	16	62034001	62035000	1000	1	1.50E-07	-0.43	19	1.9	Rbpms	Translation
DMR16:62125001	16	62125001	62127000	2000	1	2.50E-12	0.42	18	0.9	Gtf2e2	Transcription
DMR16:62255001	16	62255001	62256000	1000	1	3.40E-07	-0.43	10	1	Ubxn8	
DMR16:62319001	16	62319001	62320000	1000	1	1.40E-08	-0.53	12	1.2	RGD1560512;Tex15	
DMR16:62370001	16	62370001	62371000	1000	1	5.40E-07	-0.39	15	1.5	Tex15	
DMR16:62424001	16	62424001	62428000	4000	1	6.30E-10	0.62	78	1.95	LOC108348448;Purg	Transcription
DMR16:62482001	16	62482001	62483000	1000	1	5.50E-09	-0.55	38	3.8	Purg;Wrn	Transcription;Epigenetic
DMR16:62486001	16	62486001	62487000	1000	1	9.90E-08	0.35	9	0.9	Purg;Wrn	Transcription;Epigenetic
DMR16:62500001	16	62500001	62502000	2000	1	3.50E-07	0.63	36	1.8	Wrn	Epigenetic
DMR16:62597001	16	62597001	62603000	6000	1	1.80E-08	0.41	89	1.48	Wrn;LOC108348406	Epigenetic
DMR16:62999001	16	62999001	6.30E+07	1000	1	6.60E-07	0.44	8	0.8	Nrg1	Growth Factors
DMR16:63036001	16	63036001	63037000	1000	1	6.20E-07	-0.46	14	1.4	Nrg1	Growth Factors
DMR16:63077001	16	63077001	63078000	1000	1	3.10E-07	0.49	3	0.3	Nrg1	Growth Factors
DMR16:63507001	16	63507001	63509000	2000	1	8.30E-08	0.48	22	1.1	Nrg1	Growth Factors
DMR16:63559001	16	63559001	63562000	3000	1	2.40E-07	-0.37	18	0.6	Nrg1	Growth Factors
DMR16:63563001	16	63563001	63565000	2000	1	1.80E-08	-0.38	9	0.45	Nrg1	Growth Factors
DMR16:63724001	16	63724001	63725000	1000	1	9.90E-09	0.49	9	0.9	Nrg1	Growth Factors
DMR16:63790001	16	63790001	63791000	1000	1	5.20E-07	-0.41	26	2.6	Nrg1	Growth Factors
DMR16:63815001	16	63815001	63817000	2000	1	4.40E-07	0.54	16	0.8	Nrg1	Growth Factors

DMR16:63828001	16	63828001	63830000	2000	1	5.30E-07	0.61	75	3.75	Nrg1	Growth Factors
DMR16:63915001	16	63915001	63916000	1000	1	1.30E-07	-0.42	22	2.2	Nrg1	Growth Factors
DMR16:63954001	16	63954001	63955000	1000	1	3.00E-07	-0.33	23	2.3	Nrg1	Growth Factors
DMR16:64662001	16	64662001	64663000	1000	1	4.80E-08	-0.63	22	2.2	Fut10	Golgi
DMR16:67308001	16	67308001	67310000	2000	1	1.90E-08	-0.42	22	1.1	Unc5d	Receptor
DMR16:67447001	16	67447001	67450000	3000	1	6.40E-08	0.35	26	0.87	Unc5d	Receptor
DMR16:68485001	16	68485001	68488000	3000	1	3.30E-07	-0.44	46	1.53	RGD1562638	
DMR16:68537001	16	68537001	68538000	1000	1	9.10E-07	0.29	8	0.8	RGD1562638	
DMR16:68586001	16	68586001	68587000	1000	1	6.50E-10	-0.42	19	1.9	RGD1562638	
DMR16:68820001	16	68820001	68821000	1000	1	1.10E-12	0.88	33	3.3	Poteg	Transport
DMR16:68976001	16	68976001	68983000	7000	1	8.20E-07	-0.23	63	0.9	Eif4ebp1	Translation
DMR16:69028001	16	69028001	69031000	3000	1	4.90E-07	-0.39	45	1.5	Got1l1	Metabolism
DMR16:69084001	16	69084001	69086000	2000	1	8.00E-10	0.33	14	0.7	Rab11fip1;Brf2;Adgra2	Transcription;Signaling
DMR16:69136001	16	69136001	69137000	1000	1	9.80E-08	0.36	15	1.5	Adgra2	Signaling
DMR16:69170001	16	69170001	69174000	4000	1	2.40E-10	-0.43	52	1.3	Prosc;Erlin2	
DMR16:69250001	16	69250001	69253000	3000	1	2.90E-08	0.56	54	1.8	Zfp703;LOC108348410	
DMR16:70006001	16	70006001	70007000	1000	1	8.60E-08	0.51	22	2.2	Pasd1	
DMR16:70725001	16	70725001	70734000	9000	1	1.70E-07	-0.38	107	1.19	Hook3	
DMR16:70971001	16	70971001	70972000	1000	1	2.70E-08	0.52	8	0.8	Kcnu1	Transport
DMR16:70977001	16	70977001	70980000	3000	1	3.20E-11	0.81	60	2	Kcnu1	Transport
DMR16:71000001	16	71000001	71001000	1000	1	1.60E-11	0.79	28	2.8	Kcnu1	Transport
DMR16:71082001	16	71082001	71085000	3000	1	1.80E-07	-0.57	25	0.83	Bag4;Ddhd2	Metabolism
DMR16:71091001	16	71091001	71093000	2000	1	1.40E-08	-0.37	28	1.4	Ddhd2	Metabolism
DMR16:71104001	16	71104001	71106000	2000	1	3.40E-07	0.4	8	0.4	Ddhd2	Metabolism
DMR16:71130001	16	71130001	71132000	2000	1	2.10E-09	-0.5	33	1.65	Ddhd2;Plpp5;Whsc1l1	Metabolism;Signaling
DMR16:71134001	16	71134001	71138000	4000	2	8.50E-08	-0.5	77	1.93	Plpp5;Whsc1l1	Signaling
DMR16:71189001	16	71189001	71192000	3000	1	1.80E-09	-0.55	35	1.17	Whsc1l1	
DMR16:71202001	16	71202001	71203000	1000	1	3.40E-09	-0.44	14	1.4	Whsc1l1	
DMR16:71252001	16	71252001	71254000	2000	1	6.80E-08	-0.53	39	1.95	Letm2	Transport
DMR16:71289001	16	71289001	71292000	3000	1	7.30E-07	0.34	49	1.63	Fgfr1	Receptor
DMR16:71620001	16	71620001	71622000	2000	1	2.70E-07	-0.45	44	2.2	Tacc1	
DMR16:71646001	16	71646001	71649000	3000	1	2.50E-09	-0.45	57	1.9	Tacc1	
DMR16:71653001	16	71653001	71657000	4000	1	6.10E-09	-0.52	98	2.45	Tacc1	
DMR16:71811001	16	71811001	71813000	2000	1	1.90E-07	-0.47	25	1.25	Htra4;Tm2d2;Adam9	Protease;Protease
DMR16:71872001	16	71872001	71880000	8000	2	2.50E-11	-0.28	80	1	Adam9;Adam32	Protease
DMR16:71899001	16	71899001	71900000	1000	1	1.90E-07	-0.36	22	2.2	Adam32	Protease
DMR16:71906001	16	71906001	71908000	2000	1	6.20E-08	-0.48	31	1.55	Adam32	Protease
DMR16:71941001	16	71941001	71946000	5000	2	2.20E-07	-0.37	43	0.86	Adam32	Protease
DMR16:71981001	16	71981001	71986000	5000	1	7.90E-12	-0.49	42	0.84	Adam32	Protease
DMR16:71994001	16	71994001	71997000	3000	1	4.20E-07	-0.26	26	0.87	Adam32	Protease
DMR16:72014001	16	72014001	72016000	2000	1	2.20E-07	-0.44	33	1.65	Adam5	Protease
DMR16:72141001	16	72141001	72146000	5000	2	2.50E-14	-0.39	48	0.96	Adam3a;Adam18	Protease
DMR16:72147001	16	72147001	72150000	3000	1	1.70E-10	-0.44	19	0.63	Adam18	Protease
DMR16:72168001	16	72168001	72170000	2000	1	2.90E-07	0.59	50	2.5	Adam18	Protease
DMR16:72186001	16	72186001	72188000	2000	2	3.80E-07	-0.45	29	1.45	Adam18	Protease
DMR16:72758001	16	72758001	72760000	2000	1	5.50E-08	0.37	21	1.05	Zmat4	
DMR16:72761001	16	72761001	72762000	1000	1	3.50E-09	0.45	4	0.4	Zmat4	
DMR16:72923001	16	72923001	72924000	1000	1	8.60E-08	-0.5	24	2.4	Zmat4	
DMR16:72949001	16	72949001	72951000	2000	2	3.60E-10	-0.57	20	1	Zmat4	
DMR16:72956001	16	72956001	72959000	3000	1	1.90E-07	0.5	39	1.3	Zmat4	
DMR16:73128001	16	73128001	73130000	2000	1	1.10E-07	0.49	38	1.9	Zmat4	
DMR16:73382001	16	73382001	73384000	2000	1	5.40E-07	-0.44	50	2.5	Sfrp1	Receptor
DMR16:73565001	16	73565001	73567000	2000	1	1.30E-08	-0.36	29	1.45	Golga7	
DMR16:73587001	16	73587001	73589000	2000	2	2.30E-07	-0.36	29	1.45	Golga7;Gins4	
DMR16:73669001	16	73669001	73671000	2000	1	2.60E-08	0.45	44	2.2	Nkx6-3	Development
DMR16:73696001	16	73696001	73697000	1000	1	1.50E-07	-0.35	12	1.2	Ank1	
DMR16:73788001	16	73788001	73789000	1000	1	3.70E-07	-0.44	24	2.4	Ank1	
DMR16:73914001	16	73914001	73915000	1000	1	4.10E-08	0.64	29	2.9	Ank1	
DMR16:73971001	16	73971001	73972000	1000	1	6.00E-07	-0.54	10	1	Kat6a	Epigenetic
DMR16:74066001	16	74066001	74067000	1000	1	2.50E-09	0.55	19	1.9	LOC102556951;Ap3m2	Transport
DMR16:74084001	16	74084001	74085000	1000	1	9.50E-08	0.6	42	4.2	LOC102556951;Ap3m2	Transport
DMR16:74181001	16	74181001	74183000	2000	1	1.60E-08	0.34	17	0.85	Ikbkb;LOC108348421	Signaling
DMR16:74202001	16	74202001	74204000	2000	1	2.50E-07	0.4	36	1.8	Ikbkb	Signaling

DMR16:74250001	16	74250001	74252000	2000	1	4.70E-09	0.45	31	1.55	Polb;Dkk4	Transcription
DMR16:74271001	16	74271001	74276000	5000	1	8.80E-09	-0.53	78	1.56	Dkk4	
DMR16:74480001	16	74480001	74482000	2000	2	5.50E-09	-0.61	25	1.25	Mrps31	Translation
DMR16:74488001	16	74488001	74491000	3000	1	1.20E-07	-0.52	43	1.43	Mrps31	Translation
DMR16:74515001	16	74515001	74517000	2000	1	2.40E-07	-0.47	34	1.7	Slc25a15	Transport
DMR16:74618001	16	74618001	74622000	4000	1	1.20E-13	-0.6	35	0.88	Tpte2	Signaling
DMR16:74645001	16	74645001	74647000	2000	1	2.30E-09	-0.46	35	1.75	Tpte2;LOC102550098	Signaling
DMR16:74786001	16	74786001	74787000	1000	1	3.50E-10	0.6	34	3.4	Ckap2;Nek3	Cytoskeleton;Signaling
DMR16:74886001	16	74886001	74887000	1000	1	9.80E-09	0.46	24	2.4	Atp7b	Transport
DMR16:75014001	16	75014001	75016000	2000	1	3.90E-09	-0.31	20	1	Fam90a1	
DMR16:75129001	16	75129001	75132000	3000	1	8.00E-08	-0.48	35	1.17	Defb12	
DMR16:75187001	16	75187001	75192000	5000	1	3.40E-07	-0.27	44	0.88	Defb9;Defb10	Signaling
DMR16:75297001	16	75297001	75298000	1000	1	2.10E-07	0.42	5	0.5	Defb1	Signaling
DMR16:75349001	16	75349001	75356000	7000	1	7.70E-07	-0.26	82	1.17	Defa5;Defa24	Signaling
DMR16:75370001	16	75370001	75372000	2000	1	1.50E-08	-0.41	12	0.6	Defal1	Signaling
DMR16:75601001	16	75601001	75603000	2000	1	9.80E-07	-0.36	8	0.4	Defb5	Signaling
DMR16:75673001	16	75673001	75674000	1000	1	2.30E-07	-0.56	13	1.3	Spag11a	
DMR16:75750001	16	75750001	75752000	2000	1	6.30E-10	0.79	47	2.35	Xkr5	
DMR16:75756001	16	75756001	75758000	2000	1	2.30E-09	0.32	19	0.95	Xkr5	
DMR16:75924001	16	75924001	75926000	2000	1	3.60E-09	-0.49	52	2.6	Mcph1	DNA Repair
DMR16:75938001	16	75938001	75939000	1000	1	4.40E-08	-0.51	31	3.1	Mcph1	DNA Repair
DMR16:77352001	16	77352001	77353000	1000	1	4.50E-07	-0.63	4	0.4	Csmd1	
DMR16:77426001	16	77426001	77430000	4000	2	8.80E-07	-0.24	36	0.9	Csmd1	
DMR16:77718001	16	77718001	77719000	1000	1	7.80E-07	-0.5	6	0.6	Csmd1	
DMR16:78229001	16	78229001	78232000	3000	1	9.80E-08	-0.34	82	2.73	Csmd1	
DMR16:78244001	16	78244001	78245000	1000	1	2.00E-07	-0.35	6	0.6	Csmd1	
DMR16:78378001	16	78378001	78383000	5000	1	1.10E-07	-0.4	33	0.66	Csmd1	
DMR16:78501001	16	78501001	78505000	4000	1	4.60E-08	-0.3	22	0.55	Csmd1	
DMR16:78697001	16	78697001	78700000	3000	1	1.50E-07	-0.4	47	1.57	Csmd1	
DMR16:78810001	16	78810001	78811000	1000	1	2.70E-07	0.37	16	1.6	Csmd1	
DMR16:79587001	16	79587001	79589000	2000	2	1.50E-12	0.7	58	2.9	Myom2;LOC102551089	
DMR16:79611001	16	79611001	79613000	2000	2	2.30E-09	0.77	34	1.7	Myom2;LOC108348427	
DMR16:79644001	16	79644001	79646000	2000	1	6.60E-08	-0.41	37	1.85	Myom2	
DMR16:79759001	16	79759001	79768000	9000	1	3.30E-08	-0.49	302	3.36	Arhgef10	Transcription
DMR16:79969001	16	79969001	79970000	1000	1	3.50E-08	0.62	22	2.2	Dlgap2	Cytoskeleton
DMR16:80126001	16	80126001	80127000	1000	1	6.80E-08	0.33	6	0.6	Dlgap2	Cytoskeleton
DMR16:80137001	16	80137001	80142000	5000	1	2.10E-08	0.32	63	1.26	Dlgap2	Cytoskeleton
DMR16:80153001	16	80153001	80157000	4000	2	1.70E-09	-0.4	61	1.52	Dlgap2	Cytoskeleton
DMR16:80171001	16	80171001	80172000	1000	1	5.00E-08	0.38	8	0.8	Dlgap2	Cytoskeleton
DMR16:80218001	16	80218001	80220000	2000	1	3.70E-07	-0.44	20	1	Dlgap2	Cytoskeleton
DMR16:80258001	16	80258001	80259000	1000	1	5.50E-07	0.4	11	1.1	Dlgap2	Cytoskeleton
DMR16:80349001	16	80349001	80351000	2000	1	6.80E-08	0.36	15	0.75	Dlgap2	Cytoskeleton
DMR16:80438001	16	80438001	80440000	2000	1	8.80E-07	-0.48	57	2.85	Dlgap2	Cytoskeleton
DMR16:80517001	16	80517001	80518000	1000	1	3.20E-07	0.38	14	1.4	Dlgap2	Cytoskeleton
DMR16:80541001	16	80541001	80542000	1000	1	7.90E-08	0.47	13	1.3	Dlgap2	Cytoskeleton
DMR16:80567001	16	80567001	80571000	4000	1	2.60E-07	0.61	47	1.18	Dlgap2;LOC108348428	Cytoskeleton
DMR16:80579001	16	80579001	80581000	2000	1	5.10E-15	0.91	74	3.7	Dlgap2;LOC108348428	Cytoskeleton
DMR16:80629001	16	80629001	80632000	3000	1	5.30E-10	-0.45	75	2.5	Erich1	
DMR16:80642001	16	80642001	80646000	4000	1	2.00E-09	-0.5	105	2.62	Erich1;LOC103693997	
DMR16:80732001	16	80732001	80736000	4000	1	1.30E-07	-0.53	75	1.88	Tdrp	
DMR16:80737001	16	80737001	80740000	3000	1	2.00E-07	-0.42	51	1.7	Tdrp	
DMR16:80795001	16	80795001	80802000	7000	1	5.40E-07	-0.4	136	1.94	Fbxo25;LOC108348429	
DMR16:80823001	16	80823001	80825000	2000	1	7.40E-09	-0.57	23	1.15	Coprs	
DMR16:81077001	16	81077001	81080000	3000	1	3.70E-08	-0.48	44	1.47	Tmco3;LOC102548465;Tfdp1	Transcription
DMR16:81179001	16	81179001	81180000	1000	1	5.20E-08	0.35	14	1.4	Tmem255b	
DMR16:81246001	16	81246001	81248000	2000	1	1.50E-07	0.77	57	2.85	Gas6;LOC108349416;LOC103693998	Extracellular Matrix
DMR16:81394001	16	81394001	81396000	2000	1	1.40E-07	0.44	32	1.6	Rasa3	Signaling
DMR16:81641001	16	81641001	81642000	1000	1	3.50E-09	-0.54	35	3.5	Adprhl1;LOC102548530	Signaling
DMR16:81662001	16	81662001	81663000	1000	1	1.50E-07	0.47	14	1.4	LOC102548586;Grtp1	Signaling
DMR16:81795001	16	81795001	81800000	5000	1	1.90E-07	-0.49	108	2.16	Proz;F10;LOC108348431	Protease
DMR16:81864001	16	81864001	81865000	1000	1	5.80E-08	0.37	4	0.4	Mcf2l	Transcription
DMR16:81917001	16	81917001	81918000	1000	1	1.60E-07	0.39	12	1.2	Mcf2l	Transcription

DMR16:82038001	16	82038001	82039000	1000	1	9.50E-08	0.46	12	1.2	Atp11a	Transport
DMR16:82057001	16	82057001	82061000	4000	1	1.00E-08	-0.46	74	1.85	Atp11a	Transport
DMR16:82179001	16	82179001	82182000	3000	2	2.80E-09	0.43	40	1.33	LOC102552970;Tubgcp3	Cytoskeleton
DMR16:82993001	16	82993001	82995000	2000	1	7.10E-07	-0.42	40	2	Tex29	
DMR16:83275001	16	83275001	83279000	4000	1	1.70E-08	-0.51	93	2.33	Ing1;Cars2	Epigenetic;Translation
DMR16:83340001	16	83340001	83342000	2000	1	1.80E-08	-0.44	43	2.15	Naxd	Metabolism
DMR16:83345001	16	83345001	83347000	2000	1	1.20E-09	0.54	34	1.7	Naxd;LOC102553902	Metabolism
DMR16:83365001	16	83365001	83367000	2000	1	6.70E-09	-0.49	38	1.9	LOC102553902;Rab20	
DMR16:83392001	16	83392001	83393000	1000	1	2.00E-07	0.39	20	2	Rab20;Col4a2	Extracellular Matrix
DMR16:83395001	16	83395001	83396000	1000	1	2.70E-08	0.5	10	1	Col4a2	Extracellular Matrix
DMR16:83414001	16	83414001	83415000	1000	1	4.20E-09	0.71	31	3.1	Col4a2	Extracellular Matrix
DMR16:83491001	16	83491001	83494000	3000	1	4.30E-09	0.27	56	1.87	Col4a2	Extracellular Matrix
DMR16:83514001	16	83514001	83516000	2000	1	1.30E-09	0.47	21	1.05	Col4a2;Col4a1	Extracellular Matrix
DMR16:83589001	16	83589001	83590000	1000	1	3.30E-07	0.39	12	1.2	Col4a1	Extracellular Matrix
DMR16:83591001	16	83591001	83593000	2000	1	6.50E-14	0.64	6	0.3	Col4a1	Extracellular Matrix
DMR16:83639001	16	83639001	83643000	4000	1	7.20E-09	0.38	48	1.2	Col4a1	Extracellular Matrix
DMR16:83834001	16	83834001	83838000	4000	1	5.60E-09	0.37	45	1.12	Irs2	
DMR16:84590001	16	84590001	84593000	3000	2	9.10E-18	0.44	26	0.87	Myo16	
DMR16:84624001	16	84624001	84625000	1000	1	1.40E-08	0.43	4	0.4	Myo16;LOC103694009	
DMR16:84903001	16	84903001	84906000	3000	1	1.10E-07	-0.37	32	1.07	Myo16	
DMR16:84907001	16	84907001	84908000	1000	1	8.10E-13	0.58	7	0.7	Myo16	
DMR16:84945001	16	84945001	84947000	2000	1	4.50E-07	0.34	10	0.5	Myo16	
DMR16:85003001	16	85003001	85005000	2000	1	5.30E-07	0.46	11	0.55	Myo16	
DMR16:85049001	16	85049001	85050000	1000	1	2.80E-07	0.37	6	0.6	Myo16	
DMR16:85155001	16	85155001	85156000	1000	1	1.30E-08	0.46	6	0.6	Myo16;Marco	Extracellular Matrix
DMR16:85300001	16	85300001	85302000	2000	1	1.70E-08	0.38	31	1.55	Tnfsf13b	
DMR16:85458001	16	85458001	85461000	3000	1	2.90E-09	0.46	27	0.9	RGD1565902	
DMR16:85594001	16	85594001	85595000	1000	1	3.00E-07	0.32	4	0.4	Fam155a	
DMR16:85666001	16	85666001	85668000	2000	1	9.90E-09	0.58	38	1.9	Fam155a	
DMR16:85775001	16	85775001	85777000	2000	1	1.40E-07	-0.63	13	0.65	Fam155a	
DMR16:85780001	16	85780001	85783000	3000	2	1.80E-08	0.3	26	0.87	Fam155a	
DMR16:85821001	16	85821001	85824000	3000	1	1.10E-07	-0.42	42	1.4	Fam155a	
DMR16:85855001	16	85855001	85856000	1000	1	5.10E-09	0.62	21	2.1	Fam155a	
DMR16:85903001	16	85903001	85904000	1000	1	3.40E-08	0.67	24	2.4	Fam155a	
DMR16:85908001	16	85908001	85909000	1000	1	1.80E-10	0.51	13	1.3	Fam155a	
DMR16:85977001	16	85977001	85978000	1000	1	7.80E-10	0.58	11	1.1	Fam155a	
DMR16:85997001	16	85997001	86001000	4000	1	8.80E-07	0.47	53	1.32	Fam155a	
DMR16:86029001	16	86029001	86031000	2000	1	2.20E-07	0.3	21	1.05	Fam155a	
DMR16:86597001	16	86597001	86600000	3000	2	4.70E-12	-0.55	25	0.83	Arglu1	
DMR16:86655001	16	86655001	86660000	5000	1	1.50E-08	0.46	82	1.64	Efnb2	Signaling
DMR16:90311001	16	90311001	90317000	6000	1	8.40E-07	-0.39	22	0.37	Slc10a2	Transport
DMR17:527001	17	527001	533000	6000	1	9.70E-10	-0.28	62	1.03	Npepo	
DMR17:674001	17	674001	675000	1000	1	1.10E-14	0.56	14	1.4	Npepo	
DMR17:796001	17	796001	802000	6000	1	3.10E-07	-0.36	116	1.93	Npepo	
DMR17:1053001	17	1053001	1056000	3000	1	2.20E-09	-0.58	43	1.43	Ptch1	
DMR17:1429001	17	1429001	1430000	1000	1	9.60E-08	0.36	7	0.7	Ercc6l2	
DMR17:1582001	17	1582001	1583000	1000	1	7.70E-08	-0.39	12	1.2	Hsd17b3	
DMR17:1710001	17	1710001	1713000	3000	1	2.10E-08	-0.49	37	1.23	Habp4;Cdc14b	Metabolism;Signaling
DMR17:1728001	17	1728001	1729000	1000	1	1.00E-13	-0.55	6	0.6	Cdc14b	Signaling
DMR17:1827001	17	1827001	1828000	1000	1	1.10E-07	-0.53	14	1.4	Aaed1	
DMR17:1838001	17	1838001	1840000	2000	1	2.70E-08	0.64	30	1.5	Aaed1	
DMR17:1938001	17	1938001	1940000	2000	1	6.50E-07	0.33	30	1.5	Cdk20	Signaling
DMR17:2165001	17	2165001	2167000	2000	1	5.30E-08	0.43	28	1.4	RGD1563581	
DMR17:2818001	17	2818001	2821000	3000	1	5.70E-07	-0.4	12	0.4	LOC502109;Prss47	Protease
DMR17:3393001	17	3393001	3397000	4000	1	1.00E-09	-0.45	21	0.52	LOC102552000;Cts8	Protease
DMR17:3829001	17	3829001	3835000	6000	1	1.10E-07	-0.31	50	0.83	Ctsm	Protease
DMR17:3896001	17	3896001	3901000	5000	2	8.60E-09	-0.36	45	0.9	Ctsr	Protease
DMR17:4296001	17	4296001	4301000	5000	1	1.90E-07	-0.46	100	2	Dapk1	Signaling
DMR17:4449001	17	4449001	4450000	1000	1	1.40E-08	-0.46	33	3.3	Dapk1	Signaling
DMR17:5227001	17	5227001	5231000	4000	2	4.10E-09	-0.46	52	1.3	Zcchc6	
DMR17:5339001	17	5339001	5342000	3000	1	4.50E-09	0.3	35	1.17	Spata31d1b;Spata31d3	
DMR17:5382001	17	5382001	5385000	3000	1	5.40E-08	-0.36	60	2	Golm1	
DMR17:5449001	17	5449001	5451000	2000	1	9.70E-07	-0.38	19	0.95	Naa35	Metabolism

DMR17:5529001	17	5529001	5533000	4000	1	4.90E-08	-0.53	103	2.58	Agtpbp1	Protease
DMR17:5554001	17	5554001	5556000	2000	1	1.00E-11	-0.67	36	1.8	Agtpbp1	Protease
DMR17:5975001	17	5975001	5977000	2000	1	8.90E-16	0.81	50	2.5	Ntrk2	Receptor
DMR17:5986001	17	5986001	5988000	2000	1	7.60E-08	0.44	22	1.1	Ntrk2	Receptor
DMR17:5993001	17	5993001	5994000	1000	1	1.00E-06	0.32	16	1.6	Ntrk2	Receptor
DMR17:6018001	17	6018001	6022000	4000	1	3.20E-09	0.74	80	2	Ntrk2	Receptor
DMR17:6032001	17	6032001	6035000	3000	1	4.40E-07	0.33	47	1.57	Ntrk2	Receptor
DMR17:6089001	17	6089001	6091000	2000	1	1.40E-07	0.35	52	2.6	Ntrk2;LOC108353117	Receptor
DMR17:6113001	17	6113001	6116000	3000	1	9.50E-08	-0.41	59	1.97	Ntrk2	Receptor
DMR17:6187001	17	6187001	6189000	2000	1	2.30E-08	-0.44	40	2	Ntrk2	Receptor
DMR17:6461001	17	6461001	6465000	4000	2	9.30E-09	-0.38	78	1.95	Slc28a3	Transport
DMR17:6498001	17	6498001	6500000	2000	1	3.00E-09	0.43	55	2.75	Slc28a3	Transport
DMR17:6668001	17	6668001	6671000	3000	2	1.00E-08	-0.47	37	1.23	Rmi1;Hnrnpk;Mir7a-1	Metabolism
DMR17:6699001	17	6699001	6700000	1000	1	1.20E-07	0.56	20	2	RGD1311345;LOC103694036;Kif27	Cytoskeleton
DMR17:6769001	17	6769001	6770000	1000	1	1.30E-09	0.45	10	1	Kif27;LOC102551320;LOC102551121	Cytoskeleton
DMR17:6811001	17	6811001	6815000	4000	1	1.70E-09	-0.44	71	1.77	Gkap1	Cytoskeleton
DMR17:6856001	17	6856001	6858000	2000	2	3.70E-08	-0.53	22	1.1	Ubqln1	
DMR17:6871001	17	6871001	6874000	3000	1	1.00E-07	-0.33	69	2.3	Ubqln1	
DMR17:6931001	17	6931001	6938000	7000	2	4.40E-15	0.91	160	2.29	LOC108348619;Klhl3	Cytoskeleton
DMR17:6942001	17	6942001	6944000	2000	1	3.10E-07	0.26	29	1.45	Klhl3	Cytoskeleton
DMR17:6955001	17	6955001	6957000	2000	1	2.50E-08	0.46	69	3.45	Klhl3	Cytoskeleton
DMR17:7670001	17	7670001	7676000	6000	1	2.50E-07	0.36	63	1.05	Spock1;LOC108348496	Signaling
DMR17:7727001	17	7727001	7729000	2000	1	3.40E-08	0.36	22	1.1	Spock1	Signaling
DMR17:7755001	17	7755001	7757000	2000	1	2.00E-08	0.4	46	2.3	Spock1	Signaling
DMR17:7760001	17	7760001	7762000	2000	1	8.90E-08	0.42	38	1.9	Spock1	Signaling
DMR17:7797001	17	7797001	7799000	2000	1	3.80E-07	0.55	48	2.4	Spock1	Signaling
DMR17:7805001	17	7805001	7806000	1000	1	5.20E-14	0.72	42	4.2	Spock1	Signaling
DMR17:8275001	17	8275001	8277000	2000	1	3.00E-07	0.6	37	1.85	Trpc7	Transport
DMR17:8396001	17	8396001	8397000	1000	1	4.20E-08	0.45	11	1.1	Tgfbi	Cytoskeleton
DMR17:8398001	17	8398001	8400000	2000	1	6.70E-08	0.3	26	1.3	Tgfbi	Cytoskeleton
DMR17:8503001	17	8503001	8505000	2000	1	4.30E-07	-0.41	68	3.4	Lect2;Fbxl21	Growth Factors
DMR17:8522001	17	8522001	8524000	2000	1	5.50E-07	0.59	54	2.7	Fbxl21;LOC108348500	
DMR17:8583001	17	8583001	8584000	1000	1	1.50E-07	-0.36	21	2.1	Slc25a48	Transport
DMR17:8590001	17	8590001	8592000	2000	1	3.50E-07	0.33	25	1.25	Slc25a48	Transport
DMR17:9342001	17	9342001	9343000	1000	1	2.70E-09	0.58	27	2.7	H2afy	
DMR17:9432001	17	9432001	9433000	1000	1	5.00E-07	0.4	7	0.7	LOC103694037;Txndc15	
DMR17:9470001	17	9470001	9472000	2000	1	6.00E-07	0.51	37	1.85	RGD1566359;Ddx46	
DMR17:9495001	17	9495001	9496000	1000	1	3.30E-07	-0.43	15	1.5	Ddx46	
DMR17:9513001	17	9513001	9516000	3000	1	2.40E-07	-0.41	64	2.13	Ddx46;Camlg	
DMR17:9548001	17	9548001	9550000	2000	1	1.60E-09	-0.36	43	2.15	B4galt7	Golgi
DMR17:9593001	17	9593001	9595000	2000	2	1.10E-11	0.41	17	0.85	Fam193b	
DMR17:9673001	17	9673001	9676000	3000	1	4.80E-10	0.35	36	1.2	Pdlim7;Dbn1	Cytoskeleton;Cytoskeleto n
DMR17:9793001	17	9793001	9794000	1000	1	8.90E-09	-0.48	25	2.5	Rgs14;Lman2	Transport
DMR17:9799001	17	9799001	9801000	2000	1	2.00E-17	-0.64	28	1.4	Rgs14;Lman2	Transport
DMR17:9846001	17	9846001	9849000	3000	1	5.10E-08	-0.37	39	1.3	Prelid1;Rab24;Nsd1	
DMR17:9990001	17	9990001	9992000	2000	2	3.70E-10	0.64	60	3	LOC498705;Fgfr4	Receptor
DMR17:10007001	17	10007001	10009000	2000	2	3.40E-10	0.48	22	1.1	Fgfr4	Receptor
DMR17:10147001	17	10147001	10150000	3000	1	3.50E-08	0.41	66	2.2	Hk3;Unc5a	Signaling;Receptor
DMR17:10351001	17	10351001	10352000	1000	1	3.60E-07	0.62	25	2.5	Tspan17	
DMR17:10385001	17	10385001	10389000	4000	1	9.60E-10	0.46	74	1.85	Eif4e1b;Sncb;LOC102547505	Transport
DMR17:10415001	17	10415001	10417000	2000	1	7.00E-07	0.41	24	1.2	Gprin1;Cdhr2	Cytoskeleton
DMR17:10422001	17	10422001	10424000	2000	1	7.20E-09	0.52	27	1.35	Gprin1;Cdhr2	Cytoskeleton
DMR17:10480001	17	10480001	10481000	1000	1	6.50E-08	0.55	18	1.8	Rnf44;Faf2	
DMR17:10741001	17	10741001	10742000	1000	1	3.30E-07	0.57	14	1.4	Lnc012	
DMR17:10757001	17	10757001	10760000	3000	2	1.20E-07	0.74	74	2.47	Lnc012;Cplx2	
DMR17:10780001	17	10780001	10781000	1000	1	9.80E-07	0.36	16	1.6	Lnc012;Cplx2;LOC103694917	
DMR17:10909001	17	10909001	10910000	1000	1	2.60E-08	0.34	19	1.9	Hrh2	Signaling
DMR17:10916001	17	10916001	10917000	1000	1	5.00E-07	-0.47	18	1.8	Hrh2	Signaling
DMR17:10933001	17	10933001	10937000	4000	1	1.20E-07	0.35	22	0.55	Hrh2	Signaling
DMR17:11672001	17	11672001	11676000	4000	1	5.20E-07	0.36	53	1.32	LOC108348511;Msx2	Development
DMR17:11907001	17	11907001	11908000	1000	1	5.10E-07	-0.38	9	0.9	RGD1561671	Transcription

DMR17:11915001	17	11915001	11916000	1000	1	8.40E-11	0.69	35	3.5	RGD1561671	Transcription
DMR17:12029001	17	12029001	12030000	1000	1	1.80E-08	0.72	24	2.4	Ror2	Receptor
DMR17:12044001	17	12044001	12048000	4000	1	4.70E-07	0.34	89	2.22	Ror2	Receptor
DMR17:12060001	17	12060001	12063000	3000	1	1.20E-09	0.38	47	1.57	Ror2	Receptor
DMR17:12067001	17	12067001	12069000	2000	1	5.20E-14	-0.53	48	2.4	Ror2	Receptor
DMR17:12085001	17	12085001	12091000	6000	1	5.20E-07	-0.31	92	1.53	Ror2	Receptor
DMR17:12112001	17	12112001	12113000	1000	1	8.40E-07	0.35	14	1.4	Ror2	Receptor
DMR17:12125001	17	12125001	12126000	1000	1	2.80E-07	0.45	37	3.7	Ror2	Receptor
DMR17:12269001	17	12269001	12271000	2000	1	5.60E-07	-0.4	33	1.65	Nfil3	Transcription
DMR17:12272001	17	12272001	12274000	2000	1	5.40E-07	-0.46	29	1.45	Nfil3	Transcription
DMR17:12319001	17	12319001	12323000	4000	1	6.10E-09	-0.45	73	1.82	Auh	Metabolism
DMR17:12377001	17	12377001	12379000	2000	1	1.90E-09	-0.43	43	2.15	Auh	Metabolism
DMR17:12608001	17	12608001	12610000	2000	1	3.60E-07	0.39	20	1	Syk	
DMR17:12621001	17	12621001	12624000	3000	2	4.00E-09	-0.51	41	1.37	Syk	
DMR17:12629001	17	12629001	12630000	1000	1	3.70E-07	0.4	19	1.9	Syk	
DMR17:12735001	17	12735001	12737000	2000	1	2.20E-12	0.82	43	2.15	LOC103694041;Diras2	Signaling
DMR17:13516001	17	13516001	13520000	4000	1	4.70E-09	0.48	70	1.75	Sema4d	Signaling
DMR17:13727001	17	13727001	13730000	3000	1	1.40E-07	0.48	18	0.6	Shc3	Cytoskeleton
DMR17:13771001	17	13771001	13772000	1000	1	8.90E-08	0.68	18	1.8	Shc3	Cytoskeleton
DMR17:14075001	17	14075001	14080000	5000	1	7.60E-07	-0.46	85	1.7	Spin1	
DMR17:14459001	17	14459001	14467000	8000	1	6.20E-08	-0.45	93	1.16	LOC689437;Trnak-cuu;LOC689448;LOC689458;LOC108353119	
DMR17:14600001	17	14600001	14603000	3000	1	1.30E-07	-0.37	14	0.47	LOC108348068;Ogn	
DMR17:15085001	17	15085001	15087000	2000	1	7.50E-09	-0.49	42	2.1	LOC689316;Fbxw17	
DMR17:15566001	17	15566001	15567000	1000	1	1.30E-08	-0.54	10	1	LOC679342;Ecm2	
DMR17:15753001	17	15753001	15756000	3000	1	1.00E-06	0.31	39	1.3	Fgd3	Transcription
DMR17:15764001	17	15764001	15765000	1000	1	5.20E-08	-0.4	20	2	Fgd3	Transcription
DMR17:15886001	17	15886001	15887000	1000	1	4.50E-07	0.41	13	1.3	Ninj1	Cytoskeleton
DMR17:15963001	17	15963001	15965000	2000	1	8.20E-08	-0.36	24	1.2	Wnk2	Signaling
DMR17:16274001	17	16274001	16275000	1000	1	9.10E-07	-0.35	30	3	Phf2	
DMR17:16354001	17	16354001	16355000	1000	1	4.60E-07	-0.43	25	2.5	Ptpdc1	Signaling
DMR17:16419001	17	16419001	16420000	1000	1	1.70E-09	-0.66	10	1	Mirlet7a1;Mirlet7f1;Mirlet7f-1;Mirlet7d;Mir3596b;LOC108348525	
DMR17:16439001	17	16439001	16440000	1000	1	3.60E-07	-0.39	13	1.3	LOC108348525;Zfp169	Transcription
DMR17:16460001	17	16460001	16462000	2000	1	1.50E-09	0.73	37	1.85	Zfp169;LOC108348526	Transcription
DMR17:16682001	17	16682001	16683000	1000	1	6.90E-09	0.42	14	1.4	Id4	Transcription
DMR17:17807001	17	17807001	17808000	1000	1	1.00E-08	0.64	22	2.2	Rnf144b	Proteolysis
DMR17:17818001	17	17818001	17819000	1000	1	5.60E-08	-0.41	26	2.6	Rnf144b	Proteolysis
DMR17:17833001	17	17833001	17836000	3000	1	3.00E-08	-0.44	66	2.2	Rnf144b	Proteolysis
DMR17:17865001	17	17865001	17866000	1000	1	9.40E-09	-0.64	19	1.9	Rnf144b;LOC102555870	Proteolysis
DMR17:17922001	17	17922001	17925000	3000	1	6.50E-10	0.34	30	1	Rnf144b	Proteolysis
DMR17:17967001	17	17967001	17969000	2000	1	3.30E-07	-0.36	19	0.95	LOC103694052;Dek	Epigenetic
DMR17:18147001	17	18147001	18148000	1000	1	1.10E-07	0.52	17	1.7	LOC108348531;Kif13a	Cytoskeleton
DMR17:18199001	17	18199001	18200000	1000	1	3.10E-07	0.62	32	3.2	Kif13a	Cytoskeleton
DMR17:18291001	17	18291001	18292000	1000	1	3.90E-11	0.45	20	2	Kif13a	Cytoskeleton
DMR17:18296001	17	18296001	18297000	1000	1	3.80E-07	0.34	21	2.1	Kif13a	Cytoskeleton
DMR17:18310001	17	18310001	18311000	1000	1	1.10E-07	-0.41	23	2.3	Kif13a	Cytoskeleton
DMR17:18365001	17	18365001	18366000	1000	1	2.10E-10	-0.48	7	0.7	Nup153	Transport
DMR17:18425001	17	18425001	18426000	1000	1	1.40E-11	0.71	28	2.8	Fam8a1	
DMR17:18468001	17	18468001	18469000	1000	1	1.40E-12	-0.61	24	2.4	Cap2	Cytoskeleton
DMR17:18507001	17	18507001	18511000	4000	1	7.10E-08	-0.37	54	1.35	Cap2;LOC108353127	Cytoskeleton
DMR17:18535001	17	18535001	18538000	3000	1	9.70E-07	-0.37	54	1.8	Cap2	Cytoskeleton
DMR17:18587001	17	18587001	18590000	3000	1	5.90E-08	0.55	53	1.77	Cap2	Cytoskeleton
DMR17:19201001	17	19201001	19205000	4000	1	1.20E-07	-0.46	59	1.48	Atxn1	
DMR17:19315001	17	19315001	19316000	1000	1	1.10E-08	0.64	30	3	Atxn1	
DMR17:19330001	17	19330001	19331000	1000	1	1.60E-08	0.39	14	1.4	Atxn1	
DMR17:19445001	17	19445001	19447000	2000	1	1.60E-07	-0.35	42	2.1	Atxn1	
DMR17:19586001	17	19586001	19587000	1000	1	1.40E-09	0.52	38	3.8	Gmpr	Metabolism
DMR17:19681001	17	19681001	19683000	2000	1	2.00E-09	-0.47	31	1.55	Mylip	
DMR17:19685001	17	19685001	19686000	1000	1	8.90E-08	0.35	8	0.8	Mylip	
DMR17:19690001	17	19690001	19693000	3000	2	7.90E-10	-0.58	57	1.9	Mylip	

DMR17:21328001	17	21328001	21330000	2000	1	3.70E-12	0.46	21	1.05	Smim13	
DMR17:21429001	17	21429001	21432000	3000	1	2.10E-07	-0.39	38	1.27	Elovl2;LOC108348546;Sycp2l	Metabolism
DMR17:21436001	17	21436001	21437000	1000	1	1.40E-08	0.33	14	1.4	LOC108348546;Sycp2l	
DMR17:21467001	17	21467001	21469000	2000	1	1.50E-07	-0.47	32	1.6	Sycp2l	
DMR17:21471001	17	21471001	21472000	1000	1	1.50E-08	-0.39	14	1.4	Sycp2l	
DMR17:21494001	17	21494001	21495000	1000	1	1.10E-07	0.6	42	4.2	Sycp2l;Gcm2	
DMR17:21531001	17	21531001	21532000	1000	1	1.00E-07	-0.4	25	2.5	Mak	Signaling
DMR17:21640001	17	21640001	21644000	4000	1	9.80E-07	-0.44	57	1.43	LOC103694058;Gcnt2	Golgi
DMR17:21649001	17	21649001	21654000	5000	3	1.30E-10	0.59	75	1.5	Gcnt2	Golgi
DMR17:21725001	17	21725001	21726000	1000	1	6.70E-10	0.72	32	3.2	Gcnt6	
DMR17:22383001	17	22383001	22387000	4000	1	9.40E-07	-0.37	73	1.82	Hivep1	
DMR17:22636001	17	22636001	22637000	1000	1	1.50E-07	0.33	11	1.1	Adtrp	
DMR17:23130001	17	23130001	23132000	2000	1	3.00E-07	-0.44	31	1.55	Nedd9	
DMR17:23165001	17	23165001	23170000	5000	1	2.40E-10	0.38	61	1.22	Nedd9;LOC108353140	
DMR17:23280001	17	23280001	23281000	1000	1	3.10E-10	0.72	42	4.2	Phactr1	Signaling
DMR17:23293001	17	23293001	23295000	2000	1	2.70E-07	-0.46	35	1.75	Phactr1	Signaling
DMR17:23345001	17	23345001	23346000	1000	1	2.60E-07	0.62	18	1.8	Phactr1	Signaling
DMR17:23416001	17	23416001	23419000	3000	1	3.50E-07	-0.32	51	1.7	Phactr1	Signaling
DMR17:23445001	17	23445001	23447000	2000	1	2.90E-08	0.38	17	0.85	Phactr1	Signaling
DMR17:23509001	17	23509001	23510000	1000	1	1.60E-09	0.46	9	0.9	Phactr1	Signaling
DMR17:23511001	17	23511001	23515000	4000	1	8.40E-07	0.34	55	1.38	Phactr1	Signaling
DMR17:23527001	17	23527001	23529000	2000	2	1.10E-07	0.49	34	1.7	Phactr1	Signaling
DMR17:23641001	17	23641001	23642000	1000	1	3.00E-07	0.41	8	0.8	Phactr1	Signaling
DMR17:23884001	17	23884001	23885000	1000	1	1.40E-10	0.54	8	0.8	Gfod1	Metabolism
DMR17:23934001	17	23934001	23936000	2000	1	4.10E-08	0.42	24	1.2	Gfod1	Metabolism
DMR17:24059001	17	24059001	24060000	1000	1	4.80E-08	-0.71	4	0.4	Ranbp9	Cytoskeleton
DMR17:24083001	17	24083001	24084000	1000	1	9.30E-07	-0.46	10	1	Ranbp9	Cytoskeleton
DMR17:24272001	17	24272001	24276000	4000	2	5.40E-09	-0.39	58	1.45	Rnf182	
DMR17:24278001	17	24278001	24279000	1000	1	7.00E-10	0.37	12	1.2	Rnf182	
DMR17:24296001	17	24296001	24298000	2000	1	3.20E-09	-0.57	35	1.75	Rnf182	
DMR17:24663001	17	24663001	24665000	2000	1	2.50E-16	0.75	70	3.5	Tfap2a	Transcription
DMR17:24672001	17	24672001	24674000	2000	1	1.80E-10	-0.65	22	1.1	Tfap2a	Transcription
DMR17:25101001	17	25101001	25102000	1000	1	1.80E-07	0.43	16	1.6	Ofcc1	
DMR17:25141001	17	25141001	25144000	3000	1	2.70E-07	-0.47	53	1.77	Ofcc1	
DMR17:25182001	17	25182001	25184000	2000	1	2.20E-08	0.58	24	1.2	Ofcc1	
DMR17:25288001	17	25288001	25290000	2000	2	1.30E-09	0.41	10	0.5	Ofcc1	
DMR17:25312001	17	25312001	25313000	1000	1	6.70E-09	0.53	19	1.9	Ofcc1	
DMR17:27045001	17	27045001	27048000	3000	1	1.10E-08	-0.44	32	1.07	Bmp6	Growth Factors
DMR17:27115001	17	27115001	27118000	3000	1	7.40E-08	-0.46	52	1.73	Bmp6;LOC102556358	Growth Factors
DMR17:27247001	17	27247001	27251000	4000	1	5.40E-09	-0.57	49	1.23	Snrnp48	
DMR17:27284001	17	27284001	27286000	2000	1	5.50E-07	-0.45	26	1.3	Snrnp48;Dsp	Cytoskeleton
DMR17:27290001	17	27290001	27295000	5000	1	9.00E-11	-0.47	100	2	Dsp	Cytoskeleton
DMR17:27318001	17	27318001	27319000	1000	1	1.70E-10	0.43	17	1.7	Dsp	Cytoskeleton
DMR17:27326001	17	27326001	27328000	2000	1	8.20E-08	-0.42	34	1.7	Dsp	Cytoskeleton
DMR17:27480001	17	27480001	27482000	2000	2	1.00E-16	0.45	16	0.8	Cage1	
DMR17:27529001	17	27529001	27530000	1000	1	3.00E-08	0.35	24	2.4	Rreb1	
DMR17:27572001	17	27572001	27573000	1000	1	7.30E-07	0.35	15	1.5	Rreb1	
DMR17:27642001	17	27642001	27645000	3000	1	2.40E-08	-0.55	66	2.2	Rreb1	
DMR17:27679001	17	27679001	27681000	2000	1	5.70E-09	0.53	23	1.15	Rreb1	
DMR17:27929001	17	27929001	27932000	3000	1	1.50E-11	-0.55	76	2.53	Nrn1;LOC683105	
DMR17:27999001	17	27999001	28001000	2000	1	4.50E-07	0.38	25	1.25	Nrn1;LOC102549751;LOC108348548	
DMR17:28710001	17	28710001	28711000	1000	1	1.20E-10	0.64	8	0.8	RGD1564781	
DMR17:29049001	17	29049001	29052000	3000	1	6.90E-08	0.48	39	1.3	Fars2	Translation
DMR17:29270001	17	29270001	29274000	4000	2	7.60E-08	-0.35	36	0.9	Fars2	Translation
DMR17:29330001	17	29330001	29331000	1000	1	8.90E-08	0.36	4	0.4	Fars2	Translation
DMR17:29426001	17	29426001	29427000	1000	1	1.40E-07	0.55	13	1.3	Fars2	Translation
DMR17:29573001	17	29573001	29574000	1000	1	4.40E-07	-0.55	7	0.7	Ppp1r3g;LOC102550566	Signaling
DMR17:29660001	17	29660001	29663000	3000	2	3.40E-09	0.39	46	1.53	Cdyl	
DMR17:29708001	17	29708001	29716000	8000	1	4.80E-09	-0.45	119	1.49	Cdyl	
DMR17:29768001	17	29768001	29772000	4000	1	2.50E-10	-0.8	48	1.2	Cdyl	
DMR17:29828001	17	29828001	29831000	3000	1	1.60E-07	-0.31	36	1.2	Cdyl	
DMR17:29842001	17	29842001	29846000	4000	1	2.80E-07	0.39	39	0.98	Cdyl	

DMR17:30686001	17	30686001	30688000	2000	1	6.70E-11	-0.74	27	1.35	Fam217a;Prpf4b	Signaling
DMR17:30703001	17	30703001	30707000	4000	1	2.60E-07	-0.35	53	1.32	Prpf4b	Signaling
DMR17:30970001	17	30970001	30971000	1000	1	3.50E-08	0.45	26	2.6	Pxdc1	
DMR17:31226001	17	31226001	31228000	2000	1	9.00E-07	-0.32	32	1.6	Slc22a23	Transport
DMR17:31231001	17	31231001	31232000	1000	1	3.10E-09	0.39	6	0.6	Slc22a23	Transport
DMR17:31251001	17	31251001	31252000	1000	1	2.80E-07	0.33	12	1.2	Slc22a23	Transport
DMR17:31277001	17	31277001	31278000	1000	1	9.80E-08	0.36	4	0.4	Slc22a23	Transport
DMR17:31323001	17	31323001	31324000	1000	1	1.30E-07	0.46	17	1.7	Slc22a23	Transport
DMR17:31328001	17	31328001	31329000	1000	1	4.80E-07	0.42	4	0.4	Slc22a23	Transport
DMR17:31372001	17	31372001	31375000	3000	1	1.30E-08	0.43	43	1.43	Slc22a23	Transport
DMR17:31532001	17	31532001	31534000	2000	1	2.10E-09	-0.39	19	0.95	Bphl;Ripk1	Metabolism;Signaling
DMR17:31558001	17	31558001	31560000	2000	1	6.30E-09	-0.5	22	1.1	Ripk1	Signaling
DMR17:32171001	17	32171001	32173000	2000	1	5.10E-07	-0.38	52	2.6	Serpinb6;LOC102554685	
DMR17:32186001	17	32186001	32188000	2000	1	6.80E-07	0.34	18	0.9	Serpinb6;LOC102554685	
DMR17:32278001	17	32278001	32282000	4000	1	3.20E-09	-0.39	23	0.58	RGD1564786	
DMR17:32506001	17	32506001	32507000	1000	1	3.40E-08	-0.47	4	0.4	Serpinb9d	Protease; Proteolysis
DMR17:32629001	17	32629001	32634000	5000	1	7.70E-12	-0.48	25	0.5	RGD1562844	
DMR17:32779001	17	32779001	32781000	2000	1	1.30E-07	-0.48	9	0.45	Serpinb6b	Protease; Proteolysis
DMR17:32893001	17	32893001	32897000	4000	1	4.90E-10	-0.37	38	0.95	LOC108348553;Serpinb1a	Protease; Proteolysis
DMR17:33050001	17	33050001	33052000	2000	1	2.80E-07	0.4	13	0.65	Mylk4	Signaling
DMR17:33590001	17	33590001	33595000	5000	2	3.40E-08	-0.37	48	0.96	Gmds	Metabolism
DMR17:33656001	17	33656001	33657000	1000	1	2.10E-08	0.48	8	0.8	Gmds	Metabolism
DMR17:34143001	17	34143001	34145000	2000	1	1.10E-07	0.43	15	0.75	Foxf2	
DMR17:34712001	17	34712001	34715000	3000	2	3.40E-08	-0.42	34	1.13	Exoc2;LOC102547446	
DMR17:34748001	17	34748001	34749000	1000	1	5.00E-07	-0.54	7	0.7	Exoc2	
DMR17:34765001	17	34765001	34766000	1000	1	5.70E-08	-0.4	15	1.5	Exoc2	
DMR17:34986001	17	34986001	34988000	2000	1	2.30E-07	-0.75	23	1.15	Dusp22	Signaling
DMR17:35027001	17	35027001	35028000	1000	1	1.50E-07	-0.35	16	1.6	Dusp22	Signaling
DMR17:35940001	17	35940001	35946000	6000	1	3.80E-07	-0.27	62	1.03	Agtr1a	
DMR17:35963001	17	35963001	35968000	5000	1	6.80E-07	-0.58	38	0.76	Agtr1a	
DMR17:36186001	17	36186001	36188000	2000	1	8.60E-08	0.48	21	1.05	Mboat1	Metabolism
DMR17:36542001	17	36542001	36544000	2000	1	3.60E-07	-0.39	24	1.2	Cdkal1	
DMR17:37079001	17	37079001	37088000	9000	1	1.90E-07	-0.3	81	0.9	Cdkal1	
DMR17:37099001	17	37099001	37100000	1000	1	6.60E-08	0.41	3	0.3	Cdkal1	
DMR17:37153001	17	37153001	37155000	2000	1	8.50E-07	-0.33	22	1.1	Cdkal1	
DMR17:38322001	17	38322001	38323000	1000	1	1.50E-07	-0.47	3	0.3	Prl5a1	Hormone
DMR17:38983001	17	38983001	38984000	1000	1	6.50E-09	0.63	15	1.5	Prl7d1	Hormone
DMR17:39260001	17	39260001	39264000	4000	1	4.20E-07	-0.4	34	0.85	Prl3d4	Hormone
DMR17:39276001	17	39276001	39277000	1000	1	1.80E-16	0.86	6	0.6	Prl3d4	Hormone
DMR17:39419001	17	39419001	39426000	7000	2	7.90E-07	-0.24	88	1.26	Prl8a5	Hormone
DMR17:41942001	17	41942001	41945000	3000	1	3.60E-07	0.34	35	1.17	Dcdc2	
DMR17:41956001	17	41956001	41962000	6000	1	2.40E-07	-0.61	57	0.95	Dcdc2	
DMR17:41988001	17	41988001	41990000	2000	1	9.80E-07	-0.51	33	1.65	Dcdc2	
DMR17:42009001	17	42009001	42011000	2000	1	2.20E-07	0.43	4	0.2	Dcdc2	
DMR17:42049001	17	42049001	42056000	7000	1	6.40E-07	-0.28	89	1.27	Mrs2	Translation
DMR17:42173001	17	42173001	42179000	6000	1	4.90E-09	-0.27	60	1	RGD1307443;Rpl37-ps1	
DMR17:42314001	17	42314001	42315000	1000	1	5.30E-07	0.4	1	0.1	Gmnn;LOC102548126;Fam65b	
DMR17:42324001	17	42324001	42326000	2000	1	1.80E-08	0.55	46	2.3	LOC102548126;Fam65b	
DMR17:42484001	17	42484001	42485000	1000	1	1.50E-09	0.46	5	0.5	Fam65b	
DMR17:42606001	17	42606001	42608000	2000	1	3.30E-09	0.41	40	2	Cmah	
DMR17:42613001	17	42613001	42614000	1000	1	3.20E-08	-0.49	11	1.1	Cmah	
DMR17:42700001	17	42700001	42701000	1000	1	1.80E-07	0.44	3	0.3	Prl6a1	Hormone
DMR17:42730001	17	42730001	42732000	2000	1	2.30E-07	-0.27	24	1.2	Prl3a1	Hormone
DMR17:43149001	17	43149001	43150000	1000	1	1.40E-07	-0.49	4	0.4	Carmil1	
DMR17:43176001	17	43176001	43181000	5000	1	1.10E-09	-0.36	40	0.8	Carmil1	
DMR17:43432001	17	43432001	43434000	2000	1	3.50E-08	-0.63	13	0.65	LOC100909739;LOC102548588;Hist1h2aa;Hist1h2ba	
DMR17:43452001	17	43452001	43454000	2000	1	5.40E-08	-0.44	18	0.9	LOC100909739;Slc17a4	Transport
DMR17:43552001	17	43552001	43558000	6000	2	4.60E-11	-0.42	45	0.75	Slc17a3;Slc17a2	Transport
DMR17:43812001	17	43812001	43814000	2000	1	2.90E-07	-0.31	25	1.25	LOC102551184;LOC100910366;LOC100365043;Hist1h2ail1;Hist1h2bl;LOC108348560;Trnam-cau	Epigenetic
DMR17:43888001	17	43888001	43890000	2000	1	2.50E-08	0.4	17	0.85	Btn2a2;Btn1a1	Immune

DMR17:44403001	17	44403001	44409000	6000	5	2.80E-11	0.61	123	2.05	RGD1561897	
DMR17:44410001	17	44410001	44412000	2000	1	4.30E-13	0.62	26	1.3	RGD1561897	
DMR17:44416001	17	44416001	44419000	3000	2	5.80E-11	0.84	37	1.23	RGD1561897	
DMR17:44420001	17	44420001	44427000	7000	5	1.30E-12	0.58	193	2.76	RGD1561897	
DMR17:44614001	17	44614001	44618000	4000	1	1.00E-11	-0.44	39	0.98	Zfp184	
DMR17:44845001	17	44845001	44847000	2000	1	2.00E-08	-0.7	35	1.75	LOC100360950;Hist1h2ak;LOC108348485;Trnam-cau;Trnag-gcc;Olr1654	Epigenetic;Receptor
DMR17:45294001	17	45294001	45296000	2000	1	8.50E-08	-0.41	16	0.8	Zscan12	Transcription
DMR17:45529001	17	45529001	45530000	1000	1	1.20E-07	0.47	19	1.9	Trnar-ucg;Trnat-ugu;Trnaq-uug	
DMR17:45562001	17	45562001	45563000	1000	1	1.60E-08	0.63	20	2	Trnaa-cgc;Trnat-agu;Trnaa-agc;Trnaa-ugc	
DMR17:45585001	17	45585001	45586000	1000	1	4.40E-08	0.59	19	1.9	Trnaa-agc;Trnar-ccg	
DMR17:45648001	17	45648001	45655000	7000	1	1.80E-11	-0.24	74	1.06	LOC102547106;Trnaf-gaa;Trnae-cuc	
DMR17:45696001	17	45696001	45701000	5000	1	2.50E-07	-0.36	46	0.92	Olr1662	Receptor
DMR17:45736001	17	45736001	45738000	2000	1	6.40E-10	-0.42	16	0.8	LOC100912958;RGD1564243	Receptor
DMR17:45745001	17	45745001	45748000	3000	1	6.50E-11	-0.39	27	0.9	RGD1564243	
DMR17:45806001	17	45806001	45812000	6000	2	1.20E-07	-0.34	55	0.92	Olr1664	Receptor
DMR17:45957001	17	45957001	45963000	6000	1	7.30E-07	-0.34	42	0.7	Aoah	Metabolism
DMR17:46347001	17	46347001	46348000	1000	1	5.40E-09	0.43	5	0.5	Elmo1	Cytoskeleton
DMR17:46467001	17	46467001	46468000	1000	1	3.30E-07	0.55	4	0.4	Elmo1	Cytoskeleton
DMR17:46713001	17	46713001	46721000	8000	1	8.70E-07	-0.31	95	1.19	Elmo1	Cytoskeleton
DMR17:46832001	17	46832001	46834000	2000	1	1.80E-08	-0.49	19	0.95	Elmo1	Cytoskeleton
DMR17:47261001	17	47261001	47262000	1000	1	1.40E-09	0.56	17	1.7	Gpr141;LOC102546579	Signaling
DMR17:47291001	17	47291001	47294000	3000	1	2.60E-08	0.52	20	0.67	LOC102546469;Nme8	
DMR17:47334001	17	47334001	47335000	1000	1	2.20E-07	0.48	9	0.9	Nme8	
DMR17:47357001	17	47357001	47362000	5000	1	1.20E-09	-0.26	39	0.78	Nme8	
DMR17:48305001	17	48305001	48309000	4000	1	3.70E-10	-0.32	50	1.25	Amph	
DMR17:48381001	17	48381001	48387000	6000	3	1.40E-09	-0.62	59	0.98	Amph	
DMR17:48413001	17	48413001	48414000	1000	1	3.30E-11	-0.55	5	0.5	Amph;LOC102555260	
DMR17:48416001	17	48416001	48418000	2000	1	5.80E-08	-0.3	17	0.85	Amph;LOC102555260	
DMR17:48429001	17	48429001	48438000	9000	2	3.70E-08	-0.34	72	0.8	Amph	
DMR17:48442001	17	48442001	48444000	2000	1	2.50E-10	-0.72	9	0.45	Amph	
DMR17:48449001	17	48449001	48457000	8000	1	4.10E-08	-0.36	54	0.68	Amph	
DMR17:48459001	17	48459001	48466000	7000	2	1.40E-09	-0.36	70	1	Amph	
DMR17:49108001	17	49108001	49113000	5000	1	9.20E-14	0.63	44	0.88	Pou6f2	
DMR17:49115001	17	49115001	49116000	1000	1	1.10E-07	0.52	9	0.9	Pou6f2	
DMR17:49136001	17	49136001	49138000	2000	1	6.20E-08	-0.52	7	0.35	Pou6f2	
DMR17:49374001	17	49374001	49376000	2000	1	8.30E-11	0.43	8	0.4	Pou6f2	
DMR17:49390001	17	49390001	49393000	3000	1	6.50E-07	-0.32	21	0.7	Pou6f2	
DMR17:49394001	17	49394001	49397000	3000	1	2.90E-07	-0.29	30	1	Pou6f2	
DMR17:49464001	17	49464001	49465000	1000	1	7.10E-07	-0.5	11	1.1	Pou6f2	
DMR17:49516001	17	49516001	49517000	1000	1	2.40E-09	0.54	31	3.1	Pou6f2;Tgif2-ps1	
DMR17:49732001	17	49732001	49733000	1000	1	7.50E-08	-0.49	5	0.5	Rala	Signaling
DMR17:49985001	17	49985001	49986000	1000	1	5.50E-07	-0.53	4	0.4	Mplkip;Sugct	Transport
DMR17:50254001	17	50254001	50261000	7000	2	6.60E-07	-0.28	74	1.06	Sugct	Transport
DMR17:50307001	17	50307001	50312000	5000	1	1.80E-07	-0.48	30	0.6	Sugct	Transport
DMR17:50371001	17	50371001	50376000	5000	1	5.50E-07	-0.33	46	0.92	Sugct	Transport
DMR17:50395001	17	50395001	50399000	4000	1	4.30E-10	-0.36	35	0.88	Sugct	Transport
DMR17:50540001	17	50540001	50546000	6000	1	8.90E-08	-0.21	48	0.8	Sugct	Transport
DMR17:50563001	17	50563001	50564000	1000	1	1.80E-11	-0.47	6	0.6	Sugct	Transport
DMR17:50582001	17	50582001	50587000	5000	2	4.20E-08	-0.39	48	0.96	Sugct	Transport
DMR17:50614001	17	50614001	50619000	5000	3	8.70E-15	-0.47	45	0.9	Sugct	Transport
DMR17:50841001	17	50841001	50842000	1000	1	2.30E-07	0.47	3	0.3	Sugct	Transport
DMR17:50856001	17	50856001	50860000	4000	1	9.50E-07	0.43	40	1	Sugct	Transport
DMR17:51009001	17	51009001	51012000	3000	1	1.30E-07	-0.32	19	0.63	Sugct	Transport
DMR17:51040001	17	51040001	51041000	1000	1	2.70E-07	0.46	13	1.3	Sugct	Transport
DMR17:51918001	17	51918001	51919000	1000	1	1.30E-11	0.34	6	0.6	Inhba;LOC103694103	Growth Factors
DMR17:52317001	17	52317001	52319000	2000	1	8.40E-08	-0.37	11	0.55	LOC103694103;Gli3	Transcription
DMR17:53358001	17	53358001	53361000	3000	1	4.10E-07	-0.46	13	0.43	Hecw1	Proteolysis
DMR17:53382001	17	53382001	53383000	1000	1	3.30E-07	-0.45	2	0.2	Hecw1	Proteolysis
DMR17:53466001	17	53466001	53468000	2000	1	3.70E-08	-0.35	16	0.8	Hecw1;LOC108348567	Proteolysis

DMR17:53524001	17	53524001	53527000	3000	1	1.20E-07	-0.61	22	0.73	Hecw1	Proteolysis
DMR17:53818001	17	53818001	53823000	5000	1	1.10E-07	-0.37	49	0.98	Arid4b	Transcription
DMR17:53906001	17	53906001	53907000	1000	1	1.10E-07	-0.58	3	0.3	Arid4b	Transcription
DMR17:54319001	17	54319001	54325000	6000	1	5.20E-07	-0.4	44	0.73	Arhgap12	Signaling
DMR17:54332001	17	54332001	54334000	2000	1	5.50E-07	-0.29	14	0.7	Arhgap12	Signaling
DMR17:55665001	17	55665001	55666000	1000	1	1.50E-07	0.47	6	0.6	RGD1562037	
DMR17:56035001	17	56035001	56037000	2000	1	2.60E-08	-0.36	9	0.45	Mtpap	Metabolism
DMR17:56056001	17	56056001	56059000	3000	1	2.30E-09	-0.64	40	1.33	Mtpap	Metabolism
DMR17:57069001	17	57069001	57071000	2000	1	5.80E-07	0.42	13	0.65	Crem	
DMR17:57321001	17	57321001	57324000	3000	1	1.80E-08	-0.54	47	1.57	Epc1	Epigenetic
DMR17:57399001	17	57399001	57401000	2000	1	1.50E-10	0.37	12	0.6	Epc1	Epigenetic
DMR17:57958001	17	57958001	57959000	1000	1	5.60E-07	-0.51	8	0.8	RGD1564999;LOC102552003	
DMR17:58109001	17	58109001	58112000	3000	1	5.80E-07	0.48	38	1.27	Adarb2	Metabolism
DMR17:58118001	17	58118001	58119000	1000	1	4.00E-07	0.39	12	1.2	Adarb2	Metabolism
DMR17:60114001	17	60114001	60115000	1000	1	4.00E-09	0.47	14	1.4	Mpp7	Cytoskeleton
DMR17:60117001	17	60117001	60120000	3000	1	4.30E-09	-0.51	13	0.43	Mpp7	Cytoskeleton
DMR17:60165001	17	60165001	60166000	1000	1	3.80E-09	-0.41	3	0.3	Mpp7	Cytoskeleton
DMR17:60410001	17	60410001	60413000	3000	1	1.30E-11	-0.68	32	1.07	Armc4	
DMR17:62251001	17	62251001	62253000	2000	1	4.50E-07	-0.46	7	0.35	Fzd8	Receptor
DMR17:62361001	17	62361001	62364000	3000	2	1.70E-08	-0.46	17	0.57	Ccny	
DMR17:62410001	17	62410001	62411000	1000	1	7.40E-07	-0.38	9	0.9	Ccny	
DMR17:63269001	17	63269001	63271000	2000	1	1.40E-07	-0.38	19	0.95	Larp4b	Metabolism
DMR17:63452001	17	63452001	63453000	1000	1	7.70E-07	-0.39	6	0.6	Dip2c	
DMR17:64033001	17	64033001	64036000	3000	1	6.00E-08	-0.23	26	0.87	Chrm3	Signaling
DMR17:64283001	17	64283001	64284000	1000	1	7.40E-10	-0.46	5	0.5	Chrm3	Signaling
DMR17:64410001	17	64410001	64412000	2000	1	1.30E-08	-0.44	12	0.6	Chrm3	Signaling
DMR17:65882001	17	65882001	65884000	2000	1	1.90E-10	-0.43	18	0.9	Ryr2	Ion Channel
DMR17:66204001	17	66204001	66206000	2000	1	3.10E-08	-0.31	32	1.6	Mtr	
DMR17:66335001	17	66335001	66339000	4000	2	1.90E-07	-0.5	48	1.2	Actn2	
DMR17:69583001	17	69583001	69587000	4000	1	2.30E-07	-0.33	76	1.9	Akr1c12l1	Metabolism
DMR17:69668001	17	69668001	69671000	3000	1	6.80E-07	-0.46	8	0.27	Akr1c19	Metabolism
DMR17:69680001	17	69680001	69681000	1000	1	5.80E-07	0.39	4	0.4	Akr1c19	Metabolism
DMR17:69713001	17	69713001	69718000	5000	1	1.40E-08	-0.34	34	0.68	Akr1c13	Metabolism
DMR17:69758001	17	69758001	69759000	1000	1	1.60E-07	-0.5	15	1.5	Akr1c3	
DMR17:69847001	17	69847001	69851000	4000	2	8.50E-09	0.52	61	1.52	Akr1cl	
DMR17:69954001	17	69954001	69956000	2000	1	3.10E-08	0.37	21	1.05	Ucn3	
DMR17:69976001	17	69976001	69981000	5000	1	1.00E-07	-0.46	41	0.82	Ucn3;Tubal3	Cytoskeleton
DMR17:70211001	17	70211001	70213000	2000	1	2.70E-13	0.52	37	1.85	Rpl29-ps3;Asb13	
DMR17:70214001	17	70214001	70215000	1000	1	1.90E-08	0.38	2	0.2	Asb13	
DMR17:70219001	17	70219001	70222000	3000	1	2.60E-07	-0.3	23	0.77	Asb13	
DMR17:70231001	17	70231001	70232000	1000	1	1.50E-07	0.64	17	1.7	Asb13	
DMR17:70305001	17	70305001	70308000	3000	1	1.40E-07	-0.42	38	1.27	Fam208b;Gdi2	Signaling
DMR17:70460001	17	70460001	70462000	2000	1	3.20E-09	0.61	40	2	Il15ra	Receptor
DMR17:70490001	17	70490001	70494000	4000	1	1.50E-09	0.48	34	0.85	Il15ra;Il2ra	Receptor
DMR17:70579001	17	70579001	70582000	3000	1	2.00E-09	0.74	49	1.63	LOC102553333;Rbm17	Translation
DMR17:70994001	17	70994001	70995000	1000	1	3.30E-11	0.4	14	1.4	Prkcq	Signaling
DMR17:70996001	17	70996001	71002000	6000	1	3.90E-09	0.4	72	1.2	Prkcq	Signaling
DMR17:71003001	17	71003001	71005000	2000	2	6.80E-15	1.14	49	2.45	Prkcq	Signaling
DMR17:71026001	17	71026001	71027000	1000	1	1.30E-11	0.46	18	1.8	Prkcq	Signaling
DMR17:71074001	17	71074001	71075000	1000	1	2.50E-07	0.47	8	0.8	Prkcq	Signaling
DMR17:71174001	17	71174001	71179000	5000	2	1.50E-12	-0.58	47	0.94	RGD1564416	
DMR17:71707001	17	71707001	71713000	6000	1	1.30E-07	-0.36	55	0.92	Sfmbt2	Epigenetic
DMR17:71848001	17	71848001	71853000	5000	1	7.00E-07	-0.57	148	2.96	Sfmbt2	Epigenetic
DMR17:71862001	17	71862001	71865000	3000	1	9.20E-07	-0.49	41	1.37	Sfmbt2	Epigenetic
DMR17:72167001	17	72167001	72168000	1000	1	1.60E-14	0.4	13	1.3	Itih2	Protease; Proteolysis
DMR17:72181001	17	72181001	72185000	4000	1	1.90E-09	0.4	50	1.25	Itih2	Protease; Proteolysis
DMR17:72278001	17	72278001	72280000	2000	1	1.20E-08	-0.51	28	1.4	Taf3	
DMR17:72389001	17	72389001	72390000	1000	1	2.80E-10	-0.47	16	1.6	Taf3;LOC102546306	
DMR17:72434001	17	72434001	72441000	7000	1	3.30E-08	-0.5	90	1.29	Gata3	Transcription
DMR17:75458001	17	75458001	75463000	5000	3	1.30E-09	-0.4	43	0.86	Celf2	
DMR17:75582001	17	75582001	75583000	1000	1	2.30E-07	0.36	16	1.6	Celf2	
DMR17:75656001	17	75656001	75657000	1000	1	1.70E-08	-0.48	12	1.2	Celf2	
DMR17:75769001	17	75769001	75770000	1000	1	2.90E-09	0.32	8	0.8	Usp6nl	Signaling

DMR17:75822001	17	75822001	75825000	3000	1	2.30E-08	-0.5	48	1.6	Usp6nl	Signaling
DMR17:75829001	17	75829001	75830000	1000	1	2.00E-10	-0.53	8	0.8	Usp6nl	Signaling
DMR17:76295001	17	76295001	76298000	3000	1	1.20E-07	0.63	67	2.23	Upf2;LOC103694151;Dhtkd1	Metabolism;Metabolism
DMR17:76340001	17	76340001	76341000	1000	1	9.40E-07	0.29	15	1.5	Dhtkd1;LOC108353134	Metabolism
DMR17:76406001	17	76406001	76409000	3000	2	4.20E-09	-0.45	42	1.4	Nudt5;Cdc123	Signaling
DMR17:76429001	17	76429001	76431000	2000	1	3.30E-08	0.35	16	0.8	Cdc123	
DMR17:76542001	17	76542001	76544000	2000	1	4.60E-07	-0.48	27	1.35	Camk1d	Signaling
DMR17:76717001	17	76717001	76719000	2000	1	7.50E-07	0.24	14	0.7	Camk1d	Signaling
DMR17:76731001	17	76731001	76733000	2000	1	4.40E-11	0.41	14	0.7	Camk1d	Signaling
DMR17:76881001	17	76881001	76882000	1000	1	5.10E-09	0.4	3	0.3	Camk1d	Signaling
DMR17:76883001	17	76883001	76886000	3000	2	6.80E-11	0.68	51	1.7	Camk1d	Signaling
DMR17:76981001	17	76981001	76983000	2000	1	2.60E-10	0.48	24	1.2	Ccdc3	
DMR17:77028001	17	77028001	77030000	2000	1	1.50E-07	0.35	37	1.85	Ccdc3	
DMR17:77081001	17	77081001	77086000	5000	2	1.70E-07	-0.35	41	0.82	Ccdc3	
DMR17:77100001	17	77100001	77101000	1000	1	1.60E-07	0.37	15	1.5	Ccdc3	
DMR17:77283001	17	77283001	77285000	2000	1	3.30E-07	0.35	19	0.95	Phyh	Metabolism
DMR17:77514001	17	77514001	77518000	4000	1	4.90E-09	-0.35	49	1.23	Bend7	
DMR17:77633001	17	77633001	77637000	4000	1	2.00E-07	-0.5	79	1.98	Prpf18;LOC108348591;Frmd4a	Translation
DMR17:77667001	17	77667001	77669000	2000	1	2.10E-07	-0.43	43	2.15	Frmd4a	
DMR17:77762001	17	77762001	77763000	1000	1	3.20E-09	0.37	10	1	Frmd4a	
DMR17:77813001	17	77813001	77818000	5000	1	7.00E-07	0.36	74	1.48	Frmd4a	
DMR17:78604001	17	78604001	78605000	1000	1	1.20E-13	0.75	19	1.9	Fam107b;LOC108348592	
DMR17:78628001	17	78628001	78633000	5000	1	3.50E-07	-0.38	80	1.6	Fam107b	
DMR17:78643001	17	78643001	78646000	3000	1	1.30E-08	-0.44	56	1.87	Fam107b	
DMR17:78774001	17	78774001	78775000	1000	1	1.10E-08	0.6	9	0.9	Suv39h2;Dclre1c	Epigenetic;Transcription
DMR17:78828001	17	78828001	78830000	2000	1	7.50E-08	0.41	51	2.55	Meig1	
DMR17:78906001	17	78906001	78910000	4000	1	1.60E-07	0.39	60	1.5	Olah;Acbd7;Rpp38;Nmt2	Metabolism;Transport;Transport
DMR17:79036001	17	79036001	79039000	3000	1	7.60E-12	0.69	101	3.37	Fam171a1	
DMR17:79076001	17	79076001	79077000	1000	1	3.90E-07	-0.51	23	2.3	Fam171a1	
DMR17:79129001	17	79129001	79136000	7000	1	3.70E-07	-0.47	122	1.74	Fam171a1;LOC103694133	
DMR17:79146001	17	79146001	79148000	2000	1	4.70E-07	-0.4	43	2.15	Fam171a1	
DMR17:79342001	17	79342001	79346000	4000	1	3.70E-09	0.44	65	1.62	Itga8	Extracellular Matrix
DMR17:79361001	17	79361001	79363000	2000	1	3.10E-07	0.33	21	1.05	Itga8	Extracellular Matrix
DMR17:79374001	17	79374001	79377000	3000	1	1.90E-07	0.5	51	1.7	Itga8	Extracellular Matrix
DMR17:79485001	17	79485001	79486000	1000	1	3.90E-09	-0.6	4	0.4	Itga8	Extracellular Matrix
DMR17:79577001	17	79577001	79578000	1000	1	7.70E-07	-0.41	8	0.8	Itga8	Extracellular Matrix
DMR17:79617001	17	79617001	79618000	1000	1	4.20E-07	0.31	9	0.9	Itga8	Extracellular Matrix
DMR17:79667001	17	79667001	79668000	1000	1	1.10E-09	0.69	26	2.6	Itga8	Extracellular Matrix
DMR17:79745001	17	79745001	79747000	2000	1	3.50E-07	-0.53	17	0.85	Fam188a	
DMR17:79775001	17	79775001	79777000	2000	1	8.90E-07	0.39	32	1.6	Fam188a;LOC108353136	
DMR17:79792001	17	79792001	79794000	2000	1	6.40E-07	-0.38	27	1.35	Fam188a;LOC108353136	
DMR17:80437001	17	80437001	80439000	2000	1	6.70E-14	-0.73	27	1.35	Rsu1	Cytoskeleton
DMR17:80457001	17	80457001	80458000	1000	1	1.80E-08	0.39	13	1.3	Rsu1	Cytoskeleton
DMR17:80493001	17	80493001	80495000	2000	1	7.10E-07	0.38	11	0.55	Rsu1	Cytoskeleton
DMR17:80529001	17	80529001	80530000	1000	1	1.80E-07	-0.44	23	2.3	Rsu1	Cytoskeleton
DMR17:80545001	17	80545001	80549000	4000	1	6.30E-09	-0.46	109	2.72	Rsu1	Cytoskeleton
DMR17:80572001	17	80572001	80575000	3000	1	2.00E-07	0.62	63	2.1	Rsu1;Cubn	Cytoskeleton
DMR17:80631001	17	80631001	80635000	4000	1	3.20E-08	-0.44	47	1.18	Cubn	
DMR17:80737001	17	80737001	80738000	1000	1	3.40E-07	0.35	9	0.9	Cubn	
DMR17:80804001	17	80804001	80806000	2000	1	1.10E-11	0.89	44	2.2	Cubn;LOC102550536	
DMR17:80868001	17	80868001	80873000	5000	1	4.20E-07	0.33	49	0.98	Trdmt1;Vim	Translation
DMR17:80879001	17	80879001	80880000	1000	1	3.30E-07	-0.34	18	1.8	Vim	
DMR17:80887001	17	80887001	80892000	5000	1	3.70E-16	0.63	94	1.88	Vim	
DMR17:80959001	17	80959001	80961000	2000	1	1.40E-16	0.88	55	2.75	St8sia6	Transport
DMR17:80975001	17	80975001	80982000	7000	1	1.40E-07	-0.5	77	1.1	St8sia6	Transport
DMR17:81005001	17	81005001	81008000	3000	2	1.30E-10	-0.5	47	1.57	St8sia6	Transport
DMR17:81167001	17	81167001	81169000	2000	1	2.00E-08	0.37	31	1.55	Hacd1	
DMR17:81202001	17	81202001	81203000	1000	1	3.20E-16	0.78	21	2.1	Hacd1;Stam	Cytoskeleton
DMR17:81661001	17	81661001	81664000	3000	1	3.60E-07	0.34	42	1.4	Cacnb2	Transport
DMR17:81803001	17	81803001	81805000	2000	1	4.40E-07	-0.42	18	0.9	Cacnb2	Transport
DMR17:81843001	17	81843001	81846000	3000	1	5.00E-07	0.38	40	1.33	Cacnb2	Transport
DMR17:81916001	17	81916001	81918000	2000	1	6.70E-08	-0.57	15	0.75	Cacnb2	Transport

DMR17:81931001	17	81931001	81932000	1000	1	9.30E-07	0.43	10	1	Cacnb2	Transport
DMR17:82029001	17	82029001	82030000	1000	1	7.00E-07	-0.44	17	1.7	Cacnb2;Nsun6;LOC103694137	Transport
DMR17:82067001	17	82067001	82073000	6000	1	1.70E-08	-0.63	62	1.03	Nsun6;Arl5b	Signaling
DMR17:82421001	17	82421001	82422000	1000	1	4.90E-10	0.63	7	0.7	Malrd1	
DMR17:82571001	17	82571001	82575000	4000	1	5.60E-12	0.56	44	1.1	Malrd1	
DMR17:82582001	17	82582001	82583000	1000	1	4.10E-11	0.44	4	0.4	Malrd1	
DMR17:82591001	17	82591001	82592000	1000	1	2.30E-08	0.36	4	0.4	Malrd1	
DMR17:82757001	17	82757001	82763000	6000	1	8.70E-09	-0.4	62	1.03	Malrd1	
DMR17:83375001	17	83375001	83381000	6000	1	9.80E-11	-0.42	65	1.08	Plxdc2	
DMR17:83534001	17	83534001	83535000	1000	1	4.90E-08	0.27	28	2.8	Plxdc2	
DMR17:84924001	17	84924001	84926000	2000	1	4.90E-07	-0.33	25	1.25	Mllt10	Transcription
DMR17:84933001	17	84933001	84935000	2000	1	5.70E-07	-0.31	43	2.15	Mllt10	Transcription
DMR17:85019001	17	85019001	85020000	1000	1	2.30E-07	-0.4	4	0.4	Dnajc1	Transcription
DMR17:85098001	17	85098001	85100000	2000	1	6.00E-07	-0.47	10	0.5	Dnajc1	Transcription
DMR17:85101001	17	85101001	85104000	3000	1	4.00E-07	-0.55	25	0.83	Dnajc1	Transcription
DMR17:85116001	17	85116001	85120000	4000	1	2.90E-09	-0.62	44	1.1	Dnajc1	Transcription
DMR17:85146001	17	85146001	85148000	2000	1	1.20E-11	0.61	29	1.45	Dnajc1	Transcription
DMR17:85547001	17	85547001	85548000	1000	1	4.50E-08	-0.45	19	1.9	Pip4k2a	Signaling
DMR17:85554001	17	85554001	85555000	1000	1	3.00E-07	-0.47	25	2.5	Pip4k2a	Signaling
DMR17:85654001	17	85654001	85657000	3000	1	1.90E-07	-0.33	29	0.97	Pip4k2a	Signaling
DMR17:85713001	17	85713001	85715000	2000	1	1.10E-07	-0.61	37	1.85	Pip4k2a	Signaling
DMR17:85756001	17	85756001	85759000	3000	1	4.00E-16	0.85	62	2.07	Pip4k2a	Signaling
DMR17:85893001	17	85893001	85896000	3000	1	1.10E-09	-0.47	30	1	LOC102556022;Armc3;LOC100361451	
DMR17:85909001	17	85909001	85911000	2000	1	8.10E-08	0.49	56	2.8	Armc3;LOC100361451;LOC103694139	
DMR17:85949001	17	85949001	85952000	3000	1	5.30E-08	0.65	68	2.27	Armc3;LOC680565	
DMR17:86429001	17	86429001	86430000	1000	1	5.80E-09	-0.48	15	1.5	RGD1564650	
DMR17:87221001	17	87221001	87224000	3000	1	5.40E-13	-0.62	38	1.27	Etl4	
DMR17:87263001	17	87263001	87267000	4000	1	1.80E-07	-0.3	74	1.85	Etl4	
DMR17:87307001	17	87307001	87312000	5000	1	4.80E-07	0.33	59	1.18	Etl4	
DMR17:87319001	17	87319001	87321000	2000	1	2.40E-07	-0.43	13	0.65	Etl4	
DMR17:87358001	17	87358001	87361000	3000	1	7.40E-08	0.53	51	1.7	Etl4	
DMR17:87411001	17	87411001	87415000	4000	2	1.40E-07	-0.52	80	2	Etl4	
DMR17:87508001	17	87508001	87511000	3000	1	4.30E-07	0.26	40	1.33	Etl4	
DMR17:87676001	17	87676001	87680000	4000	1	1.10E-07	-0.63	80	2	Etl4	
DMR17:87686001	17	87686001	87687000	1000	1	1.20E-09	-0.79	22	2.2	Etl4	
DMR17:87723001	17	87723001	87729000	6000	1	5.70E-07	-0.33	135	2.25	Arhgap21	
DMR17:87762001	17	87762001	87764000	2000	2	1.20E-08	0.37	29	1.45	Arhgap21	
DMR17:87782001	17	87782001	87786000	4000	1	7.60E-09	-0.39	74	1.85	Arhgap21;LOC108348598	
DMR17:87799001	17	87799001	87800000	1000	1	5.80E-10	-0.49	21	2.1	Arhgap21	
DMR17:87943001	17	87943001	87945000	2000	1	9.60E-09	-0.39	62	3.1	Prtfdc1	Golgi
DMR17:87965001	17	87965001	87966000	1000	1	5.90E-13	0.33	12	1.2	Prtfdc1	Golgi
DMR17:88034001	17	88034001	88036000	2000	1	3.30E-08	0.51	41	2.05	Prtfdc1;LOC102550221	Golgi
DMR17:88377001	17	88377001	88380000	3000	1	3.30E-11	-0.35	24	0.8	Gpr158	Signaling
DMR17:88493001	17	88493001	88495000	2000	1	5.60E-07	-0.53	26	1.3	Gpr158	Signaling
DMR17:88522001	17	88522001	88523000	1000	1	6.10E-07	0.41	5	0.5	Gpr158	Signaling
DMR17:88943001	17	88943001	88944000	1000	1	5.70E-08	-0.49	11	1.1	Myo3a	
DMR17:89071001	17	89071001	89073000	2000	1	9.00E-07	0.34	14	0.7	Myo3a	
DMR17:89617001	17	89617001	89620000	3000	1	1.00E-09	-0.39	39	1.3	Potec	Transport
DMR17:89636001	17	89636001	89637000	1000	1	4.60E-08	-0.49	11	1.1	Potec	Transport
DMR17:89645001	17	89645001	89648000	3000	1	7.30E-08	-0.47	7	0.23	Potec	Transport
DMR17:89886001	17	89886001	89887000	1000	1	1.40E-08	0.61	27	2.7	LOC100910957;Acbd5	Transport
DMR17:90103001	17	90103001	90104000	1000	1	5.90E-10	-0.47	12	1.2	Apbb1ip	Cytoskeleton
DMR17:90144001	17	90144001	90146000	2000	1	1.30E-07	0.46	19	0.95	Apbb1ip;LOC685062	Cytoskeleton
DMR17:90379001	17	90379001	90382000	3000	1	5.00E-08	-0.45	21	0.7	Lyst	
DMR17:90478001	17	90478001	90483000	5000	2	3.30E-08	-0.35	54	1.08	Lyst	
DMR17:90484001	17	90484001	90486000	2000	2	1.10E-08	-0.34	16	0.8	Lyst	
DMR17:90774001	17	90774001	90777000	3000	1	6.60E-07	0.39	34	1.13	Ero1b	Metabolism
DMR18:25001	18	25001	29000	4000	2	1.40E-08	-0.46	16	0.4	Vom2r75	Signaling
DMR18:289001	18	289001	292000	3000	2	6.20E-10	0.81	92	3.07	Mtmr1	Signaling
DMR18:294001	18	294001	296000	2000	2	2.60E-10	0.85	84	4.2	Mtmr1	Signaling
DMR18:303001	18	303001	308000	5000	1	7.50E-10	-0.53	116	2.32	Mtmr1	Signaling

DMR18:364001	18	364001	365000	1000	1	5.60E-15	1.04	41	4.1	F8	Metabolism
DMR18:372001	18	372001	375000	3000	3	1.00E-23	1.19	145	4.83	F8	Metabolism
DMR18:377001	18	377001	379000	2000	1	1.50E-13	0.99	111	5.55	F8	Metabolism
DMR18:380001	18	380001	381000	1000	1	3.80E-07	0.46	12	1.2	F8	Metabolism
DMR18:382001	18	382001	385000	3000	1	5.10E-08	0.73	120	4	F8	Metabolism
DMR18:390001	18	390001	392000	2000	1	1.80E-08	0.35	81	4.05	F8;Fundc2	Metabolism
DMR18:394001	18	394001	397000	3000	1	1.70E-12	0.97	156	5.2	F8;Fundc2	Metabolism
DMR18:761001	18	761001	763000	2000	1	1.50E-09	0.41	23	1.15	Zfp136;LOC100360541	Transcription
DMR18:821001	18	821001	822000	1000	1	8.20E-07	0.46	18	1.8	LOC684627;RGD1561771;LOC364806	
DMR18:946001	18	946001	948000	2000	1	2.50E-07	-0.5	15	0.75	Colec12	
DMR18:1508001	18	1508001	1510000	2000	1	2.00E-07	0.34	9	0.45	Rpl36al	
DMR18:1578001	18	1578001	1579000	1000	1	4.80E-07	0.33	8	0.8	Greb1l	
DMR18:1634001	18	1634001	1637000	3000	2	6.60E-07	-0.43	9	0.3	Greb1l	
DMR18:1704001	18	1704001	1705000	1000	1	4.50E-07	-0.76	14	1.4	Greb1l	
DMR18:1736001	18	1736001	1739000	3000	1	9.50E-07	-0.57	30	1	Greb1l;LOC103694176	
DMR18:1764001	18	1764001	1767000	3000	1	2.40E-08	0.38	51	1.7	Greb1l;LOC684764	
DMR18:3417001	18	3417001	3419000	2000	1	7.30E-07	0.4	28	1.4	Cables1;Tmem241;LOC103694167	Transport
DMR18:3624001	18	3624001	3627000	3000	1	3.00E-08	-0.57	61	2.03	RGD1311805;Npc1	
DMR18:3634001	18	3634001	3639000	5000	1	5.50E-09	0.45	100	2	Npc1	
DMR18:3723001	18	3723001	3726000	3000	1	1.90E-10	0.44	44	1.47	Lama3	Extracellular Matrix
DMR18:3779001	18	3779001	3781000	2000	1	3.60E-10	0.47	26	1.3	Lama3	Extracellular Matrix
DMR18:3881001	18	3881001	3883000	2000	1	2.30E-07	-0.4	24	1.2	Lama3	Extracellular Matrix
DMR18:3971001	18	3971001	3973000	2000	1	1.80E-09	-0.72	33	1.65	Ttc39c	
DMR18:4031001	18	4031001	4039000	8000	1	4.10E-07	-0.41	132	1.65	Ttc39c	
DMR18:4047001	18	4047001	4048000	1000	1	5.10E-08	0.38	10	1	Ttc39c	
DMR18:4276001	18	4276001	4278000	2000	1	1.10E-07	0.34	30	1.5	Osbpl1a	
DMR18:4381001	18	4381001	4383000	2000	1	4.80E-08	0.33	39	1.95	Hrh4	Signaling
DMR18:4386001	18	4386001	4387000	1000	1	3.20E-10	0.6	48	4.8	Hrh4	Signaling
DMR18:5064001	18	5064001	5066000	2000	1	1.40E-07	0.49	32	1.6	Zfp521	Transcription
DMR18:5180001	18	5180001	5183000	3000	1	1.90E-09	-0.4	36	1.2	Zfp521	Transcription
DMR18:5273001	18	5273001	5276000	3000	1	2.30E-07	0.31	48	1.6	Zfp521	Transcription
DMR18:5323001	18	5323001	5325000	2000	2	1.20E-12	0.55	17	0.85	Zfp521	Transcription
DMR18:6015001	18	6015001	6018000	3000	1	3.80E-07	0.61	50	1.67	Ss18	Transcription
DMR18:6053001	18	6053001	6055000	2000	1	3.10E-10	-0.42	25	1.25	Ss18	Transcription
DMR18:6140001	18	6140001	6144000	4000	1	3.10E-08	-0.42	18	0.45	Psma8	Protease
DMR18:6367001	18	6367001	6370000	3000	1	5.20E-10	-0.65	36	1.2	Kctd1	Cytoskeleton
DMR18:6414001	18	6414001	6416000	2000	1	7.00E-07	-0.4	23	1.15	Kctd1	Cytoskeleton
DMR18:6819001	18	6819001	6827000	8000	1	6.60E-07	-0.32	101	1.26	LOC102551834;Chst9	Transport
DMR18:8191001	18	8191001	8192000	1000	1	4.90E-07	-0.51	15	1.5	Cdh2	Cytoskeleton
DMR18:8215001	18	8215001	8216000	1000	1	9.60E-08	-0.7	7	0.7	Cdh2	Cytoskeleton
DMR18:8225001	18	8225001	8228000	3000	1	1.60E-07	0.41	29	0.97	Cdh2	Cytoskeleton
DMR18:8265001	18	8265001	8269000	4000	2	3.20E-07	-0.48	53	1.32	Cdh2;LOC102552679	Cytoskeleton
DMR18:8300001	18	8300001	8303000	3000	1	1.20E-09	-0.4	41	1.37	Cdh2	Cytoskeleton
DMR18:8321001	18	8321001	8323000	2000	2	1.30E-09	0.41	15	0.75	Cdh2	Cytoskeleton
DMR18:8334001	18	8334001	8336000	2000	2	6.50E-09	-0.54	21	1.05	Cdh2	Cytoskeleton
DMR18:8375001	18	8375001	8377000	2000	1	9.50E-07	0.39	13	0.65	Cdh2	Cytoskeleton
DMR18:11789001	18	11789001	11790000	1000	1	1.40E-11	0.7	33	3.3	Dsc3	Cytoskeleton
DMR18:11866001	18	11866001	11868000	2000	1	4.00E-07	-0.37	15	0.75	Dsc2;Dsc1	Cytoskeleton
DMR18:12228001	18	12228001	12231000	3000	1	1.30E-08	0.35	46	1.53	RGD1562080	
DMR18:12278001	18	12278001	12283000	5000	1	1.50E-07	0.34	77	1.54	RGD1562080;LOC102554807	
DMR18:12286001	18	12286001	12288000	2000	1	4.00E-16	0.82	51	2.55	RGD1562080;LOC102554807	
DMR18:12293001	18	12293001	12294000	1000	1	8.70E-14	0.98	23	2.3	RGD1562080	
DMR18:12520001	18	12520001	12521000	1000	1	1.00E-08	0.4	9	0.9	Klhl14	
DMR18:12577001	18	12577001	12579000	2000	1	8.60E-08	-0.39	15	0.75	Klhl14	
DMR18:12580001	18	12580001	12583000	3000	1	2.20E-07	-0.27	43	1.43	Klhl14	
DMR18:12603001	18	12603001	12604000	1000	1	4.80E-08	0.65	33	3.3	Klhl14	
DMR18:12810001	18	12810001	12813000	3000	1	1.10E-07	-0.76	18	0.6	Ccdc178	
DMR18:12887001	18	12887001	12888000	1000	1	2.40E-07	0.32	13	1.3	Ccdc178	
DMR18:12900001	18	12900001	12906000	6000	1	2.30E-09	-0.31	50	0.83	Ccdc178	
DMR18:12942001	18	12942001	12944000	2000	1	1.10E-07	-0.47	32	1.6	Ccdc178	
DMR18:13019001	18	13019001	13020000	1000	1	1.70E-07	0.47	15	1.5	Ccdc178	
DMR18:13081001	18	13081001	13082000	1000	1	5.80E-13	0.62	17	1.7	Ccdc178	

DMR18:13131001	18	13131001	13134000	3000	1	2.40E-07	-0.54	7	0.23	Ccdc178	
DMR18:13146001	18	13146001	13150000	4000	1	1.10E-09	-0.44	21	0.52	Ccdc178	
DMR18:13198001	18	13198001	13200000	2000	1	1.70E-11	0.81	34	1.7	Ccdc178	
DMR18:13318001	18	13318001	13319000	1000	1	6.20E-07	0.29	13	1.3	Asxl3	
DMR18:13741001	18	13741001	13742000	1000	1	3.50E-07	-0.47	11	1.1	Nol4	
DMR18:13786001	18	13786001	13787000	1000	1	8.50E-07	-0.63	9	0.9	Nol4	
DMR18:13849001	18	13849001	13850000	1000	1	6.90E-09	0.68	11	1.1	Nol4	
DMR18:13890001	18	13890001	13891000	1000	1	3.10E-08	0.4	7	0.7	Nol4	
DMR18:13967001	18	13967001	13971000	4000	3	2.00E-10	-0.32	30	0.75	Nol4	
DMR18:14805001	18	14805001	14807000	2000	1	1.50E-08	-0.56	18	0.9	Mapre2	Cytoskeleton
DMR18:15023001	18	15023001	15024000	1000	1	4.40E-10	0.63	25	2.5	Mapre2	Cytoskeleton
DMR18:15081001	18	15081001	15084000	3000	1	4.40E-07	0.34	22	0.73	Mapre2;Mep1b	Cytoskeleton;Protease
DMR18:15242001	18	15242001	15244000	2000	1	1.50E-07	0.58	41	2.05	Mapre2;LOC291746	Cytoskeleton
DMR18:15245001	18	15245001	15246000	1000	1	1.20E-11	0.61	13	1.3	Mapre2;LOC291746	Cytoskeleton
DMR18:15376001	18	15376001	15377000	1000	1	7.20E-10	0.61	17	1.7	Mapre2;Trappc8	Cytoskeleton
DMR18:15404001	18	15404001	15407000	3000	1	3.70E-07	-0.44	46	1.53	Mapre2	Cytoskeleton
DMR18:15427001	18	15427001	15430000	3000	1	3.30E-07	-0.43	8	0.27	Mapre2;LOC100909691	Cytoskeleton
DMR18:15588001	18	15588001	15590000	2000	1	9.00E-07	-0.43	10	0.5	Mapre2;Dsg2	Cytoskeleton;Cytoskeleto n
DMR18:15591001	18	15591001	15592000	1000	1	9.60E-11	0.68	38	3.8	Mapre2;Dsg2	Cytoskeleton;Cytoskeleto n
DMR18:15596001	18	15596001	15598000	2000	1	4.30E-08	-0.47	57	2.85	Mapre2;Dsg2	Cytoskeleton;Cytoskeleto n
DMR18:15621001	18	15621001	15627000	6000	2	5.60E-07	-0.34	60	1	Mapre2;Dsg2	Cytoskeleton;Cytoskeleto n
DMR18:15687001	18	15687001	15692000	5000	2	5.30E-09	0.56	84	1.68	Mapre2;Dsg3;LOC108353181;LOC10 0362510	Cytoskeleton;Cytoskeleto n
DMR18:15755001	18	15755001	15756000	1000	1	4.70E-07	0.44	7	0.7	Mapre2	Cytoskeleton
DMR18:15785001	18	15785001	15787000	2000	1	6.80E-07	-0.57	24	1.2	Mapre2	Cytoskeleton
DMR18:15790001	18	15790001	15794000	4000	1	1.30E-08	-0.38	59	1.48	Mapre2	Cytoskeleton
DMR18:15912001	18	15912001	15914000	2000	1	3.40E-11	-0.53	16	0.8	LOC102552794;Zfp24	
DMR18:15917001	18	15917001	15920000	3000	1	2.30E-09	-0.54	52	1.73	LOC102552794;Zfp24	
DMR18:16133001	18	16133001	16137000	4000	2	6.10E-08	-0.34	39	0.98	Galnt1	Golgi
DMR18:16192001	18	16192001	16194000	2000	1	1.40E-07	-0.3	50	2.5	Galnt1;LOC498829	Golgi
DMR18:16388001	18	16388001	16390000	2000	1	2.90E-09	0.31	19	0.95	Mir187	
DMR18:16659001	18	16659001	16661000	2000	1	6.80E-09	-0.44	34	1.7	Fhod3	
DMR18:16674001	18	16674001	16675000	1000	1	1.20E-07	-0.45	28	2.8	Fhod3	
DMR18:16692001	18	16692001	16694000	2000	1	6.00E-07	-0.59	30	1.5	Fhod3	
DMR18:16716001	18	16716001	16718000	2000	1	6.20E-07	0.38	21	1.05	Fhod3	
DMR18:17033001	18	17033001	17035000	2000	2	9.20E-12	0.44	25	1.25	Fhod3	
DMR18:17244001	18	17244001	17245000	1000	1	4.50E-07	0.43	29	2.9	Fhod3	
DMR18:17335001	18	17335001	17336000	1000	1	2.80E-08	-0.51	18	1.8	Fhod3;Tpgs2	Cytoskeleton
DMR18:17371001	18	17371001	17372000	1000	1	7.60E-18	0.68	13	1.3	Tpgs2	Cytoskeleton
DMR18:17458001	18	17458001	17460000	2000	1	3.20E-07	-0.62	5	0.25	RGD1562608	
DMR18:17481001	18	17481001	17484000	3000	1	7.20E-08	0.36	30	1	RGD1562608	
DMR18:17638001	18	17638001	17641000	3000	1	3.30E-07	-0.52	16	0.53	RGD1562608	
DMR18:17761001	18	17761001	17763000	2000	1	2.80E-08	0.42	28	1.4	Celf4	
DMR18:17856001	18	17856001	17859000	3000	1	2.60E-08	0.36	24	0.8	Celf4;LOC108348757	
DMR18:17918001	18	17918001	17919000	1000	1	2.60E-10	0.7	27	2.7	Celf4;LOC102555477	
DMR18:17934001	18	17934001	17937000	3000	1	2.40E-08	0.47	54	1.8	Celf4;LOC291758	
DMR18:17967001	18	17967001	17969000	2000	1	7.50E-08	0.35	8	0.4	Celf4;LOC681320	
DMR18:18014001	18	18014001	18015000	1000	1	5.70E-10	-0.6	8	0.8	Celf4	
DMR18:19149001	18	19149001	19150000	1000	1	4.00E-09	0.69	28	2.8	Spata45	
DMR18:22995001	18	22995001	22997000	2000	1	3.90E-07	-0.28	32	1.6	Pik3c3	Signaling
DMR18:22999001	18	22999001	23002000	3000	1	2.50E-07	-0.46	89	2.97	Pik3c3	Signaling
DMR18:23794001	18	23794001	23797000	3000	1	8.30E-09	-0.4	21	0.7	Rit2	Signaling
DMR18:23805001	18	23805001	23807000	2000	1	6.40E-09	0.34	18	0.9	Rit2	Signaling
DMR18:24488001	18	24488001	24489000	1000	1	5.40E-07	0.44	4	0.4	Sap130	Epigenetic
DMR18:24506001	18	24506001	24512000	6000	2	1.10E-12	-0.45	46	0.77	Sap130	Epigenetic
DMR18:24695001	18	24695001	24697000	2000	1	1.70E-07	-0.37	23	1.15	Wdr33;Sft2d3	Translation
DMR18:24746001	18	24746001	24748000	2000	1	9.20E-09	0.49	29	1.45	Lims2;Gpr17;Myo7b	Cytoskeleton;Signaling;Cy toskeleton
DMR18:24764001	18	24764001	24765000	1000	1	7.50E-08	-0.48	17	1.7	Myo7b	Cytoskeleton

DMR18:24963001	18	24963001	24964000	1000	1	8.00E-08	-0.49	16	1.6	Map3k2	
DMR18:24984001	18	24984001	24985000	1000	1	3.20E-11	0.36	9	0.9	Map3k2	
DMR18:25188001	18	25188001	25189000	1000	1	8.60E-07	-0.61	8	0.8	Bin1	
DMR18:25221001	18	25221001	25222000	1000	1	6.70E-08	0.55	33	3.3	Bin1	
DMR18:25347001	18	25347001	25350000	3000	1	6.30E-08	-0.34	40	1.33	Gypc	
DMR18:25612001	18	25612001	25613000	1000	1	8.10E-07	0.42	15	1.5	Tslp	
DMR18:25882001	18	25882001	25883000	1000	1	3.60E-07	0.49	13	1.3	Camk4	Signaling
DMR18:25905001	18	25905001	25908000	3000	1	3.60E-08	-0.47	40	1.33	Camk4	Signaling
DMR18:25993001	18	25993001	25996000	3000	1	8.60E-09	-0.38	56	1.87	Stard4	
DMR18:26174001	18	26174001	26176000	2000	1	7.50E-08	0.41	33	1.65	Nrep	
DMR18:26179001	18	26179001	26184000	5000	4	8.80E-26	1.01	150	3	Nrep	
DMR18:26190001	18	26190001	26195000	5000	2	9.90E-09	0.39	88	1.76	Nrep	
DMR18:26569001	18	26569001	26570000	1000	1	2.70E-09	-0.6	23	2.3	Epb41l4a	
DMR18:26611001	18	26611001	26613000	2000	1	1.60E-08	-0.46	31	1.55	Epb41l4a	
DMR18:26656001	18	26656001	26664000	8000	1	5.40E-07	-0.41	148	1.85	Epb41l4a	
DMR18:26688001	18	26688001	26693000	5000	2	5.90E-10	-0.42	60	1.2	Epb41l4a	
DMR18:26713001	18	26713001	26716000	3000	1	7.00E-07	-0.35	48	1.6	Epb41l4a	
DMR18:26773001	18	26773001	26776000	3000	1	7.30E-11	-0.62	41	1.37	Epb41l4a	
DMR18:27070001	18	27070001	27073000	3000	1	2.10E-07	-0.52	32	1.07	Apc;LOC102549857	
DMR18:27102001	18	27102001	27106000	4000	1	2.50E-08	-0.41	90	2.25	Apc;Srp19	Transport
DMR18:27321001	18	27321001	27323000	2000	1	9.80E-07	0.44	20	1	Wnt8a	Signaling
DMR18:27341001	18	27341001	27343000	2000	1	5.90E-08	0.38	22	1.1	Wnt8a	Signaling
DMR18:27351001	18	27351001	27354000	3000	1	2.10E-11	0.36	30	1	Nme5	Signaling
DMR18:27489001	18	27489001	27491000	2000	1	1.20E-07	0.38	31	1.55	Gfra3	Receptor
DMR18:27498001	18	27498001	27500000	2000	1	4.70E-07	0.38	52	2.6	Gfra3	Receptor
DMR18:27509001	18	27509001	27511000	2000	1	3.60E-08	0.41	19	0.95	Gfra3	Receptor
DMR18:27958001	18	27958001	27959000	1000	1	3.10E-07	-0.58	9	0.9	Ctnna1	Cytoskeleton
DMR18:28125001	18	28125001	28130000	5000	1	2.20E-07	-0.34	47	0.94	Sil1	
DMR18:28142001	18	28142001	28144000	2000	1	3.00E-08	0.4	11	0.55	Sil1	
DMR18:28686001	18	28686001	28687000	1000	1	2.00E-07	0.5	16	1.6	RGD1564574	
DMR18:28834001	18	28834001	28835000	1000	1	2.00E-07	0.46	17	1.7	Psd2;Nrg2	Transcription;Growth Factors
DMR18:28922001	18	28922001	28923000	1000	1	2.60E-08	0.41	7	0.7	Nrg2;LOC103694189	Growth Factors
DMR18:28943001	18	28943001	28946000	3000	2	5.00E-10	-0.47	48	1.6	Nrg2	Growth Factors
DMR18:28952001	18	28952001	28954000	2000	1	4.40E-07	-0.56	28	1.4	Nrg2	Growth Factors
DMR18:28982001	18	28982001	28983000	1000	1	8.70E-10	0.47	10	1	Nrg2	Growth Factors
DMR18:29159001	18	29159001	29161000	2000	1	6.20E-07	-0.4	20	1	Cystm1	
DMR18:29513001	18	29513001	29515000	2000	1	1.70E-07	0.49	20	1	Apbb3;Slc35a4;RGD1565779	Transport
DMR18:29526001	18	29526001	29528000	2000	1	7.90E-07	-0.78	21	1.05	RGD1565779;LOC307495	
DMR18:30079001	18	30079001	30084000	5000	1	1.80E-08	-0.32	38	0.76	Pcdha4	Cytoskeleton
DMR18:30096001	18	30096001	30100000	4000	1	6.30E-11	-0.33	29	0.72	Pcdha4	Cytoskeleton
DMR18:30156001	18	30156001	30157000	1000	1	4.90E-07	0.61	3	0.3	Pcdha4	Cytoskeleton
DMR18:30379001	18	30379001	30380000	1000	1	5.10E-08	0.63	16	1.6	Pcdhb2;Pcdhb3;Pcdhb4	Cytoskeleton
DMR18:30494001	18	30494001	30495000	1000	1	1.00E-08	-0.79	4	0.4	Pcdhb10;Pcdhb11	Cytoskeleton
DMR18:30517001	18	30517001	30518000	1000	1	2.20E-07	0.49	30	3	LOC100360378;Pcdhb12;LOC108348233;Pcdhb14	Cytoskeleton
DMR18:30603001	18	30603001	30609000	6000	1	4.30E-09	-0.37	54	0.9	Pcdhb22	Cytoskeleton
DMR18:30844001	18	30844001	30845000	1000	1	1.90E-07	0.51	9	0.9	Pcdhga1;Pcdhga2;Pcdhga3;Pcdhga4;LOC108353166;Pcdhga5;LOC103694196	Cytoskeleton
DMR18:31067001	18	31067001	31070000	3000	1	4.80E-07	-0.41	27	0.9	Diaph1;Hdac3	
DMR18:31576001	18	31576001	31577000	1000	1	5.00E-08	-0.68	23	2.3	Ndfip1	
DMR18:31589001	18	31589001	31590000	1000	1	4.30E-07	-0.5	11	1.1	Ndfip1;LOC108348773	
DMR18:31796001	18	31796001	31798000	2000	1	3.10E-07	0.32	17	0.85	Nr3c1;LOC100911367;Arhgap26	Signaling
DMR18:31849001	18	31849001	31855000	6000	1	8.30E-07	-0.3	60	1	Nr3c1;Arhgap26	Signaling
DMR18:31895001	18	31895001	31897000	2000	1	2.30E-11	0.4	20	1	Nr3c1;Arhgap26	Signaling
DMR18:31960001	18	31960001	31963000	3000	1	1.60E-11	0.34	31	1.03	Nr3c1;Arhgap26	Signaling
DMR18:31975001	18	31975001	31978000	3000	1	2.10E-08	0.53	15	0.5	Nr3c1;Arhgap26	Signaling
DMR18:32106001	18	32106001	32108000	2000	1	2.00E-07	0.32	13	0.65	Nr3c1;Arhgap26	Signaling
DMR18:32178001	18	32178001	32180000	2000	1	6.40E-09	0.42	14	0.7	Nr3c1;Arhgap26	Signaling
DMR18:32238001	18	32238001	32243000	5000	1	6.10E-07	-0.26	43	0.86	Nr3c1;Arhgap26	Signaling
DMR18:32335001	18	32335001	32336000	1000	1	3.70E-07	-0.54	9	0.9	Nr3c1;Arhgap26;Fgf1	Signaling;Growth Factors

DMR18:32393001	18	32393001	32394000	1000	1	2.80E-16	0.93	23	2.3	Nr3c1;Arhgap26	Signaling
DMR18:32613001	18	32613001	32616000	3000	1	5.90E-07	0.63	49	1.63	Nr3c1;Spry4	Cytoskeleton
DMR18:32639001	18	32639001	32641000	2000	1	4.10E-07	-0.43	10	0.5	Nr3c1	
DMR18:32666001	18	32666001	32667000	1000	1	1.90E-07	-0.42	6	0.6	Nr3c1	
DMR18:33618001	18	33618001	33622000	4000	1	9.70E-09	-0.34	70	1.75	Kctd16	
DMR18:35790001	18	35790001	35794000	4000	1	1.90E-08	-0.4	36	0.9	Mcc	
DMR18:36216001	18	36216001	36217000	1000	1	7.40E-08	-0.43	4	0.4	Prelid2	
DMR18:36219001	18	36219001	36220000	1000	1	4.30E-07	0.45	9	0.9	Prelid2;LOC103694262	
DMR18:36388001	18	36388001	36390000	2000	1	4.60E-08	0.44	7	0.35	Sh3rf2;LOC108348774	
DMR18:36453001	18	36453001	36456000	3000	1	2.20E-07	0.39	55	1.83	Sh3rf2	
DMR18:36530001	18	36530001	36534000	4000	1	1.60E-07	-0.43	40	1	Lars	Translation
DMR18:36655001	18	36655001	36657000	2000	1	7.40E-11	-0.56	18	0.9	Rbm27	Metabolism
DMR18:36808001	18	36808001	36811000	3000	1	5.60E-08	-0.34	18	0.6	Tcerg1;LOC103694264	Transcription
DMR18:36812001	18	36812001	36813000	1000	1	2.20E-07	-0.26	9	0.9	Tcerg1	Transcription
DMR18:37033001	18	37033001	37036000	3000	1	1.70E-07	0.37	28	0.93	Ppp2r2b	Signaling
DMR18:37041001	18	37041001	37042000	1000	1	1.60E-15	-0.8	7	0.7	Ppp2r2b	Signaling
DMR18:37066001	18	37066001	37072000	6000	1	5.30E-12	-0.31	68	1.13	Ppp2r2b	Signaling
DMR18:37757001	18	37757001	37758000	1000	1	8.40E-07	-0.48	7	0.7	Dpysl3	Metabolism
DMR18:37759001	18	37759001	37760000	1000	1	4.40E-07	0.4	6	0.6	Dpysl3	Metabolism
DMR18:37791001	18	37791001	37792000	1000	1	1.60E-08	-0.53	10	1	Dpysl3;LOC108348778	Metabolism
DMR18:37880001	18	37880001	37885000	5000	2	1.90E-13	-0.39	40	0.8	Jakmip2	
DMR18:37988001	18	37988001	37992000	4000	1	1.50E-07	-0.34	41	1.02	Jakmip2	
DMR18:38038001	18	38038001	38040000	2000	1	2.70E-07	0.35	11	0.55	Jakmip2	
DMR18:38098001	18	38098001	38099000	1000	1	8.40E-07	0.54	13	1.3	Jakmip2	
DMR18:40046001	18	40046001	40048000	2000	1	4.30E-08	-0.48	16	0.8	Rps27a-ps13	
DMR18:40087001	18	40087001	40094000	7000	2	5.80E-08	-0.46	60	0.86	Trim36;LOC108348781	Proteolysis
DMR18:40119001	18	40119001	40120000	1000	1	2.20E-07	-0.53	16	1.6	Trim36	Proteolysis
DMR18:40249001	18	40249001	40253000	4000	1	6.60E-09	-0.32	38	0.95	Ccdc112	
DMR18:40712001	18	40712001	40714000	2000	1	8.60E-07	-0.33	20	1	Cdo1;LOC102547034;Atg12	Metabolism
DMR18:40876001	18	40876001	40881000	5000	3	2.60E-09	-0.77	105	2.1	LOC317165;Lvrn	Protease
DMR18:40972001	18	40972001	40975000	3000	1	4.70E-09	-0.51	23	0.77	Arl14epl	Signaling
DMR18:40986001	18	40986001	40987000	1000	1	3.00E-10	-0.48	11	1.1	Arl14epl	Signaling
DMR18:40999001	18	40999001	41003000	4000	2	4.10E-09	-0.33	32	0.8	Arl14epl	Signaling
DMR18:41072001	18	41072001	41077000	5000	2	1.50E-10	-0.5	38	0.76	Commd10	
DMR18:41116001	18	41116001	41123000	7000	1	4.00E-10	-0.41	80	1.14	Commd10	
DMR18:41129001	18	41129001	41130000	1000	1	2.30E-08	-0.39	13	1.3	Commd10	
DMR18:41332001	18	41332001	41335000	3000	3	6.00E-30	1.47	85	2.83	Sema6a	Signaling
DMR18:41932001	18	41932001	41934000	2000	1	2.00E-07	-0.36	19	0.95	LOC108348787;RGD1565083	
DMR18:41948001	18	41948001	41950000	2000	1	7.10E-07	-0.6	5	0.25	LOC108348787;RGD1565083	
DMR18:43893001	18	43893001	43899000	6000	1	5.80E-09	-0.28	63	1.05	Dtwd2;LOC103694209	
DMR18:43938001	18	43938001	43942000	4000	1	2.10E-07	-0.3	27	0.68	Dtwd2	
DMR18:43952001	18	43952001	43954000	2000	1	1.10E-07	-0.41	29	1.45	Dtwd2	
DMR18:44479001	18	44479001	44481000	2000	1	8.20E-08	0.47	16	0.8	Dmxl1	
DMR18:44508001	18	44508001	44515000	7000	2	1.30E-09	0.36	56	0.8	Dmxl1	
DMR18:44549001	18	44549001	44552000	3000	1	7.30E-13	-0.5	29	0.97	Dmxl1	
DMR18:44638001	18	44638001	44641000	3000	1	1.30E-08	-0.57	31	1.03	Dmxl1;LOC103694212	
DMR18:44777001	18	44777001	44781000	4000	1	2.50E-08	-0.51	66	1.65	Tnfaip8	
DMR18:44818001	18	44818001	44820000	2000	1	2.70E-07	-0.31	25	1.25	Hsd17b4	
DMR18:44860001	18	44860001	44865000	5000	1	1.20E-09	-0.42	25	0.5	Hsd17b4	
DMR18:45012001	18	45012001	45016000	4000	2	3.70E-08	-0.57	31	0.78	Fam170a	
DMR18:45024001	18	45024001	45025000	1000	1	1.60E-11	0.61	19	1.9	Fam170a	
DMR18:46037001	18	46037001	46041000	4000	1	4.80E-08	-0.37	43	1.07	Prr16	
DMR18:46065001	18	46065001	46067000	2000	2	1.10E-07	0.47	18	0.9	Prr16	
DMR18:46119001	18	46119001	46120000	1000	1	6.00E-07	0.44	3	0.3	Prr16	
DMR18:47301001	18	47301001	47304000	3000	1	1.20E-07	-0.4	22	0.73	Ftmt	Transport
DMR18:47449001	18	47449001	47451000	2000	1	2.00E-09	-0.41	6	0.3	Srfbp1	Metabolism
DMR18:47509001	18	47509001	47510000	1000	1	4.50E-08	0.42	2	0.2	Lox;LOC681584	Metabolism
DMR18:47552001	18	47552001	47554000	2000	1	2.80E-07	0.5	34	1.7	Lox	Metabolism
DMR18:47777001	18	47777001	47779000	2000	1	1.30E-11	0.47	20	1	Sncaip	
DMR18:48136001	18	48136001	48139000	3000	1	3.20E-10	-0.54	21	0.7	LOC100362233;Snx2	Cytoskeleton
DMR18:48168001	18	48168001	48169000	1000	1	7.10E-07	-0.46	9	0.9	Snx2	Cytoskeleton
DMR18:48193001	18	48193001	48194000	1000	1	5.50E-07	-0.51	10	1	Snx24	Cytoskeleton
DMR18:48206001	18	48206001	48207000	1000	1	2.10E-09	-0.56	12	1.2	Snx24	Cytoskeleton

DMR18:48216001	18	48216001	48218000	2000	2	3.90E-08	0.45	9	0.45	Snx24	Cytoskeleton
DMR18:48273001	18	48273001	48276000	3000	1	3.00E-08	-0.52	50	1.67	Snx24	Cytoskeleton
DMR18:48546001	18	48546001	48549000	3000	1	5.20E-08	-0.62	37	1.23	Prdm6	Transcription
DMR18:48694001	18	48694001	48695000	1000	1	4.90E-09	-0.47	11	1.1	Cep120	
DMR18:49914001	18	49914001	49919000	5000	1	1.20E-08	-0.44	86	1.72	Zfp608	
DMR18:51484001	18	51484001	51486000	2000	1	5.60E-08	-0.56	18	0.9	LOC100910311;Gramd3	
DMR18:51513001	18	51513001	51516000	3000	1	1.30E-07	-0.44	38	1.27	Gramd3	
DMR18:51568001	18	51568001	51571000	3000	1	7.90E-08	0.38	56	1.87	Gramd3	
DMR18:51664001	18	51664001	51668000	4000	1	1.10E-08	-0.41	72	1.8	Phax	Metabolism
DMR18:51685001	18	51685001	51688000	3000	1	2.40E-09	0.33	29	0.97	Phax;Tex43	Metabolism
DMR18:51828001	18	51828001	51830000	2000	1	6.60E-07	-0.33	39	1.95	Lmnb1	
DMR18:51849001	18	51849001	51855000	6000	1	6.60E-11	-0.28	63	1.05	3-Mar	
DMR18:51892001	18	51892001	51894000	2000	1	3.80E-09	0.42	24	1.2	3-Mar	
DMR18:51995001	18	51995001	51996000	1000	1	1.90E-07	0.56	16	1.6	March3;LOC102547345	
DMR18:52000001	18	52000001	52007000	7000	1	1.60E-09	-0.39	78	1.11	March3;LOC102552123	
DMR18:52314001	18	52314001	52319000	5000	1	7.90E-09	0.42	59	1.18	Megf10	Extracellular Matrix
DMR18:52440001	18	52440001	52442000	2000	1	6.00E-07	-0.4	31	1.55	Prrc1	
DMR18:52983001	18	52983001	52993000	10000	1	9.60E-07	-0.4	105	1.05	Slc12a2	Transport
DMR18:53095001	18	53095001	53097000	2000	1	2.80E-08	-0.46	36	1.8	Fbn2	Extracellular Matrix
DMR18:53944001	18	53944001	53947000	3000	1	6.90E-08	0.33	34	1.13	Adamts19	Protease
DMR18:53964001	18	53964001	53970000	6000	1	3.60E-07	-0.37	71	1.18	Adamts19	Protease
DMR18:53971001	18	53971001	53973000	2000	1	3.90E-13	0.39	18	0.9	Adamts19	Protease
DMR18:54067001	18	54067001	54072000	5000	1	5.40E-07	-0.54	57	1.14	Adamts19	Protease
DMR18:54233001	18	54233001	54238000	5000	1	3.00E-07	0.33	50	1	Chsy3l;Chsy3	Golgi
DMR18:54303001	18	54303001	54308000	5000	1	3.20E-07	-0.35	63	1.26	Chsy3	Golgi
DMR18:54322001	18	54322001	54323000	1000	1	8.00E-07	-0.3	13	1.3	Chsy3	Golgi
DMR18:55490001	18	55490001	55494000	4000	1	2.40E-11	-0.47	31	0.78	LOC103694227;RGD1309362	
DMR18:55579001	18	55579001	55580000	1000	1	9.20E-07	0.46	5	0.5	LOC102555905;MGC108823	
DMR18:55589001	18	55589001	55590000	1000	1	1.70E-08	0.5	3	0.3	LOC102555905;MGC108823	
DMR18:55637001	18	55637001	55642000	5000	1	2.50E-08	-0.34	47	0.94	RGD1305184	
DMR18:55677001	18	55677001	55683000	6000	3	1.50E-09	-0.4	60	1	RGD1305184;MGC105567	
DMR18:55697001	18	55697001	55703000	6000	1	2.70E-09	-0.43	57	0.95	MGC105567	
DMR18:55708001	18	55708001	55713000	5000	1	9.20E-09	-0.29	45	0.9	MGC105567;LOC102550835	
DMR18:55844001	18	55844001	55846000	2000	1	5.60E-07	0.5	23	1.15	Rbm22;Myoz3	Translation;Cytoskeleton
DMR18:55870001	18	55870001	55876000	6000	1	2.10E-11	0.83	141	2.35	Myoz3;Synpo	Cytoskeleton;Cytoskeleto n
DMR18:55927001	18	55927001	55929000	2000	2	2.30E-09	0.5	12	0.6	Synpo;LOC103694228	Cytoskeleton
DMR18:56057001	18	56057001	56060000	3000	1	5.70E-07	0.42	41	1.37	Rps14	Translation
DMR18:56119001	18	56119001	56120000	1000	1	1.20E-11	-0.61	16	1.6	Tcof1	Metabolism
DMR18:56225001	18	56225001	56227000	2000	1	1.10E-08	0.52	35	1.75	Camk2a	Signaling
DMR18:56278001	18	56278001	56279000	1000	1	1.80E-11	0.55	16	1.6	Camk2a	Signaling
DMR18:56317001	18	56317001	56318000	1000	1	4.80E-07	0.34	7	0.7	Slc6a7;LOC103690061	Transport
DMR18:56381001	18	56381001	56384000	3000	1	3.40E-12	0.44	67	2.23	Pdgfrb;LOC102555480	Receptor
DMR18:56548001	18	56548001	56551000	3000	1	2.10E-11	-0.37	24	0.8	Pde6a	Signaling
DMR18:57152001	18	57152001	57155000	3000	1	6.10E-07	-0.48	39	1.3	Ablim3	Cytoskeleton
DMR18:57189001	18	57189001	57190000	1000	1	3.10E-09	0.47	5	0.5	Ablim3	Cytoskeleton
DMR18:57330001	18	57330001	57331000	1000	1	1.50E-07	-0.45	12	1.2	Sh3tc2	
DMR18:57354001	18	57354001	57355000	1000	1	1.90E-11	0.52	8	0.8	Sh3tc2	
DMR18:57392001	18	57392001	57398000	6000	1	8.70E-09	-0.36	55	0.92	Sh3tc2	
DMR18:57525001	18	57525001	57528000	3000	1	1.90E-10	0.76	58	1.93	Adrb2;LOC102556293	Signaling
DMR18:57644001	18	57644001	57647000	3000	1	8.20E-10	0.45	43	1.43	Htr4;LOC108348797	Signaling
DMR18:57690001	18	57690001	57692000	2000	1	1.50E-14	0.39	2	0.1	Htr4	Signaling
DMR18:57700001	18	57700001	57701000	1000	1	6.00E-08	-0.48	10	1	Htr4	Signaling
DMR18:57715001	18	57715001	57718000	3000	1	6.20E-07	-0.52	32	1.07	Htr4	Signaling
DMR18:57818001	18	57818001	57822000	4000	1	2.00E-13	0.44	27	0.68	Htr4;Fbxo38	Signaling
DMR18:57834001	18	57834001	57838000	4000	2	2.50E-09	-0.51	58	1.45	Fbxo38	
DMR18:57923001	18	57923001	57924000	1000	1	4.10E-08	-0.39	8	0.8	Spink7	Protease; Proteolysis
DMR18:57953001	18	57953001	57958000	5000	1	1.20E-12	-0.34	40	0.8	Spink13;LOC100360147	Protease; Proteolysis
DMR18:58393001	18	58393001	58395000	2000	1	2.70E-08	0.36	11	0.55	Piezo2	
DMR18:58480001	18	58480001	58482000	2000	1	1.30E-08	0.43	23	1.15	Piezo2	
DMR18:58516001	18	58516001	58518000	2000	1	1.60E-08	0.47	16	0.8	Piezo2	
DMR18:58532001	18	58532001	58533000	1000	1	3.10E-07	0.32	9	0.9	Piezo2	
DMR18:58563001	18	58563001	58565000	2000	1	7.10E-08	-0.64	6	0.3	Piezo2	

DMR18:58628001	18	58628001	58632000	4000	1	1.20E-07	-0.4	36	0.9	Piezo2	
DMR18:59099001	18	59099001	59102000	3000	1	1.20E-09	-0.42	51	1.7	Wdr7	
DMR18:59216001	18	59216001	59220000	4000	1	2.40E-08	-0.49	52	1.3	Wdr7	
DMR18:59264001	18	59264001	59268000	4000	1	5.80E-07	-0.45	45	1.12	Wdr7	
DMR18:59292001	18	59292001	59295000	3000	1	4.40E-07	-0.43	20	0.67	Wdr7	
DMR18:59326001	18	59326001	59327000	1000	1	4.30E-08	-0.54	10	1	Wdr7	
DMR18:59373001	18	59373001	59376000	3000	1	8.90E-07	0.29	31	1.03	Wdr7	
DMR18:59392001	18	59392001	59394000	2000	1	6.10E-07	0.3	41	2.05	Wdr7	
DMR18:59962001	18	59962001	59964000	2000	1	7.20E-09	-0.45	44	2.2	Fech	Metabolism
DMR18:60027001	18	60027001	60030000	3000	1	4.60E-08	-0.3	39	1.3	Atp8b1	Transport
DMR18:60491001	18	60491001	60494000	3000	1	3.40E-07	0.36	25	0.83	Nedd4l	Proteolysis
DMR18:60571001	18	60571001	60573000	2000	1	1.10E-07	-0.41	42	2.1	Nedd4l	Proteolysis
DMR18:60600001	18	60600001	60603000	3000	1	3.10E-08	0.57	35	1.17	Nedd4l	Proteolysis
DMR18:60671001	18	60671001	60678000	7000	3	9.30E-12	-0.5	132	1.89	Nedd4l	Proteolysis
DMR18:60691001	18	60691001	60693000	2000	2	8.30E-10	0.47	19	0.95	Nedd4l	Proteolysis
DMR18:60706001	18	60706001	60707000	1000	1	8.80E-07	-0.32	21	2.1	Nedd4l	Proteolysis
DMR18:61072001	18	61072001	61074000	2000	1	2.00E-07	0.43	14	0.7	Alpk2	Signaling
DMR18:61312001	18	61312001	61314000	2000	1	4.50E-09	0.29	19	0.95	Zfp532	
DMR18:61323001	18	61323001	61326000	3000	1	4.40E-09	-0.43	80	2.67	Zfp532	
DMR18:61385001	18	61385001	61388000	3000	1	1.30E-09	-0.46	45	1.5	Oacyl	Metabolism
DMR18:61687001	18	61687001	61690000	3000	1	4.80E-08	-0.51	47	1.57	LOC108348804;Lman1	Transport
DMR18:61791001	18	61791001	61793000	2000	1	9.50E-09	-0.43	23	1.15	Ccbe1	Extracellular Matrix
DMR18:61839001	18	61839001	61840000	1000	1	1.10E-08	0.39	10	1	Ccbe1	Extracellular Matrix
DMR18:62282001	18	62282001	62287000	5000	1	1.20E-07	-0.29	64	1.28	LOC102553438;RGD1566303	
DMR18:62320001	18	62320001	62327000	7000	1	1.60E-08	-0.4	61	0.87	RGD1566303	
DMR18:63115001	18	63115001	63119000	4000	1	3.80E-08	0.33	41	1.02	Cidea	
DMR18:63196001	18	63196001	63198000	2000	1	2.20E-07	-0.59	32	1.6	LOC103694268;Prelid3a	
DMR18:63305001	18	63305001	63308000	3000	1	4.70E-10	-0.44	40	1.33	Spire1	Cytoskeleton
DMR18:63616001	18	63616001	63619000	3000	1	5.50E-07	-0.41	41	1.37	Seh1l	
DMR18:63662001	18	63662001	63664000	2000	1	3.00E-07	-0.5	21	1.05	Seh1l	
DMR18:63758001	18	63758001	63759000	1000	1	3.20E-07	-0.39	16	1.6	Ldlrad4	
DMR18:63762001	18	63762001	63763000	1000	1	4.70E-07	0.58	21	2.1	Ldlrad4	
DMR18:63772001	18	63772001	63774000	2000	1	4.20E-07	-0.39	36	1.8	Ldlrad4	
DMR18:63815001	18	63815001	63817000	2000	1	4.80E-08	-0.53	28	1.4	Ldlrad4;RGD1562758	
DMR18:63827001	18	63827001	63828000	1000	1	1.90E-08	-0.39	15	1.5	Ldlrad4;RGD1562758	
DMR18:63895001	18	63895001	63896000	1000	1	5.10E-07	-0.45	20	2	Ldlrad4	
DMR18:63903001	18	63903001	63905000	2000	1	2.70E-08	-0.48	18	0.9	Ldlrad4	
DMR18:63930001	18	63930001	63931000	1000	1	6.30E-07	-0.48	15	1.5	Ldlrad4	
DMR18:63944001	18	63944001	63945000	1000	1	7.70E-07	-0.46	11	1.1	Ldlrad4	
DMR18:64121001	18	64121001	64122000	1000	1	6.70E-07	0.34	11	1.1	Mc5r	Signaling
DMR18:65308001	18	65308001	65309000	1000	1	3.40E-07	-0.39	23	2.3	Tcf4	Transcription
DMR18:65359001	18	65359001	65360000	1000	1	1.30E-17	0.45	6	0.6	Tcf4	Transcription
DMR18:65395001	18	65395001	65397000	2000	1	7.70E-08	0.33	25	1.25	Tcf4	Transcription
DMR18:65498001	18	65498001	65500000	2000	1	2.00E-07	0.3	37	1.85	Tcf4	Transcription
DMR18:65863001	18	65863001	65866000	3000	1	8.30E-08	-0.42	14	0.47	Mbd2	
DMR18:65890001	18	65890001	65894000	4000	1	2.80E-07	0.35	52	1.3	Mbd2	
DMR18:66717001	18	66717001	66718000	1000	1	3.40E-09	-0.45	10	1	Dcc	
DMR18:66738001	18	66738001	66739000	1000	1	6.60E-13	0.64	14	1.4	Dcc	
DMR18:66854001	18	66854001	66856000	2000	1	1.20E-07	0.33	13	0.65	Dcc;LOC108348868	
DMR18:67029001	18	67029001	67031000	2000	1	4.00E-07	-0.35	29	1.45	Dcc	
DMR18:67082001	18	67082001	67083000	1000	1	8.90E-07	0.4	6	0.6	Dcc	
DMR18:67156001	18	67156001	67158000	2000	1	4.00E-07	0.41	15	0.75	Dcc	
DMR18:67458001	18	67458001	67460000	2000	1	5.60E-07	-0.22	19	0.95	Dcc	
DMR18:68399001	18	68399001	68401000	2000	1	2.40E-07	0.53	31	1.55	Ccdc68	
DMR18:68468001	18	68468001	68469000	1000	1	9.60E-07	-0.47	9	0.9	Ccdc68	
DMR18:68612001	18	68612001	68613000	1000	1	1.80E-08	-0.39	9	0.9	Rab27b	
DMR18:68998001	18	68998001	69001000	3000	1	9.50E-08	-0.48	28	0.93	Stard6	
DMR18:69632001	18	69632001	69634000	2000	1	3.10E-08	-0.45	36	1.8	Smad4	Transcription
DMR18:69663001	18	69663001	69665000	2000	1	1.90E-09	-0.5	24	1.2	Smad4	Transcription
DMR18:69698001	18	69698001	69700000	2000	1	3.30E-08	0.33	14	0.7	LOC102551667;Elac1	Signaling
DMR18:69772001	18	69772001	69773000	1000	1	4.00E-07	0.37	13	1.3	Me2	Metabolism
DMR18:69777001	18	69777001	69782000	5000	2	5.80E-11	-0.56	67	1.34	Me2	Metabolism
DMR18:69860001	18	69860001	69862000	2000	1	3.40E-10	0.76	44	2.2	Mro;LOC102551811	

DMR18:69932001	18	69932001	69937000	5000	1	2.00E-07	-0.47	117	2.34	Mapk4	Signaling
DMR18:70004001	18	70004001	70008000	4000	2	2.00E-12	-0.69	93	2.33	Mapk4	Signaling
DMR18:70049001	18	70049001	70054000	5000	1	4.50E-07	-0.58	56	1.12	Mapk4;LOC102551934	Signaling
DMR18:70249001	18	70249001	70250000	1000	1	1.20E-08	-0.36	13	1.3	LOC108348812;Mbd1	
DMR18:70317001	18	70317001	70318000	1000	1	2.80E-11	0.61	24	2.4	Cfap53;LOC108348813	
DMR18:70612001	18	70612001	70613000	1000	1	3.40E-07	-0.41	21	2.1	Myo5b	Cytoskeleton
DMR18:70638001	18	70638001	70645000	7000	1	1.00E-08	0.52	117	1.67	Myo5b	Cytoskeleton
DMR18:70722001	18	70722001	70726000	4000	1	6.70E-18	-0.54	55	1.38	Myo5b;Acaa2	Cytoskeleton;Metabolism
DMR18:71047001	18	71047001	71050000	3000	1	2.60E-09	-0.55	42	1.4	Dym	
DMR18:71207001	18	71207001	71213000	6000	1	4.90E-12	-0.47	62	1.03	Dym	
DMR18:71295001	18	71295001	71297000	2000	1	1.20E-12	0.57	26	1.3	Dym	
DMR18:71313001	18	71313001	71315000	2000	1	6.50E-10	0.48	30	1.5	Dym	
DMR18:71319001	18	71319001	71320000	1000	1	2.80E-07	0.41	11	1.1	Dym	
DMR18:71321001	18	71321001	71325000	4000	1	1.80E-10	0.59	80	2	Dym	
DMR18:71708001	18	71708001	71709000	1000	1	2.50E-07	0.46	21	2.1	Ctif	Metabolism
DMR18:72155001	18	72155001	72157000	2000	1	9.50E-08	-0.39	48	2.4	Zbtb7c;LOC102546313	
DMR18:72204001	18	72204001	72207000	3000	3	1.80E-08	0.44	42	1.4	Zbtb7c	
DMR18:72225001	18	72225001	72226000	1000	1	6.70E-07	0.28	11	1.1	Zbtb7c	
DMR18:72286001	18	72286001	72287000	1000	1	1.60E-07	-0.4	23	2.3	Zbtb7c	
DMR18:72388001	18	72388001	72390000	2000	1	3.20E-18	0.58	19	0.95	Zbtb7c	
DMR18:72414001	18	72414001	72416000	2000	1	1.20E-10	0.46	32	1.6	Zbtb7c	
DMR18:72418001	18	72418001	72419000	1000	1	9.80E-07	-0.43	3	0.3	Zbtb7c	
DMR18:72428001	18	72428001	72430000	2000	1	2.70E-11	-0.54	44	2.2	Zbtb7c	
DMR18:72440001	18	72440001	72443000	3000	1	1.10E-07	0.3	42	1.4	Zbtb7c	
DMR18:72453001	18	72453001	72456000	3000	1	1.30E-07	0.41	56	1.87	Zbtb7c	
DMR18:72560001	18	72560001	72565000	5000	1	9.60E-07	-0.36	57	1.14	Smad2;LOC102546474	Transcription
DMR18:73165001	18	73165001	73168000	3000	1	3.30E-07	-0.44	53	1.77	Skor2	
DMR18:73248001	18	73248001	73254000	6000	1	1.20E-07	-0.35	115	1.92	Hdhd2	Signaling
DMR18:73302001	18	73302001	73305000	3000	1	1.10E-07	0.34	38	1.27	Katnal2	Cytoskeleton
DMR18:73624001	18	73624001	73625000	1000	1	2.40E-10	0.68	43	4.3	St8sia5	Transport
DMR18:73686001	18	73686001	73689000	3000	1	9.20E-07	-0.41	38	1.27	Loxhd1	
DMR18:73803001	18	73803001	73808000	5000	2	2.40E-11	-0.62	66	1.32	Loxhd1	
DMR18:73817001	18	73817001	73819000	2000	1	1.80E-07	0.34	30	1.5	Loxhd1	
DMR18:73847001	18	73847001	73848000	1000	1	8.70E-07	-0.52	15	1.5	Rnf165	Proteolysis
DMR18:74160001	18	74160001	74165000	5000	1	1.70E-11	-0.49	121	2.42	Haus1;Atp5a1;Pstpip2	Cytoskeleton
DMR18:74343001	18	74343001	74345000	2000	1	6.50E-13	-0.45	16	0.8	Epg5	
DMR18:74363001	18	74363001	74367000	4000	1	3.90E-11	-0.43	79	1.98	Epg5	
DMR18:74387001	18	74387001	74389000	2000	1	9.00E-08	-0.37	21	1.05	Epg5	
DMR18:74418001	18	74418001	74419000	1000	1	2.80E-07	0.35	5	0.5	Siglec15	
DMR18:74466001	18	74466001	74469000	3000	1	7.40E-08	-0.49	35	1.17	Slc14a1	Transport
DMR18:74492001	18	74492001	74493000	1000	1	1.30E-10	0.51	21	2.1	Slc14a1;LOC102553274	Transport
DMR18:74540001	18	74540001	74542000	2000	1	2.60E-07	0.34	34	1.7	Slc14a2	Transport
DMR18:74614001	18	74614001	74615000	1000	1	2.00E-08	0.43	13	1.3	Slc14a2	Transport
DMR18:75263001	18	75263001	75264000	1000	1	3.10E-07	-0.4	9	0.9	Setbp1	Epigenetic
DMR18:75317001	18	75317001	75319000	2000	1	3.20E-07	0.39	20	1	Setbp1	Epigenetic
DMR18:75360001	18	75360001	75363000	3000	1	8.40E-08	0.4	14	0.47	Setbp1	Epigenetic
DMR18:76545001	18	76545001	76555000	10000	2	7.30E-09	-0.32	130	1.3	LOC100911388;Pard6g	Cell Junction
DMR18:76572001	18	76572001	76574000	2000	1	8.50E-07	-0.5	41	2.05	Pard6g	Cell Junction
DMR18:76700001	18	76700001	76701000	1000	1	6.30E-07	0.57	30	3	LOC108348825;LOC108348824;LOC108348740;Rbfa	
DMR18:76735001	18	76735001	76743000	8000	1	8.30E-07	-0.53	134	1.68	Txnl4a;RGD1560212;Hsbp1l1	Metabolism
DMR18:76755001	18	76755001	76758000	3000	1	8.30E-07	0.51	31	1.03	Hsbp1l1	
DMR18:76759001	18	76759001	76760000	1000	1	7.20E-07	0.48	3	0.3	Hsbp1l1;Pqlc1	
DMR18:76774001	18	76774001	76777000	3000	1	8.10E-07	0.39	74	2.47	Pqlc1	
DMR18:76783001	18	76783001	76788000	5000	1	2.70E-12	-0.52	86	1.72	Pqlc1	
DMR18:76797001	18	76797001	76801000	4000	1	4.50E-08	0.34	97	2.42	Pqlc1;Kcng2	Transport
DMR18:76850001	18	76850001	76851000	1000	1	1.60E-09	0.52	33	3.3	Kcng2	Transport
DMR18:77199001	18	77199001	77204000	5000	1	1.60E-11	0.37	107	2.14	Nfatc1	Transcription
DMR18:77333001	18	77333001	77336000	3000	1	2.30E-16	0.63	51	1.7	Atp9b	Transport
DMR18:77350001	18	77350001	77351000	1000	1	5.60E-07	0.56	8	0.8	Atp9b	Transport
DMR18:77368001	18	77368001	77369000	1000	1	5.20E-07	0.38	19	1.9	Atp9b	Transport
DMR18:77583001	18	77583001	77584000	1000	1	9.20E-10	-0.56	17	1.7	Sall3;LOC108348827	Transcription

DMR18:79241001	18	79241001	79244000	3000	1	1.60E-08	0.32	51	1.7	Galr1	Signaling
DMR18:79379001	18	79379001	79380000	1000	1	3.30E-07	0.39	20	2	Mbp	Cytoskeleton
DMR18:79422001	18	79422001	79424000	2000	1	3.40E-24	0.93	59	2.95	Mbp;LOC108348833	Cytoskeleton
DMR18:79438001	18	79438001	79441000	3000	3	1.80E-10	0.56	29	0.97	Mbp;Zfp236	Cytoskeleton;Transcription
DMR18:79443001	18	79443001	79447000	4000	1	6.30E-10	0.34	39	0.98	Mbp;Zfp236	Cytoskeleton;Transcription
DMR18:79493001	18	79493001	79496000	3000	1	7.70E-07	-0.34	41	1.37	Zfp236	Transcription
DMR18:79514001	18	79514001	79517000	3000	2	2.60E-11	0.72	15	0.5	Zfp236	Transcription
DMR18:79535001	18	79535001	79536000	1000	1	4.40E-08	0.43	13	1.3	Zfp236	Transcription
DMR18:79757001	18	79757001	79760000	3000	1	2.00E-07	0.75	87	2.9	LOC102552572;Zfp516	Transcription
DMR18:79781001	18	79781001	79786000	5000	1	3.70E-07	-0.45	116	2.32	Zfp516	Transcription
DMR18:79808001	18	79808001	79810000	2000	2	2.20E-11	0.52	15	0.75	Zfp516	Transcription
DMR18:80876001	18	80876001	80877000	1000	1	1.50E-07	0.39	10	1	Tshz1	Transcription
DMR18:80885001	18	80885001	80886000	1000	1	2.60E-07	-0.46	23	2.3	Tshz1	Transcription
DMR18:81134001	18	81134001	81138000	4000	2	2.80E-14	-0.61	52	1.3	Zfp407	Transcription
DMR18:81139001	18	81139001	81142000	3000	1	5.00E-08	-0.38	33	1.1	Zfp407	Transcription
DMR18:81338001	18	81338001	81343000	5000	1	2.40E-15	0.56	82	1.64	Zfp407	Transcription
DMR18:81359001	18	81359001	81362000	3000	1	3.10E-07	0.37	30	1	Zfp407	Transcription
DMR18:81545001	18	81545001	81546000	1000	1	2.20E-07	0.57	14	1.4	Cndp2	Protease
DMR18:81573001	18	81573001	81574000	1000	1	2.30E-07	0.37	14	1.4	Fam69c	
DMR18:81844001	18	81844001	81847000	3000	2	4.70E-07	-0.28	28	0.93	Fbxo15	
DMR18:81857001	18	81857001	81864000	7000	1	4.40E-09	-0.29	69	0.99	Fbxo15	
DMR18:83468001	18	83468001	83469000	1000	1	6.60E-08	0.47	2	0.2	Neto1	Protease
DMR18:83487001	18	83487001	83489000	2000	1	1.80E-08	0.35	16	0.8	Neto1	Protease
DMR18:86091001	18	86091001	86097000	6000	1	5.80E-08	-0.31	66	1.1	Rttn	
DMR18:86355001	18	86355001	86361000	6000	2	7.80E-08	-0.36	52	0.87	Cd226;LOC102546621	
DMR18:86431001	18	86431001	86434000	3000	1	1.20E-10	-0.45	15	0.5	Dok6	
DMR18:86781001	18	86781001	86783000	2000	1	5.10E-07	-0.41	15	0.75	Dok6	
DMR18:86795001	18	86795001	86796000	1000	1	2.30E-07	0.47	9	0.9	Dok6	
DMR19:73001	19	73001	81000	8000	2	8.60E-09	-0.31	70	0.88	Ces2a;Ces2e	Metabolism
DMR19:105001	19	105001	106000	1000	1	5.10E-10	-0.44	5	0.5	Ces2e	Metabolism
DMR19:681001	19	681001	682000	1000	1	2.50E-09	0.99	36	3.6	Nae1;Terb1	Proteolysis
DMR19:868001	19	868001	870000	2000	1	7.20E-09	0.63	40	2	Cmtm1;LOC102554842	Transport
DMR19:949001	19	949001	950000	1000	1	8.30E-11	0.61	12	1.2	Tk2;LOC108348885;LOC108348886;Bean1	Signaling
DMR19:1077001	19	1077001	1081000	4000	2	1.50E-10	0.54	75	1.88	Cdh5	Cytoskeleton
DMR19:2493001	19	2493001	2494000	1000	1	5.10E-12	0.74	8	0.8	Cdh11	Cytoskeleton
DMR19:5203001	19	5203001	5204000	1000	1	6.80E-07	0.37	8	0.8	RGD1563412	
DMR19:6023001	19	6023001	6024000	1000	1	8.60E-07	-0.47	14	1.4	LOC102547740;Cdh8	Cytoskeleton
DMR19:6081001	19	6081001	6083000	2000	1	4.60E-07	-0.44	16	0.8	Cdh8;LOC102547668	Cytoskeleton
DMR19:6105001	19	6105001	6106000	1000	1	3.50E-08	0.4	5	0.5	Cdh8;LOC102547668	Cytoskeleton
DMR19:6118001	19	6118001	6119000	1000	1	4.90E-07	-0.35	12	1.2	Cdh8	Cytoskeleton
DMR19:6236001	19	6236001	6237000	1000	1	6.90E-08	0.44	4	0.4	Cdh8	Cytoskeleton
DMR19:6287001	19	6287001	6288000	1000	1	6.30E-07	0.42	11	1.1	Cdh8	Cytoskeleton
DMR19:9442001	19	9442001	9444000	2000	1	6.30E-07	0.45	21	1.05	Rpl26-ps3	
DMR19:9604001	19	9604001	9608000	4000	2	2.00E-11	-0.45	81	2.02	Got2	Metabolism
DMR19:9672001	19	9672001	9674000	2000	1	8.50E-07	-0.5	23	1.15	Cnot1	Translation
DMR19:9747001	19	9747001	9749000	2000	1	2.00E-09	-0.5	20	1	Cnot1;Setd6	Translation;Epigenetic
DMR19:9812001	19	9812001	9815000	3000	1	2.30E-07	-0.35	56	1.87	Ndrg4;LOC688942	Protease
DMR19:9825001	19	9825001	9827000	2000	1	3.20E-07	-0.36	24	1.2	Gins3	
DMR19:9903001	19	9903001	9905000	2000	1	1.10E-07	0.33	24	1.2	Prss54;Ccdc113	Protease
DMR19:9924001	19	9924001	9928000	4000	1	1.70E-11	-0.6	96	2.4	Ccdc113	
DMR19:9998001	19	9998001	9999000	1000	1	7.30E-07	-0.55	15	1.5	Csnk2a2	
DMR19:10003001	19	10003001	10005000	2000	1	1.30E-08	-0.7	25	1.25	Csnk2a2	
DMR19:10076001	19	10076001	10080000	4000	1	5.90E-07	0.6	81	2.02	Mmp15	Protease
DMR19:10153001	19	10153001	10154000	1000	1	5.40E-08	0.42	15	1.5	Cngb1	Ion Channel
DMR19:10169001	19	10169001	10172000	3000	1	2.30E-09	0.46	29	0.97	Cngb1	Ion Channel
DMR19:10197001	19	10197001	10199000	2000	1	4.10E-07	-0.45	35	1.75	Cngb1	Ion Channel
DMR19:10339001	19	10339001	10340000	1000	1	1.20E-07	0.62	12	1.2	Kifc3;Katnb1	Cytoskeleton
DMR19:10394001	19	10394001	10395000	1000	1	3.60E-08	0.44	12	1.2	Adgrg3	Signaling
DMR19:10494001	19	10494001	10496000	2000	1	5.60E-07	0.34	27	1.35	Adgrg5	Signaling
DMR19:10517001	19	10517001	10518000	1000	1	6.20E-10	0.41	19	1.9	Adgrg5;Ccdc102a	Signaling

DMR19:10744001	19	10744001	10745000	1000	1	9.80E-07	0.59	20	2	Pllp;Arl2bp	Transport
DMR19:10746001	19	10746001	10749000	3000	1	5.30E-08	0.51	35	1.17	Pllp;Arl2bp	Transport
DMR19:10852001	19	10852001	10854000	2000	1	9.10E-07	0.45	18	0.9	Fam192a;Cpne2	
DMR19:10878001	19	10878001	10882000	4000	1	8.30E-07	0.4	73	1.82	Cpne2	
DMR19:10962001	19	10962001	10965000	3000	1	5.10E-07	0.38	43	1.43	Nlrc5	Cytoskeleton
DMR19:10986001	19	10986001	10988000	2000	1	7.90E-08	-0.41	26	1.3	Nlrc5;LOC108348891	Cytoskeleton
DMR19:11047001	19	11047001	11049000	2000	1	1.50E-09	-0.5	54	2.7	Herpud1	
DMR19:11189001	19	11189001	11194000	5000	1	3.90E-07	-0.47	71	1.42	Nup93	Transport
DMR19:11406001	19	11406001	11407000	1000	1	8.80E-07	-0.36	26	2.6	Bbs2	
DMR19:11411001	19	11411001	11413000	2000	1	4.80E-13	-0.54	35	1.75	Bbs2	
DMR19:11486001	19	11486001	11490000	4000	1	5.90E-07	-0.57	84	2.1	Amfr	Proteolysis
DMR19:11640001	19	11640001	11641000	1000	1	9.80E-08	0.38	19	1.9	Gnao1	Signaling
DMR19:11650001	19	11650001	11651000	1000	1	2.70E-08	0.38	2	0.2	Gnao1	Signaling
DMR19:12571001	19	12571001	12575000	4000	1	6.80E-08	-0.53	31	0.78	Large1	Golgi
DMR19:12654001	19	12654001	12657000	3000	1	3.30E-11	-0.53	35	1.17	Large1	Golgi
DMR19:12807001	19	12807001	12813000	6000	1	1.90E-07	0.37	64	1.07	Large1	Golgi
DMR19:12832001	19	12832001	12835000	3000	1	1.00E-08	-0.53	59	1.97	Large1;LOC108348896	Golgi
DMR19:12842001	19	12842001	12846000	4000	1	1.00E-06	0.45	12	0.3	Large1;LOC108348896	Golgi
DMR19:12858001	19	12858001	12863000	5000	1	5.30E-08	-0.39	48	0.96	Large1;LOC108348896	Golgi
DMR19:12871001	19	12871001	12878000	7000	1	9.40E-07	-0.36	111	1.59	Large1;LOC102554714	Golgi
DMR19:14357001	19	14357001	14359000	2000	1	2.10E-08	-0.6	34	1.7	Hmgxb4	
DMR19:14541001	19	14541001	14542000	1000	1	1.70E-07	0.35	16	1.6	Mcm5	Transcription
DMR19:15099001	19	15099001	15106000	7000	1	4.60E-07	-0.33	83	1.19	Ces1f	Metabolism
DMR19:15225001	19	15225001	15227000	2000	1	7.50E-13	-0.51	18	0.9	Ces1d	Metabolism
DMR19:15328001	19	15328001	15333000	5000	1	3.50E-07	-0.33	34	0.68	Ces1c;Ces1a	Metabolism
DMR19:15464001	19	15464001	15466000	2000	2	1.20E-07	-0.47	33	1.65	Lpcat2	Metabolism
DMR19:15544001	19	15544001	15546000	2000	1	4.60E-07	0.4	22	1.1	Lpcat2;Mmp2	Metabolism;Protease
DMR19:15736001	19	15736001	15738000	2000	1	1.00E-09	-0.53	47	2.35	Irx6	Development
DMR19:16357001	19	16357001	16359000	2000	1	1.40E-07	0.51	44	2.2	LOC103694293;Trnas-aga	
DMR19:16404001	19	16404001	16405000	1000	1	3.00E-08	0.44	24	2.4	Crnde	
DMR19:16775001	19	16775001	16779000	4000	1	6.10E-07	0.3	73	1.82	Fto	Metabolism
DMR19:16885001	19	16885001	16886000	1000	1	4.40E-11	-0.63	20	2	Fto	Metabolism
DMR19:16910001	19	16910001	16913000	3000	1	7.80E-08	0.36	63	2.1	Fto;LOC102546731	Metabolism
DMR19:16976001	19	16976001	16978000	2000	1	2.10E-10	-0.52	39	1.95	Fto	Metabolism
DMR19:17003001	19	17003001	17004000	1000	1	9.90E-09	0.48	23	2.3	Fto	Metabolism
DMR19:17010001	19	17010001	17012000	2000	1	3.50E-08	-0.48	28	1.4	Fto;LOC102546355	Metabolism
DMR19:17215001	19	17215001	17220000	5000	1	8.50E-08	-0.46	39	0.78	Rpgrip1l	Cytoskeleton
DMR19:17441001	19	17441001	17445000	4000	1	1.60E-09	0.49	39	0.98	Chd9	
DMR19:18110001	19	18110001	18111000	1000	1	1.80E-09	-0.5	11	1.1	Tox3	
DMR19:18156001	19	18156001	18157000	1000	1	1.40E-08	0.34	8	0.8	Tox3	
DMR19:19392001	19	19392001	19396000	4000	3	1.70E-08	0.55	62	1.55	Nod2;Snx20	Cytoskeleton
DMR19:19472001	19	19472001	19474000	2000	1	8.40E-08	0.64	19	0.95	Nkd1	
DMR19:19490001	19	19490001	19493000	3000	1	8.40E-09	0.39	41	1.37	Nkd1	
DMR19:19720001	19	19720001	19722000	2000	1	5.50E-11	0.51	24	1.2	Adcy7	
DMR19:19758001	19	19758001	19759000	1000	1	1.60E-08	0.37	5	0.5	Adcy7	
DMR19:19760001	19	19760001	19761000	1000	1	1.30E-08	-0.45	13	1.3	Adcy7	
DMR19:19811001	19	19811001	19812000	1000	1	2.50E-10	-0.49	12	1.2	Papd5	
DMR19:19905001	19	19905001	19906000	1000	1	4.30E-07	-0.44	9	0.9	Heatr3;Cnep1r1;LOC108348906	
DMR19:20249001	19	20249001	20251000	2000	1	2.70E-09	0.46	39	1.95	Zfp423	
DMR19:21232001	19	21232001	21234000	2000	1	1.40E-09	-0.51	27	1.35	N4bp1	Translation
DMR19:21241001	19	21241001	21244000	3000	1	7.70E-07	-0.42	17	0.57	N4bp1	Translation
DMR19:21558001	19	21558001	21559000	1000	1	2.80E-09	0.39	6	0.6	Abcc12	Transport
DMR19:21584001	19	21584001	21587000	3000	1	2.50E-07	0.37	25	0.83	Abcc12	Transport
DMR19:21596001	19	21596001	21604000	8000	2	4.70E-10	-0.6	135	1.69	Abcc12	Transport
DMR19:22116001	19	22116001	22119000	3000	1	3.30E-08	0.31	45	1.5	Phkb	Signaling
DMR19:22182001	19	22182001	22183000	1000	1	1.30E-07	-0.41	8	0.8	Phkb	Signaling
DMR19:22290001	19	22290001	22292000	2000	1	1.90E-07	-0.51	38	1.9	Phkb;Itfg1	Signaling
DMR19:22368001	19	22368001	22372000	4000	1	1.20E-07	-0.45	24	0.6	Itfg1	
DMR19:22439001	19	22439001	22441000	2000	1	7.50E-08	-0.33	55	2.75	Neto2	Protease
DMR19:22461001	19	22461001	22463000	2000	1	1.60E-07	-0.45	19	0.95	Neto2	Protease
DMR19:22718001	19	22718001	22725000	7000	1	3.20E-09	-0.54	85	1.21	RGD1308706;LOC100363129;LOC108348909	

DMR19:22734001	19	22734001	22738000	4000	1	3.10E-09	0.46	46	1.15	RGD1308706;LOC108348909;LOC102548593	
DMR19:23402001	19	23402001	23406000	4000	1	2.00E-08	-0.55	91	2.28	Sall1;LOC103694299	Transcription
DMR19:23945001	19	23945001	23946000	1000	1	1.50E-11	-0.57	19	1.9	Zfp330	
DMR19:24053001	19	24053001	24057000	4000	2	1.40E-17	0.64	172	4.3	Rnf150	
DMR19:24158001	19	24158001	24160000	2000	1	7.90E-07	0.34	13	0.65	Rnf150	
DMR19:24181001	19	24181001	24186000	5000	1	9.90E-08	-0.47	89	1.78	Rnf150	
DMR19:24245001	19	24245001	24247000	2000	1	1.40E-08	0.33	19	0.95	Rnf150	
DMR19:24429001	19	24429001	24430000	1000	1	5.30E-07	-0.39	22	2.2	Tbc1d9	Signaling
DMR19:24574001	19	24574001	24576000	2000	1	8.80E-07	-0.38	43	2.15	Clgn;Scoc	Transcription
DMR19:24623001	19	24623001	24624000	1000	1	2.60E-07	-0.4	2	0.2	Scoc	
DMR19:24859001	19	24859001	24860000	1000	1	4.50E-10	-0.48	31	3.1	Ddx39a;Adgre5	Signaling
DMR19:25144001	19	25144001	25148000	4000	1	2.80E-07	0.41	78	1.95	RGD1310262;Palm3;Il27ra	Receptor
DMR19:25165001	19	25165001	25168000	3000	1	2.10E-09	0.48	64	2.13	Il27ra;Rln3;LOC102555451	Receptor
DMR19:25201001	19	25201001	25203000	2000	1	3.00E-08	0.43	54	2.7	Rfx1;Dcaf15	Transcription
DMR19:25247001	19	25247001	25251000	4000	1	1.00E-07	0.44	77	1.93	Podnl1;Cc2d1a	
DMR19:25468001	19	25468001	25470000	2000	1	1.90E-07	0.44	32	1.6	Cacna1a	Transport
DMR19:25488001	19	25488001	25492000	4000	1	1.50E-09	0.53	41	1.02	Cacna1a	Transport
DMR19:25657001	19	25657001	25662000	5000	1	5.20E-07	0.35	50	1	Cacna1a	Transport
DMR19:25701001	19	25701001	25702000	1000	1	1.10E-10	-0.41	18	1.8	Cacna1a	Transport
DMR19:25707001	19	25707001	25710000	3000	1	2.00E-21	0.94	56	1.87	Cacna1a	Transport
DMR19:25725001	19	25725001	25728000	3000	1	3.30E-09	0.46	82	2.73	Cacna1a	Transport
DMR19:25757001	19	25757001	25759000	2000	1	5.80E-11	0.55	32	1.6	Cacna1a	Transport
DMR19:25827001	19	25827001	25829000	2000	1	4.00E-09	0.58	27	1.35	Lyl1;Nf1x	Transcription;Transcription
DMR19:25859001	19	25859001	25860000	1000	1	2.70E-07	0.46	10	1	Nf1x;LOC102546551	Transcription
DMR19:25941001	19	25941001	25944000	3000	1	1.60E-07	0.33	20	0.67	Dand5;Gadd45gip1;Rad23a	DNA Repair
DMR19:26047001	19	26047001	26049000	2000	1	4.60E-07	0.31	16	0.8	MAST1;Mir3550	
DMR19:26072001	19	26072001	26073000	1000	1	3.20E-08	0.43	27	2.7	LOC108348915;Rtbdn;Rnaseh2a	Translation
DMR19:26109001	19	26109001	26118000	9000	2	1.40E-07	0.43	166	1.84	Hook2;Best2	Transport;Transport
DMR19:26124001	19	26124001	26125000	1000	1	2.30E-12	0.82	28	2.8	Hook2;Best2;Asna1	Transport;Transport
DMR19:26144001	19	26144001	26147000	3000	1	3.10E-13	-0.57	49	1.63	Asna1;RGD1564093;Tnpo2	Transport
DMR19:27443001	19	27443001	27444000	1000	1	4.50E-12	0.38	11	1.1	Mylk3	Signaling
DMR19:27555001	19	27555001	27559000	4000	1	3.10E-07	-0.3	29	0.72	Olr1666	Receptor
DMR19:28688001	19	28688001	28695000	7000	2	2.10E-09	-0.38	76	1.09	LOC501317;LOC691712;RGD1562660	
DMR19:28726001	19	28726001	28732000	6000	2	6.00E-07	-0.33	50	0.83	LOC501317;LOC691712;RGD1562660;LOC100912126;LOC102550448	
DMR19:28748001	19	28748001	28751000	3000	1	1.50E-07	-0.47	15	0.5	LOC501317;LOC691712;RGD1562660	
DMR19:28822001	19	28822001	28823000	1000	1	1.20E-07	-0.58	6	0.6	LOC501317;RGD1562660	
DMR19:28827001	19	28827001	28832000	5000	1	1.40E-08	-0.49	49	0.98	LOC501317;RGD1562660	
DMR19:28875001	19	28875001	28876000	1000	1	3.50E-07	0.49	10	1	LOC501317;RGD1562660	
DMR19:29768001	19	29768001	29771000	3000	2	2.10E-16	0.42	21	0.7	Inpp4b	
DMR19:29941001	19	29941001	29947000	6000	2	1.40E-07	-0.28	59	0.98	Inpp4b	
DMR19:30022001	19	30022001	30025000	3000	1	1.70E-09	0.39	30	1	Inpp4b	
DMR19:30188001	19	30188001	30190000	2000	1	1.40E-08	0.36	15	0.75	Inpp4b	
DMR19:30311001	19	30311001	30313000	2000	1	9.40E-13	0.83	30	1.5	Inpp4b;RGD1565102	
DMR19:30786001	19	30786001	30787000	1000	1	1.80E-07	0.35	6	0.6	LOC688904;Gab1	Cytoskeleton
DMR19:30792001	19	30792001	30793000	1000	1	9.50E-07	0.48	20	2	LOC688904;Gab1	Cytoskeleton
DMR19:30833001	19	30833001	30834000	1000	1	6.10E-07	-0.36	14	1.4	Gab1	Cytoskeleton
DMR19:30841001	19	30841001	30844000	3000	1	1.00E-12	-0.59	44	1.47	Gab1	Cytoskeleton
DMR19:30850001	19	30850001	30855000	5000	1	1.90E-10	-0.54	85	1.7	Gab1	Cytoskeleton
DMR19:30862001	19	30862001	30864000	2000	1	9.00E-07	-0.33	32	1.6	Gab1	Cytoskeleton
DMR19:30962001	19	30962001	30966000	4000	1	1.90E-08	-0.46	39	0.98	Smarca5;Frem3	Epigenetic
DMR19:31020001	19	31020001	31022000	2000	1	6.40E-07	0.52	20	1	Frem3	
DMR19:31540001	19	31540001	31544000	4000	2	1.30E-09	0.44	30	0.75	Hhip	Signaling
DMR19:31588001	19	31588001	31589000	1000	1	5.30E-11	0.43	23	2.3	Hhip;LOC102549515	Signaling
DMR19:31622001	19	31622001	31625000	3000	1	2.20E-07	0.3	28	0.93	Hhip	Signaling
DMR19:31810001	19	31810001	31811000	1000	1	4.10E-08	-0.65	8	0.8	Anapc10	Proteolysis
DMR19:32210001	19	32210001	32211000	1000	1	4.00E-10	-0.49	16	1.6	Smad1	Transcription
DMR19:32214001	19	32214001	32216000	2000	1	8.20E-07	-0.37	52	2.6	Smad1	Transcription
DMR19:32331001	19	32331001	32334000	3000	2	4.10E-11	-0.61	49	1.63	Mmaa;LOC102550010	Metabolism

DMR19:32488001	19	32488001	32492000	4000	1	1.70E-08	0.49	57	1.43	Zfp827	Transcription
DMR19:32502001	19	32502001	32503000	1000	1	8.70E-07	-0.5	13	1.3	Zfp827	Transcription
DMR19:32599001	19	32599001	32600000	1000	1	2.70E-09	0.58	19	1.9	Zfp827	Transcription
DMR19:32791001	19	32791001	32792000	1000	1	6.20E-10	0.47	12	1.2	Lsm6	Translation
DMR19:32816001	19	32816001	32817000	1000	1	2.80E-09	0.47	4	0.4	RGD1564764	
DMR19:32855001	19	32855001	32856000	1000	1	1.50E-07	0.32	15	1.5	Slc10a7	Transport
DMR19:32949001	19	32949001	32952000	3000	2	5.20E-09	-0.51	50	1.67	Slc10a7	Transport
DMR19:32976001	19	32976001	32978000	2000	1	2.10E-07	-0.4	34	1.7	Slc10a7;LOC103694318	Transport
DMR19:33035001	19	33035001	33036000	1000	1	1.80E-07	0.41	14	1.4	Slc10a7	Transport
DMR19:33268001	19	33268001	33273000	5000	2	6.40E-08	-0.38	27	0.54	Ttc29	
DMR19:33275001	19	33275001	33277000	2000	1	4.40E-07	-0.46	29	1.45	Ttc29	
DMR19:33344001	19	33344001	33346000	2000	1	2.80E-09	0.51	32	1.6	Ttc29	
DMR19:33355001	19	33355001	33356000	1000	1	9.70E-07	-0.45	4	0.4	Ttc29	
DMR19:33392001	19	33392001	33393000	1000	1	1.20E-07	0.56	16	1.6	Ttc29	
DMR19:33406001	19	33406001	33408000	2000	1	2.70E-07	0.35	24	1.2	Ttc29	
DMR19:34247001	19	34247001	34252000	5000	2	6.50E-09	-0.55	80	1.6	Arhgap10	Signaling
DMR19:34290001	19	34290001	34292000	2000	1	3.20E-07	-0.54	42	2.1	Arhgap10	Signaling
DMR19:34396001	19	34396001	34399000	3000	1	3.50E-08	0.41	39	1.3	Arhgap10;Nr3c2	Signaling
DMR19:34493001	19	34493001	34497000	4000	1	1.10E-07	0.41	58	1.45	Nr3c2	
DMR19:34500001	19	34500001	34505000	5000	1	7.40E-11	-0.63	45	0.9	Nr3c2	
DMR19:34647001	19	34647001	34648000	1000	1	5.20E-08	0.45	15	1.5	Nr3c2	
DMR19:37007001	19	37007001	37010000	3000	2	2.00E-09	0.55	67	2.23	LOC108348882;RGD1564100;Slc25a36l1	
DMR19:37011001	19	37011001	37016000	5000	4	1.90E-13	0.5	105	2.1	RGD1564100;Slc25a36l1;Ces2g	Metabolism
DMR19:37038001	19	37038001	37043000	5000	2	2.90E-08	-0.44	35	0.7	Ces2g;LOC102548129;Ces2h	Metabolism
DMR19:37095001	19	37095001	37097000	2000	1	8.60E-07	0.38	23	1.15	Ces4a	Metabolism
DMR19:37289001	19	37289001	37290000	1000	1	4.90E-08	0.43	7	0.7	Lrrc29;Tmem208;Fhod1	
DMR19:37548001	19	37548001	37550000	2000	1	1.20E-12	0.61	24	1.2	Agrp;Fam65a	Signaling
DMR19:37695001	19	37695001	37699000	4000	1	7.70E-09	0.37	39	0.98	Gfod2	Metabolism
DMR19:37918001	19	37918001	37923000	5000	2	5.60E-07	0.8	129	2.58	Psmb10;Lcat;Slc12a4	Protease;Metabolism;Transport
DMR19:37951001	19	37951001	37953000	2000	1	1.50E-10	0.67	47	2.35	Dpep3	Protease
DMR19:37969001	19	37969001	37972000	3000	1	3.30E-10	0.46	45	1.5	Dpep2	Protease
DMR19:38280001	19	38280001	38282000	2000	1	1.00E-07	0.34	18	0.9	Smpd3	
DMR19:38341001	19	38341001	38342000	1000	1	9.40E-07	-0.46	17	1.7	Eif5b-ps1;LOC103690129	
DMR19:38831001	19	38831001	38835000	4000	1	6.20E-08	-0.32	70	1.75	Cdh1	Cytoskeleton
DMR19:38836001	19	38836001	38837000	1000	1	1.70E-08	-0.43	15	1.5	Cdh1;Tango6	Cytoskeleton
DMR19:38943001	19	38943001	38946000	3000	1	2.90E-07	0.4	26	0.87	Tango6	
DMR19:39002001	19	39002001	39003000	1000	1	4.20E-07	0.36	8	0.8	Tango6	
DMR19:39094001	19	39094001	39099000	5000	1	3.10E-10	-0.46	74	1.48	Chtf8;Utp4	
DMR19:39195001	19	39195001	39196000	1000	1	1.10E-09	0.42	6	0.6	Sntb2	
DMR19:39207001	19	39207001	39210000	3000	1	3.10E-07	0.38	32	1.07	Sntb2	
DMR19:39268001	19	39268001	39270000	2000	1	4.80E-09	-0.7	15	0.75	Nip7;Tmed6;Terf2	Cytoskeleton;Transport
DMR19:39591001	19	39591001	39597000	6000	1	1.50E-09	0.44	51	0.85	Wwp2	Proteolysis
DMR19:41034001	19	41034001	41036000	2000	1	7.30E-08	-0.52	27	1.35	Vac14;Hydin	Cytoskeleton
DMR19:41067001	19	41067001	41070000	3000	1	8.50E-08	-0.56	34	1.13	Hydin	
DMR19:41176001	19	41176001	41177000	1000	1	7.60E-07	0.37	20	2	Hydin	
DMR19:41241001	19	41241001	41242000	1000	1	3.10E-08	0.36	6	0.6	Hydin	
DMR19:41324001	19	41324001	41326000	2000	1	3.00E-07	-0.51	32	1.6	Hydin	
DMR19:41485001	19	41485001	41488000	3000	1	6.60E-07	0.35	25	0.83	Calb2	Signaling
DMR19:41636001	19	41636001	41637000	1000	1	2.20E-08	0.34	16	1.6	Chst4	Transport
DMR19:41676001	19	41676001	41678000	2000	1	5.70E-08	0.88	54	2.7	Tat	Metabolism
DMR19:41734001	19	41734001	41738000	4000	1	1.90E-09	0.4	31	0.78	Marveld3;Phlpp2	Cytoskeleton
DMR19:42076001	19	42076001	42077000	1000	1	1.40E-11	-0.44	15	1.5	Dhodh	Metabolism
DMR19:42090001	19	42090001	42092000	2000	1	7.10E-09	0.42	20	1	Dhodh;Hp;Txnl4b	Metabolism;Protease;Metabolism
DMR19:42141001	19	42141001	42143000	2000	1	4.10E-07	-0.48	31	1.55	Pmfbp1	
DMR19:42758001	19	42758001	42760000	2000	1	1.90E-09	0.65	88	4.4	Zfhx3	Transcription
DMR19:42782001	19	42782001	42786000	4000	1	1.30E-08	0.46	85	2.12	Zfhx3	Transcription
DMR19:42877001	19	42877001	42880000	3000	1	3.90E-07	-0.39	67	2.23	Zfhx3	Transcription
DMR19:43327001	19	43327001	43329000	2000	1	3.70E-07	0.37	23	1.15	St3gal2;Fuk	Transport
DMR19:43666001	19	43666001	43669000	3000	1	2.60E-07	-0.52	42	1.4	Wdr59	
DMR19:43686001	19	43686001	43688000	2000	1	7.80E-09	-0.6	23	1.15	Wdr59	

DMR19:43700001	19	43700001	43701000	1000	1	9.30E-10	-0.43	16	1.6	Wdr59	
DMR19:43703001	19	43703001	43705000	2000	2	6.70E-10	0.42	12	0.6	Wdr59	
DMR19:43890001	19	43890001	43895000	5000	1	7.80E-07	0.46	87	1.74	Zfp1	
DMR19:43906001	19	43906001	43911000	5000	1	2.70E-07	0.81	89	1.78	Ctrb1	Protease
DMR19:43915001	19	43915001	43917000	2000	1	7.70E-08	-0.34	40	2	Ctrb1	Protease
DMR19:43918001	19	43918001	43919000	1000	1	1.40E-12	0.61	19	1.9	Ctrb1	Protease
DMR19:44155001	19	44155001	44157000	2000	1	7.10E-13	0.72	31	1.55	Tmem231;Gabarapl2	Cytoskeleton
DMR19:44262001	19	44262001	44266000	4000	1	1.70E-07	0.44	46	1.15	RGD1563834	
DMR19:44811001	19	44811001	44815000	4000	1	9.00E-09	0.42	45	1.12	Cntnap4	
DMR19:44816001	19	44816001	44817000	1000	1	9.10E-07	0.4	13	1.3	Cntnap4	
DMR19:44853001	19	44853001	44855000	2000	1	7.30E-08	-0.44	35	1.75	Cntnap4	
DMR19:44939001	19	44939001	44942000	3000	1	8.70E-10	-0.53	61	2.03	Cntnap4	
DMR19:44943001	19	44943001	44946000	3000	1	1.60E-09	0.46	34	1.13	Cntnap4	
DMR19:45044001	19	45044001	45045000	1000	1	1.60E-07	0.37	6	0.6	Cntnap4	
DMR19:45057001	19	45057001	45059000	2000	1	1.90E-10	0.5	15	0.75	Cntnap4	
DMR19:46079001	19	46079001	46082000	3000	1	6.20E-07	-0.49	34	1.13	Adamts18	Protease
DMR19:46175001	19	46175001	46176000	1000	1	2.00E-09	-0.47	18	1.8	Adamts18	Protease
DMR19:46448001	19	46448001	46450000	2000	1	4.60E-09	0.43	13	0.65	Nudt7	Signaling
DMR19:46649001	19	46649001	46650000	1000	1	7.50E-08	0.49	3	0.3	Vat1l	
DMR19:46662001	19	46662001	46663000	1000	1	1.80E-11	0.6	23	2.3	Vat1l	
DMR19:46671001	19	46671001	46672000	1000	1	1.20E-09	0.64	19	1.9	Vat1l	
DMR19:46832001	19	46832001	46834000	2000	1	8.70E-07	0.39	21	1.05	Wwox	
DMR19:46943001	19	46943001	46944000	1000	1	1.90E-07	0.39	12	1.2	Wwox	
DMR19:47143001	19	47143001	47144000	1000	1	1.20E-08	0.49	12	1.2	Wwox	
DMR19:47159001	19	47159001	47160000	1000	1	8.40E-07	0.37	10	1	Wwox	
DMR19:47236001	19	47236001	47238000	2000	1	1.10E-10	0.62	9	0.45	Wwox	
DMR19:47293001	19	47293001	47296000	3000	1	3.40E-07	0.36	38	1.27	Wwox	
DMR19:47360001	19	47360001	47362000	2000	1	4.10E-08	0.44	15	0.75	Wwox	
DMR19:47472001	19	47472001	47473000	1000	1	3.10E-07	0.59	8	0.8	Wwox	
DMR19:47495001	19	47495001	47500000	5000	2	9.60E-09	0.65	110	2.2	Wwox	
DMR19:47582001	19	47582001	47584000	2000	1	5.60E-08	0.31	41	2.05	Wwox	
DMR19:47622001	19	47622001	47624000	2000	1	6.40E-07	-0.39	37	1.85	Wwox;LOC108348986	
DMR19:47703001	19	47703001	47705000	2000	1	6.10E-07	-0.38	22	1.1	Wwox;LOC108348985	
DMR19:49009001	19	49009001	49010000	1000	1	1.70E-07	0.63	26	2.6	Dynlrb2	Cytoskeleton
DMR19:49108001	19	49108001	49110000	2000	1	6.30E-10	0.64	52	2.6	Cdyl2	
DMR19:49142001	19	49142001	49147000	5000	2	8.20E-09	-0.38	48	0.96	Cdyl2	
DMR19:49177001	19	49177001	49180000	3000	1	2.70E-08	0.43	28	0.93	Cdyl2	
DMR19:49209001	19	49209001	49212000	3000	1	5.60E-07	0.26	40	1.33	Cdyl2	
DMR19:49609001	19	49609001	49610000	1000	1	3.60E-15	0.72	22	2.2	Pkd1l2	Transport
DMR19:49618001	19	49618001	49619000	1000	1	1.20E-08	0.41	26	2.6	Pkd1l2	Transport
DMR19:49780001	19	49780001	49784000	4000	1	3.80E-08	0.41	48	1.2	Cmip	
DMR19:49937001	19	49937001	49941000	4000	1	2.10E-07	0.37	64	1.6	Cmip	
DMR19:49968001	19	49968001	49970000	2000	1	9.40E-07	-0.4	44	2.2	Cmip	
DMR19:50008001	19	50008001	50012000	4000	2	2.40E-08	0.62	69	1.73	Cmip	
DMR19:50104001	19	50104001	50106000	2000	1	5.20E-07	0.4	13	0.65	Plcg2	Metabolism
DMR19:50115001	19	50115001	50118000	3000	1	1.10E-07	0.38	50	1.67	Plcg2	Metabolism
DMR19:50196001	19	50196001	50198000	2000	1	2.20E-10	-0.37	17	0.85	Sdr42e1	Metabolism
DMR19:50203001	19	50203001	50205000	2000	1	3.80E-11	0.77	54	2.7	Sdr42e1	Metabolism
DMR19:50214001	19	50214001	50217000	3000	1	4.90E-10	0.55	52	1.73	Sdr42e1	Metabolism
DMR19:50218001	19	50218001	50222000	4000	1	1.80E-08	0.5	76	1.9	Sdr42e1	Metabolism
DMR19:50270001	19	50270001	50274000	4000	1	1.10E-07	-0.28	33	0.82	Hsd17b2	Metabolism
DMR19:50282001	19	50282001	50283000	1000	1	3.30E-07	0.3	9	0.9	Hsd17b2	Metabolism
DMR19:50856001	19	50856001	50858000	2000	2	1.90E-09	0.44	14	0.7	Cdh13	Cytoskeleton
DMR19:50933001	19	50933001	50934000	1000	1	1.00E-07	-0.38	14	1.4	Cdh13	Cytoskeleton
DMR19:50948001	19	50948001	50952000	4000	1	7.20E-09	0.37	42	1.05	Cdh13	Cytoskeleton
DMR19:50953001	19	50953001	50955000	2000	1	9.60E-07	0.32	29	1.45	Cdh13	Cytoskeleton
DMR19:50980001	19	50980001	50983000	3000	1	6.70E-08	0.32	27	0.9	Cdh13	Cytoskeleton
DMR19:51003001	19	51003001	51004000	1000	1	4.70E-09	0.4	5	0.5	Cdh13	Cytoskeleton
DMR19:51126001	19	51126001	51129000	3000	1	1.40E-10	0.79	50	1.67	Cdh13	Cytoskeleton
DMR19:51194001	19	51194001	51196000	2000	1	2.20E-10	0.43	21	1.05	Cdh13;LOC102552714	Cytoskeleton
DMR19:51217001	19	51217001	51218000	1000	1	9.20E-07	0.53	24	2.4	Cdh13;LOC102552714	Cytoskeleton
DMR19:51291001	19	51291001	51292000	1000	1	2.80E-14	0.38	6	0.6	Cdh13	Cytoskeleton
DMR19:51396001	19	51396001	51400000	4000	2	1.00E-08	0.75	89	2.22	Cdh13	Cytoskeleton

DMR19:51430001	19	51430001	51431000	1000	1	3.10E-07	0.35	15	1.5	Cdh13	Cytoskeleton
DMR19:51437001	19	51437001	51438000	1000	1	1.20E-11	0.85	27	2.7	Cdh13	Cytoskeleton
DMR19:51450001	19	51450001	51452000	2000	1	5.90E-08	0.52	23	1.15	Cdh13	Cytoskeleton
DMR19:51549001	19	51549001	51551000	2000	1	9.70E-07	0.34	37	1.85	Cdh13	Cytoskeleton
DMR19:51594001	19	51594001	51596000	2000	1	1.70E-07	-0.45	64	3.2	Cdh13	Cytoskeleton
DMR19:51664001	19	51664001	51668000	4000	1	9.90E-10	0.42	25	0.62	Cdh13	Cytoskeleton
DMR19:51703001	19	51703001	51706000	3000	1	4.90E-08	0.66	44	1.47	Cdh13	Cytoskeleton
DMR19:51724001	19	51724001	51725000	1000	1	3.50E-09	0.36	8	0.8	Cdh13	Cytoskeleton
DMR19:51748001	19	51748001	51749000	1000	1	1.50E-09	0.48	13	1.3	Cdh13	Cytoskeleton
DMR19:51764001	19	51764001	51766000	2000	1	2.30E-08	0.44	21	1.05	Cdh13	Cytoskeleton
DMR19:51771001	19	51771001	51775000	4000	1	4.50E-08	0.54	56	1.4	Cdh13	Cytoskeleton
DMR19:51919001	19	51919001	51921000	2000	2	9.80E-09	0.35	21	1.05	Cdh13	Cytoskeleton
DMR19:51924001	19	51924001	51926000	2000	1	1.90E-09	0.46	29	1.45	Cdh13	Cytoskeleton
DMR19:52086001	19	52086001	52090000	4000	1	9.50E-08	0.7	173	4.32	Osgin1;Necab2	
DMR19:52098001	19	52098001	52099000	1000	1	2.20E-08	-0.44	15	1.5	Necab2	
DMR19:52260001	19	52260001	52264000	4000	1	3.00E-07	0.34	68	1.7	Taf1c;Adad2;Kcng4	Transcription;Metabolism;Transport
DMR19:52273001	19	52273001	52274000	1000	1	4.10E-08	0.6	45	4.5	Adad2;Kcng4	Metabolism;Transport
DMR19:52289001	19	52289001	52290000	1000	1	2.60E-07	0.56	14	1.4	Kcng4	Transport
DMR19:52433001	19	52433001	52436000	3000	1	1.00E-07	-0.42	55	1.83	Tldc1	
DMR19:52459001	19	52459001	52463000	4000	1	5.20E-07	0.37	56	1.4	Cotl1	Cytoskeleton
DMR19:52477001	19	52477001	52479000	2000	2	2.90E-16	0.44	7	0.35	Cotl1	Cytoskeleton
DMR19:52524001	19	52524001	52525000	1000	1	5.00E-10	-0.57	17	1.7	LOC103694347;Klhl36	
DMR19:52527001	19	52527001	52528000	1000	1	1.50E-08	0.54	1	0.1	Klhl36	
DMR19:52595001	19	52595001	52599000	4000	1	8.30E-08	-0.46	87	2.17	Usp10	Protease
DMR19:52678001	19	52678001	52683000	5000	1	2.50E-07	0.41	83	1.66	Crispld2	Immune
DMR19:52744001	19	52744001	52747000	3000	1	2.10E-07	-0.48	30	1	Zdhhc7	
DMR19:52799001	19	52799001	52802000	3000	1	3.60E-09	-0.49	85	2.83	RGD1304884	
DMR19:52814001	19	52814001	52817000	3000	1	6.10E-07	0.47	49	1.63	RGD1304884	
DMR19:52854001	19	52854001	52861000	7000	1	9.10E-07	0.33	102	1.46	RGD1304884	
DMR19:52887001	19	52887001	52889000	2000	1	5.70E-09	0.51	29	1.45	RGD1304884	
DMR19:52900001	19	52900001	52901000	1000	1	1.10E-11	0.35	16	1.6	RGD1304884	
DMR19:52906001	19	52906001	52910000	4000	1	1.00E-07	0.32	69	1.73	RGD1304884	
DMR19:52945001	19	52945001	52946000	1000	1	5.00E-11	0.75	40	4	RGD1304884	
DMR19:53000001	19	53000001	53008000	8000	1	1.40E-08	-0.37	189	2.36	RGD1304884;Fendrr;Foxf1	
DMR19:53099001	19	53099001	53100000	1000	1	4.40E-08	0.66	24	2.4	RGD1304884	
DMR19:53185001	19	53185001	53187000	2000	1	3.60E-08	0.44	16	0.8	RGD1304884;LOC103694348	
DMR19:53208001	19	53208001	53210000	2000	1	1.60E-07	0.57	31	1.55	RGD1304884	
DMR19:53242001	19	53242001	53244000	2000	1	1.10E-07	0.43	35	1.75	RGD1304884	
DMR19:53291001	19	53291001	53294000	3000	1	4.50E-07	0.36	51	1.7	RGD1304884	
DMR19:53381001	19	53381001	53386000	5000	1	6.60E-08	0.4	85	1.7	RGD1304884;LOC108348955	
DMR19:53391001	19	53391001	53393000	2000	1	1.40E-17	0.71	65	3.25	RGD1304884;LOC108348955	
DMR19:53417001	19	53417001	53419000	2000	1	8.60E-07	0.51	47	2.35	RGD1304884	
DMR19:53429001	19	53429001	53430000	1000	1	9.10E-10	0.71	27	2.7	RGD1304884	
DMR19:53812001	19	53812001	53813000	1000	1	1.70E-08	-0.36	24	2.4	Gse1	
DMR19:53836001	19	53836001	53839000	3000	1	1.20E-10	0.38	47	1.57	Gse1	
DMR19:53847001	19	53847001	53848000	1000	1	1.00E-08	0.44	17	1.7	Gse1	
DMR19:53853001	19	53853001	53857000	4000	1	1.10E-07	0.5	63	1.57	Gse1;LOC103694351	
DMR19:53887001	19	53887001	53893000	6000	1	2.00E-08	0.37	90	1.5	Gse1	
DMR19:53913001	19	53913001	53915000	2000	1	9.60E-08	0.43	27	1.35	Gse1;LOC102551937	
DMR19:53938001	19	53938001	53940000	2000	1	9.70E-09	0.56	27	1.35	Gse1	
DMR19:53970001	19	53970001	53971000	1000	1	5.20E-07	0.4	18	1.8	Gse1	
DMR19:53998001	19	53998001	5.40E+07	2000	1	5.90E-08	0.44	22	1.1	Gse1;LOC108348949	
DMR19:54003001	19	54003001	54004000	1000	1	5.30E-10	0.46	9	0.9	Gse1;LOC108348949	
DMR19:54012001	19	54012001	54013000	1000	1	5.40E-08	0.41	28	2.8	Gse1	
DMR19:54050001	19	54050001	54054000	4000	2	1.00E-10	0.42	45	1.12	Gse1;RGD1565689	
DMR19:54105001	19	54105001	54106000	1000	1	8.20E-09	0.59	32	3.2	Gse1	
DMR19:54115001	19	54115001	54117000	2000	1	1.70E-07	0.36	52	2.6	Gse1	
DMR19:54166001	19	54166001	54170000	4000	2	1.50E-08	0.62	64	1.6	Gins2	Transcription
DMR19:54308001	19	54308001	54313000	5000	1	2.60E-09	0.51	57	1.14	Irf8	Transcription
DMR19:54827001	19	54827001	54834000	7000	1	7.50E-16	0.4	79	1.13	Banp;Zfp469	
DMR19:54888001	19	54888001	54892000	4000	1	3.30E-07	0.36	47	1.18	Zfp469	
DMR19:54969001	19	54969001	54971000	2000	1	3.40E-07	0.43	39	1.95	Zfp469	

DMR19:55011001	19	55011001	55014000	3000	1	8.50E-07	0.41	40	1.33	Zfp469	
DMR19:55178001	19	55178001	55179000	1000	1	3.40E-08	0.5	12	1.2	Mlnr	Transcription
DMR19:55463001	19	55463001	55464000	1000	1	1.30E-09	0.47	10	1	Cbfa2t3;LOC108348958	Transcription
DMR19:55471001	19	55471001	55474000	3000	1	6.10E-09	0.47	43	1.43	Cbfa2t3	Transcription
DMR19:55592001	19	55592001	55593000	1000	1	5.40E-07	0.37	8	0.8	Acsf3	Metabolism
DMR19:55699001	19	55699001	55702000	3000	1	7.50E-07	0.29	43	1.43	Cdh15;Ankrd11	Cytoskeleton
DMR19:55801001	19	55801001	55804000	3000	1	5.90E-08	-0.42	44	1.47	Ankrd11	
DMR19:55817001	19	55817001	55820000	3000	1	2.60E-07	-0.57	36	1.2	Ankrd11	
DMR19:55821001	19	55821001	55825000	4000	1	1.30E-07	-0.41	46	1.15	Ankrd11	
DMR19:55837001	19	55837001	55838000	1000	1	2.00E-09	-0.5	7	0.7	Ankrd11	
DMR19:56336001	19	56336001	56340000	4000	1	2.70E-12	0.49	109	2.72	Gas8	Cytoskeleton
DMR19:56607001	19	56607001	56610000	3000	1	6.10E-07	0.4	42	1.4	Rab4a;Ccsap	
DMR19:56640001	19	56640001	56642000	2000	1	1.50E-07	-0.51	33	1.65	Ccsap;LOC108348965	
DMR19:56715001	19	56715001	56717000	2000	1	2.60E-09	-0.76	102	5.1	Nup133	
DMR19:56749001	19	56749001	56752000	3000	1	9.80E-07	0.44	41	1.37	Abcb10	Transport
DMR19:56774001	19	56774001	56777000	3000	1	8.60E-07	-0.43	59	1.97	Abcb10;Taf5l	Transport
DMR19:57121001	19	57121001	57122000	1000	1	3.10E-07	0.44	4	0.4	Galnt2;Pgbd5	Golgi;Epigenetic
DMR19:57133001	19	57133001	57135000	2000	1	1.10E-11	0.44	36	1.8	Pgbd5	Epigenetic
DMR19:57336001	19	57336001	57339000	3000	1	6.20E-11	0.48	46	1.53	Agt;Capn9	Protease; Proteolysis;Protease
DMR19:57348001	19	57348001	57349000	1000	1	2.20E-11	0.67	27	2.7	Capn9	Protease
DMR19:57412001	19	57412001	57415000	3000	1	6.70E-09	0.53	53	1.77	RGD1559896	
DMR19:57416001	19	57416001	57419000	3000	1	1.40E-10	0.51	36	1.2	RGD1559896	
DMR19:57509001	19	57509001	57512000	3000	1	5.60E-07	0.31	43	1.43	Fam89a	Cytoskeleton
DMR19:57544001	19	57544001	57547000	3000	1	2.10E-07	0.42	43	1.43	LOC102549725;Trim67	Proteolysis
DMR19:57599001	19	57599001	57601000	2000	1	4.00E-07	-0.39	34	1.7	Trim67;RGD1562218	Proteolysis
DMR19:57610001	19	57610001	57614000	4000	1	3.60E-09	-0.59	65	1.62	RGD1562218;Gnpat	Metabolism
DMR19:57975001	19	57975001	57976000	1000	1	1.50E-12	0.66	18	1.8	Disc1	
DMR19:58017001	19	58017001	58018000	1000	1	6.50E-07	0.44	9	0.9	Disc1	
DMR19:58409001	19	58409001	58411000	2000	1	1.90E-07	0.51	37	1.85	Sipa1l2;Set	Signaling;Epigenetic
DMR19:58598001	19	58598001	58599000	1000	1	1.20E-10	0.82	24	2.4	Pcnx2	
DMR19:58604001	19	58604001	58608000	4000	1	4.40E-12	0.55	79	1.98	Pcnx2;LOC108353198	
DMR19:58635001	19	58635001	58641000	6000	2	2.20E-08	0.4	93	1.55	Pcnx2	
DMR19:58675001	19	58675001	58676000	1000	1	9.70E-07	0.36	5	0.5	Pcnx2	
DMR19:58712001	19	58712001	58713000	1000	1	1.10E-08	-0.52	20	2	Pcnx2	
DMR19:58730001	19	58730001	58731000	1000	1	9.50E-07	0.48	13	1.3	Pcnx2	
DMR19:58864001	19	58864001	58865000	1000	1	2.60E-08	0.43	8	0.8	Kcnk1	Transport
DMR19:58997001	19	58997001	5.90E+07	3000	1	7.70E-07	0.32	46	1.53	Slc35f3	
DMR19:59037001	19	59037001	59050000	13000	2	3.60E-12	0.49	216	1.66	Slc35f3	
DMR19:59125001	19	59125001	59130000	5000	2	1.80E-07	0.43	40	0.8	Slc35f3	
DMR19:59214001	19	59214001	59219000	5000	2	8.50E-15	0.49	53	1.06	Slc35f3	
DMR19:59274001	19	59274001	59275000	1000	1	4.10E-07	-0.41	25	2.5	Slc35f3	
DMR19:59345001	19	59345001	59347000	2000	1	2.10E-08	0.64	14	0.7	Tarbp1	Epigenetic
DMR19:59374001	19	59374001	59375000	1000	1	5.30E-09	0.46	13	1.3	Tarbp1	Epigenetic
DMR19:59889001	19	59889001	59891000	2000	1	1.20E-07	-0.34	22	1.1	Rbm34	Translation
DMR19:59979001	19	59979001	59981000	2000	1	1.10E-10	0.45	26	1.3	LOC365026;Hmg1l1;LOC679596	
DMR19:60073001	19	60073001	60075000	2000	1	2.00E-13	0.51	15	0.75	Pard3	
DMR19:60081001	19	60081001	60083000	2000	1	5.80E-08	-0.4	33	1.65	Pard3	
DMR19:60153001	19	60153001	60156000	3000	1	5.90E-09	0.32	53	1.77	Pard3	
DMR19:60210001	19	60210001	60211000	1000	1	1.60E-07	-0.35	26	2.6	Pard3;LOC103694366	
DMR19:60332001	19	60332001	60335000	3000	1	5.00E-08	-0.48	65	2.17	Pard3	
DMR19:60498001	19	60498001	60499000	1000	1	2.20E-09	0.49	6	0.6	Pard3	
DMR19:60542001	19	60542001	60544000	2000	1	8.40E-11	0.52	10	0.5	Pard3	
DMR19:60581001	19	60581001	60583000	2000	1	2.60E-11	0.43	19	0.95	Pard3	
DMR19:61930001	19	61930001	61938000	8000	2	4.70E-09	-0.39	81	1.01	Ccdc7	
DMR19:61947001	19	61947001	61952000	5000	1	1.50E-07	-0.27	53	1.06	Ccdc7	
DMR19:62108001	19	62108001	62110000	2000	1	2.00E-07	-0.34	17	0.85	Ccdc7	
DMR19:62130001	19	62130001	62136000	6000	1	1.00E-07	-0.26	67	1.12	Ccdc7	
DMR20:51001	20	51001	52000	1000	1	2.40E-07	-0.37	3	0.3	2600a1a2	
DMR20:63001	20	63001	64000	1000	1	2.40E-07	0.27	22	2.2	2600a1a2	
DMR20:163001	20	163001	164000	1000	1	1.40E-08	0.34	13	1.3	RT1-CE10;LOC108353254;LOC103694368;LOC108349020	Immune

DMR20:234001	20	234001	237000	3000	1	3.00E-07	0.27	31	1.03	Tmlhe;LOC102550933	Metabolism
DMR20:253001	20	253001	257000	4000	1	1.50E-10	-0.45	41	1.02	Tmlhe;LOC102550933	Metabolism
DMR20:261001	20	261001	263000	2000	2	5.70E-22	0.64	44	2.2	Tmlhe	Metabolism
DMR20:266001	20	266001	270000	4000	2	1.20E-10	0.49	42	1.05	Tmlhe	Metabolism
DMR20:356001	20	356001	360000	4000	1	8.90E-08	-0.73	42	1.05	Olr1669-ps	
DMR20:371001	20	371001	375000	4000	1	5.50E-07	-0.33	31	0.78	Olr1669-ps	
DMR20:541001	20	541001	547000	6000	1	1.10E-07	-0.24	56	0.93	Olr1683	Receptor
DMR20:1044001	20	1044001	1048000	4000	1	2.80E-08	-0.31	37	0.92	LOC100911129;Olr1697	
DMR20:1203001	20	1203001	1207000	4000	1	8.60E-07	-0.3	30	0.75	Olr1710;Olr1711-ps	Signaling
DMR20:1767001	20	1767001	1768000	1000	1	1.40E-09	-0.56	6	0.6	Olr1736	
DMR20:1797001	20	1797001	1801000	4000	1	2.90E-11	-0.49	26	0.65	Olr1738;Olr1739	Receptor
DMR20:1950001	20	1950001	1951000	1000	1	3.70E-08	-0.5	12	1.2	Olr1750;Gabbr1	Receptor;Signaling
DMR20:1979001	20	1979001	1982000	3000	2	3.60E-09	0.65	104	3.47	Gabbr1	Signaling
DMR20:2069001	20	2069001	2073000	4000	1	3.30E-09	-0.39	26	0.65	RT1-M6-1;RT1-M6-2;Znrd1as1	Immune
DMR20:2076001	20	2076001	2077000	1000	1	4.00E-09	0.67	24	2.4	RT1-M6-2;Znrd1as1	Immune
DMR20:2175001	20	2175001	2177000	2000	1	4.30E-07	-0.37	28	1.4	Trim40;Ppp1cb-ps;Rpl39-ps;Pbp-ps;Trim10	Proteolysis
DMR20:2184001	20	2184001	2187000	3000	1	4.20E-07	-0.47	49	1.63	Trim40;Rpl39-ps;Pbp-ps;Trim10;Trim15;Trim15-ps1	Proteolysis
DMR20:2346001	20	2346001	2350000	4000	1	6.20E-07	-0.28	30	0.75	RT1-M1-5	
DMR20:2495001	20	2495001	2497000	2000	1	6.70E-08	-0.42	12	0.6	RT1-M10-1;3082ex4-5;Trim39	Proteolysis
DMR20:2547001	20	2547001	2553000	6000	1	1.40E-09	-0.36	41	0.68	RT1-P2-ps1;Rcrg1-ps40;Nerg-ps15	
DMR20:2561001	20	2561001	2564000	3000	1	2.60E-07	-0.34	23	0.77	Rcrg1-ps40;Nerg-ps15	
DMR20:2586001	20	2586001	2589000	3000	1	6.60E-07	-0.19	22	0.73	2994a1;Nerg-ps10;Nerg-ps13;Rcrg1-ps34;Rcrg1-ps24;2824a1	
DMR20:2605001	20	2605001	2608000	3000	1	1.90E-08	-0.29	20	0.67	2824a1;2661a1a2;2795a1;2886a1-a1a2	
DMR20:2865001	20	2865001	2866000	1000	1	3.80E-07	-0.52	39	3.9	Btnl8	Immune
DMR20:2982001	20	2982001	2985000	3000	1	3.00E-08	-0.53	21	0.7	2920a1a2;2846a1a2;2619a1_ps1;2927a1;Nerg-ps12;Rcrg1-ps31	
DMR20:3117001	20	3117001	3119000	2000	1	1.30E-07	0.32	19	0.95	RT1-T18;LOC108348140	
DMR20:3371001	20	3371001	3372000	1000	1	6.10E-07	-0.56	17	1.7	Atat1;LOC108353245;RGD1302996;Dhx16;Ppp1r18	Transcription
DMR20:3424001	20	3424001	3427000	3000	1	2.10E-07	-0.45	115	3.83	Mdc1;Tubb5;Flot1	Cytoskeleton
DMR20:3538001	20	3538001	3541000	3000	1	4.50E-07	0.33	31	1.03	Cb707485	
DMR20:3591001	20	3591001	3595000	4000	1	8.60E-08	0.36	89	2.22	Gtf2h4;LOC108349028;Vars2;Sfta2	Transcription;Translation;Transport
DMR20:3601001	20	3601001	3605000	4000	1	8.60E-10	0.52	68	1.7	Vars2;Sfta2;Dpcr1	Translation;Transport
DMR20:3670001	20	3670001	3671000	1000	1	6.40E-14	0.86	30	3	Mt1m	
DMR20:3705001	20	3705001	3706000	1000	1	5.60E-21	1.17	39	3.9	Cdsn	
DMR20:3813001	20	3813001	3815000	2000	1	8.10E-11	0.75	75	3.75	Ring1;Mir219-1;Hsd17b8;Slc39a7;Rxrb	Proteolysis;Metabolism;Transport;Transcription
DMR20:3855001	20	3855001	3857000	2000	1	6.10E-07	0.31	54	2.7	Col11a2;RT1-Hb-ps1	Extracellular Matrix
DMR20:3953001	20	3953001	3957000	4000	1	7.50E-09	0.54	68	1.7	RT1-DMb	Immune
DMR20:3974001	20	3974001	3975000	1000	1	9.40E-11	0.93	28	2.8	Psmb9;Tap1	Protease;Transport
DMR20:4067001	20	4067001	4069000	2000	1	6.30E-07	-0.5	50	2.5	RT1-Ba	Immune
DMR20:4074001	20	4074001	4076000	2000	1	1.20E-10	0.55	35	1.75	RT1-Ba	Immune
DMR20:4129001	20	4129001	4131000	2000	1	3.70E-08	0.55	48	2.4	RT1-Db2;Arrb2-ps;RT1-Da;Btnl2	Immune;Immune
DMR20:4165001	20	4165001	4166000	1000	1	2.80E-07	0.43	18	1.8	Btnl2;Btnl3;LOC108349035	Immune
DMR20:4171001	20	4171001	4177000	6000	2	2.10E-07	0.32	87	1.45	Btnl3;LOC108349035;Tesb	Immune
DMR20:4255001	20	4255001	4257000	2000	1	1.50E-07	-0.46	66	3.3	Btn3a2	
DMR20:4335001	20	4335001	4341000	6000	1	3.10E-07	0.33	130	2.17	Stk19;Notch4	
DMR20:4449001	20	4449001	4452000	3000	2	1.10E-09	0.58	75	2.5	Stk19	
DMR20:4463001	20	4463001	4464000	1000	1	7.00E-07	0.43	16	1.6	Stk19	
DMR20:4612001	20	4612001	4615000	3000	1	9.70E-07	0.63	98	3.27	Slc44a4;Neu1	Transport;Metabolism
DMR20:4960001	20	4960001	4961000	1000	1	1.70E-07	0.43	9	0.9	Hspa1l;LOC103694387;Lsm2	Translation
DMR20:5014001	20	5014001	5016000	2000	2	2.20E-08	0.39	37	1.85	Vars;Vwa7;Sapcd1;Msh5	Transcription
DMR20:5029001	20	5029001	5035000	6000	1	7.30E-07	-0.35	93	1.55	Vwa7;Sapcd1;Msh5;Clic1	Transcription;Transport
DMR20:5115001	20	5115001	5116000	1000	1	1.30E-07	-0.45	24	2.4	Ly6g5b;Csnk2b;Gpank1;G4;Apom;Bag6	Signaling;Binding Proteins;Transcription
DMR20:5152001	20	5152001	5155000	3000	2	1.00E-12	-0.82	84	2.8	Prrc2a;E230034O05Rik;Aif1	Metabolism;Signaling

DMR20:5329001	20	5329001	5330000	1000	1	1.40E-07	0.52	21	2.1	LOC100911886;Sacm2l-ps5;Sacm2l-ps7;Cdk2ap1-ps6	
DMR20:5425001	20	5425001	5427000	2000	1	1.90E-08	-0.44	46	2.3	RT1-A1;Vps52	Immune;Transport
DMR20:5563001	20	5563001	5564000	1000	1	8.10E-13	0.71	24	2.4	Syngap1;Zbtb9	Signaling;Cytoskeleton
DMR20:5599001	20	5599001	5604000	5000	1	3.60E-10	0.65	93	1.86	Bak1	
DMR20:5610001	20	5610001	5614000	4000	1	1.10E-09	0.69	94	2.35	Bak1;Ggnbp1	Protease
DMR20:5694001	20	5694001	5696000	2000	1	2.20E-07	0.44	31	1.55	Itpr3	Ion Channel
DMR20:5731001	20	5731001	5737000	6000	1	6.90E-08	-0.48	140	2.33	Uqcc2;Ip6k3	Transcription;Signaling
DMR20:5758001	20	5758001	5764000	6000	2	3.60E-09	0.44	28	0.47	Ip6k3;LOC108349021	Signaling
DMR20:5794001	20	5794001	5802000	8000	2	3.80E-09	0.61	139	1.74	Lemd2;Clpsl2;Clps	Signaling
DMR20:5809001	20	5809001	5810000	1000	1	5.70E-08	0.48	8	0.8	Clps;Lhfpl5	Signaling
DMR20:5875001	20	5875001	5878000	3000	1	6.50E-08	0.38	45	1.5	Srpk1;Slc26a8	Signaling;Transport
DMR20:5983001	20	5983001	5984000	1000	1	6.60E-11	-0.44	21	2.1	Mapk14	Signaling
DMR20:6044001	20	6044001	6045000	1000	1	6.70E-12	0.55	13	1.3	Brpf3	Transcription
DMR20:6112001	20	6112001	6113000	1000	1	1.00E-09	0.44	17	1.7	Pnpla1	Metabolism
DMR20:6145001	20	6145001	6147000	2000	1	3.90E-08	0.53	31	1.55	Pnpla1;LOC689561	Metabolism
DMR20:6291001	20	6291001	6296000	5000	1	2.30E-08	-0.38	73	1.46	Srsf3	Translation
DMR20:6470001	20	6470001	6473000	3000	1	4.30E-09	0.44	48	1.6	Cpne5	
DMR20:6516001	20	6516001	6518000	2000	1	3.40E-07	-0.4	58	2.9	LOC102547155;Ppil1	Transcription
DMR20:6611001	20	6611001	6612000	1000	1	6.60E-08	0.34	28	2.8	Ppil1;LOC102551791	Transcription
DMR20:6666001	20	6666001	6668000	2000	1	4.90E-08	0.45	28	1.4	Ppil1	Transcription
DMR20:6688001	20	6688001	6689000	1000	1	2.10E-07	0.38	18	1.8	Ppil1	Transcription
DMR20:6740001	20	6740001	6742000	2000	1	2.10E-07	0.47	35	1.75	Ppil1;Grm4	Transcription;Signaling
DMR20:6761001	20	6761001	6764000	3000	1	5.40E-08	0.33	54	1.8	Ppil1;Grm4	Transcription;Signaling
DMR20:6791001	20	6791001	6793000	2000	1	8.10E-09	0.45	44	2.2	Ppil1;Grm4	Transcription;Signaling
DMR20:6867001	20	6867001	6868000	1000	1	3.30E-08	-0.71	17	1.7	Ppil1;RGD735065	Transcription
DMR20:6916001	20	6916001	6917000	1000	1	2.50E-07	0.37	11	1.1	RGD735065;Pi16	Immune
DMR20:7129001	20	7129001	7130000	1000	1	6.20E-07	-0.41	29	2.9	Hmga1	Transcription
DMR20:7196001	20	7196001	7197000	1000	1	1.10E-08	0.48	24	2.4	Nudt3	Signaling
DMR20:7198001	20	7198001	7200000	2000	1	2.00E-08	-0.46	36	1.8	Nudt3	Signaling
DMR20:7206001	20	7206001	7207000	1000	1	5.40E-09	-0.61	19	1.9	Nudt3;Rps10	Signaling;Translation
DMR20:7238001	20	7238001	7240000	2000	1	1.30E-07	0.49	59	2.95	Pacsin1	Cytoskeleton
DMR20:7245001	20	7245001	7247000	2000	1	3.80E-09	0.67	46	2.3	Pacsin1	Cytoskeleton
DMR20:7313001	20	7313001	7315000	2000	1	2.80E-07	0.38	22	1.1	Spdef	Transcription
DMR20:7317001	20	7317001	7318000	1000	1	3.20E-11	0.51	7	0.7	Spdef;LOC499407	Transcription
DMR20:7519001	20	7519001	7520000	1000	1	1.30E-12	-0.57	25	2.5	Anks1a	Cytoskeleton
DMR20:7533001	20	7533001	7535000	2000	1	1.90E-07	-0.46	47	2.35	Anks1a	Cytoskeleton
DMR20:7720001	20	7720001	7723000	3000	1	7.90E-07	0.32	62	2.07	Scube3;LOC108349040	Extracellular Matrix
DMR20:7742001	20	7742001	7745000	3000	1	4.90E-07	0.34	71	2.37	Scube3;LOC108349040;Zfp523	Extracellular Matrix
DMR20:7759001	20	7759001	7761000	2000	1	5.90E-09	-0.41	33	1.65	Scube3;LOC108349040;Zfp523	Extracellular Matrix
DMR20:7794001	20	7794001	7799000	5000	1	1.20E-11	0.68	86	1.72	Zfp523;Def6	Cytoskeleton
DMR20:8043001	20	8043001	8045000	2000	1	9.10E-15	-0.85	13	0.65	Fkbp5;LOC103694392	Transcription
DMR20:8052001	20	8052001	8054000	2000	1	6.50E-08	-0.45	45	2.25	Fkbp5	Transcription
DMR20:8064001	20	8064001	8066000	2000	1	3.40E-07	0.38	30	1.5	Fkbp5	Transcription
DMR20:8121001	20	8121001	8122000	1000	1	9.30E-08	0.38	19	1.9	Armc12	
DMR20:8162001	20	8162001	8163000	1000	1	9.90E-07	-0.37	26	2.6	Pim1	Signaling
DMR20:8194001	20	8194001	8195000	1000	1	1.60E-08	0.56	39	3.9	LOC687478;LOC102547645;Tmem217	
DMR20:8519001	20	8519001	8522000	3000	1	7.00E-10	0.51	67	2.23	Mdga1	
DMR20:8533001	20	8533001	8535000	2000	1	2.00E-14	0.78	56	2.8	Mdga1	
DMR20:8539001	20	8539001	8540000	1000	1	1.00E-07	0.58	26	2.6	Mdga1	
DMR20:8541001	20	8541001	8544000	3000	1	8.70E-09	0.41	48	1.6	Mdga1	
DMR20:8687001	20	8687001	8688000	1000	1	3.70E-07	0.58	29	2.9	Zfand3	
DMR20:8702001	20	8702001	8704000	2000	1	7.40E-08	-0.47	20	1	Zfand3	
DMR20:8717001	20	8717001	8720000	3000	1	9.70E-07	-0.42	39	1.3	Zfand3	
DMR20:8731001	20	8731001	8733000	2000	1	1.20E-07	-0.43	26	1.3	Zfand3	
DMR20:8736001	20	8736001	8746000	10000	1	9.00E-07	-0.54	129	1.29	Zfand3	
DMR20:8774001	20	8774001	8780000	6000	1	3.40E-13	-0.57	90	1.5	Zfand3	
DMR20:8782001	20	8782001	8784000	2000	1	2.00E-07	-0.42	32	1.6	Zfand3	
DMR20:8836001	20	8836001	8839000	3000	1	6.80E-11	-0.62	44	1.47	Zfand3	
DMR20:8849001	20	8849001	8853000	4000	1	5.50E-08	-0.43	74	1.85	Zfand3	
DMR20:8902001	20	8902001	8905000	3000	1	1.90E-07	0.37	53	1.77	Zfand3;Btbd9	
DMR20:8989001	20	8989001	8990000	1000	1	2.70E-07	0.38	11	1.1	Btbd9	

DMR20:9004001	20	9004001	9006000	2000	1	4.70E-09	-0.41	28	1.4	Btbd9	
DMR20:9104001	20	9104001	9106000	2000	1	1.10E-07	-0.34	41	2.05	Btbd9	
DMR20:9168001	20	9168001	9170000	2000	1	4.60E-09	-0.44	0	0	Btbd9	
DMR20:9283001	20	9283001	9284000	1000	1	4.00E-08	0.42	26	2.6	Glo1	Metabolism
DMR20:9354001	20	9354001	9356000	2000	2	1.40E-07	0.72	50	2.5	Dnah8	Cytoskeleton
DMR20:9385001	20	9385001	9386000	1000	1	5.00E-08	0.52	20	2	Dnah8;LOC103694393	Cytoskeleton
DMR20:9391001	20	9391001	9394000	3000	2	3.80E-15	1.01	103	3.43	Dnah8;LOC103694393	Cytoskeleton
DMR20:9400001	20	9400001	9402000	2000	1	4.20E-16	0.85	49	2.45	Dnah8	Cytoskeleton
DMR20:9418001	20	9418001	9420000	2000	1	3.90E-16	0.83	48	2.4	Dnah8	Cytoskeleton
DMR20:9423001	20	9423001	9424000	1000	1	4.20E-10	0.79	38	3.8	Dnah8	Cytoskeleton
DMR20:9431001	20	9431001	9433000	2000	2	4.50E-12	0.95	76	3.8	Dnah8	Cytoskeleton
DMR20:9482001	20	9482001	9485000	3000	1	6.70E-08	0.7	76	2.53	Dnah8	Cytoskeleton
DMR20:9526001	20	9526001	9529000	3000	1	4.00E-08	-0.41	64	2.13	Dnah8	Cytoskeleton
DMR20:9558001	20	9558001	9560000	2000	1	6.00E-09	0.39	21	1.05	Dnah8	Cytoskeleton
DMR20:9570001	20	9570001	9572000	2000	1	1.50E-11	0.79	55	2.75	Dnah8	Cytoskeleton
DMR20:9586001	20	9586001	9587000	1000	1	6.40E-07	0.46	38	3.8	Glp1r	Receptor
DMR20:9636001	20	9636001	9638000	2000	1	7.70E-09	0.5	36	1.8	Glp1r;Umodl1	Receptor
DMR20:9642001	20	9642001	9646000	4000	1	2.60E-08	0.62	92	2.3	Umodl1	Receptor
DMR20:9662001	20	9662001	9666000	4000	1	7.00E-08	0.53	53	1.32	Umodl1	Receptor
DMR20:9667001	20	9667001	9668000	1000	1	3.00E-13	0.37	19	1.9	Umodl1	Receptor
DMR20:9865001	20	9865001	9872000	7000	1	4.40E-17	0.92	150	2.14	Tff3;Tff2	Signaling
DMR20:9873001	20	9873001	9876000	3000	1	7.00E-10	0.35	56	1.87	Tff2	Signaling
DMR20:9921001	20	9921001	9923000	2000	1	1.10E-07	0.3	25	1.25	Tmprss3	Protease
DMR20:9925001	20	9925001	9926000	1000	1	3.50E-08	-0.51	26	2.6	Tmprss3	Protease
DMR20:9946001	20	9946001	9947000	1000	1	8.30E-07	-0.48	27	2.7	Ubash3a	
DMR20:9962001	20	9962001	9964000	2000	1	7.20E-08	0.44	36	1.8	Ubash3a	
DMR20:10045001	20	10045001	10048000	3000	1	8.50E-07	0.53	38	1.27	Slc37a1	Transport
DMR20:10093001	20	10093001	10094000	1000	1	1.80E-07	0.6	27	2.7	Slc37a1;LOC108349044	Transport
DMR20:10153001	20	10153001	10154000	1000	1	1.50E-07	0.37	12	1.2	Pde9a	Signaling
DMR20:10168001	20	10168001	10170000	2000	1	1.70E-07	0.38	22	1.1	Pde9a	Signaling
DMR20:10181001	20	10181001	10183000	2000	1	5.40E-07	-0.52	50	2.5	Pde9a	Signaling
DMR20:10192001	20	10192001	10198000	6000	1	3.90E-07	0.42	110	1.83	Pde9a	Signaling
DMR20:10342001	20	10342001	10343000	1000	1	2.30E-07	-0.43	30	3	Pknox1	Development
DMR20:10374001	20	10374001	10375000	1000	1	5.10E-08	0.69	27	2.7	Cbs	Metabolism
DMR20:10668001	20	10668001	10673000	5000	1	3.30E-09	0.7	149	2.98	Sik1;LOC108349047	Signaling
DMR20:10938001	20	10938001	10939000	1000	1	2.70E-07	-0.4	26	2.6	Pdxk	Signaling
DMR20:10964001	20	10964001	10966000	2000	1	1.60E-08	0.59	56	2.8	Cstb	Protease; Proteolysis
DMR20:11132001	20	11132001	11141000	9000	1	1.30E-08	-0.46	205	2.28	Agpat3	Metabolism
DMR20:11147001	20	11147001	11153000	6000	1	7.10E-07	0.34	111	1.85	Agpat3	Metabolism
DMR20:11317001	20	11317001	11320000	3000	1	6.20E-07	-0.36	40	1.33	Icoslg	Immune
DMR20:11356001	20	11356001	11361000	5000	3	8.80E-14	0.61	100	2	Dnmt3l;Aire	Epigenetic
DMR20:11374001	20	11374001	11378000	4000	1	2.70E-07	0.38	77	1.93	Aire	
DMR20:11383001	20	11383001	11385000	2000	1	1.00E-08	-0.35	39	1.95	Aire;Pfkl	Metabolism
DMR20:11421001	20	11421001	11422000	1000	1	3.80E-07	-0.44	36	3.6	Pfkl;RGD1309594	Metabolism
DMR20:11477001	20	11477001	11478000	1000	1	2.10E-08	0.38	22	2.2	Trpm2	Transport
DMR20:11561001	20	11561001	11563000	2000	1	5.80E-09	0.64	31	1.55	RGD1561557;LOC690415;LOC690425	
DMR20:11564001	20	11564001	11567000	3000	1	4.90E-09	0.34	32	1.07	RGD1561557;LOC690415;LOC690425	
DMR20:12007001	20	12007001	12008000	1000	1	1.70E-11	-0.45	24	2.4	Adarb1	Metabolism
DMR20:12076001	20	12076001	12080000	4000	1	9.40E-07	-0.3	67	1.68	Adarb1	Metabolism
DMR20:12281001	20	12281001	12282000	1000	1	4.90E-07	-0.36	14	1.4	Col18a1	Extracellular Matrix
DMR20:12283001	20	12283001	12284000	1000	1	1.10E-08	0.48	6	0.6	Col18a1	Extracellular Matrix
DMR20:12328001	20	12328001	12329000	1000	1	7.80E-09	-0.57	14	1.4	Col18a1;Slc19a1	Extracellular Matrix;Transport
DMR20:12334001	20	12334001	12337000	3000	1	9.20E-07	-0.36	71	2.37	Col18a1;Slc19a1	Extracellular Matrix;Transport
DMR20:12360001	20	12360001	12364000	4000	1	1.70E-07	-0.47	107	2.67	Slc19a1;LOC100912209	Transport
DMR20:12602001	20	12602001	12604000	2000	1	1.20E-07	0.48	30	1.5	Pcbp3	Metabolism
DMR20:12804001	20	12804001	12805000	1000	1	6.30E-08	0.31	14	1.4	Col6a2;Ftcd	Extracellular Matrix
DMR20:12816001	20	12816001	12817000	1000	1	5.80E-07	0.56	30	3	Ftcd;Spatc1l	
DMR20:12946001	20	12946001	12949000	3000	1	2.20E-07	-0.32	71	2.37	RGD1564149;Pcnt	
DMR20:12967001	20	12967001	12968000	1000	1	5.50E-07	-0.47	25	2.5	Pcnt	

DMR20:12977001	20	12977001	12978000	1000	1	9.50E-08	-0.48	27	2.7	Pcnt	
DMR20:12997001	20	12997001	13001000	4000	1	2.10E-12	-0.55	60	1.5	Pcnt	
DMR20:13013001	20	13013001	13014000	1000	1	3.50E-10	0.6	8	0.8	Pcnt	
DMR20:13022001	20	13022001	13023000	1000	1	3.90E-07	-0.33	21	2.1	Pcnt	
DMR20:13045001	20	13045001	13049000	4000	1	6.50E-07	-0.43	81	2.02	Pcnt;Dip2a	
DMR20:13058001	20	13058001	13060000	2000	1	1.90E-11	0.39	38	1.9	Dip2a	
DMR20:13083001	20	13083001	13084000	1000	1	8.30E-07	0.38	3	0.3	Dip2a	
DMR20:13131001	20	13131001	13132000	1000	1	1.40E-08	-0.32	32	3.2	Dip2a;S100b	Signaling
DMR20:13205001	20	13205001	13207000	2000	1	2.20E-11	0.77	46	2.3	Prmt2;Mir678;LOC103694402	Golgi
DMR20:13452001	20	13452001	13456000	4000	1	3.80E-07	-0.41	67	1.68	Slc5a4	Transport
DMR20:13491001	20	13491001	13496000	5000	1	8.10E-08	-0.45	30	0.6	Slc5a4b	Transport
DMR20:13522001	20	13522001	13531000	9000	1	1.60E-16	0.96	117	1.3	Slc5a4b	Transport
DMR20:13568001	20	13568001	13573000	5000	1	5.60E-10	-0.49	137	2.74	Zfp280b	Transcription
DMR20:13591001	20	13591001	13593000	2000	1	5.70E-07	0.4	27	1.35	Zfp280b;LOC103690331	Transcription
DMR20:13639001	20	13639001	13644000	5000	1	2.70E-08	-0.48	80	1.6	RGD1563815	
DMR20:13650001	20	13650001	13653000	3000	1	8.40E-09	0.79	71	2.37	RGD1563815;LOC102552984;Vpreb3;RGD1564162	Immune
DMR20:13712001	20	13712001	13713000	1000	1	7.60E-08	0.63	24	2.4	Smarcb1;Derl3;Mif	Transcription;Transport;Metabolism
DMR20:13757001	20	13757001	13759000	2000	1	6.00E-09	0.52	48	2.4	Gstt2;LOC108349022	
DMR20:13762001	20	13762001	13763000	1000	1	3.20E-07	0.53	15	1.5	Gstt2;LOC108349022	
DMR20:13769001	20	13769001	13771000	2000	1	2.60E-07	0.39	21	1.05	Gstt2;LOC108349022;Gstt4	
DMR20:13779001	20	13779001	13784000	5000	3	3.60E-07	0.62	90	1.8	Gstt4	
DMR20:13895001	20	13895001	13896000	1000	1	1.90E-07	0.54	9	0.9	Cabin1	Signaling
DMR20:13926001	20	13926001	13928000	2000	1	8.60E-08	0.39	9	0.45	Cabin1;LOC102556116	Signaling
DMR20:13960001	20	13960001	13963000	3000	1	2.00E-07	0.38	55	1.83	Cabin1;Susd2	Signaling
DMR20:14116001	20	14116001	14118000	2000	1	2.10E-08	-0.47	19	0.95	Mir327	
DMR20:14184001	20	14184001	14187000	3000	1	1.60E-23	0.82	45	1.5	Upb1	Metabolism
DMR20:14257001	20	14257001	14258000	1000	1	2.30E-10	0.51	28	2.8	Adora2a	Signaling
DMR20:14419001	20	14419001	14420000	1000	1	9.90E-09	0.63	28	2.8	Bcr	Signaling
DMR20:14556001	20	14556001	14558000	2000	1	3.80E-08	0.48	36	1.8	Bcr;Rab36	Signaling
DMR20:14583001	20	14583001	14585000	2000	1	7.00E-15	0.68	52	2.6	Rab36;Rtdr1;Gnaz	Signaling
DMR20:14591001	20	14591001	14592000	1000	1	1.20E-17	0.9	30	3	Rtdr1;Gnaz	Signaling
DMR20:14657001	20	14657001	14660000	3000	1	6.20E-07	0.34	55	1.83	Rtdr1	
DMR20:14691001	20	14691001	14692000	1000	1	4.70E-07	0.37	5	0.5	RGD1561987	
DMR20:14700001	20	14700001	14701000	1000	1	7.20E-09	0.45	12	1.2	RGD1561987	
DMR20:14820001	20	14820001	14826000	6000	1	9.90E-08	-0.38	56	0.93	Polr3d-ps1	
DMR20:14952001	20	14952001	14953000	1000	1	1.70E-08	0.61	40	4	Pcdh15	Cytoskeleton
DMR20:14972001	20	14972001	14975000	3000	2	1.40E-09	0.55	49	1.63	Pcdh15;LOC103694424	Cytoskeleton
DMR20:15039001	20	15039001	15040000	1000	1	1.00E-07	0.48	6	0.6	Pcdh15	Cytoskeleton
DMR20:15161001	20	15161001	15164000	3000	1	1.20E-09	0.57	38	1.27	Pcdh15	Cytoskeleton
DMR20:15178001	20	15178001	15181000	3000	1	5.80E-09	0.39	42	1.4	Pcdh15	Cytoskeleton
DMR20:15281001	20	15281001	15284000	3000	1	1.70E-10	0.39	49	1.63	Pcdh15	Cytoskeleton
DMR20:15765001	20	15765001	15768000	3000	1	9.10E-07	-0.34	15	0.5	NEWGENE_1590969	
DMR20:15779001	20	15779001	15781000	2000	1	4.90E-07	-0.48	33	1.65	NEWGENE_1590969	
DMR20:15957001	20	15957001	15959000	2000	1	2.50E-07	0.59	32	1.6	NEWGENE_1590969	
DMR20:16074001	20	16074001	16078000	4000	1	2.20E-08	0.37	39	0.98	NEWGENE_1590969	
DMR20:16151001	20	16151001	16152000	1000	1	1.60E-08	0.48	4	0.4	NEWGENE_1590969	
DMR20:16169001	20	16169001	16174000	5000	1	1.70E-07	-0.5	80	1.6	NEWGENE_1590969	
DMR20:16175001	20	16175001	16176000	1000	1	2.10E-09	0.34	6	0.6	NEWGENE_1590969	
DMR20:16306001	20	16306001	16308000	2000	1	2.20E-07	0.53	49	2.45	NEWGENE_1590969	
DMR20:16366001	20	16366001	16368000	2000	1	3.10E-08	0.53	37	1.85	NEWGENE_1590969	
DMR20:16379001	20	16379001	16380000	1000	1	5.70E-07	0.36	13	1.3	NEWGENE_1590969	
DMR20:16397001	20	16397001	16399000	2000	1	9.60E-07	-0.31	12	0.6	NEWGENE_1590969	
DMR20:16435001	20	16435001	16437000	2000	1	9.80E-13	0.5	27	1.35	NEWGENE_1590969	
DMR20:16471001	20	16471001	16475000	4000	1	2.10E-08	-0.49	48	1.2	NEWGENE_1590969	
DMR20:16573001	20	16573001	16576000	3000	1	2.30E-07	-0.54	15	0.5	NEWGENE_1590969	
DMR20:16649001	20	16649001	16650000	1000	1	9.90E-08	0.69	21	2.1	NEWGENE_1590969;LOC108349009	
DMR20:16674001	20	16674001	16675000	1000	1	1.70E-07	-0.46	8	0.8	NEWGENE_1590969	
DMR20:16697001	20	16697001	16699000	2000	1	5.30E-08	-0.59	29	1.45	NEWGENE_1590969	
DMR20:16730001	20	16730001	16734000	4000	1	2.20E-07	0.3	18	0.45	NEWGENE_1590969	
DMR20:16745001	20	16745001	16746000	1000	1	2.60E-08	0.49	8	0.8	NEWGENE_1590969	

DMR20:16769001	20	16769001	16771000	2000	1	1.80E-10	-0.5	22	1.1	NEWGENE_1590969	
DMR20:16775001	20	16775001	16776000	1000	1	3.30E-07	0.4	19	1.9	NEWGENE_1590969	
DMR20:16785001	20	16785001	16789000	4000	1	6.20E-07	-0.46	43	1.07	NEWGENE_1590969	
DMR20:16800001	20	16800001	16805000	5000	1	2.20E-09	0.34	48	0.96	NEWGENE_1590969	
DMR20:17082001	20	17082001	17085000	3000	1	3.80E-08	-0.38	40	1.33	Zwint;LOC108349051	
DMR20:17748001	20	17748001	17749000	1000	1	5.80E-09	-0.52	23	2.3	RGD1559903	
DMR20:18465001	20	18465001	18468000	3000	1	7.10E-14	-0.58	63	2.1	Ipmk	Signaling
DMR20:18481001	20	18481001	18487000	6000	2	1.30E-11	-0.6	149	2.48	Ipmk;LOC103694404;Cisd1	Signaling
DMR20:18498001	20	18498001	18503000	5000	1	9.40E-07	-0.48	85	1.7	Ipmk;LOC103694404;Cisd1	Signaling
DMR20:18536001	20	18536001	18538000	2000	1	6.10E-16	0.72	38	1.9	Ube2d1	Proteolysis
DMR20:18543001	20	18543001	18547000	4000	1	7.80E-07	0.42	85	2.12	Ube2d1	Proteolysis
DMR20:18574001	20	18574001	18576000	2000	1	2.30E-07	0.4	49	2.45	Ube2d1	Proteolysis
DMR20:18841001	20	18841001	18843000	2000	1	2.20E-07	-0.44	32	1.6	Bicc1	Metabolism
DMR20:18904001	20	18904001	18905000	1000	1	2.50E-16	0.79	38	3.8	Bicc1	Metabolism
DMR20:19362001	20	19362001	19364000	2000	1	2.80E-09	-0.44	32	1.6	Phyhipl;Fam13c	
DMR20:19369001	20	19369001	19373000	4000	2	1.60E-11	0.59	81	2.02	Phyhipl;Fam13c	
DMR20:19406001	20	19406001	19407000	1000	1	4.10E-11	0.88	26	2.6	Fam13c	
DMR20:19442001	20	19442001	19445000	3000	1	5.20E-10	0.64	51	1.7	Fam13c	
DMR20:19451001	20	19451001	19453000	2000	1	9.40E-07	-0.56	31	1.55	Fam13c	
DMR20:19665001	20	19665001	19666000	1000	1	1.90E-14	0.95	27	2.7	Slc16a9	
DMR20:19728001	20	19728001	19734000	6000	1	2.10E-10	-0.5	183	3.05	Mrln;Ccdc6	
DMR20:19738001	20	19738001	19746000	8000	1	8.10E-08	-0.51	173	2.16	Ccdc6	
DMR20:19804001	20	19804001	19806000	2000	1	7.30E-13	-0.49	42	2.1	Ccdc6	
DMR20:19949001	20	19949001	19952000	3000	1	1.90E-07	-0.38	66	2.2	Ank3	
DMR20:19999001	20	19999001	20002000	3000	1	9.90E-08	-0.4	52	1.73	Ank3	
DMR20:20038001	20	20038001	20039000	1000	1	2.80E-07	-0.42	19	1.9	Ank3	
DMR20:20086001	20	20086001	20088000	2000	1	3.00E-09	0.54	30	1.5	Ank3	
DMR20:20192001	20	20192001	20196000	4000	1	3.50E-10	-0.45	66	1.65	Ank3	
DMR20:20205001	20	20205001	20207000	2000	1	3.80E-08	0.35	22	1.1	Ank3;LOC102554153	
DMR20:20239001	20	20239001	20241000	2000	1	2.60E-08	-0.53	37	1.85	Ank3;LOC108353248	
DMR20:20274001	20	20274001	20275000	1000	1	5.00E-08	0.44	38	3.8	Ank3	
DMR20:20294001	20	20294001	20296000	2000	1	6.60E-07	0.41	21	1.05	Ank3	
DMR20:20379001	20	20379001	20383000	4000	1	6.30E-07	-0.35	81	2.02	Ank3;LOC102554005;LOC102553944	
DMR20:20409001	20	20409001	20411000	2000	1	4.70E-07	-0.38	35	1.75	Ank3	
DMR20:20549001	20	20549001	20550000	1000	1	6.70E-09	0.74	28	2.8	Rpl29-ps2	
DMR20:20553001	20	20553001	20555000	2000	1	2.10E-13	0.6	66	3.3	Rpl29-ps2	
DMR20:20601001	20	20601001	20602000	1000	1	1.80E-09	0.89	16	1.6	Cdk1	Signaling
DMR20:20658001	20	20658001	20659000	1000	1	9.70E-08	0.75	49	4.9	Rhobtb1	Signaling
DMR20:20681001	20	20681001	20685000	4000	1	1.50E-07	-0.31	58	1.45	Rhobtb1	Signaling
DMR20:21270001	20	21270001	21275000	5000	1	2.00E-12	-0.5	72	1.44	RGD1564320	
DMR20:21307001	20	21307001	21308000	1000	1	5.90E-08	-0.46	15	1.5	RGD1306739	
DMR20:21358001	20	21358001	21359000	1000	1	2.10E-11	0.43	2	0.2	RGD1306739	
DMR20:21376001	20	21376001	21379000	3000	1	4.50E-07	0.42	34	1.13	RGD1306739	
DMR20:21395001	20	21395001	21396000	1000	1	8.10E-11	0.38	11	1.1	RGD1306739	
DMR20:21651001	20	21651001	21654000	3000	2	9.00E-09	-0.35	27	0.9	Tmem26	
DMR20:21770001	20	21770001	21771000	1000	1	6.40E-07	0.52	27	2.7	Arid5b	
DMR20:21777001	20	21777001	21778000	1000	1	2.60E-11	0.53	22	2.2	Arid5b	
DMR20:21799001	20	21799001	21802000	3000	1	6.60E-08	-0.43	53	1.77	Arid5b	
DMR20:21827001	20	21827001	21828000	1000	1	1.00E-10	-0.56	22	2.2	Arid5b	
DMR20:21829001	20	21829001	21831000	2000	1	1.50E-07	-0.41	52	2.6	Arid5b	
DMR20:21890001	20	21890001	21897000	7000	1	2.30E-07	0.36	81	1.16	Arid5b;LOC108349057;LOC102550601	
DMR20:21910001	20	21910001	21914000	4000	1	4.40E-09	0.53	73	1.82	LOC108349057;LOC102550601;Rtkn2	Cytoskeleton
DMR20:21937001	20	21937001	21939000	2000	1	1.70E-08	0.35	37	1.85	Rtkn2	Cytoskeleton
DMR20:21960001	20	21960001	21962000	2000	1	7.30E-10	0.69	52	2.6	Rtkn2	Cytoskeleton
DMR20:21994001	20	21994001	21995000	1000	1	3.30E-07	0.51	36	3.6	Rtkn2	Cytoskeleton
DMR20:22009001	20	22009001	22010000	1000	1	1.80E-13	0.8	25	2.5	Rtkn2	Cytoskeleton
DMR20:22084001	20	22084001	22087000	3000	1	2.10E-07	0.5	76	2.53	Zfp365	
DMR20:22730001	20	22730001	22733000	3000	1	9.20E-07	-0.52	32	1.07	Nrbf2	Transcription
DMR20:22760001	20	22760001	22763000	3000	1	2.50E-07	-0.4	51	1.7	Jmjd1c	Epigenetic
DMR20:22824001	20	22824001	22827000	3000	3	7.50E-08	-0.53	50	1.67	Jmjd1c	Epigenetic

DMR20:22837001	20	22837001	22840000	3000	1	2.30E-08	-0.62	37	1.23	Jmjd1c	Epigenetic
DMR20:22886001	20	22886001	22888000	2000	1	7.40E-10	-0.57	42	2.1	Jmjd1c;Reep3	Epigenetic;Transport
DMR20:22899001	20	22899001	22906000	7000	2	2.30E-10	-0.54	154	2.2	Jmjd1c;Reep3	Epigenetic;Transport
DMR20:22946001	20	22946001	22949000	3000	1	4.00E-07	0.28	49	1.63	Reep3	Transport
DMR20:22969001	20	22969001	22970000	1000	1	5.20E-14	0.89	32	3.2	Reep3	Transport
DMR20:22989001	20	22989001	22992000	3000	2	1.30E-07	0.42	38	1.27	Reep3	Transport
DMR20:23137001	20	23137001	23139000	2000	1	5.40E-08	0.53	41	2.05	RGD1559781	
DMR20:25085001	20	25085001	25087000	2000	1	3.00E-08	-0.56	10	0.5	Ctnna3	
DMR20:25092001	20	25092001	25097000	5000	1	7.60E-10	-0.37	44	0.88	Ctnna3	
DMR20:25134001	20	25134001	25138000	4000	1	5.50E-08	0.54	39	0.98	Ctnna3	
DMR20:25290001	20	25290001	25292000	2000	1	4.00E-08	-0.55	26	1.3	Ctnna3	
DMR20:25387001	20	25387001	25390000	3000	2	1.90E-07	-0.49	33	1.1	Ctnna3	
DMR20:25561001	20	25561001	25562000	1000	1	3.50E-09	0.51	3	0.3	Ctnna3	
DMR20:25829001	20	25829001	25834000	5000	2	2.30E-08	-0.31	39	0.78	Ctnna3	
DMR20:25857001	20	25857001	25858000	1000	1	3.00E-08	-0.34	10	1	Ctnna3	
DMR20:25959001	20	25959001	25960000	1000	1	3.20E-13	0.8	18	1.8	Ctnna3	
DMR20:26031001	20	26031001	26032000	1000	1	5.90E-07	0.36	6	0.6	Ctnna3;Lrrtm3	Receptor
DMR20:26037001	20	26037001	26040000	3000	1	2.20E-13	0.47	33	1.1	Ctnna3;Lrrtm3	Receptor
DMR20:26189001	20	26189001	26190000	1000	1	8.30E-07	0.4	13	1.3	Ctnna3	
DMR20:26296001	20	26296001	26297000	1000	1	4.20E-07	0.51	4	0.4	Ctnna3	
DMR20:26406001	20	26406001	26408000	2000	1	6.60E-07	-0.55	24	1.2	Ctnna3	
DMR20:26452001	20	26452001	26457000	5000	1	4.20E-07	-0.23	39	0.78	Ctnna3	
DMR20:26492001	20	26492001	26494000	2000	2	4.40E-13	0.64	16	0.8	Ctnna3	
DMR20:26547001	20	26547001	26549000	2000	1	3.10E-08	-0.48	24	1.2	Ctnna3;LOC108349010	
DMR20:26590001	20	26590001	26594000	4000	1	2.50E-07	0.42	31	0.78	Ctnna3	
DMR20:26622001	20	26622001	26623000	1000	1	5.00E-07	0.34	7	0.7	Ctnna3	
DMR20:26988001	20	26988001	26989000	1000	1	6.90E-09	0.33	12	1.2	Mypn	
DMR20:27045001	20	27045001	27049000	4000	1	3.50E-07	0.34	47	1.18	Mypn	
DMR20:27121001	20	27121001	27122000	1000	1	3.20E-08	0.45	18	1.8	Pbld1;Pbld2;Taf9-ps	
DMR20:27650001	20	27650001	27651000	1000	1	9.60E-11	0.43	5	0.5	Trappc3l;Fam26e	
DMR20:27792001	20	27792001	27798000	6000	1	7.50E-08	-0.26	54	0.9	Dse;LOC499444	
DMR20:27872001	20	27872001	27875000	3000	1	2.10E-07	-0.48	24	0.8	Gcc2	Cytoskeleton
DMR20:28140001	20	28140001	28144000	4000	1	4.50E-10	-0.6	45	1.12	Ccdc138	
DMR20:28392001	20	28392001	28394000	2000	1	8.40E-10	-0.44	55	2.75	Sh3rf3	
DMR20:28467001	20	28467001	28468000	1000	1	8.00E-07	-0.42	18	1.8	Sh3rf3	
DMR20:28492001	20	28492001	28494000	2000	1	2.30E-07	0.51	13	0.65	Sh3rf3	
DMR20:28531001	20	28531001	28532000	1000	1	1.00E-08	0.41	6	0.6	Sh3rf3	
DMR20:28591001	20	28591001	28597000	6000	1	7.60E-08	-0.43	61	1.02	Sh3rf3	
DMR20:28809001	20	28809001	28812000	3000	2	1.70E-11	-0.61	27	0.9	Sept10;Sowahc	
DMR20:28930001	20	28930001	28931000	1000	1	7.20E-07	-0.43	20	2	P4ha1	Golgi
DMR20:28968001	20	28968001	28977000	9000	1	7.60E-11	-0.53	143	1.59	P4ha1	Golgi
DMR20:29020001	20	29020001	29021000	1000	1	9.30E-10	0.8	33	3.3	Oit3	Receptor
DMR20:29032001	20	29032001	29034000	2000	1	5.00E-08	-0.65	33	1.65	Oit3;Mcu	Receptor
DMR20:29040001	20	29040001	29041000	1000	1	6.20E-07	0.35	9	0.9	Mcu	
DMR20:29052001	20	29052001	29053000	1000	1	3.80E-07	0.32	11	1.1	Mcu	
DMR20:29066001	20	29066001	29067000	1000	1	2.40E-07	0.38	4	0.4	Mcu	
DMR20:29091001	20	29091001	29092000	1000	1	5.20E-07	0.37	11	1.1	Mcu	
DMR20:29171001	20	29171001	29172000	1000	1	2.70E-07	-0.47	32	3.2	Mcu	
DMR20:29342001	20	29342001	29343000	1000	1	7.20E-07	-0.48	14	1.4	Micu1	Signaling
DMR20:29385001	20	29385001	29387000	2000	1	1.30E-07	-0.45	35	1.75	Micu1	Signaling
DMR20:29432001	20	29432001	29435000	3000	1	1.50E-08	-0.45	57	1.9	Micu1	Signaling
DMR20:29671001	20	29671001	29675000	4000	1	9.60E-08	0.54	68	1.7	Spock2	Extracellular Matrix
DMR20:29875001	20	29875001	29878000	3000	1	2.80E-07	0.4	61	2.03	Cdh23	Cytoskeleton
DMR20:29895001	20	29895001	29897000	2000	1	1.30E-09	0.42	15	0.75	Cdh23;Vsir	Cytoskeleton;Immune
DMR20:29975001	20	29975001	29976000	1000	1	2.50E-09	0.4	9	0.9	Cdh23	Cytoskeleton
DMR20:30091001	20	30091001	30092000	1000	1	3.00E-07	0.45	17	1.7	Cdh23	Cytoskeleton
DMR20:30160001	20	30160001	30164000	4000	1	5.70E-07	0.41	60	1.5	Cdh23	Cytoskeleton
DMR20:30211001	20	30211001	30215000	4000	3	2.70E-08	0.41	48	1.2	Cdh23	Cytoskeleton
DMR20:30281001	20	30281001	30282000	1000	1	2.80E-07	0.44	7	0.7	Slc29a3	Transport
DMR20:30289001	20	30289001	30290000	1000	1	6.90E-07	0.38	13	1.3	Slc29a3	Transport
DMR20:30787001	20	30787001	30790000	3000	1	1.80E-07	0.35	37	1.23	Tbata;LOC108349065	
DMR20:30791001	20	30791001	30794000	3000	2	1.10E-08	0.6	52	1.73	Tbata;LOC108349065	
DMR20:30803001	20	30803001	30805000	2000	1	4.00E-09	0.39	35	1.75	Tbata;LOC108349065;Adamts14	Protease

DMR20:30817001	20	30817001	30818000	1000	1	2.50E-07	0.39	13	1.3	Adamts14	Protease
DMR20:30833001	20	30833001	30835000	2000	1	4.60E-07	0.31	34	1.7	Adamts14	Protease
DMR20:30905001	20	30905001	30906000	1000	1	3.70E-07	0.34	5	0.5	Prf1	
DMR20:30990001	20	30990001	30992000	2000	1	1.90E-07	-0.38	38	1.9	Pald1	Signaling
DMR20:31169001	20	31169001	31170000	1000	1	4.80E-07	-0.39	13	1.3	Lrrc20	
DMR20:31345001	20	31345001	31349000	4000	1	1.00E-09	0.47	44	1.1	Aifm2;H2afy2	Metabolism;Epigenetic
DMR20:31350001	20	31350001	31352000	2000	1	1.10E-07	0.49	27	1.35	Aifm2;H2afy2	Metabolism;Epigenetic
DMR20:31478001	20	31478001	31479000	1000	1	2.50E-08	0.36	15	1.5	Col13a1	Extracellular Matrix
DMR20:31581001	20	31581001	31583000	2000	1	4.80E-07	0.46	17	0.85	Col13a1	Extracellular Matrix
DMR20:31809001	20	31809001	31810000	1000	1	1.80E-07	0.39	13	1.3	Tspan15	
DMR20:31846001	20	31846001	31849000	3000	1	3.00E-08	0.56	36	1.2	Tspan15	
DMR20:31850001	20	31850001	31851000	1000	1	9.40E-07	0.51	22	2.2	Tspan15	
DMR20:31911001	20	31911001	31913000	2000	1	1.60E-10	0.68	58	2.9	Tacr2;Hk1	Signaling;Signaling
DMR20:31961001	20	31961001	31962000	1000	1	2.10E-07	0.36	5	0.5	Hk1	Signaling
DMR20:31966001	20	31966001	31967000	1000	1	9.40E-08	0.38	12	1.2	Hk1	Signaling
DMR20:32069001	20	32069001	32071000	2000	1	1.10E-07	-0.43	32	1.6	Supv3l1	Transcription
DMR20:32098001	20	32098001	32099000	1000	1	3.50E-08	-0.55	9	0.9	Vps26a	Transport
DMR20:32140001	20	32140001	32141000	1000	1	8.30E-13	0.51	11	1.1	Srgn;LOC108353232	
DMR20:32181001	20	32181001	32182000	1000	1	3.20E-08	0.44	19	1.9	Kif1bp	
DMR20:32239001	20	32239001	32241000	2000	1	9.90E-09	-0.51	25	1.25	Ddx21;Ddx50	
DMR20:32322001	20	32322001	32328000	6000	1	1.90E-07	-0.36	67	1.12	Stox1	
DMR20:32396001	20	32396001	32397000	1000	1	1.50E-07	-0.6	18	1.8	Ccar1	Epigenetic
DMR20:32419001	20	32419001	32422000	3000	1	9.70E-12	-0.41	89	2.97	Ccar1	Epigenetic
DMR20:32529001	20	32529001	32530000	1000	1	4.50E-10	-0.49	5	0.5	Kpna5	Transport
DMR20:32718001	20	32718001	32719000	1000	1	7.40E-08	0.46	3	0.3	Rfx6	Transcription
DMR20:32731001	20	32731001	32732000	1000	1	2.20E-08	0.41	7	0.7	Rfx6	Transcription
DMR20:33078001	20	33078001	33081000	3000	1	8.30E-07	-0.56	58	1.93	Vgll2	Transcription
DMR20:33087001	20	33087001	33089000	2000	1	5.90E-08	0.28	18	0.9	Vgll2	Transcription
DMR20:33125001	20	33125001	33127000	2000	1	1.10E-07	0.55	40	2	Ros1	Receptor
DMR20:33129001	20	33129001	33130000	1000	1	4.10E-11	0.9	31	3.1	Ros1	Receptor
DMR20:33317001	20	33317001	33320000	3000	1	8.00E-07	-0.38	25	0.83	Ros1	Receptor
DMR20:33414001	20	33414001	33416000	2000	1	2.00E-08	-0.41	11	0.55	Dcbld1	Protease
DMR20:33781001	20	33781001	33782000	1000	1	4.60E-07	0.47	13	1.3	Slc35f1	
DMR20:33890001	20	33890001	33891000	1000	1	8.10E-07	-0.59	18	1.8	Slc35f1;LOC100911233	
DMR20:33912001	20	33912001	33915000	3000	1	4.90E-07	-0.34	99	3.3	Slc35f1;LOC100911233	
DMR20:33921001	20	33921001	33922000	1000	1	1.10E-09	-0.53	6	0.6	Slc35f1	
DMR20:33973001	20	33973001	33975000	2000	1	2.40E-07	-0.34	10	0.5	Slc35f1;LOC100911315	
DMR20:34233001	20	34233001	34234000	1000	1	2.90E-10	0.8	15	1.5	Slc35f1;LOC691731	
DMR20:34666001	20	34666001	34668000	2000	1	2.60E-07	-0.42	6	0.3	Cep85l	
DMR20:34676001	20	34676001	34679000	3000	1	7.40E-08	-0.63	22	0.73	Cep85l	
DMR20:34687001	20	34687001	34689000	2000	1	2.30E-08	0.53	29	1.45	Cep85l	
DMR20:34696001	20	34696001	34699000	3000	1	4.00E-09	0.67	56	1.87	Cep85l	
DMR20:34744001	20	34744001	34745000	1000	1	1.60E-10	0.81	30	3	Cep85l;LOC691736	
DMR20:34828001	20	34828001	34832000	4000	1	8.00E-07	-0.42	64	1.6	Mcm9	Transcription
DMR20:34955001	20	34955001	34958000	3000	2	2.10E-10	-0.54	80	2.67	Fam184a	
DMR20:35007001	20	35007001	35011000	4000	2	2.20E-12	-0.5	29	0.72	Fam184a	
DMR20:35030001	20	35030001	35031000	1000	1	1.40E-07	-0.46	11	1.1	Fam184a	
DMR20:35259001	20	35259001	35260000	1000	1	9.20E-09	-0.51	13	1.3	LOC108353234;Man1a1	Golgi
DMR20:37461001	20	37461001	37463000	2000	1	5.80E-07	-0.31	11	0.55	Tbc1d32	
DMR20:37489001	20	37489001	37490000	1000	1	7.90E-07	-0.37	13	1.3	Tbc1d32;LOC103694439	
DMR20:37865001	20	37865001	37868000	3000	1	2.80E-07	0.48	32	1.07	Gja1	Cytoskeleton
DMR20:37883001	20	37883001	37885000	2000	1	2.90E-09	-0.46	30	1.5	Gja1	Cytoskeleton
DMR20:37889001	20	37889001	37895000	6000	1	6.60E-08	0.36	76	1.27	Gja1	Cytoskeleton
DMR20:38339001	20	38339001	38341000	2000	1	4.20E-08	0.35	15	0.75	RGD1561288	
DMR20:38970001	20	38970001	38973000	3000	2	4.60E-07	-0.46	18	0.6	Hsf2;Serinc1	Transcription;Signaling
DMR20:40318001	20	40318001	40320000	2000	1	7.90E-07	0.58	18	0.9	Hs3st5;LOC102553345	Transport
DMR20:40415001	20	40415001	40419000	4000	2	7.30E-08	-0.58	45	1.12	Hs3st5	Transport
DMR20:40431001	20	40431001	40432000	1000	1	2.00E-09	0.48	4	0.4	Hs3st5	Transport
DMR20:40526001	20	40526001	40527000	1000	1	1.50E-07	0.6	21	2.1	Hs3st5	Transport
DMR20:40652001	20	40652001	40653000	1000	1	9.50E-09	0.46	11	1.1	Pkib	Signaling
DMR20:40770001	20	40770001	40772000	2000	1	1.00E-07	-0.41	22	1.1	Fabp7;Smpdl3a	Signaling
DMR20:41134001	20	41134001	41135000	1000	1	2.70E-09	0.4	2	0.2	Nt5dc1	Signaling
DMR20:43087001	20	43087001	43092000	5000	1	3.80E-10	-0.56	79	1.58	Hdac2	

DMR20:43946001	20	43946001	43948000	2000	1	8.90E-09	-0.4	17	0.85	Rfpl4b	
DMR20:43954001	20	43954001	43957000	3000	1	2.20E-09	-0.43	42	1.4	Rfpl4b	
DMR20:44064001	20	44064001	44069000	5000	1	1.10E-07	-0.44	62	1.24	Lama4	
DMR20:44204001	20	44204001	44206000	2000	2	1.20E-12	0.87	165	8.25	Lama4;Fam229b	
DMR20:44494001	20	44494001	44496000	2000	1	4.20E-08	0.39	24	1.2	Fyn	
DMR20:44575001	20	44575001	44576000	1000	1	5.30E-08	-0.59	15	1.5	Fyn	
DMR20:44594001	20	44594001	44596000	2000	1	1.70E-08	-0.34	47	2.35	Fyn;LOC108349083	
DMR20:44636001	20	44636001	44642000	6000	2	2.80E-11	0.72	171	2.85	Fyn	
DMR20:44708001	20	44708001	44710000	2000	1	1.20E-07	-0.46	39	1.95	Traf3ip2;LOC102551236	Cytoskeleton
DMR20:44724001	20	44724001	44729000	5000	1	3.40E-07	-0.47	99	1.98	Traf3ip2	Cytoskeleton
DMR20:44833001	20	44833001	44837000	4000	2	2.00E-11	-0.49	24	0.6	Rev3l	Transcription
DMR20:44852001	20	44852001	44855000	3000	1	3.60E-07	0.51	57	1.9	Rev3l	Transcription
DMR20:44899001	20	44899001	44902000	3000	1	4.30E-17	-0.64	56	1.87	Rev3l	Transcription
DMR20:44915001	20	44915001	44918000	3000	1	9.20E-07	0.4	34	1.13	Rev3l	Transcription
DMR20:45031001	20	45031001	45036000	5000	2	1.80E-14	-0.68	84	1.68	RGD1304770;RGD1561777	Transport
DMR20:45045001	20	45045001	45050000	5000	2	2.50E-09	-0.42	38	0.76	RGD1561777	Transport
DMR20:45098001	20	45098001	45100000	2000	1	9.70E-08	0.61	33	1.65	Naglt1	
DMR20:45113001	20	45113001	45114000	1000	1	3.50E-11	0.64	30	3	Naglt1;RGD1310495	Transport
DMR20:45146001	20	45146001	45148000	2000	1	2.10E-08	0.33	38	1.9	RGD1310495;Slc16a10	Transport
DMR20:45171001	20	45171001	45174000	3000	1	9.10E-07	-0.31	32	1.07	Slc16a10	Transport
DMR20:45316001	20	45316001	45317000	1000	1	2.00E-10	-0.52	34	3.4	LOC103694442;LOC108349085;Rpf2	
DMR20:45339001	20	45339001	45342000	3000	1	3.90E-09	-0.5	51	1.7	Rpf2;Gtf3c6	
DMR20:45450001	20	45450001	45455000	5000	1	5.30E-14	-0.58	38	0.76	LOC100361424;LOC100361476;Cdk19	Signaling
DMR20:45551001	20	45551001	45552000	1000	1	4.30E-10	0.44	9	0.9	Cdk19	Signaling
DMR20:45817001	20	45817001	45819000	2000	1	4.30E-08	0.37	36	1.8	Gpr6	Signaling
DMR20:45944001	20	45944001	45946000	2000	2	1.00E-10	0.33	30	1.5	Fig4	Signaling
DMR20:46052001	20	46052001	46054000	2000	1	1.10E-07	0.41	28	1.4	Fig4;Ak9	Signaling;Signaling
DMR20:46099001	20	46099001	46104000	5000	1	7.80E-07	-0.41	94	1.88	Ak9	Signaling
DMR20:46134001	20	46134001	46137000	3000	1	3.70E-16	0.86	84	2.8	Ak9	Signaling
DMR20:46144001	20	46144001	46145000	1000	1	2.40E-07	0.38	14	1.4	Ak9	Signaling
DMR20:46169001	20	46169001	46172000	3000	1	3.40E-09	0.39	58	1.93	Ak9;Zbtb24	Signaling;Transcription
DMR20:46439001	20	46439001	46442000	3000	1	6.80E-07	-0.45	58	1.93	Foxo3	
DMR20:46454001	20	46454001	46456000	2000	1	8.10E-10	-0.46	49	2.45	Foxo3	
DMR20:46471001	20	46471001	46474000	3000	1	8.00E-08	-0.53	60	2	Foxo3	
DMR20:46478001	20	46478001	46479000	1000	1	3.80E-09	-0.4	23	2.3	Foxo3	
DMR20:46487001	20	46487001	46490000	3000	1	3.10E-07	-0.53	62	2.07	Foxo3	
DMR20:46493001	20	46493001	46497000	4000	1	1.20E-07	-0.4	75	1.88	Foxo3	
DMR20:46505001	20	46505001	46507000	2000	1	2.60E-07	-0.44	99	4.95	Foxo3	
DMR20:46508001	20	46508001	46511000	3000	1	3.50E-09	-0.48	55	1.83	Foxo3	
DMR20:46600001	20	46600001	46601000	1000	1	3.50E-08	0.5	24	2.4	RGD1561147;Cep57l1	
DMR20:46743001	20	46743001	46746000	3000	1	2.70E-07	-0.52	56	1.87	Sesn1	Metabolism
DMR20:46788001	20	46788001	46790000	2000	1	1.70E-07	0.28	30	1.5	Armc2	
DMR20:46792001	20	46792001	46796000	4000	1	1.40E-09	0.39	76	1.9	Armc2	
DMR20:46800001	20	46800001	46803000	3000	1	2.60E-09	0.41	34	1.13	Armc2	
DMR20:46804001	20	46804001	46806000	2000	1	9.60E-07	0.5	25	1.25	Armc2	
DMR20:46837001	20	46837001	46838000	1000	1	5.00E-07	0.53	34	3.4	Armc2	
DMR20:46986001	20	46986001	46987000	1000	1	2.50E-07	0.32	9	0.9	RGD1560975	
DMR20:46988001	20	46988001	46991000	3000	1	3.00E-11	0.69	66	2.2	RGD1560975	
DMR20:46992001	20	46992001	46993000	1000	1	1.70E-07	0.59	19	1.9	RGD1560975	
DMR20:47076001	20	47076001	47078000	2000	1	2.10E-10	-0.47	50	2.5	Lace1	
DMR20:47086001	20	47086001	47092000	6000	1	2.20E-09	0.41	91	1.52	Lace1	
DMR20:47196001	20	47196001	47197000	1000	1	1.20E-09	0.66	23	2.3	Lace1	
DMR20:47201001	20	47201001	47208000	7000	1	5.90E-07	0.3	135	1.93	Lace1	
DMR20:47259001	20	47259001	47260000	1000	1	3.00E-07	-0.43	17	1.7	Snx3	
DMR20:47284001	20	47284001	47286000	2000	1	1.80E-07	0.41	22	1.1	Nr2e1	Transcription
DMR20:47578001	20	47578001	47589000	11000	3	1.20E-11	0.56	187	1.7	LOC102550842;LOC365595;Scml4	Epigenetic
DMR20:47592001	20	47592001	47593000	1000	1	1.40E-10	0.47	13	1.3	LOC365595;Scml4	Epigenetic
DMR20:47607001	20	47607001	47608000	1000	1	2.80E-12	0.6	14	1.4	Scml4	Epigenetic
DMR20:47613001	20	47613001	47614000	1000	1	2.80E-11	0.42	5	0.5	Scml4	Epigenetic
DMR20:47618001	20	47618001	47619000	1000	1	3.70E-09	0.46	14	1.4	Scml4	Epigenetic
DMR20:47663001	20	47663001	47666000	3000	1	1.20E-09	0.32	47	1.57	Scml4	Epigenetic

DMR20:47669001	20	47669001	47670000	1000	1	5.00E-08	0.35	13	1.3	Scml4	Epigenetic
DMR20:47765001	20	47765001	47768000	3000	1	1.40E-07	-0.45	52	1.73	Sobp	
DMR20:47802001	20	47802001	47805000	3000	2	2.60E-07	0.5	64	2.13	Sobp	
DMR20:47825001	20	47825001	47828000	3000	1	1.30E-07	0.67	94	3.13	Sobp;LOC108349095	
DMR20:47845001	20	47845001	47848000	3000	1	2.50E-10	-0.52	47	1.57	Sobp;LOC108349095	
DMR20:47868001	20	47868001	47876000	8000	1	6.70E-08	-0.39	151	1.89	Sobp	
DMR20:47888001	20	47888001	47889000	1000	1	1.30E-07	0.43	6	0.6	Sobp	
DMR20:47907001	20	47907001	47908000	1000	1	2.50E-08	-0.38	20	2	Sobp;LOC688390	
DMR20:47998001	20	47998001	48001000	3000	1	7.70E-09	-0.57	56	1.87	Pdss2	
DMR20:48079001	20	48079001	48080000	1000	1	2.40E-08	-0.46	28	2.8	Pdss2;LOC102551730	
DMR20:48099001	20	48099001	48100000	1000	1	5.10E-10	0.35	56	5.6	Pdss2	
DMR20:48122001	20	48122001	48124000	2000	1	5.70E-07	-0.44	39	1.95	Pdss2	
DMR20:48177001	20	48177001	48178000	1000	1	7.10E-10	0.33	17	1.7	Pdss2	
DMR20:48482001	20	48482001	48484000	2000	1	1.70E-08	-0.36	38	1.9	Wasf1	Cytoskeleton
DMR20:48680001	20	48680001	48682000	2000	2	5.70E-12	0.79	43	2.15	Mettl24	Epigenetic
DMR20:48689001	20	48689001	48691000	2000	1	6.00E-10	-0.47	17	0.85	Mettl24	Epigenetic
DMR20:48865001	20	48865001	48866000	1000	1	8.60E-08	-0.4	20	2	Qrsl1	Metabolism
DMR20:48931001	20	48931001	48935000	4000	1	5.30E-08	-0.42	102	2.55	Rtn4ip1;Aim1	Metabolism
DMR20:48991001	20	48991001	48992000	1000	1	1.50E-08	0.4	7	0.7	Aim1	
DMR20:48999001	20	48999001	49003000	4000	2	5.00E-08	-0.34	63	1.57	Aim1	
DMR20:49029001	20	49029001	49031000	2000	1	3.90E-08	-0.41	36	1.8	Aim1	
DMR20:49083001	20	49083001	49086000	3000	1	2.50E-11	-0.56	76	2.53	Aim1	
DMR20:49343001	20	49343001	49344000	1000	1	1.20E-12	-0.51	16	1.6	Atg5	Transport
DMR20:49355001	20	49355001	49361000	6000	1	4.70E-09	0.37	88	1.47	Atg5	Transport
DMR20:49456001	20	49456001	49459000	3000	1	1.30E-07	-0.47	38	1.27	LOC102554933;Prdm1	Transcription
DMR20:49473001	20	49473001	49475000	2000	1	1.70E-13	-0.71	40	2	Prdm1	Transcription
DMR20:49526001	20	49526001	49531000	5000	1	9.60E-07	0.54	82	1.64	Prdm1	Transcription
DMR20:50196001	20	50196001	50202000	6000	1	2.70E-08	-0.53	172	2.87	Prep	Protease
DMR20:50204001	20	50204001	50210000	6000	1	4.90E-09	-0.44	122	2.03	Prep	Protease
DMR20:50521001	20	50521001	50523000	2000	1	1.80E-08	-0.44	36	1.8	Lin28b	Metabolism
DMR20:50565001	20	50565001	50567000	2000	1	4.20E-08	-0.54	42	2.1	Lin28b	Metabolism
DMR20:50655001	20	50655001	50658000	3000	1	3.20E-07	-0.44	37	1.23	Hace1;LOC103694456	Proteolysis
DMR20:50660001	20	50660001	50662000	2000	1	2.50E-07	0.36	14	0.7	Hace1;LOC103694456	Proteolysis
DMR20:50676001	20	50676001	50680000	4000	1	6.70E-07	-0.47	82	2.05	Hace1;LOC103694456	Proteolysis
DMR20:53840001	20	53840001	53842000	2000	1	1.90E-07	0.37	21	1.05	Grik2	Receptor
DMR20:54166001	20	54166001	54168000	2000	1	1.10E-07	0.5	26	1.3	Grik2	Receptor
DMR20:54319001	20	54319001	54320000	1000	1	5.40E-08	0.4	4	0.4	Grik2	Receptor
DMR20:54377001	20	54377001	54379000	2000	1	3.70E-08	0.42	4	0.2	Grik2	Receptor
DMR20:55309001	20	55309001	55310000	1000	1	4.40E-07	0.5	9	0.9	Ascc3	Cytoskeleton
DMR20:55344001	20	55344001	55347000	3000	1	4.50E-07	-0.41	21	0.7	Ascc3	Cytoskeleton
DMR20:55534001	20	55534001	55535000	1000	1	6.10E-07	0.38	13	1.3	Ascc3	Cytoskeleton
DMR20:55547001	20	55547001	55555000	8000	1	1.60E-13	-0.55	81	1.01	Ascc3	Cytoskeleton
DMR20:55617001	20	55617001	55619000	2000	1	6.60E-08	-0.41	21	1.05	Sim1	Transcription
DMR20:56179001	20	56179001	56183000	4000	1	1.80E-10	-0.74	102	2.55	LOC103694466;LOC102556677;LOC103694460;Cd99	
DMR20:56199001	20	56199001	56201000	2000	1	3.30E-11	0.64	66	3.3	LOC103694460;Cd99	
DMRX:290001	X	290001	293000	3000	2	5.80E-27	0.78	43	1.43	Olr1756-ps	
DMRX:2122001	X	2122001	2124000	2000	1	1.80E-08	-0.29	19	0.95	Rp2	Signaling
DMRX:5805001	X	5805001	5817000	12000	3	8.70E-08	-0.35	110	0.92	LOC691655;Efhc2	Signaling
DMRX:5922001	X	5922001	5923000	1000	1	1.80E-14	0.76	9	0.9	Efhc2	Signaling
DMRX:6005001	X	6005001	6006000	1000	1	7.20E-09	0.45	11	1.1	Efhc2	Signaling
DMRX:6036001	X	6036001	6040000	4000	1	3.80E-09	-0.27	36	0.9	Efhc2	Signaling
DMRX:14097001	X	14097001	14099000	2000	1	1.10E-08	-0.41	12	0.6	Sytl5	
DMRX:14452001	X	14452001	14453000	1000	1	3.30E-07	-0.45	4	0.4	Lancl3	
DMRX:14530001	X	14530001	14531000	1000	1	8.40E-08	-0.53	25	2.5	Xk;LOC102553061	
DMRX:14717001	X	14717001	14727000	10000	3	4.30E-11	-0.39	89	0.89	RGD1561106	
DMRX:15712001	X	15712001	15713000	1000	1	5.90E-08	0.55	15	1.5	Syp;Cacna1f	Transport;Transport
DMRX:15776001	X	15776001	15778000	2000	1	4.10E-07	-0.49	21	1.05	Foxp3;Ppp1r3f	
DMRX:15948001	X	15948001	15950000	2000	2	2.90E-16	0.95	47	2.35	RGD1566093	
DMRX:16123001	X	16123001	16126000	3000	1	1.00E-10	-0.37	23	0.77	Mir362;Mir3560;Mir501;Mir500	
DMRX:16160001	X	16160001	16161000	1000	1	2.00E-08	-0.43	10	1	Clcn5	Transport
DMRX:16319001	X	16319001	16323000	4000	1	1.20E-08	-0.29	31	0.78	Ccnb3	Signaling
DMRX:16941001	X	16941001	16942000	1000	1	2.90E-08	0.37	3	0.3	RGD1563606	

DMRX:18874001	X	18874001	18875000	1000	1	3.30E-10	-0.62	15	1.5	Klf8	Transcription
DMRX:19155001	X	19155001	19156000	1000	1	3.10E-07	-0.59	4	0.4	Foxr2	
DMRX:20997001	X	20997001	21002000	5000	1	5.80E-08	-0.37	46	0.92	FAM120C	
DMRX:21146001	X	21146001	21153000	7000	1	1.40E-07	-0.38	94	1.34	Phf8	
DMRX:23425001	X	23425001	23427000	2000	1	1.50E-08	-0.53	9	0.45	Cldn34a;LOC685689	Cell Junction
DMRX:23554001	X	23554001	23556000	2000	1	8.00E-09	-0.64	13	0.65	Shroom2	Cytoskeleton
DMRX:25529001	X	25529001	25530000	1000	1	6.70E-07	0.48	23	2.3	Mid1	Proteolysis
DMRX:26381001	X	26381001	26386000	5000	1	5.20E-09	-0.31	48	0.96	Arhgap6	Signaling
DMRX:26696001	X	26696001	26698000	2000	1	4.30E-12	0.7	12	0.6	Arhgap6	Signaling
DMRX:26751001	X	26751001	26758000	7000	1	7.80E-08	-0.3	79	1.13	Arhgap6	Signaling
DMRX:27944001	X	27944001	27946000	2000	1	1.20E-07	-0.35	14	0.7	Frmpd4	
DMRX:28117001	X	28117001	28120000	3000	2	5.20E-07	-0.36	29	0.97	Frmpd4	
DMRX:28560001	X	28560001	28562000	2000	2	1.50E-07	-0.6	31	1.55	Tlr8	Receptor
DMRX:30644001	X	30644001	30647000	3000	1	1.30E-07	-0.3	26	0.87	Glra2	Ion Channel
DMRX:31752001	X	31752001	31758000	6000	1	2.40E-07	-0.34	58	0.97	Asb9;Asb11	
DMRX:31847001	X	31847001	31848000	1000	1	8.60E-08	0.65	24	2.4	Figf;LOC108349213;Pir	Transcription
DMRX:32134001	X	32134001	32137000	3000	1	2.30E-08	-0.32	29	0.97	Tmem27	
DMRX:32284001	X	32284001	32289000	5000	1	3.40E-08	-0.41	47	0.94	Ca5b	
DMRX:32382001	X	32382001	32383000	1000	1	3.30E-08	-0.68	12	1.2	Ap1s2	Transport
DMRX:33359001	X	33359001	33362000	3000	1	1.80E-14	-0.51	25	0.83	RGD1564836;LOC108349120	Transcription
DMRX:33829001	X	33829001	33831000	2000	1	7.90E-07	-0.6	17	0.85	Reps2;LOC108349215	Transport
DMRX:35366001	X	35366001	35368000	2000	1	1.80E-09	-0.35	18	0.9	Scml2	Epigenetic
DMRX:35872001	X	35872001	35878000	6000	1	1.60E-10	-0.46	63	1.05	Ppef1;LOC108349217	Signaling
DMRX:36122001	X	36122001	36123000	1000	1	8.10E-07	0.47	7	0.7	Phka2	
DMRX:36309001	X	36309001	36311000	2000	1	3.70E-09	-0.61	12	0.6	Phka2	
DMRX:36386001	X	36386001	36390000	4000	1	2.50E-09	-0.36	41	1.02	Phka2	
DMRX:36450001	X	36450001	36454000	4000	2	5.00E-14	0.72	74	1.85	Phka2;LOC108353287	
DMRX:36455001	X	36455001	36457000	2000	2	1.60E-16	0.73	40	2	Phka2;LOC108353287	
DMRX:36467001	X	36467001	36469000	2000	2	1.80E-09	0.58	54	2.7	Phka2;LOC108353287	
DMRX:39901001	X	39901001	39905000	4000	1	5.90E-08	-0.31	38	0.95	Cnksr2	
DMRX:43003001	X	43003001	43008000	5000	1	3.00E-09	-0.4	41	0.82	Spink9	
DMRX:43301001	X	43301001	43303000	2000	2	2.50E-08	0.9	58	2.9	LOC680190;RGD1566060	Cell Junction
DMRX:43865001	X	43865001	43869000	4000	1	2.80E-08	-0.58	38	0.95	Cldn34d	Cell Junction
DMRX:45088001	X	45088001	45095000	7000	2	8.30E-10	-0.32	58	0.83	Prkx	Signaling
DMRX:45111001	X	45111001	45112000	1000	1	2.70E-08	-0.87	8	0.8	Prkx	Signaling
DMRX:45403001	X	45403001	45410000	7000	1	5.00E-07	-0.28	115	1.64	Ptges3l1	Transcription
DMRX:46734001	X	46734001	46736000	2000	1	4.90E-07	0.39	15	0.75	Cfap47	
DMRX:48768001	X	48768001	48774000	6000	1	3.60E-09	-0.41	55	0.92	Tmem47	
DMRX:49648001	X	49648001	49654000	6000	2	1.20E-09	-0.31	71	1.18	Srsf8	Translation
DMRX:49701001	X	49701001	49706000	5000	1	2.30E-09	0.59	129	2.58	Srsf8;LOC103690857	Translation
DMRX:51190001	X	51190001	51192000	2000	1	1.60E-07	-0.23	25	1.25	Dmd	
DMRX:51193001	X	51193001	51195000	2000	1	5.30E-09	-0.26	17	0.85	Dmd	
DMRX:51206001	X	51206001	51207000	1000	1	1.80E-08	-0.42	6	0.6	Dmd	
DMRX:51277001	X	51277001	51278000	1000	1	7.80E-13	0.8	22	2.2	Dmd;LOC102551195	
DMRX:51318001	X	51318001	51320000	2000	1	8.20E-07	-0.31	11	0.55	Dmd;LOC102549123	
DMRX:51376001	X	51376001	51377000	1000	1	1.50E-07	0.51	6	0.6	Dmd;LOC108349159	
DMRX:51414001	X	51414001	51416000	2000	1	1.70E-12	0.55	21	1.05	Dmd;LOC103690859	
DMRX:51787001	X	51787001	51792000	5000	4	6.00E-13	0.66	277	5.54	Dmd;LOC102549011	
DMRX:51882001	X	51882001	51883000	1000	1	6.60E-07	-0.65	5	0.5	Dmd	
DMRX:52456001	X	52456001	52457000	1000	1	6.60E-07	0.32	13	1.3	Dmd	
DMRX:52502001	X	52502001	52508000	6000	1	9.80E-08	-0.41	66	1.1	Dmd	
DMRX:52701001	X	52701001	52705000	4000	1	2.40E-07	-0.28	37	0.92	Dmd	
DMRX:52793001	X	52793001	52794000	1000	1	3.00E-07	-0.27	10	1	Dmd	
DMRX:52815001	X	52815001	52821000	6000	1	1.90E-07	-0.35	54	0.9	Dmd	
DMRX:52841001	X	52841001	52845000	4000	1	1.50E-08	-0.35	44	1.1	Dmd	
DMRX:53039001	X	53039001	53048000	9000	1	1.90E-07	-0.27	92	1.02	Dmd	
DMRX:53120001	X	53120001	53121000	1000	1	9.90E-07	0.44	5	0.5	Dmd	
DMRX:53129001	X	53129001	53135000	6000	2	1.60E-07	-0.34	67	1.12	Dmd	
DMRX:53267001	X	53267001	53271000	4000	1	7.60E-09	-0.3	27	0.68	Dmd	
DMRX:53272001	X	53272001	53275000	3000	1	1.10E-07	-0.45	20	0.67	Dmd	
DMRX:53306001	X	53306001	53314000	8000	1	6.30E-07	-0.26	97	1.21	Dmd	
DMRX:53616001	X	53616001	53621000	5000	1	2.70E-07	-0.37	40	0.8	Fthl17e	Transport
DMRX:54882001	X	54882001	54884000	2000	1	3.30E-09	-0.42	14	0.7	mageb1l1;Mageb1	

DMRX:55906001	X	55906001	55908000	2000	1	9.20E-07	0.47	20	1	Il1rapl1	Receptor
DMRX:56020001	X	56020001	56023000	3000	1	7.80E-08	-0.21	28	0.93	Il1rapl1	Receptor
DMRX:56325001	X	56325001	56333000	8000	2	1.50E-08	0.44	70	0.88	Il1rapl1	Receptor
DMRX:56612001	X	56612001	56613000	1000	1	5.40E-07	-0.64	2	0.2	Il1rapl1	Receptor
DMRX:56781001	X	56781001	56784000	3000	1	7.00E-09	-0.34	26	0.87	Il1rapl1	Receptor
DMRX:56854001	X	56854001	56858000	4000	2	1.90E-09	-0.46	38	0.95	Il1rapl1	Receptor
DMRX:57710001	X	57710001	57714000	4000	2	9.40E-08	-0.29	33	0.82	RGD1565286	
DMRX:61107001	X	61107001	61108000	1000	1	1.10E-11	0.66	19	1.9	MGC114483;Mageb4	Cytoskeleton
DMRX:61149001	X	61149001	61150000	1000	1	1.70E-08	0.54	14	1.4	Mageb4	Cytoskeleton
DMRX:62853001	X	62853001	62854000	1000	1	1.30E-08	-0.69	12	1.2	Pdk3	Signaling
DMRX:63165001	X	63165001	63166000	1000	1	5.50E-12	-0.68	19	1.9	Zfx	Transcription
DMRX:64223001	X	64223001	64224000	1000	1	1.80E-10	0.81	12	1.2	Spin4	
DMRX:64295001	X	64295001	64300000	5000	1	2.20E-09	-0.44	51	1.02	Arhgef9	Transcription
DMRX:64808001	X	64808001	64810000	2000	1	4.00E-07	-0.3	16	0.8	Asb12;LOC102550262	
DMRX:65519001	X	65519001	65524000	5000	1	6.70E-07	-0.25	44	0.88	Heph	Metabolism
DMRX:65579001	X	65579001	65583000	4000	1	3.60E-07	-0.39	38	0.95	Heph	Metabolism
DMRX:66507001	X	66507001	66512000	5000	1	1.60E-07	-0.42	47	0.94	Rpl34l1;RGD1565058	
DMRX:66523001	X	66523001	66527000	4000	1	8.50E-09	-0.37	26	0.65	RGD1565058	
DMRX:68210001	X	68210001	68213000	3000	1	9.30E-09	-0.28	33	1.1	Ophn1	Signaling
DMRX:68227001	X	68227001	68230000	3000	1	8.30E-07	-0.37	28	0.93	Ophn1	Signaling
DMRX:68270001	X	68270001	68276000	6000	3	1.80E-09	-0.4	67	1.12	Ophn1	Signaling
DMRX:68627001	X	68627001	68628000	1000	1	1.40E-08	0.42	43	4.3	Yipf6	
DMRX:69825001	X	69825001	69827000	2000	1	3.20E-08	-0.46	14	0.7	Eda	
DMRX:69836001	X	69836001	69841000	5000	2	1.30E-08	-0.46	46	0.92	Eda	
DMRX:70401001	X	70401001	70405000	4000	2	6.20E-08	-0.36	35	0.88	Awat1	Metabolism
DMRX:70551001	X	70551001	70555000	4000	1	1.60E-09	-0.34	37	0.92	Kif4a;Trnaf-gaa;Gdpd2	Cytoskeleton;Signaling
DMRX:70677001	X	70677001	70682000	5000	1	2.80E-10	-0.41	46	0.92	Tex11	
DMRX:70762001	X	70762001	70764000	2000	1	1.80E-11	0.84	13	0.65	Tex11	
DMRX:70772001	X	70772001	70773000	1000	1	2.50E-07	-0.55	12	1.2	Tex11	
DMRX:70934001	X	70934001	70937000	3000	1	4.00E-08	-0.34	24	0.8	Tex11;LOC100362309	
DMRX:71130001	X	71130001	71132000	2000	1	7.90E-07	-0.54	11	0.55	Snx12;LOC102548326	
DMRX:71548001	X	71548001	71549000	1000	1	8.80E-10	-0.55	9	0.9	Ogt	Golgi
DMRX:71970001	X	71970001	71972000	2000	1	3.10E-10	-0.61	27	1.35	Nhsl2;LOC100911832	
DMRX:72682001	X	72682001	72686000	4000	3	1.60E-13	0.97	93	2.33	LOC680024;Dmrtc1c1	Transcription
DMRX:73002001	X	73002001	73003000	1000	1	6.10E-08	0.65	19	1.9	LOC100360246;RGD1563270	
DMRX:73399001	X	73399001	73402000	3000	3	2.60E-08	0.63	16	0.53	Zfp449	Transcription
DMRX:73496001	X	73496001	73498000	2000	2	2.60E-07	0.68	59	2.95	Fam9b	
DMRX:73574001	X	73574001	73576000	2000	1	8.40E-10	0.37	30	1.5	LOC100910451;LOC100363632;Fam127c;LOC100363642	
DMRX:74380001	X	74380001	74381000	1000	1	2.10E-07	-0.45	21	2.1	Jpx;LOC367858	
DMRX:74386001	X	74386001	74389000	3000	1	8.50E-11	0.45	21	0.7	Jpx	
DMRX:74651001	X	74651001	74653000	2000	1	1.80E-07	-0.55	39	1.95	Slc16a2	
DMRX:75372001	X	75372001	75373000	1000	1	9.30E-07	-0.4	14	1.4	Uprt	Signaling
DMRX:77255001	X	77255001	77259000	4000	1	9.00E-09	-0.27	36	0.9	LOC680814;Pgk1	Metabolism
DMRX:77294001	X	77294001	77296000	2000	2	2.30E-14	0.97	88	4.4	Taf9b;LOC100359505	
DMRX:77485001	X	77485001	77487000	2000	2	6.30E-07	-0.53	66	3.3	Fndc3c1	Proteolysis
DMRX:78191001	X	78191001	78193000	2000	1	7.00E-08	-0.25	17	0.85	P2ry10	Signaling
DMRX:78290001	X	78290001	78291000	1000	1	2.70E-07	-0.57	10	1	RGD1560455;LOC100909985	Signaling
DMRX:78384001	X	78384001	78388000	4000	2	4.50E-11	-0.44	38	0.95	Gpr174;LOC102552443	Signaling
DMRX:80280001	X	80280001	80281000	1000	1	4.50E-07	0.52	10	1	Sh3bgrl	
DMRX:80313001	X	80313001	80317000	4000	1	7.00E-07	-0.24	34	0.85	Sh3bgrl	
DMRX:84976001	X	84976001	84977000	1000	1	7.20E-07	0.46	8	0.8	Dach2	Transcription
DMRX:85272001	X	85272001	85276000	4000	1	3.00E-10	-0.33	31	0.78	Dach2	Transcription
DMRX:85368001	X	85368001	85369000	1000	1	2.30E-07	-0.24	6	0.6	Dach2	Transcription
DMRX:92914001	X	92914001	92920000	6000	2	2.30E-07	-0.35	47	0.78	Pcdh11x	
DMRX:93067001	X	93067001	93070000	3000	1	1.10E-08	-0.32	26	0.87	Pcdh11x;LOC100361016	Development
DMRX:93091001	X	93091001	93093000	2000	1	2.70E-10	-0.41	57	2.85	Pcdh11x	
DMRX:93124001	X	93124001	93128000	4000	1	4.20E-07	-0.26	31	0.78	Pcdh11x	
DMRX:95341001	X	95341001	95343000	2000	1	1.80E-07	-0.31	16	0.8	Olr10;Olr11	Signaling
DMRX:99763001	X	99763001	99764000	1000	1	1.30E-13	-0.35	10	1	Diaph2	
DMRX:100222001	X	100222001	100226000	4000	1	5.40E-07	-0.34	34	0.85	Diaph2	
DMRX:105002001	X	105002001	105003000	1000	1	1.70E-09	0.38	1	0.1	LOC102554368;Arl13a	
DMRX:105044001	X	105044001	105046000	2000	2	1.20E-11	-0.48	16	0.8	Arl13a	

DMRX:105488001	X	105488001	105493000	5000	1	3.70E-07	-0.33	43	0.86	Armcx4	
DMRX:106531001	X	106531001	106534000	3000	1	2.40E-07	-0.54	33	1.1	Arxes2	
DMRX:106777001	X	106777001	106778000	1000	1	7.90E-07	0.54	11	1.1	Tceal7;LOC680327	Transcription
DMRX:108419001	X	108419001	108421000	2000	2	7.70E-12	0.4	11	0.55	Il1rapl2	Receptor
DMRX:108566001	X	108566001	108567000	1000	1	5.90E-08	0.53	13	1.3	Il1rapl2	Receptor
DMRX:109017001	X	109017001	109018000	1000	1	4.40E-07	0.44	5	0.5	Il1rapl2	Receptor
DMRX:109160001	X	109160001	109166000	6000	3	2.30E-09	-0.35	53	0.88	Il1rapl2	Receptor
DMRX:109218001	X	109218001	109222000	4000	1	8.30E-08	-0.31	35	0.88	Il1rapl2	Receptor
DMRX:109264001	X	109264001	109267000	3000	1	2.00E-07	-0.52	41	1.37	Il1rapl2	Receptor
DMRX:109462001	X	109462001	109465000	3000	1	8.60E-10	-0.47	27	0.9	Il1rapl2	Receptor
DMRX:109553001	X	109553001	109555000	2000	1	9.40E-07	-0.29	15	0.75	Il1rapl2	Receptor
DMRX:109669001	X	109669001	109672000	3000	1	2.90E-07	-0.38	24	0.8	Il1rapl2;LOC681392	Receptor
DMRX:109742001	X	109742001	109745000	3000	1	2.20E-07	-0.36	26	0.87	Il1rapl2	Receptor
DMRX:109749001	X	109749001	109753000	4000	1	2.50E-07	-0.38	36	0.9	Il1rapl2	Receptor
DMRX:110213001	X	110213001	110217000	4000	2	4.60E-11	-0.33	36	0.9	Serpina7	Protease; Proteolysis
DMRX:110854001	X	110854001	110855000	1000	1	1.60E-07	0.56	11	1.1	Rnf128	
DMRX:110972001	X	110972001	110979000	7000	1	3.90E-09	-0.31	86	1.23	Tbc1d8b	Signaling
DMRX:111160001	X	111160001	111161000	1000	1	5.60E-07	-0.68	11	1.1	Morc4	
DMRX:111442001	X	111442001	111443000	1000	1	5.90E-08	-0.62	8	0.8	Pih1d3	
DMRX:111726001	X	111726001	111728000	2000	1	5.00E-09	0.47	15	0.75	Frmpd3	
DMRX:112738001	X	112738001	112742000	4000	1	2.10E-07	-0.28	31	0.78	Col4a6	Extracellular Matrix
DMRX:112870001	X	112870001	112873000	3000	1	9.90E-08	-0.49	14	0.47	Col4a5	Extracellular Matrix
DMRX:112996001	X	112996001	112998000	2000	1	8.80E-07	-0.27	17	0.85	Irs4	
DMRX:113230001	X	113230001	113231000	1000	1	7.20E-09	-0.72	13	1.3	RGD1566136	
DMRX:113408001	X	113408001	113411000	3000	1	5.30E-09	-0.33	21	0.7	Gucy2f	Signaling
DMRX:113569001	X	113569001	113575000	6000	1	1.10E-07	-0.41	63	1.05	Kcne5	Transport
DMRX:114873001	X	114873001	114874000	1000	1	2.50E-10	-0.79	4	0.4	Pak3	Signaling
DMRX:115269001	X	115269001	115270000	1000	1	1.80E-09	0.61	26	2.6	RGD1566355	
DMRX:115655001	X	115655001	115658000	3000	1	1.80E-08	-0.57	31	1.03	Trpc5	Transport
DMRX:115692001	X	115692001	115698000	6000	1	4.30E-07	-0.22	67	1.12	Trpc5;Trpc5os	Transport
DMRX:115779001	X	115779001	115780000	1000	1	8.60E-07	-0.67	14	1.4	Trpc5	Transport
DMRX:116382001	X	116382001	116387000	5000	2	6.10E-07	-0.36	37	0.74	Zcchc16	
DMRX:118145001	X	118145001	118146000	1000	1	5.90E-07	0.52	6	0.6	Htr2c	Signaling
DMRX:118245001	X	118245001	118246000	1000	1	2.50E-08	0.6	10	1	Htr2c	Signaling
DMRX:118473001	X	118473001	118475000	2000	1	1.20E-07	-0.62	5	0.25	Il13ra2	Receptor
DMRX:118719001	X	118719001	118720000	1000	1	8.50E-07	-0.39	11	1.1	Ott	
DMRX:118735001	X	118735001	118736000	1000	1	3.30E-07	-0.42	7	0.7	Ott	
DMRX:119062001	X	119062001	119063000	1000	1	1.20E-08	-0.65	16	1.6	Pls3	Cytoskeleton
DMRX:122981001	X	122981001	122984000	3000	1	1.40E-07	-0.27	29	0.97	Lonrf3	Proteolysis
DMRX:123070001	X	123070001	123076000	6000	3	3.10E-08	-0.49	65	1.08	RGD1561445	
DMRX:123308001	X	123308001	123309000	1000	1	9.10E-08	-0.62	10	1	RGD1565032	
DMRX:123318001	X	123318001	123321000	3000	1	1.60E-11	-0.34	34	1.13	RGD1565032;LOC100361883	
DMRX:123737001	X	123737001	123738000	1000	1	1.40E-12	-0.65	14	1.4	Upf3b	
DMRX:123743001	X	123743001	123744000	1000	1	1.30E-07	-0.26	7	0.7	Nkap	
DMRX:123873001	X	123873001	123878000	5000	1	6.90E-07	-0.32	47	0.94	Rhox3;Rhox2	
DMRX:123998001	X	123998001	123999000	1000	1	4.30E-08	0.73	31	3.1	Rhox2;LOC100364002	Development
DMRX:124452001	X	124452001	124455000	3000	1	1.20E-07	-0.42	30	1	Tmem255a	
DMRX:128180001	X	128180001	128184000	4000	1	1.80E-07	-0.31	44	1.1	Thoc2	Transcription
DMRX:128276001	X	128276001	128277000	1000	1	2.50E-08	0.5	3	0.3	Thoc2;LOC691151	Transcription
DMRX:128539001	X	128539001	128544000	5000	1	8.20E-07	-0.27	48	0.96	Stag2	Epigenetic
DMRX:128672001	X	128672001	128673000	1000	1	1.90E-08	-0.47	8	0.8	RGD1560119	
DMRX:128674001	X	128674001	128675000	1000	1	5.80E-08	-0.52	11	1.1	RGD1560119	
DMRX:128681001	X	128681001	128682000	1000	1	1.60E-07	0.54	14	1.4	RGD1560119	
DMRX:128683001	X	128683001	128687000	4000	2	1.10E-08	-0.35	35	0.88	RGD1560119	
DMRX:128936001	X	128936001	128937000	1000	1	4.90E-08	-0.63	13	1.3	Tenm1	
DMRX:128941001	X	128941001	128943000	2000	1	1.40E-07	0.41	22	1.1	Tenm1;LOC103694513	
DMRX:129288001	X	129288001	129293000	5000	1	4.40E-08	-0.32	39	0.78	Tenm1;LOC108349252	
DMRX:134875001	X	134875001	134876000	1000	1	2.00E-08	-0.64	11	1.1	Apln	
DMRX:136746001	X	136746001	136747000	1000	1	9.30E-09	0.6	4	0.4	Olr1765	Signaling
DMRX:136894001	X	136894001	136898000	4000	1	2.00E-09	-0.59	21	0.52	Olr1766	Receptor
DMRX:139935001	X	139935001	139936000	1000	1	3.20E-08	-0.56	8	0.8	Gpc3	
DMRX:140172001	X	140172001	140173000	1000	1	2.90E-07	-0.57	10	1	Ccdc160	
DMRX:142541001	X	142541001	142542000	1000	1	1.10E-08	0.58	10	1	Fgf13	Growth Factors

DMRX:143230001	X	143230001	143231000	1000	1	2.00E-09	-0.61	7	0.7	Mcf2	Transcription
DMRX:143376001	X	143376001	143378000	2000	1	1.40E-07	-0.5	15	0.75	Atp11c	Transport
DMRX:143414001	X	143414001	143420000	6000	1	1.80E-07	-0.29	57	0.95	Atp11c	Transport
DMRX:150478001	X	150478001	150481000	3000	1	1.20E-13	0.9	39	1.3	Olr1761-ps	
DMRX:150488001	X	150488001	150489000	1000	1	3.10E-07	0.39	7	0.7	Olr1761-ps	
DMRX:150490001	X	150490001	150494000	4000	2	4.10E-24	0.74	54	1.35	Olr1761-ps	
DMRX:150552001	X	150552001	150555000	3000	1	2.20E-07	0.37	15	0.5	Olr1770-ps;LOC103694521	
DMRX:150556001	X	150556001	150557000	1000	1	9.40E-07	0.41	5	0.5	Olr1770-ps;LOC103694521	
DMRX:152454001	X	152454001	152457000	3000	1	1.10E-09	-0.42	30	1	Gabra3	Ion Channel
DMRX:152540001	X	152540001	152545000	5000	2	2.50E-09	-0.39	46	0.92	Gabra3	Ion Channel
DMRX:152604001	X	152604001	152605000	1000	1	1.20E-07	0.53	7	0.7	Gabra3	Ion Channel
DMRX:153009001	X	153009001	153012000	3000	1	3.00E-08	-0.33	35	1.17	Zfp185	
DMRX:153920001	X	153920001	153921000	1000	1	4.10E-08	0.44	15	1.5	Aff2	Transcription
DMRX:155640001	X	155640001	155643000	3000	1	2.00E-07	-0.28	29	0.97	RGD1565338	
DMRX:155833001	X	155833001	155839000	6000	3	3.50E-11	0.43	170	2.83	Dkc1	Cell Cycle
DMRX:155840001	X	155840001	155848000	8000	3	8.40E-27	0.63	279	3.49	Dkc1	Cell Cycle
DMRX:155850001	X	155850001	155853000	3000	1	8.00E-21	0.94	187	6.23	Dkc1	Cell Cycle
DMRX:156295001	X	156295001	156302000	7000	1	6.60E-08	-0.29	104	1.49	G6pd	
DMRX:156320001	X	156320001	156321000	1000	1	1.00E-10	-0.71	10	1	Fam3a	Signaling
DMRX:157878001	X	157878001	157879000	1000	1	3.50E-07	-0.67	15	1.5	Fam122b	
DMRX:159137001	X	159137001	159138000	1000	1	3.90E-11	0.55	6	0.6	Fhl1	
DMRX:159358001	X	159358001	159360000	2000	1	8.10E-11	-0.57	17	0.85	Gpr112l	
DMRX:159546001	X	159546001	159549000	3000	1	1.00E-08	-0.45	23	0.77	Vgll1	
DMRX:159894001	X	159894001	159897000	3000	2	6.80E-08	-0.24	26	0.87	Rbmx	
DMRY:1123001	Y	1123001	1126000	3000	1	2.60E-07	-0.42	23	0.77	Kdm6a	Epigenetic
DMRY:1179001	Y	1179001	1180000	1000	1	2.30E-07	-0.56	4	0.4	Kdm6a	Epigenetic
DMRY:1315001	Y	1315001	1322000	7000	2	4.90E-08	-0.36	60	0.86	Usp9y	Protease
DMRY:1352001	Y	1352001	1358000	6000	1	4.10E-07	-0.27	54	0.9	Usp9y	Protease
DMRY:1391001	Y	1391001	1395000	4000	2	3.70E-11	-0.41	43	1.07	Usp9y	Protease
DMRY:1409001	Y	1409001	1416000	7000	1	5.50E-08	-0.43	75	1.07	Usp9y	Protease
