# Supplementary material for: Environmental induced transgenerational inheritance impacts systems epigenetics in disease etiology
Source: Sci Rep. 2022 Apr 19;12:5452. doi: 10.1038/s41598-022-09336-0 (PMC9018793; doi:10.1038/s41598-022-09336-0)
Supplement: Supplementary file 18 — Supplementary Table S10. [file 41598_2022_9336_MOESM18_ESM.pdf]

Supplemental Table S10
Pesticides DMR p<1e-06

DMR Name	Chr	Start	Stop	Length	# Sig Win	minP	maxLFC	CpG #	CpG Density	Gene Annotation	Gene Category
DMR1:1762001	1	1762001	1764000	2000	1	5.30E-07	-0.49	15	0.75	Pcmt1;Nup43	Epigenetic
DMR1:1975001	1	1975001	1978000	3000	1	2.30E-07	-0.45	57	1.9	Zc3h12d	Translation
DMR1:2566001	1	2566001	2570000	4000	1	3.20E-08	0.32	54	1.35	Ust	Transport
DMR1:2852001	1	2852001	2855000	3000	1	5.40E-07	0.37	63	2.1	Sash1	
DMR1:3840001	1	3840001	3841000	1000	1	6.20E-08	-0.45	19	1.9	RGD1560633	Translation
DMR1:4000001	1	4000001	4002000	2000	1	3.10E-08	0.31	14	0.7	Stxbp5	Transport
DMR1:4469001	1	4469001	4472000	3000	1	8.40E-07	-0.37	19	0.63	Adgb	
DMR1:4525001	1	4525001	4530000	5000	1	1.90E-07	-0.3	54	1.08	Adgb	
DMR1:5378001	1	5378001	5380000	2000	1	2.10E-09	-0.55	21	1.05	Fbxo30	
DMR1:5453001	1	5453001	5459000	6000	2	1.60E-07	-0.48	67	1.12	Epm2a	
DMR1:6440001	1	6440001	6443000	3000	1	9.60E-07	0.44	29	0.97	Utrn	
DMR1:6565001	1	6565001	6567000	2000	2	1.10E-07	-0.44	13	0.65	Utrn	
DMR1:6587001	1	6587001	6588000	1000	1	7.30E-07	-0.5	10	1	Utrn	
DMR1:6589001	1	6589001	6590000	1000	1	7.50E-07	0.33	15	1.5	Utrn	
DMR1:7055001	1	7055001	7056000	1000	1	9.30E-07	-0.45	32	3.2	Stx11	Transcription
DMR1:7231001	1	7231001	7234000	3000	1	8.60E-07	0.34	57	1.9	Plagl1	Transcription
DMR1:7287001	1	7287001	7290000	3000	1	1.60E-08	-0.49	51	1.7	Zc2hc1b	Transcription
DMR1:7421001	1	7421001	7430000	9000	1	1.20E-07	-0.38	91	1.01	Phactr2	Signaling
DMR1:7702001	1	7702001	7704000	2000	1	3.80E-07	-0.46	10	0.5	Pex3	
DMR1:8592001	1	8592001	8594000	2000	1	2.00E-07	-0.49	16	0.8	Adgrg6	Signaling
DMR1:12103001	1	12103001	12105000	2000	1	3.20E-07	-0.38	14	0.7	Mcc	
DMR1:12964001	1	12964001	12970000	6000	2	3.30E-09	-0.31	62	1.03	Txlnb	Transport
DMR1:13341001	1	13341001	13342000	1000	1	4.30E-08	0.35	15	1.5	NEWGENE_2319083	
DMR1:13390001	1	13390001	13392000	2000	1	2.60E-09	0.47	51	2.55	Ccdc28a;LOC102549233	Receptor
DMR1:13686001	1	13686001	13687000	1000	1	8.90E-07	-0.42	20	2	Nhsl1	
DMR1:13692001	1	13692001	13693000	1000	1	7.10E-10	0.65	9	0.9	Nhsl1	
DMR1:13881001	1	13881001	13883000	2000	1	6.50E-09	-0.5	29	1.45	Arfgef3	Transcription
DMR1:14108001	1	14108001	14109000	1000	1	1.20E-07	-0.58	24	2.4	RGD1560303	Signaling
DMR1:15205001	1	15205001	15207000	2000	1	9.40E-07	-0.38	23	1.15	Il20ra	Receptor
DMR1:15530001	1	15530001	15531000	1000	1	1.60E-11	0.45	31	3.1	Map3k5	Signaling
DMR1:15692001	1	15692001	15695000	3000	1	2.50E-07	0.34	60	2	Map7	Cytoskeleton
DMR1:15701001	1	15701001	15703000	2000	1	2.50E-09	0.42	67	3.35	Map7	Cytoskeleton
DMR1:15711001	1	15711001	15716000	5000	1	5.60E-09	0.42	83	1.66	Map7	Cytoskeleton
DMR1:15923001	1	15923001	15926000	3000	1	4.80E-09	0.45	55	1.83	Pde7b	Signaling
DMR1:15964001	1	15964001	15966000	2000	1	2.30E-07	0.35	34	1.7	Pde7b	Signaling
DMR1:16845001	1	16845001	16846000	1000	1	1.90E-10	0.47	13	1.3	Hbs1l	Translation
DMR1:16898001	1	16898001	16899000	1000	1	1.50E-07	-0.39	6	0.6	Hbs1l;LOC108349717	Translation
DMR1:17561001	1	17561001	17568000	7000	2	2.80E-11	-0.35	98	1.4	Ptprk;LOC102552760;LOC100911400	Signaling
DMR1:17817001	1	17817001	17819000	2000	1	1.70E-07	-0.41	34	1.7	Ptprk	Signaling
DMR1:18052001	1	18052001	18054000	2000	1	1.10E-07	0.29	18	0.9	Ptprk	Signaling
DMR1:18700001	1	18700001	18704000	4000	2	1.50E-09	-0.35	48	1.2	Lama2	Extracellular Matrix
DMR1:18994001	1	18994001	18995000	1000	1	4.40E-09	-0.63	8	0.8	Lama2	Extracellular Matrix
DMR1:19075001	1	19075001	19081000	6000	1	6.60E-08	-0.27	65	1.08	Lama2	Extracellular Matrix
DMR1:20031001	1	20031001	20032000	1000	1	6.60E-07	0.35	21	2.1	L3mbtl3	Epigenetic
DMR1:20122001	1	20122001	20123000	1000	1	4.60E-12	0.47	11	1.1	Samd3;LOC100360791	
DMR1:20929001	1	20929001	20930000	1000	1	5.80E-13	0.56	3	0.3	Epb41l2	
DMR1:20985001	1	20985001	20987000	2000	2	3.00E-11	0.43	58	2.9	Epb41l2	
DMR1:21021001	1	21021001	21022000	1000	1	3.30E-09	0.56	3	0.3	Epb41l2	
DMR1:21138001	1	21138001	21139000	1000	1	3.40E-10	0.52	34	3.4	LOC102552728;Akap7;LOC100364016	Translation
DMR1:21773001	1	21773001	21775000	2000	1	6.80E-07	0.28	10	0.5	Enpp1	
DMR1:22146001	1	22146001	22148000	2000	1	3.90E-09	-0.5	37	1.85	Moxd1	Metabolism
DMR1:22187001	1	22187001	22192000	5000	1	5.30E-07	-0.34	53	1.06	Moxd1	Metabolism
DMR1:22262001	1	22262001	22263000	1000	1	6.60E-07	0.34	16	1.6	Stx7	Transcription
DMR1:22335001	1	22335001	22340000	5000	1	2.60E-07	-0.32	38	0.76	Taar8c	Signaling
DMR1:22488001	1	22488001	22493000	5000	2	4.30E-08	-0.35	47	0.94	Taar7e;Taar7d	Signaling

DMR1:22518001	1	22518001	22520000	2000	1	2.00E-07	-0.58	18	0.9	Taar7c;Taar7b;Taar7a	Signaling
DMR1:22577001	1	22577001	22580000	3000	1	1.20E-09	-0.43	50	1.67	LOC102554023;Taar3;Taar2	Signaling
DMR1:23246001	1	23246001	23248000	2000	1	3.50E-07	-0.42	22	1.1	Eya4	
DMR1:23393001	1	23393001	23396000	3000	1	8.40E-08	0.38	33	1.1	Eya4	
DMR1:23457001	1	23457001	23459000	2000	1	4.20E-07	0.41	31	1.55	Eya4;LOC103690935	
DMR1:23593001	1	23593001	23594000	1000	1	1.80E-07	-0.38	4	0.4	Eya4	
DMR1:25743001	1	25743001	25750000	7000	3	2.00E-09	-0.36	87	1.24	Trdn	
DMR1:28549001	1	28549001	28550000	1000	1	2.60E-07	-0.5	20	2	Tpd52l1	
DMR1:29358001	1	29358001	29362000	4000	1	3.00E-10	-0.33	56	1.4	Ncoa7;LOC108348877	
DMR1:31705001	1	31705001	31707000	2000	1	1.60E-07	-0.4	29	1.45	Ahrr;Exoc3	Transcription;Transport
DMR1:31843001	1	31843001	31845000	2000	1	1.80E-07	-0.27	14	0.7	Cep72	
DMR1:32017001	1	32017001	32018000	1000	1	3.50E-08	0.46	12	1.2	Trip13	Epigenetic
DMR1:32098001	1	32098001	32099000	1000	1	7.40E-08	0.4	39	3.9	Slc12a7	Transport
DMR1:32176001	1	32176001	32178000	2000	1	7.80E-13	0.47	29	1.45	Slc12a7	Transport
DMR1:32342001	1	32342001	32344000	2000	1	3.40E-07	0.51	24	1.2	Slc6a3	Transport
DMR1:32405001	1	32405001	32406000	1000	1	2.60E-09	0.54	41	4.1	Lpcat1	Metabolism
DMR1:35083001	1	35083001	35084000	1000	1	9.20E-07	-0.41	8	0.8	Adamts16	Protease
DMR1:35151001	1	35151001	35158000	7000	2	3.80E-11	-0.35	82	1.17	Adamts16	Protease
DMR1:36361001	1	36361001	36362000	1000	1	9.00E-12	0.49	8	0.8	Srd5a1	Metabolism
DMR1:36438001	1	36438001	36445000	7000	3	8.80E-09	-0.37	77	1.1	Papd7	
DMR1:37272001	1	37272001	37273000	1000	1	2.50E-07	-0.31	16	1.6	Adcy2	
DMR1:37403001	1	37403001	37406000	3000	1	2.70E-08	-0.43	28	0.93	Adcy2	
DMR1:37579001	1	37579001	37585000	6000	1	9.10E-08	-0.32	58	0.97	Adcy2	
DMR1:37651001	1	37651001	37655000	4000	1	2.50E-10	-0.44	16	0.4	Adcy2	
DMR1:37661001	1	37661001	37664000	3000	2	1.20E-08	-0.44	28	0.93	Adcy2	
DMR1:37672001	1	37672001	37682000	10000	6	7.70E-19	0.59	69	0.69	Adcy2	
DMR1:37740001	1	37740001	37742000	2000	1	1.80E-08	-0.53	37	1.85	Fastkd3;Mtrr	Metabolism
DMR1:37767001	1	37767001	37768000	1000	1	4.90E-07	-0.47	8	0.8	Mtrr	Metabolism
DMR1:37867001	1	37867001	37870000	3000	1	2.20E-09	-0.33	50	1.67	RGD1566325;LOC103690951	
DMR1:39832001	1	39832001	39836000	4000	2	1.30E-09	-0.38	29	0.72	Ppp1r14c	Signaling
DMR1:39908001	1	39908001	39909000	1000	1	6.70E-07	-0.4	4	0.4	Ppp1r14c	Signaling
DMR1:40104001	1	40104001	40110000	6000	2	2.30E-13	-0.39	56	0.93	Iyd	Metabolism
DMR1:40290001	1	40290001	40296000	6000	1	1.90E-08	-0.4	39	0.65	LOC103690956;Plekhg1	
DMR1:40621001	1	40621001	40622000	1000	1	7.30E-08	-0.44	17	1.7	Mthfd1l	
DMR1:41255001	1	41255001	41262000	7000	2	4.80E-10	-0.5	66	0.94	Esr1	
DMR1:41427001	1	41427001	41434000	7000	2	6.60E-16	0.39	85	1.21	Esr1	
DMR1:41437001	1	41437001	41438000	1000	1	4.50E-07	0.52	4	0.4	Esr1	
DMR1:42477001	1	42477001	42478000	1000	1	2.80E-07	0.42	10	1	Fbxo5;Mtrf1l	Translation
DMR1:44289001	1	44289001	44290000	1000	1	1.70E-10	-0.7	11	1.1	Tiam2	
DMR1:44375001	1	44375001	44376000	1000	1	6.90E-07	0.39	9	0.9	Tiam2	
DMR1:44601001	1	44601001	44602000	1000	1	1.00E-09	-0.57	12	1.2	Nox3	Metabolism
DMR1:45948001	1	45948001	45952000	4000	1	2.10E-08	-0.52	39	0.98	Arid1b	
DMR1:46064001	1	46064001	46065000	1000	1	3.40E-14	0.5	15	1.5	Arid1b	
DMR1:46130001	1	46130001	46131000	1000	1	9.60E-08	0.41	22	2.2	Arid1b	
DMR1:46529001	1	46529001	46530000	1000	1	2.50E-07	0.39	12	1.2	Zdhhc14	
DMR1:46574001	1	46574001	46576000	2000	1	6.70E-09	0.36	37	1.85	Zdhhc14	
DMR1:46799001	1	46799001	46802000	3000	1	8.40E-08	-0.31	41	1.37	Snx9	Cytoskeleton
DMR1:46920001	1	46920001	46925000	5000	1	6.30E-08	0.35	90	1.8	Synj2;LOC103690968;Serac1	Signaling
DMR1:47092001	1	47092001	47094000	2000	1	1.30E-07	0.45	32	1.6	Tulp4	
DMR1:47308001	1	47308001	47309000	1000	1	4.40E-09	0.57	9	0.9	Ezr	Cytoskeleton
DMR1:47993001	1	47993001	47995000	2000	1	1.60E-08	0.27	23	1.15	Acat2	Metabolism
DMR1:48008001	1	48008001	48009000	1000	1	6.30E-07	0.43	18	1.8	Acat2l1;LOC108349718	Metabolism
DMR1:48095001	1	48095001	48096000	1000	1	5.70E-07	0.42	18	1.8	Mas1l;Mas1	Signaling
DMR1:48659001	1	48659001	48661000	2000	1	3.80E-07	-0.4	24	1.2	Map3k4	Signaling
DMR1:48680001	1	48680001	48682000	2000	1	7.00E-08	0.39	26	1.3	Map3k4	Signaling
DMR1:48710001	1	48710001	48711000	1000	1	5.80E-08	0.44	20	2	Map3k4;Agpat4	Signaling;Metabolism
DMR1:48727001	1	48727001	48728000	1000	1	2.20E-07	0.7	13	1.3	Agpat4	Metabolism
DMR1:48867001	1	48867001	48871000	4000	1	7.10E-09	0.34	63	1.57	Park2	

DMR1:48901001	1	48901001	48903000	2000	1	7.90E-07	0.42	35	1.75	Park2;LOC103690973	
DMR1:49031001	1	49031001	49032000	1000	1	3.10E-07	0.37	12	1.2	Park2	
DMR1:49113001	1	49113001	49114000	1000	1	6.30E-07	-0.41	14	1.4	Park2	
DMR1:49132001	1	49132001	49133000	1000	1	1.50E-07	0.64	25	2.5	Park2	
DMR1:49414001	1	49414001	49418000	4000	1	1.40E-08	0.42	33	0.82	Park2	
DMR1:49419001	1	49419001	49421000	2000	1	1.30E-09	-0.57	32	1.6	Park2	
DMR1:49438001	1	49438001	49443000	5000	1	5.00E-07	-0.5	21	0.42	Park2	
DMR1:49487001	1	49487001	49489000	2000	1	4.00E-08	0.43	27	1.35	Park2	
DMR1:49510001	1	49510001	49512000	2000	1	9.90E-07	0.55	14	0.7	Park2	
DMR1:49564001	1	49564001	49573000	9000	1	4.80E-07	-0.26	117	1.3	Park2	
DMR1:49915001	1	49915001	49921000	6000	3	9.40E-11	-0.4	65	1.08	Park2	
DMR1:49953001	1	49953001	49954000	1000	1	1.20E-07	-0.44	10	1	Park2;LOC108349802	
DMR1:50081001	1	50081001	50084000	3000	1	9.20E-09	0.54	28	0.93	Pacrg	
DMR1:50229001	1	50229001	50230000	1000	1	2.70E-07	0.42	12	1.2	Pacrg	
DMR1:50359001	1	50359001	50360000	1000	1	4.40E-09	0.49	10	1	Pacrg	
DMR1:50365001	1	50365001	50367000	2000	1	6.80E-07	-0.34	29	1.45	Pacrg	
DMR1:50408001	1	50408001	50410000	2000	1	1.60E-07	0.54	28	1.4	Pacrg	
DMR1:52341001	1	52341001	52344000	3000	1	6.40E-09	0.39	32	1.07	MGC94891	
DMR1:52408001	1	52408001	52411000	3000	1	4.70E-09	-0.58	52	1.73	Pde10a	Signaling
DMR1:52506001	1	52506001	52507000	1000	1	3.80E-07	0.4	19	1.9	Pde10a	Signaling
DMR1:52876001	1	52876001	52879000	3000	1	6.90E-07	0.29	45	1.5	T	
DMR1:53752001	1	53752001	53753000	1000	1	7.20E-07	-0.4	16	1.6	Afdn	Cytoskeleton
DMR1:54041001	1	54041001	54046000	5000	1	2.10E-09	0.29	34	0.68	RGD1560718;LOC102552087	Signaling
DMR1:54047001	1	54047001	54048000	1000	1	1.30E-07	0.43	7	0.7	RGD1560718;LOC102552087;LOC108349470	Signaling
DMR1:54623001	1	54623001	54627000	4000	1	3.90E-07	-0.46	29	0.72	Vom2r7	
DMR1:54846001	1	54846001	54847000	1000	1	1.30E-09	0.37	20	2	Smok2a	Signaling
DMR1:55217001	1	55217001	55226000	9000	2	1.90E-09	0.35	86	0.96	RGD1561667	Signaling
DMR1:55227001	1	55227001	55233000	6000	1	3.20E-10	0.24	38	0.63	RGD1561667	Signaling
DMR1:55234001	1	55234001	55239000	5000	2	3.50E-10	0.3	39	0.78	RGD1561667	Signaling
DMR1:55644001	1	55644001	55664000	20000	4	1.80E-11	0.71	114	0.57	LOC102556502;LOC103690993;LOC102546375;LOC108349807;RGD1561185	
DMR1:55672001	1	55672001	55674000	2000	1	1.10E-13	0.33	34	1.7	RGD1561185	
DMR1:55899001	1	55899001	55900000	1000	1	6.30E-07	0.43	10	1	Vom2r9	Signaling
DMR1:56841001	1	56841001	56844000	3000	1	1.50E-08	-0.42	26	0.87	Wdr27	
DMR1:59539001	1	59539001	59543000	4000	1	5.80E-07	-0.34	24	0.6	Vom2r-ps19	
DMR1:59957001	1	59957001	59961000	4000	1	4.20E-07	-0.43	26	0.65	Vom2r-ps23	
DMR1:60270001	1	60270001	60275000	5000	1	1.10E-07	-0.34	41	0.82	Vom1r10	Receptor
DMR1:60625001	1	60625001	60627000	2000	1	6.20E-07	-0.49	5	0.25	Vom1r15	Receptor
DMR1:60748001	1	60748001	60751000	3000	1	1.20E-07	-0.34	36	1.2	Vom1r-ps17	
DMR1:61076001	1	61076001	61079000	3000	1	1.70E-07	-0.26	25	0.83	Vom1r-ps23	
DMR1:61410001	1	61410001	61413000	3000	1	9.00E-08	-0.38	19	0.63	Zfp52	Transcription
DMR1:61511001	1	61511001	61518000	7000	3	3.80E-09	-0.54	77	1.1	LOC108348215;Znf761	Transcription
DMR1:61549001	1	61549001	61553000	4000	1	1.20E-12	0.61	29	0.72	RGD1565566	
DMR1:61615001	1	61615001	61617000	2000	1	4.80E-08	-0.43	19	0.95	Vom1r22	Receptor
DMR1:61674001	1	61674001	61676000	2000	1	1.10E-07	-0.27	22	1.1	Vom1r23	Receptor
DMR1:61688001	1	61688001	61693000	5000	1	8.40E-07	-0.34	54	1.08	Vom1r23;LOC102553001	Receptor
DMR1:62299001	1	62299001	62300000	1000	1	9.60E-07	-0.39	9	0.9	RGD1566248	
DMR1:62545001	1	62545001	62546000	1000	1	2.20E-08	-0.34	11	1.1	Zfp40	Transcription
DMR1:63344001	1	63344001	63348000	4000	1	1.60E-11	-0.49	53	1.32	Vom1r30	Receptor
DMR1:63437001	1	63437001	63442000	5000	1	2.80E-11	-0.42	43	0.86	Vom2r-ps41	
DMR1:63542001	1	63542001	63547000	5000	2	2.80E-10	-0.34	69	1.38	Vom2r-ps42;Vom2r26	Signaling
DMR1:63548001	1	63548001	63556000	8000	2	8.70E-10	-0.29	101	1.26	Vom2r26	Signaling
DMR1:63671001	1	63671001	63673000	2000	1	6.50E-10	-0.26	40	2	Lilrc2	Immune
DMR1:63674001	1	63674001	63678000	4000	1	1.40E-09	-0.32	45	1.12	Lilrc2	Immune
DMR1:63697001	1	63697001	63710000	13000	3	1.50E-08	-0.39	133	1.02	RGD1562625	
DMR1:63724001	1	63724001	63732000	8000	4	6.10E-07	-0.43	103	1.29	RGD1562625;Pirb	
DMR1:63837001	1	63837001	63838000	1000	1	2.50E-07	-0.52	6	0.6	Lilrb3l;LOC683420	Immune
DMR1:63854001	1	63854001	63861000	7000	2	1.90E-08	-0.27	88	1.26	Lilrb3l	Immune
DMR1:63892001	1	63892001	63896000	4000	1	6.90E-08	-0.32	49	1.23	Lilrb1	

DMR1:63935001	1	63935001	63941000	6000	2	3.70E-11	-0.31	82	1.37	Lilra3	
DMR1:64131001	1	64131001	64134000	3000	1	4.90E-14	0.61	43	1.43	Tmc4;Leng1;Cnot3;Mir3572	Transcription
DMR1:64387001	1	64387001	64388000	1000	1	8.40E-11	0.45	18	1.8	LOC103691003;Cacng7	Transport
DMR1:64688001	1	64688001	64691000	3000	1	4.20E-13	-0.52	18	0.6	Vom2r27	Signaling
DMR1:64839001	1	64839001	64841000	2000	1	1.10E-08	-0.48	20	1	LOC103691004;Vom2r12	
DMR1:64856001	1	64856001	64857000	1000	1	2.70E-08	-0.41	12	1.2	Vom2r12	
DMR1:65010001	1	65010001	65014000	4000	1	1.20E-08	-0.38	42	1.05	LOC108349538;Vom2r-ps26	
DMR1:65759001	1	65759001	65765000	6000	2	1.70E-09	-0.43	44	0.73	Zfp110;LOC108348438	
DMR1:65929001	1	65929001	65932000	3000	1	1.20E-12	-0.51	28	0.93	LOC108349536;Vom2r36;Vom2r33	Signaling
DMR1:65984001	1	65984001	65992000	8000	3	2.70E-11	-0.38	97	1.21	Vom2r36	Signaling
DMR1:66027001	1	66027001	66033000	6000	1	5.80E-10	-0.39	77	1.28	Vom2r36;Vom2r35;LOC108348434	Signaling
DMR1:66247001	1	66247001	66248000	1000	1	3.30E-07	-0.36	10	1	Zfp606	Transcription
DMR1:66285001	1	66285001	66291000	6000	2	9.60E-11	-0.43	65	1.08	Vom2r-ps59	
DMR1:66701001	1	66701001	66707000	6000	1	2.10E-09	-0.35	58	0.97	Vom1r-ps65	
DMR1:66858001	1	66858001	66867000	9000	2	3.20E-08	-0.43	95	1.06	Vom1r51	Receptor
DMR1:66997001	1	66997001	6.70E+07	3000	1	2.00E-08	-0.47	24	0.8	Vom1r-ps53;Vom1r-ps52	
DMR1:67193001	1	67193001	67199000	6000	2	1.20E-10	-0.43	64	1.07	Vom1r43	Receptor
DMR1:67269001	1	67269001	67271000	2000	1	6.70E-08	-0.39	17	0.85	LOC103691020;Vom1r42	
DMR1:68206001	1	68206001	68212000	6000	1	5.90E-07	-0.27	64	1.07	Vom1r-ps38;Vom1r29	Receptor
DMR1:69364001	1	69364001	69369000	5000	1	4.10E-07	-0.25	56	1.12	Nlrp4a	
DMR1:69381001	1	69381001	69385000	4000	1	5.70E-07	-0.37	59	1.48	Nlrp4a	
DMR1:69390001	1	69390001	69397000	7000	1	7.40E-10	-0.28	70	1	Nlrp4a	
DMR1:69417001	1	69417001	69421000	4000	1	5.20E-07	-0.38	32	0.8	Nlrp4a	
DMR1:69509001	1	69509001	69515000	6000	2	2.60E-09	-0.39	68	1.13	Vom2r-ps45;LOC100362711	
DMR1:69731001	1	69731001	69733000	2000	1	4.70E-10	-0.34	13	0.65	Zfp978	
DMR1:69837001	1	69837001	69839000	2000	1	1.50E-09	-0.39	15	0.75	LOC108349745;Zfp773-ps1;LOC108349540	Transcription
DMR1:69850001	1	69850001	69851000	1000	1	7.70E-07	-0.38	8	0.8	Zfp773-ps1;LOC108349540;Zfp772;LOC102547517	Transcription;Transcription
DMR1:71355001	1	71355001	71357000	2000	1	3.50E-08	0.49	21	1.05	Galp	
DMR1:71721001	1	71721001	71724000	3000	1	7.90E-09	-0.45	18	0.6	Nlrp4;RGD1564651	
DMR1:71727001	1	71727001	71731000	4000	1	1.00E-08	-0.36	40	1	RGD1564651	
DMR1:72189001	1	72189001	72192000	3000	1	2.20E-09	-0.42	19	0.63	Vom1r36;LOC108349748	Receptor
DMR1:72435001	1	72435001	72437000	2000	2	5.20E-08	0.34	36	1.8	Sbk2;Ssc5d	Signaling;Protease
DMR1:72454001	1	72454001	72456000	2000	1	5.60E-09	0.48	38	1.9	Ssc5d;Nat14;Zfp628	Protease;Metabolism;Transcription
DMR1:72461001	1	72461001	72463000	2000	1	2.20E-08	0.5	20	1	Ssc5d;Nat14;Zfp628	Protease;Metabolism;Transcription
DMR1:72466001	1	72466001	72469000	3000	1	4.70E-07	0.48	95	3.17	Ssc5d;Nat14;Zfp628	Protease;Metabolism;Transcription
DMR1:72539001	1	72539001	72541000	2000	1	3.70E-07	-0.42	36	1.8	Isoc2b;Shisa7	
DMR1:72542001	1	72542001	72544000	2000	1	2.10E-09	0.53	12	0.6	Isoc2b;Shisa7	
DMR1:72647001	1	72647001	72651000	4000	1	8.80E-08	-0.42	38	0.95	Il11;Fam71e2	Cytokine
DMR1:72965001	1	72965001	72972000	7000	1	3.70E-08	-0.38	92	1.31	Rdh13	Golgi
DMR1:73239001	1	73239001	73242000	3000	1	1.90E-07	-0.46	25	0.83	Fcar;LOC108349720	Immune
DMR1:73324001	1	73324001	73325000	1000	1	7.30E-07	-0.43	9	0.9	Kir3dl1	Immune
DMR1:73846001	1	73846001	73849000	3000	1	3.40E-09	-0.6	24	0.8	Lair1	Immune
DMR1:74711001	1	74711001	74713000	2000	1	6.40E-08	-0.63	13	0.65	Vom2r-ps54	
DMR1:74979001	1	74979001	74983000	4000	1	3.40E-11	-0.56	22	0.55	LOC100911630;LOC100911587;Vom2r31	Signaling
DMR1:75138001	1	75138001	75139000	1000	1	2.80E-08	-0.43	9	0.9	Vom1r58;Vom1r-ps66	Receptor
DMR1:75155001	1	75155001	75156000	1000	1	1.00E-09	-0.48	11	1.1	RGD1564801	Signaling
DMR1:75171001	1	75171001	75172000	1000	1	5.30E-08	-0.64	5	0.5	Vom1r59	Receptor
DMR1:75293001	1	75293001	75295000	2000	1	6.80E-12	-0.52	17	0.85	Vom1r61	Receptor
DMR1:75300001	1	75300001	75306000	6000	1	1.60E-08	-0.34	75	1.25	Vom1r61	Receptor
DMR1:75307001	1	75307001	75310000	3000	1	9.80E-08	-0.42	18	0.6	Vom1r61	Receptor

DMR1:75458001	1	75458001	75463000	5000	1	2.50E-12	-0.36	63	1.26	Pla2g4c	Metabolism
DMR1:75629001	1	75629001	75635000	6000	1	2.20E-09	-0.34	71	1.18	LOC691813;Peli1-ps1	
DMR1:75648001	1	75648001	75653000	5000	1	1.90E-09	-0.36	49	0.98	Peli1-ps1	
DMR1:75759001	1	75759001	75765000	6000	1	5.00E-07	-0.3	76	1.27	Bsph1	
DMR1:76337001	1	76337001	76339000	2000	1	4.20E-08	-0.6	15	0.75	Sult2a2;LOC100363218	
DMR1:76412001	1	76412001	76413000	1000	1	1.60E-09	-0.66	4	0.4	Sult2a2	
DMR1:76428001	1	76428001	76433000	5000	1	2.30E-07	-0.37	58	1.16	Sult2a2	
DMR1:76574001	1	76574001	76575000	1000	1	6.80E-07	-0.44	3	0.3	Sult2a6;Sult2a1	
DMR1:76680001	1	76680001	76686000	6000	2	2.70E-08	-0.48	67	1.12	Sult2a6	
DMR1:77623001	1	77623001	77628000	5000	2	7.10E-09	-0.4	21	0.42	Obox1;LOC102554917	
DMR1:77875001	1	77875001	77878000	3000	1	5.20E-09	0.59	34	1.13	Ehd2;Gltscr1;LOC103691043	Transport
DMR1:78104001	1	78104001	78107000	3000	1	9.80E-09	0.43	73	2.43	Dhx34	Transcription
DMR1:78845001	1	78845001	78847000	2000	1	3.30E-07	0.38	32	1.6	Ptgir;Calm3	Signaling;Signaling
DMR1:78965001	1	78965001	78967000	2000	1	5.60E-07	0.49	18	0.9	Ppp5c;Hif3a	Signaling;Transcription
DMR1:78971001	1	78971001	78973000	2000	1	2.30E-09	0.44	24	1.2	Ppp5c;Hif3a	Signaling;Transcription
DMR1:79016001	1	79016001	79022000	6000	1	4.90E-08	-0.33	58	0.97	Cgm4	
DMR1:79114001	1	79114001	79120000	6000	2	4.50E-14	-0.36	64	1.07	Ceacam3	Immune
DMR1:79172001	1	79172001	79176000	4000	2	6.20E-08	-0.42	44	1.1	Psgb1	
DMR1:79240001	1	79240001	79249000	9000	1	4.70E-12	-0.49	102	1.13	Psg29	
DMR1:79464001	1	79464001	79466000	2000	1	1.80E-07	-0.49	12	0.6	Igfl3	
DMR1:79748001	1	79748001	79749000	1000	1	7.50E-07	-0.41	15	1.5	LOC100362871;Micb	Immune
DMR1:79793001	1	79793001	79794000	1000	1	6.70E-07	0.47	10	1	Pglyrp1;Ccdc61	Epigenetic
DMR1:79809001	1	79809001	79813000	4000	1	1.70E-08	0.44	56	1.4	Ccdc61	Epigenetic
DMR1:79938001	1	79938001	79939000	1000	1	5.90E-08	-0.43	20	2	Foxa3;Sympk	Transcription
DMR1:80107001	1	80107001	80110000	3000	1	6.80E-07	0.31	63	2.1	Eml2	
DMR1:80171001	1	80171001	80173000	2000	1	9.80E-13	0.62	28	1.4	Opa3;Vasp	Cytoskeleton
DMR1:80261001	1	80261001	80262000	1000	1	6.20E-07	0.33	7	0.7	Ercc1;Cd3eap;Ppp1r13l	Transcription;Transcription
DMR1:80355001	1	80355001	80358000	3000	2	1.60E-18	1.26	31	1.03	Mark4	Signaling
DMR1:80684001	1	80684001	80685000	1000	1	2.30E-07	0.47	18	1.8	Bcam	Immune
DMR1:80836001	1	80836001	80841000	5000	1	4.50E-11	0.42	77	1.54	PVR;Igsf23	
DMR1:80917001	1	80917001	80918000	1000	1	2.00E-11	0.51	5	0.5	Ceacam20;Zfp180	Transcription
DMR1:81277001	1	81277001	81278000	1000	1	4.80E-07	0.38	19	1.9	Smg9	
DMR1:81290001	1	81290001	81292000	2000	2	1.40E-10	0.48	39	1.95	Smg9;Irgc	
DMR1:81351001	1	81351001	81354000	3000	1	5.60E-12	0.49	53	1.77	Plaur	Receptor
DMR1:81443001	1	81443001	81444000	1000	1	2.10E-07	0.37	14	1.4	Xrcc1;Zfp575	Transcription;Transcription
DMR1:81852001	1	81852001	81854000	2000	1	5.50E-08	0.38	57	2.85	Rabac1;Atp1a3	Transport;Transport
DMR1:81862001	1	81862001	81863000	1000	1	7.30E-09	0.4	11	1.1	Atp1a3	Transport
DMR1:81924001	1	81924001	81928000	4000	1	1.80E-07	0.39	57	1.43	Grik5	Receptor
DMR1:81933001	1	81933001	81936000	3000	2	4.50E-12	0.44	54	1.8	Grik5	Receptor
DMR1:81937001	1	81937001	81939000	2000	1	3.50E-07	0.34	42	2.1	Grik5	Receptor
DMR1:81995001	1	81995001	81998000	3000	1	8.40E-08	0.34	72	2.4	Pou2f2;LOC103691054	
DMR1:81999001	1	81999001	82001000	2000	1	1.90E-07	0.46	19	0.95	Pou2f2;LOC103691054	
DMR1:82113001	1	82113001	82119000	6000	2	8.00E-10	0.57	144	2.4	Gsk3a;Erf	Signaling;Transcription
DMR1:82141001	1	82141001	82143000	2000	1	5.80E-11	0.52	70	3.5	Cic	Transcription
DMR1:82160001	1	82160001	82161000	1000	1	1.60E-10	0.48	13	1.3	Cic;Pafah1b3;Prr19	Transcription;Golgi
DMR1:82251001	1	82251001	82254000	3000	2	5.50E-10	0.49	65	2.17	LOC102549342;Lipe	Metabolism
DMR1:82406001	1	82406001	82413000	7000	2	8.80E-18	-0.85	94	1.34	LOC102549342;Erich4;Atp5sl;B3gnt8	Golgi
DMR1:82592001	1	82592001	82595000	3000	1	4.00E-07	0.36	45	1.5	Cyp2s1	Metabolism
DMR1:82961001	1	82961001	82962000	1000	1	1.10E-07	-0.61	28	2.8	LOC102553892;LOC100911309;Cd177	
DMR1:83116001	1	83116001	83117000	1000	1	2.70E-07	-0.39	11	1.1	Cyp2b2	Metabolism
DMR1:83154001	1	83154001	83157000	3000	1	7.20E-08	-0.4	23	0.77	Cyp2b3	Metabolism
DMR1:83201001	1	83201001	83203000	2000	2	1.10E-08	-0.46	15	0.75	Cyp2b3	Metabolism
DMR1:83205001	1	83205001	83210000	5000	1	3.90E-08	-0.34	39	0.78	Cyp2b3	Metabolism
DMR1:83218001	1	83218001	83221000	3000	2	6.60E-08	-0.47	21	0.7	Cyp2b3	Metabolism
DMR1:83226001	1	83226001	83229000	3000	2	1.90E-08	-0.42	31	1.03	Cyp2b3	Metabolism
DMR1:83230001	1	83230001	83232000	2000	1	5.70E-07	-0.27	29	1.45	Cyp2b3	Metabolism
DMR1:83257001	1	83257001	83260000	3000	1	5.40E-08	-0.38	20	0.67	Eif4g2-ps2	
DMR1:83262001	1	83262001	83265000	3000	2	4.60E-12	-0.66	16	0.53	Eif4g2-ps2	

DMR1:83407001	1	83407001	83409000	2000	1	1.70E-08	-0.39	20	1	Cyp2b21	Metabolism
DMR1:83525001	1	83525001	83526000	1000	1	5.00E-07	-0.51	12	1.2	Cyp2b12	Metabolism
DMR1:83670001	1	83670001	83672000	2000	1	1.90E-12	-0.59	13	0.65	Cyp2a3	Metabolism
DMR1:83738001	1	83738001	83739000	1000	1	9.20E-07	-0.41	4	0.4	Cyp2a2	Metabolism
DMR1:83816001	1	83816001	83818000	2000	1	1.30E-08	-0.45	26	1.3	LOC108349422;Sdccag1-ps1;LOC365218	
DMR1:83924001	1	83924001	83929000	5000	2	1.90E-10	-0.38	50	1	Cyp2f4	
DMR1:83957001	1	83957001	83958000	1000	1	8.60E-07	0.51	12	1.2	Cyp2f4;Cyp2t1	Metabolism
DMR1:84066001	1	84066001	84067000	1000	1	7.40E-10	0.51	29	2.9	Adck4;Numbl	Cytoskeleton
DMR1:84073001	1	84073001	84074000	1000	1	2.20E-09	0.38	22	2.2	Adck4;Numbl;LOC100909621	Cytoskeleton
DMR1:84311001	1	84311001	84313000	2000	1	2.50E-07	0.52	51	2.55	Prx;LOC102552059	
DMR1:84323001	1	84323001	84325000	2000	1	4.50E-07	0.36	37	1.85	Prx;LOC102552059;Hipk4	
DMR1:84329001	1	84329001	84331000	2000	1	4.40E-09	0.5	13	0.65	Prx;LOC102552059;Hipk4;Pld3	Metabolism
DMR1:84666001	1	84666001	84669000	3000	1	1.30E-11	-0.48	16	0.53	LOC100910046;Zfp780b	Transcription
DMR1:85020001	1	85020001	85023000	3000	1	5.70E-07	0.32	69	2.3	Fcgbp	Extracellular Matrix
DMR1:85252001	1	85252001	85253000	1000	1	4.30E-07	0.53	6	0.6	Ifnl1	Cytokine
DMR1:86050001	1	86050001	86054000	4000	1	1.60E-09	-0.32	57	1.43	Vom1r-ps5	
DMR1:86136001	1	86136001	86142000	6000	1	9.80E-08	-0.32	66	1.1	Vom2r10	Signaling
DMR1:86775001	1	86775001	86786000	11000	1	6.70E-09	0.85	42	0.38	Vom2r7;LOC102549906	
DMR1:86938001	1	86938001	86940000	2000	1	7.60E-08	0.35	49	2.45	Sars2;LOC102550248;Ccer2;Nfkbib;Sirt2	Translation
DMR1:87145001	1	87145001	87147000	2000	1	9.00E-08	0.38	45	2.25	Actn4;LOC102550585	
DMR1:87363001	1	87363001	87365000	2000	1	1.70E-07	-0.37	30	1.5	Sipa1l3	Signaling
DMR1:87368001	1	87368001	87370000	2000	1	8.70E-09	0.42	27	1.35	Sipa1l3	Signaling
DMR1:87391001	1	87391001	87392000	1000	1	5.90E-07	0.5	13	1.3	Sipa1l3;LOC108348907	Signaling
DMR1:87442001	1	87442001	87444000	2000	1	8.10E-08	0.38	26	1.3	Sipa1l3	Signaling
DMR1:87458001	1	87458001	87459000	1000	1	4.70E-08	0.35	21	2.1	Sipa1l3	Signaling
DMR1:87639001	1	87639001	87641000	2000	1	2.40E-07	0.37	18	0.9	LOC100909892;Zfp84	Transcription
DMR1:87950001	1	87950001	87955000	5000	1	4.10E-10	0.44	66	1.32	Map4k1;Ryr1	Ion Channel
DMR1:88084001	1	88084001	88086000	2000	1	1.60E-12	0.47	28	1.4	Rasgrp4;Fam98c	Transcription;Translation
DMR1:88911001	1	88911001	88913000	2000	1	1.90E-11	0.5	43	2.15	Aplp1;Kirrel2;Nphs1	Protease; Proteolysis
DMR1:88971001	1	88971001	88973000	2000	1	3.30E-07	0.31	27	1.35	Prodh2;RGD1560986;Arhgap33	Metabolism;Signaling
DMR1:88978001	1	88978001	88981000	3000	1	1.60E-07	0.42	62	2.07	Prodh2;RGD1560986;Arhgap33	Metabolism;Signaling
DMR1:89026001	1	89026001	89030000	4000	1	9.30E-07	0.39	50	1.25	Psenen;U2af1l4;Igflr1;Kmt2b	Translation;Receptor;Epigenetic
DMR1:89156001	1	89156001	89158000	2000	1	7.70E-07	0.55	35	1.75	LOC688924;Atp4a	Transport
DMR1:89475001	1	89475001	89478000	3000	1	7.90E-08	0.4	42	1.4	Fxyd5;Fxyd7;Fxyd1	Transport
DMR1:89494001	1	89494001	89496000	2000	1	3.90E-08	0.42	34	1.7	Fxyd7;Fxyd1;Lgi4;Fxyd3	Transport
DMR1:89790001	1	89790001	89791000	1000	1	1.90E-08	-0.4	7	0.7	Apbh	
DMR1:89966001	1	89966001	89969000	3000	1	2.40E-07	0.31	47	1.57	Wtip	
DMR1:90171001	1	90171001	90174000	3000	1	6.90E-09	-0.48	30	1	RGD1308428	
DMR1:90558001	1	90558001	90559000	1000	1	9.10E-07	0.4	28	2.8	Chst8;LOC108349467	Transport
DMR1:90561001	1	90561001	90562000	1000	1	7.40E-13	0.5	32	3.2	Chst8;LOC108349467	Transport
DMR1:90835001	1	90835001	90842000	7000	2	3.70E-09	-0.35	66	0.94	Pepd	Protease
DMR1:90877001	1	90877001	90880000	3000	1	1.20E-07	0.38	31	1.03	Pepd	Protease
DMR1:91375001	1	91375001	91377000	2000	1	6.00E-07	-0.41	39	1.95	Cebpa;LOC108349562	Transcription
DMR1:91427001	1	91427001	91428000	1000	1	4.10E-09	-0.45	20	2	Slc7a10	Transport
DMR1:91444001	1	91444001	91445000	1000	1	2.30E-08	0.43	9	0.9	Slc7a10;Lrp3	Transport;Binding Proteins
DMR1:91800001	1	91800001	91801000	1000	1	1.40E-07	-0.56	7	0.7	Tdrd12	Cytoskeleton
DMR1:92731001	1	92731001	92733000	2000	2	8.10E-07	0.58	44	2.2	Tshz3	Transcription
DMR1:93689001	1	93689001	93690000	1000	1	4.10E-07	0.34	23	2.3	Zfp536	Transcription
DMR1:93700001	1	93700001	93702000	2000	1	1.40E-08	0.65	28	1.4	Zfp536	Transcription
DMR1:93747001	1	93747001	93749000	2000	1	8.60E-08	0.36	35	1.75	Zfp536	Transcription
DMR1:93766001	1	93766001	93769000	3000	1	8.90E-07	0.46	60	2	Zfp536;LOC108349103	Transcription
DMR1:93855001	1	93855001	93856000	1000	1	2.60E-12	0.58	13	1.3	Zfp536	Transcription
DMR1:93882001	1	93882001	93888000	6000	1	1.10E-08	0.5	90	1.5	Zfp536	Transcription
DMR1:93931001	1	93931001	93933000	2000	1	2.90E-07	0.37	32	1.6	Zfp536	Transcription

DMR1:93980001	1	93980001	93981000	1000	1	2.40E-10	0.41	14	1.4	Zfp536	Transcription
DMR1:94360001	1	94360001	94362000	2000	1	2.30E-08	-0.47	17	0.85	Uri1	Epigenetic
DMR1:98484001	1	98484001	98485000	1000	1	2.20E-11	0.48	5	0.5	Etfb;Cldnd2;Nkg7	Metabolism;Cytoskeleton
DMR1:98905001	1	98905001	98906000	1000	1	3.50E-08	-0.52	6	0.6	Vom2r38	
DMR1:98982001	1	98982001	98985000	3000	3	5.50E-09	-0.42	28	0.93	Vom2r38;Vom2r37	Signaling
DMR1:99041001	1	99041001	99042000	1000	1	7.50E-08	0.29	2	0.2	Vom2r38;Vom2r37;LOC100911353	Signaling
DMR1:99048001	1	99048001	99058000	10000	6	2.60E-10	0.63	95	0.95	Vom2r38;Vom2r37;LOC100911353;LOC108349760	Signaling
DMR1:99059001	1	99059001	99063000	4000	1	1.80E-08	-0.22	26	0.65	Vom2r38;Vom2r37;LOC100911353;LOC108349760	Signaling
DMR1:99075001	1	99075001	99080000	5000	2	2.50E-15	0.4	38	0.76	Vom2r38;Vom2r37;LOC108349760;LOC103691104	Signaling
DMR1:99081001	1	99081001	99085000	4000	1	2.80E-10	0.3	29	0.72	Vom2r38;Vom2r37;LOC108349760;LOC103691104	Signaling
DMR1:99116001	1	99116001	99119000	3000	1	6.60E-07	-0.31	60	2	Vom2r38;Vom2r37;LOC100912942;LOC108349568	Signaling
DMR1:100328001	1	100328001	100333000	5000	2	2.90E-09	0.47	84	1.68	Shank1	
DMR1:100475001	1	100475001	100477000	2000	1	7.30E-09	0.37	24	1.2	Lrrc4b;Aspdh;Josd2	Metabolism;Protease
DMR1:100539001	1	100539001	100540000	1000	1	6.40E-07	0.53	34	3.4	Mybpc2;Spib;Pold1	Transcription;Transcription
DMR1:100543001	1	100543001	100548000	5000	1	5.60E-12	0.57	153	3.06	Spib;Pold1;Nr1h2	Transcription;Transcription;Transcription
DMR1:100580001	1	100580001	100582000	2000	1	1.70E-07	0.43	19	0.95	Napsa	
DMR1:100630001	1	100630001	100632000	2000	1	7.30E-07	0.39	45	2.25	Myh14	
DMR1:100774001	1	100774001	100776000	2000	1	6.10E-09	0.43	30	1.5	Vrk3	Signaling
DMR1:100782001	1	100782001	100784000	2000	1	8.60E-08	0.37	32	1.6	Vrk3	Signaling
DMR1:100807001	1	100807001	100811000	4000	1	4.80E-12	0.46	50	1.25	Atf5;Nup62;Il4i1	Transcription;Transport;Metabolism
DMR1:100889001	1	100889001	100890000	1000	1	4.60E-07	-0.52	8	0.8	Med25;Fuz;Ap2a1	Transcription;Transport
DMR1:101040001	1	101040001	101045000	5000	1	1.40E-08	0.45	174	3.48	Prr12;Prrg2	Protease
DMR1:101218001	1	101218001	101221000	3000	2	6.30E-13	0.46	37	1.23	Dkkl1;Tead2;Cd37	Transcription
DMR1:101232001	1	101232001	101233000	1000	1	2.20E-07	0.39	38	3.8	Tead2;Cd37;Slc6a16	Transcription;Transport
DMR1:101242001	1	101242001	101246000	4000	2	2.80E-07	0.44	42	1.05	Cd37;Slc6a16	Transport
DMR1:101319001	1	101319001	101320000	1000	1	9.50E-07	0.51	17	1.7	Trpm4;Hrc;Ppfia3	Transport
DMR1:101358001	1	101358001	101360000	2000	1	4.60E-16	0.54	49	2.45	Ppfia3;Lin7b;Snrnp70	Cytoskeleton;Translation
DMR1:101367001	1	101367001	101368000	1000	1	4.10E-12	0.5	31	3.1	Ppfia3;Lin7b;Snrnp70	Cytoskeleton;Translation
DMR1:101637001	1	101637001	101640000	3000	1	5.60E-07	0.4	53	1.77	Mamstr;Fut2	Golgi
DMR1:101680001	1	101680001	101681000	1000	1	4.10E-07	0.45	9	0.9	Sec1;Ntn5;Car11;Dbp	Golgi;Extracellular Matrix;Transcription
DMR1:101739001	1	101739001	101741000	2000	1	3.90E-07	0.38	26	1.3	Sult2b1	Transport
DMR1:101748001	1	101748001	101751000	3000	1	1.20E-11	0.45	41	1.37	Sult2b1	Transport
DMR1:101963001	1	101963001	101965000	2000	1	1.60E-07	0.44	24	1.2	Abcc6	Transport
DMR1:102116001	1	102116001	102118000	2000	1	3.40E-09	0.48	28	1.4	Kcnj11;Abcc8	Transport;Transport
DMR1:102219001	1	102219001	102222000	3000	1	6.80E-13	0.55	61	2.03	Ush1c	Cytoskeleton
DMR1:102317001	1	102317001	102318000	1000	1	1.60E-07	0.46	16	1.6	Otog	Extracellular Matrix
DMR1:102387001	1	102387001	102390000	3000	1	2.70E-07	0.36	66	2.2	Myod1	Transcription
DMR1:102541001	1	102541001	102549000	8000	1	1.40E-07	-0.26	96	1.2	Sergef	Proteolysis
DMR1:102631001	1	102631001	102634000	3000	1	6.00E-08	0.43	52	1.73	Sergef	Proteolysis
DMR1:102639001	1	102639001	102640000	1000	1	1.30E-08	0.5	16	1.6	Sergef	Proteolysis
DMR1:102727001	1	102727001	102731000	4000	1	6.00E-08	0.34	72	1.8	LOC691143;Saal1	Binding Proteins
DMR1:102977001	1	102977001	102983000	6000	4	7.20E-10	-0.4	76	1.27	Tsg101;Uevld	Proteolysis
DMR1:103232001	1	103232001	103233000	1000	1	5.70E-08	0.33	19	1.9	Ptpn5	
DMR1:103419001	1	103419001	103425000	6000	2	2.40E-10	-0.34	75	1.25	Mrgprb4	Signaling
DMR1:103625001	1	103625001	103626000	1000	1	7.90E-07	-0.59	6	0.6	LOC100361204;Mrgprb13	
DMR1:104010001	1	104010001	104015000	5000	1	8.70E-07	-0.33	30	0.6	RGD1560730;Mrgprb2	Signaling
DMR1:104044001	1	104044001	104047000	3000	1	6.10E-07	-0.3	31	1.03	Mrgprb2	
DMR1:104960001	1	104960001	104964000	4000	1	7.90E-07	0.37	54	1.35	Dbx1	
DMR1:105412001	1	105412001	105413000	1000	1	1.10E-07	0.37	12	1.2	Nell1	Signaling
DMR1:105444001	1	105444001	105445000	1000	1	3.10E-07	-0.41	26	2.6	Nell1	Signaling
DMR1:105497001	1	105497001	105498000	1000	1	1.90E-07	0.32	18	1.8	Nell1	Signaling
DMR1:105531001	1	105531001	105538000	7000	2	2.30E-08	-0.29	72	1.03	Nell1	Signaling

DMR1:105567001	1	105567001	105571000	4000	1	3.40E-08	-0.35	48	1.2	Nell1	Signaling
DMR1:105626001	1	105626001	105630000	4000	1	2.10E-07	0.42	53	1.32	Nell1;LOC102557182	Signaling
DMR1:105818001	1	105818001	105822000	4000	1	4.70E-11	0.55	34	0.85	Nell1	Signaling
DMR1:105823001	1	105823001	105824000	1000	1	7.70E-07	0.48	7	0.7	Nell1	Signaling
DMR1:105874001	1	105874001	105877000	3000	2	1.30E-09	-0.4	27	0.9	Nell1	Signaling
DMR1:105897001	1	105897001	105898000	1000	1	3.80E-08	0.4	12	1.2	Nell1	Signaling
DMR1:106207001	1	106207001	106210000	3000	1	8.80E-07	0.3	41	1.37	Nell1	Signaling
DMR1:112448001	1	112448001	112456000	8000	1	2.70E-09	-0.32	84	1.05	Gabrg3	Ion Channel
DMR1:112783001	1	112783001	112790000	7000	2	4.60E-10	-0.47	54	0.77	Gabrg3	Ion Channel
DMR1:114087001	1	114087001	114092000	5000	1	4.00E-08	-0.39	40	0.8	Siglech	
DMR1:114430001	1	114430001	114432000	2000	1	1.00E-08	0.42	14	0.7	Nipa1;LOC108349578	
DMR1:114521001	1	114521001	114526000	5000	1	3.50E-10	-0.34	54	1.08	Herc2	Transcription
DMR1:114592001	1	114592001	114595000	3000	1	7.20E-07	-0.27	27	0.9	Herc2	Transcription
DMR1:114596001	1	114596001	114597000	1000	1	7.10E-09	-0.43	7	0.7	Herc2	Transcription
DMR1:114608001	1	114608001	114612000	4000	3	1.40E-09	-0.54	53	1.32	Herc2	Transcription
DMR1:116083001	1	116083001	116089000	6000	1	4.80E-07	-0.31	47	0.78	Atp10a	Transport
DMR1:116629001	1	116629001	116634000	5000	2	3.10E-15	0.73	35	0.7	Ube3a	Proteolysis
DMR1:123023001	1	123023001	123024000	1000	1	4.00E-09	0.61	3	0.3	Magel2	Cytoskeleton
DMR1:123335001	1	123335001	123343000	8000	2	5.10E-07	0.49	86	1.07	Vom2r-ps62	
DMR1:124634001	1	124634001	124635000	1000	1	2.70E-07	0.41	16	1.6	Otud7a	Protease
DMR1:125050001	1	125050001	125052000	2000	1	1.70E-07	0.36	37	1.85	Trpm1;Mir211	Transport
DMR1:125054001	1	125054001	125056000	2000	1	1.70E-07	0.41	27	1.35	Trpm1	Transport
DMR1:125190001	1	125190001	125193000	3000	3	1.00E-10	0.59	66	2.2	Fan1	
DMR1:125394001	1	125394001	125395000	1000	1	3.50E-12	0.52	13	1.3	Apba2	Transport
DMR1:125458001	1	125458001	125462000	4000	1	1.00E-13	0.49	101	2.52	Apba2	Transport
DMR1:125541001	1	125541001	125542000	1000	1	4.60E-12	0.51	12	1.2	Apba2	Transport
DMR1:125927001	1	125927001	125930000	3000	1	2.60E-07	-0.33	29	0.97	Fam189a1	
DMR1:125948001	1	125948001	125950000	2000	1	2.10E-07	-0.43	18	0.9	Fam189a1	
DMR1:126173001	1	126173001	126175000	2000	2	3.40E-10	-0.58	29	1.45	Tjp1	Cell Junction
DMR1:126359001	1	126359001	126363000	4000	1	4.40E-07	-0.28	41	1.02	Tjp1	Cell Junction
DMR1:126469001	1	126469001	126474000	5000	1	6.90E-07	-0.28	59	1.18	Tjp1;LOC102546680	Cell Junction
DMR1:126600001	1	126600001	126603000	3000	1	2.00E-09	0.39	62	2.07	Tm2d3;LOC108349586	
DMR1:126765001	1	126765001	126767000	2000	1	7.00E-09	0.51	32	1.6	Pcsk6;LOC108349587	Protease
DMR1:126889001	1	126889001	126895000	6000	2	7.60E-11	-0.36	64	1.07	Pcsk6	Protease
DMR1:126913001	1	126913001	126914000	1000	1	1.30E-07	0.4	18	1.8	Pcsk6	Protease
DMR1:127538001	1	127538001	127541000	3000	1	8.60E-12	0.51	59	1.97	Asb7	Transport
DMR1:127571001	1	127571001	127572000	1000	1	1.90E-07	0.42	15	1.5	Asb7	Transport
DMR1:127915001	1	127915001	127922000	7000	1	8.30E-09	-0.46	79	1.13	Adamts17	Protease
DMR1:127946001	1	127946001	127948000	2000	1	1.00E-11	0.46	31	1.55	Adamts17	Protease
DMR1:128597001	1	128597001	128600000	3000	1	1.40E-09	0.5	57	1.9	Lrrc28;Ttc23	
DMR1:128919001	1	128919001	128921000	2000	1	4.00E-07	-0.42	26	1.3	Igf1r	Receptor
DMR1:128928001	1	128928001	128933000	5000	1	6.70E-08	-0.44	85	1.7	Igf1r	Receptor
DMR1:128937001	1	128937001	128943000	6000	1	1.40E-07	0.35	116	1.93	Igf1r	Receptor
DMR1:130525001	1	130525001	130527000	2000	1	5.40E-07	-0.27	36	1.8	Olr9-ps	
DMR1:132185001	1	132185001	132186000	1000	1	1.00E-09	-0.49	12	1.2	RGD1562781	
DMR1:133529001	1	133529001	133535000	6000	1	2.30E-12	-0.35	46	0.77	Mctp2	
DMR1:134806001	1	134806001	134807000	1000	1	8.00E-08	0.38	46	4.6	Chd2	
DMR1:135177001	1	135177001	135179000	2000	1	1.80E-09	0.41	37	1.85	Fam174b;LOC103691163	
DMR1:136025001	1	136025001	136027000	2000	1	4.30E-07	-0.47	18	0.9	Slco3a1	Transport
DMR1:138163001	1	138163001	138167000	4000	1	4.20E-07	-0.29	28	0.7	Agbl1	Protease
DMR1:138194001	1	138194001	138195000	1000	1	3.20E-07	-0.49	10	1	Agbl1	Protease
DMR1:138319001	1	138319001	138326000	7000	2	6.10E-11	-0.32	79	1.13	Agbl1	Protease
DMR1:138383001	1	138383001	138385000	2000	1	1.80E-07	-0.51	10	0.5	Agbl1	Protease
DMR1:138840001	1	138840001	138846000	6000	1	4.70E-08	-0.31	50	0.83	Vom1r-ps67	
DMR1:140577001	1	140577001	140579000	2000	1	2.30E-07	0.52	41	2.05	LOC102547416;Mir7a-2;Aen	Transcription
DMR1:140617001	1	140617001	140619000	2000	1	1.10E-08	0.42	19	0.95	Isg20	Transcription
DMR1:141059001	1	141059001	141061000	2000	1	7.50E-11	0.45	27	1.35	Abhd2	Protease
DMR1:141645001	1	141645001	141647000	2000	1	6.20E-09	0.45	26	1.3	Ap3s2	Transport
DMR1:141805001	1	141805001	141808000	3000	1	4.00E-11	-0.31	37	1.23	Zfp710	Transcription
DMR1:141841001	1	141841001	141844000	3000	1	2.50E-07	0.41	52	1.73	Zfp710;LOC100911225	Transcription

DMR1:141861001	1	141861001	141862000	1000	1	3.10E-07	0.38	32	3.2	Zfp710	Transcription
DMR1:141864001	1	141864001	141867000	3000	1	9.10E-08	0.4	55	1.83	Zfp710;Idh2	Transcription;Metabolism
DMR1:142290001	1	142290001	142294000	4000	1	2.60E-07	-0.43	57	1.43	Blm	Epigenetic
DMR1:142578001	1	142578001	142579000	1000	1	1.70E-08	0.36	15	1.5	Iqgap1	Signaling
DMR1:142878001	1	142878001	142879000	1000	1	9.40E-08	0.38	12	1.2	Zfp592;Alpk3	Signaling
DMR1:142901001	1	142901001	142908000	7000	1	3.40E-09	0.38	123	1.76	Alpk3;LOC102555867	Signaling
DMR1:143054001	1	143054001	143057000	3000	1	2.20E-07	0.44	30	1	Pde8a	Signaling
DMR1:143392001	1	143392001	143393000	1000	1	1.60E-07	0.38	23	2.3	Fsd2;Whamm	Proteolysis
DMR1:143655001	1	143655001	143657000	2000	1	1.40E-10	0.4	37	1.85	Btbd1;Tm6sf1	Proteolysis
DMR1:144143001	1	144143001	144147000	4000	1	5.90E-07	0.42	39	0.98	Sh3gl3	
DMR1:144413001	1	144413001	144420000	7000	1	1.10E-08	-0.34	73	1.04	Adamtsl3	Protease
DMR1:145748001	1	145748001	145751000	3000	2	3.10E-09	-0.37	27	0.9	Tmc3	
DMR1:145858001	1	145858001	145864000	6000	2	5.40E-07	-0.3	73	1.22	Il16	Cytokine
DMR1:146293001	1	146293001	146299000	6000	2	7.70E-18	-0.48	57	0.95	Abhd17c;LOC108349171	Protease
DMR1:146528001	1	146528001	146530000	2000	1	1.30E-08	0.5	8	0.4	Arnt2	Transcription
DMR1:146825001	1	146825001	146827000	2000	1	1.00E-09	-0.59	11	0.55	Zfand6	
DMR1:147552001	1	147552001	147556000	4000	1	4.80E-07	-0.28	44	1.1	Cyp2c7;LOC100361434	Metabolism
DMR1:147715001	1	147715001	147723000	8000	1	7.90E-07	-0.28	109	1.36	Cyp2c7;Cyp2c6v1	Metabolism
DMR1:147743001	1	147743001	147746000	3000	1	2.40E-08	-0.42	25	0.83	Cyp2c7;Cyp2c6v1	Metabolism
DMR1:147783001	1	147783001	147787000	4000	2	2.40E-12	-0.44	25	0.62	Cyp2c7;Cyp2c6v1	Metabolism
DMR1:147790001	1	147790001	147800000	10000	1	6.00E-08	-0.31	49	0.49	Cyp2c7;Cyp2c6v1	Metabolism
DMR1:147847001	1	147847001	147851000	4000	1	6.40E-11	-0.44	25	0.62	Cyp2c7	Metabolism
DMR1:147892001	1	147892001	147894000	2000	1	7.70E-08	-0.35	11	0.55	Cyp2c7;LOC100911718	Metabolism
DMR1:147936001	1	147936001	147941000	5000	2	2.80E-10	-0.32	30	0.6	Cyp2c7	Metabolism
DMR1:147942001	1	147942001	147944000	2000	1	3.40E-11	-0.27	32	1.6	Cyp2c7	Metabolism
DMR1:147986001	1	147986001	147987000	1000	1	2.90E-09	-0.41	9	0.9	Cyp2c7	Metabolism
DMR1:148062001	1	148062001	148064000	2000	1	1.90E-08	-0.29	21	1.05	Cyp2c7	Metabolism
DMR1:148080001	1	148080001	148081000	1000	1	6.60E-07	0.56	1	0.1	Cyp2c7	Metabolism
DMR1:148461001	1	148461001	148464000	3000	1	2.30E-07	0.36	72	2.4	Mpp1	
DMR1:149161001	1	149161001	149166000	5000	1	1.90E-12	-0.39	44	0.88	LOC108349603;Vom2r42	Signaling
DMR1:149825001	1	149825001	149829000	4000	1	2.90E-08	-0.33	36	0.9	Olr14;Olr15-ps	Signaling
DMR1:150019001	1	150019001	150025000	6000	1	4.30E-09	-0.31	69	1.15	Olr22-ps	
DMR1:150161001	1	150161001	150168000	7000	1	3.30E-07	-0.26	99	1.41	Olr26-ps;Olr27	Signaling
DMR1:150278001	1	150278001	150281000	3000	1	7.20E-10	-0.46	27	0.9	Olr33-ps	
DMR1:150850001	1	150850001	150851000	1000	1	9.80E-07	-0.57	7	0.7	Nox4	Metabolism
DMR1:150986001	1	150986001	150989000	3000	1	7.00E-07	-0.29	25	0.83	Nox4	Metabolism
DMR1:151260001	1	151260001	151266000	6000	2	1.90E-10	-0.41	47	0.78	Grm5	Signaling
DMR1:151293001	1	151293001	151294000	1000	1	5.70E-08	-0.37	7	0.7	Grm5	Signaling
DMR1:151493001	1	151493001	151500000	7000	2	2.70E-10	-0.4	80	1.14	Grm5	Signaling
DMR1:151697001	1	151697001	151699000	2000	1	1.10E-10	-0.43	16	0.8	Grm5	Signaling
DMR1:152098001	1	152098001	152106000	8000	2	4.10E-09	-0.4	83	1.04	Rab38	
DMR1:152926001	1	152926001	152929000	3000	1	5.30E-08	-0.26	43	1.43	Tmem135	
DMR1:152930001	1	152930001	152932000	2000	1	7.60E-08	-0.36	16	0.8	Tmem135	
DMR1:152941001	1	152941001	152944000	3000	2	3.00E-07	-0.43	30	1	Tmem135	
DMR1:153038001	1	153038001	153040000	2000	1	3.40E-10	0.44	16	0.8	Tmem135	
DMR1:153058001	1	153058001	153063000	5000	1	1.10E-10	-0.41	35	0.7	Tmem135	
DMR1:153088001	1	153088001	153092000	4000	1	4.40E-07	-0.36	43	1.07	Tmem135	
DMR1:154488001	1	154488001	154494000	6000	2	6.90E-08	-0.41	64	1.07	Ccdc83	
DMR1:155565001	1	155565001	155571000	6000	2	1.40E-13	-0.38	75	1.25	Dlg2	Cytoskeleton
DMR1:155712001	1	155712001	155715000	3000	1	2.90E-07	-0.31	23	0.77	Dlg2	Cytoskeleton
DMR1:155941001	1	155941001	155943000	2000	1	3.80E-08	-0.56	21	1.05	Dlg2	Cytoskeleton
DMR1:156302001	1	156302001	156304000	2000	1	2.20E-07	-0.45	10	0.5	Dlg2;Crebzf	Cytoskeleton;Transcription
DMR1:156473001	1	156473001	156480000	7000	1	3.30E-08	-0.32	71	1.01	Dlg2;Ccdc83	Cytoskeleton
DMR1:156659001	1	156659001	156660000	1000	1	2.80E-07	0.49	4	0.4	Dlg2	Cytoskeleton
DMR1:156859001	1	156859001	156863000	4000	1	6.70E-08	-0.34	45	1.12	Dlg2	Cytoskeleton
DMR1:157485001	1	157485001	157486000	1000	1	2.30E-10	-0.63	14	1.4	Pcf11;LOC680479	Translation
DMR1:157601001	1	157601001	157607000	6000	1	7.50E-12	-0.38	69	1.15	Rab30	
DMR1:157627001	1	157627001	157628000	1000	1	1.60E-10	-0.56	13	1.3	Rab30	
DMR1:161732001	1	161732001	161733000	1000	1	6.60E-10	0.46	13	1.3	Tenm4	
DMR1:161931001	1	161931001	161932000	1000	1	4.90E-08	-0.49	10	1	Nars2	Translation
DMR1:161933001	1	161933001	161934000	1000	1	4.10E-09	-0.58	11	1.1	Nars2	Translation

DMR1:162018001	1	162018001	162024000	6000	2	2.20E-08	-0.31	69	1.15	Nars2	Translation
DMR1:162154001	1	162154001	162158000	4000	2	3.80E-12	-0.37	42	1.05	Gab2	Cytoskeleton
DMR1:162201001	1	162201001	162202000	1000	1	8.70E-07	0.31	7	0.7	Gab2	Cytoskeleton
DMR1:162475001	1	162475001	162476000	1000	1	9.00E-12	0.53	13	1.3	Ints4	
DMR1:162756001	1	162756001	162761000	5000	1	8.00E-08	-0.3	53	1.06	LOC102550562;Pak1	Signaling
DMR1:163192001	1	163192001	163198000	6000	2	3.40E-11	-0.38	68	1.13	Acer3	
DMR1:163229001	1	163229001	163234000	5000	1	6.00E-09	-0.5	24	0.48	Acer3	
DMR1:163239001	1	163239001	163245000	6000	1	1.10E-08	-0.31	65	1.08	Acer3	
DMR1:163271001	1	163271001	163273000	2000	1	3.20E-07	0.31	10	0.5	Acer3	
DMR1:164078001	1	164078001	164079000	1000	1	2.40E-10	0.46	14	1.4	Uvrag	
DMR1:164250001	1	164250001	164252000	2000	1	3.10E-08	0.39	40	2	Map6	
DMR1:164295001	1	164295001	164297000	2000	1	6.70E-07	0.43	15	0.75	Map6;Serpinh1	Protease; Proteolysis
DMR1:164325001	1	164325001	164328000	3000	1	4.50E-12	0.52	38	1.27	Gdpd5	Signaling
DMR1:164380001	1	164380001	164384000	4000	1	4.10E-07	0.37	76	1.9	Gdpd5	Signaling
DMR1:164793001	1	164793001	164797000	4000	1	3.90E-09	-0.33	34	0.85	Neu3	Metabolism
DMR1:164923001	1	164923001	164924000	1000	1	4.50E-09	0.44	14	1.4	Xrra1;Rnf169	
DMR1:165110001	1	165110001	165111000	1000	1	4.80E-12	0.54	25	2.5	Pold3	Transcription
DMR1:165283001	1	165283001	165284000	1000	1	2.50E-07	-0.46	8	0.8	Pgm2l1	Metabolism
DMR1:165317001	1	165317001	165321000	4000	1	2.30E-07	0.36	50	1.25	P4ha3	Golgi
DMR1:165917001	1	165917001	165918000	1000	1	1.80E-07	0.36	22	2.2	Mir3102	
DMR1:166171001	1	166171001	166173000	2000	1	1.10E-09	-0.45	21	1.05	Fchsd2	
DMR1:166188001	1	166188001	166190000	2000	1	9.70E-09	-0.57	12	0.6	Fchsd2	
DMR1:166240001	1	166240001	166247000	7000	4	4.70E-08	-0.29	71	1.01	Fchsd2	
DMR1:166352001	1	166352001	166361000	9000	2	1.40E-07	-0.37	117	1.3	Fchsd2	
DMR1:166438001	1	166438001	166439000	1000	1	1.20E-09	0.46	17	1.7	Stard10	
DMR1:166470001	1	166470001	166473000	3000	1	8.50E-07	0.37	45	1.5	Arap1	Signaling
DMR1:166498001	1	166498001	166503000	5000	2	5.40E-07	0.37	57	1.14	Arap1	Signaling
DMR1:166506001	1	166506001	166509000	3000	1	2.90E-07	0.34	50	1.67	Arap1	Signaling
DMR1:166535001	1	166535001	166536000	1000	1	1.60E-14	0.48	17	1.7	Arap1;Pde2a	Signaling;Signaling
DMR1:166583001	1	166583001	166585000	2000	1	1.00E-10	0.42	38	1.9	Pde2a;Mir139	Signaling
DMR1:166634001	1	166634001	166639000	5000	1	9.90E-14	-0.39	61	1.22	Pde2a	Signaling
DMR1:166780001	1	166780001	166782000	2000	1	3.00E-08	0.5	20	1	Clpb	Protease
DMR1:166850001	1	166850001	166851000	1000	1	1.90E-11	0.49	6	0.6	Clpb	Protease
DMR1:167072001	1	167072001	167076000	4000	2	3.00E-08	0.46	74	1.85	Numa1	Cytoskeleton
DMR1:167103001	1	167103001	167104000	1000	1	1.60E-24	1	13	1.3	Il18bp;Rnf121	Proteolysis
DMR1:167228001	1	167228001	167233000	5000	1	2.20E-07	-0.27	50	1	Nup98	Transport
DMR1:167692001	1	167692001	167697000	5000	2	8.80E-09	-0.31	56	1.12	Olr42-ps;Trim21	Proteolysis
DMR1:167727001	1	167727001	167730000	3000	1	8.00E-10	-0.53	18	0.6	Olr43;Olr44	Receptor
DMR1:167878001	1	167878001	167880000	2000	1	4.00E-07	0.61	6	0.3	Olr63;Olr62;Olr61	Receptor
DMR1:168103001	1	168103001	168109000	6000	1	6.70E-07	-0.3	55	0.92	Or51t1;Olr66-ps;Olr67	Receptor
DMR1:168150001	1	168150001	168157000	7000	1	3.70E-08	-0.31	68	0.97	Olr69;Olr70	Receptor
DMR1:168186001	1	168186001	168190000	4000	1	6.20E-07	-0.36	22	0.55	Olr72;Olr73-ps;Olr74	Receptor
DMR1:168252001	1	168252001	168260000	8000	1	2.40E-09	-0.43	66	0.82	Olr79	Receptor
DMR1:168274001	1	168274001	168280000	6000	1	1.10E-07	-0.4	52	0.87	Olr80	Receptor
DMR1:168627001	1	168627001	168635000	8000	2	6.20E-11	-0.47	123	1.54	Olr107;Olr108;Olr109	Receptor
DMR1:168667001	1	168667001	168671000	4000	1	1.70E-09	-0.3	37	0.92	Olr111	
DMR1:168715001	1	168715001	168720000	5000	1	1.20E-08	-0.22	79	1.58	Olr114;Olr115	Receptor
DMR1:168731001	1	168731001	168737000	6000	2	1.40E-19	-0.43	53	0.88	Olr115;LOC499215	Receptor
DMR1:168805001	1	168805001	168811000	6000	3	1.10E-08	-0.34	62	1.03	Olr119;Olr120;Olr121	Receptor
DMR1:168895001	1	168895001	168897000	2000	1	5.60E-07	-0.39	18	0.9	Olr127	Receptor
DMR1:168914001	1	168914001	168920000	6000	2	2.10E-07	-0.34	86	1.43	Olr127;Olr128	Receptor
DMR1:168947001	1	168947001	168953000	6000	1	2.60E-08	-0.3	55	0.92	Hbb-b1;LOC100134871	
DMR1:169057001	1	169057001	169061000	4000	3	8.20E-18	-0.5	51	1.27	Olr131	Receptor
DMR1:169070001	1	169070001	169071000	1000	1	4.30E-07	-0.63	6	0.6	Olr132	Receptor
DMR1:169189001	1	169189001	169192000	3000	1	1.60E-07	-0.31	23	0.77	LOC102555557;Olr139;LOC689243	Receptor
DMR1:169202001	1	169202001	169204000	2000	1	9.50E-09	-0.52	16	0.8	Olr140;Olr141	Receptor
DMR1:169214001	1	169214001	169221000	7000	1	3.30E-08	0.46	74	1.06	Olr140;Olr141;Olr142	Receptor
DMR1:169256001	1	169256001	169263000	7000	2	2.00E-09	-0.46	90	1.29	Olr144;Olr145	Receptor
DMR1:169264001	1	169264001	169269000	5000	1	6.40E-08	-0.25	47	0.94	Olr145	Receptor
DMR1:169364001	1	169364001	169371000	7000	2	2.10E-08	-0.38	87	1.24	RGD1310717;Olr149	Development;Receptor

DMR1:169377001	1	169377001	169384000	7000	1	4.50E-07	-0.29	91	1.3	Olr149;Olr150;LOC108348458	Receptor
DMR1:169579001	1	169579001	169586000	7000	1	3.80E-07	-0.25	92	1.31	Olr155;Olr154	Receptor
DMR1:169687001	1	169687001	169693000	6000	3	5.70E-11	-0.5	62	1.03	Olr162;Olr163	Receptor
DMR1:169831001	1	169831001	169837000	6000	3	3.30E-12	-0.34	75	1.25	Olr179	Receptor
DMR1:169973001	1	169973001	169974000	1000	1	2.40E-08	-0.75	5	0.5	Olr190;Olr191-ps;RGD1559839	Receptor
DMR1:170431001	1	170431001	170433000	2000	1	2.80E-07	-0.47	19	0.95	Hpx;Trim3	Protease;Proteolysis
DMR1:170448001	1	170448001	170454000	6000	1	1.40E-07	0.41	125	2.08	Trim3	Proteolysis
DMR1:170564001	1	170564001	170565000	1000	1	5.10E-07	0.55	21	2.1	Dnhd1;Rrp8	Cytoskeleton
DMR1:170595001	1	170595001	170596000	1000	1	2.80E-11	0.49	21	2.1	Ilk;Taf10;Tpp1;Dchs1	Signaling;Transcription;Protease;Cytoskeleton
DMR1:170731001	1	170731001	170732000	1000	1	1.00E-10	-0.34	16	1.6	Olr207-ps;LOC103691201	
DMR1:170906001	1	170906001	170911000	5000	2	3.00E-07	-0.32	69	1.38	Olr216-ps;LOC100911720;Olr217	Receptor
DMR1:170990001	1	170990001	170992000	2000	1	7.80E-09	-0.38	20	1	Olr221	Receptor
DMR1:171099001	1	171099001	171102000	3000	1	7.70E-12	-0.42	36	1.2	Olr227	
DMR1:171622001	1	171622001	171624000	2000	1	3.40E-07	-0.34	17	0.85	Syt9	Transport
DMR1:171727001	1	171727001	171728000	1000	1	8.90E-07	-0.38	6	0.6	Syt9	Transport
DMR1:172538001	1	172538001	172544000	6000	1	1.10E-07	-0.34	59	0.98	Olr257	Signaling
DMR1:172560001	1	172560001	172562000	2000	1	1.10E-07	0.36	28	1.4	Olr257;Olr258-ps;Olr259	Signaling
DMR1:172576001	1	172576001	172577000	1000	1	5.60E-07	-0.41	4	0.4	Olr259	Signaling
DMR1:172675001	1	172675001	172683000	8000	2	3.70E-09	-0.31	86	1.07	Olr264;Olr265-ps	Signaling
DMR1:172764001	1	172764001	172765000	1000	1	1.40E-07	-0.57	9	0.9	Olr269-ps	
DMR1:173383001	1	173383001	173388000	5000	2	1.50E-10	-0.56	44	0.88	Olr285	Receptor
DMR1:173569001	1	173569001	173571000	2000	1	3.00E-09	0.37	30	1.5	Tub	
DMR1:173665001	1	173665001	173668000	3000	1	9.10E-07	-0.34	32	1.07	Ric3	
DMR1:173892001	1	173892001	173897000	5000	1	1.10E-07	-0.31	31	0.62	Stk33	Signaling
DMR1:173999001	1	173999001	174003000	4000	1	1.70E-08	-0.36	36	0.9	Stk33	Signaling
DMR1:174123001	1	174123001	174126000	3000	1	9.70E-07	-0.26	32	1.07	Trim66;Rpl27a	Epigenetic;Translation
DMR1:174268001	1	174268001	174269000	1000	1	7.00E-07	0.38	14	1.4	St5;LOC108349575	
DMR1:174295001	1	174295001	174298000	3000	1	6.00E-09	-0.3	27	0.9	St5	
DMR1:174321001	1	174321001	174325000	4000	1	4.10E-07	-0.32	32	0.8	Akip1	
DMR1:174442001	1	174442001	174443000	1000	1	6.60E-07	-0.47	14	1.4	Scube2	Extracellular Matrix
DMR1:174470001	1	174470001	174471000	1000	1	4.00E-10	-0.42	14	1.4	Scube2	Extracellular Matrix
DMR1:174787001	1	174787001	174788000	1000	1	1.20E-07	0.47	11	1.1	Wee1	Signaling
DMR1:174878001	1	174878001	174879000	1000	1	3.60E-08	-0.4	12	1.2	Swap70	Cytoskeleton
DMR1:174941001	1	174941001	174943000	2000	1	2.30E-08	0.54	29	1.45	Sbf2	Signaling
DMR1:175129001	1	175129001	175134000	5000	3	5.60E-10	-0.33	57	1.14	Sbf2	Signaling
DMR1:175143001	1	175143001	175145000	2000	2	5.20E-09	0.39	30	1.5	Sbf2	Signaling
DMR1:175175001	1	175175001	175178000	3000	1	2.80E-07	0.56	25	0.83	Sbf2;LOC108349611	Signaling
DMR1:175271001	1	175271001	175275000	4000	1	3.80E-07	-0.36	28	0.7	Sbf2	Signaling
DMR1:175390001	1	175390001	175394000	4000	1	6.00E-07	-0.37	43	1.07	Sbf2	Signaling
DMR1:176308001	1	176308001	176309000	1000	1	3.00E-07	0.26	4	0.4	Galnt18	Golgi
DMR1:176428001	1	176428001	176435000	7000	2	3.60E-08	-0.43	97	1.39	Galnt18	Golgi
DMR1:177079001	1	177079001	177082000	3000	2	7.20E-11	0.49	34	1.13	Mical2	
DMR1:177224001	1	177224001	177226000	2000	1	3.30E-07	0.4	25	1.25	Micalcl	
DMR1:177301001	1	177301001	177302000	1000	1	2.20E-09	0.36	12	1.2	Parva	Cytoskeleton
DMR1:177363001	1	177363001	177366000	3000	1	2.50E-09	0.5	30	1	Parva	Cytoskeleton
DMR1:177392001	1	177392001	177393000	1000	1	5.40E-08	0.45	11	1.1	Parva	Cytoskeleton
DMR1:178060001	1	178060001	178061000	1000	1	1.30E-09	0.43	63	6.3	Arntl	Transcription
DMR1:178090001	1	178090001	178093000	3000	2	1.10E-08	0.49	33	1.1	Arntl	Transcription
DMR1:178660001	1	178660001	178662000	2000	1	3.00E-08	0.38	25	1.25	Spon1	Cytoskeleton
DMR1:178681001	1	178681001	178687000	6000	3	9.60E-08	-0.37	63	1.05	Spon1	Cytoskeleton
DMR1:178842001	1	178842001	178847000	5000	1	7.80E-07	0.39	59	1.18	Spon1;LOC108349620	Cytoskeleton
DMR1:178887001	1	178887001	178892000	5000	1	1.90E-08	-0.3	39	0.78	Spon1	Cytoskeleton
DMR1:182843001	1	182843001	182844000	1000	1	2.20E-13	0.36	18	1.8	Ssty1	
DMR1:182847001	1	182847001	182850000	3000	1	2.60E-07	0.24	46	1.53	Ssty1	
DMR1:183935001	1	183935001	183939000	4000	1	3.10E-08	-0.3	41	1.02	Pde3b	Signaling
DMR1:184019001	1	184019001	184022000	3000	1	3.20E-07	-0.29	21	0.7	Pde3b	Signaling
DMR1:184025001	1	184025001	184031000	6000	1	4.00E-07	-0.29	83	1.38	Pde3b	Signaling

DMR1:185756001	1	185756001	185763000	7000	1	1.50E-07	-0.29	86	1.23	Sox6	
DMR1:185915001	1	185915001	185917000	2000	1	8.30E-12	0.5	9	0.45	Sox6	
DMR1:189891001	1	189891001	189892000	1000	1	1.90E-07	0.48	6	0.6	Thumpd1;Tmem159;LOC108349820;Zp2	
DMR1:190126001	1	190126001	190127000	1000	1	7.60E-07	-0.35	12	1.2	Thumpd1;Abca15	Transport
DMR1:190261001	1	190261001	190262000	1000	1	3.00E-08	-0.27	15	1.5	Thumpd1;Abca16	Transport
DMR1:190718001	1	190718001	190721000	3000	1	2.70E-08	0.49	22	0.73	Vwa3a	
DMR1:191068001	1	191068001	191070000	2000	1	9.30E-07	0.42	27	1.35	Otoa	Cytoskeleton
DMR1:191387001	1	191387001	191389000	2000	1	2.30E-09	0.56	26	1.3	Hs3st2	Transport
DMR1:191390001	1	191390001	191396000	6000	1	1.90E-07	-0.28	85	1.42	Hs3st2	Transport
DMR1:191459001	1	191459001	191460000	1000	1	8.30E-07	-0.36	19	1.9	Hs3st2	Transport
DMR1:191462001	1	191462001	191465000	3000	2	6.70E-10	-0.47	26	0.87	Hs3st2	Transport
DMR1:191603001	1	191603001	191606000	3000	1	4.50E-08	0.37	30	1	Usp31	Protease
DMR1:191723001	1	191723001	191727000	4000	2	6.00E-09	0.64	34	0.85	Scnn1g	Transport
DMR1:191980001	1	191980001	191984000	4000	1	2.00E-10	0.39	50	1.25	Gga2	
DMR1:192352001	1	192352001	192354000	2000	1	4.90E-11	0.62	20	1	Prkcb	Signaling
DMR1:192389001	1	192389001	192391000	2000	1	2.10E-09	0.29	30	1.5	Prkcb	Signaling
DMR1:192407001	1	192407001	192408000	1000	1	8.00E-07	0.34	21	2.1	Prkcb	Signaling
DMR1:192468001	1	192468001	192471000	3000	1	2.00E-07	0.38	31	1.03	Prkcb	Signaling
DMR1:192516001	1	192516001	192520000	4000	1	2.30E-10	0.45	55	1.38	Prkcb	Signaling
DMR1:192964001	1	192964001	192965000	1000	1	2.80E-07	-0.35	17	1.7	Rbbp6	Proteolysis
DMR1:193709001	1	193709001	193713000	4000	2	3.90E-10	-0.32	38	0.95	Paip2l1	
DMR1:197030001	1	197030001	197032000	2000	1	2.40E-07	0.39	24	1.2	Il21r;Gtf3c1	Receptor;Transcription
DMR1:197279001	1	197279001	197281000	2000	1	6.40E-08	0.4	30	1.5	Gsg1l	Cytoskeleton
DMR1:197441001	1	197441001	197442000	1000	1	1.10E-08	0.43	10	1	Gsg1l	Cytoskeleton
DMR1:197458001	1	197458001	197460000	2000	1	1.20E-08	0.41	28	1.4	Gsg1l	Cytoskeleton
DMR1:197856001	1	197856001	197857000	1000	1	4.90E-10	0.45	32	3.2	Rabep2;Atp2a1	Transport
DMR1:197883001	1	197883001	197885000	2000	1	1.80E-07	0.42	39	1.95	Atp2a1;Sh2b1	Transport;Cytoskeleton
DMR1:198084001	1	198084001	198085000	1000	1	3.60E-07	-0.47	20	2	Sgf29;LOC691797	
DMR1:198245001	1	198245001	198246000	1000	1	9.50E-09	0.4	17	1.7	LOC103691235;Fam57b	
DMR1:198374001	1	198374001	198376000	2000	1	8.50E-07	0.34	34	1.7	Kctd13;Asphd1;Sez6l2	Metabolism
DMR1:198560001	1	198560001	198561000	1000	1	2.00E-07	0.41	10	1	Qprt;LOC108349646	
DMR1:198683001	1	198683001	198684000	1000	1	1.40E-09	0.45	32	3.2	Znf48;Zfp771	Transcription
DMR1:198936001	1	198936001	198938000	2000	1	5.50E-07	0.42	48	2.4	Fbrs	
DMR1:199030001	1	199030001	199031000	1000	1	1.60E-07	0.39	18	1.8	Phkg2;Ccdc189;Rnf40	Signaling
DMR1:199417001	1	199417001	199418000	1000	1	8.10E-21	0.67	15	1.5	Fus	Metabolism
DMR1:199424001	1	199424001	199425000	1000	1	7.80E-09	0.41	13	1.3	Fus	Metabolism
DMR1:199426001	1	199426001	199427000	1000	1	5.70E-07	0.37	15	1.5	Fus	Metabolism
DMR1:199455001	1	199455001	199456000	1000	1	3.60E-10	0.49	19	1.9	Trim72	Proteolysis
DMR1:199616001	1	199616001	199618000	2000	1	9.30E-10	0.41	32	1.6	Itgad;Cox6a2	Extracellular Matrix;Metabolism
DMR1:199693001	1	199693001	199695000	2000	1	5.70E-08	0.6	43	2.15	Slc5a2;RGD1310127	Transport
DMR1:199815001	1	199815001	199816000	1000	1	7.20E-07	-0.41	19	1.9	Rgs10	
DMR1:200102001	1	200102001	200103000	1000	1	6.70E-16	0.78	22	2.2	Inpp5f	Signaling
DMR1:200690001	1	200690001	200694000	4000	1	1.30E-10	0.45	96	2.4	Fgfr2	Receptor
DMR1:201322001	1	201322001	201324000	2000	1	2.50E-08	0.43	43	2.15	Tacc2	
DMR1:201346001	1	201346001	201347000	1000	1	2.10E-09	0.41	14	1.4	Btbd16	
DMR1:201422001	1	201422001	201425000	3000	1	1.00E-09	0.37	50	1.67	Plekha1	
DMR1:201927001	1	201927001	201928000	1000	1	4.30E-07	0.38	10	1	RGD1305014	
DMR1:201964001	1	201964001	201965000	1000	1	5.10E-10	0.51	7	0.7	Pstk;Ikzf5	Transcription
DMR1:202026001	1	202026001	202027000	1000	1	1.20E-08	-0.71	16	1.6	Acadsb	Metabolism
DMR1:202550001	1	202550001	202553000	3000	1	1.40E-08	0.48	26	0.87	Plpp4	Signaling
DMR1:202559001	1	202559001	202560000	1000	1	5.30E-08	0.53	6	0.6	Plpp4	Signaling
DMR1:203226001	1	203226001	203228000	2000	1	8.40E-11	0.5	22	1.1	Dmbt1	Protease
DMR1:204243001	1	204243001	204248000	5000	1	2.60E-08	0.37	91	1.82	Chst15	Transport
DMR1:204661001	1	204661001	204666000	5000	1	1.60E-10	0.47	73	1.46	Lhpp	Signaling
DMR1:204695001	1	204695001	204696000	1000	1	2.60E-07	0.42	27	2.7	Lhpp	Signaling
DMR1:204704001	1	204704001	204707000	3000	1	1.40E-09	0.5	37	1.23	Lhpp;Fam53b	Signaling
DMR1:204777001	1	204777001	204781000	4000	1	6.70E-07	0.41	39	0.98	Fam53b	
DMR1:205799001	1	205799001	205800000	1000	1	2.90E-09	0.44	10	1	Bccip;Dhx32	Transcription
DMR1:205942001	1	205942001	205949000	7000	2	3.90E-10	-0.37	77	1.1	Fank1;Adam12	Protease

DMR1:206235001	1	206235001	206241000	6000	2	6.50E-15	-0.46	64	1.07	Adam12	Protease
DMR1:206939001	1	206939001	206940000	1000	1	7.20E-11	0.46	13	1.3	Dock1	Transcription
DMR1:207153001	1	207153001	207155000	2000	1	7.70E-08	0.38	31	1.55	Dock1;Fam196a	Transcription
DMR1:207391001	1	207391001	207392000	1000	1	5.20E-10	0.51	7	0.7	Dock1;LOC103691254	Transcription
DMR1:207661001	1	207661001	207662000	1000	1	9.50E-07	0.52	12	1.2	Foxi2	Transcription
DMR1:207941001	1	207941001	207943000	2000	1	3.90E-11	0.74	21	1.05	Ptpre;LOC108349658;LOC102551963	Signaling
DMR1:209261001	1	209261001	209262000	1000	1	1.50E-07	-0.39	22	2.2	Mgmt	
DMR1:209614001	1	209614001	209615000	1000	1	5.80E-07	0.44	5	0.5	Ebf3	Transcription
DMR1:210713001	1	210713001	210715000	2000	1	2.40E-07	0.42	24	1.2	Tcerg1l	Transcription
DMR1:211537001	1	211537001	211538000	1000	1	1.10E-09	-0.44	15	1.5	Lrrc27	
DMR1:211794001	1	211794001	211799000	5000	1	1.60E-08	0.43	35	0.7	Inpp5a	Signaling
DMR1:211959001	1	211959001	211960000	1000	1	1.50E-09	0.41	35	3.5	Cfap46	
DMR1:211961001	1	211961001	211963000	2000	1	5.50E-07	0.47	26	1.3	Cfap46	
DMR1:212644001	1	212644001	212646000	2000	1	4.70E-08	-0.3	15	0.75	Olr287	Receptor
DMR1:212676001	1	212676001	212680000	4000	1	3.10E-09	0.47	49	1.23	Olr288	Receptor
DMR1:212728001	1	212728001	212729000	1000	1	6.50E-07	-0.31	23	2.3	Olr292	
DMR1:212852001	1	212852001	212858000	6000	1	3.70E-11	-0.35	64	1.07	Olr292;Olr290-ps	
DMR1:212898001	1	212898001	212905000	7000	2	4.20E-10	-0.37	93	1.33	Olr293-ps;Olr294-ps;Olr295	Signaling
DMR1:212979001	1	212979001	212986000	7000	2	1.90E-07	-0.28	78	1.11	Olr298	
DMR1:213134001	1	213134001	213142000	8000	2	4.10E-07	-0.27	90	1.12	Olr304	Signaling
DMR1:213366001	1	213366001	213371000	5000	1	2.70E-07	-0.31	60	1.2	Olr311	Signaling
DMR1:213394001	1	213394001	213402000	8000	1	8.00E-08	-0.25	85	1.06	Olr312	Signaling
DMR1:213410001	1	213410001	213414000	4000	1	8.70E-08	-0.39	37	0.92	Olr312	Signaling
DMR1:213433001	1	213433001	213437000	4000	2	3.60E-10	-0.34	51	1.27	Olr313;Olr314-ps	Signaling
DMR1:213616001	1	213616001	213621000	5000	1	5.20E-08	0.48	54	1.08	Bet1l;Ric8a;Sirt3	Transcription
DMR1:213668001	1	213668001	213677000	9000	2	7.70E-12	-0.36	109	1.21	Nlrp6;LOC108348167	
DMR1:213745001	1	213745001	213746000	1000	1	5.40E-07	0.39	25	2.5	Pgghg;Ifitm5;LOC108349669;Ifitm2	Metabolism
DMR1:214348001	1	214348001	214350000	2000	1	2.80E-08	0.35	32	1.6	Eps8l2	Cytoskeleton
DMR1:214368001	1	214368001	214370000	2000	1	1.10E-10	0.51	19	0.95	Taldo1	Metabolism
DMR1:214609001	1	214609001	214616000	7000	5	3.80E-16	0.45	149	2.13	Ap2a2;Muc6	Transport;Extracellular Matrix
DMR1:214639001	1	214639001	214641000	2000	1	3.30E-08	-0.43	24	1.2	Muc6	Extracellular Matrix
DMR1:214686001	1	214686001	214687000	1000	1	1.40E-07	0.57	5	0.5	Muc2	Extracellular Matrix
DMR1:214743001	1	214743001	214745000	2000	1	1.70E-08	0.38	22	1.1	Muc5ac	
DMR1:214793001	1	214793001	214796000	3000	2	1.20E-12	0.54	45	1.5	Muc5b	Extracellular Matrix
DMR1:215053001	1	215053001	215057000	4000	1	9.40E-08	0.64	35	0.88	Dusp8	Signaling
DMR1:215514001	1	215514001	215516000	2000	1	4.40E-07	0.34	34	1.7	Ifitm10	
DMR1:215598001	1	215598001	215599000	1000	1	5.80E-14	0.46	23	2.3	LOC102546801;Syt8	Transport
DMR1:215746001	1	215746001	215749000	3000	1	2.50E-08	0.5	88	2.93	H19;Mir675;LOC102547221;LOC102548505	
DMR1:216345001	1	216345001	216348000	3000	2	7.20E-09	0.52	49	1.63	Kcnq1	Transport
DMR1:216510001	1	216510001	216511000	1000	1	1.50E-07	-0.43	15	1.5	Kcnq1	Transport
DMR1:216548001	1	216548001	216551000	3000	1	1.40E-07	0.44	35	1.17	Kcnq1	Transport
DMR1:216604001	1	216604001	216605000	1000	1	1.00E-09	0.5	11	1.1	Kcnq1	Transport
DMR1:216877001	1	216877001	216879000	2000	1	1.70E-08	0.56	24	1.2	Osbpl5	
DMR1:217001001	1	217001001	217003000	2000	1	6.40E-07	0.4	20	1	Nadsyn1	Metabolism
DMR1:217259001	1	217259001	217260000	1000	1	6.80E-10	0.7	7	0.7	Shank2	
DMR1:217321001	1	217321001	217325000	4000	1	7.80E-07	0.33	44	1.1	Shank2	
DMR1:217673001	1	217673001	217674000	1000	1	6.00E-11	0.71	6	0.6	Ppfia1	
DMR1:217756001	1	217756001	217757000	1000	1	9.10E-08	0.4	7	0.7	Fadd;Ano1	
DMR1:217763001	1	217763001	217764000	1000	1	7.10E-08	0.47	19	1.9	Ano1	
DMR1:218413001	1	218413001	218417000	4000	1	1.60E-08	0.4	37	0.92	Tpcn2	Transport
DMR1:218619001	1	218619001	218622000	3000	1	3.10E-08	0.4	40	1.33	Cpt1a	Metabolism
DMR1:218864001	1	218864001	218866000	2000	2	2.60E-08	0.47	44	2.2	Lrp5	Receptor
DMR1:219269001	1	219269001	219271000	2000	1	5.90E-07	0.53	23	1.15	Ndufv1	Metabolism
DMR1:219309001	1	219309001	219311000	2000	1	1.20E-08	0.4	26	1.3	RGD1307603	Transport
DMR1:219353001	1	219353001	219360000	7000	1	9.80E-07	0.56	125	1.79	Cdk2ap2;Pitpnm1;Aip;LOC108349683	Signaling;Transport;Transcription

DMR1:219395001	1	219395001	219397000	2000	1	1.40E-09	0.45	26	1.3	Cabp4;Gpr152;LOC108349682;Coro1b;Ptprcap;Rps6kb2	Signaling;Cytoskeleton;Golgi
DMR1:220346001	1	220346001	220349000	3000	1	1.40E-08	0.48	49	1.63	LOC103691276;Rin1;Cd248;LOC102554272	Transcription
DMR1:220543001	1	220543001	220545000	2000	1	3.20E-07	0.33	13	0.65	Pacs1	
DMR1:220719001	1	220719001	220720000	1000	1	7.10E-10	0.52	16	1.6	Catsper1;Cst6	Transport
DMR1:220891001	1	220891001	220894000	3000	1	1.30E-09	0.4	66	2.2	Snx32;LOC102551929;LOC102556290	Cytoskeleton
DMR1:220953001	1	220953001	220954000	1000	1	5.20E-08	0.52	21	2.1	Ap5b1	
DMR1:221046001	1	221046001	221048000	2000	1	1.60E-07	0.48	17	0.85	Pcnx3;Map3k11;LOC102546344;Kcnk7	Signaling;Transport
DMR1:221121001	1	221121001	221124000	3000	1	4.00E-08	0.42	59	1.97	Ltbp3;Scyl1	Extracellular Matrix;Signaling
DMR1:221245001	1	221245001	221246000	1000	1	5.50E-08	-0.58	7	0.7	Slc25a45;Tigd3;Dpf2	Transport;Epigenetic;Epigenetic
DMR1:221417001	1	221417001	221419000	2000	1	2.00E-08	0.44	24	1.2	Syvn1;Mrpl49;Fau;Znhit2;Tm7sf2	Translation;Metabolism
DMR1:221487001	1	221487001	221489000	2000	1	3.60E-14	0.52	34	1.7	Naaladl1;Sac3d1;Snx15	Protease;Cytoskeleton;Signaling
DMR1:221682001	1	221682001	221683000	1000	1	5.30E-07	0.4	25	2.5	Cdc42bpg	Signaling
DMR1:221746001	1	221746001	221748000	2000	2	3.10E-10	0.52	52	2.6	Sf1;Pygm	Translation;Golgi
DMR1:221814001	1	221814001	221817000	3000	1	6.90E-09	0.37	41	1.37	Nrxn2	
DMR1:222232001	1	222232001	222233000	1000	1	7.50E-09	0.54	7	0.7	Plcb3;Ppp1r14b;Fkbp2;Vegfb;Dnajc4	Metabolism;Signaling;Growth Factors
DMR1:222815001	1	222815001	222822000	7000	2	1.30E-07	-0.34	78	1.11	LOC686151;RGD1563402	
DMR1:223456001	1	223456001	223458000	2000	1	4.40E-09	-0.37	18	0.9	Slc22a24	
DMR1:223473001	1	223473001	223477000	4000	2	2.20E-07	-0.34	48	1.2	Slc22a24	
DMR1:223492001	1	223492001	223494000	2000	1	3.00E-07	-0.33	16	0.8	Slc22a24	
DMR1:223513001	1	223513001	223515000	2000	1	1.10E-09	-0.47	18	0.9	Slc22a24	
DMR1:224312001	1	224312001	224318000	6000	1	1.90E-09	-0.34	64	1.07	UST4r	Transport
DMR1:224333001	1	224333001	224340000	7000	1	2.00E-10	-0.41	64	0.91	UST4r	Transport
DMR1:224352001	1	224352001	224356000	4000	2	6.10E-08	-0.47	28	0.7	UST4r	Transport
DMR1:224399001	1	224399001	224403000	4000	1	7.50E-10	-0.32	38	0.95	UST4r	Transport
DMR1:224451001	1	224451001	224452000	1000	1	2.30E-08	-0.32	8	0.8	Ust5r	Transport
DMR1:224466001	1	224466001	224473000	7000	5	1.30E-10	-0.49	67	0.96	Ust5r	Transport
DMR1:224480001	1	224480001	224488000	8000	1	5.40E-07	-0.27	86	1.07	Ust5r	Transport
DMR1:224495001	1	224495001	224497000	2000	1	4.80E-11	-0.38	17	0.85	Ust5r	Transport
DMR1:224498001	1	224498001	224499000	1000	1	1.60E-07	-0.39	11	1.1	Ust5r	Transport
DMR1:224516001	1	224516001	224521000	5000	3	1.00E-08	-0.29	61	1.22	Ust5r	Transport
DMR1:224536001	1	224536001	224542000	6000	1	7.20E-07	-0.32	72	1.2	Ust5r	Transport
DMR1:224583001	1	224583001	224587000	4000	1	3.90E-07	-0.36	34	0.85	Slc22a25	Transport
DMR1:224669001	1	224669001	224671000	2000	1	1.50E-11	-0.5	20	1	Slc22a25	Transport
DMR1:224936001	1	224936001	224937000	1000	1	2.50E-07	0.34	11	1.1	LOC690251;Wdr74;Stx5	Translation;Transcription
DMR1:225131001	1	225131001	225133000	2000	1	2.90E-09	-0.51	32	1.6	B3gat3;Rom1;Eml3;Mta2	Golgi;Development
DMR1:225297001	1	225297001	225299000	2000	1	1.30E-07	0.51	30	1.5	Ahnak	
DMR1:225344001	1	225344001	225345000	1000	1	4.30E-07	0.5	7	0.7	Ahnak	
DMR1:225349001	1	225349001	225356000	7000	2	4.20E-09	-0.37	72	1.03	Ahnak	
DMR1:225460001	1	225460001	225466000	6000	2	9.40E-13	-0.36	58	0.97	LOC108348288;RGD1566289	
DMR1:225597001	1	225597001	225598000	1000	1	4.10E-07	-0.51	5	0.5	Scgb1d2	
DMR1:226062001	1	226062001	226063000	1000	1	8.40E-08	0.42	16	1.6	Fads2	
DMR1:226275001	1	226275001	226277000	2000	1	1.60E-08	0.52	36	1.8	Myrf	
DMR1:226293001	1	226293001	226294000	1000	1	7.30E-07	-0.46	17	1.7	Myrf;Dagla	Metabolism
DMR1:226297001	1	226297001	226298000	1000	1	5.50E-07	0.47	9	0.9	Myrf;Dagla	Metabolism
DMR1:226323001	1	226323001	226326000	3000	2	7.60E-09	0.63	27	0.9	Dagla	Metabolism
DMR1:226338001	1	226338001	226339000	1000	1	8.10E-09	0.57	19	1.9	Dagla	Metabolism
DMR1:226438001	1	226438001	226442000	4000	2	4.60E-10	0.49	53	1.32	LOC108349692;Syt7	Transport
DMR1:226747001	1	226747001	226748000	1000	1	7.10E-07	-0.41	14	1.4	Vps37c	
DMR1:226756001	1	226756001	226758000	2000	1	5.20E-07	0.41	24	1.2	Vps37c	
DMR1:226849001	1	226849001	226851000	2000	1	4.30E-08	0.61	37	1.85	Cd6	Protease
DMR1:227071001	1	227071001	227073000	2000	1	5.90E-07	-0.43	25	1.25	Ms4a18	Transport

DMR1:227260001	1	227260001	227264000	4000	2	2.70E-09	-0.4	40	1	LOC102557108;Ms4a6bl	
DMR1:227565001	1	227565001	227571000	6000	3	1.60E-09	-0.33	56	0.93	Ms4a4a	Transport
DMR1:227632001	1	227632001	227636000	4000	1	2.30E-07	-0.33	38	0.95	Ms4a4c	Transport
DMR1:228025001	1	228025001	228028000	3000	1	1.70E-08	-0.51	25	0.83	Oosp1	
DMR1:228109001	1	228109001	228113000	4000	2	7.60E-09	-0.37	40	1	Gif	
DMR1:228423001	1	228423001	228424000	1000	1	8.40E-07	-0.47	13	1.3	Osbp	
DMR1:228495001	1	228495001	228501000	6000	1	6.90E-09	-0.3	61	1.02	Olr322	Receptor
DMR1:228547001	1	228547001	228548000	1000	1	3.00E-08	-0.48	11	1.1	Olr322;Olr324;Olr325;Olr326	Receptor;Signaling
DMR1:228615001	1	228615001	228621000	6000	3	2.00E-08	-0.36	70	1.17	Olr330	Signaling
DMR1:229715001	1	229715001	229716000	1000	1	8.60E-07	-0.32	6	0.6	Olr339	Receptor
DMR1:229791001	1	229791001	229792000	1000	1	3.00E-10	-0.6	9	0.9	Olr342-ps	
DMR1:229867001	1	229867001	229869000	2000	1	6.30E-11	-0.53	12	0.6	Olr347	Receptor
DMR1:229965001	1	229965001	229972000	7000	2	5.00E-09	-0.28	96	1.37	Olr350-ps	
DMR1:230289001	1	230289001	230296000	7000	1	4.40E-07	-0.3	94	1.34	LOC687097;Olr365	Receptor
DMR1:230436001	1	230436001	230439000	3000	1	8.60E-08	-0.44	26	0.87	Olr370-ps	
DMR1:230723001	1	230723001	230727000	4000	3	3.30E-10	-0.45	36	0.9	Olr382	Receptor
DMR1:230760001	1	230760001	230761000	1000	1	2.40E-07	-0.39	6	0.6	Olr383	Receptor
DMR1:233611001	1	233611001	233616000	5000	2	7.20E-07	-0.36	70	1.4	Gnaq	Signaling
DMR1:233618001	1	233618001	233619000	1000	1	5.60E-08	0.66	10	1	Gnaq	Signaling
DMR1:234443001	1	234443001	234445000	2000	1	2.40E-07	0.47	30	1.5	Rorb	Transcription
DMR1:234537001	1	234537001	234539000	2000	1	2.80E-08	0.44	15	0.75	Trpm6	Transport
DMR1:234606001	1	234606001	234609000	3000	1	8.10E-07	-0.32	24	0.8	Trpm6;LOC108349696	Transport
DMR1:235155001	1	235155001	235161000	6000	1	6.50E-10	-0.36	60	1	Gna14	Signaling
DMR1:235195001	1	235195001	235197000	2000	1	1.50E-09	-0.45	26	1.3	Gna14;LOC103691297	Signaling
DMR1:235361001	1	235361001	235364000	3000	1	7.00E-09	-0.31	21	0.7	Vps13a	
DMR1:235456001	1	235456001	235459000	3000	1	6.10E-08	-0.55	12	0.4	Vps13a	
DMR1:235503001	1	235503001	235508000	5000	1	9.50E-10	-0.34	46	0.92	Vps13a	
DMR1:235695001	1	235695001	235698000	3000	1	6.20E-10	-0.33	32	1.07	Vps13a	
DMR1:235768001	1	235768001	235771000	3000	1	2.60E-11	-0.62	22	0.73	Vps13a	
DMR1:235783001	1	235783001	235789000	6000	1	1.00E-11	-0.33	54	0.9	Vps13a	
DMR1:236268001	1	236268001	236270000	2000	1	5.30E-12	0.49	9	0.45	Pcsk5	Protease
DMR1:236770001	1	236770001	236771000	1000	1	5.40E-07	-0.45	15	1.5	Prune2	
DMR1:238505001	1	238505001	238512000	7000	2	4.30E-08	-0.32	67	0.96	Tmc1	
DMR1:238862001	1	238862001	238865000	3000	1	7.10E-08	-0.27	31	1.03	Zfand5;LOC102551544;LOC103691306	
DMR1:239212001	1	239212001	239213000	1000	1	7.90E-08	0.58	11	1.1	RGD1359158	
DMR1:239230001	1	239230001	239231000	1000	1	1.50E-07	0.62	6	0.6	RGD1359158	
DMR1:239298001	1	239298001	239299000	1000	1	5.60E-07	0.38	11	1.1	Abhd17b;LOC108348948	Protease
DMR1:239891001	1	239891001	239892000	1000	1	3.60E-07	-0.48	19	1.9	Trpm3	Transport
DMR1:240574001	1	240574001	240577000	3000	1	2.70E-07	-0.37	37	1.23	Trpm3;LOC108349824;Aldh1a7	Transport;Metabolism
DMR1:241015001	1	241015001	241020000	5000	1	8.40E-12	-0.39	55	1.1	Smc5	
DMR1:241087001	1	241087001	241088000	1000	1	1.60E-07	0.4	12	1.2	Mamdc2	
DMR1:241779001	1	241779001	241784000	5000	1	1.50E-10	-0.5	101	2.02	Apba1	Transport
DMR1:241820001	1	241820001	241821000	1000	1	6.30E-07	0.42	13	1.3	Fam189a2	
DMR1:242336001	1	242336001	242339000	3000	3	1.80E-10	-0.38	23	0.77	Pip5k1b	Signaling
DMR1:242562001	1	242562001	242565000	3000	1	6.80E-08	0.58	36	1.2	Tmem252	
DMR1:242676001	1	242676001	242680000	4000	3	3.30E-28	0.71	30	0.75	Pgm5	Metabolism
DMR1:242942001	1	242942001	242943000	1000	1	3.70E-07	0.34	5	0.5	Dock8	Transcription
DMR1:242957001	1	242957001	242958000	1000	1	8.60E-11	-0.6	12	1.2	Dock8;C1H9orf66	Transcription
DMR1:243283001	1	243283001	243284000	1000	1	3.40E-15	0.84	7	0.7	Kank1	Cytoskeleton
DMR1:244606001	1	244606001	244608000	2000	1	1.50E-07	-0.34	31	1.55	LOC108349293;Smarca2	Epigenetic
DMR1:244767001	1	244767001	244769000	2000	1	1.10E-08	0.4	24	1.2	Smarca2	Epigenetic
DMR1:244780001	1	244780001	244781000	1000	1	6.20E-07	0.4	9	0.9	Smarca2	Epigenetic
DMR1:244792001	1	244792001	244796000	4000	1	2.90E-08	-0.32	51	1.27	Smarca2	Epigenetic
DMR1:245474001	1	245474001	245476000	2000	1	6.50E-08	0.32	23	1.15	Kcnv2;Pum3	Transport
DMR1:245492001	1	245492001	245499000	7000	1	9.00E-10	-0.37	75	1.07	Pum3	
DMR1:245849001	1	245849001	245850000	1000	1	5.20E-07	-0.49	17	1.7	Rfx3	Transcription
DMR1:245956001	1	245956001	245957000	1000	1	5.40E-11	-0.36	11	1.1	Rfx3	Transcription
DMR1:245958001	1	245958001	245963000	5000	2	2.00E-08	-0.39	63	1.26	Rfx3	Transcription

DMR1:246478001	1	246478001	246479000	1000	1	7.60E-08	0.37	27	2.7	Glis3	Transcription
DMR1:246749001	1	246749001	246750000	1000	1	2.10E-08	-0.47	12	1.2	NEWGENE_1565505;LOC103691328	
DMR1:247122001	1	247122001	247123000	1000	1	8.30E-07	0.62	8	0.8	Cdc37l1	Transcription
DMR1:247126001	1	247126001	247129000	3000	1	3.80E-09	-0.47	24	0.8	Cdc37l1	Transcription
DMR1:247166001	1	247166001	247167000	1000	1	2.90E-10	-0.54	24	2.4	Ak3	Signaling
DMR1:247254001	1	247254001	247256000	2000	1	2.50E-10	0.4	27	1.35	Rcl1;Mir101-2	Metabolism
DMR1:247274001	1	247274001	247275000	1000	1	6.10E-07	0.45	11	1.1	Rcl1	Metabolism
DMR1:247408001	1	247408001	247410000	2000	1	4.60E-08	0.66	18	0.9	Jak2	
DMR1:247750001	1	247750001	247752000	2000	1	7.50E-10	-0.46	18	0.9	Ric1	
DMR1:247797001	1	247797001	247800000	3000	1	3.30E-11	0.47	44	1.47	Ermp1	Protease
DMR1:248124001	1	248124001	248126000	2000	1	1.90E-07	0.59	24	1.2	Il33	
DMR1:248181001	1	248181001	248189000	8000	1	2.00E-08	-0.29	104	1.3	Tpd52l3	
DMR1:248218001	1	248218001	248220000	2000	1	4.00E-07	0.32	40	2	LOC681078;Uhrf2	Proteolysis
DMR1:248221001	1	248221001	248225000	4000	1	9.60E-07	0.28	40	1	LOC681078;Uhrf2	Proteolysis
DMR1:248905001	1	248905001	248910000	5000	2	5.80E-08	-0.31	70	1.4	Dkk1;LOC100912084	
DMR1:249236001	1	249236001	249242000	6000	2	1.30E-07	-0.35	74	1.23	Prkg1	
DMR1:249290001	1	249290001	249291000	1000	1	1.40E-10	0.76	7	0.7	Prkg1	
DMR1:249303001	1	249303001	249305000	2000	1	9.20E-07	-0.29	37	1.85	Prkg1	
DMR1:249306001	1	249306001	249309000	3000	3	1.70E-14	-0.41	30	1	Prkg1	
DMR1:249348001	1	249348001	249353000	5000	1	1.70E-09	-0.28	72	1.44	Prkg1	
DMR1:249476001	1	249476001	249481000	5000	2	3.10E-08	-0.32	46	0.92	Prkg1	
DMR1:249637001	1	249637001	249639000	2000	1	8.30E-15	0.72	26	1.3	Prkg1	
DMR1:249709001	1	249709001	249714000	5000	2	2.10E-07	-0.27	42	0.84	Prkg1	
DMR1:249717001	1	249717001	249719000	2000	1	1.90E-10	0.38	11	0.55	Prkg1	
DMR1:249733001	1	249733001	249735000	2000	1	3.80E-07	-0.25	24	1.2	Prkg1	
DMR1:250823001	1	250823001	250825000	2000	1	6.70E-08	0.43	38	1.9	Sgms1	
DMR1:250992001	1	250992001	250994000	2000	1	6.20E-10	0.4	15	0.75	Nutf2-ps1	
DMR1:251081001	1	251081001	251084000	3000	1	7.80E-07	-0.42	27	0.9	Minpp1	Signaling
DMR1:251183001	1	251183001	251185000	2000	1	4.40E-09	0.43	32	1.6	Papss2	
DMR1:251210001	1	251210001	251211000	1000	1	1.90E-07	0.36	7	0.7	Papss2	
DMR1:251372001	1	251372001	251375000	3000	2	8.50E-09	-0.58	34	1.13	Atad1;LOC100910127	Metabolism
DMR1:251920001	1	251920001	251921000	1000	1	8.80E-09	0.52	11	1.1	Rnls	
DMR1:251954001	1	251954001	251961000	7000	1	4.30E-07	-0.29	68	0.97	Rnls	
DMR1:252077001	1	252077001	252083000	6000	3	1.80E-10	-0.41	65	1.08	Rnls	
DMR1:252273001	1	252273001	252278000	5000	2	8.20E-08	-0.29	45	0.9	Lipf	Metabolism
DMR1:253028001	1	253028001	253030000	2000	1	1.40E-07	0.35	28	1.4	Slc16a12	Transport
DMR1:253278001	1	253278001	253279000	1000	1	4.00E-09	-0.48	9	0.9	Kif20b	Cytoskeleton
DMR1:253285001	1	253285001	253293000	8000	1	5.60E-07	-0.38	98	1.23	Kif20b;Ears2l1	Cytoskeleton
DMR1:254574001	1	254574001	254578000	4000	1	5.90E-09	-0.34	30	0.75	Htr7	Signaling
DMR1:254612001	1	254612001	254614000	2000	1	4.80E-07	-0.3	14	0.7	Htr7;LOC108349452	Signaling
DMR1:254628001	1	254628001	254633000	5000	1	2.10E-07	-0.29	65	1.3	Htr7;LOC108349452	Signaling
DMR1:255015001	1	255015001	255016000	1000	1	5.90E-07	0.33	21	2.1	Pcgf5	Epigenetic
DMR1:255928001	1	255928001	255930000	2000	1	4.00E-11	0.54	57	2.85	Ide	Protease
DMR1:255980001	1	255980001	255988000	8000	2	4.60E-09	-0.32	91	1.14	Ide	Protease
DMR1:256051001	1	256051001	256058000	7000	3	1.20E-11	-0.33	84	1.2	Kif11	Cytoskeleton
DMR1:256315001	1	256315001	256318000	3000	2	6.10E-12	0.51	21	0.7	Exoc6	Transport
DMR1:256394001	1	256394001	256395000	1000	1	1.10E-10	-0.49	20	2	Cyp26a1	Metabolism
DMR1:256674001	1	256674001	256677000	3000	1	1.80E-07	0.43	41	1.37	Myof	Transport
DMR1:256703001	1	256703001	256704000	1000	1	2.10E-08	0.57	7	0.7	Myof;LOC100911555	Transport
DMR1:256869001	1	256869001	256870000	1000	1	4.00E-07	0.29	27	2.7	Pde6c	Signaling
DMR1:256900001	1	256900001	256903000	3000	2	5.00E-17	0.61	102	3.4	Fra10ac1	Signaling
DMR1:256987001	1	256987001	256988000	1000	1	3.90E-07	-0.43	12	1.2	Lgi1	
DMR1:257187001	1	257187001	257191000	4000	1	3.50E-08	0.41	42	1.05	Plce1	Metabolism
DMR1:257215001	1	257215001	257219000	4000	1	7.50E-08	-0.28	40	1	Plce1;LOC102550070	Metabolism
DMR1:257379001	1	257379001	257383000	4000	1	1.30E-07	-0.4	41	1.02	Plce1	Metabolism
DMR1:257481001	1	257481001	257482000	1000	1	1.40E-09	0.61	3	0.3	Noc3l	
DMR1:257526001	1	257526001	257527000	1000	1	1.90E-08	0.58	5	0.5	Tbc1d12	Signaling
DMR1:257993001	1	257993001	257996000	3000	1	4.80E-07	-0.3	22	0.73	Cyp2c11	Metabolism
DMR1:258784001	1	258784001	258788000	4000	2	4.90E-08	-0.48	33	0.82	Cyp2c13	Metabolism
DMR1:258804001	1	258804001	258808000	4000	1	3.20E-07	-0.27	41	1.02	Cyp2c13	Metabolism

DMR1:258844001	1	258844001	258853000	9000	1	3.10E-07	-0.43	78	0.87	Cyp2c13	Metabolism
DMR1:259380001	1	259380001	259384000	4000	1	5.20E-12	0.41	54	1.35	Sorbs1	
DMR1:259461001	1	259461001	259464000	3000	1	3.50E-07	0.34	68	2.27	Sorbs1	
DMR1:259630001	1	259630001	259634000	4000	2	1.00E-08	-0.34	46	1.15	Aldh18a1	Metabolism
DMR1:259813001	1	259813001	259815000	2000	1	5.20E-09	-0.44	21	1.05	Entpd1	Signaling
DMR1:260202001	1	260202001	260204000	2000	1	1.30E-07	0.48	27	1.35	Blnk	Cytoskeleton
DMR1:260347001	1	260347001	260348000	1000	1	1.70E-07	-0.42	26	2.6	Opalin;Tll2	Protease
DMR1:260554001	1	260554001	260557000	3000	1	7.90E-10	0.54	53	1.77	Pik3ap1	
DMR1:260646001	1	260646001	260652000	6000	1	4.50E-14	-0.39	77	1.28	Pik3ap1	
DMR1:260895001	1	260895001	260896000	1000	1	2.00E-07	0.36	29	2.9	Slit1	
DMR1:260944001	1	260944001	260946000	2000	1	3.80E-07	0.42	29	1.45	Slit1;LOC102553433	
DMR1:261010001	1	261010001	261013000	3000	1	3.40E-07	0.37	46	1.53	Arhgap19	Signaling
DMR1:261149001	1	261149001	261150000	1000	1	2.70E-08	0.36	15	1.5	Rrp12;Pgam1	Metabolism
DMR1:261282001	1	261282001	261284000	2000	1	4.40E-10	0.49	26	1.3	Ubtd1;Ankrd2;Hoga1	Proteolysis;Metabolism
DMR1:261508001	1	261508001	261509000	1000	1	3.20E-07	0.36	17	1.7	Golga7b	
DMR1:261565001	1	261565001	261567000	2000	1	3.80E-11	0.45	62	3.1	Crtac1	
DMR1:261614001	1	261614001	261616000	2000	1	3.50E-07	0.44	23	1.15	Crtac1	
DMR1:261962001	1	261962001	261963000	1000	1	5.40E-08	0.4	16	1.6	Pyroxd2	Metabolism
DMR1:262084001	1	262084001	262085000	1000	1	1.60E-08	0.43	12	1.2	Hpse2	
DMR1:262143001	1	262143001	262144000	1000	1	3.70E-11	0.63	2	0.2	Hpse2	
DMR1:262158001	1	262158001	262160000	2000	1	1.00E-08	0.45	23	1.15	Hpse2	
DMR1:262336001	1	262336001	262337000	1000	1	3.50E-08	-0.31	21	2.1	Hpse2	
DMR1:262338001	1	262338001	262342000	4000	1	6.20E-07	-0.3	39	0.98	Hpse2	
DMR1:262361001	1	262361001	262362000	1000	1	1.30E-07	0.48	10	1	Hpse2	
DMR1:262372001	1	262372001	262374000	2000	1	5.50E-07	-0.4	23	1.15	Hpse2	
DMR1:262389001	1	262389001	262393000	4000	2	9.00E-10	-0.35	56	1.4	Hpse2	
DMR1:262512001	1	262512001	262518000	6000	1	1.40E-09	-0.31	58	0.97	Hpse2	
DMR1:262534001	1	262534001	262540000	6000	2	5.80E-08	-0.34	66	1.1	Hpse2	
DMR1:262559001	1	262559001	262560000	1000	1	1.50E-08	0.55	14	1.4	Hpse2	
DMR1:262588001	1	262588001	262591000	3000	2	3.30E-10	-0.39	30	1	Hpse2	
DMR1:262724001	1	262724001	262729000	5000	2	6.20E-08	-0.42	40	0.8	Hpse2	
DMR1:262908001	1	262908001	262909000	1000	1	7.80E-07	0.25	1	0.1	Hpse2;LOC100911951	
DMR1:263019001	1	263019001	263021000	2000	1	1.30E-09	-0.57	14	0.7	Hpse2;LOC100911779;LOC100911855	
DMR1:263071001	1	263071001	263072000	1000	1	9.60E-11	-0.49	12	1.2	Hpse2	
DMR1:263105001	1	263105001	263110000	5000	1	8.80E-10	-0.39	40	0.8	Hpse2	
DMR1:263203001	1	263203001	263205000	2000	1	9.30E-08	-0.56	26	1.3	Cnnm1	
DMR1:264094001	1	264094001	264095000	1000	1	4.20E-11	0.53	16	1.6	Scd4	
DMR1:264265001	1	264265001	264269000	4000	1	2.60E-09	0.37	64	1.6	Wnt8b;Sec31b	Signaling;Transport
DMR1:264759001	1	264759001	264762000	3000	1	3.30E-09	0.62	33	1.1	Sema4g;Mrpl43;Peo1;Lzts2	Signaling;Translation
DMR1:264787001	1	264787001	264790000	3000	1	1.80E-17	0.56	54	1.8	Pdzd7;Sfxn3	Cytoskeleton;Transport
DMR1:264836001	1	264836001	264839000	3000	2	1.90E-10	0.44	41	1.37	Kazald1	
DMR1:264908001	1	264908001	264914000	6000	1	8.90E-09	-0.38	65	1.08	Tlx1	
DMR1:265255001	1	265255001	265257000	2000	1	7.60E-08	0.65	24	1.2	Btrc	Cytoskeleton
DMR1:265361001	1	265361001	265362000	1000	1	7.10E-07	0.43	8	0.8	Fbxw4	
DMR1:265557001	1	265557001	265558000	1000	1	1.00E-07	0.26	3	0.3	Kcnip2	
DMR1:266497001	1	266497001	266498000	1000	1	2.40E-08	-0.47	22	2.2	As3mt	Epigenetic
DMR1:266743001	1	266743001	266745000	2000	1	1.80E-07	0.35	40	2	Nt5c2	Signaling
DMR1:266779001	1	266779001	266781000	2000	1	2.30E-07	-0.36	32	1.6	Nt5c2;Trnar-ucu;Ina	Signaling
DMR1:266794001	1	266794001	266799000	5000	1	4.00E-09	0.41	71	1.42	Ina	
DMR1:266847001	1	266847001	266849000	2000	1	3.90E-08	0.42	14	0.7	Taf5	
DMR1:266958001	1	266958001	266961000	3000	1	9.20E-07	0.37	43	1.43	Neurl1	Proteolysis
DMR1:266975001	1	266975001	266978000	3000	1	1.00E-09	0.34	36	1.2	Neurl1	Proteolysis
DMR1:266979001	1	266979001	266980000	1000	1	4.90E-07	0.39	16	1.6	Neurl1	Proteolysis
DMR1:267024001	1	267024001	267025000	1000	1	2.80E-07	0.45	8	0.8	Neurl1	Proteolysis
DMR1:267035001	1	267035001	267037000	2000	1	8.50E-08	0.35	34	1.7	Neurl1;Sh3pxd2a	Proteolysis
DMR1:267237001	1	267237001	267240000	3000	1	4.70E-07	0.32	51	1.7	Sh3pxd2a	
DMR1:267409001	1	267409001	267410000	1000	1	4.90E-09	0.41	10	1	Slk;Col17a1	Extracellular Matrix
DMR1:267686001	1	267686001	267687000	1000	1	2.50E-10	-0.71	29	2.9	Cfap58	Development
DMR1:267802001	1	267802001	267810000	8000	1	5.70E-08	-0.29	108	1.35	Cfap58	Development

DMR1:268011001	1	268011001	268018000	7000	1	2.00E-09	-0.34	88	1.26	Sorcs3	Transport
DMR1:268030001	1	268030001	268031000	1000	1	2.00E-09	-0.56	20	2	Sorcs3	Transport
DMR1:268090001	1	268090001	268098000	8000	2	2.50E-10	-0.37	132	1.65	Sorcs3	Transport
DMR1:268240001	1	268240001	268250000	10000	1	4.70E-07	-0.29	124	1.24	Sorcs3	Transport
DMR1:268599001	1	268599001	268600000	1000	1	1.70E-09	0.48	6	0.6	Sorcs3	Transport
DMR1:269964001	1	269964001	269966000	2000	1	1.90E-08	0.66	17	0.85	Sorcs1	Transport
DMR1:270062001	1	270062001	270064000	2000	1	9.70E-09	-0.41	16	0.8	Sorcs1	Transport
DMR1:271405001	1	271405001	271410000	5000	3	4.10E-07	-0.4	61	1.22	Ccdc147	
DMR1:272896001	1	272896001	272898000	2000	1	5.90E-07	0.53	24	1.2	Olr385	
DMR1:273548001	1	273548001	273554000	6000	2	3.30E-07	-0.3	57	0.95	RGD1561333	
DMR1:273866001	1	273866001	273867000	1000	1	1.00E-07	-0.48	20	2	Add3	Cytoskeleton
DMR1:273889001	1	273889001	273890000	1000	1	6.60E-09	0.56	7	0.7	Add3	Cytoskeleton
DMR1:274042001	1	274042001	274043000	1000	1	4.70E-07	-0.36	22	2.2	Mxi1;LOC108349710	Transcription
DMR1:274241001	1	274241001	274243000	2000	1	2.10E-13	0.5	39	1.95	Dusp5	Signaling
DMR1:274420001	1	274420001	274421000	1000	1	1.60E-08	0.48	23	2.3	Rbm20	
DMR1:274720001	1	274720001	274722000	2000	1	4.50E-07	-0.51	22	1.1	Shoc2	Cytoskeleton
DMR1:274734001	1	274734001	274740000	6000	2	3.70E-13	-0.4	77	1.28	Shoc2	Cytoskeleton
DMR1:274757001	1	274757001	274759000	2000	1	4.60E-07	0.6	12	0.6	Shoc2;Adra2a	Cytoskeleton;Signaling
DMR1:275847001	1	275847001	275850000	3000	2	1.30E-08	0.3	17	0.57	Gpam;LOC102553769	Metabolism
DMR1:276223001	1	276223001	276226000	3000	1	1.90E-08	0.51	24	0.8	Gucy2g	Signaling
DMR1:276441001	1	276441001	276444000	3000	1	7.40E-09	0.46	40	1.33	Vti1a	Transcription
DMR1:276446001	1	276446001	276448000	2000	1	2.70E-09	0.4	29	1.45	Vti1a;LOC103691376	Transcription
DMR1:276726001	1	276726001	276732000	6000	2	7.60E-07	0.58	106	1.77	Tcf7l2	Transcription
DMR1:277149001	1	277149001	277150000	1000	1	9.40E-09	0.52	10	1	Nrap	
DMR1:277629001	1	277629001	277631000	2000	1	3.10E-11	-0.61	54	2.7	Ccdc186;Tdrd1	Cytoskeleton
DMR1:277655001	1	277655001	277657000	2000	1	5.50E-07	0.8	5	0.25	Tdrd1	Cytoskeleton
DMR1:277796001	1	277796001	277799000	3000	2	3.00E-08	0.45	27	0.9	Afap1l2	
DMR1:277884001	1	277884001	277885000	1000	1	3.20E-11	0.55	3	0.3	Ablim1	
DMR1:278317001	1	278317001	278319000	2000	1	3.60E-07	0.45	35	1.75	Trub1	Metabolism
DMR1:278322001	1	278322001	278323000	1000	1	3.40E-11	0.47	18	1.8	Trub1	Metabolism
DMR1:278394001	1	278394001	278398000	4000	1	2.60E-12	0.28	26	0.65	Trub1	Metabolism
DMR1:278550001	1	278550001	278552000	2000	1	6.10E-07	-0.37	21	1.05	Atrnl1	Extracellular Matrix
DMR1:278608001	1	278608001	278611000	3000	1	1.10E-10	0.67	45	1.5	Atrnl1	Extracellular Matrix
DMR1:278753001	1	278753001	278755000	2000	1	6.20E-08	-0.53	30	1.5	Atrnl1	Extracellular Matrix
DMR1:278815001	1	278815001	278819000	4000	1	4.10E-09	-0.46	76	1.9	Atrnl1;LOC108349827	Extracellular Matrix
DMR1:278822001	1	278822001	278827000	5000	1	4.80E-10	0.55	50	1	Atrnl1;LOC108349827	Extracellular Matrix
DMR1:278944001	1	278944001	278946000	2000	1	2.40E-09	0.41	18	0.9	Atrnl1	Extracellular Matrix
DMR1:279058001	1	279058001	279059000	1000	1	5.20E-13	0.44	15	1.5	Atrnl1	Extracellular Matrix
DMR1:279236001	1	279236001	279244000	8000	4	2.90E-10	-0.35	100	1.25	Gfra1;LOC102556644	Receptor
DMR1:279652001	1	279652001	279657000	5000	2	2.20E-15	0.68	86	1.72	Ccdc172;LOC108349704	
DMR1:279942001	1	279942001	279945000	3000	1	8.70E-07	0.43	30	1	LOC681006;Hspa12a	
DMR1:280171001	1	280171001	280175000	4000	2	5.90E-16	0.54	48	1.2	Shtn1	
DMR1:280410001	1	280410001	280411000	1000	1	6.90E-08	0.4	22	2.2	Slc18a2	Transport
DMR1:282145001	1	282145001	282146000	1000	1	7.60E-07	0.4	21	2.1	Nanos1;Eif3a	Metabolism;Translation
DMR1:282279001	1	282279001	282280000	1000	1	7.20E-08	-0.51	13	1.3	Grk5	Signaling
DMR2:2131001	2	2131001	2135000	4000	1	5.10E-07	-0.3	45	1.12	Rpl17-ps1	
DMR2:2533001	2	2533001	2535000	2000	1	1.20E-09	0.46	12	0.6	Ell2;LOC102549076	Transcription
DMR2:2740001	2	2740001	2743000	3000	1	5.20E-07	-0.46	9	0.3	Spata9	
DMR2:2753001	2	2753001	2758000	5000	1	1.40E-08	-0.35	44	0.88	Spata9;Rfesd	Metabolism
DMR2:2821001	2	2821001	2825000	4000	1	1.10E-07	-0.34	61	1.52	Gpr150	Signaling
DMR2:2850001	2	2850001	2851000	1000	1	5.70E-10	-0.48	5	0.5	Arsk	
DMR2:2853001	2	2853001	2857000	4000	2	1.70E-09	-0.37	38	0.95	Arsk	
DMR2:2922001	2	2922001	2927000	5000	1	7.80E-08	-0.46	51	1.02	Ttc37	
DMR2:2974001	2	2974001	2978000	4000	2	6.50E-09	-0.51	29	0.72	Ttc37	
DMR2:2979001	2	2979001	2986000	7000	3	2.10E-11	-0.33	90	1.29	Ttc37	
DMR2:3020001	2	3020001	3023000	3000	1	8.30E-08	-0.41	15	0.5	Ttc37;Fam81b	
DMR2:3076001	2	3076001	3083000	7000	2	9.60E-09	-0.48	71	1.01	Fam81b	
DMR2:3085001	2	3085001	3086000	1000	1	4.30E-07	-0.47	1	0.1	Fam81b	
DMR2:3542001	2	3542001	3544000	2000	1	2.10E-08	-0.5	7	0.35	Mctp1	
DMR2:3575001	2	3575001	3577000	2000	1	4.40E-11	-0.32	20	1	Mctp1	
DMR2:3598001	2	3598001	3605000	7000	1	8.70E-07	-0.34	92	1.31	Mctp1	

DMR2:3811001	2	3811001	3818000	7000	2	6.40E-13	-0.51	78	1.11	Mctp1	
DMR2:3926001	2	3926001	3928000	2000	1	8.80E-07	-0.5	12	0.6	Mctp1	
DMR2:3982001	2	3982001	3983000	1000	1	2.20E-07	-0.5	6	0.6	Mctp1	
DMR2:4007001	2	4007001	4011000	4000	2	7.50E-08	0.39	64	1.6	Mctp1	
DMR2:4041001	2	4041001	4043000	2000	1	6.20E-09	-0.4	16	0.8	Mctp1	
DMR2:4131001	2	4131001	4132000	1000	1	9.90E-08	-0.43	12	1.2	Slf1	
DMR2:4134001	2	4134001	4142000	8000	1	9.10E-09	-0.33	92	1.15	Slf1	
DMR2:4167001	2	4167001	4168000	1000	1	4.00E-08	-0.44	5	0.5	Slf1	
DMR2:4292001	2	4292001	4298000	6000	1	2.90E-08	-0.35	50	0.83	RGD1560883	
DMR2:4386001	2	4386001	4389000	3000	1	7.30E-09	-0.39	24	0.8	RGD1560883	
DMR2:4413001	2	4413001	4414000	1000	1	5.60E-09	-0.35	10	1	RGD1560883	
DMR2:4422001	2	4422001	4424000	2000	1	1.30E-25	-0.88	43	2.15	RGD1560883	
DMR2:4452001	2	4452001	4459000	7000	3	1.50E-10	-0.31	69	0.99	RGD1560883	
DMR2:4498001	2	4498001	4500000	2000	2	1.70E-14	0.66	0	0	RGD1560883	
DMR2:4559001	2	4559001	4562000	3000	2	3.50E-10	-0.33	31	1.03	RGD1560883	
DMR2:4674001	2	4674001	4683000	9000	1	1.30E-07	-0.41	84	0.93	RGD1560883	
DMR2:4758001	2	4758001	4764000	6000	2	3.40E-10	-0.35	78	1.3	RGD1560883	
DMR2:4793001	2	4793001	4796000	3000	1	1.60E-07	-0.33	28	0.93	Olr206	Receptor
DMR2:4809001	2	4809001	4817000	8000	3	2.20E-09	-0.39	80	1	Olr206;Olr205	Receptor
DMR2:5265001	2	5265001	5269000	4000	2	3.00E-08	-0.48	29	0.72	Fam172a	
DMR2:5320001	2	5320001	5323000	3000	2	1.80E-09	-0.39	31	1.03	Fam172a	
DMR2:5345001	2	5345001	5346000	1000	1	6.50E-07	-0.27	7	0.7	Fam172a	
DMR2:5375001	2	5375001	5377000	2000	1	1.30E-07	-0.41	13	0.65	Fam172a	
DMR2:5396001	2	5396001	5400000	4000	1	6.20E-12	-0.38	35	0.88	Fam172a	
DMR2:5415001	2	5415001	5416000	1000	1	2.80E-13	0.51	10	1	Fam172a	
DMR2:5453001	2	5453001	5456000	3000	1	3.90E-09	-0.32	29	0.97	Fam172a	
DMR2:5457001	2	5457001	5459000	2000	1	1.30E-10	-0.3	23	1.15	Fam172a	
DMR2:5514001	2	5514001	5517000	3000	2	2.00E-08	-0.41	32	1.07	Fam172a	
DMR2:5536001	2	5536001	5539000	3000	1	9.60E-07	-0.34	34	1.13	Fam172a;LOC102548021	
DMR2:9039001	2	9039001	9040000	1000	1	9.10E-08	-0.41	6	0.6	Adgrv1	Signaling
DMR2:9204001	2	9204001	9207000	3000	2	6.00E-13	-0.44	35	1.17	Adgrv1	Signaling
DMR2:11064001	2	11064001	11065000	1000	1	2.40E-12	0.75	10	1	Rps27a-ps29	
DMR2:12367001	2	12367001	12373000	6000	3	9.90E-10	-0.38	42	0.7	Tmem161b	
DMR2:13684001	2	13684001	13689000	5000	1	2.80E-09	-0.72	33	0.66	Rasa1	Signaling
DMR2:14711001	2	14711001	14714000	3000	2	1.10E-07	-0.36	25	0.83	Cox7c	Metabolism
DMR2:18054001	2	18054001	18055000	1000	1	3.80E-07	0.49	4	0.4	Edil3	Metabolism
DMR2:18077001	2	18077001	18080000	3000	1	7.10E-08	-0.34	24	0.8	Edil3	Metabolism
DMR2:18093001	2	18093001	18094000	1000	1	5.10E-07	-0.61	9	0.9	Edil3	Metabolism
DMR2:18475001	2	18475001	18481000	6000	2	4.80E-08	-0.44	69	1.15	Vcan	Extracellular Matrix
DMR2:18509001	2	18509001	18510000	1000	1	9.50E-08	-0.48	10	1	Vcan	Extracellular Matrix
DMR2:18749001	2	18749001	18754000	5000	1	7.90E-07	-0.24	82	1.64	Xrcc4	Transcription
DMR2:18830001	2	18830001	18831000	1000	1	7.20E-08	0.48	3	0.3	Xrcc4	Transcription
DMR2:18851001	2	18851001	18856000	5000	1	4.40E-09	-0.31	52	1.04	Xrcc4	Transcription
DMR2:18857001	2	18857001	18860000	3000	1	4.30E-08	-0.39	28	0.93	Xrcc4	Transcription
DMR2:18863001	2	18863001	18868000	5000	1	3.20E-07	-0.25	55	1.1	Xrcc4;LOC108350153	Transcription
DMR2:18896001	2	18896001	18897000	1000	1	3.70E-07	0.63	3	0.3	Xrcc4	Transcription
DMR2:18905001	2	18905001	18906000	1000	1	5.60E-09	-0.51	6	0.6	Xrcc4	Transcription
DMR2:19843001	2	19843001	19846000	3000	1	2.10E-12	-0.66	60	2	Atg10	Proteolysis
DMR2:19847001	2	19847001	19848000	1000	1	8.90E-08	-0.56	17	1.7	Atg10	Proteolysis
DMR2:19934001	2	19934001	19935000	1000	1	4.40E-07	0.64	1	0.1	Atg10	Proteolysis
DMR2:20011001	2	20011001	20015000	4000	1	2.00E-08	-0.29	47	1.18	Atg10	Proteolysis
DMR2:20031001	2	20031001	20037000	6000	1	2.40E-08	0.38	77	1.28	Atg10	Proteolysis
DMR2:20483001	2	20483001	20485000	2000	1	5.70E-07	-0.29	23	1.15	Ssbp2	Transcription
DMR2:20489001	2	20489001	20492000	3000	1	8.20E-08	-0.49	33	1.1	Ssbp2	Transcription
DMR2:20514001	2	20514001	20516000	2000	1	1.20E-10	0.52	29	1.45	Ssbp2	Transcription
DMR2:20594001	2	20594001	20599000	5000	4	1.70E-13	-1.31	35	0.7	Ssbp2	Transcription
DMR2:20699001	2	20699001	20703000	4000	2	8.60E-10	-0.63	71	1.77	Ssbp2	Transcription
DMR2:20714001	2	20714001	20722000	8000	4	5.50E-13	-0.38	72	0.9	Ssbp2	Transcription
DMR2:20849001	2	20849001	20851000	2000	1	9.40E-07	-0.3	25	1.25	Acot12	Metabolism
DMR2:20900001	2	20900001	20903000	3000	1	4.60E-08	0.48	40	1.33	Acot12;Zcchc9	Metabolism
DMR2:20905001	2	20905001	20906000	1000	1	5.90E-08	0.34	6	0.6	Acot12;Zcchc9	Metabolism

DMR2:21429001	2	21429001	21430000	1000	1	3.40E-11	-0.55	24	2.4	Ckmt2	Signaling
DMR2:21523001	2	21523001	21526000	3000	1	3.40E-07	0.4	44	1.47	Rasgrf2;LOC108349920	Transcription
DMR2:21936001	2	21936001	21939000	3000	1	7.50E-07	-0.4	38	1.27	Msh3;Dhfr	Transcription;Metabolism
DMR2:22037001	2	22037001	22039000	2000	2	5.70E-10	0.42	29	1.45	Fam151b	
DMR2:22115001	2	22115001	22117000	2000	1	1.40E-07	-0.49	6	0.3	Zfyve16	
DMR2:22209001	2	22209001	22210000	1000	1	2.70E-12	-0.51	30	3	Serinc5	Signaling
DMR2:22259001	2	22259001	22260000	1000	1	4.10E-08	0.45	11	1.1	Serinc5	Signaling
DMR2:22766001	2	22766001	22773000	7000	2	5.40E-08	-0.29	63	0.9	Papd4	
DMR2:22932001	2	22932001	22935000	3000	1	2.20E-07	-0.42	33	1.1	Homer1	
DMR2:22959001	2	22959001	22961000	2000	1	4.40E-07	0.36	26	1.3	Homer1	
DMR2:23428001	2	23428001	23430000	2000	1	9.30E-09	-0.44	33	1.65	Arsb	
DMR2:23740001	2	23740001	23741000	1000	1	3.90E-07	-0.35	28	2.8	Lhfpl2	
DMR2:24142001	2	24142001	24143000	1000	1	8.40E-07	-0.53	23	2.3	Ap3b1	Transport
DMR2:24154001	2	24154001	24159000	5000	2	1.40E-09	0.49	67	1.34	Ap3b1	Transport
DMR2:26170001	2	26170001	26171000	1000	1	7.00E-07	-0.4	24	2.4	Iqgap2	Signaling
DMR2:26310001	2	26310001	26312000	2000	1	2.30E-07	0.52	25	1.25	Iqgap2	Signaling
DMR2:26385001	2	26385001	26387000	2000	1	1.50E-08	0.38	30	1.5	Iqgap2	Signaling
DMR2:26516001	2	26516001	26519000	3000	1	6.10E-07	-0.28	36	1.2	Sv2c	
DMR2:26544001	2	26544001	26551000	7000	3	7.40E-15	-0.47	103	1.47	Sv2c	
DMR2:26671001	2	26671001	26674000	3000	1	6.70E-07	0.38	39	1.3	Sv2c	
DMR2:27026001	2	27026001	27027000	1000	1	1.40E-07	0.36	14	1.4	Poc5;Ankdd1b	
DMR2:27290001	2	27290001	27291000	1000	1	8.10E-07	0.42	22	2.2	Ankdd1b	
DMR2:27307001	2	27307001	27312000	5000	1	5.90E-09	0.43	87	1.74	Ankdd1b;Polk	Transcription
DMR2:27318001	2	27318001	27321000	3000	1	1.30E-08	-0.49	47	1.57	Polk	Transcription
DMR2:27371001	2	27371001	27373000	2000	1	5.60E-13	-0.48	18	0.9	Polk;Col4a3bp	Transcription
DMR2:27389001	2	27389001	27391000	2000	1	3.50E-08	-0.41	10	0.5	Col4a3bp	
DMR2:27654001	2	27654001	27661000	7000	1	5.10E-10	-0.53	79	1.13	Ankrd31	
DMR2:27676001	2	27676001	27678000	2000	1	6.60E-08	-0.45	22	1.1	Ankrd31;LOC102554085	
DMR2:27697001	2	27697001	27701000	4000	1	1.10E-09	-0.41	24	0.6	Ankrd31	
DMR2:27734001	2	27734001	27738000	4000	1	5.40E-08	-0.37	26	0.65	Ankrd31	
DMR2:27910001	2	27910001	27912000	2000	1	4.00E-08	0.39	28	1.4	Fam169a	
DMR2:28409001	2	28409001	28410000	1000	1	7.30E-08	0.51	12	1.2	LOC108349926;Btf3	Transcription
DMR2:30188001	2	30188001	30189000	1000	1	2.50E-07	0.23	4	0.4	Mccc2	Metabolism
DMR2:30235001	2	30235001	30236000	1000	1	1.20E-08	0.49	6	0.6	Mccc2	Metabolism
DMR2:30256001	2	30256001	30258000	2000	1	1.80E-09	-0.47	24	1.2	Mccc2;Bdp1	Metabolism;Transcription
DMR2:30366001	2	30366001	30367000	1000	1	1.00E-08	0.56	7	0.7	Smn1	Translation
DMR2:30384001	2	30384001	30385000	1000	1	6.00E-08	0.64	7	0.7	Naip6	
DMR2:30494001	2	30494001	30495000	1000	1	6.30E-07	-0.52	10	1	LOC102554754;Gtf2h2	Transcription
DMR2:30516001	2	30516001	30521000	5000	1	1.60E-09	-0.45	35	0.7	Gtf2h2;LOC365655;Ocln	Transcription
DMR2:30574001	2	30574001	30576000	2000	1	9.90E-10	-0.57	39	1.95	Ocln	Transcription
DMR2:30700001	2	30700001	30701000	1000	1	2.40E-08	0.37	24	2.4	Ccdc125;Cdk7	Signaling
DMR2:30807001	2	30807001	30808000	1000	1	2.00E-07	-0.43	8	0.8	Slc30a5	
DMR2:31759001	2	31759001	31763000	4000	2	1.10E-08	0.39	65	1.62	Pik3r1	Signaling
DMR2:32984001	2	32984001	32987000	3000	1	2.60E-07	-0.49	52	1.73	NEWGENE_1310139	
DMR2:33024001	2	33024001	33025000	1000	1	7.20E-07	0.42	21	2.1	NEWGENE_1310139	
DMR2:33034001	2	33034001	33035000	1000	1	6.20E-07	0.34	9	0.9	NEWGENE_1310139	
DMR2:33060001	2	33060001	33061000	1000	1	4.20E-12	0.54	13	1.3	NEWGENE_1310139	
DMR2:33125001	2	33125001	33127000	2000	1	5.30E-08	-0.31	18	0.9	NEWGENE_1310139	
DMR2:33160001	2	33160001	33163000	3000	1	7.00E-08	-0.36	53	1.77	NEWGENE_1310139	
DMR2:33182001	2	33182001	33184000	2000	1	4.30E-09	0.59	32	1.6	NEWGENE_1310139	
DMR2:33875001	2	33875001	33880000	5000	1	1.20E-07	-0.3	71	1.42	Erbin	Cytoskeleton
DMR2:33912001	2	33912001	33913000	1000	1	1.90E-07	-0.45	14	1.4	Erbin	Cytoskeleton
DMR2:34121001	2	34121001	34127000	6000	2	1.40E-09	-0.38	64	1.07	Nln	Protease
DMR2:34298001	2	34298001	34303000	5000	1	1.60E-07	0.37	48	0.96	Trim23;Ppwd1;Cenpk	Signaling;Transcription
DMR2:34387001	2	34387001	34388000	1000	1	2.30E-07	-0.49	8	0.8	Adamts6	Protease
DMR2:34408001	2	34408001	34413000	5000	2	4.70E-08	-0.41	38	0.76	Adamts6;LOC108349936	Protease
DMR2:34446001	2	34446001	34450000	4000	1	2.50E-07	-0.28	65	1.62	Adamts6	Protease
DMR2:34452001	2	34452001	34454000	2000	1	5.70E-10	-0.46	21	1.05	Adamts6	Protease
DMR2:34469001	2	34469001	34474000	5000	1	4.00E-07	-0.41	48	0.96	Adamts6	Protease
DMR2:34538001	2	34538001	34541000	3000	1	2.40E-08	-0.52	25	0.83	Adamts6	Protease
DMR2:34782001	2	34782001	34784000	2000	1	5.20E-09	-0.45	21	1.05	Cwc27	Transcription

DMR2:34804001	2	34804001	34806000	2000	1	5.30E-08	-0.39	20	1	Cwc27	Transcription
DMR2:36240001	2	36240001	36243000	3000	1	2.50E-07	-0.45	37	1.23	Htr1a	Signaling
DMR2:38213001	2	38213001	38219000	6000	2	2.60E-09	-0.33	52	0.87	Kif2a	Cytoskeleton
DMR2:39022001	2	39022001	39027000	5000	1	1.20E-09	-0.37	55	1.1	Zswim6	
DMR2:40299001	2	40299001	40300000	1000	1	3.50E-08	-0.58	2	0.2	Pde4d	Signaling
DMR2:40390001	2	40390001	40391000	1000	1	1.80E-07	-0.5	9	0.9	Pde4d;Elovl7;LOC108350211	Signaling;Metabolism
DMR2:40519001	2	40519001	40525000	6000	1	6.40E-07	-0.32	47	0.78	Pde4d	Signaling
DMR2:40656001	2	40656001	40657000	1000	1	1.70E-07	0.35	16	1.6	Pde4d	Signaling
DMR2:41133001	2	41133001	41134000	1000	1	1.50E-08	-0.46	17	1.7	Pde4d	Signaling
DMR2:41342001	2	41342001	41343000	1000	1	5.00E-07	-0.45	13	1.3	Pde4d	Signaling
DMR2:44384001	2	44384001	44386000	2000	1	1.50E-07	-0.43	11	0.55	Il31ra	Receptor
DMR2:44414001	2	44414001	44419000	5000	3	1.00E-08	-0.42	70	1.4	Il31ra	Receptor
DMR2:44828001	2	44828001	44829000	1000	1	4.70E-11	-0.4	20	2	Dhx29	Transcription
DMR2:44830001	2	44830001	44832000	2000	2	1.90E-09	-0.44	20	1	Dhx29	Transcription
DMR2:45058001	2	45058001	45060000	2000	2	1.70E-10	0.71	20	1	Gzmk	Protease
DMR2:45666001	2	45666001	45667000	1000	1	8.70E-10	-0.62	9	0.9	Arl15	
DMR2:45716001	2	45716001	45719000	3000	1	7.90E-07	0.56	53	1.77	Arl15	
DMR2:45815001	2	45815001	45822000	7000	1	3.80E-08	-0.43	106	1.51	Arl15	
DMR2:45920001	2	45920001	45922000	2000	1	2.20E-07	0.57	9	0.45	Arl15	
DMR2:45989001	2	45989001	45991000	2000	1	8.30E-08	-0.48	17	0.85	Arl15	
DMR2:46048001	2	46048001	46050000	2000	1	5.20E-07	0.42	21	1.05	Arl15	
DMR2:46470001	2	46470001	46471000	1000	1	6.00E-16	0.52	53	5.3	Ndufs4	Metabolism
DMR2:46534001	2	46534001	46535000	1000	1	2.00E-08	0.49	9	0.9	Fst	Protease; Proteolysis
DMR2:47204001	2	47204001	47208000	4000	1	9.70E-11	0.48	32	0.8	Itga1	Extracellular Matrix
DMR2:47235001	2	47235001	47236000	1000	1	2.00E-08	-0.49	14	1.4	Itga1	Extracellular Matrix
DMR2:49232001	2	49232001	49234000	2000	2	5.10E-08	-0.35	19	0.95	Parp8	
DMR2:50144001	2	50144001	50149000	5000	2	6.30E-09	-0.3	53	1.06	Hcn1	Transport
DMR2:50227001	2	50227001	50234000	7000	2	3.10E-09	-0.55	61	0.87	Hcn1	Transport
DMR2:50333001	2	50333001	50338000	5000	1	4.20E-11	-0.28	48	0.96	Hcn1	Transport
DMR2:52176001	2	52176001	52177000	1000	1	4.70E-07	-0.56	18	1.8	RGD1561520	
DMR2:53172001	2	53172001	53176000	4000	1	2.90E-09	-0.28	40	1	Ghr	Receptor
DMR2:54147001	2	54147001	54149000	2000	1	2.30E-07	-0.29	14	0.7	RGD1566344	
DMR2:54242001	2	54242001	54247000	5000	1	7.70E-08	-0.28	46	0.92	Plcxd3	
DMR2:54287001	2	54287001	54293000	6000	1	3.60E-07	-0.23	71	1.18	Plcxd3	
DMR2:54348001	2	54348001	54351000	3000	1	2.00E-07	-0.38	12	0.4	Plcxd3	
DMR2:54810001	2	54810001	54811000	1000	1	2.70E-07	-0.38	13	1.3	Card6	
DMR2:54815001	2	54815001	54817000	2000	1	5.00E-08	0.43	44	2.2	Card6	
DMR2:54884001	2	54884001	54886000	2000	1	3.70E-07	0.55	12	0.6	Prkaa1	Signaling
DMR2:55050001	2	55050001	55052000	2000	1	1.70E-07	-0.33	15	0.75	LOC103691475;Rps15al4	
DMR2:55968001	2	55968001	55976000	8000	1	8.80E-14	-0.44	71	0.89	Fyb	
DMR2:56468001	2	56468001	56469000	1000	1	2.60E-09	0.54	9	0.9	Lifr	Receptor
DMR2:56972001	2	56972001	56978000	6000	1	2.50E-08	-0.35	66	1.1	Wdr70	
DMR2:57142001	2	57142001	57144000	2000	2	9.90E-12	-0.43	15	0.75	Wdr70	
DMR2:57865001	2	57865001	57867000	2000	1	1.00E-07	0.4	28	1.4	Slc1a3	Transport
DMR2:57912001	2	57912001	57914000	2000	1	8.20E-10	-0.53	28	1.4	Slc1a3	Transport
DMR2:57916001	2	57916001	57917000	1000	1	6.90E-10	0.51	8	0.8	Slc1a3	Transport
DMR2:58276001	2	58276001	58281000	5000	1	2.20E-11	-0.31	53	1.06	Ranbp3l;LOC365693	Cytoskeleton
DMR2:58319001	2	58319001	58320000	1000	1	5.40E-07	-0.48	15	1.5	Ranbp3l	Cytoskeleton
DMR2:58952001	2	58952001	58959000	7000	2	7.30E-08	-0.28	90	1.29	Spef2	
DMR2:58960001	2	58960001	58962000	2000	1	6.40E-08	-0.27	19	0.95	Spef2	
DMR2:60193001	2	60193001	60198000	5000	2	5.10E-09	-0.36	52	1.04	Prlr	Receptor
DMR2:60422001	2	60422001	60428000	6000	2	5.00E-09	-0.37	66	1.1	Dnajc21	Transcription
DMR2:60977001	2	60977001	60980000	3000	1	2.30E-09	-0.36	27	0.9	Slc45a2	Transport
DMR2:61419001	2	61419001	61424000	5000	1	5.10E-07	-0.32	46	0.92	Tars	
DMR2:62288001	2	62288001	62291000	3000	1	1.10E-07	0.43	39	1.3	Mtmr12	Signaling
DMR2:62524001	2	62524001	62525000	1000	1	7.30E-11	0.6	7	0.7	Pdzd2	Cytokine
DMR2:62895001	2	62895001	62897000	2000	1	6.10E-07	0.41	65	3.25	RGD1306502;Drosha	Translation
DMR2:63149001	2	63149001	63155000	6000	1	5.80E-07	-0.32	74	1.23	Cdh6	Cytoskeleton
DMR2:63179001	2	63179001	63180000	1000	1	7.40E-07	-0.3	9	0.9	Cdh6	Cytoskeleton
DMR2:68905001	2	68905001	68906000	1000	1	3.80E-07	-0.5	8	0.8	Egf	Receptor

DMR2:69464001	2	69464001	69465000	1000	1	1.30E-07	-0.42	17	1.7	Cdh10;LOC103691484	Cytoskeleton
DMR2:69572001	2	69572001	69576000	4000	1	9.40E-07	-0.32	52	1.3	Cdh10	Cytoskeleton
DMR2:71332001	2	71332001	71334000	2000	1	1.90E-07	-0.4	19	0.95	Cdh12	Cytoskeleton
DMR2:71404001	2	71404001	71405000	1000	1	3.80E-07	-0.42	8	0.8	Cdh12	Cytoskeleton
DMR2:72065001	2	72065001	72066000	1000	1	4.80E-08	-0.4	7	0.7	Cdh12	Cytoskeleton
DMR2:72367001	2	72367001	72368000	1000	1	9.70E-07	-0.29	14	1.4	Cdh12	Cytoskeleton
DMR2:72401001	2	72401001	72403000	2000	1	3.50E-09	-0.61	17	0.85	Cdh12	Cytoskeleton
DMR2:72470001	2	72470001	72473000	3000	1	9.40E-12	-0.61	19	0.63	Cdh12	Cytoskeleton
DMR2:72520001	2	72520001	72525000	5000	1	2.10E-09	-0.36	81	1.62	Cdh12	Cytoskeleton
DMR2:73980001	2	73980001	73987000	7000	2	1.10E-12	-0.43	76	1.09	Cdh18	Cytoskeleton
DMR2:74113001	2	74113001	74116000	3000	1	6.40E-07	-0.36	20	0.67	Cdh18	Cytoskeleton
DMR2:74314001	2	74314001	74320000	6000	2	5.60E-10	-0.38	70	1.17	Cdh18	Cytoskeleton
DMR2:74346001	2	74346001	74349000	3000	1	2.10E-10	-0.41	37	1.23	Cdh18	Cytoskeleton
DMR2:74354001	2	74354001	74359000	5000	1	3.30E-07	-0.33	48	0.96	Cdh18	Cytoskeleton
DMR2:74514001	2	74514001	74516000	2000	1	1.80E-15	-0.38	26	1.3	Cdh18	Cytoskeleton
DMR2:78814001	2	78814001	78818000	4000	1	3.80E-14	-0.39	43	1.07	11-Mar	
DMR2:79055001	2	79055001	79058000	3000	1	3.90E-10	-0.49	18	0.6	Fbxl7	
DMR2:79324001	2	79324001	79325000	1000	1	1.40E-07	0.49	8	0.8	Fbxl7	
DMR2:79431001	2	79431001	79432000	1000	1	3.70E-07	-0.51	11	1.1	Fbxl7	
DMR2:80182001	2	80182001	80185000	3000	1	6.40E-08	0.37	50	1.67	Ankh	
DMR2:80538001	2	80538001	80541000	3000	1	1.20E-07	0.55	28	0.93	Trio	Transcription
DMR2:80559001	2	80559001	80560000	1000	1	8.80E-08	0.37	23	2.3	Trio	Transcription
DMR2:81007001	2	81007001	81008000	1000	1	3.10E-07	-0.4	12	1.2	Dnah5	Cytoskeleton
DMR2:81038001	2	81038001	81044000	6000	1	4.90E-09	-0.37	64	1.07	Dnah5	Cytoskeleton
DMR2:81275001	2	81275001	81276000	1000	1	8.60E-07	-0.51	15	1.5	Dnah5	Cytoskeleton
DMR2:81286001	2	81286001	81293000	7000	1	6.30E-07	-0.31	77	1.1	Dnah5	Cytoskeleton
DMR2:81338001	2	81338001	81342000	4000	2	2.70E-12	-0.37	37	0.92	Dnah5	Cytoskeleton
DMR2:83443001	2	83443001	83444000	1000	1	6.80E-08	-0.61	11	1.1	Ctnnd2	Cytoskeleton
DMR2:83459001	2	83459001	83466000	7000	2	3.30E-08	-0.37	82	1.17	Ctnnd2	Cytoskeleton
DMR2:83912001	2	83912001	83916000	4000	1	8.30E-08	-0.4	52	1.3	Ctnnd2	Cytoskeleton
DMR2:84319001	2	84319001	84321000	2000	1	4.20E-08	-0.53	33	1.65	Dap	
DMR2:84553001	2	84553001	84557000	4000	1	4.70E-10	-0.3	59	1.48	6-Mar	Proteolysis
DMR2:85564001	2	85564001	85565000	1000	1	2.40E-08	0.45	5	0.5	Sema5a	Signaling
DMR2:88103001	2	88103001	88104000	1000	1	2.90E-07	0.33	7	0.7	Car2	
DMR2:88124001	2	88124001	88127000	3000	1	1.70E-07	-0.67	23	0.77	Car3	
DMR2:88367001	2	88367001	88377000	10000	1	3.00E-07	0.26	87	0.87	E2f5;Lrrcc1	Transcription;Cytoskeleton
DMR2:88611001	2	88611001	88615000	4000	1	1.60E-11	-0.52	39	0.98	Slc7a12	Transport
DMR2:89131001	2	89131001	89138000	7000	2	8.70E-11	-0.39	101	1.44	Ralyl	
DMR2:89229001	2	89229001	89233000	4000	3	8.70E-10	-0.43	41	1.02	Ralyl	
DMR2:89340001	2	89340001	89346000	6000	2	2.60E-08	-0.38	56	0.93	Ralyl;LOC108349976	
DMR2:89527001	2	89527001	89529000	2000	1	1.00E-10	0.63	11	0.55	Ralyl	
DMR2:89571001	2	89571001	89573000	2000	1	2.70E-07	0.43	7	0.35	Ralyl	
DMR2:89604001	2	89604001	89610000	6000	2	4.10E-14	-0.44	65	1.08	Ralyl	
DMR2:93612001	2	93612001	93615000	3000	1	2.30E-07	-0.35	26	0.87	Chmp4c	Transport
DMR2:93645001	2	93645001	93646000	1000	1	2.30E-13	0.54	9	0.9	Chmp4c	Transport
DMR2:93795001	2	93795001	93796000	1000	1	8.70E-07	-0.42	22	2.2	Fabp4;Fabp9	
DMR2:94384001	2	94384001	94387000	3000	1	9.20E-09	-0.32	29	0.97	Zfp704	Transcription
DMR2:94975001	2	94975001	94977000	2000	1	3.10E-07	0.36	59	2.95	Tpd52	
DMR2:96622001	2	96622001	96624000	2000	1	7.50E-07	-0.41	8	0.4	Pkia	Signaling
DMR2:103945001	2	103945001	103951000	6000	3	3.50E-11	-0.43	68	1.13	Armc1	
DMR2:103963001	2	103963001	103965000	2000	1	1.20E-07	-0.5	13	0.65	Armc1	
DMR2:104765001	2	104765001	104771000	6000	2	1.70E-07	-0.3	66	1.1	Cp	Metabolism
DMR2:104820001	2	104820001	104822000	2000	1	8.10E-08	-0.35	24	1.2	Hps3	
DMR2:104858001	2	104858001	104860000	2000	1	1.10E-10	-0.54	15	0.75	Hltf	
DMR2:105002001	2	105002001	105008000	6000	1	2.20E-07	-0.37	60	1	LOC682402;Cpa3	Protease
DMR2:105052001	2	105052001	105059000	7000	2	4.20E-14	0.54	76	1.09	Cpa3;Cpb1	Protease
DMR2:105605001	2	105605001	105607000	2000	1	9.80E-07	-0.37	32	1.6	Med14-ps1	
DMR2:109080001	2	109080001	109083000	3000	1	1.80E-07	-0.29	24	0.8	Naaladl2	
DMR2:111097001	2	111097001	111105000	8000	3	2.50E-12	-0.38	101	1.26	Nlgn1	Cytoskeleton
DMR2:111636001	2	111636001	111638000	2000	1	3.40E-07	-0.44	16	0.8	Nlgn1	Cytoskeleton
DMR2:111685001	2	111685001	111688000	3000	1	2.60E-08	-0.3	29	0.97	Nlgn1	Cytoskeleton
DMR2:111889001	2	111889001	111891000	2000	1	6.00E-10	-0.56	21	1.05	Nlgn1	Cytoskeleton

DMR2:112367001	2	112367001	112373000	6000	1	7.60E-08	-0.42	73	1.22	Spata16	
DMR2:112642001	2	112642001	112647000	5000	2	1.80E-07	-0.29	50	1	Spata16	
DMR2:112779001	2	112779001	112781000	2000	1	8.20E-08	-0.39	16	0.8	Ect2	Transcription
DMR2:113170001	2	113170001	113171000	1000	1	1.40E-07	0.47	13	1.3	Fndc3b	Proteolysis
DMR2:113289001	2	113289001	113290000	1000	1	3.10E-08	-0.33	14	1.4	Fndc3b	Proteolysis
DMR2:113330001	2	113330001	113332000	2000	1	8.70E-10	0.38	24	1.2	Fndc3b	Proteolysis
DMR2:113364001	2	113364001	113365000	1000	1	2.60E-09	0.55	11	1.1	Fndc3b	Proteolysis
DMR2:114103001	2	114103001	114104000	1000	1	3.30E-07	-0.47	13	1.3	Tnik	Signaling
DMR2:114288001	2	114288001	114290000	2000	1	1.60E-10	0.46	33	1.65	Tnik	Signaling
DMR2:114326001	2	114326001	114328000	2000	1	2.30E-09	0.54	23	1.15	Tnik	Signaling
DMR2:114375001	2	114375001	114376000	1000	1	8.60E-07	-0.42	17	1.7	Tnik	Signaling
DMR2:115086001	2	115086001	115088000	2000	1	7.20E-09	-0.38	20	1	Olr1279	
DMR2:116135001	2	116135001	116139000	4000	2	1.10E-07	-0.37	37	0.92	Gpr160	Signaling
DMR2:116326001	2	116326001	116328000	2000	1	7.30E-09	0.4	31	1.55	Lrrc31	
DMR2:116513001	2	116513001	116516000	3000	1	6.40E-08	-0.42	21	0.7	Egfem1	
DMR2:116635001	2	116635001	116645000	10000	1	1.80E-07	-0.27	116	1.16	Egfem1	
DMR2:116777001	2	116777001	116780000	3000	1	1.60E-08	-0.32	19	0.63	Egfem1	
DMR2:116914001	2	116914001	116916000	2000	1	8.40E-07	-0.28	20	1	Egfem1	
DMR2:116950001	2	116950001	116957000	7000	1	6.80E-09	-0.29	76	1.09	Egfem1;LOC103691523	
DMR2:116980001	2	116980001	116984000	4000	1	2.10E-07	-0.31	37	0.92	Egfem1	
DMR2:117046001	2	117046001	117051000	5000	1	1.60E-10	-0.37	55	1.1	Egfem1	
DMR2:117502001	2	117502001	117504000	2000	2	1.20E-07	-0.43	33	1.65	Mecom	Transcription
DMR2:117848001	2	117848001	117850000	2000	1	4.30E-07	0.38	25	1.25	Mecom;LOC103691544	Transcription
DMR2:117857001	2	117857001	117859000	2000	1	2.40E-07	-0.43	31	1.55	Mecom;LOC103691544	Transcription
DMR2:118449001	2	118449001	118451000	2000	1	2.10E-09	0.65	12	0.6	Kcnmb2	Transport
DMR2:118487001	2	118487001	118496000	9000	1	5.00E-08	-0.31	116	1.29	Kcnmb2	Transport
DMR2:119300001	2	119300001	119302000	2000	1	9.20E-08	-0.37	52	2.6	Usp13	Protease
DMR2:122468001	2	122468001	122475000	7000	2	9.60E-10	0.43	79	1.13	Dcun1d1	
DMR2:122712001	2	122712001	122716000	4000	2	3.20E-09	-0.5	47	1.18	Ccdc144b	
DMR2:122939001	2	122939001	122940000	1000	1	6.50E-12	0.58	12	1.2	Qrfpr	Signaling
DMR2:123363001	2	123363001	123365000	2000	1	9.50E-07	0.4	30	1.5	Trpc3	Transport
DMR2:123436001	2	123436001	123441000	5000	1	4.10E-07	0.37	60	1.2	Trpc3;LOC365768	Transport
DMR2:123465001	2	123465001	123467000	2000	1	4.80E-09	0.37	11	0.55	Trpc3	Transport
DMR2:123624001	2	123624001	123627000	3000	1	1.50E-10	-0.31	30	1	RGD1307100	
DMR2:123645001	2	123645001	123649000	4000	1	5.00E-08	-0.28	52	1.3	RGD1307100	
DMR2:123726001	2	123726001	123729000	3000	1	1.50E-12	-0.45	29	0.97	RGD1307100	
DMR2:124029001	2	124029001	124038000	9000	1	7.40E-10	-0.35	108	1.2	Cetn4	Signaling
DMR2:124184001	2	124184001	124185000	1000	1	1.20E-08	0.39	11	1.1	Spata5;LOC108349998	
DMR2:124290001	2	124290001	124294000	4000	1	2.60E-09	-0.45	35	0.88	Spata5;LOC102546767	
DMR2:125311001	2	125311001	125312000	1000	1	2.40E-07	0.38	16	1.6	Ankrd50	Cytoskeleton
DMR2:127759001	2	127759001	127764000	5000	1	1.80E-07	-0.35	40	0.8	Mfsd8;Abhd18	
DMR2:127931001	2	127931001	127938000	7000	1	3.70E-07	-0.33	68	0.97	RGD1565989	
DMR2:128618001	2	128618001	128619000	1000	1	2.80E-09	-0.47	8	0.8	Sclt1	
DMR2:128656001	2	128656001	128664000	8000	2	3.40E-08	-0.31	102	1.27	Sclt1	
DMR2:128685001	2	128685001	128690000	5000	2	2.20E-08	-0.38	54	1.08	Sclt1;RGD1359508	
DMR2:130662001	2	130662001	130665000	3000	1	3.50E-07	-0.4	20	0.67	Pabpc4l	
DMR2:132238001	2	132238001	132239000	1000	1	1.60E-07	0.62	69	6.9	RGD1563562	Signaling
DMR2:140339001	2	140339001	140341000	2000	1	3.90E-07	0.38	31	1.55	Elf2	Transcription
DMR2:140819001	2	140819001	140825000	6000	1	1.50E-08	-0.38	44	0.73	Maml3	
DMR2:140846001	2	140846001	140853000	7000	1	8.20E-07	0.32	84	1.2	Maml3;Dusp14l1	
DMR2:141150001	2	141150001	141151000	1000	1	8.90E-07	-0.37	23	2.3	Maml3	
DMR2:142836001	2	142836001	142839000	3000	1	1.80E-07	0.42	45	1.5	Frem2	
DMR2:142859001	2	142859001	142860000	1000	1	1.60E-08	-0.4	19	1.9	Frem2	
DMR2:142895001	2	142895001	142899000	4000	2	4.40E-09	-0.28	46	1.15	Frem2	
DMR2:143483001	2	143483001	143488000	5000	1	2.10E-07	-0.31	73	1.46	Trpc4	Transport
DMR2:143834001	2	143834001	143836000	2000	1	2.30E-08	-0.36	16	0.8	Rexo1l1-ps1	Transcription
DMR2:143964001	2	143964001	143965000	1000	1	1.00E-10	-0.6	20	2	Smad9	Transcription
DMR2:143975001	2	143975001	143976000	1000	1	1.00E-10	0.33	3	0.3	Smad9	Transcription
DMR2:145100001	2	145100001	145107000	7000	1	3.30E-07	-0.26	78	1.11	Nbea	
DMR2:145124001	2	145124001	145126000	2000	1	1.90E-15	0.59	111	5.55	Nbea	
DMR2:145257001	2	145257001	145264000	7000	1	3.60E-07	-0.26	78	1.11	Nbea	

DMR2:145311001	2	145311001	145316000	5000	1	1.30E-08	-0.3	42	0.84	Nbea	
DMR2:145436001	2	145436001	145437000	1000	1	2.70E-07	-0.5	9	0.9	Nbea	
DMR2:147004001	2	147004001	147006000	2000	1	9.00E-07	0.55	11	0.55	RGD1306704	
DMR2:147462001	2	147462001	147468000	6000	1	4.20E-08	-0.3	65	1.08	RGD1563943	
DMR2:147472001	2	147472001	147473000	1000	1	5.40E-09	-0.58	3	0.3	RGD1563943	
DMR2:147483001	2	147483001	147484000	1000	1	2.60E-09	-0.55	5	0.5	RGD1563943	
DMR2:147967001	2	147967001	147974000	7000	3	1.20E-09	-0.32	72	1.03	Pfn2	Cytoskeleton
DMR2:148843001	2	148843001	148846000	3000	1	3.00E-07	-0.37	46	1.53	Erich6	
DMR2:149245001	2	149245001	149246000	1000	1	2.30E-08	0.38	16	1.6	Med12l	Transcription
DMR2:149399001	2	149399001	149400000	1000	1	3.20E-07	-0.49	32	3.2	Gpr87	Signaling
DMR2:149423001	2	149423001	149425000	2000	1	9.60E-07	0.35	21	1.05	Gpr87;P2ry13	Signaling
DMR2:149746001	2	149746001	149749000	3000	1	3.20E-08	-0.48	20	0.67	RGD1559622;LOC103691561	Metabolism
DMR2:149834001	2	149834001	149836000	2000	1	2.30E-08	-0.42	17	0.85	RGD1561998	Metabolism
DMR2:149919001	2	149919001	149920000	1000	1	3.00E-07	-0.42	11	1.1	RGD1560324	Metabolism
DMR2:150047001	2	150047001	150051000	4000	1	1.90E-07	-0.29	36	0.9	RGD1560162	
DMR2:150092001	2	150092001	150095000	3000	1	5.10E-09	-0.41	21	0.7	RGD1560162;LOC108350019	
DMR2:150715001	2	150715001	150720000	5000	1	4.40E-11	-0.34	59	1.18	Mbnl1	Translation
DMR2:152853001	2	152853001	152859000	6000	1	8.10E-09	-0.34	63	1.05	Gpr149	Signaling
DMR2:153864001	2	153864001	153865000	1000	1	1.10E-12	0.6	7	0.7	Mme	Protease
DMR2:154361001	2	154361001	154363000	2000	1	1.40E-07	-0.36	13	0.65	Plch1	Metabolism
DMR2:154386001	2	154386001	154393000	7000	2	1.90E-09	-0.46	71	1.01	Plch1	Metabolism
DMR2:154499001	2	154499001	154503000	4000	2	9.20E-11	-0.3	40	1	RGD1565059	
DMR2:154623001	2	154623001	154626000	3000	1	3.40E-08	-0.58	7	0.23	Vom2r44	Signaling
DMR2:154753001	2	154753001	154755000	2000	1	1.00E-07	-0.33	14	0.7	Vom2r46	Signaling
DMR2:155358001	2	155358001	155360000	2000	1	2.90E-08	-0.51	5	0.25	Vom2r47	Signaling
DMR2:155669001	2	155669001	155674000	5000	1	5.10E-07	-0.26	51	1.02	Kcnab1	
DMR2:155783001	2	155783001	155787000	4000	1	6.50E-08	0.57	19	0.48	Kcnab1	
DMR2:155883001	2	155883001	155888000	5000	2	1.40E-10	-0.35	41	0.82	Kcnab1	
DMR2:155989001	2	155989001	155990000	1000	1	8.80E-07	0.47	67	6.7	Kcnab1;LOC108350024	
DMR2:157544001	2	157544001	157546000	2000	1	6.70E-07	-0.36	19	0.95	Lekr1	
DMR2:157562001	2	157562001	157565000	3000	1	1.10E-07	-0.53	32	1.07	Lekr1	
DMR2:157882001	2	157882001	157883000	1000	1	6.70E-09	-0.29	12	1.2	Veph1	
DMR2:157951001	2	157951001	157956000	5000	2	2.80E-09	-0.37	63	1.26	Veph1	
DMR2:162413001	2	162413001	162421000	8000	3	1.30E-14	0.43	155	1.94	Olr1818-ps	
DMR2:164173001	2	164173001	164175000	2000	2	9.70E-12	0.45	72	3.6	Rsrc1	
DMR2:164283001	2	164283001	164285000	2000	1	2.90E-08	-0.44	18	0.9	Rsrc1	
DMR2:164378001	2	164378001	164383000	5000	1	7.90E-09	-0.3	68	1.36	Rsrc1	
DMR2:164469001	2	164469001	164473000	4000	1	1.80E-07	-0.42	25	0.62	Rsrc1	
DMR2:164500001	2	164500001	164501000	1000	1	3.90E-08	-0.56	15	1.5	Rsrc1	
DMR2:164586001	2	164586001	164593000	7000	3	1.40E-09	-0.36	97	1.39	Mlf1;Gfm1	Signaling
DMR2:164655001	2	164655001	164658000	3000	1	7.50E-07	0.43	27	0.9	Gfm1;Rarres1	Protease; Proteolysis
DMR2:164744001	2	164744001	164745000	1000	1	8.80E-09	-0.5	8	0.8	Mfsd1	
DMR2:166170001	2	166170001	166172000	2000	1	3.10E-07	-0.49	20	1	Ppm1l	Signaling
DMR2:171165001	2	171165001	171166000	1000	1	5.40E-07	0.61	20	2	Bche	Metabolism
DMR2:172331001	2	172331001	172338000	7000	1	9.50E-07	-0.27	82	1.17	Schip1	
DMR2:172655001	2	172655001	172659000	4000	2	2.20E-07	-0.31	44	1.1	Schip1;LOC100910449	
DMR2:172811001	2	172811001	172812000	1000	1	2.40E-08	0.42	11	1.1	Schip1;LOC102547484	
DMR2:172891001	2	172891001	172897000	6000	1	6.60E-07	-0.31	63	1.05	Schip1	
DMR2:173086001	2	173086001	173087000	1000	1	6.90E-07	0.43	19	1.9	Schip1	
DMR2:173097001	2	173097001	173103000	6000	1	1.60E-10	-0.54	53	0.88	Schip1	
DMR2:173444001	2	173444001	173447000	3000	1	4.40E-07	-0.48	18	0.6	LOC365821;Zbbx	
DMR2:173477001	2	173477001	173479000	2000	1	1.00E-08	-0.47	5	0.25	Zbbx	
DMR2:173766001	2	173766001	173770000	4000	1	8.40E-12	0.38	37	0.92	Wdr49;LOC102556029	
DMR2:174067001	2	174067001	174069000	2000	1	4.80E-07	-0.28	17	0.85	Serpini1	Protease; Proteolysis
DMR2:174373001	2	174373001	174380000	7000	1	3.00E-08	-0.28	88	1.26	Golim4	
DMR2:174387001	2	174387001	174393000	6000	1	1.50E-09	-0.34	65	1.08	Golim4	
DMR2:174638001	2	174638001	174642000	4000	1	2.70E-09	-0.28	39	0.98	Fstl5	Protease; Proteolysis
DMR2:174889001	2	174889001	174890000	1000	1	1.90E-11	-0.64	6	0.6	Fstl5	Protease; Proteolysis

DMR2:177643001	2	177643001	177650000	7000	2	1.40E-07	-0.3	75	1.07	LOC361966;Hsc70-ps1;LOC100363048	
DMR2:177914001	2	177914001	177917000	3000	1	4.10E-07	-0.32	31	1.03	Rapgef2	Transcription
DMR2:178723001	2	178723001	178726000	3000	1	8.30E-07	-0.29	51	1.7	Fam198b	
DMR2:179634001	2	179634001	179638000	4000	1	6.00E-07	-0.38	45	1.12	Gria2	Receptor
DMR2:179713001	2	179713001	179719000	6000	3	9.70E-11	-0.32	73	1.22	Gria2	Receptor
DMR2:179763001	2	179763001	179764000	1000	1	9.10E-07	-0.44	14	1.4	Glrb	Ion Channel
DMR2:180954001	2	180954001	180955000	1000	1	1.80E-14	0.64	3	0.3	Asic5	Transport
DMR2:182097001	2	182097001	182105000	8000	1	5.00E-07	-0.24	100	1.25	Dchs2	
DMR2:182826001	2	182826001	182837000	11000	2	6.10E-13	-0.39	133	1.21	LOC102551394;Tlr2	
DMR2:183439001	2	183439001	183445000	6000	3	1.30E-14	0.36	69	1.15	Fhdc1	
DMR2:185321001	2	185321001	185322000	1000	1	8.70E-13	-0.55	16	1.6	Sh3d19	
DMR2:185350001	2	185350001	185352000	2000	1	8.90E-12	0.46	36	1.8	Sh3d19	
DMR2:185357001	2	185357001	185362000	5000	1	1.20E-07	0.46	72	1.44	Sh3d19	
DMR2:185597001	2	185597001	185598000	1000	1	3.50E-08	0.41	25	2.5	Lrba	
DMR2:185741001	2	185741001	185745000	4000	1	8.40E-07	-0.3	30	0.75	Lrba	
DMR2:186015001	2	186015001	186017000	2000	1	3.10E-09	-0.3	20	1	Lrba	
DMR2:186425001	2	186425001	186428000	3000	1	3.50E-07	0.33	61	2.03	Kirrel	
DMR2:186430001	2	186430001	186432000	2000	1	2.70E-14	0.47	43	2.15	Kirrel	
DMR2:186910001	2	186910001	186913000	3000	1	9.10E-07	0.41	35	1.17	Etv3l;LOC108350054;LOC100361532	Transcription
DMR2:187751001	2	187751001	187754000	3000	2	4.80E-14	0.8	37	1.23	Paqr6;Bglap;Pmf1	Signaling
DMR2:188252001	2	188252001	188253000	1000	1	9.90E-08	0.61	75	7.5	Dap3;Ash1l	Translation;Epigenetic
DMR2:188580001	2	188580001	188581000	1000	1	7.60E-07	-0.4	17	1.7	Dpm3;Slc50a1;Efna1	Transport;Signaling
DMR2:188937001	2	188937001	188938000	1000	1	6.70E-07	-0.39	11	1.1	Kcnn3	Transport
DMR2:189048001	2	189048001	189049000	1000	1	6.60E-10	0.47	1	0.1	Adar	Metabolism
DMR2:189202001	2	189202001	189203000	1000	1	3.70E-07	0.37	15	1.5	She;Il6r	Receptor
DMR2:189370001	2	189370001	189371000	1000	1	8.80E-10	-0.45	7	0.7	Atp8b2	Transport
DMR2:189456001	2	189456001	189457000	1000	1	2.80E-07	-0.47	19	1.9	Tpm3;Nup210l	Cytoskeleton;Transport
DMR2:189515001	2	189515001	189517000	2000	1	4.60E-07	0.3	74	3.7	Nup210l	Transport
DMR2:189530001	2	189530001	189531000	1000	1	4.20E-07	-0.47	7	0.7	Nup210l	Transport
DMR2:189705001	2	189705001	189706000	1000	1	1.10E-09	0.59	6	0.6	Gatad2b	Transcription
DMR2:189732001	2	189732001	189733000	1000	1	8.50E-07	0.29	15	1.5	Gatad2b	Transcription
DMR2:192236001	2	192236001	192238000	2000	1	3.50E-07	-0.35	17	0.85	Pglyrp3	
DMR2:193145001	2	193145001	193147000	2000	1	7.30E-07	-0.48	15	0.75	Kprp	
DMR2:193368001	2	193368001	193369000	1000	1	1.10E-07	0.6	4	0.4	RGD1561111	
DMR2:194607001	2	194607001	194609000	2000	1	3.10E-08	-0.33	22	1.1	LOC689026;Tdpoz1	
DMR2:194872001	2	194872001	194875000	3000	2	3.90E-09	-0.54	18	0.6	RGD1566337	Proteolysis
DMR2:195043001	2	195043001	195046000	3000	1	3.40E-07	-0.31	40	1.33	RGD1563392	
DMR2:195319001	2	195319001	195321000	2000	1	3.60E-07	-0.32	29	1.45	RGD1563667	Proteolysis
DMR2:195516001	2	195516001	195517000	1000	1	9.50E-08	-0.49	32	3.2	RGD1563667;RGD1560554	Proteolysis
DMR2:195522001	2	195522001	195523000	1000	1	3.90E-08	-0.42	8	0.8	RGD1563667;RGD1560554	Proteolysis
DMR2:195529001	2	195529001	195530000	1000	1	3.90E-11	-0.52	6	0.6	RGD1563667;RGD1560554	Proteolysis
DMR2:195569001	2	195569001	195573000	4000	1	2.90E-07	0.38	49	1.23	Them4;Them5	Metabolism
DMR2:195658001	2	195658001	195660000	2000	1	7.30E-09	0.62	10	0.5	Tdrkh;LOC103691639	Cytoskeleton
DMR2:195713001	2	195713001	195715000	2000	1	6.90E-14	0.49	117	5.85	Riiad1;Celf3	
DMR2:196288001	2	196288001	196290000	2000	1	1.50E-07	0.4	29	1.45	Vps72;Tmod4	Transcription;Cytoskeleton
DMR2:196321001	2	196321001	196322000	1000	1	3.70E-07	0.57	10	1	Lysmd1;Tnfaip8l2;Sema6c	Signaling
DMR2:197702001	2	197702001	197705000	3000	2	3.00E-12	-0.39	22	0.73	Hormad1;LOC102547423	
DMR2:198091001	2	198091001	198093000	2000	1	1.20E-07	-0.46	32	1.6	RGD1562196;LOC108350069	
DMR2:198268001	2	198268001	198269000	1000	1	1.10E-08	0.37	18	1.8	Otud7b;LOC102548788	Protease
DMR2:198522001	2	198522001	198524000	2000	1	8.50E-07	-0.5	32	1.6	Trnae-uuc;Trnag-ccc	
DMR2:198590001	2	198590001	198591000	1000	1	1.80E-35	2.01	18	1.8	LOC103691644;LOC100360229;Trnah-gug;Trnan-guu;LOC108350070	
DMR2:198735001	2	198735001	198736000	1000	1	2.50E-08	-0.56	13	1.3	Ankrd34a;Lix1l	
DMR2:199160001	2	199160001	199161000	1000	1	6.70E-07	0.42	14	1.4	Gja5	Cytoskeleton
DMR2:199341001	2	199341001	199342000	1000	1	2.20E-08	0.44	17	1.7	Bcl9	

DMR2:199391001	2	199391001	199392000	1000	1	6.10E-14	0.55	9	0.9	Bcl9	
DMR2:199706001	2	199706001	199712000	6000	1	4.30E-09	-0.32	59	0.98	Chd1l	
DMR2:199774001	2	199774001	199775000	1000	1	8.00E-11	0.44	12	1.2	Chd1l	
DMR2:199854001	2	199854001	199856000	2000	1	9.70E-08	-0.33	20	1	Prkab2;Trnaq-cug;LOC102550575	Signaling
DMR2:199932001	2	199932001	199933000	1000	1	1.10E-08	0.5	22	2.2	Pde4dip	
DMR2:200226001	2	200226001	200228000	2000	1	2.60E-07	0.57	29	1.45	Notch2	Extracellular Matrix
DMR2:200238001	2	200238001	200239000	1000	1	4.70E-09	0.43	17	1.7	Notch2	Extracellular Matrix
DMR2:200248001	2	200248001	200251000	3000	1	1.70E-08	0.45	37	1.23	Notch2	Extracellular Matrix
DMR2:200407001	2	200407001	200408000	1000	1	7.70E-14	0.52	52	5.2	Reg4	
DMR2:200701001	2	200701001	200703000	2000	1	2.10E-07	-0.42	7	0.35	LOC502588;Hsd3b6	Metabolism
DMR2:200784001	2	200784001	200787000	3000	1	3.70E-07	-0.39	23	0.77	Hao2	Metabolism
DMR2:201418001	2	201418001	201424000	6000	4	1.00E-08	-0.38	78	1.3	RGD1561508	
DMR2:202176001	2	202176001	202181000	5000	1	1.80E-10	-0.36	76	1.52	Spag17	
DMR2:202372001	2	202372001	202374000	2000	1	5.60E-09	-0.47	15	0.75	Spag17	
DMR2:202417001	2	202417001	202421000	4000	1	3.60E-07	-0.31	28	0.7	Spag17	
DMR2:202811001	2	202811001	202814000	3000	1	5.80E-07	0.35	36	1.2	Fam46c	
DMR2:202948001	2	202948001	202955000	7000	2	5.80E-08	-0.32	82	1.17	Man1a2	Golgi
DMR2:203001001	2	203001001	203002000	1000	1	4.60E-07	-0.46	7	0.7	Man1a2	Golgi
DMR2:203324001	2	203324001	203326000	2000	1	7.50E-10	0.46	28	1.4	Ttf2	
DMR2:203392001	2	203392001	203394000	2000	1	4.30E-07	0.43	24	1.2	Cd101	Immune
DMR2:203857001	2	203857001	203862000	5000	1	4.50E-08	0.6	72	1.44	Igsf3	Immune
DMR2:203997001	2	203997001	2.04E+08	3000	2	5.00E-10	-0.49	62	2.07	Atp1a1	Transport
DMR2:204239001	2	204239001	204240000	1000	1	4.40E-20	1.17	2	0.2	Mab21l3	
DMR2:204250001	2	204250001	204252000	2000	1	3.70E-07	0.45	22	1.1	Mab21l3	
DMR2:204306001	2	204306001	204308000	2000	1	3.10E-07	0.35	39	1.95	Slc22a15	Transport
DMR2:204519001	2	204519001	204520000	1000	1	6.20E-07	-0.47	21	2.1	Casq2	Signaling
DMR2:205195001	2	205195001	205197000	2000	1	5.50E-07	-0.3	39	1.95	Tspan2	
DMR2:205295001	2	205295001	205297000	2000	1	4.00E-09	-0.43	12	0.6	Sycp1	
DMR2:205347001	2	205347001	205353000	6000	3	1.20E-09	-0.38	78	1.3	Sycp1	
DMR2:205355001	2	205355001	205359000	4000	1	2.50E-07	-0.29	41	1.02	Sycp1	
DMR2:205371001	2	205371001	205374000	3000	1	5.50E-07	-0.41	31	1.03	Sycp1	
DMR2:205386001	2	205386001	205387000	1000	1	1.20E-07	-0.4	13	1.3	Sycp1	
DMR2:205393001	2	205393001	205398000	5000	1	1.60E-09	-0.49	42	0.84	Sycp1	
DMR2:206217001	2	206217001	206219000	2000	1	7.00E-08	0.46	31	1.55	LOC102547265;Olfml3;Hipk1	Development
DMR2:206275001	2	206275001	206276000	1000	1	5.10E-09	-0.58	10	1	Hipk1;LOC108350077;Dclre1b	Transcription
DMR2:207387001	2	207387001	207393000	6000	2	1.80E-07	-0.35	52	0.87	St7l	
DMR2:207929001	2	207929001	207930000	1000	1	8.70E-07	0.26	19	1.9	Kcnd3	Transport
DMR2:208760001	2	208760001	208766000	6000	1	5.10E-08	-0.29	80	1.33	Chia	Metabolism
DMR2:208788001	2	208788001	208795000	7000	2	4.40E-10	-0.34	86	1.23	Chia	Metabolism
DMR2:208988001	2	208988001	208990000	2000	1	6.70E-08	-0.59	11	0.55	RGD1309110	Metabolism
DMR2:209423001	2	209423001	209427000	4000	3	1.80E-08	-0.46	34	0.85	Lrif1	
DMR2:209513001	2	209513001	209516000	3000	1	9.60E-07	-0.37	13	0.43	Cd53	
DMR2:209853001	2	209853001	209855000	2000	1	2.90E-07	0.36	19	0.95	Kcna2	Transport
DMR2:209949001	2	209949001	209956000	7000	4	9.30E-09	-0.42	80	1.14	LOC102557194;Cym	
DMR2:210853001	2	210853001	210854000	1000	1	3.90E-09	0.37	22	2.2	Ampd2	Metabolism
DMR2:210867001	2	210867001	210868000	1000	1	7.90E-11	0.39	16	1.6	Ampd2	Metabolism
DMR2:210884001	2	210884001	210890000	6000	3	8.80E-12	0.52	113	1.88	Ampd2;Gnat2;Gnai3	Metabolism;Signaling
DMR2:211121001	2	211121001	211122000	1000	1	2.60E-07	-0.52	22	2.2	Sort1	Transport
DMR2:211485001	2	211485001	211491000	6000	2	9.90E-08	-0.36	63	1.05	Clcc1;Gpsm2	Transport
DMR2:211807001	2	211807001	211810000	3000	1	8.40E-10	0.57	57	1.9	Fam102b	
DMR2:211961001	2	211961001	211962000	1000	1	3.80E-09	-0.5	9	0.9	Slc25a24	Transport
DMR2:212285001	2	212285001	212289000	4000	1	1.00E-07	-0.48	62	1.55	Vav3	
DMR2:212359001	2	212359001	212365000	6000	1	4.40E-07	-0.33	66	1.1	Vav3	
DMR2:212439001	2	212439001	212443000	4000	1	6.00E-08	-0.31	33	0.82	Vav3;Mrps17-ps1	
DMR2:212473001	2	212473001	212474000	1000	1	2.00E-07	-0.6	15	1.5	Vav3	
DMR2:212529001	2	212529001	212530000	1000	1	1.00E-08	0.63	6	0.6	Vav3	
DMR2:212547001	2	212547001	212548000	1000	1	1.90E-07	-0.55	11	1.1	Vav3	
DMR2:213027001	2	213027001	213034000	7000	2	2.50E-07	-0.28	67	0.96	Ntng1	Extracellular Matrix

DMR2:216245001	2	216245001	216246000	1000	1	9.60E-07	-0.34	19	1.9	Amy2-ps2	
DMR2:217911001	2	217911001	217914000	3000	1	3.60E-07	-0.41	23	0.77	Olfm3	Development
DMR2:218668001	2	218668001	218671000	3000	1	1.90E-08	-0.47	23	0.77	S1pr1	Signaling
DMR2:219038001	2	219038001	219040000	2000	1	7.40E-07	-0.32	25	1.25	RGD1559607	
DMR2:219522001	2	219522001	219528000	6000	1	3.20E-08	-0.27	70	1.17	Rtcd1	
DMR2:219882001	2	219882001	219887000	5000	1	1.60E-07	-0.29	45	0.9	Plppr5	Signaling
DMR2:219970001	2	219970001	219971000	1000	1	3.20E-08	-0.49	8	0.8	Plppr5	Signaling
DMR2:220030001	2	220030001	220034000	4000	1	9.00E-08	-0.29	40	1	Plppr5;LOC108348210	Signaling
DMR2:221869001	2	221869001	221875000	6000	1	1.10E-09	-0.32	71	1.18	Dpyd	Metabolism
DMR2:222194001	2	222194001	222200000	6000	2	2.80E-08	-0.28	66	1.1	Dpyd	Metabolism
DMR2:222385001	2	222385001	222391000	6000	1	5.90E-07	-0.32	74	1.23	Dpyd	Metabolism
DMR2:222432001	2	222432001	222438000	6000	2	6.90E-10	-0.31	70	1.17	Dpyd	Metabolism
DMR2:222573001	2	222573001	222575000	2000	1	3.40E-10	-0.32	15	0.75	Dpyd	Metabolism
DMR2:222667001	2	222667001	222672000	5000	3	2.50E-08	-0.36	58	1.16	Dpyd	Metabolism
DMR2:224869001	2	224869001	224871000	2000	1	2.80E-07	0.48	16	0.8	Alg14;LOC108350097	Transport
DMR2:225342001	2	225342001	225343000	1000	1	4.40E-13	0.51	16	1.6	Abcd3	Transport
DMR2:225381001	2	225381001	225382000	1000	1	8.00E-07	-0.46	19	1.9	Abcd3;LOC108350099	Transport
DMR2:225656001	2	225656001	225657000	1000	1	6.20E-07	0.4	7	0.7	Abca4	Transport
DMR2:225816001	2	225816001	225820000	4000	1	2.40E-07	-0.36	42	1.05	LOC102555028;Gclm	Metabolism
DMR2:226604001	2	226604001	226605000	1000	1	4.20E-07	0.4	24	2.4	Bcar3	
DMR2:226612001	2	226612001	226613000	1000	1	4.80E-09	0.46	25	2.5	Bcar3	
DMR2:226743001	2	226743001	226744000	1000	1	3.10E-07	-0.43	16	1.6	Fnbp1l	
DMR2:226748001	2	226748001	226750000	2000	1	3.10E-07	-0.36	19	0.95	Fnbp1l	
DMR2:227332001	2	227332001	227333000	1000	1	2.20E-08	0.44	71	7.1	Synpo2	Cytoskeleton
DMR2:227553001	2	227553001	227555000	2000	1	3.90E-08	0.35	57	2.85	Sec24d	Transport
DMR2:227702001	2	227702001	227706000	4000	1	1.00E-08	-0.44	62	1.55	Prss12	
DMR2:227740001	2	227740001	227741000	1000	1	4.80E-10	-0.52	17	1.7	Ndst3	Transport
DMR2:229217001	2	229217001	229219000	2000	1	2.10E-09	0.58	12	0.6	Ndst4	Transport
DMR2:229362001	2	229362001	229367000	5000	2	3.50E-10	-0.35	53	1.06	Ndst4	Transport
DMR2:231334001	2	231334001	231336000	2000	1	7.20E-08	-0.47	28	1.4	Ank2	
DMR2:232045001	2	232045001	232046000	1000	1	6.10E-07	0.57	27	2.7	Alpk1;LOC103691696	Signaling
DMR2:232057001	2	232057001	232060000	3000	1	1.70E-13	0.74	88	2.93	Alpk1	Signaling
DMR2:234984001	2	234984001	234990000	6000	1	7.60E-07	-0.37	52	0.87	RGD1560484	
DMR2:235280001	2	235280001	235285000	5000	2	2.60E-08	-0.37	62	1.24	Cfi	Protease
DMR2:235349001	2	235349001	235350000	1000	1	6.20E-07	-0.58	18	1.8	Casp6;Mcub	Protease
DMR2:235572001	2	235572001	235575000	3000	1	2.00E-08	0.43	45	1.5	Col25a1	Extracellular Matrix
DMR2:235610001	2	235610001	235612000	2000	1	2.20E-10	0.51	25	1.25	Col25a1	Extracellular Matrix
DMR2:235863001	2	235863001	235864000	1000	1	7.00E-08	-0.48	17	1.7	Rpl34	Translation
DMR2:236263001	2	236263001	236267000	4000	1	2.20E-08	0.71	49	1.23	Lef1;LOC103691708	Transcription
DMR2:236612001	2	236612001	236614000	2000	2	3.60E-15	0.59	20	1	Papss1	
DMR2:237230001	2	237230001	237232000	2000	1	4.80E-07	0.47	14	0.7	Dkk2	
DMR2:237818001	2	237818001	237825000	7000	1	6.50E-08	-0.28	89	1.27	Tbck	Signaling
DMR2:238232001	2	238232001	238234000	2000	1	1.20E-10	-0.52	29	1.45	Gstcd	
DMR2:238240001	2	238240001	238242000	2000	2	4.50E-13	-0.64	33	1.65	Gstcd	
DMR2:238329001	2	238329001	238336000	7000	1	4.60E-07	-0.26	74	1.06	Arhgef38	
DMR2:238688001	2	238688001	238689000	1000	1	2.60E-08	-0.46	4	0.4	Gpbp1l2	
DMR2:238699001	2	238699001	238701000	2000	1	8.30E-08	-0.38	35	1.75	Gpbp1l2	
DMR2:238723001	2	238723001	238724000	1000	1	1.20E-07	0.36	20	2	Tet2	
DMR2:240539001	2	240539001	240540000	1000	1	4.00E-08	-0.42	13	1.3	Slc9b1	
DMR2:240554001	2	240554001	240561000	7000	2	1.50E-13	0.32	61	0.87	Slc9b1	
DMR2:240593001	2	240593001	240595000	2000	2	6.30E-12	-0.38	21	1.05	Cisd2	
DMR2:240713001	2	240713001	240722000	9000	1	6.50E-07	-0.27	89	0.99	Manba	Golgi
DMR2:241467001	2	241467001	241469000	2000	1	7.90E-07	0.33	21	1.05	Bank1	
DMR2:241934001	2	241934001	241935000	1000	1	4.60E-07	-0.45	15	1.5	Ppp3ca	Signaling
DMR2:241962001	2	241962001	241963000	1000	1	5.50E-12	0.61	6	0.6	Ppp3ca	Signaling
DMR2:243209001	2	243209001	243211000	2000	1	7.40E-07	0.51	38	1.9	Dapp1	
DMR2:243356001	2	243356001	243358000	2000	1	1.30E-08	0.41	24	1.2	LOC102555814;Mttp	Transport
DMR2:243427001	2	243427001	243429000	2000	1	8.30E-07	-0.54	33	1.65	Trmt10a	Epigenetic
DMR2:243516001	2	243516001	243517000	1000	1	2.20E-07	-0.47	16	1.6	Adh7;LOC102556058	Metabolism
DMR2:243581001	2	243581001	243588000	7000	2	9.00E-11	-0.33	90	1.29	LOC102556144;Adh6a	Metabolism
DMR2:243600001	2	243600001	243601000	1000	1	8.80E-07	0.41	10	1	Adh6a	Metabolism

DMR2:243855001	2	243855001	243858000	3000	1	6.80E-10	-0.49	48	1.6	Eif4e	Translation
DMR2:244161001	2	244161001	244162000	1000	1	1.70E-09	0.49	15	1.5	Tspan5	
DMR2:244315001	2	244315001	244317000	2000	1	9.10E-11	-0.53	28	1.4	Rap1gds1	
DMR2:244542001	2	244542001	244549000	7000	2	3.60E-10	-0.36	95	1.36	Stpg2	Development
DMR2:244551001	2	244551001	244553000	2000	1	2.30E-09	-0.34	17	0.85	Stpg2	Development
DMR2:244651001	2	244651001	244655000	4000	2	3.60E-08	-0.36	39	0.98	Stpg2	Development
DMR2:244671001	2	244671001	244674000	3000	1	4.00E-07	-0.29	26	0.87	Stpg2	Development
DMR2:244680001	2	244680001	244684000	4000	1	3.30E-07	-0.3	31	0.78	Stpg2	Development
DMR2:244825001	2	244825001	244829000	4000	2	1.40E-09	-0.4	37	0.92	Stpg2;LOC100910816	Development
DMR2:244848001	2	244848001	244854000	6000	2	2.10E-13	-0.39	69	1.15	Stpg2	Development
DMR2:244855001	2	244855001	244857000	2000	1	9.10E-09	-0.39	27	1.35	Stpg2	Development
DMR2:244928001	2	244928001	244932000	4000	2	2.20E-07	-0.31	48	1.2	Stpg2	Development
DMR2:244998001	2	244998001	245005000	7000	1	1.80E-08	-0.4	78	1.11	Stpg2	Development
DMR2:247330001	2	247330001	247332000	2000	1	2.20E-09	0.53	22	1.1	Unc5c	Receptor
DMR2:248415001	2	248415001	248417000	2000	1	6.20E-10	-0.35	20	1	Gbp1	Signaling
DMR2:248717001	2	248717001	248718000	1000	1	3.50E-07	0.49	11	1.1	LOC108350125;Gtf2b	Transcription
DMR2:250589001	2	250589001	250590000	1000	1	2.70E-07	-0.46	17	1.7	Hs2st1	Transport
DMR2:250840001	2	250840001	250842000	2000	1	3.10E-08	0.4	19	0.95	Clca4	Transport
DMR2:251033001	2	251033001	251035000	2000	1	3.90E-08	0.66	22	1.1	Odf2l	
DMR2:251292001	2	251292001	251293000	1000	1	2.30E-08	0.39	16	1.6	Col24a1	Extracellular Matrix
DMR2:251630001	2	251630001	251631000	1000	1	1.80E-08	-0.51	22	2.2	Ddah1	Metabolism
DMR2:251855001	2	251855001	251857000	2000	1	2.60E-07	-0.42	23	1.15	Syde2	Signaling
DMR2:251879001	2	251879001	251880000	1000	1	5.80E-10	0.29	17	1.7	Syde2	Signaling
DMR2:251881001	2	251881001	251883000	2000	1	7.30E-08	-0.34	47	2.35	Syde2	Signaling
DMR2:251964001	2	251964001	251965000	1000	1	3.90E-08	0.47	3	0.3	Wdr63;Mcoln3	Cytoskeleton;Transport
DMR2:252455001	2	252455001	252459000	4000	1	3.30E-07	0.6	78	1.95	Dnase2b;Uox	Transcription
DMR2:252490001	2	252490001	252491000	1000	1	9.50E-07	0.33	16	1.6	Uox;LOC56764	
DMR2:252665001	2	252665001	252666000	1000	1	4.60E-09	-0.78	10	1	Prkacb	Signaling
DMR2:252840001	2	252840001	252842000	2000	1	8.50E-07	0.59	20	1	Ttll7	Cytoskeleton
DMR2:252855001	2	252855001	252856000	1000	1	6.90E-07	0.41	9	0.9	Ttll7	Cytoskeleton
DMR2:256709001	2	256709001	256710000	1000	1	9.90E-07	-0.55	14	1.4	Adgrl4	Signaling
DMR2:256891001	2	256891001	256896000	5000	1	7.60E-07	-0.34	29	0.58	Ifi44	
DMR2:256982001	2	256982001	256983000	1000	1	2.90E-07	0.53	27	2.7	Ifi44l	
DMR2:257471001	2	257471001	257473000	2000	1	4.40E-07	-0.43	29	1.45	Nexn;LOC102548862	Cytoskeleton
DMR2:258121001	2	258121001	258125000	4000	2	1.30E-10	-0.45	38	0.95	St6galnac5	
DMR2:258296001	2	258296001	258297000	1000	1	5.30E-07	0.59	13	1.3	St6galnac5	
DMR2:258632001	2	258632001	258637000	5000	2	9.10E-10	-0.4	63	1.26	St6galnac5	
DMR2:258906001	2	258906001	258908000	2000	1	2.30E-10	0.57	35	1.75	St6galnac5;Adgrl2	Signaling
DMR2:259032001	2	259032001	259033000	1000	1	2.50E-08	0.59	14	1.4	St6galnac5	
DMR2:259453001	2	259453001	259455000	2000	1	8.70E-15	0.44	59	2.95	St6galnac3	
DMR2:260322001	2	260322001	260325000	3000	2	4.30E-16	0.59	45	1.5	Slc44a5;LOC102553583	Transport
DMR2:260331001	2	260331001	260332000	1000	1	4.30E-07	0.57	12	1.2	Slc44a5;LOC102553583	Transport
DMR2:260352001	2	260352001	260354000	2000	1	5.90E-07	-0.27	25	1.25	Slc44a5	Transport
DMR2:260381001	2	260381001	260384000	3000	1	1.00E-07	-0.35	38	1.27	Slc44a5	Transport
DMR2:260386001	2	260386001	260388000	2000	1	1.10E-08	0.81	34	1.7	Slc44a5	Transport
DMR2:260582001	2	260582001	260584000	2000	1	1.00E-07	0.59	21	1.05	Lhx8	Development
DMR2:260588001	2	260588001	260590000	2000	1	6.50E-07	-0.47	34	1.7	Lhx8	Development
DMR2:260966001	2	260966001	260973000	7000	2	6.90E-08	-0.41	70	1	Erich3;LOC102555516	
DMR2:261045001	2	261045001	261048000	3000	1	3.80E-08	0.55	54	1.8	Erich3	
DMR2:261149001	2	261149001	261153000	4000	1	9.70E-09	0.35	21	0.52	LOC102555624;Tnni3k	
DMR2:261300001	2	261300001	261301000	1000	1	7.80E-07	-0.45	9	0.9	Tnni3k	
DMR2:261369001	2	261369001	261381000	12000	4	9.00E-08	0.91	368	3.07	Fpgt	Transport
DMR2:265199001	2	265199001	265205000	6000	1	2.50E-07	-0.29	48	0.8	Lrrc7	Cytoskeleton
DMR2:266405001	2	266405001	266409000	4000	3	7.30E-08	-0.32	49	1.23	Wls	
DMR3:115001	3	115001	116000	1000	1	8.80E-07	-0.36	4	0.4	Nxph2	Signaling
DMR3:195001	3	195001	198000	3000	1	6.70E-07	-0.48	23	0.77	Nxph2	Signaling
DMR3:202001	3	202001	203000	1000	1	2.40E-09	-0.43	10	1	Nxph2	Signaling
DMR3:236001	3	236001	238000	2000	1	6.90E-08	-0.54	6	0.3	Nxph2	Signaling
DMR3:239001	3	239001	240000	1000	1	1.00E-07	-0.38	12	1.2	Nxph2	Signaling
DMR3:1299001	3	1299001	1302000	3000	1	6.30E-09	-0.38	26	0.87	Il36g	Cytokine
DMR3:1349001	3	1349001	1351000	2000	1	4.40E-07	-0.35	14	0.7	RGD1559890	

DMR3:1915001	3	1915001	1916000	1000	1	1.90E-09	0.44	22	2.2	Cacna1b	Transport
DMR3:2446001	3	2446001	2448000	2000	1	6.00E-11	0.44	14	0.7	Fam166a;Tubb4b;Slc34a3;Rnf224	Cytoskeleton;Transport
DMR3:2451001	3	2451001	2454000	3000	1	2.20E-07	0.41	52	1.73	Fam166a;Tubb4b;Slc34a3;Rnf224;Cysrt1;Rnf208	Cytoskeleton;Transport
DMR3:2455001	3	2455001	2457000	2000	1	3.90E-07	0.41	31	1.55	Slc34a3;Rnf224;Cysrt1;Rnf208;Ndor1	Transport;Metabolism
DMR3:2510001	3	2510001	2515000	5000	1	3.00E-07	0.38	115	2.3	Anapc2;Tmem210;Lrrc26;Grin1	Receptor
DMR3:2559001	3	2559001	2561000	2000	2	1.30E-09	0.47	55	2.75	Man1b1;LOC103691747;Dpp7	Golgi;Protease
DMR3:2627001	3	2627001	2630000	3000	1	1.20E-10	0.51	44	1.47	Entpd2;Npdc1;LOC366006;Fut7	Signaling;Golgi
DMR3:2665001	3	2665001	2670000	5000	1	1.10E-09	0.4	146	2.92	Abca2;Clic3;RGD1306215	Transport;Transport
DMR3:2724001	3	2724001	2725000	1000	1	4.00E-07	0.39	34	3.4	Lcn12;C8g;Fbxw5	Transport
DMR3:2792001	3	2792001	2795000	3000	1	9.40E-10	0.49	46	1.53	Edf1;Mamdc4;Phpt1;RGD1560470	
DMR3:2896001	3	2896001	2898000	2000	1	1.40E-09	0.45	28	1.4	LOC108350548;Lcn6;Lcn10	Transport
DMR3:2928001	3	2928001	2929000	1000	1	4.00E-22	0.92	5	0.5	LOC100360027;Lcn11	Transport
DMR3:3272001	3	3272001	3273000	1000	1	7.90E-09	0.61	19	1.9	Glt6d1;Lcn9	Golgi;Transport
DMR3:3306001	3	3306001	3308000	2000	1	9.90E-07	0.42	29	1.45	Kcnt1	Transport
DMR3:3346001	3	3346001	3350000	4000	1	4.70E-09	0.49	76	1.9	Kcnt1	Transport
DMR3:3409001	3	3409001	3410000	1000	1	1.10E-07	-0.41	13	1.3	Camsap1	
DMR3:3530001	3	3530001	3532000	2000	1	6.80E-08	0.33	43	2.15	Nacc2	
DMR3:3550001	3	3550001	3551000	1000	1	3.70E-08	0.38	28	2.8	Nacc2	
DMR3:3565001	3	3565001	3568000	3000	1	2.00E-11	0.51	40	1.33	Nacc2	
DMR3:3696001	3	3696001	3697000	1000	1	6.30E-07	0.38	21	2.1	Qsox2;LOC103691753	Metabolism
DMR3:3869001	3	3869001	3872000	3000	1	5.30E-08	0.35	47	1.57	Sec16a	
DMR3:3907001	3	3907001	3909000	2000	1	9.30E-07	0.36	50	2.5	Notch1	Extracellular Matrix
DMR3:4024001	3	4024001	4025000	1000	1	4.10E-10	0.51	20	2	LOC108350341;Egfl7	Signaling
DMR3:4385001	3	4385001	4386000	1000	1	1.10E-07	-0.38	9	0.9	LOC100911704;Abo	Golgi
DMR3:5377001	3	5377001	5382000	5000	1	6.00E-11	-0.37	49	0.98	Abo2	Golgi
DMR3:5752001	3	5752001	5753000	1000	1	8.60E-09	0.39	12	1.2	Sardh	Metabolism
DMR3:5784001	3	5784001	5785000	1000	1	2.40E-09	0.42	16	1.6	Sardh	Metabolism
DMR3:5828001	3	5828001	5830000	2000	1	7.70E-07	0.51	46	2.3	Vav2	
DMR3:5831001	3	5831001	5832000	1000	1	4.60E-09	0.58	8	0.8	Vav2	
DMR3:5867001	3	5867001	5869000	2000	1	4.60E-08	0.46	28	1.4	Vav2	
DMR3:5923001	3	5923001	5925000	2000	1	6.20E-07	0.5	10	0.5	Vav2	
DMR3:5960001	3	5960001	5963000	3000	1	1.10E-07	0.43	59	1.97	Vav2	
DMR3:6034001	3	6034001	6035000	1000	1	1.20E-12	0.64	13	1.3	Brd3	
DMR3:6633001	3	6633001	6634000	1000	1	7.40E-09	0.43	15	1.5	Fcnb	
DMR3:7242001	3	7242001	7243000	1000	1	2.60E-07	-0.46	17	1.7	Tsc1	
DMR3:7258001	3	7258001	7261000	3000	1	1.40E-07	0.52	43	1.43	Tsc1;Spaca9	
DMR3:7291001	3	7291001	7293000	2000	1	2.60E-10	0.5	25	1.25	Ak8	Signaling
DMR3:7304001	3	7304001	7307000	3000	1	1.40E-08	0.4	40	1.33	Ak8	Signaling
DMR3:7310001	3	7310001	7312000	2000	1	3.90E-08	0.4	23	1.15	Ak8	Signaling
DMR3:7336001	3	7336001	7338000	2000	1	1.40E-09	0.53	23	1.15	Ak8	Signaling
DMR3:7593001	3	7593001	7596000	3000	1	1.60E-07	0.44	58	1.93	Cfap77	Development
DMR3:7647001	3	7647001	7648000	1000	1	7.70E-09	0.54	10	1	Ttf1	
DMR3:7727001	3	7727001	7728000	1000	1	8.30E-13	0.57	10	1	Setx	
DMR3:8232001	3	8232001	8233000	1000	1	6.80E-08	-0.28	12	1.2	Rapgef1	Transcription
DMR3:8495001	3	8495001	8496000	1000	1	2.70E-08	-0.66	12	1.2	Odf2;LOC108350347;Gle1	Translation
DMR3:8606001	3	8606001	8608000	2000	1	6.10E-09	0.48	42	2.1	Sptan1;Wdr34	Cytoskeleton
DMR3:9710001	3	9710001	9715000	5000	1	2.20E-11	0.48	87	1.74	Prrx2	
DMR3:9919001	3	9919001	9920000	1000	1	8.40E-09	0.48	14	1.4	Fnbp1	
DMR3:10167001	3	10167001	10171000	4000	1	7.80E-10	-0.36	54	1.35	Exosc2	Translation
DMR3:10354001	3	10354001	10355000	1000	1	1.50E-07	0.4	14	1.4	Ass1	Metabolism
DMR3:10451001	3	10451001	10453000	2000	1	3.50E-08	0.48	42	2.1	Hmcn2	
DMR3:10523001	3	10523001	10527000	4000	1	1.30E-08	-0.45	107	2.67	Hmcn2	
DMR3:10994001	3	10994001	10996000	2000	1	4.10E-15	0.64	35	1.75	Nup214	Transport
DMR3:11157001	3	11157001	11158000	1000	1	3.50E-09	-0.45	17	1.7	Prrc2b	Metabolism

DMR3:11301001	3	11301001	11305000	4000	2	1.30E-09	-0.44	31	0.78	LOC103691766;Swi5	Transcription
DMR3:11353001	3	11353001	11354000	1000	1	3.20E-07	-0.39	19	1.9	Dnm1	Transport
DMR3:11405001	3	11405001	11406000	1000	1	8.80E-09	0.46	15	1.5	Ciz1;RGD1561113;Lcn2	Transport
DMR3:11429001	3	11429001	11430000	1000	1	1.10E-07	0.37	24	2.4	Ptges2	Metabolism
DMR3:11823001	3	11823001	11826000	3000	1	6.70E-08	0.37	48	1.6	Ptrh1;Cfap157;Stxbp1	Metabolism;Development;Transport
DMR3:12026001	3	12026001	12027000	1000	1	9.90E-09	0.54	24	2.4	Slc2a8;Garnl3	Signaling
DMR3:12608001	3	12608001	12609000	1000	1	1.50E-09	0.55	13	1.3	Lmx1b	Development
DMR3:12778001	3	12778001	12780000	2000	1	1.80E-09	0.41	38	1.9	Mvb12b	
DMR3:13608001	3	13608001	13609000	1000	1	3.20E-07	-0.49	19	1.9	Mapkap1	Cytoskeleton
DMR3:13850001	3	13850001	13853000	3000	1	2.00E-09	0.4	42	1.4	Hspa5;Rabepk	
DMR3:14115001	3	14115001	14118000	3000	1	1.90E-10	-0.49	24	0.8	C5	
DMR3:14227001	3	14227001	14235000	8000	1	1.70E-13	-0.43	104	1.3	C5	
DMR3:14498001	3	14498001	14501000	3000	1	9.80E-09	0.37	57	1.9	Gsn	Cytoskeleton
DMR3:14509001	3	14509001	14510000	1000	1	1.90E-09	-0.5	25	2.5	Gsn;Stom	Cytoskeleton;Cytoskeleton
DMR3:14526001	3	14526001	14527000	1000	1	1.10E-09	0.39	20	2	Stom;LOC108350355	Cytoskeleton
DMR3:14661001	3	14661001	14664000	3000	1	2.40E-07	0.55	46	1.53	Ggta1	Golgi
DMR3:14680001	3	14680001	14681000	1000	1	3.40E-07	0.45	9	0.9	Ggta1	Golgi
DMR3:14939001	3	14939001	14944000	5000	1	1.80E-07	0.4	82	1.64	Dab2ip	Signaling
DMR3:14973001	3	14973001	14976000	3000	1	7.70E-07	0.34	76	2.53	Dab2ip	Signaling
DMR3:15101001	3	15101001	15103000	2000	1	1.60E-07	0.4	36	1.8	Ttll11	Cytoskeleton
DMR3:15105001	3	15105001	15107000	2000	2	5.60E-12	0.6	21	1.05	Ttll11	Cytoskeleton
DMR3:15168001	3	15168001	15172000	4000	1	3.30E-07	0.5	72	1.8	Ttll11	Cytoskeleton
DMR3:15231001	3	15231001	15235000	4000	1	6.00E-07	0.32	42	1.05	Ttll11	Cytoskeleton
DMR3:15360001	3	15360001	15363000	3000	1	1.60E-07	0.41	42	1.4	Ndufa8	Metabolism
DMR3:15612001	3	15612001	15614000	2000	1	6.10E-09	-0.42	16	0.8	Olr393-ps	
DMR3:15628001	3	15628001	15636000	8000	2	1.90E-08	-0.39	100	1.25	Olr393-ps;Olr394-ps	
DMR3:15694001	3	15694001	15701000	7000	1	2.00E-13	-0.38	84	1.2	Olr395	Receptor
DMR3:15754001	3	15754001	15757000	3000	1	2.90E-09	-0.36	24	0.8	Olr396	Receptor
DMR3:15925001	3	15925001	15926000	1000	1	3.50E-07	-0.47	9	0.9	Olr401	Receptor
DMR3:16008001	3	16008001	16013000	5000	1	5.80E-09	-0.4	39	0.78	Olr402	Receptor
DMR3:16230001	3	16230001	16238000	8000	3	2.30E-08	-0.39	83	1.04	Olr407	Receptor
DMR3:16248001	3	16248001	16254000	6000	1	3.70E-07	-0.36	60	1	Olr407	Receptor
DMR3:17637001	3	17637001	17639000	2000	1	3.40E-08	-0.47	11	0.55	RGD1564462	
DMR3:21664001	3	21664001	21665000	1000	1	2.30E-10	0.5	10	1	Rc3h2	
DMR3:21787001	3	21787001	21788000	1000	1	7.80E-07	-0.48	11	1.1	Rabgap1	Signaling
DMR3:21804001	3	21804001	21805000	1000	1	1.30E-07	-0.58	18	1.8	Rabgap1	Signaling
DMR3:22855001	3	22855001	22856000	1000	1	1.00E-08	0.54	9	0.9	Nek6	
DMR3:23436001	3	23436001	23441000	5000	1	2.80E-07	-0.32	47	0.94	Scai	
DMR3:25592001	3	25592001	25595000	3000	1	2.70E-08	-0.31	29	0.97	Lrp1b	
DMR3:25600001	3	25600001	25601000	1000	1	7.70E-08	0.59	4	0.4	Lrp1b	
DMR3:25837001	3	25837001	25839000	2000	1	4.70E-07	-0.48	11	0.55	Lrp1b	
DMR3:25883001	3	25883001	25886000	3000	1	5.20E-09	-0.52	9	0.3	Lrp1b	
DMR3:25985001	3	25985001	25986000	1000	1	1.00E-10	-0.57	9	0.9	Lrp1b	
DMR3:26214001	3	26214001	26217000	3000	1	6.40E-07	-0.29	16	0.53	Lrp1b	
DMR3:26406001	3	26406001	26411000	5000	2	1.50E-11	-0.38	53	1.06	Lrp1b	
DMR3:26885001	3	26885001	26887000	2000	1	3.80E-10	0.45	10	0.5	Lrp1b	
DMR3:26911001	3	26911001	26915000	4000	1	8.80E-09	-0.48	43	1.07	Lrp1b	
DMR3:28633001	3	28633001	28635000	2000	1	1.70E-07	0.36	25	1.25	Arhgap15	Signaling
DMR3:28715001	3	28715001	28717000	2000	1	1.20E-07	-0.43	18	0.9	Arhgap15	Signaling
DMR3:28719001	3	28719001	28722000	3000	1	6.10E-08	0.44	46	1.53	Arhgap15	Signaling
DMR3:29013001	3	29013001	29015000	2000	1	1.60E-08	-0.43	15	0.75	Arhgap15	Signaling
DMR3:29083001	3	29083001	29084000	1000	1	4.60E-08	0.41	11	1.1	Arhgap15	Signaling
DMR3:29899001	3	29899001	29901000	2000	1	9.90E-08	-0.35	33	1.65	Zeb2	Transcription
DMR3:29957001	3	29957001	29958000	1000	1	1.20E-08	-0.49	20	2	Zeb2	Transcription
DMR3:29968001	3	29968001	29972000	4000	1	1.10E-08	0.37	73	1.82	Zeb2	Transcription
DMR3:31821001	3	31821001	31823000	2000	1	7.20E-08	-0.46	9	0.45	Olr435-ps	
DMR3:33361001	3	33361001	33362000	1000	1	3.60E-07	-0.44	10	1	Mbd5	
DMR3:33377001	3	33377001	33379000	2000	2	3.60E-08	-0.35	19	0.95	Mbd5	
DMR3:33413001	3	33413001	33419000	6000	1	1.80E-08	-0.3	51	0.85	Mbd5	
DMR3:33677001	3	33677001	33682000	5000	1	4.70E-08	-0.38	49	0.98	Epc2	Epigenetic

DMR3:33693001	3	33693001	33698000	5000	3	7.20E-14	-0.38	54	1.08	Epc2	Epigenetic
DMR3:33743001	3	33743001	33744000	1000	1	3.60E-09	0.44	12	1.2	Epc2	Epigenetic
DMR3:35007001	3	35007001	35010000	3000	1	3.00E-09	0.49	48	1.6	Kif5c	
DMR3:35021001	3	35021001	35025000	4000	2	2.30E-13	0.59	95	2.38	Kif5c	
DMR3:35170001	3	35170001	35172000	2000	1	3.00E-07	0.4	28	1.4	Kif5c	
DMR3:37558001	3	37558001	37560000	2000	1	1.20E-10	0.47	23	1.15	Tnfaip6	
DMR3:37740001	3	37740001	37741000	1000	1	1.90E-09	0.47	12	1.2	Neb	
DMR3:37771001	3	37771001	37778000	7000	1	4.20E-10	-0.35	96	1.37	Neb	
DMR3:37873001	3	37873001	37874000	1000	1	1.70E-09	-0.71	11	1.1	RGD1563584	
DMR3:38015001	3	38015001	38022000	7000	2	2.10E-08	-0.32	105	1.5	Cacnb4	Transport
DMR3:38356001	3	38356001	38359000	3000	1	9.60E-14	0.48	42	1.4	RGD1564306	
DMR3:38552001	3	38552001	38554000	2000	1	9.70E-07	0.47	20	1	RGD1560248	
DMR3:40040001	3	40040001	40042000	2000	1	2.40E-08	-0.44	16	0.8	Galnt13	Golgi
DMR3:43131001	3	43131001	43134000	3000	1	6.60E-09	0.41	45	1.5	Nr4a2	Transcription
DMR3:43214001	3	43214001	43215000	1000	1	1.90E-07	-0.44	14	1.4	Gpd2	Metabolism
DMR3:43281001	3	43281001	43287000	6000	1	4.50E-10	-0.39	57	0.95	Gpd2	Metabolism
DMR3:43359001	3	43359001	43362000	3000	1	1.90E-09	0.48	43	1.43	Gpd2;LOC366056	Metabolism
DMR3:44052001	3	44052001	44057000	5000	2	7.20E-09	-0.31	56	1.12	Galnt5	Golgi
DMR3:44091001	3	44091001	44092000	1000	1	9.70E-07	-0.4	6	0.6	Ermn	
DMR3:44501001	3	44501001	44504000	3000	1	5.90E-07	-0.47	24	0.8	Acvr1	Signaling
DMR3:45124001	3	45124001	45133000	9000	2	8.90E-08	-0.42	91	1.01	Ccdc148	
DMR3:45175001	3	45175001	45178000	3000	1	2.60E-07	-0.36	27	0.9	Ccdc148	
DMR3:45297001	3	45297001	45299000	2000	1	2.00E-07	0.46	12	0.6	Pkp4	Cytoskeleton
DMR3:45373001	3	45373001	45375000	2000	2	5.20E-14	0.63	40	2	Pkp4	Cytoskeleton
DMR3:45412001	3	45412001	45413000	1000	1	3.40E-07	0.41	11	1.1	Pkp4	Cytoskeleton
DMR3:45691001	3	45691001	45693000	2000	1	6.40E-07	-0.47	23	1.15	Tanc1	
DMR3:45756001	3	45756001	45757000	1000	1	6.40E-12	0.44	27	2.7	Tanc1	
DMR3:46209001	3	46209001	46211000	2000	1	1.30E-07	0.44	14	0.7	Baz2b;LOC100361645	Epigenetic;Transcription
DMR3:46213001	3	46213001	46215000	2000	1	4.80E-09	0.44	19	0.95	Baz2b;LOC100361645	Epigenetic;Transcription
DMR3:46451001	3	46451001	46452000	1000	1	4.80E-07	0.41	8	0.8	Ly75;LOC108350581	
DMR3:46980001	3	46980001	46986000	6000	1	3.50E-09	-0.33	58	0.97	Rbms1	
DMR3:47027001	3	47027001	47028000	1000	1	1.50E-07	-0.54	16	1.6	Rbms1	
DMR3:47437001	3	47437001	47439000	2000	1	2.70E-10	-0.45	36	1.8	Tank	
DMR3:48184001	3	48184001	48185000	1000	1	1.80E-08	-0.34	12	1.2	Slc4a10	Transport
DMR3:48227001	3	48227001	48229000	2000	1	2.00E-08	0.33	23	1.15	Slc4a10	Transport
DMR3:49134001	3	49134001	49135000	1000	1	1.30E-09	-0.64	9	0.9	Kcnh7	Transport
DMR3:50054001	3	50054001	50056000	2000	1	3.20E-07	0.34	32	1.6	Fign	Cytoskeleton
DMR3:50980001	3	50980001	50981000	1000	1	1.10E-07	-0.29	10	1	Grb14	Cytoskeleton
DMR3:50984001	3	50984001	50986000	2000	1	8.90E-07	-0.4	22	1.1	Grb14	Cytoskeleton
DMR3:51003001	3	51003001	51006000	3000	1	9.20E-08	-0.39	31	1.03	Grb14;LOC102552585	Cytoskeleton
DMR3:51013001	3	51013001	51014000	1000	1	2.00E-08	0.5	8	0.8	Grb14;LOC102552585	Cytoskeleton
DMR3:51150001	3	51150001	51155000	5000	1	6.60E-07	-0.3	58	1.16	Cobll1	
DMR3:51186001	3	51186001	51192000	6000	2	1.10E-09	-0.59	94	1.57	Cobll1	
DMR3:51259001	3	51259001	51261000	2000	1	2.10E-08	0.43	20	1	Cobll1	
DMR3:51360001	3	51360001	51362000	2000	1	2.50E-14	-3.41	23	1.15	Slc38a11	Transport
DMR3:51537001	3	51537001	51539000	2000	1	1.20E-07	0.36	25	1.25	LOC102553383;Scn3a	Transport
DMR3:51791001	3	51791001	51793000	2000	1	6.60E-08	0.43	25	1.25	Scn2a	Transport
DMR3:51949001	3	51949001	51951000	2000	1	1.10E-07	-0.5	38	1.9	Csrnp3	
DMR3:52053001	3	52053001	52057000	4000	2	3.20E-12	-0.31	17	0.42	Csrnp3	
DMR3:52061001	3	52061001	52064000	3000	2	5.30E-11	0.41	49	1.63	Csrnp3	
DMR3:52076001	3	52076001	52080000	4000	1	1.70E-13	0.42	17	0.42	Csrnp3	
DMR3:52222001	3	52222001	52223000	1000	1	1.90E-07	-0.38	13	1.3	Galnt3	Golgi
DMR3:52475001	3	52475001	52479000	4000	1	3.50E-08	-0.52	28	0.7	Scn1a	Transport
DMR3:52535001	3	52535001	52536000	1000	1	3.20E-10	-0.53	13	1.3	Scn1a;LOC108350387	Transport
DMR3:52570001	3	52570001	52574000	4000	1	2.10E-09	-0.39	32	0.8	LOC108350387;Scn9a	Transport
DMR3:52607001	3	52607001	52608000	1000	1	1.90E-08	-0.51	11	1.1	LOC108350387;Scn9a	Transport
DMR3:53557001	3	53557001	53558000	1000	1	7.30E-08	-0.59	4	0.4	Xirp2	Cytoskeleton
DMR3:53912001	3	53912001	53917000	5000	1	8.30E-07	-0.39	42	0.84	B3galt1	Golgi
DMR3:54452001	3	54452001	54454000	2000	1	9.40E-07	0.38	13	0.65	Stk39	
DMR3:54531001	3	54531001	54534000	3000	1	4.30E-12	-0.41	21	0.7	Stk39	
DMR3:54618001	3	54618001	54620000	2000	1	9.60E-08	0.43	30	1.5	Stk39	

DMR3:55300001	3	55300001	55301000	1000	1	5.60E-08	-0.3	7	0.7	Cers6	
DMR3:55329001	3	55329001	55330000	1000	1	4.90E-08	0.7	6	0.6	Cers6	
DMR3:55358001	3	55358001	55362000	4000	1	8.80E-07	-0.37	62	1.55	LOC366068;Nostrin	
DMR3:56439001	3	56439001	56441000	2000	1	6.50E-07	-0.43	11	0.55	Myo3b	
DMR3:56552001	3	56552001	56553000	1000	1	4.50E-08	0.46	1	0.1	Myo3b	
DMR3:57120001	3	57120001	57121000	1000	1	2.10E-09	0.54	14	1.4	Tlk1;LOC366071	Signaling
DMR3:57680001	3	57680001	57684000	4000	1	1.30E-07	-0.44	60	1.5	Dcaf17	
DMR3:57974001	3	57974001	57976000	2000	1	6.40E-07	0.31	18	0.9	Slc25a12	Transport
DMR3:58145001	3	58145001	58146000	1000	1	8.40E-25	0.74	12	1.2	Metap1d;LOC108350393	Protease
DMR3:58674001	3	58674001	58676000	2000	1	2.80E-07	0.37	20	1	Rapgef4	Transcription
DMR3:60067001	3	60067001	60073000	6000	1	2.00E-08	-0.31	64	1.07	Gpr155	Transcription
DMR3:60090001	3	60090001	60091000	1000	1	1.90E-07	0.46	17	1.7	Gpr155	Transcription
DMR3:60623001	3	60623001	60624000	1000	1	8.90E-07	-0.45	20	2	Chn1	
DMR3:63025001	3	63025001	63028000	3000	1	9.70E-07	-0.43	38	1.27	Pde11a	Signaling
DMR3:63035001	3	63035001	63036000	1000	1	1.30E-07	-0.38	19	1.9	Pde11a	Signaling
DMR3:63109001	3	63109001	63112000	3000	1	1.90E-07	-0.24	28	0.93	Pde11a	Signaling
DMR3:63113001	3	63113001	63120000	7000	2	2.00E-11	-0.29	95	1.36	Pde11a	Signaling
DMR3:63171001	3	63171001	63178000	7000	1	3.00E-07	-0.34	82	1.17	Pde11a	Signaling
DMR3:63356001	3	63356001	63358000	2000	1	1.30E-07	-0.42	15	0.75	Osbpl6	
DMR3:63493001	3	63493001	63495000	2000	1	7.00E-10	0.47	17	0.85	Prkra	
DMR3:63673001	3	63673001	63674000	1000	1	2.90E-07	0.36	22	2.2	Ttn	
DMR3:63685001	3	63685001	63688000	3000	1	4.40E-07	-0.53	50	1.67	Ttn	
DMR3:63995001	3	63995001	63996000	1000	1	2.80E-07	-0.4	9	0.9	Ccdc141	
DMR3:64092001	3	64092001	64095000	3000	1	1.10E-07	-0.5	42	1.4	Sestd1	
DMR3:64538001	3	64538001	64539000	1000	1	5.20E-08	0.5	13	1.3	Zfp385b	
DMR3:64751001	3	64751001	64752000	1000	1	8.20E-07	-0.48	15	1.5	Cwc22	Translation
DMR3:64789001	3	64789001	64793000	4000	1	5.90E-07	-0.39	27	0.68	Cwc22;LOC295681	Translation
DMR3:66168001	3	66168001	66169000	1000	1	2.90E-08	-0.51	13	1.3	Itga4	Extracellular Matrix
DMR3:66319001	3	66319001	66324000	5000	1	5.10E-08	-0.3	57	1.14	Cerkl	Signaling
DMR3:66669001	3	66669001	66670000	1000	1	6.00E-07	-0.43	8	0.8	Ppp1r1c	Signaling
DMR3:66686001	3	66686001	66688000	2000	1	5.30E-11	0.55	26	1.3	Ppp1r1c	Signaling
DMR3:66740001	3	66740001	66745000	5000	1	8.90E-08	-0.38	45	0.9	Ppp1r1c	Signaling
DMR3:66756001	3	66756001	66757000	1000	1	1.00E-09	0.57	10	1	Ppp1r1c	Signaling
DMR3:67073001	3	67073001	67076000	3000	1	8.20E-07	-0.29	20	0.67	Pde1a	Signaling
DMR3:67524001	3	67524001	67529000	5000	1	5.00E-13	0.71	28	0.56	Dnajc10	Transcription
DMR3:67811001	3	67811001	67814000	3000	1	4.30E-07	-0.45	36	1.2	Nckap1	
DMR3:69571001	3	69571001	69572000	1000	1	8.90E-10	-0.68	21	2.1	Zfp804a	
DMR3:71745001	3	71745001	71747000	2000	1	1.20E-07	0.36	16	0.8	Calcrl	Receptor
DMR3:71783001	3	71783001	71784000	1000	1	9.70E-07	-0.26	17	1.7	Calcrl	Receptor
DMR3:71865001	3	71865001	71867000	2000	1	8.80E-08	0.52	12	0.6	Tfpi	Protease; Proteolysis
DMR3:72086001	3	72086001	72087000	1000	1	2.00E-09	0.48	6	0.6	Tmx2;Med19;Zdhhc5	Metabolism
DMR3:72281001	3	72281001	72284000	3000	1	6.60E-07	0.36	37	1.23	Rtn4rl2;LOC102554396	Receptor
DMR3:72355001	3	72355001	72362000	7000	1	4.10E-09	-0.33	88	1.26	Slc43a3;LOC366097	Transport
DMR3:72422001	3	72422001	72423000	1000	1	5.50E-07	0.46	3	0.3	P2rx3	Ion Channel
DMR3:72460001	3	72460001	72462000	2000	1	6.40E-07	0.31	31	1.55	Ssrp1;Tnks1bp1	
DMR3:72799001	3	72799001	72801000	2000	1	1.60E-07	-0.4	18	0.9	Olr441	Receptor
DMR3:73440001	3	73440001	73447000	7000	1	4.70E-07	-0.47	66	0.94	Olr478-ps;Olr479	Receptor
DMR3:73530001	3	73530001	73531000	1000	1	1.90E-07	0.57	1	0.1	Olr484	Receptor
DMR3:73547001	3	73547001	73554000	7000	3	5.40E-10	-0.37	78	1.11	Olr485;Olr486	Receptor
DMR3:73599001	3	73599001	73605000	6000	1	2.40E-07	-0.31	64	1.07	Olr489-ps	
DMR3:73693001	3	73693001	73698000	5000	1	8.70E-07	-0.27	52	1.04	Olr498-ps	
DMR3:73769001	3	73769001	73770000	1000	1	2.10E-07	-0.56	6	0.6	Olr500;Olr503-ps;Olr505	Receptor
DMR3:73805001	3	73805001	73806000	1000	1	3.50E-07	0.33	3	0.3	Olr507	Receptor
DMR3:73953001	3	73953001	73960000	7000	1	1.30E-11	-0.35	83	1.19	Olr514	Receptor
DMR3:74024001	3	74024001	74028000	4000	1	2.20E-07	-0.39	36	0.9	Olr516	Signaling
DMR3:74397001	3	74397001	74399000	2000	1	2.50E-08	-0.52	11	0.55	Olr525-ps	
DMR3:74494001	3	74494001	74495000	1000	1	3.60E-09	-0.53	6	0.6	Olr529	Receptor
DMR3:74505001	3	74505001	74508000	3000	1	9.40E-10	-0.34	19	0.63	Olr529	Receptor
DMR3:75079001	3	75079001	75083000	4000	3	2.70E-08	-0.3	41	1.02	Olr542	Signaling
DMR3:75305001	3	75305001	75307000	2000	1	2.50E-08	-0.34	15	0.75	Olr555	Receptor
DMR3:75394001	3	75394001	75399000	5000	3	4.30E-14	-0.44	42	0.84	Olr559;LOC685743	Receptor

DMR3:75493001	3	75493001	75501000	8000	2	4.40E-17	0.42	74	0.92	Olr564-ps;LOC108350559;Olr565-ps	
DMR3:75608001	3	75608001	75612000	4000	2	1.30E-10	-0.37	39	0.98	Olr571-ps	
DMR3:75642001	3	75642001	75643000	1000	1	9.20E-07	-0.25	21	2.1	Olr572-ps;LOC100361457	
DMR3:75645001	3	75645001	75648000	3000	1	8.90E-07	-0.46	30	1	Olr572-ps;LOC100361457;LOC100360176	
DMR3:75692001	3	75692001	75701000	9000	1	1.10E-09	-0.46	89	0.99	Olr574-ps	
DMR3:75799001	3	75799001	75800000	1000	1	2.20E-08	0.5	6	0.6	Olr579-ps;Olr580-ps;Olr581	Signaling
DMR3:75827001	3	75827001	75833000	6000	2	4.50E-13	-0.38	76	1.27	Olr582	Signaling
DMR3:75838001	3	75838001	75843000	5000	1	7.80E-07	-0.32	31	0.62	Olr582;Olr583	Signaling
DMR3:75852001	3	75852001	75855000	3000	1	5.40E-07	-0.36	20	0.67	Olr583	Signaling
DMR3:75994001	3	75994001	75995000	1000	1	3.70E-07	-0.58	12	1.2	Olr594;Olr595	Receptor
DMR3:76123001	3	76123001	76127000	4000	3	9.10E-19	-0.45	34	0.85	LOC102555599;Olr603-ps;Olr604	Receptor
DMR3:76306001	3	76306001	76309000	3000	1	1.00E-14	-0.48	29	0.97	Olr610	
DMR3:76478001	3	76478001	76483000	5000	1	6.20E-07	-0.37	33	0.66	Olr619;Olr620-ps	Receptor
DMR3:76684001	3	76684001	76689000	5000	1	3.30E-08	-0.33	37	0.74	Olr628-ps;Olr629	Receptor
DMR3:76909001	3	76909001	76915000	6000	1	1.70E-07	-0.34	65	1.08	Olr640	Receptor
DMR3:76936001	3	76936001	76937000	1000	1	9.50E-08	-0.51	2	0.2	Olr641	Receptor
DMR3:76987001	3	76987001	76988000	1000	1	1.30E-09	-0.56	8	0.8	Olr644-ps	
DMR3:77077001	3	77077001	77079000	2000	1	1.10E-07	-0.34	21	1.05	Olr649	Receptor
DMR3:77080001	3	77080001	77088000	8000	1	3.70E-11	-0.35	84	1.05	Olr649;Olr650	Receptor
DMR3:77375001	3	77375001	77379000	4000	1	2.00E-07	-0.28	54	1.35	LOC686900;Olr657	Receptor
DMR3:77439001	3	77439001	77446000	7000	2	3.90E-08	-0.29	89	1.27	Olr660	Receptor
DMR3:77468001	3	77468001	77473000	5000	1	5.20E-08	-0.26	87	1.74	Olr661	Receptor
DMR3:77530001	3	77530001	77531000	1000	1	1.50E-10	-0.48	6	0.6	Olr663	Receptor
DMR3:77991001	3	77991001	77995000	4000	1	2.90E-07	-0.34	43	1.07	Olr684;Olr685-ps	Receptor
DMR3:78176001	3	78176001	78183000	7000	1	6.00E-07	-0.24	78	1.11	Olr696	Receptor
DMR3:78220001	3	78220001	78223000	3000	1	2.30E-07	-0.34	25	0.83	Olr697	Receptor
DMR3:78254001	3	78254001	78255000	1000	1	5.30E-08	0.63	1	0.1	Olr700-ps	
DMR3:78762001	3	78762001	78769000	7000	1	5.60E-08	-0.28	79	1.13	Olr1258	
DMR3:78925001	3	78925001	78929000	4000	1	2.90E-10	-0.37	43	1.07	Olr730-ps	
DMR3:78930001	3	78930001	78931000	1000	1	1.20E-07	-0.25	9	0.9	Olr730-ps	
DMR3:78962001	3	78962001	78968000	6000	1	9.60E-07	-0.27	62	1.03	Olr733;Olr732-ps	Receptor
DMR3:78999001	3	78999001	79008000	9000	1	6.70E-15	-0.33	81	0.9	Olr735;Olr736	Receptor
DMR3:79025001	3	79025001	79026000	1000	1	5.20E-09	-0.5	7	0.7	Olr736	Receptor
DMR3:79174001	3	79174001	79179000	5000	1	2.00E-07	-0.41	53	1.06	Olr744;Olr745	Receptor
DMR3:79486001	3	79486001	79490000	4000	1	4.50E-08	-0.33	41	1.02	LOC102555600;LOC103691844;Nup160	Transport
DMR3:79491001	3	79491001	79493000	2000	1	7.50E-07	-0.23	45	2.25	LOC102555600;Nup160	Transport
DMR3:79910001	3	79910001	79911000	1000	1	5.40E-08	0.43	17	1.7	LOC103691845;LOC102555995;Spi1	Transcription
DMR3:80121001	3	80121001	80123000	2000	1	7.70E-09	0.58	18	0.9	RGD1309540	
DMR3:80406001	3	80406001	80410000	4000	1	3.10E-07	0.45	49	1.23	Lrp4	Binding Proteins
DMR3:80550001	3	80550001	80552000	2000	1	6.40E-07	0.47	57	2.85	F2;Znf408;Arhgap1	Protease;Signaling
DMR3:80675001	3	80675001	80678000	3000	2	3.90E-08	0.42	47	1.57	Ambra1	
DMR3:80845001	3	80845001	80848000	3000	1	9.30E-07	0.38	53	1.77	Chrm4;Mdk;Dgkz	Signaling;Growth Factors;Signaling
DMR3:81471001	3	81471001	81473000	2000	1	6.00E-07	0.32	23	1.15	Chst1	Transport
DMR3:81510001	3	81510001	81514000	4000	1	1.60E-08	0.52	73	1.82	Chst1	Transport
DMR3:82117001	3	82117001	82119000	2000	1	8.80E-11	0.53	30	1.5	Tspan18	
DMR3:82579001	3	82579001	82581000	2000	1	1.10E-07	0.41	30	1.5	Alx4	Development
DMR3:82599001	3	82599001	82601000	2000	1	2.30E-07	0.42	30	1.5	Ext2	Golgi
DMR3:83128001	3	83128001	83131000	3000	1	4.10E-07	-0.44	26	0.87	Mir129-2	
DMR3:83214001	3	83214001	83216000	2000	1	7.20E-08	-0.26	42	2.1	Ttc17	
DMR3:85583001	3	85583001	85584000	1000	1	5.70E-08	-0.33	10	1	Lrrc4c	
DMR3:85832001	3	85832001	85838000	6000	1	1.60E-07	-0.32	60	1	Lrrc4c;LOC366130	
DMR3:85855001	3	85855001	85857000	2000	1	2.70E-08	-0.57	16	0.8	Lrrc4c;LOC366130	
DMR3:86040001	3	86040001	86041000	1000	1	1.50E-09	-0.53	3	0.3	Lrrc4c	

DMR3:86590001	3	86590001	86593000	3000	1	1.50E-07	-0.53	12	0.4	Lrrc4c	
DMR3:91094001	3	91094001	91096000	2000	1	7.60E-11	0.45	75	3.75	RGD1309730	
DMR3:91357001	3	91357001	91359000	2000	1	3.20E-07	0.37	22	1.1	Prr5l	
DMR3:91388001	3	91388001	91389000	1000	1	1.50E-07	0.4	10	1	Prr5l	
DMR3:92063001	3	92063001	92067000	4000	2	2.60E-12	0.39	38	0.95	Trim44	Proteolysis
DMR3:92145001	3	92145001	92150000	5000	1	4.00E-21	1.16	57	1.14	Trim44;LOC102551100	Proteolysis
DMR3:92204001	3	92204001	92208000	4000	1	1.30E-11	0.55	34	0.85	Trim44	Proteolysis
DMR3:92437001	3	92437001	92440000	3000	1	7.40E-07	-0.35	70	2.33	Pamr1	Protease
DMR3:93001001	3	93001001	93004000	3000	1	3.00E-08	-0.42	21	0.7	Apip	Metabolism
DMR3:94374001	3	94374001	94375000	1000	1	4.90E-07	-0.37	6	0.6	Hipk3	
DMR3:94641001	3	94641001	94645000	4000	1	2.80E-09	-0.4	42	1.05	Tcp11l1	Cytoskeleton
DMR3:94724001	3	94724001	94726000	2000	1	8.30E-07	-0.39	19	0.95	Qser1	
DMR3:94880001	3	94880001	94887000	7000	2	5.10E-10	-0.36	89	1.27	Ccdc73	
DMR3:94909001	3	94909001	94911000	2000	1	4.20E-14	-0.42	17	0.85	Ccdc73	
DMR3:94921001	3	94921001	94926000	5000	3	6.10E-13	-0.47	34	0.68	Ccdc73	
DMR3:95729001	3	95729001	95730000	1000	1	3.60E-07	-0.51	14	1.4	Pax6;Elp4	
DMR3:95907001	3	95907001	95913000	6000	1	2.80E-09	-0.39	48	0.8	Elp4	
DMR3:95995001	3	95995001	95997000	2000	1	9.20E-10	0.42	29	1.45	Immp1l	Protease
DMR3:97268001	3	97268001	97271000	3000	1	2.90E-07	-0.46	39	1.3	Dcdc5	
DMR3:97364001	3	97364001	97369000	5000	1	2.50E-07	-0.3	54	1.08	Dcdc5	
DMR3:97376001	3	97376001	97379000	3000	1	2.00E-07	-0.34	21	0.7	Dcdc5	
DMR3:99459001	3	99459001	99460000	1000	1	5.40E-08	-0.5	5	0.5	Olr746-ps	
DMR3:100359001	3	100359001	100361000	2000	1	3.20E-07	-0.43	11	0.55	Mettl15;Kif18a	Epigenetic;Cytoskeleton
DMR3:101023001	3	101023001	101028000	5000	2	5.80E-08	-0.27	50	1	Lin7c	Cytoskeleton
DMR3:101048001	3	101048001	101050000	2000	1	3.00E-16	0.51	12	0.6	Lgr4	
DMR3:101073001	3	101073001	101074000	1000	1	3.20E-07	-0.42	17	1.7	Lgr4	
DMR3:101454001	3	101454001	101455000	1000	1	2.00E-10	0.4	5	0.5	Bbox1	Metabolism
DMR3:101484001	3	101484001	101487000	3000	3	1.90E-08	-0.46	17	0.57	Bbox1	Metabolism
DMR3:101975001	3	101975001	101981000	6000	1	6.80E-09	-0.39	51	0.85	Ano3	
DMR3:102058001	3	102058001	102064000	6000	2	3.00E-09	-0.36	55	0.92	Ano3	
DMR3:102303001	3	102303001	102305000	2000	1	9.70E-07	-0.37	14	0.7	Ankrd30a	
DMR3:102389001	3	102389001	102395000	6000	2	8.10E-09	-0.31	66	1.1	Ankrd30a	
DMR3:102494001	3	102494001	102496000	2000	1	6.30E-09	0.45	8	0.4	Olr750	Receptor
DMR3:102538001	3	102538001	102544000	6000	3	1.70E-10	-0.35	55	0.92	Olr752;Olr753	Receptor
DMR3:102783001	3	102783001	102786000	3000	1	2.30E-07	-0.47	9	0.3	Olr767	Receptor
DMR3:102939001	3	102939001	102940000	1000	1	2.90E-08	-0.46	8	0.8	Olr773	Receptor
DMR3:103047001	3	103047001	103051000	4000	2	3.40E-09	-0.44	33	0.82	LOC108350606;Olr775	Receptor
DMR3:103238001	3	103238001	103239000	1000	1	2.50E-09	-0.44	9	0.9	Olr786	
DMR3:103275001	3	103275001	103276000	1000	1	5.10E-08	-0.35	7	0.7	Olr787-ps	
DMR3:103389001	3	103389001	103392000	3000	2	2.20E-09	-0.46	23	0.77	Olr790	Receptor
DMR3:103549001	3	103549001	103550000	1000	1	4.80E-07	-0.31	8	0.8	Olr792	Receptor
DMR3:103644001	3	103644001	103650000	6000	1	2.60E-10	-0.32	68	1.13	Olr795;Olr796	Receptor
DMR3:103729001	3	103729001	103730000	1000	1	2.50E-07	0.34	18	1.8	Lpcat4;Nutm1	Metabolism
DMR3:103917001	3	103917001	103919000	2000	1	3.20E-07	0.51	19	0.95	Katnbl1	
DMR3:104031001	3	104031001	104032000	1000	1	3.70E-07	0.39	7	0.7	Aven	
DMR3:104678001	3	104678001	104683000	5000	2	9.40E-10	-0.43	49	0.98	Hmgn4	
DMR3:104742001	3	104742001	104748000	6000	1	8.60E-08	-0.33	43	0.72	Tmco5b;LOC102553315	
DMR3:108536001	3	108536001	108540000	4000	2	3.50E-08	-0.31	56	1.4	Tmco5a	
DMR3:108937001	3	108937001	108943000	6000	1	4.70E-08	-0.36	58	0.97	Fam98b	
DMR3:109045001	3	109045001	109046000	1000	1	6.80E-11	0.46	23	2.3	Rasgrp1	Transcription
DMR3:110557001	3	110557001	110558000	1000	1	2.70E-10	0.48	11	1.1	LOC102546973;RGD1565536;Phgr1	
DMR3:110717001	3	110717001	110718000	1000	1	1.20E-09	0.48	10	1	Bahd1	
DMR3:110726001	3	110726001	110728000	2000	1	3.40E-07	0.47	38	1.9	Bahd1;Chst14	Transport
DMR3:110898001	3	110898001	110899000	1000	1	1.00E-06	-0.46	10	1	Knl1	
DMR3:111113001	3	111113001	111115000	2000	1	5.30E-10	0.62	27	1.35	Vps18	Transport
DMR3:111843001	3	111843001	111844000	1000	1	2.00E-07	0.44	16	1.6	Pla2g4b;Sptbn5	Metabolism
DMR3:111850001	3	111850001	111851000	1000	1	6.10E-07	0.41	23	2.3	Sptbn5	
DMR3:112082001	3	112082001	112084000	2000	1	8.60E-07	0.37	29	1.45	Pla2g4f;Vps39	Metabolism
DMR3:112270001	3	112270001	112271000	1000	1	5.70E-07	0.33	13	1.3	Capn3;LOC108350446;Zfp106	Protease

DMR3:112648001	3	112648001	112651000	3000	1	6.30E-07	-0.26	29	0.97	Stard9;Cdan1	Cytoskeleton
DMR3:112903001	3	112903001	112904000	1000	1	5.60E-08	0.35	15	1.5	Ubr1	Proteolysis
DMR3:112954001	3	112954001	112958000	4000	1	4.90E-07	0.72	72	1.8	Tmem62;Ccndbp1;Epb42	Transcription;Transport
DMR3:112968001	3	112968001	112970000	2000	1	1.00E-07	-0.49	29	1.45	Ccndbp1;Epb42	Transport
DMR3:113037001	3	113037001	113039000	2000	1	1.90E-09	0.45	20	1	Tgm7l1;Tgm7	Transport
DMR3:113175001	3	113175001	113178000	3000	1	3.90E-07	0.3	19	0.63	Tp53bp1	Transcription
DMR3:113216001	3	113216001	113217000	1000	1	5.90E-07	-0.38	9	0.9	Tp53bp1	Transcription
DMR3:113460001	3	113460001	113461000	1000	1	3.10E-11	0.72	1	0.1	Wdr76	
DMR3:113472001	3	113472001	113477000	5000	1	4.10E-07	-0.46	60	1.2	Wdr76;Frmd5	
DMR3:113579001	3	113579001	113588000	9000	2	9.40E-08	-0.34	127	1.41	Frmd5	
DMR3:113888001	3	113888001	113896000	8000	3	4.90E-10	0.37	90	1.12	Casc4	
DMR3:114249001	3	114249001	114250000	1000	1	3.90E-08	0.57	5	0.5	Duoxa2;Duoxa1;Duox1	Metabolism
DMR3:114299001	3	114299001	114301000	2000	1	2.00E-07	0.42	27	1.35	Shf;LOC102557338;Trnah-gug	
DMR3:114532001	3	114532001	114534000	2000	1	4.10E-07	-0.34	12	0.6	Slc28a2	Transport
DMR3:114927001	3	114927001	114930000	3000	1	2.40E-07	-0.59	35	1.17	Sqrdl	
DMR3:114942001	3	114942001	114946000	4000	1	3.40E-07	-0.31	35	0.88	Sqrdl	
DMR3:114947001	3	114947001	114950000	3000	2	1.70E-10	-0.34	44	1.47	Sqrdl	
DMR3:117409001	3	117409001	117410000	1000	1	2.40E-07	-0.5	14	1.4	Ctxn2	
DMR3:117483001	3	117483001	117485000	2000	1	3.50E-07	0.51	17	0.85	Slc12a1;LOC102555270	Transport
DMR3:117597001	3	117597001	117599000	2000	1	4.00E-08	0.44	30	1.5	Fbn1	Extracellular Matrix
DMR3:117840001	3	117840001	117841000	1000	1	6.40E-08	0.36	16	1.6	Cep152	
DMR3:118026001	3	118026001	118029000	3000	1	1.80E-09	0.43	56	1.87	Secisbp2l	
DMR3:118117001	3	118117001	118119000	2000	1	1.70E-10	0.53	22	1.1	Cops2	
DMR3:118166001	3	118166001	118174000	8000	3	1.60E-09	-0.39	82	1.02	Galk2	Metabolism
DMR3:118224001	3	118224001	118225000	1000	1	2.10E-09	0.67	1	0.1	Galk2	Metabolism
DMR3:118286001	3	118286001	118293000	7000	1	1.00E-09	-0.34	92	1.31	Fam227b	
DMR3:118410001	3	118410001	118416000	6000	1	4.50E-09	-0.48	50	0.83	Fam227b	
DMR3:118784001	3	118784001	118785000	1000	1	5.70E-07	-0.42	12	1.2	Atp8b4	Transport
DMR3:119076001	3	119076001	119077000	1000	1	8.90E-07	0.41	14	1.4	Hdc	Metabolism
DMR3:119104001	3	119104001	119106000	2000	1	1.30E-08	0.5	20	1	Gabpb1	
DMR3:120299001	3	120299001	120300000	1000	1	4.40E-08	0.38	10	1	Mall	Transport
DMR3:120424001	3	120424001	120425000	1000	1	1.50E-10	0.5	13	1.3	Acoxl	Metabolism
DMR3:120703001	3	120703001	120710000	7000	1	6.60E-07	-0.37	85	1.21	Acoxl	Metabolism
DMR3:122011001	3	122011001	122018000	7000	1	7.00E-08	-0.42	85	1.21	RGD1566226	
DMR3:122158001	3	122158001	122159000	1000	1	9.60E-11	-0.54	14	1.4	Sirpa	Receptor
DMR3:122838001	3	122838001	122839000	1000	1	1.70E-11	0.47	16	1.6	Ebf4	Transcription
DMR3:123108001	3	123108001	123117000	9000	3	1.10E-10	-0.4	117	1.3	Oxt;Avp	Signaling
DMR3:123182001	3	123182001	123183000	1000	1	9.00E-10	-0.46	14	1.4	Lzts3	
DMR3:123565001	3	123565001	123566000	1000	1	6.40E-07	0.41	12	1.2	Atrn;Gfra4	Extracellular Matrix;Receptor
DMR3:123745001	3	123745001	123748000	3000	1	8.00E-08	-0.45	28	0.93	Cdc25b;Ap5s1	Signaling
DMR3:123786001	3	123786001	123788000	2000	1	5.20E-10	0.71	18	0.9	Mavs	
DMR3:123985001	3	123985001	123987000	2000	1	2.30E-17	0.57	33	1.65	RGD1564425	
DMR3:124572001	3	124572001	124573000	1000	1	7.70E-07	-0.35	15	1.5	Rassf2	Cytoskeleton
DMR3:127473001	3	127473001	127479000	6000	2	1.00E-08	-0.35	81	1.35	Hao1;LOC108350470	Metabolism
DMR3:127546001	3	127546001	127550000	4000	1	2.20E-07	-0.31	49	1.23	Tmx4	
DMR3:128011001	3	128011001	128018000	7000	3	4.60E-12	-0.37	99	1.41	Plcb1	Metabolism
DMR3:128287001	3	128287001	128288000	1000	1	5.90E-08	0.34	15	1.5	Plcb1	Metabolism
DMR3:128622001	3	128622001	128629000	7000	1	1.00E-07	-0.26	83	1.19	Plcb4	Metabolism
DMR3:128836001	3	128836001	128837000	1000	1	3.40E-08	-0.31	9	0.9	Plcb4	Metabolism
DMR3:128838001	3	128838001	128844000	6000	1	1.70E-07	-0.32	63	1.05	Plcb4	Metabolism
DMR3:129096001	3	129096001	129099000	3000	2	2.20E-11	-0.39	30	1	Pak7	
DMR3:129469001	3	129469001	129470000	1000	1	4.70E-13	0.73	7	0.7	Ankef1	
DMR3:129484001	3	129484001	129485000	1000	1	4.60E-07	-0.61	9	0.9	Ankef1;LOC108350472	
DMR3:129538001	3	129538001	129545000	7000	2	4.10E-08	-0.35	63	0.9	Ankef1;LOC108350472	
DMR3:129861001	3	129861001	129863000	2000	1	5.30E-12	0.61	18	0.9	LOC100361315;Mkks	Transcription
DMR3:132585001	3	132585001	132590000	5000	2	2.80E-07	-0.35	49	0.98	Sptlc3	Metabolism
DMR3:132647001	3	132647001	132651000	4000	2	8.60E-09	-0.33	30	0.75	Sptlc3	Metabolism
DMR3:133207001	3	133207001	133208000	1000	1	3.20E-07	0.61	5	0.5	Esf1	
DMR3:135734001	3	135734001	135739000	5000	1	4.10E-07	-0.4	54	1.08	Macrod2	

DMR3:136083001	3	136083001	136085000	2000	2	5.20E-10	0.44	16	0.8	Macrod2	
DMR3:136130001	3	136130001	136131000	1000	1	1.70E-08	0.36	10	1	Macrod2	
DMR3:136348001	3	136348001	136349000	1000	1	5.90E-07	0.45	6	0.6	Macrod2	
DMR3:136406001	3	136406001	136408000	2000	1	5.20E-10	-0.54	15	0.75	Macrod2	
DMR3:136799001	3	136799001	136804000	5000	1	1.70E-07	-0.3	43	0.86	Kif16b	Cytoskeleton
DMR3:136826001	3	136826001	136834000	8000	2	1.10E-08	-0.31	106	1.32	Kif16b	Cytoskeleton
DMR3:137635001	3	137635001	137639000	4000	1	8.00E-09	-0.35	32	0.8	Pcsk2;LOC100361436	Protease
DMR3:137663001	3	137663001	137669000	6000	2	1.40E-07	-0.38	50	0.83	Pcsk2;LOC108350478	Protease
DMR3:137732001	3	137732001	137733000	1000	1	6.00E-11	0.5	8	0.8	Pcsk2	Protease
DMR3:137735001	3	137735001	137736000	1000	1	9.90E-07	0.39	13	1.3	Pcsk2	Protease
DMR3:137793001	3	137793001	137794000	1000	1	6.20E-07	-0.63	11	1.1	Pcsk2	Protease
DMR3:138458001	3	138458001	138459000	1000	1	5.20E-08	0.43	14	1.4	Ovol2	
DMR3:138658001	3	138658001	138660000	2000	1	7.80E-08	-0.4	16	0.8	Dzank1	
DMR3:139477001	3	139477001	139479000	2000	1	2.80E-09	0.58	15	0.75	Slc24a3	Transport
DMR3:139810001	3	139810001	139812000	2000	1	8.00E-07	0.54	19	0.95	Slc24a3	Transport
DMR3:140091001	3	140091001	140094000	3000	1	8.90E-07	0.45	33	1.1	Rin2;LOC102555365	Transcription
DMR3:140177001	3	140177001	140179000	2000	1	3.70E-14	-0.39	54	2.7	Cfap61	
DMR3:140379001	3	140379001	140380000	1000	1	4.20E-09	0.51	6	0.6	Cfap61	
DMR3:140546001	3	140546001	140553000	7000	2	5.90E-12	-0.39	75	1.07	Ralgapa2	Signaling
DMR3:140603001	3	140603001	140604000	1000	1	3.30E-07	0.49	5	0.5	Ralgapa2	Signaling
DMR3:141108001	3	141108001	141110000	2000	2	9.80E-11	0.44	71	3.55	Kiz	
DMR3:141121001	3	141121001	141126000	5000	1	8.20E-10	-0.4	38	0.76	Kiz	
DMR3:141182001	3	141182001	141188000	6000	2	2.60E-09	-0.38	82	1.37	Kiz;LOC688692	
DMR3:141597001	3	141597001	141599000	2000	1	8.70E-08	0.47	6	0.3	Pax1	
DMR3:143044001	3	143044001	143045000	1000	1	7.30E-09	0.49	14	1.4	Napb	Transport
DMR3:143053001	3	143053001	143059000	6000	2	7.50E-10	-0.41	58	0.97	Napb	Transport
DMR3:146999001	3	146999001	1.47E+08	1000	1	3.30E-09	-0.47	6	0.6	Nsfl1c	Signaling
DMR3:147108001	3	147108001	147110000	2000	1	1.30E-08	0.39	27	1.35	Sdcbp2;Snph	Transport;Transcription
DMR3:147424001	3	147424001	147426000	2000	1	1.20E-11	0.53	19	0.95	Angpt4	Signaling
DMR3:147495001	3	147495001	147498000	3000	1	1.60E-09	0.42	50	1.67	Fam110a;LOC108350501;Slc52a3	Transport
DMR3:147509001	3	147509001	147510000	1000	1	5.20E-12	0.51	19	1.9	Slc52a3	Transport
DMR3:147515001	3	147515001	147517000	2000	1	3.40E-08	0.59	30	1.5	Slc52a3	Transport
DMR3:148181001	3	148181001	148182000	1000	1	3.50E-08	0.48	11	1.1	Hm13	Proteolysis
DMR3:148260001	3	148260001	148263000	3000	1	5.00E-11	0.57	32	1.07	Bcl2l1	
DMR3:148379001	3	148379001	148381000	2000	1	7.10E-07	0.46	24	1.2	Tpx2;LOC102553350;Mylk2	Cytoskeleton;Signaling
DMR3:148393001	3	148393001	148397000	4000	1	4.90E-07	0.33	82	2.05	LOC102553350;Mylk2;Foxs1	Signaling;Transcription
DMR3:148521001	3	148521001	148523000	2000	1	4.70E-07	0.41	29	1.45	Xkr7;LOC108350503	
DMR3:148698001	3	148698001	148699000	1000	1	3.20E-07	0.39	20	2	Tm9sf4;Tspy26	Transport;Epigenetic
DMR3:148909001	3	148909001	148911000	2000	1	1.50E-07	0.52	20	1	Asxl1;Nol4l	
DMR3:149117001	3	149117001	149119000	2000	1	8.70E-09	-0.56	29	1.45	Commd7	
DMR3:149713001	3	149713001	149714000	1000	1	1.80E-10	0.47	10	1	Bpifb5	
DMR3:149728001	3	149728001	149729000	1000	1	3.40E-14	0.74	32	3.2	Bpifb5;LOC102547046	
DMR3:149851001	3	149851001	149855000	4000	1	1.40E-08	0.39	73	1.82	Cdk5rap1	
DMR3:149859001	3	149859001	149860000	1000	1	1.70E-10	0.66	2	0.2	Cdk5rap1	
DMR3:149896001	3	149896001	149898000	2000	1	1.90E-09	0.57	18	0.9	Snta1	
DMR3:150060001	3	150060001	150061000	1000	1	8.50E-09	0.41	20	2	Necab3;RGD1561517;Actl10;E2f1	Cytoskeleton;Transcription
DMR3:150169001	3	150169001	150170000	1000	1	4.40E-12	0.66	15	1.5	Zfp341	
DMR3:150171001	3	150171001	150174000	3000	1	8.30E-07	0.33	60	2	Zfp341	
DMR3:150213001	3	150213001	150214000	1000	1	9.60E-09	0.38	13	1.3	Chmp4b	Transport
DMR3:150406001	3	150406001	150407000	1000	1	2.30E-08	-0.4	8	0.8	Eif2s2	Translation
DMR3:150522001	3	150522001	150529000	7000	1	3.10E-08	-0.34	67	0.96	Asip	Signaling
DMR3:150598001	3	150598001	150599000	1000	1	3.00E-07	0.45	4	0.4	Ahcy	Metabolism
DMR3:150747001	3	150747001	150749000	2000	1	1.20E-08	0.43	15	0.75	Itch	Proteolysis
DMR3:151134001	3	151134001	151135000	1000	1	9.00E-09	0.38	19	1.9	Myh7b;Mir499	
DMR3:151342001	3	151342001	151345000	3000	1	4.50E-07	0.35	62	2.07	Mmp24	Protease
DMR3:152748001	3	152748001	152751000	3000	1	6.00E-10	0.46	45	1.5	Dlgap4	Cytoskeleton
DMR3:152754001	3	152754001	152759000	5000	1	1.10E-07	0.37	60	1.2	Dlgap4	Cytoskeleton

DMR3:152867001	3	152867001	152869000	2000	1	7.30E-09	0.45	22	1.1	Myl9;LOC102551821	Cytoskeleton
DMR3:153030001	3	153030001	153034000	4000	1	6.50E-07	-0.72	52	1.3	Ndrg3;LOC102554288	Protease
DMR3:153059001	3	153059001	153066000	7000	2	2.70E-08	-0.79	105	1.5	Ndrg3	Protease
DMR3:153116001	3	153116001	153117000	1000	1	6.40E-07	0.48	4	0.4	Dsn1	
DMR3:153194001	3	153194001	153196000	2000	1	8.70E-09	0.43	28	1.4	Soga1;Tldc2	
DMR3:153203001	3	153203001	153204000	1000	1	9.10E-11	0.44	20	2	Tldc2;Samhd1	Metabolism
DMR3:153322001	3	153322001	153325000	3000	1	2.80E-07	-0.47	31	1.03	Rbl1;LOC108350507;Mroh8	Epigenetic
DMR3:153549001	3	153549001	153551000	2000	1	1.00E-11	0.46	19	0.95	LOC108350508;Src;LOC102552922	
DMR3:153585001	3	153585001	153587000	2000	1	6.00E-07	0.41	34	1.7	Src	
DMR3:154054001	3	154054001	154055000	1000	1	8.50E-08	-0.41	18	1.8	Nnat	
DMR3:154381001	3	154381001	154382000	1000	1	1.10E-07	0.44	17	1.7	Ctnnbl1	
DMR3:154593001	3	154593001	154595000	2000	1	6.10E-07	0.41	21	1.05	Tgm2	Transport
DMR3:154627001	3	154627001	154628000	1000	1	1.10E-07	0.46	42	4.2	Tgm2	Transport
DMR3:154776001	3	154776001	154777000	1000	1	3.40E-09	0.4	9	0.9	Bpi;Lbp	
DMR3:155024001	3	155024001	155025000	1000	1	7.90E-07	-0.43	29	2.9	Arhgap40	Signaling
DMR3:156824001	3	156824001	156832000	8000	3	5.70E-13	-0.39	102	1.27	Zhx3	Development
DMR3:157806001	3	157806001	157808000	2000	1	3.60E-07	0.46	23	1.15	Ptprt	Signaling
DMR3:157838001	3	157838001	157844000	6000	4	1.50E-14	0.66	57	0.95	Ptprt	Signaling
DMR3:158013001	3	158013001	158015000	2000	1	1.40E-08	0.37	53	2.65	Ptprt	Signaling
DMR3:158214001	3	158214001	158216000	2000	1	5.10E-07	0.38	6	0.3	Ptprt	Signaling
DMR3:159303001	3	159303001	159304000	1000	1	1.10E-07	0.38	18	1.8	LOC100362466;Srsf6	Translation
DMR3:159600001	3	159600001	159605000	5000	1	6.20E-07	0.53	79	1.58	Tox2	
DMR3:159629001	3	159629001	159631000	2000	1	3.50E-07	0.37	27	1.35	Tox2	
DMR3:159691001	3	159691001	159692000	1000	1	3.10E-08	0.4	29	2.9	Tox2	
DMR3:159826001	3	159826001	159829000	3000	1	7.70E-07	0.36	48	1.6	Gdap1l1	
DMR3:159888001	3	159888001	159891000	3000	1	1.20E-09	0.38	45	1.5	LOC102548241;R3hdml	Immune
DMR3:160261001	3	160261001	160265000	4000	1	1.00E-06	0.31	68	1.7	Rims4	Transport
DMR3:160310001	3	160310001	160314000	4000	1	2.60E-07	0.36	50	1.25	Rims4	Transport
DMR3:160458001	3	160458001	160459000	1000	1	7.40E-08	0.39	6	0.6	Tomm34;Stk4	Signaling
DMR3:160704001	3	160704001	160706000	2000	1	4.70E-07	-0.39	25	1.25	Svs6;Svs5	
DMR3:161038001	3	161038001	161039000	1000	1	4.70E-08	0.31	12	1.2	Wfdc2;Spint3;Wfdc6a;Eppin	Protease; Proteolysis
DMR3:161066001	3	161066001	161067000	1000	1	8.20E-10	0.41	13	1.3	Wfdc8;Wfdc6b	
DMR3:161179001	3	161179001	161181000	2000	1	5.20E-07	0.34	32	1.6	Spint5p	Protease; Proteolysis
DMR3:161452001	3	161452001	161456000	4000	3	4.90E-14	0.47	90	2.25	Slc12a5;Ncoa5	Transport;Transcription
DMR3:161457001	3	161457001	161462000	5000	1	6.80E-07	0.35	115	2.3	Slc12a5;Ncoa5	Transport;Transcription
DMR3:161505001	3	161505001	161506000	1000	1	2.50E-08	0.42	9	0.9	Ncoa5	Transcription
DMR3:161515001	3	161515001	161518000	3000	1	6.90E-13	0.47	50	1.67	Cd40	Receptor
DMR3:161578001	3	161578001	161580000	2000	1	3.40E-09	0.43	38	1.9	Cdh22	Cytoskeleton
DMR3:162032001	3	162032001	162036000	4000	1	1.30E-09	0.38	49	1.23	Zfp663	Transcription
DMR3:162341001	3	162341001	162342000	1000	1	6.90E-09	0.43	22	2.2	Eya2	
DMR3:162431001	3	162431001	162434000	3000	1	1.00E-07	0.56	71	2.37	Eya2	
DMR3:162805001	3	162805001	162807000	2000	1	6.70E-08	0.42	40	2	Sulf2	Metabolism
DMR3:162820001	3	162820001	162823000	3000	1	2.60E-08	0.55	43	1.43	Sulf2;LOC108350522	Metabolism
DMR3:162835001	3	162835001	162838000	3000	1	2.50E-07	0.44	50	1.67	Sulf2;LOC108350522	Metabolism
DMR3:163413001	3	163413001	163415000	2000	1	8.90E-11	0.4	37	1.85	Prex1	Transcription
DMR3:163438001	3	163438001	163439000	1000	1	9.50E-14	0.54	19	1.9	Prex1	Transcription
DMR3:163448001	3	163448001	163449000	1000	1	2.80E-10	0.45	23	2.3	Prex1	Transcription
DMR3:163578001	3	163578001	163580000	2000	1	1.90E-14	0.45	38	1.9	Arfgef2	Transcription
DMR3:163601001	3	163601001	163603000	2000	1	1.20E-07	0.33	17	0.85	Arfgef2	Transcription
DMR3:163951001	3	163951001	163955000	4000	2	9.20E-11	0.6	87	2.17	LOC102552303;Ptgis	
DMR3:164727001	3	164727001	164730000	3000	1	8.50E-12	0.48	63	2.1	Fam65c	
DMR3:164756001	3	164756001	164757000	1000	1	7.60E-12	0.48	19	1.9	Fam65c	
DMR3:164761001	3	164761001	164762000	1000	1	5.90E-10	0.41	13	1.3	Fam65c	
DMR3:164772001	3	164772001	164776000	4000	1	1.30E-08	0.51	44	1.1	Fam65c	
DMR3:164788001	3	164788001	164791000	3000	1	8.70E-14	0.62	78	2.6	Fam65c	
DMR3:164835001	3	164835001	164837000	2000	1	7.40E-07	0.34	46	2.3	Pard6b;LOC102552887	Cell Junction
DMR3:165268001	3	165268001	165271000	3000	2	3.50E-09	0.42	63	2.1	Nfatc2	Transcription
DMR3:166551001	3	166551001	166552000	1000	1	3.90E-07	-0.57	20	2	Tshz2	Transcription

DMR3:167030001	3	167030001	167031000	1000	1	5.50E-07	0.59	14	1.4	Zfp217	Transcription
DMR3:167960001	3	167960001	167961000	1000	1	6.30E-08	0.43	15	1.5	Bcas1	
DMR3:168029001	3	168029001	168031000	2000	1	5.00E-08	0.37	45	2.25	Bcas1	
DMR3:168429001	3	168429001	168432000	3000	1	2.50E-08	-0.5	42	1.4	Dok5	
DMR3:170252001	3	170252001	170254000	2000	1	9.60E-07	0.38	70	3.5	Mc3r	Signaling
DMR3:170262001	3	170262001	170263000	1000	1	2.80E-09	0.42	27	2.7	Mc3r;LOC102549927	Signaling
DMR3:170871001	3	170871001	170873000	2000	1	1.50E-08	0.45	56	2.8	Bmp7	Growth Factors
DMR3:170902001	3	170902001	170905000	3000	1	2.00E-08	0.36	54	1.8	Bmp7	Growth Factors
DMR3:171006001	3	171006001	171007000	1000	1	1.20E-07	0.41	19	1.9	Spo11;Rae1	Transcription;Metabolism
DMR3:171299001	3	171299001	171302000	3000	1	2.40E-08	0.39	69	2.3	Zbp1;Pmepa1	Transcription
DMR3:171307001	3	171307001	171308000	1000	1	1.30E-07	0.45	17	1.7	Pmepa1;LOC102547304	
DMR3:171310001	3	171310001	171312000	2000	1	1.30E-08	0.39	49	2.45	Pmepa1;LOC102547304	
DMR3:171790001	3	171790001	171791000	1000	1	9.50E-07	-0.48	24	2.4	Rab22a	
DMR3:171924001	3	171924001	171926000	2000	1	3.40E-08	-0.39	18	0.9	Apcdd1l	
DMR3:171937001	3	171937001	171938000	1000	1	4.40E-08	0.4	15	1.5	Apcdd1l	
DMR3:172170001	3	172170001	172173000	3000	1	2.80E-07	-0.36	24	0.8	Stx16	Transcription
DMR3:172429001	3	172429001	172431000	2000	1	2.40E-15	0.9	18	0.9	Gnas;LOC103692002	Signaling
DMR3:172502001	3	172502001	172503000	1000	1	9.30E-08	0.41	15	1.5	Nelfcd	
DMR3:172535001	3	172535001	172537000	2000	1	1.00E-07	0.36	26	1.3	Nelfcd;Ctsz	Protease
DMR3:172683001	3	172683001	172685000	2000	1	1.20E-07	-0.39	36	1.8	Zfp831	
DMR3:175276001	3	175276001	175280000	4000	2	8.40E-08	0.39	127	3.17	Cdh4	Cytoskeleton
DMR3:175558001	3	175558001	175559000	1000	1	5.50E-07	0.35	39	3.9	Adrm1;Lama5	Extracellular Matrix
DMR3:175697001	3	175697001	175699000	2000	1	3.10E-08	0.4	29	1.45	LOC102553093;Gata5	Transcription
DMR3:175859001	3	175859001	175865000	6000	1	1.30E-07	0.55	100	1.67	Slco4a1;LOC102552621;LOC102552550	Transport
DMR3:176111001	3	176111001	176113000	2000	2	8.80E-13	0.48	26	1.3	Col9a3	Extracellular Matrix
DMR3:176732001	3	176732001	176734000	2000	1	8.90E-08	0.42	51	2.55	Fndc11;Helz2	Transcription
DMR4:467001	4	467001	470000	3000	2	5.80E-18	0.8	42	1.4	Cnpy1	
DMR4:681001	4	681001	682000	1000	1	7.40E-07	-0.4	7	0.7	Rbm33;LOC103692014	
DMR4:1835001	4	1835001	1841000	6000	2	1.70E-09	-0.35	58	0.97	Olr1096	Receptor
DMR4:2142001	4	2142001	2144000	2000	2	6.60E-27	0.78	5	0.25	Lmbr1	Receptor
DMR4:2179001	4	2179001	2180000	1000	1	6.90E-12	-0.33	11	1.1	Lmbr1	Receptor
DMR4:2181001	4	2181001	2185000	4000	1	8.40E-09	-0.3	39	0.98	Lmbr1	Receptor
DMR4:4042001	4	4042001	4044000	2000	1	1.20E-09	0.52	19	0.95	Dpp6	Protease
DMR4:4114001	4	4114001	4119000	5000	3	2.00E-09	-0.37	43	0.86	Dpp6	Protease
DMR4:4169001	4	4169001	4171000	2000	1	3.80E-08	0.44	45	2.25	Dpp6	Protease
DMR4:4208001	4	4208001	4214000	6000	1	2.90E-09	-0.32	63	1.05	Dpp6	Protease
DMR4:4441001	4	4441001	4442000	1000	1	3.00E-07	0.41	9	0.9	Dpp6	Protease
DMR4:4887001	4	4887001	4894000	7000	1	4.20E-09	-0.34	62	0.89	Dpp6	Protease
DMR4:6019001	4	6019001	6023000	4000	1	8.20E-08	-0.32	53	1.32	RGD1560124;LOC100363537;LOC108350645	
DMR4:6337001	4	6337001	6338000	1000	1	2.20E-09	0.49	16	1.6	Galnt11;Galntl5	Golgi
DMR4:6446001	4	6446001	6448000	2000	1	6.00E-09	-0.42	26	1.3	Galntl5	Golgi
DMR4:6877001	4	6877001	6880000	3000	3	8.80E-10	0.39	58	1.93	Rheb	Signaling
DMR4:7074001	4	7074001	7082000	8000	1	1.70E-07	0.41	135	1.69	Smarcd3	Epigenetic
DMR4:7088001	4	7088001	7089000	1000	1	5.00E-08	0.54	11	1.1	Smarcd3	Epigenetic
DMR4:7224001	4	7224001	7227000	3000	2	3.30E-08	0.39	70	2.33	Agap3;LOC102554667	
DMR4:7369001	4	7369001	7370000	1000	1	1.60E-09	0.65	12	1.2	Kcnh2	Transport
DMR4:7516001	4	7516001	7518000	2000	1	1.30E-14	0.51	25	1.25	Nupl2	
DMR4:7652001	4	7652001	7654000	2000	1	3.90E-09	0.42	31	1.55	Fam126a	
DMR4:7734001	4	7734001	7735000	1000	1	4.80E-07	0.35	42	4.2	Fam126a;LOC103692024	
DMR4:8111001	4	8111001	8113000	2000	1	7.00E-13	0.61	30	1.5	Srpk2	Signaling
DMR4:9862001	4	9862001	9863000	1000	1	1.20E-07	-0.58	19	1.9	Slc26a5;Psmc2	Transport;Protease
DMR4:9866001	4	9866001	9867000	1000	1	8.50E-07	0.37	17	1.7	Slc26a5;Psmc2	Transport;Protease
DMR4:9900001	4	9900001	9902000	2000	1	1.50E-09	0.69	30	1.5	Dnajc2;Pmpcb	Transcription;Protease
DMR4:10410001	4	10410001	10412000	2000	1	8.60E-08	0.49	34	1.7	Ccdc146	Development
DMR4:10492001	4	10492001	10494000	2000	1	1.60E-10	0.57	35	1.75	Gsap	Signaling
DMR4:10620001	4	10620001	10623000	3000	1	4.40E-09	-0.59	38	1.27	LOC102556034;Ptpn12	
DMR4:10711001	4	10711001	10712000	1000	1	3.00E-07	-0.5	12	1.2	Ptpn12	
DMR4:10794001	4	10794001	10797000	3000	1	1.20E-08	0.59	29	0.97	Rsbn1l	
DMR4:10807001	4	10807001	10810000	3000	1	1.50E-08	0.35	47	1.57	Rsbn1l;Tmem60	

DMR4:11040001	4	11040001	11043000	3000	1	9.00E-11	0.42	37	1.23	Magi2	
DMR4:11087001	4	11087001	11090000	3000	1	2.30E-07	-0.44	15	0.5	Magi2	
DMR4:11329001	4	11329001	11330000	1000	1	1.20E-07	0.47	5	0.5	Magi2	
DMR4:11840001	4	11840001	11843000	3000	1	5.30E-07	-0.46	18	0.6	Magi2	
DMR4:11884001	4	11884001	11888000	4000	1	1.40E-07	-0.27	39	0.98	Magi2;LOC108350831	
DMR4:12183001	4	12183001	12185000	2000	1	8.60E-07	-0.45	6	0.3	Magi2;LOC103692037	
DMR4:12189001	4	12189001	12196000	7000	1	2.30E-11	-0.41	76	1.09	Magi2;LOC103692037	
DMR4:12248001	4	12248001	12253000	5000	2	3.00E-12	-0.35	53	1.06	Magi2	
DMR4:12400001	4	12400001	12404000	4000	2	9.70E-10	-0.32	40	1	Magi2	
DMR4:13470001	4	13470001	13474000	4000	1	1.20E-08	-0.5	40	1	Gnai1	Signaling
DMR4:14331001	4	14331001	14340000	9000	4	7.50E-10	-0.32	101	1.12	Sema3c	Signaling
DMR4:16658001	4	16658001	16661000	3000	1	7.20E-08	-0.3	32	1.07	Pclo	
DMR4:16968001	4	16968001	16975000	7000	1	4.10E-07	-0.28	74	1.06	Pclo	
DMR4:18540001	4	18540001	18541000	1000	1	1.20E-09	0.47	8	0.8	Sema3a	Signaling
DMR4:21392001	4	21392001	21393000	1000	1	4.80E-10	0.4	10	1	Grm3	Signaling
DMR4:21427001	4	21427001	21429000	2000	1	1.30E-10	0.43	19	0.95	Grm3	Signaling
DMR4:21629001	4	21629001	21633000	4000	1	4.60E-14	0.3	63	1.57	RGD1563349	
DMR4:21854001	4	21854001	21855000	1000	1	3.30E-07	0.31	10	1	Dmtf1	Transcription
DMR4:21880001	4	21880001	21882000	2000	2	7.70E-11	-0.46	27	1.35	Dmtf1;LOC108350660	Transcription
DMR4:22237001	4	22237001	22239000	2000	1	7.40E-07	0.54	24	1.2	Abcb1b	
DMR4:22255001	4	22255001	22260000	5000	3	2.00E-13	-0.39	65	1.3	Abcb1b	
DMR4:22979001	4	22979001	22981000	2000	1	1.30E-08	-0.5	28	1.4	Adam22	Protease
DMR4:22990001	4	22990001	22992000	2000	1	4.80E-09	0.47	19	0.95	Adam22	Protease
DMR4:23142001	4	23142001	23143000	1000	1	1.00E-07	-0.49	6	0.6	Steap4	
DMR4:23670001	4	23670001	23671000	1000	1	4.30E-07	-0.31	21	2.1	Zfp804b	
DMR4:23672001	4	23672001	23673000	1000	1	8.10E-08	0.38	13	1.3	Zfp804b	
DMR4:25449001	4	25449001	25455000	6000	1	6.20E-07	-0.37	75	1.25	Steap1	
DMR4:25885001	4	25885001	25892000	7000	1	1.60E-08	-0.32	86	1.23	Cdk14	Signaling
DMR4:25992001	4	25992001	2.60E+07	8000	3	8.40E-08	-0.35	87	1.09	Cdk14	Signaling
DMR4:26033001	4	26033001	26036000	3000	1	3.20E-07	-0.28	21	0.7	Cdk14	Signaling
DMR4:26202001	4	26202001	26210000	8000	1	5.80E-08	-0.3	73	0.91	Cdk14	Signaling
DMR4:26290001	4	26290001	26295000	5000	1	1.90E-08	-0.31	56	1.12	Cdk14	Signaling
DMR4:26352001	4	26352001	26353000	1000	1	4.50E-11	0.7	0	0	Cdk14	Signaling
DMR4:26461001	4	26461001	26469000	8000	1	2.20E-08	0.63	102	1.27	Fzd1	Receptor
DMR4:27209001	4	27209001	27212000	3000	1	2.30E-08	-0.55	53	1.77	Akap9	
DMR4:27299001	4	27299001	27302000	3000	1	4.50E-08	-0.47	61	2.03	Akap9	
DMR4:27374001	4	27374001	27380000	6000	2	3.70E-07	-0.33	61	1.02	Mterf1	
DMR4:27759001	4	27759001	27763000	4000	1	7.60E-11	-0.38	39	0.98	Fam133b	Metabolism
DMR4:27784001	4	27784001	27786000	2000	1	5.60E-08	-0.48	37	1.85	Cdk6	Signaling
DMR4:27803001	4	27803001	27806000	3000	1	4.50E-07	0.44	66	2.2	Cdk6	Signaling
DMR4:27863001	4	27863001	27868000	5000	1	7.70E-11	0.46	74	1.48	Cdk6	Signaling
DMR4:27948001	4	27948001	27951000	3000	1	2.50E-08	-0.45	49	1.63	Cdk6	Signaling
DMR4:28522001	4	28522001	28528000	6000	1	1.80E-07	-0.38	60	1	Vps50	
DMR4:28695001	4	28695001	28697000	2000	1	3.00E-08	-0.39	22	1.1	Calcr;LOC102551706	Receptor
DMR4:29649001	4	29649001	29651000	2000	2	5.90E-10	0.37	9	0.45	Casd1;LOC108350670	Metabolism
DMR4:29653001	4	29653001	29656000	3000	3	1.60E-10	0.39	12	0.4	Casd1;LOC108350670	Metabolism
DMR4:29750001	4	29750001	29751000	1000	1	5.50E-07	-0.42	22	2.2	Sgce	Cytoskeleton
DMR4:29770001	4	29770001	29771000	1000	1	8.30E-07	-0.37	26	2.6	Sgce	Cytoskeleton
DMR4:29788001	4	29788001	29789000	1000	1	5.40E-07	-0.52	22	2.2	Peg10	
DMR4:29978001	4	29978001	29981000	3000	1	6.60E-08	0.35	63	2.1	Ppp1r9a	
DMR4:30091001	4	30091001	30100000	9000	2	1.30E-08	-0.33	107	1.19	Ppp1r9a	
DMR4:30102001	4	30102001	30104000	2000	2	3.30E-07	0.52	11	0.55	Ppp1r9a	
DMR4:30122001	4	30122001	30123000	1000	1	2.20E-08	-0.49	11	1.1	Ppp1r9a	
DMR4:30204001	4	30204001	30205000	1000	1	6.20E-07	-0.38	6	0.6	Ppp1r9a	
DMR4:30270001	4	30270001	30272000	2000	1	5.30E-07	-0.37	37	1.85	Pon1;LOC102552373	
DMR4:30381001	4	30381001	30389000	8000	2	2.50E-07	-0.39	90	1.12	Pon2	
DMR4:30471001	4	30471001	30472000	1000	1	7.10E-11	-0.38	14	1.4	Asb4	Cytoskeleton
DMR4:30478001	4	30478001	30481000	3000	1	6.90E-15	0.59	30	1	Asb4	Cytoskeleton
DMR4:30881001	4	30881001	30883000	2000	1	4.40E-07	-0.51	8	0.4	Dync1i1	Cytoskeleton
DMR4:31118001	4	31118001	31119000	1000	1	1.40E-07	-0.53	12	1.2	Dync1i1	Cytoskeleton
DMR4:31165001	4	31165001	31166000	1000	1	1.10E-07	-0.37	17	1.7	Slc25a13	Transport

DMR4:31261001	4	31261001	31263000	2000	1	2.50E-08	-0.31	25	1.25	Slc25a13	Transport
DMR4:31374001	4	31374001	31377000	3000	1	2.70E-07	-0.38	34	1.13	Slc25a13	Transport
DMR4:31693001	4	31693001	31695000	2000	1	4.30E-07	0.33	19	0.95	Slc25a13;LOC103692067;LOC108350671	Transport
DMR4:32531001	4	32531001	32533000	2000	2	7.60E-10	-0.35	22	1.1	Sdhaf3	
DMR4:33937001	4	33937001	33938000	1000	1	2.10E-08	-0.54	7	0.7	C1galt1	Transport
DMR4:34088001	4	34088001	34090000	2000	1	7.80E-08	-0.35	19	0.95	Col28a1	Extracellular Matrix
DMR4:34408001	4	34408001	34409000	1000	1	2.00E-09	0.37	18	1.8	Glcci1	
DMR4:34440001	4	34440001	34441000	1000	1	6.10E-07	0.48	10	1	Glcci1	
DMR4:34684001	4	34684001	34685000	1000	1	9.70E-11	-0.47	15	1.5	Ica1	
DMR4:35065001	4	35065001	35071000	6000	2	1.70E-09	-0.29	53	0.88	Nxph1	Signaling
DMR4:35222001	4	35222001	35227000	5000	1	1.50E-08	-0.32	48	0.96	Nxph1	Signaling
DMR4:38219001	4	38219001	38224000	5000	1	9.60E-07	-0.24	50	1	Ndufa4	Metabolism
DMR4:38263001	4	38263001	38264000	1000	1	2.70E-07	-0.4	11	1.1	Phf14	Transcription
DMR4:38810001	4	38810001	38817000	7000	1	2.70E-08	-0.34	56	0.8	Thsd7a	Cytoskeleton
DMR4:38896001	4	38896001	38901000	5000	1	2.50E-08	-0.32	40	0.8	Thsd7a;LOC102548177	Cytoskeleton
DMR4:39715001	4	39715001	39716000	1000	1	6.60E-09	0.42	4	0.4	Vwde	
DMR4:40133001	4	40133001	40134000	1000	1	2.10E-07	-0.58	8	0.8	Bmt2	
DMR4:41369001	4	41369001	41370000	1000	1	2.50E-10	-0.57	13	1.3	Foxp2	
DMR4:41394001	4	41394001	41399000	5000	1	2.60E-08	-0.29	42	0.84	Foxp2	
DMR4:41460001	4	41460001	41461000	1000	1	1.90E-08	0.51	5	0.5	Foxp2	
DMR4:41484001	4	41484001	41487000	3000	1	4.40E-10	-0.32	34	1.13	Foxp2	
DMR4:41529001	4	41529001	41532000	3000	1	1.70E-11	-0.35	29	0.97	Foxp2	
DMR4:41668001	4	41668001	41670000	2000	1	1.70E-10	0.5	15	0.75	Foxp2	
DMR4:41682001	4	41682001	41683000	1000	1	7.30E-08	-0.45	15	1.5	Foxp2	
DMR4:41811001	4	41811001	41813000	2000	2	4.60E-10	0.4	19	0.95	Foxp2	
DMR4:42847001	4	42847001	42854000	7000	2	2.20E-08	-0.42	62	0.89	Cftr	Transport
DMR4:42995001	4	42995001	42996000	1000	1	1.60E-07	0.3	11	1.1	Cttnbp2;LOC108350672	
DMR4:44144001	4	44144001	44151000	7000	1	3.50E-07	-0.38	81	1.16	Tfec	
DMR4:44947001	4	44947001	44949000	2000	1	7.00E-07	-0.39	22	1.1	Capza2	Cytoskeleton
DMR4:45276001	4	45276001	45277000	1000	1	7.40E-07	0.42	6	0.6	Wnt2	Signaling
DMR4:48406001	4	48406001	48408000	2000	1	2.20E-07	-0.42	25	1.25	Kcnd2	Transport
DMR4:48440001	4	48440001	48442000	2000	2	1.80E-11	0.43	29	1.45	Kcnd2	Transport
DMR4:48580001	4	48580001	48585000	5000	1	3.40E-09	-0.33	46	0.92	Kcnd2	Transport
DMR4:48909001	4	48909001	48915000	6000	1	2.70E-08	-0.31	72	1.2	Tspan12	
DMR4:49972001	4	49972001	49973000	1000	1	3.30E-07	-0.49	18	1.8	Ptprz1	Signaling
DMR4:50151001	4	50151001	50153000	2000	1	2.10E-07	-0.45	16	0.8	Aass	Metabolism
DMR4:50434001	4	50434001	50436000	2000	1	5.80E-12	0.44	24	1.2	Cadps2	Transport
DMR4:50578001	4	50578001	50580000	2000	1	3.10E-07	-0.58	24	1.2	Cadps2	Transport
DMR4:50622001	4	50622001	50625000	3000	1	5.80E-08	0.43	55	1.83	Cadps2	Transport
DMR4:50639001	4	50639001	50641000	2000	1	1.40E-09	-0.56	10	0.5	Cadps2	Transport
DMR4:51580001	4	51580001	51581000	1000	1	6.60E-07	0.59	18	1.8	Iqub;Ndufa5	Metabolism
DMR4:52208001	4	52208001	52216000	8000	2	1.70E-08	-0.34	81	1.01	Spam1	Metabolism
DMR4:54522001	4	54522001	54529000	7000	2	1.80E-11	-0.35	70	1	Grm8	Signaling
DMR4:54663001	4	54663001	54665000	2000	1	1.50E-08	-0.3	28	1.4	Grm8	Signaling
DMR4:54678001	4	54678001	54681000	3000	1	1.40E-07	-0.29	27	0.9	Grm8	Signaling
DMR4:54763001	4	54763001	54768000	5000	1	7.60E-10	-0.32	44	0.88	Grm8	Signaling
DMR4:54819001	4	54819001	54821000	2000	1	8.50E-07	0.5	23	1.15	Grm8	Signaling
DMR4:54837001	4	54837001	54843000	6000	2	7.80E-12	-0.45	53	0.88	Grm8	Signaling
DMR4:54846001	4	54846001	54853000	7000	1	7.30E-07	-0.28	74	1.06	Grm8	Signaling
DMR4:55250001	4	55250001	55257000	7000	1	2.20E-07	-0.39	70	1	Grm8	Signaling
DMR4:55299001	4	55299001	55311000	12000	2	1.30E-08	-0.35	130	1.08	Grm8	Signaling
DMR4:57058001	4	57058001	57060000	2000	1	3.10E-07	-0.53	20	1	Ahcyl2	Metabolism
DMR4:57279001	4	57279001	57282000	3000	1	2.90E-07	0.47	39	1.3	Smkr1	
DMR4:57452001	4	57452001	57456000	4000	1	5.00E-12	0.55	68	1.7	Nrf1;LOC108350684;Mir182;Mir96;Mir183;Mir3553	
DMR4:57567001	4	57567001	57568000	1000	1	1.10E-08	0.37	12	1.2	Ube2h	Proteolysis
DMR4:58196001	4	58196001	58198000	2000	1	6.70E-07	0.29	27	1.35	Copg2;Tsga13	Transport
DMR4:59482001	4	59482001	59483000	1000	1	8.30E-11	0.45	12	1.2	Plxna4;LOC108350688	
DMR4:60135001	4	60135001	60136000	1000	1	6.30E-07	0.55	4	0.4	Chchd3	
DMR4:60274001	4	60274001	60279000	5000	1	2.00E-11	-0.39	49	0.98	Chchd3	

DMR4:60541001	4	60541001	60546000	5000	1	6.90E-09	-0.33	45	0.9	RGD1565435;Exoc4	Transport
DMR4:60656001	4	60656001	60657000	1000	1	3.50E-07	0.39	10	1	Exoc4	Transport
DMR4:60691001	4	60691001	60693000	2000	2	8.70E-08	0.57	4	0.2	Exoc4	Transport
DMR4:60711001	4	60711001	60713000	2000	1	1.20E-12	0.46	9	0.45	Exoc4	Transport
DMR4:60747001	4	60747001	60748000	1000	1	1.40E-14	0.5	5	0.5	Exoc4	Transport
DMR4:60814001	4	60814001	60820000	6000	2	6.70E-12	-0.37	85	1.42	Exoc4	Transport
DMR4:60889001	4	60889001	60894000	5000	2	6.80E-09	-0.33	44	0.88	Exoc4	Transport
DMR4:61124001	4	61124001	61125000	1000	1	1.40E-08	0.49	11	1.1	Exoc4	Transport
DMR4:61174001	4	61174001	61176000	2000	1	1.00E-07	-0.47	22	1.1	Exoc4	Transport
DMR4:61256001	4	61256001	61263000	7000	3	2.70E-10	-0.37	80	1.14	Exoc4	Transport
DMR4:61320001	4	61320001	61324000	4000	3	3.60E-11	-0.41	38	0.95	Exoc4	Transport
DMR4:61345001	4	61345001	61346000	1000	1	1.40E-07	0.46	7	0.7	Exoc4;LOC108350836	Transport
DMR4:62240001	4	62240001	62242000	2000	1	2.00E-08	-0.6	35	1.75	Cald1	Cytoskeleton
DMR4:62417001	4	62417001	62422000	5000	3	5.50E-11	-0.35	57	1.14	Wdr91	
DMR4:62452001	4	62452001	62453000	1000	1	3.90E-08	0.37	8	0.8	Stra8	
DMR4:62514001	4	62514001	62519000	5000	1	3.70E-07	0.36	90	1.8	RGD1565367	Transport
DMR4:62554001	4	62554001	62557000	3000	1	3.60E-08	0.42	43	1.43	Cnot4	Proteolysis
DMR4:62788001	4	62788001	62792000	4000	1	1.70E-07	0.41	55	1.38	LOC689574;Slc13a4	Transport
DMR4:64379001	4	64379001	64381000	2000	1	3.90E-07	-0.4	23	1.15	Dgki	Signaling
DMR4:64507001	4	64507001	64508000	1000	1	1.00E-09	-0.52	18	1.8	Dgki	Signaling
DMR4:64813001	4	64813001	64818000	5000	1	5.40E-10	-0.31	57	1.14	Dgki	Signaling
DMR4:64860001	4	64860001	64863000	3000	1	4.60E-07	0.38	50	1.67	Creb3l2	
DMR4:64972001	4	64972001	64973000	1000	1	7.30E-07	0.39	8	0.8	Creb3l2	
DMR4:65794001	4	65794001	65798000	4000	1	9.90E-07	-0.34	35	0.88	Atp6v0a4;Tmem213	Metabolism
DMR4:65900001	4	65900001	65903000	3000	1	1.00E-07	0.37	68	2.27	RGD1306271	
DMR4:66386001	4	66386001	66387000	1000	1	6.50E-07	0.43	7	0.7	Klrg2	Receptor
DMR4:66415001	4	66415001	66416000	1000	1	8.40E-10	0.35	19	1.9	Clec2l	
DMR4:66514001	4	66514001	66516000	2000	1	8.40E-07	0.45	10	0.5	Hipk2	
DMR4:66685001	4	66685001	66689000	4000	2	8.50E-08	0.51	51	1.27	Tbxas1	Metabolism
DMR4:66701001	4	66701001	66702000	1000	1	8.10E-08	0.41	10	1	Tbxas1	Metabolism
DMR4:67305001	4	67305001	67308000	3000	1	6.10E-07	-0.42	23	0.77	Dennd2a	
DMR4:67674001	4	67674001	67675000	1000	1	1.20E-08	0.45	12	1.2	Tmem178b	
DMR4:67890001	4	67890001	67891000	1000	1	4.70E-07	-0.41	11	1.1	Tmem178b	
DMR4:68157001	4	68157001	68162000	5000	1	1.90E-08	-0.48	40	0.8	Vom2r-ps15	
DMR4:68520001	4	68520001	68526000	6000	3	2.60E-09	-0.33	85	1.42	Agk	Signaling
DMR4:70665001	4	70665001	70674000	9000	2	7.00E-09	-0.31	98	1.09	LOC108348060;Try10	Protease
DMR4:70682001	4	70682001	70685000	3000	1	9.30E-08	-0.31	24	0.8	LOC108348060;Prss2	Protease
DMR4:70702001	4	70702001	70710000	8000	3	5.50E-11	-0.36	84	1.05	LOC108348060;Prss2	Protease
DMR4:70780001	4	70780001	70785000	5000	1	1.30E-07	-0.29	41	0.82	LOC108348060;Prss1	Protease
DMR4:71071001	4	71071001	71077000	6000	2	2.00E-14	-0.5	98	1.63	RGD1562066	Signaling
DMR4:71378001	4	71378001	71381000	3000	2	1.00E-08	-0.32	48	1.6	Sval1	
DMR4:71609001	4	71609001	71616000	7000	1	4.10E-10	-0.33	97	1.39	Gstk1	Transport
DMR4:71634001	4	71634001	71640000	6000	2	6.50E-12	-0.39	85	1.42	Gstk1;LOC100360758;Tmem139	Transport
DMR4:71643001	4	71643001	71645000	2000	1	1.60E-08	-0.41	26	1.3	LOC100360758;Tmem139;Casp2	Protease
DMR4:71976001	4	71976001	71978000	2000	1	3.00E-09	-0.34	16	0.8	Olr804	Receptor
DMR4:72154001	4	72154001	72160000	6000	2	2.00E-14	-0.44	74	1.23	Tcaf1	
DMR4:72277001	4	72277001	72278000	1000	1	2.90E-07	0.47	1	0.1	Olr808	Receptor
DMR4:72553001	4	72553001	72557000	4000	1	8.20E-07	-0.32	37	0.92	Olr816	Receptor
DMR4:72573001	4	72573001	72576000	3000	1	4.80E-07	-0.29	25	0.83	Olr816	Receptor
DMR4:72591001	4	72591001	72598000	7000	2	1.50E-07	-0.29	73	1.04	Olr437;Olr818	Receptor
DMR4:72674001	4	72674001	72679000	5000	1	1.10E-07	-0.31	41	0.82	Olr821	Receptor
DMR4:72780001	4	72780001	72786000	6000	1	1.30E-07	-0.29	69	1.15	Tpk1	Signaling
DMR4:75152001	4	75152001	75156000	4000	1	2.70E-07	-0.34	44	1.1	Cntnap2	
DMR4:75462001	4	75462001	75466000	4000	1	4.50E-08	-0.33	42	1.05	Cntnap2	
DMR4:75804001	4	75804001	75805000	1000	1	1.20E-07	-0.44	8	0.8	Cntnap2	
DMR4:75929001	4	75929001	75935000	6000	1	1.40E-07	-0.36	62	1.03	Cntnap2	
DMR4:76223001	4	76223001	76224000	1000	1	3.00E-07	-0.5	1	0.1	Cntnap2	
DMR4:76303001	4	76303001	76310000	7000	1	6.00E-08	-0.3	61	0.87	Cntnap2	
DMR4:76520001	4	76520001	76523000	3000	1	4.00E-07	-0.27	48	1.6	Cntnap2	

DMR4:76856001	4	76856001	76861000	5000	2	6.70E-09	-0.36	41	0.82	Cntnap2	
DMR4:76948001	4	76948001	76955000	7000	1	4.90E-08	-0.3	80	1.14	Cntnap2	
DMR4:77939001	4	77939001	77946000	7000	2	5.70E-07	-0.36	64	0.91	LOC108350703;LOC681173;Trnac-gca	
DMR4:77949001	4	77949001	77951000	2000	1	8.80E-11	-0.55	11	0.55	LOC108350703;Trnac-gca	
DMR4:78123001	4	78123001	78124000	1000	1	4.10E-10	0.57	8	0.8	Sspo	Extracellular Matrix
DMR4:78648001	4	78648001	78653000	5000	1	7.10E-08	-0.29	80	1.6	Svs1	Metabolism
DMR4:78654001	4	78654001	78655000	1000	1	1.70E-07	-0.41	12	1.2	Svs1	Metabolism
DMR4:78725001	4	78725001	78727000	2000	1	6.40E-07	-0.37	15	0.75	Gpnmb;Malsu1	Signaling
DMR4:79140001	4	79140001	79142000	2000	1	5.90E-07	-0.38	8	0.4	Stk31	
DMR4:79313001	4	79313001	79319000	6000	1	6.90E-10	-0.34	55	0.92	RGD1564712	
DMR4:81294001	4	81294001	81295000	1000	1	8.20E-07	-0.37	26	2.6	RGD1561341	
DMR4:81870001	4	81870001	81872000	2000	1	9.70E-08	0.34	26	1.3	Skap2	Cytoskeleton
DMR4:83625001	4	83625001	83626000	1000	1	1.10E-07	-0.45	11	1.1	Creb5	Transcription
DMR4:84014001	4	84014001	84019000	5000	1	8.20E-10	-0.37	57	1.14	Cpvl	
DMR4:84447001	4	84447001	84448000	1000	1	6.00E-07	-0.41	12	1.2	Chn2	
DMR4:84789001	4	84789001	84790000	1000	1	6.50E-09	-0.48	6	0.6	Plekha8;LOC102554291;LOC103692135	Transport
DMR4:85039001	4	85039001	85045000	6000	1	3.70E-07	-0.31	65	1.08	Znrf2	Proteolysis
DMR4:85067001	4	85067001	85069000	2000	1	5.70E-07	-0.36	22	1.1	Znrf2	Proteolysis
DMR4:85607001	4	85607001	85609000	2000	1	8.60E-07	0.34	29	1.45	Ghrhr;LOC102551107	Receptor
DMR4:85685001	4	85685001	85686000	1000	1	1.10E-09	0.63	12	1.2	Adcyap1r1	Receptor
DMR4:86614001	4	86614001	86616000	2000	1	9.80E-08	-0.26	51	2.55	Pde1c	Signaling
DMR4:86710001	4	86710001	86717000	7000	1	4.60E-08	-0.32	59	0.84	Pde1c	Signaling
DMR4:86940001	4	86940001	86944000	4000	2	9.00E-09	-0.4	43	1.07	RGD1565107	
DMR4:87158001	4	87158001	87163000	5000	1	1.80E-07	-0.31	79	1.58	Fkbp9	
DMR4:87228001	4	87228001	87230000	2000	1	5.60E-07	0.37	19	0.95	Fkbp9;Nt5c3a	Metabolism
DMR4:87283001	4	87283001	87289000	6000	1	1.90E-07	-0.28	72	1.2	Nt5c3a;LOC103692137;LOC688782	Metabolism
DMR4:87427001	4	87427001	87431000	4000	1	1.70E-07	-0.31	43	1.07	Vom1r68	Receptor
DMR4:87606001	4	87606001	87608000	2000	1	1.50E-07	-0.39	4	0.2	Vom1r71	Receptor
DMR4:87863001	4	87863001	87864000	1000	1	1.80E-09	-0.32	14	1.4	Vom1r76;LOC689033;Vom1r77	Receptor
DMR4:87971001	4	87971001	87973000	2000	1	7.50E-08	-0.4	15	0.75	Vom1r79	Receptor
DMR4:87988001	4	87988001	87991000	3000	1	9.60E-11	-0.37	44	1.47	Vom1r79	Receptor
DMR4:88129001	4	88129001	88135000	6000	1	7.00E-09	-0.34	59	0.98	Vom1r82;Vom1r-ps73	Receptor
DMR4:88228001	4	88228001	88231000	3000	1	2.00E-07	-0.29	35	1.17	Vom1r85	Receptor
DMR4:88336001	4	88336001	88341000	5000	2	4.60E-11	-0.43	54	1.08	Vom1r87	Receptor
DMR4:88381001	4	88381001	88384000	3000	1	6.40E-10	-0.36	27	0.9	Vom1r-ps111	
DMR4:88387001	4	88387001	88400000	13000	1	3.10E-08	-0.39	134	1.03	Vom1r-ps111	
DMR4:88509001	4	88509001	88510000	1000	1	2.60E-07	-0.3	19	1.9	Vopp1	
DMR4:88568001	4	88568001	88573000	5000	1	3.70E-07	-0.29	41	0.82	Lancl2;LOC103695213	
DMR4:88635001	4	88635001	88637000	2000	1	9.60E-11	0.52	20	1	Herc6	Proteolysis
DMR4:88654001	4	88654001	88660000	6000	1	5.40E-11	-0.38	57	0.95	Herc6	Proteolysis
DMR4:88688001	4	88688001	88689000	1000	1	7.30E-08	0.45	2	0.2	LOC500148;Ppm1k	
DMR4:89719001	4	89719001	89725000	6000	4	4.00E-08	-0.4	57	0.95	Gprin3	
DMR4:89788001	4	89788001	89789000	1000	1	2.60E-07	0.49	7	0.7	Gprin3	
DMR4:90876001	4	90876001	90877000	1000	1	2.80E-07	0.48	8	0.8	Snca	Transport
DMR4:91242001	4	91242001	91243000	1000	1	1.80E-07	-0.54	14	1.4	LOC108350812;Ccser1	
DMR4:91298001	4	91298001	91301000	3000	1	6.80E-18	0.65	21	0.7	Ccser1	
DMR4:91637001	4	91637001	91638000	1000	1	8.50E-07	0.56	7	0.7	Ccser1;LOC103692146	
DMR4:91833001	4	91833001	91840000	7000	1	4.50E-07	-0.26	87	1.24	Ccser1	
DMR4:92219001	4	92219001	92220000	1000	1	2.80E-09	-0.7	13	1.3	Ccser1	
DMR4:92324001	4	92324001	92333000	9000	2	1.70E-13	0.5	76	0.84	Ccser1	
DMR4:92390001	4	92390001	92392000	2000	1	4.40E-08	-0.26	13	0.65	Ccser1	
DMR4:92461001	4	92461001	92463000	2000	2	9.10E-08	-0.34	20	1	Ccser1	
DMR4:94058001	4	94058001	94061000	3000	1	5.60E-09	-0.33	22	0.73	Grid2	Receptor
DMR4:94200001	4	94200001	94201000	1000	1	7.70E-08	-0.56	7	0.7	Grid2	Receptor
DMR4:94911001	4	94911001	94912000	1000	1	2.70E-08	-0.66	8	0.8	Grid2	Receptor
DMR4:94959001	4	94959001	94964000	5000	1	1.90E-09	-0.31	49	0.98	Grid2	Receptor
DMR4:95032001	4	95032001	95033000	1000	1	5.30E-07	-0.54	10	1	Grid2	Receptor

DMR4:95147001	4	95147001	95148000	1000	1	5.20E-10	-0.39	13	1.3	Grid2	Receptor
DMR4:95245001	4	95245001	95249000	4000	1	4.50E-12	-0.41	32	0.8	Grid2	Receptor
DMR4:95278001	4	95278001	95284000	6000	2	7.10E-10	-0.33	60	1	Grid2	Receptor
DMR4:96464001	4	96464001	96468000	4000	1	7.70E-07	0.36	31	0.78	Tnip3	
DMR4:96822001	4	96822001	96825000	3000	1	2.90E-07	-0.29	28	0.93	RSA-14-44	Signaling
DMR4:97323001	4	97323001	97326000	3000	2	7.50E-12	0.55	48	1.6	RGD1564699	
DMR4:97653001	4	97653001	97657000	4000	1	3.60E-08	-0.32	53	1.32	Gng12	Signaling
DMR4:98216001	4	98216001	98222000	6000	2	1.40E-08	-0.36	72	1.2	Il23r	Receptor
DMR4:98287001	4	98287001	98292000	5000	1	1.80E-08	-0.48	36	0.72	Il23r	Receptor
DMR4:98603001	4	98603001	98609000	6000	1	3.60E-07	-0.34	69	1.15	Rpia	Metabolism
DMR4:99154001	4	99154001	99164000	10000	1	3.90E-10	-0.32	150	1.5	Krcc1;LOC102552663;LOC108350716	
DMR4:99267001	4	99267001	99268000	1000	1	3.30E-07	0.35	11	1.1	Rmnd5a	
DMR4:99389001	4	99389001	99395000	6000	1	7.00E-09	-0.26	80	1.33	Rnf103	Proteolysis
DMR4:99419001	4	99419001	99422000	3000	1	3.00E-07	-0.65	39	1.3	Rnf103	Proteolysis
DMR4:99687001	4	99687001	99688000	1000	1	4.30E-07	-0.57	10	1	Reep1	Transport
DMR4:99721001	4	99721001	99722000	1000	1	6.20E-09	0.49	17	1.7	Reep1	Transport
DMR4:100107001	4	100107001	100110000	3000	1	2.20E-07	0.64	51	1.7	Atoh8	Transcription
DMR4:100189001	4	100189001	100192000	3000	1	5.20E-08	-0.41	37	1.23	Usp39	Protease
DMR4:100290001	4	100290001	100292000	2000	1	2.00E-07	0.41	26	1.3	Ggcx;Mat2a	Metabolism
DMR4:100492001	4	100492001	100496000	4000	2	6.60E-13	0.49	55	1.38	Tgoln2;LOC102549526;Tcf7l1	Transcription
DMR4:100784001	4	100784001	100785000	1000	1	8.30E-07	0.5	3	0.3	Kcmf1	Proteolysis
DMR4:100978001	4	100978001	100979000	1000	1	2.50E-13	0.5	2	0.2	Dnah6	Cytoskeleton
DMR4:101003001	4	101003001	101005000	2000	1	2.40E-07	0.44	27	1.35	Dnah6	Cytoskeleton
DMR4:101138001	4	101138001	101142000	4000	1	2.20E-09	0.38	60	1.5	Dnah6	Cytoskeleton
DMR4:101147001	4	101147001	101149000	2000	1	4.40E-08	-0.6	29	1.45	Dnah6	Cytoskeleton
DMR4:101174001	4	101174001	101175000	1000	1	1.40E-08	0.4	12	1.2	Suclg1	Metabolism
DMR4:101212001	4	101212001	101213000	1000	1	3.00E-09	0.39	11	1.1	Suclg1	Metabolism
DMR4:108245001	4	108245001	108249000	4000	1	8.20E-07	-0.33	38	0.95	Ctnna2	Cytoskeleton
DMR4:108804001	4	108804001	108808000	4000	1	1.10E-07	-0.32	40	1	Ctnna2	Cytoskeleton
DMR4:108857001	4	108857001	108858000	1000	1	2.90E-07	-0.4	10	1	Ctnna2	Cytoskeleton
DMR4:108880001	4	108880001	108886000	6000	1	1.30E-07	-0.29	58	0.97	Ctnna2	Cytoskeleton
DMR4:108934001	4	108934001	108938000	4000	1	1.30E-10	-0.35	36	0.9	Ctnna2	Cytoskeleton
DMR4:109503001	4	109503001	109506000	3000	1	5.20E-08	-0.34	20	0.67	Reg1a	
DMR4:110731001	4	110731001	110733000	2000	1	1.30E-08	0.59	51	2.55	Lrrtm4	Receptor
DMR4:111115001	4	111115001	111116000	1000	1	9.80E-07	-0.43	7	0.7	Lrrtm4	Receptor
DMR4:112796001	4	112796001	112797000	1000	1	5.80E-08	0.34	22	2.2	Eva1a	
DMR4:113272001	4	113272001	113273000	1000	1	1.30E-07	0.5	16	1.6	Tacr1	Signaling
DMR4:113730001	4	113730001	113731000	1000	1	2.00E-07	0.37	9	0.9	Sema4f	Signaling
DMR4:113776001	4	113776001	113777000	1000	1	2.00E-08	-0.55	12	1.2	M1ap	
DMR4:113800001	4	113800001	113802000	2000	1	9.10E-10	-0.53	33	1.65	M1ap;LOC680041	
DMR4:113841001	4	113841001	113842000	1000	1	1.80E-07	-0.4	14	1.4	M1ap	
DMR4:115087001	4	115087001	115090000	3000	1	1.10E-10	0.68	23	0.77	Tet3	
DMR4:115105001	4	115105001	115106000	1000	1	4.80E-08	0.34	19	1.9	Tet3	
DMR4:115173001	4	115173001	115175000	2000	1	3.60E-08	0.38	53	2.65	Dguok	Signaling
DMR4:115758001	4	115758001	115762000	4000	1	1.30E-13	0.54	49	1.23	Dysf	Transport
DMR4:115809001	4	115809001	115815000	6000	1	1.90E-07	0.52	76	1.27	Dysf	Transport
DMR4:115852001	4	115852001	115853000	1000	1	1.80E-08	-0.45	20	2	Dysf	Transport
DMR4:116720001	4	116720001	116724000	4000	1	1.10E-10	-0.36	53	1.32	Exoc6b	Transport
DMR4:117478001	4	117478001	117486000	8000	2	8.00E-08	-0.32	92	1.15	Alms1;Nat8f3	Metabolism
DMR4:117954001	4	117954001	117955000	1000	1	9.20E-07	-0.41	15	1.5	Tgfa	Growth Factors
DMR4:118424001	4	118424001	118426000	2000	1	2.70E-07	0.38	15	0.75	Asprv1	
DMR4:118452001	4	118452001	118454000	2000	1	2.40E-08	0.4	58	2.9	Mxd1	Transcription
DMR4:118956001	4	118956001	118957000	1000	1	1.60E-08	0.28	11	1.1	Antxr1	Cytoskeleton
DMR4:119071001	4	119071001	119074000	3000	1	8.60E-13	0.53	44	1.47	Antxr1	Cytoskeleton
DMR4:119099001	4	119099001	119101000	2000	1	1.70E-07	-0.37	27	1.35	Antxr1	Cytoskeleton
DMR4:119515001	4	119515001	119519000	4000	1	1.60E-07	0.33	75	1.88	Aplf	
DMR4:119546001	4	119546001	119548000	2000	1	1.20E-08	-0.45	28	1.4	Aplf	
DMR4:119640001	4	119640001	119642000	2000	1	5.60E-09	0.44	28	1.4	LOC100910604;Efcc1	Translation
DMR4:119870001	4	119870001	119871000	1000	1	2.20E-08	-0.65	10	1	Hmces;LOC108350727	

DMR4:120565001	4	120565001	120566000	1000	1	9.40E-10	-0.62	17	1.7	Kbtbd12	Cytoskeleton
DMR4:121333001	4	121333001	121334000	1000	1	3.20E-11	0.55	10	1	Chchd6	
DMR4:121574001	4	121574001	121577000	3000	2	6.10E-10	-0.39	21	0.7	Chchd6	
DMR4:121835001	4	121835001	121842000	7000	1	8.40E-07	-0.26	63	0.9	Vom1r-ps77	
DMR4:121855001	4	121855001	121860000	5000	2	2.50E-07	-0.31	53	1.06	Vom1r-ps77	
DMR4:122079001	4	122079001	122085000	6000	1	9.00E-10	-0.35	64	1.07	Vom1r-ps84;Vom1r-ps85	
DMR4:122676001	4	122676001	122678000	2000	1	4.90E-07	0.64	28	1.4	Nup210	Transport
DMR4:122827001	4	122827001	122829000	2000	1	3.80E-09	0.44	15	0.75	Fbln2	Extracellular Matrix
DMR4:122872001	4	122872001	122874000	2000	1	9.30E-09	0.28	16	0.8	Fbln2	Extracellular Matrix
DMR4:123450001	4	123450001	123454000	4000	2	7.70E-10	0.45	90	2.25	Iqsec1	Transcription
DMR4:123914001	4	123914001	123915000	1000	1	3.00E-09	0.46	11	1.1	Fgd5	Transcription
DMR4:124515001	4	124515001	124517000	2000	1	8.80E-08	-0.42	25	1.25	Prickle2	Cytoskeleton
DMR4:125864001	4	125864001	125866000	2000	1	5.10E-07	-0.47	24	1.2	Magi1	
DMR4:127602001	4	127602001	127609000	7000	3	3.70E-10	-0.42	66	0.94	Suclg2	Metabolism
DMR4:127667001	4	127667001	127673000	6000	2	1.90E-15	-0.34	78	1.3	Suclg2	Metabolism
DMR4:127687001	4	127687001	127693000	6000	1	9.70E-08	-0.27	51	0.85	Suclg2	Metabolism
DMR4:129242001	4	129242001	129245000	3000	1	2.40E-09	-0.47	22	0.73	Fam19a4	
DMR4:129352001	4	129352001	129359000	7000	2	2.40E-17	-0.44	86	1.23	Fam19a4	
DMR4:129360001	4	129360001	129364000	4000	1	7.90E-11	-0.38	41	1.02	Fam19a4	
DMR4:129384001	4	129384001	129386000	2000	2	1.20E-15	0.61	20	1	Fam19a4	
DMR4:129529001	4	129529001	129530000	1000	1	5.80E-07	0.54	10	1	Tmf1	
DMR4:129837001	4	129837001	129839000	2000	1	3.30E-07	0.51	31	1.55	Frmd4b	
DMR4:130194001	4	130194001	130195000	1000	1	1.80E-07	0.56	4	0.4	Mitf	
DMR4:130404001	4	130404001	130411000	7000	1	2.80E-08	-0.33	90	1.29	Mitf	
DMR4:131505001	4	131505001	131506000	1000	1	7.60E-08	0.54	7	0.7	Foxp1	
DMR4:131575001	4	131575001	131577000	2000	1	5.30E-07	-0.38	28	1.4	Foxp1	
DMR4:131653001	4	131653001	131654000	1000	1	7.70E-07	0.51	9	0.9	Foxp1	
DMR4:131697001	4	131697001	131698000	1000	1	1.10E-10	0.45	14	1.4	Foxp1	
DMR4:131862001	4	131862001	131865000	3000	1	3.90E-11	0.47	44	1.47	Foxp1	
DMR4:133056001	4	133056001	133057000	1000	1	9.80E-07	-0.42	15	1.5	Shq1	
DMR4:133090001	4	133090001	133091000	1000	1	6.10E-08	-0.5	9	0.9	Shq1	
DMR4:133758001	4	133758001	133759000	1000	1	1.10E-08	0.42	18	1.8	Pdzrn3	
DMR4:133786001	4	133786001	133787000	1000	1	1.30E-07	0.36	6	0.6	Pdzrn3	
DMR4:133867001	4	133867001	133868000	1000	1	1.50E-07	0.45	6	0.6	Pdzrn3	
DMR4:135005001	4	135005001	135012000	7000	1	1.60E-09	-0.33	65	0.93	Cntn3	
DMR4:135083001	4	135083001	135084000	1000	1	1.20E-08	0.4	4	0.4	Cntn3	
DMR4:136622001	4	136622001	136624000	2000	1	4.80E-07	0.55	12	0.6	Cntn6	
DMR4:136628001	4	136628001	136632000	4000	1	4.50E-08	-0.36	43	1.07	Cntn6	
DMR4:136882001	4	136882001	136887000	5000	2	6.60E-11	-0.37	42	0.84	Cntn6	
DMR4:137770001	4	137770001	137777000	7000	1	2.80E-07	-0.23	77	1.1	Cntn4	Cytoskeleton
DMR4:138043001	4	138043001	138048000	5000	2	3.90E-10	-0.31	48	0.96	Cntn4	Cytoskeleton
DMR4:138645001	4	138645001	138648000	3000	1	6.70E-07	-0.38	28	0.93	Cntn4	Cytoskeleton
DMR4:138777001	4	138777001	138778000	1000	1	5.30E-07	-0.52	7	0.7	Cntn4	Cytoskeleton
DMR4:140148001	4	140148001	140150000	2000	1	9.30E-08	-0.47	16	0.8	Sumf1	
DMR4:140211001	4	140211001	140213000	2000	1	4.30E-09	-0.35	28	1.4	Sumf1	
DMR4:140536001	4	140536001	140539000	3000	1	5.20E-07	-0.52	29	0.97	Itpr1	Ion Channel
DMR4:140543001	4	140543001	140544000	1000	1	9.60E-07	-0.4	22	2.2	Itpr1	Ion Channel
DMR4:142554001	4	142554001	142555000	1000	1	3.80E-09	-0.62	12	1.2	Grm7	Signaling
DMR4:142717001	4	142717001	142721000	4000	2	1.90E-09	-0.36	38	0.95	Grm7	Signaling
DMR4:143000001	4	143000001	143004000	4000	1	3.40E-11	-0.47	30	0.75	Grm7	Signaling
DMR4:143100001	4	143100001	143107000	7000	2	2.40E-13	-0.39	75	1.07	Grm7	Signaling
DMR4:143375001	4	143375001	143376000	1000	1	8.40E-07	0.55	3	0.3	Grm7	Signaling
DMR4:144567001	4	144567001	144572000	5000	1	5.80E-09	-0.37	55	1.1	Rad18	Proteolysis
DMR4:144652001	4	144652001	144654000	2000	1	5.70E-09	0.33	41	2.05	LOC102548080;Srgap3	Signaling
DMR4:144713001	4	144713001	144714000	1000	1	2.50E-07	-0.44	9	0.9	Srgap3	Signaling
DMR4:144824001	4	144824001	144825000	1000	1	6.10E-07	0.6	8	0.8	Srgap3	Signaling
DMR4:144859001	4	144859001	144860000	1000	1	1.70E-07	-0.5	13	1.3	Srgap3	Signaling
DMR4:144981001	4	144981001	144983000	2000	1	1.50E-09	-0.56	32	1.6	LOC102548337;Thumpd3	Epigenetic
DMR4:145112001	4	145112001	145114000	2000	1	1.20E-07	0.5	14	0.7	LOC108350744;Lhfpl4	
DMR4:145243001	4	145243001	145245000	2000	1	2.40E-08	0.34	20	1	Mtmr14;Cpne9	Signaling
DMR4:145401001	4	145401001	145402000	1000	1	1.80E-08	0.55	6	0.6	LOC100361844;Jagn1	

DMR4:145731001	4	145731001	145733000	2000	1	2.30E-09	0.54	22	1.1	Atp2b2	Transport
DMR4:146254001	4	146254001	146256000	2000	1	1.00E-10	0.54	25	1.25	Slc6a1	Transport
DMR4:146672001	4	146672001	146673000	1000	1	1.10E-07	0.49	9	0.9	Atg7	Proteolysis
DMR4:146803001	4	146803001	146804000	1000	1	2.20E-07	0.36	17	1.7	Vgll4	Transcription
DMR4:146808001	4	146808001	146810000	2000	1	3.80E-07	0.36	42	2.1	Vgll4	Transcription
DMR4:147111001	4	147111001	147118000	7000	1	1.00E-07	-0.26	80	1.14	Syn2;LOC100365210;LOC100911724	Transport
DMR4:147498001	4	147498001	147499000	1000	1	4.70E-13	0.67	7	0.7	Tsen2;Mkrn2os	Transcription
DMR4:148129001	4	148129001	148138000	9000	2	7.70E-10	-0.35	105	1.17	Fam21c	
DMR4:148280001	4	148280001	148285000	5000	1	5.90E-14	-0.42	56	1.12	Zfand4;March8	
DMR4:148327001	4	148327001	148328000	1000	1	1.20E-08	0.38	8	0.8	8-Mar	
DMR4:148397001	4	148397001	148398000	1000	1	3.80E-07	0.43	11	1.1	March8;Alox5	Metabolism
DMR4:148512001	4	148512001	148513000	1000	1	3.00E-09	-0.73	4	0.4	Olr824	Receptor
DMR4:148556001	4	148556001	148558000	2000	2	8.40E-10	-0.38	19	0.95	Olr826	Receptor
DMR4:148600001	4	148600001	148606000	6000	2	1.40E-10	-0.47	68	1.13	Olr827	Receptor
DMR4:148642001	4	148642001	148645000	3000	1	7.20E-07	-0.41	14	0.47	Olr829	Receptor
DMR4:148673001	4	148673001	148674000	1000	1	1.20E-07	-0.48	18	1.8	Olr830	Receptor
DMR4:148679001	4	148679001	148682000	3000	1	2.60E-07	-0.32	25	0.83	Olr831	Receptor
DMR4:148686001	4	148686001	148688000	2000	1	5.00E-07	-0.38	21	1.05	Olr831	Receptor
DMR4:150071001	4	150071001	150072000	1000	1	2.30E-07	0.34	18	1.8	Rasgef1a	Transcription
DMR4:150083001	4	150083001	150086000	3000	1	9.90E-07	0.5	30	1	Rasgef1a	Transcription
DMR4:150474001	4	150474001	150478000	4000	1	1.70E-09	-0.43	62	1.55	Bms1;Zfp9	
DMR4:150574001	4	150574001	150575000	1000	1	2.40E-07	0.36	12	1.2	Ankrd26	
DMR4:150585001	4	150585001	150586000	1000	1	9.40E-09	-0.45	10	1	Ankrd26	
DMR4:150605001	4	150605001	150606000	1000	1	1.20E-08	0.42	4	0.4	Ankrd26	
DMR4:150651001	4	150651001	150652000	1000	1	7.20E-07	0.35	20	2	Cacna1c	Transport
DMR4:151165001	4	151165001	151166000	1000	1	7.60E-07	0.33	13	1.3	Cacna1c	Transport
DMR4:152079001	4	152079001	152080000	1000	1	9.60E-07	0.36	16	1.6	Erc1	Transport
DMR4:152184001	4	152184001	152187000	3000	1	8.80E-08	-0.34	24	0.8	Erc1	Transport
DMR4:152337001	4	152337001	152342000	5000	1	3.60E-08	-0.36	38	0.76	Erc1;LOC102552689	Transport
DMR4:152750001	4	152750001	152754000	4000	2	1.70E-07	0.51	64	1.6	B4galnt3	Golgi
DMR4:153393001	4	153393001	153394000	1000	1	8.90E-08	0.51	6	0.6	Bcl2l13	
DMR4:153494001	4	153494001	153495000	1000	1	1.20E-07	0.49	17	1.7	Mical3	
DMR4:153601001	4	153601001	153603000	2000	1	4.90E-10	0.45	35	1.75	Mical3	
DMR4:153829001	4	153829001	153833000	4000	1	1.30E-08	-0.29	74	1.85	Usp18;LOC102553072	Protease
DMR4:153954001	4	153954001	153956000	2000	2	5.80E-14	0.56	35	1.75	Iqsec3	Transcription
DMR4:154230001	4	154230001	154236000	6000	1	4.80E-07	-0.39	48	0.8	Mug2	Protease; Proteolysis
DMR4:154876001	4	154876001	154878000	2000	1	8.80E-11	-0.35	31	1.55	Cpamd8	
DMR4:154943001	4	154943001	154949000	6000	1	2.10E-09	-0.4	66	1.1	Cpamd8;LOC102554538	
DMR4:154955001	4	154955001	154960000	5000	1	9.00E-10	-0.34	36	0.72	Cpamd8	
DMR4:154989001	4	154989001	154994000	5000	1	9.00E-14	-0.45	45	0.9	Cpamd8;LOC108350758	
DMR4:155077001	4	155077001	155083000	6000	3	3.70E-10	-0.43	68	1.13	M6pr	Transport
DMR4:155426001	4	155426001	155429000	3000	1	5.00E-10	0.59	37	1.23	Gdf3;Dppa3	Growth Factors
DMR4:155675001	4	155675001	155677000	2000	1	5.80E-07	-0.5	22	1.1	Foxj2;C3ar1	Signaling
DMR4:155788001	4	155788001	155791000	3000	1	5.20E-07	-0.29	28	0.93	Clec4a2	Transport
DMR4:155798001	4	155798001	155803000	5000	2	2.10E-10	-0.39	42	0.84	Clec4a2	Transport
DMR4:155810001	4	155810001	155813000	3000	1	6.70E-07	-0.28	27	0.9	Clec4a2	Transport
DMR4:155933001	4	155933001	155934000	1000	1	1.50E-07	-0.49	10	1	Clec4a3	Transport
DMR4:155940001	4	155940001	155942000	2000	1	1.90E-07	0.33	24	1.2	Clec4a1	Transport
DMR4:156132001	4	156132001	156139000	7000	2	3.80E-10	-0.36	103	1.47	Clec4b2	Transport
DMR4:156322001	4	156322001	156324000	2000	1	3.60E-07	-0.4	20	1	LOC108350823;Vom2r48	Signaling
DMR4:156488001	4	156488001	156493000	5000	2	2.50E-07	-0.38	36	0.72	Vom2r50	Signaling
DMR4:156540001	4	156540001	156543000	3000	1	9.80E-07	-0.36	25	0.83	Vom2r51	Signaling
DMR4:156828001	4	156828001	156830000	2000	1	1.80E-10	-0.4	35	1.75	RGD1307916	
DMR4:156915001	4	156915001	156919000	4000	1	4.00E-09	-0.5	16	0.4	RGD1307916	
DMR4:156932001	4	156932001	156936000	4000	1	6.10E-07	-0.43	38	0.95	RGD1307916	
DMR4:156947001	4	156947001	156948000	1000	1	1.10E-07	-0.52	9	0.9	RGD1307916	
DMR4:157083001	4	157083001	157085000	2000	1	2.10E-17	0.62	24	1.2	Clstn3;LOC102553636	Transport
DMR4:157086001	4	157086001	157087000	1000	1	7.60E-20	0.78	0	0	Clstn3;LOC102553636	Transport
DMR4:157105001	4	157105001	157107000	2000	1	8.20E-07	0.41	29	1.45	LOC102553636;C1rl	Protease
DMR4:157145001	4	157145001	157147000	2000	1	3.80E-07	0.39	17	0.85	C1r;C1s	Protease
DMR4:157515001	4	157515001	157518000	3000	1	6.00E-07	0.35	32	1.07	Pianp;Zfp384	

DMR4:157573001	4	157573001	157574000	1000	1	8.90E-08	0.34	14	1.4	LOC102554095;Ing4;Acrbp;Lpar5	Epigenetic;Signaling
DMR4:157663001	4	157663001	157667000	4000	1	2.30E-10	0.45	84	2.1	Nop2;Iffo1;Gapdh	Metabolism
DMR4:157743001	4	157743001	157745000	2000	1	2.60E-08	0.4	22	1.1	Vamp1;Tapbpl;Cd27;LOC102555485	Immune
DMR4:157884001	4	157884001	157885000	1000	1	8.60E-10	0.42	14	1.4	Tnfrsf1a;Plekhg6	Receptor
DMR4:157971001	4	157971001	157973000	2000	1	6.60E-10	0.42	39	1.95	Cd9	
DMR4:158085001	4	158085001	158087000	2000	1	8.70E-10	0.45	28	1.4	Vwf	
DMR4:158125001	4	158125001	158127000	2000	1	4.90E-09	0.46	30	1.5	Vwf	
DMR4:158212001	4	158212001	158213000	1000	1	7.90E-07	0.46	12	1.2	Vwf;Ano2	
DMR4:159287001	4	159287001	159288000	1000	1	6.00E-08	-0.49	16	1.6	Kcna6	Transport
DMR4:159362001	4	159362001	159363000	1000	1	1.10E-08	-0.52	18	1.8	LOC103690194;Ndufa9	Metabolism
DMR4:159648001	4	159648001	159652000	4000	1	8.80E-07	-0.39	51	1.27	Tigar	Signaling
DMR4:160042001	4	160042001	160044000	2000	1	6.30E-09	0.43	30	1.5	Parp11	
DMR4:160165001	4	160165001	160167000	2000	1	3.70E-07	0.52	19	0.95	Cracr2a	
DMR4:160322001	4	160322001	160323000	1000	1	8.20E-07	0.33	20	2	Prmt8	Golgi
DMR4:160548001	4	160548001	160554000	6000	1	7.10E-07	0.35	133	2.22	Tspan9	
DMR4:160642001	4	160642001	160644000	2000	1	2.10E-09	0.5	28	1.4	Tspan9	
DMR4:162735001	4	162735001	162739000	4000	1	1.30E-07	-0.26	57	1.43	Ly49i7	
DMR4:163076001	4	163076001	163079000	3000	1	5.60E-09	-0.34	26	0.87	RGD1564770	
DMR4:163330001	4	163330001	163337000	7000	2	1.70E-07	-0.34	73	1.04	Klre1	
DMR4:163340001	4	163340001	163341000	1000	1	4.60E-12	0.59	8	0.8	Klre1	
DMR4:163371001	4	163371001	163375000	4000	2	1.70E-10	-0.39	43	1.07	Klrd1	
DMR4:163376001	4	163376001	163382000	6000	1	8.70E-09	-0.38	49	0.82	Klrd1	
DMR4:163911001	4	163911001	163915000	4000	1	9.00E-08	-0.36	36	0.9	Ly49si2	
DMR4:164381001	4	164381001	164384000	3000	1	4.50E-08	-0.38	30	1	Klra22	
DMR4:164472001	4	164472001	164474000	2000	1	1.20E-07	-0.47	13	0.65	Ly49s4	
DMR4:165443001	4	165443001	165449000	6000	1	2.60E-11	-0.44	50	0.83	Klra1	
DMR4:165463001	4	165463001	165466000	3000	1	3.50E-09	-0.31	31	1.03	Klra1	
DMR4:165776001	4	165776001	165783000	7000	2	1.80E-09	-0.33	62	0.89	Tas2r107;Tas2r106	Receptor
DMR4:165970001	4	165970001	165976000	6000	1	3.30E-08	-0.34	67	1.12	LOC500342;Prb1	
DMR4:166272001	4	166272001	166274000	2000	1	1.90E-09	-0.33	29	1.45	Prp15	Signaling
DMR4:166928001	4	166928001	166933000	5000	1	7.70E-09	-0.31	49	0.98	Tas2r102	Receptor
DMR4:167077001	4	167077001	167078000	1000	1	5.40E-09	-0.38	13	1.3	Tas2r125	Receptor
DMR4:167463001	4	167463001	167469000	6000	1	4.40E-08	-0.33	48	0.8	Grpcb	Signaling
DMR4:167487001	4	167487001	167490000	3000	1	3.10E-07	-0.41	17	0.57	Grpcb	Signaling
DMR4:168194001	4	168194001	168196000	2000	1	5.40E-09	0.39	35	1.75	LOC108350780;Lrp6	Binding Proteins
DMR4:168301001	4	168301001	168302000	1000	1	2.00E-08	0.34	12	1.2	Lrp6	Binding Proteins
DMR4:168358001	4	168358001	168359000	1000	1	1.90E-08	0.4	21	2.1	Mansc1	
DMR4:168361001	4	168361001	168367000	6000	1	1.00E-08	0.38	112	1.87	Mansc1	
DMR4:169012001	4	169012001	169013000	1000	1	7.90E-11	0.56	12	1.2	Fam234b;Gsg1	Cytoskeleton
DMR4:169645001	4	169645001	169647000	2000	1	1.30E-07	0.37	29	1.45	Grin2b	Receptor
DMR4:169721001	4	169721001	169723000	2000	1	5.60E-07	0.54	18	0.9	Grin2b	Receptor
DMR4:169758001	4	169758001	169759000	1000	1	2.90E-08	0.4	4	0.4	Grin2b	Receptor
DMR4:170548001	4	170548001	170550000	2000	1	1.70E-07	0.34	25	1.25	Atf7ip	Transcription
DMR4:170612001	4	170612001	170614000	2000	1	5.30E-12	0.44	28	1.4	Plbd1	Metabolism
DMR4:170652001	4	170652001	170655000	3000	1	2.40E-07	-0.4	17	0.57	Gucy2c	Signaling
DMR4:170714001	4	170714001	170715000	1000	1	8.00E-13	-0.68	19	1.9	Gucy2c;LOC103692245	Signaling
DMR4:171379001	4	171379001	171381000	2000	1	1.40E-07	-0.45	43	2.15	Ptpro	Receptor
DMR4:171639001	4	171639001	171641000	2000	1	3.00E-07	-0.36	43	2.15	Eps8	Cytoskeleton
DMR4:171781001	4	171781001	171783000	2000	1	8.20E-08	0.35	45	2.25	Dera	Metabolism
DMR4:172041001	4	172041001	172042000	1000	1	2.60E-07	-0.45	15	1.5	Slc15a5	Transport
DMR4:172047001	4	172047001	172049000	2000	1	6.40E-07	-0.63	29	1.45	Slc15a5	Transport
DMR4:172111001	4	172111001	172115000	4000	1	1.20E-08	-0.55	49	1.23	Mgst1	Transport
DMR4:173827001	4	173827001	173828000	1000	1	4.70E-08	-0.36	12	1.2	Pik3c2g	Signaling
DMR4:173875001	4	173875001	173884000	9000	2	7.40E-11	-0.33	151	1.68	Pik3c2g	Signaling
DMR4:173904001	4	173904001	173910000	6000	3	8.60E-13	-0.45	73	1.22	Pik3c2g	Signaling
DMR4:173921001	4	173921001	173925000	4000	1	4.90E-08	-0.31	37	0.92	Pik3c2g	Signaling
DMR4:173976001	4	173976001	173982000	6000	1	6.40E-07	-0.28	63	1.05	Pik3c2g	Signaling
DMR4:174081001	4	174081001	174082000	1000	1	2.50E-07	0.34	9	0.9	Pik3c2g	Signaling
DMR4:174158001	4	174158001	174160000	2000	1	5.00E-07	0.4	41	2.05	Plcz1	Metabolism

DMR4:174682001	4	174682001	174684000	2000	1	3.10E-15	0.62	28	1.4	Plekha5	
DMR4:174843001	4	174843001	174848000	5000	1	6.70E-07	-0.37	59	1.18	Aebp2	
DMR4:174852001	4	174852001	174856000	4000	1	2.50E-09	0.73	51	1.27	Aebp2	
DMR4:174866001	4	174866001	174868000	2000	1	3.00E-07	0.43	17	0.85	Aebp2	
DMR4:175673001	4	175673001	175674000	1000	1	1.20E-18	-0.93	13	1.3	Pde3a	Signaling
DMR4:175822001	4	175822001	175827000	5000	1	2.20E-10	-0.3	44	0.88	LOC103692254;Slco1b2	Transport
DMR4:175974001	4	175974001	175978000	4000	1	6.60E-09	-0.34	35	0.88	Slco1a2	
DMR4:176165001	4	176165001	176166000	1000	1	8.70E-10	-0.33	17	1.7	Slco1a1	Transport
DMR4:176167001	4	176167001	176170000	3000	1	2.80E-07	-0.31	27	0.9	Slco1a1	Transport
DMR4:176495001	4	176495001	176500000	5000	3	5.20E-09	-0.35	43	0.86	Slco1a5;Iapp	Transport;Hormone
DMR4:176998001	4	176998001	177001000	3000	1	3.70E-09	0.52	30	1	Cmas	Metabolism
DMR4:177163001	4	177163001	177164000	1000	1	5.20E-07	0.33	25	2.5	St8sia1	Transport
DMR4:177242001	4	177242001	177244000	2000	1	8.30E-08	-0.4	24	1.2	C2cd5	
DMR4:178186001	4	178186001	178188000	2000	1	1.10E-08	-0.61	39	1.95	Sox5	
DMR4:178212001	4	178212001	178215000	3000	1	8.00E-07	0.34	42	1.4	Sox5	
DMR4:178522001	4	178522001	178525000	3000	1	3.30E-08	-0.5	41	1.37	Sox5	
DMR4:178665001	4	178665001	178666000	1000	1	7.80E-08	-0.52	15	1.5	Sox5	
DMR4:178972001	4	178972001	178974000	2000	1	4.20E-07	0.6	47	2.35	Sox5	
DMR4:179404001	4	179404001	179406000	2000	1	3.80E-08	0.49	25	1.25	Lrmp	
DMR4:179440001	4	179440001	179443000	3000	1	6.60E-07	0.32	152	5.07	Lrmp;Casc1	
DMR4:179796001	4	179796001	179802000	6000	1	3.30E-09	-0.31	84	1.4	Lmntd1;LOC108350787	
DMR4:179816001	4	179816001	179819000	3000	1	1.30E-07	0.37	48	1.6	Lmntd1	
DMR4:179896001	4	179896001	179901000	5000	1	8.00E-09	0.36	112	2.24	Lmntd1;LOC108350789;Tuba3b	
DMR4:180469001	4	180469001	180473000	4000	1	4.30E-09	0.46	58	1.45	Itpr2	Ion Channel
DMR4:180610001	4	180610001	180611000	1000	1	6.20E-07	-0.42	22	2.2	Itpr2	Ion Channel
DMR4:180803001	4	180803001	180805000	2000	1	3.00E-07	-0.41	32	1.6	Itpr2	Ion Channel
DMR4:181296001	4	181296001	181298000	2000	1	1.20E-10	0.55	66	3.3	Ppfibp1	
DMR4:181465001	4	181465001	181466000	1000	1	9.40E-08	0.34	16	1.6	Mrps35;Mansc4	Translation
DMR4:181666001	4	181666001	181669000	3000	1	1.30E-13	0.52	127	4.23	Pthlh	Hormone
DMR4:181886001	4	181886001	181888000	2000	2	3.50E-10	0.4	52	2.6	Ccdc91	
DMR4:182017001	4	182017001	182020000	3000	2	4.70E-12	0.44	41	1.37	Ccdc91	
DMR4:182034001	4	182034001	182035000	1000	1	7.90E-08	-0.37	17	1.7	Ccdc91	
DMR4:183607001	4	183607001	183610000	3000	1	6.10E-10	-0.49	24	0.8	Dennd5b	
DMR4:183646001	4	183646001	183652000	6000	2	4.00E-11	-0.36	77	1.28	Etfbkmt	Epigenetic
DMR4:184098001	4	184098001	184104000	6000	1	6.50E-08	-0.43	68	1.13	Bicd1;LOC100362344	Translation
DMR5:563001	5	563001	564000	1000	1	3.30E-11	-0.58	7	0.7	Cct6a-ps3	
DMR5:653001	5	653001	658000	5000	1	1.10E-12	-0.35	54	1.08	Crispld1	Immune
DMR5:1737001	5	1737001	1741000	4000	1	1.30E-07	-0.36	40	1	Rps4x-ps8;LOC679619	
DMR5:2011001	5	2011001	2012000	1000	1	7.20E-08	-0.44	8	0.8	Tmem70	
DMR5:2481001	5	2481001	2482000	1000	1	2.00E-09	0.59	3	0.3	Stau2;LOC102555040	
DMR5:2614001	5	2614001	2617000	3000	1	2.00E-09	0.51	88	2.93	Rdh10	
DMR5:3060001	5	3060001	3062000	2000	1	1.60E-08	-0.4	40	2	Kcnb2	Transport
DMR5:3077001	5	3077001	3080000	3000	1	4.80E-10	-0.57	18	0.6	Kcnb2	Transport
DMR5:3131001	5	3131001	3134000	3000	1	6.30E-07	0.35	36	1.2	Kcnb2	Transport
DMR5:3167001	5	3167001	3175000	8000	2	8.30E-15	-0.46	89	1.11	Kcnb2	Transport
DMR5:3213001	5	3213001	3214000	1000	1	5.60E-07	-0.52	5	0.5	Kcnb2	Transport
DMR5:3269001	5	3269001	3271000	2000	1	3.20E-09	-0.31	23	1.15	Kcnb2	Transport
DMR5:3301001	5	3301001	3303000	2000	1	5.10E-07	0.47	18	0.9	Kcnb2	Transport
DMR5:3369001	5	3369001	3374000	5000	1	9.50E-07	-0.29	44	0.88	Kcnb2	Transport
DMR5:3418001	5	3418001	3419000	1000	1	6.20E-10	0.61	28	2.8	Kcnb2	Transport
DMR5:4284001	5	4284001	4287000	3000	1	4.10E-07	-0.51	17	0.57	Eya1	
DMR5:4290001	5	4290001	4294000	4000	1	4.90E-10	-0.38	38	0.95	Eya1	
DMR5:4444001	5	4444001	4445000	1000	1	3.50E-08	0.55	5	0.5	Eya1	
DMR5:4450001	5	4450001	4452000	2000	1	6.70E-07	0.52	19	0.95	Eya1	
DMR5:4884001	5	4884001	4888000	4000	1	1.70E-07	-0.29	29	0.72	Rbpjl2	Transcription
DMR5:5549001	5	5549001	5552000	3000	1	8.20E-07	-0.38	41	1.37	Ncoa2	Epigenetic
DMR5:5561001	5	5561001	5562000	1000	1	3.60E-12	0.45	18	1.8	Ncoa2	Epigenetic
DMR5:5980001	5	5980001	5982000	2000	1	7.70E-07	-0.3	37	1.85	Slco5a1	Transport
DMR5:6955001	5	6955001	6964000	9000	4	1.80E-09	-0.37	79	0.88	RGD1564053	
DMR5:6988001	5	6988001	6994000	6000	1	2.10E-10	-0.51	57	0.95	RGD1564053	

DMR5:7045001	5	7045001	7046000	1000	1	2.70E-07	-0.35	22	2.2	RGD1564053	
DMR5:7160001	5	7160001	7163000	3000	1	1.90E-07	0.42	22	0.73	RGD1564053	
DMR5:7318001	5	7318001	7319000	1000	1	6.50E-08	-0.49	9	0.9	RGD1564053	
DMR5:7329001	5	7329001	7330000	1000	1	1.90E-07	0.35	13	1.3	RGD1564053	
DMR5:7400001	5	7400001	7402000	2000	1	3.70E-07	-0.42	12	0.6	RGD1564053	
DMR5:7474001	5	7474001	7475000	1000	1	7.40E-11	0.44	3	0.3	RGD1564053	
DMR5:8276001	5	8276001	8281000	5000	2	2.80E-13	-0.38	37	0.74	Cpa6	Protease
DMR5:8297001	5	8297001	8302000	5000	2	1.30E-08	-0.4	43	0.86	Cpa6	Protease
DMR5:8510001	5	8510001	8511000	1000	1	1.80E-07	-0.46	16	1.6	Cpa6	Protease
DMR5:8785001	5	8785001	8790000	5000	1	5.70E-08	-0.57	66	1.32	Cspp1	Cell Cycle
DMR5:8891001	5	8891001	8892000	1000	1	4.80E-07	0.41	33	3.3	Cops5	Translation
DMR5:9005001	5	9005001	9008000	3000	1	5.10E-09	-0.55	28	0.93	Mcmdc2;LOC102555954	Transcription
DMR5:10113001	5	10113001	10117000	4000	1	3.40E-08	-0.33	42	1.05	Sntg1	
DMR5:10267001	5	10267001	10270000	3000	1	4.30E-08	-0.32	20	0.67	Sntg1	
DMR5:10421001	5	10421001	10423000	2000	1	1.30E-07	-0.3	19	0.95	Sntg1	
DMR5:12456001	5	12456001	12461000	5000	2	5.00E-07	-0.32	38	0.76	St18	Transcription
DMR5:12495001	5	12495001	12496000	1000	1	1.40E-09	0.59	11	1.1	St18	Transcription
DMR5:12738001	5	12738001	12740000	2000	1	4.10E-10	-0.35	15	0.75	St18	Transcription
DMR5:14401001	5	14401001	14407000	6000	1	2.30E-07	-0.28	52	0.87	Rgs20	Signaling
DMR5:14485001	5	14485001	14486000	1000	1	1.00E-06	-0.43	12	1.2	Rgs20	Signaling
DMR5:14584001	5	14584001	14585000	1000	1	2.80E-07	-0.6	14	1.4	Lypla1;LOC103692292	Metabolism
DMR5:15082001	5	15082001	15083000	1000	1	8.90E-07	-0.43	7	0.7	Cyld-ps1	
DMR5:15776001	5	15776001	15781000	5000	1	1.80E-12	-0.42	47	0.94	Xkr4	
DMR5:15995001	5	15995001	15996000	1000	1	6.50E-07	0.38	10	1	Xkr4	
DMR5:16089001	5	16089001	16095000	6000	1	3.80E-07	-0.31	48	0.8	Xkr4	
DMR5:16146001	5	16146001	16148000	2000	1	3.30E-07	-0.3	19	0.95	Xkr4	
DMR5:16397001	5	16397001	16403000	6000	2	2.50E-10	-0.38	60	1	Tmem68	
DMR5:16498001	5	16498001	16501000	3000	1	1.40E-07	0.37	28	0.93	RGD1564981	
DMR5:16504001	5	16504001	16506000	2000	1	4.10E-07	-0.5	17	0.85	RGD1564981	
DMR5:16756001	5	16756001	16763000	7000	1	1.70E-11	-0.4	78	1.11	Mos;LOC685119;LOC100364265	Signaling;Epigenetic
DMR5:16808001	5	16808001	16819000	11000	2	1.70E-09	-0.34	126	1.15	Plag1	Transcription
DMR5:16823001	5	16823001	16825000	2000	1	7.50E-09	0.43	30	1.5	Plag1	Transcription
DMR5:18979001	5	18979001	18985000	6000	1	5.90E-08	-0.3	61	1.02	RGD1565372	
DMR5:21315001	5	21315001	21316000	1000	1	1.30E-07	0.39	10	1	Car8	
DMR5:21866001	5	21866001	21868000	2000	1	3.40E-07	-0.39	37	1.85	Chd7;LOC108350929	
DMR5:21872001	5	21872001	21874000	2000	1	4.00E-07	-0.39	37	1.85	Chd7;LOC108350929	
DMR5:21917001	5	21917001	21918000	1000	1	4.40E-07	0.41	34	3.4	Chd7	
DMR5:21924001	5	21924001	21927000	3000	1	2.00E-07	0.29	63	2.1	Chd7	
DMR5:22480001	5	22480001	22481000	1000	1	5.90E-09	-0.4	16	1.6	Clvs1	Transport
DMR5:22507001	5	22507001	22515000	8000	3	2.30E-14	-0.38	92	1.15	Clvs1	Transport
DMR5:22516001	5	22516001	22517000	1000	1	7.70E-09	-0.57	14	1.4	Clvs1	Transport
DMR5:22680001	5	22680001	22683000	3000	1	9.00E-13	-0.44	49	1.63	Asph	Golgi
DMR5:24605001	5	24605001	24606000	1000	1	1.20E-10	0.44	17	1.7	Esrp1	Translation
DMR5:24881001	5	24881001	24886000	5000	1	2.30E-13	-0.48	81	1.62	RGD1559441	
DMR5:24906001	5	24906001	24907000	1000	1	1.10E-10	-0.5	16	1.6	RGD1559441	
DMR5:25070001	5	25070001	25077000	7000	3	3.50E-09	-0.42	75	1.07	RGD1559904	
DMR5:25432001	5	25432001	25433000	1000	1	2.00E-07	0.29	3	0.3	Cdh17	Cytoskeleton
DMR5:26558001	5	26558001	26559000	1000	1	2.20E-07	-0.42	6	0.6	Triqk	
DMR5:27998001	5	27998001	28002000	4000	1	5.20E-12	-0.43	40	1	Slc26a7	Transport
DMR5:28013001	5	28013001	28018000	5000	1	1.50E-07	-0.35	49	0.98	Slc26a7	Transport
DMR5:28040001	5	28040001	28045000	5000	1	9.30E-07	-0.24	47	0.94	Slc26a7	Transport
DMR5:28067001	5	28067001	28071000	4000	1	1.90E-07	-0.35	36	0.9	Slc26a7	Transport
DMR5:28242001	5	28242001	28248000	6000	2	1.60E-09	-0.36	52	0.87	Lrrc69	Cytoskeleton
DMR5:28591001	5	28591001	28596000	5000	1	1.70E-10	-0.36	31	0.62	Necab1;LOC108350937	
DMR5:28613001	5	28613001	28618000	5000	2	8.10E-08	-0.31	50	1	Necab1	
DMR5:28661001	5	28661001	28666000	5000	1	1.30E-10	-0.38	52	1.04	Necab1	
DMR5:28738001	5	28738001	28739000	1000	1	8.10E-08	0.47	13	1.3	Necab1	
DMR5:30651001	5	30651001	30658000	7000	1	3.80E-10	-0.32	79	1.13	RGD1565988	
DMR5:32597001	5	32597001	32602000	5000	1	3.40E-08	-0.38	40	0.8	Cnbd1	
DMR5:32662001	5	32662001	32664000	2000	1	6.10E-07	0.56	9	0.45	Cnbd1	

DMR5:32771001	5	32771001	32776000	5000	1	8.80E-07	-0.29	47	0.94	Cnbd1	
DMR5:32922001	5	32922001	32926000	4000	1	3.60E-08	-0.33	40	1	Cnbd1	
DMR5:33263001	5	33263001	33266000	3000	1	2.90E-07	-0.72	19	0.63	Cngb3	Ion Channel
DMR5:33472001	5	33472001	33477000	5000	2	9.00E-15	-0.45	64	1.28	Cngb3	Ion Channel
DMR5:33532001	5	33532001	33533000	1000	1	6.10E-07	0.54	8	0.8	Cpne3	
DMR5:34227001	5	34227001	34230000	3000	1	1.60E-07	-0.4	25	0.83	Nkain3	
DMR5:34318001	5	34318001	34320000	2000	1	3.60E-11	-0.51	19	0.95	Nkain3	
DMR5:34453001	5	34453001	34459000	6000	2	7.70E-08	-0.29	69	1.15	Nkain3	
DMR5:34681001	5	34681001	34682000	1000	1	1.30E-15	-0.77	12	1.2	Nkain3	
DMR5:34779001	5	34779001	34784000	5000	1	3.20E-10	-0.34	38	0.76	Nkain3	
DMR5:35905001	5	35905001	35914000	9000	3	9.10E-09	-0.43	94	1.04	Tstd3;Usp45	Transport;Protease
DMR5:39071001	5	39071001	39075000	4000	1	1.10E-07	-0.35	58	1.45	Klhl32	
DMR5:39103001	5	39103001	39105000	2000	1	8.80E-08	-0.32	15	0.75	Klhl32	
DMR5:39192001	5	39192001	39193000	1000	1	3.30E-07	0.46	1	0.1	Klhl32	
DMR5:39203001	5	39203001	39206000	3000	1	1.10E-07	0.41	38	1.27	Klhl32	
DMR5:40857001	5	40857001	40858000	1000	1	9.00E-11	0.64	6	0.6	Manea	
DMR5:47928001	5	47928001	47930000	2000	1	7.30E-08	-0.44	19	0.95	Mdn1	
DMR5:48327001	5	48327001	48328000	1000	1	8.60E-08	0.58	9	0.9	Gabrr2	Ion Channel
DMR5:48758001	5	48758001	48764000	6000	1	4.90E-07	-0.29	66	1.1	Rngtt	Translation
DMR5:49298001	5	49298001	49302000	4000	1	6.70E-07	-0.24	42	1.05	Cnr1	Signaling
DMR5:50158001	5	50158001	50164000	6000	3	1.20E-11	-0.37	73	1.22	Cfap206	
DMR5:50187001	5	50187001	50189000	2000	1	2.40E-07	0.47	32	1.6	Cfap206	
DMR5:50197001	5	50197001	50205000	8000	2	3.20E-13	-0.38	74	0.92	Cfap206;RGD1563056	
DMR5:50284001	5	50284001	50286000	2000	1	2.10E-07	-0.59	18	0.9	Zfp292	Transcription
DMR5:50374001	5	50374001	50375000	1000	1	7.30E-09	0.54	54	5.4	Cga;LOC102547206;LOC108350953	Hormone
DMR5:50582001	5	50582001	50586000	4000	1	1.70E-07	-0.47	62	1.55	Mob3b	Signaling
DMR5:51540001	5	51540001	51545000	5000	3	7.60E-12	-0.34	60	1.2	Lingo2	
DMR5:56522001	5	56522001	56523000	1000	1	4.50E-07	-0.37	20	2	Ddx58	
DMR5:56556001	5	56556001	56559000	3000	1	9.00E-08	-0.58	73	2.43	Topors;LOC102552818;Ndufb6	Proteolysis;Metabolism
DMR5:57303001	5	57303001	57304000	1000	1	1.60E-08	0.46	12	1.2	Nfx1	Transcription
DMR5:57362001	5	57362001	57364000	2000	1	6.60E-07	0.31	35	1.75	Aqp7	Transport
DMR5:58085001	5	58085001	58086000	1000	1	7.40E-12	0.57	6	0.6	Cntfr	Receptor
DMR5:58114001	5	58114001	58118000	4000	1	6.00E-07	0.48	86	2.15	Dctn3;Arid3c;Sigmar1	Cytoskeleton;Transcription;Receptor
DMR5:58175001	5	58175001	58176000	1000	1	1.90E-07	-0.42	12	1.2	Ccl27;LOC102547621;Ccl19;LOC689481	Growth Factors;Growth Factors
DMR5:58275001	5	58275001	58281000	6000	1	7.00E-11	-0.37	51	0.85	Fam205a	
DMR5:58588001	5	58588001	58590000	2000	1	1.60E-08	-0.31	19	0.95	Unc13b	
DMR5:58620001	5	58620001	58621000	1000	1	6.20E-07	-0.6	9	0.9	Unc13b	
DMR5:58760001	5	58760001	58761000	1000	1	8.50E-08	-0.47	18	1.8	Atp8b5p	Transport
DMR5:58788001	5	58788001	58789000	1000	1	3.20E-07	-0.48	11	1.1	Atp8b5p	Transport
DMR5:58941001	5	58941001	58943000	2000	1	3.00E-07	0.36	42	2.1	LOC103692345;Tesk1;Cd72	
DMR5:58957001	5	58957001	58961000	4000	3	1.70E-09	-0.35	50	1.25	Cd72;RGD1560723	
DMR5:59040001	5	59040001	59042000	2000	1	2.60E-08	0.42	35	1.75	Tln1	
DMR5:59176001	5	59176001	59179000	3000	1	4.40E-10	0.41	47	1.57	Tmem8b	Cytoskeleton
DMR5:59296001	5	59296001	59297000	1000	1	1.90E-07	0.6	20	2	Olr838	Receptor
DMR5:59301001	5	59301001	59304000	3000	1	1.90E-08	-0.44	16	0.53	Olr838;Olr839	Receptor
DMR5:59554001	5	59554001	59559000	5000	1	5.40E-08	-0.6	59	1.18	Gne	Transcription
DMR5:60048001	5	60048001	60050000	2000	1	1.70E-09	0.44	26	1.3	Pax5	
DMR5:60057001	5	60057001	60058000	1000	1	4.60E-07	0.49	9	0.9	Pax5	
DMR5:60067001	5	60067001	60069000	2000	1	2.30E-14	0.54	37	1.85	Pax5	
DMR5:60588001	5	60588001	60592000	4000	1	3.50E-08	0.46	84	2.1	Polr1e;Fbxo10	Transcription;Proteolysis
DMR5:60610001	5	60610001	60611000	1000	1	9.10E-14	0.49	16	1.6	Fbxo10	Proteolysis
DMR5:60619001	5	60619001	60622000	3000	1	3.50E-07	0.31	37	1.23	Fbxo10	Proteolysis
DMR5:60638001	5	60638001	60642000	4000	1	3.20E-07	-0.56	66	1.65	Fbxo10	Proteolysis
DMR5:60982001	5	60982001	60988000	6000	1	2.60E-08	0.46	102	1.7	Shb	
DMR5:60989001	5	60989001	60990000	1000	1	6.70E-07	0.59	12	1.2	Shb	
DMR5:60992001	5	60992001	60995000	3000	1	4.00E-07	0.41	66	2.2	Shb	

DMR5:61475001	5	61475001	61476000	1000	1	5.00E-09	0.6	3	0.3	RGD1305807;Ccdc180	
DMR5:61529001	5	61529001	61532000	3000	2	1.80E-08	-0.47	24	0.8	Ccdc180	
DMR5:61533001	5	61533001	61535000	2000	1	2.10E-07	-0.3	29	1.45	Ccdc180	
DMR5:61536001	5	61536001	61537000	1000	1	3.50E-07	0.38	18	1.8	Ccdc180	
DMR5:61784001	5	61784001	61787000	3000	1	1.90E-19	-0.47	24	0.8	Xpa;LOC102550553	DNA Repair
DMR5:62023001	5	62023001	62025000	2000	1	4.00E-08	0.4	25	1.25	Hemgn	
DMR5:62289001	5	62289001	62290000	1000	1	2.50E-08	0.36	10	1	Gabbr2	Signaling
DMR5:62339001	5	62339001	62341000	2000	1	6.90E-10	0.41	25	1.25	Gabbr2	Signaling
DMR5:62357001	5	62357001	62359000	2000	1	3.80E-08	-0.49	30	1.5	Gabbr2	Signaling
DMR5:62360001	5	62360001	62362000	2000	1	9.70E-09	0.39	48	2.4	Gabbr2	Signaling
DMR5:62489001	5	62489001	62491000	2000	1	5.30E-09	-0.52	36	1.8	Gabbr2	Signaling
DMR5:62503001	5	62503001	62504000	1000	1	3.60E-07	0.35	28	2.8	Gabbr2	Signaling
DMR5:62633001	5	62633001	62635000	2000	1	8.10E-10	0.69	23	1.15	Anks6	Metabolism
DMR5:63869001	5	63869001	63871000	2000	1	7.10E-10	-0.57	8	0.4	Stx17	Transcription
DMR5:63893001	5	63893001	63894000	1000	1	7.40E-08	0.36	10	1	Stx17	Transcription
DMR5:63957001	5	63957001	63962000	5000	1	1.90E-07	-0.31	46	0.92	Erp44	
DMR5:64095001	5	64095001	64096000	1000	1	1.10E-07	0.6	4	0.4	Invs	
DMR5:64805001	5	64805001	64807000	2000	1	6.90E-08	-0.66	30	1.5	Zfp189;Aldob	Metabolism
DMR5:64980001	5	64980001	64981000	1000	1	4.90E-07	-0.49	6	0.6	Grin3a	Receptor
DMR5:68806001	5	68806001	68810000	4000	1	1.20E-08	-0.34	46	1.15	Olr848	Receptor
DMR5:68864001	5	68864001	68869000	5000	1	2.10E-08	-0.33	40	0.8	Olr848	Receptor
DMR5:68900001	5	68900001	68904000	4000	1	4.40E-10	-0.31	39	0.98	Olr848;RGD1309291	Receptor;Proteolysis
DMR5:69938001	5	69938001	69939000	1000	1	9.00E-07	0.49	6	0.6	Abca1	Transport
DMR5:70274001	5	70274001	70278000	4000	1	6.30E-07	-0.35	49	1.23	Slc44a1	Transport
DMR5:70391001	5	70391001	70393000	2000	1	8.40E-07	-0.39	36	1.8	Slc44a1	Transport
DMR5:70494001	5	70494001	70496000	2000	1	4.30E-09	-0.38	27	1.35	Fsd1l	Proteolysis
DMR5:71750001	5	71750001	71751000	1000	1	1.30E-10	0.41	70	7	Zfp462	Transcription
DMR5:73996001	5	73996001	73998000	2000	1	7.90E-07	0.44	34	1.7	Tmem245;Frrs1l	
DMR5:74034001	5	74034001	74040000	6000	2	1.90E-10	0.37	193	3.22	Frrs1l;Epb41l4b	
DMR5:74298001	5	74298001	74300000	2000	1	8.10E-07	0.36	28	1.4	Ptpn3;LOC108350978	Signaling
DMR5:75406001	5	75406001	75407000	1000	1	1.80E-07	0.41	6	0.6	Musk	Receptor
DMR5:75431001	5	75431001	75432000	1000	1	3.40E-08	-0.43	9	0.9	Musk;LOC102550203	Receptor
DMR5:75457001	5	75457001	75461000	4000	1	2.30E-13	-0.42	69	1.73	Musk	Receptor
DMR5:75487001	5	75487001	75489000	2000	1	6.10E-07	-0.47	24	1.2	Musk	Receptor
DMR5:75656001	5	75656001	75658000	2000	1	2.20E-07	0.35	41	2.05	Lpar1	Signaling
DMR5:76069001	5	76069001	76070000	1000	1	2.80E-07	-0.47	9	0.9	Cct6a-ps4	
DMR5:76950001	5	76950001	76957000	7000	3	4.90E-09	-0.34	87	1.24	RGD1310951	
DMR5:76987001	5	76987001	76989000	2000	1	3.70E-07	-0.35	19	0.95	RGD1310951	
DMR5:77062001	5	77062001	77063000	1000	1	3.90E-08	0.43	29	2.9	Snx30	Cytoskeleton
DMR5:77123001	5	77123001	77127000	4000	2	5.20E-14	-0.4	37	0.92	Snx30	Cytoskeleton
DMR5:77776001	5	77776001	77778000	2000	1	4.20E-09	-0.39	18	0.9	Mup5	Transport
DMR5:77795001	5	77795001	77797000	2000	1	6.50E-11	0.31	13	0.65	Mup5	Transport
DMR5:77863001	5	77863001	77870000	7000	1	1.90E-10	-0.36	100	1.43	Mup5;Zfp37	Transport;Transcription
DMR5:77888001	5	77888001	77889000	1000	1	2.50E-08	0.38	9	0.9	Mup5;Zfp37	Transport;Transcription
DMR5:78241001	5	78241001	78242000	1000	1	2.80E-07	0.6	7	0.7	Slc31a1	Transport
DMR5:78545001	5	78545001	78546000	1000	1	3.30E-09	0.43	10	1	Rgs3	
DMR5:78971001	5	78971001	78973000	2000	1	2.10E-07	0.39	28	1.4	Zfp618;Ambp	Transcription
DMR5:79224001	5	79224001	79226000	2000	1	5.70E-10	0.53	17	0.85	Akna;LOC102547858;Whrn	Cytoskeleton
DMR5:79249001	5	79249001	79250000	1000	1	1.50E-09	0.72	6	0.6	Whrn	Cytoskeleton
DMR5:79256001	5	79256001	79257000	1000	1	6.90E-07	0.4	9	0.9	Whrn	Cytoskeleton
DMR5:79277001	5	79277001	79280000	3000	1	5.10E-08	0.57	37	1.23	Whrn	Cytoskeleton
DMR5:79693001	5	79693001	79694000	1000	1	6.00E-07	-0.41	10	1	Tnfsf8	
DMR5:81249001	5	81249001	81250000	1000	1	2.40E-07	0.46	2	0.2	Astn2	
DMR5:81269001	5	81269001	81270000	1000	1	3.90E-07	0.5	3	0.3	Astn2	
DMR5:81396001	5	81396001	81398000	2000	1	1.00E-07	-0.49	36	1.8	Astn2	
DMR5:81540001	5	81540001	81541000	1000	1	7.10E-09	-0.46	8	0.8	Astn2	
DMR5:81689001	5	81689001	81690000	1000	1	6.80E-07	-0.38	9	0.9	Astn2	
DMR5:81776001	5	81776001	81777000	1000	1	3.40E-07	-0.26	29	2.9	Astn2	
DMR5:81828001	5	81828001	81836000	8000	2	6.90E-08	-0.35	87	1.09	Astn2	
DMR5:82011001	5	82011001	82017000	6000	2	1.40E-11	-0.39	52	0.87	Astn2	
DMR5:84937001	5	84937001	84941000	4000	2	1.10E-07	-0.3	40	1	Brinp1	

DMR5:86411001	5	86411001	86412000	1000	1	1.30E-07	0.5	8	0.8	Cdk5rap2	
DMR5:86595001	5	86595001	86601000	6000	1	7.40E-09	-0.31	63	1.05	Megf9	Extracellular Matrix
DMR5:90046001	5	90046001	90053000	7000	1	7.10E-07	-0.4	71	1.01	Frmd3;LOC108350987	
DMR5:90141001	5	90141001	90142000	1000	1	5.20E-16	-0.69	8	0.8	Frmd3	
DMR5:90190001	5	90190001	90196000	6000	1	8.40E-11	-0.3	67	1.12	Frmd3;LOC688541	
DMR5:90202001	5	90202001	90204000	2000	1	1.30E-07	-0.45	12	0.6	Frmd3	
DMR5:90215001	5	90215001	90220000	5000	1	6.70E-10	-0.36	54	1.08	Frmd3	
DMR5:90408001	5	90408001	90410000	2000	1	1.50E-07	-0.45	7	0.35	Rasef	
DMR5:90949001	5	90949001	90952000	3000	1	9.90E-07	-0.48	24	0.8	Kdm4c	
DMR5:98504001	5	98504001	98508000	4000	1	1.50E-07	-0.33	36	0.9	Lurap1l	
DMR5:100507001	5	100507001	100508000	1000	1	7.50E-07	-0.51	20	2	Nfib	Transcription
DMR5:100565001	5	100565001	100566000	1000	1	6.60E-08	0.5	4	0.4	Nfib	Transcription
DMR5:101631001	5	101631001	101633000	2000	1	5.10E-07	-0.41	19	0.95	Ccdc171	
DMR5:101811001	5	101811001	101813000	2000	1	2.10E-09	-0.35	21	1.05	Ccdc171	
DMR5:101814001	5	101814001	101815000	1000	1	1.00E-09	-0.34	15	1.5	Ccdc171	
DMR5:101827001	5	101827001	101828000	1000	1	7.90E-07	0.31	13	1.3	Ccdc171	
DMR5:101883001	5	101883001	101886000	3000	1	3.80E-07	-0.36	17	0.57	Ccdc171	
DMR5:103859001	5	103859001	103863000	4000	1	2.00E-08	-0.32	31	0.78	Adamtsl1	
DMR5:103871001	5	103871001	103875000	4000	1	9.80E-09	-0.32	57	1.43	Adamtsl1	
DMR5:103950001	5	103950001	103957000	7000	2	8.10E-08	-0.27	78	1.11	Adamtsl1	
DMR5:103965001	5	103965001	103967000	2000	1	2.20E-07	0.44	15	0.75	Adamtsl1	
DMR5:104031001	5	104031001	104032000	1000	1	1.20E-08	-0.41	11	1.1	Adamtsl1	
DMR5:104056001	5	104056001	104064000	8000	1	7.00E-08	-0.29	90	1.12	Adamtsl1	
DMR5:104301001	5	104301001	104306000	5000	2	1.10E-11	-0.37	50	1	Adamtsl1	
DMR5:104504001	5	104504001	104508000	4000	1	1.40E-11	-0.32	41	1.02	Adamtsl1	
DMR5:104716001	5	104716001	104717000	1000	1	4.00E-08	0.44	10	1	Adamtsl1	
DMR5:104809001	5	104809001	104816000	7000	2	5.80E-20	0.93	87	1.24	Fam154a	
DMR5:104925001	5	104925001	104929000	4000	1	7.70E-08	-0.41	42	1.05	Fam154a	
DMR5:105401001	5	105401001	105403000	2000	2	2.90E-08	0.54	26	1.3	Slc24a2	Transport
DMR5:106535001	5	106535001	106536000	1000	1	1.40E-07	-0.48	22	2.2	Focad	
DMR5:106591001	5	106591001	106598000	7000	1	2.10E-08	-0.4	62	0.89	Focad	
DMR5:106654001	5	106654001	106659000	5000	1	5.90E-07	-0.36	51	1.02	Focad	
DMR5:106929001	5	106929001	106932000	3000	1	2.60E-07	-0.41	11	0.37	Ifna4	Immune
DMR5:106941001	5	106941001	106942000	1000	1	5.60E-07	-0.42	12	1.2	Ifna5	
DMR5:106950001	5	106950001	106951000	1000	1	2.20E-11	0.63	27	2.7	Ifna5;LOC690903	
DMR5:106991001	5	106991001	106996000	5000	1	4.40E-07	-0.27	50	1	RGD1561246	
DMR5:107435001	5	107435001	107440000	5000	2	1.00E-12	0.62	42	0.84	RGD1564637;Ifna16l1	Immune
DMR5:110328001	5	110328001	110329000	1000	1	1.40E-08	0.49	5	0.5	Zfp352	Transcription
DMR5:113637001	5	113637001	113642000	5000	1	2.50E-09	-0.33	68	1.36	Ift74	
DMR5:114569001	5	114569001	114570000	1000	1	2.70E-08	-0.47	8	0.8	Fggy	Metabolism
DMR5:114571001	5	114571001	114574000	3000	1	7.80E-09	-0.32	32	1.07	Fggy	Metabolism
DMR5:114740001	5	114740001	114741000	1000	1	4.90E-07	-0.27	16	1.6	Fggy;LOC103692418	Metabolism
DMR5:114804001	5	114804001	114806000	2000	1	4.20E-12	0.71	21	1.05	Fggy;LOC103692418	Metabolism
DMR5:114963001	5	114963001	114966000	3000	1	6.50E-08	-0.41	15	0.5	Hook1	Transport
DMR5:115103001	5	115103001	115106000	3000	1	1.40E-07	-0.33	30	1	Cyp2j10	Metabolism
DMR5:116985001	5	116985001	116987000	2000	1	1.00E-07	-0.41	29	1.45	Tm2d1	
DMR5:117196001	5	117196001	117200000	4000	1	3.50E-09	-0.5	45	1.12	Patj	
DMR5:117293001	5	117293001	117294000	1000	1	1.90E-07	-0.43	15	1.5	Patj	
DMR5:117644001	5	117644001	117649000	5000	3	6.10E-09	-0.47	31	0.62	Dock7	Transcription
DMR5:118426001	5	118426001	118430000	4000	1	9.20E-07	-0.37	37	0.92	Alg6	Golgi
DMR5:118445001	5	118445001	118448000	3000	1	5.10E-08	-0.47	30	1	Alg6	Golgi
DMR5:118642001	5	118642001	118643000	1000	1	4.40E-07	-0.66	15	1.5	Ube2u	
DMR5:118738001	5	118738001	118740000	2000	2	4.30E-07	0.37	25	1.25	Pgm1	Metabolism
DMR5:119816001	5	119816001	119818000	2000	1	1.50E-09	0.56	21	1.05	Cachd1	Transport
DMR5:119835001	5	119835001	119837000	2000	1	1.50E-08	0.58	15	0.75	Cachd1	Transport
DMR5:120366001	5	120366001	120368000	2000	1	3.30E-07	-0.56	27	1.35	Dnajc6	Transport
DMR5:120443001	5	120443001	120445000	2000	1	5.70E-09	-0.43	20	1	Dnajc6	Transport
DMR5:120472001	5	120472001	120480000	8000	2	1.00E-09	-0.35	80	1	Dnajc6	Transport
DMR5:121856001	5	121856001	121862000	6000	1	2.40E-07	-0.29	56	0.93	Pde4b	Signaling
DMR5:121898001	5	121898001	121903000	5000	3	7.80E-11	-0.4	32	0.64	Pde4b	Signaling
DMR5:122565001	5	122565001	122566000	1000	1	1.60E-07	-0.53	11	1.1	Wdr78	Cytoskeleton

DMR5:122570001	5	122570001	122574000	4000	2	6.20E-08	-0.31	42	1.05	Wdr78	Cytoskeleton
DMR5:122598001	5	122598001	122603000	5000	2	2.20E-08	-0.46	72	1.44	Wdr78	Cytoskeleton
DMR5:122774001	5	122774001	122780000	6000	1	5.20E-09	-0.31	61	1.02	RGD1562532	
DMR5:122887001	5	122887001	122890000	3000	3	9.50E-09	-0.51	21	0.7	Oma1	Protease
DMR5:123166001	5	123166001	123167000	1000	1	1.50E-09	-0.6	13	1.3	Dab1	Cytoskeleton
DMR5:123279001	5	123279001	123280000	1000	1	3.30E-07	-0.45	7	0.7	Dab1	Cytoskeleton
DMR5:123431001	5	123431001	123432000	1000	1	9.20E-08	-0.4	13	1.3	Dab1	Cytoskeleton
DMR5:124400001	5	124400001	124401000	1000	1	1.10E-08	-0.5	22	2.2	C8a	
DMR5:124407001	5	124407001	124409000	2000	1	4.90E-07	-0.28	21	1.05	C8a	
DMR5:124769001	5	124769001	124772000	3000	1	2.50E-07	0.33	33	1.1	Plpp3;LOC102549668	Signaling
DMR5:124775001	5	124775001	124776000	1000	1	1.20E-07	0.6	25	2.5	Plpp3;LOC102549668	Signaling
DMR5:126061001	5	126061001	126065000	4000	1	7.10E-12	0.45	52	1.3	Pcsk9;Bsnd	Protease
DMR5:126650001	5	126650001	126651000	1000	1	6.20E-07	0.37	14	1.4	Ssbp3;Mrpl37	Transcription;Translation
DMR5:127091001	5	127091001	127093000	2000	1	9.00E-09	0.4	50	2.5	Glis1	Transcription
DMR5:127110001	5	127110001	127111000	1000	1	3.40E-07	0.36	10	1	Glis1;LOC102551042	Transcription
DMR5:127188001	5	127188001	127190000	2000	1	1.80E-11	0.48	37	1.85	Glis1	Transcription
DMR5:127398001	5	127398001	127399000	1000	1	2.20E-07	-0.35	25	2.5	Lrp8	Binding Proteins
DMR5:127618001	5	127618001	127620000	2000	1	6.40E-07	0.37	26	1.3	Slc1a7;LOC102552895	Transport
DMR5:127657001	5	127657001	127660000	3000	1	1.90E-08	0.38	33	1.1	Scp2	Transport
DMR5:127745001	5	127745001	127747000	2000	1	5.40E-07	-0.36	22	1.1	Scp2	Transport
DMR5:127820001	5	127820001	127821000	1000	1	8.80E-07	-0.44	19	1.9	Zyg11a	
DMR5:127975001	5	127975001	127977000	2000	1	6.60E-12	0.6	17	0.85	Fam159a	
DMR5:128422001	5	128422001	128424000	2000	1	2.90E-08	0.37	21	1.05	Btf3l4	Transcription
DMR5:129039001	5	129039001	129043000	4000	1	1.50E-08	0.33	65	1.62	Ttc39a	
DMR5:129303001	5	129303001	129306000	3000	1	4.90E-09	-0.38	31	1.03	Rnf11	
DMR5:129415001	5	129415001	129422000	7000	4	7.90E-10	-0.39	69	0.99	Faf1	
DMR5:129503001	5	129503001	129505000	2000	1	3.60E-10	-0.44	17	0.85	Faf1	
DMR5:129559001	5	129559001	129565000	6000	1	8.90E-07	-0.45	72	1.2	Faf1	
DMR5:129651001	5	129651001	129657000	6000	2	4.40E-07	-0.33	60	1	Faf1	
DMR5:129676001	5	129676001	129677000	1000	1	5.20E-07	0.43	10	1	Faf1	
DMR5:129682001	5	129682001	129685000	3000	1	3.60E-12	-0.42	35	1.17	Faf1	
DMR5:129693001	5	129693001	129698000	5000	1	1.10E-09	-0.33	51	1.02	Faf1	
DMR5:130298001	5	130298001	130299000	1000	1	6.90E-07	-0.44	5	0.5	Agbl4	Protease
DMR5:131566001	5	131566001	131568000	2000	1	2.10E-07	-0.32	39	1.95	LOC100911395;RGD1565822;LOC102547169	
DMR5:133259001	5	133259001	133260000	1000	1	1.00E-07	0.49	4	0.4	Trabd2b	Protease
DMR5:133337001	5	133337001	133338000	1000	1	5.50E-11	-1.27	12	1.2	Trabd2b	Protease
DMR5:133391001	5	133391001	133393000	2000	1	6.50E-10	0.45	16	0.8	Trabd2b	Protease
DMR5:133880001	5	133880001	133884000	4000	2	6.10E-14	0.5	46	1.15	Tal1	Transcription
DMR5:133966001	5	133966001	133969000	3000	1	4.60E-09	-0.49	29	0.97	Cyp4x1;Cyp4a8	Metabolism
DMR5:133978001	5	133978001	133979000	1000	1	3.50E-07	0.48	4	0.4	Cyp4a8	
DMR5:134104001	5	134104001	134108000	4000	1	3.80E-07	-0.31	43	1.07	RGD1562603	
DMR5:134341001	5	134341001	134342000	1000	1	2.10E-07	0.45	19	1.9	Faah	Metabolism
DMR5:134626001	5	134626001	134628000	2000	1	8.90E-13	0.59	8	0.4	Efcab14;Tex38	
DMR5:134659001	5	134659001	134662000	3000	3	1.60E-12	0.54	60	2	Atpaf1;LOC103692454	Transcription
DMR5:134725001	5	134725001	134731000	6000	1	1.20E-08	0.36	97	1.62	Mknk1;Kncn	Signaling
DMR5:134776001	5	134776001	134780000	4000	2	2.10E-07	0.35	63	1.57	Dmbx1	Development
DMR5:134912001	5	134912001	134913000	1000	1	9.50E-07	-0.44	17	1.7	Nsun4;LOC366449;Uqcrh	Epigenetic;Metabolism
DMR5:135921001	5	135921001	135922000	1000	1	5.80E-08	0.64	5	0.5	Eif2b3	Translation
DMR5:136020001	5	136020001	136021000	1000	1	8.70E-07	-0.42	14	1.4	Best4;Rps8;Kif2c	Transport;Translation;Cytoskeleton
DMR5:136088001	5	136088001	136089000	1000	1	5.70E-12	0.48	27	2.7	RGD1563714	
DMR5:136141001	5	136141001	136145000	4000	1	4.80E-07	0.39	62	1.55	Rnf220	
DMR5:136230001	5	136230001	136233000	3000	2	3.90E-18	0.56	64	2.13	Rnf220	
DMR5:136591001	5	136591001	136598000	7000	1	8.50E-08	0.4	65	0.93	Klf17	Transcription
DMR5:136847001	5	136847001	136848000	1000	1	5.90E-08	0.4	14	1.4	St3gal3	Transport
DMR5:136885001	5	136885001	136888000	3000	1	1.10E-08	0.41	73	2.43	St3gal3	Transport
DMR5:136939001	5	136939001	136941000	2000	1	4.20E-07	-0.42	46	2.3	St3gal3	Transport
DMR5:137071001	5	137071001	137072000	1000	1	1.10E-07	0.35	21	2.1	Ptprf	Signaling
DMR5:137079001	5	137079001	137081000	2000	1	5.50E-07	0.44	29	1.45	Ptprf	Signaling
DMR5:137092001	5	137092001	137094000	2000	1	6.20E-09	0.38	30	1.5	Ptprf;LOC102553423	Signaling

DMR5:137098001	5	137098001	137101000	3000	1	5.40E-10	0.4	34	1.13	Ptprf;LOC102553423	Signaling
DMR5:137112001	5	137112001	137114000	2000	1	6.80E-13	0.5	41	2.05	Ptprf;LOC102553423	Signaling
DMR5:137305001	5	137305001	137308000	3000	1	3.60E-07	0.33	70	2.33	Tie1	Receptor
DMR5:137390001	5	137390001	137391000	1000	1	6.40E-07	0.4	20	2	Cfap57	
DMR5:137824001	5	137824001	137828000	4000	1	9.00E-10	-0.41	38	0.95	Olr865	Receptor
DMR5:137835001	5	137835001	137838000	3000	1	1.30E-08	-0.35	21	0.7	Olr866	Receptor
DMR5:137938001	5	137938001	137940000	2000	1	9.40E-09	-0.57	19	0.95	Olr869;LOC679953;Olr870-ps	Receptor
DMR5:137949001	5	137949001	137952000	3000	1	3.30E-10	-0.54	16	0.53	Olr870-ps;Olr871-ps	
DMR5:137971001	5	137971001	137979000	8000	3	3.20E-10	-0.34	60	0.75	Olr872-ps	
DMR5:138299001	5	138299001	138304000	5000	1	3.60E-07	0.35	81	1.62	P3h1;Cldn19	Extracellular Matrix;Cell Junction
DMR5:138326001	5	138326001	138328000	2000	1	8.80E-08	0.35	28	1.4	Ybx1	
DMR5:138364001	5	138364001	138365000	1000	1	2.10E-10	-0.6	20	2	Ppih;LOC103692462;Ccdc30	Transcription
DMR5:138455001	5	138455001	138458000	3000	1	1.80E-07	0.4	46	1.53	Ccdc30;Ppcs	Transport
DMR5:138491001	5	138491001	138493000	2000	1	7.50E-13	0.47	32	1.6	Zmynd12	
DMR5:138978001	5	138978001	138979000	1000	1	3.30E-11	0.4	12	1.2	Hivep3	
DMR5:139071001	5	139071001	139073000	2000	1	3.70E-07	0.37	54	2.7	Hivep3	
DMR5:139201001	5	139201001	139203000	2000	1	1.70E-13	0.57	32	1.6	Foxo6	
DMR5:139211001	5	139211001	139215000	4000	1	2.40E-08	0.37	56	1.4	Foxo6	
DMR5:139408001	5	139408001	139413000	5000	3	2.90E-10	-0.4	42	0.84	Scmh1	Epigenetic
DMR5:139656001	5	139656001	139657000	1000	1	3.60E-08	0.52	13	1.3	Kcnq4	Transport
DMR5:139659001	5	139659001	139660000	1000	1	9.90E-08	0.37	19	1.9	Kcnq4	Transport
DMR5:139786001	5	139786001	139787000	1000	1	2.60E-08	0.36	21	2.1	Rims3	Transport
DMR5:139886001	5	139886001	139887000	1000	1	9.30E-07	0.35	19	1.9	Smap2	
DMR5:140108001	5	140108001	140109000	1000	1	2.50E-08	0.55	12	1.2	Rlf	Transcription
DMR5:140872001	5	140872001	140874000	2000	1	1.70E-07	0.54	18	0.9	Hpcal4	
DMR5:140947001	5	140947001	140953000	6000	1	1.50E-11	0.64	51	0.85	Heyl;LOC108351027	Transcription
DMR5:141063001	5	141063001	141064000	1000	1	6.90E-07	-0.57	18	1.8	Macf1	Cytoskeleton
DMR5:141456001	5	141456001	141457000	1000	1	3.10E-07	0.47	26	2.6	Rhbdl2	
DMR5:142647001	5	142647001	142648000	1000	1	2.00E-07	0.46	12	1.2	LOC102548116;Pou3f1	
DMR5:142693001	5	142693001	142696000	3000	1	3.10E-08	0.47	74	2.47	Fhl3;Sf3a3	Transcription;Translation
DMR5:143641001	5	143641001	143644000	3000	1	6.00E-09	0.37	37	1.23	Grik3;LOC108351030	Receptor
DMR5:143714001	5	143714001	143717000	3000	1	3.90E-09	0.38	58	1.93	Grik3	Receptor
DMR5:144251001	5	144251001	144252000	1000	1	2.00E-07	0.36	21	2.1	Map7d1	Cytoskeleton
DMR5:144309001	5	144309001	144310000	1000	1	6.40E-08	0.36	16	1.6	Col8a2	Extracellular Matrix
DMR5:144775001	5	144775001	144778000	3000	1	1.20E-10	0.44	62	2.07	Ncdn;LOC100294508	Cytoskeleton
DMR5:144866001	5	144866001	144870000	4000	1	1.60E-09	0.4	53	1.32	LOC100294508;Zmym4	Transcription
DMR5:145261001	5	145261001	145264000	3000	1	1.50E-07	0.38	56	1.87	Dlgap3	Cytoskeleton
DMR5:145292001	5	145292001	145296000	4000	2	1.20E-08	0.4	53	1.32	Dlgap3	Cytoskeleton
DMR5:145378001	5	145378001	145379000	1000	1	2.90E-10	0.57	13	1.3	Gja4	Cytoskeleton
DMR5:146235001	5	146235001	146236000	1000	1	1.40E-07	0.53	14	1.4	Csmd2	
DMR5:146445001	5	146445001	146446000	1000	1	7.50E-08	-0.55	18	1.8	Csmd2;Hmgb4	
DMR5:146536001	5	146536001	146538000	2000	1	2.80E-08	0.38	38	1.9	Csmd2	
DMR5:146572001	5	146572001	146575000	3000	1	3.80E-13	0.51	50	1.67	Csmd2	
DMR5:146857001	5	146857001	146861000	4000	2	2.30E-10	0.52	80	2	Phc2	Epigenetic
DMR5:146877001	5	146877001	146880000	3000	1	4.80E-08	0.34	48	1.6	Phc2	Epigenetic
DMR5:147119001	5	147119001	147121000	2000	1	1.90E-09	0.46	34	1.7	Azin2	Metabolism
DMR5:147141001	5	147141001	147143000	2000	1	4.30E-17	0.51	47	2.35	Azin2	Metabolism
DMR5:147316001	5	147316001	147317000	1000	1	4.60E-12	0.48	19	1.9	Fndc5	
DMR5:147425001	5	147425001	147429000	4000	1	3.60E-11	0.38	78	1.95	RGD1561149	
DMR5:147541001	5	147541001	147542000	1000	1	2.30E-07	-0.47	13	1.3	Rbbp4;Zbtb8os	
DMR5:147567001	5	147567001	147573000	6000	1	1.10E-08	0.37	104	1.73	Zbtb8a;LOC108351038	Cytoskeleton
DMR5:147723001	5	147723001	147725000	2000	1	1.60E-07	-0.46	45	2.25	Marcksl1;Hdac1	
DMR5:147785001	5	147785001	147789000	4000	1	3.40E-07	0.32	64	1.6	Lck;Fam167b;Eif3i	Translation
DMR5:148206001	5	148206001	148213000	7000	1	8.60E-09	0.39	124	1.77	Adgrb2	Signaling
DMR5:148237001	5	148237001	148238000	1000	1	1.20E-09	0.49	10	1	Adgrb2	Signaling
DMR5:148251001	5	148251001	148254000	3000	1	1.30E-07	0.36	63	2.1	Col16a1	Extracellular Matrix
DMR5:148295001	5	148295001	148297000	2000	1	6.40E-07	0.43	32	1.6	Col16a1	Extracellular Matrix
DMR5:148385001	5	148385001	148391000	6000	2	5.70E-08	0.41	105	1.75	Tinagl1	Protease

DMR5:148627001	5	148627001	148628000	1000	1	7.50E-07	0.32	13	1.3	Nkain1	
DMR5:148940001	5	148940001	148944000	4000	3	6.80E-09	0.63	47	1.18	Sdc3	Receptor
DMR5:149048001	5	149048001	149049000	1000	1	1.40E-13	0.72	19	1.9	Laptm5	Transport
DMR5:149052001	5	149052001	149056000	4000	2	5.00E-15	0.55	63	1.57	Laptm5	Transport
DMR5:151199001	5	151199001	151200000	1000	1	5.40E-08	0.33	28	2.8	Fgr;Ahdc1	
DMR5:151218001	5	151218001	151223000	5000	1	7.20E-10	0.4	92	1.84	Ahdc1	
DMR5:151261001	5	151261001	151265000	4000	1	5.20E-07	0.4	142	3.55	Ahdc1	
DMR5:151618001	5	151618001	151620000	2000	1	1.30E-09	0.41	32	1.6	Slc9a1	Transport
DMR5:152216001	5	152216001	152218000	2000	1	1.80E-09	-0.56	41	2.05	Dhdds	Metabolism
DMR5:152436001	5	152436001	152437000	1000	1	1.80E-08	0.34	6	0.6	Umodl;Catsper4	Receptor
DMR5:152467001	5	152467001	152469000	2000	1	1.60E-07	0.48	40	2	Cnksr1;LOC108351043;Zfp593;Fam110d	Transcription
DMR5:152568001	5	152568001	152570000	2000	1	6.20E-10	0.44	28	1.4	Slc30a2;Extl1	Transport;Golgi
DMR5:152786001	5	152786001	152789000	3000	1	1.40E-08	0.44	70	2.33	Man1c1	Golgi
DMR5:152851001	5	152851001	152853000	2000	1	8.20E-07	0.45	34	1.7	Man1c1	Golgi
DMR5:152956001	5	152956001	152959000	3000	1	1.00E-11	0.48	64	2.13	Ldlrap1	Cytoskeleton
DMR5:152977001	5	152977001	152979000	2000	1	9.50E-08	0.44	25	1.25	Ldlrap1	Cytoskeleton
DMR5:153223001	5	153223001	153224000	1000	1	5.50E-07	0.28	22	2.2	Rhd;Tmem50a	Transport
DMR5:153255001	5	153255001	153256000	1000	1	2.00E-09	0.38	16	1.6	Tmem50a;Rsrp1	
DMR5:153615001	5	153615001	153617000	2000	1	3.10E-09	0.39	21	1.05	Clic4	Transport
DMR5:153679001	5	153679001	153681000	2000	1	1.10E-07	0.38	37	1.85	Srrm1	Translation
DMR5:153736001	5	153736001	153737000	1000	1	2.40E-12	0.46	23	2.3	Ncmap	Cytoskeleton
DMR5:153811001	5	153811001	153813000	2000	1	2.70E-07	-0.58	57	2.85	Nipal3	
DMR5:153881001	5	153881001	153883000	2000	1	2.60E-11	0.43	36	1.8	Stpg1	Development
DMR5:153922001	5	153922001	153924000	2000	1	1.50E-07	0.45	58	2.9	Grhl3	Transcription
DMR5:154130001	5	154130001	154131000	1000	1	7.00E-10	0.52	14	1.4	Myom3	
DMR5:154230001	5	154230001	154233000	3000	1	2.60E-07	0.43	65	2.17	Pnrc2;Cnr2	Signaling
DMR5:154499001	5	154499001	154501000	2000	1	1.30E-08	0.56	33	1.65	Id3	Transcription
DMR5:154565001	5	154565001	154569000	4000	1	3.40E-08	0.39	60	1.5	Asap3	
DMR5:155050001	5	155050001	155051000	1000	1	2.60E-08	0.35	22	2.2	Ephb2	Receptor
DMR5:155300001	5	155300001	155307000	7000	1	4.50E-07	0.43	152	2.17	Epha8	Receptor
DMR5:155312001	5	155312001	155316000	4000	1	2.90E-07	0.34	75	1.88	Epha8	Receptor
DMR5:155670001	5	155670001	155673000	3000	1	1.60E-12	0.5	76	2.53	Wnt4	Signaling
DMR5:155876001	5	155876001	155880000	4000	1	1.80E-09	0.46	62	1.55	Hspg2	
DMR5:155891001	5	155891001	155896000	5000	1	4.00E-07	0.34	102	2.04	Hspg2	
DMR5:155898001	5	155898001	155900000	2000	1	1.40E-07	0.43	22	1.1	Hspg2	
DMR5:155904001	5	155904001	155906000	2000	1	5.30E-11	0.53	21	1.05	Hspg2;Ldlrad2	
DMR5:156102001	5	156102001	156103000	1000	1	1.80E-11	0.51	19	1.9	Alpl	Signaling
DMR5:156123001	5	156123001	156124000	1000	1	9.20E-07	-0.36	19	1.9	Alpl	Signaling
DMR5:156223001	5	156223001	156225000	2000	1	7.10E-08	0.42	28	1.4	Ece1	Protease
DMR5:156242001	5	156242001	156243000	1000	1	5.50E-09	0.46	18	1.8	Ece1	Protease
DMR5:156580001	5	156580001	156581000	1000	1	8.10E-07	0.48	18	1.8	LOC102553854;Hp1bp3	Cytoskeleton
DMR5:156618001	5	156618001	156620000	2000	1	2.10E-09	0.42	48	2.4	Hp1bp3;Sh2d5;Kif17	Cytoskeleton;Cytoskeleton
DMR5:156637001	5	156637001	156638000	1000	1	7.30E-09	0.69	11	1.1	Kif17	Cytoskeleton
DMR5:156658001	5	156658001	156660000	2000	1	3.00E-08	0.45	46	2.3	Kif17;Ddost	Cytoskeleton;Golgi
DMR5:156871001	5	156871001	156872000	1000	1	3.90E-07	0.32	11	1.1	Mul1;Camk2n1;LOC102555044	Signaling
DMR5:157420001	5	157420001	157422000	2000	1	4.00E-09	0.58	30	1.5	Rnf186;Tmco4	
DMR5:157499001	5	157499001	157500000	1000	1	2.10E-07	0.45	10	1	Tmco4;Htr6	Signaling
DMR5:157762001	5	157762001	157763000	1000	1	3.30E-08	0.42	18	1.8	Pqlc2;Akr7a2;LOC108351049	Metabolism
DMR5:158048001	5	158048001	158051000	3000	1	3.50E-07	0.43	58	1.93	Iffo2;Mir6215	Metabolism
DMR5:158500001	5	158500001	158501000	1000	1	2.10E-08	0.43	17	1.7	LOC108351052;Igsf21	
DMR5:158542001	5	158542001	158544000	2000	1	5.10E-08	0.39	43	2.15	Igsf21;LOC102547437	
DMR5:158551001	5	158551001	158554000	3000	1	1.30E-08	0.46	76	2.53	Igsf21;LOC102547437	
DMR5:158636001	5	158636001	158637000	1000	1	1.30E-07	-0.39	17	1.7	Igsf21	
DMR5:158649001	5	158649001	158652000	3000	1	8.40E-08	0.47	68	2.27	Igsf21	
DMR5:159017001	5	159017001	159018000	1000	1	3.00E-07	-0.37	21	2.1	Arhgef10l	Transcription
DMR5:159033001	5	159033001	159034000	1000	1	2.80E-07	0.49	11	1.1	Arhgef10l	Transcription
DMR5:159060001	5	159060001	159062000	2000	1	2.80E-07	0.5	45	2.25	Arhgef10l	Transcription
DMR5:159070001	5	159070001	159073000	3000	1	4.70E-11	0.48	62	2.07	Arhgef10l	Transcription

DMR5:159345001	5	159345001	159346000	1000	1	7.20E-11	0.47	11	1.1	Padi3;Padi1	
DMR5:159357001	5	159357001	159359000	2000	1	3.40E-09	0.41	40	2	Padi1;Trnaa-ggc	
DMR5:159480001	5	159480001	159482000	2000	1	2.10E-24	0.74	23	1.15	Padi2;Sdhb	Metabolism
DMR5:159678001	5	159678001	159680000	2000	1	4.60E-09	0.49	24	1.2	Fbxo42	
DMR5:159702001	5	159702001	159703000	1000	1	2.50E-07	-0.49	19	1.9	Fbxo42	
DMR5:159729001	5	159729001	159730000	1000	1	4.20E-08	0.42	11	1.1	Fbxo42;Rsg1	
DMR5:159748001	5	159748001	159752000	4000	1	1.10E-11	0.39	55	1.38	Trnai-aau;LOC108351054;Arhgef19	Transcription
DMR5:159772001	5	159772001	159774000	2000	1	4.20E-09	0.4	42	2.1	Arhgef19	Transcription
DMR5:159781001	5	159781001	159783000	2000	1	5.20E-09	0.65	29	1.45	Arhgef19	Transcription
DMR5:159867001	5	159867001	159871000	4000	1	1.10E-07	0.47	75	1.88	Epha2	Receptor
DMR5:159969001	5	159969001	159971000	2000	1	5.90E-08	0.42	35	1.75	Clcnkb;Hspb7	Transport
DMR5:159989001	5	159989001	159990000	1000	1	4.60E-08	0.48	12	1.2	RGD1566083;Zbtb17	Transcription
DMR5:160169001	5	160169001	160170000	1000	1	2.60E-13	0.71	23	2.3	Tmem82;Slc25a34	
DMR5:160183001	5	160183001	160187000	4000	1	5.60E-07	0.38	102	2.55	Tmem82;Slc25a34;Plekhm2	
DMR5:160210001	5	160210001	160214000	4000	1	3.90E-08	0.4	59	1.48	Plekhm2	
DMR5:160330001	5	160330001	160333000	3000	1	1.00E-10	0.52	37	1.23	Agmat;Dnajc16	Metabolism;Transcription
DMR5:160461001	5	160461001	160465000	4000	1	3.10E-09	0.45	89	2.22	Fhad1	
DMR5:160469001	5	160469001	160471000	2000	1	2.10E-07	0.36	48	2.4	Fhad1	
DMR5:160491001	5	160491001	160493000	2000	2	3.70E-10	0.46	33	1.65	Fhad1	
DMR5:160599001	5	160599001	160603000	4000	1	9.20E-08	0.36	60	1.5	Tmem51	
DMR5:160891001	5	160891001	160892000	1000	1	1.20E-07	0.38	13	1.3	Kazn	
DMR5:161071001	5	161071001	161073000	2000	1	3.90E-09	0.56	24	1.2	Kazn	
DMR5:161250001	5	161250001	161251000	1000	1	1.50E-08	0.43	29	2.9	Kazn;LOC102554076	
DMR5:161287001	5	161287001	161289000	2000	1	2.20E-07	-0.42	39	1.95	Kazn	
DMR5:161390001	5	161390001	161391000	1000	1	2.20E-07	-0.32	12	1.2	Kazn	
DMR5:161464001	5	161464001	161466000	2000	1	1.00E-13	0.47	20	1	Kazn	
DMR5:161680001	5	161680001	161685000	5000	1	8.40E-08	-0.54	100	2	Kazn	
DMR5:161940001	5	161940001	161942000	2000	1	6.20E-12	0.53	27	1.35	Pdpn	
DMR5:162162001	5	162162001	162164000	2000	1	1.80E-07	0.46	21	1.05	Pramef27	
DMR5:162744001	5	162744001	162747000	3000	1	9.70E-08	-0.31	18	0.6	RGD1559644	Metabolism
DMR5:162799001	5	162799001	162802000	3000	1	7.20E-07	0.23	39	1.3	LOC102550246;Dhrs3	
DMR5:163191001	5	163191001	163192000	1000	1	7.30E-07	0.46	3	0.3	Tnfrsf8	Receptor
DMR5:164411001	5	164411001	164413000	2000	1	1.80E-07	-0.36	30	1.5	LOC108351057;RGD1565622	
DMR5:164797001	5	164797001	164800000	3000	1	1.00E-09	0.44	40	1.33	Nppb;Nppa	
DMR5:164852001	5	164852001	164853000	1000	1	3.40E-11	0.6	19	1.9	Clcn6;Mthfr	Transport;Metabolism
DMR5:164927001	5	164927001	164928000	1000	1	2.50E-10	0.51	17	1.7	Draxin;LOC108351058	
DMR5:164940001	5	164940001	164942000	2000	1	1.60E-09	0.48	26	1.3	Draxin;Mad2l2	
DMR5:165360001	5	165360001	165363000	3000	1	1.30E-09	0.46	58	1.93	Mtor	Signaling
DMR5:165414001	5	165414001	165417000	3000	1	1.90E-07	0.4	45	1.5	Srm;Masp2	Protease
DMR5:165727001	5	165727001	165728000	1000	1	5.80E-07	0.41	13	1.3	Casz1;LOC103692511	Transcription
DMR5:165740001	5	165740001	165744000	4000	1	8.20E-10	0.5	85	2.12	Casz1;LOC103692511	Transcription
DMR5:165796001	5	165796001	165798000	2000	1	1.40E-07	0.4	41	2.05	Pex14	Transport
DMR5:165807001	5	165807001	165811000	4000	1	3.40E-07	0.33	93	2.33	Pex14	Transport
DMR5:166471001	5	166471001	166475000	4000	1	3.20E-07	0.46	62	1.55	Ctnnbip1	
DMR5:166597001	5	166597001	166598000	1000	1	5.60E-10	0.49	29	2.9	Clstn1;Pik3cd	Transport;Signaling
DMR5:166684001	5	166684001	166685000	1000	1	1.80E-09	0.48	23	2.3	Tmem201	
DMR5:167031001	5	167031001	167032000	1000	1	1.40E-08	0.45	10	1	H6pd;LOC108351062	Metabolism
DMR5:167126001	5	167126001	167129000	3000	1	3.40E-07	0.39	57	1.9	Gpr157	Signaling
DMR5:167209001	5	167209001	167210000	1000	1	5.60E-10	0.43	26	2.6	Slc2a7	
DMR5:167452001	5	167452001	167454000	2000	1	2.60E-09	0.39	10	0.5	Rere	
DMR5:167592001	5	167592001	167595000	3000	1	1.80E-07	0.35	41	1.37	Rere	
DMR5:167688001	5	167688001	167689000	1000	1	3.10E-07	0.48	18	1.8	Slc45a1	Transport
DMR5:168096001	5	168096001	168099000	3000	1	4.20E-09	0.4	60	2	Per3	Transcription
DMR5:168239001	5	168239001	168240000	1000	1	6.50E-07	0.33	22	2.2	Camta1	Transcription
DMR5:168448001	5	168448001	168449000	1000	1	3.90E-07	0.44	23	2.3	Camta1	Transcription
DMR5:168542001	5	168542001	168545000	3000	1	1.30E-07	0.38	93	3.1	Camta1;LOC102546564	Transcription
DMR5:168666001	5	168666001	168668000	2000	2	1.30E-09	0.59	55	2.75	Camta1	Transcription
DMR5:168709001	5	168709001	168713000	4000	1	4.80E-07	0.43	75	1.88	Camta1	Transcription

DMR5:168778001	5	168778001	168780000	2000	1	2.30E-10	0.46	27	1.35	Camta1	Transcription
DMR5:168870001	5	168870001	168872000	2000	1	3.60E-07	0.4	33	1.65	Camta1	Transcription
DMR5:169152001	5	169152001	169157000	5000	1	2.00E-07	0.39	108	2.16	Dnajc11;Thap3;Phf13	Transcription
DMR5:169162001	5	169162001	169165000	3000	1	7.20E-09	0.5	62	2.07	Dnajc11;Thap3;Phf13	Transcription
DMR5:169286001	5	169286001	169288000	2000	1	5.50E-07	0.37	48	2.4	Plekhg5;Tnfrsf25;Espn	Receptor;Cytoskeleton
DMR5:169423001	5	169423001	169425000	2000	1	9.90E-07	0.39	39	1.95	Acot7	Metabolism
DMR5:169500001	5	169500001	169501000	1000	1	9.40E-08	0.56	10	1	Rnf207;Rpl22	Translation
DMR5:169534001	5	169534001	169540000	6000	1	7.90E-07	0.35	116	1.93	Chd5	
DMR5:169576001	5	169576001	169580000	4000	1	1.10E-07	0.42	80	2	Chd5;Kcnab2	
DMR5:169640001	5	169640001	169643000	3000	2	4.10E-09	0.52	80	2.67	Kcnab2;LOC500594;Nphp4	Translation
DMR5:170667001	5	170667001	170669000	2000	1	9.10E-07	0.38	50	2.5	Ajap1	
DMR5:171290001	5	171290001	171292000	2000	1	4.20E-07	0.41	30	1.5	Cep104;Lrrc47	Translation
DMR5:171409001	5	171409001	171412000	3000	2	9.10E-09	0.49	72	2.4	Tp73	Transcription
DMR5:171449001	5	171449001	171451000	2000	1	8.30E-09	0.44	40	2	Wrap73;Tprg1l	
DMR5:171598001	5	171598001	171599000	1000	1	8.00E-08	0.54	11	1.1	Megf6	Extracellular Matrix
DMR5:171686001	5	171686001	171688000	2000	1	1.50E-07	0.42	81	4.05	Prdm16	Transcription
DMR5:172069001	5	172069001	172070000	1000	1	6.00E-16	0.57	15	1.5	Actrt2	Cytoskeleton
DMR5:172250001	5	172250001	172251000	1000	1	8.40E-07	0.37	12	1.2	Ttc34	
DMR5:172387001	5	172387001	172391000	4000	1	5.90E-08	0.49	107	2.67	Pank4;Plch2	Signaling;Metabolism
DMR5:172419001	5	172419001	172423000	4000	1	1.20E-09	0.49	65	1.62	Plch2	Metabolism
DMR5:172445001	5	172445001	172448000	3000	1	6.60E-08	0.38	35	1.17	Plch2	Metabolism
DMR5:172560001	5	172560001	172561000	1000	1	2.00E-09	0.46	26	2.6	Ski	
DMR5:172579001	5	172579001	172581000	2000	1	2.10E-08	0.42	15	0.75	Ski	
DMR5:172692001	5	172692001	172694000	2000	1	5.70E-07	0.36	31	1.55	Prkcz	Signaling
DMR5:172829001	5	172829001	172836000	7000	1	6.60E-07	-0.33	66	0.94	Cfap74	
DMR5:172884001	5	172884001	172886000	2000	1	6.60E-07	0.36	31	1.55	Cfap74;Tmem52	
DMR5:173162001	5	173162001	173163000	1000	1	1.90E-07	-0.63	11	1.1	Ssu72;LOC103692527	Signaling
DMR5:173526001	5	173526001	173528000	2000	1	3.60E-10	-0.41	26	1.3	Ttll10	
DMR5:173670001	5	173670001	173672000	2000	1	8.30E-07	0.36	20	1	Noc2l;Samd11	Epigenetic
DMR6:883001	6	883001	886000	3000	2	1.80E-07	0.35	55	1.83	Crim1	
DMR6:1107001	6	1107001	1111000	4000	2	1.50E-07	0.32	50	1.25	Vit	Extracellular Matrix
DMR6:1131001	6	1131001	1134000	3000	1	6.60E-08	-0.35	29	0.97	Vit	Extracellular Matrix
DMR6:1297001	6	1297001	1298000	1000	1	2.60E-12	0.56	22	2.2	Strn	
DMR6:1361001	6	1361001	1362000	1000	1	9.20E-11	0.63	0	0	Heatr5b;LOC103692530	
DMR6:1685001	6	1685001	1687000	2000	1	1.70E-08	0.47	33	1.65	Qpct	Transport
DMR6:2927001	6	2927001	2930000	3000	2	1.70E-13	0.52	38	1.27	Dhx57	Transcription
DMR6:3107001	6	3107001	3109000	2000	1	3.50E-07	0.4	41	2.05	Arhgef33;Sos1	Transcription
DMR6:3178001	6	3178001	3180000	2000	1	1.60E-07	0.46	16	0.8	Sos1	Transcription
DMR6:3377001	6	3377001	3378000	1000	1	1.30E-07	-0.37	3	0.3	Map4k3;LOC102551744;LOC103692535	
DMR6:3698001	6	3698001	3702000	4000	1	2.70E-11	0.45	62	1.55	Tmem178a	
DMR6:3726001	6	3726001	3727000	1000	1	2.70E-09	0.38	15	1.5	Tmem178a	
DMR6:3750001	6	3750001	3757000	7000	1	8.60E-07	0.38	73	1.04	Thumpd2	Epigenetic
DMR6:3758001	6	3758001	3765000	7000	3	6.20E-11	-0.42	84	1.2	Thumpd2	Epigenetic
DMR6:6708001	6	6708001	6709000	1000	1	1.30E-08	-0.49	13	1.3	Cox7a2l;LOC108351165	Metabolism
DMR6:6911001	6	6911001	6914000	3000	1	1.60E-11	0.47	50	1.67	Mta3	Development
DMR6:7639001	6	7639001	7646000	7000	1	5.60E-10	-0.33	97	1.39	Thada	Cytoskeleton
DMR6:7681001	6	7681001	7683000	2000	1	7.50E-09	-0.46	15	0.75	Thada	Cytoskeleton
DMR6:7920001	6	7920001	7923000	3000	1	5.80E-10	0.75	43	1.43	Dync2li1	Cytoskeleton
DMR6:7943001	6	7943001	7944000	1000	1	7.10E-08	0.38	28	2.8	Dync2li1;Abcg5	Cytoskeleton;Transport
DMR6:8355001	6	8355001	8356000	1000	1	2.40E-07	-0.57	14	1.4	Prepl;Camkmt	Protease;Golgi
DMR6:8549001	6	8549001	8550000	1000	1	8.20E-07	-0.59	16	1.6	Camkmt	Golgi
DMR6:8665001	6	8665001	8667000	2000	1	1.30E-07	0.36	25	1.25	Camkmt	Golgi
DMR6:8668001	6	8668001	8672000	4000	1	3.20E-08	-0.59	72	1.8	Camkmt	Golgi
DMR6:9344001	6	9344001	9346000	2000	2	2.50E-11	0.49	92	4.6	Srbd1	Translation
DMR6:9540001	6	9540001	9541000	1000	1	4.50E-08	-0.36	10	1	Prkce	Signaling
DMR6:9544001	6	9544001	9546000	2000	1	9.40E-10	0.62	23	1.15	Prkce	Signaling
DMR6:9655001	6	9655001	9656000	1000	1	6.20E-08	0.49	12	1.2	Prkce	Signaling
DMR6:9736001	6	9736001	9738000	2000	1	2.50E-08	0.57	29	1.45	Prkce	Signaling
DMR6:9910001	6	9910001	9913000	3000	1	8.40E-10	0.52	60	2	Prkce;LOC102557388	Signaling
DMR6:10325001	6	10325001	10327000	2000	1	1.80E-07	-0.38	38	1.9	Epas1	Transcription

DMR6:10344001	6	10344001	10345000	1000	1	2.50E-12	0.62	32	3.2	Epas1	Transcription
DMR6:10481001	6	10481001	10482000	1000	1	2.50E-09	0.46	21	2.1	Tmem247	
DMR6:10551001	6	10551001	10552000	1000	1	8.50E-07	0.36	19	1.9	Rhoq	Signaling
DMR6:10603001	6	10603001	10604000	1000	1	8.40E-07	0.35	10	1	Pigf;Cript	Golgi
DMR6:10963001	6	10963001	10968000	5000	1	1.20E-13	0.58	90	1.8	Ttc7a	
DMR6:11226001	6	11226001	11228000	2000	1	1.10E-07	-0.4	37	1.85	Msh2;LOC103692555	Transcription
DMR6:11378001	6	11378001	11379000	1000	1	9.20E-12	0.69	7	0.7	Kcnk12	Transport
DMR6:11641001	6	11641001	11644000	3000	1	2.40E-07	-0.37	66	2.2	Msh6	Transcription
DMR6:11651001	6	11651001	11652000	1000	1	1.50E-14	0.48	27	2.7	Msh6	Transcription
DMR6:12208001	6	12208001	12209000	1000	1	1.80E-07	-0.49	20	2	Foxn2;LOC102546644	
DMR6:12307001	6	12307001	12310000	3000	1	3.80E-09	0.41	81	2.7	Ppp1r21	
DMR6:12362001	6	12362001	12363000	1000	1	2.90E-07	0.35	19	1.9	Ston1	Transport
DMR6:12372001	6	12372001	12376000	4000	1	1.50E-12	0.56	94	2.35	Ston1	Transport
DMR6:12408001	6	12408001	12413000	5000	1	4.30E-10	0.48	33	0.66	Gtf2a1l	Transcription
DMR6:12482001	6	12482001	12484000	2000	1	1.30E-11	0.29	39	1.95	Lhcgr	Signaling
DMR6:14167001	6	14167001	14170000	3000	1	3.60E-08	-0.41	39	1.3	Nrxn1	
DMR6:14315001	6	14315001	14316000	1000	1	1.60E-07	-0.53	17	1.7	Nrxn1;LOC103692560	
DMR6:14389001	6	14389001	14391000	2000	1	1.40E-07	0.63	22	1.1	Nrxn1	
DMR6:14487001	6	14487001	14489000	2000	1	2.90E-08	0.42	38	1.9	Nrxn1;LOC102550556	
DMR6:14891001	6	14891001	14897000	6000	1	2.50E-08	-0.31	58	0.97	Nrxn1	
DMR6:15014001	6	15014001	15021000	7000	3	2.00E-10	-0.36	84	1.2	Nrxn1	
DMR6:15200001	6	15200001	15204000	4000	1	4.20E-09	-0.47	36	0.9	Nrxn1	
DMR6:18897001	6	18897001	18900000	3000	1	2.80E-07	-0.28	27	0.9	Alkbh8	
DMR6:18998001	6	18998001	19002000	4000	1	4.50E-08	-0.34	30	0.75	Cwf19l2	
DMR6:19015001	6	19015001	19017000	2000	1	8.50E-11	-0.37	15	0.75	Cwf19l2;LOC102554832	
DMR6:21474001	6	21474001	21475000	1000	1	4.30E-07	0.54	6	0.6	Ltbp1	Extracellular Matrix
DMR6:21836001	6	21836001	21839000	3000	1	1.90E-09	0.51	21	0.7	Ttc27	
DMR6:21889001	6	21889001	21891000	2000	1	2.50E-07	-0.44	27	1.35	Ttc27;Birc6	
DMR6:22033001	6	22033001	22034000	1000	1	1.50E-08	-0.64	15	1.5	Birc6	
DMR6:22159001	6	22159001	22167000	8000	3	2.80E-10	-0.47	100	1.25	Nlrc4	
DMR6:22218001	6	22218001	22219000	1000	1	4.20E-10	0.81	2	0.2	Slc30a6;LOC103692563	Transport
DMR6:23216001	6	23216001	23219000	3000	1	3.30E-07	0.47	31	1.03	LOC108351181;Clip4	Transcription
DMR6:23326001	6	23326001	23327000	1000	1	9.30E-09	0.54	9	0.9	Clip4	Transcription
DMR6:23451001	6	23451001	23453000	2000	1	1.20E-07	0.36	22	1.1	Wdr43	
DMR6:24485001	6	24485001	24491000	6000	1	8.00E-07	-0.29	68	1.13	Lclat1	Metabolism
DMR6:25738001	6	25738001	25743000	5000	1	2.30E-07	-0.32	79	1.58	Bre	
DMR6:26644001	6	26644001	26646000	2000	1	4.50E-07	0.36	26	1.3	Slc30a3	Transport
DMR6:26669001	6	26669001	26670000	1000	1	1.10E-07	0.41	14	1.4	Cad	Metabolism
DMR6:27006001	6	27006001	27011000	5000	1	6.80E-10	0.5	46	0.92	Dpysl5	Metabolism
DMR6:27331001	6	27331001	27332000	1000	1	1.10E-09	0.52	17	1.7	RGD1559683;Otof	Transport
DMR6:27338001	6	27338001	27340000	2000	1	3.90E-07	-0.39	25	1.25	Otof	Transport
DMR6:27418001	6	27418001	27419000	1000	1	2.70E-08	0.38	11	1.1	Otof;Drc1	Transport
DMR6:27646001	6	27646001	27647000	1000	1	4.00E-07	-0.39	8	0.8	Garem2;LOC503104	
DMR6:27790001	6	27790001	27791000	1000	1	1.50E-07	0.54	14	1.4	Kif3c	Cytoskeleton
DMR6:28273001	6	28273001	28274000	1000	1	1.60E-08	0.41	15	1.5	Dnmt3a	Epigenetic
DMR6:28375001	6	28375001	28377000	2000	1	1.40E-07	0.38	29	1.45	RGD1565766;Pomc	
DMR6:28613001	6	28613001	28615000	2000	1	8.40E-10	0.41	19	0.95	Adcy3	
DMR6:28725001	6	28725001	28726000	1000	1	9.30E-07	-0.4	9	0.9	Ncoa1	Epigenetic
DMR6:28798001	6	28798001	28800000	2000	1	8.70E-10	0.45	13	0.65	Ncoa1	Epigenetic
DMR6:28805001	6	28805001	28812000	7000	2	5.40E-09	-0.38	69	0.99	Ncoa1;LOC103692576	Epigenetic
DMR6:28824001	6	28824001	28829000	5000	2	4.10E-11	-0.48	42	0.84	Ncoa1;LOC103692576	Epigenetic
DMR6:28892001	6	28892001	28896000	4000	1	8.70E-10	-0.43	37	0.92	Ncoa1	Epigenetic
DMR6:29188001	6	29188001	29191000	3000	1	5.10E-09	0.62	45	1.5	Klhl29	Cytoskeleton
DMR6:30115001	6	30115001	30118000	3000	1	3.30E-07	0.42	41	1.37	Itsn2	Transport
DMR6:33179001	6	33179001	33180000	1000	1	7.90E-07	-0.44	28	2.8	Apob	Binding Proteins
DMR6:33480001	6	33480001	33485000	5000	1	4.80E-08	-0.31	41	0.82	Ldah	Metabolism
DMR6:33918001	6	33918001	33920000	2000	1	5.70E-07	0.34	27	1.35	Sdc1	Receptor
DMR6:34119001	6	34119001	34121000	2000	1	2.10E-07	-0.27	32	1.6	Wdr35	
DMR6:36874001	6	36874001	36875000	1000	1	1.00E-08	-0.43	15	1.5	Msgn1	
DMR6:38095001	6	38095001	38097000	2000	1	5.60E-08	0.36	23	1.15	Trnav-uac	
DMR6:38534001	6	38534001	38536000	2000	1	9.70E-09	-0.5	15	0.75	Nbas	

DMR6:38574001	6	38574001	38577000	3000	3	2.80E-07	-0.4	29	0.97	Nbas;LOC108351202	
DMR6:38686001	6	38686001	38691000	5000	2	5.10E-10	-0.34	67	1.34	Nbas	
DMR6:41786001	6	41786001	41788000	2000	1	1.40E-12	0.51	14	0.7	LOC102550954;Lpin1	
DMR6:43041001	6	43041001	43043000	2000	1	1.10E-09	0.46	43	2.15	Hpcal1	
DMR6:43225001	6	43225001	43226000	1000	1	4.30E-08	0.43	19	1.9	Asap2	
DMR6:44330001	6	44330001	44333000	3000	1	1.30E-09	0.5	48	1.6	Kidins220	
DMR6:48552001	6	48552001	48553000	1000	1	4.10E-07	0.59	4	0.4	Myt1l	Transcription
DMR6:49030001	6	49030001	49035000	5000	2	2.10E-07	-0.31	44	0.88	Tpo	Metabolism
DMR6:50520001	6	50520001	50528000	8000	1	4.30E-13	0.56	144	1.8	Lamb1	Extracellular Matrix
DMR6:50898001	6	50898001	50906000	8000	2	5.10E-10	-0.41	89	1.11	Bcap29	Transport
DMR6:50980001	6	50980001	50984000	4000	1	6.30E-09	-0.31	47	1.18	Cog5	
DMR6:51067001	6	51067001	51068000	1000	1	6.20E-07	0.5	6	0.6	Cog5	
DMR6:51319001	6	51319001	51322000	3000	1	1.50E-07	0.35	27	0.9	Prkar2b	Signaling
DMR6:51336001	6	51336001	51337000	1000	1	7.60E-07	-0.5	17	1.7	Prkar2b	Signaling
DMR6:52528001	6	52528001	52530000	2000	1	6.50E-07	0.37	44	2.2	Atxn7l1	
DMR6:52556001	6	52556001	52558000	2000	1	2.30E-07	0.33	24	1.2	Atxn7l1	
DMR6:53581001	6	53581001	53584000	3000	1	2.10E-08	0.49	40	1.33	Hdac9	
DMR6:53670001	6	53670001	53672000	2000	1	2.10E-07	-0.43	16	0.8	Hdac9	
DMR6:53680001	6	53680001	53686000	6000	2	1.90E-09	-0.35	53	0.88	Hdac9	
DMR6:54019001	6	54019001	54020000	1000	1	3.30E-08	0.63	6	0.6	Hdac9	
DMR6:54129001	6	54129001	54131000	2000	1	1.00E-08	0.5	13	0.65	Hdac9	
DMR6:54175001	6	54175001	54180000	5000	1	9.00E-08	-0.34	58	1.16	Hdac9	
DMR6:54203001	6	54203001	54204000	1000	1	6.30E-07	0.33	11	1.1	Hdac9	
DMR6:54234001	6	54234001	54235000	1000	1	3.60E-07	0.37	15	1.5	Hdac9	
DMR6:54408001	6	54408001	54416000	8000	2	1.40E-08	-0.33	92	1.15	Prps1l1	Signaling
DMR6:54991001	6	54991001	54994000	3000	1	2.30E-07	0.4	53	1.77	Ahr;LOC102554773	Transcription
DMR6:55368001	6	55368001	55369000	1000	1	9.10E-08	0.45	7	0.7	Agr3	Metabolism
DMR6:55396001	6	55396001	55401000	5000	1	8.50E-08	-0.35	41	0.82	Agr3;LOC100361632	Metabolism
DMR6:55613001	6	55613001	55614000	1000	1	3.20E-07	0.51	10	1	Bzw2	Transcription
DMR6:55625001	6	55625001	55628000	3000	1	3.80E-09	0.43	54	1.8	Bzw2	Transcription
DMR6:55667001	6	55667001	55668000	1000	1	2.00E-09	0.47	10	1	Ankmy2;LOC102556505	
DMR6:55997001	6	55997001	55998000	1000	1	4.90E-07	-0.42	12	1.2	Ispd	
DMR6:56096001	6	56096001	56101000	5000	2	6.80E-10	-0.44	46	0.92	Ispd	
DMR6:56613001	6	56613001	56617000	4000	2	5.20E-10	-0.4	37	0.92	Meox2	Development
DMR6:56664001	6	56664001	56665000	1000	1	6.80E-07	0.45	4	0.4	Meox2	Development
DMR6:56723001	6	56723001	56727000	4000	1	5.90E-09	-0.4	33	0.82	Vom2r49	Signaling
DMR6:57115001	6	57115001	57119000	4000	1	9.10E-10	-0.34	45	1.12	Agmo	
DMR6:57970001	6	57970001	57975000	5000	1	2.00E-09	-0.33	60	1.2	Dgkb	Signaling
DMR6:57986001	6	57986001	57992000	6000	2	2.60E-12	-0.4	52	0.87	Dgkb	Signaling
DMR6:58150001	6	58150001	58151000	1000	1	2.20E-07	0.49	6	0.6	Dgkb	Signaling
DMR6:58192001	6	58192001	58198000	6000	1	3.30E-10	-0.32	66	1.1	Dgkb	Signaling
DMR6:58264001	6	58264001	58271000	7000	3	4.50E-12	-0.55	85	1.21	Dgkb	Signaling
DMR6:60062001	6	60062001	60063000	1000	1	3.20E-08	-0.54	14	1.4	Scin;LOC103692615	Cytoskeleton
DMR6:60285001	6	60285001	60286000	1000	1	2.30E-20	0.61	16	1.6	Zfp277	Transcription
DMR6:60460001	6	60460001	60461000	1000	1	4.60E-07	0.38	8	0.8	Dock4	Transcription
DMR6:60649001	6	60649001	60652000	3000	1	1.80E-07	0.32	41	1.37	Dock4	Transcription
DMR6:61050001	6	61050001	61052000	2000	1	1.20E-09	-0.34	19	0.95	Immp2l;LOC102552766	
DMR6:61549001	6	61549001	61551000	2000	1	1.50E-12	-0.54	29	1.45	Immp2l	
DMR6:61621001	6	61621001	61622000	1000	1	2.20E-07	-0.52	4	0.4	Immp2l	
DMR6:61822001	6	61822001	61827000	5000	1	8.60E-08	-0.3	54	1.08	Immp2l	
DMR6:64414001	6	64414001	64416000	2000	1	1.00E-07	-0.5	32	1.6	Nrcam	Cytoskeleton
DMR6:64472001	6	64472001	64478000	6000	3	3.20E-07	-0.34	66	1.1	Nrcam	Cytoskeleton
DMR6:64782001	6	64782001	64786000	4000	2	1.00E-08	-0.43	44	1.1	Nrcam;LOC100911922	Cytoskeleton
DMR6:71209001	6	71209001	71216000	7000	1	1.20E-44	0.6	72	1.03	Prkd1	Signaling
DMR6:71219001	6	71219001	71221000	2000	1	5.60E-07	-0.47	24	1.2	Prkd1	Signaling
DMR6:71273001	6	71273001	71275000	2000	1	2.60E-07	-0.56	23	1.15	Prkd1	Signaling
DMR6:72690001	6	72690001	72696000	6000	3	9.80E-08	-0.33	66	1.1	Heatr5a	
DMR6:72743001	6	72743001	72745000	2000	1	3.40E-08	-0.51	20	1	Heatr5a;LOC108351334	
DMR6:72800001	6	72800001	72803000	3000	1	8.30E-07	-0.29	42	1.4	Gpr33	Signaling
DMR6:73753001	6	73753001	73754000	1000	1	3.60E-07	-0.5	10	1	Akap6	
DMR6:73878001	6	73878001	73879000	1000	1	2.70E-09	-0.51	11	1.1	Akap6	

DMR6:73931001	6	73931001	73937000	6000	2	6.50E-09	-0.35	68	1.13	Akap6	
DMR6:73968001	6	73968001	73971000	3000	1	6.90E-07	-0.46	52	1.73	Akap6	
DMR6:74110001	6	74110001	74111000	1000	1	8.50E-11	-0.61	20	2	Npas3	
DMR6:74389001	6	74389001	74390000	1000	1	3.40E-09	0.5	6	0.6	Npas3	
DMR6:74789001	6	74789001	74790000	1000	1	1.60E-08	-0.6	18	1.8	Npas3	
DMR6:74872001	6	74872001	74876000	4000	2	1.50E-13	-0.42	35	0.88	Npas3	
DMR6:75078001	6	75078001	75083000	5000	1	7.80E-08	0.51	59	1.18	Egln3	
DMR6:76105001	6	76105001	76111000	6000	1	1.50E-09	-0.34	67	1.12	RGD1305089	
DMR6:76114001	6	76114001	76116000	2000	1	5.10E-09	0.46	16	0.8	RGD1305089	
DMR6:76435001	6	76435001	76441000	6000	2	1.10E-07	-0.3	66	1.1	Ralgapa1	Signaling
DMR6:77431001	6	77431001	77433000	2000	1	3.00E-09	0.41	28	1.4	Nkx2-1;LOC102552092	Development
DMR6:77688001	6	77688001	77694000	6000	2	4.80E-07	-0.38	67	1.12	Slc25a21;LOC102554460	Transport
DMR6:77696001	6	77696001	77697000	1000	1	1.00E-07	0.5	6	0.6	Slc25a21	Transport
DMR6:77760001	6	77760001	77763000	3000	1	1.30E-09	-0.38	28	0.93	Slc25a21;LOC108351240	Transport
DMR6:77836001	6	77836001	77841000	5000	1	1.70E-07	-0.26	47	0.94	Slc25a21	Transport
DMR6:77976001	6	77976001	77978000	2000	1	9.00E-07	-0.37	16	0.8	Slc25a21	Transport
DMR6:78033001	6	78033001	78037000	4000	1	1.80E-07	-0.36	40	1	Slc25a21	Transport
DMR6:78100001	6	78100001	78102000	2000	1	2.00E-07	0.33	20	1	Slc25a21	Transport
DMR6:78130001	6	78130001	78136000	6000	1	2.50E-08	-0.28	41	0.68	Slc25a21	Transport
DMR6:78305001	6	78305001	78309000	4000	2	1.40E-11	-0.42	29	0.72	Mipol1	
DMR6:78422001	6	78422001	78424000	2000	1	7.90E-07	-0.31	22	1.1	Mipol1	
DMR6:78425001	6	78425001	78426000	1000	1	6.10E-13	-0.46	15	1.5	Mipol1	
DMR6:78733001	6	78733001	78735000	2000	1	1.90E-07	0.41	10	0.5	RGD1560556	
DMR6:78738001	6	78738001	78740000	2000	1	6.90E-09	-0.46	11	0.55	RGD1560556	
DMR6:83171001	6	83171001	83173000	2000	1	2.20E-08	-0.26	18	0.9	Lrfn5	
DMR6:83287001	6	83287001	83288000	1000	1	3.50E-07	-0.4	7	0.7	Lrfn5	
DMR6:84102001	6	84102001	84106000	4000	1	4.60E-08	-0.44	28	0.7	Cct6a-ps11	
DMR6:84117001	6	84117001	84118000	1000	1	8.70E-09	-0.55	7	0.7	Cct6a-ps11	
DMR6:84128001	6	84128001	84131000	3000	1	4.10E-09	-0.33	30	1	Cct6a-ps11	
DMR6:86233001	6	86233001	86240000	7000	3	7.10E-12	-0.51	59	0.84	Fscb	
DMR6:86751001	6	86751001	86753000	2000	1	1.30E-08	-0.57	11	0.55	Fam179b	
DMR6:88493001	6	88493001	88494000	1000	1	2.90E-08	-0.57	10	1	Mdga2	
DMR6:88511001	6	88511001	88516000	5000	2	2.30E-14	-0.39	55	1.1	Mdga2	
DMR6:88567001	6	88567001	88574000	7000	1	7.40E-09	-0.3	78	1.11	Mdga2;LOC108351351	
DMR6:88692001	6	88692001	88696000	4000	2	1.40E-12	-0.31	46	1.15	Mdga2	
DMR6:88773001	6	88773001	88775000	2000	1	4.30E-07	-0.38	17	0.85	Mdga2;LOC103692647	
DMR6:91564001	6	91564001	91565000	1000	1	3.10E-08	0.46	18	1.8	Klhdc1	
DMR6:91886001	6	91886001	91889000	3000	1	3.70E-07	0.34	44	1.47	Vcpkmt;Sos2	Transcription
DMR6:92130001	6	92130001	92133000	3000	1	4.30E-10	0.56	31	1.03	Cdkl1;Map4k5	Signaling
DMR6:92137001	6	92137001	92140000	3000	1	2.40E-09	-0.56	48	1.6	Map4k5	
DMR6:92413001	6	92413001	92419000	6000	1	2.60E-07	-0.28	68	1.13	LOC500665;Nin	
DMR6:93758001	6	93758001	93763000	5000	1	3.30E-07	-0.29	52	1.04	Dact1	
DMR6:95020001	6	95020001	95021000	1000	1	6.20E-16	0.65	7	0.7	Rtn1	
DMR6:95139001	6	95139001	95140000	1000	1	1.10E-08	0.42	27	2.7	Rtn1	
DMR6:95224001	6	95224001	95226000	2000	1	1.00E-07	0.61	25	1.25	Lrrc9	
DMR6:95270001	6	95270001	95272000	2000	1	9.30E-07	-0.39	23	1.15	Lrrc9	
DMR6:95350001	6	95350001	95351000	1000	1	2.20E-07	0.46	9	0.9	LOC102549502;Pcnx4	
DMR6:95470001	6	95470001	95474000	4000	1	5.00E-08	-0.41	40	1	Dhrs7	Metabolism
DMR6:95475001	6	95475001	95476000	1000	1	1.50E-07	-0.29	11	1.1	Dhrs7	Metabolism
DMR6:96143001	6	96143001	96146000	3000	2	6.30E-10	-0.51	22	0.73	Mnat1	Transcription
DMR6:96446001	6	96446001	96449000	3000	1	9.30E-07	-0.36	33	1.1	Tmem30b	
DMR6:96626001	6	96626001	96627000	1000	1	2.00E-07	0.37	12	1.2	Prkch;LOC108351320	Signaling
DMR6:97098001	6	97098001	97103000	5000	2	1.80E-14	-0.4	47	0.94	Syt16;LOC102550910	
DMR6:97156001	6	97156001	97159000	3000	1	8.90E-09	-0.58	32	1.07	Syt16	
DMR6:98276001	6	98276001	98277000	1000	1	1.10E-07	0.65	7	0.7	Rhoj	Signaling
DMR6:98329001	6	98329001	98332000	3000	1	5.70E-13	0.63	43	1.43	Rhoj	Signaling
DMR6:98425001	6	98425001	98426000	1000	1	1.70E-10	0.42	12	1.2	Ppp2r5e	Signaling
DMR6:99041001	6	99041001	99044000	3000	1	4.50E-07	0.35	76	2.53	Syne2	
DMR6:99687001	6	99687001	99688000	1000	1	4.10E-07	-0.49	21	2.1	Sptb	
DMR6:99696001	6	99696001	99697000	1000	1	5.70E-08	0.43	13	1.3	Sptb	
DMR6:99733001	6	99733001	99735000	2000	1	4.50E-10	0.41	25	1.25	Sptb	

DMR6:99865001	6	99865001	99867000	2000	1	2.50E-07	0.39	27	1.35	Rab15;LOC102546646	
DMR6:99910001	6	99910001	99911000	1000	1	9.90E-09	0.51	8	0.8	Fntb	Metabolism
DMR6:100376001	6	100376001	100382000	6000	2	2.50E-10	-0.5	62	1.03	Fut8	Golgi
DMR6:100413001	6	100413001	100419000	6000	1	3.30E-08	-0.32	67	1.12	Fut8	Golgi
DMR6:100475001	6	100475001	100481000	6000	2	1.20E-07	-0.28	71	1.18	Fut8	Golgi
DMR6:101295001	6	101295001	101299000	4000	1	8.10E-07	-0.34	40	1	RGD1562540	
DMR6:101354001	6	101354001	101357000	3000	1	7.10E-09	-0.44	26	0.87	Gphn	
DMR6:101420001	6	101420001	101421000	1000	1	1.90E-07	-0.43	11	1.1	Gphn;LOC102547341	
DMR6:101543001	6	101543001	101545000	2000	1	1.70E-09	-0.52	14	0.7	Gphn	
DMR6:101704001	6	101704001	101707000	3000	2	6.50E-12	-0.41	26	0.87	Gphn	
DMR6:101729001	6	101729001	101730000	1000	1	3.60E-13	0.67	7	0.7	Gphn	
DMR6:101814001	6	101814001	101818000	4000	1	7.70E-07	-0.29	38	0.95	Gphn	
DMR6:101819001	6	101819001	101828000	9000	2	9.30E-12	-0.38	141	1.57	Gphn	
DMR6:101846001	6	101846001	101849000	3000	1	2.20E-07	-0.49	12	0.4	Gphn	
DMR6:101872001	6	101872001	101878000	6000	1	1.40E-07	-0.44	35	0.58	Fam71d;LOC100909680	
DMR6:101889001	6	101889001	101895000	6000	1	8.40E-09	-0.39	52	0.87	Fam71d	
DMR6:101947001	6	101947001	101951000	4000	1	3.70E-12	-0.39	40	1	Mpp5	Cytoskeleton
DMR6:102018001	6	102018001	102024000	6000	1	2.40E-08	-0.32	66	1.1	Mpp5;Atp6v1d	Cytoskeleton;Metabolism
DMR6:102495001	6	102495001	102499000	4000	1	2.10E-07	-0.4	36	0.9	Rad51b	Transcription
DMR6:102838001	6	102838001	102840000	2000	1	7.40E-07	0.46	18	0.9	Rad51b	Transcription
DMR6:103378001	6	103378001	103381000	3000	1	7.00E-11	0.55	54	1.8	Actn1	
DMR6:103387001	6	103387001	103388000	1000	1	7.10E-13	0.69	20	2	Actn1	
DMR6:103448001	6	103448001	103450000	2000	1	9.10E-08	0.36	35	1.75	Actn1;LOC100359669	
DMR6:104348001	6	104348001	104350000	2000	2	1.30E-09	0.63	12	0.6	Plekhd1	Cytoskeleton
DMR6:104834001	6	104834001	104835000	1000	1	6.10E-08	0.49	9	0.9	Smoc1	Signaling
DMR6:105319001	6	105319001	105321000	2000	1	4.80E-07	-0.43	35	1.75	Med6	Transcription
DMR6:105479001	6	105479001	105481000	2000	1	5.00E-07	0.55	31	1.55	Map3k9	Signaling
DMR6:106485001	6	106485001	106488000	3000	1	5.10E-13	0.66	37	1.23	Rgs6	
DMR6:106491001	6	106491001	106492000	1000	1	2.30E-08	0.47	9	0.9	Rgs6	
DMR6:106538001	6	106538001	106540000	2000	1	5.30E-07	0.4	30	1.5	Rgs6	
DMR6:106691001	6	106691001	106693000	2000	1	4.90E-09	0.41	26	1.3	Dpf3	Epigenetic
DMR6:106844001	6	106844001	106846000	2000	1	3.20E-09	0.39	43	2.15	Dpf3	Epigenetic
DMR6:106886001	6	106886001	106889000	3000	1	1.90E-08	0.49	40	1.33	Dpf3	Epigenetic
DMR6:107004001	6	107004001	107006000	2000	1	1.00E-07	0.38	42	2.1	Dcaf4	
DMR6:107675001	6	107675001	107676000	1000	1	2.70E-11	0.62	10	1	Elmsan1	
DMR6:108275001	6	108275001	108277000	2000	1	4.30E-07	-0.41	23	1.15	Vsx2	Development
DMR6:108375001	6	108375001	108377000	2000	1	2.00E-09	0.43	23	1.15	Vrtn	
DMR6:108475001	6	108475001	108476000	1000	1	1.80E-07	0.34	13	1.3	Npc2	
DMR6:108621001	6	108621001	108623000	2000	1	2.00E-09	0.48	18	0.9	Arel1	Proteolysis
DMR6:108896001	6	108896001	108897000	1000	1	1.40E-07	-0.46	15	1.5	Ylpm1	Metabolism
DMR6:109067001	6	109067001	109070000	3000	1	1.00E-07	0.35	37	1.23	Mlh3	Transcription
DMR6:109844001	6	109844001	109846000	2000	1	1.20E-08	0.47	15	0.75	Ttll5	Cytoskeleton
DMR6:109891001	6	109891001	109892000	1000	1	6.30E-09	-0.48	12	1.2	Ttll5	Cytoskeleton
DMR6:110365001	6	110365001	110367000	2000	1	2.00E-09	0.38	29	1.45	Esrrb	
DMR6:110418001	6	110418001	110421000	3000	1	3.80E-11	0.48	31	1.03	Esrrb	
DMR6:110448001	6	110448001	110451000	3000	1	6.00E-08	0.39	50	1.67	Esrrb	
DMR6:110645001	6	110645001	110646000	1000	1	1.70E-08	0.47	14	1.4	Vash1;Angel1	Translation
DMR6:110737001	6	110737001	110738000	1000	1	4.50E-09	0.46	26	2.6	Lrrc74a	
DMR6:111004001	6	111004001	111006000	2000	1	1.30E-07	0.34	32	1.6	Zdhhc22	
DMR6:111537001	6	111537001	111539000	2000	1	2.70E-07	-0.37	29	1.45	RGD1563496;RGD1561148; LOC100910634;LOC503053	
DMR6:111688001	6	111688001	111689000	1000	1	3.10E-07	0.5	8	0.8	Adck1	Transport
DMR6:111736001	6	111736001	111738000	2000	1	8.60E-07	0.46	12	0.6	Adck1	Transport
DMR6:112246001	6	112246001	112247000	1000	1	2.60E-07	0.36	19	1.9	Nrxn3	
DMR6:112390001	6	112390001	112392000	2000	1	3.30E-07	0.4	24	1.2	Nrxn3	
DMR6:112424001	6	112424001	112430000	6000	4	5.60E-10	-0.42	69	1.15	Nrxn3	
DMR6:112652001	6	112652001	112653000	1000	1	5.10E-07	-0.38	10	1	Nrxn3	
DMR6:112710001	6	112710001	112711000	1000	1	1.30E-09	0.64	11	1.1	Nrxn3	
DMR6:113101001	6	113101001	113107000	6000	2	2.60E-07	-0.29	80	1.33	Nrxn3	
DMR6:113262001	6	113262001	113263000	1000	1	6.20E-07	0.45	7	0.7	Nrxn3	
DMR6:113322001	6	113322001	113323000	1000	1	1.90E-07	0.55	2	0.2	Nrxn3	

DMR6:113412001	6	113412001	113418000	6000	1	7.00E-08	-0.38	83	1.38	Nrxn3	
DMR6:113522001	6	113522001	113523000	1000	1	1.10E-07	-0.5	12	1.2	Nrxn3	
DMR6:113718001	6	113718001	113722000	4000	2	5.70E-08	-0.43	34	0.85	Nrxn3	
DMR6:114069001	6	114069001	114070000	1000	1	8.00E-08	-0.5	18	1.8	Nrxn3	
DMR6:114858001	6	114858001	114862000	4000	1	6.80E-08	-0.36	57	1.43	Cep128	
DMR6:114890001	6	114890001	114893000	3000	1	5.30E-09	-0.48	27	0.9	Cep128	
DMR6:115210001	6	115210001	115215000	5000	1	1.30E-08	-0.35	56	1.12	Tshr	Signaling
DMR6:115285001	6	115285001	115287000	2000	1	1.50E-07	0.54	12	0.6	Tshr	Signaling
DMR6:115416001	6	115416001	115417000	1000	1	2.50E-07	0.41	19	1.9	Ston2	Transport
DMR6:115489001	6	115489001	115495000	6000	2	7.80E-13	-0.37	64	1.07	Ston2;LOC102550912	Transport
DMR6:115533001	6	115533001	115534000	1000	1	3.10E-09	0.32	6	0.6	Ston2	Transport
DMR6:122600001	6	122600001	122601000	1000	1	7.70E-09	-0.44	8	0.8	Spata7	
DMR6:122691001	6	122691001	122697000	6000	2	1.70E-09	-0.39	53	0.88	Ptpn21	Signaling
DMR6:122774001	6	122774001	122775000	1000	1	7.00E-11	-0.51	12	1.2	Zc3h14;Eml5	
DMR6:122825001	6	122825001	122827000	2000	1	6.90E-07	-0.41	15	0.75	Eml5	
DMR6:122884001	6	122884001	122888000	4000	1	5.70E-08	-0.3	31	0.78	Eml5	
DMR6:123214001	6	123214001	123218000	4000	1	3.40E-08	0.39	64	1.6	Foxn3	
DMR6:123247001	6	123247001	123248000	1000	1	4.80E-10	0.61	13	1.3	Foxn3	
DMR6:123426001	6	123426001	123429000	3000	1	5.50E-07	0.58	33	1.1	Foxn3	
DMR6:123551001	6	123551001	123552000	1000	1	5.40E-09	0.38	19	1.9	Foxn3	
DMR6:123579001	6	123579001	123580000	1000	1	6.90E-07	0.36	12	1.2	Foxn3;LOC102550560	
DMR6:124035001	6	124035001	124039000	4000	1	2.00E-12	0.52	50	1.25	Kcnk13	Transport
DMR6:124445001	6	124445001	124446000	1000	1	2.20E-14	0.45	6	0.6	Ttc7b	
DMR6:125174001	6	125174001	125176000	2000	1	5.60E-07	-0.4	24	1.2	Catsperb	
DMR6:125258001	6	125258001	125260000	2000	2	1.40E-08	-0.42	12	0.6	Catsperb;LOC100360199	
DMR6:125294001	6	125294001	125295000	1000	1	1.20E-07	-0.68	16	1.6	Catsperb	
DMR6:125519001	6	125519001	125522000	3000	1	3.50E-07	-0.37	23	0.77	Tc2n	
DMR6:125544001	6	125544001	125546000	2000	1	5.40E-09	-0.44	17	0.85	Tc2n	
DMR6:125603001	6	125603001	125610000	7000	1	8.10E-07	-0.3	76	1.09	Tc2n	
DMR6:125862001	6	125862001	125863000	1000	1	1.00E-09	-0.5	19	1.9	Atxn3;Ndufb1;LOC103692691;LOC100911956;Cpsf2	Epigenetic;Metabolism
DMR6:126009001	6	126009001	126010000	1000	1	6.70E-08	0.33	12	1.2	Slc24a4	Transport
DMR6:126031001	6	126031001	126032000	1000	1	1.90E-08	0.51	16	1.6	Slc24a4	Transport
DMR6:126085001	6	126085001	126086000	1000	1	1.20E-10	0.56	21	2.1	Slc24a4	Transport
DMR6:126230001	6	126230001	126232000	2000	1	1.80E-07	0.48	20	1	Rin3	Transcription
DMR6:126360001	6	126360001	126361000	1000	1	1.30E-08	-0.46	15	1.5	Golga5	
DMR6:126825001	6	126825001	126827000	2000	1	5.60E-07	0.3	50	2.5	Unc79	
DMR6:127002001	6	127002001	127007000	5000	2	4.40E-08	0.4	121	2.42	Unc79	
DMR6:127050001	6	127050001	127051000	1000	1	2.20E-10	0.53	17	1.7	Unc79	
DMR6:127342001	6	127342001	127345000	3000	1	5.20E-08	0.31	55	1.83	Ifi27;Ifi27l2b	
DMR6:127424001	6	127424001	127427000	3000	1	1.50E-08	-0.61	28	0.93	Ppp4r4	Signaling
DMR6:127690001	6	127690001	127692000	2000	1	7.30E-08	0.37	26	1.3	Serpina9;LOC108351294;Serpina12	Protease; Proteolysis
DMR6:127825001	6	127825001	127829000	4000	2	1.20E-09	-0.31	56	1.4	Serpina3m	Protease; Proteolysis
DMR6:128672001	6	128672001	128674000	2000	1	4.40E-07	0.59	17	0.85	Syne3	
DMR6:129413001	6	129413001	129415000	2000	1	5.80E-07	0.43	32	1.6	Bdkrb2;LOC103692699	Signaling
DMR6:131882001	6	131882001	131884000	2000	1	4.20E-07	0.39	49	2.45	Bcl11b	Transcription
DMR6:132112001	6	132112001	132113000	1000	1	5.40E-08	0.45	17	1.7	Ccnk;Ccdc85c	Signaling
DMR6:132145001	6	132145001	132147000	2000	2	2.70E-16	0.7	41	2.05	Ccdc85c	
DMR6:132222001	6	132222001	132223000	1000	1	1.60E-08	0.46	20	2	Hhipl1	Signaling
DMR6:132270001	6	132270001	132271000	1000	1	7.10E-09	0.46	29	2.9	Cyp46a1	Metabolism
DMR6:132366001	6	132366001	132369000	3000	1	2.80E-07	0.35	58	1.93	Eml1	
DMR6:132594001	6	132594001	132595000	1000	1	6.00E-07	0.37	18	1.8	Evl;Degs2	Cytoskeleton;Metabolism
DMR6:132749001	6	132749001	132752000	3000	1	1.30E-07	0.38	46	1.53	Slc25a29;Mir345	Transport
DMR6:133699001	6	133699001	133705000	6000	2	1.80E-09	0.41	74	1.23	RGD1566401;Mir673;Mir493;Mir337;Mir3544;Mir540;Mir665;Rtl1;Mir431;Mir433;Mir127	
DMR6:133736001	6	133736001	133741000	5000	1	7.50E-12	0.51	93	1.86	Mir341;Mir1188;Mir370	
DMR6:133850001	6	133850001	133852000	2000	1	1.90E-07	-0.5	13	0.65	Mir379;Mir411;Mir299b;Mir299;Mir3579;Mir380;Mir323;Mir758	

DMR6:133873001	6	133873001	133875000	2000	1	2.40E-07	0.32	40	2	Mir494;Mir1193;Mir666;Mir543;Mir495;Mir667;Mir376c;Mir376b;Mir3595;Mir376a;Mir300;Mir381;Mir487b;Mir3576;Mir539;Mir6331;Mir544;Mir3592;Mir382;Mir134	
DMR6:133891001	6	133891001	133894000	3000	1	7.30E-08	0.37	63	2.1	Mir3592;Mir382;Mir134;Mir668;Mir485;Mir154;Mir496;Mir377;Mir541;Mir3581;Mir409;Mir412;Mir3578;Mir369;Mir410;Mir3072	
DMR6:133904001	6	133904001	133905000	1000	1	1.30E-08	0.42	10	1	Mir410;Mir3072	
DMR6:134830001	6	134830001	134833000	3000	1	4.70E-10	-0.5	57	1.9	Ppp2r5c;LOC102547242;LOC102547175	Signaling
DMR6:134866001	6	134866001	134867000	1000	1	4.10E-08	0.4	45	4.5	Ppp2r5c	Signaling
DMR6:134904001	6	134904001	134905000	1000	1	6.30E-08	0.43	10	1	Ppp2r5c	Signaling
DMR6:135120001	6	135120001	135123000	3000	1	2.10E-11	-0.56	62	2.07	Hsp90aa1;LOC102549239	Signaling
DMR6:135202001	6	135202001	135204000	2000	1	3.40E-07	0.5	21	1.05	Wdr20	
DMR6:135481001	6	135481001	135482000	1000	1	6.20E-07	0.49	25	2.5	Rcor1	
DMR6:135720001	6	135720001	135724000	4000	1	1.30E-07	0.48	60	1.5	Traf3;Amn	Cytoskeleton
DMR6:135808001	6	135808001	135809000	1000	1	8.30E-11	0.41	26	2.6	Cdc42bpb	Signaling
DMR6:136544001	6	136544001	136546000	2000	1	6.30E-10	0.5	31	1.55	Tdrd9;LOC691437;Rd3l	Transcription
DMR6:136568001	6	136568001	136570000	2000	1	2.00E-12	0.58	68	3.4	Tdrd9;LOC102548527	Transcription
DMR6:136625001	6	136625001	136627000	2000	1	4.20E-07	0.37	65	3.25	Tdrd9	Transcription
DMR6:136639001	6	136639001	136640000	1000	1	8.60E-10	-0.5	21	2.1	Tdrd9	Transcription
DMR6:137162001	6	137162001	137165000	3000	1	9.40E-08	0.47	61	2.03	Inf2	
DMR6:137180001	6	137180001	137182000	2000	1	7.70E-07	0.34	40	2	Inf2;Adssl1	Metabolism
DMR6:137319001	6	137319001	137321000	2000	1	2.60E-07	-0.45	42	2.1	Cep170b;LOC102552635;Pld4	Cytoskeleton;Metabolism
DMR6:137387001	6	137387001	137388000	1000	1	1.70E-07	0.51	20	2	Ahnak2;RGD1307315;Cdca4	Transcription
DMR6:137667001	6	137667001	137668000	1000	1	3.10E-09	0.44	9	0.9	Gpr132	Signaling
DMR6:137713001	6	137713001	137721000	8000	1	7.00E-07	0.36	247	3.09	Jag2	
DMR6:137722001	6	137722001	137725000	3000	1	4.40E-09	0.44	65	2.17	Jag2	
DMR6:137846001	6	137846001	137847000	1000	1	4.50E-07	-0.36	29	2.9	Pacs2	
DMR6:137883001	6	137883001	137885000	2000	1	6.90E-11	0.51	32	1.6	Pacs2;Tex22	
DMR6:137948001	6	137948001	137951000	3000	1	7.50E-07	0.38	63	2.1	Mta1;Crip2;Crip1	Development;Cytoskeleton
DMR6:138444001	6	138444001	138446000	2000	1	3.90E-10	-0.35	40	2	Ighv;Adam6	
DMR6:139169001	6	139169001	139170000	1000	1	7.70E-07	-0.41	8	0.8	Ighg	
DMR6:140923001	6	140923001	140926000	3000	2	1.90E-07	-0.32	23	0.77	RGD1560842	
DMR6:144087001	6	144087001	144090000	3000	1	1.70E-07	0.61	29	0.97	Wdr60	Cytoskeleton
DMR6:144129001	6	144129001	144135000	6000	1	8.60E-07	-0.29	59	0.98	Wdr60	Cytoskeleton
DMR6:144306001	6	144306001	144307000	1000	1	4.10E-07	0.35	12	1.2	Ncapg2	
DMR6:144320001	6	144320001	144325000	5000	2	5.00E-09	-0.38	46	0.92	Ncapg2	
DMR6:144557001	6	144557001	144559000	2000	1	1.90E-07	-0.47	23	1.15	Ptprn2	Signaling
DMR6:144637001	6	144637001	144642000	5000	2	1.20E-12	-0.36	46	0.92	Ptprn2	Signaling
DMR6:144795001	6	144795001	144796000	1000	1	1.20E-09	0.53	9	0.9	Ptprn2	Signaling
DMR6:145052001	6	145052001	145056000	4000	1	1.50E-09	0.45	48	1.2	Ptprn2	Signaling
DMR6:145453001	6	145453001	145454000	1000	1	4.10E-07	-0.46	19	1.9	Rapgef5	Transcription
DMR6:145603001	6	145603001	145608000	5000	1	8.60E-08	0.4	77	1.54	Rapgef5	Transcription
DMR6:145756001	6	145756001	145760000	4000	1	6.10E-07	-0.29	60	1.5	Cdca7l	
DMR6:145767001	6	145767001	145769000	2000	2	3.50E-09	0.53	32	1.6	Cdca7l;Rpl32-ps3	
DMR6:145926001	6	145926001	145927000	1000	1	1.80E-10	0.46	26	2.6	Dnah11	Cytoskeleton
DMR6:145953001	6	145953001	145956000	3000	1	4.90E-08	0.37	45	1.5	Dnah11	Cytoskeleton
DMR6:146157001	6	146157001	146164000	7000	1	9.00E-07	-0.23	72	1.03	Sp4	Transcription
DMR6:146205001	6	146205001	146206000	1000	1	8.90E-08	0.49	4	0.4	Sp4	Transcription
DMR6:146906001	6	146906001	146907000	1000	1	5.50E-10	-0.64	5	0.5	Abcb5	Transport
DMR7:2110001	7	2110001	2112000	2000	1	7.50E-09	-0.45	72	3.6	LOC103692755;Rab1-ps1	
DMR7:2466001	7	2466001	2468000	2000	1	1.70E-10	0.36	19	0.95	Naca	Transcription
DMR7:2645001	7	2645001	2650000	5000	1	9.00E-07	0.4	15	0.3	Mip;Timeless	Transport
DMR7:2926001	7	2926001	2929000	3000	1	1.50E-08	0.47	34	1.13	Esyt1;LOC102552826	

DMR7:2984001	7	2984001	2985000	1000	1	9.40E-07	-0.52	19	1.9	Pa2g4;Erbb3	Protease;Receptor
DMR7:3197001	7	3197001	3198000	1000	1	9.20E-15	0.54	14	1.4	Pym1	
DMR7:3323001	7	3323001	3324000	1000	1	8.70E-07	0.41	16	1.6	Gdf11;LOC102553109;Cd63	Growth Factors
DMR7:3416001	7	3416001	3420000	4000	1	5.40E-08	-0.39	32	0.8	Olr875	Signaling
DMR7:3441001	7	3441001	3443000	2000	1	3.60E-07	-0.38	17	0.85	Olr877	Receptor
DMR7:4525001	7	4525001	4527000	2000	1	2.20E-07	-0.29	38	1.9	Olr908	
DMR7:5146001	7	5146001	5147000	1000	1	8.20E-07	-0.32	17	1.7	Olr893-ps	
DMR7:5216001	7	5216001	5218000	2000	1	9.10E-09	0.56	37	1.85	Olr897-ps	
DMR7:5241001	7	5241001	5246000	5000	1	3.80E-11	-0.38	44	0.88	Olr898	Receptor
DMR7:5302001	7	5302001	5309000	7000	2	5.20E-12	-0.4	73	1.04	Olr900-ps	
DMR7:5350001	7	5350001	5355000	5000	1	5.20E-07	-0.34	43	0.86	Olr902-ps	
DMR7:5428001	7	5428001	5430000	2000	1	2.30E-08	-0.33	27	1.35	Olr904-ps;Olr920	
DMR7:5443001	7	5443001	5448000	5000	1	2.00E-09	-0.42	35	0.7	Olr905	Receptor
DMR7:5755001	7	5755001	5756000	1000	1	2.00E-07	0.52	5	0.5	Olr1024	
DMR7:6490001	7	6490001	6491000	1000	1	9.70E-07	0.58	5	0.5	Olr887-ps	
DMR7:6575001	7	6575001	6580000	5000	1	1.70E-07	-0.28	78	1.56	Olr966-ps	
DMR7:6829001	7	6829001	6836000	7000	1	8.00E-07	-0.3	87	1.24	Olr1021-ps	
DMR7:7763001	7	7763001	7765000	2000	1	4.50E-08	-0.4	13	0.65	Olr1029;Olr1030-ps	Receptor
DMR7:8266001	7	8266001	8268000	2000	1	4.70E-09	-0.54	19	0.95	Olr1048-ps	
DMR7:8893001	7	8893001	8894000	1000	1	9.50E-07	-0.69	4	0.4	Olr935-ps	
DMR7:9030001	7	9030001	9033000	3000	1	3.00E-07	-0.31	24	0.8	Olr930-ps	
DMR7:9034001	7	9034001	9036000	2000	1	1.40E-10	0.25	32	1.6	Olr930-ps	
DMR7:9419001	7	9419001	9421000	2000	1	5.70E-10	-0.48	23	1.15	Olr1068	Signaling
DMR7:9582001	7	9582001	9589000	7000	1	9.70E-07	-0.27	57	0.81	Olr1071	Signaling
DMR7:9609001	7	9609001	9620000	11000	2	3.20E-09	-0.33	105	0.95	Olr1072	Signaling
DMR7:9638001	7	9638001	9640000	2000	1	7.90E-07	-0.41	12	0.6	Olr1072	Signaling
DMR7:11060001	7	11060001	11062000	2000	1	5.20E-07	-0.42	35	1.75	Gna15	Signaling
DMR7:11123001	7	11123001	11125000	2000	1	3.90E-07	0.38	33	1.65	Celf5;LOC102552420	
DMR7:11154001	7	11154001	11155000	1000	1	2.80E-08	0.43	28	2.8	LOC102552420;Nfic	Transcription
DMR7:11158001	7	11158001	11162000	4000	1	3.70E-11	0.44	118	2.95	Nfic	Transcription
DMR7:11208001	7	11208001	11211000	3000	2	1.40E-17	0.6	75	2.5	LOC102552770;Fzr1;Mfsd12;LOC690617	Proteolysis
DMR7:11252001	7	11252001	11253000	1000	1	1.70E-13	0.64	25	2.5	Hmg20b;LOC102552827;Gipc3;Tbxa2r;Cactin	Cytoskeleton;Signaling
DMR7:11284001	7	11284001	11287000	3000	1	8.30E-12	0.49	38	1.27	Pip5k1c;Tjp3	Signaling;Cell Junction
DMR7:11358001	7	11358001	11359000	1000	1	1.00E-07	0.49	5	0.5	Zfr2;Atcay	Metabolism
DMR7:11531001	7	11531001	11532000	1000	1	4.70E-10	0.55	15	1.5	Sgta	
DMR7:11536001	7	11536001	11539000	3000	1	2.10E-07	0.41	63	2.1	Sgta;Slc39a3	Transport
DMR7:11725001	7	11725001	11726000	1000	1	4.00E-07	0.63	25	2.5	Sppl2b;Lsm7	Proteolysis;Translation
DMR7:11778001	7	11778001	11780000	2000	1	1.00E-11	0.61	31	1.55	Jsrp1;Amh;Sf3a2;Plekhj1;Dot1l	Translation;Transport;Epigenetic
DMR7:11940001	7	11940001	11943000	3000	1	6.00E-08	0.41	98	3.27	Btbd2;Csnk1g2	Proteolysis;Signaling
DMR7:12036001	7	12036001	12042000	6000	1	1.40E-09	0.5	173	2.88	Rexo1;Atp8b3	Transcription;Transport
DMR7:12062001	7	12062001	12063000	1000	1	9.40E-07	0.44	26	2.6	Atp8b3	Transport
DMR7:12072001	7	12072001	12074000	2000	1	3.50E-09	0.4	15	0.75	Atp8b3	Transport
DMR7:12902001	7	12902001	12904000	2000	1	1.50E-07	0.46	34	1.7	Gzmm;Cdc34;Tpgs1	Protease;Cytoskeleton
DMR7:13027001	7	13027001	13029000	2000	1	4.30E-07	0.4	28	1.4	Theg	
DMR7:13052001	7	13052001	13054000	2000	1	7.70E-08	0.64	36	1.8	Mier2;Plpp2	Development;Signaling
DMR7:13095001	7	13095001	13097000	2000	1	4.20E-07	-0.39	41	2.05	Giot1	
DMR7:13136001	7	13136001	13139000	3000	1	5.10E-11	-0.32	31	1.03	LOC100910608;Vom2r53	Signaling
DMR7:13171001	7	13171001	13173000	2000	1	5.00E-08	-0.56	10	0.5	Vom2r53	Signaling
DMR7:13435001	7	13435001	13441000	6000	2	5.00E-08	-0.31	77	1.28	Olr1076	Receptor
DMR7:13451001	7	13451001	13455000	4000	2	7.30E-12	-0.44	35	0.88	Olr1077	Receptor
DMR7:13469001	7	13469001	13474000	5000	1	3.10E-07	-0.33	39	0.78	Olr1077;Olr1078	Receptor
DMR7:13502001	7	13502001	13508000	6000	1	8.60E-10	-0.4	67	1.12	Olr1078;Olr1080-ps	Receptor
DMR7:13562001	7	13562001	13569000	7000	4	4.00E-09	-0.33	73	1.04	Olr1082	Receptor
DMR7:13619001	7	13619001	13625000	6000	1	7.60E-08	-0.45	56	0.93	Olr1084	Receptor
DMR7:13634001	7	13634001	13647000	13000	2	3.20E-07	-0.29	166	1.28	Olr1084	Receptor
DMR7:13707001	7	13707001	13709000	2000	1	3.40E-07	-0.37	10	0.5	Olr1086	Receptor
DMR7:14096001	7	14096001	14103000	7000	2	9.20E-12	-0.33	87	1.24	LOC108351408;Olr1088	

DMR7:14122001	7	14122001	14124000	2000	1	3.80E-08	-0.47	17	0.85	Olr1088	
DMR7:14228001	7	14228001	14231000	3000	1	3.40E-07	0.41	37	1.23	Brd4	
DMR7:15251001	7	15251001	15254000	3000	1	1.70E-07	-0.67	38	1.27	LOC102548621;Zfp472	
DMR7:15328001	7	15328001	15330000	2000	1	5.40E-09	0.41	22	1.1	RGD1560314	
DMR7:16416001	7	16416001	16422000	6000	3	1.40E-08	-0.42	62	1.03	Olr1056-ps	
DMR7:16945001	7	16945001	16956000	11000	1	1.70E-08	-0.26	96	0.87	Vom2r54	Signaling
DMR7:16975001	7	16975001	16977000	2000	1	1.60E-10	-0.33	18	0.9	Vom2r54	Signaling
DMR7:17055001	7	17055001	17062000	7000	3	1.10E-13	-0.45	73	1.04	Vom2r55	
DMR7:17342001	7	17342001	17348000	6000	1	9.70E-08	-0.41	59	0.98	Vom2r-ps82;Vom2r-ps83	
DMR7:17484001	7	17484001	17486000	2000	1	7.50E-07	-0.42	8	0.4	Vom1r-ps93	
DMR7:17770001	7	17770001	17771000	1000	1	4.10E-07	-0.35	9	0.9	Vom2r-ps85	
DMR7:17861001	7	17861001	17867000	6000	1	1.00E-07	-0.28	62	1.03	Zscan4f	Transcription
DMR7:17887001	7	17887001	17890000	3000	1	3.50E-08	-0.31	36	1.2	Vom1r-ps94	
DMR7:18060001	7	18060001	18067000	7000	1	5.50E-09	-0.31	78	1.11	Vom1r107	Receptor
DMR7:18119001	7	18119001	18120000	1000	1	1.30E-09	-0.47	7	0.7	Vom1r108	Receptor
DMR7:18253001	7	18253001	18259000	6000	1	6.90E-08	-0.31	56	0.93	Vom1r-ps102;LOC108351411	
DMR7:18264001	7	18264001	18271000	7000	1	1.70E-11	-0.34	66	0.94	LOC108351411;RGD1565010	
DMR7:18282001	7	18282001	18285000	3000	1	2.20E-07	-0.31	43	1.43	RGD1565010;LOC108351412	
DMR7:18297001	7	18297001	18302000	5000	1	9.60E-08	-0.29	61	1.22	LOC108351412;Actl9	Cytoskeleton
DMR7:18436001	7	18436001	18439000	3000	1	2.40E-07	0.42	40	1.33	Adamts10;Myo1f	Protease;Cytoskeleton
DMR7:18473001	7	18473001	18475000	2000	1	6.00E-11	0.44	24	1.2	Myo1f;LOC102547064	Cytoskeleton
DMR7:18690001	7	18690001	18691000	1000	1	1.10E-07	0.46	21	2.1	Kank3;Rps28;Ndufa7;Cd320	Cytoskeleton;Translation;Metabolism;Binding Proteins
DMR7:19067001	7	19067001	19071000	4000	1	8.00E-07	-0.41	32	0.8	Vom2r57	Signaling
DMR7:20093001	7	20093001	20095000	2000	2	1.40E-16	0.63	14	0.7	RGD1565071	Signaling
DMR7:20376001	7	20376001	20379000	3000	2	7.30E-09	-0.54	27	0.9	RGD1564409;LOC100912403;LOC102552467;LOC103692792	
DMR7:22921001	7	22921001	22925000	4000	1	4.20E-08	-0.46	29	0.72	Olr1099-ps;Vom2r-ps87	
DMR7:23482001	7	23482001	23483000	1000	1	3.50E-10	0.42	16	1.6	Syn3	Transport
DMR7:23729001	7	23729001	23730000	1000	1	1.90E-07	0.4	0	0	Syn3	Transport
DMR7:23887001	7	23887001	23890000	3000	1	1.10E-09	-0.45	43	1.43	Bpifc	
DMR7:23897001	7	23897001	23898000	1000	1	5.70E-07	-0.39	12	1.2	Bpifc	
DMR7:23930001	7	23930001	23931000	1000	1	6.40E-07	0.43	8	0.8	Rtcb	
DMR7:23934001	7	23934001	23941000	7000	2	6.10E-11	-0.42	87	1.24	Rtcb;Ascl4	Transcription
DMR7:24001001	7	24001001	24002000	1000	1	1.90E-07	0.4	22	2.2	Pwp1	Epigenetic
DMR7:24095001	7	24095001	24098000	3000	1	9.30E-09	0.39	47	1.57	Btbd11	Cytoskeleton
DMR7:24235001	7	24235001	24236000	1000	1	1.20E-08	0.45	16	1.6	Btbd11	Cytoskeleton
DMR7:24633001	7	24633001	24634000	1000	1	4.00E-07	-0.42	17	1.7	Cry1;Mterf2	DNA Repair
DMR7:25841001	7	25841001	25845000	4000	2	9.10E-12	0.53	52	1.3	LOC100910996;Rfx4	Transcription
DMR7:25927001	7	25927001	25928000	1000	1	9.80E-07	0.31	19	1.9	LOC100910996;LOC108351424;Ric8b	
DMR7:25976001	7	25976001	25978000	2000	1	1.70E-07	0.42	20	1	LOC100910996;Ric8b	
DMR7:26280001	7	26280001	26282000	2000	1	1.90E-08	0.37	39	1.95	Appl2	Cytoskeleton
DMR7:26565001	7	26565001	26566000	1000	1	3.40E-10	-0.51	18	1.8	Slc41a2;LOC103692827	Transport
DMR7:26669001	7	26669001	26670000	1000	1	2.00E-10	0.44	20	2	Chst11	Transport
DMR7:27327001	7	27327001	27329000	2000	1	2.70E-12	0.52	37	1.85	Nt5dc3	Signaling
DMR7:27399001	7	27399001	27401000	2000	1	4.00E-07	-0.36	49	2.45	Stab2	Transport
DMR7:27722001	7	27722001	27723000	1000	1	1.40E-10	0.57	8	0.8	RGD1560034	
DMR7:27780001	7	27780001	27781000	1000	1	3.70E-07	0.3	16	1.6	RGD1560034	
DMR7:28096001	7	28096001	28100000	4000	1	1.40E-08	-0.39	62	1.55	Pah	
DMR7:28136001	7	28136001	28137000	1000	1	4.80E-09	-0.56	7	0.7	Pah;LOC100359965	
DMR7:28908001	7	28908001	28912000	4000	1	3.90E-09	0.54	57	1.43	Dram1	
DMR7:29058001	7	29058001	29059000	1000	1	3.70E-08	-0.4	14	1.4	Chpt1	Transport
DMR7:29330001	7	29330001	29331000	1000	1	1.40E-08	0.42	6	0.6	Utp20	
DMR7:29365001	7	29365001	29369000	4000	1	6.60E-07	0.37	59	1.48	Utp20	
DMR7:30010001	7	30010001	30011000	1000	1	1.40E-07	-0.56	14	1.4	Ano4;Nr1h4	Transcription
DMR7:30340001	7	30340001	30341000	1000	1	1.30E-07	0.36	24	2.4	Scyl2	Signaling
DMR7:30564001	7	30564001	30569000	5000	3	3.90E-12	-0.43	49	0.98	Anks1b	Cytoskeleton

DMR7:30577001	7	30577001	30582000	5000	1	1.10E-09	-0.34	48	0.96	Anks1b	Cytoskeleton
DMR7:31007001	7	31007001	31009000	2000	1	1.20E-07	-0.36	21	1.05	Anks1b	Cytoskeleton
DMR7:31313001	7	31313001	31320000	7000	2	6.60E-09	-0.36	84	1.2	Anks1b	Cytoskeleton
DMR7:31555001	7	31555001	31559000	4000	1	1.00E-09	0.47	81	2.02	Anks1b	Cytoskeleton
DMR7:31576001	7	31576001	31577000	1000	1	7.60E-07	0.34	17	1.7	Anks1b	Cytoskeleton
DMR7:33671001	7	33671001	33672000	1000	1	3.50E-10	0.68	5	0.5	RGD1565866	
DMR7:33765001	7	33765001	33767000	2000	1	6.20E-08	0.4	32	1.6	RGD1565866	
DMR7:34027001	7	34027001	34030000	3000	1	5.30E-07	-0.37	39	1.3	Cdk17	Signaling
DMR7:34611001	7	34611001	34612000	1000	1	7.10E-07	-0.56	24	2.4	Ntn4;LOC100362560	Extracellular Matrix
DMR7:34794001	7	34794001	34795000	1000	1	8.90E-09	-0.47	16	1.6	Metap2	Protease
DMR7:35102001	7	35102001	35105000	3000	1	2.10E-07	0.6	31	1.03	Nr2c1	Transcription
DMR7:35152001	7	35152001	35160000	8000	1	6.60E-10	-0.32	104	1.3	Ndufa12	Metabolism
DMR7:35451001	7	35451001	35452000	1000	1	6.30E-07	-0.4	31	3.1	Tmcc3	
DMR7:35491001	7	35491001	35492000	1000	1	5.10E-09	0.37	10	1	Tmcc3	
DMR7:35830001	7	35830001	35834000	4000	1	4.70E-07	-0.44	21	0.52	Cep83	
DMR7:37148001	7	37148001	37149000	1000	1	7.90E-07	0.43	4	0.4	Eea1;LOC102547143	Transport
DMR7:37177001	7	37177001	37184000	7000	2	2.30E-09	-0.44	86	1.23	Eea1;Plekhg7	Transport
DMR7:40274001	7	40274001	40281000	7000	2	8.70E-08	-0.44	68	0.97	Cep290	
DMR7:41170001	7	41170001	41173000	3000	1	1.70E-07	-0.54	43	1.43	Atp2b1	Transport
DMR7:41222001	7	41222001	41224000	2000	1	1.10E-11	-0.56	37	1.85	Atp2b1;LOC102553961	Transport
DMR7:41469001	7	41469001	41470000	1000	1	1.20E-07	-0.37	21	2.1	Dusp6	Signaling
DMR7:43320001	7	43320001	43321000	1000	1	4.70E-07	0.39	7	0.7	Mgat4c	Transport
DMR7:43557001	7	43557001	43561000	4000	1	1.90E-07	-0.47	21	0.52	Mgat4c	Transport
DMR7:43574001	7	43574001	43575000	1000	1	6.40E-07	-0.43	6	0.6	Mgat4c	Transport
DMR7:43637001	7	43637001	43640000	3000	1	3.20E-08	-0.37	21	0.7	Mgat4c	Transport
DMR7:43872001	7	43872001	43880000	8000	1	5.50E-10	-0.48	93	1.16	Mgat4c	Transport
DMR7:44004001	7	44004001	44005000	1000	1	7.20E-07	0.43	10	1	Mgat4c	Transport
DMR7:44157001	7	44157001	44158000	1000	1	1.70E-08	0.53	3	0.3	Rassf9	Cytoskeleton
DMR7:45113001	7	45113001	45114000	1000	1	3.80E-07	-0.58	8	0.8	Lrriq1	
DMR7:47351001	7	47351001	47358000	7000	1	8.00E-08	-0.38	73	1.04	Tmtc2	Golgi
DMR7:47377001	7	47377001	47378000	1000	1	2.80E-07	0.47	8	0.8	Tmtc2	Golgi
DMR7:47458001	7	47458001	47464000	6000	1	2.70E-07	-0.3	66	1.1	Tmtc2	Golgi
DMR7:48629001	7	48629001	48633000	4000	1	2.20E-08	-0.33	36	0.9	Ppfia2	
DMR7:49083001	7	49083001	49086000	3000	1	2.80E-08	-0.29	29	0.97	Acss3	Metabolism
DMR7:49087001	7	49087001	49092000	5000	3	2.70E-10	-0.34	45	0.9	Acss3	Metabolism
DMR7:49160001	7	49160001	49168000	8000	2	6.80E-10	-0.33	75	0.94	Acss3	Metabolism
DMR7:49170001	7	49170001	49173000	3000	1	6.70E-08	0.57	21	0.7	Acss3	Metabolism
DMR7:49489001	7	49489001	49495000	6000	2	5.50E-09	-0.3	55	0.92	Lin7a	Cytoskeleton
DMR7:49827001	7	49827001	49828000	1000	1	8.70E-08	-0.47	18	1.8	Ptprq	Receptor
DMR7:49874001	7	49874001	49878000	4000	1	3.40E-07	-0.38	42	1.05	Ptprq;LOC103690159	Receptor
DMR7:50312001	7	50312001	50318000	6000	1	5.60E-07	-0.27	93	1.55	Syt1	Transport
DMR7:50401001	7	50401001	50402000	1000	1	2.70E-09	-0.55	13	1.3	Syt1;LOC108351599;LOC108351598	Transport
DMR7:50424001	7	50424001	50427000	3000	2	1.60E-10	-0.41	25	0.83	Syt1	Transport
DMR7:50471001	7	50471001	50474000	3000	1	1.30E-07	-0.39	21	0.7	Syt1	Transport
DMR7:50475001	7	50475001	50477000	2000	1	2.90E-07	-0.25	26	1.3	Syt1	Transport
DMR7:50559001	7	50559001	50567000	8000	3	6.40E-08	-0.54	77	0.96	Syt1	Transport
DMR7:51355001	7	51355001	51357000	2000	1	3.60E-10	-0.44	33	1.65	Pawr	
DMR7:51831001	7	51831001	51832000	1000	1	8.40E-07	-0.42	8	0.8	Otogl	Extracellular Matrix
DMR7:51845001	7	51845001	51846000	1000	1	8.40E-09	-0.48	9	0.9	Otogl	Extracellular Matrix
DMR7:52409001	7	52409001	52412000	3000	1	6.60E-08	-0.37	22	0.73	Nav3	
DMR7:53264001	7	53264001	53267000	3000	1	8.70E-07	-0.38	52	1.73	E2f7	Transcription
DMR7:53720001	7	53720001	53721000	1000	1	6.00E-07	-0.4	15	1.5	Zdhhc17;LOC108351448	
DMR7:54804001	7	54804001	54807000	3000	1	1.10E-07	-0.35	21	0.7	Glipr1l2	Immune
DMR7:54840001	7	54840001	54847000	7000	1	5.50E-10	-0.26	83	1.19	Glipr1l1	Immune
DMR7:57367001	7	57367001	57368000	1000	1	1.30E-07	-0.43	5	0.5	Trhde	Protease
DMR7:57594001	7	57594001	57597000	3000	1	1.10E-07	-0.46	43	1.43	Trhde	Protease
DMR7:58050001	7	58050001	58051000	1000	1	1.20E-11	0.47	11	1.1	Tph2	
DMR7:58502001	7	58502001	58503000	1000	1	2.40E-09	0.5	7	0.7	Lgr5	Signaling
DMR7:58834001	7	58834001	58838000	4000	2	2.30E-09	0.47	33	0.82	Tspan8	
DMR7:59239001	7	59239001	59249000	10000	2	1.90E-07	-0.4	89	0.89	Ptprr	

DMR7:59592001	7	59592001	59594000	2000	1	7.80E-08	-0.47	11	0.55	Cnot2	Transcription
DMR7:59976001	7	59976001	59978000	2000	1	6.60E-07	0.44	29	1.45	Rab3ip;Best3	Transcription;Transport
DMR7:60079001	7	60079001	60082000	3000	1	8.50E-09	-0.34	32	1.07	LOC689014;Lrrc10	
DMR7:60324001	7	60324001	60328000	4000	1	2.30E-07	0.4	35	0.88	Lyc2;Lyz2	
DMR7:60402001	7	60402001	60403000	1000	1	5.40E-08	0.44	15	1.5	Cpsf6	Translation
DMR7:60427001	7	60427001	60428000	1000	1	4.20E-07	0.36	19	1.9	Cpsf6	Translation
DMR7:60716001	7	60716001	60717000	1000	1	7.80E-15	0.56	7	0.7	Cpm;Mdm2	Protease;Epigenetic
DMR7:60774001	7	60774001	60775000	1000	1	1.70E-08	-0.42	19	1.9	LOC108351461;Slc35e3;Nup107	Transport;Transport
DMR7:60816001	7	60816001	60817000	1000	1	5.00E-07	0.5	12	1.2	Nup107	Transport
DMR7:61167001	7	61167001	61170000	3000	1	6.30E-07	0.36	56	1.87	LOC102551023;Mdm1	
DMR7:61781001	7	61781001	61786000	5000	1	9.50E-08	-0.38	92	1.84	Dyrk2	
DMR7:62902001	7	62902001	62903000	1000	1	3.50E-07	-0.43	24	2.4	Msrb3;Styxl2	Metabolism
DMR7:63167001	7	63167001	63170000	3000	1	6.80E-08	-0.31	30	1	Wif1	Signaling
DMR7:63542001	7	63542001	63543000	1000	1	3.10E-07	0.35	15	1.5	Rassf3	Cytoskeleton
DMR7:64079001	7	64079001	64081000	2000	1	3.20E-07	0.51	16	0.8	Srgap1	Signaling
DMR7:64190001	7	64190001	64193000	3000	1	6.90E-10	0.64	26	0.87	Srgap1	Signaling
DMR7:64396001	7	64396001	64397000	1000	1	5.70E-08	-0.51	5	0.5	Vps54-ps1	
DMR7:64746001	7	64746001	64748000	2000	1	5.80E-07	-0.41	27	1.35	Grip1	
DMR7:64854001	7	64854001	64859000	5000	1	2.40E-10	-0.54	75	1.5	Grip1;Helb	Transcription
DMR7:64864001	7	64864001	64865000	1000	1	1.20E-08	0.39	12	1.2	Grip1;Helb	Transcription
DMR7:64915001	7	64915001	64917000	2000	1	7.10E-07	0.42	43	2.15	Irak3	
DMR7:64994001	7	64994001	64996000	2000	1	6.80E-08	0.43	39	1.95	Tmbim4;Llph	
DMR7:65152001	7	65152001	65153000	1000	1	3.10E-11	0.8	2	0.2	Hmga2	Transcription
DMR7:65221001	7	65221001	65223000	2000	1	4.90E-07	-0.39	30	1.5	Hmga2;LOC102549373;LOC103690417	Transcription
DMR7:66063001	7	66063001	66066000	3000	2	7.00E-08	0.61	10	0.33	Fam19a2	
DMR7:66311001	7	66311001	66314000	3000	1	1.30E-08	0.39	30	1	Fam19a2	
DMR7:66723001	7	66723001	66725000	2000	1	2.20E-09	-0.47	21	1.05	Mon2;LOC108351468;LOC680056	Transcription
DMR7:67039001	7	67039001	67042000	3000	1	4.90E-18	0.6	66	2.2	Ppm1h	Signaling
DMR7:70314001	7	70314001	70316000	2000	1	1.10E-07	0.49	32	1.6	Avil;Tsfm;Mettl21b	Cytoskeleton;Translation
DMR7:70359001	7	70359001	70362000	3000	1	2.80E-09	0.43	46	1.53	March9;Cdk4;Tspan31;LOC103692874;Agap2	Signaling
DMR7:70518001	7	70518001	70520000	2000	2	4.10E-09	0.43	126	6.3	Kif5a	Cytoskeleton
DMR7:70859001	7	70859001	70863000	4000	1	1.50E-13	0.47	74	1.85	Lrp1	Binding Proteins
DMR7:70867001	7	70867001	70872000	5000	2	2.20E-10	0.46	98	1.96	Lrp1	Binding Proteins
DMR7:71021001	7	71021001	71025000	4000	1	3.40E-07	0.38	62	1.55	Myo1a;Tac3	Cytoskeleton
DMR7:71791001	7	71791001	71797000	6000	2	3.70E-11	0.37	69	1.15	Cpq	Protease
DMR7:71806001	7	71806001	71811000	5000	1	2.40E-07	-0.33	44	0.88	Cpq;LOC103692875	Protease
DMR7:71958001	7	71958001	71960000	2000	1	1.00E-09	0.25	15	0.75	Cpq	Protease
DMR7:72148001	7	72148001	72152000	4000	1	3.50E-10	-0.32	48	1.2	Cpq	Protease
DMR7:72168001	7	72168001	72176000	8000	1	5.20E-10	-0.33	80	1	Cpq	Protease
DMR7:73003001	7	73003001	73005000	2000	1	1.50E-10	0.61	29	1.45	Matn2	Extracellular Matrix
DMR7:73011001	7	73011001	73018000	7000	2	9.80E-16	-0.41	76	1.09	Matn2	Extracellular Matrix
DMR7:73039001	7	73039001	73040000	1000	1	1.10E-07	0.56	16	1.6	Matn2	Extracellular Matrix
DMR7:73457001	7	73457001	73464000	7000	3	1.70E-08	-0.34	68	0.97	Nipal2	
DMR7:73552001	7	73552001	73554000	2000	1	2.20E-09	0.67	26	1.3	Kcns2	Transport
DMR7:73645001	7	73645001	73646000	1000	1	3.10E-07	-0.4	8	0.8	Stk3	Signaling
DMR7:73656001	7	73656001	73663000	7000	2	2.70E-08	-0.34	77	1.1	Stk3	Signaling
DMR7:73723001	7	73723001	73729000	6000	1	5.20E-07	-0.24	71	1.18	Stk3	Signaling
DMR7:73851001	7	73851001	73858000	7000	2	5.50E-09	-0.34	55	0.79	Stk3	Signaling
DMR7:74189001	7	74189001	74197000	8000	1	4.30E-07	-0.35	62	0.78	Vps13b;LOC103692884	
DMR7:74243001	7	74243001	74244000	1000	1	2.80E-07	0.45	31	3.1	Vps13b	
DMR7:74298001	7	74298001	74300000	2000	1	3.40E-10	-0.38	21	1.05	Vps13b	
DMR7:74512001	7	74512001	74513000	1000	1	3.10E-08	0.44	16	1.6	Vps13b	
DMR7:74631001	7	74631001	74635000	4000	1	8.60E-07	-0.29	61	1.52	Vps13b	
DMR7:74813001	7	74813001	74815000	2000	1	8.00E-09	-0.53	17	0.85	Rgs22	
DMR7:74836001	7	74836001	74837000	1000	1	5.20E-15	0.51	7	0.7	Rgs22	
DMR7:74842001	7	74842001	74844000	2000	1	1.20E-08	-0.31	18	0.9	Rgs22	
DMR7:74920001	7	74920001	74923000	3000	1	7.80E-09	-0.39	28	0.93	Fbxo43	

DMR7:76080001	7	76080001	76081000	1000	1	5.80E-07	-0.4	18	1.8	Grhl2	Transcription
DMR7:76244001	7	76244001	76246000	2000	1	2.50E-07	0.37	27	1.35	Ncald;LOC102549684	
DMR7:76512001	7	76512001	76517000	5000	1	7.70E-10	-0.44	45	0.9	Ncald	
DMR7:76761001	7	76761001	76762000	1000	1	1.30E-07	0.31	22	2.2	Rrm2b	Metabolism
DMR7:76874001	7	76874001	76876000	2000	1	9.50E-07	-0.39	36	1.8	Ubr5	Proteolysis
DMR7:77159001	7	77159001	77160000	1000	1	5.30E-07	0.43	18	1.8	LOC108351483;Klf10;LOC102550687	Transcription
DMR7:77890001	7	77890001	77896000	6000	2	3.80E-07	-0.28	66	1.1	Fzd6	Receptor
DMR7:78138001	7	78138001	78139000	1000	1	3.40E-09	-0.48	5	0.5	Rims2	Transport
DMR7:78154001	7	78154001	78158000	4000	1	4.00E-09	-0.48	25	0.62	Rims2	Transport
DMR7:78196001	7	78196001	78197000	1000	1	3.10E-07	0.34	9	0.9	Rims2	Transport
DMR7:78259001	7	78259001	78267000	8000	2	3.40E-08	-0.3	81	1.01	Rims2	Transport
DMR7:78393001	7	78393001	78397000	4000	3	3.20E-10	-0.4	58	1.45	Rims2	Transport
DMR7:78791001	7	78791001	78794000	3000	1	4.10E-08	0.33	40	1.33	Dpys	Metabolism
DMR7:79621001	7	79621001	79623000	2000	1	1.10E-07	-0.43	21	1.05	Zfpm2	Transcription
DMR7:79974001	7	79974001	79975000	1000	1	2.90E-09	-0.52	8	0.8	Zfpm2	Transcription
DMR7:80409001	7	80409001	80411000	2000	1	2.40E-07	0.39	22	1.1	Oxr1	
DMR7:80695001	7	80695001	80697000	2000	2	5.10E-09	0.46	21	1.05	Oxr1	
DMR7:80702001	7	80702001	80704000	2000	1	1.10E-09	0.52	16	0.8	Oxr1	
DMR7:80757001	7	80757001	80759000	2000	1	4.50E-08	-0.4	22	1.1	Oxr1	
DMR7:81446001	7	81446001	81448000	2000	1	6.40E-07	0.49	17	0.85	Angpt1	Signaling
DMR7:81474001	7	81474001	81480000	6000	2	2.10E-11	-0.34	63	1.05	Angpt1	Signaling
DMR7:81482001	7	81482001	81484000	2000	1	5.40E-08	0.63	9	0.45	Angpt1	Signaling
DMR7:82010001	7	82010001	82011000	1000	1	1.70E-07	0.44	9	0.9	Rspo2	
DMR7:82066001	7	82066001	82067000	1000	1	2.00E-11	0.46	2	0.2	Rspo2	
DMR7:83404001	7	83404001	83411000	7000	2	2.80E-11	-0.34	76	1.09	Pkhd1l1	
DMR7:83519001	7	83519001	83525000	6000	2	9.60E-08	-0.43	58	0.97	Pkhd1l1	
DMR7:86982001	7	86982001	86984000	2000	1	5.10E-10	-0.44	19	0.95	Csmd3	
DMR7:87455001	7	87455001	87462000	7000	1	9.80E-09	-0.36	77	1.1	Csmd3	
DMR7:87534001	7	87534001	87539000	5000	2	5.10E-10	-0.35	71	1.42	Csmd3	
DMR7:87608001	7	87608001	87612000	4000	1	5.50E-11	-0.42	31	0.78	Csmd3	
DMR7:87712001	7	87712001	87713000	1000	1	2.90E-08	-0.31	26	2.6	Csmd3	
DMR7:87714001	7	87714001	87718000	4000	1	7.60E-10	-0.38	39	0.98	Csmd3	
DMR7:87751001	7	87751001	87755000	4000	1	4.00E-07	-0.43	40	1	Csmd3	
DMR7:87823001	7	87823001	87828000	5000	1	4.60E-08	-0.31	59	1.18	Csmd3	
DMR7:88068001	7	88068001	88069000	1000	1	1.50E-10	-0.67	11	1.1	Csmd3	
DMR7:90303001	7	90303001	90305000	2000	1	2.30E-11	0.57	27	1.35	Trps1	Transcription
DMR7:90322001	7	90322001	90323000	1000	1	4.50E-07	-0.43	11	1.1	Trps1	Transcription
DMR7:92754001	7	92754001	92756000	2000	1	2.30E-08	-0.49	30	1.5	Ext1	Golgi
DMR7:93022001	7	93022001	93029000	7000	1	1.40E-08	-0.29	71	1.01	Samd12	
DMR7:93033001	7	93033001	93040000	7000	2	5.80E-13	-0.37	75	1.07	Samd12	
DMR7:93198001	7	93198001	93205000	7000	4	1.10E-08	-0.41	62	0.89	Samd12	
DMR7:93267001	7	93267001	93273000	6000	2	1.90E-07	-0.45	62	1.03	Samd12	
DMR7:93994001	7	93994001	9.40E+07	6000	1	6.10E-10	-0.32	49	0.82	Colec10	Transport
DMR7:94014001	7	94014001	94018000	4000	2	2.90E-08	-0.41	37	0.92	Colec10	Transport
DMR7:94824001	7	94824001	94829000	5000	1	2.20E-07	-0.3	57	1.14	Deptor;LOC108351561	Transcription
DMR7:94965001	7	94965001	94966000	1000	1	1.10E-11	0.56	4	0.4	Deptor	Transcription
DMR7:95168001	7	95168001	95170000	2000	1	2.60E-08	-0.32	13	0.65	Col14a1	Extracellular Matrix
DMR7:95231001	7	95231001	95237000	6000	1	5.10E-07	0.23	68	1.13	Col14a1	Extracellular Matrix
DMR7:95326001	7	95326001	95327000	1000	1	5.00E-08	-0.25	11	1.1	Mtbp	
DMR7:95328001	7	95328001	95331000	3000	1	8.00E-07	-0.48	28	0.93	Mtbp	
DMR7:97073001	7	97073001	97078000	5000	1	9.60E-07	-0.29	62	1.24	Slc22a22	Transport
DMR7:98065001	7	98065001	98067000	2000	1	4.40E-09	0.66	25	1.25	Fbxo32	
DMR7:98475001	7	98475001	98481000	6000	3	3.90E-10	-0.37	76	1.27	Fer1l6	Transport
DMR7:98838001	7	98838001	98839000	1000	1	8.10E-07	0.45	11	1.1	Mtss1	Cytoskeleton
DMR7:99674001	7	99674001	99675000	1000	1	3.90E-07	-0.41	10	1	RGD1564420;Nsmce2	
DMR7:99731001	7	99731001	99733000	2000	1	1.40E-07	0.43	27	1.35	Nsmce2	
DMR7:99792001	7	99792001	99794000	2000	2	3.00E-07	0.49	57	2.85	Nsmce2	
DMR7:102674001	7	102674001	102681000	7000	2	3.90E-14	-0.39	63	0.9	Pvt1	
DMR7:104790001	7	104790001	104795000	5000	1	3.60E-07	-0.28	68	1.36	Asap1	
DMR7:105364001	7	105364001	105373000	9000	1	1.60E-08	-0.36	100	1.11	Adcy8	

DMR7:105518001	7	105518001	105523000	5000	1	4.10E-07	-0.3	54	1.08	Adcy8	
DMR7:107031001	7	107031001	107033000	2000	1	2.80E-12	0.53	18	0.9	Trnak-cuu	
DMR7:107527001	7	107527001	107528000	1000	1	2.50E-08	0.48	6	0.6	Tg	Cytoskeleton
DMR7:107575001	7	107575001	107578000	3000	1	8.60E-08	-0.33	20	0.67	Tg;Sla	Cytoskeleton
DMR7:107707001	7	107707001	107709000	2000	2	4.50E-08	0.45	16	0.8	Wisp1	
DMR7:107741001	7	107741001	107745000	4000	1	1.50E-07	0.52	47	1.18	Ndrg1	Protease
DMR7:107888001	7	107888001	107889000	1000	1	2.10E-09	-0.43	10	1	St3gal1	Transport
DMR7:108763001	7	108763001	108765000	2000	1	6.00E-10	0.68	21	1.05	Phf20l1	
DMR7:109101001	7	109101001	109102000	1000	1	9.40E-07	0.41	7	0.7	Zfat	Transcription
DMR7:109408001	7	109408001	109409000	1000	1	8.40E-09	0.42	7	0.7	RGD1564548	Epigenetic
DMR7:110025001	7	110025001	110026000	1000	1	4.60E-11	0.46	10	1	Khdrbs3	Translation
DMR7:111729001	7	111729001	111734000	5000	1	2.90E-08	-0.52	39	0.78	Rps19l1	Translation
DMR7:112746001	7	112746001	112748000	2000	1	4.10E-07	0.56	10	0.5	Fam135b	
DMR7:113110001	7	113110001	113111000	1000	1	1.20E-08	0.36	11	1.1	Col22a1	Extracellular Matrix
DMR7:113945001	7	113945001	113947000	2000	1	2.20E-08	-0.56	9	0.45	Kcnk9	Transport
DMR7:113978001	7	113978001	113979000	1000	1	4.30E-07	-0.38	8	0.8	Trappc9	
DMR7:114328001	7	114328001	114329000	1000	1	2.50E-07	0.42	10	1	Chrac1	Transcription
DMR7:114964001	7	114964001	114966000	2000	1	2.50E-07	0.63	26	1.3	Ptp4a3	Signaling
DMR7:115866001	7	115866001	115867000	1000	1	1.80E-07	0.55	9	0.9	LOC102550355;Mroh4	
DMR7:115920001	7	115920001	115923000	3000	1	6.10E-09	0.36	29	0.97	Arc;LOC103692938	
DMR7:115972001	7	115972001	115973000	1000	1	2.10E-07	0.58	9	0.9	Slurp1;Lypd2;RGD1308195;Lynx1	
DMR7:116648001	7	116648001	116649000	1000	1	2.30E-09	0.44	13	1.3	Zfp41	Transcription
DMR7:117106001	7	117106001	117107000	1000	1	1.70E-07	0.4	15	1.5	RGD1563870;Scrib	Cytoskeleton
DMR7:117242001	7	117242001	117245000	3000	2	1.20E-07	0.45	96	3.2	Plec	Cytoskeleton
DMR7:117258001	7	117258001	117260000	2000	1	6.30E-07	0.42	47	2.35	Plec	Cytoskeleton
DMR7:117451001	7	117451001	117452000	1000	1	6.00E-10	-0.53	15	1.5	Mroh1	
DMR7:117523001	7	117523001	117526000	3000	1	8.20E-09	0.49	55	1.83	Bop1;Scx	Translation;Transcription
DMR7:117662001	7	117662001	117664000	2000	1	2.00E-08	0.45	50	2.5	Adck5;Cpsf1	Transport;Translation
DMR7:117775001	7	117775001	117777000	2000	1	1.40E-07	0.6	16	0.8	Mfsd3;Recql4;Lrrc14;Lrrc24;MGC94207	Transport;Epigenetic;Immune
DMR7:118862001	7	118862001	118864000	2000	1	1.10E-08	0.34	27	1.35	Apol11a;LOC102552424	Binding Proteins
DMR7:118930001	7	118930001	118933000	3000	1	3.00E-07	-0.46	35	1.17	LOC100911761;Apol9a	Binding Proteins
DMR7:119249001	7	119249001	119253000	4000	1	1.40E-07	0.35	57	1.43	Cacng2	Transport
DMR7:119397001	7	119397001	119398000	1000	1	2.10E-09	0.45	38	3.8	Ift27	
DMR7:119399001	7	119399001	119402000	3000	1	1.10E-11	0.51	69	2.3	Ift27	
DMR7:119664001	7	119664001	119665000	1000	1	2.30E-08	0.46	16	1.6	Kctd17;Tmprss6	Protease
DMR7:119902001	7	119902001	119905000	3000	1	1.10E-10	0.46	40	1.33	Elfn2;LOC102549375	Receptor
DMR7:120054001	7	120054001	120061000	7000	1	4.90E-08	0.33	75	1.07	LOC108351511;Cdc42ep1	
DMR7:120136001	7	120136001	120138000	2000	1	3.80E-07	0.36	39	1.95	LOC108351513;Sh3bp1;Pdxp	Signaling
DMR7:120187001	7	120187001	120191000	4000	1	3.70E-07	0.32	80	2	Triobp	Cytoskeleton
DMR7:120319001	7	120319001	120322000	3000	1	7.80E-07	-0.35	51	1.7	Eif3l	Translation
DMR7:120497001	7	120497001	120501000	4000	1	3.20E-08	0.31	59	1.48	Slc16a8;Baiap2l2	Transport;Cytoskeleton
DMR7:120517001	7	120517001	120518000	1000	1	8.60E-07	0.34	19	1.9	Baiap2l2;Pla2g6	Cytoskeleton;Metabolism
DMR7:120619001	7	120619001	120622000	3000	1	2.90E-09	0.47	53	1.77	Tmem184b	Transport
DMR7:120721001	7	120721001	120722000	1000	1	1.90E-14	0.55	16	1.6	Kcnj4	Transport
DMR7:121021001	7	121021001	121023000	2000	1	2.40E-07	0.39	35	1.75	Nptxr	
DMR7:121234001	7	121234001	121235000	1000	1	9.60E-11	0.56	16	1.6	Pdgfb;LOC108351514	Growth Factors
DMR7:121333001	7	121333001	121335000	2000	1	9.80E-12	0.48	34	1.7	Syngr1;LOC103692946	Transport
DMR7:121523001	7	121523001	121524000	1000	1	2.70E-07	0.42	7	0.7	Cacna1i	Transport
DMR7:121849001	7	121849001	121852000	3000	1	3.50E-08	0.41	41	1.37	Grap2	
DMR7:121942001	7	121942001	121944000	2000	1	2.80E-08	-0.45	23	1.15	Tnrc6b;Rpl26-ps1	Metabolism
DMR7:122164001	7	122164001	122165000	1000	1	3.50E-08	0.35	15	1.5	Adsl;LOC100362980	Metabolism
DMR7:122631001	7	122631001	122632000	1000	1	8.10E-07	0.48	9	0.9	St13;Xpnpep3	Protease
DMR7:122991001	7	122991001	122996000	5000	1	1.90E-12	0.47	110	2.2	Zc3h7b	Metabolism
DMR7:123010001	7	123010001	123012000	2000	1	2.10E-07	0.36	50	2.5	Zc3h7b	Metabolism
DMR7:123396001	7	123396001	123399000	3000	2	7.10E-09	0.48	100	3.33	Srebf2	
DMR7:123960001	7	123960001	123961000	1000	1	4.60E-08	0.4	10	1	Serhl2	Metabolism
DMR7:124037001	7	124037001	124038000	1000	1	2.20E-07	0.45	12	1.2	Cyb5r3	Metabolism
DMR7:124236001	7	124236001	124237000	1000	1	1.80E-07	0.66	7	0.7	Pacsin2	Cytoskeleton

DMR7:124355001	7	124355001	124358000	3000	1	2.10E-07	0.47	54	1.8	Ttll1	Cytoskeleton
DMR7:124552001	7	124552001	124555000	3000	2	4.40E-09	0.55	35	1.17	Scube1	Extracellular Matrix
DMR7:124579001	7	124579001	124580000	1000	1	9.80E-11	0.62	8	0.8	Scube1	Extracellular Matrix
DMR7:124582001	7	124582001	124586000	4000	2	8.80E-08	0.51	51	1.27	Scube1	Extracellular Matrix
DMR7:124667001	7	124667001	124668000	1000	1	5.20E-07	0.56	11	1.1	Mpped1	Metabolism
DMR7:124788001	7	124788001	124791000	3000	1	8.90E-07	0.56	42	1.4	Efcab6;LOC102549885	Signaling
DMR7:125036001	7	125036001	125037000	1000	1	1.50E-07	0.45	14	1.4	Pnpla3	Metabolism
DMR7:125308001	7	125308001	125309000	1000	1	2.20E-08	0.35	11	1.1	Parvg;LOC108351521	Cytoskeleton
DMR7:125395001	7	125395001	125400000	5000	1	9.60E-08	0.44	51	1.02	RGD1566029	
DMR7:125401001	7	125401001	125403000	2000	1	9.50E-10	0.52	29	1.45	RGD1566029	
DMR7:125577001	7	125577001	125582000	5000	1	8.10E-07	0.47	90	1.8	Prr5	
DMR7:125599001	7	125599001	125600000	1000	1	1.40E-09	0.44	19	1.9	Prr5	
DMR7:125657001	7	125657001	125658000	1000	1	5.80E-07	0.52	10	1	Phf21b	
DMR7:125787001	7	125787001	125789000	2000	1	5.70E-09	0.34	44	2.2	Arhgap8;LOC102550959	Signaling
DMR7:125879001	7	125879001	125881000	2000	1	2.70E-07	0.4	35	1.75	RGD1304694	
DMR7:126435001	7	126435001	126440000	5000	1	9.80E-10	0.51	64	1.28	Wnt7b	Signaling
DMR7:126841001	7	126841001	126844000	3000	1	9.20E-12	0.47	49	1.63	Celsr1	Cytoskeleton
DMR7:126863001	7	126863001	126865000	2000	1	6.60E-10	0.48	18	0.9	Celsr1	Cytoskeleton
DMR7:126890001	7	126890001	126895000	5000	1	9.20E-13	0.46	81	1.62	Celsr1	Cytoskeleton
DMR7:127026001	7	127026001	127027000	1000	1	3.50E-10	0.43	24	2.4	Cerk	Signaling
DMR7:127031001	7	127031001	127032000	1000	1	5.10E-07	0.37	21	2.1	Cerk	Signaling
DMR7:127175001	7	127175001	127176000	1000	1	1.30E-08	0.51	11	1.1	Tbc1d22a	Signaling
DMR7:127221001	7	127221001	127223000	2000	1	5.10E-07	0.5	12	0.6	Tbc1d22a	Signaling
DMR7:128691001	7	128691001	128692000	1000	1	9.60E-07	0.52	7	0.7	Fam19a5	
DMR7:129693001	7	129693001	129695000	2000	2	3.80E-07	0.54	14	0.7	Brd1	Transcription
DMR7:129822001	7	129822001	129823000	1000	1	2.90E-08	0.48	10	1	Alg12;Creld2	Golgi;Extracellular Matrix
DMR7:130051001	7	130051001	130053000	2000	1	3.30E-07	0.4	57	2.85	Panx2;Trabd	
DMR7:130167001	7	130167001	130168000	1000	1	2.00E-11	0.41	20	2	Plxnb2;LOC102549176;Dennd6b	
DMR7:130226001	7	130226001	130227000	1000	1	3.50E-12	0.65	7	0.7	Ppp6r2	Signaling
DMR7:130333001	7	130333001	130334000	1000	1	3.60E-07	0.26	10	1	Lmf2;Ncaph2;Tymp	Golgi
DMR7:130468001	7	130468001	130469000	1000	1	1.80E-07	-0.37	15	1.5	Shank3	
DMR7:130514001	7	130514001	130517000	3000	1	6.00E-09	0.65	39	1.3	Shank3	
DMR7:130799001	7	130799001	130800000	1000	1	7.10E-07	-0.38	20	2	Syt10	Transport
DMR7:131806001	7	131806001	131808000	2000	1	2.50E-08	0.4	33	1.65	Cpne8	
DMR7:132572001	7	132572001	132578000	6000	1	5.20E-07	-0.33	67	1.12	Slc2a13	Transport
DMR7:132619001	7	132619001	132623000	4000	1	7.30E-07	-0.31	38	0.95	Slc2a13	Transport
DMR7:132654001	7	132654001	132657000	3000	2	4.70E-09	-0.39	30	1	Slc2a13	Transport
DMR7:132980001	7	132980001	132981000	1000	1	6.80E-09	0.5	7	0.7	Lrrk2;Hmgb1-ps3	Cytoskeleton
DMR7:133000001	7	133000001	133001000	1000	1	6.10E-09	0.51	4	0.4	Lrrk2	Cytoskeleton
DMR7:133121001	7	133121001	133135000	14000	1	2.00E-08	0.43	208	1.49	Muc19;LOC108351530	
DMR7:133316001	7	133316001	133317000	1000	1	3.80E-07	-0.49	24	2.4	Cntn1	
DMR7:133473001	7	133473001	133474000	1000	1	2.30E-11	0.32	15	1.5	Cntn1	
DMR7:133526001	7	133526001	133528000	2000	1	3.20E-07	0.62	24	1.2	Cntn1	
DMR7:133989001	7	133989001	133991000	2000	1	2.10E-07	-0.41	38	1.9	Pdzrn4	
DMR7:134615001	7	134615001	134616000	1000	1	7.40E-10	-0.5	13	1.3	Pphln1;LOC108351532	
DMR7:134644001	7	134644001	134648000	4000	1	9.80E-09	0.4	50	1.25	Pphln1;LOC108351532	
DMR7:134767001	7	134767001	134771000	4000	2	1.40E-13	0.52	62	1.55	Prickle1	Cytoskeleton
DMR7:135903001	7	135903001	135905000	2000	1	2.50E-09	0.26	32	1.6	Tmem117	
DMR7:135965001	7	135965001	135966000	1000	1	2.90E-07	0.37	21	2.1	Tmem117	
DMR7:136034001	7	136034001	136047000	13000	3	1.50E-07	0.36	198	1.52	Tmem117;Muc19l1;LOC108351540	
DMR7:136153001	7	136153001	136154000	1000	1	4.60E-07	0.36	6	0.6	Tmem117	
DMR7:136372001	7	136372001	136375000	3000	1	5.70E-07	0.3	31	1.03	Tmem117	
DMR7:136387001	7	136387001	136393000	6000	1	1.10E-07	-0.36	60	1	Tmem117	
DMR7:136623001	7	136623001	136624000	1000	1	1.40E-07	0.38	15	1.5	Nell2	Signaling
DMR7:137002001	7	137002001	137007000	5000	1	6.40E-07	-0.6	68	1.36	Dbx2	
DMR7:137289001	7	137289001	137290000	1000	1	1.20E-08	0.38	15	1.5	Ano6	
DMR7:137319001	7	137319001	137322000	3000	1	1.70E-07	0.35	59	1.97	Ano6	
DMR7:137743001	7	137743001	137747000	4000	1	7.70E-08	-0.37	27	0.68	Arid2	
DMR7:137822001	7	137822001	137825000	3000	1	3.10E-08	-0.53	24	0.8	Scaf11	

DMR7:139198001	7	139198001	139199000	1000	1	8.50E-14	0.58	11	1.1	Rpap3;Endou	
DMR7:139245001	7	139245001	139247000	2000	1	2.00E-10	0.44	49	2.45	Rapgef3;LOC102548155	Transcription
DMR7:139294001	7	139294001	139297000	3000	1	2.00E-08	0.44	87	2.9	Hdac7	Epigenetic
DMR7:139317001	7	139317001	139318000	1000	1	5.10E-08	0.5	35	3.5	Hdac7;LOC108351548	Epigenetic
DMR7:139468001	7	139468001	139470000	2000	1	1.30E-07	0.44	53	2.65	Col2a1	Extracellular Matrix
DMR7:139677001	7	139677001	139679000	2000	1	6.80E-08	0.29	23	1.15	Senp1	Protease
DMR7:139720001	7	139720001	139722000	2000	2	2.50E-08	0.48	15	0.75	Pfkm;Asb8	Metabolism
DMR7:139865001	7	139865001	139866000	1000	1	9.30E-09	-0.5	9	0.9	LOC100911698;H1fnt	
DMR7:139945001	7	139945001	139946000	1000	1	6.50E-07	0.41	13	1.3	Olr1106;Olr1105	Receptor
DMR7:140065001	7	140065001	140072000	7000	3	5.20E-12	-0.41	88	1.26	Olr1108;Olr1109-ps	Receptor
DMR7:140094001	7	140094001	140096000	2000	1	2.60E-07	-0.38	41	2.05	Lalba	
DMR7:140528001	7	140528001	140530000	2000	2	6.40E-13	0.53	53	2.65	Kmt2d	
DMR7:140538001	7	140538001	140540000	2000	1	2.70E-13	0.58	36	1.8	Kmt2d	
DMR7:140954001	7	140954001	140957000	3000	1	4.70E-07	0.34	35	1.17	Fam186b;Prpf40b	
DMR7:141146001	7	141146001	141148000	2000	1	6.50E-08	0.38	27	1.35	Bcdin3d	
DMR7:141175001	7	141175001	141176000	1000	1	2.60E-08	0.45	22	2.2	Faim2	
DMR7:141372001	7	141372001	141373000	1000	1	7.70E-09	0.41	12	1.2	Smarcd1;Gpd1;Cox14	Epigenetic;Metabolism;Transcription
DMR7:141386001	7	141386001	141387000	1000	1	2.50E-07	0.46	4	0.4	Gpd1;Cox14;Cers5	Metabolism;Transcription
DMR7:141749001	7	141749001	141751000	2000	1	3.70E-09	0.53	52	2.6	Dip2b	
DMR7:141755001	7	141755001	141757000	2000	1	1.20E-10	-0.54	32	1.6	Dip2b	
DMR7:141829001	7	141829001	141832000	3000	1	6.40E-10	0.44	69	2.3	Dip2b	
DMR7:141930001	7	141930001	141932000	2000	1	6.20E-07	0.46	23	1.15	Atf1	
DMR7:142036001	7	142036001	142039000	3000	2	4.60E-08	0.53	50	1.67	Slc11a2	Transport
DMR7:142329001	7	142329001	142330000	1000	1	2.00E-08	0.47	9	0.9	Galnt6	Golgi
DMR7:142568001	7	142568001	142569000	1000	1	2.60E-10	0.47	24	2.4	Scn8a	Transport
DMR7:142595001	7	142595001	142599000	4000	1	1.40E-08	0.39	82	2.05	Scn8a	Transport
DMR7:142637001	7	142637001	142640000	3000	1	1.10E-07	-0.41	30	1	Scn8a	Transport
DMR7:142704001	7	142704001	142709000	5000	1	2.50E-07	0.33	95	1.9	Fignl2	
DMR7:142903001	7	142903001	142905000	2000	1	3.20E-08	0.4	29	1.45	Nr4a1	Transcription
DMR7:142914001	7	142914001	142920000	6000	2	1.00E-07	0.38	137	2.28	Nr4a1;Atg101	Transcription
DMR7:142994001	7	142994001	142995000	1000	1	3.00E-07	0.32	20	2	Krt80	
DMR7:143068001	7	143068001	143071000	3000	1	6.90E-08	0.36	61	2.03	Krt7;Krt83	
DMR7:143223001	7	143223001	143224000	1000	1	1.90E-10	0.49	26	2.6	Kb15;Krt75;LOC108351555	
DMR7:143413001	7	143413001	143420000	7000	1	1.70E-07	0.73	134	1.91	Krt73;Krt2	
DMR7:143421001	7	143421001	143425000	4000	1	8.10E-08	0.42	39	0.98	Krt2	
DMR7:143534001	7	143534001	143537000	3000	1	1.30E-16	0.7	45	1.5	Krt79;Krt78	
DMR7:143550001	7	143550001	143551000	1000	1	4.30E-07	0.38	17	1.7	Krt78	
DMR7:143598001	7	143598001	143599000	1000	1	3.10E-07	0.35	25	2.5	Krt8	
DMR7:143715001	7	143715001	143718000	3000	1	4.70E-07	0.4	60	2	Tns2;Spryd3	Cytoskeleton;Cytoskeleton
DMR7:143762001	7	143762001	143765000	3000	1	9.30E-08	0.45	71	2.37	Igfbp6;LOC102551965;Soat2	Protease; Proteolysis;Metabolism
DMR7:143787001	7	143787001	143791000	4000	1	2.10E-09	0.49	38	0.95	Csad	Metabolism
DMR7:143855001	7	143855001	143863000	8000	2	2.30E-16	0.48	165	2.06	Rarg	Transcription
DMR7:143964001	7	143964001	143968000	4000	1	5.90E-08	0.51	56	1.4	Aaas;Sp7	Transcription
DMR7:144352001	7	144352001	144354000	2000	1	7.00E-08	0.48	35	1.75	Cistr	
DMR7:144777001	7	144777001	144778000	1000	1	1.90E-07	0.41	21	2.1	Smug1	Epigenetic
DMR7:144928001	7	144928001	144929000	1000	1	3.20E-07	0.35	8	0.8	Copz1;Gpr84	Transport;Signaling
DMR7:145143001	7	145143001	145145000	2000	1	2.50E-13	0.57	20	1	Pde1b;Ppp1r1a;LOC102547969	Signaling;Signaling
DMR8:673001	8	673001	678000	5000	2	2.00E-07	-0.32	48	0.96	Gucy1a2	Signaling
DMR8:700001	8	700001	702000	2000	1	8.80E-07	-0.5	12	0.6	Gucy1a2	Signaling
DMR8:725001	8	725001	726000	1000	1	1.20E-09	-0.39	19	1.9	Gucy1a2	Signaling
DMR8:802001	8	802001	807000	5000	1	1.60E-07	-0.29	60	1.2	Gucy1a2	Signaling
DMR8:862001	8	862001	866000	4000	1	2.60E-07	-0.27	36	0.9	Gucy1a2	Signaling
DMR8:1492001	8	1492001	1498000	6000	1	8.40E-09	-0.34	69	1.15	LOC102549625;Msantd4	
DMR8:1510001	8	1510001	1512000	2000	1	6.10E-07	-0.45	44	2.2	LOC102549625;Msantd4	
DMR8:1613001	8	1613001	1620000	7000	1	2.90E-07	-0.3	64	0.91	Gria4	Receptor
DMR8:1847001	8	1847001	1849000	2000	1	3.60E-08	0.44	17	0.85	Gria4	Receptor
DMR8:1954001	8	1954001	1957000	3000	1	3.60E-15	-0.51	26	0.87	Gria4	Receptor
DMR8:4078001	8	4078001	4083000	5000	1	6.80E-12	-0.37	51	1.02	Actl9b	

DMR8:4656001	8	4656001	4658000	2000	1	1.50E-07	-0.49	7	0.35	Pdgfd	Growth Factors
DMR8:5362001	8	5362001	5367000	5000	1	4.30E-08	-0.34	35	0.7	Dync2h1	Cytoskeleton
DMR8:5388001	8	5388001	5390000	2000	1	3.20E-10	0.52	25	1.25	Dync2h1	Cytoskeleton
DMR8:5724001	8	5724001	5730000	6000	2	1.70E-07	-0.36	50	0.83	Mmp1;Mmp10	Protease
DMR8:5836001	8	5836001	5838000	2000	1	1.40E-08	0.49	18	0.9	Mmp20	Protease
DMR8:5975001	8	5975001	5978000	3000	3	9.00E-10	0.45	184	6.13	LOC102556477;Tmem123	
DMR8:6005001	8	6005001	6011000	6000	1	3.00E-07	-0.27	66	1.1	Tmem123;Birc2	Protease; Proteolysis
DMR8:6061001	8	6061001	6062000	1000	1	6.90E-09	0.35	13	1.3	Birc3;LOC100361957	Protease; Proteolysis
DMR8:7161001	8	7161001	7162000	1000	1	5.10E-09	0.46	14	1.4	Pgr	
DMR8:7870001	8	7870001	7871000	1000	1	4.50E-07	0.54	1	0.1	Cntn5	
DMR8:8159001	8	8159001	8162000	3000	1	5.10E-08	-0.39	27	0.9	Cntn5	
DMR8:8799001	8	8799001	8804000	5000	2	2.80E-09	-0.36	42	0.84	Cntn5	
DMR8:12859001	8	12859001	12860000	1000	1	5.00E-09	0.4	9	0.9	Sesn3	Metabolism
DMR8:12893001	8	12893001	12894000	1000	1	6.40E-08	0.41	14	1.4	Endod1	
DMR8:13235001	8	13235001	13238000	3000	1	1.20E-10	-0.4	55	1.83	Piwil4;LOC102549248	Translation
DMR8:13539001	8	13539001	13541000	2000	1	5.40E-07	-0.4	16	0.8	Med17	Transcription
DMR8:13577001	8	13577001	13581000	4000	1	2.40E-09	0.4	45	1.12	Med17;Panx1	Transcription
DMR8:13628001	8	13628001	13629000	1000	1	3.80E-08	0.34	27	2.7	Med17;Hephl1	Transcription;Metabolism
DMR8:13710001	8	13710001	13711000	1000	1	3.30E-09	0.38	7	0.7	Med17;LOC108351808	Transcription
DMR8:14143001	8	14143001	14144000	1000	1	1.90E-13	0.57	13	1.3	Deup1	Cytoskeleton
DMR8:14247001	8	14247001	14252000	5000	1	3.50E-11	0.51	86	1.72	Slc36a4	Transport
DMR8:14258001	8	14258001	14260000	2000	1	4.80E-07	0.4	47	2.35	Slc36a4	Transport
DMR8:14694001	8	14694001	14695000	1000	1	2.00E-11	0.59	6	0.6	Fat3	Cytoskeleton
DMR8:14767001	8	14767001	14768000	1000	1	1.50E-07	-0.4	7	0.7	Fat3	Cytoskeleton
DMR8:15936001	8	15936001	15941000	5000	2	5.30E-09	-0.29	74	1.48	LOC108351816;Nek2l1	Signaling
DMR8:18402001	8	18402001	18408000	6000	2	1.70E-08	-0.31	62	1.03	Zfp558	
DMR8:18543001	8	18543001	18548000	5000	1	1.60E-10	-0.41	53	1.06	Muc16	
DMR8:18564001	8	18564001	18567000	3000	1	1.50E-08	-0.51	12	0.4	Muc16	
DMR8:18801001	8	18801001	18802000	1000	1	1.00E-09	0.48	1	0.1	Olr1124	Receptor
DMR8:18897001	8	18897001	18900000	3000	1	1.80E-07	-0.37	12	0.4	Olr1126	Receptor
DMR8:18946001	8	18946001	18953000	7000	1	9.00E-08	-0.25	75	1.07	Olr1131-ps	
DMR8:19024001	8	19024001	19025000	1000	1	6.90E-08	-0.29	8	0.8	Olr1132	Receptor
DMR8:19026001	8	19026001	19030000	4000	1	4.70E-07	-0.29	78	1.95	Olr1132;Olr1133-ps	Receptor
DMR8:19089001	8	19089001	19093000	4000	1	6.50E-09	-0.32	47	1.18	Olr1135	Receptor
DMR8:19194001	8	19194001	19198000	4000	1	9.20E-07	-0.41	42	1.05	Olr1140-ps	
DMR8:19374001	8	19374001	19380000	6000	2	5.80E-08	-0.31	59	0.98	Olr1145	Receptor
DMR8:19725001	8	19725001	19729000	4000	1	4.10E-07	-0.35	45	1.12	Olr1153-ps	
DMR8:19741001	8	19741001	19746000	5000	1	2.90E-07	-0.29	58	1.16	Olr1154-ps	
DMR8:19776001	8	19776001	19779000	3000	1	4.20E-13	-0.49	30	1	Olr1155	Receptor
DMR8:19839001	8	19839001	19846000	7000	1	2.30E-08	-0.29	83	1.19	Olr1157-ps	
DMR8:19895001	8	19895001	19901000	6000	1	5.60E-08	-0.31	64	1.07	Zfp317	
DMR8:20179001	8	20179001	20180000	1000	1	5.10E-08	-0.47	5	0.5	Olr1163;RGD1563757	Receptor
DMR8:20250001	8	20250001	20257000	7000	1	5.90E-08	-0.41	66	0.94	Olr1166	Receptor
DMR8:20451001	8	20451001	20455000	4000	1	8.80E-07	-0.28	35	0.88	Olr1172	
DMR8:21003001	8	21003001	21005000	2000	1	5.40E-07	-0.32	30	1.5	Olfr873	Receptor
DMR8:21471001	8	21471001	21473000	2000	1	1.20E-09	0.45	13	0.65	Zfp26;Zfp426	Transcription
DMR8:21998001	8	21998001	22001000	3000	1	6.70E-08	0.44	38	1.27	S1pr2	Signaling
DMR8:22224001	8	22224001	22225000	1000	1	1.80E-10	0.81	9	0.9	Pde4a	Signaling
DMR8:22302001	8	22302001	22303000	1000	1	4.10E-08	0.56	12	1.2	Atg4d;Kri1	Protease
DMR8:22354001	8	22354001	22356000	2000	1	1.00E-07	0.37	25	1.25	Slc44a2	Transport
DMR8:22366001	8	22366001	22367000	1000	1	2.20E-07	0.43	12	1.2	Slc44a2	Transport
DMR8:23351001	8	23351001	23357000	6000	2	3.10E-11	0.45	9	0.15	Anln	Cytoskeleton
DMR8:23730001	8	23730001	23732000	2000	1	1.50E-07	0.49	37	1.85	Bbs9	
DMR8:24576001	8	24576001	24578000	2000	1	1.30E-08	0.36	20	1	Bmper	Extracellular Matrix
DMR8:25396001	8	25396001	25397000	1000	1	2.60E-07	-0.52	7	0.7	Npsr1	Signaling
DMR8:25585001	8	25585001	25588000	3000	1	6.50E-08	0.41	36	1.2	Dpy19l1	
DMR8:25636001	8	25636001	25640000	4000	3	6.70E-17	0.62	103	2.58	Dpy19l1	
DMR8:25641001	8	25641001	25643000	2000	1	2.20E-08	0.36	28	1.4	Dpy19l1	
DMR8:25762001	8	25762001	25769000	7000	2	4.10E-07	-0.33	70	1	Dpy19l2	
DMR8:27782001	8	27782001	27784000	2000	1	3.70E-11	0.51	26	1.3	B3gat1	Golgi
DMR8:27804001	8	27804001	27805000	1000	1	1.00E-06	0.37	27	2.7	B3gat1;Glb1l2	Golgi;Metabolism

DMR8:27890001	8	27890001	27892000	2000	1	1.50E-07	-0.48	9	0.45	Glb1l3	Metabolism
DMR8:27981001	8	27981001	27988000	7000	1	8.10E-14	-0.4	63	0.9	Bin2a	Metabolism
DMR8:28099001	8	28099001	28100000	1000	1	1.50E-09	-0.43	12	1.2	Ncapd3	Epigenetic
DMR8:28850001	8	28850001	28851000	1000	1	4.90E-07	-0.41	11	1.1	Opcml	Immune
DMR8:29202001	8	29202001	29204000	2000	1	1.40E-08	0.55	14	0.7	Opcml	Immune
DMR8:29338001	8	29338001	29339000	1000	1	3.40E-10	0.48	7	0.7	Opcml	Immune
DMR8:29373001	8	29373001	29376000	3000	1	4.90E-08	-0.34	32	1.07	Opcml	Immune
DMR8:29463001	8	29463001	29464000	1000	1	2.50E-07	-0.36	17	1.7	Opcml	Immune
DMR8:29615001	8	29615001	29617000	2000	1	5.40E-12	0.5	17	0.85	Opcml	Immune
DMR8:29645001	8	29645001	29646000	1000	1	4.00E-07	-0.6	11	1.1	Opcml	Immune
DMR8:30101001	8	30101001	30102000	1000	1	7.40E-15	0.62	9	0.9	Ntm	Immune
DMR8:30285001	8	30285001	30286000	1000	1	4.40E-11	0.65	4	0.4	Ntm	Immune
DMR8:30391001	8	30391001	30397000	6000	2	1.40E-07	-0.35	66	1.1	Ntm	Immune
DMR8:30468001	8	30468001	30472000	4000	2	1.00E-07	0.36	51	1.27	Ntm	Immune
DMR8:30561001	8	30561001	30564000	3000	1	8.50E-09	0.45	35	1.17	Ntm	Immune
DMR8:30676001	8	30676001	30682000	6000	3	5.90E-09	-0.33	91	1.52	Ntm	Immune
DMR8:30748001	8	30748001	30749000	1000	1	4.90E-07	0.5	8	0.8	Ntm	Immune
DMR8:30905001	8	30905001	30908000	3000	1	1.10E-07	0.43	30	1	Ntm	Immune
DMR8:32193001	8	32193001	32200000	7000	2	2.80E-10	-0.33	74	1.06	Zbtb44	Transcription
DMR8:32443001	8	32443001	32444000	1000	1	1.60E-08	0.51	3	0.3	Prdm10	Transcription
DMR8:32945001	8	32945001	32948000	3000	1	2.10E-07	0.36	45	1.5	Barx2;LOC108351669	Development
DMR8:32984001	8	32984001	32985000	1000	1	7.60E-08	0.34	25	2.5	Barx2	Development
DMR8:33261001	8	33261001	33262000	1000	1	6.70E-13	0.46	13	1.3	Arhgap32	Signaling
DMR8:33547001	8	33547001	33550000	3000	1	8.50E-10	0.48	30	1	Fli1	Transcription
DMR8:33638001	8	33638001	33640000	2000	1	4.30E-07	0.42	34	1.7	Fli1	Transcription
DMR8:33812001	8	33812001	33814000	2000	1	9.60E-07	0.38	34	1.7	Ets1	Transcription
DMR8:33849001	8	33849001	33850000	1000	1	6.90E-09	-0.42	16	1.6	Ets1	Transcription
DMR8:35735001	8	35735001	35736000	1000	1	1.30E-08	0.54	12	1.2	Kirrel3	
DMR8:36034001	8	36034001	36036000	2000	1	5.70E-07	-0.49	24	1.2	Kirrel3	
DMR8:36070001	8	36070001	36073000	3000	1	2.60E-08	0.48	42	1.4	Kirrel3	
DMR8:36198001	8	36198001	36199000	1000	1	5.50E-11	0.49	8	0.8	Kirrel3	
DMR8:36399001	8	36399001	36401000	2000	1	1.30E-07	0.6	12	0.6	Tirap;Foxred1;Srpra	Metabolism;Signaling
DMR8:36653001	8	36653001	36654000	1000	1	2.50E-08	0.34	18	1.8	Cdon	
DMR8:36824001	8	36824001	36829000	5000	3	6.40E-12	-0.39	56	1.12	Pate2;Pate4	
DMR8:37100001	8	37100001	37105000	5000	3	2.20E-10	-0.33	45	0.9	Pate-f	
DMR8:37816001	8	37816001	37818000	2000	1	3.80E-08	-0.66	21	1.05	RGD1560348	
DMR8:37878001	8	37878001	37883000	5000	1	6.40E-07	-0.31	28	0.56	RGD1560348	
DMR8:38058001	8	38058001	38059000	1000	1	1.80E-09	-0.32	8	0.8	Trnaa-ugc	
DMR8:39762001	8	39762001	39763000	1000	1	3.90E-07	-0.45	18	1.8	Ccdc15	
DMR8:40090001	8	40090001	40091000	1000	1	7.30E-10	0.49	7	0.7	Siae	Metabolism
DMR8:40224001	8	40224001	40229000	5000	1	1.40E-07	-0.32	39	0.78	Olr1195	Receptor
DMR8:40395001	8	40395001	40401000	6000	1	1.50E-10	-0.43	61	1.02	Olr1202	Receptor
DMR8:42312001	8	42312001	42318000	6000	1	6.40E-08	-0.29	62	1.03	Olr1239;Olr1240;Olr1241	Receptor
DMR8:43219001	8	43219001	43223000	4000	2	5.90E-10	-0.38	63	1.57	Olr1303	
DMR8:43553001	8	43553001	43557000	4000	1	6.40E-07	-0.36	29	0.72	Olr1316	Receptor
DMR8:43572001	8	43572001	43574000	2000	1	1.30E-08	-0.36	19	0.95	Olr1316;Olr1317-ps	Receptor
DMR8:43682001	8	43682001	43688000	6000	1	4.20E-09	-0.33	60	1	Olr1323	Receptor
DMR8:43722001	8	43722001	43723000	1000	1	7.70E-08	-0.36	12	1.2	Olr1325	Receptor
DMR8:43876001	8	43876001	43883000	7000	2	1.40E-11	-0.42	106	1.51	Olr1337	Receptor
DMR8:43930001	8	43930001	43936000	6000	1	1.60E-10	-0.34	74	1.23	Olr1339	Receptor
DMR8:44048001	8	44048001	44050000	2000	1	7.60E-08	0.58	31	1.55	Olr1341;Zfp202	Receptor;Transcription
DMR8:44197001	8	44197001	44202000	5000	1	1.20E-08	0.41	95	1.9	Gramd1b	
DMR8:44211001	8	44211001	44212000	1000	1	1.10E-11	0.48	10	1	Gramd1b	
DMR8:44221001	8	44221001	44222000	1000	1	7.80E-07	0.38	12	1.2	Gramd1b	
DMR8:44390001	8	44390001	44391000	1000	1	1.60E-07	0.37	16	1.6	Gramd1b;LOC102553969	
DMR8:46838001	8	46838001	46839000	1000	1	3.10E-07	0.36	11	1.1	Grik4	Receptor
DMR8:46910001	8	46910001	46912000	2000	1	2.30E-08	0.41	32	1.6	Grik4	Receptor
DMR8:46925001	8	46925001	46931000	6000	2	7.80E-08	0.42	81	1.35	Grik4	Receptor
DMR8:47191001	8	47191001	47199000	8000	2	5.70E-10	0.46	135	1.69	Grik4	Receptor
DMR8:47243001	8	47243001	47244000	1000	1	7.20E-07	0.42	9	0.9	Grik4;LOC102549942	Receptor
DMR8:47334001	8	47334001	47335000	1000	1	5.90E-07	-0.5	17	1.7	Arhgef12	Transcription

DMR8:47471001	8	47471001	47473000	2000	1	2.10E-09	0.44	121	6.05	Pou2f3	
DMR8:48084001	8	48084001	48087000	3000	1	3.30E-07	0.39	40	1.33	Nectin1	
DMR8:48129001	8	48129001	48133000	4000	1	2.30E-10	0.65	56	1.4	Nectin1	
DMR8:48462001	8	48462001	48465000	3000	1	2.10E-08	0.39	49	1.63	LOC103693069;Mcam	Immune
DMR8:48585001	8	48585001	48587000	2000	2	2.60E-10	0.65	56	2.8	Ccdc153;Pdzd3;Nlrx1	Cytoskeleton
DMR8:48598001	8	48598001	48600000	2000	1	6.10E-10	0.44	24	1.2	Nlrx1;Abcg4	Cytoskeleton;Transport
DMR8:48811001	8	48811001	48815000	4000	1	1.30E-07	0.5	46	1.15	Bcl9l	
DMR8:49009001	8	49009001	49015000	6000	1	4.80E-07	0.41	107	1.78	Phldb1	
DMR8:49161001	8	49161001	49162000	1000	1	2.10E-07	-0.46	20	2	Kmt2a	Epigenetic
DMR8:49360001	8	49360001	49361000	1000	1	1.30E-10	0.42	16	1.6	Mpzl2;Mpzl3	Cytoskeleton
DMR8:49680001	8	49680001	49682000	2000	1	2.60E-07	0.35	32	1.6	Fxyd6	Transport
DMR8:49702001	8	49702001	49704000	2000	1	8.00E-07	0.56	34	1.7	Fxyd6;Fxyd2	Transport
DMR8:49844001	8	49844001	49845000	1000	1	3.20E-07	0.32	25	2.5	Dscaml1	Cytoskeleton
DMR8:49968001	8	49968001	49970000	2000	1	5.00E-07	0.35	45	2.25	Dscaml1	Cytoskeleton
DMR8:50072001	8	50072001	50074000	2000	1	1.80E-07	0.39	30	1.5	Cep164	Cytoskeleton
DMR8:50075001	8	50075001	50078000	3000	1	2.90E-07	0.47	75	2.5	Cep164	Cytoskeleton
DMR8:50404001	8	50404001	50405000	1000	1	3.10E-10	0.42	12	1.2	Sik3	Signaling
DMR8:50407001	8	50407001	50408000	1000	1	1.40E-09	-0.53	17	1.7	Sik3	Signaling
DMR8:50488001	8	50488001	50489000	1000	1	7.40E-07	0.45	19	1.9	Sik3	Signaling
DMR8:50579001	8	50579001	50581000	2000	1	3.30E-07	0.47	18	0.9	Zfp259;Bud13	
DMR8:51951001	8	51951001	51953000	2000	1	1.90E-09	-0.53	34	1.7	Cadm1;LOC102550632	
DMR8:51959001	8	51959001	51962000	3000	1	1.90E-11	0.47	59	1.97	Cadm1;LOC102550632	
DMR8:52157001	8	52157001	52159000	2000	1	1.90E-07	-0.54	22	1.1	Cadm1	
DMR8:52758001	8	52758001	52759000	1000	1	2.30E-08	0.45	18	1.8	Nxpe1	
DMR8:52769001	8	52769001	52770000	1000	1	1.20E-07	-0.39	13	1.3	Nxpe1	
DMR8:52929001	8	52929001	52930000	1000	1	2.50E-07	-0.5	20	2	Nnmt	Epigenetic
DMR8:52997001	8	52997001	5.30E+07	3000	1	6.90E-08	-0.28	54	1.8	Zbtb16	Transcription
DMR8:53042001	8	53042001	53043000	1000	1	3.40E-09	0.42	24	2.4	Zbtb16	Transcription
DMR8:53436001	8	53436001	53438000	2000	1	3.90E-07	-0.37	29	1.45	Tmprss5	Protease
DMR8:53771001	8	53771001	53774000	3000	2	1.60E-07	0.39	34	1.13	Ttc12	
DMR8:53832001	8	53832001	53833000	1000	1	1.70E-11	0.7	5	0.5	LOC102556026;Ncam1	
DMR8:53837001	8	53837001	53838000	1000	1	5.10E-07	-0.53	12	1.2	LOC102556026;Ncam1	
DMR8:53841001	8	53841001	53843000	2000	1	2.10E-07	0.34	34	1.7	Ncam1	
DMR8:53914001	8	53914001	53915000	1000	1	8.50E-08	-0.59	8	0.8	Ncam1	
DMR8:55041001	8	55041001	55043000	2000	1	7.60E-07	-0.47	19	0.95	Sdhd;Timm8b;LOC100151767;Pih1d2	Metabolism
DMR8:55130001	8	55130001	55131000	1000	1	9.10E-08	0.37	9	0.9	Dixdc1	Cytoskeleton
DMR8:55232001	8	55232001	55234000	2000	1	1.80E-09	0.41	20	1	Alg9	Golgi
DMR8:55259001	8	55259001	55260000	1000	1	4.00E-07	0.37	15	1.5	Alg9	Golgi
DMR8:55333001	8	55333001	55336000	3000	1	7.60E-07	0.45	28	0.93	Sik2	Signaling
DMR8:56220001	8	56220001	56223000	3000	1	1.10E-07	0.46	34	1.13	Arhgap20	Signaling
DMR8:56268001	8	56268001	56278000	10000	1	2.00E-07	-0.35	99	0.99	Arhgap20	Signaling
DMR8:56390001	8	56390001	56391000	1000	1	2.30E-13	0.49	9	0.9	Fdx1	Metabolism
DMR8:57898001	8	57898001	57901000	3000	1	8.80E-15	0.55	51	1.7	Exph5;LOC108351708	
DMR8:57966001	8	57966001	57969000	3000	1	4.60E-07	0.32	26	0.87	Exph5;Kdelc2	
DMR8:58070001	8	58070001	58071000	1000	1	8.40E-08	-0.38	21	2.1	Atm	Signaling
DMR8:59618001	8	59618001	59619000	1000	1	1.40E-08	0.52	5	0.5	Chrnb4	Ion Channel
DMR8:59758001	8	59758001	59761000	3000	2	4.70E-09	-0.36	33	1.1	Ube2q2;Fbxo22	Proteolysis
DMR8:59815001	8	59815001	59818000	3000	1	4.40E-11	0.52	37	1.23	Nrg4;LOC100360079	Growth Factors
DMR8:60193001	8	60193001	60194000	1000	1	2.20E-07	-0.52	12	1.2	Scaper	
DMR8:60248001	8	60248001	60249000	1000	1	7.20E-08	-0.37	23	2.3	Scaper	
DMR8:60265001	8	60265001	60268000	3000	1	1.40E-10	-0.48	28	0.93	Scaper	
DMR8:60358001	8	60358001	60360000	2000	1	2.70E-08	-0.42	20	1	Scaper;Nxpe2	
DMR8:60399001	8	60399001	60403000	4000	1	5.30E-10	-0.46	42	1.05	Scaper;LOC102554336;LOC108348227	
DMR8:60427001	8	60427001	60429000	2000	1	2.40E-08	-0.4	26	1.3	Scaper	
DMR8:60504001	8	60504001	60511000	7000	1	6.60E-08	-0.29	52	0.74	Scaper	
DMR8:60600001	8	60600001	60602000	2000	1	3.10E-07	-0.27	22	1.1	Scaper;RGD1563578	
DMR8:60662001	8	60662001	60665000	3000	1	3.90E-11	-1.28	33	1.1	RGD1563578	
DMR8:60779001	8	60779001	60781000	2000	1	4.30E-12	0.33	16	0.8	Pstpip1	Cytoskeleton
DMR8:60909001	8	60909001	60910000	1000	1	1.10E-08	0.41	12	1.2	Peak1	Signaling

DMR8:61450001	8	61450001	61452000	2000	1	3.90E-08	0.39	25	1.25	Lingo1	Receptor
DMR8:62235001	8	62235001	62238000	3000	1	1.50E-12	0.51	31	1.03	Scamp5	Transport
DMR8:62376001	8	62376001	62377000	1000	1	1.10E-09	0.55	17	1.7	Scamp2;Ulk3;Cplx3;LOC103693104;Lman1l	Transport;Signaling;Transport
DMR8:62378001	8	62378001	62379000	1000	1	2.70E-15	0.55	16	1.6	Scamp2;Ulk3;Cplx3;LOC103693104;Lman1l	Transport;Signaling;Transport
DMR8:62411001	8	62411001	62414000	3000	1	2.10E-07	0.44	30	1	Csk	
DMR8:62505001	8	62505001	62507000	2000	1	1.80E-07	0.55	7	0.35	Edc3	
DMR8:62605001	8	62605001	62609000	4000	1	1.30E-10	-0.45	57	1.43	Arid3b	Transcription
DMR8:62625001	8	62625001	62629000	4000	1	1.30E-21	0.49	51	1.27	Arid3b	Transcription
DMR8:62811001	8	62811001	62813000	2000	1	9.50E-07	0.55	22	1.1	Cyp11a1;Ccdc33	
DMR8:63041001	8	63041001	63043000	2000	1	5.60E-07	0.35	28	1.4	Pml;Stoml1	Cytoskeleton
DMR8:63139001	8	63139001	63143000	4000	2	8.80E-09	0.46	54	1.35	Tbc1d21	Signaling
DMR8:63373001	8	63373001	63375000	2000	1	6.30E-08	0.77	15	0.75	Nptn	Cytoskeleton
DMR8:63531001	8	63531001	63532000	1000	1	2.90E-09	0.41	18	1.8	Rec114	
DMR8:63537001	8	63537001	63540000	3000	1	7.60E-07	0.44	23	0.77	Rec114	
DMR8:63614001	8	63614001	63617000	3000	2	1.50E-09	0.46	52	1.73	Hcn4	Transport
DMR8:64197001	8	64197001	64199000	2000	1	5.20E-09	0.26	12	0.6	Arih1	Proteolysis
DMR8:64226001	8	64226001	64227000	1000	1	2.30E-09	-0.54	13	1.3	Arih1	Proteolysis
DMR8:64376001	8	64376001	64377000	1000	1	1.60E-11	0.49	11	1.1	Celf6	
DMR8:64407001	8	64407001	64410000	3000	1	5.60E-07	-0.35	24	0.8	Celf6	
DMR8:64432001	8	64432001	64433000	1000	1	3.30E-07	-0.42	18	1.8	Celf6;Parp6	
DMR8:64447001	8	64447001	64449000	2000	1	1.70E-09	0.47	20	1	Parp6	
DMR8:64584001	8	64584001	64587000	3000	1	6.20E-09	-0.37	19	0.63	Myo9a;LOC108351717	
DMR8:64675001	8	64675001	64676000	1000	1	1.70E-10	-0.43	11	1.1	Myo9a;LOC102546766	
DMR8:65142001	8	65142001	65145000	3000	1	2.50E-12	-0.32	31	1.03	Thsd4	
DMR8:65191001	8	65191001	65194000	3000	1	4.60E-07	0.35	32	1.07	Thsd4	
DMR8:65263001	8	65263001	65269000	6000	4	5.90E-11	-0.36	60	1	Thsd4	
DMR8:66948001	8	66948001	66949000	1000	1	6.80E-07	0.38	23	2.3	Paqr5	Signaling
DMR8:67384001	8	67384001	67387000	3000	1	2.70E-07	0.52	38	1.27	Coro2b	Cytoskeleton
DMR8:67469001	8	67469001	67473000	4000	1	5.20E-07	0.35	52	1.3	Coro2b	Cytoskeleton
DMR8:68151001	8	68151001	68152000	1000	1	6.30E-08	0.4	8	0.8	Map2k5	Signaling
DMR8:68254001	8	68254001	68255000	1000	1	1.70E-09	0.55	6	0.6	Map2k5	Signaling
DMR8:68302001	8	68302001	68303000	1000	1	6.70E-07	0.62	6	0.6	LOC108351724;RGD1309779	
DMR8:68431001	8	68431001	68432000	1000	1	9.10E-08	0.46	9	0.9	Iqch;LOC103693117	
DMR8:68558001	8	68558001	68562000	4000	1	4.20E-08	0.38	52	1.3	Aagab;Smad3	Transcription
DMR8:68583001	8	68583001	68585000	2000	1	9.60E-08	0.39	19	0.95	Smad3	Transcription
DMR8:68895001	8	68895001	68899000	4000	1	3.40E-11	0.51	88	2.2	Smad6	Transcription
DMR8:69266001	8	69266001	69268000	2000	1	3.00E-08	-0.48	19	0.95	Map2k1	Signaling
DMR8:69523001	8	69523001	69527000	4000	1	3.10E-08	-0.53	29	0.72	Map2k1	Signaling
DMR8:69599001	8	69599001	69602000	3000	1	7.60E-07	-0.47	23	0.77	Map2k1;Tmem185a	Signaling
DMR8:69878001	8	69878001	69881000	3000	1	2.90E-11	0.45	28	0.93	Megf11	Extracellular Matrix
DMR8:69942001	8	69942001	69944000	2000	1	4.40E-07	0.37	21	1.05	Megf11	Extracellular Matrix
DMR8:70017001	8	70017001	70018000	1000	1	3.80E-07	0.38	16	1.6	Megf11;LOC102548470	Extracellular Matrix
DMR8:70285001	8	70285001	70287000	2000	1	2.50E-09	0.43	27	1.35	LOC102548661;Dennd4a	
DMR8:70393001	8	70393001	70402000	9000	1	2.30E-09	-0.43	86	0.96	Dennd4a;Slc24a1	Transport
DMR8:71481001	8	71481001	71486000	5000	1	1.90E-11	-0.39	42	0.84	Trip4	Transcription
DMR8:71526001	8	71526001	71527000	1000	1	4.90E-07	0.39	6	0.6	Trip4;Ns5atp9;Csnk1g1	Transcription;Signaling
DMR8:71588001	8	71588001	71590000	2000	1	6.30E-08	-0.51	17	0.85	Csnk1g1	Signaling
DMR8:72144001	8	72144001	72147000	3000	1	8.10E-08	-0.36	23	0.77	Herc1	Transcription
DMR8:72170001	8	72170001	72172000	2000	1	1.20E-07	0.59	15	0.75	Herc1	Transcription
DMR8:72404001	8	72404001	72406000	2000	1	1.00E-10	0.53	44	2.2	Car12	
DMR8:72807001	8	72807001	72808000	1000	1	3.10E-07	0.49	11	1.1	Tpm1	Cytoskeleton
DMR8:73007001	8	73007001	73008000	1000	1	2.10E-12	0.57	10	1	Tln2	
DMR8:73009001	8	73009001	73011000	2000	1	1.90E-07	0.47	22	1.1	Tln2	
DMR8:73079001	8	73079001	73081000	2000	1	7.50E-12	0.54	20	1	Tln2	
DMR8:73087001	8	73087001	73092000	5000	1	2.50E-07	0.35	64	1.28	Tln2	
DMR8:73210001	8	73210001	73211000	1000	1	6.10E-09	0.45	26	2.6	Tln2	
DMR8:73345001	8	73345001	73347000	2000	1	2.30E-09	0.4	26	1.3	Tln2	
DMR8:73402001	8	73402001	73409000	7000	1	1.20E-07	-0.28	97	1.39	Tln2	

DMR8:73581001	8	73581001	73583000	2000	1	2.60E-07	0.28	15	0.75	LOC108351734;C2cd4b	
DMR8:73804001	8	73804001	73811000	7000	1	4.30E-07	-0.75	191	2.73	Vps13c	Transport
DMR8:76454001	8	76454001	76455000	1000	1	9.60E-07	-0.45	12	1.2	LOC108351740;Gcnt3	Golgi
DMR8:76989001	8	76989001	76991000	2000	1	4.20E-07	0.49	17	0.85	Sltm	
DMR8:77015001	8	77015001	77016000	1000	1	6.90E-07	-0.41	23	2.3	Sltm	
DMR8:77124001	8	77124001	77125000	1000	1	3.30E-07	-0.48	6	0.6	Adam10	Protease
DMR8:77158001	8	77158001	77160000	2000	1	5.00E-08	-0.45	8	0.4	Adam10	Protease
DMR8:77392001	8	77392001	77394000	2000	1	5.30E-07	-0.64	33	1.65	Lipc	Metabolism
DMR8:77561001	8	77561001	77563000	2000	1	2.40E-08	0.6	16	0.8	Aqp9	Transport
DMR8:77581001	8	77581001	77585000	4000	1	9.40E-08	0.26	44	1.1	Aqp9	Transport
DMR8:77695001	8	77695001	77696000	1000	1	1.60E-09	0.59	2	0.2	Aldh1a2	Metabolism
DMR8:77710001	8	77710001	77712000	2000	1	1.10E-07	0.41	19	0.95	Aldh1a2	Metabolism
DMR8:78026001	8	78026001	78028000	2000	1	9.20E-08	0.52	20	1	Myzap	
DMR8:78070001	8	78070001	78071000	1000	1	6.90E-10	0.5	11	1.1	Myzap	
DMR8:78123001	8	78123001	78126000	3000	1	1.10E-09	0.56	30	1	Cgnl1	
DMR8:78452001	8	78452001	78454000	2000	1	6.40E-10	-0.47	22	1.1	Tcf12	Transcription
DMR8:78468001	8	78468001	78469000	1000	1	4.20E-10	-0.56	9	0.9	Tcf12	Transcription
DMR8:79053001	8	79053001	79054000	1000	1	1.90E-07	0.43	19	1.9	LOC103693138;Mns1	Development
DMR8:79117001	8	79117001	79119000	2000	1	8.40E-10	0.63	19	0.95	Tex9;LOC102554871;LOC103693140	Cytoskeleton
DMR8:79248001	8	79248001	79250000	2000	1	2.30E-09	0.67	20	1	Rfx7	Transcription
DMR8:79875001	8	79875001	79880000	5000	1	6.80E-11	-0.29	46	0.92	LOC102554551;RGD1564166	
DMR8:79914001	8	79914001	79916000	2000	1	4.30E-08	0.62	20	1	LOC102554551;RGD1564166	
DMR8:80304001	8	80304001	80305000	1000	1	1.50E-08	-0.46	11	1.1	Unc13c	
DMR8:81942001	8	81942001	81945000	3000	1	1.70E-07	-0.45	32	1.07	Fam214a;Arpp19	
DMR8:82131001	8	82131001	82137000	6000	1	2.80E-11	-0.38	70	1.17	Myo5a	Cytoskeleton
DMR8:82493001	8	82493001	82495000	2000	2	1.30E-33	0.84	23	1.15	Tmod3	Cytoskeleton
DMR8:82522001	8	82522001	82524000	2000	1	1.40E-07	0.49	17	0.85	Tmod2	Cytoskeleton
DMR8:82884001	8	82884001	82886000	2000	1	4.30E-07	0.64	16	0.8	Bmp5	Growth Factors
DMR8:83171001	8	83171001	83174000	3000	1	1.50E-08	-0.39	46	1.53	Hmgcll1	Metabolism
DMR8:83641001	8	83641001	83645000	4000	1	4.40E-07	0.46	82	2.05	Fam83b	
DMR8:83682001	8	83682001	83684000	2000	1	1.70E-09	0.67	15	0.75	Fam83b	
DMR8:84137001	8	84137001	84138000	1000	1	2.60E-08	-0.61	15	1.5	Tinag	Protease
DMR8:84451001	8	84451001	84454000	3000	1	3.30E-08	-0.53	37	1.23	Mlip	
DMR8:84508001	8	84508001	84513000	5000	2	2.60E-07	-0.31	78	1.56	Mlip	
DMR8:84599001	8	84599001	84601000	2000	1	4.60E-09	-0.42	13	0.65	Mlip	
DMR8:84772001	8	84772001	84773000	1000	1	1.30E-12	0.5	21	2.1	Lrrc1	
DMR8:84802001	8	84802001	84803000	1000	1	5.60E-07	-0.35	22	2.2	Lrrc1	
DMR8:85247001	8	85247001	85249000	2000	2	2.20E-14	0.46	37	1.85	Elovl5	Metabolism
DMR8:85265001	8	85265001	85270000	5000	1	4.40E-07	-0.49	63	1.26	Elovl5	Metabolism
DMR8:85291001	8	85291001	85293000	2000	1	9.70E-10	0.46	24	1.2	Elovl5	Metabolism
DMR8:85701001	8	85701001	85703000	2000	1	2.10E-07	0.54	46	2.3	Dppa5	
DMR8:85911001	8	85911001	85913000	2000	2	6.20E-10	0.51	15	0.75	Slc17a5	Transport
DMR8:85963001	8	85963001	85971000	8000	2	1.20E-08	-0.41	96	1.2	Cd109	Protease; Proteolysis
DMR8:87098001	8	87098001	87100000	2000	1	6.40E-07	-0.5	30	1.5	Col12a1	
DMR8:87126001	8	87126001	87129000	3000	1	2.10E-09	-0.53	49	1.63	Col12a1	
DMR8:87298001	8	87298001	87303000	5000	1	1.80E-10	-0.34	63	1.26	Filip1	
DMR8:87413001	8	87413001	87415000	2000	1	9.00E-07	0.3	28	1.4	Filip1	
DMR8:87479001	8	87479001	87480000	1000	1	3.40E-11	0.41	14	1.4	Senp6	Protease
DMR8:87764001	8	87764001	87765000	1000	1	9.60E-07	0.37	29	2.9	Impg1	Extracellular Matrix
DMR8:87794001	8	87794001	87800000	6000	1	8.60E-07	-0.28	59	0.98	Impg1	Extracellular Matrix
DMR8:87854001	8	87854001	87861000	7000	4	3.20E-09	-0.5	75	1.07	Impg1	Extracellular Matrix
DMR8:90312001	8	90312001	90314000	2000	1	9.60E-07	-0.46	12	0.6	RGD1562068	
DMR8:90368001	8	90368001	90374000	6000	1	3.50E-07	-0.41	63	1.05	Irak1bp1	
DMR8:90666001	8	90666001	90669000	3000	1	4.00E-08	0.37	27	0.9	Hmgn3	Epigenetic
DMR8:90670001	8	90670001	90676000	6000	1	4.40E-08	-0.27	64	1.07	Hmgn3	Epigenetic
DMR8:91109001	8	91109001	91111000	2000	1	1.70E-07	-0.45	19	0.95	LOC102554622;Sh3bgrl2;LOC102554552	
DMR8:91162001	8	91162001	91164000	2000	1	5.20E-10	-0.71	31	1.55	Sh3bgrl2	

DMR8:91412001	8	91412001	91414000	2000	1	2.40E-07	-0.46	36	1.8	Ttk;LOC108351756	Signaling
DMR8:91614001	8	91614001	91619000	5000	2	2.20E-12	-0.44	61	1.22	Bckdhb	Metabolism
DMR8:93470001	8	93470001	93472000	2000	2	2.30E-07	-0.52	41	2.05	RGD1564645	
DMR8:94033001	8	94033001	94041000	8000	1	8.70E-08	-0.41	60	0.75	Ube3d;LOC102551813	Proteolysis
DMR8:94137001	8	94137001	94142000	5000	1	8.10E-07	-0.27	39	0.78	Dopey1	
DMR8:94325001	8	94325001	94326000	1000	1	2.70E-14	0.5	19	1.9	Me1	Metabolism
DMR8:94516001	8	94516001	94522000	6000	1	2.60E-09	-0.32	64	1.07	Snap91	Transport
DMR8:94797001	8	94797001	94799000	2000	1	6.30E-07	-0.43	27	1.35	RGD1559935;Mrap2	
DMR8:95888001	8	95888001	95889000	1000	1	9.00E-08	0.49	4	0.4	RGD1561192	
DMR8:95991001	8	95991001	95992000	1000	1	8.10E-10	0.41	24	2.4	Nt5e	Signaling
DMR8:96414001	8	96414001	96418000	4000	1	7.50E-11	-0.44	28	0.7	RGD1560775	
DMR8:96421001	8	96421001	96423000	2000	1	2.30E-09	-0.39	17	0.85	RGD1560775	
DMR8:96447001	8	96447001	96452000	5000	1	9.00E-07	-0.32	43	0.86	RGD1560775	
DMR8:96474001	8	96474001	96478000	4000	1	1.30E-07	-0.29	34	0.85	RGD1560775	
DMR8:96504001	8	96504001	96512000	8000	2	4.20E-11	-0.48	79	0.99	RGD1560775	
DMR8:96524001	8	96524001	96528000	4000	1	2.40E-07	-0.5	39	0.98	RGD1560775	
DMR8:97313001	8	97313001	97315000	2000	1	1.30E-12	0.46	26	1.3	Rasgrf1	Transcription
DMR8:97362001	8	97362001	97369000	7000	1	3.60E-07	-0.25	75	1.07	Rasgrf1	Transcription
DMR8:97602001	8	97602001	97604000	2000	1	5.80E-09	0.41	40	2	Tbc1d2b	Signaling
DMR8:97624001	8	97624001	97625000	1000	1	6.40E-07	0.35	43	4.3	Tbc1d2b	Signaling
DMR8:100069001	8	100069001	100071000	2000	1	1.30E-08	-0.57	29	1.45	Plod2	Golgi
DMR8:102876001	8	102876001	102882000	6000	1	1.00E-07	-0.32	63	1.05	Slc9a9	Transport
DMR8:103510001	8	103510001	103517000	7000	1	4.40E-10	-0.32	94	1.34	Trpc1	Transport
DMR8:103846001	8	103846001	103850000	4000	2	4.40E-09	-0.38	34	0.85	Xrn1	Transcription
DMR8:104153001	8	104153001	104156000	3000	1	1.60E-07	-0.48	44	1.47	Tfdp2;LOC102550734	Transcription
DMR8:104966001	8	104966001	104968000	2000	1	3.60E-10	0.41	27	1.35	Slc25a36	Transport
DMR8:105371001	8	105371001	105374000	3000	1	1.50E-10	0.47	38	1.27	Clstn2	Transport
DMR8:105669001	8	105669001	105674000	5000	3	2.00E-08	-0.34	60	1.2	Clstn2;LOC100909709	Transport
DMR8:105682001	8	105682001	105686000	4000	1	4.50E-07	-0.35	40	1	Clstn2	Transport
DMR8:105995001	8	105995001	105997000	2000	1	8.50E-10	0.39	14	0.7	Clstn2	Transport
DMR8:106421001	8	106421001	106423000	2000	1	7.90E-15	0.66	24	1.2	Nmnat3	Metabolism
DMR8:106434001	8	106434001	106435000	1000	1	1.20E-07	0.32	20	2	Nmnat3	Metabolism
DMR8:106543001	8	106543001	106545000	2000	1	1.10E-11	0.43	35	1.75	RGD1565403	
DMR8:107306001	8	107306001	107308000	2000	2	6.30E-07	0.49	28	1.4	Pik3cb	Signaling
DMR8:107329001	8	107329001	107331000	2000	1	6.20E-07	-0.4	11	0.55	Pik3cb	Signaling
DMR8:107369001	8	107369001	107371000	2000	1	2.30E-14	0.68	12	0.6	Pik3cb	Signaling
DMR8:107637001	8	107637001	107639000	2000	1	6.80E-09	0.51	31	1.55	Mras	Signaling
DMR8:107645001	8	107645001	107648000	3000	1	8.20E-07	0.28	54	1.8	Mras	Signaling
DMR8:107727001	8	107727001	107728000	1000	1	4.90E-07	0.51	11	1.1	Nme9;Armc8	
DMR8:107808001	8	107808001	107810000	2000	1	1.10E-08	-0.4	28	1.4	Armc8	
DMR8:107933001	8	107933001	107936000	3000	1	3.50E-07	0.4	54	1.8	Cldn18	Cell Junction
DMR8:108813001	8	108813001	108820000	7000	1	1.50E-07	-0.47	86	1.23	Nck1	Cytoskeleton
DMR8:109010001	8	109010001	109017000	7000	2	5.90E-08	-0.36	78	1.11	Stag1	Epigenetic
DMR8:109028001	8	109028001	109034000	6000	1	4.70E-07	-0.31	77	1.28	Stag1	Epigenetic
DMR8:109133001	8	109133001	109135000	2000	2	5.80E-08	0.5	8	0.4	Stag1	Epigenetic
DMR8:109256001	8	109256001	109260000	4000	1	1.30E-10	-0.53	37	0.92	Stag1	Epigenetic
DMR8:109403001	8	109403001	109404000	1000	1	2.10E-08	-0.49	35	3.5	Pccb	Metabolism
DMR8:109579001	8	109579001	109582000	3000	1	1.80E-08	-0.43	20	0.67	Ppp2r3a	Signaling
DMR8:109596001	8	109596001	109604000	8000	1	1.50E-07	-0.38	98	1.23	Ppp2r3a	Signaling
DMR8:110425001	8	110425001	110436000	11000	1	5.80E-10	-0.35	124	1.13	Ephb1	Receptor
DMR8:110583001	8	110583001	110585000	2000	1	9.30E-09	0.54	17	0.85	Ephb1	Receptor
DMR8:110805001	8	110805001	110806000	1000	1	7.90E-09	0.37	17	1.7	Ephb1	Receptor
DMR8:111013001	8	111013001	111016000	3000	1	3.00E-07	0.44	23	0.77	Ky	
DMR8:111236001	8	111236001	111237000	1000	1	8.90E-09	0.38	13	1.3	Amotl2;LOC102549628	
DMR8:111558001	8	111558001	111560000	2000	1	4.40E-08	0.48	26	1.3	Slco2a1	Transport
DMR8:111670001	8	111670001	111672000	2000	1	3.60E-09	0.41	22	1.1	Rab6b;Srprb	Transport
DMR8:111674001	8	111674001	111675000	1000	1	1.90E-08	0.38	12	1.2	Rab6b;Srprb	Transport
DMR8:111715001	8	111715001	111716000	1000	1	5.30E-07	0.41	11	1.1	Tf	Transport
DMR8:112197001	8	112197001	112199000	2000	1	8.60E-08	0.36	39	1.95	Tmem108	
DMR8:112244001	8	112244001	112245000	1000	1	2.20E-12	-0.55	17	1.7	Tmem108	
DMR8:113319001	8	113319001	113321000	2000	1	1.40E-16	0.51	42	2.1	Cpne4	

DMR8:113423001	8	113423001	113424000	1000	1	7.60E-08	0.54	15	1.5	Cpne4	
DMR8:113858001	8	113858001	113859000	1000	1	2.10E-08	0.59	12	1.2	Nek11	Signaling
DMR8:113973001	8	113973001	113974000	1000	1	1.70E-07	0.5	6	0.6	Atp2c1;LOC102551490	Transport
DMR8:115135001	8	115135001	115136000	1000	1	8.00E-07	0.36	16	1.6	Rpl29;Acy1;Abhd14a	Translation
DMR8:115226001	8	115226001	115229000	3000	1	7.40E-09	0.38	53	1.77	Iqcf3	
DMR8:115657001	8	115657001	115659000	2000	1	3.20E-07	0.46	11	0.55	Dock3	Transcription
DMR8:115709001	8	115709001	115716000	7000	1	1.10E-07	-0.24	75	1.07	Dock3	Transcription
DMR8:115717001	8	115717001	115720000	3000	1	7.20E-07	-0.29	51	1.7	Dock3	Transcription
DMR8:115723001	8	115723001	115736000	13000	2	6.50E-09	-0.45	128	0.98	Dock3	Transcription
DMR8:115761001	8	115761001	115765000	4000	1	2.40E-07	-0.34	37	0.92	Dock3	Transcription
DMR8:115777001	8	115777001	115780000	3000	1	3.60E-08	0.41	24	0.8	Dock3	Transcription
DMR8:115856001	8	115856001	115858000	2000	1	7.40E-09	-0.48	10	0.5	Dock3	Transcription
DMR8:115900001	8	115900001	115904000	4000	2	2.30E-08	-0.43	26	0.65	Dock3	Transcription
DMR8:115917001	8	115917001	115920000	3000	2	6.50E-10	-0.44	15	0.5	Dock3;LOC102550698	Transcription
DMR8:115960001	8	115960001	115962000	2000	1	6.70E-11	-0.47	9	0.45	Dock3	Transcription
DMR8:116016001	8	116016001	116018000	2000	1	1.00E-14	0.62	25	1.25	Mapkapk3	Signaling
DMR8:116169001	8	116169001	116172000	3000	1	1.00E-11	0.52	49	1.63	Cacna2d2	Transport
DMR8:116277001	8	116277001	116279000	2000	1	1.50E-07	0.44	44	2.2	Cacna2d2	Transport
DMR8:116338001	8	116338001	116340000	2000	1	1.40E-08	0.47	35	1.75	Hyal2;Hyal1;Nat6;Hyal3;Ifrd2;Lsmem2	Metabolism
DMR8:116354001	8	116354001	116356000	2000	1	6.60E-07	0.43	62	3.1	Ifrd2;Lsmem2;Sema3b	Signaling
DMR8:116449001	8	116449001	116453000	4000	1	1.10E-10	0.43	89	2.22	Sema3f	Signaling
DMR8:116899001	8	116899001	116902000	3000	1	1.00E-08	0.44	53	1.77	Bsn	
DMR8:116982001	8	116982001	116984000	2000	1	2.50E-07	0.43	47	2.35	Dag1	Cytoskeleton
DMR8:117610001	8	117610001	117611000	1000	1	1.00E-10	0.39	15	1.5	Nckipsd;Celsr3	Cytoskeleton;Cytoskeleton
DMR8:117652001	8	117652001	117655000	3000	1	4.80E-11	0.48	68	2.27	Celsr3;Slc26a6;LOC108351778;Tmem89	Cytoskeleton;Transport
DMR8:117668001	8	117668001	117669000	1000	1	1.90E-08	0.4	14	1.4	Slc26a6;LOC108351778;Tmem89	Transport
DMR8:117698001	8	117698001	117702000	4000	1	6.80E-07	0.36	94	2.35	Uqcrc1;Col7a1;Mir711	Protease;Extracellular Matrix
DMR8:117790001	8	117790001	117792000	2000	1	9.50E-07	0.32	42	2.1	LOC100911077;LOC108351779;Shisa5;Trex1;Atrip	Cytoskeleton
DMR8:117843001	8	117843001	117845000	2000	1	5.10E-07	0.36	41	2.05	Plxnb1	
DMR8:118172001	8	118172001	118173000	1000	1	1.30E-07	0.42	9	0.9	Dhx30	Transcription
DMR8:118593001	8	118593001	118594000	1000	1	6.10E-07	-0.37	14	1.4	Scap	
DMR8:118629001	8	118629001	118633000	4000	1	3.30E-13	0.64	99	2.48	Scap;Ptpn23	Transport
DMR8:118996001	8	118996001	118998000	2000	1	6.50E-07	0.41	27	1.35	Pth1r	Receptor
DMR8:119029001	8	119029001	119031000	2000	1	4.00E-07	0.43	22	1.1	Myl3	Cytoskeleton
DMR8:119162001	8	119162001	119163000	1000	1	1.30E-10	0.44	21	2.1	Tmie;Als2cl	
DMR8:119406001	8	119406001	119408000	2000	2	3.20E-09	0.36	17	0.85	Lrrfip2	Transcription
DMR8:119698001	8	119698001	119699000	1000	1	3.90E-08	0.43	5	0.5	Dclk3;LOC102552650	Signaling
DMR8:119702001	8	119702001	119703000	1000	1	5.90E-07	-0.43	26	2.6	Dclk3;LOC102552650	Signaling
DMR8:121957001	8	121957001	121960000	3000	1	5.50E-07	0.42	40	1.33	Pdcd6ip	Transport
DMR8:121976001	8	121976001	121979000	3000	1	4.10E-07	0.43	31	1.03	Pdcd6ip;LOC100910424	Transport
DMR8:122071001	8	122071001	122074000	3000	1	2.00E-08	0.38	35	1.17	Clasp2	Cytoskeleton
DMR8:122115001	8	122115001	122117000	2000	1	5.10E-08	-0.5	31	1.55	Clasp2	Cytoskeleton
DMR8:122135001	8	122135001	122137000	2000	1	2.20E-07	-0.37	19	0.95	Clasp2	Cytoskeleton
DMR8:122392001	8	122392001	122395000	3000	1	9.80E-09	-0.36	44	1.47	Crtap	
DMR8:122411001	8	122411001	122413000	2000	1	8.40E-08	-0.3	37	1.85	Crtap;LOC108348127	
DMR8:122829001	8	122829001	122832000	3000	2	5.90E-16	0.62	48	1.6	Cmtm7	Transport
DMR8:122846001	8	122846001	122847000	1000	1	2.10E-07	0.5	15	1.5	Cmtm7;Cmtm8	Transport
DMR8:122885001	8	122885001	122886000	1000	1	6.60E-07	0.33	20	2	Cmtm8	Transport
DMR8:123033001	8	123033001	123037000	4000	1	2.60E-09	0.57	76	1.9	LOC108351788;Osbpl10	
DMR8:123094001	8	123094001	123096000	2000	1	2.30E-09	0.37	45	2.25	Osbpl10	
DMR8:123111001	8	123111001	123113000	2000	1	5.00E-08	0.74	40	2	Osbpl10	
DMR8:124031001	8	124031001	124035000	4000	1	2.30E-07	0.41	52	1.3	Gadl1	
DMR8:124887001	8	124887001	124888000	1000	1	7.40E-08	-0.6	19	1.9	Rbms3	
DMR8:124964001	8	124964001	124965000	1000	1	5.00E-07	0.49	2	0.2	Rbms3	
DMR8:125023001	8	125023001	125025000	2000	1	3.40E-07	-0.4	24	1.2	Rbms3	
DMR8:125036001	8	125036001	125037000	1000	1	6.60E-07	0.36	11	1.1	Rbms3	

DMR8:126145001	8	126145001	126147000	2000	1	7.10E-07	0.3	36	1.8	Zcwpw2	
DMR8:126264001	8	126264001	126265000	1000	1	6.70E-09	0.4	17	1.7	Zcwpw2	
DMR8:126408001	8	126408001	126409000	1000	1	2.40E-07	-0.46	15	1.5	Zcwpw2;Azi2	
DMR8:126481001	8	126481001	126482000	1000	1	1.10E-08	0.35	34	3.4	Cmc1	
DMR8:127546001	8	127546001	127548000	2000	1	4.40E-10	0.54	29	1.45	Ctdspl;Itga9	Extracellular Matrix
DMR8:127643001	8	127643001	127645000	2000	1	1.70E-07	-0.41	41	2.05	Ctdspl;LOC108351797	
DMR8:127661001	8	127661001	127662000	1000	1	7.80E-07	0.57	30	3	Ctdspl;LOC108351797	
DMR8:127758001	8	127758001	127760000	2000	1	1.10E-11	0.45	49	2.45	Vill;Plcd1	Cytoskeleton;Metabolism
DMR8:127782001	8	127782001	127784000	2000	2	3.40E-11	0.69	51	2.55	Plcd1;LOC102548352;Dlec1	Metabolism
DMR8:127814001	8	127814001	127816000	2000	1	8.00E-07	0.38	51	2.55	Dlec1	
DMR8:127895001	8	127895001	127897000	2000	1	1.40E-07	0.42	22	1.1	Slc22a13;LOC685081	Transport
DMR8:128058001	8	128058001	128060000	2000	1	6.30E-10	0.35	43	2.15	Xylb	Metabolism
DMR8:128167001	8	128167001	128175000	8000	1	4.50E-12	0.46	238	2.98	Scn5a	Transport
DMR8:128311001	8	128311001	128314000	3000	1	1.80E-11	0.42	68	2.27	Scn10a	Transport
DMR8:128536001	8	128536001	128538000	2000	1	8.80E-07	-0.43	45	2.25	Scn11a;LOC108351800	Transport
DMR8:128994001	8	128994001	128995000	1000	1	2.20E-07	-0.54	17	1.7	Myrip	Cytoskeleton
DMR8:129060001	8	129060001	129062000	2000	1	9.10E-10	0.48	20	1	Myrip	Cytoskeleton
DMR8:129233001	8	129233001	129234000	1000	1	1.20E-11	0.5	7	0.7	Entpd3;Rpl14	Signaling;Translation
DMR8:129241001	8	129241001	129244000	3000	2	3.50E-11	0.48	50	1.67	Entpd3;Rpl14	Signaling;Translation
DMR8:129709001	8	129709001	129711000	2000	1	7.50E-07	0.45	24	1.2	Ulk4	Signaling
DMR8:129850001	8	129850001	129853000	3000	1	6.00E-08	0.37	59	1.97	Ulk4	Signaling
DMR8:129958001	8	129958001	129959000	1000	1	6.20E-08	0.35	21	2.1	Trak1	Transport
DMR8:130055001	8	130055001	130057000	2000	1	6.00E-10	0.49	41	2.05	Trak1	Transport
DMR8:130107001	8	130107001	130108000	1000	1	1.80E-08	0.47	16	1.6	Trak1	Transport
DMR8:130234001	8	130234001	130235000	1000	1	3.60E-07	0.6	15	1.5	Lyzl4	
DMR8:130319001	8	130319001	130320000	1000	1	5.30E-08	0.38	23	2.3	Vipr1;Sec22c	Receptor
DMR8:130327001	8	130327001	130331000	4000	3	6.50E-09	0.58	69	1.73	Sec22c	
DMR8:130433001	8	130433001	130435000	2000	1	2.00E-08	0.71	36	1.8	Hhatl;Ccdc13	Metabolism
DMR8:130471001	8	130471001	130472000	1000	1	4.90E-07	0.35	17	1.7	Ccdc13	
DMR8:130807001	8	130807001	130809000	2000	1	2.90E-09	0.47	24	1.2	Snrk;Ano10	Signaling
DMR8:130928001	8	130928001	130929000	1000	1	1.70E-07	-0.57	18	1.8	Ano10;LOC108351805	
DMR8:131832001	8	131832001	131834000	2000	1	4.90E-10	0.56	11	0.55	Topaz1	
DMR8:131941001	8	131941001	131943000	2000	1	3.30E-07	0.42	21	1.05	Zfp167	
DMR8:132019001	8	132019001	132023000	4000	1	6.80E-08	-0.35	39	0.98	Zfp105;LOC688614;LOC367195;RGD1311745	Transcription;Translation
DMR8:132124001	8	132124001	132131000	7000	1	7.20E-15	-0.45	62	0.89	Tgm4	Transport
DMR8:132191001	8	132191001	132192000	1000	1	2.10E-08	0.48	7	0.7	Zdhhc3	
DMR8:132689001	8	132689001	132695000	6000	2	5.10E-12	-0.31	63	1.05	RGD1566368	
DMR8:132924001	8	132924001	132927000	3000	1	2.20E-07	-0.61	44	1.47	Xcr1;LOC688866	
DMR9:39001	9	39001	40000	1000	1	1.70E-07	-0.4	26	2.6	Efhb	Signaling
DMR9:50001	9	50001	51000	1000	1	6.00E-10	0.41	11	1.1	Efhb	Signaling
DMR9:67001	9	67001	74000	7000	1	6.80E-07	-0.29	101	1.44	Efhb	Signaling
DMR9:1321001	9	1321001	1323000	2000	1	5.10E-08	-0.4	10	0.5	Tbc1d5	Signaling
DMR9:1403001	9	1403001	1404000	1000	1	1.40E-13	0.37	10	1	Tbc1d5;LOC108348193	Signaling
DMR9:1425001	9	1425001	1428000	3000	1	6.50E-07	-0.41	23	0.77	Tbc1d5;LOC108348193	Signaling
DMR9:1464001	9	1464001	1468000	4000	1	1.30E-07	-0.37	39	0.98	Tbc1d5	Signaling
DMR9:1498001	9	1498001	1502000	4000	1	2.70E-08	-0.34	56	1.4	Tbc1d5	Signaling
DMR9:1556001	9	1556001	1562000	6000	4	3.00E-11	-0.4	51	0.85	Tbc1d5	Signaling
DMR9:1736001	9	1736001	1738000	2000	1	8.10E-07	-0.44	24	1.2	Tbc1d5	Signaling
DMR9:3131001	9	3131001	3132000	1000	1	2.40E-10	-0.51	17	1.7	Kcnh8;LOC103690491	Transport
DMR9:3215001	9	3215001	3219000	4000	1	1.00E-08	-0.32	32	0.8	Kcnh8;LOC102551325	Transport
DMR9:3252001	9	3252001	3253000	1000	1	1.90E-07	-0.41	6	0.6	Kcnh8	Transport
DMR9:3481001	9	3481001	3483000	2000	1	6.20E-07	-0.29	19	0.95	Kcnh8	Transport
DMR9:3537001	9	3537001	3540000	3000	1	2.00E-07	-0.38	14	0.47	Kcnh8	Transport
DMR9:4164001	9	4164001	4166000	2000	1	8.50E-07	0.56	5	0.25	Sult1c2a	Transport
DMR9:4169001	9	4169001	4172000	3000	1	2.90E-07	-0.3	16	0.53	Sult1c2a	Transport
DMR9:4267001	9	4267001	4275000	8000	1	8.30E-07	0.37	54	0.68	RGD1562392	Transport
DMR9:4406001	9	4406001	4408000	2000	1	4.80E-18	-0.54	14	0.7	RGD1562392	Transport
DMR9:4414001	9	4414001	4416000	2000	1	1.90E-08	-0.51	16	0.8	RGD1562392;Sgo1	Transport;Cell Cycle
DMR9:4854001	9	4854001	4858000	4000	1	7.70E-07	0.32	16	0.4	RGD1559960	Transport

DMR9:5219001	9	5219001	5221000	2000	1	2.30E-09	-0.28	28	1.4	RGD1560901	
DMR9:8105001	9	8105001	8107000	2000	1	6.50E-09	-0.6	10	0.5	Adgre4	Signaling
DMR9:8130001	9	8130001	8132000	2000	1	6.50E-10	0.38	29	1.45	Adgre4	Signaling
DMR9:8154001	9	8154001	8157000	3000	1	1.60E-11	-0.47	14	0.47	Adgre4	Signaling
DMR9:9302001	9	9302001	9310000	8000	1	1.00E-07	-0.28	81	1.01	Vom2r-ps140	
DMR9:9425001	9	9425001	9426000	1000	1	4.50E-07	-0.28	11	1.1	Adgre1	Signaling
DMR9:9427001	9	9427001	9429000	2000	1	1.30E-07	-0.34	18	0.9	Adgre1	Signaling
DMR9:9460001	9	9460001	9463000	3000	1	5.80E-11	-0.39	26	0.87	Adgre1	Signaling
DMR9:9513001	9	9513001	9516000	3000	1	1.70E-09	-0.48	19	0.63	Adgre1	Signaling
DMR9:9620001	9	9620001	9621000	1000	1	3.20E-08	0.47	15	1.5	Vav1	
DMR9:9658001	9	9658001	9660000	2000	1	1.50E-07	0.5	41	2.05	Vav1	
DMR9:9711001	9	9711001	9716000	5000	2	2.60E-09	0.44	105	2.1	Trip10;Gpr108;C3	Signaling;Protease; Proteolysis
DMR9:9726001	9	9726001	9727000	1000	1	8.10E-08	0.43	18	1.8	C3	Protease; Proteolysis
DMR9:9772001	9	9772001	9774000	2000	1	9.10E-07	-0.41	8	0.4	Tnfsf14	
DMR9:9874001	9	9874001	9875000	1000	1	5.70E-07	0.57	11	1.1	Brcc3	Translation
DMR9:10121001	9	10121001	10123000	2000	1	7.60E-07	-0.37	21	1.05	Mllt1	Transcription
DMR9:10156001	9	10156001	10157000	1000	1	4.00E-07	0.55	10	1	Mllt1;LOC316124	Transcription;Metabolism
DMR9:10237001	9	10237001	10238000	1000	1	4.60E-10	0.49	2	0.2	Rfx2	Transcription
DMR9:10247001	9	10247001	10248000	1000	1	1.90E-11	0.47	16	1.6	Rfx2	Transcription
DMR9:10353001	9	10353001	10354000	1000	1	2.50E-07	0.34	17	1.7	Ranbp3	Cytoskeleton
DMR9:10401001	9	10401001	10402000	1000	1	2.00E-11	0.49	8	0.8	Catsperd	
DMR9:10452001	9	10452001	10454000	2000	1	1.00E-07	0.4	53	2.65	LOC301124;Safb	
DMR9:10507001	9	10507001	10510000	3000	2	4.00E-12	0.62	30	1	Tincr;LOC102548161	
DMR9:10589001	9	10589001	10592000	3000	1	7.80E-09	0.43	94	3.13	Ptprs	Signaling
DMR9:10610001	9	10610001	10613000	3000	1	2.60E-08	0.36	37	1.23	Ptprs	Signaling
DMR9:10659001	9	10659001	10661000	2000	1	2.40E-12	0.53	30	1.5	Kdm4b	Epigenetic
DMR9:10852001	9	10852001	10854000	2000	1	8.70E-08	0.42	14	0.7	LOC103693194;Dpp9	Protease
DMR9:11163001	9	11163001	11170000	7000	3	3.20E-11	0.68	75	1.07	Uxs1	Metabolism
DMR9:13100001	9	13100001	13106000	6000	1	2.30E-07	0.31	113	1.88	Kif6	Cytoskeleton
DMR9:13144001	9	13144001	13146000	2000	1	4.00E-13	0.56	31	1.55	Kif6;LOC108351886	Cytoskeleton
DMR9:13201001	9	13201001	13203000	2000	1	8.30E-08	0.52	11	0.55	Kif6	Cytoskeleton
DMR9:13911001	9	13911001	13914000	3000	1	1.10E-08	0.49	37	1.23	Lrfn2;LOC108351889	
DMR9:14001001	9	14001001	14002000	1000	1	1.80E-12	0.46	18	1.8	Lrfn2	
DMR9:14571001	9	14571001	14574000	3000	1	1.20E-07	-0.43	31	1.03	Nfya	Transcription
DMR9:14585001	9	14585001	14587000	2000	1	8.40E-07	0.38	15	0.75	Nfya	Transcription
DMR9:14784001	9	14784001	14787000	3000	2	7.10E-09	0.46	38	1.27	Ncr2	
DMR9:15102001	9	15102001	15105000	3000	1	5.40E-07	0.32	65	2.17	Foxp4	
DMR9:15114001	9	15114001	15118000	4000	1	8.20E-07	0.33	77	1.93	Foxp4	
DMR9:15157001	9	15157001	15161000	4000	1	7.20E-10	-0.4	23	0.58	Mdfi	
DMR9:15163001	9	15163001	15164000	1000	1	6.50E-08	0.44	13	1.3	Mdfi	
DMR9:15210001	9	15210001	15213000	3000	2	1.80E-09	0.44	59	1.97	Tfeb	
DMR9:15548001	9	15548001	15551000	3000	1	1.30E-09	0.49	65	2.17	RGD1561662	
DMR9:15647001	9	15647001	15648000	1000	1	1.50E-09	0.6	10	1	Mrps10;Trerf1	Translation
DMR9:15707001	9	15707001	15710000	3000	1	1.00E-07	0.39	54	1.8	Trerf1	
DMR9:15715001	9	15715001	15717000	2000	1	1.60E-09	0.46	47	2.35	Trerf1	
DMR9:15871001	9	15871001	15872000	1000	1	3.80E-07	0.38	17	1.7	Trerf1	
DMR9:16133001	9	16133001	16136000	3000	1	1.40E-07	-0.48	58	1.93	Prph2;LOC100911489	
DMR9:16180001	9	16180001	16181000	1000	1	4.50E-08	0.49	16	1.6	Prph2	
DMR9:16256001	9	16256001	16259000	3000	1	3.90E-08	0.49	72	2.4	Prph2;LOC102555538;LOC102555622	
DMR9:16473001	9	16473001	16479000	6000	1	8.90E-09	0.43	85	1.42	Gltscr1l	
DMR9:16624001	9	16624001	16627000	3000	1	3.50E-11	0.46	70	2.33	Mea1;Klhdc3;Rrp36;LOC680835	
DMR9:16641001	9	16641001	16643000	2000	1	2.10E-07	0.32	50	2.5	LOC680835;Mrpl2;Klc4	Translation;Cytoskeleton
DMR9:16837001	9	16837001	16844000	7000	1	1.90E-07	0.38	149	2.13	Cul9;LOC100910410;Dnph1	Metabolism
DMR9:16904001	9	16904001	16907000	3000	1	2.20E-07	-0.49	53	1.77	Ttbk1	Signaling
DMR9:16923001	9	16923001	16924000	1000	1	1.80E-09	0.46	34	3.4	Slc22a7;Crip3	Transport;Cytoskeleton
DMR9:17111001	9	17111001	17112000	1000	1	5.30E-07	0.46	13	1.3	Tjap1;Lrrc73;Yipf3;Polr1c	Cell Junction;Transcription
DMR9:17179001	9	17179001	17180000	1000	1	2.90E-08	0.44	18	1.8	Polh;LOC103690509	Transcription

DMR9:17740001	9	17740001	17741000	1000	1	6.00E-09	0.44	24	2.4	Capn11	Protease
DMR9:17751001	9	17751001	17754000	3000	1	1.50E-07	0.36	43	1.43	Capn11	Protease
DMR9:17796001	9	17796001	17797000	1000	1	2.50E-09	0.49	9	0.9	Slc29a1	Transport
DMR9:17821001	9	17821001	17823000	2000	1	3.80E-07	0.36	35	1.75	Hsp90ab1;Slc35b2;Nfkbie	Signaling;Transport;Transport
DMR9:18483001	9	18483001	18485000	2000	1	1.30E-11	0.55	15	0.75	Supt3h;Znrd1-as1	Transcription
DMR9:18646001	9	18646001	18647000	1000	1	3.50E-08	0.5	15	1.5	Runx2	Transcription
DMR9:18692001	9	18692001	18694000	2000	1	2.50E-09	0.47	34	1.7	Runx2	Transcription
DMR9:18709001	9	18709001	18710000	1000	1	4.00E-07	0.3	16	1.6	Runx2	Transcription
DMR9:18765001	9	18765001	18770000	5000	1	4.90E-10	0.45	89	1.78	Runx2	Transcription
DMR9:19140001	9	19140001	19141000	1000	1	7.40E-07	0.38	12	1.2	Clic5	Transport
DMR9:19255001	9	19255001	19257000	2000	1	7.30E-08	0.48	12	0.6	Clic5	Transport
DMR9:19843001	9	19843001	19848000	5000	1	2.00E-07	0.41	76	1.52	Cyp39a1	Metabolism
DMR9:20142001	9	20142001	20144000	2000	1	1.90E-07	-0.32	18	0.9	Adgrf5	
DMR9:20823001	9	20823001	20825000	2000	1	3.20E-10	-0.38	22	1.1	Cd2ap	
DMR9:20991001	9	20991001	20998000	7000	1	1.80E-10	-0.38	83	1.19	Adgrf4	
DMR9:21236001	9	21236001	21237000	1000	1	7.50E-07	-0.34	9	0.9	Ptchd4	Signaling
DMR9:21902001	9	21902001	21906000	4000	2	1.10E-11	-0.48	20	0.5	Olr1828-ps	
DMR9:23445001	9	23445001	23449000	4000	2	2.50E-11	-0.36	32	0.8	LOC108351992;Cyp2ac1	
DMR9:26298001	9	26298001	26299000	1000	1	3.40E-07	-0.44	18	1.8	Pkhd1	
DMR9:26606001	9	26606001	26608000	2000	1	5.30E-08	-0.4	23	1.15	Pkhd1	
DMR9:26850001	9	26850001	26852000	2000	1	2.80E-11	0.47	24	1.2	Il17a;LOC688876	
DMR9:26940001	9	26940001	26941000	1000	1	1.30E-07	0.34	11	1.1	Mcm3;LOC100362372	Transcription
DMR9:27145001	9	27145001	27148000	3000	1	3.40E-09	0.53	26	0.87	Tram2	
DMR9:27168001	9	27168001	27169000	1000	1	6.70E-07	0.32	23	2.3	Tram2	
DMR9:27582001	9	27582001	27584000	2000	1	3.20E-07	-0.43	13	0.65	Kcnq5	Transport
DMR9:27959001	9	27959001	27965000	6000	1	3.50E-12	-0.41	51	0.85	Kcnq5	Transport
DMR9:28063001	9	28063001	28065000	2000	1	3.50E-07	-0.42	19	0.95	Kcnq5	Transport
DMR9:28103001	9	28103001	28108000	5000	1	1.20E-07	-0.27	59	1.18	Kcnq5	Transport
DMR9:28542001	9	28542001	28544000	2000	1	5.40E-08	-0.42	28	1.4	Rims1	Transport
DMR9:28643001	9	28643001	28648000	5000	1	2.80E-08	-0.41	73	1.46	Rims1	Transport
DMR9:28730001	9	28730001	28731000	1000	1	2.50E-08	-0.44	17	1.7	Rims1	Transport
DMR9:28845001	9	28845001	28847000	2000	1	1.90E-07	0.54	12	0.6	Rims1	Transport
DMR9:30412001	9	30412001	30421000	9000	1	1.90E-07	-0.33	104	1.16	Col9a1	Extracellular Matrix
DMR9:30552001	9	30552001	30553000	1000	1	3.40E-07	-0.53	23	2.3	Col19a1	Extracellular Matrix
DMR9:30561001	9	30561001	30566000	5000	1	6.40E-07	-0.49	62	1.24	Col19a1	Extracellular Matrix
DMR9:30580001	9	30580001	30583000	3000	1	1.50E-07	-0.29	22	0.73	Col19a1	Extracellular Matrix
DMR9:30748001	9	30748001	30749000	1000	1	1.40E-07	-0.4	16	1.6	Col19a1	Extracellular Matrix
DMR9:31560001	9	31560001	31562000	2000	1	8.60E-07	-0.54	20	1	Adgrb3	Signaling
DMR9:31699001	9	31699001	31702000	3000	2	4.70E-08	0.55	18	0.6	Adgrb3	Signaling
DMR9:31734001	9	31734001	31735000	1000	1	1.30E-10	0.49	10	1	Adgrb3	Signaling
DMR9:31843001	9	31843001	31844000	1000	1	3.20E-07	-0.43	6	0.6	Adgrb3	Signaling
DMR9:31972001	9	31972001	31975000	3000	1	8.00E-07	-0.42	31	1.03	Adgrb3	Signaling
DMR9:37090001	9	37090001	37091000	1000	1	5.90E-07	-0.45	8	0.8	Phf3	Transcription
DMR9:37092001	9	37092001	37097000	5000	1	8.90E-13	-0.39	48	0.96	Phf3	Transcription
DMR9:37463001	9	37463001	37464000	1000	1	1.00E-08	0.42	8	0.8	Lgsn	Metabolism
DMR9:37786001	9	37786001	37788000	2000	1	5.00E-08	0.45	45	2.25	Ccdc115;Trnae-uuc;LOC102551518	
DMR9:38130001	9	38130001	38131000	1000	1	5.20E-07	-0.35	22	2.2	Dst	Cytoskeleton
DMR9:38187001	9	38187001	38190000	3000	1	1.30E-07	-0.49	43	1.43	Dst;LOC102551615	Cytoskeleton
DMR9:38270001	9	38270001	38272000	2000	1	1.80E-11	-0.57	27	1.35	Dst	Cytoskeleton
DMR9:38418001	9	38418001	38421000	3000	1	5.00E-08	-0.37	24	0.8	Zfp451	Transcription
DMR9:38623001	9	38623001	38627000	4000	1	2.30E-09	-0.44	69	1.73	Prim2	Cell Cycle
DMR9:38639001	9	38639001	38642000	3000	1	3.60E-07	-0.49	35	1.17	Prim2	Cell Cycle
DMR9:39634001	9	39634001	39636000	2000	1	1.30E-07	-0.53	17	0.85	Khdrbs2	Translation
DMR9:39668001	9	39668001	39669000	1000	1	2.00E-07	-0.59	15	1.5	Khdrbs2	Translation
DMR9:39864001	9	39864001	39868000	4000	2	4.80E-10	-0.36	37	0.92	Khdrbs2	Translation
DMR9:39894001	9	39894001	39897000	3000	1	1.40E-07	0.64	35	1.17	Khdrbs2	Translation
DMR9:40001001	9	40001001	40005000	4000	1	4.10E-09	0.59	31	0.78	Khdrbs2	Translation
DMR9:40982001	9	40982001	40985000	3000	1	4.50E-08	-0.51	41	1.37	Ptpn18	
DMR9:41164001	9	41164001	41166000	2000	1	4.00E-09	0.54	17	0.85	RGD1562461;Arhgef4	Transcription

DMR9:41267001	9	41267001	41268000	1000	1	5.20E-07	-0.36	15	1.5	Arhgef4	Transcription
DMR9:41304001	9	41304001	41306000	2000	1	8.40E-12	0.54	39	1.95	Arhgef4;Fam168b	Transcription;Cytoskeleton
DMR9:42878001	9	42878001	42879000	1000	1	9.00E-09	0.43	9	0.9	Arid5a	Transcription
DMR9:42882001	9	42882001	42884000	2000	1	5.80E-09	0.47	40	2	Arid5a	Transcription
DMR9:42890001	9	42890001	42891000	1000	1	7.70E-07	0.34	14	1.4	Arid5a;Kansl3	Transcription
DMR9:42954001	9	42954001	42956000	2000	1	4.10E-07	0.32	7	0.35	Fer1l5	Transport
DMR9:42977001	9	42977001	42979000	2000	1	7.50E-07	0.35	31	1.55	Fer1l5	Transport
DMR9:42992001	9	42992001	42995000	3000	1	3.70E-08	0.39	47	1.57	Fer1l5	Transport
DMR9:43161001	9	43161001	43165000	4000	1	7.60E-10	0.49	50	1.25	Fam178b	
DMR9:43504001	9	43504001	43505000	1000	1	6.70E-09	0.49	11	1.1	Tmem131;LOC102554557	
DMR9:43835001	9	43835001	43839000	4000	1	8.20E-07	0.74	26	0.65	Cnga3	Ion Channel
DMR9:44037001	9	44037001	44040000	3000	1	1.90E-08	-0.59	50	1.67	Unc50;Mgat4a	Transport
DMR9:44068001	9	44068001	44070000	2000	1	7.70E-08	0.32	29	1.45	Mgat4a	Transport
DMR9:44247001	9	44247001	44249000	2000	1	1.50E-07	-0.36	40	2	RGD1310819	
DMR9:44253001	9	44253001	44255000	2000	1	1.20E-09	0.5	29	1.45	RGD1310819	
DMR9:44262001	9	44262001	44264000	2000	1	9.30E-07	0.37	41	2.05	RGD1310819	
DMR9:44353001	9	44353001	44354000	1000	1	1.10E-09	-0.58	10	1	Tsga10	Epigenetic
DMR9:44648001	9	44648001	44650000	2000	1	4.60E-07	0.35	69	3.45	Txndc9;Eif5b	Signaling;Translation
DMR9:44792001	9	44792001	44793000	1000	1	1.80E-07	-0.42	16	1.6	Rev1	Transcription
DMR9:44794001	9	44794001	44799000	5000	1	8.20E-13	0.61	57	1.14	Rev1	Transcription
DMR9:45096001	9	45096001	45097000	1000	1	5.40E-07	0.56	7	0.7	Aff3	Transcription
DMR9:45109001	9	45109001	45110000	1000	1	7.20E-12	0.72	7	0.7	Aff3	Transcription
DMR9:46374001	9	46374001	46376000	2000	1	1.20E-07	0.51	18	0.9	Creg2	
DMR9:46461001	9	46461001	46465000	4000	1	8.90E-10	0.48	82	2.05	Rfx8	Transcription
DMR9:46470001	9	46470001	46472000	2000	1	4.30E-10	0.45	33	1.65	Rfx8;LOC103690520;LOC108351918	Transcription
DMR9:46710001	9	46710001	46711000	1000	1	7.50E-07	0.5	9	0.9	Map4k4	Signaling
DMR9:46865001	9	46865001	46868000	3000	1	2.10E-08	0.31	29	0.97	Il1r2	Receptor
DMR9:46969001	9	46969001	46971000	2000	1	5.70E-08	0.38	34	1.7	Il1r1	Receptor
DMR9:47071001	9	47071001	47076000	5000	1	2.00E-07	-0.37	50	1	Il1rl2	Receptor
DMR9:47377001	9	47377001	47380000	3000	1	2.50E-07	-0.35	31	1.03	LOC103690524;Slc9a2	Transport
DMR9:47397001	9	47397001	47399000	2000	1	4.00E-08	0.35	16	0.8	Slc9a2;LOC103690525	Transport
DMR9:49791001	9	49791001	49793000	2000	1	8.60E-08	-0.48	24	1.2	Arpc5l-ps1	
DMR9:49853001	9	49853001	49854000	1000	1	8.20E-07	0.39	20	2	Tgfbrap1	
DMR9:50933001	9	50933001	50934000	1000	1	5.40E-14	0.5	6	0.6	Ercc5	Transcription
DMR9:50976001	9	50976001	50979000	3000	1	2.10E-08	0.39	19	0.63	Ercc5	Transcription
DMR9:51118001	9	51118001	51123000	5000	1	3.70E-14	-0.38	41	0.82	RGD1562067	
DMR9:51361001	9	51361001	51365000	4000	1	9.50E-11	-0.31	39	0.98	Gulp1	Cytoskeleton
DMR9:51430001	9	51430001	51432000	2000	1	2.40E-08	-0.42	21	1.05	Gulp1	Cytoskeleton
DMR9:51442001	9	51442001	51444000	2000	1	1.40E-07	-0.4	14	0.7	Gulp1	Cytoskeleton
DMR9:51533001	9	51533001	51534000	1000	1	6.70E-07	-0.58	3	0.3	Gulp1	Cytoskeleton
DMR9:52178001	9	52178001	52183000	5000	2	5.40E-11	-0.4	44	0.88	Col5a2	Extracellular Matrix
DMR9:53111001	9	53111001	53112000	1000	1	9.20E-07	-0.45	23	2.3	LOC100911809;Ormdl1;Pms1	Transcription
DMR9:53168001	9	53168001	53170000	2000	1	1.40E-14	0.59	12	0.6	Pms1	Transcription
DMR9:53435001	9	53435001	53438000	3000	1	1.10E-07	-0.54	25	0.83	LOC685203;LOC100912473;Hibch	Cytoskeleton;Metabolism
DMR9:54332001	9	54332001	54333000	1000	1	1.40E-12	0.55	83	8.3	Stat1;Stat4	Transcription
DMR9:54770001	9	54770001	54771000	1000	1	2.00E-07	-0.48	12	1.2	Myo1b	Cytoskeleton
DMR9:55525001	9	55525001	55534000	9000	5	1.70E-15	-0.47	102	1.13	Tmeff2;LOC108351929	
DMR9:59560001	9	59560001	59562000	2000	1	3.70E-08	0.47	44	2.2	Dnah7	Cytoskeleton
DMR9:60146001	9	60146001	60149000	3000	1	5.00E-07	-0.32	27	0.9	Dnah7	Cytoskeleton
DMR9:60226001	9	60226001	60232000	6000	2	1.60E-07	-0.37	74	1.23	Dnah7	Cytoskeleton
DMR9:60516001	9	60516001	60518000	2000	1	1.60E-07	0.46	25	1.25	Hecw2	Proteolysis
DMR9:60927001	9	60927001	60931000	4000	1	6.40E-07	-0.39	27	0.68	Ccdc150;LOC108351931	
DMR9:60939001	9	60939001	60940000	1000	1	4.00E-08	0.42	21	2.1	Ccdc150	
DMR9:61305001	9	61305001	61313000	8000	1	1.00E-07	-0.3	103	1.29	Ankrd44	Cytoskeleton
DMR9:61403001	9	61403001	61405000	2000	1	8.00E-07	0.67	23	1.15	Ankrd44	Cytoskeleton
DMR9:61494001	9	61494001	61500000	6000	1	3.70E-10	-0.35	66	1.1	Ankrd44	Cytoskeleton
DMR9:61552001	9	61552001	61558000	6000	2	2.50E-16	-0.4	63	1.05	Tmem258b	
DMR9:61849001	9	61849001	61851000	2000	1	2.40E-11	-0.55	16	0.8	Boll	Metabolism

DMR9:61949001	9	61949001	61953000	4000	1	1.90E-07	-0.31	56	1.4	Boll	Metabolism
DMR9:62076001	9	62076001	62080000	4000	1	5.90E-07	-0.39	30	0.75	Plcl1	Metabolism
DMR9:62084001	9	62084001	62085000	1000	1	7.60E-08	0.51	4	0.4	Plcl1	Metabolism
DMR9:62171001	9	62171001	62172000	1000	1	1.30E-08	-0.48	17	1.7	Plcl1	Metabolism
DMR9:62337001	9	62337001	62340000	3000	1	1.50E-07	-0.32	25	0.83	Plcl1;LOC103690535	Metabolism
DMR9:63463001	9	63463001	63466000	3000	1	4.80E-08	0.45	30	1	Satb2	Epigenetic
DMR9:63593001	9	63593001	63595000	2000	1	7.60E-07	0.37	24	1.2	Satb2	Epigenetic
DMR9:63630001	9	63630001	63631000	1000	1	5.20E-08	0.53	7	0.7	Satb2;LOC102549320	Epigenetic
DMR9:64812001	9	64812001	64813000	1000	1	4.90E-08	0.37	27	2.7	Spats2l	
DMR9:64879001	9	64879001	64880000	1000	1	4.00E-09	-0.42	12	1.2	Kctd18;LOC102550420	Cytoskeleton
DMR9:65579001	9	65579001	65581000	2000	1	4.00E-09	-0.43	31	1.55	Cflar	Protease
DMR9:65645001	9	65645001	65646000	1000	1	3.10E-07	0.41	11	1.1	Casp8	Protease
DMR9:65752001	9	65752001	65757000	5000	1	1.50E-09	0.44	51	1.02	Trak2;Stradb	Transport;Signaling
DMR9:65875001	9	65875001	65876000	1000	1	1.10E-07	-0.41	7	0.7	Als2cr11;Tmem237	
DMR9:65977001	9	65977001	65979000	2000	1	2.10E-09	0.7	24	1.2	Als2	
DMR9:66340001	9	66340001	66341000	1000	1	6.40E-11	0.51	14	1.4	RGD1562029	
DMR9:66868001	9	66868001	66875000	7000	1	1.10E-07	-0.29	74	1.06	Wdr12;Carf	Transcription
DMR9:66979001	9	66979001	66982000	3000	1	5.70E-08	-0.55	9	0.3	Nbeal1	
DMR9:67110001	9	67110001	67111000	1000	1	8.30E-08	-0.44	14	1.4	Nbeal1;LOC108351938	
DMR9:67133001	9	67133001	67135000	2000	1	2.40E-08	-0.44	45	2.25	Cyp20a1	Metabolism
DMR9:67178001	9	67178001	67185000	7000	2	3.50E-09	-0.31	93	1.33	Cyp20a1	Metabolism
DMR9:67324001	9	67324001	67326000	2000	1	4.60E-10	0.58	28	1.4	Raph1	Cytoskeleton
DMR9:67336001	9	67336001	67339000	3000	1	1.70E-07	-0.36	27	0.9	Raph1	Cytoskeleton
DMR9:68455001	9	68455001	68459000	4000	2	7.10E-13	-0.44	37	0.92	Pard3b;LOC103690539	
DMR9:68571001	9	68571001	68572000	1000	1	1.50E-08	-0.49	4	0.4	Pard3b	
DMR9:69278001	9	69278001	69280000	2000	2	3.90E-11	0.45	69	3.45	Pard3b	
DMR9:69425001	9	69425001	69428000	3000	1	8.20E-08	0.43	47	1.57	Pard3b	
DMR9:69603001	9	69603001	69604000	1000	1	2.80E-07	0.34	8	0.8	Nrp2	
DMR9:70000001	9	70000001	70002000	2000	1	1.20E-07	0.41	29	1.45	Gpr1	Signaling
DMR9:70346001	9	70346001	70347000	1000	1	3.30E-08	-0.46	2	0.2	Dytn	Proteolysis
DMR9:70390001	9	70390001	70397000	7000	2	4.00E-09	-0.31	79	1.13	Dytn;Mdh1b	Proteolysis;Metabolism
DMR9:70410001	9	70410001	70413000	3000	1	2.00E-09	-0.56	17	0.57	Mdh1b	Metabolism
DMR9:70414001	9	70414001	70417000	3000	1	2.00E-07	-0.35	15	0.5	Mdh1b	Metabolism
DMR9:70781001	9	70781001	70783000	2000	1	2.00E-08	0.42	25	1.25	Klf7	
DMR9:71550001	9	71550001	71551000	1000	1	1.30E-07	0.61	10	1	Plekhm3	
DMR9:71859001	9	71859001	71860000	1000	1	2.40E-07	0.4	5	0.5	Cryga	
DMR9:72065001	9	72065001	72066000	1000	1	1.60E-08	0.53	4	0.4	Pth2r	Receptor
DMR9:72137001	9	72137001	72143000	6000	2	6.80E-11	-0.43	67	1.12	Pth2r	Receptor
DMR9:72168001	9	72168001	72176000	8000	1	1.40E-10	-0.36	82	1.02	Pth2r	Receptor
DMR9:73829001	9	73829001	73833000	4000	1	2.80E-09	-0.32	53	1.32	Kansl1l;Acadl	Metabolism
DMR9:73957001	9	73957001	73958000	1000	1	7.80E-08	-0.51	16	1.6	Myl1	Cytoskeleton
DMR9:75250001	9	75250001	75252000	2000	1	6.60E-09	-0.49	15	0.75	Erbb4	Receptor
DMR9:75397001	9	75397001	75403000	6000	2	3.60E-11	-0.37	68	1.13	Erbb4	Receptor
DMR9:75432001	9	75432001	75440000	8000	1	1.30E-07	-0.33	110	1.38	Erbb4	Receptor
DMR9:75974001	9	75974001	75976000	2000	2	1.20E-07	-0.38	25	1.25	Erbb4	Receptor
DMR9:76007001	9	76007001	76008000	1000	1	1.30E-08	-0.46	11	1.1	Erbb4	Receptor
DMR9:76134001	9	76134001	76136000	2000	1	1.40E-07	-0.31	15	0.75	Erbb4	Receptor
DMR9:76760001	9	76760001	76763000	3000	1	8.30E-10	-0.61	50	1.67	Ikzf2	Transcription
DMR9:77232001	9	77232001	77233000	1000	1	4.40E-07	-0.42	13	1.3	Spag16;LOC102550938	Cytoskeleton
DMR9:77512001	9	77512001	77520000	8000	3	5.90E-08	-0.43	81	1.01	Spag16	Cytoskeleton
DMR9:77662001	9	77662001	77664000	2000	1	5.40E-07	0.49	18	0.9	Spag16	Cytoskeleton
DMR9:77679001	9	77679001	77685000	6000	2	1.20E-08	-0.34	61	1.02	Spag16	Cytoskeleton
DMR9:77687001	9	77687001	77691000	4000	1	4.10E-08	-0.29	52	1.3	Spag16	Cytoskeleton
DMR9:78546001	9	78546001	78547000	1000	1	2.70E-11	0.51	13	1.3	Abca12	Transport
DMR9:78632001	9	78632001	78633000	1000	1	8.00E-07	0.31	5	0.5	Abca12	Transport
DMR9:78681001	9	78681001	78683000	2000	1	7.10E-11	0.42	32	1.6	Abca12	Transport
DMR9:79480001	9	79480001	79482000	2000	1	5.90E-07	-0.4	16	0.8	Mreg	
DMR9:79508001	9	79508001	79509000	1000	1	4.10E-11	0.42	14	1.4	Mreg	
DMR9:79552001	9	79552001	79554000	2000	1	6.20E-07	0.62	25	1.25	Mreg	
DMR9:79567001	9	79567001	79574000	7000	3	1.30E-09	-0.38	87	1.24	Mreg;LOC108351947	
DMR9:79758001	9	79758001	79761000	3000	1	1.10E-07	0.65	25	0.83	Xrcc5;LOC108351949	Epigenetic

DMR9:79852001	9	79852001	79858000	6000	1	1.40E-08	-0.31	72	1.2	4-Mar	Proteolysis
DMR9:81656001	9	81656001	81660000	4000	1	3.70E-08	0.65	45	1.12	Catip;Slc11a1	Transport
DMR9:81715001	9	81715001	81718000	3000	1	1.10E-08	0.49	28	0.93	Vil1;Usp37	Cytoskeleton;Protease
DMR9:82300001	9	82300001	82302000	2000	1	1.50E-07	0.5	14	0.7	Nhej1	
DMR9:82566001	9	82566001	82567000	1000	1	1.10E-07	0.69	12	1.2	Des;LOC102550477;Speg	
DMR9:82623001	9	82623001	82625000	2000	1	1.40E-11	0.5	42	2.1	Speg;Gmppa	Transport
DMR9:82636001	9	82636001	82640000	4000	1	3.60E-07	0.4	67	1.68	Speg;Gmppa;LOC108351955;Asic4	Transport;Transport
DMR9:82684001	9	82684001	82686000	2000	1	3.20E-08	0.37	52	2.6	Obsl1;Tmem198	
DMR9:83260001	9	83260001	83262000	2000	1	7.60E-07	-0.52	33	1.65	Epha4;LOC102551060	Receptor
DMR9:84315001	9	84315001	84317000	2000	1	2.60E-07	0.37	44	2.2	Sgpp2;Farsb	Signaling;Translation
DMR9:85348001	9	85348001	85350000	2000	1	4.20E-07	-0.45	34	1.7	RGD1566037	
DMR9:85402001	9	85402001	85403000	1000	1	6.10E-10	0.62	3	0.3	Ap1s3	Transport
DMR9:85475001	9	85475001	85477000	2000	1	5.50E-08	0.34	26	1.3	LOC108351959;Wdfy1	
DMR9:86456001	9	86456001	86458000	2000	1	2.60E-07	0.46	15	0.75	Dock10	
DMR9:86492001	9	86492001	86499000	7000	2	2.50E-08	-0.31	80	1.14	Dock10	
DMR9:88060001	9	88060001	88061000	1000	1	3.90E-07	0.34	19	1.9	Irs1	
DMR9:88118001	9	88118001	88119000	1000	1	8.00E-10	0.49	7	0.7	Rhbdd1	Protease
DMR9:89085001	9	89085001	89086000	1000	1	7.90E-07	0.43	3	0.3	Sphkap	Cytoskeleton
DMR9:89184001	9	89184001	89185000	1000	1	4.80E-07	-0.52	12	1.2	Sphkap	Cytoskeleton
DMR9:91656001	9	91656001	91662000	6000	3	8.10E-11	-0.36	72	1.2	Pid1	
DMR9:92270001	9	92270001	92271000	1000	1	6.20E-09	-0.48	15	1.5	Dner	
DMR9:92589001	9	92589001	92591000	2000	1	7.60E-08	0.51	23	1.15	Sp110	
DMR9:93189001	9	93189001	93190000	1000	1	3.10E-07	0.53	7	0.7	Armc9	Cytoskeleton
DMR9:93196001	9	93196001	93197000	1000	1	8.00E-07	-0.37	21	2.1	Armc9	Cytoskeleton
DMR9:93276001	9	93276001	93278000	2000	2	1.10E-09	0.42	31	1.55	Armc9	Cytoskeleton
DMR9:93291001	9	93291001	93293000	2000	1	6.80E-09	0.39	30	1.5	Armc9;LOC102548542	Cytoskeleton
DMR9:93872001	9	93872001	93873000	1000	1	4.30E-08	-0.43	9	0.9	Dis3l2	Transcription
DMR9:93909001	9	93909001	93915000	6000	3	1.00E-13	-0.51	71	1.18	Dis3l2	Transcription
DMR9:94042001	9	94042001	94045000	3000	1	8.40E-08	-0.33	32	1.07	Dis3l2;Rpl21-ps1	Transcription
DMR9:94130001	9	94130001	94133000	3000	1	1.40E-09	0.61	61	2.03	Dis3l2	Transcription
DMR9:94190001	9	94190001	94193000	3000	1	3.80E-09	0.4	77	2.57	Alppl2;Alpp;Alpi	Signaling
DMR9:94268001	9	94268001	94269000	1000	1	6.80E-11	0.56	15	1.5	Prss56	Protease
DMR9:94399001	9	94399001	94401000	2000	1	8.40E-07	0.35	19	0.95	Efhd1	Signaling
DMR9:94706001	9	94706001	94709000	3000	1	2.00E-07	-0.44	48	1.6	Neu2	Metabolism
DMR9:95326001	9	95326001	95327000	1000	1	2.20E-13	0.55	21	2.1	Mroh2a	
DMR9:95328001	9	95328001	95329000	1000	1	4.80E-07	0.45	13	1.3	Mroh2a	
DMR9:96203001	9	96203001	96204000	1000	1	3.20E-07	-0.63	17	1.7	Sh3bp4	
DMR9:96857001	9	96857001	96858000	1000	1	9.70E-07	0.48	13	1.3	Agap1	
DMR9:96913001	9	96913001	96916000	3000	1	1.10E-13	0.72	41	1.37	Agap1	
DMR9:97010001	9	97010001	97011000	1000	1	6.80E-07	0.45	16	1.6	Agap1	
DMR9:97212001	9	97212001	97213000	1000	1	2.40E-07	0.31	19	1.9	Iqca1	
DMR9:97294001	9	97294001	97296000	2000	1	4.30E-07	0.35	18	0.9	Iqca1	
DMR9:98457001	9	98457001	98460000	3000	1	1.30E-07	0.51	53	1.77	Scly;Espnl	Metabolism;Cytoskeleton
DMR9:98497001	9	98497001	98500000	3000	1	6.70E-09	0.61	55	1.83	Klhl30;Erfe	Cytoskeleton;Hormone
DMR9:99058001	9	99058001	99059000	1000	1	6.60E-10	0.49	10	1	Hdac4	
DMR9:99690001	9	99690001	99691000	1000	1	1.10E-07	0.62	27	2.7	Ppp1r7;Olr1345	Signaling;Signaling
DMR9:100012001	9	100012001	100021000	9000	1	5.00E-07	0.36	169	1.88	Ppp1r7;Gpc1;Mir149;Ankmy1	Signaling
DMR9:100041001	9	100041001	100042000	1000	1	1.00E-06	0.34	15	1.5	Ppp1r7;Ankmy1	Signaling
DMR9:100204001	9	100204001	100205000	1000	1	8.80E-10	0.48	33	3.3	Ppp1r7;Kif1a	Signaling;Cytoskeleton
DMR9:100343001	9	100343001	100345000	2000	2	1.80E-09	0.4	18	0.9	Ppp1r7;Crocc2	Signaling
DMR9:100354001	9	100354001	100356000	2000	1	2.30E-09	0.43	37	1.85	Ppp1r7;Crocc2	Signaling
DMR9:100940001	9	100940001	100943000	3000	1	3.30E-08	0.43	74	2.47	Dtymk;Ing5	Signaling;Epigenetic
DMR9:104471001	9	104471001	104477000	6000	1	2.30E-07	-0.37	37	0.62	Slco6b1	Transport
DMR9:104722001	9	104722001	104728000	6000	1	6.80E-07	-0.38	69	1.15	Slco6d1;LOC102554041	Transport
DMR9:104852001	9	104852001	104853000	1000	1	3.70E-07	-0.63	9	0.9	Slco6d1	Transport
DMR9:105537001	9	105537001	105545000	8000	1	1.80E-08	-0.32	73	0.91	LOC102554561;RGD1560925	
DMR9:105702001	9	105702001	105704000	2000	1	9.80E-08	-0.47	27	1.35	Nudt12	Metabolism
DMR9:110047001	9	110047001	110049000	2000	1	3.20E-08	0.41	25	1.25	Efna5	Signaling

DMR9:110727001	9	110727001	110729000	2000	1	4.90E-08	0.34	30	1.5	Fbxl17	Metabolism
DMR9:111015001	9	111015001	111021000	6000	3	2.30E-08	-0.41	63	1.05	Pam	Metabolism
DMR9:111141001	9	111141001	111143000	2000	1	8.40E-08	-0.47	38	1.9	Pam	Metabolism
DMR9:111625001	9	111625001	111628000	3000	1	3.10E-07	0.48	85	2.83	Fer	
DMR9:111741001	9	111741001	111742000	1000	1	3.10E-10	-0.33	17	1.7	Fer	
DMR9:111743001	9	111743001	111747000	4000	1	1.70E-09	-0.38	39	0.98	Fer	
DMR9:111826001	9	111826001	111827000	1000	1	1.00E-09	-0.51	14	1.4	Fer	
DMR9:112311001	9	112311001	112312000	1000	1	8.30E-08	0.49	40	4	Man2a1	
DMR9:113363001	9	113363001	113365000	2000	1	8.40E-08	-0.42	37	1.85	Txndc2;Rab31	Metabolism
DMR9:113428001	9	113428001	113431000	3000	1	9.40E-10	-0.6	73	2.43	Rab31	
DMR9:113439001	9	113439001	113441000	2000	1	3.60E-13	0.42	95	4.75	Rab31	
DMR9:113487001	9	113487001	113488000	1000	1	1.10E-07	0.35	19	1.9	Rab31	
DMR9:113521001	9	113521001	113522000	1000	1	1.20E-12	0.43	15	1.5	Ppp4r1;LOC102552879	Signaling
DMR9:113659001	9	113659001	113660000	1000	1	3.90E-11	0.53	70	7	Ankrd12	
DMR9:113701001	9	113701001	113703000	2000	1	3.80E-08	0.66	35	1.75	Twsg1	
DMR9:113911001	9	113911001	113916000	5000	1	8.90E-09	-0.3	62	1.24	Wash1;LOC102553152	
DMR9:113937001	9	113937001	113938000	1000	1	1.20E-08	0.36	4	0.4	Wash1;LOC102553152;LOC102553382	
DMR9:115031001	9	115031001	115032000	1000	1	4.30E-07	0.51	5	0.5	Ptprm	Signaling
DMR9:116203001	9	116203001	116204000	1000	1	6.10E-07	0.34	9	0.9	Arhgap28	Signaling
DMR9:116571001	9	116571001	116574000	3000	1	9.00E-07	-0.4	20	0.67	L3mbtl4	
DMR9:117397001	9	117397001	117403000	6000	2	4.80E-09	0.33	29	0.48	Epb41l3;LOC100361186	
DMR9:117404001	9	117404001	117406000	2000	1	7.00E-07	-0.25	13	0.65	Epb41l3;LOC100361186;LOC689002	
DMR9:117585001	9	117585001	117588000	3000	1	1.20E-07	0.34	58	1.93	Epb41l3	
DMR9:118272001	9	118272001	118274000	2000	1	1.80E-09	-0.43	19	0.95	Dlgap1	Cytoskeleton
DMR9:118526001	9	118526001	118532000	6000	1	6.30E-14	0.62	51	0.85	Dlgap1	Cytoskeleton
DMR9:118783001	9	118783001	118790000	7000	1	7.40E-10	-0.32	78	1.11	Dlgap1	Cytoskeleton
DMR9:118940001	9	118940001	118941000	1000	1	2.50E-08	0.57	1	0.1	Dlgap1	Cytoskeleton
DMR9:119688001	9	119688001	119689000	1000	1	3.70E-07	-0.48	3	0.3	Smchd1;LOC102556146	
DMR9:119756001	9	119756001	119759000	3000	2	6.10E-10	-0.52	24	0.8	Smchd1	
DMR9:121849001	9	121849001	121853000	4000	1	3.90E-08	-0.33	46	1.15	Yes1	
DMR10:738001	10	738001	741000	3000	1	3.40E-08	-0.3	32	1.07	LOC103693254;Fopnl	
DMR10:825001	10	825001	831000	6000	3	2.10E-10	-0.36	76	1.27	LOC103693254;Myh11	
DMR10:885001	10	885001	890000	5000	1	1.20E-09	-0.38	42	0.84	LOC103693254;Nde1	
DMR10:2922001	10	2922001	2924000	2000	1	1.20E-13	0.4	28	1.4	Shisa9	
DMR10:2987001	10	2987001	2989000	2000	1	7.50E-07	-0.24	42	2.1	Shisa9	
DMR10:3247001	10	3247001	3254000	7000	2	1.40E-10	-0.31	82	1.17	Pdxdc1	
DMR10:3726001	10	3726001	3727000	1000	1	7.00E-09	0.45	13	1.3	Cpped1	
DMR10:3745001	10	3745001	3751000	6000	1	2.70E-07	-0.32	69	1.15	Cpped1	
DMR10:4010001	10	4010001	4011000	1000	1	1.50E-13	-0.53	10	1	Snx29	Cytoskeleton
DMR10:4013001	10	4013001	4016000	3000	1	2.30E-07	-0.35	36	1.2	Snx29;LOC108352047	Cytoskeleton
DMR10:4526001	10	4526001	4529000	3000	1	3.10E-09	-0.38	41	1.37	Zc3h7a	Metabolism
DMR10:4766001	10	4766001	4767000	1000	1	1.40E-07	0.38	15	1.5	Litaf;LOC103693267;LOC102552269	Cytoskeleton
DMR10:5036001	10	5036001	5039000	3000	1	1.20E-07	-0.3	48	1.6	Clec16a	
DMR10:5215001	10	5215001	5216000	1000	1	1.30E-08	0.39	16	1.6	Dexi;Ciita	
DMR10:5235001	10	5235001	5237000	2000	1	2.40E-09	0.55	22	1.1	Ciita	
DMR10:5314001	10	5314001	5321000	7000	2	6.60E-08	0.39	73	1.04	Tvp23a	
DMR10:5474001	10	5474001	5476000	2000	1	1.80E-09	0.53	29	1.45	Emp2;Atf7ip2	Cytoskeleton;Transcription
DMR10:5729001	10	5729001	5733000	4000	4	9.50E-11	-2.56	75	1.88	Grin2a	Receptor
DMR10:5765001	10	5765001	5766000	1000	1	1.70E-09	0.45	15	1.5	Grin2a	Receptor
DMR10:5950001	10	5950001	5952000	2000	1	4.20E-08	-0.47	17	0.85	Grin2a	Receptor
DMR10:5973001	10	5973001	5976000	3000	1	4.00E-14	-1.12	21	0.7	Grin2a	Receptor
DMR10:6040001	10	6040001	6041000	1000	1	3.70E-08	0.39	20	2	Grin2a	Receptor
DMR10:7143001	10	7143001	7146000	3000	1	2.60E-07	0.48	37	1.23	Abat	Metabolism
DMR10:7208001	10	7208001	7209000	1000	1	6.20E-10	0.59	14	1.4	Abat	Metabolism
DMR10:7300001	10	7300001	7303000	3000	1	9.20E-09	0.38	35	1.17	Tmem114;LOC102546970	
DMR10:8332001	10	8332001	8333000	1000	1	6.80E-08	0.42	17	1.7	Rbfox1	Translation
DMR10:8427001	10	8427001	8429000	2000	1	1.30E-07	-0.46	53	2.65	Rbfox1	Translation
DMR10:9912001	10	9912001	9914000	2000	1	1.40E-07	0.43	16	0.8	Rbfox1	Translation

DMR10:10014001	10	10014001	10015000	1000	1	2.00E-08	0.4	9	0.9	Rbfox1	Translation
DMR10:10641001	10	10641001	10642000	1000	1	9.70E-07	0.49	17	1.7	Ppl	Cytoskeleton
DMR10:10703001	10	10703001	10705000	2000	1	1.20E-08	0.38	19	0.95	Ubn1	Cytoskeleton
DMR10:11251001	10	11251001	11255000	4000	1	5.70E-08	0.4	50	1.25	Srl	Transport
DMR10:11292001	10	11292001	11293000	1000	1	3.00E-12	0.63	56	5.6	Srl;LOC103693274	Transport
DMR10:11661001	10	11661001	11663000	2000	1	1.20E-07	-0.44	18	0.9	Crebbp	Epigenetic
DMR10:11739001	10	11739001	11740000	1000	1	4.20E-07	0.46	7	0.7	Trap1	Signaling
DMR10:11839001	10	11839001	11841000	2000	1	9.40E-12	0.67	21	1.05	Nlrc3;Cluap1	Cytoskeleton
DMR10:12287001	10	12287001	12291000	4000	2	1.10E-07	-0.3	49	1.23	Olr1354-ps;Olr1525;Olr1359-ps	Receptor
DMR10:12356001	10	12356001	12362000	6000	1	2.30E-07	-0.29	56	0.93	Olr1362	Receptor
DMR10:12497001	10	12497001	12500000	3000	1	9.60E-07	-0.38	13	0.43	Olr1368;Olr1369	Receptor
DMR10:12577001	10	12577001	12584000	7000	1	1.50E-07	-0.34	86	1.23	Olr1372	Receptor
DMR10:12652001	10	12652001	12660000	8000	1	9.60E-08	-0.33	103	1.29	Olr1375	Receptor
DMR10:12661001	10	12661001	12667000	6000	1	3.10E-08	-0.26	63	1.05	Olr1376	Receptor
DMR10:12704001	10	12704001	12705000	1000	1	2.60E-08	0.4	10	1	Olr1377-ps	
DMR10:12709001	10	12709001	12710000	1000	1	2.90E-07	-0.39	6	0.6	Olr1377-ps	
DMR10:12714001	10	12714001	12726000	12000	1	2.90E-09	-0.4	137	1.14	Olr1377-ps;Olr1378	Receptor
DMR10:12764001	10	12764001	12767000	3000	1	4.20E-08	-0.26	30	1	LOC680641;Olr1379-ps;Olr1380	Receptor
DMR10:12784001	10	12784001	12795000	11000	2	2.30E-08	-0.38	92	0.84	Olr1380;Rps4x-ps5	Receptor
DMR10:12952001	10	12952001	12954000	2000	1	6.20E-07	0.38	23	1.15	Mmp25	
DMR10:13552001	10	13552001	13558000	6000	2	5.70E-09	0.48	128	2.13	Atp6v0c;Tbc1d24	Metabolism;Signaling
DMR10:13662001	10	13662001	13663000	1000	1	5.90E-08	0.48	5	0.5	Abca17	Transport
DMR10:14226001	10	14226001	14227000	1000	1	5.00E-07	0.32	14	1.4	Hagh	
DMR10:14433001	10	14433001	14435000	2000	1	5.50E-09	0.48	23	1.15	Ift140;Tmem204	Development
DMR10:14482001	10	14482001	14484000	2000	1	6.70E-07	0.39	27	1.35	Telo2;Ptx4;Clcn7	Transport
DMR10:14566001	10	14566001	14567000	1000	1	2.10E-08	0.5	16	1.6	Unkl	
DMR10:14764001	10	14764001	14766000	2000	1	7.90E-08	0.39	27	1.35	Cacna1h	Transport
DMR10:14934001	10	14934001	14935000	1000	1	1.30E-07	0.44	36	3.6	Sox8;LOC102548300	
DMR10:15207001	10	15207001	15209000	2000	1	6.50E-08	0.39	46	2.3	Jmjd8;Stub1;Rhbdl1;Rhot2;Wdr90	Golgi;Proteolysis;Signaling
DMR10:15418001	10	15418001	15420000	2000	1	3.90E-07	0.38	35	1.75	Rab11fip3;LOC102549079	
DMR10:15532001	10	15532001	15533000	1000	1	5.50E-07	-0.59	15	1.5	LOC108352066;Luc7l	
DMR10:16054001	10	16054001	16058000	4000	1	1.30E-07	-0.54	59	1.48	Cpeb4	Translation
DMR10:16672001	10	16672001	16675000	3000	1	2.10E-08	0.33	36	1.2	Bnip1	
DMR10:16894001	10	16894001	16896000	2000	1	4.70E-10	0.44	56	2.8	Ergic1	
DMR10:17200001	10	17200001	17201000	1000	1	5.80E-08	0.48	6	0.6	LOC688443;Sh3pxd2b	
DMR10:17461001	10	17461001	17464000	3000	2	2.00E-16	0.88	25	0.83	Stk10	
DMR10:17522001	10	17522001	17524000	2000	1	2.90E-07	-0.38	36	1.8	Stk10	
DMR10:18068001	10	18068001	18069000	1000	1	3.90E-07	0.41	13	1.3	Fgf18	Growth Factors
DMR10:18319001	10	18319001	18320000	1000	1	7.60E-07	-0.48	12	1.2	Ranbp17	Transport
DMR10:18330001	10	18330001	18332000	2000	1	3.10E-09	-0.51	29	1.45	Ranbp17	Transport
DMR10:18387001	10	18387001	18389000	2000	1	2.20E-07	-0.38	15	0.75	Ranbp17	Transport
DMR10:18477001	10	18477001	18483000	6000	3	3.90E-10	-0.39	64	1.07	Gabrp	Ion Channel
DMR10:18600001	10	18600001	18602000	2000	1	7.50E-07	0.51	17	0.85	Kcnip1;LOC108352197	
DMR10:18793001	10	18793001	18794000	1000	1	1.50E-09	0.59	15	1.5	Kcnip1	
DMR10:18804001	10	18804001	18807000	3000	2	1.70E-08	-0.95	36	1.2	Kcnip1	
DMR10:18891001	10	18891001	18893000	2000	1	2.00E-09	0.43	31	1.55	Kcnip1	
DMR10:19516001	10	19516001	19517000	1000	1	9.60E-08	0.35	29	2.9	Dock2	
DMR10:19541001	10	19541001	19544000	3000	1	1.80E-08	0.6	45	1.5	Dock2	
DMR10:19568001	10	19568001	19570000	2000	1	1.30E-11	-0.61	9	0.45	Dock2	
DMR10:19632001	10	19632001	19634000	2000	1	2.50E-07	0.4	18	0.9	Spdl1	Transport
DMR10:20176001	10	20176001	20179000	3000	1	1.00E-06	0.36	53	1.77	Slit3	
DMR10:20227001	10	20227001	20232000	5000	1	8.70E-07	0.5	57	1.14	Slit3	
DMR10:20243001	10	20243001	20245000	2000	1	8.40E-07	-0.39	37	1.85	Slit3	
DMR10:20250001	10	20250001	20252000	2000	1	1.80E-09	-0.55	34	1.7	Slit3	
DMR10:20354001	10	20354001	20355000	1000	1	3.20E-09	0.4	19	1.9	Slit3	
DMR10:20631001	10	20631001	20632000	1000	1	8.20E-07	0.38	8	0.8	Fbll1;Rars	Translation
DMR10:20816001	10	20816001	20817000	1000	1	1.10E-07	-0.45	22	2.2	Wwc1	
DMR10:21023001	10	21023001	21026000	3000	1	4.10E-07	-0.34	27	0.9	Tenm2	

DMR10:21203001	10	21203001	21205000	2000	1	6.10E-11	0.46	35	1.75	Tenm2	
DMR10:21348001	10	21348001	21349000	1000	1	4.50E-11	-0.6	15	1.5	Tenm2	
DMR10:23679001	10	23679001	23680000	1000	1	1.90E-07	0.54	6	0.6	Ebf1	Transcription
DMR10:23773001	10	23773001	23775000	2000	1	1.70E-07	-0.5	21	1.05	Ebf1	Transcription
DMR10:27138001	10	27138001	27139000	1000	1	1.90E-07	-0.53	13	1.3	Gabrg2;LOC102548672	Ion Channel
DMR10:27186001	10	27186001	27189000	3000	1	4.60E-07	-0.29	28	0.93	Gabrg2	Ion Channel
DMR10:27834001	10	27834001	27840000	6000	2	2.20E-08	-0.32	60	1	Gabra6	Ion Channel
DMR10:28540001	10	28540001	28547000	7000	1	1.70E-16	-0.53	65	0.93	Atp10b	Transport
DMR10:28614001	10	28614001	28615000	1000	1	1.00E-28	1.63	16	1.6	Atp10b	Transport
DMR10:28816001	10	28816001	28822000	6000	1	4.90E-10	-0.33	86	1.43	Atp10b	Transport
DMR10:28934001	10	28934001	28936000	2000	1	1.00E-07	0.61	23	1.15	Trnai-aau	
DMR10:29171001	10	29171001	29172000	1000	1	2.40E-09	0.49	12	1.2	Ccnjl	Signaling
DMR10:29319001	10	29319001	29320000	1000	1	7.20E-11	-0.53	25	2.5	Pwwp2a	Epigenetic
DMR10:31083001	10	31083001	31085000	2000	1	9.20E-09	-0.79	15	0.75	Sox30	Transcription
DMR10:31304001	10	31304001	31309000	5000	1	5.10E-07	0.35	76	1.52	Cyfip2	Cytoskeleton
DMR10:31483001	10	31483001	31484000	1000	1	2.70E-07	0.33	20	2	Itk	
DMR10:31517001	10	31517001	31520000	3000	1	1.60E-16	0.53	37	1.23	Fam71b	
DMR10:31523001	10	31523001	31526000	3000	1	1.30E-12	-0.51	52	1.73	Med7	Immune
DMR10:31749001	10	31749001	31752000	3000	2	2.30E-07	-0.35	17	0.57	Timd2	
DMR10:31843001	10	31843001	31844000	1000	1	3.30E-07	0.45	7	0.7	Havcr1	
DMR10:32172001	10	32172001	32176000	4000	2	1.40E-08	-0.32	43	1.07	Sgcd	Cytoskeleton
DMR10:34310001	10	34310001	34312000	2000	1	4.70E-07	-0.36	13	0.65	Olr1383;LOC690160;RGD1560464	Receptor
DMR10:34372001	10	34372001	34378000	6000	2	2.00E-07	-0.28	54	0.9	LOC690222;Olr1386	Receptor
DMR10:34582001	10	34582001	34583000	1000	1	3.50E-09	-0.42	8	0.8	Olr1389;Olr1390-ps	Receptor
DMR10:34652001	10	34652001	34656000	4000	1	8.80E-09	-0.3	37	0.92	Olr1392;Olr1393	Receptor
DMR10:34678001	10	34678001	34682000	4000	1	1.30E-07	-0.29	38	0.95	Olr1394	Receptor
DMR10:34808001	10	34808001	34812000	4000	3	6.20E-09	-0.36	48	1.2	Olr1401	Receptor
DMR10:35030001	10	35030001	35032000	2000	1	3.60E-11	0.51	24	1.2	Trappc2b	
DMR10:35781001	10	35781001	35783000	2000	1	2.50E-07	0.35	23	1.15	Maml1	Transcription
DMR10:35886001	10	35886001	35887000	1000	1	1.30E-07	0.34	16	1.6	Hnrnph1;Rufy1	Translation
DMR10:36160001	10	36160001	36162000	2000	2	3.10E-15	0.43	21	1.05	Adamts2	Protease
DMR10:36217001	10	36217001	36222000	5000	1	2.90E-09	0.34	66	1.32	Adamts2	Protease
DMR10:36224001	10	36224001	36226000	2000	1	3.60E-09	0.53	18	0.9	Adamts2	Protease
DMR10:36258001	10	36258001	36260000	2000	1	5.50E-09	0.42	30	1.5	Adamts2	Protease
DMR10:36373001	10	36373001	36375000	2000	1	4.70E-08	0.34	41	2.05	Grm6	Signaling
DMR10:36427001	10	36427001	36435000	8000	1	7.90E-07	-0.39	110	1.38	Znf354b	
DMR10:37150001	10	37150001	37152000	2000	2	1.90E-08	-0.44	15	0.75	Sec24a	Transport
DMR10:37280001	10	37280001	37282000	2000	1	2.60E-09	0.53	41	2.05	Jade2	Transcription
DMR10:38152001	10	38152001	38155000	3000	2	1.70E-08	0.43	58	1.93	Fstl4	Protease; Proteolysis
DMR10:38368001	10	38368001	38370000	2000	1	5.00E-13	0.51	25	1.25	Fstl4	Protease; Proteolysis
DMR10:38435001	10	38435001	38437000	2000	1	4.50E-08	0.55	15	0.75	Fstl4	Protease; Proteolysis
DMR10:38496001	10	38496001	38498000	2000	1	4.40E-07	-0.29	21	1.05	Fstl4	Protease; Proteolysis
DMR10:38529001	10	38529001	38533000	4000	1	3.30E-07	0.35	74	1.85	Fstl4;LOC108352083	Protease; Proteolysis
DMR10:38662001	10	38662001	38664000	2000	1	4.30E-09	-0.47	40	2	LOC103693336;Zcchc10	
DMR10:39720001	10	39720001	39721000	1000	1	6.90E-09	0.39	17	1.7	Acsl6;Meikin	Metabolism
DMR10:39900001	10	39900001	39903000	3000	1	4.10E-08	-0.27	29	0.97	Rapgef6;LOC108352084	Transcription
DMR10:40771001	10	40771001	40774000	3000	1	7.10E-08	0.39	57	1.9	Sparc;LOC108352218	Extracellular Matrix
DMR10:40792001	10	40792001	40794000	2000	1	3.00E-09	0.58	23	1.15	Atox1	
DMR10:40905001	10	40905001	40909000	4000	2	1.10E-09	-0.39	49	1.23	Glra1;LOC103693333	Ion Channel
DMR10:43103001	10	43103001	43105000	2000	1	2.30E-07	0.37	26	1.3	Galnt10;LOC102553586	Golgi
DMR10:43170001	10	43170001	43172000	2000	1	1.20E-09	0.45	40	2	Galnt10	Golgi
DMR10:43203001	10	43203001	43204000	1000	1	1.60E-07	0.45	19	1.9	Galnt10	Golgi
DMR10:43210001	10	43210001	43211000	1000	1	1.00E-07	0.45	16	1.6	Galnt10	Golgi
DMR10:43506001	10	43506001	43509000	3000	1	2.20E-07	0.39	44	1.47	Larp1;Faxdc2	Metabolism;Metabolism
DMR10:43578001	10	43578001	43579000	1000	1	3.40E-08	0.36	17	1.7	Gemin5	
DMR10:43986001	10	43986001	43994000	8000	1	4.70E-08	-0.26	93	1.16	Olr1417	Receptor
DMR10:44016001	10	44016001	44020000	4000	1	4.10E-07	-0.31	34	0.85	Olr1418	Receptor
DMR10:44063001	10	44063001	44065000	2000	2	1.30E-07	-0.39	10	0.5	Olr1420-ps	
DMR10:44073001	10	44073001	44075000	2000	1	1.10E-08	-0.42	13	0.65	Olr1421	Receptor
DMR10:44128001	10	44128001	44134000	6000	1	7.10E-11	-0.4	55	0.92	Olr1423	Receptor

DMR10:44135001	10	44135001	44137000	2000	1	6.50E-09	-0.48	12	0.6	Olr1424	Receptor
DMR10:44340001	10	44340001	44343000	3000	1	7.80E-08	-0.26	38	1.27	Olr1434;Olr1435	Receptor
DMR10:44669001	10	44669001	44675000	6000	2	1.60E-08	-0.38	42	0.7	RGD1559534;Olr1450	Receptor
DMR10:44783001	10	44783001	44785000	2000	1	4.30E-07	-0.43	8	0.4	Olr1458;Olr1459	Signaling
DMR10:45148001	10	45148001	45150000	2000	1	2.90E-07	-0.33	20	1	LOC691460;RGD1560444;Olr1461	Signaling
DMR10:45244001	10	45244001	45251000	7000	1	5.30E-08	-0.38	53	0.76	Zfp39;Btnl10	Transcription;Immune
DMR10:45293001	10	45293001	45295000	2000	1	5.40E-07	-0.42	20	1	Hist3h2ba;Hist3h2bb;Hist3h2a;Hist3h3	Epigenetic
DMR10:45379001	10	45379001	45387000	8000	2	4.70E-07	0.48	189	2.36	Obscn	
DMR10:45457001	10	45457001	45459000	2000	1	3.00E-08	0.31	39	1.95	Obscn	
DMR10:45492001	10	45492001	45499000	7000	2	2.50E-07	-0.32	85	1.21	Obscn;Iba57	
DMR10:45669001	10	45669001	45671000	2000	1	7.90E-07	0.39	14	0.7	Wnt9a	Signaling
DMR10:45786001	10	45786001	45788000	2000	1	2.40E-07	-0.43	29	1.45	Snap47	Transcription
DMR10:46166001	10	46166001	46168000	2000	1	5.70E-09	0.41	41	2.05	Flcn;LOC100359474	
DMR10:46199001	10	46199001	46200000	1000	1	2.50E-09	-0.51	19	1.9	Cops3	Protease
DMR10:46230001	10	46230001	46231000	1000	1	4.90E-09	0.41	13	1.3	Nt5m	
DMR10:46396001	10	46396001	46397000	1000	1	1.80E-07	0.42	13	1.3	Pemt	Epigenetic
DMR10:46512001	10	46512001	46515000	3000	1	2.80E-10	0.49	48	1.6	Rai1	Transcription
DMR10:46536001	10	46536001	46538000	2000	1	1.70E-07	0.58	24	1.2	Rai1	Transcription
DMR10:46539001	10	46539001	46541000	2000	1	6.20E-08	0.36	25	1.25	Rai1	Transcription
DMR10:46777001	10	46777001	46778000	1000	1	1.70E-07	0.49	3	0.3	Atpaf2;Gid4	Transcription
DMR10:46841001	10	46841001	46843000	2000	1	1.50E-07	0.65	62	3.1	Drg2;Myo15a	Cytoskeleton
DMR10:46855001	10	46855001	46858000	3000	1	6.60E-07	0.56	31	1.03	Myo15a	Cytoskeleton
DMR10:46944001	10	46944001	46945000	1000	1	1.00E-09	0.57	12	1.2	Llgl1	Transport
DMR10:46952001	10	46952001	46955000	3000	1	7.80E-08	0.47	57	1.9	Llgl1;Flii	Transport;Cytoskeleton
DMR10:47483001	10	47483001	47484000	1000	1	4.10E-08	0.38	18	1.8	Aldh3a1	Metabolism
DMR10:47485001	10	47485001	47486000	1000	1	3.40E-07	-0.4	17	1.7	Aldh3a1	Metabolism
DMR10:47508001	10	47508001	47510000	2000	1	6.00E-07	0.4	36	1.8	Aldh3a1;RGD1566257	Metabolism
DMR10:47555001	10	47555001	47559000	4000	1	5.40E-08	0.42	39	0.98	Aldh3a2	Metabolism
DMR10:48252001	10	48252001	48254000	2000	1	4.40E-09	-0.55	29	1.45	Specc1;LOC102551978	
DMR10:48314001	10	48314001	48316000	2000	1	4.30E-11	0.49	12	0.6	Specc1	
DMR10:49163001	10	49163001	49165000	2000	1	1.40E-07	-0.51	35	1.75	Trnaw-cca	
DMR10:49355001	10	49355001	49359000	4000	2	1.00E-07	-0.35	52	1.3	LOC102552952;Cdrt4	
DMR10:49575001	10	49575001	49577000	2000	1	5.80E-07	-0.51	22	1.1	Pmp22;LOC108352093	Cytoskeleton
DMR10:51498001	10	51498001	51500000	2000	2	8.10E-17	0.68	19	0.95	Elac2;Arhgap44	Signaling
DMR10:51647001	10	51647001	51651000	4000	1	1.20E-08	0.37	57	1.43	Arhgap44	
DMR10:51662001	10	51662001	51664000	2000	1	1.30E-07	-0.43	32	1.6	Arhgap44	
DMR10:52204001	10	52204001	52210000	6000	2	5.10E-12	-0.41	58	0.97	Map2k4	Signaling
DMR10:52353001	10	52353001	52354000	1000	1	2.90E-07	0.46	7	0.7	Zfp18;Dnah9	Cytoskeleton
DMR10:52477001	10	52477001	52483000	6000	1	5.00E-09	-0.27	58	0.97	Dnah9	Cytoskeleton
DMR10:52584001	10	52584001	52587000	3000	1	2.50E-09	0.58	14	0.47	Dnah9	Cytoskeleton
DMR10:54081001	10	54081001	54083000	2000	1	5.30E-09	0.53	13	0.65	Gas7	Cytoskeleton
DMR10:54099001	10	54099001	54102000	3000	1	5.00E-07	0.56	33	1.1	Gas7	Cytoskeleton
DMR10:54154001	10	54154001	54157000	3000	3	9.40E-13	0.59	36	1.2	Gas7	Cytoskeleton
DMR10:54214001	10	54214001	54220000	6000	2	2.50E-14	0.61	79	1.32	Gas7	Cytoskeleton
DMR10:54228001	10	54228001	54229000	1000	1	2.10E-07	0.56	6	0.6	Gas7	Cytoskeleton
DMR10:54364001	10	54364001	54365000	1000	1	1.20E-08	0.71	8	0.8	Dhrs7c	
DMR10:54399001	10	54399001	54401000	2000	2	6.50E-12	0.5	18	0.9	Usp43	Protease
DMR10:54500001	10	54500001	54503000	3000	1	2.70E-09	0.46	41	1.37	Cfap52;Stx8	Transcription
DMR10:54549001	10	54549001	54552000	3000	1	1.20E-13	0.51	42	1.4	Stx8	Transcription
DMR10:54682001	10	54682001	54683000	1000	1	3.30E-07	0.36	10	1	Stx8	Transcription
DMR10:54877001	10	54877001	54880000	3000	1	9.30E-07	0.38	39	1.3	Ntn1	Extracellular Matrix
DMR10:55188001	10	55188001	55191000	3000	1	6.70E-07	-0.48	48	1.6	Ccdc42	
DMR10:55371001	10	55371001	55373000	2000	1	8.00E-08	0.38	35	1.75	Myh10	
DMR10:55410001	10	55410001	55411000	1000	1	8.60E-10	0.46	13	1.3	Myh10;Ndel1	
DMR10:55470001	10	55470001	55473000	3000	1	6.40E-07	-0.39	54	1.8	Rnf222	
DMR10:55508001	10	55508001	55510000	2000	1	5.60E-09	0.45	30	1.5	Odf4;Rps4x-ps7	Cytoskeleton
DMR10:55543001	10	55543001	55546000	3000	1	4.50E-09	0.42	56	1.87	Arhgef15;Slc25a35	Transcription
DMR10:55699001	10	55699001	55700000	1000	1	2.30E-07	-0.43	14	1.4	Per1;Trnat-agu;Trnas-cga;Hes7;Trnag-gcc;LOC103693354	Transcription;Transcription

DMR10:55935001	10	55935001	55938000	3000	1	6.20E-07	-0.5	44	1.47	Cntrob;Trappc1;Kcnab3;RGD1563441;Chd3	Transport
DMR10:56006001	10	56006001	56010000	4000	1	2.70E-12	0.52	108	2.7	Cyb5d1;Naa38;Tmem88;Kdm6b	Translation;Epigenetic
DMR10:56122001	10	56122001	56125000	3000	1	1.50E-08	0.46	68	2.27	Dnah2	Cytoskeleton
DMR10:57261001	10	57261001	57263000	2000	1	1.00E-07	0.34	20	1	Rpl36a-ps2;Gp1ba;Slc25a11;Rnf167	Receptor;Transport
DMR10:57429001	10	57429001	57430000	1000	1	1.90E-07	0.32	8	0.8	LOC108352100;Scimp	
DMR10:57627001	10	57627001	57628000	1000	1	1.30E-11	0.57	12	1.2	C1qbp;Dhx33	Transcription
DMR10:57682001	10	57682001	57683000	1000	1	1.50E-07	-0.26	18	1.8	Nlrp1a	
DMR10:57708001	10	57708001	57709000	1000	1	4.30E-07	-0.31	18	1.8	Nlrp1a	
DMR10:57710001	10	57710001	57712000	2000	1	1.40E-08	-0.3	13	0.65	Nlrp1a	
DMR10:58658001	10	58658001	58659000	1000	1	8.50E-07	0.36	17	1.7	Pitpnm3	Metabolism
DMR10:58828001	10	58828001	58829000	1000	1	1.00E-06	0.52	6	0.6	Slc13a5	Transport
DMR10:58958001	10	58958001	58959000	1000	1	6.40E-07	0.42	16	1.6	Smtnl2	
DMR10:59012001	10	59012001	59015000	3000	1	6.60E-09	0.5	36	1.2	Mybbp1a;Spns2	Epigenetic;Transport
DMR10:59217001	10	59217001	59219000	2000	1	1.90E-07	-0.52	12	0.6	Ube2g1	
DMR10:59299001	10	59299001	59300000	1000	1	1.80E-07	-0.45	8	0.8	Ankfy1	Transport
DMR10:59377001	10	59377001	59382000	5000	1	8.30E-08	-0.3	38	0.76	Zzef1;LOC102548894	
DMR10:59809001	10	59809001	59810000	1000	1	1.10E-08	0.72	9	0.9	Trpv1	Transport
DMR10:59926001	10	59926001	59928000	2000	1	3.40E-09	0.62	8	0.4	Olr1466;Olr1467	Receptor
DMR10:60118001	10	60118001	60122000	4000	1	4.90E-08	-0.35	38	0.95	Olr1474	Receptor
DMR10:60123001	10	60123001	60126000	3000	1	5.40E-08	-0.32	72	2.4	Olr1474	Receptor
DMR10:60268001	10	60268001	60272000	4000	1	9.30E-08	-0.38	36	0.9	Olr1485;Olr1484-ps	
DMR10:60304001	10	60304001	60305000	1000	1	7.20E-12	-0.39	26	2.6	Olr1486	Receptor
DMR10:60924001	10	60924001	60929000	5000	1	5.30E-11	-0.36	57	1.14	Olr1508-ps	
DMR10:60949001	10	60949001	60955000	6000	3	3.80E-13	-0.44	71	1.18	Olr1509;Olr1510-ps;LOC497952	Receptor
DMR10:61342001	10	61342001	61346000	4000	1	2.10E-07	0.4	60	1.5	Rap1gap2	Signaling
DMR10:61697001	10	61697001	61698000	1000	1	4.10E-07	0.42	33	3.3	Mnt;Sgsm2	Transcription;Signaling
DMR10:61756001	10	61756001	61757000	1000	1	4.40E-08	0.42	18	1.8	Tsr1;Srr	Metabolism
DMR10:62171001	10	62171001	62174000	3000	1	7.00E-07	-0.53	46	1.53	Rpa1;LOC102554043	Transcription
DMR10:62250001	10	62250001	62252000	2000	1	1.40E-09	0.4	13	0.65	Serpinf1	Protease; Proteolysis
DMR10:62270001	10	62270001	62271000	1000	1	1.40E-07	0.59	6	0.6	Serpinf2;Wdr81	Protease; Proteolysis
DMR10:62595001	10	62595001	62596000	1000	1	8.90E-09	0.48	7	0.7	Taok1	Signaling
DMR10:62780001	10	62780001	62782000	2000	1	9.80E-10	0.42	22	1.1	Ssh2	Signaling
DMR10:63989001	10	63989001	63991000	2000	1	4.90E-07	0.39	24	1.2	Rph3al;LOC102549294	
DMR10:64008001	10	64008001	64011000	3000	1	3.10E-11	0.48	25	0.83	Rph3al;LOC102549294	
DMR10:64504001	10	64504001	64505000	1000	1	2.10E-07	-0.51	15	1.5	Nxn	Metabolism
DMR10:64603001	10	64603001	64605000	2000	1	1.80E-08	0.48	37	1.85	Abr	Signaling
DMR10:64615001	10	64615001	64616000	1000	1	2.90E-10	0.51	13	1.3	Abr	Signaling
DMR10:64617001	10	64617001	64623000	6000	1	4.20E-07	0.49	74	1.23	Abr	Signaling
DMR10:64984001	10	64984001	64987000	3000	1	1.10E-08	0.62	78	2.6	LOC103693392;Myo18a;LOC102557169	
DMR10:65012001	10	65012001	65014000	2000	1	4.80E-10	0.53	41	2.05	Myo18a	
DMR10:65666001	10	65666001	65667000	1000	1	8.40E-09	0.54	10	1	Slc13a2	Transport
DMR10:66318001	10	66318001	66322000	4000	1	7.30E-07	-0.31	62	1.55	LOC497963;Ksr1	Signaling
DMR10:66400001	10	66400001	66401000	1000	1	2.50E-08	0.47	14	1.4	Ksr1	Signaling
DMR10:67047001	10	67047001	67049000	2000	1	6.80E-07	0.32	24	1.2	Rab11fip4;LOC102550168;Trnat-cgu	
DMR10:67532001	10	67532001	67534000	2000	1	4.30E-11	0.56	49	2.45	Adap2;Rnf135	
DMR10:67541001	10	67541001	67545000	4000	1	2.40E-11	0.57	43	1.07	Rnf135	
DMR10:67714001	10	67714001	67715000	1000	1	4.80E-15	0.55	12	1.2	Rhbdl3	
DMR10:68347001	10	68347001	68348000	1000	1	2.60E-07	0.38	18	1.8	Asic2	Transport
DMR10:68379001	10	68379001	68381000	2000	1	5.60E-07	0.39	26	1.3	Asic2	Transport
DMR10:68719001	10	68719001	68720000	1000	1	1.00E-07	0.39	7	0.7	Asic2	Transport
DMR10:68761001	10	68761001	68762000	1000	1	6.40E-08	0.47	7	0.7	Asic2	Transport
DMR10:68907001	10	68907001	68909000	2000	1	4.80E-08	0.69	30	1.5	Asic2	Transport
DMR10:69067001	10	69067001	69068000	1000	1	3.60E-07	0.4	8	0.8	Asic2;LOC103693402	Transport
DMR10:70086001	10	70086001	70089000	3000	1	6.70E-07	0.41	34	1.13	Cct6b;LOC108352113	Translation

DMR10:70119001	10	70119001	70123000	4000	1	8.10E-07	-0.58	65	1.62	Cct6b;Zfp830;LOC103693403	Translation
DMR10:70360001	10	70360001	70366000	6000	2	5.80E-12	-0.4	56	0.93	LOC108352183;Slfn2	
DMR10:70457001	10	70457001	70461000	4000	1	3.30E-07	-0.27	68	1.7	Slfn3	
DMR10:70895001	10	70895001	70899000	4000	1	1.50E-07	-0.5	47	1.18	Ccl4	Growth Factors
DMR10:71670001	10	71670001	71675000	5000	1	8.90E-07	-0.46	62	1.24	Acaca	
DMR10:71778001	10	71778001	71780000	2000	1	7.30E-07	0.52	43	2.15	Aatf	Epigenetic
DMR10:73043001	10	73043001	73045000	2000	1	8.20E-10	0.7	8	0.4	Bcas3	
DMR10:73222001	10	73222001	73223000	1000	1	3.30E-08	0.43	15	1.5	Bcas3	
DMR10:73326001	10	73326001	73327000	1000	1	6.40E-07	-0.45	12	1.2	Tbx4	Transcription
DMR10:73540001	10	73540001	73541000	1000	1	1.50E-07	0.38	16	1.6	Brip1	Epigenetic
DMR10:73607001	10	73607001	73611000	4000	1	4.70E-08	-0.5	60	1.5	Brip1	Epigenetic
DMR10:74111001	10	74111001	74112000	1000	1	2.70E-07	0.65	5	0.5	Dhx40	Transcription
DMR10:74514001	10	74514001	74515000	1000	1	3.50E-07	-0.49	10	1	Trim37	Proteolysis
DMR10:74670001	10	74670001	74671000	1000	1	3.20E-07	-0.48	20	2	Ppm1e	Signaling
DMR10:75021001	10	75021001	75023000	2000	1	7.00E-07	0.34	41	2.05	Rnf43;Supt4h1	Transcription
DMR10:75046001	10	75046001	75048000	2000	1	4.90E-09	0.4	17	0.85	Supt4h1;Mir142;Tspoap1	Transcription
DMR10:75186001	10	75186001	75188000	2000	1	7.60E-08	0.49	23	1.15	Olr1521;Olr1522	Receptor
DMR10:75377001	10	75377001	75380000	3000	1	5.40E-07	0.35	36	1.2	Vezf1	Transcription
DMR10:75607001	10	75607001	75610000	3000	1	9.00E-07	0.27	28	0.93	Ccdc182	
DMR10:75945001	10	75945001	75946000	1000	1	1.30E-08	0.39	13	1.3	Msi2	
DMR10:76152001	10	76152001	76154000	2000	1	4.90E-10	0.69	38	1.9	Akap1	Cytoskeleton
DMR10:76171001	10	76171001	76173000	2000	1	6.00E-07	0.4	23	1.15	Akap1	Cytoskeleton
DMR10:76222001	10	76222001	76223000	1000	1	2.20E-07	0.4	0	0	LOC303431;Scpep1	Protease
DMR10:77909001	10	77909001	77913000	4000	1	6.40E-07	0.48	58	1.45	Hlf	Transcription
DMR10:77920001	10	77920001	77923000	3000	1	5.50E-07	-0.44	46	1.53	Hlf	Transcription
DMR10:78118001	10	78118001	78120000	2000	1	1.40E-10	0.57	50	2.5	Stxbp4;Cox11	Transcription
DMR10:78128001	10	78128001	78130000	2000	1	4.80E-07	-0.41	42	2.1	Stxbp4;Cox11	Transcription
DMR10:78139001	10	78139001	78141000	2000	1	2.00E-09	0.32	18	0.9	Stxbp4;Cox11	Transcription
DMR10:78148001	10	78148001	78150000	2000	2	1.50E-12	0.5	32	1.6	Stxbp4;Cox11	Transcription
DMR10:82743001	10	82743001	82744000	1000	1	9.60E-09	0.45	17	1.7	LOC102556395;Col1a1	Extracellular Matrix
DMR10:82848001	10	82848001	82849000	1000	1	7.50E-07	0.38	19	1.9	Samd14;Pdk2;Itga3	Signaling;Extracellular Matrix
DMR10:82929001	10	82929001	82931000	2000	1	2.00E-07	-0.47	30	1.5	Dlx3	Development
DMR10:82940001	10	82940001	82942000	2000	1	1.60E-07	0.38	42	2.1	Dlx3	Development
DMR10:83074001	10	83074001	83076000	2000	1	1.30E-07	0.39	33	1.65	LOC103693422;Tac4	
DMR10:83117001	10	83117001	83118000	1000	1	1.30E-09	0.37	8	0.8	Kat7	Epigenetic
DMR10:83327001	10	83327001	83329000	2000	1	7.80E-07	0.38	31	1.55	Nxph3	Signaling
DMR10:84155001	10	84155001	84157000	2000	1	9.60E-13	0.43	28	1.4	Hoxb5os;Hoxb6;Hoxb5;Mir10a	Development
DMR10:84305001	10	84305001	84307000	2000	1	2.10E-07	0.43	37	1.85	Skap1	Cytoskeleton
DMR10:84361001	10	84361001	84368000	7000	1	3.20E-08	-0.41	70	1	Skap1	Cytoskeleton
DMR10:84861001	10	84861001	84863000	2000	1	1.20E-08	-0.39	18	0.9	LOC102552748;Prr15l	
DMR10:84971001	10	84971001	84975000	4000	1	2.40E-11	0.48	54	1.35	Sp6;Scrn2;Lrrc46;Mrpl10	Transcription;Signaling;Translation
DMR10:85464001	10	85464001	85468000	4000	2	1.20E-09	0.47	102	2.55	Srcin1	
DMR10:85780001	10	85780001	85782000	2000	1	7.00E-10	0.38	33	1.65	Lasp1;LOC108352127	
DMR10:86069001	10	86069001	86070000	1000	1	9.90E-08	0.39	15	1.5	Fbxl20	
DMR10:86340001	10	86340001	86342000	2000	1	1.30E-07	0.44	69	3.45	Stard3;Tcap;Pnmt;Pgap3	Epigenetic
DMR10:86474001	10	86474001	86478000	4000	1	2.50E-11	-0.52	84	2.1	Ikzf3;LOC108352128	Transcription
DMR10:86568001	10	86568001	86570000	2000	1	2.00E-08	0.78	61	3.05	Lrrc3c;Gsdma;LOC108352129	
DMR10:86679001	10	86679001	86685000	6000	1	1.20E-08	0.39	122	2.03	Thra;Nr1d1	Transcription
DMR10:86799001	10	86799001	86800000	1000	1	6.20E-08	0.5	2	0.2	Wipf2	Cytoskeleton
DMR10:86807001	10	86807001	86808000	1000	1	2.80E-07	0.35	15	1.5	Wipf2	Cytoskeleton
DMR10:86861001	10	86861001	86865000	4000	1	1.30E-09	0.43	77	1.93	Rara	Transcription
DMR10:86958001	10	86958001	86959000	1000	1	8.90E-10	0.43	22	2.2	Igfbp4	Protease; Proteolysis
DMR10:87656001	10	87656001	87657000	1000	1	2.10E-07	-0.55	7	0.7	Cct6a-ps2	
DMR10:88116001	10	88116001	88118000	2000	1	4.50E-08	0.38	24	1.2	Krt9;Krt14	
DMR10:88142001	10	88142001	88146000	4000	1	4.20E-08	0.4	69	1.73	Ka11;Krt16	
DMR10:88556001	10	88556001	88558000	2000	1	3.80E-07	0.34	37	1.85	Zfp385c	

DMR10:88596001	10	88596001	88599000	3000	1	1.10E-08	0.39	59	1.97	Zfp385c;Dhx58	
DMR10:88824001	10	88824001	88827000	3000	1	2.80E-08	0.41	32	1.07	Stat3	Transcription
DMR10:88871001	10	88871001	88873000	2000	1	1.90E-07	0.38	33	1.65	Ptrf	
DMR10:88916001	10	88916001	88918000	2000	1	6.20E-08	-0.48	23	1.15	Atp6v0a1	Metabolism
DMR10:88955001	10	88955001	88962000	7000	1	6.50E-09	0.42	93	1.33	Atp6v0a1	Metabolism
DMR10:89024001	10	89024001	89026000	2000	1	1.90E-07	0.32	31	1.55	Fam134c;Tubg1	Cytoskeleton
DMR10:89035001	10	89035001	89038000	3000	1	2.90E-07	0.37	72	2.4	Fam134c;Tubg1	Cytoskeleton
DMR10:89938001	10	89938001	89940000	2000	1	2.40E-09	0.38	76	3.8	RGD1565533;Mpp3	Cytoskeleton
DMR10:89983001	10	89983001	89988000	5000	1	2.50E-08	-0.33	83	1.66	Cd300lg	Immune
DMR10:90170001	10	90170001	90171000	1000	1	1.10E-07	0.38	16	1.6	Hdac5	
DMR10:90203001	10	90203001	90209000	6000	1	4.20E-07	0.35	99	1.65	LOC303566;Asb16	Transport
DMR10:90255001	10	90255001	90258000	3000	1	8.80E-07	0.36	87	2.9	Ubtf	Transcription
DMR10:90346001	10	90346001	90352000	6000	1	4.90E-07	0.44	133	2.22	Pno1-ps1;LOC108352138;Rundc3a;Slc25a39	
DMR10:90423001	10	90423001	90427000	4000	2	2.50E-08	0.48	99	2.48	Itga2b;Gpatch8	Extracellular Matrix
DMR10:90722001	10	90722001	90724000	2000	1	1.90E-08	0.46	30	1.5	Dbf4b;Adam11	Protease
DMR10:90735001	10	90735001	90737000	2000	1	2.80E-07	0.33	31	1.55	Adam11	Protease
DMR10:90739001	10	90739001	90742000	3000	1	4.50E-07	0.51	58	1.93	Adam11;LOC102547852	Protease
DMR10:90830001	10	90830001	90831000	1000	1	1.90E-07	0.62	11	1.1	Gjc1	Cytoskeleton
DMR10:91018001	10	91018001	91020000	2000	1	1.10E-11	0.44	42	2.1	Kif18b	Cytoskeleton
DMR10:91168001	10	91168001	91169000	1000	1	9.00E-11	0.45	16	1.6	Nmt1;Plcd3	Transport;Metabolism
DMR10:91247001	10	91247001	91249000	2000	1	1.30E-09	0.45	14	0.7	Fmnl1	
DMR10:91256001	10	91256001	91259000	3000	1	3.90E-07	0.53	34	1.13	Fmnl1	
DMR10:91266001	10	91266001	91269000	3000	2	1.90E-12	0.5	66	2.2	Fmnl1	
DMR10:91789001	10	91789001	91794000	5000	1	3.90E-11	0.51	97	1.94	Wnt9b	Signaling
DMR10:91887001	10	91887001	91889000	2000	1	1.40E-07	-0.46	42	2.1	Nsf	Transport
DMR10:91893001	10	91893001	91895000	2000	1	1.20E-08	-0.49	29	1.45	Nsf	Transport
DMR10:91996001	10	91996001	91997000	1000	1	3.60E-09	0.41	14	1.4	Nsf	Transport
DMR10:92278001	10	92278001	92280000	2000	1	1.10E-07	0.37	31	1.55	LOC108352187;Mapt	
DMR10:92368001	10	92368001	92369000	1000	1	8.70E-07	-0.36	20	2	Mapt	
DMR10:92410001	10	92410001	92411000	1000	1	3.40E-07	0.57	5	0.5	Kansl1	
DMR10:92606001	10	92606001	92607000	1000	1	3.70E-13	0.5	16	1.6	Cdc27	Proteolysis
DMR10:92622001	10	92622001	92625000	3000	1	8.10E-07	0.32	42	1.4	Myl4	Cytoskeleton
DMR10:92723001	10	92723001	92732000	9000	1	9.40E-07	-0.3	81	0.9	Itgb3	Extracellular Matrix
DMR10:92737001	10	92737001	92743000	6000	2	1.60E-09	-0.29	68	1.13	Itgb3	Extracellular Matrix
DMR10:92813001	10	92813001	92815000	2000	1	2.10E-08	-0.48	22	1.1	LOC102552446;Efcab13	
DMR10:93365001	10	93365001	93366000	1000	1	2.20E-07	0.43	11	1.1	Mettl2b	Epigenetic
DMR10:93516001	10	93516001	93517000	1000	1	2.40E-07	0.52	12	1.2	Tlk2;Mrc2	Signaling
DMR10:93613001	10	93613001	93615000	2000	1	8.10E-07	0.33	37	1.85	10-Mar	
DMR10:93661001	10	93661001	93662000	1000	1	7.40E-13	0.57	28	2.8	10-Mar	
DMR10:93985001	10	93985001	93989000	4000	1	8.80E-13	-0.39	54	1.35	Tanc2	
DMR10:94000001	10	94000001	94004000	4000	2	2.20E-07	-0.29	45	1.12	Tanc2	
DMR10:94192001	10	94192001	94193000	1000	1	4.20E-07	0.57	9	0.9	Ace;Ace3;LOC102556428	Protease
DMR10:94202001	10	94202001	94204000	2000	1	3.20E-12	0.46	37	1.85	Ace;Ace3;LOC102556428;Kcnh6;LOC108352145	Protease;Transport
DMR10:94467001	10	94467001	94469000	2000	1	4.10E-07	0.37	35	1.75	Smarcd2;Tcam1	Epigenetic
DMR10:94521001	10	94521001	94523000	2000	1	1.90E-07	0.47	27	1.35	Scn4a;LOC108352146	Transport
DMR10:94544001	10	94544001	94546000	2000	1	3.10E-09	0.44	30	1.5	Scn4a;LOC108352146	Transport
DMR10:94735001	10	94735001	94737000	2000	1	5.10E-10	0.52	31	1.55	Tex2	
DMR10:94768001	10	94768001	94772000	4000	1	2.80E-09	0.53	73	1.82	Tex2	
DMR10:94897001	10	94897001	94899000	2000	2	6.40E-14	0.47	32	1.6	Pecam1	Immune
DMR10:95074001	10	95074001	95076000	2000	1	8.70E-08	-0.46	21	1.05	Smurf2	Proteolysis
DMR10:95315001	10	95315001	95319000	4000	1	2.90E-08	0.44	51	1.27	Bptf	
DMR10:95490001	10	95490001	95491000	1000	1	1.50E-13	0.47	19	1.9	Pitpnc1	
DMR10:95504001	10	95504001	95505000	1000	1	2.00E-07	0.36	27	2.7	Pitpnc1;LOC102549520	
DMR10:95799001	10	95799001	95800000	1000	1	3.50E-09	-0.43	25	2.5	Helz	
DMR10:95867001	10	95867001	95869000	2000	1	3.10E-12	0.43	24	1.2	Helz	
DMR10:95952001	10	95952001	95954000	2000	1	2.10E-09	0.45	43	2.15	Cacng4	Transport
DMR10:95977001	10	95977001	95979000	2000	1	4.60E-08	0.38	41	2.05	Cacng4;LOC108352150	Transport
DMR10:95993001	10	95993001	95997000	4000	2	3.40E-08	0.43	70	1.75	Cacng4;LOC108352150	Transport
DMR10:96347001	10	96347001	96348000	1000	1	1.30E-08	-0.47	5	0.5	Prkca	Signaling

DMR10:96471001	10	96471001	96474000	3000	1	7.70E-12	0.56	38	1.27	Prkca	Signaling
DMR10:96743001	10	96743001	96755000	12000	4	1.00E-11	-0.45	213	1.77	Cep112	
DMR10:97028001	10	97028001	97030000	2000	1	6.00E-07	0.41	23	1.15	Cep112	
DMR10:97086001	10	97086001	97087000	1000	1	3.20E-07	-0.45	15	1.5	Cep112	
DMR10:97224001	10	97224001	97227000	3000	1	2.20E-08	0.43	60	2	Axin2	Cytoskeleton
DMR10:97685001	10	97685001	97688000	3000	1	3.00E-07	0.36	33	1.1	Gna13	Signaling
DMR10:97738001	10	97738001	97745000	7000	1	3.60E-07	0.45	136	1.94	Arsg;Slc16a6	Metabolism;Transport
DMR10:97994001	10	97994001	97996000	2000	1	2.30E-12	-0.36	19	0.95	Fam20a	
DMR10:98587001	10	98587001	98592000	5000	1	4.00E-07	-0.33	49	0.98	Abca5	Transport
DMR10:98595001	10	98595001	98601000	6000	1	4.60E-07	0.42	68	1.13	Abca5	Transport
DMR10:98722001	10	98722001	98723000	1000	1	2.80E-07	0.33	22	2.2	Map2k6	Signaling
DMR10:98725001	10	98725001	98728000	3000	1	2.20E-15	0.44	39	1.3	Map2k6	Signaling
DMR10:101690001	10	101690001	101691000	1000	1	8.30E-07	0.5	12	1.2	LOC102549836;Mir297	
DMR10:101804001	10	101804001	101805000	1000	1	5.40E-07	0.49	17	1.7	Slc39a11	Transport
DMR10:101956001	10	101956001	101958000	2000	2	7.80E-09	0.41	26	1.3	Slc39a11	Transport
DMR10:102088001	10	102088001	102090000	2000	1	4.80E-07	0.32	30	1.5	Slc39a11	Transport
DMR10:102224001	10	102224001	102226000	2000	1	4.10E-08	0.42	19	0.95	Cpsf4l	
DMR10:102254001	10	102254001	102257000	3000	1	5.20E-10	0.49	33	1.1	Cdc42ep4	
DMR10:102357001	10	102357001	102359000	2000	1	8.40E-07	0.41	36	1.8	Sdk2	
DMR10:102462001	10	102462001	102466000	4000	1	1.20E-10	0.42	80	2	Sdk2	
DMR10:102506001	10	102506001	102507000	1000	1	3.30E-07	0.46	12	1.2	Sdk2	
DMR10:102526001	10	102526001	102529000	3000	1	5.20E-07	0.35	74	2.47	Sdk2;LOC102547687	
DMR10:102530001	10	102530001	102531000	1000	1	1.50E-08	0.47	22	2.2	Sdk2;LOC102547687	
DMR10:102549001	10	102549001	102550000	1000	1	1.80E-08	0.41	21	2.1	Sdk2;LOC102547687	
DMR10:103277001	10	103277001	103278000	1000	1	3.00E-08	0.4	15	1.5	Dnai2	Cytoskeleton
DMR10:103448001	10	103448001	103450000	2000	1	8.40E-07	0.38	26	1.3	LOC108352159;Cd300a	Immune
DMR10:103488001	10	103488001	103490000	2000	1	8.20E-07	-0.38	23	1.15	Cd300lb;RGD1559458	Immune
DMR10:103763001	10	103763001	103767000	4000	1	3.70E-10	0.51	63	1.57	Tmem104	
DMR10:103869001	10	103869001	103870000	1000	1	5.40E-10	0.73	43	4.3	Ush1g;Otop2	Transport
DMR10:104223001	10	104223001	104224000	1000	1	1.60E-07	0.33	12	1.2	Grb2	
DMR10:104341001	10	104341001	104343000	2000	1	2.70E-08	0.42	43	2.15	Mir3577;Caskin2	Cytoskeleton
DMR10:104350001	10	104350001	104352000	2000	1	6.90E-17	0.61	31	1.55	Mir3577;Caskin2;Tsen54	Cytoskeleton;Translation
DMR10:104537001	10	104537001	104542000	5000	1	1.80E-10	0.65	107	2.14	Itgb4	Extracellular Matrix
DMR10:104627001	10	104627001	104628000	1000	1	9.20E-07	0.48	17	1.7	Unc13d;Wbp2	
DMR10:105363001	10	105363001	105367000	4000	1	8.90E-07	0.45	63	1.57	Rnf157	Proteolysis
DMR10:105395001	10	105395001	105398000	3000	1	4.80E-15	0.66	37	1.23	LOC102554855;Ubald2;Qrich2	
DMR10:105405001	10	105405001	105407000	2000	1	2.40E-09	0.47	57	2.85	Ubald2;Qrich2	
DMR10:105428001	10	105428001	105429000	1000	1	1.30E-07	-0.46	16	1.6	Qrich2;LOC102550872;Prpsap1	Signaling
DMR10:105575001	10	105575001	105582000	7000	1	1.80E-09	0.53	181	2.59	RGD1559519;Aanat;Rhbdf2	Metabolism;Protease
DMR10:105612001	10	105612001	105615000	3000	1	8.00E-11	0.46	48	1.6	Cygb	
DMR10:105654001	10	105654001	105656000	2000	2	1.70E-08	0.44	56	2.8	LOC100912247;LOC108352161;St6galnac2	
DMR10:105658001	10	105658001	105661000	3000	1	6.20E-08	0.48	57	1.9	LOC100912247;LOC108352161;St6galnac2	
DMR10:105702001	10	105702001	105704000	2000	1	5.20E-09	0.31	37	1.85	St6galnac1	
DMR10:105763001	10	105763001	105765000	2000	1	9.00E-09	0.39	34	1.7	Mxra7;LOC103693479	
DMR10:106102001	10	106102001	106104000	2000	1	5.50E-09	0.44	43	2.15	Sec14l1;LOC102547461	
DMR10:106301001	10	106301001	106303000	2000	1	1.40E-08	0.46	51	2.55	9-Sep	
DMR10:106869001	10	106869001	106872000	3000	1	3.10E-07	0.52	49	1.63	Birc5;Tmem235;LOC688286	Metabolism
DMR10:107037001	10	107037001	107040000	3000	1	1.30E-07	0.41	90	3	Pgs1;Dnah17	Transport;Cytoskeleton
DMR10:107096001	10	107096001	107098000	2000	1	4.90E-07	0.33	41	2.05	Dnah17	Cytoskeleton
DMR10:107100001	10	107100001	107101000	1000	1	2.40E-09	0.49	25	2.5	Dnah17	Cytoskeleton
DMR10:107118001	10	107118001	107121000	3000	1	6.00E-09	0.5	58	1.93	Dnah17	Cytoskeleton
DMR10:107231001	10	107231001	107232000	1000	1	1.50E-07	0.41	15	1.5	Cyth1	Transcription
DMR10:107303001	10	107303001	107305000	2000	1	3.10E-12	0.48	23	1.15	Usp36	Protease
DMR10:107337001	10	107337001	107339000	2000	1	2.50E-09	0.46	56	2.8	Timp2	Protease; Proteolysis
DMR10:107414001	10	107414001	107417000	3000	1	4.30E-10	0.52	52	1.73	Lgals3bp	
DMR10:107462001	10	107462001	107464000	2000	1	2.10E-12	0.5	36	1.8	LOC102548791;C1qtnf1	

DMR10:107469001	10	107469001	107472000	3000	1	5.70E-08	0.36	65	2.17	C1qtnf1	
DMR10:107509001	10	107509001	107511000	2000	1	1.70E-07	0.35	32	1.6	Engase;Rbfox3	Metabolism;Translation
DMR10:107539001	10	107539001	107540000	1000	1	4.00E-07	0.41	35	3.5	Rbfox3	Translation
DMR10:107554001	10	107554001	107559000	5000	1	1.60E-07	0.38	109	2.18	Rbfox3	Translation
DMR10:107588001	10	107588001	107591000	3000	1	3.30E-08	0.33	33	1.1	Rbfox3	Translation
DMR10:107689001	10	107689001	107690000	1000	1	1.20E-07	0.55	9	0.9	Rbfox3	Translation
DMR10:107790001	10	107790001	107792000	2000	1	8.80E-07	0.37	54	2.7	Rbfox3	Translation
DMR10:108053001	10	108053001	108055000	2000	1	2.20E-08	0.58	45	2.25	LOC108348054;Ccdc40	
DMR10:108098001	10	108098001	108101000	3000	1	3.60E-10	0.46	37	1.23	Ccdc40;Enpp7	
DMR10:108327001	10	108327001	108329000	2000	1	3.80E-07	0.52	26	1.3	Ccdc40;Tbc1d16	Signaling
DMR10:108341001	10	108341001	108342000	1000	1	2.70E-07	0.41	19	1.9	Ccdc40	
DMR10:108456001	10	108456001	108457000	1000	1	1.90E-07	0.55	1	0.1	Card14	
DMR10:108650001	10	108650001	108653000	3000	1	5.00E-07	0.4	45	1.5	Endov	
DMR10:108864001	10	108864001	108868000	4000	1	8.30E-07	0.41	66	1.65	Rptor	
DMR10:109101001	10	109101001	109102000	1000	1	1.70E-10	0.54	15	1.5	LOC102555825;LOC108352166;Baiap2	Cytoskeleton
DMR10:109253001	10	109253001	109255000	2000	1	2.90E-08	0.41	42	2.1	Cep131;Mir3594;Mir3562	
DMR10:109414001	10	109414001	109416000	2000	1	3.90E-07	0.41	23	1.15	Bahcc1	Transcription
DMR10:109461001	10	109461001	109465000	4000	1	1.50E-09	0.57	86	2.15	Bahcc1	Transcription
DMR10:109477001	10	109477001	109482000	5000	1	8.90E-07	0.44	155	3.1	Bahcc1	Transcription
DMR10:109635001	10	109635001	109637000	2000	1	7.40E-09	0.45	20	1	Pde6g;Oxld1;Ccdc137;Arl16;Hgs	Signaling;Metabolism
DMR10:110147001	10	110147001	110149000	2000	1	3.80E-08	0.46	49	2.45	Slc16a3;Csnk1d	Transport;Signaling
DMR10:110348001	10	110348001	110353000	5000	1	2.60E-07	0.51	52	1.04	Tex19.1	
DMR10:110548001	10	110548001	110549000	1000	1	9.40E-08	-0.54	7	0.7	Wdr45b;Rab40b	
DMR10:110660001	10	110660001	110663000	3000	1	7.50E-07	0.39	23	0.77	Tbcd	Transcription
DMR10:110692001	10	110692001	110693000	1000	1	2.70E-09	0.32	19	1.9	Tbcd;Znf750	Transcription
DMR10:111429001	10	111429001	111436000	7000	2	4.20E-09	-0.32	79	1.13	Vom2r-ps3	
DMR11:1578001	11	1578001	1580000	2000	1	3.40E-07	-0.42	13	0.65	Csnka2ip	
DMR11:4267001	11	4267001	4274000	7000	2	1.20E-07	-0.28	82	1.17	Cadm2	
DMR11:7240001	11	7240001	7244000	4000	1	8.20E-08	-0.27	39	0.98	Gbe1	Metabolism
DMR11:7302001	11	7302001	7304000	2000	1	6.50E-10	0.34	27	1.35	Gbe1;LOC108352291	Metabolism
DMR11:9076001	11	9076001	9077000	1000	1	6.50E-07	0.37	9	0.9	Robo1	
DMR11:9312001	11	9312001	9318000	6000	1	2.80E-07	-0.28	62	1.03	Robo1	
DMR11:9365001	11	9365001	9366000	1000	1	2.90E-11	-0.72	8	0.8	Robo1	
DMR11:9750001	11	9750001	9751000	1000	1	2.20E-09	0.36	13	1.3	Robo1	
DMR11:9879001	11	9879001	9885000	6000	2	1.10E-07	-0.3	61	1.02	Robo1	
DMR11:9949001	11	9949001	9951000	2000	1	3.40E-09	0.42	20	1	Robo1	
DMR11:11098001	11	11098001	11101000	3000	1	1.20E-15	0.58	51	1.7	Robo2	
DMR11:11148001	11	11148001	11149000	1000	1	1.40E-07	-0.42	7	0.7	Robo2	
DMR11:11513001	11	11513001	11514000	1000	1	3.70E-07	-0.46	10	1	Robo2	
DMR11:11701001	11	11701001	11702000	1000	1	7.10E-07	0.39	16	1.6	Robo2	
DMR11:11703001	11	11703001	11704000	1000	1	5.30E-07	0.45	7	0.7	Robo2	
DMR11:11830001	11	11830001	11837000	7000	1	2.50E-08	-0.4	79	1.13	Robo2	
DMR11:11898001	11	11898001	11901000	3000	1	6.40E-07	-0.49	35	1.17	Robo2	
DMR11:12050001	11	12050001	12056000	6000	1	6.50E-07	-0.4	44	0.73	Robo2;LOC108352380	
DMR11:12410001	11	12410001	12411000	1000	1	3.50E-09	-0.62	9	0.9	Robo2	
DMR11:12519001	11	12519001	12521000	2000	1	2.90E-07	-0.43	11	0.55	Robo2	
DMR11:13977001	11	13977001	13978000	1000	1	2.30E-07	-0.5	7	0.7	Lipi	Metabolism
DMR11:14013001	11	14013001	14015000	2000	1	8.60E-07	-0.57	43	2.15	Rbm11	Metabolism
DMR11:14329001	11	14329001	14330000	1000	1	5.10E-08	0.47	11	1.1	Samsn1	
DMR11:14403001	11	14403001	14406000	3000	1	5.70E-07	-0.43	48	1.6	Samsn1	
DMR11:15475001	11	15475001	15480000	5000	1	2.60E-08	-0.3	62	1.24	Usp25	Protease
DMR11:15502001	11	15502001	15503000	1000	1	1.90E-07	-0.58	10	1	Usp25	Protease
DMR11:17094001	11	17094001	17096000	2000	1	5.20E-07	-0.48	36	1.8	RGD1563888	
DMR11:17586001	11	17586001	17590000	4000	2	3.60E-10	-0.34	50	1.25	Tmprss15	Protease
DMR11:17591001	11	17591001	17592000	1000	1	9.60E-08	-0.42	9	0.9	Tmprss15	Protease
DMR11:17608001	11	17608001	17609000	1000	1	3.10E-07	-0.55	10	1	Tmprss15	Protease
DMR11:19758001	11	19758001	19760000	2000	1	2.20E-08	-0.55	6	0.3	Ncam2;LOC681289	
DMR11:19771001	11	19771001	19772000	1000	1	1.90E-24	0.64	13	1.3	Ncam2;LOC681289	
DMR11:19832001	11	19832001	19835000	3000	1	3.00E-08	-0.29	32	1.07	Ncam2	

DMR11:20082001	11	20082001	20084000	2000	2	2.60E-10	-0.41	19	0.95	Ncam2	
DMR11:20664001	11	20664001	20665000	1000	1	3.40E-07	-0.44	6	0.6	Ncam2	
DMR11:24434001	11	24434001	24435000	1000	1	3.10E-10	0.75	7	0.7	App	Protease; Proteolysis
DMR11:24645001	11	24645001	24648000	3000	2	7.30E-12	-0.37	28	0.93	App	Protease; Proteolysis
DMR11:25058001	11	25058001	25060000	2000	1	3.80E-08	-0.65	20	1	Cyyr1	
DMR11:27035001	11	27035001	27039000	4000	1	1.30E-08	0.48	41	1.02	Ltn1	Proteolysis
DMR11:27088001	11	27088001	27089000	1000	1	4.40E-08	-0.4	21	2.1	Ltn1;Rwdd2b	Proteolysis
DMR11:27149001	11	27149001	27153000	4000	1	2.60E-08	-0.37	44	1.1	Cct8	Translation
DMR11:28119001	11	28119001	28124000	5000	1	6.80E-07	-0.31	49	0.98	Grik1;LOC108352301	Receptor
DMR11:28127001	11	28127001	28129000	2000	1	7.20E-07	-0.52	19	0.95	Grik1;LOC108352301	Receptor
DMR11:29971001	11	29971001	29972000	1000	1	2.50E-07	-0.31	20	2	Tiam1	
DMR11:30065001	11	30065001	30066000	1000	1	5.60E-08	0.42	26	2.6	Tiam1	
DMR11:30597001	11	30597001	30604000	7000	1	1.10E-09	0.39	104	1.49	Hunk	
DMR11:30611001	11	30611001	30614000	3000	1	2.10E-08	-0.49	44	1.47	Hunk	
DMR11:30669001	11	30669001	30671000	2000	1	1.10E-09	0.29	24	1.2	Hunk	
DMR11:30883001	11	30883001	30884000	1000	1	8.70E-10	0.46	1	0.1	Mis18a;LOC100911990	
DMR11:31012001	11	31012001	31013000	1000	1	2.30E-12	0.43	9	0.9	Eva1c	
DMR11:31086001	11	31086001	31087000	1000	1	1.10E-07	0.37	11	1.1	RGD1306954	
DMR11:31120001	11	31120001	31125000	5000	1	1.10E-07	-0.41	53	1.06	Synj1	Signaling
DMR11:31382001	11	31382001	31384000	2000	1	9.40E-07	0.33	22	1.1	Olig2	Transcription
DMR11:31840001	11	31840001	31842000	2000	1	8.60E-09	-0.67	32	1.6	Son;Donson;Cryzl1	Translation;Metabolism
DMR11:31877001	11	31877001	31878000	1000	1	2.00E-08	0.42	8	0.8	Cryzl1	Metabolism
DMR11:31884001	11	31884001	31886000	2000	2	4.80E-13	0.62	27	1.35	Cryzl1	Metabolism
DMR11:32783001	11	32783001	32785000	2000	1	7.90E-07	-0.34	38	1.9	Runx1	Transcription
DMR11:33940001	11	33940001	33941000	1000	1	1.10E-11	0.41	11	1.1	Dopey2;LOC103693520	
DMR11:34663001	11	34663001	34665000	2000	1	3.10E-11	0.59	21	1.05	Ttc3	Proteolysis
DMR11:34675001	11	34675001	34677000	2000	1	2.00E-11	0.58	12	0.6	Ttc3	Proteolysis
DMR11:34745001	11	34745001	34746000	1000	1	2.50E-08	0.31	6	0.6	Ttc3;LOC108352310;LOC102549457	Proteolysis
DMR11:35123001	11	35123001	35125000	2000	1	2.70E-09	0.39	21	1.05	Kcnj6	Transport
DMR11:35546001	11	35546001	35547000	1000	1	2.00E-13	0.53	28	2.8	LOC102550135;Kcnj15	Transport
DMR11:35578001	11	35578001	35579000	1000	1	8.10E-07	0.34	22	2.2	Kcnj15	Transport
DMR11:35591001	11	35591001	35594000	3000	1	3.20E-07	0.4	44	1.47	Kcnj15	Transport
DMR11:35595001	11	35595001	35597000	2000	1	1.00E-07	0.44	29	1.45	Kcnj15	Transport
DMR11:35650001	11	35650001	35652000	2000	1	3.90E-09	0.63	27	1.35	Erg	Transcription
DMR11:36347001	11	36347001	36351000	4000	1	3.30E-07	-0.36	61	1.52	Psmg1;Brwd1	Transcription
DMR11:36529001	11	36529001	36530000	1000	1	3.70E-10	0.45	5	0.5	Lca5l	
DMR11:36778001	11	36778001	36779000	1000	1	5.20E-08	-0.55	20	2	Igsf5	Immune
DMR11:37081001	11	37081001	37084000	3000	1	7.30E-07	0.6	32	1.07	Dscam	Cytoskeleton
DMR11:37460001	11	37460001	37461000	1000	1	1.50E-08	0.52	5	0.5	Dscam	Cytoskeleton
DMR11:38531001	11	38531001	38533000	2000	1	2.50E-07	-0.34	19	0.95	LOC108352317;Nsun3	Epigenetic
DMR11:38584001	11	38584001	38585000	1000	1	5.40E-07	-0.41	26	2.6	Nsun3;LOC102554704	Epigenetic
DMR11:38747001	11	38747001	38753000	6000	1	1.30E-07	-0.27	62	1.03	Zfp758	
DMR11:41887001	11	41887001	41889000	2000	1	8.80E-10	-0.7	15	0.75	Epha6;LOC102556000	Receptor
DMR11:42206001	11	42206001	42207000	1000	1	6.80E-09	0.5	14	1.4	Epha6	Receptor
DMR11:42563001	11	42563001	42568000	5000	1	4.60E-08	-0.36	50	1	Epha6	Receptor
DMR11:42630001	11	42630001	42631000	1000	1	7.50E-07	-0.39	19	1.9	Epha6	Receptor
DMR11:42654001	11	42654001	42657000	3000	1	1.20E-08	-0.34	38	1.27	Epha6	Receptor
DMR11:42843001	11	42843001	42846000	3000	1	7.40E-07	0.43	36	1.2	Epha6	Receptor
DMR11:43100001	11	43100001	43105000	5000	1	4.40E-09	-0.37	50	1	Gabrr3	Ion Channel
DMR11:43106001	11	43106001	43112000	6000	1	1.10E-08	0.39	82	1.37	Gabrr3	Ion Channel
DMR11:43134001	11	43134001	43140000	6000	2	5.90E-08	-0.4	76	1.27	Olr1528;Olr1529	Signaling
DMR11:43321001	11	43321001	43326000	5000	1	5.10E-07	-0.44	41	0.82	Olr1540	Signaling
DMR11:43366001	11	43366001	43368000	2000	1	8.10E-08	-0.41	21	1.05	Olr1542	Signaling
DMR11:43436001	11	43436001	43437000	1000	1	3.00E-07	-0.34	13	1.3	Olr1545	Signaling
DMR11:43534001	11	43534001	43544000	10000	2	4.90E-09	-0.47	114	1.14	Olr1552-ps	
DMR11:43844001	11	43844001	43847000	3000	1	1.80E-08	-0.44	31	1.03	Olr1563	
DMR11:43872001	11	43872001	43875000	3000	1	2.60E-11	-0.37	26	0.87	Olr1561	Signaling
DMR11:45020001	11	45020001	45028000	8000	2	5.90E-10	-0.4	95	1.19	Cmss1	Metabolism
DMR11:45100001	11	45100001	45101000	1000	1	3.90E-10	0.41	14	1.4	Cmss1;Filip1l	Metabolism
DMR11:45447001	11	45447001	45450000	3000	2	2.10E-07	-0.46	53	1.77	Tbc1d23;LOC108352326	

DMR11:45647001	11	45647001	45648000	1000	1	3.10E-07	0.4	10	1	Olr1534-ps	
DMR11:45656001	11	45656001	45660000	4000	1	2.70E-08	-0.31	42	1.05	Olr1534-ps;Olr1535	Signaling
DMR11:46067001	11	46067001	46070000	3000	1	6.70E-07	-0.43	44	1.47	Tmem45al	
DMR11:46138001	11	46138001	46144000	6000	1	4.50E-07	-0.46	58	0.97	Adgrg7	Signaling
DMR11:46237001	11	46237001	46238000	1000	1	1.10E-08	0.44	10	1	Abi3bp	
DMR11:46642001	11	46642001	46643000	1000	1	2.10E-07	0.34	10	1	Impg2	Extracellular Matrix
DMR11:46921001	11	46921001	46924000	3000	1	1.10E-07	-0.34	25	0.83	Senp7	Protease
DMR11:46937001	11	46937001	46941000	4000	2	3.40E-08	-0.32	43	1.07	Senp7	Protease
DMR11:46978001	11	46978001	46980000	2000	2	4.20E-10	-0.44	17	0.85	Senp7	Protease
DMR11:47043001	11	47043001	47045000	2000	1	5.80E-09	0.43	22	1.1	Senp7;LOC102552047;Trmt10c	Protease;Epigenetic
DMR11:47230001	11	47230001	47231000	1000	1	5.30E-12	0.65	32	3.2	Nxpe3	
DMR11:50874001	11	50874001	50875000	1000	1	4.00E-07	0.59	0	0	Alcam	Immune
DMR11:51155001	11	51155001	51161000	6000	2	1.10E-09	-0.37	76	1.27	Cblb	Metabolism
DMR11:53656001	11	53656001	53662000	6000	1	6.30E-07	-0.37	45	0.75	Ift57	Development
DMR11:53706001	11	53706001	53711000	5000	1	1.60E-09	-0.34	63	1.26	Ift57	Development
DMR11:53712001	11	53712001	53717000	5000	2	6.20E-11	-0.35	48	0.96	Ift57	Development
DMR11:54653001	11	54653001	54656000	3000	1	7.00E-10	-0.31	24	0.8	Trat1	Immune
DMR11:54878001	11	54878001	54883000	5000	1	3.20E-13	-0.36	62	1.24	Morc1	
DMR11:55021001	11	55021001	55025000	4000	1	5.20E-07	-0.39	37	0.92	Morc1	
DMR11:55485001	11	55485001	55486000	1000	1	1.00E-07	-0.52	4	0.4	Olr1453	Receptor
DMR11:57237001	11	57237001	57238000	1000	1	4.50E-08	-0.54	13	1.3	Plcxd2	
DMR11:59336001	11	59336001	59337000	1000	1	2.00E-07	-0.36	12	1.2	Lsamp	Immune
DMR11:60260001	11	60260001	60262000	2000	1	1.00E-08	-0.6	16	0.8	Slc9c1;LOC685706;LOC685716	Transport
DMR11:61125001	11	61125001	61127000	2000	1	1.60E-08	0.45	16	0.8	Boc	
DMR11:61136001	11	61136001	61137000	1000	1	3.10E-07	0.37	15	1.5	Boc	
DMR11:61301001	11	61301001	61305000	4000	2	6.60E-11	-0.38	48	1.2	Spice1	
DMR11:61326001	11	61326001	61327000	1000	1	1.80E-07	-0.42	13	1.3	Sidt1	
DMR11:61719001	11	61719001	61720000	1000	1	3.00E-08	-0.55	5	0.5	Ccdc191	
DMR11:62123001	11	62123001	62124000	1000	1	5.70E-07	0.39	13	1.3	Zbtb20	Transcription
DMR11:62400001	11	62400001	62402000	2000	1	2.70E-07	-0.26	29	1.45	Zbtb20	Transcription
DMR11:62482001	11	62482001	62488000	6000	3	9.10E-28	0.46	66	1.1	Zbtb20	Transcription
DMR11:62630001	11	62630001	62632000	2000	1	3.30E-08	0.43	29	1.45	Gramd1c	
DMR11:64431001	11	64431001	64433000	2000	1	7.70E-07	-0.44	12	0.6	Igsf11	Immune
DMR11:64545001	11	64545001	64546000	1000	1	2.30E-07	-0.48	27	2.7	Upk1b	
DMR11:64618001	11	64618001	64619000	1000	1	2.00E-07	0.33	14	1.4	Arhgap31	Signaling
DMR11:64738001	11	64738001	64739000	1000	1	7.40E-08	-0.54	9	0.9	Tmem39a	
DMR11:64804001	11	64804001	64806000	2000	1	6.40E-07	0.35	20	1	Timmdc1;Cd80	Immune
DMR11:64862001	11	64862001	64863000	1000	1	1.90E-09	0.49	22	2.2	Cd80;LOC108352341;Adprh	Immune;Metabolism
DMR11:64957001	11	64957001	64958000	1000	1	1.90E-07	0.4	8	0.8	Popdc2;Cox17	Transcription
DMR11:64961001	11	64961001	64963000	2000	1	3.00E-22	1.89	15	0.75	Popdc2;Cox17	Transcription
DMR11:65015001	11	65015001	65019000	4000	1	1.40E-10	0.48	45	1.12	Maats1;Nr1i2	Transcription
DMR11:65027001	11	65027001	65029000	2000	1	2.90E-08	0.41	36	1.8	Maats1;Nr1i2	Transcription
DMR11:66806001	11	66806001	66807000	1000	1	6.20E-08	-0.45	25	2.5	Golgb1	
DMR11:66829001	11	66829001	66836000	7000	3	1.40E-10	-0.46	68	0.97	Golgb1;LOC108352342;Iqcb1	
DMR11:66911001	11	66911001	66912000	1000	1	9.30E-09	-0.93	24	2.4	Eaf2	
DMR11:67186001	11	67186001	67188000	2000	1	1.10E-07	-0.43	27	1.35	Casr	Signaling
DMR11:67250001	11	67250001	67251000	1000	1	8.50E-07	0.35	6	0.6	Casr	Signaling
DMR11:67268001	11	67268001	67269000	1000	1	4.60E-08	0.48	17	1.7	Casr	Signaling
DMR11:67572001	11	67572001	67575000	3000	1	7.30E-07	-0.27	41	1.37	Csta;RGD1565665;LOC100911878;RGD1565297	Protease; Proteolysis
DMR11:67579001	11	67579001	67580000	1000	1	3.90E-11	-0.58	6	0.6	RGD1565665;LOC100911878;RGD1565297	
DMR11:68379001	11	68379001	68380000	1000	1	5.10E-08	0.42	10	1	Sema5b	Signaling
DMR11:68386001	11	68386001	68387000	1000	1	3.30E-09	0.5	10	1	Sema5b	Signaling
DMR11:68422001	11	68422001	68423000	1000	1	2.90E-08	-0.46	19	1.9	Sema5b	Signaling
DMR11:68669001	11	68669001	68671000	2000	1	1.20E-10	-0.37	24	1.2	Sec22a	
DMR11:68715001	11	68715001	68717000	2000	1	1.10E-08	0.42	39	1.95	LOC102551038;Adcy5	

DMR11:68895001	11	68895001	68896000	1000	1	5.20E-07	0.55	5	0.5	Hacd2	
DMR11:69247001	11	69247001	69249000	2000	1	3.40E-10	-0.58	31	1.55	Mylk	
DMR11:69519001	11	69519001	69520000	1000	1	1.70E-08	0.4	12	1.2	Kalrn	Transcription
DMR11:69521001	11	69521001	69523000	2000	1	3.60E-08	0.38	21	1.05	Kalrn	Transcription
DMR11:69829001	11	69829001	69831000	2000	1	7.70E-08	0.44	26	1.3	Kalrn	Transcription
DMR11:69870001	11	69870001	69871000	1000	1	2.60E-07	-0.49	10	1	Kalrn	Transcription
DMR11:69886001	11	69886001	69887000	1000	1	1.30E-07	-0.42	22	2.2	Kalrn	Transcription
DMR11:69991001	11	69991001	69995000	4000	1	5.20E-08	0.35	62	1.55	Kalrn;LOC103693587	Transcription
DMR11:70535001	11	70535001	70536000	1000	1	3.40E-10	0.52	9	0.9	Zfp148	
DMR11:70580001	11	70580001	70584000	4000	1	1.10E-07	-0.53	48	1.2	Zfp148	
DMR11:70921001	11	70921001	70925000	4000	1	3.10E-13	-0.49	37	0.92	Lmln	Protease
DMR11:70996001	11	70996001	70998000	2000	1	2.10E-10	0.4	36	1.8	Iqcg	Cytoskeleton
DMR11:71034001	11	71034001	71037000	3000	2	7.20E-15	0.49	52	1.73	Lrch3	
DMR11:71090001	11	71090001	71091000	1000	1	7.70E-07	-0.51	12	1.2	Lrch3	
DMR11:71150001	11	71150001	71153000	3000	1	1.00E-07	0.51	59	1.97	Rubcn	
DMR11:72279001	11	72279001	72280000	1000	1	2.30E-09	0.41	9	0.9	Dlg1	Cytoskeleton
DMR11:72336001	11	72336001	72337000	1000	1	5.50E-08	-0.47	11	1.1	Dlg1	Cytoskeleton
DMR11:72583001	11	72583001	72589000	6000	1	8.30E-09	0.39	110	1.83	Bdh1	Metabolism
DMR11:72602001	11	72602001	72603000	1000	1	3.40E-09	0.34	40	4	Bdh1	Metabolism
DMR11:73180001	11	73180001	73182000	2000	1	1.60E-07	0.37	33	1.65	Acap2	
DMR11:73335001	11	73335001	73338000	3000	1	9.90E-08	-0.49	39	1.3	Xxylt1;LOC102553680	Transport
DMR11:73744001	11	73744001	73746000	2000	2	8.80E-09	0.45	27	1.35	Tmem44;LOC102548792	
DMR11:73754001	11	73754001	73755000	1000	1	1.80E-09	0.48	16	1.6	Tmem44;LOC102548792	
DMR11:74030001	11	74030001	74031000	1000	1	3.20E-08	0.48	26	2.6	Lrrc15	
DMR11:75526001	11	75526001	75527000	1000	1	5.10E-07	0.41	16	1.6	Mb21d2	
DMR11:75661001	11	75661001	75663000	2000	1	5.00E-10	0.43	21	1.05	Fgf12	Growth Factors
DMR11:75756001	11	75756001	75757000	1000	1	7.10E-10	0.49	12	1.2	Fgf12	Growth Factors
DMR11:76157001	11	76157001	76158000	1000	1	5.80E-07	0.36	12	1.2	Fgf12	Growth Factors
DMR11:78451001	11	78451001	78452000	1000	1	3.20E-10	-0.59	13	1.3	Tp63	Transcription
DMR11:79300001	11	79300001	79307000	7000	2	2.40E-08	-0.34	103	1.47	Lpp	Signaling
DMR11:79319001	11	79319001	79322000	3000	2	2.80E-10	-0.41	36	1.2	Lpp	Signaling
DMR11:79427001	11	79427001	79429000	2000	1	8.20E-07	0.49	13	0.65	Lpp	Signaling
DMR11:79560001	11	79560001	79562000	2000	1	7.10E-11	-0.59	24	1.2	Lpp	Signaling
DMR11:79830001	11	79830001	79831000	1000	1	8.10E-07	-0.42	21	2.1	Lpp;Lppos	Signaling
DMR11:80305001	11	80305001	80312000	7000	2	7.30E-08	-0.29	64	0.91	Rtp2	
DMR11:80936001	11	80936001	80937000	1000	1	5.20E-07	-0.44	16	1.6	St6gal1	Transport
DMR11:81961001	11	81961001	81962000	1000	1	1.50E-07	0.5	51	5.1	LOC102552590;Dgkg	Signaling
DMR11:82021001	11	82021001	82024000	3000	1	4.20E-11	0.46	69	2.3	Dgkg	Signaling
DMR11:82055001	11	82055001	82058000	3000	1	1.90E-08	0.45	75	2.5	Dgkg	Signaling
DMR11:82124001	11	82124001	82126000	2000	1	9.90E-08	-0.56	22	1.1	Dgkg	Signaling
DMR11:82167001	11	82167001	82168000	1000	1	8.20E-08	0.44	18	1.8	Dgkg	Signaling
DMR11:82217001	11	82217001	82222000	5000	1	1.20E-07	0.37	68	1.36	Etv5	Transcription
DMR11:82670001	11	82670001	82673000	3000	1	8.20E-07	0.41	42	1.4	Senp2;Liph	Protease;Metabolism
DMR11:83200001	11	83200001	83202000	2000	1	1.20E-07	0.32	29	1.45	Vps8	Cytoskeleton
DMR11:83210001	11	83210001	83211000	1000	1	3.50E-07	0.32	14	1.4	Vps8	Cytoskeleton
DMR11:83224001	11	83224001	83226000	2000	1	5.70E-13	0.51	14	0.7	Vps8	Cytoskeleton
DMR11:83235001	11	83235001	83236000	1000	1	4.40E-13	0.46	9	0.9	Vps8	Cytoskeleton
DMR11:84042001	11	84042001	84045000	3000	1	2.30E-09	0.39	59	1.97	Abcf3;LOC102551795;Ap2m1;Dvl3	Translation;Transport;Cytoskeleton
DMR11:84052001	11	84052001	84054000	2000	1	4.50E-10	0.48	43	2.15	Ap2m1;Dvl3	Transport;Cytoskeleton
DMR11:84103001	11	84103001	84104000	1000	1	9.90E-07	0.45	7	0.7	Rps15al2	
DMR11:84434001	11	84434001	84435000	1000	1	9.00E-11	0.38	16	1.6	Yeats2;Abcc5	Transcription;Transport
DMR11:84464001	11	84464001	84466000	2000	1	2.90E-07	0.37	18	0.9	Yeats2;Abcc5	Transcription;Transport
DMR11:84496001	11	84496001	84499000	3000	1	2.20E-09	0.45	53	1.77	Yeats2;Abcc5;Cyp2ab1	Transcription;Transport;Metabolism
DMR11:84565001	11	84565001	84566000	1000	1	7.20E-08	-0.47	7	0.7	Yeats2	Transcription
DMR11:84775001	11	84775001	84777000	2000	1	6.30E-08	0.34	19	0.95	Klhl6;LOC102547727	Cytoskeleton
DMR11:85145001	11	85145001	85152000	7000	2	6.00E-09	-0.4	72	1.03	Olr1565	Receptor
DMR11:85274001	11	85274001	85280000	6000	1	1.20E-07	-0.38	57	0.95	Olr1567;Olr1568	
DMR11:85296001	11	85296001	85297000	1000	1	1.10E-08	-0.51	3	0.3	Olr1568	
DMR11:86158001	11	86158001	86162000	4000	1	6.00E-07	-0.42	52	1.3	Hira	Epigenetic

DMR11:86203001	11	86203001	86207000	4000	1	9.40E-07	-0.52	22	0.55	Hira;LOC108352361	Epigenetic
DMR11:86316001	11	86316001	86319000	3000	3	3.10E-11	0.37	21	0.7	Ufd1l;Cdc45	Cell Cycle
DMR11:86617001	11	86617001	86624000	7000	2	5.40E-10	-0.33	59	0.84	Gnb1l	Cytoskeleton
DMR11:86758001	11	86758001	86761000	3000	2	4.00E-09	0.44	78	2.6	Arvcf	Cytoskeleton
DMR11:86782001	11	86782001	86784000	2000	2	1.60E-08	0.39	27	1.35	Arvcf;Tango2	Cytoskeleton
DMR11:86909001	11	86909001	86914000	5000	3	2.20E-07	0.47	136	2.72	Zdhhc8;Ccdc188;LOC102555338	
DMR11:86985001	11	86985001	86992000	7000	1	4.50E-07	0.4	133	1.9	Rtn4r	
DMR11:87232001	11	87232001	87233000	1000	1	5.80E-07	0.39	18	1.8	Gsc2;Dgcr14;Tssk2;Tssk1b;Dgcr2	Development;Signaling
DMR11:87557001	11	87557001	87560000	3000	2	5.60E-20	0.68	52	1.73	Ccdc74a;Med15	
DMR11:87949001	11	87949001	87952000	3000	1	6.80E-07	-0.4	48	1.6	Pi4ka	Signaling
DMR11:88215001	11	88215001	88218000	3000	1	9.90E-14	0.7	27	0.9	Mapk1	Signaling
DMR11:88305001	11	88305001	88306000	1000	1	1.30E-16	0.54	10	1	Ppm1f	Signaling
DMR11:88332001	11	88332001	88333000	1000	1	4.50E-15	0.5	10	1	Ppm1f	Signaling
DMR11:88455001	11	88455001	88462000	7000	2	1.80E-08	-0.33	92	1.31	Spag6	Cytoskeleton
DMR11:88497001	11	88497001	88503000	6000	2	9.80E-11	-0.44	79	1.32	Spag6	Cytoskeleton
DMR11:88934001	11	88934001	88936000	2000	1	9.50E-07	0.51	16	0.8	Pkp2;LOC103693606	
DMR11:89288001	11	89288001	89291000	3000	1	1.00E-07	-0.5	9	0.3	Mzt2b;Prkdc;LOC102550200	Signaling
DMR11:89426001	11	89426001	89427000	1000	1	4.40E-11	-0.35	17	1.7	Prkdc	Signaling
DMR11:89491001	11	89491001	89497000	6000	3	2.10E-08	-0.39	62	1.03	Prkdc	Signaling
DMR11:89683001	11	89683001	89687000	4000	1	9.70E-07	-0.36	54	1.35	RGD1560337	
DMR12:498001	12	498001	5.00E+05	2000	1	2.60E-07	0.34	35	1.75	Zar1l;Brca2	DNA Repair
DMR12:1011001	12	1011001	1014000	3000	1	1.60E-08	0.42	57	1.9	LOC102548964;Stard13	Signaling
DMR12:1054001	12	1054001	1056000	2000	1	1.10E-07	0.37	29	1.45	Stard13	Signaling
DMR12:1124001	12	1124001	1130000	6000	1	4.20E-10	0.45	121	2.02	Stard13	Signaling
DMR12:1232001	12	1232001	1235000	3000	1	1.30E-07	0.44	49	1.63	Stard13;LOC108352405	Signaling
DMR12:1508001	12	1508001	1514000	6000	1	4.90E-08	0.28	111	1.85	LOC103693612;Rn5s	
DMR12:1804001	12	1804001	1805000	1000	1	4.50E-07	0.44	11	1.1	Insr	Receptor
DMR12:1807001	12	1807001	1808000	1000	1	1.90E-08	0.35	10	1	Insr	Receptor
DMR12:1995001	12	1995001	1997000	2000	2	6.50E-12	0.51	44	2.2	Arhgef18;Pex11g	
DMR12:2088001	12	2088001	2089000	1000	1	1.30E-10	0.36	17	1.7	Pnpla6	Metabolism
DMR12:2105001	12	2105001	2106000	1000	1	6.60E-07	-0.35	10	1	Pnpla6	Metabolism
DMR12:2107001	12	2107001	2110000	3000	1	2.30E-09	-0.33	29	0.97	Pnpla6	Metabolism
DMR12:2655001	12	2655001	2656000	1000	1	6.50E-07	0.5	11	1.1	LOC103690865;Cd209f	Transport
DMR12:2660001	12	2660001	2661000	1000	1	3.40E-09	0.6	12	1.2	LOC103690865;Cd209f	Transport
DMR12:2812001	12	2812001	2813000	1000	1	9.30E-08	-0.46	10	1	LOC108352406;Cd209b	
DMR12:4138001	12	4138001	4143000	5000	2	4.10E-08	-0.37	41	0.82	Vom2r60;LOC102555699	Signaling
DMR12:4317001	12	4317001	4322000	5000	1	2.00E-07	-0.34	71	1.42	Vom2r60	Signaling
DMR12:4329001	12	4329001	4335000	6000	3	2.00E-10	-0.37	62	1.03	Vom2r60	Signaling
DMR12:4355001	12	4355001	4361000	6000	1	6.20E-07	-0.3	53	0.88	Vom2r60	Signaling
DMR12:4421001	12	4421001	4423000	2000	1	7.10E-07	-0.28	16	0.8	Vom2r60	Signaling
DMR12:4439001	12	4439001	4440000	1000	1	4.10E-07	0.43	5	0.5	Vom2r60;Cers4	Signaling
DMR12:4786001	12	4786001	4789000	3000	1	2.90E-07	-0.27	22	0.73	Vom2r-ps96	
DMR12:5496001	12	5496001	5497000	1000	1	5.80E-12	-0.49	19	1.9	Zfp958	Transcription
DMR12:5588001	12	5588001	5592000	4000	1	7.70E-10	0.51	73	1.82	Fry	Cytoskeleton
DMR12:5643001	12	5643001	5645000	2000	1	8.30E-11	0.47	31	1.55	Fry	Cytoskeleton
DMR12:5819001	12	5819001	5821000	2000	1	1.70E-07	0.39	44	2.2	Fry	Cytoskeleton
DMR12:5969001	12	5969001	5970000	1000	1	1.40E-08	-0.38	27	2.7	Lnc001	
DMR12:5992001	12	5992001	5995000	3000	1	1.80E-08	-0.42	52	1.73	Lnc001;LOC100361192	
DMR12:6018001	12	6018001	6019000	1000	1	6.00E-08	0.34	14	1.4	Rxfp2;LOC102549667	Signaling
DMR12:6048001	12	6048001	6049000	1000	1	3.70E-07	0.33	23	2.3	Rxfp2	Signaling
DMR12:6733001	12	6733001	6734000	1000	1	6.10E-10	0.42	7	0.7	Medag	
DMR12:6920001	12	6920001	6921000	1000	1	5.10E-07	-0.41	20	2	LOC103691071;Trnan-guu;Uspl1	Protease
DMR12:8118001	12	8118001	8119000	1000	1	5.10E-22	0.85	15	1.5	Slc7a1;Mtus2	Transport
DMR12:8310001	12	8310001	8312000	2000	1	1.90E-07	-0.43	16	0.8	Mtus2	
DMR12:8494001	12	8494001	8497000	3000	1	6.00E-09	0.41	44	1.47	Mtus2	
DMR12:8749001	12	8749001	8750000	1000	1	3.30E-10	0.77	35	3.5	Slc46a3;Pomp	Transcription
DMR12:9166001	12	9166001	9167000	1000	1	3.20E-11	0.52	16	1.6	Flt1	Receptor

DMR12:9488001	12	9488001	9490000	2000	1	3.40E-10	0.5	21	1.05	Pdx1	
DMR12:9798001	12	9798001	9799000	1000	1	2.10E-07	-0.4	13	1.3	Lnx2;LOC103691017	
DMR12:9985001	12	9985001	9986000	1000	1	5.70E-07	0.4	20	2	Rasl11a	
DMR12:10324001	12	10324001	10325000	1000	1	1.80E-10	0.65	21	2.1	Wasf3	Cytoskeleton
DMR12:10517001	12	10517001	10520000	3000	1	1.50E-07	0.55	97	3.23	Cdk8	
DMR12:10552001	12	10552001	10555000	3000	1	5.00E-08	0.68	34	1.13	Cdk8	
DMR12:10629001	12	10629001	10633000	4000	1	6.80E-07	-0.34	27	0.68	Cyp3a18	Metabolism
DMR12:10712001	12	10712001	10714000	2000	1	6.30E-07	-0.22	34	1.7	Cyp3a73	
DMR12:11193001	12	11193001	11195000	2000	1	1.30E-07	-0.41	40	2	Zfp394;Atp5j2;Cpsf4	Translation
DMR12:11337001	12	11337001	11338000	1000	1	9.10E-08	0.49	9	0.9	LOC108352477;Kpna7	Transport
DMR12:11483001	12	11483001	11484000	1000	1	7.00E-08	-0.47	25	2.5	Smurf1	Proteolysis
DMR12:11686001	12	11686001	11687000	1000	1	3.00E-07	-0.39	8	0.8	Cyp3a2	Metabolism
DMR12:11828001	12	11828001	11831000	3000	1	7.20E-08	0.31	36	1.2	Tmem130	Signaling
DMR12:12194001	12	12194001	12196000	2000	1	4.80E-07	-0.44	36	1.8	LOC100360200;RGD1565131	
DMR12:12328001	12	12328001	12330000	2000	1	1.10E-07	0.37	27	1.35	Baiap2l1;Bri3;LOC102554669	Cytoskeleton
DMR12:12774001	12	12774001	12776000	2000	1	1.80E-09	0.45	26	1.3	Eif2ak1	Signaling
DMR12:12886001	12	12886001	12887000	1000	1	2.40E-07	0.49	20	2	Usp42;Cyth3	Protease;Transcription
DMR12:13418001	12	13418001	13419000	1000	1	1.90E-12	0.55	10	1	LOC102546559;Rbak	Transcription
DMR12:13747001	12	13747001	13749000	2000	1	3.00E-07	0.51	34	1.7	Fbxl18	
DMR12:13917001	12	13917001	13920000	3000	2	2.10E-08	0.38	74	2.47	Tnrc18;Slc29a4	Transcription;Transport
DMR12:14052001	12	14052001	14054000	2000	1	1.20E-07	0.37	35	1.75	Mmd2	Signaling
DMR12:14474001	12	14474001	14475000	1000	1	7.70E-09	0.41	22	2.2	Sdk1	
DMR12:14843001	12	14843001	14845000	2000	1	8.80E-07	0.61	49	2.45	Sdk1	
DMR12:15130001	12	15130001	15131000	1000	1	2.60E-07	0.38	8	0.8	Sdk1	
DMR12:15252001	12	15252001	15253000	1000	1	1.70E-07	-0.39	12	1.2	Sdk1	
DMR12:15370001	12	15370001	15371000	1000	1	1.80E-07	-0.5	15	1.5	Sdk1	
DMR12:15953001	12	15953001	15954000	1000	1	1.80E-07	0.56	6	0.6	Gna12	Signaling
DMR12:16064001	12	16064001	16065000	1000	1	2.00E-08	0.43	19	1.9	Iqce	
DMR12:16099001	12	16099001	16100000	1000	1	3.20E-08	0.48	16	1.6	Ttyh3	Transport
DMR12:16104001	12	16104001	16112000	8000	2	3.10E-07	0.35	159	1.99	Ttyh3;Lfng	Transport;Golgi
DMR12:16728001	12	16728001	16731000	3000	1	1.10E-15	0.69	55	1.83	Mad1l1	
DMR12:16761001	12	16761001	16763000	2000	2	2.90E-10	0.4	34	1.7	Elfn1;LOC108352426	Receptor
DMR12:16940001	12	16940001	16942000	2000	1	7.20E-07	0.4	23	1.15	Mafk;LOC102546414;Ints1	Transcription
DMR12:16985001	12	16985001	16986000	1000	1	5.30E-09	0.42	10	1	Micall2	
DMR12:17696001	12	17696001	17697000	1000	1	2.60E-13	0.73	19	1.9	Prkar1b	Signaling
DMR12:17755001	12	17755001	17756000	1000	1	7.20E-07	-0.41	30	3	Pdgfa	Growth Factors
DMR12:17953001	12	17953001	17954000	1000	1	7.50E-10	0.42	15	1.5	Fam20c	
DMR12:18100001	12	18100001	18103000	3000	2	3.20E-11	0.72	43	1.43	RGD1566386	Transcription
DMR12:18119001	12	18119001	18120000	1000	1	5.80E-08	-0.4	14	1.4	RGD1566386	Transcription
DMR12:18364001	12	18364001	18366000	2000	1	4.70E-07	-0.35	19	0.95	LOC100911071;RGD1560474	
DMR12:18622001	12	18622001	18624000	2000	1	8.70E-07	-0.39	16	0.8	RGD1560288	
DMR12:19022001	12	19022001	19026000	4000	1	1.50E-07	-0.38	30	0.75	Vamp7	Transcription
DMR12:19090001	12	19090001	19092000	2000	1	1.10E-07	-0.41	23	1.15	Cyp3a9	Metabolism
DMR12:19151001	12	19151001	19153000	2000	1	2.90E-10	-0.52	14	0.7	Rpl31l4	Translation
DMR12:19540001	12	19540001	19541000	1000	1	1.60E-08	0.25	6	0.6	LOC102551138;Nxpe5l1	
DMR12:19634001	12	19634001	19645000	11000	2	4.50E-12	-0.37	114	1.04	Stag3	Epigenetic
DMR12:19689001	12	19689001	19692000	3000	1	3.80E-09	-0.55	16	0.53	Pvrig;LOC108352428	
DMR12:20431001	12	20431001	20435000	4000	1	3.40E-07	-0.25	47	1.18	Vom2r-ps101	
DMR12:20957001	12	20957001	20959000	2000	1	2.10E-11	-0.4	22	1.1	Vom2r64	Signaling
DMR12:20981001	12	20981001	20986000	5000	1	1.00E-11	-0.32	45	0.9	Vom2r64	Signaling
DMR12:22116001	12	22116001	22117000	1000	1	9.50E-09	0.43	16	1.6	Agfg2;Irs3;Sap25	Epigenetic
DMR12:22185001	12	22185001	22186000	1000	1	3.70E-09	0.39	17	1.7	Tfr2;Actl6b	Protease;Cytoskeleton
DMR12:22217001	12	22217001	22220000	3000	1	1.30E-08	0.68	87	2.9	Actl6b;LOC108352483;Gnb2	Cytoskeleton;Signaling
DMR12:22294001	12	22294001	22297000	3000	1	1.50E-07	0.34	45	1.5	Zan	
DMR12:22425001	12	22425001	22427000	2000	1	9.90E-08	0.35	27	1.35	Ephb4;LOC103691283;Slc12a9	Receptor;Transport
DMR12:22467001	12	22467001	22469000	2000	2	8.30E-08	0.37	31	1.55	Srrt;Ufsp1;Ache	Metabolism

DMR12:22758001	12	22758001	22762000	4000	1	2.60E-10	0.55	44	1.1	Cldn15;Fis1	Cell Junction
DMR12:22766001	12	22766001	22768000	2000	1	1.40E-07	0.38	43	2.15	Fis1	
DMR12:23011001	12	23011001	23015000	4000	1	5.40E-08	0.41	72	1.8	Myl10	Cytoskeleton
DMR12:23172001	12	23172001	23175000	3000	1	4.60E-07	-0.42	23	0.77	Cux1	Development
DMR12:23622001	12	23622001	23623000	1000	1	2.70E-07	0.32	19	1.9	Rasa4	Signaling
DMR12:23879001	12	23879001	23880000	1000	1	2.40E-10	0.44	14	1.4	Srrm3	
DMR12:24221001	12	24221001	24223000	2000	1	6.20E-12	0.46	32	1.6	Hip1	Cytoskeleton
DMR12:24228001	12	24228001	24229000	1000	1	1.10E-07	0.41	13	1.3	Hip1	Cytoskeleton
DMR12:24289001	12	24289001	24293000	4000	1	1.60E-11	1.01	97	2.42	Hip1	Cytoskeleton
DMR12:24424001	12	24424001	24425000	1000	1	5.10E-08	0.55	6	0.6	Fkbp6	Transcription
DMR12:24548001	12	24548001	24550000	2000	1	4.20E-07	-0.39	33	1.65	Bcl7b	
DMR12:24661001	12	24661001	24663000	2000	1	2.00E-07	0.35	14	0.7	Vps37d;Dnajc30;Wbscr22	Transcription
DMR12:24801001	12	24801001	24805000	4000	1	8.10E-09	0.4	53	1.32	Wbscr28	
DMR12:24971001	12	24971001	24972000	1000	1	5.80E-08	0.45	20	2	LOC102553484;Eln	Development
DMR12:25197001	12	25197001	25199000	2000	1	1.30E-07	0.34	45	2.25	Clip2	Transcription
DMR12:28765001	12	28765001	28766000	1000	1	3.10E-07	-0.43	9	0.9	Wbscr17	
DMR12:28783001	12	28783001	28785000	2000	1	4.70E-12	0.51	64	3.2	Wbscr17	
DMR12:29142001	12	29142001	29144000	2000	1	9.60E-11	0.43	32	1.6	Wbscr17	
DMR12:29214001	12	29214001	29217000	3000	1	4.30E-09	-0.49	69	2.3	Wbscr17;LOC103690026	
DMR12:29615001	12	29615001	29622000	7000	1	7.00E-08	-0.29	84	1.2	Caln1;LOC108352439	
DMR12:29623001	12	29623001	29624000	1000	1	4.60E-07	0.37	15	1.5	Caln1;LOC108352439	
DMR12:29670001	12	29670001	29671000	1000	1	1.80E-10	-0.53	15	1.5	Caln1	
DMR12:29746001	12	29746001	29747000	1000	1	5.00E-07	0.35	22	2.2	Caln1	
DMR12:29857001	12	29857001	29859000	2000	1	1.30E-10	-0.62	46	2.3	Tyw1	Metabolism
DMR12:29896001	12	29896001	29897000	1000	1	2.10E-09	0.44	21	2.1	Tyw1;LOC103691321	Metabolism
DMR12:30002001	12	30002001	30004000	2000	1	4.50E-07	0.34	24	1.2	Rabgef1	Transcription
DMR12:30224001	12	30224001	30226000	2000	1	3.30E-11	0.52	33	1.65	Gusb;Vkorc1l1	Metabolism
DMR12:30538001	12	30538001	30540000	2000	1	4.50E-07	0.4	34	1.7	Gbas	
DMR12:30669001	12	30669001	30670000	1000	1	5.00E-08	0.36	25	2.5	Zfp11	
DMR12:31446001	12	31446001	31448000	2000	1	2.80E-08	-0.51	43	2.15	Rimbp2	
DMR12:31488001	12	31488001	31489000	1000	1	1.80E-07	0.43	14	1.4	Rimbp2	
DMR12:31606001	12	31606001	31608000	2000	1	1.10E-09	0.47	30	1.5	Rimbp2;Piwil1	Translation
DMR12:31935001	12	31935001	31937000	2000	2	8.40E-15	0.68	36	1.8	Tmem132d	
DMR12:32132001	12	32132001	32133000	1000	1	4.50E-09	0.85	28	2.8	Tmem132d	
DMR12:32180001	12	32180001	32182000	2000	1	8.30E-07	0.34	35	1.75	Tmem132d	
DMR12:32382001	12	32382001	32386000	4000	1	1.10E-10	-0.39	55	1.38	Tmem132d	
DMR12:32552001	12	32552001	32554000	2000	1	3.00E-07	0.42	35	1.75	Tmem132d	
DMR12:32671001	12	32671001	32672000	1000	1	9.30E-07	0.33	12	1.2	Glt1d1	
DMR12:32692001	12	32692001	32693000	1000	1	6.80E-08	0.48	22	2.2	Glt1d1	
DMR12:36267001	12	36267001	36270000	3000	1	6.40E-07	0.28	44	1.47	Tmem132b	
DMR12:36359001	12	36359001	36360000	1000	1	8.40E-08	0.44	16	1.6	Tmem132b	
DMR12:36682001	12	36682001	36685000	3000	1	1.10E-08	0.46	51	1.7	Scarb1	Transport
DMR12:36961001	12	36961001	36963000	2000	1	2.60E-08	0.4	55	2.75	Ncor2	Epigenetic
DMR12:36972001	12	36972001	36973000	1000	1	2.50E-07	0.36	17	1.7	Ncor2	Epigenetic
DMR12:37011001	12	37011001	37013000	2000	1	7.40E-07	0.38	36	1.8	Ncor2	Epigenetic
DMR12:37018001	12	37018001	37020000	2000	1	2.60E-12	0.49	57	2.85	Ncor2	Epigenetic
DMR12:37021001	12	37021001	37025000	4000	1	2.00E-09	0.47	126	3.15	Ncor2	Epigenetic
DMR12:37029001	12	37029001	37033000	4000	2	2.90E-09	0.54	105	2.62	Ncor2;Fam101a	Epigenetic
DMR12:37043001	12	37043001	37046000	3000	1	4.80E-07	0.36	48	1.6	Ncor2;Fam101a	Epigenetic
DMR12:37307001	12	37307001	37308000	1000	1	4.50E-08	0.49	12	1.2	Dnah10	
DMR12:37309001	12	37309001	37311000	2000	1	2.70E-07	0.43	31	1.55	Dnah10	
DMR12:37326001	12	37326001	37328000	2000	1	1.30E-10	0.47	129	6.45	Dnah10;LOC103691347	
DMR12:37578001	12	37578001	37579000	1000	1	3.80E-08	-0.53	16	1.6	Kmt5a	Golgi
DMR12:37591001	12	37591001	37592000	1000	1	9.70E-08	0.4	13	1.3	Kmt5a;Sbno1	Golgi
DMR12:37807001	12	37807001	37810000	3000	1	1.80E-07	0.34	59	1.97	Pitpnm2	Transport
DMR12:37875001	12	37875001	37877000	2000	1	8.70E-07	0.44	25	1.25	Pitpnm2	Transport
DMR12:37895001	12	37895001	37901000	6000	1	1.70E-12	0.58	163	2.72	Pitpnm2;Arl6ip4;Ogfod2;LOC103691351	Transport;Metabolism
DMR12:38196001	12	38196001	38197000	1000	1	4.40E-11	0.5	13	1.3	Kntc1	
DMR12:38407001	12	38407001	38408000	1000	1	1.20E-07	0.32	18	1.8	Clip1	Transcription
DMR12:38843001	12	38843001	38848000	5000	1	1.30E-07	0.45	126	2.52	Hpd;LOC100359816	Metabolism

DMR12:39093001	12	39093001	39096000	3000	1	1.40E-07	-0.46	62	2.07	Kdm2b	
DMR12:39126001	12	39126001	39127000	1000	1	4.00E-08	-0.53	23	2.3	Kdm2b	
DMR12:40049001	12	40049001	40053000	4000	1	2.20E-10	0.43	76	1.9	Cux2	Development
DMR12:40110001	12	40110001	40111000	1000	1	2.20E-07	0.37	16	1.6	Cux2	Development
DMR12:40122001	12	40122001	40123000	1000	1	8.70E-09	0.45	18	1.8	Cux2	Development
DMR12:40193001	12	40193001	40196000	3000	1	8.10E-08	0.39	53	1.77	Cux2	Development
DMR12:40204001	12	40204001	40205000	1000	1	2.60E-07	0.38	22	2.2	Cux2	Development
DMR12:40660001	12	40660001	40661000	1000	1	3.10E-07	-0.49	15	1.5	Naa25;LOC100364561	Metabolism
DMR12:40671001	12	40671001	40672000	1000	1	1.20E-07	0.49	10	1	Naa25;LOC100364561	Metabolism
DMR12:40790001	12	40790001	40792000	2000	1	6.70E-07	0.35	39	1.95	Hectd4	Proteolysis
DMR12:41147001	12	41147001	41150000	3000	1	6.80E-07	0.36	46	1.53	Rph3a;Oas1i	Metabolism
DMR12:41363001	12	41363001	41365000	2000	1	4.90E-11	0.62	34	1.7	Oas3;Oas2	Metabolism
DMR12:41403001	12	41403001	41406000	3000	1	1.30E-15	0.49	37	1.23	Dtx1	Proteolysis
DMR12:41502001	12	41502001	41504000	2000	1	1.50E-11	0.58	24	1.2	Iqcd;Tpcn1	Transport
DMR12:41519001	12	41519001	41522000	3000	1	1.50E-10	0.4	58	1.93	Tpcn1	Transport
DMR12:41654001	12	41654001	41655000	1000	1	8.30E-07	0.52	9	0.9	Sdsl;Lhx5	Metabolism;Development
DMR12:41660001	12	41660001	41661000	1000	1	4.20E-11	0.61	19	1.9	Lhx5;LOC108352452	Development
DMR12:41904001	12	41904001	41905000	1000	1	8.60E-07	0.35	19	1.9	Rbm19	Translation
DMR12:41934001	12	41934001	41937000	3000	1	7.10E-08	0.42	55	1.83	Rbm19	Translation
DMR12:42074001	12	42074001	42076000	2000	1	4.60E-07	0.37	32	1.6	Tbx5	Transcription
DMR12:42086001	12	42086001	42088000	2000	1	1.00E-06	0.34	43	2.15	Tbx5;LOC102549962	Transcription
DMR12:42482001	12	42482001	42484000	2000	1	4.40E-07	0.4	89	4.45	Tbx3	Transcription
DMR12:43519001	12	43519001	43522000	3000	1	1.90E-08	0.49	47	1.57	Med13l	
DMR12:44020001	12	44020001	44023000	3000	1	1.70E-07	0.54	31	1.03	Hrk	
DMR12:44052001	12	44052001	44056000	4000	1	5.10E-07	0.73	64	1.6	Fbxw8	
DMR12:44071001	12	44071001	44075000	4000	2	5.90E-07	0.36	73	1.82	Fbxw8	
DMR12:44226001	12	44226001	44228000	2000	1	3.10E-10	0.61	40	2	Nos1	Metabolism
DMR12:44268001	12	44268001	44270000	2000	1	4.30E-09	0.39	31	1.55	Nos1;Fbxo21	Metabolism
DMR12:44731001	12	44731001	44732000	1000	1	1.30E-09	0.41	9	0.9	Ksr2	Signaling
DMR12:44762001	12	44762001	44763000	1000	1	2.50E-07	0.38	21	2.1	Ksr2	Signaling
DMR12:45079001	12	45079001	45080000	1000	1	1.10E-09	0.34	3	0.3	Taok3	Signaling
DMR12:45771001	12	45771001	45774000	3000	1	3.50E-07	0.46	53	1.77	Srrm4	
DMR12:45877001	12	45877001	45878000	1000	1	9.10E-07	0.47	14	1.4	Srrm4	
DMR12:46089001	12	46089001	46097000	8000	1	4.10E-14	0.5	137	1.71	Ccdc60	
DMR12:46114001	12	46114001	46116000	2000	1	1.10E-08	0.35	29	1.45	Ccdc60	
DMR12:46129001	12	46129001	46136000	7000	1	2.60E-07	0.31	108	1.54	Ccdc60	
DMR12:46144001	12	46144001	46149000	5000	1	8.10E-07	0.33	74	1.48	Ccdc60	
DMR12:46171001	12	46171001	46172000	1000	1	1.40E-07	0.51	9	0.9	Ccdc60	
DMR12:46378001	12	46378001	46381000	3000	1	1.70E-10	0.5	61	2.03	Cit	Signaling
DMR12:46437001	12	46437001	46440000	3000	1	2.90E-09	0.41	49	1.63	Cit	Signaling
DMR12:46670001	12	46670001	46673000	3000	1	2.30E-08	-0.44	47	1.57	Bicdl1	Transport
DMR12:46752001	12	46752001	46756000	4000	1	8.60E-09	0.33	91	2.28	Gcn1l1	
DMR12:46801001	12	46801001	46806000	5000	2	6.20E-08	0.37	102	2.04	Rplp0;Pxn;LOC108352464	Translation;Cytoskeleton
DMR12:46877001	12	46877001	46879000	2000	1	5.50E-12	-0.67	37	1.85	Sirt4;Pla2g1b	Metabolism
DMR12:47117001	12	47117001	47122000	5000	1	8.00E-08	0.39	70	1.4	Rnf10	
DMR12:47446001	12	47446001	47448000	2000	1	1.10E-07	0.39	41	2.05	RGD1311899;Oasl	Metabolism
DMR12:47731001	12	47731001	47732000	1000	1	1.10E-07	0.52	12	1.2	Trpv4	Transport
DMR12:47747001	12	47747001	47751000	4000	2	2.70E-08	0.45	152	3.8	Trpv4;Fam222a	Transport
DMR12:47922001	12	47922001	47924000	2000	1	2.70E-11	0.51	83	4.15	Mvk;Mmab	Metabolism;Metabolism
DMR12:47948001	12	47948001	47951000	3000	1	6.10E-08	0.42	56	1.87	Ube3b	Proteolysis
DMR12:48011001	12	48011001	48013000	2000	1	2.00E-11	0.44	67	3.35	Kctd10;LOC108352465	Cytoskeleton
DMR12:48044001	12	48044001	48045000	1000	1	3.00E-07	0.56	24	2.4	Myo1h	
DMR12:48317001	12	48317001	48323000	6000	1	9.00E-08	0.39	118	1.97	Usp30;Svop	Protease;Transport
DMR12:48354001	12	48354001	48359000	5000	2	6.60E-08	0.36	70	1.4	Svop;Dao	Transport;Metabolism
DMR12:48798001	12	48798001	48799000	1000	1	7.20E-08	0.39	15	1.5	Cmklr1	Signaling
DMR12:49392001	12	49392001	49393000	1000	1	2.60E-09	0.4	19	1.9	Sgsm1;Tmem211	Signaling
DMR12:49407001	12	49407001	49408000	1000	1	2.60E-11	0.45	6	0.6	Tmem211;LOC108352470	
DMR12:49483001	12	49483001	49486000	3000	1	4.60E-09	0.62	68	2.27	RGD1306556	
DMR12:49487001	12	49487001	49489000	2000	2	1.30E-08	0.5	28	1.4	RGD1306556	
DMR12:49499001	12	49499001	49503000	4000	1	3.70E-07	0.32	88	2.2	RGD1306556;LOC102552141	

DMR12:49622001	12	49622001	49624000	2000	1	1.50E-08	0.52	22	1.1	Grk3	Signaling
DMR12:49812001	12	49812001	49814000	2000	1	3.60E-09	0.55	12	0.6	Myo18b	
DMR12:49877001	12	49877001	49879000	2000	1	6.70E-07	0.35	28	1.4	Myo18b	
DMR12:50222001	12	50222001	50226000	4000	1	7.00E-11	0.52	66	1.65	Sez6l;LOC102552378	
DMR12:50369001	12	50369001	50371000	2000	1	1.00E-11	0.6	24	1.2	Tpst2	Transport
DMR12:50456001	12	50456001	50459000	3000	1	6.90E-07	0.35	36	1.2	LOC103691455;Miat	
DMR12:51351001	12	51351001	51352000	1000	1	1.10E-07	-0.51	9	0.9	Pitpnb;LOC102554233	Transport
DMR12:51400001	12	51400001	51404000	4000	1	1.10E-14	0.61	71	1.77	Ttc28	Cytoskeleton
DMR12:51408001	12	51408001	51410000	2000	1	3.70E-10	0.6	41	2.05	Ttc28	Cytoskeleton
DMR12:51426001	12	51426001	51432000	6000	1	1.50E-11	0.59	90	1.5	Ttc28	Cytoskeleton
DMR12:51492001	12	51492001	51494000	2000	2	4.00E-11	0.44	27	1.35	Ttc28;LOC102553561	Cytoskeleton
DMR12:51525001	12	51525001	51527000	2000	1	1.80E-07	0.5	32	1.6	Ttc28	Cytoskeleton
DMR12:51540001	12	51540001	51542000	2000	1	6.00E-08	0.38	32	1.6	Ttc28;LOC102553486	Cytoskeleton
DMR12:51625001	12	51625001	51626000	1000	1	7.30E-09	-0.5	9	0.9	Ttc28	Cytoskeleton
DMR12:51655001	12	51655001	51656000	1000	1	3.60E-13	0.42	26	2.6	Ttc28	Cytoskeleton
DMR12:51722001	12	51722001	51723000	1000	1	2.00E-18	0.61	16	1.6	Ttc28;LOC108352475;LOC102553196	Cytoskeleton
DMR12:51729001	12	51729001	51730000	1000	1	1.50E-10	0.67	21	2.1	Ttc28;LOC102553196	Cytoskeleton
DMR12:51734001	12	51734001	51736000	2000	2	7.60E-13	0.48	25	1.25	Ttc28;LOC102553196	Cytoskeleton
DMR12:51749001	12	51749001	51752000	3000	1	1.00E-26	0.68	39	1.3	Ttc28	Cytoskeleton
DMR12:51756001	12	51756001	51757000	1000	1	1.60E-09	0.77	0	0	Ttc28	Cytoskeleton
DMR12:51798001	12	51798001	51805000	7000	1	4.60E-07	-0.31	78	1.11	Ttc28	Cytoskeleton
DMR13:239001	13	239001	242000	3000	1	1.60E-08	-0.31	24	0.8	Cntnap5a	
DMR13:249001	13	249001	255000	6000	2	2.20E-10	-0.34	65	1.08	Cntnap5a	
DMR13:6694001	13	6694001	6697000	3000	1	8.10E-07	-0.53	10	0.33	Cntnap5c	
DMR13:6739001	13	6739001	6745000	6000	1	1.70E-09	0.47	44	0.73	Cntnap5c	
DMR13:7063001	13	7063001	7067000	4000	1	4.90E-10	-0.41	34	0.85	Cntnap5c	
DMR13:7070001	13	7070001	7071000	1000	1	5.90E-11	0.65	9	0.9	Cntnap5c	
DMR13:7224001	13	7224001	7226000	2000	1	2.50E-07	0.57	6	0.3	Cntnap5c	
DMR13:7353001	13	7353001	7357000	4000	1	4.90E-07	-0.25	36	0.9	Cntnap5c	
DMR13:7394001	13	7394001	7395000	1000	1	1.30E-09	-0.53	12	1.2	Cntnap5c	
DMR13:7455001	13	7455001	7459000	4000	1	1.70E-07	-0.36	39	0.98	Cntnap5c	
DMR13:7491001	13	7491001	7492000	1000	1	6.30E-08	-0.43	10	1	Cntnap5c	
DMR13:7517001	13	7517001	7522000	5000	1	6.60E-14	0.42	50	1	Cntnap5c	
DMR13:19217001	13	19217001	19220000	3000	1	7.60E-10	-0.44	28	0.93	LOC100362382;RGD1561440	
DMR13:21801001	13	21801001	21805000	4000	1	2.40E-10	-0.35	42	1.05	Cntnap5b	
DMR13:21806001	13	21806001	21807000	1000	1	3.90E-10	-0.34	14	1.4	Cntnap5b	
DMR13:21871001	13	21871001	21872000	1000	1	4.90E-09	-0.36	12	1.2	Cntnap5b	
DMR13:22064001	13	22064001	22067000	3000	3	2.30E-11	-0.43	29	0.97	Cntnap5b	
DMR13:22097001	13	22097001	22104000	7000	1	1.80E-07	-0.44	85	1.21	Cntnap5b	
DMR13:22136001	13	22136001	22139000	3000	1	1.50E-07	-0.39	29	0.97	Cntnap5b	
DMR13:22264001	13	22264001	22267000	3000	2	1.10E-11	-0.38	26	0.87	Cntnap5b	
DMR13:22447001	13	22447001	22450000	3000	1	1.90E-07	-0.55	10	0.33	Cntnap5b	
DMR13:24760001	13	24760001	24767000	7000	1	1.20E-10	-0.34	59	0.84	Cdh20	Cytoskeleton
DMR13:24851001	13	24851001	24853000	2000	1	9.20E-11	-0.39	49	2.45	Cdh20	Cytoskeleton
DMR13:25203001	13	25203001	25204000	1000	1	7.90E-10	0.49	13	1.3	Rnf152	
DMR13:25521001	13	25521001	25522000	1000	1	1.40E-07	0.37	7	0.7	Pign	Extracellular Matrix
DMR13:25554001	13	25554001	25561000	7000	1	4.90E-07	-0.29	82	1.17	Pign	Extracellular Matrix
DMR13:25666001	13	25666001	25669000	3000	2	9.80E-13	-0.58	26	0.87	Pign;RGD1307235	Extracellular Matrix
DMR13:25761001	13	25761001	25766000	5000	1	4.40E-07	-0.29	63	1.26	RGD1307235	
DMR13:26039001	13	26039001	26040000	1000	1	2.70E-07	-0.46	23	2.3	Zcchc2	
DMR13:26180001	13	26180001	26182000	2000	1	1.00E-08	0.3	20	1	Phlpp1	Cytoskeleton
DMR13:26223001	13	26223001	26224000	1000	1	2.40E-09	0.42	8	0.8	Phlpp1	Cytoskeleton
DMR13:26252001	13	26252001	26254000	2000	1	3.10E-08	-0.43	28	1.4	Phlpp1	Cytoskeleton
DMR13:26317001	13	26317001	26320000	3000	1	1.40E-09	0.51	31	1.03	Phlpp1	Cytoskeleton
DMR13:26327001	13	26327001	26336000	9000	3	9.80E-11	-0.44	95	1.06	Phlpp1	Cytoskeleton
DMR13:26714001	13	26714001	26716000	2000	1	3.30E-07	-0.4	29	1.45	Bcl2	
DMR13:26740001	13	26740001	26743000	3000	3	1.60E-13	0.66	96	3.2	Bcl2	
DMR13:26928001	13	26928001	26929000	1000	1	3.80E-09	-0.31	11	1.1	Serpinb5	Protease; Proteolysis
DMR13:26962001	13	26962001	26963000	1000	1	1.20E-07	0.5	6	0.6	Serpinb12	Protease; Proteolysis

DMR13:27267001	13	27267001	27268000	1000	1	8.00E-07	-0.39	6	0.6	Serpinb11;LOC680414	Protease; Proteolysis
DMR13:27864001	13	27864001	27867000	3000	2	1.40E-12	-0.55	20	0.67	Serpinb8	Protease; Proteolysis
DMR13:32457001	13	32457001	32459000	2000	1	1.10E-07	-0.43	15	0.75	Cdh19	Cytoskeleton
DMR13:34230001	13	34230001	34234000	4000	2	5.50E-10	-0.37	59	1.48	Tsn	
DMR13:34893001	13	34893001	34894000	1000	1	1.60E-07	0.5	11	1.1	Gli2	Transcription
DMR13:35055001	13	35055001	35056000	1000	1	4.50E-07	0.33	11	1.1	Gli2	Transcription
DMR13:35645001	13	35645001	35646000	1000	1	4.50E-08	-0.37	17	1.7	Epb4.1l5	
DMR13:37508001	13	37508001	37514000	6000	2	1.80E-09	-0.35	57	0.95	Ddx18	
DMR13:39481001	13	39481001	39482000	1000	1	1.70E-07	-0.42	11	1.1	Dpp10	Protease
DMR13:39483001	13	39483001	39487000	4000	1	8.10E-07	-0.28	44	1.1	Dpp10	Protease
DMR13:39894001	13	39894001	39895000	1000	1	1.80E-07	-0.39	10	1	Dpp10	Protease
DMR13:40099001	13	40099001	40100000	1000	1	3.90E-07	0.48	7	0.7	Dpp10	Protease
DMR13:40207001	13	40207001	40212000	5000	1	2.90E-18	-0.54	39	0.78	Dpp10	Protease
DMR13:40323001	13	40323001	40324000	1000	1	1.70E-10	-0.33	13	1.3	Dpp10	Protease
DMR13:41093001	13	41093001	41095000	2000	1	4.50E-07	-0.31	11	0.55	Dpp10	Protease
DMR13:41120001	13	41120001	41127000	7000	3	1.00E-08	-0.44	101	1.44	Dpp10	Protease
DMR13:42127001	13	42127001	42128000	1000	1	1.60E-08	0.62	19	1.9	Gpr39	Signaling
DMR13:42130001	13	42130001	42139000	9000	3	1.30E-10	-0.35	88	0.98	Gpr39	Signaling
DMR13:42425001	13	42425001	42432000	7000	2	5.60E-08	-0.39	91	1.3	Nckap5	
DMR13:42624001	13	42624001	42628000	4000	3	4.80E-12	-0.44	47	1.18	Nckap5	
DMR13:42790001	13	42790001	42793000	3000	1	7.30E-08	-0.29	55	1.83	Nckap5	
DMR13:42966001	13	42966001	42967000	1000	1	3.10E-14	0.69	4	0.4	Nckap5	
DMR13:44222001	13	44222001	44224000	2000	1	1.10E-07	0.38	24	1.2	Tmem163	
DMR13:44645001	13	44645001	44646000	1000	1	8.90E-08	0.35	12	1.2	Rab3gap1	Signaling
DMR13:44673001	13	44673001	44674000	1000	1	3.00E-09	0.43	55	5.5	Zranb3	Transcription
DMR13:44687001	13	44687001	44688000	1000	1	9.00E-07	0.4	25	2.5	Zranb3	Transcription
DMR13:45133001	13	45133001	45135000	2000	1	2.00E-07	0.35	27	1.35	Dars	
DMR13:45321001	13	45321001	45322000	1000	1	3.10E-08	-0.43	18	1.8	Cxcr4	
DMR13:46398001	13	46398001	46405000	7000	2	4.90E-10	-0.33	87	1.24	Thsd7b	Cytoskeleton
DMR13:46545001	13	46545001	46546000	1000	1	4.40E-08	0.58	5	0.5	Thsd7b	Cytoskeleton
DMR13:46740001	13	46740001	46745000	5000	2	3.20E-07	-0.3	55	1.1	Thsd7b	Cytoskeleton
DMR13:47272001	13	47272001	47277000	5000	1	6.10E-07	-0.27	55	1.1	Zp3r	
DMR13:47306001	13	47306001	47309000	3000	1	2.10E-07	-0.34	25	0.83	Zp3r	
DMR13:47449001	13	47449001	47450000	1000	1	7.70E-07	0.5	10	1	Pfkfb2;Yod1;LOC498222	Metabolism;Protease
DMR13:47586001	13	47586001	47588000	2000	1	9.00E-09	0.41	46	2.3	Pigr	Immune
DMR13:48598001	13	48598001	48601000	3000	1	7.00E-07	-0.41	41	1.37	Pm20d1;Slc41a1	Metabolism;Transport
DMR13:49084001	13	49084001	49085000	1000	1	7.00E-07	0.36	33	3.3	Klhdc8a;Nuak2	Signaling
DMR13:49105001	13	49105001	49107000	2000	2	5.30E-08	0.61	35	1.75	Nuak2	Signaling
DMR13:49127001	13	49127001	49129000	2000	1	8.80E-08	-0.46	19	0.95	Tmcc2	
DMR13:49286001	13	49286001	49291000	5000	1	3.40E-08	0.43	99	1.98	Rbbp5;Tmem81;Cntn2	
DMR13:49329001	13	49329001	49331000	2000	1	1.60E-07	0.42	24	1.2	Nfasc	
DMR13:49397001	13	49397001	49401000	4000	1	1.30E-08	0.4	63	1.57	Nfasc	
DMR13:49477001	13	49477001	49478000	1000	1	6.10E-07	-0.32	17	1.7	Nfasc	
DMR13:49863001	13	49863001	49865000	2000	1	7.70E-11	0.44	27	1.35	Pik3c2b	Signaling
DMR13:49879001	13	49879001	49881000	2000	1	5.10E-07	0.38	27	1.35	Pik3c2b	Signaling
DMR13:50102001	13	50102001	50103000	1000	1	7.20E-07	0.34	14	1.4	Atp2b4	Transport
DMR13:50473001	13	50473001	50475000	2000	1	5.70E-07	0.4	39	1.95	Sox13;Etnk2	Signaling
DMR13:50557001	13	50557001	50561000	4000	2	2.70E-10	0.45	89	2.22	Golt1a;Plekha6	Metabolism
DMR13:50724001	13	50724001	50726000	2000	1	2.30E-08	0.34	41	2.05	Optc	Extracellular Matrix
DMR13:50754001	13	50754001	50756000	2000	1	7.20E-07	0.38	35	1.75	Optc;Prelp;LOC102557118	Extracellular Matrix
DMR13:51085001	13	51085001	51087000	2000	1	1.90E-08	0.54	53	2.65	Adora1	Signaling
DMR13:51152001	13	51152001	51153000	1000	1	2.00E-07	0.41	21	2.1	Ppfia4	
DMR13:51158001	13	51158001	51160000	2000	1	2.10E-11	0.45	34	1.7	Ppfia4	
DMR13:51171001	13	51171001	51174000	3000	1	5.40E-08	0.61	66	2.2	Ppfia4	
DMR13:51713001	13	51713001	51716000	3000	1	1.50E-07	0.42	45	1.5	Ppp1r12b	Signaling
DMR13:51744001	13	51744001	51746000	2000	1	4.50E-12	0.51	52	2.6	Ppp1r12b	Signaling
DMR13:52155001	13	52155001	52158000	3000	1	3.10E-07	0.37	42	1.4	Lmod1	Cytoskeleton
DMR13:52172001	13	52172001	52174000	2000	1	3.70E-07	0.33	32	1.6	Lmod1	Cytoskeleton
DMR13:52220001	13	52220001	52221000	1000	1	6.70E-12	0.63	6	0.6	Ipo9	Transport
DMR13:52260001	13	52260001	52263000	3000	1	1.00E-08	0.5	43	1.43	Ipo9;Nav1	Transport
DMR13:52282001	13	52282001	52286000	4000	1	5.50E-08	-0.51	65	1.62	Nav1	

DMR13:52685001	13	52685001	52686000	1000	1	5.10E-11	0.5	10	1	Tnnt2	Cytoskeleton
DMR13:52873001	13	52873001	52874000	1000	1	3.40E-07	0.39	18	1.8	Tmem9	
DMR13:53015001	13	53015001	53018000	3000	1	1.00E-07	0.38	55	1.83	Kif21b	Cytoskeleton
DMR13:53030001	13	53030001	53032000	2000	1	9.60E-07	0.49	31	1.55	Kif21b;LOC498236	Cytoskeleton
DMR13:53452001	13	53452001	53454000	2000	1	1.20E-07	-0.5	31	1.55	RGD1562613	
DMR13:53814001	13	53814001	53815000	1000	1	1.40E-07	-0.51	17	1.7	Nr5a2	Transcription
DMR13:55558001	13	55558001	55559000	1000	1	2.80E-09	-0.4	17	1.7	Nek7	Signaling
DMR13:55846001	13	55846001	55847000	1000	1	2.50E-07	-0.4	12	1.2	Lhx9	Development
DMR13:56013001	13	56013001	56014000	1000	1	4.00E-07	-0.43	17	1.7	Dennd1b	
DMR13:56110001	13	56110001	56114000	4000	1	2.10E-07	-0.5	21	0.52	Dennd1b	
DMR13:56173001	13	56173001	56174000	1000	1	2.70E-07	-0.44	17	1.7	Dennd1b	
DMR13:56549001	13	56549001	56550000	1000	1	7.90E-07	-0.55	17	1.7	Zbtb41;Aspm	Transcription
DMR13:56568001	13	56568001	56574000	6000	1	9.90E-07	-0.28	72	1.2	Aspm	
DMR13:56668001	13	56668001	56669000	1000	1	7.10E-09	-0.38	9	0.9	Cfhr2	
DMR13:57023001	13	57023001	57026000	3000	1	5.50E-07	-0.33	23	0.77	Cfh	
DMR13:57050001	13	57050001	57054000	4000	1	1.40E-07	-0.24	54	1.35	Cfh	
DMR13:57239001	13	57239001	57240000	1000	1	9.20E-07	-0.43	12	1.2	Kcnt2	Transport
DMR13:57324001	13	57324001	57326000	2000	1	2.20E-12	-0.48	16	0.8	Kcnt2	Transport
DMR13:60502001	13	60502001	60504000	2000	1	8.00E-07	-0.4	26	1.3	Cdc73;LOC108352545	
DMR13:60585001	13	60585001	60587000	2000	1	2.50E-07	-0.5	9	0.45	Uchl5	Protease
DMR13:62652001	13	62652001	62657000	5000	2	1.40E-08	-0.31	61	1.22	RGD1565548	
DMR13:63690001	13	63690001	63692000	2000	1	1.20E-07	-0.47	12	0.6	Brinp3	
DMR13:63747001	13	63747001	63753000	6000	1	4.00E-10	-0.36	69	1.15	Brinp3	
DMR13:63878001	13	63878001	63880000	2000	1	2.50E-08	-0.28	18	0.9	Brinp3	
DMR13:67540001	13	67540001	67541000	1000	1	4.10E-07	-0.38	9	0.9	Pdc	
DMR13:67632001	13	67632001	67633000	1000	1	7.60E-08	-0.49	14	1.4	Tpr	Transport
DMR13:67640001	13	67640001	67645000	5000	3	1.20E-15	-0.49	49	0.98	Tpr	Transport
DMR13:67820001	13	67820001	67821000	1000	1	7.30E-07	0.4	16	1.6	Hmcn1	
DMR13:68113001	13	68113001	68115000	2000	1	6.40E-12	-0.38	18	0.9	Hmcn1	
DMR13:68137001	13	68137001	68138000	1000	1	3.30E-10	0.47	5	0.5	Hmcn1	
DMR13:68235001	13	68235001	68238000	3000	1	4.80E-07	-0.34	11	0.37	Hmcn1	
DMR13:68709001	13	68709001	68710000	1000	1	1.40E-10	-0.58	17	1.7	Ivns1abp	Cytoskeleton
DMR13:68975001	13	68975001	68978000	3000	1	1.10E-07	0.39	45	1.5	Fam129a	
DMR13:69037001	13	69037001	69039000	2000	1	5.50E-07	0.58	18	0.9	Fam129a	
DMR13:69046001	13	69046001	69047000	1000	1	1.30E-09	0.45	16	1.6	Fam129a	
DMR13:69284001	13	69284001	69285000	1000	1	9.50E-08	0.35	19	1.9	RGD1309104	
DMR13:69353001	13	69353001	69356000	3000	1	3.90E-08	0.35	59	1.97	RGD1309104	
DMR13:69784001	13	69784001	69790000	6000	2	4.70E-10	-0.35	71	1.18	Tsen15;Colgalt2	Golgi
DMR13:69930001	13	69930001	69932000	2000	1	3.50E-07	0.43	33	1.65	Rgl1	Transcription
DMR13:69961001	13	69961001	69962000	1000	1	4.00E-07	0.35	10	1	Rgl1;LOC102551528	Transcription
DMR13:70033001	13	70033001	70037000	4000	1	9.40E-07	0.34	54	1.35	Rgl1	Transcription
DMR13:70981001	13	70981001	70982000	1000	1	4.50E-09	0.57	17	1.7	Npl	Metabolism
DMR13:71107001	13	71107001	71109000	2000	1	1.00E-07	0.34	31	1.55	Rgs8	Signaling
DMR13:71112001	13	71112001	71114000	2000	1	1.00E-10	0.53	34	1.7	Rgs8	Signaling
DMR13:71140001	13	71140001	71143000	3000	1	6.70E-12	0.53	59	1.97	Rgs8;LOC108352555	Signaling
DMR13:71526001	13	71526001	71529000	3000	1	9.20E-08	0.59	24	0.8	Trnaw-cca	
DMR13:71942001	13	71942001	71943000	1000	1	1.10E-07	0.46	8	0.8	Cacna1e;LOC108352605	Transport
DMR13:72006001	13	72006001	72008000	2000	1	5.30E-07	0.43	13	0.65	Cacna1e	Transport
DMR13:72016001	13	72016001	72021000	5000	2	5.50E-07	-0.28	48	0.96	Cacna1e	Transport
DMR13:72053001	13	72053001	72054000	1000	1	1.60E-08	0.54	3	0.3	Cacna1e	Transport
DMR13:72270001	13	72270001	72271000	1000	1	4.30E-07	-0.6	15	1.5	Cacna1e	Transport
DMR13:72416001	13	72416001	72418000	2000	1	8.90E-08	0.34	20	1	Cacna1e	Transport
DMR13:72781001	13	72781001	72783000	2000	1	1.20E-10	0.42	27	1.35	Mr1;LOC102552997	Immune
DMR13:72817001	13	72817001	72820000	3000	2	5.50E-10	0.51	80	2.67	Stx6	Transcription
DMR13:72947001	13	72947001	72948000	1000	1	4.20E-08	-0.43	26	2.6	Xpr1;LOC108352558	Transport
DMR13:73144001	13	73144001	73145000	1000	1	9.20E-11	-0.62	17	1.7	RGD1561635	
DMR13:73154001	13	73154001	73157000	3000	1	5.80E-07	0.57	43	1.43	RGD1561635	
DMR13:73236001	13	73236001	73239000	3000	1	4.10E-07	-0.44	62	2.07	Acbd6;LOC100526819	
DMR13:73306001	13	73306001	73307000	1000	1	9.00E-07	-0.34	22	2.2	Acbd6	
DMR13:73317001	13	73317001	73318000	1000	1	2.60E-07	0.35	18	1.8	Acbd6	
DMR13:73423001	13	73423001	73424000	1000	1	1.10E-10	0.46	17	1.7	LOC102554128;Qsox1	Metabolism

DMR13:73425001	13	73425001	73429000	4000	1	5.70E-10	0.61	52	1.3	LOC102554128;Qsox1	Metabolism
DMR13:73591001	13	73591001	73593000	2000	1	1.10E-11	-0.58	18	0.9	Cep350	Cytoskeleton
DMR13:73868001	13	73868001	73869000	1000	1	9.60E-09	-0.52	26	2.6	LOC679963;Tdrd5	Cytoskeleton
DMR13:74217001	13	74217001	74220000	3000	1	3.20E-08	-0.49	19	0.63	Abl2	
DMR13:74263001	13	74263001	74264000	1000	1	2.00E-07	0.42	7	0.7	Abl2;Tor3a	Transcription
DMR13:74284001	13	74284001	74288000	4000	1	7.20E-09	0.46	60	1.5	Abl2;Tor3a;Fam20b	Transcription
DMR13:74336001	13	74336001	74337000	1000	1	6.70E-07	-0.43	17	1.7	Abl2;Fam20b	
DMR13:74486001	13	74486001	74487000	1000	1	6.00E-08	0.59	9	0.9	Ralgps2	Transcription
DMR13:74501001	13	74501001	74508000	7000	2	1.80E-08	0.52	67	0.96	Ralgps2	Transcription
DMR13:74521001	13	74521001	74522000	1000	1	6.10E-07	-0.54	9	0.9	Ralgps2	Transcription
DMR13:74796001	13	74796001	74797000	1000	1	2.40E-08	0.42	10	1	Rasal2;LOC100912060	Signaling
DMR13:74900001	13	74900001	74905000	5000	1	4.40E-09	-0.37	66	1.32	Rasal2	Signaling
DMR13:74939001	13	74939001	74940000	1000	1	1.30E-07	0.54	12	1.2	Rasal2;LOC108352559	Signaling
DMR13:75195001	13	75195001	75197000	2000	1	7.10E-08	0.34	24	1.2	Sec16b	
DMR13:76016001	13	76016001	76017000	1000	1	4.70E-08	-0.52	12	1.2	Brinp2	
DMR13:76261001	13	76261001	76263000	2000	1	7.70E-08	-0.44	17	0.85	Astn1	
DMR13:76637001	13	76637001	76642000	5000	1	3.90E-08	-0.29	53	1.06	Pappa2	
DMR13:76643001	13	76643001	76644000	1000	1	2.60E-07	-0.3	9	0.9	Pappa2	
DMR13:78234001	13	78234001	78239000	5000	1	1.50E-08	-0.3	51	1.02	Rabgap1l	Signaling
DMR13:78290001	13	78290001	78293000	3000	2	7.40E-10	-0.38	46	1.53	Rabgap1l	Signaling
DMR13:78422001	13	78422001	78426000	4000	2	3.30E-08	-0.46	62	1.55	Rabgap1l	Signaling
DMR13:78456001	13	78456001	78460000	4000	1	5.70E-08	-0.33	36	0.9	Rabgap1l	Signaling
DMR13:78511001	13	78511001	78514000	3000	1	5.80E-09	-0.37	25	0.83	Rabgap1l	Signaling
DMR13:78556001	13	78556001	78561000	5000	2	2.50E-11	-0.4	50	1	Rabgap1l	Signaling
DMR13:78562001	13	78562001	78563000	1000	1	2.60E-09	-0.33	15	1.5	Rabgap1l	Signaling
DMR13:78737001	13	78737001	78738000	1000	1	1.40E-07	-0.42	14	1.4	Rc3h1	
DMR13:78741001	13	78741001	78743000	2000	1	1.50E-07	-0.48	11	0.55	Rc3h1;LOC108352560	
DMR13:79053001	13	79053001	79058000	5000	2	8.90E-13	-0.4	44	0.88	Slc9c2	
DMR13:79737001	13	79737001	79738000	1000	1	3.40E-07	0.53	7	0.7	Suco	Development
DMR13:79773001	13	79773001	79776000	3000	1	7.80E-11	-0.56	17	0.57	Suco	Development
DMR13:80299001	13	80299001	80300000	1000	1	2.00E-07	0.51	7	0.7	Dnm3	Transport
DMR13:80384001	13	80384001	80386000	2000	1	6.90E-13	-0.44	11	0.55	Dnm3;LOC102554073;LOC102555077	Transport
DMR13:80778001	13	80778001	80779000	1000	1	3.00E-08	0.62	13	1.3	Fmo2;LOC498264	Metabolism
DMR13:80793001	13	80793001	80800000	7000	1	6.80E-09	-0.25	91	1.3	LOC498264;Fmo6	Metabolism
DMR13:81687001	13	81687001	81688000	1000	1	5.40E-07	-0.54	12	1.2	Gorab	
DMR13:82026001	13	82026001	82028000	2000	1	8.50E-07	0.52	11	0.55	Mettl11b	Epigenetic
DMR13:82123001	13	82123001	82130000	7000	1	2.50E-11	-0.39	59	0.84	Kifap3	Cytoskeleton
DMR13:82201001	13	82201001	82206000	5000	1	3.70E-07	0.39	45	0.9	Kifap3	Cytoskeleton
DMR13:82246001	13	82246001	82248000	2000	1	1.10E-07	0.35	17	0.85	Scyl3;LOC498265	Signaling
DMR13:82584001	13	82584001	82586000	2000	1	4.20E-08	0.36	28	1.4	Ccdc181;Blzf1	Transcription
DMR13:82653001	13	82653001	82656000	3000	1	5.10E-07	-0.41	39	1.3	Nme7	Signaling
DMR13:83510001	13	83510001	83511000	1000	1	3.70E-07	0.36	12	1.2	Tiprl	
DMR13:83641001	13	83641001	83642000	1000	1	7.10E-07	0.34	12	1.2	Dcaf6	Proteolysis
DMR13:83858001	13	83858001	83860000	2000	1	9.00E-08	0.51	21	1.05	Trnap-cgg;Rcsd1	Cytoskeleton
DMR13:84056001	13	84056001	84058000	2000	1	7.00E-07	-0.43	23	1.15	Cd247	Immune
DMR13:84329001	13	84329001	84330000	1000	1	9.10E-07	-0.36	15	1.5	Dusp27;Gpa33	
DMR13:84430001	13	84430001	84431000	1000	1	5.20E-08	-0.54	3	0.3	Mael;LOC681179	
DMR13:84461001	13	84461001	84465000	4000	1	2.70E-07	-0.34	32	0.8	Mael;Ildr2	Immune
DMR13:84518001	13	84518001	84519000	1000	1	7.60E-07	0.36	6	0.6	Ildr2	Immune
DMR13:84689001	13	84689001	84692000	3000	2	9.70E-08	-0.41	50	1.67	Fmo13	Metabolism
DMR13:85363001	13	85363001	85366000	3000	1	3.10E-07	0.36	53	1.77	RGD1563679	
DMR13:85422001	13	85422001	85424000	2000	1	9.20E-07	0.35	36	1.8	Uck2	Signaling
DMR13:86394001	13	86394001	86395000	1000	1	4.70E-07	0.57	5	0.5	Pbx1	Development
DMR13:86568001	13	86568001	86569000	1000	1	5.00E-07	0.47	8	0.8	Pbx1	Development
DMR13:87828001	13	87828001	87830000	2000	1	9.50E-12	0.48	18	0.9	Nuf2	Cytoskeleton
DMR13:88356001	13	88356001	88358000	2000	2	4.90E-14	0.5	18	0.9	Ddr2	Receptor
DMR13:89004001	13	89004001	89010000	6000	1	1.60E-07	0.41	87	1.45	Olfml2b	Development
DMR13:89079001	13	89079001	89080000	1000	1	9.30E-08	0.34	16	1.6	Atf6	
DMR13:89082001	13	89082001	89087000	5000	1	7.30E-14	-0.43	41	0.82	Atf6	
DMR13:89110001	13	89110001	89115000	5000	2	2.50E-11	-0.37	46	0.92	Atf6	

DMR13:89274001	13	89274001	89277000	3000	1	7.50E-07	0.52	40	1.33	Dusp12;Fcrlb	Signaling;Immune
DMR13:89348001	13	89348001	89351000	3000	1	8.60E-09	0.39	55	1.83	Fcgr2b	
DMR13:89441001	13	89441001	89448000	7000	1	3.00E-08	0.37	262	3.74	LOC108348047;Trnae-cuc;Trnag-ucc;Trnad-guc;Trnal-cag;Trnag-gcc;LOC102557067	
DMR13:89472001	13	89472001	89474000	2000	2	1.10E-07	0.48	24	1.2	Znrd1-ps1;Trnal-cag;Trnad-guc;Trnag-ucc;Trnae-cuc;Trnav-cac;Cfap126	
DMR13:89780001	13	89780001	89784000	4000	1	5.80E-10	0.44	49	1.23	Nectin4;Arhgap30	Signaling
DMR13:90003001	13	90003001	90005000	2000	1	4.20E-07	0.42	16	0.8	Cd244;LOC103692282;Ly9	Immune
DMR13:90326001	13	90326001	90327000	1000	1	4.60E-08	0.41	11	1.1	Slamf6	Immune
DMR13:90328001	13	90328001	90330000	2000	1	5.40E-07	-0.43	20	1	Slamf6;LOC688740	Immune
DMR13:90382001	13	90382001	90384000	2000	1	6.90E-07	0.42	93	4.65	Vangl2	
DMR13:90763001	13	90763001	90764000	1000	1	2.10E-07	-0.51	14	1.4	Kcnj10;Pigm	Transport;Golgi
DMR13:90856001	13	90856001	90858000	2000	1	1.00E-08	0.39	39	1.95	Slamf9;LOC108352575	Immune
DMR13:91016001	13	91016001	91017000	1000	1	6.70E-08	0.57	19	1.9	Dusp23	Signaling
DMR13:91205001	13	91205001	91209000	4000	1	1.30E-09	0.43	94	2.35	LOC103693683;Trnag-ucc;LOC108352576;Trnag-gcc	
DMR13:91942001	13	91942001	91943000	1000	1	9.10E-07	0.36	10	1	Aim2	Transcription
DMR13:92313001	13	92313001	92320000	7000	3	2.20E-10	-0.46	84	1.2	Spta1	
DMR13:92635001	13	92635001	92636000	1000	1	7.30E-07	0.54	10	1	Fmn2	
DMR13:94052001	13	94052001	94053000	1000	1	2.70E-08	0.48	10	1	Pld5	Metabolism
DMR13:94056001	13	94056001	94058000	2000	1	1.10E-14	0.47	69	3.45	Pld5	Metabolism
DMR13:94239001	13	94239001	94240000	1000	1	2.70E-07	-0.63	6	0.6	Pld5	Metabolism
DMR13:94867001	13	94867001	94869000	2000	1	8.20E-09	-0.48	24	1.2	Cep170	Cytoskeleton
DMR13:94961001	13	94961001	94964000	3000	1	7.10E-11	0.42	35	1.17	Sdccag8	
DMR13:95145001	13	95145001	95147000	2000	2	1.20E-11	-0.53	13	0.65	Akt3	Signaling
DMR13:95169001	13	95169001	95170000	1000	1	7.90E-12	0.65	8	0.8	Akt3	Signaling
DMR13:95176001	13	95176001	95182000	6000	2	3.70E-07	-0.32	64	1.07	Akt3	Signaling
DMR13:95206001	13	95206001	95207000	1000	1	5.30E-07	0.44	8	0.8	Akt3	Signaling
DMR13:95305001	13	95305001	95308000	3000	1	3.40E-07	-0.38	25	0.83	Akt3	Signaling
DMR13:95409001	13	95409001	95414000	5000	1	2.80E-08	-0.29	50	1	Rpl30l1	Translation
DMR13:95952001	13	95952001	95954000	2000	1	7.90E-11	0.44	22	1.1	Adss;RGD1565309	Metabolism
DMR13:96185001	13	96185001	96187000	2000	1	5.20E-08	0.4	37	1.85	RGD1563812	
DMR13:96866001	13	96866001	96867000	1000	1	2.20E-10	0.36	9	0.9	Kif26b	Cytoskeleton
DMR13:96936001	13	96936001	96940000	4000	1	4.60E-08	0.35	83	2.08	Kif26b;LOC102553287	Cytoskeleton
DMR13:97094001	13	97094001	97097000	3000	1	9.80E-09	0.39	40	1.33	Kif26b	Cytoskeleton
DMR13:97347001	13	97347001	97351000	4000	1	2.40E-08	0.34	84	2.1	Smyd3	
DMR13:97362001	13	97362001	97363000	1000	1	3.60E-07	-0.42	20	2	Smyd3	
DMR13:97379001	13	97379001	97380000	1000	1	7.90E-07	0.32	7	0.7	Smyd3	
DMR13:97569001	13	97569001	97571000	2000	1	1.10E-14	0.63	44	2.2	Smyd3	
DMR13:98484001	13	98484001	98487000	3000	1	5.50E-07	0.28	47	1.57	Adck3	
DMR13:98848001	13	98848001	98850000	2000	1	1.40E-08	0.41	36	1.8	Parp1	Transcription
DMR13:99029001	13	99029001	99030000	1000	1	2.40E-07	0.53	4	0.4	Acbd3	
DMR13:99240001	13	99240001	99243000	3000	1	6.30E-08	0.44	54	1.8	Tmem63a	
DMR13:99294001	13	99294001	99297000	3000	1	7.60E-09	0.66	32	1.07	Ephx1	Metabolism
DMR13:99653001	13	99653001	99654000	1000	1	8.30E-07	0.38	17	1.7	Cnih3	Transport
DMR13:99732001	13	99732001	99734000	2000	1	3.10E-09	0.38	26	1.3	Cnih3	Transport
DMR13:99739001	13	99739001	99742000	3000	1	4.90E-08	-0.4	38	1.27	Cnih3	Transport
DMR13:99759001	13	99759001	99760000	1000	1	4.70E-08	0.33	22	2.2	Cnih3	Transport
DMR13:100115001	13	100115001	100121000	6000	2	6.40E-10	-0.37	60	1	Eif3e	Translation
DMR13:100166001	13	100166001	100167000	1000	1	4.60E-08	-0.45	18	1.8	Eif3e	Translation
DMR13:100297001	13	100297001	100300000	3000	1	8.50E-07	0.41	44	1.47	Enah	Cytoskeleton
DMR13:100316001	13	100316001	100318000	2000	1	1.50E-09	0.47	70	3.5	Enah	Cytoskeleton
DMR13:100353001	13	100353001	100354000	1000	1	5.90E-15	0.65	10	1	Enah	Cytoskeleton
DMR13:100564001	13	100564001	100565000	1000	1	4.30E-09	0.52	12	1.2	Srp9	Metabolism
DMR13:100852001	13	100852001	100853000	1000	1	1.90E-09	0.28	11	1.1	Tp53bp2	Signaling
DMR13:100886001	13	100886001	100887000	1000	1	9.90E-09	0.42	26	2.6	Capn2	Protease
DMR13:101372001	13	101372001	101373000	1000	1	1.50E-08	0.39	15	1.5	Tlr5	
DMR13:101526001	13	101526001	101527000	1000	1	7.50E-11	0.5	8	0.8	Disp1	

DMR13:101546001	13	101546001	101547000	1000	1	9.40E-12	0.48	21	2.1	Disp1	
DMR13:101552001	13	101552001	101553000	1000	1	4.10E-16	0.7	4	0.4	Disp1	
DMR13:101793001	13	101793001	101794000	1000	1	3.40E-08	0.36	25	2.5	Taf1a	
DMR13:101827001	13	101827001	101828000	1000	1	4.30E-07	0.54	18	1.8	Hhipl2	Signaling
DMR13:102631001	13	102631001	102633000	2000	1	3.70E-07	0.46	55	2.75	Hlx	Development
DMR13:102786001	13	102786001	102790000	4000	1	4.50E-07	0.39	64	1.6	RGD1310587	
DMR13:103331001	13	103331001	103332000	1000	1	5.60E-07	-0.44	14	1.4	Eprs	Translation
DMR13:105076001	13	105076001	105083000	7000	3	7.70E-08	-0.3	71	1.01	Tgfb2	Growth Factors
DMR13:105093001	13	105093001	105100000	7000	2	1.70E-07	-0.28	83	1.19	Tgfb2	Growth Factors
DMR13:105480001	13	105480001	105487000	7000	2	7.60E-10	-0.45	92	1.31	Spata17	
DMR13:105493001	13	105493001	105498000	5000	1	4.50E-08	0.4	85	1.7	Spata17	
DMR13:105548001	13	105548001	105549000	1000	1	1.20E-11	0.56	17	1.7	Spata17	
DMR13:105665001	13	105665001	105670000	5000	1	3.10E-11	-0.42	47	0.94	Spata17	
DMR13:106235001	13	106235001	106236000	1000	1	7.10E-08	0.48	4	0.4	Esrrg	
DMR13:106281001	13	106281001	106282000	1000	1	1.10E-09	0.52	32	3.2	Esrrg	
DMR13:106492001	13	106492001	106495000	3000	1	1.20E-07	0.5	44	1.47	Esrrg	
DMR13:106581001	13	106581001	106582000	1000	1	1.30E-07	0.34	13	1.3	Esrrg	
DMR13:106612001	13	106612001	106615000	3000	1	5.20E-07	0.35	39	1.3	Esrrg	
DMR13:106828001	13	106828001	106829000	1000	1	2.30E-07	0.35	14	1.4	Ush2a	Extracellular Matrix
DMR13:107059001	13	107059001	107060000	1000	1	1.60E-08	0.53	9	0.9	Ush2a	Extracellular Matrix
DMR13:107071001	13	107071001	107072000	1000	1	8.60E-09	0.52	23	2.3	Ush2a	Extracellular Matrix
DMR13:107170001	13	107170001	107173000	3000	1	4.00E-08	-0.46	41	1.37	Ush2a	Extracellular Matrix
DMR13:107236001	13	107236001	107238000	2000	1	8.40E-08	0.59	21	1.05	Ush2a	Extracellular Matrix
DMR13:107292001	13	107292001	107294000	2000	1	6.30E-07	0.57	29	1.45	Ush2a	Extracellular Matrix
DMR13:107358001	13	107358001	107361000	3000	1	3.00E-07	-0.34	45	1.5	Ush2a	Extracellular Matrix
DMR13:107700001	13	107700001	107702000	2000	1	1.60E-08	0.37	22	1.1	Kcnk2	Transport
DMR13:107868001	13	107868001	107870000	2000	1	9.90E-07	0.31	27	1.35	Kcnk2	Transport
DMR13:108441001	13	108441001	108442000	1000	1	4.20E-07	-0.43	19	1.9	Prox1;LOC498308	Development
DMR13:108705001	13	108705001	108707000	2000	1	4.70E-09	0.39	32	1.6	Ptpn14	Signaling
DMR13:108762001	13	108762001	108763000	1000	1	8.30E-07	0.37	25	2.5	Ptpn14	Signaling
DMR13:109390001	13	109390001	109396000	6000	2	1.40E-08	-0.33	79	1.32	Rps6kc1	Signaling
DMR13:109439001	13	109439001	109442000	3000	1	3.50E-10	0.44	63	2.1	Rps6kc1	Signaling
DMR13:109583001	13	109583001	109584000	1000	1	7.70E-10	0.45	11	1.1	Rps6kc1;LOC108348225	Signaling
DMR13:110275001	13	110275001	110276000	1000	1	7.20E-10	0.41	15	1.5	Ints7;LOC108352606	
DMR13:110278001	13	110278001	110281000	3000	1	3.20E-09	0.46	43	1.43	Ints7;LOC108352606	
DMR13:110464001	13	110464001	110465000	1000	1	6.20E-12	0.49	28	2.8	Lpgat1;LOC102555670	Metabolism
DMR13:110667001	13	110667001	110669000	2000	1	3.20E-07	0.29	40	2	Slc30a1	
DMR13:110765001	13	110765001	110767000	2000	1	1.10E-10	0.43	27	1.35	Traf5	Cytoskeleton
DMR13:110848001	13	110848001	110851000	3000	1	5.70E-07	0.33	62	2.07	Rcor3	
DMR13:111282001	13	111282001	111284000	2000	1	5.80E-09	0.47	27	1.35	Hhat	Metabolism
DMR13:111336001	13	111336001	111343000	7000	2	1.70E-11	-0.35	79	1.13	Hhat	Metabolism
DMR13:111442001	13	111442001	111443000	1000	1	4.80E-08	-0.51	22	2.2	Hhat	Metabolism
DMR13:111701001	13	111701001	111703000	2000	1	1.40E-07	0.45	19	0.95	Syt14	
DMR13:111740001	13	111740001	111743000	3000	2	1.80E-07	-0.45	23	0.77	Syt14	
DMR13:111926001	13	111926001	111932000	6000	2	3.00E-08	-0.35	64	1.07	Traf3ip3	Cytoskeleton
DMR13:112060001	13	112060001	112061000	1000	1	2.70E-07	0.56	14	1.4	Lamb3	Extracellular Matrix
DMR13:112104001	13	112104001	112106000	2000	1	8.40E-08	0.35	9	0.45	Camk1g	Signaling
DMR13:113578001	13	113578001	113584000	6000	1	6.90E-07	-0.31	68	1.13	Plxna2	
DMR14:446001	14	446001	449000	3000	2	5.70E-08	0.54	68	2.27	RGD1564814;LOC100912107	
DMR14:517001	14	517001	518000	1000	1	2.80E-07	-0.6	10	1	LOC100912233;RGD1562932	
DMR14:519001	14	519001	522000	3000	2	2.20E-08	-0.64	12	0.4	LOC100912233;RGD1562932	
DMR14:529001	14	529001	531000	2000	1	1.60E-07	-0.66	18	0.9	RGD1562932	
DMR14:610001	14	610001	616000	6000	2	2.80E-11	-0.38	65	1.08	RGD1563483	
DMR14:684001	14	684001	687000	3000	2	7.90E-13	-0.69	13	0.43	RGD1561977	
DMR14:957001	14	957001	962000	5000	1	3.00E-12	-0.37	48	0.96	Vom2r-ps116	
DMR14:1392001	14	1392001	1395000	3000	1	6.50E-07	-0.45	25	0.83	Vom2r66	
DMR14:1656001	14	1656001	1661000	5000	2	6.00E-08	-0.38	43	0.86	Ccdc18	Cytoskeleton
DMR14:2159001	14	2159001	2164000	5000	1	1.70E-08	-0.28	82	1.64	Gak	Transport

DMR14:2264001	14	2264001	2265000	1000	1	1.50E-08	-0.47	5	0.5	Pcgf3	Epigenetic
DMR14:2585001	14	2585001	2587000	2000	1	7.90E-07	-0.53	18	0.9	Ccdc18	Cytoskeleton
DMR14:2662001	14	2662001	2664000	2000	1	8.20E-07	-0.37	26	1.3	Mtf2	Epigenetic
DMR14:3018001	14	3018001	3019000	1000	1	2.10E-08	0.38	9	0.9	Evi5	Signaling
DMR14:3028001	14	3028001	3032000	4000	1	1.10E-13	0.58	47	1.18	Evi5	Signaling
DMR14:3169001	14	3169001	3171000	2000	1	1.00E-12	0.46	29	1.45	Rpap2	
DMR14:3304001	14	3304001	3308000	4000	1	2.60E-07	0.5	65	1.62	Btbd8	
DMR14:3869001	14	3869001	3872000	3000	1	2.00E-07	0.43	55	1.83	Hfm1	Transcription
DMR14:3899001	14	3899001	3901000	2000	1	8.00E-07	-0.52	24	1.2	Hfm1	Transcription
DMR14:3923001	14	3923001	3924000	1000	1	1.40E-08	0.46	10	1	Hfm1	Transcription
DMR14:5104001	14	5104001	5106000	2000	1	6.90E-07	-0.53	54	2.7	Lrrc8d	Cytoskeleton
DMR14:5186001	14	5186001	5190000	4000	1	5.50E-07	-0.42	67	1.68	Lrrc8d	Cytoskeleton
DMR14:5361001	14	5361001	5363000	2000	1	2.80E-08	-0.4	23	1.15	Lrrc8c	Cytoskeleton
DMR14:5380001	14	5380001	5387000	7000	2	2.00E-08	-0.32	89	1.27	Lrrc8c;LOC108352696	Cytoskeleton
DMR14:5819001	14	5819001	5822000	3000	1	2.20E-07	-0.3	34	1.13	Abcg3l3	Transport
DMR14:5861001	14	5861001	5867000	6000	1	2.30E-09	-0.47	38	0.63	Abcg3l3;LOC100359633;LOC100912618;LOC102554096	Transport;Proteolysis
DMR14:5900001	14	5900001	5902000	2000	1	1.10E-08	-0.44	36	1.8	Abcg3l3;LOC100909418	Transport
DMR14:7037001	14	7037001	7040000	3000	1	5.60E-08	0.4	47	1.57	LOC100359907;Nudt9	Metabolism
DMR14:7120001	14	7120001	7121000	1000	1	4.00E-08	0.6	5	0.5	Hsd17b13	
DMR14:7234001	14	7234001	7235000	1000	1	8.90E-07	0.31	21	2.1	Aff1	Transcription
DMR14:7308001	14	7308001	7311000	3000	1	3.10E-08	0.4	45	1.5	Aff1	Transcription
DMR14:7697001	14	7697001	7698000	1000	1	9.10E-08	0.51	18	1.8	Ptpn13	Signaling
DMR14:8211001	14	8211001	8213000	2000	1	2.60E-08	-0.51	22	1.1	Mapk10	Signaling
DMR14:8247001	14	8247001	8249000	2000	1	5.50E-07	-0.42	25	1.25	Mapk10	Signaling
DMR14:8274001	14	8274001	8280000	6000	1	7.60E-08	-0.42	69	1.15	Mapk10	Signaling
DMR14:8289001	14	8289001	8290000	1000	1	1.10E-07	0.38	13	1.3	Mapk10	Signaling
DMR14:8321001	14	8321001	8325000	4000	1	8.50E-09	-0.41	40	1	Mapk10	Signaling
DMR14:8591001	14	8591001	8592000	1000	1	1.50E-13	0.49	19	1.9	Arhgap24	
DMR14:9030001	14	9030001	9032000	2000	1	3.50E-12	-0.61	20	1	RGD1560931	
DMR14:9189001	14	9189001	9192000	3000	2	4.80E-10	0.5	54	1.8	Wdfy3	
DMR14:9408001	14	9408001	9411000	3000	1	6.40E-09	0.48	63	2.1	Cds1	Transport
DMR14:9413001	14	9413001	9415000	2000	1	9.20E-07	0.38	25	1.25	Cds1	Transport
DMR14:9431001	14	9431001	9432000	1000	1	1.40E-11	0.52	33	3.3	Cds1	Transport
DMR14:9435001	14	9435001	9440000	5000	1	8.70E-07	0.43	83	1.66	Cds1	Transport
DMR14:9452001	14	9452001	9455000	3000	1	1.60E-07	0.33	71	2.37	Cds1;LOC108352705	Transport
DMR14:10449001	14	10449001	10452000	3000	1	2.50E-07	-0.44	58	1.93	Fam175a;Mrps18c;Helq	Translation
DMR14:10472001	14	10472001	10474000	2000	1	5.40E-13	0.56	46	2.3	Helq	
DMR14:10783001	14	10783001	10785000	2000	1	5.70E-12	0.44	22	1.1	Lin54	
DMR14:11126001	14	11126001	11127000	1000	1	5.20E-15	0.7	9	0.9	Tmem150c	
DMR14:11835001	14	11835001	11836000	1000	1	4.90E-08	-0.45	7	0.7	Rasgef1b	Transcription
DMR14:12313001	14	12313001	12314000	1000	1	7.70E-07	-0.56	21	2.1	Prkg2	Signaling
DMR14:14509001	14	14509001	14511000	2000	1	9.90E-07	0.41	34	1.7	Fras1	
DMR14:14730001	14	14730001	14731000	1000	1	4.80E-07	0.38	7	0.7	Fras1	
DMR14:15050001	14	15050001	15054000	4000	1	4.90E-09	-0.33	55	1.38	Cnot6l	Translation
DMR14:15428001	14	15428001	15430000	2000	1	4.40E-07	-0.52	18	0.9	LOC100909497;RGD1561699;LOC108348100	
DMR14:15734001	14	15734001	15740000	6000	1	1.80E-08	-0.28	50	0.83	RGD1559999;RGD1561226	
DMR14:15819001	14	15819001	15820000	1000	1	5.10E-07	-0.46	8	0.8	RGD1559999	
DMR14:16786001	14	16786001	16788000	2000	1	9.70E-09	-0.57	37	1.85	Shroom3;LOC108352720	Cytoskeleton
DMR14:16800001	14	16800001	16803000	3000	1	1.60E-13	0.53	49	1.63	Shroom3	Cytoskeleton
DMR14:16981001	14	16981001	16983000	2000	1	3.50E-07	-0.7	31	1.55	Ccdc158;Stbd1;Fam47e	
DMR14:18084001	14	18084001	18085000	1000	1	3.40E-08	-0.46	27	2.7	Parm1	
DMR14:18539001	14	18539001	18540000	1000	1	2.80E-17	1.04	9	0.9	Areg	Growth Factors
DMR14:19015001	14	19015001	19017000	2000	1	1.70E-07	0.52	13	0.65	Rassf6	Cytoskeleton
DMR14:19090001	14	19090001	19094000	4000	1	1.80E-08	0.34	60	1.5	Afm	Transport
DMR14:19100001	14	19100001	19101000	1000	1	1.10E-11	-0.39	15	1.5	Afm	Transport
DMR14:19452001	14	19452001	19455000	3000	1	1.50E-07	-0.5	14	0.47	Cox18	Transport
DMR14:20687001	14	20687001	20689000	2000	1	9.10E-07	-0.36	30	1.5	Slc4a4	Transport
DMR14:20733001	14	20733001	20741000	8000	2	5.60E-08	-0.35	90	1.12	Slc4a4	Transport

DMR14:20809001	14	20809001	20812000	3000	1	9.50E-07	-0.39	38	1.27	Slc4a4	Transport
DMR14:20996001	14	20996001	20997000	1000	1	1.20E-08	-0.48	10	1	Mob1b	Signaling
DMR14:21086001	14	21086001	21087000	1000	1	2.40E-07	-0.66	6	0.6	Rufy3	
DMR14:21538001	14	21538001	21539000	1000	1	4.20E-08	-0.4	11	1.1	Smr3b	
DMR14:21546001	14	21546001	21552000	6000	1	2.80E-07	-0.31	57	0.95	Smr3b;Vcsa2	Protease; Proteolysis
DMR14:21571001	14	21571001	21576000	5000	1	1.10E-07	-0.3	49	0.98	Smr3b;Vcsa2	Protease; Proteolysis
DMR14:21577001	14	21577001	21581000	4000	1	8.80E-08	-0.28	42	1.05	Smr3b	
DMR14:21598001	14	21598001	21601000	3000	1	1.30E-07	-0.31	25	0.83	Smr3b;LOC108352726;LOC108352824	
DMR14:22219001	14	22219001	22225000	6000	2	1.20E-09	-0.38	57	0.95	Ugt2a1	
DMR14:22571001	14	22571001	22575000	4000	1	2.40E-07	-0.31	46	1.15	Ugt2b35	
DMR14:22611001	14	22611001	22618000	7000	1	2.30E-12	-0.41	75	1.07	Ugt2b7	
DMR14:22761001	14	22761001	22767000	6000	1	1.80E-07	-0.35	61	1.02	RGD1565664	
DMR14:23156001	14	23156001	23157000	1000	1	3.60E-07	0.53	15	1.5	Tmprss11g	Protease
DMR14:23340001	14	23340001	23344000	4000	1	1.00E-07	-0.32	43	1.07	Tmprss11d	Protease
DMR14:23478001	14	23478001	23479000	1000	1	1.20E-09	-0.45	19	1.9	LOC103692776;Gnrhr	Signaling
DMR14:23561001	14	23561001	23568000	7000	3	3.30E-10	-0.38	84	1.2	Uba6;Stap1	Proteolysis;Cytoskeleton
DMR14:24039001	14	24039001	24040000	1000	1	3.10E-09	0.66	20	2	Tmprss11f;LOC100364381	Protease
DMR14:24057001	14	24057001	24061000	4000	3	1.00E-08	-0.4	37	0.92	Tmprss11f;LOC100364381	Protease
DMR14:25636001	14	25636001	25640000	4000	1	3.20E-07	-0.29	31	0.78	Epha5	Receptor
DMR14:25690001	14	25690001	25691000	1000	1	1.20E-07	-0.55	6	0.6	Epha5	Receptor
DMR14:28440001	14	28440001	28442000	2000	1	1.20E-07	0.39	17	0.85	Adgrl3	Signaling
DMR14:28578001	14	28578001	28579000	1000	1	4.40E-07	-0.38	9	0.9	Adgrl3	Signaling
DMR14:33449001	14	33449001	33451000	2000	1	6.80E-08	0.49	22	1.1	Arl9;LOC108352736;Srp72	
DMR14:33632001	14	33632001	33633000	1000	1	1.60E-08	-0.53	16	1.6	Aasdh	Metabolism
DMR14:33638001	14	33638001	33639000	1000	1	8.90E-12	0.5	9	0.9	Aasdh;LOC108352737	Metabolism
DMR14:33671001	14	33671001	33673000	2000	2	2.90E-17	0.62	66	3.3	RGD1311575	
DMR14:33720001	14	33720001	33725000	5000	1	4.60E-08	0.65	87	1.74	RGD1311575;LOC108352738	
DMR14:34051001	14	34051001	34052000	1000	1	8.30E-15	0.54	10	1	Cep135	Epigenetic
DMR14:34457001	14	34457001	34458000	1000	1	3.70E-07	-0.42	13	1.3	Clock	
DMR14:34576001	14	34576001	34583000	7000	2	5.10E-11	-0.35	86	1.23	Srd5a3	
DMR14:34787001	14	34787001	34788000	1000	1	5.60E-07	-0.41	28	2.8	Kdr	Receptor
DMR14:35538001	14	35538001	35539000	1000	1	2.20E-09	-0.56	24	2.4	Pdgfra	Receptor
DMR14:35565001	14	35565001	35567000	2000	1	5.00E-10	-0.5	43	2.15	Pdgfra;LOC102554024	Receptor
DMR14:36085001	14	36085001	36087000	2000	1	3.90E-10	0.38	21	1.05	Lnx1	
DMR14:36101001	14	36101001	36104000	3000	1	1.30E-07	0.41	46	1.53	Lnx1	
DMR14:36313001	14	36313001	36318000	5000	1	8.20E-07	-0.31	50	1	Scfd2	Transport
DMR14:36329001	14	36329001	36331000	2000	1	3.00E-13	0.52	11	0.55	Scfd2	Transport
DMR14:36334001	14	36334001	36336000	2000	1	3.50E-07	-0.46	16	0.8	Scfd2	Transport
DMR14:36349001	14	36349001	36351000	2000	1	1.20E-07	0.45	19	0.95	Scfd2	Transport
DMR14:36397001	14	36397001	36398000	1000	1	7.40E-08	0.54	6	0.6	Scfd2	Transport
DMR14:36526001	14	36526001	36531000	5000	1	5.70E-08	-0.37	34	0.68	Scfd2	Transport
DMR14:37147001	14	37147001	37149000	2000	1	7.90E-18	0.64	39	1.95	Lrrc66	Receptor
DMR14:37275001	14	37275001	37277000	2000	1	1.90E-07	0.37	41	2.05	Dcun1d4	
DMR14:37450001	14	37450001	37451000	1000	1	7.10E-07	-0.45	14	1.4	Ociad2;Ociad1	Transport
DMR14:37640001	14	37640001	37641000	1000	1	9.20E-07	0.34	11	1.1	Fryl	Cytoskeleton
DMR14:37846001	14	37846001	37848000	2000	1	3.10E-08	-0.43	23	1.15	Slain2	
DMR14:37879001	14	37879001	37881000	2000	1	3.60E-07	0.38	29	1.45	Slain2;LOC102556376	
DMR14:37950001	14	37950001	37952000	2000	1	1.00E-10	-0.52	22	1.1	Tec	
DMR14:38095001	14	38095001	38097000	2000	1	3.50E-07	0.42	35	1.75	Txk;Nipal1	
DMR14:38339001	14	38339001	38345000	6000	2	2.10E-09	-0.35	65	1.08	Corin	Binding Proteins
DMR14:38368001	14	38368001	38375000	7000	1	7.90E-07	-0.36	92	1.31	Corin;LOC680377;LOC102550273	Binding Proteins
DMR14:38853001	14	38853001	38857000	4000	2	9.20E-09	-0.39	43	1.07	Gabrb1	Ion Channel
DMR14:38999001	14	38999001	39006000	7000	1	9.00E-09	-0.31	72	1.03	Gabrb1	Ion Channel
DMR14:39374001	14	39374001	39375000	1000	1	9.20E-12	0.59	19	1.9	Cox7b2	Metabolism
DMR14:39691001	14	39691001	39692000	1000	1	2.60E-10	-0.54	7	0.7	Gabra2	Ion Channel
DMR14:41682001	14	41682001	41690000	8000	1	1.30E-07	-0.3	86	1.07	Grxcr1	Metabolism
DMR14:41988001	14	41988001	41990000	2000	1	1.50E-07	0.29	41	2.05	Atp8a1	Transport
DMR14:42454001	14	42454001	42456000	2000	1	2.60E-07	0.4	30	1.5	LOC108352852;Slc30a9	Transport

DMR14:42495001	14	42495001	42498000	3000	1	3.40E-09	-0.41	29	0.97	Slc30a9	Transport
DMR14:42504001	14	42504001	42506000	2000	1	4.30E-09	-0.59	12	0.6	Slc30a9	Transport
DMR14:43125001	14	43125001	43127000	2000	1	3.20E-07	0.36	24	1.2	Uchl1	Protease
DMR14:43848001	14	43848001	43858000	10000	1	2.70E-08	-0.34	105	1.05	Rbm47	Metabolism
DMR14:44555001	14	44555001	44556000	1000	1	1.70E-09	0.45	5	0.5	Klb;LOC108352748	
DMR14:44809001	14	44809001	44811000	2000	1	7.90E-07	0.37	33	1.65	LOC100910864;Klhl5	Cytoskeleton
DMR14:44969001	14	44969001	44970000	1000	1	3.80E-08	0.38	3	0.3	Fam114a1;LOC108352749	
DMR14:45151001	14	45151001	45152000	1000	1	1.50E-07	0.63	12	1.2	Klf3	Transcription
DMR14:45697001	14	45697001	45701000	4000	2	1.40E-09	0.42	73	1.82	Tbc1d1	Signaling
DMR14:46155001	14	46155001	46161000	6000	2	6.00E-12	-0.44	67	1.12	Nwd2	
DMR14:48616001	14	48616001	48622000	6000	3	5.80E-08	-0.36	60	1	LOC685935;Dthd1	
DMR14:48748001	14	48748001	48752000	4000	1	5.90E-07	-0.31	39	0.98	Arap2	Signaling
DMR14:53861001	14	53861001	53864000	3000	1	5.20E-08	-0.3	28	0.93	Vom1r-ps103	
DMR14:54905001	14	54905001	54907000	2000	1	5.80E-10	0.42	14	0.7	Pcdh7;LOC103693027	Cytoskeleton
DMR14:54924001	14	54924001	54930000	6000	2	1.80E-13	-0.46	79	1.32	Pcdh7;LOC103693027	Cytoskeleton
DMR14:58872001	14	58872001	58875000	3000	2	4.70E-11	-0.38	29	0.97	RGD1562755	
DMR14:59442001	14	59442001	59443000	1000	1	2.10E-07	0.47	10	1	Tbc1d19	
DMR14:59772001	14	59772001	59773000	1000	1	8.90E-08	-0.41	13	1.3	Rbpj;LOC108352754	Transcription
DMR14:60905001	14	60905001	60908000	3000	1	9.00E-10	0.29	32	1.07	Ccdc149	
DMR14:60958001	14	60958001	60960000	2000	1	7.00E-07	0.6	71	3.55	Ccdc149;Sod3	Metabolism
DMR14:61968001	14	61968001	61970000	2000	1	4.00E-07	-0.46	10	0.5	Kctd8	
DMR14:62083001	14	62083001	62085000	2000	1	3.90E-07	-0.32	18	0.9	Kctd8	
DMR14:62297001	14	62297001	62298000	1000	1	7.90E-20	0.97	22	2.2	Vom1r-ps31	
DMR14:64690001	14	64690001	64693000	3000	1	6.70E-09	0.38	32	1.07	Adgra3	Signaling
DMR14:64760001	14	64760001	64768000	8000	1	5.50E-07	-0.32	112	1.4	Adgra3	Signaling
DMR14:65769001	14	65769001	65770000	1000	1	3.80E-11	0.54	4	0.4	Kcnip4	
DMR14:66349001	14	66349001	66352000	3000	1	2.50E-07	-0.44	26	0.87	Kcnip4	
DMR14:66731001	14	66731001	66740000	9000	2	5.30E-08	-0.3	100	1.11	Kcnip4;Pacrgl	
DMR14:66753001	14	66753001	66754000	1000	1	1.60E-07	0.49	7	0.7	Kcnip4;Pacrgl	
DMR14:66974001	14	66974001	66976000	2000	1	8.00E-07	-0.58	32	1.6	Slit2	
DMR14:67153001	14	67153001	67154000	1000	1	3.80E-07	-0.45	24	2.4	Slit2	
DMR14:69757001	14	69757001	69763000	6000	2	4.80E-09	-0.32	59	0.98	Lcorl	Transcription
DMR14:70070001	14	70070001	70077000	7000	2	2.30E-15	-0.42	82	1.17	Fam184b	
DMR14:71725001	14	71725001	71726000	1000	1	1.40E-09	0.55	18	1.8	Cd38	Metabolism
DMR14:71763001	14	71763001	71769000	6000	1	3.50E-08	-0.31	66	1.1	Cd38	Metabolism
DMR14:72898001	14	72898001	72900000	2000	1	9.50E-07	-0.39	21	1.05	Rab5al1;RGD1565560	
DMR14:73676001	14	73676001	73677000	1000	1	2.60E-08	-0.57	4	0.4	RGD1561694	
DMR14:73913001	14	73913001	73916000	3000	1	3.30E-09	-0.38	30	1	Rab28	
DMR14:73942001	14	73942001	73946000	4000	1	4.10E-09	-0.35	36	0.9	Rab28	
DMR14:77726001	14	77726001	77728000	2000	1	5.40E-13	0.54	9	0.45	Msx1	Development
DMR14:77866001	14	77866001	77868000	2000	1	3.60E-10	0.46	19	0.95	Stk32b	Signaling
DMR14:77879001	14	77879001	77880000	1000	1	8.80E-07	-0.51	15	1.5	Stk32b	Signaling
DMR14:78082001	14	78082001	78083000	1000	1	1.50E-08	0.51	17	1.7	Stk32b;LOC102553032	Signaling
DMR14:78128001	14	78128001	78129000	1000	1	2.30E-15	0.49	7	0.7	Evc2;LOC102553293	
DMR14:78149001	14	78149001	78150000	1000	1	1.30E-07	-0.42	16	1.6	Evc2	
DMR14:78234001	14	78234001	78236000	2000	1	5.50E-10	0.63	17	0.85	Evc	
DMR14:78475001	14	78475001	78477000	2000	1	2.90E-07	-0.39	31	1.55	Jakmip1	
DMR14:78722001	14	78722001	78723000	1000	1	6.40E-10	0.54	18	1.8	Ppp2r2c	Signaling
DMR14:79698001	14	79698001	79699000	1000	1	1.30E-09	0.51	28	2.8	Sorcs2	Transport
DMR14:79720001	14	79720001	79724000	4000	2	1.00E-13	0.48	58	1.45	Sorcs2;Psapl1	Transport;Cytoskeleton
DMR14:79849001	14	79849001	79850000	1000	1	4.60E-07	0.53	5	0.5	Sorcs2	Transport
DMR14:80107001	14	80107001	80109000	2000	1	2.80E-08	-0.46	24	1.2	Ablim2	Cytoskeleton
DMR14:80275001	14	80275001	80278000	3000	1	2.30E-08	0.45	40	1.33	Htra3	Protease
DMR14:80304001	14	80304001	80307000	3000	1	9.00E-09	0.61	27	0.9	Acox3	Metabolism
DMR14:80323001	14	80323001	80328000	5000	5	1.50E-10	0.39	70	1.4	Acox3	Metabolism
DMR14:80343001	14	80343001	80351000	8000	1	9.30E-10	-0.46	101	1.26	Acox3	Metabolism
DMR14:80925001	14	80925001	80926000	1000	1	1.50E-09	0.53	9	0.9	Lrpap1;Dok7	Transcription
DMR14:80934001	14	80934001	80935000	1000	1	2.70E-08	0.55	25	2.5	Dok7	
DMR14:80936001	14	80936001	80942000	6000	2	2.80E-07	0.49	58	0.97	Dok7	
DMR14:81207001	14	81207001	81210000	3000	2	7.50E-08	-0.53	27	0.9	Htt	
DMR14:81228001	14	81228001	81230000	2000	1	9.00E-08	0.36	29	1.45	Htt	

DMR14:81480001	14	81480001	81484000	4000	1	6.40E-07	0.37	45	1.12	Sh3bp2;Tnip2	
DMR14:81704001	14	81704001	81706000	2000	1	1.70E-07	0.35	29	1.45	Cfap99	Development
DMR14:81710001	14	81710001	81713000	3000	2	3.00E-12	0.42	39	1.3	Cfap99	Development
DMR14:81828001	14	81828001	81829000	1000	1	2.10E-10	0.49	15	1.5	Mxd4;LOC108352782;Poln;Haus3	Transcription;Transcription
DMR14:81864001	14	81864001	81869000	5000	1	1.30E-08	-0.32	51	1.02	Poln;Haus3	Transcription
DMR14:82280001	14	82280001	82282000	2000	1	3.00E-10	0.44	31	1.55	Fgfr3	Receptor
DMR14:82701001	14	82701001	82704000	3000	1	2.70E-07	0.35	31	1.03	Maea	
DMR14:83131001	14	83131001	83134000	3000	1	1.90E-08	0.59	26	0.87	Depdc5	Signaling
DMR14:83202001	14	83202001	83203000	1000	1	2.40E-08	-0.73	12	1.2	Depdc5	Signaling
DMR14:83608001	14	83608001	83610000	2000	1	3.00E-08	-0.41	42	2.1	Limk2;LOC102546870	
DMR14:83770001	14	83770001	83771000	1000	1	7.20E-08	0.41	21	2.1	Smtn	
DMR14:84018001	14	84018001	84020000	2000	1	3.90E-08	0.43	27	1.35	Osbp2;LOC108352785	
DMR14:84040001	14	84040001	84043000	3000	1	3.40E-07	-0.42	38	1.27	Osbp2;LOC108352785	
DMR14:84115001	14	84115001	84116000	1000	1	2.60E-08	0.37	21	2.1	Osbp2;LOC102556795	
DMR14:84221001	14	84221001	84223000	2000	1	1.20E-09	0.58	37	1.85	Pes1;Gal3st1	Metabolism;Transport
DMR14:84631001	14	84631001	84634000	3000	1	1.40E-08	0.41	38	1.27	Hormad2	
DMR14:84650001	14	84650001	84651000	1000	1	3.10E-07	0.48	6	0.6	Hormad2	
DMR14:84693001	14	84693001	84695000	2000	1	6.10E-07	0.3	48	2.4	Mtmr3	Signaling
DMR14:84758001	14	84758001	84759000	1000	1	5.90E-07	-0.47	11	1.1	Mtmr3	Signaling
DMR14:84969001	14	84969001	84971000	2000	1	7.90E-07	0.43	65	3.25	Zmat5;Cabp7	
DMR14:85247001	14	85247001	85248000	1000	1	2.80E-07	0.43	11	1.1	Ap1b1	Transport
DMR14:85384001	14	85384001	85392000	8000	1	4.30E-08	0.41	132	1.65	Emid1	
DMR14:85446001	14	85446001	85447000	1000	1	8.90E-07	0.43	16	1.6	LOC102557376;Kremen1	Receptor
DMR14:85571001	14	85571001	85573000	2000	1	2.40E-08	-0.34	13	0.65	Znrf3	
DMR14:85915001	14	85915001	85923000	8000	1	5.80E-10	-0.38	91	1.14	Ankrd36;LOC102550119	
DMR14:86037001	14	86037001	86039000	2000	2	5.50E-10	0.67	15	0.75	Dbnl;Pgam2	Cytoskeleton;Metabolism
DMR14:86056001	14	86056001	86057000	1000	1	1.30E-07	-0.52	23	2.3	Pgam2	Metabolism
DMR14:86127001	14	86127001	86136000	9000	2	5.30E-08	-0.38	98	1.09	Pold2;LOC108352788;Myl7	Transcription;Cytoskeleton
DMR14:86163001	14	86163001	86164000	1000	1	3.70E-08	0.4	21	2.1	Gck	Signaling
DMR14:86180001	14	86180001	86182000	2000	1	5.40E-11	0.42	31	1.55	Gck	Signaling
DMR14:86183001	14	86183001	86185000	2000	1	3.00E-07	0.5	17	0.85	Gck	Signaling
DMR14:86209001	14	86209001	86210000	1000	1	2.60E-09	0.4	16	1.6	Ykt6;Camk2b;LOC108352789	Signaling
DMR14:86214001	14	86214001	86218000	4000	1	1.30E-07	0.45	74	1.85	Ykt6;Camk2b;LOC108352789	Signaling
DMR14:86219001	14	86219001	86221000	2000	1	1.40E-13	0.64	44	2.2	Camk2b;LOC108352789	Signaling
DMR14:86694001	14	86694001	86696000	2000	1	6.00E-08	-0.4	36	1.8	H2afv	Epigenetic
DMR14:86851001	14	86851001	86853000	2000	1	8.10E-08	0.42	50	2.5	Ccm2;Nacad	Transcription
DMR14:86892001	14	86892001	86893000	1000	1	4.40E-09	0.59	3	0.3	Tbrg4;Wap	Protease; Proteolysis
DMR14:87338001	14	87338001	87339000	1000	1	3.70E-07	0.42	6	0.6	Adcy1	
DMR14:88710001	14	88710001	88712000	2000	1	6.60E-09	0.5	44	2.2	Tns3	Cytoskeleton
DMR14:89047001	14	89047001	89049000	2000	1	3.50E-08	-0.38	34	1.7	Pkd1l1	Transport
DMR14:89201001	14	89201001	89202000	1000	1	2.40E-08	0.4	14	1.4	Pkd1l1;Hus1	Transport;Transcription
DMR14:89242001	14	89242001	89249000	7000	1	1.40E-09	-0.34	76	1.09	Sun3;LOC688553	Cytoskeleton
DMR14:89315001	14	89315001	89317000	2000	1	3.70E-07	0.61	43	2.15	Upp1	Signaling
DMR14:89549001	14	89549001	89554000	5000	2	5.10E-09	-0.33	52	1.04	Abca13	Transport
DMR14:89715001	14	89715001	89722000	7000	2	1.80E-11	-0.42	92	1.31	Abca13	Transport
DMR14:91366001	14	91366001	91368000	2000	1	3.40E-07	-0.57	15	0.75	Zpbp	
DMR14:91412001	14	91412001	91414000	2000	1	5.00E-08	-0.38	14	0.7	Zpbp	
DMR14:91544001	14	91544001	91549000	5000	1	1.40E-09	-0.34	69	1.38	Zpbp;RGD1309870	
DMR14:91816001	14	91816001	91818000	2000	1	4.00E-08	0.42	41	2.05	Ikzf1	Transcription
DMR14:91830001	14	91830001	91831000	1000	1	4.50E-10	0.71	6	0.6	Ikzf1	Transcription
DMR14:91964001	14	91964001	91967000	3000	1	7.00E-07	0.6	51	1.7	Ddc	Metabolism
DMR14:92014001	14	92014001	92018000	4000	2	1.20E-08	0.45	53	1.32	LOC103693175;Grb10	Cytoskeleton
DMR14:92024001	14	92024001	92026000	2000	1	1.20E-10	0.45	44	2.2	LOC103693175;Grb10	Cytoskeleton
DMR14:92442001	14	92442001	92444000	2000	1	1.10E-08	-0.58	24	1.2	Cobl;LOC103693778;LOC102553003	
DMR14:99986001	14	99986001	99987000	1000	1	2.50E-07	0.43	5	0.5	Egfr	Receptor
DMR14:100084001	14	100084001	100089000	5000	1	1.00E-07	-0.31	44	0.88	Egfr	Receptor
DMR14:100385001	14	100385001	100388000	3000	1	1.40E-07	0.65	35	1.17	Wdr92	Metabolism

DMR14:101758001	14	101758001	101761000	3000	2	7.00E-09	-0.35	23	0.77	Olr1827-ps	
DMR14:102100001	14	102100001	102104000	4000	1	2.30E-09	0.41	79	1.98	Olr1806-ps	
DMR14:102118001	14	102118001	102119000	1000	1	6.30E-07	-0.4	5	0.5	Olr1806-ps	
DMR14:104282001	14	104282001	104283000	1000	1	2.20E-07	0.33	13	1.3	Spred2	Cytoskeleton
DMR14:104444001	14	104444001	104445000	1000	1	3.60E-08	0.77	28	2.8	LOC100359517;Rpl38-ps2	
DMR14:104468001	14	104468001	104469000	1000	1	1.60E-07	0.45	6	0.6	Rab1a	
DMR14:104575001	14	104575001	104577000	2000	1	2.80E-10	0.44	24	1.2	Slc1a4	Transport
DMR14:104827001	14	104827001	104829000	2000	1	1.60E-12	0.52	13	0.65	Sertad2	Transcription
DMR14:106618001	14	106618001	106620000	2000	1	5.70E-07	-0.35	13	0.65	Wdpcp	
DMR14:106644001	14	106644001	106645000	1000	1	4.70E-07	-0.43	12	1.2	Wdpcp	
DMR14:107031001	14	107031001	107035000	4000	1	5.40E-09	0.43	28	0.7	Ehbp1;LOC108352811;LOC102550584	
DMR14:107043001	14	107043001	107046000	3000	1	5.60E-08	-0.51	47	1.57	Ehbp1;LOC102550584	
DMR14:107274001	14	107274001	107276000	2000	1	4.10E-09	-0.4	35	1.75	Tmem17	
DMR14:108074001	14	108074001	108077000	3000	1	4.60E-07	0.39	28	0.93	Usp34;LOC108352814	Protease
DMR14:108267001	14	108267001	108268000	1000	1	1.00E-07	0.4	20	2	Usp34;Ahsa2	Protease;Transcription
DMR14:108436001	14	108436001	108437000	1000	1	2.20E-07	0.38	21	2.1	Pus10	
DMR14:108621001	14	108621001	108625000	4000	1	9.40E-09	-0.4	42	1.05	LOC108352863;Papolg	Translation
DMR14:108659001	14	108659001	108661000	2000	1	8.90E-07	-0.52	31	1.55	Papolg	Translation
DMR14:108877001	14	108877001	108879000	2000	1	2.40E-08	0.48	40	2	Bcl11a	Transcription
DMR14:108929001	14	108929001	108930000	1000	1	6.80E-07	-0.41	21	2.1	Bcl11a;LOC102557119	Transcription
DMR14:110834001	14	110834001	110841000	7000	1	2.80E-08	-0.32	83	1.19	Vrk2	Signaling
DMR14:113122001	14	113122001	113123000	1000	1	2.30E-10	-0.47	3	0.3	LOC102550342;Mir216a;Mir217	
DMR14:113193001	14	113193001	113198000	5000	1	8.40E-07	-0.27	75	1.5	Efemp1	Extracellular Matrix
DMR14:113805001	14	113805001	113807000	2000	1	5.20E-08	-0.57	9	0.45	Ccdc88a	Transport
DMR14:113850001	14	113850001	113851000	1000	1	1.70E-08	-0.47	11	1.1	Ccdc88a;LOC679625	Transport
DMR14:113881001	14	113881001	113883000	2000	1	8.10E-14	-0.46	19	0.95	Ccdc88a	Transport
DMR14:113887001	14	113887001	113888000	1000	1	6.50E-07	-0.37	15	1.5	Ccdc88a	Transport
DMR14:113894001	14	113894001	113896000	2000	2	3.70E-08	-0.49	11	0.55	Ccdc88a	Transport
DMR14:113999001	14	113999001	114004000	5000	3	7.40E-12	-0.46	47	0.94	Clhc1	Transport
DMR14:114254001	14	114254001	114258000	4000	1	3.40E-07	-0.34	38	0.95	Eml6	
DMR14:114902001	14	114902001	114906000	4000	1	2.30E-07	-0.3	47	1.18	Acyp2	Signaling
DMR14:115243001	14	115243001	115244000	1000	1	9.10E-09	-0.46	9	0.9	Psme4	
DMR14:115309001	14	115309001	115313000	4000	1	4.10E-09	-0.32	48	1.2	Erlec1	
DMR15:472001	15	472001	473000	1000	1	8.10E-15	0.58	1	0.1	Kcnma1	Transport
DMR15:2819001	15	2819001	2820000	1000	1	1.40E-08	0.46	14	1.4	Kat6b;LOC103693812	Epigenetic
DMR15:2933001	15	2933001	2936000	3000	1	8.00E-10	0.47	47	1.57	Kat6b	Epigenetic
DMR15:3036001	15	3036001	3037000	1000	1	7.90E-10	0.4	30	3	Adk	
DMR15:3300001	15	3300001	3304000	4000	1	9.40E-10	-0.34	62	1.55	Adk	
DMR15:3462001	15	3462001	3467000	5000	1	6.80E-11	0.46	55	1.1	Ap3m1;Vcl	Transport
DMR15:3470001	15	3470001	3473000	3000	1	8.70E-09	0.56	40	1.33	Vcl	
DMR15:4576001	15	4576001	4578000	2000	1	2.40E-10	0.54	28	1.4	Kcnk5	Transport
DMR15:4605001	15	4605001	4606000	1000	1	4.60E-12	0.45	26	2.6	Kcnk5	Transport
DMR15:4811001	15	4811001	4812000	1000	1	8.00E-08	0.49	19	1.9	Gng2	Signaling
DMR15:4935001	15	4935001	4936000	1000	1	2.20E-07	0.27	8	0.8	Nid2	Signaling
DMR15:4943001	15	4943001	4948000	5000	1	5.30E-08	0.23	48	0.96	Nid2	Signaling
DMR15:5470001	15	5470001	5474000	4000	1	3.60E-09	-0.28	37	0.92	Spetex-2F;LOC102547093	
DMR15:5490001	15	5490001	5491000	1000	1	2.90E-11	-0.38	15	1.5	Spetex-2F;LOC102547093;LOC108352879	
DMR15:5545001	15	5545001	5547000	2000	1	1.30E-09	-0.46	20	1	Spetex-2C;LOC102549465	
DMR15:5562001	15	5562001	5563000	1000	1	1.10E-07	-0.45	14	1.4	Spetex-2C	
DMR15:5623001	15	5623001	5625000	2000	1	9.20E-09	-0.6	25	1.25	Spetex-2C;LOC108352878	
DMR15:6332001	15	6332001	6334000	2000	1	6.30E-08	-0.61	22	1.1	Zfp385d	
DMR15:6578001	15	6578001	6582000	4000	1	2.40E-07	-0.42	21	0.52	Zfp385d	
DMR15:6637001	15	6637001	6644000	7000	1	1.30E-08	-0.35	81	1.16	Zfp385d	
DMR15:6728001	15	6728001	6729000	1000	1	1.50E-08	0.59	7	0.7	Zfp385d	
DMR15:6850001	15	6850001	6853000	3000	1	3.20E-10	-0.47	23	0.77	Zfp385d	
DMR15:6965001	15	6965001	6969000	4000	1	2.60E-07	-0.42	34	0.85	Zfp385d	
DMR15:7032001	15	7032001	7034000	2000	1	9.60E-09	-0.41	8	0.4	Zfp385d	
DMR15:7140001	15	7140001	7141000	1000	1	2.40E-15	0.59	9	0.9	Zfp385d	

DMR15:7511001	15	7511001	7517000	6000	3	2.30E-07	-0.31	62	1.03	Ube2e2	Proteolysis
DMR15:7537001	15	7537001	7542000	5000	1	1.10E-09	-0.57	37	0.74	Ube2e2	Proteolysis
DMR15:7578001	15	7578001	7579000	1000	1	4.50E-07	-0.41	5	0.5	Ube2e2	Proteolysis
DMR15:7622001	15	7622001	7628000	6000	2	2.50E-10	-0.41	69	1.15	Ube2e2;LOC100366184	Proteolysis
DMR15:7686001	15	7686001	7687000	1000	1	7.90E-10	0.44	21	2.1	Ube2e2;LOC103693837	Proteolysis
DMR15:7723001	15	7723001	7724000	1000	1	1.70E-10	0.46	10	1	Ube2e2	Proteolysis
DMR15:7729001	15	7729001	7731000	2000	1	5.00E-13	0.67	34	1.7	Ube2e2	Proteolysis
DMR15:7848001	15	7848001	7850000	2000	1	7.70E-10	0.44	27	1.35	Ube2e2;LOC102551991	Proteolysis
DMR15:8715001	15	8715001	8721000	6000	1	4.50E-08	-0.33	68	1.13	Nr1d2	Transcription
DMR15:8968001	15	8968001	8972000	4000	1	1.70E-11	0.6	61	1.52	Thrb	Transcription
DMR15:9007001	15	9007001	9018000	11000	2	4.50E-08	-0.28	142	1.29	Thrb	Transcription
DMR15:10003001	15	10003001	10004000	1000	1	4.20E-07	0.39	10	1	Rarb	Transcription
DMR15:10161001	15	10161001	10162000	1000	1	5.80E-07	-0.43	7	0.7	Rarb	Transcription
DMR15:10228001	15	10228001	10230000	2000	1	3.80E-07	0.3	15	0.75	Rarb	Transcription
DMR15:11649001	15	11649001	11651000	2000	1	5.90E-07	-0.54	44	2.2	Nek10;LOC498453	Signaling
DMR15:11697001	15	11697001	11699000	2000	1	8.50E-07	0.38	12	0.6	Nek10	Signaling
DMR15:11817001	15	11817001	11821000	4000	1	2.10E-08	-0.3	50	1.25	Nek10;LOC103693313	Signaling
DMR15:11916001	15	11916001	11918000	2000	1	3.10E-11	-0.51	24	1.2	Slc4a7	Transport
DMR15:11919001	15	11919001	11920000	1000	1	8.50E-08	-0.49	10	1	Slc4a7	Transport
DMR15:12427001	15	12427001	12429000	2000	1	2.50E-14	0.54	26	1.3	Atxn7;LOC108352888	
DMR15:12456001	15	12456001	12457000	1000	1	4.60E-08	0.41	11	1.1	Atxn7	
DMR15:12488001	15	12488001	12490000	2000	1	4.50E-09	0.41	72	3.6	Atxn7	
DMR15:12498001	15	12498001	12499000	1000	1	8.30E-07	0.33	12	1.2	Atxn7	
DMR15:12524001	15	12524001	12526000	2000	1	3.20E-09	0.44	19	0.95	Atxn7	
DMR15:12939001	15	12939001	12946000	7000	1	1.40E-11	-0.33	100	1.43	Ptprg	Signaling
DMR15:12966001	15	12966001	12970000	4000	1	1.40E-09	0.5	43	1.07	Ptprg	Signaling
DMR15:13066001	15	13066001	13067000	1000	1	2.50E-10	0.51	8	0.8	Ptprg	Signaling
DMR15:13413001	15	13413001	13419000	6000	1	4.60E-07	-0.44	92	1.53	Ptprg	Signaling
DMR15:13569001	15	13569001	13571000	2000	1	7.00E-08	0.49	16	0.8	Ptprg	Signaling
DMR15:14461001	15	14461001	14466000	5000	2	8.10E-10	-0.33	47	0.94	RGD1559573	
DMR15:14742001	15	14742001	14743000	1000	1	2.30E-13	0.54	22	2.2	Synpr	Transport
DMR15:15351001	15	15351001	15354000	3000	1	8.70E-08	0.35	40	1.33	Cadps	Transport
DMR15:15731001	15	15731001	15732000	1000	1	2.80E-07	0.37	7	0.7	Fhit	Signaling
DMR15:16417001	15	16417001	16418000	1000	1	8.20E-07	-0.39	19	1.9	Fhit	Signaling
DMR15:16434001	15	16434001	16435000	1000	1	3.70E-17	0.95	34	3.4	Fhit	Signaling
DMR15:16449001	15	16449001	16450000	1000	1	1.00E-07	-0.47	12	1.2	Fhit	Signaling
DMR15:16568001	15	16568001	16569000	1000	1	1.40E-10	-0.58	24	2.4	Fhit	Signaling
DMR15:16814001	15	16814001	16819000	5000	1	1.30E-07	-0.29	41	0.82	Fhit	Signaling
DMR15:18327001	15	18327001	18328000	1000	1	1.90E-08	0.36	11	1.1	Fam3d	Signaling
DMR15:18402001	15	18402001	18404000	2000	1	1.90E-08	0.52	21	1.05	Fam107a	
DMR15:18497001	15	18497001	18500000	3000	2	8.30E-14	0.64	38	1.27	Kctd6;LOC103693296	Cytoskeleton
DMR15:18600001	15	18600001	18603000	3000	1	3.80E-07	0.57	66	2.2	Pxk	
DMR15:18784001	15	18784001	18785000	1000	1	3.90E-11	0.62	12	1.2	Flnb	
DMR15:19525001	15	19525001	19531000	6000	2	9.40E-09	-0.33	77	1.28	Txndc16	Metabolism
DMR15:19620001	15	19620001	19621000	1000	1	9.80E-08	0.46	16	1.6	Gpr137c;Ero1a	Metabolism
DMR15:19708001	15	19708001	19709000	1000	1	1.30E-07	0.31	13	1.3	Styx;LOC689246	
DMR15:19800001	15	19800001	19802000	2000	1	4.20E-08	0.35	33	1.65	Fermt2	
DMR15:19814001	15	19814001	19816000	2000	1	9.60E-08	0.36	44	2.2	Fermt2	
DMR15:20025001	15	20025001	20026000	1000	1	1.40E-08	-0.38	21	2.1	Ddhd1;LOC108352895	Metabolism
DMR15:23690001	15	23690001	23691000	1000	1	7.30E-07	-0.48	23	2.3	Samd4a	
DMR15:23782001	15	23782001	23783000	1000	1	2.80E-17	0.56	14	1.4	Samd4a	
DMR15:23787001	15	23787001	23789000	2000	1	3.10E-07	0.55	37	1.85	Samd4a	
DMR15:23860001	15	23860001	23861000	1000	1	5.60E-13	0.59	24	2.4	Samd4a	
DMR15:24270001	15	24270001	24273000	3000	1	9.90E-07	0.34	46	1.53	Fbxo34	
DMR15:24294001	15	24294001	24297000	3000	1	6.30E-07	0.5	30	1	Fbxo34	
DMR15:24519001	15	24519001	24520000	1000	1	7.70E-07	-0.49	15	1.5	Ktn1	
DMR15:24986001	15	24986001	24988000	2000	1	3.60E-07	0.35	37	1.85	Peli2	Proteolysis
DMR15:25039001	15	25039001	25041000	2000	1	1.10E-07	0.33	43	2.15	Peli2	Proteolysis
DMR15:25093001	15	25093001	25096000	3000	1	7.40E-07	-0.35	31	1.03	Peli2	Proteolysis
DMR15:25905001	15	25905001	25906000	1000	1	1.20E-08	0.4	9	0.9	Exoc5	Transport
DMR15:26055001	15	26055001	26057000	2000	2	3.60E-10	0.44	19	0.95	Naa30	Metabolism

DMR15:26171001	15	26171001	26173000	2000	1	4.50E-11	0.46	25	1.25	Slc35f4	
DMR15:26246001	15	26246001	26248000	2000	1	2.50E-07	0.55	10	0.5	Slc35f4;LOC102556222	
DMR15:27353001	15	27353001	27354000	1000	1	2.30E-08	-0.37	13	1.3	Olr1619	Signaling
DMR15:27387001	15	27387001	27394000	7000	1	8.70E-08	-0.31	75	1.07	Olr1620;LOC690055	Signaling
DMR15:27972001	15	27972001	27977000	5000	1	2.90E-07	-0.38	90	1.8	LOC102553566;Rnase9;Rnase11;Rnase12	
DMR15:28144001	15	28144001	28147000	3000	1	3.00E-09	0.44	52	1.73	Rnase2	
DMR15:28163001	15	28163001	28166000	3000	1	9.60E-07	-0.41	74	2.47	Rnase2	
DMR15:28218001	15	28218001	28221000	3000	1	5.10E-07	-0.3	65	2.17	Ear11	
DMR15:28322001	15	28322001	28324000	2000	1	1.00E-09	0.49	34	1.7	Ndrg2;Tppp2;Rnase13	Protease;Cytoskeleton
DMR15:28589001	15	28589001	28591000	2000	1	1.00E-07	0.4	26	1.3	Supt16h	Epigenetic
DMR15:28828001	15	28828001	28832000	4000	2	2.40E-09	-0.31	44	1.1	Olr1641	Receptor
DMR15:30069001	15	30069001	30072000	3000	1	1.50E-07	-0.67	20	0.67	RGD1561152;LOC103690223	
DMR15:30087001	15	30087001	30091000	4000	1	4.70E-08	-0.48	53	1.32	RGD1561152;LOC103690223	
DMR15:31368001	15	31368001	31373000	5000	2	2.60E-07	-0.62	19	0.38	LOC102554516;LOC103693848;RGD1564188	
DMR15:31575001	15	31575001	31577000	2000	1	4.70E-07	-0.39	15	0.75	LOC103693857;RGD1563477	
DMR15:31588001	15	31588001	31589000	1000	1	2.40E-09	-0.49	7	0.7	LOC103693857;RGD1563477	
DMR15:32409001	15	32409001	32413000	4000	1	1.00E-09	-0.32	41	1.02	LOC685078;LOC102556949;LOC103693549;RGD1560771	
DMR15:32917001	15	32917001	32919000	2000	1	2.20E-08	-0.49	23	1.15	RGD1566241;Olr1646	Receptor
DMR15:33128001	15	33128001	33129000	1000	1	4.20E-08	0.66	5	0.5	Rem2	
DMR15:33398001	15	33398001	33399000	1000	1	5.80E-07	0.4	17	1.7	Slc7a8	Transport
DMR15:33672001	15	33672001	33673000	1000	1	5.00E-08	-0.53	20	2	Ngdn	Metabolism
DMR15:33822001	15	33822001	33824000	2000	1	8.40E-10	0.52	34	1.7	RGD1564324	Metabolism
DMR15:34172001	15	34172001	34175000	3000	1	1.50E-07	0.42	55	1.83	Dhrs4;Carmil3;LOC102552093	Metabolism
DMR15:34189001	15	34189001	34194000	5000	2	2.00E-08	0.39	118	2.36	Carmil3;LOC102552093;Cpne6;Nrl	Transcription
DMR15:34419001	15	34419001	34421000	2000	1	3.50E-10	0.5	14	0.7	Dhrs1	Metabolism
DMR15:34657001	15	34657001	34663000	6000	1	2.20E-08	-0.32	70	1.17	Mcpt10;Mcpt8l3	Protease
DMR15:34686001	15	34686001	34689000	3000	2	1.20E-09	-0.32	27	0.9	LOC498518;Mcpt8	Protease
DMR15:34704001	15	34704001	34708000	4000	1	5.50E-07	-0.39	53	1.32	Mcpt8	Protease
DMR15:35152001	15	35152001	35156000	4000	2	1.40E-19	0.64	86	2.15	Gzmn;LOC691670	Protease
DMR15:35188001	15	35188001	35193000	5000	1	3.40E-07	0.36	21	0.42	Gzmf;LOC691677	Protease
DMR15:35407001	15	35407001	35412000	5000	3	3.00E-08	-0.37	54	1.08	Gzmb	Protease
DMR15:36463001	15	36463001	36467000	4000	2	8.80E-07	-0.31	38	0.95	Gzmbl2	
DMR15:36628001	15	36628001	36634000	6000	2	2.30E-07	-0.36	53	0.88	Rnf17	
DMR15:36753001	15	36753001	36756000	3000	1	9.70E-09	0.4	18	0.6	Rnf17;Cenpj	Cytoskeleton
DMR15:36763001	15	36763001	36770000	7000	1	1.30E-10	-0.36	70	1	Cenpj	Cytoskeleton
DMR15:37135001	15	37135001	37137000	2000	1	2.50E-09	0.46	26	1.3	RGD1563527	
DMR15:37143001	15	37143001	37148000	5000	1	4.30E-09	-0.38	37	0.74	RGD1563527;Zmym2	Transcription
DMR15:37576001	15	37576001	37577000	1000	1	7.20E-10	0.38	13	1.3	Cryl1	Metabolism
DMR15:37640001	15	37640001	37642000	2000	1	2.30E-08	0.39	32	1.6	Cryl1	Metabolism
DMR15:37747001	15	37747001	37748000	1000	1	5.70E-07	0.38	10	1	Ift88	
DMR15:37911001	15	37911001	37916000	5000	1	2.80E-09	-0.42	45	0.9	Xpo4	Transport
DMR15:37957001	15	37957001	37959000	2000	1	1.10E-08	0.48	15	0.75	Lats2	Signaling
DMR15:38236001	15	38236001	38241000	5000	2	6.60E-09	-0.48	51	1.02	Micu2	Signaling
DMR15:38821001	15	38821001	38822000	1000	1	2.40E-08	-0.51	7	0.7	Phf11;Setdb2	Transcription;Epigenetic
DMR15:39221001	15	39221001	39230000	9000	2	1.60E-12	-0.41	117	1.3	Phf11;Setdb2	Transcription;Epigenetic
DMR15:39609001	15	39609001	39612000	3000	1	7.40E-08	0.61	1	0.03	Phf11;Setdb2;RGD1563302;LOC102552417	Transcription;Epigenetic
DMR15:39755001	15	39755001	39756000	1000	1	2.80E-07	-0.44	6	0.6	Setdb2;Cab39l	Epigenetic
DMR15:40015001	15	40015001	40016000	1000	1	3.90E-07	0.56	15	1.5	Atp8a2	Transport
DMR15:40241001	15	40241001	40246000	5000	1	6.90E-09	-0.39	44	0.88	Atp8a2	Transport
DMR15:40397001	15	40397001	40399000	2000	1	5.60E-08	0.36	18	0.9	Atp8a2	Transport
DMR15:41032001	15	41032001	41034000	2000	1	5.30E-08	0.35	17	0.85	Spata13	Transcription

DMR15:41045001	15	41045001	41048000	3000	1	3.60E-09	0.41	43	1.43	Spata13	Transcription
DMR15:41053001	15	41053001	41055000	2000	1	3.30E-12	0.46	40	2	Spata13	Transcription
DMR15:41105001	15	41105001	41108000	3000	1	8.90E-12	0.44	112	3.73	Mipep	Protease
DMR15:41184001	15	41184001	41185000	1000	1	2.70E-08	0.56	6	0.6	Mipep	Protease
DMR15:41187001	15	41187001	41189000	2000	1	8.90E-09	0.43	36	1.8	Mipep	Protease
DMR15:41709001	15	41709001	41710000	1000	1	2.50E-07	0.35	10	1	Kpna3	Transport
DMR15:41725001	15	41725001	41730000	5000	1	1.30E-07	-0.28	50	1	Kpna3;LOC100911441	Transport
DMR15:41860001	15	41860001	41862000	2000	1	4.10E-07	-0.35	18	0.9	Srpyd7	
DMR15:42620001	15	42620001	42621000	1000	1	4.10E-12	0.42	9	0.9	Scara3;Clu	Extracellular Matrix
DMR15:42722001	15	42722001	42725000	3000	2	3.00E-08	0.47	49	1.63	LOC102555773;Adam2	Protease
DMR15:42729001	15	42729001	42730000	1000	1	1.80E-07	0.35	21	2.1	LOC102555773;Adam2	Protease
DMR15:43710001	15	43710001	43711000	1000	1	1.30E-11	-0.6	14	1.4	Ppp2r2a	Signaling
DMR15:44603001	15	44603001	44607000	4000	1	6.70E-07	0.35	53	1.32	Dock5	Transcription
DMR15:45367001	15	45367001	45369000	2000	1	1.00E-08	0.45	26	1.3	Dleu7	
DMR15:45477001	15	45477001	45481000	4000	1	2.40E-08	-0.42	61	1.52	Rnaseh2b;Gucy1b2	Signaling
DMR15:45533001	15	45533001	45535000	2000	1	2.50E-09	0.41	18	0.9	Gucy1b2	Signaling
DMR15:46353001	15	46353001	46354000	1000	1	4.70E-07	0.59	3	0.3	Fdft1	Transport
DMR15:46596001	15	46596001	46598000	2000	1	7.70E-08	-0.34	41	2.05	Blk	
DMR15:47143001	15	47143001	47145000	2000	1	5.60E-09	0.51	27	1.35	Xkr6	
DMR15:47650001	15	47650001	47651000	1000	1	2.10E-07	0.45	5	0.5	Msra	Metabolism
DMR15:47794001	15	47794001	47795000	1000	1	6.90E-09	0.54	11	1.1	Msra	Metabolism
DMR15:48433001	15	48433001	48434000	1000	1	1.50E-08	0.36	20	2	Extl3	Golgi
DMR15:48628001	15	48628001	48630000	2000	1	5.90E-07	-0.38	37	1.85	Fzd3;LOC108352940	Receptor
DMR15:48654001	15	48654001	48655000	1000	1	6.60E-07	-0.66	9	0.9	Fzd3	Receptor
DMR15:51047001	15	51047001	51055000	8000	3	6.90E-14	0.65	81	1.01	Nkx2-6	Development
DMR15:51111001	15	51111001	51117000	6000	1	1.60E-07	-0.44	38	0.63	Slc25a37	
DMR15:51281001	15	51281001	51283000	2000	1	1.60E-10	-0.5	21	1.05	Loxl2	Metabolism
DMR15:51538001	15	51538001	51542000	4000	1	2.90E-07	0.49	45	1.12	Pebp4	
DMR15:51575001	15	51575001	51576000	1000	1	1.50E-09	0.36	11	1.1	Pebp4	
DMR15:51770001	15	51770001	51772000	2000	1	7.50E-07	0.47	14	0.7	Egr3	Transcription
DMR15:51861001	15	51861001	51864000	3000	1	1.70E-08	0.42	45	1.5	Pdlim2;Sorbs3	Cytoskeleton
DMR15:52012001	15	52012001	52013000	1000	1	4.30E-09	0.41	22	2.2	Slc39a14	Transport
DMR15:52577001	15	52577001	52582000	5000	2	2.30E-11	0.42	36	0.72	Gfra2	Receptor
DMR15:52618001	15	52618001	52620000	2000	1	2.60E-07	0.41	11	0.55	Gfra2	Receptor
DMR15:52847001	15	52847001	52852000	5000	2	2.70E-07	-0.31	60	1.2	Dpm3-ps1	
DMR15:54369001	15	54369001	54375000	6000	1	3.50E-08	-0.3	93	1.55	Fndc3a	Proteolysis
DMR15:54422001	15	54422001	54424000	2000	1	8.90E-10	-0.53	11	0.55	Fndc3a	Proteolysis
DMR15:54504001	15	54504001	54506000	2000	1	1.10E-07	-0.27	27	1.35	Fndc3a	Proteolysis
DMR15:54508001	15	54508001	54512000	4000	2	5.70E-09	-0.35	67	1.68	Fndc3a	Proteolysis
DMR15:54523001	15	54523001	54526000	3000	1	2.20E-10	-0.36	29	0.97	Fndc3a	Proteolysis
DMR15:54539001	15	54539001	54540000	1000	1	5.00E-07	-0.53	5	0.5	Fndc3a	Proteolysis
DMR15:55087001	15	55087001	55088000	1000	1	2.40E-10	0.48	13	1.3	Rb1	Epigenetic
DMR15:55466001	15	55466001	55467000	1000	1	6.00E-08	0.5	7	0.7	Nudt15;Trnae-uuc;Sucla2	Metabolism
DMR15:56682001	15	56682001	56685000	3000	1	1.70E-13	0.76	36	1.2	Htr2a	Signaling
DMR15:56722001	15	56722001	56724000	2000	1	3.70E-08	0.46	25	1.25	Htr2a	Signaling
DMR15:57138001	15	57138001	57139000	1000	1	1.30E-07	-0.35	5	0.5	Lrrc63	Cytoskeleton
DMR15:57302001	15	57302001	57303000	1000	1	1.30E-08	-0.47	15	1.5	Cpb2;LOC691812	Protease
DMR15:58233001	15	58233001	58234000	1000	1	5.00E-11	0.76	10	1	Trnae-uuc	
DMR15:58644001	15	58644001	58646000	2000	1	2.30E-07	0.36	19	0.95	Tsc22d1	
DMR15:59155001	15	59155001	59156000	1000	1	9.70E-07	-0.46	16	1.6	LOC108353005;Ccdc122	
DMR15:59437001	15	59437001	59439000	2000	1	1.50E-07	-0.42	42	2.1	Enox1	Metabolism
DMR15:59524001	15	59524001	59525000	1000	1	5.90E-07	-0.36	14	1.4	Enox1	Metabolism
DMR15:59584001	15	59584001	59586000	2000	1	8.80E-10	-0.4	157	7.85	Enox1	Metabolism
DMR15:59714001	15	59714001	59715000	1000	1	4.20E-08	-0.62	21	2.1	Enox1	Metabolism
DMR15:59996001	15	59996001	59998000	2000	1	5.00E-09	0.46	12	0.6	Dnajc15	
DMR15:60057001	15	60057001	60062000	5000	1	1.40E-11	-0.37	60	1.2	Dnajc15	
DMR15:60155001	15	60155001	60157000	2000	1	7.10E-07	0.39	77	3.85	Epsti1	
DMR15:60278001	15	60278001	60283000	5000	1	1.20E-09	-0.33	43	0.86	Fam216b	
DMR15:60890001	15	60890001	60892000	2000	1	3.60E-08	0.35	47	2.35	Dgkh	Signaling
DMR15:60908001	15	60908001	60910000	2000	1	5.00E-08	0.39	51	2.55	Dgkh	Signaling
DMR15:61355001	15	61355001	61357000	2000	1	9.80E-17	0.5	24	1.2	Vwa8	

DMR15:61735001	15	61735001	61741000	6000	1	1.00E-10	-0.37	69	1.15	Kbtbd6;Wbp4	Cytoskeleton
DMR15:62187001	15	62187001	62191000	4000	2	8.30E-09	-0.35	44	1.1	Pcdh8	Cytoskeleton
DMR15:69958001	15	69958001	69961000	3000	1	3.40E-09	-0.51	29	0.97	Diaph3	
DMR15:70030001	15	70030001	70033000	3000	1	9.80E-09	-0.31	37	1.23	Diaph3	
DMR15:70122001	15	70122001	70125000	3000	1	2.40E-08	0.48	40	1.33	Diaph3	
DMR15:70153001	15	70153001	70154000	1000	1	2.00E-08	-0.49	11	1.1	Diaph3	
DMR15:70160001	15	70160001	70161000	1000	1	6.90E-08	-0.4	11	1.1	Diaph3	
DMR15:70180001	15	70180001	70183000	3000	1	8.70E-15	0.48	41	1.37	Diaph3	
DMR15:70293001	15	70293001	70296000	3000	1	3.10E-09	-0.44	31	1.03	Diaph3	
DMR15:70778001	15	70778001	70786000	8000	1	2.40E-10	-0.4	78	0.98	Tdrd3	Translation
DMR15:71768001	15	71768001	71769000	1000	1	1.60E-07	-0.47	8	0.8	Pcdh20	Cytoskeleton
DMR15:76891001	15	76891001	76893000	2000	1	8.40E-08	-0.42	30	1.5	Pcdh9	Cytoskeleton
DMR15:76922001	15	76922001	76929000	7000	2	1.10E-09	-0.33	70	1	Pcdh9	Cytoskeleton
DMR15:77493001	15	77493001	77494000	1000	1	9.50E-07	-0.58	12	1.2	Pcdh9	Cytoskeleton
DMR15:77527001	15	77527001	77534000	7000	2	9.60E-11	-0.35	78	1.11	Pcdh9	Cytoskeleton
DMR15:77615001	15	77615001	77617000	2000	1	2.10E-09	0.53	16	0.8	Pcdh9	Cytoskeleton
DMR15:77633001	15	77633001	77635000	2000	1	1.70E-07	-0.6	27	1.35	Pcdh9	Cytoskeleton
DMR15:80516001	15	80516001	80518000	2000	1	2.30E-07	-0.29	19	0.95	Klhl1	Cytoskeleton
DMR15:80578001	15	80578001	80582000	4000	2	3.60E-15	-0.41	39	0.98	Klhl1	Cytoskeleton
DMR15:80650001	15	80650001	80656000	6000	1	7.50E-07	-0.32	59	0.98	Klhl1	Cytoskeleton
DMR15:80723001	15	80723001	80730000	7000	3	1.90E-09	-0.39	74	1.06	Klhl1	Cytoskeleton
DMR15:82209001	15	82209001	82210000	1000	1	1.10E-08	-0.64	15	1.5	Dach1	Transcription
DMR15:83638001	15	83638001	83640000	2000	1	2.80E-07	0.3	25	1.25	Pibf1	
DMR15:84329001	15	84329001	84331000	2000	2	1.70E-14	0.55	19	0.95	Klf12;LOC103693766	Transcription
DMR15:84618001	15	84618001	84622000	4000	1	3.10E-07	-0.47	52	1.3	Klf12	Transcription
DMR15:84662001	15	84662001	84663000	1000	1	3.90E-08	-0.42	10	1	Klf12	Transcription
DMR15:86017001	15	86017001	86020000	3000	1	4.70E-09	0.48	37	1.23	Tbc1d4	Signaling
DMR15:86054001	15	86054001	86057000	3000	1	9.30E-08	-0.38	32	1.07	Tbc1d4	Signaling
DMR15:86608001	15	86608001	86609000	1000	1	2.00E-07	-0.42	17	1.7	LOC290444;RGD1562819	
DMR15:87755001	15	87755001	87759000	4000	1	4.30E-10	-0.42	50	1.25	Scel	
DMR15:87872001	15	87872001	87874000	2000	1	2.00E-08	-0.45	24	1.2	Slain1	
DMR15:87893001	15	87893001	87894000	1000	1	9.00E-15	0.55	21	2.1	Slain1	
DMR15:87899001	15	87899001	87901000	2000	1	1.80E-07	0.4	19	0.95	Slain1	
DMR15:93689001	15	93689001	93690000	1000	1	1.20E-09	-0.45	13	1.3	Mycbp2	Proteolysis
DMR15:95501001	15	95501001	95505000	4000	1	4.00E-07	-0.32	54	1.35	Slitrk6	
DMR15:100394001	15	100394001	100399000	5000	2	8.20E-09	-0.32	59	1.18	Gpc5	
DMR15:100473001	15	100473001	100478000	5000	1	8.10E-09	-0.4	43	0.86	Gpc5	
DMR15:100726001	15	100726001	100727000	1000	1	8.90E-07	-0.54	4	0.4	Gpc5	
DMR15:100890001	15	100890001	100892000	2000	1	3.50E-07	0.41	25	1.25	Gpc5	
DMR15:101086001	15	101086001	101087000	1000	1	9.50E-07	0.49	9	0.9	Gpc5	
DMR15:101169001	15	101169001	101170000	1000	1	7.10E-09	0.43	9	0.9	Gpc5	
DMR15:101347001	15	101347001	101350000	3000	1	2.20E-07	-0.31	39	1.3	Gpc5	
DMR15:102313001	15	102313001	102315000	2000	1	6.90E-09	0.56	22	1.1	Gpc6	
DMR15:102472001	15	102472001	102474000	2000	2	2.00E-08	0.38	26	1.3	Gpc6	
DMR15:102603001	15	102603001	102604000	1000	1	3.00E-08	0.39	19	1.9	Gpc6	
DMR15:102722001	15	102722001	102723000	1000	1	2.70E-07	-0.55	10	1	Gpc6	
DMR15:102875001	15	102875001	102876000	1000	1	1.70E-07	-0.42	25	2.5	Gpc6	
DMR15:102955001	15	102955001	102956000	1000	1	4.20E-08	0.58	5	0.5	Gpc6	
DMR15:102962001	15	102962001	102964000	2000	1	5.80E-07	0.55	27	1.35	Gpc6	
DMR15:103775001	15	103775001	103776000	1000	1	1.50E-08	0.41	18	1.8	Abcc4	Transport
DMR15:103864001	15	103864001	103867000	3000	1	2.30E-09	0.4	52	1.73	Abcc4	Transport
DMR15:103894001	15	103894001	103897000	3000	1	1.30E-07	0.4	62	2.07	Abcc4	Transport
DMR15:103913001	15	103913001	103918000	5000	3	1.20E-09	-0.39	45	0.9	Abcc4	Transport
DMR15:104051001	15	104051001	104053000	2000	1	8.20E-09	0.6	22	1.1	Cldn10	Cell Junction
DMR15:104154001	15	104154001	104156000	2000	1	8.90E-07	0.31	47	2.35	Dzip1	
DMR15:104203001	15	104203001	104208000	5000	1	1.20E-07	-0.29	53	1.06	Dnajc3	
DMR15:104402001	15	104402001	104403000	1000	1	1.40E-12	-0.77	10	1	Uggt2	
DMR15:104412001	15	104412001	104414000	2000	1	7.10E-07	0.4	24	1.2	Uggt2	
DMR15:104477001	15	104477001	104479000	2000	1	6.40E-07	-0.46	33	1.65	Hs6st3	
DMR15:104639001	15	104639001	104646000	7000	4	2.20E-09	-0.36	70	1	Hs6st3	
DMR15:104953001	15	104953001	104954000	1000	1	8.80E-07	-0.44	6	0.6	Hs6st3	

DMR15:105042001	15	105042001	105043000	1000	1	5.10E-07	-0.38	16	1.6	Hs6st3	
DMR15:105202001	15	105202001	105206000	4000	1	1.50E-08	-0.43	66	1.65	Hs6st3	
DMR15:105229001	15	105229001	105230000	1000	1	2.90E-09	0.46	7	0.7	Hs6st3;LOC102556639	
DMR15:105772001	15	105772001	105773000	1000	1	2.00E-08	-0.48	28	2.8	Mbnl2	Translation
DMR15:106643001	15	106643001	106645000	2000	1	7.10E-07	0.36	28	1.4	Stk24	
DMR15:106658001	15	106658001	106661000	3000	2	1.50E-22	0.67	75	2.5	Stk24	
DMR15:106685001	15	106685001	106688000	3000	2	6.30E-08	0.47	44	1.47	Stk24	
DMR15:107955001	15	107955001	107956000	1000	1	7.70E-14	0.53	23	2.3	Dock9	Transcription
DMR15:107963001	15	107963001	107964000	1000	1	9.20E-10	-0.36	25	2.5	Dock9	Transcription
DMR15:107978001	15	107978001	107980000	2000	1	6.30E-07	0.37	24	1.2	Dock9	Transcription
DMR15:108077001	15	108077001	108080000	3000	2	8.40E-13	0.59	36	1.2	Dock9	Transcription
DMR15:108165001	15	108165001	108168000	3000	1	2.80E-08	0.46	34	1.13	Dock9	Transcription
DMR15:108220001	15	108220001	108221000	1000	1	8.60E-11	0.59	7	0.7	Dock9	Transcription
DMR15:108379001	15	108379001	108382000	3000	1	8.00E-08	0.48	24	0.8	Ubac2;Gpr183	Signaling
DMR15:108545001	15	108545001	108546000	1000	1	6.30E-07	0.37	12	1.2	Tm9sf2;LOC102555195	Transport
DMR15:108653001	15	108653001	108655000	2000	2	5.70E-16	0.72	4	0.2	Clybl	Metabolism
DMR15:108687001	15	108687001	108689000	2000	1	1.10E-08	0.48	17	0.85	Clybl	Metabolism
DMR15:108794001	15	108794001	108798000	4000	1	1.60E-07	0.62	60	1.5	Clybl	Metabolism
DMR15:108957001	15	108957001	108958000	1000	1	5.90E-08	-0.4	11	1.1	Pcca	Metabolism
DMR15:109034001	15	109034001	109036000	2000	1	2.80E-07	0.43	27	1.35	Pcca	Metabolism
DMR15:109043001	15	109043001	109044000	1000	1	4.50E-09	0.36	10	1	Pcca	Metabolism
DMR15:109880001	15	109880001	109883000	3000	1	5.50E-07	-0.42	12	0.4	Nalcn	Transport
DMR15:110055001	15	110055001	110059000	4000	2	4.20E-12	-0.36	37	0.92	Nalcn	Transport
DMR15:110211001	15	110211001	110215000	4000	4	1.50E-08	-0.46	33	0.82	Itgbl1	Extracellular Matrix
DMR15:110405001	15	110405001	110408000	3000	1	5.80E-09	0.59	23	0.77	Fgf14	Growth Factors
DMR15:110430001	15	110430001	110438000	8000	1	3.40E-08	-0.27	101	1.26	Fgf14	Growth Factors
DMR15:110508001	15	110508001	110512000	4000	3	1.50E-09	-0.31	39	0.98	Fgf14	Growth Factors
DMR15:110715001	15	110715001	110716000	1000	1	8.10E-11	0.52	4	0.4	Fgf14	Growth Factors
DMR16:1741001	16	1741001	1743000	2000	1	8.00E-11	0.52	27	1.35	Zmiz1;LOC102553140	
DMR16:1819001	16	1819001	1820000	1000	1	4.00E-07	0.33	16	1.6	Zmiz1;Mir3075	
DMR16:2399001	16	2399001	2405000	6000	2	4.70E-09	-0.3	77	1.28	Dnah12	Cytoskeleton
DMR16:2715001	16	2715001	2718000	3000	1	9.90E-09	0.47	38	1.27	Il17rd	Receptor
DMR16:2820001	16	2820001	2823000	3000	2	3.70E-15	0.53	33	1.1	Arhgef3;LOC108353070	Transcription
DMR16:2894001	16	2894001	2897000	3000	1	1.80E-08	0.36	56	1.87	Arhgef3	Transcription
DMR16:3009001	16	3009001	3010000	1000	1	3.30E-07	0.54	6	0.6	Arhgef3	Transcription
DMR16:3281001	16	3281001	3284000	3000	1	1.10E-07	-0.31	29	0.97	Erc2	Transport
DMR16:3359001	16	3359001	3360000	1000	1	2.40E-07	0.36	14	1.4	Erc2;LOC108348452;LOC102553494	Transport
DMR16:3442001	16	3442001	3443000	1000	1	4.70E-08	0.6	7	0.7	Erc2	Transport
DMR16:3843001	16	3843001	3845000	2000	1	6.20E-09	0.43	21	1.05	Erc2;Plac9	Transport
DMR16:3871001	16	3871001	3878000	7000	3	4.80E-08	0.39	115	1.64	Erc2;Plac9;Anxa11	Transport;Signaling
DMR16:3879001	16	3879001	3882000	3000	1	7.40E-07	0.37	40	1.33	Erc2;Anxa11	Transport;Signaling
DMR16:3986001	16	3986001	3987000	1000	1	9.50E-07	-0.6	17	1.7	Erc2	Transport
DMR16:5210001	16	5210001	5211000	1000	1	3.30E-08	0.56	6	0.6	Cacna2d3;LOC108348455	Transport
DMR16:5673001	16	5673001	5674000	1000	1	3.00E-10	0.35	18	1.8	Cacna2d3	Transport
DMR16:5676001	16	5676001	5677000	1000	1	1.40E-07	0.28	7	0.7	Cacna2d3	Transport
DMR16:5699001	16	5699001	5702000	3000	1	1.70E-08	0.65	44	1.47	Cacna2d3	Transport
DMR16:6125001	16	6125001	6127000	2000	1	2.80E-09	0.46	47	2.35	Cacna1d	Transport
DMR16:6169001	16	6169001	6174000	5000	1	5.60E-07	0.4	86	1.72	Cacna1d;LOC108348454	Transport
DMR16:6290001	16	6290001	6292000	2000	1	1.20E-12	-0.65	25	1.25	Cacna1d;LOC103693892	Transport
DMR16:6363001	16	6363001	6365000	2000	1	1.10E-09	0.48	55	2.75	Cacna1d	Transport
DMR16:6549001	16	6549001	6552000	3000	1	4.40E-09	-0.5	41	1.37	Dcp1a	Translation
DMR16:6663001	16	6663001	6664000	1000	1	1.90E-08	0.36	8	0.8	Prkcd	Signaling
DMR16:7140001	16	7140001	7141000	1000	1	9.20E-07	-0.46	17	1.7	Pbrm1	Epigenetic
DMR16:7168001	16	7168001	7169000	1000	1	8.00E-08	-0.37	10	1	Pbrm1	Epigenetic
DMR16:7220001	16	7220001	7225000	5000	1	1.10E-08	0.41	157	3.14	Smim4;Nt5dc2;Stab1	Signaling;Transport
DMR16:7240001	16	7240001	7243000	3000	1	9.70E-14	0.61	78	2.6	Stab1	Transport
DMR16:7302001	16	7302001	7303000	1000	1	4.50E-07	-0.63	20	2	Tnnc1;Sema3g	Cytoskeleton;Signaling
DMR16:7322001	16	7322001	7323000	1000	1	2.40E-09	0.62	8	0.8	Sema3g;Phf7	Signaling;Transcription
DMR16:7351001	16	7351001	7355000	4000	1	7.30E-09	0.4	97	2.42	Bap1;Dnah1	Protease;Cytoskeleton
DMR16:7358001	16	7358001	7361000	3000	1	5.70E-12	0.49	68	2.27	Dnah1	Cytoskeleton

DMR16:7363001	16	7363001	7367000	4000	1	3.70E-07	0.36	84	2.1	Dnah1	Cytoskeleton
DMR16:7372001	16	7372001	7373000	1000	1	6.50E-09	0.56	19	1.9	Dnah1	Cytoskeleton
DMR16:7675001	16	7675001	7676000	1000	1	9.90E-07	0.43	7	0.7	Colq;LOC108353071	Extracellular Matrix
DMR16:8212001	16	8212001	8215000	3000	1	5.00E-07	0.36	40	1.33	Dph3;Oxnad1	Metabolism
DMR16:8269001	16	8269001	8273000	4000	1	2.10E-07	0.52	59	1.48	Msmb	Signaling
DMR16:8770001	16	8770001	8771000	1000	1	7.60E-07	0.43	3	0.3	Ercc6	
DMR16:9335001	16	9335001	9336000	1000	1	8.40E-09	0.46	14	1.4	Wdfy4	
DMR16:9439001	16	9439001	9444000	5000	1	1.10E-07	0.37	85	1.7	Wdfy4	
DMR16:9458001	16	9458001	9465000	7000	1	2.30E-17	0.66	121	1.73	Arhgap22	
DMR16:9506001	16	9506001	9507000	1000	1	5.90E-07	0.57	3	0.3	Arhgap22	
DMR16:10406001	16	10406001	10408000	2000	1	2.10E-07	0.45	24	1.2	Antxrl;Anxa8	Cytoskeleton;Signaling
DMR16:10709001	16	10709001	10713000	4000	1	1.10E-10	0.43	59	1.48	Fam25a;Sncg	Transport
DMR16:10945001	16	10945001	10947000	2000	1	6.20E-07	0.38	27	1.35	Ldb3;Opn4	Cytoskeleton;Signaling
DMR16:11184001	16	11184001	11186000	2000	1	9.40E-07	0.43	31	1.55	Grid1	Receptor
DMR16:11241001	16	11241001	11246000	5000	1	2.50E-10	0.47	69	1.38	Grid1;Mir346	Receptor
DMR16:11262001	16	11262001	11263000	1000	1	1.50E-11	0.53	6	0.6	Grid1	Receptor
DMR16:11540001	16	11540001	11543000	3000	1	1.40E-08	-0.34	43	1.43	Grid1	Receptor
DMR16:11931001	16	11931001	11933000	2000	1	1.10E-08	0.53	26	1.3	Grid1	Receptor
DMR16:11937001	16	11937001	11938000	1000	1	1.20E-07	0.51	5	0.5	Grid1	Receptor
DMR16:14185001	16	14185001	14188000	3000	1	8.40E-09	-0.54	48	1.6	Ccser2	
DMR16:14192001	16	14192001	14193000	1000	1	1.70E-07	0.37	23	2.3	Ccser2	
DMR16:16132001	16	16132001	16134000	2000	1	1.90E-18	0.91	24	1.2	Nrg3	Growth Factors
DMR16:16179001	16	16179001	16180000	1000	1	1.80E-07	0.36	4	0.4	Nrg3	Growth Factors
DMR16:16451001	16	16451001	16452000	1000	1	2.20E-07	0.57	5	0.5	Nrg3	Growth Factors
DMR16:16482001	16	16482001	16484000	2000	1	2.20E-07	0.5	14	0.7	Nrg3	Growth Factors
DMR16:16548001	16	16548001	16549000	1000	1	4.30E-07	0.44	5	0.5	Nrg3	Growth Factors
DMR16:16620001	16	16620001	16621000	1000	1	6.40E-11	-0.69	10	1	Nrg3	Growth Factors
DMR16:16764001	16	16764001	16765000	1000	1	8.90E-08	0.45	14	1.4	Nrg3	Growth Factors
DMR16:16868001	16	16868001	16871000	3000	1	1.10E-11	-0.59	14	0.47	Nrg3	Growth Factors
DMR16:17686001	16	17686001	17695000	9000	1	3.30E-07	-0.38	110	1.22	Sh2d4b	Immune
DMR16:17704001	16	17704001	17706000	2000	1	3.00E-08	0.49	8	0.4	Sh2d4b	Immune
DMR16:18885001	16	18885001	18886000	1000	1	4.80E-08	0.39	18	1.8	Nwd1	
DMR16:19077001	16	19077001	19078000	1000	1	3.70E-08	0.45	22	2.2	LOC103693914;Cherp;RGD 1311847	Metabolism
DMR16:19336001	16	19336001	19337000	1000	1	1.50E-09	0.51	12	1.2	Cib3;Hsh2d	Immune
DMR16:19631001	16	19631001	19633000	2000	1	8.00E-09	0.38	29	1.45	Zfp709	Transcription
DMR16:19937001	16	19937001	19940000	3000	1	7.20E-09	0.65	48	1.6	LOC108353095;Bst2	
DMR16:20139001	16	20139001	20142000	3000	1	3.70E-07	0.39	63	2.1	B3gnt3;LOC108348389;Fch o1	Golgi;Cytoskeleton
DMR16:20162001	16	20162001	20164000	2000	1	4.80E-08	0.43	26	1.3	Fcho1;LOC108348388;LOC1 08348387	Cytoskeleton
DMR16:20339001	16	20339001	20341000	2000	2	5.30E-08	0.48	47	2.35	Kcnn1	Transport
DMR16:20363001	16	20363001	20365000	2000	1	1.20E-08	0.39	30	1.5	Arrdc2;Il12rb1	Receptor
DMR16:20458001	16	20458001	20462000	4000	2	3.10E-07	0.59	70	1.75	Pde4c;LOC102547294	Signaling
DMR16:20471001	16	20471001	20475000	4000	1	2.10E-07	0.39	95	2.38	Pde4c;LOC102547294	Signaling
DMR16:20679001	16	20679001	20680000	1000	1	4.00E-08	0.45	16	1.6	Uba52;RGD1566239;Crlf1	Receptor
DMR16:20745001	16	20745001	20748000	3000	3	7.10E-12	0.58	67	2.23	Crtc1;LOC102549994	Transcription
DMR16:21112001	16	21112001	21113000	1000	1	4.40E-07	-0.28	10	1	Sugp1	Translation
DMR16:21216001	16	21216001	21218000	2000	2	2.20E-07	0.46	28	1.4	Gatad2a	Transcription
DMR16:21950001	16	21950001	21956000	6000	1	2.00E-09	0.51	30	0.5	RGD1563748;RGD1564941	
DMR16:22006001	16	22006001	22011000	5000	2	2.20E-09	-0.45	46	0.92	RGD1563748;RGD1564941	
DMR16:22013001	16	22013001	22022000	9000	2	2.90E-08	0.34	135	1.5	RGD1563748;RGD1564941	
DMR16:22030001	16	22030001	22033000	3000	2	3.80E-11	-0.32	33	1.1	RGD1563748;RGD1564941	
DMR16:22036001	16	22036001	22039000	3000	1	1.70E-07	0.26	16	0.53	RGD1563748;RGD1564941	
DMR16:22045001	16	22045001	22047000	2000	1	5.80E-16	0.38	3	0.15	RGD1563748;RGD1564941	

DMR16:22048001	16	22048001	22049000	1000	1	2.10E-09	-0.29	4	0.4	RGD1563748;RGD1564941	
DMR16:22053001	16	22053001	22057000	4000	2	1.40E-08	0.46	54	1.35	RGD1563748;RGD1564941	
DMR16:22071001	16	22071001	22075000	4000	3	3.60E-12	0.37	33	0.82	RGD1563748;RGD1564941	
DMR16:22076001	16	22076001	22092000	16000	1	3.40E-10	0.27	111	0.69	RGD1563748;RGD1564941	
DMR16:22115001	16	22115001	22117000	2000	2	3.20E-12	0.29	29	1.45	RGD1563748;RGD1564941;LOC103693919;LOC108353083	
DMR16:22118001	16	22118001	22123000	5000	2	9.30E-18	0.45	36	0.72	RGD1563748;RGD1564941;LOC108353083	
DMR16:22124001	16	22124001	22131000	7000	1	1.40E-08	0.27	36	0.51	RGD1563748;RGD1564941;LOC108353083;LOC108353082	
DMR16:22553001	16	22553001	22555000	2000	1	9.10E-11	-0.63	32	1.6	Lpl	Metabolism
DMR16:22654001	16	22654001	22657000	3000	1	2.10E-08	0.4	18	0.6	Ints10	
DMR16:22792001	16	22792001	22793000	1000	1	2.70E-07	-0.56	22	2.2	Csgalnact1;LOC680966	Golgi
DMR16:23133001	16	23133001	23135000	2000	1	2.60E-10	0.68	18	0.9	Sh2d4a	Immune
DMR16:23233001	16	23233001	23236000	3000	1	5.20E-07	0.47	19	0.63	Psd3	Transcription
DMR16:23314001	16	23314001	23317000	3000	3	1.10E-23	0.77	68	2.27	Psd3	Transcription
DMR16:23350001	16	23350001	23355000	5000	1	1.70E-11	-0.4	56	1.12	Psd3	Transcription
DMR16:23385001	16	23385001	23386000	1000	1	3.30E-08	-0.58	10	1	Psd3	Transcription
DMR16:23398001	16	23398001	23399000	1000	1	4.00E-07	0.5	8	0.8	Psd3	Transcription
DMR16:23759001	16	23759001	23761000	2000	1	3.10E-07	0.37	19	0.95	Psd3	Transcription
DMR16:23787001	16	23787001	23789000	2000	1	3.60E-13	0.89	12	0.6	Psd3	Transcription
DMR16:24785001	16	24785001	24788000	3000	1	1.70E-07	0.61	42	1.4	Npy1r;Npy5r	Signaling
DMR16:25106001	16	25106001	25109000	3000	3	4.20E-14	-0.36	44	1.47	March1;LOC103693923	
DMR16:25209001	16	25209001	25214000	5000	1	8.20E-07	-0.37	43	0.86	1-Mar	
DMR16:25215001	16	25215001	25216000	1000	1	1.50E-07	0.48	6	0.6	1-Mar	
DMR16:25233001	16	25233001	25239000	6000	1	2.90E-07	-0.25	57	0.95	1-Mar	
DMR16:25330001	16	25330001	25331000	1000	1	2.60E-07	0.62	5	0.5	1-Mar	
DMR16:25716001	16	25716001	25722000	6000	1	8.30E-09	-0.34	59	0.98	1-Mar	
DMR16:25760001	16	25760001	25765000	5000	1	9.30E-10	-0.39	63	1.26	March1;Gapdh-ps2	
DMR16:25776001	16	25776001	25778000	2000	1	4.90E-08	-0.39	16	0.8	March1;Gapdh-ps2	
DMR16:27534001	16	27534001	27536000	2000	1	9.20E-08	0.4	21	1.05	Tll1	Protease
DMR16:28511001	16	28511001	28514000	3000	1	5.50E-07	-0.36	25	0.83	Spock3	Extracellular Matrix
DMR16:28621001	16	28621001	28622000	1000	1	2.80E-09	-0.65	6	0.6	Spock3	Extracellular Matrix
DMR16:28688001	16	28688001	28690000	2000	1	1.30E-08	-0.36	23	1.15	Spock3	Extracellular Matrix
DMR16:29848001	16	29848001	29854000	6000	2	2.80E-14	-0.49	49	0.82	Ddx60	
DMR16:32083001	16	32083001	32085000	2000	1	2.30E-07	-0.4	34	1.7	Sh3rf1	
DMR16:32530001	16	32530001	32532000	2000	1	1.40E-07	-0.5	29	1.45	Clcn3;Hpf1	Transport;Epigenetic
DMR16:32763001	16	32763001	32764000	1000	1	2.70E-07	0.46	8	0.8	Mfap3l	
DMR16:32838001	16	32838001	32844000	6000	2	2.90E-07	-0.35	67	1.12	Aadat	Metabolism
DMR16:34613001	16	34613001	34622000	9000	1	2.30E-07	-0.29	101	1.12	Galntl6	Golgi
DMR16:34628001	16	34628001	34633000	5000	1	3.00E-08	-0.29	53	1.06	Galntl6	Golgi
DMR16:34847001	16	34847001	34851000	4000	2	1.40E-09	-0.34	56	1.4	Galntl6	Golgi
DMR16:34919001	16	34919001	34926000	7000	3	3.80E-08	-0.39	76	1.09	Galntl6	Golgi
DMR16:35104001	16	35104001	35110000	6000	3	4.00E-08	0.43	67	1.12	Galntl6	Golgi
DMR16:35145001	16	35145001	35148000	3000	1	2.10E-08	0.53	61	2.03	Galntl6	Golgi
DMR16:35171001	16	35171001	35176000	5000	2	1.60E-09	-0.31	49	0.98	Galntl6	Golgi
DMR16:35658001	16	35658001	35662000	4000	1	3.00E-07	-0.29	54	1.35	Galntl6	Golgi
DMR16:35676001	16	35676001	35679000	3000	1	6.00E-08	-0.3	29	0.97	Galntl6	Golgi
DMR16:35680001	16	35680001	35681000	1000	1	3.10E-09	-0.39	24	2.4	Galntl6	Golgi
DMR16:35709001	16	35709001	35716000	7000	1	7.40E-08	-0.35	79	1.13	Galntl6	Golgi
DMR16:35718001	16	35718001	35721000	3000	1	2.50E-10	-0.39	28	0.93	Galntl6	Golgi
DMR16:37483001	16	37483001	37486000	3000	1	4.10E-08	-0.25	28	0.93	Hpgd	Metabolism
DMR16:37748001	16	37748001	37779000	31000	18	5.10E-22	0.37	281	0.91	Glra3	Ion Channel
DMR16:37780001	16	37780001	37789000	9000	1	4.50E-07	-0.35	104	1.16	Glra3	Ion Channel
DMR16:37918001	16	37918001	37919000	1000	1	6.20E-08	-0.47	8	0.8	Glra3	Ion Channel
DMR16:39823001	16	39823001	39826000	3000	1	2.40E-12	-0.36	27	0.9	Wdr17	

DMR16:39996001	16	39996001	39997000	1000	1	3.30E-07	0.53	5	0.5	Asb5	
DMR16:40450001	16	40450001	40454000	4000	2	2.30E-08	-0.4	33	0.82	Vegfc	Growth Factors
DMR16:41095001	16	41095001	41102000	7000	1	5.90E-08	-0.27	84	1.2	Neil3	Epigenetic
DMR16:46550001	16	46550001	46553000	3000	1	5.70E-08	-0.45	22	0.73	Tenm3;LOC108353111	
DMR16:46581001	16	46581001	46584000	3000	1	6.50E-07	0.29	62	2.07	Tenm3	
DMR16:46627001	16	46627001	46628000	1000	1	5.40E-07	-0.43	15	1.5	Tenm3	
DMR16:46740001	16	46740001	46743000	3000	1	1.70E-08	-0.51	56	1.87	Tenm3	
DMR16:47727001	16	47727001	47730000	3000	1	8.90E-09	0.52	31	1.03	Ing2	Epigenetic
DMR16:48142001	16	48142001	48149000	7000	1	4.40E-07	0.38	121	1.73	Stox2	
DMR16:48222001	16	48222001	48223000	1000	1	2.00E-07	-0.47	25	2.5	Stox2	
DMR16:48333001	16	48333001	48338000	5000	2	4.70E-07	-0.32	55	1.1	LOC103693959;Enpp6	
DMR16:49290001	16	49290001	49296000	6000	3	7.20E-09	-0.33	65	1.08	Cfap97	
DMR16:50063001	16	50063001	50069000	6000	3	1.90E-10	-0.36	64	1.07	Fam149a	
DMR16:50363001	16	50363001	50366000	3000	1	4.90E-07	0.4	57	1.9	Mtnr1a;Fat1	Signaling;Cytoskeleton
DMR16:50387001	16	50387001	50390000	3000	1	2.50E-07	0.35	62	2.07	Fat1	Cytoskeleton
DMR16:50479001	16	50479001	50480000	1000	1	2.90E-07	-0.42	15	1.5	Fat1	Cytoskeleton
DMR16:50481001	16	50481001	50483000	2000	1	6.50E-08	0.52	20	1	Fat1	Cytoskeleton
DMR16:51752001	16	51752001	51753000	1000	1	2.80E-07	-0.46	17	1.7	Adam34	Protease
DMR16:52650001	16	52650001	52655000	5000	2	1.50E-11	-0.36	63	1.26	Rps27a-ps15	
DMR16:54296001	16	54296001	54298000	2000	1	5.00E-08	0.55	13	0.65	Mtus1;LOC103693968	
DMR16:54416001	16	54416001	54421000	5000	1	1.20E-09	0.34	69	1.38	Pdgfrl;LOC690241;LOC100910853	Receptor
DMR16:54442001	16	54442001	54443000	1000	1	2.50E-08	-0.44	16	1.6	Pdgfrl	Receptor
DMR16:54496001	16	54496001	54497000	1000	1	8.30E-08	0.55	3	0.3	Slc7a2	Transport
DMR16:54641001	16	54641001	54643000	2000	1	4.90E-07	-0.36	14	0.7	Adam24;LOC100911065	Protease
DMR16:54808001	16	54808001	54814000	6000	2	5.00E-07	-0.41	69	1.15	Mtmr7	Signaling
DMR16:54879001	16	54879001	54885000	6000	2	8.30E-12	-0.53	67	1.12	Vps37a	
DMR16:54945001	16	54945001	54949000	4000	1	9.90E-11	-0.32	53	1.32	Zdhhc2	
DMR16:54954001	16	54954001	54957000	3000	1	7.00E-11	-0.51	25	0.83	Zdhhc2	
DMR16:55084001	16	55084001	55085000	1000	1	5.30E-07	0.65	7	0.7	Micu3	Signaling
DMR16:56847001	16	56847001	56848000	1000	1	5.20E-09	-0.45	9	0.9	Msr1	Protease
DMR16:56887001	16	56887001	56892000	5000	3	1.20E-07	-0.3	52	1.04	Msr1	Protease
DMR16:57565001	16	57565001	57570000	5000	1	4.60E-09	-0.4	46	0.92	Sgcz	
DMR16:57802001	16	57802001	57807000	5000	1	1.50E-07	-0.37	47	0.94	Sgcz	
DMR16:57919001	16	57919001	57920000	1000	1	3.20E-07	-0.34	12	1.2	Sgcz	
DMR16:58018001	16	58018001	58019000	1000	1	7.80E-08	0.54	7	0.7	Sgcz	
DMR16:58813001	16	58813001	58814000	1000	1	6.80E-09	0.46	14	1.4	Dlc1	Signaling
DMR16:60116001	16	60116001	60117000	1000	1	9.70E-11	0.46	15	1.5	Mfhas1	Cytoskeleton
DMR16:60335001	16	60335001	60337000	2000	1	3.00E-07	0.41	26	1.3	Eri1	Transcription
DMR16:61803001	16	61803001	61805000	2000	1	1.40E-12	0.87	20	1	Mboat4;Dctn6	Metabolism;Cytoskeleton
DMR16:62531001	16	62531001	62532000	1000	1	4.70E-08	0.53	15	1.5	Wrn	Epigenetic
DMR16:62538001	16	62538001	62539000	1000	1	4.90E-11	-0.52	15	1.5	Wrn	Epigenetic
DMR16:62982001	16	62982001	62984000	2000	2	1.90E-11	0.55	21	1.05	Nrg1	Growth Factors
DMR16:63873001	16	63873001	63874000	1000	1	2.50E-08	-0.75	11	1.1	Nrg1	Growth Factors
DMR16:63877001	16	63877001	63878000	1000	1	4.40E-08	-0.63	14	1.4	Nrg1	Growth Factors
DMR16:63939001	16	63939001	63940000	1000	1	4.10E-07	-0.42	19	1.9	Nrg1	Growth Factors
DMR16:64055001	16	64055001	64056000	1000	1	4.70E-09	0.42	20	2	Nrg1	Growth Factors
DMR16:67211001	16	67211001	67212000	1000	1	9.00E-08	0.52	8	0.8	Unc5d	Receptor
DMR16:67267001	16	67267001	67268000	1000	1	1.20E-07	0.57	5	0.5	Unc5d	Receptor
DMR16:67359001	16	67359001	67360000	1000	1	8.10E-08	-0.44	23	2.3	Unc5d	Receptor
DMR16:67361001	16	67361001	67362000	1000	1	6.20E-07	0.41	10	1	Unc5d	Receptor
DMR16:68564001	16	68564001	68565000	1000	1	3.20E-07	-0.36	18	1.8	RGD1562638	
DMR16:68586001	16	68586001	68587000	1000	1	7.00E-07	-0.36	19	1.9	RGD1562638	
DMR16:68749001	16	68749001	68750000	1000	1	3.70E-07	-0.46	4	0.4	Poteg	Transport
DMR16:68796001	16	68796001	68802000	6000	1	8.20E-07	-0.28	57	0.95	Poteg	Transport
DMR16:68886001	16	68886001	68892000	6000	2	1.10E-07	-0.35	79	1.32	Chrnb3	Ion Channel
DMR16:68977001	16	68977001	68984000	7000	2	1.60E-11	-0.39	77	1.1	Eif4ebp1	Translation
DMR16:71259001	16	71259001	71260000	1000	1	1.50E-09	0.37	35	3.5	Letm2;Fgfr1	Transport;Receptor
DMR16:71289001	16	71289001	71291000	2000	1	2.70E-12	0.51	32	1.6	Fgfr1	Receptor
DMR16:71307001	16	71307001	71310000	3000	1	4.30E-10	0.53	56	1.87	Fgfr1	Receptor
DMR16:71733001	16	71733001	71735000	2000	1	7.90E-08	0.41	28	1.4	Plekha2	

DMR16:71754001	16	71754001	71755000	1000	1	5.80E-07	-0.68	11	1.1	Plekha2	
DMR16:71803001	16	71803001	71806000	3000	1	4.00E-09	0.51	39	1.3	Htra4;Tm2d2;Adam9	Protease;Protease
DMR16:71877001	16	71877001	71880000	3000	3	3.20E-09	-0.28	31	1.03	Adam9;Adam32	Protease
DMR16:71883001	16	71883001	71886000	3000	1	4.10E-08	0.67	29	0.97	Adam9;Adam32	Protease
DMR16:71899001	16	71899001	71901000	2000	2	1.50E-09	-0.47	31	1.55	Adam32	Protease
DMR16:71995001	16	71995001	72001000	6000	1	1.60E-07	-0.3	84	1.4	Adam32;Adam5	Protease
DMR16:72037001	16	72037001	72041000	4000	1	5.70E-08	-0.43	29	0.72	Adam5	Protease
DMR16:72141001	16	72141001	72146000	5000	2	2.40E-09	-0.31	48	0.96	Adam3a;Adam18	Protease
DMR16:72797001	16	72797001	72799000	2000	2	3.90E-12	0.45	105	5.25	Zmat4	
DMR16:72880001	16	72880001	72882000	2000	1	7.50E-08	-0.43	26	1.3	Zmat4;LOC103693991	
DMR16:73368001	16	73368001	73370000	2000	1	4.30E-09	0.35	40	2	Sfrp1	Receptor
DMR16:73631001	16	73631001	73632000	1000	1	2.30E-07	0.42	16	1.6	Gpat4;LOC108348420	Metabolism
DMR16:73640001	16	73640001	73641000	1000	1	1.10E-07	0.45	21	2.1	Gpat4;LOC108348420	Metabolism
DMR16:73930001	16	73930001	73933000	3000	1	2.30E-08	-0.46	36	1.2	Kat6a	Epigenetic
DMR16:74202001	16	74202001	74204000	2000	1	1.20E-10	0.49	36	1.8	Ikbkb	Signaling
DMR16:74336001	16	74336001	74337000	1000	1	2.20E-09	0.43	16	1.6	Slc20a2	Transport
DMR16:74574001	16	74574001	74575000	1000	1	3.80E-08	-0.6	9	0.9	Tpte2	Signaling
DMR16:74620001	16	74620001	74623000	3000	1	1.40E-08	-0.51	41	1.37	Tpte2	Signaling
DMR16:74764001	16	74764001	74766000	2000	2	3.10E-10	0.44	23	1.15	Ckap2	Cytoskeleton
DMR16:74885001	16	74885001	74887000	2000	1	2.00E-08	0.45	35	1.75	Atp7b	Transport
DMR16:74955001	16	74955001	74957000	2000	1	8.20E-07	0.52	12	0.6	LOC108353085;Ccdc70	
DMR16:75022001	16	75022001	75023000	1000	1	6.50E-07	-0.37	3	0.3	Fam90a1;Defb13	
DMR16:75103001	16	75103001	75104000	1000	1	6.40E-07	-0.4	13	1.3	Defb37;Defb15	Signaling
DMR16:75134001	16	75134001	75140000	6000	2	2.70E-10	-0.33	66	1.1	Spag11bl;Defb11	Signaling
DMR16:75185001	16	75185001	75193000	8000	1	5.50E-08	-0.31	98	1.23	Defb9;Defb10	Signaling
DMR16:75254001	16	75254001	75260000	6000	1	4.70E-09	-0.34	54	0.9	Defb50	
DMR16:75287001	16	75287001	75288000	1000	1	1.30E-07	-0.58	10	1	Defb1	Signaling
DMR16:75602001	16	75602001	75605000	3000	1	8.10E-08	-0.3	56	1.87	Defb5	Signaling
DMR16:75606001	16	75606001	75610000	4000	1	2.30E-07	0.36	41	1.02	Defb5	Signaling
DMR16:75630001	16	75630001	75634000	4000	1	5.10E-07	0.35	26	0.65	Defb4	Signaling
DMR16:75994001	16	75994001	75999000	5000	1	1.00E-09	0.46	77	1.54	Mcph1;Angpt2	DNA Repair;Signaling
DMR16:77426001	16	77426001	77431000	5000	2	1.10E-07	-0.3	52	1.04	Csmd1	
DMR16:77503001	16	77503001	77508000	5000	1	6.70E-09	-0.35	66	1.32	Csmd1	
DMR16:77560001	16	77560001	77561000	1000	1	6.60E-08	-0.33	6	0.6	Csmd1	
DMR16:77718001	16	77718001	77719000	1000	1	3.90E-07	-0.55	6	0.6	Csmd1	
DMR16:78107001	16	78107001	78108000	1000	1	1.90E-11	-0.38	10	1	Csmd1	
DMR16:78380001	16	78380001	78385000	5000	1	2.50E-08	-0.35	40	0.8	Csmd1	
DMR16:80126001	16	80126001	80128000	2000	1	5.00E-07	0.32	28	1.4	Dlgap2	Cytoskeleton
DMR16:80406001	16	80406001	80407000	1000	1	6.20E-07	0.37	11	1.1	Dlgap2	Cytoskeleton
DMR16:80486001	16	80486001	80487000	1000	1	6.90E-08	-0.56	10	1	Dlgap2;LOC102547542	Cytoskeleton
DMR16:80517001	16	80517001	80518000	1000	1	2.00E-14	0.56	14	1.4	Dlgap2	Cytoskeleton
DMR16:80630001	16	80630001	80632000	2000	1	9.40E-07	-0.37	44	2.2	Erich1	
DMR16:80638001	16	80638001	80639000	1000	1	1.60E-08	0.39	21	2.1	Erich1;LOC103693997	
DMR16:80790001	16	80790001	80791000	1000	1	2.30E-08	0.42	31	3.1	Fbxo25;LOC108348429	
DMR16:80999001	16	80999001	81001000	2000	1	2.40E-07	0.42	44	2.2	Dcun1d2	
DMR16:81173001	16	81173001	81176000	3000	2	3.10E-17	0.72	46	1.53	Grk1;Tmem255b	Signaling
DMR16:81363001	16	81363001	81365000	2000	1	1.60E-07	0.42	39	1.95	Rasa3	Signaling
DMR16:81715001	16	81715001	81717000	2000	1	1.80E-10	0.55	35	1.75	Lamp1;Cul4a	Transport;Proteolysis
DMR16:82030001	16	82030001	82032000	2000	2	2.80E-15	0.5	118	5.9	Atp11a	Transport
DMR16:82206001	16	82206001	82208000	2000	1	6.50E-07	0.4	22	1.1	Tubgcp3	Cytoskeleton
DMR16:82219001	16	82219001	82220000	1000	1	1.30E-10	0.48	22	2.2	Tubgcp3	Cytoskeleton
DMR16:82986001	16	82986001	82988000	2000	1	1.10E-09	0.54	28	1.4	Tex29	
DMR16:83021001	16	83021001	83023000	2000	1	6.80E-08	0.39	39	1.95	Arhgef7	Transcription
DMR16:83491001	16	83491001	83493000	2000	1	1.70E-08	0.29	33	1.65	Col4a2	Extracellular Matrix
DMR16:83551001	16	83551001	83553000	2000	1	1.40E-07	0.4	50	2.5	Col4a1	Extracellular Matrix
DMR16:83592001	16	83592001	83593000	1000	1	2.20E-10	0.57	4	0.4	Col4a1	Extracellular Matrix
DMR16:83639001	16	83639001	83642000	3000	1	5.20E-07	0.31	40	1.33	Col4a1	Extracellular Matrix
DMR16:84787001	16	84787001	84789000	2000	1	1.50E-08	0.63	4	0.2	Myo16	
DMR16:85053001	16	85053001	85054000	1000	1	6.30E-12	0.48	19	1.9	Myo16	
DMR16:85100001	16	85100001	85101000	1000	1	2.40E-07	0.45	18	1.8	Myo16	
DMR16:85104001	16	85104001	85106000	2000	1	5.80E-08	0.45	27	1.35	Myo16;Marco	Extracellular Matrix

DMR16:85308001	16	85308001	85310000	2000	1	5.30E-07	-0.44	24	1.2	Tnfsf13b;Abhd13	Protease
DMR16:85345001	16	85345001	85346000	1000	1	9.70E-07	0.53	7	0.7	Lig4	Transcription
DMR16:85574001	16	85574001	85575000	1000	1	8.00E-09	0.33	16	1.6	Fam155a	
DMR16:85685001	16	85685001	85690000	5000	1	1.40E-08	-0.53	86	1.72	Fam155a	
DMR17:427001	17	427001	429000	2000	1	6.20E-07	0.66	14	0.7	Fbp1	Metabolism
DMR17:527001	17	527001	535000	8000	2	1.40E-10	-0.34	82	1.02	Npepo	
DMR17:1053001	17	1053001	1054000	1000	1	3.00E-12	0.45	19	1.9	Ptch1	
DMR17:1064001	17	1064001	1066000	2000	1	1.70E-10	0.52	20	1	Ptch1	
DMR17:1364001	17	1364001	1369000	5000	1	1.40E-07	-0.27	59	1.18	Ercc6l2	
DMR17:1639001	17	1639001	1640000	1000	1	5.40E-07	-0.4	12	1.2	Slc35d2	Transport
DMR17:3395001	17	3395001	3396000	1000	1	8.60E-08	-0.45	6	0.6	LOC102552000;Cts8	Protease
DMR17:3829001	17	3829001	3832000	3000	1	6.50E-07	-0.31	23	0.77	Ctsm	Protease
DMR17:3895001	17	3895001	3903000	8000	1	1.10E-08	-0.28	86	1.07	Ctsr	Protease
DMR17:5217001	17	5217001	5219000	2000	1	3.60E-10	-0.45	29	1.45	Zcchc6	
DMR17:5229001	17	5229001	5230000	1000	1	1.50E-07	-0.44	13	1.3	Zcchc6	
DMR17:5321001	17	5321001	5324000	3000	3	2.00E-09	0.42	81	2.7	LOC102547665;LOC102547829;Spata31d1d;Spata31d1b	
DMR17:5576001	17	5576001	5577000	1000	1	3.00E-07	0.41	32	3.2	Agtpbp1	Protease
DMR17:6650001	17	6650001	6651000	1000	1	5.80E-12	-0.5	17	1.7	Rmi1	
DMR17:6814001	17	6814001	6815000	1000	1	1.20E-07	-0.39	28	2.8	Gkap1	Cytoskeleton
DMR17:6832001	17	6832001	6833000	1000	1	5.60E-07	0.49	7	0.7	Gkap1;Ubqln1	Cytoskeleton
DMR17:7669001	17	7669001	7672000	3000	1	4.60E-08	0.43	35	1.17	Spock1;LOC108348496	Signaling
DMR17:7705001	17	7705001	7706000	1000	1	3.10E-13	0.49	17	1.7	Spock1	Signaling
DMR17:8916001	17	8916001	8917000	1000	1	7.30E-08	-0.72	17	1.7	Catsper3	Transport
DMR17:9593001	17	9593001	9595000	2000	1	2.10E-09	0.37	17	0.85	Fam193b	
DMR17:9658001	17	9658001	9661000	3000	1	4.10E-08	0.38	68	2.27	Pdlim7	Cytoskeleton
DMR17:9788001	17	9788001	9789000	1000	1	2.00E-09	0.47	10	1	Rgs14;Lman2	Transport
DMR17:9799001	17	9799001	9801000	2000	1	7.90E-13	-0.58	28	1.4	Rgs14;Lman2	Transport
DMR17:9808001	17	9808001	9815000	7000	2	3.90E-13	0.56	150	2.14	Lman2	Transport
DMR17:9828001	17	9828001	9829000	1000	1	6.40E-09	0.43	15	1.5	LOC102550438;Mxd3;Prelid1;Rab24	Transcription
DMR17:10146001	17	10146001	10148000	2000	1	2.60E-08	0.46	57	2.85	Hk3;Unc5a	Signaling;Receptor
DMR17:10155001	17	10155001	10161000	6000	1	1.90E-10	0.41	139	2.32	Hk3;Unc5a	Signaling;Receptor
DMR17:10374001	17	10374001	10377000	3000	1	6.90E-09	0.5	46	1.53	Tspan17;Eif4e1b;Sncb	Transport
DMR17:10467001	17	10467001	10472000	5000	1	9.60E-10	0.47	112	2.24	Rnf44	
DMR17:10482001	17	10482001	10483000	1000	1	5.30E-23	0.62	12	1.2	Rnf44;Faf2	
DMR17:11987001	17	11987001	11988000	1000	1	2.10E-07	0.47	19	1.9	Ror2	Receptor
DMR17:13595001	17	13595001	13596000	1000	1	8.60E-09	0.46	4	0.4	Secisbp2;Cks2	Cytoskeleton
DMR17:14071001	17	14071001	14075000	4000	1	2.00E-07	0.58	49	1.23	Spin1	
DMR17:14464001	17	14464001	14465000	1000	1	4.00E-07	-0.37	13	1.3	LOC689437;Trnak-cuu;LOC689448;LOC689458;LOC108353119	
DMR17:15441001	17	15441001	15444000	3000	1	1.30E-08	-0.45	20	0.67	LOC679342;Ogn	
DMR17:15562001	17	15562001	15564000	2000	1	9.40E-07	-0.41	18	0.9	LOC679342;Ecm2	
DMR17:15786001	17	15786001	15789000	3000	1	2.30E-08	0.47	59	1.97	Fgd3	Transcription
DMR17:15790001	17	15790001	15792000	2000	1	2.70E-07	0.43	29	1.45	Fgd3	Transcription
DMR17:15793001	17	15793001	15797000	4000	1	6.10E-14	0.49	42	1.05	Fgd3	Transcription
DMR17:15890001	17	15890001	15893000	3000	1	1.70E-08	0.67	47	1.57	Ninj1	Cytoskeleton
DMR17:15992001	17	15992001	15995000	3000	1	1.30E-09	0.47	77	2.57	Wnk2	Signaling
DMR17:16148001	17	16148001	16150000	2000	1	3.00E-07	0.36	46	2.3	Fam120a	
DMR17:16479001	17	16479001	16480000	1000	1	7.30E-08	-0.51	14	1.4	Zfp169	Transcription
DMR17:18015001	17	18015001	18016000	1000	1	5.00E-08	-0.54	29	2.9	Kdm1b	Metabolism
DMR17:18019001	17	18019001	18022000	3000	1	1.40E-07	0.36	62	2.07	Kdm1b;Tpmt	Metabolism;Epigenetic
DMR17:18101001	17	18101001	18102000	1000	1	1.20E-07	-0.39	21	2.1	RGD1564366	
DMR17:18143001	17	18143001	18144000	1000	1	3.10E-07	0.37	19	1.9	LOC108348531;Kif13a	Cytoskeleton
DMR17:18227001	17	18227001	18228000	1000	1	2.30E-07	0.36	22	2.2	Kif13a	Cytoskeleton
DMR17:18310001	17	18310001	18311000	1000	1	2.30E-07	-0.41	23	2.3	Kif13a	Cytoskeleton
DMR17:19329001	17	19329001	19332000	3000	1	2.80E-11	0.41	52	1.73	Atxn1	
DMR17:19556001	17	19556001	19559000	3000	1	2.70E-07	0.35	52	1.73	Gmpr	Metabolism
DMR17:20116001	17	20116001	20117000	1000	1	2.40E-07	0.4	7	0.7	Dtnbp1	
DMR17:20221001	17	20221001	20223000	2000	1	3.40E-08	0.39	25	1.25	Jarid2	Epigenetic

DMR17:21549001	17	21549001	21552000	3000	1	1.80E-11	0.37	44	1.47	Mak	Signaling
DMR17:21651001	17	21651001	21654000	3000	1	8.50E-07	0.37	57	1.9	Gcnt2	Golgi
DMR17:21744001	17	21744001	21746000	2000	1	8.60E-07	-0.43	32	1.6	Gcnt6	
DMR17:22617001	17	22617001	22618000	1000	1	2.70E-11	0.61	4	0.4	Adtrp	
DMR17:23390001	17	23390001	23393000	3000	1	8.50E-08	0.55	34	1.13	Phactr1	Signaling
DMR17:23487001	17	23487001	23492000	5000	1	1.30E-08	0.61	70	1.4	Phactr1	Signaling
DMR17:23552001	17	23552001	23553000	1000	1	1.70E-11	0.44	7	0.7	Phactr1	Signaling
DMR17:23997001	17	23997001	2.40E+07	3000	1	1.10E-07	0.43	41	1.37	Sirt5	
DMR17:24425001	17	24425001	24427000	2000	1	4.60E-11	0.58	33	1.65	Cd83	
DMR17:26485001	17	26485001	26488000	3000	1	2.00E-07	0.57	20	0.67	Slc35b3	Transport
DMR17:26827001	17	26827001	26831000	4000	1	4.00E-14	0.58	62	1.55	Bloc1s5	
DMR17:27317001	17	27317001	27319000	2000	1	1.40E-09	0.45	38	1.9	Dsp	Cytoskeleton
DMR17:27442001	17	27442001	27444000	2000	1	4.00E-07	0.34	25	1.25	Riok1;Cage1	Signaling
DMR17:27615001	17	27615001	27616000	1000	1	4.00E-07	0.38	11	1.1	Rreb1	
DMR17:27965001	17	27965001	27966000	1000	1	3.10E-07	0.36	11	1.1	Nrn1	
DMR17:28093001	17	28093001	28099000	6000	2	4.30E-08	-0.29	68	1.13	Ly86	
DMR17:28533001	17	28533001	28534000	1000	1	9.00E-09	0.4	6	0.6	F13a1	Transport
DMR17:29006001	17	29006001	29007000	1000	1	4.40E-07	0.59	10	1	Fars2	Translation
DMR17:29041001	17	29041001	29046000	5000	1	7.90E-09	-0.27	53	1.06	Fars2	Translation
DMR17:29269001	17	29269001	29273000	4000	1	6.40E-07	-0.35	36	0.9	Fars2	Translation
DMR17:29426001	17	29426001	29427000	1000	1	4.70E-07	0.57	13	1.3	Fars2	Translation
DMR17:29692001	17	29692001	29694000	2000	1	9.00E-07	0.35	21	1.05	Cdyl	
DMR17:29829001	17	29829001	29831000	2000	1	4.40E-09	-0.37	28	1.4	Cdyl	
DMR17:30565001	17	30565001	30567000	2000	1	1.80E-14	0.59	24	1.2	Eci2	Transport
DMR17:30978001	17	30978001	30981000	3000	1	1.20E-07	0.62	11	0.37	Pxdc1	
DMR17:30984001	17	30984001	30985000	1000	1	9.00E-07	0.32	9	0.9	Pxdc1	
DMR17:31415001	17	31415001	31417000	2000	1	5.50E-07	0.4	26	1.3	Psmg4	Transcription
DMR17:32242001	17	32242001	32243000	1000	1	8.80E-11	0.47	7	0.7	RGD1564786	
DMR17:32283001	17	32283001	32284000	1000	1	1.80E-07	0.4	13	1.3	RGD1564786	
DMR17:32291001	17	32291001	32293000	2000	1	8.40E-11	-0.44	13	0.65	RGD1564786	
DMR17:32432001	17	32432001	32438000	6000	1	7.20E-08	-0.33	58	0.97	Serpinb6e	
DMR17:33400001	17	33400001	33401000	1000	1	1.10E-07	0.32	13	1.3	Gmds	Metabolism
DMR17:33524001	17	33524001	33526000	2000	1	7.20E-08	0.53	18	0.9	Gmds	Metabolism
DMR17:33589001	17	33589001	33596000	7000	4	7.40E-11	0.66	70	1	Gmds	Metabolism
DMR17:33736001	17	33736001	33737000	1000	1	3.10E-07	0.36	14	1.4	Gmds	Metabolism
DMR17:34679001	17	34679001	34683000	4000	1	1.80E-07	-0.29	34	0.85	Exoc2;LOC103690165	Transcription
DMR17:34699001	17	34699001	34701000	2000	1	5.90E-07	-0.27	23	1.15	Exoc2;LOC103690165	Transcription
DMR17:34838001	17	34838001	34839000	1000	1	5.20E-08	0.42	6	0.6	Exoc2	
DMR17:34882001	17	34882001	34883000	1000	1	2.20E-11	0.53	4	0.4	Irf4	Transcription
DMR17:35943001	17	35943001	35947000	4000	2	1.10E-08	-0.36	53	1.32	Agtr1a	
DMR17:36393001	17	36393001	36394000	1000	1	1.20E-07	0.4	15	1.5	E2f3;LOC103694081	Transcription
DMR17:36632001	17	36632001	36639000	7000	1	2.80E-07	-0.29	69	0.99	Cdkal1	
DMR17:37132001	17	37132001	37138000	6000	1	2.20E-07	-0.27	96	1.6	Cdkal1	
DMR17:38840001	17	38840001	38842000	2000	1	1.90E-12	-0.41	18	0.9	Prl2c1	Hormone
DMR17:38843001	17	38843001	38846000	3000	1	9.40E-08	-0.32	21	0.7	Prl2c1	Hormone
DMR17:39231001	17	39231001	39238000	7000	2	4.70E-09	-0.25	79	1.13	Prl8a9	Hormone
DMR17:39276001	17	39276001	39277000	1000	1	6.70E-10	0.73	6	0.6	Prl3d4	Hormone
DMR17:39405001	17	39405001	39408000	3000	1	2.50E-08	-0.44	17	0.57	Prl8a5	Hormone
DMR17:39421001	17	39421001	39426000	5000	3	2.20E-12	-0.35	67	1.34	Prl8a5	Hormone
DMR17:39783001	17	39783001	39785000	2000	1	1.10E-09	-0.58	9	0.45	Prl3d1	Hormone
DMR17:41955001	17	41955001	41956000	1000	1	2.90E-14	0.51	33	3.3	Dcdc2	
DMR17:42171001	17	42171001	42178000	7000	2	6.70E-08	-0.39	75	1.07	LOC103694089;RGD1307443;Rpl37-ps1	
DMR17:42375001	17	42375001	42377000	2000	1	4.20E-09	0.46	17	0.85	Fam65b	
DMR17:42412001	17	42412001	42414000	2000	2	3.00E-12	0.74	17	0.85	Fam65b	
DMR17:42575001	17	42575001	42581000	6000	1	5.10E-07	-0.27	48	0.8	LOC102552189;Cmah	
DMR17:42606001	17	42606001	42608000	2000	1	4.40E-15	0.53	40	2	Cmah	
DMR17:42612001	17	42612001	42614000	2000	1	5.60E-09	-0.54	21	1.05	Cmah	
DMR17:42728001	17	42728001	42733000	5000	2	2.80E-19	-0.47	40	0.8	Prl3a1	Hormone
DMR17:43176001	17	43176001	43181000	5000	1	7.30E-08	-0.33	40	0.8	Carmil1	
DMR17:43553001	17	43553001	43559000	6000	1	1.60E-07	-0.31	71	1.18	Slc17a3;Slc17a2	Transport

DMR17:44362001	17	44362001	44363000	1000	1	8.00E-08	0.48	13	1.3	Trnas-gcu	
DMR17:44404001	17	44404001	44409000	5000	1	3.40E-07	0.37	114	2.28	RGD1561897	
DMR17:44416001	17	44416001	44419000	3000	1	2.80E-07	0.71	37	1.23	RGD1561897	
DMR17:44498001	17	44498001	44499000	1000	1	6.80E-07	0.46	13	1.3	Trnas-gcu	
DMR17:45008001	17	45008001	45013000	5000	2	1.40E-07	-0.36	46	0.92	Olr1658	Receptor
DMR17:45158001	17	45158001	45164000	6000	3	4.00E-13	-0.4	68	1.13	Zkscan4	Transcription
DMR17:45656001	17	45656001	45662000	6000	1	7.50E-08	-0.28	71	1.18	Olr1660	Receptor
DMR17:45695001	17	45695001	45701000	6000	1	9.90E-12	-0.49	51	0.85	Olr1662	Receptor
DMR17:45807001	17	45807001	45815000	8000	2	3.90E-07	-0.33	87	1.09	Olr1664	Receptor
DMR17:45856001	17	45856001	45865000	9000	1	6.00E-07	-0.37	60	0.67	Aoah	Metabolism
DMR17:45901001	17	45901001	45903000	2000	1	1.20E-14	-0.39	22	1.1	Aoah	Metabolism
DMR17:45929001	17	45929001	45931000	2000	1	1.10E-07	-0.44	23	1.15	Aoah	Metabolism
DMR17:46587001	17	46587001	46589000	2000	1	9.50E-07	0.49	15	0.75	Elmo1	Cytoskeleton
DMR17:46619001	17	46619001	46621000	2000	1	5.30E-08	0.42	6	0.3	Elmo1	Cytoskeleton
DMR17:46714001	17	46714001	46720000	6000	1	1.30E-09	-0.33	67	1.12	Elmo1	Cytoskeleton
DMR17:47358001	17	47358001	47362000	4000	2	7.20E-12	-0.36	35	0.88	Nme8	
DMR17:48307001	17	48307001	48309000	2000	1	6.10E-14	-0.41	34	1.7	Amph	
DMR17:48432001	17	48432001	48439000	7000	1	6.60E-09	-0.44	72	1.03	Amph	
DMR17:48458001	17	48458001	48466000	8000	2	6.50E-08	-0.31	76	0.95	Amph	
DMR17:48730001	17	48730001	48732000	2000	1	3.80E-07	-0.33	15	0.75	Vps41	Transport
DMR17:49262001	17	49262001	49263000	1000	1	3.10E-08	0.53	7	0.7	Pou6f2	
DMR17:49337001	17	49337001	49339000	2000	1	3.20E-07	-0.46	19	0.95	Pou6f2	
DMR17:49358001	17	49358001	49363000	5000	1	1.90E-07	-0.26	53	1.06	Pou6f2	
DMR17:49394001	17	49394001	49397000	3000	1	6.00E-08	-0.33	30	1	Pou6f2	
DMR17:49907001	17	49907001	49908000	1000	1	3.00E-07	0.35	10	1	Cdk13	Signaling
DMR17:50021001	17	50021001	50022000	1000	1	2.50E-10	-0.5	9	0.9	Sugct	Transport
DMR17:50211001	17	50211001	50213000	2000	1	1.90E-07	0.68	9	0.45	Sugct	Transport
DMR17:50253001	17	50253001	50260000	7000	3	8.80E-13	-0.42	67	0.96	Sugct	Transport
DMR17:50336001	17	50336001	50338000	2000	1	8.20E-14	0.53	12	0.6	Sugct	Transport
DMR17:50372001	17	50372001	50378000	6000	1	1.90E-07	-0.3	49	0.82	Sugct	Transport
DMR17:50395001	17	50395001	50399000	4000	2	2.50E-08	-0.35	35	0.88	Sugct	Transport
DMR17:50529001	17	50529001	50533000	4000	1	1.30E-10	-0.31	47	1.18	Sugct	Transport
DMR17:50553001	17	50553001	50559000	6000	1	4.80E-08	-0.32	65	1.08	Sugct	Transport
DMR17:50593001	17	50593001	50594000	1000	1	2.70E-07	-0.3	19	1.9	Sugct	Transport
DMR17:50609001	17	50609001	50611000	2000	1	1.40E-14	0.71	24	1.2	Sugct	Transport
DMR17:50614001	17	50614001	50620000	6000	1	5.90E-07	-0.31	69	1.15	Sugct	Transport
DMR17:50661001	17	50661001	50662000	1000	1	2.30E-07	-0.5	8	0.8	Sugct	Transport
DMR17:51917001	17	51917001	51919000	2000	1	3.80E-07	0.27	20	1	Inhba;LOC103694103	Growth Factors
DMR17:53033001	17	53033001	53036000	3000	1	7.80E-09	-0.54	28	0.93	Hspd1-ps3	
DMR17:53446001	17	53446001	53447000	1000	1	1.50E-10	-0.43	14	1.4	Hecw1	Proteolysis
DMR17:53515001	17	53515001	53520000	5000	3	1.70E-09	-0.35	74	1.48	Hecw1	Proteolysis
DMR17:53524001	17	53524001	53528000	4000	1	9.20E-08	-0.32	42	1.05	Hecw1	Proteolysis
DMR17:53606001	17	53606001	53611000	5000	1	1.10E-09	-0.37	46	0.92	Hecw1;LOC103694104	Proteolysis
DMR17:53618001	17	53618001	53622000	4000	1	2.60E-07	-0.31	37	0.92	Hecw1;LOC100912163	Proteolysis
DMR17:53816001	17	53816001	53824000	8000	1	3.40E-09	-0.38	125	1.56	Arid4b	Transcription
DMR17:54075001	17	54075001	54078000	3000	1	9.20E-09	-0.44	21	0.7	B3galnt2	Golgi
DMR17:54332001	17	54332001	54335000	3000	1	9.50E-08	-0.32	36	1.2	Arhgap12	Signaling
DMR17:55244001	17	55244001	55245000	1000	1	7.20E-08	0.46	14	1.4	Svil	Cytoskeleton
DMR17:55718001	17	55718001	55719000	1000	1	9.70E-07	0.32	25	2.5	RGD1562037;LOC102557539	
DMR17:58106001	17	58106001	58108000	2000	1	6.10E-07	0.59	8	0.4	Adarb2	Metabolism
DMR17:58197001	17	58197001	58198000	1000	1	6.90E-08	0.48	14	1.4	Adarb2	Metabolism
DMR17:58262001	17	58262001	58263000	1000	1	4.10E-08	-0.45	10	1	Adarb2	Metabolism
DMR17:59717001	17	59717001	59723000	6000	2	1.10E-11	-0.44	54	0.9	RGD1560860	
DMR17:59744001	17	59744001	59745000	1000	1	8.40E-08	-0.57	20	2	RGD1560860	
DMR17:60063001	17	60063001	60067000	4000	1	8.10E-09	-0.44	19	0.48	LOC108348580;Mpp7	Cytoskeleton
DMR17:60213001	17	60213001	60214000	1000	1	8.10E-07	-0.42	14	1.4	Mpp7	Cytoskeleton
DMR17:60421001	17	60421001	60425000	4000	1	1.70E-07	-0.29	37	0.92	Armc4	
DMR17:60428001	17	60428001	60431000	3000	1	8.90E-10	0.51	48	1.6	Armc4	
DMR17:62332001	17	62332001	62334000	2000	1	3.30E-08	-0.47	25	1.25	Ccny	
DMR17:62366001	17	62366001	62367000	1000	1	2.50E-09	-0.64	12	1.2	Ccny	

DMR17:62380001	17	62380001	62381000	1000	1	2.50E-08	0.45	10	1	Ccny	
DMR17:62410001	17	62410001	62411000	1000	1	1.20E-08	-0.51	9	0.9	Ccny	
DMR17:62432001	17	62432001	62434000	2000	1	1.20E-08	0.47	12	0.6	Ccny	
DMR17:63855001	17	63855001	63856000	1000	1	1.60E-10	0.51	10	1	Zmynd11;LOC108353151	Transcription
DMR17:64032001	17	64032001	64037000	5000	2	2.30E-08	-0.35	52	1.04	Chrm3	Signaling
DMR17:64304001	17	64304001	64311000	7000	1	1.60E-07	-0.3	81	1.16	Chrm3	Signaling
DMR17:64459001	17	64459001	64460000	1000	1	3.10E-07	0.44	10	1	Chrm3	Signaling
DMR17:65880001	17	65880001	65887000	7000	1	1.80E-08	-0.44	74	1.06	Ryr2	Ion Channel
DMR17:66205001	17	66205001	66207000	2000	1	9.50E-07	-0.28	17	0.85	Mtr	
DMR17:66272001	17	66272001	66274000	2000	1	1.60E-07	-0.29	36	1.8	Mtr	
DMR17:66534001	17	66534001	66536000	2000	1	6.30E-09	-0.4	34	1.7	Anlnl1	Cytoskeleton
DMR17:66537001	17	66537001	66539000	2000	1	2.10E-10	-0.45	20	1	Anlnl1	Cytoskeleton
DMR17:67214001	17	67214001	67220000	6000	1	3.40E-11	-0.4	56	0.93	Moap1-ps1	
DMR17:69480001	17	69480001	69487000	7000	2	3.30E-12	-0.4	113	1.61	RGD1564865	Metabolism
DMR17:69744001	17	69744001	69752000	8000	3	1.30E-08	-0.32	113	1.41	Akr1c3	
DMR17:69830001	17	69830001	69832000	2000	1	8.80E-11	0.51	21	1.05	Akr1c14;Akr1cl	
DMR17:69839001	17	69839001	69841000	2000	2	1.50E-10	0.42	9	0.45	Akr1cl	
DMR17:70219001	17	70219001	70220000	1000	1	7.10E-08	-0.33	8	0.8	Asb13	
DMR17:70686001	17	70686001	70687000	1000	1	9.70E-08	-0.44	16	1.6	Pfkfb3	Metabolism
DMR17:71172001	17	71172001	71179000	7000	1	8.20E-10	-0.35	78	1.11	RGD1564416	
DMR17:71182001	17	71182001	71185000	3000	1	7.90E-07	-0.41	16	0.53	RGD1564416	
DMR17:71708001	17	71708001	71713000	5000	1	1.00E-13	-0.48	50	1	Sfmbt2	Epigenetic
DMR17:72254001	17	72254001	72255000	1000	1	5.60E-07	0.41	6	0.6	Taf3	
DMR17:72459001	17	72459001	72460000	1000	1	3.30E-07	0.64	6	0.6	Gata3	Transcription
DMR17:75458001	17	75458001	75464000	6000	1	5.70E-08	-0.3	56	0.93	Celf2	
DMR17:76020001	17	76020001	76021000	1000	1	1.20E-07	0.35	23	2.3	Echdc3	
DMR17:76092001	17	76092001	76094000	2000	1	1.00E-06	0.39	33	1.65	Proser2	
DMR17:76132001	17	76132001	76133000	1000	1	1.80E-09	0.4	22	2.2	Proser2	
DMR17:76325001	17	76325001	76326000	1000	1	7.30E-07	-0.46	12	1.2	Dhtkd1	Metabolism
DMR17:76452001	17	76452001	76456000	4000	1	1.60E-07	0.42	72	1.8	Cdc123	
DMR17:76731001	17	76731001	76733000	2000	2	1.60E-09	0.51	14	0.7	Camk1d	Signaling
DMR17:77460001	17	77460001	77462000	2000	1	2.70E-07	-0.44	57	2.85	Bend7	
DMR17:77474001	17	77474001	77475000	1000	1	2.50E-07	-0.38	24	2.4	Bend7	
DMR17:77814001	17	77814001	77817000	3000	1	7.20E-07	0.38	38	1.27	Frmd4a	
DMR17:77873001	17	77873001	77874000	1000	1	3.50E-08	-0.54	17	1.7	Frmd4a	
DMR17:78516001	17	78516001	78518000	2000	1	9.30E-08	0.42	40	2	Fam107b	
DMR17:78522001	17	78522001	78524000	2000	1	1.30E-10	0.56	32	1.6	Fam107b	
DMR17:78604001	17	78604001	78606000	2000	1	4.20E-07	0.55	27	1.35	Fam107b;LOC108348592	
DMR17:78792001	17	78792001	78797000	5000	1	1.60E-15	0.54	100	2	Suv39h2;Dclre1c;LOC100125598;LOC108353135	Epigenetic;Transcription
DMR17:78885001	17	78885001	78889000	4000	1	7.60E-08	-0.4	75	1.88	Rpl34-ps1;Olah	Metabolism
DMR17:78894001	17	78894001	78895000	1000	1	2.30E-07	0.38	12	1.2	Olah;Acbd7	Metabolism;Transport
DMR17:79094001	17	79094001	79098000	4000	1	6.60E-10	0.52	110	2.75	Fam171a1	
DMR17:79377001	17	79377001	79379000	2000	1	9.30E-09	0.41	35	1.75	Itga8	Extracellular Matrix
DMR17:79407001	17	79407001	79408000	1000	1	6.50E-09	-0.5	9	0.9	Itga8	Extracellular Matrix
DMR17:79613001	17	79613001	79616000	3000	1	5.90E-09	0.64	26	0.87	Itga8	Extracellular Matrix
DMR17:79776001	17	79776001	79777000	1000	1	6.10E-09	0.52	7	0.7	Fam188a;LOC108353136	
DMR17:80953001	17	80953001	80955000	2000	1	2.70E-07	0.56	15	0.75	St8sia6	Transport
DMR17:80976001	17	80976001	80980000	4000	1	2.40E-08	-0.27	57	1.43	St8sia6	Transport
DMR17:81005001	17	81005001	81007000	2000	1	2.30E-08	-0.38	37	1.85	St8sia6	Transport
DMR17:81009001	17	81009001	81014000	5000	1	1.10E-08	-0.33	60	1.2	St8sia6	Transport
DMR17:81493001	17	81493001	81494000	1000	1	2.00E-11	-0.34	11	1.1	Slc39a12	Transport
DMR17:81521001	17	81521001	81528000	7000	1	3.10E-07	-0.33	76	1.09	Slc39a12	Transport
DMR17:81874001	17	81874001	81875000	1000	1	1.10E-07	-0.39	11	1.1	Cacnb2	Transport
DMR17:82421001	17	82421001	82422000	1000	1	6.40E-08	0.6	7	0.7	Malrd1	
DMR17:82499001	17	82499001	82501000	2000	1	1.70E-07	-0.39	16	0.8	Malrd1	
DMR17:82569001	17	82569001	82573000	4000	2	2.00E-20	0.74	42	1.05	Malrd1	
DMR17:82574001	17	82574001	82576000	2000	1	6.10E-09	-0.29	30	1.5	Malrd1	
DMR17:82757001	17	82757001	82763000	6000	1	3.50E-07	-0.28	62	1.03	Malrd1	
DMR17:82846001	17	82846001	82848000	2000	1	3.50E-08	-0.34	11	0.55	Malrd1	
DMR17:82941001	17	82941001	82943000	2000	1	4.90E-09	0.58	9	0.45	Malrd1	

DMR17:83301001	17	83301001	83306000	5000	1	7.80E-10	-0.33	64	1.28	Plxdc2	
DMR17:83377001	17	83377001	83382000	5000	1	4.00E-09	-0.31	58	1.16	Plxdc2	
DMR17:84923001	17	84923001	84926000	3000	1	4.90E-08	-0.41	34	1.13	Mllt10	Transcription
DMR17:84955001	17	84955001	84956000	1000	1	5.50E-12	0.58	14	1.4	Mllt10	Transcription
DMR17:84981001	17	84981001	84982000	1000	1	2.00E-08	0.42	14	1.4	Mllt10;Dnajc1	Transcription;Transcription
DMR17:85654001	17	85654001	85659000	5000	1	1.50E-08	-0.37	58	1.16	Pip4k2a	Signaling
DMR17:85894001	17	85894001	85896000	2000	1	2.90E-08	-0.41	27	1.35	LOC102556022;Armc3;LOC100361451	
DMR17:86075001	17	86075001	86077000	2000	1	5.00E-11	0.48	33	1.65	Msrb2	Metabolism
DMR17:87307001	17	87307001	87312000	5000	1	5.80E-09	0.55	59	1.18	Etl4	
DMR17:87386001	17	87386001	87388000	2000	1	1.50E-10	0.54	26	1.3	Etl4	
DMR17:87464001	17	87464001	87465000	1000	1	9.90E-07	0.58	1	0.1	Etl4	
DMR17:87685001	17	87685001	87688000	3000	1	6.80E-07	-0.73	55	1.83	Etl4	
DMR17:87975001	17	87975001	87978000	3000	1	3.30E-07	-0.38	50	1.67	Prtfdc1	Golgi
DMR17:88088001	17	88088001	88089000	1000	1	5.20E-08	-0.57	15	1.5	Enkur;LOC108348599;Thnsl1	
DMR17:88296001	17	88296001	88299000	3000	1	1.90E-07	-0.3	32	1.07	Gpr158	Signaling
DMR17:88367001	17	88367001	88369000	2000	1	1.10E-08	-0.39	18	0.9	Gpr158	Signaling
DMR17:88377001	17	88377001	88384000	7000	2	8.30E-09	-0.33	72	1.03	Gpr158	Signaling
DMR17:88999001	17	88999001	89005000	6000	3	5.90E-11	-0.36	66	1.1	Myo3a	
DMR17:89029001	17	89029001	89030000	1000	1	6.50E-12	0.46	2	0.2	Myo3a	
DMR17:89219001	17	89219001	89220000	1000	1	2.30E-07	0.35	3	0.3	Gad2	Metabolism
DMR17:89668001	17	89668001	89671000	3000	1	2.60E-09	-0.39	22	0.73	Potec	Transport
DMR17:90379001	17	90379001	90382000	3000	1	4.80E-07	-0.46	21	0.7	Lyst	
DMR17:90476001	17	90476001	90487000	11000	2	3.20E-09	-0.36	132	1.2	Lyst	
DMR17:90530001	17	90530001	90531000	1000	1	1.20E-07	-0.4	9	0.9	Lyst	
DMR17:90686001	17	90686001	90687000	1000	1	5.30E-07	0.38	9	0.9	Gpr137b	
DMR18:755001	18	755001	756000	1000	1	2.20E-07	-0.5	5	0.5	Zfp136;LOC100360541	Transcription
DMR18:763001	18	763001	769000	6000	1	1.70E-09	-0.39	65	1.08	Zfp136;LOC100360541	Transcription
DMR18:913001	18	913001	914000	1000	1	3.90E-08	-0.55	3	0.3	Colec12	
DMR18:1009001	18	1009001	1011000	2000	1	7.10E-07	-0.53	14	0.7	Colec12	
DMR18:1134001	18	1134001	1135000	1000	1	7.60E-08	0.35	10	1	LOC684667;Thoc1	Metabolism
DMR18:1356001	18	1356001	1358000	2000	1	5.20E-07	-0.42	12	0.6	Rock1	Signaling
DMR18:1495001	18	1495001	1498000	3000	1	7.10E-07	-0.3	29	0.97	Rpl36al	
DMR18:1612001	18	1612001	1613000	1000	1	3.20E-10	-0.67	6	0.6	Greb1l	
DMR18:1633001	18	1633001	1636000	3000	1	9.10E-07	-0.6	11	0.37	Greb1l	
DMR18:2008001	18	2008001	2009000	1000	1	5.20E-09	0.54	3	0.3	Mib1	Proteolysis
DMR18:2023001	18	2023001	2024000	1000	1	1.60E-09	0.56	10	1	Mib1	Proteolysis
DMR18:3463001	18	3463001	3467000	4000	1	3.30E-08	0.45	47	1.18	Tmem241	Transport
DMR18:3635001	18	3635001	3637000	2000	2	1.30E-09	0.46	34	1.7	Npc1	
DMR18:3678001	18	3678001	3679000	1000	1	5.80E-10	0.46	13	1.3	Ankrd29	
DMR18:3771001	18	3771001	3773000	2000	1	1.30E-12	0.47	13	0.65	Lama3	Extracellular Matrix
DMR18:3967001	18	3967001	3969000	2000	1	7.70E-07	0.35	23	1.15	Ttc39c	
DMR18:4030001	18	4030001	4032000	2000	1	5.40E-08	0.39	35	1.75	Ttc39c	
DMR18:4188001	18	4188001	4190000	2000	1	1.30E-09	0.58	26	1.3	Osbpl1a	
DMR18:4276001	18	4276001	4278000	2000	1	1.90E-18	0.55	30	1.5	Osbpl1a	
DMR18:4282001	18	4282001	4283000	1000	1	2.90E-13	0.56	17	1.7	Osbpl1a	
DMR18:5133001	18	5133001	5136000	3000	1	9.40E-08	-0.51	58	1.93	Zfp521	Transcription
DMR18:5158001	18	5158001	5162000	4000	1	1.40E-10	0.5	56	1.4	Zfp521	Transcription
DMR18:5234001	18	5234001	5235000	1000	1	4.10E-07	0.41	8	0.8	Zfp521	Transcription
DMR18:6075001	18	6075001	6079000	4000	1	5.90E-08	-0.39	80	2	Ss18	Transcription
DMR18:6140001	18	6140001	6143000	3000	1	1.40E-08	-0.48	15	0.5	Psma8	Protease
DMR18:6239001	18	6239001	6240000	1000	1	2.40E-07	-0.49	8	0.8	Taf4b	Transcription
DMR18:6819001	18	6819001	6825000	6000	2	4.10E-11	-0.47	76	1.27	LOC102551834;Chst9	Transport
DMR18:6950001	18	6950001	6952000	2000	1	5.40E-09	0.37	37	1.85	Chst9	Transport
DMR18:12264001	18	12264001	12266000	2000	1	2.40E-08	0.49	20	1	RGD1562080;LOC102554807	
DMR18:12898001	18	12898001	12905000	7000	1	5.70E-09	-0.33	79	1.13	Ccdc178	
DMR18:13146001	18	13146001	13149000	3000	1	3.00E-07	-0.39	16	0.53	Ccdc178	
DMR18:13705001	18	13705001	13706000	1000	1	6.50E-08	0.57	4	0.4	Nol4	
DMR18:13966001	18	13966001	13970000	4000	1	2.90E-10	-0.3	51	1.27	Nol4	

DMR18:14541001	18	14541001	14544000	3000	2	1.30E-10	-0.49	26	0.87	LOC102556080;Dtna	Proteolysis
DMR18:15390001	18	15390001	15393000	3000	1	3.20E-07	-0.54	57	1.9	Mapre2	Cytoskeleton
DMR18:15427001	18	15427001	15430000	3000	1	4.20E-07	-0.45	8	0.27	Mapre2;LOC100909691	Cytoskeleton
DMR18:15620001	18	15620001	15629000	9000	1	8.10E-10	-0.43	119	1.32	Mapre2;Dsg2;LOC102551904	Cytoskeleton;Cytoskeleton
DMR18:16183001	18	16183001	16184000	1000	1	5.70E-07	0.41	17	1.7	Galnt1;LOC498829	Golgi
DMR18:16516001	18	16516001	16517000	1000	1	2.00E-07	0.41	9	0.9	Slc39a6	Transport
DMR18:16534001	18	16534001	16535000	1000	1	5.10E-11	0.5	24	2.4	Slc39a6;Elp2	Transport
DMR18:17002001	18	17002001	17003000	1000	1	4.50E-07	0.41	10	1	Fhod3	
DMR18:17062001	18	17062001	17064000	2000	1	1.90E-07	0.35	35	1.75	Fhod3	
DMR18:17154001	18	17154001	17155000	1000	1	7.10E-09	-0.52	15	1.5	Fhod3	
DMR18:17464001	18	17464001	17469000	5000	2	3.10E-08	-0.35	48	0.96	RGD1562608	
DMR18:17523001	18	17523001	17528000	5000	2	1.60E-07	-0.26	47	0.94	RGD1562608	
DMR18:17588001	18	17588001	17594000	6000	2	1.60E-10	-0.35	59	0.98	RGD1562608	
DMR18:17638001	18	17638001	17641000	3000	1	8.20E-07	-0.52	16	0.53	RGD1562608	
DMR18:17828001	18	17828001	17830000	2000	1	5.00E-07	0.36	33	1.65	Celf4	
DMR18:22850001	18	22850001	22854000	4000	1	2.00E-08	-0.34	43	1.07	RGD1564026	
DMR18:23024001	18	23024001	23025000	1000	1	1.90E-07	0.35	12	1.2	Pik3c3	Signaling
DMR18:23795001	18	23795001	23799000	4000	1	6.80E-08	-0.42	36	0.9	Rit2	Signaling
DMR18:23919001	18	23919001	23922000	3000	1	3.20E-09	-0.35	18	0.6	Rit2	Signaling
DMR18:24500001	18	24500001	24501000	1000	1	4.80E-14	0.4	11	1.1	Sap130	Epigenetic
DMR18:24664001	18	24664001	24667000	3000	1	6.90E-07	-0.43	63	2.1	Wdr33	Translation
DMR18:24764001	18	24764001	24765000	1000	1	4.30E-08	-0.5	17	1.7	Myo7b	Cytoskeleton
DMR18:24901001	18	24901001	24902000	1000	1	5.10E-08	0.38	14	1.4	Iws1	
DMR18:25212001	18	25212001	25215000	3000	1	2.20E-07	0.44	61	2.03	Bin1	
DMR18:25851001	18	25851001	25853000	2000	1	3.60E-11	0.45	41	2.05	Camk4	Signaling
DMR18:25905001	18	25905001	25906000	1000	1	6.60E-07	-0.46	11	1.1	Camk4	Signaling
DMR18:26179001	18	26179001	26183000	4000	2	3.20E-10	0.66	132	3.3	Nrep	
DMR18:26688001	18	26688001	26694000	6000	2	6.50E-11	-0.39	71	1.18	Epb41l4a	
DMR18:26724001	18	26724001	26726000	2000	1	4.20E-07	0.34	44	2.2	Epb41l4a	
DMR18:27069001	18	27069001	27072000	3000	1	5.00E-07	-0.55	27	0.9	Apc;LOC102549857	
DMR18:27127001	18	27127001	27129000	2000	1	2.60E-10	0.43	37	1.85	Srp19;Reep5;LOC102549915	Transport;Transport
DMR18:27131001	18	27131001	27133000	2000	2	8.50E-07	0.36	44	2.2	Srp19;Reep5;LOC102549915	Transport;Transport
DMR18:27624001	18	27624001	27625000	1000	1	3.60E-07	-0.43	25	2.5	Kdm3b;Reep2	Epigenetic;Transport
DMR18:27665001	18	27665001	27667000	2000	1	3.70E-08	-0.42	38	1.9	Egr1	Transcription
DMR18:27992001	18	27992001	27994000	2000	1	8.90E-07	0.38	22	1.1	Ctnna1	Cytoskeleton
DMR18:28126001	18	28126001	28132000	6000	1	4.70E-09	-0.33	65	1.08	Sil1	
DMR18:28422001	18	28422001	28425000	3000	1	2.30E-07	0.38	76	2.53	Paip2;Slc23a1	Transport
DMR18:28527001	18	28527001	28530000	3000	1	1.20E-11	0.62	29	0.97	Tmem173	
DMR18:28944001	18	28944001	28946000	2000	1	8.50E-08	-0.42	29	1.45	Nrg2	Growth Factors
DMR18:30053001	18	30053001	30057000	4000	1	9.20E-07	-0.51	17	0.42	Pcdha4	Cytoskeleton
DMR18:30058001	18	30058001	30059000	1000	1	6.70E-08	-0.53	5	0.5	Pcdha4	Cytoskeleton
DMR18:30078001	18	30078001	30084000	6000	2	1.20E-07	-0.34	48	0.8	Pcdha4	Cytoskeleton
DMR18:30096001	18	30096001	30099000	3000	1	3.10E-11	-0.37	23	0.77	Pcdha4	Cytoskeleton
DMR18:30158001	18	30158001	30161000	3000	1	6.60E-08	-0.44	15	0.5	Pcdha4	Cytoskeleton
DMR18:30402001	18	30402001	30410000	8000	1	2.20E-16	-0.43	88	1.1	Pcdhb5;Pcdhb6	Cytoskeleton
DMR18:30445001	18	30445001	30446000	1000	1	8.70E-08	-0.49	9	0.9	Pcdhb8	Cytoskeleton
DMR18:30602001	18	30602001	30608000	6000	1	1.50E-08	-0.4	69	1.15	Pcdhb22	Cytoskeleton
DMR18:30795001	18	30795001	30796000	1000	1	2.90E-11	0.52	13	1.3	RGD1563159	
DMR18:30990001	18	30990001	30991000	1000	1	8.30E-08	-0.47	9	0.9	Diaph1	
DMR18:31330001	18	31330001	31336000	6000	1	8.90E-07	0.31	103	1.72	Pcdh1	Cytoskeleton
DMR18:31599001	18	31599001	31600000	1000	1	7.90E-07	0.38	7	0.7	Ndfip1;LOC108348773	
DMR18:31973001	18	31973001	31974000	1000	1	3.20E-10	0.59	1	0.1	Nr3c1;Arhgap26	Signaling
DMR18:31976001	18	31976001	31978000	2000	1	1.30E-11	0.63	11	0.55	Nr3c1;Arhgap26	Signaling
DMR18:32231001	18	32231001	32234000	3000	1	3.20E-07	-0.45	28	0.93	Nr3c1;Arhgap26	Signaling
DMR18:32238001	18	32238001	32245000	7000	2	7.10E-11	-0.37	62	0.89	Nr3c1;Arhgap26	Signaling
DMR18:32356001	18	32356001	32358000	2000	1	1.80E-09	0.48	27	1.35	Nr3c1;Arhgap26;Fgf1	Signaling;Growth Factors
DMR18:32404001	18	32404001	32405000	1000	1	1.30E-07	0.44	12	1.2	Nr3c1;Arhgap26	Signaling
DMR18:32694001	18	32694001	32695000	1000	1	2.20E-07	-0.62	9	0.9	Nr3c1	

DMR18:33470001	18	33470001	33476000	6000	1	2.50E-08	-0.32	56	0.93	Kctd16	
DMR18:33614001	18	33614001	33616000	2000	1	3.30E-09	0.48	28	1.4	Kctd16	
DMR18:35505001	18	35505001	35507000	2000	1	1.80E-09	-0.38	21	1.05	Dcp2	
DMR18:37066001	18	37066001	37071000	5000	2	2.50E-17	-0.41	50	1	Ppp2r2b	Signaling
DMR18:37208001	18	37208001	37209000	1000	1	7.00E-09	0.63	46	4.6	Ppp2r2b	Signaling
DMR18:37855001	18	37855001	37859000	4000	1	1.50E-07	-0.42	38	0.95	LOC108348779;Jakmip2	
DMR18:37879001	18	37879001	37885000	6000	1	3.50E-10	-0.47	62	1.03	Jakmip2	
DMR18:37988001	18	37988001	37992000	4000	1	2.00E-10	-0.4	41	1.02	Jakmip2	
DMR18:38098001	18	38098001	38099000	1000	1	1.10E-11	0.76	13	1.3	Jakmip2	
DMR18:40045001	18	40045001	40047000	2000	1	7.80E-10	-0.44	17	0.85	Rps27a-ps13	
DMR18:40193001	18	40193001	40196000	3000	1	9.30E-07	-0.36	13	0.43	Pggt1b	Metabolism
DMR18:40246001	18	40246001	40253000	7000	2	4.00E-09	-0.29	73	1.04	Ccdc112	
DMR18:40986001	18	40986001	40987000	1000	1	2.60E-08	-0.44	11	1.1	Arl14epl	Signaling
DMR18:40998001	18	40998001	41003000	5000	3	2.10E-09	-0.36	55	1.1	Arl14epl	Signaling
DMR18:41074001	18	41074001	41077000	3000	1	2.80E-08	-0.29	27	0.9	Commd10	
DMR18:41390001	18	41390001	41391000	1000	1	1.50E-12	0.53	40	4	Sema6a	Signaling
DMR18:43893001	18	43893001	43899000	6000	2	1.10E-08	-0.31	63	1.05	Dtwd2;LOC103694209	
DMR18:43939001	18	43939001	43942000	3000	1	3.40E-07	-0.31	23	0.77	Dtwd2	
DMR18:43952001	18	43952001	43955000	3000	1	1.80E-08	-0.45	40	1.33	Dtwd2	
DMR18:44501001	18	44501001	44503000	2000	1	6.90E-07	-0.45	12	0.6	Dmxl1	
DMR18:44509001	18	44509001	44515000	6000	2	3.40E-11	-0.4	45	0.75	Dmxl1	
DMR18:44548001	18	44548001	44553000	5000	2	4.70E-11	-0.34	60	1.2	Dmxl1	
DMR18:44619001	18	44619001	44622000	3000	1	4.00E-07	-0.39	14	0.47	Dmxl1	
DMR18:44769001	18	44769001	44771000	2000	1	7.70E-07	-0.41	22	1.1	Tnfaip8	
DMR18:45013001	18	45013001	45016000	3000	1	5.50E-08	-0.28	27	0.9	Fam170a	
DMR18:46037001	18	46037001	46041000	4000	1	1.90E-07	-0.28	43	1.07	Prr16	
DMR18:46047001	18	46047001	46048000	1000	1	2.60E-07	-0.54	15	1.5	Prr16	
DMR18:46087001	18	46087001	46093000	6000	1	3.20E-09	-0.31	69	1.15	Prr16	
DMR18:47289001	18	47289001	47293000	4000	1	2.00E-07	-0.3	43	1.07	Ftmt	Transport
DMR18:47533001	18	47533001	47535000	2000	2	1.40E-18	0.54	118	5.9	Lox	Metabolism
DMR18:48297001	18	48297001	48298000	1000	1	5.20E-09	0.38	15	1.5	Snx24	Cytoskeleton
DMR18:48456001	18	48456001	48458000	2000	1	2.90E-07	0.37	20	1	Prdm6	Transcription
DMR18:48459001	18	48459001	48460000	1000	1	4.30E-07	0.34	13	1.3	Prdm6	Transcription
DMR18:51211001	18	51211001	51212000	1000	1	2.00E-07	0.6	8	0.8	RGD1560341	
DMR18:51518001	18	51518001	51519000	1000	1	3.90E-07	-0.4	19	1.9	Gramd3	
DMR18:51531001	18	51531001	51532000	1000	1	2.10E-07	-0.44	20	2	Gramd3	
DMR18:51850001	18	51850001	51856000	6000	1	2.30E-13	-0.35	66	1.1	3-Mar	
DMR18:52000001	18	52000001	52006000	6000	1	3.70E-07	-0.29	64	1.07	March3;LOC102552123	
DMR18:52206001	18	52206001	52207000	1000	1	6.50E-07	-0.34	5	0.5	LOC108348736;Megf10	Extracellular Matrix
DMR18:52317001	18	52317001	52318000	1000	1	5.10E-08	0.44	9	0.9	Megf10	Extracellular Matrix
DMR18:53186001	18	53186001	53189000	3000	1	7.60E-07	0.36	43	1.43	Fbn2	Extracellular Matrix
DMR18:53215001	18	53215001	53216000	1000	1	1.40E-07	-0.56	23	2.3	Fbn2	Extracellular Matrix
DMR18:53732001	18	53732001	53734000	2000	1	2.20E-11	0.49	30	1.5	Isoc1	Metabolism
DMR18:54113001	18	54113001	54115000	2000	1	4.50E-07	0.63	17	0.85	RGD1312005	
DMR18:55635001	18	55635001	55640000	5000	2	2.30E-10	-0.39	52	1.04	RGD1305184	
DMR18:55678001	18	55678001	55684000	6000	2	1.40E-10	-0.42	62	1.03	RGD1305184;MGC105567	
DMR18:55871001	18	55871001	55873000	2000	1	2.80E-09	0.36	68	3.4	Myoz3;Synpo	Cytoskeleton;Cytoskeleton
DMR18:55995001	18	55995001	55998000	3000	1	2.90E-08	0.37	39	1.3	Ndst1	Transport
DMR18:56202001	18	56202001	56203000	1000	1	1.10E-07	0.35	13	1.3	Camk2a	Signaling
DMR18:56455001	18	56455001	56456000	1000	1	2.90E-07	0.55	7	0.7	Csf1r;Hmgxb3	Receptor
DMR18:56548001	18	56548001	56552000	4000	1	3.60E-08	-0.32	48	1.2	Pde6a	Signaling
DMR18:56559001	18	56559001	56562000	3000	1	1.40E-07	0.42	43	1.43	Pde6a	Signaling
DMR18:57072001	18	57072001	57074000	2000	1	7.70E-15	0.51	24	1.2	Afap1l1	
DMR18:57212001	18	57212001	57213000	1000	1	6.20E-08	0.54	6	0.6	Ablim3	Cytoskeleton
DMR18:57392001	18	57392001	57399000	7000	2	4.60E-07	-0.3	79	1.13	Sh3tc2	
DMR18:57680001	18	57680001	57681000	1000	1	5.10E-08	-0.46	15	1.5	Htr4;LOC108348797	Signaling
DMR18:57818001	18	57818001	57819000	1000	1	3.80E-11	0.4	1	0.1	Htr4;Fbxo38	Signaling
DMR18:57953001	18	57953001	57959000	6000	1	1.80E-10	-0.34	48	0.8	Spink13;LOC100360147;Spink10	Protease; Proteolysis
DMR18:58075001	18	58075001	58077000	2000	1	4.60E-11	0.68	24	1.2	Spink13	Protease; Proteolysis
DMR18:59148001	18	59148001	59154000	6000	2	1.50E-08	-0.36	61	1.02	Wdr7	
DMR18:59202001	18	59202001	59203000	1000	1	8.00E-08	-0.48	14	1.4	Wdr7	

DMR18:59217001	18	59217001	59220000	3000	1	1.40E-09	-0.35	41	1.37	Wdr7	
DMR18:59347001	18	59347001	59350000	3000	1	7.90E-07	0.37	41	1.37	Wdr7	
DMR18:59384001	18	59384001	59386000	2000	1	3.20E-08	0.55	25	1.25	Wdr7	
DMR18:60495001	18	60495001	60498000	3000	1	1.30E-07	-0.38	50	1.67	Nedd4l	Proteolysis
DMR18:60634001	18	60634001	60636000	2000	1	4.60E-09	0.39	35	1.75	Nedd4l;LOC108353172	Proteolysis
DMR18:60646001	18	60646001	60647000	1000	1	2.10E-07	0.4	16	1.6	Nedd4l	Proteolysis
DMR18:60704001	18	60704001	60706000	2000	1	1.40E-07	0.37	23	1.15	Nedd4l	Proteolysis
DMR18:61001001	18	61001001	61003000	2000	2	2.20E-10	0.42	35	1.75	Alpk2;LOC108348802	Signaling
DMR18:61073001	18	61073001	61075000	2000	1	1.50E-09	0.38	15	0.75	Alpk2	Signaling
DMR18:61127001	18	61127001	61128000	1000	1	1.60E-08	-0.57	21	2.1	Malt1	Protease
DMR18:61340001	18	61340001	61341000	1000	1	9.70E-07	-0.45	16	1.6	Zfp532	
DMR18:61421001	18	61421001	61426000	5000	1	1.90E-07	0.53	82	1.64	Oacyl	Metabolism
DMR18:61437001	18	61437001	61442000	5000	1	3.30E-07	-0.41	52	1.04	Oacyl	Metabolism
DMR18:61714001	18	61714001	61716000	2000	1	4.20E-12	-0.5	15	0.75	Lman1	Transport
DMR18:61892001	18	61892001	61893000	1000	1	6.30E-07	0.39	22	2.2	Ccbe1	Extracellular Matrix
DMR18:62283001	18	62283001	62287000	4000	1	4.70E-08	-0.32	32	0.8	LOC102553438;RGD1566303	
DMR18:63778001	18	63778001	63782000	4000	1	1.90E-07	-0.32	68	1.7	Ldlrad4	
DMR18:64171001	18	64171001	64173000	2000	1	1.10E-07	0.39	21	1.05	Mc2r	Signaling
DMR18:65290001	18	65290001	65291000	1000	1	2.20E-07	-0.39	20	2	Tcf4	Transcription
DMR18:65359001	18	65359001	65360000	1000	1	5.30E-07	0.29	6	0.6	Tcf4	Transcription
DMR18:65508001	18	65508001	65513000	5000	1	3.50E-09	-0.38	90	1.8	Tcf4	Transcription
DMR18:66680001	18	66680001	66683000	3000	1	4.00E-08	0.37	43	1.43	Dcc	
DMR18:66846001	18	66846001	66849000	3000	1	9.60E-11	-0.35	25	0.83	Dcc;LOC108348868	
DMR18:66954001	18	66954001	66955000	1000	1	6.70E-10	-0.59	17	1.7	Dcc	
DMR18:67082001	18	67082001	67084000	2000	1	6.90E-07	0.5	10	0.5	Dcc	
DMR18:67454001	18	67454001	67460000	6000	1	4.60E-17	-0.38	69	1.15	Dcc	
DMR18:68399001	18	68399001	68401000	2000	1	4.90E-08	0.59	31	1.55	Ccdc68	
DMR18:69006001	18	69006001	69008000	2000	1	1.20E-07	0.37	20	1	Stard6	
DMR18:70258001	18	70258001	70259000	1000	1	2.40E-10	0.47	8	0.8	Mbd1;Cfap53	
DMR18:70310001	18	70310001	70312000	2000	2	1.90E-15	0.46	35	1.75	Cfap53;LOC108348813	
DMR18:70324001	18	70324001	70326000	2000	1	5.10E-07	-0.31	37	1.85	Cfap53	
DMR18:70684001	18	70684001	70685000	1000	1	1.60E-07	-0.47	20	2	Myo5b	Cytoskeleton
DMR18:70728001	18	70728001	70730000	2000	1	3.90E-07	0.46	27	1.35	Myo5b;Acaa2	Cytoskeleton;Metabolism
DMR18:70984001	18	70984001	70985000	1000	1	8.50E-08	0.53	9	0.9	RGD1562987	
DMR18:71086001	18	71086001	71088000	2000	1	2.80E-07	0.53	26	1.3	Dym	
DMR18:71187001	18	71187001	71192000	5000	1	4.50E-10	0.41	88	1.76	Dym	
DMR18:71193001	18	71193001	71195000	2000	1	2.30E-10	0.39	55	2.75	Dym	
DMR18:71207001	18	71207001	71211000	4000	1	7.20E-09	-0.43	35	0.88	Dym	
DMR18:71314001	18	71314001	71321000	7000	4	1.90E-08	0.43	98	1.4	Dym	
DMR18:71405001	18	71405001	71410000	5000	2	8.90E-08	0.47	76	1.52	Smad7	Transcription
DMR18:71464001	18	71464001	71469000	5000	2	2.50E-08	0.43	115	2.3	Ctif	Metabolism
DMR18:72455001	18	72455001	72456000	1000	1	1.70E-07	0.42	8	0.8	Zbtb7c	
DMR18:73136001	18	73136001	73138000	2000	1	7.10E-07	-0.36	16	0.8	Skor2	
DMR18:73806001	18	73806001	73809000	3000	1	1.30E-08	-0.55	43	1.43	Loxhd1	
DMR18:74336001	18	74336001	74338000	2000	2	3.00E-25	0.82	35	1.75	Epg5	
DMR18:74391001	18	74391001	74393000	2000	1	6.50E-07	0.3	54	2.7	Epg5	
DMR18:74411001	18	74411001	74413000	2000	1	7.70E-07	0.49	28	1.4	Siglec15	
DMR18:74417001	18	74417001	74422000	5000	1	6.60E-08	0.36	50	1	Siglec15	
DMR18:74625001	18	74625001	74626000	1000	1	5.00E-11	0.49	14	1.4	Slc14a2	Transport
DMR18:74780001	18	74780001	74781000	1000	1	2.80E-07	-0.38	19	1.9	Slc14a2;LOC102553338	Transport
DMR18:75424001	18	75424001	75426000	2000	1	2.20E-09	-0.5	36	1.8	Setbp1	Epigenetic
DMR18:76549001	18	76549001	76553000	4000	2	3.30E-11	-0.37	43	1.07	LOC100911388;Pard6g	Cell Junction
DMR18:76671001	18	76671001	76672000	1000	1	8.50E-07	-0.36	16	1.6	Adnp2	
DMR18:76786001	18	76786001	76787000	1000	1	1.90E-09	-0.44	21	2.1	Pqlc1	
DMR18:76795001	18	76795001	76798000	3000	1	2.50E-10	0.39	96	3.2	Pqlc1	
DMR18:76957001	18	76957001	76959000	2000	1	2.70E-08	0.34	39	1.95	Ctdp1	Signaling
DMR18:77197001	18	77197001	77202000	5000	2	1.40E-08	0.43	83	1.66	Nfatc1	Transcription
DMR18:77289001	18	77289001	77290000	1000	1	2.90E-08	0.34	32	3.2	Nfatc1	Transcription
DMR18:77457001	18	77457001	77464000	7000	3	7.30E-09	-0.4	83	1.19	Atp9b;LOC108348826	Transport
DMR18:79390001	18	79390001	79391000	1000	1	6.50E-10	0.4	8	0.8	Mbp	Cytoskeleton

DMR18:79425001	18	79425001	79428000	3000	1	1.50E-07	0.43	47	1.57	Mbp	Cytoskeleton
DMR18:79523001	18	79523001	79524000	1000	1	3.90E-08	0.38	20	2	Zfp236	Transcription
DMR18:79569001	18	79569001	79572000	3000	1	1.10E-09	-0.5	18	0.6	RGD1562171	
DMR18:79808001	18	79808001	79810000	2000	2	3.60E-08	0.42	15	0.75	Zfp516	Transcription
DMR18:81332001	18	81332001	81333000	1000	1	1.20E-08	0.37	19	1.9	Zfp407	Transcription
DMR18:81813001	18	81813001	81814000	1000	1	3.40E-07	0.37	12	1.2	Fbxo15;Timm21	
DMR18:81845001	18	81845001	81850000	5000	2	8.50E-13	-0.42	67	1.34	Fbxo15	
DMR18:81858001	18	81858001	81864000	6000	1	1.10E-07	-0.29	52	0.87	Fbxo15	
DMR18:83494001	18	83494001	83496000	2000	1	1.20E-10	-0.49	15	0.75	Neto1	Protease
DMR18:83540001	18	83540001	83541000	1000	1	7.20E-09	0.55	8	0.8	Neto1	Protease
DMR18:86090001	18	86090001	86097000	7000	3	1.00E-09	-0.49	79	1.13	Rttn	
DMR18:86177001	18	86177001	86182000	5000	1	2.00E-13	-0.36	57	1.14	Rttn	
DMR18:86353001	18	86353001	86363000	10000	1	3.00E-11	-0.39	89	0.89	Cd226;LOC102546621	
DMR18:86431001	18	86431001	86432000	1000	1	1.60E-07	-0.41	6	0.6	Dok6	
DMR18:86780001	18	86780001	86783000	3000	1	1.00E-08	-0.31	20	0.67	Dok6	
DMR19:74001	19	74001	78000	4000	4	1.40E-15	-0.39	42	1.05	Ces2a;Ces2e	Metabolism
DMR19:108001	19	108001	109000	1000	1	2.20E-09	0.57	5	0.5	Ces2e;LOC689453	Metabolism
DMR19:634001	19	634001	637000	3000	1	4.50E-07	0.33	26	0.87	Car7	
DMR19:891001	19	891001	892000	1000	1	2.10E-12	0.57	2	0.2	Cmtm2a;LOC102555510	Transport
DMR19:2541001	19	2541001	2542000	1000	1	1.20E-07	0.63	4	0.4	Cdh11	Cytoskeleton
DMR19:8949001	19	8949001	8950000	1000	1	1.70E-08	-0.62	16	1.6	Polr2l-ps1	
DMR19:9733001	19	9733001	9737000	4000	1	2.70E-10	-0.32	56	1.4	Cnot1	Translation
DMR19:9781001	19	9781001	9785000	4000	1	4.00E-11	0.53	62	1.55	Ndrg4	Protease
DMR19:10084001	19	10084001	10085000	1000	1	5.50E-09	0.49	25	2.5	Mmp15	Protease
DMR19:10339001	19	10339001	10340000	1000	1	2.40E-08	0.69	12	1.2	Kifc3;Katnb1	Cytoskeleton
DMR19:10413001	19	10413001	10414000	1000	1	2.10E-07	0.38	42	4.2	Adgrg3;Adgrg1	Signaling
DMR19:10623001	19	10623001	10625000	2000	1	3.50E-10	0.36	32	1.6	Ccl17;LOC307650	Growth Factors
DMR19:10681001	19	10681001	10684000	3000	1	2.00E-07	0.35	51	1.7	Ccl22	Growth Factors
DMR19:10726001	19	10726001	10728000	2000	1	3.50E-09	-0.76	31	1.55	LOC102550889;Trnal-cag;Pllp	Transport
DMR19:10746001	19	10746001	10747000	1000	1	6.10E-08	0.52	17	1.7	Pllp;Arl2bp	Transport
DMR19:10753001	19	10753001	10754000	1000	1	3.10E-08	0.38	13	1.3	Pllp;Arl2bp	Transport
DMR19:10761001	19	10761001	10763000	2000	1	4.30E-07	0.58	42	2.1	Pllp;Arl2bp;Rspry1	Transport
DMR19:10775001	19	10775001	10776000	1000	1	2.20E-07	0.37	26	2.6	Rspry1	
DMR19:10865001	19	10865001	10866000	1000	1	2.50E-08	0.4	25	2.5	Fam192a;Cpne2	
DMR19:10920001	19	10920001	10922000	2000	1	2.70E-10	-0.34	43	2.15	Nlrc5	Cytoskeleton
DMR19:10951001	19	10951001	10952000	1000	1	5.80E-12	0.74	11	1.1	Nlrc5	Cytoskeleton
DMR19:10963001	19	10963001	10965000	2000	2	7.60E-16	0.6	29	1.45	Nlrc5	Cytoskeleton
DMR19:11496001	19	11496001	11497000	1000	1	8.90E-07	0.35	15	1.5	Amfr	Proteolysis
DMR19:11520001	19	11520001	11521000	1000	1	6.40E-10	0.47	18	1.8	Gnao1	Signaling
DMR19:11640001	19	11640001	11641000	1000	1	2.80E-10	0.46	19	1.9	Gnao1	Signaling
DMR19:11658001	19	11658001	11660000	2000	1	5.40E-09	0.44	18	0.9	Gnao1;LOC108348879	Signaling
DMR19:12571001	19	12571001	12574000	3000	2	2.20E-07	-0.48	22	0.73	Large1	Golgi
DMR19:12581001	19	12581001	12585000	4000	1	7.50E-10	-0.48	33	0.82	Large1	Golgi
DMR19:12632001	19	12632001	12634000	2000	1	2.20E-07	0.57	25	1.25	Large1	Golgi
DMR19:12858001	19	12858001	12859000	1000	1	6.10E-07	-0.39	14	1.4	Large1;LOC108348896	Golgi
DMR19:15214001	19	15214001	15216000	2000	2	1.20E-09	0.81	16	0.8	Ces1d	Metabolism
DMR19:15425001	19	15425001	15426000	1000	1	1.50E-08	0.64	6	0.6	Slc6a2	Transport
DMR19:16863001	19	16863001	16865000	2000	1	1.90E-07	0.38	25	1.25	Fto	Metabolism
DMR19:17081001	19	17081001	17085000	4000	1	2.20E-07	0.39	61	1.52	Fto	Metabolism
DMR19:17325001	19	17325001	17326000	1000	1	1.50E-07	0.42	14	1.4	Rbl2	Epigenetic
DMR19:17511001	19	17511001	17513000	2000	1	2.10E-07	-0.5	20	1	Chd9	
DMR19:19379001	19	19379001	19381000	2000	2	1.00E-09	0.41	14	0.7	Nod2	Cytoskeleton
DMR19:19426001	19	19426001	19427000	1000	1	9.90E-07	0.6	28	2.8	Nkd1	
DMR19:20177001	19	20177001	20180000	3000	1	1.40E-07	0.4	46	1.53	Zfp423	
DMR19:20240001	19	20240001	20241000	1000	1	3.20E-09	0.44	15	1.5	Zfp423	
DMR19:20330001	19	20330001	20337000	7000	1	2.50E-08	0.37	141	2.01	Zfp423	
DMR19:21249001	19	21249001	21250000	1000	1	7.40E-07	-0.36	9	0.9	N4bp1	Translation
DMR19:21419001	19	21419001	21420000	1000	1	1.60E-08	0.44	31	3.1	Lonp2	Protease
DMR19:22082001	19	22082001	22083000	1000	1	2.50E-09	0.39	13	1.3	Phkb	Signaling
DMR19:22103001	19	22103001	22105000	2000	1	1.90E-07	-0.44	21	1.05	Phkb	Signaling

DMR19:22130001	19	22130001	22133000	3000	1	4.00E-08	-0.58	47	1.57	Phkb	Signaling
DMR19:22182001	19	22182001	22184000	2000	1	9.20E-11	-0.5	20	1	Phkb	Signaling
DMR19:22572001	19	22572001	22573000	1000	1	6.60E-11	0.43	12	1.2	Dnaja2	Transcription
DMR19:23610001	19	23610001	23611000	1000	1	1.70E-09	0.44	72	7.2	Il15	Cytokine
DMR19:24054001	19	24054001	24058000	4000	2	5.70E-09	0.45	195	4.88	Rnf150	
DMR19:24387001	19	24387001	24388000	1000	1	7.70E-07	0.42	8	0.8	Tbc1d9	Signaling
DMR19:24395001	19	24395001	24397000	2000	1	9.60E-09	0.46	38	1.9	Tbc1d9	Signaling
DMR19:24493001	19	24493001	24496000	3000	1	5.60E-07	0.3	41	1.37	Elmod2;Mgat4d	Cytoskeleton;Transport
DMR19:24655001	19	24655001	24661000	6000	1	4.80E-07	-0.26	68	1.13	Olr1667	
DMR19:24737001	19	24737001	24738000	1000	1	6.20E-07	-0.43	17	1.7	Tecr;Rps2-ps7;Dnajb1	Metabolism;Transcription
DMR19:24790001	19	24790001	24793000	3000	1	1.50E-14	0.72	60	2	Gipc1;Ptger1	Cytoskeleton;Signaling
DMR19:25054001	19	25054001	25058000	4000	1	3.40E-08	0.39	51	1.27	Adgrl1	Signaling
DMR19:25073001	19	25073001	25076000	3000	1	6.60E-07	-0.41	34	1.13	Adgrl1;Asf1b	Signaling;Epigenetic
DMR19:25205001	19	25205001	25207000	2000	1	3.30E-07	0.38	39	1.95	Rfx1;Dcaf15	Transcription
DMR19:25247001	19	25247001	25249000	2000	1	9.90E-12	0.58	44	2.2	Podnl1;Cc2d1a	
DMR19:25338001	19	25338001	25340000	2000	1	1.20E-08	0.38	41	2.05	Zswim4	
DMR19:25825001	19	25825001	25828000	3000	1	9.60E-11	0.68	43	1.43	Lyl1;Nf1x	Transcription;Transcription
DMR19:26056001	19	26056001	26057000	1000	1	4.50E-07	-0.56	7	0.7	MAST1;LOC108348915;Rtbdn	
DMR19:27432001	19	27432001	27434000	2000	1	6.30E-07	-0.44	13	0.65	Mylk3	Signaling
DMR19:27554001	19	27554001	27559000	5000	1	5.50E-09	-0.37	42	0.84	Olr1666	Receptor
DMR19:28659001	19	28659001	28661000	2000	1	1.90E-15	0.62	37	1.85	LOC501317;LOC691712;RGD1562660	
DMR19:28684001	19	28684001	28686000	2000	1	9.00E-07	-0.29	29	1.45	LOC501317;LOC691712;RGD1562660	
DMR19:28688001	19	28688001	28694000	6000	2	2.50E-07	-0.34	67	1.12	LOC501317;LOC691712;RGD1562660	
DMR19:28726001	19	28726001	28728000	2000	1	7.80E-08	-0.38	20	1	LOC501317;LOC691712;RGD1562660;LOC100912126;LOC102550448	
DMR19:28729001	19	28729001	28733000	4000	1	2.50E-07	-0.33	44	1.1	LOC501317;LOC691712;RGD1562660;LOC100912126;LOC102550448	
DMR19:28856001	19	28856001	28863000	7000	4	4.90E-13	0.63	51	0.73	LOC501317;RGD1562660	
DMR19:29750001	19	29750001	29753000	3000	1	2.90E-07	0.39	45	1.5	Inpp4b	
DMR19:29758001	19	29758001	29765000	7000	2	1.80E-08	-0.33	59	0.84	Inpp4b	
DMR19:29769001	19	29769001	29771000	2000	1	1.60E-11	0.37	11	0.55	Inpp4b	
DMR19:29942001	19	29942001	29947000	5000	1	6.00E-08	-0.28	50	1	Inpp4b	
DMR19:30038001	19	30038001	30043000	5000	1	3.80E-16	0.43	40	0.8	Inpp4b	
DMR19:30827001	19	30827001	30830000	3000	1	4.60E-07	0.36	40	1.33	Gab1	Cytoskeleton
DMR19:30961001	19	30961001	30964000	3000	1	4.20E-07	-0.48	30	1	Smarca5;Frem3	Epigenetic
DMR19:32510001	19	32510001	32511000	1000	1	5.30E-07	0.36	12	1.2	Zfp827	Transcription
DMR19:32574001	19	32574001	32576000	2000	1	2.10E-08	0.53	16	0.8	Zfp827	Transcription
DMR19:32922001	19	32922001	32925000	3000	1	2.60E-11	0.6	27	0.9	Slc10a7	Transport
DMR19:33347001	19	33347001	33350000	3000	1	1.60E-09	0.37	36	1.2	Ttc29	
DMR19:33371001	19	33371001	33377000	6000	2	4.40E-11	-0.42	58	0.97	Ttc29	
DMR19:33403001	19	33403001	33405000	2000	1	2.70E-08	0.35	41	2.05	Ttc29	
DMR19:34256001	19	34256001	34257000	1000	1	8.80E-07	-0.36	15	1.5	Arhgap10	Signaling
DMR19:34405001	19	34405001	34406000	1000	1	3.30E-08	0.42	13	1.3	Arhgap10;Nr3c2	Signaling
DMR19:34472001	19	34472001	34473000	1000	1	2.60E-14	-0.7	16	1.6	Nr3c2	
DMR19:34598001	19	34598001	34599000	1000	1	3.90E-08	0.54	6	0.6	Nr3c2	
DMR19:34620001	19	34620001	34621000	1000	1	7.30E-08	-0.5	19	1.9	Nr3c2;Rps27a-ps6	
DMR19:37289001	19	37289001	37290000	1000	1	4.70E-07	0.45	7	0.7	Lrrc29;Tmem208;Fhod1	
DMR19:37424001	19	37424001	37425000	1000	1	1.90E-10	0.51	17	1.7	Lrrc36;Tppp3;Zdhhc1	Cytoskeleton
DMR19:37752001	19	37752001	37753000	1000	1	4.10E-07	-0.55	11	1.1	Ranbp10;LOC108348930	Cytoskeleton
DMR19:38419001	19	38419001	38420000	1000	1	3.10E-07	0.39	18	1.8	Nob1;Nqo1	Metabolism
DMR19:38469001	19	38469001	38471000	2000	1	5.30E-08	0.49	13	0.65	Nfat5	Transcription
DMR19:38892001	19	38892001	38894000	2000	1	8.90E-07	0.4	25	1.25	Tango6	
DMR19:38986001	19	38986001	38987000	1000	1	3.70E-07	0.4	22	2.2	Tango6	
DMR19:38993001	19	38993001	38994000	1000	1	4.90E-07	-0.45	8	0.8	Tango6	
DMR19:39094001	19	39094001	39099000	5000	1	3.00E-09	-0.39	74	1.48	Chtf8;Utp4	
DMR19:39163001	19	39163001	39164000	1000	1	8.50E-08	0.36	29	2.9	Sntb2	

DMR19:39592001	19	39592001	39596000	4000	2	5.20E-11	0.47	34	0.85	Wwp2	Proteolysis
DMR19:39625001	19	39625001	39627000	2000	1	3.50E-07	0.44	28	1.4	RGD1562833	
DMR19:42074001	19	42074001	42077000	3000	1	3.80E-08	-0.41	36	1.2	Dhodh	Metabolism
DMR19:42759001	19	42759001	42762000	3000	1	4.80E-09	0.39	84	2.8	Zfhx3	Transcription
DMR19:42805001	19	42805001	42806000	1000	1	4.50E-09	0.46	19	1.9	Zfhx3	Transcription
DMR19:43351001	19	43351001	43353000	2000	1	5.90E-08	0.34	44	2.2	Fuk;Cog4	Transport
DMR19:43858001	19	43858001	43859000	1000	1	2.90E-09	0.45	9	0.9	Ldhd;Zfp1	Metabolism
DMR19:44087001	19	44087001	44090000	3000	1	9.70E-07	-0.39	39	1.3	Cfdp1;Tmem170a	
DMR19:44102001	19	44102001	44103000	1000	1	4.90E-07	-0.69	12	1.2	Tmem170a	
DMR19:44181001	19	44181001	44182000	1000	1	1.80E-09	0.41	20	2	Gabarapl2;Adat1	Cytoskeleton;Translation
DMR19:44218001	19	44218001	44220000	2000	1	2.80E-09	-0.49	32	1.6	Adat1;Kars	Translation
DMR19:46743001	19	46743001	46744000	1000	1	1.50E-07	0.41	16	1.6	Clec3a	Signaling
DMR19:46756001	19	46756001	46757000	1000	1	1.00E-09	0.37	4	0.4	Wwox	
DMR19:46793001	19	46793001	46796000	3000	1	1.50E-09	0.47	43	1.43	Wwox	
DMR19:46983001	19	46983001	46986000	3000	1	1.60E-11	0.52	60	2	Wwox	
DMR19:47000001	19	47000001	47002000	2000	1	6.40E-07	0.57	19	0.95	Wwox	
DMR19:47441001	19	47441001	47442000	1000	1	7.00E-07	0.46	6	0.6	Wwox	
DMR19:47498001	19	47498001	47500000	2000	1	2.30E-07	0.32	58	2.9	Wwox	
DMR19:47540001	19	47540001	47542000	2000	1	4.10E-08	0.41	45	2.25	Wwox	
DMR19:49107001	19	49107001	49110000	3000	1	2.00E-13	0.87	76	2.53	Cdyl2	
DMR19:49537001	19	49537001	49541000	4000	1	1.80E-07	0.52	68	1.7	Gcsh;Pkd1l2	Transport
DMR19:49548001	19	49548001	49554000	6000	1	4.30E-10	0.51	111	1.85	Pkd1l2	Transport
DMR19:49618001	19	49618001	49620000	2000	1	4.70E-14	0.56	57	2.85	Pkd1l2	Transport
DMR19:49682001	19	49682001	49684000	2000	1	4.70E-07	0.34	35	1.75	Bco1;Gan	Metabolism;Cytoskeleton
DMR19:49751001	19	49751001	49752000	1000	1	7.10E-07	0.34	20	2	Gan	Cytoskeleton
DMR19:49937001	19	49937001	49938000	1000	1	1.30E-07	0.41	16	1.6	Cmip	
DMR19:49963001	19	49963001	49964000	1000	1	7.70E-10	0.42	20	2	Cmip	
DMR19:49991001	19	49991001	5.00E+07	9000	2	7.90E-09	0.59	169	1.88	Cmip	
DMR19:50087001	19	50087001	50091000	4000	1	3.20E-07	0.42	65	1.62	Plcg2	Metabolism
DMR19:50364001	19	50364001	50367000	3000	1	6.10E-07	0.36	55	1.83	Mphosph6	
DMR19:50989001	19	50989001	50991000	2000	1	6.50E-07	0.43	31	1.55	Cdh13	Cytoskeleton
DMR19:51087001	19	51087001	51089000	2000	1	6.00E-07	0.38	28	1.4	Cdh13	Cytoskeleton
DMR19:51338001	19	51338001	51340000	2000	1	3.60E-11	0.46	38	1.9	Cdh13	Cytoskeleton
DMR19:51396001	19	51396001	51398000	2000	1	3.40E-16	0.58	29	1.45	Cdh13	Cytoskeleton
DMR19:51748001	19	51748001	51750000	2000	1	3.40E-10	0.52	20	1	Cdh13	Cytoskeleton
DMR19:51871001	19	51871001	51874000	3000	1	9.50E-07	0.42	24	0.8	Cdh13	Cytoskeleton
DMR19:51891001	19	51891001	51893000	2000	1	7.30E-08	0.77	19	0.95	Cdh13	Cytoskeleton
DMR19:52053001	19	52053001	52064000	11000	1	2.40E-07	0.43	126	1.15	Mlycd;Osgin1	
DMR19:52092001	19	52092001	52094000	2000	1	2.70E-07	0.44	20	1	Osgin1;Necab2	
DMR19:52122001	19	52122001	52128000	6000	1	2.80E-08	0.33	118	1.97	Necab2;Slc38a8	Transport
DMR19:52131001	19	52131001	52132000	1000	1	4.70E-08	0.43	12	1.2	Slc38a8	Transport
DMR19:52241001	19	52241001	52243000	2000	1	4.50E-09	0.45	27	1.35	Dnaaf1;LOC102546392;Taf1c	Signaling;Transcription
DMR19:52397001	19	52397001	52401000	4000	1	9.10E-07	0.58	74	1.85	Atp2c2;LOC103694346;Tldc1	Transport
DMR19:52409001	19	52409001	52416000	7000	1	8.80E-12	0.45	122	1.74	Atp2c2;LOC103694346;Tldc1	Transport
DMR19:52480001	19	52480001	52481000	1000	1	1.60E-07	0.34	37	3.7	Cotl1	Cytoskeleton
DMR19:52696001	19	52696001	52698000	2000	1	9.20E-08	0.35	24	1.2	Crispld2	Immune
DMR19:52715001	19	52715001	52717000	2000	1	6.20E-07	0.37	30	1.5	Crispld2	Immune
DMR19:52845001	19	52845001	52847000	2000	1	6.90E-07	0.33	16	0.8	RGD1304884	
DMR19:52854001	19	52854001	52856000	2000	1	5.30E-09	0.34	41	2.05	RGD1304884	
DMR19:52880001	19	52880001	52881000	1000	1	1.50E-10	0.67	6	0.6	RGD1304884	
DMR19:52900001	19	52900001	52901000	1000	1	2.70E-07	0.28	16	1.6	RGD1304884	
DMR19:52912001	19	52912001	52914000	2000	1	2.90E-08	0.38	20	1	RGD1304884	
DMR19:53079001	19	53079001	53083000	4000	1	1.50E-08	0.41	51	1.27	RGD1304884	
DMR19:53391001	19	53391001	53393000	2000	1	1.30E-09	0.44	65	3.25	RGD1304884;LOC108348955	
DMR19:53395001	19	53395001	53397000	2000	1	9.60E-09	0.43	45	2.25	RGD1304884;LOC108348955	

DMR19:53649001	19	53649001	53651000	2000	1	1.00E-07	0.43	63	3.15	RGD1304884;Map1lc3b;Zcchc14	Cytoskeleton
DMR19:53834001	19	53834001	53839000	5000	1	4.30E-08	0.43	84	1.68	Gse1	
DMR19:54023001	19	54023001	54026000	3000	1	8.00E-12	0.45	51	1.7	Gse1;LOC102551873	
DMR19:54027001	19	54027001	54028000	1000	1	3.70E-07	0.52	12	1.2	Gse1;LOC102551873	
DMR19:54050001	19	54050001	54053000	3000	1	6.20E-09	0.43	38	1.27	Gse1;RGD1565689	
DMR19:54055001	19	54055001	54057000	2000	1	2.60E-07	0.38	20	1	Gse1;RGD1565689	
DMR19:54077001	19	54077001	54078000	1000	1	3.00E-07	0.4	33	3.3	Gse1;RGD1565689	
DMR19:54157001	19	54157001	54158000	1000	1	5.40E-09	0.58	17	1.7	Gse1;Gins2	Transcription
DMR19:54572001	19	54572001	54578000	6000	1	5.30E-08	0.4	154	2.57	Jph3	
DMR19:54686001	19	54686001	54687000	1000	1	6.20E-08	0.39	14	1.4	LOC108348956;Slc7a5	Transport
DMR19:54735001	19	54735001	54737000	2000	1	3.40E-07	0.45	19	0.95	Ca5a	
DMR19:54827001	19	54827001	54831000	4000	1	4.00E-16	0.41	30	0.75	Banp	
DMR19:54917001	19	54917001	54921000	4000	1	4.70E-07	0.32	64	1.6	Zfp469	
DMR19:54937001	19	54937001	54940000	3000	1	3.20E-09	0.37	67	2.23	Zfp469	
DMR19:54955001	19	54955001	54959000	4000	1	2.50E-07	0.4	75	1.88	Zfp469	
DMR19:54962001	19	54962001	54964000	2000	1	7.90E-11	0.5	27	1.35	Zfp469	
DMR19:54996001	19	54996001	54998000	2000	1	5.60E-10	0.46	42	2.1	Zfp469	
DMR19:55108001	19	55108001	55110000	2000	1	1.50E-11	0.86	8	0.4	Zfpm1	Transcription
DMR19:55232001	19	55232001	55236000	4000	1	9.20E-07	0.32	64	1.6	Zc3h18	
DMR19:55451001	19	55451001	55456000	5000	2	4.20E-07	0.46	126	2.52	Cbfa2t3;LOC108348958	Transcription
DMR19:55460001	19	55460001	55462000	2000	1	1.40E-08	0.56	39	1.95	Cbfa2t3;LOC108348958	Transcription
DMR19:55764001	19	55764001	55765000	1000	1	6.10E-07	0.35	20	2	Ankrd11	
DMR19:55883001	19	55883001	55885000	2000	1	3.40E-07	-0.39	39	1.95	LOC108348981;LOC102551789;Spg7	Protease
DMR19:55901001	19	55901001	55903000	2000	1	8.20E-07	0.38	28	1.4	Spg7	Protease
DMR19:55936001	19	55936001	55940000	4000	1	7.00E-07	0.36	73	1.82	Cpne7	
DMR19:56278001	19	56278001	56283000	5000	1	9.10E-09	-0.45	78	1.56	Afg3l1	Protease
DMR19:56336001	19	56336001	56340000	4000	4	6.00E-17	0.63	109	2.72	Gas8	Cytoskeleton
DMR19:56602001	19	56602001	56603000	1000	1	3.90E-07	0.39	18	1.8	Rab4a	
DMR19:56703001	19	56703001	56704000	1000	1	1.00E-11	0.57	4	0.4	Nup133	
DMR19:57128001	19	57128001	57129000	1000	1	2.80E-07	0.46	28	2.8	Pgbd5	Epigenetic
DMR19:57160001	19	57160001	57163000	3000	1	1.10E-08	0.38	56	1.87	Pgbd5	Epigenetic
DMR19:57336001	19	57336001	57344000	8000	1	2.00E-09	0.44	127	1.59	Agt;Capn9	Protease; Proteolysis;Protease
DMR19:57367001	19	57367001	57368000	1000	1	1.50E-07	0.4	8	0.8	Capn9	Protease
DMR19:57416001	19	57416001	57420000	4000	1	6.20E-10	0.52	46	1.15	RGD1559896;Ttc13	
DMR19:57474001	19	57474001	57475000	1000	1	1.60E-10	0.46	31	3.1	Ttc13;Arv1	
DMR19:57581001	19	57581001	57586000	5000	1	2.20E-08	0.47	107	2.14	Trim67	Proteolysis
DMR19:57975001	19	57975001	57976000	1000	1	5.70E-15	0.75	18	1.8	Disc1	
DMR19:58236001	19	58236001	58239000	3000	1	5.30E-10	0.51	66	2.2	Sipa1l2	Signaling
DMR19:58260001	19	58260001	58263000	3000	1	8.40E-09	0.44	46	1.53	Sipa1l2	Signaling
DMR19:59216001	19	59216001	59219000	3000	1	1.20E-07	0.36	18	0.6	Slc35f3	
DMR19:59979001	19	59979001	59981000	2000	1	3.90E-07	0.39	26	1.3	LOC365026;Hmg1l1;LOC679596	
DMR19:60068001	19	60068001	60069000	1000	1	8.10E-10	0.43	20	2	Pard3	
DMR19:60467001	19	60467001	60468000	1000	1	1.00E-08	-0.54	10	1	Pard3	
DMR19:61948001	19	61948001	61951000	3000	1	8.40E-07	-0.31	29	0.97	Ccdc7	
DMR19:62109001	19	62109001	62115000	6000	2	1.20E-09	-0.32	76	1.27	Ccdc7	
DMR19:62130001	19	62130001	62136000	6000	3	1.20E-08	-0.3	67	1.12	Ccdc7	
DMR20:42001	20	42001	43000	1000	1	6.40E-07	0.34	13	1.3	Olr1679	Receptor
DMR20:253001	20	253001	258000	5000	1	1.40E-09	-0.44	45	0.9	Tmlhe;LOC102550933	Metabolism
DMR20:324001	20	324001	330000	6000	1	1.10E-12	-0.34	66	1.1	LOC100362712;Olr1668;LOC108353249	Receptor
DMR20:357001	20	357001	361000	4000	1	1.40E-10	-0.35	39	0.98	Olr1669-ps	
DMR20:369001	20	369001	377000	8000	1	1.90E-07	-0.33	78	0.98	Olr1669-ps;Olr1670	Receptor
DMR20:504001	20	504001	506000	2000	2	6.00E-14	0.72	18	0.9	Olr1680;Olr1681	
DMR20:890001	20	890001	891000	1000	1	3.30E-07	-0.55	7	0.7	Olr1685-ps	
DMR20:1067001	20	1067001	1074000	7000	1	1.40E-07	-0.56	60	0.86	Olr1698-ps;Olr1699	
DMR20:1159001	20	1159001	1163000	4000	2	5.40E-10	-0.35	47	1.18	Olr1707;Olr1708	Signaling
DMR20:1188001	20	1188001	1191000	3000	1	2.00E-09	-0.42	15	0.5	Olr1709;Olr1710	Signaling

DMR20:1204001	20	1204001	1207000	3000	1	3.60E-09	-0.36	24	0.8	Olr1710;Olr1711-ps	Signaling
DMR20:1230001	20	1230001	1237000	7000	2	3.20E-15	-0.35	91	1.3	Olr1712-ps	
DMR20:1727001	20	1727001	1730000	3000	1	1.90E-08	-0.3	32	1.07	Olr1734	
DMR20:1739001	20	1739001	1740000	1000	1	3.80E-11	0.5	4	0.4	Olr1734;Olr1735	
DMR20:1799001	20	1799001	1801000	2000	1	5.70E-07	-0.23	19	0.95	Olr1739	Receptor
DMR20:1803001	20	1803001	1804000	1000	1	5.30E-08	-0.41	8	0.8	Olr1739	Receptor
DMR20:2068001	20	2068001	2074000	6000	1	4.20E-08	-0.31	49	0.82	RT1-M6-1;RT1-M6-2;Znrd1as1	Immune
DMR20:2260001	20	2260001	2261000	1000	1	1.00E-07	0.54	12	1.2	RT1-M10-4-ps	
DMR20:2478001	20	2478001	2482000	4000	1	7.00E-08	-0.29	40	1	RT1-M10-1	
DMR20:2529001	20	2529001	2533000	4000	1	1.00E-08	-0.36	20	0.5	RT1-P2-ps1	
DMR20:2535001	20	2535001	2536000	1000	1	3.80E-08	0.42	8	0.8	RT1-P2-ps1	
DMR20:2548001	20	2548001	2550000	2000	1	7.30E-07	-0.32	11	0.55	RT1-P2-ps1;Rcrg1-ps40;Nerg-ps15	
DMR20:2591001	20	2591001	2593000	2000	1	8.60E-07	-0.55	12	0.6	2994a1;Nerg-ps10;Nerg-ps13;Rcrg1-ps34;Rcrg1-ps24;2824a1;2661a1a2;2795a1	
DMR20:3410001	20	3410001	3411000	1000	1	1.80E-07	0.33	18	1.8	Nrm;Mdc1	
DMR20:3447001	20	3447001	3448000	1000	1	3.40E-07	0.36	9	0.9	Flot1;Ier3	
DMR20:3536001	20	3536001	3537000	1000	1	1.70E-08	0.4	18	1.8	Cb707485	
DMR20:3570001	20	3570001	3572000	2000	1	4.20E-07	0.38	52	2.6	Ddr1	Receptor
DMR20:3612001	20	3612001	3614000	2000	1	6.80E-07	0.41	36	1.8	Sfta2;Dpcr1	Transport
DMR20:3633001	20	3633001	3637000	4000	2	2.10E-10	0.38	52	1.3	CB741658	
DMR20:3726001	20	3726001	3730000	4000	2	1.00E-10	0.46	75	1.88	Psors1c2;Cchcr1	
DMR20:3746001	20	3746001	3750000	4000	1	4.10E-11	0.45	92	2.3	Cchcr1;Tcf19;Pou5f1	Transcription
DMR20:4193001	20	4193001	4194000	1000	1	2.60E-08	0.34	27	2.7	Tesb	
DMR20:4219001	20	4219001	4224000	5000	1	9.30E-07	0.38	54	1.08	Tesb;LOC103694377	
DMR20:4379001	20	4379001	4381000	2000	2	9.00E-09	0.52	46	2.3	Stk19;Agpat1;Egfl8;Ppt2	Metabolism;Signaling;Golgi
DMR20:4382001	20	4382001	4383000	1000	1	3.00E-09	0.52	20	2	Stk19;Agpat1;Egfl8;Ppt2;Prrt1	Metabolism;Signaling;Golgi
DMR20:4400001	20	4400001	4402000	2000	1	7.10E-09	0.39	42	2.1	Stk19;Ppt2;Prrt1;Fkbpl	Golgi;Transcription
DMR20:4495001	20	4495001	4496000	1000	1	3.80E-07	0.45	12	1.2	Stk19;Tnxa-ps1;Cyp21a1;LOC103689965	Protease; Proteolysis
DMR20:4541001	20	4541001	4545000	4000	1	6.10E-12	0.48	103	2.58	Nelfe;Cfb;C2	
DMR20:4582001	20	4582001	4589000	7000	2	1.20E-08	0.44	244	3.49	LOC102553878;Zbtb12;Ehmt2;Slc44a4	Transcription;Transport
DMR20:4673001	20	4673001	4675000	2000	1	1.60E-07	0.31	15	0.75	RT1-CE6;LOC103694379	
DMR20:4811001	20	4811001	4812000	1000	1	7.90E-08	0.4	13	1.3	Ddx39b;Atp6v1g2	Metabolism
DMR20:4856001	20	4856001	4858000	2000	1	1.70E-07	0.46	63	3.15	Lta;LOC103694380;LOC103694381	
DMR20:4983001	20	4983001	4985000	2000	1	1.30E-07	-0.47	47	2.35	Rpl13-ps1;LOC108349032;Vars	
DMR20:5005001	20	5005001	5008000	3000	1	9.40E-10	0.58	90	3	Vars;Vwa7	
DMR20:5015001	20	5015001	5021000	6000	1	2.80E-07	0.41	86	1.43	Vars;Vwa7;Sapcd1;Msh5	Transcription
DMR20:5059001	20	5059001	5061000	2000	1	2.30E-07	0.39	38	1.9	Clic1;Ddah2;G6b;Ly6g6c;Ly6g6d;Ly6g6e	Transport;Metabolism
DMR20:5089001	20	5089001	5092000	3000	1	7.00E-08	0.42	54	1.8	Abhd16a;Ly6g5c	Protease
DMR20:5552001	20	5552001	5557000	5000	1	9.70E-07	0.33	142	2.84	Syngap1;Zbtb9	Signaling;Cytoskeleton
DMR20:5625001	20	5625001	5628000	3000	1	1.10E-08	0.51	68	2.27	Bak1;Ggnbp1	Protease
DMR20:5654001	20	5654001	5658000	4000	3	1.80E-12	0.49	67	1.68	Itpr3	Ion Channel
DMR20:5687001	20	5687001	5694000	7000	4	3.50E-13	0.49	163	2.33	Itpr3	Ion Channel
DMR20:5797001	20	5797001	5798000	1000	1	5.80E-07	0.41	12	1.2	Clpsl2;Clps	Signaling
DMR20:5887001	20	5887001	5888000	1000	1	9.30E-07	0.37	20	2	Slc26a8	Transport
DMR20:5981001	20	5981001	5982000	1000	1	3.60E-07	0.33	14	1.4	Mapk14	Signaling
DMR20:5983001	20	5983001	5984000	1000	1	6.30E-08	-0.37	21	2.1	Mapk14	Signaling
DMR20:6044001	20	6044001	6045000	1000	1	1.20E-07	0.51	13	1.3	Brpf3	Transcription
DMR20:6106001	20	6106001	6107000	1000	1	9.20E-07	-0.43	19	1.9	Pnpla1	Metabolism
DMR20:6246001	20	6246001	6248000	2000	1	1.90E-08	0.35	15	0.75	Stk38	Signaling
DMR20:6392001	20	6392001	6394000	2000	1	5.10E-08	0.47	34	1.7	Rab44	

DMR20:6520001	20	6520001	6523000	3000	1	9.30E-07	0.39	38	1.27	Ppil1	Transcription
DMR20:6595001	20	6595001	6596000	1000	1	4.80E-12	0.47	84	8.4	Ppil1;LOC102551729;LOC102551791	Transcription
DMR20:6637001	20	6637001	6638000	1000	1	2.50E-07	0.4	16	1.6	Ppil1	Transcription
DMR20:6666001	20	6666001	6668000	2000	1	3.00E-10	0.55	28	1.4	Ppil1	Transcription
DMR20:6669001	20	6669001	6671000	2000	1	9.20E-07	0.34	40	2	Ppil1	Transcription
DMR20:6678001	20	6678001	6681000	3000	1	9.10E-08	0.38	50	1.67	Ppil1	Transcription
DMR20:6690001	20	6690001	6693000	3000	1	7.80E-07	0.3	63	2.1	Ppil1	Transcription
DMR20:6741001	20	6741001	6742000	1000	1	1.10E-12	0.66	14	1.4	Ppil1;Grm4	Transcription;Signaling
DMR20:6745001	20	6745001	6749000	4000	1	3.50E-08	0.38	87	2.17	Ppil1;Grm4	Transcription;Signaling
DMR20:6874001	20	6874001	6876000	2000	1	1.40E-07	0.47	32	1.6	Ppil1;RGD735065	Transcription
DMR20:6901001	20	6901001	6903000	2000	1	8.00E-11	0.49	36	1.8	RGD735065	
DMR20:6910001	20	6910001	6911000	1000	1	5.60E-07	0.32	22	2.2	RGD735065	
DMR20:6958001	20	6958001	6960000	2000	1	7.00E-07	0.3	42	2.1	Mtch1;LOC102552169	Transport
DMR20:7316001	20	7316001	7320000	4000	1	8.20E-10	0.49	43	1.07	Spdef;LOC499407	Transcription
DMR20:7525001	20	7525001	7528000	3000	1	3.00E-07	0.37	55	1.83	Anks1a	Cytoskeleton
DMR20:7532001	20	7532001	7535000	3000	1	8.40E-08	-0.51	66	2.2	Anks1a	Cytoskeleton
DMR20:7720001	20	7720001	7723000	3000	1	1.40E-09	0.42	62	2.07	Scube3;LOC108349040	Extracellular Matrix
DMR20:7731001	20	7731001	7733000	2000	1	4.10E-08	0.43	29	1.45	Scube3;LOC108349040	Extracellular Matrix
DMR20:7778001	20	7778001	7783000	5000	5	4.90E-11	0.69	169	3.38	Zfp523;Def6	Cytoskeleton
DMR20:7852001	20	7852001	7853000	1000	1	1.50E-07	0.5	24	2.4	Ppard	Transcription
DMR20:7881001	20	7881001	7883000	2000	1	6.20E-07	0.33	48	2.4	Ppard;Fance	Transcription
DMR20:8227001	20	8227001	8231000	4000	1	4.40E-07	0.4	78	1.95	Tbc1d22b	Signaling
DMR20:8270001	20	8270001	8273000	3000	1	9.30E-07	0.38	66	2.2	Tbc1d22b	Signaling
DMR20:8297001	20	8297001	8298000	1000	1	2.40E-08	0.5	18	1.8	Rnf8	Proteolysis
DMR20:8340001	20	8340001	8341000	1000	1	5.90E-07	0.35	17	1.7	Cmtr1	
DMR20:8566001	20	8566001	8568000	2000	1	2.50E-08	0.36	40	2	Mdga1	
DMR20:8790001	20	8790001	8791000	1000	1	3.10E-08	0.39	41	4.1	Zfand3	
DMR20:8801001	20	8801001	8805000	4000	1	2.70E-07	0.51	71	1.77	Zfand3	
DMR20:8997001	20	8997001	8998000	1000	1	6.80E-07	0.38	21	2.1	Btbd9	
DMR20:9023001	20	9023001	9029000	6000	1	2.00E-16	0.54	100	1.67	Btbd9	
DMR20:9156001	20	9156001	9159000	3000	1	4.50E-12	0.46	59	1.97	Btbd9	
DMR20:9219001	20	9219001	9221000	2000	1	1.20E-07	-0.5	33	1.65	Btbd9	
DMR20:9258001	20	9258001	9260000	2000	1	1.70E-12	0.58	19	0.95	Btbd9;LOC102548069	
DMR20:9586001	20	9586001	9588000	2000	1	1.70E-10	0.64	53	2.65	Glp1r	Receptor
DMR20:9592001	20	9592001	9595000	3000	2	9.30E-09	0.4	57	1.9	Glp1r	Receptor
DMR20:9836001	20	9836001	9837000	1000	1	3.10E-12	0.46	18	1.8	Abcg1	Transport
DMR20:9900001	20	9900001	9901000	1000	1	4.70E-08	0.46	14	1.4	Tff1;Tmprss3	Signaling;Protease
DMR20:9953001	20	9953001	9954000	1000	1	2.40E-08	0.54	10	1	Ubash3a	
DMR20:10192001	20	10192001	10193000	1000	1	2.00E-07	0.44	20	2	Pde9a	Signaling
DMR20:10255001	20	10255001	10256000	1000	1	4.40E-08	0.41	14	1.4	Wdr4;Ndufv3	Translation;Metabolism
DMR20:10342001	20	10342001	10343000	1000	1	4.70E-07	-0.44	30	3	Pknox1	Development
DMR20:10815001	20	10815001	10816000	1000	1	4.90E-09	0.5	34	3.4	Hsf2bp	
DMR20:11419001	20	11419001	11420000	1000	1	2.80E-09	0.51	22	2.2	Pfkl;RGD1309594	Metabolism
DMR20:11442001	20	11442001	11445000	3000	1	7.80E-10	0.39	47	1.57	Trpm2	Transport
DMR20:11555001	20	11555001	11556000	1000	1	7.90E-09	0.52	14	1.4	LOC690386;RGD1561557;LOC690415	
DMR20:11752001	20	11752001	11753000	1000	1	7.20E-09	0.39	22	2.2	Pttg1ip	
DMR20:11780001	20	11780001	11784000	4000	1	7.80E-07	0.36	85	2.12	Itgb2	Extracellular Matrix
DMR20:11883001	20	11883001	11885000	2000	2	1.50E-20	0.5	40	2	Fam207a	
DMR20:12001001	20	12001001	12002000	1000	1	3.80E-09	-0.55	14	1.4	Adarb1	Metabolism
DMR20:12076001	20	12076001	12079000	3000	1	2.40E-09	-0.41	59	1.97	Adarb1	Metabolism
DMR20:12313001	20	12313001	12315000	2000	1	3.90E-07	0.43	26	1.3	Col18a1	Extracellular Matrix
DMR20:12340001	20	12340001	12343000	3000	1	1.50E-09	0.46	86	2.87	Col18a1;Slc19a1	Extracellular Matrix;Transport
DMR20:12602001	20	12602001	12604000	2000	1	4.20E-07	0.49	30	1.5	Pcbp3	Metabolism
DMR20:12919001	20	12919001	12921000	2000	1	5.10E-07	-0.51	39	1.95	Mcm3ap;Ybey;RGD1564149	Cytoskeleton
DMR20:13061001	20	13061001	13064000	3000	1	1.20E-07	0.5	48	1.6	Dip2a	
DMR20:13220001	20	13220001	13221000	1000	1	1.00E-07	0.41	13	1.3	Prmt2;Mir678;LOC103694402	Golgi

DMR20:13506001	20	13506001	13507000	1000	1	4.80E-09	-0.53	15	1.5	Slc5a4b	Transport
DMR20:13560001	20	13560001	13563000	3000	1	1.30E-07	-0.37	30	1	Zfp280b	Transcription
DMR20:13819001	20	13819001	13821000	2000	1	5.10E-07	0.46	43	2.15	Gstt1;Gstt3;Ddt	Metabolism
DMR20:13926001	20	13926001	13928000	2000	2	6.20E-10	0.46	9	0.45	Cabin1;LOC102556116	Signaling
DMR20:13953001	20	13953001	13955000	2000	1	4.00E-10	0.49	23	1.15	Cabin1	Signaling
DMR20:14046001	20	14046001	14048000	2000	1	1.20E-08	0.37	25	1.25	Ggt1;Lrrc75b	Protease
DMR20:14533001	20	14533001	14534000	1000	1	4.40E-08	0.34	19	1.9	Bcr	Signaling
DMR20:14594001	20	14594001	14602000	8000	1	7.10E-11	0.57	224	2.8	Rtdr1;Gnaz	Signaling
DMR20:14621001	20	14621001	14626000	5000	2	1.10E-07	0.39	122	2.44	Rtdr1;Gnaz	Signaling
DMR20:14630001	20	14630001	14633000	3000	2	4.10E-08	0.4	70	2.33	Rtdr1;Gnaz	Signaling
DMR20:14660001	20	14660001	14663000	3000	1	3.20E-09	0.43	52	1.73	Rtdr1	
DMR20:14984001	20	14984001	14990000	6000	2	4.60E-08	-0.37	75	1.25	Pcdh15	Cytoskeleton
DMR20:15056001	20	15056001	15059000	3000	1	1.40E-11	-0.33	27	0.9	Pcdh15	Cytoskeleton
DMR20:15141001	20	15141001	15143000	2000	1	6.70E-08	0.56	18	0.9	Pcdh15	Cytoskeleton
DMR20:15157001	20	15157001	15158000	1000	1	1.30E-07	-0.41	18	1.8	Pcdh15	Cytoskeleton
DMR20:15866001	20	15866001	15873000	7000	2	2.10E-12	-0.38	77	1.1	NEWGENE_1590969	
DMR20:15906001	20	15906001	15907000	1000	1	9.30E-08	0.37	16	1.6	NEWGENE_1590969	
DMR20:16105001	20	16105001	16106000	1000	1	6.20E-10	0.54	6	0.6	NEWGENE_1590969	
DMR20:16313001	20	16313001	16314000	1000	1	5.40E-07	-0.52	10	1	NEWGENE_1590969	
DMR20:16441001	20	16441001	16448000	7000	1	8.50E-10	-0.36	96	1.37	NEWGENE_1590969	
DMR20:16450001	20	16450001	16452000	2000	1	4.60E-09	0.49	15	0.75	NEWGENE_1590969	
DMR20:16770001	20	16770001	16771000	1000	1	6.00E-08	-0.44	16	1.6	NEWGENE_1590969	
DMR20:18822001	20	18822001	18826000	4000	1	1.20E-07	0.43	55	1.38	Bicc1	Metabolism
DMR20:18904001	20	18904001	18907000	3000	1	4.90E-08	0.59	83	2.77	Bicc1	Metabolism
DMR20:19466001	20	19466001	19467000	1000	1	3.50E-10	0.44	20	2	Fam13c	
DMR20:19737001	20	19737001	19740000	3000	1	1.20E-10	0.41	75	2.5	Ccdc6	
DMR20:19807001	20	19807001	19808000	1000	1	5.10E-13	0.67	6	0.6	Ccdc6	
DMR20:19833001	20	19833001	19834000	1000	1	6.60E-07	-0.38	9	0.9	Ccdc6	
DMR20:20043001	20	20043001	20045000	2000	1	4.90E-08	0.38	22	1.1	Ank3	
DMR20:20359001	20	20359001	20360000	1000	1	7.80E-07	0.41	9	0.9	Ank3;LOC102554005	
DMR20:21385001	20	21385001	21389000	4000	1	8.40E-08	-0.3	60	1.5	RGD1306739	
DMR20:21991001	20	21991001	21993000	2000	1	2.00E-07	-0.47	35	1.75	Rtkn2	Cytoskeleton
DMR20:22075001	20	22075001	22079000	4000	1	3.20E-13	0.46	63	1.57	Zfp365	
DMR20:22969001	20	22969001	22971000	2000	1	1.90E-09	0.76	46	2.3	Reep3	Transport
DMR20:23472001	20	23472001	23473000	1000	1	1.80E-07	-0.49	21	2.1	RGD1566373	Translation
DMR20:25092001	20	25092001	25099000	7000	2	6.20E-09	-0.42	66	0.94	Ctnna3	
DMR20:25855001	20	25855001	25858000	3000	1	5.10E-14	-0.5	18	0.6	Ctnna3	
DMR20:25937001	20	25937001	25940000	3000	1	6.70E-07	0.46	26	0.87	Ctnna3	
DMR20:26025001	20	26025001	26027000	2000	1	9.00E-07	-0.44	27	1.35	Ctnna3;Lrrtm3	Receptor
DMR20:26075001	20	26075001	26076000	1000	1	5.70E-11	0.47	18	1.8	Ctnna3;Lrrtm3	Receptor
DMR20:26452001	20	26452001	26457000	5000	2	2.50E-07	-0.32	39	0.78	Ctnna3	
DMR20:26792001	20	26792001	26793000	1000	1	4.20E-12	-0.72	11	1.1	Herc4	Proteolysis
DMR20:27098001	20	27098001	27099000	1000	1	1.30E-08	0.29	23	2.3	LOC100362906;Pbld1	
DMR20:27746001	20	27746001	27750000	4000	1	9.20E-10	-0.41	46	1.15	Dse	
DMR20:27794001	20	27794001	27797000	3000	1	5.10E-12	-0.37	32	1.07	Dse	
DMR20:27843001	20	27843001	27845000	2000	1	7.80E-10	-0.66	18	0.9	Gcc2	Cytoskeleton
DMR20:27967001	20	27967001	27969000	2000	1	1.80E-07	0.47	11	0.55	Lims1	Cytoskeleton
DMR20:28120001	20	28120001	28123000	3000	1	1.10E-13	0.64	42	1.4	Ccdc138	
DMR20:28522001	20	28522001	28523000	1000	1	2.60E-13	0.46	11	1.1	Sh3rf3	
DMR20:28593001	20	28593001	28596000	3000	1	1.10E-07	0.44	32	1.07	Sh3rf3	
DMR20:29628001	20	29628001	29631000	3000	1	2.60E-07	0.51	36	1.2	Ascc1	
DMR20:29673001	20	29673001	29675000	2000	1	2.70E-09	0.65	30	1.5	Spock2	Extracellular Matrix
DMR20:29861001	20	29861001	29864000	3000	1	3.60E-08	0.37	56	1.87	Psap;Cdh23	Cytoskeleton;Cytoskeleton
DMR20:29959001	20	29959001	29960000	1000	1	1.50E-07	0.4	16	1.6	Cdh23;LOC100361018	Cytoskeleton
DMR20:29963001	20	29963001	29965000	2000	1	2.10E-11	0.64	35	1.75	Cdh23;LOC100361018	Cytoskeleton
DMR20:30133001	20	30133001	30135000	2000	2	8.70E-08	0.57	34	1.7	Cdh23	Cytoskeleton
DMR20:30193001	20	30193001	30196000	3000	1	3.80E-07	0.43	37	1.23	Cdh23	Cytoskeleton
DMR20:30341001	20	30341001	30350000	9000	1	6.30E-07	0.49	177	1.97	Unc5b	Receptor
DMR20:30353001	20	30353001	30354000	1000	1	3.70E-09	0.47	13	1.3	Unc5b	Receptor
DMR20:30355001	20	30355001	30357000	2000	1	1.90E-07	0.55	37	1.85	Unc5b	Receptor
DMR20:30363001	20	30363001	30365000	2000	1	7.10E-10	0.45	36	1.8	Unc5b	Receptor

DMR20:30838001	20	30838001	30839000	1000	1	7.80E-07	0.41	15	1.5	Adamts14	Protease
DMR20:30846001	20	30846001	30847000	1000	1	4.60E-09	0.41	11	1.1	Adamts14	Protease
DMR20:30871001	20	30871001	30875000	4000	1	6.40E-07	0.35	58	1.45	Adamts14	Protease
DMR20:30921001	20	30921001	30922000	1000	1	7.80E-07	0.4	11	1.1	Prf1	
DMR20:31030001	20	31030001	31034000	4000	2	6.90E-08	-0.57	77	1.93	Nodal	Growth Factors
DMR20:31093001	20	31093001	31094000	1000	1	5.50E-11	0.4	11	1.1	Lrrc20;LOC365572	
DMR20:31103001	20	31103001	31106000	3000	1	1.80E-08	0.46	44	1.47	Lrrc20	
DMR20:31202001	20	31202001	31203000	1000	1	3.10E-08	0.42	19	1.9	Lrrc20	
DMR20:31584001	20	31584001	31586000	2000	1	1.20E-08	0.51	21	1.05	Col13a1	Extracellular Matrix
DMR20:32314001	20	32314001	32316000	2000	1	1.80E-08	0.37	40	2	Stox1	
DMR20:32324001	20	32324001	32325000	1000	1	3.30E-10	-0.48	11	1.1	Stox1	
DMR20:32420001	20	32420001	32421000	1000	1	1.80E-07	-0.33	38	3.8	Ccar1	Epigenetic
DMR20:32562001	20	32562001	32566000	4000	1	7.00E-07	-0.32	37	0.92	Kpna5;LOC103694421	Transport
DMR20:33087001	20	33087001	33090000	3000	1	1.10E-07	0.31	33	1.1	Vgll2	Transcription
DMR20:33168001	20	33168001	33170000	2000	1	1.30E-07	-0.3	33	1.65	Ros1;LOC100361475;LOC103694423	Receptor
DMR20:33317001	20	33317001	33320000	3000	1	4.90E-11	-0.58	25	0.83	Ros1	Receptor
DMR20:33414001	20	33414001	33416000	2000	1	6.70E-08	-0.49	11	0.55	Dcbld1	Protease
DMR20:33766001	20	33766001	33767000	1000	1	3.30E-08	-0.41	8	0.8	Slc35f1	
DMR20:33949001	20	33949001	33951000	2000	1	5.10E-07	0.46	35	1.75	Slc35f1;LOC103689994;LOC102547010	
DMR20:34250001	20	34250001	34251000	1000	1	1.70E-09	0.49	12	1.2	Slc35f1	
DMR20:34793001	20	34793001	34795000	2000	1	4.70E-07	0.55	28	1.4	RGD1562458	
DMR20:34871001	20	34871001	34875000	4000	2	3.70E-09	0.47	46	1.15	Mcm9	Transcription
DMR20:34883001	20	34883001	34884000	1000	1	4.50E-08	-0.49	16	1.6	Mcm9	Transcription
DMR20:34970001	20	34970001	34972000	2000	1	1.50E-07	0.48	15	0.75	Fam184a	
DMR20:35007001	20	35007001	35010000	3000	1	2.80E-08	-0.41	23	0.77	Fam184a	
DMR20:37479001	20	37479001	37486000	7000	1	4.20E-07	-0.3	78	1.11	Tbc1d32;LOC103694439	
DMR20:37570001	20	37570001	37573000	3000	1	1.30E-07	-0.3	29	0.97	Tbc1d32;Msl3l2	Epigenetic
DMR20:38970001	20	38970001	38972000	2000	1	6.30E-11	-0.61	15	0.75	Hsf2;Serinc1	Transcription;Signaling
DMR20:40355001	20	40355001	40357000	2000	1	3.50E-12	0.46	17	0.85	Hs3st5;LOC102553345	Transport
DMR20:40415001	20	40415001	40416000	1000	1	7.70E-07	-0.34	24	2.4	Hs3st5	Transport
DMR20:41151001	20	41151001	41152000	1000	1	1.10E-07	-0.64	10	1	Nt5dc1	Signaling
DMR20:41219001	20	41219001	41225000	6000	1	6.40E-11	-0.37	66	1.1	Nt5dc1	Signaling
DMR20:43946001	20	43946001	43948000	2000	1	3.90E-09	-0.45	17	0.85	Rfpl4b	
DMR20:44264001	20	44264001	44266000	2000	1	9.40E-08	-0.5	25	1.25	Wisp3	
DMR20:44545001	20	44545001	44548000	3000	1	3.40E-10	0.45	66	2.2	Fyn	
DMR20:44855001	20	44855001	44857000	2000	1	2.60E-12	0.45	16	0.8	Rev3l	Transcription
DMR20:44899001	20	44899001	44901000	2000	1	4.10E-08	-0.43	46	2.3	Rev3l	Transcription
DMR20:45006001	20	45006001	45008000	2000	1	2.40E-10	0.55	31	1.55	Mfsd4b;RGD1304770	Transport
DMR20:45045001	20	45045001	45051000	6000	2	3.10E-12	-0.44	50	0.83	RGD1561777	Transport
DMR20:45085001	20	45085001	45090000	5000	1	2.30E-07	-0.28	51	1.02	Naglt1	
DMR20:45154001	20	45154001	45155000	1000	1	7.30E-07	0.57	12	1.2	Slc16a10	Transport
DMR20:45349001	20	45349001	45350000	1000	1	9.40E-13	0.51	3	0.3	Rpf2;Gtf3c6	
DMR20:45448001	20	45448001	45455000	7000	1	1.20E-09	-0.39	60	0.86	LOC100361424;LOC100361476;Cdk19	Signaling
DMR20:45550001	20	45550001	45552000	2000	1	4.30E-07	0.38	32	1.6	Cdk19	Signaling
DMR20:45944001	20	45944001	45946000	2000	1	8.90E-07	0.27	30	1.5	Fig4	Signaling
DMR20:45976001	20	45976001	45978000	2000	1	6.00E-07	0.35	42	2.1	Fig4	Signaling
DMR20:46016001	20	46016001	46018000	2000	2	8.20E-14	0.56	152	7.6	Fig4	Signaling
DMR20:46019001	20	46019001	46020000	1000	1	4.50E-09	0.47	9	0.9	Fig4	Signaling
DMR20:46083001	20	46083001	46084000	1000	1	5.80E-09	0.45	16	1.6	Ak9	Signaling
DMR20:46440001	20	46440001	46442000	2000	1	1.10E-11	-0.45	38	1.9	Foxo3	
DMR20:46801001	20	46801001	46803000	2000	1	2.10E-12	0.49	30	1.5	Armc2	
DMR20:47101001	20	47101001	47104000	3000	1	2.90E-07	0.43	46	1.53	Lace1	
DMR20:47530001	20	47530001	47531000	1000	1	1.50E-10	0.51	85	8.5	Sec63	Cytoskeleton
DMR20:47549001	20	47549001	47552000	3000	1	7.40E-07	-0.45	22	0.73	Sec63	Cytoskeleton
DMR20:47607001	20	47607001	47608000	1000	1	3.20E-10	0.6	14	1.4	Scml4	Epigenetic
DMR20:47654001	20	47654001	47655000	1000	1	4.00E-09	0.39	13	1.3	Scml4	Epigenetic
DMR20:48099001	20	48099001	48100000	1000	1	1.20E-12	0.42	56	5.6	Pdss2	
DMR20:48902001	20	48902001	48904000	2000	1	6.10E-07	0.32	53	2.65	Rtn4ip1	Metabolism

DMR20:49017001	20	49017001	49021000	4000	1	1.30E-08	0.41	57	1.43	Aim1	
DMR20:49093001	20	49093001	49096000	3000	1	1.90E-09	-0.38	53	1.77	Aim1	
DMR20:49295001	20	49295001	49297000	2000	1	1.60E-07	-0.59	24	1.2	LOC102554815;LOC103694449;Atg5	Transport
DMR20:50231001	20	50231001	50236000	5000	4	2.10E-20	1.09	133	2.66	Prep	Protease
DMR20:50272001	20	50272001	50274000	2000	1	9.90E-10	0.61	19	0.95	Prep	Protease
DMR20:50524001	20	50524001	50526000	2000	1	9.70E-10	-0.57	31	1.55	Lin28b	Metabolism
DMR20:50536001	20	50536001	50538000	2000	1	5.90E-11	0.4	79	3.95	Lin28b	Metabolism
DMR20:50705001	20	50705001	50707000	2000	2	2.00E-12	0.54	49	2.45	Hace1	Proteolysis
DMR20:50769001	20	50769001	50770000	1000	1	3.20E-09	-0.56	26	2.6	Hace1	Proteolysis
DMR20:52779001	20	52779001	52781000	2000	1	2.50E-08	-0.35	20	1	Hspd1-ps19	
DMR20:54167001	20	54167001	54168000	1000	1	1.50E-09	0.57	9	0.9	Grik2	Receptor
DMR20:54191001	20	54191001	54192000	1000	1	6.30E-10	-0.37	12	1.2	Grik2	Receptor
DMR20:54267001	20	54267001	54269000	2000	1	5.50E-07	-0.43	5	0.25	Grik2	Receptor
DMR20:55333001	20	55333001	55338000	5000	1	5.70E-07	-0.3	53	1.06	Ascc3	Cytoskeleton
DMR20:55547001	20	55547001	55551000	4000	1	3.50E-07	-0.27	40	1	Ascc3	Cytoskeleton
DMRX:969001	X	969001	975000	6000	3	1.20E-08	0.43	65	1.08	Zfp182	
DMRX:980001	X	980001	981000	1000	1	4.00E-07	-0.52	11	1.1	Zfp182	
DMRX:1863001	X	1863001	1868000	5000	1	2.60E-07	-0.45	59	1.18	RGD1564855	
DMRX:2122001	X	2122001	2124000	2000	1	1.10E-09	-0.33	19	0.95	Rp2	Signaling
DMRX:2592001	X	2592001	2597000	5000	1	5.60E-08	-0.34	70	1.4	Slc9a7	Transport
DMRX:3577001	X	3577001	3579000	2000	2	4.40E-07	-0.53	21	1.05	RGD1561575	
DMRX:5811001	X	5811001	5817000	6000	3	1.30E-13	-0.47	59	0.98	Efhc2	Signaling
DMRX:5922001	X	5922001	5923000	1000	1	5.60E-13	0.75	9	0.9	Efhc2	Signaling
DMRX:10387001	X	10387001	10391000	4000	1	3.10E-07	-0.31	47	1.18	Ddx3x	
DMRX:11070001	X	11070001	11076000	6000	1	5.10E-08	-0.27	69	1.15	Med14;RGD1565685	Transcription
DMRX:13249001	X	13249001	13255000	6000	1	8.60E-07	-0.28	88	1.47	Tspan7	
DMRX:13885001	X	13885001	13891000	6000	1	1.30E-08	-0.3	61	1.02	Sytl5	
DMRX:14095001	X	14095001	14099000	4000	1	6.90E-07	-0.39	33	0.82	Sytl5	
DMRX:14396001	X	14396001	14401000	5000	2	2.20E-08	-0.36	34	0.68	Lancl3	
DMRX:14482001	X	14482001	14487000	5000	2	2.90E-08	-0.33	47	0.94	Lancl3;LOC108349207	
DMRX:14717001	X	14717001	14723000	6000	2	9.50E-11	-0.38	64	1.07	RGD1561106	
DMRX:16320001	X	16320001	16322000	2000	1	2.90E-07	-0.27	22	1.1	Ccnb3	Signaling
DMRX:16515001	X	16515001	16517000	2000	1	2.10E-07	-0.27	22	1.1	Dgkk	Signaling
DMRX:16951001	X	16951001	16953000	2000	1	1.80E-09	-0.45	24	1.2	RGD1563606	
DMRX:18882001	X	18882001	18885000	3000	1	7.30E-07	-0.34	24	0.8	Klf8	Transcription
DMRX:20784001	X	20784001	20786000	2000	1	4.00E-07	-0.49	18	0.9	FAM120C	
DMRX:20956001	X	20956001	20960000	4000	1	1.40E-09	-0.45	40	1	FAM120C	
DMRX:20996001	X	20996001	21003000	7000	1	5.00E-07	-0.36	84	1.2	FAM120C	
DMRX:21146001	X	21146001	21153000	7000	2	1.10E-08	-0.35	94	1.34	Phf8	
DMRX:23548001	X	23548001	23552000	4000	3	5.40E-10	-0.55	35	0.88	Shroom2	Cytoskeleton
DMRX:24995001	X	24995001	24999000	4000	1	1.20E-07	-0.32	37	0.92	Wwc3	
DMRX:25529001	X	25529001	25530000	1000	1	1.80E-07	0.51	23	2.3	Mid1	Proteolysis
DMRX:26381001	X	26381001	26388000	7000	2	2.90E-08	-0.42	93	1.33	Arhgap6	Signaling
DMRX:26751001	X	26751001	26756000	5000	1	8.50E-10	-0.36	55	1.1	Arhgap6	Signaling
DMRX:27608001	X	27608001	27610000	2000	1	2.40E-07	-0.38	42	2.1	Frmpd4	
DMRX:27924001	X	27924001	27925000	1000	1	3.90E-07	-0.46	7	0.7	Frmpd4	
DMRX:27944001	X	27944001	27947000	3000	2	6.90E-08	-0.41	24	0.8	Frmpd4	
DMRX:28011001	X	28011001	28014000	3000	1	3.30E-10	-0.37	30	1	Frmpd4	
DMRX:30645001	X	30645001	30647000	2000	1	3.60E-08	-0.34	14	0.7	Glra2	Ion Channel
DMRX:31137001	X	31137001	31138000	1000	1	5.50E-10	-0.65	18	1.8	Fancb;Mospd2	
DMRX:31752001	X	31752001	31757000	5000	2	5.70E-07	-0.32	50	1	Asb9;Asb11	
DMRX:32079001	X	32079001	32080000	1000	1	6.40E-08	0.39	7	0.7	Ace2	Protease
DMRX:35361001	X	35361001	35368000	7000	1	3.80E-09	-0.31	91	1.3	Scml2	Epigenetic
DMRX:35550001	X	35550001	35552000	2000	1	4.80E-07	-0.53	29	1.45	Cdkl5	Signaling
DMRX:35872001	X	35872001	35879000	7000	1	3.20E-10	-0.35	87	1.24	Ppef1;LOC108349217	Signaling
DMRX:36386001	X	36386001	36389000	3000	2	1.60E-11	-0.46	30	1	Phka2	
DMRX:36424001	X	36424001	36425000	1000	1	8.70E-07	-0.66	6	0.6	Phka2	
DMRX:36446001	X	36446001	36449000	3000	1	4.50E-13	0.45	46	1.53	Phka2	
DMRX:36450001	X	36450001	36457000	7000	2	8.00E-10	0.4	117	1.67	Phka2;LOC108353287	
DMRX:36484001	X	36484001	36488000	4000	1	3.60E-08	-0.47	17	0.42	Phka2	

DMRX:38806001	X	38806001	38811000	5000	2	1.20E-10	-0.32	68	1.36	RGD1563991	
DMRX:39899001	X	39899001	39905000	6000	1	6.60E-12	-0.4	60	1	Cnksr2	
DMRX:40478001	X	40478001	40483000	5000	1	5.10E-15	-0.34	55	1.1	Phex	Protease
DMRX:43578001	X	43578001	43582000	4000	1	7.80E-07	-0.26	52	1.3	Acot9	Metabolism
DMRX:43866001	X	43866001	43869000	3000	1	1.40E-07	-0.58	21	0.7	Cldn34d	Cell Junction
DMRX:44595001	X	44595001	44596000	1000	1	2.00E-07	-0.43	13	1.3	RGD1562775	
DMRX:45086001	X	45086001	45091000	5000	1	2.60E-07	-0.25	61	1.22	Prkx	Signaling
DMRX:45403001	X	45403001	45407000	4000	1	1.40E-08	-0.48	40	1	Ptges3l1	Transcription
DMRX:45421001	X	45421001	45423000	2000	1	5.10E-07	0.37	44	2.2	Ptges3l1;Sts	Transcription;Metabolism
DMRX:45686001	X	45686001	45692000	6000	3	3.60E-07	-0.4	63	1.05	Prrg1	Protease
DMRX:46966001	X	46966001	46970000	4000	1	5.60E-10	-0.5	39	0.98	Mageb16	Cytoskeleton
DMRX:48768001	X	48768001	48774000	6000	1	1.80E-08	-0.32	55	0.92	Tmem47	
DMRX:49649001	X	49649001	49655000	6000	2	7.10E-10	-0.4	54	0.9	Srsf8	Translation
DMRX:49714001	X	49714001	49715000	1000	1	4.20E-10	-0.33	12	1.2	Srsf8;LOC103690857	Translation
DMRX:51189001	X	51189001	51192000	3000	1	8.00E-07	-0.23	54	1.8	Dmd	
DMRX:51405001	X	51405001	51410000	5000	1	7.40E-08	-0.33	35	0.7	Dmd;LOC103690859	
DMRX:51414001	X	51414001	51415000	1000	1	4.70E-08	0.45	13	1.3	Dmd;LOC103690859	
DMRX:51528001	X	51528001	51534000	6000	2	1.40E-09	-0.35	59	0.98	Dmd	
DMRX:51884001	X	51884001	51891000	7000	3	8.70E-12	-0.4	88	1.26	Dmd	
DMRX:51896001	X	51896001	51902000	6000	2	5.70E-12	-0.46	62	1.03	Dmd	
DMRX:52430001	X	52430001	52435000	5000	2	3.10E-10	-0.35	46	0.92	Dmd	
DMRX:52436001	X	52436001	52437000	1000	1	8.80E-09	-0.5	12	1.2	Dmd	
DMRX:52502001	X	52502001	52508000	6000	2	2.10E-12	-0.36	66	1.1	Dmd	
DMRX:52700001	X	52700001	52706000	6000	1	4.90E-08	-0.31	65	1.08	Dmd	
DMRX:52786001	X	52786001	52795000	9000	2	3.10E-07	-0.38	97	1.08	Dmd	
DMRX:52814001	X	52814001	52820000	6000	1	3.20E-07	-0.31	69	1.15	Dmd	
DMRX:52841001	X	52841001	52845000	4000	2	1.10E-08	-0.41	44	1.1	Dmd	
DMRX:52846001	X	52846001	52848000	2000	1	1.20E-07	-0.29	21	1.05	Dmd	
DMRX:53007001	X	53007001	53013000	6000	1	2.10E-07	-0.39	66	1.1	Dmd	
DMRX:53130001	X	53130001	53135000	5000	3	1.80E-08	-0.34	62	1.24	Dmd	
DMRX:53205001	X	53205001	53207000	2000	1	8.90E-07	-0.34	19	0.95	Dmd	
DMRX:53615001	X	53615001	53621000	6000	1	5.10E-07	-0.35	58	0.97	Fthl17e	Transport
DMRX:55549001	X	55549001	55554000	5000	2	1.10E-09	-0.35	49	0.98	Il1rapl1	Receptor
DMRX:56019001	X	56019001	56023000	4000	1	3.40E-10	-0.27	43	1.07	Il1rapl1	Receptor
DMRX:56325001	X	56325001	56333000	8000	3	9.30E-12	-0.39	70	0.88	Il1rapl1	Receptor
DMRX:56387001	X	56387001	56389000	2000	2	1.50E-07	-0.35	23	1.15	Il1rapl1	Receptor
DMRX:56570001	X	56570001	56571000	1000	1	4.60E-07	-0.49	12	1.2	Il1rapl1	Receptor
DMRX:56781001	X	56781001	56784000	3000	1	2.30E-12	-0.42	26	0.87	Il1rapl1	Receptor
DMRX:57708001	X	57708001	57714000	6000	1	8.90E-12	-0.37	63	1.05	RGD1565286	
DMRX:59118001	X	59118001	59122000	4000	3	2.50E-11	-0.45	45	1.12	RGD1563978	
DMRX:60838001	X	60838001	60840000	2000	1	3.40E-07	-0.41	20	1	Mageb5	Cytoskeleton
DMRX:64223001	X	64223001	64224000	1000	1	6.00E-07	0.67	12	1.2	Spin4	
DMRX:64294001	X	64294001	64300000	6000	1	6.60E-08	-0.3	64	1.07	Arhgef9	Transcription
DMRX:64335001	X	64335001	64339000	4000	1	4.70E-07	-0.31	39	0.98	Arhgef9	Transcription
DMRX:64348001	X	64348001	64350000	2000	1	2.50E-07	-0.37	18	0.9	Arhgef9	Transcription
DMRX:64808001	X	64808001	64810000	2000	1	5.30E-09	-0.35	16	0.8	Asb12;LOC102550262	
DMRX:65518001	X	65518001	65520000	2000	1	2.10E-07	-0.43	23	1.15	Heph	Metabolism
DMRX:65521001	X	65521001	65525000	4000	2	5.00E-08	-0.34	47	1.18	Heph	Metabolism
DMRX:65578001	X	65578001	65582000	4000	2	8.80E-11	-0.49	41	1.02	Heph	Metabolism
DMRX:66504001	X	66504001	66505000	1000	1	5.30E-08	-0.71	6	0.6	Rpl34l1	
DMRX:66506001	X	66506001	66511000	5000	1	1.50E-07	-0.31	50	1	Rpl34l1;RGD1565058	
DMRX:66524001	X	66524001	66528000	4000	1	3.60E-07	-0.3	48	1.2	RGD1565058	
DMRX:68211001	X	68211001	68218000	7000	1	1.50E-07	-0.26	82	1.17	Ophn1	Signaling
DMRX:68235001	X	68235001	68236000	1000	1	6.30E-07	-0.55	8	0.8	Ophn1	Signaling
DMRX:68269001	X	68269001	68276000	7000	3	1.00E-12	-0.4	77	1.1	Ophn1	Signaling
DMRX:68316001	X	68316001	68317000	1000	1	7.70E-08	-0.61	12	1.2	Ophn1	Signaling
DMRX:68358001	X	68358001	68364000	6000	2	1.00E-11	-0.34	87	1.45	Ophn1	Signaling
DMRX:68485001	X	68485001	68489000	4000	1	4.60E-08	-0.31	48	1.2	Ophn1;LOC102551847	Signaling
DMRX:69835001	X	69835001	69839000	4000	2	5.00E-08	-0.29	55	1.38	Eda	
DMRX:70398001	X	70398001	70404000	6000	2	5.10E-09	-0.42	76	1.27	Awat1	Metabolism
DMRX:70550001	X	70550001	70555000	5000	2	6.50E-08	-0.32	53	1.06	Kif4a;Trnaf-gaa;Gdpd2	Cytoskeleton;Signaling

DMRX:70677001	X	70677001	70680000	3000	1	2.60E-11	-0.41	24	0.8	Tex11	
DMRX:70691001	X	70691001	70692000	1000	1	2.20E-09	-0.45	8	0.8	Tex11	
DMRX:70762001	X	70762001	70763000	1000	1	7.70E-17	1.04	6	0.6	Tex11	
DMRX:70772001	X	70772001	70773000	1000	1	4.00E-12	-0.76	12	1.2	Tex11	
DMRX:71955001	X	71955001	71956000	1000	1	7.90E-08	0.59	15	1.5	Nhsl2;LOC100911832	
DMRX:72485001	X	72485001	72486000	1000	1	5.10E-07	-0.45	22	2.2	Phka1	Signaling
DMRX:73399001	X	73399001	73402000	3000	3	7.60E-16	0.89	16	0.53	Zfp449	Transcription
DMRX:74020001	X	74020001	74022000	2000	1	4.70E-07	-0.29	13	0.65	RGD1562521;LOC103690866	
DMRX:74971001	X	74971001	74973000	2000	1	1.10E-07	-0.6	17	0.85	RGD1561931;Mir672	
DMRX:75522001	X	75522001	75524000	2000	1	5.30E-07	-0.59	7	0.35	Zdhhc15	
DMRX:75801001	X	75801001	75804000	3000	1	3.20E-08	-0.36	38	1.27	Magee2	Cytoskeleton
DMRX:76876001	X	76876001	76877000	1000	1	8.60E-07	-0.59	11	1.1	Atrx	Transcription
DMRX:78176001	X	78176001	78182000	6000	4	5.50E-12	-0.43	65	1.08	P2ry10	Signaling
DMRX:80313001	X	80313001	80318000	5000	1	1.30E-07	-0.28	55	1.1	Sh3bgrl	
DMRX:83091001	X	83091001	83092000	1000	1	1.10E-08	-0.35	12	1.2	Hdx	
DMRX:83940001	X	83940001	83941000	1000	1	2.10E-07	-0.42	14	1.4	RGD1561958	
DMRX:86187001	X	86187001	86189000	2000	1	4.60E-07	-0.3	14	0.7	Klhl4	Cytoskeleton
DMRX:87725001	X	87725001	87728000	3000	1	7.00E-09	-0.39	24	0.8	RGD1560954	
DMRX:92804001	X	92804001	92808000	4000	1	4.70E-09	-0.37	51	1.27	Pcdh11x	
DMRX:92844001	X	92844001	92846000	2000	1	4.40E-08	-0.3	21	1.05	Pcdh11x	
DMRX:93124001	X	93124001	93130000	6000	2	1.50E-07	-0.29	95	1.58	Pcdh11x	
DMRX:95341001	X	95341001	95343000	2000	1	5.90E-07	-0.32	16	0.8	Olr10;Olr11	Signaling
DMRX:96539001	X	96539001	96540000	1000	1	4.00E-07	-0.5	7	0.7	RGD1563104	Metabolism
DMRX:99762001	X	99762001	99767000	5000	2	6.70E-11	0.6	66	1.32	Diaph2	
DMRX:100334001	X	100334001	100336000	2000	1	8.80E-11	-0.37	22	1.1	Diaph2	
DMRX:104246001	X	104246001	104251000	5000	1	3.50E-07	-0.31	44	0.88	Rps27a-ps4	
DMRX:105490001	X	105490001	105493000	3000	1	1.20E-07	-0.4	26	0.87	Armcx4	
DMRX:105766001	X	105766001	105767000	1000	1	1.40E-08	-0.5	5	0.5	Nxf2	Metabolism
DMRX:106167001	X	106167001	106169000	2000	2	2.20E-13	0.44	16	0.8	Tcp11x2	Cytoskeleton
DMRX:106172001	X	106172001	106185000	13000	9	7.50E-15	0.48	55	0.42	Tcp11x2	Cytoskeleton
DMRX:106530001	X	106530001	106534000	4000	1	3.40E-11	-0.4	44	1.1	Arxes2	
DMRX:107414001	X	107414001	107416000	2000	1	9.70E-07	-0.33	23	1.15	Morf4l2;LOC102555540;Glra4	Epigenetic;Ion Channel
DMRX:107880001	X	107880001	107886000	6000	3	6.30E-11	-0.37	67	1.12	RGD1561860	
DMRX:108443001	X	108443001	108446000	3000	1	1.50E-10	-0.37	41	1.37	Il1rapl2	Receptor
DMRX:109160001	X	109160001	109169000	9000	4	8.90E-10	-0.41	76	0.84	Il1rapl2	Receptor
DMRX:109218001	X	109218001	109223000	5000	2	1.00E-07	-0.34	54	1.08	Il1rapl2	Receptor
DMRX:109428001	X	109428001	109433000	5000	1	4.80E-08	-0.37	70	1.4	Il1rapl2	Receptor
DMRX:109553001	X	109553001	109555000	2000	1	2.00E-15	-0.52	15	0.75	Il1rapl2	Receptor
DMRX:109742001	X	109742001	109746000	4000	2	5.30E-08	-0.45	38	0.95	Il1rapl2	Receptor
DMRX:109750001	X	109750001	109752000	2000	1	7.90E-09	-0.45	20	1	Il1rapl2	Receptor
DMRX:109825001	X	109825001	109826000	1000	1	1.70E-07	-0.63	9	0.9	Il1rapl2	Receptor
DMRX:109827001	X	109827001	109828000	1000	1	3.30E-08	0.58	3	0.3	Il1rapl2	Receptor
DMRX:110514001	X	110514001	110516000	2000	1	1.90E-07	-0.45	20	1	Trap1a	
DMRX:110972001	X	110972001	110979000	7000	1	1.20E-07	-0.31	86	1.23	Tbc1d8b	Signaling
DMRX:111272001	X	111272001	111273000	1000	1	4.20E-08	-0.61	7	0.7	Rbm41	
DMRX:112738001	X	112738001	112743000	5000	1	1.30E-10	-0.38	55	1.1	Col4a6	Extracellular Matrix
DMRX:112995001	X	112995001	112998000	3000	1	4.20E-07	-0.29	37	1.23	Irs4	
DMRX:113408001	X	113408001	113412000	4000	1	3.50E-08	-0.44	36	0.9	Gucy2f	Signaling
DMRX:113569001	X	113569001	113575000	6000	1	3.10E-07	-0.28	63	1.05	Kcne5	Transport
DMRX:114031001	X	114031001	114032000	1000	1	4.00E-08	-0.36	14	1.4	Tmem164	
DMRX:115079001	X	115079001	115080000	1000	1	1.10E-07	-0.73	5	0.5	Capn6	Protease
DMRX:115693001	X	115693001	115697000	4000	1	6.30E-12	-0.34	43	1.07	Trpc5;Trpc5os	Transport
DMRX:116381001	X	116381001	116387000	6000	1	2.70E-07	-0.35	51	0.85	Zcchc16	
DMRX:118215001	X	118215001	118222000	7000	1	2.00E-09	-0.3	76	1.09	Htr2c	Signaling
DMRX:120909001	X	120909001	120910000	1000	1	6.90E-07	-0.38	11	1.1	Wdr44	
DMRX:122633001	X	122633001	122635000	2000	1	3.20E-08	-0.31	20	1	Dock11	Transcription
DMRX:122761001	X	122761001	122762000	1000	1	3.40E-07	-0.54	16	1.6	Il13ra1	
DMRX:122979001	X	122979001	122980000	1000	1	6.90E-07	-0.29	10	1	Lonrf3	Proteolysis
DMRX:122981001	X	122981001	122984000	3000	2	8.70E-11	-0.36	29	0.97	Lonrf3	Proteolysis

DMRX:123061001	X	123061001	123062000	1000	1	7.30E-08	-0.71	14	1.4	RGD1561445	
DMRX:123070001	X	123070001	123076000	6000	1	8.90E-07	-0.35	65	1.08	RGD1561445	
DMRX:123314001	X	123314001	123322000	8000	3	2.40E-13	-0.4	83	1.04	RGD1565032;LOC100361883	
DMRX:123737001	X	123737001	123745000	8000	3	3.90E-09	-0.55	110	1.38	Upf3b;Nkap	
DMRX:123788001	X	123788001	123790000	2000	1	8.70E-07	0.56	21	1.05	Akap14	Cytoskeleton
DMRX:124452001	X	124452001	124455000	3000	1	1.40E-08	-0.47	30	1	Tmem255a	
DMRX:124651001	X	124651001	124652000	1000	1	2.20E-07	-0.68	11	1.1	Atp1b4	Transport
DMRX:128171001	X	128171001	128172000	1000	1	4.90E-11	-0.75	11	1.1	Thoc2;LOC108349251	Transcription
DMRX:128178001	X	128178001	128179000	1000	1	2.70E-07	-0.27	14	1.4	Thoc2	Transcription
DMRX:128180001	X	128180001	128182000	2000	1	2.50E-08	-0.35	22	1.1	Thoc2	Transcription
DMRX:128539001	X	128539001	128544000	5000	3	4.10E-12	-0.4	48	0.96	Stag2	Epigenetic
DMRX:128676001	X	128676001	128678000	2000	1	1.90E-07	-0.4	27	1.35	RGD1560119	
DMRX:128681001	X	128681001	128688000	7000	3	8.00E-13	-0.42	77	1.1	RGD1560119	
DMRX:129229001	X	129229001	129232000	3000	1	7.10E-07	-0.26	27	0.9	Tenm1	
DMRX:129287001	X	129287001	129291000	4000	1	7.80E-07	-0.32	53	1.32	Tenm1;LOC108349252	
DMRX:135181001	X	135181001	135182000	1000	1	1.80E-07	-0.54	12	1.2	Bcorl1	
DMRX:136142001	X	136142001	136146000	4000	1	3.30E-07	-0.36	45	1.12	Enox2;LOC103690897	Metabolism
DMRX:136746001	X	136746001	136747000	1000	1	1.20E-15	0.83	4	0.4	Olr1765	Signaling
DMRX:136815001	X	136815001	136819000	4000	1	1.40E-09	-0.34	45	1.12	Igsf1	Immune
DMRX:138368001	X	138368001	138369000	1000	1	3.80E-07	-0.31	18	1.8	Mbnl3	Translation
DMRX:139290001	X	139290001	139291000	1000	1	2.70E-09	-0.38	22	2.2	MGC114492;LOC102550607	Cytoskeleton
DMRX:139756001	X	139756001	139762000	6000	1	1.30E-10	-0.36	65	1.08	Gpc3	
DMRX:142298001	X	142298001	142300000	2000	1	5.10E-07	-0.26	20	1	Fgf13	Growth Factors
DMRX:143388001	X	143388001	143390000	2000	2	8.40E-12	0.61	48	2.4	Atp11c	Transport
DMRX:143415001	X	143415001	143417000	2000	1	5.70E-08	-0.31	20	1	Atp11c	Transport
DMRX:143418001	X	143418001	143419000	1000	1	2.60E-10	-0.36	18	1.8	Atp11c	Transport
DMRX:143449001	X	143449001	143450000	1000	1	1.20E-07	-0.45	11	1.1	Atp11c	Transport
DMRX:152203001	X	152203001	152209000	6000	2	6.30E-07	-0.33	68	1.13	Magea4	Cytoskeleton
DMRX:152454001	X	152454001	152457000	3000	1	5.20E-07	-0.35	30	1	Gabra3	Ion Channel
DMRX:152538001	X	152538001	152547000	9000	1	3.50E-07	-0.3	108	1.2	Gabra3	Ion Channel
DMRX:152580001	X	152580001	152582000	2000	1	6.20E-10	-0.36	21	1.05	Gabra3;Mir105	Ion Channel
DMRX:153008001	X	153008001	153010000	2000	1	4.70E-07	0.45	34	1.7	Zfp185	
DMRX:153800001	X	153800001	153803000	3000	1	1.20E-08	-0.38	45	1.5	Aff2	Transcription
DMRX:154636001	X	154636001	154638000	2000	1	1.70E-08	0.44	8	0.4	Fmr1nb	
DMRX:155636001	X	155636001	155642000	6000	1	1.50E-08	-0.32	56	0.93	RGD1565338	
DMRX:155840001	X	155840001	155848000	8000	3	3.70E-09	0.41	279	3.49	Dkc1	Cell Cycle
DMRX:156218001	X	156218001	156222000	4000	1	1.40E-08	-0.49	56	1.4	Olr1768;LOC690348	Receptor
DMRX:156296001	X	156296001	156303000	7000	2	4.70E-09	-0.37	110	1.57	G6pd	
DMRX:157282001	X	157282001	157283000	1000	1	6.90E-12	0.5	6	0.6	Atp2b3	Transport
DMRX:159363001	X	159363001	159365000	2000	1	3.80E-10	-0.49	15	0.75	Gpr112l	
DMRX:159816001	X	159816001	159823000	7000	3	1.90E-08	-0.33	81	1.16	Arhgef6	Transcription
DMRX:159892001	X	159892001	159897000	5000	2	3.60E-09	-0.32	44	0.88	Rbmx	
DMRX:159898001	X	159898001	159900000	2000	1	2.80E-09	-0.33	38	1.9	Rbmx	
DMRY:907001	Y	907001	909000	2000	1	9.70E-07	-0.41	14	0.7	Eif2s3y	Translation
DMRY:1133001	Y	1133001	1139000	6000	1	2.70E-08	-0.52	45	0.75	Kdm6a	Epigenetic
DMRY:1154001	Y	1154001	1161000	7000	2	2.00E-07	-0.37	79	1.13	Kdm6a	Epigenetic
DMRY:1242001	Y	1242001	1246000	4000	1	3.00E-07	-0.3	50	1.25	Ddx3	
DMRY:1315001	Y	1315001	1322000	7000	1	1.50E-10	-0.47	60	0.86	Usp9y	Protease
DMRY:1353001	Y	1353001	1357000	4000	2	1.20E-07	-0.3	31	0.78	Usp9y	Protease
DMRY:1364001	Y	1364001	1365000	1000	1	4.70E-07	-0.57	7	0.7	Usp9y	Protease
DMRY:1391001	Y	1391001	1395000	4000	2	7.80E-08	-0.41	43	1.07	Usp9y	Protease
DMRY:1409001	Y	1409001	1416000	7000	1	6.20E-08	-0.36	75	1.07	Usp9y	Protease
