# Supplementary material for: Environmental induced transgenerational inheritance impacts systems epigenetics in disease etiology
Source: Sci Rep. 2022 Apr 19;12:5452. doi: 10.1038/s41598-022-09336-0 (PMC9018793; doi:10.1038/s41598-022-09336-0)
Supplement: Supplementary file 20 — Supplementary Table S12. [file 41598_2022_9336_MOESM20_ESM.pdf]

Supplemental Table S12
Dioxin DMR p<1e-06

DMR Name	Chr	Start	Stop	Length	# Sig Win	minP	maxLFC	CpG #	CpG Density	Gene Annotation	Gene Category
DMR1:545001	1	545001	548000	3000	1	1.20E-10	0.79	24	0.8	Vom2r-ps5;LOC102556463	
DMR1:562001	1	562001	563000	1000	1	9.00E-19	-0.66	7	0.7	Vom2r-ps5	
DMR1:649001	1	649001	650000	1000	1	3.10E-08	-0.44	19	1.9	Vom2r-ps5	
DMR1:656001	1	656001	660000	4000	3	3.30E-08	-0.46	150	3.75	Vom2r-ps5	
DMR1:680001	1	680001	685000	5000	1	8.70E-08	-0.41	57	1.14	Vom2r-ps5	
DMR1:1478001	1	1478001	1479000	1000	1	5.60E-09	0.22	3	0.3	Raet1d	
DMR1:1689001	1	1689001	1693000	4000	1	8.30E-07	-0.32	108	2.7	LOC108348623;Lrp11	
DMR1:1806001	1	1806001	1807000	1000	1	6.80E-09	-0.65	15	1.5	Lats1	Signaling
DMR1:1889001	1	1889001	1890000	1000	1	9.70E-07	0.36	26	2.6	Ginm1;RGD1563636;Ppil4	Transcription
DMR1:1925001	1	1925001	1926000	1000	1	2.20E-07	0.42	10	1	Ppil4	Transcription
DMR1:1945001	1	1945001	1949000	4000	1	2.00E-07	0.32	57	1.43	Zc3h12d	Translation
DMR1:2335001	1	2335001	2337000	2000	1	5.90E-08	0.44	18	0.9	Ust;LOC108348767	Transport
DMR1:2346001	1	2346001	2349000	3000	1	3.40E-07	-0.36	51	1.7	Ust;LOC108348767	Transport
DMR1:2378001	1	2378001	2380000	2000	1	4.50E-08	0.5	39	1.95	Ust	Transport
DMR1:2505001	1	2505001	2506000	1000	1	4.40E-07	-0.49	9	0.9	Ust	Transport
DMR1:2596001	1	2596001	2599000	3000	1	6.80E-15	0.97	45	1.5	Ust	Transport
DMR1:2636001	1	2636001	2637000	1000	1	1.00E-11	-0.49	21	2.1	Ust;LOC365036	Transport
DMR1:2776001	1	2776001	2781000	5000	2	9.30E-12	0.86	94	1.88	Sash1	
DMR1:2789001	1	2789001	2792000	3000	1	2.80E-12	0.68	72	2.4	Sash1	
DMR1:2797001	1	2797001	2798000	1000	1	3.70E-07	-0.41	19	1.9	Sash1	
DMR1:2811001	1	2811001	2812000	1000	1	1.20E-09	0.75	42	4.2	Sash1	
DMR1:2856001	1	2856001	2861000	5000	1	2.40E-11	-0.41	85	1.7	Sash1	
DMR1:2898001	1	2898001	2901000	3000	1	3.00E-07	-0.61	44	1.47	Sash1	
DMR1:2907001	1	2907001	2908000	1000	1	6.60E-07	-0.37	18	1.8	Sash1	
DMR1:2910001	1	2910001	2914000	4000	1	6.50E-07	-0.36	65	1.62	Sash1	
DMR1:2957001	1	2957001	2961000	4000	1	5.00E-12	0.8	54	1.35	Sash1	
DMR1:3855001	1	3855001	3856000	1000	1	8.10E-10	0.64	26	2.6	RGD1560633;Stxbp5	Translation;Transport
DMR1:3973001	1	3973001	3978000	5000	1	1.00E-10	0.46	40	0.8	Stxbp5	Transport
DMR1:4447001	1	4447001	4449000	2000	1	2.40E-07	0.59	40	2	Adgb;LOC102555130	
DMR1:4578001	1	4578001	4579000	1000	1	7.90E-07	-0.54	9	0.9	Adgb	
DMR1:4595001	1	4595001	4596000	1000	1	5.80E-13	0.77	26	2.6	Adgb	
DMR1:4755001	1	4755001	4756000	1000	1	2.60E-13	0.9	23	2.3	Grm1	Signaling
DMR1:4786001	1	4786001	4787000	1000	1	5.00E-22	1.39	32	3.2	Grm1	Signaling
DMR1:4872001	1	4872001	4873000	1000	1	1.40E-07	0.6	7	0.7	Grm1	Signaling
DMR1:4875001	1	4875001	4876000	1000	1	8.50E-10	-0.6	1	0.1	Grm1	Signaling
DMR1:4906001	1	4906001	4907000	1000	1	1.30E-09	0.49	10	1	Grm1	Signaling
DMR1:5027001	1	5027001	5029000	2000	1	3.60E-08	0.56	38	1.9	Grm1	Signaling
DMR1:5123001	1	5123001	5125000	2000	1	1.30E-07	0.58	37	1.85	Grm1	Signaling
DMR1:5127001	1	5127001	5130000	3000	1	3.00E-13	0.72	36	1.2	Grm1	Signaling
DMR1:5510001	1	5510001	5511000	1000	1	8.90E-10	0.93	29	2.9	Epm2a	
DMR1:6443001	1	6443001	6444000	1000	1	2.20E-07	0.61	21	2.1	Utrn	
DMR1:6514001	1	6514001	6519000	5000	1	5.20E-07	-0.45	73	1.46	Utrn	
DMR1:6596001	1	6596001	6602000	6000	2	2.10E-08	0.36	81	1.35	Utrn	
DMR1:6631001	1	6631001	6632000	1000	1	1.50E-07	0.51	13	1.3	Utrn	
DMR1:6633001	1	6633001	6635000	2000	1	1.10E-07	0.44	23	1.15	Utrn	
DMR1:6685001	1	6685001	6686000	1000	1	5.60E-12	0.33	12	1.2	Utrn	
DMR1:6783001	1	6783001	6784000	1000	1	2.10E-07	-0.45	13	1.3	Utrn	
DMR1:6874001	1	6874001	6876000	2000	1	1.70E-08	-0.55	39	1.95	Utrn	
DMR1:6879001	1	6879001	6881000	2000	1	3.60E-09	-0.45	15	0.75	Utrn	
DMR1:6895001	1	6895001	6896000	1000	1	2.60E-07	-0.31	20	2	Utrn	
DMR1:6899001	1	6899001	6900000	1000	1	5.70E-10	-0.46	15	1.5	Utrn	
DMR1:7015001	1	7015001	7016000	1000	1	1.40E-07	0.63	22	2.2	Trnal-uaa	
DMR1:7030001	1	7030001	7036000	6000	1	6.20E-12	0.82	120	2	Stx11	Transcription
DMR1:7047001	1	7047001	7049000	2000	1	1.20E-16	1	47	2.35	Stx11	Transcription
DMR1:7052001	1	7052001	7054000	2000	1	1.30E-09	-0.4	27	1.35	Stx11	Transcription
DMR1:7092001	1	7092001	7095000	3000	1	3.40E-07	0.49	57	1.9	Sf3b5	Translation
DMR1:7283001	1	7283001	7285000	2000	2	1.80E-08	-0.56	27	1.35	Zc2hc1b	Transcription
DMR1:7312001	1	7312001	7314000	2000	1	1.90E-07	-0.49	20	1	Zc2hc1b;Ltv1	Transcription

DMR1:7438001	1	7438001	7443000	5000	1	3.40E-07	0.39	82	1.64	Phactr2	Signaling
DMR1:7595001	1	7595001	7601000	6000	1	4.40E-08	-0.42	98	1.63	Phactr2	Signaling
DMR1:7602001	1	7602001	7605000	3000	1	2.90E-09	0.38	25	0.83	Phactr2	Signaling
DMR1:7626001	1	7626001	7628000	2000	1	6.60E-08	-0.39	27	1.35	Phactr2;LOC103690919	Signaling
DMR1:7637001	1	7637001	7640000	3000	1	4.10E-10	0.45	52	1.73	Phactr2;LOC103690919	Signaling
DMR1:7651001	1	7651001	7652000	1000	1	4.10E-09	0.67	32	3.2	LOC103690919;Fuca2	Metabolism
DMR1:7653001	1	7653001	7654000	1000	1	9.70E-07	-0.35	6	0.6	LOC103690919;Fuca2	Metabolism
DMR1:7664001	1	7664001	7667000	3000	1	1.90E-10	1	90	3	Fuca2	Metabolism
DMR1:7673001	1	7673001	7676000	3000	1	7.60E-07	-0.38	57	1.9	Fuca2;Pex3	Metabolism
DMR1:7681001	1	7681001	7683000	2000	1	7.00E-08	-0.44	29	1.45	Fuca2;Pex3	Metabolism
DMR1:7746001	1	7746001	7747000	1000	1	1.80E-14	0.81	29	2.9	Adat2	Metabolism
DMR1:7802001	1	7802001	7805000	3000	1	1.00E-08	0.74	50	1.67	RGD1563591	
DMR1:7860001	1	7860001	7863000	3000	1	2.10E-09	-0.44	59	1.97	Aig1	
DMR1:7882001	1	7882001	7886000	4000	1	2.60E-12	-0.46	69	1.73	Aig1	
DMR1:7895001	1	7895001	7896000	1000	1	2.20E-08	-0.59	7	0.7	Aig1	
DMR1:7923001	1	7923001	7926000	3000	1	6.10E-11	-0.52	36	1.2	Aig1	
DMR1:7959001	1	7959001	7965000	6000	1	6.60E-11	-0.41	102	1.7	Aig1	
DMR1:7999001	1	7999001	8001000	2000	1	1.80E-14	0.79	42	2.1	Aig1	
DMR1:8011001	1	8011001	8016000	5000	1	1.70E-07	-0.37	118	2.36	Aig1	
DMR1:8026001	1	8026001	8028000	2000	1	4.30E-08	-0.41	62	3.1	Aig1	
DMR1:8133001	1	8133001	8137000	4000	1	4.90E-07	-0.36	70	1.75	Hivep2	
DMR1:8146001	1	8146001	8151000	5000	1	4.50E-08	-0.47	89	1.78	Hivep2	
DMR1:8173001	1	8173001	8175000	2000	1	7.30E-07	-0.36	23	1.15	Hivep2	
DMR1:8177001	1	8177001	8180000	3000	1	4.10E-15	-0.57	59	1.97	Hivep2	
DMR1:8204001	1	8204001	8205000	1000	1	6.60E-10	-0.47	7	0.7	Hivep2;LOC102551141;LOC108348466	
DMR1:8208001	1	8208001	8211000	3000	1	3.50E-07	-0.54	49	1.63	Hivep2;LOC102551141;LOC108348466	
DMR1:8212001	1	8212001	8215000	3000	1	4.30E-07	-0.41	69	2.3	Hivep2;LOC102551141;LOC108348466	
DMR1:8239001	1	8239001	8242000	3000	1	7.50E-07	-0.36	64	2.13	Hivep2	
DMR1:8270001	1	8270001	8276000	6000	1	7.10E-07	-0.38	117	1.95	Hivep2	
DMR1:8315001	1	8315001	8316000	1000	1	3.80E-17	0.88	29	2.9	Hivep2	
DMR1:8338001	1	8338001	8339000	1000	1	4.20E-08	-0.48	14	1.4	Hivep2	
DMR1:8592001	1	8592001	8594000	2000	1	4.20E-07	-0.56	16	0.8	Adgrg6	Signaling
DMR1:8714001	1	8714001	8715000	1000	1	4.50E-13	0.88	14	1.4	Adgrg6	Signaling
DMR1:12116001	1	12116001	12118000	2000	1	1.00E-08	-0.48	14	0.7	Mcc	
DMR1:12906001	1	12906001	12909000	3000	1	1.60E-07	-0.37	123	4.1	LOC102554594;Txlnb	Transport
DMR1:12919001	1	12919001	12922000	3000	1	1.60E-12	0.59	51	1.7	LOC102554594;Txlnb	Transport
DMR1:13053001	1	13053001	13056000	3000	1	2.80E-07	-0.41	29	0.97	Heca	
DMR1:13165001	1	13165001	13167000	2000	1	6.50E-07	-0.46	38	1.9	LOC108349444;Abracl	
DMR1:13168001	1	13168001	13172000	4000	1	1.90E-08	-0.35	66	1.65	LOC108349444;Abracl	
DMR1:13461001	1	13461001	13465000	4000	3	5.10E-08	-0.68	60	1.5	LOC102549339;Nhsl1	
DMR1:13551001	1	13551001	13553000	2000	1	5.20E-07	-0.51	35	1.75	Nhsl1	
DMR1:13562001	1	13562001	13563000	1000	1	4.50E-08	0.52	20	2	Nhsl1	
DMR1:13569001	1	13569001	13578000	9000	1	4.10E-10	-0.51	190	2.11	Nhsl1	
DMR1:13630001	1	13630001	13631000	1000	1	7.30E-14	-0.63	38	3.8	Nhsl1	
DMR1:13633001	1	13633001	13634000	1000	1	8.00E-08	-0.33	21	2.1	Nhsl1	
DMR1:13640001	1	13640001	13643000	3000	3	3.10E-08	-0.46	83	2.77	Nhsl1	
DMR1:13662001	1	13662001	13666000	4000	1	2.00E-07	-0.34	73	1.82	Nhsl1	
DMR1:13680001	1	13680001	13681000	1000	1	2.60E-07	-0.47	7	0.7	Nhsl1	
DMR1:13683001	1	13683001	13685000	2000	1	8.80E-09	-0.56	32	1.6	Nhsl1	
DMR1:13700001	1	13700001	13709000	9000	1	4.50E-10	0.59	207	2.3	Nhsl1;Hebp2	
DMR1:13854001	1	13854001	13855000	1000	1	4.90E-15	0.81	20	2	Arfgef3	Transcription
DMR1:13880001	1	13880001	13883000	3000	1	5.10E-07	-0.37	44	1.47	Arfgef3	Transcription
DMR1:13886001	1	13886001	13887000	1000	1	1.50E-12	0.83	22	2.2	Arfgef3	Transcription
DMR1:14404001	1	14404001	14406000	2000	2	1.30E-15	0.81	65	3.25	Tnfaip3	Protease
DMR1:14421001	1	14421001	14423000	2000	1	3.40E-07	0.49	11	0.55	Tnfaip3	Protease
DMR1:15084001	1	15084001	15088000	4000	1	1.10E-09	0.5	65	1.62	Ifngr1;Il22ra2	Receptor
DMR1:15101001	1	15101001	15103000	2000	1	3.90E-17	1.04	43	2.15	Il22ra2	Receptor
DMR1:15112001	1	15112001	15115000	3000	1	8.90E-08	-0.41	49	1.63	Il22ra2	Receptor
DMR1:15200001	1	15200001	15201000	1000	1	3.60E-07	0.32	13	1.3	Il20ra	Receptor

DMR1:15214001	1	15214001	15215000	1000	1	1.70E-07	0.73	12	1.2	Il20ra	Receptor
DMR1:15295001	1	15295001	15298000	3000	1	4.80E-09	0.68	28	0.93	Slc35d3	Transport
DMR1:15314001	1	15314001	15325000	11000	2	2.50E-10	0.36	80	0.73	Pex7	
DMR1:15442001	1	15442001	15443000	1000	1	1.00E-14	0.91	26	2.6	Map3k5	Signaling
DMR1:15498001	1	15498001	15500000	2000	1	5.50E-09	-0.37	26	1.3	Map3k5	Signaling
DMR1:15513001	1	15513001	15516000	3000	1	4.30E-07	-0.31	55	1.83	Map3k5	Signaling
DMR1:15521001	1	15521001	15525000	4000	1	3.00E-08	-0.36	91	2.28	Map3k5	Signaling
DMR1:15550001	1	15550001	15551000	1000	1	2.90E-09	-0.37	24	2.4	Map3k5;LOC102547350	Signaling
DMR1:15573001	1	15573001	15574000	1000	1	2.70E-07	-0.42	5	0.5	Map3k5	Signaling
DMR1:15629001	1	15629001	15633000	4000	1	4.00E-08	-0.49	87	2.17	Map7;LOC108349472	Cytoskeleton
DMR1:15646001	1	15646001	15649000	3000	1	6.70E-09	-0.36	54	1.8	Map7	Cytoskeleton
DMR1:15677001	1	15677001	15679000	2000	1	8.60E-12	-0.57	53	2.65	Map7	Cytoskeleton
DMR1:15696001	1	15696001	15697000	1000	1	8.10E-15	-0.57	24	2.4	Map7	Cytoskeleton
DMR1:15703001	1	15703001	15706000	3000	1	4.50E-08	0.55	41	1.37	Map7	Cytoskeleton
DMR1:15746001	1	15746001	15747000	1000	1	1.80E-08	-0.43	19	1.9	Map7	Cytoskeleton
DMR1:15793001	1	15793001	15795000	2000	1	2.70E-11	-0.58	38	1.9	Bclaf1	
DMR1:15840001	1	15840001	15844000	4000	2	2.10E-13	-0.5	80	2	Mtfr2	
DMR1:15893001	1	15893001	15894000	1000	1	2.80E-15	0.9	46	4.6	Pde7b	Signaling
DMR1:15897001	1	15897001	15898000	1000	1	2.70E-09	0.39	13	1.3	Pde7b	Signaling
DMR1:15901001	1	15901001	15902000	1000	1	1.40E-07	-0.49	5	0.5	Pde7b	Signaling
DMR1:15961001	1	15961001	15963000	2000	1	5.10E-08	-0.38	28	1.4	Pde7b	Signaling
DMR1:15973001	1	15973001	15979000	6000	1	1.30E-07	-0.42	87	1.45	Pde7b	Signaling
DMR1:15997001	1	15997001	15999000	2000	1	3.20E-15	1	62	3.1	Pde7b	Signaling
DMR1:16030001	1	16030001	16032000	2000	1	2.70E-09	-0.39	26	1.3	Pde7b	Signaling
DMR1:16056001	1	16056001	16058000	2000	1	1.20E-09	0.66	16	0.8	Pde7b	Signaling
DMR1:16096001	1	16096001	16098000	2000	1	1.80E-09	0.67	32	1.6	Pde7b	Signaling
DMR1:16100001	1	16100001	16101000	1000	1	1.50E-11	0.72	19	1.9	Pde7b	Signaling
DMR1:16102001	1	16102001	16105000	3000	2	8.70E-11	1.03	78	2.6	Pde7b	Signaling
DMR1:16120001	1	16120001	16122000	2000	1	1.20E-09	-0.55	15	0.75	Pde7b	Signaling
DMR1:16171001	1	16171001	16173000	2000	1	1.20E-09	0.42	22	1.1	Pde7b	Signaling
DMR1:16175001	1	16175001	16179000	4000	1	2.70E-16	1.08	72	1.8	Pde7b	Signaling
DMR1:16647001	1	16647001	16649000	2000	1	2.30E-07	0.6	27	1.35	RGD1560020_predicted	
DMR1:16693001	1	16693001	16694000	1000	1	1.70E-08	0.48	20	2	RGD1560020_predicted	
DMR1:16843001	1	16843001	16846000	3000	1	1.50E-09	-0.46	36	1.2	Hbs1l	Translation
DMR1:16918001	1	16918001	16919000	1000	1	1.30E-11	-0.42	13	1.3	Aldh8a1	Metabolism
DMR1:16920001	1	16920001	16922000	2000	1	3.70E-10	0.54	38	1.9	Aldh8a1	Metabolism
DMR1:16936001	1	16936001	16938000	2000	1	7.40E-08	0.37	43	2.15	Aldh8a1	Metabolism
DMR1:17200001	1	17200001	17201000	1000	1	3.00E-07	0.52	50	5	Themis	
DMR1:17227001	1	17227001	17229000	2000	1	1.20E-07	0.51	23	1.15	Themis	
DMR1:17253001	1	17253001	17254000	1000	1	1.40E-07	-0.58	10	1	Themis;LOC108349008;LOC102552380	
DMR1:17339001	1	17339001	17340000	1000	1	4.70E-07	0.44	18	1.8	Themis	
DMR1:17449001	1	17449001	17451000	2000	1	4.00E-15	0.84	41	2.05	Ptprk	Signaling
DMR1:17538001	1	17538001	17541000	3000	1	5.70E-07	0.52	55	1.83	Ptprk	Signaling
DMR1:17662001	1	17662001	17663000	1000	1	2.10E-07	0.28	16	1.6	Ptprk	Signaling
DMR1:17872001	1	17872001	17873000	1000	1	3.20E-15	1.12	24	2.4	Ptprk	Signaling
DMR1:17930001	1	17930001	17931000	1000	1	1.10E-07	-0.43	24	2.4	Ptprk	Signaling
DMR1:18014001	1	18014001	18015000	1000	1	6.00E-14	-0.53	18	1.8	Ptprk	Signaling
DMR1:18053001	1	18053001	18054000	1000	1	3.50E-09	0.29	10	1	Ptprk	Signaling
DMR1:18700001	1	18700001	18707000	7000	1	6.00E-09	-0.3	68	0.97	Lama2	Extracellular Matrix
DMR1:18708001	1	18708001	18709000	1000	1	5.00E-08	-0.59	5	0.5	Lama2	Extracellular Matrix
DMR1:18886001	1	18886001	18887000	1000	1	1.80E-12	0.45	8	0.8	Lama2	Extracellular Matrix
DMR1:18960001	1	18960001	18963000	3000	1	2.60E-07	-0.4	44	1.47	Lama2	Extracellular Matrix
DMR1:19018001	1	19018001	19021000	3000	1	3.00E-07	-0.33	17	0.57	Lama2	Extracellular Matrix
DMR1:19063001	1	19063001	19064000	1000	1	1.80E-07	0.4	11	1.1	Lama2	Extracellular Matrix
DMR1:19121001	1	19121001	19122000	1000	1	1.60E-07	-0.43	24	2.4	Lama2	Extracellular Matrix
DMR1:19123001	1	19123001	19124000	1000	1	2.50E-07	-0.53	18	1.8	Lama2	Extracellular Matrix
DMR1:20007001	1	20007001	20008000	1000	1	4.10E-21	-0.6	18	1.8	L3mbtl3	Epigenetic
DMR1:20047001	1	20047001	20048000	1000	1	7.30E-08	-0.43	22	2.2	L3mbtl3	Epigenetic
DMR1:20057001	1	20057001	20059000	2000	1	4.10E-12	-0.45	30	1.5	L3mbtl3	Epigenetic
DMR1:20088001	1	20088001	20090000	2000	1	3.10E-07	-0.4	30	1.5	L3mbtl3	Epigenetic
DMR1:20117001	1	20117001	20123000	6000	1	6.20E-35	0.83	55	0.92	Samd3;LOC100360791	

DMR1:20409001	1	20409001	20411000	2000	2	6.60E-10	0.82	66	3.3	Tmem200a;RGD1559962	
DMR1:21016001	1	21016001	21018000	2000	1	2.90E-07	-0.44	17	0.85	Epb41l2	
DMR1:21021001	1	21021001	21022000	1000	1	4.80E-07	0.44	3	0.3	Epb41l2	
DMR1:21122001	1	21122001	21124000	2000	2	2.10E-08	0.66	41	2.05	LOC102552728;Akap7	Translation
DMR1:21237001	1	21237001	21238000	1000	1	3.40E-10	-0.49	15	1.5	Akap7	Translation
DMR1:21254001	1	21254001	21257000	3000	1	2.40E-07	-0.37	39	1.3	Akap7	Translation
DMR1:21548001	1	21548001	21550000	2000	1	1.60E-07	-0.46	34	1.7	Med23	Transcription
DMR1:21597001	1	21597001	21599000	2000	2	6.10E-09	-0.58	7	0.35	Med23	Transcription
DMR1:21670001	1	21670001	21672000	2000	1	6.10E-10	-0.55	39	1.95	Enpp3	
DMR1:21675001	1	21675001	21678000	3000	1	2.10E-13	-0.65	43	1.43	Enpp3	
DMR1:21771001	1	21771001	21772000	1000	1	9.30E-10	-0.51	16	1.6	Enpp1	
DMR1:21773001	1	21773001	21778000	5000	2	7.00E-12	-0.5	61	1.22	Enpp1	
DMR1:21861001	1	21861001	21866000	5000	1	1.40E-14	-0.55	55	1.1	Ctgf;LOC108349248;LOC103690930	
DMR1:22238001	1	22238001	22241000	3000	2	2.70E-11	-0.75	18	0.6	Stx7	Transcription
DMR1:22261001	1	22261001	22264000	3000	1	1.30E-07	-0.34	60	2	Stx7	Transcription
DMR1:22319001	1	22319001	22322000	3000	1	8.30E-11	0.8	72	2.4	Taar9	Signaling
DMR1:22647001	1	22647001	22649000	2000	1	5.40E-09	0.54	21	1.05	Vnn3	Metabolism
DMR1:22671001	1	22671001	22680000	9000	1	3.40E-08	-0.39	173	1.92	Vnn3;LOC108349832	Metabolism
DMR1:22729001	1	22729001	22733000	4000	1	4.20E-07	-0.3	27	0.68	Slc18b1	Transport
DMR1:23331001	1	23331001	23334000	3000	2	8.10E-08	-0.53	17	0.57	Eya4	
DMR1:23343001	1	23343001	23344000	1000	1	4.90E-07	0.4	7	0.7	Eya4	
DMR1:23539001	1	23539001	23542000	3000	2	4.00E-12	-0.73	15	0.5	Eya4;LOC100910446	Transcription
DMR1:23593001	1	23593001	23594000	1000	1	4.80E-08	-0.36	4	0.4	Eya4	
DMR1:23917001	1	23917001	23918000	1000	1	3.40E-16	-0.57	5	0.5	Tcf21	Transcription
DMR1:23975001	1	23975001	23977000	2000	1	9.10E-09	0.59	41	2.05	Tbpl1	Transcription
DMR1:24022001	1	24022001	24024000	2000	1	6.70E-07	0.34	34	1.7	Slc2a12	
DMR1:24042001	1	24042001	24044000	2000	1	1.90E-08	-0.45	39	1.95	Slc2a12	
DMR1:24208001	1	24208001	24211000	3000	2	3.30E-11	0.76	64	2.13	Sgk1	Signaling
DMR1:25174001	1	25174001	25175000	1000	1	3.10E-10	0.85	18	1.8	Clvs2	Transport
DMR1:25176001	1	25176001	25178000	2000	1	2.50E-08	-0.52	15	0.75	Clvs2	Transport
DMR1:25472001	1	25472001	25473000	1000	1	2.00E-07	-0.52	8	0.8	Trdn	
DMR1:25701001	1	25701001	25707000	6000	3	8.00E-15	-0.41	53	0.88	Trdn	
DMR1:25743001	1	25743001	25745000	2000	2	1.90E-08	0.29	35	1.75	Trdn	
DMR1:25746001	1	25746001	25749000	3000	1	4.40E-09	-0.28	30	1	Trdn	
DMR1:28593001	1	28593001	28595000	2000	1	4.80E-07	-0.44	31	1.55	Hddc2	
DMR1:29199001	1	29199001	29200000	1000	1	7.10E-17	1.16	34	3.4	Hey2	Transcription
DMR1:29219001	1	29219001	29220000	1000	1	4.10E-07	-0.37	16	1.6	Trnae-cuc;Ncoa7	
DMR1:29329001	1	29329001	29333000	4000	1	2.60E-09	0.85	60	1.5	Ncoa7	
DMR1:29410001	1	29410001	29411000	1000	1	6.00E-08	0.38	8	0.8	Hint3	DNA Repair
DMR1:31639001	1	31639001	31642000	3000	1	6.70E-25	0.55	40	1.33	Ahrr	Transcription
DMR1:31713001	1	31713001	31715000	2000	1	3.20E-08	-0.5	36	1.8	Exoc3	Transport
DMR1:31858001	1	31858001	31859000	1000	1	3.80E-07	0.5	13	1.3	Cep72;Tppp	Cytoskeleton
DMR1:31873001	1	31873001	31875000	2000	1	2.40E-11	0.71	32	1.6	Cep72;Tppp	Cytoskeleton
DMR1:31877001	1	31877001	31879000	2000	1	3.40E-09	0.66	24	1.2	Tppp	Cytoskeleton
DMR1:31880001	1	31880001	31881000	1000	1	9.80E-07	-0.38	30	3	Tppp	Cytoskeleton
DMR1:31914001	1	31914001	31916000	2000	1	4.60E-07	-0.43	18	0.9	Zdhhc11	
DMR1:32123001	1	32123001	32128000	5000	1	8.10E-07	0.45	61	1.22	Slc12a7	Transport
DMR1:32232001	1	32232001	32235000	3000	2	9.30E-09	0.66	44	1.47	Slc6a18;LOC103690946	Transport
DMR1:32263001	1	32263001	32264000	1000	1	2.30E-08	0.43	16	1.6	Tert	Transcription
DMR1:32273001	1	32273001	32274000	1000	1	3.60E-08	0.62	14	1.4	Tert;Clptm1l	Transcription
DMR1:32407001	1	32407001	32413000	6000	1	4.40E-07	0.44	64	1.07	Lpcat1	Metabolism
DMR1:33269001	1	33269001	33270000	1000	1	1.80E-10	0.61	11	1.1	Irx2;LOC103690947	Development
DMR1:33281001	1	33281001	33282000	1000	1	1.10E-07	0.47	14	1.4	Irx2;LOC103690947	Development
DMR1:33925001	1	33925001	33926000	1000	1	1.50E-07	-0.53	7	0.7	Irx1	Development
DMR1:35058001	1	35058001	35059000	1000	1	8.40E-09	0.4	11	1.1	Adamts16	Protease
DMR1:35151001	1	35151001	35155000	4000	1	6.80E-11	-0.43	44	1.1	Adamts16	Protease
DMR1:35294001	1	35294001	35297000	3000	1	9.90E-07	0.51	35	1.17	Ice1	Golgi
DMR1:36212001	1	36212001	36213000	1000	1	2.20E-07	-0.37	16	1.6	Ube2ql1	Proteolysis
DMR1:36361001	1	36361001	36362000	1000	1	5.50E-10	0.4	8	0.8	Srd5a1	Metabolism
DMR1:37208001	1	37208001	37209000	1000	1	3.30E-13	-0.64	0	0	Adcy2	
DMR1:37244001	1	37244001	37245000	1000	1	1.40E-09	0.46	7	0.7	Adcy2	

DMR1:37271001	1	37271001	37276000	5000	1	1.80E-07	0.31	45	0.9	Adcy2	
DMR1:37352001	1	37352001	37357000	5000	1	2.50E-08	0.25	62	1.24	Adcy2	
DMR1:37469001	1	37469001	37471000	2000	2	1.90E-16	1.09	46	2.3	Adcy2	
DMR1:37563001	1	37563001	37564000	1000	1	1.80E-10	-0.72	7	0.7	Adcy2;LOC100362709	
DMR1:37661001	1	37661001	37663000	2000	1	3.00E-07	-0.34	17	0.85	Adcy2	
DMR1:37675001	1	37675001	37684000	9000	1	5.70E-09	-0.38	60	0.67	Adcy2	
DMR1:37767001	1	37767001	37768000	1000	1	9.30E-07	-0.4	8	0.8	Mtrr	Metabolism
DMR1:37889001	1	37889001	37891000	2000	1	1.50E-07	-0.49	15	0.75	RGD1566325;LOC103690951	
DMR1:37999001	1	37999001	3.80E+07	1000	1	3.20E-07	-0.44	13	1.3	Zfp748	
DMR1:38517001	1	38517001	38519000	2000	2	6.90E-25	1.41	90	4.5	RGD1564382;LOC102551340	Transcription
DMR1:39831001	1	39831001	39836000	5000	1	1.90E-08	-0.32	37	0.74	Ppp1r14c	Signaling
DMR1:39841001	1	39841001	39843000	2000	1	3.30E-08	-0.45	14	0.7	Ppp1r14c	Signaling
DMR1:40313001	1	40313001	40314000	1000	1	1.10E-08	0.37	0	0	Plekhg1	
DMR1:40428001	1	40428001	40430000	2000	1	1.70E-08	-0.59	12	0.6	Plekhg1	
DMR1:40514001	1	40514001	40515000	1000	1	9.30E-07	0.54	26	2.6	Plekhg1	
DMR1:40620001	1	40620001	40623000	3000	1	7.80E-10	-0.66	42	1.4	Mthfd1l;LOC102546677	
DMR1:40883001	1	40883001	40887000	4000	2	2.00E-08	-0.38	44	1.1	Akap12	Cytoskeleton
DMR1:40977001	1	40977001	40982000	5000	1	5.60E-07	-0.4	53	1.06	Rmnd1;Armt1	
DMR1:41045001	1	41045001	41049000	4000	1	2.60E-08	-0.39	29	0.72	Ccdc170	
DMR1:41215001	1	41215001	41216000	1000	1	3.50E-10	0.78	25	2.5	Esr1	
DMR1:41221001	1	41221001	41224000	3000	1	1.40E-07	0.43	41	1.37	Esr1	
DMR1:41265001	1	41265001	41266000	1000	1	9.90E-14	0.65	21	2.1	Esr1	
DMR1:41413001	1	41413001	41414000	1000	1	7.00E-08	0.36	3	0.3	Esr1	
DMR1:41428001	1	41428001	41433000	5000	1	2.50E-25	0.49	52	1.04	Esr1	
DMR1:41469001	1	41469001	41470000	1000	1	4.10E-08	-0.61	9	0.9	Esr1	
DMR1:41518001	1	41518001	41519000	1000	1	5.90E-07	-0.47	5	0.5	Esr1	
DMR1:41609001	1	41609001	41610000	1000	1	5.80E-08	-0.42	18	1.8	Syne1	
DMR1:41935001	1	41935001	41936000	1000	1	5.70E-10	0.52	9	0.9	Syne1	
DMR1:43486001	1	43486001	43488000	2000	1	8.80E-10	-0.56	14	0.7	Oprm1	Signaling
DMR1:43590001	1	43590001	43592000	2000	1	9.90E-09	-0.45	27	1.35	Oprm1;Ipcef1	Signaling
DMR1:43714001	1	43714001	43715000	1000	1	9.00E-07	-0.46	12	1.2	Oprm1;Ipcef1	Signaling
DMR1:43934001	1	43934001	43936000	2000	1	6.30E-15	0.73	40	2	RGD1560234	
DMR1:44071001	1	44071001	44073000	2000	1	1.10E-07	-0.51	10	0.5	Scaf8	Translation
DMR1:44312001	1	44312001	44314000	2000	1	1.90E-08	-0.42	35	1.75	Tiam2	
DMR1:44334001	1	44334001	44335000	1000	1	1.30E-07	0.35	13	1.3	Tiam2	
DMR1:44341001	1	44341001	44343000	2000	1	1.10E-08	0.27	15	0.75	Tiam2	
DMR1:44352001	1	44352001	44354000	2000	1	3.80E-07	0.27	26	1.3	Tiam2	
DMR1:44383001	1	44383001	44384000	1000	1	3.20E-07	-0.34	22	2.2	Tiam2	
DMR1:44446001	1	44446001	44448000	2000	1	9.00E-08	0.44	35	1.75	Tfb1m;Cldn20;LOC102547288	Epigenetic;Cell Junction
DMR1:44538001	1	44538001	44543000	5000	1	6.90E-07	-0.33	41	0.82	LOC103690965;Nox3	Metabolism
DMR1:44577001	1	44577001	44579000	2000	1	1.80E-10	0.52	27	1.35	Nox3	Metabolism
DMR1:44587001	1	44587001	44590000	3000	1	7.50E-08	0.4	18	0.6	Nox3	Metabolism
DMR1:45960001	1	45960001	45963000	3000	1	2.20E-07	-0.4	49	1.63	Arid1b	
DMR1:45986001	1	45986001	45989000	3000	1	7.50E-11	-0.51	29	0.97	Arid1b	
DMR1:46003001	1	46003001	46005000	2000	1	6.20E-13	-0.63	29	1.45	Arid1b	
DMR1:46298001	1	46298001	46300000	2000	1	3.50E-08	-0.44	41	2.05	Tmem242;Ldhal6b	
DMR1:46307001	1	46307001	46308000	1000	1	2.90E-07	0.46	16	1.6	Tmem242;Ldhal6b	
DMR1:46324001	1	46324001	46327000	3000	2	3.80E-07	0.48	35	1.17	Tmem242	
DMR1:46459001	1	46459001	46460000	1000	1	4.50E-08	0.37	16	1.6	Zdhhc14	
DMR1:46461001	1	46461001	46464000	3000	2	9.10E-11	0.6	34	1.13	Zdhhc14	
DMR1:46541001	1	46541001	46544000	3000	2	7.80E-17	0.98	62	2.07	Zdhhc14	
DMR1:46816001	1	46816001	46818000	2000	1	2.30E-09	-0.55	39	1.95	Snx9	Cytoskeleton
DMR1:46905001	1	46905001	46907000	2000	1	4.80E-08	0.49	20	1	Synj2;LOC103690968	Signaling
DMR1:46993001	1	46993001	46997000	4000	1	9.90E-07	-0.39	51	1.27	Gtf2h5;LOC102551377;Tulp4	Transcription
DMR1:47085001	1	47085001	47086000	1000	1	8.30E-07	0.32	16	1.6	Tulp4	
DMR1:47122001	1	47122001	47125000	3000	1	3.70E-07	-0.34	58	1.93	Tulp4	
DMR1:47138001	1	47138001	47139000	1000	1	5.30E-07	-0.36	14	1.4	Tulp4	
DMR1:47167001	1	47167001	47172000	5000	1	4.70E-07	0.56	82	1.64	Tmem181	
DMR1:47179001	1	47179001	47180000	1000	1	6.90E-07	0.54	13	1.3	Tmem181	
DMR1:47195001	1	47195001	47196000	1000	1	1.80E-10	0.49	10	1	Tmem181	
DMR1:47199001	1	47199001	47200000	1000	1	6.70E-07	0.5	16	1.6	Tmem181;Dynlt1	Cytoskeleton

DMR1:47270001	1	47270001	47273000	3000	1	2.70E-07	-0.28	58	1.93	Sytl3	
DMR1:47275001	1	47275001	47277000	2000	1	7.10E-11	0.45	16	0.8	Sytl3	
DMR1:47327001	1	47327001	47330000	3000	1	5.10E-11	-0.53	49	1.63	Ezr	Cytoskeleton
DMR1:47415001	1	47415001	47416000	1000	1	5.60E-08	0.65	15	1.5	Rsph3	Development
DMR1:47424001	1	47424001	47425000	1000	1	7.70E-11	0.69	19	1.9	Rsph3	Development
DMR1:47586001	1	47586001	47588000	2000	1	6.40E-14	0.96	39	1.95	RGD1560015	
DMR1:47650001	1	47650001	47652000	2000	1	2.80E-09	0.85	40	2	Fndc1	
DMR1:47655001	1	47655001	47656000	1000	1	1.80E-21	1.22	39	3.9	Fndc1	
DMR1:47927001	1	47927001	47931000	4000	1	1.50E-10	-0.46	57	1.43	Sod2	Metabolism
DMR1:47989001	1	47989001	47994000	5000	1	4.00E-16	0.34	44	0.88	Acat2	Metabolism
DMR1:47996001	1	47996001	47997000	1000	1	4.10E-10	0.92	38	3.8	Acat2	Metabolism
DMR1:48008001	1	48008001	48011000	3000	2	9.70E-14	0.61	41	1.37	Acat2l1;LOC108349718	Metabolism
DMR1:48012001	1	48012001	48016000	4000	2	5.70E-09	-0.45	68	1.7	Acat2l1;LOC108349718;Tcp1	Metabolism;Translation
DMR1:48040001	1	48040001	48042000	2000	1	1.20E-07	0.63	41	2.05	Tcp1;Mrpl18;Pnldc1	Translation;Translation;Translation
DMR1:48084001	1	48084001	48085000	1000	1	3.20E-13	-0.42	15	1.5	Mas1l;Mas1	Signaling
DMR1:48119001	1	48119001	48120000	1000	1	3.50E-07	0.5	18	1.8	Mas1l	
DMR1:48130001	1	48130001	48132000	2000	1	2.10E-09	-0.45	32	1.6	Mas1l	
DMR1:48204001	1	48204001	48206000	2000	2	3.30E-16	1.08	99	4.95	Mas1l;Igf2r	Transport
DMR1:48245001	1	48245001	48248000	3000	1	8.50E-09	0.42	41	1.37	Igf2r	Transport
DMR1:48304001	1	48304001	48306000	2000	1	1.00E-07	0.39	33	1.65	Slc22a1	Transport
DMR1:48308001	1	48308001	48312000	4000	1	1.20E-08	0.76	61	1.52	Slc22a1;Slc22a2	Transport
DMR1:48331001	1	48331001	48334000	3000	1	9.10E-13	-0.54	18	0.6	Slc22a2	Transport
DMR1:48483001	1	48483001	48484000	1000	1	4.30E-08	0.54	11	1.1	Slc22a3	Transport
DMR1:48496001	1	48496001	48497000	1000	1	4.70E-07	0.34	19	1.9	Slc22a3	Transport
DMR1:48650001	1	48650001	48651000	1000	1	1.80E-08	0.62	10	1	Map3k4	Signaling
DMR1:48714001	1	48714001	48718000	4000	1	1.40E-08	-0.43	96	2.4	Map3k4;Agpat4	Signaling;Metabolism
DMR1:48908001	1	48908001	48911000	3000	1	6.10E-08	0.56	43	1.43	Park2;LOC103690973	
DMR1:48919001	1	48919001	48920000	1000	1	9.10E-08	0.28	10	1	Park2	
DMR1:48938001	1	48938001	48939000	1000	1	1.50E-07	0.34	10	1	Park2	
DMR1:48940001	1	48940001	48946000	6000	1	2.30E-13	0.71	107	1.78	Park2	
DMR1:48952001	1	48952001	48954000	2000	1	3.00E-14	0.83	42	2.1	Park2	
DMR1:48966001	1	48966001	48969000	3000	1	9.90E-13	1.04	50	1.67	Park2	
DMR1:49037001	1	49037001	49038000	1000	1	2.70E-12	0.67	16	1.6	Park2	
DMR1:49047001	1	49047001	49048000	1000	1	1.10E-07	-0.45	24	2.4	Park2	
DMR1:49092001	1	49092001	49093000	1000	1	2.30E-08	0.65	15	1.5	Park2	
DMR1:49150001	1	49150001	49152000	2000	1	7.20E-08	0.5	29	1.45	Park2	
DMR1:49346001	1	49346001	49348000	2000	1	1.10E-08	0.43	17	0.85	Park2	
DMR1:49373001	1	49373001	49375000	2000	1	6.60E-08	0.57	22	1.1	Park2	
DMR1:49422001	1	49422001	49423000	1000	1	8.90E-11	0.36	2	0.2	Park2	
DMR1:49448001	1	49448001	49450000	2000	1	1.40E-10	0.73	42	2.1	Park2	
DMR1:49746001	1	49746001	49748000	2000	1	5.50E-07	0.49	17	0.85	Park2	
DMR1:49795001	1	49795001	49797000	2000	2	7.90E-13	-0.65	24	1.2	Park2	
DMR1:49914001	1	49914001	49920000	6000	1	9.80E-08	-0.28	73	1.22	Park2	
DMR1:50041001	1	50041001	50042000	1000	1	5.70E-07	-0.38	5	0.5	Park2	
DMR1:50113001	1	50113001	50116000	3000	1	6.90E-08	-0.45	48	1.6	Pacrg	
DMR1:50173001	1	50173001	50175000	2000	1	9.40E-07	-0.55	11	0.55	Pacrg;LOC102552213	
DMR1:50243001	1	50243001	50245000	2000	1	2.60E-08	-0.53	16	0.8	Pacrg	
DMR1:50246001	1	50246001	50248000	2000	1	2.10E-07	0.41	22	1.1	Pacrg	
DMR1:50273001	1	50273001	50275000	2000	1	9.70E-07	0.4	28	1.4	Pacrg	
DMR1:50358001	1	50358001	50360000	2000	1	2.50E-08	0.4	26	1.3	Pacrg	
DMR1:51047001	1	51047001	51048000	1000	1	1.30E-08	-0.45	8	0.8	Qk	
DMR1:51619001	1	51619001	51622000	3000	2	2.20E-11	0.82	93	3.1	LOC108348950;Pabpc6	
DMR1:52344001	1	52344001	52345000	1000	1	2.10E-07	-0.43	12	1.2	MGC94891	
DMR1:52366001	1	52366001	52368000	2000	1	4.70E-07	0.38	28	1.4	Pde10a	Signaling
DMR1:52481001	1	52481001	52483000	2000	1	4.80E-08	0.55	31	1.55	Pde10a	Signaling
DMR1:53328001	1	53328001	53333000	5000	1	4.00E-10	0.43	93	1.86	Rps6ka2	Golgi
DMR1:53341001	1	53341001	53347000	6000	2	7.20E-08	0.34	69	1.15	Rps6ka2	Golgi
DMR1:53403001	1	53403001	53404000	1000	1	4.10E-08	0.48	9	0.9	Rps6ka2	Golgi
DMR1:53405001	1	53405001	53406000	1000	1	5.80E-08	0.5	20	2	Rps6ka2	Golgi
DMR1:53424001	1	53424001	53425000	1000	1	8.90E-07	0.31	21	2.1	Rps6ka2	Golgi

DMR1:53484001	1	53484001	53487000	3000	1	1.00E-14	0.79	44	1.47	Rps6ka2	Golgi
DMR1:53558001	1	53558001	53561000	3000	1	6.00E-10	0.39	38	1.27	Tcp10b;Ttll2	Cytoskeleton
DMR1:53846001	1	53846001	53848000	2000	1	7.90E-08	-0.44	18	0.9	Afdn;LOC103690979	Cytoskeleton
DMR1:53853001	1	53853001	53855000	2000	1	4.40E-10	-0.47	23	1.15	Afdn	Cytoskeleton
DMR1:54023001	1	54023001	54025000	2000	1	1.30E-08	-0.51	22	1.1	RGD1560718	Signaling
DMR1:54028001	1	54028001	54031000	3000	1	6.60E-07	0.42	29	0.97	RGD1560718	Signaling
DMR1:54041001	1	54041001	54042000	1000	1	2.30E-10	0.29	5	0.5	RGD1560718	Signaling
DMR1:54047001	1	54047001	54048000	1000	1	1.70E-09	0.42	7	0.7	RGD1560718;LOC102552087;LOC108349470	Signaling
DMR1:54850001	1	54850001	54853000	3000	1	1.80E-08	0.18	36	1.2	Smok2a	Signaling
DMR1:55218001	1	55218001	55219000	1000	1	2.00E-08	0.3	15	1.5	RGD1561667	Signaling
DMR1:55232001	1	55232001	55233000	1000	1	6.40E-19	0.29	14	1.4	RGD1561667	Signaling
DMR1:55235001	1	55235001	55238000	3000	2	5.80E-09	0.26	28	0.93	RGD1561667	Signaling
DMR1:55662001	1	55662001	55664000	2000	1	4.60E-07	0.24	17	0.85	LOC102556502;LOC103690993;LOC102546375;LOC108349807;RGD1561185	
DMR1:55671001	1	55671001	55673000	2000	1	3.60E-16	0.32	36	1.8	LOC108349807;RGD1561185	
DMR1:55898001	1	55898001	55900000	2000	1	4.90E-17	0.63	18	0.9	Vom2r9	Signaling
DMR1:56173001	1	56173001	56174000	1000	1	7.30E-09	0.47	10	1	Dact2	
DMR1:56303001	1	56303001	56307000	4000	1	1.40E-09	0.4	33	0.82	Smoc2	Signaling
DMR1:56665001	1	56665001	56666000	1000	1	1.70E-07	-0.31	24	2.4	Thbs2	
DMR1:56683001	1	56683001	56684000	1000	1	2.30E-08	-0.49	19	1.9	Thbs2	
DMR1:56921001	1	56921001	56923000	2000	1	3.20E-07	0.46	23	1.15	Wdr27	
DMR1:57014001	1	57014001	57017000	3000	1	1.50E-09	0.54	31	1.03	Ermard	
DMR1:57329001	1	57329001	57331000	2000	2	4.60E-11	-0.51	36	1.8	Dll1	
DMR1:57377001	1	57377001	57379000	2000	1	1.00E-07	-0.36	27	1.35	Fam120b	
DMR1:57704001	1	57704001	57706000	2000	1	7.60E-09	-0.48	24	1.2	Chd1	
DMR1:57712001	1	57712001	57715000	3000	1	6.80E-16	-0.67	31	1.03	Chd1	
DMR1:57716001	1	57716001	57718000	2000	2	4.40E-19	-0.67	31	1.55	Chd1	
DMR1:57798001	1	57798001	57800000	2000	1	1.30E-10	-0.6	29	1.45	Rgmb	
DMR1:59169001	1	59169001	59170000	1000	1	1.70E-10	-0.64	7	0.7	Lix1;LOC102552556	
DMR1:59201001	1	59201001	59202000	1000	1	1.70E-15	0.76	27	2.7	Lix1	
DMR1:59699001	1	59699001	59701000	2000	1	8.10E-07	0.74	44	2.2	LOC688452;Mir99b;Mir3596c;Mirlet7e;Mir125a	
DMR1:59818001	1	59818001	59821000	3000	1	1.10E-12	-0.45	23	0.77	Fpr2	Signaling
DMR1:60091001	1	60091001	60092000	1000	1	3.70E-10	-0.45	5	0.5	Vom1r7	Receptor
DMR1:60122001	1	60122001	60125000	3000	1	1.00E-07	-0.36	23	0.77	Vom1r8	Receptor
DMR1:60548001	1	60548001	60549000	1000	1	4.70E-08	-0.43	36	3.6	Vom1r-ps11	
DMR1:60687001	1	60687001	60690000	3000	1	2.90E-09	-0.41	24	0.8	Vom1r-ps15	
DMR1:60771001	1	60771001	60773000	2000	1	1.20E-09	-0.65	5	0.25	Vom1r-ps18	
DMR1:61077001	1	61077001	61080000	3000	2	3.40E-08	0.35	32	1.07	Vom1r-ps23	
DMR1:61097001	1	61097001	61100000	3000	1	9.30E-09	-0.39	50	1.67	Vom1r19	Receptor
DMR1:61332001	1	61332001	61334000	2000	1	2.30E-09	-0.5	8	0.4	Zfp51	Transcription
DMR1:61511001	1	61511001	61517000	6000	1	2.70E-08	-0.29	68	1.13	LOC108348215;Znf761	Transcription
DMR1:61553001	1	61553001	61554000	1000	1	3.10E-07	0.42	27	2.7	RGD1565566	
DMR1:61693001	1	61693001	61697000	4000	1	6.30E-08	-0.36	29	0.72	Vom1r23;LOC102553001	Receptor
DMR1:62492001	1	62492001	62493000	1000	1	7.70E-13	-0.54	31	3.1	Vom2r80	Signaling
DMR1:63550001	1	63550001	63555000	5000	1	7.50E-27	0.44	41	0.82	Vom2r26	Signaling
DMR1:63560001	1	63560001	63561000	1000	1	2.10E-43	0.76	23	2.3	Vom2r26	Signaling
DMR1:63563001	1	63563001	63566000	3000	1	9.90E-35	0.48	25	0.83	Vom2r26	Signaling
DMR1:63811001	1	63811001	63812000	1000	1	6.40E-08	-0.59	5	0.5	Lilrb3l;LOC683420	Immune
DMR1:63856001	1	63856001	63861000	5000	1	1.90E-07	-0.23	43	0.86	Lilrb3l	Immune
DMR1:64187001	1	64187001	64191000	4000	1	7.30E-10	-0.42	54	1.35	Oscar	Immune
DMR1:64314001	1	64314001	64315000	1000	1	1.80E-08	-0.47	7	0.7	Cacng6	Transport
DMR1:64327001	1	64327001	64329000	2000	1	1.10E-07	-0.31	22	1.1	Cacng6;Cacng8;Mir935	Transport
DMR1:64382001	1	64382001	64383000	1000	1	1.70E-07	0.65	13	1.3	LOC103691003;Cacng7	Transport
DMR1:64415001	1	64415001	64418000	3000	1	4.40E-07	0.48	29	0.97	Cacng7;Prkcg	Transport;Signaling
DMR1:64479001	1	64479001	64483000	4000	1	3.40E-11	0.6	55	1.38	Olr1l;LOC103689958;Olr386	Receptor
DMR1:64519001	1	64519001	64520000	1000	1	7.60E-08	0.4	6	0.6	Nlrp12	
DMR1:64533001	1	64533001	64534000	1000	1	7.00E-07	0.49	10	1	Nlrp12;Mir295-1;Mir294;Mir293;Mir291b;Mir292;Mir291a;Mir290	
DMR1:65605001	1	65605001	65608000	3000	1	1.50E-07	0.62	67	2.23	Zfp446;LOC102552250;Zfp324	

DMR1:65763001	1	65763001	65764000	1000	1	1.80E-10	-0.46	7	0.7	Zfp110;LOC108348438	
DMR1:65771001	1	65771001	65774000	3000	1	5.80E-12	-0.41	63	2.1	LOC108348438;Zfp329	Transcription
DMR1:65946001	1	65946001	65947000	1000	1	1.80E-08	-0.75	0	0	Vom2r36;Vom2r33	Signaling
DMR1:65977001	1	65977001	65978000	1000	1	3.90E-07	0.28	26	2.6	Vom2r36	Signaling
DMR1:65980001	1	65980001	65989000	9000	1	8.20E-13	-0.41	81	0.9	Vom2r36	Signaling
DMR1:65996001	1	65996001	65999000	3000	1	5.50E-08	-0.36	27	0.9	Vom2r36;Vom2r35	Signaling
DMR1:66026001	1	66026001	66030000	4000	1	1.80E-07	-0.41	44	1.1	Vom2r36;Vom2r35;LOC108348434	Signaling
DMR1:66070001	1	66070001	66071000	1000	1	8.30E-08	0.28	6	0.6	Vom2r36	Signaling
DMR1:66120001	1	66120001	66125000	5000	2	2.90E-07	0.46	113	2.26	Vom2r36;LOC108349537;LOC108349534;Mir295-2	Signaling
DMR1:66369001	1	66369001	66371000	2000	1	9.90E-08	-0.47	8	0.4	Vom1r-ps63	
DMR1:66401001	1	66401001	66403000	2000	1	2.80E-08	-0.37	15	0.75	Vom1r53;Vom1r-ps61	Receptor
DMR1:66677001	1	66677001	66678000	1000	1	9.00E-08	0.42	13	1.3	Vom1r57	Receptor
DMR1:66701001	1	66701001	66707000	6000	1	1.00E-08	-0.34	58	0.97	Vom1r-ps65	
DMR1:67055001	1	67055001	67058000	3000	1	4.50E-07	-0.35	30	1	Vom1r46	Receptor
DMR1:67119001	1	67119001	67124000	5000	1	6.20E-07	-0.51	24	0.48	Vom1r45	Receptor
DMR1:67143001	1	67143001	67146000	3000	1	9.80E-09	-0.57	14	0.47	Vom1r45	Receptor
DMR1:67297001	1	67297001	67300000	3000	1	2.10E-07	-0.62	9	0.3	LOC103691020;Vom1r42	
DMR1:67337001	1	67337001	67338000	1000	1	1.10E-18	1.3	31	3.1	Vom1r-ps49;LOC100362054	Transcription
DMR1:69367001	1	69367001	69369000	2000	1	2.50E-07	-0.24	28	1.4	Nlrp4a	
DMR1:69394001	1	69394001	69397000	3000	2	1.70E-13	-0.48	30	1	Nlrp4a	
DMR1:69784001	1	69784001	69785000	1000	1	3.00E-11	-0.45	6	0.6	Mfsd14a;Hiat1-ps1	
DMR1:69850001	1	69850001	69851000	1000	1	8.60E-07	-0.35	8	0.8	Zfp773-ps1;LOC108349540;Zfp772;LOC102547517	Transcription;Transcription
DMR1:69865001	1	69865001	69866000	1000	1	4.30E-10	0.86	18	1.8	Zfp772;LOC102547517;LOC102547412	Transcription
DMR1:70037001	1	70037001	70040000	3000	1	8.90E-07	-0.33	38	1.27	Usp29;LOC102547903	Protease
DMR1:70135001	1	70135001	70137000	2000	1	9.00E-09	-0.6	18	0.9	LOC102547903;Mir3099	
DMR1:70230001	1	70230001	70231000	1000	1	3.10E-14	1.02	44	4.4	Peg3;Apeg3	Transcription
DMR1:70238001	1	70238001	70240000	2000	1	3.80E-08	-0.42	10	0.5	Peg3;Apeg3;Zim1	Transcription
DMR1:71125001	1	71125001	71131000	6000	1	5.60E-07	-0.28	80	1.33	Smim17	
DMR1:71182001	1	71182001	71185000	3000	1	3.80E-07	-0.51	70	2.33	Zfp28;Zfp78	Transcription
DMR1:71231001	1	71231001	71237000	6000	1	1.20E-08	-0.58	51	0.85	LOC102548162;Zfp583	
DMR1:71350001	1	71350001	71352000	2000	2	1.70E-16	0.65	41	2.05	Zscan5b;Galp	Transcription
DMR1:71383001	1	71383001	71386000	3000	1	1.40E-12	0.87	88	2.93	Galp;Zfp444	Transcription
DMR1:71565001	1	71565001	71566000	1000	1	4.20E-08	-0.41	4	0.4	Vom2r-ps118	
DMR1:71667001	1	71667001	71669000	2000	1	8.40E-07	-0.34	19	0.95	Nlrp4	
DMR1:71682001	1	71682001	71684000	2000	1	2.20E-08	-0.4	35	1.75	Nlrp4	
DMR1:71709001	1	71709001	71710000	1000	1	8.60E-12	0.93	18	1.8	Nlrp4;RGD1564651	
DMR1:71781001	1	71781001	71784000	3000	1	2.90E-07	0.5	28	0.93	Obox2	Development
DMR1:72202001	1	72202001	72204000	2000	1	1.00E-07	0.5	43	2.15	Vom1r36;LOC108349748;Vom1r37	Receptor
DMR1:72365001	1	72365001	72367000	2000	1	3.20E-13	0.78	81	4.05	Zfp865;LOC100911196;Zfp524	Transcription
DMR1:72466001	1	72466001	72469000	3000	1	7.70E-11	0.71	95	3.17	Ssc5d;Nat14;Zfp628	Protease;Metabolism;Transcription
DMR1:72533001	1	72533001	72534000	1000	1	4.10E-09	-0.55	3	0.3	Isoc2b	
DMR1:72543001	1	72543001	72545000	2000	1	1.20E-09	0.63	14	0.7	Isoc2b;Shisa7	
DMR1:72723001	1	72723001	72726000	3000	1	4.00E-08	-0.69	35	1.17	Brsk1;LOC108349434;Hspbp1	Signaling
DMR1:72865001	1	72865001	72868000	3000	1	2.00E-12	0.89	45	1.5	Ptprh;Syt5;LOC108349515;Dnaaf3	Signaling;Transport;Transcription
DMR1:72878001	1	72878001	72879000	1000	1	8.70E-13	0.88	34	3.4	Dnaaf3;Tnni3	Transcription;Cytoskeleton
DMR1:72883001	1	72883001	72884000	1000	1	1.40E-13	0.96	30	3	Dnaaf3;Tnni3;Tnnt1	Transcription;Cytoskeleton;Cytoskeleton
DMR1:72934001	1	72934001	72935000	1000	1	1.10E-08	0.46	24	2.4	Ppp1r12c;Eps8l1	Signaling;Cytoskeleton
DMR1:72936001	1	72936001	72939000	3000	1	6.30E-18	1.26	76	2.53	Ppp1r12c;Eps8l1	Signaling;Cytoskeleton
DMR1:72951001	1	72951001	72953000	2000	1	2.20E-07	-0.48	9	0.45	Eps8l1;Rdh13	Cytoskeleton;Golgi
DMR1:72962001	1	72962001	72963000	1000	1	8.60E-09	0.62	16	1.6	Rdh13	Golgi
DMR1:72966001	1	72966001	72971000	5000	1	1.90E-09	-0.38	47	0.94	Rdh13	Golgi
DMR1:73014001	1	73014001	73018000	4000	1	4.50E-07	0.41	24	0.6	Gp6	Immune

DMR1:73750001	1	73750001	73752000	2000	1	8.90E-11	0.71	26	1.3	Ttyh1;Lair1	Transport;Immune
DMR1:74430001	1	74430001	74432000	2000	1	1.40E-07	0.24	21	1.05	Vom2r-ps51	
DMR1:74994001	1	74994001	74995000	1000	1	1.80E-07	0.48	5	0.5	Vom2r31	Signaling
DMR1:75140001	1	75140001	75143000	3000	1	2.20E-08	0.33	27	0.9	Vom1r58;Vom1r-ps66;RGD1564801	Receptor;Signaling
DMR1:75336001	1	75336001	75338000	2000	1	3.20E-12	0.79	59	2.95	Vom1r62;RGD1310257	Receptor
DMR1:75347001	1	75347001	75348000	1000	1	2.10E-08	0.58	18	1.8	RGD1310257;Lig1	Methylation
DMR1:75434001	1	75434001	75439000	5000	1	7.70E-07	-0.27	40	0.8	Pla2g4c	Metabolism
DMR1:75455001	1	75455001	75464000	9000	1	2.80E-07	-0.24	123	1.37	Pla2g4c	Metabolism
DMR1:75748001	1	75748001	75749000	1000	1	4.30E-07	-0.72	4	0.4	Bsph1	
DMR1:76650001	1	76650001	76652000	2000	1	2.60E-09	0.66	39	1.95	Sult2a6;Srp72-ps1	
DMR1:77113001	1	77113001	77115000	2000	1	3.60E-07	-0.32	87	4.35	RGD1565590	
DMR1:77850001	1	77850001	77851000	1000	1	1.60E-09	0.59	13	1.3	Gltscr2;Ehd2	Transport
DMR1:77869001	1	77869001	77871000	2000	2	7.20E-20	1.14	68	3.4	Ehd2;Gltscr1;LOC103691043	Transport
DMR1:77875001	1	77875001	77876000	1000	1	1.60E-09	0.61	9	0.9	Ehd2;Gltscr1;LOC103691043	Transport
DMR1:77928001	1	77928001	77930000	2000	1	1.30E-08	-0.46	27	1.35	Gltscr1	
DMR1:77974001	1	77974001	77975000	1000	1	2.20E-08	0.71	14	1.4	Zfp541	
DMR1:78043001	1	78043001	78044000	1000	1	5.20E-09	-0.37	15	1.5	Slc8a2	Transport
DMR1:78052001	1	78052001	78053000	1000	1	1.70E-07	0.64	27	2.7	Slc8a2	Transport
DMR1:78065001	1	78065001	78067000	2000	1	4.00E-07	-0.45	21	1.05	Meis3;Dhx34	Development;Transcription
DMR1:78109001	1	78109001	78112000	3000	1	7.80E-14	0.8	62	2.07	Dhx34	Transcription
DMR1:78183001	1	78183001	78187000	4000	2	1.30E-11	0.71	83	2.08	C5ar2;LOC684925;C5ar1	Signaling
DMR1:78275001	1	78275001	78276000	1000	1	3.70E-08	0.6	17	1.7	Bbc3;Sae1	Proteolysis
DMR1:78374001	1	78374001	78377000	3000	1	6.60E-11	0.69	48	1.6	Zc3h4	Transcription
DMR1:78382001	1	78382001	78384000	2000	1	6.20E-08	0.41	23	1.15	Zc3h4	Transcription
DMR1:78395001	1	78395001	78397000	2000	1	8.60E-07	-0.29	41	2.05	Zc3h4	Transcription
DMR1:78422001	1	78422001	78423000	1000	1	2.80E-08	0.45	15	1.5	Tmem160;Npas1	Transcription
DMR1:78436001	1	78436001	78438000	2000	1	5.20E-07	0.45	29	1.45	Npas1	Transcription
DMR1:78448001	1	78448001	78450000	2000	1	2.90E-07	-0.35	23	1.15	Npas1;Arhgap35	Transcription;Signaling
DMR1:78571001	1	78571001	78573000	2000	1	1.00E-06	-0.35	81	4.05	Arhgap35	Signaling
DMR1:78618001	1	78618001	78622000	4000	1	8.70E-08	-0.5	54	1.35	LOC102550403;LOC108349752;Ceacam9	
DMR1:78641001	1	78641001	78643000	2000	1	3.60E-10	0.49	42	2.1	LOC108349752;Ceacam9	
DMR1:78771001	1	78771001	78774000	3000	1	1.70E-13	0.66	75	2.5	Strn4;Prkd2	Signaling
DMR1:78816001	1	78816001	78817000	1000	1	3.30E-07	0.43	9	0.9	Dact3;Gng8	Signaling
DMR1:78877001	1	78877001	78878000	1000	1	2.40E-09	0.89	47	4.7	Pnmal2;LOC103691045;Pnmal1	
DMR1:78887001	1	78887001	78889000	2000	2	2.20E-10	0.62	29	1.45	Pnmal2;LOC103691045;Pnmal1	
DMR1:78975001	1	78975001	78979000	4000	1	5.90E-07	0.44	42	1.05	Ppp5c;Hif3a	Signaling;Transcription
DMR1:79006001	1	79006001	79007000	1000	1	3.80E-08	-0.37	21	2.1	Hif3a	Transcription
DMR1:79075001	1	79075001	79076000	1000	1	5.00E-07	-0.48	2	0.2	Psg16	
DMR1:79195001	1	79195001	79197000	2000	1	8.00E-07	0.29	14	0.7	Psgb1	
DMR1:79677001	1	79677001	79678000	1000	1	8.50E-08	0.53	12	1.2	LOC292672;Psg19	Immune
DMR1:79756001	1	79756001	79759000	3000	1	1.10E-09	-0.36	37	1.23	LOC100362871;Micb	Immune
DMR1:79766001	1	79766001	79768000	2000	1	2.80E-07	-0.38	15	0.75	Micb	Immune
DMR1:79771001	1	79771001	79773000	2000	1	2.10E-14	0.97	48	2.4	Micb	Immune
DMR1:79888001	1	79888001	79891000	3000	2	2.60E-07	0.49	39	1.3	Nanos2;Mypop	Metabolism;Transcription
DMR1:79976001	1	79976001	79979000	3000	1	6.70E-07	0.55	52	1.73	Rsph6a;LOC103691057;LOC108349062;Dmwd;Dmpk	Cytoskeleton;Signaling
DMR1:79995001	1	79995001	79998000	3000	1	4.00E-10	0.78	61	2.03	Dmwd;Dmpk;Six5;LOC102553424	Signaling;Development
DMR1:80011001	1	80011001	80013000	2000	1	8.50E-07	0.35	30	1.5	Six5;LOC102553424	Development
DMR1:80026001	1	80026001	80027000	1000	1	2.60E-16	1	67	6.7	LOC102553424;Fbxo46	
DMR1:80071001	1	80071001	80072000	1000	1	1.40E-09	0.72	32	3.2	Gipr	Receptor
DMR1:80111001	1	80111001	80115000	4000	2	6.20E-14	0.72	62	1.55	Eml2	
DMR1:80169001	1	80169001	80170000	1000	1	8.50E-11	0.57	35	3.5	Opa3;Vasp	Cytoskeleton
DMR1:80282001	1	80282001	80290000	8000	2	7.30E-09	0.59	170	2.12	Ppp1r13l;Ercc2;Mir343	Epigenetic
DMR1:80324001	1	80324001	80327000	3000	2	1.60E-08	0.61	54	1.8	Klc3;Ckm;Mark4	Cytoskeleton;Signaling;Signaling

DMR1:80355001	1	80355001	80358000	3000	2	2.40E-10	0.86	31	1.03	Mark4	Signaling
DMR1:80395001	1	80395001	80397000	2000	1	1.60E-10	0.68	35	1.75	Exoc3l2	Transport
DMR1:80492001	1	80492001	80496000	4000	1	4.50E-09	0.5	70	1.75	Gemin7;Zfp296;Clasrp	Transcription;Translation
DMR1:80646001	1	80646001	80650000	4000	1	5.10E-09	0.48	57	1.43	Nectin2	
DMR1:80655001	1	80655001	80656000	1000	1	6.40E-14	0.74	35	3.5	Nectin2	
DMR1:80676001	1	80676001	80679000	3000	2	8.80E-14	0.73	77	2.57	Nectin2;Bcam	Immune
DMR1:80731001	1	80731001	80732000	1000	1	8.90E-10	0.75	38	3.8	Bcl3	Transport
DMR1:80734001	1	80734001	80736000	2000	1	6.80E-09	0.53	29	1.45	Bcl3	Transport
DMR1:80744001	1	80744001	80745000	1000	1	2.50E-07	-0.44	45	4.5	Bcl3	Transport
DMR1:80753001	1	80753001	80757000	4000	1	1.30E-07	0.67	64	1.6	Bcl3	Transport
DMR1:80851001	1	80851001	80853000	2000	1	2.30E-11	0.77	44	2.2	Igsf23;LOC108349553	
DMR1:80892001	1	80892001	80893000	1000	1	2.90E-08	-0.41	23	2.3	Ceacam20;LOC102548132	
DMR1:80932001	1	80932001	80933000	1000	1	4.20E-07	0.65	18	1.8	Zfp180;LOC108348994	Transcription
DMR1:80947001	1	80947001	80948000	1000	1	1.20E-10	0.89	26	2.6	Zfp180;Zfp112	Transcription
DMR1:80962001	1	80962001	80963000	1000	1	5.10E-08	0.32	9	0.9	Zfp112;Znf235	Transcription
DMR1:81115001	1	81115001	81118000	3000	1	3.90E-09	0.71	53	1.77	Zfp61	Transcription
DMR1:81137001	1	81137001	81140000	3000	1	1.20E-07	-0.35	89	2.97	Zfp61;Zfp94	Transcription
DMR1:81185001	1	81185001	81190000	5000	2	2.90E-11	0.5	126	2.52	Tescl	
DMR1:81200001	1	81200001	81206000	6000	2	4.50E-09	0.73	107	1.78	Tescl;Lypd5	Receptor
DMR1:81331001	1	81331001	81332000	1000	1	1.40E-08	0.42	12	1.2	Plaur	Receptor
DMR1:81334001	1	81334001	81336000	2000	1	5.90E-10	0.38	25	1.25	Plaur	Receptor
DMR1:81397001	1	81397001	81399000	2000	2	4.60E-18	1.05	68	3.4	Irgq;Pinlyp	Cytoskeleton
DMR1:81407001	1	81407001	81410000	3000	1	2.90E-07	-0.39	73	2.43	Irgq;Pinlyp;Xrcc1	Cytoskeleton;Transcription
DMR1:81447001	1	81447001	81449000	2000	1	1.10E-17	1.14	66	3.3	Xrcc1;Zfp575;Ethe1	Transcription;Transcription;Metabolism
DMR1:81484001	1	81484001	81485000	1000	1	1.10E-07	0.63	27	2.7	Phldb3;LOC108349554	
DMR1:81635001	1	81635001	81638000	3000	1	2.20E-07	-0.49	39	1.3	LOC100909700;LOC108349555;LOC100910734;Ceacam4	
DMR1:81876001	1	81876001	81880000	4000	1	3.00E-11	0.69	68	1.7	Atp1a3;Grik5	Transport;Receptor
DMR1:82018001	1	82018001	82021000	3000	1	7.20E-07	0.46	61	2.03	Pou2f2;LOC103691054	
DMR1:82044001	1	82044001	82053000	9000	2	4.90E-10	-0.43	287	3.19	Pou2f2	
DMR1:82056001	1	82056001	82059000	3000	1	1.90E-07	0.6	55	1.83	Pou2f2	
DMR1:82070001	1	82070001	82073000	3000	1	1.60E-11	0.49	51	1.7	Dedd2	
DMR1:82127001	1	82127001	82131000	4000	1	1.90E-09	0.77	67	1.68	Erf;Cic	Transcription;Transcription
DMR1:82169001	1	82169001	82171000	2000	1	4.70E-10	0.69	36	1.8	Cic;Pafah1b3;Prr19;Tmem145	Transcription;Golgi
DMR1:82242001	1	82242001	82244000	2000	1	7.30E-11	0.88	43	2.15	Megf8;Cnfn;LOC102549342;Lipe	Extracellular Matrix;Metabolism
DMR1:82249001	1	82249001	82250000	1000	1	1.60E-12	0.91	21	2.1	LOC102549342;Lipe	Metabolism
DMR1:82323001	1	82323001	82331000	8000	1	1.10E-07	-0.35	203	2.54	LOC102549342;Ceacam1	
DMR1:82410001	1	82410001	82412000	2000	1	2.80E-07	-0.32	28	1.4	LOC102549342;Erich4;Atp5sl;B3gnt8	Golgi
DMR1:82422001	1	82422001	82423000	1000	1	4.70E-07	0.49	24	2.4	Atp5sl;B3gnt8;Bckdha	Golgi;Metabolism
DMR1:82498001	1	82498001	82499000	1000	1	1.60E-10	-0.43	12	1.2	Tgfb1;Ccdc97	Growth Factors
DMR1:82608001	1	82608001	82609000	1000	1	1.40E-08	0.57	19	1.9	Cyp2s1	Metabolism
DMR1:83226001	1	83226001	83231000	5000	2	3.20E-08	-0.39	45	0.9	Cyp2b3	Metabolism
DMR1:83261001	1	83261001	83265000	4000	1	8.40E-07	-0.37	29	0.72	Eif4g2-ps2	
DMR1:83519001	1	83519001	83521000	2000	1	1.10E-07	-0.53	4	0.2	Cyp2b12	Metabolism
DMR1:83942001	1	83942001	83947000	5000	2	7.80E-15	1.05	117	2.34	Cyp2f4	
DMR1:83948001	1	83948001	83950000	2000	1	7.30E-08	0.6	27	1.35	Cyp2f4	
DMR1:84108001	1	84108001	84109000	1000	1	1.20E-13	0.67	20	2	LOC108348928;Ltbp4	Extracellular Matrix
DMR1:84111001	1	84111001	84112000	1000	1	5.80E-10	0.87	36	3.6	LOC108348928;Ltbp4	Extracellular Matrix
DMR1:84116001	1	84116001	84123000	7000	3	9.10E-18	1.07	144	2.06	Ltbp4	Extracellular Matrix
DMR1:84136001	1	84136001	84141000	5000	2	1.10E-12	0.99	144	2.88	Ltbp4	Extracellular Matrix
DMR1:84151001	1	84151001	84152000	1000	1	1.50E-20	1.21	25	2.5	Ltbp4;Shkbp1	Extracellular Matrix;Cytoskeleton
DMR1:84157001	1	84157001	84158000	1000	1	9.90E-07	0.64	26	2.6	Ltbp4;Shkbp1	Extracellular Matrix;Cytoskeleton
DMR1:84174001	1	84174001	84175000	1000	1	4.60E-07	0.5	13	1.3	Shkbp1;Sptbn4	Cytoskeleton
DMR1:84216001	1	84216001	84217000	1000	1	3.60E-07	0.46	7	0.7	Sptbn4	

DMR1:84228001	1	84228001	84229000	1000	1	1.00E-15	1.18	32	3.2	Sptbn4	
DMR1:84249001	1	84249001	84251000	2000	1	1.50E-08	0.54	39	1.95	Sptbn4;Blvrb	Metabolism
DMR1:84259001	1	84259001	84260000	1000	1	5.50E-07	0.43	13	1.3	Sptbn4;Blvrb	Metabolism
DMR1:84320001	1	84320001	84321000	1000	1	1.80E-09	0.69	25	2.5	Prx;LOC102552059;Hipk4	
DMR1:84335001	1	84335001	84337000	2000	1	5.10E-12	0.72	71	3.55	Hipk4;Pld3	Metabolism
DMR1:84338001	1	84338001	84342000	4000	1	2.40E-08	0.51	74	1.85	Hipk4;Pld3	Metabolism
DMR1:84833001	1	84833001	84837000	4000	2	3.10E-12	-0.6	23	0.58	Wdr87	
DMR1:84839001	1	84839001	84842000	3000	2	2.20E-13	-0.48	37	1.23	Wdr87	
DMR1:85026001	1	85026001	85029000	3000	2	1.50E-13	-0.51	63	2.1	Fcgbp	Extracellular Matrix
DMR1:85042001	1	85042001	85050000	8000	1	2.70E-07	-0.32	144	1.8	Fcgbp;Fcgbpl1	Extracellular Matrix
DMR1:85070001	1	85070001	85076000	6000	2	1.80E-18	1.3	125	2.08	Fcgbpl1	Extracellular Matrix
DMR1:85119001	1	85119001	85122000	3000	1	2.70E-07	0.44	66	2.2	Fbl;Dyrk1b	Translation
DMR1:85140001	1	85140001	85142000	2000	1	2.30E-08	-0.56	14	0.7	Acp7	Signaling
DMR1:85221001	1	85221001	85222000	1000	1	1.20E-08	-0.59	5	0.5	Nccrp1;Sycn	
DMR1:85230001	1	85230001	85233000	3000	2	1.40E-08	0.63	42	1.4	Sycn;Ifnl3	Cytokine
DMR1:85355001	1	85355001	85356000	1000	1	2.10E-10	0.53	21	2.1	Samd4b;Paf1	
DMR1:85516001	1	85516001	85517000	1000	1	2.80E-10	0.63	44	4.4	Eid2b;Eid2	
DMR1:86095001	1	86095001	86097000	2000	1	2.80E-08	-0.36	9	0.45	Vom2r10	Signaling
DMR1:86315001	1	86315001	86321000	6000	1	6.10E-07	-0.54	31	0.52	Vom2r-ps22	
DMR1:86352001	1	86352001	86353000	1000	1	1.30E-07	-0.48	2	0.2	Vom1r2	Receptor
DMR1:86601001	1	86601001	86603000	2000	1	5.50E-07	-0.47	5	0.25	Vom2r11	Signaling
DMR1:86878001	1	86878001	86882000	4000	1	1.40E-07	-0.38	50	1.25	LOC108349512;Fbxo27	
DMR1:86940001	1	86940001	86944000	4000	1	8.30E-12	0.64	78	1.95	Sars2;LOC102550248;Ccer2;Nfkbib;Sirt2	Translation
DMR1:86982001	1	86982001	86984000	2000	1	2.20E-08	-0.45	29	1.45	Rinl	Transcription
DMR1:86999001	1	86999001	87003000	4000	1	2.50E-07	-0.41	79	1.98	Hnrnpl;Ech1	Metabolism
DMR1:87044001	1	87044001	87048000	4000	1	2.80E-13	0.88	102	2.55	Lgals7	Extracellular Matrix
DMR1:87052001	1	87052001	87053000	1000	1	2.80E-10	0.61	24	2.4	Lgals7	Extracellular Matrix
DMR1:87069001	1	87069001	87071000	2000	1	1.40E-07	0.36	37	1.85	Capn12;LOC102550499;Actn4	Protease
DMR1:87138001	1	87138001	87139000	1000	1	2.00E-07	-0.38	15	1.5	Actn4;LOC102550585	
DMR1:87145001	1	87145001	87147000	2000	2	1.80E-10	-0.5	45	2.25	Actn4;LOC102550585	
DMR1:87157001	1	87157001	87160000	3000	1	2.60E-16	-0.67	71	2.37	Actn4;LOC102550585;LOC100909725	
DMR1:87210001	1	87210001	87212000	2000	1	1.20E-08	-0.5	17	0.85	Spint2	Protease; Proteolysis
DMR1:87239001	1	87239001	87240000	1000	1	7.80E-09	0.56	25	2.5	Ppp1r14a;Dpf1	Signaling;Epigenetic
DMR1:87255001	1	87255001	87258000	3000	1	5.30E-07	-0.39	86	2.87	Dpf1;Sipa1l3	Epigenetic;Signaling
DMR1:87352001	1	87352001	87353000	1000	1	2.10E-08	-0.53	30	3	Sipa1l3	Signaling
DMR1:87417001	1	87417001	87422000	5000	1	6.30E-08	-0.41	101	2.02	Sipa1l3;LOC108348907	Signaling
DMR1:87458001	1	87458001	87467000	9000	2	7.20E-16	-0.66	164	1.82	Sipa1l3	Signaling
DMR1:87477001	1	87477001	87478000	1000	1	1.70E-09	0.82	39	3.9	Sipa1l3;NEWGENE_1585333	Signaling
DMR1:87653001	1	87653001	87658000	5000	2	3.10E-08	-0.47	104	2.08	Zfp84;LOC102547413	Transcription
DMR1:87773001	1	87773001	87774000	1000	1	7.20E-07	-0.41	13	1.3	Zfp27	
DMR1:87958001	1	87958001	87960000	2000	1	5.00E-08	0.59	38	1.9	Map4k1;Ryr1	Ion Channel
DMR1:87965001	1	87965001	87966000	1000	1	2.40E-12	0.77	21	2.1	Map4k1;Ryr1	Ion Channel
DMR1:87984001	1	87984001	87988000	4000	1	3.50E-11	0.77	84	2.1	Ryr1	Ion Channel
DMR1:87994001	1	87994001	87995000	1000	1	2.30E-09	0.72	28	2.8	Ryr1	Ion Channel
DMR1:88011001	1	88011001	88016000	5000	1	5.20E-10	-0.36	93	1.86	Ryr1	Ion Channel
DMR1:88018001	1	88018001	88021000	3000	1	2.80E-10	0.65	64	2.13	Ryr1	Ion Channel
DMR1:88036001	1	88036001	88038000	2000	1	6.70E-08	0.4	45	2.25	Ryr1	Ion Channel
DMR1:88115001	1	88115001	88116000	1000	1	2.40E-18	1.21	43	4.3	Spred3;Ggn;Psmd8	Cytoskeleton;Protease
DMR1:88159001	1	88159001	88162000	3000	1	8.40E-10	-0.51	70	2.33	Catsperg1;Kcnk6	Transport
DMR1:88359001	1	88359001	88360000	1000	1	1.80E-07	-0.45	19	1.9	Zfp14	
DMR1:88554001	1	88554001	88555000	1000	1	3.50E-09	-0.54	13	1.3	Zfp420;LOC102550277	
DMR1:88600001	1	88600001	88602000	2000	1	1.40E-14	1.08	59	2.95	Zfp382;LOC100912964	
DMR1:88642001	1	88642001	88643000	1000	1	1.90E-09	-0.51	24	2.4	Cox7a1;LOC100912380;LOC108349558;LOC687527	
DMR1:88721001	1	88721001	88722000	1000	1	2.50E-07	0.51	18	1.8	Wdr62	
DMR1:88821001	1	88821001	88825000	4000	1	1.60E-12	0.83	123	3.08	Lrfn3	
DMR1:88829001	1	88829001	88831000	2000	1	5.80E-11	0.74	29	1.45	Lrfn3	
DMR1:88969001	1	88969001	88973000	4000	1	4.80E-10	0.67	71	1.77	Prodh2;RGD1560986;Arhgap33	Metabolism;Signaling

DMR1:88974001	1	88974001	88976000	2000	1	1.10E-07	0.51	37	1.85	Prodh2;RGD1560986;Arhgap33	Metabolism;Signaling
DMR1:88995001	1	88995001	88998000	3000	1	5.70E-12	0.78	57	1.9	Arhgap33;Proser3	Signaling
DMR1:89028001	1	89028001	89029000	1000	1	1.80E-07	0.46	15	1.5	U2af1l4;Igflr1;Kmt2b	Translation;Receptor;Epigenetic
DMR1:89053001	1	89053001	89054000	1000	1	6.90E-08	-0.64	21	2.1	Zbtb32;Upk1a	Transcription
DMR1:89094001	1	89094001	89097000	3000	1	1.00E-08	0.62	43	1.43	LOC688869;Etv2;Rbm42	Transcription
DMR1:89131001	1	89131001	89133000	2000	1	1.80E-07	0.48	24	1.2	Haus5;LOC100912333;LOC688924	
DMR1:89156001	1	89156001	89158000	2000	1	5.20E-07	0.59	35	1.75	LOC688924;Atp4a	Transport
DMR1:89165001	1	89165001	89166000	1000	1	3.00E-10	0.68	43	4.3	Atp4a	Transport
DMR1:89235001	1	89235001	89236000	1000	1	2.20E-16	1.15	26	2.6	Dmkn;Krtdap	
DMR1:89259001	1	89259001	89262000	3000	2	2.10E-10	0.93	61	2.03	LOC100361079;Ffar2	Translation
DMR1:89280001	1	89280001	89281000	1000	1	5.00E-08	0.53	13	1.3	Ffar2;LOC102554169	
DMR1:89312001	1	89312001	89318000	6000	2	1.50E-15	0.94	117	1.95	Ffar3;Ffar1;Cd22	
DMR1:89346001	1	89346001	89347000	1000	1	2.70E-09	0.78	24	2.4	LOC102554355;Mag;LOC108349549	Immune
DMR1:89420001	1	89420001	89422000	2000	1	4.20E-07	0.45	26	1.3	Fam187b	Immune
DMR1:89502001	1	89502001	89503000	1000	1	2.30E-08	0.56	20	2	Lgi4;Fxyd3	Transport
DMR1:89533001	1	89533001	89535000	2000	1	4.40E-10	0.81	44	2.2	Hpn	Protease
DMR1:89629001	1	89629001	89634000	5000	2	1.10E-10	0.84	80	1.6	LOC108349560;RGD1561430	
DMR1:89644001	1	89644001	89646000	2000	1	1.70E-07	0.69	28	1.4	RGD1561430;RGD1563307	Epigenetic
DMR1:89733001	1	89733001	89734000	1000	1	1.70E-08	-0.42	11	1.1	Scgb2b2	
DMR1:89946001	1	89946001	89947000	1000	1	9.60E-07	0.37	12	1.2	LOC494538;Scgb1b30	
DMR1:89984001	1	89984001	89987000	3000	1	4.50E-08	-0.45	19	0.63	Wtip	
DMR1:90002001	1	90002001	90003000	1000	1	9.60E-08	0.39	11	1.1	Wtip;Uba2	Proteolysis
DMR1:90073001	1	90073001	90075000	2000	1	9.20E-13	-0.47	44	2.2	Gpi	Metabolism
DMR1:90533001	1	90533001	90534000	1000	1	8.30E-09	-0.39	6	0.6	Chst8	Transport
DMR1:90596001	1	90596001	90598000	2000	1	1.80E-07	0.34	26	1.3	Chst8	Transport
DMR1:90616001	1	90616001	90617000	1000	1	3.30E-16	0.73	24	2.4	Chst8	Transport
DMR1:90641001	1	90641001	90642000	1000	1	6.40E-09	-0.47	30	3	Chst8	Transport
DMR1:90657001	1	90657001	90664000	7000	2	2.20E-17	1.08	141	2.01	Chst8	Transport
DMR1:90726001	1	90726001	90729000	3000	1	6.20E-07	-0.4	49	1.63	RGD1560088	
DMR1:91083001	1	91083001	91084000	1000	1	4.40E-07	-0.46	25	2.5	Pepd;LOC687508	Protease;Metabolism
DMR1:91193001	1	91193001	91195000	2000	2	4.60E-07	-0.5	29	1.45	Pepd	Protease
DMR1:91221001	1	91221001	91225000	4000	1	3.10E-08	0.51	43	1.07	Pepd;LOC100912350	Protease
DMR1:91236001	1	91236001	91239000	3000	2	4.20E-08	0.52	42	1.4	Pepd	Protease
DMR1:91255001	1	91255001	91259000	4000	1	2.30E-07	-0.31	86	2.15	Pepd	Protease
DMR1:91442001	1	91442001	91443000	1000	1	5.30E-07	0.57	18	1.8	Slc7a10;Lrp3	Transport;Binding Proteins
DMR1:91463001	1	91463001	91466000	3000	1	4.20E-07	0.41	42	1.4	Lrp3	Binding Proteins
DMR1:91467001	1	91467001	91471000	4000	1	5.40E-07	0.4	72	1.8	Lrp3	Binding Proteins
DMR1:91472001	1	91472001	91474000	2000	1	2.70E-07	-0.27	40	2	Lrp3	Binding Proteins
DMR1:91497001	1	91497001	91501000	4000	1	3.80E-07	-0.36	74	1.85	Wdr88	
DMR1:91526001	1	91526001	91528000	2000	1	2.00E-13	0.96	50	2.5	Wdr88;Gpatch1	
DMR1:91540001	1	91540001	91541000	1000	1	2.80E-08	0.62	12	1.2	Gpatch1	
DMR1:91569001	1	91569001	91571000	2000	1	2.00E-08	0.49	22	1.1	Gpatch1	
DMR1:91598001	1	91598001	91599000	1000	1	3.40E-11	0.59	21	2.1	LOC687679;Rhpn2	Cytoskeleton
DMR1:91606001	1	91606001	91609000	3000	1	8.60E-07	0.52	33	1.1	Rhpn2	Cytoskeleton
DMR1:91620001	1	91620001	91621000	1000	1	2.40E-11	0.38	10	1	Rhpn2	Cytoskeleton
DMR1:91852001	1	91852001	91853000	1000	1	4.70E-07	0.63	16	1.6	Nudt19;Rgs9bp;Ankrd27	
DMR1:91957001	1	91957001	91959000	2000	1	2.30E-08	-0.46	49	2.45	Ankrd27;Pdcd5	Signaling
DMR1:91991001	1	91991001	91994000	3000	1	3.20E-09	0.78	52	1.73	Dpy19l3	
DMR1:91998001	1	91998001	91999000	1000	1	2.60E-11	0.82	21	2.1	Dpy19l3	
DMR1:92051001	1	92051001	92052000	1000	1	4.20E-07	-0.39	15	1.5	Dpy19l3	
DMR1:92061001	1	92061001	92063000	2000	1	4.50E-14	-0.47	49	2.45	Dpy19l3	
DMR1:92101001	1	92101001	92102000	1000	1	2.10E-07	-0.59	6	0.6	Zfp507	Transcription
DMR1:92702001	1	92702001	92704000	2000	1	3.00E-07	-0.42	31	1.55	Tshz3	Transcription
DMR1:92728001	1	92728001	92730000	2000	1	2.90E-07	0.37	34	1.7	Tshz3	Transcription
DMR1:92761001	1	92761001	92766000	5000	2	2.10E-10	-0.5	102	2.04	Tshz3;LOC108349564	Transcription
DMR1:92770001	1	92770001	92771000	1000	1	9.00E-07	-0.43	13	1.3	Tshz3;LOC108349564	Transcription
DMR1:93233001	1	93233001	93235000	2000	1	6.50E-09	0.52	35	1.75	RGD1562402	Translation

DMR1:93251001	1	93251001	93252000	1000	1	4.40E-10	0.39	7	0.7	RGD1562402	Translation
DMR1:93720001	1	93720001	93725000	5000	1	1.00E-07	0.5	65	1.3	Zfp536	Transcription
DMR1:93730001	1	93730001	93732000	2000	1	2.60E-09	0.6	48	2.4	Zfp536	Transcription
DMR1:93739001	1	93739001	93742000	3000	1	1.60E-13	0.51	58	1.93	Zfp536	Transcription
DMR1:93753001	1	93753001	93756000	3000	1	2.50E-12	0.87	76	2.53	Zfp536	Transcription
DMR1:93757001	1	93757001	93758000	1000	1	6.30E-10	-0.72	5	0.5	Zfp536	Transcription
DMR1:93783001	1	93783001	93785000	2000	1	9.00E-08	-0.31	32	1.6	Zfp536;LOC108349103	Transcription
DMR1:93811001	1	93811001	93813000	2000	1	4.00E-08	0.45	42	2.1	Zfp536;LOC108349103	Transcription
DMR1:93883001	1	93883001	93887000	4000	1	1.20E-07	-0.36	48	1.2	Zfp536	Transcription
DMR1:93895001	1	93895001	93898000	3000	1	9.50E-09	-0.42	54	1.8	Zfp536	Transcription
DMR1:93942001	1	93942001	93947000	5000	1	4.50E-10	-0.45	93	1.86	Zfp536	Transcription
DMR1:93963001	1	93963001	93970000	7000	1	7.70E-15	0.92	124	1.77	Zfp536	Transcription
DMR1:93973001	1	93973001	93975000	2000	1	1.50E-14	1.09	44	2.2	Zfp536	Transcription
DMR1:94029001	1	94029001	94031000	2000	2	3.90E-07	0.51	29	1.45	Zfp536	Transcription
DMR1:94155001	1	94155001	94156000	1000	1	5.20E-09	0.52	30	3	Zfp536	Transcription
DMR1:94336001	1	94336001	94338000	2000	1	1.50E-10	-0.43	26	1.3	LOC108349449;Uri1	Epigenetic
DMR1:94614001	1	94614001	94615000	1000	1	3.30E-10	-0.46	18	1.8	Plekhf1;LOC108349450	
DMR1:97705001	1	97705001	97706000	1000	1	9.20E-10	-0.48	5	0.5	LOC690265;MGC114499	
DMR1:98405001	1	98405001	98408000	3000	1	5.80E-08	-0.37	18	0.6	Cd33;Siglecl1	Immune
DMR1:98466001	1	98466001	98467000	1000	1	2.50E-07	0.42	15	1.5	Iglon5;Vsig10l;Etfb	Immune;Metabolism
DMR1:98473001	1	98473001	98475000	2000	1	1.30E-07	0.43	24	1.2	Vsig10l;Etfb	Metabolism
DMR1:98513001	1	98513001	98514000	1000	1	2.50E-07	0.42	15	1.5	C1H19orf84;LOC102550061;Siglec10	Immune
DMR1:98560001	1	98560001	98563000	3000	2	2.60E-15	-0.98	11	0.37	Siglec5	Immune
DMR1:98978001	1	98978001	98986000	8000	2	1.90E-08	-0.38	89	1.11	Vom2r38;Vom2r37	Signaling
DMR1:99028001	1	99028001	99029000	1000	1	1.90E-11	0.64	10	1	Vom2r38;Vom2r37	Signaling
DMR1:99053001	1	99053001	99057000	4000	4	7.90E-12	0.45	49	1.23	Vom2r38;Vom2r37;LOC100911353;LOC108349760	Signaling
DMR1:99079001	1	99079001	99080000	1000	1	1.40E-13	0.29	19	1.9	Vom2r38;Vom2r37;LOC108349760;LOC103691104	Signaling
DMR1:99081001	1	99081001	99083000	2000	1	2.00E-07	0.32	17	0.85	Vom2r38;Vom2r37;LOC108349760;LOC103691104	Signaling
DMR1:99116001	1	99116001	99118000	2000	1	1.40E-08	-0.31	30	1.5	Vom2r38;Vom2r37;LOC100912942;LOC108349568	Signaling
DMR1:99573001	1	99573001	99583000	10000	1	5.00E-10	0.45	131	1.31	Ceacam18;Siglec8	Immune
DMR1:99595001	1	99595001	99596000	1000	1	1.40E-07	0.58	15	1.5	Siglec8;Ctu1	Immune
DMR1:99602001	1	99602001	99603000	1000	1	1.50E-13	1.03	51	5.1	Ctu1	
DMR1:99664001	1	99664001	99666000	2000	1	1.00E-09	-0.61	15	0.75	Klk13	Protease
DMR1:99736001	1	99736001	99739000	3000	1	6.00E-12	0.85	40	1.33	Klk9;Klk8	Protease
DMR1:99745001	1	99745001	99748000	3000	1	2.60E-07	0.37	56	1.87	Klk8;Klk7	Protease
DMR1:99820001	1	99820001	99821000	1000	1	4.50E-08	0.6	22	2.2	Klk4	Protease
DMR1:100133001	1	100133001	100134000	1000	1	8.10E-07	-0.28	10	1	LOC690730;Klk1;LOC108349762;Klk1c3	Protease
DMR1:100166001	1	100166001	100168000	2000	1	2.60E-16	0.69	27	1.35	Klk1c3;LOC108348116;LOC108349569;LOC103691133	Protease
DMR1:100210001	1	100210001	100212000	2000	1	1.10E-07	0.38	36	1.8	Klk1b3;LOC102552731;LOC102552485;Fv1	
DMR1:100244001	1	100244001	100247000	3000	1	3.70E-10	-0.45	48	1.6	Acpt;LOC103691109;LOC102552286;Gpr32	
DMR1:100250001	1	100250001	100251000	1000	1	4.10E-11	0.93	24	2.4	Gpr32	
DMR1:100404001	1	100404001	100406000	2000	1	1.60E-07	0.62	57	2.85	Syt3	Transport
DMR1:100446001	1	100446001	100450000	4000	1	1.80E-07	-0.52	73	1.82	Lrrc4b	
DMR1:100584001	1	100584001	100586000	2000	2	4.20E-15	0.77	42	2.1	Napsa;Kcnc3	Transport
DMR1:100598001	1	100598001	100599000	1000	1	5.30E-09	0.7	38	3.8	Napsa;Kcnc3;Myh14	Transport
DMR1:100637001	1	100637001	100641000	4000	1	2.00E-07	0.35	91	2.28	Myh14	
DMR1:100656001	1	100656001	100660000	4000	1	6.60E-09	0.56	56	1.4	Myh14	
DMR1:100731001	1	100731001	100734000	3000	1	7.80E-09	-0.39	16	0.53	Izumo2;Zfp473	Transcription
DMR1:100743001	1	100743001	100745000	2000	1	6.10E-08	0.51	23	1.15	Zfp473	Transcription
DMR1:100804001	1	100804001	100805000	1000	1	2.10E-08	0.47	9	0.9	Atf5;Nup62;Il4i1	Transcription;Transport;Metabolism
DMR1:100847001	1	100847001	100848000	1000	1	9.90E-09	0.54	14	1.4	Tbc1d17;Akt1s1;Pnkp	Signaling
DMR1:100944001	1	100944001	100945000	1000	1	2.40E-11	0.44	13	1.3	Tsks	

DMR1:100947001	1	100947001	100948000	1000	1	1.00E-07	0.59	17	1.7	Tsks;Cpt1c	Metabolism
DMR1:100949001	1	100949001	100954000	5000	2	1.10E-13	0.95	97	1.94	Tsks;Cpt1c	Metabolism
DMR1:101002001	1	101002001	101003000	1000	1	3.80E-09	-0.53	85	8.5	Bcl2l12;Irf3;Scaf1;Rras	Transcription;Translation;Signaling
DMR1:101076001	1	101076001	101080000	4000	1	4.80E-09	0.64	78	1.95	Nosip;Rcn3;Fcgrt	Signaling;Immune
DMR1:101153001	1	101153001	101157000	4000	1	4.70E-08	0.51	57	1.43	Aldh16a1;Pih1d1;LOC108349571;Slc17a7	Metabolism;Transport
DMR1:101164001	1	101164001	101166000	2000	1	6.50E-07	-0.37	46	2.3	Pih1d1;LOC108349571;Slc17a7;Gfy;Pth2	Transport
DMR1:101169001	1	101169001	101171000	2000	1	3.90E-07	0.42	54	2.7	LOC108349571;Slc17a7;Gfy;Pth2;Ccdc155	Transport
DMR1:101229001	1	101229001	101231000	2000	1	2.50E-09	0.53	45	2.25	Tead2;Cd37;Slc6a16	Transcription;Transport
DMR1:101237001	1	101237001	101238000	1000	1	2.30E-17	1.01	34	3.4	Tead2;Cd37;Slc6a16	Transcription;Transport
DMR1:101291001	1	101291001	101292000	1000	1	1.70E-08	0.55	23	2.3	LOC108349572;Trpm4	Transport
DMR1:101294001	1	101294001	101300000	6000	2	3.40E-09	0.75	132	2.2	LOC108349572;Trpm4	Transport
DMR1:101325001	1	101325001	101327000	2000	1	4.90E-09	-0.36	41	2.05	Trpm4;Hrc;Ppfia3	Transport
DMR1:101345001	1	101345001	101347000	2000	1	4.50E-10	0.57	49	2.45	Ppfia3	
DMR1:101367001	1	101367001	101369000	2000	1	3.00E-08	-0.41	73	3.65	Ppfia3;Lin7b;Snrnp70	Cytoskeleton;Translation
DMR1:101411001	1	101411001	101416000	5000	1	7.30E-11	0.67	102	2.04	Kcna7;Ntf4;Lhb;Ruvbl2	Transport;Growth Factors;Hormone
DMR1:101426001	1	101426001	101427000	1000	1	1.90E-08	-0.53	28	2.8	Ruvbl2;Gys1	
DMR1:101503001	1	101503001	101504000	1000	1	7.90E-16	1.18	28	2.8	Tulp2;Nucb1;Ppp1r15a	Signaling
DMR1:101535001	1	101535001	101538000	3000	1	2.20E-07	0.67	62	2.07	Plekha4;Hsd17b14	Metabolism
DMR1:101563001	1	101563001	101564000	1000	1	6.00E-07	-0.35	18	1.8	LOC102556190;Bcat2	Metabolism
DMR1:101582001	1	101582001	101583000	1000	1	1.20E-10	0.73	18	1.8	Bcat2	Metabolism
DMR1:101586001	1	101586001	101588000	2000	1	1.90E-07	0.45	38	1.9	Fgf21	Growth Factors
DMR1:101632001	1	101632001	101634000	2000	1	2.80E-15	1.06	54	2.7	Mamstr;Fut2	Golgi
DMR1:101675001	1	101675001	101677000	2000	1	1.40E-07	0.45	22	1.1	Sec1;Ntn5;Car11	Golgi;Extracellular Matrix
DMR1:101698001	1	101698001	101699000	1000	1	3.90E-09	-0.49	21	2.1	Dbp;Sphk2;Rpl18;Fam83e;Spaca4	Transcription;Signaling;Translation
DMR1:101727001	1	101727001	101729000	2000	1	1.60E-07	-0.37	32	1.6	Sult2b1	Transport
DMR1:101779001	1	101779001	101783000	4000	2	2.80E-28	1.54	108	2.7	Sult2b1;Lmtk3	Transport
DMR1:101784001	1	101784001	101786000	2000	1	5.60E-19	1.22	57	2.85	Sult2b1;Lmtk3	Transport
DMR1:101787001	1	101787001	101788000	1000	1	2.20E-09	0.69	59	5.9	Lmtk3;Cyth2	Transcription
DMR1:101789001	1	101789001	101791000	2000	1	1.90E-16	1.12	57	2.85	Lmtk3;Cyth2	Transcription
DMR1:101824001	1	101824001	101828000	4000	1	8.50E-07	0.51	43	1.07	Grwd1;Grin2d	Receptor
DMR1:101848001	1	101848001	101849000	1000	1	1.20E-12	0.67	40	4	Grin2d	Receptor
DMR1:101911001	1	101911001	101912000	1000	1	8.70E-12	0.62	35	3.5	Emp3;Ccdc114	Cytoskeleton
DMR1:101925001	1	101925001	101927000	2000	2	3.00E-12	0.82	43	2.15	Ccdc114	
DMR1:101940001	1	101940001	101942000	2000	1	7.00E-07	-0.4	55	2.75	Ccdc114	
DMR1:101959001	1	101959001	101963000	4000	1	5.50E-07	0.43	68	1.7	Abcc6	Transport
DMR1:102040001	1	102040001	102044000	4000	1	4.40E-08	0.53	76	1.9	Nomo1	
DMR1:102066001	1	102066001	102069000	3000	1	2.30E-07	0.4	69	2.3	Nomo1;LOC103691113;Ncr3lg1	
DMR1:102070001	1	102070001	102075000	5000	1	4.40E-10	-0.37	45	0.9	Nomo1;Ncr3lg1	
DMR1:102100001	1	102100001	102109000	9000	3	2.40E-16	1.03	221	2.46	Ncr3lg1;LOC102550828;Kcnj11;Abcc8	Transport;Transport
DMR1:102185001	1	102185001	102187000	2000	2	4.40E-13	0.85	15	0.75	Abcc8	Transport
DMR1:102223001	1	102223001	102225000	2000	1	5.50E-07	0.44	31	1.55	Ush1c	Cytoskeleton
DMR1:102242001	1	102242001	102245000	3000	1	7.80E-08	0.58	49	1.63	Ush1c	Cytoskeleton
DMR1:102295001	1	102295001	102296000	1000	1	4.40E-08	-0.4	15	1.5	Otog;LOC103691114	Extracellular Matrix
DMR1:102431001	1	102431001	102435000	4000	1	2.30E-16	-0.64	81	2.02	Kcnc1	Transport
DMR1:102445001	1	102445001	102455000	10000	3	7.30E-10	0.75	210	2.1	Kcnc1;Sergef	Transport;Proteolysis
DMR1:102541001	1	102541001	102550000	9000	1	6.20E-08	-0.34	113	1.26	Sergef	Proteolysis
DMR1:102730001	1	102730001	102733000	3000	2	9.30E-08	-0.42	72	2.4	LOC691143;Saal1	Binding Proteins
DMR1:102742001	1	102742001	102744000	2000	1	7.80E-07	-0.36	43	2.15	LOC691143;Saal1	Binding Proteins
DMR1:102765001	1	102765001	102767000	2000	1	4.70E-11	-0.46	88	4.4	LOC108349573;Saa4	
DMR1:102961001	1	102961001	102963000	2000	1	9.70E-12	-0.59	27	1.35	Tsg101	Proteolysis

DMR1:103218001	1	103218001	103219000	1000	1	1.00E-09	-0.74	3	0.3	Ptpn5	
DMR1:103236001	1	103236001	103237000	1000	1	1.00E-10	-0.54	21	2.1	Ptpn5	
DMR1:103297001	1	103297001	103300000	3000	2	1.40E-18	1.03	64	2.13	Mrgprx3	Signaling
DMR1:103732001	1	103732001	103735000	3000	1	3.70E-07	-0.41	11	0.37	Mrgprx2	Signaling
DMR1:104170001	1	104170001	104172000	2000	1	7.40E-07	-0.42	23	1.15	Csrp3	Cytoskeleton
DMR1:104212001	1	104212001	104214000	2000	1	1.20E-10	0.31	7	0.35	E2f8	Transcription
DMR1:104630001	1	104630001	104635000	5000	1	3.70E-09	-0.47	69	1.38	Nav2	
DMR1:104640001	1	104640001	104643000	3000	1	1.40E-07	-0.43	57	1.9	Nav2	
DMR1:104773001	1	104773001	104775000	2000	2	6.30E-15	0.43	43	2.15	Nav2	
DMR1:104815001	1	104815001	104816000	1000	1	7.60E-08	-0.41	12	1.2	Nav2	
DMR1:104842001	1	104842001	104844000	2000	1	4.40E-07	-0.33	38	1.9	Nav2	
DMR1:104856001	1	104856001	104859000	3000	1	7.80E-10	-0.48	63	2.1	Nav2	
DMR1:105166001	1	105166001	105168000	2000	1	1.10E-07	0.54	21	1.05	Prmt3	Golgi
DMR1:105184001	1	105184001	105187000	3000	1	2.50E-07	0.41	31	1.03	Prmt3	Golgi
DMR1:105196001	1	105196001	105199000	3000	1	8.00E-08	0.5	34	1.13	Prmt3	Golgi
DMR1:105350001	1	105350001	105352000	2000	1	3.50E-08	-0.47	14	0.7	Nell1	Signaling
DMR1:105477001	1	105477001	105480000	3000	1	1.90E-07	-0.38	30	1	Nell1	Signaling
DMR1:105589001	1	105589001	105590000	1000	1	4.20E-07	0.44	13	1.3	Nell1	Signaling
DMR1:105776001	1	105776001	105780000	4000	1	6.60E-10	-0.53	58	1.45	Nell1	Signaling
DMR1:105896001	1	105896001	105897000	1000	1	2.20E-08	-0.53	5	0.5	Nell1	Signaling
DMR1:106006001	1	106006001	106009000	3000	1	2.30E-07	0.32	32	1.07	Nell1	Signaling
DMR1:106032001	1	106032001	106034000	2000	1	3.00E-09	-0.57	15	0.75	Nell1	Signaling
DMR1:106163001	1	106163001	106168000	5000	1	6.80E-08	0.53	54	1.08	Nell1	Signaling
DMR1:106984001	1	106984001	106985000	1000	1	5.00E-07	0.47	6	0.6	Slc17a6	Transport
DMR1:107241001	1	107241001	107242000	1000	1	2.20E-07	0.55	13	1.3	Fancf;Gas2	
DMR1:111867001	1	111867001	111869000	2000	1	7.80E-08	-0.45	6	0.3	Luzp2	
DMR1:112372001	1	112372001	112374000	2000	1	7.90E-07	-0.67	11	0.55	Gabrg3	Ion Channel
DMR1:112427001	1	112427001	112428000	1000	1	1.40E-07	-0.6	6	0.6	Gabrg3	Ion Channel
DMR1:112785001	1	112785001	112788000	3000	1	4.40E-07	-0.36	25	0.83	Gabrg3	Ion Channel
DMR1:112805001	1	112805001	112807000	2000	1	3.00E-08	-0.5	16	0.8	Gabrg3	Ion Channel
DMR1:113179001	1	113179001	113181000	2000	1	3.60E-07	0.44	27	1.35	Gabrb3	Ion Channel
DMR1:113476001	1	113476001	113477000	1000	1	1.20E-07	0.65	11	1.1	Luzp2	
DMR1:114089001	1	114089001	114093000	4000	1	7.10E-07	-0.33	40	1	Siglech	
DMR1:114139001	1	114139001	114141000	2000	1	4.60E-10	-0.43	21	1.05	Siglech;LOC108349722	
DMR1:114267001	1	114267001	114268000	1000	1	4.80E-15	0.8	22	2.2	Cyfip1	Cytoskeleton
DMR1:114532001	1	114532001	114534000	2000	1	3.70E-08	-0.6	13	0.65	Herc2	Transcription
DMR1:116008001	1	116008001	116009000	1000	1	1.10E-08	-0.39	8	0.8	Atp10a	Transport
DMR1:116083001	1	116083001	116086000	3000	1	4.50E-07	-0.27	27	0.9	Atp10a	Transport
DMR1:116114001	1	116114001	116115000	1000	1	2.50E-11	0.71	16	1.6	Atp10a	Transport
DMR1:116632001	1	116632001	116634000	2000	1	2.20E-26	0.93	19	0.95	Ube3a	Proteolysis
DMR1:116666001	1	116666001	116669000	3000	1	3.50E-07	-0.53	10	0.33	Ube3a;LOC103691125	Proteolysis
DMR1:120234001	1	120234001	120235000	1000	1	3.30E-08	-0.63	2	0.2	RGD1560408	
DMR1:123924001	1	123924001	123926000	2000	1	3.30E-11	-0.48	20	1	Chrna7	Ion Channel
DMR1:123978001	1	123978001	123979000	1000	1	1.70E-09	0.66	7	0.7	Chrna7	Ion Channel
DMR1:124022001	1	124022001	124026000	4000	1	5.10E-08	-0.5	23	0.58	Chrna7	Ion Channel
DMR1:124484001	1	124484001	124486000	2000	2	1.10E-19	-0.89	13	0.65	Otud7a;Hmgn5b	Protease
DMR1:124508001	1	124508001	124510000	2000	1	4.30E-07	0.43	21	1.05	Otud7a	Protease
DMR1:124784001	1	124784001	124786000	2000	1	4.40E-07	-0.47	23	1.15	Klf13	Transcription
DMR1:125033001	1	125033001	125036000	3000	1	3.80E-10	-0.53	36	1.2	Trpm1;Mir211	Transport
DMR1:125084001	1	125084001	125085000	1000	1	2.90E-16	-0.67	13	1.3	Trpm1	Transport
DMR1:125091001	1	125091001	125093000	2000	1	9.00E-07	-0.42	20	1	Trpm1	Transport
DMR1:125246001	1	125246001	125248000	2000	1	2.00E-08	0.7	31	1.55	Mcee	Metabolism
DMR1:125398001	1	125398001	125402000	4000	1	1.80E-08	-0.36	57	1.43	Apba2	Transport
DMR1:125546001	1	125546001	125548000	2000	1	2.00E-07	0.51	29	1.45	Apba2;Fam189a1	Transport
DMR1:125552001	1	125552001	125554000	2000	1	2.10E-10	0.67	28	1.4	Apba2;Fam189a1	Transport
DMR1:125636001	1	125636001	125640000	4000	1	1.50E-10	0.58	69	1.73	Fam189a1	
DMR1:125669001	1	125669001	125671000	2000	1	5.00E-07	-0.48	38	1.9	Fam189a1;Ndnl2	
DMR1:125750001	1	125750001	125751000	1000	1	4.80E-15	0.91	30	3	Fam189a1	
DMR1:125754001	1	125754001	125756000	2000	1	1.60E-11	-0.49	44	2.2	Fam189a1	
DMR1:125780001	1	125780001	125786000	6000	1	4.50E-08	-0.57	60	1	Fam189a1	
DMR1:125788001	1	125788001	125790000	2000	1	3.20E-07	-0.35	33	1.65	Fam189a1	
DMR1:125948001	1	125948001	125950000	2000	1	2.20E-08	-0.41	18	0.9	Fam189a1	

DMR1:126194001	1	126194001	126195000	1000	1	1.70E-07	-0.39	15	1.5	Tjp1	Cell Junction
DMR1:126344001	1	126344001	126345000	1000	1	8.90E-10	-0.52	23	2.3	Tjp1	Cell Junction
DMR1:126371001	1	126371001	126376000	5000	2	4.10E-14	0.84	84	1.68	Tjp1	Cell Junction
DMR1:126385001	1	126385001	126387000	2000	1	2.30E-07	-0.35	52	2.6	Tjp1	Cell Junction
DMR1:126390001	1	126390001	126391000	1000	1	4.90E-08	-0.41	15	1.5	Tjp1	Cell Junction
DMR1:126413001	1	126413001	126418000	5000	1	2.40E-08	-0.41	52	1.04	Tjp1	Cell Junction
DMR1:126436001	1	126436001	126442000	6000	1	4.60E-07	-0.33	76	1.27	Tjp1	Cell Junction
DMR1:126545001	1	126545001	126546000	1000	1	9.20E-07	0.38	15	1.5	Tarsl2	Translation
DMR1:126752001	1	126752001	126754000	2000	1	5.10E-07	-0.37	34	1.7	Pcsk6	Protease
DMR1:126757001	1	126757001	126763000	6000	1	2.20E-07	0.59	74	1.23	Pcsk6;LOC108349587	Protease
DMR1:126775001	1	126775001	126776000	1000	1	8.00E-10	0.39	14	1.4	Pcsk6;LOC108349587	Protease
DMR1:126789001	1	126789001	126792000	3000	1	6.30E-07	0.38	47	1.57	Pcsk6	Protease
DMR1:126889001	1	126889001	126895000	6000	2	1.70E-08	-0.39	64	1.07	Pcsk6	Protease
DMR1:127242001	1	127242001	127243000	1000	1	3.10E-10	-0.67	6	0.6	Lrrk1	Signaling
DMR1:127247001	1	127247001	127248000	1000	1	1.20E-07	0.46	17	1.7	Lrrk1	Signaling
DMR1:127260001	1	127260001	127262000	2000	1	3.60E-10	-0.57	42	2.1	Lrrk1	Signaling
DMR1:127267001	1	127267001	127270000	3000	1	7.00E-07	-0.45	46	1.53	Lrrk1	Signaling
DMR1:127285001	1	127285001	127286000	1000	1	1.30E-07	-0.33	17	1.7	Lrrk1	Signaling
DMR1:127308001	1	127308001	127309000	1000	1	1.90E-09	-0.46	17	1.7	Lrrk1;Aldh1a3	Signaling;Metabolism
DMR1:127535001	1	127535001	127536000	1000	1	1.90E-07	-0.4	12	1.2	Asb7	Transport
DMR1:127589001	1	127589001	127590000	1000	1	3.10E-18	-0.75	23	2.3	Asb7;Lins1	Transport
DMR1:127808001	1	127808001	127811000	3000	2	4.20E-08	0.46	40	1.33	Adamts17	Protease
DMR1:127916001	1	127916001	127920000	4000	1	5.00E-07	-0.36	27	0.68	Adamts17	Protease
DMR1:127925001	1	127925001	127927000	2000	1	4.60E-08	-0.36	21	1.05	Adamts17	Protease
DMR1:127962001	1	127962001	127965000	3000	1	8.10E-08	0.47	34	1.13	Adamts17	Protease
DMR1:128009001	1	128009001	128013000	4000	2	7.90E-08	0.53	50	1.25	Adamts17	Protease
DMR1:128028001	1	128028001	128032000	4000	1	7.70E-08	0.58	54	1.35	Adamts17	Protease
DMR1:128196001	1	128196001	128197000	1000	1	9.10E-12	0.61	20	2	Lysmd4	
DMR1:128255001	1	128255001	128256000	1000	1	2.10E-11	-0.47	9	0.9	Mef2a	Transcription
DMR1:128598001	1	128598001	128600000	2000	1	4.20E-07	-0.35	32	1.6	Lrrc28;Ttc23	
DMR1:128607001	1	128607001	128611000	4000	1	1.70E-11	-0.46	66	1.65	Lrrc28;Ttc23;LOC108349234	
DMR1:128620001	1	128620001	128622000	2000	1	1.60E-07	0.34	29	1.45	Ttc23;LOC108349234	
DMR1:128651001	1	128651001	128653000	2000	1	4.80E-08	-0.47	36	1.8	Ttc23	
DMR1:128696001	1	128696001	128699000	3000	1	2.80E-07	-0.32	75	2.5	Ttc23;Synm	
DMR1:128927001	1	128927001	128933000	6000	2	1.40E-10	-0.42	113	1.88	Igf1r	Receptor
DMR1:128941001	1	128941001	128944000	3000	2	2.50E-14	-0.62	88	2.93	Igf1r	Receptor
DMR1:128949001	1	128949001	128951000	2000	1	3.30E-14	-0.72	35	1.75	Igf1r	Receptor
DMR1:128962001	1	128962001	128964000	2000	1	2.80E-09	-0.36	23	1.15	Igf1r	Receptor
DMR1:128967001	1	128967001	128970000	3000	1	2.70E-09	-0.52	31	1.03	Igf1r	Receptor
DMR1:128971001	1	128971001	128972000	1000	1	2.10E-08	0.49	16	1.6	Igf1r	Receptor
DMR1:128985001	1	128985001	128987000	2000	1	3.00E-07	-0.36	45	2.25	Igf1r	Receptor
DMR1:129021001	1	129021001	129022000	1000	1	1.70E-07	-0.45	16	1.6	Igf1r	Receptor
DMR1:129045001	1	129045001	129051000	6000	2	2.10E-09	-0.45	106	1.77	Igf1r	Receptor
DMR1:129058001	1	129058001	129060000	2000	1	2.20E-07	-0.4	38	1.9	Igf1r	Receptor
DMR1:129093001	1	129093001	129099000	6000	1	4.40E-08	-0.38	99	1.65	Igf1r	Receptor
DMR1:129105001	1	129105001	129106000	1000	1	4.00E-09	-0.64	9	0.9	Igf1r	Receptor
DMR1:129164001	1	129164001	129165000	1000	1	5.80E-08	-0.51	36	3.6	Igf1r	Receptor
DMR1:129201001	1	129201001	129202000	1000	1	7.10E-07	0.45	13	1.3	Igf1r	Receptor
DMR1:129253001	1	129253001	129255000	2000	2	2.60E-11	0.95	56	2.8	Pgpep1l;Fam169b	Protease
DMR1:129759001	1	129759001	129760000	1000	1	2.30E-08	0.34	9	0.9	Arrdc4	
DMR1:133330001	1	133330001	133331000	1000	1	6.40E-08	0.5	15	1.5	Mctp2	
DMR1:133471001	1	133471001	133473000	2000	1	5.10E-14	0.76	41	2.05	Mctp2	
DMR1:133529001	1	133529001	133535000	6000	1	1.80E-08	-0.4	46	0.77	Mctp2	
DMR1:134830001	1	134830001	134834000	4000	1	3.70E-09	-0.43	36	0.9	Chd2	
DMR1:135103001	1	135103001	135106000	3000	1	1.40E-10	-0.45	28	0.93	Fam174b	
DMR1:135135001	1	135135001	135136000	1000	1	4.50E-07	-0.56	8	0.8	Fam174b	
DMR1:135172001	1	135172001	135175000	3000	1	3.00E-07	0.49	45	1.5	Fam174b	
DMR1:135381001	1	135381001	135382000	1000	1	1.00E-07	-0.48	10	1	St8sia2	Transport
DMR1:136015001	1	136015001	136017000	2000	1	2.70E-07	0.48	29	1.45	Slco3a1	Transport
DMR1:136702001	1	136702001	136704000	2000	2	2.80E-08	-0.63	14	0.7	Sv2b	
DMR1:136751001	1	136751001	136752000	1000	1	9.70E-08	0.47	11	1.1	Sv2b	
DMR1:136804001	1	136804001	136806000	2000	2	9.10E-09	-0.52	24	1.2	Sv2b	

DMR1:136826001	1	136826001	136829000	3000	1	5.40E-12	0.51	35	1.17	Sv2b	
DMR1:136830001	1	136830001	136832000	2000	1	3.80E-18	1.25	35	1.75	Sv2b;LOC108349288	
DMR1:137290001	1	137290001	137291000	1000	1	4.00E-07	-0.36	12	1.2	Akap13	
DMR1:137812001	1	137812001	137813000	1000	1	2.70E-07	0.41	13	1.3	Agbl1	Protease
DMR1:137909001	1	137909001	137916000	7000	1	4.70E-11	0.66	74	1.06	Agbl1;LOC108349814	Protease
DMR1:138320001	1	138320001	138326000	6000	1	2.40E-07	-0.23	66	1.1	Agbl1	Protease
DMR1:139957001	1	139957001	139958000	1000	1	3.70E-08	0.63	6	0.6	Ntrk3	Receptor
DMR1:140222001	1	140222001	140223000	1000	1	3.40E-13	0.66	16	1.6	Ntrk3	Receptor
DMR1:140473001	1	140473001	140475000	2000	1	6.00E-09	-0.43	29	1.45	Mrpl46;Mrps11	Translation
DMR1:140520001	1	140520001	140521000	1000	1	2.90E-07	-0.43	27	2.7	Det1	Proteolysis
DMR1:140804001	1	140804001	140805000	1000	1	4.50E-07	0.55	14	1.4	Acan	Extracellular Matrix
DMR1:140840001	1	140840001	140842000	2000	1	1.90E-07	0.42	25	1.25	Hapln3;Mfge8	Extracellular Matrix;Metabolism
DMR1:141002001	1	141002001	141004000	2000	1	7.20E-08	-0.39	35	1.75	LOC108348916;Abhd2	Protease
DMR1:141163001	1	141163001	141164000	1000	1	6.20E-10	0.47	10	1	Fanci;Polg	Transcription
DMR1:141187001	1	141187001	141188000	1000	1	2.90E-12	0.7	60	6	Polg;Trnar-ucg	Transcription
DMR1:141478001	1	141478001	141479000	1000	1	8.90E-07	-0.41	18	1.8	Plin1;Pex11a;Wdr93	Metabolism
DMR1:141542001	1	141542001	141544000	2000	1	1.50E-07	0.63	22	1.1	Mesp1;Mesp2	
DMR1:141554001	1	141554001	141557000	3000	1	2.90E-08	0.42	28	0.93	Mesp2;Anpep	Protease
DMR1:141561001	1	141561001	141564000	3000	1	1.00E-07	0.43	45	1.5	Mesp2;Anpep	Protease
DMR1:141588001	1	141588001	141592000	4000	2	2.30E-19	1.27	71	1.77	Anpep	Protease
DMR1:141788001	1	141788001	141790000	2000	1	2.90E-08	-0.37	31	1.55	Zfp710	Transcription
DMR1:141842001	1	141842001	141844000	2000	1	2.70E-11	-0.53	30	1.5	Zfp710;LOC100911225	Transcription
DMR1:141872001	1	141872001	141873000	1000	1	3.80E-07	-0.38	16	1.6	Zfp710;Idh2	Transcription;Metabolism
DMR1:141898001	1	141898001	141900000	2000	1	3.30E-08	0.5	37	1.85	Idh2	Metabolism
DMR1:141983001	1	141983001	141984000	1000	1	5.50E-07	-0.58	10	1	Sema4b	Signaling
DMR1:142284001	1	142284001	142288000	4000	1	7.20E-07	0.58	33	0.82	Blm	Epigenetic
DMR1:142579001	1	142579001	142581000	2000	1	4.50E-08	-0.42	27	1.35	Iqgap1	Signaling
DMR1:142589001	1	142589001	142595000	6000	2	8.40E-08	-0.41	92	1.53	Iqgap1	Signaling
DMR1:142610001	1	142610001	142613000	3000	1	4.40E-10	-0.42	52	1.73	Iqgap1	Signaling
DMR1:142756001	1	142756001	142757000	1000	1	1.00E-07	-0.41	11	1.1	Sec11a	Protease
DMR1:142913001	1	142913001	142917000	4000	1	3.00E-14	-0.47	69	1.73	Alpk3;LOC102555867	Signaling
DMR1:142920001	1	142920001	142921000	1000	1	1.90E-09	0.72	16	1.6	Alpk3;LOC102555804	Signaling
DMR1:142931001	1	142931001	142933000	2000	1	3.70E-07	0.53	25	1.25	Alpk3;LOC102555804	Signaling
DMR1:142934001	1	142934001	142935000	1000	1	1.80E-10	0.58	16	1.6	Alpk3;LOC102555804	Signaling
DMR1:142979001	1	142979001	142981000	2000	1	1.00E-08	-0.44	25	1.25	Slc28a1	Transport
DMR1:142995001	1	142995001	142998000	3000	1	2.60E-07	0.48	49	1.63	Slc28a1	Transport
DMR1:143028001	1	143028001	143030000	2000	1	6.60E-08	0.47	17	0.85	Pde8a	Signaling
DMR1:143039001	1	143039001	143040000	1000	1	8.90E-07	-0.56	11	1.1	Pde8a	Signaling
DMR1:143101001	1	143101001	143103000	2000	1	1.00E-07	-0.45	24	1.2	Pde8a	Signaling
DMR1:143394001	1	143394001	143395000	1000	1	8.70E-07	0.36	10	1	Fsd2;Whamm	Proteolysis
DMR1:143457001	1	143457001	143459000	2000	1	2.40E-07	-0.4	49	2.45	Homer2	
DMR1:143532001	1	143532001	143534000	2000	1	3.40E-09	-0.43	30	1.5	Homer2	
DMR1:143582001	1	143582001	143583000	1000	1	2.00E-09	0.65	19	1.9	Fam103a1	
DMR1:143643001	1	143643001	143645000	2000	1	1.80E-08	-0.39	26	1.3	Btbd1	Proteolysis
DMR1:143716001	1	143716001	143718000	2000	1	5.30E-07	-0.52	16	0.8	Hdgfrp3	
DMR1:144168001	1	144168001	144171000	3000	1	1.10E-07	-0.33	41	1.37	Sh3gl3	
DMR1:144173001	1	144173001	144176000	3000	1	8.40E-08	-0.44	42	1.4	Sh3gl3	
DMR1:144352001	1	144352001	144353000	1000	1	2.90E-07	0.63	13	1.3	Adamtsl3	Protease
DMR1:144414001	1	144414001	144419000	5000	2	4.30E-07	-0.3	39	0.78	Adamtsl3	Protease
DMR1:144456001	1	144456001	144457000	1000	1	3.00E-08	-0.56	7	0.7	Adamtsl3	Protease
DMR1:144534001	1	144534001	144535000	1000	1	1.50E-07	0.42	11	1.1	Adamtsl3	Protease
DMR1:145739001	1	145739001	145741000	2000	1	7.20E-11	-0.68	8	0.4	Tmc3	
DMR1:145795001	1	145795001	145798000	3000	1	6.90E-10	-0.39	25	0.83	Il16;LOC103691182	Cytokine
DMR1:145815001	1	145815001	145816000	1000	1	7.60E-07	-0.39	9	0.9	Il16;LOC103691182	Cytokine
DMR1:145827001	1	145827001	145829000	2000	1	6.80E-09	0.5	21	1.05	Il16	Cytokine
DMR1:145931001	1	145931001	145933000	2000	1	1.10E-07	-0.51	28	1.4	Cfap161	Development
DMR1:146195001	1	146195001	146197000	2000	1	1.80E-07	0.53	13	0.65	Cemip	
DMR1:146293001	1	146293001	146298000	5000	1	2.40E-07	-0.37	55	1.1	Abhd17c;LOC108349171	Protease
DMR1:146434001	1	146434001	146435000	1000	1	2.40E-08	0.43	6	0.6	Arnt2	Transcription
DMR1:146436001	1	146436001	146438000	2000	1	9.20E-07	0.44	13	0.65	Arnt2	Transcription

DMR1:146525001	1	146525001	146526000	1000	1	5.20E-11	-0.47	11	1.1	Arnt2	Transcription
DMR1:146755001	1	146755001	146757000	2000	1	6.50E-09	-0.48	16	0.8	Zfand6	
DMR1:146784001	1	146784001	146785000	1000	1	1.50E-07	-0.59	4	0.4	Zfand6	
DMR1:147047001	1	147047001	147049000	2000	1	8.90E-11	0.69	33	1.65	Xlr3a	
DMR1:147440001	1	147440001	147443000	3000	1	4.60E-08	-0.39	21	0.7	Cyp2c7	Metabolism
DMR1:147535001	1	147535001	147538000	3000	1	7.00E-09	0.25	32	1.07	Cyp2c7;LOC100361434	Metabolism
DMR1:147561001	1	147561001	147563000	2000	1	7.40E-08	-0.36	6	0.3	Cyp2c7;LOC100361434	Metabolism
DMR1:147567001	1	147567001	147572000	5000	2	5.10E-10	-0.38	25	0.5	Cyp2c7;LOC100361434	Metabolism
DMR1:147599001	1	147599001	147600000	1000	1	5.60E-07	0.39	19	1.9	Cyp2c7;LOC100361434	Metabolism
DMR1:147603001	1	147603001	147604000	1000	1	3.80E-17	-0.47	4	0.4	Cyp2c7;LOC100361434	Metabolism
DMR1:147610001	1	147610001	147612000	2000	1	4.80E-23	0.75	20	1	Cyp2c7	Metabolism
DMR1:147655001	1	147655001	147656000	1000	1	9.70E-07	-0.27	11	1.1	Cyp2c7	Metabolism
DMR1:147720001	1	147720001	147721000	1000	1	3.00E-07	-0.27	7	0.7	Cyp2c7;Cyp2c6v1	Metabolism
DMR1:147839001	1	147839001	147843000	4000	1	5.10E-09	-0.33	16	0.4	Cyp2c7	Metabolism
DMR1:147848001	1	147848001	147851000	3000	1	1.30E-08	-0.47	21	0.7	Cyp2c7	Metabolism
DMR1:148065001	1	148065001	148067000	2000	1	1.10E-12	-0.54	18	0.9	Cyp2c7	Metabolism
DMR1:148091001	1	148091001	148096000	5000	1	5.30E-08	-0.43	19	0.38	Cyp2c7	Metabolism
DMR1:148415001	1	148415001	148418000	3000	3	1.80E-17	0.8	131	4.37	Vbp1	Transcription
DMR1:148428001	1	148428001	148431000	3000	3	1.10E-21	1.12	224	7.47	Vbp1	Transcription
DMR1:148446001	1	148446001	148448000	2000	1	1.20E-11	0.71	65	3.25	Vbp1;LOC102546990;Mpp1	Transcription
DMR1:148459001	1	148459001	148460000	1000	1	4.00E-15	0.52	31	3.1	Mpp1	
DMR1:149498001	1	149498001	149499000	1000	1	1.60E-07	-0.36	6	0.6	Vom2r43	Signaling
DMR1:149538001	1	149538001	149540000	2000	1	8.50E-07	-0.61	6	0.3	Vom2r43	Signaling
DMR1:150278001	1	150278001	150280000	2000	1	4.60E-08	-0.35	14	0.7	Olr33-ps	
DMR1:150403001	1	150403001	150405000	2000	1	4.60E-07	-0.4	11	0.55	Folh1;Vom2r-ps66	Protease
DMR1:150923001	1	150923001	150925000	2000	1	8.60E-08	0.49	18	0.9	Nox4	Metabolism
DMR1:151034001	1	151034001	151036000	2000	1	1.80E-07	-0.41	7	0.35	Tyr	Metabolism
DMR1:151050001	1	151050001	151052000	2000	1	5.70E-08	-0.6	11	0.55	Tyr	Metabolism
DMR1:151099001	1	151099001	151101000	2000	1	6.30E-10	-0.61	22	1.1	Tyr	Metabolism
DMR1:151261001	1	151261001	151266000	5000	1	1.00E-08	-0.3	38	0.76	Grm5	Signaling
DMR1:151349001	1	151349001	151351000	2000	1	2.50E-08	-0.51	12	0.6	Grm5;LOC102555017	Signaling
DMR1:151452001	1	151452001	151454000	2000	2	3.30E-10	-0.74	8	0.4	Grm5	Signaling
DMR1:151495001	1	151495001	151500000	5000	1	8.80E-09	-0.44	49	0.98	Grm5	Signaling
DMR1:151604001	1	151604001	151605000	1000	1	4.90E-07	-0.34	7	0.7	Grm5	Signaling
DMR1:151657001	1	151657001	151659000	2000	2	6.60E-10	-0.65	6	0.3	Grm5	Signaling
DMR1:151697001	1	151697001	151699000	2000	1	6.90E-08	-0.34	16	0.8	Grm5	Signaling
DMR1:151776001	1	151776001	151777000	1000	1	2.30E-07	-0.52	3	0.3	Grm5	Signaling
DMR1:151948001	1	151948001	151950000	2000	1	2.00E-07	0.61	16	0.8	Ctsc	Protease
DMR1:152924001	1	152924001	152926000	2000	2	7.00E-13	-0.46	17	0.85	Tmem135	
DMR1:153081001	1	153081001	153082000	1000	1	2.50E-07	-0.5	13	1.3	Tmem135	
DMR1:153953001	1	153953001	153955000	2000	1	4.30E-10	0.5	17	0.85	Me3	Metabolism
DMR1:154086001	1	154086001	154088000	2000	1	1.70E-11	-0.74	14	0.7	Ccdc81	
DMR1:154091001	1	154091001	154096000	5000	1	1.70E-07	-0.32	70	1.4	Ccdc81	
DMR1:154097001	1	154097001	154098000	1000	1	8.20E-07	-0.4	1	0.1	Ccdc81	
DMR1:154482001	1	154482001	154484000	2000	1	1.10E-09	0.79	32	1.6	Ccdc83	
DMR1:154488001	1	154488001	154492000	4000	1	9.60E-08	-0.38	35	0.88	Ccdc83	
DMR1:155101001	1	155101001	155102000	1000	1	1.00E-08	0.74	16	1.6	Dlg2	Cytoskeleton
DMR1:155185001	1	155185001	155186000	1000	1	2.70E-10	-0.44	8	0.8	Dlg2	Cytoskeleton
DMR1:155238001	1	155238001	155239000	1000	1	1.10E-07	0.53	9	0.9	Dlg2	Cytoskeleton
DMR1:155342001	1	155342001	155344000	2000	1	6.80E-09	-0.41	17	0.85	Dlg2	Cytoskeleton
DMR1:155529001	1	155529001	155530000	1000	1	2.30E-13	0.61	0	0	Dlg2	Cytoskeleton
DMR1:156474001	1	156474001	156479000	5000	1	7.20E-12	-0.45	43	0.86	Dlg2;Ccdc83	Cytoskeleton
DMR1:156499001	1	156499001	156500000	1000	1	1.40E-08	0.4	10	1	Dlg2;Ccdc83	Cytoskeleton
DMR1:156748001	1	156748001	156749000	1000	1	7.80E-07	-0.39	8	0.8	Dlg2	Cytoskeleton
DMR1:156875001	1	156875001	156876000	1000	1	6.20E-07	0.45	8	0.8	Dlg2	Cytoskeleton
DMR1:156962001	1	156962001	156963000	1000	1	3.90E-10	0.77	12	1.2	Dlg2	Cytoskeleton
DMR1:156976001	1	156976001	156977000	1000	1	7.10E-07	-0.3	9	0.9	Dlg2	Cytoskeleton
DMR1:157394001	1	157394001	157398000	4000	1	2.90E-16	0.34	42	1.05	LOC100361001;Ccdc90b	
DMR1:157599001	1	157599001	157607000	8000	1	2.20E-08	-0.28	98	1.23	Rab30	
DMR1:157642001	1	157642001	157643000	1000	1	1.50E-08	0.7	33	3.3	Rab30;LOC103691196	
DMR1:157742001	1	157742001	157743000	1000	1	2.10E-07	-0.46	2	0.2	Prcp	Protease
DMR1:157755001	1	157755001	157757000	2000	1	8.70E-09	0.8	29	1.45	Prcp	Protease

DMR1:161378001	1	161378001	161383000	5000	1	5.80E-07	0.44	53	1.06	Tenm4	
DMR1:161556001	1	161556001	161558000	2000	1	9.50E-07	0.51	14	0.7	Tenm4	
DMR1:161765001	1	161765001	161769000	4000	1	3.50E-08	-0.53	58	1.45	Tenm4	
DMR1:161815001	1	161815001	161818000	3000	1	4.80E-08	0.38	44	1.47	Tenm4	
DMR1:161826001	1	161826001	161827000	1000	1	5.70E-07	0.46	8	0.8	Tenm4	
DMR1:161878001	1	161878001	161880000	2000	1	8.60E-08	0.39	30	1.5	Tenm4	
DMR1:162002001	1	162002001	162003000	1000	1	8.60E-07	-0.36	13	1.3	Nars2;LOC108349194	Translation
DMR1:162018001	1	162018001	162024000	6000	2	7.30E-12	-0.36	69	1.15	Nars2	Translation
DMR1:162399001	1	162399001	162402000	3000	1	6.20E-08	-0.5	38	1.27	RGD1562118;LOC102557251	
DMR1:162660001	1	162660001	162662000	2000	1	8.80E-07	-0.34	29	1.45	Rsf1	Epigenetic
DMR1:162891001	1	162891001	162892000	1000	1	5.80E-09	0.63	10	1	Pak1;Gdpd4	Signaling;Signaling
DMR1:162912001	1	162912001	162913000	1000	1	3.20E-08	0.5	21	2.1	Gdpd4	Signaling
DMR1:162969001	1	162969001	162971000	2000	1	7.90E-10	0.57	38	1.9	Gdpd4	Signaling
DMR1:163121001	1	163121001	163123000	2000	1	1.10E-07	-0.4	20	1	Capn5	Protease
DMR1:163229001	1	163229001	163234000	5000	1	1.90E-07	-0.37	24	0.48	Acer3	
DMR1:163239001	1	163239001	163242000	3000	1	3.50E-07	-0.36	22	0.73	Acer3	
DMR1:163243001	1	163243001	163244000	1000	1	2.50E-14	-0.38	8	0.8	Acer3	
DMR1:163272001	1	163272001	163274000	2000	1	8.00E-13	0.38	14	0.7	Acer3	
DMR1:163672001	1	163672001	163673000	1000	1	8.20E-08	-0.3	13	1.3	LOC108349776;Thap12	
DMR1:163802001	1	163802001	163809000	7000	2	1.00E-10	0.6	118	1.69	Wnt11;RGD1561870	Signaling;Translation
DMR1:163855001	1	163855001	163857000	2000	1	7.40E-08	0.59	29	1.45	Uvrag	
DMR1:164027001	1	164027001	164028000	1000	1	8.90E-08	-0.52	9	0.9	Uvrag	
DMR1:164087001	1	164087001	164089000	2000	1	2.00E-07	-0.43	25	1.25	Uvrag	
DMR1:164196001	1	164196001	164199000	3000	1	1.60E-15	0.72	59	1.97	Mogat2	Metabolism
DMR1:164357001	1	164357001	164363000	6000	1	1.40E-07	0.49	70	1.17	Gdpd5	Signaling
DMR1:164378001	1	164378001	164379000	1000	1	3.30E-07	-0.49	14	1.4	Gdpd5	Signaling
DMR1:164429001	1	164429001	164430000	1000	1	3.40E-08	0.59	24	2.4	Klhl35;Rps3	Cytoskeleton;Translation
DMR1:164451001	1	164451001	164453000	2000	1	2.10E-09	0.61	39	1.95	Rps3;LOC108349777;LOC100912335	Translation
DMR1:164507001	1	164507001	164510000	3000	1	8.10E-07	0.36	25	0.83	LOC100912335;Arrb1;Mir326	Cytoskeleton
DMR1:164540001	1	164540001	164542000	2000	1	6.70E-07	0.39	29	1.45	Arrb1	Cytoskeleton
DMR1:164543001	1	164543001	164549000	6000	1	4.60E-08	0.53	92	1.53	Arrb1	Cytoskeleton
DMR1:164559001	1	164559001	164560000	1000	1	2.20E-07	0.42	10	1	Arrb1	Cytoskeleton
DMR1:164793001	1	164793001	164797000	4000	2	2.10E-10	-0.43	34	0.85	Neu3	Metabolism
DMR1:164868001	1	164868001	164869000	1000	1	5.50E-07	-0.32	19	1.9	Xrra1;LOC102547456	
DMR1:164966001	1	164966001	164968000	2000	1	1.20E-08	-0.34	18	0.9	Rnf169	
DMR1:165034001	1	165034001	165035000	1000	1	1.40E-07	-0.6	10	1	Chrdl2	
DMR1:165040001	1	165040001	165043000	3000	1	9.20E-07	-0.38	50	1.67	Chrdl2	
DMR1:165198001	1	165198001	165199000	1000	1	7.80E-09	-0.42	16	1.6	LOC100912071;Kcne3	Transport
DMR1:165327001	1	165327001	165331000	4000	1	3.10E-07	0.34	34	0.85	P4ha3;Ppme1	Golgi
DMR1:165519001	1	165519001	165520000	1000	1	5.30E-07	0.36	11	1.1	Ucp2;Dnajb13;LOC103691200	Transport;Transcription
DMR1:165544001	1	165544001	165547000	3000	1	2.80E-13	1.05	42	1.4	LOC103691200;Coa4	Transcription
DMR1:165596001	1	165596001	165597000	1000	1	7.70E-07	-0.41	10	1	Mrpl48;LOC102548233	Translation
DMR1:165686001	1	165686001	165687000	1000	1	9.80E-07	-0.41	9	0.9	Plekhb1;LOC108349605	
DMR1:165841001	1	165841001	165847000	6000	1	3.20E-07	-0.36	68	1.13	Fam168a	Cytoskeleton
DMR1:165897001	1	165897001	165899000	2000	1	4.50E-13	0.65	20	1	Relt	Receptor
DMR1:165906001	1	165906001	165907000	1000	1	6.10E-07	0.49	8	0.8	Relt	Receptor
DMR1:165910001	1	165910001	165913000	3000	1	8.70E-08	0.62	40	1.33	Relt;Mir3102	Receptor
DMR1:165917001	1	165917001	165921000	4000	1	5.90E-07	0.42	99	2.48	Mir3102	
DMR1:165973001	1	165973001	165975000	2000	1	6.00E-22	1.15	68	3.4	P2ry6	Signaling
DMR1:165987001	1	165987001	165989000	2000	1	4.00E-09	0.49	28	1.4	P2ry6	Signaling
DMR1:166030001	1	166030001	166034000	4000	1	3.10E-12	0.9	92	2.3	P2ry2;LOC108349453	Signaling
DMR1:166046001	1	166046001	166050000	4000	1	2.70E-07	0.43	60	1.5	P2ry2;LOC108349453	Signaling
DMR1:166132001	1	166132001	166134000	2000	1	9.90E-08	0.55	23	1.15	Fchsd2	
DMR1:166390001	1	166390001	166391000	1000	1	2.20E-07	-0.35	5	0.5	Fchsd2	
DMR1:166414001	1	166414001	166418000	4000	1	4.60E-13	-0.43	59	1.48	Atg16l2;LOC102546541	
DMR1:166486001	1	166486001	166491000	5000	2	3.60E-12	-0.46	65	1.3	Arap1	Signaling
DMR1:166619001	1	166619001	166621000	2000	1	4.10E-10	0.46	33	1.65	Pde2a	Signaling
DMR1:166795001	1	166795001	166799000	4000	1	6.50E-07	0.36	35	0.88	Clpb	Protease
DMR1:166894001	1	166894001	166895000	1000	1	2.10E-07	0.42	30	3	Phox2a;Inppl1	Development

DMR1:166899001	1	166899001	166904000	5000	1	3.20E-10	0.55	90	1.8	Phox2a;Inppl1;LOC103691225	Development
DMR1:166941001	1	166941001	166946000	5000	1	1.80E-11	-0.5	56	1.12	Folr2;Folr1	
DMR1:167103001	1	167103001	167104000	1000	1	1.60E-49	1.28	13	1.3	Il18bp;Rnf121	Proteolysis
DMR1:167135001	1	167135001	167136000	1000	1	3.30E-08	-0.68	4	0.4	Rnf121	Proteolysis
DMR1:167147001	1	167147001	167149000	2000	1	1.50E-07	-0.46	19	0.95	Rnf121;LOC102549471	Proteolysis
DMR1:167169001	1	167169001	167170000	1000	1	3.90E-07	0.5	14	1.4	Rnf121;LOC102549471;LOC108348605	Proteolysis
DMR1:167196001	1	167196001	167199000	3000	1	2.30E-07	0.5	43	1.43	Trpc2;Art5;Art1;Chrna10	Transport;Transport;Ion Channel
DMR1:167327001	1	167327001	167330000	3000	1	1.70E-08	-0.42	38	1.27	Pgap2;Rhog	Golgi;Signaling
DMR1:167344001	1	167344001	167347000	3000	1	2.90E-09	-0.36	38	1.27	Pgap2;Rhog;LOC108348579	Golgi;Signaling
DMR1:167603001	1	167603001	167606000	3000	1	2.70E-11	0.75	59	1.97	Olr40	Receptor
DMR1:167683001	1	167683001	167686000	3000	1	1.80E-10	0.79	36	1.2	Olr42-ps;Trim21	Proteolysis
DMR1:167720001	1	167720001	167721000	1000	1	5.40E-11	0.82	20	2	Olr43	Receptor
DMR1:167762001	1	167762001	167763000	1000	1	3.20E-08	-0.63	4	0.4	Olr46;Bcl2l1-ps1;Olr47	Receptor
DMR1:167765001	1	167765001	167766000	1000	1	1.90E-13	0.82	34	3.4	Olr46;Bcl2l1-ps1;Olr47	Receptor
DMR1:167775001	1	167775001	167776000	1000	1	3.00E-07	0.59	14	1.4	Bcl2l1-ps1;Olr47	Receptor
DMR1:167912001	1	167912001	167913000	1000	1	1.20E-07	-0.48	5	0.5	Olr59;Olr58-ps	
DMR1:167967001	1	167967001	167969000	2000	1	3.30E-11	0.4	18	0.9	Olr54-ps;Olr53	Receptor
DMR1:168186001	1	168186001	168187000	1000	1	2.60E-07	-0.31	6	0.6	Olr72;Olr73-ps;Olr74	Receptor
DMR1:168252001	1	168252001	168256000	4000	1	2.00E-07	-0.35	39	0.98	Olr79	Receptor
DMR1:168420001	1	168420001	168421000	1000	1	9.00E-08	-0.57	1	0.1	Olr89;Olr90-ps	Receptor
DMR1:168586001	1	168586001	168587000	1000	1	6.00E-12	0.67	18	1.8	Olr103;Olr104	Receptor
DMR1:168627001	1	168627001	168632000	5000	1	6.60E-10	-0.43	46	0.92	Olr107;Olr108	Receptor
DMR1:168674001	1	168674001	168676000	2000	1	8.40E-18	0.61	20	1	Olr111	
DMR1:168731001	1	168731001	168732000	1000	1	6.20E-08	0.37	10	1	Olr115;LOC499215	Receptor
DMR1:168733001	1	168733001	168737000	4000	3	4.30E-10	-0.26	35	0.88	Olr115;LOC499215	Receptor
DMR1:168758001	1	168758001	168759000	1000	1	7.00E-10	-0.38	26	2.6	Olr116-ps;Olr117-ps;LOC108349608	
DMR1:168873001	1	168873001	168875000	2000	1	9.50E-07	-0.57	7	0.35	Olr125;Olr126	Receptor
DMR1:169012001	1	169012001	169013000	1000	1	1.00E-08	-0.59	6	0.6	Hbe1	
DMR1:169058001	1	169058001	169063000	5000	2	9.20E-13	-0.37	39	0.78	Olr131	Receptor
DMR1:169137001	1	169137001	169139000	2000	1	3.10E-08	-0.4	16	0.8	Olr136;LOC499219	Receptor;Transcription
DMR1:169214001	1	169214001	169222000	8000	1	2.00E-14	0.62	85	1.06	Olr140;Olr141;Olr142	Receptor
DMR1:169365001	1	169365001	169372000	7000	1	2.00E-07	-0.37	63	0.9	RGD1310717;Olr149	Development;Receptor
DMR1:169580001	1	169580001	169583000	3000	1	1.70E-10	-0.41	23	0.77	Olr155;Olr154	Receptor
DMR1:169584001	1	169584001	169585000	1000	1	1.20E-07	-0.24	11	1.1	Olr155;Olr154	Receptor
DMR1:169616001	1	169616001	169617000	1000	1	3.80E-07	0.54	21	2.1	Olr157	
DMR1:169640001	1	169640001	169641000	1000	1	1.90E-07	0.56	9	0.9	Olr158;Olr159	Receptor
DMR1:169745001	1	169745001	169746000	1000	1	5.30E-09	0.52	27	2.7	Olr164	Receptor
DMR1:169790001	1	169790001	169792000	2000	1	3.70E-07	-0.39	5	0.25	Olr166-ps;Olr167	Receptor
DMR1:169837001	1	169837001	169838000	1000	1	8.30E-07	0.31	20	2	Olr179	Receptor
DMR1:169929001	1	169929001	169932000	3000	1	6.80E-08	-0.33	31	1.03	Olr186;Olr187-ps	Receptor
DMR1:170052001	1	170052001	170053000	1000	1	5.20E-09	-0.49	8	0.8	Olr195-ps	
DMR1:170073001	1	170073001	170074000	1000	1	4.60E-09	0.58	16	1.6	Olr196	Receptor
DMR1:170076001	1	170076001	170082000	6000	2	8.50E-08	-0.4	56	0.93	Olr196;Olr197	Receptor
DMR1:170098001	1	170098001	170100000	2000	1	4.30E-07	-0.46	9	0.45	Olr197;Olr198	Receptor
DMR1:170131001	1	170131001	170134000	3000	1	3.60E-09	-0.32	18	0.6	Olr199	Receptor
DMR1:170138001	1	170138001	170140000	2000	1	4.50E-08	-0.57	7	0.35	Olr200	Receptor
DMR1:170271001	1	170271001	170273000	2000	1	2.30E-16	0.76	44	2.2	LOC108349007;Cckbr	Signaling
DMR1:170319001	1	170319001	170320000	1000	1	2.80E-07	0.67	15	1.5	Prkcdbp	
DMR1:170441001	1	170441001	170443000	2000	1	6.80E-10	0.51	23	1.15	Hpx;Trim3	Protease;Proteolysis
DMR1:170545001	1	170545001	170547000	2000	1	2.10E-11	-0.67	3	0.15	Dnhd1	Cytoskeleton
DMR1:170564001	1	170564001	170565000	1000	1	2.00E-10	0.69	21	2.1	Dnhd1;Rrp8	Cytoskeleton
DMR1:170605001	1	170605001	170606000	1000	1	1.20E-07	0.63	15	1.5	Dchs1	Cytoskeleton
DMR1:170615001	1	170615001	170617000	2000	1	8.60E-08	-0.34	30	1.5	Dchs1	Cytoskeleton
DMR1:170642001	1	170642001	170644000	2000	1	6.50E-11	-0.45	16	0.8	Mrpl17	Translation
DMR1:170806001	1	170806001	170810000	4000	1	1.40E-07	-0.34	23	0.58	Olr210;Olr211	Receptor
DMR1:170941001	1	170941001	170944000	3000	1	1.40E-08	-0.46	11	0.37	Olr219	Receptor
DMR1:170991001	1	170991001	170995000	4000	1	7.50E-08	-0.42	24	0.6	Olr221	Receptor
DMR1:171099001	1	171099001	171101000	2000	1	4.50E-07	-0.3	13	0.65	Olr227	
DMR1:171188001	1	171188001	171189000	1000	1	2.90E-09	0.72	13	1.3	Olr230;Olr231;Olr232	Receptor

DMR1:171425001	1	171425001	171426000	1000	1	1.20E-07	0.49	10	1	Rbmxl2	
DMR1:171589001	1	171589001	171591000	2000	1	1.40E-08	-0.4	28	1.4	LOC100912015;Syt9	Transport
DMR1:171622001	1	171622001	171624000	2000	1	4.60E-09	-0.39	17	0.85	Syt9	Transport
DMR1:171641001	1	171641001	171642000	1000	1	1.60E-07	0.58	13	1.3	Syt9	Transport
DMR1:171711001	1	171711001	171713000	2000	1	3.00E-12	0.93	30	1.5	Syt9	Transport
DMR1:171723001	1	171723001	171724000	1000	1	2.10E-07	0.34	14	1.4	Syt9	Transport
DMR1:171727001	1	171727001	171728000	1000	1	8.20E-07	-0.33	6	0.6	Syt9	Transport
DMR1:171789001	1	171789001	171791000	2000	1	3.40E-07	-0.61	17	0.85	Olfml1	Development
DMR1:171814001	1	171814001	171815000	1000	1	7.70E-08	0.5	10	1	Olfml1;Ppfibp2	Development
DMR1:171916001	1	171916001	171919000	3000	1	4.30E-07	-0.39	34	1.13	Ppfibp2	
DMR1:171925001	1	171925001	171926000	1000	1	1.20E-08	0.46	9	0.9	Ppfibp2	
DMR1:172032001	1	172032001	172035000	3000	1	7.50E-07	-0.44	8	0.27	Ovch2	Protease
DMR1:172039001	1	172039001	172040000	1000	1	6.40E-07	-0.4	2	0.2	Ovch2	Protease
DMR1:172078001	1	172078001	172085000	7000	1	6.00E-07	-0.33	77	1.1	Olr239;Olr240	Signaling
DMR1:172681001	1	172681001	172682000	1000	1	6.90E-07	-0.25	6	0.6	Olr265-ps	
DMR1:173567001	1	173567001	173568000	1000	1	3.80E-07	0.39	16	1.6	Tub	
DMR1:173614001	1	173614001	173615000	1000	1	1.90E-09	0.48	10	1	Tub	
DMR1:173618001	1	173618001	173619000	1000	1	9.90E-08	0.43	22	2.2	Tub	
DMR1:173664001	1	173664001	173668000	4000	1	4.10E-08	-0.33	49	1.23	Ric3	
DMR1:173773001	1	173773001	173774000	1000	1	2.50E-07	-0.41	14	1.4	Lmo1	
DMR1:173893001	1	173893001	173897000	4000	1	2.60E-08	-0.43	29	0.72	Stk33	Signaling
DMR1:174023001	1	174023001	174025000	2000	2	1.70E-08	-0.5	10	0.5	Stk33	Signaling
DMR1:174201001	1	174201001	174203000	2000	1	4.90E-07	-0.43	13	0.65	St5	
DMR1:174264001	1	174264001	174265000	1000	1	3.90E-08	-0.57	4	0.4	St5;LOC108349575	
DMR1:174429001	1	174429001	174432000	3000	1	3.30E-07	-0.29	45	1.5	Scube2	Extracellular Matrix
DMR1:174470001	1	174470001	174472000	2000	1	9.70E-07	-0.29	30	1.5	Scube2	Extracellular Matrix
DMR1:174873001	1	174873001	174874000	1000	1	5.80E-12	-0.74	12	1.2	Swap70	Cytoskeleton
DMR1:174877001	1	174877001	174880000	3000	1	4.70E-10	-0.43	45	1.5	Swap70	Cytoskeleton
DMR1:174900001	1	174900001	174902000	2000	2	1.00E-10	-0.52	20	1	Swap70	Cytoskeleton
DMR1:174932001	1	174932001	174934000	2000	1	3.10E-09	-0.4	21	1.05	Swap70	Cytoskeleton
DMR1:175021001	1	175021001	175024000	3000	1	7.20E-10	-0.36	83	2.77	Sbf2	Signaling
DMR1:175107001	1	175107001	175113000	6000	1	5.80E-09	-0.27	73	1.22	Sbf2	Signaling
DMR1:175132001	1	175132001	175134000	2000	1	1.10E-08	-0.3	20	1	Sbf2	Signaling
DMR1:175278001	1	175278001	175280000	2000	1	2.80E-07	-0.48	10	0.5	Sbf2	Signaling
DMR1:175327001	1	175327001	175328000	1000	1	1.20E-07	-0.57	5	0.5	Sbf2	Signaling
DMR1:175373001	1	175373001	175375000	2000	1	9.10E-07	-0.38	23	1.15	Sbf2	Signaling
DMR1:175592001	1	175592001	175596000	4000	1	2.20E-07	0.5	47	1.18	LOC103691204;Ampd3	Metabolism
DMR1:175610001	1	175610001	175613000	3000	1	6.80E-09	0.44	49	1.63	Ampd3	Metabolism
DMR1:175616001	1	175616001	175619000	3000	1	3.50E-09	0.7	73	2.43	Ampd3	Metabolism
DMR1:175723001	1	175723001	175725000	2000	1	2.30E-17	-0.63	18	0.9	Mrvi1;LOC103691205	Cytoskeleton
DMR1:175811001	1	175811001	175813000	2000	1	1.00E-08	-0.48	24	1.2	Mrvi1	Cytoskeleton
DMR1:175892001	1	175892001	175893000	1000	1	8.10E-12	-0.57	11	1.1	Eif4g2	Translation
DMR1:176077001	1	176077001	176079000	2000	1	1.40E-12	0.85	36	1.8	RGD1566189	
DMR1:176084001	1	176084001	176085000	1000	1	2.60E-09	-0.35	15	1.5	RGD1566189	
DMR1:176307001	1	176307001	176309000	2000	1	3.20E-14	0.36	24	1.2	Galnt18	Golgi
DMR1:176338001	1	176338001	176339000	1000	1	6.00E-08	0.45	14	1.4	Galnt18	Golgi
DMR1:176375001	1	176375001	176376000	1000	1	1.80E-09	0.66	7	0.7	Galnt18	Golgi
DMR1:176380001	1	176380001	176382000	2000	1	3.40E-07	0.37	23	1.15	Galnt18	Golgi
DMR1:176454001	1	176454001	176455000	1000	1	3.00E-07	0.43	12	1.2	Galnt18	Golgi
DMR1:176498001	1	176498001	176500000	2000	1	5.80E-16	-0.58	27	1.35	Galnt18	Golgi
DMR1:176905001	1	176905001	176906000	1000	1	8.00E-08	-0.53	12	1.2	Usp47	Protease
DMR1:177301001	1	177301001	177302000	1000	1	3.70E-13	0.4	12	1.2	Parva	Cytoskeleton
DMR1:177317001	1	177317001	177319000	2000	1	1.10E-07	-0.54	32	1.6	Parva	Cytoskeleton
DMR1:177413001	1	177413001	177414000	1000	1	9.40E-07	0.42	12	1.2	Parva	Cytoskeleton
DMR1:178693001	1	178693001	178698000	5000	1	6.10E-10	0.5	67	1.34	Spon1	Cytoskeleton
DMR1:178776001	1	178776001	178780000	4000	1	4.80E-10	-0.42	55	1.38	Spon1	Cytoskeleton
DMR1:178887001	1	178887001	178894000	7000	1	5.00E-07	0.35	64	0.91	Spon1	Cytoskeleton
DMR1:178912001	1	178912001	178914000	2000	1	2.70E-07	-0.47	4	0.2	Spon1	Cytoskeleton
DMR1:178965001	1	178965001	178966000	1000	1	2.90E-07	-0.38	15	1.5	Rras2	Signaling
DMR1:178988001	1	178988001	178989000	1000	1	4.80E-07	0.48	13	1.3	Rras2	Signaling
DMR1:182062001	1	182062001	182064000	2000	2	1.40E-28	1.02	34	1.7	Ythdc2	Transcription
DMR1:182067001	1	182067001	182071000	4000	4	3.40E-32	1.06	69	1.73	Ythdc2	Transcription

DMR1:182843001	1	182843001	182855000	12000	4	1.00E-10	-0.59	139	1.16	Ssty1	
DMR1:183672001	1	183672001	183673000	1000	1	2.60E-07	0.42	18	1.8	RGD1565301	
DMR1:183677001	1	183677001	183679000	2000	1	9.70E-08	0.59	33	1.65	RGD1565301	
DMR1:183681001	1	183681001	183682000	1000	1	1.10E-07	0.44	11	1.1	RGD1565301	
DMR1:184025001	1	184025001	184028000	3000	1	3.10E-07	-0.3	26	0.87	Pde3b	Signaling
DMR1:184035001	1	184035001	184038000	3000	1	5.40E-10	-0.39	44	1.47	Pde3b	Signaling
DMR1:184402001	1	184402001	184403000	1000	1	2.20E-07	-0.41	10	1	Insc	
DMR1:185443001	1	185443001	185444000	1000	1	1.10E-08	0.59	9	0.9	Plekha7	
DMR1:185460001	1	185460001	185461000	1000	1	5.80E-08	-0.47	17	1.7	Plekha7	
DMR1:185499001	1	185499001	185502000	3000	1	2.00E-07	0.49	52	1.73	Plekha7	
DMR1:185691001	1	185691001	185692000	1000	1	4.90E-09	0.31	9	0.9	Sox6	
DMR1:185756001	1	185756001	185759000	3000	1	2.80E-08	-0.4	22	0.73	Sox6	
DMR1:185819001	1	185819001	185821000	2000	1	2.00E-07	-0.41	14	0.7	Sox6	
DMR1:186028001	1	186028001	186030000	2000	1	5.00E-10	-0.44	15	0.75	Sox6	
DMR1:186193001	1	186193001	186197000	4000	1	8.70E-09	0.5	62	1.55	Sox6	
DMR1:187034001	1	187034001	187035000	1000	1	9.20E-10	0.67	10	1	Xylt1	Transport
DMR1:187220001	1	187220001	187222000	2000	1	5.50E-07	0.5	28	1.4	Xylt1	Transport
DMR1:187243001	1	187243001	187246000	3000	1	1.20E-07	0.41	46	1.53	Xylt1	Transport
DMR1:187785001	1	187785001	187786000	1000	1	1.50E-07	-0.37	21	2.1	Arl6ip1;Smg1	
DMR1:187886001	1	187886001	187887000	1000	1	1.80E-10	-0.49	13	1.3	Smg1	
DMR1:188017001	1	188017001	188020000	3000	1	4.20E-07	0.51	46	1.53	Clec19a	
DMR1:188141001	1	188141001	188142000	1000	1	1.10E-11	0.74	50	5	Itpripl2	
DMR1:188169001	1	188169001	188171000	2000	1	3.20E-09	0.58	33	1.65	LOC108349625;Coq7	
DMR1:188253001	1	188253001	188254000	1000	1	6.20E-08	-0.44	11	1.1	Tmc7	
DMR1:188282001	1	188282001	188284000	2000	1	1.20E-07	-0.41	25	1.25	Tmc5	
DMR1:188681001	1	188681001	188682000	1000	1	1.70E-07	-0.42	10	1	Iqck;Gprc5b	Signaling
DMR1:189158001	1	189158001	189159000	1000	1	3.00E-14	0.61	9	0.9	Gp2	Receptor
DMR1:189190001	1	189190001	189192000	2000	2	2.70E-13	-0.61	10	0.5	Gp2;Umod	Receptor
DMR1:189198001	1	189198001	189200000	2000	1	4.60E-08	0.57	23	1.15	Umod;Pdilt	Receptor;Transcription
DMR1:189312001	1	189312001	189315000	3000	1	1.10E-07	-0.55	26	0.87	Acsm2a	
DMR1:189448001	1	189448001	189449000	1000	1	5.70E-08	0.42	13	1.3	Acsm4;Thumpd1	Metabolism
DMR1:189594001	1	189594001	189595000	1000	1	3.30E-07	0.47	11	1.1	Thumpd1;Exnef	
DMR1:189814001	1	189814001	189815000	1000	1	9.40E-09	0.6	9	0.9	Thumpd1;Dnah3	
DMR1:189998001	1	189998001	189999000	1000	1	1.90E-07	0.36	11	1.1	Thumpd1;Abca14	Transport
DMR1:190260001	1	190260001	190262000	2000	1	2.10E-07	-0.23	26	1.3	Thumpd1;Abca16	Transport
DMR1:190684001	1	190684001	190685000	1000	1	1.50E-08	-0.53	7	0.7	LOC102555900;Vwa3a	
DMR1:190734001	1	190734001	190737000	3000	1	4.40E-09	0.42	35	1.17	Vwa3a	
DMR1:191025001	1	191025001	191026000	1000	1	3.40E-14	0.95	27	2.7	Otoa	Cytoskeleton
DMR1:191032001	1	191032001	191033000	1000	1	1.10E-10	0.44	4	0.4	Otoa	Cytoskeleton
DMR1:191097001	1	191097001	191098000	1000	1	8.30E-07	-0.47	14	1.4	Otoa	Cytoskeleton
DMR1:191393001	1	191393001	191398000	5000	1	1.10E-08	0.35	40	0.8	Hs3st2	Transport
DMR1:191444001	1	191444001	191445000	1000	1	8.90E-07	0.54	26	2.6	Hs3st2	Transport
DMR1:191447001	1	191447001	191448000	1000	1	1.80E-07	0.37	11	1.1	Hs3st2	Transport
DMR1:191473001	1	191473001	191474000	1000	1	8.20E-10	0.66	24	2.4	Hs3st2	Transport
DMR1:191839001	1	191839001	191841000	2000	1	1.20E-07	0.36	20	1	LOC102549682;Scnn1b	Transport
DMR1:192170001	1	192170001	192172000	2000	1	8.30E-10	0.69	45	2.25	Chp2	
DMR1:192290001	1	192290001	192293000	3000	2	5.10E-10	0.56	27	0.9	Prkcb	Signaling
DMR1:192302001	1	192302001	192303000	1000	1	1.00E-08	0.44	12	1.2	Prkcb	Signaling
DMR1:192305001	1	192305001	192307000	2000	1	3.60E-09	-0.49	24	1.2	Prkcb	Signaling
DMR1:192352001	1	192352001	192355000	3000	1	7.90E-07	0.46	33	1.1	Prkcb	Signaling
DMR1:192388001	1	192388001	192391000	3000	1	5.80E-11	0.29	41	1.37	Prkcb	Signaling
DMR1:192546001	1	192546001	192554000	8000	1	6.10E-09	0.6	99	1.24	Prkcb	Signaling
DMR1:192706001	1	192706001	192709000	3000	1	2.10E-08	0.62	54	1.8	Cacng3	Transport
DMR1:192927001	1	192927001	192928000	1000	1	5.80E-09	0.65	12	1.2	Rbbp6	Proteolysis
DMR1:192961001	1	192961001	192963000	2000	1	4.80E-15	-0.5	42	2.1	Rbbp6	Proteolysis
DMR1:192964001	1	192964001	192966000	2000	1	1.50E-09	-0.38	29	1.45	Rbbp6	Proteolysis
DMR1:192980001	1	192980001	192983000	3000	1	1.30E-07	-0.4	22	0.73	Tnrc6a	Metabolism
DMR1:192987001	1	192987001	192988000	1000	1	8.10E-09	-0.32	7	0.7	Tnrc6a	Metabolism
DMR1:193002001	1	193002001	193004000	2000	1	1.60E-17	0.72	28	1.4	Tnrc6a	Metabolism
DMR1:193044001	1	193044001	193045000	1000	1	3.60E-07	-0.32	21	2.1	Tnrc6a	Metabolism
DMR1:193112001	1	193112001	193113000	1000	1	7.80E-08	-0.41	16	1.6	Tnrc6a	Metabolism
DMR1:193209001	1	193209001	193210000	1000	1	1.20E-09	-0.37	14	1.4	Slc5a11	Transport

DMR1:193231001	1	193231001	193233000	2000	1	5.80E-07	-0.43	5	0.25	Slc5a11;Arhgap17	Transport
DMR1:193882001	1	193882001	193883000	1000	1	1.40E-09	-0.54	7	0.7	Hs3st4	
DMR1:193988001	1	193988001	193991000	3000	1	4.50E-07	0.61	35	1.17	Hs3st4	
DMR1:194161001	1	194161001	194166000	5000	1	1.20E-07	0.32	55	1.1	Hs3st4	
DMR1:194212001	1	194212001	194213000	1000	1	2.00E-11	-0.49	10	1	Hs3st4	
DMR1:194248001	1	194248001	194249000	1000	1	3.40E-10	-0.66	57	5.7	Hs3st4	
DMR1:195095001	1	195095001	195096000	1000	1	6.20E-10	0.77	27	2.7	Snurf;Snrpn	Translation
DMR1:196847001	1	196847001	196848000	1000	1	1.00E-08	0.47	15	1.5	Kdm8;Nsmce1	
DMR1:196878001	1	196878001	196883000	5000	1	2.60E-13	-0.53	70	1.4	Nsmce1;LOC103691233	
DMR1:196991001	1	196991001	196994000	3000	1	4.30E-07	0.43	34	1.13	Il21r	Receptor
DMR1:197025001	1	197025001	197026000	1000	1	9.30E-09	-0.56	8	0.8	Il21r;Gtf3c1	Receptor;Transcription
DMR1:197036001	1	197036001	197037000	1000	1	5.70E-07	0.5	9	0.9	Gtf3c1	Transcription
DMR1:197051001	1	197051001	197052000	1000	1	1.90E-08	0.59	8	0.8	Gtf3c1	Transcription
DMR1:197301001	1	197301001	197302000	1000	1	5.40E-08	-0.46	14	1.4	Gsg1l	Cytoskeleton
DMR1:197317001	1	197317001	197322000	5000	1	5.60E-07	-0.49	57	1.14	Gsg1l	Cytoskeleton
DMR1:197325001	1	197325001	197326000	1000	1	7.50E-07	0.44	10	1	Gsg1l	Cytoskeleton
DMR1:197347001	1	197347001	197350000	3000	1	6.40E-07	0.51	43	1.43	Gsg1l	Cytoskeleton
DMR1:197478001	1	197478001	197482000	4000	1	4.40E-12	0.61	45	1.12	Gsg1l	Cytoskeleton
DMR1:197545001	1	197545001	197548000	3000	1	1.50E-07	-0.44	25	0.83	Xpo6	
DMR1:197759001	1	197759001	197761000	2000	1	2.70E-07	0.44	25	1.25	Lat	
DMR1:197771001	1	197771001	197775000	4000	1	2.30E-10	0.62	83	2.08	Lat;Spns1	Transport
DMR1:197804001	1	197804001	197805000	1000	1	1.80E-08	0.56	22	2.2	Nfatc2ip;LOC100361060	
DMR1:197999001	1	197999001	198002000	3000	1	1.70E-11	-0.52	41	1.37	Cln3;Apobr;Il27	Transport;Cytokine
DMR1:198124001	1	198124001	198128000	4000	1	8.30E-12	0.87	104	2.6	Slx1b;RGD1565363;Coro1a	Transcription;Cytoskeleton
DMR1:198426001	1	198426001	198428000	2000	1	6.80E-07	0.4	30	1.5	LOC103691236;Mvp	Cytoskeleton
DMR1:198453001	1	198453001	198454000	1000	1	1.30E-09	0.54	9	0.9	Mvp;Pagr1;Prrt2;Maz;Kif22	Cytoskeleton;Transcription;Cytoskeleton
DMR1:198630001	1	198630001	198632000	2000	1	1.10E-07	0.44	26	1.3	Cd2bp2;Tbc1d10b	Translation;Signaling
DMR1:198708001	1	198708001	198709000	1000	1	1.90E-09	0.54	11	1.1	Zfp771;Dctpp1	Transcription;Signaling
DMR1:199020001	1	199020001	199023000	3000	1	4.20E-10	-0.45	54	1.8	Tmem265;Phkg2;Ccdc189	Signaling
DMR1:199044001	1	199044001	199046000	2000	1	2.30E-07	-0.5	51	2.55	Ccdc189;Rnf40;Zfp629	
DMR1:199072001	1	199072001	199074000	2000	1	4.50E-11	-0.48	30	1.5	Zfp629	
DMR1:199203001	1	199203001	199208000	5000	1	5.00E-07	-0.34	73	1.46	LOC102554987;Fbxl19;Orai3	Transport
DMR1:199248001	1	199248001	199250000	2000	1	3.70E-07	0.62	22	1.1	Setd1b;Hsd3b7;Stx1b	Epigenetic;Metabolism;Transcription
DMR1:199307001	1	199307001	199309000	2000	1	4.50E-09	0.51	21	1.05	Zfp668	Transcription
DMR1:199350001	1	199350001	199351000	1000	1	5.00E-10	0.51	24	2.4	Vkorc1;Bckdk;Kat8	Metabolism;Signaling;Epigenetic
DMR1:199362001	1	199362001	199364000	2000	1	1.60E-07	-0.42	24	1.2	Bckdk;Kat8;Prss8	Signaling;Epigenetic;Protease
DMR1:199383001	1	199383001	199386000	3000	1	2.70E-07	0.62	47	1.57	Prss8;Prss36	Protease
DMR1:199393001	1	199393001	199394000	1000	1	1.40E-13	1.07	35	3.5	Prss36	Protease
DMR1:199513001	1	199513001	199515000	2000	1	2.00E-07	-0.36	31	1.55	Itgam	
DMR1:199659001	1	199659001	199662000	3000	1	1.20E-08	-0.4	80	2.67	LOC103691238;Armc5;Tgfb1i1	Transcription;Cytoskeleton
DMR1:199684001	1	199684001	199689000	5000	1	3.90E-07	0.62	98	1.96	Slc5a2;RGD1310127	Transport
DMR1:199727001	1	199727001	199729000	2000	1	4.30E-07	0.32	18	0.9	Ahsp	Transcription
DMR1:199964001	1	199964001	199965000	1000	1	5.40E-11	0.81	37	3.7	Bag3	
DMR1:200107001	1	200107001	200109000	2000	2	1.50E-07	0.7	66	3.3	Inpp5f;Mcmbp	Signaling
DMR1:200672001	1	200672001	200677000	5000	1	1.50E-07	-0.41	98	1.96	Fgfr2	Receptor
DMR1:200690001	1	200690001	200695000	5000	1	2.70E-10	-0.53	120	2.4	Fgfr2	Receptor
DMR1:201119001	1	201119001	201122000	3000	2	1.90E-07	-0.46	50	1.67	Nsmce4a	
DMR1:201244001	1	201244001	201245000	1000	1	4.90E-13	-0.54	15	1.5	Tacc2;LOC103691241	
DMR1:201318001	1	201318001	201319000	1000	1	9.20E-08	-0.5	10	1	Tacc2	
DMR1:201320001	1	201320001	201321000	1000	1	8.20E-07	-0.36	21	2.1	Tacc2	
DMR1:201367001	1	201367001	201370000	3000	1	4.30E-15	0.5	17	0.57	Btbd16	
DMR1:201473001	1	201473001	201476000	3000	1	5.50E-07	-0.37	34	1.13	Plekha1	
DMR1:201496001	1	201496001	201498000	2000	2	1.30E-16	0.72	33	1.65	Htra1	Protease
DMR1:201890001	1	201890001	201891000	1000	1	6.30E-07	-0.42	14	1.4	Cuzd1	Receptor
DMR1:202439001	1	202439001	202440000	1000	1	6.30E-07	0.48	15	1.5	Plpp4	Signaling
DMR1:202449001	1	202449001	202451000	2000	1	1.70E-07	0.52	25	1.25	Plpp4	Signaling

DMR1:203191001	1	203191001	203192000	1000	1	3.20E-15	0.59	18	1.8	Dmbt1	Protease
DMR1:204253001	1	204253001	204254000	1000	1	3.30E-07	0.47	12	1.2	Chst15	Transport
DMR1:204272001	1	204272001	204274000	2000	1	1.40E-08	0.46	24	1.2	Chst15	Transport
DMR1:204279001	1	204279001	204280000	1000	1	8.30E-09	0.57	10	1	Chst15	Transport
DMR1:204553001	1	204553001	204554000	1000	1	8.00E-07	0.56	19	1.9	Oat	Metabolism
DMR1:204583001	1	204583001	204586000	3000	1	2.00E-07	-0.39	41	1.37	Oat	Metabolism
DMR1:204671001	1	204671001	204675000	4000	1	4.00E-09	0.67	58	1.45	Lhpp	Signaling
DMR1:204763001	1	204763001	204764000	1000	1	3.90E-11	0.68	10	1	Fam53b	
DMR1:204896001	1	204896001	204897000	1000	1	1.10E-09	0.66	22	2.2	Fam175b;RGD1565272	
DMR1:204933001	1	204933001	204937000	4000	1	7.40E-08	0.61	111	2.78	Zranb1	Protease
DMR1:205008001	1	205008001	205009000	1000	1	3.40E-07	-0.44	6	0.6	Zranb1;Ctbp2	Protease;Transcription
DMR1:205943001	1	205943001	205948000	5000	1	4.70E-10	-0.42	40	0.8	Fank1;Adam12	Protease
DMR1:205974001	1	205974001	205976000	2000	1	7.50E-07	0.38	34	1.7	Adam12	Protease
DMR1:205997001	1	205997001	206002000	5000	1	7.00E-07	0.48	51	1.02	Adam12	Protease
DMR1:206046001	1	206046001	206048000	2000	1	3.50E-08	-0.43	22	1.1	Adam12	Protease
DMR1:206107001	1	206107001	206110000	3000	1	2.70E-12	0.52	32	1.07	Adam12;LOC108349655	Protease
DMR1:206138001	1	206138001	206139000	1000	1	4.60E-09	0.48	12	1.2	Adam12	Protease
DMR1:206220001	1	206220001	206223000	3000	1	1.20E-07	0.46	36	1.2	Adam12	Protease
DMR1:206237001	1	206237001	206241000	4000	1	7.60E-08	-0.28	42	1.05	Adam12	Protease
DMR1:206269001	1	206269001	206270000	1000	1	2.30E-09	0.81	29	2.9	Adam12	Protease
DMR1:207028001	1	207028001	207029000	1000	1	1.90E-07	0.56	20	2	Dock1	Transcription
DMR1:207055001	1	207055001	207057000	2000	1	3.40E-08	0.56	20	1	Dock1	Transcription
DMR1:207252001	1	207252001	207253000	1000	1	3.20E-18	0.59	0	0	Dock1	Transcription
DMR1:207940001	1	207940001	207942000	2000	2	2.10E-20	0.87	22	1.1	Ptpre;LOC108349658;LOC102551963	Signaling
DMR1:207990001	1	207990001	207991000	1000	1	9.80E-07	0.36	11	1.1	Ptpre;Mki67	Signaling
DMR1:209227001	1	209227001	209228000	1000	1	8.90E-07	0.58	16	1.6	Mgmt	
DMR1:209283001	1	209283001	209286000	3000	1	2.00E-07	0.4	46	1.53	Mgmt	
DMR1:209297001	1	209297001	209298000	1000	1	1.50E-07	0.53	13	1.3	Mgmt	
DMR1:209307001	1	209307001	209311000	4000	1	4.30E-08	-0.31	76	1.9	Mgmt	
DMR1:209319001	1	209319001	209322000	3000	1	2.80E-07	0.57	44	1.47	Mgmt	
DMR1:209458001	1	209458001	209459000	1000	1	1.30E-08	0.55	15	1.5	Mgmt	
DMR1:210618001	1	210618001	210619000	1000	1	9.80E-08	0.44	15	1.5	Tcerg1l	Transcription
DMR1:211185001	1	211185001	211186000	1000	1	2.70E-07	-0.33	18	1.8	Mapk1ip1	
DMR1:211381001	1	211381001	211383000	2000	1	8.60E-09	0.54	23	1.15	Jakmip3	
DMR1:211387001	1	211387001	211388000	1000	1	3.50E-11	0.68	16	1.6	Jakmip3	
DMR1:211431001	1	211431001	211432000	1000	1	4.30E-07	0.48	14	1.4	Jakmip3;Dpysl4;Stk32c	Metabolism;Signaling
DMR1:211507001	1	211507001	211509000	2000	1	6.10E-07	-0.36	25	1.25	Stk32c	Signaling
DMR1:211571001	1	211571001	211573000	2000	1	1.70E-07	0.52	33	1.65	Lrrc27;Pwwp2b	Epigenetic
DMR1:211794001	1	211794001	211799000	5000	1	2.20E-10	0.47	35	0.7	Inpp5a	Signaling
DMR1:211800001	1	211800001	211801000	1000	1	3.80E-16	0.65	14	1.4	Inpp5a	Signaling
DMR1:211828001	1	211828001	211829000	1000	1	3.70E-11	0.8	14	1.4	Inpp5a	Signaling
DMR1:211905001	1	211905001	211906000	1000	1	1.50E-07	0.47	15	1.5	Inpp5a	Signaling
DMR1:211927001	1	211927001	211929000	2000	1	6.60E-08	0.52	30	1.5	Inpp5a;Nkx6-2	Signaling;Development
DMR1:211956001	1	211956001	211957000	1000	1	6.30E-07	0.51	16	1.6	Cfap46	
DMR1:211960001	1	211960001	211964000	4000	1	7.00E-08	0.53	57	1.43	Cfap46	
DMR1:211965001	1	211965001	211967000	2000	1	5.80E-08	0.52	21	1.05	Cfap46	
DMR1:211974001	1	211974001	211976000	2000	1	7.30E-07	0.45	26	1.3	Cfap46	
DMR1:212249001	1	212249001	212251000	2000	1	4.70E-14	0.96	45	2.25	Kndc1	Transcription
DMR1:212334001	1	212334001	212339000	5000	1	2.80E-10	0.57	65	1.3	Adam8;Tubgcp2;LOC102554107	Protease;Cytoskeleton
DMR1:212518001	1	212518001	212520000	2000	1	1.00E-08	0.52	16	0.8	LOC680741;Msx3;LOC102551319	Development
DMR1:212528001	1	212528001	212530000	2000	1	1.50E-08	-0.69	22	1.1	LOC102551319;Caly	
DMR1:212536001	1	212536001	212537000	1000	1	8.40E-08	0.43	14	1.4	LOC102551319;Caly	
DMR1:212697001	1	212697001	212698000	1000	1	4.80E-07	0.38	12	1.2	Cd163l1	
DMR1:212852001	1	212852001	212857000	5000	1	9.00E-10	-0.39	44	0.88	Olr292;Olr290-ps	
DMR1:212910001	1	212910001	212912000	2000	1	5.20E-10	0.55	14	0.7	Olr294-ps;Olr295	Signaling
DMR1:212951001	1	212951001	212953000	2000	1	3.50E-07	-0.35	10	0.5	Olr297	
DMR1:213184001	1	213184001	213186000	2000	1	4.70E-09	0.58	12	0.6	Olr306	Signaling
DMR1:213198001	1	213198001	213199000	1000	1	5.90E-08	-0.48	5	0.5	Olr306	Signaling

DMR1:213366001	1	213366001	213370000	4000	1	2.00E-08	-0.3	41	1.02	Olr311	Signaling
DMR1:213411001	1	213411001	213416000	5000	2	5.50E-13	0.61	35	0.7	Olr312	Signaling
DMR1:213528001	1	213528001	213529000	1000	1	2.70E-08	0.35	3	0.3	Cyp2e1;Syce1;LOC103691262	Metabolism
DMR1:213669001	1	213669001	213676000	7000	1	5.60E-07	-0.27	79	1.13	Nlrp6;LOC108348167	
DMR1:213739001	1	213739001	213742000	3000	1	3.40E-07	0.46	44	1.47	LOC102555474;Pgghg;Ifitm5;LOC108349669;Ifitm2	Metabolism
DMR1:213909001	1	213909001	213910000	1000	1	4.60E-12	0.61	8	0.8	Sigirr;Ano9	Receptor
DMR1:214245001	1	214245001	214246000	1000	1	2.20E-07	-0.37	22	2.2	Phrf1;Irf7;Cdhr5	Transcription;Cytoskeleton
DMR1:214258001	1	214258001	214259000	1000	1	9.80E-07	0.47	13	1.3	Phrf1;Irf7;Cdhr5;Sct	Transcription;Cytoskeleton
DMR1:214282001	1	214282001	214289000	7000	1	9.30E-07	0.48	71	1.01	Sct;Drd4;Deaf1	Signaling;Transcription
DMR1:214350001	1	214350001	214353000	3000	2	2.70E-10	0.51	61	2.03	Eps8l2	Cytoskeleton
DMR1:214357001	1	214357001	214358000	1000	1	8.70E-09	0.53	13	1.3	Eps8l2	Cytoskeleton
DMR1:214720001	1	214720001	214723000	3000	1	8.40E-07	0.48	51	1.7	Muc5ac	
DMR1:214746001	1	214746001	214750000	4000	2	1.30E-09	0.48	75	1.88	Muc5ac	
DMR1:214769001	1	214769001	214770000	1000	1	7.80E-07	-0.37	15	1.5	Muc5b	Extracellular Matrix
DMR1:214851001	1	214851001	214852000	1000	1	2.70E-10	-0.48	10	1	Tollip	
DMR1:214937001	1	214937001	214939000	2000	1	3.20E-07	0.61	28	1.4	Brsk2;LOC103691267	Signaling
DMR1:215126001	1	215126001	215128000	2000	2	9.20E-16	0.59	12	0.6	LOC108349786;Krtap5-1;LOC103691269	
DMR1:215134001	1	215134001	215137000	3000	1	1.30E-11	0.51	22	0.73	LOC108349786;Krtap5-1;LOC103691269	
DMR1:215608001	1	215608001	215612000	4000	2	4.20E-09	0.56	66	1.65	LOC102546801;Syt8;Tnni2	Transport;Cytoskeleton
DMR1:215641001	1	215641001	215647000	6000	1	1.70E-08	0.57	82	1.37	Lsp1	Cytoskeleton
DMR1:215660001	1	215660001	215662000	2000	1	1.10E-07	0.53	32	1.6	Lsp1;Prr33;Tnnt3	Cytoskeleton;Cytoskeleton
DMR1:215719001	1	215719001	215721000	2000	1	9.40E-11	0.67	30	1.5	Mrpl23	Translation
DMR1:215745001	1	215745001	215748000	3000	1	2.20E-12	0.58	76	2.53	H19;Mir675;LOC102547221;LOC102548505	
DMR1:215823001	1	215823001	215825000	2000	1	8.00E-07	0.36	21	1.05	Igf2;Mir483	Growth Factors
DMR1:215835001	1	215835001	215837000	2000	1	1.90E-08	-0.81	146	7.3	Igf2;Mir483;LOC100909737;LOC102548568	Growth Factors
DMR1:215842001	1	215842001	215846000	4000	1	3.30E-10	0.66	63	1.57	Igf2;Mir483;LOC100909737;LOC102548568	Growth Factors
DMR1:215848001	1	215848001	215849000	1000	1	7.10E-11	0.53	8	0.8	Igf2;LOC100909737;LOC102548568;Ins2	Growth Factors
DMR1:216080001	1	216080001	216081000	1000	1	2.10E-11	0.68	24	2.4	Th	
DMR1:216084001	1	216084001	216085000	1000	1	7.40E-09	0.45	15	1.5	Th	
DMR1:216185001	1	216185001	216192000	7000	1	1.00E-07	-0.33	90	1.29	Tspan32	
DMR1:216263001	1	216263001	216264000	1000	1	1.10E-09	0.63	16	1.6	Cd81;Tssc4;Trpm5	Transport
DMR1:216274001	1	216274001	216277000	3000	1	9.20E-08	0.42	37	1.23	Trpm5	Transport
DMR1:216319001	1	216319001	216321000	2000	1	1.90E-08	-0.41	27	1.35	Kcnq1	Transport
DMR1:216342001	1	216342001	216344000	2000	1	3.40E-07	0.6	26	1.3	Kcnq1	Transport
DMR1:216369001	1	216369001	216371000	2000	1	4.40E-09	0.61	18	0.9	Kcnq1	Transport
DMR1:216372001	1	216372001	216375000	3000	1	5.20E-12	0.73	34	1.13	Kcnq1	Transport
DMR1:216384001	1	216384001	216385000	1000	1	2.40E-07	0.35	7	0.7	Kcnq1	Transport
DMR1:216523001	1	216523001	216524000	1000	1	7.80E-09	0.46	13	1.3	Kcnq1	Transport
DMR1:216573001	1	216573001	216579000	6000	1	1.10E-07	0.4	67	1.12	Kcnq1	Transport
DMR1:216611001	1	216611001	216614000	3000	1	1.00E-08	0.59	46	1.53	Kcnq1	Transport
DMR1:216636001	1	216636001	216638000	2000	1	6.00E-07	0.51	29	1.45	Kcnq1	Transport
DMR1:216718001	1	216718001	216719000	1000	1	1.20E-07	0.55	9	0.9	Nap1l4	Epigenetic
DMR1:216814001	1	216814001	216815000	1000	1	4.00E-07	0.52	13	1.3	Tnfrsf26	Receptor
DMR1:216878001	1	216878001	216879000	1000	1	8.80E-10	0.56	10	1	Osbpl5	
DMR1:216975001	1	216975001	216976000	1000	1	3.40E-07	-0.48	19	1.9	Mrgpre;Nadsyn1	Signaling;Metabolism
DMR1:217196001	1	217196001	217197000	1000	1	5.90E-07	0.61	13	1.3	Shank2	
DMR1:217201001	1	217201001	217205000	4000	2	2.40E-08	0.58	58	1.45	Shank2	
DMR1:217237001	1	217237001	217241000	4000	2	2.40E-08	0.55	56	1.4	Shank2	
DMR1:217273001	1	217273001	217275000	2000	1	1.00E-10	0.4	34	1.7	Shank2	
DMR1:217308001	1	217308001	217311000	3000	2	7.70E-09	0.53	39	1.3	Shank2	
DMR1:217323001	1	217323001	217325000	2000	1	1.40E-10	0.62	26	1.3	Shank2	
DMR1:217340001	1	217340001	217341000	1000	1	1.50E-08	0.56	12	1.2	Shank2	

DMR1:217412001	1	217412001	217414000	2000	1	5.20E-10	0.53	28	1.4	Shank2	
DMR1:217463001	1	217463001	217464000	1000	1	2.00E-09	0.47	19	1.9	Shank2	
DMR1:217466001	1	217466001	217468000	2000	1	1.90E-08	-0.38	32	1.6	Shank2	
DMR1:217488001	1	217488001	217491000	3000	1	2.50E-07	0.56	60	2	Shank2	
DMR1:217494001	1	217494001	217495000	1000	1	3.90E-07	0.4	7	0.7	Shank2	
DMR1:217521001	1	217521001	217522000	1000	1	3.30E-09	0.64	16	1.6	Shank2	
DMR1:217542001	1	217542001	217543000	1000	1	6.50E-09	0.53	7	0.7	Shank2	
DMR1:217590001	1	217590001	217591000	1000	1	7.10E-07	0.37	24	2.4	Shank2	
DMR1:217774001	1	217774001	217777000	3000	1	1.80E-12	0.71	57	1.9	Ano1	
DMR1:217794001	1	217794001	217797000	3000	1	6.50E-08	0.48	45	1.5	Ano1	
DMR1:218031001	1	218031001	218032000	1000	1	2.30E-07	0.49	9	0.9	Fgf4	Growth Factors
DMR1:218036001	1	218036001	218039000	3000	1	8.20E-07	0.58	44	1.47	Fgf4	Growth Factors
DMR1:218051001	1	218051001	218052000	1000	1	5.60E-07	0.49	11	1.1	Fgf19	Growth Factors
DMR1:218409001	1	218409001	218412000	3000	1	5.50E-07	0.46	37	1.23	Tpcn2	Transport
DMR1:218419001	1	218419001	218421000	2000	1	2.40E-10	0.68	29	1.45	Tpcn2	Transport
DMR1:218456001	1	218456001	218457000	1000	1	8.30E-07	-0.37	10	1	Tpcn2;Mrgprf	Transport;Signaling
DMR1:218513001	1	218513001	218514000	1000	1	2.70E-07	0.51	18	1.8	Ighmbp2	Transcription
DMR1:218547001	1	218547001	218550000	3000	1	5.40E-11	0.57	47	1.57	Mrpl21	Translation
DMR1:218585001	1	218585001	218590000	5000	1	1.70E-09	0.43	44	0.88	Cpt1a	Metabolism
DMR1:218700001	1	218700001	218701000	1000	1	2.10E-08	-0.43	18	1.8	Ppp6r3	Signaling
DMR1:218847001	1	218847001	218849000	2000	1	2.20E-07	0.45	33	1.65	Lrp5	Receptor
DMR1:218935001	1	218935001	218936000	1000	1	8.90E-07	-0.44	11	1.1	RGD1311946	
DMR1:219022001	1	219022001	219023000	1000	1	5.40E-08	-0.69	1	0.1	Kmt5b	
DMR1:219128001	1	219128001	219131000	3000	1	6.00E-08	0.54	37	1.23	Chka;Tcirg1	Signaling;Metabolism
DMR1:219177001	1	219177001	219179000	2000	2	3.00E-10	0.63	61	3.05	Aldh3b1;Unc93b1	Metabolism
DMR1:219226001	1	219226001	219230000	4000	1	8.80E-07	-0.36	79	1.98	Aldh3b2;Acy3;Tbx10	Transcription
DMR1:219242001	1	219242001	219244000	2000	1	1.40E-07	0.38	35	1.75	Acy3;Tbx10;LOC103691273;Nudt8;Doc2g	Transcription;Signaling
DMR1:219284001	1	219284001	219286000	2000	1	6.10E-09	0.56	22	1.1	Gstp1	Transport
DMR1:219298001	1	219298001	219300000	2000	1	3.50E-07	0.36	16	0.8	Gstp1;RGD1307603	Transport
DMR1:219324001	1	219324001	219325000	1000	1	1.20E-08	-0.42	12	1.2	Cabp2	
DMR1:219331001	1	219331001	219333000	2000	1	6.70E-07	0.36	28	1.4	Cabp2	
DMR1:219425001	1	219425001	219426000	1000	1	7.20E-07	0.5	11	1.1	Carns1;Tbc1d10c	Signaling
DMR1:219451001	1	219451001	219455000	4000	1	2.00E-08	0.6	43	1.07	Ppp1ca;Rad9a	Signaling;DNA Repair
DMR1:219512001	1	219512001	219514000	2000	1	8.40E-07	0.46	25	1.25	LOC103691274;Ssh3;Ankrd13d	Signaling
DMR1:219602001	1	219602001	219604000	2000	1	4.70E-08	-0.4	23	1.15	Kdm2a	
DMR1:219674001	1	219674001	219677000	3000	1	2.10E-07	0.47	34	1.13	Rhod	Signaling
DMR1:219703001	1	219703001	219705000	2000	1	8.60E-14	-0.56	22	1.1	Syt12	Transport
DMR1:219770001	1	219770001	219772000	2000	1	5.20E-11	-0.62	8	0.4	Pc	Metabolism
DMR1:219902001	1	219902001	219906000	4000	1	5.90E-07	-0.55	31	0.78	RGD1563323	
DMR1:220046001	1	220046001	220048000	2000	1	5.60E-07	-0.32	30	1.5	Rbm4;Rbm14	Translation
DMR1:220098001	1	220098001	220102000	4000	1	1.30E-07	-0.57	53	1.32	Ccs;Ccdc87	Metabolism
DMR1:220107001	1	220107001	220110000	3000	1	1.70E-07	-0.4	33	1.1	Ccdc87;Ctsf	Protease
DMR1:220127001	1	220127001	220131000	4000	1	1.50E-07	0.47	59	1.48	Ctsf;Actn3;Zdhhc24	Protease
DMR1:220135001	1	220135001	220136000	1000	1	3.40E-07	0.52	10	1	Actn3;Zdhhc24	
DMR1:220676001	1	220676001	220677000	1000	1	1.30E-13	1.01	47	4.7	Gal3st3	Transport
DMR1:220678001	1	220678001	220679000	1000	1	2.10E-09	-0.43	2	0.2	Gal3st3	Transport
DMR1:220684001	1	220684001	220686000	2000	1	4.10E-09	0.56	27	1.35	Gal3st3	Transport
DMR1:220738001	1	220738001	220740000	2000	1	1.50E-07	0.43	25	1.25	Cst6;Banf1;Eif1ad	Translation
DMR1:220769001	1	220769001	220771000	2000	1	1.00E-07	-0.4	57	2.85	LOC103691278;Sart1;LOC102555676;Tsga10ip	Extracellular Matrix
DMR1:220796001	1	220796001	220798000	2000	1	4.70E-07	-0.39	24	1.2	Tsga10ip;Drap1;Bles03	Transcription
DMR1:220829001	1	220829001	220831000	2000	1	3.20E-08	0.61	24	1.2	Fosl1;Ccdc85b;Fibp	Transcription
DMR1:220891001	1	220891001	220894000	3000	1	1.70E-10	-0.41	66	2.2	Snx32;LOC102551929;LOC102556290	Cytoskeleton
DMR1:221075001	1	221075001	221080000	5000	1	3.70E-10	0.42	66	1.32	Ehbp1l1;Fam89b;Sssca1	Cytoskeleton
DMR1:221108001	1	221108001	221109000	1000	1	1.60E-11	0.78	33	3.3	Ltbp3;Scyl1	Extracellular Matrix;Signaling
DMR1:221134001	1	221134001	221136000	2000	1	8.80E-13	0.56	34	1.7	Scyl1	Signaling
DMR1:221287001	1	221287001	221290000	3000	1	4.90E-07	-0.39	27	0.9	Cdc42ep2;LOC108348875;Pola2	Transcription
DMR1:221349001	1	221349001	221352000	3000	1	9.30E-09	0.37	47	1.57	Slc22a20;Capn1	Transport;Protease

DMR1:221380001	1	221380001	221382000	2000	1	5.30E-08	0.57	22	1.1	Capn1;LOC108349685	Protease
DMR1:221506001	1	221506001	221509000	3000	1	3.00E-10	-0.43	47	1.57	Snx15;Arl2	Signaling
DMR1:221618001	1	221618001	221623000	5000	1	5.70E-07	0.45	99	1.98	Atg2a	
DMR1:221624001	1	221624001	221625000	1000	1	4.50E-09	0.44	21	2.1	Atg2a;Mir194-2;Mir192	
DMR1:221650001	1	221650001	221654000	4000	2	5.70E-11	0.65	41	1.02	Ehd1	Transport
DMR1:221720001	1	221720001	221723000	3000	1	8.60E-07	0.5	34	1.13	Men1;Map4k2;LOC108349687	
DMR1:221756001	1	221756001	221758000	2000	1	1.80E-07	0.5	35	1.75	Sf1;Pygm	Translation;Golgi
DMR1:221785001	1	221785001	221788000	3000	2	1.40E-13	0.79	66	2.2	Rasgrp2;Nrxn2	Transcription
DMR1:221837001	1	221837001	221839000	2000	1	1.90E-09	-0.34	35	1.75	Nrxn2	
DMR1:221848001	1	221848001	221849000	1000	1	1.40E-07	0.55	10	1	Nrxn2	
DMR1:222090001	1	222090001	222091000	1000	1	8.10E-08	0.53	26	2.6	Rps6ka4	Golgi
DMR1:222100001	1	222100001	222109000	9000	1	1.80E-07	0.5	179	1.99	Rps6ka4;Ccdc88b	Golgi;Transport
DMR1:222115001	1	222115001	222117000	2000	1	4.90E-11	0.81	64	3.2	Ccdc88b	Transport
DMR1:222156001	1	222156001	222159000	3000	1	5.20E-13	0.92	43	1.43	Prdx5;Trmt112;Esrra	Metabolism
DMR1:222484001	1	222484001	222487000	3000	1	9.90E-10	-0.45	57	1.9	Naa40;LOC102551606	
DMR1:222588001	1	222588001	222590000	2000	1	2.60E-09	-0.58	42	2.1	Mark2	Signaling
DMR1:222821001	1	222821001	222823000	2000	1	4.40E-08	0.59	24	1.2	LOC686151;RGD1563402	
DMR1:224324001	1	224324001	224326000	2000	1	2.20E-07	-0.35	48	2.4	UST4r	Transport
DMR1:224467001	1	224467001	224473000	6000	1	6.50E-07	-0.25	59	0.98	Ust5r	Transport
DMR1:224493001	1	224493001	224495000	2000	1	5.70E-08	0.23	29	1.45	Ust5r	Transport
DMR1:224806001	1	224806001	224809000	3000	1	7.10E-11	-0.42	25	0.83	Slc22a8	Transport
DMR1:224864001	1	224864001	224865000	1000	1	7.80E-08	-0.43	7	0.7	Chrm1	Signaling
DMR1:224895001	1	224895001	224897000	2000	1	3.90E-07	0.58	14	0.7	Chrm1;Slc3a2	Signaling
DMR1:224999001	1	224999001	2.25E+08	1000	1	6.60E-08	0.56	14	1.4	Polr2g;Zbtb3;Ttc9c	Transcription;Transcription;Transcription
DMR1:225069001	1	225069001	225070000	1000	1	2.20E-08	-0.34	18	1.8	Ubxn1;Uqcc3	Transcription
DMR1:225142001	1	225142001	225145000	3000	1	2.10E-08	-0.4	51	1.7	Eml3;Mta2;Tut1	Development;Metabolism
DMR1:225176001	1	225176001	225177000	1000	1	1.10E-07	0.5	10	1	Eef1g;Ahnak	
DMR1:225183001	1	225183001	225185000	2000	1	5.40E-07	0.64	50	2.5	Eef1g;Ahnak	
DMR1:225258001	1	225258001	225259000	1000	1	5.80E-14	-0.49	10	1	Ahnak	
DMR1:225284001	1	225284001	225286000	2000	1	9.70E-07	-0.31	23	1.15	Ahnak;Scgb1a1	Growth Factors
DMR1:225293001	1	225293001	225295000	2000	1	1.20E-08	-0.43	35	1.75	Ahnak;Scgb1a1	Growth Factors
DMR1:225350001	1	225350001	225355000	5000	1	1.30E-07	-0.29	37	0.74	Ahnak	
DMR1:225363001	1	225363001	225367000	4000	1	9.40E-07	-0.34	34	0.85	Ahnak	
DMR1:225368001	1	225368001	225370000	2000	1	9.80E-08	-0.36	77	3.85	Ahnak	
DMR1:225460001	1	225460001	225466000	6000	1	2.90E-08	-0.29	58	0.97	LOC108348288;RGD1566289	
DMR1:225471001	1	225471001	225473000	2000	1	2.20E-09	-0.36	16	0.8	RGD1566289	
DMR1:225584001	1	225584001	225587000	3000	1	3.90E-07	-0.61	7	0.23	RGD1566289;LOC690454;Scgb1d2	
DMR1:225597001	1	225597001	225598000	1000	1	1.10E-07	-0.47	5	0.5	Scgb1d2	
DMR1:225694001	1	225694001	225696000	2000	1	1.80E-07	-0.68	6	0.3	Psbpc2	Transport
DMR1:225964001	1	225964001	225966000	2000	1	1.00E-07	-0.44	22	1.1	Fads2;LOC102552566	
DMR1:225967001	1	225967001	225970000	3000	1	7.90E-08	-0.46	36	1.2	Fads2;LOC102552566	
DMR1:225989001	1	225989001	225991000	2000	2	2.20E-07	-0.43	30	1.5	Fads2;LOC102552566	
DMR1:226025001	1	226025001	226030000	5000	2	6.30E-08	-0.34	78	1.56	Fads2;LOC102552566;Fth1;Best1;LOC102554361	Transport;Transport
DMR1:226087001	1	226087001	226088000	1000	1	5.60E-08	-0.57	9	0.9	Fads2;Rab3il1;Fads3	Transcription
DMR1:226123001	1	226123001	226126000	3000	1	1.10E-07	-0.51	33	1.1	Fads2	
DMR1:226133001	1	226133001	226137000	4000	2	5.70E-15	-0.58	55	1.38	Fads2	
DMR1:226139001	1	226139001	226142000	3000	2	1.80E-11	-0.47	43	1.43	Fads2	
DMR1:226282001	1	226282001	226287000	5000	1	9.20E-07	0.49	73	1.46	Myrf	
DMR1:226324001	1	226324001	226327000	3000	1	1.40E-08	0.48	28	0.93	Dagla	Metabolism
DMR1:226354001	1	226354001	226356000	2000	1	6.90E-08	0.56	19	0.95	Dagla;LOC100911105;LOC102552972;LOC108349691	Metabolism
DMR1:226514001	1	226514001	226515000	1000	1	1.70E-07	0.39	17	1.7	Ppp1r32	Signaling
DMR1:226553001	1	226553001	226556000	3000	1	6.10E-09	-0.49	26	0.87	Sdhaf2	
DMR1:226692001	1	226692001	226694000	2000	1	6.70E-12	-0.58	11	0.55	Ddb1;Vwce	Translation
DMR1:226750001	1	226750001	226751000	1000	1	1.70E-07	-0.42	13	1.3	Vps37c	
DMR1:226850001	1	226850001	226851000	1000	1	1.80E-09	0.63	14	1.4	Cd6	Protease
DMR1:226886001	1	226886001	226888000	2000	1	2.40E-07	0.51	31	1.55	Cd6;Slc15a3	Protease;Transport
DMR1:226903001	1	226903001	226908000	5000	1	6.20E-08	0.35	57	1.14	Slc15a3;Tmem132a	Transport

DMR1:226909001	1	226909001	226912000	3000	1	1.10E-10	0.7	40	1.33	Slc15a3;Tmem132a	Transport
DMR1:226942001	1	226942001	226944000	2000	1	3.10E-10	-0.45	12	0.6	Tmem109;Prpf19	Translation
DMR1:226968001	1	226968001	226970000	2000	1	1.30E-10	0.49	26	1.3	Prpf19;Zp1;Ptgdr2	Translation;Signaling
DMR1:227013001	1	227013001	227018000	5000	1	6.00E-09	0.48	59	1.18	Ms4a10	Transport
DMR1:227036001	1	227036001	227039000	3000	1	5.40E-09	0.6	45	1.5	Ms4a10;Ms4a15	Transport
DMR1:227134001	1	227134001	227135000	1000	1	3.00E-07	-0.33	1	0.1	Ms4a8	Transport
DMR1:227363001	1	227363001	227366000	3000	1	3.10E-10	-0.59	14	0.47	LOC100912336;Ms4a13-ps1	Transport
DMR1:228371001	1	228371001	228373000	2000	1	1.40E-08	-0.46	21	1.05	Patl1;LOC103691294	Translation
DMR1:229045001	1	229045001	229047000	2000	1	2.90E-07	-0.47	14	0.7	Glyatl1	Metabolism
DMR1:229206001	1	229206001	229207000	1000	1	1.70E-12	0.83	23	2.3	RGD1305704	
DMR1:229473001	1	229473001	229474000	1000	1	2.80E-08	-0.64	5	0.5	Olr387	Signaling
DMR1:229525001	1	229525001	229527000	2000	1	5.00E-08	-0.46	3	0.15	Glyatl2	
DMR1:229715001	1	229715001	229716000	1000	1	1.60E-08	-0.37	6	0.6	Olr339	Receptor
DMR1:229753001	1	229753001	229754000	1000	1	3.00E-07	-0.43	9	0.9	Olr341;Olr340	Receptor
DMR1:229856001	1	229856001	229860000	4000	2	3.10E-10	-0.5	59	1.48	Olr346	Receptor
DMR1:230088001	1	230088001	230092000	4000	2	3.70E-07	-0.41	37	0.92	Olr358;Olr359-ps	Receptor
DMR1:230151001	1	230151001	230158000	7000	1	7.70E-08	0.27	67	0.96	Olr361	Receptor
DMR1:230835001	1	230835001	230837000	2000	1	3.80E-09	-0.48	9	0.45	Vom2r-ps69	
DMR1:231469001	1	231469001	231470000	1000	1	7.10E-10	-0.53	11	1.1	Tle4	Transcription
DMR1:231535001	1	231535001	231536000	1000	1	1.20E-12	-0.63	9	0.9	Tle4	Transcription
DMR1:231541001	1	231541001	231547000	6000	1	1.90E-10	-0.45	68	1.13	Tle4	Transcription
DMR1:233178001	1	233178001	233179000	1000	1	4.40E-14	-0.66	23	2.3	Cep78	
DMR1:233397001	1	233397001	233401000	4000	1	3.10E-09	-0.52	34	0.85	Gnaq	Signaling
DMR1:233415001	1	233415001	233417000	2000	1	2.40E-09	-0.58	17	0.85	Gnaq	Signaling
DMR1:233613001	1	233613001	233615000	2000	1	6.20E-07	-0.3	18	0.9	Gnaq	Signaling
DMR1:234297001	1	234297001	234300000	3000	1	5.90E-07	-0.39	26	0.87	Rorb;LOC108349695	Transcription
DMR1:234413001	1	234413001	234414000	1000	1	3.30E-09	0.55	20	2	Rorb	Transcription
DMR1:234506001	1	234506001	234510000	4000	1	8.90E-09	-0.36	32	0.8	Trpm6	Transport
DMR1:234615001	1	234615001	234617000	2000	2	5.40E-17	0.45	9	0.45	Trpm6;LOC108349696	Transport
DMR1:234724001	1	234724001	234729000	5000	1	1.40E-07	0.42	64	1.28	Nmrk1;LOC108349697	Signaling
DMR1:235155001	1	235155001	235160000	5000	1	7.70E-11	-0.46	44	0.88	Gna14	Signaling
DMR1:235169001	1	235169001	235171000	2000	1	6.10E-07	-0.39	32	1.6	Gna14	Signaling
DMR1:235361001	1	235361001	235365000	4000	2	3.00E-08	-0.3	31	0.78	Vps13a	
DMR1:235456001	1	235456001	235457000	1000	1	9.80E-07	-0.45	4	0.4	Vps13a	
DMR1:235535001	1	235535001	235537000	2000	1	1.10E-07	-0.49	20	1	Vps13a	
DMR1:235622001	1	235622001	235625000	3000	1	2.50E-09	0.45	64	2.13	Vps13a	
DMR1:235799001	1	235799001	235800000	1000	1	6.70E-10	0.66	5	0.5	Vps13a	
DMR1:235806001	1	235806001	235807000	1000	1	1.80E-11	-0.49	14	1.4	Vps13a	
DMR1:236137001	1	236137001	236138000	1000	1	5.10E-08	-0.42	5	0.5	Pcsk5	Protease
DMR1:236268001	1	236268001	236270000	2000	1	1.40E-11	0.45	9	0.45	Pcsk5	Protease
DMR1:236320001	1	236320001	236321000	1000	1	1.50E-09	0.44	11	1.1	Pcsk5	Protease
DMR1:236322001	1	236322001	236323000	1000	1	1.40E-07	-0.55	4	0.4	Pcsk5	Protease
DMR1:236566001	1	236566001	236570000	4000	1	3.90E-07	0.61	42	1.05	Gcnt1	Golgi
DMR1:236575001	1	236575001	236577000	2000	1	1.20E-16	-0.45	21	1.05	Gcnt1	Golgi
DMR1:236771001	1	236771001	236772000	1000	1	2.00E-07	-0.38	29	2.9	Prune2	
DMR1:236889001	1	236889001	236891000	2000	1	2.00E-07	-0.39	20	1	Prune2	
DMR1:238403001	1	238403001	238404000	1000	1	6.10E-08	0.78	21	2.1	Tmc1	
DMR1:238467001	1	238467001	238468000	1000	1	7.90E-08	0.45	15	1.5	Tmc1	
DMR1:238483001	1	238483001	238484000	1000	1	6.30E-08	-0.69	7	0.7	Tmc1	
DMR1:239208001	1	239208001	239210000	2000	1	9.00E-07	-0.45	24	1.2	RGD1359158	
DMR1:239457001	1	239457001	239458000	1000	1	7.90E-08	-0.4	25	2.5	Tmem2	
DMR1:239459001	1	239459001	239460000	1000	1	1.10E-11	0.84	35	3.5	Tmem2	
DMR1:239765001	1	239765001	239766000	1000	1	1.30E-08	-0.67	6	0.6	Trpm3	Transport
DMR1:240322001	1	240322001	240325000	3000	1	4.00E-08	-0.34	24	0.8	Trpm3	Transport
DMR1:240373001	1	240373001	240374000	1000	1	5.80E-08	-0.43	9	0.9	Trpm3;LOC102552001	Transport
DMR1:240574001	1	240574001	240576000	2000	1	1.30E-07	0.36	19	0.95	Trpm3;LOC108349824;Aldh1a7	Transport;Metabolism
DMR1:240670001	1	240670001	240671000	1000	1	7.00E-09	0.58	22	2.2	Trpm3	Transport
DMR1:240685001	1	240685001	240686000	1000	1	6.80E-08	0.35	14	1.4	Trpm3	Transport
DMR1:240754001	1	240754001	240759000	5000	1	9.70E-07	0.38	74	1.48	Trpm3;LOC103691311	Transport
DMR1:241064001	1	241064001	241066000	2000	1	1.30E-08	0.57	30	1.5	Mamdc2	
DMR1:241068001	1	241068001	241069000	1000	1	4.50E-07	-0.38	20	2	Mamdc2	

DMR1:241641001	1	241641001	241643000	2000	1	2.00E-08	0.34	4	0.2	Apba1	Transport
DMR1:241658001	1	241658001	241660000	2000	2	1.40E-07	-0.4	20	1	Apba1	Transport
DMR1:241802001	1	241802001	241804000	2000	1	5.40E-09	-0.42	27	1.35	Apba1	Transport
DMR1:241814001	1	241814001	241818000	4000	1	7.90E-07	-0.35	40	1	Fam189a2	
DMR1:241846001	1	241846001	241847000	1000	1	1.60E-07	-0.53	12	1.2	Fam189a2	
DMR1:241855001	1	241855001	241858000	3000	1	2.10E-10	-0.42	41	1.37	Fam189a2	
DMR1:241939001	1	241939001	241942000	3000	1	2.50E-08	-0.38	48	1.6	Tjp2	Cell Junction
DMR1:241956001	1	241956001	241960000	4000	1	4.80E-07	-0.38	76	1.9	Tjp2	Cell Junction
DMR1:241983001	1	241983001	241985000	2000	1	2.90E-07	-0.45	32	1.6	Tjp2	Cell Junction
DMR1:242172001	1	242172001	242175000	3000	3	6.60E-27	1.12	51	1.7	Pip5k1b	Signaling
DMR1:242195001	1	242195001	242198000	3000	1	3.00E-08	-0.41	48	1.6	Pip5k1b	Signaling
DMR1:242210001	1	242210001	242212000	2000	1	3.00E-18	1.15	59	2.95	Pip5k1b	Signaling
DMR1:242216001	1	242216001	242219000	3000	2	2.40E-08	-0.44	38	1.27	Pip5k1b	Signaling
DMR1:242228001	1	242228001	242230000	2000	1	1.70E-07	0.49	48	2.4	Pip5k1b	Signaling
DMR1:242262001	1	242262001	242264000	2000	1	9.50E-08	-0.35	40	2	Pip5k1b	Signaling
DMR1:242326001	1	242326001	242327000	1000	1	1.10E-09	-0.44	22	2.2	Pip5k1b	Signaling
DMR1:242349001	1	242349001	242352000	3000	1	4.70E-10	-0.54	64	2.13	Pip5k1b	Signaling
DMR1:242363001	1	242363001	242366000	3000	1	3.70E-09	-0.42	40	1.33	Pip5k1b;Fam122a	Signaling
DMR1:242390001	1	242390001	242392000	2000	1	1.30E-12	-0.56	23	1.15	Pip5k1b	Signaling
DMR1:242408001	1	242408001	242411000	3000	1	4.00E-13	-0.67	59	1.97	Pip5k1b	Signaling
DMR1:242412001	1	242412001	242419000	7000	4	1.80E-15	-0.73	130	1.86	Pip5k1b	Signaling
DMR1:242675001	1	242675001	242679000	4000	3	6.80E-18	0.49	20	0.5	Pgm5	Metabolism
DMR1:242752001	1	242752001	242754000	2000	1	7.50E-07	-0.48	21	1.05	Pgm5	Metabolism
DMR1:242770001	1	242770001	242771000	1000	1	3.50E-07	-0.68	5	0.5	Pgm5	Metabolism
DMR1:243027001	1	243027001	243031000	4000	1	3.60E-08	-0.43	47	1.18	Dock8	Transcription
DMR1:243046001	1	243046001	243047000	1000	1	9.00E-10	-0.51	18	1.8	Dock8	Transcription
DMR1:243283001	1	243283001	243284000	1000	1	1.10E-16	0.84	7	0.7	Kank1	Cytoskeleton
DMR1:243315001	1	243315001	243317000	2000	2	2.20E-07	-0.31	40	2	Kank1;LOC102554752	Cytoskeleton
DMR1:243489001	1	243489001	243492000	3000	1	5.60E-07	-0.34	58	1.93	Dmrt1	Transcription
DMR1:243511001	1	243511001	243512000	1000	1	6.30E-09	-0.44	16	1.6	Dmrt1	Transcription
DMR1:243592001	1	243592001	243593000	1000	1	1.10E-08	-0.48	15	1.5	Dmrt1;Dmrt3	Transcription
DMR1:244734001	1	244734001	244738000	4000	1	1.20E-12	-0.62	66	1.65	Smarca2	Epigenetic
DMR1:244781001	1	244781001	244782000	1000	1	3.70E-11	-0.41	19	1.9	Smarca2	Epigenetic
DMR1:244793001	1	244793001	244799000	6000	1	1.30E-18	1.16	117	1.95	Smarca2	Epigenetic
DMR1:245245001	1	245245001	245246000	1000	1	2.00E-11	0.5	17	1.7	Vldlr	Binding Proteins
DMR1:245259001	1	245259001	245260000	1000	1	4.60E-08	0.58	14	1.4	Vldlr	Binding Proteins
DMR1:245869001	1	245869001	245870000	1000	1	8.60E-08	0.6	6	0.6	Rfx3	Transcription
DMR1:246475001	1	246475001	246476000	1000	1	4.00E-08	-0.36	16	1.6	Glis3	Transcription
DMR1:246568001	1	246568001	246573000	5000	1	2.50E-09	-0.49	75	1.5	Glis3;LOC102556263	Transcription
DMR1:246574001	1	246574001	246577000	3000	1	1.60E-07	-0.29	42	1.4	Glis3;LOC102556263	Transcription
DMR1:246697001	1	246697001	246698000	1000	1	1.80E-08	-0.41	18	1.8	NEWGENE_1565505;LOC102550766	
DMR1:246739001	1	246739001	246742000	3000	1	2.70E-10	-0.56	55	1.83	NEWGENE_1565505;LOC103691328	
DMR1:246746001	1	246746001	246747000	1000	1	2.60E-07	-0.38	20	2	NEWGENE_1565505;LOC103691328	
DMR1:246748001	1	246748001	246750000	2000	1	1.80E-07	-0.4	22	1.1	NEWGENE_1565505;LOC103691328	
DMR1:246964001	1	246964001	246966000	2000	1	1.60E-14	-0.53	25	1.25	LOC102546345;Slc1a1	Transport
DMR1:246971001	1	246971001	246974000	3000	1	1.10E-07	0.65	44	1.47	Slc1a1	Transport
DMR1:246986001	1	246986001	246988000	2000	1	1.50E-08	-0.5	48	2.4	Slc1a1	Transport
DMR1:246995001	1	246995001	246999000	4000	1	2.60E-07	0.54	61	1.52	Slc1a1	Transport
DMR1:247018001	1	247018001	247020000	2000	1	3.60E-11	0.56	26	1.3	Slc1a1	Transport
DMR1:247099001	1	247099001	247102000	3000	1	8.50E-10	0.37	32	1.07	LOC680727;RGD1565057;Cdc37l1	Transcription
DMR1:247118001	1	247118001	247120000	2000	1	1.70E-07	-0.65	17	0.85	RGD1565057;Cdc37l1	Transcription
DMR1:247217001	1	247217001	247220000	3000	1	1.30E-09	-0.64	36	1.2	Rcl1	Metabolism
DMR1:247417001	1	247417001	247420000	3000	1	3.00E-10	-0.5	32	1.07	Jak2	
DMR1:247468001	1	247468001	247470000	2000	1	3.60E-08	0.58	37	1.85	Insl6;LOC102546806	Hormone
DMR1:247478001	1	247478001	247480000	2000	1	1.00E-07	0.51	24	1.2	Insl6;LOC102546806;Rln1	Hormone
DMR1:247483001	1	247483001	247485000	2000	1	1.30E-09	0.8	45	2.25	Insl6;LOC102546806;Rln1	Hormone
DMR1:247487001	1	247487001	247489000	2000	1	8.00E-09	-0.44	20	1	LOC102546806;Rln1	Hormone

DMR1:247528001	1	247528001	247531000	3000	1	2.40E-07	0.33	33	1.1	Cd274	Immune
DMR1:247534001	1	247534001	247537000	3000	1	3.80E-07	-0.38	46	1.53	Cd274	Immune
DMR1:247577001	1	247577001	247578000	1000	1	1.20E-08	-0.5	2	0.2	Pdcd1lg2;LOC102547032	Immune
DMR1:247628001	1	247628001	247631000	3000	1	6.60E-09	0.34	28	0.93	Pdcd1lg2	Immune
DMR1:247902001	1	247902001	247903000	1000	1	1.20E-08	-0.36	19	1.9	RGD1311595	
DMR1:248112001	1	248112001	248114000	2000	1	1.00E-06	-0.39	31	1.55	Il33	
DMR1:248124001	1	248124001	248126000	2000	2	1.70E-10	0.66	24	1.2	Il33	
DMR1:248146001	1	248146001	248147000	1000	1	2.00E-08	0.52	17	1.7	Il33	
DMR1:248188001	1	248188001	248189000	1000	1	4.00E-12	0.81	30	3	Tpd52l3	
DMR1:248245001	1	248245001	248246000	1000	1	6.30E-09	-0.53	9	0.9	Uhrf2	Proteolysis
DMR1:248307001	1	248307001	248309000	2000	1	9.20E-08	-0.35	30	1.5	Gldc	Metabolism
DMR1:248368001	1	248368001	248370000	2000	1	1.70E-09	-0.48	34	1.7	Gldc	Metabolism
DMR1:249054001	1	249054001	249056000	2000	1	4.70E-07	-0.56	10	0.5	Prkg1	
DMR1:249304001	1	249304001	249309000	5000	1	6.40E-10	-0.4	43	0.86	Prkg1	
DMR1:249476001	1	249476001	249480000	4000	1	4.80E-10	-0.47	33	0.82	Prkg1	
DMR1:249595001	1	249595001	249600000	5000	2	1.90E-14	0.9	74	1.48	Prkg1	
DMR1:249605001	1	249605001	249607000	2000	1	2.10E-08	0.36	31	1.55	Prkg1	
DMR1:249641001	1	249641001	249642000	1000	1	9.80E-09	-0.44	11	1.1	Prkg1	
DMR1:249650001	1	249650001	249651000	1000	1	8.50E-07	-0.6	6	0.6	Prkg1	
DMR1:250052001	1	250052001	250053000	1000	1	3.00E-07	-0.37	11	1.1	Prkg1	
DMR1:250094001	1	250094001	250097000	3000	1	1.10E-12	-0.53	32	1.07	Prkg1	
DMR1:250166001	1	250166001	250169000	3000	1	3.30E-11	-0.47	28	0.93	Prkg1	
DMR1:250279001	1	250279001	250281000	2000	1	7.10E-08	-0.42	21	1.05	Prkg1	
DMR1:250904001	1	250904001	250906000	2000	1	3.60E-07	-0.3	23	1.15	Sgms1	
DMR1:250926001	1	250926001	250928000	2000	1	1.30E-10	-0.43	23	1.15	Sgms1	
DMR1:251052001	1	251052001	251054000	2000	1	1.20E-09	-0.43	31	1.55	Minpp1	Signaling
DMR1:251075001	1	251075001	251080000	5000	1	6.80E-07	0.59	78	1.56	Minpp1	Signaling
DMR1:251146001	1	251146001	251147000	1000	1	5.00E-08	0.53	22	2.2	Papss2	
DMR1:251192001	1	251192001	251196000	4000	1	1.10E-07	0.43	38	0.95	Papss2	
DMR1:251209001	1	251209001	251211000	2000	1	1.20E-11	0.88	28	1.4	Papss2	
DMR1:251216001	1	251216001	251220000	4000	1	3.20E-08	-0.38	50	1.25	Papss2	
DMR1:251341001	1	251341001	251344000	3000	1	2.90E-08	-0.44	29	0.97	Atad1;Cyp2b15;LOC100910127	Metabolism
DMR1:251358001	1	251358001	251360000	2000	1	5.40E-08	-0.49	18	0.9	Atad1;LOC100910127	Metabolism
DMR1:251388001	1	251388001	251390000	2000	1	7.80E-13	-0.55	19	0.95	Atad1	
DMR1:251602001	1	251602001	251606000	4000	1	7.40E-07	-0.47	76	1.9	RGD1565695	
DMR1:251827001	1	251827001	251831000	4000	1	3.60E-08	-0.52	32	0.8	Rnls	
DMR1:251954001	1	251954001	251956000	2000	1	7.50E-08	-0.3	25	1.25	Rnls	
DMR1:252358001	1	252358001	252359000	1000	1	2.10E-12	-0.66	6	0.6	Lipk	Metabolism
DMR1:252455001	1	252455001	252456000	1000	1	2.90E-12	0.83	24	2.4	Ankrd22	
DMR1:252464001	1	252464001	252466000	2000	2	1.80E-11	-0.64	15	0.75	Ankrd22	
DMR1:252480001	1	252480001	252481000	1000	1	5.20E-09	-0.62	2	0.2	Stambpl1	Protease
DMR1:252594001	1	252594001	252595000	1000	1	1.60E-07	0.31	8	0.8	Fas	Receptor
DMR1:252857001	1	252857001	252860000	3000	1	1.00E-08	0.43	26	0.87	Lipa	Metabolism
DMR1:252895001	1	252895001	252897000	2000	1	1.90E-08	-0.48	19	0.95	Lipa;Ifit2;Ifit3	Metabolism
DMR1:253008001	1	253008001	253009000	1000	1	8.40E-10	-0.49	14	1.4	Slc16a12	Transport
DMR1:253151001	1	253151001	253154000	3000	1	4.70E-09	0.72	37	1.23	Pank1	Signaling
DMR1:253165001	1	253165001	253166000	1000	1	1.30E-09	-0.4	15	1.5	Pank1	Signaling
DMR1:253255001	1	253255001	253256000	1000	1	7.90E-11	-0.49	11	1.1	Kif20b	Cytoskeleton
DMR1:253285001	1	253285001	253288000	3000	1	1.50E-09	-0.4	30	1	Kif20b;Ears2l1	Cytoskeleton
DMR1:253304001	1	253304001	253306000	2000	1	1.40E-07	0.54	29	1.45	Ears2l1	
DMR1:254548001	1	254548001	254550000	2000	1	5.80E-07	-0.4	22	1.1	Htr7	Signaling
DMR1:254605001	1	254605001	254607000	2000	1	1.80E-11	0.53	28	1.4	Htr7	Signaling
DMR1:254627001	1	254627001	254634000	7000	1	7.70E-08	-0.32	84	1.2	Htr7;LOC108349452	Signaling
DMR1:254673001	1	254673001	254674000	1000	1	2.60E-07	0.46	21	2.1	Htr7	Signaling
DMR1:254737001	1	254737001	254738000	1000	1	2.00E-08	-0.46	4	0.4	Ankrd1	
DMR1:255047001	1	255047001	255048000	1000	1	4.00E-07	-0.53	10	1	Pcgf5	Epigenetic
DMR1:255070001	1	255070001	255072000	2000	1	3.40E-07	-0.35	29	1.45	Pcgf5	Epigenetic
DMR1:255209001	1	255209001	255211000	2000	1	2.30E-09	-0.54	12	0.6	Hectd2	Proteolysis
DMR1:255546001	1	255546001	255549000	3000	1	2.90E-07	0.55	59	1.97	Fgfbp3	
DMR1:255645001	1	255645001	255648000	3000	1	7.80E-10	-0.43	36	1.2	Btaf1	Epigenetic
DMR1:255660001	1	255660001	255665000	5000	1	5.30E-13	-0.61	106	2.12	Btaf1;Cpeb3	Epigenetic;Translation

DMR1:255729001	1	255729001	255731000	2000	1	6.20E-11	-0.64	20	1	Cpeb3	Translation
DMR1:255781001	1	255781001	255782000	1000	1	1.90E-07	-0.6	6	0.6	Cpeb3	Translation
DMR1:255869001	1	255869001	255873000	4000	1	2.70E-09	0.63	58	1.45	5-Mar	Proteolysis
DMR1:255934001	1	255934001	255935000	1000	1	4.50E-07	-0.44	20	2	Ide;LOC103691353	Protease
DMR1:256052001	1	256052001	256057000	5000	2	5.80E-08	-0.27	46	0.92	Kif11	Cytoskeleton
DMR1:256253001	1	256253001	256255000	2000	1	2.40E-07	-0.38	31	1.55	Exoc6	Transport
DMR1:256264001	1	256264001	256265000	1000	1	9.30E-09	-0.35	8	0.8	Exoc6	Transport
DMR1:256360001	1	256360001	256362000	2000	1	5.80E-07	-0.44	18	0.9	Exoc6;Cyp26c1	Transport;Metabolism
DMR1:256379001	1	256379001	256380000	1000	1	8.70E-09	0.68	12	1.2	Exoc6;Cyp26c1;LOC102549441;Cyp26a1	Transport;Metabolism
DMR1:256644001	1	256644001	256645000	1000	1	2.00E-07	0.35	24	2.4	Myof	Transport
DMR1:256782001	1	256782001	256785000	3000	1	3.80E-07	0.43	36	1.2	Ffar4	Signaling
DMR1:256819001	1	256819001	256820000	1000	1	3.40E-07	-0.6	6	0.6	Rbp4;Pde6c	Transport;Signaling
DMR1:256821001	1	256821001	256822000	1000	1	7.40E-09	0.51	20	2	Rbp4;Pde6c	Transport;Signaling
DMR1:256832001	1	256832001	256833000	1000	1	1.00E-07	0.56	15	1.5	Pde6c	Signaling
DMR1:256852001	1	256852001	256854000	2000	1	7.10E-10	0.58	54	2.7	Pde6c	Signaling
DMR1:256953001	1	256953001	256955000	2000	1	1.90E-09	0.45	38	1.9	RGD1561251;Lgi1;LOC103691354	
DMR1:256967001	1	256967001	256968000	1000	1	2.90E-08	-0.47	13	1.3	Lgi1;LOC103691354	
DMR1:256992001	1	256992001	256993000	1000	1	4.50E-11	0.54	13	1.3	Lgi1	
DMR1:257285001	1	257285001	257287000	2000	1	3.10E-07	-0.37	41	2.05	Plce1	Metabolism
DMR1:257336001	1	257336001	257337000	1000	1	5.50E-11	-0.41	14	1.4	Plce1	Metabolism
DMR1:257375001	1	257375001	257376000	1000	1	3.30E-10	-0.72	5	0.5	Plce1	Metabolism
DMR1:257382001	1	257382001	257384000	2000	1	6.20E-07	-0.35	12	0.6	Plce1	Metabolism
DMR1:257451001	1	257451001	257452000	1000	1	1.50E-07	0.45	18	1.8	Plce1	Metabolism
DMR1:257509001	1	257509001	257510000	1000	1	2.10E-07	0.42	9	0.9	Tbc1d12	Signaling
DMR1:258111001	1	258111001	258113000	2000	1	3.30E-07	-0.5	5	0.25	Cyp2c24	Metabolism
DMR1:258884001	1	258884001	258885000	1000	1	3.70E-08	-0.4	19	1.9	Cyp2c13	Metabolism
DMR1:259264001	1	259264001	259267000	3000	1	1.90E-11	0.8	39	1.3	Cyp2c22	
DMR1:259317001	1	259317001	259318000	1000	1	5.30E-08	-0.56	4	0.4	Pdlim1	Cytoskeleton
DMR1:259410001	1	259410001	259411000	1000	1	2.30E-08	0.66	22	2.2	Sorbs1	
DMR1:259412001	1	259412001	259413000	1000	1	3.50E-11	0.74	24	2.4	Sorbs1	
DMR1:259422001	1	259422001	259423000	1000	1	5.00E-08	-0.34	24	2.4	Sorbs1	
DMR1:259424001	1	259424001	259426000	2000	1	2.60E-07	-0.39	25	1.25	Sorbs1	
DMR1:259485001	1	259485001	259486000	1000	1	2.30E-08	0.47	9	0.9	Sorbs1	
DMR1:259696001	1	259696001	259698000	2000	2	1.50E-07	-0.62	20	1	Tctn3;Entpd1;LOC100361643	Signaling
DMR1:259756001	1	259756001	259758000	2000	1	6.40E-09	-0.44	35	1.75	Entpd1	Signaling
DMR1:259798001	1	259798001	259802000	4000	1	5.80E-07	-0.51	80	2	Entpd1	Signaling
DMR1:260070001	1	260070001	260074000	4000	1	2.00E-07	-0.63	43	1.07	Cc2d2b	
DMR1:260201001	1	260201001	260204000	3000	2	8.30E-10	0.77	50	1.67	Blnk	Cytoskeleton
DMR1:260213001	1	260213001	260214000	1000	1	6.40E-11	0.7	15	1.5	Blnk	Cytoskeleton
DMR1:260289001	1	260289001	260290000	1000	1	9.20E-08	0.47	22	2.2	Dntt	Transcription
DMR1:260333001	1	260333001	260334000	1000	1	5.00E-08	-0.38	15	1.5	Opalin	
DMR1:260374001	1	260374001	260381000	7000	1	3.00E-07	-0.36	126	1.8	Tll2	Protease
DMR1:260449001	1	260449001	260452000	3000	1	8.60E-11	0.66	38	1.27	Tll2	Protease
DMR1:260477001	1	260477001	260479000	2000	1	2.60E-08	-0.48	34	1.7	Tm9sf3	Transport
DMR1:260582001	1	260582001	260586000	4000	1	3.40E-10	-0.83	41	1.02	Pik3ap1	
DMR1:260597001	1	260597001	260605000	8000	2	3.20E-07	-0.45	142	1.77	Pik3ap1	
DMR1:260616001	1	260616001	260620000	4000	2	1.40E-07	-0.38	65	1.62	Pik3ap1	
DMR1:260632001	1	260632001	260635000	3000	1	4.60E-08	-0.35	52	1.73	Pik3ap1	
DMR1:260819001	1	260819001	260820000	1000	1	5.30E-10	-0.46	19	1.9	Lcor	
DMR1:260842001	1	260842001	260844000	2000	1	5.40E-08	0.56	50	2.5	Lcor;Slit1	
DMR1:260877001	1	260877001	260880000	3000	1	4.80E-09	-0.45	46	1.53	Slit1	
DMR1:260916001	1	260916001	260920000	4000	1	1.20E-08	-0.44	72	1.8	Slit1	
DMR1:260954001	1	260954001	260956000	2000	2	3.20E-09	-0.35	37	1.85	Slit1;LOC102553433	
DMR1:260996001	1	260996001	2.61E+08	4000	1	9.90E-09	0.51	75	1.88	Slit1	
DMR1:261062001	1	261062001	261066000	4000	1	5.50E-09	0.52	59	1.48	LOC102553733;Frat1	
DMR1:261074001	1	261074001	261075000	1000	1	6.70E-11	0.82	36	3.6	LOC102553733;Frat1	
DMR1:261093001	1	261093001	261098000	5000	1	9.40E-14	-0.56	70	1.4	Frat2;Rrp12	
DMR1:261127001	1	261127001	261128000	1000	1	5.20E-07	-0.41	17	1.7	Rrp12	
DMR1:261141001	1	261141001	261142000	1000	1	2.50E-20	0.45	12	1.2	Rrp12	
DMR1:261241001	1	261241001	261242000	1000	1	8.40E-08	-0.5	14	1.4	Ubtd1	Proteolysis

DMR1:261252001	1	261252001	261257000	5000	1	3.40E-08	0.62	41	0.82	Ubtd1	Proteolysis
DMR1:261270001	1	261270001	261271000	1000	1	2.00E-09	0.51	16	1.6	Ubtd1	Proteolysis
DMR1:261395001	1	261395001	261396000	1000	1	9.40E-11	0.66	26	2.6	Marveld1	
DMR1:261464001	1	261464001	261466000	2000	1	8.70E-08	-0.5	24	1.2	RGD1563029	
DMR1:261491001	1	261491001	261496000	5000	1	9.00E-11	-0.45	59	1.18	LOC102547392;Golga7b	
DMR1:261509001	1	261509001	261513000	4000	1	1.70E-09	0.5	79	1.98	Golga7b	
DMR1:261580001	1	261580001	261582000	2000	1	3.40E-07	0.39	48	2.4	Crtac1	
DMR1:261589001	1	261589001	261594000	5000	1	8.60E-08	-0.39	89	1.78	Crtac1	
DMR1:261633001	1	261633001	261636000	3000	1	5.10E-09	-0.57	46	1.53	Crtac1	
DMR1:261646001	1	261646001	261648000	2000	1	1.10E-07	-0.37	60	3	Crtac1	
DMR1:261656001	1	261656001	261657000	1000	1	5.10E-08	-0.43	30	3	Crtac1	
DMR1:261666001	1	261666001	261667000	1000	1	1.20E-07	0.52	12	1.2	Crtac1	
DMR1:261961001	1	261961001	261968000	7000	1	1.30E-08	0.4	104	1.49	Pyroxd2	Metabolism
DMR1:262055001	1	262055001	262058000	3000	1	5.00E-08	-0.44	24	0.8	LOC108349471;Hpse2;LOC102554961	
DMR1:262128001	1	262128001	262129000	1000	1	9.60E-09	-0.44	18	1.8	Hpse2	
DMR1:262169001	1	262169001	262170000	1000	1	2.30E-10	0.45	10	1	Hpse2	
DMR1:262186001	1	262186001	262188000	2000	1	5.80E-13	-0.4	43	2.15	Hpse2	
DMR1:262224001	1	262224001	262228000	4000	1	8.20E-10	-0.4	28	0.7	Hpse2;LOC102555060;LOC108349826	
DMR1:262512001	1	262512001	262516000	4000	2	2.00E-07	-0.36	44	1.1	Hpse2	
DMR1:262517001	1	262517001	262519000	2000	1	2.20E-13	0.4	29	1.45	Hpse2	
DMR1:262588001	1	262588001	262591000	3000	1	7.40E-07	-0.27	30	1	Hpse2	
DMR1:262622001	1	262622001	262625000	3000	1	4.00E-09	-0.39	32	1.07	Hpse2	
DMR1:262664001	1	262664001	262665000	1000	1	7.60E-07	-0.32	8	0.8	Hpse2	
DMR1:262908001	1	262908001	262909000	1000	1	3.90E-11	0.31	1	0.1	Hpse2;LOC100911951	
DMR1:263247001	1	263247001	263254000	7000	1	3.70E-07	0.5	151	2.16	Cnnm1;Got1	Metabolism
DMR1:263381001	1	263381001	263386000	5000	1	5.90E-08	0.32	75	1.5	Nkx2-3	Development
DMR1:263423001	1	263423001	263424000	1000	1	2.50E-07	-0.36	20	2	Slc25a28	
DMR1:263452001	1	263452001	263460000	8000	1	4.50E-15	-0.55	126	1.57	Entpd7	Signaling
DMR1:263562001	1	263562001	263567000	5000	2	2.90E-10	0.71	97	1.94	LOC102546509;Abcc2	Transport
DMR1:263570001	1	263570001	263574000	4000	1	2.80E-10	-0.45	57	1.43	Abcc2	Transport
DMR1:263596001	1	263596001	263597000	1000	1	3.70E-18	1	29	2.9	Abcc2	Transport
DMR1:263601001	1	263601001	263603000	2000	1	1.60E-08	-0.41	34	1.7	Abcc2	Transport
DMR1:263665001	1	263665001	263667000	2000	1	3.00E-09	-0.52	50	2.5	Dnmbp	
DMR1:263679001	1	263679001	263683000	4000	1	3.60E-08	-0.35	74	1.85	Dnmbp	
DMR1:263689001	1	263689001	263690000	1000	1	6.30E-11	-0.44	22	2.2	Dnmbp	
DMR1:263705001	1	263705001	263710000	5000	1	1.20E-10	0.75	81	1.62	Dnmbp	
DMR1:263723001	1	263723001	263726000	3000	1	1.90E-07	0.33	46	1.53	Dnmbp;Cpn1	Protease
DMR1:263761001	1	263761001	263762000	1000	1	2.20E-08	0.47	26	2.6	Cpn1;LOC102554493	Protease
DMR1:263765001	1	263765001	263766000	1000	1	5.60E-08	0.42	21	2.1	Cpn1;LOC102554493	Protease
DMR1:263798001	1	263798001	263799000	1000	1	5.60E-10	0.56	22	2.2	Cyp2c23	Metabolism
DMR1:263932001	1	263932001	263935000	3000	1	1.00E-10	0.54	42	1.4	Pkd2l1	Transport
DMR1:263943001	1	263943001	263944000	1000	1	9.70E-07	0.43	14	1.4	Pkd2l1	Transport
DMR1:263968001	1	263968001	263969000	1000	1	6.20E-07	0.41	12	1.2	Pkd2l1;LOC681458	Transport
DMR1:264063001	1	264063001	264066000	3000	1	1.50E-08	-0.38	62	2.07	LOC108349475;Scd2	
DMR1:264067001	1	264067001	264070000	3000	1	9.30E-07	-0.39	74	2.47	LOC108349475;Scd2	
DMR1:264071001	1	264071001	264073000	2000	1	2.10E-08	-0.47	43	2.15	Scd2	
DMR1:264111001	1	264111001	264112000	1000	1	1.40E-07	0.47	14	1.4	Scd4	
DMR1:264164001	1	264164001	264167000	3000	1	4.10E-09	-0.48	63	2.1	Scd	
DMR1:264236001	1	264236001	264238000	2000	1	1.00E-09	0.4	33	1.65	Wnt8b	Signaling
DMR1:264240001	1	264240001	264246000	6000	1	9.70E-14	-0.53	78	1.3	Wnt8b	Signaling
DMR1:264270001	1	264270001	264274000	4000	1	1.70E-10	-0.5	37	0.92	Wnt8b;Sec31b	Signaling;Transport
DMR1:264275001	1	264275001	264276000	1000	1	2.10E-07	-0.44	20	2	Wnt8b;Sec31b	Signaling;Transport
DMR1:264316001	1	264316001	264319000	3000	2	2.40E-23	0.9	33	1.1	Hif1an	
DMR1:264517001	1	264517001	264523000	6000	2	5.30E-13	-0.47	124	2.07	Pax2	
DMR1:264549001	1	264549001	264554000	5000	2	1.80E-10	-0.48	83	1.66	Pax2	
DMR1:264555001	1	264555001	264558000	3000	2	5.10E-08	-0.51	44	1.47	Pax2	
DMR1:264559001	1	264559001	264561000	2000	2	2.40E-12	-0.54	23	1.15	Pax2	
DMR1:264583001	1	264583001	264585000	2000	2	2.90E-15	1.09	123	6.15	Pax2	
DMR1:264775001	1	264775001	264778000	3000	1	4.10E-13	0.68	78	2.6	Lzts2;Pdzd7	Cytoskeleton
DMR1:264782001	1	264782001	264786000	4000	1	4.80E-09	0.63	89	2.22	Lzts2;Pdzd7	Cytoskeleton

DMR1:264793001	1	264793001	264795000	2000	1	6.80E-10	0.84	37	1.85	Pdzd7;Sfxn3	Cytoskeleton;Transport
DMR1:264906001	1	264906001	264907000	1000	1	7.20E-07	0.29	6	0.6	Tlx1	
DMR1:264908001	1	264908001	264914000	6000	1	1.30E-07	-0.33	65	1.08	Tlx1	
DMR1:265092001	1	265092001	265095000	3000	1	2.80E-07	-0.52	11	0.37	Btrc	Cytoskeleton
DMR1:265163001	1	265163001	265165000	2000	1	3.20E-08	-0.39	27	1.35	Btrc	Cytoskeleton
DMR1:265244001	1	265244001	265245000	1000	1	5.80E-07	0.37	13	1.3	Btrc	Cytoskeleton
DMR1:265300001	1	265300001	265302000	2000	1	5.70E-09	-0.42	30	1.5	Poll;Dpcd	Transcription
DMR1:265335001	1	265335001	265336000	1000	1	2.90E-08	0.6	14	1.4	Fbxw4	
DMR1:265557001	1	265557001	265558000	1000	1	3.80E-12	0.3	3	0.3	Kcnip2	
DMR1:265664001	1	265664001	265666000	2000	1	5.80E-15	-0.74	17	0.85	C1H10orf76	
DMR1:266020001	1	266020001	266022000	2000	1	6.40E-08	0.37	24	1.2	Gbf1	Transcription
DMR1:266048001	1	266048001	266051000	3000	1	4.70E-09	0.68	43	1.43	LOC102551485;Nfkb2;Psd	Transcription;Transcription
DMR1:266053001	1	266053001	266056000	3000	1	2.90E-08	0.42	88	2.93	LOC102551485;Nfkb2;Psd	Transcription;Transcription
DMR1:266057001	1	266057001	266061000	4000	1	2.00E-09	0.64	88	2.2	LOC102551485;Nfkb2;Psd	Transcription;Transcription
DMR1:266071001	1	266071001	266074000	3000	1	1.50E-10	0.69	57	1.9	Psd;Fbxl15;Cuedc2	Transcription
DMR1:266214001	1	266214001	266215000	1000	1	1.30E-09	0.5	12	1.2	Sufu	Cytoskeleton
DMR1:266302001	1	266302001	266307000	5000	2	1.10E-07	-0.4	49	0.98	Arl3;LOC108349707	
DMR1:266361001	1	266361001	266369000	8000	2	3.50E-08	-0.51	161	2.01	Wbp1l	
DMR1:266372001	1	266372001	266374000	2000	1	3.70E-15	-0.57	46	2.3	Wbp1l	
DMR1:266381001	1	266381001	266384000	3000	2	2.40E-08	-0.36	52	1.73	Wbp1l	
DMR1:266389001	1	266389001	266394000	5000	2	6.50E-09	-0.4	79	1.58	Wbp1l	
DMR1:266481001	1	266481001	266482000	1000	1	1.20E-10	-0.5	15	1.5	As3mt	Epigenetic
DMR1:266541001	1	266541001	266543000	2000	1	2.80E-13	-0.51	26	1.3	Cnnm2	
DMR1:266580001	1	266580001	266581000	1000	1	3.00E-08	0.4	15	1.5	Cnnm2	
DMR1:266627001	1	266627001	266631000	4000	1	3.40E-08	-0.42	61	1.52	Cnnm2	
DMR1:266807001	1	266807001	266808000	1000	1	2.90E-07	-0.37	29	2.9	Pcgf6	Epigenetic
DMR1:266832001	1	266832001	266835000	3000	2	1.30E-08	-0.42	44	1.47	Pcgf6;Taf5	Epigenetic
DMR1:266864001	1	266864001	266865000	1000	1	4.60E-08	-0.48	9	0.9	Taf5;Usmg5;Pdcd11	Metabolism
DMR1:266884001	1	266884001	266887000	3000	1	9.00E-16	-0.65	29	0.97	Pdcd11	Metabolism
DMR1:266925001	1	266925001	266926000	1000	1	3.40E-08	0.54	26	2.6	Calhm1;Calhm3	
DMR1:266934001	1	266934001	266940000	6000	1	1.70E-09	-0.52	83	1.38	Calhm3	
DMR1:266992001	1	266992001	266994000	2000	1	5.00E-08	-0.3	17	0.85	Neurl1	Proteolysis
DMR1:267015001	1	267015001	267017000	2000	1	2.50E-08	0.33	30	1.5	Neurl1;LOC108349530	Proteolysis
DMR1:267036001	1	267036001	267039000	3000	2	7.60E-09	0.58	66	2.2	Neurl1;Sh3pxd2a	Proteolysis
DMR1:267052001	1	267052001	267053000	1000	1	7.60E-08	0.49	16	1.6	Sh3pxd2a	
DMR1:267145001	1	267145001	267148000	3000	1	1.00E-09	0.51	43	1.43	Sh3pxd2a	
DMR1:267154001	1	267154001	267155000	1000	1	1.20E-09	0.61	23	2.3	Sh3pxd2a	
DMR1:267199001	1	267199001	267200000	1000	1	8.10E-07	0.48	12	1.2	Sh3pxd2a	
DMR1:267223001	1	267223001	267226000	3000	1	1.40E-08	-0.4	54	1.8	Sh3pxd2a	
DMR1:267284001	1	267284001	267287000	3000	1	4.30E-09	0.59	42	1.4	Obfc1;LOC108349533	
DMR1:267429001	1	267429001	267430000	1000	1	2.50E-08	0.66	18	1.8	Col17a1	Extracellular Matrix
DMR1:267440001	1	267440001	267441000	1000	1	1.40E-07	-0.53	28	2.8	Col17a1;LOC103691371	Extracellular Matrix
DMR1:267442001	1	267442001	267443000	1000	1	1.50E-08	0.33	20	2	Col17a1;LOC103691371	Extracellular Matrix
DMR1:267461001	1	267461001	267465000	4000	1	7.30E-08	-0.35	84	2.1	Col17a1;LOC103691371	Extracellular Matrix
DMR1:267467001	1	267467001	267468000	1000	1	2.00E-10	0.64	15	1.5	Col17a1;LOC103691371	Extracellular Matrix
DMR1:267541001	1	267541001	267545000	4000	1	1.60E-09	-0.39	83	2.08	Cfap43	
DMR1:267563001	1	267563001	267566000	3000	2	1.40E-07	0.55	79	2.63	Cfap43	
DMR1:267574001	1	267574001	267576000	2000	1	2.20E-07	0.37	46	2.3	Cfap43	
DMR1:267720001	1	267720001	267724000	4000	2	4.50E-10	-0.55	80	2	Cfap58;LOC108349708	Development
DMR1:267808001	1	267808001	267809000	1000	1	5.90E-07	-0.28	10	1	Cfap58	Development
DMR1:268004001	1	268004001	268008000	4000	1	2.10E-08	0.33	37	0.92	Sorcs3	Transport
DMR1:268012001	1	268012001	268017000	5000	2	4.30E-07	-0.3	42	0.84	Sorcs3	Transport
DMR1:268028001	1	268028001	268029000	1000	1	5.80E-10	-0.48	7	0.7	Sorcs3	Transport
DMR1:268064001	1	268064001	268066000	2000	1	8.70E-08	-0.35	41	2.05	Sorcs3	Transport
DMR1:268139001	1	268139001	268140000	1000	1	8.60E-08	-0.54	9	0.9	Sorcs3	Transport
DMR1:268183001	1	268183001	268187000	4000	1	2.30E-08	-0.55	44	1.1	Sorcs3	Transport
DMR1:268322001	1	268322001	268327000	5000	2	1.00E-12	0.72	77	1.54	Sorcs3	Transport
DMR1:268380001	1	268380001	268381000	1000	1	2.80E-08	0.53	13	1.3	Sorcs3	Transport
DMR1:268524001	1	268524001	268525000	1000	1	2.30E-07	-0.59	11	1.1	Sorcs3	Transport

DMR1:268569001	1	268569001	268570000	1000	1	1.80E-07	0.5	9	0.9	Sorcs3	Transport
DMR1:268578001	1	268578001	268579000	1000	1	4.20E-07	0.34	11	1.1	Sorcs3	Transport
DMR1:268581001	1	268581001	268582000	1000	1	8.90E-08	0.53	10	1	Sorcs3	Transport
DMR1:269964001	1	269964001	269967000	3000	1	9.40E-08	0.59	34	1.13	Sorcs1	Transport
DMR1:270059001	1	270059001	270064000	5000	1	7.50E-07	-0.31	38	0.76	Sorcs1	Transport
DMR1:270291001	1	270291001	270293000	2000	1	1.70E-11	-0.6	17	0.85	Sorcs1	Transport
DMR1:270431001	1	270431001	270432000	1000	1	2.20E-08	-0.45	7	0.7	Sorcs1	Transport
DMR1:271323001	1	271323001	271327000	4000	2	3.60E-12	-0.57	82	2.05	Ccdc147;LOC108349546	
DMR1:271344001	1	271344001	271347000	3000	1	9.00E-07	0.53	62	2.07	Ccdc147	
DMR1:272861001	1	272861001	272862000	1000	1	1.10E-08	-0.5	12	1.2	Olr385	
DMR1:272868001	1	272868001	272869000	1000	1	5.50E-08	0.44	16	1.6	Olr385	
DMR1:273548001	1	273548001	273556000	8000	1	1.10E-07	-0.29	89	1.11	RGD1561333	
DMR1:273740001	1	273740001	273742000	2000	1	1.40E-07	-0.37	26	1.3	Xpnpep1	Protease
DMR1:273752001	1	273752001	273755000	3000	1	4.00E-09	-0.43	78	2.6	Xpnpep1;LOC102550358	Protease
DMR1:273965001	1	273965001	273966000	1000	1	9.00E-10	-0.42	14	1.4	Add3	Cytoskeleton
DMR1:273970001	1	273970001	273975000	5000	1	5.70E-08	0.42	83	1.66	Add3	Cytoskeleton
DMR1:274064001	1	274064001	274066000	2000	1	1.70E-11	-0.54	55	2.75	Mxi1;LOC108349609	Transcription
DMR1:274069001	1	274069001	274072000	3000	1	9.10E-10	-0.42	49	1.63	Mxi1;LOC108349609	Transcription
DMR1:274084001	1	274084001	274088000	4000	1	5.80E-11	-0.46	101	2.52	Mxi1;LOC108349609;Smndc1	Transcription;Translation
DMR1:274248001	1	274248001	274249000	1000	1	1.50E-07	-0.66	5	0.5	Dusp5	Signaling
DMR1:274297001	1	274297001	274299000	2000	2	6.60E-12	-0.57	25	1.25	Rps12l2	
DMR1:274304001	1	274304001	274307000	3000	2	1.00E-08	0.58	45	1.5	Rps12l2;Smc3	
DMR1:274392001	1	274392001	274395000	3000	2	8.80E-10	0.81	88	2.93	Rbm20	
DMR1:274396001	1	274396001	274399000	3000	1	6.00E-07	0.43	66	2.2	Rbm20	
DMR1:274416001	1	274416001	274419000	3000	1	6.00E-07	0.51	38	1.27	Rbm20	
DMR1:274420001	1	274420001	274421000	1000	1	3.60E-07	0.39	23	2.3	Rbm20	
DMR1:274489001	1	274489001	274491000	2000	1	2.80E-07	-0.37	27	1.35	Rbm20	
DMR1:274493001	1	274493001	274496000	3000	2	2.50E-09	-0.54	58	1.93	Rbm20	
DMR1:274517001	1	274517001	274520000	3000	1	2.00E-10	0.75	88	2.93	Rbm20	
DMR1:274566001	1	274566001	274569000	3000	2	1.80E-07	0.48	48	1.6	Rbm20	
DMR1:274616001	1	274616001	274618000	2000	1	6.80E-07	-0.37	20	1	Pdcd4	Translation
DMR1:274720001	1	274720001	274722000	2000	1	4.20E-08	-0.59	22	1.1	Shoc2	Cytoskeleton
DMR1:274735001	1	274735001	274736000	1000	1	5.40E-09	-0.26	8	0.8	Shoc2	Cytoskeleton
DMR1:274753001	1	274753001	274756000	3000	1	3.40E-09	0.47	30	1	Shoc2	Cytoskeleton
DMR1:275831001	1	275831001	275834000	3000	2	4.60E-12	0.9	78	2.6	LOC108349650;Gpam	Metabolism
DMR1:275880001	1	275880001	275882000	2000	1	1.40E-08	-0.31	23	1.15	Gpam	Metabolism
DMR1:275883001	1	275883001	275886000	3000	1	6.50E-07	-0.39	28	0.93	Gpam	Metabolism
DMR1:275958001	1	275958001	275959000	1000	1	5.90E-07	0.49	24	2.4	Tectb	Receptor
DMR1:275981001	1	275981001	275982000	1000	1	3.10E-07	-0.66	4	0.4	Tectb;LOC108348158	Receptor
DMR1:275983001	1	275983001	275989000	6000	3	1.20E-12	-0.69	106	1.77	Tectb;LOC108348158	Receptor
DMR1:276174001	1	276174001	276182000	8000	1	1.30E-11	-0.5	147	1.84	Gucy2g	Signaling
DMR1:276202001	1	276202001	276205000	3000	1	3.60E-07	0.57	63	2.1	Gucy2g	Signaling
DMR1:276223001	1	276223001	276225000	2000	1	1.90E-08	0.49	16	0.8	Gucy2g	Signaling
DMR1:276330001	1	276330001	276332000	2000	1	3.60E-07	0.5	24	1.2	Vti1a	Transcription
DMR1:276350001	1	276350001	276351000	1000	1	2.00E-10	-0.49	7	0.7	Vti1a	Transcription
DMR1:276725001	1	276725001	276729000	4000	1	1.40E-19	1.05	64	1.6	Tcf7l2	Transcription
DMR1:277068001	1	277068001	277070000	2000	1	1.30E-09	-0.52	48	2.4	LOC102546997;LOC102546765;Habp2	Protease
DMR1:277075001	1	277075001	277076000	1000	1	3.30E-07	0.46	7	0.7	Habp2	Protease
DMR1:277164001	1	277164001	277166000	2000	1	6.40E-07	0.32	22	1.1	Nrap	
DMR1:277176001	1	277176001	277178000	2000	1	1.40E-16	0.83	52	2.6	Nrap	
DMR1:277212001	1	277212001	277216000	4000	1	2.80E-08	0.34	69	1.73	Casp7	Protease
DMR1:277217001	1	277217001	277219000	2000	1	1.10E-08	-0.46	29	1.45	Casp7	Protease
DMR1:277227001	1	277227001	277231000	4000	1	2.70E-09	-0.41	100	2.5	Casp7	Protease
DMR1:277251001	1	277251001	277252000	1000	1	4.90E-07	-0.33	21	2.1	Casp7;Plekhs1	Protease
DMR1:277327001	1	277327001	277329000	2000	1	4.90E-10	0.53	23	1.15	LOC103691381;LOC680527;Dclre1a	Transcription
DMR1:277425001	1	277425001	277428000	3000	2	1.60E-08	-0.42	42	1.4	Nhlrc2	
DMR1:277641001	1	277641001	277643000	2000	1	1.00E-26	1.42	69	3.45	Ccdc186;Tdrd1	Cytoskeleton
DMR1:277660001	1	277660001	277661000	1000	1	7.00E-08	-0.44	15	1.5	Tdrd1	Cytoskeleton

DMR1:277693001	1	277693001	277695000	2000	1	1.20E-09	-0.52	43	2.15	Tdrd1;Vwa2	Cytoskeleton;Extracellular Matrix
DMR1:277704001	1	277704001	277705000	1000	1	9.30E-08	0.39	8	0.8	Vwa2	Extracellular Matrix
DMR1:277706001	1	277706001	277707000	1000	1	1.30E-07	-0.44	14	1.4	Vwa2	Extracellular Matrix
DMR1:277869001	1	277869001	277872000	3000	1	1.10E-11	-0.44	53	1.77	Ablim1	
DMR1:277907001	1	277907001	277915000	8000	1	7.50E-11	-0.57	101	1.26	Ablim1;LOC103691383	
DMR1:277983001	1	277983001	277985000	2000	1	1.30E-13	0.83	43	2.15	Ablim1	
DMR1:278008001	1	278008001	278010000	2000	1	1.20E-07	0.35	38	1.9	Ablim1;LOC102550100	
DMR1:278188001	1	278188001	278189000	1000	1	2.40E-13	0.74	17	1.7	LOC102550567;Fam160b1	
DMR1:278391001	1	278391001	278393000	2000	1	2.00E-08	-0.3	6	0.3	Trub1	Metabolism
DMR1:278394001	1	278394001	278398000	4000	2	5.80E-13	0.37	26	0.65	Trub1	Metabolism
DMR1:278408001	1	278408001	278409000	1000	1	4.80E-07	-0.46	31	3.1	Trub1	Metabolism
DMR1:278424001	1	278424001	278428000	4000	1	2.10E-08	-0.4	78	1.95	Trub1;LOC108349700	Metabolism
DMR1:278792001	1	278792001	278794000	2000	1	1.60E-07	-0.34	30	1.5	Atrnl1	Extracellular Matrix
DMR1:278937001	1	278937001	278938000	1000	1	2.50E-07	0.61	31	3.1	Atrnl1	Extracellular Matrix
DMR1:279058001	1	279058001	279060000	2000	1	5.00E-11	0.36	29	1.45	Atrnl1	Extracellular Matrix
DMR1:279193001	1	279193001	279197000	4000	2	3.90E-08	0.55	38	0.95	Gfra1	Receptor
DMR1:279237001	1	279237001	279243000	6000	1	7.50E-07	-0.23	59	0.98	Gfra1;LOC102556644	Receptor
DMR1:279865001	1	279865001	279867000	2000	1	3.00E-08	-0.42	35	1.75	Pnliprp1	Metabolism
DMR1:279894001	1	279894001	279897000	3000	1	9.00E-07	0.42	30	1	Pnliprp2	Metabolism
DMR1:279946001	1	279946001	279948000	2000	1	5.10E-08	0.51	27	1.35	LOC681006;Hspa12a	
DMR1:279949001	1	279949001	279952000	3000	1	1.80E-07	0.38	73	2.43	LOC681006;Hspa12a	
DMR1:280000001	1	280000001	280003000	3000	1	4.40E-09	0.6	66	2.2	Hspa12a;LOC103691386	
DMR1:280010001	1	280010001	280013000	3000	1	2.00E-08	0.39	32	1.07	Hspa12a;LOC103691386	
DMR1:280029001	1	280029001	280032000	3000	1	1.70E-07	0.53	29	0.97	Hspa12a;LOC100363557	
DMR1:280063001	1	280063001	280064000	1000	1	4.20E-07	0.4	18	1.8	Hspa12a	
DMR1:280171001	1	280171001	280174000	3000	1	8.10E-08	0.32	39	1.3	Shtn1	
DMR1:280326001	1	280326001	280327000	1000	1	5.00E-08	-0.4	16	1.6	Vax1	Development
DMR1:280386001	1	280386001	280391000	5000	1	4.40E-12	-0.78	49	0.98	Kcnk18;Slc18a2	Transport;Transport
DMR1:280393001	1	280393001	280395000	2000	1	1.60E-08	0.56	20	1	Kcnk18;Slc18a2	Transport;Transport
DMR1:280481001	1	280481001	280483000	2000	1	2.10E-07	-0.44	23	1.15	Pdzd8	Cytoskeleton
DMR1:280639001	1	280639001	280642000	3000	2	1.60E-09	-0.47	42	1.4	LOC103691392;Emx2	Development
DMR1:281395001	1	281395001	281398000	3000	1	1.60E-12	-0.58	32	1.07	Fam204a;LOC108349713	
DMR1:281748001	1	281748001	281749000	1000	1	3.60E-07	0.51	11	1.1	Prlhr	Signaling
DMR1:282145001	1	282145001	282151000	6000	1	9.20E-11	-0.57	76	1.27	Nanos1;Eif3a	Metabolism;Translation
DMR1:282152001	1	282152001	282153000	1000	1	9.00E-13	-0.53	15	1.5	Eif3a	Translation
DMR2:1397001	2	1397001	1401000	4000	4	1.90E-20	0.86	94	2.35	LOC102548635;Erap1	Protease
DMR2:1456001	2	1456001	1459000	3000	1	9.50E-07	-0.65	13	0.43	Erap1;Cast	Protease;Protease; Proteolysis
DMR2:1477001	2	1477001	1479000	2000	1	4.40E-07	-0.54	8	0.4	Cast	Protease; Proteolysis
DMR2:1523001	2	1523001	1525000	2000	1	3.70E-08	-0.34	50	2.5	Cast	Protease; Proteolysis
DMR2:2913001	2	2913001	2915000	2000	1	3.50E-09	-0.5	8	0.4	Ttc37	
DMR2:2946001	2	2946001	2948000	2000	1	1.10E-08	0.42	60	3	Ttc37	
DMR2:2979001	2	2979001	2985000	6000	1	3.90E-10	-0.3	66	1.1	Ttc37	
DMR2:3085001	2	3085001	3086000	1000	1	3.50E-07	-0.44	1	0.1	Fam81b	
DMR2:3600001	2	3600001	3605000	5000	1	3.60E-09	-0.3	81	1.62	Mctp1	
DMR2:3631001	2	3631001	3633000	2000	1	2.60E-07	0.54	17	0.85	Mctp1	
DMR2:3864001	2	3864001	3866000	2000	1	1.40E-07	-0.55	15	0.75	Mctp1	
DMR2:3894001	2	3894001	3897000	3000	1	6.90E-07	-0.57	12	0.4	Mctp1	
DMR2:4023001	2	4023001	4024000	1000	1	8.20E-08	-0.44	4	0.4	Mctp1	
DMR2:4042001	2	4042001	4043000	1000	1	5.40E-09	-0.38	7	0.7	Mctp1	
DMR2:4138001	2	4138001	4140000	2000	1	1.90E-08	-0.31	17	0.85	Slf1	
DMR2:4241001	2	4241001	4242000	1000	1	1.60E-12	-0.86	0	0	RGD1560883;LOC102546510	
DMR2:4422001	2	4422001	4425000	3000	2	7.00E-30	-0.84	57	1.9	RGD1560883	
DMR2:4496001	2	4496001	4497000	1000	1	6.40E-08	0.33	7	0.7	RGD1560883	
DMR2:4675001	2	4675001	4679000	4000	1	4.60E-08	-0.37	37	0.92	RGD1560883	
DMR2:4697001	2	4697001	4699000	2000	1	5.90E-07	-0.39	17	0.85	RGD1560883	
DMR2:4724001	2	4724001	4726000	2000	1	3.00E-07	-0.35	16	0.8	RGD1560883	
DMR2:4748001	2	4748001	4749000	1000	1	3.10E-09	0.48	7	0.7	RGD1560883	
DMR2:4814001	2	4814001	4816000	2000	1	6.50E-13	-0.47	18	0.9	Olr205	Receptor
DMR2:4995001	2	4995001	5.00E+06	5000	1	8.60E-07	-0.34	42	0.84	Fam172a	

DMR2:5347001	2	5347001	5350000	3000	1	7.40E-07	0.2	31	1.03	Fam172a	
DMR2:5396001	2	5396001	5402000	6000	1	1.00E-07	-0.28	47	0.78	Fam172a	
DMR2:5459001	2	5459001	5460000	1000	1	5.70E-11	0.36	14	1.4	Fam172a	
DMR2:5471001	2	5471001	5474000	3000	1	3.00E-07	-0.44	24	0.8	Fam172a	
DMR2:5521001	2	5521001	5522000	1000	1	2.60E-07	-0.39	7	0.7	Fam172a;LOC102548021	
DMR2:8959001	2	8959001	8964000	5000	1	6.20E-12	0.79	56	1.12	Adgrv1	Signaling
DMR2:9026001	2	9026001	9033000	7000	1	1.70E-07	-0.28	63	0.9	Adgrv1	Signaling
DMR2:9165001	2	9165001	9169000	4000	1	4.10E-07	-0.45	59	1.48	Adgrv1	Signaling
DMR2:9204001	2	9204001	9207000	3000	1	3.50E-07	-0.29	35	1.17	Adgrv1	Signaling
DMR2:9300001	2	9300001	9302000	2000	1	2.20E-07	0.5	28	1.4	Adgrv1	Signaling
DMR2:9320001	2	9320001	9321000	1000	1	1.50E-07	0.39	8	0.8	Adgrv1	Signaling
DMR2:9451001	2	9451001	9453000	2000	1	6.60E-07	-0.38	26	1.3	Adgrv1	Signaling
DMR2:11064001	2	11064001	11067000	3000	1	1.70E-31	1.24	16	0.53	Rps27a-ps29	
DMR2:11729001	2	11729001	11731000	2000	1	3.90E-07	-0.35	36	1.8	Mef2c	Transcription
DMR2:11739001	2	11739001	11741000	2000	1	9.40E-07	-0.57	14	0.7	Mef2c	Transcription
DMR2:11889001	2	11889001	11890000	1000	1	6.90E-09	-0.51	4	0.4	Mir3597-2;Mir9-2	
DMR2:12280001	2	12280001	12282000	2000	1	8.70E-08	-0.42	27	1.35	Tmem161b	
DMR2:13671001	2	13671001	13672000	1000	1	1.80E-07	0.4	4	0.4	Rasa1	Signaling
DMR2:14711001	2	14711001	14714000	3000	2	1.80E-09	-0.37	25	0.83	Cox7c	Metabolism
DMR2:17624001	2	17624001	17625000	1000	1	2.10E-08	0.47	16	1.6	Edil3	Metabolism
DMR2:18491001	2	18491001	18492000	1000	1	1.30E-07	0.72	24	2.4	Vcan	Extracellular Matrix
DMR2:18498001	2	18498001	18499000	1000	1	5.50E-07	0.66	15	1.5	Vcan	Extracellular Matrix
DMR2:18505001	2	18505001	18506000	1000	1	4.00E-08	0.32	8	0.8	Vcan	Extracellular Matrix
DMR2:18574001	2	18574001	18576000	2000	1	3.30E-07	-0.38	25	1.25	Vcan	Extracellular Matrix
DMR2:18597001	2	18597001	18599000	2000	2	2.50E-11	-0.64	11	0.55	Vcan	Extracellular Matrix
DMR2:18894001	2	18894001	18895000	1000	1	6.60E-11	-0.4	6	0.6	Xrcc4	Transcription
DMR2:19843001	2	19843001	19846000	3000	1	2.60E-11	-0.58	60	2	Atg10	Proteolysis
DMR2:19894001	2	19894001	19895000	1000	1	6.40E-16	0.55	7	0.7	Atg10	Proteolysis
DMR2:19938001	2	19938001	19940000	2000	1	7.20E-09	-0.44	22	1.1	Atg10	Proteolysis
DMR2:20059001	2	20059001	20060000	1000	1	2.60E-07	0.57	24	2.4	Atg10	Proteolysis
DMR2:20093001	2	20093001	20094000	1000	1	5.80E-16	0.8	17	1.7	Atg10	Proteolysis
DMR2:20458001	2	20458001	20464000	6000	1	4.30E-07	-0.46	50	0.83	Ssbp2	Transcription
DMR2:20486001	2	20486001	20487000	1000	1	8.60E-07	-0.35	10	1	Ssbp2	Transcription
DMR2:20653001	2	20653001	20657000	4000	1	2.10E-07	0.43	32	0.8	Ssbp2	Transcription
DMR2:20711001	2	20711001	20713000	2000	1	6.50E-08	0.57	22	1.1	Ssbp2	Transcription
DMR2:20864001	2	20864001	20865000	1000	1	4.60E-08	0.4	14	1.4	Acot12	Metabolism
DMR2:20872001	2	20872001	20876000	4000	1	5.00E-08	-0.39	76	1.9	Acot12	Metabolism
DMR2:21432001	2	21432001	21433000	1000	1	2.30E-09	0.64	25	2.5	Ckmt2	Signaling
DMR2:21447001	2	21447001	21450000	3000	1	1.10E-07	-0.62	39	1.3	Ckmt2	Signaling
DMR2:21522001	2	21522001	21523000	1000	1	5.20E-07	0.51	12	1.2	Rasgrf2;LOC108349920	Transcription
DMR2:21699001	2	21699001	21700000	1000	1	4.40E-09	0.54	52	5.2	Rasgrf2	Transcription
DMR2:21937001	2	21937001	21938000	1000	1	9.90E-08	-0.38	9	0.9	Msh3;Dhfr	Transcription;Metabolism
DMR2:22038001	2	22038001	22042000	4000	1	1.50E-07	0.45	101	2.52	Fam151b	
DMR2:22246001	2	22246001	22248000	2000	2	1.20E-07	-0.4	28	1.4	Serinc5	Signaling
DMR2:22319001	2	22319001	22325000	6000	1	3.30E-08	0.6	98	1.63	Serinc5;LOC108350165	Signaling
DMR2:22449001	2	22449001	22451000	2000	1	1.90E-10	0.5	50	2.5	Mtx3;LOC108350222	
DMR2:22696001	2	22696001	22697000	1000	1	1.10E-14	0.64	2	0.2	Cmya5	Proteolysis
DMR2:22716001	2	22716001	22718000	2000	1	9.30E-10	-0.4	27	1.35	Cmya5	Proteolysis
DMR2:22736001	2	22736001	22739000	3000	1	2.80E-07	-0.49	21	0.7	Cmya5;Papd4	Proteolysis
DMR2:22922001	2	22922001	22925000	3000	1	1.30E-09	-0.38	39	1.3	Homer1	
DMR2:23004001	2	23004001	23007000	3000	1	4.40E-07	-0.37	39	1.3	Homer1	
DMR2:23236001	2	23236001	23239000	3000	2	1.40E-09	0.58	71	2.37	Bhmt	Epigenetic
DMR2:23251001	2	23251001	23253000	2000	2	1.10E-18	1.08	51	2.55	Bhmt;LOC103691423	Epigenetic
DMR2:23273001	2	23273001	23274000	1000	1	7.20E-12	0.54	16	1.6	Bhmt2	Epigenetic
DMR2:23288001	2	23288001	23289000	1000	1	6.20E-07	0.59	24	2.4	Bhmt2;Dmgdh	Epigenetic;Metabolism
DMR2:23417001	2	23417001	23419000	2000	1	2.00E-09	-0.45	41	2.05	Arsb	
DMR2:23423001	2	23423001	23425000	2000	1	3.00E-09	-0.38	57	2.85	Arsb	
DMR2:23447001	2	23447001	23450000	3000	1	1.20E-07	-0.39	52	1.73	Arsb	
DMR2:23532001	2	23532001	23534000	2000	1	2.90E-09	-0.51	25	1.25	Arsb	
DMR2:23550001	2	23550001	23553000	3000	1	3.80E-07	0.32	23	0.77	Arsb	

DMR2:23782001	2	23782001	23784000	2000	1	4.00E-08	-0.34	39	1.95	Lhfpl2	
DMR2:23787001	2	23787001	23791000	4000	1	7.30E-09	-0.39	61	1.52	Lhfpl2	
DMR2:23873001	2	23873001	23877000	4000	1	4.50E-12	-0.5	75	1.88	Scamp1	Transport
DMR2:23912001	2	23912001	23917000	5000	1	1.20E-08	-0.47	81	1.62	Scamp1	Transport
DMR2:24016001	2	24016001	24017000	1000	1	1.30E-10	0.88	21	2.1	Ap3b1	Transport
DMR2:24018001	2	24018001	24019000	1000	1	5.80E-07	0.49	11	1.1	Ap3b1	Transport
DMR2:24029001	2	24029001	24031000	2000	1	4.50E-07	-0.39	23	1.15	Ap3b1	Transport
DMR2:24034001	2	24034001	24035000	1000	1	3.00E-09	-0.39	19	1.9	Ap3b1	Transport
DMR2:24036001	2	24036001	24038000	2000	1	2.80E-09	-0.43	31	1.55	Ap3b1	Transport
DMR2:24093001	2	24093001	24096000	3000	2	7.10E-09	-0.6	45	1.5	Ap3b1	Transport
DMR2:24657001	2	24657001	24659000	2000	1	3.30E-07	-0.39	26	1.3	Wdr41	
DMR2:24677001	2	24677001	24679000	2000	1	3.70E-07	0.51	45	2.25	Wdr41	
DMR2:24830001	2	24830001	24831000	1000	1	2.50E-08	0.48	17	1.7	Pde8b	Signaling
DMR2:24897001	2	24897001	24899000	2000	1	1.60E-14	0.89	48	2.4	Pde8b	Signaling
DMR2:25082001	2	25082001	25085000	3000	1	1.40E-07	-0.46	40	1.33	Aggf1	
DMR2:25134001	2	25134001	25136000	2000	1	5.80E-07	-0.38	40	2	Crhbp	
DMR2:25158001	2	25158001	25160000	2000	1	2.00E-13	-0.48	32	1.6	Crhbp	
DMR2:25186001	2	25186001	25188000	2000	1	3.00E-17	1.11	53	2.65	S100z	Signaling
DMR2:25240001	2	25240001	25241000	1000	1	1.90E-07	-0.32	17	1.7	F2rl1;LOC102550768	Signaling
DMR2:25242001	2	25242001	25245000	3000	1	7.70E-10	0.8	64	2.13	F2rl1;LOC102550768	Signaling
DMR2:25732001	2	25732001	25733000	1000	1	2.90E-08	-0.41	13	1.3	Arhgef28	
DMR2:25745001	2	25745001	25747000	2000	1	4.80E-08	-0.43	43	2.15	Arhgef28	
DMR2:25795001	2	25795001	25796000	1000	1	2.50E-08	-0.46	24	2.4	Arhgef28	
DMR2:25799001	2	25799001	25803000	4000	2	1.10E-07	-0.41	76	1.9	Arhgef28	
DMR2:25815001	2	25815001	25817000	2000	1	1.60E-11	-0.46	27	1.35	Arhgef28	
DMR2:25836001	2	25836001	25845000	9000	1	5.30E-09	-0.38	140	1.56	Arhgef28	
DMR2:25846001	2	25846001	25848000	2000	1	2.40E-07	-0.39	24	1.2	Arhgef28	
DMR2:25865001	2	25865001	25866000	1000	1	3.90E-09	-0.38	21	2.1	Arhgef28	
DMR2:25906001	2	25906001	25909000	3000	1	2.80E-07	-0.5	54	1.8	Arhgef28	
DMR2:25913001	2	25913001	25915000	2000	1	1.20E-10	-0.43	28	1.4	Arhgef28	
DMR2:25956001	2	25956001	25957000	1000	1	2.50E-11	-0.5	26	2.6	Arhgef28	
DMR2:25987001	2	25987001	25991000	4000	2	1.70E-19	1.14	70	1.75	Arhgef28	
DMR2:26010001	2	26010001	26012000	2000	1	2.20E-11	-0.45	45	2.25	Arhgef28;RGD1566212	
DMR2:26068001	2	26068001	26073000	5000	1	1.10E-07	0.45	65	1.3	RGD1566212	
DMR2:26076001	2	26076001	26077000	1000	1	7.80E-11	0.71	29	2.9	RGD1566212	
DMR2:26112001	2	26112001	26114000	2000	1	9.90E-09	-0.37	26	1.3	F2r	Signaling
DMR2:26121001	2	26121001	26123000	2000	1	1.80E-16	1.1	77	3.85	F2r	Signaling
DMR2:26213001	2	26213001	26215000	2000	1	1.10E-08	-0.35	53	2.65	Iqgap2	Signaling
DMR2:26242001	2	26242001	26246000	4000	1	9.60E-08	-0.4	71	1.77	Iqgap2;F2rl2	Signaling;Signaling
DMR2:26440001	2	26440001	26441000	1000	1	2.50E-09	0.56	27	2.7	Iqgap2	Signaling
DMR2:26544001	2	26544001	26552000	8000	1	3.60E-09	-0.35	122	1.52	Sv2c	
DMR2:26572001	2	26572001	26573000	1000	1	1.10E-07	-0.49	16	1.6	Sv2c	
DMR2:26619001	2	26619001	26620000	1000	1	2.30E-08	-0.41	7	0.7	Sv2c	
DMR2:26686001	2	26686001	26688000	2000	1	2.80E-14	0.68	40	2	Sv2c	
DMR2:27040001	2	27040001	27042000	2000	1	3.30E-07	0.31	35	1.75	Poc5;Ankdd1b	
DMR2:27123001	2	27123001	27124000	1000	1	1.80E-12	-0.89	0	0	Ankdd1b;Arhgef26	Transcription
DMR2:27208001	2	27208001	27210000	2000	1	2.90E-09	-0.57	26	1.3	Ankdd1b;Arhgef26	Transcription
DMR2:27220001	2	27220001	27221000	1000	1	1.90E-07	-0.5	15	1.5	Ankdd1b;Arhgef26	Transcription
DMR2:27290001	2	27290001	27294000	4000	1	2.30E-07	-0.44	54	1.35	Ankdd1b	
DMR2:27333001	2	27333001	27334000	1000	1	3.00E-07	-0.35	21	2.1	Polk	Transcription
DMR2:27507001	2	27507001	27511000	4000	1	8.10E-07	-0.37	84	2.1	Hmgcr	Metabolism
DMR2:27677001	2	27677001	27679000	2000	1	2.50E-08	-0.41	14	0.7	Ankrd31;LOC102554085	
DMR2:28054001	2	28054001	28058000	4000	1	4.70E-08	-0.45	95	2.38	Enc1	
DMR2:28346001	2	28346001	28347000	1000	1	7.70E-07	0.55	25	2.5	LOC108349925;Utp15	Translation
DMR2:29068001	2	29068001	29071000	3000	1	3.70E-08	0.45	45	1.5	Tnpo1	Transport
DMR2:29468001	2	29468001	29469000	1000	1	5.40E-11	-0.61	4	0.4	Zfp366;LOC108349928	Transcription
DMR2:29572001	2	29572001	29573000	1000	1	1.30E-07	-0.36	13	1.3	Ptcd2	
DMR2:29623001	2	29623001	29626000	3000	1	1.20E-12	-0.64	40	1.33	Mrps27	Translation
DMR2:29677001	2	29677001	29682000	5000	1	4.40E-07	-0.44	117	2.34	Map1b	Cytoskeleton
DMR2:29685001	2	29685001	29689000	4000	2	4.10E-14	-0.51	144	3.6	Map1b	Cytoskeleton
DMR2:29754001	2	29754001	29757000	3000	1	1.30E-15	-0.57	56	1.87	Map1b	Cytoskeleton
DMR2:30113001	2	30113001	30116000	3000	2	7.40E-14	0.9	66	2.2	LOC680951;Cartpt	

DMR2:30173001	2	30173001	30176000	3000	1	2.00E-08	-0.39	48	1.6	Mccc2	Metabolism
DMR2:30188001	2	30188001	30189000	1000	1	5.60E-18	0.37	4	0.4	Mccc2	Metabolism
DMR2:30242001	2	30242001	30243000	1000	1	2.60E-09	-0.38	14	1.4	Mccc2;Bdp1	Metabolism;Transcription
DMR2:30292001	2	30292001	30295000	3000	1	7.30E-07	-0.34	39	1.3	Bdp1	Transcription
DMR2:30328001	2	30328001	30330000	2000	1	1.50E-07	-0.37	27	1.35	Bdp1	Transcription
DMR2:30386001	2	30386001	30388000	2000	1	2.30E-07	-0.31	35	1.75	Naip6	
DMR2:30392001	2	30392001	30393000	1000	1	9.00E-07	0.46	17	1.7	Naip6	
DMR2:30428001	2	30428001	30429000	1000	1	4.80E-10	-0.58	3	0.3	Naip6;Naip5	
DMR2:30516001	2	30516001	30520000	4000	1	1.80E-08	-0.39	23	0.58	Gtf2h2;LOC365655;Ocln	Transcription
DMR2:30582001	2	30582001	30584000	2000	1	1.40E-09	0.56	37	1.85	Ocln	Transcription
DMR2:30625001	2	30625001	30630000	5000	1	5.90E-15	0.8	131	2.62	LOC102557393;Marveld2;Rad17	Transcription
DMR2:30631001	2	30631001	30632000	1000	1	3.50E-07	-0.53	18	1.8	LOC102557393;Marveld2;Rad17	Transcription
DMR2:30686001	2	30686001	30688000	2000	1	4.60E-09	0.58	55	2.75	Ak6;Ccdc125	Epigenetic
DMR2:30850001	2	30850001	30851000	1000	1	4.50E-08	-0.38	9	0.9	Slc30a5	
DMR2:31746001	2	31746001	31754000	8000	2	2.50E-11	-0.57	152	1.9	Pik3r1	Signaling
DMR2:31767001	2	31767001	31771000	4000	2	2.00E-09	-0.45	65	1.62	Pik3r1	Signaling
DMR2:31772001	2	31772001	31775000	3000	1	1.20E-07	-0.41	73	2.43	Pik3r1	Signaling
DMR2:31778001	2	31778001	31782000	4000	1	3.80E-09	-0.43	91	2.28	Pik3r1	Signaling
DMR2:31805001	2	31805001	31806000	1000	1	1.60E-07	-0.36	17	1.7	Pik3r1	Signaling
DMR2:31832001	2	31832001	31834000	2000	1	9.20E-08	0.31	34	1.7	Pik3r1;LOC103691440;LOC103691441	Signaling
DMR2:32474001	2	32474001	32475000	1000	1	5.30E-08	0.53	14	1.4	Mast4;LOC108349933	Signaling
DMR2:32490001	2	32490001	32491000	1000	1	4.10E-07	-0.41	26	2.6	Mast4;LOC108349933	Signaling
DMR2:32825001	2	32825001	32827000	2000	2	7.00E-14	0.91	45	2.25	LOC108349935;Cd180	Receptor
DMR2:32866001	2	32866001	32868000	2000	1	5.90E-07	-0.39	48	2.4	NEWGENE_1310139	
DMR2:33068001	2	33068001	33070000	2000	1	2.50E-07	-0.56	25	1.25	NEWGENE_1310139	
DMR2:33095001	2	33095001	33097000	2000	1	2.70E-07	-0.31	46	2.3	NEWGENE_1310139	
DMR2:33198001	2	33198001	33199000	1000	1	1.70E-08	-0.36	14	1.4	NEWGENE_1310139	
DMR2:33208001	2	33208001	33209000	1000	1	1.60E-07	0.65	17	1.7	NEWGENE_1310139	
DMR2:33239001	2	33239001	33240000	1000	1	9.80E-07	-0.38	18	1.8	NEWGENE_1310139	
DMR2:33257001	2	33257001	33262000	5000	2	6.50E-12	0.63	71	1.42	NEWGENE_1310139	
DMR2:33292001	2	33292001	33293000	1000	1	1.50E-07	-0.38	23	2.3	NEWGENE_1310139	
DMR2:33301001	2	33301001	33304000	3000	2	3.50E-12	0.76	65	2.17	NEWGENE_1310139	
DMR2:33334001	2	33334001	33335000	1000	1	1.80E-07	-0.41	17	1.7	NEWGENE_1310139;LOC103695096	
DMR2:34114001	2	34114001	34115000	1000	1	3.40E-08	-0.51	10	1	Nln	Protease
DMR2:34300001	2	34300001	34303000	3000	1	1.30E-07	-0.48	28	0.93	Ppwd1;Cenpk	Transcription
DMR2:34453001	2	34453001	34454000	1000	1	6.70E-08	-0.36	11	1.1	Adamts6	Protease
DMR2:34470001	2	34470001	34473000	3000	1	2.50E-07	-0.37	27	0.9	Adamts6	Protease
DMR2:34594001	2	34594001	34595000	1000	1	5.90E-10	0.7	18	1.8	Adamts6	Protease
DMR2:34706001	2	34706001	34708000	2000	1	4.20E-08	0.41	28	1.4	LOC108349938;Cwc27	Transcription
DMR2:34947001	2	34947001	34948000	1000	1	1.30E-09	-0.49	20	2	Srek1ip1;Fam159b	
DMR2:35082001	2	35082001	35083000	1000	1	1.80E-08	-0.36	14	1.4	Rgs7bp	
DMR2:35096001	2	35096001	35098000	2000	2	7.00E-11	-0.58	24	1.2	Rgs7bp	
DMR2:35602001	2	35602001	35603000	1000	1	8.90E-19	0.97	32	3.2	Rgs7bp	
DMR2:35628001	2	35628001	35630000	2000	2	1.30E-20	1.12	44	2.2	Rgs7bp	
DMR2:35876001	2	35876001	35879000	3000	1	1.10E-07	-0.48	43	1.43	Rnf180	Proteolysis
DMR2:38193001	2	38193001	38194000	1000	1	2.40E-08	0.53	7	0.7	Kif2a	Cytoskeleton
DMR2:38195001	2	38195001	38197000	2000	1	3.90E-08	-0.42	15	0.75	Kif2a	Cytoskeleton
DMR2:38991001	2	38991001	38992000	1000	1	1.20E-08	-0.53	10	1	Zswim6	
DMR2:39326001	2	39326001	39328000	2000	1	6.20E-09	-0.49	34	1.7	Smim15;Ndufaf2	Transcription
DMR2:39348001	2	39348001	39349000	1000	1	2.70E-07	-0.41	17	1.7	Ndufaf2	Transcription
DMR2:39428001	2	39428001	39431000	3000	1	2.10E-07	0.44	31	1.03	Ndufaf2;Ercc8	Transcription;Transcription
DMR2:40013001	2	40013001	40017000	4000	2	4.60E-12	-0.67	37	0.92	Depdc1b	Cytoskeleton
DMR2:40025001	2	40025001	40026000	1000	1	1.30E-08	0.47	17	1.7	Depdc1b	Cytoskeleton
DMR2:40067001	2	40067001	40068000	1000	1	3.10E-07	0.55	17	1.7	Depdc1b	Cytoskeleton
DMR2:40369001	2	40369001	40371000	2000	1	3.30E-09	-0.43	19	0.95	Pde4d;Elovl7;LOC108350211	Signaling;Metabolism
DMR2:40383001	2	40383001	40384000	1000	1	2.50E-10	0.69	22	2.2	Pde4d;Elovl7;LOC108350211	Signaling;Metabolism

DMR2:40772001	2	40772001	40775000	3000	2	5.30E-07	0.5	44	1.47	Pde4d	Signaling
DMR2:40782001	2	40782001	40784000	2000	1	6.20E-08	0.34	22	1.1	Pde4d	Signaling
DMR2:40892001	2	40892001	40893000	1000	1	9.10E-08	-0.34	17	1.7	Pde4d;LOC108350210	Signaling
DMR2:40916001	2	40916001	40919000	3000	1	3.20E-07	0.43	27	0.9	Pde4d;LOC108350210	Signaling
DMR2:41031001	2	41031001	41032000	1000	1	5.60E-08	-0.49	18	1.8	Pde4d	Signaling
DMR2:41053001	2	41053001	41060000	7000	1	5.40E-07	0.39	74	1.06	Pde4d	Signaling
DMR2:41206001	2	41206001	41209000	3000	1	1.50E-08	-0.46	29	0.97	Pde4d	Signaling
DMR2:41576001	2	41576001	41577000	1000	1	1.10E-09	0.8	17	1.7	Rab3c	
DMR2:41779001	2	41779001	41781000	2000	1	7.70E-09	-0.41	14	0.7	Rab3c	
DMR2:41862001	2	41862001	41863000	1000	1	2.80E-07	-0.48	16	1.6	Gapt	
DMR2:43359001	2	43359001	43362000	3000	1	1.70E-08	-0.66	55	1.83	Map3k1	
DMR2:43375001	2	43375001	43376000	1000	1	1.60E-07	-0.57	13	1.3	Map3k1	
DMR2:43830001	2	43830001	43833000	3000	1	2.90E-08	0.78	50	1.67	Ankrd55	Cytoskeleton
DMR2:43872001	2	43872001	43874000	2000	1	9.70E-08	0.31	25	1.25	Ankrd55	Cytoskeleton
DMR2:43982001	2	43982001	43983000	1000	1	2.00E-07	-0.53	18	1.8	Ankrd55;LOC100912399	Cytoskeleton;Signaling
DMR2:44070001	2	44070001	44072000	2000	1	1.40E-15	-0.58	40	2	Ankrd55	Cytoskeleton
DMR2:44170001	2	44170001	44173000	3000	1	7.50E-07	-0.3	60	2	Ankrd55;LOC103691456	Cytoskeleton
DMR2:44282001	2	44282001	44283000	1000	1	6.80E-08	-0.37	11	1.1	Il6st	Receptor
DMR2:44323001	2	44323001	44324000	1000	1	1.90E-07	0.59	11	1.1	Il6st;Il31ra	Receptor
DMR2:44407001	2	44407001	44408000	1000	1	6.60E-09	-0.46	12	1.2	Il31ra	Receptor
DMR2:44449001	2	44449001	44452000	3000	1	1.20E-07	-0.36	26	0.87	Ddx4	
DMR2:44536001	2	44536001	44538000	2000	1	7.10E-07	-0.37	24	1.2	Slc38a9	Transport
DMR2:44553001	2	44553001	44556000	3000	1	4.60E-07	-0.47	24	0.8	Slc38a9;LOC102551224	Transport
DMR2:44561001	2	44561001	44564000	3000	1	3.00E-10	-0.57	42	1.4	Slc38a9;LOC102551224	Transport
DMR2:44591001	2	44591001	44592000	1000	1	2.50E-11	-0.46	28	2.8	Slc38a9	Transport
DMR2:44708001	2	44708001	44711000	3000	1	2.10E-08	-0.48	41	1.37	Plpp1	Signaling
DMR2:44778001	2	44778001	44779000	1000	1	3.00E-07	-0.58	4	0.4	Skiv2l2;Dhx29	Transcription
DMR2:44828001	2	44828001	44832000	4000	1	5.60E-07	-0.28	55	1.38	Dhx29	Transcription
DMR2:44843001	2	44843001	44845000	2000	1	3.10E-07	-0.34	21	1.05	Dhx29	Transcription
DMR2:44902001	2	44902001	44904000	2000	1	1.70E-12	0.59	25	1.25	Mir449c;Mir449a;Gpx8;LOC108350166	Metabolism
DMR2:44914001	2	44914001	44916000	2000	1	3.80E-09	-0.42	38	1.9	Gpx8;LOC108350166;Cdc20b	Metabolism
DMR2:44990001	2	44990001	44993000	3000	2	1.50E-10	0.69	41	1.37	Gzma	Protease
DMR2:45073001	2	45073001	45074000	1000	1	2.00E-07	0.38	16	1.6	Gzmk	Protease
DMR2:45174001	2	45174001	45175000	1000	1	2.50E-07	0.57	34	3.4	RGD1561161	
DMR2:45446001	2	45446001	45447000	1000	1	1.90E-10	0.63	30	3	Snx18	Cytoskeleton
DMR2:45462001	2	45462001	45472000	10000	3	9.90E-10	-0.47	156	1.56	Snx18	Cytoskeleton
DMR2:45484001	2	45484001	45485000	1000	1	3.20E-08	-0.6	19	1.9	Snx18;LOC108349945	Cytoskeleton
DMR2:45486001	2	45486001	45488000	2000	1	1.40E-07	-0.48	28	1.4	Snx18;LOC108349945	Cytoskeleton
DMR2:45663001	2	45663001	45667000	4000	1	9.80E-07	0.33	45	1.12	Arl15	
DMR2:45675001	2	45675001	45678000	3000	1	4.30E-15	-0.57	54	1.8	Arl15	
DMR2:45724001	2	45724001	45725000	1000	1	3.60E-07	-0.28	15	1.5	Arl15	
DMR2:45727001	2	45727001	45729000	2000	1	1.20E-07	-0.44	36	1.8	Arl15	
DMR2:45730001	2	45730001	45731000	1000	1	3.00E-08	-0.39	16	1.6	Arl15	
DMR2:45738001	2	45738001	45739000	1000	1	8.90E-08	-0.56	11	1.1	Arl15	
DMR2:45817001	2	45817001	45818000	1000	1	8.20E-08	-0.38	25	2.5	Arl15	
DMR2:45826001	2	45826001	45829000	3000	1	8.80E-08	-0.46	41	1.37	Arl15	
DMR2:45958001	2	45958001	45959000	1000	1	2.00E-07	-0.38	19	1.9	Arl15;LOC103691458	
DMR2:46045001	2	46045001	46053000	8000	1	2.00E-08	0.46	154	1.93	Arl15	
DMR2:46136001	2	46136001	46138000	2000	1	2.20E-11	0.54	16	0.8	Olr1262	Receptor
DMR2:46530001	2	46530001	46532000	2000	1	3.00E-08	0.32	28	1.4	Fst	Protease; Proteolysis
DMR2:47137001	2	47137001	47138000	1000	1	6.20E-07	0.35	13	1.3	Itga1	Extracellular Matrix
DMR2:47159001	2	47159001	47162000	3000	1	4.30E-10	0.59	54	1.8	Itga1	Extracellular Matrix
DMR2:47198001	2	47198001	47200000	2000	1	8.10E-07	-0.35	22	1.1	Itga1	Extracellular Matrix
DMR2:47235001	2	47235001	47236000	1000	1	8.30E-07	-0.42	14	1.4	Itga1	Extracellular Matrix
DMR2:49134001	2	49134001	49137000	3000	1	3.70E-12	-0.59	32	1.07	Parp8	
DMR2:49276001	2	49276001	49278000	2000	1	3.00E-08	0.46	27	1.35	Parp8	
DMR2:49287001	2	49287001	49288000	1000	1	8.20E-10	0.58	12	1.2	Parp8	
DMR2:49303001	2	49303001	49305000	2000	1	3.90E-08	0.46	50	2.5	Parp8	
DMR2:49316001	2	49316001	49329000	13000	1	1.00E-06	0.28	410	3.15	Parp8;LOC100910954	
DMR2:49332001	2	49332001	49334000	2000	2	1.00E-16	0.78	31	1.55	Parp8;LOC100910954	
DMR2:49336001	2	49336001	49338000	2000	2	2.40E-14	0.68	36	1.8	Parp8;LOC100910954	

DMR2:49352001	2	49352001	49367000	15000	8	3.00E-15	0.51	444	2.96	Parp8;LOC100910954	
DMR2:49368001	2	49368001	49369000	1000	1	1.30E-18	0.56	18	1.8	Parp8;LOC100910954	
DMR2:49370001	2	49370001	49372000	2000	2	1.10E-22	0.8	42	2.1	Parp8;LOC100910954	
DMR2:49460001	2	49460001	49462000	2000	1	2.40E-12	-0.54	42	2.1	Parp8	
DMR2:50147001	2	50147001	50150000	3000	1	6.70E-07	-0.32	28	0.93	Hcn1	Transport
DMR2:50185001	2	50185001	50187000	2000	1	4.00E-09	-0.38	13	0.65	Hcn1	Transport
DMR2:50267001	2	50267001	50268000	1000	1	2.80E-08	-0.64	7	0.7	Hcn1	Transport
DMR2:50430001	2	50430001	50432000	2000	1	4.70E-09	-0.42	18	0.9	Hcn1	Transport
DMR2:50498001	2	50498001	50500000	2000	1	2.90E-08	0.71	34	1.7	Hcn1	Transport
DMR2:52159001	2	52159001	52166000	7000	1	1.00E-07	-0.53	24	0.34	RGD1561520	
DMR2:52339001	2	52339001	52341000	2000	1	4.60E-07	-0.4	36	1.8	RGD1306227	
DMR2:52396001	2	52396001	52398000	2000	1	8.00E-07	0.52	37	1.85	Ccl28;LOC102547360	Growth Factors
DMR2:52400001	2	52400001	52401000	1000	1	2.20E-07	0.56	10	1	Ccl28;LOC102547360;LOC103691467	Growth Factors
DMR2:52408001	2	52408001	52409000	1000	1	1.20E-07	-0.78	3	0.3	Ccl28;LOC102547360;LOC103691467	Growth Factors
DMR2:52458001	2	52458001	52465000	7000	1	3.40E-16	1.25	92	1.31	Nim1k	Signaling
DMR2:52487001	2	52487001	52488000	1000	1	1.70E-08	-0.69	6	0.6	Nim1k	Signaling
DMR2:53140001	2	53140001	53141000	1000	1	5.00E-08	0.77	28	2.8	Ccdc152;LOC103691472;Ghr	Receptor
DMR2:53232001	2	53232001	53233000	1000	1	6.10E-07	0.36	2	0.2	Ghr	Receptor
DMR2:53864001	2	53864001	53866000	2000	1	2.10E-07	-0.43	27	1.35	Oxct1	Transport
DMR2:53894001	2	53894001	53896000	2000	1	1.90E-07	-0.45	25	1.25	Oxct1	Transport
DMR2:53908001	2	53908001	53910000	2000	1	2.40E-07	-0.3	39	1.95	Oxct1	Transport
DMR2:53913001	2	53913001	53916000	3000	2	4.60E-10	-0.47	53	1.77	Oxct1	Transport
DMR2:54200001	2	54200001	54202000	2000	1	4.10E-10	-0.62	15	0.75	LOC108349953;Plcxd3	
DMR2:54458001	2	54458001	54460000	2000	1	1.10E-12	-0.46	18	0.9	C6	
DMR2:54663001	2	54663001	54664000	1000	1	9.20E-07	0.56	19	1.9	Mroh2b	
DMR2:54712001	2	54712001	54717000	5000	1	5.20E-07	0.36	71	1.42	C7	
DMR2:54746001	2	54746001	54751000	5000	1	1.30E-07	-0.35	45	0.9	C7	
DMR2:54761001	2	54761001	54764000	3000	2	1.40E-11	-0.55	27	0.9	C7	
DMR2:54817001	2	54817001	54820000	3000	1	8.70E-13	0.84	39	1.3	Card6	
DMR2:55801001	2	55801001	55802000	1000	1	8.80E-07	-0.4	10	1	C9	
DMR2:55847001	2	55847001	55849000	2000	1	2.80E-08	0.53	32	1.6	Fyb	
DMR2:55921001	2	55921001	55923000	2000	1	3.20E-07	-0.75	16	0.8	Fyb	
DMR2:55958001	2	55958001	55960000	2000	1	3.30E-07	-0.59	11	0.55	Fyb	
DMR2:55969001	2	55969001	55971000	2000	1	8.80E-10	-0.33	15	0.75	Fyb	
DMR2:56154001	2	56154001	56155000	1000	1	1.00E-08	-0.41	22	2.2	Osmr	Receptor
DMR2:56171001	2	56171001	56175000	4000	2	1.20E-07	0.6	54	1.35	Osmr	Receptor
DMR2:56420001	2	56420001	56422000	2000	1	1.60E-07	-0.58	23	1.15	Lifr	Receptor
DMR2:56462001	2	56462001	56466000	4000	1	8.90E-08	-0.36	69	1.73	Lifr	Receptor
DMR2:56585001	2	56585001	56587000	2000	1	4.10E-08	-0.41	23	1.15	Egflam	Extracellular Matrix
DMR2:56664001	2	56664001	56666000	2000	1	2.60E-08	-0.5	29	1.45	Egflam	Extracellular Matrix
DMR2:56907001	2	56907001	56908000	1000	1	8.30E-09	-0.5	5	0.5	Gdnf	Growth Factors
DMR2:56973001	2	56973001	56975000	2000	1	2.10E-07	-0.27	18	0.9	Wdr70	
DMR2:57097001	2	57097001	57099000	2000	1	5.00E-08	-0.39	29	1.45	Wdr70	
DMR2:57215001	2	57215001	57217000	2000	1	3.80E-07	0.44	22	1.1	Nup155	Transport
DMR2:57297001	2	57297001	57300000	3000	2	1.10E-07	0.54	39	1.3	RGD1310081;LOC365694	
DMR2:57518001	2	57518001	57520000	2000	1	1.00E-09	-0.5	23	1.15	Nipbl	Epigenetic
DMR2:57583001	2	57583001	57584000	1000	1	3.60E-07	-0.44	13	1.3	Nipbl	Epigenetic
DMR2:57866001	2	57866001	57871000	5000	1	3.00E-20	1	76	1.52	Slc1a3	Transport
DMR2:57878001	2	57878001	57880000	2000	1	9.50E-08	0.49	32	1.6	Slc1a3	Transport
DMR2:57892001	2	57892001	57898000	6000	1	7.90E-08	-0.45	75	1.25	Slc1a3	Transport
DMR2:57943001	2	57943001	57946000	3000	2	2.30E-09	-0.55	44	1.47	Slc1a3;LOC108349962	Transport
DMR2:58226001	2	58226001	58227000	1000	1	9.60E-17	1.05	34	3.4	Ranbp3l;LOC679871	Cytoskeleton
DMR2:58252001	2	58252001	58253000	1000	1	1.50E-07	-0.45	2	0.2	Ranbp3l	Cytoskeleton
DMR2:58318001	2	58318001	58320000	2000	1	9.20E-10	-0.57	21	1.05	Ranbp3l	Cytoskeleton
DMR2:58344001	2	58344001	58347000	3000	1	1.60E-08	0.65	40	1.33	Ranbp3l;LOC108350155	Cytoskeleton
DMR2:58353001	2	58353001	58357000	4000	1	1.50E-11	-0.43	40	1	Ranbp3l;LOC108350155	Cytoskeleton
DMR2:58362001	2	58362001	58363000	1000	1	5.30E-11	-0.49	9	0.9	Ranbp3l;LOC108350155	Cytoskeleton
DMR2:58391001	2	58391001	58393000	2000	1	2.70E-07	-0.33	19	0.95	Ranbp3l	Cytoskeleton
DMR2:58470001	2	58470001	58471000	1000	1	8.10E-07	-0.33	17	1.7	Nadk2	
DMR2:58511001	2	58511001	58512000	1000	1	3.50E-07	-0.33	22	2.2	Nadk2;Skp2	

DMR2:58518001	2	58518001	58522000	4000	1	3.50E-10	-0.43	70	1.75	Skp2	
DMR2:58545001	2	58545001	58548000	3000	1	6.30E-12	0.43	44	1.47	Lmbrd2	
DMR2:58597001	2	58597001	58599000	2000	1	1.80E-08	-0.4	17	0.85	Lmbrd2	
DMR2:58954001	2	58954001	58958000	4000	1	3.80E-07	-0.23	36	0.9	Spef2	
DMR2:60204001	2	60204001	60205000	1000	1	1.20E-07	-0.37	21	2.1	Prlr	Receptor
DMR2:60281001	2	60281001	60282000	1000	1	8.40E-07	0.45	14	1.4	Prlr	Receptor
DMR2:60378001	2	60378001	60379000	1000	1	2.20E-10	0.68	15	1.5	Agxt2	
DMR2:60386001	2	60386001	60389000	3000	1	3.60E-08	-0.53	38	1.27	Agxt2	
DMR2:60494001	2	60494001	60495000	1000	1	3.30E-10	-0.42	25	2.5	LOC108349965;Ttc23l	
DMR2:60501001	2	60501001	60505000	4000	1	4.70E-08	-0.37	21	0.52	Ttc23l	
DMR2:60545001	2	60545001	60547000	2000	1	1.90E-07	0.54	29	1.45	Ttc23l;Rai14	
DMR2:60574001	2	60574001	60576000	2000	1	2.00E-10	-0.45	27	1.35	Rai14	
DMR2:60657001	2	60657001	60659000	2000	1	5.50E-08	-0.48	41	2.05	Rai14	
DMR2:61058001	2	61058001	61059000	1000	1	1.70E-07	-0.67	6	0.6	Adamts12	Protease
DMR2:61276001	2	61276001	61278000	2000	1	2.40E-08	-0.56	13	0.65	Adamts12	Protease
DMR2:61299001	2	61299001	61300000	1000	1	1.20E-07	-0.59	9	0.9	Adamts12	Protease
DMR2:61411001	2	61411001	61413000	2000	1	4.70E-08	-0.39	29	1.45	Tars	
DMR2:61928001	2	61928001	61933000	5000	1	2.20E-07	-0.41	68	1.36	Npr3	Receptor
DMR2:62350001	2	62350001	62351000	1000	1	1.60E-11	0.86	21	2.1	Golph3	
DMR2:62386001	2	62386001	62388000	2000	1	6.60E-07	-0.43	28	1.4	Golph3	
DMR2:62464001	2	62464001	62465000	1000	1	4.10E-09	-0.47	14	1.4	Pdzd2	Cytokine
DMR2:62880001	2	62880001	62882000	2000	1	1.30E-10	0.41	17	0.85	RGD1306502;Drosha	Translation
DMR2:63006001	2	63006001	63009000	3000	1	5.60E-09	-0.44	48	1.6	Drosha	Translation
DMR2:63113001	2	63113001	63115000	2000	1	6.30E-09	-0.35	19	0.95	LOC103695100;Cdh6	Cytoskeleton
DMR2:63133001	2	63133001	63136000	3000	2	2.40E-07	-0.48	51	1.7	Cdh6	Cytoskeleton
DMR2:63145001	2	63145001	63147000	2000	2	6.20E-08	-0.6	8	0.4	Cdh6	Cytoskeleton
DMR2:63178001	2	63178001	63182000	4000	1	1.30E-07	-0.3	28	0.7	Cdh6	Cytoskeleton
DMR2:68823001	2	68823001	68825000	2000	1	2.00E-08	-0.4	44	2.2	Egf	Receptor
DMR2:68861001	2	68861001	68862000	1000	1	2.50E-07	-0.54	12	1.2	Egf	Receptor
DMR2:69473001	2	69473001	69475000	2000	1	1.40E-10	0.29	19	0.95	Cdh10;LOC103691484	Cytoskeleton
DMR2:69570001	2	69570001	69572000	2000	1	3.50E-07	-0.4	20	1	Cdh10	Cytoskeleton
DMR2:71455001	2	71455001	71456000	1000	1	2.70E-10	0.67	9	0.9	Cdh12	Cytoskeleton
DMR2:72059001	2	72059001	72061000	2000	1	4.00E-08	-0.45	7	0.35	Cdh12	Cytoskeleton
DMR2:72266001	2	72266001	72267000	1000	1	8.60E-07	0.56	5	0.5	Cdh12	Cytoskeleton
DMR2:72519001	2	72519001	72521000	2000	1	7.20E-07	-0.37	18	0.9	Cdh12	Cytoskeleton
DMR2:72527001	2	72527001	72528000	1000	1	1.50E-09	0.41	8	0.8	Cdh12	Cytoskeleton
DMR2:73745001	2	73745001	73746000	1000	1	3.40E-09	0.47	7	0.7	Cdh18	Cytoskeleton
DMR2:73760001	2	73760001	73761000	1000	1	1.80E-10	-0.56	6	0.6	Cdh18	Cytoskeleton
DMR2:74085001	2	74085001	74086000	1000	1	6.60E-07	0.49	10	1	Cdh18;LOC102554526	Cytoskeleton
DMR2:74113001	2	74113001	74116000	3000	1	1.80E-13	0.52	20	0.67	Cdh18	Cytoskeleton
DMR2:74223001	2	74223001	74227000	4000	1	5.30E-08	-0.35	36	0.9	Cdh18	Cytoskeleton
DMR2:74315001	2	74315001	74320000	5000	1	2.60E-07	-0.43	47	0.94	Cdh18	Cytoskeleton
DMR2:74555001	2	74555001	74556000	1000	1	6.80E-08	0.65	13	1.3	Cdh18	Cytoskeleton
DMR2:74564001	2	74564001	74565000	1000	1	1.30E-11	-0.5	6	0.6	Cdh18	Cytoskeleton
DMR2:77637001	2	77637001	77640000	3000	1	1.70E-07	-0.55	23	0.77	Basp1	
DMR2:78027001	2	78027001	78030000	3000	1	1.80E-07	-0.39	49	1.63	Myo10	
DMR2:79206001	2	79206001	79208000	2000	1	3.40E-25	0.92	14	0.7	Fbxl7	
DMR2:79278001	2	79278001	79279000	1000	1	1.10E-10	-0.69	3	0.3	Fbxl7	
DMR2:79324001	2	79324001	79325000	1000	1	9.50E-07	0.42	8	0.8	Fbxl7	
DMR2:79367001	2	79367001	79368000	1000	1	1.60E-08	0.39	26	2.6	Fbxl7	
DMR2:79419001	2	79419001	79422000	3000	1	3.30E-07	-0.45	38	1.27	Fbxl7	
DMR2:80487001	2	80487001	80488000	1000	1	2.00E-09	0.61	48	4.8	Trio	Transcription
DMR2:80511001	2	80511001	80513000	2000	1	9.10E-13	-0.45	52	2.6	Trio	Transcription
DMR2:80757001	2	80757001	80758000	1000	1	1.80E-08	-0.42	8	0.8	Trio	Transcription
DMR2:80960001	2	80960001	80962000	2000	1	2.60E-17	0.97	33	1.65	Dnah5	Cytoskeleton
DMR2:81017001	2	81017001	81018000	1000	1	1.30E-08	-0.63	4	0.4	Dnah5	Cytoskeleton
DMR2:81077001	2	81077001	81078000	1000	1	2.20E-08	-0.52	12	1.2	Dnah5	Cytoskeleton
DMR2:81291001	2	81291001	81294000	3000	1	1.80E-07	-0.36	20	0.67	Dnah5	Cytoskeleton
DMR2:81339001	2	81339001	81343000	4000	1	3.60E-11	-0.33	27	0.68	Dnah5	Cytoskeleton
DMR2:83442001	2	83442001	83444000	2000	1	8.10E-08	-0.63	14	0.7	Ctnnd2	Cytoskeleton
DMR2:83460001	2	83460001	83462000	2000	1	7.60E-07	-0.22	22	1.1	Ctnnd2	Cytoskeleton
DMR2:83509001	2	83509001	83512000	3000	1	7.20E-10	-0.64	13	0.43	Ctnnd2	Cytoskeleton

DMR2:83875001	2	83875001	83877000	2000	1	1.80E-07	-0.46	16	0.8	Ctnnd2	Cytoskeleton
DMR2:83957001	2	83957001	83958000	1000	1	1.50E-07	-0.41	17	1.7	Ctnnd2	Cytoskeleton
DMR2:83980001	2	83980001	83982000	2000	1	6.70E-07	0.43	21	1.05	Ctnnd2	Cytoskeleton
DMR2:84415001	2	84415001	84416000	1000	1	3.00E-07	0.51	13	1.3	Ankrd33b	
DMR2:85368001	2	85368001	85369000	1000	1	2.70E-07	-0.41	4	0.4	LOC100909539;Sema5a	Signaling
DMR2:85533001	2	85533001	85535000	2000	1	1.20E-08	-0.44	13	0.65	Sema5a	Signaling
DMR2:85768001	2	85768001	85770000	2000	1	6.80E-09	0.64	39	1.95	Sema5a	Signaling
DMR2:85783001	2	85783001	85784000	1000	1	3.30E-07	-0.57	5	0.5	Sema5a	Signaling
DMR2:86975001	2	86975001	86976000	1000	1	8.10E-07	-0.52	7	0.7	LOC108349973;Zfp458	Transcription
DMR2:89256001	2	89256001	89261000	5000	1	1.30E-08	-0.31	45	0.9	Ralyl	
DMR2:89347001	2	89347001	89348000	1000	1	3.30E-11	-0.86	5	0.5	Ralyl;LOC108349976	
DMR2:89359001	2	89359001	89362000	3000	1	1.20E-11	-0.51	19	0.63	Ralyl	
DMR2:89542001	2	89542001	89543000	1000	1	6.70E-10	-0.6	2	0.2	Ralyl	
DMR2:89663001	2	89663001	89666000	3000	1	3.70E-16	0.77	35	1.17	Ralyl;LOC103695106	
DMR2:89771001	2	89771001	89772000	1000	1	9.40E-14	0.63	15	1.5	Ralyl	
DMR2:93503001	2	93503001	93509000	6000	1	3.20E-07	-0.29	67	1.12	LOC103691511;Snx16	
DMR2:93578001	2	93578001	93579000	1000	1	5.00E-07	-0.38	13	1.3	Chmp4c	Transport
DMR2:94963001	2	94963001	94967000	4000	1	5.30E-13	-0.47	47	1.18	Tpd52	
DMR2:95036001	2	95036001	95041000	5000	1	3.20E-08	-0.37	68	1.36	Tpd52	
DMR2:95317001	2	95317001	95319000	2000	1	2.00E-07	0.67	23	1.15	Hey1;LOC108349980	Transcription
DMR2:95433001	2	95433001	95435000	2000	1	1.70E-10	0.63	28	1.4	Stmn2	
DMR2:95459001	2	95459001	95462000	3000	1	1.10E-13	0.76	46	1.53	Stmn2	
DMR2:96597001	2	96597001	96598000	1000	1	6.00E-07	0.34	21	2.1	Pkia	Signaling
DMR2:96651001	2	96651001	96652000	1000	1	8.00E-07	-0.48	13	1.3	Pkia	Signaling
DMR2:98426001	2	98426001	98428000	2000	1	2.30E-11	0.65	29	1.45	Zfhx4	Transcription
DMR2:100309001	2	100309001	100310000	1000	1	3.30E-09	0.37	12	1.2	Hnf4g	Transcription
DMR2:100381001	2	100381001	100384000	3000	1	2.40E-07	-0.48	19	0.63	Hnf4g	Transcription
DMR2:100665001	2	100665001	100670000	5000	2	7.20E-08	0.36	50	1	Ythdf3	
DMR2:103945001	2	103945001	103950000	5000	1	4.30E-07	-0.22	58	1.16	Armc1	
DMR2:103963001	2	103963001	103965000	2000	2	1.30E-15	-0.68	13	0.65	Armc1	
DMR2:104429001	2	104429001	104430000	1000	1	2.50E-07	-0.63	11	1.1	Trim55	Proteolysis
DMR2:104879001	2	104879001	104880000	1000	1	1.30E-08	0.47	21	2.1	Hltf	
DMR2:104898001	2	104898001	104899000	1000	1	2.80E-07	-0.55	6	0.6	Hltf	
DMR2:105021001	2	105021001	105026000	5000	1	1.00E-09	-0.4	49	0.98	Cpa3	Protease
DMR2:105048001	2	105048001	105049000	1000	1	3.90E-08	-0.58	3	0.3	Cpa3	Protease
DMR2:105081001	2	105081001	105082000	1000	1	2.20E-07	-0.39	8	0.8	Cpb1	Protease
DMR2:107252001	2	107252001	107253000	1000	1	2.10E-08	-0.43	7	0.7	Tbl1xr1	
DMR2:107346001	2	107346001	107347000	1000	1	6.00E-07	-0.41	11	1.1	Tbl1xr1	
DMR2:109336001	2	109336001	109338000	2000	1	3.10E-07	0.6	33	1.65	Naaladl2	
DMR2:109434001	2	109434001	109435000	1000	1	2.50E-07	-0.48	5	0.5	Naaladl2	
DMR2:110005001	2	110005001	110006000	1000	1	2.20E-08	-0.42	7	0.7	Naaladl2	
DMR2:110028001	2	110028001	110029000	1000	1	5.70E-09	0.46	19	1.9	Naaladl2	
DMR2:110073001	2	110073001	110074000	1000	1	1.40E-07	-0.63	2	0.2	Naaladl2	
DMR2:110105001	2	110105001	110107000	2000	2	1.40E-07	-0.51	15	0.75	Naaladl2	
DMR2:110305001	2	110305001	110306000	1000	1	9.50E-23	1.38	10	1	Naaladl2;LOC499584	
DMR2:111320001	2	111320001	111321000	1000	1	5.60E-07	0.45	17	1.7	Nlgn1	Cytoskeleton
DMR2:111515001	2	111515001	111517000	2000	1	8.50E-08	-0.37	18	0.9	Nlgn1;LOC108350212	Cytoskeleton
DMR2:111645001	2	111645001	111647000	2000	1	6.50E-07	-0.64	11	0.55	Nlgn1	Cytoskeleton
DMR2:111685001	2	111685001	111688000	3000	1	2.00E-08	-0.37	29	0.97	Nlgn1	Cytoskeleton
DMR2:111739001	2	111739001	111740000	1000	1	3.70E-11	-0.45	6	0.6	Nlgn1	Cytoskeleton
DMR2:111763001	2	111763001	111765000	2000	1	7.10E-09	-0.61	13	0.65	Nlgn1	Cytoskeleton
DMR2:111917001	2	111917001	111918000	1000	1	2.90E-07	-0.47	7	0.7	Nlgn1	Cytoskeleton
DMR2:112328001	2	112328001	112330000	2000	1	4.50E-09	-0.31	13	0.65	Spata16	
DMR2:112351001	2	112351001	112352000	1000	1	2.50E-08	0.5	15	1.5	Spata16	
DMR2:112354001	2	112354001	112356000	2000	1	3.60E-08	-0.3	20	1	Spata16	
DMR2:112367001	2	112367001	112372000	5000	1	8.80E-07	-0.35	43	0.86	Spata16	
DMR2:112488001	2	112488001	112490000	2000	1	2.60E-08	-0.64	14	0.7	Spata16	
DMR2:112560001	2	112560001	112563000	3000	1	1.80E-07	-0.38	40	1.33	Spata16	
DMR2:112644001	2	112644001	112648000	4000	1	4.00E-14	0.51	39	0.98	Spata16	
DMR2:112794001	2	112794001	112795000	1000	1	3.40E-08	0.54	16	1.6	Ect2	Transcription
DMR2:113022001	2	113022001	113023000	1000	1	4.30E-08	-0.55	8	0.8	Tnfsf10	
DMR2:113057001	2	113057001	113058000	1000	1	8.10E-08	-0.54	5	0.5	LOC103691520;Ghsr	Signaling

DMR2:113134001	2	113134001	113136000	2000	1	9.40E-07	-0.47	21	1.05	Fndc3b	Proteolysis
DMR2:113168001	2	113168001	113170000	2000	1	2.00E-07	-0.61	22	1.1	Fndc3b	Proteolysis
DMR2:113331001	2	113331001	113334000	3000	1	1.80E-09	0.34	34	1.13	Fndc3b	Proteolysis
DMR2:113400001	2	113400001	113401000	1000	1	1.70E-11	-0.53	19	1.9	Fndc3b	Proteolysis
DMR2:113640001	2	113640001	113646000	6000	1	4.00E-09	-0.43	89	1.48	Pld1	Metabolism
DMR2:113678001	2	113678001	113680000	2000	1	8.50E-07	0.6	55	2.75	Pld1	Metabolism
DMR2:113792001	2	113792001	113793000	1000	1	2.80E-07	0.4	8	0.8	Pld1	Metabolism
DMR2:113972001	2	113972001	113975000	3000	1	3.00E-08	0.58	54	1.8	Tnik	Signaling
DMR2:115342001	2	115342001	115343000	1000	1	3.10E-07	0.53	11	1.1	Eif5a2	Translation
DMR2:115365001	2	115365001	115367000	2000	2	3.20E-11	-0.47	19	0.95	Rpl22l1	Translation
DMR2:115781001	2	115781001	115782000	1000	1	2.70E-08	0.39	15	1.5	Slc7a14	Transport
DMR2:115788001	2	115788001	115789000	1000	1	8.70E-07	-0.42	27	2.7	Slc7a14	Transport
DMR2:115820001	2	115820001	115826000	6000	1	7.70E-17	1.17	94	1.57	Cldn11	Cell Junction
DMR2:115829001	2	115829001	115833000	4000	1	8.30E-14	1.06	83	2.08	Cldn11	Cell Junction
DMR2:115960001	2	115960001	115961000	1000	1	7.10E-07	-0.33	7	0.7	Prkci	Signaling
DMR2:116121001	2	116121001	116123000	2000	1	4.20E-07	-0.38	8	0.4	Gpr160	Signaling
DMR2:116135001	2	116135001	116139000	4000	1	2.50E-08	-0.31	37	0.92	Gpr160	Signaling
DMR2:116277001	2	116277001	116278000	1000	1	1.20E-08	-0.59	9	0.9	Samd7	Epigenetic
DMR2:116311001	2	116311001	116313000	2000	1	2.40E-09	0.71	32	1.6	Lrrc31	
DMR2:116334001	2	116334001	116335000	1000	1	4.60E-08	0.51	10	1	Lrrc31	
DMR2:116356001	2	116356001	116358000	2000	1	7.10E-07	-0.6	47	2.35	Lrriq4	Cytoskeleton
DMR2:116514001	2	116514001	116516000	2000	1	3.60E-08	-0.39	11	0.55	Egfem1	
DMR2:116583001	2	116583001	116584000	1000	1	3.00E-08	-0.51	5	0.5	Egfem1	
DMR2:116600001	2	116600001	116602000	2000	1	4.70E-10	-0.55	19	0.95	Egfem1	
DMR2:116638001	2	116638001	116641000	3000	1	9.10E-08	-0.39	26	0.87	Egfem1	
DMR2:116854001	2	116854001	116855000	1000	1	4.60E-09	0.45	11	1.1	Egfem1	
DMR2:116879001	2	116879001	116880000	1000	1	3.90E-08	-0.61	2	0.2	Egfem1	
DMR2:116941001	2	116941001	116942000	1000	1	1.70E-10	-0.58	12	1.2	Egfem1;LOC103691523	
DMR2:116951001	2	116951001	116954000	3000	1	8.50E-10	-0.3	26	0.87	Egfem1;LOC103691523	
DMR2:117463001	2	117463001	117464000	1000	1	7.50E-10	0.63	35	3.5	Mecom	Transcription
DMR2:117514001	2	117514001	117515000	1000	1	2.20E-09	-0.37	15	1.5	Mecom	Transcription
DMR2:117738001	2	117738001	117741000	3000	1	8.00E-10	0.7	68	2.27	Mecom;LOC103691544	Transcription
DMR2:117777001	2	117777001	117780000	3000	1	4.90E-07	-0.39	31	1.03	Mecom;LOC103691544	Transcription
DMR2:117785001	2	117785001	117788000	3000	1	1.20E-07	-0.35	39	1.3	Mecom;LOC103691544	Transcription
DMR2:117960001	2	117960001	117962000	2000	1	9.60E-08	0.57	31	1.55	Mecom;LOC103691544	Transcription
DMR2:117968001	2	117968001	117969000	1000	1	6.50E-09	0.65	4	0.4	Mecom;LOC103691544	Transcription
DMR2:117976001	2	117976001	117977000	1000	1	4.90E-08	-0.59	6	0.6	Mecom	Transcription
DMR2:118380001	2	118380001	118383000	3000	1	1.60E-10	-0.44	28	0.93	Kcnmb2;LOC102548736	Transport
DMR2:118443001	2	118443001	118445000	2000	1	6.50E-07	0.65	24	1.2	Kcnmb2	Transport
DMR2:118721001	2	118721001	118722000	1000	1	9.80E-16	0.92	35	3.5	Zmat3	
DMR2:120520001	2	120520001	120522000	2000	2	1.10E-15	0.92	55	2.75	Dnajc19;LOC100366044;Fxr1	Translation
DMR2:120552001	2	120552001	120554000	2000	1	3.50E-12	-0.5	19	0.95	Fxr1	Translation
DMR2:120580001	2	120580001	120581000	1000	1	1.80E-09	0.45	13	1.3	Fxr1	Translation
DMR2:122565001	2	122565001	122566000	1000	1	9.10E-11	-0.4	18	1.8	Mccc1	Metabolism
DMR2:122699001	2	122699001	122700000	1000	1	6.00E-07	-0.44	8	0.8	Mccc1;Ccdc144b	Metabolism
DMR2:122742001	2	122742001	122743000	1000	1	6.60E-09	-0.38	9	0.9	Ccdc144b;RGD1561709	
DMR2:122749001	2	122749001	122753000	4000	1	2.50E-08	-0.36	47	1.18	Ccdc144b	
DMR2:122863001	2	122863001	122864000	1000	1	3.70E-14	0.93	31	3.1	Rslcan18;LOC102556166;LOC108349996	
DMR2:122878001	2	122878001	122880000	2000	2	1.70E-18	1.07	56	2.8	Rslcan18;LOC108349996;Arse	Metabolism
DMR2:122881001	2	122881001	122884000	3000	3	2.10E-13	1.03	94	3.13	Rslcan18;Arse;Qrfpr	Metabolism;Signaling
DMR2:122940001	2	122940001	122944000	4000	1	4.90E-08	-0.42	66	1.65	Qrfpr	Signaling
DMR2:122945001	2	122945001	122947000	2000	1	3.20E-07	-0.38	9	0.45	Qrfpr	Signaling
DMR2:123176001	2	123176001	123177000	1000	1	6.70E-08	-0.52	7	0.7	Anxa5	Signaling
DMR2:123378001	2	123378001	123379000	1000	1	1.50E-07	0.52	35	3.5	Trpc3	Transport
DMR2:123420001	2	123420001	123421000	1000	1	7.20E-07	0.29	18	1.8	Trpc3;LOC365768	Transport
DMR2:123436001	2	123436001	123440000	4000	1	1.90E-08	-0.3	37	0.92	Trpc3;LOC365768	Transport
DMR2:123591001	2	123591001	123596000	5000	1	5.20E-09	-0.33	43	0.86	RGD1307100	
DMR2:123790001	2	123790001	123792000	2000	2	2.00E-23	1.47	69	3.45	Adad1	Metabolism
DMR2:124072001	2	124072001	124073000	1000	1	2.80E-08	-0.45	12	1.2	Fgf2	Growth Factors
DMR2:124132001	2	124132001	124133000	1000	1	1.30E-07	0.33	3	0.3	Fgf2;Nudt6	Growth Factors;Signaling

DMR2:124291001	2	124291001	124294000	3000	1	3.20E-07	-0.36	26	0.87	Spata5;LOC102546767	
DMR2:124409001	2	124409001	124411000	2000	1	1.50E-08	-0.37	28	1.4	Spry1	Cytoskeleton
DMR2:124414001	2	124414001	124416000	2000	1	4.00E-07	-0.35	43	2.15	Spry1	Cytoskeleton
DMR2:125256001	2	125256001	125259000	3000	1	4.20E-07	-0.62	21	0.7	RGD1564378	
DMR2:125777001	2	125777001	125778000	1000	1	1.50E-09	-0.49	16	1.6	Fat4	Cytoskeleton
DMR2:125831001	2	125831001	125834000	3000	1	1.60E-07	0.37	26	0.87	Fat4	Cytoskeleton
DMR2:127539001	2	127539001	127540000	1000	1	1.70E-08	0.43	11	1.1	LOC100360560;Slc25a31	Transport
DMR2:127777001	2	127777001	127779000	2000	1	5.30E-20	1.42	37	1.85	Mfsd8;Abhd18;LOC365778	
DMR2:128454001	2	128454001	128455000	1000	1	7.80E-19	1.3	29	2.9	Jade1	Transcription
DMR2:128657001	2	128657001	128665000	8000	1	1.40E-07	-0.28	93	1.16	Sclt1	
DMR2:132234001	2	132234001	132235000	1000	1	9.40E-09	0.46	9	0.9	RGD1563562	Signaling
DMR2:139459001	2	139459001	139461000	2000	1	5.60E-09	-0.38	22	1.1	Slc7a11	Transport
DMR2:139532001	2	139532001	139534000	2000	1	2.20E-07	-0.35	12	0.6	Slc7a11	Transport
DMR2:140309001	2	140309001	140312000	3000	1	9.90E-07	-0.37	51	1.7	Noct;Elf2	Translation;Transcription
DMR2:140344001	2	140344001	140348000	4000	1	8.50E-09	0.43	68	1.7	Elf2	Transcription
DMR2:140372001	2	140372001	140378000	6000	1	3.90E-07	-0.36	100	1.67	Elf2	Transcription
DMR2:140599001	2	140599001	140602000	3000	1	7.10E-07	-0.36	50	1.67	Setd7	Epigenetic
DMR2:140733001	2	140733001	140735000	2000	1	1.90E-12	0.68	52	2.6	Mgst2;Maml3	Transport
DMR2:140806001	2	140806001	140807000	1000	1	4.80E-07	0.45	14	1.4	Maml3;LOC679491	
DMR2:140958001	2	140958001	140960000	2000	1	6.80E-09	0.63	36	1.8	Maml3	
DMR2:140998001	2	140998001	1.41E+08	2000	1	1.00E-08	-0.46	46	2.3	Maml3	
DMR2:141017001	2	141017001	141021000	4000	1	1.50E-08	-0.53	64	1.6	Maml3	
DMR2:141023001	2	141023001	141026000	3000	1	1.20E-13	0.82	69	2.3	Maml3	
DMR2:141027001	2	141027001	141029000	2000	1	4.10E-11	-0.5	33	1.65	Maml3	
DMR2:141061001	2	141061001	141063000	2000	1	7.80E-07	0.47	35	1.75	Maml3	
DMR2:141208001	2	141208001	141213000	5000	2	7.80E-09	-0.45	69	1.38	Maml3	
DMR2:141268001	2	141268001	141269000	1000	1	3.20E-08	-0.44	20	2	Maml3	
DMR2:141270001	2	141270001	141272000	2000	1	2.20E-09	-0.45	41	2.05	Maml3	
DMR2:142325001	2	142325001	142326000	1000	1	2.40E-10	-0.52	26	2.6	Lhfp	
DMR2:142373001	2	142373001	142375000	2000	1	4.50E-07	-0.42	25	1.25	Lhfp	
DMR2:142413001	2	142413001	142414000	1000	1	9.70E-09	-0.35	17	1.7	Lhfp	
DMR2:142424001	2	142424001	142426000	2000	1	1.10E-15	0.89	43	2.15	Lhfp	
DMR2:142453001	2	142453001	142457000	4000	1	1.80E-09	-0.53	37	0.92	Lhfp	
DMR2:142702001	2	142702001	142704000	2000	1	3.30E-17	0.93	67	3.35	Proser1	
DMR2:142738001	2	142738001	142740000	2000	1	1.20E-12	0.87	40	2	Frem2	
DMR2:142793001	2	142793001	142794000	1000	1	1.00E-08	0.52	18	1.8	Frem2	
DMR2:142798001	2	142798001	142802000	4000	1	2.20E-07	0.49	67	1.68	Frem2	
DMR2:143425001	2	143425001	143426000	1000	1	1.70E-07	-0.39	17	1.7	Trpc4	Transport
DMR2:143482001	2	143482001	143487000	5000	1	5.20E-09	-0.32	43	0.86	Trpc4	Transport
DMR2:143516001	2	143516001	143517000	1000	1	5.70E-10	-0.66	10	1	Trpc4	Transport
DMR2:143566001	2	143566001	143567000	1000	1	2.20E-09	0.46	10	1	Trpc4	Transport
DMR2:143661001	2	143661001	143663000	2000	1	1.10E-07	0.43	35	1.75	Postn	Cytoskeleton
DMR2:143675001	2	143675001	143676000	1000	1	4.90E-07	-0.31	12	1.2	Postn	Cytoskeleton
DMR2:143964001	2	143964001	143965000	1000	1	7.70E-07	-0.4	20	2	Smad9	Transcription
DMR2:143975001	2	143975001	143976000	1000	1	5.20E-14	0.34	3	0.3	Smad9	Transcription
DMR2:144012001	2	144012001	144015000	3000	1	3.10E-10	0.76	50	1.67	Smad9;Rfxap	Transcription
DMR2:144450001	2	144450001	144452000	2000	1	6.50E-09	-0.33	23	1.15	Ccna1	Signaling
DMR2:144525001	2	144525001	144527000	2000	1	1.40E-07	-0.49	15	0.75	Spg20	
DMR2:144756001	2	144756001	144757000	1000	1	3.80E-07	0.37	16	1.6	Dclk1	Signaling
DMR2:144807001	2	144807001	144808000	1000	1	8.60E-13	0.85	21	2.1	Dclk1;LOC102552324	Signaling
DMR2:144823001	2	144823001	144827000	4000	1	1.60E-08	0.62	64	1.6	Dclk1	Signaling
DMR2:144922001	2	144922001	144923000	1000	1	7.80E-14	0.8	25	2.5	Dclk1	Signaling
DMR2:145099001	2	145099001	145105000	6000	1	2.00E-11	0.42	57	0.95	Nbea	
DMR2:145186001	2	145186001	145187000	1000	1	2.10E-08	-0.48	11	1.1	Nbea;Mab21l1	
DMR2:145433001	2	145433001	145434000	1000	1	2.50E-07	-0.4	8	0.8	Nbea	
DMR2:145453001	2	145453001	145456000	3000	1	4.00E-07	-0.32	29	0.97	Nbea	
DMR2:147568001	2	147568001	147570000	2000	1	9.90E-07	-0.35	18	0.9	Wwtr1	Transcription
DMR2:147814001	2	147814001	147817000	3000	1	4.20E-07	0.56	31	1.03	Ankub1;Rpl36a-ps1	
DMR2:147825001	2	147825001	147828000	3000	1	6.00E-07	-0.39	44	1.47	Ankub1;Rpl36a-ps1	
DMR2:147844001	2	147844001	147847000	3000	1	2.60E-10	0.83	40	1.33	Rnf13	
DMR2:148807001	2	148807001	148812000	5000	2	3.40E-07	-0.55	33	0.66	Erich6	

DMR2:148835001	2	148835001	148836000	1000	1	2.20E-07	-0.36	14	1.4	Erich6	
DMR2:149239001	2	149239001	149241000	2000	2	1.50E-07	-0.39	40	2	Med12l	Transcription
DMR2:149294001	2	149294001	149300000	6000	1	4.90E-07	-0.35	55	0.92	Med12l	Transcription
DMR2:149347001	2	149347001	149349000	2000	1	1.70E-12	-0.67	33	1.65	P2ry14	Signaling
DMR2:149435001	2	149435001	149436000	1000	1	3.50E-10	0.49	12	1.2	P2ry13;P2ry12	Signaling
DMR2:150000001	2	150000001	150001000	1000	1	8.50E-07	0.48	13	1.3	RGD1560324	Metabolism
DMR2:152859001	2	152859001	152860000	1000	1	9.70E-07	0.43	13	1.3	Gpr149	Signaling
DMR2:153905001	2	153905001	153907000	2000	1	1.50E-07	0.42	37	1.85	LOC108350023;RGD1560703	
DMR2:154263001	2	154263001	154264000	1000	1	1.10E-08	-0.46	9	0.9	Plch1	Metabolism
DMR2:155763001	2	155763001	155770000	7000	1	6.10E-07	-0.35	79	1.13	Kcnab1	
DMR2:155783001	2	155783001	155785000	2000	1	6.10E-12	0.7	10	0.5	Kcnab1	
DMR2:155879001	2	155879001	155882000	3000	1	6.70E-07	-0.49	10	0.33	Kcnab1	
DMR2:155989001	2	155989001	155990000	1000	1	1.00E-07	0.49	67	6.7	Kcnab1;LOC108350024	
DMR2:157335001	2	157335001	157336000	1000	1	2.90E-07	-0.44	9	0.9	Tiparp;LOC108350031	
DMR2:157471001	2	157471001	157473000	2000	1	3.90E-07	-0.53	23	1.15	Lekr1	
DMR2:157542001	2	157542001	157543000	1000	1	2.20E-07	-0.46	4	0.4	Lekr1	
DMR2:157879001	2	157879001	157881000	2000	1	7.60E-07	0.27	36	1.8	Veph1	
DMR2:158069001	2	158069001	158070000	1000	1	8.60E-07	-0.39	10	1	Veph1	
DMR2:161925001	2	161925001	161926000	1000	1	1.70E-07	-0.53	7	0.7	Olr1847-ps	
DMR2:162412001	2	162412001	162418000	6000	3	6.10E-16	0.49	121	2.02	Olr1818-ps	
DMR2:164143001	2	164143001	164145000	2000	1	1.60E-07	-0.56	7	0.35	Rsrc1	
DMR2:164445001	2	164445001	164448000	3000	1	2.10E-07	-0.42	35	1.17	Rsrc1	
DMR2:164586001	2	164586001	164592000	6000	1	4.20E-07	-0.35	68	1.13	Mlf1;Gfm1	Signaling
DMR2:164772001	2	164772001	164775000	3000	1	2.00E-07	-0.43	26	0.87	Mfsd1;LOC310487	
DMR2:165067001	2	165067001	165069000	2000	1	3.90E-11	0.43	37	1.85	Il12a	
DMR2:165753001	2	165753001	165754000	1000	1	2.60E-09	0.46	22	2.2	Arl14	Signaling
DMR2:165784001	2	165784001	165785000	1000	1	3.90E-08	0.66	13	1.3	Arl14	Signaling
DMR2:165843001	2	165843001	165844000	1000	1	2.30E-07	-0.44	8	0.8	NEWGENE_1585613	
DMR2:165987001	2	165987001	165990000	3000	1	1.30E-07	-0.52	45	1.5	Ppm1l	Signaling
DMR2:166146001	2	166146001	166147000	1000	1	3.90E-07	-0.43	14	1.4	Ppm1l	Signaling
DMR2:166196001	2	166196001	166197000	1000	1	3.60E-07	0.49	13	1.3	Ppm1l	Signaling
DMR2:166205001	2	166205001	166210000	5000	1	3.00E-07	-0.32	78	1.56	Ppm1l	Signaling
DMR2:166399001	2	166399001	166400000	1000	1	1.10E-09	-0.42	23	2.3	Nmd3	
DMR2:171165001	2	171165001	171166000	1000	1	2.00E-12	0.74	20	2	Bche	Metabolism
DMR2:172334001	2	172334001	172338000	4000	1	4.40E-07	-0.37	39	0.98	Schip1	
DMR2:172357001	2	172357001	172359000	2000	1	4.80E-09	-0.44	34	1.7	Schip1	
DMR2:172365001	2	172365001	172369000	4000	1	1.60E-08	-0.37	49	1.23	Schip1	
DMR2:172435001	2	172435001	172438000	3000	1	4.90E-07	-0.43	44	1.47	Schip1	
DMR2:172626001	2	172626001	172627000	1000	1	6.30E-09	-0.48	14	1.4	Schip1	
DMR2:172891001	2	172891001	172896000	5000	1	2.30E-08	-0.38	50	1	Schip1	
DMR2:173054001	2	173054001	173056000	2000	1	1.40E-07	-0.6	8	0.4	Schip1	
DMR2:173057001	2	173057001	173058000	1000	1	4.90E-09	-0.73	1	0.1	Schip1	
DMR2:173079001	2	173079001	173080000	1000	1	1.10E-07	0.56	11	1.1	Schip1	
DMR2:173086001	2	173086001	173088000	2000	1	3.50E-07	-0.5	39	1.95	Schip1	
DMR2:173765001	2	173765001	173767000	2000	1	2.50E-25	0.54	39	1.95	Wdr49;LOC102556029	
DMR2:174090001	2	174090001	174093000	3000	2	3.60E-09	-0.73	12	0.4	Serpini1	Protease; Proteolysis
DMR2:174100001	2	174100001	174101000	1000	1	5.20E-07	-0.62	3	0.3	Serpini1	Protease; Proteolysis
DMR2:174330001	2	174330001	174332000	2000	1	1.00E-07	-0.38	26	1.3	Golim4	
DMR2:174373001	2	174373001	174377000	4000	1	9.50E-09	-0.35	45	1.12	Golim4	
DMR2:174565001	2	174565001	174566000	1000	1	1.80E-07	-0.48	8	0.8	Fstl5	Protease; Proteolysis
DMR2:174613001	2	174613001	174614000	1000	1	3.30E-08	-0.49	4	0.4	Fstl5	Protease; Proteolysis
DMR2:174908001	2	174908001	174909000	1000	1	6.30E-09	-0.84	9	0.9	Fstl5	Protease; Proteolysis
DMR2:177937001	2	177937001	177940000	3000	1	4.10E-08	-0.56	10	0.33	Rapgef2	Transcription
DMR2:177999001	2	177999001	178003000	4000	1	2.10E-08	0.48	56	1.4	Rapgef2;LOC103691592	Transcription
DMR2:178221001	2	178221001	178222000	1000	1	7.70E-07	-0.38	13	1.3	LOC499643;LOC108350042;LOC365827;Fnip2	
DMR2:178407001	2	178407001	178411000	4000	1	1.10E-07	-0.42	58	1.45	Rxfp1	Signaling
DMR2:178451001	2	178451001	178456000	5000	1	2.50E-08	0.56	52	1.04	Rxfp1	Signaling
DMR2:178462001	2	178462001	178463000	1000	1	3.50E-07	0.48	14	1.4	Rxfp1	Signaling
DMR2:178473001	2	178473001	178478000	5000	1	2.10E-07	-0.41	30	0.6	Rxfp1	Signaling
DMR2:178583001	2	178583001	178584000	1000	1	2.50E-07	-0.49	22	2.2	Tmem144	
DMR2:178620001	2	178620001	178624000	4000	1	6.10E-08	-0.43	41	1.02	Tmem144;LOC102556854	

DMR2:179604001	2	179604001	179606000	2000	2	2.50E-11	-0.59	5	0.25	Gria2	Receptor
DMR2:180024001	2	180024001	180026000	2000	1	4.60E-12	-0.53	7	0.35	Pdgfc	Growth Factors
DMR2:180027001	2	180027001	180029000	2000	1	3.70E-07	-0.49	17	0.85	Pdgfc	Growth Factors
DMR2:180951001	2	180951001	180955000	4000	2	1.90E-11	-0.7	19	0.48	Asic5	Transport
DMR2:180997001	2	180997001	180998000	1000	1	5.80E-07	0.44	14	1.4	Gucy1b3	
DMR2:181100001	2	181100001	181101000	1000	1	5.00E-08	0.71	9	0.9	Gucy1a3	
DMR2:181336001	2	181336001	181337000	1000	1	2.40E-08	0.49	13	1.3	Map9	
DMR2:181355001	2	181355001	181356000	1000	1	9.60E-12	0.89	25	2.5	Map9	
DMR2:181914001	2	181914001	181915000	1000	1	1.20E-07	-0.32	11	1.1	Lrat;LOC108350045	Metabolism
DMR2:182099001	2	182099001	182104000	5000	2	7.50E-08	-0.32	48	0.96	Dchs2	
DMR2:182156001	2	182156001	182158000	2000	1	1.70E-11	-0.51	28	1.4	Dchs2	
DMR2:182190001	2	182190001	182191000	1000	1	5.10E-08	0.42	6	0.6	Dchs2	
DMR2:182261001	2	182261001	182265000	4000	1	9.60E-08	-0.38	61	1.52	Dchs2	
DMR2:183064001	2	183064001	183065000	1000	1	1.40E-07	-0.37	12	1.2	LOC103691596;Mnd1	
DMR2:183230001	2	183230001	183231000	1000	1	7.20E-07	-0.44	13	1.3	Trim2;LOC102550635	Proteolysis
DMR2:183439001	2	183439001	183444000	5000	2	2.90E-18	0.4	41	0.82	Fhdc1	
DMR2:183461001	2	183461001	183464000	3000	2	1.60E-12	-0.47	60	2	Fhdc1	
DMR2:183584001	2	183584001	183587000	3000	2	8.70E-10	-0.64	28	0.93	Arfip1	Transport
DMR2:183675001	2	183675001	183678000	3000	1	4.00E-11	-0.44	36	1.2	Tmem154;LOC108350047	
DMR2:184275001	2	184275001	184278000	3000	1	1.10E-14	-0.54	34	1.13	Fbxw7	Proteolysis
DMR2:184962001	2	184962001	184965000	3000	1	6.50E-07	-0.39	42	1.4	Fam160a1	
DMR2:185291001	2	185291001	185292000	1000	1	7.50E-07	-0.46	15	1.5	Sh3d19;Prss48	Protease
DMR2:185321001	2	185321001	185322000	1000	1	7.20E-07	-0.32	16	1.6	Sh3d19	
DMR2:185417001	2	185417001	185424000	7000	1	1.80E-07	0.54	105	1.5	Sh3d19	
DMR2:185453001	2	185453001	185458000	5000	3	2.60E-16	-0.62	87	1.74	Rps3a	Translation
DMR2:185740001	2	185740001	185745000	5000	1	3.80E-08	-0.35	44	0.88	Lrba	
DMR2:185760001	2	185760001	185763000	3000	1	6.30E-12	-0.47	30	1	Lrba	
DMR2:185852001	2	185852001	185853000	1000	1	2.60E-12	0.75	27	2.7	Lrba;Mab21l2	
DMR2:185854001	2	185854001	185855000	1000	1	2.40E-14	0.91	19	1.9	Lrba;Mab21l2	
DMR2:185940001	2	185940001	185941000	1000	1	4.30E-07	-0.49	6	0.6	Lrba	
DMR2:186076001	2	186076001	186079000	3000	1	2.80E-12	0.81	38	1.27	Lrba	
DMR2:186198001	2	186198001	186200000	2000	1	3.20E-07	-0.38	19	0.95	Dclk2	Signaling
DMR2:186447001	2	186447001	186450000	3000	1	7.80E-09	0.67	48	1.6	Kirrel	
DMR2:186483001	2	186483001	186484000	1000	1	1.50E-07	0.46	20	2	Kirrel	
DMR2:186914001	2	186914001	186916000	2000	1	8.40E-08	0.43	19	0.95	Etv3l;LOC108350054;LOC100361532	Transcription
DMR2:187021001	2	187021001	187023000	2000	1	4.20E-08	-0.42	25	1.25	Arhgef11	Transcription
DMR2:187124001	2	187124001	187127000	3000	1	5.80E-09	0.44	44	1.47	Lrrc71;Pear1	Signaling
DMR2:187179001	2	187179001	187181000	2000	1	4.90E-14	-0.7	26	1.3	Insrr	Receptor
DMR2:187409001	2	187409001	187410000	1000	1	1.50E-07	-0.41	13	1.3	Hapln2;Gpatch4	Extracellular Matrix;Metabolism
DMR2:187483001	2	187483001	187486000	3000	1	2.20E-10	-0.43	51	1.7	Iqgap3	Signaling
DMR2:187733001	2	187733001	187734000	1000	1	1.70E-07	0.53	8	0.8	Smg5;Paqr6;Bglap	Metabolism;Signaling
DMR2:187805001	2	187805001	187810000	5000	1	3.10E-07	-0.39	74	1.48	Sema4a	Signaling
DMR2:187846001	2	187846001	187847000	1000	1	4.30E-08	0.55	40	4	Lmna	
DMR2:188055001	2	188055001	188056000	1000	1	1.10E-07	-0.46	7	0.7	RGD1565775;LOC108350056	
DMR2:188191001	2	188191001	188193000	2000	1	1.80E-07	-0.36	26	1.3	Gon4l	Transcription
DMR2:188308001	2	188308001	188311000	3000	1	1.30E-09	-0.52	29	0.97	Ash1l	Epigenetic
DMR2:188372001	2	188372001	188373000	1000	1	3.40E-07	-0.44	14	1.4	Ash1l;LOC108350157	Epigenetic
DMR2:188524001	2	188524001	188525000	1000	1	2.10E-07	0.58	9	0.9	Gba;Mtx1;Thbs3	
DMR2:188592001	2	188592001	188594000	2000	1	1.90E-07	-0.38	43	2.15	Dpm3;Slc50a1;Efna1;LOC102550325	Transport;Signaling
DMR2:188705001	2	188705001	188709000	4000	2	2.80E-13	1.08	97	2.42	Dcst2;Zbtb7b	
DMR2:188766001	2	188766001	188767000	1000	1	7.90E-07	-0.47	14	1.4	Shc1;Pygo2;Pbxip1	Cytoskeleton
DMR2:188768001	2	188768001	188772000	4000	1	1.30E-08	0.48	48	1.2	Pygo2;Pbxip1	
DMR2:188789001	2	188789001	188792000	3000	1	2.00E-08	0.52	32	1.07	Pmvk	Signaling
DMR2:188845001	2	188845001	188846000	1000	1	8.60E-07	-0.49	13	1.3	Kcnn3	Transport
DMR2:188863001	2	188863001	188865000	2000	1	1.80E-10	0.48	29	1.45	Kcnn3	Transport
DMR2:188907001	2	188907001	188908000	1000	1	5.30E-07	0.5	15	1.5	Kcnn3	Transport
DMR2:188982001	2	188982001	188983000	1000	1	6.60E-07	0.39	15	1.5	Kcnn3	Transport
DMR2:189047001	2	189047001	189049000	2000	1	3.00E-10	0.43	9	0.45	Adar	Metabolism

DMR2:189092001	2	189092001	189094000	2000	2	6.10E-10	0.77	62	3.1	Adar;Chrnb2	Metabolism;Ion Channel
DMR2:189617001	2	189617001	189620000	3000	1	5.30E-08	-0.37	36	1.2	Slc39a1;Crtc2;Dennd4b	Transport;Transcription
DMR2:189989001	2	189989001	189992000	3000	1	8.30E-07	0.4	35	1.17	S100a3;LOC100910139;S100a4;S100a5	Signaling
DMR2:191949001	2	191949001	191950000	1000	1	1.40E-07	-0.48	2	0.2	Pglyrp3;LOC100911387	
DMR2:191969001	2	191969001	191971000	2000	1	5.60E-09	-0.42	57	2.85	Pglyrp3;LOC108350060	
DMR2:192090001	2	192090001	192091000	1000	1	7.80E-07	0.47	11	1.1	Pglyrp3	
DMR2:192779001	2	192779001	192780000	1000	1	2.10E-07	-0.57	3	0.3	Ivl;Smcp	
DMR2:193133001	2	193133001	193134000	1000	1	9.90E-30	1.34	42	4.2	Kprp	
DMR2:193368001	2	193368001	193369000	1000	1	5.70E-07	0.52	4	0.4	RGD1561111	
DMR2:193572001	2	193572001	193575000	3000	2	3.80E-45	1.56	100	3.33	LOC108350062;Flg	
DMR2:194018001	2	194018001	194020000	2000	1	7.60E-08	-0.51	5	0.25	RGD1564513	
DMR2:194372001	2	194372001	194374000	2000	1	1.20E-10	-0.45	6	0.3	LOC102548071;RGD1563451	Proteolysis
DMR2:194512001	2	194512001	194513000	1000	1	9.00E-11	-0.49	1	0.1	LOC365852;LOC100363449;RGD1560510	Proteolysis
DMR2:194872001	2	194872001	194875000	3000	1	2.90E-13	-0.56	18	0.6	RGD1566337	Proteolysis
DMR2:194925001	2	194925001	194929000	4000	1	2.10E-08	-0.43	24	0.6	RGD1562104;RGD1564386	
DMR2:195258001	2	195258001	195260000	2000	1	1.10E-08	-0.34	8	0.4	RGD1563667	Proteolysis
DMR2:195273001	2	195273001	195276000	3000	1	2.70E-08	-0.34	12	0.4	RGD1563667	Proteolysis
DMR2:195529001	2	195529001	195531000	2000	1	2.50E-10	-0.44	8	0.4	RGD1563667;RGD1560554	Proteolysis
DMR2:195562001	2	195562001	195564000	2000	1	3.80E-07	-0.38	21	1.05	RGD1563667;RGD1560554;Them4	Proteolysis;Metabolism
DMR2:195623001	2	195623001	195626000	3000	1	8.50E-07	0.44	35	1.17	Rorc;Lingo4	Transcription;Receptor
DMR2:195640001	2	195640001	195643000	3000	1	6.20E-09	0.41	52	1.73	Rorc;Lingo4;Tdrkh	Transcription;Receptor;Cytoskeleton
DMR2:195713001	2	195713001	195716000	3000	1	1.60E-08	0.35	131	4.37	Riiad1;Celf3	
DMR2:195847001	2	195847001	195848000	1000	1	1.30E-09	-0.38	34	3.4	RGD1560263	
DMR2:196013001	2	196013001	196014000	1000	1	1.80E-07	-0.38	21	2.1	Pogz;LOC108350064	Transcription
DMR2:196140001	2	196140001	196141000	1000	1	5.70E-09	-0.6	35	3.5	Selenbp1;LOC108350066;Pi4kb	Immune;Signaling
DMR2:196148001	2	196148001	196150000	2000	1	1.30E-09	0.4	27	1.35	Selenbp1;LOC108350066;Pi4kb	Immune;Signaling
DMR2:196269001	2	196269001	196270000	1000	1	1.70E-07	-0.45	15	1.5	Pip5k1a;Vps72	Signaling;Transcription
DMR2:196293001	2	196293001	196294000	1000	1	2.70E-18	1.11	33	3.3	Vps72;Tmod4;Scnm1	Transcription;Cytoskeleton;Transport
DMR2:196336001	2	196336001	196338000	2000	1	6.10E-07	-0.33	21	1.05	Tnfaip8l2;Sema6c	Signaling
DMR2:196404001	2	196404001	196406000	2000	1	6.40E-07	-0.47	32	1.6	Mllt11;Cdc42se1;RGD1359334;Bnipl	
DMR2:196414001	2	196414001	196415000	1000	1	2.50E-11	0.8	27	2.7	Cdc42se1;RGD1359334;Bnipl	
DMR2:196498001	2	196498001	196501000	3000	1	1.20E-07	-0.49	41	1.37	Cers2;Setdb1	Epigenetic
DMR2:196808001	2	196808001	196809000	1000	1	4.80E-07	-0.53	10	1	Olr1864-ps	
DMR2:196960001	2	196960001	196970000	10000	1	1.40E-13	-0.41	136	1.36	RGD1566258	
DMR2:197659001	2	197659001	197661000	2000	1	4.70E-07	-0.46	31	1.55	Ctss	Protease
DMR2:197663001	2	197663001	197668000	5000	1	3.00E-12	-0.56	85	1.7	Ctss	Protease
DMR2:197682001	2	197682001	197683000	1000	1	5.60E-15	0.96	26	2.6	Ctss;Hormad1;LOC102547423	Protease
DMR2:197805001	2	197805001	197808000	3000	1	3.90E-08	0.45	57	1.9	Adamtsl4	Protease
DMR2:197856001	2	197856001	197860000	4000	2	3.00E-11	0.59	94	2.35	Ecm1;Tars2	Translation
DMR2:197993001	2	197993001	197999000	6000	1	5.70E-07	-0.4	74	1.23	Ciart;LOC108348218;Aph1a	Transcription;Protease
DMR2:198044001	2	198044001	198046000	2000	1	4.00E-09	-0.41	26	1.3	Anp32e	Epigenetic
DMR2:198086001	2	198086001	198087000	1000	1	1.20E-08	0.63	20	2	RGD1562196;LOC108350069	
DMR2:198121001	2	198121001	198122000	1000	1	5.60E-07	0.51	19	1.9	Plekho1;Vps45	Transport
DMR2:198182001	2	198182001	198184000	2000	1	1.10E-07	-0.42	32	1.6	Vps45	Transport
DMR2:198323001	2	198323001	198327000	4000	1	4.00E-08	0.74	74	1.85	Sf3b4;Sv2a	Translation
DMR2:198457001	2	198457001	198458000	1000	1	5.30E-11	0.53	15	1.5	LOC100359862;Trnag-ccc	
DMR2:198502001	2	198502001	198504000	2000	1	1.70E-08	-0.4	31	1.55	Trnan-guu;LOC690204;Trnaq-cug;Trnae-uuc	
DMR2:198564001	2	198564001	198565000	1000	1	1.80E-07	0.58	25	2.5	Trnag-ccc;Trnak-cuu	
DMR2:198581001	2	198581001	198582000	1000	1	4.50E-07	-0.3	15	1.5	Trnah-gug;LOC103691644;LOC100360229	

DMR2:198590001	2	198590001	198592000	2000	1	6.20E-31	1.78	35	1.75	LOC103691644;LOC100360229;Trnah-gug;Trnan-guu;LOC108350070	
DMR2:198676001	2	198676001	198677000	1000	1	1.50E-08	-0.37	14	1.4	LOC103691645;Txnip	
DMR2:198688001	2	198688001	198689000	1000	1	3.90E-07	-0.5	22	2.2	LOC103691645;Txnip;Polr3gl	Transcription
DMR2:198742001	2	198742001	198746000	4000	1	9.10E-11	-0.49	46	1.15	Lix1l;Rbm8a	
DMR2:198902001	2	198902001	198905000	3000	1	2.80E-12	-0.75	11	0.37	Rnf115	Proteolysis
DMR2:198924001	2	198924001	198928000	4000	1	8.00E-07	-0.32	30	0.75	Rnf115;Cd160	Proteolysis
DMR2:198940001	2	198940001	198942000	2000	1	7.60E-09	-0.49	34	1.7	Cd160	
DMR2:198948001	2	198948001	198951000	3000	1	3.90E-07	0.44	57	1.9	Cd160	
DMR2:198998001	2	198998001	199001000	3000	1	5.80E-08	-0.36	63	2.1	Pdzk1;Gpr89b	Signaling
DMR2:199044001	2	199044001	199047000	3000	1	5.00E-07	0.34	15	0.5	Gpr89b;Gja8	Signaling;Cytoskeleton
DMR2:199329001	2	199329001	199330000	1000	1	1.60E-10	-0.5	10	1	Bcl9	
DMR2:199343001	2	199343001	199344000	1000	1	7.10E-08	-0.36	21	2.1	Bcl9	
DMR2:199360001	2	199360001	199361000	1000	1	9.50E-07	-0.46	11	1.1	Bcl9	
DMR2:199362001	2	199362001	199366000	4000	1	1.60E-07	-0.36	82	2.05	Bcl9	
DMR2:199381001	2	199381001	199382000	1000	1	3.10E-07	0.76	30	3	Bcl9	
DMR2:199405001	2	199405001	199414000	9000	1	1.10E-17	1.15	176	1.96	Bcl9;LOC102549654	
DMR2:199532001	2	199532001	199533000	1000	1	6.10E-10	-0.52	14	1.4	Olr390	Signaling
DMR2:199707001	2	199707001	199711000	4000	1	5.50E-07	-0.25	30	0.75	Chd1l	
DMR2:199712001	2	199712001	199713000	1000	1	4.40E-07	0.38	21	2.1	Chd1l	
DMR2:199757001	2	199757001	199758000	1000	1	2.10E-08	-0.37	14	1.4	Chd1l	
DMR2:199766001	2	199766001	199767000	1000	1	7.30E-08	0.47	22	2.2	Chd1l	
DMR2:199781001	2	199781001	199787000	6000	1	1.10E-07	-0.39	46	0.77	Chd1l;Fmo5	Metabolism
DMR2:199822001	2	199822001	199823000	1000	1	1.30E-12	0.76	17	1.7	Fmo5;Prkab2	Metabolism;Signaling
DMR2:199829001	2	199829001	199830000	1000	1	5.00E-07	0.39	7	0.7	Fmo5;Prkab2	Metabolism;Signaling
DMR2:199850001	2	199850001	199856000	6000	1	3.50E-08	-0.31	58	0.97	Prkab2;Trnaq-cug;LOC102550575	Signaling
DMR2:199863001	2	199863001	199867000	4000	2	2.40E-11	-0.42	32	0.8	Trnaq-cug;LOC102550575;Pde4dip	
DMR2:199953001	2	199953001	199954000	1000	1	3.70E-09	-0.61	16	1.6	Pde4dip	
DMR2:199971001	2	199971001	199974000	3000	2	6.90E-15	-0.49	30	1	Pde4dip	
DMR2:200260001	2	200260001	200262000	2000	1	2.80E-09	-0.43	47	2.35	Notch2	Extracellular Matrix
DMR2:200387001	2	200387001	200389000	2000	1	2.40E-09	-0.79	21	1.05	Reg4	
DMR2:200515001	2	200515001	200519000	4000	2	4.40E-11	-0.44	54	1.35	Phgdh	Metabolism
DMR2:200618001	2	200618001	200620000	2000	1	4.70E-09	-0.47	37	1.85	LOC102550892;Hsd3b5	Metabolism
DMR2:201393001	2	201393001	201394000	1000	1	3.00E-07	0.38	12	1.2	Tbx15;LOC102553016;LOC500213	Transcription
DMR2:202192001	2	202192001	202194000	2000	1	1.10E-09	-0.44	24	1.2	Spag17	
DMR2:202281001	2	202281001	202282000	1000	1	1.60E-12	0.9	30	3	Spag17	
DMR2:202302001	2	202302001	202303000	1000	1	7.80E-09	-0.41	13	1.3	Spag17	
DMR2:202417001	2	202417001	202420000	3000	1	2.10E-08	0.3	21	0.7	Spag17	
DMR2:202437001	2	202437001	202439000	2000	1	4.00E-08	-0.48	31	1.55	Spag17;Wdr3	
DMR2:202998001	2	202998001	202999000	1000	1	1.60E-09	-0.47	12	1.2	Man1a2	Golgi
DMR2:203250001	2	203250001	203252000	2000	1	1.60E-07	-0.42	6	0.3	Vtcn1	Immune
DMR2:203397001	2	203397001	203399000	2000	1	1.40E-08	-0.49	22	1.1	Cd101	Immune
DMR2:203434001	2	203434001	203435000	1000	1	5.80E-11	-0.56	14	1.4	Ptgfrn	Immune
DMR2:203471001	2	203471001	203473000	2000	1	2.40E-10	0.61	43	2.15	Ptgfrn;LOC108350075	Immune
DMR2:203487001	2	203487001	203490000	3000	2	2.90E-07	-0.36	62	2.07	Ptgfrn	Immune
DMR2:203681001	2	203681001	203684000	3000	1	3.90E-09	-0.58	44	1.47	Cd2	Immune
DMR2:203837001	2	203837001	203841000	4000	2	2.60E-07	-0.49	60	1.5	Igsf3	Immune
DMR2:204248001	2	204248001	204251000	3000	1	4.60E-12	0.55	44	1.47	Mab21l3	
DMR2:204421001	2	204421001	204423000	2000	1	2.60E-07	-0.32	33	1.65	Nhlh2	Transcription
DMR2:204509001	2	204509001	204512000	3000	1	4.40E-10	0.49	46	1.53	Casq2	Signaling
DMR2:204602001	2	204602001	204603000	1000	1	7.20E-11	0.73	28	2.8	Vangl1	
DMR2:204948001	2	204948001	204950000	2000	1	4.20E-13	0.53	21	1.05	Ngf	Growth Factors
DMR2:205151001	2	205151001	205153000	2000	1	9.90E-08	-0.47	17	0.85	LOC108350076;Tspan2	
DMR2:205195001	2	205195001	205197000	2000	1	4.10E-08	-0.33	39	1.95	Tspan2	
DMR2:205348001	2	205348001	205353000	5000	1	2.30E-08	-0.3	43	0.86	Sycp1	
DMR2:205375001	2	205375001	205377000	2000	1	2.60E-08	-0.37	22	1.1	Sycp1	
DMR2:205393001	2	205393001	205398000	5000	1	3.30E-09	-0.42	42	0.84	Sycp1	
DMR2:205448001	2	205448001	205452000	4000	1	4.10E-07	0.36	95	2.38	Nr1h5	Transcription

DMR2:205453001	2	205453001	205454000	1000	1	8.40E-07	-0.52	4	0.4	Nr1h5	Transcription
DMR2:205581001	2	205581001	205583000	2000	1	2.70E-07	-0.46	26	1.3	Ampd1;Dennd2c	Metabolism
DMR2:205670001	2	205670001	205671000	1000	1	1.80E-10	-0.45	20	2	Dennd2c;Bcas2	Translation
DMR2:205679001	2	205679001	205681000	2000	1	8.00E-08	0.4	15	0.75	Bcas2;LOC691020	Translation
DMR2:205822001	2	205822001	205823000	1000	1	4.50E-07	-0.56	4	0.4	Trim33	Epigenetic
DMR2:206113001	2	206113001	206114000	1000	1	9.80E-08	-0.36	17	1.7	Syt6	Transport
DMR2:206581001	2	206581001	206582000	1000	1	5.70E-09	-0.64	3	0.3	Magi3	
DMR2:206589001	2	206589001	206591000	2000	1	2.80E-07	-0.46	10	0.5	Magi3	
DMR2:206626001	2	206626001	206629000	3000	1	2.00E-07	-0.32	45	1.5	Magi3	
DMR2:206889001	2	206889001	206893000	4000	1	7.30E-07	0.43	41	1.02	LOC103691658;RGD1564469	
DMR2:207497001	2	207497001	207502000	5000	1	1.10E-07	-0.41	73	1.46	Cttnbp2nl;LOC103691661	
DMR2:208024001	2	208024001	208025000	1000	1	2.00E-07	-0.64	5	0.5	Kcnd3	Transport
DMR2:208045001	2	208045001	208048000	3000	1	2.60E-07	-0.49	29	0.97	Kcnd3	Transport
DMR2:208072001	2	208072001	208073000	1000	1	5.10E-09	-0.68	11	1.1	Kcnd3	Transport
DMR2:208087001	2	208087001	208091000	4000	1	1.20E-08	-0.5	54	1.35	Kcnd3	Transport
DMR2:208162001	2	208162001	208166000	4000	1	2.00E-07	0.59	56	1.4	Ddx20;Fam212b	
DMR2:208183001	2	208183001	208184000	1000	1	2.00E-07	0.52	21	2.1	Fam212b;Rap1a	Signaling
DMR2:208212001	2	208212001	208214000	2000	1	8.70E-08	-0.36	20	1	Rap1a;LOC108350082	Signaling
DMR2:208760001	2	208760001	208765000	5000	1	9.70E-09	-0.3	43	0.86	Chia	Metabolism
DMR2:208789001	2	208789001	208794000	5000	1	5.60E-08	-0.32	48	0.96	Chia	Metabolism
DMR2:209416001	2	209416001	209417000	1000	1	5.50E-07	-0.44	6	0.6	Lrif1	
DMR2:209513001	2	209513001	209518000	5000	1	8.20E-09	-0.43	25	0.5	Cd53	
DMR2:209761001	2	209761001	209764000	3000	1	6.00E-08	-0.37	23	0.77	LOC108350087;Kcna3;LOC103691668	Transport
DMR2:209830001	2	209830001	209832000	2000	1	4.10E-07	-0.34	22	1.1	LOC108350088;Kcna2	Transport
DMR2:209957001	2	209957001	209959000	2000	1	8.90E-07	-0.33	27	1.35	LOC102557194;Cym	
DMR2:210227001	2	210227001	210228000	1000	1	2.70E-11	0.67	18	1.8	Kcnc4	Transport
DMR2:210282001	2	210282001	210284000	2000	1	5.60E-07	-0.51	43	2.15	Slc6a17	Transport
DMR2:210529001	2	210529001	210531000	2000	1	1.00E-13	0.89	33	1.65	Csf1	
DMR2:210548001	2	210548001	210550000	2000	1	1.40E-08	-0.49	16	0.8	Csf1	
DMR2:210677001	2	210677001	210679000	2000	1	6.40E-07	-0.32	22	1.1	Eps8l3;Gstm3	Cytoskeleton
DMR2:210712001	2	210712001	210713000	1000	1	5.70E-09	0.64	23	2.3	Gstm6l;Gstm7	Transport
DMR2:210810001	2	210810001	210813000	3000	1	4.90E-10	-0.43	37	1.23	Gstm1	Transport
DMR2:210836001	2	210836001	210837000	1000	1	9.80E-07	-0.5	16	1.6	Gstm4	Transport
DMR2:210861001	2	210861001	210866000	5000	1	2.70E-09	0.36	108	2.16	Ampd2	Metabolism
DMR2:210884001	2	210884001	210888000	4000	1	5.70E-07	-0.45	74	1.85	Ampd2;Gnat2;Gnai3	Metabolism;Signaling
DMR2:210915001	2	210915001	210918000	3000	1	3.60E-07	-0.59	22	0.73	Gnai3	Signaling
DMR2:210989001	2	210989001	210990000	1000	1	1.70E-07	0.49	11	1.1	Amigo1;Cyb561d1;Atxn7l2	
DMR2:211083001	2	211083001	211087000	4000	1	3.70E-09	-0.46	54	1.35	Psma5;Sort1	Protease;Transport
DMR2:211200001	2	211200001	211201000	1000	1	8.50E-07	-0.34	23	2.3	Celsr2;LOC108350091	Cytoskeleton
DMR2:211401001	2	211401001	211403000	2000	1	4.80E-08	-0.29	14	0.7	Wdr47	
DMR2:211635001	2	211635001	211637000	2000	1	3.80E-07	-0.34	23	1.15	Stxbp3	Transport
DMR2:211707001	2	211707001	211709000	2000	1	5.30E-12	-0.47	44	2.2	Prpf38b	
DMR2:211711001	2	211711001	211712000	1000	1	5.80E-07	-0.43	17	1.7	Prpf38b	
DMR2:211794001	2	211794001	211795000	1000	1	6.70E-10	-0.45	19	1.9	Fam102b	
DMR2:211807001	2	211807001	211808000	1000	1	3.50E-12	0.75	25	2.5	Fam102b	
DMR2:211839001	2	211839001	211840000	1000	1	1.80E-07	-0.42	16	1.6	Fam102b	
DMR2:211891001	2	211891001	211892000	1000	1	1.80E-08	0.63	24	2.4	Slc25a54	Transport
DMR2:211973001	2	211973001	211975000	2000	2	1.20E-10	0.68	29	1.45	Slc25a24	Transport
DMR2:212244001	2	212244001	212245000	1000	1	1.90E-07	-0.38	3	0.3	Vav3	
DMR2:212253001	2	212253001	212254000	1000	1	1.10E-07	-0.42	9	0.9	Vav3	
DMR2:212435001	2	212435001	212436000	1000	1	3.90E-13	0.77	27	2.7	Vav3;Mrps17-ps1	
DMR2:212544001	2	212544001	212548000	4000	1	6.70E-07	-0.48	48	1.2	Vav3	
DMR2:213035001	2	213035001	213037000	2000	1	1.50E-07	0.47	25	1.25	Ntng1	Extracellular Matrix
DMR2:213165001	2	213165001	213166000	1000	1	9.70E-15	0.88	19	1.9	Prmt6	Golgi
DMR2:216440001	2	216440001	216441000	1000	1	7.50E-08	0.63	10	1	Amy1a	Metabolism
DMR2:216889001	2	216889001	216890000	1000	1	2.90E-09	-0.46	9	0.9	Col11a1	Extracellular Matrix
DMR2:216912001	2	216912001	216913000	1000	1	6.00E-07	-0.55	9	0.9	Col11a1	Extracellular Matrix
DMR2:217057001	2	217057001	217058000	1000	1	3.10E-11	0.43	10	1	Col11a1	Extracellular Matrix
DMR2:218668001	2	218668001	218671000	3000	2	1.20E-08	-0.41	23	0.77	S1pr1	Signaling
DMR2:219052001	2	219052001	219053000	1000	1	5.40E-08	-0.52	12	1.2	RGD1559607;Rps27a-ps24	
DMR2:219329001	2	219329001	219330000	1000	1	2.90E-08	0.65	13	1.3	Cdc14a	Signaling

DMR2:219359001	2	219359001	219363000	4000	1	1.80E-14	-0.53	73	1.82	Cdc14a	Signaling
DMR2:219454001	2	219454001	219455000	1000	1	3.10E-08	0.44	16	1.6	Cdc14a	Signaling
DMR2:219581001	2	219581001	219583000	2000	1	1.60E-10	-0.46	16	0.8	Dbt	Metabolism
DMR2:219716001	2	219716001	219719000	3000	1	8.10E-07	-0.49	19	0.63	LOC102548510;Slc35a3	Transport
DMR2:219734001	2	219734001	219735000	1000	1	2.50E-07	-0.56	7	0.7	Slc35a3	Transport
DMR2:219779001	2	219779001	219780000	1000	1	2.00E-07	-0.47	6	0.6	Agl	
DMR2:219885001	2	219885001	219887000	2000	1	6.40E-07	-0.27	19	0.95	Plppr5	Signaling
DMR2:219988001	2	219988001	219990000	2000	1	3.70E-09	-0.44	20	1	Plppr5	Signaling
DMR2:220337001	2	220337001	220338000	1000	1	5.20E-08	0.53	11	1.1	Plppr4	Signaling
DMR2:220419001	2	220419001	220422000	3000	1	1.50E-08	-0.46	29	0.97	LOC102548573;Frrs1	Metabolism
DMR2:220437001	2	220437001	220438000	1000	1	8.00E-07	0.47	16	1.6	Frrs1	Metabolism
DMR2:220512001	2	220512001	220513000	1000	1	5.90E-08	-0.55	14	1.4	Palmd	
DMR2:221083001	2	221083001	221084000	1000	1	3.20E-09	0.75	23	2.3	Snx7	Cytoskeleton
DMR2:222226001	2	222226001	222228000	2000	1	1.70E-12	0.57	19	0.95	Dpyd	Metabolism
DMR2:222385001	2	222385001	222391000	6000	1	2.90E-09	-0.36	74	1.23	Dpyd	Metabolism
DMR2:222397001	2	222397001	222399000	2000	1	2.70E-07	-0.7	10	0.5	Dpyd	Metabolism
DMR2:222407001	2	222407001	222411000	4000	3	2.30E-18	0.42	33	0.82	Dpyd	Metabolism
DMR2:222638001	2	222638001	222642000	4000	1	9.10E-07	0.27	59	1.48	Dpyd	Metabolism
DMR2:222668001	2	222668001	222672000	4000	1	9.70E-12	-0.41	42	1.05	Dpyd	Metabolism
DMR2:223267001	2	223267001	223269000	2000	1	6.90E-07	-0.31	20	1	Ptbp2	
DMR2:224719001	2	224719001	224720000	1000	1	1.50E-07	-0.33	20	2	Rwdd3	
DMR2:224818001	2	224818001	224820000	2000	1	3.10E-07	-0.33	30	1.5	Tmem56	
DMR2:224847001	2	224847001	224848000	1000	1	5.20E-20	1.33	32	3.2	Tmem56;Alg14	Transport
DMR2:224882001	2	224882001	224885000	3000	1	7.80E-08	-0.38	39	1.3	Alg14;LOC108350097	Transport
DMR2:224908001	2	224908001	224916000	8000	1	2.50E-07	-0.43	97	1.21	Alg14	Transport
DMR2:225093001	2	225093001	225096000	3000	1	3.70E-07	-0.38	50	1.67	Slc44a3;LOC108350098	Transport
DMR2:225337001	2	225337001	225338000	1000	1	4.70E-09	-0.69	18	1.8	Abcd3	Transport
DMR2:225385001	2	225385001	225387000	2000	1	7.80E-08	-0.42	26	1.3	Abcd3;LOC108350099	Transport
DMR2:225662001	2	225662001	225665000	3000	1	5.30E-08	0.3	21	0.7	Abca4	Transport
DMR2:225670001	2	225670001	225673000	3000	1	1.80E-07	-0.51	41	1.37	Abca4	Transport
DMR2:225690001	2	225690001	225693000	3000	1	6.10E-07	0.42	46	1.53	Abca4	Transport
DMR2:225712001	2	225712001	225714000	2000	1	3.40E-09	0.44	37	1.85	Abca4	Transport
DMR2:225715001	2	225715001	225719000	4000	1	1.80E-10	0.62	69	1.73	Abca4	Transport
DMR2:225816001	2	225816001	225819000	3000	1	5.10E-09	-0.38	28	0.93	LOC102555028;Gclm	Metabolism
DMR2:226586001	2	226586001	226589000	3000	1	2.00E-08	-0.37	40	1.33	Bcar3	
DMR2:226619001	2	226619001	226620000	1000	1	4.20E-08	-0.54	20	2	Bcar3	
DMR2:226644001	2	226644001	226646000	2000	1	5.20E-19	-0.58	38	1.9	Bcar3;LOC108350102	
DMR2:226656001	2	226656001	226660000	4000	1	1.80E-09	0.64	91	2.28	Bcar3;LOC108350102	
DMR2:226704001	2	226704001	226706000	2000	1	2.10E-07	-0.62	15	0.75	Fnbp1l	
DMR2:226751001	2	226751001	226754000	3000	1	1.90E-08	-0.36	25	0.83	Fnbp1l	
DMR2:226910001	2	226910001	226911000	1000	1	8.00E-07	-0.27	6	0.6	Pde5a	Signaling
DMR2:226912001	2	226912001	226916000	4000	1	6.10E-13	0.54	33	0.82	Pde5a	Signaling
DMR2:226934001	2	226934001	226935000	1000	1	7.70E-14	-0.55	9	0.9	Pde5a	Signaling
DMR2:226967001	2	226967001	226974000	7000	1	3.00E-07	-0.34	143	2.04	Pde5a	Signaling
DMR2:226979001	2	226979001	226981000	2000	1	5.60E-07	-0.41	34	1.7	Pde5a	Signaling
DMR2:227005001	2	227005001	227007000	2000	1	5.10E-08	-0.66	24	1.2	Pde5a	Signaling
DMR2:227008001	2	227008001	227009000	1000	1	1.70E-08	0.48	15	1.5	Pde5a	Signaling
DMR2:227013001	2	227013001	227015000	2000	1	1.20E-07	-0.57	28	1.4	Pde5a	Signaling
DMR2:227040001	2	227040001	227045000	5000	1	7.20E-09	0.7	105	2.1	Pde5a	Signaling
DMR2:227119001	2	227119001	227122000	3000	1	7.70E-07	-0.37	25	0.83	Usp53	Protease
DMR2:227255001	2	227255001	227260000	5000	2	3.00E-07	-0.46	55	1.1	Synpo2	Cytoskeleton
DMR2:227268001	2	227268001	227272000	4000	1	1.80E-10	0.71	61	1.52	Synpo2	Cytoskeleton
DMR2:227273001	2	227273001	227278000	5000	1	5.90E-09	-0.42	49	0.98	Synpo2	Cytoskeleton
DMR2:227294001	2	227294001	227296000	2000	2	3.70E-20	1.3	55	2.75	Synpo2	Cytoskeleton
DMR2:227318001	2	227318001	227320000	2000	1	6.90E-20	-0.62	23	1.15	Synpo2	Cytoskeleton
DMR2:227371001	2	227371001	227372000	1000	1	6.10E-07	-0.35	16	1.6	Synpo2	Cytoskeleton
DMR2:227471001	2	227471001	227472000	1000	1	3.60E-14	-0.53	16	1.6	Sec24d	Transport
DMR2:227481001	2	227481001	227482000	1000	1	3.40E-08	0.47	16	1.6	Sec24d	Transport
DMR2:227485001	2	227485001	227486000	1000	1	2.00E-09	-0.42	24	2.4	Sec24d	Transport
DMR2:227499001	2	227499001	227501000	2000	1	4.10E-08	-0.43	20	1	Sec24d	Transport
DMR2:227580001	2	227580001	227582000	2000	1	6.50E-10	-0.68	26	1.3	Mettl14	Translation
DMR2:227814001	2	227814001	227815000	1000	1	4.80E-10	-0.5	15	1.5	Ndst3	Transport

DMR2:227818001	2	227818001	227820000	2000	1	2.50E-07	0.39	37	1.85	Ndst3	Transport
DMR2:227847001	2	227847001	227850000	3000	1	2.50E-07	-0.34	57	1.9	Ndst3	Transport
DMR2:229181001	2	229181001	229183000	2000	1	8.40E-07	-0.49	13	0.65	Ndst4	Transport
DMR2:229391001	2	229391001	229392000	1000	1	1.90E-08	-0.53	7	0.7	Ndst4	Transport
DMR2:229423001	2	229423001	229429000	6000	1	5.90E-07	-0.31	51	0.85	Ndst4	Transport
DMR2:229473001	2	229473001	229475000	2000	1	8.90E-17	0.99	27	1.35	Ndst4	Transport
DMR2:230157001	2	230157001	230158000	1000	1	3.30E-07	-0.48	10	1	Arsj	
DMR2:230191001	2	230191001	230196000	5000	1	5.70E-07	0.58	98	1.96	Arsj;LOC103689977;Mcub	
DMR2:230204001	2	230204001	230206000	2000	1	4.70E-09	-0.48	22	1.1	Arsj;LOC103689977;Mcub	
DMR2:230256001	2	230256001	230259000	3000	2	6.50E-08	0.59	34	1.13	Arsj;Mcub	
DMR2:230266001	2	230266001	230268000	2000	1	9.00E-11	0.71	19	0.95	Arsj;Mcub	
DMR2:230385001	2	230385001	230386000	1000	1	6.20E-08	0.5	21	2.1	Arsj;LOC103691692	
DMR2:230398001	2	230398001	230400000	2000	2	8.40E-14	0.86	38	1.9	Arsj	
DMR2:230462001	2	230462001	230466000	4000	1	3.10E-08	0.57	56	1.4	Arsj;LOC102553088	
DMR2:230483001	2	230483001	230484000	1000	1	9.30E-11	-0.56	27	2.7	Arsj;LOC102553088	
DMR2:230648001	2	230648001	230651000	3000	1	4.00E-10	0.47	34	1.13	Arsj	
DMR2:230920001	2	230920001	230923000	3000	1	6.20E-08	-0.38	44	1.47	Camk2d	Signaling
DMR2:230943001	2	230943001	230945000	2000	1	2.00E-08	-0.48	25	1.25	Camk2d	Signaling
DMR2:231020001	2	231020001	231024000	4000	1	1.40E-09	0.61	37	0.92	Camk2d	Signaling
DMR2:231232001	2	231232001	231236000	4000	1	3.30E-07	-0.42	64	1.6	Ank2	
DMR2:231249001	2	231249001	231252000	3000	1	3.70E-09	-0.39	68	2.27	Ank2	
DMR2:231261001	2	231261001	231263000	2000	1	1.60E-08	-0.58	22	1.1	Ank2	
DMR2:231300001	2	231300001	231304000	4000	1	1.30E-07	-0.44	80	2	Ank2	
DMR2:231338001	2	231338001	231341000	3000	1	2.60E-15	-0.62	35	1.17	Ank2	
DMR2:231405001	2	231405001	231406000	1000	1	1.90E-15	-0.59	11	1.1	Ank2	
DMR2:231417001	2	231417001	231419000	2000	1	9.10E-07	-0.43	37	1.85	Ank2	
DMR2:231422001	2	231422001	231424000	2000	1	3.40E-07	-0.32	26	1.3	Ank2	
DMR2:231428001	2	231428001	231429000	1000	1	7.20E-10	-0.42	17	1.7	Ank2	
DMR2:231516001	2	231516001	231517000	1000	1	2.20E-08	-0.37	16	1.6	Ank2	
DMR2:231520001	2	231520001	231521000	1000	1	3.40E-07	0.45	21	2.1	Ank2	
DMR2:231871001	2	231871001	231872000	1000	1	9.00E-07	-0.43	10	1	Larp7;Zgrf1	Metabolism
DMR2:231875001	2	231875001	231878000	3000	1	6.80E-07	-0.36	49	1.63	Larp7;Zgrf1	Metabolism
DMR2:231926001	2	231926001	231927000	1000	1	4.60E-09	-0.57	8	0.8	Zgrf1	
DMR2:231971001	2	231971001	231973000	2000	1	1.10E-11	-0.63	19	0.95	Neurog2	Transcription
DMR2:232008001	2	232008001	232010000	2000	1	5.50E-07	0.36	38	1.9	Alpk1	Signaling
DMR2:232016001	2	232016001	232018000	2000	1	1.80E-09	0.4	20	1	Alpk1	Signaling
DMR2:232059001	2	232059001	232062000	3000	1	1.80E-09	-0.39	60	2	Alpk1	Signaling
DMR2:232066001	2	232066001	232069000	3000	1	1.70E-07	-0.35	62	2.07	Alpk1	Signaling
DMR2:232118001	2	232118001	232122000	4000	2	5.70E-13	-0.84	29	0.72	Alpk1;Tifa	Signaling
DMR2:232156001	2	232156001	232159000	3000	1	5.10E-07	-0.32	49	1.63	Ap1ar	
DMR2:232314001	2	232314001	232315000	1000	1	1.60E-07	0.54	28	2.8	RGD1565088	
DMR2:233734001	2	233734001	233735000	1000	1	1.90E-07	-0.66	6	0.6	Enpep	Protease
DMR2:234182001	2	234182001	234183000	1000	1	4.80E-07	-0.48	22	2.2	Elovl6	Metabolism
DMR2:234206001	2	234206001	234207000	1000	1	4.10E-11	-0.54	16	1.6	Elovl6	Metabolism
DMR2:234240001	2	234240001	234242000	2000	2	2.20E-08	-0.55	35	1.75	Elovl6	Metabolism
DMR2:235202001	2	235202001	235205000	3000	1	4.00E-09	-0.48	46	1.53	Lrit3	
DMR2:235301001	2	235301001	235302000	1000	1	4.40E-07	-0.4	15	1.5	Cfi;Pla2g12a	Protease;Metabolism
DMR2:235582001	2	235582001	235584000	2000	1	2.80E-07	-0.63	28	1.4	Col25a1	Extracellular Matrix
DMR2:235639001	2	235639001	235640000	1000	1	1.80E-08	-0.48	28	2.8	Col25a1	Extracellular Matrix
DMR2:235692001	2	235692001	235695000	3000	1	4.00E-09	0.41	57	1.9	Col25a1	Extracellular Matrix
DMR2:235742001	2	235742001	235743000	1000	1	1.90E-07	0.55	29	2.9	Etnppl	
DMR2:236254001	2	236254001	236257000	3000	1	7.80E-14	0.85	56	1.87	Lef1;LOC103691708	Transcription
DMR2:236264001	2	236264001	236267000	3000	1	2.60E-08	0.65	38	1.27	Lef1;LOC103691708	Transcription
DMR2:236355001	2	236355001	236357000	2000	1	2.60E-11	0.78	36	1.8	Lef1;Hadh	Transcription
DMR2:236425001	2	236425001	236429000	4000	1	3.10E-08	-0.37	94	2.35	Cyp2u1;LOC102552472	Metabolism
DMR2:236473001	2	236473001	236474000	1000	1	9.60E-07	-0.41	15	1.5	Sgms2;LOC108350108	
DMR2:236498001	2	236498001	236503000	5000	1	7.90E-10	-0.6	92	1.84	Sgms2;LOC108350108;LOC108350109	
DMR2:236523001	2	236523001	236529000	6000	1	8.40E-08	-0.51	111	1.85	Sgms2;LOC108350109;LOC108350110	
DMR2:236534001	2	236534001	236535000	1000	1	1.50E-10	-0.7	5	0.5	Sgms2;LOC108350110	
DMR2:236610001	2	236610001	236614000	4000	2	9.30E-09	0.38	41	1.02	Papss1	

DMR2:237190001	2	237190001	237192000	2000	1	4.80E-08	0.5	22	1.1	Dkk2	
DMR2:237738001	2	237738001	237739000	1000	1	3.30E-08	-0.46	18	1.8	Aimp1	Translation
DMR2:237753001	2	237753001	237755000	2000	1	3.00E-08	-0.39	17	0.85	Aimp1;Tbck	Translation;Signaling
DMR2:237887001	2	237887001	237888000	1000	1	6.90E-14	0.36	27	2.7	Tbck	Signaling
DMR2:237940001	2	237940001	237942000	2000	1	1.50E-08	-0.7	11	0.55	Tbck	Signaling
DMR2:237964001	2	237964001	237966000	2000	1	3.50E-07	-0.28	32	1.6	Tbck;LOC680335;LOC102556143	Signaling
DMR2:238066001	2	238066001	238067000	1000	1	1.00E-07	0.54	23	2.3	Npnt;LOC365942	
DMR2:238107001	2	238107001	238108000	1000	1	4.10E-09	0.64	24	2.4	LOC102548236;Gstcd	
DMR2:238232001	2	238232001	238234000	2000	1	3.50E-14	-0.58	29	1.45	Gstcd	
DMR2:238240001	2	238240001	238242000	2000	2	5.20E-20	-0.74	33	1.65	Gstcd	
DMR2:238255001	2	238255001	238256000	1000	1	7.80E-12	-0.44	14	1.4	Gstcd;Ints12	
DMR2:238541001	2	238541001	238543000	2000	1	2.70E-09	-0.45	44	2.2	Ppa2	Signaling
DMR2:238571001	2	238571001	238572000	1000	1	2.30E-12	0.93	26	2.6	Ppa2	Signaling
DMR2:238584001	2	238584001	238585000	1000	1	6.10E-19	0.85	20	2	Ppa2	Signaling
DMR2:238594001	2	238594001	238596000	2000	1	1.10E-07	-0.4	35	1.75	Ppa2	Signaling
DMR2:238709001	2	238709001	238714000	5000	2	6.10E-17	1	73	1.46	Tet2	
DMR2:240121001	2	240121001	240122000	1000	1	4.00E-07	-0.37	17	1.7	Tacr3	Signaling
DMR2:240417001	2	240417001	240418000	1000	1	5.80E-07	0.42	11	1.1	Cenpe	
DMR2:240468001	2	240468001	240470000	2000	1	6.70E-07	-0.33	43	2.15	Bdh2	Metabolism
DMR2:240508001	2	240508001	240510000	2000	1	5.50E-11	0.81	53	2.65	Slc9b2	
DMR2:240521001	2	240521001	240523000	2000	2	5.00E-07	-0.44	27	1.35	Slc9b2;Slc9b1	
DMR2:240560001	2	240560001	240561000	1000	1	4.60E-22	0.37	10	1	Slc9b1	
DMR2:240688001	2	240688001	240690000	2000	1	2.60E-07	0.48	24	1.2	Manba	Golgi
DMR2:240730001	2	240730001	240734000	4000	1	3.20E-08	-0.47	54	1.35	Manba	Golgi
DMR2:240772001	2	240772001	240774000	2000	1	1.50E-07	-0.31	24	1.2	Nfkb1	Transcription
DMR2:240824001	2	240824001	240830000	6000	1	5.60E-07	-0.48	131	2.18	Nfkb1	Transcription
DMR2:240883001	2	240883001	240884000	1000	1	2.20E-10	-0.5	24	2.4	Nfkb1	Transcription
DMR2:240886001	2	240886001	240888000	2000	1	2.60E-07	-0.41	30	1.5	Nfkb1	Transcription
DMR2:240892001	2	240892001	240893000	1000	1	3.20E-11	0.46	4	0.4	Nfkb1	Transcription
DMR2:241047001	2	241047001	241048000	1000	1	3.70E-07	0.34	29	2.9	Slc39a8	Transport
DMR2:241451001	2	241451001	241453000	2000	1	5.20E-10	0.69	20	1	Bank1	
DMR2:242623001	2	242623001	242625000	2000	2	3.50E-10	-0.58	7	0.35	Emcn	
DMR2:242684001	2	242684001	242686000	2000	1	6.00E-07	0.59	36	1.8	Emcn;LOC108350120	
DMR2:243145001	2	243145001	243146000	1000	1	4.00E-08	-0.48	20	2	Dnajb14	
DMR2:243153001	2	243153001	243155000	2000	1	4.10E-09	-0.43	43	2.15	Dnajb14;Lamtor3	Cytoskeleton
DMR2:243215001	2	243215001	243217000	2000	1	6.20E-10	-0.49	38	1.9	Dapp1	
DMR2:243219001	2	243219001	243221000	2000	1	1.60E-07	0.71	37	1.85	Dapp1;LOC102555357	
DMR2:243385001	2	243385001	243386000	1000	1	9.40E-07	-0.34	15	1.5	Mttp	Transport
DMR2:243472001	2	243472001	243473000	1000	1	2.50E-07	0.49	16	1.6	RGD1309170	
DMR2:243474001	2	243474001	243477000	3000	1	5.20E-11	0.88	58	1.93	RGD1309170	
DMR2:243526001	2	243526001	243527000	1000	1	8.50E-11	-0.56	6	0.6	Adh7;LOC102556058	Metabolism
DMR2:243582001	2	243582001	243585000	3000	1	2.30E-08	-0.27	25	0.83	LOC102556144;Adh6a	Metabolism
DMR2:243600001	2	243600001	243601000	1000	1	1.10E-13	0.58	10	1	Adh6a	Metabolism
DMR2:243659001	2	243659001	243662000	3000	1	3.70E-07	0.55	29	0.97	Adh6	Metabolism
DMR2:243685001	2	243685001	243691000	6000	1	3.30E-08	-0.42	88	1.47	Adh6	Metabolism
DMR2:243693001	2	243693001	243694000	1000	1	2.60E-08	0.58	13	1.3	Adh6;Adh4	Metabolism
DMR2:243745001	2	243745001	243748000	3000	1	2.30E-07	-0.41	42	1.4	Adh5;Metap1	Metabolism;Protease
DMR2:243808001	2	243808001	243810000	2000	1	5.50E-08	-0.36	39	1.95	LOC102556448;Eif4e;Mir1956	Translation
DMR2:244060001	2	244060001	244062000	2000	1	7.70E-08	-0.39	46	2.3	LOC102549290;Tspan5	
DMR2:244116001	2	244116001	244120000	4000	1	7.40E-13	-0.52	97	2.42	Tspan5	
DMR2:244162001	2	244162001	244164000	2000	1	8.00E-07	-0.44	30	1.5	Tspan5	
DMR2:244166001	2	244166001	244169000	3000	1	3.10E-07	-0.38	42	1.4	Tspan5	
DMR2:244259001	2	244259001	244261000	2000	1	1.70E-10	-0.44	50	2.5	Rap1gds1	
DMR2:244316001	2	244316001	244319000	3000	1	1.60E-10	-0.47	37	1.23	Rap1gds1	
DMR2:244355001	2	244355001	244356000	1000	1	8.00E-09	-0.41	15	1.5	Rap1gds1	
DMR2:244685001	2	244685001	244689000	4000	1	1.50E-12	0.4	87	2.17	Stpg2	Development
DMR2:244737001	2	244737001	244741000	4000	1	1.90E-07	0.28	24	0.6	Stpg2	Development
DMR2:244926001	2	244926001	244927000	1000	1	9.50E-08	-0.33	14	1.4	Stpg2	Development
DMR2:247042001	2	247042001	247044000	2000	1	8.70E-07	0.43	28	1.4	Unc5c	Receptor
DMR2:247254001	2	247254001	247255000	1000	1	1.40E-07	-0.52	4	0.4	Unc5c	Receptor
DMR2:247321001	2	247321001	247323000	2000	1	1.50E-07	-0.39	12	0.6	Unc5c	Receptor

DMR2:247330001	2	247330001	247332000	2000	1	7.00E-07	0.38	22	1.1	Unc5c	Receptor
DMR2:247526001	2	247526001	247527000	1000	1	1.30E-13	-0.64	11	1.1	Bmpr1b;LOC108350217	Signaling
DMR2:247539001	2	247539001	247540000	1000	1	3.80E-07	-0.42	14	1.4	Bmpr1b	Signaling
DMR2:247549001	2	247549001	247551000	2000	1	1.00E-06	-0.4	17	0.85	Bmpr1b	Signaling
DMR2:247867001	2	247867001	247873000	6000	1	1.30E-11	-0.57	54	0.9	Pdlim5	Cytoskeleton
DMR2:248197001	2	248197001	248199000	2000	1	4.50E-07	-0.39	16	0.8	Gbp5	Signaling
DMR2:248677001	2	248677001	248678000	1000	1	3.70E-07	-0.35	8	0.8	Kyat3	Metabolism
DMR2:248691001	2	248691001	248693000	2000	2	4.90E-21	1.22	43	2.15	Kyat3	Metabolism
DMR2:248702001	2	248702001	248703000	1000	1	9.00E-07	-0.34	28	2.8	LOC108350125;Gtf2b	Transcription
DMR2:250208001	2	250208001	250209000	1000	1	7.40E-07	-0.57	3	0.3	Lmo4	
DMR2:250213001	2	250213001	250219000	6000	1	1.10E-09	0.62	106	1.77	Lmo4	
DMR2:250226001	2	250226001	250228000	2000	1	7.90E-09	-0.53	40	2	Lmo4;LOC102549255	
DMR2:250488001	2	250488001	250490000	2000	1	3.40E-08	-0.58	38	1.9	Hs2st1	Transport
DMR2:250764001	2	250764001	250765000	1000	1	1.40E-16	1.07	24	2.4	Clca2	Transport
DMR2:250789001	2	250789001	250793000	4000	1	1.80E-14	-0.48	80	2	Clca4l;LOC108350129;LOC102549543	Transport
DMR2:250874001	2	250874001	250877000	3000	1	1.50E-08	-0.38	38	1.27	Clca4	Transport
DMR2:250894001	2	250894001	250897000	3000	1	6.40E-07	0.46	59	1.97	Clca1	Transport
DMR2:250920001	2	250920001	250921000	1000	1	3.80E-07	-0.36	8	0.8	Clca1;LOC108350130	Transport
DMR2:250988001	2	250988001	250990000	2000	1	3.50E-07	-0.39	24	1.2	Clca5;LOC103691724;Odf2l	Transport
DMR2:251022001	2	251022001	251025000	3000	1	2.10E-07	-0.34	53	1.77	Odf2l	
DMR2:251033001	2	251033001	251035000	2000	1	8.10E-08	0.56	22	1.1	Odf2l	
DMR2:251219001	2	251219001	251221000	2000	1	6.00E-11	-0.45	52	2.6	Col24a1	Extracellular Matrix
DMR2:251259001	2	251259001	251263000	4000	1	2.10E-07	-0.4	58	1.45	Col24a1	Extracellular Matrix
DMR2:251274001	2	251274001	251275000	1000	1	2.20E-09	-0.64	1	0.1	Col24a1	Extracellular Matrix
DMR2:251397001	2	251397001	251400000	3000	1	5.60E-08	-0.54	39	1.3	Col24a1	Extracellular Matrix
DMR2:251415001	2	251415001	251418000	3000	1	3.30E-07	0.35	31	1.03	Col24a1	Extracellular Matrix
DMR2:251534001	2	251534001	251536000	2000	1	7.80E-08	0.76	50	2.5	Cyr61;LOC102553018	
DMR2:251622001	2	251622001	251626000	4000	1	5.10E-17	1.02	89	2.22	Ddah1	Metabolism
DMR2:251718001	2	251718001	251720000	2000	1	5.50E-09	-0.36	36	1.8	Ddah1;LOC103691727	Metabolism
DMR2:251727001	2	251727001	251729000	2000	1	2.80E-08	-0.37	33	1.65	Ddah1;LOC103691727	Metabolism
DMR2:251864001	2	251864001	251867000	3000	1	4.30E-08	-0.36	52	1.73	Syde2	Signaling
DMR2:251884001	2	251884001	251888000	4000	1	1.20E-07	-0.33	94	2.35	Syde2	Signaling
DMR2:251900001	2	251900001	251904000	4000	2	1.10E-13	-0.72	71	1.77	Syde2;Wdr63	Signaling;Cytoskeleton
DMR2:251927001	2	251927001	251928000	1000	1	2.20E-16	0.92	36	3.6	Wdr63;Mcoln3	Cytoskeleton;Transport
DMR2:251964001	2	251964001	251965000	1000	1	2.50E-15	0.62	3	0.3	Wdr63;Mcoln3	Cytoskeleton;Transport
DMR2:251969001	2	251969001	251970000	1000	1	5.20E-11	-0.39	19	1.9	Wdr63;Mcoln3	Cytoskeleton;Transport
DMR2:252006001	2	252006001	252007000	1000	1	6.90E-08	0.34	15	1.5	Mcoln3	Transport
DMR2:252075001	2	252075001	252076000	1000	1	7.80E-08	-0.44	9	0.9	Mcoln2	Transport
DMR2:252091001	2	252091001	252092000	1000	1	1.20E-10	0.76	15	1.5	Lpar3	Signaling
DMR2:252100001	2	252100001	252104000	4000	1	5.10E-08	-0.4	85	2.12	Lpar3	Signaling
DMR2:252149001	2	252149001	252151000	2000	1	1.70E-07	0.46	35	1.75	Lpar3	Signaling
DMR2:252168001	2	252168001	252169000	1000	1	4.20E-08	0.64	18	1.8	Lpar3	Signaling
DMR2:252434001	2	252434001	252435000	1000	1	4.70E-09	-0.6	5	0.5	Dnase2b	Transcription
DMR2:252436001	2	252436001	252439000	3000	1	1.50E-07	-0.43	53	1.77	Dnase2b	Transcription
DMR2:252628001	2	252628001	252630000	2000	1	1.50E-08	-0.6	16	0.8	Prkacb	Signaling
DMR2:252665001	2	252665001	252667000	2000	1	7.90E-07	-0.59	14	0.7	Prkacb	Signaling
DMR2:252787001	2	252787001	252792000	5000	1	6.60E-08	-0.47	130	2.6	Ttll7	Cytoskeleton
DMR2:252824001	2	252824001	252825000	1000	1	4.70E-10	0.37	9	0.9	Ttll7	Cytoskeleton
DMR2:255934001	2	255934001	255936000	2000	1	5.20E-08	-0.59	4	0.2	RGD1566247	
DMR2:256148001	2	256148001	256149000	1000	1	1.40E-11	-0.55	6	0.6	Calm-ps1	
DMR2:256652001	2	256652001	256653000	1000	1	8.40E-08	0.51	12	1.2	Adgrl4	Signaling
DMR2:256949001	2	256949001	256950000	1000	1	4.30E-08	-0.46	16	1.6	Ifi44l	
DMR2:257293001	2	257293001	257294000	1000	1	1.40E-11	0.93	34	3.4	Gipc2	Cytoskeleton
DMR2:257380001	2	257380001	257382000	2000	2	2.30E-16	1.16	37	1.85	Gipc2	Cytoskeleton
DMR2:257405001	2	257405001	257407000	2000	1	7.00E-08	-0.6	27	1.35	LOC108350143;Dnajb4	Transcription
DMR2:257464001	2	257464001	257468000	4000	1	1.00E-07	-0.5	41	1.02	Nexn;LOC102548862	Cytoskeleton
DMR2:257482001	2	257482001	257484000	2000	2	3.30E-11	0.75	34	1.7	Nexn;Miga1	Cytoskeleton
DMR2:257485001	2	257485001	257487000	2000	1	2.10E-07	0.65	53	2.65	Nexn;Miga1	Cytoskeleton
DMR2:257935001	2	257935001	257939000	4000	1	1.30E-07	-0.62	65	1.62	Pigk	
DMR2:257942001	2	257942001	257943000	1000	1	1.10E-08	-0.57	29	2.9	Pigk	
DMR2:257978001	2	257978001	257980000	2000	1	6.70E-09	0.48	45	2.25	Pigk	

DMR2:258110001	2	258110001	258111000	1000	1	1.40E-08	-0.54	10	1	St6galnac5	
DMR2:258121001	2	258121001	258125000	4000	1	9.90E-07	-0.32	38	0.95	St6galnac5	
DMR2:258179001	2	258179001	258182000	3000	1	2.70E-17	0.82	47	1.57	St6galnac5	
DMR2:258321001	2	258321001	258323000	2000	1	5.20E-07	-0.37	24	1.2	St6galnac5	
DMR2:258404001	2	258404001	258407000	3000	2	2.30E-17	1.24	60	2	St6galnac5	
DMR2:258435001	2	258435001	258438000	3000	1	2.10E-12	0.97	58	1.93	St6galnac5	
DMR2:258494001	2	258494001	258495000	1000	1	1.20E-07	-0.37	13	1.3	St6galnac5	
DMR2:258504001	2	258504001	258505000	1000	1	1.90E-11	0.75	13	1.3	St6galnac5	
DMR2:258632001	2	258632001	258636000	4000	1	7.10E-08	-0.27	30	0.75	St6galnac5	
DMR2:258640001	2	258640001	258641000	1000	1	1.80E-07	-0.48	19	1.9	St6galnac5	
DMR2:258666001	2	258666001	258668000	2000	2	2.00E-10	0.81	46	2.3	St6galnac5	
DMR2:258734001	2	258734001	258737000	3000	1	9.40E-15	0.88	44	1.47	St6galnac5	
DMR2:258816001	2	258816001	258821000	5000	1	1.50E-07	-0.45	83	1.66	St6galnac5;Adgrl2	Signaling
DMR2:258831001	2	258831001	258833000	2000	1	6.50E-13	-0.43	41	2.05	St6galnac5;Adgrl2	Signaling
DMR2:258849001	2	258849001	258851000	2000	1	2.20E-07	-0.34	59	2.95	St6galnac5;Adgrl2	Signaling
DMR2:258885001	2	258885001	258886000	1000	1	8.70E-07	0.33	26	2.6	St6galnac5;Adgrl2	Signaling
DMR2:258898001	2	258898001	258902000	4000	1	3.70E-08	0.46	74	1.85	St6galnac5;Adgrl2	Signaling
DMR2:259032001	2	259032001	259033000	1000	1	5.20E-10	0.6	14	1.4	St6galnac5	
DMR2:259035001	2	259035001	259039000	4000	1	2.70E-08	0.46	43	1.07	St6galnac5	
DMR2:259118001	2	259118001	259119000	1000	1	6.20E-07	0.36	8	0.8	St6galnac5	
DMR2:259144001	2	259144001	259148000	4000	2	2.80E-08	-0.6	54	1.35	St6galnac5	
DMR2:259375001	2	259375001	259377000	2000	1	7.30E-07	0.64	57	2.85	St6galnac3	
DMR2:259427001	2	259427001	259431000	4000	1	6.40E-08	0.54	83	2.08	St6galnac3	
DMR2:259476001	2	259476001	259479000	3000	1	4.60E-07	0.53	51	1.7	St6galnac3	
DMR2:259518001	2	259518001	259519000	1000	1	1.00E-09	-0.44	15	1.5	St6galnac3	
DMR2:259579001	2	259579001	259580000	1000	1	5.60E-11	0.89	20	2	St6galnac3	
DMR2:259616001	2	259616001	259619000	3000	1	1.70E-08	0.42	53	1.77	St6galnac3	
DMR2:259716001	2	259716001	259718000	2000	2	5.30E-08	-0.63	20	1	St6galnac3;LOC108350218	
DMR2:259860001	2	259860001	259868000	8000	1	1.60E-07	0.56	121	1.51	St6galnac3	
DMR2:259904001	2	259904001	259907000	3000	1	4.40E-10	0.6	45	1.5	St6galnac3;LOC103691740	
DMR2:259913001	2	259913001	259916000	3000	2	1.70E-13	0.92	94	3.13	St6galnac3;LOC103691740	
DMR2:260113001	2	260113001	260116000	3000	1	3.80E-10	-0.75	59	1.97	Msh4;Rabggtb;Acadm	Transcription;Metabolism;Metabolism
DMR2:260192001	2	260192001	260195000	3000	2	2.00E-08	0.49	39	1.3	Slc44a5	Transport
DMR2:260258001	2	260258001	260263000	5000	1	5.30E-07	0.41	93	1.86	Slc44a5	Transport
DMR2:260269001	2	260269001	260275000	6000	1	3.30E-07	0.44	112	1.87	Slc44a5	Transport
DMR2:260292001	2	260292001	260294000	2000	1	5.40E-10	0.62	49	2.45	Slc44a5	Transport
DMR2:260300001	2	260300001	260303000	3000	2	1.30E-16	1.04	81	2.7	Slc44a5	Transport
DMR2:260331001	2	260331001	260332000	1000	1	3.90E-20	1	12	1.2	Slc44a5;LOC102553583	Transport
DMR2:260352001	2	260352001	260355000	3000	1	1.70E-07	-0.28	46	1.53	Slc44a5	Transport
DMR2:260386001	2	260386001	260387000	1000	1	2.50E-11	0.89	20	2	Slc44a5	Transport
DMR2:260402001	2	260402001	260403000	1000	1	2.20E-07	0.55	21	2.1	Slc44a5	Transport
DMR2:260434001	2	260434001	260435000	1000	1	5.20E-07	-0.48	16	1.6	Slc44a5	Transport
DMR2:260477001	2	260477001	260479000	2000	1	2.50E-14	1.02	44	2.2	Slc44a5	Transport
DMR2:260480001	2	260480001	260481000	1000	1	1.80E-12	0.79	20	2	Slc44a5	Transport
DMR2:260855001	2	260855001	260857000	2000	1	2.80E-07	0.33	16	0.8	Tyw3	Epigenetic
DMR2:260909001	2	260909001	260910000	1000	1	1.30E-07	-0.33	18	1.8	Cryz	Metabolism
DMR2:260951001	2	260951001	260952000	1000	1	3.80E-07	0.35	14	1.4	Erich3;LOC102555516	
DMR2:261044001	2	261044001	261048000	4000	2	1.80E-10	0.62	69	1.73	Erich3	
DMR2:261135001	2	261135001	261138000	3000	1	1.10E-07	-0.47	53	1.77	LOC102555624;Tnni3k	
DMR2:261340001	2	261340001	261343000	3000	2	2.40E-08	-0.41	35	1.17	Tnni3k	
DMR2:261369001	2	261369001	261381000	12000	12	5.00E-15	1.29	368	3.07	Fpgt	Transport
DMR2:261402001	2	261402001	261403000	1000	1	1.50E-07	-0.79	35	3.5	Fpgt;Lrriq3	Transport
DMR2:262947001	2	262947001	262949000	2000	1	4.70E-12	0.5	24	1.2	Negr1	Immune
DMR2:263005001	2	263005001	263006000	1000	1	1.10E-15	0.88	23	2.3	Negr1	Immune
DMR2:263156001	2	263156001	263158000	2000	1	7.50E-09	-0.41	29	1.45	Negr1	Immune
DMR2:263465001	2	263465001	263466000	1000	1	7.10E-09	0.55	10	1	Negr1	Immune
DMR2:263628001	2	263628001	263631000	3000	1	8.20E-07	-0.3	23	0.77	Negr1	Immune
DMR2:263966001	2	263966001	263967000	1000	1	7.60E-07	-0.38	12	1.2	Ptger3	Signaling
DMR2:264699001	2	264699001	264704000	5000	1	1.90E-08	-0.65	27	0.54	Depdc1	Cytoskeleton
DMR2:265190001	2	265190001	265191000	1000	1	4.90E-07	-0.4	14	1.4	Lrrc7	Cytoskeleton
DMR3:18001	3	18001	20000	2000	2	2.00E-12	0.52	47	2.35	RGD1562465	

DMR3:236001	3	236001	238000	2000	1	4.10E-07	-0.44	6	0.3	Nxph2	Signaling
DMR3:239001	3	239001	243000	4000	1	5.70E-11	-0.4	39	0.98	Nxph2	Signaling
DMR3:254001	3	254001	258000	4000	3	9.80E-11	0.52	95	2.38	Nxph2;LOC679914	Signaling
DMR3:260001	3	260001	261000	1000	1	7.00E-15	0.86	24	2.4	Nxph2;LOC679914	Signaling
DMR3:268001	3	268001	271000	3000	1	9.40E-07	-0.52	8	0.27	Nxph2;LOC679914	Signaling
DMR3:1298001	3	1298001	1301000	3000	1	2.10E-11	-0.4	23	0.77	Il36g	Cytokine
DMR3:1511001	3	1511001	1512000	1000	1	4.40E-07	0.54	21	2.1	Psd4	Transcription
DMR3:1537001	3	1537001	1539000	2000	2	2.90E-10	-0.57	16	0.8	Pax8;LOC102549321	
DMR3:1566001	3	1566001	1567000	1000	1	1.90E-07	-0.49	5	0.5	Pax8	
DMR3:1579001	3	1579001	1581000	2000	1	1.30E-08	-0.41	18	0.9	Pax8	
DMR3:1743001	3	1743001	1744000	1000	1	2.90E-18	0.95	31	3.1	Cacna1b	Transport
DMR3:1804001	3	1804001	1807000	3000	1	1.00E-08	-0.44	60	2	Cacna1b	Transport
DMR3:1830001	3	1830001	1832000	2000	1	2.70E-07	-0.42	25	1.25	Cacna1b	Transport
DMR3:1840001	3	1840001	1841000	1000	1	2.20E-07	0.51	23	2.3	Cacna1b	Transport
DMR3:2005001	3	2005001	2007000	2000	1	7.80E-07	-0.52	13	0.65	Ehmt1	
DMR3:2418001	3	2418001	2420000	2000	2	1.80E-13	-0.84	13	0.65	Tor4a;Nelfb	Transcription
DMR3:2433001	3	2433001	2434000	1000	1	5.00E-12	0.93	34	3.4	Nelfb;Stpg3;Fam166a;Tubb4b	Development;Cytoskeleton
DMR3:2466001	3	2466001	2468000	2000	1	9.40E-08	0.46	34	1.7	Rnf224;Cysrt1;Rnf208;Ndor1;Tmem203	Metabolism
DMR3:2528001	3	2528001	2529000	1000	1	3.20E-11	0.64	27	2.7	Grin1	Receptor
DMR3:2573001	3	2573001	2574000	1000	1	4.00E-07	-0.52	20	2	Man1b1;Dpp7;Uap1l1	Golgi;Protease
DMR3:2605001	3	2605001	2607000	2000	1	2.60E-08	-0.49	31	1.55	Sapcd2	
DMR3:2638001	3	2638001	2643000	5000	1	1.30E-07	0.42	55	1.1	Npdc1;LOC366006;Fut7;Abca2	Golgi;Transport
DMR3:2645001	3	2645001	2646000	1000	1	2.50E-10	0.7	48	4.8	Fut7;Abca2	Golgi;Transport
DMR3:2664001	3	2664001	2665000	1000	1	5.00E-07	-0.39	40	4	Abca2	Transport
DMR3:2800001	3	2800001	2801000	1000	1	1.30E-12	1.01	46	4.6	Mamdc4;Phpt1;RGD1560470;Rabl6	Signaling
DMR3:2883001	3	2883001	2887000	4000	1	2.40E-11	-0.42	55	1.38	Lcn8;Lcn5;LOC108350548;Lcn6;Lcn10	Transport
DMR3:3332001	3	3332001	3335000	3000	1	3.90E-12	0.6	45	1.5	Kcnt1	Transport
DMR3:3344001	3	3344001	3351000	7000	3	2.60E-12	0.6	111	1.59	Kcnt1	Transport
DMR3:3354001	3	3354001	3359000	5000	1	1.80E-11	0.6	110	2.2	Kcnt1	Transport
DMR3:3384001	3	3384001	3390000	6000	1	5.80E-08	-0.4	151	2.52	Camsap1;LOC100360117	
DMR3:3424001	3	3424001	3427000	3000	1	4.00E-12	-0.52	31	1.03	Camsap1;LOC102550543	
DMR3:3454001	3	3454001	3456000	2000	1	8.40E-08	0.43	23	1.15	LOC102552327;Ubac1	
DMR3:3478001	3	3478001	3479000	1000	1	3.00E-07	-0.56	10	1	Ubac1	
DMR3:3514001	3	3514001	3516000	2000	1	3.40E-08	0.45	47	2.35	Nacc2	
DMR3:3538001	3	3538001	3542000	4000	2	1.20E-10	0.53	85	2.12	Nacc2	
DMR3:3732001	3	3732001	3737000	5000	1	6.90E-10	0.72	88	1.76	Ccdc187	Cytoskeleton
DMR3:3770001	3	3770001	3775000	5000	1	2.90E-07	0.55	57	1.14	Gpsm1	
DMR3:3804001	3	3804001	3807000	3000	1	2.10E-07	0.55	45	1.5	Gpsm1;Dnlz;Card9;LOC108350340;Snapc4	
DMR3:3824001	3	3824001	3825000	1000	1	3.30E-10	0.57	14	1.4	Snapc4;Sdccag3;Pmpca	Protease
DMR3:3847001	3	3847001	3849000	2000	1	7.40E-08	0.55	26	1.3	Pmpca;Inpp5e;Sec16a	Protease;Signaling
DMR3:3900001	3	3900001	3902000	2000	1	3.20E-09	0.45	26	1.3	Sec16a;Notch1	Extracellular Matrix
DMR3:4034001	3	4034001	4036000	2000	1	7.90E-07	0.49	37	1.85	LOC108350341;Egfl7;Mir126a;Mir126;Agpat2	Signaling;Metabolism
DMR3:4040001	3	4040001	4041000	1000	1	1.30E-07	0.57	15	1.5	Egfl7;Mir126a;Mir126;Agpat2	Signaling;Metabolism
DMR3:4086001	3	4086001	4088000	2000	1	8.30E-07	0.38	22	1.1	Fam69b;LOC100911266;LOC681186	
DMR3:4090001	3	4090001	4091000	1000	1	2.10E-07	0.53	18	1.8	Fam69b;LOC100911266;LOC681186	
DMR3:5383001	3	5383001	5385000	2000	2	3.40E-11	0.69	48	2.4	Abo2	Golgi
DMR3:5537001	3	5537001	5539000	2000	1	7.30E-07	-0.37	35	1.75	Adamts13	Protease
DMR3:5601001	3	5601001	5602000	1000	1	6.70E-07	0.46	20	2	Tmem8c	
DMR3:5609001	3	5609001	5612000	3000	1	7.50E-12	0.69	60	2	Tmem8c	
DMR3:5640001	3	5640001	5643000	3000	1	4.10E-07	-0.35	40	1.33	Adamtsl2	Protease
DMR3:5651001	3	5651001	5653000	2000	1	3.40E-08	0.43	15	0.75	Adamtsl2;Fam163b	Protease
DMR3:5714001	3	5714001	5716000	2000	2	1.40E-07	0.43	36	1.8	Dbh	Metabolism
DMR3:5825001	3	5825001	5827000	2000	1	4.30E-08	0.54	48	2.4	Vav2	
DMR3:5828001	3	5828001	5832000	4000	1	9.30E-07	0.48	72	1.8	Vav2	

DMR3:5837001	3	5837001	5840000	3000	1	2.50E-08	0.56	59	1.97	Vav2	
DMR3:5847001	3	5847001	5849000	2000	1	4.00E-07	0.57	31	1.55	Vav2	
DMR3:5885001	3	5885001	5886000	1000	1	3.50E-09	0.57	11	1.1	Vav2	
DMR3:5953001	3	5953001	5957000	4000	1	4.70E-07	-0.39	59	1.48	Vav2	
DMR3:6268001	3	6268001	6269000	1000	1	8.90E-08	0.57	17	1.7	Rxra	Transcription
DMR3:6436001	3	6436001	6437000	1000	1	3.00E-09	0.37	7	0.7	Col5a1	Extracellular Matrix
DMR3:6440001	3	6440001	6441000	1000	1	5.40E-07	-0.3	20	2	Col5a1	Extracellular Matrix
DMR3:6463001	3	6463001	6468000	5000	1	3.60E-07	0.42	70	1.4	Col5a1	Extracellular Matrix
DMR3:6493001	3	6493001	6496000	3000	1	1.70E-07	0.45	54	1.8	Col5a1	Extracellular Matrix
DMR3:6507001	3	6507001	6508000	1000	1	1.10E-08	0.52	14	1.4	Col5a1	Extracellular Matrix
DMR3:6511001	3	6511001	6514000	3000	1	5.80E-08	0.49	40	1.33	Col5a1	Extracellular Matrix
DMR3:6525001	3	6525001	6529000	4000	1	4.90E-08	0.42	49	1.23	Col5a1	Extracellular Matrix
DMR3:6532001	3	6532001	6534000	2000	1	5.00E-07	0.58	23	1.15	Col5a1	Extracellular Matrix
DMR3:6558001	3	6558001	6560000	2000	1	1.50E-07	0.57	21	1.05	Col5a1	Extracellular Matrix
DMR3:6609001	3	6609001	6610000	1000	1	3.50E-07	-0.48	22	2.2	Fcnb	
DMR3:6788001	3	6788001	6791000	3000	1	8.30E-07	0.4	41	1.37	Olfm1	Development
DMR3:7119001	3	7119001	7123000	4000	1	8.80E-07	0.45	86	2.15	Ralgds	Transcription
DMR3:7131001	3	7131001	7138000	7000	1	1.10E-07	0.44	121	1.73	Ralgds;Cel;Gtf3c5	Transcription;Cytoskeleton;Transcription
DMR3:7268001	3	7268001	7273000	5000	1	7.60E-07	0.45	72	1.44	Tsc1;Spaca9;Ak8	Signaling
DMR3:7286001	3	7286001	7288000	2000	1	1.20E-07	0.65	26	1.3	Spaca9;Ak8	Signaling
DMR3:7386001	3	7386001	7388000	2000	1	2.80E-07	-0.48	46	2.3	Ak8	Signaling
DMR3:7504001	3	7504001	7505000	1000	1	6.70E-07	0.36	16	1.6	Barhl1;Cfap77	Development;Development
DMR3:7564001	3	7564001	7569000	5000	1	2.10E-08	0.47	75	1.5	Cfap77	Development
DMR3:7744001	3	7744001	7746000	2000	1	3.30E-10	0.56	32	1.6	Ntng2	Extracellular Matrix
DMR3:7775001	3	7775001	7776000	1000	1	2.30E-12	0.86	36	3.6	Ntng2	Extracellular Matrix
DMR3:7960001	3	7960001	7961000	1000	1	1.80E-07	0.59	20	2	Med27	Transcription
DMR3:8014001	3	8014001	8016000	2000	1	4.40E-07	0.45	28	1.4	Med27	Transcription
DMR3:8042001	3	8042001	8043000	1000	1	6.40E-13	0.5	11	1.1	Med27	Transcription
DMR3:8184001	3	8184001	8185000	1000	1	3.60E-07	0.52	15	1.5	Rapgef1	Transcription
DMR3:8189001	3	8189001	8191000	2000	1	4.70E-08	0.57	34	1.7	Rapgef1	Transcription
DMR3:8288001	3	8288001	8290000	2000	1	2.70E-07	0.47	23	1.15	Rapgef1	Transcription
DMR3:8296001	3	8296001	8301000	5000	1	7.10E-08	0.57	61	1.22	Rapgef1	Transcription
DMR3:8302001	3	8302001	8304000	2000	1	3.70E-08	0.52	34	1.7	Rapgef1;LOC102546892	Transcription
DMR3:8584001	3	8584001	8587000	3000	1	8.60E-07	-0.4	41	1.37	Sptan1	
DMR3:8644001	3	8644001	8651000	7000	1	7.10E-08	0.44	135	1.93	Pkn3;Zdhhc12	
DMR3:8731001	3	8731001	8735000	4000	1	4.90E-10	-0.57	28	0.7	Endog;LOC499770	
DMR3:8777001	3	8777001	8780000	3000	1	1.70E-08	0.48	46	1.53	Kyat1	Metabolism
DMR3:9040001	3	9040001	9042000	2000	1	8.20E-07	0.37	32	1.6	Ier5l	
DMR3:9253001	3	9253001	9254000	1000	1	3.90E-07	0.37	21	2.1	Aif1l	Signaling
DMR3:9266001	3	9266001	9268000	2000	1	2.80E-07	0.6	31	1.55	Aif1l;Lamc3	Signaling;Extracellular Matrix
DMR3:9292001	3	9292001	9295000	3000	1	7.20E-09	0.44	56	1.87	Lamc3	Extracellular Matrix
DMR3:9620001	3	9620001	9621000	1000	1	8.70E-07	0.51	25	2.5	RGD1311084	
DMR3:9695001	3	9695001	9696000	1000	1	9.60E-08	0.52	16	1.6	Prrx2	
DMR3:9722001	3	9722001	9727000	5000	1	2.80E-07	-0.52	74	1.48	Prrx2;Ptges	Transport
DMR3:9827001	3	9827001	9832000	5000	1	8.30E-15	-0.56	78	1.56	RGD1305178;Usp20	Protease
DMR3:9836001	3	9836001	9839000	3000	1	9.80E-08	0.36	43	1.43	Usp20	Protease
DMR3:9852001	3	9852001	9853000	1000	1	2.80E-08	0.51	16	1.6	Usp20	Protease
DMR3:9867001	3	9867001	9873000	6000	1	9.30E-13	0.89	143	2.38	Fnbp1	
DMR3:9886001	3	9886001	9891000	5000	1	4.10E-11	-0.39	116	2.32	Fnbp1	
DMR3:9994001	3	9994001	9997000	3000	1	2.50E-08	-0.46	45	1.5	Fibcd1l1	
DMR3:10315001	3	10315001	10318000	3000	2	3.90E-11	0.7	53	1.77	Ass1	Metabolism
DMR3:10369001	3	10369001	10370000	1000	1	2.50E-09	0.52	12	1.2	Ass1	Metabolism
DMR3:10399001	3	10399001	10401000	2000	1	1.20E-08	0.6	24	1.2	Hmcn2	
DMR3:10525001	3	10525001	10527000	2000	1	1.90E-10	-0.47	55	2.75	Hmcn2	
DMR3:10557001	3	10557001	10558000	1000	1	1.60E-08	-0.45	38	3.8	LOC108350350;Ncs1	
DMR3:10597001	3	10597001	10601000	4000	1	1.80E-13	0.49	62	1.55	Ncs1;LOC103691761	
DMR3:11024001	3	11024001	11026000	2000	1	3.50E-07	-0.38	47	2.35	Nup214	Transport
DMR3:11074001	3	11074001	11076000	2000	1	6.10E-09	0.51	19	0.95	Nup214;LOC102555629	Transport

DMR3:11080001	3	11080001	11083000	3000	1	2.20E-07	0.45	65	2.17	Nup214;LOC102555629;Fam78a	Transport
DMR3:11094001	3	11094001	11098000	4000	1	3.20E-07	0.44	63	1.57	Fam78a	
DMR3:11116001	3	11116001	11118000	2000	1	1.20E-09	0.67	26	1.3	Plpp7	Signaling
DMR3:11128001	3	11128001	11129000	1000	1	1.80E-08	0.58	20	2	Plpp7	Signaling
DMR3:11169001	3	11169001	11177000	8000	2	5.50E-16	-0.68	188	2.35	Prrc2b	Metabolism
DMR3:11194001	3	11194001	11196000	2000	1	6.80E-08	-0.4	46	2.3	Prrc2b	Metabolism
DMR3:11369001	3	11369001	11371000	2000	1	7.30E-07	0.5	39	1.95	Dnm1	Transport
DMR3:11394001	3	11394001	11396000	2000	1	5.40E-07	-0.33	31	1.55	Ciz1	
DMR3:11402001	3	11402001	11404000	2000	1	1.40E-09	0.54	34	1.7	Ciz1;RGD1561113	
DMR3:11624001	3	11624001	11628000	4000	1	6.30E-09	0.52	71	1.77	St6galnac4;St6galnac6	
DMR3:11636001	3	11636001	11637000	1000	1	6.90E-09	0.64	26	2.6	St6galnac6	
DMR3:11707001	3	11707001	11709000	2000	1	2.40E-13	0.86	45	2.25	Eng;Fpgs	Receptor;Metabolism
DMR3:11800001	3	11800001	11802000	2000	1	9.70E-07	0.39	19	0.95	Sh2d3c;Tor2a;Ttc16;Ptrh1	Transcription;Metabolism
DMR3:11809001	3	11809001	11811000	2000	1	5.00E-08	0.52	36	1.8	Tor2a;Ttc16;Ptrh1;Cfap157	Transcription;Metabolism;Development
DMR3:11913001	3	11913001	11915000	2000	2	3.10E-11	-0.56	35	1.75	Fam129b	
DMR3:11949001	3	11949001	11951000	2000	1	5.00E-07	-0.36	41	2.05	Fam129b	
DMR3:11962001	3	11962001	11965000	3000	1	9.70E-08	-0.46	59	1.97	Fam129b;Lrsam1	
DMR3:11986001	3	11986001	11988000	2000	1	1.30E-07	0.65	42	2.1	Lrsam1	
DMR3:12022001	3	12022001	12023000	1000	1	7.20E-09	0.55	12	1.2	Slc2a8	
DMR3:12057001	3	12057001	12061000	4000	1	1.80E-07	0.59	62	1.55	Garnl3	Signaling
DMR3:12191001	3	12191001	12192000	1000	1	9.10E-07	0.5	12	1.2	Ralgps1	Transcription
DMR3:12672001	3	12672001	12676000	4000	1	3.30E-09	-0.54	60	1.5	Lmx1b	Development
DMR3:12767001	3	12767001	12773000	6000	2	2.60E-08	-0.42	114	1.9	Mvb12b	
DMR3:12821001	3	12821001	12824000	3000	1	2.10E-08	0.64	35	1.17	Mvb12b	
DMR3:13239001	3	13239001	13240000	1000	1	2.60E-07	0.56	11	1.1	Pbx3	Development
DMR3:13337001	3	13337001	13338000	1000	1	1.90E-08	-0.45	6	0.6	Pbx3	Development
DMR3:13513001	3	13513001	13515000	2000	1	1.20E-10	-0.67	13	0.65	Mapkap1;LOC100911753	Cytoskeleton
DMR3:13741001	3	13741001	13745000	4000	1	3.90E-07	-0.37	55	1.38	Gapvd1	Transcription
DMR3:13840001	3	13840001	13843000	3000	1	3.20E-12	-0.54	40	1.33	Hspa5;Rabepk	
DMR3:13871001	3	13871001	13872000	1000	1	2.20E-07	0.61	12	1.2	Rabepk;Fbxw2	
DMR3:13921001	3	13921001	13925000	4000	1	3.80E-07	-0.39	75	1.88	Psmd5	Protease
DMR3:14006001	3	14006001	14007000	1000	1	1.20E-07	0.31	12	1.2	Traf1	Cytoskeleton
DMR3:14045001	3	14045001	14048000	3000	1	2.50E-08	-0.36	43	1.43	C5	
DMR3:14063001	3	14063001	14065000	2000	1	8.20E-09	0.45	21	1.05	C5	
DMR3:14066001	3	14066001	14068000	2000	1	4.90E-15	0.71	25	1.25	C5	
DMR3:14208001	3	14208001	14210000	2000	1	1.30E-09	0.64	47	2.35	LOC100912221;C5	
DMR3:14213001	3	14213001	14219000	6000	1	6.50E-12	0.58	78	1.3	LOC100912221;C5	
DMR3:14231001	3	14231001	14234000	3000	1	2.70E-07	-0.3	20	0.67	C5	
DMR3:14347001	3	14347001	14350000	3000	1	6.00E-10	-0.42	43	1.43	Cntrl	
DMR3:14479001	3	14479001	14487000	8000	1	1.20E-08	0.51	153	1.91	Gsn	Cytoskeleton
DMR3:14488001	3	14488001	14490000	2000	1	2.50E-11	0.43	20	1	Gsn	Cytoskeleton
DMR3:14509001	3	14509001	14511000	2000	1	4.50E-07	0.47	38	1.9	Gsn;Stom	Cytoskeleton;Cytoskeleton
DMR3:14539001	3	14539001	14542000	3000	1	1.90E-07	0.4	41	1.37	Stom	Cytoskeleton
DMR3:14567001	3	14567001	14569000	2000	1	1.00E-12	1.03	47	2.35	Ggta1l1;LOC102548933	Golgi
DMR3:14614001	3	14614001	14616000	2000	1	4.00E-08	-0.47	29	1.45	LOC502618;Ggta1	Golgi
DMR3:14659001	3	14659001	14664000	5000	1	3.40E-07	0.36	69	1.38	Ggta1	Golgi
DMR3:14675001	3	14675001	14677000	2000	1	1.90E-09	0.42	40	2	Ggta1	Golgi
DMR3:14708001	3	14708001	14709000	1000	1	2.00E-07	0.54	13	1.3	Ggta1	Golgi
DMR3:14885001	3	14885001	14889000	4000	2	4.70E-09	0.44	44	1.1	Dab2ip	Signaling
DMR3:14894001	3	14894001	14898000	4000	1	1.20E-07	0.46	45	1.12	Dab2ip	Signaling
DMR3:14927001	3	14927001	14930000	3000	1	3.10E-07	0.61	39	1.3	Dab2ip;LOC108350356	Signaling
DMR3:15025001	3	15025001	15031000	6000	2	1.70E-08	0.57	55	0.92	Dab2ip	Signaling
DMR3:15100001	3	15100001	15107000	7000	1	5.50E-07	-0.4	104	1.49	Ttll11	Cytoskeleton
DMR3:15122001	3	15122001	15124000	2000	1	3.30E-10	-0.45	57	2.85	Ttll11	Cytoskeleton
DMR3:15163001	3	15163001	15168000	5000	1	2.40E-08	-0.39	96	1.92	Ttll11	Cytoskeleton
DMR3:15199001	3	15199001	15201000	2000	1	2.50E-07	0.44	44	2.2	Ttll11;LOC108350357	Cytoskeleton
DMR3:15305001	3	15305001	15307000	2000	1	5.40E-07	-0.37	32	1.6	Ttll11	Cytoskeleton
DMR3:15356001	3	15356001	15357000	1000	1	9.50E-07	-0.38	22	2.2	LOC108350358;Ndufa8	Metabolism

DMR3:15647001	3	15647001	15649000	2000	1	2.00E-07	-0.29	16	0.8	Olr394-ps	
DMR3:15684001	3	15684001	15685000	1000	1	2.70E-10	-0.69	2	0.2	Olr395	Receptor
DMR3:15698001	3	15698001	15700000	2000	1	5.30E-08	-0.26	16	0.8	Olr395	Receptor
DMR3:15772001	3	15772001	15773000	1000	1	3.90E-09	0.5	63	6.3	Klf5-ps1	
DMR3:16043001	3	16043001	16048000	5000	1	2.30E-11	-0.42	45	0.9	Olr403	Receptor
DMR3:21564001	3	21564001	21566000	2000	2	8.30E-20	1.24	59	2.95	LOC100911251;Klf5-ps2	
DMR3:21694001	3	21694001	21696000	2000	1	4.90E-07	-0.41	20	1	Zbtb6;LOC108350365;Zbtb26;Rabgap1	Transcription;Signaling
DMR3:21759001	3	21759001	21760000	1000	1	3.70E-07	-0.47	7	0.7	Rabgap1;Gpr21	Signaling;Signaling
DMR3:21866001	3	21866001	21871000	5000	1	1.70E-07	-0.62	19	0.38	Strbp	Metabolism
DMR3:21984001	3	21984001	21985000	1000	1	8.20E-14	0.85	25	2.5	RGD1561137	
DMR3:22038001	3	22038001	22039000	1000	1	3.50E-18	1.05	35	3.5	Crb2	Cytoskeleton
DMR3:22053001	3	22053001	22054000	1000	1	9.00E-07	0.5	21	2.1	Crb2;Dennd1a	Cytoskeleton
DMR3:22072001	3	22072001	22073000	1000	1	3.50E-07	0.56	9	0.9	Dennd1a	
DMR3:22083001	3	22083001	22084000	1000	1	1.10E-12	0.6	12	1.2	Dennd1a	
DMR3:22116001	3	22116001	22117000	1000	1	2.10E-07	0.56	12	1.2	Dennd1a	
DMR3:22328001	3	22328001	22329000	1000	1	1.80E-07	0.36	8	0.8	Dennd1a	
DMR3:22443001	3	22443001	22445000	2000	1	4.70E-07	-0.54	10	0.5	Dennd1a	
DMR3:22467001	3	22467001	22468000	1000	1	2.90E-08	-0.52	6	0.6	Dennd1a	
DMR3:22623001	3	22623001	22624000	1000	1	9.50E-17	-0.93	2	0.2	Lhx2	Development
DMR3:22848001	3	22848001	22849000	1000	1	1.20E-09	0.45	10	1	Nek6	
DMR3:22851001	3	22851001	22852000	1000	1	7.10E-07	0.45	22	2.2	Nek6	
DMR3:23014001	3	23014001	23015000	1000	1	2.20E-09	0.78	28	2.8	Nr5a1	Transcription
DMR3:23020001	3	23020001	23021000	1000	1	4.20E-07	0.54	17	1.7	Nr5a1;LOC102554503;Nr6a1	Transcription
DMR3:25277001	3	25277001	25279000	2000	1	1.80E-07	-0.44	13	0.65	Lrp1b	
DMR3:25708001	3	25708001	25709000	1000	1	1.30E-07	-0.6	3	0.3	Lrp1b	
DMR3:25883001	3	25883001	25886000	3000	1	1.30E-08	-0.64	9	0.3	Lrp1b	
DMR3:26402001	3	26402001	26405000	3000	1	1.20E-07	0.35	34	1.13	Lrp1b	
DMR3:26443001	3	26443001	26444000	1000	1	2.30E-07	0.57	12	1.2	Lrp1b	
DMR3:26461001	3	26461001	26463000	2000	1	2.80E-07	-0.51	4	0.2	Lrp1b	
DMR3:26621001	3	26621001	26626000	5000	1	5.60E-07	-0.33	50	1	Lrp1b	
DMR3:27005001	3	27005001	27007000	2000	1	2.00E-07	-0.51	7	0.35	Lrp1b	
DMR3:27161001	3	27161001	27162000	1000	1	1.30E-15	0.96	11	1.1	Lrp1b	
DMR3:27345001	3	27345001	27346000	1000	1	1.20E-07	0.57	14	1.4	Lrp1b	
DMR3:28486001	3	28486001	28488000	2000	1	6.50E-08	0.49	23	1.15	Kynu	Metabolism
DMR3:28626001	3	28626001	28627000	1000	1	5.50E-10	0.45	18	1.8	Arhgap15	Signaling
DMR3:28690001	3	28690001	28692000	2000	1	8.00E-10	0.77	30	1.5	Arhgap15	Signaling
DMR3:28733001	3	28733001	28734000	1000	1	7.00E-07	-0.46	11	1.1	Arhgap15	Signaling
DMR3:28774001	3	28774001	28776000	2000	1	6.50E-09	-0.48	35	1.75	Arhgap15	Signaling
DMR3:28788001	3	28788001	28789000	1000	1	9.90E-08	-0.5	10	1	Arhgap15	Signaling
DMR3:28875001	3	28875001	28876000	1000	1	1.80E-09	-0.42	11	1.1	Arhgap15	Signaling
DMR3:28943001	3	28943001	28945000	2000	1	1.10E-08	-0.49	20	1	Arhgap15	Signaling
DMR3:29064001	3	29064001	29067000	3000	1	1.50E-07	-0.33	29	0.97	Arhgap15	Signaling
DMR3:29109001	3	29109001	29110000	1000	1	3.10E-07	-0.36	9	0.9	Arhgap15	Signaling
DMR3:29111001	3	29111001	29112000	1000	1	2.00E-07	-0.55	7	0.7	Arhgap15	Signaling
DMR3:29127001	3	29127001	29130000	3000	1	4.50E-07	0.49	39	1.3	Arhgap15	Signaling
DMR3:29182001	3	29182001	29183000	1000	1	5.30E-07	-0.38	13	1.3	Arhgap15	Signaling
DMR3:29515001	3	29515001	29516000	1000	1	4.90E-08	-0.82	2	0.2	Gtdc1;LOC108350367	Golgi
DMR3:29646001	3	29646001	29647000	1000	1	3.90E-09	0.43	8	0.8	Gtdc1	Golgi
DMR3:29669001	3	29669001	29672000	3000	1	1.50E-07	-0.37	36	1.2	Gtdc1	Golgi
DMR3:29853001	3	29853001	29855000	2000	1	1.00E-08	0.53	29	1.45	Zeb2	Transcription
DMR3:29862001	3	29862001	29863000	1000	1	7.20E-08	0.73	39	3.9	Zeb2	Transcription
DMR3:29928001	3	29928001	29932000	4000	1	8.80E-07	0.43	59	1.48	Zeb2	Transcription
DMR3:33356001	3	33356001	33358000	2000	1	3.50E-07	-0.37	19	0.95	Mbd5	
DMR3:33396001	3	33396001	33398000	2000	1	5.80E-08	-0.55	30	1.5	Mbd5	
DMR3:33651001	3	33651001	33652000	1000	1	2.40E-08	-0.44	8	0.8	LOC108350553;Epc2	Epigenetic
DMR3:33676001	3	33676001	33681000	5000	1	1.70E-07	-0.38	48	0.96	Epc2	Epigenetic
DMR3:33691001	3	33691001	33699000	8000	1	5.40E-20	0.74	89	1.11	Epc2	Epigenetic
DMR3:33707001	3	33707001	33709000	2000	2	1.30E-09	-0.53	15	0.75	Epc2	Epigenetic
DMR3:33719001	3	33719001	33720000	1000	1	6.80E-08	-0.45	9	0.9	Epc2	Epigenetic
DMR3:35081001	3	35081001	35082000	1000	1	1.90E-07	0.62	15	1.5	Kif5c	
DMR3:35205001	3	35205001	35207000	2000	1	3.50E-07	-0.41	32	1.6	Kif5c;LOC102556679	

DMR3:35420001	3	35420001	35422000	2000	1	2.90E-07	-0.4	16	0.8	Lypd6b	
DMR3:35540001	3	35540001	35541000	1000	1	5.70E-11	-0.42	25	2.5	Ns5atp4	
DMR3:35600001	3	35600001	35603000	3000	1	3.00E-08	-0.65	25	0.83	Lypd6	
DMR3:35668001	3	35668001	35670000	2000	1	7.10E-07	-0.43	23	1.15	Lypd6;LOC102555146	
DMR3:35697001	3	35697001	35702000	5000	1	6.80E-07	-0.34	84	1.68	Lypd6	
DMR3:35703001	3	35703001	35705000	2000	1	2.30E-11	-0.45	28	1.4	Lypd6	
DMR3:37149001	3	37149001	37150000	1000	1	1.30E-07	0.66	25	2.5	Tas2r134	Receptor
DMR3:37476001	3	37476001	37478000	2000	1	8.60E-12	-0.48	33	1.65	Nmi	Transcription
DMR3:37774001	3	37774001	37777000	3000	1	6.30E-07	-0.3	30	1	Neb	
DMR3:37834001	3	37834001	37835000	1000	1	5.00E-08	0.4	11	1.1	Neb	
DMR3:37941001	3	37941001	37944000	3000	1	3.40E-12	-0.41	28	0.93	Arl5a;Cacnb4	Signaling;Transport
DMR3:38003001	3	38003001	38004000	1000	1	3.10E-08	-0.35	22	2.2	Cacnb4	Transport
DMR3:38013001	3	38013001	38016000	3000	1	3.80E-11	-0.44	53	1.77	Cacnb4	Transport
DMR3:38070001	3	38070001	38074000	4000	1	1.10E-08	0.68	70	1.75	Cacnb4	Transport
DMR3:38143001	3	38143001	38146000	3000	1	2.50E-07	-0.39	67	2.23	Cacnb4	Transport
DMR3:38159001	3	38159001	38162000	3000	1	4.10E-08	0.52	38	1.27	Cacnb4	Transport
DMR3:38170001	3	38170001	38172000	2000	1	1.10E-09	-0.52	29	1.45	Cacnb4	Transport
DMR3:38197001	3	38197001	38200000	3000	1	9.60E-09	-0.69	19	0.63	Cacnb4	Transport
DMR3:38204001	3	38204001	38206000	2000	1	1.70E-07	0.59	58	2.9	Cacnb4	Transport
DMR3:38402001	3	38402001	38403000	1000	1	2.50E-07	0.52	12	1.2	RGD1560248	
DMR3:38411001	3	38411001	38412000	1000	1	2.20E-10	-0.48	19	1.9	RGD1560248	
DMR3:38597001	3	38597001	38599000	2000	1	1.90E-09	-0.61	39	1.95	RGD1560248	
DMR3:38629001	3	38629001	38633000	4000	1	1.80E-08	0.41	36	0.9	RGD1560248	
DMR3:38640001	3	38640001	38641000	1000	1	2.50E-07	-0.5	15	1.5	RGD1560248	
DMR3:38707001	3	38707001	38708000	1000	1	2.10E-08	-0.32	12	1.2	Prpf40a	
DMR3:39973001	3	39973001	39974000	1000	1	3.30E-09	-0.44	13	1.3	Galnt13	Golgi
DMR3:40010001	3	40010001	40012000	2000	1	6.10E-07	-0.47	12	0.6	Galnt13	Golgi
DMR3:40040001	3	40040001	40042000	2000	1	1.40E-07	-0.34	16	0.8	Galnt13	Golgi
DMR3:40120001	3	40120001	40121000	1000	1	7.20E-07	0.58	4	0.4	Galnt13	Golgi
DMR3:40258001	3	40258001	40259000	1000	1	4.10E-08	0.55	6	0.6	Galnt13	Golgi
DMR3:40270001	3	40270001	40272000	2000	1	9.40E-07	-0.42	7	0.35	Galnt13	Golgi
DMR3:40630001	3	40630001	40632000	2000	1	6.10E-10	0.63	18	0.9	Galnt13	Golgi
DMR3:41017001	3	41017001	41018000	1000	1	4.80E-08	-0.54	11	1.1	LOC108350374;Kcnj3	Transport
DMR3:41035001	3	41035001	41037000	2000	1	4.10E-10	-0.47	20	1	Kcnj3	Transport
DMR3:41146001	3	41146001	41147000	1000	1	1.50E-07	-0.44	4	0.4	Kcnj3	Transport
DMR3:43347001	3	43347001	43349000	2000	1	1.80E-07	-0.36	49	2.45	Gpd2	Metabolism
DMR3:43355001	3	43355001	43356000	1000	1	2.30E-07	-0.4	19	1.9	Gpd2	Metabolism
DMR3:44178001	3	44178001	44179000	1000	1	3.00E-18	-0.61	23	2.3	Cytip	
DMR3:44303001	3	44303001	44305000	2000	1	2.20E-09	-0.68	18	0.9	Acvr1c	Signaling
DMR3:44310001	3	44310001	44312000	2000	1	1.60E-07	0.46	35	1.75	Acvr1c	Signaling
DMR3:44436001	3	44436001	44439000	3000	1	6.30E-13	-0.44	31	1.03	Acvr1	Signaling
DMR3:44503001	3	44503001	44504000	1000	1	3.20E-07	-0.45	11	1.1	Acvr1	Signaling
DMR3:44538001	3	44538001	44539000	1000	1	8.20E-08	-0.35	16	1.6	Acvr1;LOC103691813	Signaling
DMR3:44993001	3	44993001	44994000	1000	1	5.70E-07	-0.36	4	0.4	Ccdc148	
DMR3:45080001	3	45080001	45081000	1000	1	6.40E-09	0.78	22	2.2	Ccdc148	
DMR3:45123001	3	45123001	45125000	2000	1	4.00E-08	-0.4	20	1	Ccdc148	
DMR3:45128001	3	45128001	45129000	1000	1	7.90E-10	-0.28	7	0.7	Ccdc148	
DMR3:45291001	3	45291001	45292000	1000	1	1.80E-08	-0.51	10	1	Pkp4	Cytoskeleton
DMR3:45344001	3	45344001	45350000	6000	1	2.50E-09	0.54	96	1.6	Pkp4	Cytoskeleton
DMR3:45673001	3	45673001	45674000	1000	1	1.30E-07	-0.55	7	0.7	Tanc1	
DMR3:45689001	3	45689001	45690000	1000	1	1.10E-07	-0.46	21	2.1	Tanc1	
DMR3:45787001	3	45787001	45789000	2000	1	5.20E-07	-0.37	41	2.05	Tanc1	
DMR3:45796001	3	45796001	45797000	1000	1	9.60E-08	-0.36	25	2.5	Tanc1	
DMR3:45826001	3	45826001	45832000	6000	1	1.50E-07	-0.39	113	1.88	Tanc1	
DMR3:45845001	3	45845001	45847000	2000	1	1.10E-08	-0.43	45	2.25	Tanc1	
DMR3:45978001	3	45978001	45984000	6000	1	1.80E-07	-0.44	120	2	Baz2b	Epigenetic
DMR3:46165001	3	46165001	46168000	3000	2	5.10E-07	-0.34	49	1.63	Baz2b	Epigenetic
DMR3:46220001	3	46220001	46226000	6000	1	4.50E-09	-0.48	36	0.6	Baz2b;LOC100361645;LOC102551618	Epigenetic;Transcription
DMR3:46264001	3	46264001	46266000	2000	1	1.10E-12	-0.47	34	1.7	Baz2b	Epigenetic
DMR3:46338001	3	46338001	46340000	2000	1	4.00E-13	0.66	18	0.9	Cd302	
DMR3:46364001	3	46364001	46366000	2000	1	2.90E-15	0.81	48	2.4	Cd302;Mir6216;Ly75	

DMR3:46373001	3	46373001	46374000	1000	1	7.00E-07	-0.3	20	2	Ly75	
DMR3:46416001	3	46416001	46417000	1000	1	4.10E-07	0.45	12	1.2	Ly75	
DMR3:46512001	3	46512001	46515000	3000	1	1.70E-08	0.72	62	2.07	LOC108350581;Pla2r1	
DMR3:46524001	3	46524001	46527000	3000	1	6.70E-07	-0.4	30	1	Pla2r1	
DMR3:46708001	3	46708001	46711000	3000	1	9.50E-08	0.59	50	1.67	Itgb6	Extracellular Matrix
DMR3:46726001	3	46726001	46728000	2000	1	8.00E-07	-0.46	42	2.1	Itgb6	Extracellular Matrix
DMR3:46730001	3	46730001	46732000	2000	2	2.30E-08	-0.38	34	1.7	Itgb6;LOC108350379	Extracellular Matrix
DMR3:46752001	3	46752001	46756000	4000	1	6.30E-07	-0.36	53	1.32	Itgb6;LOC108350379	Extracellular Matrix
DMR3:46834001	3	46834001	46835000	1000	1	6.20E-08	0.63	16	1.6	Rbms1	
DMR3:46853001	3	46853001	46855000	2000	1	5.20E-08	-0.42	49	2.45	Rbms1	
DMR3:46936001	3	46936001	46938000	2000	1	4.70E-09	-0.52	18	0.9	Rbms1	
DMR3:46982001	3	46982001	46985000	3000	1	6.60E-08	-0.28	30	1	Rbms1	
DMR3:47014001	3	47014001	47015000	1000	1	8.20E-07	0.56	14	1.4	Rbms1	
DMR3:47444001	3	47444001	47447000	3000	2	7.70E-08	-0.4	25	0.83	Tank	
DMR3:47448001	3	47448001	47449000	1000	1	1.20E-11	0.89	20	2	Tank	
DMR3:47516001	3	47516001	47517000	1000	1	6.20E-07	0.57	25	2.5	Tank	
DMR3:48045001	3	48045001	48046000	1000	1	2.00E-08	-0.45	13	1.3	Slc4a10	Transport
DMR3:48268001	3	48268001	48270000	2000	1	4.30E-11	-0.61	46	2.3	Slc4a10	Transport
DMR3:48487001	3	48487001	48489000	2000	1	3.00E-10	-0.51	12	0.6	Fap	Protease
DMR3:48531001	3	48531001	48535000	4000	1	8.30E-12	0.59	47	1.18	Fap	Protease
DMR3:48691001	3	48691001	48692000	1000	1	7.70E-07	-0.44	15	1.5	Kcnh7	Transport
DMR3:48781001	3	48781001	48782000	1000	1	4.50E-07	0.51	18	1.8	Kcnh7	Transport
DMR3:48869001	3	48869001	48870000	1000	1	5.30E-08	0.46	11	1.1	Kcnh7	Transport
DMR3:49994001	3	49994001	49995000	1000	1	1.20E-19	1.05	31	3.1	Fign	Cytoskeleton
DMR3:51001001	3	51001001	51003000	2000	1	4.30E-08	0.49	36	1.8	Grb14	Cytoskeleton
DMR3:51150001	3	51150001	51157000	7000	2	2.50E-08	-0.3	82	1.17	Cobll1	
DMR3:51260001	3	51260001	51261000	1000	1	7.80E-07	0.33	5	0.5	Cobll1	
DMR3:51594001	3	51594001	51595000	1000	1	7.40E-09	-0.41	14	1.4	Scn3a	Transport
DMR3:51605001	3	51605001	51606000	1000	1	3.10E-07	0.41	8	0.8	Scn3a	Transport
DMR3:51608001	3	51608001	51610000	2000	1	4.80E-07	0.35	40	2	Scn3a	Transport
DMR3:51683001	3	51683001	51684000	1000	1	2.00E-14	0.81	23	2.3	Scn2a	Transport
DMR3:51758001	3	51758001	51759000	1000	1	3.30E-13	0.93	20	2	Scn2a	Transport
DMR3:51815001	3	51815001	51816000	1000	1	7.90E-07	-0.47	7	0.7	Scn2a	Transport
DMR3:51928001	3	51928001	51929000	1000	1	4.90E-07	-0.48	5	0.5	Csrnp3	
DMR3:51994001	3	51994001	51997000	3000	1	1.60E-07	0.75	53	1.77	Csrnp3	
DMR3:52042001	3	52042001	52044000	2000	1	1.70E-08	0.38	24	1.2	Csrnp3	
DMR3:52055001	3	52055001	52058000	3000	1	2.30E-10	-0.25	19	0.63	Csrnp3	
DMR3:52061001	3	52061001	52063000	2000	1	2.60E-17	0.59	44	2.2	Csrnp3	
DMR3:52065001	3	52065001	52071000	6000	3	1.60E-17	0.72	48	0.8	Csrnp3	
DMR3:52192001	3	52192001	52193000	1000	1	1.90E-07	-0.49	18	1.8	Galnt3	Golgi
DMR3:52282001	3	52282001	52284000	2000	1	1.60E-07	-0.41	25	1.25	Ttc21b	
DMR3:52315001	3	52315001	52317000	2000	1	2.60E-07	-0.37	30	1.5	Ttc21b;LOC108350386	
DMR3:52426001	3	52426001	52428000	2000	1	2.00E-08	-0.53	5	0.25	Scn1a	Transport
DMR3:52442001	3	52442001	52447000	5000	1	2.60E-10	-0.43	81	1.62	Scn1a	Transport
DMR3:52476001	3	52476001	52479000	3000	1	3.90E-08	-0.53	17	0.57	Scn1a	Transport
DMR3:52601001	3	52601001	52602000	1000	1	4.40E-08	0.52	7	0.7	LOC108350387;Scn9a	Transport
DMR3:52658001	3	52658001	52659000	1000	1	2.60E-11	0.55	11	1.1	Scn9a	Transport
DMR3:52998001	3	52998001	52999000	1000	1	1.10E-09	0.79	24	2.4	RGD1560831	
DMR3:53589001	3	53589001	53590000	1000	1	2.30E-08	-0.41	18	1.8	Xirp2	Cytoskeleton
DMR3:53611001	3	53611001	53612000	1000	1	1.70E-09	-0.53	9	0.9	Xirp2	Cytoskeleton
DMR3:53642001	3	53642001	53643000	1000	1	1.60E-08	0.79	22	2.2	Xirp2	Cytoskeleton
DMR3:53760001	3	53760001	53762000	2000	1	9.70E-13	0.8	29	1.45	B3galt1	Golgi
DMR3:53916001	3	53916001	53919000	3000	1	2.20E-08	-0.57	19	0.63	B3galt1	Golgi
DMR3:54056001	3	54056001	54059000	3000	1	3.20E-08	-0.27	22	0.73	B3galt1	Golgi
DMR3:54251001	3	54251001	54253000	2000	1	4.00E-10	0.5	27	1.35	B3galt1	Golgi
DMR3:54259001	3	54259001	54260000	1000	1	7.00E-12	0.84	14	1.4	B3galt1	Golgi
DMR3:54381001	3	54381001	54383000	2000	1	9.40E-07	0.53	33	1.65	Stk39;LOC100911723	
DMR3:54505001	3	54505001	54507000	2000	1	4.50E-07	-0.34	26	1.3	Stk39	
DMR3:54515001	3	54515001	54516000	1000	1	2.80E-09	-0.54	13	1.3	Stk39	
DMR3:54530001	3	54530001	54534000	4000	1	7.10E-07	-0.26	35	0.88	Stk39	
DMR3:54544001	3	54544001	54545000	1000	1	2.90E-08	-0.54	20	2	Stk39	
DMR3:54560001	3	54560001	54563000	3000	2	2.00E-12	-0.47	51	1.7	Stk39	

DMR3:54565001	3	54565001	54570000	5000	1	9.70E-09	-0.44	106	2.12	Stk39	
DMR3:54593001	3	54593001	54598000	5000	1	1.30E-08	-0.42	78	1.56	Stk39	
DMR3:55179001	3	55179001	55180000	1000	1	5.20E-08	-0.38	12	1.2	Cers6	
DMR3:55210001	3	55210001	55211000	1000	1	3.50E-09	-0.37	28	2.8	Cers6	
DMR3:55398001	3	55398001	55399000	1000	1	3.20E-07	-0.57	9	0.9	Nostrin	
DMR3:55557001	3	55557001	55560000	3000	1	4.40E-07	0.38	32	1.07	Abcb11	Transport
DMR3:55589001	3	55589001	55592000	3000	1	1.80E-12	-0.53	40	1.33	Abcb11	Transport
DMR3:55664001	3	55664001	55665000	1000	1	4.80E-07	-0.46	10	1	Lrp2	
DMR3:55690001	3	55690001	55694000	4000	1	9.10E-16	-0.49	85	2.12	Lrp2	
DMR3:55723001	3	55723001	55724000	1000	1	6.20E-11	-0.56	16	1.6	Lrp2	
DMR3:55779001	3	55779001	55782000	3000	2	2.60E-08	-0.6	37	1.23	Lrp2	
DMR3:55824001	3	55824001	55825000	1000	1	8.20E-07	0.51	24	2.4	Lrp2	
DMR3:55986001	3	55986001	55988000	2000	1	1.40E-08	-0.39	33	1.65	Ppig;Ccdc173	Transcription
DMR3:56069001	3	56069001	56073000	4000	1	2.80E-10	-0.6	66	1.65	Klhl23	Cytoskeleton
DMR3:56137001	3	56137001	56139000	2000	1	8.20E-07	-0.44	14	0.7	Ubr3	Proteolysis
DMR3:56191001	3	56191001	56192000	1000	1	7.60E-10	-0.57	4	0.4	Ubr3	Proteolysis
DMR3:56207001	3	56207001	56208000	1000	1	8.20E-12	-0.53	24	2.4	Ubr3	Proteolysis
DMR3:56419001	3	56419001	56421000	2000	1	5.70E-08	0.43	29	1.45	Myo3b	
DMR3:56438001	3	56438001	56441000	3000	1	3.20E-07	-0.41	15	0.5	Myo3b	
DMR3:56492001	3	56492001	56495000	3000	1	2.20E-08	-0.66	12	0.4	Myo3b	
DMR3:56499001	3	56499001	56501000	2000	1	9.70E-07	-0.4	24	1.2	Myo3b	
DMR3:56513001	3	56513001	56514000	1000	1	1.50E-08	0.61	26	2.6	Myo3b	
DMR3:56528001	3	56528001	56532000	4000	1	4.80E-07	-0.5	42	1.05	Myo3b	
DMR3:56533001	3	56533001	56535000	2000	1	2.90E-07	0.3	21	1.05	Myo3b	
DMR3:56603001	3	56603001	56606000	3000	1	3.60E-07	-0.45	47	1.57	Myo3b	
DMR3:56638001	3	56638001	56640000	2000	1	1.00E-11	0.45	18	0.9	Myo3b	
DMR3:56641001	3	56641001	56643000	2000	1	4.50E-09	-0.67	22	1.1	Myo3b	
DMR3:56712001	3	56712001	56715000	3000	1	5.40E-09	-0.44	36	1.2	Myo3b	
DMR3:56716001	3	56716001	56718000	2000	1	4.20E-07	-0.39	50	2.5	Myo3b	
DMR3:56726001	3	56726001	56727000	1000	1	4.10E-12	-0.67	3	0.3	Myo3b	
DMR3:56799001	3	56799001	56801000	2000	2	1.70E-12	-0.66	17	0.85	Erich2;LOC103691815	
DMR3:56838001	3	56838001	56839000	1000	1	1.90E-07	-0.46	17	1.7	Erich2	
DMR3:57017001	3	57017001	57018000	1000	1	1.30E-10	-1	14	1.4	Tlk1	Signaling
DMR3:57139001	3	57139001	57147000	8000	1	3.60E-11	-0.52	121	1.51	Tlk1	Signaling
DMR3:57185001	3	57185001	57189000	4000	1	3.20E-07	-0.42	65	1.62	Tlk1;RGD1565767	Signaling;Translation
DMR3:57195001	3	57195001	57197000	2000	1	4.40E-07	-0.37	55	2.75	Tlk1;RGD1565767	Signaling;Translation
DMR3:57213001	3	57213001	57214000	1000	1	5.20E-07	-0.37	11	1.1	Tlk1	Signaling
DMR3:57250001	3	57250001	57251000	1000	1	9.20E-07	0.66	33	3.3	Tlk1;Mettl8	Signaling
DMR3:57323001	3	57323001	57325000	2000	1	6.80E-09	-0.58	27	1.35	Tlk1;Mettl8;LOC103690017	Signaling
DMR3:57975001	3	57975001	57977000	2000	1	7.50E-07	-0.3	23	1.15	Slc25a12	Transport
DMR3:58105001	3	58105001	58106000	1000	1	1.50E-11	0.56	16	1.6	Metap1d	Protease
DMR3:58124001	3	58124001	58125000	1000	1	3.30E-07	-0.35	15	1.5	Metap1d	Protease
DMR3:58648001	3	58648001	58653000	5000	1	5.40E-10	-0.47	44	0.88	Rapgef4	Transcription
DMR3:58705001	3	58705001	58706000	1000	1	5.90E-08	0.48	29	2.9	Rapgef4	Transcription
DMR3:58760001	3	58760001	58761000	1000	1	5.30E-12	0.59	13	1.3	Rapgef4	Transcription
DMR3:58798001	3	58798001	58800000	2000	1	5.90E-08	0.54	22	1.1	Rapgef4	Transcription
DMR3:58808001	3	58808001	58809000	1000	1	7.00E-10	0.68	25	2.5	Rapgef4	Transcription
DMR3:58817001	3	58817001	58821000	4000	2	4.50E-07	-0.4	46	1.15	Rapgef4	Transcription
DMR3:58839001	3	58839001	58843000	4000	1	6.30E-08	-0.4	66	1.65	Rapgef4	Transcription
DMR3:58876001	3	58876001	58877000	1000	1	1.10E-08	-0.38	18	1.8	Rapgef4;LOC103695171	Transcription
DMR3:58899001	3	58899001	58901000	2000	1	9.50E-12	0.62	37	1.85	Rapgef4;LOC103695171	Transcription
DMR3:58962001	3	58962001	58966000	4000	1	2.10E-10	-0.46	80	2	Zak	
DMR3:58992001	3	58992001	58993000	1000	1	6.90E-13	-0.64	23	2.3	Zak	
DMR3:59402001	3	59402001	59404000	2000	1	1.40E-08	0.5	34	1.7	Eno1-ps1	
DMR3:59592001	3	59592001	59593000	1000	1	2.40E-10	-0.4	17	1.7	LOC108350397;RGD1563716	
DMR3:59597001	3	59597001	59600000	3000	1	6.90E-07	-0.37	64	2.13	LOC108350397;RGD1563716	
DMR3:60158001	3	60158001	60159000	1000	1	2.20E-22	1.13	38	3.8	Wipf1	Cytoskeleton
DMR3:60167001	3	60167001	60169000	2000	1	1.20E-13	0.97	27	1.35	Wipf1	Cytoskeleton
DMR3:60176001	3	60176001	60178000	2000	1	9.20E-07	-0.31	40	2	Wipf1	Cytoskeleton
DMR3:60206001	3	60206001	60207000	1000	1	1.50E-10	0.85	26	2.6	Wipf1	Cytoskeleton
DMR3:60608001	3	60608001	60611000	3000	1	4.50E-07	-0.35	29	0.97	Chn1	
DMR3:60655001	3	60655001	60657000	2000	1	8.60E-09	-0.65	40	2	Chn1	

DMR3:61560001	3	61560001	61563000	3000	2	6.10E-08	-0.49	48	1.6	LOC502637;Evx2	
DMR3:61617001	3	61617001	61619000	2000	1	8.50E-11	0.78	27	1.35	Hoxd10;Hoxd9;Hoxd8	Development
DMR3:61644001	3	61644001	61646000	2000	1	3.30E-14	-0.67	49	2.45	Hoxd4;Mir10b;Hoxd3	
DMR3:61652001	3	61652001	61653000	1000	1	3.80E-09	-0.65	10	1	Hoxd4;Mir10b;Hoxd3	
DMR3:61767001	3	61767001	61770000	3000	1	6.80E-10	-0.45	16	0.53	Mtx2	
DMR3:61778001	3	61778001	61779000	1000	1	9.40E-09	0.66	18	1.8	Mtx2	
DMR3:62498001	3	62498001	62499000	1000	1	8.40E-07	-0.36	23	2.3	Hnrnpa3;Nfe2l2	Transcription
DMR3:62753001	3	62753001	62754000	1000	1	5.10E-07	0.51	14	1.4	Agps;Ttc30b	
DMR3:62834001	3	62834001	62836000	2000	1	8.30E-08	-0.37	49	2.45	Pde11a	Signaling
DMR3:62837001	3	62837001	62838000	1000	1	5.60E-07	-0.31	30	3	Pde11a	Signaling
DMR3:62895001	3	62895001	62897000	2000	1	4.60E-09	-0.54	44	2.2	Pde11a	Signaling
DMR3:62914001	3	62914001	62915000	1000	1	4.30E-09	0.69	14	1.4	Pde11a	Signaling
DMR3:63050001	3	63050001	63051000	1000	1	1.40E-07	-0.41	14	1.4	Pde11a	Signaling
DMR3:63116001	3	63116001	63120000	4000	2	1.30E-07	-0.34	41	1.02	Pde11a	Signaling
DMR3:63141001	3	63141001	63147000	6000	1	5.60E-10	0.86	64	1.07	Pde11a	Signaling
DMR3:63172001	3	63172001	63177000	5000	2	5.00E-09	-0.36	43	0.86	Pde11a	Signaling
DMR3:63194001	3	63194001	63197000	3000	1	4.60E-14	1.01	48	1.6	Pde11a;Cyct;LOC678730	Signaling
DMR3:63206001	3	63206001	63208000	2000	1	9.20E-07	-0.39	20	1	Pde11a;Cyct;LOC678730	Signaling
DMR3:63230001	3	63230001	63233000	3000	3	1.20E-17	1.03	68	2.27	Rbm45	
DMR3:63236001	3	63236001	63237000	1000	1	9.10E-09	0.28	7	0.7	Rbm45	
DMR3:63273001	3	63273001	63274000	1000	1	9.70E-11	0.44	12	1.2	Osbpl6	
DMR3:63279001	3	63279001	63280000	1000	1	8.90E-07	-0.46	12	1.2	Osbpl6	
DMR3:63316001	3	63316001	63318000	2000	1	6.80E-11	-0.48	38	1.9	Osbpl6	
DMR3:63344001	3	63344001	63350000	6000	1	1.20E-07	-0.38	107	1.78	Osbpl6	
DMR3:63496001	3	63496001	63497000	1000	1	7.80E-07	-0.32	19	1.9	Prkra	
DMR3:63546001	3	63546001	63548000	2000	1	2.30E-08	-0.56	19	0.95	Plekha3	
DMR3:63565001	3	63565001	63568000	3000	1	1.50E-09	-0.5	68	2.27	Plekha3;Ttn	
DMR3:63571001	3	63571001	63574000	3000	1	3.30E-08	-0.4	140	4.67	Ttn	
DMR3:63805001	3	63805001	63807000	2000	1	3.30E-07	-0.41	53	2.65	Ttn	
DMR3:63976001	3	63976001	63981000	5000	1	9.70E-10	-0.51	48	0.96	Ccdc141	
DMR3:63997001	3	63997001	63999000	2000	1	1.00E-08	-0.56	15	0.75	Ccdc141	
DMR3:64019001	3	64019001	64023000	4000	2	3.90E-08	-0.41	69	1.73	Ccdc141	
DMR3:64042001	3	64042001	64044000	2000	1	5.40E-10	-0.44	22	1.1	Sestd1	
DMR3:64056001	3	64056001	64060000	4000	1	5.30E-07	-0.31	56	1.4	Sestd1	
DMR3:64126001	3	64126001	64132000	6000	2	6.40E-11	-0.69	86	1.43	Sestd1	
DMR3:64268001	3	64268001	64270000	2000	1	2.00E-07	0.39	32	1.6	Zfp385b	
DMR3:64287001	3	64287001	64289000	2000	1	2.10E-07	-0.44	13	0.65	Zfp385b	
DMR3:64431001	3	64431001	64433000	2000	1	3.00E-07	-0.35	23	1.15	Zfp385b	
DMR3:64462001	3	64462001	64464000	2000	1	2.60E-08	0.53	26	1.3	Zfp385b	
DMR3:64517001	3	64517001	64519000	2000	1	4.20E-08	-0.42	23	1.15	Zfp385b	
DMR3:65666001	3	65666001	65669000	3000	1	8.30E-08	-0.44	59	1.97	RGD1564319	Signaling
DMR3:65839001	3	65839001	65841000	2000	1	7.40E-12	-0.49	13	0.65	Ube2e3	Proteolysis
DMR3:66296001	3	66296001	66297000	1000	1	3.60E-07	0.58	14	1.4	Cerkl	Signaling
DMR3:66306001	3	66306001	66308000	2000	2	1.40E-17	0.85	31	1.55	Cerkl	Signaling
DMR3:66411001	3	66411001	66413000	2000	1	8.00E-08	-0.47	22	1.1	LOC102553021;Neurod1;LOC108350408	Transcription
DMR3:66686001	3	66686001	66687000	1000	1	1.50E-09	0.45	10	1	Ppp1r1c	Signaling
DMR3:66800001	3	66800001	66803000	3000	1	3.20E-07	0.52	24	0.8	Pde1a	Signaling
DMR3:66901001	3	66901001	66904000	3000	1	2.00E-10	-0.48	38	1.27	Pde1a	Signaling
DMR3:67060001	3	67060001	67063000	3000	1	9.20E-10	-0.6	37	1.23	Pde1a	Signaling
DMR3:67070001	3	67070001	67075000	5000	1	1.30E-11	-0.39	58	1.16	Pde1a	Signaling
DMR3:67527001	3	67527001	67529000	2000	1	3.00E-12	0.62	12	0.6	Dnajc10	Transcription
DMR3:67539001	3	67539001	67540000	1000	1	1.60E-08	-0.44	12	1.2	Dnajc10	Transcription
DMR3:67557001	3	67557001	67560000	3000	1	7.60E-07	-0.38	26	0.87	Dnajc10	Transcription
DMR3:67567001	3	67567001	67569000	2000	1	2.30E-07	-0.55	16	0.8	Dnajc10	Transcription
DMR3:67670001	3	67670001	67673000	3000	1	7.30E-09	0.73	30	1	Frzb	Receptor
DMR3:67726001	3	67726001	67728000	2000	1	5.60E-07	-0.49	25	1.25	Nckap1	
DMR3:71036001	3	71036001	71038000	2000	1	9.70E-07	-0.37	25	1.25	Zc3h15	Transcription
DMR3:71196001	3	71196001	71200000	4000	1	4.90E-08	-0.38	45	1.12	Itgav	Extracellular Matrix
DMR3:71201001	3	71201001	71203000	2000	1	1.90E-09	-0.36	43	2.15	Itgav;Fam171b	Extracellular Matrix
DMR3:71271001	3	71271001	71272000	1000	1	1.20E-07	-0.39	11	1.1	Fam171b;LOC108350410;Zswim2	Proteolysis

DMR3:71855001	3	71855001	71857000	2000	1	8.50E-09	0.49	45	2.25	Calcrl;Tfpi	Receptor;Protease; Proteolysis
DMR3:72060001	3	72060001	72062000	2000	2	2.80E-13	0.82	48	2.4	Ctnnd1;Btbd18;RGD1563348	Cytoskeleton
DMR3:72113001	3	72113001	72114000	1000	1	4.10E-07	-0.47	11	1.1	Zdhhc5	
DMR3:72134001	3	72134001	72136000	2000	1	5.80E-07	-0.44	24	1.2	Clp1;Ypel4;Mir130a;Mir3590	Translation
DMR3:72360001	3	72360001	72361000	1000	1	4.80E-07	-0.26	9	0.9	Slc43a3;LOC366097	Transport
DMR3:72471001	3	72471001	72475000	4000	2	5.40E-12	-0.54	64	1.6	Tnks1bp1	
DMR3:72589001	3	72589001	72591000	2000	1	3.70E-07	-0.38	15	0.75	LOC102554972;Lrrc55	
DMR3:72602001	3	72602001	72603000	1000	1	3.00E-11	0.67	24	2.4	Lrrc55	
DMR3:72608001	3	72608001	72611000	3000	1	4.70E-14	-0.51	25	0.83	Lrrc55	
DMR3:72807001	3	72807001	72810000	3000	1	2.90E-08	0.58	24	0.8	Olr441;Olr442	Receptor
DMR3:72905001	3	72905001	72906000	1000	1	9.50E-10	-0.42	6	0.6	Fads2l1	
DMR3:73018001	3	73018001	73019000	1000	1	5.10E-07	0.36	9	0.9	Olr447	Signaling
DMR3:73586001	3	73586001	73587000	1000	1	5.10E-11	0.47	14	1.4	Olr488;Olr489-ps	Receptor
DMR3:73764001	3	73764001	73765000	1000	1	3.20E-07	0.56	14	1.4	Olr500;Olr502;Olr503-ps	Receptor
DMR3:73831001	3	73831001	73837000	6000	1	9.50E-07	-0.26	51	0.85	Olr508	Receptor
DMR3:74023001	3	74023001	74028000	5000	1	1.20E-07	-0.35	41	0.82	Olr516	Signaling
DMR3:74364001	3	74364001	74369000	5000	1	7.00E-09	-0.39	51	1.02	Olr518	Signaling
DMR3:74467001	3	74467001	74468000	1000	1	5.90E-07	0.51	19	1.9	Olr528	Receptor
DMR3:74518001	3	74518001	74519000	1000	1	2.90E-07	-0.47	7	0.7	Olr530	Receptor
DMR3:75305001	3	75305001	75307000	2000	1	2.90E-08	-0.29	15	0.75	Olr555	Receptor
DMR3:75414001	3	75414001	75416000	2000	1	9.40E-11	0.69	41	2.05	LOC685743;Olr560;LOC685772	Receptor
DMR3:75422001	3	75422001	75428000	6000	1	4.10E-09	0.37	55	0.92	Olr560;LOC685772;Olr561	Receptor
DMR3:75495001	3	75495001	75501000	6000	1	6.00E-22	0.44	64	1.07	Olr564-ps;LOC108350559;Olr565-ps	
DMR3:75633001	3	75633001	75634000	1000	1	2.60E-07	-0.5	2	0.2	Olr572-ps;LOC100361457	
DMR3:75692001	3	75692001	75700000	8000	1	3.80E-07	0.37	67	0.84	Olr574-ps	
DMR3:75727001	3	75727001	75730000	3000	1	2.30E-07	0.37	31	1.03	Olr576;Olr577	Signaling;Receptor
DMR3:75747001	3	75747001	75748000	1000	1	6.90E-07	0.54	15	1.5	Olr578	Signaling
DMR3:75828001	3	75828001	75834000	6000	1	6.00E-08	-0.26	52	0.87	Olr582	Signaling
DMR3:75837001	3	75837001	75839000	2000	1	4.40E-09	0.41	24	1.2	Olr582;Olr583	Signaling
DMR3:75840001	3	75840001	75841000	1000	1	2.20E-07	-0.27	8	0.8	Olr582;Olr583	Signaling
DMR3:75896001	3	75896001	75897000	1000	1	1.60E-12	0.5	14	1.4	Olr585-ps;Olr586	Signaling
DMR3:75940001	3	75940001	75943000	3000	1	7.00E-07	-0.39	24	0.8	Olr588;Olr589-ps	Receptor
DMR3:76022001	3	76022001	76027000	5000	1	1.30E-08	-0.26	42	0.84	Olr596	Signaling
DMR3:76090001	3	76090001	76091000	1000	1	7.50E-07	-0.38	6	0.6	Olr600-ps;Olr601	Receptor
DMR3:76149001	3	76149001	76151000	2000	2	2.30E-09	0.74	20	1	Olr605-ps	
DMR3:76545001	3	76545001	76548000	3000	1	5.20E-09	-0.59	12	0.4	Olr621	Receptor
DMR3:76984001	3	76984001	76988000	4000	1	4.80E-09	-0.33	52	1.3	Olr644-ps	
DMR3:77084001	3	77084001	77090000	6000	1	1.10E-07	-0.27	66	1.1	Olr650	Receptor
DMR3:77496001	3	77496001	77497000	1000	1	6.70E-08	-0.67	0	0	Olr662	Receptor
DMR3:78257001	3	78257001	78258000	1000	1	4.40E-07	0.49	9	0.9	Olr700-ps	
DMR3:78286001	3	78286001	78290000	4000	1	1.20E-08	-0.51	12	0.3	Olr702	Receptor
DMR3:78355001	3	78355001	78356000	1000	1	3.70E-10	0.59	7	0.7	Olr714	Receptor
DMR3:78416001	3	78416001	78422000	6000	2	1.90E-22	0.55	69	1.15	Olr704;Olr705	Receptor
DMR3:78549001	3	78549001	78550000	1000	1	3.40E-14	-0.59	6	0.6	Olr710;Olr711	Receptor
DMR3:78703001	3	78703001	78707000	4000	2	1.00E-07	0.36	33	0.82	Olr719-ps;Olr720	Receptor
DMR3:78776001	3	78776001	78778000	2000	1	3.00E-07	0.42	22	1.1	Olr1258	
DMR3:78845001	3	78845001	78846000	1000	1	3.60E-07	-0.55	5	0.5	Olr725;Olr726	Receptor
DMR3:78925001	3	78925001	78929000	4000	2	1.40E-08	-0.32	43	1.07	Olr730-ps	
DMR3:79014001	3	79014001	79015000	1000	1	3.10E-07	-0.65	2	0.2	Olr735;Olr736	Receptor
DMR3:79261001	3	79261001	79263000	2000	1	1.20E-07	-0.38	23	1.15	Ptprj	Signaling
DMR3:79485001	3	79485001	79492000	7000	1	7.40E-07	-0.34	68	0.97	LOC102555600;LOC103691844; Nup160	Transport
DMR3:79547001	3	79547001	79550000	3000	1	9.10E-08	-0.46	42	1.4	Nup160	Transport
DMR3:80303001	3	80303001	80305000	2000	1	1.20E-07	-0.56	18	0.9	RGD1309540	
DMR3:80377001	3	80377001	80378000	1000	1	8.80E-08	0.5	20	2	Lrp4	Binding Proteins
DMR3:80380001	3	80380001	80382000	2000	1	2.70E-08	-0.4	50	2.5	Lrp4	Binding Proteins
DMR3:80669001	3	80669001	80671000	2000	1	4.30E-08	-0.54	19	0.95	Ambra1	
DMR3:80727001	3	80727001	80729000	2000	1	2.40E-11	-0.56	9	0.45	Ambra1	

DMR3:80849001	3	80849001	80852000	3000	1	3.70E-07	0.38	63	2.1	Chrm4;Mdk;Dgkz	Signaling;Growth Factors;Signaling
DMR3:80887001	3	80887001	80888000	1000	1	7.20E-10	0.54	21	2.1	Dgkz;LOC103691847;Creb3l1	Signaling
DMR3:80937001	3	80937001	80939000	2000	1	4.70E-07	0.35	19	0.95	LOC103691847;Creb3l1	
DMR3:81357001	3	81357001	81358000	1000	1	2.50E-10	-0.45	17	1.7	Slc35c1	Transport
DMR3:81430001	3	81430001	81433000	3000	1	1.10E-07	0.72	53	1.77	LOC108350417;Chst1	Transport
DMR3:81451001	3	81451001	81452000	1000	1	8.20E-12	0.62	10	1	Chst1	Transport
DMR3:81513001	3	81513001	81515000	2000	1	9.50E-11	-0.43	29	1.45	Chst1	Transport
DMR3:81867001	3	81867001	81868000	1000	1	1.20E-11	0.56	11	1.1	Prdm11	Transcription
DMR3:81887001	3	81887001	81891000	4000	1	3.50E-09	0.59	45	1.12	Prdm11;LOC102547078	Transcription
DMR3:82091001	3	82091001	82094000	3000	1	1.50E-09	0.57	33	1.1	Tp53i11;Tspan18	
DMR3:82104001	3	82104001	82106000	2000	1	3.10E-10	0.69	20	1	Tp53i11;Tspan18	
DMR3:82109001	3	82109001	82111000	2000	1	2.20E-08	0.45	25	1.25	Tspan18	
DMR3:82136001	3	82136001	82137000	1000	1	4.30E-08	-0.65	3	0.3	Tspan18	
DMR3:82146001	3	82146001	82150000	4000	1	4.80E-09	-0.52	43	1.07	Tspan18	
DMR3:82170001	3	82170001	82171000	1000	1	2.20E-12	-0.49	17	1.7	Tspan18	
DMR3:82208001	3	82208001	82209000	1000	1	2.60E-09	0.49	16	1.6	Tspan18	
DMR3:82550001	3	82550001	82551000	1000	1	9.90E-08	0.47	28	2.8	Alx4	Development
DMR3:82557001	3	82557001	82561000	4000	1	8.60E-09	0.49	37	0.92	Alx4	Development
DMR3:82643001	3	82643001	82644000	1000	1	2.80E-08	0.44	16	1.6	Ext2	Golgi
DMR3:82659001	3	82659001	82660000	1000	1	1.10E-09	-0.41	13	1.3	Ext2	Golgi
DMR3:82720001	3	82720001	82723000	3000	1	9.20E-07	-0.3	63	2.1	Ext2	Golgi
DMR3:82728001	3	82728001	82729000	1000	1	2.10E-07	-0.45	14	1.4	Ext2	Golgi
DMR3:83035001	3	83035001	83037000	2000	1	1.60E-15	-0.52	21	1.05	Hsd17b12	
DMR3:83299001	3	83299001	83302000	3000	1	2.20E-07	0.38	30	1	Ttc17;Api5	
DMR3:83322001	3	83322001	83324000	2000	1	1.50E-09	0.66	21	1.05	Api5	
DMR3:85897001	3	85897001	85898000	1000	1	1.60E-09	-0.44	6	0.6	Lrrc4c	
DMR3:86150001	3	86150001	86151000	1000	1	6.20E-08	0.48	10	1	Lrrc4c	
DMR3:86158001	3	86158001	86159000	1000	1	1.20E-11	-0.66	2	0.2	Lrrc4c	
DMR3:86239001	3	86239001	86241000	2000	1	3.80E-10	-0.43	15	0.75	Lrrc4c	
DMR3:86462001	3	86462001	86464000	2000	1	5.90E-17	-0.69	10	0.5	Lrrc4c	
DMR3:86529001	3	86529001	86531000	2000	2	5.70E-17	-0.75	4	0.2	Lrrc4c	
DMR3:86613001	3	86613001	86614000	1000	1	3.30E-08	0.66	17	1.7	Lrrc4c;LOC100362626	
DMR3:86682001	3	86682001	86684000	2000	1	2.20E-12	-0.65	18	0.9	Lrrc4c	
DMR3:91178001	3	91178001	91179000	1000	1	5.90E-07	-0.49	0	0	RGD1309730	
DMR3:91405001	3	91405001	91406000	1000	1	4.80E-07	-0.46	15	1.5	Prr5l	
DMR3:91440001	3	91440001	91441000	1000	1	2.10E-11	0.85	27	2.7	Prr5l	
DMR3:91466001	3	91466001	91469000	3000	1	3.90E-10	-0.65	32	1.07	Prr5l;Commd9	
DMR3:91482001	3	91482001	91484000	2000	1	7.70E-08	-0.49	27	1.35	Commd9	
DMR3:91723001	3	91723001	91725000	2000	1	1.40E-12	-0.42	39	1.95	Ldlrad3;LOC108350421	Binding Proteins
DMR3:91824001	3	91824001	91830000	6000	1	9.60E-11	-0.53	76	1.27	Ldlrad3	Binding Proteins
DMR3:92145001	3	92145001	92147000	2000	1	6.00E-07	0.35	35	1.75	Trim44;LOC102551100	Proteolysis
DMR3:92200001	3	92200001	92204000	4000	1	6.00E-13	0.34	53	1.32	Trim44	Proteolysis
DMR3:92205001	3	92205001	92208000	3000	1	1.70E-07	0.39	24	0.8	Trim44	Proteolysis
DMR3:92458001	3	92458001	92459000	1000	1	3.60E-07	-0.48	6	0.6	Pamr1	Protease
DMR3:92569001	3	92569001	92570000	1000	1	7.10E-09	0.5	9	0.9	Slc1a2	Transport
DMR3:92960001	3	92960001	92962000	2000	1	7.50E-07	-0.48	17	0.85	Apip	Metabolism
DMR3:92979001	3	92979001	92981000	2000	1	6.10E-13	0.61	26	1.3	Apip	Metabolism
DMR3:93338001	3	93338001	93339000	1000	1	1.60E-09	0.53	14	1.4	Elf5	Transcription
DMR3:93639001	3	93639001	93642000	3000	1	3.10E-09	0.69	46	1.53	Abtb2	Cytoskeleton
DMR3:93769001	3	93769001	93771000	2000	1	4.60E-10	0.74	27	1.35	Caprin1;LOC108350425	Metabolism
DMR3:93994001	3	93994001	93996000	2000	1	2.00E-07	-0.51	18	0.9	Fbxo3	
DMR3:93997001	3	93997001	93999000	2000	1	2.30E-08	-0.46	19	0.95	Fbxo3	
DMR3:94634001	3	94634001	94635000	1000	1	3.30E-07	-0.37	19	1.9	Tcp11l1	Cytoskeleton
DMR3:94642001	3	94642001	94649000	7000	1	1.20E-09	-0.44	70	1	Tcp11l1	Cytoskeleton
DMR3:94664001	3	94664001	94665000	1000	1	5.00E-07	-0.48	4	0.4	Tcp11l1;Depdc7	Cytoskeleton;Cytoskeleton
DMR3:94831001	3	94831001	94834000	3000	2	2.20E-09	0.67	50	1.67	RGD1560017	
DMR3:94849001	3	94849001	94850000	1000	1	5.70E-07	0.57	19	1.9	Ccdc73	
DMR3:94880001	3	94880001	94887000	7000	1	5.10E-07	-0.31	89	1.27	Ccdc73	
DMR3:94921001	3	94921001	94926000	5000	1	1.30E-12	-0.47	34	0.68	Ccdc73	
DMR3:95148001	3	95148001	95151000	3000	1	9.60E-07	0.61	59	1.97	Wt1	Transcription

DMR3:95153001	3	95153001	95154000	1000	1	2.20E-08	-0.57	5	0.5	Wt1	Transcription
DMR3:95710001	3	95710001	95711000	1000	1	2.80E-07	-0.36	18	1.8	Pax6	
DMR3:95780001	3	95780001	95781000	1000	1	3.60E-10	0.51	11	1.1	Elp4	
DMR3:95909001	3	95909001	95912000	3000	1	9.70E-07	-0.29	24	0.8	Elp4	
DMR3:97363001	3	97363001	97364000	1000	1	7.30E-07	0.27	25	2.5	Dcdc5	
DMR3:97365001	3	97365001	97369000	4000	1	1.60E-07	-0.4	38	0.95	Dcdc5	
DMR3:97803001	3	97803001	97806000	3000	1	5.90E-12	-0.62	76	2.53	Mpped2	Metabolism
DMR3:97882001	3	97882001	97883000	1000	1	1.20E-09	-0.48	26	2.6	Mpped2	Metabolism
DMR3:98298001	3	98298001	98300000	2000	2	5.90E-15	1.08	46	2.3	Kcna4	Transport
DMR3:100197001	3	100197001	100198000	1000	1	8.60E-07	0.25	4	0.4	Mettl15	Epigenetic
DMR3:100296001	3	100296001	100299000	3000	1	1.40E-07	0.53	31	1.03	Mettl15	Epigenetic
DMR3:101047001	3	101047001	101048000	1000	1	9.30E-09	-0.33	11	1.1	Lgr4	
DMR3:101049001	3	101049001	101050000	1000	1	8.70E-14	0.42	5	0.5	Lgr4	
DMR3:101085001	3	101085001	101088000	3000	1	1.70E-11	-0.42	47	1.57	Lgr4	
DMR3:101092001	3	101092001	101093000	1000	1	1.50E-08	-0.62	15	1.5	Lgr4	
DMR3:101195001	3	101195001	101199000	4000	1	8.60E-07	-0.31	30	0.75	Ccdc34	
DMR3:101453001	3	101453001	101455000	2000	1	2.40E-14	0.43	19	0.95	Bbox1	Metabolism
DMR3:102086001	3	102086001	102088000	2000	1	8.00E-08	-0.55	34	1.7	Ano3	
DMR3:102287001	3	102287001	102288000	1000	1	2.10E-07	-0.43	1	0.1	LOC102555149;Ankrd30a	
DMR3:102363001	3	102363001	102364000	1000	1	1.20E-11	-0.55	0	0	Ankrd30a	
DMR3:102662001	3	102662001	102665000	3000	1	4.90E-07	0.43	30	1	Olr758;RGD1561276;Olr760	Receptor
DMR3:102748001	3	102748001	102750000	2000	1	3.90E-10	0.57	38	1.9	Olr766	Receptor
DMR3:102809001	3	102809001	102810000	1000	1	1.50E-08	-0.6	2	0.2	Olr768	Receptor
DMR3:102939001	3	102939001	102940000	1000	1	2.40E-10	-0.43	8	0.8	Olr773	Receptor
DMR3:103006001	3	103006001	103007000	1000	1	2.20E-07	-0.48	5	0.5	Olr774	Receptor
DMR3:103103001	3	103103001	103104000	1000	1	3.20E-08	-0.67	0	0	LOC100363400;Olr778	Receptor
DMR3:103273001	3	103273001	103276000	3000	1	1.70E-07	-0.31	32	1.07	Olr787-ps	
DMR3:103848001	3	103848001	103850000	2000	2	1.10E-09	0.41	24	1.2	Slc12a6;Emc4	Transport
DMR3:104005001	3	104005001	104007000	2000	1	2.30E-10	0.76	18	0.9	Chrm5;Aven	Signaling
DMR3:104138001	3	104138001	104139000	1000	1	6.70E-07	-0.44	10	1	Ryr3	Ion Channel
DMR3:104213001	3	104213001	104215000	2000	1	8.00E-14	0.79	30	1.5	Ryr3;LOC103691859	Ion Channel
DMR3:104248001	3	104248001	104249000	1000	1	5.40E-07	-0.4	19	1.9	Ryr3	Ion Channel
DMR3:104307001	3	104307001	104310000	3000	1	8.30E-14	1.12	40	1.33	Ryr3	Ion Channel
DMR3:104656001	3	104656001	104660000	4000	1	2.30E-14	-0.54	46	1.15	Ryr3	Ion Channel
DMR3:104673001	3	104673001	104683000	10000	1	3.20E-07	-0.27	117	1.17	Ryr3;Hmgn4	Ion Channel
DMR3:104743001	3	104743001	104749000	6000	1	2.10E-07	-0.4	46	0.77	Tmco5b;LOC102553315	
DMR3:104772001	3	104772001	104774000	2000	1	9.70E-07	-0.42	29	1.45	Tmco5b;LOC102553315;Fmn1	
DMR3:104857001	3	104857001	104858000	1000	1	1.80E-08	-0.63	18	1.8	Fmn1	
DMR3:105013001	3	105013001	105014000	1000	1	7.40E-17	-0.61	11	1.1	Fmn1	
DMR3:105220001	3	105220001	105221000	1000	1	2.70E-08	-0.42	10	1	Grem1	
DMR3:106053001	3	106053001	106054000	1000	1	2.10E-09	0.52	20	2	Dph6;LOC108350438	
DMR3:107137001	3	107137001	107138000	1000	1	8.20E-08	0.6	14	1.4	RGD1563680	
DMR3:107202001	3	107202001	107204000	2000	1	1.40E-09	-0.54	18	0.9	RGD1563680	
DMR3:107312001	3	107312001	107314000	2000	1	2.60E-07	0.3	18	0.9	RGD1563680	
DMR3:107347001	3	107347001	107348000	1000	1	2.80E-07	-0.41	10	1	RGD1563680	
DMR3:108538001	3	108538001	108539000	1000	1	2.40E-07	-0.29	7	0.7	Tmco5a	
DMR3:108937001	3	108937001	108943000	6000	1	7.80E-08	-0.24	58	0.97	Fam98b	
DMR3:108954001	3	108954001	108955000	1000	1	3.50E-11	-0.4	6	0.6	Fam98b	
DMR3:110000001	3	110000001	110004000	4000	1	3.40E-09	-0.41	51	1.27	Fsip1	
DMR3:110072001	3	110072001	110074000	2000	1	3.20E-08	0.33	11	0.55	Gpr176	Signaling
DMR3:110094001	3	110094001	110097000	3000	1	2.00E-09	-0.52	37	1.23	Gpr176	Signaling
DMR3:110125001	3	110125001	110126000	1000	1	4.00E-09	0.4	12	1.2	Gpr176	Signaling
DMR3:110168001	3	110168001	110171000	3000	1	2.90E-07	0.44	37	1.23	Eif2ak4	Signaling
DMR3:110684001	3	110684001	110688000	4000	1	5.20E-07	0.46	56	1.4	Ivd	Metabolism
DMR3:110735001	3	110735001	110736000	1000	1	2.70E-11	0.71	13	1.3	Bahd1;Chst14	Transport
DMR3:110742001	3	110742001	110745000	3000	2	3.70E-11	-0.82	15	0.5	Bahd1;Chst14	Transport
DMR3:111023001	3	111023001	111024000	1000	1	2.30E-08	0.55	11	1.1	Dnajc17;Zfyve19;Ppp1r14d	Transcription;Signaling
DMR3:111052001	3	111052001	111056000	4000	1	1.00E-08	-0.38	45	1.12	Spint1	Protease; Proteolysis
DMR3:111087001	3	111087001	111088000	1000	1	3.90E-09	0.79	26	2.6	Rhov;Vps18	Signaling;Transport
DMR3:111130001	3	111130001	111131000	1000	1	6.30E-07	0.55	14	1.4	LOC103691871;Dll4	
DMR3:111469001	3	111469001	111470000	1000	1	2.50E-07	0.55	27	2.7	Ndufaf1;Rtf1	
DMR3:111513001	3	111513001	111515000	2000	1	5.80E-07	0.31	25	1.25	Rtf1	

DMR3:111550001	3	111550001	111555000	5000	1	9.60E-07	0.48	99	1.98	Itpka;Ltk	Signaling;Receptor
DMR3:111557001	3	111557001	111558000	1000	1	2.70E-12	0.71	10	1	Itpka;Ltk;Rpap1	Signaling;Receptor
DMR3:111606001	3	111606001	111608000	2000	1	9.60E-07	0.48	38	1.9	Tyro3	Receptor
DMR3:111837001	3	111837001	111840000	3000	1	2.40E-10	0.62	46	1.53	Pla2g4b;Sptbn5	Metabolism
DMR3:111860001	3	111860001	111861000	1000	1	2.70E-07	0.4	16	1.6	Sptbn5	
DMR3:111864001	3	111864001	111870000	6000	1	6.90E-07	0.37	135	2.25	Sptbn5;Ehd4	Transport
DMR3:111873001	3	111873001	111874000	1000	1	2.20E-08	0.65	18	1.8	Sptbn5;Ehd4	Transport
DMR3:111923001	3	111923001	111924000	1000	1	6.90E-08	-0.55	7	0.7	Ehd4	Transport
DMR3:112097001	3	112097001	112099000	2000	1	1.70E-07	0.57	25	1.25	Vps39;LOC691532	
DMR3:112242001	3	112242001	112243000	1000	1	6.30E-11	0.63	15	1.5	Capn3	Protease
DMR3:112649001	3	112649001	112653000	4000	1	4.10E-07	0.56	54	1.35	Stard9;Cdan1	Cytoskeleton
DMR3:112871001	3	112871001	112873000	2000	1	2.00E-07	-0.59	25	1.25	Ubr1	Proteolysis
DMR3:113027001	3	113027001	113029000	2000	1	3.50E-07	-0.48	12	0.6	Tgm7l1	
DMR3:113030001	3	113030001	113032000	2000	1	2.00E-07	-0.6	4	0.2	Tgm7l1;Tgm7	Transport
DMR3:113111001	3	113111001	113113000	2000	1	3.40E-11	-0.42	41	2.05	Tgm7;Adal;LOC108350448	Transport;Metabolism
DMR3:113259001	3	113259001	113260000	1000	1	9.80E-09	0.44	10	1	Tp53bp1;Map1a	Transcription;Cytoskeleton
DMR3:113299001	3	113299001	113302000	3000	1	8.40E-07	-0.54	32	1.07	Ppip5k1	Signaling
DMR3:113303001	3	113303001	113304000	1000	1	3.40E-07	-0.6	4	0.4	Ppip5k1	Signaling
DMR3:113320001	3	113320001	113322000	2000	1	6.00E-07	-0.3	41	2.05	Ppip5k1;Ckmt1b;Strc	Signaling;Cytoskeleton
DMR3:113360001	3	113360001	113362000	2000	2	3.20E-08	-0.44	24	1.2	Catsper2	Transport
DMR3:113460001	3	113460001	113461000	1000	1	2.00E-11	0.64	1	0.1	Wdr76	
DMR3:113489001	3	113489001	113490000	1000	1	2.60E-09	0.47	20	2	Wdr76;Frmd5	
DMR3:113579001	3	113579001	113580000	1000	1	2.50E-09	-0.59	18	1.8	Frmd5	
DMR3:113597001	3	113597001	113598000	1000	1	3.80E-07	-0.48	6	0.6	Frmd5	
DMR3:113852001	3	113852001	113853000	1000	1	1.70E-07	0.58	16	1.6	Casc4	
DMR3:113859001	3	113859001	113860000	1000	1	1.50E-09	0.75	22	2.2	Casc4	
DMR3:114008001	3	114008001	114009000	1000	1	1.20E-07	-0.46	6	0.6	Eif3j;Spg11	Translation
DMR3:114034001	3	114034001	114035000	1000	1	3.80E-08	0.44	21	2.1	Spg11	
DMR3:114088001	3	114088001	114091000	3000	2	1.10E-07	-0.6	49	1.63	B2m	Immune
DMR3:114115001	3	114115001	114118000	3000	1	2.30E-11	-0.53	28	0.93	Trim69	Proteolysis
DMR3:114142001	3	114142001	114144000	2000	1	2.90E-09	0.56	22	1.1	Terb2	
DMR3:114230001	3	114230001	114232000	2000	1	4.80E-07	0.44	21	1.05	Duox2;Duoxa2;Duoxa1	Metabolism
DMR3:114245001	3	114245001	114250000	5000	1	7.00E-07	0.5	49	0.98	Duox2;Duoxa2;Duoxa1;Duox1	Metabolism
DMR3:114289001	3	114289001	114291000	2000	1	2.30E-08	0.42	22	1.1	Duox1;Shf	Metabolism
DMR3:114562001	3	114562001	114564000	2000	1	2.20E-07	-0.41	62	3.1	Slc28a2	Transport
DMR3:114576001	3	114576001	114577000	1000	1	9.30E-07	-0.3	9	0.9	Slc28a2	Transport
DMR3:114725001	3	114725001	114727000	2000	1	2.50E-07	-0.47	27	1.35	Gatm	Transport
DMR3:114764001	3	114764001	114771000	7000	1	1.80E-07	-0.34	106	1.51	Spata5l1;MGC105649;Mir147	
DMR3:114802001	3	114802001	114803000	1000	1	7.40E-11	0.49	11	1.1	Slc30a4	Transport
DMR3:114831001	3	114831001	114832000	1000	1	1.90E-07	0.48	16	1.6	Slc30a4;LOC108350451	Transport
DMR3:114942001	3	114942001	114948000	6000	2	2.30E-07	-0.31	47	0.78	Sqrdl	
DMR3:116935001	3	116935001	116937000	2000	1	4.10E-09	-0.38	29	1.45	Sema6d	Signaling
DMR3:116947001	3	116947001	116951000	4000	1	7.80E-16	-0.66	109	2.72	Sema6d	Signaling
DMR3:117450001	3	117450001	117452000	2000	1	1.20E-09	0.73	32	1.6	Slc12a1	Transport
DMR3:117672001	3	117672001	117673000	1000	1	7.20E-09	0.69	20	2	Fbn1	Extracellular Matrix
DMR3:117752001	3	117752001	117755000	3000	2	8.30E-08	-0.38	56	1.87	Fbn1	Extracellular Matrix
DMR3:117827001	3	117827001	117830000	3000	1	7.00E-07	-0.41	62	2.07	LOC102555758;Cep152	
DMR3:117886001	3	117886001	117888000	2000	1	6.40E-09	-0.45	20	1	Cep152;Shc4	Cytoskeleton
DMR3:117907001	3	117907001	117908000	1000	1	1.40E-11	-0.49	16	1.6	Shc4;LOC108350457	Cytoskeleton
DMR3:117962001	3	117962001	117964000	2000	2	2.60E-12	-0.87	7	0.35	Shc4	Cytoskeleton
DMR3:117971001	3	117971001	117977000	6000	1	6.70E-08	0.45	89	1.48	Shc4	Cytoskeleton
DMR3:117979001	3	117979001	117980000	1000	1	6.50E-07	-0.4	24	2.4	Shc4	Cytoskeleton
DMR3:118109001	3	118109001	118110000	1000	1	2.60E-08	-0.6	13	1.3	Cops2	
DMR3:118143001	3	118143001	118145000	2000	1	1.90E-07	-0.43	37	1.85	Cops2;Galk2	Metabolism
DMR3:118149001	3	118149001	118151000	2000	1	9.30E-10	-0.77	11	0.55	Galk2	Metabolism
DMR3:118244001	3	118244001	118246000	2000	1	2.10E-07	-0.7	10	0.5	Galk2	Metabolism
DMR3:118290001	3	118290001	118293000	3000	1	4.10E-09	-0.3	25	0.83	Fam227b	
DMR3:118643001	3	118643001	118644000	1000	1	5.90E-08	-0.62	2	0.2	Atp8b4	Transport
DMR3:118675001	3	118675001	118676000	1000	1	9.90E-17	1.37	8	0.8	Atp8b4	Transport
DMR3:118789001	3	118789001	118790000	1000	1	9.30E-09	0.64	8	0.8	Atp8b4	Transport
DMR3:118934001	3	118934001	118935000	1000	1	2.00E-07	-0.55	10	1	Atp8b4	Transport

DMR3:119079001	3	119079001	119082000	3000	1	2.40E-13	0.91	42	1.4	Hdc;Gabpb1	Metabolism
DMR3:119231001	3	119231001	119232000	1000	1	4.90E-07	-0.3	22	2.2	Usp8;Usp50	Protease
DMR3:119318001	3	119318001	119320000	2000	1	1.10E-07	-0.33	33	1.65	Trpm7	Transport
DMR3:119541001	3	119541001	119545000	4000	1	4.80E-08	-0.58	39	0.98	Ap4e1;Blvra	Transport;Metabolism
DMR3:119767001	3	119767001	119770000	3000	1	4.20E-11	0.54	25	0.83	Dusp2	Signaling
DMR3:120299001	3	120299001	120303000	4000	1	4.80E-08	0.34	43	1.07	Mall	Transport
DMR3:120414001	3	120414001	120416000	2000	1	4.80E-09	0.56	30	1.5	Bub1;Acoxl	Signaling;Metabolism
DMR3:120624001	3	120624001	120625000	1000	1	3.40E-07	0.51	17	1.7	Acoxl	Metabolism
DMR3:120626001	3	120626001	120630000	4000	1	4.10E-07	0.52	39	0.98	Acoxl	Metabolism
DMR3:120656001	3	120656001	120657000	1000	1	5.50E-08	0.63	14	1.4	Acoxl	Metabolism
DMR3:120704001	3	120704001	120709000	5000	1	1.10E-07	-0.36	42	0.84	Acoxl	Metabolism
DMR3:120731001	3	120731001	120734000	3000	1	4.30E-10	0.73	36	1.2	Acoxl;Bcl2l11	Metabolism
DMR3:121273001	3	121273001	121277000	4000	1	6.70E-09	-0.44	85	2.12	Mertk	Receptor
DMR3:121322001	3	121322001	121326000	4000	2	3.80E-13	-0.68	44	1.1	Mertk	Receptor
DMR3:121340001	3	121340001	121347000	7000	1	2.30E-07	0.39	92	1.31	Mertk	Receptor
DMR3:121401001	3	121401001	121403000	2000	1	5.40E-08	0.47	29	1.45	Tmem87b;Fbln7	Signaling;Extracellular Matrix
DMR3:121426001	3	121426001	121431000	5000	1	1.90E-09	-0.44	49	0.98	Fbln7	Extracellular Matrix
DMR3:121613001	3	121613001	121615000	2000	1	9.20E-07	-0.39	25	1.25	Ttl	
DMR3:121671001	3	121671001	121673000	2000	1	1.60E-08	-0.44	17	0.85	Chchd5	
DMR3:121963001	3	121963001	121969000	6000	2	2.30E-08	-0.43	48	0.8	RGD1566226	
DMR3:121970001	3	121970001	121973000	3000	2	1.80E-12	-0.57	13	0.43	RGD1566226	
DMR3:121987001	3	121987001	121991000	4000	1	7.30E-10	-0.59	16	0.4	RGD1566226	
DMR3:122144001	3	122144001	122147000	3000	1	2.80E-09	-0.42	43	1.43	Sirpa	Receptor
DMR3:122546001	3	122546001	122549000	3000	1	6.00E-09	0.62	26	0.87	LOC108350463;Tgm3	Transport
DMR3:122579001	3	122579001	122581000	2000	1	7.60E-10	0.7	47	2.35	Tgm3;LOC103691886	Transport
DMR3:122665001	3	122665001	122666000	1000	1	3.10E-07	0.42	10	1	Tgm6	Transport
DMR3:122687001	3	122687001	122689000	2000	1	7.70E-07	0.43	23	1.15	Tgm6;Snrpb	Transport;Translation
DMR3:122881001	3	122881001	122883000	2000	1	8.80E-10	0.6	40	2	Ebf4	Transcription
DMR3:122884001	3	122884001	122887000	3000	1	9.50E-10	0.72	48	1.6	Ebf4	Transcription
DMR3:122907001	3	122907001	122910000	3000	1	2.40E-09	0.73	49	1.63	Cpxm1	Protease
DMR3:122922001	3	122922001	122923000	1000	1	2.10E-07	0.51	10	1	Cpxm1;LOC100365450;Tmem239	Protease
DMR3:122929001	3	122929001	122931000	2000	1	2.30E-10	0.73	33	1.65	Cpxm1;LOC100365450;Tmem239	Protease
DMR3:122965001	3	122965001	122967000	2000	1	4.00E-08	0.46	32	1.6	Vps16;Ptpra	Transport;Signaling
DMR3:123110001	3	123110001	123116000	6000	1	6.90E-07	-0.25	77	1.28	Oxt;Avp	Signaling
DMR3:123178001	3	123178001	123179000	1000	1	9.70E-09	0.65	17	1.7	Ubox5;Fastkd5;Lzts3	
DMR3:123227001	3	123227001	123229000	2000	1	2.60E-08	0.52	56	2.8	Itpa;Slc4a11	Signaling;Transport
DMR3:123332001	3	123332001	123334000	2000	1	1.80E-07	-0.61	25	1.25	RGD1565616	
DMR3:123618001	3	123618001	123621000	3000	1	4.60E-08	0.55	56	1.87	Siglec1	
DMR3:123622001	3	123622001	123624000	2000	1	9.50E-08	-0.49	27	1.35	Siglec1	
DMR3:123667001	3	123667001	123669000	2000	1	2.80E-08	0.5	3	0.15	LOC296165;Hspa12b	
DMR3:123683001	3	123683001	123685000	2000	1	1.70E-08	0.62	27	1.35	Hspa12b;RGD1311739	
DMR3:123736001	3	123736001	123739000	3000	1	1.10E-07	0.54	41	1.37	Cdc25b	Signaling
DMR3:123756001	3	123756001	123759000	3000	1	1.20E-07	0.45	38	1.27	Ap5s1	
DMR3:124101001	3	124101001	124105000	4000	1	8.50E-09	-0.41	70	1.75	Smox	Metabolism
DMR3:124525001	3	124525001	124527000	2000	1	2.60E-09	-0.44	38	1.9	Prnp	Signaling
DMR3:124650001	3	124650001	124651000	1000	1	9.70E-07	0.47	18	1.8	Slc23a2	Transport
DMR3:124808001	3	124808001	124809000	1000	1	3.00E-08	0.42	29	2.9	Slc23a2	Transport
DMR3:124833001	3	124833001	124834000	1000	1	9.70E-08	0.36	14	1.4	Slc23a2	Transport
DMR3:124892001	3	124892001	124894000	2000	1	6.50E-10	-0.47	25	1.25	Pcna;Cds2	Transcription;Transport
DMR3:125008001	3	125008001	125011000	3000	2	2.80E-09	0.59	60	2	Prokr2	Signaling
DMR3:125159001	3	125159001	125160000	1000	1	1.10E-10	0.73	27	2.7	Gpcpd1	Signaling
DMR3:125173001	3	125173001	125176000	3000	1	1.20E-10	0.58	27	0.9	Gpcpd1	Signaling
DMR3:125420001	3	125420001	125421000	1000	1	4.50E-10	-0.7	4	0.4	Chgb	
DMR3:125439001	3	125439001	125440000	1000	1	1.80E-16	0.91	26	2.6	Chgb	
DMR3:127474001	3	127474001	127477000	3000	1	9.20E-08	-0.27	29	0.97	Hao1;LOC108350470	Metabolism
DMR3:127541001	3	127541001	127543000	2000	1	3.70E-07	-0.47	8	0.4	Tmx4	
DMR3:128084001	3	128084001	128085000	1000	1	5.00E-08	0.52	13	1.3	Plcb1	Metabolism
DMR3:128313001	3	128313001	128314000	1000	1	8.80E-07	0.53	14	1.4	Plcb1	Metabolism

DMR3:128346001	3	128346001	128347000	1000	1	1.70E-11	-0.68	1	0.1	Plcb1	Metabolism
DMR3:128615001	3	128615001	128618000	3000	1	5.80E-07	0.37	35	1.17	Plcb4	Metabolism
DMR3:128663001	3	128663001	128665000	2000	1	7.00E-18	-0.58	34	1.7	Plcb4	Metabolism
DMR3:128676001	3	128676001	128677000	1000	1	4.10E-07	0.37	5	0.5	Plcb4	Metabolism
DMR3:128692001	3	128692001	128697000	5000	1	4.50E-07	0.35	101	2.02	Plcb4;LOC102549547	Metabolism
DMR3:128708001	3	128708001	128710000	2000	1	2.80E-07	-0.36	27	1.35	Plcb4	Metabolism
DMR3:128973001	3	128973001	128975000	2000	1	4.30E-07	-0.53	16	0.8	Plcb4	Metabolism
DMR3:129095001	3	129095001	129099000	4000	1	9.20E-11	-0.38	38	0.95	Pak7	
DMR3:129141001	3	129141001	129143000	2000	1	3.60E-10	0.56	23	1.15	Pak7	
DMR3:129469001	3	129469001	129470000	1000	1	5.30E-15	0.77	7	0.7	Ankef1	
DMR3:129971001	3	129971001	129973000	2000	1	1.40E-09	-0.68	13	0.65	Slx4ip	
DMR3:130109001	3	130109001	130111000	2000	1	3.40E-07	-0.52	25	1.25	Jag1	
DMR3:131351001	3	131351001	131352000	1000	1	7.20E-09	-0.56	28	2.8	LOC108350568;Btbd3	Proteolysis
DMR3:132584001	3	132584001	132588000	4000	1	7.60E-08	-0.33	32	0.8	Sptlc3	Metabolism
DMR3:132647001	3	132647001	132651000	4000	1	5.80E-07	-0.24	30	0.75	Sptlc3	Metabolism
DMR3:132811001	3	132811001	132812000	1000	1	3.40E-11	-0.57	6	0.6	Ism1;LOC500653	
DMR3:132959001	3	132959001	132961000	2000	1	1.00E-08	0.4	6	0.3	Tasp1	Protease
DMR3:134699001	3	134699001	134701000	2000	1	3.10E-09	0.56	29	1.45	Flrt3	
DMR3:135846001	3	135846001	135847000	1000	1	8.00E-07	-0.53	3	0.3	Macrod2	
DMR3:135957001	3	135957001	135958000	1000	1	1.50E-12	-0.65	5	0.5	Macrod2	
DMR3:136130001	3	136130001	136131000	1000	1	7.30E-08	0.32	10	1	Macrod2	
DMR3:136262001	3	136262001	136263000	1000	1	1.80E-07	0.55	25	2.5	Macrod2	
DMR3:136295001	3	136295001	136297000	2000	1	8.10E-07	0.35	27	1.35	Macrod2	
DMR3:136406001	3	136406001	136409000	3000	1	4.90E-08	-0.41	19	0.63	Macrod2	
DMR3:136792001	3	136792001	136795000	3000	1	3.80E-07	-0.38	30	1	Kif16b	Cytoskeleton
DMR3:137783001	3	137783001	137785000	2000	1	2.70E-07	-0.51	29	1.45	Pcsk2	Protease
DMR3:137880001	3	137880001	137882000	2000	1	9.10E-10	-0.6	2	0.1	Pcsk2	Protease
DMR3:137885001	3	137885001	137886000	1000	1	1.10E-10	-0.48	21	2.1	Pcsk2	Protease
DMR3:138093001	3	138093001	138095000	2000	2	2.70E-11	-0.58	8	0.4	Rrbp1	Cytoskeleton
DMR3:138163001	3	138163001	138164000	1000	1	2.50E-07	-0.42	2	0.2	Banf2;LOC108350479	
DMR3:138554001	3	138554001	138557000	3000	1	7.00E-07	0.54	26	0.87	Kat14	
DMR3:138843001	3	138843001	138846000	3000	1	5.40E-08	0.49	26	0.87	Dtd1;LOC102550869;LOC102550813	Metabolism
DMR3:138894001	3	138894001	138896000	2000	1	1.50E-07	0.53	20	1	Dtd1	Metabolism
DMR3:139499001	3	139499001	139501000	2000	1	1.20E-08	0.42	14	0.7	Slc24a3	Transport
DMR3:139766001	3	139766001	139767000	1000	1	3.80E-11	-0.37	15	1.5	Slc24a3	Transport
DMR3:139816001	3	139816001	139817000	1000	1	3.10E-08	0.52	21	2.1	Slc24a3	Transport
DMR3:140091001	3	140091001	140093000	2000	1	7.20E-07	0.43	18	0.9	Rin2;LOC102555365	Transcription
DMR3:140177001	3	140177001	140178000	1000	1	4.00E-10	-0.3	10	1	Cfap61	
DMR3:140223001	3	140223001	140224000	1000	1	7.60E-12	0.66	14	1.4	Cfap61	
DMR3:140274001	3	140274001	140276000	2000	1	3.90E-09	-0.5	42	2.1	Cfap61;LOC108350485	
DMR3:140306001	3	140306001	140308000	2000	1	1.30E-07	-0.36	30	1.5	Cfap61	
DMR3:140380001	3	140380001	140381000	1000	1	1.90E-09	-0.45	21	2.1	Cfap61	
DMR3:140467001	3	140467001	140470000	3000	1	4.30E-09	-0.43	50	1.67	Ralgapa2;LOC102556455	Signaling
DMR3:140660001	3	140660001	140662000	2000	1	1.70E-12	-0.51	24	1.2	Ralgapa2	Signaling
DMR3:141116001	3	141116001	141118000	2000	1	1.50E-07	-0.38	17	0.85	Kiz	
DMR3:141121001	3	141121001	141126000	5000	1	4.80E-09	-0.33	38	0.76	Kiz	
DMR3:141324001	3	141324001	141325000	1000	1	9.70E-09	-0.52	7	0.7	LOC102547081;Amd1-ps3	
DMR3:142397001	3	142397001	142398000	1000	1	9.10E-08	-0.52	12	1.2	Foxa2	Transcription
DMR3:142761001	3	142761001	142763000	2000	1	1.40E-07	-0.6	11	0.55	Thbd	Receptor
DMR3:143098001	3	143098001	143100000	2000	1	3.60E-09	-0.49	14	0.7	Cstl1;Cst11;LOC102548897;LOC257643	
DMR3:143120001	3	143120001	143121000	1000	1	1.30E-08	0.43	13	1.3	LOC257643;Cst12;Cst8	
DMR3:143571001	3	143571001	143573000	2000	1	7.80E-08	-0.65	5	0.25	RGD1559924	
DMR3:145033001	3	145033001	145034000	1000	1	1.50E-07	0.65	23	2.3	Syndig1	
DMR3:145075001	3	145075001	145076000	1000	1	2.50E-07	-0.53	4	0.4	Syndig1;LOC108350492	
DMR3:145158001	3	145158001	145159000	1000	1	3.70E-08	-0.46	7	0.7	Syndig1	
DMR3:145654001	3	145654001	145657000	3000	1	9.40E-07	-0.54	16	0.53	LOC102554315;Agfg1-ps1	Transcription
DMR3:146317001	3	146317001	146319000	2000	1	5.10E-07	-0.31	18	0.9	RGD1565143;LOC296255	
DMR3:146410001	3	146410001	146412000	2000	1	2.00E-07	-0.41	18	0.9	Apmap;Acss1	Metabolism
DMR3:146576001	3	146576001	146578000	2000	1	8.60E-07	0.51	32	1.6	Entpd6;Pygb	Signaling;Golgi
DMR3:146596001	3	146596001	146599000	3000	1	8.60E-10	-0.55	29	0.97	Pygb	Golgi

DMR3:146600001	3	146600001	146602000	2000	1	1.30E-07	-0.63	6	0.3	Pygb	Golgi
DMR3:146650001	3	146650001	146653000	3000	1	4.00E-08	-0.57	32	1.07	Abhd12	Protease
DMR3:146707001	3	146707001	146708000	1000	1	6.20E-08	-0.38	11	1.1	Gins1;LOC102547581	Transcription
DMR3:146720001	3	146720001	146722000	2000	1	3.20E-08	0.61	28	1.4	Gins1;Ninl	Transcription
DMR3:146971001	3	146971001	146974000	3000	1	2.10E-09	-0.42	29	0.97	LOC108350571;LOC499916;LOC102548446;Nsfl1c	Signaling
DMR3:147082001	3	147082001	147087000	5000	1	1.70E-07	-0.26	48	0.96	Sdcbp2	Transport
DMR3:147137001	3	147137001	147138000	1000	1	6.90E-07	0.4	11	1.1	Snph	Transcription
DMR3:147193001	3	147193001	147194000	1000	1	1.60E-14	0.65	17	1.7	Rad21l1;Tmem74bos	
DMR3:147417001	3	147417001	147420000	3000	1	6.90E-07	0.49	42	1.4	Angpt4	Signaling
DMR3:147489001	3	147489001	147491000	2000	1	4.00E-15	0.65	43	2.15	Fam110a;LOC108350501	
DMR3:147597001	3	147597001	147598000	1000	1	2.50E-10	0.91	100	10	Scrt2	Transcription
DMR3:147703001	3	147703001	147705000	2000	1	5.90E-08	0.48	21	1.05	Csnk2a1	
DMR3:147771001	3	147771001	147772000	1000	1	1.20E-12	0.75	37	3.7	Tbc1d20	Signaling
DMR3:147956001	3	147956001	147961000	5000	1	1.60E-07	0.32	87	1.74	Defb20;Defb22	
DMR3:148006001	3	148006001	148010000	4000	1	1.50E-08	-0.61	51	1.27	LOC100911269;Defb29	
DMR3:148068001	3	148068001	148069000	1000	1	4.40E-10	0.88	24	2.4	Defb27	Signaling
DMR3:148243001	3	148243001	148246000	3000	1	8.70E-09	0.67	34	1.13	Cox4i2	Metabolism
DMR3:148311001	3	148311001	148312000	1000	1	7.30E-08	-0.5	24	2.4	Bcl2l1	
DMR3:148390001	3	148390001	148395000	5000	2	1.20E-08	0.51	82	1.64	LOC102553350;Mylk2	Signaling
DMR3:148406001	3	148406001	148407000	1000	1	9.00E-07	0.45	16	1.6	Mylk2;Foxs1	Signaling;Transcription
DMR3:148566001	3	148566001	148567000	1000	1	1.00E-08	0.55	16	1.6	Ccm2l	
DMR3:148662001	3	148662001	148664000	2000	1	3.50E-09	-0.57	21	1.05	Tm9sf4	Transport
DMR3:148744001	3	148744001	148746000	2000	1	1.50E-09	0.64	20	1	Pofut1	Golgi
DMR3:148804001	3	148804001	148805000	1000	1	8.00E-08	0.61	14	1.4	Kif3b	Cytoskeleton
DMR3:148834001	3	148834001	148837000	3000	1	1.40E-08	-0.41	26	0.87	LOC102554727;Asxl1	
DMR3:149212001	3	149212001	149213000	1000	1	3.50E-12	0.62	17	1.7	Dnmt3b-ps1;Mapre1	Cytoskeleton
DMR3:149293001	3	149293001	149297000	4000	1	2.50E-11	0.7	57	1.43	Efcab8	
DMR3:149310001	3	149310001	149313000	3000	2	1.10E-07	0.44	59	1.97	Efcab8	
DMR3:149341001	3	149341001	149342000	1000	1	3.40E-09	0.51	11	1.1	Sun5	Cytoskeleton
DMR3:149382001	3	149382001	149384000	2000	1	1.70E-07	-0.33	23	1.15	Bpifb2;Bpifb6	
DMR3:149387001	3	149387001	149390000	3000	1	7.10E-10	0.65	47	1.57	Bpifb2;Bpifb6	
DMR3:149702001	3	149702001	149704000	2000	1	1.50E-08	0.55	28	1.4	Bpifb1;Bpifb5	
DMR3:149856001	3	149856001	149858000	2000	1	1.00E-07	0.65	34	1.7	Cdk5rap1	
DMR3:149928001	3	149928001	149932000	4000	1	6.60E-10	-0.53	43	1.07	Cbfa2t2	Transcription
DMR3:150005001	3	150005001	150006000	1000	1	8.40E-07	0.45	7	0.7	Cbfa2t2	Transcription
DMR3:150140001	3	150140001	150145000	5000	1	5.60E-07	-0.37	62	1.24	Zfp341;LOC103691953;Trnaa-agc;LOC103691939	
DMR3:150210001	3	150210001	150215000	5000	1	1.40E-07	-0.4	71	1.42	Chmp4b	Transport
DMR3:150406001	3	150406001	150407000	1000	1	3.80E-13	-0.48	8	0.8	Eif2s2	Translation
DMR3:150499001	3	150499001	150501000	2000	1	2.50E-10	0.69	47	2.35	Asip	Signaling
DMR3:150533001	3	150533001	150536000	3000	2	1.70E-16	0.93	59	1.97	Asip	Signaling
DMR3:150571001	3	150571001	150572000	1000	1	2.20E-15	0.96	25	2.5	Asip	Signaling
DMR3:150590001	3	150590001	150591000	1000	1	5.10E-07	0.43	21	2.1	Ahcy	Metabolism
DMR3:150599001	3	150599001	150602000	3000	1	2.40E-07	-0.36	30	1	Ahcy	Metabolism
DMR3:150605001	3	150605001	150609000	4000	2	3.60E-13	0.46	58	1.45	Ahcy	Metabolism
DMR3:150665001	3	150665001	150667000	2000	1	1.90E-07	-0.36	24	1.2	LOC102549926;Itch	Proteolysis
DMR3:151021001	3	151021001	151024000	3000	1	2.30E-14	1	74	2.47	Ggt7;Acss2	Protease;Metabolism
DMR3:151042001	3	151042001	151043000	1000	1	8.70E-08	-0.36	9	0.9	Acss2	Metabolism
DMR3:151058001	3	151058001	151059000	1000	1	9.00E-07	-0.65	4	0.4	Acss2	Metabolism
DMR3:151082001	3	151082001	151083000	1000	1	2.80E-09	-0.48	9	0.9	Acss2;Gss	Metabolism
DMR3:151129001	3	151129001	151130000	1000	1	9.50E-10	0.75	17	1.7	Myh7b;Mir499	
DMR3:151136001	3	151136001	151137000	1000	1	5.70E-10	0.87	27	2.7	Myh7b;Mir499	
DMR3:151144001	3	151144001	151145000	1000	1	1.60E-07	0.65	24	2.4	Myh7b;Mir499;Trpc4ap	
DMR3:151148001	3	151148001	151152000	4000	1	2.10E-08	0.66	97	2.42	Myh7b;Mir499;Trpc4ap	
DMR3:151281001	3	151281001	151283000	2000	1	2.60E-09	-0.4	31	1.55	Procr	Signaling
DMR3:151295001	3	151295001	151297000	2000	1	3.90E-15	-0.57	40	2	Procr	Signaling
DMR3:151342001	3	151342001	151346000	4000	1	6.10E-07	-0.36	74	1.85	Mmp24	Protease
DMR3:151368001	3	151368001	151369000	1000	1	4.90E-09	-0.42	12	1.2	Eif6;Fam83c	Translation
DMR3:151370001	3	151370001	151372000	2000	1	9.00E-19	0.91	48	2.4	Eif6;Fam83c	Translation
DMR3:151443001	3	151443001	151444000	1000	1	1.10E-07	-0.43	21	2.1	Uqcc1	Transcription
DMR3:151536001	3	151536001	151541000	5000	1	1.30E-15	-0.7	103	2.06	Cep250;LOC102550306	Epigenetic

DMR3:151579001	3	151579001	151581000	2000	1	6.80E-07	-0.41	41	2.05	Fer1l4	Transport
DMR3:152307001	3	152307001	152310000	3000	1	1.10E-08	-0.55	47	1.57	Phf20	
DMR3:152406001	3	152406001	152408000	2000	1	5.70E-11	0.8	40	2	Cnbd2	
DMR3:152455001	3	152455001	152458000	3000	1	9.30E-08	-0.32	39	1.3	Cnbd2	
DMR3:152767001	3	152767001	152769000	2000	1	3.50E-08	-0.34	44	2.2	Dlgap4	Cytoskeleton
DMR3:152944001	3	152944001	152947000	3000	1	3.80E-08	0.4	40	1.33	RGD1307752;Sla2	Signaling
DMR3:152996001	3	152996001	152997000	1000	1	1.40E-09	0.46	18	1.8	Sla2;Ndrg3	Signaling;Protease
DMR3:153144001	3	153144001	153145000	1000	1	1.20E-07	0.48	13	1.3	Soga1	
DMR3:153185001	3	153185001	153186000	1000	1	2.50E-07	-0.4	21	2.1	Soga1;Tldc2	
DMR3:153197001	3	153197001	153199000	2000	1	3.60E-10	0.5	20	1	Soga1;Tldc2	
DMR3:153347001	3	153347001	153349000	2000	1	7.00E-09	0.47	25	1.25	Mroh8	
DMR3:153427001	3	153427001	153429000	2000	1	2.00E-07	-0.36	26	1.3	Rpn2	Golgi
DMR3:153581001	3	153581001	153582000	1000	1	2.40E-08	0.6	48	4.8	Src	
DMR3:154044001	3	154044001	154046000	2000	1	6.40E-08	0.53	88	4.4	LOC100911299;Blcap;Nnat	
DMR3:154218001	3	154218001	154219000	1000	1	1.70E-12	-0.54	20	2	Ctnnbl1	
DMR3:154304001	3	154304001	154306000	2000	1	9.70E-09	0.36	22	1.1	Ctnnbl1	
DMR3:154372001	3	154372001	154375000	3000	1	4.40E-12	0.76	36	1.2	Ctnnbl1	
DMR3:154560001	3	154560001	154563000	3000	1	5.50E-07	0.42	47	1.57	Rprd1b;LOC108350509	Signaling
DMR3:154616001	3	154616001	154619000	3000	1	1.00E-06	-0.31	36	1.2	Tgm2	Transport
DMR3:154726001	3	154726001	154728000	2000	1	7.60E-07	0.5	19	0.95	RGD1563354	Transcription
DMR3:154754001	3	154754001	154755000	1000	1	1.30E-08	-0.4	11	1.1	Bpi	
DMR3:154762001	3	154762001	154763000	1000	1	2.70E-07	-0.45	16	1.6	Bpi	
DMR3:154773001	3	154773001	154774000	1000	1	2.90E-11	0.54	12	1.2	Bpi	
DMR3:154800001	3	154800001	154805000	5000	2	4.10E-09	0.47	67	1.34	Lbp	
DMR3:154821001	3	154821001	154824000	3000	1	4.50E-07	0.34	45	1.5	Lbp;LOC680485;LOC100363723	
DMR3:154869001	3	154869001	154870000	1000	1	1.10E-07	-0.4	27	2.7	Snhg11	
DMR3:154972001	3	154972001	154974000	2000	1	1.30E-07	-0.51	16	0.8	Ralgapb	
DMR3:155045001	3	155045001	155046000	1000	1	2.50E-07	-0.54	18	1.8	Arhgap40	Signaling
DMR3:155131001	3	155131001	155132000	1000	1	5.50E-09	0.5	16	1.6	Actr5	Cytoskeleton
DMR3:155169001	3	155169001	155171000	2000	1	1.60E-08	-0.52	52	2.6	Ppp1r16b	Signaling
DMR3:155189001	3	155189001	155190000	1000	1	4.20E-07	0.68	20	2	Ppp1r16b;RGD1563145	Signaling
DMR3:155192001	3	155192001	155194000	2000	1	1.60E-08	0.56	66	3.3	Ppp1r16b;RGD1563145	Signaling
DMR3:155235001	3	155235001	155237000	2000	1	4.60E-07	-0.45	31	1.55	Ppp1r16b	Signaling
DMR3:156826001	3	156826001	156832000	6000	2	2.60E-09	-0.35	59	0.98	Zhx3	Development
DMR3:156899001	3	156899001	156902000	3000	1	3.60E-08	0.56	59	1.97	Lpin3;LOC102551402;Emilin3	
DMR3:157538001	3	157538001	157539000	1000	1	8.40E-07	0.59	8	0.8	Ptprt	Signaling
DMR3:157554001	3	157554001	157556000	2000	1	5.80E-07	0.43	15	0.75	Ptprt	Signaling
DMR3:157564001	3	157564001	157565000	1000	1	6.90E-22	1.13	23	2.3	Ptprt	Signaling
DMR3:157618001	3	157618001	157620000	2000	1	2.40E-07	-0.27	20	1	Ptprt	Signaling
DMR3:157628001	3	157628001	157632000	4000	2	2.30E-10	-0.76	27	0.68	Ptprt;LOC108350516	Signaling
DMR3:157718001	3	157718001	157721000	3000	1	2.00E-07	0.39	33	1.1	Ptprt	Signaling
DMR3:157799001	3	157799001	157801000	2000	1	9.20E-08	-0.34	37	1.85	Ptprt	Signaling
DMR3:157824001	3	157824001	157825000	1000	1	1.10E-07	0.52	9	0.9	Ptprt	Signaling
DMR3:158013001	3	158013001	158015000	2000	2	2.40E-08	0.36	53	2.65	Ptprt	Signaling
DMR3:158054001	3	158054001	158057000	3000	1	3.60E-09	0.56	50	1.67	Ptprt	Signaling
DMR3:159303001	3	159303001	159304000	1000	1	1.90E-19	0.6	18	1.8	LOC100362466;Srsf6	Translation
DMR3:159406001	3	159406001	159407000	1000	1	8.10E-07	0.57	11	1.1	Ift52	
DMR3:159573001	3	159573001	159581000	8000	1	2.40E-09	0.62	130	1.62	Tox2	
DMR3:159591001	3	159591001	159592000	1000	1	1.00E-13	0.82	35	3.5	Tox2	
DMR3:159594001	3	159594001	159595000	1000	1	1.10E-08	-0.43	15	1.5	Tox2	
DMR3:159603001	3	159603001	159607000	4000	1	1.10E-11	0.72	73	1.82	Tox2	
DMR3:159624001	3	159624001	159627000	3000	1	5.60E-10	0.6	48	1.6	Tox2	
DMR3:159647001	3	159647001	159648000	1000	1	7.10E-11	0.7	27	2.7	Tox2	
DMR3:159653001	3	159653001	159656000	3000	1	1.90E-18	0.91	54	1.8	Tox2	
DMR3:159671001	3	159671001	159674000	3000	1	8.70E-20	0.89	51	1.7	Tox2	
DMR3:159680001	3	159680001	159685000	5000	1	1.20E-12	0.66	88	1.76	Tox2	
DMR3:159692001	3	159692001	159696000	4000	2	2.30E-12	-0.73	50	1.25	Tox2	
DMR3:159709001	3	159709001	159713000	4000	1	6.70E-07	0.36	76	1.9	Jph2	
DMR3:159721001	3	159721001	159724000	3000	1	3.90E-09	0.37	54	1.8	Jph2	
DMR3:159728001	3	159728001	159730000	2000	1	8.50E-07	0.4	32	1.6	Jph2	
DMR3:159763001	3	159763001	159764000	1000	1	9.70E-10	0.58	13	1.3	Jph2	

DMR3:159774001	3	159774001	159776000	2000	1	9.00E-19	1.16	47	2.35	Jph2;Oser1	
DMR3:159817001	3	159817001	159818000	1000	1	3.90E-07	-0.43	15	1.5	Gdap1l1	
DMR3:159864001	3	159864001	159866000	2000	1	5.30E-07	0.41	25	1.25	Fitm2;LOC103691964	
DMR3:159908001	3	159908001	159913000	5000	4	1.10E-15	1.06	130	2.6	LOC102548241;R3hdml;Hnf4a	Immune;Transcription
DMR3:159930001	3	159930001	159937000	7000	1	4.40E-10	0.4	93	1.33	Hnf4a	Transcription
DMR3:160104001	3	160104001	160105000	1000	1	5.60E-10	0.57	9	0.9	Pkig	Signaling
DMR3:160107001	3	160107001	160112000	5000	2	8.60E-17	-0.56	65	1.3	Pkig;Ada	Signaling;Metabolism
DMR3:160120001	3	160120001	160121000	1000	1	3.00E-11	0.63	11	1.1	Pkig;Ada	Signaling;Metabolism
DMR3:160123001	3	160123001	160125000	2000	1	8.80E-07	0.35	36	1.8	Pkig;Ada	Signaling;Metabolism
DMR3:160441001	3	160441001	160443000	2000	1	2.50E-07	0.35	46	2.3	Pabpc1l;Tomm34	
DMR3:160444001	3	160444001	160445000	1000	1	9.30E-09	0.52	16	1.6	Pabpc1l;Tomm34	
DMR3:160477001	3	160477001	160480000	3000	2	2.30E-12	-0.53	62	2.07	Stk4	Signaling
DMR3:160510001	3	160510001	160511000	1000	1	2.10E-09	-0.46	22	2.2	Stk4	Signaling
DMR3:160613001	3	160613001	160615000	2000	1	3.50E-08	-0.52	38	1.9	Wfdc15b	Protease; Proteolysis
DMR3:160837001	3	160837001	160838000	1000	1	7.00E-07	0.63	17	1.7	Matn4	Extracellular Matrix
DMR3:160854001	3	160854001	160855000	1000	1	7.70E-08	0.6	24	2.4	Matn4;Rbpjl	Extracellular Matrix;Transcription
DMR3:160856001	3	160856001	160857000	1000	1	3.70E-08	0.53	13	1.3	Matn4;Rbpjl	Extracellular Matrix;Transcription
DMR3:160862001	3	160862001	160864000	2000	1	7.20E-14	0.72	63	3.15	Matn4;Rbpjl;Sdc4	Extracellular Matrix;Transcription;Receptor
DMR3:160871001	3	160871001	160872000	1000	1	3.00E-07	0.59	35	3.5	Rbpjl;Sdc4	Transcription;Receptor
DMR3:160876001	3	160876001	160877000	1000	1	7.60E-07	0.43	11	1.1	Sdc4	Receptor
DMR3:160956001	3	160956001	160958000	2000	1	1.50E-09	0.49	29	1.45	Pigt	
DMR3:160986001	3	160986001	160991000	5000	1	2.00E-10	0.82	87	1.74	RGD1309998	
DMR3:160999001	3	160999001	1.61E+08	1000	1	5.40E-11	-0.62	5	0.5	RGD1309998	
DMR3:161069001	3	161069001	161071000	2000	1	5.90E-08	-0.48	24	1.2	Wfdc8;Wfdc6b;LOC100362743	
DMR3:161079001	3	161079001	161080000	1000	1	4.80E-07	-0.32	15	1.5	Wfdc6b;LOC100362743	
DMR3:161093001	3	161093001	161094000	1000	1	3.50E-07	-0.44	18	1.8	Wfdc16	Protease; Proteolysis
DMR3:161112001	3	161112001	161114000	2000	1	7.70E-08	-0.51	30	1.5	Wfdc9;Wfdc10	Protease; Proteolysis
DMR3:161135001	3	161135001	161140000	5000	1	6.10E-09	-0.39	89	1.78	Wfdc11;LOC108350573;Wfdc13	Protease; Proteolysis
DMR3:161156001	3	161156001	161158000	2000	1	5.00E-07	0.53	43	2.15	Wfdc13;Spint4	Protease; Proteolysis
DMR3:161184001	3	161184001	161186000	2000	1	4.60E-07	-0.34	37	1.85	Spint5p;LOC108350622;Wfdc3	Protease; Proteolysis
DMR3:161230001	3	161230001	161232000	2000	1	2.20E-08	0.51	26	1.3	Dnttip1;Ube2c	Transcription;Proteolysis
DMR3:161258001	3	161258001	161261000	3000	1	8.00E-10	0.67	69	2.3	Snx21;Acot8	Cytoskeleton;Metabolism
DMR3:161301001	3	161301001	161303000	2000	1	5.30E-07	0.48	43	2.15	Zswim1;Spata25;Neurl2;Ctsa;Pltp	Proteolysis;Protease
DMR3:161326001	3	161326001	161327000	1000	1	2.30E-08	0.44	13	1.3	Pltp	
DMR3:161333001	3	161333001	161335000	2000	1	6.20E-08	0.33	19	0.95	Pcif1;LOC103691999	
DMR3:161410001	3	161410001	161412000	2000	1	5.70E-07	0.31	29	1.45	Mmp9	Protease
DMR3:161450001	3	161450001	161451000	1000	1	4.90E-08	0.48	10	1	Slc12a5	Transport
DMR3:161524001	3	161524001	161529000	5000	1	4.90E-08	0.47	88	1.76	Cd40	Receptor
DMR3:161683001	3	161683001	161684000	1000	1	8.20E-07	0.43	7	0.7	Cdh22	Cytoskeleton
DMR3:161792001	3	161792001	161796000	4000	1	5.30E-09	0.43	59	1.48	Cdh22;Slc35c2	Cytoskeleton;Transport
DMR3:161802001	3	161802001	161806000	4000	1	2.00E-07	0.41	77	1.93	Cdh22;Slc35c2;Elmo2	Cytoskeleton;Transport;Cytoskeleton
DMR3:161870001	3	161870001	161872000	2000	1	7.70E-08	0.68	53	2.65	Cdh22	Cytoskeleton
DMR3:161891001	3	161891001	161892000	1000	1	2.40E-10	0.62	24	2.4	Cdh22	Cytoskeleton
DMR3:161893001	3	161893001	161895000	2000	1	3.80E-10	0.48	35	1.75	Cdh22	Cytoskeleton
DMR3:162022001	3	162022001	162024000	2000	1	6.80E-07	0.51	45	2.25	Zfp663	Transcription
DMR3:162028001	3	162028001	162032000	4000	1	4.30E-10	-0.5	88	2.2	Zfp663	Transcription
DMR3:162046001	3	162046001	162047000	1000	1	1.30E-12	0.74	28	2.8	Zfp663;LOC685574	Transcription
DMR3:162075001	3	162075001	162078000	3000	1	4.50E-09	0.62	93	3.1	LOC685574;Zfp334;Slc13a3	Transport
DMR3:162101001	3	162101001	162103000	2000	1	2.40E-07	0.39	28	1.4	Slc13a3	Transport
DMR3:162141001	3	162141001	162146000	5000	1	6.80E-09	0.53	74	1.48	Slc13a3	Transport
DMR3:162157001	3	162157001	162160000	3000	1	1.70E-07	0.63	28	0.93	Slc13a3;LOC685619	Transport;Metabolism
DMR3:162185001	3	162185001	162186000	1000	1	8.70E-08	-0.47	9	0.9	Slc2a10	

DMR3:162289001	3	162289001	162291000	2000	1	3.40E-08	-0.46	47	2.35	Eya2	
DMR3:162308001	3	162308001	162312000	4000	2	6.80E-10	0.36	61	1.52	Eya2	
DMR3:162327001	3	162327001	162329000	2000	1	4.20E-11	0.55	28	1.4	Eya2	
DMR3:162360001	3	162360001	162362000	2000	1	3.10E-11	-0.43	41	2.05	Eya2	
DMR3:162396001	3	162396001	162399000	3000	1	6.10E-09	0.65	51	1.7	Eya2	
DMR3:162413001	3	162413001	162416000	3000	1	7.10E-08	0.42	38	1.27	Eya2	
DMR3:162432001	3	162432001	162436000	4000	1	4.50E-08	0.38	113	2.83	Eya2	
DMR3:162441001	3	162441001	162443000	2000	1	1.30E-10	-0.38	42	2.1	Eya2	
DMR3:162448001	3	162448001	162451000	3000	1	3.80E-07	-0.51	38	1.27	Eya2	
DMR3:162457001	3	162457001	162460000	3000	1	2.90E-08	0.44	50	1.67	Eya2	
DMR3:162538001	3	162538001	162540000	2000	1	4.70E-08	-0.37	60	3	Zmynd8	
DMR3:162557001	3	162557001	162558000	1000	1	6.20E-08	0.57	15	1.5	Zmynd8	
DMR3:162719001	3	162719001	162721000	2000	1	3.50E-08	-0.5	20	1	Ncoa3	Epigenetic
DMR3:162807001	3	162807001	162809000	2000	1	2.70E-07	0.34	39	1.95	Sulf2	Metabolism
DMR3:162810001	3	162810001	162812000	2000	1	7.90E-09	0.59	29	1.45	Sulf2	Metabolism
DMR3:162827001	3	162827001	162830000	3000	1	2.10E-07	0.41	53	1.77	Sulf2;LOC108350522	Metabolism
DMR3:162856001	3	162856001	162861000	5000	1	3.80E-07	0.52	75	1.5	Sulf2	Metabolism
DMR3:163329001	3	163329001	163332000	3000	1	4.30E-09	0.49	79	2.63	Prex1	Transcription
DMR3:163337001	3	163337001	163340000	3000	1	6.00E-07	-0.36	43	1.43	Prex1	Transcription
DMR3:163341001	3	163341001	163348000	7000	1	2.60E-07	0.42	136	1.94	Prex1	Transcription
DMR3:163362001	3	163362001	163364000	2000	1	5.50E-09	0.63	37	1.85	Prex1	Transcription
DMR3:163393001	3	163393001	163396000	3000	1	2.10E-09	0.6	46	1.53	Prex1	Transcription
DMR3:163407001	3	163407001	163410000	3000	1	1.40E-07	0.41	56	1.87	Prex1	Transcription
DMR3:163434001	3	163434001	163437000	3000	2	7.70E-11	0.57	46	1.53	Prex1	Transcription
DMR3:163472001	3	163472001	163476000	4000	1	9.70E-07	0.38	54	1.35	Prex1	Transcription
DMR3:163568001	3	163568001	163571000	3000	2	1.40E-08	-0.5	96	3.2	Arfgef2	Transcription
DMR3:163791001	3	163791001	163794000	3000	1	9.60E-08	0.47	39	1.3	Ddx27;Znfx1	
DMR3:163911001	3	163911001	163915000	4000	1	6.60E-08	0.37	66	1.65	Kcnb1	Transport
DMR3:163934001	3	163934001	163935000	1000	1	1.00E-08	0.41	18	1.8	Kcnb1;LOC102552303	Transport
DMR3:164267001	3	164267001	164269000	2000	1	2.60E-07	0.49	22	1.1	Rnf114;Snai1	Transcription
DMR3:164270001	3	164270001	164274000	4000	2	7.30E-11	0.9	59	1.48	Rnf114;Snai1	Transcription
DMR3:164344001	3	164344001	164345000	1000	1	2.70E-07	0.49	28	2.8	LOC103691970;Ube2v1	
DMR3:164666001	3	164666001	164669000	3000	1	7.70E-07	-0.51	42	1.4	Ptpn1	Signaling
DMR3:164719001	3	164719001	164726000	7000	1	6.70E-19	0.91	128	1.83	Ptpn1;Fam65c	Signaling
DMR3:164740001	3	164740001	164745000	5000	2	3.20E-07	0.46	82	1.64	Fam65c	
DMR3:164756001	3	164756001	164757000	1000	1	2.60E-13	0.47	19	1.9	Fam65c	
DMR3:164760001	3	164760001	164763000	3000	1	6.70E-09	0.35	35	1.17	Fam65c	
DMR3:164788001	3	164788001	164791000	3000	1	4.70E-10	0.68	78	2.6	Fam65c	
DMR3:164792001	3	164792001	164794000	2000	1	6.90E-07	0.52	33	1.65	Fam65c	
DMR3:164842001	3	164842001	164844000	2000	1	9.80E-09	-0.46	44	2.2	Pard6b;LOC102552887	Cell Junction
DMR3:164945001	3	164945001	164947000	2000	1	3.40E-07	-0.45	30	1.5	Adnp	
DMR3:165021001	3	165021001	165024000	3000	1	3.00E-18	1.02	53	1.77	Kcng1	Transport
DMR3:165027001	3	165027001	165030000	3000	1	7.00E-11	0.46	71	2.37	Kcng1	Transport
DMR3:165239001	3	165239001	165242000	3000	2	1.20E-09	0.88	45	1.5	LOC102554533;Nfatc2	Transcription
DMR3:165333001	3	165333001	165334000	1000	1	1.60E-12	0.6	14	1.4	Nfatc2	Transcription
DMR3:165338001	3	165338001	165340000	2000	2	1.60E-16	1.23	78	3.9	Nfatc2	Transcription
DMR3:165341001	3	165341001	165351000	10000	1	1.20E-11	0.66	179	1.79	Nfatc2	Transcription
DMR3:165377001	3	165377001	165381000	4000	1	1.00E-10	0.48	81	2.02	Nfatc2;LOC102554478	Transcription
DMR3:165384001	3	165384001	165388000	4000	3	2.10E-08	0.68	59	1.48	Nfatc2;LOC102554478	Transcription
DMR3:165395001	3	165395001	165396000	1000	1	8.80E-07	0.52	13	1.3	Atp9a	Transport
DMR3:165420001	3	165420001	165423000	3000	2	7.90E-08	0.39	69	2.3	Atp9a	Transport
DMR3:165427001	3	165427001	165431000	4000	1	3.90E-08	-0.47	87	2.17	Atp9a	Transport
DMR3:165692001	3	165692001	165693000	1000	1	4.00E-08	0.5	28	2.8	Zfp93	
DMR3:165708001	3	165708001	165710000	2000	1	3.30E-10	0.65	58	2.9	Zfp93;Zfp64	Transcription
DMR3:165712001	3	165712001	165714000	2000	1	6.00E-09	0.62	58	2.9	Zfp64	Transcription
DMR3:165732001	3	165732001	165737000	5000	2	2.40E-09	0.57	91	1.82	Zfp64	Transcription
DMR3:166478001	3	166478001	166482000	4000	2	4.80E-11	0.76	85	2.12	LOC103691977;Tshz2	Transcription
DMR3:166489001	3	166489001	166492000	3000	1	2.20E-07	0.29	28	0.93	Tshz2	Transcription
DMR3:166494001	3	166494001	166496000	2000	1	6.00E-08	-0.34	29	1.45	Tshz2	Transcription
DMR3:166518001	3	166518001	166520000	2000	1	4.20E-07	-0.32	60	3	Tshz2	Transcription
DMR3:166529001	3	166529001	166533000	4000	1	1.00E-07	0.44	105	2.62	Tshz2;LOC102556304	Transcription
DMR3:166567001	3	166567001	166570000	3000	1	1.10E-08	-0.52	68	2.27	Tshz2	Transcription

DMR3:166580001	3	166580001	166586000	6000	2	2.20E-09	0.48	170	2.83	Tshz2	Transcription
DMR3:166604001	3	166604001	166608000	4000	3	7.60E-22	1.02	77	1.93	Tshz2	Transcription
DMR3:166667001	3	166667001	166670000	3000	1	4.50E-07	-0.37	62	2.07	Tshz2	Transcription
DMR3:166672001	3	166672001	166675000	3000	1	5.30E-09	-0.49	76	2.53	Tshz2	Transcription
DMR3:166676001	3	166676001	166678000	2000	1	2.50E-07	-0.32	32	1.6	Tshz2	Transcription
DMR3:166682001	3	166682001	166684000	2000	1	5.00E-07	-0.43	33	1.65	Tshz2	Transcription
DMR3:166687001	3	166687001	166688000	1000	1	2.20E-09	0.37	20	2	Tshz2	Transcription
DMR3:166737001	3	166737001	166738000	1000	1	1.00E-11	0.61	18	1.8	Tshz2	Transcription
DMR3:166751001	3	166751001	166753000	2000	2	8.60E-10	-0.38	61	3.05	Tshz2	Transcription
DMR3:166969001	3	166969001	166973000	4000	2	1.30E-14	1.21	98	2.45	Zfp217	Transcription
DMR3:167021001	3	167021001	167024000	3000	2	2.10E-10	-0.51	52	1.73	Zfp217	Transcription
DMR3:167970001	3	167970001	167972000	2000	1	8.20E-22	1.14	54	2.7	Bcas1	
DMR3:168003001	3	168003001	168008000	5000	1	3.00E-09	-0.54	86	1.72	Bcas1	
DMR3:168032001	3	168032001	168036000	4000	1	3.00E-09	0.56	74	1.85	Bcas1	
DMR3:168381001	3	168381001	168383000	2000	1	2.20E-09	0.62	32	1.6	Dok5	
DMR3:168398001	3	168398001	168403000	5000	2	1.40E-21	1.06	91	1.82	Dok5	
DMR3:168410001	3	168410001	168413000	3000	1	1.60E-07	0.67	54	1.8	Dok5	
DMR3:168415001	3	168415001	168419000	4000	1	9.50E-10	-0.68	51	1.27	Dok5	
DMR3:168425001	3	168425001	168428000	3000	1	6.50E-07	-0.34	45	1.5	Dok5	
DMR3:168447001	3	168447001	168451000	4000	1	6.90E-07	-0.31	52	1.3	Dok5	
DMR3:168492001	3	168492001	168500000	8000	1	6.40E-07	0.58	137	1.71	Dok5;LOC102552784	
DMR3:170042001	3	170042001	170043000	1000	1	1.50E-09	0.49	8	0.8	Cbln4	
DMR3:170045001	3	170045001	170046000	1000	1	9.90E-07	0.49	29	2.9	Cbln4	
DMR3:170249001	3	170249001	170250000	1000	1	2.80E-10	-0.54	22	2.2	Mc3r	Signaling
DMR3:170257001	3	170257001	170262000	5000	1	4.40E-07	0.38	67	1.34	Mc3r;LOC102549927	Signaling
DMR3:170387001	3	170387001	170390000	3000	1	2.20E-08	-0.42	67	2.23	Aurka;Cstf1;Cass4	Signaling
DMR3:170960001	3	170960001	170962000	2000	1	1.00E-07	-0.36	55	2.75	Bmp7	Growth Factors
DMR3:170988001	3	170988001	170990000	2000	1	2.10E-10	-0.39	41	2.05	Spo11	Transcription
DMR3:171001001	3	171001001	171003000	2000	1	8.20E-07	0.49	43	2.15	Spo11;Rae1	Transcription;Metabolism
DMR3:171146001	3	171146001	171147000	1000	1	3.00E-09	0.39	11	1.1	LOC102550488;Ctcfl	Transcription
DMR3:171167001	3	171167001	171168000	1000	1	2.90E-09	0.64	27	2.7	Ctcfl;LOC103691985	Transcription
DMR3:171212001	3	171212001	171217000	5000	1	3.40E-09	0.67	118	2.36	Pck1	Signaling
DMR3:171218001	3	171218001	171219000	1000	1	6.40E-08	0.63	26	2.6	Pck1	Signaling
DMR3:171270001	3	171270001	171273000	3000	1	1.90E-12	0.74	38	1.27	Zbp1	Transcription
DMR3:171274001	3	171274001	171276000	2000	2	3.50E-10	0.65	38	1.9	Zbp1	Transcription
DMR3:171882001	3	171882001	171884000	2000	1	2.60E-08	0.62	47	2.35	Apcdd1l	
DMR3:171885001	3	171885001	171886000	1000	1	2.80E-08	0.44	26	2.6	Apcdd1l	
DMR3:171890001	3	171890001	171892000	2000	1	7.00E-08	0.44	30	1.5	Apcdd1l	
DMR3:171893001	3	171893001	171896000	3000	2	3.20E-09	0.46	55	1.83	Apcdd1l	
DMR3:171906001	3	171906001	171907000	1000	1	2.30E-11	-0.44	13	1.3	Apcdd1l	
DMR3:171918001	3	171918001	171920000	2000	1	1.20E-08	-0.51	23	1.15	Apcdd1l	
DMR3:171925001	3	171925001	171932000	7000	2	1.50E-10	-0.57	92	1.31	Apcdd1l	
DMR3:171939001	3	171939001	171940000	1000	1	6.10E-08	0.37	8	0.8	Apcdd1l	
DMR3:171952001	3	171952001	171954000	2000	1	2.60E-10	0.5	25	1.25	Apcdd1l;LOC102548149	
DMR3:171962001	3	171962001	171964000	2000	1	1.90E-11	0.78	44	2.2	Apcdd1l;LOC102548149	
DMR3:172190001	3	172190001	172193000	3000	1	1.20E-10	-0.57	82	2.73	Stx16;Npepl1	Transcription;Protease
DMR3:172368001	3	172368001	172369000	1000	1	6.80E-08	-0.31	26	2.6	Mir298;Gnas;LOC102548488	Signaling
DMR3:172372001	3	172372001	172374000	2000	1	5.30E-10	0.63	73	3.65	Gnas;LOC102548488	Signaling
DMR3:172377001	3	172377001	172378000	1000	1	8.10E-13	0.83	36	3.6	Gnas;LOC102548488	Signaling
DMR3:172404001	3	172404001	172405000	1000	1	4.60E-08	0.42	17	1.7	Gnas;LOC103692002	Signaling
DMR3:172434001	3	172434001	172436000	2000	1	2.00E-09	0.81	46	2.3	Gnas	Signaling
DMR3:172438001	3	172438001	172440000	2000	1	3.40E-12	0.88	37	1.85	Gnas	Signaling
DMR3:172549001	3	172549001	172554000	5000	2	5.20E-16	1.08	89	1.78	Tubb1;Atp5e	Cytoskeleton
DMR3:173788001	3	173788001	173790000	2000	1	6.20E-08	0.52	39	1.95	Phactr3	Signaling
DMR3:173852001	3	173852001	173856000	4000	1	3.80E-07	0.33	54	1.35	Phactr3	Signaling
DMR3:173972001	3	173972001	173974000	2000	1	1.20E-07	0.54	33	1.65	Cdh26	Cytoskeleton
DMR3:173979001	3	173979001	173982000	3000	1	7.00E-07	0.47	22	0.73	Cdh26	Cytoskeleton
DMR3:174010001	3	174010001	174014000	4000	1	6.00E-08	0.59	51	1.27	Cdh26	Cytoskeleton
DMR3:175228001	3	175228001	175229000	1000	1	1.80E-08	0.63	9	0.9	Cdh4	Cytoskeleton
DMR3:175249001	3	175249001	175251000	2000	1	2.20E-08	0.42	26	1.3	Cdh4	Cytoskeleton
DMR3:175278001	3	175278001	175282000	4000	1	4.10E-09	0.56	107	2.67	Cdh4	Cytoskeleton

DMR3:175283001	3	175283001	175284000	1000	1	8.30E-07	-0.33	15	1.5	Cdh4	Cytoskeleton
DMR3:175422001	3	175422001	175424000	2000	1	1.40E-08	-0.39	37	1.85	Lsm14b;Psma7;Ss18l1	Metabolism;Protease;Transcription
DMR3:175444001	3	175444001	175448000	4000	1	6.40E-08	-0.41	79	1.98	Ss18l1;Mtg2	Transcription
DMR3:175568001	3	175568001	175570000	2000	1	1.10E-10	0.68	54	2.7	Lama5	Extracellular Matrix
DMR3:175593001	3	175593001	175595000	2000	1	9.20E-09	0.58	29	1.45	Lama5	Extracellular Matrix
DMR3:175610001	3	175610001	175611000	1000	1	1.10E-09	0.79	13	1.3	Lama5	Extracellular Matrix
DMR3:175656001	3	175656001	175658000	2000	1	3.70E-09	0.46	26	1.3	LOC103691993;Rbbp8nl	Transcription
DMR3:175668001	3	175668001	175674000	6000	1	3.30E-09	0.53	44	0.73	Rbbp8nl	Transcription
DMR3:175763001	3	175763001	175765000	2000	1	1.40E-07	0.6	25	1.25	LOC102552888;Mir1b	
DMR3:175860001	3	175860001	175867000	7000	1	1.30E-08	0.43	134	1.91	Slco4a1;LOC102552621;LOC102552550	Transport
DMR3:176042001	3	176042001	176044000	2000	1	3.40E-13	0.73	57	2.85	Ntsr1	Signaling
DMR3:176156001	3	176156001	176158000	2000	1	9.30E-10	0.79	41	2.05	LOC108350539;LOC690498;Dido1	Transcription
DMR3:176164001	3	176164001	176165000	1000	1	5.80E-11	0.85	40	4	LOC108350539;Dido1	Transcription
DMR3:176277001	3	176277001	176280000	3000	1	3.50E-07	-0.61	41	1.37	Bhlhe23	Transcription
DMR3:176466001	3	176466001	176467000	1000	1	1.10E-11	0.65	24	2.4	Nkain4;Arfgap1	Signaling
DMR3:176524001	3	176524001	176526000	2000	1	7.30E-13	0.63	32	1.6	Col20a1;LOC102546950;Chrna4	Extracellular Matrix;Ion Channel
DMR3:176527001	3	176527001	176531000	4000	1	2.70E-11	-0.51	42	1.05	Col20a1;LOC102546950;Chrna4	Extracellular Matrix;Ion Channel
DMR3:176591001	3	176591001	176593000	2000	1	1.60E-07	0.59	49	2.45	Kcnq2	Transport
DMR3:176597001	3	176597001	176599000	2000	1	2.10E-07	0.42	40	2	Kcnq2	Transport
DMR3:176643001	3	176643001	176644000	1000	1	4.10E-07	0.48	10	1	Kcnq2	Transport
DMR3:176665001	3	176665001	176666000	1000	1	3.70E-07	0.62	20	2	Eef1a2	Translation
DMR3:176667001	3	176667001	176668000	1000	1	1.10E-09	0.63	14	1.4	Eef1a2	Translation
DMR3:176711001	3	176711001	176714000	3000	1	6.30E-09	0.51	43	1.43	Ptk6;Srms;LOC108350541;Fndc11	
DMR3:176741001	3	176741001	176743000	2000	1	2.80E-07	0.61	38	1.9	Helz2	Transcription
DMR3:176768001	3	176768001	176769000	1000	1	9.00E-09	-0.52	6	0.6	Gmeb2	
DMR3:176880001	3	176880001	176882000	2000	1	3.90E-07	0.56	27	1.35	Zgpat;Lime1;Zbtb46	Cytoskeleton;Cytoskeleton
DMR3:176925001	3	176925001	176929000	4000	1	3.90E-08	0.49	56	1.4	Zbtb46	Cytoskeleton
DMR3:177102001	3	177102001	177106000	4000	1	1.10E-10	-0.49	33	0.82	Samd10;Prpf6	Translation
DMR3:177234001	3	177234001	177236000	2000	1	4.80E-07	0.34	30	1.5	Oprl1	Signaling
DMR4:718001	4	718001	719000	1000	1	3.00E-10	0.93	63	6.3	Shh;LOC108350640;LOC102546975	
DMR4:1454001	4	1454001	1455000	1000	1	6.00E-11	0.75	16	1.6	Olr1233;Olr1234	Receptor
DMR4:1475001	4	1475001	1476000	1000	1	7.70E-08	0.53	16	1.6	Olr1235;Olr1236	Receptor
DMR4:1836001	4	1836001	1841000	5000	1	8.40E-08	-0.29	47	0.94	Olr1096	Receptor
DMR4:3993001	4	3993001	3994000	1000	1	6.90E-08	0.61	9	0.9	Paxip1	
DMR4:4076001	4	4076001	4079000	3000	1	5.20E-07	0.54	41	1.37	Dpp6	Protease
DMR4:4082001	4	4082001	4083000	1000	1	2.40E-21	0.99	5	0.5	Dpp6	Protease
DMR4:4114001	4	4114001	4120000	6000	2	9.00E-08	-0.31	54	0.9	Dpp6	Protease
DMR4:4164001	4	4164001	4165000	1000	1	8.70E-08	0.45	12	1.2	Dpp6	Protease
DMR4:4298001	4	4298001	4300000	2000	1	7.40E-11	0.51	29	1.45	Dpp6	Protease
DMR4:4372001	4	4372001	4373000	1000	1	1.50E-07	-0.39	8	0.8	Dpp6	Protease
DMR4:4753001	4	4753001	4754000	1000	1	2.10E-08	-0.49	4	0.4	Dpp6	Protease
DMR4:4778001	4	4778001	4781000	3000	2	4.50E-08	-0.55	24	0.8	Dpp6	Protease
DMR4:4831001	4	4831001	4833000	2000	1	1.50E-07	0.4	26	1.3	Dpp6	Protease
DMR4:4845001	4	4845001	4847000	2000	1	3.50E-07	-0.48	18	0.9	Dpp6	Protease
DMR4:4888001	4	4888001	4891000	3000	1	2.60E-09	-0.46	21	0.7	Dpp6	Protease
DMR4:6016001	4	6016001	6019000	3000	1	7.40E-10	-0.54	36	1.2	RGD1560124	
DMR4:6099001	4	6099001	6100000	1000	1	1.90E-11	-0.63	6	0.6	Kmt2c	
DMR4:6132001	4	6132001	6133000	1000	1	6.80E-07	-0.48	12	1.2	Kmt2c	
DMR4:6286001	4	6286001	6288000	2000	1	2.60E-09	-0.47	29	1.45	Kmt2c;Galnt11	Golgi
DMR4:6305001	4	6305001	6306000	1000	1	2.00E-07	-0.34	21	2.1	Galnt11;LOC108350646	Golgi
DMR4:6589001	4	6589001	6590000	1000	1	9.50E-08	0.42	18	1.8	Prkag2	Signaling
DMR4:6609001	4	6609001	6611000	2000	1	2.10E-08	0.7	24	1.2	Prkag2	Signaling
DMR4:6720001	4	6720001	6721000	1000	1	3.70E-07	0.36	14	1.4	Prkag2;LOC102554193	Signaling
DMR4:6755001	4	6755001	6757000	2000	1	9.10E-07	-0.36	49	2.45	Prkag2	Signaling

DMR4:6877001	4	6877001	6880000	3000	3	4.90E-13	0.41	58	1.93	Rheb	Signaling
DMR4:6895001	4	6895001	6898000	3000	1	5.90E-08	0.48	57	1.9	Crygn	
DMR4:6917001	4	6917001	6919000	2000	1	3.60E-07	0.37	37	1.85	Crygn	
DMR4:6942001	4	6942001	6946000	4000	2	1.10E-08	0.59	59	1.48	Wdr86;Nub1	
DMR4:7077001	4	7077001	7078000	1000	1	2.70E-07	-0.5	6	0.6	Smarcd3	Epigenetic
DMR4:7132001	4	7132001	7134000	2000	1	9.50E-08	0.4	29	1.45	Abcf2	Translation
DMR4:7145001	4	7145001	7150000	5000	2	1.80E-13	0.7	110	2.2	Abcf2;Iqca1l;Asb10	Translation;Transport
DMR4:7220001	4	7220001	7221000	1000	1	1.30E-08	0.57	33	3.3	Agap3;LOC102554667	
DMR4:7292001	4	7292001	7295000	3000	2	1.50E-16	0.91	62	2.07	Slc4a2;Cdk5;Asic3;Abcb8	Transport;Signaling;Transport;Transport
DMR4:7325001	4	7325001	7326000	1000	1	1.10E-10	0.79	33	3.3	Atg9b;Nos3	Metabolism
DMR4:7331001	4	7331001	7336000	5000	1	4.70E-07	0.29	66	1.32	Atg9b;Nos3	Metabolism
DMR4:7337001	4	7337001	7340000	3000	1	5.30E-08	0.62	69	2.3	Nos3	Metabolism
DMR4:7347001	4	7347001	7349000	2000	1	2.90E-08	0.37	16	0.8	Nos3;Kcnh2	Metabolism;Transport
DMR4:7382001	4	7382001	7385000	3000	1	3.80E-10	0.55	75	2.5	Kcnh2	Transport
DMR4:7397001	4	7397001	7399000	2000	1	7.30E-07	-0.7	15	0.75	Kcnh2	Transport
DMR4:7559001	4	7559001	7560000	1000	1	8.60E-08	-0.55	7	0.7	Klhl7;LOC100364673	Cytoskeleton
DMR4:7574001	4	7574001	7576000	2000	1	3.00E-11	-0.43	27	1.35	Klhl7;LOC100364673;LOC108350648	Cytoskeleton
DMR4:7588001	4	7588001	7591000	3000	1	3.80E-08	-0.75	9	0.3	Klhl7;LOC108350648	Cytoskeleton
DMR4:7698001	4	7698001	7699000	1000	1	5.20E-08	-0.37	15	1.5	Fam126a;LOC108350649	
DMR4:7719001	4	7719001	7722000	3000	1	1.00E-07	-0.42	54	1.8	Fam126a	
DMR4:7762001	4	7762001	7767000	5000	1	1.40E-09	0.53	65	1.3	Fam126a;LOC103692024	
DMR4:7774001	4	7774001	7776000	2000	1	1.30E-08	0.39	33	1.65	Fam126a;LOC103692024	
DMR4:7893001	4	7893001	7895000	2000	1	2.80E-07	-0.28	45	2.25	Rint1;Pus7	
DMR4:7923001	4	7923001	7924000	1000	1	2.80E-08	-0.37	22	2.2	Pus7	
DMR4:7937001	4	7937001	7939000	2000	1	4.80E-08	-0.4	23	1.15	Pus7	
DMR4:8067001	4	8067001	8068000	1000	1	9.10E-08	-0.62	15	1.5	Srpk2	Signaling
DMR4:8138001	4	8138001	8140000	2000	1	5.10E-07	-0.46	33	1.65	Srpk2	Signaling
DMR4:9482001	4	9482001	9483000	1000	1	3.90E-07	-0.43	22	2.2	Reln	Extracellular Matrix
DMR4:9494001	4	9494001	9498000	4000	1	1.40E-10	-0.43	53	1.32	Reln	Extracellular Matrix
DMR4:9542001	4	9542001	9544000	2000	1	1.40E-07	-0.33	26	1.3	Reln	Extracellular Matrix
DMR4:9641001	4	9641001	9643000	2000	1	6.90E-11	-0.51	40	2	Reln	Extracellular Matrix
DMR4:9701001	4	9701001	9705000	4000	2	9.80E-14	-0.71	101	2.52	Reln	Extracellular Matrix
DMR4:9783001	4	9783001	9790000	7000	1	9.10E-09	0.39	85	1.21	Reln;Slc26a5	Extracellular Matrix;Transport
DMR4:9815001	4	9815001	9817000	2000	1	5.80E-07	-0.32	29	1.45	Slc26a5;LOC102555727	Transport
DMR4:9833001	4	9833001	9834000	1000	1	5.50E-19	1	32	3.2	Slc26a5	Transport
DMR4:9886001	4	9886001	9890000	4000	1	1.40E-11	-0.71	65	1.62	Psmc2;Dnajc2	Protease;Transcription
DMR4:9896001	4	9896001	9899000	3000	1	3.00E-10	-0.38	46	1.53	Dnajc2;Pmpcb	Transcription;Protease
DMR4:9968001	4	9968001	9973000	5000	1	2.90E-07	-0.41	75	1.5	Napepld	Metabolism
DMR4:10012001	4	10012001	10013000	1000	1	1.30E-12	0.96	35	3.5	Napepld;Armc10;Fbxl13	Metabolism
DMR4:10107001	4	10107001	10109000	2000	1	4.70E-07	-0.4	47	2.35	Fbxl13;Lrrc17	Receptor
DMR4:10118001	4	10118001	10120000	2000	1	4.30E-08	0.47	44	2.2	Fbxl13;Lrrc17	Receptor
DMR4:10132001	4	10132001	10135000	3000	2	2.70E-16	1	59	1.97	Fbxl13;Lrrc17	Receptor
DMR4:10139001	4	10139001	10142000	3000	1	4.20E-13	0.45	37	1.23	Fbxl13;Lrrc17	Receptor
DMR4:10143001	4	10143001	10144000	1000	1	7.30E-08	0.44	16	1.6	Fbxl13;Lrrc17	Receptor
DMR4:10153001	4	10153001	10154000	1000	1	3.80E-09	0.44	16	1.6	Fbxl13	
DMR4:10280001	4	10280001	10281000	1000	1	2.60E-07	-0.62	4	0.4	Ccdc146	Development
DMR4:10411001	4	10411001	10412000	1000	1	4.20E-11	0.52	13	1.3	Ccdc146	Development
DMR4:10620001	4	10620001	10626000	6000	1	1.80E-08	0.41	79	1.32	LOC102556034;Ptpn12	
DMR4:10772001	4	10772001	10773000	1000	1	4.00E-08	-0.45	17	1.7	Rsbn1l	
DMR4:10989001	4	10989001	10990000	1000	1	3.20E-09	0.6	26	2.6	LOC102556088;Magi2	
DMR4:10999001	4	10999001	1.10E+07	1000	1	8.20E-18	1.13	21	2.1	Magi2	
DMR4:11038001	4	11038001	11041000	3000	1	7.00E-09	-0.36	34	1.13	Magi2	
DMR4:11061001	4	11061001	11067000	6000	1	4.50E-10	-0.48	99	1.65	Magi2	
DMR4:11087001	4	11087001	11088000	1000	1	1.40E-08	-0.42	4	0.4	Magi2	
DMR4:11116001	4	11116001	11117000	1000	1	1.40E-16	0.59	29	2.9	Magi2	
DMR4:11118001	4	11118001	11120000	2000	1	2.90E-09	0.62	35	1.75	Magi2	
DMR4:11140001	4	11140001	11142000	2000	1	3.10E-14	1.13	60	3	Magi2	
DMR4:11159001	4	11159001	11160000	1000	1	2.20E-08	0.53	22	2.2	Magi2	
DMR4:11182001	4	11182001	11183000	1000	1	2.60E-28	1.38	30	3	Magi2	

DMR4:11239001	4	11239001	11241000	2000	2	5.20E-07	0.58	38	1.9	Magi2	
DMR4:11265001	4	11265001	11266000	1000	1	8.80E-07	-0.42	1	0.1	Magi2	
DMR4:11395001	4	11395001	11398000	3000	1	6.70E-08	-0.46	44	1.47	Magi2;LOC100909926	
DMR4:11564001	4	11564001	11567000	3000	1	2.80E-08	0.32	36	1.2	Magi2	
DMR4:11605001	4	11605001	11606000	1000	1	9.10E-10	-0.45	8	0.8	Magi2	
DMR4:11683001	4	11683001	11685000	2000	1	8.40E-07	-0.49	11	0.55	Magi2	
DMR4:11780001	4	11780001	11783000	3000	1	5.30E-14	0.76	30	1	Magi2	
DMR4:11825001	4	11825001	11827000	2000	1	1.80E-07	-0.49	9	0.45	Magi2	
DMR4:11863001	4	11863001	11865000	2000	1	9.30E-09	0.68	27	1.35	Magi2	
DMR4:11877001	4	11877001	11878000	1000	1	2.10E-11	0.72	18	1.8	Magi2;LOC108350831	
DMR4:11903001	4	11903001	11904000	1000	1	8.00E-08	-0.46	7	0.7	Magi2;LOC108350830	
DMR4:11981001	4	11981001	11982000	1000	1	2.30E-09	-0.48	7	0.7	Magi2	
DMR4:12187001	4	12187001	12189000	2000	2	2.40E-10	0.74	36	1.8	Magi2;LOC103692037	
DMR4:12190001	4	12190001	12193000	3000	1	3.10E-07	-0.28	28	0.93	Magi2;LOC103692037	
DMR4:12283001	4	12283001	12285000	2000	1	4.60E-07	0.49	37	1.85	Magi2	
DMR4:12400001	4	12400001	12404000	4000	2	3.30E-10	-0.38	40	1	Magi2	
DMR4:12478001	4	12478001	12480000	2000	2	4.40E-10	-0.72	8	0.4	Magi2;LOC102547160;LOC100365492	
DMR4:13451001	4	13451001	13454000	3000	1	1.20E-08	0.39	43	1.43	Gnai1	Signaling
DMR4:13819001	4	13819001	13821000	2000	1	9.60E-12	0.6	28	1.4	Gnat3	Signaling
DMR4:14118001	4	14118001	14120000	2000	1	4.80E-09	0.5	44	2.2	RGD1565355;LOC108350652	
DMR4:14122001	4	14122001	14123000	1000	1	1.40E-14	0.87	33	3.3	RGD1565355;LOC108350652	
DMR4:14170001	4	14170001	14172000	2000	2	7.10E-09	-0.54	10	0.5	Cd36	Transport
DMR4:14333001	4	14333001	14336000	3000	1	1.30E-08	-0.28	32	1.07	Sema3c	Signaling
DMR4:14415001	4	14415001	14416000	1000	1	6.60E-07	0.46	16	1.6	Sema3c	Signaling
DMR4:14444001	4	14444001	14445000	1000	1	2.30E-09	-0.49	6	0.6	Sema3c	Signaling
DMR4:15949001	4	15949001	15950000	1000	1	2.80E-07	-0.46	13	1.3	Cacna2d1	Transport
DMR4:16012001	4	16012001	16013000	1000	1	5.40E-13	0.76	12	1.2	Cacna2d1	Transport
DMR4:16029001	4	16029001	16031000	2000	1	1.30E-07	-0.47	35	1.75	Cacna2d1	Transport
DMR4:16121001	4	16121001	16122000	1000	1	9.20E-08	-0.49	10	1	Cacna2d1	Transport
DMR4:16136001	4	16136001	16137000	1000	1	5.20E-12	-0.46	13	1.3	Cacna2d1	Transport
DMR4:16499001	4	16499001	16500000	1000	1	2.80E-07	-0.53	4	0.4	Pclo	
DMR4:16539001	4	16539001	16540000	1000	1	2.90E-09	-0.41	10	1	Pclo	
DMR4:16607001	4	16607001	16608000	1000	1	5.40E-08	0.63	15	1.5	Pclo	
DMR4:16646001	4	16646001	16648000	2000	1	3.40E-11	0.73	33	1.65	Pclo	
DMR4:16652001	4	16652001	16655000	3000	2	6.00E-12	0.96	76	2.53	Pclo	
DMR4:17030001	4	17030001	17032000	2000	1	3.20E-07	0.4	17	0.85	Pclo	
DMR4:17383001	4	17383001	17385000	2000	1	2.10E-08	0.52	11	0.55	Sema3e	Signaling
DMR4:18302001	4	18302001	18303000	1000	1	6.70E-07	-0.36	9	0.9	Sema3a	Signaling
DMR4:18426001	4	18426001	18427000	1000	1	9.50E-07	0.43	8	0.8	Sema3a	Signaling
DMR4:18444001	4	18444001	18445000	1000	1	2.20E-07	-0.36	15	1.5	Sema3a	Signaling
DMR4:18451001	4	18451001	18452000	1000	1	2.40E-08	-0.49	8	0.8	Sema3a	Signaling
DMR4:18501001	4	18501001	18503000	2000	1	2.20E-09	0.69	30	1.5	Sema3a	Signaling
DMR4:19227001	4	19227001	19228000	1000	1	1.10E-07	0.41	7	0.7	Sema3d	Signaling
DMR4:19292001	4	19292001	19293000	1000	1	9.80E-07	-0.38	16	1.6	Sema3d	Signaling
DMR4:21630001	4	21630001	21632000	2000	1	3.00E-28	0.43	19	0.95	RGD1563349	
DMR4:21726001	4	21726001	21727000	1000	1	1.60E-08	-0.48	15	1.5	RGD1563349;LOC108350658	
DMR4:21753001	4	21753001	21754000	1000	1	1.90E-08	-0.35	12	1.2	RGD1563349;LOC108350658;LOC102555663	
DMR4:21879001	4	21879001	21882000	3000	2	6.50E-10	-0.43	42	1.4	Dmtf1;LOC108350660	Transcription
DMR4:22222001	4	22222001	22223000	1000	1	1.80E-07	-0.51	17	1.7	LOC103692045;Abcb1b	
DMR4:22255001	4	22255001	22261000	6000	1	5.10E-09	-0.28	71	1.18	Abcb1b	
DMR4:22332001	4	22332001	22334000	2000	1	8.20E-16	0.85	35	1.75	Abcb1a	
DMR4:22351001	4	22351001	22352000	1000	1	2.00E-07	-0.36	11	1.1	Abcb1a	
DMR4:22660001	4	22660001	22661000	1000	1	1.90E-09	-0.44	8	0.8	Slc25a40;LOC103690019	
DMR4:23014001	4	23014001	23017000	3000	1	1.30E-07	-0.41	75	2.5	Sri	Cytoskeleton
DMR4:23025001	4	23025001	23029000	4000	1	1.70E-07	-0.45	38	0.95	Sri	Cytoskeleton
DMR4:24068001	4	24068001	24069000	1000	1	2.30E-07	-0.71	46	4.6	Zfp804b	
DMR4:24093001	4	24093001	24094000	1000	1	8.10E-08	-0.63	1	0.1	Zfp804b	
DMR4:24149001	4	24149001	24150000	1000	1	5.30E-09	0.72	20	2	Zfp804b	
DMR4:25011001	4	25011001	25012000	1000	1	2.00E-10	0.78	20	2	RGD1561754	
DMR4:25449001	4	25449001	25455000	6000	1	3.50E-07	-0.37	75	1.25	Steap1	

DMR4:25528001	4	25528001	25531000	3000	1	1.60E-07	-0.37	29	0.97	Steap2;Cfap69	Development
DMR4:25545001	4	25545001	25548000	3000	1	2.10E-07	0.43	99	3.3	Cfap69;LOC103692047	Development
DMR4:25624001	4	25624001	25627000	3000	1	6.70E-07	0.59	62	2.07	Gtpbp10	
DMR4:25816001	4	25816001	25819000	3000	1	3.70E-16	0.93	46	1.53	Cdk14	Signaling
DMR4:25886001	4	25886001	25892000	6000	1	5.90E-07	-0.35	65	1.08	Cdk14	Signaling
DMR4:25903001	4	25903001	25905000	2000	1	1.70E-10	0.66	26	1.3	Cdk14	Signaling
DMR4:25945001	4	25945001	25946000	1000	1	1.30E-07	-0.5	22	2.2	Cdk14	Signaling
DMR4:26025001	4	26025001	26027000	2000	2	1.60E-07	-0.38	30	1.5	Cdk14	Signaling
DMR4:26035001	4	26035001	26037000	2000	1	5.60E-11	0.34	24	1.2	Cdk14	Signaling
DMR4:26115001	4	26115001	26116000	1000	1	2.60E-07	-0.45	14	1.4	Cdk14	Signaling
DMR4:26204001	4	26204001	26208000	4000	1	2.20E-09	-0.43	39	0.98	Cdk14	Signaling
DMR4:26265001	4	26265001	26268000	3000	1	5.60E-07	-0.4	23	0.77	Cdk14;LOC102549548	Signaling
DMR4:26422001	4	26422001	26425000	3000	1	7.20E-07	-0.44	52	1.73	Cdk14	Signaling
DMR4:27164001	4	27164001	27166000	2000	1	1.10E-07	0.29	29	1.45	LOC108350665;Cyp51	
DMR4:27179001	4	27179001	27183000	4000	1	1.50E-13	-0.44	95	2.38	LOC108350665;Cyp51	
DMR4:27270001	4	27270001	27272000	2000	1	9.80E-07	-0.33	33	1.65	Akap9	
DMR4:27280001	4	27280001	27282000	2000	1	1.30E-07	-0.4	28	1.4	Akap9	
DMR4:27298001	4	27298001	27302000	4000	2	2.00E-08	-0.57	73	1.82	Akap9	
DMR4:27311001	4	27311001	27313000	2000	1	2.60E-07	-0.41	42	2.1	Akap9	
DMR4:27367001	4	27367001	27370000	3000	1	4.20E-08	-0.43	14	0.47	Mterf1	
DMR4:27417001	4	27417001	27420000	3000	1	4.90E-15	-0.58	26	0.87	Lrrd1	Cytoskeleton
DMR4:27619001	4	27619001	27621000	2000	1	9.10E-10	0.7	24	1.2	RGD1306626	
DMR4:27803001	4	27803001	27807000	4000	2	7.30E-08	-0.48	80	2	Cdk6	Signaling
DMR4:27867001	4	27867001	27871000	4000	2	5.90E-09	-0.46	74	1.85	Cdk6	Signaling
DMR4:27891001	4	27891001	27893000	2000	1	9.10E-09	-0.47	47	2.35	Cdk6;LOC102550744	Signaling
DMR4:27908001	4	27908001	27912000	4000	1	5.40E-09	-0.5	94	2.35	Cdk6;LOC102550744	Signaling
DMR4:27948001	4	27948001	27949000	1000	1	2.70E-07	-0.38	11	1.1	Cdk6	Signaling
DMR4:28214001	4	28214001	28215000	1000	1	2.00E-08	-0.42	13	1.3	RGD1565361	
DMR4:28235001	4	28235001	28238000	3000	1	5.00E-07	-0.53	19	0.63	RGD1565361;LOC100911141	
DMR4:28295001	4	28295001	28297000	2000	1	5.10E-07	-0.37	48	2.4	LOC103690231;LOC100359932;RGD1563091	
DMR4:28402001	4	28402001	28403000	1000	1	1.00E-12	0.61	18	1.8	Hepacam2	
DMR4:28405001	4	28405001	28406000	1000	1	7.90E-12	0.76	20	2	Hepacam2	
DMR4:28451001	4	28451001	28455000	4000	1	1.90E-08	-0.52	55	1.38	Vps50	
DMR4:28522001	4	28522001	28528000	6000	1	2.50E-08	-0.45	60	1	Vps50	
DMR4:28686001	4	28686001	28688000	2000	1	5.20E-07	0.56	20	1	Calcr	Receptor
DMR4:28704001	4	28704001	28708000	4000	2	1.80E-11	0.66	58	1.45	Calcr;LOC102551706	Receptor
DMR4:28950001	4	28950001	28952000	2000	1	1.70E-07	0.44	27	1.35	Tfpi2	Protease; Proteolysis
DMR4:29097001	4	29097001	29098000	1000	1	4.60E-17	1.05	17	1.7	Bet1	Transcription
DMR4:29718001	4	29718001	29719000	1000	1	5.00E-10	0.53	12	1.2	Sgce	Cytoskeleton
DMR4:29747001	4	29747001	29748000	1000	1	3.80E-07	-0.56	14	1.4	Sgce	Cytoskeleton
DMR4:29750001	4	29750001	29752000	2000	1	9.20E-09	-0.43	36	1.8	Sgce	Cytoskeleton
DMR4:30254001	4	30254001	30255000	1000	1	5.00E-07	0.48	18	1.8	Ppp1r9a;Pon1	
DMR4:30316001	4	30316001	30317000	1000	1	2.80E-07	0.47	12	1.2	Pon3	
DMR4:30468001	4	30468001	30473000	5000	2	6.60E-11	0.7	84	1.68	Asb4	Cytoskeleton
DMR4:30476001	4	30476001	30479000	3000	1	9.50E-09	-0.45	32	1.07	Asb4	Cytoskeleton
DMR4:30492001	4	30492001	30493000	1000	1	3.50E-09	0.68	27	2.7	Asb4	Cytoskeleton
DMR4:30545001	4	30545001	30547000	2000	1	3.00E-11	-0.56	27	1.35	LOC102552448;Pdk4	Signaling
DMR4:30564001	4	30564001	30566000	2000	1	1.80E-08	-0.55	10	0.5	Pdk4	Signaling
DMR4:31021001	4	31021001	31022000	1000	1	1.60E-07	0.56	17	1.7	Dync1i1	Cytoskeleton
DMR4:31088001	4	31088001	31090000	2000	1	3.10E-11	-0.41	23	1.15	Dync1i1	Cytoskeleton
DMR4:31118001	4	31118001	31119000	1000	1	2.00E-07	-0.44	12	1.2	Dync1i1	Cytoskeleton
DMR4:31197001	4	31197001	31200000	3000	1	5.30E-10	0.36	21	0.7	Slc25a13	Transport
DMR4:31316001	4	31316001	31319000	3000	1	1.80E-09	-0.48	22	0.73	Slc25a13	Transport
DMR4:31398001	4	31398001	31400000	2000	1	6.10E-07	-0.75	6	0.3	Slc25a13;LOC100912034	Transport
DMR4:31431001	4	31431001	31432000	1000	1	1.50E-11	0.92	15	1.5	Slc25a13	Transport
DMR4:31503001	4	31503001	31504000	1000	1	1.40E-07	-0.4	11	1.1	Slc25a13	Transport
DMR4:31580001	4	31580001	31582000	2000	2	2.10E-08	-0.56	15	0.75	Slc25a13	Transport
DMR4:31611001	4	31611001	31614000	3000	1	9.40E-07	-0.38	34	1.13	Slc25a13	Transport
DMR4:31683001	4	31683001	31687000	4000	1	3.00E-10	-0.39	50	1.25	Slc25a13;LOC103692067	Transport
DMR4:31736001	4	31736001	31742000	6000	1	9.50E-14	0.54	43	0.72	Slc25a13	Transport
DMR4:31743001	4	31743001	31744000	1000	1	7.20E-09	-0.46	13	1.3	Slc25a13	Transport

DMR4:31746001	4	31746001	31751000	5000	1	2.10E-07	-0.51	53	1.06	Slc25a13	Transport
DMR4:32091001	4	32091001	32093000	2000	2	7.00E-11	0.81	39	1.95	Shfm1	
DMR4:32377001	4	32377001	32380000	3000	1	8.50E-10	-0.45	34	1.13	LOC100912227;Dlx6;Dlx5	Development
DMR4:32531001	4	32531001	32534000	3000	2	6.40E-10	0.33	33	1.1	Sdhaf3	
DMR4:33740001	4	33740001	33742000	2000	2	2.10E-11	-0.57	16	0.8	Asns	Metabolism
DMR4:33910001	4	33910001	33911000	1000	1	5.00E-11	-0.41	10	1	C1galt1	Transport
DMR4:34069001	4	34069001	34070000	1000	1	4.90E-07	0.53	7	0.7	Col28a1	Extracellular Matrix
DMR4:34635001	4	34635001	34636000	1000	1	2.60E-07	0.56	30	3	Ica1	
DMR4:34642001	4	34642001	34643000	1000	1	8.60E-10	0.41	17	1.7	Ica1	
DMR4:34718001	4	34718001	34720000	2000	1	1.30E-09	-0.55	14	0.7	Ica1	
DMR4:34999001	4	34999001	35001000	2000	1	7.80E-08	-0.55	14	0.7	Nxph1	Signaling
DMR4:35003001	4	35003001	35005000	2000	1	5.10E-07	0.44	20	1	Nxph1	Signaling
DMR4:35101001	4	35101001	35102000	1000	1	3.60E-15	1.08	24	2.4	Nxph1	Signaling
DMR4:35274001	4	35274001	35275000	1000	1	7.20E-07	-0.39	6	0.6	Nxph1	Signaling
DMR4:37554001	4	37554001	37556000	2000	1	2.70E-07	-0.27	19	0.95	RGD1562387	
DMR4:38225001	4	38225001	38226000	1000	1	2.30E-07	-0.64	3	0.3	Ndufa4	Metabolism
DMR4:38242001	4	38242001	38243000	1000	1	2.60E-11	-0.5	6	0.6	Ndufa4;Phf14	Metabolism;Transcription
DMR4:38810001	4	38810001	38812000	2000	1	6.60E-08	-0.32	18	0.9	Thsd7a	Cytoskeleton
DMR4:38961001	4	38961001	38963000	2000	1	7.50E-07	0.45	17	0.85	Thsd7a	Cytoskeleton
DMR4:39624001	4	39624001	39626000	2000	1	1.20E-08	-0.34	65	3.25	Vwde	
DMR4:39666001	4	39666001	39667000	1000	1	1.00E-11	0.67	23	2.3	Vwde	
DMR4:39755001	4	39755001	39756000	1000	1	1.40E-11	-0.44	19	1.9	Tas2r7l	Receptor
DMR4:40038001	4	40038001	40039000	1000	1	4.10E-09	-0.43	4	0.4	Tmem168	
DMR4:41394001	4	41394001	41402000	8000	1	1.10E-07	0.23	93	1.16	Foxp2	
DMR4:41473001	4	41473001	41474000	1000	1	7.70E-07	0.35	9	0.9	Foxp2	
DMR4:41480001	4	41480001	41484000	4000	2	3.90E-10	-0.37	39	0.98	Foxp2	
DMR4:41492001	4	41492001	41493000	1000	1	8.10E-07	-0.46	3	0.3	Foxp2	
DMR4:41522001	4	41522001	41524000	2000	1	1.80E-08	-0.38	33	1.65	Foxp2	
DMR4:41530001	4	41530001	41533000	3000	1	6.20E-09	-0.28	28	0.93	Foxp2	
DMR4:41577001	4	41577001	41578000	1000	1	5.10E-07	-0.35	15	1.5	Foxp2	
DMR4:41584001	4	41584001	41585000	1000	1	6.30E-08	0.61	14	1.4	Foxp2	
DMR4:41602001	4	41602001	41604000	2000	1	7.90E-07	-0.4	17	0.85	Foxp2	
DMR4:41622001	4	41622001	41624000	2000	1	6.00E-07	0.5	28	1.4	Foxp2	
DMR4:41665001	4	41665001	41667000	2000	2	4.30E-08	-0.43	9	0.45	Foxp2	
DMR4:41676001	4	41676001	41677000	1000	1	8.20E-08	0.51	12	1.2	Foxp2	
DMR4:41685001	4	41685001	41686000	1000	1	2.30E-13	0.56	15	1.5	Foxp2	
DMR4:42197001	4	42197001	42200000	3000	1	2.40E-07	0.49	36	1.2	Rbmxl1;Mdfic	
DMR4:42239001	4	42239001	42240000	1000	1	3.00E-08	-0.61	13	1.3	Mdfic	
DMR4:42250001	4	42250001	42251000	1000	1	9.00E-07	-0.43	16	1.6	Mdfic	
DMR4:42781001	4	42781001	42782000	1000	1	1.00E-07	0.35	14	1.4	Cftr	Transport
DMR4:42825001	4	42825001	42827000	2000	1	5.50E-15	-0.81	19	0.95	Cftr	Transport
DMR4:43139001	4	43139001	43141000	2000	1	2.10E-08	0.67	36	1.8	RGD1566227	
DMR4:43905001	4	43905001	43907000	2000	1	9.10E-08	0.49	29	1.45	RGD1562178	
DMR4:44133001	4	44133001	44134000	1000	1	8.70E-09	0.73	22	2.2	Tfec	
DMR4:44336001	4	44336001	44341000	5000	2	4.10E-08	0.45	91	1.82	Tes	Cytoskeleton
DMR4:44347001	4	44347001	44354000	7000	2	7.50E-07	0.58	116	1.66	Tes	Cytoskeleton
DMR4:44367001	4	44367001	44369000	2000	1	2.80E-12	-0.66	12	0.6	Tes	Cytoskeleton
DMR4:44582001	4	44582001	44583000	1000	1	6.60E-07	-0.51	6	0.6	LOC102550644;Cav2	Cytoskeleton
DMR4:44781001	4	44781001	44785000	4000	1	3.30E-10	-0.44	82	2.05	Met	Receptor
DMR4:45022001	4	45022001	45023000	1000	1	4.80E-08	-0.53	23	2.3	ST7	
DMR4:45037001	4	45037001	45042000	5000	1	5.50E-07	-0.35	39	0.78	ST7	
DMR4:45069001	4	45069001	45074000	5000	1	3.00E-09	-0.37	46	0.92	ST7	
DMR4:45199001	4	45199001	45200000	1000	1	1.90E-07	0.34	7	0.7	ST7	
DMR4:45257001	4	45257001	45258000	1000	1	1.20E-07	0.43	13	1.3	ST7	
DMR4:45382001	4	45382001	45384000	2000	1	8.80E-10	-0.48	10	0.5	Asz1	
DMR4:45389001	4	45389001	45390000	1000	1	7.00E-08	-0.38	9	0.9	Asz1	
DMR4:45412001	4	45412001	45413000	1000	1	5.60E-11	-0.63	3	0.3	Asz1	
DMR4:48360001	4	48360001	48364000	4000	1	6.40E-10	-0.57	36	0.9	Kcnd2	Transport
DMR4:48440001	4	48440001	48442000	2000	2	8.20E-27	0.74	29	1.45	Kcnd2	Transport
DMR4:48453001	4	48453001	48457000	4000	1	8.50E-08	-0.36	39	0.98	Kcnd2	Transport
DMR4:48621001	4	48621001	48622000	1000	1	2.00E-07	0.51	10	1	Kcnd2	Transport

DMR4:48884001	4	48884001	48886000	2000	1	4.00E-08	-0.36	13	0.65	Tspan12	
DMR4:48897001	4	48897001	48898000	1000	1	5.40E-07	-0.42	22	2.2	Tspan12	
DMR4:48910001	4	48910001	48915000	5000	3	1.70E-08	-0.42	39	0.78	Tspan12	
DMR4:48935001	4	48935001	48936000	1000	1	4.20E-07	-0.45	13	1.3	Tspan12;LOC100359702	
DMR4:48940001	4	48940001	48942000	2000	1	8.50E-07	-0.43	29	1.45	Tspan12;LOC100359702	
DMR4:48948001	4	48948001	48950000	2000	1	1.10E-13	-0.49	19	0.95	Tspan12	
DMR4:49011001	4	49011001	49012000	1000	1	4.30E-08	0.49	13	1.3	Ing3	Epigenetic
DMR4:49060001	4	49060001	49061000	1000	1	1.90E-10	-0.52	15	1.5	Cped1	
DMR4:49072001	4	49072001	49075000	3000	2	1.40E-09	0.75	67	2.23	Cped1	
DMR4:49118001	4	49118001	49120000	2000	1	6.90E-09	-0.46	15	0.75	Cped1	
DMR4:49183001	4	49183001	49185000	2000	1	1.70E-08	0.56	42	2.1	Cped1	
DMR4:49190001	4	49190001	49192000	2000	1	3.20E-07	-0.34	29	1.45	Cped1	
DMR4:49193001	4	49193001	49194000	1000	1	1.30E-13	1.1	31	3.1	Cped1	
DMR4:49306001	4	49306001	49307000	1000	1	2.00E-09	0.48	12	1.2	Cped1	
DMR4:49320001	4	49320001	49322000	2000	1	3.80E-07	-0.55	37	1.85	Cped1	
DMR4:49372001	4	49372001	49373000	1000	1	6.40E-10	0.7	14	1.4	Wnt16	Signaling
DMR4:49404001	4	49404001	49410000	6000	1	5.60E-07	-0.41	141	2.35	Fam3c;LOC103692083	Signaling
DMR4:50362001	4	50362001	50365000	3000	2	1.70E-09	-0.57	18	0.6	Cadps2	Transport
DMR4:50474001	4	50474001	50476000	2000	1	2.50E-09	-0.4	40	2	Cadps2	Transport
DMR4:50575001	4	50575001	50577000	2000	1	4.40E-09	-0.45	24	1.2	Cadps2	Transport
DMR4:50606001	4	50606001	50608000	2000	1	3.20E-09	0.52	24	1.2	Cadps2	Transport
DMR4:50622001	4	50622001	50623000	1000	1	2.60E-07	0.56	27	2.7	Cadps2	Transport
DMR4:50791001	4	50791001	50792000	1000	1	4.90E-08	0.48	12	1.2	Cadps2	Transport
DMR4:50801001	4	50801001	50806000	5000	1	1.20E-08	-0.47	74	1.48	Cadps2	Transport
DMR4:50868001	4	50868001	50869000	1000	1	1.70E-11	0.81	17	1.7	Cadps2;LOC108350675	Transport
DMR4:50993001	4	50993001	50994000	1000	1	1.40E-09	0.77	21	2.1	Tas2r118	
DMR4:51144001	4	51144001	51148000	4000	1	1.00E-07	0.68	51	1.27	Slc13a1	Transport
DMR4:51170001	4	51170001	51171000	1000	1	2.70E-07	0.56	7	0.7	Slc13a1	Transport
DMR4:51199001	4	51199001	51200000	1000	1	2.80E-08	-0.43	10	1	Slc13a1;LOC102555859	Transport
DMR4:51580001	4	51580001	51581000	1000	1	1.50E-07	0.65	18	1.8	Iqub;Ndufa5	Metabolism
DMR4:51628001	4	51628001	51629000	1000	1	1.10E-21	0.8	24	2.4	Asb15;LOC108350676	
DMR4:51631001	4	51631001	51632000	1000	1	5.00E-12	0.71	13	1.3	Asb15;LOC108350676	
DMR4:51637001	4	51637001	51644000	7000	1	2.60E-07	-0.45	141	2.01	Asb15;LOC108350676	
DMR4:51721001	4	51721001	51724000	3000	2	1.20E-10	-0.64	15	0.5	Wasl	
DMR4:51823001	4	51823001	51824000	1000	1	1.50E-08	0.71	34	3.4	Gpr37	Signaling
DMR4:51842001	4	51842001	51843000	1000	1	1.30E-09	0.57	18	1.8	Gpr37	Signaling
DMR4:51883001	4	51883001	51886000	3000	1	6.70E-09	-0.55	16	0.53	Pot1	Transcription
DMR4:52238001	4	52238001	52240000	2000	1	2.00E-09	0.43	24	1.2	Hyal5	Metabolism
DMR4:52303001	4	52303001	52304000	1000	1	4.70E-07	-0.53	14	1.4	Hyal5	Metabolism
DMR4:52344001	4	52344001	52345000	1000	1	3.40E-08	-0.7	3	0.3	Tmem229a	
DMR4:54522001	4	54522001	54529000	7000	2	3.00E-07	-0.36	70	1	Grm8	Signaling
DMR4:54620001	4	54620001	54623000	3000	1	3.20E-08	-0.37	28	0.93	Grm8	Signaling
DMR4:54635001	4	54635001	54636000	1000	1	5.70E-10	0.7	18	1.8	Grm8	Signaling
DMR4:54661001	4	54661001	54665000	4000	1	9.50E-07	-0.22	40	1	Grm8	Signaling
DMR4:54747001	4	54747001	54755000	8000	2	1.90E-17	-0.6	79	0.99	Grm8	Signaling
DMR4:54764001	4	54764001	54771000	7000	1	3.30E-10	0.49	77	1.1	Grm8	Signaling
DMR4:54838001	4	54838001	54842000	4000	1	1.60E-08	-0.32	38	0.95	Grm8	Signaling
DMR4:55012001	4	55012001	55015000	3000	2	3.00E-08	-0.57	10	0.33	Grm8	Signaling
DMR4:55252001	4	55252001	55257000	5000	1	4.80E-10	-0.42	34	0.68	Grm8	Signaling
DMR4:55419001	4	55419001	55420000	1000	1	6.70E-07	0.25	12	1.2	Grm8	Signaling
DMR4:55721001	4	55721001	55722000	1000	1	3.00E-08	0.59	18	1.8	Gcc1;Arf5;Fscn3	Signaling;Cytoskeleton
DMR4:55788001	4	55788001	55790000	2000	1	1.20E-12	-0.53	17	0.85	Snd1	
DMR4:55908001	4	55908001	55911000	3000	2	4.10E-14	-0.59	21	0.7	Snd1	
DMR4:55968001	4	55968001	55970000	2000	1	1.50E-07	-0.37	24	1.2	Snd1	
DMR4:56008001	4	56008001	56009000	1000	1	7.70E-07	-0.5	10	1	Snd1	
DMR4:56094001	4	56094001	56095000	1000	1	1.80E-07	0.54	10	1	Snd1	
DMR4:56146001	4	56146001	56147000	1000	1	1.90E-07	-0.44	11	1.1	Snd1	
DMR4:56391001	4	56391001	56392000	1000	1	6.20E-12	0.75	21	2.1	Rbm28	
DMR4:56424001	4	56424001	56426000	2000	1	7.30E-07	-0.49	12	0.6	Rbm28	
DMR4:56428001	4	56428001	56432000	4000	1	2.90E-07	-0.29	69	1.73	Rbm28	
DMR4:56451001	4	56451001	56453000	2000	1	4.40E-07	0.5	38	1.9	Prrt4	
DMR4:56456001	4	56456001	56459000	3000	1	6.30E-07	-0.32	17	0.57	Prrt4	

DMR4:56557001	4	56557001	56560000	3000	1	3.00E-08	0.6	54	1.8	Hilpda;Fam71f2;LOC103692085	
DMR4:56614001	4	56614001	56617000	3000	1	2.10E-17	1.18	63	2.1	Fam71f1;Calu	Signaling
DMR4:56669001	4	56669001	56670000	1000	1	5.60E-14	-0.58	4	0.4	Trnap-agg;Ccdc136	Cytoskeleton
DMR4:56774001	4	56774001	56775000	1000	1	2.20E-26	1.13	36	3.6	Kcp	
DMR4:56781001	4	56781001	56786000	5000	1	1.10E-09	0.61	68	1.36	Kcp	
DMR4:56791001	4	56791001	56792000	1000	1	5.70E-07	0.4	15	1.5	Kcp	
DMR4:56811001	4	56811001	56815000	4000	1	2.50E-09	-0.54	45	1.12	Irf5;Tnpo3	Transcription;Transport
DMR4:56900001	4	56900001	56906000	6000	1	1.70E-07	0.32	60	1	Tnpo3	Transport
DMR4:57085001	4	57085001	57086000	1000	1	2.70E-11	0.85	17	1.7	Ahcyl2;LOC108350683	Metabolism
DMR4:57185001	4	57185001	57186000	1000	1	2.40E-07	-0.35	10	1	Ahcyl2	Metabolism
DMR4:57501001	4	57501001	57503000	2000	1	4.40E-14	0.84	40	2	Ube2h	Proteolysis
DMR4:57553001	4	57553001	57555000	2000	1	1.60E-08	-0.35	22	1.1	Ube2h	Proteolysis
DMR4:57770001	4	57770001	57772000	2000	1	4.30E-07	0.5	34	1.7	Klhdc10;LOC108350685	
DMR4:57860001	4	57860001	57862000	2000	1	8.90E-09	0.63	63	3.15	Cpa2	Protease
DMR4:57873001	4	57873001	57875000	2000	1	4.10E-10	-0.72	9	0.45	Cpa2;Cpa4	Protease
DMR4:57906001	4	57906001	57912000	6000	1	3.80E-07	-0.38	95	1.58	Cpa4;LOC108350686;LOC102553416	Protease
DMR4:57943001	4	57943001	57946000	3000	1	5.00E-08	0.43	48	1.6	Cpa5;Cpa1	Protease
DMR4:58045001	4	58045001	58049000	4000	2	1.80E-20	1.07	101	2.52	Mest	Protease
DMR4:58076001	4	58076001	58078000	2000	1	2.50E-10	-0.43	13	0.65	Copg2	Transport
DMR4:58110001	4	58110001	58111000	1000	1	1.40E-08	-0.54	4	0.4	Copg2	Transport
DMR4:58216001	4	58216001	58223000	7000	3	6.60E-10	-0.48	115	1.64	Tsga13	
DMR4:58235001	4	58235001	58239000	4000	1	4.80E-07	-0.34	47	1.18	LOC103692110;Klf14	Transcription
DMR4:58336001	4	58336001	58338000	2000	1	3.70E-07	-0.44	37	1.85	Mir3556a;Mir29a;Mir3587;Mir29b1	
DMR4:58790001	4	58790001	58791000	1000	1	9.80E-09	0.57	10	1	Mkln1	
DMR4:59437001	4	59437001	59440000	3000	1	1.90E-11	0.79	33	1.1	Plxna4;LOC108350688	
DMR4:59482001	4	59482001	59483000	1000	1	1.20E-07	0.36	12	1.2	Plxna4;LOC108350688	
DMR4:59862001	4	59862001	59863000	1000	1	3.10E-13	-0.5	14	1.4	Plxna4	
DMR4:60118001	4	60118001	60119000	1000	1	1.20E-07	-0.43	1	0.1	Chchd3	
DMR4:60274001	4	60274001	60279000	5000	1	5.60E-07	-0.33	49	0.98	Chchd3	
DMR4:60287001	4	60287001	60290000	3000	1	9.10E-07	-0.46	18	0.6	Chchd3	
DMR4:60359001	4	60359001	60360000	1000	1	2.40E-08	0.77	28	2.8	Chchd3	
DMR4:60541001	4	60541001	60543000	2000	2	7.50E-09	-0.36	16	0.8	RGD1565435;Exoc4	Transport
DMR4:60574001	4	60574001	60577000	3000	1	1.20E-07	-0.42	18	0.6	Exoc4	Transport
DMR4:60742001	4	60742001	60744000	2000	1	6.70E-09	0.48	20	1	Exoc4	Transport
DMR4:60879001	4	60879001	60881000	2000	1	1.10E-07	-0.39	21	1.05	Exoc4	Transport
DMR4:60952001	4	60952001	60954000	2000	1	1.50E-07	-0.36	14	0.7	Exoc4	Transport
DMR4:61119001	4	61119001	61120000	1000	1	1.70E-07	0.25	24	2.4	Exoc4	Transport
DMR4:61256001	4	61256001	61263000	7000	1	3.10E-09	-0.31	80	1.14	Exoc4	Transport
DMR4:61321001	4	61321001	61325000	4000	1	2.00E-08	-0.36	43	1.07	Exoc4	Transport
DMR4:61361001	4	61361001	61362000	1000	1	6.90E-13	-0.47	21	2.1	Exoc4;LOC108350836	Transport
DMR4:61414001	4	61414001	61415000	1000	1	8.20E-08	-0.43	8	0.8	LOC689234;Lrguk	Signaling
DMR4:61420001	4	61420001	61422000	2000	1	1.00E-07	-0.33	41	2.05	Lrguk	Signaling
DMR4:61423001	4	61423001	61425000	2000	1	1.70E-12	0.53	51	2.55	Lrguk	Signaling
DMR4:61439001	4	61439001	61440000	1000	1	3.00E-08	-0.4	17	1.7	Lrguk	Signaling
DMR4:61480001	4	61480001	61481000	1000	1	2.40E-14	-0.67	14	1.4	Lrguk	Signaling
DMR4:61549001	4	61549001	61550000	1000	1	7.60E-07	-0.41	20	2	Lrguk;Slc35b4	Signaling;Transport
DMR4:61771001	4	61771001	61772000	1000	1	1.80E-08	-0.4	17	1.7	Akr1b8	Metabolism
DMR4:62105001	4	62105001	62108000	3000	1	2.20E-09	-0.46	43	1.43	Cald1	Cytoskeleton
DMR4:62132001	4	62132001	62138000	6000	2	2.90E-09	-0.52	45	0.75	Cald1	Cytoskeleton
DMR4:62186001	4	62186001	62189000	3000	1	1.30E-09	-0.41	52	1.73	Cald1	Cytoskeleton
DMR4:62243001	4	62243001	62246000	3000	2	2.10E-13	-0.46	64	2.13	Cald1	Cytoskeleton
DMR4:62269001	4	62269001	62272000	3000	1	2.30E-07	0.59	36	1.2	Cald1	Cytoskeleton
DMR4:62281001	4	62281001	62282000	1000	1	7.00E-08	0.63	24	2.4	Cald1;LOC108350691	Cytoskeleton
DMR4:62284001	4	62284001	62285000	1000	1	1.90E-09	0.45	13	1.3	Cald1;LOC108350691;Agbl3	Cytoskeleton;Protease
DMR4:62381001	4	62381001	62384000	3000	1	8.00E-07	-0.34	53	1.77	Agbl3;Tmem140;LOC500077	Protease
DMR4:62385001	4	62385001	62388000	3000	2	3.50E-08	0.7	81	2.7	Agbl3;Tmem140;LOC500077	Protease
DMR4:62417001	4	62417001	62422000	5000	2	2.30E-09	-0.36	57	1.14	Wdr91	
DMR4:62451001	4	62451001	62452000	1000	1	6.50E-24	1.23	34	3.4	Stra8	

DMR4:62484001	4	62484001	62486000	2000	2	6.50E-10	-0.44	6	0.3	Stra8;RGD1565367;LOC103692099	Transport
DMR4:62496001	4	62496001	62497000	1000	1	9.00E-07	0.4	23	2.3	RGD1565367;LOC103692099	Transport
DMR4:62521001	4	62521001	62526000	5000	1	4.50E-08	-0.37	73	1.46	RGD1565367	Transport
DMR4:62535001	4	62535001	62536000	1000	1	1.20E-09	0.88	33	3.3	RGD1565367	Transport
DMR4:62666001	4	62666001	62669000	3000	1	3.90E-16	-0.54	34	1.13	Cnot4;LOC102553126	Proteolysis
DMR4:62699001	4	62699001	62701000	2000	1	9.40E-15	0.79	38	1.9	Nup205	
DMR4:62785001	4	62785001	62787000	2000	1	4.40E-07	-0.41	40	2	RGD1564627;LOC689574;Slc13a4	Transport
DMR4:62794001	4	62794001	62796000	2000	1	7.00E-08	-0.45	39	1.95	LOC689574;Slc13a4	Transport
DMR4:63342001	4	63342001	63343000	1000	1	1.10E-10	0.77	22	2.2	Mir490	
DMR4:64365001	4	64365001	64366000	1000	1	1.90E-13	0.98	24	2.4	Dgki	Signaling
DMR4:64475001	4	64475001	64477000	2000	1	9.60E-08	-0.36	29	1.45	Dgki	Signaling
DMR4:64584001	4	64584001	64585000	1000	1	1.30E-12	-0.59	19	1.9	Dgki	Signaling
DMR4:64664001	4	64664001	64665000	1000	1	6.10E-07	-0.36	8	0.8	Dgki	Signaling
DMR4:64745001	4	64745001	64747000	2000	1	7.00E-10	-0.41	30	1.5	Dgki	Signaling
DMR4:64780001	4	64780001	64782000	2000	1	7.70E-08	-0.53	28	1.4	Dgki	Signaling
DMR4:64858001	4	64858001	64862000	4000	1	1.00E-06	0.41	64	1.6	Creb3l2	
DMR4:64909001	4	64909001	64911000	2000	1	2.90E-07	-0.34	29	1.45	Creb3l2	
DMR4:64967001	4	64967001	64970000	3000	1	8.00E-10	-0.59	51	1.7	Creb3l2	
DMR4:65120001	4	65120001	65121000	1000	1	4.30E-08	-0.42	10	1	Akr1d1	Metabolism
DMR4:65710001	4	65710001	65711000	1000	1	1.20E-09	0.69	19	1.9	Svopl	Transport
DMR4:65743001	4	65743001	65745000	2000	1	5.10E-09	0.48	26	1.3	Svopl;Atp6v0a4	Transport;Metabolism
DMR4:65761001	4	65761001	65762000	1000	1	3.80E-07	0.4	17	1.7	Atp6v0a4	Metabolism
DMR4:65888001	4	65888001	65890000	2000	1	3.60E-07	-0.35	32	1.6	RGD1306271	
DMR4:65909001	4	65909001	65911000	2000	1	1.70E-09	0.45	26	1.3	RGD1306271	
DMR4:65920001	4	65920001	65923000	3000	2	2.10E-14	-0.55	51	1.7	RGD1306271	
DMR4:65933001	4	65933001	65934000	1000	1	1.90E-08	-0.4	16	1.6	RGD1306271	
DMR4:65967001	4	65967001	65969000	2000	1	8.70E-10	-0.4	20	1	RGD1306271	
DMR4:66091001	4	66091001	66093000	2000	1	7.40E-10	-0.57	16	0.8	Ttc26;LOC108350693	
DMR4:66259001	4	66259001	66260000	1000	1	8.20E-09	-0.42	18	1.8	Ubn2;LOC100362586	Cytoskeleton
DMR4:66261001	4	66261001	66264000	3000	1	2.00E-07	-0.43	49	1.63	Ubn2;LOC100362586	Cytoskeleton
DMR4:66336001	4	66336001	66337000	1000	1	1.00E-07	0.66	59	5.9	Luc7l2;LOC103692107;LOC108350694	
DMR4:66349001	4	66349001	66351000	2000	1	2.60E-08	-0.42	26	1.3	Luc7l2;LOC108350694	
DMR4:66422001	4	66422001	66425000	3000	1	1.70E-07	0.39	45	1.5	Clec2l	
DMR4:66469001	4	66469001	66470000	1000	1	8.00E-07	0.48	10	1	Hipk2	
DMR4:66556001	4	66556001	66557000	1000	1	8.70E-11	0.35	10	1	Hipk2;LOC103692101	
DMR4:66654001	4	66654001	66656000	2000	1	1.70E-07	0.47	29	1.45	Tbxas1	Metabolism
DMR4:66671001	4	66671001	66673000	2000	1	2.70E-08	-0.53	44	2.2	Tbxas1	Metabolism
DMR4:66686001	4	66686001	66689000	3000	3	7.30E-10	0.54	36	1.2	Tbxas1	Metabolism
DMR4:66701001	4	66701001	66704000	3000	1	1.70E-09	0.46	34	1.13	Tbxas1	Metabolism
DMR4:66740001	4	66740001	66742000	2000	1	1.50E-08	0.53	40	2	Tbxas1	Metabolism
DMR4:66755001	4	66755001	66757000	2000	1	2.40E-09	0.49	19	0.95	Tbxas1	Metabolism
DMR4:66793001	4	66793001	66794000	1000	1	5.40E-09	0.33	11	1.1	Tbxas1	Metabolism
DMR4:66836001	4	66836001	66837000	1000	1	3.60E-07	-0.45	21	2.1	Tbxas1;Parp12	Metabolism
DMR4:67129001	4	67129001	67131000	2000	1	4.50E-07	-0.35	39	1.95	Slc37a3	Transport
DMR4:67299001	4	67299001	67302000	3000	1	2.00E-07	-0.42	56	1.87	Dennd2a	
DMR4:67347001	4	67347001	67350000	3000	1	4.20E-07	0.47	45	1.5	Dennd2a;LOC102554568;Adck2	
DMR4:67842001	4	67842001	67843000	1000	1	1.80E-07	-0.66	1	0.1	Tmem178b	
DMR4:67863001	4	67863001	67864000	1000	1	5.30E-14	0.88	19	1.9	Tmem178b	
DMR4:67981001	4	67981001	67982000	1000	1	3.10E-07	0.39	12	1.2	Tmem178b	
DMR4:68492001	4	68492001	68493000	1000	1	1.90E-07	-0.37	13	1.3	Agk	Signaling
DMR4:68638001	4	68638001	68642000	4000	1	1.60E-07	-0.53	35	0.88	Wee2;Ssbp1;Tas2r137	Signaling;Transcription
DMR4:68810001	4	68810001	68812000	2000	1	4.50E-10	-0.64	11	0.55	Olr799;Clec5a	Signaling
DMR4:68823001	4	68823001	68824000	1000	1	2.30E-08	-0.38	7	0.7	Clec5a;Tas2r138	
DMR4:69107001	4	69107001	69108000	1000	1	1.50E-07	0.3	2	0.2	Mgam;Moxd2	Metabolism;Metabolism
DMR4:70912001	4	70912001	70913000	1000	1	4.70E-09	0.69	33	3.3	Ephb6;Trpv6	Receptor;Transport
DMR4:70914001	4	70914001	70917000	3000	1	3.20E-08	0.41	51	1.7	Ephb6;Trpv6	Receptor;Transport
DMR4:70921001	4	70921001	70922000	1000	1	1.30E-07	-0.39	8	0.8	Ephb6;Trpv6	Receptor;Transport

DMR4:71378001	4	71378001	71380000	2000	1	1.80E-07	-0.24	15	0.75	Sval1	
DMR4:71634001	4	71634001	71639000	5000	1	1.40E-07	-0.3	44	0.88	Gstk1;LOC100360758;Tmem139	Transport
DMR4:71664001	4	71664001	71665000	1000	1	6.40E-07	-0.38	7	0.7	Casp2;Clcn1	Protease;Transport
DMR4:71735001	4	71735001	71736000	1000	1	8.90E-07	0.52	13	1.3	Zyx	Cytoskeleton
DMR4:71771001	4	71771001	71772000	1000	1	9.40E-09	-0.51	14	1.4	Epha1	Receptor
DMR4:71791001	4	71791001	71792000	1000	1	9.50E-08	-0.55	9	0.9	LOC103690198;Tas2r135	Receptor
DMR4:71861001	4	71861001	71863000	2000	1	6.30E-08	0.33	11	0.55	Olr801	Receptor
DMR4:72155001	4	72155001	72160000	5000	1	2.30E-09	-0.38	50	1	Tcaf1	
DMR4:72281001	4	72281001	72284000	3000	1	2.30E-08	0.39	26	0.87	Olr808;LOC103690255	Receptor
DMR4:72538001	4	72538001	72540000	2000	1	2.20E-07	-0.43	20	1	Olr815-ps	
DMR4:72829001	4	72829001	72831000	2000	1	5.50E-09	-0.44	17	0.85	Tpk1	Signaling
DMR4:73066001	4	73066001	73070000	4000	1	9.60E-07	-0.53	15	0.38	Tpk1	Signaling
DMR4:75200001	4	75200001	75202000	2000	1	3.40E-07	-0.45	12	0.6	Cntnap2	
DMR4:75429001	4	75429001	75430000	1000	1	2.50E-09	0.6	15	1.5	Cntnap2	
DMR4:75592001	4	75592001	75593000	1000	1	5.70E-07	0.41	12	1.2	Cntnap2	
DMR4:75741001	4	75741001	75742000	1000	1	6.40E-07	0.56	6	0.6	Cntnap2	
DMR4:75774001	4	75774001	75775000	1000	1	7.30E-10	0.42	40	4	Cntnap2	
DMR4:75843001	4	75843001	75844000	1000	1	2.60E-07	-0.4	8	0.8	Cntnap2;LOC103692121	
DMR4:76221001	4	76221001	76224000	3000	1	3.40E-08	-0.47	9	0.3	Cntnap2	
DMR4:76247001	4	76247001	76248000	1000	1	4.80E-07	-0.41	6	0.6	Cntnap2	
DMR4:76305001	4	76305001	76309000	4000	1	3.70E-10	-0.35	27	0.68	Cntnap2	
DMR4:76747001	4	76747001	76748000	1000	1	5.70E-08	0.68	19	1.9	Cntnap2	
DMR4:76761001	4	76761001	76762000	1000	1	7.90E-08	-0.73	43	4.3	Cntnap2	
DMR4:76835001	4	76835001	76837000	2000	1	3.00E-07	0.43	16	0.8	Cntnap2	
DMR4:77001001	4	77001001	77002000	1000	1	3.20E-07	-0.26	8	0.8	Cntnap2;LOC102553884	
DMR4:77483001	4	77483001	77486000	3000	1	8.90E-07	-0.33	36	1.2	Pdia4;Zfp786	Transcription;Transcription
DMR4:77556001	4	77556001	77560000	4000	1	4.20E-07	-0.42	46	1.15	Zfp398;Zfp282	Transcription
DMR4:77574001	4	77574001	77576000	2000	1	1.40E-11	-0.45	23	1.15	Zfp282	Transcription
DMR4:77849001	4	77849001	77851000	2000	1	6.30E-11	-0.46	16	0.8	Trnac-gca	
DMR4:78070001	4	78070001	78072000	2000	2	7.10E-20	1.53	114	5.7	Zfp467;Sspo	Extracellular Matrix
DMR4:78076001	4	78076001	78078000	2000	1	8.90E-07	-0.58	109	5.45	Zfp467;Sspo	Extracellular Matrix
DMR4:78123001	4	78123001	78124000	1000	1	1.30E-08	0.48	8	0.8	Sspo	Extracellular Matrix
DMR4:78179001	4	78179001	78182000	3000	1	6.40E-07	-0.36	35	1.17	Atp6v0e2;Lrrc61	Metabolism;Cytoskeleton
DMR4:78236001	4	78236001	78237000	1000	1	3.00E-08	0.52	31	3.1	LOC100363281;Repin1;Zfp775	Transcription
DMR4:78268001	4	78268001	78269000	1000	1	2.00E-08	0.41	13	1.3	Zfp775;RGD1559747	
DMR4:78618001	4	78618001	78622000	4000	1	3.10E-07	0.65	47	1.18	Doxl2	Metabolism
DMR4:78798001	4	78798001	78800000	2000	2	2.20E-09	0.61	57	2.85	Igf2bp3	Metabolism
DMR4:78913001	4	78913001	78914000	1000	1	2.60E-07	-0.34	9	0.9	Tra2a	
DMR4:78976001	4	78976001	78979000	3000	1	2.40E-10	0.81	52	1.73	Ccdc126	
DMR4:78989001	4	78989001	78991000	2000	1	6.70E-07	-0.3	32	1.6	Ccdc126	
DMR4:79073001	4	79073001	79074000	1000	1	1.70E-09	0.54	8	0.8	Stk31	
DMR4:79861001	4	79861001	79864000	3000	1	2.70E-07	-0.3	30	1	Mpp6	Cytoskeleton
DMR4:80037001	4	80037001	80038000	1000	1	3.80E-09	-0.56	20	2	Osbpl3	
DMR4:80150001	4	80150001	80152000	2000	1	1.40E-07	-0.45	30	1.5	Osbpl3	
DMR4:81018001	4	81018001	81020000	2000	1	8.20E-08	0.59	25	1.25	Mir148a	
DMR4:81195001	4	81195001	81197000	2000	2	2.00E-14	0.8	38	1.9	Nfe2l3	Transcription
DMR4:81294001	4	81294001	81297000	3000	1	2.40E-10	-0.41	64	2.13	RGD1561341	
DMR4:81302001	4	81302001	81303000	1000	1	1.10E-09	0.61	24	2.4	RGD1561341;Snx10	
DMR4:81360001	4	81360001	81363000	3000	2	2.30E-13	0.4	13	0.43	Snx10	
DMR4:81370001	4	81370001	81372000	2000	1	3.30E-09	-0.49	50	2.5	Snx10	
DMR4:81807001	4	81807001	81810000	3000	1	1.60E-11	-0.58	54	1.8	Skap2	Cytoskeleton
DMR4:81834001	4	81834001	81836000	2000	1	1.50E-07	-0.49	28	1.4	Skap2	Cytoskeleton
DMR4:81880001	4	81880001	81881000	1000	1	8.40E-08	-0.52	8	0.8	Skap2	Cytoskeleton
DMR4:82138001	4	82138001	82139000	1000	1	1.60E-10	0.82	27	2.7	Hotairm1;Hoxa2;Hoxa3;LOC102554823	
DMR4:82161001	4	82161001	82164000	3000	1	2.50E-11	-0.63	56	1.87	Hoxa3;Hoxa4;LOC102547399;Hoxaas3;LOC103689922;Hoxa6	Development

DMR4:82652001	4	82652001	82654000	2000	1	9.40E-08	-0.47	26	1.3	Hibadh;LOC685406;LOC685391	Metabolism
DMR4:83021001	4	83021001	83022000	1000	1	1.60E-07	0.46	7	0.7	Jazf1	
DMR4:83046001	4	83046001	83047000	1000	1	2.30E-11	-0.49	18	1.8	Jazf1	
DMR4:83120001	4	83120001	83121000	1000	1	2.00E-08	-0.5	7	0.7	Jazf1;LOC108350711	
DMR4:83489001	4	83489001	83491000	2000	1	1.80E-09	-0.57	29	1.45	Creb5	Transcription
DMR4:83698001	4	83698001	83699000	1000	1	2.00E-07	0.46	9	0.9	Creb5	Transcription
DMR4:83722001	4	83722001	83726000	4000	1	2.50E-07	-0.46	56	1.4	Creb5	Transcription
DMR4:83838001	4	83838001	83839000	1000	1	1.90E-08	0.62	22	2.2	Creb5	Transcription
DMR4:84073001	4	84073001	84074000	1000	1	5.30E-09	-0.46	9	0.9	Cpvl;LOC108350713	
DMR4:84655001	4	84655001	84657000	2000	1	5.20E-07	0.7	34	1.7	Wipf3;LOC103692134	Cytoskeleton
DMR4:84747001	4	84747001	84748000	1000	1	4.40E-07	-0.55	5	0.5	Scrn1;LOC102553885;Fkbp14	Transcription
DMR4:84829001	4	84829001	84832000	3000	1	2.10E-07	0.64	60	2	Plekha8	Transport
DMR4:84871001	4	84871001	84872000	1000	1	8.40E-07	-0.37	15	1.5	Mturn	
DMR4:85038001	4	85038001	85043000	5000	1	1.60E-11	0.56	61	1.22	Znrf2	Proteolysis
DMR4:85067001	4	85067001	85068000	1000	1	3.90E-07	-0.35	9	0.9	Znrf2	Proteolysis
DMR4:85191001	4	85191001	85192000	1000	1	1.70E-07	-0.42	9	0.9	Ggct	
DMR4:85288001	4	85288001	85290000	2000	1	8.20E-15	0.83	26	1.3	Crhr2	Receptor
DMR4:85302001	4	85302001	85304000	2000	1	2.10E-07	0.56	23	1.15	Crhr2	Receptor
DMR4:85439001	4	85439001	85441000	2000	1	4.30E-08	-0.45	43	2.15	Fam188b	
DMR4:85555001	4	85555001	85558000	3000	1	2.20E-07	0.52	42	1.4	LOC108350810;Aqp1	Transport
DMR4:85607001	4	85607001	85608000	1000	1	3.70E-07	0.44	23	2.3	Ghrhr;LOC102551107	Receptor
DMR4:85913001	4	85913001	85914000	1000	1	5.40E-08	0.65	20	2	Neurod6	Transcription
DMR4:86231001	4	86231001	86232000	1000	1	3.70E-08	0.4	11	1.1	Ccdc129	
DMR4:86369001	4	86369001	86370000	1000	1	3.80E-08	0.56	10	1	Pde1c;LOC102551243	Signaling
DMR4:86601001	4	86601001	86603000	2000	1	2.60E-09	-0.59	15	0.75	Pde1c	Signaling
DMR4:87160001	4	87160001	87164000	4000	1	4.30E-08	0.21	33	0.82	Fkbp9	
DMR4:87303001	4	87303001	87308000	5000	2	1.10E-08	-0.36	32	0.64	LOC688782;Vom1r65	Receptor
DMR4:87320001	4	87320001	87321000	1000	1	3.60E-08	-0.43	4	0.4	Vom1r65;LOC108350861	Receptor
DMR4:87606001	4	87606001	87608000	2000	1	1.80E-11	-0.47	4	0.2	Vom1r71	Receptor
DMR4:87692001	4	87692001	87694000	2000	1	9.60E-11	-0.53	6	0.3	Vom1r72	Receptor
DMR4:87759001	4	87759001	87762000	3000	1	2.10E-08	-0.37	30	1	Vom1r73	Receptor
DMR4:87862001	4	87862001	87866000	4000	1	6.60E-07	0.21	61	1.52	Vom1r76;LOC689033;Vom1r77	Receptor
DMR4:88055001	4	88055001	88056000	1000	1	1.00E-07	0.51	11	1.1	Vom1r81	Receptor
DMR4:88097001	4	88097001	88098000	1000	1	4.30E-11	0.6	5	0.5	Vom1r82	Receptor
DMR4:88234001	4	88234001	88235000	1000	1	6.80E-08	-0.59	3	0.3	Vom1r85	Receptor
DMR4:88654001	4	88654001	88659000	5000	1	3.30E-09	-0.31	37	0.74	Herc6	Proteolysis
DMR4:89093001	4	89093001	89096000	3000	1	5.20E-07	-0.34	29	0.97	Herc3	Proteolysis
DMR4:89250001	4	89250001	89253000	3000	1	9.50E-07	-0.29	27	0.9	Fam13a	
DMR4:90846001	4	90846001	90847000	1000	1	9.20E-09	-0.42	9	0.9	Snca	Transport
DMR4:91299001	4	91299001	91301000	2000	1	2.00E-33	0.83	17	0.85	Ccser1	
DMR4:91449001	4	91449001	91450000	1000	1	2.70E-07	0.49	7	0.7	Ccser1	
DMR4:91499001	4	91499001	91504000	5000	1	6.20E-08	0.35	55	1.1	Ccser1	
DMR4:91722001	4	91722001	91723000	1000	1	4.00E-09	-0.51	5	0.5	Ccser1	
DMR4:91825001	4	91825001	91826000	1000	1	9.40E-07	0.44	11	1.1	Ccser1	
DMR4:91833001	4	91833001	91839000	6000	1	1.50E-07	-0.32	64	1.07	Ccser1	
DMR4:92320001	4	92320001	92322000	2000	1	6.50E-07	0.47	20	1	Ccser1	
DMR4:92324001	4	92324001	92325000	1000	1	3.10E-10	0.38	3	0.3	Ccser1	
DMR4:92329001	4	92329001	92333000	4000	1	4.80E-09	-0.39	38	0.95	Ccser1	
DMR4:92458001	4	92458001	92459000	1000	1	4.60E-07	0.48	13	1.3	Ccser1	
DMR4:92492001	4	92492001	92493000	1000	1	3.30E-08	-0.59	5	0.5	Ccser1	
DMR4:92516001	4	92516001	92518000	2000	1	1.00E-07	0.47	28	1.4	Ccser1	
DMR4:94060001	4	94060001	94062000	2000	1	9.70E-07	-0.25	24	1.2	Grid2	Receptor
DMR4:94120001	4	94120001	94122000	2000	1	3.20E-07	-0.41	25	1.25	Grid2	Receptor
DMR4:94178001	4	94178001	94179000	1000	1	3.50E-07	-0.51	5	0.5	Grid2	Receptor
DMR4:94333001	4	94333001	94334000	1000	1	4.60E-07	-0.54	3	0.3	Grid2	Receptor
DMR4:94715001	4	94715001	94716000	1000	1	3.20E-07	0.49	13	1.3	Grid2	Receptor
DMR4:94978001	4	94978001	94979000	1000	1	2.30E-07	0.59	16	1.6	Grid2	Receptor
DMR4:95221001	4	95221001	95223000	2000	1	1.70E-08	-0.59	6	0.3	Grid2	Receptor
DMR4:95236001	4	95236001	95239000	3000	2	3.50E-18	-0.64	30	1	Grid2	Receptor
DMR4:95261001	4	95261001	95262000	1000	1	1.80E-07	-0.36	11	1.1	Grid2	Receptor

DMR4:96711001	4	96711001	96713000	2000	1	5.10E-07	0.5	22	1.1	Prdm5	Transcription
DMR4:97323001	4	97323001	97325000	2000	2	3.60E-17	0.61	27	1.35	RGD1564699	
DMR4:97687001	4	97687001	97688000	1000	1	3.90E-09	-0.37	13	1.3	Gng12;LOC103692153	Signaling
DMR4:98031001	4	98031001	98033000	2000	2	1.40E-07	-0.52	29	1.45	Serbp1	Metabolism
DMR4:98217001	4	98217001	98222000	5000	1	2.90E-07	-0.34	40	0.8	Il23r	Receptor
DMR4:98563001	4	98563001	98566000	3000	1	4.30E-07	0.4	29	0.97	Rpia	Metabolism
DMR4:98655001	4	98655001	98656000	1000	1	1.90E-07	-0.37	8	0.8	LOC102549525;Eif2ak3	Signaling
DMR4:98688001	4	98688001	98692000	4000	1	1.20E-08	-0.42	73	1.82	Eif2ak3	Signaling
DMR4:98727001	4	98727001	98729000	2000	1	1.20E-07	0.42	29	1.45	Tex37	
DMR4:98782001	4	98782001	98785000	3000	1	2.30E-08	0.59	36	1.2	Foxi3	Transcription
DMR4:99001001	4	99001001	99003000	2000	1	1.60E-09	-0.44	24	1.2	Thnsl2	
DMR4:99146001	4	99146001	99147000	1000	1	3.00E-11	-0.53	6	0.6	Krcc1	
DMR4:99216001	4	99216001	99220000	4000	1	5.40E-09	-0.5	46	1.15	LOC500198;LOC108350717;Cd8a	
DMR4:99286001	4	99286001	99287000	1000	1	7.10E-07	-0.39	12	1.2	Rmnd5a	
DMR4:99302001	4	99302001	99305000	3000	1	5.20E-07	-0.55	20	0.67	Rmnd5a	
DMR4:99447001	4	99447001	99448000	1000	1	1.90E-07	-0.47	13	1.3	Chmp3	Transport
DMR4:99654001	4	99654001	99656000	2000	1	8.50E-08	0.35	23	1.15	Reep1	Transport
DMR4:99782001	4	99782001	99783000	1000	1	7.20E-07	-0.4	5	0.5	Immt	
DMR4:99987001	4	99987001	99989000	2000	1	9.20E-09	0.44	40	2	St3gal5	Transport
DMR4:100066001	4	100066001	100067000	1000	1	3.20E-09	0.51	15	1.5	Atoh8	Transcription
DMR4:100188001	4	100188001	100195000	7000	1	8.50E-13	-0.53	68	0.97	Usp39	Protease
DMR4:100497001	4	100497001	100502000	5000	1	7.10E-07	-0.28	99	1.98	LOC102549526;Tcf7l1	Transcription
DMR4:100510001	4	100510001	100511000	1000	1	5.30E-08	-0.42	14	1.4	Tcf7l1	Transcription
DMR4:100978001	4	100978001	100980000	2000	1	1.70E-10	0.39	16	0.8	Dnah6	Cytoskeleton
DMR4:101007001	4	101007001	101009000	2000	1	4.40E-12	0.67	26	1.3	Dnah6	Cytoskeleton
DMR4:101044001	4	101044001	101045000	1000	1	3.00E-07	-0.45	16	1.6	Dnah6	Cytoskeleton
DMR4:101070001	4	101070001	101071000	1000	1	4.70E-07	-0.46	21	2.1	Dnah6	Cytoskeleton
DMR4:101077001	4	101077001	101078000	1000	1	7.80E-07	0.46	6	0.6	Dnah6	Cytoskeleton
DMR4:101174001	4	101174001	101175000	1000	1	8.00E-18	0.54	12	1.2	Suclg1	Metabolism
DMR4:101190001	4	101190001	101191000	1000	1	3.40E-10	-0.68	3	0.3	Suclg1	Metabolism
DMR4:101390001	4	101390001	101391000	1000	1	3.30E-09	-0.59	6	0.6	RGD1562515	Cytoskeleton
DMR4:107947001	4	107947001	107948000	1000	1	9.00E-11	0.57	13	1.3	Ctnna2	Cytoskeleton
DMR4:108068001	4	108068001	108070000	2000	1	5.30E-11	0.4	17	0.85	Ctnna2	Cytoskeleton
DMR4:108218001	4	108218001	108219000	1000	1	9.50E-07	0.42	3	0.3	Ctnna2	Cytoskeleton
DMR4:108225001	4	108225001	108226000	1000	1	3.80E-07	0.39	11	1.1	Ctnna2	Cytoskeleton
DMR4:108568001	4	108568001	108569000	1000	1	3.10E-10	-0.58	6	0.6	Ctnna2	Cytoskeleton
DMR4:108659001	4	108659001	108662000	3000	1	1.20E-07	0.64	40	1.33	Ctnna2	Cytoskeleton
DMR4:108691001	4	108691001	108692000	1000	1	8.60E-08	-0.52	13	1.3	Ctnna2	Cytoskeleton
DMR4:108846001	4	108846001	108847000	1000	1	3.90E-07	0.37	13	1.3	Ctnna2	Cytoskeleton
DMR4:108934001	4	108934001	108938000	4000	1	1.00E-08	-0.32	36	0.9	Ctnna2	Cytoskeleton
DMR4:108966001	4	108966001	108967000	1000	1	2.00E-08	-0.56	3	0.3	Ctnna2	Cytoskeleton
DMR4:109014001	4	109014001	109016000	2000	1	2.40E-07	-0.46	18	0.9	Ctnna2	Cytoskeleton
DMR4:109503001	4	109503001	109504000	1000	1	2.70E-07	-0.3	8	0.8	Reg1a	
DMR4:110950001	4	110950001	110952000	2000	1	2.00E-08	-0.48	9	0.45	Lrrtm4	Receptor
DMR4:111096001	4	111096001	111097000	1000	1	3.60E-09	-0.6	3	0.3	Lrrtm4	Receptor
DMR4:111127001	4	111127001	111128000	1000	1	9.90E-09	0.49	9	0.9	Lrrtm4	Receptor
DMR4:112713001	4	112713001	112714000	1000	1	6.10E-09	-0.5	19	1.9	Mrpl19;Eva1a	Translation
DMR4:113283001	4	113283001	113286000	3000	1	2.30E-08	-0.3	29	0.97	Tacr1	Signaling
DMR4:113553001	4	113553001	113554000	1000	1	3.10E-09	-0.37	15	1.5	Hk2	Signaling
DMR4:113615001	4	113615001	113616000	1000	1	1.50E-08	-0.71	1	0.1	Hk2	Signaling
DMR4:113742001	4	113742001	113743000	1000	1	4.40E-07	0.4	10	1	Sema4f	Signaling
DMR4:113748001	4	113748001	113749000	1000	1	6.90E-07	-0.34	14	1.4	Sema4f	Signaling
DMR4:113772001	4	113772001	113773000	1000	1	3.60E-10	1	25	2.5	Sema4f;M1ap	Signaling
DMR4:113841001	4	113841001	113842000	1000	1	6.20E-08	-0.43	14	1.4	M1ap	
DMR4:113847001	4	113847001	113849000	2000	1	4.40E-08	-0.51	21	1.05	M1ap	
DMR4:113869001	4	113869001	113871000	2000	1	1.40E-07	-0.48	28	1.4	M1ap;Dok1;Loxl3	Metabolism
DMR4:114832001	4	114832001	114833000	1000	1	8.50E-07	-0.63	5	0.5	NEWGENE_1562258;LOC108350720;Rtkn	Cytoskeleton
DMR4:114940001	4	114940001	114942000	2000	1	3.90E-07	0.33	21	1.05	Slc4a5	Transport
DMR4:114956001	4	114956001	114958000	2000	1	4.50E-07	0.45	34	1.7	Slc4a5	Transport
DMR4:115065001	4	115065001	115066000	1000	1	1.90E-07	-0.35	32	3.2	Bola3;Tet3	

DMR4:115097001	4	115097001	115099000	2000	1	2.80E-07	0.5	31	1.55	Tet3	
DMR4:115269001	4	115269001	115270000	1000	1	6.80E-07	-0.32	23	2.3	Stambp	Protease
DMR4:115399001	4	115399001	115400000	1000	1	4.90E-09	-0.47	14	1.4	Vax2;LOC108350721	Development
DMR4:115430001	4	115430001	115431000	1000	1	5.00E-08	0.54	13	1.3	Atp6v1b1;Ankrd53	Metabolism
DMR4:115432001	4	115432001	115434000	2000	1	9.70E-08	0.35	29	1.45	Atp6v1b1;Ankrd53	Metabolism
DMR4:115444001	4	115444001	115445000	1000	1	3.90E-07	0.45	10	1	Atp6v1b1;Ankrd53;Tex261	Metabolism
DMR4:115738001	4	115738001	115740000	2000	1	1.30E-07	-0.54	20	1	Dysf	Transport
DMR4:115761001	4	115761001	115763000	2000	1	6.50E-16	0.6	32	1.6	Dysf	Transport
DMR4:115772001	4	115772001	115773000	1000	1	4.80E-08	-0.54	13	1.3	Dysf	Transport
DMR4:115792001	4	115792001	115797000	5000	2	5.50E-08	0.47	74	1.48	Dysf	Transport
DMR4:115802001	4	115802001	115805000	3000	1	1.40E-09	-0.41	57	1.9	Dysf	Transport
DMR4:115864001	4	115864001	115866000	2000	1	1.10E-08	0.51	24	1.2	Dysf	Transport
DMR4:116371001	4	116371001	116373000	2000	1	9.80E-08	-0.45	11	0.55	Exoc6b	Transport
DMR4:116746001	4	116746001	116749000	3000	1	2.30E-09	-0.41	17	0.57	Exoc6b	Transport
DMR4:116760001	4	116760001	116762000	2000	1	1.40E-09	-0.55	14	0.7	Exoc6b	Transport
DMR4:116767001	4	116767001	116768000	1000	1	2.60E-07	-0.46	6	0.6	Exoc6b	Transport
DMR4:116921001	4	116921001	116923000	2000	1	3.40E-08	-0.49	25	1.25	Spr	Metabolism
DMR4:117115001	4	117115001	117119000	4000	2	8.40E-09	0.59	48	1.2	Sfxn5;Rab11fip5	Transport
DMR4:117204001	4	117204001	117205000	1000	1	2.50E-09	0.44	22	2.2	Noto	Development
DMR4:117258001	4	117258001	117259000	1000	1	7.20E-07	0.44	11	1.1	Cct7;Fbxo41	Translation
DMR4:117261001	4	117261001	117262000	1000	1	1.50E-08	0.59	21	2.1	Cct7;Fbxo41	Translation
DMR4:117480001	4	117480001	117485000	5000	1	1.20E-07	-0.39	39	0.78	Alms1;Nat8f3	Metabolism
DMR4:117530001	4	117530001	117535000	5000	1	2.90E-08	-0.54	72	1.44	Nat8f5;Nat8	Metabolism
DMR4:117675001	4	117675001	117676000	1000	1	1.60E-07	0.53	12	1.2	Figla	Transcription
DMR4:117707001	4	117707001	117708000	1000	1	2.00E-07	0.35	9	0.9	Add2	Cytoskeleton
DMR4:117892001	4	117892001	117893000	1000	1	1.20E-07	-0.42	24	2.4	Add2	Cytoskeleton
DMR4:118010001	4	118010001	118012000	2000	1	6.20E-07	-0.58	21	1.05	Tgfa	Growth Factors
DMR4:118262001	4	118262001	118265000	3000	1	4.90E-08	-0.43	31	1.03	RGD1306746	
DMR4:118424001	4	118424001	118426000	2000	1	5.40E-08	0.44	15	0.75	Asprv1	
DMR4:118435001	4	118435001	118436000	1000	1	9.90E-07	0.42	16	1.6	Asprv1	
DMR4:118479001	4	118479001	118483000	4000	1	7.40E-08	-0.48	58	1.45	Mxd1;Snrnp27	Transcription
DMR4:118495001	4	118495001	118496000	1000	1	8.60E-08	-0.51	13	1.3	Snrnp27;Gmcl1	Proteolysis
DMR4:118570001	4	118570001	118571000	1000	1	4.10E-07	0.34	2	0.2	Anxa4	Signaling
DMR4:118599001	4	118599001	118603000	4000	1	9.80E-07	0.48	43	1.07	Anxa4;LOC103692178	Signaling
DMR4:118673001	4	118673001	118675000	2000	1	9.40E-08	-0.41	20	1	Aak1	Signaling
DMR4:118837001	4	118837001	118839000	2000	1	3.60E-08	0.56	27	1.35	Nfu1	
DMR4:118956001	4	118956001	118958000	2000	1	2.70E-15	0.37	21	1.05	Antxr1	Cytoskeleton
DMR4:119018001	4	119018001	119021000	3000	2	2.30E-09	0.67	24	0.8	Antxr1	Cytoskeleton
DMR4:119095001	4	119095001	119098000	3000	1	2.30E-09	-0.4	46	1.53	Antxr1	Cytoskeleton
DMR4:119145001	4	119145001	119146000	1000	1	2.10E-07	0.34	9	0.9	Gkn2;Gkn1	
DMR4:119217001	4	119217001	119219000	2000	1	1.50E-08	-0.37	38	1.9	Bmp10	Growth Factors
DMR4:119221001	4	119221001	119222000	1000	1	9.50E-12	-0.54	11	1.1	Bmp10	Growth Factors
DMR4:119261001	4	119261001	119263000	2000	1	4.10E-09	0.61	29	1.45	Arhgap25	
DMR4:119320001	4	119320001	119321000	1000	1	1.30E-09	0.84	28	2.8	Arhgap25	
DMR4:119326001	4	119326001	119329000	3000	1	2.90E-08	-0.48	66	2.2	Arhgap25	
DMR4:119332001	4	119332001	119333000	1000	1	8.60E-07	0.52	20	2	Arhgap25	
DMR4:119399001	4	119399001	119400000	1000	1	3.90E-08	-0.52	5	0.5	Vom1r101	Receptor
DMR4:119515001	4	119515001	119518000	3000	1	4.10E-07	-0.4	59	1.97	Aplf	
DMR4:119640001	4	119640001	119645000	5000	1	6.90E-08	0.56	66	1.32	LOC100910604;Efcc1	Translation
DMR4:119664001	4	119664001	119667000	3000	1	1.50E-14	-0.68	32	1.07	Efcc1	Translation
DMR4:120000001	4	120000001	120002000	2000	1	4.30E-10	-0.38	28	1.4	Rpn1	Golgi
DMR4:120126001	4	120126001	120127000	1000	1	6.10E-19	0.79	31	3.1	Gata2	Transcription
DMR4:120131001	4	120131001	120132000	1000	1	1.60E-07	0.67	27	2.7	Gata2	Transcription
DMR4:120136001	4	120136001	120139000	3000	1	1.10E-08	0.58	71	2.37	Gata2	Transcription
DMR4:120177001	4	120177001	120179000	2000	1	4.60E-07	0.56	36	1.8	Tcp1-ps1	
DMR4:120189001	4	120189001	120192000	3000	2	4.90E-09	0.49	31	1.03	Tcp1-ps1;Eefsec	Translation
DMR4:120199001	4	120199001	120200000	1000	1	1.90E-09	0.53	9	0.9	Eefsec	Translation
DMR4:120201001	4	120201001	120202000	1000	1	8.70E-14	0.75	6	0.6	Eefsec	Translation
DMR4:120231001	4	120231001	120234000	3000	1	1.50E-07	0.54	28	0.93	Eefsec	Translation
DMR4:120237001	4	120237001	120238000	1000	1	4.40E-09	0.64	6	0.6	Eefsec	Translation
DMR4:120496001	4	120496001	120499000	3000	1	7.00E-07	0.47	55	1.83	Kbtbd12	Cytoskeleton
DMR4:120577001	4	120577001	120578000	1000	1	1.70E-08	-0.5	16	1.6	Kbtbd12	Cytoskeleton

DMR4:120676001	4	120676001	120677000	1000	1	3.60E-08	-0.37	15	1.5	Mgll	Metabolism
DMR4:120678001	4	120678001	120682000	4000	1	1.10E-08	-0.4	71	1.77	Mgll	Metabolism
DMR4:120749001	4	120749001	120750000	1000	1	1.40E-07	-0.41	24	2.4	Mgll;LOC102554878	Metabolism
DMR4:120789001	4	120789001	120790000	1000	1	1.50E-08	-0.44	18	1.8	Abtb1;Podxl2	
DMR4:121035001	4	121035001	121036000	1000	1	5.30E-08	0.29	15	1.5	Prr20e	
DMR4:121222001	4	121222001	121225000	3000	1	2.40E-10	0.63	50	1.67	Plxna1	
DMR4:121234001	4	121234001	121237000	3000	1	6.00E-07	0.55	55	1.83	Plxna1	
DMR4:121342001	4	121342001	121343000	1000	1	2.10E-10	0.66	8	0.8	Chchd6	
DMR4:121436001	4	121436001	121439000	3000	1	2.10E-08	-0.54	12	0.4	Chchd6;LOC108350729	
DMR4:121836001	4	121836001	121840000	4000	1	3.30E-07	-0.32	26	0.65	Vom1r-ps77	
DMR4:121867001	4	121867001	121869000	2000	1	9.70E-11	0.36	19	0.95	Vom1r96	Receptor
DMR4:122120001	4	122120001	122123000	3000	1	2.90E-07	-0.39	16	0.53	Vom1r103	
DMR4:122158001	4	122158001	122161000	3000	1	4.10E-09	-0.42	26	0.87	Vom1r104	
DMR4:122636001	4	122636001	122637000	1000	1	6.60E-09	-0.39	29	2.9	LOC108350819;Nup210	Transport
DMR4:122727001	4	122727001	122731000	4000	1	6.10E-10	0.52	50	1.25	Nup210	Transport
DMR4:122872001	4	122872001	122874000	2000	1	2.10E-10	0.28	16	0.8	Fbln2	Extracellular Matrix
DMR4:122991001	4	122991001	122995000	4000	1	5.60E-09	0.57	61	1.52	Wnt7a	Signaling
DMR4:123015001	4	123015001	123017000	2000	1	2.70E-10	0.43	9	0.45	Wnt7a	Signaling
DMR4:123018001	4	123018001	123022000	4000	1	1.20E-07	0.51	45	1.12	Wnt7a	Signaling
DMR4:123023001	4	123023001	123025000	2000	1	4.90E-07	0.47	39	1.95	Wnt7a	Signaling
DMR4:123026001	4	123026001	123029000	3000	1	6.00E-09	0.54	33	1.1	Wnt7a	Signaling
DMR4:123126001	4	123126001	123127000	1000	1	2.70E-07	0.42	14	1.4	Chchd4;Tmem43;Xpc	DNA Repair
DMR4:123137001	4	123137001	123139000	2000	1	1.00E-06	0.41	40	2	Tmem43;Xpc	DNA Repair
DMR4:123434001	4	123434001	123437000	3000	1	1.10E-07	0.47	30	1	Iqsec1	Transcription
DMR4:123441001	4	123441001	123444000	3000	1	7.20E-10	0.59	32	1.07	Iqsec1	Transcription
DMR4:123462001	4	123462001	123463000	1000	1	5.20E-08	0.41	12	1.2	Iqsec1;Slc41a3	Transcription;Transport
DMR4:123537001	4	123537001	123539000	2000	1	4.60E-09	0.6	28	1.4	Aldh1l1	Metabolism
DMR4:123573001	4	123573001	123576000	3000	1	5.60E-07	-0.4	32	1.07	Aldh1l1;LOC108350870;Grip2	Metabolism
DMR4:123645001	4	123645001	123646000	1000	1	6.90E-10	0.49	18	1.8	Grip2;Slc6a6	Transport
DMR4:123686001	4	123686001	123687000	1000	1	1.10E-08	0.58	12	1.2	Slc6a6	Transport
DMR4:123719001	4	123719001	123720000	1000	1	2.40E-07	0.6	7	0.7	Slc6a6;LOC102548902	Transport
DMR4:123750001	4	123750001	123755000	5000	2	1.50E-07	0.52	59	1.18	Ccdc174;LOC108350801	
DMR4:123828001	4	123828001	123830000	2000	1	7.40E-08	0.48	25	1.25	RGD1560289	
DMR4:123896001	4	123896001	123899000	3000	2	5.90E-09	0.47	39	1.3	Fgd5	Transcription
DMR4:123959001	4	123959001	123961000	2000	2	6.30E-09	0.63	13	0.65	Fgd5	Transcription
DMR4:124254001	4	124254001	124255000	1000	1	8.20E-08	-0.41	17	1.7	Prickle2	Cytoskeleton
DMR4:124480001	4	124480001	124483000	3000	1	8.20E-07	0.54	14	0.47	Prickle2	Cytoskeleton
DMR4:124667001	4	124667001	124671000	4000	1	1.80E-12	0.71	67	1.68	Adamts9	Protease
DMR4:124770001	4	124770001	124771000	1000	1	3.20E-13	-0.79	5	0.5	Adamts9	Protease
DMR4:124800001	4	124800001	124801000	1000	1	7.80E-07	-0.39	23	2.3	Adamts9	Protease
DMR4:125688001	4	125688001	125689000	1000	1	5.80E-07	0.65	27	2.7	Magi1;LOC102556496	
DMR4:125758001	4	125758001	125762000	4000	1	1.80E-09	-0.49	38	0.95	Magi1	
DMR4:125969001	4	125969001	125970000	1000	1	3.70E-07	-0.31	22	2.2	Magi1	
DMR4:126005001	4	126005001	126008000	3000	1	1.10E-08	0.38	37	1.23	Magi1;LOC102556397	
DMR4:126059001	4	126059001	126060000	1000	1	8.40E-07	-0.35	17	1.7	Magi1	
DMR4:126180001	4	126180001	126181000	1000	1	2.50E-08	-0.43	19	1.9	Magi1	
DMR4:126187001	4	126187001	126190000	3000	1	3.20E-07	-0.41	31	1.03	Magi1	
DMR4:127574001	4	127574001	127577000	3000	1	8.00E-08	-0.42	37	1.23	Suclg2	Metabolism
DMR4:127667001	4	127667001	127674000	7000	1	3.20E-07	-0.24	94	1.34	Suclg2	Metabolism
DMR4:127688001	4	127688001	127691000	3000	1	9.70E-09	-0.32	30	1	Suclg2	Metabolism
DMR4:127728001	4	127728001	127729000	1000	1	2.10E-08	-0.65	9	0.9	Suclg2	Metabolism
DMR4:127978001	4	127978001	127980000	2000	1	6.20E-07	-0.33	24	1.2	RGD1564292	
DMR4:128891001	4	128891001	128892000	1000	1	5.30E-13	0.86	30	3	Fam19a1	
DMR4:129355001	4	129355001	129358000	3000	1	2.30E-07	-0.35	28	0.93	Fam19a4	
DMR4:129360001	4	129360001	129363000	3000	1	3.50E-08	-0.39	27	0.9	Fam19a4	
DMR4:129392001	4	129392001	129394000	2000	1	7.70E-11	-0.6	26	1.3	Fam19a4	
DMR4:129468001	4	129468001	129469000	1000	1	7.60E-12	-0.55	17	1.7	Eogt	Golgi
DMR4:129676001	4	129676001	129679000	3000	1	1.70E-07	-0.45	62	2.07	Frmd4b;LOC108350736	
DMR4:129681001	4	129681001	129684000	3000	1	1.30E-13	-0.68	76	2.53	Frmd4b;LOC108350736	
DMR4:129692001	4	129692001	129695000	3000	1	1.40E-10	-0.43	62	2.07	Frmd4b;LOC108350736	
DMR4:129736001	4	129736001	129737000	1000	1	1.60E-09	-0.39	16	1.6	Frmd4b	

DMR4:129758001	4	129758001	129762000	4000	1	3.90E-08	0.56	45	1.12	Frmd4b	
DMR4:129795001	4	129795001	129796000	1000	1	1.20E-09	0.63	13	1.3	Frmd4b	
DMR4:129802001	4	129802001	129804000	2000	1	1.10E-08	-0.34	40	2	Frmd4b	
DMR4:129821001	4	129821001	129823000	2000	1	6.70E-09	-0.47	42	2.1	Frmd4b	
DMR4:129882001	4	129882001	129887000	5000	1	6.70E-07	0.3	107	2.14	Frmd4b;LOC108350735	
DMR4:129943001	4	129943001	129946000	3000	1	1.40E-07	0.32	29	0.97	Frmd4b	
DMR4:129980001	4	129980001	129983000	3000	1	2.90E-07	0.52	35	1.17	Frmd4b	
DMR4:130327001	4	130327001	130328000	1000	1	1.50E-08	-0.54	4	0.4	Mitf	
DMR4:130353001	4	130353001	130354000	1000	1	1.10E-07	0.36	10	1	Mitf	
DMR4:130405001	4	130405001	130409000	4000	1	1.40E-09	-0.39	37	0.92	Mitf	
DMR4:131366001	4	131366001	131369000	3000	1	5.90E-11	-0.48	77	2.57	Foxp1	
DMR4:131416001	4	131416001	131417000	1000	1	7.00E-07	0.53	13	1.3	Foxp1	
DMR4:131479001	4	131479001	131481000	2000	1	7.00E-07	-0.37	29	1.45	Foxp1	
DMR4:131530001	4	131530001	131538000	8000	1	4.10E-10	-0.5	140	1.75	Foxp1	
DMR4:131709001	4	131709001	131711000	2000	2	3.80E-10	-0.46	47	2.35	Foxp1	
DMR4:131812001	4	131812001	131813000	1000	1	8.50E-07	-0.34	19	1.9	Foxp1	
DMR4:131838001	4	131838001	131841000	3000	1	3.70E-07	-0.43	46	1.53	Foxp1	
DMR4:131862001	4	131862001	131863000	1000	1	1.80E-08	0.36	12	1.2	Foxp1	
DMR4:131868001	4	131868001	131869000	1000	1	6.10E-07	-0.42	14	1.4	Foxp1	
DMR4:132295001	4	132295001	132299000	4000	1	2.30E-10	0.76	66	1.65	LOC102555186;RGD1560513	
DMR4:133052001	4	133052001	133054000	2000	1	1.20E-09	-0.43	14	0.7	Shq1	
DMR4:133274001	4	133274001	133276000	2000	1	1.70E-08	-0.56	30	1.5	Ppp4r2	
DMR4:133326001	4	133326001	133330000	4000	1	8.60E-07	-0.43	56	1.4	Ppp4r2	
DMR4:133847001	4	133847001	133848000	1000	1	2.70E-07	-0.37	17	1.7	Pdzrn3	
DMR4:134782001	4	134782001	134783000	1000	1	3.40E-09	-0.61	11	1.1	Cntn3	
DMR4:134785001	4	134785001	134787000	2000	1	4.80E-09	-0.57	9	0.45	Cntn3	
DMR4:134819001	4	134819001	134820000	1000	1	1.40E-07	0.35	13	1.3	Cntn3	
DMR4:135036001	4	135036001	135038000	2000	1	1.40E-09	-0.45	20	1	Cntn3	
DMR4:135129001	4	135129001	135130000	1000	1	3.10E-10	0.51	12	1.2	Cntn3	
DMR4:136677001	4	136677001	136678000	1000	1	3.10E-07	0.49	12	1.2	Cntn6	
DMR4:136887001	4	136887001	136888000	1000	1	4.30E-07	0.43	23	2.3	Cntn6	
DMR4:137771001	4	137771001	137777000	6000	1	1.50E-10	-0.37	56	0.93	Cntn4	Cytoskeleton
DMR4:138071001	4	138071001	138072000	1000	1	1.30E-08	-0.68	7	0.7	Cntn4	Cytoskeleton
DMR4:138754001	4	138754001	138755000	1000	1	2.90E-07	-0.45	5	0.5	Cntn4	Cytoskeleton
DMR4:138787001	4	138787001	138788000	1000	1	1.80E-07	0.54	7	0.7	Cntn4;Il5ra	Cytoskeleton;Receptor
DMR4:139666001	4	139666001	139667000	1000	1	5.20E-07	-0.53	10	1	Lrrn1	Receptor
DMR4:140148001	4	140148001	140150000	2000	1	2.00E-08	-0.45	16	0.8	Sumf1	
DMR4:140212001	4	140212001	140213000	1000	1	7.90E-09	-0.35	7	0.7	Sumf1	
DMR4:140365001	4	140365001	140368000	3000	1	4.60E-09	-0.52	32	1.07	Itpr1	Ion Channel
DMR4:140415001	4	140415001	140416000	1000	1	3.50E-07	-0.54	5	0.5	Itpr1	Ion Channel
DMR4:140479001	4	140479001	140480000	1000	1	4.10E-07	-0.58	17	1.7	Itpr1	Ion Channel
DMR4:140543001	4	140543001	140544000	1000	1	2.70E-09	-0.45	22	2.2	Itpr1	Ion Channel
DMR4:142716001	4	142716001	142722000	6000	1	2.50E-07	-0.27	71	1.18	Grm7	Signaling
DMR4:142815001	4	142815001	142816000	1000	1	1.60E-09	0.83	16	1.6	Grm7	Signaling
DMR4:142843001	4	142843001	142844000	1000	1	3.40E-09	-0.44	10	1	Grm7	Signaling
DMR4:143206001	4	143206001	143209000	3000	1	1.20E-07	-0.49	25	0.83	Grm7	Signaling
DMR4:144541001	4	144541001	144543000	2000	1	5.40E-07	0.48	27	1.35	Rad18	Proteolysis
DMR4:144692001	4	144692001	144694000	2000	1	2.20E-08	0.47	21	1.05	LOC102548080;Srgap3	Signaling
DMR4:144742001	4	144742001	144745000	3000	1	9.40E-07	-0.32	41	1.37	Srgap3	Signaling
DMR4:144751001	4	144751001	144752000	1000	1	5.30E-07	-0.64	9	0.9	Srgap3	Signaling
DMR4:144772001	4	144772001	144773000	1000	1	6.30E-07	0.59	11	1.1	Srgap3	Signaling
DMR4:144852001	4	144852001	144853000	1000	1	1.20E-10	0.76	26	2.6	Srgap3	Signaling
DMR4:145120001	4	145120001	145122000	2000	1	6.10E-09	0.6	46	2.3	LOC108350744;Lhfpl4	
DMR4:145139001	4	145139001	145140000	1000	1	1.40E-08	-0.5	12	1.2	Lhfpl4	
DMR4:145270001	4	145270001	145272000	2000	1	1.00E-08	-0.46	30	1.5	Cpne9;Brpf1	Transcription
DMR4:145400001	4	145400001	145402000	2000	1	1.50E-09	0.54	41	2.05	Cidec;LOC100361844;Jagn1	
DMR4:145452001	4	145452001	145456000	4000	1	5.00E-13	0.9	65	1.62	Creld1;Prrt3	Extracellular Matrix
DMR4:145544001	4	145544001	145545000	1000	1	2.60E-09	0.6	17	1.7	Fancd2;Fancd2os	
DMR4:145551001	4	145551001	145552000	1000	1	1.20E-07	0.52	15	1.5	Fancd2;Fancd2os;Brk1	
DMR4:145560001	4	145560001	145562000	2000	1	9.40E-08	-0.33	39	1.95	Fancd2;Fancd2os;Brk1	
DMR4:145596001	4	145596001	145598000	2000	2	8.50E-08	-0.49	23	1.15	Vhl;Irak2	Proteolysis
DMR4:145610001	4	145610001	145612000	2000	1	2.00E-08	-0.44	28	1.4	Irak2	

DMR4:145726001	4	145726001	145729000	3000	2	1.60E-09	0.42	34	1.13	Atp2b2	Transport
DMR4:145779001	4	145779001	145780000	1000	1	8.60E-10	0.66	16	1.6	Atp2b2	Transport
DMR4:145839001	4	145839001	145840000	1000	1	8.80E-08	0.5	12	1.2	Atp2b2	Transport
DMR4:145891001	4	145891001	145893000	2000	1	1.50E-07	0.35	15	0.75	Atp2b2	Transport
DMR4:146232001	4	146232001	146233000	1000	1	7.60E-07	-0.41	7	0.7	Slc6a11	Transport
DMR4:146420001	4	146420001	146423000	3000	1	6.30E-07	0.4	32	1.07	Hrh1	Signaling
DMR4:146561001	4	146561001	146564000	3000	3	3.00E-26	1.05	71	2.37	LOC108350747;Atg7	Proteolysis
DMR4:146568001	4	146568001	146569000	1000	1	1.60E-10	-0.47	5	0.5	LOC108350747;Atg7	Proteolysis
DMR4:146607001	4	146607001	146608000	1000	1	7.10E-07	-0.52	6	0.6	Atg7	Proteolysis
DMR4:146737001	4	146737001	146740000	3000	1	3.10E-08	-0.47	22	0.73	Atg7	Proteolysis
DMR4:146793001	4	146793001	146795000	2000	1	2.50E-09	-0.62	20	1	Vgll4	Transcription
DMR4:146803001	4	146803001	146807000	4000	1	2.50E-07	-0.47	81	2.02	Vgll4	Transcription
DMR4:146833001	4	146833001	146835000	2000	1	6.10E-08	-0.41	35	1.75	Vgll4	Transcription
DMR4:146849001	4	146849001	146851000	2000	1	3.10E-09	-0.53	20	1	Vgll4	Transcription
DMR4:146852001	4	146852001	146855000	3000	1	1.50E-07	-0.44	46	1.53	Vgll4	Transcription
DMR4:146857001	4	146857001	146860000	3000	1	3.30E-07	-0.46	21	0.7	Vgll4	Transcription
DMR4:147111001	4	147111001	147117000	6000	2	1.60E-09	-0.37	71	1.18	Syn2;LOC100365210;LOC100911724	Transport
DMR4:147137001	4	147137001	147140000	3000	2	4.40E-07	-0.5	20	0.67	Syn2	Transport
DMR4:147692001	4	147692001	147693000	1000	1	3.00E-07	0.66	11	1.1	Cand2	Proteolysis
DMR4:147694001	4	147694001	147697000	3000	1	3.60E-10	0.47	49	1.63	Cand2	Proteolysis
DMR4:147846001	4	147846001	147848000	2000	1	2.30E-11	0.79	34	1.7	Rho;H1foo;Plxnd1	Signaling;Epigenetic
DMR4:147930001	4	147930001	147932000	2000	1	3.40E-09	-0.52	13	0.65	Tmcc1;LOC680385	
DMR4:147997001	4	147997001	148001000	4000	1	1.40E-07	-0.33	35	0.88	Tmcc1	
DMR4:148155001	4	148155001	148157000	2000	1	5.20E-08	-0.68	26	1.3	Fam21c	
DMR4:148291001	4	148291001	148293000	2000	1	6.80E-08	-0.39	19	0.95	8-Mar	
DMR4:148327001	4	148327001	148328000	1000	1	6.40E-13	0.43	8	0.8	8-Mar	
DMR4:148436001	4	148436001	148437000	1000	1	5.40E-07	-0.6	5	0.5	Alox5;LOC108350749	Metabolism
DMR4:148458001	4	148458001	148460000	2000	2	5.60E-07	-0.49	6	0.3	Olr823	
DMR4:148524001	4	148524001	148526000	2000	1	1.60E-13	-0.61	4	0.2	Olr824	Receptor
DMR4:148598001	4	148598001	148599000	1000	1	1.90E-08	-0.39	7	0.7	Olr827	Receptor
DMR4:148664001	4	148664001	148666000	2000	1	3.70E-20	1.19	43	2.15	Olr830	Receptor
DMR4:148681001	4	148681001	148684000	3000	2	2.90E-12	-0.6	22	0.73	Olr831	Receptor
DMR4:148710001	4	148710001	148712000	2000	1	3.70E-21	1.08	43	2.15	Olr832;Trnac-gca	Receptor
DMR4:148787001	4	148787001	148788000	1000	1	9.70E-07	0.54	16	1.6	Rassf4;LOC500300	Cytoskeleton
DMR4:148838001	4	148838001	148839000	1000	1	4.70E-07	-0.53	5	0.5	Tmem72	
DMR4:149866001	4	149866001	149868000	2000	1	9.00E-07	0.32	21	1.05	Zfp637	Transcription
DMR4:149946001	4	149946001	149948000	2000	1	1.40E-11	0.73	61	3.05	Hnrnpf	Translation
DMR4:149972001	4	149972001	149974000	2000	1	1.20E-08	-0.38	10	0.5	Hnrnpf	Translation
DMR4:150068001	4	150068001	150072000	4000	1	2.60E-10	0.4	57	1.43	Rasgef1a	Transcription
DMR4:150120001	4	150120001	150123000	3000	1	1.20E-07	0.34	44	1.47	Rasgef1a	Transcription
DMR4:150218001	4	150218001	150221000	3000	1	8.30E-07	0.38	43	1.43	Ret	Receptor
DMR4:150223001	4	150223001	150227000	4000	1	6.40E-08	0.53	66	1.65	Ret	Receptor
DMR4:150247001	4	150247001	150253000	6000	1	4.20E-08	0.47	91	1.52	Ret	Receptor
DMR4:150493001	4	150493001	150495000	2000	1	6.70E-07	-0.42	22	1.1	Zfp9	
DMR4:150549001	4	150549001	150550000	1000	1	3.40E-08	-0.44	31	3.1	Ankrd26	
DMR4:150579001	4	150579001	150582000	3000	1	4.50E-08	-0.32	53	1.77	Ankrd26	
DMR4:150667001	4	150667001	150670000	3000	1	4.90E-09	0.39	32	1.07	Cacna1c	Transport
DMR4:150747001	4	150747001	150749000	2000	1	2.60E-07	0.43	26	1.3	Cacna1c	Transport
DMR4:150806001	4	150806001	150807000	1000	1	6.50E-07	-0.43	21	2.1	Cacna1c	Transport
DMR4:150865001	4	150865001	150868000	3000	1	1.10E-07	-0.36	68	2.27	Cacna1c	Transport
DMR4:150964001	4	150964001	150966000	2000	1	5.70E-12	0.72	21	1.05	Cacna1c	Transport
DMR4:151104001	4	151104001	151105000	1000	1	1.40E-08	-0.5	14	1.4	Cacna1c;LOC108350845	Transport
DMR4:151178001	4	151178001	151180000	2000	1	1.20E-09	0.57	41	2.05	Cacna1c;LOC108350846	Transport
DMR4:151218001	4	151218001	151221000	3000	1	5.20E-09	-0.57	26	0.87	Cacna1c	Transport
DMR4:151324001	4	151324001	151325000	1000	1	4.10E-07	-0.36	12	1.2	Cacna2d4	Transport
DMR4:151332001	4	151332001	151337000	5000	1	2.30E-08	0.39	73	1.46	Cacna2d4	Transport
DMR4:151392001	4	151392001	151396000	4000	1	8.00E-07	0.45	74	1.85	Cacna2d4;Lrtm2	Transport
DMR4:151423001	4	151423001	151424000	1000	1	8.10E-07	-0.45	5	0.5	Adipor2	Signaling
DMR4:151436001	4	151436001	151439000	3000	1	3.00E-07	0.28	15	0.5	Adipor2	Signaling
DMR4:151483001	4	151483001	151486000	3000	1	2.10E-07	-0.38	41	1.37	Adipor2	Signaling
DMR4:151513001	4	151513001	151516000	3000	1	6.30E-07	0.5	49	1.63	Wnt5b	Signaling
DMR4:152162001	4	152162001	152166000	4000	2	1.80E-09	-0.44	37	0.92	Erc1	Transport

DMR4:152184001	4	152184001	152188000	4000	1	5.20E-08	-0.34	31	0.78	Erc1	Transport
DMR4:152303001	4	152303001	152305000	2000	1	4.20E-07	-0.34	27	1.35	Erc1	Transport
DMR4:152336001	4	152336001	152341000	5000	1	1.50E-07	-0.38	42	0.84	Erc1;LOC102552689	Transport
DMR4:152434001	4	152434001	152437000	3000	1	5.50E-07	-0.44	43	1.43	Rad52	Transcription
DMR4:152477001	4	152477001	152481000	4000	1	6.20E-09	-0.45	41	1.02	Wnk1	Signaling
DMR4:152642001	4	152642001	152644000	2000	1	5.40E-07	-0.47	13	0.65	Ninj2	Cytoskeleton
DMR4:152666001	4	152666001	152668000	2000	1	1.70E-11	-0.52	35	1.75	Ninj2	Cytoskeleton
DMR4:152703001	4	152703001	152705000	2000	2	9.40E-08	-0.6	13	0.65	Ninj2;LOC681016	Cytoskeleton
DMR4:152749001	4	152749001	152753000	4000	2	5.70E-09	0.67	81	2.02	B4galnt3	Golgi
DMR4:152780001	4	152780001	152782000	2000	1	7.10E-07	0.32	11	0.55	B4galnt3	Golgi
DMR4:152783001	4	152783001	152786000	3000	1	8.40E-09	0.52	38	1.27	B4galnt3	Golgi
DMR4:152799001	4	152799001	152802000	3000	1	1.90E-11	0.51	37	1.23	B4galnt3	Golgi
DMR4:152823001	4	152823001	152826000	3000	1	1.10E-07	-0.35	51	1.7	B4galnt3	Golgi
DMR4:152843001	4	152843001	152845000	2000	1	1.60E-07	-0.56	48	2.4	B4galnt3	Golgi
DMR4:152938001	4	152938001	152939000	1000	1	1.30E-07	0.46	10	1	Kdm5a	Epigenetic
DMR4:153031001	4	153031001	153032000	1000	1	5.70E-08	0.29	9	0.9	Cecr6	
DMR4:153398001	4	153398001	153399000	1000	1	5.70E-07	-0.45	7	0.7	Bcl2l13	
DMR4:153421001	4	153421001	153422000	1000	1	1.90E-09	0.61	14	1.4	Bcl2l13	
DMR4:153466001	4	153466001	153467000	1000	1	6.20E-12	0.66	25	2.5	Bid	
DMR4:153610001	4	153610001	153612000	2000	1	6.80E-07	-0.37	31	1.55	Mical3	
DMR4:153634001	4	153634001	153635000	1000	1	1.80E-10	-0.5	18	1.8	Mical3	
DMR4:153640001	4	153640001	153647000	7000	1	4.50E-07	-0.37	113	1.61	Mical3	
DMR4:153739001	4	153739001	153741000	2000	1	8.50E-07	0.64	44	2.2	Pex26	Transcription
DMR4:153798001	4	153798001	153802000	4000	1	1.00E-07	-0.41	59	1.48	Tuba8	Cytoskeleton
DMR4:153836001	4	153836001	153838000	2000	1	1.60E-13	0.91	28	1.4	Usp18;LOC102553072	Protease
DMR4:153893001	4	153893001	153894000	1000	1	2.10E-07	-0.38	15	1.5	Slc6a13	Transport
DMR4:154001001	4	154001001	154003000	2000	1	3.10E-08	0.64	52	2.6	Iqsec3	Transcription
DMR4:154018001	4	154018001	154020000	2000	1	8.40E-11	-0.66	10	0.5	Iqsec3	Transcription
DMR4:154026001	4	154026001	154031000	5000	1	1.50E-09	-0.51	90	1.8	Iqsec3	Transcription
DMR4:154113001	4	154113001	154114000	1000	1	2.10E-10	0.56	17	1.7	RGD1564159	
DMR4:154211001	4	154211001	154212000	1000	1	7.30E-07	-0.34	7	0.7	Mug2	Protease; Proteolysis
DMR4:154230001	4	154230001	154233000	3000	2	2.50E-16	0.53	18	0.6	Mug2	Protease; Proteolysis
DMR4:154249001	4	154249001	154252000	3000	1	9.10E-08	-0.42	21	0.7	Mug2	Protease; Proteolysis
DMR4:154268001	4	154268001	154269000	1000	1	3.70E-09	-0.69	4	0.4	Mug2	Protease; Proteolysis
DMR4:154861001	4	154861001	154864000	3000	1	6.80E-07	-0.34	51	1.7	Cpamd8	
DMR4:154890001	4	154890001	154892000	2000	1	1.40E-13	0.54	35	1.75	Cpamd8	
DMR4:154942001	4	154942001	154946000	4000	1	8.70E-08	-0.27	71	1.77	Cpamd8;LOC102554538	
DMR4:155055001	4	155055001	155057000	2000	2	1.60E-11	0.74	42	2.1	Klrg1	Receptor
DMR4:155076001	4	155076001	155083000	7000	2	2.00E-08	-0.4	85	1.21	M6pr	Transport
DMR4:155121001	4	155121001	155123000	2000	1	4.10E-11	-0.43	40	2	Phc1	Epigenetic
DMR4:155312001	4	155312001	155313000	1000	1	9.40E-08	-0.35	22	2.2	Mfap5	
DMR4:155324001	4	155324001	155330000	6000	2	2.30E-09	0.77	103	1.72	Mfap5	
DMR4:155344001	4	155344001	155346000	2000	1	1.60E-09	-0.7	18	0.9	Mfap5	
DMR4:155356001	4	155356001	155359000	3000	1	4.40E-10	-0.48	55	1.83	Aicda	Translation
DMR4:155361001	4	155361001	155363000	2000	2	4.50E-12	0.89	43	2.15	Aicda	Translation
DMR4:155368001	4	155368001	155369000	1000	1	2.90E-13	0.69	17	1.7	Aicda	Translation
DMR4:155413001	4	155413001	155414000	1000	1	3.90E-11	-0.53	21	2.1	Apobec1;Gdf3	Translation;Growth Factors
DMR4:155568001	4	155568001	155570000	2000	1	8.10E-07	-0.69	27	1.35	Slc2a3	
DMR4:155649001	4	155649001	155652000	3000	1	8.40E-08	0.43	34	1.13	Foxj2	
DMR4:155672001	4	155672001	155675000	3000	1	1.20E-11	0.81	67	2.23	Foxj2;C3ar1	Signaling
DMR4:156133001	4	156133001	156138000	5000	1	5.80E-11	-0.43	55	1.1	Clec4b2	Transport
DMR4:156322001	4	156322001	156324000	2000	1	4.90E-08	-0.36	20	1	LOC108350823;Vom2r48	Signaling
DMR4:156535001	4	156535001	156537000	2000	1	2.10E-08	-0.38	17	0.85	Vom2r51	Signaling
DMR4:156602001	4	156602001	156603000	1000	1	1.80E-09	0.36	15	1.5	Vom2r52	Signaling
DMR4:156720001	4	156720001	156721000	1000	1	2.30E-07	-0.38	10	1	RGD1561715	
DMR4:156754001	4	156754001	156755000	1000	1	2.00E-13	0.96	23	2.3	Cd163	Protease
DMR4:156775001	4	156775001	156777000	2000	1	8.10E-08	-0.35	12	0.6	Cd163	Protease
DMR4:156975001	4	156975001	156976000	1000	1	4.40E-09	-0.44	5	0.5	LOC108350759;Pex5	Transport
DMR4:157083001	4	157083001	157085000	2000	1	3.10E-11	0.46	24	1.2	Clstn3;LOC102553636	Transport
DMR4:157086001	4	157086001	157088000	2000	1	5.40E-08	0.45	23	1.15	Clstn3;LOC102553636	Transport
DMR4:157113001	4	157113001	157114000	1000	1	7.10E-14	-0.54	13	1.3	C1rl	Protease

DMR4:157123001	4	157123001	157124000	1000	1	1.60E-07	0.57	17	1.7	C1rl;LOC102553847;C1r;LOC108350760	Protease
DMR4:157141001	4	157141001	157143000	2000	1	4.30E-10	0.57	26	1.3	C1r;C1s	Protease
DMR4:157146001	4	157146001	157155000	9000	1	6.00E-10	-0.53	123	1.37	C1r;C1s	Protease
DMR4:157171001	4	157171001	157174000	3000	1	6.20E-07	0.3	39	1.3	Lpcat3	Metabolism
DMR4:157367001	4	157367001	157373000	6000	1	4.00E-10	0.81	111	1.85	Gnb3;P3h3;Gpr162;Cd4	Signaling;Extracellular Matrix;Signaling
DMR4:157374001	4	157374001	157376000	2000	1	3.30E-07	0.4	112	5.6	P3h3;Gpr162;Cd4	Extracellular Matrix;Signaling
DMR4:157378001	4	157378001	157381000	3000	1	8.90E-07	-0.41	103	3.43	P3h3;Gpr162;Cd4	Extracellular Matrix;Signaling
DMR4:157416001	4	157416001	157419000	3000	2	8.00E-07	-0.37	39	1.3	Cd4;Lag3	Receptor
DMR4:157430001	4	157430001	157432000	2000	1	3.50E-09	0.51	40	2	Lag3;Ptms;LOC102553981	Receptor
DMR4:157434001	4	157434001	157438000	4000	1	4.10E-07	-0.4	68	1.7	Lag3;Ptms;LOC102553981	Receptor
DMR4:157447001	4	157447001	157448000	1000	1	1.00E-12	-0.49	31	3.1	Ptms;LOC102553981;Mlf2	Signaling
DMR4:157509001	4	157509001	157511000	2000	2	1.40E-14	-0.75	48	2.4	Pianp	
DMR4:157518001	4	157518001	157520000	2000	1	1.70E-07	-0.44	18	0.9	Pianp;Zfp384	
DMR4:157616001	4	157616001	157619000	3000	3	5.20E-16	-0.6	70	2.33	Chd4	
DMR4:157629001	4	157629001	157631000	2000	1	5.20E-07	-0.39	31	1.55	Chd4	
DMR4:157731001	4	157731001	157732000	1000	1	2.20E-07	-0.46	11	1.1	Vamp1;Tapbpl	Immune
DMR4:157743001	4	157743001	157745000	2000	2	1.20E-14	0.5	22	1.1	Vamp1;Tapbpl;Cd27;LOC102555485	Immune
DMR4:157748001	4	157748001	157755000	7000	2	1.00E-12	0.61	128	1.83	Tapbpl;Cd27;LOC102555485	Immune
DMR4:157818001	4	157818001	157820000	2000	1	2.80E-07	-0.36	40	2	Ltbr	Receptor
DMR4:157876001	4	157876001	157877000	1000	1	1.40E-09	0.66	32	3.2	Tnfrsf1a;Plekhg6	Receptor
DMR4:158180001	4	158180001	158182000	2000	1	1.10E-07	-0.4	36	1.8	Vwf	
DMR4:158195001	4	158195001	158197000	2000	1	3.30E-07	-0.54	17	0.85	Vwf	
DMR4:158198001	4	158198001	158201000	3000	1	4.20E-10	0.52	36	1.2	Vwf	
DMR4:158212001	4	158212001	158214000	2000	1	2.60E-07	0.49	32	1.6	Vwf;Ano2	
DMR4:158218001	4	158218001	158219000	1000	1	6.40E-08	-0.4	9	0.9	Vwf;Ano2	
DMR4:158469001	4	158469001	158472000	3000	1	5.70E-07	0.51	32	1.07	Ano2	
DMR4:158713001	4	158713001	158715000	2000	1	6.40E-08	-0.45	27	1.35	Ntf3	Growth Factors
DMR4:159076001	4	159076001	159078000	2000	1	3.20E-09	0.69	37	1.85	Kcna5	Transport
DMR4:159181001	4	159181001	159186000	5000	1	6.30E-08	-0.37	92	1.84	Kcna1	Transport
DMR4:159271001	4	159271001	159273000	2000	1	2.80E-08	0.32	17	0.85	Kcna6	Transport
DMR4:159380001	4	159380001	159382000	2000	1	1.20E-11	-0.51	29	1.45	Ndufa9	Metabolism
DMR4:159423001	4	159423001	159424000	1000	1	9.50E-07	-0.32	11	1.1	Akap3;Dyrk4	Cytoskeleton
DMR4:159428001	4	159428001	159431000	3000	2	1.00E-09	0.52	33	1.1	Akap3;Dyrk4	Cytoskeleton
DMR4:159531001	4	159531001	159537000	6000	1	6.40E-11	-0.48	103	1.72	RGD1564885;LOC689087;RGD1559795	
DMR4:159538001	4	159538001	159541000	3000	1	3.10E-10	0.86	47	1.57	LOC689087;RGD1559795	
DMR4:159564001	4	159564001	159567000	3000	1	2.10E-08	0.38	53	1.77	Fgf6	Growth Factors
DMR4:159658001	4	159658001	159660000	2000	1	2.10E-09	0.68	36	1.8	Tigar	Signaling
DMR4:159690001	4	159690001	159694000	4000	2	2.00E-18	1.04	83	2.08	Ccnd2	Signaling
DMR4:159705001	4	159705001	159706000	1000	1	2.40E-08	-0.5	20	2	Ccnd2	Signaling
DMR4:159707001	4	159707001	159711000	4000	1	3.10E-09	0.67	73	1.82	Ccnd2	Signaling
DMR4:160161001	4	160161001	160163000	2000	1	2.00E-07	-0.55	9	0.45	Cracr2a	
DMR4:160187001	4	160187001	160193000	6000	1	2.00E-09	-0.42	81	1.35	Cracr2a	
DMR4:160253001	4	160253001	160254000	1000	1	6.00E-08	0.34	17	1.7	Prmt8	Golgi
DMR4:160293001	4	160293001	160295000	2000	1	8.60E-07	0.49	28	1.4	Prmt8	Golgi
DMR4:160509001	4	160509001	160510000	1000	1	1.10E-08	0.59	12	1.2	Tspan11;LOC108350762	
DMR4:160514001	4	160514001	160515000	1000	1	7.20E-07	0.55	14	1.4	Tspan11;LOC108350762	
DMR4:160573001	4	160573001	160579000	6000	1	2.90E-07	0.6	83	1.38	Tspan9	
DMR4:160613001	4	160613001	160616000	3000	1	3.80E-07	-0.52	27	0.9	Tspan9	
DMR4:160673001	4	160673001	160675000	2000	1	2.50E-07	-0.49	28	1.4	Tspan9	
DMR4:160721001	4	160721001	160722000	1000	1	4.70E-08	-0.37	16	1.6	Tspan9	
DMR4:160728001	4	160728001	160731000	3000	1	2.70E-10	-0.46	42	1.4	Tspan9	
DMR4:161595001	4	161595001	161597000	2000	1	6.00E-12	0.75	17	0.85	Tead4	Transcription
DMR4:161602001	4	161602001	161603000	1000	1	7.20E-13	0.86	25	2.5	Tead4	Transcription
DMR4:161621001	4	161621001	161623000	2000	1	7.00E-07	0.44	26	1.3	Tead4;LOC100362138	Transcription
DMR4:161650001	4	161650001	161652000	2000	1	1.50E-10	0.71	29	1.45	Tulp3	

DMR4:161692001	4	161692001	161696000	4000	1	7.90E-07	0.44	57	1.43	Rhno1;Foxm1;LOC108350769;LOC100362909	
DMR4:161813001	4	161813001	161816000	3000	1	5.70E-09	-0.44	36	1.2	Pzp	
DMR4:161824001	4	161824001	161827000	3000	1	5.90E-07	-0.44	56	1.87	Pzp	
DMR4:161888001	4	161888001	161891000	3000	1	5.00E-07	-0.45	35	1.17	A2ml1	Protease; Proteolysis
DMR4:161907001	4	161907001	161909000	2000	1	5.30E-09	-0.45	52	2.6	A2ml1	Protease; Proteolysis
DMR4:161911001	4	161911001	161914000	3000	1	3.80E-07	-0.35	61	2.03	A2ml1	Protease; Proteolysis
DMR4:162027001	4	162027001	162030000	3000	1	6.60E-10	-0.4	52	1.73	Klrb1b;LOC108350770	Receptor
DMR4:162302001	4	162302001	162304000	2000	1	4.80E-14	-0.73	25	1.25	Clec2d;LOC102547086	
DMR4:162817001	4	162817001	162819000	2000	1	3.90E-07	0.36	13	0.65	Clec2h;LOC102553980	
DMR4:163092001	4	163092001	163093000	1000	1	1.60E-09	-0.58	6	0.6	RGD1564770	
DMR4:163118001	4	163118001	163119000	1000	1	1.50E-11	0.71	25	2.5	Clec12a;Chtop	Metabolism
DMR4:163330001	4	163330001	163337000	7000	2	4.00E-08	-0.33	73	1.04	Klre1	
DMR4:163519001	4	163519001	163520000	1000	1	4.10E-11	0.47	6	0.6	Klri1	
DMR4:163912001	4	163912001	163914000	2000	1	1.80E-07	-0.33	20	1	Ly49si2	
DMR4:164381001	4	164381001	164383000	2000	1	4.90E-07	-0.32	16	0.8	Klra22	
DMR4:164472001	4	164472001	164474000	2000	1	5.50E-07	-0.37	13	0.65	Ly49s4	
DMR4:165435001	4	165435001	165436000	1000	1	1.70E-07	-0.53	1	0.1	Klra1	
DMR4:165467001	4	165467001	165468000	1000	1	1.20E-07	-0.38	7	0.7	Klra1	
DMR4:165525001	4	165525001	165526000	1000	1	8.30E-12	-0.57	11	1.1	Magohb;Styk1	
DMR4:165533001	4	165533001	165534000	1000	1	7.40E-07	0.45	16	1.6	Styk1	
DMR4:165635001	4	165635001	165636000	1000	1	5.70E-07	-0.44	8	0.8	Ybx3	
DMR4:165765001	4	165765001	165770000	5000	1	5.30E-07	-0.43	25	0.5	Tas2r107	Receptor
DMR4:166017001	4	166017001	166019000	2000	1	1.70E-09	-0.64	9	0.45	Prpmp5	
DMR4:166273001	4	166273001	166274000	1000	1	2.60E-09	-0.32	4	0.4	Prp15	Signaling
DMR4:167073001	4	167073001	167074000	1000	1	1.40E-12	0.37	20	2	Tas2r125	Receptor
DMR4:167075001	4	167075001	167078000	3000	1	8.90E-09	-0.34	32	1.07	Tas2r125	Receptor
DMR4:167463001	4	167463001	167469000	6000	1	4.80E-12	0.32	48	0.8	Grpcb	Signaling
DMR4:167755001	4	167755001	167761000	6000	1	1.30E-10	-0.42	121	2.02	Etv6	Transcription
DMR4:167824001	4	167824001	167830000	6000	2	6.70E-11	-0.52	136	2.27	Etv6	Transcription
DMR4:167941001	4	167941001	167942000	1000	1	3.20E-09	-0.48	16	1.6	Etv6	Transcription
DMR4:167953001	4	167953001	167955000	2000	1	1.90E-09	0.67	44	2.2	Etv6	Transcription
DMR4:168145001	4	168145001	168149000	4000	1	5.00E-07	-0.55	75	1.88	Bcl2l14	
DMR4:168153001	4	168153001	168157000	4000	1	6.70E-07	-0.42	50	1.25	Bcl2l14	
DMR4:168164001	4	168164001	168168000	4000	1	2.30E-07	-0.35	53	1.32	Bcl2l14	
DMR4:168290001	4	168290001	168292000	2000	1	1.40E-07	-0.5	22	1.1	Lrp6	Binding Proteins
DMR4:168296001	4	168296001	168298000	2000	1	2.90E-12	-0.52	27	1.35	Lrp6	Binding Proteins
DMR4:168356001	4	168356001	168357000	1000	1	2.50E-10	-0.4	17	1.7	Mansc1	
DMR4:168372001	4	168372001	168373000	1000	1	6.40E-09	0.46	28	2.8	Mansc1	
DMR4:168378001	4	168378001	168382000	4000	1	1.40E-11	0.85	74	1.85	Mansc1	
DMR4:168405001	4	168405001	168406000	1000	1	3.80E-09	-0.57	20	2	Borcs5	
DMR4:168410001	4	168410001	168412000	2000	1	5.00E-07	-0.53	12	0.6	Borcs5	
DMR4:168488001	4	168488001	168490000	2000	1	5.90E-16	-0.58	21	1.05	Dusp16	Signaling
DMR4:168592001	4	168592001	168593000	1000	1	2.10E-08	-0.44	19	1.9	Crebl2	
DMR4:168622001	4	168622001	168623000	1000	1	3.10E-07	-0.37	20	2	Crebl2;LOC108350781	
DMR4:168685001	4	168685001	168686000	1000	1	7.50E-07	-0.39	21	2.1	Cdkn1b	Signaling
DMR4:168793001	4	168793001	168796000	3000	1	6.40E-10	-0.53	40	1.33	Ddx47	
DMR4:168843001	4	168843001	168846000	3000	1	3.80E-07	0.53	38	1.27	Gprc5a	Signaling
DMR4:168914001	4	168914001	168917000	3000	2	5.90E-13	0.65	65	2.17	Hebp1	
DMR4:168992001	4	168992001	168994000	2000	1	6.60E-07	0.44	24	1.2	Fam234b	
DMR4:169015001	4	169015001	169017000	2000	1	7.10E-07	0.52	46	2.3	Fam234b;Gsg1	Cytoskeleton
DMR4:169036001	4	169036001	169037000	1000	1	7.30E-10	0.67	24	2.4	Fam234b;Gsg1	Cytoskeleton
DMR4:169060001	4	169060001	169066000	6000	2	2.80E-12	0.98	111	1.85	Fam234b	
DMR4:169552001	4	169552001	169555000	3000	1	9.20E-09	-0.46	49	1.63	Grin2b	Receptor
DMR4:169559001	4	169559001	169563000	4000	2	2.30E-24	1.29	125	3.12	Grin2b	Receptor
DMR4:169583001	4	169583001	169586000	3000	1	3.10E-09	-0.49	27	0.9	Grin2b	Receptor
DMR4:169720001	4	169720001	169722000	2000	1	1.60E-09	-0.43	19	0.95	Grin2b	Receptor
DMR4:169738001	4	169738001	169739000	1000	1	8.40E-08	0.38	14	1.4	Grin2b	Receptor
DMR4:169781001	4	169781001	169785000	4000	1	2.10E-09	-0.52	57	1.43	Grin2b	Receptor
DMR4:169795001	4	169795001	169796000	1000	1	1.00E-16	1.07	19	1.9	Grin2b	Receptor
DMR4:169807001	4	169807001	169813000	6000	1	1.40E-07	-0.43	85	1.42	Grin2b	Receptor
DMR4:169839001	4	169839001	169841000	2000	1	2.70E-09	-0.46	32	1.6	Grin2b	Receptor

DMR4:169868001	4	169868001	169869000	1000	1	2.00E-08	0.65	30	3	Grin2b	Receptor
DMR4:169879001	4	169879001	169880000	1000	1	1.50E-07	-0.32	20	2	Grin2b	Receptor
DMR4:169887001	4	169887001	169891000	4000	1	2.60E-13	1.02	73	1.82	Grin2b	Receptor
DMR4:169920001	4	169920001	169926000	6000	1	3.20E-09	0.67	104	1.73	Grin2b	Receptor
DMR4:169947001	4	169947001	169952000	5000	1	1.70E-08	-0.36	73	1.46	Grin2b	Receptor
DMR4:170682001	4	170682001	170684000	2000	1	3.20E-08	0.41	47	2.35	Gucy2c	Signaling
DMR4:170696001	4	170696001	170698000	2000	1	1.50E-09	-0.53	19	0.95	Gucy2c	Signaling
DMR4:170724001	4	170724001	170731000	7000	1	2.10E-07	-0.52	90	1.29	Gucy2c;LOC103692245	Signaling
DMR4:170751001	4	170751001	170753000	2000	1	1.30E-09	0.61	49	2.45	RGD1562378	
DMR4:170779001	4	170779001	170781000	2000	1	2.40E-07	-0.51	46	2.3	Wbp11;LOC103690365	
DMR4:170926001	4	170926001	170928000	2000	1	2.90E-07	-0.33	44	2.2	Arhgdib	Signaling
DMR4:170932001	4	170932001	170934000	2000	1	4.10E-09	-0.44	34	1.7	Arhgdib	Signaling
DMR4:171067001	4	171067001	171071000	4000	1	1.10E-12	1.13	116	2.9	LOC102551070;Rerg	Signaling
DMR4:171079001	4	171079001	171082000	3000	2	5.90E-13	0.69	70	2.33	Rerg	Signaling
DMR4:171100001	4	171100001	171101000	1000	1	9.50E-09	-0.38	13	1.3	Rerg	Signaling
DMR4:171126001	4	171126001	171129000	3000	1	6.50E-10	0.64	41	1.37	Rerg	Signaling
DMR4:171268001	4	171268001	171271000	3000	1	2.90E-07	-0.32	57	1.9	Ptpro	Receptor
DMR4:171273001	4	171273001	171275000	2000	1	8.60E-07	0.27	16	0.8	Ptpro	Receptor
DMR4:171313001	4	171313001	171315000	2000	1	1.50E-07	-0.37	18	0.9	Ptpro	Receptor
DMR4:171324001	4	171324001	171326000	2000	1	4.50E-07	-0.3	38	1.9	Ptpro	Receptor
DMR4:171340001	4	171340001	171343000	3000	1	7.70E-11	0.69	43	1.43	Ptpro	Receptor
DMR4:171376001	4	171376001	171380000	4000	1	7.00E-07	-0.38	110	2.75	Ptpro	Receptor
DMR4:171455001	4	171455001	171461000	6000	2	6.60E-08	-0.37	123	2.05	Ptpro	Receptor
DMR4:171477001	4	171477001	171483000	6000	2	3.20E-09	-0.41	120	2	Eps8	Cytoskeleton
DMR4:171541001	4	171541001	171543000	2000	1	2.70E-10	-0.49	37	1.85	Eps8	Cytoskeleton
DMR4:171550001	4	171550001	171553000	3000	2	4.40E-08	-0.47	57	1.9	Eps8	Cytoskeleton
DMR4:171561001	4	171561001	171564000	3000	2	7.90E-08	-0.43	59	1.97	Eps8	Cytoskeleton
DMR4:171582001	4	171582001	171585000	3000	1	8.90E-07	-0.41	58	1.93	Eps8	Cytoskeleton
DMR4:171626001	4	171626001	171636000	10000	4	7.20E-10	-0.72	167	1.67	Eps8	Cytoskeleton
DMR4:171640001	4	171640001	171642000	2000	1	1.50E-11	-0.46	39	1.95	Eps8	Cytoskeleton
DMR4:171959001	4	171959001	171963000	4000	2	2.40E-09	0.53	65	1.62	Slc15a5	Transport
DMR4:171967001	4	171967001	171969000	2000	1	4.90E-08	0.48	51	2.55	Slc15a5	Transport
DMR4:171991001	4	171991001	171994000	3000	1	2.90E-07	-0.47	40	1.33	Slc15a5	Transport
DMR4:172012001	4	172012001	172015000	3000	1	2.50E-08	0.35	32	1.07	Slc15a5	Transport
DMR4:172016001	4	172016001	172017000	1000	1	1.00E-26	1.25	29	2.9	Slc15a5	Transport
DMR4:172060001	4	172060001	172063000	3000	1	3.50E-09	-0.39	17	0.57	Slc15a5	Transport
DMR4:172069001	4	172069001	172071000	2000	1	7.90E-08	0.55	40	2	Slc15a5	Transport
DMR4:172072001	4	172072001	172073000	1000	1	1.80E-13	0.88	26	2.6	Slc15a5	Transport
DMR4:172140001	4	172140001	172141000	1000	1	3.60E-10	-0.74	4	0.4	Mgst1	Transport
DMR4:172719001	4	172719001	172722000	3000	1	6.40E-07	-0.43	13	0.43	Igbp1b	Transcription
DMR4:172967001	4	172967001	172969000	2000	1	1.60E-07	0.48	44	2.2	Lmo3	
DMR4:172977001	4	172977001	172979000	2000	1	3.30E-22	1.1	43	2.15	Lmo3	
DMR4:173009001	4	173009001	173010000	1000	1	1.40E-07	-0.55	3	0.3	Lmo3	
DMR4:173646001	4	173646001	173648000	2000	1	2.50E-09	-0.45	42	2.1	Rergl	
DMR4:173860001	4	173860001	173862000	2000	1	6.10E-17	1	26	1.3	Pik3c2g	Signaling
DMR4:173904001	4	173904001	173909000	5000	2	1.10E-11	-0.38	50	1	Pik3c2g	Signaling
DMR4:173976001	4	173976001	173981000	5000	1	1.30E-08	-0.39	48	0.96	Pik3c2g	Signaling
DMR4:174012001	4	174012001	174014000	2000	1	2.50E-12	-0.57	23	1.15	Pik3c2g	Signaling
DMR4:174085001	4	174085001	174086000	1000	1	7.70E-07	0.43	15	1.5	Pik3c2g	Signaling
DMR4:174102001	4	174102001	174104000	2000	1	4.90E-07	-0.37	26	1.3	Pik3c2g	Signaling
DMR4:174121001	4	174121001	174124000	3000	1	2.00E-08	0.49	37	1.23	Plcz1	Metabolism
DMR4:174161001	4	174161001	174162000	1000	1	3.00E-07	0.28	4	0.4	Plcz1	Metabolism
DMR4:174614001	4	174614001	174616000	2000	1	8.60E-07	-0.3	19	0.95	Plekha5	
DMR4:174638001	4	174638001	174640000	2000	1	2.60E-07	-0.47	11	0.55	Plekha5	
DMR4:174787001	4	174787001	174790000	3000	1	7.50E-09	0.59	52	1.73	Aebp2	
DMR4:174842001	4	174842001	174843000	1000	1	9.40E-10	0.52	19	1.9	Aebp2	
DMR4:174861001	4	174861001	174863000	2000	1	3.80E-11	-0.76	19	0.95	Aebp2	
DMR4:175427001	4	175427001	175428000	1000	1	1.20E-07	0.69	24	2.4	Pde3a	Signaling
DMR4:175451001	4	175451001	175455000	4000	2	1.60E-15	0.8	72	1.8	Pde3a	Signaling
DMR4:175491001	4	175491001	175492000	1000	1	2.00E-08	-0.47	16	1.6	Pde3a	Signaling
DMR4:175514001	4	175514001	175515000	1000	1	1.70E-10	-0.51	24	2.4	Pde3a	Signaling
DMR4:175629001	4	175629001	175633000	4000	2	1.10E-08	0.31	28	0.7	Pde3a	Signaling

DMR4:175652001	4	175652001	175653000	1000	1	1.80E-17	1.13	26	2.6	Pde3a	Signaling
DMR4:175655001	4	175655001	175656000	1000	1	1.80E-09	0.52	5	0.5	Pde3a	Signaling
DMR4:175713001	4	175713001	175718000	5000	2	3.90E-08	-0.52	61	1.22	Pde3a	Signaling
DMR4:175751001	4	175751001	175752000	1000	1	6.60E-07	0.52	13	1.3	Slco1c1	Transport
DMR4:175822001	4	175822001	175828000	6000	2	3.60E-08	-0.39	62	1.03	LOC103692254;Slco1b2	Transport
DMR4:176150001	4	176150001	176152000	2000	1	6.40E-07	-0.4	16	0.8	Slco1a1	Transport
DMR4:176165001	4	176165001	176172000	7000	2	2.30E-08	-0.69	61	0.87	Slco1a1	Transport
DMR4:176267001	4	176267001	176268000	1000	1	1.10E-07	0.51	8	0.8	Slc21a4	
DMR4:176387001	4	176387001	176388000	1000	1	1.10E-08	-0.4	4	0.4	Slco1a6	Transport
DMR4:176436001	4	176436001	176438000	2000	1	4.60E-08	-0.33	73	3.65	Slco1a5	Transport
DMR4:176495001	4	176495001	176499000	4000	2	2.50E-08	-0.29	36	0.9	Slco1a5	Transport
DMR4:176516001	4	176516001	176518000	2000	1	1.90E-08	-0.48	43	2.15	Slco1a5;Iapp	Transport;Hormone
DMR4:176628001	4	176628001	176629000	1000	1	8.00E-07	-0.39	4	0.4	Spx;Gys2	
DMR4:176710001	4	176710001	176712000	2000	1	3.20E-08	0.52	63	3.15	Ldhb	Metabolism
DMR4:176715001	4	176715001	176717000	2000	1	1.10E-07	-0.39	16	0.8	Ldhb	Metabolism
DMR4:176808001	4	176808001	176810000	2000	1	1.10E-08	0.38	15	0.75	Abcc9	Transport
DMR4:176818001	4	176818001	176819000	1000	1	3.00E-07	0.45	10	1	Abcc9	Transport
DMR4:176890001	4	176890001	176894000	4000	1	1.30E-08	0.51	78	1.95	Abcc9	Transport
DMR4:176897001	4	176897001	176898000	1000	1	1.20E-07	-0.7	6	0.6	Abcc9	Transport
DMR4:177018001	4	177018001	177020000	2000	1	5.60E-07	0.47	52	2.6	Cmas;RGD1561551	Metabolism;Transport
DMR4:177060001	4	177060001	177066000	6000	1	3.60E-08	0.44	85	1.42	St8sia1	Transport
DMR4:177101001	4	177101001	177102000	1000	1	8.40E-08	0.4	21	2.1	St8sia1	Transport
DMR4:177161001	4	177161001	177162000	1000	1	1.30E-17	1.13	31	3.1	St8sia1	Transport
DMR4:177238001	4	177238001	177240000	2000	1	1.70E-08	0.48	26	1.3	C2cd5	
DMR4:177337001	4	177337001	177339000	2000	1	3.70E-10	0.66	46	2.3	C2cd5	
DMR4:177399001	4	177399001	177401000	2000	1	7.40E-07	-0.35	25	1.25	Etnk1	Signaling
DMR4:177453001	4	177453001	177455000	2000	1	6.50E-07	0.47	43	2.15	Etnk1	Signaling
DMR4:178067001	4	178067001	178070000	3000	1	1.60E-13	0.92	82	2.73	Sox5	
DMR4:178143001	4	178143001	178148000	5000	1	2.00E-09	0.46	92	1.84	Sox5	
DMR4:178154001	4	178154001	178156000	2000	1	8.60E-08	0.69	35	1.75	Sox5	
DMR4:178165001	4	178165001	178168000	3000	1	7.50E-08	-0.47	55	1.83	Sox5	
DMR4:178170001	4	178170001	178172000	2000	1	2.40E-07	0.64	41	2.05	Sox5	
DMR4:178184001	4	178184001	178185000	1000	1	9.20E-08	-0.55	3	0.3	Sox5	
DMR4:178192001	4	178192001	178195000	3000	2	7.20E-15	1.05	66	2.2	Sox5	
DMR4:178205001	4	178205001	178210000	5000	3	6.60E-12	0.87	111	2.22	Sox5	
DMR4:178230001	4	178230001	178233000	3000	1	9.50E-07	0.32	55	1.83	Sox5	
DMR4:178243001	4	178243001	178244000	1000	1	5.60E-08	0.33	9	0.9	Sox5	
DMR4:178254001	4	178254001	178256000	2000	2	3.60E-10	-0.36	25	1.25	Sox5	
DMR4:178285001	4	178285001	178286000	1000	1	3.80E-08	-0.36	11	1.1	Sox5	
DMR4:178290001	4	178290001	178292000	2000	1	7.50E-09	0.36	31	1.55	Sox5	
DMR4:178303001	4	178303001	178305000	2000	1	5.90E-09	0.34	18	0.9	Sox5	
DMR4:178368001	4	178368001	178372000	4000	1	1.90E-07	-0.39	94	2.35	Sox5	
DMR4:178397001	4	178397001	178399000	2000	1	8.50E-08	-0.33	31	1.55	Sox5	
DMR4:178446001	4	178446001	178448000	2000	1	3.90E-07	0.45	19	0.95	Sox5	
DMR4:178500001	4	178500001	178502000	2000	1	3.30E-08	-0.38	56	2.8	Sox5	
DMR4:178539001	4	178539001	178542000	3000	1	1.70E-07	-0.43	72	2.4	Sox5	
DMR4:178562001	4	178562001	178564000	2000	1	1.30E-08	0.61	44	2.2	Sox5	
DMR4:178696001	4	178696001	178697000	1000	1	3.90E-08	-0.41	18	1.8	Sox5	
DMR4:178763001	4	178763001	178765000	2000	2	3.00E-15	1.13	50	2.5	Sox5	
DMR4:178769001	4	178769001	178772000	3000	1	3.30E-10	0.67	49	1.63	Sox5	
DMR4:178793001	4	178793001	178796000	3000	2	6.00E-23	1.18	74	2.47	Sox5	
DMR4:178918001	4	178918001	178922000	4000	1	7.10E-08	0.65	70	1.75	Sox5	
DMR4:178928001	4	178928001	178929000	1000	1	1.20E-08	0.32	15	1.5	Sox5	
DMR4:178960001	4	178960001	178965000	5000	1	5.30E-17	0.9	88	1.76	Sox5	
DMR4:178972001	4	178972001	178975000	3000	1	1.10E-21	1.06	62	2.07	Sox5	
DMR4:178980001	4	178980001	178983000	3000	1	8.20E-15	0.74	50	1.67	Sox5	
DMR4:179001001	4	179001001	179002000	1000	1	4.60E-16	0.8	28	2.8	Sox5	
DMR4:179009001	4	179009001	179012000	3000	1	4.40E-10	0.47	76	2.53	Sox5	
DMR4:179022001	4	179022001	179025000	3000	1	2.10E-10	0.66	52	1.73	Sox5	
DMR4:179273001	4	179273001	179276000	3000	1	1.60E-08	0.58	60	2	Bcat1	Metabolism
DMR4:179309001	4	179309001	179312000	3000	1	1.30E-07	0.44	35	1.17	Bcat1	Metabolism
DMR4:179335001	4	179335001	179336000	1000	1	9.30E-10	0.56	32	3.2	Bcat1	Metabolism

DMR4:179340001	4	179340001	179343000	3000	1	6.90E-07	0.61	39	1.3	Bcat1	Metabolism
DMR4:179461001	4	179461001	179464000	3000	1	1.20E-07	-0.4	61	2.03	Casc1	
DMR4:179521001	4	179521001	179523000	2000	1	1.20E-14	0.99	42	2.1	Kras	Signaling
DMR4:179673001	4	179673001	179674000	1000	1	3.00E-23	0.9	12	1.2	Lmntd1	
DMR4:179675001	4	179675001	179676000	1000	1	1.90E-19	0.81	9	0.9	Lmntd1	
DMR4:179677001	4	179677001	179685000	8000	6	4.20E-30	0.89	105	1.31	Lmntd1	
DMR4:179686001	4	179686001	179687000	1000	1	1.90E-07	0.59	31	3.1	Lmntd1	
DMR4:179704001	4	179704001	179705000	1000	1	3.60E-07	0.4	20	2	Lmntd1	
DMR4:179734001	4	179734001	179736000	2000	1	1.10E-09	0.43	25	1.25	Lmntd1;LOC108350788	
DMR4:179749001	4	179749001	179750000	1000	1	3.00E-10	-0.52	21	2.1	Lmntd1;LOC108350788;LOC108350787	
DMR4:179758001	4	179758001	179760000	2000	1	2.10E-09	-0.53	50	2.5	Lmntd1;LOC108350788;LOC108350787	
DMR4:179776001	4	179776001	179778000	2000	1	1.70E-08	-0.47	35	1.75	Lmntd1;LOC108350787	
DMR4:179862001	4	179862001	179864000	2000	1	9.00E-10	-0.62	32	1.6	Lmntd1	
DMR4:180083001	4	180083001	180088000	5000	1	1.70E-08	-0.55	80	1.6	Rassf8	Cytoskeleton
DMR4:180136001	4	180136001	180142000	6000	4	2.30E-27	0.87	112	1.87	Rassf8;LOC100912509	Cytoskeleton
DMR4:180454001	4	180454001	180459000	5000	1	1.90E-08	0.58	53	1.06	Itpr2	Ion Channel
DMR4:180471001	4	180471001	180473000	2000	1	2.80E-11	0.68	37	1.85	Itpr2	Ion Channel
DMR4:180483001	4	180483001	180485000	2000	1	3.90E-08	0.75	44	2.2	Itpr2	Ion Channel
DMR4:180506001	4	180506001	180509000	3000	1	9.20E-15	0.81	48	1.6	Itpr2	Ion Channel
DMR4:180535001	4	180535001	180540000	5000	1	2.00E-12	-0.53	93	1.86	Itpr2	Ion Channel
DMR4:180614001	4	180614001	180618000	4000	2	8.30E-09	0.67	106	2.65	Itpr2	Ion Channel
DMR4:180630001	4	180630001	180633000	3000	1	4.30E-07	-0.32	96	3.2	Itpr2	Ion Channel
DMR4:180679001	4	180679001	180681000	2000	1	7.10E-19	0.89	46	2.3	Itpr2	Ion Channel
DMR4:180759001	4	180759001	180760000	1000	1	2.60E-20	1.36	31	3.1	Itpr2	Ion Channel
DMR4:180859001	4	180859001	180860000	1000	1	1.20E-11	-0.66	10	1	Asun	
DMR4:180931001	4	180931001	180934000	3000	1	1.00E-07	-0.37	55	1.83	Tm7sf3	
DMR4:181269001	4	181269001	181272000	3000	1	7.40E-14	-0.55	51	1.7	Smco2	
DMR4:181273001	4	181273001	181275000	2000	1	7.80E-15	1	36	1.8	Smco2	
DMR4:181280001	4	181280001	181282000	2000	1	4.30E-07	0.47	30	1.5	Ppfibp1	
DMR4:181393001	4	181393001	181395000	2000	1	3.60E-08	-0.35	29	1.45	Ppfibp1;LOC108350794	
DMR4:181404001	4	181404001	181407000	3000	1	1.90E-08	-0.39	46	1.53	Ppfibp1;LOC108350794	
DMR4:181483001	4	181483001	181485000	2000	1	7.80E-11	0.86	22	1.1	Mansc4;Klhl42	
DMR4:181490001	4	181490001	181491000	1000	1	2.10E-07	-0.36	14	1.4	Klhl42	
DMR4:181683001	4	181683001	181684000	1000	1	1.20E-12	0.99	25	2.5	Pthlh;LOC108350879	Hormone
DMR4:181864001	4	181864001	181866000	2000	1	1.90E-14	0.79	47	2.35	Ccdc91	
DMR4:181881001	4	181881001	181882000	1000	1	9.10E-07	-0.39	14	1.4	Ccdc91	
DMR4:181909001	4	181909001	181911000	2000	1	2.10E-09	-0.53	39	1.95	Ccdc91	
DMR4:182018001	4	182018001	182020000	2000	2	8.30E-13	0.42	24	1.2	Ccdc91	
DMR4:182473001	4	182473001	182477000	4000	1	2.90E-07	0.35	44	1.1	Far2	
DMR4:182478001	4	182478001	182480000	2000	1	8.30E-07	-0.42	31	1.55	Far2	
DMR4:182503001	4	182503001	182506000	3000	1	3.00E-08	-0.4	29	0.97	Far2	
DMR4:182532001	4	182532001	182535000	3000	2	9.80E-10	-0.51	32	1.07	Far2	
DMR4:182543001	4	182543001	182545000	2000	2	4.60E-13	-0.47	46	2.3	Far2	
DMR4:182583001	4	182583001	182584000	1000	1	5.10E-07	-0.47	12	1.2	Ergic2	
DMR4:182595001	4	182595001	182596000	1000	1	7.40E-07	-0.43	8	0.8	Ergic2;LOC102554859	
DMR4:182798001	4	182798001	182800000	2000	1	3.20E-08	-0.37	22	1.1	Tmtc1	
DMR4:183391001	4	183391001	183393000	2000	1	9.50E-08	-0.33	25	1.25	LOC108350880;Fam60a	
DMR4:183477001	4	183477001	183478000	1000	1	2.40E-07	-0.53	4	0.4	Dennd5b	
DMR4:183546001	4	183546001	183548000	2000	1	7.90E-08	0.35	15	0.75	Dennd5b	
DMR4:183551001	4	183551001	183552000	1000	1	2.10E-08	-0.4	6	0.6	Dennd5b	
DMR4:183646001	4	183646001	183648000	2000	1	3.80E-09	-0.3	39	1.95	Etfbkmt	Epigenetic
DMR4:184034001	4	184034001	184036000	2000	1	2.00E-09	-0.68	22	1.1	Bicd1	
DMR4:184172001	4	184172001	184175000	3000	3	4.50E-10	0.66	48	1.6	Bicd1;LOC102547920	
DMR5:651001	5	651001	655000	4000	1	1.90E-07	0.43	30	0.75	Crispld1	Immune
DMR5:1331001	5	1331001	1332000	1000	1	2.30E-07	-0.53	12	1.2	Gdap1	
DMR5:1434001	5	1434001	1436000	2000	1	6.10E-07	-0.31	10	0.5	Jph1;LOC102552140	
DMR5:1441001	5	1441001	1442000	1000	1	4.00E-08	0.53	9	0.9	Jph1;LOC102552140	
DMR5:2634001	5	2634001	2635000	1000	1	7.20E-09	-0.42	22	2.2	Rdh10;Rpl7;LOC102546601;LOC100912291	Translation
DMR5:2817001	5	2817001	2820000	3000	1	8.20E-08	0.44	49	1.63	Sbspon	

DMR5:2821001	5	2821001	2823000	2000	1	2.90E-08	-0.42	19	0.95	Sbspon	
DMR5:2876001	5	2876001	2877000	1000	1	1.90E-09	-0.48	10	1	Terf1	
DMR5:3131001	5	3131001	3132000	1000	1	4.40E-07	0.58	10	1	Kcnb2	Transport
DMR5:3166001	5	3166001	3174000	8000	1	3.20E-07	-0.26	85	1.06	Kcnb2	Transport
DMR5:3269001	5	3269001	3270000	1000	1	4.50E-07	-0.26	7	0.7	Kcnb2	Transport
DMR5:3301001	5	3301001	3306000	5000	1	4.30E-07	-0.39	117	2.34	Kcnb2	Transport
DMR5:3399001	5	3399001	3401000	2000	1	1.80E-08	0.55	36	1.8	Kcnb2	Transport
DMR5:3953001	5	3953001	3955000	2000	1	7.10E-12	0.86	34	1.7	LOC102555885;Msc	Transcription
DMR5:4285001	5	4285001	4287000	2000	1	4.30E-07	-0.48	11	0.55	Eya1	
DMR5:4384001	5	4384001	4386000	2000	1	5.00E-08	-0.55	16	0.8	Eya1	
DMR5:5218001	5	5218001	5221000	3000	2	2.90E-08	-0.52	45	1.5	Tram1	
DMR5:5539001	5	5539001	5543000	4000	1	2.80E-07	-0.55	60	1.5	Ncoa2	Epigenetic
DMR5:5568001	5	5568001	5572000	4000	2	2.10E-08	-0.37	52	1.3	Ncoa2;Mir378b	Epigenetic
DMR5:5647001	5	5647001	5648000	1000	1	4.10E-09	-0.36	17	1.7	Ncoa2	Epigenetic
DMR5:5658001	5	5658001	5660000	2000	1	3.10E-07	0.61	36	1.8	Ncoa2	Epigenetic
DMR5:5679001	5	5679001	5683000	4000	1	9.00E-07	0.5	94	2.35	Ncoa2	Epigenetic
DMR5:5737001	5	5737001	5738000	1000	1	1.80E-07	0.76	27	2.7	Prdm14	Transcription
DMR5:5858001	5	5858001	5859000	1000	1	1.00E-09	0.47	21	2.1	Slco5a1	Transport
DMR5:5895001	5	5895001	5896000	1000	1	1.50E-14	-0.64	15	1.5	Slco5a1	Transport
DMR5:5949001	5	5949001	5954000	5000	1	1.80E-07	-0.57	53	1.06	Slco5a1	Transport
DMR5:5964001	5	5964001	5965000	1000	1	7.20E-11	-0.57	6	0.6	Slco5a1	Transport
DMR5:6024001	5	6024001	6028000	4000	1	8.30E-08	-0.52	70	1.75	Sulf1	Metabolism
DMR5:6100001	5	6100001	6103000	3000	1	2.60E-07	-0.42	47	1.57	Sulf1	Metabolism
DMR5:6955001	5	6955001	6962000	7000	1	3.40E-07	-0.23	56	0.8	RGD1564053	
DMR5:6988001	5	6988001	6994000	6000	1	4.10E-07	-0.36	57	0.95	RGD1564053	
DMR5:7108001	5	7108001	7109000	1000	1	4.20E-08	-0.51	6	0.6	RGD1564053	
DMR5:7187001	5	7187001	7188000	1000	1	3.10E-07	0.5	16	1.6	RGD1564053	
DMR5:7474001	5	7474001	7475000	1000	1	1.00E-11	0.41	3	0.3	RGD1564053	
DMR5:7735001	5	7735001	7737000	2000	1	1.90E-08	0.53	19	0.95	Prex2	Transcription
DMR5:7917001	5	7917001	7918000	1000	1	1.40E-07	0.49	15	1.5	Prex2	Transcription
DMR5:8207001	5	8207001	8208000	1000	1	5.20E-07	-0.31	55	5.5	Cpa6	Protease
DMR5:8276001	5	8276001	8281000	5000	1	1.40E-07	-0.26	37	0.74	Cpa6	Protease
DMR5:8297001	5	8297001	8302000	5000	1	9.80E-12	-0.47	43	0.86	Cpa6	Protease
DMR5:8327001	5	8327001	8328000	1000	1	3.80E-08	0.63	13	1.3	Cpa6	Protease
DMR5:8506001	5	8506001	8507000	1000	1	3.20E-13	0.57	13	1.3	Cpa6	Protease
DMR5:8531001	5	8531001	8532000	1000	1	2.70E-07	-0.35	11	1.1	Cpa6	Protease
DMR5:8560001	5	8560001	8564000	4000	1	1.30E-19	1.18	74	1.85	Cpa6	Protease
DMR5:8572001	5	8572001	8574000	2000	1	5.00E-17	0.83	34	1.7	Cpa6	Protease
DMR5:8756001	5	8756001	8757000	1000	1	1.80E-07	0.46	17	1.7	Arfgef1;Cspp1	Transcription;Cell Cycle
DMR5:8774001	5	8774001	8779000	5000	1	2.90E-09	-0.5	112	2.24	Cspp1	Cell Cycle
DMR5:8832001	5	8832001	8833000	1000	1	2.90E-07	-0.44	13	1.3	Cspp1	Cell Cycle
DMR5:8905001	5	8905001	8907000	2000	1	1.50E-08	-0.51	17	0.85	Ppp1r42	Signaling
DMR5:8944001	5	8944001	8946000	2000	1	5.10E-08	-0.4	19	0.95	Ppp1r42	Signaling
DMR5:10112001	5	10112001	10116000	4000	1	3.60E-12	-0.47	37	0.92	Sntg1	
DMR5:10169001	5	10169001	10171000	2000	1	2.70E-07	-0.5	12	0.6	Sntg1	
DMR5:10201001	5	10201001	10203000	2000	1	6.00E-07	-0.61	7	0.35	Sntg1	
DMR5:10372001	5	10372001	10375000	3000	1	1.20E-07	0.44	19	0.63	Sntg1	
DMR5:12473001	5	12473001	12475000	2000	1	9.30E-08	0.53	20	1	St18	Transcription
DMR5:13047001	5	13047001	13049000	2000	1	7.50E-07	-0.32	16	0.8	Rb1cc1	Cytoskeleton
DMR5:14476001	5	14476001	14477000	1000	1	4.90E-08	-0.48	8	0.8	Rgs20	Signaling
DMR5:14544001	5	14544001	14546000	2000	1	3.90E-07	-0.4	12	0.6	Tcea1	Transcription
DMR5:15061001	5	15061001	15064000	3000	1	8.70E-10	-0.58	7	0.23	Rp1	
DMR5:15828001	5	15828001	15830000	2000	1	6.30E-07	-0.39	7	0.35	Xkr4	
DMR5:16040001	5	16040001	16041000	1000	1	6.30E-09	-0.51	6	0.6	Xkr4	
DMR5:16090001	5	16090001	16093000	3000	1	1.80E-07	-0.39	25	0.83	Xkr4	
DMR5:16120001	5	16120001	16121000	1000	1	5.10E-12	0.96	18	1.8	Xkr4	
DMR5:16146001	5	16146001	16150000	4000	1	8.60E-08	0.27	34	0.85	Xkr4	
DMR5:16396001	5	16396001	16403000	7000	1	2.20E-10	0.41	82	1.17	Tmem68	
DMR5:16504001	5	16504001	16506000	2000	1	1.90E-07	-0.54	17	0.85	RGD1564981	
DMR5:16581001	5	16581001	16582000	1000	1	8.80E-08	0.51	14	1.4	Lyn	
DMR5:16628001	5	16628001	16630000	2000	1	6.90E-11	-0.62	39	1.95	Lyn	

DMR5:16713001	5	16713001	16716000	3000	2	3.10E-08	-0.56	24	0.8	Rps20	Translation
DMR5:16756001	5	16756001	16762000	6000	1	1.20E-07	-0.28	72	1.2	Mos;LOC685119;LOC100364265	Signaling;Epigenetic
DMR5:16931001	5	16931001	16933000	2000	1	1.10E-08	0.46	29	1.45	Sdr16c5	
DMR5:17395001	5	17395001	17397000	2000	1	3.10E-10	-0.88	6	0.3	RGD1563405	
DMR5:17400001	5	17400001	17401000	1000	1	1.80E-17	1.28	22	2.2	RGD1563405	
DMR5:18838001	5	18838001	18839000	1000	1	9.70E-07	-0.42	17	1.7	Fam110b	
DMR5:18967001	5	18967001	18968000	1000	1	3.80E-07	0.45	12	1.2	LOC100909800;RGD1565372	
DMR5:18986001	5	18986001	18987000	1000	1	5.50E-10	-0.71	4	0.4	RGD1565372	
DMR5:20067001	5	20067001	20068000	1000	1	2.10E-10	0.8	21	2.1	Tox	
DMR5:20164001	5	20164001	20165000	1000	1	7.80E-13	-0.56	6	0.6	Tox	
DMR5:21048001	5	21048001	21049000	1000	1	5.10E-08	0.43	7	0.7	Gm5471	
DMR5:21340001	5	21340001	21344000	4000	1	6.60E-09	-0.41	48	1.2	Car8	
DMR5:21661001	5	21661001	21662000	1000	1	1.90E-11	-0.57	15	1.5	Rab2a	
DMR5:21833001	5	21833001	21834000	1000	1	1.00E-08	-0.41	29	2.9	Chd7	
DMR5:21855001	5	21855001	21857000	2000	2	1.70E-10	-0.48	46	2.3	Chd7	
DMR5:21949001	5	21949001	21950000	1000	1	2.60E-12	0.84	50	5	Chd7	
DMR5:21952001	5	21952001	21956000	4000	1	2.90E-11	0.58	48	1.2	Chd7	
DMR5:22412001	5	22412001	22416000	4000	1	4.10E-07	-0.58	43	1.07	Clvs1	Transport
DMR5:22423001	5	22423001	22424000	1000	1	6.70E-07	-0.31	14	1.4	Clvs1	Transport
DMR5:22485001	5	22485001	22487000	2000	2	9.70E-11	0.53	46	2.3	Clvs1	Transport
DMR5:22508001	5	22508001	22512000	4000	1	6.80E-09	0.41	38	0.95	Clvs1	Transport
DMR5:22565001	5	22565001	22566000	1000	1	6.30E-08	-0.4	7	0.7	Clvs1	Transport
DMR5:22680001	5	22680001	22681000	1000	1	2.50E-16	-0.46	14	1.4	Asph	Golgi
DMR5:23061001	5	23061001	23066000	5000	1	1.90E-07	-0.46	49	0.98	Gdf6	Growth Factors
DMR5:24158001	5	24158001	24159000	1000	1	1.60E-07	-0.45	17	1.7	MGC94199	
DMR5:24583001	5	24583001	24584000	1000	1	4.60E-07	-0.34	13	1.3	Esrp1	Translation
DMR5:24609001	5	24609001	24612000	3000	2	1.60E-13	-0.63	63	2.1	Esrp1	Translation
DMR5:24759001	5	24759001	24762000	3000	1	6.50E-07	-0.27	23	0.77	LOC103695239;LOC103692302;RGD1564278	
DMR5:24905001	5	24905001	24906000	1000	1	3.80E-12	0.72	44	4.4	RGD1559441	
DMR5:25336001	5	25336001	25337000	1000	1	7.50E-10	0.82	16	1.6	Gem	
DMR5:25432001	5	25432001	25433000	1000	1	1.40E-10	0.31	3	0.3	Cdh17	Cytoskeleton
DMR5:25743001	5	25743001	25744000	1000	1	4.20E-08	0.52	12	1.2	Fam92a1	
DMR5:27293001	5	27293001	27294000	1000	1	7.40E-10	0.65	20	2	Runx1t1	Transcription
DMR5:27389001	5	27389001	27390000	1000	1	1.50E-08	0.34	8	0.8	Runx1t1	Transcription
DMR5:27406001	5	27406001	27407000	1000	1	1.80E-08	0.42	10	1	Runx1t1	Transcription
DMR5:27409001	5	27409001	27410000	1000	1	4.30E-08	0.52	11	1.1	Runx1t1	Transcription
DMR5:28286001	5	28286001	28289000	3000	1	1.80E-12	0.95	52	1.73	Lrrc69	Cytoskeleton
DMR5:28356001	5	28356001	28358000	2000	1	1.40E-08	0.74	29	1.45	Otud6b	Protease
DMR5:28529001	5	28529001	28534000	5000	1	4.20E-07	-0.3	46	0.92	Necab1	
DMR5:28591001	5	28591001	28592000	1000	1	1.70E-08	-0.4	2	0.2	Necab1;LOC108350937	
DMR5:28612001	5	28612001	28618000	6000	1	2.90E-07	-0.24	57	0.95	Necab1	
DMR5:28728001	5	28728001	28729000	1000	1	7.00E-08	0.47	21	2.1	Necab1	
DMR5:28841001	5	28841001	28842000	1000	1	3.30E-13	-0.6	10	1	Tmem64	
DMR5:28853001	5	28853001	28854000	1000	1	6.60E-09	-0.55	19	1.9	Tmem64;LOC108350938	
DMR5:28862001	5	28862001	28864000	2000	1	1.10E-07	-0.38	16	0.8	Tmem64;LOC108350938	
DMR5:28881001	5	28881001	28883000	2000	1	1.30E-14	0.89	32	1.6	Tmem64	
DMR5:29846001	5	29846001	29847000	1000	1	5.90E-07	0.53	13	1.3	Ripk2	Signaling
DMR5:29880001	5	29880001	29882000	2000	1	1.60E-08	-0.66	7	0.35	Ripk2	Signaling
DMR5:32617001	5	32617001	32619000	2000	2	1.60E-12	-0.67	9	0.45	Cnbd1	
DMR5:32641001	5	32641001	32642000	1000	1	6.40E-07	-0.47	5	0.5	Cnbd1	
DMR5:32706001	5	32706001	32710000	4000	1	7.50E-07	-0.56	29	0.72	Cnbd1	
DMR5:32744001	5	32744001	32745000	1000	1	4.60E-07	-0.47	5	0.5	Cnbd1	
DMR5:32843001	5	32843001	32844000	1000	1	2.10E-10	-0.53	4	0.4	Cnbd1	
DMR5:33280001	5	33280001	33281000	1000	1	6.40E-09	0.51	13	1.3	Cngb3	Ion Channel
DMR5:33285001	5	33285001	33286000	1000	1	1.50E-15	0.95	17	1.7	Cngb3	Ion Channel
DMR5:33321001	5	33321001	33322000	1000	1	2.70E-08	0.45	26	2.6	Cngb3	Ion Channel
DMR5:33436001	5	33436001	33437000	1000	1	8.00E-08	0.39	14	1.4	Cngb3	Ion Channel
DMR5:33472001	5	33472001	33476000	4000	1	4.00E-07	-0.25	39	0.98	Cngb3	Ion Channel
DMR5:33865001	5	33865001	33869000	4000	1	1.20E-07	-0.5	46	1.15	Atp6v0d2	Metabolism
DMR5:34022001	5	34022001	34024000	2000	2	1.00E-08	-0.56	8	0.4	Ttpa	Transport

DMR5:34069001	5	34069001	34070000	1000	1	3.00E-12	0.74	14	1.4	Ggh	Protease
DMR5:34254001	5	34254001	34256000	2000	1	2.50E-07	0.36	35	1.75	Nkain3	
DMR5:34585001	5	34585001	34588000	3000	1	8.90E-07	-0.47	21	0.7	Nkain3;LOC103692317	
DMR5:34615001	5	34615001	34620000	5000	1	1.30E-09	0.27	51	1.02	Nkain3	
DMR5:34771001	5	34771001	34772000	1000	1	4.20E-07	0.51	11	1.1	Nkain3	
DMR5:36062001	5	36062001	36064000	2000	1	2.90E-07	-0.31	22	1.1	Coq3	Epigenetic
DMR5:36068001	5	36068001	36069000	1000	1	4.80E-07	0.61	16	1.6	Faxc	
DMR5:36133001	5	36133001	36134000	1000	1	2.50E-15	-0.74	13	1.3	Faxc	
DMR5:39035001	5	39035001	39036000	1000	1	5.80E-07	-0.28	18	1.8	Klhl32	
DMR5:39075001	5	39075001	39077000	2000	2	1.00E-08	0.37	20	1	Klhl32	
DMR5:39325001	5	39325001	39327000	2000	1	6.50E-08	-0.62	8	0.4	Gpr63	Signaling
DMR5:39651001	5	39651001	39652000	1000	1	5.40E-10	-0.66	3	0.3	Ufl1	Proteolysis
DMR5:40246001	5	40246001	40250000	4000	2	1.40E-12	-0.57	37	0.92	Fut9	Golgi
DMR5:40837001	5	40837001	40838000	1000	1	8.40E-07	-0.48	0	0	Manea	
DMR5:43617001	5	43617001	43618000	1000	1	1.50E-18	1.1	19	1.9	Epha7	Receptor
DMR5:43690001	5	43690001	43691000	1000	1	3.60E-08	-0.6	14	1.4	Epha7	Receptor
DMR5:47449001	5	47449001	47451000	2000	1	1.10E-11	0.87	47	2.35	LOC108350945;LOC102551110;Bach2	
DMR5:47474001	5	47474001	47477000	3000	1	1.90E-07	-0.4	57	1.9	Bach2	
DMR5:47627001	5	47627001	47631000	4000	1	1.30E-07	-0.33	64	1.6	Bach2	
DMR5:47650001	5	47650001	47653000	3000	1	1.10E-08	-0.37	63	2.1	Bach2	
DMR5:47710001	5	47710001	47711000	1000	1	5.30E-09	0.74	26	2.6	Bach2	
DMR5:47715001	5	47715001	47716000	1000	1	7.70E-08	-0.44	18	1.8	Bach2	
DMR5:47755001	5	47755001	47759000	4000	1	1.00E-11	-0.53	83	2.08	Bach2	
DMR5:47797001	5	47797001	47802000	5000	1	9.90E-07	0.57	102	2.04	Bach2	
DMR5:47898001	5	47898001	47900000	2000	1	6.00E-08	-0.45	20	1	Casp8ap2;Mdn1	Signaling
DMR5:48135001	5	48135001	48138000	3000	1	2.70E-11	-1.24	38	1.27	Ankrd6	
DMR5:48163001	5	48163001	48165000	2000	1	9.60E-07	-0.41	32	1.6	Ankrd6	
DMR5:48236001	5	48236001	48237000	1000	1	3.00E-07	-0.41	22	2.2	Rragd	Signaling
DMR5:48348001	5	48348001	48349000	1000	1	2.00E-07	0.3	15	1.5	Gabrr2;LOC102555980	Ion Channel
DMR5:48421001	5	48421001	48423000	2000	1	4.10E-12	0.84	40	2	Gabrr1;Pm20d2	Ion Channel;Protease
DMR5:48470001	5	48470001	48472000	2000	1	9.40E-08	-0.4	32	1.6	Srsf12	Translation
DMR5:48495001	5	48495001	48496000	1000	1	6.70E-07	-0.42	2	0.2	Pnrc1	
DMR5:48513001	5	48513001	48516000	3000	1	1.20E-07	-0.35	67	2.23	Pnrc1;LOC102556064	
DMR5:48550001	5	48550001	48552000	2000	1	1.00E-18	0.69	35	1.75	LOC108350948;Rngtt	Translation
DMR5:49298001	5	49298001	49301000	3000	1	4.60E-07	-0.34	30	1	Cnr1	Signaling
DMR5:49416001	5	49416001	49417000	1000	1	4.60E-11	0.58	5	0.5	LOC103692331;Spaca1	
DMR5:49438001	5	49438001	49439000	1000	1	1.00E-07	0.53	13	1.3	Spaca1	
DMR5:50137001	5	50137001	50139000	2000	1	2.00E-07	0.41	25	1.25	Rars2;Slc35a1;Cfap206	Translation;Transport
DMR5:50161001	5	50161001	50164000	3000	1	2.10E-07	-0.38	30	1	Cfap206	
DMR5:50167001	5	50167001	50168000	1000	1	1.60E-07	-0.59	4	0.4	Cfap206	
DMR5:50199001	5	50199001	50204000	5000	1	4.20E-09	-0.27	57	1.14	Cfap206;RGD1563056	
DMR5:50307001	5	50307001	50309000	2000	1	6.80E-07	-0.43	28	1.4	Zfp292	Transcription
DMR5:50428001	5	50428001	50431000	3000	1	7.80E-07	-0.34	11	0.37	Mob3b	Signaling
DMR5:50479001	5	50479001	50480000	1000	1	3.60E-07	-0.52	21	2.1	Mob3b;LOC102549496	Signaling
DMR5:50503001	5	50503001	50504000	1000	1	1.20E-07	-0.54	19	1.9	Mob3b	Signaling
DMR5:50512001	5	50512001	50514000	2000	1	3.80E-08	-0.52	51	2.55	Mob3b	Signaling
DMR5:50552001	5	50552001	50554000	2000	2	4.70E-12	-0.46	55	2.75	Mob3b;LOC688867	Signaling
DMR5:50586001	5	50586001	50587000	1000	1	7.50E-19	1.04	22	2.2	Mob3b	Signaling
DMR5:50617001	5	50617001	50619000	2000	1	8.20E-12	-0.55	33	1.65	Mob3b	Signaling
DMR5:50640001	5	50640001	50641000	1000	1	5.60E-13	0.67	19	1.9	Mob3b;Ifnk;LOC102549230	Signaling;Immune
DMR5:51372001	5	51372001	51374000	2000	1	3.50E-07	0.58	28	1.4	Capns1-ps1	
DMR5:51468001	5	51468001	51469000	1000	1	2.90E-13	0.83	15	1.5	Lingo2	
DMR5:51539001	5	51539001	51544000	5000	1	6.90E-07	-0.26	51	1.02	Lingo2	
DMR5:52474001	5	52474001	52475000	1000	1	4.20E-08	0.75	13	1.3	Mir873	
DMR5:56462001	5	56462001	56466000	4000	1	1.00E-11	-0.56	71	1.77	Aco1	Metabolism
DMR5:56555001	5	56555001	56560000	5000	1	2.70E-11	-0.49	110	2.2	Topors;LOC102552818;Ndufb6	Proteolysis;Metabolism
DMR5:57000001	5	57000001	57002000	2000	1	2.60E-08	-0.44	24	1.2	Aptx	DNA Repair
DMR5:57020001	5	57020001	57021000	1000	1	1.40E-07	-0.45	17	1.7	Dnaja1;Mir207	Transcription
DMR5:57142001	5	57142001	57146000	4000	1	2.10E-09	-0.56	66	1.65	B4galt1	
DMR5:57151001	5	57151001	57154000	3000	1	9.30E-10	-0.52	41	1.37	B4galt1	

DMR5:57166001	5	57166001	57167000	1000	1	4.10E-13	-0.55	13	1.3	B4galt1	
DMR5:57303001	5	57303001	57304000	1000	1	1.40E-17	0.61	12	1.2	Nfx1	Transcription
DMR5:57364001	5	57364001	57368000	4000	1	8.60E-07	-0.33	73	1.82	Aqp7	Transport
DMR5:57370001	5	57370001	57372000	2000	1	6.60E-11	0.56	30	1.5	Aqp7	Transport
DMR5:57430001	5	57430001	57431000	1000	1	7.00E-07	0.4	19	1.9	Aqp3	Transport
DMR5:57439001	5	57439001	57441000	2000	1	1.50E-13	0.85	43	2.15	Aqp3;Nol6	Transport;Metabolism
DMR5:57442001	5	57442001	57445000	3000	1	2.40E-10	-0.44	37	1.23	Nol6	Metabolism
DMR5:57497001	5	57497001	57498000	1000	1	5.50E-08	-0.39	18	1.8	Ube2r2	
DMR5:57527001	5	57527001	57528000	1000	1	3.50E-07	-0.37	16	1.6	Ube2r2;Ubap2	
DMR5:57529001	5	57529001	57532000	3000	1	4.60E-07	-0.3	41	1.37	Ube2r2;Ubap2	
DMR5:57729001	5	57729001	57730000	1000	1	9.20E-10	0.69	19	1.9	LOC690990;Ubap1	
DMR5:57742001	5	57742001	57743000	1000	1	8.80E-09	0.36	6	0.6	Ubap1	
DMR5:57898001	5	57898001	57901000	3000	1	1.40E-09	0.55	63	2.1	RGD1561916;LOC102553821;Fam219a	
DMR5:58181001	5	58181001	58183000	2000	2	6.70E-11	0.63	57	2.85	LOC102547621;Ccl19;LOC689481	Growth Factors
DMR5:58365001	5	58365001	58369000	4000	1	8.10E-07	-0.38	70	1.75	Phf24	Signaling
DMR5:58876001	5	58876001	58879000	3000	2	4.40E-11	-0.44	89	2.97	Rusc2;Fam166b	
DMR5:58880001	5	58880001	58882000	2000	1	3.30E-09	0.59	67	3.35	Rusc2;Fam166b	
DMR5:58934001	5	58934001	58935000	1000	1	5.60E-10	-0.55	16	1.6	LOC103692345;Tesk1;Cd72	
DMR5:58952001	5	58952001	58960000	8000	2	2.50E-09	-0.42	88	1.1	Tesk1;Cd72;RGD1560723	
DMR5:58964001	5	58964001	58965000	1000	1	1.30E-07	0.42	15	1.5	RGD1560723	
DMR5:58986001	5	58986001	58987000	1000	1	8.80E-08	0.57	27	2.7	Sit1;Ccdc107	Cytoskeleton
DMR5:59058001	5	59058001	59062000	4000	1	5.60E-17	-0.51	54	1.35	Tln1;Creb3;Gba2	Metabolism
DMR5:59129001	5	59129001	59130000	1000	1	6.30E-07	0.5	35	3.5	Npr2	Signaling
DMR5:59203001	5	59203001	59204000	1000	1	2.50E-09	-0.39	21	2.1	Tmem8b;Olr833;Olr834	Cytoskeleton;Receptor
DMR5:59235001	5	59235001	59237000	2000	1	8.90E-08	0.42	24	1.2	Hrct1	
DMR5:59290001	5	59290001	59291000	1000	1	4.90E-07	0.49	25	2.5	Olr838	Receptor
DMR5:59429001	5	59429001	59431000	2000	1	1.40E-07	-0.61	24	1.2	Glipr2	Immune
DMR5:59482001	5	59482001	59485000	3000	1	1.10E-09	0.78	48	1.6	Clta	Transport
DMR5:59492001	5	59492001	59494000	2000	1	1.00E-07	-0.38	31	1.55	Clta	Transport
DMR5:59537001	5	59537001	59540000	3000	2	9.10E-12	-0.49	77	2.57	Gne	Transcription
DMR5:59548001	5	59548001	59554000	6000	4	4.90E-14	-0.58	117	1.95	Gne	Transcription
DMR5:59555001	5	59555001	59559000	4000	2	3.20E-09	-0.42	52	1.3	Gne	Transcription
DMR5:60111001	5	60111001	60114000	3000	2	1.80E-10	-0.44	76	2.53	Pax5	
DMR5:60152001	5	60152001	60154000	2000	1	5.90E-09	0.44	30	1.5	Pax5	
DMR5:60187001	5	60187001	60189000	2000	1	7.50E-07	-0.41	26	1.3	Pax5	
DMR5:60342001	5	60342001	60345000	3000	2	2.30E-10	-0.82	20	0.67	Zcchc7	
DMR5:60521001	5	60521001	60523000	2000	1	3.90E-07	0.39	33	1.65	Grhpr	Metabolism
DMR5:60584001	5	60584001	60587000	3000	1	4.20E-09	-0.41	43	1.43	Polr1e;Fbxo10	Transcription;Proteolysis
DMR5:60602001	5	60602001	60604000	2000	1	1.50E-10	-0.35	41	2.05	Fbxo10	Proteolysis
DMR5:60609001	5	60609001	60611000	2000	1	3.80E-08	-0.44	34	1.7	Fbxo10	Proteolysis
DMR5:60619001	5	60619001	60622000	3000	2	2.20E-11	-0.5	37	1.23	Fbxo10	Proteolysis
DMR5:60726001	5	60726001	60729000	3000	1	8.30E-08	-0.53	26	0.87	Frmpd1	
DMR5:60754001	5	60754001	60761000	7000	2	1.70E-07	0.37	115	1.64	Frmpd1	
DMR5:60987001	5	60987001	60990000	3000	1	1.20E-08	0.63	42	1.4	Shb	
DMR5:61019001	5	61019001	61021000	2000	1	4.00E-07	-0.38	43	2.15	Shb	
DMR5:61493001	5	61493001	61494000	1000	1	1.90E-10	0.63	26	2.6	Ccdc180	
DMR5:61497001	5	61497001	61504000	7000	1	2.50E-08	-0.43	122	1.74	Ccdc180	
DMR5:61663001	5	61663001	61667000	4000	1	2.30E-09	0.26	61	1.52	Tmod1	Cytoskeleton
DMR5:61730001	5	61730001	61733000	3000	1	4.00E-07	-0.38	25	0.83	Tstd2;Ncbp1	Transport;Translation
DMR5:62023001	5	62023001	62026000	3000	1	6.90E-07	-0.35	46	1.53	Hemgn	
DMR5:62090001	5	62090001	62093000	3000	1	8.70E-12	-0.47	36	1.2	Anp32b	Epigenetic
DMR5:62113001	5	62113001	62114000	1000	1	7.00E-07	-0.32	26	2.6	Nans	Metabolism
DMR5:62199001	5	62199001	62202000	3000	1	2.00E-07	-0.41	47	1.57	Coro2a;LOC108350963	Cytoskeleton
DMR5:62249001	5	62249001	62251000	2000	2	1.60E-11	-0.51	34	1.7	Tbc1d2	Signaling
DMR5:62364001	5	62364001	62367000	3000	1	4.80E-10	0.5	46	1.53	Gabbr2	Signaling
DMR5:62368001	5	62368001	62369000	1000	1	2.30E-07	-0.37	20	2	Gabbr2	Signaling
DMR5:62469001	5	62469001	62472000	3000	1	9.90E-18	0.88	65	2.17	Gabbr2	Signaling
DMR5:62490001	5	62490001	62491000	1000	1	1.70E-09	-0.38	8	0.8	Gabbr2	Signaling
DMR5:62545001	5	62545001	62546000	1000	1	3.90E-07	0.49	16	1.6	Gabbr2;LOC102550819	Signaling

DMR5:62547001	5	62547001	62548000	1000	1	2.30E-15	0.58	30	3	Gabbr2;LOC102550819	Signaling
DMR5:62575001	5	62575001	62577000	2000	1	5.10E-08	-0.41	27	1.35	Gabbr2	Signaling
DMR5:62585001	5	62585001	62591000	6000	1	1.30E-07	0.54	118	1.97	Gabbr2	Signaling
DMR5:62634001	5	62634001	62636000	2000	1	2.80E-09	0.6	23	1.15	Anks6	Metabolism
DMR5:62756001	5	62756001	62757000	1000	1	5.10E-08	-0.37	18	1.8	Galnt12	Golgi
DMR5:62872001	5	62872001	62873000	1000	1	1.10E-08	-0.64	1	0.1	Col15a1	Extracellular Matrix
DMR5:63104001	5	63104001	63105000	1000	1	7.80E-07	-0.53	6	0.6	Tgfbr1	Signaling
DMR5:63194001	5	63194001	63196000	2000	1	2.00E-09	-0.39	35	1.75	Alg2;Sec61b	Transport;Transport
DMR5:63205001	5	63205001	63206000	1000	1	4.20E-08	-0.36	11	1.1	Sec61b;LOC108350964	Transport
DMR5:63772001	5	63772001	63775000	3000	2	3.80E-09	0.59	31	1.03	Nr4a3	Transcription
DMR5:63869001	5	63869001	63871000	2000	1	8.90E-12	-0.55	8	0.4	Stx17	Transcription
DMR5:64009001	5	64009001	64010000	1000	1	1.70E-07	0.52	11	1.1	Erp44	
DMR5:64106001	5	64106001	64107000	1000	1	1.50E-09	-0.49	11	1.1	Invs;LOC102551711	
DMR5:64216001	5	64216001	64218000	2000	1	6.80E-07	-0.54	17	0.85	Tex10;LOC108350966	
DMR5:64271001	5	64271001	64275000	4000	1	1.10E-19	0.97	72	1.8	Msantd3	
DMR5:64314001	5	64314001	64318000	4000	2	6.40E-12	-0.47	43	1.07	Msantd3;LOC108350968;Tmeff1	
DMR5:64368001	5	64368001	64369000	1000	1	1.10E-07	-0.35	25	2.5	Tmeff1	
DMR5:66313001	5	66313001	66314000	1000	1	9.50E-08	-0.35	4	0.4	Cylc2	Development
DMR5:67866001	5	67866001	67867000	1000	1	7.60E-09	0.55	13	1.3	Plppr1	Signaling
DMR5:68713001	5	68713001	68715000	2000	1	3.60E-10	-0.49	12	0.6	Olr848;Smc2	Receptor
DMR5:68901001	5	68901001	68903000	2000	2	1.00E-08	-0.35	16	0.8	Olr848;RGD1309291	Receptor;Proteolysis
DMR5:68992001	5	68992001	68993000	1000	1	9.80E-07	-0.54	3	0.3	Olr848	Receptor
DMR5:69027001	5	69027001	69029000	2000	1	3.80E-11	0.56	23	1.15	Olr848	Receptor
DMR5:69049001	5	69049001	69051000	2000	1	2.50E-07	0.57	25	1.25	Olr848	Receptor
DMR5:69118001	5	69118001	69119000	1000	1	5.50E-08	0.5	12	1.2	Olr848	Receptor
DMR5:69789001	5	69789001	69792000	3000	1	1.50E-07	0.48	26	0.87	Olr853	Receptor
DMR5:69807001	5	69807001	69808000	1000	1	3.40E-08	-0.57	3	0.3	Nipsnap3a	
DMR5:69872001	5	69872001	69875000	3000	1	3.90E-08	-0.46	50	1.67	Abca1	Transport
DMR5:69930001	5	69930001	69934000	4000	1	1.10E-09	-0.44	73	1.82	Abca1	Transport
DMR5:69938001	5	69938001	69941000	3000	1	2.60E-07	-0.32	29	0.97	Abca1	Transport
DMR5:70321001	5	70321001	70323000	2000	2	6.70E-09	-0.44	62	3.1	Slc44a1;LOC102551474	Transport
DMR5:70494001	5	70494001	70495000	1000	1	6.80E-07	-0.3	7	0.7	Fsd1l	Proteolysis
DMR5:70532001	5	70532001	70533000	1000	1	3.90E-09	-0.43	21	2.1	Fktn	
DMR5:70746001	5	70746001	70748000	2000	1	1.50E-09	-0.56	15	0.75	RGD1564956	
DMR5:74036001	5	74036001	74042000	6000	2	6.90E-08	0.3	178	2.97	Frrs1l;Epb41l4b	
DMR5:74118001	5	74118001	74120000	2000	1	1.30E-07	0.61	65	3.25	Epb41l4b	
DMR5:74347001	5	74347001	74351000	4000	1	5.90E-07	-0.32	72	1.8	Ptpn3	Signaling
DMR5:74772001	5	74772001	74774000	2000	1	7.00E-27	0.79	37	1.85	Palm2	
DMR5:74876001	5	74876001	74878000	2000	1	4.10E-07	-0.46	38	1.9	Akap2	
DMR5:74892001	5	74892001	74894000	2000	1	4.40E-07	-0.38	12	0.6	Akap2	
DMR5:74950001	5	74950001	74951000	1000	1	4.30E-09	-0.38	21	2.1	Akap2	
DMR5:74960001	5	74960001	74961000	1000	1	4.00E-07	0.35	17	1.7	Akap2	
DMR5:75047001	5	75047001	75048000	1000	1	3.40E-10	0.57	25	2.5	Txn1	
DMR5:75153001	5	75153001	75155000	2000	1	2.20E-10	-0.44	42	2.1	LOC103692362;Svep1;LOC102546532	
DMR5:75219001	5	75219001	75221000	2000	1	1.60E-11	0.8	45	2.25	Svep1	
DMR5:75243001	5	75243001	75244000	1000	1	2.70E-18	0.5	12	1.2	Svep1	
DMR5:75257001	5	75257001	75259000	2000	1	1.30E-10	-0.41	32	1.6	Svep1	
DMR5:75275001	5	75275001	75277000	2000	1	8.40E-07	-0.41	25	1.25	Svep1	
DMR5:75297001	5	75297001	75300000	3000	1	9.90E-08	0.52	34	1.13	Svep1	
DMR5:75393001	5	75393001	75395000	2000	1	5.90E-13	-0.55	27	1.35	Musk	Receptor
DMR5:75456001	5	75456001	75460000	4000	1	1.70E-07	-0.29	57	1.43	Musk	Receptor
DMR5:75492001	5	75492001	75493000	1000	1	7.00E-15	0.9	23	2.3	Musk	Receptor
DMR5:75501001	5	75501001	75502000	1000	1	4.00E-08	0.57	10	1	Musk	Receptor
DMR5:75552001	5	75552001	75554000	2000	1	6.00E-11	-0.59	4	0.2	Lpar1	Signaling
DMR5:75609001	5	75609001	75611000	2000	1	2.90E-18	0.97	40	2	Lpar1	Signaling
DMR5:75667001	5	75667001	75671000	4000	2	2.20E-12	-0.49	73	1.82	Lpar1	Signaling
DMR5:75673001	5	75673001	75677000	4000	2	1.60E-08	-0.62	112	2.8	Lpar1	Signaling
DMR5:76060001	5	76060001	76062000	2000	1	3.40E-07	-0.46	19	0.95	Hspd1-ps10;Cct6a-ps4	
DMR5:76112001	5	76112001	76114000	2000	1	7.50E-12	-0.5	37	1.85	Zfp483;Ptgr1	Metabolism
DMR5:76419001	5	76419001	76420000	1000	1	3.30E-10	-0.48	17	1.7	Ugcg	Golgi

DMR5:76537001	5	76537001	76539000	2000	1	7.10E-07	-0.35	43	2.15	Susd1	Extracellular Matrix
DMR5:76609001	5	76609001	76611000	2000	1	4.40E-07	-0.31	38	1.9	Susd1	Extracellular Matrix
DMR5:76666001	5	76666001	76668000	2000	1	6.00E-08	0.41	21	1.05	Ptbp3	
DMR5:76762001	5	76762001	76763000	1000	1	1.10E-07	-0.46	12	1.2	Ptbp3	
DMR5:76879001	5	76879001	76882000	3000	1	1.00E-07	-0.5	29	0.97	RGD1310951	
DMR5:76897001	5	76897001	76900000	3000	1	2.40E-10	-0.47	37	1.23	RGD1310951	
DMR5:76950001	5	76950001	76955000	5000	2	8.30E-09	-0.39	47	0.94	RGD1310951	
DMR5:76956001	5	76956001	76957000	1000	1	7.30E-07	-0.44	14	1.4	RGD1310951	
DMR5:77037001	5	77037001	77038000	1000	1	6.40E-08	-0.47	7	0.7	Inip	
DMR5:77072001	5	77072001	77075000	3000	1	7.80E-07	-0.35	48	1.6	Snx30	Cytoskeleton
DMR5:77122001	5	77122001	77129000	7000	1	8.60E-08	-0.29	96	1.37	Snx30	Cytoskeleton
DMR5:77789001	5	77789001	77791000	2000	1	4.90E-08	-0.32	14	0.7	Mup5	Transport
DMR5:77888001	5	77888001	77889000	1000	1	4.50E-07	0.34	9	0.9	Mup5;Zfp37	Transport;Transcription
DMR5:78122001	5	78122001	78124000	2000	1	4.80E-07	-0.42	27	1.35	Slc31a2;Fkbp15	Transport
DMR5:78346001	5	78346001	78347000	1000	1	1.10E-07	0.46	22	2.2	Bspry	Proteolysis
DMR5:78424001	5	78424001	78425000	1000	1	4.40E-10	-0.55	7	0.7	Rgs3	
DMR5:78457001	5	78457001	78462000	5000	1	3.10E-08	-0.45	76	1.52	Rgs3	
DMR5:78534001	5	78534001	78536000	2000	1	1.30E-07	-0.54	14	0.7	Rgs3	
DMR5:78538001	5	78538001	78539000	1000	1	2.20E-08	-0.46	30	3	Rgs3	
DMR5:78564001	5	78564001	78565000	1000	1	7.80E-07	0.59	21	2.1	Rgs3	
DMR5:78882001	5	78882001	78883000	1000	1	1.90E-07	0.58	11	1.1	Zfp618	Transcription
DMR5:78895001	5	78895001	78896000	1000	1	5.50E-07	0.57	12	1.2	Zfp618	Transcription
DMR5:79121001	5	79121001	79122000	1000	1	1.70E-10	0.53	19	1.9	Col27a1	Extracellular Matrix
DMR5:79155001	5	79155001	79156000	1000	1	4.90E-08	0.51	17	1.7	Col27a1	Extracellular Matrix
DMR5:79235001	5	79235001	79236000	1000	1	6.30E-07	0.39	24	2.4	Akna;Whrn	Cytoskeleton
DMR5:79237001	5	79237001	79239000	2000	1	1.70E-07	0.59	52	2.6	Akna;Whrn	Cytoskeleton
DMR5:79362001	5	79362001	79364000	2000	1	2.10E-07	-0.41	30	1.5	Atp6v1g1	Metabolism
DMR5:79386001	5	79386001	79387000	1000	1	1.90E-09	-0.46	10	1	Tmem268	
DMR5:79691001	5	79691001	79693000	2000	1	1.00E-07	0.52	32	1.6	Tnfsf8	
DMR5:81094001	5	81094001	81095000	1000	1	7.40E-08	0.54	19	1.9	Pappa	
DMR5:81139001	5	81139001	81141000	2000	1	1.40E-08	-0.57	9	0.45	Pappa	
DMR5:81228001	5	81228001	81230000	2000	2	8.70E-11	-0.56	8	0.4	Astn2	
DMR5:81238001	5	81238001	81239000	1000	1	8.30E-09	0.49	15	1.5	Astn2	
DMR5:81396001	5	81396001	81397000	1000	1	3.40E-09	-0.45	12	1.2	Astn2	
DMR5:81644001	5	81644001	81647000	3000	1	2.40E-07	-0.44	22	0.73	Astn2	
DMR5:81778001	5	81778001	81782000	4000	2	2.70E-10	-0.39	36	0.9	Astn2	
DMR5:84937001	5	84937001	84940000	3000	1	1.00E-07	-0.36	27	0.9	Brinp1	
DMR5:85022001	5	85022001	85023000	1000	1	1.90E-07	-0.62	3	0.3	Brinp1	
DMR5:86541001	5	86541001	86542000	1000	1	6.60E-07	-0.42	5	0.5	Cdk5rap2	
DMR5:90068001	5	90068001	90069000	1000	1	2.90E-07	-0.3	9	0.9	Frmd3	
DMR5:90141001	5	90141001	90142000	1000	1	6.50E-07	-0.36	8	0.8	Frmd3	
DMR5:90215001	5	90215001	90220000	5000	1	1.20E-09	-0.43	54	1.08	Frmd3	
DMR5:90227001	5	90227001	90231000	4000	1	3.60E-07	-0.38	38	0.95	Frmd3	
DMR5:90425001	5	90425001	90426000	1000	1	2.50E-09	-0.37	12	1.2	Rasef	
DMR5:90807001	5	90807001	90808000	1000	1	8.30E-07	-0.49	4	0.4	Dppa3-ps2;Kdm4c	
DMR5:91566001	5	91566001	91569000	3000	2	5.40E-08	0.48	16	0.53	Vom2r-ps76	
DMR5:91584001	5	91584001	91586000	2000	1	2.30E-14	-0.69	4	0.2	Vom2r-ps76	
DMR5:92936001	5	92936001	92937000	1000	1	9.80E-07	-0.43	8	0.8	Ptprd	Signaling
DMR5:93067001	5	93067001	93068000	1000	1	1.80E-07	0.42	13	1.3	Ptprd	Signaling
DMR5:98504001	5	98504001	98509000	5000	1	3.70E-07	-0.31	45	0.9	Lurap1l	
DMR5:99171001	5	99171001	99173000	2000	2	3.30E-11	-0.71	7	0.35	RGD1306186	
DMR5:99418001	5	99418001	99421000	3000	1	1.50E-07	0.6	42	1.4	Mpdz	
DMR5:100461001	5	100461001	100462000	1000	1	2.60E-08	-0.52	19	1.9	Nfib	Transcription
DMR5:100507001	5	100507001	100509000	2000	1	3.00E-07	-0.42	37	1.85	Nfib	Transcription
DMR5:100601001	5	100601001	100604000	3000	1	1.10E-07	-0.35	39	1.3	Nfib	Transcription
DMR5:100995001	5	100995001	100996000	1000	1	3.90E-07	0.38	13	1.3	Cer1	
DMR5:101009001	5	101009001	101010000	1000	1	1.20E-11	0.7	20	2	Cer1;Frem1	
DMR5:101294001	5	101294001	101296000	2000	1	9.10E-07	-0.39	22	1.1	Ttc39b	
DMR5:101415001	5	101415001	101416000	1000	1	9.20E-07	0.44	12	1.2	Ttc39b	
DMR5:101564001	5	101564001	101565000	1000	1	1.70E-08	-0.46	5	0.5	Snapc3;Psip1;LOC103692397	Transcription;Transcrip tion

DMR5:101631001	5	101631001	101634000	3000	1	4.20E-07	-0.35	28	0.93	Ccdc171	
DMR5:101767001	5	101767001	101769000	2000	1	9.90E-07	-0.42	11	0.55	Ccdc171	
DMR5:101771001	5	101771001	101775000	4000	2	8.90E-08	0.46	34	0.85	Ccdc171	
DMR5:101936001	5	101936001	101937000	1000	1	1.00E-15	0.98	20	2	Ccdc171	
DMR5:102541001	5	102541001	102543000	2000	1	2.00E-09	-0.44	25	1.25	Bnc2	Transcription
DMR5:102551001	5	102551001	102552000	1000	1	3.00E-08	0.59	11	1.1	Bnc2	Transcription
DMR5:102557001	5	102557001	102558000	1000	1	2.60E-08	-0.51	0	0	Bnc2	Transcription
DMR5:102560001	5	102560001	102561000	1000	1	1.30E-07	0.55	13	1.3	Bnc2	Transcription
DMR5:103813001	5	103813001	103816000	3000	1	2.50E-09	-0.74	15	0.5	Adamtsl1	
DMR5:103919001	5	103919001	103921000	2000	1	8.10E-10	-0.52	15	0.75	Adamtsl1	
DMR5:103937001	5	103937001	103939000	2000	1	9.70E-09	-0.45	15	0.75	Adamtsl1	
DMR5:103951001	5	103951001	103957000	6000	2	3.30E-10	-0.26	62	1.03	Adamtsl1	
DMR5:104134001	5	104134001	104136000	2000	1	7.20E-09	-0.56	20	1	Adamtsl1	
DMR5:104301001	5	104301001	104305000	4000	1	1.80E-09	-0.31	34	0.85	Adamtsl1	
DMR5:104414001	5	104414001	104415000	1000	1	1.60E-07	-0.65	4	0.4	Adamtsl1	
DMR5:104453001	5	104453001	104454000	1000	1	2.80E-11	0.74	14	1.4	Adamtsl1	
DMR5:104481001	5	104481001	104483000	2000	1	3.00E-07	-0.34	29	1.45	Adamtsl1	
DMR5:104503001	5	104503001	104507000	4000	1	5.10E-07	0.34	38	0.95	Adamtsl1	
DMR5:104588001	5	104588001	104590000	2000	1	1.10E-07	-0.59	12	0.6	Adamtsl1	
DMR5:104764001	5	104764001	104766000	2000	1	5.30E-08	-0.37	50	2.5	Fam154a	
DMR5:104810001	5	104810001	104816000	6000	1	1.60E-31	1.12	54	0.9	Fam154a	
DMR5:104896001	5	104896001	104898000	2000	1	6.40E-13	0.84	33	1.65	Fam154a	
DMR5:104919001	5	104919001	104922000	3000	2	3.30E-09	0.6	50	1.67	Fam154a	
DMR5:105279001	5	105279001	105281000	2000	1	1.30E-09	-0.51	23	1.15	Acer2	
DMR5:105379001	5	105379001	105380000	1000	1	8.20E-09	-0.54	11	1.1	Slc24a2	Transport
DMR5:105416001	5	105416001	105417000	1000	1	6.50E-08	0.37	5	0.5	Slc24a2	Transport
DMR5:105471001	5	105471001	105472000	1000	1	1.10E-07	0.46	17	1.7	Slc24a2	Transport
DMR5:105587001	5	105587001	105588000	1000	1	5.30E-10	0.62	17	1.7	Slc24a2	Transport
DMR5:106534001	5	106534001	106536000	2000	1	2.80E-07	-0.44	27	1.35	Focad	
DMR5:106693001	5	106693001	106696000	3000	1	5.30E-07	0.46	32	1.07	Focad	
DMR5:106950001	5	106950001	106951000	1000	1	3.70E-25	0.89	27	2.7	Ifna5;LOC690903	
DMR5:106960001	5	106960001	106962000	2000	1	6.40E-07	-0.45	13	0.65	Ifna5;LOC690903	
DMR5:106991001	5	106991001	106996000	5000	1	4.90E-07	-0.24	50	1	RGD1561246	
DMR5:107241001	5	107241001	107242000	1000	1	2.80E-09	-0.7	3	0.3	Ifne	Immune
DMR5:107315001	5	107315001	107316000	1000	1	8.80E-08	-0.48	8	0.8	LOC680462;Klhl9	
DMR5:107324001	5	107324001	107325000	1000	1	4.70E-07	-0.58	22	2.2	LOC680462;Klhl9;LOC108350997	
DMR5:107362001	5	107362001	107364000	2000	1	6.10E-07	-0.27	17	0.85	RGD1559679;LOC100912314	Immune
DMR5:107422001	5	107422001	107424000	2000	1	6.00E-19	0.6	24	1.2	Ifna2;RGD1564637	Immune
DMR5:107436001	5	107436001	107440000	4000	1	6.10E-21	0.77	35	0.88	RGD1564637;Ifna16l1	Immune
DMR5:107813001	5	107813001	107814000	1000	1	9.90E-07	-0.52	2	0.2	Cdkn2a	
DMR5:108757001	5	108757001	108758000	1000	1	1.40E-12	0.78	18	1.8	Zfp353;LOC500497	Transcription
DMR5:109583001	5	109583001	109584000	1000	1	5.30E-11	0.52	9	0.9	Elavl2	Translation
DMR5:109641001	5	109641001	109643000	2000	1	5.00E-08	-0.52	20	1	Elavl2	Translation
DMR5:113724001	5	113724001	113726000	2000	2	3.20E-07	0.53	29	1.45	Tek	Receptor
DMR5:114616001	5	114616001	114617000	1000	1	6.30E-16	0.32	21	2.1	Fggy	Metabolism
DMR5:114817001	5	114817001	114819000	2000	1	1.70E-09	-0.71	5	0.25	Fggy;LOC103692418	Metabolism
DMR5:115392001	5	115392001	115394000	2000	1	4.80E-08	-0.44	19	0.95	RGD1560146	
DMR5:116419001	5	116419001	116424000	5000	1	8.10E-07	-0.4	88	1.76	Nfia	Transcription
DMR5:116428001	5	116428001	116431000	3000	1	9.40E-08	-0.34	46	1.53	Nfia	Transcription
DMR5:116432001	5	116432001	116436000	4000	1	1.10E-08	-0.36	67	1.68	Nfia	Transcription
DMR5:116498001	5	116498001	116499000	1000	1	3.30E-09	-0.39	15	1.5	Nfia	Transcription
DMR5:116562001	5	116562001	116564000	2000	1	1.20E-09	-0.57	35	1.75	Nfia	Transcription
DMR5:116613001	5	116613001	116617000	4000	1	1.20E-07	-0.37	50	1.25	Nfia	Transcription
DMR5:116714001	5	116714001	116716000	2000	1	8.20E-09	-0.42	26	1.3	Nfia	Transcription
DMR5:117012001	5	117012001	117015000	3000	1	2.60E-10	-0.54	30	1	Tm2d1	
DMR5:117069001	5	117069001	117070000	1000	1	1.80E-07	0.56	8	0.8	Patj	
DMR5:117230001	5	117230001	117231000	1000	1	1.40E-07	0.53	14	1.4	Patj	
DMR5:117354001	5	117354001	117357000	3000	1	3.70E-18	0.83	65	2.17	L1td1	Epigenetic
DMR5:117616001	5	117616001	117618000	2000	1	8.40E-09	-0.48	13	0.65	Dock7	Transcription
DMR5:117634001	5	117634001	117637000	3000	1	1.50E-09	-0.57	45	1.5	Dock7	Transcription
DMR5:117645001	5	117645001	117647000	2000	1	4.00E-09	-0.37	14	0.7	Dock7	Transcription

DMR5:117648001	5	117648001	117649000	1000	1	1.30E-11	0.33	2	0.2	Dock7	Transcription
DMR5:117685001	5	117685001	117686000	1000	1	8.40E-10	-0.53	11	1.1	Dock7	Transcription
DMR5:117714001	5	117714001	117716000	2000	1	2.00E-07	-0.39	26	1.3	Dock7;Angptl3	Transcription;Signaling
DMR5:117772001	5	117772001	117780000	8000	3	1.50E-10	-0.55	94	1.18	Dock7;LOC100366073	Transcription
DMR5:117787001	5	117787001	117789000	2000	1	8.70E-12	-0.5	32	1.6	Dock7;LOC100366073	Transcription
DMR5:118356001	5	118356001	118357000	1000	1	3.50E-07	0.46	10	1	LOC102556374;Foxd3	Transcription
DMR5:118407001	5	118407001	118411000	4000	1	5.80E-12	0.74	66	1.65	Alg6	Golgi
DMR5:118427001	5	118427001	118430000	3000	1	1.30E-07	-0.31	30	1	Alg6	Golgi
DMR5:118510001	5	118510001	118512000	2000	1	8.40E-08	0.51	19	0.95	Itgb3bp	
DMR5:118591001	5	118591001	118594000	3000	1	4.80E-07	-0.58	27	0.9	Ube2u	
DMR5:118764001	5	118764001	118767000	3000	1	4.60E-07	-0.36	50	1.67	Pgm1	Metabolism
DMR5:118802001	5	118802001	118803000	1000	1	2.40E-07	0.52	14	1.4	Pgm1	Metabolism
DMR5:118881001	5	118881001	118883000	2000	1	3.40E-08	-0.48	13	0.65	Ror1	Receptor
DMR5:118929001	5	118929001	118930000	1000	1	3.30E-10	0.56	25	2.5	Ror1	Receptor
DMR5:119058001	5	119058001	119059000	1000	1	1.50E-07	-0.44	16	1.6	Ror1	Receptor
DMR5:119734001	5	119734001	119737000	3000	2	8.40E-07	-0.43	38	1.27	Cachd1	Transport
DMR5:119822001	5	119822001	119829000	7000	2	1.00E-08	-0.56	98	1.4	Cachd1	Transport
DMR5:119910001	5	119910001	119913000	3000	1	1.20E-16	0.78	44	1.47	Raver2;LOC103692426	Metabolism
DMR5:119947001	5	119947001	119948000	1000	1	1.10E-07	-0.44	8	0.8	Raver2	Metabolism
DMR5:119964001	5	119964001	119966000	2000	1	2.30E-07	-0.57	27	1.35	Raver2	Metabolism
DMR5:119974001	5	119974001	119977000	3000	2	1.00E-10	-0.71	47	1.57	Raver2;Jak1	Metabolism
DMR5:120092001	5	120092001	120094000	2000	1	4.10E-07	-0.45	15	0.75	Jak1	
DMR5:120266001	5	120266001	120268000	2000	1	2.30E-08	-0.41	31	1.55	Ak4	Signaling
DMR5:120295001	5	120295001	120298000	3000	1	5.00E-07	-0.34	29	0.97	Ak4	Signaling
DMR5:120452001	5	120452001	120455000	3000	1	2.10E-10	-0.54	47	1.57	Dnajc6	Transport
DMR5:120463001	5	120463001	120464000	1000	1	1.70E-10	0.73	16	1.6	Dnajc6	Transport
DMR5:120472001	5	120472001	120478000	6000	1	3.10E-17	-0.42	62	1.03	Dnajc6	Transport
DMR5:120480001	5	120480001	120485000	5000	1	7.50E-09	0.48	43	0.86	Dnajc6	Transport
DMR5:121286001	5	121286001	121289000	3000	1	5.80E-08	-0.7	13	0.43	Olr1778-ps	
DMR5:121854001	5	121854001	121856000	2000	2	2.20E-16	0.48	40	2	Pde4b	Signaling
DMR5:121992001	5	121992001	121993000	1000	1	4.30E-08	-0.76	0	0	Pde4b	Signaling
DMR5:122027001	5	122027001	122028000	1000	1	7.10E-09	-0.37	10	1	Pde4b	Signaling
DMR5:122036001	5	122036001	122037000	1000	1	6.30E-07	0.56	10	1	Pde4b;LOC108351007	Signaling
DMR5:122119001	5	122119001	122122000	3000	1	7.90E-07	-0.35	50	1.67	Pde4b	Signaling
DMR5:122425001	5	122425001	122428000	3000	1	1.10E-10	-0.55	26	0.87	Sgip1;LOC102548308	Cytoskeleton
DMR5:122522001	5	122522001	122529000	7000	1	5.20E-08	-0.5	99	1.41	Tctex1d1;LOC103692428;Insl5	Cytoskeleton
DMR5:122560001	5	122560001	122562000	2000	1	1.80E-08	-0.31	25	1.25	Wdr78	Cytoskeleton
DMR5:122571001	5	122571001	122576000	5000	1	4.40E-07	-0.22	43	0.86	Wdr78	Cytoskeleton
DMR5:122586001	5	122586001	122589000	3000	1	1.40E-07	-0.41	47	1.57	Wdr78	Cytoskeleton
DMR5:122660001	5	122660001	122661000	1000	1	5.30E-07	-0.51	5	0.5	Mier1	Development
DMR5:122701001	5	122701001	122704000	3000	1	3.40E-08	0.43	39	1.3	Mier1;Slc35d1	Development;Transport
DMR5:122761001	5	122761001	122763000	2000	1	6.90E-09	-0.73	10	0.5	RGD1562532	
DMR5:122886001	5	122886001	122890000	4000	1	5.20E-08	-0.34	26	0.65	Oma1	Protease
DMR5:123244001	5	123244001	123245000	1000	1	3.60E-09	-0.65	6	0.6	Dab1	Cytoskeleton
DMR5:123617001	5	123617001	123618000	1000	1	2.10E-08	0.44	23	2.3	Dab1	Cytoskeleton
DMR5:123665001	5	123665001	123666000	1000	1	2.60E-07	0.5	4	0.4	Dab1	Cytoskeleton
DMR5:123794001	5	123794001	123795000	1000	1	3.90E-07	0.64	9	0.9	Dab1	Cytoskeleton
DMR5:123800001	5	123800001	123803000	3000	1	8.80E-08	-0.32	30	1	Dab1	Cytoskeleton
DMR5:123809001	5	123809001	123810000	1000	1	5.90E-07	0.52	8	0.8	Dab1	Cytoskeleton
DMR5:123973001	5	123973001	123974000	1000	1	4.60E-07	-0.36	17	1.7	Dab1	Cytoskeleton
DMR5:124241001	5	124241001	124244000	3000	1	3.90E-10	-0.5	29	0.97	Dab1	Cytoskeleton
DMR5:124316001	5	124316001	124319000	3000	1	2.20E-11	0.63	36	1.2	C8b	
DMR5:125921001	5	125921001	125924000	3000	1	3.90E-09	-0.38	38	1.27	Usp24	Protease
DMR5:125927001	5	125927001	125928000	1000	1	1.20E-07	-0.39	10	1	Usp24	Protease
DMR5:126042001	5	126042001	126047000	5000	2	4.60E-08	0.62	73	1.46	Pcsk9	Protease
DMR5:126058001	5	126058001	126060000	2000	1	1.30E-08	0.48	18	0.9	Pcsk9	Protease
DMR5:126210001	5	126210001	126213000	3000	1	1.70E-08	0.5	59	1.97	Lexm;Ttc22	
DMR5:126300001	5	126300001	126302000	2000	1	2.20E-10	0.57	39	1.95	Mroh7	
DMR5:126304001	5	126304001	126305000	1000	1	1.10E-07	0.53	11	1.1	Mroh7	
DMR5:126341001	5	126341001	126347000	6000	2	1.20E-08	0.63	82	1.37	Fam151a;Acot11	Metabolism
DMR5:126367001	5	126367001	126369000	2000	1	1.20E-08	0.59	41	2.05	Acot11	Metabolism

DMR5:126384001	5	126384001	126389000	5000	1	1.10E-07	0.34	80	1.6	Acot11	Metabolism
DMR5:126400001	5	126400001	126401000	1000	1	7.90E-10	0.65	16	1.6	Acot11	Metabolism
DMR5:126685001	5	126685001	126686000	1000	1	1.90E-07	0.56	15	1.5	Cyb5rl;LOC103692436;LOC100363867	Metabolism
DMR5:126697001	5	126697001	126698000	1000	1	1.40E-07	0.47	19	1.9	LOC103692436;LOC100363867;Cdcp2	Protease
DMR5:126717001	5	126717001	126719000	2000	1	7.60E-07	0.47	26	1.3	Cdcp2	Protease
DMR5:126826001	5	126826001	126829000	3000	1	1.90E-07	-0.37	34	1.13	Ldlrad1;Lrrc42	
DMR5:126995001	5	126995001	126996000	1000	1	1.70E-10	-0.46	15	1.5	Ndc1	
DMR5:127054001	5	127054001	127056000	2000	1	2.50E-07	0.34	27	1.35	Glis1	Transcription
DMR5:127096001	5	127096001	127098000	2000	1	3.00E-09	0.4	26	1.3	Glis1	Transcription
DMR5:127100001	5	127100001	127101000	1000	1	6.60E-07	0.44	14	1.4	Glis1	Transcription
DMR5:127121001	5	127121001	127123000	2000	1	1.80E-07	0.65	27	1.35	Glis1;LOC102551042	Transcription
DMR5:127150001	5	127150001	127156000	6000	2	2.80E-07	0.54	95	1.58	Glis1	Transcription
DMR5:127162001	5	127162001	127163000	1000	1	5.70E-09	0.45	14	1.4	Glis1	Transcription
DMR5:127164001	5	127164001	127170000	6000	1	7.70E-07	0.49	89	1.48	Glis1	Transcription
DMR5:127171001	5	127171001	127174000	3000	1	4.00E-07	0.53	39	1.3	Glis1	Transcription
DMR5:127204001	5	127204001	127207000	3000	1	6.90E-09	0.38	34	1.13	Glis1	Transcription
DMR5:127214001	5	127214001	127216000	2000	1	2.00E-09	0.56	28	1.4	Glis1	Transcription
DMR5:127219001	5	127219001	127222000	3000	1	6.90E-09	0.44	39	1.3	Glis1	Transcription
DMR5:127233001	5	127233001	127234000	1000	1	1.40E-07	0.41	9	0.9	Glis1	Transcription
DMR5:127238001	5	127238001	127239000	1000	1	3.90E-09	0.57	12	1.2	Glis1	Transcription
DMR5:127243001	5	127243001	127245000	2000	1	1.40E-08	-0.45	15	0.75	Glis1	Transcription
DMR5:127274001	5	127274001	127275000	1000	1	2.20E-09	0.67	27	2.7	Dmrtb1	Transcription
DMR5:127447001	5	127447001	127449000	2000	1	3.80E-07	0.44	40	2	Lrp8	Binding Proteins
DMR5:127565001	5	127565001	127570000	5000	1	8.70E-11	0.55	72	1.44	Slc1a7	Transport
DMR5:127596001	5	127596001	127600000	4000	2	1.20E-09	0.54	75	1.88	Slc1a7	Transport
DMR5:127623001	5	127623001	127625000	2000	2	2.50E-08	0.68	56	2.8	Slc1a7;LOC102552895	Transport
DMR5:127660001	5	127660001	127661000	1000	1	7.40E-12	-0.52	25	2.5	Scp2	Transport
DMR5:127665001	5	127665001	127667000	2000	1	3.00E-07	-0.52	20	1	Scp2	Transport
DMR5:127670001	5	127670001	127671000	1000	1	2.30E-09	-0.41	16	1.6	Scp2	Transport
DMR5:127724001	5	127724001	127726000	2000	1	2.50E-10	-0.51	28	1.4	Scp2	Transport
DMR5:127763001	5	127763001	127764000	1000	1	5.70E-07	0.32	18	1.8	Echdc2	Metabolism
DMR5:127773001	5	127773001	127774000	1000	1	1.60E-14	0.79	24	2.4	Echdc2	Metabolism
DMR5:127870001	5	127870001	127872000	2000	1	5.70E-10	-0.45	20	1	Zyg11b	
DMR5:127928001	5	127928001	127929000	1000	1	2.90E-07	-0.49	9	0.9	Coa7	
DMR5:127995001	5	127995001	127998000	3000	1	1.00E-10	-0.55	25	0.83	Gpx7	Metabolism
DMR5:128229001	5	128229001	128230000	1000	1	1.40E-07	0.51	16	1.6	Cc2d1b;Zfyve9	
DMR5:128263001	5	128263001	128264000	1000	1	5.50E-07	0.53	9	0.9	Zfyve9	
DMR5:128274001	5	128274001	128278000	4000	1	6.70E-11	0.77	68	1.7	Zfyve9	
DMR5:128320001	5	128320001	128321000	1000	1	3.60E-09	-0.47	7	0.7	Zfyve9	
DMR5:128523001	5	128523001	128525000	2000	1	1.30E-07	0.44	26	1.3	Rab3b	
DMR5:128694001	5	128694001	128696000	2000	1	1.10E-13	-0.56	35	1.75	Nrdc;Osbpl9	Protease
DMR5:128709001	5	128709001	128711000	2000	1	1.20E-07	-0.41	19	0.95	Osbpl9	
DMR5:128795001	5	128795001	128797000	2000	1	6.80E-08	-0.51	39	1.95	Osbpl9	
DMR5:128957001	5	128957001	128959000	2000	1	1.60E-08	-0.56	12	0.6	Eps15	Transport
DMR5:129417001	5	129417001	129422000	5000	1	6.50E-10	-0.3	49	0.98	Faf1	
DMR5:129562001	5	129562001	129566000	4000	1	8.40E-07	-0.37	39	0.98	Faf1	
DMR5:129588001	5	129588001	129589000	1000	1	6.60E-08	-0.47	5	0.5	Faf1	
DMR5:129679001	5	129679001	129685000	6000	1	1.70E-08	0.25	63	1.05	Faf1	
DMR5:129693001	5	129693001	129696000	3000	1	3.70E-08	-0.35	27	0.9	Faf1	
DMR5:130075001	5	130075001	130076000	1000	1	2.70E-07	-0.34	15	1.5	Elavl4	Translation
DMR5:130084001	5	130084001	130087000	3000	2	6.30E-09	-0.5	50	1.67	Elavl4	Translation
DMR5:130305001	5	130305001	130306000	1000	1	1.90E-08	-0.42	7	0.7	Agbl4	Protease
DMR5:133230001	5	133230001	133231000	1000	1	4.20E-08	-0.44	21	2.1	Trabd2b;LOC103692452	Protease
DMR5:133232001	5	133232001	133237000	5000	1	1.70E-07	0.51	71	1.42	Trabd2b;LOC103692452	Protease
DMR5:133290001	5	133290001	133291000	1000	1	1.30E-07	0.45	12	1.2	Trabd2b	Protease
DMR5:133293001	5	133293001	133295000	2000	2	6.20E-08	0.56	18	0.9	Trabd2b	Protease
DMR5:133364001	5	133364001	133365000	1000	1	3.50E-07	0.5	17	1.7	Trabd2b	Protease
DMR5:133382001	5	133382001	133383000	1000	1	1.60E-07	0.34	13	1.3	Trabd2b	Protease
DMR5:133385001	5	133385001	133387000	2000	1	1.60E-11	0.6	19	0.95	Trabd2b	Protease
DMR5:133412001	5	133412001	133414000	2000	1	8.50E-08	0.43	20	1	Trabd2b	Protease

DMR5:133442001	5	133442001	133445000	3000	1	3.00E-07	-0.4	34	1.13	Trabd2b	Protease
DMR5:134106001	5	134106001	134107000	1000	1	8.00E-08	-0.33	8	0.8	RGD1562603	
DMR5:134208001	5	134208001	134214000	6000	2	2.60E-07	0.43	66	1.1	Cyp4a2;LOC108351019	Metabolism
DMR5:134452001	5	134452001	134459000	7000	3	2.80E-11	-0.43	58	0.83	LOC102554130;LOC103690754;Cyp4a3	
DMR5:134495001	5	134495001	134496000	1000	1	5.90E-07	-0.37	12	1.2	Cyp4a1	
DMR5:134657001	5	134657001	134662000	5000	1	6.80E-08	-0.38	74	1.48	Atpaf1;LOC103692454	Transcription
DMR5:135132001	5	135132001	135133000	1000	1	1.20E-10	-0.4	12	1.2	Pik3r3	Signaling
DMR5:135206001	5	135206001	135208000	2000	1	2.40E-08	-0.31	34	1.7	Mast2	Signaling
DMR5:135260001	5	135260001	135261000	1000	1	2.80E-08	-0.38	9	0.9	Mast2	Signaling
DMR5:135401001	5	135401001	135402000	1000	1	2.80E-07	-0.53	7	0.7	Tmem69;Gpbp1l1	
DMR5:135495001	5	135495001	135497000	2000	1	7.70E-10	-0.46	17	0.85	Akr1a1	Metabolism
DMR5:135550001	5	135550001	135551000	1000	1	1.70E-09	-0.53	2	0.2	Prdx1;Mmachc	Metabolism
DMR5:135575001	5	135575001	135577000	2000	1	5.80E-08	-0.43	14	0.7	Ccdc163;Tesk2	
DMR5:135836001	5	135836001	135839000	3000	1	1.10E-08	-0.44	35	1.17	Zswim5	
DMR5:135844001	5	135844001	135846000	2000	1	3.40E-08	-0.38	25	1.25	Zswim5;Urod	Epigenetic
DMR5:135969001	5	135969001	135971000	2000	1	1.30E-08	-0.41	30	1.5	Ptch2	
DMR5:136038001	5	136038001	136040000	2000	1	1.50E-07	0.42	31	1.55	Kif2c	Cytoskeleton
DMR5:136090001	5	136090001	136094000	4000	1	2.90E-08	0.41	60	1.5	RGD1563714	
DMR5:136127001	5	136127001	136128000	1000	1	1.20E-07	0.48	32	3.2	Tmem53;Rnf220	
DMR5:136169001	5	136169001	136170000	1000	1	1.10E-08	0.41	12	1.2	Rnf220	
DMR5:136179001	5	136179001	136183000	4000	1	1.30E-07	-0.4	93	2.33	Rnf220	
DMR5:136227001	5	136227001	136229000	2000	1	2.70E-08	0.47	20	1	Rnf220	
DMR5:136230001	5	136230001	136233000	3000	1	3.30E-20	0.78	64	2.13	Rnf220	
DMR5:136235001	5	136235001	136239000	4000	1	5.70E-10	-0.5	58	1.45	Rnf220	
DMR5:136591001	5	136591001	136595000	4000	1	8.00E-07	0.34	48	1.2	Klf17	Transcription
DMR5:136610001	5	136610001	136614000	4000	1	5.10E-09	0.74	61	1.52	Klf17	Transcription
DMR5:136657001	5	136657001	136660000	3000	1	6.90E-11	0.52	55	1.83	Slc6a9	Transport
DMR5:136684001	5	136684001	136686000	2000	1	8.80E-09	0.44	38	1.9	Slc6a9;Ccdc24	Transport
DMR5:136711001	5	136711001	136712000	1000	1	7.00E-09	0.34	26	2.6	Slc6a9;Ccdc24;B4galt2;Atp6v0b	Transport;Golgi;Metabolism
DMR5:136844001	5	136844001	136847000	3000	1	1.40E-07	-0.45	72	2.4	St3gal3;LOC102553361	Transport
DMR5:136877001	5	136877001	136878000	1000	1	3.00E-10	-0.44	18	1.8	St3gal3	Transport
DMR5:136887001	5	136887001	136891000	4000	1	4.70E-07	-0.4	77	1.93	St3gal3	Transport
DMR5:136905001	5	136905001	136910000	5000	1	1.70E-07	-0.42	102	2.04	St3gal3	Transport
DMR5:137031001	5	137031001	137033000	2000	1	1.60E-07	-0.4	29	1.45	Ptprf	Signaling
DMR5:137095001	5	137095001	137098000	3000	1	1.30E-07	-0.52	68	2.27	Ptprf;LOC102553423	Signaling
DMR5:137220001	5	137220001	137226000	6000	1	1.10E-10	-0.43	126	2.1	Szt2	
DMR5:137280001	5	137280001	137281000	1000	1	2.10E-07	0.42	25	2.5	Mpl;LOC102554074	Receptor
DMR5:137377001	5	137377001	137378000	1000	1	3.50E-07	0.52	10	1	Tmem125;Cfap57	
DMR5:137392001	5	137392001	137396000	4000	1	3.00E-07	0.39	62	1.55	Cfap57	
DMR5:137824001	5	137824001	137826000	2000	1	6.50E-07	-0.31	20	1	Olr865	Receptor
DMR5:137853001	5	137853001	137856000	3000	1	7.90E-07	0.24	29	0.97	Olr866	Receptor
DMR5:137983001	5	137983001	137984000	1000	1	2.20E-09	-0.5	5	0.5	Olr872-ps	
DMR5:138190001	5	138190001	138194000	4000	1	4.30E-08	-0.56	61	1.52	Slc2a1	
DMR5:138240001	5	138240001	138244000	4000	1	6.40E-13	-0.62	48	1.2	Ermap;Svbp	Immune
DMR5:138291001	5	138291001	138293000	2000	1	2.70E-07	-0.45	44	2.2	P3h1;Cldn19	Extracellular Matrix;Cell Junction
DMR5:138405001	5	138405001	138407000	2000	1	5.70E-07	0.51	32	1.6	Ccdc30;LOC102552311	
DMR5:138421001	5	138421001	138422000	1000	1	8.80E-07	0.48	10	1	Ccdc30;LOC102552311	
DMR5:138456001	5	138456001	138460000	4000	2	1.00E-08	-0.44	76	1.9	Ccdc30;Ppcs	Transport
DMR5:138491001	5	138491001	138494000	3000	1	8.40E-09	-0.4	43	1.43	Zmynd12	
DMR5:138699001	5	138699001	138703000	4000	2	8.80E-10	-0.85	33	0.82	Guca2b	Signaling
DMR5:138957001	5	138957001	138958000	1000	1	2.40E-14	0.68	14	1.4	Hivep3	
DMR5:139084001	5	139084001	139087000	3000	1	6.00E-07	0.45	49	1.63	Hivep3	
DMR5:139096001	5	139096001	139100000	4000	1	1.10E-09	0.49	67	1.68	Edn2	Hormone
DMR5:139107001	5	139107001	139109000	2000	1	5.10E-08	0.46	21	1.05	Edn2	Hormone
DMR5:139207001	5	139207001	139208000	1000	1	1.00E-14	0.98	31	3.1	Foxo6	
DMR5:139218001	5	139218001	139222000	4000	2	2.50E-09	0.56	46	1.15	Foxo6	
DMR5:139408001	5	139408001	139413000	5000	2	1.10E-08	-0.36	42	0.84	Scmh1	Epigenetic
DMR5:139469001	5	139469001	139470000	1000	1	1.50E-07	0.6	16	1.6	Scmh1;Slfnl1;Ctps1	Epigenetic;Metabolism

DMR5:139494001	5	139494001	139496000	2000	1	4.40E-07	-0.43	27	1.35	Ctps1	Metabolism
DMR5:139615001	5	139615001	139616000	1000	1	1.10E-08	0.52	20	2	Kcnq4	Transport
DMR5:139626001	5	139626001	139627000	1000	1	2.20E-21	1.18	25	2.5	Kcnq4	Transport
DMR5:139646001	5	139646001	139652000	6000	1	9.20E-08	0.5	96	1.6	Kcnq4	Transport
DMR5:139965001	5	139965001	139967000	2000	1	6.60E-07	0.52	34	1.7	Col9a2;LOC108351024	Extracellular Matrix
DMR5:139973001	5	139973001	139974000	1000	1	5.80E-08	0.51	11	1.1	Col9a2;LOC108351024;LOC108351025;Zmpste24	Extracellular Matrix;Protease
DMR5:140532001	5	140532001	140534000	2000	1	5.00E-10	-0.51	20	1	Ppt1	Golgi
DMR5:140583001	5	140583001	140585000	2000	1	3.70E-08	-0.34	21	1.05	Cap1	Cytoskeleton
DMR5:140592001	5	140592001	140596000	4000	2	5.70E-10	0.58	40	1	Cap1	Cytoskeleton
DMR5:140645001	5	140645001	140648000	3000	1	1.70E-09	0.58	53	1.77	Mfsd2a;LOC100909490	
DMR5:140667001	5	140667001	140669000	2000	1	2.50E-10	0.5	26	1.3	Mfsd2a	
DMR5:140893001	5	140893001	140896000	3000	1	5.10E-07	-0.41	45	1.5	Nt5c1a	
DMR5:140947001	5	140947001	140953000	6000	2	1.70E-08	0.52	51	0.85	Heyl;LOC108351027	Transcription
DMR5:140979001	5	140979001	140980000	1000	1	4.20E-15	0.95	73	7.3	Pabpc4	
DMR5:141031001	5	141031001	141033000	2000	1	8.40E-07	0.5	24	1.2	Bmp8a;Macf1	Growth Factors;Cytoskeleton
DMR5:141206001	5	141206001	141209000	3000	1	3.90E-07	-0.42	44	1.47	Macf1	Cytoskeleton
DMR5:141303001	5	141303001	141304000	1000	1	7.30E-10	0.43	9	0.9	Macf1	Cytoskeleton
DMR5:141325001	5	141325001	141326000	1000	1	7.50E-07	0.41	11	1.1	Macf1	Cytoskeleton
DMR5:141368001	5	141368001	141371000	3000	1	8.90E-08	0.34	36	1.2	Macf1;LOC108351106	Cytoskeleton
DMR5:141429001	5	141429001	141430000	1000	1	3.90E-08	-0.56	17	1.7	Akirin1	
DMR5:141433001	5	141433001	141435000	2000	1	1.20E-07	0.45	11	0.55	Akirin1	
DMR5:141452001	5	141452001	141453000	1000	1	3.40E-08	-0.48	12	1.2	Rhbdl2	
DMR5:141505001	5	141505001	141507000	2000	1	7.70E-07	-0.44	15	0.75	Rhbdl2;LOC103692469	
DMR5:141588001	5	141588001	141590000	2000	1	6.90E-07	0.51	36	1.8	Rragc	Signaling
DMR5:141823001	5	141823001	141831000	8000	1	1.20E-07	-0.4	103	1.29	Lnc081	
DMR5:142700001	5	142700001	142701000	1000	1	1.80E-08	-0.42	13	1.3	Fhl3;Sf3a3;LOC103692473	Transcription;Translation
DMR5:142879001	5	142879001	142881000	2000	2	4.00E-18	1.06	58	2.9	Epha10;LOC108351028	Receptor
DMR5:143054001	5	143054001	143055000	1000	1	8.80E-07	0.58	10	1	Gnl2;Dnali1;Snip1	Cytoskeleton;Translation
DMR5:143094001	5	143094001	143095000	1000	1	2.80E-08	-0.44	6	0.6	Meaf6	
DMR5:143110001	5	143110001	143113000	3000	1	6.90E-08	0.46	51	1.7	Meaf6;Zc3h12a	Translation
DMR5:143129001	5	143129001	143130000	1000	1	8.60E-08	-0.47	20	2	Zc3h12a	Translation
DMR5:143542001	5	143542001	143543000	1000	1	4.10E-09	-0.47	14	1.4	Grik3	Receptor
DMR5:143577001	5	143577001	143578000	1000	1	1.80E-09	0.66	12	1.2	Grik3	Receptor
DMR5:143677001	5	143677001	143680000	3000	1	8.30E-07	0.48	47	1.57	Grik3;LOC108351030	Receptor
DMR5:143709001	5	143709001	143714000	5000	1	2.20E-10	-0.48	99	1.98	Grik3	Receptor
DMR5:144023001	5	144023001	144024000	1000	1	3.10E-07	0.47	14	1.4	Csf3r	Receptor
DMR5:144028001	5	144028001	144030000	2000	1	1.00E-07	0.35	17	0.85	Csf3r	Receptor
DMR5:144060001	5	144060001	144062000	2000	1	2.30E-08	0.42	20	1	Csf3r;Mrps15;Oscp1	Receptor;Translation
DMR5:144190001	5	144190001	144192000	2000	1	5.30E-13	-0.57	25	1.25	Thrap3	Metabolism
DMR5:144251001	5	144251001	144253000	2000	1	3.70E-07	-0.4	59	2.95	Map7d1	Cytoskeleton
DMR5:144276001	5	144276001	144279000	3000	1	4.70E-20	0.92	54	1.8	Map7d1;Trappc3	Cytoskeleton
DMR5:144296001	5	144296001	144299000	3000	1	4.00E-07	-0.56	29	0.97	Trappc3;Col8a2	Extracellular Matrix
DMR5:144343001	5	144343001	144345000	2000	1	8.80E-10	0.66	44	2.2	Col8a2;Adprhl2;Tekt2	Extracellular Matrix;Metabolism;Cytoskeleton
DMR5:144767001	5	144767001	144768000	1000	1	5.90E-12	-0.51	14	1.4	Tfap2e;Ncdn	Transcription;Cytoskeleton
DMR5:144874001	5	144874001	144876000	2000	1	5.40E-10	-0.48	30	1.5	LOC100294508;Zmym4	Transcription
DMR5:145223001	5	145223001	145227000	4000	1	1.50E-07	-0.45	46	1.15	Dlgap3	Cytoskeleton
DMR5:145300001	5	145300001	145301000	1000	1	3.10E-07	0.53	12	1.2	Dlgap3	Cytoskeleton
DMR5:145302001	5	145302001	145303000	1000	1	6.50E-12	0.65	16	1.6	Dlgap3;Smim12	Cytoskeleton
DMR5:145408001	5	145408001	145409000	1000	1	8.20E-16	0.82	27	2.7	Gjb4	Cytoskeleton
DMR5:145417001	5	145417001	145419000	2000	2	5.10E-10	0.6	69	3.45	Gjb4;Gjb5	Cytoskeleton
DMR5:145448001	5	145448001	145451000	3000	1	2.00E-07	0.62	45	1.5	Gjb5	Cytoskeleton
DMR5:146130001	5	146130001	146132000	2000	1	1.50E-07	0.49	23	1.15	Csmd2	
DMR5:146163001	5	146163001	146164000	1000	1	1.80E-08	-0.38	14	1.4	Csmd2	
DMR5:146172001	5	146172001	146175000	3000	1	8.50E-07	0.42	46	1.53	Csmd2	
DMR5:146176001	5	146176001	146178000	2000	1	7.90E-07	-0.3	35	1.75	Csmd2	

DMR5:146354001	5	146354001	146355000	1000	1	4.40E-08	-0.54	8	0.8	Csmd2	
DMR5:146403001	5	146403001	146409000	6000	2	4.10E-08	-0.42	40	0.67	Csmd2	
DMR5:146418001	5	146418001	146422000	4000	1	8.90E-09	-0.47	57	1.43	Csmd2	
DMR5:146425001	5	146425001	146427000	2000	1	3.10E-08	-0.51	25	1.25	Csmd2	
DMR5:146460001	5	146460001	146463000	3000	1	3.70E-07	-0.4	41	1.37	Csmd2	
DMR5:146492001	5	146492001	146493000	1000	1	1.00E-06	0.46	22	2.2	Csmd2	
DMR5:146494001	5	146494001	146496000	2000	1	5.90E-13	0.68	38	1.9	Csmd2	
DMR5:146547001	5	146547001	146548000	1000	1	1.60E-13	-0.55	9	0.9	Csmd2	
DMR5:146560001	5	146560001	146562000	2000	1	1.50E-08	-0.35	24	1.2	Csmd2	
DMR5:146575001	5	146575001	146576000	1000	1	7.10E-12	0.7	22	2.2	Csmd2	
DMR5:146582001	5	146582001	146583000	1000	1	2.10E-07	0.46	9	0.9	Csmd2	
DMR5:146599001	5	146599001	146600000	1000	1	3.00E-07	-0.39	27	2.7	Csmd2	
DMR5:146610001	5	146610001	146612000	2000	1	1.40E-07	-0.52	49	2.45	Csmd2	
DMR5:146644001	5	146644001	146647000	3000	2	4.40E-10	-0.47	65	2.17	Csmd2	
DMR5:146673001	5	146673001	146674000	1000	1	9.00E-13	0.7	12	1.2	Csmd2	
DMR5:146689001	5	146689001	146690000	1000	1	1.90E-07	0.39	9	0.9	Csmd2	
DMR5:146720001	5	146720001	146722000	2000	1	5.50E-09	0.47	50	2.5	Csmd2	
DMR5:146734001	5	146734001	146736000	2000	1	5.10E-07	0.47	49	2.45	Csmd2	
DMR5:146742001	5	146742001	146747000	5000	1	2.40E-08	-0.35	91	1.82	Csmd2	
DMR5:146754001	5	146754001	146755000	1000	1	6.70E-11	0.75	25	2.5	Zscan20	Transcription
DMR5:146823001	5	146823001	146825000	2000	2	2.60E-13	0.62	30	1.5	Phc2	Epigenetic
DMR5:146835001	5	146835001	146842000	7000	1	3.20E-11	-0.45	125	1.79	Phc2	Epigenetic
DMR5:146909001	5	146909001	146910000	1000	1	3.40E-07	-0.42	22	2.2	Phc2	Epigenetic
DMR5:147116001	5	147116001	147124000	8000	1	3.00E-07	0.41	146	1.82	Azin2	Metabolism
DMR5:147266001	5	147266001	147268000	2000	1	2.50E-07	-0.43	41	2.05	Rnf19b	Proteolysis
DMR5:147376001	5	147376001	147378000	2000	2	9.90E-10	-0.56	17	0.85	S100pbp;Yars	
DMR5:147418001	5	147418001	147422000	4000	1	3.20E-07	-0.41	69	1.73	RGD1561149	
DMR5:147425001	5	147425001	147428000	3000	1	1.30E-07	-0.33	66	2.2	RGD1561149	
DMR5:147429001	5	147429001	147430000	1000	1	1.90E-08	-0.43	21	2.1	RGD1561149	
DMR5:147477001	5	147477001	147481000	4000	2	3.40E-09	0.73	68	1.7	Sync	
DMR5:147482001	5	147482001	147484000	2000	1	5.50E-14	0.78	59	2.95	Sync	
DMR5:147570001	5	147570001	147572000	2000	1	8.50E-07	-0.34	30	1.5	Zbtb8a;LOC108351038	Cytoskeleton
DMR5:147584001	5	147584001	147586000	2000	1	4.70E-17	-0.75	23	1.15	Zbtb8a;LOC108351038	Cytoskeleton
DMR5:147593001	5	147593001	147595000	2000	2	1.80E-08	0.71	42	2.1	Zbtb8a;LOC108351038	Cytoskeleton
DMR5:147648001	5	147648001	147650000	2000	1	1.90E-09	0.61	31	1.55	LOC108351038;Zbtb8b	Cytoskeleton
DMR5:148051001	5	148051001	148052000	1000	1	9.10E-11	-0.44	19	1.9	Ptp4a2	Signaling
DMR5:148209001	5	148209001	148211000	2000	1	9.90E-09	0.38	35	1.75	Adgrb2	Signaling
DMR5:148217001	5	148217001	148218000	1000	1	4.10E-11	0.52	31	3.1	Adgrb2	Signaling
DMR5:148283001	5	148283001	148288000	5000	1	1.90E-08	0.45	119	2.38	Col16a1	Extracellular Matrix
DMR5:148305001	5	148305001	148308000	3000	1	1.80E-08	-0.62	31	1.03	Col16a1	Extracellular Matrix
DMR5:148324001	5	148324001	148325000	1000	1	4.20E-09	-0.39	9	0.9	Pef1	
DMR5:148352001	5	148352001	148354000	2000	1	7.10E-10	0.46	34	1.7	Pef1;Hcrtr1	Signaling
DMR5:148357001	5	148357001	148361000	4000	1	1.50E-07	0.35	49	1.23	Hcrtr1	Signaling
DMR5:148362001	5	148362001	148369000	7000	1	3.30E-07	-0.39	148	2.11	Hcrtr1	Signaling
DMR5:148380001	5	148380001	148381000	1000	1	1.30E-07	0.47	30	3	Tinagl1	Protease
DMR5:148439001	5	148439001	148440000	1000	1	4.90E-07	-0.45	21	2.1	RGD1562036	Metabolism
DMR5:148453001	5	148453001	148455000	2000	1	6.90E-07	0.49	16	0.8	RGD1562036;LOC102552786	Metabolism
DMR5:148477001	5	148477001	148478000	1000	1	1.40E-07	0.45	22	2.2	LOC102552786;Serinc2	Signaling
DMR5:148481001	5	148481001	148482000	1000	1	3.30E-16	1.11	31	3.1	Serinc2	Signaling
DMR5:148501001	5	148501001	148506000	5000	3	5.20E-11	-0.73	70	1.4	Serinc2	Signaling
DMR5:148529001	5	148529001	148530000	1000	1	3.40E-08	0.89	30	3	Fabp3;Zcchc17	
DMR5:148539001	5	148539001	148542000	3000	1	1.60E-07	-0.48	42	1.4	Fabp3;Zcchc17	
DMR5:148548001	5	148548001	148549000	1000	1	2.60E-08	-0.41	17	1.7	Zcchc17	
DMR5:148564001	5	148564001	148568000	4000	2	8.70E-10	-0.5	78	1.95	Zcchc17;Snrnp40	
DMR5:148663001	5	148663001	148667000	4000	1	3.00E-09	-0.44	64	1.6	Nkain1;LOC103692481	
DMR5:148886001	5	148886001	148887000	1000	1	3.20E-13	-0.58	4	0.4	Pum1	Metabolism
DMR5:149011001	5	149011001	149013000	2000	1	6.40E-07	0.35	19	0.95	Laptm5	Transport
DMR5:149027001	5	149027001	149030000	3000	1	5.90E-12	0.68	58	1.93	Laptm5	Transport
DMR5:149077001	5	149077001	149080000	3000	1	3.20E-08	0.46	68	2.27	Laptm5;Matn1	Transport;Extracellular Matrix
DMR5:149949001	5	149949001	149950000	1000	1	3.70E-08	0.48	27	2.7	Ptpru	Signaling
DMR5:149977001	5	149977001	149981000	4000	1	1.80E-11	0.64	81	2.02	Ptpru	Signaling

DMR5:150059001	5	150059001	150063000	4000	1	1.10E-08	-0.42	126	3.15	Srsf4	Translation
DMR5:150107001	5	150107001	150109000	2000	1	5.00E-07	-0.28	40	2	Epb41	
DMR5:150120001	5	150120001	150122000	2000	1	1.10E-07	-0.41	26	1.3	Epb41	
DMR5:150312001	5	150312001	150315000	3000	2	1.30E-10	0.75	53	1.77	Oprd1	Signaling
DMR5:150333001	5	150333001	150334000	1000	1	6.60E-10	-0.51	12	1.2	Oprd1	Signaling
DMR5:150404001	5	150404001	150405000	1000	1	9.50E-14	0.84	34	3.4	Gmeb1	
DMR5:150582001	5	150582001	150584000	2000	1	3.10E-07	-0.46	40	2	Phactr4	Signaling
DMR5:150599001	5	150599001	150601000	2000	1	1.30E-07	-0.43	23	1.15	Phactr4	Signaling
DMR5:150614001	5	150614001	150615000	1000	1	3.30E-07	-0.47	4	0.4	Phactr4	Signaling
DMR5:150649001	5	150649001	150651000	2000	1	7.00E-10	-0.4	42	2.1	Med18	Transcription
DMR5:150771001	5	150771001	150773000	2000	1	2.00E-08	0.52	37	1.85	Ptafr	Signaling
DMR5:150908001	5	150908001	150909000	1000	1	3.90E-07	-0.28	22	2.2	Eya3;Xkr8	
DMR5:150916001	5	150916001	150919000	3000	2	1.30E-20	1	52	1.73	Eya3;Xkr8;Smpdl3b	Signaling
DMR5:150936001	5	150936001	150945000	9000	1	1.00E-10	-0.42	146	1.62	Smpdl3b	Signaling
DMR5:150961001	5	150961001	150964000	3000	1	1.90E-07	-0.38	42	1.4	Rpa2;Themis2	
DMR5:151039001	5	151039001	151041000	2000	1	1.50E-12	-0.48	21	1.05	Ppp1r8;Stx12	Translation;Transcription
DMR5:151090001	5	151090001	151091000	1000	1	2.60E-07	-0.43	4	0.4	Fam76a	
DMR5:151202001	5	151202001	151203000	1000	1	2.90E-10	0.97	33	3.3	Fgr;Ahdc1	
DMR5:151321001	5	151321001	151322000	1000	1	1.80E-07	-0.49	17	1.7	Wasf2	Cytoskeleton
DMR5:151395001	5	151395001	151396000	1000	1	2.10E-14	0.92	29	2.9	Wasf2;Gpr3;Cd164l2	Cytoskeleton;Signaling
DMR5:151441001	5	151441001	151444000	3000	2	1.00E-11	0.72	87	2.9	Map3k6;Sytl1	Signaling
DMR5:151450001	5	151450001	151452000	2000	1	1.30E-07	0.58	42	2.1	Map3k6;Sytl1;Tmem222	Signaling
DMR5:151600001	5	151600001	151601000	1000	1	4.20E-07	0.55	20	2	Slc9a1	Transport
DMR5:151734001	5	151734001	151738000	4000	1	7.90E-08	0.35	43	1.07	LOC103690094;Kdf1	
DMR5:151774001	5	151774001	151777000	3000	1	1.40E-08	0.51	43	1.43	Nudc;Nr0b2	Cytoskeleton;Transcription
DMR5:151826001	5	151826001	151827000	1000	1	8.50E-07	0.56	11	1.1	Sfn;Zdhhc18	Cytoskeleton
DMR5:151858001	5	151858001	151860000	2000	1	8.60E-09	-0.39	34	1.7	Zdhhc18;LOC366479	
DMR5:151894001	5	151894001	151895000	1000	1	7.00E-07	0.44	18	1.8	Pigv;Arid1a	Golgi
DMR5:151930001	5	151930001	151932000	2000	1	9.50E-07	-0.38	24	1.2	Arid1a	
DMR5:151951001	5	151951001	151953000	2000	1	2.40E-08	-0.44	22	1.1	Arid1a	
DMR5:151983001	5	151983001	151986000	3000	1	3.80E-07	0.43	26	0.87	Arid1a	
DMR5:152193001	5	152193001	152194000	1000	1	8.20E-11	-0.57	14	1.4	LOC683456;LOC689346;Hmgn2;Dhdds	Epigenetic;Metabolism
DMR5:152277001	5	152277001	152278000	1000	1	5.30E-09	-0.38	26	2.6	Zfp683	
DMR5:152292001	5	152292001	152294000	2000	1	9.00E-07	0.43	25	1.25	Zfp683;Aim1l	
DMR5:152325001	5	152325001	152328000	3000	1	4.70E-08	0.56	69	2.3	Aim1l;Cd52;LOC100360087;Ubxn11	Signaling
DMR5:152443001	5	152443001	152444000	1000	1	2.20E-07	-0.35	15	1.5	Catsper4;Cnksr1	
DMR5:152535001	5	152535001	152538000	3000	1	2.00E-07	0.47	37	1.23	Trim63	Proteolysis
DMR5:152552001	5	152552001	152554000	2000	1	4.40E-07	0.61	33	1.65	Trim63;Slc30a2	Proteolysis;Transport
DMR5:152570001	5	152570001	152571000	1000	1	1.20E-08	-0.38	20	2	Slc30a2;Extl1	Transport;Golgi
DMR5:152576001	5	152576001	152579000	3000	1	8.40E-08	0.61	51	1.7	Slc30a2;Extl1	Transport;Golgi
DMR5:152580001	5	152580001	152583000	3000	1	1.90E-07	0.45	37	1.23	Slc30a2;Extl1	Transport;Golgi
DMR5:152609001	5	152609001	152611000	2000	1	3.10E-07	-0.53	28	1.4	Pafah2	Golgi
DMR5:152692001	5	152692001	152694000	2000	1	8.10E-07	0.55	31	1.55	Stmn1;LOC108351044	
DMR5:152718001	5	152718001	152721000	3000	1	9.30E-12	0.65	78	2.6	LOC102546455;Paqr7;Aunip	Signaling
DMR5:152760001	5	152760001	152761000	1000	1	4.20E-16	1	32	3.2	Sepn1	
DMR5:152764001	5	152764001	152767000	3000	2	1.50E-10	0.57	42	1.4	Sepn1;Man1c1	Golgi
DMR5:152781001	5	152781001	152783000	2000	1	6.40E-09	0.49	38	1.9	Man1c1	Golgi
DMR5:152830001	5	152830001	152832000	2000	1	5.20E-07	-0.36	40	2	Man1c1	Golgi
DMR5:152871001	5	152871001	152873000	2000	1	8.20E-07	0.4	27	1.35	Man1c1	Golgi
DMR5:152889001	5	152889001	152893000	4000	1	1.70E-07	0.32	52	1.3	Man1c1	Golgi
DMR5:153233001	5	153233001	153235000	2000	2	4.60E-10	0.69	47	2.35	Rhd;Tmem50a	Transport
DMR5:153249001	5	153249001	153250000	1000	1	4.60E-08	0.47	24	2.4	Tmem50a	
DMR5:153509001	5	153509001	153510000	1000	1	1.00E-07	-0.58	37	3.7	Runx3	Transcription
DMR5:153541001	5	153541001	153544000	3000	2	2.90E-07	0.45	57	1.9	Runx3	Transcription
DMR5:153693001	5	153693001	153695000	2000	1	1.00E-08	-0.41	42	2.1	Srrm1	Translation
DMR5:153782001	5	153782001	153787000	5000	1	5.00E-10	-0.55	95	1.9	Rcan3	Signaling
DMR5:153824001	5	153824001	153826000	2000	1	1.20E-11	0.63	29	1.45	Nipal3;Stpg1	Development
DMR5:154031001	5	154031001	154034000	3000	1	1.20E-11	0.49	61	2.03	Ifnlr1	Receptor

DMR5:154051001	5	154051001	154054000	3000	1	1.00E-07	0.3	48	1.6	Ifnlr1	Receptor
DMR5:154107001	5	154107001	154112000	5000	2	2.60E-08	0.48	82	1.64	Il22ra1;Myom3	Receptor
DMR5:154126001	5	154126001	154128000	2000	1	6.30E-07	0.35	32	1.6	Myom3	
DMR5:154232001	5	154232001	154233000	1000	1	3.90E-09	-0.46	20	2	Pnrc2;Cnr2	Signaling
DMR5:154298001	5	154298001	154302000	4000	1	3.10E-08	0.46	80	2	Hmgcl;Gale	Metabolism;Metabolism
DMR5:154312001	5	154312001	154316000	4000	2	4.40E-09	0.7	111	2.78	Hmgcl;Gale;Lypla2;Pithd1	Metabolism;Metabolism;Metabolism;Metabolism
DMR5:154391001	5	154391001	154393000	2000	1	1.90E-09	-0.44	31	1.55	Rpl11;LOC102551799	Translation
DMR5:154434001	5	154434001	154438000	4000	1	5.40E-07	0.56	73	1.82	RGD1563135	
DMR5:154527001	5	154527001	154530000	3000	1	3.40E-08	-0.42	55	1.83	E2f2	Transcription
DMR5:154647001	5	154647001	154648000	1000	1	8.40E-07	-0.39	19	1.9	RGD1564482;Zfp46	
DMR5:154661001	5	154661001	154664000	3000	1	8.50E-09	-0.46	57	1.9	Zfp46;Hnrnpr	Metabolism
DMR5:154766001	5	154766001	154771000	5000	1	3.30E-16	0.82	70	1.4	Htr1d	Signaling
DMR5:154800001	5	154800001	154803000	3000	1	4.30E-17	0.94	68	2.27	Htr1d	Signaling
DMR5:154899001	5	154899001	154900000	1000	1	3.80E-12	-0.49	25	2.5	Luzp1;LOC103692495;Kdm1a	Metabolism
DMR5:154952001	5	154952001	154953000	1000	1	5.40E-08	-0.36	11	1.1	Kdm1a	Metabolism
DMR5:154992001	5	154992001	154993000	1000	1	6.50E-07	0.36	11	1.1	RGD1561426	
DMR5:155039001	5	155039001	155042000	3000	1	1.80E-14	0.92	60	2	Ephb2	Receptor
DMR5:155096001	5	155096001	155097000	1000	1	2.30E-08	0.4	4	0.4	Ephb2	Receptor
DMR5:155121001	5	155121001	155125000	4000	1	1.50E-13	0.74	100	2.5	Ephb2	Receptor
DMR5:155139001	5	155139001	155140000	1000	1	6.40E-08	0.73	30	3	Ephb2	Receptor
DMR5:155142001	5	155142001	155143000	1000	1	7.30E-08	0.51	11	1.1	Ephb2	Receptor
DMR5:155252001	5	155252001	155255000	3000	1	8.40E-08	0.5	49	1.63	C1qb;C1qc;C1qa	
DMR5:155298001	5	155298001	155299000	1000	1	1.60E-07	0.44	24	2.4	Epha8	Receptor
DMR5:155765001	5	155765001	155770000	5000	1	3.20E-09	0.43	71	1.42	Cela3b	Protease
DMR5:155842001	5	155842001	155846000	4000	1	4.70E-08	0.48	70	1.75	Hspg2	
DMR5:155871001	5	155871001	155875000	4000	1	1.50E-07	0.51	63	1.57	Hspg2	
DMR5:155885001	5	155885001	155889000	4000	1	6.00E-07	0.42	100	2.5	Hspg2	
DMR5:155905001	5	155905001	155910000	5000	1	3.70E-07	0.39	93	1.86	Hspg2;Ldlrad2	
DMR5:156049001	5	156049001	156051000	2000	1	1.60E-09	0.5	27	1.35	Rap1gap	Signaling
DMR5:156075001	5	156075001	156077000	2000	1	4.00E-09	-0.76	17	0.85	Rap1gap;Alpl	Signaling;Signaling
DMR5:156089001	5	156089001	156090000	1000	1	6.30E-08	0.39	18	1.8	Alpl	Signaling
DMR5:156108001	5	156108001	156114000	6000	2	7.70E-09	0.59	108	1.8	Alpl	Signaling
DMR5:156234001	5	156234001	156238000	4000	1	1.50E-08	-0.4	72	1.8	Ece1	Protease
DMR5:156345001	5	156345001	156353000	8000	3	4.70E-16	-0.7	135	1.69	Eif4g3	Translation
DMR5:156443001	5	156443001	156445000	2000	1	7.80E-12	-0.47	29	1.45	Eif4g3	Translation
DMR5:156456001	5	156456001	156457000	1000	1	6.10E-09	0.5	22	2.2	Eif4g3	Translation
DMR5:156579001	5	156579001	156581000	2000	1	6.40E-15	0.67	30	1.5	LOC102553854;Hp1bp3	Cytoskeleton
DMR5:156621001	5	156621001	156624000	3000	1	3.40E-08	0.42	63	2.1	Sh2d5;Kif17	Cytoskeleton;Cytoskeleton
DMR5:156641001	5	156641001	156643000	2000	1	5.60E-08	0.45	42	2.1	Kif17	Cytoskeleton
DMR5:156730001	5	156730001	156732000	2000	1	2.60E-07	0.47	26	1.3	Cda	Metabolism
DMR5:156741001	5	156741001	156744000	3000	1	7.30E-08	-0.48	40	1.33	Cda	Metabolism
DMR5:156863001	5	156863001	156865000	2000	1	6.40E-08	0.55	34	1.7	Mul1	
DMR5:156996001	5	156996001	157001000	5000	1	8.40E-09	0.52	71	1.42	LOC108351047;Vwa5b1	
DMR5:157169001	5	157169001	157172000	3000	1	5.40E-08	0.51	53	1.77	Ubxn10;Pla2g2c	Metabolism
DMR5:157244001	5	157244001	157245000	1000	1	1.80E-07	-0.37	16	1.6	Pla2g5	Metabolism
DMR5:157267001	5	157267001	157271000	4000	1	1.30E-09	-0.59	46	1.15	Pla2g5	Metabolism
DMR5:157330001	5	157330001	157334000	4000	2	2.40E-11	0.87	78	1.95	Pla2g2e	
DMR5:157433001	5	157433001	157436000	3000	1	2.60E-07	0.48	45	1.5	Tmco4	
DMR5:157506001	5	157506001	157508000	2000	1	1.80E-12	0.7	40	2	Tmco4;Htr6	Signaling
DMR5:157693001	5	157693001	157694000	1000	1	8.00E-08	-0.53	20	2	Capzb	Cytoskeleton
DMR5:157740001	5	157740001	157743000	3000	1	1.30E-12	-0.57	60	2	Capzb;Pqlc2	Cytoskeleton
DMR5:158034001	5	158034001	158035000	1000	1	1.90E-07	0.35	15	1.5	Iffo2	Metabolism
DMR5:158106001	5	158106001	158107000	1000	1	2.90E-07	0.53	32	3.2	Aldh4a1	Metabolism
DMR5:158135001	5	158135001	158137000	2000	1	4.90E-08	0.51	49	2.45	Tas1r2;LOC108351051	Signaling
DMR5:158200001	5	158200001	158202000	2000	1	7.00E-12	-0.57	20	1	Pax7	
DMR5:158293001	5	158293001	158298000	5000	2	1.20E-07	0.56	49	0.98	Pax7	
DMR5:158306001	5	158306001	158308000	2000	1	6.80E-09	-0.53	33	1.65	Pax7	
DMR5:158514001	5	158514001	158515000	1000	1	2.10E-08	0.45	24	2.4	LOC108351052;Igsf21	

DMR5:158592001	5	158592001	158593000	1000	1	1.30E-09	0.54	13	1.3	Igsf21	
DMR5:158622001	5	158622001	158625000	3000	1	3.10E-08	0.58	57	1.9	Igsf21	
DMR5:158633001	5	158633001	158634000	1000	1	6.00E-10	0.6	12	1.2	Igsf21	
DMR5:159009001	5	159009001	159010000	1000	1	1.80E-08	0.63	32	3.2	Arhgef10l	Transcription
DMR5:159017001	5	159017001	159020000	3000	1	1.20E-08	-0.4	76	2.53	Arhgef10l	Transcription
DMR5:159058001	5	159058001	159059000	1000	1	7.40E-07	0.34	20	2	Arhgef10l	Transcription
DMR5:159060001	5	159060001	159062000	2000	1	1.10E-14	0.78	45	2.25	Arhgef10l	Transcription
DMR5:159226001	5	159226001	159227000	1000	1	9.10E-07	0.43	19	1.9	Rcc2	
DMR5:159256001	5	159256001	159259000	3000	1	2.70E-08	0.42	51	1.7	Padi6;Padi4	
DMR5:159262001	5	159262001	159265000	3000	1	1.90E-10	0.54	53	1.77	Padi6;Padi4	
DMR5:159288001	5	159288001	159296000	8000	1	7.40E-08	0.43	135	1.69	Padi4	
DMR5:159297001	5	159297001	159299000	2000	1	1.50E-10	0.67	40	2	Padi4;Padi3	
DMR5:159306001	5	159306001	159311000	5000	1	3.60E-07	0.42	80	1.6	Padi3	
DMR5:159318001	5	159318001	159324000	6000	1	4.90E-08	0.37	117	1.95	Padi3	
DMR5:159336001	5	159336001	159340000	4000	1	3.80E-08	0.49	57	1.43	Padi3;Padi1	
DMR5:159341001	5	159341001	159343000	2000	1	7.40E-07	0.55	28	1.4	Padi3;Padi1	
DMR5:159344001	5	159344001	159347000	3000	1	1.30E-13	0.67	45	1.5	Padi3;Padi1	
DMR5:159355001	5	159355001	159362000	7000	1	6.10E-09	0.53	129	1.84	Padi1;Trnaa-ggc	
DMR5:159364001	5	159364001	159367000	3000	1	4.90E-11	0.69	42	1.4	Padi1;Trnaa-ggc	
DMR5:159378001	5	159378001	159382000	4000	1	3.60E-07	-0.36	56	1.4	Padi1	
DMR5:159523001	5	159523001	159534000	11000	1	3.90E-07	0.48	241	2.19	Atp13a2;Mfap2;Crocc	Epigenetic
DMR5:159580001	5	159580001	159582000	2000	1	4.30E-08	-0.37	32	1.6	Crocc;Necap2	Epigenetic;Transport
DMR5:159621001	5	159621001	159626000	5000	1	5.10E-09	0.39	86	1.72	Spata21	Signaling
DMR5:159645001	5	159645001	159646000	1000	1	5.30E-08	-0.4	11	1.1	Spata21;Szrd1	Signaling
DMR5:159739001	5	159739001	159742000	3000	2	8.70E-15	1.06	76	2.53	Fbxo42;Rsg1	
DMR5:159764001	5	159764001	159765000	1000	1	1.80E-10	0.7	55	5.5	Trnai-aau;LOC108351054;Arhgef19	Transcription
DMR5:159766001	5	159766001	159767000	1000	1	3.90E-07	0.54	30	3	LOC108351054;Arhgef19	Transcription
DMR5:159775001	5	159775001	159777000	2000	1	6.40E-10	0.71	45	2.25	Arhgef19	Transcription
DMR5:159782001	5	159782001	159783000	1000	1	5.60E-13	0.75	16	1.6	Arhgef19	Transcription
DMR5:159835001	5	159835001	159837000	2000	1	1.50E-07	0.52	45	2.25	LOC103692501;Epha2	Receptor
DMR5:159841001	5	159841001	159842000	1000	1	2.00E-09	0.81	28	2.8	LOC103692501;Epha2	Receptor
DMR5:159844001	5	159844001	159846000	2000	1	6.60E-08	-0.38	69	3.45	LOC103692501;Epha2	Receptor
DMR5:159877001	5	159877001	159884000	7000	2	5.10E-14	0.76	120	1.71	Epha2	Receptor
DMR5:159916001	5	159916001	159918000	2000	1	5.00E-15	0.76	41	2.05	Fam131c	
DMR5:159933001	5	159933001	159937000	4000	1	1.40E-08	0.39	61	1.52	Fam131c;Clcnka	Transport
DMR5:159938001	5	159938001	159946000	8000	1	5.40E-07	0.49	155	1.94	Fam131c;Clcnka;Clcnkb	Transport
DMR5:159956001	5	159956001	159958000	2000	1	6.50E-11	0.86	52	2.6	Clcnka;Clcnkb	Transport
DMR5:159960001	5	159960001	159963000	3000	1	7.60E-07	0.41	50	1.67	Clcnkb;Hspb7	Transport
DMR5:160024001	5	160024001	160029000	5000	1	3.50E-07	-0.38	149	2.98	Zbtb17;Spen	Transcription;Metabolism
DMR5:160042001	5	160042001	160044000	2000	1	5.80E-09	-0.53	26	1.3	Spen	Metabolism
DMR5:160168001	5	160168001	160170000	2000	1	1.20E-10	0.59	35	1.75	Fblim1;Tmem82;Slc25a34	Signaling
DMR5:160185001	5	160185001	160187000	2000	1	1.60E-11	0.6	51	2.55	Tmem82;Slc25a34;Plekhm2	
DMR5:160217001	5	160217001	160218000	1000	1	4.00E-07	0.53	15	1.5	Plekhm2	
DMR5:160231001	5	160231001	160234000	3000	1	1.40E-07	0.64	58	1.93	Ddi2	Proteolysis
DMR5:160278001	5	160278001	160279000	1000	1	8.80E-11	-0.61	16	1.6	Ddi2	Proteolysis
DMR5:160340001	5	160340001	160342000	2000	2	7.60E-11	-0.52	25	1.25	Dnajc16	Transcription
DMR5:160366001	5	160366001	160370000	4000	2	4.60E-08	0.5	47	1.18	Casp9;Cela2a	Protease;Protease
DMR5:160455001	5	160455001	160456000	1000	1	7.00E-10	0.62	18	1.8	Fhad1	
DMR5:160510001	5	160510001	160511000	1000	1	7.40E-07	0.56	21	2.1	Fhad1	
DMR5:160520001	5	160520001	160521000	1000	1	7.20E-12	0.37	13	1.3	Fhad1	
DMR5:160612001	5	160612001	160614000	2000	1	1.50E-09	-0.39	32	1.6	Tmem51	
DMR5:160762001	5	160762001	160767000	5000	2	7.80E-10	-0.45	91	1.82	Kazn	
DMR5:160780001	5	160780001	160782000	2000	1	6.50E-17	0.92	49	2.45	Kazn	
DMR5:160785001	5	160785001	160786000	1000	1	1.00E-07	0.5	17	1.7	Kazn	
DMR5:160794001	5	160794001	160796000	2000	1	5.60E-08	0.64	44	2.2	Kazn	
DMR5:160906001	5	160906001	160907000	1000	1	6.50E-09	0.55	13	1.3	Kazn	
DMR5:160930001	5	160930001	160933000	3000	1	1.90E-12	-0.46	46	1.53	Kazn	
DMR5:160946001	5	160946001	160947000	1000	1	6.80E-09	-0.7	3	0.3	Kazn	
DMR5:160984001	5	160984001	160985000	1000	1	4.40E-07	-0.42	24	2.4	Kazn	
DMR5:160987001	5	160987001	160988000	1000	1	2.60E-10	0.39	9	0.9	Kazn	

DMR5:161071001	5	161071001	161075000	4000	2	5.70E-17	0.66	44	1.1	Kazn	
DMR5:161082001	5	161082001	161086000	4000	1	6.70E-08	-0.57	98	2.45	Kazn	
DMR5:161185001	5	161185001	161190000	5000	1	1.40E-07	0.5	65	1.3	Kazn	
DMR5:161195001	5	161195001	161197000	2000	1	6.10E-07	0.43	14	0.7	Kazn	
DMR5:161284001	5	161284001	161289000	5000	1	1.80E-08	-0.43	79	1.58	Kazn	
DMR5:161290001	5	161290001	161292000	2000	2	3.40E-12	-0.77	9	0.45	Kazn	
DMR5:161316001	5	161316001	161318000	2000	1	5.10E-08	-0.36	52	2.6	Kazn	
DMR5:161334001	5	161334001	161340000	6000	2	1.80E-12	0.59	107	1.78	Kazn	
DMR5:161401001	5	161401001	161402000	1000	1	7.90E-07	0.45	10	1	Kazn;LOC100362622	
DMR5:161405001	5	161405001	161406000	1000	1	4.10E-12	-0.73	11	1.1	Kazn;LOC100362622	
DMR5:161466001	5	161466001	161468000	2000	1	2.50E-09	-0.39	48	2.4	Kazn	
DMR5:161475001	5	161475001	161476000	1000	1	2.70E-08	0.82	22	2.2	Kazn	
DMR5:161518001	5	161518001	161519000	1000	1	1.10E-11	-0.58	3	0.3	Kazn	
DMR5:161566001	5	161566001	161569000	3000	1	6.40E-09	0.54	65	2.17	Kazn	
DMR5:161604001	5	161604001	161606000	2000	1	4.80E-09	0.39	29	1.45	Kazn	
DMR5:161614001	5	161614001	161617000	3000	2	3.50E-18	0.82	62	2.07	Kazn	
DMR5:161618001	5	161618001	161621000	3000	1	1.90E-13	-0.66	40	1.33	Kazn	
DMR5:161645001	5	161645001	161648000	3000	1	7.40E-09	0.75	34	1.13	Kazn	
DMR5:161673001	5	161673001	161674000	1000	1	2.70E-11	0.54	24	2.4	Kazn	
DMR5:161675001	5	161675001	161677000	2000	1	2.60E-07	0.56	41	2.05	Kazn	
DMR5:161699001	5	161699001	161702000	3000	1	1.20E-10	-0.64	50	1.67	Kazn	
DMR5:161719001	5	161719001	161724000	5000	1	5.80E-08	0.36	68	1.36	Kazn;RGD1560231	
DMR5:161853001	5	161853001	161855000	2000	1	6.20E-08	-0.4	37	1.85	Prdm2	Transcription
DMR5:162155001	5	162155001	162159000	4000	1	9.50E-27	1.26	82	2.05	Pramef27	
DMR5:162311001	5	162311001	162315000	4000	1	6.20E-07	-0.46	48	1.2	Aadacl3	Metabolism
DMR5:162320001	5	162320001	162321000	1000	1	5.50E-07	-0.56	1	0.1	Aadacl3	Metabolism
DMR5:162378001	5	162378001	162379000	1000	1	1.30E-18	1.18	29	2.9	LOC691162;Pramef12	
DMR5:162412001	5	162412001	162414000	2000	1	1.50E-08	0.52	22	1.1	Pramef20;Pramef25	
DMR5:162755001	5	162755001	162757000	2000	1	3.30E-08	-0.35	31	1.55	RGD1559644	Metabolism
DMR5:162991001	5	162991001	162995000	4000	1	3.50E-07	-0.61	43	1.07	Vps13d	Transport
DMR5:163091001	5	163091001	163092000	1000	1	2.50E-07	-0.43	12	1.2	Vps13d	Transport
DMR5:163127001	5	163127001	163129000	2000	1	3.60E-14	0.78	51	2.55	Vps13d;LOC102556804;Tnfrsf1b	Transport;Receptor
DMR5:163174001	5	163174001	163176000	2000	2	1.10E-14	-0.52	32	1.6	Tnfrsf1b	Receptor
DMR5:163200001	5	163200001	163201000	1000	1	5.20E-07	-0.61	7	0.7	Tnfrsf8	Receptor
DMR5:163212001	5	163212001	163215000	3000	1	8.60E-08	0.43	36	1.2	Tnfrsf8	Receptor
DMR5:163217001	5	163217001	163220000	3000	1	3.80E-07	0.41	35	1.17	Tnfrsf8	Receptor
DMR5:164800001	5	164800001	164802000	2000	1	5.30E-09	-0.53	24	1.2	Nppb;Nppa;Clcn6	Transport
DMR5:164845001	5	164845001	164848000	3000	1	7.90E-07	-0.4	68	2.27	Clcn6;Mthfr	Transport;Metabolism
DMR5:164870001	5	164870001	164876000	6000	1	1.00E-11	-0.42	86	1.43	Mthfr;LOC103692509;LOC102551309	Metabolism
DMR5:164877001	5	164877001	164880000	3000	1	5.50E-08	0.48	53	1.77	LOC102551309;Agtrap	
DMR5:164930001	5	164930001	164933000	3000	3	1.20E-15	0.87	60	2	Draxin;LOC108351058	
DMR5:164987001	5	164987001	164990000	3000	1	9.60E-07	-0.32	58	1.93	Fbxo2	
DMR5:165402001	5	165402001	165404000	2000	1	1.40E-08	0.57	22	1.1	Exosc10;Srm	
DMR5:165690001	5	165690001	165692000	2000	1	3.70E-10	-0.5	42	2.1	Casz1	Transcription
DMR5:165697001	5	165697001	165699000	2000	1	1.60E-10	0.56	46	2.3	Casz1	Transcription
DMR5:165775001	5	165775001	165776000	1000	1	4.90E-07	0.61	27	2.7	Casz1;Pex14	Transcription;Transport
DMR5:165828001	5	165828001	165829000	1000	1	6.30E-08	-0.42	16	1.6	Pex14	Transport
DMR5:165897001	5	165897001	165898000	1000	1	3.50E-09	-0.34	19	1.9	Pex14	Transport
DMR5:165942001	5	165942001	165944000	2000	1	3.00E-08	-0.41	47	2.35	Dffa;Cort;Apitd1	Signaling
DMR5:165946001	5	165946001	165947000	1000	1	2.20E-08	-0.39	31	3.1	Cort;Apitd1	Signaling
DMR5:165957001	5	165957001	165959000	2000	2	7.50E-13	0.8	52	2.6	Apitd1;Pgd	Metabolism
DMR5:165999001	5	165999001	1.66E+08	1000	1	7.30E-09	0.58	18	1.8	Kif1b	Cytoskeleton
DMR5:166036001	5	166036001	166038000	2000	1	6.30E-07	-0.37	48	2.4	Kif1b	Cytoskeleton
DMR5:166236001	5	166236001	166237000	1000	1	2.50E-07	-0.53	20	2	Ube4b	Proteolysis
DMR5:166285001	5	166285001	166286000	1000	1	1.60E-08	0.4	23	2.3	LOC103692512;Rbp7;LOC691196	Metabolism
DMR5:166409001	5	166409001	166410000	1000	1	1.80E-08	-0.36	23	2.3	Nmnat1	Metabolism
DMR5:166423001	5	166423001	166425000	2000	2	9.00E-09	0.6	61	3.05	Nmnat1;LOC103692520;Lzic	Metabolism
DMR5:166448001	5	166448001	166450000	2000	1	8.90E-07	-0.33	37	1.85	Lzic	

DMR5:166507001	5	166507001	166509000	2000	1	2.80E-08	0.34	20	1	Ctnnbip1	
DMR5:166519001	5	166519001	166522000	3000	1	1.10E-11	0.56	41	1.37	Ctnnbip1	
DMR5:166550001	5	166550001	166554000	4000	1	2.50E-08	-0.39	91	2.28	Clstn1	Transport
DMR5:166608001	5	166608001	166611000	3000	1	7.60E-14	0.97	76	2.53	Clstn1;Pik3cd	Transport;Signaling
DMR5:166659001	5	166659001	166665000	6000	2	1.70E-14	0.95	71	1.18	Tmem201	
DMR5:166672001	5	166672001	166675000	3000	1	4.30E-11	0.65	48	1.6	Tmem201	
DMR5:166691001	5	166691001	166693000	2000	1	4.80E-08	0.52	31	1.55	Tmem201;Slc25a33	Transport
DMR5:166735001	5	166735001	166740000	5000	1	8.00E-07	0.55	88	1.76	Slc25a33;LOC102548220	Transport
DMR5:166924001	5	166924001	166927000	3000	1	4.20E-07	-0.43	55	1.83	Spsb1	
DMR5:166981001	5	166981001	166985000	4000	1	2.10E-11	0.83	80	2	Spsb1;LOC108351061;H6pd	Metabolism
DMR5:167082001	5	167082001	167085000	3000	2	4.20E-13	0.59	48	1.6	LOC103692514;Mir34a	
DMR5:167175001	5	167175001	167177000	2000	1	2.40E-07	0.4	39	1.95	Slc2a5;LOC103692515	
DMR5:167202001	5	167202001	167203000	1000	1	1.60E-09	0.59	26	2.6	Slc2a7	
DMR5:167238001	5	167238001	167241000	3000	1	3.70E-11	0.56	56	1.87	Car6	
DMR5:167249001	5	167249001	167251000	2000	1	3.70E-12	0.48	48	2.4	Car6	
DMR5:167345001	5	167345001	167347000	2000	1	5.50E-07	-0.42	13	0.65	Rere;LOC103692516	
DMR5:167380001	5	167380001	167382000	2000	1	5.80E-10	-0.54	29	1.45	Rere;LOC108351064	
DMR5:167391001	5	167391001	167395000	4000	2	3.70E-13	-0.45	64	1.6	Rere;Mir6332	
DMR5:167429001	5	167429001	167430000	1000	1	7.70E-07	-0.38	21	2.1	Rere	
DMR5:167453001	5	167453001	167459000	6000	1	4.60E-10	-0.42	77	1.28	Rere	
DMR5:167525001	5	167525001	167527000	2000	1	7.80E-07	-0.33	42	2.1	Rere	
DMR5:167553001	5	167553001	167555000	2000	1	2.90E-10	-0.37	26	1.3	Rere;LOC108351072	
DMR5:167688001	5	167688001	167689000	1000	1	6.50E-09	0.52	18	1.8	Slc45a1	Transport
DMR5:167701001	5	167701001	167703000	2000	1	7.40E-08	0.28	37	1.85	Slc45a1	Transport
DMR5:167947001	5	167947001	167950000	3000	1	4.50E-09	-0.49	42	1.4	LOC102556220;Errfi1	
DMR5:167970001	5	167970001	167971000	1000	1	3.50E-07	0.29	15	1.5	Errfi1;LOC102556316	
DMR5:168028001	5	168028001	168030000	2000	2	2.40E-13	0.86	53	2.65	Tnfrsf9	Receptor
DMR5:168032001	5	168032001	168034000	2000	1	1.70E-07	-0.42	27	1.35	Tnfrsf9	Receptor
DMR5:168118001	5	168118001	168122000	4000	2	1.40E-07	-0.37	103	2.58	Per3;Vamp3	Transcription
DMR5:168148001	5	168148001	168149000	1000	1	9.50E-11	-0.68	10	1	Camta1	Transcription
DMR5:168173001	5	168173001	168175000	2000	1	9.90E-08	0.39	36	1.8	Camta1	Transcription
DMR5:168178001	5	168178001	168183000	5000	2	4.00E-15	0.61	119	2.38	Camta1	Transcription
DMR5:168292001	5	168292001	168293000	1000	1	8.40E-09	0.63	19	1.9	Camta1	Transcription
DMR5:168357001	5	168357001	168358000	1000	1	9.40E-07	0.36	17	1.7	Camta1	Transcription
DMR5:168382001	5	168382001	168386000	4000	1	9.70E-07	-0.38	81	2.02	Camta1	Transcription
DMR5:168401001	5	168401001	168405000	4000	1	5.20E-08	0.44	55	1.38	Camta1	Transcription
DMR5:168409001	5	168409001	168412000	3000	2	8.40E-16	1.04	59	1.97	Camta1	Transcription
DMR5:168431001	5	168431001	168432000	1000	1	5.00E-10	0.76	26	2.6	Camta1	Transcription
DMR5:168437001	5	168437001	168439000	2000	1	1.00E-07	0.33	36	1.8	Camta1	Transcription
DMR5:168442001	5	168442001	168445000	3000	1	1.20E-07	0.49	50	1.67	Camta1	Transcription
DMR5:168452001	5	168452001	168457000	5000	1	3.10E-10	0.72	118	2.36	Camta1	Transcription
DMR5:168471001	5	168471001	168472000	1000	1	1.30E-07	0.45	16	1.6	Camta1;LOC102546456	Transcription
DMR5:168474001	5	168474001	168475000	1000	1	4.40E-08	-0.39	56	5.6	Camta1;LOC102546456	Transcription
DMR5:168517001	5	168517001	168518000	1000	1	9.00E-10	0.79	34	3.4	Camta1	Transcription
DMR5:168542001	5	168542001	168544000	2000	2	1.80E-19	1.11	65	3.25	Camta1;LOC102546564	Transcription
DMR5:168545001	5	168545001	168549000	4000	1	6.40E-07	0.38	96	2.4	Camta1;LOC102546564	Transcription
DMR5:168552001	5	168552001	168553000	1000	1	1.70E-07	0.6	11	1.1	Camta1;LOC102546564	Transcription
DMR5:168555001	5	168555001	168558000	3000	1	3.70E-12	0.94	81	2.7	Camta1;LOC102546564	Transcription
DMR5:168569001	5	168569001	168570000	1000	1	7.00E-09	0.39	16	1.6	Camta1	Transcription
DMR5:168607001	5	168607001	168608000	1000	1	9.70E-07	0.53	34	3.4	Camta1	Transcription
DMR5:168666001	5	168666001	168668000	2000	2	4.60E-14	0.69	55	2.75	Camta1	Transcription
DMR5:168670001	5	168670001	168671000	1000	1	5.30E-21	1.12	36	3.6	Camta1	Transcription
DMR5:168732001	5	168732001	168734000	2000	2	2.40E-14	0.92	61	3.05	Camta1	Transcription
DMR5:168738001	5	168738001	168740000	2000	1	3.70E-08	0.35	29	1.45	Camta1	Transcription
DMR5:168786001	5	168786001	168788000	2000	1	5.50E-07	0.38	42	2.1	Camta1	Transcription
DMR5:168791001	5	168791001	168792000	1000	1	7.60E-10	0.7	32	3.2	Camta1	Transcription
DMR5:168793001	5	168793001	168795000	2000	2	9.30E-13	0.8	39	1.95	Camta1	Transcription
DMR5:168796001	5	168796001	168798000	2000	1	3.50E-07	0.35	24	1.2	Camta1	Transcription
DMR5:168829001	5	168829001	168831000	2000	1	1.80E-12	0.71	31	1.55	Camta1	Transcription
DMR5:168837001	5	168837001	168839000	2000	1	1.00E-10	0.71	49	2.45	Camta1;LOC102546752	Transcription
DMR5:168853001	5	168853001	168855000	2000	1	2.60E-07	0.38	60	3	Camta1;LOC102546752	Transcription
DMR5:168873001	5	168873001	168875000	2000	1	1.00E-08	0.71	59	2.95	Camta1	Transcription

DMR5:168880001	5	168880001	168884000	4000	1	9.60E-15	0.78	75	1.88	Camta1	Transcription
DMR5:168895001	5	168895001	168896000	1000	1	4.60E-27	1.32	29	2.9	Camta1	Transcription
DMR5:169249001	5	169249001	169250000	1000	1	8.50E-15	0.86	29	2.9	Plekhg5	
DMR5:169280001	5	169280001	169281000	1000	1	5.20E-07	0.54	40	4	Plekhg5;Tnfrsf25	Receptor
DMR5:169345001	5	169345001	169347000	2000	1	8.20E-10	0.51	42	2.1	Hes2	Transcription
DMR5:169397001	5	169397001	169398000	1000	1	2.00E-09	0.65	11	1.1	Acot7	Metabolism
DMR5:169427001	5	169427001	169429000	2000	1	7.90E-07	0.45	23	1.15	Acot7	Metabolism
DMR5:169432001	5	169432001	169434000	2000	1	1.70E-07	0.63	28	1.4	Acot7	Metabolism
DMR5:169497001	5	169497001	169499000	2000	1	4.10E-07	0.56	57	2.85	Rnf207;Rpl22	Translation
DMR5:169529001	5	169529001	169533000	4000	1	5.30E-11	-0.46	73	1.82	Chd5	
DMR5:169597001	5	169597001	169598000	1000	1	2.10E-07	0.55	10	1	Kcnab2	
DMR5:169615001	5	169615001	169622000	7000	1	4.90E-10	0.6	144	2.06	Kcnab2	
DMR5:169675001	5	169675001	169677000	2000	1	7.10E-12	-0.58	59	2.95	Nphp4	
DMR5:169722001	5	169722001	169724000	2000	1	9.40E-07	-0.55	39	1.95	Nphp4	
DMR5:170622001	5	170622001	170625000	3000	1	6.00E-08	-0.4	59	1.97	Ajap1	
DMR5:170635001	5	170635001	170636000	1000	1	5.10E-07	0.53	11	1.1	Ajap1	
DMR5:170641001	5	170641001	170643000	2000	1	2.30E-07	-0.44	30	1.5	Ajap1	
DMR5:170680001	5	170680001	170686000	6000	1	1.40E-08	0.55	134	2.23	Ajap1	
DMR5:171345001	5	171345001	171347000	2000	1	7.60E-08	-0.32	58	2.9	LOC102551562;Tp73	Transcription
DMR5:171362001	5	171362001	171363000	1000	1	2.80E-08	0.67	14	1.4	LOC102551562;Tp73	Transcription
DMR5:171635001	5	171635001	171637000	2000	1	2.40E-10	0.6	33	1.65	Arhgef16	Transcription
DMR5:171661001	5	171661001	171665000	4000	1	2.40E-07	-0.37	94	2.35	Prdm16	Transcription
DMR5:171670001	5	171670001	171672000	2000	1	1.40E-08	0.4	19	0.95	Prdm16	Transcription
DMR5:171673001	5	171673001	171674000	1000	1	1.20E-18	0.95	38	3.8	Prdm16	Transcription
DMR5:171675001	5	171675001	171677000	2000	1	6.20E-08	0.45	40	2	Prdm16	Transcription
DMR5:171715001	5	171715001	171718000	3000	1	8.10E-07	0.4	52	1.73	Prdm16	Transcription
DMR5:172087001	5	172087001	172088000	1000	1	1.10E-12	0.73	23	2.3	Actrt2	Cytoskeleton
DMR5:172251001	5	172251001	172253000	2000	1	1.40E-09	0.74	31	1.55	Ttc34	
DMR5:172340001	5	172340001	172344000	4000	1	1.60E-07	0.57	69	1.73	Tnfrsf14	Receptor
DMR5:172389001	5	172389001	172394000	5000	1	1.90E-10	0.58	111	2.22	Pank4;Plch2	Signaling;Metabolism
DMR5:172403001	5	172403001	172406000	3000	1	2.30E-08	0.41	82	2.73	Plch2	Metabolism
DMR5:172409001	5	172409001	172415000	6000	1	1.70E-07	0.45	110	1.83	Plch2	Metabolism
DMR5:172456001	5	172456001	172459000	3000	2	3.70E-09	-0.79	41	1.37	Plch2	Metabolism
DMR5:172514001	5	172514001	172517000	3000	1	7.50E-08	-0.38	38	1.27	Morn1;LOC108351067	Signaling
DMR5:172529001	5	172529001	172536000	7000	2	8.50E-11	0.6	117	1.67	Morn1;LOC108351067	Signaling
DMR5:172573001	5	172573001	172574000	1000	1	8.60E-07	-0.37	17	1.7	Ski	
DMR5:172579001	5	172579001	172581000	2000	1	3.20E-09	0.37	15	0.75	Ski	
DMR5:172606001	5	172606001	172608000	2000	1	3.60E-10	-0.48	36	1.8	Ski	
DMR5:172659001	5	172659001	172661000	2000	1	1.80E-07	-0.46	58	2.9	Faap20;Prkcz	Signaling
DMR5:172814001	5	172814001	172816000	2000	1	1.30E-09	0.5	24	1.2	Gabrd;Cfap74	Ion Channel
DMR5:172857001	5	172857001	172859000	2000	1	1.90E-08	-0.46	22	1.1	Cfap74	
DMR5:172868001	5	172868001	172870000	2000	1	5.10E-08	-0.28	19	0.95	Cfap74	
DMR5:172880001	5	172880001	172881000	1000	1	4.40E-08	0.52	16	1.6	Cfap74;Tmem52	
DMR5:172883001	5	172883001	172890000	7000	1	5.00E-10	0.4	134	1.91	Cfap74;Tmem52	
DMR5:172898001	5	172898001	172899000	1000	1	1.30E-14	0.82	31	3.1	Tmem52	
DMR5:173079001	5	173079001	173080000	1000	1	6.70E-08	0.6	20	2	Cdk11b;LOC108351073;Mmp23;Mib2	Signaling;Protease;Proteolysis
DMR5:173227001	5	173227001	173229000	2000	1	5.90E-07	-0.43	20	1	Vwa1;Tmem88b;Ankrd65	Extracellular Matrix
DMR5:173290001	5	173290001	173291000	1000	1	1.70E-13	1.07	55	5.5	Mxra8;Dvl1	Cytoskeleton
DMR5:173431001	5	173431001	173435000	4000	1	1.20E-07	-0.39	40	1	B3galt6;Sdf4	Golgi;Signaling
DMR5:173539001	5	173539001	173541000	2000	1	1.20E-08	-0.45	16	0.8	RGD1311517	
DMR5:173566001	5	173566001	173568000	2000	1	3.60E-07	0.43	38	1.9	RGD1311517;Rnf223	
DMR5:173648001	5	173648001	173649000	1000	1	1.80E-13	0.74	14	1.4	Perm1;Plekhn1;Klhl17	Cytoskeleton
DMR5:173655001	5	173655001	173659000	4000	2	8.70E-08	0.43	97	2.42	Perm1;Plekhn1;Klhl17;Noc2l	Cytoskeleton
DMR5:173687001	5	173687001	173689000	2000	1	3.70E-10	0.55	26	1.3	Samd11	Epigenetic
DMR5:173698001	5	173698001	173700000	2000	1	6.10E-07	0.45	17	0.85	Samd11	Epigenetic
DMR6:780001	6	780001	784000	4000	2	5.10E-15	-0.6	41	1.02	Crim1	
DMR6:785001	6	785001	786000	1000	1	6.00E-09	-0.46	16	1.6	Crim1	
DMR6:830001	6	830001	834000	4000	2	4.20E-13	-0.54	74	1.85	Crim1	
DMR6:879001	6	879001	882000	3000	1	1.40E-07	0.55	55	1.83	Crim1	
DMR6:897001	6	897001	903000	6000	1	1.20E-07	0.44	113	1.88	Crim1	
DMR6:1103001	6	1103001	1109000	6000	2	6.50E-10	0.35	96	1.6	Vit	Extracellular Matrix

DMR6:1147001	6	1147001	1154000	7000	2	3.90E-09	-0.59	88	1.26	Vit	Extracellular Matrix
DMR6:1214001	6	1214001	1217000	3000	1	6.90E-11	0.39	31	1.03	Vit	Extracellular Matrix
DMR6:1265001	6	1265001	1266000	1000	1	9.70E-08	-0.42	12	1.2	Strn	
DMR6:1290001	6	1290001	1292000	2000	1	3.10E-08	-0.48	20	1	Strn	
DMR6:1307001	6	1307001	1311000	4000	1	1.10E-08	-0.42	43	1.07	Strn	
DMR6:1386001	6	1386001	1388000	2000	1	3.00E-07	-0.45	19	0.95	Heatr5b	
DMR6:1493001	6	1493001	1494000	1000	1	7.40E-16	0.95	29	2.9	Sult6b1	Transport
DMR6:1531001	6	1531001	1534000	3000	1	1.40E-12	-0.54	66	2.2	Cebpz;Ndufaf7	Transcription
DMR6:1536001	6	1536001	1537000	1000	1	7.60E-07	-0.31	15	1.5	Cebpz;Ndufaf7;Prkd3	Transcription;Signaling
DMR6:1649001	6	1649001	1650000	1000	1	1.40E-07	-0.5	3	0.3	Qpct	Transport
DMR6:1660001	6	1660001	1661000	1000	1	2.50E-10	-0.5	4	0.4	Qpct	Transport
DMR6:1664001	6	1664001	1665000	1000	1	2.60E-13	0.91	19	1.9	Qpct	Transport
DMR6:1685001	6	1685001	1687000	2000	1	4.70E-08	-0.45	33	1.65	Qpct	Transport
DMR6:2231001	6	2231001	2234000	3000	2	8.60E-08	0.51	62	2.07	Rmdn2	
DMR6:2560001	6	2560001	2561000	1000	1	7.20E-08	-0.33	14	1.4	Atl2	Signaling
DMR6:2618001	6	2618001	2620000	2000	1	3.60E-11	0.9	48	2.4	Atl2	Signaling
DMR6:2916001	6	2916001	2917000	1000	1	9.70E-12	0.78	41	4.1	Gemin6;Dhx57	Transcription
DMR6:2991001	6	2991001	2992000	1000	1	7.60E-07	0.51	18	1.8	LOC500607;Arhgef33	Transcription
DMR6:3020001	6	3020001	3022000	2000	1	6.40E-07	-0.35	29	1.45	Arhgef33	Transcription
DMR6:3125001	6	3125001	3128000	3000	1	7.00E-07	-0.5	46	1.53	Sos1	Transcription
DMR6:3237001	6	3237001	3240000	3000	1	3.10E-07	0.67	29	0.97	Cdkl4	Signaling
DMR6:3263001	6	3263001	3266000	3000	1	5.90E-07	-0.46	37	1.23	Cdkl4	Signaling
DMR6:3269001	6	3269001	3271000	2000	2	1.10E-08	0.94	45	2.25	Cdkl4;Map4k3	Signaling
DMR6:3281001	6	3281001	3283000	2000	1	4.10E-09	-0.45	39	1.95	Cdkl4;Map4k3	Signaling
DMR6:3414001	6	3414001	3417000	3000	1	2.20E-08	-0.29	25	0.83	Map4k3	
DMR6:3661001	6	3661001	3662000	1000	1	1.40E-19	0.97	38	3.8	Tmem178a	
DMR6:3685001	6	3685001	3688000	3000	1	2.80E-14	-0.61	53	1.77	Tmem178a	
DMR6:3689001	6	3689001	3691000	2000	1	4.00E-08	-0.44	36	1.8	Tmem178a	
DMR6:3715001	6	3715001	3716000	1000	1	5.40E-11	-0.46	25	2.5	Tmem178a	
DMR6:3760001	6	3760001	3761000	1000	1	4.90E-09	-0.31	10	1	Thumpd2	Epigenetic
DMR6:4296001	6	4296001	4297000	1000	1	2.50E-07	0.27	5	0.5	Slc8a1	Transport
DMR6:4362001	6	4362001	4365000	3000	1	1.40E-09	0.45	48	1.6	Slc8a1	Transport
DMR6:4419001	6	4419001	4421000	2000	1	2.80E-08	-0.55	36	1.8	Slc8a1	Transport
DMR6:4558001	6	4558001	4560000	2000	1	1.10E-07	-0.35	25	1.25	Slc8a1	Transport
DMR6:6710001	6	6710001	6711000	1000	1	8.20E-07	-0.59	23	2.3	Cox7a2l;LOC108351165	Metabolism
DMR6:6937001	6	6937001	6938000	1000	1	2.40E-07	-0.36	20	2	Mta3	Development
DMR6:6968001	6	6968001	6969000	1000	1	8.50E-07	0.31	24	2.4	Mta3	Development
DMR6:7004001	6	7004001	7007000	3000	2	1.20E-14	1.06	86	2.87	Mta3	Development
DMR6:7011001	6	7011001	7013000	2000	1	2.80E-08	-0.44	35	1.75	Mta3	Development
DMR6:7015001	6	7015001	7019000	4000	1	5.80E-11	-0.62	86	2.15	Mta3	Development
DMR6:7047001	6	7047001	7051000	4000	1	1.40E-09	0.61	59	1.48	Haao	Metabolism
DMR6:7056001	6	7056001	7058000	2000	1	8.00E-18	0.88	51	2.55	Haao	Metabolism
DMR6:7612001	6	7612001	7614000	2000	1	7.20E-12	-0.53	26	1.3	Thada	Cytoskeleton
DMR6:7627001	6	7627001	7628000	1000	1	6.30E-07	-0.38	10	1	Thada	Cytoskeleton
DMR6:7639001	6	7639001	7645000	6000	1	3.60E-08	-0.27	76	1.27	Thada	Cytoskeleton
DMR6:7836001	6	7836001	7837000	1000	1	4.70E-09	0.58	16	1.6	Plekhh2	
DMR6:7843001	6	7843001	7844000	1000	1	5.20E-07	0.56	21	2.1	Plekhh2	
DMR6:7850001	6	7850001	7855000	5000	1	7.30E-07	-0.36	68	1.36	Plekhh2	
DMR6:7856001	6	7856001	7859000	3000	2	1.60E-09	-0.57	60	2	Plekhh2	
DMR6:7962001	6	7962001	7964000	2000	1	1.40E-10	0.66	28	1.4	Abcg5;Abcg8	Transport
DMR6:7969001	6	7969001	7972000	3000	1	1.10E-12	0.64	72	2.4	Abcg5;Abcg8	Transport
DMR6:8225001	6	8225001	8226000	1000	1	2.30E-07	-0.46	8	0.8	Ppm1b	
DMR6:8249001	6	8249001	8251000	2000	1	2.10E-08	-0.46	28	1.4	Ppm1b	
DMR6:8298001	6	8298001	8299000	1000	1	1.50E-11	0.82	30	3	Slc3a1	Metabolism
DMR6:8317001	6	8317001	8318000	1000	1	1.10E-07	-0.36	26	2.6	Slc3a1;Prepl	Metabolism;Protease
DMR6:8328001	6	8328001	8329000	1000	1	3.10E-09	0.47	11	1.1	Slc3a1;Prepl;LOC103692550	Metabolism;Protease
DMR6:8377001	6	8377001	8380000	3000	1	7.70E-07	-0.38	28	0.93	Camkmt	Golgi
DMR6:8471001	6	8471001	8473000	2000	1	3.40E-12	-0.55	25	1.25	Camkmt	Golgi
DMR6:8605001	6	8605001	8606000	1000	1	8.60E-08	-0.4	22	2.2	Camkmt	Golgi
DMR6:8698001	6	8698001	8699000	1000	1	2.40E-08	-0.41	18	1.8	Camkmt	Golgi
DMR6:8737001	6	8737001	8738000	1000	1	3.30E-07	0.5	13	1.3	Camkmt	Golgi
DMR6:8739001	6	8739001	8742000	3000	1	1.50E-09	0.63	34	1.13	Camkmt	Golgi

DMR6:8875001	6	8875001	8877000	2000	1	1.40E-13	-0.74	45	2.25	LOC108351167;Six3	Development
DMR6:9374001	6	9374001	9376000	2000	1	2.80E-07	0.38	24	1.2	Srbd1	Translation
DMR6:9487001	6	9487001	9488000	1000	1	2.40E-07	-0.42	16	1.6	Prkce	Signaling
DMR6:9490001	6	9490001	9492000	2000	1	2.40E-07	-0.35	42	2.1	Prkce	Signaling
DMR6:9528001	6	9528001	9529000	1000	1	2.90E-07	-0.34	30	3	Prkce	Signaling
DMR6:9533001	6	9533001	9534000	1000	1	9.20E-08	-0.44	15	1.5	Prkce	Signaling
DMR6:9560001	6	9560001	9561000	1000	1	2.70E-08	0.34	6	0.6	Prkce	Signaling
DMR6:9590001	6	9590001	9592000	2000	1	2.70E-08	0.53	21	1.05	Prkce	Signaling
DMR6:9613001	6	9613001	9614000	1000	1	3.50E-10	-0.57	20	2	Prkce;LOC102551687	Signaling
DMR6:9623001	6	9623001	9631000	8000	2	7.60E-08	-0.41	139	1.74	Prkce;LOC102551687	Signaling
DMR6:9757001	6	9757001	9761000	4000	1	2.00E-07	-0.4	90	2.25	Prkce;RGD1562146	Signaling
DMR6:9781001	6	9781001	9783000	2000	1	5.00E-09	-0.36	45	2.25	Prkce	Signaling
DMR6:9790001	6	9790001	9793000	3000	1	5.60E-12	-0.46	35	1.17	Prkce	Signaling
DMR6:9798001	6	9798001	9802000	4000	1	5.30E-09	-0.36	67	1.68	Prkce	Signaling
DMR6:9838001	6	9838001	9839000	1000	1	1.10E-10	0.32	17	1.7	Prkce	Signaling
DMR6:9874001	6	9874001	9876000	2000	1	1.60E-07	-0.4	26	1.3	Prkce	Signaling
DMR6:9939001	6	9939001	9942000	3000	1	6.70E-07	0.58	52	1.73	Prkce	Signaling
DMR6:10302001	6	10302001	10305000	3000	1	3.80E-08	0.45	36	1.2	Epas1	Transcription
DMR6:10386001	6	10386001	10388000	2000	1	2.20E-07	-0.37	57	2.85	Epas1	Transcription
DMR6:10483001	6	10483001	10485000	2000	1	3.90E-19	1.07	46	2.3	Tmem247	
DMR6:10513001	6	10513001	10514000	1000	1	3.80E-07	-0.55	10	1	Atp6v1e2	
DMR6:10544001	6	10544001	10545000	1000	1	1.60E-07	-0.41	30	3	Rhoq	Signaling
DMR6:10667001	6	10667001	10668000	1000	1	1.60E-11	-0.6	21	2.1	LOC103692551;Socs5	Signaling
DMR6:10710001	6	10710001	10711000	1000	1	1.90E-07	0.42	10	1	Socs5	Signaling
DMR6:10875001	6	10875001	10881000	6000	2	2.40E-09	0.54	99	1.65	Mcfd2;LOC103692553	Transport
DMR6:10885001	6	10885001	10886000	1000	1	2.20E-07	0.37	11	1.1	Mcfd2;LOC103692553	Transport
DMR6:10917001	6	10917001	10919000	2000	1	3.30E-15	0.9	56	2.8	Ttc7a	
DMR6:10951001	6	10951001	10952000	1000	1	2.50E-08	0.47	15	1.5	Ttc7a	
DMR6:11311001	6	11311001	11317000	6000	2	1.60E-09	0.51	96	1.6	Epcam	
DMR6:11361001	6	11361001	11364000	3000	2	6.40E-19	1.17	131	4.37	Kcnk12	Transport
DMR6:11376001	6	11376001	11377000	1000	1	1.60E-22	1.24	23	2.3	Kcnk12	Transport
DMR6:11420001	6	11420001	11422000	2000	1	3.30E-07	0.38	42	2.1	Kcnk12;LOC103689957	Transport
DMR6:11500001	6	11500001	11501000	1000	1	8.10E-07	0.41	14	1.4	Kcnk12	Transport
DMR6:11686001	6	11686001	11688000	2000	1	6.60E-08	-0.43	33	1.65	Fbxo11	Proteolysis
DMR6:11703001	6	11703001	11704000	1000	1	6.40E-13	-0.57	14	1.4	Fbxo11	Proteolysis
DMR6:12322001	6	12322001	12324000	2000	2	6.40E-08	0.55	34	1.7	Ppp1r21;LOC103692558;Ston1	Transport
DMR6:12406001	6	12406001	12411000	5000	1	3.40E-07	-0.38	34	0.68	Gtf2a1l	Transcription
DMR6:12415001	6	12415001	12416000	1000	1	5.50E-11	0.74	36	3.6	Gtf2a1l	Transcription
DMR6:12442001	6	12442001	12443000	1000	1	6.90E-08	0.6	26	2.6	Gtf2a1l;LOC102549561	Transcription
DMR6:12461001	6	12461001	12462000	1000	1	3.80E-09	-0.56	3	0.3	Gtf2a1l	Transcription
DMR6:12915001	6	12915001	12916000	1000	1	5.90E-07	-0.64	0	0	Fshr	Signaling
DMR6:12982001	6	12982001	12983000	1000	1	8.10E-07	0.27	22	2.2	Fshr	Signaling
DMR6:13886001	6	13886001	13888000	2000	1	6.20E-11	0.76	37	1.85	Nrxn1	
DMR6:14388001	6	14388001	14390000	2000	1	1.30E-14	0.93	26	1.3	Nrxn1	
DMR6:14487001	6	14487001	14490000	3000	1	5.10E-09	0.6	41	1.37	Nrxn1;LOC102550556	
DMR6:14717001	6	14717001	14718000	1000	1	4.50E-15	1.12	29	2.9	Nrxn1	
DMR6:14756001	6	14756001	14757000	1000	1	5.90E-09	0.58	13	1.3	Nrxn1;LOC103692561	
DMR6:14854001	6	14854001	14856000	2000	1	6.80E-07	-0.34	17	0.85	Nrxn1	
DMR6:14890001	6	14890001	14892000	2000	1	5.10E-08	-0.48	18	0.9	Nrxn1	
DMR6:14957001	6	14957001	14959000	2000	1	1.40E-10	0.44	17	0.85	Nrxn1	
DMR6:15136001	6	15136001	15137000	1000	1	5.70E-09	-0.63	0	0	Nrxn1	
DMR6:18349001	6	18349001	18351000	2000	1	1.30E-14	0.97	42	2.1	Crnkl1;Crnkl1-ps1	Translation
DMR6:18835001	6	18835001	18839000	4000	2	8.10E-09	0.72	138	3.45	Mtm1	Signaling
DMR6:21041001	6	21041001	21044000	3000	1	1.50E-11	-0.52	49	1.63	Fam98a	Translation
DMR6:21243001	6	21243001	21244000	1000	1	3.50E-07	0.47	7	0.7	Ltbp1	Extracellular Matrix
DMR6:21254001	6	21254001	21256000	2000	1	2.40E-07	-0.41	15	0.75	Ltbp1	Extracellular Matrix
DMR6:21304001	6	21304001	21305000	1000	1	3.40E-07	-0.59	4	0.4	Ltbp1	Extracellular Matrix
DMR6:21430001	6	21430001	21432000	2000	1	1.20E-07	-0.61	11	0.55	Ltbp1	Extracellular Matrix
DMR6:21492001	6	21492001	21494000	2000	1	8.60E-07	0.4	13	0.65	Ltbp1	Extracellular Matrix
DMR6:21551001	6	21551001	21552000	1000	1	2.90E-15	0.79	29	2.9	Ltbp1	Extracellular Matrix
DMR6:21555001	6	21555001	21557000	2000	1	5.90E-07	0.36	16	0.8	Ltbp1	Extracellular Matrix
DMR6:21587001	6	21587001	21596000	9000	1	9.60E-07	-0.4	152	1.69	Ltbp1	Extracellular Matrix

DMR6:21608001	6	21608001	21610000	2000	1	6.10E-13	0.9	47	2.35	Ltbp1	Extracellular Matrix
DMR6:21731001	6	21731001	21733000	2000	1	1.00E-13	-0.57	42	2.1	Ttc27	
DMR6:21803001	6	21803001	21806000	3000	1	9.90E-09	-0.39	65	2.17	Ttc27;LOC108351178	
DMR6:21832001	6	21832001	21833000	1000	1	5.10E-08	0.36	9	0.9	Ttc27	
DMR6:21894001	6	21894001	21896000	2000	1	1.50E-07	-0.46	20	1	Birc6	
DMR6:22156001	6	22156001	22158000	2000	1	3.30E-07	0.66	27	1.35	Nlrc4	
DMR6:22170001	6	22170001	22172000	2000	1	7.80E-07	0.51	36	1.8	Nlrc4	
DMR6:22696001	6	22696001	22697000	1000	1	2.30E-07	0.53	16	1.6	Alk	Receptor
DMR6:22738001	6	22738001	22740000	2000	1	1.80E-11	-0.47	15	0.75	Alk	Receptor
DMR6:22817001	6	22817001	22818000	1000	1	2.70E-07	0.41	8	0.8	Alk	Receptor
DMR6:22941001	6	22941001	22944000	3000	1	4.70E-07	0.38	35	1.17	Alk	Receptor
DMR6:22945001	6	22945001	22946000	1000	1	4.40E-07	0.39	8	0.8	Alk	Receptor
DMR6:22984001	6	22984001	22985000	1000	1	4.90E-09	-0.56	2	0.2	Alk;LOC108351180	Receptor
DMR6:23030001	6	23030001	23031000	1000	1	2.90E-07	-0.45	6	0.6	Alk	Receptor
DMR6:23045001	6	23045001	23047000	2000	1	3.10E-16	1.02	37	1.85	Alk	Receptor
DMR6:23066001	6	23066001	23067000	1000	1	7.00E-07	-0.36	15	1.5	Alk	Receptor
DMR6:23163001	6	23163001	23168000	5000	1	1.00E-06	0.61	69	1.38	Alk	Receptor
DMR6:23199001	6	23199001	23202000	3000	2	1.40E-15	0.9	65	2.17	Alk;LOC108351181	Receptor
DMR6:23209001	6	23209001	23210000	1000	1	1.40E-11	0.87	21	2.1	Alk;LOC108351181	Receptor
DMR6:23316001	6	23316001	23317000	1000	1	1.10E-07	0.55	30	3	Clip4	Transcription
DMR6:23379001	6	23379001	23380000	1000	1	4.00E-07	0.39	16	1.6	Fam179a	
DMR6:23390001	6	23390001	23391000	1000	1	1.10E-10	-0.63	6	0.6	Fam179a	
DMR6:23492001	6	23492001	23494000	2000	1	7.00E-07	-0.51	20	1	Trnac-gca;Spdya	
DMR6:24144001	6	24144001	24147000	3000	1	1.90E-09	-0.47	71	2.37	Lbh	
DMR6:24154001	6	24154001	24156000	2000	1	7.20E-08	0.38	30	1.5	Lbh	
DMR6:24165001	6	24165001	24168000	3000	1	1.80E-08	-0.41	64	2.13	Lbh	
DMR6:24181001	6	24181001	24182000	1000	1	1.50E-07	-0.35	22	2.2	Lbh	
DMR6:24386001	6	24386001	24389000	3000	1	4.80E-07	-0.28	34	1.13	Lclat1	Metabolism
DMR6:24440001	6	24440001	24441000	1000	1	1.90E-08	0.61	14	1.4	Lclat1	Metabolism
DMR6:24570001	6	24570001	24571000	1000	1	2.80E-07	-0.6	18	1.8	LOC685881;Capn13	Protease
DMR6:24577001	6	24577001	24581000	4000	2	3.10E-17	1.07	71	1.77	Capn13	Protease
DMR6:24638001	6	24638001	24641000	3000	1	3.20E-16	0.82	29	0.97	Capn13;LOC102554046	Protease
DMR6:24923001	6	24923001	24925000	2000	1	3.00E-12	0.73	28	1.4	Galnt14	Golgi
DMR6:24953001	6	24953001	24956000	3000	1	7.00E-08	-0.44	27	0.9	Galnt14	Golgi
DMR6:24991001	6	24991001	24992000	1000	1	4.70E-08	0.53	11	1.1	Galnt14	Golgi
DMR6:25180001	6	25180001	25181000	1000	1	5.90E-07	-0.38	25	2.5	Xdh	Metabolism
DMR6:25211001	6	25211001	25212000	1000	1	1.20E-07	-0.4	20	2	Xdh	Metabolism
DMR6:25364001	6	25364001	25367000	3000	1	2.00E-07	0.35	43	1.43	Plb1	Metabolism
DMR6:25490001	6	25490001	25494000	4000	1	1.30E-11	-0.65	66	1.65	Plb1	Metabolism
DMR6:25515001	6	25515001	25518000	3000	1	7.10E-09	-0.79	33	1.1	Plb1	Metabolism
DMR6:25659001	6	25659001	25660000	1000	1	2.10E-07	0.47	13	1.3	Bre	
DMR6:25886001	6	25886001	25887000	1000	1	1.90E-09	-0.5	25	2.5	Bre	
DMR6:25943001	6	25943001	25944000	1000	1	5.20E-08	-0.49	11	1.1	Bre	
DMR6:26101001	6	26101001	26104000	3000	1	4.10E-08	0.48	39	1.3	Bre;Rbks	Metabolism
DMR6:26117001	6	26117001	26119000	2000	1	2.10E-09	0.55	25	1.25	Bre;Rbks	Metabolism
DMR6:26222001	6	26222001	26223000	1000	1	3.90E-07	-0.34	26	2.6	Slc4a1ap	Translation
DMR6:26273001	6	26273001	26274000	1000	1	8.20E-08	0.51	21	2.1	Gpn1;RGD1560110	Signaling
DMR6:26286001	6	26286001	26287000	1000	1	6.40E-07	0.5	33	3.3	RGD1560110;Zfp512	
DMR6:26316001	6	26316001	26318000	2000	1	3.50E-07	-0.4	41	2.05	Zfp512;LOC102556504	
DMR6:26371001	6	26371001	26373000	2000	1	2.80E-07	0.64	40	2	Gckr	Signaling
DMR6:26626001	6	26626001	26628000	2000	1	4.10E-10	-0.42	25	1.25	Trim54;Dnajc5g;Slc30a3	Proteolysis;Transcription;Transport
DMR6:26768001	6	26768001	26770000	2000	1	1.40E-07	0.62	46	2.3	Tcf23	Transcription
DMR6:26785001	6	26785001	26786000	1000	1	4.30E-07	-0.38	19	1.9	Prr30;Preb;Abhd1	Transcription;Protease
DMR6:26837001	6	26837001	26838000	1000	1	6.20E-11	-0.54	14	1.4	Emilin1;LOC103692571;Ost4;Agbl5	Golgi;Protease
DMR6:26921001	6	26921001	26923000	2000	1	9.20E-14	0.81	41	2.05	Mapre3;LOC108351189;LOC108351188	Cytoskeleton
DMR6:27077001	6	27077001	27080000	3000	1	3.90E-08	0.47	49	1.63	LOC103692572;Cenpa	Epigenetic
DMR6:27082001	6	27082001	27084000	2000	1	6.10E-07	0.33	36	1.8	Cenpa	Epigenetic
DMR6:27099001	6	27099001	27101000	2000	1	3.90E-09	0.75	20	1	Slc35f6	
DMR6:27158001	6	27158001	27162000	4000	1	1.90E-07	0.31	45	1.12	Kcnk3	Transport

DMR6:27170001	6	27170001	27172000	2000	1	5.50E-07	0.49	21	1.05	Kcnk3	Transport
DMR6:27195001	6	27195001	27197000	2000	1	8.80E-07	0.48	31	1.55	Kcnk3	Transport
DMR6:27233001	6	27233001	27238000	5000	1	3.60E-09	0.46	77	1.54	Cib4	
DMR6:27246001	6	27246001	27247000	1000	1	5.40E-08	-0.43	9	0.9	Cib4	
DMR6:27275001	6	27275001	27277000	2000	1	3.80E-08	0.64	43	2.15	Cib4	
DMR6:27286001	6	27286001	27288000	2000	1	7.20E-08	0.49	29	1.45	Cib4	
DMR6:27302001	6	27302001	27306000	4000	1	4.30E-07	0.43	48	1.2	Cib4;RGD1559683	
DMR6:27339001	6	27339001	27340000	1000	1	4.20E-13	0.78	15	1.5	Otof	Transport
DMR6:27363001	6	27363001	27364000	1000	1	4.80E-09	0.61	19	1.9	Otof	Transport
DMR6:27401001	6	27401001	27407000	6000	1	2.70E-07	-0.29	123	2.05	Otof	Transport
DMR6:27408001	6	27408001	27409000	1000	1	9.80E-08	0.44	17	1.7	Otof	Transport
DMR6:27489001	6	27489001	27490000	1000	1	1.80E-10	-0.59	8	0.8	Seli	
DMR6:27632001	6	27632001	27633000	1000	1	2.00E-09	0.66	24	2.4	Hadha;Garem2	
DMR6:27653001	6	27653001	27656000	3000	1	8.90E-09	0.66	42	1.4	Garem2;LOC503104	
DMR6:27984001	6	27984001	27987000	3000	1	1.40E-07	-0.46	33	1.1	Dtnb	Proteolysis
DMR6:28086001	6	28086001	28089000	3000	1	1.60E-09	-0.39	42	1.4	Dtnb	Proteolysis
DMR6:28108001	6	28108001	28113000	5000	1	3.50E-07	0.36	65	1.3	Dtnb	Proteolysis
DMR6:28132001	6	28132001	28134000	2000	1	2.80E-09	-0.49	28	1.4	Dtnb	Proteolysis
DMR6:28404001	6	28404001	28409000	5000	1	2.10E-11	0.63	96	1.92	Efr3b	
DMR6:28437001	6	28437001	28440000	3000	2	5.20E-09	0.78	75	2.5	Efr3b	
DMR6:28474001	6	28474001	28475000	1000	1	1.20E-10	0.65	22	2.2	Efr3b	
DMR6:28553001	6	28553001	28557000	4000	1	9.50E-13	0.37	59	1.48	Dnajc27;LOC689056	
DMR6:28572001	6	28572001	28573000	1000	1	5.50E-12	0.79	42	4.2	Adcy3	
DMR6:28670001	6	28670001	28672000	2000	1	1.80E-09	-0.49	23	1.15	Cenpo;Ptrhd1;Ncoa1	Epigenetic
DMR6:28805001	6	28805001	28811000	6000	1	8.10E-07	-0.29	57	0.95	Ncoa1;LOC103692576	Epigenetic
DMR6:28825001	6	28825001	28828000	3000	1	5.80E-10	-0.41	24	0.8	Ncoa1;LOC103692576	Epigenetic
DMR6:28868001	6	28868001	28869000	1000	1	1.50E-08	-0.41	16	1.6	Ncoa1	Epigenetic
DMR6:28899001	6	28899001	28900000	1000	1	5.80E-11	-0.67	9	0.9	Ncoa1	Epigenetic
DMR6:28941001	6	28941001	28943000	2000	2	2.40E-12	0.61	39	1.95	Ncoa1	Epigenetic
DMR6:29200001	6	29200001	29203000	3000	1	4.90E-08	0.6	38	1.27	Klhl29	Cytoskeleton
DMR6:29269001	6	29269001	29271000	2000	1	4.40E-12	-0.47	25	1.25	Klhl29	Cytoskeleton
DMR6:29456001	6	29456001	29457000	1000	1	4.80E-08	0.47	7	0.7	Klhl29	Cytoskeleton
DMR6:29974001	6	29974001	29975000	1000	1	6.50E-07	-0.45	8	0.8	Wdcp;Fkbp1b	Transcription
DMR6:30053001	6	30053001	30055000	2000	1	4.30E-09	-0.52	17	0.85	Fam228b	
DMR6:33506001	6	33506001	33507000	1000	1	1.90E-07	0.7	21	2.1	Gdf7	Growth Factors
DMR6:33870001	6	33870001	33872000	2000	1	6.70E-07	0.54	44	2.2	Pum2	Metabolism
DMR6:34158001	6	34158001	34166000	8000	4	6.50E-13	-0.77	143	1.79	Wdr35;Ttc32	
DMR6:34171001	6	34171001	34172000	1000	1	1.80E-10	0.66	29	2.9	Ttc32	
DMR6:35316001	6	35316001	35317000	1000	1	5.90E-12	0.75	27	2.7	Osr1	Transcription
DMR6:35320001	6	35320001	35322000	2000	1	1.00E-12	0.99	72	3.6	Osr1	Transcription
DMR6:36101001	6	36101001	36102000	1000	1	8.70E-07	-0.4	7	0.7	LOC102547504;Nt5c1b;Rdh14	Metabolism
DMR6:36958001	6	36958001	36960000	2000	1	9.30E-08	-0.42	18	0.9	Smc6	Transcription
DMR6:36974001	6	36974001	36975000	1000	1	6.00E-07	-0.5	12	1.2	Smc6	Transcription
DMR6:38059001	6	38059001	38062000	3000	1	7.70E-10	-0.68	23	0.77	RGD1565679	
DMR6:38090001	6	38090001	38092000	2000	2	2.80E-11	-0.67	8	0.4	Trnav-uac	
DMR6:38213001	6	38213001	38215000	2000	1	5.70E-07	0.39	29	1.45	Mycn	Transcription
DMR6:38223001	6	38223001	38225000	2000	1	8.00E-09	-0.43	68	3.4	Mycn	Transcription
DMR6:38234001	6	38234001	38237000	3000	1	1.70E-07	0.59	53	1.77	Mycn;LOC103692611	Transcription
DMR6:38416001	6	38416001	38418000	2000	1	7.40E-08	0.47	28	1.4	Ddx1	
DMR6:38470001	6	38470001	38472000	2000	1	3.80E-08	-0.54	8	0.4	Nbas	
DMR6:38573001	6	38573001	38574000	1000	1	1.70E-10	-0.36	16	1.6	Nbas;LOC108351202	
DMR6:38575001	6	38575001	38577000	2000	1	4.70E-07	-0.35	17	0.85	Nbas;LOC108351202	
DMR6:38604001	6	38604001	38606000	2000	1	6.90E-09	-0.5	9	0.45	Nbas	
DMR6:41849001	6	41849001	41851000	2000	1	7.60E-07	0.41	25	1.25	Lpin1	
DMR6:41899001	6	41899001	41900000	1000	1	6.80E-08	-0.48	18	1.8	Lpin1	
DMR6:41965001	6	41965001	41969000	4000	1	8.70E-07	0.54	72	1.8	Greb1	
DMR6:42303001	6	42303001	42305000	2000	1	8.10E-11	0.62	25	1.25	Pqlc3;LOC690276	
DMR6:42570001	6	42570001	42571000	1000	1	8.90E-07	-0.42	15	1.5	Pdia6	Transcription
DMR6:42708001	6	42708001	42709000	1000	1	1.60E-12	-0.59	20	2	Nol10	
DMR6:42730001	6	42730001	42732000	2000	1	5.10E-07	-0.49	24	1.2	Nol10	
DMR6:42949001	6	42949001	42955000	6000	2	5.80E-08	0.45	77	1.28	LOC100909414;RGD1563157	
DMR6:43043001	6	43043001	43044000	1000	1	1.50E-10	-0.5	17	1.7	Hpcal1	

DMR6:43102001	6	43102001	43106000	4000	1	4.10E-08	0.43	73	1.82	Hpcal1;LOC108351210	
DMR6:43278001	6	43278001	43280000	2000	1	1.20E-11	-0.5	35	1.75	Asap2;LOC103692603	
DMR6:43282001	6	43282001	43284000	2000	1	4.40E-10	-0.41	35	1.75	Asap2;LOC103692603	
DMR6:43634001	6	43634001	43636000	2000	1	8.40E-12	-0.55	30	1.5	Taf1b	
DMR6:43754001	6	43754001	43755000	1000	1	3.90E-07	0.54	16	1.6	Grhl1	Transcription
DMR6:43785001	6	43785001	43786000	1000	1	9.00E-07	-0.4	19	1.9	Grhl1	Transcription
DMR6:43845001	6	43845001	43846000	1000	1	3.70E-07	0.47	17	1.7	Klf11;Cys1	Transcription
DMR6:44026001	6	44026001	44027000	1000	1	9.60E-08	-0.47	20	2	Mboat2	Metabolism
DMR6:44089001	6	44089001	44092000	3000	1	8.30E-08	0.55	30	1	Mboat2	Metabolism
DMR6:44106001	6	44106001	44108000	2000	1	3.20E-08	-0.44	37	1.85	Mboat2	Metabolism
DMR6:45590001	6	45590001	45593000	3000	1	4.50E-07	0.47	36	1.2	Rnf144a	Proteolysis
DMR6:45630001	6	45630001	45632000	2000	1	7.80E-08	0.52	39	1.95	Rnf144a;LOC108351212	Proteolysis
DMR6:47785001	6	47785001	47787000	2000	1	1.80E-08	0.42	14	0.7	Dcdc2c	
DMR6:47814001	6	47814001	47820000	6000	1	8.40E-08	-0.34	47	0.78	Dcdc2c;Allc	
DMR6:47832001	6	47832001	47833000	1000	1	1.10E-07	0.48	7	0.7	Allc	
DMR6:47906001	6	47906001	47908000	2000	1	4.20E-07	-0.56	9	0.45	Rps7;Rnaseh1	Translation;Translation
DMR6:48568001	6	48568001	48569000	1000	1	6.10E-12	-0.58	10	1	Myt1l;LOC103692607	Transcription
DMR6:48765001	6	48765001	48766000	1000	1	5.60E-10	0.66	26	2.6	Myt1l	Transcription
DMR6:48976001	6	48976001	48977000	1000	1	2.30E-08	0.57	6	0.6	Pxdn	Metabolism
DMR6:49060001	6	49060001	49061000	1000	1	1.20E-23	1.35	31	3.1	Tpo	Metabolism
DMR6:49159001	6	49159001	49160000	1000	1	7.10E-11	-0.65	10	1	Sntg2	
DMR6:49234001	6	49234001	49236000	2000	1	2.10E-09	-0.4	24	1.2	Sntg2	
DMR6:49264001	6	49264001	49266000	2000	1	1.70E-09	-0.42	15	0.75	Sntg2	
DMR6:49331001	6	49331001	49333000	2000	2	1.00E-07	-0.51	6	0.3	Sntg2	
DMR6:49528001	6	49528001	49529000	1000	1	1.40E-10	0.69	7	0.7	Tmem18	
DMR6:50518001	6	50518001	50521000	3000	1	1.10E-07	-0.38	52	1.73	Lamb1	Extracellular Matrix
DMR6:50620001	6	50620001	50621000	1000	1	1.60E-07	-0.6	2	0.2	Dld	Metabolism
DMR6:50747001	6	50747001	50749000	2000	1	1.20E-08	0.7	45	2.25	Slc26a3	Transport
DMR6:50802001	6	50802001	50803000	1000	1	2.30E-17	-0.69	18	1.8	LOC102555378;Slc26a4	Transport
DMR6:50902001	6	50902001	50904000	2000	2	4.10E-08	-0.35	20	1	Bcap29	Transport
DMR6:51480001	6	51480001	51481000	1000	1	9.00E-10	0.72	18	1.8	Pik3cg	Signaling
DMR6:51511001	6	51511001	51512000	1000	1	6.00E-07	0.31	12	1.2	Pik3cg	Signaling
DMR6:52574001	6	52574001	52575000	1000	1	2.70E-08	-0.34	17	1.7	Atxn7l1	
DMR6:52605001	6	52605001	52612000	7000	1	1.40E-12	-0.56	111	1.59	Atxn7l1;LOC102549100	
DMR6:53404001	6	53404001	53405000	1000	1	1.10E-08	-0.62	16	1.6	Twist1	Transcription
DMR6:53512001	6	53512001	53514000	2000	1	2.20E-08	0.43	19	0.95	Hdac9	
DMR6:53558001	6	53558001	53559000	1000	1	4.70E-07	-0.58	4	0.4	Hdac9	
DMR6:53670001	6	53670001	53672000	2000	1	8.90E-08	-0.39	16	0.8	Hdac9	
DMR6:53899001	6	53899001	53901000	2000	2	4.50E-17	-0.73	38	1.9	Hdac9	
DMR6:53980001	6	53980001	53982000	2000	1	5.70E-07	-0.49	20	1	Hdac9	
DMR6:54177001	6	54177001	54178000	1000	1	8.90E-08	-0.32	9	0.9	Hdac9	
DMR6:54179001	6	54179001	54183000	4000	1	4.10E-09	0.23	53	1.32	Hdac9	
DMR6:54318001	6	54318001	54321000	3000	1	4.40E-07	-0.37	20	0.67	Hdac9	
DMR6:54502001	6	54502001	54503000	1000	1	2.80E-10	-0.39	11	1.1	Snx13	Cytoskeleton
DMR6:55002001	6	55002001	55005000	3000	1	1.70E-09	0.58	30	1	Ahr;LOC102554773	Transcription
DMR6:55390001	6	55390001	55391000	1000	1	1.40E-10	-0.48	11	1.1	Agr3;LOC100361632	Metabolism
DMR6:55702001	6	55702001	55703000	1000	1	9.20E-07	-0.44	21	2.1	Lrrc72;LOC102546494	
DMR6:55740001	6	55740001	55742000	2000	1	7.10E-07	-0.4	28	1.4	Lrrc72	
DMR6:56095001	6	56095001	56102000	7000	2	1.90E-12	-0.46	80	1.14	Ispd	
DMR6:56612001	6	56612001	56617000	5000	1	1.50E-10	-0.39	53	1.06	Meox2	Development
DMR6:56674001	6	56674001	56675000	1000	1	8.40E-07	-0.58	0	0	Meox2	Development
DMR6:56743001	6	56743001	56745000	2000	1	1.70E-07	-0.55	3	0.15	Vom2r49	Signaling
DMR6:57050001	6	57050001	57051000	1000	1	1.70E-07	-0.59	1	0.1	Agmo	
DMR6:57064001	6	57064001	57066000	2000	2	7.50E-12	-0.71	4	0.2	Agmo	
DMR6:57406001	6	57406001	57407000	1000	1	3.70E-07	-0.57	2	0.2	RGD1563613	
DMR6:57653001	6	57653001	57656000	3000	1	4.40E-08	-0.36	32	1.07	Dgkb	Signaling
DMR6:57747001	6	57747001	57748000	1000	1	4.90E-09	0.62	12	1.2	Dgkb	Signaling
DMR6:57941001	6	57941001	57942000	1000	1	9.10E-07	0.57	11	1.1	Dgkb	Signaling
DMR6:57970001	6	57970001	57971000	1000	1	8.10E-07	0.35	12	1.2	Dgkb	Signaling
DMR6:58217001	6	58217001	58219000	2000	1	9.30E-07	0.33	17	0.85	Dgkb	Signaling
DMR6:58276001	6	58276001	58278000	2000	1	1.70E-09	0.66	27	1.35	Dgkb	Signaling

DMR6:60044001	6	60044001	60045000	1000	1	1.20E-08	0.66	6	0.6	Scin;LOC103692615	Cytoskeleton
DMR6:60144001	6	60144001	60146000	2000	1	2.10E-08	-0.39	24	1.2	Ifrd1	
DMR6:60296001	6	60296001	60297000	1000	1	2.20E-07	0.45	10	1	Zfp277	Transcription
DMR6:60351001	6	60351001	60354000	3000	1	3.30E-15	0.48	10	0.33	Zfp277	Transcription
DMR6:60379001	6	60379001	60380000	1000	1	2.40E-13	-0.67	9	0.9	Zfp277;Dock4	Transcription;Transcription
DMR6:60537001	6	60537001	60540000	3000	1	9.00E-10	-0.42	44	1.47	Dock4	Transcription
DMR6:60589001	6	60589001	60590000	1000	1	9.40E-07	-0.6	5	0.5	Dock4	Transcription
DMR6:60645001	6	60645001	60647000	2000	1	4.10E-09	-0.34	29	1.45	Dock4	Transcription
DMR6:61318001	6	61318001	61319000	1000	1	6.60E-08	-0.65	10	1	Immp2l	
DMR6:61404001	6	61404001	61405000	1000	1	6.50E-07	-0.48	21	2.1	Immp2l;Lrrn3	Receptor
DMR6:61534001	6	61534001	61536000	2000	1	1.40E-08	0.66	39	1.95	Immp2l	
DMR6:61609001	6	61609001	61610000	1000	1	4.10E-22	1.23	20	2	Immp2l	
DMR6:61825001	6	61825001	61827000	2000	1	3.90E-07	-0.35	17	0.85	Immp2l	
DMR6:64227001	6	64227001	64228000	1000	1	1.80E-08	-0.52	3	0.3	LOC103692619;Pnpla8	Metabolism
DMR6:64249001	6	64249001	64251000	2000	1	4.40E-07	-0.32	61	3.05	Pnpla8	Metabolism
DMR6:64806001	6	64806001	64807000	1000	1	6.50E-07	-0.32	2	0.2	Nrcam	Cytoskeleton
DMR6:65108001	6	65108001	65109000	1000	1	1.20E-08	-0.39	12	1.2	Stxbp6	Transcription
DMR6:65165001	6	65165001	65166000	1000	1	8.80E-09	-0.55	5	0.5	Stxbp6	Transcription
DMR6:66980001	6	66980001	66981000	1000	1	2.20E-14	0.72	7	0.7	Nova1	Metabolism
DMR6:67067001	6	67067001	67069000	2000	1	7.10E-07	-0.43	12	0.6	Nova1	Metabolism
DMR6:71109001	6	71109001	71113000	4000	1	1.10E-07	-0.44	26	0.65	Prkd1	Signaling
DMR6:71209001	6	71209001	71215000	6000	1	2.90E-30	0.47	61	1.02	Prkd1	Signaling
DMR6:71244001	6	71244001	71246000	2000	1	1.90E-12	-0.47	16	0.8	Prkd1	Signaling
DMR6:72137001	6	72137001	72139000	2000	1	1.00E-11	-0.46	23	1.15	Scfd1;LOC680823	Transport
DMR6:72206001	6	72206001	72207000	1000	1	3.70E-07	-0.5	1	0.1	Scfd1	Transport
DMR6:72425001	6	72425001	72426000	1000	1	5.80E-07	-0.4	5	0.5	Strn3	
DMR6:72441001	6	72441001	72442000	1000	1	1.30E-10	-0.48	12	1.2	Strn3	
DMR6:72517001	6	72517001	72518000	1000	1	4.40E-11	-0.55	19	1.9	Hectd1	Proteolysis
DMR6:72638001	6	72638001	72639000	1000	1	3.30E-09	-0.55	21	2.1	Heatr5a	
DMR6:72689001	6	72689001	72695000	6000	1	1.50E-08	-0.34	56	0.93	Heatr5a	
DMR6:72792001	6	72792001	72793000	1000	1	3.30E-07	-0.41	6	0.6	Dtd2	Metabolism
DMR6:72896001	6	72896001	72899000	3000	1	7.30E-10	0.53	49	1.63	Nubpl	
DMR6:73155001	6	73155001	73156000	1000	1	1.80E-08	0.34	3	0.3	Nubpl	
DMR6:73336001	6	73336001	73339000	3000	1	3.90E-11	0.84	53	1.77	Arhgap5	Signaling
DMR6:73601001	6	73601001	73602000	1000	1	2.60E-07	0.61	22	2.2	Akap6	
DMR6:73604001	6	73604001	73606000	2000	1	1.40E-15	0.94	27	1.35	Akap6	
DMR6:73635001	6	73635001	73637000	2000	1	3.50E-07	-0.57	15	0.75	Akap6	
DMR6:73652001	6	73652001	73653000	1000	1	2.20E-12	1.09	27	2.7	Akap6	
DMR6:73732001	6	73732001	73735000	3000	1	7.50E-09	0.47	56	1.87	Akap6	
DMR6:73818001	6	73818001	73819000	1000	1	5.50E-07	-0.37	20	2	Akap6	
DMR6:73823001	6	73823001	73826000	3000	1	1.70E-11	0.47	46	1.53	Akap6	
DMR6:73931001	6	73931001	73934000	3000	1	8.70E-07	-0.28	30	1	Akap6	
DMR6:73998001	6	73998001	7.40E+07	2000	1	8.70E-07	-0.34	31	1.55	Akap6	
DMR6:74119001	6	74119001	74121000	2000	1	6.40E-20	1.29	45	2.25	Npas3	
DMR6:74122001	6	74122001	74123000	1000	1	9.40E-13	0.79	15	1.5	Npas3	
DMR6:74144001	6	74144001	74147000	3000	1	2.30E-07	0.35	50	1.67	Npas3	
DMR6:74442001	6	74442001	74443000	1000	1	1.40E-07	-0.45	12	1.2	Npas3	
DMR6:74681001	6	74681001	74682000	1000	1	6.60E-11	0.61	17	1.7	Npas3	
DMR6:74715001	6	74715001	74718000	3000	1	4.50E-07	0.39	52	1.73	Npas3	
DMR6:75072001	6	75072001	75075000	3000	1	6.50E-09	0.69	58	1.93	Egln3	
DMR6:75081001	6	75081001	75083000	2000	1	3.00E-10	0.31	12	0.6	Egln3	
DMR6:75084001	6	75084001	75085000	1000	1	7.90E-07	-0.39	19	1.9	Egln3	
DMR6:75428001	6	75428001	75431000	3000	1	1.10E-08	0.55	45	1.5	Rps10l1	Translation
DMR6:75434001	6	75434001	75436000	2000	1	1.40E-07	0.5	21	1.05	Rps10l1	Translation
DMR6:75528001	6	75528001	75531000	3000	1	2.30E-11	-0.46	39	1.3	Sptssa	Golgi
DMR6:75547001	6	75547001	75548000	1000	1	1.70E-07	-0.38	11	1.1	Sptssa	Golgi
DMR6:75616001	6	75616001	75618000	2000	2	7.50E-18	0.99	55	2.75	LOC102548522;Snx6	Cytoskeleton
DMR6:75621001	6	75621001	75622000	1000	1	3.00E-11	0.87	32	3.2	LOC102548522;Snx6	Cytoskeleton
DMR6:75623001	6	75623001	75627000	4000	2	1.70E-11	1.03	142	3.55	LOC102548522;Snx6	Cytoskeleton
DMR6:75772001	6	75772001	75775000	3000	1	1.30E-12	-0.48	49	1.63	Cfl2	Cytoskeleton
DMR6:75938001	6	75938001	75939000	1000	1	8.40E-14	-0.68	12	1.2	RGD1304624	Epigenetic

DMR6:76090001	6	76090001	76092000	2000	1	7.40E-10	-0.43	32	1.6	RGD1305089	
DMR6:76096001	6	76096001	76097000	1000	1	1.00E-06	-0.42	10	1	RGD1305089	
DMR6:76101001	6	76101001	76104000	3000	1	6.10E-07	-0.34	40	1.33	RGD1305089	
DMR6:76173001	6	76173001	76175000	2000	1	1.40E-08	0.58	24	1.2	RGD1305089;Psma6	Protease
DMR6:76199001	6	76199001	76202000	3000	1	5.10E-07	-0.35	27	0.9	Psma6	Protease
DMR6:76359001	6	76359001	76361000	2000	1	2.90E-09	0.68	18	0.9	Aldoart2	Metabolism
DMR6:76467001	6	76467001	76468000	1000	1	2.10E-09	-0.38	5	0.5	Ralgapa1	Signaling
DMR6:76652001	6	76652001	76653000	1000	1	1.10E-12	0.88	45	4.5	Ralgapa1;LOC100359668;Mir3593	Signaling;Transport
DMR6:77416001	6	77416001	77420000	4000	2	2.10E-10	-0.77	102	2.55	LOC100911923;Nkx2-1;LOC102552092	Development
DMR6:77616001	6	77616001	77617000	1000	1	3.20E-08	-0.6	6	0.6	Pax9;Slc25a21	Transport
DMR6:77702001	6	77702001	77706000	4000	1	6.80E-07	0.32	55	1.38	Slc25a21	Transport
DMR6:77744001	6	77744001	77745000	1000	1	1.00E-13	1.03	19	1.9	Slc25a21;LOC108351240	Transport
DMR6:77836001	6	77836001	77842000	6000	1	6.00E-09	-0.27	54	0.9	Slc25a21	Transport
DMR6:77897001	6	77897001	77899000	2000	2	1.10E-13	0.9	36	1.8	Slc25a21;LOC103692639	Transport
DMR6:77964001	6	77964001	77967000	3000	1	8.50E-07	-0.42	12	0.4	Slc25a21	Transport
DMR6:77976001	6	77976001	77979000	3000	1	3.60E-08	-0.43	26	0.87	Slc25a21	Transport
DMR6:78033001	6	78033001	78037000	4000	2	3.90E-07	-0.35	40	1	Slc25a21	Transport
DMR6:78101001	6	78101001	78103000	2000	1	2.00E-08	0.31	15	0.75	Slc25a21;LOC102552151	Transport
DMR6:78327001	6	78327001	78328000	1000	1	1.10E-08	0.55	17	1.7	Mipol1	
DMR6:78425001	6	78425001	78426000	1000	1	1.70E-09	-0.35	15	1.5	Mipol1	
DMR6:78526001	6	78526001	78530000	4000	1	1.20E-08	-0.38	52	1.3	Foxa1;LOC102553136	Transcription
DMR6:78619001	6	78619001	78622000	3000	1	2.80E-09	-0.38	41	1.37	RGD1560556	
DMR6:78633001	6	78633001	78634000	1000	1	3.60E-07	-0.39	29	2.9	RGD1560556	
DMR6:78706001	6	78706001	78708000	2000	1	1.80E-09	-0.59	21	1.05	RGD1560556;LOC366632	
DMR6:78713001	6	78713001	78714000	1000	1	1.60E-07	-0.49	10	1	RGD1560556;LOC366632	
DMR6:78781001	6	78781001	78788000	7000	1	5.40E-09	-0.42	66	0.94	RGD1560556	
DMR6:80067001	6	80067001	80069000	2000	2	2.00E-24	1.41	95	4.75	Sec23a;LOC102553646	Transport
DMR6:80087001	6	80087001	80090000	3000	1	5.80E-07	-0.39	40	1.33	Sec23a	Transport
DMR6:80161001	6	80161001	80164000	3000	2	8.60E-08	-0.42	28	0.93	Trappc6b;Pnn	
DMR6:80292001	6	80292001	80293000	1000	1	1.20E-08	-0.52	2	0.2	LOC100912115;Fbxo33	
DMR6:83281001	6	83281001	83282000	1000	1	3.50E-09	-0.62	1	0.1	Lrfn5	
DMR6:84103001	6	84103001	84106000	3000	1	3.00E-08	-0.4	19	0.63	Cct6a-ps11	
DMR6:86703001	6	86703001	86706000	3000	1	5.20E-08	-0.36	36	1.2	Klhl28;Fam179b	Cytoskeleton
DMR6:86922001	6	86922001	86923000	1000	1	8.80E-08	-0.42	8	0.8	Mis18bp1;LOC100360647	
DMR6:88511001	6	88511001	88515000	4000	1	1.90E-07	-0.27	32	0.8	Mdga2	
DMR6:88612001	6	88612001	88613000	1000	1	2.40E-09	0.46	27	2.7	Mdga2	
DMR6:88615001	6	88615001	88617000	2000	1	8.90E-08	0.45	25	1.25	Mdga2	
DMR6:88691001	6	88691001	88693000	2000	1	1.30E-13	0.55	22	1.1	Mdga2	
DMR6:88773001	6	88773001	88777000	4000	1	2.00E-07	-0.36	42	1.05	Mdga2;LOC103692647	
DMR6:88878001	6	88878001	88879000	1000	1	4.00E-09	-0.38	8	0.8	Mdga2	
DMR6:89231001	6	89231001	89232000	1000	1	4.10E-09	0.64	9	0.9	Mdga2	
DMR6:91491001	6	91491001	91492000	1000	1	6.80E-07	-0.39	16	1.6	Dnaaf2;Pole2	Transcription
DMR6:91589001	6	91589001	91594000	5000	1	1.30E-09	-0.62	61	1.22	Klhdc1;LOC102547663;Klhdc2	
DMR6:91991001	6	91991001	91992000	1000	1	1.40E-10	-0.5	14	1.4	Sos2;LOC103692654	Transcription
DMR6:92021001	6	92021001	92022000	1000	1	1.10E-11	0.56	13	1.3	LOC103692650;L2hgdh	Metabolism
DMR6:92607001	6	92607001	92608000	1000	1	2.80E-07	0.49	21	2.1	Pygl	Golgi
DMR6:92631001	6	92631001	92633000	2000	1	5.30E-07	-0.35	83	4.15	Pygl	Golgi
DMR6:92638001	6	92638001	92639000	1000	1	3.80E-08	0.37	15	1.5	Pygl	Golgi
DMR6:92644001	6	92644001	92645000	1000	1	3.80E-08	0.65	23	2.3	Pygl	Golgi
DMR6:92666001	6	92666001	92667000	1000	1	1.30E-07	-0.46	27	2.7	Trim9	Proteolysis
DMR6:92697001	6	92697001	92700000	3000	1	1.50E-07	-0.46	44	1.47	Trim9	Proteolysis
DMR6:92737001	6	92737001	92739000	2000	1	1.50E-07	-0.38	30	1.5	Trim9	Proteolysis
DMR6:93324001	6	93324001	93326000	2000	2	3.00E-07	0.43	29	1.45	Frmd6	
DMR6:93340001	6	93340001	93342000	2000	1	7.40E-07	-0.43	38	1.9	Frmd6	
DMR6:93361001	6	93361001	93363000	2000	1	2.40E-11	0.83	34	1.7	Frmd6	
DMR6:93511001	6	93511001	93514000	3000	1	1.50E-07	-0.37	40	1.33	Arid4a	Transcription
DMR6:93521001	6	93521001	93523000	2000	1	1.20E-10	-0.5	65	3.25	Arid4a	Transcription
DMR6:93526001	6	93526001	93528000	2000	1	9.80E-08	0.4	23	1.15	Arid4a;LOC102552632	Transcription
DMR6:93760001	6	93760001	93763000	3000	1	9.50E-07	-0.26	30	1	Dact1	
DMR6:93778001	6	93778001	93780000	2000	1	1.30E-08	-0.48	17	0.85	RGD1559545	

DMR6:94493001	6	94493001	94496000	3000	1	1.20E-07	-0.44	34	1.13	Ppm1a;LOC108351251	
DMR6:94717001	6	94717001	94720000	3000	1	5.20E-09	-0.44	37	1.23	Daam1	
DMR6:94736001	6	94736001	94738000	2000	1	5.60E-15	1.06	47	2.35	Daam1;LOC108351252	
DMR6:94896001	6	94896001	94901000	5000	1	6.30E-09	-0.5	40	0.8	Ccdc175	
DMR6:94951001	6	94951001	94954000	3000	1	2.90E-08	0.38	48	1.6	Rtn1	
DMR6:94964001	6	94964001	94967000	3000	1	1.20E-09	0.76	55	1.83	Rtn1	
DMR6:95270001	6	95270001	95272000	2000	1	3.30E-07	-0.39	23	1.15	Lrrc9	
DMR6:95284001	6	95284001	95285000	1000	1	1.40E-07	-0.46	8	0.8	Lrrc9;LOC102549502	
DMR6:95348001	6	95348001	95351000	3000	1	5.80E-07	0.44	23	0.77	LOC102549502;Pcnx4	
DMR6:95428001	6	95428001	95431000	3000	1	2.20E-07	-0.34	61	2.03	Pcnx4;Dhrs7	Metabolism
DMR6:95471001	6	95471001	95473000	2000	1	7.80E-08	-0.39	15	0.75	Dhrs7	Metabolism
DMR6:95982001	6	95982001	95984000	2000	1	3.30E-07	0.38	22	1.1	LOC690390;Six4	Development
DMR6:95988001	6	95988001	95990000	2000	2	9.70E-11	0.73	50	2.5	Six4;Mnat1	Development;Transcription
DMR6:96178001	6	96178001	96179000	1000	1	6.10E-11	0.6	16	1.6	Trmt5;Slc38a6	Epigenetic;Transport
DMR6:96201001	6	96201001	96204000	3000	1	1.40E-07	-0.38	45	1.5	Slc38a6;LOC688981	Transport;Translation
DMR6:96528001	6	96528001	96533000	5000	1	7.60E-09	-0.39	103	2.06	Prkch	Signaling
DMR6:96658001	6	96658001	96663000	5000	1	1.10E-08	-0.64	47	0.94	Prkch;LOC108351320	Signaling
DMR6:96860001	6	96860001	96863000	3000	1	4.00E-07	0.55	46	1.53	Hif1a;Snapc1	Transcription
DMR6:97121001	6	97121001	97123000	2000	1	8.40E-09	0.52	13	0.65	Syt16;LOC102550910	
DMR6:97130001	6	97130001	97133000	3000	1	6.10E-10	0.82	53	1.77	Syt16;LOC102550910	
DMR6:97200001	6	97200001	97203000	3000	1	5.90E-11	-0.58	80	2.67	Syt16;LOC108351259	
DMR6:97934001	6	97934001	97937000	3000	2	6.20E-10	-0.47	39	1.3	Kcnh5	Transport
DMR6:98282001	6	98282001	98284000	2000	1	1.90E-07	-0.54	24	1.2	Rhoj	Signaling
DMR6:98317001	6	98317001	98318000	1000	1	6.10E-07	-0.37	14	1.4	Rhoj	Signaling
DMR6:98353001	6	98353001	98355000	2000	1	1.00E-12	1.06	39	1.95	Rhoj	Signaling
DMR6:98434001	6	98434001	98436000	2000	1	2.20E-07	0.53	24	1.2	Ppp2r5e	Signaling
DMR6:98451001	6	98451001	98453000	2000	1	9.00E-07	-0.37	30	1.5	Ppp2r5e;LOC108351261	Signaling
DMR6:98577001	6	98577001	98579000	2000	1	4.20E-09	0.68	37	1.85	Ppp2r5e	Signaling
DMR6:98671001	6	98671001	98672000	1000	1	3.40E-07	0.5	30	3	LOC102555381;Sgpp1	Signaling
DMR6:98882001	6	98882001	98885000	3000	2	1.10E-12	-0.69	61	2.03	Syne2	
DMR6:98887001	6	98887001	98890000	3000	1	2.80E-11	-0.51	58	1.93	Syne2	
DMR6:98891001	6	98891001	98899000	8000	1	1.50E-07	-0.33	130	1.62	Syne2	
DMR6:98939001	6	98939001	98940000	1000	1	2.90E-07	-0.4	14	1.4	Syne2	
DMR6:98956001	6	98956001	98959000	3000	1	3.40E-08	-0.45	59	1.97	Syne2	
DMR6:98960001	6	98960001	98963000	3000	1	6.00E-07	-0.44	24	0.8	Syne2	
DMR6:99080001	6	99080001	99082000	2000	1	2.10E-07	-0.44	42	2.1	Syne2	
DMR6:99087001	6	99087001	99090000	3000	1	5.60E-07	-0.34	56	1.87	Syne2	
DMR6:99121001	6	99121001	99124000	3000	1	4.60E-11	-0.43	63	2.1	Syne2	
DMR6:99195001	6	99195001	99197000	2000	1	6.70E-12	-0.68	18	0.9	Esr2	
DMR6:99223001	6	99223001	99224000	1000	1	5.90E-07	0.45	11	1.1	Esr2	
DMR6:99263001	6	99263001	99265000	2000	1	8.80E-07	0.49	25	1.25	Tex21	
DMR6:99346001	6	99346001	99349000	3000	1	9.40E-08	0.49	41	1.37	Mthfd1;Akap5	Cytoskeleton
DMR6:99468001	6	99468001	99470000	2000	1	1.00E-07	-0.33	23	1.15	Ppp1r36;LOC102556012	
DMR6:99646001	6	99646001	99649000	3000	1	4.40E-07	-0.4	51	1.7	Plekhg3;Sptb	
DMR6:99682001	6	99682001	99683000	1000	1	2.70E-08	0.34	21	2.1	Sptb	
DMR6:99762001	6	99762001	99763000	1000	1	3.70E-07	-0.38	15	1.5	Sptb	
DMR6:99765001	6	99765001	99768000	3000	1	4.90E-09	-0.44	58	1.93	Sptb	
DMR6:99784001	6	99784001	99785000	1000	1	2.40E-07	0.62	20	2	Sptb	
DMR6:99857001	6	99857001	99858000	1000	1	1.10E-10	-0.44	11	1.1	Rab15	
DMR6:99872001	6	99872001	99874000	2000	1	3.90E-07	0.46	22	1.1	Rab15;LOC102546646;LOC108348171;Fntb	Metabolism
DMR6:99961001	6	99961001	99962000	1000	1	6.00E-08	0.64	15	1.5	Fntb	Metabolism
DMR6:99976001	6	99976001	99981000	5000	2	1.90E-09	-0.7	49	0.98	Fntb;Max	Metabolism;Transcription
DMR6:100322001	6	100322001	100323000	1000	1	4.90E-07	-0.56	7	0.7	Fut8	Golgi
DMR6:101303001	6	101303001	101304000	1000	1	8.30E-09	0.65	15	1.5	RGD1562540	
DMR6:101351001	6	101351001	101355000	4000	2	8.00E-12	-0.52	21	0.52	Gphn	
DMR6:101359001	6	101359001	101363000	4000	2	3.10E-10	-0.3	56	1.4	Gphn	
DMR6:101405001	6	101405001	101408000	3000	1	1.50E-08	-0.44	20	0.67	Gphn;LOC108351264;LOC102547341	
DMR6:101704001	6	101704001	101707000	3000	1	4.30E-09	-0.38	26	0.87	Gphn	

DMR6:101813001	6	101813001	101814000	1000	1	8.50E-09	0.3	6	0.6	Gphn	
DMR6:101887001	6	101887001	101895000	8000	1	2.10E-09	0.48	75	0.94	Fam71d	
DMR6:101904001	6	101904001	101905000	1000	1	1.20E-08	-0.41	15	1.5	Fam71d	
DMR6:101927001	6	101927001	101929000	2000	1	5.70E-11	-0.5	20	1	LOC108351336;Mpp5	Cytoskeleton
DMR6:101965001	6	101965001	101966000	1000	1	6.00E-12	-0.53	6	0.6	Mpp5	Cytoskeleton
DMR6:101973001	6	101973001	101974000	1000	1	1.50E-08	-0.44	13	1.3	Mpp5	Cytoskeleton
DMR6:102018001	6	102018001	102020000	2000	1	2.20E-09	-0.44	19	0.95	Mpp5	Cytoskeleton
DMR6:102032001	6	102032001	102034000	2000	1	3.50E-07	-0.51	21	1.05	Mpp5;Atp6v1d	Cytoskeleton;Metabolism
DMR6:102207001	6	102207001	102209000	2000	1	2.30E-09	-0.53	24	1.2	LOC108351265;Plekhh1	
DMR6:102256001	6	102256001	102259000	3000	1	2.40E-07	0.48	49	1.63	Plekhh1;LOC102547892;Pigh	Golgi
DMR6:102690001	6	102690001	102691000	1000	1	1.40E-08	-0.41	19	1.9	Rad51b	Transcription
DMR6:102732001	6	102732001	102735000	3000	1	6.60E-07	-0.3	24	0.8	Rad51b	Transcription
DMR6:103367001	6	103367001	103369000	2000	1	2.80E-07	0.59	33	1.65	Actn1	
DMR6:103474001	6	103474001	103476000	2000	1	6.60E-08	-0.47	7	0.35	Actn1	
DMR6:103568001	6	103568001	103569000	1000	1	2.30E-09	-0.62	13	1.3	Dcaf5;Scarna3	Proteolysis
DMR6:103640001	6	103640001	103641000	1000	1	3.10E-17	1.2	42	4.2	RGD1562299;RGD1561024	
DMR6:103708001	6	103708001	103711000	3000	1	2.40E-09	0.54	56	1.87	LOC679790;RGD1560857	
DMR6:103794001	6	103794001	103799000	5000	1	7.40E-10	0.48	38	0.76	LOC100911208;LOC680798;RGD1566331;LOC680819	
DMR6:103820001	6	103820001	103821000	1000	1	1.60E-14	0.83	17	1.7	LOC100911289;RGD1565752;Mphosph6-ps1;LOC103692709;LOC685142	
DMR6:103823001	6	103823001	103827000	4000	1	6.20E-07	0.55	57	1.43	LOC100911289;RGD1565752;Mphosph6-ps1;LOC103692709;LOC685142	
DMR6:104065001	6	104065001	104070000	5000	1	1.40E-07	0.57	67	1.34	Galnt16	Golgi
DMR6:104106001	6	104106001	104109000	3000	1	9.70E-07	-0.43	28	0.93	Galnt16	Golgi
DMR6:104234001	6	104234001	104238000	4000	2	5.00E-11	-0.51	48	1.2	Galnt16	Golgi
DMR6:104612001	6	104612001	104616000	4000	2	5.90E-08	-0.44	78	1.95	Srsf5;Slc10a1	Translation;Transport
DMR6:104805001	6	104805001	104808000	3000	1	2.20E-07	-0.56	33	1.1	Smoc1	Signaling
DMR6:104924001	6	104924001	104925000	1000	1	3.70E-07	-0.57	5	0.5	Slc8a3	Transport
DMR6:105058001	6	105058001	105059000	1000	1	5.50E-07	-0.37	19	1.9	Slc8a3	Transport
DMR6:105236001	6	105236001	105237000	1000	1	3.80E-07	-0.37	7	0.7	Synj2bp	Cytoskeleton
DMR6:105251001	6	105251001	105252000	1000	1	2.80E-08	-0.53	6	0.6	Synj2bp;LOC103690066	Cytoskeleton
DMR6:105262001	6	105262001	105264000	2000	1	8.40E-07	-0.29	25	1.25	Synj2bp;LOC103690066	Cytoskeleton
DMR6:105307001	6	105307001	105308000	1000	1	1.70E-07	-0.36	19	1.9	LOC103690152;Med6	Transcription
DMR6:105388001	6	105388001	105390000	2000	1	1.90E-07	-0.43	25	1.25	Ttc9	Transcription
DMR6:105632001	6	105632001	105634000	2000	2	5.00E-15	-0.7	14	0.7	Pcnx1	
DMR6:105690001	6	105690001	105692000	2000	1	9.10E-07	-0.38	37	1.85	Pcnx1	
DMR6:105696001	6	105696001	105700000	4000	1	6.10E-07	-0.35	58	1.45	Pcnx1	
DMR6:106316001	6	106316001	106317000	1000	1	3.00E-08	0.31	6	0.6	Rgs6	
DMR6:106391001	6	106391001	106399000	8000	1	4.10E-12	-0.54	110	1.38	Rgs6	
DMR6:106415001	6	106415001	106418000	3000	1	8.10E-10	0.55	57	1.9	Rgs6	
DMR6:106487001	6	106487001	106492000	5000	1	2.20E-12	0.57	52	1.04	Rgs6	
DMR6:106519001	6	106519001	106522000	3000	1	1.10E-07	0.48	38	1.27	Rgs6	
DMR6:106576001	6	106576001	106580000	4000	1	1.00E-08	-0.43	63	1.57	Rgs6;LOC103692676	
DMR6:106684001	6	106684001	106686000	2000	1	7.70E-08	0.66	38	1.9	Dpf3	Epigenetic
DMR6:106784001	6	106784001	106790000	6000	1	6.50E-09	0.73	103	1.72	Dpf3	Epigenetic
DMR6:106912001	6	106912001	106915000	3000	1	8.80E-07	-0.4	49	1.63	Dpf3	Epigenetic
DMR6:106922001	6	106922001	106927000	5000	1	2.20E-07	0.59	74	1.48	Dpf3	Epigenetic
DMR6:106929001	6	106929001	106931000	2000	1	9.10E-07	0.35	19	0.95	Dpf3	Epigenetic
DMR6:106977001	6	106977001	106978000	1000	1	2.20E-26	1.08	25	2.5	Dpf3	Epigenetic
DMR6:106995001	6	106995001	106998000	3000	1	8.90E-09	-0.44	31	1.03	Dcaf4	
DMR6:107037001	6	107037001	107038000	1000	1	3.70E-08	0.53	19	1.9	Dcaf4;Zfyve1	
DMR6:107147001	6	107147001	107148000	1000	1	7.60E-10	-0.42	30	3	Rbm25	
DMR6:107257001	6	107257001	107261000	4000	2	3.40E-24	1.29	105	2.62	Papln	Protease
DMR6:107295001	6	107295001	107296000	1000	1	7.40E-07	-0.48	7	0.7	Numb	Cytoskeleton
DMR6:107451001	6	107451001	107453000	2000	1	1.50E-12	0.91	38	1.9	Heatr4;Acot2	Metabolism;Metabolism
DMR6:107500001	6	107500001	107502000	2000	1	2.60E-07	-0.29	35	1.75	Acot1;LOC299190	Metabolism

DMR6:107560001	6	107560001	107562000	2000	1	9.10E-09	-0.39	19	0.95	Acot5;LOC299192	Metabolism
DMR6:107590001	6	107590001	107594000	4000	1	4.00E-15	-0.52	62	1.55	Acot6;LOC108351272;Dnal1	Metabolism;Cytoskeleton
DMR6:107653001	6	107653001	107655000	2000	1	3.80E-07	-0.38	35	1.75	Elmsan1	
DMR6:108034001	6	108034001	108035000	1000	1	3.00E-07	-0.38	19	1.9	Ptgr2;Zfp410	Metabolism;Transcription
DMR6:108162001	6	108162001	108163000	1000	1	8.20E-11	-0.48	8	0.8	Aldh6a1;Lin52	Metabolism
DMR6:108186001	6	108186001	108188000	2000	1	5.60E-09	-0.56	8	0.4	Lin52	
DMR6:108268001	6	108268001	108271000	3000	1	7.50E-10	0.82	64	2.13	Lin52	
DMR6:108299001	6	108299001	108302000	3000	1	2.20E-09	0.61	40	1.33	Vsx2	Development
DMR6:108306001	6	108306001	108307000	1000	1	5.20E-14	0.72	27	2.7	Vsx2;Abcd4	Development;Transport
DMR6:108315001	6	108315001	108318000	3000	1	2.20E-07	0.4	53	1.77	Vsx2;Abcd4	Development;Transport
DMR6:108392001	6	108392001	108398000	6000	2	2.00E-08	0.67	90	1.5	LOC108351273;Syndig1l	
DMR6:108458001	6	108458001	108464000	6000	1	8.60E-08	0.37	75	1.25	Npc2	
DMR6:108512001	6	108512001	108515000	3000	2	5.10E-07	0.65	47	1.57	Ltbp2	Extracellular Matrix
DMR6:108854001	6	108854001	108855000	1000	1	7.10E-08	0.36	9	0.9	Ylpm1	Metabolism
DMR6:108858001	6	108858001	108860000	2000	2	3.50E-09	-0.4	43	2.15	Ylpm1	Metabolism
DMR6:108866001	6	108866001	108868000	2000	1	3.40E-08	0.68	25	1.25	Ylpm1	Metabolism
DMR6:108920001	6	108920001	108921000	1000	1	7.50E-12	0.77	20	2	Prox2	Development
DMR6:108943001	6	108943001	108944000	1000	1	2.30E-08	0.5	13	1.3	Dlst	Transport
DMR6:108991001	6	108991001	108992000	1000	1	2.80E-15	-0.74	0	0	Pgf	Growth Factors
DMR6:109199001	6	109199001	109203000	4000	1	5.20E-07	-0.42	58	1.45	Tmed10	Transport
DMR6:109450001	6	109450001	109455000	5000	1	4.90E-08	-0.45	61	1.22	Jdp2	Transcription
DMR6:109679001	6	109679001	109682000	3000	1	1.60E-07	0.46	31	1.03	Flvcr2;RGD1310769	Transport
DMR6:109740001	6	109740001	109742000	2000	1	8.80E-14	-0.46	34	1.7	Ttll5	Cytoskeleton
DMR6:109880001	6	109880001	109882000	2000	1	9.00E-10	0.4	23	1.15	Ttll5	Cytoskeleton
DMR6:109973001	6	109973001	109975000	2000	1	4.70E-07	0.35	16	0.8	Ift43	
DMR6:109990001	6	109990001	109992000	2000	1	1.10E-09	0.52	34	1.7	Ift43	
DMR6:110015001	6	110015001	110017000	2000	1	9.60E-07	0.44	27	1.35	Ift43	
DMR6:110441001	6	110441001	110442000	1000	1	4.30E-20	0.89	32	3.2	Esrrb	
DMR6:110634001	6	110634001	110636000	2000	1	2.40E-09	0.72	47	2.35	Vash1;Angel1	Translation
DMR6:110745001	6	110745001	110747000	2000	1	7.50E-07	-0.5	34	1.7	Lrrc74a	
DMR6:110754001	6	110754001	110758000	4000	1	8.40E-07	-0.39	48	1.2	Lrrc74a	
DMR6:110774001	6	110774001	110778000	4000	2	6.30E-11	0.61	70	1.75	Lrrc74a	
DMR6:111139001	6	111139001	111140000	1000	1	1.50E-07	0.5	15	1.5	Ngb;Pomt2	Transport;Transport
DMR6:111297001	6	111297001	111299000	2000	1	1.80E-09	-0.68	31	1.55	Vipas39;Ahsa1	Transcription
DMR6:111388001	6	111388001	111391000	3000	1	5.70E-07	-0.4	23	0.77	Sptlc2	Metabolism
DMR6:111540001	6	111540001	111542000	2000	1	4.90E-07	0.46	40	2	RGD1563496;RGD1561148;LOC100910634;LOC503053	
DMR6:111691001	6	111691001	111692000	1000	1	9.80E-08	0.47	6	0.6	Adck1	Transport
DMR6:111736001	6	111736001	111738000	2000	1	3.00E-13	0.58	12	0.6	Adck1	Transport
DMR6:112203001	6	112203001	112204000	1000	1	4.40E-08	0.6	23	2.3	Nrxn3	
DMR6:112424001	6	112424001	112429000	5000	1	1.00E-06	-0.34	48	0.96	Nrxn3	
DMR6:112641001	6	112641001	112644000	3000	1	1.20E-09	-0.58	19	0.63	Nrxn3	
DMR6:112646001	6	112646001	112653000	7000	1	2.80E-22	0.34	86	1.23	Nrxn3	
DMR6:112666001	6	112666001	112667000	1000	1	1.70E-09	-0.47	5	0.5	Nrxn3	
DMR6:112952001	6	112952001	112953000	1000	1	3.00E-07	0.52	13	1.3	Nrxn3	
DMR6:113104001	6	113104001	113107000	3000	1	6.90E-07	-0.25	27	0.9	Nrxn3	
DMR6:113112001	6	113112001	113113000	1000	1	6.20E-08	-0.34	9	0.9	Nrxn3	
DMR6:113550001	6	113550001	113552000	2000	1	4.90E-08	0.54	12	0.6	Nrxn3	
DMR6:113667001	6	113667001	113669000	2000	2	4.70E-12	-0.58	12	0.6	Nrxn3	
DMR6:113671001	6	113671001	113672000	1000	1	1.70E-10	-0.52	7	0.7	Nrxn3	
DMR6:114032001	6	114032001	114037000	5000	1	1.50E-08	-0.54	41	0.82	Nrxn3	
DMR6:114466001	6	114466001	114468000	2000	1	1.30E-07	-0.55	8	0.4	Dio2	
DMR6:114844001	6	114844001	114845000	1000	1	1.70E-07	-0.46	7	0.7	Cep128	
DMR6:114858001	6	114858001	114862000	4000	1	3.60E-07	-0.31	57	1.43	Cep128	
DMR6:114897001	6	114897001	114898000	1000	1	3.20E-07	-0.42	4	0.4	Cep128	
DMR6:114967001	6	114967001	114968000	1000	1	4.40E-07	-0.41	11	1.1	Cep128	
DMR6:115211001	6	115211001	115214000	3000	1	8.30E-07	-0.29	36	1.2	Tshr	Signaling
DMR6:115242001	6	115242001	115246000	4000	1	3.30E-11	-0.42	45	1.12	Tshr	Signaling

DMR6:115421001	6	115421001	115423000	2000	1	9.40E-07	-0.37	35	1.75	Ston2;LOC102551017	Transport
DMR6:115490001	6	115490001	115495000	5000	1	2.30E-08	-0.36	47	0.94	Ston2;LOC102550912	Transport
DMR6:115511001	6	115511001	115514000	3000	1	5.70E-07	-0.47	47	1.57	Ston2	Transport
DMR6:115518001	6	115518001	115519000	1000	1	5.20E-07	-0.35	17	1.7	Ston2	Transport
DMR6:115574001	6	115574001	115576000	2000	1	9.10E-07	-0.37	45	2.25	Sel1l	
DMR6:115609001	6	115609001	115610000	1000	1	2.40E-07	-0.33	21	2.1	Sel1l	
DMR6:119506001	6	119506001	119509000	3000	1	1.80E-08	0.49	52	1.73	LOC108351288;Flrt2	
DMR6:119607001	6	119607001	119608000	1000	1	2.40E-17	1.06	27	2.7	Flrt2	
DMR6:122184001	6	122184001	122185000	1000	1	1.60E-08	0.65	17	1.7	Galc	Metabolism
DMR6:122455001	6	122455001	122456000	1000	1	3.50E-08	-0.46	12	1.2	Kcnk10	Transport
DMR6:122516001	6	122516001	122517000	1000	1	8.40E-20	-0.58	8	0.8	Kcnk10	Transport
DMR6:122626001	6	122626001	122627000	1000	1	3.90E-07	-0.41	17	1.7	Spata7	
DMR6:122628001	6	122628001	122629000	1000	1	1.10E-07	-0.34	15	1.5	Spata7	
DMR6:122662001	6	122662001	122664000	2000	1	1.00E-07	-0.39	53	2.65	Ptpn21	Signaling
DMR6:122711001	6	122711001	122713000	2000	1	7.00E-11	-0.62	18	0.9	Ptpn21	Signaling
DMR6:122715001	6	122715001	122716000	1000	1	8.70E-08	-0.34	13	1.3	Ptpn21	Signaling
DMR6:122854001	6	122854001	122855000	1000	1	1.60E-09	-0.57	3	0.3	Eml5	
DMR6:122885001	6	122885001	122887000	2000	1	2.20E-08	-0.27	15	0.75	Eml5	
DMR6:123226001	6	123226001	123227000	1000	1	9.20E-08	-0.38	11	1.1	Foxn3	
DMR6:123244001	6	123244001	123245000	1000	1	9.50E-08	0.38	18	1.8	Foxn3	
DMR6:123269001	6	123269001	123271000	2000	1	4.30E-09	-0.47	48	2.4	Foxn3	
DMR6:123354001	6	123354001	123356000	2000	1	9.40E-07	-0.35	42	2.1	Foxn3;LOC102550790	
DMR6:123412001	6	123412001	123416000	4000	1	1.30E-11	0.59	60	1.5	Foxn3	
DMR6:123436001	6	123436001	123437000	1000	1	4.50E-08	0.38	13	1.3	Foxn3	
DMR6:123468001	6	123468001	123469000	1000	1	4.00E-08	0.35	22	2.2	Foxn3	
DMR6:123488001	6	123488001	123492000	4000	1	6.10E-13	0.51	77	1.93	Foxn3;LOC103692688	
DMR6:123495001	6	123495001	123497000	2000	1	9.80E-07	-0.47	24	1.2	Foxn3;LOC103692688	
DMR6:123540001	6	123540001	123543000	3000	1	3.90E-07	0.53	45	1.5	Foxn3	
DMR6:123775001	6	123775001	123778000	3000	1	5.70E-09	-0.38	49	1.63	Efcab11	Signaling
DMR6:123819001	6	123819001	123820000	1000	1	1.30E-08	0.57	13	1.3	Efcab11	Signaling
DMR6:123977001	6	123977001	123983000	6000	1	1.70E-11	-0.38	89	1.48	Kcnk13	Transport
DMR6:124079001	6	124079001	124081000	2000	1	2.30E-16	0.94	44	2.2	Kcnk13	Transport
DMR6:124231001	6	124231001	124232000	1000	1	2.80E-08	-0.52	5	0.5	Calm1	Signaling
DMR6:124306001	6	124306001	124308000	2000	1	3.00E-08	0.46	26	1.3	Ttc7b	
DMR6:124337001	6	124337001	124338000	1000	1	5.00E-07	-0.34	13	1.3	Ttc7b	
DMR6:124394001	6	124394001	124395000	1000	1	4.00E-10	-0.48	13	1.3	Ttc7b	
DMR6:124435001	6	124435001	124436000	1000	1	4.40E-07	0.6	12	1.2	Ttc7b	
DMR6:124445001	6	124445001	124446000	1000	1	1.50E-13	0.4	6	0.6	Ttc7b	
DMR6:124476001	6	124476001	124479000	3000	1	1.80E-15	-0.65	46	1.53	Ttc7b	
DMR6:124528001	6	124528001	124530000	2000	1	1.90E-07	0.41	85	4.25	Ttc7b	
DMR6:124717001	6	124717001	124719000	2000	1	2.20E-09	-0.39	32	1.6	Rps6ka5	Golgi
DMR6:124741001	6	124741001	124743000	2000	1	3.40E-11	-0.62	32	1.6	Rps6ka5	Golgi
DMR6:124782001	6	124782001	124785000	3000	2	1.10E-09	-0.5	34	1.13	RGD1311756	
DMR6:124829001	6	124829001	124831000	2000	1	6.60E-07	-0.47	30	1.5	RGD1311756	
DMR6:124921001	6	124921001	124923000	2000	1	5.10E-07	-0.38	45	2.25	Ccdc88c	Transport
DMR6:124942001	6	124942001	124944000	2000	1	6.00E-08	0.47	54	2.7	Ccdc88c	Transport
DMR6:124977001	6	124977001	124980000	3000	1	6.60E-08	-0.37	58	1.93	Ccdc88c	Transport
DMR6:125007001	6	125007001	125010000	3000	1	1.80E-08	-0.36	48	1.6	Ccdc88c	Transport
DMR6:125360001	6	125360001	125364000	4000	2	6.10E-25	0.6	55	1.38	Catsperb	
DMR6:125368001	6	125368001	125369000	1000	1	2.90E-09	0.42	15	1.5	Catsperb	
DMR6:125450001	6	125450001	125453000	3000	2	4.80E-19	0.49	48	1.6	Tc2n	
DMR6:125455001	6	125455001	125463000	8000	3	2.40E-09	-0.36	84	1.05	Tc2n	
DMR6:125464001	6	125464001	125466000	2000	2	1.10E-09	-0.54	17	0.85	Tc2n	
DMR6:125478001	6	125478001	125480000	2000	1	1.60E-10	-0.45	50	2.5	Tc2n	
DMR6:125487001	6	125487001	125488000	1000	1	7.00E-18	-0.65	18	1.8	Tc2n	
DMR6:125495001	6	125495001	125499000	4000	1	4.90E-07	-0.37	61	1.52	Tc2n	
DMR6:125500001	6	125500001	125502000	2000	2	5.60E-11	-0.47	83	4.15	Tc2n	
DMR6:125506001	6	125506001	125509000	3000	1	3.20E-08	-0.27	48	1.6	Tc2n	
DMR6:125510001	6	125510001	125511000	1000	1	4.80E-10	0.86	26	2.6	Tc2n	
DMR6:125512001	6	125512001	125515000	3000	2	3.60E-10	-0.3	50	1.67	Tc2n	
DMR6:125654001	6	125654001	125655000	1000	1	4.20E-10	-0.45	12	1.2	Fbln5	Extracellular Matrix
DMR6:125666001	6	125666001	125668000	2000	1	1.50E-09	-0.37	31	1.55	Fbln5	Extracellular Matrix

DMR6:125676001	6	125676001	125682000	6000	1	1.00E-07	-0.38	62	1.03	Fbln5	Extracellular Matrix
DMR6:125705001	6	125705001	125710000	5000	1	1.80E-09	0.54	86	1.72	Fbln5	Extracellular Matrix
DMR6:125726001	6	125726001	125731000	5000	1	9.20E-09	-0.47	64	1.28	Fbln5	Extracellular Matrix
DMR6:125889001	6	125889001	125892000	3000	1	1.30E-09	-0.5	56	1.87	Cpsf2	
DMR6:126098001	6	126098001	126100000	2000	1	2.90E-07	0.43	22	1.1	Slc24a4	Transport
DMR6:126118001	6	126118001	126120000	2000	2	4.40E-10	0.75	40	2	Slc24a4	Transport
DMR6:126193001	6	126193001	126199000	6000	1	4.20E-07	-0.5	81	1.35	Rin3	Transcription
DMR6:126257001	6	126257001	126259000	2000	2	1.10E-11	0.73	55	2.75	Rin3	Transcription
DMR6:126311001	6	126311001	126314000	3000	1	1.50E-07	-0.45	42	1.4	Lgmn	Protease
DMR6:126360001	6	126360001	126364000	4000	1	1.20E-11	-0.44	42	1.05	Golga5	
DMR6:126503001	6	126503001	126504000	1000	1	1.00E-12	0.57	21	2.1	Itpk1	Signaling
DMR6:126539001	6	126539001	126542000	3000	1	5.80E-07	0.51	69	2.3	Itpk1;LOC102548189	Signaling
DMR6:126546001	6	126546001	126547000	1000	1	3.70E-07	-0.35	15	1.5	Itpk1;LOC102548189;LOC103692695	Signaling
DMR6:126730001	6	126730001	126732000	2000	1	7.50E-10	-0.57	13	0.65	Btbd7	
DMR6:126752001	6	126752001	126753000	1000	1	2.90E-08	-0.54	3	0.3	Btbd7	
DMR6:126766001	6	126766001	126768000	2000	1	3.00E-11	0.99	49	2.45	Cox8c	Metabolism
DMR6:126860001	6	126860001	126863000	3000	1	9.50E-10	-0.44	60	2	Unc79	
DMR6:126876001	6	126876001	126880000	4000	1	4.60E-07	-0.42	84	2.1	Unc79	
DMR6:126898001	6	126898001	126900000	2000	1	6.20E-07	-0.45	33	1.65	Unc79;LOC108351291	
DMR6:126916001	6	126916001	126918000	2000	2	1.10E-11	0.83	47	2.35	Unc79;LOC108351291	
DMR6:126919001	6	126919001	126921000	2000	1	3.30E-15	-0.74	40	2	Unc79;LOC108351291	
DMR6:126928001	6	126928001	126933000	5000	5	1.60E-10	-0.51	38	0.76	Unc79	
DMR6:126945001	6	126945001	126949000	4000	2	6.50E-10	-0.49	82	2.05	Unc79	
DMR6:127022001	6	127022001	127024000	2000	1	3.50E-08	-0.43	48	2.4	Unc79	
DMR6:127087001	6	127087001	127088000	1000	1	2.00E-07	0.51	17	1.7	Prima1	
DMR6:127125001	6	127125001	127127000	2000	1	5.00E-08	0.68	36	1.8	Prima1	
DMR6:127222001	6	127222001	127223000	1000	1	5.30E-07	-0.41	20	2	Asb2	
DMR6:127234001	6	127234001	127236000	2000	1	2.70E-07	-0.59	30	1.5	Asb2	
DMR6:127271001	6	127271001	127274000	3000	1	1.40E-08	0.56	58	1.93	LOC102549032;Otub2	Protease
DMR6:127317001	6	127317001	127318000	1000	1	7.80E-09	-0.44	20	2	Ddx24;Ifi27	
DMR6:127324001	6	127324001	127325000	1000	1	6.90E-07	-0.36	8	0.8	Ddx24;Ifi27	
DMR6:127346001	6	127346001	127347000	1000	1	2.60E-07	-0.39	7	0.7	Ifi27l2b	
DMR6:127426001	6	127426001	127427000	1000	1	8.00E-07	-0.42	5	0.5	Ppp4r4	Signaling
DMR6:127696001	6	127696001	127697000	1000	1	8.40E-08	0.54	19	1.9	LOC108351294;Serpina12	Protease; Proteolysis
DMR6:128086001	6	128086001	128091000	5000	2	1.40E-16	0.98	87	1.74	Serpina3n;LOC690435	Protease; Proteolysis
DMR6:128379001	6	128379001	128382000	3000	1	2.80E-11	-0.48	69	2.3	Dicer1	
DMR6:128392001	6	128392001	128393000	1000	1	7.30E-07	-0.37	22	2.2	Dicer1	
DMR6:128517001	6	128517001	128519000	2000	1	8.90E-08	-0.38	44	2.2	Clmn	
DMR6:128539001	6	128539001	128541000	2000	1	1.30E-08	-0.48	28	1.4	Clmn	
DMR6:128710001	6	128710001	128714000	4000	1	1.60E-08	-0.5	65	1.62	Syne3	
DMR6:128899001	6	128899001	128901000	2000	1	1.10E-07	-0.52	19	0.95	Tcl1a;LOC108351373;LOC108351374	
DMR6:128902001	6	128902001	128905000	3000	1	1.90E-09	-0.39	65	2.17	Tcl1a;LOC108351373;LOC108351374	
DMR6:129419001	6	129419001	129423000	4000	1	8.80E-09	0.51	67	1.68	Bdkrb2;LOC103692699	Signaling
DMR6:129446001	6	129446001	129447000	1000	1	2.20E-08	0.38	13	1.3	Bdkrb1;Atg2b	Signaling
DMR6:129598001	6	129598001	129602000	4000	2	6.70E-23	1.18	82	2.05	Ak7;Papola	Signaling;Translation
DMR6:131818001	6	131818001	131825000	7000	1	1.20E-15	1.13	170	2.43	Bcl11b	Transcription
DMR6:131833001	6	131833001	131834000	1000	1	2.20E-10	0.71	28	2.8	Bcl11b	Transcription
DMR6:131836001	6	131836001	131837000	1000	1	4.60E-18	1.12	34	3.4	Bcl11b	Transcription
DMR6:131838001	6	131838001	131840000	2000	1	1.20E-13	0.83	91	4.55	Bcl11b	Transcription
DMR6:131849001	6	131849001	131851000	2000	1	9.40E-07	-0.29	65	3.25	Bcl11b	Transcription
DMR6:131865001	6	131865001	131869000	4000	1	2.60E-07	0.48	72	1.8	Bcl11b;LOC103692704	Transcription
DMR6:131884001	6	131884001	131886000	2000	1	1.90E-07	-0.39	51	2.55	Bcl11b	Transcription
DMR6:131896001	6	131896001	131900000	4000	2	7.10E-08	-0.37	100	2.5	Bcl11b;LOC103692702	Transcription
DMR6:132157001	6	132157001	132161000	4000	1	3.10E-07	-0.41	102	2.55	Ccdc85c	
DMR6:132215001	6	132215001	132218000	3000	2	3.90E-08	0.63	48	1.6	Hhipl1	Signaling
DMR6:132231001	6	132231001	132234000	3000	1	6.10E-11	0.66	66	2.2	Hhipl1;Cyp46a1	Signaling;Metabolism
DMR6:132257001	6	132257001	132260000	3000	1	2.20E-08	0.42	37	1.23	Cyp46a1	Metabolism
DMR6:132389001	6	132389001	132390000	1000	1	4.90E-08	-0.43	17	1.7	Eml1	
DMR6:132397001	6	132397001	132401000	4000	1	2.00E-07	-0.32	56	1.4	Eml1	

DMR6:132428001	6	132428001	132430000	2000	1	2.30E-08	-0.63	39	1.95	Eml1	
DMR6:132435001	6	132435001	132436000	1000	1	6.10E-09	0.62	21	2.1	Eml1	
DMR6:132473001	6	132473001	132474000	1000	1	4.60E-07	-0.39	12	1.2	Evl	Cytoskeleton
DMR6:132585001	6	132585001	132586000	1000	1	6.60E-08	0.59	16	1.6	Evl;Degs2	Cytoskeleton;Metabolism
DMR6:132590001	6	132590001	132591000	1000	1	2.10E-08	0.65	22	2.2	Evl;Degs2	Cytoskeleton;Metabolism
DMR6:132612001	6	132612001	132614000	2000	1	6.40E-08	-0.41	29	1.45	Degs2	Metabolism
DMR6:132742001	6	132742001	132746000	4000	1	6.50E-09	-0.39	58	1.45	Slc25a29;Mir345	Transport
DMR6:132876001	6	132876001	132877000	1000	1	7.90E-09	0.54	14	1.4	Wdr25	Cytoskeleton
DMR6:132897001	6	132897001	132904000	7000	1	1.40E-08	0.47	133	1.9	Wdr25	Cytoskeleton
DMR6:132931001	6	132931001	132933000	2000	1	3.30E-07	0.65	37	1.85	Wdr25;Begain	Cytoskeleton;Cell Junction
DMR6:132937001	6	132937001	132938000	1000	1	3.70E-08	0.67	26	2.6	Wdr25;Begain	Cytoskeleton;Cell Junction
DMR6:132940001	6	132940001	132941000	1000	1	8.70E-15	0.83	28	2.8	Wdr25;Begain	Cytoskeleton;Cell Junction
DMR6:132961001	6	132961001	132963000	2000	1	8.40E-10	0.68	23	1.15	Begain	Cell Junction
DMR6:132973001	6	132973001	132975000	2000	1	2.50E-11	0.64	32	1.6	Begain	Cell Junction
DMR6:133565001	6	133565001	133568000	3000	1	7.40E-07	0.59	73	2.43	Dlk1	Signaling
DMR6:133586001	6	133586001	133594000	8000	2	4.50E-07	0.63	123	1.54	Dlk1	Signaling
DMR6:133683001	6	133683001	133684000	1000	1	1.40E-07	0.41	20	2	RGD1566401;Mir770;Mir673	
DMR6:133727001	6	133727001	133729000	2000	1	4.80E-10	-0.49	53	2.65	Rtl1;Mir341;Mir1188	
DMR6:133873001	6	133873001	133875000	2000	1	2.10E-12	0.41	40	2	Mir494;Mir1193;Mir666;Mir543;Mir495;Mir667;Mir376c;Mir376b;Mir3595;Mir376a;Mir300;Mir381;Mir487b;Mir3576;Mir539;Mir6331;Mir544;Mir3592;Mir382;Mir134	
DMR6:134821001	6	134821001	134824000	3000	2	2.70E-07	0.46	54	1.8	Ppp2r5c	Signaling
DMR6:134841001	6	134841001	134842000	1000	1	6.40E-08	0.4	17	1.7	Ppp2r5c;LOC102547242;LOC102547175	Signaling
DMR6:134844001	6	134844001	134848000	4000	2	1.10E-10	-0.57	88	2.2	Ppp2r5c;LOC102547242;LOC102547175	Signaling
DMR6:135000001	6	135000001	135004000	4000	1	2.20E-07	0.46	49	1.23	Dync1h1	Cytoskeleton
DMR6:135017001	6	135017001	135018000	1000	1	6.40E-08	-0.49	28	2.8	Dync1h1;LOC103690170	Cytoskeleton
DMR6:135151001	6	135151001	135153000	2000	1	5.50E-08	0.34	22	1.1	Wdr20	
DMR6:135279001	6	135279001	135284000	5000	1	6.60E-07	0.57	71	1.42	LOC108351302;Zfp839;LOC103692717;Cinp	
DMR6:135328001	6	135328001	135329000	1000	1	3.20E-08	-0.44	26	2.6	Tecpr2	
DMR6:135402001	6	135402001	135404000	2000	1	5.60E-07	0.57	39	1.95	Tecpr2;Ankrd9	
DMR6:135409001	6	135409001	135412000	3000	2	2.90E-12	0.74	112	3.73	Tecpr2;Ankrd9	
DMR6:135500001	6	135500001	135506000	6000	2	1.70E-10	-0.57	84	1.4	Rcor1	
DMR6:135555001	6	135555001	135557000	2000	1	2.70E-07	0.49	38	1.9	Rcor1;LOC102547964	
DMR6:135619001	6	135619001	135620000	1000	1	4.00E-07	-0.7	38	3.8	Traf3	Cytoskeleton
DMR6:135668001	6	135668001	135669000	1000	1	1.40E-09	-0.47	7	0.7	Traf3	Cytoskeleton
DMR6:135771001	6	135771001	135772000	1000	1	1.20E-14	-0.52	29	2.9	Cdc42bpb	Signaling
DMR6:135816001	6	135816001	135818000	2000	1	4.40E-10	-0.43	45	2.25	Cdc42bpb	Signaling
DMR6:135852001	6	135852001	135856000	4000	2	7.90E-17	1.01	66	1.65	RGD1560608	
DMR6:135892001	6	135892001	135893000	1000	1	3.00E-11	0.64	14	1.4	Tnfaip2	Transport
DMR6:135911001	6	135911001	135916000	5000	1	6.30E-12	0.62	60	1.2	Tnfaip2	Transport
DMR6:136328001	6	136328001	136330000	2000	1	4.70E-07	0.53	39	1.95	Klc1	Cytoskeleton
DMR6:136354001	6	136354001	136355000	1000	1	6.80E-07	0.33	19	1.9	Klc1	Cytoskeleton
DMR6:136421001	6	136421001	136425000	4000	2	4.40E-09	0.43	64	1.6	Ppp1r13b	Signaling
DMR6:136549001	6	136549001	136550000	1000	1	3.00E-07	-0.49	23	2.3	Tdrd9;LOC691437;Rd3l	Transcription
DMR6:136652001	6	136652001	136654000	2000	1	1.10E-08	-0.45	47	2.35	Tdrd9	Transcription
DMR6:136672001	6	136672001	136677000	5000	1	5.70E-07	0.67	75	1.5	Aspg	
DMR6:136731001	6	136731001	136733000	2000	1	7.70E-07	0.43	45	2.25	Kif26a	Cytoskeleton
DMR6:136734001	6	136734001	136739000	5000	1	3.00E-10	0.64	103	2.06	Kif26a	Cytoskeleton
DMR6:136764001	6	136764001	136765000	1000	1	9.40E-10	-0.51	19	1.9	Kif26a	Cytoskeleton
DMR6:137059001	6	137059001	137065000	6000	2	3.30E-10	-0.7	77	1.28	LOC691485;Tmem179	
DMR6:137679001	6	137679001	137681000	2000	1	7.00E-07	0.51	42	2.1	Gpr132	Signaling

DMR6:139404001	6	139404001	139407000	3000	1	6.50E-08	-0.41	22	0.73	Ighg;LOC100912547;LOC108351311	
DMR6:139785001	6	139785001	139787000	2000	1	1.10E-07	-0.42	10	0.5	Ighg	
DMR6:140943001	6	140943001	140944000	1000	1	1.40E-11	-0.64	2	0.2	RGD1560842;LOC100360610;LOC100360661	
DMR6:142489001	6	142489001	142491000	2000	1	8.90E-09	-0.44	92	4.6	LOC691919;LOC108351379;RGD1560697	
DMR6:144109001	6	144109001	144110000	1000	1	2.00E-08	-0.54	16	1.6	Wdr60	Cytoskeleton
DMR6:144111001	6	144111001	144113000	2000	1	3.30E-10	0.8	40	2	Wdr60	Cytoskeleton
DMR6:144128001	6	144128001	144134000	6000	1	4.90E-07	-0.41	49	0.82	Wdr60	Cytoskeleton
DMR6:144148001	6	144148001	144149000	1000	1	1.20E-09	-0.48	7	0.7	Esyt2	
DMR6:144191001	6	144191001	144193000	2000	1	9.80E-07	-0.34	12	0.6	Esyt2;LOC691128	
DMR6:144321001	6	144321001	144324000	3000	1	2.20E-08	-0.34	30	1	Ncapg2	
DMR6:144427001	6	144427001	144429000	2000	1	9.90E-07	-0.48	31	1.55	Ptprn2	Signaling
DMR6:144448001	6	144448001	144449000	1000	1	8.60E-08	-0.52	6	0.6	Ptprn2	Signaling
DMR6:144492001	6	144492001	144497000	5000	1	9.80E-13	-0.47	69	1.38	Ptprn2	Signaling
DMR6:144595001	6	144595001	144598000	3000	1	2.10E-10	-0.44	23	0.77	Ptprn2	Signaling
DMR6:144610001	6	144610001	144614000	4000	1	1.00E-08	0.6	40	1	Ptprn2	Signaling
DMR6:144637001	6	144637001	144642000	5000	1	7.20E-08	-0.37	46	0.92	Ptprn2	Signaling
DMR6:145125001	6	145125001	145128000	3000	1	5.90E-07	-0.33	45	1.5	Ptprn2	Signaling
DMR6:145384001	6	145384001	145387000	3000	1	4.60E-08	0.62	33	1.1	Rapgef5	Transcription
DMR6:145440001	6	145440001	145442000	2000	1	3.20E-07	-0.44	32	1.6	Rapgef5	Transcription
DMR6:145531001	6	145531001	145533000	2000	2	8.10E-14	-0.54	21	1.05	Rapgef5;LOC108351316;LOC102556588	Transcription
DMR6:145787001	6	145787001	145789000	2000	1	4.10E-08	0.46	17	0.85	Cdca7l;Rpl32-ps3;Dnah11	Cytoskeleton
DMR6:145817001	6	145817001	145819000	2000	1	8.60E-09	-0.45	34	1.7	Dnah11	Cytoskeleton
DMR6:145910001	6	145910001	145911000	1000	1	1.50E-08	-0.62	5	0.5	Dnah11	Cytoskeleton
DMR6:145983001	6	145983001	145984000	1000	1	7.20E-08	0.55	11	1.1	Dnah11	Cytoskeleton
DMR6:146023001	6	146023001	146024000	1000	1	1.90E-08	-0.41	14	1.4	Dnah11	Cytoskeleton
DMR6:146100001	6	146100001	146101000	1000	1	1.60E-07	0.53	6	0.6	Dnah11	Cytoskeleton
DMR6:146403001	6	146403001	146404000	1000	1	7.40E-09	0.69	13	1.3	RGD1562420	Translation
DMR6:147321001	6	147321001	147323000	2000	1	7.60E-09	-0.46	23	1.15	Macc1	
DMR7:1120001	7	1120001	1122000	2000	1	5.30E-07	-0.32	71	3.55	Arl13b	
DMR7:2741001	7	2741001	2743000	2000	1	4.90E-07	-0.31	42	2.1	Pan2;Cnpy2;Cs	Transcription;Transport
DMR7:2811001	7	2811001	2815000	4000	1	3.70E-07	0.5	59	1.48	Ankrd52;Slc39a5;Nabp2	Cytoskeleton;Transport;Metabolism
DMR7:2991001	7	2991001	2992000	1000	1	1.80E-13	0.75	49	4.9	Pa2g4;Erbb3	Protease;Receptor
DMR7:3059001	7	3059001	3065000	6000	2	6.80E-10	0.6	110	1.83	Rps26;Ikzf4;LOC103692757	Translation;Transcription
DMR7:3126001	7	3126001	3128000	2000	1	1.80E-07	0.5	25	1.25	Rab5b;Cdk2;Pmel	Signaling;Signaling
DMR7:3192001	7	3192001	3194000	2000	1	2.40E-14	-0.55	42	2.1	Pym1	
DMR7:3211001	7	3211001	3213000	2000	1	1.50E-08	-0.57	13	0.65	Mmp19	Protease
DMR7:3324001	7	3324001	3326000	2000	1	4.30E-08	0.3	20	1	Gdf11;LOC102553109;Cd63;Rdh5	Growth Factors;Metabolism
DMR7:3383001	7	3383001	3384000	1000	1	4.60E-08	0.48	16	1.6	Itga7;Mettl7b	Extracellular Matrix;Epigenetic
DMR7:3414001	7	3414001	3416000	2000	1	2.70E-07	-0.33	13	0.65	Olr875	Signaling
DMR7:3452001	7	3452001	3453000	1000	1	1.30E-20	0.95	23	2.3	Olr877	Receptor
DMR7:3742001	7	3742001	3743000	1000	1	4.50E-07	-0.55	3	0.3	Olr880	Receptor
DMR7:3798001	7	3798001	3801000	3000	1	1.20E-13	0.53	18	0.6	Olr881	Receptor
DMR7:3823001	7	3823001	3827000	4000	1	1.70E-07	-0.39	39	0.98	Olr882-ps	
DMR7:4065001	7	4065001	4066000	1000	1	1.40E-07	-0.6	9	0.9	Olr907	
DMR7:4341001	7	4341001	4342000	1000	1	7.60E-19	0.95	20	2	Olr986-ps	
DMR7:5210001	7	5210001	5223000	13000	12	4.80E-24	1.02	239	1.84	Olr897-ps	
DMR7:5346001	7	5346001	5348000	2000	2	7.40E-13	0.5	16	0.8	Olr902-ps	
DMR7:5713001	7	5713001	5714000	1000	1	8.40E-14	0.7	6	0.6	Olr913-ps	
DMR7:5755001	7	5755001	5756000	1000	1	5.30E-11	0.58	5	0.5	Olr1024	
DMR7:5847001	7	5847001	5849000	2000	1	2.50E-10	0.42	54	2.7	Olr968-ps	
DMR7:6090001	7	6090001	6091000	1000	1	3.80E-08	-0.69	2	0.2	Olr1006	Receptor
DMR7:7043001	7	7043001	7044000	1000	1	3.60E-08	-0.4	58	5.8	Olr1011-ps;LOC103692769	
DMR7:7087001	7	7087001	7088000	1000	1	7.60E-07	0.38	7	0.7	Olr1013-ps	

DMR7:7703001	7	7703001	7705000	2000	1	5.50E-07	-0.32	45	2.25	Olr1028-ps	
DMR7:7749001	7	7749001	7750000	1000	1	5.60E-07	0.43	13	1.3	LOC100909402;Olr1029	Receptor
DMR7:7764001	7	7764001	7765000	1000	1	4.60E-07	-0.31	11	1.1	Olr1030-ps	
DMR7:8556001	7	8556001	8561000	5000	1	4.00E-11	0.42	45	0.9	Olr1041-ps;Olr1042-ps	
DMR7:9034001	7	9034001	9036000	2000	1	8.20E-23	0.38	32	1.6	Olr930-ps	
DMR7:9334001	7	9334001	9335000	1000	1	9.70E-09	-0.36	19	1.9	Olr1066-ps	
DMR7:9615001	7	9615001	9620000	5000	1	1.50E-08	-0.39	44	0.88	Olr1072	Signaling
DMR7:9719001	7	9719001	9722000	3000	1	9.00E-12	-0.57	28	0.93	Neurod4	Transcription
DMR7:10949001	7	10949001	10952000	3000	1	3.00E-10	0.74	52	1.73	Sirt6;LOC102551996	
DMR7:10959001	7	10959001	10962000	3000	1	6.30E-07	-0.43	68	2.27	LOC102551996;Tle6	Transcription
DMR7:11129001	7	11129001	11142000	13000	1	5.40E-07	0.33	92	0.71	Celf5;LOC102552420;Nfic	Transcription
DMR7:11175001	7	11175001	11176000	1000	1	5.70E-07	0.6	17	1.7	Nfic;Smim24	Transcription
DMR7:11226001	7	11226001	11227000	1000	1	5.20E-10	-0.46	28	2.8	Mfsd12;LOC690617	
DMR7:11240001	7	11240001	11241000	1000	1	1.50E-09	0.48	29	2.9	Hmg20b;LOC102552827;Gipc3	Cytoskeleton
DMR7:11252001	7	11252001	11253000	1000	1	1.40E-09	0.53	25	2.5	Hmg20b;LOC102552827;Gipc3;Tbxa2r;Cactin	Cytoskeleton;Signaling
DMR7:11272001	7	11272001	11273000	1000	1	4.70E-07	0.52	17	1.7	Cactin;Pip5k1c	Cytoskeleton;Signaling
DMR7:11313001	7	11313001	11319000	6000	1	6.20E-10	0.71	196	3.27	Tjp3;Apba3;Mrpl54;Matk	Cell Junction;Transport;Translation
DMR7:11335001	7	11335001	11336000	1000	1	8.90E-08	0.62	16	1.6	Matk;Zfr2	Metabolism
DMR7:11347001	7	11347001	11352000	5000	2	7.30E-10	0.68	90	1.8	Zfr2;Atcay	Metabolism
DMR7:11358001	7	11358001	11359000	1000	1	1.50E-08	0.45	5	0.5	Zfr2;Atcay	Metabolism
DMR7:11383001	7	11383001	11387000	4000	2	4.60E-09	0.59	102	2.55	Atcay;Nmrk2;Dapk3	Signaling;Signaling
DMR7:11427001	7	11427001	11428000	1000	1	5.50E-12	0.88	91	9.1	Pias4;Zbtb7a	
DMR7:11530001	7	11530001	11533000	3000	1	1.50E-07	0.43	44	1.47	Sgta	
DMR7:11617001	7	11617001	11624000	7000	1	4.60E-07	0.43	90	1.29	Gng7	Signaling
DMR7:11655001	7	11655001	11656000	1000	1	3.70E-09	0.49	18	1.8	Gadd45b;Lmnb2	
DMR7:11680001	7	11680001	11681000	1000	1	1.90E-09	0.63	47	4.7	Lmnb2;Timm13;Tmprss9	Protease
DMR7:11897001	7	11897001	11898000	1000	1	5.80E-07	-0.43	15	1.5	Mob3a;LOC108351611	Signaling
DMR7:11929001	7	11929001	11933000	4000	1	6.00E-08	-0.62	117	2.92	Mknk2;Btbd2	Signaling;Proteolysis
DMR7:11945001	7	11945001	11948000	3000	1	6.00E-16	0.85	168	5.6	Btbd2;Csnk1g2	Proteolysis;Signaling
DMR7:12048001	7	12048001	12049000	1000	1	4.90E-10	0.7	19	1.9	Rexo1;Atp8b3	Transcription;Transport
DMR7:12061001	7	12061001	12064000	3000	2	1.00E-22	0.79	77	2.57	Atp8b3	Transport
DMR7:12161001	7	12161001	12163000	2000	1	3.50E-07	0.5	47	2.35	Tcf3;Uqcr11	Transcription;Metabolism
DMR7:12199001	7	12199001	12202000	3000	1	7.30E-07	0.39	68	2.27	Mex3d	Metabolism
DMR7:12209001	7	12209001	12211000	2000	1	3.80E-08	-0.57	5	0.25	Plk5	Signaling
DMR7:12322001	7	12322001	12323000	1000	1	9.50E-07	0.38	17	1.7	Dazap1;Gamt;Ndufs7;Trnan-guu;Trnaf-gaa;Mum1	Epigenetic;Metabolism
DMR7:12436001	7	12436001	12437000	1000	1	1.70E-07	0.5	32	3.2	Midn;Atp5d;Cbarp;Stk11	Transport
DMR7:12441001	7	12441001	12442000	1000	1	2.60E-07	0.55	33	3.3	Atp5d;Cbarp;Stk11	Transport
DMR7:12587001	7	12587001	12588000	1000	1	1.70E-16	0.9	34	3.4	Arhgap45;Arid3a;LOC103692777	Transcription
DMR7:12590001	7	12590001	12594000	4000	1	4.50E-07	-0.44	102	2.55	Arhgap45;Arid3a	Transcription
DMR7:12630001	7	12630001	12633000	3000	1	2.20E-15	0.93	67	2.23	Arhgap45;Med16;Cfd;Elane	Protease
DMR7:12640001	7	12640001	12644000	4000	2	1.40E-08	0.53	82	2.05	Arhgap45;Med16;Cfd;Elane;Prtn3;Plppr3	Protease;Signaling
DMR7:12653001	7	12653001	12655000	2000	1	1.50E-07	0.64	42	2.1	Arhgap45;Prtn3;Plppr3;Ptbp1	Protease;Signaling
DMR7:12666001	7	12666001	12670000	4000	2	1.40E-15	-0.56	121	3.02	Arhgap45;Plppr3;Ptbp1	Signaling
DMR7:12684001	7	12684001	12686000	2000	1	3.60E-07	0.51	39	1.95	Arhgap45;Misp	
DMR7:12726001	7	12726001	12730000	4000	1	3.20E-08	0.46	77	1.93	Arhgap45;Palm	
DMR7:12731001	7	12731001	12733000	2000	1	8.80E-17	0.9	70	3.5	Arhgap45;Palm;Abca7	Transport
DMR7:12739001	7	12739001	12742000	3000	1	4.80E-07	0.51	72	2.4	Arhgap45;Abca7	Transport
DMR7:12754001	7	12754001	12755000	1000	1	4.20E-08	-0.89	76	7.6	Abca7;Grin3b	Transport;Receptor
DMR7:12771001	7	12771001	12774000	3000	2	1.40E-12	0.84	166	5.53	Abca7;Grin3b;Tmem259;Cnn2	Transport;Receptor;Cytoskeleton;Cytoskeleton
DMR7:12796001	7	12796001	12798000	2000	1	3.00E-08	-0.48	21	1.05	Cnn2;Prss57;Fstl3	Cytoskeleton;Protease; Protease; Proteolysis
DMR7:12846001	7	12846001	12851000	5000	2	1.50E-12	0.77	194	3.88	Polrmt;Hcn2	Transcription;Transport

DMR7:12852001	7	12852001	12854000	2000	1	8.50E-09	0.5	83	4.15	Polrmt;Hcn2	Transcription;Transport
DMR7:12893001	7	12893001	12896000	3000	1	4.50E-07	0.47	54	1.8	Gzmm;Cdc34	Protease
DMR7:12930001	7	12930001	12931000	1000	1	4.70E-07	-0.31	13	1.3	Madcam1	
DMR7:12943001	7	12943001	12947000	4000	1	6.20E-07	0.5	72	1.8	Odf3l2;Shc2	Development;Cytoskeleton
DMR7:12958001	7	12958001	12960000	2000	1	3.60E-07	0.69	44	2.2	Shc2	Cytoskeleton
DMR7:13016001	7	13016001	13019000	3000	2	6.70E-15	0.92	69	2.3	Theg	
DMR7:13026001	7	13026001	13029000	3000	1	8.80E-07	0.41	46	1.53	Theg	
DMR7:13050001	7	13050001	13051000	1000	1	5.40E-07	0.43	23	2.3	Mier2	Development
DMR7:13077001	7	13077001	13078000	1000	1	5.60E-07	-0.44	7	0.7	Plpp2	Signaling
DMR7:13171001	7	13171001	13173000	2000	1	5.70E-07	-0.5	10	0.5	Vom2r53	Signaling
DMR7:13186001	7	13186001	13188000	2000	1	2.10E-08	0.5	34	1.7	Vom2r53	Signaling
DMR7:13431001	7	13431001	13432000	1000	1	2.40E-08	-0.62	7	0.7	Olr1076	Receptor
DMR7:13435001	7	13435001	13440000	5000	1	6.60E-07	-0.27	53	1.06	Olr1076	Receptor
DMR7:13540001	7	13540001	13544000	4000	1	9.50E-08	0.55	71	1.77	Olr1081;LOC108351571;Olr1082	Receptor
DMR7:13554001	7	13554001	13555000	1000	1	4.70E-07	-0.52	1	0.1	LOC108351571;Olr1082	Receptor
DMR7:13622001	7	13622001	13625000	3000	1	3.20E-07	-0.34	24	0.8	Olr1084	Receptor
DMR7:13729001	7	13729001	13733000	4000	2	9.20E-10	-0.54	26	0.65	Slc1a6	Transport
DMR7:13757001	7	13757001	13758000	1000	1	1.40E-07	0.46	29	2.9	Slc1a6;LOC102549402	Transport
DMR7:13917001	7	13917001	13920000	3000	3	1.10E-25	1.2	86	2.87	Ccdc105	
DMR7:13946001	7	13946001	13950000	4000	1	3.80E-07	-0.36	67	1.68	Casp14	Protease
DMR7:13988001	7	13988001	13994000	6000	1	3.00E-07	-0.53	94	1.57	LOC102555134;Olr1087	Receptor
DMR7:14058001	7	14058001	14061000	3000	1	1.00E-08	-0.44	35	1.17	Ilvbl;LOC687281;LOC314605	Metabolism
DMR7:14110001	7	14110001	14111000	1000	1	2.20E-08	0.48	17	1.7	Olr1088	
DMR7:14138001	7	14138001	14140000	2000	1	1.20E-07	0.52	48	2.4	Olr1089-ps;Notch3	
DMR7:14162001	7	14162001	14163000	1000	1	4.30E-18	1	33	3.3	Notch3	
DMR7:14176001	7	14176001	14177000	1000	1	3.00E-13	0.83	35	3.5	Notch3	
DMR7:14197001	7	14197001	14202000	5000	1	3.10E-08	0.59	78	1.56	Notch3	
DMR7:14205001	7	14205001	14206000	1000	1	3.70E-10	0.65	25	2.5	Ephx3	Metabolism
DMR7:14244001	7	14244001	14249000	5000	1	8.10E-08	-0.56	53	1.06	Brd4	
DMR7:14346001	7	14346001	14347000	1000	1	2.60E-07	-0.57	15	1.5	Akap8;Akap8l	Cytoskeleton
DMR7:14388001	7	14388001	14389000	1000	1	5.30E-09	0.45	15	1.5	Wiz	Transcription
DMR7:14412001	7	14412001	14415000	3000	1	2.70E-15	0.58	85	2.83	Wiz;Rasal3	Transcription;Signaling
DMR7:14448001	7	14448001	14450000	2000	1	9.80E-07	0.4	65	3.25	Cyp4f39	Metabolism
DMR7:14477001	7	14477001	14478000	1000	1	2.40E-09	0.5	23	2.3	Cyp4f39;LOC102555438	Metabolism
DMR7:14485001	7	14485001	14488000	3000	1	8.70E-12	0.53	50	1.67	Cyp4f39;LOC102555438	Metabolism
DMR7:14504001	7	14504001	14506000	2000	1	8.60E-08	-0.33	47	2.35	Cyp4f39	Metabolism
DMR7:14515001	7	14515001	14516000	1000	1	4.30E-16	0.93	22	2.2	Cyp4f39	Metabolism
DMR7:14523001	7	14523001	14527000	4000	1	3.70E-11	0.61	86	2.15	Cyp4f39;Cyp4f17	Metabolism
DMR7:15236001	7	15236001	15241000	5000	1	8.20E-07	-0.43	38	0.76	LOC102548621;Zfp472	
DMR7:15435001	7	15435001	15438000	3000	1	4.40E-09	-0.4	28	0.93	Zfp563	
DMR7:15481001	7	15481001	15482000	1000	1	8.00E-09	0.56	31	3.1	Morc2b	
DMR7:15703001	7	15703001	15704000	1000	1	1.60E-09	-0.4	5	0.5	Olr1094-ps;Olr1092	
DMR7:16420001	7	16420001	16423000	3000	1	7.40E-07	-0.18	33	1.1	Olr1056-ps	
DMR7:16951001	7	16951001	16954000	3000	1	6.20E-08	-0.33	26	0.87	Vom2r54	Signaling
DMR7:17056001	7	17056001	17060000	4000	2	2.40E-07	-0.3	38	0.95	Vom2r55	
DMR7:17961001	7	17961001	17966000	5000	1	8.50E-08	0.52	103	2.06	Vom1r106;Vom1r-ps96	Receptor
DMR7:18173001	7	18173001	18175000	2000	1	3.40E-09	-0.48	15	0.75	LOC691480;LOC103690176;Vom1r-ps97;Zfp494	Transcription
DMR7:18224001	7	18224001	18225000	1000	1	9.50E-09	-0.44	7	0.7	Vom1r-ps100;Vom1r-ps101	
DMR7:18257001	7	18257001	18259000	2000	1	9.00E-09	0.43	12	0.6	Vom1r-ps102;LOC108351411	
DMR7:18476001	7	18476001	18478000	2000	1	6.00E-07	-0.49	11	0.55	Myo1f;LOC102547064	Cytoskeleton
DMR7:18509001	7	18509001	18513000	4000	1	4.00E-08	-0.46	36	0.9	Zfp414;Pram1;Hnrnpm	Cytoskeleton;Translation
DMR7:18900001	7	18900001	18905000	5000	1	9.50E-07	-0.29	32	0.64	Vom2r56;Olr1097-ps	
DMR7:19100001	7	19100001	19101000	1000	1	2.40E-07	-0.42	5	0.5	Vom2r57	Signaling
DMR7:20092001	7	20092001	20093000	1000	1	2.40E-07	-0.62	4	0.4	RGD1565071	Signaling
DMR7:20123001	7	20123001	20124000	1000	1	8.60E-08	-0.36	7	0.7	RGD1565071;LOC103690279	Signaling
DMR7:20257001	7	20257001	20259000	2000	2	3.40E-10	-0.45	10	0.5	LOC100912242;RGD1564409	
DMR7:20260001	7	20260001	20262000	2000	1	1.80E-08	0.42	22	1.1	LOC100912242;RGD1564409	

DMR7:20310001	7	20310001	20312000	2000	1	2.30E-11	0.29	16	0.8	RGD1564409	
DMR7:20341001	7	20341001	20342000	1000	1	9.50E-07	0.46	18	1.8	RGD1564409;LOC100912315;LOC103690230	
DMR7:20360001	7	20360001	20363000	3000	1	3.60E-12	0.41	24	0.8	RGD1564409;LOC100912403	
DMR7:20369001	7	20369001	20372000	3000	2	6.40E-21	1.46	76	2.53	RGD1564409;LOC100912403	
DMR7:21603001	7	21603001	21606000	3000	1	6.60E-18	1.1	47	1.57	Olr1829-ps	
DMR7:22921001	7	22921001	22925000	4000	1	1.40E-07	-0.48	29	0.72	Olr1099-ps;Vom2r-ps87	
DMR7:23525001	7	23525001	23528000	3000	1	2.30E-08	-0.42	37	1.23	Syn3	Transport
DMR7:23709001	7	23709001	23711000	2000	1	1.40E-07	0.42	30	1.5	Syn3	Transport
DMR7:23807001	7	23807001	23809000	2000	1	1.90E-07	0.56	28	1.4	Syn3;LOC102551348;Fbxo7	Transport
DMR7:23837001	7	23837001	23838000	1000	1	5.50E-07	-0.44	2	0.2	Fbxo7	
DMR7:23848001	7	23848001	23850000	2000	1	6.60E-08	-0.36	30	1.5	Fbxo7;Bpifc	
DMR7:23887001	7	23887001	23889000	2000	1	1.10E-07	-0.35	21	1.05	Bpifc	
DMR7:23936001	7	23936001	23940000	4000	2	3.50E-10	-0.41	36	0.9	Rtcb;Ascl4	Transcription
DMR7:24111001	7	24111001	24113000	2000	1	5.50E-10	-0.5	14	0.7	Btbd11	Cytoskeleton
DMR7:24142001	7	24142001	24144000	2000	2	2.20E-07	0.59	39	1.95	Btbd11	Cytoskeleton
DMR7:24180001	7	24180001	24181000	1000	1	3.50E-09	0.64	21	2.1	Btbd11	Cytoskeleton
DMR7:24223001	7	24223001	24228000	5000	2	2.50E-08	0.49	86	1.72	Btbd11;Ybx1-ps4	Cytoskeleton
DMR7:24287001	7	24287001	24291000	4000	1	4.20E-08	0.31	76	1.9	Btbd11	Cytoskeleton
DMR7:24319001	7	24319001	24320000	1000	1	7.70E-09	0.66	25	2.5	Btbd11	Cytoskeleton
DMR7:24536001	7	24536001	24537000	1000	1	4.10E-10	-0.41	18	1.8	Cry1	DNA Repair
DMR7:24543001	7	24543001	24545000	2000	1	1.50E-07	-0.37	18	0.9	Cry1	DNA Repair
DMR7:24578001	7	24578001	24583000	5000	1	5.20E-08	-0.4	66	1.32	Cry1;LOC102551657	DNA Repair
DMR7:24737001	7	24737001	24738000	1000	1	5.90E-10	0.5	21	2.1	Polr3b	Transcription
DMR7:24943001	7	24943001	24946000	3000	1	6.70E-11	-0.43	87	2.9	Ckap4	
DMR7:25120001	7	25120001	25121000	1000	1	5.00E-08	0.6	18	1.8	Nuak1	Signaling
DMR7:25870001	7	25870001	25871000	1000	1	5.20E-09	0.36	8	0.8	LOC100910996;Rfx4	Transcription
DMR7:25888001	7	25888001	25892000	4000	1	9.60E-07	-0.44	85	2.12	LOC100910996;Rfx4	Transcription
DMR7:26023001	7	26023001	26024000	1000	1	6.70E-13	0.69	23	2.3	LOC100910996;Ric8b	
DMR7:26132001	7	26132001	26134000	2000	1	1.90E-09	0.54	26	1.3	LOC100910996;Fhl4	
DMR7:26286001	7	26286001	26289000	3000	1	1.90E-07	0.32	13	0.43	Appl2	Cytoskeleton
DMR7:26528001	7	26528001	26531000	3000	1	3.90E-08	-0.35	58	1.93	Slc41a2	Transport
DMR7:26546001	7	26546001	26548000	2000	1	7.20E-08	-0.39	49	2.45	Slc41a2;LOC103692827	Transport
DMR7:26559001	7	26559001	26562000	3000	1	4.00E-07	-0.34	54	1.8	Slc41a2;LOC103692827	Transport
DMR7:26575001	7	26575001	26581000	6000	1	2.50E-10	-0.38	107	1.78	Slc41a2	Transport
DMR7:26634001	7	26634001	26636000	2000	1	2.20E-08	0.6	28	1.4	Chst11	Transport
DMR7:26728001	7	26728001	26730000	2000	1	1.00E-08	0.47	40	2	Chst11	Transport
DMR7:26755001	7	26755001	26756000	1000	1	1.30E-10	0.88	19	1.9	Chst11	Transport
DMR7:26785001	7	26785001	26786000	1000	1	2.40E-07	-0.43	26	2.6	Chst11	Transport
DMR7:26812001	7	26812001	26814000	2000	1	6.50E-08	-0.44	36	1.8	Chst11	Transport
DMR7:26894001	7	26894001	26895000	1000	1	1.30E-10	0.7	11	1.1	Chst11	Transport
DMR7:26973001	7	26973001	26976000	3000	1	7.70E-09	-0.4	50	1.67	Txnrd1;Eid3;LOC108351425	Metabolism
DMR7:27087001	7	27087001	27088000	1000	1	1.90E-09	-0.53	20	2	Nfyb	Transcription
DMR7:27152001	7	27152001	27153000	1000	1	4.60E-09	-0.35	25	2.5	Glt8d2	Golgi
DMR7:27161001	7	27161001	27164000	3000	1	3.70E-12	0.78	75	2.5	Glt8d2	Golgi
DMR7:27203001	7	27203001	27207000	4000	1	5.40E-10	-0.39	71	1.77	Glt8d2;Tdg	Golgi;Epigenetic
DMR7:27326001	7	27326001	27327000	1000	1	2.10E-08	-0.41	26	2.6	Nt5dc3	Signaling
DMR7:27403001	7	27403001	27404000	1000	1	4.20E-11	0.7	29	2.9	Stab2	Transport
DMR7:27436001	7	27436001	27439000	3000	2	2.50E-16	0.83	61	2.03	Stab2;LOC102550284	Transport
DMR7:27485001	7	27485001	27488000	3000	1	4.60E-07	0.54	46	1.53	Stab2	Transport
DMR7:27507001	7	27507001	27508000	1000	1	2.60E-12	0.66	16	1.6	Stab2	Transport
DMR7:27547001	7	27547001	27549000	2000	1	2.70E-08	0.6	31	1.55	Stab2	Transport
DMR7:27705001	7	27705001	27706000	1000	1	3.30E-07	0.73	32	3.2	RGD1560034	
DMR7:28094001	7	28094001	28095000	1000	1	1.00E-11	0.81	11	1.1	Pah	
DMR7:28096001	7	28096001	28101000	5000	3	6.80E-16	0.89	73	1.46	Pah	
DMR7:28753001	7	28753001	28755000	2000	1	6.00E-07	0.63	38	1.9	Nup37;Ccdc53	Transport
DMR7:28894001	7	28894001	28896000	2000	1	9.10E-07	0.42	35	1.75	LOC108351579;Dram1	
DMR7:28917001	7	28917001	28920000	3000	1	1.30E-08	0.73	71	2.37	Dram1	
DMR7:28980001	7	28980001	28981000	1000	1	7.80E-07	0.44	12	1.2	Gnptab	
DMR7:29053001	7	29053001	29059000	6000	1	4.50E-07	-0.34	63	1.05	Chpt1;Sycp3	Transport
DMR7:29077001	7	29077001	29082000	5000	1	2.20E-09	-0.53	58	1.16	Chpt1;LOC102554140;Mybpc1	Transport
DMR7:29104001	7	29104001	29107000	3000	2	1.50E-13	-0.5	45	1.5	Mybpc1	

DMR7:29116001	7	29116001	29118000	2000	1	7.20E-07	0.5	26	1.3	Mybpc1	
DMR7:29119001	7	29119001	29121000	2000	1	4.00E-10	0.72	43	2.15	Mybpc1	
DMR7:29166001	7	29166001	29171000	5000	1	8.50E-12	0.77	102	2.04	Mybpc1	
DMR7:29218001	7	29218001	29220000	2000	1	7.80E-08	0.49	31	1.55	Spic	Transcription
DMR7:29350001	7	29350001	29352000	2000	1	8.20E-10	-0.4	33	1.65	Utp20	
DMR7:29422001	7	29422001	29426000	4000	1	5.90E-08	0.34	44	1.1	Slc5a8	Transport
DMR7:29436001	7	29436001	29438000	2000	1	2.30E-09	0.59	43	2.15	Slc5a8	Transport
DMR7:29641001	7	29641001	29643000	2000	1	2.50E-07	0.58	42	2.1	Ano4;LOC108351429	
DMR7:29695001	7	29695001	29697000	2000	1	1.20E-08	-0.57	9	0.45	Ano4	
DMR7:29699001	7	29699001	29702000	3000	1	5.60E-08	-0.53	32	1.07	Ano4	
DMR7:29743001	7	29743001	29744000	1000	1	2.00E-09	-0.62	5	0.5	Ano4	
DMR7:29748001	7	29748001	29750000	2000	1	2.40E-10	-0.45	25	1.25	Ano4	
DMR7:29877001	7	29877001	29878000	1000	1	4.40E-07	-0.51	6	0.6	Ano4;LOC102554867	
DMR7:29892001	7	29892001	29894000	2000	1	2.30E-09	0.61	38	1.9	Ano4;LOC102554867	
DMR7:29900001	7	29900001	29902000	2000	1	1.30E-15	1	34	1.7	Ano4;LOC102554867;LOC1009 09955	
DMR7:30083001	7	30083001	30086000	3000	1	1.30E-18	-1.02	26	0.87	Nr1h4;LOC102548681	Transcription
DMR7:30123001	7	30123001	30124000	1000	1	7.40E-07	-0.46	23	2.3	Nr1h4;LOC100910056	Transcription
DMR7:30138001	7	30138001	30139000	1000	1	3.90E-10	-0.73	5	0.5	Nr1h4;LOC100910056	Transcription
DMR7:30276001	7	30276001	30279000	3000	1	7.30E-10	0.47	49	1.63	Slc17a8	Transport
DMR7:30370001	7	30370001	30371000	1000	1	9.10E-07	0.46	10	1	Actr6	Cytoskeleton
DMR7:30443001	7	30443001	30444000	1000	1	2.00E-07	-0.59	20	2	Uhrf1bp1l	
DMR7:30458001	7	30458001	30459000	1000	1	2.30E-07	-0.32	13	1.3	Uhrf1bp1l	
DMR7:30563001	7	30563001	30565000	2000	1	5.30E-08	0.32	39	1.95	Anks1b	Cytoskeleton
DMR7:30579001	7	30579001	30582000	3000	2	4.30E-07	-0.29	26	0.87	Anks1b	Cytoskeleton
DMR7:30708001	7	30708001	30710000	2000	1	5.40E-07	-0.43	38	1.9	Anks1b	Cytoskeleton
DMR7:31022001	7	31022001	31025000	3000	1	9.90E-10	0.62	46	1.53	Anks1b	Cytoskeleton
DMR7:31117001	7	31117001	31118000	1000	1	2.40E-13	1.03	20	2	Anks1b;LOC108351597	Cytoskeleton
DMR7:31157001	7	31157001	31158000	1000	1	2.20E-08	0.57	27	2.7	Anks1b	Cytoskeleton
DMR7:31176001	7	31176001	31179000	3000	1	4.20E-12	-0.55	33	1.1	Anks1b	Cytoskeleton
DMR7:31263001	7	31263001	31267000	4000	1	2.80E-14	-0.59	87	2.17	Anks1b	Cytoskeleton
DMR7:31281001	7	31281001	31284000	3000	1	2.20E-07	-0.43	44	1.47	Anks1b	Cytoskeleton
DMR7:31379001	7	31379001	31380000	1000	1	2.10E-07	0.55	15	1.5	Anks1b	Cytoskeleton
DMR7:31403001	7	31403001	31406000	3000	2	2.50E-09	0.75	45	1.5	Anks1b	Cytoskeleton
DMR7:31425001	7	31425001	31429000	4000	1	4.80E-15	1.05	86	2.15	Anks1b	Cytoskeleton
DMR7:31583001	7	31583001	31585000	2000	1	1.00E-07	0.38	40	2	Anks1b;LOC102547065	Cytoskeleton
DMR7:31592001	7	31592001	31597000	5000	1	8.20E-07	-0.31	54	1.08	Anks1b;LOC102547065	Cytoskeleton
DMR7:31628001	7	31628001	31631000	3000	1	1.30E-08	0.53	81	2.7	Anks1b	Cytoskeleton
DMR7:31632001	7	31632001	31635000	3000	2	7.40E-09	-0.42	89	2.97	Anks1b	Cytoskeleton
DMR7:31662001	7	31662001	31665000	3000	1	6.10E-08	0.58	43	1.43	Anks1b	Cytoskeleton
DMR7:31680001	7	31680001	31681000	1000	1	2.30E-08	0.54	40	4	Anks1b	Cytoskeleton
DMR7:31683001	7	31683001	31687000	4000	1	1.10E-07	0.47	72	1.8	Anks1b	Cytoskeleton
DMR7:31808001	7	31808001	31811000	3000	1	2.30E-09	0.73	50	1.67	Ikbip;Slc25a3	Transport
DMR7:31825001	7	31825001	31826000	1000	1	4.00E-07	0.62	17	1.7	Slc25a3	Transport
DMR7:31893001	7	31893001	31894000	1000	1	1.70E-08	0.77	24	2.4	LOC103692821;Trnaw-cca;Trnad-guc	
DMR7:32684001	7	32684001	32686000	2000	1	8.60E-07	-0.41	15	0.75	RGD1565429	
DMR7:32894001	7	32894001	32896000	2000	1	4.60E-08	-0.48	22	1.1	Mir135a	
DMR7:33644001	7	33644001	33645000	1000	1	3.30E-08	-0.43	5	0.5	RGD1565866	
DMR7:33671001	7	33671001	33673000	2000	1	2.00E-08	0.59	19	0.95	RGD1565866	
DMR7:33689001	7	33689001	33691000	2000	1	2.40E-08	-0.58	19	0.95	RGD1565866	
DMR7:33692001	7	33692001	33694000	2000	1	8.00E-08	-0.44	25	1.25	RGD1565866	
DMR7:33872001	7	33872001	33874000	2000	2	3.80E-09	0.74	57	2.85	RGD1565866	
DMR7:34032001	7	34032001	34033000	1000	1	9.00E-10	-0.68	16	1.6	Cdk17	Signaling
DMR7:34037001	7	34037001	34040000	3000	1	6.90E-07	-0.47	46	1.53	Cdk17	Signaling
DMR7:34072001	7	34072001	34074000	2000	2	3.70E-08	-0.53	35	1.75	Cdk17	Signaling
DMR7:34302001	7	34302001	34303000	1000	1	2.20E-10	-0.43	26	2.6	Lta4h	
DMR7:34330001	7	34330001	34332000	2000	1	7.00E-10	-0.56	27	1.35	Lta4h;LOC102553539;Hal	Metabolism
DMR7:34347001	7	34347001	34352000	5000	2	3.30E-07	-0.39	75	1.5	Hal	Metabolism
DMR7:34365001	7	34365001	34366000	1000	1	3.20E-09	0.54	27	2.7	Hal;Amdhd1	Metabolism;Protease
DMR7:34486001	7	34486001	34487000	1000	1	2.90E-08	-0.42	17	1.7	Ccdc38;Snrpf	Translation
DMR7:34525001	7	34525001	34529000	4000	1	7.70E-09	-0.41	89	2.22	Ntn4	Extracellular Matrix

DMR7:34614001	7	34614001	34617000	3000	1	3.70E-08	0.62	89	2.97	Ntn4;LOC100362560	Extracellular Matrix
DMR7:34628001	7	34628001	34629000	1000	1	9.80E-07	0.58	21	2.1	Ntn4	Extracellular Matrix
DMR7:34747001	7	34747001	34748000	1000	1	2.10E-13	0.62	35	3.5	Usp44	Protease
DMR7:34793001	7	34793001	34795000	2000	1	1.30E-11	-0.51	35	1.75	Metap2	Protease
DMR7:34972001	7	34972001	34977000	5000	1	5.90E-08	-0.36	84	1.68	Fgd6	Transcription
DMR7:34996001	7	34996001	3.50E+07	4000	1	8.00E-08	-0.56	44	1.1	Fgd6	Transcription
DMR7:35030001	7	35030001	35033000	3000	1	2.90E-07	-0.41	51	1.7	Fgd6	Transcription
DMR7:35040001	7	35040001	35044000	4000	1	3.60E-09	-0.42	70	1.75	Fgd6	Transcription
DMR7:35282001	7	35282001	35287000	5000	1	3.80E-07	-0.39	93	1.86	Tmcc3	
DMR7:35313001	7	35313001	35315000	2000	1	1.20E-07	-0.43	17	0.85	Tmcc3	
DMR7:35370001	7	35370001	35372000	2000	1	1.50E-07	-0.42	44	2.2	Tmcc3	
DMR7:35436001	7	35436001	35437000	1000	1	1.10E-08	-0.44	19	1.9	Tmcc3	
DMR7:35455001	7	35455001	35457000	2000	1	1.80E-07	0.54	50	2.5	Tmcc3	
DMR7:35503001	7	35503001	35505000	2000	1	8.30E-08	-0.37	34	1.7	Tmcc3	
DMR7:35709001	7	35709001	35711000	2000	2	2.40E-13	-0.83	2	0.1	LOC100912201;Cep83os	
DMR7:36007001	7	36007001	36009000	2000	2	5.10E-16	1.14	42	2.1	Plxnc1	
DMR7:36486001	7	36486001	36488000	2000	2	2.50E-07	0.38	45	2.25	Socs2	Signaling
DMR7:36489001	7	36489001	36492000	3000	1	7.00E-13	-0.66	61	2.03	Socs2	Signaling
DMR7:36493001	7	36493001	36495000	2000	1	1.10E-09	0.62	31	1.55	Socs2	Signaling
DMR7:36509001	7	36509001	36510000	1000	1	6.00E-07	0.41	14	1.4	Socs2	Signaling
DMR7:36835001	7	36835001	36838000	3000	1	1.90E-11	0.6	41	1.37	Ndufa13;LOC108351434	Metabolism
DMR7:37134001	7	37134001	37136000	2000	1	8.20E-07	0.52	18	0.9	Eea1;LOC102547143	Transport
DMR7:37207001	7	37207001	37208000	1000	1	1.80E-19	0.62	26	2.6	Plekhg7	
DMR7:37219001	7	37219001	37220000	1000	1	8.50E-07	-0.34	39	3.9	Plekhg7	
DMR7:37809001	7	37809001	37812000	3000	1	2.50E-07	-0.36	30	1	LOC102550725;Btg1	
DMR7:38957001	7	38957001	38958000	1000	1	1.70E-07	-0.42	6	0.6	Ccer1	
DMR7:38967001	7	38967001	38968000	1000	1	4.10E-08	-0.59	6	0.6	Ccer1	
DMR7:41107001	7	41107001	41109000	2000	1	9.60E-09	-0.4	21	1.05	Atp2b1	Transport
DMR7:41141001	7	41141001	41144000	3000	1	5.00E-09	-0.36	43	1.43	Atp2b1	Transport
DMR7:41166001	7	41166001	41168000	2000	1	3.00E-08	-0.39	36	1.8	Atp2b1	Transport
DMR7:41200001	7	41200001	41202000	2000	1	7.20E-10	-0.56	51	2.55	Atp2b1	Transport
DMR7:41209001	7	41209001	41214000	5000	1	8.10E-10	-0.46	134	2.68	Atp2b1	Transport
DMR7:41314001	7	41314001	41315000	1000	1	3.30E-07	-0.4	12	1.2	Poc1b;Galnt4	
DMR7:41367001	7	41367001	41369000	2000	1	1.60E-09	-0.55	27	1.35	Poc1b	
DMR7:41411001	7	41411001	41412000	1000	1	6.00E-14	0.95	28	2.8	Poc1b;LOC108351444	
DMR7:42016001	7	42016001	42017000	1000	1	2.70E-09	-0.46	16	1.6	RGD1565753	
DMR7:42309001	7	42309001	42313000	4000	1	7.00E-07	0.37	60	1.5	Kitlg	
DMR7:42356001	7	42356001	42358000	2000	2	3.30E-13	1.11	48	2.4	Kitlg;LOC108351446	
DMR7:43416001	7	43416001	43417000	1000	1	5.50E-07	-0.47	4	0.4	Mgat4c	Transport
DMR7:43663001	7	43663001	43664000	1000	1	8.80E-07	-0.66	0	0	Mgat4c	Transport
DMR7:43873001	7	43873001	43877000	4000	1	8.50E-07	-0.28	59	1.48	Mgat4c	Transport
DMR7:44159001	7	44159001	44162000	3000	1	4.90E-07	-0.48	35	1.17	Rassf9	Cytoskeleton
DMR7:45372001	7	45372001	45373000	1000	1	2.10E-12	-0.44	13	1.3	Slc6a15	Transport
DMR7:45380001	7	45380001	45381000	1000	1	2.60E-10	0.73	31	3.1	Slc6a15	Transport
DMR7:47230001	7	47230001	47236000	6000	1	3.30E-08	0.48	52	0.87	Tmtc2	Golgi
DMR7:47353001	7	47353001	47358000	5000	1	1.70E-08	-0.36	50	1	Tmtc2	Golgi
DMR7:47476001	7	47476001	47477000	1000	1	8.10E-21	1.31	25	2.5	Tmtc2	Golgi
DMR7:48561001	7	48561001	48564000	3000	2	1.80E-08	-0.53	17	0.57	Ppfia2	
DMR7:48725001	7	48725001	48727000	2000	1	8.30E-10	0.67	35	1.75	Ppfia2	
DMR7:48933001	7	48933001	48936000	3000	1	5.80E-07	-0.5	10	0.33	Ppfia2	
DMR7:48977001	7	48977001	48978000	1000	1	4.60E-08	0.71	19	1.9	Ppfia2	
DMR7:48994001	7	48994001	48996000	2000	1	6.40E-07	-0.42	22	1.1	Ppfia2	
DMR7:49087001	7	49087001	49090000	3000	1	1.30E-08	-0.29	30	1	Acss3	Metabolism
DMR7:49189001	7	49189001	49192000	3000	1	1.10E-11	-0.43	31	1.03	Acss3	Metabolism
DMR7:49255001	7	49255001	49258000	3000	2	5.80E-08	-0.41	24	0.8	Acss3	Metabolism
DMR7:49719001	7	49719001	49720000	1000	1	1.60E-07	0.42	14	1.4	Myf5	Transcription
DMR7:49838001	7	49838001	49839000	1000	1	2.50E-17	0.86	9	0.9	Ptprq	Receptor
DMR7:50132001	7	50132001	50133000	1000	1	1.50E-08	0.52	13	1.3	Syt1	Transport
DMR7:50283001	7	50283001	50284000	1000	1	6.90E-11	0.57	19	1.9	Syt1;LOC103692848	Transport
DMR7:50316001	7	50316001	50318000	2000	1	7.80E-12	0.42	47	2.35	Syt1	Transport
DMR7:50494001	7	50494001	50495000	1000	1	7.50E-10	0.54	10	1	Syt1	Transport
DMR7:50519001	7	50519001	50520000	1000	1	3.60E-09	-0.44	24	2.4	Syt1	Transport

DMR7:51264001	7	51264001	51266000	2000	1	6.30E-11	-0.68	30	1.5	Pawr	
DMR7:51282001	7	51282001	51283000	1000	1	8.20E-08	-0.52	24	2.4	Pawr;LOC102552256	
DMR7:51359001	7	51359001	51362000	3000	1	1.20E-10	-0.44	45	1.5	Pawr	
DMR7:52167001	7	52167001	52168000	1000	1	2.10E-07	0.54	24	2.4	Nav3	
DMR7:52182001	7	52182001	52183000	1000	1	2.00E-07	-0.44	5	0.5	Nav3	
DMR7:52204001	7	52204001	52207000	3000	1	7.90E-14	0.58	41	1.37	Nav3	
DMR7:52208001	7	52208001	52209000	1000	1	5.50E-08	0.62	29	2.9	Nav3	
DMR7:52248001	7	52248001	52249000	1000	1	1.20E-15	0.94	23	2.3	Nav3	
DMR7:52309001	7	52309001	52313000	4000	1	7.50E-08	0.45	38	0.95	Nav3	
DMR7:53628001	7	53628001	53629000	1000	1	4.60E-08	0.67	17	1.7	Csrp2	Cytoskeleton
DMR7:53636001	7	53636001	53639000	3000	1	2.00E-08	-0.42	54	1.8	Csrp2	Cytoskeleton
DMR7:53869001	7	53869001	53872000	3000	1	1.70E-11	-0.62	31	1.03	LOC100361991;Osbpl8	
DMR7:53874001	7	53874001	53875000	1000	1	1.60E-09	-0.5	6	0.6	LOC100361991;Osbpl8	
DMR7:53878001	7	53878001	53879000	1000	1	1.70E-08	-0.45	87	8.7	LOC100361991;Osbpl8	
DMR7:54038001	7	54038001	54039000	1000	1	6.10E-07	-0.46	14	1.4	Osbpl8;Bbs10	
DMR7:54208001	7	54208001	54210000	2000	1	5.90E-08	-0.31	32	1.6	LOC103692852;Nap1l1	Epigenetic
DMR7:54826001	7	54826001	54827000	1000	1	1.30E-12	0.98	26	2.6	Glipr1l2;Glipr1l1	Immune
DMR7:54840001	7	54840001	54842000	2000	1	3.90E-09	-0.22	28	1.4	Glipr1l1	Immune
DMR7:54867001	7	54867001	54868000	1000	1	2.00E-08	-0.4	6	0.6	Caps2	Signaling
DMR7:54988001	7	54988001	54989000	1000	1	8.00E-07	0.32	14	1.4	Kcnc2	Transport
DMR7:55129001	7	55129001	55131000	2000	1	1.70E-07	-0.36	80	4	Kcnc2;LOC103692854	Transport
DMR7:55142001	7	55142001	55144000	2000	1	7.50E-07	-0.34	21	1.05	Kcnc2	Transport
DMR7:55591001	7	55591001	55592000	1000	1	7.90E-09	-0.51	12	1.2	Atxn7l3b	
DMR7:57519001	7	57519001	57522000	3000	1	6.10E-08	-0.58	12	0.4	Trhde	Protease
DMR7:57616001	7	57616001	57619000	3000	1	2.40E-12	0.98	47	1.57	Trhde	Protease
DMR7:57680001	7	57680001	57683000	3000	2	3.70E-13	0.81	52	1.73	Trhde	Protease
DMR7:58072001	7	58072001	58073000	1000	1	4.70E-10	0.34	8	0.8	Tph2	
DMR7:58079001	7	58079001	58081000	2000	1	2.00E-07	-0.41	44	2.2	Tph2	
DMR7:58100001	7	58100001	58103000	3000	2	7.70E-19	1.18	42	1.4	Tph2;LOC103692856	
DMR7:58119001	7	58119001	58121000	2000	1	5.20E-09	-0.44	27	1.35	Tph2	
DMR7:58145001	7	58145001	58147000	2000	1	5.00E-08	-0.44	55	2.75	Tph2	
DMR7:58224001	7	58224001	58225000	1000	1	2.80E-12	0.61	23	2.3	Tbc1d15	Signaling
DMR7:58438001	7	58438001	58439000	1000	1	3.00E-10	0.52	27	2.7	LOC100362230;Lgr5	Signaling
DMR7:58465001	7	58465001	58466000	1000	1	7.80E-08	0.58	23	2.3	Lgr5	Signaling
DMR7:58518001	7	58518001	58520000	2000	1	9.10E-08	0.42	24	1.2	Lgr5	Signaling
DMR7:58804001	7	58804001	58805000	1000	1	1.10E-09	0.62	27	2.7	Tspan8	
DMR7:58829001	7	58829001	58831000	2000	1	3.10E-07	0.58	33	1.65	Tspan8	
DMR7:59092001	7	59092001	59095000	3000	1	1.00E-09	0.67	69	2.3	Ptprr	
DMR7:59137001	7	59137001	59141000	4000	1	1.80E-23	1.34	68	1.7	Ptprr;Taf7l-ps1	
DMR7:59239001	7	59239001	59246000	7000	1	5.80E-07	-0.34	69	0.99	Ptprr	
DMR7:59426001	7	59426001	59429000	3000	1	7.00E-15	-0.53	50	1.67	Ptprb	Receptor
DMR7:59464001	7	59464001	59465000	1000	1	5.40E-08	-0.43	8	0.8	Kcnmb4	Transport
DMR7:59477001	7	59477001	59478000	1000	1	6.40E-07	0.43	19	1.9	Kcnmb4	Transport
DMR7:59513001	7	59513001	59514000	1000	1	4.80E-07	0.42	21	2.1	Kcnmb4	Transport
DMR7:59595001	7	59595001	59596000	1000	1	7.20E-08	-0.48	11	1.1	Cnot2	Transcription
DMR7:59603001	7	59603001	59605000	2000	2	1.10E-08	-0.51	11	0.55	Cnot2	Transcription
DMR7:59836001	7	59836001	59837000	1000	1	4.40E-13	0.72	30	3	Myrfl	
DMR7:59873001	7	59873001	59879000	6000	1	2.00E-09	0.63	96	1.6	Myrfl	
DMR7:59881001	7	59881001	59882000	1000	1	1.70E-07	-0.56	5	0.5	Myrfl	
DMR7:59895001	7	59895001	59897000	2000	1	3.70E-07	0.57	35	1.75	Myrfl	
DMR7:59913001	7	59913001	59919000	6000	1	1.40E-07	0.41	95	1.58	Myrfl;LOC688961;Rab3ip	Transcription
DMR7:59950001	7	59950001	59951000	1000	1	5.40E-07	-0.44	20	2	Rab3ip	Transcription
DMR7:59952001	7	59952001	59954000	2000	1	2.30E-10	-0.47	33	1.65	Rab3ip	Transcription
DMR7:59972001	7	59972001	59973000	1000	1	2.40E-10	0.6	9	0.9	Rab3ip;Best3	Transcription;Transport
DMR7:59988001	7	59988001	59991000	3000	1	3.40E-13	0.91	71	2.37	Best3	Transport
DMR7:60047001	7	60047001	60048000	1000	1	2.60E-07	-0.5	19	1.9	Best3	Transport
DMR7:60124001	7	60124001	60125000	1000	1	3.30E-07	0.48	11	1.1	Cct2;Frs2	Translation
DMR7:60179001	7	60179001	60181000	2000	2	6.40E-09	-0.63	11	0.55	Frs2	
DMR7:60211001	7	60211001	60218000	7000	1	3.80E-07	0.46	106	1.51	Frs2	
DMR7:60333001	7	60333001	60334000	1000	1	1.10E-08	-0.44	19	1.9	Lyc2;Lyz2	
DMR7:60344001	7	60344001	60345000	1000	1	7.60E-08	-0.47	9	0.9	Lyz2	

DMR7:60667001	7	60667001	60669000	2000	1	7.90E-07	-0.32	46	2.3	Cpm	Protease
DMR7:60670001	7	60670001	60675000	5000	1	6.10E-08	-0.36	91	1.82	Cpm	Protease
DMR7:60769001	7	60769001	60776000	7000	2	1.30E-07	-0.36	102	1.46	LOC108351461;Slc35e3;Nup107	Transport;Transport
DMR7:61157001	7	61157001	61158000	1000	1	9.10E-12	0.69	21	2.1	LOC102551023;Mdm1	
DMR7:61205001	7	61205001	61207000	2000	1	1.00E-12	-0.51	37	1.85	Mdm1	
DMR7:61782001	7	61782001	61787000	5000	1	1.30E-10	-0.45	94	1.88	Dyrk2	
DMR7:61807001	7	61807001	61809000	2000	1	3.30E-07	-0.39	34	1.7	Dyrk2	
DMR7:62121001	7	62121001	62123000	2000	1	3.20E-11	-0.54	23	1.15	LOC102551387;Cand1	Proteolysis
DMR7:62900001	7	62900001	62902000	2000	1	1.30E-09	-0.4	54	2.7	Msrb3;Styxl2	Metabolism
DMR7:62946001	7	62946001	62948000	2000	1	7.30E-24	1.29	56	2.8	Msrb3	Metabolism
DMR7:62966001	7	62966001	62967000	1000	1	2.50E-17	1	33	3.3	Msrb3;Lemd3	Metabolism
DMR7:62975001	7	62975001	62980000	5000	1	2.60E-07	0.4	76	1.52	Msrb3;Lemd3	Metabolism
DMR7:63082001	7	63082001	63086000	4000	1	1.00E-08	-0.52	39	0.98	Wif1	Signaling
DMR7:63113001	7	63113001	63114000	1000	1	4.30E-17	1.07	16	1.6	Wif1	Signaling
DMR7:63133001	7	63133001	63136000	3000	1	2.10E-11	-0.49	34	1.13	Wif1	Signaling
DMR7:63164001	7	63164001	63166000	2000	1	2.40E-07	-0.53	26	1.3	Wif1	Signaling
DMR7:63366001	7	63366001	63367000	1000	1	7.80E-10	0.77	28	2.8	Tbc1d30	Signaling
DMR7:63397001	7	63397001	63400000	3000	1	1.40E-08	0.55	46	1.53	Tbc1d30	Signaling
DMR7:63419001	7	63419001	63422000	3000	2	9.40E-09	-0.41	49	1.63	Tbc1d30;LOC102548194	Signaling
DMR7:63753001	7	63753001	63755000	2000	2	9.50E-08	0.49	32	1.6	RGD1561648	
DMR7:63932001	7	63932001	63933000	1000	1	3.70E-09	0.49	8	0.8	RGD1565498	
DMR7:63992001	7	63992001	63995000	3000	1	9.30E-07	0.4	54	1.8	Srgap1	Signaling
DMR7:64032001	7	64032001	64033000	1000	1	3.80E-10	-0.49	27	2.7	Srgap1	Signaling
DMR7:64116001	7	64116001	64118000	2000	1	3.80E-08	0.5	24	1.2	Srgap1	Signaling
DMR7:64188001	7	64188001	64191000	3000	1	8.40E-09	0.53	30	1	Srgap1	Signaling
DMR7:64249001	7	64249001	64250000	1000	1	7.90E-07	0.53	14	1.4	Srgap1	Signaling
DMR7:64348001	7	64348001	64349000	1000	1	5.60E-14	0.89	28	2.8	Tmem5	
DMR7:64671001	7	64671001	64673000	2000	1	4.20E-07	-0.3	40	2	Grip1	
DMR7:64732001	7	64732001	64733000	1000	1	4.20E-10	0.46	24	2.4	Grip1	
DMR7:64764001	7	64764001	64765000	1000	1	5.90E-08	0.63	27	2.7	Grip1	
DMR7:64856001	7	64856001	64857000	1000	1	5.00E-07	-0.38	17	1.7	Grip1;Helb	Transcription
DMR7:64914001	7	64914001	64917000	3000	2	9.90E-09	0.44	65	2.17	Irak3	
DMR7:64928001	7	64928001	64929000	1000	1	1.60E-09	-0.49	35	3.5	Irak3	
DMR7:64941001	7	64941001	64942000	1000	1	2.10E-08	0.67	23	2.3	Irak3	
DMR7:65159001	7	65159001	65160000	1000	1	1.60E-08	0.63	22	2.2	Hmga2	Transcription
DMR7:65166001	7	65166001	65167000	1000	1	1.30E-07	0.47	12	1.2	Hmga2	Transcription
DMR7:65178001	7	65178001	65179000	1000	1	9.50E-09	-0.48	15	1.5	Hmga2;LOC102549373	Transcription
DMR7:65194001	7	65194001	65195000	1000	1	1.50E-09	0.44	19	1.9	Hmga2;LOC102549373	Transcription
DMR7:66084001	7	66084001	66085000	1000	1	1.50E-12	0.55	18	1.8	Fam19a2	
DMR7:66219001	7	66219001	66221000	2000	1	3.90E-07	-0.53	27	1.35	Fam19a2	
DMR7:66276001	7	66276001	66277000	1000	1	4.60E-07	-0.37	20	2	Fam19a2	
DMR7:66354001	7	66354001	66356000	2000	2	3.60E-11	0.44	17	0.85	Fam19a2	
DMR7:66430001	7	66430001	66433000	3000	1	4.50E-07	0.45	37	1.23	Fam19a2	
DMR7:66463001	7	66463001	66465000	2000	1	1.10E-07	-0.45	23	1.15	Fam19a2	
DMR7:66590001	7	66590001	66591000	1000	1	9.00E-16	-0.6	25	2.5	Usp15	Protease
DMR7:66636001	7	66636001	66639000	3000	1	1.10E-12	-0.64	23	0.77	Usp15	Protease
DMR7:66769001	7	66769001	66771000	2000	1	7.60E-07	-0.55	20	1	Mon2	Transcription
DMR7:66855001	7	66855001	66858000	3000	1	8.10E-07	-0.34	59	1.97	Ppm1h	Signaling
DMR7:66865001	7	66865001	66866000	1000	1	4.70E-09	-0.46	17	1.7	Ppm1h	Signaling
DMR7:66870001	7	66870001	66873000	3000	1	4.90E-07	-0.59	22	0.73	Ppm1h	Signaling
DMR7:66879001	7	66879001	66880000	1000	1	6.70E-09	0.74	26	2.6	Ppm1h	Signaling
DMR7:66898001	7	66898001	66899000	1000	1	1.20E-09	-0.34	19	1.9	Ppm1h	Signaling
DMR7:66905001	7	66905001	66910000	5000	1	4.50E-08	-0.37	97	1.94	Ppm1h	Signaling
DMR7:66949001	7	66949001	66952000	3000	1	2.70E-08	-0.49	40	1.33	Ppm1h	Signaling
DMR7:67000001	7	67000001	67001000	1000	1	1.50E-07	-0.37	18	1.8	Ppm1h	Signaling
DMR7:67061001	7	67061001	67062000	1000	1	1.40E-07	-0.53	11	1.1	Ppm1h	Signaling
DMR7:67097001	7	67097001	67098000	1000	1	9.10E-07	-0.37	18	1.8	Ppm1h	Signaling
DMR7:69205001	7	69205001	69207000	2000	1	2.80E-09	-0.54	19	0.95	LOC103692872;Lrig3	
DMR7:69228001	7	69228001	69230000	2000	1	2.50E-13	-0.58	32	1.6	Lrig3	
DMR7:70276001	7	70276001	70277000	1000	1	7.10E-10	-0.52	10	1	LOC102552534;LOC108351472;Ctdsp2	

DMR7:70457001	7	70457001	70462000	5000	2	1.30E-16	0.94	131	2.62	B4galnt1;Slc26a10;Arhgef25	Transport;Transcription
DMR7:70477001	7	70477001	70478000	1000	1	9.10E-08	0.57	18	1.8	Slc26a10;Arhgef25;Dtx3;Pip4k2c	Transport;Transcription;Proteolysis;Signaling
DMR7:70518001	7	70518001	70522000	4000	1	2.40E-07	0.34	170	4.25	Kif5a	Cytoskeleton
DMR7:70550001	7	70550001	70552000	2000	1	1.50E-08	0.61	26	1.3	Kif5a;Dctn2	Cytoskeleton
DMR7:70621001	7	70621001	70623000	2000	2	9.90E-16	1.02	54	2.7	Arhgap9;Gli1	Signaling;Transcription
DMR7:70708001	7	70708001	70710000	2000	1	1.10E-10	-0.53	30	1.5	R3hdm2	
DMR7:70836001	7	70836001	70841000	5000	1	5.70E-07	-0.4	102	2.04	Shmt2;Nxph4;Lrp1	Epigenetic;Signaling;Binding Proteins
DMR7:70869001	7	70869001	70872000	3000	1	1.80E-07	0.51	38	1.27	Lrp1	Binding Proteins
DMR7:70893001	7	70893001	70898000	5000	1	3.10E-13	0.72	95	1.9	Lrp1	Binding Proteins
DMR7:70965001	7	70965001	70967000	2000	1	8.40E-09	-0.48	47	2.35	Stat6;Nab2	Transcription
DMR7:71120001	7	71120001	71122000	2000	1	8.80E-07	-0.41	13	0.65	Rdh7	Metabolism
DMR7:71152001	7	71152001	71154000	2000	1	4.70E-07	-0.37	18	0.9	Rdh7;Sdr9c7	Metabolism
DMR7:71155001	7	71155001	71157000	2000	2	2.00E-09	0.58	39	1.95	Rdh7;Sdr9c7	Metabolism
DMR7:71166001	7	71166001	71169000	3000	1	2.20E-11	0.54	31	1.03	Rdh7;Sdr9c7	Metabolism
DMR7:71305001	7	71305001	71309000	4000	1	1.00E-11	-0.41	46	1.15	Ptdss1	Transport
DMR7:71402001	7	71402001	71404000	2000	1	5.60E-08	0.49	13	0.65	Esco2-ps1	
DMR7:71661001	7	71661001	71665000	4000	1	1.30E-07	-0.41	23	0.58	Sdc2	Receptor
DMR7:71669001	7	71669001	71671000	2000	1	7.20E-08	0.4	38	1.9	Sdc2;LOC102556409	Receptor
DMR7:71788001	7	71788001	71789000	1000	1	5.00E-10	-0.52	13	1.3	Cpq	Protease
DMR7:71796001	7	71796001	71797000	1000	1	6.70E-31	0.59	20	2	Cpq	Protease
DMR7:71856001	7	71856001	71857000	1000	1	4.90E-07	-0.43	8	0.8	Cpq	Protease
DMR7:71958001	7	71958001	71960000	2000	2	8.00E-30	0.44	15	0.75	Cpq	Protease
DMR7:72146001	7	72146001	72152000	6000	1	6.30E-08	-0.33	65	1.08	Cpq	Protease
DMR7:72913001	7	72913001	72917000	4000	1	2.10E-09	-0.45	42	1.05	Laptm4b	Transport
DMR7:73237001	7	73237001	73238000	1000	1	1.70E-07	-0.39	26	2.6	Erich5;LOC103692882;LOC108351477	
DMR7:73248001	7	73248001	73249000	1000	1	4.10E-09	0.54	24	2.4	Erich5;LOC103692882;LOC108351477;Rida	Metabolism
DMR7:73328001	7	73328001	73333000	5000	1	7.90E-12	-0.5	141	2.82	LOC102554988;Nipal2	
DMR7:73410001	7	73410001	73411000	1000	1	1.70E-10	0.52	2	0.2	Nipal2	
DMR7:73552001	7	73552001	73554000	2000	2	6.90E-08	0.54	26	1.3	Kcns2	Transport
DMR7:73657001	7	73657001	73662000	5000	1	3.50E-11	-0.4	44	0.88	Stk3	Signaling
DMR7:73674001	7	73674001	73675000	1000	1	2.20E-11	-0.53	10	1	Stk3	Signaling
DMR7:73723001	7	73723001	73728000	5000	1	9.00E-07	-0.23	45	0.9	Stk3	Signaling
DMR7:73781001	7	73781001	73784000	3000	1	6.50E-07	-0.3	20	0.67	Stk3	Signaling
DMR7:73857001	7	73857001	73858000	1000	1	1.70E-09	-0.46	6	0.6	Stk3	Signaling
DMR7:73866001	7	73866001	73867000	1000	1	4.60E-07	-0.47	8	0.8	Stk3	Signaling
DMR7:73878001	7	73878001	73880000	2000	2	2.50E-09	-0.48	21	1.05	Stk3	Signaling
DMR7:74058001	7	74058001	74063000	5000	1	3.80E-08	-0.39	54	1.08	Osr2	Transcription
DMR7:74198001	7	74198001	74200000	2000	1	1.40E-09	-0.49	21	1.05	Vps13b;LOC103692884	
DMR7:74283001	7	74283001	74284000	1000	1	3.10E-11	0.69	18	1.8	Vps13b	
DMR7:74327001	7	74327001	74328000	1000	1	3.40E-07	0.48	25	2.5	Vps13b;LOC680989	
DMR7:74506001	7	74506001	74507000	1000	1	1.50E-11	-0.64	5	0.5	Vps13b	
DMR7:74604001	7	74604001	74605000	1000	1	8.10E-08	-0.57	5	0.5	Vps13b	
DMR7:74609001	7	74609001	74610000	1000	1	1.90E-09	-0.81	7	0.7	Vps13b	
DMR7:74651001	7	74651001	74656000	5000	1	1.70E-07	-0.38	37	0.74	Vps13b	
DMR7:74757001	7	74757001	74758000	1000	1	1.00E-08	0.66	11	1.1	LOC102555166;RGD1565056	
DMR7:74837001	7	74837001	74841000	4000	1	2.10E-10	-0.48	40	1	Rgs22	
DMR7:74895001	7	74895001	74897000	2000	1	2.60E-08	0.67	33	1.65	Rgs22	
DMR7:74922001	7	74922001	74923000	1000	1	1.10E-07	-0.34	10	1	Fbxo43	
DMR7:74980001	7	74980001	74981000	1000	1	2.80E-08	-0.72	23	2.3	Polr2k	Transcription
DMR7:75274001	7	75274001	75275000	1000	1	8.30E-08	0.48	17	1.7	LOC103692885;Ankrd46	
DMR7:75281001	7	75281001	75283000	2000	1	3.00E-11	-0.45	28	1.4	LOC103692885;Ankrd46	
DMR7:75307001	7	75307001	75309000	2000	1	3.20E-09	0.53	23	1.15	Ankrd46;Snx31	Cytoskeleton
DMR7:75313001	7	75313001	75315000	2000	1	7.30E-11	-0.4	16	0.8	Snx31	Cytoskeleton
DMR7:75588001	7	75588001	75590000	2000	1	4.80E-10	-0.72	18	0.9	Ywhaz	Cytoskeleton
DMR7:75594001	7	75594001	75595000	1000	1	4.20E-10	-0.57	24	2.4	Ywhaz	Cytoskeleton
DMR7:76087001	7	76087001	76089000	2000	1	2.00E-11	0.86	32	1.6	Grhl2	Transcription
DMR7:76150001	7	76150001	76155000	5000	1	4.90E-08	-0.4	59	1.18	Grhl2	Transcription

DMR7:76253001	7	76253001	76257000	4000	2	1.80E-08	0.58	67	1.68	Ncald;LOC102549684	
DMR7:76316001	7	76316001	76317000	1000	1	1.80E-10	0.75	22	2.2	Ncald	
DMR7:76324001	7	76324001	76326000	2000	1	8.00E-11	0.34	17	0.85	Ncald	
DMR7:76442001	7	76442001	76443000	1000	1	1.10E-08	0.58	8	0.8	Ncald	
DMR7:76535001	7	76535001	76536000	1000	1	1.20E-08	0.55	16	1.6	Ncald	
DMR7:76537001	7	76537001	76538000	1000	1	9.00E-07	0.5	3	0.3	Ncald	
DMR7:76610001	7	76610001	76612000	2000	1	8.20E-12	0.58	32	1.6	Ncald;LOC102549311	
DMR7:76751001	7	76751001	76753000	2000	1	9.30E-08	-0.43	18	0.9	Rrm2b	Metabolism
DMR7:76860001	7	76860001	76862000	2000	1	3.10E-08	-0.46	28	1.4	Ubr5	Proteolysis
DMR7:77669001	7	77669001	77671000	2000	1	2.30E-10	0.84	55	2.75	Atp6v1c1	Metabolism
DMR7:77756001	7	77756001	77758000	2000	1	1.40E-09	0.42	20	1	Baalc	
DMR7:77803001	7	77803001	77804000	1000	1	5.80E-11	0.73	19	1.9	Baalc	
DMR7:77832001	7	77832001	77834000	2000	1	1.20E-09	0.53	27	1.35	Baalc	
DMR7:77891001	7	77891001	77896000	5000	2	6.90E-10	-0.4	42	0.84	Fzd6	Receptor
DMR7:77909001	7	77909001	77912000	3000	1	6.10E-07	-0.38	37	1.23	Fzd6	Receptor
DMR7:77966001	7	77966001	77967000	1000	1	8.90E-08	0.57	52	5.2	Cthrc1	Metabolism
DMR7:78240001	7	78240001	78243000	3000	1	7.50E-08	0.51	65	2.17	Rims2	Transport
DMR7:78260001	7	78260001	78265000	5000	1	6.90E-08	-0.3	46	0.92	Rims2	Transport
DMR7:78300001	7	78300001	78301000	1000	1	1.00E-08	0.5	14	1.4	Rims2;LOC103692901	Transport
DMR7:78765001	7	78765001	78767000	2000	2	1.00E-11	0.45	24	1.2	LOC103690326;Dpys	Metabolism
DMR7:78782001	7	78782001	78786000	4000	2	8.60E-08	-0.34	65	1.62	Dpys	Metabolism
DMR7:78833001	7	78833001	78834000	1000	1	2.40E-07	-0.41	7	0.7	Dpys;LOC103692892	Metabolism
DMR7:78884001	7	78884001	78886000	2000	1	1.50E-12	-0.53	26	1.3	Lrp12	Binding Proteins
DMR7:78917001	7	78917001	78920000	3000	1	1.70E-09	-0.5	33	1.1	Lrp12	Binding Proteins
DMR7:78983001	7	78983001	78984000	1000	1	2.90E-08	-0.32	13	1.3	RGD1565735	
DMR7:79467001	7	79467001	79468000	1000	1	6.50E-07	-0.69	3	0.3	LOC108351485;Zfpm2	Transcription
DMR7:79497001	7	79497001	79498000	1000	1	3.00E-16	0.63	17	1.7	Zfpm2	Transcription
DMR7:79774001	7	79774001	79776000	2000	1	8.00E-08	-0.4	28	1.4	Zfpm2	Transcription
DMR7:79829001	7	79829001	79830000	1000	1	2.40E-10	-0.71	27	2.7	Zfpm2	Transcription
DMR7:79929001	7	79929001	79930000	1000	1	3.20E-07	0.34	15	1.5	Zfpm2	Transcription
DMR7:80449001	7	80449001	80451000	2000	1	3.90E-07	0.46	11	0.55	Oxr1;LOC108351486	
DMR7:80714001	7	80714001	80716000	2000	1	1.20E-08	-0.57	23	1.15	Oxr1	
DMR7:80764001	7	80764001	80765000	1000	1	3.90E-07	-0.35	6	0.6	Oxr1	
DMR7:81329001	7	81329001	81330000	1000	1	6.10E-07	-0.58	4	0.4	LOC108351488;Angpt1	Signaling
DMR7:81353001	7	81353001	81354000	1000	1	1.70E-12	0.51	25	2.5	Angpt1	Signaling
DMR7:81414001	7	81414001	81417000	3000	1	1.50E-09	-0.54	19	0.63	Angpt1	Signaling
DMR7:81571001	7	81571001	81572000	1000	1	8.30E-07	0.41	13	1.3	Angpt1	Signaling
DMR7:82005001	7	82005001	82006000	1000	1	8.80E-07	-0.61	11	1.1	Rspo2	
DMR7:82374001	7	82374001	82375000	1000	1	2.30E-14	0.93	31	3.1	Emc2	Transcription
DMR7:83104001	7	83104001	83106000	2000	1	1.30E-07	0.53	21	1.05	Trhr	Transcription
DMR7:83435001	7	83435001	83438000	3000	1	1.90E-07	-0.6	13	0.43	Pkhd1l1	
DMR7:83522001	7	83522001	83525000	3000	1	2.10E-07	-0.36	28	0.93	Pkhd1l1	
DMR7:83588001	7	83588001	83589000	1000	1	5.20E-08	-0.47	10	1	Ebag9	
DMR7:83670001	7	83670001	83671000	1000	1	2.80E-08	0.71	21	2.1	Sybu	Transcription
DMR7:83682001	7	83682001	83683000	1000	1	2.20E-07	0.68	19	1.9	RGD1309489	
DMR7:83783001	7	83783001	83784000	1000	1	5.20E-08	-0.44	7	0.7	RGD1309489	
DMR7:83795001	7	83795001	83799000	4000	1	6.50E-08	-0.43	14	0.35	RGD1309489	
DMR7:86803001	7	86803001	86805000	2000	1	8.00E-07	-0.56	7	0.35	Csmd3	
DMR7:86865001	7	86865001	86866000	1000	1	9.70E-26	1.54	30	3	Csmd3;LOC103692900	
DMR7:86988001	7	86988001	86989000	1000	1	8.30E-07	0.32	19	1.9	Csmd3	
DMR7:87054001	7	87054001	87055000	1000	1	1.70E-08	0.52	27	2.7	Csmd3	
DMR7:87130001	7	87130001	87131000	1000	1	1.90E-09	-0.75	7	0.7	Csmd3	
DMR7:87178001	7	87178001	87179000	1000	1	3.20E-07	-0.64	3	0.3	Csmd3	
DMR7:87369001	7	87369001	87370000	1000	1	2.30E-07	-0.68	4	0.4	Csmd3	
DMR7:87461001	7	87461001	87462000	1000	1	1.60E-07	-0.41	2	0.2	Csmd3	
DMR7:87533001	7	87533001	87539000	6000	1	1.70E-08	-0.28	79	1.32	Csmd3	
DMR7:87606001	7	87606001	87611000	5000	1	3.30E-07	-0.34	54	1.08	Csmd3	
DMR7:87678001	7	87678001	87679000	1000	1	5.10E-08	-0.52	4	0.4	Csmd3	
DMR7:87879001	7	87879001	87881000	2000	1	9.10E-12	0.85	33	1.65	Csmd3	
DMR7:87888001	7	87888001	87889000	1000	1	2.50E-07	0.5	19	1.9	Csmd3	
DMR7:87936001	7	87936001	87937000	1000	1	1.70E-14	0.72	25	2.5	Csmd3	
DMR7:87938001	7	87938001	87940000	2000	1	2.90E-07	-0.44	11	0.55	Csmd3	

DMR7:87991001	7	87991001	87993000	2000	1	1.80E-07	0.64	49	2.45	Csmd3	
DMR7:88010001	7	88010001	88013000	3000	1	5.40E-10	-0.48	22	0.73	Csmd3	
DMR7:88018001	7	88018001	88023000	5000	1	2.30E-10	-0.45	19	0.38	Csmd3	
DMR7:88670001	7	88670001	88671000	1000	1	4.20E-07	0.45	15	1.5	Olr1100-ps	
DMR7:92676001	7	92676001	92680000	4000	1	1.30E-07	-0.5	48	1.2	Ext1	Golgi
DMR7:93024001	7	93024001	93028000	4000	1	5.30E-07	-0.34	39	0.98	Samd12	
DMR7:93034001	7	93034001	93039000	5000	1	1.80E-09	-0.31	41	0.82	Samd12	
DMR7:93981001	7	93981001	93983000	2000	1	9.80E-07	-0.59	10	0.5	Colec10	Transport
DMR7:93996001	7	93996001	9.40E+07	4000	1	5.40E-07	-0.24	32	0.8	Colec10	Transport
DMR7:94013001	7	94013001	94019000	6000	1	3.00E-07	-0.36	55	0.92	Colec10	Transport
DMR7:94498001	7	94498001	94500000	2000	1	8.20E-09	-0.65	13	0.65	Enpp2	
DMR7:94557001	7	94557001	94558000	1000	1	2.90E-12	0.95	27	2.7	Enpp2	
DMR7:94718001	7	94718001	94719000	1000	1	2.60E-12	-0.57	13	1.3	Taf2	Transcription
DMR7:94765001	7	94765001	94766000	1000	1	6.10E-07	-0.4	21	2.1	Taf2;Dscc1	Transcription;Cell Cycle
DMR7:94784001	7	94784001	94785000	1000	1	2.80E-08	-0.45	10	1	Dscc1;H3f3c;LOC299934	Cell Cycle;Epigenetic
DMR7:95066001	7	95066001	95068000	2000	1	5.90E-09	0.55	22	1.1	Col14a1;LOC103692916	Extracellular Matrix
DMR7:95232001	7	95232001	95233000	1000	1	9.70E-12	0.32	8	0.8	Col14a1	Extracellular Matrix
DMR7:95313001	7	95313001	95314000	1000	1	6.90E-08	-0.65	1	0.1	Mrpl13;Mtbp	Translation
DMR7:95346001	7	95346001	95347000	1000	1	2.70E-11	-0.46	20	2	Mtbp	
DMR7:95666001	7	95666001	95667000	1000	1	7.70E-07	0.35	6	0.6	Sntb1;LOC102552118	
DMR7:97038001	7	97038001	97040000	2000	1	4.90E-08	-0.36	5	0.25	LOC103692918;Slc22a22	Transport
DMR7:97654001	7	97654001	97656000	2000	1	8.10E-10	0.72	30	1.5	Zhx2	Development
DMR7:97693001	7	97693001	97694000	1000	1	5.00E-07	-0.4	36	3.6	Zhx2;LOC102553429	Development
DMR7:97695001	7	97695001	97701000	6000	1	6.60E-13	-0.54	107	1.78	Zhx2;LOC102553429	Development
DMR7:97813001	7	97813001	97815000	2000	1	1.80E-07	-0.32	44	2.2	Tbc1d31;LOC102553732	
DMR7:97842001	7	97842001	97843000	1000	1	3.10E-12	0.81	24	2.4	Tbc1d31;Trnam-cau	
DMR7:97852001	7	97852001	97853000	1000	1	2.50E-07	0.34	11	1.1	Trnam-cau;Fam83a	
DMR7:97856001	7	97856001	97857000	1000	1	3.80E-08	0.61	22	2.2	Fam83a	
DMR7:97860001	7	97860001	97862000	2000	1	3.90E-07	0.44	17	0.85	Fam83a	
DMR7:97973001	7	97973001	97974000	1000	1	2.50E-10	-0.43	18	1.8	Wdyhv1	
DMR7:97995001	7	97995001	97996000	1000	1	5.70E-07	0.55	10	1	Atad2	Epigenetic
DMR7:98262001	7	98262001	98266000	4000	1	5.10E-07	-0.33	34	0.85	Anxa13	Signaling
DMR7:98280001	7	98280001	98281000	1000	1	3.80E-09	-0.42	12	1.2	Anxa13	Signaling
DMR7:98405001	7	98405001	98406000	1000	1	5.40E-07	0.5	3	0.3	Fer1l6	Transport
DMR7:98691001	7	98691001	98693000	2000	1	6.00E-07	-0.59	25	1.25	Tmem65	
DMR7:98894001	7	98894001	98900000	6000	1	1.60E-08	-0.41	111	1.85	Mtss1	Cytoskeleton
DMR7:98904001	7	98904001	98906000	2000	1	1.70E-08	-0.4	36	1.8	Mtss1	Cytoskeleton
DMR7:99731001	7	99731001	99733000	2000	2	2.00E-12	0.58	27	1.35	Nsmce2	
DMR7:99943001	7	99943001	99945000	2000	1	1.10E-09	0.46	33	1.65	LOC108351497;LOC690120;Trib1	Signaling
DMR7:102647001	7	102647001	102648000	1000	1	4.60E-21	1.11	26	2.6	Pvt1;LOC108351503	
DMR7:102676001	7	102676001	102682000	6000	1	3.30E-07	-0.25	64	1.07	Pvt1	
DMR7:102738001	7	102738001	102740000	2000	1	4.40E-08	0.58	20	1	Pvt1	
DMR7:104499001	7	104499001	104501000	2000	1	1.90E-10	0.52	27	1.35	Gsdmc;RGD1359449	
DMR7:106564001	7	106564001	106565000	1000	1	4.90E-07	0.31	10	1	Efr3a	
DMR7:106717001	7	106717001	106718000	1000	1	5.20E-07	0.44	23	2.3	Kcnq3	Transport
DMR7:107527001	7	107527001	107528000	1000	1	7.60E-07	0.39	6	0.6	Tg	Cytoskeleton
DMR7:107575001	7	107575001	107578000	3000	1	7.80E-11	-0.37	20	0.67	Tg;Sla	Cytoskeleton
DMR7:107781001	7	107781001	107783000	2000	1	1.20E-07	-0.58	13	0.65	Ndrg1;LOC108351506	Protease
DMR7:107892001	7	107892001	107893000	1000	1	3.10E-10	0.56	8	0.8	St3gal1	Transport
DMR7:108763001	7	108763001	108765000	2000	1	1.10E-17	0.79	21	1.05	Phf20l1	
DMR7:109123001	7	109123001	109125000	2000	1	1.50E-08	0.42	15	0.75	Zfat	Transcription
DMR7:109145001	7	109145001	109146000	1000	1	7.30E-07	0.42	8	0.8	Zfat	Transcription
DMR7:109156001	7	109156001	109163000	7000	1	3.90E-07	0.37	92	1.31	Zfat	Transcription
DMR7:109173001	7	109173001	109175000	2000	1	1.50E-07	0.47	18	0.9	Zfat	Transcription
DMR7:109194001	7	109194001	109198000	4000	1	7.70E-07	0.91	35	0.88	Zfat	Transcription
DMR7:110025001	7	110025001	110026000	1000	1	1.10E-11	0.46	10	1	Khdrbs3	Translation
DMR7:110029001	7	110029001	110030000	1000	1	9.60E-07	0.4	14	1.4	Khdrbs3	Translation
DMR7:110095001	7	110095001	110097000	2000	1	4.50E-08	-0.43	28	1.4	Khdrbs3	Translation
DMR7:110177001	7	110177001	110180000	3000	1	6.50E-09	-0.48	55	1.83	Khdrbs3;LOC108351507	Translation
DMR7:111729001	7	111729001	111730000	1000	1	3.70E-11	0.46	7	0.7	Rps19l1	Translation

DMR7:112655001	7	112655001	112657000	2000	1	3.60E-10	-0.59	7	0.35	Trnap-agg	
DMR7:112822001	7	112822001	112823000	1000	1	4.30E-10	-0.56	5	0.5	Fam135b	
DMR7:113110001	7	113110001	113111000	1000	1	6.20E-09	0.34	11	1.1	Col22a1	Extracellular Matrix
DMR7:113185001	7	113185001	113187000	2000	1	3.40E-07	-0.52	20	1	Col22a1	Extracellular Matrix
DMR7:113248001	7	113248001	113249000	1000	1	4.00E-08	-0.43	9	0.9	Col22a1	Extracellular Matrix
DMR7:113943001	7	113943001	113944000	1000	1	3.60E-08	-0.67	2	0.2	Kcnk9	Transport
DMR7:114125001	7	114125001	114127000	2000	2	4.00E-08	0.52	46	2.3	Trappc9	
DMR7:114171001	7	114171001	114172000	1000	1	4.20E-08	-0.62	6	0.6	Trappc9	
DMR7:114184001	7	114184001	114185000	1000	1	9.60E-08	0.41	25	2.5	Trappc9	
DMR7:114292001	7	114292001	114296000	4000	1	1.60E-08	0.34	62	1.55	Trappc9	
DMR7:114348001	7	114348001	114349000	1000	1	2.20E-09	0.38	12	1.2	Ago2	Translation
DMR7:114817001	7	114817001	114820000	3000	1	4.00E-07	0.59	45	1.5	Dennd3;Slc45a4	Transport
DMR7:114896001	7	114896001	114899000	3000	1	4.70E-07	0.62	37	1.23	Slc45a4	Transport
DMR7:114955001	7	114955001	114957000	2000	1	1.60E-07	0.43	22	1.1	Gpr20	
DMR7:114964001	7	114964001	114966000	2000	1	1.60E-08	0.64	26	1.3	Ptp4a3	Signaling
DMR7:114985001	7	114985001	114986000	1000	1	9.50E-07	0.44	14	1.4	Ptp4a3	Signaling
DMR7:114995001	7	114995001	114999000	4000	1	2.90E-09	0.6	87	2.17	Ptp4a3;Mroh5	Signaling
DMR7:115139001	7	115139001	115140000	1000	1	1.50E-08	0.46	17	1.7	RGD1562139	
DMR7:115143001	7	115143001	115144000	1000	1	2.80E-07	0.48	6	0.6	RGD1562139	
DMR7:115838001	7	115838001	115842000	4000	1	1.20E-08	-0.46	47	1.18	Adgrb1;LOC102550505;LOC102550355;Mroh4	Signaling
DMR7:115893001	7	115893001	115897000	4000	1	6.10E-07	-0.37	44	1.1	Mroh4	
DMR7:115907001	7	115907001	115910000	3000	1	6.50E-20	0.89	56	1.87	Arc;LOC103692938	
DMR7:115913001	7	115913001	115914000	1000	1	1.10E-07	0.51	9	0.9	Arc;LOC103692938	
DMR7:115916001	7	115916001	115917000	1000	1	1.40E-07	0.51	15	1.5	Arc;LOC103692938	
DMR7:115961001	7	115961001	115963000	2000	1	2.40E-08	0.64	29	1.45	Them6;Slurp1;Lypd2	
DMR7:115971001	7	115971001	115972000	1000	1	1.60E-08	0.55	12	1.2	Slurp1;Lypd2;RGD1308195	
DMR7:116438001	7	116438001	116439000	1000	1	9.30E-10	0.59	22	2.2	LOC108351510;Ly6l	
DMR7:116598001	7	116598001	116599000	1000	1	9.90E-08	-0.39	6	0.6	Ly6h	
DMR7:116650001	7	116650001	116652000	2000	1	4.20E-10	0.44	26	1.3	Zfp41	Transcription
DMR7:116690001	7	116690001	116694000	4000	1	1.70E-07	0.53	67	1.68	Top1mt	Transcription
DMR7:116772001	7	116772001	116775000	3000	1	4.70E-07	-0.32	38	1.27	Mafa	Transcription
DMR7:116811001	7	116811001	116813000	2000	1	5.70E-08	-0.6	12	0.6	Zc3h3	
DMR7:116893001	7	116893001	116896000	3000	2	2.70E-09	0.59	46	1.53	Gsdmd	
DMR7:116898001	7	116898001	116899000	1000	1	6.90E-07	0.35	9	0.9	Gsdmd	
DMR7:116900001	7	116900001	116901000	1000	1	1.30E-07	0.52	12	1.2	Gsdmd	
DMR7:116916001	7	116916001	116918000	2000	1	7.50E-16	0.89	70	3.5	Mroh6;Naprt	Golgi
DMR7:117075001	7	117075001	117076000	1000	1	3.90E-08	0.49	16	1.6	Fam83h;RGD1563870	
DMR7:117096001	7	117096001	117099000	3000	2	3.30E-10	-0.59	49	1.63	RGD1563870;Scrib	Cytoskeleton
DMR7:117148001	7	117148001	117150000	2000	1	7.50E-11	0.64	49	2.45	Puf60;Nrbp2	Translation;Signaling
DMR7:117173001	7	117173001	117174000	1000	1	8.60E-08	0.52	11	1.1	Eppk1;LOC680875	Cytoskeleton
DMR7:117235001	7	117235001	117236000	1000	1	3.20E-07	0.45	31	3.1	Plec	Cytoskeleton
DMR7:117237001	7	117237001	117242000	5000	3	2.00E-08	-0.49	300	6	Plec	Cytoskeleton
DMR7:117301001	7	117301001	117303000	2000	1	3.40E-09	-0.47	27	1.35	Plec;Parp10;Grina	Cytoskeleton
DMR7:117326001	7	117326001	117328000	2000	1	5.80E-09	-0.47	34	1.7	LOC102549107;Spatc1	
DMR7:117435001	7	117435001	117438000	3000	1	1.70E-09	0.51	63	2.1	Wdr97;Hgh1;Tssk5;Mroh1	Signaling
DMR7:117537001	7	117537001	117538000	1000	1	9.30E-11	-0.63	19	1.9	Bop1;Hsf1	Translation;Transcription
DMR7:117562001	7	117562001	117563000	1000	1	1.70E-07	0.45	16	1.6	Hsf1;Dgat1	Transcription;Metabolism
DMR7:117597001	7	117597001	117601000	4000	1	5.70E-14	0.63	76	1.9	Scrt1;Tmem249;Fbxl6;Slc52a2;LOC108351609	Transcription;Transport
DMR7:117666001	7	117666001	117670000	4000	1	3.10E-07	0.54	74	1.85	Adck5;Cpsf1;Slc39a4	Transport;Translation;Transport
DMR7:117754001	7	117754001	117757000	3000	2	1.60E-08	0.6	54	1.8	Ppp1r16a;Gpt;Mfsd3;Recql4	Signaling;Metabolism;Transport;Epigenetic
DMR7:117760001	7	117760001	117762000	2000	1	7.30E-09	0.49	71	3.55	Ppp1r16a;Gpt;Mfsd3;Recql4	Signaling;Metabolism;Transport;Epigenetic
DMR7:117790001	7	117790001	117791000	1000	1	3.10E-10	0.63	18	1.8	Lrrc24;MGC94207;Arhgap39	Immune
DMR7:117816001	7	117816001	117817000	1000	1	1.00E-10	0.59	14	1.4	Arhgap39	
DMR7:117833001	7	117833001	117836000	3000	1	2.40E-07	-0.37	47	1.57	Arhgap39	
DMR7:118227001	7	118227001	118229000	2000	1	1.50E-07	-0.51	37	1.85	Rbfox2	Translation

DMR7:118403001	7	118403001	118404000	1000	1	1.20E-09	0.38	11	1.1	Rbfox2	Translation
DMR7:118685001	7	118685001	118687000	2000	1	1.90E-07	-0.43	24	1.2	Apol3	Binding Proteins
DMR7:119273001	7	119273001	119276000	3000	1	1.30E-09	0.39	30	1	Cacng2	Transport
DMR7:119288001	7	119288001	119291000	3000	1	3.60E-07	-0.34	55	1.83	Cacng2	Transport
DMR7:119313001	7	119313001	119315000	2000	1	7.70E-09	-0.38	50	2.5	Cacng2	Transport
DMR7:119434001	7	119434001	119437000	3000	1	3.10E-07	0.45	41	1.37	Pvalb	Signaling
DMR7:119506001	7	119506001	119507000	1000	1	4.80E-07	-0.52	5	0.5	Ncf4	
DMR7:119631001	7	119631001	119634000	3000	1	3.80E-09	0.66	40	1.33	Tst;Mpst	Transport
DMR7:119644001	7	119644001	119645000	1000	1	6.70E-09	-0.53	6	0.6	Kctd17	
DMR7:119705001	7	119705001	119710000	5000	2	1.50E-09	0.53	66	1.32	LOC102547102;Il2rb	Receptor
DMR7:119712001	7	119712001	119713000	1000	1	8.20E-08	0.48	12	1.2	Il2rb	Receptor
DMR7:119743001	7	119743001	119746000	3000	1	4.70E-07	-0.39	43	1.43	C1qtnf6	
DMR7:119763001	7	119763001	119764000	1000	1	1.40E-08	0.72	53	5.3	Sstr3;Rac2	Signaling;Signaling
DMR7:119804001	7	119804001	119806000	2000	1	1.10E-08	0.53	42	2.1	Rac2	Signaling
DMR7:119814001	7	119814001	119818000	4000	1	4.60E-16	1.09	97	2.42	Cyth4	Transcription
DMR7:119970001	7	119970001	119973000	3000	2	9.50E-11	-0.48	25	0.83	Mfng	Golgi
DMR7:120005001	7	120005001	120007000	2000	1	1.40E-07	0.35	30	1.5	Mfng;Card10	Golgi
DMR7:120054001	7	120054001	120057000	3000	1	1.40E-07	0.49	35	1.17	LOC108351511;Cdc42ep1	
DMR7:120065001	7	120065001	120069000	4000	1	2.60E-07	-0.31	101	2.52	Cdc42ep1;Lgals2	Extracellular Matrix
DMR7:120146001	7	120146001	120147000	1000	1	6.50E-07	-0.47	13	1.3	LOC108351513;Sh3bp1;Pdxp;Lgals1	Signaling;Extracellular Matrix
DMR7:120171001	7	120171001	120172000	1000	1	7.00E-07	0.45	14	1.4	Nol12;Triobp	Cytoskeleton
DMR7:120190001	7	120190001	120192000	2000	1	2.90E-10	0.64	54	2.7	Triobp	Cytoskeleton
DMR7:120250001	7	120250001	120253000	3000	1	3.00E-07	0.5	50	1.67	H1f0	
DMR7:120335001	7	120335001	120340000	5000	1	2.60E-07	0.53	71	1.42	Eif3l;Micall1	Translation
DMR7:120388001	7	120388001	120390000	2000	1	3.20E-08	0.51	22	1.1	RGD1359634;Polr2f;Sox10	Transcription
DMR7:120595001	7	120595001	120598000	3000	1	1.40E-07	0.58	43	1.43	Maff;Tmem184b	Transcription;Transport
DMR7:120659001	7	120659001	120662000	3000	1	1.70E-09	-0.44	79	2.63	Csnk1e	Signaling
DMR7:120677001	7	120677001	120680000	3000	1	1.00E-08	0.47	92	3.07	Csnk1e	Signaling
DMR7:120683001	7	120683001	120685000	2000	1	2.10E-09	0.39	31	1.55	Csnk1e	Signaling
DMR7:120724001	7	120724001	120726000	2000	1	1.40E-08	0.49	32	1.6	Kcnj4	Transport
DMR7:120813001	7	120813001	120814000	1000	1	6.10E-10	0.43	16	1.6	Dmc1	Transcription
DMR7:120838001	7	120838001	120840000	2000	1	5.60E-17	1.06	58	2.9	Dmc1;Fam227a	Transcription
DMR7:121127001	7	121127001	121129000	2000	1	3.80E-23	1.1	42	2.1	Apobec3b;Cbx7	Proteolysis
DMR7:121144001	7	121144001	121145000	1000	1	3.20E-14	0.77	15	1.5	Cbx7	Proteolysis
DMR7:121162001	7	121162001	121164000	2000	1	4.50E-09	-0.43	20	1	Cbx7	Proteolysis
DMR7:121207001	7	121207001	121210000	3000	2	3.20E-18	1.28	96	3.2	Pdgfb	Growth Factors
DMR7:121235001	7	121235001	121237000	2000	1	2.10E-07	0.54	29	1.45	Pdgfb;LOC108351514	Growth Factors
DMR7:121285001	7	121285001	121288000	3000	1	4.30E-07	0.59	43	1.43	LOC108351514;LOC102547448;Rpl3	Translation
DMR7:121380001	7	121380001	121381000	1000	1	3.10E-07	0.59	17	1.7	Tab1	Signaling
DMR7:121432001	7	121432001	121433000	1000	1	7.00E-11	0.71	31	3.1	Mgat3	Golgi
DMR7:121482001	7	121482001	121483000	1000	1	1.60E-08	-0.53	9	0.9	Mief1;Atf4;Rps19bp1	Transcription
DMR7:121492001	7	121492001	121493000	1000	1	4.10E-07	0.29	11	1.1	Atf4;Rps19bp1	Transcription
DMR7:121514001	7	121514001	121518000	4000	2	2.20E-07	0.52	55	1.38	Cacna1i	Transport
DMR7:121563001	7	121563001	121564000	1000	1	8.00E-14	0.66	10	1	Cacna1i	Transport
DMR7:121591001	7	121591001	121592000	1000	1	1.20E-08	0.48	24	2.4	Cacna1i	Transport
DMR7:121598001	7	121598001	121599000	1000	1	2.40E-08	0.52	18	1.8	Cacna1i	Transport
DMR7:121609001	7	121609001	121612000	3000	1	4.10E-09	0.58	42	1.4	Cacna1i	Transport
DMR7:121614001	7	121614001	121615000	1000	1	5.30E-10	0.65	14	1.4	Cacna1i	Transport
DMR7:121663001	7	121663001	121665000	2000	1	3.80E-07	0.45	42	2.1	LOC108351515;Enthd1	
DMR7:121781001	7	121781001	121783000	2000	1	3.50E-08	0.52	28	1.4	Enthd1;LOC102550152;Grap2	
DMR7:121831001	7	121831001	121832000	1000	1	7.90E-07	-0.4	18	1.8	Grap2	
DMR7:121943001	7	121943001	121945000	2000	1	1.70E-07	-0.62	28	1.4	Tnrc6b;Rpl26-ps1	Metabolism
DMR7:121952001	7	121952001	121955000	3000	1	6.00E-09	-0.36	26	0.87	Tnrc6b;Rpl26-ps1	Metabolism
DMR7:121987001	7	121987001	121989000	2000	2	6.10E-11	-0.46	28	1.4	Tnrc6b	Metabolism
DMR7:122111001	7	122111001	122112000	1000	1	2.50E-11	-0.48	12	1.2	Tnrc6b	Metabolism
DMR7:122160001	7	122160001	122161000	1000	1	2.00E-07	-0.3	26	2.6	Adsl;LOC100362980	Metabolism
DMR7:122237001	7	122237001	122238000	1000	1	1.90E-07	0.36	20	2	Sgsm3;Mkl1	Signaling
DMR7:122307001	7	122307001	122310000	3000	1	3.20E-09	-0.4	32	1.07	Mkl1	
DMR7:122354001	7	122354001	122355000	1000	1	1.60E-07	-0.4	28	2.8	Mkl1	

DMR7:122371001	7	122371001	122373000	2000	1	2.50E-09	-0.58	25	1.25	Mkl1	
DMR7:122409001	7	122409001	122412000	3000	1	8.90E-08	-0.4	48	1.6	Mkl1	
DMR7:122545001	7	122545001	122547000	2000	1	3.50E-10	-0.48	26	1.3	Slc25a17	
DMR7:122579001	7	122579001	122581000	2000	1	1.70E-09	-0.5	27	1.35	Slc25a17;St13	
DMR7:122643001	7	122643001	122644000	1000	1	1.30E-07	-0.45	21	2.1	St13;Xpnpep3;Dnajb7	Protease
DMR7:122716001	7	122716001	122717000	1000	1	6.50E-08	0.63	26	2.6	Rbx1	Proteolysis
DMR7:122719001	7	122719001	122720000	1000	1	1.50E-08	-0.39	15	1.5	Rbx1	Proteolysis
DMR7:122857001	7	122857001	122858000	1000	1	2.10E-09	-0.62	12	1.2	Ep300	
DMR7:123050001	7	123050001	123051000	1000	1	4.60E-07	0.5	8	0.8	Tef	Transcription
DMR7:123061001	7	123061001	123063000	2000	1	5.40E-08	-0.41	29	1.45	Tef	Transcription
DMR7:123293001	7	123293001	123295000	2000	1	3.20E-23	1.35	46	2.3	Nhp2l1;LOC100362109	
DMR7:123300001	7	123300001	123303000	3000	1	5.30E-09	-0.4	58	1.93	LOC100362109;Mei1	
DMR7:123310001	7	123310001	123313000	3000	1	8.70E-08	-0.48	42	1.4	LOC100362109;Mei1	
DMR7:123383001	7	123383001	123388000	5000	2	1.20E-11	-0.47	73	1.46	Ccdc134;Srebf2	
DMR7:123397001	7	123397001	123400000	3000	1	7.60E-08	-0.4	110	3.67	Srebf2	
DMR7:123441001	7	123441001	123442000	1000	1	1.40E-10	0.64	28	2.8	Srebf2;Mir33;Shisa8	
DMR7:123450001	7	123450001	123454000	4000	1	4.40E-11	0.79	71	1.77	Shisa8;Tnfrsf13c	Receptor
DMR7:123457001	7	123457001	123458000	1000	1	2.10E-07	0.46	7	0.7	Tnfrsf13c;Cenpm	Receptor
DMR7:123483001	7	123483001	123487000	4000	1	8.20E-10	-0.66	61	1.52	Cenpm;LOC688613;LOC108351520	
DMR7:123540001	7	123540001	123541000	1000	1	1.70E-09	-0.45	17	1.7	Wbp2nl	
DMR7:123620001	7	123620001	123622000	2000	1	2.20E-07	0.32	24	1.2	LOC103692951;Cyp2d5;Cyp2d1	Metabolism
DMR7:123857001	7	123857001	123860000	3000	1	2.00E-07	0.3	51	1.7	Nfam1	
DMR7:123891001	7	123891001	123892000	1000	1	7.20E-07	0.38	12	1.2	Nfam1	
DMR7:124086001	7	124086001	124089000	3000	1	3.50E-10	0.6	60	2	A4galt	Transport
DMR7:124267001	7	124267001	124268000	1000	1	6.70E-07	-0.47	11	1.1	Pacsin2	Cytoskeleton
DMR7:124274001	7	124274001	124276000	2000	1	2.20E-07	-0.34	23	1.15	Pacsin2	Cytoskeleton
DMR7:124344001	7	124344001	124345000	1000	1	4.30E-08	0.37	19	1.9	Ttll1	Cytoskeleton
DMR7:124377001	7	124377001	124378000	1000	1	1.50E-07	-0.35	30	3	Ttll1	Cytoskeleton
DMR7:124515001	7	124515001	124517000	2000	1	7.10E-09	0.51	31	1.55	Scube1	Extracellular Matrix
DMR7:124518001	7	124518001	124519000	1000	1	4.70E-07	0.45	10	1	Scube1	Extracellular Matrix
DMR7:124552001	7	124552001	124555000	3000	1	5.30E-09	0.43	35	1.17	Scube1	Extracellular Matrix
DMR7:124566001	7	124566001	124569000	3000	1	2.90E-08	0.38	34	1.13	Scube1	Extracellular Matrix
DMR7:124579001	7	124579001	124580000	1000	1	3.60E-08	0.51	8	0.8	Scube1	Extracellular Matrix
DMR7:124594001	7	124594001	124596000	2000	1	8.80E-07	0.42	30	1.5	Scube1	Extracellular Matrix
DMR7:124602001	7	124602001	124604000	2000	1	2.10E-07	0.33	28	1.4	Scube1	Extracellular Matrix
DMR7:124610001	7	124610001	124612000	2000	1	2.90E-10	0.8	34	1.7	Scube1	Extracellular Matrix
DMR7:124629001	7	124629001	124632000	3000	1	5.60E-07	0.49	49	1.63	Scube1;LOC108351624	Extracellular Matrix
DMR7:124669001	7	124669001	124670000	1000	1	9.40E-07	0.48	13	1.3	Mpped1	Metabolism
DMR7:124677001	7	124677001	124682000	5000	1	5.70E-07	-0.4	64	1.28	Mpped1	Metabolism
DMR7:124815001	7	124815001	124817000	2000	1	4.80E-07	0.32	25	1.25	Efcab6	Signaling
DMR7:124863001	7	124863001	124864000	1000	1	5.50E-09	-0.42	12	1.2	Efcab6	Signaling
DMR7:124913001	7	124913001	124914000	1000	1	1.10E-07	-0.44	19	1.9	Efcab6	Signaling
DMR7:124973001	7	124973001	124976000	3000	1	4.80E-07	0.42	39	1.3	Sult4a1	Transport
DMR7:124997001	7	124997001	125001000	4000	1	3.10E-07	0.48	84	2.1	Pnpla5	Metabolism
DMR7:125047001	7	125047001	125052000	5000	1	7.90E-12	-0.43	85	1.7	Pnpla3;Samm50	Metabolism
DMR7:125082001	7	125082001	125083000	1000	1	4.80E-09	0.63	18	1.8	Samm50	
DMR7:125187001	7	125187001	125190000	3000	2	5.80E-14	0.77	73	2.43	Parvb	Cytoskeleton
DMR7:125230001	7	125230001	125231000	1000	1	1.80E-08	0.41	16	1.6	Parvb	Cytoskeleton
DMR7:125244001	7	125244001	125247000	3000	3	8.70E-33	1.22	18	0.6	Parvb	Cytoskeleton
DMR7:125252001	7	125252001	125255000	3000	1	4.20E-07	-0.41	64	2.13	Parvb	Cytoskeleton
DMR7:125308001	7	125308001	125310000	2000	1	7.10E-09	0.32	26	1.3	Parvg;LOC108351521	Cytoskeleton
DMR7:125376001	7	125376001	125378000	2000	1	5.30E-07	0.53	35	1.75	RGD1566029	
DMR7:125401001	7	125401001	125403000	2000	1	3.90E-07	0.41	29	1.45	RGD1566029	
DMR7:125461001	7	125461001	125462000	1000	1	2.50E-10	0.54	15	1.5	Ldoc1l	
DMR7:125465001	7	125465001	125466000	1000	1	2.30E-11	0.59	43	4.3	Ldoc1l	
DMR7:125502001	7	125502001	125504000	2000	1	3.90E-08	0.52	44	2.2	RGD1560568	
DMR7:125597001	7	125597001	125599000	2000	2	4.70E-10	-0.57	31	1.55	Prr5	
DMR7:125665001	7	125665001	125666000	1000	1	6.00E-07	-0.4	4	0.4	Phf21b	
DMR7:125692001	7	125692001	125694000	2000	1	5.90E-07	0.4	36	1.8	Phf21b	
DMR7:125713001	7	125713001	125716000	3000	1	2.70E-07	0.45	52	1.73	Phf21b	

DMR7:125720001	7	125720001	125722000	2000	1	2.80E-15	1.11	56	2.8	Phf21b	
DMR7:125724001	7	125724001	125729000	5000	1	7.40E-08	-0.43	114	2.28	Phf21b;Arhgap8	Signaling
DMR7:125771001	7	125771001	125773000	2000	1	6.90E-07	0.41	23	1.15	Arhgap8	Signaling
DMR7:125848001	7	125848001	125849000	1000	1	5.40E-07	0.41	14	1.4	Npap60;RGD1304694	Cytoskeleton
DMR7:125865001	7	125865001	125866000	1000	1	2.60E-07	0.45	17	1.7	RGD1304694;Mir1249	
DMR7:125917001	7	125917001	125918000	1000	1	2.80E-09	0.53	20	2	Upk3a	
DMR7:125921001	7	125921001	125923000	2000	1	1.20E-12	0.59	27	1.35	Upk3a	
DMR7:126159001	7	126159001	126162000	3000	1	7.40E-07	0.53	33	1.1	Fbln1	Extracellular Matrix
DMR7:126165001	7	126165001	126170000	5000	1	5.00E-07	0.3	90	1.8	Fbln1	Extracellular Matrix
DMR7:126173001	7	126173001	126181000	8000	2	3.70E-09	0.69	115	1.44	Fbln1	Extracellular Matrix
DMR7:126258001	7	126258001	126260000	2000	1	6.80E-08	-0.37	30	1.5	Atxn10	
DMR7:126265001	7	126265001	126267000	2000	1	2.80E-10	-0.41	18	0.9	Atxn10	
DMR7:126275001	7	126275001	126276000	1000	1	1.40E-07	-0.37	12	1.2	Atxn10	
DMR7:126416001	7	126416001	126419000	3000	1	4.50E-07	0.58	32	1.07	Wnt7b	Signaling
DMR7:126469001	7	126469001	126471000	2000	1	4.90E-08	0.48	15	0.75	Wnt7b	Signaling
DMR7:126626001	7	126626001	126629000	3000	1	4.20E-07	-0.36	34	1.13	Ppara	Transcription
DMR7:126681001	7	126681001	126682000	1000	1	3.90E-09	0.64	25	2.5	Ppara;Cdpf1	Transcription
DMR7:126688001	7	126688001	126690000	2000	1	1.10E-07	0.51	30	1.5	Ppara;Cdpf1;Pkdrej	Transcription;Transport
DMR7:126715001	7	126715001	126717000	2000	1	5.00E-08	0.53	24	1.2	Ttc38	
DMR7:126804001	7	126804001	126808000	4000	1	3.00E-09	0.45	73	1.82	Celsr1	Cytoskeleton
DMR7:126822001	7	126822001	126826000	4000	1	3.80E-09	0.34	54	1.35	Celsr1	Cytoskeleton
DMR7:126905001	7	126905001	126906000	1000	1	4.10E-08	0.42	7	0.7	Celsr1	Cytoskeleton
DMR7:126947001	7	126947001	126948000	1000	1	3.90E-07	0.34	16	1.6	Gramd4	
DMR7:127004001	7	127004001	127005000	1000	1	6.90E-11	0.62	16	1.6	Gramd4	
DMR7:127038001	7	127038001	127040000	2000	1	1.00E-10	0.72	45	2.25	Cerk	Signaling
DMR7:127219001	7	127219001	127220000	1000	1	1.70E-07	-0.37	13	1.3	Tbc1d22a	Signaling
DMR7:128624001	7	128624001	128629000	5000	1	1.50E-07	0.45	64	1.28	Fam19a5	
DMR7:128674001	7	128674001	128675000	1000	1	1.90E-08	0.56	7	0.7	Fam19a5	
DMR7:128695001	7	128695001	128698000	3000	1	4.30E-07	0.33	52	1.73	Fam19a5	
DMR7:129719001	7	129719001	129721000	2000	1	8.90E-12	-0.55	51	2.55	Brd1	Transcription
DMR7:129851001	7	129851001	129853000	2000	1	2.70E-08	0.47	30	1.5	Pim3	Signaling
DMR7:129900001	7	129900001	129902000	2000	1	8.90E-08	0.42	25	1.25	Ttll8	
DMR7:129919001	7	129919001	129921000	2000	1	1.30E-08	-0.4	42	2.1	Ttll8	
DMR7:130018001	7	130018001	130021000	3000	1	3.80E-09	0.51	44	1.47	Mov10l1	
DMR7:130065001	7	130065001	130067000	2000	1	5.20E-07	0.44	30	1.5	Trabd;Selo	
DMR7:130088001	7	130088001	130092000	4000	1	8.20E-07	0.37	48	1.2	Selo;Tubgcp6	Cytoskeleton
DMR7:130096001	7	130096001	130099000	3000	1	6.20E-09	0.58	27	0.9	Tubgcp6;Hdac10	Cytoskeleton
DMR7:130191001	7	130191001	130192000	1000	1	6.20E-07	-0.36	16	1.6	Ppp6r2	Signaling
DMR7:130308001	7	130308001	130309000	1000	1	4.30E-07	0.42	14	1.4	Adm2;LOC102549998;Miox	Hormone;Metabolism
DMR7:130435001	7	130435001	130441000	6000	1	4.50E-09	0.55	124	2.07	Mapk8ip2;Arsa	Cytoskeleton;Metabolism
DMR7:130491001	7	130491001	130493000	2000	1	1.20E-07	-0.39	43	2.15	Shank3	
DMR7:130503001	7	130503001	130505000	2000	1	6.30E-09	-0.31	38	1.9	Shank3	
DMR7:130761001	7	130761001	130762000	1000	1	1.90E-07	-0.54	6	0.6	Syt10	Transport
DMR7:130808001	7	130808001	130809000	1000	1	3.20E-08	-0.37	15	1.5	Syt10	Transport
DMR7:131624001	7	131624001	131626000	2000	2	2.90E-09	0.74	51	2.55	Cpne8	
DMR7:131676001	7	131676001	131678000	2000	1	6.10E-14	-0.87	9	0.45	Cpne8	
DMR7:131682001	7	131682001	131684000	2000	1	6.10E-07	0.36	18	0.9	Cpne8	
DMR7:131739001	7	131739001	131740000	1000	1	1.70E-08	0.4	9	0.9	Cpne8	
DMR7:131782001	7	131782001	131784000	2000	1	3.60E-13	0.86	47	2.35	Cpne8	
DMR7:132118001	7	132118001	132122000	4000	1	2.10E-12	-0.56	78	1.95	Kif21a	Cytoskeleton
DMR7:132160001	7	132160001	132162000	2000	1	2.10E-07	-0.33	23	1.15	Kif21a	Cytoskeleton
DMR7:132164001	7	132164001	132166000	2000	1	1.70E-09	-0.51	45	2.25	Kif21a	Cytoskeleton
DMR7:132436001	7	132436001	132438000	2000	1	1.40E-07	0.41	42	2.1	Slc2a13	Transport
DMR7:132567001	7	132567001	132572000	5000	1	1.50E-11	0.58	51	1.02	Slc2a13	Transport
DMR7:132619001	7	132619001	132624000	5000	1	7.20E-07	-0.29	54	1.08	Slc2a13	Transport
DMR7:132658001	7	132658001	132660000	2000	1	2.20E-10	0.39	19	0.95	Slc2a13	Transport
DMR7:132664001	7	132664001	132666000	2000	1	6.90E-10	0.63	50	2.5	Slc2a13	Transport
DMR7:132856001	7	132856001	132857000	1000	1	4.70E-07	0.58	11	1.1	LOC102552227;Lrrk2	Cytoskeleton
DMR7:132895001	7	132895001	132897000	2000	1	3.80E-08	0.41	29	1.45	Lrrk2	Cytoskeleton
DMR7:132933001	7	132933001	132935000	2000	1	5.20E-08	0.74	36	1.8	Lrrk2	Cytoskeleton

DMR7:132949001	7	132949001	132951000	2000	1	2.90E-07	0.4	27	1.35	Lrrk2	Cytoskeleton
DMR7:133062001	7	133062001	133063000	1000	1	2.30E-11	-0.36	14	1.4	Muc19;Smgc	
DMR7:133092001	7	133092001	133094000	2000	1	9.90E-07	0.62	36	1.8	Muc19	
DMR7:133107001	7	133107001	133109000	2000	1	5.70E-09	-0.46	22	1.1	Muc19;LOC108351530	
DMR7:133121001	7	133121001	133124000	3000	1	2.70E-07	0.31	33	1.1	Muc19;LOC108351530	
DMR7:133293001	7	133293001	133295000	2000	1	1.60E-07	0.3	21	1.05	Cntn1	
DMR7:133307001	7	133307001	133309000	2000	1	4.30E-07	0.42	13	0.65	Cntn1	
DMR7:133514001	7	133514001	133515000	1000	1	4.90E-07	0.43	7	0.7	Cntn1	
DMR7:133896001	7	133896001	133899000	3000	1	2.40E-07	-0.37	45	1.5	Pdzrn4	
DMR7:133907001	7	133907001	133908000	1000	1	1.80E-08	0.48	8	0.8	Pdzrn4	
DMR7:133986001	7	133986001	133988000	2000	1	2.60E-07	0.48	50	2.5	Pdzrn4	
DMR7:134488001	7	134488001	134490000	2000	1	1.40E-08	-0.4	21	1.05	Gxylt1;LOC103692961	
DMR7:134728001	7	134728001	134729000	1000	1	1.80E-07	-0.35	13	1.3	Prickle1	Cytoskeleton
DMR7:134774001	7	134774001	134775000	1000	1	7.10E-09	-0.36	23	2.3	Prickle1	Cytoskeleton
DMR7:135460001	7	135460001	135462000	2000	1	4.70E-07	-0.39	52	2.6	Adamts20	Protease
DMR7:135463001	7	135463001	135465000	2000	1	2.50E-08	0.55	30	1.5	Adamts20	Protease
DMR7:135492001	7	135492001	135493000	1000	1	1.80E-15	0.94	23	2.3	Adamts20	Protease
DMR7:135514001	7	135514001	135515000	1000	1	2.00E-08	0.42	35	3.5	Adamts20	Protease
DMR7:135527001	7	135527001	135528000	1000	1	2.40E-19	1.15	42	4.2	Adamts20	Protease
DMR7:135549001	7	135549001	135551000	2000	2	2.10E-09	0.51	59	2.95	Adamts20	Protease
DMR7:135562001	7	135562001	135564000	2000	1	1.60E-08	0.47	20	1	Adamts20	Protease
DMR7:135763001	7	135763001	135764000	1000	1	3.00E-16	1.07	31	3.1	LOC108351537;LOC108351538;Pus7l	
DMR7:135854001	7	135854001	135861000	7000	1	9.50E-09	-0.62	90	1.29	Twf1;LOC108351539;Ndufb4-ps1	Cytoskeleton
DMR7:135903001	7	135903001	135905000	2000	1	2.30E-09	0.24	32	1.6	Tmem117	
DMR7:135913001	7	135913001	135916000	3000	1	1.40E-07	-0.42	78	2.6	Tmem117	
DMR7:135919001	7	135919001	135924000	5000	1	2.60E-08	-0.38	112	2.24	Tmem117	
DMR7:135931001	7	135931001	135932000	1000	1	2.90E-11	-0.54	30	3	Tmem117	
DMR7:135933001	7	135933001	135935000	2000	1	1.50E-15	0.83	41	2.05	Tmem117	
DMR7:136117001	7	136117001	136121000	4000	1	4.80E-07	0.47	46	1.15	Tmem117	
DMR7:136157001	7	136157001	136159000	2000	2	3.40E-11	0.76	39	1.95	Tmem117	
DMR7:136187001	7	136187001	136189000	2000	1	7.10E-07	0.3	21	1.05	Tmem117	
DMR7:136260001	7	136260001	136261000	1000	1	2.00E-14	1	17	1.7	Tmem117;LOC103690215;LOC102546578	
DMR7:136288001	7	136288001	136289000	1000	1	2.40E-07	0.57	12	1.2	Tmem117	
DMR7:136297001	7	136297001	136298000	1000	1	3.50E-10	0.78	20	2	Tmem117	
DMR7:136306001	7	136306001	136308000	2000	1	9.40E-07	0.39	22	1.1	Tmem117	
DMR7:136317001	7	136317001	136318000	1000	1	1.40E-07	0.64	24	2.4	Tmem117	
DMR7:136324001	7	136324001	136329000	5000	1	5.50E-07	0.32	69	1.38	Tmem117	
DMR7:136338001	7	136338001	136341000	3000	1	1.60E-12	0.79	77	2.57	Tmem117	
DMR7:136388001	7	136388001	136393000	5000	1	2.10E-07	-0.35	51	1.02	Tmem117	
DMR7:136512001	7	136512001	136517000	5000	2	2.10E-17	0.91	94	1.88	Nell2	Signaling
DMR7:136555001	7	136555001	136556000	1000	1	1.40E-07	0.46	20	2	Nell2	Signaling
DMR7:136629001	7	136629001	136630000	1000	1	5.70E-10	0.62	12	1.2	Nell2;LOC102546804	Signaling
DMR7:136659001	7	136659001	136661000	2000	1	1.50E-09	-0.48	17	0.85	Nell2;LOC102546804	Signaling
DMR7:136717001	7	136717001	136718000	1000	1	3.10E-12	-0.73	1	0.1	Nell2	Signaling
DMR7:136755001	7	136755001	136758000	3000	2	2.40E-17	1	99	3.3	Nell2	Signaling
DMR7:136848001	7	136848001	136850000	2000	1	8.30E-07	-0.43	34	1.7	Nell2	Signaling
DMR7:136989001	7	136989001	136993000	4000	1	1.20E-09	-0.45	83	2.08	Dbx2	
DMR7:137002001	7	137002001	137004000	2000	2	3.90E-15	-0.63	9	0.45	Dbx2	
DMR7:137005001	7	137005001	137007000	2000	1	5.70E-07	0.45	40	2	Dbx2	
DMR7:137312001	7	137312001	137314000	2000	1	1.70E-10	-0.48	41	2.05	Ano6	
DMR7:137339001	7	137339001	137340000	1000	1	7.20E-10	-0.42	15	1.5	Ano6	
DMR7:137343001	7	137343001	137344000	1000	1	4.80E-14	0.68	30	3	Ano6	
DMR7:137701001	7	137701001	137704000	3000	1	7.80E-07	0.46	45	1.5	Arid2	
DMR7:137772001	7	137772001	137776000	4000	1	5.20E-07	-0.33	60	1.5	Arid2	
DMR7:137805001	7	137805001	137807000	2000	1	6.80E-08	-0.52	29	1.45	Arid2;Scaf11	
DMR7:137862001	7	137862001	137864000	2000	2	9.40E-10	0.8	46	2.3	Scaf11;LOC108351542	
DMR7:137907001	7	137907001	137909000	2000	1	3.30E-07	-0.44	21	1.05	RGD1564963	
DMR7:137966001	7	137966001	137967000	1000	1	7.50E-14	0.94	21	2.1	Slc38a1	Transport
DMR7:138009001	7	138009001	138011000	2000	1	6.40E-11	0.71	49	2.45	Slc38a1	Transport

DMR7:138015001	7	138015001	138025000	10000	2	3.40E-17	0.96	224	2.24	Slc38a1	Transport
DMR7:138029001	7	138029001	138030000	1000	1	4.80E-10	0.79	33	3.3	Slc38a1	Transport
DMR7:138035001	7	138035001	138038000	3000	1	1.70E-09	0.71	71	2.37	Slc38a1;LOC108351543	Transport
DMR7:138082001	7	138082001	138084000	2000	1	8.40E-09	0.7	57	2.85	Slc38a2	Transport
DMR7:138093001	7	138093001	138100000	7000	4	1.50E-14	-0.89	199	2.84	Slc38a2	Transport
DMR7:138453001	7	138453001	138454000	1000	1	8.60E-11	0.86	23	2.3	LOC108351545;Slc38a4	Transport
DMR7:138459001	7	138459001	138463000	4000	1	5.30E-07	-0.48	85	2.12	LOC108351545;Slc38a4;LOC108351546	Transport
DMR7:138464001	7	138464001	138465000	1000	1	7.50E-16	0.95	34	3.4	Slc38a4;LOC108351546	Transport
DMR7:138467001	7	138467001	138468000	1000	1	1.00E-19	0.82	22	2.2	Slc38a4;LOC108351546	Transport
DMR7:138483001	7	138483001	138488000	5000	2	1.50E-07	-0.39	113	2.26	Slc38a4;LOC108351546	Transport
DMR7:138494001	7	138494001	138497000	3000	1	2.30E-15	1.31	82	2.73	Slc38a4	Transport
DMR7:138502001	7	138502001	138509000	7000	1	4.70E-08	0.55	141	2.01	Slc38a4	Transport
DMR7:138705001	7	138705001	138707000	2000	1	3.90E-08	-0.44	83	4.15	Amigo2;LOC102551484;Pced1b	
DMR7:138740001	7	138740001	138744000	4000	1	1.80E-08	0.57	84	2.1	LOC102551484;Pced1b	
DMR7:138785001	7	138785001	138787000	2000	1	9.70E-21	1.19	54	2.7	LOC102551484;Pced1b	
DMR7:138825001	7	138825001	138827000	2000	1	7.00E-10	-0.43	26	1.3	LOC102551484;Pced1b	
DMR7:138830001	7	138830001	138836000	6000	1	7.50E-09	-0.42	134	2.23	LOC102551484;Pced1b	
DMR7:138838001	7	138838001	138839000	1000	1	1.50E-13	0.98	41	4.1	Pced1b	
DMR7:138889001	7	138889001	138892000	3000	1	2.10E-13	0.82	57	1.9	RGD1565798	
DMR7:138903001	7	138903001	138913000	10000	2	5.30E-08	0.61	139	1.39	RGD1565798	
DMR7:139194001	7	139194001	139196000	2000	1	3.20E-08	-0.36	49	2.45	Rpap3;Endou	
DMR7:139198001	7	139198001	139199000	1000	1	8.60E-07	0.37	11	1.1	Rpap3;Endou	
DMR7:139268001	7	139268001	139271000	3000	2	1.90E-13	0.8	61	2.03	LOC102548155;Slc48a1;Hdac7	Transport;Epigenetic
DMR7:139279001	7	139279001	139282000	3000	1	2.80E-11	0.7	44	1.47	Slc48a1;Hdac7	Transport;Epigenetic
DMR7:139289001	7	139289001	139290000	1000	1	4.20E-08	0.47	34	3.4	Slc48a1;Hdac7	Transport;Epigenetic
DMR7:139302001	7	139302001	139303000	1000	1	9.00E-07	0.34	21	2.1	Hdac7	Epigenetic
DMR7:139334001	7	139334001	139336000	2000	1	4.80E-07	0.43	27	1.35	LOC108351548;LOC103692974;Vdr	Transcription
DMR7:139339001	7	139339001	139341000	2000	1	7.50E-08	0.44	21	1.05	LOC108351548;LOC103692974;Vdr	Transcription
DMR7:139351001	7	139351001	139352000	1000	1	3.80E-09	0.64	29	2.9	Vdr	Transcription
DMR7:139399001	7	139399001	139400000	1000	1	1.70E-07	-0.36	24	2.4	Vdr	Transcription
DMR7:139447001	7	139447001	139452000	5000	1	3.10E-07	-0.4	131	2.62	Tmem106c;Col2a1	Extracellular Matrix
DMR7:139457001	7	139457001	139460000	3000	2	2.70E-07	0.46	51	1.7	Tmem106c;Col2a1	Extracellular Matrix
DMR7:139736001	7	139736001	139741000	5000	2	6.60E-10	0.68	95	1.9	Asb8	
DMR7:139744001	7	139744001	139746000	2000	1	5.60E-18	0.95	30	1.5	Asb8	
DMR7:139779001	7	139779001	139782000	3000	2	6.80E-11	0.67	63	2.1	Or10ad1	
DMR7:139789001	7	139789001	139792000	3000	2	1.10E-08	-0.52	43	1.43	Or10ad1	
DMR7:139902001	7	139902001	139904000	2000	1	2.90E-07	-0.39	65	3.25	Zfp641	
DMR7:139918001	7	139918001	139922000	4000	1	9.70E-07	-0.42	47	1.18	Olr1104	Receptor
DMR7:139929001	7	139929001	139930000	1000	1	1.40E-27	1.36	35	3.5	Olr1104	Receptor
DMR7:139935001	7	139935001	139938000	3000	1	2.80E-09	0.73	35	1.17	Olr1104;Olr1106	Receptor
DMR7:139939001	7	139939001	139940000	1000	1	7.90E-07	-0.32	21	2.1	Olr1104;Olr1106	Receptor
DMR7:139972001	7	139972001	139973000	1000	1	5.10E-07	0.41	13	1.3	Olr1106	Receptor
DMR7:139989001	7	139989001	139994000	5000	1	1.60E-07	-0.3	92	1.84	Olr1106;LOC680590	Receptor
DMR7:140019001	7	140019001	140022000	3000	1	1.40E-07	-0.37	47	1.57	LOC680590;Olr1107	Receptor
DMR7:140031001	7	140031001	140033000	2000	1	5.70E-21	1.4	53	2.65	Olr1107	Receptor
DMR7:140050001	7	140050001	140056000	6000	2	8.00E-15	0.84	134	2.23	LOC680635;Olr1108	Receptor
DMR7:140116001	7	140116001	140119000	3000	1	1.30E-09	0.61	48	1.6	Olr1111;LOC103692975	Receptor
DMR7:140301001	7	140301001	140305000	4000	1	2.80E-07	-0.35	56	1.4	Adcy6;Cacnb3	Transport
DMR7:140307001	7	140307001	140309000	2000	1	2.10E-08	0.54	45	2.25	Cacnb3	Transport
DMR7:140310001	7	140310001	140311000	1000	1	1.70E-07	0.52	10	1	Cacnb3	Transport
DMR7:140334001	7	140334001	140340000	6000	2	4.00E-08	0.74	121	2.02	Cacnb3;Ddx23;Rnd1	Transport;Signaling
DMR7:140346001	7	140346001	140349000	3000	1	6.10E-07	0.42	41	1.37	Ddx23;Rnd1	Signaling
DMR7:140357001	7	140357001	140359000	2000	1	1.50E-07	0.62	34	1.7	Rnd1	Signaling
DMR7:140372001	7	140372001	140375000	3000	1	4.40E-07	-0.34	46	1.53	LOC103692978;Ccdc65	
DMR7:140392001	7	140392001	140394000	2000	1	4.30E-09	0.64	36	1.8	LOC103692978;Ccdc65;Fkbp11	
DMR7:140409001	7	140409001	140414000	5000	1	1.90E-11	-0.43	64	1.28	Fkbp11;Arf3	Signaling
DMR7:140420001	7	140420001	140422000	2000	1	7.20E-07	-0.38	27	1.35	Arf3	Signaling
DMR7:140439001	7	140439001	140440000	1000	1	1.10E-09	0.53	20	2	Arf3;Wnt10b	Signaling;Signaling

DMR7:140473001	7	140473001	140477000	4000	1	2.30E-07	0.68	89	2.22	Wnt10b;Wnt1;Ddn;LOC102555574	Signaling
DMR7:140503001	7	140503001	140505000	2000	1	6.40E-09	-0.42	37	1.85	Prkag1;Kmt2d	Signaling
DMR7:140585001	7	140585001	140586000	1000	1	7.30E-07	0.47	12	1.2	Dhh;Lmbr1l	Receptor
DMR7:140588001	7	140588001	140593000	5000	1	8.40E-07	0.3	77	1.54	Dhh;Lmbr1l;LOC102556368	Receptor
DMR7:140606001	7	140606001	140608000	2000	1	2.00E-14	0.7	24	1.2	Lmbr1l;LOC102556368;LOC680861;Tuba1b	Receptor;Cytoskeleton
DMR7:140708001	7	140708001	140710000	2000	2	2.50E-29	1.35	50	2.5	Tuba1c	Cytoskeleton
DMR7:140751001	7	140751001	140752000	1000	1	9.20E-08	0.64	24	2.4	LOC102546594;Prph;Troap	
DMR7:140765001	7	140765001	140766000	1000	1	8.30E-10	0.6	26	2.6	Troap;C1ql4	
DMR7:140943001	7	140943001	140944000	1000	1	4.60E-07	-0.4	21	2.1	Fam186b	
DMR7:140950001	7	140950001	140952000	2000	1	5.60E-10	0.67	30	1.5	Fam186b;Prpf40b	
DMR7:141336001	7	141336001	141340000	4000	1	2.40E-09	-0.33	81	2.02	Asic1	Transport
DMR7:141371001	7	141371001	141376000	5000	2	2.60E-12	0.86	102	2.04	Smarcd1;Gpd1;Cox14	Epigenetic;Metabolism;Transcription
DMR7:141429001	7	141429001	141431000	2000	1	5.50E-12	1.09	69	3.45	Cers5;Lima1	Cytoskeleton
DMR7:141435001	7	141435001	141439000	4000	1	6.60E-07	-0.34	81	2.02	Lima1	Cytoskeleton
DMR7:141445001	7	141445001	141450000	5000	1	3.10E-09	-0.35	90	1.8	Lima1	Cytoskeleton
DMR7:141456001	7	141456001	141458000	2000	1	4.50E-09	0.58	48	2.4	Lima1	Cytoskeleton
DMR7:141484001	7	141484001	141487000	3000	1	1.30E-07	-0.39	60	2	Lima1	Cytoskeleton
DMR7:141488001	7	141488001	141490000	2000	1	7.70E-07	-0.37	24	1.2	Lima1	Cytoskeleton
DMR7:141494001	7	141494001	141496000	2000	1	9.40E-10	-0.5	32	1.6	Lima1	Cytoskeleton
DMR7:141504001	7	141504001	141507000	3000	1	1.60E-07	-0.34	49	1.63	Lima1	Cytoskeleton
DMR7:141514001	7	141514001	141517000	3000	1	9.60E-10	-0.42	49	1.63	Lima1	Cytoskeleton
DMR7:141527001	7	141527001	141528000	1000	1	9.30E-10	-0.75	11	1.1	Lima1	Cytoskeleton
DMR7:141538001	7	141538001	141542000	4000	1	1.10E-10	-0.7	86	2.15	Lima1	Cytoskeleton
DMR7:141795001	7	141795001	141797000	2000	1	1.60E-09	-0.65	28	1.4	Dip2b;LOC102550811	
DMR7:141839001	7	141839001	141842000	3000	1	9.60E-09	0.4	44	1.47	Dip2b	
DMR7:142100001	7	142100001	142105000	5000	1	4.50E-07	0.41	83	1.66	LOC108351554;Letmd1	Transport
DMR7:142193001	7	142193001	142194000	1000	1	5.70E-13	0.63	4	0.4	Pou6f1	
DMR7:142314001	7	142314001	142319000	5000	1	8.10E-08	0.55	96	1.92	Bin2;Cela1;Galnt6	Protease;Golgi
DMR7:142363001	7	142363001	142367000	4000	1	8.80E-09	0.54	89	2.22	Galnt6	Golgi
DMR7:142368001	7	142368001	142369000	1000	1	1.20E-13	0.81	28	2.8	Galnt6	Golgi
DMR7:142371001	7	142371001	142375000	4000	2	3.20E-09	0.33	43	1.07	Galnt6	Golgi
DMR7:142388001	7	142388001	142390000	2000	1	1.50E-09	0.64	38	1.9	Slc4a8	Transport
DMR7:142429001	7	142429001	142439000	10000	4	1.70E-13	-0.58	257	2.57	Slc4a8	Transport
DMR7:142474001	7	142474001	142480000	6000	1	2.50E-08	0.65	112	1.87	Slc4a8;LOC108351586	Transport
DMR7:142581001	7	142581001	142582000	1000	1	7.60E-16	0.91	29	2.9	Scn8a	Transport
DMR7:142607001	7	142607001	142609000	2000	1	7.10E-08	-0.39	51	2.55	Scn8a	Transport
DMR7:142650001	7	142650001	142653000	3000	1	3.50E-22	0.98	58	1.93	Scn8a	Transport
DMR7:142670001	7	142670001	142671000	1000	1	3.50E-11	0.62	12	1.2	Scn8a	Transport
DMR7:142690001	7	142690001	142693000	3000	1	1.90E-07	0.54	58	1.93	Scn8a;Fignl2	Transport
DMR7:142696001	7	142696001	142699000	3000	2	8.20E-10	0.83	79	2.63	Fignl2	
DMR7:142776001	7	142776001	142779000	3000	2	6.90E-10	0.71	58	1.93	Acvrl1	Signaling
DMR7:142808001	7	142808001	142811000	3000	2	2.20E-14	-0.56	49	1.63	Acvr1b	Signaling
DMR7:142843001	7	142843001	142845000	2000	1	7.50E-08	-0.39	41	2.05	Acvr1b;LOC103692983	Signaling
DMR7:142900001	7	142900001	142902000	2000	1	2.40E-08	0.55	32	1.6	LOC102556412;Nr4a1	Transcription
DMR7:142918001	7	142918001	142920000	2000	1	2.70E-10	0.47	55	2.75	Nr4a1;Atg101	Transcription
DMR7:142943001	7	142943001	142945000	2000	1	1.10E-07	0.46	25	1.25	Atg101;LOC103692985	
DMR7:142991001	7	142991001	142993000	2000	1	5.60E-08	0.54	27	1.35	Krt80	
DMR7:143017001	7	143017001	143018000	1000	1	1.80E-07	0.53	12	1.2	Krt80	
DMR7:143070001	7	143070001	143074000	4000	1	5.10E-07	0.46	58	1.45	Krt7;Krt83	
DMR7:143137001	7	143137001	143138000	1000	1	3.00E-07	0.57	22	2.2	Krt86;Kb23	
DMR7:143140001	7	143140001	143141000	1000	1	8.70E-11	0.8	24	2.4	Kb23	
DMR7:143144001	7	143144001	143147000	3000	1	7.30E-09	0.49	43	1.43	Kb23	
DMR7:143163001	7	143163001	143168000	5000	1	1.60E-10	0.68	92	1.84	Krt85	
DMR7:143169001	7	143169001	143173000	4000	1	1.70E-07	0.43	42	1.05	Krt85;Krt84	
DMR7:143194001	7	143194001	143198000	4000	2	1.00E-09	0.59	59	1.48	Krt84;Krt82	
DMR7:143212001	7	143212001	143216000	4000	1	7.70E-11	0.7	70	1.75	Krt82;Kb15;Krt75;LOC108351555	
DMR7:143273001	7	143273001	143275000	2000	1	5.00E-08	0.61	22	1.1	RGD1562494	
DMR7:143345001	7	143345001	143346000	1000	1	3.00E-07	0.42	13	1.3	Krt71	

DMR7:143397001	7	143397001	143399000	2000	1	3.90E-08	0.67	33	1.65	Krt72;Krt73	
DMR7:143405001	7	143405001	143408000	3000	1	7.00E-12	0.55	61	2.03	Krt73;Krt2	
DMR7:143416001	7	143416001	143420000	4000	2	7.50E-19	1.2	77	1.93	Krt73;Krt2	
DMR7:143445001	7	143445001	143447000	2000	2	1.80E-10	0.44	13	0.65	LOC300249;Krt1	
DMR7:143589001	7	143589001	143593000	4000	1	3.50E-09	0.68	71	1.77	LOC103692986;Krt8	
DMR7:143611001	7	143611001	143613000	2000	1	9.00E-10	-0.69	20	1	Krt8	
DMR7:143637001	7	143637001	143638000	1000	1	3.40E-11	-0.6	7	0.7	Krt18	
DMR7:143706001	7	143706001	143709000	3000	1	4.40E-07	0.48	49	1.63	Eif4b;LOC108351556;Tns2	Translation;Cytoskeleton
DMR7:143748001	7	143748001	143749000	1000	1	5.30E-07	0.54	20	2	Spryd3;Igfbp6;LOC102551965;Soat2	Cytoskeleton;Protease; Proteolysis;Metabolism
DMR7:143755001	7	143755001	143759000	4000	1	2.20E-07	0.62	54	1.35	Igfbp6;LOC102551965;Soat2	Protease; Proteolysis;Metabolism
DMR7:143764001	7	143764001	143768000	4000	1	8.90E-07	0.43	85	2.12	Igfbp6;LOC102551965;Soat2	Protease; Proteolysis;Metabolism
DMR7:143790001	7	143790001	143791000	1000	1	8.70E-11	0.5	11	1.1	Csad	Metabolism
DMR7:143797001	7	143797001	143799000	2000	1	3.90E-09	-0.45	21	1.05	Csad	Metabolism
DMR7:143811001	7	143811001	143815000	4000	1	8.90E-08	-0.39	65	1.62	Csad;Znf740;Itgb7	Metabolism;Transcription;Extracellular Matrix
DMR7:143827001	7	143827001	143828000	1000	1	6.20E-08	0.47	33	3.3	Znf740;Itgb7	Transcription;Extracellular Matrix
DMR7:143842001	7	143842001	143844000	2000	1	9.20E-07	0.35	27	1.35	Itgb7;Rarg	Extracellular Matrix;Transcription
DMR7:143874001	7	143874001	143877000	3000	1	7.90E-09	0.44	57	1.9	LOC102552775;Mfsd5	Transport
DMR7:144255001	7	144255001	144257000	2000	1	6.80E-07	0.37	26	1.3	Atp5g2	
DMR7:144557001	7	144557001	144559000	2000	1	1.90E-08	-0.41	20	1	Hoxc12;Hoxc11	Development
DMR7:144572001	7	144572001	144574000	2000	2	5.30E-12	-0.53	29	1.45	Hoxc11;Hoxc10	Development
DMR7:144579001	7	144579001	144583000	4000	1	4.80E-08	-0.57	39	0.98	Hoxc10;Mir196a;LOC103692987;Hoxc9	Development
DMR7:144598001	7	144598001	144601000	3000	1	3.10E-13	-0.51	46	1.53	LOC103692987;Hoxc9;Hoxc8	
DMR7:144640001	7	144640001	144641000	1000	1	4.10E-09	0.67	27	2.7	Hoxc5;Hoxc4	Development
DMR7:144765001	7	144765001	144768000	3000	2	3.30E-08	0.56	38	1.27	LOC103692988;Smug1	Epigenetic
DMR7:144926001	7	144926001	144929000	3000	2	1.40E-09	0.67	45	1.5	Copz1;Gpr84	Transport;Signaling
DMR7:144940001	7	144940001	144941000	1000	1	1.30E-20	1.27	42	4.2	Gpr84;Zfp385a	Signaling
DMR7:144991001	7	144991001	144993000	2000	1	4.00E-07	0.66	41	2.05	Itga5	Extracellular Matrix
DMR7:145075001	7	145075001	145077000	2000	1	7.80E-08	-0.44	15	0.75	LOC102547836;Nckap1l	
DMR7:145149001	7	145149001	145151000	2000	1	6.70E-08	0.44	19	0.95	Pde1b;Ppp1r1a;LOC102547969	Signaling;Signaling
DMR7:145160001	7	145160001	145161000	1000	1	2.20E-08	0.46	9	0.9	Ppp1r1a;LOC102547969	Signaling
DMR8:451001	8	451001	454000	3000	1	4.90E-17	0.5	40	1.33	Gucy1a2;LOC690804	Signaling
DMR8:493001	8	493001	495000	2000	1	2.70E-07	-0.51	8	0.4	Gucy1a2	Signaling
DMR8:636001	8	636001	637000	1000	1	5.00E-09	0.5	11	1.1	Gucy1a2	Signaling
DMR8:700001	8	700001	703000	3000	1	3.20E-10	-0.58	18	0.6	Gucy1a2	Signaling
DMR8:862001	8	862001	866000	4000	1	1.30E-07	-0.39	36	0.9	Gucy1a2	Signaling
DMR8:891001	8	891001	892000	1000	1	8.10E-08	-0.49	7	0.7	Gucy1a2	Signaling
DMR8:1446001	8	1446001	1447000	1000	1	1.60E-09	-0.46	13	1.3	Aasdhppt;Kbtbd3	Transport;Cytoskeleton
DMR8:1461001	8	1461001	1465000	4000	1	3.60E-10	0.31	34	0.85	Kbtbd3	Cytoskeleton
DMR8:1546001	8	1546001	1548000	2000	1	2.60E-08	-0.48	5	0.25	Gria4	Receptor
DMR8:1832001	8	1832001	1833000	1000	1	6.40E-10	-0.45	3	0.3	Gria4	Receptor
DMR8:2644001	8	2644001	2646000	2000	1	2.10E-10	-0.49	8	0.4	Casp4;LOC102557277	Protease
DMR8:4564001	8	4564001	4565000	1000	1	2.90E-07	0.5	13	1.3	Pdgfd;LOC108351647	Growth Factors
DMR8:4597001	8	4597001	4599000	2000	1	4.30E-07	-0.53	10	0.5	Pdgfd	Growth Factors
DMR8:4643001	8	4643001	4644000	1000	1	5.60E-07	0.64	26	2.6	Pdgfd	Growth Factors
DMR8:5225001	8	5225001	5226000	1000	1	3.50E-07	-0.41	6	0.6	Dync2h1	Cytoskeleton
DMR8:5593001	8	5593001	5594000	1000	1	1.70E-07	-0.49	5	0.5	Mmp12	Protease
DMR8:5702001	8	5702001	5705000	3000	1	3.30E-07	0.32	36	1.2	Mmp3;Mmp1	Protease
DMR8:5815001	8	5815001	5816000	1000	1	8.10E-07	0.39	20	2	Mmp20	Protease
DMR8:5831001	8	5831001	5834000	3000	1	2.20E-09	0.57	48	1.6	Mmp20	Protease
DMR8:5845001	8	5845001	5847000	2000	1	3.80E-07	0.59	23	1.15	Mmp20	Protease

DMR8:6159001	8	6159001	6161000	2000	1	1.20E-07	-0.44	30	1.5	Yap1	Transcription
DMR8:6298001	8	6298001	6300000	2000	1	2.50E-15	0.62	32	1.6	Cep126	Transcription
DMR8:6828001	8	6828001	6830000	2000	2	8.50E-08	-0.67	26	1.3	Trpc6	Transport
DMR8:7269001	8	7269001	7271000	2000	1	1.20E-12	0.83	31	1.55	Arhgap42	Signaling
DMR8:7357001	8	7357001	7358000	1000	1	1.60E-07	-0.41	9	0.9	Arhgap42	Signaling
DMR8:8159001	8	8159001	8162000	3000	1	2.10E-08	-0.49	27	0.9	Cntn5	
DMR8:8172001	8	8172001	8173000	1000	1	1.00E-10	0.61	7	0.7	Cntn5	
DMR8:8233001	8	8233001	8234000	1000	1	7.00E-07	0.55	16	1.6	Cntn5	
DMR8:8416001	8	8416001	8418000	2000	2	4.60E-09	0.58	15	0.75	Cntn5	
DMR8:8599001	8	8599001	8600000	1000	1	1.30E-11	-0.84	5	0.5	Cntn5;LOC108351832	
DMR8:8800001	8	8800001	8804000	4000	1	1.70E-07	-0.31	32	0.8	Cntn5	
DMR8:10853001	8	10853001	10854000	1000	1	7.60E-08	-0.63	7	0.7	Olr1763-ps	
DMR8:10866001	8	10866001	10867000	1000	1	2.90E-07	-0.58	3	0.3	Olr1763-ps	
DMR8:11980001	8	11980001	11984000	4000	2	2.40E-12	0.54	41	1.02	Maml2	Transcription
DMR8:12035001	8	12035001	12038000	3000	1	5.60E-08	-0.41	63	2.1	Maml2	Transcription
DMR8:12060001	8	12060001	12063000	3000	1	7.60E-09	-0.5	59	1.97	Maml2	Transcription
DMR8:12125001	8	12125001	12127000	2000	1	4.00E-07	-0.46	30	1.5	Maml2	Transcription
DMR8:12185001	8	12185001	12187000	2000	1	7.80E-08	-0.41	41	2.05	Maml2	Transcription
DMR8:12816001	8	12816001	12819000	3000	1	5.90E-08	-0.48	32	1.07	Sesn3	Metabolism
DMR8:12932001	8	12932001	12933000	1000	1	2.20E-13	0.86	36	3.6	Endod1	
DMR8:13045001	8	13045001	13047000	2000	1	8.90E-10	0.55	35	1.75	Amotl1	
DMR8:13158001	8	13158001	13159000	1000	1	3.90E-08	0.63	19	1.9	Amotl1	
DMR8:13231001	8	13231001	13237000	6000	1	4.10E-07	-0.36	100	1.67	Piwil4;LOC102549248	Translation
DMR8:13249001	8	13249001	13253000	4000	1	1.00E-11	0.7	64	1.6	Piwil4;LOC102549248;Fut4	Translation;Golgi
DMR8:13380001	8	13380001	13384000	4000	1	5.50E-07	0.52	65	1.62	Gpr83	Signaling
DMR8:13406001	8	13406001	13408000	2000	2	1.50E-08	0.53	51	2.55	LOC102549408;Dpp3l;Izumo1r	
DMR8:13418001	8	13418001	13420000	2000	1	4.60E-09	0.45	28	1.4	Dpp3l;Izumo1r;LOC102549753	
DMR8:13567001	8	13567001	13568000	1000	1	2.10E-08	-0.56	23	2.3	Med17;Panx1	Transcription
DMR8:13620001	8	13620001	13624000	4000	1	1.00E-08	-0.48	67	1.68	Med17;Hephl1	Transcription;Metabolism
DMR8:13635001	8	13635001	13636000	1000	1	3.10E-11	0.61	29	2.9	Med17;Hephl1	Transcription;Metabolism
DMR8:13663001	8	13663001	13665000	2000	1	7.80E-09	0.49	30	1.5	Med17;Hephl1;RGD1562937	Transcription;Metabolism
DMR8:13689001	8	13689001	13692000	3000	1	9.90E-13	-0.61	26	0.87	Med17;Hephl1	Transcription;Metabolism
DMR8:13700001	8	13700001	13703000	3000	1	5.00E-08	-0.26	24	0.8	Med17;LOC108351808	Transcription
DMR8:13791001	8	13791001	13792000	1000	1	3.10E-15	0.46	14	1.4	Med17;LOC102551966;Vstm5	Transcription;Immune
DMR8:14025001	8	14025001	14026000	1000	1	3.60E-10	0.4	20	2	Smco4	
DMR8:14048001	8	14048001	14057000	9000	1	2.10E-10	-0.6	151	1.68	Smco4	
DMR8:14074001	8	14074001	14077000	3000	1	2.10E-07	-0.39	46	1.53	Smco4	
DMR8:14437001	8	14437001	14443000	6000	1	3.80E-07	0.43	111	1.85	Fat3	Cytoskeleton
DMR8:14499001	8	14499001	14500000	1000	1	1.70E-11	0.46	8	0.8	Fat3	Cytoskeleton
DMR8:14590001	8	14590001	14592000	2000	1	1.90E-07	0.5	28	1.4	Fat3	Cytoskeleton
DMR8:14700001	8	14700001	14705000	5000	1	2.60E-08	0.26	45	0.9	Fat3	Cytoskeleton
DMR8:14845001	8	14845001	14848000	3000	1	4.00E-07	0.35	57	1.9	Fat3	Cytoskeleton
DMR8:17520001	8	17520001	17521000	1000	1	5.70E-08	0.55	13	1.3	Naalad2;LOC690305	Protease
DMR8:17924001	8	17924001	17925000	1000	1	1.10E-07	-0.5	7	0.7	Mbd3l2	
DMR8:18497001	8	18497001	18499000	2000	1	9.80E-07	-0.5	5	0.25	Muc16	
DMR8:18544001	8	18544001	18548000	4000	1	7.20E-07	-0.28	42	1.05	Muc16	
DMR8:18574001	8	18574001	18577000	3000	2	9.70E-10	-0.58	13	0.43	Muc16	
DMR8:18618001	8	18618001	18619000	1000	1	3.60E-07	-0.47	1	0.1	Muc16	
DMR8:18723001	8	18723001	18725000	2000	1	5.00E-08	-0.65	7	0.35	Olr1121	Receptor
DMR8:18947001	8	18947001	18951000	4000	1	5.00E-08	-0.37	35	0.88	Olr1131-ps	
DMR8:18979001	8	18979001	18980000	1000	1	7.90E-08	-0.42	2	0.2	Olr1130	Receptor
DMR8:19011001	8	19011001	19014000	3000	1	4.40E-08	-0.47	10	0.33	Olr1132	Receptor
DMR8:19031001	8	19031001	19033000	2000	1	2.00E-08	-0.44	76	3.8	Olr1133-ps;Olr1134-ps	
DMR8:19376001	8	19376001	19379000	3000	1	1.90E-07	-0.38	28	0.93	Olr1145	Receptor
DMR8:19801001	8	19801001	19803000	2000	1	6.10E-07	-0.51	15	0.75	Olr1156	Receptor
DMR8:19895001	8	19895001	19900000	5000	2	4.50E-09	-0.32	44	0.88	Zfp317	
DMR8:20234001	8	20234001	20241000	7000	2	1.50E-08	0.42	174	2.49	Olr1165;LOC108351836	Receptor
DMR8:21002001	8	21002001	21005000	3000	1	2.40E-07	-0.29	36	1.2	Olr1187-ps;Olfr873	Receptor

DMR8:21686001	8	21686001	21688000	2000	1	4.80E-08	0.6	26	1.3	Pin1;Olfm2	Transcription;Development
DMR8:21712001	8	21712001	21715000	3000	1	3.50E-07	0.49	57	1.9	Olfm2	Development
DMR8:21757001	8	21757001	21760000	3000	1	4.00E-08	0.48	42	1.4	Olfm2;LOC102552496	Development
DMR8:21807001	8	21807001	21809000	2000	1	5.90E-08	0.46	26	1.3	Col5a3	Extracellular Matrix
DMR8:21948001	8	21948001	21950000	2000	1	4.20E-09	0.49	38	1.9	Dnmt1	Epigenetic
DMR8:21974001	8	21974001	21978000	4000	1	1.20E-16	0.91	86	2.15	Dnmt1;LOC367036;S1pr2	Epigenetic;Signaling
DMR8:22016001	8	22016001	22017000	1000	1	6.30E-07	0.41	11	1.1	Mrpl4	Translation
DMR8:22043001	8	22043001	22047000	4000	1	9.00E-11	0.52	67	1.68	LOC108351839;Icam1;Icam4;Icam5	
DMR8:22056001	8	22056001	22058000	2000	1	6.90E-10	0.83	54	2.7	Icam1;Icam4;Icam5	
DMR8:22108001	8	22108001	22109000	1000	1	4.60E-07	-0.44	9	0.9	Raver1;Tyk2	Metabolism
DMR8:22221001	8	22221001	22223000	2000	1	9.00E-09	0.53	30	1.5	Pde4a	Signaling
DMR8:22224001	8	22224001	22226000	2000	1	4.10E-11	0.82	40	2	Pde4a	Signaling
DMR8:22251001	8	22251001	22253000	2000	1	5.60E-14	0.95	58	2.9	Keap1	Cytoskeleton
DMR8:22267001	8	22267001	22270000	3000	1	7.10E-08	0.64	89	2.97	Keap1;S1pr5	Cytoskeleton;Signaling
DMR8:22282001	8	22282001	22283000	1000	1	5.00E-07	0.54	21	2.1	S1pr5;Atg4d	Signaling;Protease
DMR8:22338001	8	22338001	22339000	1000	1	9.10E-07	0.49	20	2	Ap1m2	Transport
DMR8:22373001	8	22373001	22376000	3000	1	5.20E-07	0.47	72	2.4	Slc44a2	Transport
DMR8:22448001	8	22448001	22454000	6000	1	3.70E-07	0.51	103	1.72	Ilf3;Qtrt1;Dnm2	Metabolism;Translation;Transport
DMR8:22746001	8	22746001	22748000	2000	1	3.30E-09	0.46	32	1.6	Smarca4;Ldlr	Epigenetic;Binding Proteins
DMR8:22756001	8	22756001	22758000	2000	1	2.20E-07	-0.32	37	1.85	Ldlr	Binding Proteins
DMR8:22765001	8	22765001	22768000	3000	1	1.20E-13	0.49	42	1.4	Ldlr	Binding Proteins
DMR8:22803001	8	22803001	22807000	4000	1	3.40E-07	0.43	69	1.73	Kank2	Cytoskeleton
DMR8:22817001	8	22817001	22819000	2000	2	3.60E-17	0.73	88	4.4	Kank2;Dock6	Cytoskeleton;Transcription
DMR8:22846001	8	22846001	22848000	2000	1	6.90E-15	0.63	50	2.5	Dock6;Angptl8	Transcription
DMR8:22942001	8	22942001	22945000	3000	1	3.60E-13	0.92	64	2.13	Tmem205;Ccdc159;Plppr2	Signaling
DMR8:22950001	8	22950001	22954000	4000	1	3.80E-07	0.52	70	1.75	Ccdc159;Plppr2	Signaling
DMR8:22957001	8	22957001	22959000	2000	2	1.20E-15	1.12	76	3.8	Plppr2;Swsap1	Signaling
DMR8:23010001	8	23010001	23012000	2000	1	2.80E-07	-0.66	6	0.3	Rgl3;Ccdc151;Prkcsh	Transcription;Signaling
DMR8:23023001	8	23023001	23027000	4000	1	5.30E-09	0.58	59	1.48	Ccdc151;Prkcsh;Elavl3	Signaling;Translation
DMR8:23038001	8	23038001	23041000	3000	1	3.10E-07	0.39	40	1.33	Elavl3	Translation
DMR8:23127001	8	23127001	23129000	2000	1	3.20E-10	0.48	26	1.3	Cnn1;Elof1	Cytoskeleton
DMR8:23140001	8	23140001	23143000	3000	1	3.50E-12	0.85	53	1.77	Elof1;Acp5	Signaling
DMR8:23490001	8	23490001	23491000	1000	1	8.70E-14	0.99	28	2.8	Rp9;Bbs9	
DMR8:23532001	8	23532001	23534000	2000	1	1.80E-12	0.81	38	1.9	Bbs9	
DMR8:23655001	8	23655001	23657000	2000	1	9.70E-08	-0.49	44	2.2	Bbs9;LOC103693028	
DMR8:23666001	8	23666001	23667000	1000	1	1.20E-09	0.78	17	1.7	Bbs9;LOC103693028	
DMR8:23781001	8	23781001	23783000	2000	1	1.00E-07	-0.4	39	1.95	Bbs9	
DMR8:23803001	8	23803001	23805000	2000	1	4.20E-07	-0.43	30	1.5	Bbs9	
DMR8:23845001	8	23845001	23846000	1000	1	1.60E-08	-0.57	6	0.6	Bbs9	
DMR8:23937001	8	23937001	23939000	2000	1	2.10E-07	-0.4	39	1.95	Bbs9	
DMR8:23974001	8	23974001	23975000	1000	1	4.70E-08	-0.4	21	2.1	Bbs9	
DMR8:24018001	8	24018001	24019000	1000	1	1.50E-09	-0.51	7	0.7	Bbs9	
DMR8:24057001	8	24057001	24058000	1000	1	1.50E-07	0.47	12	1.2	Bbs9	
DMR8:24402001	8	24402001	24404000	2000	1	8.00E-08	-0.43	48	2.4	Bmper	Extracellular Matrix
DMR8:24493001	8	24493001	24495000	2000	1	2.00E-18	0.58	19	0.95	Bmper	Extracellular Matrix
DMR8:25285001	8	25285001	25286000	1000	1	7.80E-08	0.37	24	2.4	Npsr1	Signaling
DMR8:25314001	8	25314001	25315000	1000	1	1.10E-07	-0.65	7	0.7	Npsr1	Signaling
DMR8:25371001	8	25371001	25374000	3000	1	6.80E-08	-0.39	17	0.57	Npsr1	Signaling
DMR8:25380001	8	25380001	25383000	3000	2	1.30E-10	-0.55	27	0.9	Npsr1	Signaling
DMR8:25642001	8	25642001	25643000	1000	1	1.50E-08	0.46	8	0.8	Dpy19l1	
DMR8:25665001	8	25665001	25666000	1000	1	5.80E-09	0.38	12	1.2	Dpy19l1	
DMR8:25762001	8	25762001	25766000	4000	1	2.60E-08	-0.37	32	0.8	Dpy19l2	
DMR8:25767001	8	25767001	25768000	1000	1	3.20E-07	-0.26	5	0.5	Dpy19l2	
DMR8:25815001	8	25815001	25817000	2000	1	1.80E-08	-0.45	10	0.5	Dpy19l2	
DMR8:26261001	8	26261001	26263000	2000	1	2.80E-07	-0.36	20	1	LOC108351663;Herpud2	
DMR8:26657001	8	26657001	26665000	8000	1	1.30E-08	-0.36	94	1.18	Eepd1	
DMR8:26705001	8	26705001	26706000	1000	1	7.70E-07	0.61	12	1.2	Eepd1	

DMR8:26740001	8	26740001	26741000	1000	1	5.90E-07	0.48	12	1.2	Eepd1	
DMR8:27804001	8	27804001	27806000	2000	1	1.30E-07	-0.35	52	2.6	B3gat1;Glb1l2	Golgi;Metabolism
DMR8:27831001	8	27831001	27834000	3000	1	5.50E-08	-0.39	26	0.87	Glb1l2	Metabolism
DMR8:27973001	8	27973001	27979000	6000	1	8.10E-08	-0.35	86	1.43	Bin2a	Metabolism
DMR8:28080001	8	28080001	28082000	2000	1	1.10E-07	-0.34	40	2	Vps26b;Ncapd3	Transport;Epigenetic
DMR8:28086001	8	28086001	28087000	1000	1	2.20E-07	-0.32	19	1.9	Ncapd3	Epigenetic
DMR8:28098001	8	28098001	28100000	2000	1	4.10E-07	-0.33	27	1.35	Ncapd3	Epigenetic
DMR8:28354001	8	28354001	28356000	2000	1	3.60E-07	-0.43	19	0.95	Igsf9b	
DMR8:28385001	8	28385001	28388000	3000	1	6.60E-19	1.12	83	2.77	Igsf9b	
DMR8:28463001	8	28463001	28464000	1000	1	6.00E-07	-0.71	9	0.9	Spata19	
DMR8:28988001	8	28988001	28993000	5000	2	2.10E-10	0.91	63	1.26	Opcml	Immune
DMR8:29048001	8	29048001	29053000	5000	1	1.80E-07	0.67	64	1.28	Opcml	Immune
DMR8:29093001	8	29093001	29094000	1000	1	4.90E-13	0.54	11	1.1	Opcml	Immune
DMR8:29353001	8	29353001	29354000	1000	1	2.90E-12	0.66	12	1.2	Opcml	Immune
DMR8:29615001	8	29615001	29616000	1000	1	3.10E-17	0.59	8	0.8	Opcml	Immune
DMR8:29831001	8	29831001	29832000	1000	1	2.30E-08	0.63	13	1.3	Opcml	Immune
DMR8:30089001	8	30089001	30091000	2000	1	5.40E-08	0.45	14	0.7	Ntm	Immune
DMR8:30096001	8	30096001	30097000	1000	1	7.70E-08	-0.37	15	1.5	Ntm	Immune
DMR8:30249001	8	30249001	30250000	1000	1	1.90E-08	0.52	11	1.1	Ntm	Immune
DMR8:30324001	8	30324001	30326000	2000	1	9.40E-11	-0.39	28	1.4	Ntm	Immune
DMR8:30395001	8	30395001	30398000	3000	1	1.30E-10	-0.39	29	0.97	Ntm	Immune
DMR8:30481001	8	30481001	30485000	4000	1	2.00E-07	-0.31	44	1.1	Ntm	Immune
DMR8:30555001	8	30555001	30557000	2000	1	1.00E-09	-0.75	59	2.95	Ntm	Immune
DMR8:30673001	8	30673001	30674000	1000	1	3.00E-07	0.63	20	2	Ntm	Immune
DMR8:30733001	8	30733001	30738000	5000	1	5.00E-08	0.53	79	1.58	Ntm	Immune
DMR8:31044001	8	31044001	31045000	1000	1	2.70E-08	0.38	14	1.4	Ntm	Immune
DMR8:32001001	8	32001001	32002000	1000	1	9.50E-07	0.36	15	1.5	Adamts15	Protease
DMR8:32033001	8	32033001	32035000	2000	1	7.30E-09	0.4	32	1.6	Adamts8	Protease
DMR8:32215001	8	32215001	32216000	1000	1	9.80E-10	-0.44	18	1.8	Zbtb44	Transcription
DMR8:32239001	8	32239001	32243000	4000	1	9.80E-08	-0.43	84	2.1	St14	Protease
DMR8:32248001	8	32248001	32249000	1000	1	8.70E-07	0.5	15	1.5	St14	Protease
DMR8:32252001	8	32252001	32255000	3000	1	1.40E-08	0.66	67	2.23	St14	Protease
DMR8:32310001	8	32310001	32312000	2000	1	1.10E-07	-0.4	37	1.85	Aplp2	Protease; Proteolysis
DMR8:32576001	8	32576001	32580000	4000	1	2.10E-08	-0.51	50	1.25	Tmem45b	
DMR8:32583001	8	32583001	32584000	1000	1	1.70E-11	0.64	22	2.2	Tmem45b	
DMR8:32950001	8	32950001	32952000	2000	1	1.10E-09	0.87	52	2.6	Barx2;LOC108351669	Development
DMR8:32979001	8	32979001	32981000	2000	1	8.10E-08	0.41	31	1.55	Barx2	Development
DMR8:33135001	8	33135001	33137000	2000	1	1.20E-08	0.51	20	1	LOC102551288;Arhgap32	Signaling
DMR8:33234001	8	33234001	33238000	4000	1	4.70E-07	-0.42	61	1.52	Arhgap32	Signaling
DMR8:33239001	8	33239001	33240000	1000	1	2.40E-08	-0.54	24	2.4	Arhgap32	Signaling
DMR8:33261001	8	33261001	33264000	3000	1	8.60E-12	0.37	46	1.53	Arhgap32	Signaling
DMR8:33272001	8	33272001	33273000	1000	1	1.50E-07	-0.52	20	2	Arhgap32	Signaling
DMR8:33328001	8	33328001	33329000	1000	1	2.00E-09	-0.44	13	1.3	Arhgap32	Signaling
DMR8:33368001	8	33368001	33372000	4000	1	4.70E-09	-0.45	76	1.9	Arhgap32	Signaling
DMR8:33390001	8	33390001	33393000	3000	1	1.40E-09	-0.57	52	1.73	Arhgap32;LOC103693036	Signaling
DMR8:33439001	8	33439001	33441000	2000	1	1.00E-07	0.48	22	1.1	Kcnj5	Transport
DMR8:33464001	8	33464001	33465000	1000	1	4.30E-08	0.57	16	1.6	Kcnj5	Transport
DMR8:33513001	8	33513001	33516000	3000	1	5.70E-07	-0.4	40	1.33	Kcnj1	Transport
DMR8:33763001	8	33763001	33765000	2000	1	4.30E-11	0.52	22	1.1	Ets1	Transcription
DMR8:33766001	8	33766001	33767000	1000	1	1.40E-12	0.83	41	4.1	Ets1	Transcription
DMR8:33813001	8	33813001	33815000	2000	1	7.90E-07	-0.36	36	1.8	Ets1	Transcription
DMR8:33817001	8	33817001	33819000	2000	1	5.10E-07	-0.4	28	1.4	Ets1;LOC102552230	Transcription
DMR8:33864001	8	33864001	33866000	2000	1	2.10E-07	-0.49	31	1.55	Ets1	Transcription
DMR8:35722001	8	35722001	35725000	3000	1	7.80E-10	0.71	93	3.1	Kirrel3;LOC102555390	
DMR8:35765001	8	35765001	35767000	2000	1	7.30E-07	0.55	15	0.75	Kirrel3	
DMR8:35796001	8	35796001	35797000	1000	1	2.30E-07	0.39	13	1.3	Kirrel3	
DMR8:35836001	8	35836001	35838000	2000	1	7.50E-07	-0.36	34	1.7	Kirrel3	
DMR8:35999001	8	35999001	3.60E+07	1000	1	2.90E-07	0.36	16	1.6	Kirrel3	
DMR8:36001001	8	36001001	36003000	2000	1	9.80E-10	0.58	26	1.3	Kirrel3	
DMR8:36040001	8	36040001	36041000	1000	1	1.20E-07	-0.35	13	1.3	Kirrel3	
DMR8:36087001	8	36087001	36091000	4000	1	3.90E-07	0.39	75	1.88	Kirrel3	
DMR8:36119001	8	36119001	36120000	1000	1	1.70E-09	-0.5	6	0.6	Kirrel3	

DMR8:36149001	8	36149001	36150000	1000	1	1.80E-07	-0.58	10	1	Kirrel3;LOC108351671	
DMR8:36194001	8	36194001	36199000	5000	1	1.10E-11	0.84	71	1.42	Kirrel3	
DMR8:36264001	8	36264001	36266000	2000	1	9.10E-08	0.56	24	1.2	Kirrel3;St3gal4	Transport
DMR8:36274001	8	36274001	36275000	1000	1	1.90E-07	0.52	9	0.9	St3gal4	Transport
DMR8:36348001	8	36348001	36349000	1000	1	6.00E-07	-0.58	0	0	Dcps	Metabolism
DMR8:36667001	8	36667001	36668000	1000	1	6.80E-07	-0.33	27	2.7	Cdon	
DMR8:36677001	8	36677001	36679000	2000	1	9.70E-07	-0.41	14	0.7	Cdon	
DMR8:36694001	8	36694001	36696000	2000	1	7.20E-10	-0.44	32	1.6	Cdon;LOC102548010	
DMR8:36748001	8	36748001	36749000	1000	1	4.70E-07	-0.51	20	2	Ddx25	
DMR8:36786001	8	36786001	36788000	2000	1	1.90E-07	-0.27	13	0.65	Pate2	
DMR8:36820001	8	36820001	36821000	1000	1	2.90E-13	-0.63	3	0.3	Pate2;Pate4	
DMR8:36824001	8	36824001	36828000	4000	1	5.20E-07	-0.23	32	0.8	Pate2;Pate4	
DMR8:37100001	8	37100001	37106000	6000	1	5.10E-11	-0.33	72	1.2	Pate-f	
DMR8:37823001	8	37823001	37827000	4000	1	4.50E-07	-0.47	25	0.62	RGD1560348	
DMR8:38058001	8	38058001	38060000	2000	1	5.70E-07	-0.25	14	0.7	Trnaa-ugc	
DMR8:38061001	8	38061001	38065000	4000	1	3.70E-10	0.73	63	1.57	Trnaa-ugc	
DMR8:39318001	8	39318001	39319000	1000	1	1.20E-10	-0.46	14	1.4	Fez1	
DMR8:39322001	8	39322001	39326000	4000	1	4.70E-10	0.39	41	1.02	Fez1	
DMR8:39389001	8	39389001	39391000	2000	1	3.90E-08	-0.41	32	1.6	Pknox2	Development
DMR8:39403001	8	39403001	39405000	2000	1	3.30E-08	0.62	36	1.8	Pknox2	Development
DMR8:39426001	8	39426001	39429000	3000	2	4.10E-08	-0.54	26	0.87	Pknox2	Development
DMR8:39444001	8	39444001	39447000	3000	1	2.50E-08	0.59	55	1.83	Pknox2	Development
DMR8:39533001	8	39533001	39538000	5000	1	1.50E-07	0.44	78	1.56	Pknox2;LOC108351675	Development
DMR8:39551001	8	39551001	39553000	2000	1	2.80E-07	0.51	28	1.4	Pknox2;LOC108351675	Development
DMR8:39699001	8	39699001	39702000	3000	1	1.20E-11	-0.46	48	1.6	Tmem218;Slc37a2	Transport
DMR8:39738001	8	39738001	39739000	1000	1	7.20E-07	0.4	29	2.9	Slc37a2	Transport
DMR8:39767001	8	39767001	39768000	1000	1	4.20E-10	-0.54	5	0.5	Ccdc15	
DMR8:39783001	8	39783001	39784000	1000	1	4.30E-10	0.4	8	0.8	Ccdc15	
DMR8:39813001	8	39813001	39816000	3000	2	1.70E-11	-0.57	25	0.83	Ccdc15	
DMR8:39845001	8	39845001	39846000	1000	1	3.50E-11	-0.58	8	0.8	Hepacam	Immune
DMR8:39848001	8	39848001	39850000	2000	1	4.00E-09	0.66	42	2.1	Hepacam	Immune
DMR8:39912001	8	39912001	39914000	2000	1	6.30E-09	-0.45	34	1.7	Robo3	
DMR8:39915001	8	39915001	39918000	3000	1	1.40E-08	0.55	38	1.27	Robo3	
DMR8:39923001	8	39923001	39925000	2000	2	3.00E-10	-0.56	22	1.1	Robo3	
DMR8:39989001	8	39989001	39992000	3000	1	1.40E-08	0.49	42	1.4	Msantd2;LOC102549473;Esam	
DMR8:40176001	8	40176001	40183000	7000	1	2.80E-07	-0.36	50	0.71	LOC103693044;LOC108351822;Olr1194	Receptor
DMR8:40271001	8	40271001	40272000	1000	1	1.00E-07	-0.68	1	0.1	Olr1197	Receptor
DMR8:40660001	8	40660001	40662000	2000	1	1.60E-09	-0.65	11	0.55	LOC103693052;Olr1210-ps	
DMR8:40678001	8	40678001	40680000	2000	1	2.20E-07	-0.48	6	0.3	Olr1212-ps	
DMR8:42149001	8	42149001	42150000	1000	1	1.10E-07	0.51	8	0.8	RGD1563738;LOC108351680	
DMR8:42289001	8	42289001	42290000	1000	1	1.70E-10	-0.6	3	0.3	Olr1237;Olr1238	Receptor
DMR8:42427001	8	42427001	42428000	1000	1	8.00E-08	0.31	12	1.2	Olr1257	Signaling
DMR8:42451001	8	42451001	42455000	4000	1	5.60E-07	-0.32	32	0.8	Vwa5a	
DMR8:43195001	8	43195001	43197000	2000	1	5.70E-07	-0.46	8	0.4	Olr1302	Receptor
DMR8:43219001	8	43219001	43223000	4000	1	1.30E-12	-0.41	63	1.57	Olr1303	
DMR8:43338001	8	43338001	43340000	2000	1	2.40E-08	-0.49	12	0.6	RGD1311744;Olr1308	Receptor
DMR8:43367001	8	43367001	43369000	2000	1	3.40E-10	-0.64	7	0.35	Olr1310-ps	
DMR8:43555001	8	43555001	43557000	2000	1	2.80E-09	-0.4	13	0.65	Olr1316	Receptor
DMR8:43603001	8	43603001	43606000	3000	1	8.70E-09	-0.44	10	0.33	Olr1319	Receptor
DMR8:43722001	8	43722001	43723000	1000	1	4.10E-10	-0.38	12	1.2	Olr1325	Receptor
DMR8:44201001	8	44201001	44205000	4000	1	2.10E-08	-0.45	72	1.8	Gramd1b	
DMR8:44214001	8	44214001	44216000	2000	1	6.70E-08	-0.36	37	1.85	Gramd1b	
DMR8:44217001	8	44217001	44219000	2000	1	1.30E-08	-0.44	26	1.3	Gramd1b	
DMR8:44276001	8	44276001	44277000	1000	1	5.10E-07	-0.34	16	1.6	Gramd1b	
DMR8:44303001	8	44303001	44305000	2000	1	1.80E-07	-0.4	42	2.1	Gramd1b;LOC103693058	
DMR8:44379001	8	44379001	44384000	5000	1	6.90E-13	-0.55	56	1.12	Gramd1b	
DMR8:44389001	8	44389001	44392000	3000	1	2.30E-08	-0.42	44	1.47	Gramd1b;LOC102553969	
DMR8:44875001	8	44875001	44877000	2000	1	2.90E-09	0.41	34	1.7	Clmp	
DMR8:44878001	8	44878001	44883000	5000	1	2.30E-19	1.02	80	1.6	Clmp	
DMR8:44910001	8	44910001	44912000	2000	1	2.90E-11	-0.5	41	2.05	Clmp	

DMR8:44921001	8	44921001	44922000	1000	1	4.80E-08	-0.61	12	1.2	Clmp	
DMR8:44940001	8	44940001	44943000	3000	1	2.00E-07	0.29	49	1.63	Clmp	
DMR8:45121001	8	45121001	45122000	1000	1	1.80E-09	0.73	39	3.9	RGD1309108	
DMR8:45163001	8	45163001	45164000	1000	1	2.40E-08	0.38	12	1.2	Crtam	
DMR8:45239001	8	45239001	45240000	1000	1	4.30E-07	-0.51	6	0.6	Ubash3b	
DMR8:45279001	8	45279001	45281000	2000	1	8.00E-08	0.41	23	1.15	Ubash3b	
DMR8:46230001	8	46230001	46233000	3000	1	8.70E-08	-0.35	55	1.83	Sorl1;LOC102547257	Transport
DMR8:46255001	8	46255001	46260000	5000	1	6.30E-08	0.35	70	1.4	Sorl1;LOC102547257	Transport
DMR8:46267001	8	46267001	46268000	1000	1	1.10E-07	0.44	25	2.5	Sorl1	Transport
DMR8:46521001	8	46521001	46522000	1000	1	3.10E-07	-0.4	22	2.2	Sc5d	Metabolism
DMR8:46596001	8	46596001	46601000	5000	1	2.40E-13	-0.81	73	1.46	Tecta	
DMR8:46626001	8	46626001	46629000	3000	1	3.50E-07	0.36	53	1.77	Tecta	
DMR8:46658001	8	46658001	46661000	3000	1	5.10E-08	0.41	37	1.23	Tecta	
DMR8:46674001	8	46674001	46679000	5000	1	6.70E-08	-0.33	55	1.1	Tecta	
DMR8:46758001	8	46758001	46759000	1000	1	7.30E-10	-0.46	18	1.8	Tbcel;LOC102549755	Transcription
DMR8:46794001	8	46794001	46795000	1000	1	4.50E-09	0.73	21	2.1	Grik4	Receptor
DMR8:46879001	8	46879001	46880000	1000	1	6.30E-08	-0.35	15	1.5	Grik4	Receptor
DMR8:46884001	8	46884001	46893000	9000	2	2.50E-08	0.47	157	1.74	Grik4	Receptor
DMR8:46899001	8	46899001	46900000	1000	1	1.50E-08	0.47	20	2	Grik4	Receptor
DMR8:46967001	8	46967001	46972000	5000	1	4.90E-07	0.42	57	1.14	Grik4	Receptor
DMR8:47015001	8	47015001	47017000	2000	1	1.60E-08	0.63	29	1.45	Grik4	Receptor
DMR8:47070001	8	47070001	47073000	3000	1	3.20E-07	0.35	46	1.53	Grik4	Receptor
DMR8:47129001	8	47129001	47132000	3000	1	1.20E-07	0.43	44	1.47	Grik4	Receptor
DMR8:47183001	8	47183001	47186000	3000	1	1.10E-07	0.57	35	1.17	Grik4	Receptor
DMR8:47207001	8	47207001	47210000	3000	1	1.30E-07	-0.55	34	1.13	Grik4	Receptor
DMR8:47222001	8	47222001	47227000	5000	1	8.10E-09	0.34	90	1.8	Grik4	Receptor
DMR8:47228001	8	47228001	47229000	1000	1	4.50E-07	0.6	11	1.1	Grik4	Receptor
DMR8:47241001	8	47241001	47244000	3000	1	1.70E-09	0.48	41	1.37	Grik4;LOC102549942	Receptor
DMR8:47245001	8	47245001	47247000	2000	1	5.30E-11	-0.52	28	1.4	Grik4;LOC102549942	Receptor
DMR8:47248001	8	47248001	47251000	3000	1	1.40E-08	-0.47	84	2.8	LOC102549942;Arhgef12	Transcription
DMR8:47275001	8	47275001	47277000	2000	1	1.20E-07	-0.4	27	1.35	Arhgef12	Transcription
DMR8:47299001	8	47299001	47301000	2000	1	1.40E-07	-0.48	37	1.85	Arhgef12	Transcription
DMR8:47381001	8	47381001	47385000	4000	1	6.00E-07	-0.47	52	1.3	Arhgef12	Transcription
DMR8:47493001	8	47493001	47495000	2000	1	3.00E-08	0.49	37	1.85	Pou2f3	
DMR8:47522001	8	47522001	47524000	2000	1	3.20E-09	0.72	32	1.6	Oaf	
DMR8:47663001	8	47663001	47666000	3000	1	1.30E-07	-0.35	54	1.8	Trim29	
DMR8:48087001	8	48087001	48091000	4000	1	8.00E-12	0.73	52	1.3	Nectin1	
DMR8:48134001	8	48134001	48137000	3000	1	1.40E-08	0.42	53	1.77	Nectin1	
DMR8:48177001	8	48177001	48179000	2000	1	3.10E-11	0.56	38	1.9	Nectin1;LOC102547347	
DMR8:48406001	8	48406001	48407000	1000	1	3.30E-10	0.5	17	1.7	Usp2	Protease
DMR8:48466001	8	48466001	48467000	1000	1	6.00E-08	0.65	19	1.9	LOC103693069;Mcam	Immune
DMR8:48468001	8	48468001	48469000	1000	1	6.20E-11	0.7	21	2.1	LOC103693069;Mcam	Immune
DMR8:48511001	8	48511001	48514000	3000	1	5.60E-08	-0.46	34	1.13	Cbl	Metabolism
DMR8:48520001	8	48520001	48523000	3000	1	4.70E-09	-0.53	25	0.83	Cbl	Metabolism
DMR8:48527001	8	48527001	48532000	5000	1	1.10E-13	-0.64	48	0.96	Cbl	Metabolism
DMR8:48553001	8	48553001	48554000	1000	1	2.40E-08	-0.48	11	1.1	Cbl	Metabolism
DMR8:48591001	8	48591001	48592000	1000	1	5.90E-08	0.56	14	1.4	Pdzd3;Nlrx1	Cytoskeleton
DMR8:48612001	8	48612001	48613000	1000	1	9.00E-07	0.51	12	1.2	Abcg4	Transport
DMR8:48617001	8	48617001	48619000	2000	1	1.70E-07	0.48	18	0.9	Abcg4;Hinfp	Transport;Transcription
DMR8:48737001	8	48737001	48739000	2000	1	8.00E-18	0.79	28	1.4	Trappc4;Rps25;Ccdc84;Mettl9-ps1	Transport;Translation
DMR8:48794001	8	48794001	48797000	3000	3	1.60E-07	-0.65	14	0.47	Bcl9l	
DMR8:48851001	8	48851001	48852000	1000	1	9.40E-07	-0.42	7	0.7	Cxcr5	
DMR8:48914001	8	48914001	48915000	1000	1	6.40E-07	0.26	13	1.3	Ddx6	
DMR8:48927001	8	48927001	48929000	2000	2	3.70E-09	-0.54	22	1.1	Ddx6	
DMR8:49042001	8	49042001	49044000	2000	1	1.30E-16	0.98	60	3	Phldb1;Arcn1	Transport
DMR8:49136001	8	49136001	49139000	3000	1	1.40E-09	-0.45	102	3.4	Kmt2a	Epigenetic
DMR8:49404001	8	49404001	49405000	1000	1	5.80E-07	-0.41	13	1.3	Jaml;LOC108351692	Cytoskeleton
DMR8:49420001	8	49420001	49423000	3000	1	5.90E-08	0.42	41	1.37	LOC108351692;Scn2b	Cytoskeleton
DMR8:49469001	8	49469001	49471000	2000	1	9.10E-09	-0.38	27	1.35	Tmprss4	Protease
DMR8:49636001	8	49636001	49638000	2000	1	5.80E-10	0.4	26	1.3	Tmprss13	Protease

DMR8:49696001	8	49696001	49697000	1000	1	6.40E-09	0.54	23	2.3	Fxyd6	Transport
DMR8:49771001	8	49771001	49773000	2000	1	1.00E-07	0.57	28	1.4	Dscaml1	Cytoskeleton
DMR8:49876001	8	49876001	49879000	3000	1	6.50E-08	0.5	32	1.07	Dscaml1	Cytoskeleton
DMR8:49900001	8	49900001	49902000	2000	1	5.60E-08	0.58	29	1.45	Dscaml1	Cytoskeleton
DMR8:49905001	8	49905001	49910000	5000	1	1.00E-08	0.4	66	1.32	Dscaml1	Cytoskeleton
DMR8:49940001	8	49940001	49942000	2000	1	8.50E-10	0.56	35	1.75	Dscaml1	Cytoskeleton
DMR8:49986001	8	49986001	49992000	6000	1	1.40E-07	0.58	86	1.43	Dscaml1	Cytoskeleton
DMR8:49994001	8	49994001	49996000	2000	1	7.90E-08	0.45	20	1	Dscaml1	Cytoskeleton
DMR8:50007001	8	50007001	50011000	4000	1	8.40E-10	0.46	85	2.12	Dscaml1	Cytoskeleton
DMR8:50057001	8	50057001	50059000	2000	1	1.70E-07	-0.39	19	0.95	Dscaml1	Cytoskeleton
DMR8:50071001	8	50071001	50072000	1000	1	8.10E-08	0.48	22	2.2	Cep164	Cytoskeleton
DMR8:50073001	8	50073001	50077000	4000	1	3.40E-10	0.53	94	2.35	Cep164	Cytoskeleton
DMR8:50095001	8	50095001	50098000	3000	1	7.50E-11	0.47	37	1.23	Cep164	Cytoskeleton
DMR8:50137001	8	50137001	50139000	2000	1	1.80E-07	0.55	47	2.35	Cep164;Bace1	Cytoskeleton;Protease
DMR8:50148001	8	50148001	50149000	1000	1	3.20E-13	-0.47	14	1.4	Bace1	Protease
DMR8:50380001	8	50380001	50381000	1000	1	3.80E-07	-0.34	19	1.9	Sik3	Signaling
DMR8:50385001	8	50385001	50387000	2000	1	2.20E-07	0.47	24	1.2	Sik3	Signaling
DMR8:50530001	8	50530001	50533000	3000	1	1.00E-07	0.51	42	1.4	Sik3;Apoa1;Apoc3;Apoa4	Signaling;Binding Proteins
DMR8:51848001	8	51848001	51850000	2000	1	6.30E-10	0.58	32	1.6	LOC102551354;Cadm1	
DMR8:51902001	8	51902001	51903000	1000	1	3.20E-08	-0.48	24	2.4	Cadm1	
DMR8:52019001	8	52019001	52021000	2000	1	1.20E-08	-0.34	57	2.85	Cadm1	
DMR8:52037001	8	52037001	52038000	1000	1	2.80E-07	-0.35	24	2.4	Cadm1;LOC103693073	
DMR8:52112001	8	52112001	52113000	1000	1	9.40E-08	0.36	17	1.7	Cadm1	
DMR8:52129001	8	52129001	52130000	1000	1	7.40E-07	0.36	15	1.5	Cadm1	
DMR8:52197001	8	52197001	52201000	4000	1	5.30E-07	0.49	76	1.9	Cadm1	
DMR8:52717001	8	52717001	52721000	4000	1	1.50E-10	-0.54	83	2.08	Nxpe4	
DMR8:52755001	8	52755001	52756000	1000	1	4.20E-10	0.34	6	0.6	Nxpe1	
DMR8:52795001	8	52795001	52799000	4000	1	3.20E-10	0.73	72	1.8	Rexo2	
DMR8:52982001	8	52982001	52986000	4000	1	2.70E-10	0.57	57	1.43	Zbtb16	Transcription
DMR8:53098001	8	53098001	53101000	3000	1	5.80E-07	0.39	60	2	Zbtb16	Transcription
DMR8:53155001	8	53155001	53158000	3000	1	2.10E-08	-0.44	29	0.97	Zbtb16	Transcription
DMR8:53209001	8	53209001	53211000	2000	1	1.20E-10	0.49	28	1.4	Htr3a	Ion Channel
DMR8:53227001	8	53227001	53229000	2000	1	6.20E-07	0.66	51	2.55	Htr3a	Ion Channel
DMR8:53250001	8	53250001	53253000	3000	1	5.60E-09	0.45	35	1.17	Htr3b	Ion Channel
DMR8:53424001	8	53424001	53428000	4000	1	6.60E-11	0.53	58	1.45	Tmprss5	Protease
DMR8:53827001	8	53827001	53828000	1000	1	1.30E-07	-0.42	9	0.9	Ttc12;LOC102555969;LOC102556026;Ncam1	
DMR8:53832001	8	53832001	53833000	1000	1	1.70E-07	0.5	5	0.5	LOC102556026;Ncam1	
DMR8:53841001	8	53841001	53844000	3000	1	9.10E-07	0.35	50	1.67	Ncam1	
DMR8:53845001	8	53845001	53846000	1000	1	5.60E-07	0.44	14	1.4	Ncam1	
DMR8:54055001	8	54055001	54057000	2000	1	1.30E-17	0.82	23	1.15	Ncam1	
DMR8:54996001	8	54996001	54997000	1000	1	4.90E-23	1.15	43	4.3	Bco2;Tex12	Metabolism
DMR8:55070001	8	55070001	55072000	2000	1	3.00E-07	-0.47	20	1	Pih1d2;Dlat	Metabolism
DMR8:55175001	8	55175001	55176000	1000	1	5.40E-08	0.39	21	2.1	LOC689959;RGD1564937;Hspb2;Cryab;LOC100359423	Signaling
DMR8:55178001	8	55178001	55180000	2000	1	4.00E-11	-0.54	30	1.5	LOC689959;RGD1564937;Hspb2;Cryab;LOC100359423	Signaling
DMR8:55232001	8	55232001	55234000	2000	1	2.00E-21	0.58	20	1	Alg9	Golgi
DMR8:55268001	8	55268001	55273000	5000	1	7.20E-11	-0.53	84	1.68	Alg9;LOC108351703;Ppp2r1b	Golgi;Translation;Signaling
DMR8:55395001	8	55395001	55398000	3000	1	1.50E-10	-0.63	19	0.63	Sik2	Signaling
DMR8:55502001	8	55502001	55504000	2000	1	1.30E-08	-0.46	22	1.1	Btg4;Mir34c;Mir34b;LOC102553270	
DMR8:55531001	8	55531001	55532000	1000	1	3.70E-07	0.5	13	1.3	LOC102553270;RGD1562811	
DMR8:56208001	8	56208001	56211000	3000	1	2.80E-07	-0.4	50	1.67	Arhgap20	Signaling
DMR8:56269001	8	56269001	56270000	1000	1	6.40E-07	-0.3	8	0.8	Arhgap20	Signaling
DMR8:57984001	8	57984001	57985000	1000	1	6.00E-15	0.91	10	1	Kdelc2;RGD1311251	
DMR8:58025001	8	58025001	58027000	2000	1	1.60E-12	-0.45	27	1.35	Atm	Signaling
DMR8:58070001	8	58070001	58073000	3000	1	7.80E-08	-0.36	44	1.47	Atm	Signaling
DMR8:58093001	8	58093001	58095000	2000	1	3.40E-07	-0.39	28	1.4	Atm	Signaling
DMR8:58353001	8	58353001	58354000	1000	1	1.40E-08	-0.56	13	1.3	Slc35f2	

DMR8:58355001	8	58355001	58361000	6000	1	1.40E-09	-0.49	81	1.35	Slc35f2	
DMR8:58421001	8	58421001	58426000	5000	2	3.40E-13	0.68	67	1.34	Sln	
DMR8:58496001	8	58496001	58497000	1000	1	9.80E-09	-0.58	6	0.6	Elmod1	Cytoskeleton
DMR8:58498001	8	58498001	58501000	3000	1	1.10E-11	-0.53	40	1.33	Elmod1	Cytoskeleton
DMR8:58530001	8	58530001	58531000	1000	1	5.00E-08	0.69	27	2.7	Elmod1	Cytoskeleton
DMR8:58620001	8	58620001	58622000	2000	1	1.90E-08	0.66	38	1.9	Tnfaip8l3	
DMR8:58652001	8	58652001	58655000	3000	1	7.70E-07	-0.5	32	1.07	Tnfaip8l3	
DMR8:58894001	8	58894001	58895000	1000	1	6.30E-07	-0.36	25	2.5	Gldn	Development
DMR8:58913001	8	58913001	58914000	1000	1	5.70E-08	-0.4	22	2.2	Gldn	Development
DMR8:58922001	8	58922001	58924000	2000	1	9.40E-08	-0.58	11	0.55	Gldn;Dmxl2	Development
DMR8:59003001	8	59003001	59006000	3000	1	7.70E-07	-0.38	3	0.1	Dmxl2	
DMR8:59101001	8	59101001	59102000	1000	1	1.80E-07	0.59	10	1	Sh2d7	Immune
DMR8:59141001	8	59141001	59143000	2000	1	1.30E-08	0.46	8	0.4	Cib2	
DMR8:59247001	8	59247001	59250000	3000	2	3.40E-11	0.7	63	2.1	Acsbg1	Metabolism
DMR8:59445001	8	59445001	59448000	3000	1	3.90E-08	-0.32	33	1.1	Ireb2	Metabolism
DMR8:59519001	8	59519001	59520000	1000	1	9.60E-10	0.4	4	0.4	Hykk	
DMR8:59539001	8	59539001	59540000	1000	1	6.10E-10	-0.56	17	1.7	Hykk;Psma4	Protease
DMR8:59558001	8	59558001	59559000	1000	1	3.70E-09	-0.44	9	0.9	Chrna5	Ion Channel
DMR8:59577001	8	59577001	59579000	2000	1	5.70E-11	-0.45	42	2.1	Chrna5;LOC690408	Ion Channel
DMR8:59749001	8	59749001	59750000	1000	1	7.80E-09	0.39	6	0.6	Ube2q2	Proteolysis
DMR8:59758001	8	59758001	59763000	5000	1	3.50E-08	0.31	55	1.1	Ube2q2;Fbxo22	Proteolysis
DMR8:60061001	8	60061001	60062000	1000	1	1.20E-13	-0.51	6	0.6	Etfa	Metabolism
DMR8:60096001	8	60096001	60097000	1000	1	4.20E-11	0.61	23	2.3	Etfa;Bola2	Metabolism
DMR8:60101001	8	60101001	60103000	2000	1	4.90E-07	0.49	29	1.45	Bola2	
DMR8:60318001	8	60318001	60319000	1000	1	2.70E-08	-0.57	6	0.6	Scaper	
DMR8:60358001	8	60358001	60363000	5000	1	1.50E-09	-0.45	72	1.44	Scaper;Nxpe2;LOC108351711;LOC499996	
DMR8:60367001	8	60367001	60368000	1000	1	1.10E-07	-0.44	7	0.7	Scaper;Nxpe2;LOC108351711;LOC499996	
DMR8:60504001	8	60504001	60507000	3000	1	1.60E-07	-0.31	28	0.93	Scaper	
DMR8:60595001	8	60595001	60596000	1000	1	9.40E-07	-0.37	8	0.8	Scaper	
DMR8:60600001	8	60600001	60601000	1000	1	5.80E-09	-0.29	14	1.4	Scaper;RGD1563578	
DMR8:60623001	8	60623001	60626000	3000	1	1.60E-08	-0.33	18	0.6	RGD1563578;LOC691004	
DMR8:60678001	8	60678001	60680000	2000	1	4.00E-07	0.34	26	1.3	RGD1563578;Dppa3l1	
DMR8:60700001	8	60700001	60703000	3000	1	1.10E-12	-0.46	116	3.87	LOC103690189;Rcn2	Signaling
DMR8:60723001	8	60723001	60724000	1000	1	9.30E-07	-0.36	12	1.2	Rcn2	Signaling
DMR8:60779001	8	60779001	60784000	5000	2	7.40E-12	0.29	69	1.38	Pstpip1	Cytoskeleton
DMR8:60831001	8	60831001	60835000	4000	1	1.10E-07	0.62	62	1.55	Tspan3;LOC108351849	
DMR8:60837001	8	60837001	60838000	1000	1	4.30E-12	0.75	20	2	Tspan3;LOC108351849	
DMR8:61138001	8	61138001	61140000	2000	1	4.60E-07	-0.52	17	0.85	Hmg20a	
DMR8:61273001	8	61273001	61276000	3000	2	6.00E-20	1.25	114	3.8	Lingo1	Receptor
DMR8:61332001	8	61332001	61337000	5000	1	8.20E-07	0.47	97	1.94	Lingo1	Receptor
DMR8:61343001	8	61343001	61344000	1000	1	1.10E-07	0.55	12	1.2	Lingo1	Receptor
DMR8:61413001	8	61413001	61418000	5000	4	3.60E-09	0.59	56	1.12	Lingo1;LOC100360369	Receptor
DMR8:61542001	8	61542001	61547000	5000	1	5.80E-12	0.51	48	0.96	Odf3l1;Cspg4	Development
DMR8:61552001	8	61552001	61556000	4000	1	7.50E-09	0.65	134	3.35	Odf3l1;Cspg4	Development
DMR8:61774001	8	61774001	61775000	1000	1	1.20E-07	-0.45	13	1.3	Sin3a	Epigenetic
DMR8:61790001	8	61790001	61791000	1000	1	3.00E-07	-0.55	13	1.3	Sin3a	Epigenetic
DMR8:61815001	8	61815001	61817000	2000	1	1.50E-07	0.48	47	2.35	Man2c1;Neil1;LOC100911806	Epigenetic
DMR8:61933001	8	61933001	61934000	1000	1	5.90E-08	0.51	9	0.9	RGD1305464	
DMR8:62117001	8	62117001	62120000	3000	1	6.20E-07	0.56	46	1.53	NEWGENE_1306267	
DMR8:62329001	8	62329001	62330000	1000	1	3.90E-13	0.67	12	1.2	Fam219b;Mpi	Metabolism
DMR8:62395001	8	62395001	62397000	2000	1	9.50E-10	0.55	29	1.45	Cplx3;LOC103693104;Lman1l;Csk	Transport
DMR8:62425001	8	62425001	62428000	3000	1	1.00E-13	-0.54	45	1.5	Csk	
DMR8:62429001	8	62429001	62432000	3000	1	2.60E-07	0.61	39	1.3	Csk	
DMR8:62571001	8	62571001	62572000	1000	1	3.40E-09	0.61	10	1	LOC103690224;LOC102552873;Arid3b	Transcription
DMR8:62602001	8	62602001	62604000	2000	1	1.00E-07	-0.36	27	1.35	Arid3b	Transcription
DMR8:62624001	8	62624001	62627000	3000	1	1.90E-25	0.49	16	0.53	Arid3b	Transcription
DMR8:62798001	8	62798001	62800000	2000	1	2.20E-11	0.61	41	2.05	Cyp11a1;Ccdc33	
DMR8:62836001	8	62836001	62837000	1000	1	9.10E-09	0.44	2	0.2	Ccdc33	

DMR8:62853001	8	62853001	62855000	2000	1	3.20E-08	0.39	24	1.2	Ccdc33	
DMR8:62862001	8	62862001	62864000	2000	1	1.30E-12	0.63	30	1.5	Ccdc33	
DMR8:62909001	8	62909001	62911000	2000	1	7.20E-07	0.44	27	1.35	Ccdc33	
DMR8:62942001	8	62942001	62943000	1000	1	1.70E-08	0.52	21	2.1	Stra6;Islr	Immune
DMR8:62956001	8	62956001	62958000	2000	2	1.10E-09	-0.53	23	1.15	Islr	Immune
DMR8:62982001	8	62982001	62984000	2000	1	3.70E-07	0.45	53	2.65	Islr2;LOC102553472	Immune
DMR8:63291001	8	63291001	63292000	1000	1	7.90E-07	0.54	14	1.4	RGD1562618	
DMR8:63531001	8	63531001	63534000	3000	1	5.20E-08	0.81	59	1.97	Rec114	
DMR8:63537001	8	63537001	63538000	1000	1	7.10E-08	0.44	6	0.6	Rec114	
DMR8:63626001	8	63626001	63629000	3000	1	1.30E-12	0.78	43	1.43	Hcn4	Transport
DMR8:63636001	8	63636001	63637000	1000	1	3.50E-19	1.13	33	3.3	Hcn4	Transport
DMR8:63702001	8	63702001	63703000	1000	1	4.30E-10	-0.59	5	0.5	Neo1	
DMR8:63707001	8	63707001	63709000	2000	1	3.00E-09	-0.38	19	0.95	Neo1	
DMR8:63734001	8	63734001	63735000	1000	1	5.70E-10	-0.56	8	0.8	Neo1	
DMR8:64110001	8	64110001	64111000	1000	1	7.40E-31	1.53	49	4.9	Adpgk;Bbs4;LOC108351713	Signaling
DMR8:64112001	8	64112001	64114000	2000	1	2.40E-07	0.57	30	1.5	Adpgk;Bbs4;LOC108351713	Signaling
DMR8:64197001	8	64197001	64198000	1000	1	5.40E-16	0.31	8	0.8	Arih1	Proteolysis
DMR8:64241001	8	64241001	64242000	1000	1	1.30E-14	-0.64	7	0.7	Arih1	Proteolysis
DMR8:64302001	8	64302001	64303000	1000	1	1.30E-09	-0.67	5	0.5	LOC103693108;Tmem202;LOC108351715	Cytoskeleton
DMR8:64650001	8	64650001	64651000	1000	1	3.40E-10	0.52	10	1	Myo9a	
DMR8:64804001	8	64804001	64805000	1000	1	1.60E-07	0.53	15	1.5	Nr2e3;Thsd4	Transcription
DMR8:64810001	8	64810001	64813000	3000	1	3.40E-08	0.42	40	1.33	Nr2e3;Thsd4	Transcription
DMR8:64905001	8	64905001	64906000	1000	1	1.70E-07	0.53	16	1.6	Thsd4	
DMR8:64907001	8	64907001	64909000	2000	1	3.10E-07	0.75	40	2	Thsd4	
DMR8:65033001	8	65033001	65035000	2000	1	1.80E-08	0.37	35	1.75	Thsd4	
DMR8:65119001	8	65119001	65121000	2000	1	8.10E-19	1	42	2.1	Thsd4	
DMR8:65148001	8	65148001	65149000	1000	1	1.60E-07	0.49	9	0.9	Thsd4	
DMR8:65159001	8	65159001	65161000	2000	2	8.80E-09	-0.5	18	0.9	Thsd4	
DMR8:65173001	8	65173001	65174000	1000	1	2.10E-09	0.54	8	0.8	Thsd4	
DMR8:65244001	8	65244001	65245000	1000	1	3.20E-07	-0.36	15	1.5	Thsd4	
DMR8:65263001	8	65263001	65269000	6000	2	3.50E-07	-0.26	60	1	Thsd4	
DMR8:65351001	8	65351001	65353000	2000	1	4.20E-09	-0.48	40	2	Thsd4	
DMR8:65490001	8	65490001	65493000	3000	1	8.10E-08	0.48	17	0.57	Lrrc49	Signaling
DMR8:65536001	8	65536001	65537000	1000	1	3.80E-10	0.55	13	1.3	Lrrc49	Signaling
DMR8:65573001	8	65573001	65575000	2000	1	3.00E-08	-0.49	14	0.7	Lrrc49	Signaling
DMR8:65752001	8	65752001	65754000	2000	1	8.80E-09	-0.47	30	1.5	Uaca	
DMR8:65763001	8	65763001	65768000	5000	2	1.30E-10	-0.47	127	2.54	Uaca	
DMR8:65779001	8	65779001	65782000	3000	1	2.50E-09	-0.41	43	1.43	Uaca	
DMR8:66288001	8	66288001	66289000	1000	1	2.20E-08	0.64	8	0.8	Tle3	Transcription
DMR8:66931001	8	66931001	66932000	1000	1	9.90E-08	0.58	22	2.2	Paqr5	Signaling
DMR8:66954001	8	66954001	66956000	2000	1	1.10E-08	-0.48	23	1.15	Paqr5	Signaling
DMR8:66962001	8	66962001	66966000	4000	1	1.20E-11	0.33	67	1.68	Paqr5	Signaling
DMR8:67053001	8	67053001	67054000	1000	1	2.40E-07	0.44	6	0.6	Glce	Metabolism
DMR8:67232001	8	67232001	67235000	3000	1	5.10E-11	1.01	79	2.63	Spesp1	
DMR8:67384001	8	67384001	67387000	3000	1	6.50E-08	0.45	38	1.27	Coro2b	Cytoskeleton
DMR8:67449001	8	67449001	67454000	5000	1	1.10E-09	0.53	67	1.34	Coro2b	Cytoskeleton
DMR8:67494001	8	67494001	67497000	3000	1	1.40E-17	0.9	65	2.17	Coro2b;LOC103693114	Cytoskeleton
DMR8:67498001	8	67498001	67504000	6000	2	2.30E-10	0.55	100	1.67	Coro2b	Cytoskeleton
DMR8:68115001	8	68115001	68117000	2000	1	1.90E-08	-0.44	40	2	Map2k5	Signaling
DMR8:68240001	8	68240001	68243000	3000	1	3.10E-10	0.59	30	1	Map2k5	Signaling
DMR8:68390001	8	68390001	68391000	1000	1	4.30E-07	0.54	13	1.3	LOC103693118;Iqch;LOC102547003	
DMR8:68467001	8	68467001	68468000	1000	1	6.30E-08	0.58	10	1	Iqch	
DMR8:68494001	8	68494001	68495000	1000	1	2.10E-07	0.37	12	1.2	Iqch	
DMR8:68633001	8	68633001	68638000	5000	1	5.60E-09	0.47	65	1.3	Smad3	Transcription
DMR8:68657001	8	68657001	68660000	3000	1	3.60E-07	-0.46	69	2.3	Smad3	Transcription
DMR8:68898001	8	68898001	68900000	2000	2	4.40E-15	0.78	68	3.4	Smad6	Transcription
DMR8:69790001	8	69790001	69796000	6000	1	2.70E-12	0.7	71	1.18	Dis3l	Transcription
DMR8:69893001	8	69893001	69894000	1000	1	3.90E-08	0.54	10	1	Megf11	Extracellular Matrix
DMR8:69908001	8	69908001	69912000	4000	1	1.90E-08	-0.7	55	1.38	Megf11	Extracellular Matrix
DMR8:69914001	8	69914001	69916000	2000	1	9.40E-07	0.36	33	1.65	Megf11	Extracellular Matrix

DMR8:70057001	8	70057001	70060000	3000	1	6.90E-07	0.41	42	1.4	Megf11	Extracellular Matrix
DMR8:70070001	8	70070001	70073000	3000	1	2.10E-08	0.43	43	1.43	Megf11	Extracellular Matrix
DMR8:70074001	8	70074001	70076000	2000	1	5.30E-08	0.43	32	1.6	Megf11	Extracellular Matrix
DMR8:70093001	8	70093001	70095000	2000	1	7.30E-10	-0.48	19	0.95	Megf11	Extracellular Matrix
DMR8:70102001	8	70102001	70103000	1000	1	1.20E-07	0.36	18	1.8	Megf11	Extracellular Matrix
DMR8:70149001	8	70149001	70150000	1000	1	6.20E-07	0.45	9	0.9	Megf11	Extracellular Matrix
DMR8:70313001	8	70313001	70314000	1000	1	3.30E-07	-0.39	10	1	Dennd4a	
DMR8:70433001	8	70433001	70436000	3000	1	1.20E-08	0.72	55	1.83	Slc24a1;LOC103693120	Transport
DMR8:70472001	8	70472001	70474000	2000	1	3.80E-08	-0.42	24	1.2	Vwa9;Hacd3	
DMR8:70769001	8	70769001	70771000	2000	1	2.80E-07	-0.38	31	1.55	Cilp	
DMR8:70894001	8	70894001	70898000	4000	1	4.80E-07	0.37	61	1.52	Ubap1l;Kbtbd13	
DMR8:70923001	8	70923001	70927000	4000	1	1.00E-11	0.66	49	1.23	Rasl12;Slc51b	Signaling;Transport
DMR8:71015001	8	71015001	71016000	1000	1	2.50E-07	0.52	10	1	Spg21	
DMR8:71026001	8	71026001	71028000	2000	1	7.30E-08	-0.54	17	0.85	Spg21;Ankdd1a	
DMR8:71103001	8	71103001	71107000	4000	1	5.60E-07	0.56	55	1.38	Plekho2	
DMR8:71111001	8	71111001	71114000	3000	1	8.00E-08	-0.45	49	1.63	Plekho2	
DMR8:71126001	8	71126001	71128000	2000	1	5.80E-07	0.42	32	1.6	Plekho2;Pif1	Epigenetic
DMR8:71214001	8	71214001	71215000	1000	1	1.60E-08	0.62	14	1.4	LOC103693122;Oaz2	Signaling
DMR8:71339001	8	71339001	71341000	2000	1	1.70E-07	-0.4	14	0.7	Zfp609	
DMR8:71410001	8	71410001	71412000	2000	1	7.10E-11	0.65	15	0.75	Trip4;LOC108351729	Transcription
DMR8:71428001	8	71428001	71431000	3000	2	1.30E-09	0.8	84	2.8	Trip4;LOC108351729	Transcription
DMR8:71434001	8	71434001	71439000	5000	1	5.00E-07	0.45	49	0.98	Trip4;LOC108351729;LOC108351728	Transcription
DMR8:71443001	8	71443001	71446000	3000	1	7.80E-08	-0.58	23	0.77	Trip4;LOC108351728	Transcription
DMR8:71468001	8	71468001	71469000	1000	1	9.80E-07	-0.42	12	1.2	Trip4	Transcription
DMR8:71480001	8	71480001	71489000	9000	2	2.90E-07	-0.3	66	0.73	Trip4	Transcription
DMR8:71694001	8	71694001	71696000	2000	2	6.50E-16	-0.54	23	1.15	Csnk1g1	Signaling
DMR8:71733001	8	71733001	71736000	3000	1	1.40E-07	-0.43	29	0.97	Ppib;Snx22;LOC108351730;Snx1	Transcription;Cytoskeleton
DMR8:71903001	8	71903001	71904000	1000	1	3.90E-09	-0.53	8	0.8	Dapk2	Signaling
DMR8:72164001	8	72164001	72165000	1000	1	7.60E-09	0.39	8	0.8	Herc1	Transcription
DMR8:72499001	8	72499001	72501000	2000	1	9.00E-08	-0.3	18	0.9	LOC691658;Aph1b	Protease
DMR8:72528001	8	72528001	72530000	2000	1	4.20E-07	0.64	31	1.55	Aph1b	Protease
DMR8:72710001	8	72710001	72715000	5000	1	4.90E-08	-0.66	120	2.4	Rab8b	
DMR8:72815001	8	72815001	72817000	2000	1	4.80E-15	-0.55	32	1.6	Tpm1	Cytoskeleton
DMR8:72822001	8	72822001	72827000	5000	1	5.80E-11	-0.43	108	2.16	Tpm1	Cytoskeleton
DMR8:73006001	8	73006001	73011000	5000	1	1.40E-13	0.58	60	1.2	Tln2	
DMR8:73025001	8	73025001	73029000	4000	1	2.40E-07	-0.34	53	1.32	Tln2;Mir190	
DMR8:73338001	8	73338001	73341000	3000	1	6.00E-07	0.38	46	1.53	Tln2	
DMR8:73342001	8	73342001	73344000	2000	1	7.20E-07	0.56	20	1	Tln2	
DMR8:73581001	8	73581001	73584000	3000	1	1.10E-12	0.37	33	1.1	LOC108351734;C2cd4b	
DMR8:73689001	8	73689001	73691000	2000	1	6.40E-11	-0.49	19	0.95	C2cd4a;Vps13c	Transport
DMR8:73707001	8	73707001	73711000	4000	1	3.80E-09	-0.39	48	1.2	Vps13c	Transport
DMR8:73722001	8	73722001	73726000	4000	2	1.50E-09	-0.46	60	1.5	Vps13c	Transport
DMR8:73750001	8	73750001	73752000	2000	1	5.70E-07	-0.45	27	1.35	Vps13c	Transport
DMR8:73791001	8	73791001	73792000	1000	1	2.80E-07	0.4	6	0.6	Vps13c	Transport
DMR8:73844001	8	73844001	73845000	1000	1	1.10E-07	-0.53	12	1.2	Vps13c	Transport
DMR8:75624001	8	75624001	75625000	1000	1	3.20E-17	1.16	21	2.1	Rora;Ice2	Transcription
DMR8:75683001	8	75683001	75686000	3000	1	1.20E-07	0.4	24	0.8	Anxa2	Signaling
DMR8:75713001	8	75713001	75715000	2000	1	6.00E-07	-0.43	53	2.65	Anxa2	Signaling
DMR8:75985001	8	75985001	75986000	1000	1	4.30E-07	0.29	13	1.3	Foxb1	
DMR8:76005001	8	76005001	76006000	1000	1	1.80E-08	0.54	15	1.5	Foxb1	
DMR8:76452001	8	76452001	76456000	4000	1	9.20E-07	0.44	44	1.1	LOC108351740;Gcnt3	Golgi
DMR8:76586001	8	76586001	76588000	2000	1	8.50E-10	0.64	80	4	Fam81a	
DMR8:76790001	8	76790001	76793000	3000	1	1.40E-08	0.53	33	1.1	Myo1e	Cytoskeleton
DMR8:76919001	8	76919001	76921000	2000	1	4.80E-08	-0.53	14	0.7	Rnf111	
DMR8:77004001	8	77004001	77006000	2000	1	1.40E-09	-0.44	26	1.3	Sltm	
DMR8:77017001	8	77017001	77019000	2000	1	1.60E-07	-0.35	49	2.45	Sltm	
DMR8:77091001	8	77091001	77093000	2000	1	1.70E-07	-0.44	17	0.85	Fam63b;LOC102551907	
DMR8:77098001	8	77098001	77099000	1000	1	2.10E-10	0.54	23	2.3	Fam63b;Adam10	Protease
DMR8:77103001	8	77103001	77105000	2000	1	2.60E-07	-0.38	17	0.85	Adam10	Protease
DMR8:77273001	8	77273001	77274000	1000	1	2.60E-08	-0.44	3	0.3	Lipc	Metabolism

DMR8:77305001	8	77305001	77306000	1000	1	1.50E-07	0.38	12	1.2	Lipc	Metabolism
DMR8:77323001	8	77323001	77326000	3000	1	2.80E-07	0.45	52	1.73	Lipc	Metabolism
DMR8:77340001	8	77340001	77343000	3000	1	3.30E-08	-0.35	44	1.47	Lipc	Metabolism
DMR8:77390001	8	77390001	77392000	2000	1	6.70E-09	0.75	50	2.5	Lipc	Metabolism
DMR8:77563001	8	77563001	77566000	3000	1	2.60E-07	-0.34	45	1.5	Aqp9	Transport
DMR8:77637001	8	77637001	77639000	2000	1	2.40E-13	0.91	42	2.1	Aldh1a2	Metabolism
DMR8:77709001	8	77709001	77711000	2000	1	2.40E-09	0.52	20	1	Aldh1a2	Metabolism
DMR8:77977001	8	77977001	77981000	4000	1	2.40E-08	-0.29	45	1.12	Polr2m	
DMR8:77989001	8	77989001	77990000	1000	1	1.20E-13	-0.62	9	0.9	Polr2m	
DMR8:77998001	8	77998001	7.80E+07	2000	1	3.80E-08	0.79	25	1.25	Polr2m;Myzap	
DMR8:78084001	8	78084001	78085000	1000	1	3.70E-08	-0.43	22	2.2	Myzap	
DMR8:78104001	8	78104001	78108000	4000	1	3.00E-08	0.69	68	1.7	Myzap;LOC108351825	
DMR8:78137001	8	78137001	78138000	1000	1	7.40E-10	-0.42	12	1.2	Cgnl1	
DMR8:78179001	8	78179001	78180000	1000	1	2.50E-12	0.4	11	1.1	Cgnl1	
DMR8:78211001	8	78211001	78213000	2000	1	4.80E-07	-0.33	19	0.95	Cgnl1	
DMR8:78227001	8	78227001	78228000	1000	1	6.50E-10	-0.46	13	1.3	Cgnl1	
DMR8:78258001	8	78258001	78263000	5000	1	9.60E-08	0.36	51	1.02	Cgnl1	
DMR8:78406001	8	78406001	78408000	2000	2	3.20E-08	0.44	7	0.35	Tcf12	Transcription
DMR8:78452001	8	78452001	78455000	3000	1	9.50E-07	-0.34	25	0.83	Tcf12	Transcription
DMR8:78581001	8	78581001	78582000	1000	1	2.10E-08	-0.51	7	0.7	Tcf12	Transcription
DMR8:78912001	8	78912001	78913000	1000	1	3.70E-07	-0.36	7	0.7	Zfp280d	Transcription
DMR8:78918001	8	78918001	78920000	2000	1	2.90E-08	-0.35	12	0.6	Zfp280d	Transcription
DMR8:79064001	8	79064001	79068000	4000	2	6.60E-14	-0.6	56	1.4	Mns1	Development
DMR8:79225001	8	79225001	79226000	1000	1	1.30E-08	-0.62	5	0.5	Rfx7	Transcription
DMR8:79355001	8	79355001	79356000	1000	1	8.00E-11	-0.44	25	2.5	Nedd4	Proteolysis
DMR8:79520001	8	79520001	79522000	2000	1	9.70E-11	-0.57	11	0.55	Prtg	
DMR8:79528001	8	79528001	79531000	3000	1	2.80E-12	0.74	62	2.07	Prtg	
DMR8:79624001	8	79624001	79626000	2000	1	2.30E-11	0.75	51	2.55	Pygo1	
DMR8:79640001	8	79640001	79641000	1000	1	3.50E-07	0.41	24	2.4	Dyx1c1	
DMR8:79766001	8	79766001	79769000	3000	1	9.10E-07	0.46	48	1.6	Rab27a	
DMR8:79879001	8	79879001	79880000	1000	1	1.40E-07	-0.23	10	1	LOC102554551;RGD1564166	
DMR8:80193001	8	80193001	80194000	1000	1	3.40E-11	0.49	9	0.9	LOC108351745;Unc13c	
DMR8:80195001	8	80195001	80196000	1000	1	1.50E-16	0.88	17	1.7	LOC108351745;Unc13c	
DMR8:80315001	8	80315001	80316000	1000	1	8.30E-11	-0.41	6	0.6	Unc13c	
DMR8:80373001	8	80373001	80375000	2000	1	3.80E-10	-0.49	27	1.35	Unc13c	
DMR8:80379001	8	80379001	80380000	1000	1	7.30E-07	0.49	8	0.8	Unc13c	
DMR8:80482001	8	80482001	80486000	4000	1	8.50E-07	0.61	56	1.4	Unc13c	
DMR8:80974001	8	80974001	80976000	2000	1	5.40E-07	0.48	25	1.25	Wdr72	
DMR8:81755001	8	81755001	81756000	1000	1	4.50E-08	-0.49	14	1.4	LOC102549203;LOC102549114;<br>Onecut1	Development
DMR8:81798001	8	81798001	81800000	2000	1	1.20E-07	0.75	34	1.7	Onecut1	Development
DMR8:82135001	8	82135001	82136000	1000	1	6.60E-10	-0.34	11	1.1	Myo5a	Cytoskeleton
DMR8:82180001	8	82180001	82182000	2000	1	2.70E-07	0.45	20	1	Myo5c	Cytoskeleton
DMR8:82294001	8	82294001	82295000	1000	1	1.80E-09	0.61	16	1.6	Gnb5;Bcl2l10	Signaling
DMR8:82319001	8	82319001	82320000	1000	1	1.70E-07	-0.38	9	0.9	Mapk6	Signaling
DMR8:82385001	8	82385001	82387000	2000	1	5.30E-08	-0.36	55	2.75	Leo1	Transcription
DMR8:82431001	8	82431001	82432000	1000	1	3.40E-08	0.45	18	1.8	LOC100912347;Tmod3	Cytoskeleton
DMR8:82443001	8	82443001	82444000	1000	1	3.30E-08	0.43	16	1.6	Tmod3	Cytoskeleton
DMR8:82476001	8	82476001	82480000	4000	1	3.00E-08	-0.42	45	1.12	Tmod3	Cytoskeleton
DMR8:82620001	8	82620001	82622000	2000	1	3.20E-13	0.72	32	1.6	Scg3	
DMR8:82627001	8	82627001	82628000	1000	1	3.00E-07	0.47	10	1	Scg3	
DMR8:82678001	8	82678001	82679000	1000	1	1.10E-07	0.49	10	1	Bmp5	Growth Factors
DMR8:82814001	8	82814001	82818000	4000	1	2.90E-19	1.34	61	1.52	Bmp5	Growth Factors
DMR8:82827001	8	82827001	82830000	3000	1	7.10E-15	-0.59	24	0.8	Bmp5	Growth Factors
DMR8:82841001	8	82841001	82844000	3000	1	7.30E-07	0.46	29	0.97	Bmp5	Growth Factors
DMR8:82889001	8	82889001	82891000	2000	1	1.70E-08	0.57	30	1.5	Bmp5	Growth Factors
DMR8:82932001	8	82932001	82933000	1000	1	7.00E-07	-0.42	6	0.6	Bmp5	Growth Factors
DMR8:83083001	8	83083001	83084000	1000	1	7.00E-09	-0.65	6	0.6	Hmgcll1	Metabolism
DMR8:83092001	8	83092001	83094000	2000	1	3.30E-16	-0.75	30	1.5	Hmgcll1	Metabolism
DMR8:83375001	8	83375001	83378000	3000	1	2.60E-07	0.42	36	1.2	Hcrtr2	Signaling
DMR8:84139001	8	84139001	84142000	3000	1	3.20E-08	0.43	36	1.2	Tinag	Protease
DMR8:84319001	8	84319001	84321000	2000	1	1.20E-08	-0.51	19	0.95	Tinag	Protease

DMR8:84494001	8	84494001	84496000	2000	1	4.70E-08	0.45	31	1.55	Mlip	
DMR8:84565001	8	84565001	84567000	2000	1	1.40E-14	0.84	34	1.7	Mlip	
DMR8:84640001	8	84640001	84641000	1000	1	3.20E-08	0.67	22	2.2	Mlip	
DMR8:84642001	8	84642001	84644000	2000	1	3.00E-07	0.41	31	1.55	Mlip	
DMR8:84742001	8	84742001	84743000	1000	1	1.80E-07	-0.41	21	2.1	Lrrc1	
DMR8:84946001	8	84946001	84947000	1000	1	4.10E-08	0.55	22	2.2	Klhl31	
DMR8:85079001	8	85079001	85080000	1000	1	4.10E-07	-0.42	13	1.3	Gclc	Metabolism
DMR8:85369001	8	85369001	85370000	1000	1	3.70E-07	-0.34	20	2	Gcm1;LOC691940	
DMR8:85377001	8	85377001	85380000	3000	1	3.30E-08	-0.36	39	1.3	Gcm1;LOC691940;Fbxo9	
DMR8:85545001	8	85545001	85546000	1000	1	1.30E-12	0.81	34	3.4	Gsta5	Transport
DMR8:85701001	8	85701001	85703000	2000	1	1.40E-09	0.76	46	2.3	Dppa5	
DMR8:85839001	8	85839001	85841000	2000	1	3.40E-07	-0.46	43	2.15	Mto1;Eef1a1	Translation
DMR8:85952001	8	85952001	85953000	1000	1	2.60E-09	0.53	22	2.2	Cd109	Protease; Proteolysis
DMR8:85981001	8	85981001	85982000	1000	1	2.30E-07	-0.53	5	0.5	Cd109	Protease; Proteolysis
DMR8:85983001	8	85983001	85984000	1000	1	1.50E-07	-0.45	6	0.6	Cd109	Protease; Proteolysis
DMR8:86021001	8	86021001	86023000	2000	1	6.20E-10	-0.59	12	0.6	Cd109	Protease; Proteolysis
DMR8:86049001	8	86049001	86050000	1000	1	6.40E-07	0.24	4	0.4	Cd109	Protease; Proteolysis
DMR8:87271001	8	87271001	87273000	2000	2	1.70E-25	1.23	67	3.35	Filip1	
DMR8:87297001	8	87297001	87298000	1000	1	3.00E-09	0.63	32	3.2	Filip1	
DMR8:87299001	8	87299001	87303000	4000	1	4.00E-07	-0.29	36	0.9	Filip1	
DMR8:87359001	8	87359001	87361000	2000	1	9.10E-09	-0.4	29	1.45	Filip1	
DMR8:87509001	8	87509001	87512000	3000	1	1.20E-07	-0.53	29	0.97	Senp6	Protease
DMR8:87590001	8	87590001	87591000	1000	1	9.20E-08	-0.42	22	2.2	Myo6	Cytoskeleton
DMR8:87596001	8	87596001	87597000	1000	1	2.90E-12	-0.53	18	1.8	Myo6	Cytoskeleton
DMR8:87607001	8	87607001	87613000	6000	1	3.10E-10	-0.55	72	1.2	Myo6	Cytoskeleton
DMR8:87708001	8	87708001	87711000	3000	1	8.10E-08	-0.36	29	0.97	Myo6	Cytoskeleton
DMR8:87725001	8	87725001	87727000	2000	1	3.20E-09	0.54	40	2	Myo6;Impg1	Cytoskeleton;Extracellular Matrix
DMR8:87793001	8	87793001	87797000	4000	1	5.10E-07	0.3	53	1.32	Impg1	Extracellular Matrix
DMR8:87798001	8	87798001	87799000	1000	1	9.20E-09	-0.38	7	0.7	Impg1	Extracellular Matrix
DMR8:87820001	8	87820001	87822000	2000	1	1.80E-12	0.74	52	2.6	Impg1	Extracellular Matrix
DMR8:87852001	8	87852001	87860000	8000	1	3.00E-08	-0.26	90	1.12	Impg1	Extracellular Matrix
DMR8:87870001	8	87870001	87871000	1000	1	4.90E-09	-0.63	4	0.4	Impg1	Extracellular Matrix
DMR8:89469001	8	89469001	89470000	1000	1	4.50E-07	-0.36	11	1.1	Mei4;LOC102553441	
DMR8:89480001	8	89480001	89482000	2000	1	2.00E-12	-0.5	17	0.85	Mei4;LOC102553441	
DMR8:90392001	8	90392001	90393000	1000	1	9.60E-07	-0.41	12	1.2	Phip	
DMR8:90467001	8	90467001	90468000	1000	1	7.00E-08	-0.59	6	0.6	Phip	
DMR8:90671001	8	90671001	90672000	1000	1	6.00E-08	-0.4	6	0.6	Hmgn3	Epigenetic
DMR8:90971001	8	90971001	90974000	3000	1	9.70E-11	-0.59	32	1.07	Lca5;LOC103690486	
DMR8:91109001	8	91109001	91112000	3000	1	4.80E-08	-0.43	32	1.07	LOC102554622;Sh3bgrl2;LOC102554552	
DMR8:91169001	8	91169001	91170000	1000	1	3.10E-07	0.43	13	1.3	Sh3bgrl2	
DMR8:91571001	8	91571001	91572000	1000	1	2.60E-11	0.48	21	2.1	Bckdhb	Metabolism
DMR8:91581001	8	91581001	91582000	1000	1	2.00E-08	-0.41	10	1	Bckdhb	Metabolism
DMR8:91614001	8	91614001	91619000	5000	1	2.00E-07	-0.38	61	1.22	Bckdhb	Metabolism
DMR8:91820001	8	91820001	91821000	1000	1	6.40E-08	0.56	28	2.8	RGD1560917	Translation
DMR8:93473001	8	93473001	93474000	1000	1	1.40E-07	0.4	11	1.1	RGD1564645	
DMR8:93488001	8	93488001	93489000	1000	1	7.90E-10	0.68	11	1.1	Tpbg	
DMR8:93941001	8	93941001	93943000	2000	1	3.20E-07	-0.57	14	0.7	Ube3d	Proteolysis
DMR8:94033001	8	94033001	94043000	10000	1	3.40E-09	0.74	96	0.96	Ube3d;LOC102551813	Proteolysis
DMR8:94074001	8	94074001	94077000	3000	1	5.30E-08	-0.38	45	1.5	Ube3d	Proteolysis
DMR8:94096001	8	94096001	94097000	1000	1	1.90E-10	0.73	24	2.4	Ube3d	Proteolysis
DMR8:94141001	8	94141001	94142000	1000	1	2.90E-07	-0.44	7	0.7	Dopey1	
DMR8:94281001	8	94281001	94282000	1000	1	3.20E-08	-0.42	2	0.2	Me1	Metabolism
DMR8:94474001	8	94474001	94476000	2000	1	4.30E-07	-0.35	50	2.5	Snap91	Transport
DMR8:94672001	8	94672001	94673000	1000	1	7.90E-07	0.48	5	0.5	Ripply2	
DMR8:94712001	8	94712001	94714000	2000	1	4.80E-07	-0.33	22	1.1	Cyb5r4	Metabolism
DMR8:94823001	8	94823001	94825000	2000	1	1.00E-08	0.5	22	1.1	Mrap2;LOC103690445	
DMR8:94910001	8	94910001	94911000	1000	1	2.50E-09	-0.47	10	1	Cep162	
DMR8:95393001	8	95393001	95394000	1000	1	1.00E-08	-0.65	16	1.6	Tbx18;LOC100909636	Transcription
DMR8:95896001	8	95896001	95898000	2000	1	9.00E-15	1.01	36	1.8	RGD1561192	
DMR8:95970001	8	95970001	95972000	2000	1	1.30E-07	-0.4	30	1.5	Nt5e	Signaling

DMR8:96053001	8	96053001	96055000	2000	1	4.30E-07	-0.5	12	0.6	Snx14	Cytoskeleton
DMR8:96449001	8	96449001	96452000	3000	1	9.30E-07	-0.37	31	1.03	RGD1560775	
DMR8:96479001	8	96479001	96484000	5000	1	1.80E-07	0.38	40	0.8	RGD1560775	
DMR8:96586001	8	96586001	96587000	1000	1	1.90E-08	-0.4	24	2.4	Mthfs	Metabolism
DMR8:97095001	8	97095001	97098000	3000	2	1.10E-07	-0.59	22	0.73	Ankrd34c	
DMR8:97166001	8	97166001	97167000	1000	1	4.20E-08	0.66	12	1.2	Mir184	
DMR8:97281001	8	97281001	97283000	2000	1	3.00E-07	-0.56	5	0.25	Rasgrf1	Transcription
DMR8:97355001	8	97355001	97358000	3000	1	2.60E-14	-0.57	20	0.67	Rasgrf1	Transcription
DMR8:97363001	8	97363001	97367000	4000	1	5.80E-09	-0.27	40	1	Rasgrf1	Transcription
DMR8:97597001	8	97597001	97598000	1000	1	5.50E-09	-0.59	18	1.8	Tbc1d2b	Signaling
DMR8:99893001	8	99893001	99895000	2000	1	3.50E-07	-0.3	18	0.9	Plscr4	Transport
DMR8:99969001	8	99969001	99971000	2000	1	1.10E-07	-0.43	8	0.4	LOC108351766;Plod2	Golgi
DMR8:102302001	8	102302001	102304000	2000	1	6.50E-07	-0.57	27	1.35	LOC102552777;Slc9a9	Transport
DMR8:102370001	8	102370001	102372000	2000	1	4.20E-07	0.33	31	1.55	Slc9a9	Transport
DMR8:102614001	8	102614001	102615000	1000	1	4.90E-07	0.55	41	4.1	Slc9a9	Transport
DMR8:102790001	8	102790001	102792000	2000	1	6.00E-07	0.48	19	0.95	Slc9a9	Transport
DMR8:102826001	8	102826001	102827000	1000	1	1.80E-07	-0.41	12	1.2	Slc9a9	Transport
DMR8:102830001	8	102830001	102831000	1000	1	6.20E-07	-0.58	1	0.1	Slc9a9	Transport
DMR8:102875001	8	102875001	102882000	7000	1	6.80E-07	-0.38	76	1.09	Slc9a9	Transport
DMR8:102943001	8	102943001	102945000	2000	1	7.10E-10	0.68	27	1.35	Slc9a9	Transport
DMR8:103011001	8	103011001	103012000	1000	1	2.70E-13	-0.48	6	0.6	Slc9a9	Transport
DMR8:103240001	8	103240001	103242000	2000	1	3.80E-08	-0.39	35	1.75	U2surp	Translation
DMR8:103603001	8	103603001	103609000	6000	2	3.40E-08	-0.39	96	1.6	Pls1	Cytoskeleton
DMR8:103664001	8	103664001	103666000	2000	1	2.30E-11	0.64	12	0.6	Pls1;Atr	Cytoskeleton;Signaling
DMR8:104102001	8	104102001	104103000	1000	1	6.70E-08	0.35	11	1.1	Tfdp2	Transcription
DMR8:104341001	8	104341001	104343000	2000	1	2.40E-08	0.66	34	1.7	Rnf7	Proteolysis
DMR8:104442001	8	104442001	104443000	1000	1	9.90E-07	0.33	20	2	Rasa2	Signaling
DMR8:104558001	8	104558001	104559000	1000	1	6.10E-07	0.39	14	1.4	Zbtb38	Transcription
DMR8:104568001	8	104568001	104569000	1000	1	3.00E-16	0.98	24	2.4	Zbtb38	Transcription
DMR8:104590001	8	104590001	104592000	2000	1	1.50E-09	0.87	75	3.75	Zbtb38;LOC100909637	Transcription
DMR8:104600001	8	104600001	104603000	3000	1	1.30E-09	-0.53	55	1.83	Zbtb38;LOC100909637	Transcription
DMR8:104618001	8	104618001	104622000	4000	2	3.40E-11	-0.58	84	2.1	Zbtb38	Transcription
DMR8:104658001	8	104658001	104662000	4000	1	8.70E-07	-0.32	43	1.07	Zbtb38	Transcription
DMR8:104741001	8	104741001	104742000	1000	1	4.10E-10	0.62	20	2	Pxylp1	Signaling
DMR8:104752001	8	104752001	104759000	7000	1	8.40E-13	-0.49	129	1.84	Pxylp1	Signaling
DMR8:104761001	8	104761001	104763000	2000	1	2.90E-07	-0.43	26	1.3	Pxylp1	Signaling
DMR8:104769001	8	104769001	104772000	3000	1	1.70E-07	0.52	48	1.6	Pxylp1	Signaling
DMR8:104786001	8	104786001	104788000	2000	1	6.50E-08	-0.49	21	1.05	Pxylp1	Signaling
DMR8:104840001	8	104840001	104844000	4000	1	1.90E-07	0.38	75	1.88	Spsb4	
DMR8:104891001	8	104891001	104892000	1000	1	3.80E-07	0.57	23	2.3	Spsb4	
DMR8:104947001	8	104947001	104949000	2000	1	2.70E-07	0.59	33	1.65	RGD1561847	
DMR8:105212001	8	105212001	105214000	2000	2	1.30E-07	-0.5	24	1.2	Trim42	
DMR8:105426001	8	105426001	105428000	2000	1	8.50E-07	0.47	20	1	Clstn2	Transport
DMR8:105696001	8	105696001	105697000	1000	1	6.10E-15	-0.66	5	0.5	Clstn2	Transport
DMR8:105700001	8	105700001	105701000	1000	1	1.60E-08	-0.37	9	0.9	Clstn2	Transport
DMR8:106433001	8	106433001	106435000	2000	1	1.10E-07	0.28	37	1.85	Nmnat3	Metabolism
DMR8:106489001	8	106489001	106490000	1000	1	2.30E-12	0.69	24	2.4	Rbp2	
DMR8:107200001	8	107200001	107202000	2000	1	5.70E-08	-0.47	56	2.8	LOC102547261;Foxl2	Transcription
DMR8:107377001	8	107377001	107380000	3000	1	3.10E-07	-0.32	50	1.67	Pik3cb	Signaling
DMR8:107386001	8	107386001	107390000	4000	1	2.40E-07	-0.49	42	1.05	Pik3cb	Signaling
DMR8:107555001	8	107555001	107557000	2000	1	8.00E-10	0.57	20	1	Cep70;Esyt3	
DMR8:107563001	8	107563001	107564000	1000	1	6.40E-08	0.62	20	2	Esyt3	
DMR8:107644001	8	107644001	107645000	1000	1	1.50E-08	0.47	17	1.7	Mras	Signaling
DMR8:107688001	8	107688001	107689000	1000	1	1.30E-08	0.4	13	1.3	Nme9	
DMR8:107732001	8	107732001	107735000	3000	1	6.40E-07	-0.36	59	1.97	Armc8	
DMR8:107879001	8	107879001	107883000	4000	1	4.70E-08	-0.39	72	1.8	LOC102547387;Dzip1l	
DMR8:107901001	8	107901001	107904000	3000	1	4.00E-09	0.52	47	1.57	Dzip1l	
DMR8:107920001	8	107920001	107924000	4000	1	6.50E-14	0.87	66	1.65	Dzip1l;Cldn18	Cell Junction
DMR8:107927001	8	107927001	107928000	1000	1	3.40E-11	0.66	25	2.5	Cldn18	Cell Junction
DMR8:107970001	8	107970001	107973000	3000	1	6.20E-07	-0.39	40	1.33	Cldn18	Cell Junction
DMR8:108117001	8	108117001	108121000	4000	1	1.10E-10	0.75	57	1.43	Sox14	Development
DMR8:108770001	8	108770001	108772000	2000	1	1.80E-07	0.35	19	0.95	Il20rb	Receptor

DMR8:108794001	8	108794001	108795000	1000	1	3.60E-09	-0.5	10	1	Nck1	Cytoskeleton
DMR8:108813001	8	108813001	108815000	2000	1	4.20E-13	0.81	51	2.55	Nck1	Cytoskeleton
DMR8:108816001	8	108816001	108818000	2000	1	1.10E-09	-0.44	14	0.7	Nck1	Cytoskeleton
DMR8:109010001	8	109010001	109014000	4000	1	7.20E-07	-0.35	35	0.88	Stag1	Epigenetic
DMR8:109015001	8	109015001	109018000	3000	1	1.10E-16	0.46	41	1.37	Stag1	Epigenetic
DMR8:109058001	8	109058001	109063000	5000	1	9.10E-08	-0.37	36	0.72	Stag1	Epigenetic
DMR8:109180001	8	109180001	109182000	2000	1	7.60E-09	-0.62	11	0.55	Stag1	Epigenetic
DMR8:109256001	8	109256001	109258000	2000	2	4.60E-07	-0.39	19	0.95	Stag1	Epigenetic
DMR8:109259001	8	109259001	109260000	1000	1	1.40E-07	-0.39	13	1.3	Stag1	Epigenetic
DMR8:109357001	8	109357001	109362000	5000	1	2.90E-08	0.34	45	0.9	LOC686039;Pccb	Metabolism
DMR8:109398001	8	109398001	109400000	2000	1	1.90E-08	-0.53	26	1.3	Pccb	Metabolism
DMR8:109446001	8	109446001	109448000	2000	2	1.20E-08	-0.34	36	1.8	Msl2	
DMR8:109597001	8	109597001	109601000	4000	1	2.60E-07	-0.35	34	0.85	Ppp2r3a	Signaling
DMR8:110423001	8	110423001	110424000	1000	1	3.00E-10	0.62	18	1.8	Ephb1	Receptor
DMR8:110430001	8	110430001	110435000	5000	1	1.40E-08	-0.32	39	0.78	Ephb1	Receptor
DMR8:110474001	8	110474001	110477000	3000	1	9.00E-07	-0.32	29	0.97	Ephb1	Receptor
DMR8:110480001	8	110480001	110481000	1000	1	4.70E-09	-0.71	1	0.1	Ephb1	Receptor
DMR8:110623001	8	110623001	110625000	2000	1	2.40E-12	-0.46	26	1.3	Ephb1	Receptor
DMR8:110804001	8	110804001	110807000	3000	1	5.20E-26	0.6	34	1.13	Ephb1	Receptor
DMR8:110818001	8	110818001	110821000	3000	1	9.60E-16	0.91	44	1.47	Ephb1	Receptor
DMR8:111036001	8	111036001	111038000	2000	1	7.30E-08	0.43	24	1.2	RGD1565365	
DMR8:111341001	8	111341001	111343000	2000	1	1.70E-07	-0.5	16	0.8	Ryk	Receptor
DMR8:111384001	8	111384001	111386000	2000	1	6.70E-08	-0.49	29	1.45	Ryk	Receptor
DMR8:111573001	8	111573001	111576000	3000	1	2.90E-07	0.43	43	1.43	Slco2a1	Transport
DMR8:111579001	8	111579001	111580000	1000	1	1.20E-09	-0.64	6	0.6	Slco2a1	Transport
DMR8:111623001	8	111623001	111624000	1000	1	2.50E-07	0.53	12	1.2	Rab6b	
DMR8:111645001	8	111645001	111649000	4000	1	1.80E-08	0.53	80	2	Rab6b	
DMR8:111699001	8	111699001	111700000	1000	1	1.10E-09	0.64	13	1.3	Tf	Transport
DMR8:111759001	8	111759001	111760000	1000	1	5.20E-07	0.6	25	2.5	RGD1310507	Transport
DMR8:111800001	8	111800001	111801000	1000	1	3.60E-09	0.45	12	1.2	Topbp1	Transcription
DMR8:111983001	8	111983001	111984000	1000	1	1.40E-07	0.47	26	2.6	Tmem108	
DMR8:112070001	8	112070001	112071000	1000	1	2.00E-09	0.38	14	1.4	Tmem108	
DMR8:112138001	8	112138001	112140000	2000	1	9.00E-07	-0.39	26	1.3	Tmem108	
DMR8:112196001	8	112196001	112197000	1000	1	3.70E-07	-0.58	8	0.8	Tmem108	
DMR8:112394001	8	112394001	112395000	1000	1	5.50E-12	0.79	28	2.8	Rpl27-ps1	
DMR8:112529001	8	112529001	112531000	2000	1	1.10E-13	0.74	47	2.35	Nphp3	
DMR8:112601001	8	112601001	112602000	1000	1	9.50E-09	-0.36	16	1.6	Uba5;Acad11	Proteolysis;Metabolism
DMR8:112707001	8	112707001	112708000	1000	1	1.90E-07	0.39	18	1.8	Dnajc13	Transcription
DMR8:112819001	8	112819001	112820000	1000	1	8.50E-14	1.03	34	3.4	Dnajc13	Transcription
DMR8:112827001	8	112827001	112829000	2000	1	1.50E-08	-0.45	34	1.7	Dnajc13;Acpp	Transcription
DMR8:112866001	8	112866001	112871000	5000	2	5.70E-08	-0.56	112	2.24	Acpp	
DMR8:112876001	8	112876001	112878000	2000	1	2.00E-07	-0.43	41	2.05	Acpp	
DMR8:112887001	8	112887001	112889000	2000	1	6.50E-07	-0.38	26	1.3	Acpp	
DMR8:112890001	8	112890001	112899000	9000	3	1.30E-19	-0.62	148	1.64	Acpp	
DMR8:112942001	8	112942001	112943000	1000	1	7.20E-07	0.51	5	0.5	Trnac-gca	
DMR8:112951001	8	112951001	112954000	3000	2	2.00E-15	0.91	45	1.5	Trnac-gca;Trnae-uuc	
DMR8:113158001	8	113158001	113159000	1000	1	7.60E-08	-0.31	14	1.4	Cpne4	
DMR8:113167001	8	113167001	113168000	1000	1	2.70E-08	-0.47	23	2.3	Cpne4	
DMR8:113210001	8	113210001	113212000	2000	1	2.70E-07	0.42	31	1.55	Cpne4	
DMR8:113579001	8	113579001	113581000	2000	1	5.90E-07	0.43	29	1.45	Cpne4	
DMR8:113602001	8	113602001	113603000	1000	1	4.40E-07	0.42	11	1.1	Mrpl3	Translation
DMR8:113760001	8	113760001	113762000	2000	1	2.90E-10	0.34	23	1.15	Nek11;LOC102551579	Signaling
DMR8:113805001	8	113805001	113809000	4000	1	1.60E-08	-0.5	43	1.07	Nek11;LOC108351775	Signaling
DMR8:113894001	8	113894001	113895000	1000	1	7.10E-07	0.63	1	0.1	Nek11	Signaling
DMR8:113915001	8	113915001	113918000	3000	1	1.60E-09	-0.44	40	1.33	Nek11	Signaling
DMR8:113982001	8	113982001	113984000	2000	1	9.80E-08	-0.58	18	0.9	Atp2c1;LOC102551490	Transport
DMR8:114047001	8	114047001	114050000	3000	1	3.70E-07	-0.41	72	2.4	Atp2c1	Transport
DMR8:114335001	8	114335001	114337000	2000	1	5.10E-07	-0.43	20	1	Pik3r4	Signaling
DMR8:114400001	8	114400001	114401000	1000	1	7.90E-07	0.51	26	2.6	Col6a6	
DMR8:114522001	8	114522001	114523000	1000	1	6.40E-07	-0.57	9	0.9	Col6a5	
DMR8:114604001	8	114604001	114606000	2000	1	3.60E-08	-0.36	23	1.15	Col6a5	

DMR8:114608001	8	114608001	114609000	1000	1	5.10E-08	0.48	17	1.7	Col6a5	
DMR8:114999001	8	114999001	115001000	2000	1	6.10E-08	-0.37	25	1.25	Poc1a	
DMR8:115214001	8	115214001	115215000	1000	1	5.00E-07	-0.46	26	2.6	Iqcf5	
DMR8:115343001	8	115343001	115345000	2000	1	1.00E-08	0.49	42	2.1	Grm2;LOC108351776	Signaling
DMR8:115359001	8	115359001	115361000	2000	1	7.60E-07	0.36	27	1.35	Grm2;LOC108351776;Tex264;LOC108351777	Signaling
DMR8:115464001	8	115464001	115466000	2000	1	8.80E-07	-0.49	15	0.75	Rad54l2	
DMR8:115718001	8	115718001	115719000	1000	1	1.60E-08	-0.32	10	1	Dock3	Transcription
DMR8:115762001	8	115762001	115764000	2000	1	2.20E-07	-0.31	15	0.75	Dock3	Transcription
DMR8:115856001	8	115856001	115858000	2000	1	3.30E-07	-0.38	10	0.5	Dock3	Transcription
DMR8:115882001	8	115882001	115884000	2000	1	6.60E-08	-0.45	7	0.35	Dock3	Transcription
DMR8:115984001	8	115984001	115986000	2000	1	2.30E-09	-0.55	19	0.95	Dock3	Transcription
DMR8:116024001	8	116024001	116026000	2000	1	2.70E-08	-0.41	20	1	Mapkapk3	Signaling
DMR8:116074001	8	116074001	116075000	1000	1	9.00E-11	-0.53	7	0.7	Hemk1	Golgi
DMR8:116086001	8	116086001	116087000	1000	1	7.30E-08	0.5	12	1.2	Hemk1;RGD1307461	Golgi
DMR8:116129001	8	116129001	116130000	1000	1	6.00E-07	0.65	27	2.7	Trnap-agg	
DMR8:116181001	8	116181001	116182000	1000	1	5.30E-07	-0.32	22	2.2	Cacna2d2	Transport
DMR8:116280001	8	116280001	116282000	2000	1	8.80E-08	0.47	46	2.3	Cacna2d2;Tmem115	Transport;Transport
DMR8:116304001	8	116304001	116306000	2000	1	6.70E-08	0.39	39	1.95	Tmem115;Cyb561d2;Nprl2;Zmynd10;Rassf1	Transport;Signaling;Cytoskeleton
DMR8:116354001	8	116354001	116356000	2000	1	4.50E-15	0.62	62	3.1	Ifrd2;Lsmem2;Sema3b	Signaling
DMR8:116450001	8	116450001	116453000	3000	1	6.20E-07	0.39	67	2.23	Sema3f	Signaling
DMR8:116698001	8	116698001	116704000	6000	1	4.00E-11	0.72	93	1.55	Mst1r;Actl11	Receptor;Cytoskeleton
DMR8:116725001	8	116725001	116729000	4000	1	2.00E-07	0.55	79	1.98	Camkv;Traip	Signaling;Proteolysis
DMR8:116758001	8	116758001	116762000	4000	1	8.60E-08	0.6	65	1.62	Traip;Uba7;Fam212a;Cdhr4	Proteolysis
DMR8:116853001	8	116853001	116854000	1000	1	1.70E-07	-0.66	3	0.3	Gmppb;Rnf123;Mst1;Apeh	Transport;Protease
DMR8:116858001	8	116858001	116859000	1000	1	4.10E-11	0.76	23	2.3	Gmppb;Rnf123;Mst1;Apeh	Transport;Protease
DMR8:116883001	8	116883001	116885000	2000	1	1.40E-11	0.81	83	4.15	Bsn	
DMR8:116983001	8	116983001	116985000	2000	1	4.20E-09	-0.4	54	2.7	Dag1	Cytoskeleton
DMR8:117201001	8	117201001	117205000	4000	1	5.20E-07	-0.36	113	2.83	Ccdc36;LOC680045	
DMR8:117215001	8	117215001	117217000	2000	1	4.60E-16	1.02	62	3.1	Ccdc36;LOC680045	
DMR8:117230001	8	117230001	117233000	3000	1	2.90E-08	0.59	56	1.87	LOC680045;Klhdc8b;Ccdc71	
DMR8:117309001	8	117309001	117311000	2000	1	2.20E-10	-0.49	20	1	Qars;Qrich1	Transcription
DMR8:117361001	8	117361001	117364000	3000	1	5.50E-07	-0.48	86	2.87	Impdh2;Ndufaf3;Mir3555;Mir191;Mir425;Dalrd3;Wdr6;P4htm	Metabolism;Golgi
DMR8:117394001	8	117394001	117395000	1000	1	2.60E-08	0.48	16	1.6	Arih2	Proteolysis
DMR8:117460001	8	117460001	117461000	1000	1	3.30E-09	-0.38	24	2.4	Slc25a20	Transport
DMR8:117548001	8	117548001	117549000	1000	1	2.30E-07	-0.42	13	1.3	Prkar2a	Signaling
DMR8:117706001	8	117706001	117708000	2000	1	5.10E-09	0.5	26	1.3	Col7a1;Mir711	Extracellular Matrix
DMR8:117728001	8	117728001	117733000	5000	1	6.60E-08	0.39	43	0.86	Col7a1;Ucn2;Pfkfb4	Extracellular Matrix;Metabolism
DMR8:117756001	8	117756001	117758000	2000	1	6.40E-07	-0.44	55	2.75	Pfkfb4	Metabolism
DMR8:117830001	8	117830001	117833000	3000	1	1.20E-09	0.46	54	1.8	Ccdc51;Tma7;Plxnb1	
DMR8:118115001	8	118115001	118118000	3000	1	1.90E-07	-0.39	36	1.2	Map4	
DMR8:118149001	8	118149001	118150000	1000	1	1.60E-07	-0.34	17	1.7	Map4	
DMR8:118269001	8	118269001	118270000	1000	1	4.20E-09	0.37	6	0.6	Smarcc1	Epigenetic
DMR8:118273001	8	118273001	118275000	2000	1	6.60E-17	-0.67	33	1.65	Smarcc1	Epigenetic
DMR8:118592001	8	118592001	118594000	2000	1	5.30E-09	-0.42	31	1.55	Scap	
DMR8:118678001	8	118678001	118679000	1000	1	4.60E-17	-0.71	14	1.4	Klhl18	Cytoskeleton
DMR8:118741001	8	118741001	118742000	1000	1	1.30E-09	0.5	7	0.7	Klhl18;Kif9	Cytoskeleton;Cytoskeleton
DMR8:118777001	8	118777001	118779000	2000	1	1.20E-07	0.41	24	1.2	Kif9	Cytoskeleton
DMR8:118824001	8	118824001	118826000	2000	1	1.80E-08	-0.4	35	1.75	Setd2	Epigenetic
DMR8:118891001	8	118891001	118892000	1000	1	4.30E-09	0.58	16	1.6	Setd2;LOC108351781;Nradd;Nbeal2	Epigenetic;Receptor
DMR8:118895001	8	118895001	118897000	2000	1	6.00E-07	0.49	48	2.4	Setd2;LOC108351781;Nradd;Nbeal2	Epigenetic;Receptor
DMR8:118898001	8	118898001	118900000	2000	1	3.70E-11	0.6	68	3.4	Setd2;Nradd;Nbeal2	Epigenetic;Receptor
DMR8:118913001	8	118913001	118914000	1000	1	6.40E-09	0.57	15	1.5	Nbeal2	
DMR8:118994001	8	118994001	118995000	1000	1	3.40E-07	0.45	16	1.6	Pth1r	Receptor
DMR8:119019001	8	119019001	119021000	2000	1	6.50E-07	0.49	22	1.1	Pth1r;Myl3	Receptor;Cytoskeleton

DMR8:119059001	8	119059001	119065000	6000	2	1.00E-09	0.66	92	1.53	Prss42	Protease
DMR8:119091001	8	119091001	119097000	6000	1	5.70E-12	0.67	118	1.97	Prss44;LOC103690462;LOC100 360828	Protease
DMR8:119099001	8	119099001	119101000	2000	1	6.50E-13	0.77	51	2.55	Prss44;LOC103690462;LOC100 360828	Protease
DMR8:119164001	8	119164001	119169000	5000	2	8.00E-10	0.62	107	2.14	Tmie;Als2cl	
DMR8:119173001	8	119173001	119175000	2000	1	6.90E-10	0.61	35	1.75	Als2cl	
DMR8:119179001	8	119179001	119180000	1000	1	5.40E-07	0.34	16	1.6	Als2cl	
DMR8:119221001	8	119221001	119225000	4000	1	1.40E-07	0.51	83	2.08	RGD1564138;Tdgf1;Lrrc2	Signaling;Cytoskeleton
DMR8:119259001	8	119259001	119260000	1000	1	9.50E-08	0.59	36	3.6	Lrrc2;Rtp3	Cytoskeleton
DMR8:119323001	8	119323001	119327000	4000	1	8.80E-08	-0.41	95	2.38	Ltf;Ccrl2	Transport
DMR8:119332001	8	119332001	119334000	2000	2	2.10E-13	0.81	48	2.4	Ccrl2	
DMR8:119509001	8	119509001	119512000	3000	1	2.30E-07	0.34	35	1.17	Mlh1	Transcription
DMR8:119561001	8	119561001	119565000	4000	1	2.20E-07	-0.47	49	1.23	Trank1	
DMR8:119718001	8	119718001	119719000	1000	1	2.90E-13	-0.62	20	2	Dclk3	Signaling
DMR8:119722001	8	119722001	119723000	1000	1	2.90E-12	0.82	25	2.5	Dclk3	Signaling
DMR8:119731001	8	119731001	119735000	4000	1	1.40E-12	1.03	82	2.05	Dclk3	Signaling
DMR8:119744001	8	119744001	119747000	3000	1	1.60E-07	-0.66	26	0.87	Dclk3;LOC108351782	Signaling
DMR8:119846001	8	119846001	119847000	1000	1	2.10E-08	-0.4	15	1.5	Stac	
DMR8:119861001	8	119861001	119865000	4000	1	5.60E-07	-0.4	45	1.12	Stac	
DMR8:119872001	8	119872001	119873000	1000	1	1.80E-07	-0.35	8	0.8	Stac	
DMR8:120343001	8	120343001	120344000	1000	1	1.10E-07	0.41	6	0.6	Arpp21	
DMR8:120381001	8	120381001	120386000	5000	1	6.70E-10	0.57	77	1.54	Arpp21;Mir128-2	
DMR8:120398001	8	120398001	120399000	1000	1	6.40E-08	-0.55	3	0.3	Arpp21	
DMR8:120435001	8	120435001	120438000	3000	1	1.30E-07	-0.52	54	1.8	Arpp21;LOC108351783	
DMR8:120480001	8	120480001	120481000	1000	1	4.60E-16	1.02	24	2.4	Arpp21	
DMR8:122069001	8	122069001	122073000	4000	1	1.50E-07	-0.39	37	0.92	Clasp2	Cytoskeleton
DMR8:122076001	8	122076001	122078000	2000	1	2.50E-07	-0.45	26	1.3	Clasp2	Cytoskeleton
DMR8:122118001	8	122118001	122121000	3000	1	1.30E-07	0.5	32	1.07	Clasp2	Cytoskeleton
DMR8:122142001	8	122142001	122143000	1000	1	4.20E-07	-0.39	14	1.4	Clasp2	Cytoskeleton
DMR8:122263001	8	122263001	122266000	3000	1	1.10E-07	0.42	69	2.3	Fbxl2	
DMR8:122323001	8	122323001	122325000	2000	1	3.10E-07	0.63	26	1.3	LOC108351786;Susd5	
DMR8:122327001	8	122327001	122329000	2000	1	6.90E-11	0.39	26	1.3	LOC108351786;Susd5	
DMR8:122355001	8	122355001	122359000	4000	1	3.70E-12	0.52	45	1.12	Susd5	
DMR8:122367001	8	122367001	122368000	1000	1	7.90E-09	0.53	31	3.1	Susd5	
DMR8:122449001	8	122449001	122450000	1000	1	4.50E-07	-0.4	11	1.1	Glb1;Tmppe	Metabolism
DMR8:122460001	8	122460001	122463000	3000	1	1.60E-09	-0.48	46	1.53	Glb1	Metabolism
DMR8:122487001	8	122487001	122489000	2000	1	1.00E-14	0.86	50	2.5	Glb1	Metabolism
DMR8:122522001	8	122522001	122526000	4000	1	8.50E-11	0.88	68	1.7	Ccr4	
DMR8:122545001	8	122545001	122546000	1000	1	1.30E-07	0.39	22	2.2	Ccr4;Trim71	Proteolysis
DMR8:122553001	8	122553001	122558000	5000	1	2.70E-07	0.33	156	3.12	Trim71	Proteolysis
DMR8:122560001	8	122560001	122564000	4000	1	5.60E-08	-0.37	56	1.4	Trim71	Proteolysis
DMR8:122792001	8	122792001	122795000	3000	1	5.10E-08	-0.44	43	1.43	Cmtm6	Transport
DMR8:122798001	8	122798001	122799000	1000	1	1.10E-07	-0.39	12	1.2	Cmtm6	Transport
DMR8:122808001	8	122808001	122811000	3000	1	2.80E-07	0.48	36	1.2	Cmtm6;Cmtm7	Transport
DMR8:122855001	8	122855001	122857000	2000	1	4.50E-07	-0.35	56	2.8	Cmtm8	Transport
DMR8:123071001	8	123071001	123072000	1000	1	2.10E-10	0.77	34	3.4	Osbpl10;LOC108351789	
DMR8:123112001	8	123112001	123113000	1000	1	4.10E-25	1.29	32	3.2	Osbpl10	
DMR8:123154001	8	123154001	123155000	1000	1	4.20E-10	0.52	21	2.1	Osbpl10	
DMR8:123212001	8	123212001	123213000	1000	1	3.60E-07	0.36	11	1.1	Osbpl10	
DMR8:123215001	8	123215001	123217000	2000	1	6.00E-10	-0.42	26	1.3	Osbpl10	
DMR8:123218001	8	123218001	123221000	3000	1	1.50E-07	-0.35	49	1.63	Osbpl10	
DMR8:123351001	8	123351001	123353000	2000	1	1.60E-09	-0.43	34	1.7	Stt3b	Golgi
DMR8:123983001	8	123983001	123984000	1000	1	6.20E-11	-0.77	6	0.6	Gadl1	
DMR8:124032001	8	124032001	124034000	2000	1	9.00E-07	-0.35	26	1.3	Gadl1	
DMR8:124049001	8	124049001	124052000	3000	2	1.90E-11	0.84	44	1.47	Gadl1	
DMR8:124179001	8	124179001	124180000	1000	1	7.20E-08	0.64	14	1.4	Gadl1;LOC102546889	
DMR8:124225001	8	124225001	124229000	4000	1	7.50E-08	0.3	25	0.62	Gadl1	
DMR8:124265001	8	124265001	124266000	1000	1	5.00E-09	0.58	23	2.3	Gadl1	
DMR8:124294001	8	124294001	124296000	2000	1	5.20E-09	-0.32	39	1.95	Gadl1	
DMR8:124297001	8	124297001	124300000	3000	1	9.30E-12	0.74	68	2.27	Gadl1	
DMR8:124333001	8	124333001	124336000	3000	1	9.20E-07	0.36	47	1.57	Tgfbr2	Signaling

DMR8:124338001	8	124338001	124341000	3000	1	6.10E-13	0.39	45	1.5	Tgfbr2	Signaling
DMR8:124369001	8	124369001	124373000	4000	1	1.20E-08	-0.4	63	1.57	Tgfbr2	Signaling
DMR8:124397001	8	124397001	124398000	1000	1	6.40E-09	0.76	28	2.8	Tgfbr2	Signaling
DMR8:124405001	8	124405001	124408000	3000	1	7.40E-07	0.31	36	1.2	Tgfbr2	Signaling
DMR8:124834001	8	124834001	124836000	2000	1	8.70E-09	0.58	54	2.7	Rbms3	
DMR8:124916001	8	124916001	124917000	1000	1	9.30E-08	-0.36	12	1.2	Rbms3	
DMR8:124921001	8	124921001	124922000	1000	1	1.10E-08	-0.37	18	1.8	Rbms3	
DMR8:124945001	8	124945001	124946000	1000	1	2.10E-17	0.92	17	1.7	Rbms3	
DMR8:125023001	8	125023001	125024000	1000	1	2.30E-10	-0.42	8	0.8	Rbms3	
DMR8:125112001	8	125112001	125115000	3000	1	2.70E-08	0.46	38	1.27	Rbms3	
DMR8:125116001	8	125116001	125121000	5000	2	2.30E-09	-0.55	61	1.22	Rbms3	
DMR8:125169001	8	125169001	125171000	2000	1	1.10E-08	-0.52	26	1.3	Rbms3	
DMR8:125302001	8	125302001	125304000	2000	1	6.10E-08	-0.65	22	1.1	Rbms3	
DMR8:125349001	8	125349001	125350000	1000	1	4.80E-08	-0.41	18	1.8	Rbms3	
DMR8:125356001	8	125356001	125358000	2000	1	4.40E-07	0.56	19	0.95	Rbms3	
DMR8:125386001	8	125386001	125390000	4000	2	1.80E-12	0.67	84	2.1	Rbms3	
DMR8:125419001	8	125419001	125421000	2000	1	1.50E-07	0.33	33	1.65	Rbms3	
DMR8:125430001	8	125430001	125432000	2000	1	8.00E-11	-0.57	14	0.7	Rbms3	
DMR8:125437001	8	125437001	125440000	3000	2	3.90E-12	0.89	74	2.47	Rbms3	
DMR8:125445001	8	125445001	125447000	2000	2	5.60E-22	0.66	11	0.55	Rbms3	
DMR8:125495001	8	125495001	125496000	1000	1	4.40E-24	1.24	25	2.5	Rbms3	
DMR8:125508001	8	125508001	125515000	7000	1	2.50E-08	0.32	89	1.27	Rbms3	
DMR8:125546001	8	125546001	125553000	7000	1	3.60E-12	-0.74	91	1.3	Rbms3;LOC102550774	
DMR8:126168001	8	126168001	126171000	3000	2	4.20E-09	0.57	44	1.47	Zcwpw2	
DMR8:126172001	8	126172001	126173000	1000	1	3.30E-07	0.41	18	1.8	Zcwpw2	
DMR8:126179001	8	126179001	126181000	2000	1	5.00E-07	0.5	38	1.9	Zcwpw2	
DMR8:126186001	8	126186001	126191000	5000	2	1.60E-09	0.67	69	1.38	Zcwpw2	
DMR8:126284001	8	126284001	126286000	2000	1	5.10E-07	0.42	24	1.2	Zcwpw2	
DMR8:127072001	8	127072001	127076000	4000	1	3.10E-08	-0.44	68	1.7	Ctdspl	
DMR8:127096001	8	127096001	127097000	1000	1	3.90E-08	0.44	9	0.9	Ctdspl	
DMR8:127223001	8	127223001	127228000	5000	3	4.90E-17	-0.78	127	2.54	Ctdspl;Golga4	Transport
DMR8:127252001	8	127252001	127256000	4000	1	4.30E-12	-0.54	82	2.05	Ctdspl;Golga4	Transport
DMR8:127262001	8	127262001	127264000	2000	1	3.70E-07	-0.55	19	0.95	Ctdspl;Itga9	Extracellular Matrix
DMR8:127272001	8	127272001	127273000	1000	1	1.00E-07	0.66	19	1.9	Ctdspl;Itga9	Extracellular Matrix
DMR8:127278001	8	127278001	127282000	4000	1	4.00E-08	0.46	62	1.55	Ctdspl;Itga9	Extracellular Matrix
DMR8:127333001	8	127333001	127335000	2000	1	5.70E-10	0.54	52	2.6	Ctdspl;Itga9	Extracellular Matrix
DMR8:127369001	8	127369001	127371000	2000	1	5.80E-08	0.33	37	1.85	Ctdspl;Itga9	Extracellular Matrix
DMR8:127480001	8	127480001	127482000	2000	1	1.90E-09	0.39	29	1.45	Ctdspl;Itga9	Extracellular Matrix
DMR8:127512001	8	127512001	127515000	3000	1	2.20E-10	0.39	35	1.17	Ctdspl;Itga9	Extracellular Matrix
DMR8:127528001	8	127528001	127536000	8000	2	3.70E-08	0.49	105	1.31	Ctdspl;Itga9	Extracellular Matrix
DMR8:127542001	8	127542001	127545000	3000	1	3.90E-07	0.63	51	1.7	Ctdspl;Itga9	Extracellular Matrix
DMR8:127547001	8	127547001	127548000	1000	1	1.90E-08	0.47	11	1.1	Ctdspl;Itga9	Extracellular Matrix
DMR8:127565001	8	127565001	127568000	3000	1	1.90E-07	0.32	55	1.83	Ctdspl;Itga9	Extracellular Matrix
DMR8:127611001	8	127611001	127612000	1000	1	6.60E-07	-0.38	22	2.2	Ctdspl	
DMR8:127628001	8	127628001	127629000	1000	1	8.60E-07	0.37	16	1.6	Ctdspl	
DMR8:127630001	8	127630001	127632000	2000	1	1.30E-07	-0.42	54	2.7	Ctdspl	
DMR8:127635001	8	127635001	127639000	4000	1	2.10E-09	-0.47	72	1.8	Ctdspl	
DMR8:127661001	8	127661001	127663000	2000	1	3.70E-08	0.63	46	2.3	Ctdspl;LOC108351797	
DMR8:127666001	8	127666001	127670000	4000	2	1.20E-08	-0.36	70	1.75	Ctdspl;LOC108351797	
DMR8:127683001	8	127683001	127684000	1000	1	2.70E-09	-0.42	33	3.3	Ctdspl;LOC108351798	
DMR8:127696001	8	127696001	127698000	2000	1	1.00E-06	-0.33	57	2.85	Ctdspl;LOC108351798	
DMR8:127745001	8	127745001	127746000	1000	1	1.30E-10	0.6	34	3.4	Vill;Plcd1	Cytoskeleton;Metabolism
DMR8:127759001	8	127759001	127761000	2000	1	4.10E-09	0.39	35	1.75	Vill;Plcd1	Cytoskeleton;Metabolism
DMR8:127782001	8	127782001	127785000	3000	1	3.00E-09	0.54	70	2.33	Plcd1;LOC102548352;Dlec1	Metabolism
DMR8:127809001	8	127809001	127811000	2000	1	3.20E-09	0.48	45	2.25	Dlec1	
DMR8:127835001	8	127835001	127836000	1000	1	6.80E-08	0.62	32	3.2	Dlec1;Acaa1b	Metabolism
DMR8:127859001	8	127859001	127860000	1000	1	4.10E-08	-0.54	3	0.3	Slc22a14	
DMR8:127864001	8	127864001	127866000	2000	2	8.00E-09	0.51	22	1.1	Slc22a14	
DMR8:127943001	8	127943001	127945000	2000	1	6.30E-07	-0.39	29	1.45	Oxsr1	
DMR8:127967001	8	127967001	127970000	3000	1	2.40E-10	-0.49	43	1.43	Oxsr1;LOC102553059	

DMR8:128023001	8	128023001	128024000	1000	1	5.40E-09	0.48	15	1.5	Myd88;Acaa1	Cytoskeleton
DMR8:128035001	8	128035001	128038000	3000	2	1.50E-07	0.39	55	1.83	Myd88;Acaa1;Xylb	Cytoskeleton;Metabolism
DMR8:128082001	8	128082001	128084000	2000	1	2.30E-09	0.71	64	3.2	Xylb;Acvr2b	Metabolism;Signaling
DMR8:128112001	8	128112001	128114000	2000	1	2.50E-18	0.91	69	3.45	Acvr2b	Signaling
DMR8:128197001	8	128197001	128199000	2000	1	2.20E-09	0.42	39	1.95	Scn5a	Transport
DMR8:128246001	8	128246001	128247000	1000	1	2.50E-08	0.68	33	3.3	Scn5a	Transport
DMR8:128261001	8	128261001	128266000	5000	1	2.20E-13	0.7	106	2.12	Scn5a	Transport
DMR8:128267001	8	128267001	128275000	8000	1	1.60E-08	-0.4	150	1.88	Scn5a	Transport
DMR8:128292001	8	128292001	128297000	5000	2	5.10E-10	0.35	75	1.5	Scn10a	Transport
DMR8:128303001	8	128303001	128310000	7000	2	2.00E-08	-0.51	115	1.64	Scn10a	Transport
DMR8:128370001	8	128370001	128374000	4000	1	8.40E-11	-0.44	72	1.8	Scn10a	Transport
DMR8:128381001	8	128381001	128385000	4000	1	2.40E-13	-0.78	64	1.6	Scn10a	Transport
DMR8:128391001	8	128391001	128393000	2000	1	2.70E-10	-0.49	18	0.9	Scn10a	Transport
DMR8:128413001	8	128413001	128414000	1000	1	2.10E-07	-0.43	17	1.7	Scn10a	Transport
DMR8:128419001	8	128419001	128420000	1000	1	8.70E-12	-0.42	15	1.5	Scn10a	Transport
DMR8:128426001	8	128426001	128427000	1000	1	9.20E-08	0.43	19	1.9	Scn10a	Transport
DMR8:128466001	8	128466001	128467000	1000	1	6.50E-21	1.27	24	2.4	Scn11a	Transport
DMR8:128482001	8	128482001	128483000	1000	1	7.80E-11	0.83	26	2.6	Scn11a	Transport
DMR8:128508001	8	128508001	128511000	3000	1	2.30E-07	0.59	57	1.9	Scn11a	Transport
DMR8:128591001	8	128591001	128594000	3000	1	2.60E-07	0.5	40	1.33	Wdr48	
DMR8:128612001	8	128612001	128613000	1000	1	8.80E-09	0.52	13	1.3	Wdr48;Gorasp1;Ttc21a	
DMR8:128623001	8	128623001	128625000	2000	1	1.80E-07	0.4	39	1.95	Gorasp1;Ttc21a	
DMR8:128646001	8	128646001	128649000	3000	2	9.10E-10	0.48	66	2.2	Ttc21a	
DMR8:128655001	8	128655001	128657000	2000	1	2.60E-08	0.42	31	1.55	Ttc21a;Csrnp1	
DMR8:128673001	8	128673001	128675000	2000	1	8.80E-16	1.02	39	1.95	Csrnp1;LOC108351801	
DMR8:128701001	8	128701001	128705000	4000	1	8.30E-08	0.49	57	1.43	Xirp1	Cytoskeleton
DMR8:128709001	8	128709001	128711000	2000	1	3.60E-07	0.44	28	1.4	Xirp1	Cytoskeleton
DMR8:128729001	8	128729001	128731000	2000	1	9.20E-07	0.32	34	1.7	Cx3cr1	
DMR8:128772001	8	128772001	128773000	1000	1	2.20E-07	0.54	25	2.5	Ccr8	
DMR8:128807001	8	128807001	128810000	3000	1	5.70E-07	-0.47	49	1.63	Slc25a38;Rpsa;LOC103690472	Transport;Translation
DMR8:128842001	8	128842001	128843000	1000	1	2.20E-07	0.55	27	2.7	Mobp	Cytoskeleton
DMR8:128855001	8	128855001	128861000	6000	1	2.90E-11	0.82	110	1.83	Mobp;LOC102549288	Cytoskeleton
DMR8:129093001	8	129093001	129096000	3000	3	5.80E-11	0.56	34	1.13	Myrip	Cytoskeleton
DMR8:129170001	8	129170001	129171000	1000	1	2.70E-09	0.52	11	1.1	Eif1b;LOC295574	Translation
DMR8:129189001	8	129189001	129193000	4000	1	1.00E-08	0.64	63	1.57	LOC295574;LOC367190;Entpd3	Signaling
DMR8:129194001	8	129194001	129196000	2000	1	1.10E-16	0.8	46	2.3	LOC367190;Entpd3	Signaling
DMR8:129375001	8	129375001	129376000	1000	1	2.70E-08	0.55	16	1.6	Snrpel1	
DMR8:129680001	8	129680001	129681000	1000	1	6.50E-07	0.47	10	1	Ulk4	Signaling
DMR8:129690001	8	129690001	129695000	5000	1	8.00E-07	0.3	67	1.34	Ulk4	Signaling
DMR8:129697001	8	129697001	129701000	4000	1	9.00E-07	0.34	55	1.38	Ulk4	Signaling
DMR8:129723001	8	129723001	129724000	1000	1	9.00E-09	-0.46	18	1.8	Ulk4	Signaling
DMR8:129752001	8	129752001	129754000	2000	1	5.50E-10	0.39	40	2	Ulk4	Signaling
DMR8:129775001	8	129775001	129777000	2000	1	2.10E-11	0.8	46	2.3	Ulk4	Signaling
DMR8:129845001	8	129845001	129846000	1000	1	7.70E-08	0.56	12	1.2	Ulk4	Signaling
DMR8:129868001	8	129868001	129874000	6000	2	7.90E-22	0.75	145	2.42	Ulk4	Signaling
DMR8:129937001	8	129937001	129941000	4000	3	2.90E-16	0.86	83	2.08	Trak1	Transport
DMR8:129949001	8	129949001	129953000	4000	1	3.90E-07	-0.38	89	2.22	Trak1	Transport
DMR8:130082001	8	130082001	130084000	2000	1	3.90E-07	0.26	47	2.35	Trak1	Transport
DMR8:130112001	8	130112001	130115000	3000	1	3.50E-07	0.52	50	1.67	Trak1;Cck	Transport
DMR8:130221001	8	130221001	130224000	3000	1	1.80E-11	0.6	41	1.37	Lyzl4	
DMR8:130233001	8	130233001	130235000	2000	1	3.20E-13	0.8	35	1.75	Lyzl4	
DMR8:130237001	8	130237001	130239000	2000	1	1.00E-07	0.5	23	1.15	Lyzl4	
DMR8:130325001	8	130325001	130328000	3000	1	2.40E-08	0.52	28	0.93	Sec22c	
DMR8:130380001	8	130380001	130383000	3000	2	2.50E-08	-0.43	29	0.97	Nktr	Transcription
DMR8:130445001	8	130445001	130448000	3000	1	1.40E-07	0.53	48	1.6	Ccdc13	
DMR8:130598001	8	130598001	130599000	1000	1	3.40E-09	0.53	20	2	Fam198a	
DMR8:130614001	8	130614001	130616000	2000	1	7.50E-08	0.41	45	2.25	Fam198a;Pomgnt2	Golgi
DMR8:130816001	8	130816001	130817000	1000	1	5.50E-07	-0.4	16	1.6	Ano10;LOC103690476	
DMR8:130970001	8	130970001	130972000	2000	1	4.50E-10	0.5	29	1.45	LOC102552874;Abhd5	

DMR8:131722001	8	131722001	131724000	2000	1	1.10E-07	0.43	30	1.5	LOC103690480;LOC103690478;Mir138-1	
DMR8:131790001	8	131790001	131791000	1000	1	1.10E-08	0.48	11	1.1	Topaz1;LOC102550701	
DMR8:131821001	8	131821001	131826000	5000	1	5.60E-08	0.6	54	1.08	Topaz1	
DMR8:131895001	8	131895001	131896000	1000	1	2.90E-07	-0.43	5	0.5	Zfp445;LOC102552507	Transcription
DMR8:131940001	8	131940001	131944000	4000	1	2.30E-09	0.48	44	1.1	Zfp167	
DMR8:132115001	8	132115001	132117000	2000	1	8.50E-11	0.44	13	0.65	Tmem42;Tgm4	Transport
DMR8:132118001	8	132118001	132120000	2000	1	1.10E-08	0.48	28	1.4	Tmem42;Tgm4	Transport
DMR8:132121001	8	132121001	132123000	2000	1	3.40E-12	0.74	37	1.85	Tgm4	Transport
DMR8:132162001	8	132162001	132164000	2000	1	3.80E-08	0.56	25	1.25	Tgm4;Zdhhc3	Transport
DMR8:132355001	8	132355001	132356000	1000	1	1.20E-07	0.72	18	1.8	Tmem158	
DMR8:132576001	8	132576001	132577000	1000	1	9.00E-07	0.53	11	1.1	Limd1	Transcription
DMR8:132688001	8	132688001	132695000	7000	1	7.00E-13	-0.41	80	1.14	RGD1566368	
DMR9:49001	9	49001	51000	2000	1	2.00E-11	0.4	19	0.95	Efhb	Signaling
DMR9:55001	9	55001	56000	1000	1	4.80E-11	0.61	10	1	Efhb	Signaling
DMR9:1194001	9	1194001	1196000	2000	1	2.50E-09	-0.64	13	0.65	Tbc1d5	Signaling
DMR9:1198001	9	1198001	1201000	3000	2	4.10E-08	-0.66	19	0.63	Tbc1d5	Signaling
DMR9:1258001	9	1258001	1260000	2000	1	6.70E-07	-0.34	17	0.85	Tbc1d5	Signaling
DMR9:1320001	9	1320001	1323000	3000	1	3.10E-09	-0.38	13	0.43	Tbc1d5	Signaling
DMR9:1409001	9	1409001	1412000	3000	1	7.20E-09	0.25	23	0.77	Tbc1d5;LOC108348193	Signaling
DMR9:1459001	9	1459001	1462000	3000	1	4.90E-07	-0.37	30	1	Tbc1d5	Signaling
DMR9:1479001	9	1479001	1481000	2000	1	4.40E-07	0.53	17	0.85	Tbc1d5	Signaling
DMR9:1498001	9	1498001	1503000	5000	1	2.90E-07	-0.23	87	1.74	Tbc1d5	Signaling
DMR9:1537001	9	1537001	1539000	2000	1	9.70E-19	0.75	32	1.6	Tbc1d5	Signaling
DMR9:1701001	9	1701001	1702000	1000	1	2.30E-08	0.43	14	1.4	Tbc1d5	Signaling
DMR9:1840001	9	1840001	1842000	2000	1	6.00E-09	-0.4	69	3.45	Plcl2	Metabolism
DMR9:2228001	9	2228001	2233000	5000	1	2.60E-09	-0.42	84	1.68	Satb1	Epigenetic
DMR9:2237001	9	2237001	2239000	2000	1	2.90E-08	0.53	25	1.25	Satb1	Epigenetic
DMR9:2252001	9	2252001	2256000	4000	1	6.90E-07	-0.33	62	1.55	Satb1	Epigenetic
DMR9:2272001	9	2272001	2274000	2000	1	1.40E-10	0.45	47	2.35	Satb1	Epigenetic
DMR9:2277001	9	2277001	2278000	1000	1	9.60E-09	0.57	18	1.8	Satb1	Epigenetic
DMR9:2281001	9	2281001	2283000	2000	1	9.10E-07	-0.41	22	1.1	Satb1	Epigenetic
DMR9:3215001	9	3215001	3219000	4000	1	1.00E-07	-0.27	32	0.8	Kcnh8;LOC102551325	Transport
DMR9:3472001	9	3472001	3475000	3000	1	3.90E-10	0.52	31	1.03	Kcnh8	Transport
DMR9:3477001	9	3477001	3478000	1000	1	1.00E-07	0.43	14	1.4	Kcnh8	Transport
DMR9:3481001	9	3481001	3483000	2000	1	3.70E-24	0.47	19	0.95	Kcnh8	Transport
DMR9:4172001	9	4172001	4175000	3000	1	5.60E-16	0.38	38	1.27	Sult1c2a	Transport
DMR9:4267001	9	4267001	4269000	2000	1	9.60E-07	-0.22	21	1.05	RGD1562392	Transport
DMR9:4371001	9	4371001	4376000	5000	2	4.90E-10	0.42	33	0.66	RGD1562392;LOC100359586	Transport
DMR9:4407001	9	4407001	4409000	2000	1	2.00E-11	-0.38	17	0.85	RGD1562392	Transport
DMR9:4476001	9	4476001	4479000	3000	1	2.90E-08	0.53	28	0.93	RGD1562392;Kat2b	Transport
DMR9:4484001	9	4484001	4487000	3000	1	3.00E-08	0.58	54	1.8	RGD1562392;Kat2b	Transport
DMR9:4524001	9	4524001	4527000	3000	1	9.40E-09	0.64	49	1.63	RGD1562392	Transport
DMR9:4667001	9	4667001	4668000	1000	1	6.00E-08	0.29	5	0.5	Sult1c2	Transport
DMR9:4856001	9	4856001	4857000	1000	1	1.50E-08	0.35	5	0.5	RGD1559960	Transport
DMR9:4861001	9	4861001	4862000	1000	1	6.70E-07	-0.36	0	0	RGD1559960	Transport
DMR9:5219001	9	5219001	5224000	5000	1	4.40E-07	0.25	54	1.08	RGD1560901	
DMR9:7770001	9	7770001	7772000	2000	1	4.00E-12	-0.59	11	0.55	Vom2r77	Signaling
DMR9:7876001	9	7876001	7878000	2000	1	1.70E-07	-0.57	6	0.3	Pot1b	Transcription
DMR9:8047001	9	8047001	8048000	1000	1	8.80E-09	-0.56	1	0.1	LOC100911278;Adgre4	Signaling
DMR9:8154001	9	8154001	8157000	3000	2	3.30E-08	-0.44	14	0.47	Adgre4	Signaling
DMR9:9138001	9	9138001	9139000	1000	1	1.90E-07	0.33	8	0.8	MGC116197	
DMR9:9425001	9	9425001	9430000	5000	1	7.10E-11	0.35	49	0.98	Adgre1	Signaling
DMR9:9462001	9	9462001	9464000	2000	1	4.60E-07	-0.26	13	0.65	Adgre1	Signaling
DMR9:9504001	9	9504001	9506000	2000	1	3.20E-07	-0.53	9	0.45	Adgre1	Signaling
DMR9:9644001	9	9644001	9646000	2000	1	3.80E-09	0.65	45	2.25	Vav1	
DMR9:9752001	9	9752001	9753000	1000	1	3.30E-09	0.62	17	1.7	C3;Tnfsf14	Protease; Proteolysis
DMR9:9840001	9	9840001	9842000	2000	1	3.70E-08	0.48	31	1.55	Cd70	
DMR9:9903001	9	9903001	9904000	1000	1	3.20E-09	0.46	8	0.8	Tnfsf9	
DMR9:9967001	9	9967001	9969000	2000	1	2.40E-14	0.74	51	2.55	LOC108351881;Tubb4a;Dennd1c	Cytoskeleton

DMR9:9971001	9	9971001	9974000	3000	1	1.10E-08	0.58	27	0.9	LOC108351881;Tubb4a;Dennd1c;Crb3	Cytoskeleton
DMR9:10122001	9	10122001	10123000	1000	1	5.00E-08	-0.37	13	1.3	Mllt1	Transcription
DMR9:10151001	9	10151001	10153000	2000	1	1.20E-10	0.62	34	1.7	Mllt1;LOC316124	Transcription;Metabolism
DMR9:10169001	9	10169001	10171000	2000	1	1.20E-07	0.66	50	2.5	LOC316124;Acsbg2	Metabolism
DMR9:10203001	9	10203001	10205000	2000	1	6.90E-07	0.37	14	0.7	Acsbg2;LOC501280	Metabolism
DMR9:10208001	9	10208001	10209000	1000	1	3.70E-09	0.72	35	3.5	Acsbg2;LOC501280;Rfx2	Metabolism;Transcription
DMR9:10249001	9	10249001	10250000	1000	1	1.90E-07	-0.37	11	1.1	Rfx2	Transcription
DMR9:10272001	9	10272001	10277000	5000	2	4.00E-09	0.61	82	1.64	Rfx2;Prr22	Transcription
DMR9:10392001	9	10392001	10397000	5000	1	1.30E-07	0.36	61	1.22	Catsperd	
DMR9:10398001	9	10398001	10399000	1000	1	3.50E-07	0.52	8	0.8	Catsperd	
DMR9:10538001	9	10538001	10539000	1000	1	6.90E-08	0.68	16	1.6	Znrf4	
DMR9:10597001	9	10597001	10599000	2000	1	8.00E-07	0.59	30	1.5	Ptprs	Signaling
DMR9:10601001	9	10601001	10606000	5000	1	4.20E-08	0.6	50	1	Ptprs	Signaling
DMR9:10607001	9	10607001	10610000	3000	1	6.20E-08	0.53	42	1.4	Ptprs	Signaling
DMR9:10611001	9	10611001	10614000	3000	1	7.80E-08	0.45	38	1.27	Ptprs	Signaling
DMR9:10615001	9	10615001	10617000	2000	1	3.00E-08	-0.4	33	1.65	Ptprs	Signaling
DMR9:10663001	9	10663001	10665000	2000	1	7.70E-08	0.41	26	1.3	Kdm4b	Epigenetic
DMR9:10709001	9	10709001	10711000	2000	1	2.60E-07	0.45	18	0.9	Kdm4b	Epigenetic
DMR9:10877001	9	10877001	10882000	5000	2	3.20E-09	0.42	79	1.58	Dpp9;Mydgf	Protease;Growth Factors
DMR9:10908001	9	10908001	10909000	1000	1	1.80E-08	-0.62	4	0.4	Tnfaip8l1	
DMR9:10961001	9	10961001	10962000	1000	1	1.10E-08	0.53	11	1.1	Sema6b;Lrg1;Plin5;Plin4	Signaling;Cytoskeleton
DMR9:11095001	9	11095001	11097000	2000	1	9.00E-09	0.69	59	2.95	Shd;Ccdc94	
DMR9:11153001	9	11153001	11156000	3000	2	4.00E-08	0.51	60	2	LOC102555289;Uxs1	Metabolism
DMR9:12659001	9	12659001	12660000	1000	1	2.00E-09	-0.52	14	1.4	Dazl	Metabolism
DMR9:12765001	9	12765001	12769000	4000	1	2.50E-07	0.25	58	1.45	Rftn1	
DMR9:12772001	9	12772001	12775000	3000	1	1.80E-08	0.5	36	1.2	Rftn1	
DMR9:12784001	9	12784001	12786000	2000	1	3.60E-07	-0.37	45	2.25	Rftn1	
DMR9:12858001	9	12858001	12862000	4000	1	1.70E-07	0.31	93	2.33	Rftn1	
DMR9:12897001	9	12897001	12898000	1000	1	5.20E-10	-0.52	7	0.7	Rftn1	
DMR9:13051001	9	13051001	13052000	1000	1	4.50E-07	0.54	15	1.5	Kif6	Cytoskeleton
DMR9:13070001	9	13070001	13071000	1000	1	3.80E-20	1.09	32	3.2	Kif6	Cytoskeleton
DMR9:13176001	9	13176001	13178000	2000	1	7.00E-09	0.67	47	2.35	Kif6	Cytoskeleton
DMR9:13241001	9	13241001	13244000	3000	2	1.70E-08	-0.48	33	1.1	Kif6	Cytoskeleton
DMR9:13521001	9	13521001	13523000	2000	1	3.60E-08	0.4	21	1.05	Mocs1	
DMR9:13860001	9	13860001	13863000	3000	1	6.60E-12	0.37	33	1.1	Lrfn2	
DMR9:13876001	9	13876001	13877000	1000	1	8.60E-17	0.55	32	3.2	Lrfn2	
DMR9:13885001	9	13885001	13886000	1000	1	1.10E-10	0.46	25	2.5	Lrfn2	
DMR9:13899001	9	13899001	13901000	2000	1	1.00E-06	0.45	16	0.8	Lrfn2;LOC108351889	
DMR9:13943001	9	13943001	13946000	3000	1	5.20E-10	0.53	52	1.73	Lrfn2	
DMR9:14485001	9	14485001	14486000	1000	1	1.60E-08	0.71	18	1.8	Unc5cl	Receptor
DMR9:14618001	9	14618001	14620000	2000	1	7.90E-11	0.61	27	1.35	Trem2;LOC102548435	Immune
DMR9:14641001	9	14641001	14642000	1000	1	4.70E-07	0.65	24	2.4	LOC102548435;RGD1307182	
DMR9:14797001	9	14797001	14798000	1000	1	1.90E-13	0.35	15	1.5	Ncr2	
DMR9:15122001	9	15122001	15126000	4000	2	1.30E-08	0.66	71	1.77	Foxp4	
DMR9:15131001	9	15131001	15137000	6000	2	5.70E-16	0.74	102	1.7	Foxp4	
DMR9:15159001	9	15159001	15162000	3000	1	1.80E-08	-0.34	20	0.67	Mdfi	
DMR9:15175001	9	15175001	15179000	4000	1	4.60E-09	0.67	54	1.35	Mdfi	
DMR9:15189001	9	15189001	15190000	1000	1	3.30E-09	0.66	21	2.1	Mdfi	
DMR9:15236001	9	15236001	15240000	4000	1	4.90E-09	0.39	84	2.1	Tfeb	
DMR9:15271001	9	15271001	15272000	1000	1	9.20E-10	0.49	17	1.7	Tfeb;Pgc;LOC102554309	Proteolysis
DMR9:15390001	9	15390001	15391000	1000	1	9.80E-10	0.66	11	1.1	Med20;Bysl	Proteolysis
DMR9:15503001	9	15503001	15510000	7000	1	2.40E-07	-0.47	112	1.6	LOC103690500;Taf8	
DMR9:15516001	9	15516001	15518000	2000	1	7.10E-10	-0.46	47	2.35	LOC103690500;Taf8	
DMR9:15555001	9	15555001	15559000	4000	2	2.20E-08	0.53	85	2.12	RGD1561662	
DMR9:15612001	9	15612001	15614000	2000	2	2.60E-07	0.61	35	1.75	LOC100912849;Guca1a;Guca1b	
DMR9:15637001	9	15637001	15641000	4000	1	1.90E-09	0.58	81	2.02	Guca1b;Mrps10	Translation
DMR9:15649001	9	15649001	15655000	6000	1	9.60E-08	-0.48	115	1.92	Mrps10;Trerf1	Translation

DMR9:15679001	9	15679001	15680000	1000	1	1.30E-07	0.44	15	1.5	Trerf1	
DMR9:15690001	9	15690001	15691000	1000	1	7.10E-11	0.92	36	3.6	Trerf1	
DMR9:15741001	9	15741001	15745000	4000	1	1.80E-09	-0.48	50	1.25	Trerf1	
DMR9:15750001	9	15750001	15751000	1000	1	5.10E-10	0.48	36	3.6	Trerf1	
DMR9:15866001	9	15866001	15873000	7000	2	1.50E-09	0.77	146	2.09	Trerf1	
DMR9:16014001	9	16014001	16016000	2000	1	3.00E-10	-0.49	35	1.75	Ubr2	Proteolysis
DMR9:16120001	9	16120001	16124000	4000	1	4.40E-11	0.7	64	1.6	Prph2	
DMR9:16175001	9	16175001	16176000	1000	1	8.40E-07	0.47	11	1.1	Prph2	
DMR9:16304001	9	16304001	16306000	2000	1	7.30E-08	-0.49	38	1.9	Prph2;LOC102555718;LOC100911564	Epigenetic
DMR9:16410001	9	16410001	16411000	1000	1	2.40E-08	-0.36	16	1.6	Tbcc;Gltscr1l	Transcription
DMR9:16412001	9	16412001	16415000	3000	1	2.30E-15	-0.58	83	2.77	Tbcc;Gltscr1l	Transcription
DMR9:16537001	9	16537001	16539000	2000	1	6.10E-08	0.45	28	1.4	Ptcra;Cnpy3	
DMR9:16621001	9	16621001	16623000	2000	1	3.30E-07	-0.52	18	0.9	Mea1;Klhdc3;Rrp36;LOC680835	
DMR9:16624001	9	16624001	16626000	2000	1	1.40E-10	0.46	49	2.45	Mea1;Klhdc3;Rrp36;LOC680835	
DMR9:16640001	9	16640001	16641000	1000	1	4.10E-08	-0.4	39	3.9	LOC680835;Mrpl2;Klc4	Translation;Cytoskeleton
DMR9:16671001	9	16671001	16676000	5000	1	1.50E-07	-0.46	115	2.3	Klc4;Ptk7	Cytoskeleton;Receptor
DMR9:16905001	9	16905001	16907000	2000	1	2.60E-07	0.35	30	1.5	Ttbk1	Signaling
DMR9:16951001	9	16951001	16952000	1000	1	6.70E-10	-0.48	15	1.5	Zfp318	
DMR9:16972001	9	16972001	16976000	4000	1	2.50E-09	-0.62	31	0.78	Zfp318	
DMR9:17058001	9	17058001	17061000	3000	1	6.70E-07	0.61	67	2.23	Abcc10;Dlk2	Transport
DMR9:17145001	9	17145001	17149000	4000	1	7.80E-07	-0.38	56	1.4	Xpo5	Transport
DMR9:17192001	9	17192001	17193000	1000	1	1.10E-09	0.54	23	2.3	Polh;LOC103690509;Gtpbp2	Transcription;Translation
DMR9:17682001	9	17682001	17683000	1000	1	1.20E-07	0.42	15	1.5	Mrpl14	Translation
DMR9:17705001	9	17705001	17707000	2000	1	3.90E-07	-0.37	45	2.25	Mrpl14;Tmem63b	Translation
DMR9:17708001	9	17708001	17712000	4000	1	2.00E-07	-0.6	41	1.02	Mrpl14;Tmem63b	Translation
DMR9:17719001	9	17719001	17725000	6000	1	6.20E-14	0.69	134	2.23	Tmem63b;Capn11	Protease
DMR9:17756001	9	17756001	17757000	1000	1	2.30E-12	0.79	26	2.6	Capn11	Protease
DMR9:17850001	9	17850001	17851000	1000	1	1.60E-12	0.91	30	3	Tmem151b;Tcte1	Cytoskeleton
DMR9:17857001	9	17857001	17858000	1000	1	9.60E-23	1.16	38	3.8	Tcte1	Cytoskeleton
DMR9:17878001	9	17878001	17879000	1000	1	3.40E-09	0.51	12	1.2	Aars2;LOC108351892	Translation
DMR9:17904001	9	17904001	17906000	2000	1	1.20E-08	-0.46	41	2.05	Spats1	
DMR9:17915001	9	17915001	17917000	2000	1	2.10E-07	0.37	26	1.3	Spats1	
DMR9:18478001	9	18478001	18480000	2000	1	1.20E-08	-0.48	12	0.6	Supt3h;Znrd1-as1	Transcription
DMR9:18615001	9	18615001	18617000	2000	1	1.60E-09	0.66	32	1.6	Runx2	Transcription
DMR9:18636001	9	18636001	18637000	1000	1	3.10E-07	0.66	34	3.4	Runx2	Transcription
DMR9:18650001	9	18650001	18657000	7000	1	5.50E-09	-0.38	102	1.46	Runx2	Transcription
DMR9:18690001	9	18690001	18692000	2000	1	9.40E-07	-0.38	22	1.1	Runx2	Transcription
DMR9:18703001	9	18703001	18705000	2000	2	2.30E-12	-0.49	45	2.25	Runx2	Transcription
DMR9:18762001	9	18762001	18765000	3000	1	1.00E-06	-0.34	52	1.73	Runx2	Transcription
DMR9:19110001	9	19110001	19112000	2000	2	6.60E-10	-0.56	10	0.5	LOC108351894;Clic5	Transport
DMR9:19139001	9	19139001	19142000	3000	1	1.80E-08	0.52	44	1.47	Clic5	Transport
DMR9:19257001	9	19257001	19260000	3000	1	4.40E-07	-0.44	32	1.07	Clic5	Transport
DMR9:19264001	9	19264001	19265000	1000	1	7.80E-16	0.97	20	2	Clic5	Transport
DMR9:19313001	9	19313001	19315000	2000	1	1.20E-25	-0.63	30	1.5	Clic5	Transport
DMR9:19686001	9	19686001	19687000	1000	1	1.10E-07	-0.43	11	1.1	Rcan2	Signaling
DMR9:19857001	9	19857001	19861000	4000	1	1.40E-07	-0.44	37	0.92	Cyp39a1	Metabolism
DMR9:19872001	9	19872001	19873000	1000	1	5.90E-09	-0.66	7	0.7	Cyp39a1;Slc25a27	Metabolism;Transport
DMR9:19888001	9	19888001	19889000	1000	1	2.40E-07	-0.44	18	1.8	Cyp39a1;Slc25a27	Metabolism;Transport
DMR9:19894001	9	19894001	19899000	5000	2	5.60E-08	0.57	91	1.82	Slc25a27	Transport
DMR9:19919001	9	19919001	19923000	4000	1	6.30E-08	0.7	116	2.9	LOC108351896;Tdrd6	Cytoskeleton
DMR9:20060001	9	20060001	20063000	3000	1	4.70E-10	0.45	28	0.93	Mep1a	Protease
DMR9:20119001	9	20119001	20125000	6000	3	5.10E-07	-0.41	67	1.12	Adgrf5	
DMR9:20188001	9	20188001	20190000	2000	1	1.50E-07	0.48	25	1.25	Adgrf5	
DMR9:20342001	9	20342001	20346000	4000	1	3.70E-08	-0.67	32	0.8	Adgrf1	
DMR9:20382001	9	20382001	20385000	3000	1	1.00E-07	0.56	63	2.1	Adgrf1	
DMR9:20823001	9	20823001	20825000	2000	1	1.40E-07	-0.29	22	1.1	Cd2ap	
DMR9:20893001	9	20893001	20895000	2000	1	4.70E-08	-0.59	23	1.15	Adgrf2	

DMR9:20912001	9	20912001	20916000	4000	1	5.40E-07	0.37	42	1.05	Adgrf2	
DMR9:20988001	9	20988001	20989000	1000	1	2.90E-08	0.54	20	2	Adgrf4	
DMR9:21062001	9	21062001	21064000	2000	1	1.10E-11	0.77	47	2.35	Opn5	Signaling
DMR9:21269001	9	21269001	21270000	1000	1	2.70E-07	-0.48	4	0.4	Ptchd4	Signaling
DMR9:21904001	9	21904001	21908000	4000	2	2.40E-09	-0.36	34	0.85	Olr1828-ps	
DMR9:23483001	9	23483001	23486000	3000	1	9.50E-07	-0.48	25	0.83	Rhag	Transport
DMR9:23489001	9	23489001	23490000	1000	1	1.10E-09	0.69	18	1.8	Rhag	Transport
DMR9:24519001	9	24519001	24521000	2000	1	4.50E-08	0.46	43	2.15	Defb16-ps;Defb17	
DMR9:25293001	9	25293001	25296000	3000	1	2.60E-08	-0.48	39	1.3	Tfap2d	Transcription
DMR9:25314001	9	25314001	25316000	2000	1	4.90E-07	-0.45	30	1.5	Tfap2d	Transcription
DMR9:25355001	9	25355001	25356000	1000	1	1.50E-07	0.55	19	1.9	Tfap2d	Transcription
DMR9:25360001	9	25360001	25363000	3000	1	1.00E-07	-0.51	14	0.47	Tfap2d	Transcription
DMR9:25439001	9	25439001	25440000	1000	1	1.20E-08	0.69	23	2.3	Tfap2b	Transcription
DMR9:26298001	9	26298001	26299000	1000	1	2.80E-08	-0.44	18	1.8	Pkhd1	
DMR9:26307001	9	26307001	26308000	1000	1	8.70E-07	-0.37	16	1.6	Pkhd1	
DMR9:26746001	9	26746001	26753000	7000	1	7.60E-10	-0.59	52	0.74	Pkhd1	
DMR9:26850001	9	26850001	26854000	4000	1	4.20E-07	-0.35	56	1.4	Il17a;LOC688876	
DMR9:26885001	9	26885001	26886000	1000	1	6.90E-08	0.5	15	1.5	Il17f	
DMR9:27001001	9	27001001	27002000	1000	1	1.50E-09	-0.43	18	1.8	Paqr8	Signaling
DMR9:27055001	9	27055001	27060000	5000	1	9.80E-11	0.54	135	2.7	Paqr8;LOC102549795;Efhc1	Signaling;Signaling
DMR9:27065001	9	27065001	27067000	2000	1	3.00E-08	-0.43	24	1.2	LOC102549795;Efhc1	Signaling
DMR9:27115001	9	27115001	27116000	1000	1	1.30E-10	0.65	15	1.5	Efhc1;Tram2	Signaling
DMR9:27120001	9	27120001	27121000	1000	1	8.80E-07	-0.35	31	3.1	Tram2	
DMR9:27147001	9	27147001	27149000	2000	1	9.70E-09	0.47	26	1.3	Tram2	
DMR9:27161001	9	27161001	27164000	3000	1	7.60E-08	0.44	40	1.33	Tram2	
DMR9:27174001	9	27174001	27175000	1000	1	1.20E-07	-0.44	28	2.8	Tram2	
DMR9:27323001	9	27323001	27325000	2000	1	4.10E-12	0.43	21	1.05	RGD1562107;LOC102550123;Tmem14a;LOC103693210	Transport
DMR9:27337001	9	27337001	27339000	2000	1	5.40E-10	0.76	32	1.6	LOC102550123;Tmem14a;LOC103693210	
DMR9:27567001	9	27567001	27570000	3000	1	8.20E-22	1.22	57	1.9	Kcnq5	Transport
DMR9:27582001	9	27582001	27583000	1000	1	3.50E-08	-0.41	6	0.6	Kcnq5	Transport
DMR9:27644001	9	27644001	27646000	2000	1	3.10E-07	-0.56	12	0.6	Kcnq5	Transport
DMR9:27748001	9	27748001	27749000	1000	1	1.80E-10	-0.71	5	0.5	Kcnq5	Transport
DMR9:27765001	9	27765001	27770000	5000	1	3.60E-10	-0.39	56	1.12	Kcnq5	Transport
DMR9:27842001	9	27842001	27844000	2000	1	2.10E-10	0.52	40	2	Kcnq5	Transport
DMR9:27994001	9	27994001	27996000	2000	1	7.00E-07	-0.36	27	1.35	Kcnq5	Transport
DMR9:28004001	9	28004001	28006000	2000	1	3.80E-08	0.47	87	4.35	Kcnq5	Transport
DMR9:28108001	9	28108001	28109000	1000	1	1.00E-07	-0.45	21	2.1	Kcnq5	Transport
DMR9:28439001	9	28439001	28441000	2000	1	3.50E-07	0.36	44	2.2	Rims1	Transport
DMR9:28512001	9	28512001	28515000	3000	1	8.70E-08	-0.41	52	1.73	Rims1	Transport
DMR9:28629001	9	28629001	28631000	2000	1	9.60E-08	0.45	35	1.75	Rims1	Transport
DMR9:28688001	9	28688001	28692000	4000	1	1.80E-08	-0.37	39	0.98	Rims1	Transport
DMR9:28825001	9	28825001	28827000	2000	1	9.30E-07	-0.54	22	1.1	Rims1	Transport
DMR9:28874001	9	28874001	28875000	1000	1	6.70E-10	-0.5	18	1.8	Rims1	Transport
DMR9:30046001	9	30046001	30048000	2000	1	4.40E-08	0.56	34	1.7	B3gat2	Golgi
DMR9:30303001	9	30303001	30304000	1000	1	1.30E-07	-0.49	7	0.7	Fam135a	
DMR9:30436001	9	30436001	30438000	2000	1	3.50E-07	-0.4	43	2.15	Col9a1	Extracellular Matrix
DMR9:30468001	9	30468001	30469000	1000	1	2.20E-07	-0.49	11	1.1	Col9a1	Extracellular Matrix
DMR9:30493001	9	30493001	30494000	1000	1	8.00E-07	-0.58	3	0.3	Col9a1	Extracellular Matrix
DMR9:30614001	9	30614001	30615000	1000	1	8.90E-12	0.8	21	2.1	Col19a1;LOC103690510	Extracellular Matrix
DMR9:30658001	9	30658001	30660000	2000	2	2.50E-15	-0.55	20	1	Col19a1	Extracellular Matrix
DMR9:30818001	9	30818001	30820000	2000	1	4.80E-09	0.56	27	1.35	Col19a1	Extracellular Matrix
DMR9:30862001	9	30862001	30864000	2000	2	1.50E-13	0.92	37	1.85	Col19a1	Extracellular Matrix
DMR9:30934001	9	30934001	30935000	1000	1	1.10E-07	0.56	10	1	Lmbrd1	Transport
DMR9:31330001	9	31330001	31331000	1000	1	2.60E-10	0.82	33	3.3	Adgrb3	Signaling
DMR9:31399001	9	31399001	31402000	3000	1	2.70E-08	0.51	41	1.37	Adgrb3	Signaling
DMR9:31622001	9	31622001	31624000	2000	1	3.60E-07	0.49	23	1.15	Adgrb3	Signaling
DMR9:31719001	9	31719001	31721000	2000	1	2.70E-11	-0.58	31	1.55	Adgrb3	Signaling
DMR9:31777001	9	31777001	31779000	2000	1	9.30E-10	-0.48	19	0.95	Adgrb3	Signaling
DMR9:31822001	9	31822001	31823000	1000	1	2.90E-09	0.46	34	3.4	Adgrb3	Signaling
DMR9:31825001	9	31825001	31826000	1000	1	2.30E-07	-0.56	9	0.9	Adgrb3	Signaling

DMR9:31827001	9	31827001	31828000	1000	1	1.40E-10	-0.44	14	1.4	Adgrb3	Signaling
DMR9:31853001	9	31853001	31855000	2000	1	2.20E-07	-0.62	13	0.65	Adgrb3	Signaling
DMR9:31909001	9	31909001	31911000	2000	1	2.30E-12	0.52	24	1.2	Adgrb3	Signaling
DMR9:31929001	9	31929001	31932000	3000	1	1.00E-08	-0.39	44	1.47	Adgrb3	Signaling
DMR9:35803001	9	35803001	35804000	1000	1	1.40E-08	0.5	9	0.9	RGD1559965	
DMR9:37075001	9	37075001	37076000	1000	1	9.40E-08	-0.32	20	2	Phf3	Transcription
DMR9:37090001	9	37090001	37097000	7000	3	9.90E-12	-0.55	60	0.86	Phf3	Transcription
DMR9:37467001	9	37467001	37468000	1000	1	3.90E-07	0.42	18	1.8	Lgsn	Metabolism
DMR9:37784001	9	37784001	37785000	1000	1	1.90E-08	-0.48	27	2.7	Imp4;Ccdc115;Trnae-uuc;LOC102551518	Translation
DMR9:37897001	9	37897001	37901000	4000	1	7.50E-10	-0.52	58	1.45	Dst	Cytoskeleton
DMR9:37902001	9	37902001	37905000	3000	2	8.30E-08	-0.38	81	2.7	Dst	Cytoskeleton
DMR9:37968001	9	37968001	37975000	7000	1	2.90E-09	-0.68	198	2.83	Dst	Cytoskeleton
DMR9:38001001	9	38001001	38003000	2000	1	1.90E-10	-0.56	47	2.35	Dst	Cytoskeleton
DMR9:38007001	9	38007001	38010000	3000	1	4.40E-07	-0.42	81	2.7	Dst	Cytoskeleton
DMR9:38011001	9	38011001	38014000	3000	1	9.80E-07	-0.35	79	2.63	Dst	Cytoskeleton
DMR9:38030001	9	38030001	38034000	4000	3	2.40E-09	-0.49	185	4.62	Dst	Cytoskeleton
DMR9:38043001	9	38043001	38046000	3000	2	9.20E-08	-0.39	100	3.33	Dst	Cytoskeleton
DMR9:38052001	9	38052001	38054000	2000	1	1.30E-07	-0.38	55	2.75	Dst	Cytoskeleton
DMR9:38055001	9	38055001	38057000	2000	1	2.50E-07	-0.35	49	2.45	Dst	Cytoskeleton
DMR9:38066001	9	38066001	38073000	7000	1	8.20E-07	-0.35	125	1.79	Dst	Cytoskeleton
DMR9:38086001	9	38086001	38093000	7000	3	6.30E-15	-0.63	157	2.24	Dst	Cytoskeleton
DMR9:38134001	9	38134001	38135000	1000	1	1.60E-07	-0.34	26	2.6	Dst	Cytoskeleton
DMR9:38246001	9	38246001	38251000	5000	1	2.50E-07	-0.33	90	1.8	Dst	Cytoskeleton
DMR9:38253001	9	38253001	38255000	2000	1	1.10E-09	-0.32	37	1.85	Dst	Cytoskeleton
DMR9:38288001	9	38288001	38290000	2000	1	3.90E-08	-0.43	43	2.15	Dst;Bend6	Cytoskeleton
DMR9:38301001	9	38301001	38302000	1000	1	5.00E-07	-0.39	18	1.8	Dst;Bend6	Cytoskeleton
DMR9:38536001	9	38536001	38537000	1000	1	2.80E-09	0.67	17	1.7	Prim2	Cell Cycle
DMR9:38603001	9	38603001	38604000	1000	1	2.00E-09	-0.33	18	1.8	Prim2	Cell Cycle
DMR9:38655001	9	38655001	38660000	5000	1	2.30E-10	0.42	78	1.56	Prim2	Cell Cycle
DMR9:39589001	9	39589001	39590000	1000	1	4.70E-19	1.09	23	2.3	Khdrbs2	Translation
DMR9:39719001	9	39719001	39721000	2000	1	9.70E-07	0.33	35	1.75	Khdrbs2	Translation
DMR9:40982001	9	40982001	40984000	2000	2	1.40E-07	-0.49	32	1.6	Ptpn18	
DMR9:41006001	9	41006001	41007000	1000	1	1.10E-11	0.61	25	2.5	Prss39;LOC102554718	
DMR9:41078001	9	41078001	41080000	2000	1	1.10E-09	0.64	40	2	Prss40;Amer3	Protease
DMR9:41099001	9	41099001	41102000	3000	1	3.90E-08	0.39	35	1.17	Amer3	
DMR9:41151001	9	41151001	41153000	2000	1	1.50E-13	0.74	36	1.8	LOC108351913;RGD1562461;Arhgef4	Transcription
DMR9:41175001	9	41175001	41178000	3000	1	1.20E-09	-0.41	45	1.5	Arhgef4	Transcription
DMR9:41271001	9	41271001	41273000	2000	1	1.70E-08	0.56	24	1.2	Arhgef4	Transcription
DMR9:41339001	9	41339001	41341000	2000	1	1.10E-08	0.45	19	0.95	Fam168b;Plekhb2	Cytoskeleton
DMR9:42641001	9	42641001	42642000	1000	1	1.00E-09	0.45	15	1.5	Hs6st1	Transport
DMR9:42707001	9	42707001	42713000	6000	1	6.20E-10	-0.38	50	0.83	Uggt1	Golgi
DMR9:42815001	9	42815001	42820000	5000	1	6.60E-13	0.72	98	1.96	Uggt1;LOC102553975;LOC100909803	Golgi
DMR9:42836001	9	42836001	42837000	1000	1	6.90E-11	0.79	21	2.1	Neurl3;LOC102554222	Proteolysis
DMR9:42891001	9	42891001	42897000	6000	1	1.80E-07	-0.32	116	1.93	Arid5a;Kansl3	Transcription
DMR9:42992001	9	42992001	42996000	4000	1	1.70E-07	0.49	61	1.52	Fer1l5	Transport
DMR9:43131001	9	43131001	43132000	1000	1	6.50E-08	0.69	19	1.9	Ankrd39;Sema4c	Signaling;Signaling
DMR9:43144001	9	43144001	43146000	2000	1	3.30E-07	0.52	21	1.05	Sema4c;Fam178b	Signaling
DMR9:43240001	9	43240001	43243000	3000	1	6.20E-08	0.46	33	1.1	Fam178b	
DMR9:43339001	9	43339001	43341000	2000	1	7.60E-08	0.51	59	2.95	Zap70	
DMR9:43348001	9	43348001	43350000	2000	2	1.20E-16	0.95	65	3.25	Zap70;Tmem131	
DMR9:43351001	9	43351001	43354000	3000	2	4.20E-14	1.17	68	2.27	Zap70;Tmem131	
DMR9:43358001	9	43358001	43360000	2000	1	2.80E-09	-0.39	49	2.45	Zap70;Tmem131	
DMR9:43383001	9	43383001	43384000	1000	1	8.80E-07	-0.33	22	2.2	Tmem131	
DMR9:43390001	9	43390001	43392000	2000	1	3.40E-09	-0.46	35	1.75	Tmem131	
DMR9:43504001	9	43504001	43505000	1000	1	3.50E-11	0.5	11	1.1	Tmem131;LOC102554557	
DMR9:43606001	9	43606001	43607000	1000	1	8.80E-10	0.62	16	1.6	Vwa3b	
DMR9:43648001	9	43648001	43649000	1000	1	3.30E-07	0.53	11	1.1	Vwa3b	
DMR9:43691001	9	43691001	43694000	3000	1	2.20E-08	-0.49	31	1.03	Vwa3b;LOC102554996	
DMR9:43713001	9	43713001	43716000	3000	1	1.20E-09	0.47	45	1.5	Vwa3b	

DMR9:43786001	9	43786001	43789000	3000	2	8.40E-19	0.93	68	2.27	Vwa3b	
DMR9:43806001	9	43806001	43807000	1000	1	2.30E-10	-0.79	3	0.3	Cnga3	Ion Channel
DMR9:43904001	9	43904001	43905000	1000	1	4.30E-09	0.61	9	0.9	Inpp4a	
DMR9:43917001	9	43917001	43918000	1000	1	3.30E-07	0.53	17	1.7	Inpp4a	
DMR9:44135001	9	44135001	44136000	1000	1	2.20E-07	0.64	19	1.9	Mgat4a	Transport
DMR9:44231001	9	44231001	44233000	2000	1	1.10E-08	-0.42	41	2.05	RGD1310819	
DMR9:44234001	9	44234001	44236000	2000	1	4.30E-09	-0.35	28	1.4	RGD1310819	
DMR9:44238001	9	44238001	44241000	3000	1	4.10E-07	-0.37	70	2.33	RGD1310819	
DMR9:44247001	9	44247001	44250000	3000	1	5.30E-10	-0.39	54	1.8	RGD1310819	
DMR9:44257001	9	44257001	44260000	3000	1	2.10E-07	-0.52	36	1.2	RGD1310819	
DMR9:44270001	9	44270001	44272000	2000	1	7.40E-09	-0.57	19	0.95	RGD1310819	
DMR9:44282001	9	44282001	44285000	3000	1	4.40E-07	-0.43	67	2.23	RGD1310819	
DMR9:44387001	9	44387001	44388000	1000	1	2.00E-07	-0.42	17	1.7	Tsga10	Epigenetic
DMR9:44439001	9	44439001	44441000	2000	1	1.40E-08	-0.36	76	3.8	Tsga10	Epigenetic
DMR9:44507001	9	44507001	44508000	1000	1	4.20E-08	-0.49	11	1.1	Lyg2	
DMR9:44525001	9	44525001	44526000	1000	1	4.00E-09	-0.57	11	1.1	Lyg1	
DMR9:44681001	9	44681001	44684000	3000	2	1.40E-11	-0.44	44	1.47	Eif5b	Translation
DMR9:44791001	9	44791001	44795000	4000	1	3.80E-10	-0.45	38	0.95	Rev1	Transcription
DMR9:44840001	9	44840001	44844000	4000	1	4.80E-08	0.86	70	1.75	Aff3	Transcription
DMR9:44909001	9	44909001	44912000	3000	1	2.50E-07	-0.4	62	2.07	Aff3	Transcription
DMR9:44974001	9	44974001	44975000	1000	1	2.60E-07	0.43	16	1.6	Aff3	Transcription
DMR9:45011001	9	45011001	45013000	2000	1	3.30E-07	0.55	28	1.4	Aff3	Transcription
DMR9:45051001	9	45051001	45052000	1000	1	2.90E-07	0.49	17	1.7	Aff3	Transcription
DMR9:45141001	9	45141001	45142000	1000	1	1.20E-07	0.62	8	0.8	Aff3	Transcription
DMR9:45151001	9	45151001	45152000	1000	1	1.60E-11	0.76	11	1.1	Aff3	Transcription
DMR9:45195001	9	45195001	45197000	2000	1	1.60E-07	0.35	25	1.25	Aff3	Transcription
DMR9:45255001	9	45255001	45257000	2000	1	1.20E-08	-0.43	37	1.85	Aff3	Transcription
DMR9:45285001	9	45285001	45286000	1000	1	6.00E-08	0.61	20	2	Aff3	Transcription
DMR9:45503001	9	45503001	45504000	1000	1	1.30E-09	0.43	2	0.2	Lonrf2	Proteolysis
DMR9:45533001	9	45533001	45534000	1000	1	2.20E-08	0.5	14	1.4	Chst10	Transport
DMR9:45595001	9	45595001	45596000	1000	1	5.80E-14	0.83	17	1.7	NMS	
DMR9:45675001	9	45675001	45679000	4000	2	4.80E-10	-0.59	104	2.6	Pdcl3	Epigenetic
DMR9:45956001	9	45956001	45958000	2000	1	1.10E-09	0.45	14	0.7	Npas2	
DMR9:46033001	9	46033001	46034000	1000	1	4.00E-18	1.04	16	1.6	Npas2	
DMR9:46089001	9	46089001	46091000	2000	1	1.00E-08	-0.43	24	1.2	Npas2;Rpl31;Tbc1d8	Translation;Signaling
DMR9:46134001	9	46134001	46136000	2000	1	5.10E-08	-0.39	33	1.65	Tbc1d8	Signaling
DMR9:46140001	9	46140001	46141000	1000	1	1.50E-12	-0.46	11	1.1	Tbc1d8	Signaling
DMR9:46373001	9	46373001	46375000	2000	1	2.00E-16	0.87	39	1.95	Creg2	
DMR9:46380001	9	46380001	46383000	3000	1	6.90E-11	0.62	42	1.4	Creg2	
DMR9:46384001	9	46384001	46385000	1000	1	1.60E-07	0.53	12	1.2	Creg2	
DMR9:46475001	9	46475001	46478000	3000	1	7.40E-07	0.54	62	2.07	Rfx8;LOC103690520;LOC108351918	Transcription
DMR9:46655001	9	46655001	46656000	1000	1	9.60E-07	0.41	11	1.1	Map4k4	Signaling
DMR9:46713001	9	46713001	46714000	1000	1	6.70E-07	-0.38	23	2.3	Map4k4	Signaling
DMR9:46774001	9	46774001	46777000	3000	1	9.30E-07	-0.52	41	1.37	Map4k4	Signaling
DMR9:46863001	9	46863001	46864000	1000	1	1.60E-12	-0.68	17	1.7	Il1r2	Receptor
DMR9:46992001	9	46992001	46994000	2000	1	1.70E-07	-0.33	42	2.1	Il1r1	Receptor
DMR9:47004001	9	47004001	47005000	1000	1	2.40E-07	0.47	12	1.2	Il1r1	Receptor
DMR9:47022001	9	47022001	47024000	2000	1	6.70E-08	-0.52	48	2.4	Il1r1	Receptor
DMR9:47040001	9	47040001	47042000	2000	1	3.70E-07	-0.49	20	1	Il1r1;Il1rl2	Receptor
DMR9:47094001	9	47094001	47100000	6000	1	2.10E-07	0.71	86	1.43	Il1rl2	Receptor
DMR9:47134001	9	47134001	47136000	2000	1	1.00E-14	0.91	30	1.5	Il1rl1	Receptor
DMR9:47172001	9	47172001	47174000	2000	1	5.10E-07	-0.38	8	0.4	Il1rl1	Receptor
DMR9:47249001	9	47249001	47254000	5000	1	2.20E-08	-0.38	65	1.3	Il18rap	Receptor
DMR9:47265001	9	47265001	47267000	2000	1	2.10E-08	-0.48	10	0.5	Il18rap	Receptor
DMR9:47377001	9	47377001	47380000	3000	1	3.90E-08	-0.33	31	1.03	LOC103690524;Slc9a2	Transport
DMR9:47397001	9	47397001	47398000	1000	1	3.60E-17	0.46	11	1.1	Slc9a2;LOC103690525	Transport
DMR9:47537001	9	47537001	47538000	1000	1	7.30E-14	0.93	17	1.7	Tmem182	
DMR9:49408001	9	49408001	49410000	2000	1	3.40E-09	0.67	27	1.35	RGD1562818	
DMR9:49449001	9	49449001	49451000	2000	2	6.20E-17	-0.6	25	1.25	Linc01158	
DMR9:49839001	9	49839001	49840000	1000	1	6.60E-10	0.56	20	2	Gpr45	Signaling
DMR9:49886001	9	49886001	49888000	2000	2	5.80E-09	-0.57	7	0.35	Tgfbrap1	

DMR9:49935001	9	49935001	49938000	3000	1	7.30E-07	-0.36	41	1.37	Fhl2	Transcription
DMR9:50372001	9	50372001	50373000	1000	1	6.70E-07	0.44	17	1.7	Nck2	Cytoskeleton
DMR9:50546001	9	50546001	50547000	1000	1	1.00E-09	-0.49	17	1.7	RGD1305645	
DMR9:50932001	9	50932001	50934000	2000	1	3.10E-18	0.5	12	0.6	Ercc5	Transcription
DMR9:50947001	9	50947001	50952000	5000	1	5.70E-07	-0.25	71	1.42	Ercc5	Transcription
DMR9:50992001	9	50992001	50993000	1000	1	1.60E-07	0.51	25	2.5	Mettl21cl1	
DMR9:51118001	9	51118001	51121000	3000	2	8.40E-09	-0.47	26	0.87	RGD1562067	
DMR9:51362001	9	51362001	51364000	2000	1	3.70E-07	-0.34	17	0.85	Gulp1	Cytoskeleton
DMR9:52145001	9	52145001	52146000	1000	1	8.10E-10	-0.69	4	0.4	Col5a2	Extracellular Matrix
DMR9:52179001	9	52179001	52183000	4000	1	5.40E-07	-0.29	40	1	Col5a2	Extracellular Matrix
DMR9:52408001	9	52408001	52411000	3000	1	2.10E-10	-0.57	24	0.8	Vom1r50;Vom1r-ps54	Receptor
DMR9:52428001	9	52428001	52429000	1000	1	3.30E-07	0.5	18	1.8	Vom1r-ps54	
DMR9:53168001	9	53168001	53170000	2000	1	3.80E-16	0.57	12	0.6	Pms1	Transcription
DMR9:53496001	9	53496001	53500000	4000	1	3.10E-10	0.43	34	0.85	Hibch	Metabolism
DMR9:53530001	9	53530001	53533000	3000	1	5.60E-07	-0.35	26	0.87	Hibch	Metabolism
DMR9:53612001	9	53612001	53613000	1000	1	5.20E-07	0.4	10	1	Bnip3-ps1	
DMR9:53640001	9	53640001	53641000	1000	1	2.00E-09	-0.5	11	1.1	Mfsd6	
DMR9:53665001	9	53665001	53667000	2000	1	4.40E-07	-0.43	11	0.55	Mfsd6	
DMR9:53737001	9	53737001	53738000	1000	1	9.60E-09	-0.37	15	1.5	Nemp2;LOC100912307	
DMR9:54323001	9	54323001	54325000	2000	1	3.10E-11	-0.55	41	2.05	Stat1	Transcription
DMR9:54369001	9	54369001	54370000	1000	1	2.40E-16	0.77	26	2.6	Stat4	
DMR9:54736001	9	54736001	54738000	2000	1	4.80E-11	-0.54	31	1.55	Myo1b	Cytoskeleton
DMR9:55067001	9	55067001	55068000	1000	1	3.30E-07	0.45	11	1.1	Nabp1;RGD1561618	Metabolism
DMR9:55505001	9	55505001	55507000	2000	1	1.20E-08	-0.45	21	1.05	Tmeff2;LOC108351929;LOC102550993	
DMR9:55528001	9	55528001	55533000	5000	2	9.00E-17	-0.51	47	0.94	Tmeff2	
DMR9:55646001	9	55646001	55648000	2000	1	8.20E-09	-0.52	8	0.4	Tmeff2	
DMR9:59561001	9	59561001	59562000	1000	1	1.00E-15	0.61	34	3.4	Dnah7	Cytoskeleton
DMR9:60014001	9	60014001	60016000	2000	1	3.20E-12	-0.46	30	1.5	Slc39a10	Transport
DMR9:60079001	9	60079001	60081000	2000	1	1.10E-07	0.22	13	0.65	Slc39a10;Dnah7	Transport;Cytoskeleton
DMR9:60089001	9	60089001	60090000	1000	1	4.00E-11	0.35	27	2.7	Dnah7	Cytoskeleton
DMR9:60108001	9	60108001	60110000	2000	1	9.70E-10	-0.48	28	1.4	Dnah7	Cytoskeleton
DMR9:60111001	9	60111001	60112000	1000	1	5.70E-09	0.35	7	0.7	Dnah7	Cytoskeleton
DMR9:60184001	9	60184001	60187000	3000	1	2.90E-07	-0.36	27	0.9	Dnah7	Cytoskeleton
DMR9:60302001	9	60302001	60303000	1000	1	4.20E-07	-0.55	5	0.5	Dnah7	Cytoskeleton
DMR9:60442001	9	60442001	60444000	2000	1	8.10E-08	0.3	17	0.85	Hecw2	Proteolysis
DMR9:60542001	9	60542001	60546000	4000	1	5.70E-07	-0.42	50	1.25	Hecw2	Proteolysis
DMR9:60905001	9	60905001	60907000	2000	1	5.40E-09	-0.46	31	1.55	Ccdc150	
DMR9:60923001	9	60923001	60925000	2000	1	1.60E-07	-0.38	25	1.25	Ccdc150;LOC108351931	
DMR9:60929001	9	60929001	60931000	2000	1	4.90E-08	-0.38	13	0.65	Ccdc150;LOC108351931	
DMR9:61024001	9	61024001	61026000	2000	2	3.70E-08	-0.44	11	0.55	Gtf3c3	Transcription
DMR9:61043001	9	61043001	61044000	1000	1	2.90E-07	-0.32	17	1.7	Gtf3c3;LOC686030	Transcription
DMR9:61065001	9	61065001	61069000	4000	2	3.30E-08	-0.36	59	1.48	Pgap1	Transport
DMR9:61234001	9	61234001	61237000	3000	1	3.20E-07	-0.41	50	1.67	Ankrd44	Cytoskeleton
DMR9:61368001	9	61368001	61370000	2000	1	5.10E-07	-0.44	8	0.4	Ankrd44	Cytoskeleton
DMR9:61373001	9	61373001	61374000	1000	1	3.80E-08	0.49	20	2	Ankrd44	Cytoskeleton
DMR9:61405001	9	61405001	61406000	1000	1	8.30E-07	0.43	14	1.4	Ankrd44	Cytoskeleton
DMR9:61493001	9	61493001	61500000	7000	1	9.70E-07	-0.34	81	1.16	Ankrd44	Cytoskeleton
DMR9:61902001	9	61902001	61904000	2000	1	5.80E-08	-0.44	13	0.65	Boll	Metabolism
DMR9:62054001	9	62054001	62057000	3000	1	1.90E-09	-0.45	46	1.53	Plcl1	Metabolism
DMR9:62130001	9	62130001	62131000	1000	1	5.80E-08	-0.37	24	2.4	Plcl1	Metabolism
DMR9:62175001	9	62175001	62176000	1000	1	7.20E-10	-0.53	17	1.7	Plcl1	Metabolism
DMR9:62188001	9	62188001	62190000	2000	2	1.80E-10	-0.42	26	1.3	Plcl1	Metabolism
DMR9:62261001	9	62261001	62264000	3000	1	2.20E-08	-0.36	54	1.8	Plcl1	Metabolism
DMR9:62301001	9	62301001	62306000	5000	1	1.90E-10	-0.45	47	0.94	Plcl1;LOC103690536;LOC103690535	Metabolism
DMR9:63471001	9	63471001	63477000	6000	1	1.10E-07	0.51	49	0.82	Satb2	Epigenetic
DMR9:63547001	9	63547001	63552000	5000	2	2.20E-14	-0.71	53	1.06	Satb2	Epigenetic
DMR9:63646001	9	63646001	63648000	2000	1	6.20E-11	-0.48	25	1.25	Satb2;LOC102549320;LOC108351934	Epigenetic
DMR9:64673001	9	64673001	64676000	3000	1	5.40E-08	-0.65	30	1	Spats2l	
DMR9:64767001	9	64767001	64768000	1000	1	5.20E-07	-0.31	17	1.7	Spats2l	

DMR9:64851001	9	64851001	64853000	2000	1	1.10E-08	-0.45	25	1.25	Spats2l;Kctd18	Cytoskeleton
DMR9:65058001	9	65058001	65061000	3000	1	2.70E-08	-0.4	41	1.37	Aox3	Metabolism
DMR9:65100001	9	65100001	65101000	1000	1	3.90E-09	-0.47	3	0.3	Aox3;Aox4	Metabolism
DMR9:65210001	9	65210001	65211000	1000	1	5.30E-07	0.33	16	1.6	Aox2;LOC108351937	Metabolism
DMR9:65381001	9	65381001	65384000	3000	1	6.70E-08	-0.3	39	1.3	Orc2	Cell Cycle
DMR9:65619001	9	65619001	65620000	1000	1	1.50E-08	-0.36	12	1.2	Casp8	Protease
DMR9:65676001	9	65676001	65679000	3000	2	8.30E-09	-0.53	30	1	Als2cr12	
DMR9:65750001	9	65750001	65755000	5000	1	8.80E-08	-0.37	52	1.04	Trak2;Stradb	Transport;Signaling
DMR9:65767001	9	65767001	65769000	2000	2	4.80E-10	-0.65	25	1.25	Trak2;Stradb	Transport;Signaling
DMR9:65811001	9	65811001	65813000	2000	1	2.40E-07	-0.45	8	0.4	LOC102552195;Als2cr11	
DMR9:65875001	9	65875001	65877000	2000	1	2.70E-07	-0.38	17	0.85	Als2cr11;Tmem237	
DMR9:65912001	9	65912001	65913000	1000	1	2.90E-07	-0.35	12	1.2	Tmem237;Mpp4	Cytoskeleton
DMR9:66037001	9	66037001	66038000	1000	1	4.10E-09	0.36	30	3	Als2;RGD1562399	
DMR9:66048001	9	66048001	66050000	2000	1	3.90E-07	-0.4	30	1.5	RGD1562399;Fzd7	Receptor
DMR9:66368001	9	66368001	66370000	2000	1	3.00E-07	0.57	25	1.25	RGD1562029	
DMR9:66371001	9	66371001	66374000	3000	2	8.00E-08	-0.44	54	1.8	RGD1562029	
DMR9:66434001	9	66434001	66435000	1000	1	8.70E-13	0.87	19	1.9	RGD1562029	
DMR9:66811001	9	66811001	66813000	2000	1	1.50E-09	0.37	20	1	Ica1l	
DMR9:66869001	9	66869001	66875000	6000	1	1.40E-07	0.34	55	0.92	Wdr12;Carf	Transcription
DMR9:67110001	9	67110001	67111000	1000	1	8.50E-07	-0.36	14	1.4	Nbeal1;LOC108351938	
DMR9:67310001	9	67310001	67312000	2000	1	1.10E-07	-0.66	15	0.75	Raph1	Cytoskeleton
DMR9:67359001	9	67359001	67360000	1000	1	6.50E-13	0.58	10	1	Raph1	Cytoskeleton
DMR9:67535001	9	67535001	67537000	2000	1	5.70E-09	0.56	25	1.25	Cd28	Immune
DMR9:67577001	9	67577001	67580000	3000	1	2.80E-08	0.46	28	0.93	Cd28	Immune
DMR9:67776001	9	67776001	67778000	2000	1	1.50E-08	-0.53	36	1.8	Icos	
DMR9:68455001	9	68455001	68459000	4000	1	7.80E-07	-0.3	37	0.92	Pard3b;LOC103690539	
DMR9:68533001	9	68533001	68535000	2000	1	5.60E-08	-0.58	3	0.15	Pard3b	
DMR9:68619001	9	68619001	68620000	1000	1	2.60E-07	0.45	9	0.9	Pard3b	
DMR9:68637001	9	68637001	68639000	2000	1	1.90E-07	0.36	18	0.9	Pard3b	
DMR9:68680001	9	68680001	68681000	1000	1	3.40E-11	0.53	6	0.6	Pard3b	
DMR9:68916001	9	68916001	68918000	2000	1	6.60E-08	0.46	20	1	Pard3b	
DMR9:69181001	9	69181001	69183000	2000	1	9.10E-07	-0.33	14	0.7	Pard3b;LOC108352006	
DMR9:69208001	9	69208001	69209000	1000	1	1.00E-08	-0.56	9	0.9	Pard3b;LOC108352006	
DMR9:69489001	9	69489001	69491000	2000	1	3.90E-12	-0.39	22	1.1	Nrp2	
DMR9:69589001	9	69589001	69592000	3000	1	5.50E-09	0.6	55	1.83	Nrp2	
DMR9:69865001	9	69865001	69867000	2000	1	9.00E-07	-0.45	18	0.9	Ino80d	
DMR9:69976001	9	69976001	69977000	1000	1	7.00E-18	1.01	32	3.2	Gpr1	Signaling
DMR9:70138001	9	70138001	70139000	1000	1	9.30E-09	-0.7	5	0.5	Adam23	Protease
DMR9:70168001	9	70168001	70171000	3000	1	1.70E-07	-0.35	23	0.77	Adam23	Protease
DMR9:70256001	9	70256001	70258000	2000	1	9.10E-07	-0.38	34	1.7	Adam23	Protease
DMR9:70265001	9	70265001	70266000	1000	1	2.10E-09	0.43	10	1	Adam23	Protease
DMR9:70352001	9	70352001	70354000	2000	1	4.60E-09	0.65	44	2.2	Dytn	Proteolysis
DMR9:70391001	9	70391001	70396000	5000	2	1.70E-09	-0.39	46	0.92	Dytn;Mdh1b	Proteolysis;Metabolism
DMR9:70414001	9	70414001	70415000	1000	1	2.40E-09	-0.36	5	0.5	Mdh1b	Metabolism
DMR9:71265001	9	71265001	71266000	1000	1	5.60E-07	0.49	8	0.8	Creb1	
DMR9:71614001	9	71614001	71617000	3000	2	1.60E-08	-0.44	46	1.53	Plekhm3	
DMR9:71767001	9	71767001	71768000	1000	1	1.30E-12	1.05	29	2.9	Cryge;LOC102552040;Crygd	
DMR9:71788001	9	71788001	71790000	2000	1	5.00E-09	0.8	41	2.05	Crygd;Crygc;Crygb	
DMR9:71824001	9	71824001	71826000	2000	1	3.10E-08	-0.56	21	1.05	Cryga	
DMR9:71827001	9	71827001	71828000	1000	1	3.20E-19	1.28	41	4.1	Cryga	
DMR9:71883001	9	71883001	71886000	3000	1	1.40E-07	0.6	29	0.97	Idh1	Metabolism
DMR9:71984001	9	71984001	71990000	6000	1	2.40E-08	-0.39	67	1.12	Pikfyve	Signaling
DMR9:72051001	9	72051001	72053000	2000	1	5.00E-07	0.56	29	1.45	LOC108351946;Pth2r	Receptor
DMR9:72137001	9	72137001	72143000	6000	1	2.70E-14	0.29	67	1.12	Pth2r	Receptor
DMR9:73518001	9	73518001	73519000	1000	1	2.10E-08	-0.48	8	0.8	Unc80	
DMR9:73825001	9	73825001	73828000	3000	1	3.80E-08	0.58	31	1.03	Kansl1l;Acadl	Metabolism
DMR9:73836001	9	73836001	73837000	1000	1	6.20E-08	-0.47	20	2	Acadl	Metabolism
DMR9:73838001	9	73838001	73842000	4000	1	1.40E-08	-0.46	49	1.23	Acadl	Metabolism
DMR9:73846001	9	73846001	73847000	1000	1	5.60E-07	0.42	17	1.7	Acadl	Metabolism
DMR9:74011001	9	74011001	74013000	2000	1	3.70E-07	-0.38	24	1.2	Lancl1	
DMR9:75397001	9	75397001	75403000	6000	1	7.10E-08	-0.38	68	1.13	Erbb4	Receptor

DMR9:75433001	9	75433001	75436000	3000	1	2.60E-07	-0.3	29	0.97	Erbb4	Receptor
DMR9:75863001	9	75863001	75864000	1000	1	5.00E-07	-0.56	3	0.3	Erbb4	Receptor
DMR9:75973001	9	75973001	75975000	2000	1	5.10E-07	-0.32	16	0.8	Erbb4	Receptor
DMR9:76073001	9	76073001	76076000	3000	1	2.00E-17	1.13	57	1.9	Erbb4	Receptor
DMR9:76120001	9	76120001	76124000	4000	1	4.60E-11	0.65	54	1.35	Erbb4	Receptor
DMR9:76439001	9	76439001	76440000	1000	1	4.10E-12	0.8	23	2.3	RGD1562431	
DMR9:76441001	9	76441001	76443000	2000	1	1.10E-15	0.67	37	1.85	RGD1562431	
DMR9:77088001	9	77088001	77090000	2000	1	1.70E-07	-0.64	3	0.15	Spag16	Cytoskeleton
DMR9:77391001	9	77391001	77393000	2000	1	4.90E-12	-0.55	20	1	Spag16	Cytoskeleton
DMR9:77596001	9	77596001	77598000	2000	1	8.10E-08	-0.63	14	0.7	Spag16	Cytoskeleton
DMR9:77662001	9	77662001	77665000	3000	2	1.20E-08	0.54	25	0.83	Spag16	Cytoskeleton
DMR9:77679001	9	77679001	77685000	6000	1	1.00E-11	-0.4	61	1.02	Spag16	Cytoskeleton
DMR9:77773001	9	77773001	77774000	1000	1	2.60E-15	0.82	18	1.8	Spag16	Cytoskeleton
DMR9:77900001	9	77900001	77906000	6000	1	1.60E-10	0.48	59	0.98	Vwc2l	
DMR9:79566001	9	79566001	79574000	8000	1	2.20E-08	-0.31	102	1.27	Mreg;LOC108351947	
DMR9:79945001	9	79945001	79946000	1000	1	2.20E-08	-0.46	9	0.9	Smarcal1	Transcription
DMR9:80115001	9	80115001	80118000	3000	1	3.70E-07	0.52	61	2.03	LOC102548066;Igfbp2	Protease; Proteolysis
DMR9:80126001	9	80126001	80127000	1000	1	1.10E-08	-0.43	22	2.2	Igfbp2	Protease; Proteolysis
DMR9:80129001	9	80129001	80130000	1000	1	2.90E-08	-0.39	24	2.4	Igfbp2	Protease; Proteolysis
DMR9:80135001	9	80135001	80138000	3000	2	1.50E-07	0.56	52	1.73	Igfbp2	Protease; Proteolysis
DMR9:80148001	9	80148001	80151000	3000	1	1.70E-07	-0.42	60	2	Igfbp2;Igfbp5	Protease; Proteolysis
DMR9:80156001	9	80156001	80158000	2000	1	3.10E-14	0.78	32	1.6	Igfbp5	Protease; Proteolysis
DMR9:81208001	9	81208001	81209000	1000	1	2.80E-07	0.36	14	1.4	Tns1	Cytoskeleton
DMR9:81237001	9	81237001	81238000	1000	1	1.40E-07	0.44	16	1.6	Tns1	Cytoskeleton
DMR9:81293001	9	81293001	81299000	6000	1	2.60E-09	0.61	76	1.27	Tns1	Cytoskeleton
DMR9:81345001	9	81345001	81347000	2000	2	5.10E-09	0.61	30	1.5	Tns1;LOC102548628	Cytoskeleton
DMR9:81348001	9	81348001	81350000	2000	1	9.70E-08	0.42	26	1.3	Tns1;LOC102548628	Cytoskeleton
DMR9:81414001	9	81414001	81417000	3000	1	3.40E-07	0.41	32	1.07	Rufy4	
DMR9:81601001	9	81601001	81602000	1000	1	6.90E-08	0.3	17	1.7	Pnkd	
DMR9:81691001	9	81691001	81694000	3000	1	2.30E-07	0.46	25	0.83	Vil1	Cytoskeleton
DMR9:81903001	9	81903001	81907000	4000	1	9.00E-07	0.53	54	1.35	Stk36	Signaling
DMR9:81963001	9	81963001	81968000	5000	1	6.30E-08	-0.42	47	0.94	Ttll4;Cyp27a1	Cytoskeleton;Metabolism
DMR9:81990001	9	81990001	81993000	3000	1	1.00E-09	0.6	39	1.3	Cyp27a1;Prkag3	Metabolism;Signaling
DMR9:82044001	9	82044001	82045000	1000	1	4.80E-10	0.74	34	3.4	Wnt6;Wnt10a	Signaling
DMR9:82242001	9	82242001	82243000	1000	1	3.50E-10	0.55	13	1.3	Nhej1;LOC100361833	
DMR9:82259001	9	82259001	82260000	1000	1	1.20E-07	-0.4	12	1.2	Nhej1;LOC100361833	
DMR9:82483001	9	82483001	82490000	7000	3	4.70E-09	0.5	108	1.54	Resp18	
DMR9:82558001	9	82558001	82561000	3000	1	7.50E-10	0.54	70	2.33	Des;LOC102550477	
DMR9:82579001	9	82579001	82582000	3000	1	8.40E-08	0.48	69	2.3	LOC102550477;Speg	
DMR9:82606001	9	82606001	82609000	3000	1	1.20E-08	0.46	55	1.83	Speg	
DMR9:82623001	9	82623001	82628000	5000	2	4.00E-11	0.65	116	2.32	Speg;Gmppa	Transport
DMR9:82702001	9	82702001	82708000	6000	1	4.60E-08	-0.47	81	1.35	Obsl1;Inha	Growth Factors
DMR9:83188001	9	83188001	83189000	1000	1	1.50E-18	1.11	23	2.3	Epha4	Receptor
DMR9:83194001	9	83194001	83195000	1000	1	2.70E-10	0.51	7	0.7	Epha4	Receptor
DMR9:83209001	9	83209001	83210000	1000	1	9.80E-07	-0.41	17	1.7	Epha4	Receptor
DMR9:83232001	9	83232001	83236000	4000	1	5.80E-09	-0.42	55	1.38	Epha4	Receptor
DMR9:83242001	9	83242001	83247000	5000	1	2.40E-16	1.16	93	1.86	Epha4	Receptor
DMR9:84041001	9	84041001	84042000	1000	1	3.80E-07	0.38	6	0.6	Pax3	
DMR9:84048001	9	84048001	84052000	4000	3	2.30E-10	0.74	65	1.62	Pax3	
DMR9:84064001	9	84064001	84065000	1000	1	1.10E-07	0.5	19	1.9	Pax3	
DMR9:84107001	9	84107001	84109000	2000	1	6.60E-07	-0.49	61	3.05	Pax3	
DMR9:84273001	9	84273001	84275000	2000	1	4.70E-07	0.44	17	0.85	Sgpp2	Signaling
DMR9:84292001	9	84292001	84294000	2000	1	2.50E-07	0.41	27	1.35	Sgpp2	Signaling
DMR9:84571001	9	84571001	84573000	2000	1	5.50E-16	-0.53	32	1.6	Acsl3	Metabolism
DMR9:84693001	9	84693001	84694000	1000	1	3.90E-09	-0.54	18	1.8	LOC108351957;Kcne4	Transport
DMR9:85403001	9	85403001	85406000	3000	1	2.20E-09	-0.52	59	1.97	Ap1s3	Transport
DMR9:85410001	9	85410001	85414000	4000	1	5.90E-07	-0.52	58	1.45	Ap1s3	Transport
DMR9:85558001	9	85558001	85560000	2000	1	8.70E-07	0.34	19	0.95	LOC108352030;Serpine2	Protease; Proteolysis
DMR9:85564001	9	85564001	85567000	3000	1	2.90E-08	0.5	38	1.27	Serpine2	Protease; Proteolysis
DMR9:85598001	9	85598001	85599000	1000	1	2.60E-07	0.39	10	1	Serpine2	Protease; Proteolysis
DMR9:86257001	9	86257001	86262000	5000	1	2.00E-08	-0.41	55	1.1	RGD1563713	

DMR9:86378001	9	86378001	86381000	3000	1	4.20E-07	-0.49	26	0.87	Dock10	
DMR9:86516001	9	86516001	86518000	2000	1	4.00E-10	0.7	37	1.85	Dock10	
DMR9:87023001	9	87023001	87028000	5000	2	9.90E-23	1.37	74	1.48	NEWGENE_1305560	
DMR9:88139001	9	88139001	88143000	4000	1	6.20E-13	-0.85	33	0.82	Rhbdd1;LOC108351962	Protease
DMR9:88169001	9	88169001	88172000	3000	1	6.00E-11	0.49	35	1.17	Rhbdd1	Protease
DMR9:88207001	9	88207001	88208000	1000	1	7.70E-07	0.46	15	1.5	Rhbdd1	Protease
DMR9:88221001	9	88221001	88223000	2000	1	1.00E-09	0.67	24	1.2	Rhbdd1	Protease
DMR9:88225001	9	88225001	88227000	2000	1	1.50E-12	0.85	40	2	Rhbdd1	Protease
DMR9:88397001	9	88397001	88401000	4000	1	1.90E-13	-0.69	71	1.77	Col4a3;LOC102554007;LOC103693235	Extracellular Matrix
DMR9:88409001	9	88409001	88412000	3000	1	2.40E-07	-0.32	38	1.27	Col4a3;LOC103693235	Extracellular Matrix
DMR9:88488001	9	88488001	88489000	1000	1	2.50E-08	-0.67	7	0.7	Col4a3;Mff	Extracellular Matrix
DMR9:88600001	9	88600001	88601000	1000	1	5.20E-09	-0.51	17	1.7	Agfg1	
DMR9:88648001	9	88648001	88650000	2000	1	1.30E-12	0.45	2	0.1	Agfg1	
DMR9:88663001	9	88663001	88664000	1000	1	4.50E-08	-0.47	17	1.7	Agfg1	
DMR9:88999001	9	88999001	89001000	2000	1	1.90E-09	-0.53	12	0.6	Daw1	
DMR9:91656001	9	91656001	91662000	6000	1	3.20E-08	-0.25	72	1.2	Pid1	
DMR9:91665001	9	91665001	91669000	4000	1	1.10E-08	-0.51	58	1.45	Pid1	
DMR9:91968001	9	91968001	91972000	4000	1	7.20E-07	0.62	56	1.4	Dner	
DMR9:92005001	9	92005001	92007000	2000	1	1.30E-09	0.55	20	1	Dner;LOC102550479	
DMR9:92011001	9	92011001	92014000	3000	1	4.50E-10	0.63	28	0.93	Dner;LOC102550479	
DMR9:92022001	9	92022001	92026000	4000	1	3.00E-11	-0.65	40	1	Dner	
DMR9:92052001	9	92052001	92054000	2000	1	4.50E-09	-0.52	6	0.3	Dner	
DMR9:92120001	9	92120001	92122000	2000	1	3.50E-07	0.42	21	1.05	Dner	
DMR9:92223001	9	92223001	92224000	1000	1	1.20E-09	0.77	16	1.6	Dner	
DMR9:92289001	9	92289001	92290000	1000	1	4.10E-12	0.91	37	3.7	Dner	
DMR9:92372001	9	92372001	92373000	1000	1	5.40E-07	-0.37	8	0.8	Trip12	Proteolysis
DMR9:92475001	9	92475001	92476000	1000	1	4.00E-07	-0.41	11	1.1	Fbxo36	
DMR9:92685001	9	92685001	92687000	2000	1	2.20E-07	0.47	27	1.35	Sp100	
DMR9:92716001	9	92716001	92718000	2000	2	2.50E-10	0.55	172	8.6	Sp100	
DMR9:92776001	9	92776001	92778000	2000	1	2.40E-09	0.72	35	1.75	RGD1563917;LOC103690562	
DMR9:92830001	9	92830001	92832000	2000	1	1.10E-16	1.06	36	1.8	Cab39	
DMR9:92974001	9	92974001	92978000	4000	2	1.20E-08	-0.4	56	1.4	Gpr55	Signaling
DMR9:93036001	9	93036001	93037000	1000	1	1.00E-08	-0.44	21	2.1	Spata3	
DMR9:93047001	9	93047001	93049000	2000	1	1.90E-10	-0.47	19	0.95	Spata3	
DMR9:93181001	9	93181001	93183000	2000	1	6.20E-07	-0.34	29	1.45	Armc9	Cytoskeleton
DMR9:93286001	9	93286001	93287000	1000	1	4.50E-08	0.57	10	1	Armc9	Cytoskeleton
DMR9:93369001	9	93369001	93370000	1000	1	5.50E-07	-0.35	14	1.4	Ncl	
DMR9:93374001	9	93374001	93375000	1000	1	3.00E-08	-0.47	7	0.7	Ncl	
DMR9:93787001	9	93787001	93789000	2000	2	5.00E-07	-0.54	7	0.35	Dis3l2	Transcription
DMR9:93907001	9	93907001	93915000	8000	1	6.30E-08	0.46	103	1.29	Dis3l2	Transcription
DMR9:93967001	9	93967001	93973000	6000	1	1.90E-07	-0.36	68	1.13	Dis3l2	Transcription
DMR9:94002001	9	94002001	94004000	2000	1	5.10E-08	-0.41	17	0.85	Dis3l2;LOC100363496	Transcription
DMR9:94202001	9	94202001	94204000	2000	1	1.40E-12	0.86	33	1.65	Alpp;Alpi	Signaling
DMR9:94231001	9	94231001	94235000	4000	2	1.80E-26	1.1	74	1.85	Akp3;Ecel1	Signaling;Protease
DMR9:94520001	9	94520001	94522000	2000	1	4.00E-07	-0.39	27	1.35	Gigyf2	
DMR9:94707001	9	94707001	94709000	2000	1	6.00E-07	-0.38	32	1.6	Neu2	Metabolism
DMR9:94734001	9	94734001	94735000	1000	1	9.10E-18	1	23	2.3	Neu2	Metabolism
DMR9:94758001	9	94758001	94759000	1000	1	1.70E-07	-0.44	10	1	Inpp5d	
DMR9:94763001	9	94763001	94764000	1000	1	6.40E-08	-0.45	21	2.1	Inpp5d	
DMR9:94765001	9	94765001	94770000	5000	1	4.10E-11	0.86	103	2.06	Inpp5d	
DMR9:95247001	9	95247001	95248000	1000	1	1.10E-08	-0.45	8	0.8	Ugt1a9;Ugt1a8;Ugt1a7c;Ugt1a6;LOC108351970;Ugt1a5	Golgi
DMR9:95332001	9	95332001	95335000	3000	1	5.00E-08	-0.39	42	1.4	Mroh2a	
DMR9:95340001	9	95340001	95344000	4000	1	7.00E-09	0.68	70	1.75	Mroh2a;Hjurp	
DMR9:95415001	9	95415001	95417000	2000	1	7.10E-07	0.52	20	1	Trpm8	Transport
DMR9:95453001	9	95453001	95455000	2000	1	6.10E-07	0.49	45	2.25	Trpm8	Transport
DMR9:95510001	9	95510001	95512000	2000	1	1.40E-09	-0.43	21	1.05	Spp2	
DMR9:96915001	9	96915001	96916000	1000	1	7.70E-08	0.52	1	0.1	Agap1	
DMR9:97024001	9	97024001	97025000	1000	1	3.30E-09	-0.52	10	1	Agap1	
DMR9:97034001	9	97034001	97035000	1000	1	9.70E-09	-0.41	15	1.5	Agap1	
DMR9:97115001	9	97115001	97116000	1000	1	7.00E-07	-0.33	13	1.3	Asb18	Transport

DMR9:97126001	9	97126001	97128000	2000	1	1.40E-08	0.5	40	2	Asb18	Transport
DMR9:97159001	9	97159001	97160000	1000	1	1.00E-07	0.61	19	1.9	Asb18;LOC102554789	Transport
DMR9:97188001	9	97188001	97190000	2000	1	8.40E-10	-0.59	14	0.7	Iqca1	
DMR9:97191001	9	97191001	97194000	3000	1	9.10E-12	-0.66	50	1.67	Iqca1	
DMR9:97216001	9	97216001	97217000	1000	1	2.00E-08	-0.34	20	2	Iqca1	
DMR9:97288001	9	97288001	97289000	1000	1	3.50E-08	-0.59	12	1.2	Iqca1	
DMR9:97343001	9	97343001	97346000	3000	1	9.50E-07	-0.33	43	1.43	Ackr3	
DMR9:97991001	9	97991001	97992000	1000	1	2.90E-08	-0.53	6	0.6	Col6a3	
DMR9:98005001	9	98005001	98006000	1000	1	8.40E-08	-0.44	18	1.8	Col6a3	
DMR9:98076001	9	98076001	98077000	1000	1	2.10E-07	-0.5	26	2.6	Mlph	Cytoskeleton
DMR9:98183001	9	98183001	98184000	1000	1	4.70E-12	-0.46	9	0.9	Lrrfip1	Transcription
DMR9:98267001	9	98267001	98272000	5000	1	2.40E-10	0.75	96	1.92	Lrrfip1;Rbm44	Transcription
DMR9:98317001	9	98317001	98318000	1000	1	2.80E-07	0.36	22	2.2	Ramp1	Receptor
DMR9:98346001	9	98346001	98348000	2000	1	2.10E-08	-0.39	43	2.15	Ramp1	Receptor
DMR9:98481001	9	98481001	98482000	1000	1	2.50E-08	0.55	18	1.8	Espnl;Klhl30	Cytoskeleton;Cytoskeleton
DMR9:98483001	9	98483001	98484000	1000	1	4.10E-07	0.53	36	3.6	Espnl;Klhl30	Cytoskeleton;Cytoskeleton
DMR9:98944001	9	98944001	98945000	1000	1	1.20E-08	0.62	20	2	Twist2	Transcription
DMR9:98969001	9	98969001	98971000	2000	2	7.80E-08	0.39	24	1.2	Twist2;Rps27a-ps9	Transcription
DMR9:98972001	9	98972001	98973000	1000	1	2.20E-07	-0.42	11	1.1	Twist2;Rps27a-ps9	Transcription
DMR9:99060001	9	99060001	99061000	1000	1	4.80E-09	0.47	8	0.8	Hdac4	
DMR9:99288001	9	99288001	99291000	3000	1	3.30E-07	0.49	31	1.03	Hdac4	
DMR9:99590001	9	99590001	99592000	2000	1	1.10E-14	0.82	33	1.65	Ppp1r7	Signaling
DMR9:99700001	9	99700001	99703000	3000	1	5.90E-07	-0.44	22	0.73	Ppp1r7	Signaling
DMR9:99765001	9	99765001	99766000	1000	1	4.60E-07	-0.34	15	1.5	Ppp1r7;Olr1351	Signaling;Signaling
DMR9:99871001	9	99871001	99872000	1000	1	4.00E-08	-0.49	3	0.3	Ppp1r7	Signaling
DMR9:99897001	9	99897001	99898000	1000	1	6.40E-07	0.51	8	0.8	Ppp1r7;LOC108352010	Signaling
DMR9:99958001	9	99958001	99959000	1000	1	2.00E-10	0.59	17	1.7	Ppp1r7	Signaling
DMR9:99992001	9	99992001	99993000	1000	1	3.40E-08	0.48	14	1.4	Ppp1r7;Gpc1	Signaling
DMR9:100012001	9	100012001	100015000	3000	1	3.80E-08	0.65	78	2.6	Ppp1r7;Gpc1;Mir149	Signaling
DMR9:100069001	9	100069001	100071000	2000	1	1.10E-07	0.61	33	1.65	Ppp1r7;Ankmy1;Dusp28;Rnpepl1	Signaling
DMR9:100133001	9	100133001	100137000	4000	1	5.30E-10	0.56	66	1.65	Ppp1r7;Gpr35	Signaling;Signaling
DMR9:100156001	9	100156001	100157000	1000	1	8.80E-07	0.48	32	3.2	Ppp1r7;Aqp12a	Signaling
DMR9:100173001	9	100173001	100175000	2000	1	2.00E-07	0.47	32	1.6	Ppp1r7;Aqp12a;Kif1a	Signaling;Cytoskeleton
DMR9:100192001	9	100192001	100194000	2000	1	1.70E-07	0.47	32	1.6	Ppp1r7;Kif1a	Signaling;Cytoskeleton
DMR9:100195001	9	100195001	100199000	4000	1	1.00E-07	0.39	62	1.55	Ppp1r7;Kif1a	Signaling;Cytoskeleton
DMR9:100221001	9	100221001	100224000	3000	2	5.80E-08	-0.39	62	2.07	Ppp1r7;Kif1a	Signaling;Cytoskeleton
DMR9:100247001	9	100247001	100251000	4000	1	5.40E-07	0.52	59	1.48	Ppp1r7;Kif1a	Signaling;Cytoskeleton
DMR9:100281001	9	100281001	100291000	10000	2	1.70E-08	0.58	141	1.41	Ppp1r7;Agxt;RGD1563692	Signaling;Metabolism
DMR9:100326001	9	100326001	100331000	5000	2	1.30E-09	0.61	76	1.52	Ppp1r7;Crocc2	Signaling
DMR9:100373001	9	100373001	100375000	2000	2	9.30E-08	0.54	29	1.45	Ppp1r7;Crocc2;Sned1	Signaling
DMR9:100400001	9	100400001	100404000	4000	1	8.90E-07	0.48	79	1.98	Ppp1r7;Sned1;LOC108352012	Signaling
DMR9:100466001	9	100466001	100467000	1000	1	9.20E-09	0.49	11	1.1	Ppp1r7;Pask	Signaling;Signaling
DMR9:100558001	9	100558001	100560000	2000	1	2.90E-07	0.35	19	0.95	Ano7;Hdlbp	Metabolism
DMR9:100737001	9	100737001	100738000	1000	1	1.50E-12	-0.5	6	0.6	Farp2	
DMR9:100745001	9	100745001	100747000	2000	1	1.60E-11	-0.47	19	0.95	Farp2	
DMR9:100756001	9	100756001	100757000	1000	1	1.10E-08	0.62	13	1.3	Farp2	
DMR9:100785001	9	100785001	100786000	1000	1	7.70E-07	0.34	14	1.4	Stk25	
DMR9:100845001	9	100845001	100848000	3000	1	2.20E-11	0.5	71	2.37	Bok;Thap4	
DMR9:100935001	9	100935001	100937000	2000	1	8.00E-08	0.27	28	1.4	Dtymk;Ing5	Signaling;Epigenetic
DMR9:100990001	9	100990001	100992000	2000	1	1.30E-08	0.55	43	2.15	Gal3st2	Transport
DMR9:101268001	9	101268001	101269000	1000	1	1.50E-08	-0.69	4	0.4	Olr178	Receptor
DMR9:104394001	9	104394001	104395000	1000	1	2.40E-10	0.77	20	2	Slco6b1	Transport
DMR9:104445001	9	104445001	104446000	1000	1	8.60E-07	-0.57	1	0.1	Slco6b1	Transport
DMR9:104722001	9	104722001	104725000	3000	1	2.50E-10	0.3	37	1.23	Slco6d1;LOC102554041	Transport
DMR9:104852001	9	104852001	104853000	1000	1	9.50E-08	-0.57	9	0.9	Slco6d1	Transport
DMR9:105553001	9	105553001	105554000	1000	1	3.40E-09	-0.46	9	0.9	RGD1560925	
DMR9:105596001	9	105596001	105599000	3000	1	2.40E-08	0.74	61	2.03	RGD1560925	
DMR9:105611001	9	105611001	105612000	1000	1	2.40E-07	0.53	24	2.4	RGD1560925	
DMR9:105688001	9	105688001	105690000	2000	1	1.30E-12	0.53	42	2.1	Nudt12	Metabolism

DMR9:110059001	9	110059001	110062000	3000	1	1.70E-08	-0.62	35	1.17	Efna5	Signaling
DMR9:110174001	9	110174001	110175000	1000	1	4.80E-07	-0.34	42	4.2	Efna5	Signaling
DMR9:110200001	9	110200001	110202000	2000	1	5.00E-08	-0.54	23	1.15	Efna5	Signaling
DMR9:110225001	9	110225001	110227000	2000	1	3.70E-07	0.49	27	1.35	Efna5	Signaling
DMR9:110229001	9	110229001	110233000	4000	1	1.50E-10	-0.55	98	2.45	Efna5	Signaling
DMR9:110234001	9	110234001	110236000	2000	1	4.70E-11	-0.54	53	2.65	Efna5	Signaling
DMR9:110269001	9	110269001	110271000	2000	1	8.30E-07	-0.52	44	2.2	Efna5	Signaling
DMR9:110302001	9	110302001	110305000	3000	1	1.60E-08	-0.43	49	1.63	Efna5	Signaling
DMR9:110315001	9	110315001	110317000	2000	1	4.40E-08	0.5	23	1.15	Efna5	Signaling
DMR9:110889001	9	110889001	110892000	3000	1	7.50E-10	-0.53	54	1.8	Fbxl17	Metabolism
DMR9:110916001	9	110916001	110918000	2000	1	4.00E-20	-0.69	43	2.15	Fbxl17	Metabolism
DMR9:110925001	9	110925001	110930000	5000	2	3.60E-08	-0.44	89	1.78	Fbxl17	Metabolism
DMR9:111041001	9	111041001	111043000	2000	1	1.20E-09	-0.45	33	1.65	Pam	Metabolism
DMR9:111069001	9	111069001	111072000	3000	1	3.20E-07	-0.4	59	1.97	Pam	Metabolism
DMR9:111139001	9	111139001	111142000	3000	1	1.60E-07	-0.41	46	1.53	Pam	Metabolism
DMR9:111153001	9	111153001	111155000	2000	1	3.80E-07	-0.32	29	1.45	Pam	Metabolism
DMR9:111186001	9	111186001	111187000	1000	1	7.60E-08	0.65	20	2	Pam	Metabolism
DMR9:111322001	9	111322001	111323000	1000	1	4.30E-07	0.67	25	2.5	RGD1562136	
DMR9:111573001	9	111573001	111576000	3000	1	1.20E-07	-0.52	29	0.97	Fer	
DMR9:111625001	9	111625001	111628000	3000	2	5.70E-19	0.74	85	2.83	Fer	
DMR9:111699001	9	111699001	111700000	1000	1	7.40E-30	0.97	2	0.2	Fer	
DMR9:111854001	9	111854001	111855000	1000	1	1.70E-07	0.46	18	1.8	Fer	
DMR9:112037001	9	112037001	112039000	2000	1	5.10E-08	-0.39	16	0.8	Pja2	Proteolysis
DMR9:112300001	9	112300001	112307000	7000	2	1.80E-08	-0.41	127	1.81	Man2a1	
DMR9:112317001	9	112317001	112321000	4000	1	2.00E-07	-0.33	89	2.22	Man2a1	
DMR9:112323001	9	112323001	112326000	3000	2	1.30E-10	-0.48	78	2.6	Man2a1	
DMR9:112330001	9	112330001	112332000	2000	1	9.20E-08	0.31	55	2.75	Man2a1	
DMR9:112376001	9	112376001	112378000	2000	1	2.40E-08	-0.46	18	0.9	Man2a1	
DMR9:112386001	9	112386001	112390000	4000	2	7.30E-13	-0.55	48	1.2	Man2a1	
DMR9:112400001	9	112400001	112401000	1000	1	5.40E-08	-0.4	16	1.6	Man2a1	
DMR9:112406001	9	112406001	112407000	1000	1	2.20E-08	-0.43	34	3.4	Man2a1	
DMR9:112998001	9	112998001	112999000	1000	1	1.20E-07	-0.33	19	1.9	Tmem232	
DMR9:113028001	9	113028001	113031000	3000	1	2.40E-11	1	63	2.1	Tmem232	
DMR9:113038001	9	113038001	113041000	3000	2	1.70E-11	0.83	49	1.63	Tmem232	
DMR9:113056001	9	113056001	113060000	4000	1	3.70E-12	0.44	63	1.57	Tmem232	
DMR9:113123001	9	113123001	113124000	1000	1	9.90E-13	0.87	19	1.9	Tmem232	
DMR9:113126001	9	113126001	113129000	3000	1	1.10E-22	0.63	60	2	Tmem232;LOC102552718	
DMR9:113170001	9	113170001	113172000	2000	1	3.10E-07	0.43	44	2.2	Tmem232	
DMR9:113182001	9	113182001	113183000	1000	1	2.50E-12	0.82	21	2.1	Tmem232	
DMR9:113218001	9	113218001	113219000	1000	1	4.50E-09	0.59	13	1.3	Tmem232	
DMR9:113242001	9	113242001	113243000	1000	1	1.30E-07	0.61	35	3.5	Tmem232	
DMR9:113264001	9	113264001	113265000	1000	1	2.60E-07	-0.46	13	1.3	Tmem232	
DMR9:113352001	9	113352001	113355000	3000	1	3.20E-09	0.39	51	1.7	Txndc2	Metabolism
DMR9:113363001	9	113363001	113365000	2000	1	3.10E-16	-0.59	37	1.85	Txndc2;Rab31	Metabolism
DMR9:113417001	9	113417001	113418000	1000	1	1.70E-07	-0.34	27	2.7	Rab31	
DMR9:113662001	9	113662001	113665000	3000	3	3.40E-13	-0.49	59	1.97	Ankrd12	
DMR9:113695001	9	113695001	113698000	3000	1	2.20E-10	0.67	69	2.3	Twsg1	
DMR9:113725001	9	113725001	113729000	4000	2	3.10E-08	-0.5	67	1.68	Twsg1	
DMR9:113912001	9	113912001	113916000	4000	1	3.70E-07	-0.25	49	1.23	Wash1;LOC102553152	
DMR9:113918001	9	113918001	113920000	2000	1	3.00E-08	0.39	31	1.55	Wash1;LOC102553152	
DMR9:114115001	9	114115001	114120000	5000	1	2.60E-10	-0.37	83	1.66	LOC103693248;Ddx11	Epigenetic
DMR9:114493001	9	114493001	114494000	1000	1	1.50E-09	-0.45	37	3.7	Mtcl1	
DMR9:114544001	9	114544001	114546000	2000	2	1.70E-27	1.37	63	3.15	Mtcl1	
DMR9:114584001	9	114584001	114586000	2000	1	1.50E-07	-0.39	42	2.1	Mtcl1	
DMR9:114589001	9	114589001	114591000	2000	1	3.20E-07	0.44	46	2.3	Mtcl1	
DMR9:114605001	9	114605001	114608000	3000	1	6.40E-07	-0.38	79	2.63	Mtcl1	
DMR9:114707001	9	114707001	114708000	1000	1	2.30E-08	-0.39	21	2.1	Rab12;LOC108352037	
DMR9:114866001	9	114866001	114870000	4000	1	1.10E-08	-0.54	107	2.67	Ptprm	Signaling
DMR9:114874001	9	114874001	114879000	5000	1	1.00E-10	-0.53	96	1.92	Ptprm	Signaling
DMR9:114970001	9	114970001	114973000	3000	1	1.80E-09	-0.41	22	0.73	Ptprm	Signaling
DMR9:115287001	9	115287001	115288000	1000	1	5.20E-09	0.55	20	2	Ptprm	Signaling
DMR9:115311001	9	115311001	115315000	4000	1	4.90E-07	0.54	67	1.68	Ptprm	Signaling

DMR9:115319001	9	115319001	115322000	3000	1	1.40E-07	0.51	59	1.97	Ptprm	Signaling
DMR9:115336001	9	115336001	115339000	3000	1	7.70E-09	-0.43	57	1.9	Ptprm	Signaling
DMR9:115425001	9	115425001	115430000	5000	1	7.40E-09	-0.45	62	1.24	Ptprm	Signaling
DMR9:115524001	9	115524001	115527000	3000	1	1.50E-12	-0.61	52	1.73	Ptprm	Signaling
DMR9:115533001	9	115533001	115535000	2000	1	4.00E-07	0.28	26	1.3	Ptprm	Signaling
DMR9:115563001	9	115563001	115564000	1000	1	7.50E-07	0.58	14	1.4	Ptprm	Signaling
DMR9:115845001	9	115845001	115847000	2000	1	6.60E-08	0.74	47	2.35	Lrrc30	Cytoskeleton
DMR9:115857001	9	115857001	115863000	6000	4	7.20E-14	0.84	112	1.87	Lrrc30	Cytoskeleton
DMR9:115970001	9	115970001	115972000	2000	1	3.00E-09	0.43	35	1.75	Lama1	Extracellular Matrix
DMR9:116028001	9	116028001	116031000	3000	1	1.60E-09	0.57	55	1.83	Lama1	Extracellular Matrix
DMR9:116036001	9	116036001	116039000	3000	1	9.30E-09	0.55	51	1.7	Lama1	Extracellular Matrix
DMR9:116060001	9	116060001	116061000	1000	1	1.60E-10	0.68	21	2.1	Arhgap28	Signaling
DMR9:116062001	9	116062001	116065000	3000	2	2.10E-20	1.18	67	2.23	Arhgap28	Signaling
DMR9:116066001	9	116066001	116070000	4000	1	6.80E-08	0.37	47	1.18	Arhgap28	Signaling
DMR9:116077001	9	116077001	116080000	3000	1	3.10E-08	0.32	32	1.07	Arhgap28	Signaling
DMR9:116088001	9	116088001	116089000	1000	1	3.20E-09	0.49	22	2.2	Arhgap28	Signaling
DMR9:116136001	9	116136001	116143000	7000	1	8.20E-07	-0.38	122	1.74	Arhgap28	Signaling
DMR9:116557001	9	116557001	116559000	2000	1	5.20E-08	-0.53	17	0.85	L3mbtl4	
DMR9:117051001	9	117051001	117052000	1000	1	7.90E-07	0.46	7	0.7	Tmem200c	
DMR9:117320001	9	117320001	117322000	2000	1	1.70E-07	-0.37	41	2.05	Epb41l3	
DMR9:117338001	9	117338001	117340000	2000	1	8.70E-07	0.34	28	1.4	Epb41l3	
DMR9:117361001	9	117361001	117363000	2000	1	6.30E-07	0.52	46	2.3	Epb41l3	
DMR9:117385001	9	117385001	117387000	2000	1	7.10E-17	1.01	59	2.95	Epb41l3	
DMR9:117403001	9	117403001	117406000	3000	1	3.60E-08	-0.27	19	0.63	Epb41l3;LOC100361186;LOC689002	
DMR9:117409001	9	117409001	117410000	1000	1	9.90E-08	-0.35	9	0.9	Epb41l3;LOC100361186;LOC689002	
DMR9:117416001	9	117416001	117418000	2000	1	6.20E-10	-0.44	8	0.4	Epb41l3;LOC689002	
DMR9:117564001	9	117564001	117566000	2000	1	1.50E-16	0.95	36	1.8	Epb41l3	
DMR9:117602001	9	117602001	117607000	5000	1	2.90E-15	-0.69	87	1.74	Epb41l3	
DMR9:117671001	9	117671001	117674000	3000	1	3.00E-11	-0.44	54	1.8	Epb41l3	
DMR9:117732001	9	117732001	117734000	2000	1	2.90E-07	-0.33	38	1.9	LOC102552880;Zfp161	Transcription
DMR9:117741001	9	117741001	117744000	3000	2	7.10E-13	-0.51	101	3.37	LOC102552880;Zfp161	Transcription
DMR9:117750001	9	117750001	117752000	2000	1	9.40E-13	0.92	61	3.05	LOC102552880;Zfp161	Transcription
DMR9:117817001	9	117817001	117818000	1000	1	4.20E-11	0.93	32	3.2	Akain1;LOC108351986	
DMR9:117844001	9	117844001	117846000	2000	1	4.80E-10	-0.69	29	1.45	Akain1	
DMR9:118272001	9	118272001	118273000	1000	1	6.50E-07	-0.33	7	0.7	Dlgap1	Cytoskeleton
DMR9:118289001	9	118289001	118291000	2000	1	1.70E-09	0.58	36	1.8	Dlgap1	Cytoskeleton
DMR9:118471001	9	118471001	118472000	1000	1	6.60E-08	0.6	6	0.6	Dlgap1	Cytoskeleton
DMR9:118528001	9	118528001	118531000	3000	1	4.50E-22	0.71	25	0.83	Dlgap1	Cytoskeleton
DMR9:118749001	9	118749001	118750000	1000	1	9.90E-13	0.66	20	2	Dlgap1	Cytoskeleton
DMR9:118752001	9	118752001	118755000	3000	1	3.70E-08	-0.49	15	0.5	Dlgap1	Cytoskeleton
DMR9:118784001	9	118784001	118790000	6000	1	3.50E-08	-0.25	55	0.92	Dlgap1	Cytoskeleton
DMR9:118940001	9	118940001	118941000	1000	1	3.00E-07	0.52	1	0.1	Dlgap1	Cytoskeleton
DMR9:118979001	9	118979001	118982000	3000	1	7.30E-08	-0.33	33	1.1	Dlgap1	Cytoskeleton
DMR9:118986001	9	118986001	118987000	1000	1	6.70E-11	0.88	21	2.1	Dlgap1	Cytoskeleton
DMR9:119033001	9	119033001	119034000	1000	1	7.20E-09	0.65	22	2.2	Dlgap1	Cytoskeleton
DMR9:119063001	9	119063001	119066000	3000	1	1.00E-10	0.31	21	0.7	Dlgap1	Cytoskeleton
DMR9:119175001	9	119175001	119176000	1000	1	9.10E-10	-0.45	19	1.9	LOC108351987;Tgif1;LOC102555570	Development
DMR9:119181001	9	119181001	119182000	1000	1	5.90E-07	-0.48	15	1.5	Tgif1;LOC102555570	Development
DMR9:119297001	9	119297001	119298000	1000	1	4.90E-08	0.52	18	1.8	Myl12b	Cytoskeleton
DMR9:119300001	9	119300001	119302000	2000	1	1.70E-11	-0.74	22	1.1	Myl12b	Cytoskeleton
DMR9:119325001	9	119325001	119326000	1000	1	7.50E-08	-0.5	17	1.7	Myl12b;Myl12a;LOC102555687	Cytoskeleton
DMR9:119428001	9	119428001	119430000	2000	1	1.00E-08	0.76	45	2.25	Myom1	
DMR9:119601001	9	119601001	119602000	1000	1	6.60E-07	-0.55	9	0.9	Lpin2;Emilin2	
DMR9:119773001	9	119773001	119775000	2000	1	1.60E-08	-0.53	7	0.35	Smchd1	
DMR9:119798001	9	119798001	119803000	5000	1	9.90E-08	-0.33	44	0.88	Smchd1	
DMR9:120099001	9	120099001	120100000	1000	1	8.70E-08	0.59	19	1.9	Fam114a1l1	
DMR9:121455001	9	121455001	121457000	2000	1	1.00E-08	0.65	33	1.65	LOC103690596;Ppidl1	Transcription
DMR9:121851001	9	121851001	121852000	1000	1	7.80E-10	-0.37	4	0.4	Yes1	

DMR10:735001	10	735001	742000	7000	1	7.70E-07	-0.33	66	0.94	LOC103693254;Fopnl	
DMR10:835001	10	835001	837000	2000	1	3.50E-10	0.38	9	0.45	LOC103693254;Myh11	
DMR10:885001	10	885001	890000	5000	1	4.80E-08	-0.37	42	0.84	LOC103693254;Nde1	
DMR10:1221001	10	1221001	1222000	1000	1	3.10E-07	0.53	11	1.1	Mkl2	
DMR10:2077001	10	2077001	2078000	1000	1	2.50E-07	0.44	14	1.4	RGD1562055	
DMR10:2780001	10	2780001	2782000	2000	1	2.30E-08	0.6	32	1.6	Shisa9	
DMR10:2988001	10	2988001	2993000	5000	1	3.80E-08	-0.35	45	0.9	Shisa9	
DMR10:3025001	10	3025001	3029000	4000	1	7.50E-09	0.4	32	0.8	Shisa9	
DMR10:3249001	10	3249001	3252000	3000	1	3.30E-07	-0.39	28	0.93	Pdxdc1	
DMR10:3311001	10	3311001	3314000	3000	2	7.70E-10	-0.79	16	0.53	Pdxdc1;LOC100362572	
DMR10:3701001	10	3701001	3703000	2000	1	3.90E-11	-0.56	27	1.35	Cpped1	
DMR10:3756001	10	3756001	3761000	5000	1	1.90E-07	0.46	42	0.84	Cpped1	
DMR10:3770001	10	3770001	3774000	4000	1	4.70E-07	-0.33	68	1.7	Cpped1	
DMR10:3839001	10	3839001	3840000	1000	1	4.70E-09	0.63	19	1.9	Snx29	Cytoskeleton
DMR10:3876001	10	3876001	3878000	2000	1	6.40E-07	0.45	21	1.05	Snx29	Cytoskeleton
DMR10:4012001	10	4012001	4014000	2000	1	1.10E-10	-0.4	17	0.85	Snx29	Cytoskeleton
DMR10:4015001	10	4015001	4021000	6000	1	1.70E-08	0.52	64	1.07	Snx29;LOC108352047	Cytoskeleton
DMR10:4111001	10	4111001	4113000	2000	1	7.90E-07	-0.43	18	0.9	Snx29	Cytoskeleton
DMR10:4178001	10	4178001	4179000	1000	1	9.80E-08	0.42	15	1.5	Snx29	Cytoskeleton
DMR10:4526001	10	4526001	4529000	3000	1	5.10E-07	-0.28	41	1.37	Zc3h7a	Metabolism
DMR10:4747001	10	4747001	4749000	2000	1	5.20E-11	-0.54	44	2.2	LOC108352050;Litaf	Cytoskeleton
DMR10:5037001	10	5037001	5038000	1000	1	7.40E-09	-0.31	19	1.9	Clec16a	
DMR10:5087001	10	5087001	5090000	3000	1	1.40E-11	0.62	29	0.97	Clec16a	
DMR10:5234001	10	5234001	5241000	7000	1	1.90E-08	0.34	83	1.19	Ciita	
DMR10:5300001	10	5300001	5302000	2000	1	1.30E-08	0.58	45	2.25	Tvp23a	
DMR10:5734001	10	5734001	5735000	1000	1	4.40E-12	0.69	24	2.4	Grin2a	Receptor
DMR10:5745001	10	5745001	5746000	1000	1	1.90E-07	0.38	8	0.8	Grin2a	Receptor
DMR10:5782001	10	5782001	5783000	1000	1	3.00E-07	-0.51	2	0.2	Grin2a	Receptor
DMR10:5820001	10	5820001	5824000	4000	1	9.50E-11	0.72	57	1.43	Grin2a	Receptor
DMR10:5878001	10	5878001	5880000	2000	1	6.20E-09	0.69	29	1.45	Grin2a	Receptor
DMR10:5938001	10	5938001	5941000	3000	1	3.30E-07	-0.32	28	0.93	Grin2a	Receptor
DMR10:5968001	10	5968001	5969000	1000	1	8.70E-10	0.55	12	1.2	Grin2a	Receptor
DMR10:5979001	10	5979001	5980000	1000	1	2.70E-10	-0.6	5	0.5	Grin2a	Receptor
DMR10:6929001	10	6929001	6932000	3000	2	4.40E-17	1.06	59	1.97	Usp7	Protease
DMR10:6989001	10	6989001	6990000	1000	1	2.00E-07	-0.5	9	0.9	Usp7	Protease
DMR10:7116001	10	7116001	7117000	1000	1	8.40E-07	0.43	14	1.4	Abat	Metabolism
DMR10:7142001	10	7142001	7151000	9000	2	3.40E-08	0.54	132	1.47	Abat	Metabolism
DMR10:7208001	10	7208001	7209000	1000	1	7.40E-11	0.58	14	1.4	Abat	Metabolism
DMR10:7250001	10	7250001	7251000	1000	1	1.10E-07	0.43	20	2	Mettl22	Epigenetic
DMR10:8425001	10	8425001	8429000	4000	1	2.90E-15	0.96	92	2.3	Rbfox1	Translation
DMR10:8442001	10	8442001	8443000	1000	1	5.20E-07	0.43	17	1.7	Rbfox1	Translation
DMR10:8490001	10	8490001	8491000	1000	1	4.70E-07	0.44	18	1.8	Rbfox1	Translation
DMR10:8612001	10	8612001	8614000	2000	1	2.90E-08	-0.51	8	0.4	Rbfox1	Translation
DMR10:8636001	10	8636001	8638000	2000	1	2.00E-09	-0.38	20	1	Rbfox1	Translation
DMR10:8720001	10	8720001	8723000	3000	2	3.50E-09	-0.72	15	0.5	Rbfox1	Translation
DMR10:8780001	10	8780001	8782000	2000	1	3.60E-08	-0.39	15	0.75	Rbfox1	Translation
DMR10:9041001	10	9041001	9043000	2000	1	4.10E-08	-0.41	14	0.7	Rbfox1	Translation
DMR10:9053001	10	9053001	9057000	4000	1	1.80E-07	-0.6	29	0.72	Rbfox1	Translation
DMR10:9063001	10	9063001	9064000	1000	1	5.10E-07	-0.55	7	0.7	Rbfox1	Translation
DMR10:9106001	10	9106001	9109000	3000	1	1.10E-07	-0.58	13	0.43	Rbfox1	Translation
DMR10:9134001	10	9134001	9136000	2000	1	4.20E-07	-0.59	13	0.65	Rbfox1	Translation
DMR10:9198001	10	9198001	9199000	1000	1	1.40E-07	-0.55	6	0.6	Rbfox1	Translation
DMR10:9250001	10	9250001	9251000	1000	1	9.50E-07	-0.57	2	0.2	Rbfox1	Translation
DMR10:9296001	10	9296001	9297000	1000	1	3.40E-07	0.45	12	1.2	Rbfox1	Translation
DMR10:9565001	10	9565001	9567000	2000	2	2.20E-10	-0.74	7	0.35	Rbfox1	Translation
DMR10:9620001	10	9620001	9624000	4000	2	8.10E-15	-0.57	41	1.02	Rbfox1	Translation
DMR10:9780001	10	9780001	9782000	2000	1	2.00E-08	0.59	20	1	Rbfox1	Translation
DMR10:9814001	10	9814001	9817000	3000	1	4.90E-08	0.43	50	1.67	Rbfox1	Translation
DMR10:10027001	10	10027001	10029000	2000	1	8.80E-07	0.48	24	1.2	Rbfox1	Translation
DMR10:10145001	10	10145001	10146000	1000	1	6.80E-07	0.53	9	0.9	Rbfox1	Translation
DMR10:10171001	10	10171001	10172000	1000	1	2.80E-07	-0.4	29	2.9	Rbfox1	Translation
DMR10:10177001	10	10177001	10179000	2000	2	5.30E-13	0.37	23	1.15	Rbfox1	Translation

DMR10:10229001	10	10229001	10233000	4000	3	1.00E-13	-0.8	47	1.18	Rbfox1	Translation
DMR10:10359001	10	10359001	10365000	6000	1	2.20E-07	-0.3	59	0.98	Rbfox1	Translation
DMR10:10380001	10	10380001	10381000	1000	1	8.80E-07	0.39	14	1.4	Rbfox1	Translation
DMR10:10400001	10	10400001	10401000	1000	1	2.10E-07	-0.37	25	2.5	Rbfox1	Translation
DMR10:10410001	10	10410001	10412000	2000	1	4.80E-11	0.68	48	2.4	Rbfox1	Translation
DMR10:10423001	10	10423001	10424000	1000	1	6.00E-07	-0.35	28	2.8	Rbfox1	Translation
DMR10:10594001	10	10594001	10597000	3000	1	4.50E-07	0.55	36	1.2	Sec14l5	
DMR10:10607001	10	10607001	10610000	3000	1	2.40E-11	0.62	66	2.2	Sec14l5	
DMR10:10624001	10	10624001	10628000	4000	1	3.90E-08	-0.48	50	1.25	Sec14l5	
DMR10:10646001	10	10646001	10649000	3000	1	6.00E-07	0.43	36	1.2	Ppl	Cytoskeleton
DMR10:10653001	10	10653001	10655000	2000	1	8.70E-07	0.31	28	1.4	Ppl	Cytoskeleton
DMR10:10669001	10	10669001	10672000	3000	1	2.00E-08	0.47	53	1.77	Ppl	Cytoskeleton
DMR10:10778001	10	10778001	10780000	2000	1	2.00E-10	0.3	17	0.85	Smim22;Sept12	
DMR10:10783001	10	10783001	10786000	3000	1	3.30E-09	0.42	50	1.67	Sept12;LOC360479	
DMR10:10793001	10	10793001	10794000	1000	1	6.40E-07	0.44	12	1.2	Sept12;LOC360479	
DMR10:10875001	10	10875001	10876000	1000	1	7.90E-09	-0.44	20	2	Mgrn1;LOC679823;LOC363543	Proteolysis
DMR10:11009001	10	11009001	11010000	1000	1	3.10E-09	-0.42	14	1.4	Hmox2	Metabolism
DMR10:11070001	10	11070001	11072000	2000	1	3.50E-07	0.47	18	0.9	Dnaja3	Transcription
DMR10:11209001	10	11209001	11212000	3000	3	1.00E-09	0.71	65	2.17	Tfap4	Transcription
DMR10:11235001	10	11235001	11241000	6000	1	5.40E-13	0.91	100	1.67	Srl	Transport
DMR10:11244001	10	11244001	11246000	2000	2	2.10E-15	-0.57	33	1.65	Srl	Transport
DMR10:11264001	10	11264001	11267000	3000	1	2.00E-09	0.61	62	2.07	Srl	Transport
DMR10:11280001	10	11280001	11282000	2000	2	1.10E-11	0.8	51	2.55	Srl;LOC103693274	Transport
DMR10:11426001	10	11426001	11428000	2000	1	3.20E-16	-0.69	34	1.7	Adcy9	
DMR10:11518001	10	11518001	11519000	1000	1	4.80E-14	0.91	17	1.7	Adcy9;LOC102551062	
DMR10:11676001	10	11676001	11678000	2000	1	5.10E-08	-0.55	17	0.85	Crebbp	Epigenetic
DMR10:11762001	10	11762001	11763000	1000	1	6.20E-09	0.64	12	1.2	Trap1;Dnase1	Signaling
DMR10:11886001	10	11886001	11888000	2000	1	9.70E-09	0.42	29	1.45	Cluap1;RGD1561796;Naa60;LOC102551430;LOC103690212	Metabolism
DMR10:12053001	10	12053001	12056000	3000	2	2.10E-09	-0.49	56	1.87	Mefv	Proteolysis
DMR10:12212001	10	12212001	12213000	1000	1	4.60E-07	-0.69	3	0.3	Olr1365	Receptor
DMR10:12342001	10	12342001	12347000	5000	1	4.50E-09	-0.39	59	1.18	Olr1361	Receptor
DMR10:12540001	10	12540001	12541000	1000	1	4.30E-07	-0.45	8	0.8	Olr1371-ps	
DMR10:12695001	10	12695001	12700000	5000	1	1.10E-10	-0.44	17	0.34	Olr1377-ps	
DMR10:12828001	10	12828001	12833000	5000	1	9.80E-08	0.28	62	1.24	Olr1382;LOC102553477	Receptor
DMR10:12909001	10	12909001	12910000	1000	1	3.10E-16	1.16	57	5.7	Zfp13	Transcription
DMR10:12930001	10	12930001	12931000	1000	1	4.30E-07	0.46	23	2.3	Zscan10	
DMR10:12933001	10	12933001	12935000	2000	1	6.80E-09	0.62	52	2.6	Zscan10	
DMR10:12938001	10	12938001	12941000	3000	1	3.30E-12	0.93	92	3.07	Zscan10	
DMR10:12986001	10	12986001	12987000	1000	1	3.10E-17	0.85	19	1.9	Bicdl2;Thoc6;Hcfc1r1;Tnfrsf12a	Transport;Receptor
DMR10:13078001	10	13078001	13080000	2000	1	7.60E-11	0.84	63	3.15	Pkmyt1;Paqr4;Kremen2;RGD1561157;Flywch1	Signaling;Signaling;Receptor
DMR10:13143001	10	13143001	13149000	6000	2	1.80E-13	-0.48	64	1.07	Srrm2	
DMR10:13157001	10	13157001	13162000	5000	1	8.50E-10	-0.44	146	2.92	Srrm2;Tceb2	
DMR10:13171001	10	13171001	13175000	4000	1	6.60E-16	0.88	67	1.68	Srrm2;Tceb2;Prss33;Prss41	Protease
DMR10:13186001	10	13186001	13187000	1000	1	8.80E-07	0.46	31	3.1	Prss33;Prss41;Prss32	Protease
DMR10:13214001	10	13214001	13215000	1000	1	1.70E-08	0.64	30	3	Prss21;LOC108352060	Protease
DMR10:13221001	10	13221001	13223000	2000	1	2.20E-07	0.36	34	1.7	Prss21;LOC108352060;Csap1	Protease
DMR10:13346001	10	13346001	13348000	2000	1	7.30E-08	0.32	28	1.4	Prss30	Protease
DMR10:13352001	10	13352001	13354000	2000	2	6.20E-14	-0.51	32	1.6	Prss30	Protease
DMR10:13366001	10	13366001	13367000	1000	1	5.40E-16	1.05	26	2.6	Prss22	Protease
DMR10:13374001	10	13374001	13375000	1000	1	4.10E-08	-0.39	14	1.4	Prss22	Protease
DMR10:13378001	10	13378001	13381000	3000	1	3.90E-09	-0.43	65	2.17	Prss22	Protease
DMR10:13398001	10	13398001	13402000	4000	1	2.40E-10	-0.45	49	1.23	Prss27	Protease
DMR10:13436001	10	13436001	13437000	1000	1	5.40E-11	-0.66	7	0.7	Kctd5;Pdpk1	Signaling
DMR10:13627001	10	13627001	13628000	1000	1	1.90E-09	0.7	24	2.4	Ccnf;Abca17	Signaling;Transport
DMR10:13636001	10	13636001	13638000	2000	1	1.30E-10	-0.48	23	1.15	Abca17	Transport
DMR10:13717001	10	13717001	13718000	1000	1	1.50E-09	-0.39	17	1.7	Abca17;Abca3	Transport
DMR10:13879001	10	13879001	13880000	1000	1	1.10E-10	0.64	22	2.2	Caskin1;Traf7	Cytoskeleton
DMR10:13901001	10	13901001	13902000	1000	1	2.60E-11	0.56	17	1.7	Traf7;LOC102551976;Rab26	
DMR10:13965001	10	13965001	13966000	1000	1	5.40E-07	0.54	15	1.5	Pkd1;Tsc2	Signaling

DMR10:13976001	10	13976001	13977000	1000	1	3.00E-12	0.74	10	1	Tsc2	Signaling
DMR10:14011001	10	14011001	14013000	2000	1	8.00E-09	0.51	38	1.9	Nthl1;Slc9a3r2;Npw	Epigenetic
DMR10:14166001	10	14166001	14170000	4000	1	2.50E-09	-0.34	73	1.82	Meiob	
DMR10:14244001	10	14244001	14246000	2000	1	9.50E-10	0.45	30	1.5	Igfals;Nubp2;Spsb3;Eme2	Receptor
DMR10:14386001	10	14386001	14388000	2000	2	4.90E-08	-0.32	86	4.3	Ift140	Development
DMR10:14485001	10	14485001	14487000	2000	1	5.50E-08	0.44	41	2.05	Telo2;Ptx4;Clcn7	Transport
DMR10:14568001	10	14568001	14569000	1000	1	6.10E-07	0.42	10	1	Unkl	
DMR10:14603001	10	14603001	14605000	2000	1	1.40E-17	1.07	66	3.3	Unkl;Gnptg;Tsr3;Baiap3;Mir3547	Signaling
DMR10:14651001	10	14651001	14653000	2000	1	3.40E-09	0.68	21	1.05	Prss34	Protease
DMR10:14687001	10	14687001	14688000	1000	1	2.60E-10	0.61	7	0.7	Prss29;RGD1559662	Protease
DMR10:14719001	10	14719001	14721000	2000	1	3.50E-08	-0.56	23	1.15	Tpsb2;Tpsg1;Cacna1h	Protease;Transport
DMR10:14759001	10	14759001	14761000	2000	1	7.20E-12	0.65	29	1.45	Cacna1h	Transport
DMR10:14767001	10	14767001	14770000	3000	1	4.70E-07	0.48	33	1.1	Cacna1h	Transport
DMR10:14773001	10	14773001	14779000	6000	1	6.10E-07	0.43	123	2.05	Cacna1h	Transport
DMR10:14782001	10	14782001	14784000	2000	1	6.40E-08	0.49	18	0.9	Cacna1h	Transport
DMR10:14844001	10	14844001	14847000	3000	1	3.90E-09	0.41	46	1.53	Sstr5	Signaling
DMR10:14856001	10	14856001	14858000	2000	1	4.70E-08	0.54	26	1.3	Sstr5	Signaling
DMR10:14934001	10	14934001	14935000	1000	1	3.60E-07	0.4	36	3.6	Sox8;LOC102548300	
DMR10:14974001	10	14974001	14979000	5000	1	6.80E-08	0.61	81	1.62	Lmf1	
DMR10:15026001	10	15026001	15028000	2000	1	3.10E-09	0.56	34	1.7	Lmf1	
DMR10:15076001	10	15076001	15079000	3000	1	7.40E-08	-0.35	42	1.4	LOC108352063;Gng13	Signaling
DMR10:15097001	10	15097001	15098000	1000	1	6.90E-07	0.45	13	1.3	LOC108352063;Gng13;Chtf18;Rpusd1;Mslnl	Signaling;Transcription;Translation;Cytoskeleton
DMR10:15127001	10	15127001	15129000	2000	1	7.60E-11	0.55	28	1.4	Mslnl;Msln	Cytoskeleton
DMR10:15277001	10	15277001	15280000	3000	1	3.90E-07	-0.43	43	1.43	Rab40c;Pigq	Golgi
DMR10:15309001	10	15309001	15311000	2000	1	1.10E-10	0.74	37	1.85	Pigq;Nhlrc4;Prr35;LOC108352065;Capn15	Golgi;Protease
DMR10:15321001	10	15321001	15324000	3000	1	2.10E-10	0.49	68	2.27	Prr35;LOC108352065;Capn15	Protease
DMR10:15359001	10	15359001	15361000	2000	1	1.50E-09	-0.41	38	1.9	Rab11fip3	
DMR10:15474001	10	15474001	15476000	2000	1	5.90E-15	-0.98	8	0.4	Nme4;LOC103693300;Tmem8a	Cytoskeleton
DMR10:15725001	10	15725001	15726000	1000	1	4.20E-10	0.93	29	2.9	RGD1560789	
DMR10:15868001	10	15868001	15870000	2000	1	2.80E-13	1.01	56	2.8	Nsg2	
DMR10:15876001	10	15876001	15877000	1000	1	2.50E-07	-0.38	24	2.4	Nsg2	
DMR10:15960001	10	15960001	15962000	2000	1	5.40E-20	1.08	64	3.2	RGD1311343	
DMR10:15988001	10	15988001	15991000	3000	1	4.90E-08	-0.49	46	1.53	Cpeb4	Translation
DMR10:15995001	10	15995001	15999000	4000	1	1.30E-08	-0.4	53	1.32	Cpeb4	Translation
DMR10:16008001	10	16008001	16009000	1000	1	1.90E-14	-0.69	8	0.8	Cpeb4	Translation
DMR10:16553001	10	16553001	16556000	3000	1	2.30E-11	0.63	74	2.47	Stc2	Hormone
DMR10:16905001	10	16905001	16906000	1000	1	2.40E-07	-0.41	20	2	Ergic1	
DMR10:16972001	10	16972001	16973000	1000	1	5.50E-08	0.45	24	2.4	LOC102552476;Dusp1	Signaling
DMR10:17048001	10	17048001	17050000	2000	2	1.20E-09	0.66	52	2.6	Neurl1b	Proteolysis
DMR10:17198001	10	17198001	17201000	3000	2	1.20E-08	-0.54	39	1.3	LOC688443;Sh3pxd2b	
DMR10:17438001	10	17438001	17443000	5000	1	1.60E-07	0.51	100	2	Stk10	
DMR10:17461001	10	17461001	17464000	3000	2	2.00E-09	0.57	25	0.83	Stk10	
DMR10:17477001	10	17477001	17478000	1000	1	2.60E-09	-0.53	23	2.3	Stk10	
DMR10:17483001	10	17483001	17485000	2000	1	1.40E-07	0.46	36	1.8	Stk10	
DMR10:17491001	10	17491001	17494000	3000	1	3.90E-07	0.6	97	3.23	Stk10	
DMR10:17556001	10	17556001	17557000	1000	1	8.60E-07	-0.37	15	1.5	Fbxw11	
DMR10:18046001	10	18046001	18047000	1000	1	8.20E-10	0.88	26	2.6	Fgf18	Growth Factors
DMR10:18100001	10	18100001	18101000	1000	1	6.00E-11	0.47	28	2.8	Npm1;LOC102546482	Transcription
DMR10:18153001	10	18153001	18154000	1000	1	1.90E-11	0.58	16	1.6	LOC108352199;Ranbp17	Transport
DMR10:18157001	10	18157001	18158000	1000	1	7.30E-08	0.45	10	1	Ranbp17	Transport
DMR10:18242001	10	18242001	18243000	1000	1	6.10E-07	-0.35	7	0.7	Ranbp17	Transport
DMR10:18333001	10	18333001	18336000	3000	1	6.80E-14	-0.59	38	1.27	Ranbp17	Transport
DMR10:18354001	10	18354001	18359000	5000	1	3.00E-17	-0.85	48	0.96	Ranbp17	Transport
DMR10:18416001	10	18416001	18417000	1000	1	6.60E-07	0.32	32	3.2	Ranbp17	Transport
DMR10:18444001	10	18444001	18445000	1000	1	6.10E-09	0.7	31	3.1	Ranbp17	Transport
DMR10:18455001	10	18455001	18457000	2000	1	9.20E-09	-0.38	41	2.05	Ranbp17	Transport
DMR10:18478001	10	18478001	18483000	5000	2	7.70E-11	-0.36	47	0.94	Gabrp	Ion Channel

DMR10:18643001	10	18643001	18645000	2000	2	1.90E-16	0.69	61	3.05	Kcnip1;LOC102547076	
DMR10:18670001	10	18670001	18671000	1000	1	1.10E-07	0.7	13	1.3	Kcnip1	
DMR10:18685001	10	18685001	18687000	2000	1	3.70E-21	1.38	49	2.45	Kcnip1	
DMR10:18747001	10	18747001	18750000	3000	1	9.80E-07	-0.4	48	1.6	Kcnip1	
DMR10:18793001	10	18793001	18798000	5000	1	6.00E-07	0.5	79	1.58	Kcnip1	
DMR10:18799001	10	18799001	18803000	4000	1	7.30E-07	0.62	76	1.9	Kcnip1	
DMR10:18834001	10	18834001	18836000	2000	1	5.20E-08	0.5	35	1.75	Kcnip1	
DMR10:18854001	10	18854001	18855000	1000	1	1.90E-07	0.36	12	1.2	Kcnip1	
DMR10:18868001	10	18868001	18871000	3000	1	6.80E-07	-0.4	39	1.3	Kcnip1	
DMR10:18889001	10	18889001	18892000	3000	1	1.90E-09	0.72	50	1.67	Kcnip1	
DMR10:19026001	10	19026001	19029000	3000	1	6.90E-09	-0.44	43	1.43	Lcp2	Cytoskeleton
DMR10:19151001	10	19151001	19154000	3000	1	4.80E-11	0.81	52	1.73	Foxi1	Transcription
DMR10:19271001	10	19271001	19273000	2000	1	4.20E-08	-0.43	44	2.2	Dock2;Fam196b	
DMR10:19278001	10	19278001	19281000	3000	1	3.40E-07	-0.31	49	1.63	Dock2;Fam196b	
DMR10:19317001	10	19317001	19318000	1000	1	2.30E-08	-0.41	13	1.3	Dock2;Fam196b	
DMR10:19367001	10	19367001	19369000	2000	1	2.80E-10	0.42	50	2.5	Dock2;Fam196b	
DMR10:19393001	10	19393001	19394000	1000	1	4.10E-10	-0.73	6	0.6	Dock2;Fam196b	
DMR10:19411001	10	19411001	19412000	1000	1	5.60E-08	0.38	11	1.1	Dock2	
DMR10:19470001	10	19470001	19471000	1000	1	1.20E-09	-0.49	11	1.1	Dock2	
DMR10:19526001	10	19526001	19528000	2000	1	4.50E-09	0.4	30	1.5	Dock2	
DMR10:19914001	10	19914001	19916000	2000	1	1.30E-07	-0.33	33	1.65	LOC108352201;Slit3	
DMR10:19948001	10	19948001	19952000	4000	1	3.50E-16	-0.66	63	1.57	Slit3	
DMR10:19958001	10	19958001	19960000	2000	1	3.90E-07	0.49	34	1.7	Slit3	
DMR10:19974001	10	19974001	19977000	3000	1	1.40E-07	-0.37	51	1.7	Slit3	
DMR10:20018001	10	20018001	20020000	2000	1	1.20E-07	-0.32	27	1.35	Slit3	
DMR10:20257001	10	20257001	20262000	5000	1	5.80E-08	-0.38	98	1.96	Slit3	
DMR10:20284001	10	20284001	20291000	7000	1	9.30E-07	-0.35	150	2.14	Slit3	
DMR10:20350001	10	20350001	20351000	1000	1	1.80E-09	-0.55	27	2.7	Slit3	
DMR10:20455001	10	20455001	20457000	2000	1	1.10E-07	-0.33	38	1.9	Slit3	
DMR10:20480001	10	20480001	20481000	1000	1	3.20E-07	0.4	12	1.2	Slit3	
DMR10:20516001	10	20516001	20517000	1000	1	8.80E-07	0.41	26	2.6	Slit3	
DMR10:20518001	10	20518001	20520000	2000	1	4.40E-07	0.53	45	2.25	Slit3	
DMR10:20582001	10	20582001	20585000	3000	1	6.10E-08	-0.43	33	1.1	Pank3	Signaling
DMR10:20664001	10	20664001	20667000	3000	1	5.10E-19	0.85	75	2.5	Rars;Wwc1	
DMR10:20716001	10	20716001	20717000	1000	1	1.00E-07	-0.44	28	2.8	Wwc1	
DMR10:20750001	10	20750001	20759000	9000	2	2.30E-09	-0.44	196	2.18	Wwc1	
DMR10:20847001	10	20847001	20848000	1000	1	6.60E-08	0.48	18	1.8	Tenm2	
DMR10:20875001	10	20875001	20876000	1000	1	2.70E-08	0.5	33	3.3	Tenm2	
DMR10:20903001	10	20903001	20906000	3000	1	4.20E-11	-0.47	72	2.4	Tenm2	
DMR10:20978001	10	20978001	20983000	5000	1	1.30E-07	-0.37	81	1.62	Tenm2;LOC108352202	
DMR10:20985001	10	20985001	20988000	3000	1	4.90E-08	0.43	63	2.1	Tenm2;LOC108352202	
DMR10:21061001	10	21061001	21062000	1000	1	2.50E-09	-0.5	11	1.1	Tenm2	
DMR10:21118001	10	21118001	21120000	2000	1	8.20E-08	-0.55	18	0.9	Tenm2	
DMR10:21125001	10	21125001	21127000	2000	1	7.20E-08	-0.41	25	1.25	Tenm2	
DMR10:21135001	10	21135001	21140000	5000	1	8.80E-07	-0.35	75	1.5	Tenm2	
DMR10:21143001	10	21143001	21146000	3000	1	7.10E-07	0.45	43	1.43	Tenm2	
DMR10:21154001	10	21154001	21156000	2000	1	6.80E-10	-0.46	51	2.55	Tenm2	
DMR10:21165001	10	21165001	21167000	2000	1	7.00E-07	-0.38	40	2	Tenm2	
DMR10:21244001	10	21244001	21245000	1000	1	1.50E-12	0.81	33	3.3	Tenm2	
DMR10:21363001	10	21363001	21366000	3000	1	1.30E-14	0.92	56	1.87	Tenm2	
DMR10:21404001	10	21404001	21405000	1000	1	3.60E-14	-0.65	10	1	Tenm2	
DMR10:21420001	10	21420001	21424000	4000	1	2.70E-09	0.54	68	1.7	Tenm2	
DMR10:21492001	10	21492001	21493000	1000	1	9.00E-10	0.7	20	2	Tenm2	
DMR10:21594001	10	21594001	21596000	2000	1	5.80E-08	0.67	43	2.15	Tenm2	
DMR10:21637001	10	21637001	21638000	1000	1	4.10E-08	0.6	18	1.8	Tenm2	
DMR10:21676001	10	21676001	21681000	5000	1	9.60E-08	-0.4	90	1.8	Tenm2	
DMR10:21726001	10	21726001	21727000	1000	1	3.30E-09	0.64	35	3.5	Tenm2	
DMR10:21734001	10	21734001	21736000	2000	1	4.70E-08	-0.37	33	1.65	Tenm2	
DMR10:21789001	10	21789001	21790000	1000	1	2.00E-17	1.3	25	2.5	Tenm2	
DMR10:21796001	10	21796001	21799000	3000	1	2.70E-13	0.32	31	1.03	Tenm2	
DMR10:23684001	10	23684001	23686000	2000	1	6.70E-07	-0.29	34	1.7	Ebf1	Transcription
DMR10:23695001	10	23695001	23696000	1000	1	4.60E-07	-0.46	8	0.8	Ebf1	Transcription

DMR10:23780001	10	23780001	23782000	2000	1	7.90E-07	0.46	31	1.55	Ebf1	Transcription
DMR10:23812001	10	23812001	23814000	2000	1	3.50E-07	-0.37	40	2	Ebf1	Transcription
DMR10:23815001	10	23815001	23817000	2000	1	3.80E-10	-0.65	34	1.7	Ebf1	Transcription
DMR10:23869001	10	23869001	23873000	4000	1	2.20E-07	-0.3	69	1.73	Ebf1	Transcription
DMR10:23912001	10	23912001	23914000	2000	1	8.00E-10	0.56	31	1.55	Ebf1	Transcription
DMR10:23929001	10	23929001	23932000	3000	1	3.50E-09	0.38	37	1.23	Ebf1	Transcription
DMR10:24042001	10	24042001	24043000	1000	1	6.90E-09	0.67	9	0.9	Ebf1	Transcription
DMR10:24059001	10	24059001	24060000	1000	1	3.40E-07	-0.38	9	0.9	Ebf1	Transcription
DMR10:28096001	10	28096001	28098000	2000	1	9.90E-07	-0.48	15	0.75	Gabrb2	Ion Channel
DMR10:28135001	10	28135001	28136000	1000	1	3.80E-08	0.64	13	1.3	Gabrb2	Ion Channel
DMR10:28573001	10	28573001	28578000	5000	1	4.20E-08	-0.58	56	1.12	Atp10b	Transport
DMR10:28614001	10	28614001	28615000	1000	1	1.10E-25	1.51	16	1.6	Atp10b	Transport
DMR10:28688001	10	28688001	28691000	3000	2	1.10E-07	-0.6	34	1.13	Atp10b	Transport
DMR10:28861001	10	28861001	28862000	1000	1	9.80E-07	0.5	12	1.2	Atp10b	Transport
DMR10:29030001	10	29030001	29034000	4000	1	2.70E-07	-0.38	54	1.35	Pttg1;Slu7	Translation
DMR10:29077001	10	29077001	29080000	3000	1	3.90E-08	0.43	41	1.37	C1qtnf2	
DMR10:29127001	10	29127001	29132000	5000	1	7.20E-07	0.37	61	1.22	Ccnjl	Signaling
DMR10:29333001	10	29333001	29334000	1000	1	3.50E-10	-0.49	13	1.3	Pwwp2a;Ttc1	Epigenetic
DMR10:30027001	10	30027001	30030000	3000	1	4.90E-07	-0.4	49	1.63	Il12b	Receptor
DMR10:30056001	10	30056001	30062000	6000	1	1.70E-10	-0.47	105	1.75	Il12b	Receptor
DMR10:30193001	10	30193001	30195000	2000	1	3.20E-08	0.32	0	0	Rnf145	
DMR10:30847001	10	30847001	30849000	2000	1	2.80E-11	0.72	34	1.7	LOC102550742;Clint1	
DMR10:30883001	10	30883001	30886000	3000	2	3.30E-08	-0.38	59	1.97	LOC102550742;Clint1	
DMR10:30897001	10	30897001	30900000	3000	1	1.20E-09	-0.5	46	1.53	LOC102550742;Clint1	
DMR10:30913001	10	30913001	30915000	2000	1	5.30E-11	-0.62	40	2	Clint1	
DMR10:31021001	10	31021001	31025000	4000	1	2.30E-07	0.6	80	2	Lsm11	Translation
DMR10:31064001	10	31064001	31068000	4000	1	9.30E-12	-0.77	32	0.8	Sox30	Transcription
DMR10:31071001	10	31071001	31072000	1000	1	1.50E-07	-0.59	2	0.2	Sox30	Transcription
DMR10:31159001	10	31159001	31162000	3000	1	8.90E-08	0.58	50	1.67	Adam19	Protease
DMR10:31259001	10	31259001	31262000	3000	1	9.10E-07	-0.54	41	1.37	Nipal4	
DMR10:31360001	10	31360001	31361000	1000	1	2.00E-07	0.48	16	1.6	Cyfip2	Cytoskeleton
DMR10:31478001	10	31478001	31479000	1000	1	3.10E-11	-0.57	7	0.7	Itk	
DMR10:31496001	10	31496001	31499000	3000	1	1.90E-09	-0.47	40	1.33	Itk;Fam71b	
DMR10:31523001	10	31523001	31526000	3000	1	1.40E-09	-0.4	52	1.73	Med7	Immune
DMR10:31749001	10	31749001	31751000	2000	1	1.10E-08	-0.35	13	0.65	Timd2	
DMR10:31804001	10	31804001	31806000	2000	1	3.20E-07	-0.42	9	0.45	Timd2;Havcr1	
DMR10:31898001	10	31898001	31899000	1000	1	4.40E-07	-0.43	4	0.4	Timd4	
DMR10:32145001	10	32145001	32147000	2000	1	8.90E-07	0.46	25	1.25	Sgcd	Cytoskeleton
DMR10:32156001	10	32156001	32157000	1000	1	1.90E-07	0.35	8	0.8	Sgcd	Cytoskeleton
DMR10:32178001	10	32178001	32181000	3000	1	1.00E-08	0.51	19	0.63	Sgcd	Cytoskeleton
DMR10:32234001	10	32234001	32235000	1000	1	2.20E-09	0.58	11	1.1	Sgcd	Cytoskeleton
DMR10:32244001	10	32244001	32246000	2000	1	2.20E-07	0.48	18	0.9	Sgcd	Cytoskeleton
DMR10:34185001	10	34185001	34186000	1000	1	7.50E-08	0.42	36	3.6	Trnak-cuu;Trnaa-ugc;Trim7	Proteolysis
DMR10:34191001	10	34191001	34192000	1000	1	7.70E-07	-0.5	21	2.1	Trnak-cuu;Trnaa-ugc;Trim7	Proteolysis
DMR10:34193001	10	34193001	34194000	1000	1	9.70E-07	-0.36	11	1.1	Trim7;Trnat-ugu	Proteolysis
DMR10:34254001	10	34254001	34255000	1000	1	6.20E-07	-0.36	8	0.8	RGD1559575	
DMR10:34372001	10	34372001	34377000	5000	1	1.40E-07	0.17	42	0.84	LOC690222;Olr1386	Receptor
DMR10:34393001	10	34393001	34394000	1000	1	1.40E-08	-0.79	12	1.2	Olr1386;LOC684471;Tpcr12	Receptor
DMR10:34442001	10	34442001	34443000	1000	1	3.20E-10	0.81	26	2.6	Btnl9	Immune
DMR10:34582001	10	34582001	34583000	1000	1	8.10E-08	-0.35	8	0.8	Olr1389;Olr1390-ps	Receptor
DMR10:34774001	10	34774001	34775000	1000	1	5.10E-07	-0.41	2	0.2	Olr1400	Receptor
DMR10:34808001	10	34808001	34814000	6000	3	2.70E-07	0.39	84	1.4	Olr1401	Receptor
DMR10:35013001	10	35013001	35014000	1000	1	6.30E-10	-0.54	26	2.6	N4bp3	
DMR10:35030001	10	35030001	35032000	2000	1	3.40E-11	0.45	24	1.2	Trappc2b	
DMR10:35106001	10	35106001	35109000	3000	1	4.10E-09	0.58	49	1.63	Flt4	Receptor
DMR10:35132001	10	35132001	35135000	3000	1	3.00E-11	0.92	53	1.77	Scgb3a1;Cnot6	Translation
DMR10:35277001	10	35277001	35279000	2000	1	2.50E-08	0.61	38	1.9	Gfpt2	Metabolism
DMR10:35397001	10	35397001	35398000	1000	1	3.30E-08	0.5	18	1.8	Rasgef1c	Transcription
DMR10:35457001	10	35457001	35462000	5000	1	2.80E-07	0.33	70	1.4	Rasgef1c	Transcription
DMR10:35538001	10	35538001	35539000	1000	1	4.20E-07	0.47	16	1.6	Rnf130	
DMR10:35667001	10	35667001	35668000	1000	1	1.30E-07	0.51	18	1.8	Tbc1d9b	Signaling
DMR10:35733001	10	35733001	35735000	2000	2	1.30E-07	0.46	49	2.45	Mgat4b;Ltc4s	Transport

DMR10:35855001	10	35855001	35856000	1000	1	4.50E-08	-0.39	20	2	Cby3	
DMR10:35916001	10	35916001	35918000	2000	2	8.00E-08	-0.52	67	3.35	Rufy1	
DMR10:36105001	10	36105001	36106000	1000	1	6.50E-07	0.45	11	1.1	Adamts2	Protease
DMR10:36107001	10	36107001	36108000	1000	1	3.70E-07	0.62	17	1.7	Adamts2	Protease
DMR10:36160001	10	36160001	36162000	2000	2	6.00E-10	0.33	21	1.05	Adamts2	Protease
DMR10:36169001	10	36169001	36171000	2000	2	2.10E-09	0.59	49	2.45	Adamts2	Protease
DMR10:36432001	10	36432001	36435000	3000	1	4.20E-08	-0.39	25	0.83	Znf354b	
DMR10:37148001	10	37148001	37152000	4000	1	8.40E-07	-0.32	37	0.92	Sec24a	Transport
DMR10:37279001	10	37279001	37281000	2000	1	5.30E-07	0.37	44	2.2	Jade2	Transcription
DMR10:37582001	10	37582001	37584000	2000	1	3.90E-09	0.54	40	2	RGD1306484	
DMR10:37648001	10	37648001	37650000	2000	1	5.00E-09	0.49	30	1.5	Tcf7	Transcription
DMR10:38135001	10	38135001	38137000	2000	1	3.20E-13	-0.48	29	1.45	Fstl4	Protease; Proteolysis
DMR10:38182001	10	38182001	38185000	3000	1	9.70E-07	0.56	33	1.1	Fstl4	Protease; Proteolysis
DMR10:38191001	10	38191001	38193000	2000	1	1.40E-07	0.57	25	1.25	Fstl4	Protease; Proteolysis
DMR10:38196001	10	38196001	38197000	1000	1	1.30E-09	0.53	18	1.8	Fstl4	Protease; Proteolysis
DMR10:38199001	10	38199001	38202000	3000	1	2.40E-08	0.61	52	1.73	Fstl4	Protease; Proteolysis
DMR10:38282001	10	38282001	38286000	4000	1	1.60E-08	0.41	49	1.23	Fstl4	Protease; Proteolysis
DMR10:38334001	10	38334001	38339000	5000	1	2.80E-08	0.58	64	1.28	Fstl4	Protease; Proteolysis
DMR10:38342001	10	38342001	38348000	6000	1	1.10E-12	0.58	92	1.53	Fstl4	Protease; Proteolysis
DMR10:38352001	10	38352001	38359000	7000	1	8.70E-10	-0.38	88	1.26	Fstl4	Protease; Proteolysis
DMR10:38404001	10	38404001	38406000	2000	1	1.70E-07	0.42	32	1.6	Fstl4	Protease; Proteolysis
DMR10:38436001	10	38436001	38449000	13000	2	6.80E-07	0.55	178	1.37	Fstl4	Protease; Proteolysis
DMR10:38494001	10	38494001	38498000	4000	1	1.00E-08	-0.31	32	0.8	Fstl4	Protease; Proteolysis
DMR10:38595001	10	38595001	38598000	3000	1	3.40E-07	-0.43	46	1.53	Hspa4	
DMR10:38676001	10	38676001	38677000	1000	1	2.80E-08	-0.38	9	0.9	Zcchc10	
DMR10:38785001	10	38785001	38788000	3000	1	5.50E-07	-0.35	22	0.73	Uqcrq;Gdf9	Metabolism;Growth Factors
DMR10:38803001	10	38803001	38805000	2000	1	1.30E-07	0.55	29	1.45	Gdf9;Shroom1	Growth Factors;Cytoskeleton
DMR10:38867001	10	38867001	38869000	2000	1	1.00E-07	-0.39	26	1.3	8-Sep	
DMR10:38892001	10	38892001	38894000	2000	1	5.40E-09	0.35	41	2.05	8-Sep	
DMR10:38986001	10	38986001	38988000	2000	1	2.70E-09	0.59	67	3.35	Il13	
DMR10:39415001	10	39415001	39420000	5000	1	2.50E-08	-0.44	55	1.1	Pdlim4	Cytoskeleton
DMR10:39687001	10	39687001	39689000	2000	1	9.90E-14	-0.81	40	2	Acsl6	Metabolism
DMR10:39704001	10	39704001	39706000	2000	1	4.70E-12	0.63	48	2.4	Acsl6	Metabolism
DMR10:39726001	10	39726001	39727000	1000	1	5.30E-08	0.72	26	2.6	Acsl6;Meikin	Metabolism
DMR10:39728001	10	39728001	39729000	1000	1	9.50E-07	-0.47	11	1.1	Meikin	
DMR10:39811001	10	39811001	39812000	1000	1	1.10E-11	-0.5	7	0.7	Fnip1	
DMR10:39824001	10	39824001	39826000	2000	1	5.50E-07	-0.44	9	0.45	Fnip1	
DMR10:39899001	10	39899001	39906000	7000	1	1.40E-07	-0.24	68	0.97	Rapgef6;LOC108352084	Transcription
DMR10:40112001	10	40112001	40114000	2000	1	8.10E-07	-0.4	20	1	Cdc42se2	
DMR10:40189001	10	40189001	40192000	3000	2	6.50E-14	-0.72	39	1.3	Lyrm7	Transcription
DMR10:40210001	10	40210001	40211000	1000	1	1.00E-10	-0.51	7	0.7	Lyrm7;Hint1	Transcription;Signaling
DMR10:40426001	10	40426001	40427000	1000	1	7.50E-07	0.4	13	1.3	Ccdc69	
DMR10:40471001	10	40471001	40476000	5000	1	9.10E-07	0.41	46	0.92	Slc36a3;LOC103693327	Transport
DMR10:40514001	10	40514001	40516000	2000	1	1.10E-07	-0.62	17	0.85	Slc36a2	Transport
DMR10:40534001	10	40534001	40537000	3000	1	3.90E-07	0.53	49	1.63	Slc36a2;Slc36a1	Transport
DMR10:40584001	10	40584001	40585000	1000	1	2.30E-10	0.78	29	2.9	Fat2	Cytoskeleton
DMR10:40596001	10	40596001	40598000	2000	1	3.80E-09	0.32	25	1.25	Fat2	Cytoskeleton
DMR10:40599001	10	40599001	40603000	4000	1	3.40E-07	0.57	52	1.3	Fat2	Cytoskeleton
DMR10:40784001	10	40784001	40789000	5000	1	2.80E-07	0.48	65	1.3	Atox1	
DMR10:40846001	10	40846001	40847000	1000	1	1.10E-07	-0.5	8	0.8	G3bp1;Glra1	Metabolism;Ion Channel
DMR10:40854001	10	40854001	40856000	2000	2	2.90E-14	0.81	51	2.55	G3bp1;Glra1	Metabolism;Ion Channel
DMR10:40889001	10	40889001	40890000	1000	1	1.30E-09	-0.44	17	1.7	Glra1;LOC103693333	Ion Channel
DMR10:42453001	10	42453001	42455000	2000	1	2.50E-08	0.36	6	0.3	Gria1	Receptor
DMR10:42486001	10	42486001	42487000	1000	1	2.60E-07	0.46	8	0.8	Gria1	Receptor
DMR10:42628001	10	42628001	42630000	2000	1	4.10E-07	0.41	40	2	Gria1	Receptor
DMR10:43073001	10	43073001	43077000	4000	1	1.80E-07	0.56	75	1.88	Galnt10;LOC103693329	Golgi
DMR10:43078001	10	43078001	43081000	3000	1	1.30E-08	0.52	36	1.2	Galnt10;LOC103693329	Golgi
DMR10:43209001	10	43209001	43211000	2000	1	2.40E-09	0.45	35	1.75	Galnt10	Golgi
DMR10:43251001	10	43251001	43253000	2000	2	1.90E-09	0.63	57	2.85	Hand1	Transcription

DMR10:43515001	10	43515001	43517000	2000	1	5.10E-07	0.3	23	1.15	Faxdc2	Metabolism
DMR10:43610001	10	43610001	43611000	1000	1	1.50E-07	0.47	11	1.1	Mrpl22;LOC102555634	Translation
DMR10:43986001	10	43986001	43990000	4000	1	9.00E-07	-0.29	42	1.05	Olr1417	Receptor
DMR10:44127001	10	44127001	44134000	7000	1	1.10E-08	0.45	72	1.03	Olr1423	Receptor
DMR10:44233001	10	44233001	44237000	4000	1	7.90E-07	-0.52	28	0.7	Olr1431;LOC691352	Receptor;Cytoskeleton
DMR10:44258001	10	44258001	44260000	2000	1	3.40E-07	0.32	16	0.8	Olr1432	Signaling
DMR10:44268001	10	44268001	44269000	1000	1	3.30E-09	0.51	34	3.4	Trim58	Proteolysis
DMR10:44282001	10	44282001	44283000	1000	1	2.20E-18	1.03	28	2.8	Trim58;Olr1433	Proteolysis;Receptor
DMR10:44293001	10	44293001	44294000	1000	1	2.20E-07	0.51	27	2.7	Olr1433	Receptor
DMR10:44339001	10	44339001	44343000	4000	2	3.80E-08	-0.26	49	1.23	Olr1434;Olr1435	Receptor
DMR10:44661001	10	44661001	44668000	7000	4	4.00E-09	-0.49	30	0.43	Olr1449;RGD1559534	Receptor
DMR10:44783001	10	44783001	44785000	2000	1	1.60E-09	-0.47	8	0.4	Olr1458;Olr1459	Signaling
DMR10:45283001	10	45283001	45287000	4000	1	3.00E-08	0.4	43	1.07	Rnf187;Hist3h2ba;Hist3h2bb	Proteolysis;Epigenetic
DMR10:45303001	10	45303001	45305000	2000	1	2.00E-08	0.63	61	3.05	Hist3h2bb;Hist3h2a;Hist3h3;Trim17	Epigenetic;Proteolysis
DMR10:45347001	10	45347001	45350000	3000	1	2.10E-08	0.6	41	1.37	LOC108352087;LOC100911111;Obscn	
DMR10:45468001	10	45468001	45471000	3000	1	4.10E-07	0.32	36	1.2	Obscn	
DMR10:45483001	10	45483001	45484000	1000	1	5.50E-13	0.67	17	1.7	Obscn	
DMR10:45496001	10	45496001	45499000	3000	1	2.30E-10	-0.43	32	1.07	Iba57	
DMR10:45529001	10	45529001	45531000	2000	1	8.60E-07	0.44	14	0.7	Gjc2;Guk1	Cytoskeleton;Signaling
DMR10:45617001	10	45617001	45619000	2000	1	8.20E-11	0.68	16	0.8	Wnt3a	Signaling
DMR10:45714001	10	45714001	45717000	3000	1	5.50E-08	0.33	39	1.3	Prss38	Protease
DMR10:45785001	10	45785001	45788000	3000	1	1.50E-10	0.66	36	1.2	Snap47	Transcription
DMR10:45822001	10	45822001	45823000	1000	1	3.60E-11	0.75	34	3.4	Zfp867;LOC108352088;LOC100912120	
DMR10:46156001	10	46156001	46157000	1000	1	9.00E-07	0.51	10	1	Flcn	
DMR10:46704001	10	46704001	46705000	1000	1	2.00E-07	-0.41	18	1.8	Tom1l2;LOC108352089;Drc3	Signaling
DMR10:46804001	10	46804001	46805000	1000	1	1.70E-09	0.57	11	1.1	Gid4;Rps4x-ps6	
DMR10:46841001	10	46841001	46846000	5000	3	4.50E-30	1.35	121	2.42	Drg2;Myo15a	Cytoskeleton
DMR10:46863001	10	46863001	46865000	2000	1	9.20E-08	0.49	28	1.4	Myo15a	Cytoskeleton
DMR10:46869001	10	46869001	46871000	2000	1	3.40E-07	0.53	26	1.3	Myo15a	Cytoskeleton
DMR10:46882001	10	46882001	46883000	1000	1	1.80E-07	0.56	22	2.2	Myo15a	Cytoskeleton
DMR10:46938001	10	46938001	46939000	1000	1	3.60E-07	0.41	17	1.7	Llgl1	Transport
DMR10:46957001	10	46957001	46958000	1000	1	6.50E-10	-0.53	22	2.2	Llgl1;Flii	Transport;Cytoskeleton
DMR10:47063001	10	47063001	47064000	1000	1	1.10E-10	0.72	25	2.5	Shmt1;RGD1559667;Dhrs7b	Epigenetic;Metabolism
DMR10:47100001	10	47100001	47103000	3000	1	4.50E-07	-0.32	22	0.73	Dhrs7b	Metabolism
DMR10:47308001	10	47308001	47314000	6000	1	7.60E-13	-0.56	94	1.57	Kcnj12	Transport
DMR10:47315001	10	47315001	47316000	1000	1	2.40E-08	-0.43	16	1.6	Kcnj12	Transport
DMR10:47493001	10	47493001	47497000	4000	1	1.40E-07	0.54	82	2.05	Aldh3a1	Metabolism
DMR10:47524001	10	47524001	47529000	5000	1	2.10E-07	0.62	93	1.86	RGD1566257;Aldh3a2	Metabolism
DMR10:47582001	10	47582001	47584000	2000	1	5.20E-09	0.45	15	0.75	Slc47a2	Transport
DMR10:47625001	10	47625001	47629000	4000	1	6.20E-10	0.74	68	1.7	Slc47a2;Slc47a1	Transport
DMR10:47717001	10	47717001	47721000	4000	3	9.60E-16	0.85	79	1.98	LOC108352219;Rnf112	Signaling
DMR10:47950001	10	47950001	47953000	3000	1	7.30E-07	0.5	62	2.07	Grap;Slc5a10;Fam83g	Transport
DMR10:48078001	10	48078001	48080000	2000	1	4.70E-08	-0.61	18	0.9	Ulk2	Signaling
DMR10:48084001	10	48084001	48085000	1000	1	5.90E-07	0.44	9	0.9	Ulk2	Signaling
DMR10:48162001	10	48162001	48165000	3000	1	3.70E-08	0.42	28	0.93	Akap10	Cytoskeleton
DMR10:48242001	10	48242001	48244000	2000	1	9.50E-07	-0.53	20	1	Specc1;LOC102551978	
DMR10:48255001	10	48255001	48257000	2000	1	2.00E-08	-0.41	32	1.6	Specc1;LOC102551978	
DMR10:48263001	10	48263001	48264000	1000	1	2.50E-07	-0.35	10	1	Specc1	
DMR10:48282001	10	48282001	48284000	2000	1	9.90E-08	-0.41	17	0.85	Specc1	
DMR10:48419001	10	48419001	48420000	1000	1	1.20E-07	-0.35	15	1.5	Specc1	
DMR10:48808001	10	48808001	48813000	5000	1	6.90E-12	-0.6	43	0.86	Pigl	Metabolism
DMR10:48904001	10	48904001	48905000	1000	1	1.20E-10	0.77	19	1.9	Trpv2	Transport
DMR10:48914001	10	48914001	48919000	5000	1	5.50E-09	0.56	65	1.3	Trpv2;LOC102552369	Transport
DMR10:48974001	10	48974001	48977000	3000	1	4.50E-08	0.44	51	1.7	Mmgt2	
DMR10:49304001	10	49304001	49306000	2000	1	9.10E-09	-0.5	27	1.35	Fbxw10;Tvp23b	Proteolysis
DMR10:49376001	10	49376001	49380000	4000	1	1.20E-08	-0.34	53	1.32	LOC102552952;Cdrt4	
DMR10:49568001	10	49568001	49570000	2000	2	3.60E-10	0.74	45	2.25	Pmp22	Cytoskeleton
DMR10:50440001	10	50440001	50441000	1000	1	5.40E-10	0.51	11	1.1	Cox10	Metabolism

DMR10:50453001	10	50453001	50454000	1000	1	1.60E-08	0.54	12	1.2	Cox10	Metabolism
DMR10:50512001	10	50512001	50513000	1000	1	6.60E-08	0.46	10	1	Cox10	Metabolism
DMR10:50930001	10	50930001	50932000	2000	1	5.10E-08	0.52	16	0.8	Hs3st3a1	Transport
DMR10:50933001	10	50933001	50935000	2000	1	5.00E-08	-0.5	16	0.8	Hs3st3a1	Transport
DMR10:51496001	10	51496001	51497000	1000	1	5.50E-07	0.59	14	1.4	Elac2;Arhgap44	Signaling
DMR10:51566001	10	51566001	51569000	3000	2	1.60E-13	0.89	68	2.27	Arhgap44	
DMR10:51662001	10	51662001	51666000	4000	1	1.30E-09	-0.44	63	1.57	Arhgap44	
DMR10:51694001	10	51694001	51697000	3000	1	3.90E-07	0.5	39	1.3	Myocd	Transcription
DMR10:51756001	10	51756001	51759000	3000	1	3.40E-08	0.5	27	0.9	Myocd	Transcription
DMR10:52206001	10	52206001	52210000	4000	2	3.90E-10	-0.66	44	1.1	Map2k4	Signaling
DMR10:52305001	10	52305001	52307000	2000	1	6.10E-07	-0.39	15	0.75	Map2k4	Signaling
DMR10:52344001	10	52344001	52346000	2000	1	5.10E-08	-0.37	21	1.05	Zfp18;Dnah9	Cytoskeleton
DMR10:52482001	10	52482001	52484000	2000	1	8.60E-08	-0.22	25	1.25	Dnah9	Cytoskeleton
DMR10:52742001	10	52742001	52743000	1000	1	1.90E-10	-0.69	3	0.3	Shisa6	
DMR10:52754001	10	52754001	52756000	2000	1	2.80E-08	-0.47	16	0.8	Shisa6	
DMR10:52769001	10	52769001	52771000	2000	1	1.80E-07	-0.45	17	0.85	Shisa6	
DMR10:53591001	10	53591001	53593000	2000	1	2.80E-20	-0.73	23	1.15	Adprm;Sco1	Metabolism
DMR10:53623001	10	53623001	53625000	2000	1	3.90E-16	0.87	47	2.35	Myh3	
DMR10:53635001	10	53635001	53638000	3000	1	1.90E-18	1.13	98	3.27	Myh3	
DMR10:53640001	10	53640001	53643000	3000	1	1.80E-07	-0.44	104	3.47	Myh3	
DMR10:53718001	10	53718001	53719000	1000	1	9.90E-09	0.6	18	1.8	Myh2	
DMR10:53735001	10	53735001	53736000	1000	1	7.10E-11	0.76	28	2.8	Myh2;Myh1	
DMR10:53744001	10	53744001	53745000	1000	1	2.90E-07	0.49	16	1.6	Myh2;Myh1;LOC102555216	
DMR10:53755001	10	53755001	53757000	2000	1	8.40E-08	0.69	49	2.45	Myh1;LOC102555216	
DMR10:54014001	10	54014001	54017000	3000	1	1.80E-07	0.53	32	1.07	Gas7	Cytoskeleton
DMR10:54077001	10	54077001	54079000	2000	1	5.00E-07	0.48	13	0.65	Gas7	Cytoskeleton
DMR10:54142001	10	54142001	54145000	3000	1	2.70E-08	0.52	38	1.27	Gas7	Cytoskeleton
DMR10:54328001	10	54328001	54330000	2000	1	4.10E-07	0.47	21	1.05	Glp2r;Gsg1l2	Receptor
DMR10:54412001	10	54412001	54414000	2000	1	1.50E-09	-0.55	29	1.45	Usp43	Protease
DMR10:54461001	10	54461001	54464000	3000	1	9.20E-07	-0.39	28	0.93	Usp43;LOC100911946;Cfap52	Protease
DMR10:54580001	10	54580001	54582000	2000	1	3.30E-09	0.53	38	1.9	Stx8	Transcription
DMR10:54668001	10	54668001	54671000	3000	1	2.30E-09	-0.43	53	1.77	Stx8	Transcription
DMR10:54775001	10	54775001	54776000	1000	1	1.30E-08	0.43	13	1.3	Ntn1	Extracellular Matrix
DMR10:54805001	10	54805001	54806000	1000	1	9.30E-10	0.58	14	1.4	Ntn1	Extracellular Matrix
DMR10:55057001	10	55057001	55063000	6000	1	2.30E-08	0.58	98	1.63	Pik3r5;Trnat-ugu	Signaling
DMR10:55076001	10	55076001	55081000	5000	1	2.20E-10	0.37	102	2.04	Pik3r5;Pik3r6	Signaling
DMR10:55113001	10	55113001	55115000	2000	1	4.50E-16	0.96	36	1.8	Pik3r6	Signaling
DMR10:55167001	10	55167001	55170000	3000	1	3.10E-07	0.52	38	1.27	Ccdc42	
DMR10:55222001	10	55222001	55224000	2000	2	2.90E-13	0.76	64	3.2	RGD1564148	
DMR10:55269001	10	55269001	55274000	5000	1	2.50E-14	-0.68	66	1.32	Myh10	
DMR10:55374001	10	55374001	55376000	2000	1	2.30E-07	-0.35	40	2	Myh10	
DMR10:55536001	10	55536001	55539000	3000	2	1.70E-11	0.77	65	2.17	Arhgef15	Transcription
DMR10:55543001	10	55543001	55545000	2000	1	3.90E-08	0.44	41	2.05	Arhgef15;Slc25a35	Transcription
DMR10:55566001	10	55566001	55569000	3000	1	7.10E-11	-0.51	33	1.1	Slc25a35;Rangrf;Pfas	
DMR10:55677001	10	55677001	55679000	2000	1	3.00E-07	-0.45	15	0.75	Vamp2;Per1	Transcription
DMR10:55707001	10	55707001	55708000	1000	1	1.00E-10	0.8	42	4.2	Trnat-agu;Trnas-cga;Hes7;Trnag-gcc;LOC103693354;Trnar-ucu;Trnal-uag;Trnaq-cug;Trnak-uuu;Aloxe3	Transcription;Metabolism
DMR10:55719001	10	55719001	55722000	3000	1	6.40E-12	0.88	70	2.33	Hes7;Trnar-ucu;Trnal-uag;Trnaq-cug;Trnak-uuu;Aloxe3	Transcription;Metabolism
DMR10:55725001	10	55725001	55731000	6000	2	2.80E-09	0.72	99	1.65	Aloxe3;LOC103693355;LOC100359495	Metabolism
DMR10:55841001	10	55841001	55845000	4000	1	3.60E-16	1.02	83	2.08	Gucy2e	Signaling
DMR10:55848001	10	55848001	55849000	1000	1	3.80E-07	0.47	18	1.8	Gucy2e	Signaling
DMR10:55934001	10	55934001	55937000	3000	1	9.10E-07	0.38	46	1.53	Cntrob;Trappc1;Kcnab3;RGD1563441;Chd3	Transport
DMR10:56001001	10	56001001	56002000	1000	1	1.40E-08	0.67	67	6.7	Cyb5d1;Naa38;Tmem88;Kdm6b	Translation;Epigenetic
DMR10:56093001	10	56093001	56096000	3000	1	2.60E-10	0.52	54	1.8	Dnah2;LOC102557224	Cytoskeleton
DMR10:56097001	10	56097001	56101000	4000	1	4.40E-07	0.43	74	1.85	Dnah2;LOC102557224	Cytoskeleton

DMR10:56142001	10	56142001	56145000	3000	2	9.20E-11	0.35	25	0.83	Dnah2	Cytoskeleton
DMR10:56383001	10	56383001	56385000	2000	1	2.40E-10	0.49	46	2.3	Zbtb4;Chrnb1	Transcription;Ion Channel
DMR10:56428001	10	56428001	56430000	2000	1	4.40E-13	0.72	53	2.65	LOC102546484;LOC497938;Spem1;Nlgn2	Cytoskeleton
DMR10:56431001	10	56431001	56438000	7000	1	1.90E-07	0.58	139	1.99	LOC102546484;LOC497938;Spem1;Nlgn2;Tmem256	Cytoskeleton
DMR10:56507001	10	56507001	56508000	1000	1	6.70E-08	-0.41	14	1.4	Acap1;LOC497940;Neurl4	Signaling;Proteolysis
DMR10:56513001	10	56513001	56514000	1000	1	4.20E-09	-0.48	18	1.8	Acap1;LOC497940;Neurl4	Signaling;Proteolysis
DMR10:56555001	10	56555001	56557000	2000	1	3.30E-07	0.46	35	1.75	Ybx2;Slc2a4;LOC102555366	
DMR10:56635001	10	56635001	56637000	2000	1	6.10E-08	0.5	51	2.55	Dlg4;LOC102546779	Cytoskeleton
DMR10:56670001	10	56670001	56671000	1000	1	7.20E-12	0.89	19	1.9	Asgr1	Transport
DMR10:56768001	10	56768001	56769000	1000	1	1.20E-28	1.2	23	2.3	Clec10a	Transport
DMR10:56841001	10	56841001	56846000	5000	1	1.30E-07	0.54	70	1.4	Slc16a13;Bcl6b;RGD1308134;Mir497;Mir195;Rnasek;Alox12	Transport;Transcription;Epigenetic;Metabolism
DMR10:56861001	10	56861001	56863000	2000	1	4.40E-08	-0.43	36	1.8	Alox12	Metabolism
DMR10:56930001	10	56930001	56934000	4000	1	5.10E-08	0.79	74	1.85	Alox12e	Metabolism
DMR10:56962001	10	56962001	56966000	4000	1	1.20E-09	-0.52	95	2.38	Alox15	Metabolism
DMR10:57070001	10	57070001	57071000	1000	1	2.10E-17	1.11	41	4.1	Cxcl16;Zmynd15	Growth Factors
DMR10:57078001	10	57078001	57081000	3000	1	4.60E-09	-0.39	52	1.73	Zmynd15	
DMR10:57163001	10	57163001	57166000	3000	1	9.50E-07	0.38	48	1.6	LOC100361777;Pld2	Metabolism
DMR10:57229001	10	57229001	57238000	9000	1	1.50E-09	0.59	152	1.69	Mink1;Chrne;LOC687707	Signaling;Ion Channel
DMR10:57313001	10	57313001	57314000	1000	1	4.50E-07	0.47	7	0.7	Camta2;Inca1;Kif1c	Transcription;Cytoskeleton
DMR10:57349001	10	57349001	57354000	5000	2	1.70E-22	1.09	100	2	Kif1c	Cytoskeleton
DMR10:57406001	10	57406001	57409000	3000	1	3.80E-07	0.37	48	1.6	LOC108352100;Scimp	
DMR10:57416001	10	57416001	57419000	3000	2	3.70E-10	-0.56	34	1.13	LOC108352100;Scimp	
DMR10:58368001	10	58368001	58372000	4000	2	2.10E-08	0.53	64	1.6	Wscd1	
DMR10:58634001	10	58634001	58636000	2000	2	1.10E-10	0.68	26	1.3	Aipl1;Pitpnm3	Transcription;Metabolism
DMR10:58641001	10	58641001	58643000	2000	1	6.70E-12	0.56	28	1.4	Aipl1;Pitpnm3	Transcription;Metabolism
DMR10:58651001	10	58651001	58652000	1000	1	4.80E-07	0.48	5	0.5	Pitpnm3	Metabolism
DMR10:58663001	10	58663001	58664000	1000	1	5.10E-09	0.51	10	1	Pitpnm3	Metabolism
DMR10:58681001	10	58681001	58683000	2000	1	4.20E-09	-0.45	34	1.7	Pitpnm3	Metabolism
DMR10:58813001	10	58813001	58815000	2000	1	1.30E-08	0.44	32	1.6	Slc13a5	Transport
DMR10:58835001	10	58835001	58837000	2000	1	4.30E-08	-0.41	34	1.7	Slc13a5	Transport
DMR10:58875001	10	58875001	58878000	3000	1	3.80E-07	0.58	57	1.9	LOC102548703;XAF1;Fbxo39;LOC108352104	Proteolysis
DMR10:58904001	10	58904001	58905000	1000	1	1.40E-07	0.53	13	1.3	Tekt1;LOC103693361	Cytoskeleton
DMR10:58916001	10	58916001	58920000	4000	1	1.70E-08	-0.42	53	1.32	Tekt1;LOC103693361	Cytoskeleton
DMR10:58932001	10	58932001	58933000	1000	1	9.50E-08	-0.57	26	2.6	Tekt1	Cytoskeleton
DMR10:58955001	10	58955001	58958000	3000	1	1.10E-09	0.39	42	1.4	Smtnl2	
DMR10:58994001	10	58994001	58997000	3000	1	4.20E-07	0.49	27	0.9	Ggt6;Mybbp1a	Metabolism;Epigenetic
DMR10:59058001	10	59058001	59062000	4000	1	5.60E-08	0.53	49	1.23	Spns2;Spns3	Transport
DMR10:59218001	10	59218001	59219000	1000	1	1.50E-08	-0.53	7	0.7	Ube2g1	
DMR10:59270001	10	59270001	59271000	1000	1	9.20E-07	-0.64	1	0.1	Ankfy1	Transport
DMR10:59344001	10	59344001	59346000	2000	1	2.20E-07	0.56	19	0.95	Cyb5d2	Receptor
DMR10:59378001	10	59378001	59381000	3000	1	1.10E-07	-0.35	26	0.87	Zzef1;LOC102548894	
DMR10:59496001	10	59496001	59497000	1000	1	7.50E-08	0.56	13	1.3	Zzef1	
DMR10:59522001	10	59522001	59523000	1000	1	5.20E-09	0.49	15	1.5	Atp2a3	Transport
DMR10:59544001	10	59544001	59545000	1000	1	2.40E-09	0.67	25	2.5	Atp2a3	Transport
DMR10:59591001	10	59591001	59593000	2000	1	3.00E-08	0.59	30	1.5	P2rx1;Camkk1	Ion Channel;Signaling
DMR10:59675001	10	59675001	59677000	2000	1	2.70E-08	0.48	47	2.35	Itgae	Extracellular Matrix
DMR10:59808001	10	59808001	59811000	3000	1	3.50E-08	0.65	43	1.43	Trpv1	Transport
DMR10:59817001	10	59817001	59818000	1000	1	1.60E-07	0.54	12	1.2	Trpv1	Transport
DMR10:59915001	10	59915001	59919000	4000	1	5.20E-10	-0.58	14	0.35	Spata22;Olr1466	
DMR10:59945001	10	59945001	59947000	2000	1	3.50E-16	0.76	26	1.3	Olr1467;LOC103693362;Olr1468	Receptor
DMR10:60016001	10	60016001	60017000	1000	1	3.80E-07	-0.47	3	0.3	Olr1481;Olr1471	Receptor

DMR10:60158001	10	60158001	60159000	1000	1	2.80E-07	-0.57	2	0.2	Olr1475	
DMR10:60173001	10	60173001	60174000	1000	1	4.60E-11	0.51	26	2.6	Olr1476-ps	
DMR10:60178001	10	60178001	60180000	2000	1	5.50E-07	0.4	29	1.45	Olr1476-ps	
DMR10:60950001	10	60950001	60955000	5000	2	1.70E-07	-0.38	49	0.98	Olr1509;Olr1510-ps;LOC497952	Receptor
DMR10:60999001	10	60999001	6.10E+07	1000	1	5.20E-07	-0.57	3	0.3	Olr1512	Receptor
DMR10:61156001	10	61156001	61159000	3000	1	7.80E-07	-0.5	9	0.3	RGD1563134;Olr1520;Olr1519	Receptor
DMR10:61225001	10	61225001	61226000	1000	1	2.10E-07	-0.44	16	1.6	Rap1gap2	Signaling
DMR10:61265001	10	61265001	61266000	1000	1	8.30E-07	0.39	11	1.1	Rap1gap2	Signaling
DMR10:61288001	10	61288001	61290000	2000	1	4.80E-07	-0.38	35	1.75	Rap1gap2	Signaling
DMR10:61319001	10	61319001	61321000	2000	2	8.90E-12	-0.52	41	2.05	Rap1gap2	Signaling
DMR10:61396001	10	61396001	61397000	1000	1	4.10E-08	-0.47	12	1.2	Rap1gap2;RGD1562276	Signaling
DMR10:61414001	10	61414001	61416000	2000	1	4.80E-07	-0.35	40	2	RGD1562276;LOC103693364	
DMR10:61427001	10	61427001	61428000	1000	1	9.50E-08	-0.71	3	0.3	RGD1562276;LOC103693364;Cluh	Translation
DMR10:61442001	10	61442001	61444000	2000	1	9.10E-07	0.45	40	2	Cluh	Translation
DMR10:61530001	10	61530001	61532000	2000	1	8.80E-10	-0.58	35	1.75	Pafah1b1;Smg6	Metabolism
DMR10:61633001	10	61633001	61634000	1000	1	4.40E-08	-0.42	5	0.5	Mettl16	Epigenetic
DMR10:61720001	10	61720001	61721000	1000	1	5.60E-07	0.43	15	1.5	Sgsm2	Signaling
DMR10:61775001	10	61775001	61777000	2000	2	1.20E-11	-0.65	35	1.75	Srr;Smg6	Metabolism;Metabolism
DMR10:61979001	10	61979001	61981000	2000	1	8.40E-09	-0.56	26	1.3	Smg6	Metabolism
DMR10:61993001	10	61993001	61997000	4000	1	1.60E-09	0.55	67	1.68	Smg6	Metabolism
DMR10:62170001	10	62170001	62174000	4000	1	1.20E-08	-0.57	60	1.5	Rpa1;LOC102554043	Transcription
DMR10:62250001	10	62250001	62252000	2000	1	3.40E-07	0.3	13	0.65	Serpinf1	Protease; Proteolysis
DMR10:62689001	10	62689001	62690000	1000	1	5.10E-14	0.63	32	3.2	Ankrd13b;Coro6	Cytoskeleton
DMR10:62692001	10	62692001	62693000	1000	1	1.30E-12	0.87	27	2.7	Ankrd13b;Coro6;Ssh2	Cytoskeleton;Signaling
DMR10:62696001	10	62696001	62697000	1000	1	7.30E-09	0.57	19	1.9	Ankrd13b;Coro6;Ssh2	Cytoskeleton;Signaling
DMR10:62779001	10	62779001	62783000	4000	1	9.60E-08	-0.42	39	0.98	Ssh2	Signaling
DMR10:62842001	10	62842001	62844000	2000	1	8.50E-08	-0.52	4	0.2	Ssh2	Signaling
DMR10:62970001	10	62970001	62971000	1000	1	2.30E-12	-0.63	8	0.8	LOC102556087;Efcab5	Signaling
DMR10:62975001	10	62975001	62976000	1000	1	5.10E-13	-0.52	16	1.6	LOC102556087;Efcab5	Signaling
DMR10:63039001	10	63039001	63040000	1000	1	8.00E-10	0.6	19	1.9	Efcab5	Signaling
DMR10:63081001	10	63081001	63082000	1000	1	1.40E-07	0.3	20	2	Efcab5	Signaling
DMR10:63127001	10	63127001	63128000	1000	1	7.90E-11	-0.59	9	0.9	Nsrp1	
DMR10:63431001	10	63431001	63432000	1000	1	1.30E-10	-0.48	33	3.3	LOC102546407;Gosr1;LOC102556493	Transcription
DMR10:63470001	10	63470001	63473000	3000	1	6.30E-10	0.59	35	1.17	Tusc5	
DMR10:63491001	10	63491001	63492000	1000	1	5.30E-08	0.55	22	2.2	Tusc5;Bhlha9	Transcription
DMR10:63663001	10	63663001	63666000	3000	2	1.80E-11	0.56	59	1.97	Prpf8;Rilp;Scarf1	Translation;Transport
DMR10:63688001	10	63688001	63693000	5000	1	1.80E-11	0.61	63	1.26	Scarf1;Slc43a2	Transport
DMR10:63732001	10	63732001	63736000	4000	1	1.80E-07	-0.46	65	1.62	Pitpna	Transport
DMR10:63862001	10	63862001	63863000	1000	1	7.30E-13	0.82	21	2.1	Crk	Cytoskeleton
DMR10:63921001	10	63921001	63922000	1000	1	1.10E-09	-0.4	14	1.4	Ywhae;Doc2b	Cytoskeleton
DMR10:63941001	10	63941001	63945000	4000	1	1.80E-07	0.4	49	1.23	Doc2b	
DMR10:63990001	10	63990001	63991000	1000	1	2.60E-07	0.36	17	1.7	Rph3al;LOC102549294	
DMR10:64077001	10	64077001	64080000	3000	1	1.20E-07	0.42	41	1.37	Rph3al;LOC102549419	
DMR10:64108001	10	64108001	64109000	1000	1	3.70E-08	-0.53	11	1.1	Rph3al	
DMR10:64167001	10	64167001	64169000	2000	1	5.90E-09	0.69	38	1.9	RGD1565611	
DMR10:64177001	10	64177001	64179000	2000	1	4.90E-09	0.59	38	1.9	RGD1565611;LOC102547232	
DMR10:64194001	10	64194001	64195000	1000	1	9.90E-07	0.39	24	2.4	LOC102547232;Fam101b	
DMR10:64419001	10	64419001	64423000	4000	2	6.80E-10	-0.4	65	1.62	Nxn	Metabolism
DMR10:64514001	10	64514001	64516000	2000	1	1.90E-07	-0.35	41	2.05	Nxn;LOC108352108	Metabolism
DMR10:64602001	10	64602001	64608000	6000	1	2.40E-08	0.42	98	1.63	Abr	Signaling
DMR10:64805001	10	64805001	64806000	1000	1	8.70E-07	0.43	9	0.9	Phf12	Epigenetic
DMR10:64887001	10	64887001	64889000	2000	1	5.70E-08	0.43	28	1.4	Sez6	
DMR10:64892001	10	64892001	64894000	2000	2	1.40E-09	-0.6	26	1.3	Sez6	
DMR10:64901001	10	64901001	64904000	3000	1	3.60E-08	0.32	29	0.97	Sez6	
DMR10:64964001	10	64964001	64965000	1000	1	2.30E-07	0.48	10	1	Pipox;LOC103693392	Metabolism
DMR10:64968001	10	64968001	64970000	2000	1	3.60E-17	0.96	48	2.4	Pipox;LOC103693392;Myo18a	Metabolism
DMR10:64980001	10	64980001	64981000	1000	1	2.40E-09	0.58	20	2	LOC103693392;Myo18a	

DMR10:64984001	10	64984001	64986000	2000	2	6.00E-10	0.63	66	3.3	LOC103693392;Myo18a;LOC102557169	
DMR10:65058001	10	65058001	65060000	2000	1	5.10E-08	0.51	32	1.6	Myo18a	
DMR10:65079001	10	65079001	65080000	1000	1	1.80E-08	0.69	19	1.9	Myo18a	
DMR10:65167001	10	65167001	65168000	1000	1	8.10E-10	-0.42	9	0.9	Cryba1;Nufip2	
DMR10:65288001	10	65288001	65290000	2000	1	1.90E-09	0.6	16	0.8	Eral1;Mir451a;Mir144	Metabolism
DMR10:65307001	10	65307001	65309000	2000	1	2.60E-07	0.62	39	1.95	Flot2	
DMR10:65358001	10	65358001	65359000	1000	1	8.30E-07	-0.36	17	1.7	Fam222b	
DMR10:65363001	10	65363001	65365000	2000	1	1.40E-07	-0.53	15	0.75	Fam222b	
DMR10:65666001	10	65666001	65667000	1000	1	2.90E-08	0.48	10	1	Slc13a2	Transport
DMR10:65681001	10	65681001	65682000	1000	1	6.30E-07	0.54	20	2	Slc13a2	Transport
DMR10:65751001	10	65751001	65753000	2000	1	5.70E-09	0.54	42	2.1	Slc46a1;Sarm1	Cytoskeleton
DMR10:65756001	10	65756001	65757000	1000	1	2.50E-07	0.37	16	1.6	Sarm1	Cytoskeleton
DMR10:65796001	10	65796001	65797000	1000	1	8.10E-07	0.42	19	1.9	Poldip2;Tnfaip1;Ift20	Cytoskeleton
DMR10:65811001	10	65811001	65813000	2000	1	5.20E-08	-0.49	22	1.1	Tnfaip1;Ift20;Tmem97	Cytoskeleton
DMR10:65832001	10	65832001	65834000	2000	1	3.30E-11	0.55	18	0.9	LOC100361067;Nlk	Signaling
DMR10:65840001	10	65840001	65841000	1000	1	1.20E-08	-0.43	19	1.9	LOC100361067;Nlk	Signaling
DMR10:65966001	10	65966001	65967000	1000	1	6.10E-10	0.77	18	1.8	Nlk	Signaling
DMR10:65971001	10	65971001	65972000	1000	1	3.60E-10	0.45	13	1.3	Nlk	Signaling
DMR10:66183001	10	66183001	66185000	2000	1	3.90E-15	0.54	22	1.1	Nos2	Metabolism
DMR10:66228001	10	66228001	66229000	1000	1	1.50E-13	0.71	17	1.7	Nos2;Lgals5	Metabolism;Extracellular Matrix
DMR10:66317001	10	66317001	66320000	3000	1	4.80E-07	-0.27	28	0.93	LOC497963;Ksr1	Signaling
DMR10:66356001	10	66356001	66357000	1000	1	5.30E-07	0.47	14	1.4	Ksr1	Signaling
DMR10:66404001	10	66404001	66408000	4000	1	5.70E-08	0.5	64	1.6	Ksr1	Signaling
DMR10:66410001	10	66410001	66412000	2000	1	3.10E-10	0.44	24	1.2	Ksr1	Signaling
DMR10:66857001	10	66857001	66858000	1000	1	5.20E-07	0.46	9	0.9	Nf1;Omg;Evi2b	Signaling
DMR10:66937001	10	66937001	66941000	4000	1	1.10E-07	0.42	61	1.52	Nf1;LOC103693399;Rab11fip4	Signaling
DMR10:66953001	10	66953001	66954000	1000	1	5.70E-07	0.43	11	1.1	Rab11fip4	
DMR10:67247001	10	67247001	67250000	3000	3	2.30E-21	0.76	20	0.67	LOC103693400;LOC363652;Utp6	Metabolism
DMR10:67496001	10	67496001	67497000	1000	1	6.30E-07	0.51	12	1.2	LOC686099;Adap2	
DMR10:67558001	10	67558001	67559000	1000	1	2.10E-09	-0.59	9	0.9	Rnf135;LOC108352109;Rhot1	Signaling
DMR10:67668001	10	67668001	67669000	1000	1	1.20E-08	0.41	5	0.5	Rhbdl3	
DMR10:67679001	10	67679001	67684000	5000	1	2.20E-08	0.62	74	1.48	Rhbdl3	
DMR10:67847001	10	67847001	67850000	3000	1	3.20E-09	0.49	23	0.77	Psmd11	
DMR10:68001001	10	68001001	68002000	1000	1	3.80E-07	0.49	13	1.3	Myo1d	Cytoskeleton
DMR10:68030001	10	68030001	68031000	1000	1	1.40E-07	0.39	13	1.3	Myo1d	Cytoskeleton
DMR10:68145001	10	68145001	68147000	2000	1	5.90E-14	-0.55	33	1.65	Myo1d	Cytoskeleton
DMR10:68274001	10	68274001	68275000	1000	1	5.00E-08	0.44	14	1.4	Asic2	Transport
DMR10:68411001	10	68411001	68412000	1000	1	2.40E-10	-0.48	17	1.7	Asic2	Transport
DMR10:68522001	10	68522001	68524000	2000	1	4.50E-07	-0.33	30	1.5	Asic2	Transport
DMR10:68526001	10	68526001	68528000	2000	1	1.30E-08	-0.53	25	1.25	Asic2	Transport
DMR10:68550001	10	68550001	68555000	5000	1	6.40E-07	0.48	63	1.26	Asic2;LOC108352209;LOC688465	Transport
DMR10:68594001	10	68594001	68598000	4000	1	4.30E-14	1	61	1.52	Asic2;LOC108352207	Transport
DMR10:68616001	10	68616001	68618000	2000	1	1.80E-07	0.75	47	2.35	Asic2	Transport
DMR10:68700001	10	68700001	68701000	1000	1	3.50E-08	-0.61	5	0.5	Asic2	Transport
DMR10:68803001	10	68803001	68805000	2000	2	6.20E-09	-0.51	27	1.35	Asic2	Transport
DMR10:68895001	10	68895001	68896000	1000	1	8.80E-07	0.39	8	0.8	Asic2	Transport
DMR10:68955001	10	68955001	68957000	2000	1	4.40E-07	0.57	29	1.45	Asic2;LOC103693401	Transport
DMR10:68991001	10	68991001	68993000	2000	1	4.10E-10	-0.46	43	2.15	Asic2	Transport
DMR10:69739001	10	69739001	69743000	4000	1	1.60E-07	0.47	50	1.25	Tmem132e	
DMR10:69752001	10	69752001	69754000	2000	1	2.30E-07	0.4	30	1.5	Tmem132e	
DMR10:69776001	10	69776001	69779000	3000	1	5.80E-07	0.5	26	0.87	Tmem132e	
DMR10:69793001	10	69793001	69796000	3000	1	2.10E-11	0.69	75	2.5	Tmem132e	
DMR10:69798001	10	69798001	69799000	1000	1	5.70E-09	0.53	10	1	Tmem132e	
DMR10:70109001	10	70109001	70110000	1000	1	4.30E-11	0.73	28	2.8	Cct6b;Zfp830	Translation
DMR10:70137001	10	70137001	70138000	1000	1	2.10E-09	-0.45	18	1.8	LOC103693403;LOC102547954;Lig3	Methylation
DMR10:70152001	10	70152001	70153000	1000	1	6.80E-08	0.65	10	1	Lig3;Rffl	Methylation;Proteolysis

DMR10:70188001	10	70188001	70193000	5000	1	1.70E-10	-0.49	64	1.28	Rffl	Proteolysis
DMR10:70230001	10	70230001	70231000	1000	1	2.30E-07	0.36	12	1.2	Rffl;Rad51d	Proteolysis;Transcription
DMR10:70351001	10	70351001	70357000	6000	4	1.20E-12	-0.8	26	0.43	Slfn13;LOC108352183	
DMR10:70370001	10	70370001	70373000	3000	1	1.60E-09	-0.46	37	1.23	LOC108352183;Slfn2	
DMR10:70405001	10	70405001	70410000	5000	1	4.50E-08	-0.34	60	1.2	Slfn1;Slfn4	
DMR10:70458001	10	70458001	70460000	2000	1	1.00E-06	-0.24	19	0.95	Slfn3	
DMR10:70493001	10	70493001	70494000	1000	1	1.30E-07	-0.6	6	0.6	Slfn14	
DMR10:70495001	10	70495001	70497000	2000	1	5.30E-08	-0.51	36	1.8	Slfn14;LOC100909755	
DMR10:70570001	10	70570001	70572000	2000	1	2.60E-08	-0.43	25	1.25	Ap2b1	Transport
DMR10:70600001	10	70600001	70602000	2000	1	2.90E-10	-0.53	11	0.55	Ap2b1	Transport
DMR10:70654001	10	70654001	70658000	4000	2	3.80E-07	-0.47	39	0.98	Gas2l2;LOC689039;Mmp28	Protease
DMR10:70872001	10	70872001	70874000	2000	1	2.50E-07	-0.61	15	0.75	Ccl3;Ccl4	Growth Factors
DMR10:71146001	10	71146001	71150000	4000	1	7.20E-13	0.44	51	1.27	Hnf1b	Transcription
DMR10:71246001	10	71246001	71247000	1000	1	1.30E-11	0.46	15	1.5	Ddx52	
DMR10:71633001	10	71633001	71635000	2000	1	5.70E-07	-0.4	18	0.9	Acaca	
DMR10:71733001	10	71733001	71737000	4000	1	8.70E-09	0.57	73	1.82	Aatf	Epigenetic
DMR10:72143001	10	72143001	72148000	5000	1	8.30E-15	0.77	91	1.82	Mrm1;Dhrs11;LOC102553386;Ggnbp2	Epigenetic;Metabolism
DMR10:72905001	10	72905001	72907000	2000	2	5.80E-09	-0.46	23	1.15	Bcas3;Ppm1d	
DMR10:73043001	10	73043001	73044000	1000	1	2.40E-10	0.65	2	0.2	Bcas3	
DMR10:73164001	10	73164001	73167000	3000	1	4.90E-07	0.42	35	1.17	Bcas3	
DMR10:73208001	10	73208001	73211000	3000	1	2.60E-07	0.43	33	1.1	Bcas3	
DMR10:73350001	10	73350001	73351000	1000	1	1.60E-07	0.56	10	1	Tbx4	Transcription
DMR10:73527001	10	73527001	73529000	2000	1	2.90E-07	-0.42	32	1.6	Brip1	Epigenetic
DMR10:73572001	10	73572001	73575000	3000	1	5.60E-07	0.55	37	1.23	Brip1	Epigenetic
DMR10:73741001	10	73741001	73743000	2000	1	2.80E-09	-0.56	13	0.65	Med13	
DMR10:73842001	10	73842001	73843000	1000	1	4.80E-08	-0.42	11	1.1	Rps6kb1	Golgi
DMR10:73873001	10	73873001	73875000	2000	1	4.80E-09	-0.45	29	1.45	Rps6kb1;Tubd1	Golgi;Cytoskeleton
DMR10:74055001	10	74055001	74058000	3000	1	1.70E-10	-0.7	15	0.5	Cltc	Transport
DMR10:74242001	10	74242001	74246000	4000	1	4.40E-09	-0.41	63	1.57	Ypel2	
DMR10:74264001	10	74264001	74265000	1000	1	6.70E-07	-0.38	14	1.4	Ypel2	
DMR10:74335001	10	74335001	74337000	2000	1	4.80E-07	-0.35	46	2.3	Gdpd1	Metabolism
DMR10:74368001	10	74368001	74370000	2000	1	8.20E-14	0.53	22	1.1	Gdpd1;Smg8	Metabolism
DMR10:74531001	10	74531001	74532000	1000	1	4.60E-09	-0.68	9	0.9	Trim37	Proteolysis
DMR10:74599001	10	74599001	74601000	2000	1	2.10E-07	-0.35	21	1.05	Ppm1e	Signaling
DMR10:74688001	10	74688001	74689000	1000	1	7.50E-08	-0.46	18	1.8	Ppm1e;Rad51c	Signaling;Transcription
DMR10:74858001	10	74858001	74864000	6000	2	7.40E-10	0.56	77	1.28	Tex14;Sept4	Cytoskeleton
DMR10:74905001	10	74905001	74907000	2000	1	5.70E-08	-0.37	41	2.05	Mtmr4;Hsf5	Signaling;Transcription
DMR10:74962001	10	74962001	74963000	1000	1	5.00E-09	-0.46	11	1.1	Hsf5;Rnf43	Transcription
DMR10:74985001	10	74985001	74991000	6000	1	2.50E-08	-0.42	83	1.38	Rnf43	
DMR10:75005001	10	75005001	75007000	2000	1	1.90E-08	-0.46	39	1.95	Rnf43	
DMR10:75054001	10	75054001	75057000	3000	1	1.20E-08	0.45	53	1.77	Mir142;Tspoap1	
DMR10:75080001	10	75080001	75081000	1000	1	1.70E-07	-0.44	13	1.3	Tspoap1;Mpo	Metabolism
DMR10:75606001	10	75606001	75610000	4000	1	3.30E-17	0.41	43	1.07	Ccdc182	
DMR10:75789001	10	75789001	75790000	1000	1	1.20E-07	0.4	19	1.9	Msi2;LOC103693413	
DMR10:75906001	10	75906001	75909000	3000	1	4.70E-07	-0.44	53	1.77	Msi2	
DMR10:75917001	10	75917001	75920000	3000	1	1.60E-07	-0.42	47	1.57	Msi2	
DMR10:75939001	10	75939001	75941000	2000	1	5.20E-07	-0.36	41	2.05	Msi2	
DMR10:76028001	10	76028001	76032000	4000	1	3.90E-11	0.36	79	1.98	Msi2;LOC102549226	
DMR10:76043001	10	76043001	76045000	2000	2	2.60E-07	-0.41	34	1.7	Msi2;LOC102549226;LOC108352117	
DMR10:76144001	10	76144001	76147000	3000	1	8.50E-10	0.6	50	1.67	Akap1	Cytoskeleton
DMR10:77487001	10	77487001	77488000	1000	1	1.40E-08	-0.44	22	2.2	Pctp	
DMR10:77522001	10	77522001	77526000	4000	1	2.80E-07	0.42	67	1.68	Pctp	
DMR10:77540001	10	77540001	77542000	2000	1	5.20E-15	0.91	34	1.7	Pctp;Tmem100;LOC103693415	
DMR10:77543001	10	77543001	77544000	1000	1	5.00E-07	0.64	21	2.1	Pctp;Tmem100;LOC103693415	
DMR10:77678001	10	77678001	77680000	2000	1	5.10E-07	-0.39	32	1.6	Mmd	Signaling
DMR10:77684001	10	77684001	77687000	3000	1	1.80E-07	-0.55	35	1.17	Mmd	Signaling
DMR10:77744001	10	77744001	77745000	1000	1	3.10E-08	-0.42	8	0.8	Mmd	Signaling

DMR10:77762001	10	77762001	77764000	2000	2	1.50E-08	-0.46	67	3.35	Mmd;LOC108352118	Signaling
DMR10:77839001	10	77839001	77841000	2000	1	2.30E-13	0.67	40	2	LOC103693417;Hlf;LOC103693416	Transcription
DMR10:77876001	10	77876001	77880000	4000	1	1.60E-08	0.44	65	1.62	Hlf	Transcription
DMR10:78006001	10	78006001	78010000	4000	2	8.50E-10	0.48	69	1.73	Stxbp4	
DMR10:78065001	10	78065001	78066000	1000	1	1.30E-12	0.76	13	1.3	Stxbp4	
DMR10:78103001	10	78103001	78104000	1000	1	2.30E-07	0.58	33	3.3	Stxbp4;Cox11	Transcription
DMR10:78128001	10	78128001	78131000	3000	1	4.00E-07	-0.35	56	1.87	Stxbp4;Cox11	Transcription
DMR10:78139001	10	78139001	78141000	2000	2	9.60E-14	0.38	18	0.9	Stxbp4;Cox11	Transcription
DMR10:78148001	10	78148001	78150000	2000	2	6.30E-12	0.4	32	1.6	Stxbp4;Cox11	Transcription
DMR10:78177001	10	78177001	78184000	7000	1	1.20E-11	-0.47	148	2.11	Stxbp4;Cox11;LOC103693419;Tom1l1	Transcription
DMR10:78202001	10	78202001	78203000	1000	1	4.60E-07	-0.31	31	3.1	Tom1l1;LOC100363423	
DMR10:78205001	10	78205001	78209000	4000	3	4.80E-10	-0.46	85	2.12	Tom1l1;LOC100363423	
DMR10:79087001	10	79087001	79088000	1000	1	2.50E-08	-0.35	16	1.6	Kif2b	Cytoskeleton
DMR10:80823001	10	80823001	80826000	3000	1	1.70E-07	-0.84	22	0.73	Car10	
DMR10:80959001	10	80959001	80960000	1000	1	1.70E-07	-0.46	10	1	Car10;LOC100911361	Translation
DMR10:80992001	10	80992001	80993000	1000	1	4.50E-08	0.46	20	2	Car10	
DMR10:81090001	10	81090001	81091000	1000	1	1.30E-07	0.58	16	1.6	Car10	
DMR10:81254001	10	81254001	81257000	3000	1	6.60E-10	0.6	55	1.83	Car10	
DMR10:81570001	10	81570001	81571000	1000	1	8.90E-07	-0.35	12	1.2	Utp18	
DMR10:81576001	10	81576001	81578000	2000	1	3.10E-07	-0.58	6	0.3	Utp18	
DMR10:81585001	10	81585001	81587000	2000	2	2.20E-17	-0.51	31	1.55	Utp18;Mbtd1	Epigenetic
DMR10:81623001	10	81623001	81625000	2000	1	6.10E-16	-0.55	21	1.05	Mbtd1	Epigenetic
DMR10:81901001	10	81901001	81904000	3000	1	4.40E-07	0.45	47	1.57	LOC100912941;Tob1	
DMR10:81943001	10	81943001	81946000	3000	2	6.60E-08	0.4	55	1.83	Wfikkn2	
DMR10:81947001	10	81947001	81948000	1000	1	2.30E-07	-0.39	18	1.8	Wfikkn2	
DMR10:82006001	10	82006001	82007000	1000	1	3.00E-08	-0.41	45	4.5	Luc7l3	
DMR10:82030001	10	82030001	82031000	1000	1	1.20E-08	-0.4	9	0.9	Luc7l3;Ankrd40	
DMR10:82037001	10	82037001	82038000	1000	1	1.50E-08	-0.47	8	0.8	Luc7l3;Ankrd40;Abcc3	Transport
DMR10:82094001	10	82094001	82096000	2000	1	5.10E-08	0.59	29	1.45	Abcc3	Transport
DMR10:82118001	10	82118001	82120000	2000	2	4.60E-10	-0.48	21	1.05	Abcc3;Cacna1g	Transport;Transport
DMR10:82173001	10	82173001	82174000	1000	1	6.00E-07	-0.45	5	0.5	Cacna1g	Transport
DMR10:82284001	10	82284001	82291000	7000	2	5.30E-11	0.48	139	1.99	Rsad1;Acsf2;Chad	Metabolism;Receptor
DMR10:82293001	10	82293001	82296000	3000	1	6.60E-11	0.48	44	1.47	Acsf2;Chad	Metabolism;Receptor
DMR10:82338001	10	82338001	82344000	6000	1	4.90E-09	-0.35	57	0.95	LOC102555790;Lrrc59	Cytoskeleton
DMR10:82388001	10	82388001	82390000	2000	1	5.20E-12	0.57	45	2.25	Mrpl27;Xylt2	Translation;Transport
DMR10:82419001	10	82419001	82423000	4000	1	9.90E-13	0.8	64	1.6	Trnar-ccu	
DMR10:82433001	10	82433001	82440000	7000	2	9.30E-19	0.76	127	1.81	Trnar-ccu	
DMR10:82754001	10	82754001	82755000	1000	1	3.40E-07	0.51	17	1.7	Col1a1	Extracellular Matrix
DMR10:82756001	10	82756001	82763000	7000	3	1.20E-10	0.69	121	1.73	Col1a1;Sgca	Extracellular Matrix;Cytoskeleton
DMR10:82841001	10	82841001	82843000	2000	1	4.60E-08	0.39	15	0.75	Samd14;Pdk2	Signaling
DMR10:82868001	10	82868001	82869000	1000	1	7.80E-08	0.5	21	2.1	Itga3	Extracellular Matrix
DMR10:82939001	10	82939001	82941000	2000	1	3.70E-11	0.55	79	3.95	Dlx3	Development
DMR10:83334001	10	83334001	83336000	2000	2	8.30E-09	0.54	37	1.85	Nxph3	Signaling
DMR10:83390001	10	83390001	83392000	2000	1	2.00E-07	0.47	44	2.2	Ngfr	Receptor
DMR10:83556001	10	83556001	83560000	4000	2	2.20E-11	-0.5	53	1.32	Zfp652	
DMR10:83763001	10	83763001	83766000	3000	2	2.90E-08	-0.54	24	0.8	Igf2bp1	Metabolism
DMR10:83882001	10	83882001	83886000	4000	1	1.90E-07	-0.5	45	1.12	Ube2z;Atp5g1	
DMR10:84158001	10	84158001	84160000	2000	1	3.90E-12	-0.67	32	1.6	Hoxb5os;Hoxb6;Hoxb5;Mir10a;Hoxb4	Development
DMR10:84170001	10	84170001	84175000	5000	2	1.50E-10	-0.53	61	1.22	Mir10a;Hoxb4;Hoxb3	
DMR10:84216001	10	84216001	84219000	3000	1	8.70E-08	0.58	38	1.27	Hoxb1	Development
DMR10:84325001	10	84325001	84328000	3000	2	2.30E-09	0.67	43	1.43	Skap1	Cytoskeleton
DMR10:84335001	10	84335001	84337000	2000	1	6.40E-07	-0.35	53	2.65	Skap1	Cytoskeleton
DMR10:84358001	10	84358001	84359000	1000	1	9.60E-17	1.09	27	2.7	Skap1	Cytoskeleton
DMR10:84361001	10	84361001	84367000	6000	1	3.80E-08	-0.36	63	1.05	Skap1	Cytoskeleton
DMR10:84386001	10	84386001	84391000	5000	3	1.10E-11	-0.55	116	2.32	Skap1	Cytoskeleton
DMR10:84392001	10	84392001	84396000	4000	1	1.10E-12	-0.56	90	2.25	Skap1	Cytoskeleton
DMR10:84399001	10	84399001	84406000	7000	2	1.60E-09	-0.51	162	2.31	Skap1	Cytoskeleton
DMR10:84418001	10	84418001	84419000	1000	1	2.60E-08	-0.47	22	2.2	Skap1;LOC102551165	Cytoskeleton

DMR10:84444001	10	84444001	84447000	3000	1	2.50E-09	-0.39	45	1.5	Skap1	Cytoskeleton
DMR10:84450001	10	84450001	84453000	3000	2	1.40E-12	-0.59	77	2.57	Skap1	Cytoskeleton
DMR10:84472001	10	84472001	84475000	3000	2	1.80E-13	-0.61	76	2.53	Skap1	Cytoskeleton
DMR10:84491001	10	84491001	84494000	3000	1	8.10E-08	-0.37	68	2.27	Skap1	Cytoskeleton
DMR10:84495001	10	84495001	84496000	1000	1	1.70E-07	-0.33	20	2	Skap1	Cytoskeleton
DMR10:84544001	10	84544001	84548000	4000	1	5.70E-09	-0.37	67	1.68	Skap1	Cytoskeleton
DMR10:84560001	10	84560001	84563000	3000	1	7.80E-10	0.55	42	1.4	Skap1	Cytoskeleton
DMR10:84576001	10	84576001	84577000	1000	1	3.40E-11	0.79	28	2.8	Skap1	Cytoskeleton
DMR10:84586001	10	84586001	84587000	1000	1	1.40E-09	0.65	37	3.7	Skap1	Cytoskeleton
DMR10:84598001	10	84598001	84607000	9000	4	3.40E-12	-0.49	173	1.92	Skap1	Cytoskeleton
DMR10:84642001	10	84642001	84646000	4000	1	6.60E-13	-0.45	66	1.65	Snx11	
DMR10:84722001	10	84722001	84725000	3000	1	2.00E-09	0.5	35	1.17	Copz2;Mir152	Transport
DMR10:84772001	10	84772001	84773000	1000	1	9.80E-10	0.59	12	1.2	Cdk5rap3	
DMR10:84860001	10	84860001	84863000	3000	1	1.10E-07	-0.35	30	1	LOC102552748;Prr15l	
DMR10:84878001	10	84878001	84879000	1000	1	6.90E-07	-0.42	20	2	Prr15l;Pnpo;LOC102547955	Metabolism
DMR10:84883001	10	84883001	84884000	1000	1	5.50E-08	0.39	11	1.1	Prr15l;Pnpo;LOC102547955;Sp2	Metabolism;Transcription
DMR10:84926001	10	84926001	84927000	1000	1	1.10E-07	0.68	28	2.8	Sp2;LOC102547278;LOC108352124	Transcription
DMR10:84997001	10	84997001	85001000	4000	1	2.50E-11	-0.44	71	1.77	Osbpl7	
DMR10:85022001	10	85022001	85024000	2000	1	1.60E-09	0.43	26	1.3	Tbx21	Transcription
DMR10:85040001	10	85040001	85041000	1000	1	2.40E-07	0.48	15	1.5	Tbx21	Transcription
DMR10:85062001	10	85062001	85063000	1000	1	1.20E-10	0.78	30	3	Tbkbp1	
DMR10:85293001	10	85293001	85296000	3000	2	7.80E-15	0.9	66	2.2	Gpr179;LOC108352126;Socs7	Signaling;Signaling
DMR10:85397001	10	85397001	85400000	3000	2	1.30E-07	0.38	46	1.53	LOC102557607;Arhgap23	
DMR10:85450001	10	85450001	85457000	7000	2	4.10E-10	0.74	117	1.67	Arhgap23;Srcin1	
DMR10:85476001	10	85476001	85477000	1000	1	7.90E-10	0.53	28	2.8	Srcin1	
DMR10:85494001	10	85494001	85496000	2000	1	1.10E-14	0.82	51	2.55	Srcin1	
DMR10:85499001	10	85499001	85501000	2000	1	1.80E-09	0.63	29	1.45	Srcin1	
DMR10:85509001	10	85509001	85510000	1000	1	6.90E-08	0.5	17	1.7	Srcin1	
DMR10:85681001	10	85681001	85683000	2000	1	1.90E-11	0.66	24	1.2	Pip4k2b;Cwc25	Signaling
DMR10:85730001	10	85730001	85732000	2000	1	1.80E-10	-0.51	18	0.9	Rpl23	Translation
DMR10:85779001	10	85779001	85783000	4000	1	2.30E-07	0.38	76	1.9	Lasp1;LOC108352127	
DMR10:85800001	10	85800001	85801000	1000	1	6.20E-09	0.6	16	1.6	Fbxo47	
DMR10:85823001	10	85823001	85824000	1000	1	1.80E-07	0.51	5	0.5	Fbxo47	
DMR10:85885001	10	85885001	85889000	4000	1	4.90E-07	0.46	63	1.57	Plxdc1	
DMR10:85910001	10	85910001	85911000	1000	1	8.00E-07	0.47	15	1.5	Plxdc1	
DMR10:85921001	10	85921001	85924000	3000	1	1.90E-09	-0.43	75	2.5	Plxdc1	
DMR10:85925001	10	85925001	85927000	2000	1	7.00E-08	0.58	26	1.3	Plxdc1	
DMR10:86006001	10	86006001	86009000	3000	1	1.70E-07	0.49	35	1.17	Stac2	
DMR10:86210001	10	86210001	86211000	1000	1	9.10E-07	-0.52	4	0.4	Cdk12	Signaling
DMR10:86306001	10	86306001	86311000	5000	1	9.60E-13	-0.42	94	1.88	Ppp1r1b;Stard3	Signaling
DMR10:86420001	10	86420001	86424000	4000	2	2.20E-13	0.69	101	2.52	Ikzf3	Transcription
DMR10:86441001	10	86441001	86446000	5000	1	2.00E-08	-0.41	79	1.58	Ikzf3	Transcription
DMR10:86452001	10	86452001	86453000	1000	1	2.90E-08	-0.35	20	2	Ikzf3	Transcription
DMR10:86472001	10	86472001	86476000	4000	1	2.60E-07	-0.42	80	2	Ikzf3;LOC108352128	Transcription
DMR10:86568001	10	86568001	86570000	2000	2	5.30E-23	1.26	61	3.05	Lrrc3c;Gsdma;LOC108352129	
DMR10:86714001	10	86714001	86715000	1000	1	1.30E-18	-0.74	14	1.4	LOC108352130;Msl1;Casc3	Epigenetic
DMR10:86782001	10	86782001	86784000	2000	2	4.10E-09	-0.47	37	1.85	Wipf2	Cytoskeleton
DMR10:86815001	10	86815001	86816000	1000	1	1.20E-09	-0.41	24	2.4	Wipf2;Cdc6	Cytoskeleton;Cell Cycle
DMR10:86968001	10	86968001	86972000	4000	1	2.10E-07	0.64	79	1.98	Igfbp4;Tns4	Protease; Proteolysis;Cytoskeleton
DMR10:87005001	10	87005001	87008000	3000	1	5.20E-11	0.7	63	2.1	Tns4	Cytoskeleton
DMR10:87055001	10	87055001	87060000	5000	1	2.60E-08	0.71	126	2.52	Ccr7	
DMR10:87178001	10	87178001	87183000	5000	1	1.80E-09	-0.43	69	1.38	Krt24	
DMR10:87194001	10	87194001	87197000	3000	1	8.30E-07	-0.38	67	2.23	Krt24	
DMR10:87198001	10	87198001	87205000	7000	2	2.00E-15	0.92	99	1.41	Krt24	
DMR10:87228001	10	87228001	87229000	1000	1	1.10E-10	0.66	20	2	Krt25	
DMR10:87238001	10	87238001	87241000	3000	2	2.70E-11	-0.43	40	1.33	Krt25;Krt26	
DMR10:87248001	10	87248001	87249000	1000	1	6.90E-11	0.67	30	3	Krt25;Krt26;Krt27	
DMR10:87284001	10	87284001	87287000	3000	1	1.70E-10	0.84	70	2.33	Krt28;LOC108352132;Krt10	

DMR10:87332001	10	87332001	87336000	4000	1	2.30E-17	1.09	92	2.3	Krt12;Krt20;LOC103695157	
DMR10:87339001	10	87339001	87346000	7000	1	3.60E-07	-0.36	140	2	Krt12;Krt20;LOC103695157	
DMR10:87382001	10	87382001	87384000	2000	1	2.60E-09	0.49	43	2.15	Krt23	
DMR10:87406001	10	87406001	87407000	1000	1	3.40E-07	0.52	28	2.8	Krt23;LOC108352133	
DMR10:87414001	10	87414001	87416000	2000	1	1.10E-07	-0.35	28	1.4	Krt23;LOC108352133	
DMR10:87474001	10	87474001	87475000	1000	1	1.40E-10	-0.73	4	0.4	LOC108352226;Krtap3-3;LOC680043;Krtap3-2;Krtap3-1	
DMR10:87492001	10	87492001	87493000	1000	1	1.10E-07	-0.35	20	2	Krtap3-1;Krtap1-5;Krtap1-1	
DMR10:87917001	10	87917001	87919000	2000	1	6.40E-09	-0.61	18	0.9	Krtap16-1;LOC102554371	
DMR10:87929001	10	87929001	87933000	4000	1	6.60E-13	-0.48	49	1.23	Krtap16-1;LOC102554371;LOC501727	
DMR10:87961001	10	87961001	87963000	2000	1	1.60E-07	0.45	31	1.55	Krt34;Krt31	
DMR10:87983001	10	87983001	87984000	1000	1	3.20E-07	-0.44	7	0.7	Krt32	
DMR10:87985001	10	87985001	87986000	1000	1	1.50E-08	0.53	17	1.7	Krt32	
DMR10:88011001	10	88011001	88015000	4000	2	7.80E-12	0.64	30	0.75	Krt35;Krt36	
DMR10:88061001	10	88061001	88063000	2000	1	2.40E-08	0.34	21	1.05	Krt19;LOC102554449	
DMR10:88142001	10	88142001	88153000	11000	1	1.70E-08	0.27	174	1.58	Ka11;Krt16;Krt17	
DMR10:88155001	10	88155001	88163000	8000	1	5.10E-09	0.48	110	1.38	Krt16;Krt17;Krt42	
DMR10:88281001	10	88281001	88282000	1000	1	3.00E-10	0.57	16	1.6	Jup	
DMR10:88285001	10	88285001	88287000	2000	1	1.20E-07	0.4	31	1.55	Jup	
DMR10:88288001	10	88288001	88295000	7000	2	1.00E-18	0.93	163	2.33	Jup	
DMR10:88363001	10	88363001	88364000	1000	1	8.00E-11	0.73	33	3.3	Nt5c3b;Klhl10	Metabolism;Cytoskeleton
DMR10:88409001	10	88409001	88412000	3000	1	1.50E-07	-0.35	28	0.93	Acly	Transport
DMR10:88568001	10	88568001	88569000	1000	1	2.10E-09	0.53	25	2.5	Zfp385c	
DMR10:88601001	10	88601001	88604000	3000	1	6.00E-11	0.62	48	1.6	Zfp385c;Dhx58;Kat2a	
DMR10:88662001	10	88662001	88665000	3000	1	2.90E-07	-0.4	60	2	Kcnh4;LOC108352136;Hcrt;Ghdc	Transport
DMR10:88834001	10	88834001	88835000	1000	1	1.80E-07	-0.45	18	1.8	Stat3	Transcription
DMR10:88883001	10	88883001	88884000	1000	1	3.40E-08	0.52	8	0.8	Ptrf	
DMR10:88908001	10	88908001	88910000	2000	1	4.10E-09	-0.49	23	1.15	Atp6v0a1	Metabolism
DMR10:89045001	10	89045001	89048000	3000	1	4.10E-11	-0.66	36	1.2	Tubg1	Cytoskeleton
DMR10:89078001	10	89078001	89080000	2000	1	3.70E-25	1.42	82	4.1	Tubg2;Plekhh3;Ccr10;Cntnap1	Cytoskeleton
DMR10:89088001	10	89088001	89089000	1000	1	7.70E-09	0.8	23	2.3	Plekhh3;Ccr10;Cntnap1	
DMR10:89326001	10	89326001	89327000	1000	1	6.00E-07	-0.38	12	1.2	Aarsd1;Ptges3l	Metabolism;Transcription
DMR10:89393001	10	89393001	89398000	5000	1	3.00E-08	0.61	67	1.34	Brca1	Proteolysis
DMR10:89429001	10	89429001	89430000	1000	1	2.50E-07	0.41	17	1.7	Brca1	Proteolysis
DMR10:89574001	10	89574001	89575000	1000	1	5.80E-10	0.74	12	1.2	Arl4d	Signaling
DMR10:89579001	10	89579001	89580000	1000	1	1.40E-07	0.52	37	3.7	Arl4d	Signaling
DMR10:89655001	10	89655001	89658000	3000	1	8.80E-08	-0.35	58	1.93	Dhx8	Transcription
DMR10:89945001	10	89945001	89948000	3000	1	1.20E-10	-0.48	55	1.83	Mpp3	Cytoskeleton
DMR10:89961001	10	89961001	89963000	2000	1	2.00E-07	0.31	31	1.55	Mpp3	Cytoskeleton
DMR10:89981001	10	89981001	89988000	7000	2	2.40E-12	0.53	99	1.41	Cd300lg	Immune
DMR10:90017001	10	90017001	90018000	1000	1	8.10E-07	-0.48	7	0.7	Mpp2	Cytoskeleton
DMR10:90053001	10	90053001	90056000	3000	1	4.80E-07	0.38	49	1.63	Ppy;Pyy	Signaling
DMR10:90077001	10	90077001	90081000	4000	1	1.90E-07	0.42	85	2.12	Nags	
DMR10:90312001	10	90312001	90314000	2000	1	6.80E-08	0.34	31	1.55	Slc4a1	Transport
DMR10:90318001	10	90318001	90319000	1000	1	9.30E-07	0.35	5	0.5	Slc4a1	Transport
DMR10:90425001	10	90425001	90427000	2000	1	2.30E-10	-0.42	69	3.45	Itga2b;Gpatch8	Extracellular Matrix
DMR10:90623001	10	90623001	90624000	1000	1	1.10E-20	1.47	69	6.9	Meioc	
DMR10:90646001	10	90646001	90647000	1000	1	7.20E-08	-0.57	13	1.3	Meioc;Ccdc43	
DMR10:90770001	10	90770001	90772000	2000	1	1.20E-07	0.51	52	2.6	LOC102550942;Gjc1	Cytoskeleton
DMR10:90927001	10	90927001	90929000	2000	1	5.90E-07	0.34	31	1.55	Higd1b;Eftud2	Translation
DMR10:90951001	10	90951001	90952000	1000	1	7.50E-09	0.41	14	1.4	Eftud2	Translation
DMR10:90989001	10	90989001	90991000	2000	2	3.60E-07	0.68	64	3.2	Eftud2;Ccdc103;FAM187A;Gfap	Translation
DMR10:91000001	10	91000001	91003000	3000	1	7.40E-07	0.54	38	1.27	FAM187A;Gfap;Kif18b	Cytoskeleton
DMR10:91007001	10	91007001	91009000	2000	1	1.40E-08	0.56	22	1.1	Gfap;Kif18b	Cytoskeleton
DMR10:91279001	10	91279001	91281000	2000	1	1.00E-07	0.46	33	1.65	Fmnl1;LOC100361655	Signaling
DMR10:91308001	10	91308001	91310000	2000	1	4.80E-11	0.44	35	1.75	Spata32;Map3k14	Signaling

DMR10:91418001	10	91418001	91419000	1000	1	3.00E-07	0.45	21	2.1	Arhgap27	Signaling
DMR10:91440001	10	91440001	91444000	4000	1	5.50E-09	-0.34	81	2.02	Arhgap27;Plekhm1	Signaling
DMR10:91719001	10	91719001	91721000	2000	1	1.80E-07	-0.62	26	1.3	Rprml	
DMR10:91740001	10	91740001	91742000	2000	1	2.30E-07	-0.44	30	1.5	Gosr2	Transcription
DMR10:91831001	10	91831001	91833000	2000	1	3.90E-10	0.61	45	2.25	Wnt3	Signaling
DMR10:91887001	10	91887001	91889000	2000	1	3.40E-10	-0.49	42	2.1	Nsf	Transport
DMR10:91956001	10	91956001	91957000	1000	1	2.30E-13	-0.51	29	2.9	Nsf	Transport
DMR10:91994001	10	91994001	91996000	2000	1	3.60E-07	-0.29	36	1.8	Nsf	Transport
DMR10:92204001	10	92204001	92207000	3000	1	9.70E-08	0.44	27	0.9	Crhr1	Receptor
DMR10:92226001	10	92226001	92228000	2000	1	5.70E-07	0.49	42	2.1	Crhr1	Receptor
DMR10:92239001	10	92239001	92242000	3000	1	1.30E-07	0.39	38	1.27	Crhr1;Sppl2c	Receptor;Proteolysis
DMR10:92247001	10	92247001	92250000	3000	1	8.10E-10	-0.44	45	1.5	Sppl2c	Proteolysis
DMR10:92301001	10	92301001	92303000	2000	2	7.90E-08	0.64	37	1.85	Mapt;LOC100912629	
DMR10:92331001	10	92331001	92332000	1000	1	4.40E-08	0.55	12	1.2	Mapt	
DMR10:92341001	10	92341001	92344000	3000	1	1.20E-10	0.67	58	1.93	Mapt;LOC102549019	
DMR10:92360001	10	92360001	92363000	3000	1	1.70E-07	0.33	48	1.6	Mapt	
DMR10:92635001	10	92635001	92636000	1000	1	1.70E-10	0.66	34	3.4	Myl4	Cytoskeleton
DMR10:92680001	10	92680001	92682000	2000	1	1.30E-08	-0.4	44	2.2	Itgb3	Extracellular Matrix
DMR10:92840001	10	92840001	92841000	1000	1	1.20E-07	0.45	12	1.2	Efcab13	
DMR10:92845001	10	92845001	92847000	2000	1	2.40E-07	-0.45	37	1.85	Efcab13	
DMR10:92939001	10	92939001	92944000	5000	1	3.30E-07	0.36	70	1.4	Efcab13	
DMR10:93077001	10	93077001	93078000	1000	1	5.20E-07	-0.51	15	1.5	Efcab13	
DMR10:93295001	10	93295001	93299000	4000	1	4.10E-07	0.43	67	1.68	Efcab3	Signaling
DMR10:93521001	10	93521001	93522000	1000	1	6.90E-09	0.6	25	2.5	Mrc2	
DMR10:93525001	10	93525001	93527000	2000	1	1.20E-07	-0.44	41	2.05	Mrc2	
DMR10:93531001	10	93531001	93532000	1000	1	5.00E-07	0.55	31	3.1	Mrc2	
DMR10:93542001	10	93542001	93545000	3000	1	2.00E-08	-0.46	62	2.07	Mrc2	
DMR10:93549001	10	93549001	93552000	3000	1	1.10E-07	0.67	59	1.97	Mrc2	
DMR10:93569001	10	93569001	93571000	2000	1	4.30E-14	0.92	48	2.4	Mrc2	
DMR10:93691001	10	93691001	93693000	2000	2	3.00E-11	-0.41	28	1.4	March10;LOC108352144	
DMR10:93987001	10	93987001	93988000	1000	1	3.90E-08	-0.28	10	1	Tanc2	
DMR10:94034001	10	94034001	94035000	1000	1	3.90E-07	0.44	8	0.8	Tanc2	
DMR10:94145001	10	94145001	94146000	1000	1	8.30E-07	-0.4	17	1.7	Cyb561	Metabolism
DMR10:94152001	10	94152001	94154000	2000	1	8.80E-11	-0.6	36	1.8	Cyb561	Metabolism
DMR10:94215001	10	94215001	94218000	3000	1	1.50E-08	-0.47	64	2.13	Ace;Ace3;LOC102556428;Kcnh6;LOC108352145	Protease;Transport
DMR10:94357001	10	94357001	94358000	1000	1	2.20E-08	0.51	12	1.2	Map3k3;Limd2;Strada	Cytoskeleton;Signaling
DMR10:94403001	10	94403001	94404000	1000	1	9.10E-15	-0.52	15	1.5	Ccdc47;Ddx42	
DMR10:94437001	10	94437001	94441000	4000	1	2.60E-24	-0.87	64	1.6	Ddx42;Ftsj3;Psmc5	Epigenetic;Protease
DMR10:94442001	10	94442001	94444000	2000	1	6.00E-07	-0.31	36	1.8	Ddx42;Ftsj3;Psmc5;Smarcd2	Epigenetic;Protease;Epigenetic
DMR10:94579001	10	94579001	94587000	8000	1	3.50E-09	-0.42	145	1.81	Prr29;Icam2;Ern1	Translation
DMR10:94640001	10	94640001	94641000	1000	1	5.60E-10	-0.49	5	0.5	Ern1	Translation
DMR10:94692001	10	94692001	94695000	3000	1	3.80E-08	0.59	44	1.47	Ern1;LOC102546698;Tex2	Translation
DMR10:94942001	10	94942001	94945000	3000	1	2.10E-11	-0.42	55	1.83	Milr1	Immune
DMR10:95030001	10	95030001	95032000	2000	2	2.80E-09	0.34	27	1.35	Smurf2	Proteolysis
DMR10:95075001	10	95075001	95077000	2000	1	7.90E-08	-0.42	26	1.3	Smurf2	Proteolysis
DMR10:95226001	10	95226001	95229000	3000	1	9.70E-10	0.66	54	1.8	Trnar-ccg	
DMR10:95327001	10	95327001	95328000	1000	1	5.90E-07	-0.44	17	1.7	Bptf	
DMR10:95332001	10	95332001	95334000	2000	1	5.10E-09	-0.59	19	0.95	Bptf	
DMR10:95430001	10	95430001	95433000	3000	2	3.20E-15	0.96	78	2.6	Pitpnc1	
DMR10:95462001	10	95462001	95463000	1000	1	5.80E-17	-0.55	17	1.7	Pitpnc1	
DMR10:95477001	10	95477001	95481000	4000	1	8.20E-07	0.46	83	2.08	Pitpnc1	
DMR10:95535001	10	95535001	95537000	2000	1	2.90E-07	0.53	38	1.9	Pitpnc1	
DMR10:95596001	10	95596001	95599000	3000	1	9.20E-07	0.51	54	1.8	Pitpnc1	
DMR10:95666001	10	95666001	95667000	1000	1	1.00E-07	0.62	31	3.1	Pitpnc1;RGD1359290	Translation
DMR10:95668001	10	95668001	95672000	4000	1	3.30E-08	0.54	82	2.05	Pitpnc1	
DMR10:95703001	10	95703001	95704000	1000	1	7.40E-10	0.49	22	2.2	Pitpnc1;LOC688936;Psmd12	Protease
DMR10:95942001	10	95942001	95944000	2000	1	2.60E-18	1.19	46	2.3	Cacng1	Transport
DMR10:95961001	10	95961001	95965000	4000	1	1.40E-07	0.52	66	1.65	Cacng4	Transport
DMR10:96112001	10	96112001	96113000	1000	1	3.80E-10	0.77	20	2	Cacng5	Transport
DMR10:96144001	10	96144001	96149000	5000	1	3.60E-07	-0.67	59	1.18	Cacng5	Transport

DMR10:96203001	10	96203001	96204000	1000	1	3.50E-11	0.48	21	2.1	Prkca	Signaling
DMR10:96217001	10	96217001	96220000	3000	1	4.70E-08	0.6	36	1.2	Prkca	Signaling
DMR10:96225001	10	96225001	96234000	9000	3	1.70E-16	0.82	169	1.88	Prkca	Signaling
DMR10:96242001	10	96242001	96243000	1000	1	8.00E-08	-0.67	6	0.6	Prkca	Signaling
DMR10:96254001	10	96254001	96257000	3000	1	1.50E-07	0.4	39	1.3	Prkca	Signaling
DMR10:96373001	10	96373001	96377000	4000	1	3.80E-09	0.46	49	1.23	Prkca	Signaling
DMR10:96517001	10	96517001	96521000	4000	1	9.50E-08	0.41	65	1.62	Prkca	Signaling
DMR10:96550001	10	96550001	96554000	4000	1	8.10E-07	0.33	63	1.57	Prkca	Signaling
DMR10:96572001	10	96572001	96576000	4000	1	6.40E-09	0.54	55	1.38	Prkca	Signaling
DMR10:96645001	10	96645001	96646000	1000	1	2.10E-11	-0.66	10	1	Apoh	
DMR10:96704001	10	96704001	96706000	2000	1	7.70E-07	-0.45	25	1.25	Cep112	
DMR10:96767001	10	96767001	96770000	3000	1	1.20E-09	-0.45	69	2.3	Cep112	
DMR10:96945001	10	96945001	96948000	3000	2	1.30E-09	0.75	41	1.37	Cep112;LOC108352152	
DMR10:97501001	10	97501001	97503000	2000	1	6.80E-09	0.37	26	1.3	RGD1565459;Rgs9	
DMR10:97574001	10	97574001	97575000	1000	1	2.70E-07	0.39	16	1.6	Rgs9	
DMR10:97659001	10	97659001	97662000	3000	1	7.40E-09	-0.46	39	1.3	Gna13	Signaling
DMR10:97698001	10	97698001	97702000	4000	1	3.40E-07	0.46	58	1.45	Amz2	
DMR10:97729001	10	97729001	97732000	3000	1	1.70E-08	-0.36	49	1.63	Amz2;Arsg;Slc16a6	Metabolism;Transport
DMR10:97798001	10	97798001	97799000	1000	1	1.30E-12	-0.79	6	0.6	Arsg	Metabolism
DMR10:97826001	10	97826001	97827000	1000	1	1.30E-08	-0.45	20	2	Arsg	Metabolism
DMR10:97832001	10	97832001	97834000	2000	1	8.80E-07	0.26	35	1.75	Arsg	Metabolism
DMR10:97856001	10	97856001	97859000	3000	1	5.20E-07	-0.45	63	2.1	Arsg;Wipi1	Metabolism
DMR10:97862001	10	97862001	97863000	1000	1	1.30E-07	0.49	21	2.1	Arsg;Wipi1	Metabolism
DMR10:97964001	10	97964001	97966000	2000	1	1.50E-11	0.76	56	2.8	Prkar1a;Fam20a	Signaling
DMR10:97971001	10	97971001	97978000	7000	1	1.30E-09	0.74	105	1.5	Fam20a	
DMR10:98025001	10	98025001	98027000	2000	1	2.80E-07	0.68	34	1.7	Fam20a	
DMR10:98360001	10	98360001	98361000	1000	1	6.50E-10	-0.49	5	0.5	Abca8a	Transport
DMR10:98466001	10	98466001	98469000	3000	1	3.60E-11	-0.43	50	1.67	Abca9;Abca6	Transport
DMR10:98481001	10	98481001	98485000	4000	1	9.20E-07	0.52	71	1.77	Abca9;Abca6	Transport
DMR10:98488001	10	98488001	98490000	2000	1	2.30E-08	-0.44	44	2.2	Abca6	Transport
DMR10:98497001	10	98497001	98498000	1000	1	8.20E-09	-0.34	23	2.3	Abca6	Transport
DMR10:98540001	10	98540001	98542000	2000	1	3.20E-08	0.47	51	2.55	Abca6	Transport
DMR10:98550001	10	98550001	98553000	3000	1	3.20E-07	-0.33	111	3.7	Abca6	Transport
DMR10:98584001	10	98584001	98592000	8000	1	2.00E-08	-0.52	72	0.9	Abca5	Transport
DMR10:98716001	10	98716001	98718000	2000	1	6.30E-16	0.98	54	2.7	Map2k6	Signaling
DMR10:98726001	10	98726001	98734000	8000	2	8.80E-15	-0.44	135	1.69	Map2k6	Signaling
DMR10:98788001	10	98788001	98790000	2000	1	1.20E-08	0.5	25	1.25	Map2k6	Signaling
DMR10:98805001	10	98805001	98806000	1000	1	1.50E-19	0.86	26	2.6	Map2k6	Signaling
DMR10:99317001	10	99317001	99322000	5000	2	1.40E-11	-0.55	63	1.26	Kcnj16	Transport
DMR10:99362001	10	99362001	99363000	1000	1	5.50E-12	0.63	13	1.3	Kcnj16	Transport
DMR10:99372001	10	99372001	99374000	2000	1	6.00E-07	-0.39	39	1.95	Kcnj16	Transport
DMR10:99444001	10	99444001	99447000	3000	1	1.80E-12	0.81	55	1.83	Kcnj2	Transport
DMR10:101692001	10	101692001	101695000	3000	2	2.10E-11	0.82	69	2.3	LOC102549836;Mir297	
DMR10:101764001	10	101764001	101767000	3000	1	7.20E-10	0.79	36	1.2	Slc39a11	Transport
DMR10:101800001	10	101800001	101803000	3000	1	2.00E-07	0.51	47	1.57	Slc39a11	Transport
DMR10:101831001	10	101831001	101834000	3000	1	7.90E-09	-0.69	71	2.37	Slc39a11	Transport
DMR10:101837001	10	101837001	101841000	4000	2	5.90E-09	-0.56	114	2.85	Slc39a11	Transport
DMR10:101895001	10	101895001	101903000	8000	1	6.90E-09	0.49	160	2	Slc39a11;LOC108352158	Transport
DMR10:101912001	10	101912001	101915000	3000	1	9.50E-07	-0.31	65	2.17	Slc39a11	Transport
DMR10:101929001	10	101929001	101930000	1000	1	1.20E-10	-0.49	37	3.7	Slc39a11	Transport
DMR10:101970001	10	101970001	101972000	2000	1	4.10E-08	0.44	19	0.95	Slc39a11	Transport
DMR10:101973001	10	101973001	101975000	2000	1	4.50E-09	-0.36	60	3	Slc39a11	Transport
DMR10:101981001	10	101981001	101982000	1000	1	3.70E-09	-0.4	25	2.5	Slc39a11	Transport
DMR10:102025001	10	102025001	102026000	1000	1	3.80E-13	0.71	33	3.3	Slc39a11	Transport
DMR10:102060001	10	102060001	102063000	3000	1	6.90E-09	0.51	58	1.93	Slc39a11	Transport
DMR10:102086001	10	102086001	102087000	1000	1	3.40E-07	-0.37	17	1.7	Slc39a11	Transport
DMR10:102096001	10	102096001	102097000	1000	1	3.80E-07	0.53	18	1.8	Slc39a11	Transport
DMR10:102125001	10	102125001	102126000	1000	1	1.40E-07	0.48	14	1.4	Slc39a11	Transport
DMR10:102182001	10	102182001	102183000	1000	1	3.00E-07	-0.43	11	1.1	Cog1;Fam104a	
DMR10:102299001	10	102299001	102301000	2000	1	5.30E-08	0.49	39	1.95	Sdk2	
DMR10:102303001	10	102303001	102304000	1000	1	3.40E-13	0.69	30	3	Sdk2	
DMR10:102312001	10	102312001	102315000	3000	1	1.80E-10	0.64	45	1.5	Sdk2	

DMR10:102338001	10	102338001	102340000	2000	1	6.90E-07	-0.49	34	1.7	Sdk2	
DMR10:102393001	10	102393001	102397000	4000	2	1.30E-12	1.02	97	2.42	Sdk2	
DMR10:102432001	10	102432001	102436000	4000	1	1.20E-07	0.55	57	1.43	Sdk2	
DMR10:102454001	10	102454001	102456000	2000	1	6.50E-15	0.79	34	1.7	Sdk2	
DMR10:102460001	10	102460001	102462000	2000	1	6.30E-13	-0.8	16	0.8	Sdk2	
DMR10:102490001	10	102490001	102494000	4000	1	3.00E-08	0.45	47	1.18	Sdk2	
DMR10:102495001	10	102495001	102497000	2000	1	5.00E-10	-0.54	38	1.9	Sdk2	
DMR10:102523001	10	102523001	102525000	2000	1	1.90E-07	0.47	25	1.25	Sdk2	
DMR10:103191001	10	103191001	103192000	1000	1	6.70E-12	0.64	28	2.8	Rpl38	Translation
DMR10:103296001	10	103296001	103298000	2000	1	3.10E-12	0.67	40	2	Dnai2	Cytoskeleton
DMR10:103303001	10	103303001	103304000	1000	1	6.70E-09	0.48	21	2.1	Dnai2;Kif19	Cytoskeleton;Cytoskeleton
DMR10:103311001	10	103311001	103313000	2000	1	7.20E-09	0.47	27	1.35	Dnai2;Kif19;LOC102548333	Cytoskeleton;Cytoskeleton
DMR10:103316001	10	103316001	103318000	2000	1	1.60E-07	0.59	27	1.35	Kif19;LOC102548333;LOC103693476	Cytoskeleton
DMR10:103442001	10	103442001	103446000	4000	2	4.20E-09	-0.58	63	1.57	LOC108352159;Cd300a	Immune
DMR10:103448001	10	103448001	103450000	2000	1	1.90E-07	0.36	26	1.3	LOC108352159;Cd300a	Immune
DMR10:103461001	10	103461001	103465000	4000	1	2.80E-15	0.8	74	1.85	Cd300a;Cd300lb	Immune
DMR10:103567001	10	103567001	103568000	1000	1	2.30E-13	-0.63	18	1.8	Cd300c	
DMR10:103573001	10	103573001	103574000	1000	1	5.20E-07	0.52	4	0.4	Cd300c;Cd300le	Immune
DMR10:103576001	10	103576001	103577000	1000	1	2.10E-12	0.86	23	2.3	Cd300le	Immune
DMR10:103581001	10	103581001	103582000	1000	1	4.20E-07	0.37	21	2.1	Cd300le	Immune
DMR10:103604001	10	103604001	103606000	2000	1	5.10E-07	-0.46	46	2.3	Cd300e;LOC363703	Immune
DMR10:103635001	10	103635001	103638000	3000	1	5.40E-07	-0.39	54	1.8	LOC103693477;Rab37	
DMR10:103669001	10	103669001	103670000	1000	1	4.20E-07	0.67	24	2.4	Rab37;Cd300lf	Immune
DMR10:103706001	10	103706001	103709000	3000	1	4.10E-09	0.56	38	1.27	Rab37;Slc9a3r1	
DMR10:103757001	10	103757001	103762000	5000	2	9.80E-10	0.47	99	1.98	Tmem104	
DMR10:103786001	10	103786001	103787000	1000	1	1.40E-08	0.51	16	1.6	Tmem104;LOC102554289	
DMR10:103817001	10	103817001	103820000	3000	1	8.40E-14	0.7	74	2.47	Grin2c;Fdxr	Receptor;Metabolism
DMR10:103824001	10	103824001	103825000	1000	1	3.50E-07	0.39	8	0.8	Grin2c;Fdxr;Fads6	Receptor;Metabolism
DMR10:103896001	10	103896001	103897000	1000	1	3.60E-07	0.65	34	3.4	Otop3;Hid1	Transport
DMR10:103898001	10	103898001	103904000	6000	1	6.20E-08	0.57	114	1.9	Otop3;Hid1	Transport
DMR10:103905001	10	103905001	103909000	4000	1	1.10E-11	0.97	78	1.95	Otop3;Hid1	Transport
DMR10:103952001	10	103952001	103955000	3000	1	1.20E-07	-0.41	42	1.4	Cdr2l;Mrpl58;Trnar-ccu	Translation
DMR10:103957001	10	103957001	103959000	2000	1	3.50E-07	0.37	19	0.95	Cdr2l;Mrpl58;Trnar-ccu;Trnar-ucg;Atp5h	Translation
DMR10:104008001	10	104008001	104013000	5000	1	1.50E-12	0.87	81	1.62	Trim80;Slc16a5	Transport
DMR10:104094001	10	104094001	104096000	2000	1	9.80E-07	0.38	26	1.3	Sumo2	
DMR10:104100001	10	104100001	104101000	1000	1	4.60E-07	0.58	21	2.1	Sumo2	
DMR10:104133001	10	104133001	104134000	1000	1	3.10E-10	0.48	11	1.1	Nup85;Gga3	Development
DMR10:104249001	10	104249001	104251000	2000	1	4.70E-11	-0.49	31	1.55	Grb2	
DMR10:104370001	10	104370001	104372000	2000	1	2.40E-07	-0.58	37	1.85	Tsen54;Llgl2	Translation;Transport
DMR10:104434001	10	104434001	104437000	3000	2	1.20E-12	0.84	89	2.97	LOC690323;Recql5	Epigenetic
DMR10:104440001	10	104440001	104443000	3000	1	2.20E-10	0.64	59	1.97	LOC690323;Recql5;Smim5	Epigenetic
DMR10:104492001	10	104492001	104494000	2000	1	3.90E-07	0.32	30	1.5	Recql5;Sap30bp	Epigenetic;Transcription
DMR10:104516001	10	104516001	104522000	6000	1	8.20E-08	0.7	141	2.35	Sap30bp;LOC100360679;Itgb4	Transcription;Extracellular Matrix
DMR10:104527001	10	104527001	104529000	2000	1	4.40E-09	0.53	48	2.4	LOC100360679;Itgb4	Extracellular Matrix
DMR10:104538001	10	104538001	104546000	8000	1	5.80E-12	0.66	159	1.99	Itgb4	Extracellular Matrix
DMR10:104823001	10	104823001	104824000	1000	1	2.80E-08	0.52	8	0.8	RGD1562156	
DMR10:104861001	10	104861001	104862000	1000	1	1.40E-07	0.27	7	0.7	Cd300ld	
DMR10:104875001	10	104875001	104880000	5000	1	2.90E-08	-0.33	46	0.92	Cd300ld	
DMR10:104934001	10	104934001	104935000	1000	1	5.90E-07	-0.4	9	0.9	RGD1562667	Immune
DMR10:104994001	10	104994001	104995000	1000	1	1.20E-07	0.64	20	2	RGD1564512;RGD1561778	
DMR10:105038001	10	105038001	105041000	3000	1	1.10E-07	-0.37	56	1.87	RGD1565046	
DMR10:105084001	10	105084001	105089000	5000	2	3.10E-09	0.46	109	2.18	Ten1	
DMR10:105282001	10	105282001	105283000	1000	1	8.30E-08	0.36	18	1.8	Foxj1;LOC108352160;Rnf157	Proteolysis
DMR10:105300001	10	105300001	105302000	2000	1	3.30E-07	0.57	37	1.85	LOC108352160;Rnf157	Proteolysis
DMR10:105373001	10	105373001	105375000	2000	1	7.50E-09	-0.42	33	1.65	Rnf157	Proteolysis
DMR10:105378001	10	105378001	105380000	2000	1	1.10E-08	0.57	36	1.8	Rnf157;LOC102554855	Proteolysis

DMR10:105454001	10	105454001	105456000	2000	1	5.10E-07	-0.36	33	1.65	Prpsap1	Signaling
DMR10:105517001	10	105517001	105518000	1000	1	3.30E-08	0.4	15	1.5	Ube2o	
DMR10:105576001	10	105576001	105578000	2000	1	4.00E-07	0.42	46	2.3	Aanat;Rhbdf2	Metabolism;Protease
DMR10:105613001	10	105613001	105614000	1000	1	1.40E-34	0.75	17	1.7	Cygb	
DMR10:105619001	10	105619001	105622000	3000	1	1.30E-10	0.58	67	2.23	Cygb;Prcd	
DMR10:105669001	10	105669001	105671000	2000	2	4.80E-19	0.78	57	2.85	St6galnac2	
DMR10:105678001	10	105678001	105682000	4000	1	2.50E-08	0.79	72	1.8	St6galnac2	
DMR10:105733001	10	105733001	105734000	1000	1	3.30E-07	0.31	17	1.7	St6galnac1;Mxra7	
DMR10:105746001	10	105746001	105748000	2000	1	4.50E-08	0.59	46	2.3	Mxra7;LOC103693479	
DMR10:105769001	10	105769001	105770000	1000	1	4.70E-07	0.41	24	2.4	Mxra7	
DMR10:105775001	10	105775001	105776000	1000	1	2.70E-14	1.06	32	3.2	Mxra7;Jmjd6	Golgi
DMR10:105876001	10	105876001	105878000	2000	1	6.60E-18	0.87	60	3	Mgat5b;LOC108352162	Golgi
DMR10:105897001	10	105897001	105902000	5000	1	8.60E-07	-0.39	90	1.8	Mgat5b	Golgi
DMR10:105935001	10	105935001	105936000	1000	1	2.70E-08	-0.41	33	3.3	Mgat5b	Golgi
DMR10:106067001	10	106067001	106069000	2000	1	4.50E-10	-0.55	40	2	Sec14l1	
DMR10:106234001	10	106234001	106235000	1000	1	2.20E-08	-0.43	18	1.8	9-Sep	
DMR10:106721001	10	106721001	106722000	1000	1	7.60E-08	0.49	18	1.8	Tnrc6c	Metabolism
DMR10:106781001	10	106781001	106782000	1000	1	2.30E-08	0.62	33	3.3	Tmc6;Tmc8	
DMR10:106821001	10	106821001	106826000	5000	2	1.80E-09	0.39	88	1.76	Syngr2;Tk1;Afmid	Transport;Signaling;Metabolism
DMR10:107008001	10	107008001	107014000	6000	1	7.00E-09	0.49	91	1.52	Pgs1	Transport
DMR10:107028001	10	107028001	107032000	4000	1	5.20E-08	0.56	89	2.22	Pgs1;Dnah17	Transport;Cytoskeleton
DMR10:107045001	10	107045001	107052000	7000	1	1.80E-09	-0.61	141	2.01	Dnah17	Cytoskeleton
DMR10:107058001	10	107058001	107061000	3000	1	2.80E-07	0.63	82	2.73	Dnah17	Cytoskeleton
DMR10:107304001	10	107304001	107306000	2000	1	3.50E-09	0.39	25	1.25	Usp36	Protease
DMR10:107328001	10	107328001	107335000	7000	1	2.00E-07	0.59	90	1.29	Usp36;Timp2	Protease;Protease; Proteolysis
DMR10:107339001	10	107339001	107341000	2000	1	1.60E-07	0.42	31	1.55	Timp2	Protease; Proteolysis
DMR10:107344001	10	107344001	107345000	1000	1	1.60E-07	0.43	10	1	Timp2	Protease; Proteolysis
DMR10:107364001	10	107364001	107366000	2000	1	8.00E-14	0.68	47	2.35	Timp2;Cep295nl	Protease; Proteolysis
DMR10:107418001	10	107418001	107422000	4000	3	6.10E-14	0.89	73	1.82	Lgals3bp	
DMR10:107453001	10	107453001	107458000	5000	1	4.50E-08	0.56	97	1.94	Cant1;LOC102548791;C1qtnf1	Signaling
DMR10:107501001	10	107501001	107502000	1000	1	7.90E-08	0.47	14	1.4	Engase	Metabolism
DMR10:107518001	10	107518001	107526000	8000	2	4.60E-12	-0.63	136	1.7	Engase;Rbfox3	Metabolism;Translation
DMR10:107547001	10	107547001	107548000	1000	1	5.00E-07	0.55	17	1.7	Rbfox3	Translation
DMR10:107559001	10	107559001	107561000	2000	1	3.50E-09	0.54	39	1.95	Rbfox3	Translation
DMR10:107588001	10	107588001	107591000	3000	2	1.50E-10	0.49	33	1.1	Rbfox3	Translation
DMR10:107593001	10	107593001	107594000	1000	1	3.90E-09	0.51	19	1.9	Rbfox3	Translation
DMR10:107642001	10	107642001	107644000	2000	1	1.40E-16	1.01	32	1.6	Rbfox3;LOC100363156	Translation
DMR10:107649001	10	107649001	107652000	3000	1	7.70E-08	0.48	10	0.33	Rbfox3	Translation
DMR10:107653001	10	107653001	107657000	4000	1	9.00E-07	0.38	65	1.62	Rbfox3	Translation
DMR10:107659001	10	107659001	107664000	5000	2	6.90E-08	0.55	76	1.52	Rbfox3	Translation
DMR10:107744001	10	107744001	107745000	1000	1	4.10E-09	0.65	16	1.6	Rbfox3	Translation
DMR10:107825001	10	107825001	107827000	2000	1	8.20E-10	0.72	22	1.1	Rbfox3	Translation
DMR10:108064001	10	108064001	108068000	4000	1	6.70E-07	0.43	67	1.68	Ccdc40	
DMR10:108139001	10	108139001	108141000	2000	1	1.30E-09	0.47	35	1.75	Ccdc40;Cbx2;Cbx8	Epigenetic
DMR10:108190001	10	108190001	108192000	2000	1	1.00E-07	0.49	80	4	Ccdc40;Cbx4;LOC103693490	Proteolysis
DMR10:108209001	10	108209001	108210000	1000	1	6.20E-07	0.35	5	0.5	Ccdc40;LOC103693490	
DMR10:108256001	10	108256001	108259000	3000	1	2.90E-10	0.73	51	1.7	Ccdc40;Tbc1d16	Signaling
DMR10:108288001	10	108288001	108292000	4000	1	7.30E-07	-0.34	117	2.92	Ccdc40;Tbc1d16	Signaling
DMR10:108346001	10	108346001	108348000	2000	1	1.40E-07	0.7	32	1.6	Ccdc40	
DMR10:108482001	10	108482001	108484000	2000	1	8.00E-07	0.34	36	1.8	Sgsh;Slc26a11	Transport
DMR10:108489001	10	108489001	108490000	1000	1	2.30E-09	0.5	14	1.4	Sgsh;Slc26a11	Transport
DMR10:108695001	10	108695001	108696000	1000	1	4.10E-07	0.49	13	1.3	Nptx1;LOC102551433	
DMR10:108804001	10	108804001	108805000	1000	1	7.60E-08	-0.42	13	1.3	Rptor	
DMR10:108984001	10	108984001	108986000	2000	1	2.50E-07	0.45	29	1.45	Rptor	
DMR10:109150001	10	109150001	109152000	2000	2	8.70E-09	0.53	38	1.9	Baiap2;LOC102555951	Cytoskeleton
DMR10:109225001	10	109225001	109227000	2000	1	3.20E-07	0.5	55	2.75	Aatk	Signaling
DMR10:109228001	10	109228001	109230000	2000	2	4.50E-07	0.36	20	1	Aatk	Signaling
DMR10:109304001	10	109304001	109306000	2000	1	7.70E-08	0.38	19	0.95	Slc38a10	Transport
DMR10:109321001	10	109321001	109322000	1000	1	2.80E-07	0.49	19	1.9	Slc38a10	Transport

DMR10:109428001	10	109428001	109431000	3000	1	2.30E-09	-0.42	75	2.5	Bahcc1	Transcription
DMR10:109434001	10	109434001	109435000	1000	1	1.80E-09	0.58	16	1.6	Bahcc1	Transcription
DMR10:109443001	10	109443001	109446000	3000	1	9.00E-15	0.8	57	1.9	Bahcc1	Transcription
DMR10:109456001	10	109456001	109459000	3000	2	2.40E-14	0.52	48	1.6	Bahcc1	Transcription
DMR10:109537001	10	109537001	109539000	2000	1	5.70E-08	0.55	24	1.2	Fscn2;Faap100	Cytoskeleton
DMR10:109542001	10	109542001	109543000	1000	1	8.70E-07	0.44	27	2.7	Fscn2;Faap100	Cytoskeleton
DMR10:109624001	10	109624001	109625000	1000	1	9.90E-07	0.46	19	1.9	Tspan10;Pde6g;Oxld1;Ccdc137	Signaling;Metabolism
DMR10:109696001	10	109696001	109699000	3000	1	3.40E-07	0.44	26	0.87	LOC103693493;Gcgr	Receptor
DMR10:109729001	10	109729001	109731000	2000	1	5.80E-07	0.55	38	1.9	Mcrip1;LOC108352191;Ppp1r27;P4hb	Signaling;Transcription
DMR10:109764001	10	109764001	109765000	1000	1	7.40E-09	0.43	14	1.4	Arhgdia;Alyref;Anapc11	Signaling;Metabolism;Proteolysis
DMR10:109806001	10	109806001	109808000	2000	1	7.80E-07	-0.46	54	2.7	Sirt7;Mafg;Pycr1	Transcription;Metabolism
DMR10:109825001	10	109825001	109826000	1000	1	4.90E-10	0.55	13	1.3	Pycr1;Myadml2;Notum	Metabolism
DMR10:109828001	10	109828001	109829000	1000	1	5.20E-09	-0.72	3	0.3	Pycr1;Myadml2;Notum	Metabolism
DMR10:109905001	10	109905001	109907000	2000	1	9.80E-10	0.68	38	1.9	Lrrc45;LOC103693494;LOC103689934;Rac3;Dcxr;LOC688320	Signaling;Metabolism
DMR10:109912001	10	109912001	109913000	1000	1	3.30E-08	0.56	20	2	LOC103689934;Rac3;Dcxr;LOC688320	Signaling;Metabolism
DMR10:110038001	10	110038001	110039000	1000	1	8.80E-07	0.46	8	0.8	Ccdc57	
DMR10:110058001	10	110058001	110059000	1000	1	1.90E-08	-0.43	18	1.8	Ccdc57	
DMR10:110222001	10	110222001	110224000	2000	1	1.70E-07	0.49	38	1.9	Cd7	
DMR10:110238001	10	110238001	110239000	1000	1	2.20E-07	-0.5	5	0.5	Cd7	
DMR10:110252001	10	110252001	110254000	2000	1	9.10E-07	0.43	31	1.55	LOC108352230;Sectm1b;Sectm1a	
DMR10:110346001	10	110346001	110347000	1000	1	4.10E-08	0.72	35	3.5	Tex19.1	
DMR10:110377001	10	110377001	110379000	2000	1	2.70E-13	0.65	28	1.4	Ogfod3	Golgi
DMR10:110438001	10	110438001	110440000	2000	1	2.60E-07	0.51	27	1.35	LOC619574;LOC691137;Narf	Metabolism
DMR10:110462001	10	110462001	110463000	1000	1	3.60E-07	0.54	14	1.4	Narf;Foxk2	Metabolism
DMR10:110607001	10	110607001	110609000	2000	1	4.40E-08	-0.42	32	1.6	Fn3krp	Signaling
DMR10:110692001	10	110692001	110694000	2000	1	8.80E-07	0.24	36	1.8	Tbcd;Znf750	Transcription
DMR10:110706001	10	110706001	110707000	1000	1	2.80E-08	-0.49	21	2.1	Tbcd;Znf750	Transcription
DMR10:110791001	10	110791001	110795000	4000	1	2.20E-11	-0.45	38	0.95	Tbcd;B3gntl1	Transcription;Golgi
DMR11:585001	11	585001	586000	1000	1	1.10E-08	-0.49	0	0	Epha3	Receptor
DMR11:723001	11	723001	725000	2000	1	6.40E-09	-0.54	19	0.95	Epha3	Receptor
DMR11:737001	11	737001	739000	2000	1	3.00E-09	-0.61	11	0.55	Epha3	Receptor
DMR11:784001	11	784001	786000	2000	1	1.70E-08	-0.39	17	0.85	Epha3	Receptor
DMR11:1437001	11	1437001	1439000	2000	2	3.60E-19	1.25	42	2.1	Csnka2ip	
DMR11:1519001	11	1519001	1521000	2000	1	3.40E-09	-0.72	10	0.5	Csnka2ip	
DMR11:1578001	11	1578001	1579000	1000	1	3.50E-08	-0.4	7	0.7	Csnka2ip	
DMR11:2813001	11	2813001	2814000	1000	1	4.10E-14	1.12	46	4.6	RGD1561853	
DMR11:4268001	11	4268001	4274000	6000	3	8.60E-08	-0.35	72	1.2	Cadm2	
DMR11:4399001	11	4399001	4401000	2000	1	1.30E-07	-0.55	12	0.6	Cadm2	
DMR11:7302001	11	7302001	7305000	3000	2	5.40E-13	0.37	34	1.13	Gbe1;LOC108352291	Metabolism
DMR11:7318001	11	7318001	7319000	1000	1	8.30E-07	-0.39	9	0.9	Gbe1	Metabolism
DMR11:9266001	11	9266001	9267000	1000	1	4.40E-10	-0.53	6	0.6	Robo1	
DMR11:9274001	11	9274001	9276000	2000	1	5.40E-07	-0.52	16	0.8	Robo1	
DMR11:9316001	11	9316001	9317000	1000	1	1.50E-07	-0.26	5	0.5	Robo1	
DMR11:9399001	11	9399001	9400000	1000	1	7.50E-15	0.85	3	0.3	Robo1	
DMR11:9425001	11	9425001	9429000	4000	2	2.30E-11	0.88	75	1.88	Robo1	
DMR11:9497001	11	9497001	9499000	2000	1	3.00E-12	0.73	26	1.3	Robo1	
DMR11:9538001	11	9538001	9540000	2000	1	3.40E-07	-0.51	11	0.55	Robo1	
DMR11:9553001	11	9553001	9554000	1000	1	3.30E-07	-0.53	11	1.1	Robo1	
DMR11:9631001	11	9631001	9632000	1000	1	4.60E-08	0.73	25	2.5	Robo1;LOC102551469	
DMR11:9650001	11	9650001	9651000	1000	1	1.10E-07	-0.5	1	0.1	Robo1;LOC102551469	
DMR11:9744001	11	9744001	9745000	1000	1	3.30E-09	0.51	14	1.4	Robo1	
DMR11:9812001	11	9812001	9814000	2000	1	1.10E-10	0.64	36	1.8	Robo1	
DMR11:9864001	11	9864001	9865000	1000	1	6.70E-09	0.59	23	2.3	Robo1;LOC108352378	
DMR11:9876001	11	9876001	9883000	7000	2	8.20E-08	0.43	91	1.3	Robo1	
DMR11:10067001	11	10067001	10069000	2000	1	9.70E-12	-0.48	37	1.85	Robo1	
DMR11:10088001	11	10088001	10092000	4000	1	8.50E-09	-0.32	78	1.95	Robo1	

DMR11:10119001	11	10119001	10121000	2000	1	6.50E-07	-0.37	26	1.3	Robo1	
DMR11:10124001	11	10124001	10125000	1000	1	4.10E-09	-0.45	21	2.1	Robo1	
DMR11:11115001	11	11115001	11116000	1000	1	1.90E-08	-0.64	5	0.5	Robo2	
DMR11:11236001	11	11236001	11239000	3000	1	3.00E-11	-0.61	29	0.97	Robo2	
DMR11:11245001	11	11245001	11247000	2000	2	4.10E-07	-0.49	14	0.7	Robo2	
DMR11:11574001	11	11574001	11575000	1000	1	6.50E-11	-0.51	16	1.6	Robo2	
DMR11:11576001	11	11576001	11577000	1000	1	6.40E-21	0.95	15	1.5	Robo2	
DMR11:11797001	11	11797001	11800000	3000	1	2.30E-08	-0.47	27	0.9	Robo2	
DMR11:11873001	11	11873001	11874000	1000	1	2.40E-07	-0.59	7	0.7	Robo2	
DMR11:11912001	11	11912001	11914000	2000	1	5.80E-07	0.38	14	0.7	Robo2	
DMR11:11936001	11	11936001	11937000	1000	1	1.40E-11	-0.64	5	0.5	Robo2	
DMR11:12012001	11	12012001	12014000	2000	1	1.40E-08	-0.52	6	0.3	Robo2	
DMR11:12272001	11	12272001	12276000	4000	1	9.70E-10	0.62	39	0.98	Robo2;LOC680624	
DMR11:12465001	11	12465001	12466000	1000	1	9.00E-07	-0.54	3	0.3	Robo2	
DMR11:12482001	11	12482001	12483000	1000	1	1.50E-07	-0.44	2	0.2	Robo2	
DMR11:12499001	11	12499001	12501000	2000	1	1.40E-14	0.39	16	0.8	Robo2	
DMR11:13968001	11	13968001	13969000	1000	1	2.10E-07	0.64	12	1.2	Lipi	Metabolism
DMR11:14238001	11	14238001	14243000	5000	1	5.60E-07	-0.23	43	0.86	Samsn1	
DMR11:14365001	11	14365001	14367000	2000	1	3.60E-07	-0.44	15	0.75	Samsn1	
DMR11:14383001	11	14383001	14384000	1000	1	1.00E-10	0.63	10	1	Samsn1	
DMR11:14410001	11	14410001	14411000	1000	1	1.30E-07	-0.73	8	0.8	Samsn1	
DMR11:14415001	11	14415001	14417000	2000	1	4.70E-08	-0.3	22	1.1	Samsn1	
DMR11:14687001	11	14687001	14688000	1000	1	5.50E-07	-0.33	20	2	Nrip1	
DMR11:14696001	11	14696001	14697000	1000	1	1.20E-08	-0.42	15	1.5	Nrip1	
DMR11:14712001	11	14712001	14716000	4000	2	2.40E-07	-0.38	68	1.7	Nrip1	
DMR11:14719001	11	14719001	14725000	6000	1	1.00E-07	-0.53	73	1.22	Nrip1	
DMR11:14726001	11	14726001	14729000	3000	1	2.60E-07	-0.37	53	1.77	Nrip1	
DMR11:14731001	11	14731001	14736000	5000	3	7.00E-13	-0.73	81	1.62	Nrip1	
DMR11:14737001	11	14737001	14740000	3000	2	7.60E-09	-0.54	51	1.7	Nrip1	
DMR11:15457001	11	15457001	15462000	5000	1	8.40E-07	-0.34	81	1.62	Usp25	Protease
DMR11:15477001	11	15477001	15478000	1000	1	2.20E-07	-0.29	7	0.7	Usp25	Protease
DMR11:15506001	11	15506001	15507000	1000	1	3.20E-09	-0.7	2	0.2	Usp25	Protease
DMR11:16060001	11	16060001	16061000	1000	1	1.80E-07	-0.51	10	1	Mir99a;Mirlet7c-1	
DMR11:16088001	11	16088001	16089000	1000	1	1.80E-08	-0.41	9	0.9	Mir3588;Mir125b-2	
DMR11:17576001	11	17576001	17577000	1000	1	1.80E-08	-0.61	4	0.4	Tmprss15	Protease
DMR11:17598001	11	17598001	17599000	1000	1	1.30E-11	0.62	21	2.1	Tmprss15	Protease
DMR11:19670001	11	19670001	19671000	1000	1	1.20E-07	-0.43	7	0.7	Ncam2	
DMR11:19688001	11	19688001	19689000	1000	1	6.40E-09	0.79	13	1.3	Ncam2	
DMR11:19770001	11	19770001	19772000	2000	1	1.10E-28	0.66	14	0.7	Ncam2;LOC681289	
DMR11:20014001	11	20014001	20015000	1000	1	5.00E-08	-0.64	4	0.4	Ncam2	
DMR11:20083001	11	20083001	20084000	1000	1	2.90E-07	-0.35	8	0.8	Ncam2	
DMR11:20420001	11	20420001	20422000	2000	1	3.10E-08	-0.49	13	0.65	Ncam2	
DMR11:24230001	11	24230001	24231000	1000	1	1.20E-08	-0.67	4	0.4	Jam2	
DMR11:24309001	11	24309001	24310000	1000	1	6.10E-07	-0.34	19	1.9	Gabpa	Transcription
DMR11:24479001	11	24479001	24482000	3000	1	1.30E-15	0.92	73	2.43	App	Protease; Proteolysis
DMR11:24617001	11	24617001	24619000	2000	1	4.10E-11	-0.64	10	0.5	App	Protease; Proteolysis
DMR11:24643001	11	24643001	24648000	5000	1	4.90E-10	-0.4	47	0.94	App	Protease; Proteolysis
DMR11:25077001	11	25077001	25078000	1000	1	1.10E-10	0.81	19	1.9	Cyyr1	
DMR11:25464001	11	25464001	25465000	1000	1	3.00E-07	-0.69	4	0.4	Adamts5;LOC102552725	Protease
DMR11:27010001	11	27010001	27011000	1000	1	4.90E-08	-0.33	24	2.4	N6amt1	Epigenetic
DMR11:27219001	11	27219001	27221000	2000	1	1.60E-12	0.42	12	0.6	Map3k7cl	
DMR11:27225001	11	27225001	27229000	4000	2	1.70E-08	0.45	48	1.2	Map3k7cl	
DMR11:27255001	11	27255001	27257000	2000	1	6.00E-08	0.7	32	1.6	Map3k7cl	
DMR11:27403001	11	27403001	27405000	2000	1	1.60E-07	-0.37	18	0.9	Bach1	
DMR11:27987001	11	27987001	27988000	1000	1	3.40E-08	0.47	5	0.5	Grik1	Receptor
DMR11:27997001	11	27997001	27998000	1000	1	1.60E-07	-0.63	2	0.2	Grik1	Receptor
DMR11:28009001	11	28009001	28010000	1000	1	2.00E-13	0.75	22	2.2	Grik1	Receptor
DMR11:28050001	11	28050001	28051000	1000	1	9.10E-07	0.46	9	0.9	Grik1;LOC102549158	Receptor
DMR11:28064001	11	28064001	28065000	1000	1	5.30E-07	-0.45	6	0.6	Grik1;LOC102549158	Receptor
DMR11:28131001	11	28131001	28132000	1000	1	2.30E-08	-0.41	4	0.4	Grik1;LOC108352301	Receptor
DMR11:28138001	11	28138001	28139000	1000	1	1.50E-08	0.63	13	1.3	Grik1;LOC108352301	Receptor

DMR11:28732001	11	28732001	28734000	2000	1	4.90E-07	0.51	24	1.2	LOC100363287;Krtap14l;Krtap13-2	Development
DMR11:28786001	11	28786001	28787000	1000	1	6.40E-07	-0.37	7	0.7	Krtap14;Krtap15-1	Development
DMR11:29649001	11	29649001	29651000	2000	1	1.00E-07	-0.35	32	1.6	LOC100359671;Krtap7-1	
DMR11:29932001	11	29932001	29936000	4000	1	2.30E-09	0.61	100	2.5	Tiam1	
DMR11:29963001	11	29963001	29965000	2000	1	1.10E-16	-0.51	60	3	Tiam1	
DMR11:30574001	11	30574001	30578000	4000	1	1.80E-08	-0.31	84	2.1	Hunk	
DMR11:30582001	11	30582001	30584000	2000	1	3.20E-07	-0.37	35	1.75	Hunk	
DMR11:30609001	11	30609001	30610000	1000	1	2.70E-08	-0.45	16	1.6	Hunk	
DMR11:30619001	11	30619001	30623000	4000	1	1.10E-07	0.48	62	1.55	Hunk	
DMR11:30627001	11	30627001	30633000	6000	1	2.70E-07	0.57	82	1.37	Hunk	
DMR11:30669001	11	30669001	30671000	2000	1	2.90E-15	0.33	24	1.2	Hunk	
DMR11:30997001	11	30997001	31001000	4000	1	9.10E-07	0.42	56	1.4	Eva1c	
DMR11:31013001	11	31013001	31014000	1000	1	7.10E-10	-0.5	42	4.2	Eva1c	
DMR11:31063001	11	31063001	31066000	3000	1	3.70E-07	0.53	51	1.7	Eva1c	
DMR11:31082001	11	31082001	31083000	1000	1	1.20E-08	0.4	12	1.2	Eva1c	
DMR11:31113001	11	31113001	31114000	1000	1	8.90E-07	0.41	13	1.3	RGD1306954;Synj1	Signaling
DMR11:31179001	11	31179001	31180000	1000	1	2.10E-07	-0.51	4	0.4	Synj1;Paxbp1	Signaling;Epigenetic
DMR11:31240001	11	31240001	31243000	3000	1	1.40E-07	-0.6	28	0.93	RGD1562726	
DMR11:31537001	11	31537001	31539000	2000	1	3.00E-09	0.5	37	1.85	Ifnar2	Receptor
DMR11:31632001	11	31632001	31634000	2000	2	2.70E-09	-0.52	26	1.3	Ifnar1	Receptor
DMR11:31668001	11	31668001	31673000	5000	1	8.50E-09	0.77	66	1.32	Ifnar1;LOC363746	Receptor
DMR11:31699001	11	31699001	31700000	1000	1	3.20E-07	-0.36	15	1.5	Ifngr2	Receptor
DMR11:31735001	11	31735001	31738000	3000	1	8.30E-07	-0.36	48	1.6	Tmem50b	
DMR11:31817001	11	31817001	31819000	2000	1	2.80E-10	-0.71	42	2.1	Son	Translation
DMR11:31939001	11	31939001	31941000	2000	1	3.70E-08	-0.4	24	1.2	Itsn1	Transport
DMR11:31943001	11	31943001	31946000	3000	1	2.60E-10	-0.55	46	1.53	Itsn1	Transport
DMR11:31994001	11	31994001	31995000	1000	1	5.00E-08	-0.45	25	2.5	Itsn1	Transport
DMR11:32012001	11	32012001	32013000	1000	1	1.00E-07	-0.39	11	1.1	Itsn1	Transport
DMR11:32053001	11	32053001	32060000	7000	1	3.90E-07	0.4	139	1.99	Itsn1	Transport
DMR11:32062001	11	32062001	32067000	5000	1	1.00E-07	-0.35	81	1.62	Itsn1	Transport
DMR11:32259001	11	32259001	32262000	3000	1	7.00E-07	-0.38	37	1.23	Mrps6	Translation
DMR11:32269001	11	32269001	32272000	3000	1	8.10E-07	-0.48	38	1.27	Mrps6	Translation
DMR11:32430001	11	32430001	32433000	3000	2	1.50E-09	0.61	38	1.27	LOC102548387;Kcne2	Transport
DMR11:32434001	11	32434001	32442000	8000	2	4.80E-09	-0.37	132	1.65	LOC102548387;Kcne2;Smim11	Transport
DMR11:32455001	11	32455001	32457000	2000	1	5.40E-07	0.37	40	2	Kcne2;Smim11;LOC498063	Transport
DMR11:32520001	11	32520001	32526000	6000	1	9.30E-12	-0.47	70	1.17	Kcne1	Transport
DMR11:32764001	11	32764001	32765000	1000	1	2.00E-07	0.4	15	1.5	Runx1	Transcription
DMR11:32769001	11	32769001	32771000	2000	1	8.30E-20	1.11	70	3.5	Runx1	Transcription
DMR11:32846001	11	32846001	32854000	8000	1	7.00E-10	-0.44	181	2.26	Runx1	Transcription
DMR11:32875001	11	32875001	32877000	2000	1	4.20E-07	0.36	25	1.25	Runx1	Transcription
DMR11:32887001	11	32887001	32889000	2000	1	4.90E-11	0.57	26	1.3	Runx1	Transcription
DMR11:32952001	11	32952001	32955000	3000	1	3.00E-10	-0.62	36	1.2	Runx1	Transcription
DMR11:33786001	11	33786001	33787000	1000	1	2.00E-07	0.41	19	1.9	Setd4	Epigenetic
DMR11:33835001	11	33835001	33837000	2000	1	2.40E-07	0.43	28	1.4	Trub2-ps1;LOC100909699;LOC108348130	
DMR11:33846001	11	33846001	33850000	4000	1	4.30E-07	0.42	44	1.1	LOC100909699;LOC108348130;Trub2-ps2;LOC100909756	
DMR11:33864001	11	33864001	33865000	1000	1	5.10E-08	0.51	8	0.8	Trub2-ps2;LOC100909756;LOC102556347	Metabolism
DMR11:33900001	11	33900001	33901000	1000	1	1.70E-07	-0.72	3	0.3	LOC100360601;Cbr3	Metabolism
DMR11:33905001	11	33905001	33906000	1000	1	2.00E-15	-0.53	17	1.7	Cbr3	Metabolism
DMR11:34123001	11	34123001	34126000	3000	1	3.60E-09	-0.46	39	1.3	Chaf1b;Cldn14	Epigenetic;Cell Junction
DMR11:34151001	11	34151001	34153000	2000	1	1.40E-07	-0.41	21	1.05	Cldn14	Cell Junction
DMR11:34326001	11	34326001	34328000	2000	2	9.90E-10	-0.61	56	2.8	Sim2	Transcription
DMR11:34336001	11	34336001	34337000	1000	1	1.10E-08	0.56	17	1.7	Sim2	Transcription
DMR11:34407001	11	34407001	34408000	1000	1	8.00E-07	0.41	14	1.4	Hlcs	Metabolism
DMR11:34435001	11	34435001	34439000	4000	1	5.20E-07	-0.47	83	2.08	Hlcs;LOC103693521;LOC108352309	Metabolism

DMR11:34504001	11	34504001	34505000	1000	1	2.90E-07	-0.34	16	1.6	Hlcs	Metabolism
DMR11:34527001	11	34527001	34528000	1000	1	1.40E-09	-0.45	12	1.2	Hlcs	Metabolism
DMR11:34596001	11	34596001	34597000	1000	1	1.50E-07	-0.49	17	1.7	Pigp;Ttc3	Transport;Proteolysis
DMR11:34744001	11	34744001	34748000	4000	1	1.30E-07	0.27	38	0.95	Ttc3;LOC108352310;LOC102549457	Proteolysis
DMR11:34786001	11	34786001	34787000	1000	1	9.10E-11	-0.44	13	1.3	Dscr3;LOC102549666;LOC108352311	
DMR11:34892001	11	34892001	34894000	2000	1	1.60E-09	-0.52	25	1.25	LOC103693522;Dyrk1a	
DMR11:34901001	11	34901001	34903000	2000	1	4.20E-08	-0.39	21	1.05	LOC103693522;Dyrk1a	
DMR11:35014001	11	35014001	35016000	2000	1	3.30E-07	0.45	39	1.95	Kcnj6	Transport
DMR11:35017001	11	35017001	35020000	3000	2	1.10E-21	0.8	62	2.07	Kcnj6	Transport
DMR11:35047001	11	35047001	35048000	1000	1	3.40E-08	0.6	22	2.2	Kcnj6;LOC102550239	Transport
DMR11:35051001	11	35051001	35053000	2000	2	8.30E-09	0.54	29	1.45	Kcnj6;LOC102550239	Transport
DMR11:35172001	11	35172001	35174000	2000	1	2.40E-08	0.52	25	1.25	Kcnj6	Transport
DMR11:35188001	11	35188001	35190000	2000	2	5.20E-12	0.57	28	1.4	Kcnj6	Transport
DMR11:35195001	11	35195001	35201000	6000	2	8.00E-12	-0.43	102	1.7	Kcnj6	Transport
DMR11:35212001	11	35212001	35213000	1000	1	2.80E-07	0.53	14	1.4	Kcnj6	Transport
DMR11:35267001	11	35267001	35268000	1000	1	7.10E-07	0.45	7	0.7	Kcnj6	Transport
DMR11:35563001	11	35563001	35565000	2000	1	7.90E-07	-0.37	20	1	Kcnj15	Transport
DMR11:35590001	11	35590001	35592000	2000	1	2.60E-07	-0.48	38	1.9	Kcnj15	Transport
DMR11:35641001	11	35641001	35644000	3000	1	1.70E-07	0.32	43	1.43	Erg	Transcription
DMR11:35645001	11	35645001	35647000	2000	1	5.40E-07	-0.35	35	1.75	Erg	Transcription
DMR11:35752001	11	35752001	35753000	1000	1	5.50E-11	0.43	15	1.5	Erg	Transcription
DMR11:36320001	11	36320001	36321000	1000	1	5.40E-10	-0.78	4	0.4	Psmg1	Transcription
DMR11:36347001	11	36347001	36349000	2000	1	3.30E-10	-0.41	21	1.05	Psmg1;Brwd1	Transcription
DMR11:36588001	11	36588001	36589000	1000	1	4.80E-08	0.45	38	3.8	Sh3bgr	
DMR11:36643001	11	36643001	36644000	1000	1	7.60E-08	0.44	17	1.7	B3galt5	Golgi
DMR11:36853001	11	36853001	36854000	1000	1	4.80E-08	-0.5	3	0.3	LOC108352315;Pcp4	
DMR11:36872001	11	36872001	36873000	1000	1	1.60E-10	-0.56	0	0	Pcp4	
DMR11:37038001	11	37038001	37039000	1000	1	6.70E-07	0.24	6	0.6	Dscam	Cytoskeleton
DMR11:37133001	11	37133001	37136000	3000	1	2.10E-07	0.36	14	0.47	Dscam	Cytoskeleton
DMR11:37250001	11	37250001	37251000	1000	1	1.70E-09	0.62	26	2.6	Dscam	Cytoskeleton
DMR11:37276001	11	37276001	37278000	2000	1	5.30E-07	0.46	25	1.25	Dscam	Cytoskeleton
DMR11:37301001	11	37301001	37302000	1000	1	5.30E-09	0.6	12	1.2	Dscam;LOC102551331	Cytoskeleton
DMR11:37559001	11	37559001	37560000	1000	1	2.30E-07	-0.57	5	0.5	Dscam	Cytoskeleton
DMR11:37608001	11	37608001	37612000	4000	1	3.70E-11	-0.72	18	0.45	Dscam	Cytoskeleton
DMR11:37813001	11	37813001	37815000	2000	1	7.20E-07	0.54	27	1.35	Bace2	Protease
DMR11:37882001	11	37882001	37884000	2000	1	2.20E-07	-0.56	17	0.85	Bace2;Mx1	Protease;Transport
DMR11:38086001	11	38086001	38089000	3000	1	5.50E-08	-0.37	42	1.4	Tmprss2	Protease
DMR11:38335001	11	38335001	38336000	1000	1	1.00E-08	0.58	12	1.2	Prdm15	Transcription
DMR11:38347001	11	38347001	38349000	2000	1	2.00E-08	-0.42	34	1.7	Prdm15;LOC108352316;C2cd2	Transcription
DMR11:38373001	11	38373001	38374000	1000	1	3.80E-09	-0.51	11	1.1	LOC108352316;C2cd2	
DMR11:38532001	11	38532001	38534000	2000	1	3.70E-07	-0.31	25	1.25	LOC108352317;Nsun3	Epigenetic
DMR11:38548001	11	38548001	38550000	2000	1	4.40E-28	0.78	34	1.7	LOC108352317;Nsun3;LOC103693528	Epigenetic
DMR11:38719001	11	38719001	38721000	2000	1	6.10E-10	-0.66	8	0.4	LOC100910184;Zfp758	
DMR11:41906001	11	41906001	41907000	1000	1	2.40E-10	-0.48	12	1.2	Epha6	Receptor
DMR11:42102001	11	42102001	42103000	1000	1	3.10E-07	-0.42	16	1.6	Epha6	Receptor
DMR11:42190001	11	42190001	42191000	1000	1	9.20E-14	0.63	14	1.4	Epha6	Receptor
DMR11:42265001	11	42265001	42266000	1000	1	2.00E-08	-0.9	2	0.2	Epha6	Receptor
DMR11:42315001	11	42315001	42316000	1000	1	2.80E-07	-0.31	18	1.8	Epha6	Receptor
DMR11:42405001	11	42405001	42406000	1000	1	1.30E-12	0.97	17	1.7	Epha6	Receptor
DMR11:42554001	11	42554001	42556000	2000	2	3.10E-10	0.47	18	0.9	Epha6	Receptor
DMR11:42566001	11	42566001	42567000	1000	1	5.90E-07	-0.31	11	1.1	Epha6	Receptor
DMR11:42602001	11	42602001	42604000	2000	1	5.00E-08	-0.4	19	0.95	Epha6	Receptor
DMR11:42615001	11	42615001	42617000	2000	2	1.50E-09	0.61	43	2.15	Epha6	Receptor
DMR11:42950001	11	42950001	42952000	2000	1	8.70E-09	0.41	28	1.4	Crybg3	
DMR11:43134001	11	43134001	43140000	6000	1	8.40E-07	-0.29	76	1.27	Olr1528;Olr1529	Signaling
DMR11:43286001	11	43286001	43287000	1000	1	1.90E-08	-0.57	2	0.2	Olr1538	Signaling
DMR11:43289001	11	43289001	43295000	6000	2	5.00E-14	-0.28	50	0.83	Olr1538	Signaling
DMR11:43505001	11	43505001	43506000	1000	1	9.00E-08	-0.49	4	0.4	Olr1550-ps;Olr1551	Signaling
DMR11:43714001	11	43714001	43717000	3000	1	7.70E-07	-0.41	65	2.17	Olr1560;LOC108352374	Signaling

DMR11:44050001	11	44050001	44052000	2000	1	1.00E-07	0.49	24	1.2	Cpox	Metabolism
DMR11:44179001	11	44179001	44181000	2000	1	4.80E-07	-0.36	37	1.85	St3gal6	Transport
DMR11:44201001	11	44201001	44204000	3000	1	5.80E-11	-0.5	45	1.5	St3gal6	Transport
DMR11:44221001	11	44221001	44223000	2000	1	3.40E-15	-0.6	35	1.75	St3gal6;LOC108352323	Transport
DMR11:44986001	11	44986001	44987000	1000	1	2.60E-07	-0.47	11	1.1	Col8a1	Extracellular Matrix
DMR11:44995001	11	44995001	44998000	3000	1	1.10E-09	0.76	53	1.77	Col8a1	Extracellular Matrix
DMR11:45006001	11	45006001	45008000	2000	2	1.50E-07	0.47	48	2.4	Col8a1	Extracellular Matrix
DMR11:45021001	11	45021001	45026000	5000	1	1.70E-08	-0.32	44	0.88	Cmss1	Metabolism
DMR11:45115001	11	45115001	45117000	2000	1	7.90E-07	-0.46	23	1.15	Cmss1;Filip1l	Metabolism
DMR11:45306001	11	45306001	45308000	2000	1	1.40E-07	-0.44	26	1.3	Cmss1	Metabolism
DMR11:45363001	11	45363001	45364000	1000	1	1.80E-08	-0.37	15	1.5	Tmem30c;LOC108352325	
DMR11:45473001	11	45473001	45474000	1000	1	8.30E-18	-0.75	8	0.8	Nit2;Tomm70	Metabolism;Transport
DMR11:45496001	11	45496001	45497000	1000	1	4.60E-09	-0.43	12	1.2	Tomm70	Transport
DMR11:45909001	11	45909001	45913000	4000	1	3.60E-10	-0.45	143	3.58	Lnp1;LOC103693553	
DMR11:46055001	11	46055001	46058000	3000	1	5.00E-09	0.74	59	1.97	Tmem45al	
DMR11:46139001	11	46139001	46141000	2000	1	1.10E-10	-0.53	12	0.6	Adgrg7	Signaling
DMR11:46299001	11	46299001	46300000	1000	1	1.00E-07	-0.58	4	0.4	Abi3bp	
DMR11:46313001	11	46313001	46314000	1000	1	4.70E-11	0.56	16	1.6	Abi3bp	
DMR11:46369001	11	46369001	46370000	1000	1	3.60E-11	0.77	18	1.8	Abi3bp	
DMR11:46625001	11	46625001	46626000	1000	1	4.30E-07	0.46	11	1.1	Impg2	Extracellular Matrix
DMR11:46641001	11	46641001	46643000	2000	1	7.10E-08	0.34	31	1.55	Impg2	Extracellular Matrix
DMR11:46923001	11	46923001	46924000	1000	1	1.10E-07	-0.35	5	0.5	Senp7	Protease
DMR11:47091001	11	47091001	47092000	1000	1	8.90E-10	-0.52	10	1	Zbtb11;LOC108352369	Transcription
DMR11:47230001	11	47230001	47231000	1000	1	2.50E-07	0.47	32	3.2	Nxpe3	
DMR11:47262001	11	47262001	47264000	2000	1	1.80E-08	-0.49	30	1.5	Nfkbiz	Signaling
DMR11:50804001	11	50804001	50805000	1000	1	8.90E-08	-0.42	8	0.8	Alcam	Immune
DMR11:51156001	11	51156001	51161000	5000	1	1.30E-08	-0.33	60	1.2	Cblb	Metabolism
DMR11:51196001	11	51196001	51197000	1000	1	2.10E-13	-0.55	10	1	Cblb	Metabolism
DMR11:53193001	11	53193001	53194000	1000	1	7.90E-09	-0.47	14	1.4	Bbx	Transcription
DMR11:53197001	11	53197001	53199000	2000	1	8.30E-10	-0.49	38	1.9	Bbx	Transcription
DMR11:53526001	11	53526001	53529000	3000	1	1.20E-07	-0.45	44	1.47	Cd47	Cytoskeleton
DMR11:53558001	11	53558001	53561000	3000	1	3.10E-07	-0.38	37	1.23	Cd47	Cytoskeleton
DMR11:53566001	11	53566001	53573000	7000	4	1.20E-09	-0.49	155	2.21	Cd47	Cytoskeleton
DMR11:54918001	11	54918001	54919000	1000	1	4.30E-09	0.39	21	2.1	Morc1	
DMR11:55044001	11	55044001	55045000	1000	1	4.70E-17	1.22	26	2.6	Morc1;LOC108352393	
DMR11:57237001	11	57237001	57238000	1000	1	1.80E-09	-0.57	13	1.3	Plcxd2	
DMR11:57385001	11	57385001	57386000	1000	1	2.00E-07	0.5	12	1.2	Phldb2;LOC102552450	
DMR11:58692001	11	58692001	58694000	2000	1	4.60E-10	0.49	27	1.35	Gap43	
DMR11:58796001	11	58796001	58797000	1000	1	5.20E-10	0.62	20	2	Lsamp	Immune
DMR11:58875001	11	58875001	58876000	1000	1	9.30E-07	-0.33	19	1.9	Lsamp	Immune
DMR11:59123001	11	59123001	59125000	2000	1	8.10E-07	-0.28	20	1	Lsamp	Immune
DMR11:59332001	11	59332001	59334000	2000	1	4.30E-16	0.86	26	1.3	Lsamp	Immune
DMR11:60038001	11	60038001	60040000	2000	1	8.60E-08	-0.5	10	0.5	Phldb2	
DMR11:60108001	11	60108001	60111000	3000	1	6.90E-07	-0.42	41	1.37	Tmprss7	Protease
DMR11:60260001	11	60260001	60262000	2000	1	5.10E-12	-0.62	16	0.8	Slc9c1;LOC685706;LOC685716	Transport
DMR11:60652001	11	60652001	60654000	2000	1	1.70E-07	-0.51	10	0.5	Ccdc80	
DMR11:60823001	11	60823001	60826000	3000	1	5.20E-09	0.49	41	1.37	Cd200r1l	Receptor
DMR11:61076001	11	61076001	61077000	1000	1	1.30E-13	0.99	25	2.5	Boc	
DMR11:61143001	11	61143001	61147000	4000	1	9.80E-07	-0.38	64	1.6	Boc	
DMR11:61207001	11	61207001	61208000	1000	1	2.40E-07	-0.31	14	1.4	Cfap44	
DMR11:61214001	11	61214001	61215000	1000	1	4.60E-12	0.67	21	2.1	Cfap44	
DMR11:61216001	11	61216001	61217000	1000	1	2.40E-21	0.93	25	2.5	Cfap44	
DMR11:61251001	11	61251001	61252000	1000	1	9.40E-07	0.51	16	1.6	Spice1	
DMR11:61330001	11	61330001	61335000	5000	1	1.90E-09	-0.41	60	1.2	Sidt1	
DMR11:61364001	11	61364001	61365000	1000	1	4.40E-16	-0.61	14	1.4	Sidt1	
DMR11:61578001	11	61578001	61579000	1000	1	1.70E-07	0.44	12	1.2	Atp6v1a	Metabolism
DMR11:61629001	11	61629001	61630000	1000	1	7.00E-08	-0.53	9	0.9	Gramd1c;LOC108352337;LOC102557200	
DMR11:61694001	11	61694001	61695000	1000	1	3.20E-07	-0.41	7	0.7	Ccdc191	
DMR11:61755001	11	61755001	61759000	4000	2	6.30E-10	0.6	70	1.75	Ccdc191;Qtrt2	Translation
DMR11:61766001	11	61766001	61771000	5000	1	3.10E-07	-0.34	68	1.36	Qtrt2	Translation
DMR11:61839001	11	61839001	61841000	2000	1	9.50E-12	0.71	42	2.1	Drd3	Signaling

DMR11:61842001	11	61842001	61845000	3000	1	3.30E-11	0.74	50	1.67	Drd3	Signaling
DMR11:61863001	11	61863001	61865000	2000	1	3.90E-09	0.6	29	1.45	Drd3	Signaling
DMR11:61960001	11	61960001	61961000	1000	1	7.80E-08	0.38	12	1.2	Tigit	
DMR11:61968001	11	61968001	61971000	3000	1	3.20E-07	0.43	42	1.4	Tigit	
DMR11:62033001	11	62033001	62036000	3000	2	4.00E-08	-0.56	28	0.93	Zbtb20	Transcription
DMR11:62055001	11	62055001	62056000	1000	1	1.30E-08	-0.36	12	1.2	Zbtb20	Transcription
DMR11:62061001	11	62061001	62066000	5000	1	1.10E-09	-0.49	76	1.52	Zbtb20	Transcription
DMR11:62119001	11	62119001	62122000	3000	1	2.40E-09	-0.58	35	1.17	Zbtb20	Transcription
DMR11:62222001	11	62222001	62225000	3000	1	5.50E-07	-0.45	31	1.03	Zbtb20	Transcription
DMR11:62381001	11	62381001	62386000	5000	1	5.60E-12	-0.64	82	1.64	Zbtb20	Transcription
DMR11:62422001	11	62422001	62424000	2000	2	7.60E-10	-0.53	12	0.6	Zbtb20	Transcription
DMR11:62482001	11	62482001	62487000	5000	2	3.10E-29	0.46	48	0.96	Zbtb20	Transcription
DMR11:62640001	11	62640001	62641000	1000	1	2.80E-08	-0.55	8	0.8	Gramd1c	
DMR11:64288001	11	64288001	64289000	1000	1	3.30E-19	0.82	66	6.6	Igsf11	Immune
DMR11:64412001	11	64412001	64414000	2000	1	2.10E-07	-0.33	46	2.3	Igsf11;LOC108352340	Immune
DMR11:64428001	11	64428001	64430000	2000	1	5.80E-11	0.91	35	1.75	Igsf11	Immune
DMR11:64518001	11	64518001	64519000	1000	1	1.70E-07	-0.32	12	1.2	RGD1306995;Upk1b	
DMR11:64598001	11	64598001	64599000	1000	1	6.90E-08	0.67	24	2.4	Arhgap31	Signaling
DMR11:64660001	11	64660001	64662000	2000	1	3.60E-07	0.63	40	2	Arhgap31	Signaling
DMR11:64727001	11	64727001	64730000	3000	1	9.10E-07	-0.29	50	1.67	Tmem39a	
DMR11:64909001	11	64909001	64911000	2000	1	1.10E-08	0.4	29	1.45	Pla1a;Popdc2	Metabolism
DMR11:64939001	11	64939001	64940000	1000	1	1.20E-07	-0.35	26	2.6	Popdc2	
DMR11:64961001	11	64961001	64963000	2000	1	1.40E-12	1.32	15	0.75	Popdc2;Cox17	Transcription
DMR11:65019001	11	65019001	65021000	2000	1	7.70E-07	-0.56	33	1.65	Maats1;Nr1i2	Transcription
DMR11:65032001	11	65032001	65034000	2000	1	1.50E-08	-0.64	18	0.9	Nr1i2	Transcription
DMR11:65041001	11	65041001	65043000	2000	1	2.40E-07	-0.6	16	0.8	Nr1i2	Transcription
DMR11:65323001	11	65323001	65324000	1000	1	9.70E-07	0.42	6	0.6	Gpr156	Signaling
DMR11:65358001	11	65358001	65359000	1000	1	1.30E-08	0.55	25	2.5	Gpr156	Signaling
DMR11:65761001	11	65761001	65763000	2000	1	3.60E-12	-0.53	19	0.95	Lrrc58;LOC102552404	Cytoskeleton
DMR11:65780001	11	65780001	65782000	2000	1	3.80E-09	-0.33	34	1.7	LOC102552404;Fstl1	Protease; Proteolysis
DMR11:65881001	11	65881001	65884000	3000	1	2.10E-07	-0.36	52	1.73	RGD1565413	
DMR11:65964001	11	65964001	65965000	1000	1	7.50E-10	-0.43	10	1	LOC102552623;Ndufb4;RGD1561232	Metabolism
DMR11:65978001	11	65978001	65980000	2000	1	4.20E-09	0.51	39	1.95	RGD1561232;Hgd	Metabolism
DMR11:66014001	11	66014001	66019000	5000	1	1.40E-07	-0.37	41	0.82	Hgd	Metabolism
DMR11:66043001	11	66043001	66046000	3000	2	4.50E-08	-0.55	24	0.8	Hgd;Rabl3	Metabolism
DMR11:66070001	11	66070001	66076000	6000	1	2.20E-07	-0.43	70	1.17	Rabl3;Gtf2e1	Transcription
DMR11:66737001	11	66737001	66742000	5000	1	1.60E-09	-0.4	75	1.5	Fbxo40;Hcls1	Cytoskeleton
DMR11:66775001	11	66775001	66776000	1000	1	2.50E-07	-0.39	25	2.5	Golgb1	
DMR11:66777001	11	66777001	66785000	8000	4	2.60E-13	-0.62	129	1.61	Golgb1	
DMR11:66911001	11	66911001	66912000	1000	1	2.30E-08	-0.81	24	2.4	Eaf2	
DMR11:66931001	11	66931001	66933000	2000	1	4.40E-08	-0.38	33	1.65	Eaf2;Slc15a2	Transport
DMR11:66967001	11	66967001	66969000	2000	1	7.20E-07	-0.33	10	0.5	Slc15a2	Transport
DMR11:67007001	11	67007001	67009000	2000	1	2.40E-08	0.61	30	1.5	Ildr1	Immune
DMR11:67018001	11	67018001	67022000	4000	1	4.80E-13	0.91	87	2.17	Ildr1	Immune
DMR11:67058001	11	67058001	67062000	4000	1	5.50E-14	-0.55	84	2.1	Cd86	Immune
DMR11:67092001	11	67092001	67093000	1000	1	4.30E-10	0.85	27	2.7	Cd86	Immune
DMR11:67127001	11	67127001	67131000	4000	1	3.70E-10	0.45	19	0.48	Cd86	Immune
DMR11:67196001	11	67196001	67199000	3000	1	2.30E-08	0.6	54	1.8	Casr	Signaling
DMR11:67215001	11	67215001	67218000	3000	1	2.60E-10	0.65	54	1.8	Casr	Signaling
DMR11:67237001	11	67237001	67241000	4000	1	6.50E-16	1.05	71	1.77	Casr	Signaling
DMR11:67268001	11	67268001	67270000	2000	1	1.30E-12	0.57	30	1.5	Casr	Signaling
DMR11:68145001	11	68145001	68150000	5000	1	7.50E-12	-0.46	86	1.72	Parp14;Hspbap1	
DMR11:68174001	11	68174001	68175000	1000	1	6.20E-09	0.4	14	1.4	Hspbap1	
DMR11:68207001	11	68207001	68209000	2000	1	1.10E-09	-0.44	28	1.4	Hspbap1;Dirc2;LOC108352343	
DMR11:68253001	11	68253001	68255000	2000	1	8.20E-09	0.43	25	1.25	Dirc2	
DMR11:68277001	11	68277001	68278000	1000	1	4.70E-08	0.6	19	1.9	Dirc2	
DMR11:68307001	11	68307001	68309000	2000	1	9.70E-12	0.78	43	2.15	Sema5b	Signaling
DMR11:68314001	11	68314001	68316000	2000	1	5.90E-10	-0.49	42	2.1	Sema5b	Signaling
DMR11:68321001	11	68321001	68322000	1000	1	9.40E-09	0.36	1	0.1	Sema5b	Signaling
DMR11:68325001	11	68325001	68326000	1000	1	2.30E-08	0.69	27	2.7	Sema5b	Signaling
DMR11:68374001	11	68374001	68378000	4000	1	3.40E-07	-0.4	53	1.32	Sema5b	Signaling

DMR11:68379001	11	68379001	68380000	1000	1	6.40E-08	0.39	10	1	Sema5b	Signaling
DMR11:68408001	11	68408001	68409000	1000	1	3.10E-07	0.41	17	1.7	Sema5b	Signaling
DMR11:68432001	11	68432001	68434000	2000	2	2.00E-09	0.71	55	2.75	Sema5b	Signaling
DMR11:68440001	11	68440001	68441000	1000	1	2.80E-07	-0.34	16	1.6	Sema5b	Signaling
DMR11:68504001	11	68504001	68505000	1000	1	1.10E-07	-0.61	16	1.6	Pdia5	Transcription
DMR11:68525001	11	68525001	68526000	1000	1	1.90E-07	-0.57	14	1.4	Pdia5	Transcription
DMR11:68575001	11	68575001	68578000	3000	1	7.80E-09	0.41	66	2.2	Pdia5	Transcription
DMR11:68715001	11	68715001	68717000	2000	1	3.00E-09	-0.41	39	1.95	LOC102551038;Adcy5	
DMR11:68730001	11	68730001	68732000	2000	1	2.30E-08	0.56	19	0.95	Adcy5	
DMR11:68777001	11	68777001	68780000	3000	1	3.40E-07	0.57	43	1.43	Adcy5;LOC108352344	
DMR11:68814001	11	68814001	68817000	3000	1	8.50E-08	0.5	59	1.97	Adcy5	
DMR11:69177001	11	69177001	69178000	1000	1	6.30E-07	0.48	13	1.3	Mylk;LOC102548830	
DMR11:69273001	11	69273001	69276000	3000	1	2.70E-10	0.76	39	1.3	Ccdc14	
DMR11:69348001	11	69348001	69352000	4000	1	2.40E-07	-0.33	54	1.35	Ropn1	
DMR11:69595001	11	69595001	69596000	1000	1	8.90E-13	0.79	11	1.1	Kalrn	Transcription
DMR11:69654001	11	69654001	69657000	3000	1	3.10E-07	-0.48	47	1.57	Kalrn	Transcription
DMR11:69732001	11	69732001	69735000	3000	2	4.00E-10	-0.42	45	1.5	Kalrn	Transcription
DMR11:69776001	11	69776001	69785000	9000	1	1.40E-12	0.67	136	1.51	Kalrn	Transcription
DMR11:69885001	11	69885001	69887000	2000	1	2.40E-07	0.34	35	1.75	Kalrn	Transcription
DMR11:70009001	11	70009001	70012000	3000	1	4.90E-07	0.31	45	1.5	Kalrn;LOC103693587	Transcription
DMR11:70024001	11	70024001	70027000	3000	1	2.90E-12	0.9	54	1.8	Kalrn;Umps	Transcription;Transport
DMR11:70112001	11	70112001	70114000	2000	1	1.80E-07	-0.48	43	2.15	Itgb5	
DMR11:70272001	11	70272001	70276000	4000	2	3.00E-08	-0.55	72	1.8	Heg1	
DMR11:70299001	11	70299001	70301000	2000	1	2.60E-09	0.55	55	2.75	Heg1	
DMR11:70326001	11	70326001	70328000	2000	1	1.40E-07	-0.41	24	1.2	Heg1	
DMR11:70455001	11	70455001	70456000	1000	1	4.80E-07	0.51	29	2.9	Slc12a8	Transport
DMR11:70458001	11	70458001	70460000	2000	1	7.70E-09	-0.52	24	1.2	Slc12a8	Transport
DMR11:70491001	11	70491001	70495000	4000	2	3.10E-14	0.89	76	1.9	Slc12a8	Transport
DMR11:70581001	11	70581001	70584000	3000	1	6.20E-09	-0.58	38	1.27	Zfp148	
DMR11:70922001	11	70922001	70926000	4000	1	5.30E-11	-0.42	52	1.3	Lmln	Protease
DMR11:71002001	11	71002001	71003000	1000	1	1.80E-07	0.31	6	0.6	Iqcg;Lrch3	Cytoskeleton
DMR11:71004001	11	71004001	71005000	1000	1	2.20E-09	-0.45	22	2.2	Iqcg;Lrch3	Cytoskeleton
DMR11:71218001	11	71218001	71221000	3000	1	3.10E-07	-0.36	58	1.93	LOC100910650;LOC103693588;Muc20	
DMR11:71238001	11	71238001	71239000	1000	1	1.30E-07	0.48	25	2.5	Muc20;Muc4	
DMR11:71258001	11	71258001	71262000	4000	3	2.70E-11	0.56	80	2	Muc4	
DMR11:71380001	11	71380001	71384000	4000	1	1.50E-09	0.61	38	0.95	Tnk2	
DMR11:71385001	11	71385001	71387000	2000	1	2.40E-07	-0.5	38	1.9	Tnk2	
DMR11:71480001	11	71480001	71481000	1000	1	9.60E-08	-0.58	4	0.4	LOC102547162;Zdhhc19	
DMR11:71486001	11	71486001	71488000	2000	1	7.40E-07	-0.55	17	0.85	LOC102547162;Zdhhc19	
DMR11:71513001	11	71513001	71516000	3000	1	3.80E-09	0.65	56	1.87	Zdhhc19	
DMR11:71519001	11	71519001	71520000	1000	1	1.60E-07	0.79	35	3.5	Zdhhc19	
DMR11:71532001	11	71532001	71533000	1000	1	5.10E-09	0.4	11	1.1	Zdhhc19;Slc51a	Transport
DMR11:71572001	11	71572001	71573000	1000	1	2.90E-08	-0.42	14	1.4	Pcyt1a	Transport
DMR11:72128001	11	72128001	72130000	2000	1	1.40E-14	0.99	38	1.9	LOC102549772;Meltf	Transport
DMR11:72148001	11	72148001	72154000	6000	3	8.70E-09	0.64	112	1.87	Meltf	Transport
DMR11:72285001	11	72285001	72288000	3000	1	3.20E-08	-0.36	37	1.23	Dlg1	Cytoskeleton
DMR11:72627001	11	72627001	72629000	2000	1	1.00E-09	0.76	41	2.05	Bdh1	Metabolism
DMR11:72718001	11	72718001	72719000	1000	1	3.10E-12	0.8	25	2.5	Apod	Binding Proteins
DMR11:72734001	11	72734001	72735000	1000	1	2.90E-08	-0.64	23	2.3	Apod	Binding Proteins
DMR11:72976001	11	72976001	72977000	1000	1	7.00E-12	-0.7	19	1.9	Ppp1r2	Signaling
DMR11:72981001	11	72981001	72982000	1000	1	6.80E-09	-0.53	19	1.9	Ppp1r2	Signaling
DMR11:73101001	11	73101001	73104000	3000	2	2.50E-11	-0.5	24	0.8	Acap2	
DMR11:73221001	11	73221001	73223000	2000	1	8.00E-07	0.4	37	1.85	Xxylt1	Transport
DMR11:73262001	11	73262001	73264000	2000	1	3.60E-09	-0.53	43	2.15	Xxylt1	Transport
DMR11:73711001	11	73711001	73716000	5000	1	4.20E-07	-0.53	59	1.18	Lsg1;LOC103693591;LOC108352351	
DMR11:73727001	11	73727001	73728000	1000	1	9.80E-07	-0.4	21	2.1	Lsg1;Tmem44	
DMR11:73736001	11	73736001	73738000	2000	1	2.70E-07	0.48	35	1.75	Tmem44;LOC102548792	
DMR11:73742001	11	73742001	73744000	2000	1	3.10E-09	-0.45	33	1.65	Tmem44;LOC102548792	
DMR11:73748001	11	73748001	73752000	4000	2	1.10E-07	0.49	87	2.17	Tmem44;LOC102548792	

DMR11:73761001	11	73761001	73763000	2000	1	7.80E-07	-0.38	35	1.75	Tmem44	
DMR11:73961001	11	73961001	73962000	1000	1	2.80E-07	-0.36	16	1.6	Atp13a3	
DMR11:74018001	11	74018001	74021000	3000	1	8.20E-09	0.58	42	1.4	Gp5	
DMR11:74038001	11	74038001	74043000	5000	1	1.90E-07	-0.38	109	2.18	Lrrc15	
DMR11:74704001	11	74704001	74710000	6000	1	5.90E-09	-0.42	112	1.87	Opa1	Transport
DMR11:74874001	11	74874001	74877000	3000	1	1.10E-10	0.6	51	1.7	Atp13a4;LOC100360218;LOC102552083	Receptor
DMR11:74905001	11	74905001	74906000	1000	1	4.70E-08	-0.39	9	0.9	Atp13a4	
DMR11:75142001	11	75142001	75143000	1000	1	8.90E-07	-0.3	16	1.6	Hrasls	
DMR11:75477001	11	75477001	75480000	3000	1	1.60E-07	-0.39	48	1.6	Mb21d2	
DMR11:75499001	11	75499001	75501000	2000	1	3.40E-07	-0.44	44	2.2	Mb21d2	
DMR11:75529001	11	75529001	75531000	2000	1	1.10E-08	-0.4	51	2.55	Mb21d2	
DMR11:75640001	11	75640001	75642000	2000	2	1.20E-07	-0.4	18	0.9	Fgf12	Growth Factors
DMR11:75829001	11	75829001	75830000	1000	1	2.30E-07	0.38	15	1.5	Fgf12	Growth Factors
DMR11:75939001	11	75939001	75940000	1000	1	5.10E-09	-0.43	15	1.5	Fgf12	Growth Factors
DMR11:76012001	11	76012001	76013000	1000	1	3.10E-08	-0.39	11	1.1	Fgf12	Growth Factors
DMR11:76740001	11	76740001	76741000	1000	1	4.40E-11	0.76	39	3.9	Ccdc50	
DMR11:76807001	11	76807001	76808000	1000	1	3.50E-14	-0.47	15	1.5	Ccdc50;LOC102548576;LOC102557186	
DMR11:76841001	11	76841001	76842000	1000	1	3.00E-07	0.55	15	1.5	Uts2b;Ostn	
DMR11:77516001	11	77516001	77518000	2000	1	8.80E-07	-0.45	17	0.85	Il1rap	Receptor
DMR11:77675001	11	77675001	77677000	2000	1	5.70E-07	-0.4	13	0.65	Cldn16	Cell Junction
DMR11:77699001	11	77699001	77701000	2000	1	1.70E-07	-0.38	18	0.9	Cldn16	Cell Junction
DMR11:77710001	11	77710001	77711000	1000	1	2.00E-16	0.93	29	2.9	Cldn16	Cell Junction
DMR11:77871001	11	77871001	77872000	1000	1	1.30E-07	-0.6	1	0.1	RGD1561729	
DMR11:78068001	11	78068001	78070000	2000	1	1.30E-07	-0.37	16	0.8	P3h2;LOC103690757	Extracellular Matrix
DMR11:78091001	11	78091001	78093000	2000	1	2.60E-08	-0.46	32	1.6	P3h2	Extracellular Matrix
DMR11:78161001	11	78161001	78162000	1000	1	2.80E-08	0.62	7	0.7	P3h2;LOC102554639	Extracellular Matrix
DMR11:78260001	11	78260001	78261000	1000	1	2.00E-08	0.69	29	2.9	Tp63	Transcription
DMR11:78367001	11	78367001	78368000	1000	1	4.00E-07	-0.46	9	0.9	Tp63	Transcription
DMR11:78376001	11	78376001	78378000	2000	1	3.60E-07	-0.44	17	0.85	Tp63	Transcription
DMR11:78392001	11	78392001	78394000	2000	1	5.70E-08	0.35	17	0.85	Tp63	Transcription
DMR11:78723001	11	78723001	78725000	2000	1	8.40E-07	-0.61	5	0.25	LOC679233;Tprg1	
DMR11:78762001	11	78762001	78764000	2000	1	3.60E-07	0.29	24	1.2	Tprg1;LOC102554536	
DMR11:78775001	11	78775001	78776000	1000	1	1.20E-08	0.68	15	1.5	Tprg1;LOC102554536	
DMR11:78780001	11	78780001	78781000	1000	1	1.40E-10	0.91	17	1.7	Tprg1	
DMR11:78788001	11	78788001	78789000	1000	1	6.40E-07	-0.3	6	0.6	Tprg1	
DMR11:79426001	11	79426001	79431000	5000	2	1.60E-14	0.66	55	1.1	Lpp	Signaling
DMR11:79446001	11	79446001	79448000	2000	1	7.00E-07	-0.33	22	1.1	Lpp	Signaling
DMR11:79457001	11	79457001	79460000	3000	1	2.40E-07	0.42	53	1.77	Lpp	Signaling
DMR11:79560001	11	79560001	79562000	2000	1	8.40E-11	-0.5	24	1.2	Lpp	Signaling
DMR11:79567001	11	79567001	79569000	2000	1	2.30E-07	-0.38	28	1.4	Lpp	Signaling
DMR11:79646001	11	79646001	79647000	1000	1	3.70E-12	-0.54	9	0.9	Lpp	Signaling
DMR11:79734001	11	79734001	79739000	5000	2	4.50E-10	-0.43	84	1.68	Lpp	Signaling
DMR11:80271001	11	80271001	80273000	2000	1	3.20E-11	0.74	46	2.3	Bcl6	Transcription
DMR11:80741001	11	80741001	80743000	2000	1	4.20E-07	0.53	32	1.6	LOC102552752;Masp1	Protease
DMR11:80927001	11	80927001	80928000	1000	1	3.30E-07	0.51	8	0.8	St6gal1	Transport
DMR11:81329001	11	81329001	81333000	4000	3	7.40E-13	0.82	75	1.88	Adipoq	
DMR11:81338001	11	81338001	81339000	1000	1	3.10E-10	-0.53	18	1.8	Adipoq	
DMR11:81373001	11	81373001	81376000	3000	1	9.90E-13	-0.58	35	1.17	Rfc4;Eif4a2	Transcription
DMR11:81447001	11	81447001	81449000	2000	1	4.40E-08	0.28	21	1.05	Kng1l1	
DMR11:81626001	11	81626001	81628000	2000	1	9.20E-08	-0.32	9	0.45	Hrg	Protease; Proteolysis
DMR11:81638001	11	81638001	81641000	3000	1	5.70E-07	-0.34	34	1.13	Hrg;Fetub	Protease; Proteolysis
DMR11:81661001	11	81661001	81662000	1000	1	3.70E-08	0.69	22	2.2	Fetub	Protease; Proteolysis
DMR11:81715001	11	81715001	81717000	2000	1	6.40E-18	0.92	41	2.05	Ahsg;LOC103690621	Protease; Proteolysis
DMR11:81803001	11	81803001	81807000	4000	1	2.00E-07	0.51	68	1.7	Tbccd1;Crygs	
DMR11:81965001	11	81965001	81966000	1000	1	2.20E-07	-0.36	20	2	LOC102552590;Dgkg	Signaling
DMR11:81975001	11	81975001	81979000	4000	1	1.10E-09	0.34	69	1.73	LOC102552590;Dgkg	Signaling
DMR11:81980001	11	81980001	81984000	4000	3	7.20E-16	-0.83	65	1.62	LOC102552590;Dgkg	Signaling
DMR11:81985001	11	81985001	81987000	2000	2	2.20E-09	-0.44	44	2.2	LOC102552590;Dgkg	Signaling
DMR11:81998001	11	81998001	81999000	1000	1	1.70E-11	0.78	33	3.3	Dgkg	Signaling
DMR11:82021001	11	82021001	82024000	3000	1	5.60E-08	-0.38	69	2.3	Dgkg	Signaling

DMR11:82043001	11	82043001	82044000	1000	1	4.50E-09	0.58	17	1.7	Dgkg	Signaling
DMR11:82084001	11	82084001	82085000	1000	1	1.30E-07	0.59	21	2.1	Dgkg	Signaling
DMR11:82160001	11	82160001	82161000	1000	1	3.90E-16	0.99	32	3.2	Dgkg	Signaling
DMR11:82171001	11	82171001	82174000	3000	1	2.00E-14	0.8	64	2.13	Dgkg	Signaling
DMR11:82202001	11	82202001	82204000	2000	1	6.70E-08	-0.44	47	2.35	Etv5	Transcription
DMR11:82216001	11	82216001	82218000	2000	1	2.70E-07	-0.36	35	1.75	Etv5	Transcription
DMR11:82237001	11	82237001	82238000	1000	1	7.00E-07	0.52	18	1.8	Etv5	Transcription
DMR11:82364001	11	82364001	82367000	3000	1	5.30E-19	0.93	75	2.5	LOC691006;RGD1559972;Tra2b	
DMR11:82457001	11	82457001	82460000	3000	1	4.10E-09	-0.39	49	1.63	Igf2bp2	Metabolism
DMR11:82613001	11	82613001	82614000	1000	1	1.30E-08	-0.47	23	2.3	Igf2bp2	Metabolism
DMR11:82638001	11	82638001	82642000	4000	1	3.10E-07	-0.37	54	1.35	Senp2	Protease
DMR11:82690001	11	82690001	82691000	1000	1	8.80E-07	0.52	25	2.5	Liph	Metabolism
DMR11:82753001	11	82753001	82754000	1000	1	6.60E-09	0.72	28	2.8	Map3k13	Signaling
DMR11:82907001	11	82907001	82908000	1000	1	6.40E-07	-0.35	15	1.5	Map3k13;LOC100912571	Signaling;Translation
DMR11:82950001	11	82950001	82951000	1000	1	5.90E-08	0.5	27	2.7	Ehhadh	Metabolism
DMR11:82957001	11	82957001	82959000	2000	1	6.10E-09	0.62	35	1.75	Ehhadh	Metabolism
DMR11:83062001	11	83062001	83069000	7000	1	5.80E-08	-0.35	101	1.44	RGD1562339	
DMR11:83077001	11	83077001	83079000	2000	1	3.30E-10	0.5	21	1.05	RGD1562339	
DMR11:83089001	11	83089001	83091000	2000	1	4.90E-07	0.36	33	1.65	RGD1562339	
DMR11:83126001	11	83126001	83129000	3000	1	1.90E-10	0.55	32	1.07	Vps8;LOC108352385	Cytoskeleton
DMR11:83167001	11	83167001	83168000	1000	1	1.40E-12	-0.59	20	2	Vps8	Cytoskeleton
DMR11:83223001	11	83223001	83227000	4000	1	3.20E-09	0.38	30	0.75	Vps8	Cytoskeleton
DMR11:83250001	11	83250001	83251000	1000	1	2.10E-13	0.77	25	2.5	Vps8	Cytoskeleton
DMR11:83530001	11	83530001	83533000	3000	1	3.40E-10	-0.42	84	2.8	Ephb3	Receptor
DMR11:83967001	11	83967001	83968000	1000	1	3.50E-10	0.54	31	3.1	Ece2;Camk2n2;LOC102551435	Protease;Signaling
DMR11:84006001	11	84006001	84007000	1000	1	4.10E-11	0.87	50	5	Vwa5b2;LOC102551647	
DMR11:84054001	11	84054001	84056000	2000	1	5.00E-10	0.47	23	1.15	Ap2m1;Dvl3	Transport;Cytoskeleton
DMR11:84057001	11	84057001	84060000	3000	1	1.30E-08	0.5	53	1.77	Ap2m1;Dvl3	Transport;Cytoskeleton
DMR11:84518001	11	84518001	84519000	1000	1	3.10E-10	-0.7	12	1.2	Yeats2;Cyp2ab1;Parl	Transcription;Metabolism;Protease
DMR11:84562001	11	84562001	84563000	1000	1	1.40E-09	-0.43	7	0.7	Yeats2	Transcription
DMR11:84587001	11	84587001	84592000	5000	2	1.80E-13	-0.53	84	1.68	Yeats2	Transcription
DMR11:84776001	11	84776001	84777000	1000	1	9.90E-08	0.31	4	0.4	Klhl6;LOC102547727	Cytoskeleton
DMR11:84909001	11	84909001	84910000	1000	1	4.90E-07	-0.5	2	0.2	B3gnt5	Golgi
DMR11:84952001	11	84952001	84953000	1000	1	1.90E-15	-0.55	3	0.3	Lamp3	Transport
DMR11:86269001	11	86269001	86271000	2000	1	5.10E-07	-0.65	9	0.45	Hira;Mrpl40	Epigenetic;Translation
DMR11:86316001	11	86316001	86319000	3000	1	2.20E-07	0.26	21	0.7	Ufd1l;Cdc45	Cell Cycle
DMR11:86333001	11	86333001	86336000	3000	1	2.40E-07	0.36	45	1.5	Ufd1l;Cdc45	Cell Cycle
DMR11:86358001	11	86358001	86360000	2000	1	2.20E-07	0.59	22	1.1	Cdc45;Cldn5	Cell Cycle;Cell Junction
DMR11:86508001	11	86508001	86510000	2000	1	3.90E-07	0.42	33	1.65	5-Sep	
DMR11:86514001	11	86514001	86515000	1000	1	4.90E-07	0.6	7	0.7	Sept5;Gp1bb	
DMR11:86529001	11	86529001	86531000	2000	1	5.00E-11	0.64	38	1.9	Sept5;Gp1bb	
DMR11:86562001	11	86562001	86565000	3000	1	1.20E-08	0.5	36	1.2	Tbx1	Transcription
DMR11:86586001	11	86586001	86592000	6000	3	1.40E-09	0.61	104	1.73	Gnb1l	Cytoskeleton
DMR11:86599001	11	86599001	86600000	1000	1	9.30E-09	0.53	13	1.3	Gnb1l	Cytoskeleton
DMR11:86601001	11	86601001	86603000	2000	1	9.10E-07	0.44	26	1.3	Gnb1l	Cytoskeleton
DMR11:86617001	11	86617001	86621000	4000	1	7.70E-10	-0.33	34	0.85	Gnb1l	Cytoskeleton
DMR11:86622001	11	86622001	86623000	1000	1	3.20E-07	0.42	10	1	Gnb1l	Cytoskeleton
DMR11:86637001	11	86637001	86638000	1000	1	1.40E-07	-0.39	15	1.5	Gnb1l	Cytoskeleton
DMR11:86668001	11	86668001	86671000	3000	1	2.50E-08	0.61	31	1.03	Txnrd2	Metabolism
DMR11:86688001	11	86688001	86697000	9000	1	3.10E-07	0.36	138	1.53	Txnrd2	Metabolism
DMR11:86817001	11	86817001	86818000	1000	1	9.80E-08	0.61	13	1.3	Tango2;Mir185	
DMR11:86822001	11	86822001	86824000	2000	1	7.40E-07	0.53	22	1.1	Tango2;Mir185	
DMR11:86918001	11	86918001	86919000	1000	1	3.30E-07	0.55	21	2.1	Zdhhc8;Ccdc188;LOC102555338	
DMR11:87022001	11	87022001	87023000	1000	1	2.20E-12	0.74	13	1.3	Rtn4r	
DMR11:87049001	11	87049001	87050000	1000	1	2.50E-07	0.4	11	1.1	Prodh1	Metabolism
DMR11:87072001	11	87072001	87074000	2000	1	3.20E-07	-0.46	37	1.85	Prodh1;Dgcr6	Metabolism
DMR11:87084001	11	87084001	87085000	1000	1	9.30E-08	0.43	3	0.3	Prodh1;Dgcr6;LOC108352363	Metabolism
DMR11:87157001	11	87157001	87159000	2000	2	5.00E-17	1.2	46	2.3	LOC103690707;Vpreb2	Immune

DMR11:87366001	11	87366001	87367000	1000	1	5.40E-07	0.47	12	1.2	Crkl;Aifm3	Cytoskeleton;Metabolism
DMR11:87405001	11	87405001	87407000	2000	1	1.50E-07	-0.32	33	1.65	Lztr1;Thap7	Transcription
DMR11:87658001	11	87658001	87660000	2000	1	1.10E-12	0.82	26	1.3	Klhl22;LOC287947	
DMR11:87664001	11	87664001	87667000	3000	1	1.20E-07	-0.32	21	0.7	Klhl22;LOC287947	
DMR11:87979001	11	87979001	87981000	2000	1	7.40E-07	0.46	33	1.65	Pi4ka;Tmem191c;Hic2	Signaling;Transcription
DMR11:88113001	11	88113001	88116000	3000	1	4.80E-07	-0.42	117	3.9	Sdf2l1	Transport
DMR11:88133001	11	88133001	88135000	2000	1	3.50E-08	0.34	20	1	Sdf2l1;RGD1308065;Mir301b;Mir130b	Transport
DMR11:88317001	11	88317001	88319000	2000	1	1.20E-09	0.6	36	1.8	Ppm1f	Signaling
DMR11:88331001	11	88331001	88333000	2000	1	2.20E-10	0.37	22	1.1	Ppm1f	Signaling
DMR11:88376001	11	88376001	88377000	1000	1	5.40E-19	1.18	27	2.7	Top3b;Vpreb1;LOC100361992	Transcription;Immune
DMR11:88433001	11	88433001	88440000	7000	1	8.40E-07	-0.5	68	0.97	Spag6	Cytoskeleton
DMR11:89230001	11	89230001	89236000	6000	1	2.20E-07	-0.47	64	1.07	Spidr	Transcription
DMR11:89326001	11	89326001	89327000	1000	1	6.40E-08	-0.29	16	1.6	Prkdc;LOC103690762	Signaling
DMR11:89493001	11	89493001	89498000	5000	1	2.40E-07	-0.32	47	0.94	Prkdc	Signaling
DMR11:89691001	11	89691001	89694000	3000	1	1.40E-08	0.64	17	0.57	RGD1560337	
DMR12:578001	12	578001	579000	1000	1	7.10E-07	0.39	18	1.8	N4bp2l1;LOC100909860	
DMR12:581001	12	581001	583000	2000	1	6.80E-09	0.39	16	0.8	N4bp2l1;LOC100909860	
DMR12:684001	12	684001	687000	3000	1	8.80E-08	-0.45	51	1.7	Pds5b	Epigenetic
DMR12:736001	12	736001	737000	1000	1	6.10E-08	0.59	16	1.6	Pds5b	Epigenetic
DMR12:760001	12	760001	763000	3000	1	4.30E-12	-0.49	29	0.97	Pds5b	Epigenetic
DMR12:801001	12	801001	803000	2000	1	2.50E-07	0.39	32	1.6	Pds5b	Epigenetic
DMR12:937001	12	937001	938000	1000	1	1.70E-11	0.79	21	2.1	Kl	Metabolism
DMR12:959001	12	959001	961000	2000	1	2.40E-08	-0.5	26	1.3	Kl	Metabolism
DMR12:978001	12	978001	980000	2000	1	6.10E-08	0.58	33	1.65	Kl	Metabolism
DMR12:981001	12	981001	983000	2000	2	1.90E-14	1.04	50	2.5	Kl	Metabolism
DMR12:985001	12	985001	987000	2000	2	6.50E-16	1.06	66	3.3	Kl;LOC102548964	Metabolism
DMR12:1025001	12	1025001	1026000	1000	1	2.60E-08	0.52	28	2.8	Stard13	Signaling
DMR12:1081001	12	1081001	1082000	1000	1	3.90E-08	-0.49	10	1	Stard13	Signaling
DMR12:1104001	12	1104001	1106000	2000	1	7.70E-20	1.09	46	2.3	Stard13	Signaling
DMR12:1113001	12	1113001	1115000	2000	1	1.00E-08	0.41	34	1.7	Stard13	Signaling
DMR12:1128001	12	1128001	1129000	1000	1	1.70E-07	-0.36	27	2.7	Stard13	Signaling
DMR12:1148001	12	1148001	1152000	4000	1	1.00E-07	-0.45	85	2.12	Stard13	Signaling
DMR12:1181001	12	1181001	1184000	3000	1	2.50E-09	-0.52	62	2.07	Stard13	Signaling
DMR12:1207001	12	1207001	1208000	1000	1	3.60E-10	0.65	34	3.4	Stard13	Signaling
DMR12:1255001	12	1255001	1259000	4000	1	4.50E-14	0.8	85	2.12	Stard13;LOC108352405	Signaling
DMR12:1294001	12	1294001	1299000	5000	2	1.00E-14	0.85	107	2.14	Stard13	Signaling
DMR12:1329001	12	1329001	1331000	2000	1	4.80E-08	0.7	35	1.75	Stard13;LOC103693611	Signaling
DMR12:1341001	12	1341001	1342000	1000	1	5.90E-08	-0.59	10	1	Stard13;LOC103693611;LOC102549089	Signaling
DMR12:1412001	12	1412001	1414000	2000	1	4.90E-07	0.48	39	1.95	Stard13	Signaling
DMR12:1450001	12	1450001	1451000	1000	1	4.00E-10	0.63	19	1.9	Rfc3	Transcription
DMR12:1504001	12	1504001	1506000	2000	1	8.10E-10	0.37	43	2.15	LOC103693612;Rn5s	
DMR12:1508001	12	1508001	1517000	9000	3	3.70E-17	0.4	139	1.54	LOC103693612;Rn5s	
DMR12:1672001	12	1672001	1673000	1000	1	1.10E-09	-0.37	25	2.5	Insr	Receptor
DMR12:1677001	12	1677001	1680000	3000	1	5.50E-07	-0.38	41	1.37	Insr	Receptor
DMR12:1682001	12	1682001	1684000	2000	1	1.90E-10	0.79	48	2.4	Insr	Receptor
DMR12:1725001	12	1725001	1727000	2000	1	4.30E-09	0.72	33	1.65	Insr	Receptor
DMR12:1739001	12	1739001	1740000	1000	1	1.10E-07	-0.35	21	2.1	Insr	Receptor
DMR12:1747001	12	1747001	1751000	4000	1	5.00E-09	-0.55	50	1.25	Insr	Receptor
DMR12:1774001	12	1774001	1782000	8000	1	5.20E-07	-0.38	90	1.12	Insr	Receptor
DMR12:1788001	12	1788001	1791000	3000	1	2.80E-09	-0.4	49	1.63	Insr	Receptor
DMR12:1884001	12	1884001	1890000	6000	1	4.60E-10	0.48	116	1.93	Arhgef18	
DMR12:1937001	12	1937001	1938000	1000	1	8.80E-11	-0.41	25	2.5	Arhgef18	
DMR12:2044001	12	2044001	2045000	1000	1	8.50E-11	0.75	17	1.7	Zfp358;Mcoln1	Transcription;Transport
DMR12:2084001	12	2084001	2086000	2000	1	1.10E-09	0.61	54	2.7	Pnpla6	Metabolism
DMR12:2087001	12	2087001	2091000	4000	1	9.00E-09	0.4	105	2.62	Pnpla6	Metabolism
DMR12:2105001	12	2105001	2106000	1000	1	1.40E-09	-0.38	10	1	Pnpla6	Metabolism
DMR12:2139001	12	2139001	2142000	3000	1	4.70E-07	-0.37	40	1.33	Camsap3	
DMR12:2374001	12	2374001	2376000	2000	1	2.40E-08	0.46	29	1.45	Cd209e	Transport

DMR12:2465001	12	2465001	2466000	1000	1	8.70E-10	-0.48	7	0.7	LOC102552452;Elavl1	Translation
DMR12:2575001	12	2575001	2576000	1000	1	3.80E-08	0.38	14	1.4	Lrrc8e;Prr36	Cytoskeleton
DMR12:2577001	12	2577001	2583000	6000	1	3.00E-07	-0.53	184	3.07	Lrrc8e;Prr36;Evi5l	Cytoskeleton;Signaling
DMR12:2834001	12	2834001	2835000	1000	1	3.40E-07	0.35	10	1	Cd209b;Cd209c	Transport
DMR12:3923001	12	3923001	3924000	1000	1	1.10E-17	1.1	39	3.9	Vom2r-ps94;RGD1562451;Vom2r-ps95	
DMR12:4104001	12	4104001	4107000	3000	1	2.20E-07	-0.27	38	1.27	Vom2r60;LOC688812	Signaling
DMR12:4111001	12	4111001	4113000	2000	1	3.60E-10	-0.49	19	0.95	Vom2r60;LOC688812	Signaling
DMR12:4442001	12	4442001	4445000	3000	1	7.80E-09	-0.32	34	1.13	Vom2r60;Cers4	Signaling
DMR12:4451001	12	4451001	4452000	1000	1	2.10E-08	-0.51	10	1	Cers4	
DMR12:5477001	12	5477001	5479000	2000	1	2.30E-09	-0.56	47	2.35	Zfp958	Transcription
DMR12:5484001	12	5484001	5485000	1000	1	1.50E-07	0.38	24	2.4	Zfp958	Transcription
DMR12:5493001	12	5493001	5497000	4000	1	7.30E-09	-0.38	51	1.27	Zfp958	Transcription
DMR12:5585001	12	5585001	5587000	2000	1	4.20E-07	-0.45	38	1.9	Fry	Cytoskeleton
DMR12:5602001	12	5602001	5605000	3000	1	2.30E-07	-0.41	47	1.57	Fry	Cytoskeleton
DMR12:5643001	12	5643001	5645000	2000	2	2.60E-16	0.48	31	1.55	Fry	Cytoskeleton
DMR12:5682001	12	5682001	5684000	2000	1	4.30E-07	-0.39	49	2.45	Fry	Cytoskeleton
DMR12:5694001	12	5694001	5696000	2000	1	1.80E-10	0.7	38	1.9	Fry	Cytoskeleton
DMR12:5725001	12	5725001	5726000	1000	1	5.00E-15	1.01	25	2.5	Fry	Cytoskeleton
DMR12:5746001	12	5746001	5747000	1000	1	3.60E-13	0.59	26	2.6	Fry	Cytoskeleton
DMR12:5813001	12	5813001	5814000	1000	1	2.20E-07	0.7	19	1.9	Fry	Cytoskeleton
DMR12:5991001	12	5991001	5995000	4000	1	5.50E-07	-0.34	71	1.77	Lnc001;LOC100361192	
DMR12:6028001	12	6028001	6033000	5000	2	2.20E-10	0.53	103	2.06	Rxfp2;LOC102549667	Signaling
DMR12:6035001	12	6035001	6040000	5000	1	4.10E-19	1	92	1.84	Rxfp2	Signaling
DMR12:6042001	12	6042001	6044000	2000	1	4.40E-15	0.87	36	1.8	Rxfp2	Signaling
DMR12:6045001	12	6045001	6051000	6000	1	3.10E-08	-0.56	98	1.63	Rxfp2	Signaling
DMR12:6062001	12	6062001	6064000	2000	1	2.90E-10	-0.56	42	2.1	Rxfp2	Signaling
DMR12:6070001	12	6070001	6071000	1000	1	5.70E-18	1.22	25	2.5	Rxfp2	Signaling
DMR12:6074001	12	6074001	6075000	1000	1	2.00E-14	1.2	33	3.3	Rxfp2	Signaling
DMR12:6270001	12	6270001	6271000	1000	1	5.30E-11	0.63	38	3.8	Wdr95	
DMR12:6272001	12	6272001	6275000	3000	1	8.80E-10	0.58	34	1.13	Wdr95	
DMR12:6345001	12	6345001	6351000	6000	1	4.90E-10	0.52	102	1.7	Hsph1;LOC108352411	
DMR12:6396001	12	6396001	6402000	6000	1	8.20E-14	-0.49	117	1.95	B3glct	Golgi
DMR12:6431001	12	6431001	6434000	3000	1	6.50E-10	0.56	51	1.7	B3glct	Golgi
DMR12:6437001	12	6437001	6441000	4000	1	6.20E-10	0.67	106	2.65	B3glct;LOC103690977	Golgi
DMR12:6442001	12	6442001	6446000	4000	3	2.20E-13	0.8	82	2.05	B3glct;LOC103690977	Golgi
DMR12:6450001	12	6450001	6452000	2000	1	4.50E-09	0.35	24	1.2	B3glct;LOC103690977	Golgi
DMR12:6461001	12	6461001	6465000	4000	2	4.10E-10	0.51	81	2.02	B3glct	Golgi
DMR12:6471001	12	6471001	6473000	2000	2	3.00E-07	0.57	46	2.3	B3glct	Golgi
DMR12:6479001	12	6479001	6481000	2000	1	2.30E-08	-0.34	26	1.3	B3glct	Golgi
DMR12:6484001	12	6484001	6485000	1000	1	9.10E-14	0.86	36	3.6	B3glct	Golgi
DMR12:6685001	12	6685001	6686000	1000	1	7.70E-07	-0.39	16	1.6	Tex26	
DMR12:6691001	12	6691001	6694000	3000	1	9.50E-10	-0.48	46	1.53	Tex26	
DMR12:6709001	12	6709001	6715000	6000	1	2.10E-07	-0.39	90	1.5	Tex26;Medag	
DMR12:6850001	12	6850001	6852000	2000	1	2.00E-15	0.87	40	2	Alox5ap	Transport
DMR12:6855001	12	6855001	6856000	1000	1	7.40E-11	0.59	40	4	Alox5ap	Transport
DMR12:6882001	12	6882001	6884000	2000	1	8.40E-18	0.99	44	2.2	Alox5ap	Transport
DMR12:7177001	12	7177001	7178000	1000	1	4.50E-10	-0.62	19	1.9	LOC102552453;Rpl35a	Translation
DMR12:7874001	12	7874001	7877000	3000	2	9.60E-13	-0.62	28	0.93	Ubl3	Proteolysis
DMR12:7898001	12	7898001	7900000	2000	1	3.30E-10	-0.49	14	0.7	Ubl3	Proteolysis
DMR12:8078001	12	8078001	8081000	3000	1	1.10E-07	-0.37	64	2.13	Slc7a1	Transport
DMR12:8118001	12	8118001	8119000	1000	1	6.00E-11	0.57	15	1.5	Slc7a1;Mtus2	Transport
DMR12:8124001	12	8124001	8129000	5000	1	3.60E-10	0.66	84	1.68	Mtus2	
DMR12:8227001	12	8227001	8231000	4000	1	7.00E-07	-0.33	70	1.75	Mtus2	
DMR12:8246001	12	8246001	8248000	2000	1	2.80E-10	-0.48	27	1.35	Mtus2	
DMR12:8257001	12	8257001	8259000	2000	1	4.90E-07	-0.36	29	1.45	Mtus2	
DMR12:8274001	12	8274001	8275000	1000	1	1.20E-09	-0.47	19	1.9	Mtus2	
DMR12:8290001	12	8290001	8295000	5000	1	2.20E-10	0.76	93	1.86	Mtus2	
DMR12:8338001	12	8338001	8341000	3000	2	9.30E-10	0.46	48	1.6	Mtus2	
DMR12:8349001	12	8349001	8351000	2000	1	9.70E-17	1.07	33	1.65	Mtus2	
DMR12:8356001	12	8356001	8358000	2000	1	5.90E-07	0.49	37	1.85	Mtus2	

DMR12:8359001	12	8359001	8360000	1000	1	4.80E-09	0.49	21	2.1	Mtus2	
DMR12:8381001	12	8381001	8383000	2000	1	4.60E-10	0.5	49	2.45	Mtus2	
DMR12:8389001	12	8389001	8390000	1000	1	2.80E-07	0.49	15	1.5	Mtus2	
DMR12:8411001	12	8411001	8416000	5000	2	4.30E-18	1.09	109	2.18	Mtus2	
DMR12:8418001	12	8418001	8421000	3000	2	1.20E-12	0.86	71	2.37	Mtus2	
DMR12:8424001	12	8424001	8425000	1000	1	4.60E-10	-0.38	15	1.5	Mtus2	
DMR12:8449001	12	8449001	8450000	1000	1	1.90E-09	0.54	29	2.9	Mtus2	
DMR12:8471001	12	8471001	8473000	2000	1	5.10E-12	0.9	49	2.45	Mtus2	
DMR12:9045001	12	9045001	9048000	3000	1	4.10E-07	0.41	24	0.8	Flt1	Receptor
DMR12:9077001	12	9077001	9079000	2000	1	1.30E-08	0.43	28	1.4	Flt1;LOC102547693	Receptor
DMR12:9119001	12	9119001	9124000	5000	1	6.20E-10	-0.5	92	1.84	Flt1	Receptor
DMR12:9145001	12	9145001	9146000	1000	1	3.10E-11	0.59	32	3.2	Flt1	Receptor
DMR12:9159001	12	9159001	9161000	2000	1	6.00E-10	0.65	46	2.3	Flt1	Receptor
DMR12:9255001	12	9255001	9258000	3000	1	7.60E-07	-0.41	38	1.27	Pan3	Transcription
DMR12:9353001	12	9353001	9355000	2000	1	9.80E-07	0.33	25	1.25	LOC363870;Flt3	Receptor
DMR12:9388001	12	9388001	9391000	3000	1	4.40E-11	0.73	76	2.53	Flt3	Receptor
DMR12:9429001	12	9429001	9431000	2000	1	1.10E-07	0.49	45	2.25	Flt3	Receptor
DMR12:9435001	12	9435001	9437000	2000	1	3.20E-08	0.51	35	1.75	Flt3;Urad	Receptor;Metabolism
DMR12:9478001	12	9478001	9480000	2000	2	1.90E-10	0.92	37	1.85	Cdx2	Development
DMR12:9726001	12	9726001	9727000	1000	1	5.80E-07	-0.41	22	2.2	Polr1d;Lnx2	
DMR12:9752001	12	9752001	9756000	4000	2	2.60E-15	-0.69	74	1.85	Lnx2	
DMR12:9796001	12	9796001	9799000	3000	1	3.40E-08	-0.39	45	1.5	Lnx2;LOC103691017	
DMR12:9849001	12	9849001	9852000	3000	1	1.40E-07	-0.41	56	1.87	Mtif3;Gtf3a	Translation;Transcription
DMR12:9980001	12	9980001	9981000	1000	1	1.40E-07	0.48	9	0.9	Rasl11a	
DMR12:10009001	12	10009001	10010000	1000	1	3.20E-08	0.6	30	3	Rpl21;LOC102549133	Translation
DMR12:10091001	12	10091001	10092000	1000	1	3.30E-07	0.44	15	1.5	Usp12	Protease
DMR12:10245001	12	10245001	10248000	3000	1	1.80E-10	-0.52	33	1.1	Gpr12	Signaling
DMR12:10324001	12	10324001	10327000	3000	1	5.30E-15	0.74	56	1.87	Wasf3	Cytoskeleton
DMR12:10369001	12	10369001	10372000	3000	1	6.60E-11	-0.5	44	1.47	Wasf3	Cytoskeleton
DMR12:10506001	12	10506001	10507000	1000	1	3.50E-08	-0.43	20	2	Cdk8	
DMR12:10583001	12	10583001	10584000	1000	1	1.80E-09	-0.61	20	2	Rnf6	
DMR12:10641001	12	10641001	10644000	3000	2	4.80E-15	0.8	42	1.4	Cyp3a18	Metabolism
DMR12:10655001	12	10655001	10656000	1000	1	7.20E-08	0.67	16	1.6	Cyp3a18	Metabolism
DMR12:11151001	12	11151001	11152000	1000	1	6.10E-07	-0.31	9	0.9	Zfp655;Zkscan5	
DMR12:11301001	12	11301001	11307000	6000	1	1.50E-07	0.63	94	1.57	Arpc1a;Rps15al1	Cytoskeleton
DMR12:11348001	12	11348001	11351000	3000	2	9.60E-08	0.62	49	1.63	LOC108352477;Kpna7	Transport
DMR12:11373001	12	11373001	11374000	1000	1	1.90E-09	-0.42	5	0.5	Kpna7	Transport
DMR12:11381001	12	11381001	11384000	3000	2	2.40E-10	-0.52	34	1.13	Kpna7	Transport
DMR12:11461001	12	11461001	11464000	3000	1	5.80E-11	0.5	40	1.33	Smurf1	Proteolysis
DMR12:11483001	12	11483001	11484000	1000	1	1.90E-08	-0.45	25	2.5	Smurf1	Proteolysis
DMR12:11614001	12	11614001	11617000	3000	1	1.10E-07	-0.35	51	1.7	Trrap	
DMR12:11845001	12	11845001	11848000	3000	1	1.20E-07	0.6	54	1.8	Tmem130	Signaling
DMR12:12009001	12	12009001	12013000	4000	1	3.00E-09	0.83	40	1	Nptx2	
DMR12:12287001	12	12287001	12288000	1000	1	8.90E-07	-0.32	16	1.6	Baiap2l1;LOC103693623	Cytoskeleton
DMR12:12329001	12	12329001	12330000	1000	1	2.00E-09	-0.48	17	1.7	Baiap2l1;Bri3;LOC102554669	Cytoskeleton
DMR12:12547001	12	12547001	12550000	3000	1	4.80E-07	-0.43	37	1.23	Lmtk2	Receptor
DMR12:12593001	12	12593001	12604000	11000	3	3.40E-11	0.69	221	2.01	Ocm2	
DMR12:12606001	12	12606001	12609000	3000	1	3.80E-14	0.7	78	2.6	Ocm2	
DMR12:12664001	12	12664001	12665000	1000	1	2.50E-07	-0.33	13	1.3	Ccz1b;Rsph10b	
DMR12:12880001	12	12880001	12887000	7000	1	5.20E-12	0.5	147	2.1	Usp42;Cyth3	Protease;Transcription
DMR12:13072001	12	13072001	13074000	2000	1	7.40E-09	0.66	34	1.7	Fam220a	
DMR12:13076001	12	13076001	13078000	2000	1	7.80E-09	0.76	52	2.6	Fam220a	
DMR12:13184001	12	13184001	13185000	1000	1	8.20E-08	0.49	19	1.9	Kdelr2	Transport
DMR12:13223001	12	13223001	13225000	2000	1	4.10E-09	0.54	24	1.2	Grid2ip	
DMR12:13232001	12	13232001	13233000	1000	1	4.30E-10	0.69	13	1.3	Grid2ip	
DMR12:13238001	12	13238001	13239000	1000	1	1.30E-09	0.48	21	2.1	Grid2ip	
DMR12:13259001	12	13259001	13260000	1000	1	1.10E-07	0.57	11	1.1	Grid2ip;LOC102555005	
DMR12:13338001	12	13338001	13343000	5000	1	2.90E-07	0.49	80	1.6	Zfp853;Zfp316	Transcription
DMR12:13379001	12	13379001	13381000	2000	1	4.10E-08	-0.47	44	2.2	Zfp12;Spdye4	
DMR12:13654001	12	13654001	13655000	1000	1	2.70E-07	0.64	12	1.2	Fscn1	Cytoskeleton
DMR12:13656001	12	13656001	13657000	1000	1	4.90E-12	0.64	26	2.6	Fscn1	Cytoskeleton

DMR12:13746001	12	13746001	13747000	1000	1	5.70E-07	-0.37	26	2.6	Fbxl18	
DMR12:13755001	12	13755001	13756000	1000	1	3.10E-07	0.3	21	2.1	Fbxl18	
DMR12:13820001	12	13820001	13823000	3000	1	8.30E-09	-0.47	52	1.73	Tnrc18	Transcription
DMR12:13887001	12	13887001	13888000	1000	1	3.10E-09	-0.42	23	2.3	Tnrc18	Transcription
DMR12:13914001	12	13914001	13915000	1000	1	4.30E-09	0.47	22	2.2	Tnrc18;Slc29a4	Transcription;Transport
DMR12:13920001	12	13920001	13921000	1000	1	1.30E-10	-0.41	26	2.6	Tnrc18;Slc29a4	Transcription;Transport
DMR12:14024001	12	14024001	14026000	2000	1	5.20E-09	-0.62	24	1.2	Mmd2	Signaling
DMR12:14078001	12	14078001	14082000	4000	1	5.90E-07	0.35	46	1.15	Mmd2	Signaling
DMR12:14122001	12	14122001	14124000	2000	1	3.50E-10	0.6	19	0.95	Radil;Papolb	
DMR12:14177001	12	14177001	14178000	1000	1	9.00E-08	0.35	15	1.5	Ap5z1;Foxk1	
DMR12:14205001	12	14205001	14209000	4000	1	6.40E-07	0.43	72	1.8	Foxk1;LOC103693620	
DMR12:14434001	12	14434001	14438000	4000	1	7.30E-07	-0.46	50	1.25	Sdk1	
DMR12:14454001	12	14454001	14458000	4000	2	2.20E-17	1.06	86	2.15	Sdk1	
DMR12:14488001	12	14488001	14489000	1000	1	1.20E-08	0.54	18	1.8	Sdk1	
DMR12:14520001	12	14520001	14522000	2000	1	1.60E-13	0.54	33	1.65	Sdk1	
DMR12:14532001	12	14532001	14533000	1000	1	1.40E-09	0.58	7	0.7	Sdk1	
DMR12:14593001	12	14593001	14595000	2000	1	9.50E-08	0.38	25	1.25	Sdk1	
DMR12:14663001	12	14663001	14665000	2000	1	3.40E-07	0.49	33	1.65	Sdk1	
DMR12:14669001	12	14669001	14673000	4000	1	2.10E-08	0.41	51	1.27	Sdk1	
DMR12:14678001	12	14678001	14682000	4000	1	3.50E-09	-0.6	56	1.4	Sdk1	
DMR12:14693001	12	14693001	14695000	2000	1	1.30E-09	0.69	24	1.2	Sdk1	
DMR12:14697001	12	14697001	14702000	5000	1	9.30E-08	0.43	62	1.24	Sdk1	
DMR12:14713001	12	14713001	14714000	1000	1	1.20E-07	0.54	13	1.3	Sdk1	
DMR12:14784001	12	14784001	14785000	1000	1	3.10E-08	-0.38	20	2	Sdk1	
DMR12:14843001	12	14843001	14844000	1000	1	3.30E-14	0.9	24	2.4	Sdk1	
DMR12:14856001	12	14856001	14857000	1000	1	1.30E-09	0.61	16	1.6	Sdk1	
DMR12:14918001	12	14918001	14920000	2000	1	8.80E-10	0.4	20	1	Sdk1	
DMR12:15074001	12	15074001	15076000	2000	1	7.10E-10	-0.45	39	1.95	Sdk1	
DMR12:15089001	12	15089001	15091000	2000	1	1.60E-14	0.87	40	2	Sdk1	
DMR12:15103001	12	15103001	15107000	4000	1	1.80E-07	0.61	66	1.65	Sdk1	
DMR12:15108001	12	15108001	15109000	1000	1	2.10E-10	0.55	11	1.1	Sdk1	
DMR12:15117001	12	15117001	15120000	3000	1	8.70E-07	0.38	44	1.47	Sdk1	
DMR12:15202001	12	15202001	15206000	4000	1	4.80E-08	-0.38	36	0.9	Sdk1;LOC108352492	
DMR12:15244001	12	15244001	15246000	2000	1	7.50E-08	-0.36	40	2	Sdk1	
DMR12:15270001	12	15270001	15272000	2000	1	5.80E-07	-0.41	28	1.4	Sdk1	
DMR12:15379001	12	15379001	15382000	3000	2	1.60E-09	-0.58	34	1.13	Sdk1	
DMR12:15393001	12	15393001	15394000	1000	1	1.60E-07	-0.4	10	1	Sdk1	
DMR12:15410001	12	15410001	15411000	1000	1	1.80E-08	-0.5	19	1.9	Sdk1	
DMR12:15768001	12	15768001	15769000	1000	1	6.40E-11	0.8	23	2.3	Card11	
DMR12:15803001	12	15803001	15805000	2000	1	9.50E-14	0.87	40	2	Card11	
DMR12:15814001	12	15814001	15816000	2000	1	4.20E-16	0.81	41	2.05	Card11	
DMR12:15974001	12	15974001	15977000	3000	1	4.60E-09	0.45	41	1.37	Gna12;Amz1	Signaling
DMR12:16033001	12	16033001	16036000	3000	1	6.60E-11	0.74	58	1.93	Brat1;Iqce	
DMR12:16061001	12	16061001	16067000	6000	1	8.60E-07	0.48	112	1.87	Iqce	
DMR12:16116001	12	16116001	16120000	4000	1	2.70E-07	0.46	58	1.45	Ttyh3;Lfng	Transport;Golgi
DMR12:16164001	12	16164001	16167000	3000	3	1.20E-08	0.71	67	2.23	Grifin	Extracellular Matrix
DMR12:16179001	12	16179001	16182000	3000	1	3.60E-15	-0.61	29	0.97	Grifin	Extracellular Matrix
DMR12:16312001	12	16312001	16317000	5000	1	5.20E-13	0.85	79	1.58	Eif3b;LOC108352425	Translation
DMR12:16334001	12	16334001	16337000	3000	1	3.10E-07	0.39	24	0.8	LOC108352425;Snx8	Cytoskeleton
DMR12:16347001	12	16347001	16348000	1000	1	8.40E-07	0.3	10	1	LOC108352425;Snx8	Cytoskeleton
DMR12:16440001	12	16440001	16442000	2000	1	5.90E-08	0.43	21	1.05	Mad1l1	
DMR12:16467001	12	16467001	16473000	6000	1	1.60E-07	0.33	97	1.62	Mad1l1	
DMR12:16537001	12	16537001	16538000	1000	1	2.30E-08	0.5	13	1.3	Mad1l1	
DMR12:16603001	12	16603001	16605000	2000	1	2.10E-07	0.44	21	1.05	Mad1l1	
DMR12:16618001	12	16618001	16619000	1000	1	5.50E-11	-0.54	12	1.2	Mad1l1	
DMR12:16624001	12	16624001	16629000	5000	1	3.30E-08	0.57	62	1.24	Mad1l1	
DMR12:16652001	12	16652001	16654000	2000	1	8.60E-07	0.37	37	1.85	Mad1l1	
DMR12:16917001	12	16917001	16922000	5000	2	3.20E-15	0.83	118	2.36	Tmem184a;Mafk	Transport;Transcription
DMR12:16968001	12	16968001	16971000	3000	1	8.50E-08	0.61	60	2	Ints1	

DMR12:17013001	12	17013001	17015000	2000	1	5.50E-08	0.54	30	1.5	Micall2	
DMR12:17186001	12	17186001	17188000	2000	1	7.50E-07	-0.62	96	4.8	Uncx	Development
DMR12:17353001	12	17353001	17356000	3000	1	5.10E-07	0.45	44	1.47	LOC498154;Gpr146	Signaling
DMR12:17378001	12	17378001	17380000	2000	1	3.70E-07	0.54	55	2.75	LOC498154;Mir339;Cyp2w1	Metabolism
DMR12:17646001	12	17646001	17650000	4000	1	2.50E-08	0.57	68	1.7	Prkar1b	Signaling
DMR12:17711001	12	17711001	17719000	8000	1	4.30E-11	0.7	86	1.07	Prkar1b;LOC103691050	Signaling
DMR12:17917001	12	17917001	17921000	4000	1	2.20E-07	0.38	49	1.23	Fam20c	
DMR12:17927001	12	17927001	17930000	3000	1	3.30E-10	0.4	34	1.13	Fam20c	
DMR12:17960001	12	17960001	17962000	2000	1	5.00E-09	0.53	18	0.9	Fam20c	
DMR12:17974001	12	17974001	17977000	3000	1	2.30E-07	0.42	37	1.23	Fam20c	
DMR12:18173001	12	18173001	18175000	2000	1	3.30E-08	0.63	24	1.2	Zfp68;RGD1562569	
DMR12:18477001	12	18477001	18480000	3000	2	7.70E-13	-0.49	52	1.73	Zfp157	Transcription
DMR12:18492001	12	18492001	18494000	2000	2	2.10E-07	-0.4	21	1.05	Zfp157	Transcription
DMR12:18518001	12	18518001	18520000	2000	2	1.20E-13	1.04	67	3.35	Dhrsx;Asmt;Akap17a	Metabolism;Epigenetic
DMR12:18522001	12	18522001	18523000	1000	1	5.80E-16	1.12	26	2.6	Dhrsx;Asmt;Akap17a;Asmtl	Metabolism;Epigenetic
DMR12:18525001	12	18525001	18528000	3000	3	7.80E-24	1.23	116	3.87	Dhrsx;Asmt;Akap17a;Asmtl;Il3ra	Metabolism;Epigenetic
DMR12:18529001	12	18529001	18530000	1000	1	2.40E-14	0.75	40	4	Dhrsx;Asmt;Akap17a;Asmtl;Il3ra	Metabolism;Epigenetic
DMR12:18537001	12	18537001	18538000	1000	1	1.70E-11	0.8	34	3.4	Asmt;Akap17a;Asmtl;Il3ra	Epigenetic
DMR12:18934001	12	18934001	18935000	1000	1	4.50E-07	-0.42	12	1.2	LOC102554070;Spry3	Cytoskeleton
DMR12:18936001	12	18936001	18940000	4000	3	7.40E-23	1.37	138	3.45	LOC102554070;Spry3	Cytoskeleton
DMR12:18943001	12	18943001	18946000	3000	2	6.10E-07	-0.37	24	0.8	Spry3	Cytoskeleton
DMR12:19006001	12	19006001	19009000	3000	2	2.60E-15	-0.58	73	2.43	Vamp7	Transcription
DMR12:19022001	12	19022001	19026000	4000	1	3.90E-08	-0.37	30	0.75	Vamp7	Transcription
DMR12:19083001	12	19083001	19086000	3000	1	2.80E-12	-0.43	28	0.93	Cyp3a9	Metabolism
DMR12:19090001	12	19090001	19091000	1000	1	2.60E-07	-0.32	10	1	Cyp3a9	Metabolism
DMR12:19282001	12	19282001	19284000	2000	1	8.90E-07	-0.4	37	1.85	Zscan21;LOC103691091;Zfp113	Transcription
DMR12:19419001	12	19419001	19421000	2000	2	5.50E-13	0.7	32	1.6	Nxpe5	
DMR12:19429001	12	19429001	19432000	3000	1	3.20E-22	0.75	29	0.97	Nxpe5	
DMR12:19510001	12	19510001	19514000	4000	1	4.00E-14	-0.83	35	0.88	LOC103690351;LOC102551138;Nxpe5l1	
DMR12:19529001	12	19529001	19532000	3000	2	2.20E-10	0.76	39	1.3	LOC102551138;Nxpe5l1	
DMR12:19533001	12	19533001	19536000	3000	1	1.20E-07	0.3	73	2.43	LOC102551138;Nxpe5l1	
DMR12:19554001	12	19554001	19555000	1000	1	3.00E-13	0.9	16	1.6	RGD1562319	
DMR12:19565001	12	19565001	19569000	4000	1	1.00E-15	0.9	57	1.43	RGD1562319;Lamtor4;RGD1305455	
DMR12:19636001	12	19636001	19641000	5000	1	2.00E-07	0.18	42	0.84	Stag3	Epigenetic
DMR12:19940001	12	19940001	19942000	2000	1	6.40E-11	-0.53	62	3.1	Vom2r62	Signaling
DMR12:20980001	12	20980001	20987000	7000	4	2.20E-11	-0.35	70	1	Vom2r64	Signaling
DMR12:21829001	12	21829001	21831000	2000	2	1.30E-11	-0.5	35	1.75	Cldn3;LOC108352482	Cell Junction
DMR12:21885001	12	21885001	21890000	5000	1	1.20E-07	0.41	38	0.76	RGD1561143;RGD1566006;LOC103693637;LOC363897	Immune
DMR12:22086001	12	22086001	22088000	2000	1	1.80E-07	-0.44	55	2.75	LOC108352431;Agfg2	
DMR12:22118001	12	22118001	22119000	1000	1	3.00E-16	0.93	28	2.8	Agfg2;Irs3;Sap25;Lrch4	Epigenetic
DMR12:22124001	12	22124001	22125000	1000	1	2.60E-07	0.58	17	1.7	Agfg2;Irs3;Sap25;Lrch4	Epigenetic
DMR12:22140001	12	22140001	22141000	1000	1	5.00E-07	0.41	10	1	Lrch4;Fbxo24;LOC108352432	
DMR12:22156001	12	22156001	22157000	1000	1	1.60E-07	0.48	33	3.3	Fbxo24;LOC108352432;Pcolce;Mospd3	Protease;Transport
DMR12:22182001	12	22182001	22183000	1000	1	8.20E-07	0.44	29	2.9	Tfr2	Protease
DMR12:22218001	12	22218001	22220000	2000	1	8.20E-19	1.02	65	3.25	Actl6b;LOC108352483;Gnb2	Cytoskeleton;Signaling
DMR12:22224001	12	22224001	22226000	2000	1	8.90E-09	0.52	44	2.2	LOC108352483;Gnb2;Gigyf1	Signaling
DMR12:22267001	12	22267001	22273000	6000	1	3.60E-07	0.55	104	1.73	Pop7;Epo	Translation
DMR12:22297001	12	22297001	22300000	3000	1	4.60E-10	0.61	33	1.1	Zan	
DMR12:22320001	12	22320001	22321000	1000	1	2.70E-07	-0.31	18	1.8	Zan	
DMR12:22335001	12	22335001	22337000	2000	1	2.10E-10	-0.46	37	1.85	Zan	
DMR12:22347001	12	22347001	22350000	3000	1	2.00E-08	0.4	55	1.83	Zan	
DMR12:22362001	12	22362001	22366000	4000	1	2.40E-08	-0.38	79	1.98	Zan	
DMR12:22378001	12	22378001	22382000	4000	1	2.80E-07	-0.4	59	1.48	Zan	

DMR12:22409001	12	22409001	22411000	2000	2	5.80E-08	0.67	47	2.35	Ephb4	Receptor
DMR12:22416001	12	22416001	22418000	2000	1	9.70E-07	-0.42	52	2.6	Ephb4	Receptor
DMR12:22474001	12	22474001	22476000	2000	1	3.20E-08	0.61	35	1.75	Srrt;Ufsp1;Ache	Metabolism
DMR12:22549001	12	22549001	22553000	4000	1	4.00E-15	-0.56	55	1.38	Muc3	
DMR12:22636001	12	22636001	22637000	1000	1	3.80E-08	-0.41	20	2	Serpine1	Protease; Proteolysis
DMR12:22669001	12	22669001	22673000	4000	1	6.60E-09	0.42	73	1.82	Ap1s1;Vgf;LOC102551204	Transport;Signaling
DMR12:22835001	12	22835001	22836000	1000	1	3.60E-09	0.97	62	6.2	Col26a1	
DMR12:22838001	12	22838001	22840000	2000	1	6.00E-07	0.46	34	1.7	Col26a1	
DMR12:22844001	12	22844001	22847000	3000	1	7.80E-08	0.56	53	1.77	Col26a1	
DMR12:22893001	12	22893001	22894000	1000	1	4.90E-07	0.41	13	1.3	Col26a1	
DMR12:22900001	12	22900001	22903000	3000	2	2.20E-13	0.78	78	2.6	Col26a1	
DMR12:22950001	12	22950001	22952000	2000	1	2.60E-10	0.47	37	1.85	Col26a1	
DMR12:22963001	12	22963001	22965000	2000	2	1.10E-09	0.69	48	2.4	Col26a1	
DMR12:23003001	12	23003001	23005000	2000	1	2.30E-08	0.4	30	1.5	Myl10	Cytoskeleton
DMR12:23037001	12	23037001	23039000	2000	2	2.40E-23	1.52	48	2.4	Myl10	Cytoskeleton
DMR12:23044001	12	23044001	23046000	2000	2	1.30E-09	0.76	49	2.45	Myl10	Cytoskeleton
DMR12:23064001	12	23064001	23070000	6000	1	2.40E-09	0.48	85	1.42	Myl10	Cytoskeleton
DMR12:23082001	12	23082001	23083000	1000	1	3.50E-08	0.41	27	2.7	Myl10	Cytoskeleton
DMR12:23203001	12	23203001	23205000	2000	1	9.70E-07	-0.38	19	0.95	Cux1	Development
DMR12:23242001	12	23242001	23245000	3000	1	5.70E-07	-0.53	45	1.5	Cux1	Development
DMR12:23252001	12	23252001	23253000	1000	1	7.20E-07	0.66	12	1.2	Cux1	Development
DMR12:23325001	12	23325001	23328000	3000	1	3.70E-07	0.41	26	0.87	Cux1	Development
DMR12:23382001	12	23382001	23383000	1000	1	1.10E-08	0.38	16	1.6	Cux1	Development
DMR12:23481001	12	23481001	23482000	1000	1	1.20E-07	0.42	19	1.9	Sh2b2	Cytoskeleton
DMR12:23565001	12	23565001	23570000	5000	1	4.00E-14	0.67	85	1.7	Orai2;Alkbh4;Lrwd1	Transport;Metabolism; Extracellular Matrix
DMR12:23602001	12	23602001	23603000	1000	1	1.80E-14	0.61	13	1.3	Polr2j;Rasa4	Transcription;Signaling
DMR12:23605001	12	23605001	23606000	1000	1	2.20E-17	1.21	31	3.1	Rasa4	Signaling
DMR12:23652001	12	23652001	23656000	4000	2	1.70E-09	0.73	83	2.08	Upk3bl	
DMR12:23657001	12	23657001	23661000	4000	1	5.70E-08	0.37	67	1.68	Upk3bl	
DMR12:23680001	12	23680001	23682000	2000	1	2.50E-10	0.59	39	1.95	Upk3b;Dtx2	Proteolysis
DMR12:23737001	12	23737001	23739000	2000	1	6.50E-10	0.48	32	1.6	Dtx2;Zp3	Proteolysis
DMR12:23740001	12	23740001	23743000	3000	1	2.60E-10	0.79	46	1.53	Zp3;Ssc4d	Protease
DMR12:23771001	12	23771001	23772000	1000	1	9.30E-07	-0.35	10	1	Ssc4d	Protease
DMR12:23883001	12	23883001	23886000	3000	1	5.30E-16	0.88	47	1.57	Srrm3	
DMR12:23914001	12	23914001	23915000	1000	1	5.50E-07	0.47	25	2.5	Srrm3	
DMR12:23918001	12	23918001	23920000	2000	1	9.00E-08	0.48	37	1.85	Srrm3;LOC108352435	
DMR12:23929001	12	23929001	23930000	1000	1	1.60E-09	0.73	22	2.2	Srrm3;LOC108352435	
DMR12:24106001	12	24106001	24108000	2000	1	2.30E-07	0.4	60	3	Rhbdd2	Protease
DMR12:24124001	12	24124001	24125000	1000	1	2.90E-08	0.32	7	0.7	Ccl24	Growth Factors
DMR12:24162001	12	24162001	24164000	2000	1	2.60E-09	0.63	29	1.45	Ccl24;Ccl26;LOC108352436;LOC363903	Growth Factors
DMR12:24192001	12	24192001	24194000	2000	1	4.00E-09	0.52	40	2	Hip1	Cytoskeleton
DMR12:24210001	12	24210001	24211000	1000	1	4.10E-11	0.5	6	0.6	Hip1	Cytoskeleton
DMR12:24234001	12	24234001	24236000	2000	2	5.20E-11	0.62	27	1.35	Hip1	Cytoskeleton
DMR12:24254001	12	24254001	24256000	2000	1	3.20E-12	0.67	44	2.2	Hip1	Cytoskeleton
DMR12:24265001	12	24265001	24267000	2000	1	2.10E-07	0.6	36	1.8	Hip1	Cytoskeleton
DMR12:24268001	12	24268001	24271000	3000	1	6.80E-07	0.52	46	1.53	Hip1	Cytoskeleton
DMR12:24289001	12	24289001	24294000	5000	2	4.10E-08	0.75	114	2.28	Hip1	Cytoskeleton
DMR12:24368001	12	24368001	24370000	2000	1	1.70E-07	0.34	28	1.4	Trim50;Fkbp6	Proteolysis;Transcription
DMR12:24424001	12	24424001	24426000	2000	1	2.50E-12	0.64	14	0.7	Fkbp6	Transcription
DMR12:24482001	12	24482001	24484000	2000	1	1.60E-08	0.59	42	2.1	Fzd9;Baz1b	Receptor
DMR12:24548001	12	24548001	24550000	2000	1	1.50E-08	-0.4	33	1.65	Bcl7b	
DMR12:24562001	12	24562001	24564000	2000	1	1.10E-08	0.54	27	1.35	Bcl7b;Tbl2	
DMR12:24607001	12	24607001	24609000	2000	1	6.90E-08	0.51	32	1.6	Mlxipl	Transcription
DMR12:24643001	12	24643001	24645000	2000	1	6.30E-07	-0.63	28	1.4	Vps37d	
DMR12:24652001	12	24652001	24654000	2000	1	8.20E-08	-0.42	35	1.75	Vps37d	
DMR12:24779001	12	24779001	24780000	1000	1	2.30E-11	0.78	31	3.1	Wbscr27;LOC103691506	
DMR12:24813001	12	24813001	24816000	3000	1	3.30E-07	0.47	78	2.6	Wbscr28	
DMR12:24989001	12	24989001	24990000	1000	1	2.50E-09	0.46	14	1.4	LOC102553484;Eln	Development
DMR12:25012001	12	25012001	25013000	1000	1	1.70E-07	0.57	15	1.5	Eln	Development

DMR12:25016001	12	25016001	25018000	2000	1	7.10E-08	0.41	24	1.2	Eln	Development
DMR12:25044001	12	25044001	25049000	5000	1	4.70E-09	0.46	85	1.7	Limk1	
DMR12:25061001	12	25061001	25063000	2000	1	1.10E-07	0.35	37	1.85	Limk1	
DMR12:25164001	12	25164001	25168000	4000	3	9.90E-17	1.14	101	2.52	LOC363906;Syna;Clip2	Transcription
DMR12:25170001	12	25170001	25172000	2000	1	6.10E-07	0.56	23	1.15	Syna;Clip2	Transcription
DMR12:25233001	12	25233001	25235000	2000	1	6.20E-11	0.73	41	2.05	Clip2	Transcription
DMR12:25312001	12	25312001	25314000	2000	1	3.90E-09	0.65	47	2.35	Gtf2ird1	Transcription
DMR12:25407001	12	25407001	25410000	3000	1	2.70E-07	-0.59	37	1.23	Gtf2i	Transcription
DMR12:25419001	12	25419001	25422000	3000	1	1.40E-10	-0.61	61	2.03	Gtf2i	Transcription
DMR12:25430001	12	25430001	25431000	1000	1	7.00E-07	-0.43	28	2.8	Gtf2i	Transcription
DMR12:25507001	12	25507001	25510000	3000	1	9.70E-11	-0.71	49	1.63	LOC103691302;Ncf1;Gtf2ird2	
DMR12:25525001	12	25525001	25528000	3000	1	2.20E-09	0.46	50	1.67	Gtf2ird2	
DMR12:25531001	12	25531001	25534000	3000	1	2.20E-10	0.81	39	1.3	Gtf2ird2	
DMR12:25583001	12	25583001	25590000	7000	1	8.40E-07	0.31	102	1.46	Rcc1l;Gatsl2	
DMR12:25613001	12	25613001	25618000	5000	1	4.90E-10	0.39	83	1.66	Gatsl2	
DMR12:26815001	12	26815001	26816000	1000	1	1.10E-10	0.47	17	1.7	LOC108352438;Mir6322	
DMR12:26817001	12	26817001	26821000	4000	1	2.20E-08	0.34	58	1.45	LOC108352438;Mir6322	
DMR12:28383001	12	28383001	28384000	1000	1	6.30E-13	0.6	19	1.9	Wbscr17	
DMR12:28412001	12	28412001	28414000	2000	1	2.00E-10	-0.47	54	2.7	Wbscr17	
DMR12:28569001	12	28569001	28571000	2000	1	1.80E-07	-0.41	22	1.1	Wbscr17	
DMR12:28577001	12	28577001	28579000	2000	1	6.00E-07	-0.36	12	0.6	Wbscr17	
DMR12:28584001	12	28584001	28586000	2000	2	2.60E-12	0.72	51	2.55	Wbscr17	
DMR12:28603001	12	28603001	28604000	1000	1	2.50E-07	-0.36	14	1.4	Wbscr17	
DMR12:28653001	12	28653001	28654000	1000	1	2.30E-10	0.46	12	1.2	Wbscr17	
DMR12:28677001	12	28677001	28679000	2000	1	3.50E-07	0.65	40	2	Wbscr17	
DMR12:28687001	12	28687001	28689000	2000	1	1.70E-08	-0.6	13	0.65	Wbscr17	
DMR12:28702001	12	28702001	28703000	1000	1	9.00E-09	-0.59	5	0.5	Wbscr17	
DMR12:28725001	12	28725001	28726000	1000	1	7.90E-17	1.02	22	2.2	Wbscr17	
DMR12:28760001	12	28760001	28761000	1000	1	2.40E-07	-0.57	3	0.3	Wbscr17	
DMR12:28810001	12	28810001	28813000	3000	1	8.90E-12	0.51	54	1.8	Wbscr17	
DMR12:28833001	12	28833001	28836000	3000	1	1.10E-16	-0.66	25	0.83	Wbscr17	
DMR12:28844001	12	28844001	28848000	4000	2	4.70E-09	0.73	72	1.8	Wbscr17	
DMR12:28857001	12	28857001	28859000	2000	1	4.90E-07	0.32	16	0.8	Wbscr17	
DMR12:28876001	12	28876001	28878000	2000	1	1.10E-07	0.37	29	1.45	Wbscr17	
DMR12:28914001	12	28914001	28916000	2000	1	1.20E-09	0.52	17	0.85	Wbscr17	
DMR12:29019001	12	29019001	29021000	2000	1	2.20E-12	0.7	49	2.45	Wbscr17;LOC100912262	
DMR12:29167001	12	29167001	29169000	2000	1	1.10E-07	0.67	51	2.55	Wbscr17	
DMR12:29254001	12	29254001	29256000	2000	1	1.20E-17	1.01	52	2.6	Wbscr17	
DMR12:29306001	12	29306001	29307000	1000	1	1.10E-12	1.03	25	2.5	Caln1	
DMR12:29327001	12	29327001	29328000	1000	1	1.90E-08	0.49	25	2.5	Caln1	
DMR12:29339001	12	29339001	29345000	6000	2	1.60E-18	0.85	116	1.93	Caln1	
DMR12:29380001	12	29380001	29382000	2000	1	4.20E-19	0.97	48	2.4	Caln1	
DMR12:29389001	12	29389001	29390000	1000	1	4.80E-07	0.51	21	2.1	Caln1	
DMR12:29428001	12	29428001	29431000	3000	1	1.10E-08	-0.37	39	1.3	Caln1	
DMR12:29432001	12	29432001	29433000	1000	1	4.10E-11	0.78	24	2.4	Caln1	
DMR12:29452001	12	29452001	29454000	2000	1	6.70E-07	-0.39	23	1.15	Caln1	
DMR12:29478001	12	29478001	29480000	2000	1	3.80E-07	0.48	35	1.75	Caln1	
DMR12:29543001	12	29543001	29544000	1000	1	8.00E-07	0.46	10	1	Caln1	
DMR12:29562001	12	29562001	29563000	1000	1	4.10E-10	0.74	16	1.6	Caln1	
DMR12:29657001	12	29657001	29659000	2000	1	2.90E-13	-0.59	30	1.5	Caln1	
DMR12:29663001	12	29663001	29665000	2000	1	4.90E-07	0.32	21	1.05	Caln1	
DMR12:29741001	12	29741001	29745000	4000	1	2.60E-07	0.58	63	1.57	Caln1	
DMR12:29783001	12	29783001	29785000	2000	1	2.10E-07	-0.57	25	1.25	Caln1	
DMR12:29857001	12	29857001	29859000	2000	1	9.90E-09	-0.5	46	2.3	Tyw1	Metabolism
DMR12:29915001	12	29915001	29918000	3000	1	6.80E-09	0.58	65	2.17	Tyw1;Sbds	Metabolism;Metabolism
DMR12:29954001	12	29954001	29956000	2000	1	6.60E-09	-0.38	27	1.35	Tmem248;Rabgef1	Transcription
DMR12:30001001	12	30001001	30003000	2000	1	6.50E-11	-0.46	22	1.1	Rabgef1	Transcription
DMR12:30007001	12	30007001	30009000	2000	1	3.50E-07	0.53	25	1.25	Rabgef1	Transcription
DMR12:30063001	12	30063001	30064000	1000	1	1.00E-08	0.48	31	3.1	Tpst1	Transport
DMR12:30096001	12	30096001	30101000	5000	1	3.50E-07	0.38	77	1.54	Tpst1	Transport
DMR12:30123001	12	30123001	30125000	2000	1	3.30E-10	0.66	53	2.65	Crcp	Signaling

DMR12:30126001	12	30126001	30131000	5000	1	3.20E-10	-0.55	71	1.42	Crcp	Signaling
DMR12:30138001	12	30138001	30141000	3000	1	1.50E-07	0.3	46	1.53	Crcp	Signaling
DMR12:30187001	12	30187001	30189000	2000	1	9.60E-11	0.75	38	1.9	Asl;LOC501835	Metabolism
DMR12:30497001	12	30497001	30499000	2000	1	2.90E-09	-0.52	25	1.25	Sumf2;Cct6a	Translation
DMR12:30519001	12	30519001	30523000	4000	1	7.90E-08	-0.37	72	1.8	Psph;Gbas	Signaling
DMR12:30651001	12	30651001	30652000	1000	1	1.00E-11	0.73	18	1.8	LOC108352487;LOC108352440;Zfp11	
DMR12:30701001	12	30701001	30702000	1000	1	2.60E-07	0.48	24	2.4	Mmp17	Protease
DMR12:30720001	12	30720001	30721000	1000	1	3.20E-15	0.6	11	1.1	Mmp17	Protease
DMR12:30808001	12	30808001	30809000	1000	1	2.90E-07	-0.39	25	2.5	Sfswap	Translation
DMR12:31182001	12	31182001	31185000	3000	1	1.50E-08	0.42	39	1.3	Adgrd1	Signaling
DMR12:31186001	12	31186001	31193000	7000	3	1.20E-08	0.54	133	1.9	Adgrd1	Signaling
DMR12:31195001	12	31195001	31197000	2000	1	6.90E-10	-0.5	60	3	Adgrd1	Signaling
DMR12:31228001	12	31228001	31232000	4000	2	2.40E-07	-0.37	71	1.77	Adgrd1	Signaling
DMR12:31236001	12	31236001	31240000	4000	2	5.70E-20	1.01	80	2	Adgrd1	Signaling
DMR12:31334001	12	31334001	31335000	1000	1	4.60E-14	0.98	21	2.1	Ran;Stx2	Signaling;Transcription
DMR12:31394001	12	31394001	31396000	2000	1	1.30E-07	-0.66	31	1.55	Rimbp2	
DMR12:31410001	12	31410001	31413000	3000	2	3.70E-12	0.61	61	2.03	Rimbp2	
DMR12:31425001	12	31425001	31428000	3000	2	1.40E-07	-0.4	27	0.9	Rimbp2	
DMR12:31431001	12	31431001	31434000	3000	1	3.20E-08	-0.51	35	1.17	Rimbp2	
DMR12:31450001	12	31450001	31456000	6000	1	7.30E-08	0.47	115	1.92	Rimbp2	
DMR12:31462001	12	31462001	31463000	1000	1	7.00E-09	0.58	11	1.1	Rimbp2	
DMR12:31491001	12	31491001	31492000	1000	1	7.70E-07	0.44	15	1.5	Rimbp2	
DMR12:31497001	12	31497001	31498000	1000	1	1.60E-08	0.38	12	1.2	Rimbp2	
DMR12:31500001	12	31500001	31502000	2000	1	2.10E-07	-0.4	21	1.05	Rimbp2	
DMR12:31526001	12	31526001	31527000	1000	1	2.70E-08	0.52	13	1.3	Rimbp2	
DMR12:31547001	12	31547001	31550000	3000	1	1.50E-10	0.46	52	1.73	Rimbp2	
DMR12:31580001	12	31580001	31581000	1000	1	6.20E-19	1.04	24	2.4	Rimbp2	
DMR12:31588001	12	31588001	31589000	1000	1	4.10E-10	0.8	24	2.4	Rimbp2	
DMR12:31594001	12	31594001	31599000	5000	1	1.40E-07	-0.48	76	1.52	Rimbp2;Piwil1	Translation
DMR12:31606001	12	31606001	31608000	2000	1	6.30E-07	0.4	30	1.5	Rimbp2;Piwil1	Translation
DMR12:31768001	12	31768001	31771000	3000	1	4.30E-09	0.7	44	1.47	Fzd10	
DMR12:31943001	12	31943001	31945000	2000	2	3.40E-08	-0.49	15	0.75	Tmem132d	
DMR12:31949001	12	31949001	31950000	1000	1	2.60E-07	0.29	14	1.4	Tmem132d	
DMR12:31975001	12	31975001	31977000	2000	1	9.50E-07	0.44	27	1.35	Tmem132d	
DMR12:32002001	12	32002001	32003000	1000	1	3.20E-07	0.57	20	2	Tmem132d	
DMR12:32105001	12	32105001	32113000	8000	1	2.00E-07	0.31	75	0.94	Tmem132d	
DMR12:32132001	12	32132001	32133000	1000	1	2.10E-17	1.2	28	2.8	Tmem132d	
DMR12:32177001	12	32177001	32182000	5000	1	2.00E-07	-0.37	74	1.48	Tmem132d	
DMR12:32289001	12	32289001	32291000	2000	1	8.60E-07	0.55	37	1.85	Tmem132d	
DMR12:32466001	12	32466001	32469000	3000	2	4.00E-13	0.67	50	1.67	Tmem132d	
DMR12:32489001	12	32489001	32494000	5000	2	3.20E-11	0.73	94	1.88	Tmem132d	
DMR12:32504001	12	32504001	32505000	1000	1	2.80E-11	-0.58	23	2.3	Tmem132d	
DMR12:32565001	12	32565001	32567000	2000	1	3.90E-17	0.96	40	2	Tmem132d	
DMR12:32692001	12	32692001	32698000	6000	1	2.40E-09	0.48	99	1.65	Glt1d1	
DMR12:32729001	12	32729001	32736000	7000	1	5.10E-09	0.43	126	1.8	Glt1d1	
DMR12:32804001	12	32804001	32807000	3000	1	1.10E-07	0.52	38	1.27	Tmem132c	
DMR12:32826001	12	32826001	32830000	4000	1	3.80E-10	0.56	65	1.62	Tmem132c;LOC108352443	
DMR12:32844001	12	32844001	32846000	2000	1	1.70E-09	0.44	25	1.25	Tmem132c;LOC108352443	
DMR12:32849001	12	32849001	32850000	1000	1	6.20E-11	0.76	22	2.2	Tmem132c	
DMR12:32875001	12	32875001	32879000	4000	1	1.60E-08	0.37	55	1.38	Tmem132c	
DMR12:32907001	12	32907001	32911000	4000	1	4.60E-13	0.7	51	1.27	Tmem132c;LOC108352442	
DMR12:32918001	12	32918001	32920000	2000	1	9.80E-09	0.6	33	1.65	Tmem132c;LOC108352442	
DMR12:32960001	12	32960001	32961000	1000	1	9.00E-11	0.71	33	3.3	Tmem132c	
DMR12:32971001	12	32971001	32975000	4000	2	9.20E-12	0.56	86	2.15	Tmem132c	
DMR12:33025001	12	33025001	33029000	4000	1	9.00E-07	-0.46	73	1.82	Tmem132c	
DMR12:33065001	12	33065001	33067000	2000	1	3.00E-08	-0.36	16	0.8	Tmem132c	
DMR12:33078001	12	33078001	33082000	4000	1	4.70E-08	0.49	39	0.98	Tmem132c	
DMR12:33121001	12	33121001	33123000	2000	1	4.50E-07	0.38	26	1.3	Tmem132c	
DMR12:33178001	12	33178001	33179000	1000	1	8.60E-16	0.86	28	2.8	RGD1559821	
DMR12:34000001	12	34000001	34005000	5000	1	1.20E-07	0.45	65	1.3	Frg1l1	
DMR12:35977001	12	35977001	35981000	4000	1	1.70E-07	0.59	82	2.05	Tmem132b	

DMR12:36030001	12	36030001	36032000	2000	1	6.80E-09	0.75	37	1.85	Tmem132b	
DMR12:36046001	12	36046001	36047000	1000	1	4.80E-11	0.82	28	2.8	Tmem132b	
DMR12:36051001	12	36051001	36057000	6000	2	9.60E-12	0.68	122	2.03	Tmem132b	
DMR12:36082001	12	36082001	36083000	1000	1	6.80E-10	0.54	19	1.9	Tmem132b	
DMR12:36120001	12	36120001	36123000	3000	2	6.30E-08	-0.35	104	3.47	Tmem132b	
DMR12:36126001	12	36126001	36133000	7000	1	7.30E-09	-0.37	76	1.09	Tmem132b	
DMR12:36251001	12	36251001	36254000	3000	1	4.00E-07	0.57	65	2.17	Tmem132b	
DMR12:36277001	12	36277001	36280000	3000	1	2.20E-08	-0.64	20	0.67	Tmem132b	
DMR12:36290001	12	36290001	36293000	3000	2	5.00E-10	0.77	48	1.6	Tmem132b	
DMR12:36380001	12	36380001	36381000	1000	1	1.70E-08	0.56	9	0.9	Tmem132b	
DMR12:36397001	12	36397001	36399000	2000	1	5.70E-11	0.74	55	2.75	Tmem132b	
DMR12:36513001	12	36513001	36519000	6000	1	2.60E-11	0.78	149	2.48	Aacs	Metabolism
DMR12:36584001	12	36584001	36587000	3000	1	2.10E-10	-0.44	62	2.07	Bri3bp;Dhx37	Transcription
DMR12:36652001	12	36652001	36657000	5000	1	7.90E-07	-0.4	137	2.74	Ubc	
DMR12:36714001	12	36714001	36721000	7000	1	1.10E-08	0.46	109	1.56	Scarb1	Transport
DMR12:36765001	12	36765001	36766000	1000	1	4.30E-08	0.53	14	1.4	Scarb1	Transport
DMR12:36870001	12	36870001	36871000	1000	1	3.80E-07	0.54	20	2	Ncor2	Epigenetic
DMR12:36990001	12	36990001	36995000	5000	1	9.10E-09	0.54	140	2.8	Ncor2	Epigenetic
DMR12:37048001	12	37048001	37049000	1000	1	4.10E-09	0.56	18	1.8	Fam101a	
DMR12:37271001	12	37271001	37273000	2000	1	6.40E-09	0.7	50	2.5	Dnah10	
DMR12:37283001	12	37283001	37284000	1000	1	7.50E-07	0.53	18	1.8	Dnah10	
DMR12:37413001	12	37413001	37415000	2000	1	3.60E-07	-0.56	19	0.95	Tctn2	
DMR12:37578001	12	37578001	37580000	2000	1	9.70E-07	-0.43	22	1.1	Kmt5a	Golgi
DMR12:37627001	12	37627001	37630000	3000	1	4.50E-07	-0.32	48	1.6	Sbno1	
DMR12:37652001	12	37652001	37655000	3000	1	7.20E-09	-0.47	56	1.87	Sbno1	
DMR12:37709001	12	37709001	37710000	1000	1	1.90E-07	0.33	20	2	LOC108348169;Mphosph9	
DMR12:37812001	12	37812001	37813000	1000	1	2.60E-09	-0.55	14	1.4	Pitpnm2	Transport
DMR12:37905001	12	37905001	37906000	1000	1	2.00E-09	-0.55	25	2.5	Pitpnm2;Arl6ip4;Ogfod2;LOC103691351	Transport;Metabolism
DMR12:37921001	12	37921001	37923000	2000	1	2.10E-07	-0.39	33	1.65	LOC103691351;Abcb9	Transport
DMR12:37929001	12	37929001	37933000	4000	1	5.10E-07	0.39	113	2.83	Abcb9	Transport
DMR12:38016001	12	38016001	38017000	1000	1	6.90E-07	-0.4	37	3.7	Vps37b;Hip1r	Cytoskeleton
DMR12:38195001	12	38195001	38198000	3000	1	1.90E-07	0.37	35	1.17	Kntc1	
DMR12:38226001	12	38226001	38227000	1000	1	2.80E-08	0.36	25	2.5	Kntc1;LOC103693647	
DMR12:38283001	12	38283001	38285000	2000	2	1.90E-15	-0.62	33	1.65	Kntc1;Rsrc2	
DMR12:38297001	12	38297001	38298000	1000	1	6.90E-17	-0.59	13	1.3	Rsrc2;Zcchc8	Metabolism
DMR12:38299001	12	38299001	38301000	2000	1	8.60E-09	-0.38	31	1.55	Rsrc2;Zcchc8	Metabolism
DMR12:38340001	12	38340001	38343000	3000	1	5.80E-16	0.84	56	1.87	LOC100359550;Clip1	Transcription
DMR12:38414001	12	38414001	38420000	6000	1	1.30E-11	-0.44	151	2.52	Clip1	Transcription
DMR12:38431001	12	38431001	38435000	4000	1	1.10E-11	0.73	74	1.85	Clip1	Transcription
DMR12:38523001	12	38523001	38524000	1000	1	1.00E-08	0.6	10	1	Lrrc43;Il31	Signaling
DMR12:38531001	12	38531001	38533000	2000	1	8.40E-07	0.56	46	2.3	Lrrc43;Il31	Signaling
DMR12:38657001	12	38657001	38658000	1000	1	8.40E-07	-0.31	28	2.8	Bcl7a	
DMR12:38659001	12	38659001	38662000	3000	3	8.70E-16	1.07	94	3.13	Bcl7a	
DMR12:38695001	12	38695001	38696000	1000	1	1.40E-07	0.52	19	1.9	Bcl7a	
DMR12:38718001	12	38718001	38720000	2000	1	1.60E-09	-0.47	31	1.55	Wdr66	
DMR12:38759001	12	38759001	38761000	2000	1	2.20E-07	-0.36	39	1.95	Wdr66	
DMR12:38780001	12	38780001	38782000	2000	1	6.30E-07	-0.42	44	2.2	Wdr66;Psmd9	Protease
DMR12:38809001	12	38809001	38810000	1000	1	6.20E-09	-0.38	19	1.9	Psmd9	Protease
DMR12:38827001	12	38827001	38830000	3000	1	5.00E-07	0.4	55	1.83	LOC102547495;Hpd	Metabolism
DMR12:38832001	12	38832001	38835000	3000	1	1.50E-07	0.43	43	1.43	LOC102547495;Hpd;LOC100359816	Metabolism
DMR12:38943001	12	38943001	38944000	1000	1	1.10E-09	0.47	17	1.7	LOC687612;Morn3	
DMR12:39002001	12	39002001	39005000	3000	1	3.20E-07	-0.41	58	1.93	Orai1;LOC102546488	Transport
DMR12:39041001	12	39041001	39042000	1000	1	6.40E-07	-0.69	4	0.4	Kdm2b	
DMR12:39104001	12	39104001	39106000	2000	1	6.20E-08	-0.36	36	1.8	Kdm2b	
DMR12:39115001	12	39115001	39122000	7000	1	1.30E-07	-0.39	119	1.7	Kdm2b	
DMR12:39155001	12	39155001	39156000	1000	1	3.40E-09	-0.34	20	2	Kdm2b;Rnf34	Proteolysis
DMR12:39196001	12	39196001	39200000	4000	1	1.10E-11	0.87	60	1.5	Anapc5	
DMR12:39224001	12	39224001	39225000	1000	1	6.70E-09	-0.52	30	3	Anapc5	
DMR12:39259001	12	39259001	39262000	3000	1	3.30E-10	0.57	72	2.4	Camkk2	
DMR12:39296001	12	39296001	39297000	1000	1	9.60E-10	0.58	18	1.8	Camkk2	

DMR12:39302001	12	39302001	39304000	2000	1	2.00E-13	0.75	55	2.75	Camkk2;P2rx4	Ion Channel
DMR12:39328001	12	39328001	39330000	2000	1	8.20E-18	-0.75	48	2.4	P2rx4;LOC501838	Ion Channel
DMR12:39494001	12	39494001	39496000	2000	1	3.30E-08	-0.32	44	2.2	Ift81;LOC108352446	
DMR12:39622001	12	39622001	39627000	5000	2	1.50E-14	-0.52	95	1.9	Anapc7;LOC100363066	Proteolysis
DMR12:39645001	12	39645001	39647000	2000	2	7.00E-09	0.6	32	1.6	Anapc7;Arpc3;LOC102548903;LOC108352447	Proteolysis;Cytoskeleton
DMR12:39658001	12	39658001	39660000	2000	1	9.60E-07	-0.36	42	2.1	Arpc3;LOC102548903;LOC108352447	Cytoskeleton
DMR12:39691001	12	39691001	39692000	1000	1	1.90E-07	-0.38	15	1.5	Fam216a;Vps29;Rad9b	Transport;DNA Repair
DMR12:39778001	12	39778001	39782000	4000	1	7.40E-09	0.48	68	1.7	Pptc7;Tctn1	Signaling
DMR12:39787001	12	39787001	39788000	1000	1	2.10E-09	-0.42	17	1.7	Tctn1	
DMR12:39834001	12	39834001	39836000	2000	2	3.30E-13	0.67	40	2	Tctn1;Hvcn1	
DMR12:39837001	12	39837001	39838000	1000	1	2.70E-15	0.76	29	2.9	Tctn1;Hvcn1	
DMR12:39902001	12	39902001	39908000	6000	1	2.40E-07	0.44	106	1.77	LOC102549705;Ccdc63	
DMR12:40029001	12	40029001	40032000	3000	1	2.20E-10	0.62	36	1.2	Cux2	Development
DMR12:40039001	12	40039001	40042000	3000	1	1.30E-12	0.55	79	2.63	Cux2	Development
DMR12:40049001	12	40049001	40051000	2000	1	1.70E-07	0.44	34	1.7	Cux2	Development
DMR12:40079001	12	40079001	40083000	4000	1	6.40E-07	-0.3	108	2.7	Cux2	Development
DMR12:40089001	12	40089001	40092000	3000	1	7.70E-07	0.47	81	2.7	Cux2	Development
DMR12:40095001	12	40095001	40099000	4000	1	1.30E-08	0.67	97	2.42	Cux2	Development
DMR12:40135001	12	40135001	40137000	2000	1	4.20E-08	0.33	25	1.25	Cux2	Development
DMR12:40273001	12	40273001	40274000	1000	1	8.50E-07	-0.39	12	1.2	Sh2b3;Atxn2	Cytoskeleton;Metabolism
DMR12:40314001	12	40314001	40315000	1000	1	1.00E-10	-0.47	15	1.5	Atxn2;LOC102556457	Metabolism
DMR12:40343001	12	40343001	40345000	2000	1	3.80E-08	-0.4	29	1.45	Atxn2	Metabolism
DMR12:40406001	12	40406001	40407000	1000	1	1.40E-07	-0.37	21	2.1	Brap	
DMR12:40420001	12	40420001	40421000	1000	1	1.60E-07	-0.38	17	1.7	Brap;Acad10	Metabolism
DMR12:40467001	12	40467001	40468000	1000	1	5.80E-07	-0.47	16	1.6	Acad10;Aldh2	Metabolism
DMR12:40505001	12	40505001	40507000	2000	1	4.40E-07	0.38	20	1	Aldh2;Mapkapk5	Metabolism;Signaling
DMR12:40727001	12	40727001	40735000	8000	2	1.30E-12	-0.55	158	1.98	Hectd4	Proteolysis
DMR12:41085001	12	41085001	41086000	1000	1	2.40E-09	0.66	28	2.8	Rph3a	
DMR12:41096001	12	41096001	41098000	2000	1	7.00E-10	0.63	39	1.95	Rph3a	
DMR12:41144001	12	41144001	41146000	2000	1	4.30E-08	0.36	41	2.05	Rph3a;Oas1i	Metabolism
DMR12:41203001	12	41203001	41205000	2000	1	2.60E-09	0.54	19	0.95	Oas1a;Oas1h	Metabolism
DMR12:41213001	12	41213001	41214000	1000	1	4.40E-07	0.44	15	1.5	Oas1a;Oas1h	Metabolism
DMR12:41251001	12	41251001	41253000	2000	1	9.40E-08	-0.66	18	0.9	Oas1b	Metabolism
DMR12:41325001	12	41325001	41326000	1000	1	3.90E-13	0.62	25	2.5	Oas3	Metabolism
DMR12:41413001	12	41413001	41414000	1000	1	1.00E-10	0.65	31	3.1	Dtx1;Rasal1	Proteolysis;Signaling
DMR12:41478001	12	41478001	41482000	4000	2	1.80E-09	0.55	62	1.55	Cfap73;Ddx54;Rita1;Iqcd	
DMR12:41560001	12	41560001	41561000	1000	1	1.90E-07	0.5	21	2.1	Tpcn1;Slc8b1	Transport;Transport
DMR12:41620001	12	41620001	41626000	6000	2	7.80E-11	0.68	114	1.9	Plbd2;Sds	Metabolism;Metabolism
DMR12:41633001	12	41633001	41634000	1000	1	4.50E-10	0.48	4	0.4	Sds;Sdsl	Metabolism
DMR12:41642001	12	41642001	41644000	2000	2	3.30E-10	-0.69	29	1.45	Sdsl	Metabolism
DMR12:41945001	12	41945001	41951000	6000	1	1.50E-07	0.58	99	1.65	Rbm19	Translation
DMR12:41953001	12	41953001	41961000	8000	1	4.10E-08	0.48	136	1.7	Rbm19	Translation
DMR12:41977001	12	41977001	41978000	1000	1	2.00E-07	0.5	18	1.8	Rbm19	Translation
DMR12:41984001	12	41984001	41986000	2000	1	1.80E-07	0.38	38	1.9	Rbm19	Translation
DMR12:42051001	12	42051001	42052000	1000	1	2.50E-17	1.24	36	3.6	Tbx5	Transcription
DMR12:42082001	12	42082001	42085000	3000	1	4.60E-07	0.48	35	1.17	Tbx5;LOC102549962	Transcription
DMR12:42129001	12	42129001	42130000	1000	1	5.80E-12	0.98	32	3.2	Tbx5	Transcription
DMR12:42131001	12	42131001	42134000	3000	1	2.20E-09	-0.56	29	0.97	Tbx5	Transcription
DMR12:42145001	12	42145001	42147000	2000	1	3.70E-20	1.01	36	1.8	Tbx5	Transcription
DMR12:42152001	12	42152001	42155000	3000	1	1.90E-07	0.34	42	1.4	Tbx5	Transcription
DMR12:42483001	12	42483001	42485000	2000	2	5.30E-11	-0.57	79	3.95	Tbx3	Transcription
DMR12:42486001	12	42486001	42491000	5000	3	7.10E-13	-0.66	99	1.98	Tbx3	Transcription
DMR12:43514001	12	43514001	43517000	3000	1	8.10E-09	-0.44	42	1.4	Med13l	
DMR12:43518001	12	43518001	43523000	5000	1	4.00E-09	-0.42	72	1.44	Med13l	
DMR12:43547001	12	43547001	43548000	1000	1	1.20E-09	-0.44	12	1.2	Med13l	
DMR12:43555001	12	43555001	43558000	3000	1	1.70E-07	0.42	28	0.93	Med13l	
DMR12:43942001	12	43942001	43944000	2000	1	4.60E-07	0.51	33	1.65	RGD1562310;Rnft2	
DMR12:44146001	12	44146001	44150000	4000	1	3.70E-09	0.41	85	2.12	Fbxw8;Tesc	

DMR12:44163001	12	44163001	44164000	1000	1	5.90E-11	0.47	13	1.3	Tesc	
DMR12:44171001	12	44171001	44172000	1000	1	4.10E-12	0.71	25	2.5	Tesc	
DMR12:44223001	12	44223001	44225000	2000	1	1.60E-07	0.57	34	1.7	Nos1	Metabolism
DMR12:44236001	12	44236001	44239000	3000	1	1.50E-12	0.6	42	1.4	Nos1;Fbxo21	Metabolism
DMR12:44282001	12	44282001	44285000	3000	1	3.90E-07	0.44	43	1.43	Nos1;Fbxo21	Metabolism
DMR12:44330001	12	44330001	44332000	2000	1	5.90E-11	0.75	45	2.25	Nos1	Metabolism
DMR12:44364001	12	44364001	44367000	3000	1	1.40E-10	0.48	54	1.8	Nos1	Metabolism
DMR12:44388001	12	44388001	44389000	1000	1	5.40E-08	0.43	17	1.7	Nos1	Metabolism
DMR12:44412001	12	44412001	44414000	2000	1	8.10E-08	-0.4	8	0.4	Nos1	Metabolism
DMR12:44550001	12	44550001	44551000	1000	1	6.20E-07	-0.36	10	1	Ksr2	Signaling
DMR12:44624001	12	44624001	44625000	1000	1	1.90E-07	0.39	1	0.1	Ksr2	Signaling
DMR12:44761001	12	44761001	44762000	1000	1	2.80E-07	0.52	27	2.7	Ksr2	Signaling
DMR12:44768001	12	44768001	44770000	2000	2	2.10E-08	0.65	44	2.2	Ksr2	Signaling
DMR12:44781001	12	44781001	44783000	2000	1	4.70E-08	0.6	34	1.7	Ksr2	Signaling
DMR12:44852001	12	44852001	44857000	5000	1	6.80E-12	0.93	93	1.86	Ksr2	Signaling
DMR12:44867001	12	44867001	44870000	3000	1	5.90E-11	0.71	44	1.47	Ksr2	Signaling
DMR12:44885001	12	44885001	44886000	1000	1	5.00E-09	0.75	26	2.6	Ksr2;LOC102553848	Signaling
DMR12:44904001	12	44904001	44907000	3000	1	2.00E-07	0.37	34	1.13	Ksr2	Signaling
DMR12:45002001	12	45002001	45004000	2000	1	1.50E-09	-0.54	21	1.05	Vsig10	
DMR12:45009001	12	45009001	45012000	3000	1	1.70E-08	0.52	80	2.67	Vsig10	
DMR12:45062001	12	45062001	45063000	1000	1	2.70E-07	-0.32	19	1.9	Taok3	Signaling
DMR12:45076001	12	45076001	45077000	1000	1	5.50E-07	0.48	11	1.1	Taok3	Signaling
DMR12:45078001	12	45078001	45080000	2000	1	1.50E-12	0.35	19	0.95	Taok3	Signaling
DMR12:45818001	12	45818001	45821000	3000	1	8.20E-14	1.03	92	3.07	Srrm4	
DMR12:45830001	12	45830001	45834000	4000	1	6.10E-11	0.74	85	2.12	Srrm4	
DMR12:45899001	12	45899001	45903000	4000	1	1.40E-12	0.83	72	1.8	Hspb8	
DMR12:46076001	12	46076001	46080000	4000	1	4.90E-16	0.62	106	2.65	Ccdc60	
DMR12:46092001	12	46092001	46095000	3000	1	7.20E-10	-0.5	55	1.83	Ccdc60	
DMR12:46182001	12	46182001	46185000	3000	1	4.30E-08	-0.68	24	0.8	Ccdc60	
DMR12:46195001	12	46195001	46196000	1000	1	7.30E-07	0.53	20	2	Ccdc60	
DMR12:46282001	12	46282001	46285000	3000	1	2.50E-08	-0.38	45	1.5	Tmem233	
DMR12:46293001	12	46293001	46298000	5000	1	6.10E-13	0.89	95	1.9	Tmem233	
DMR12:46306001	12	46306001	46312000	6000	1	3.40E-13	0.82	110	1.83	Tmem233;Prkab1	Signaling
DMR12:46327001	12	46327001	46333000	6000	2	5.00E-08	-0.41	131	2.18	Prkab1;LOC108352463;Cit	Signaling;Signaling
DMR12:46378001	12	46378001	46379000	1000	1	8.20E-09	0.48	29	2.9	Cit	Signaling
DMR12:46430001	12	46430001	46433000	3000	1	2.20E-07	-0.36	81	2.7	Cit	Signaling
DMR12:46497001	12	46497001	46510000	13000	1	7.50E-07	0.46	245	1.88	Cit;LOC363929	Signaling
DMR12:46631001	12	46631001	46632000	1000	1	4.70E-18	-0.66	26	2.6	Bicdl1	Transport
DMR12:46647001	12	46647001	46651000	4000	1	7.10E-07	-0.33	70	1.75	Bicdl1	Transport
DMR12:46692001	12	46692001	46698000	6000	1	1.50E-07	-0.4	145	2.42	Bicdl1;Rab35	Transport
DMR12:46722001	12	46722001	46728000	6000	1	3.60E-11	0.52	85	1.42	Rab35;Gcn1l1	
DMR12:46824001	12	46824001	46825000	1000	1	9.60E-09	-0.46	26	2.6	Pxn	Cytoskeleton
DMR12:46834001	12	46834001	46838000	4000	1	2.40E-08	-0.42	105	2.62	Pxn	Cytoskeleton
DMR12:46872001	12	46872001	46875000	3000	2	6.70E-13	0.66	90	3	Sirt4;Pla2g1b	Metabolism
DMR12:46879001	12	46879001	46880000	1000	1	6.60E-08	0.68	29	2.9	Sirt4;Pla2g1b	Metabolism
DMR12:47054001	12	47054001	47058000	4000	1	5.00E-07	-0.34	66	1.65	Srsf9	Translation
DMR12:47143001	12	47143001	47144000	1000	1	4.90E-08	0.6	23	2.3	Rnf10;Pop5	Transport
DMR12:47176001	12	47176001	47178000	2000	1	2.80E-15	1	55	2.75	LOC102548179;Cabp1	
DMR12:47180001	12	47180001	47183000	3000	1	7.20E-10	0.72	58	1.93	LOC102548179;Cabp1	
DMR12:47189001	12	47189001	47191000	2000	1	8.30E-07	0.44	34	1.7	LOC102548179;Cabp1	
DMR12:47194001	12	47194001	47196000	2000	2	1.60E-07	0.66	49	2.45	LOC102548179;Cabp1	
DMR12:47214001	12	47214001	47215000	1000	1	2.40E-07	0.6	20	2	Cabp1;LOC102548245;Mlec	
DMR12:47299001	12	47299001	47300000	1000	1	9.90E-08	-0.44	25	2.5	Sppl3	Proteolysis
DMR12:47396001	12	47396001	47398000	2000	1	9.80E-10	-0.87	5	0.25	LOC102546369;Hnf1a	Transcription
DMR12:47424001	12	47424001	47426000	2000	1	3.60E-26	1.5	88	4.4	Hnf1a;RGD1311899	Transcription
DMR12:47557001	12	47557001	47560000	3000	1	4.50E-09	-0.44	64	2.13	LOC102546787;RGD1560398;Ankrd13a	
DMR12:47561001	12	47561001	47564000	3000	2	1.80E-08	-0.49	63	2.1	LOC102546787;RGD1560398;Ankrd13a	
DMR12:47566001	12	47566001	47569000	3000	2	4.00E-11	-0.5	79	2.63	RGD1560398;Ankrd13a	
DMR12:47610001	12	47610001	47614000	4000	1	1.00E-07	-0.48	88	2.2	Git2	

DMR12:47651001	12	47651001	47653000	2000	1	5.00E-07	0.56	50	2.5	Tchp;LOC102547088;LOC103691428	
DMR12:47665001	12	47665001	47666000	1000	1	1.00E-09	0.74	28	2.8	LOC103691428;Gltp	
DMR12:47733001	12	47733001	47734000	1000	1	3.00E-07	0.61	23	2.3	Trpv4;Fam222a	Transport
DMR12:47735001	12	47735001	47738000	3000	1	6.30E-09	0.48	64	2.13	Trpv4;Fam222a	Transport
DMR12:47747001	12	47747001	47753000	6000	2	6.50E-10	0.56	194	3.23	Trpv4;Fam222a	Transport
DMR12:47760001	12	47760001	47762000	2000	1	4.00E-07	0.36	28	1.4	Fam222a	
DMR12:47773001	12	47773001	47781000	8000	3	1.30E-08	0.67	169	2.11	Fam222a	
DMR12:47904001	12	47904001	47910000	6000	1	2.50E-07	0.49	146	2.43	Mvk	Metabolism
DMR12:47966001	12	47966001	47969000	3000	1	3.60E-10	-0.38	59	1.97	Ube3b	Proteolysis
DMR12:47980001	12	47980001	47981000	1000	1	2.30E-11	-0.44	20	2	Ube3b	Proteolysis
DMR12:48067001	12	48067001	48070000	3000	1	3.80E-08	-0.42	58	1.93	Myo1h	
DMR12:48135001	12	48135001	48140000	5000	1	1.90E-07	0.62	95	1.9	Acacb	
DMR12:48170001	12	48170001	48173000	3000	1	3.60E-12	0.81	68	2.27	Acacb	
DMR12:48177001	12	48177001	48178000	1000	1	2.40E-08	0.7	22	2.2	Acacb	
DMR12:48179001	12	48179001	48180000	1000	1	4.90E-07	0.42	19	1.9	Acacb	
DMR12:48189001	12	48189001	48191000	2000	1	3.00E-07	0.54	23	1.15	Acacb	
DMR12:48192001	12	48192001	48195000	3000	1	6.60E-08	0.35	46	1.53	Acacb	
DMR12:48196001	12	48196001	48197000	1000	1	3.60E-07	0.63	16	1.6	Acacb	
DMR12:48228001	12	48228001	48235000	7000	1	4.60E-13	0.82	129	1.84	Acacb	
DMR12:48299001	12	48299001	48301000	2000	1	1.20E-10	0.81	46	2.3	Usp30;Svop	Protease;Transport
DMR12:48302001	12	48302001	48303000	1000	1	4.60E-07	0.54	18	1.8	Usp30;Svop	Protease;Transport
DMR12:48332001	12	48332001	48333000	1000	1	4.00E-09	0.46	21	2.1	Svop	Transport
DMR12:48355001	12	48355001	48357000	2000	1	1.10E-07	0.33	20	1	Svop;Dao	Transport;Metabolism
DMR12:48371001	12	48371001	48373000	2000	1	4.40E-10	-0.5	46	2.3	Dao	Metabolism
DMR12:48392001	12	48392001	48396000	4000	1	1.50E-09	0.55	58	1.45	Ssh1	Signaling
DMR12:48405001	12	48405001	48407000	2000	1	2.60E-09	-0.52	33	1.65	Ssh1	Signaling
DMR12:48412001	12	48412001	48417000	5000	1	7.10E-07	0.52	131	2.62	Ssh1	Signaling
DMR12:48486001	12	48486001	48487000	1000	1	6.40E-10	-0.48	16	1.6	Coro1c	Cytoskeleton
DMR12:48494001	12	48494001	48495000	1000	1	1.30E-09	-0.38	16	1.6	Coro1c	Cytoskeleton
DMR12:48544001	12	48544001	48548000	4000	1	5.60E-08	0.44	90	2.25	Coro1c	Cytoskeleton
DMR12:48736001	12	48736001	48742000	6000	1	9.70E-07	0.46	86	1.43	Cmklr1	Signaling
DMR12:48744001	12	48744001	48748000	4000	2	2.00E-13	1.03	85	2.12	Cmklr1	Signaling
DMR12:48761001	12	48761001	48763000	2000	1	4.10E-10	0.85	49	2.45	Cmklr1	Signaling
DMR12:48773001	12	48773001	48775000	2000	1	1.80E-14	0.95	62	3.1	Cmklr1	Signaling
DMR12:48796001	12	48796001	48799000	3000	1	2.50E-09	0.4	47	1.57	Cmklr1	Signaling
DMR12:48825001	12	48825001	48830000	5000	1	1.70E-13	0.73	112	2.24	Wscd2	
DMR12:49375001	12	49375001	49378000	3000	1	1.60E-08	-0.38	52	1.73	Sgsm1	Signaling
DMR12:49379001	12	49379001	49380000	1000	1	1.80E-07	0.41	15	1.5	Sgsm1	Signaling
DMR12:49387001	12	49387001	49389000	2000	1	6.60E-10	0.56	30	1.5	Sgsm1	Signaling
DMR12:49407001	12	49407001	49408000	1000	1	3.20E-18	0.52	6	0.6	Tmem211;LOC108352470	
DMR12:49430001	12	49430001	49431000	1000	1	9.50E-07	0.34	4	0.4	RGD1306556	
DMR12:49441001	12	49441001	49442000	1000	1	1.20E-10	0.55	47	4.7	RGD1306556	
DMR12:49472001	12	49472001	49474000	2000	1	3.80E-13	1	45	2.25	RGD1306556	
DMR12:49568001	12	49568001	49572000	4000	1	6.70E-08	0.64	64	1.6	RGD1306556;Crybb3;Crybb2	
DMR12:49596001	12	49596001	49598000	2000	1	5.30E-07	0.43	12	0.6	Crybb2	
DMR12:49717001	12	49717001	49722000	5000	1	3.40E-08	0.56	83	1.66	Grk3	Signaling
DMR12:49779001	12	49779001	49781000	2000	1	9.30E-15	0.9	49	2.45	Myo18b	
DMR12:49797001	12	49797001	49798000	1000	1	2.10E-28	1.21	36	3.6	Myo18b	
DMR12:49812001	12	49812001	49814000	2000	1	6.40E-10	0.51	12	0.6	Myo18b	
DMR12:49824001	12	49824001	49830000	6000	2	2.30E-12	-0.48	98	1.63	Myo18b	
DMR12:49877001	12	49877001	49878000	1000	1	8.50E-08	0.37	9	0.9	Myo18b	
DMR12:49899001	12	49899001	49900000	1000	1	5.60E-07	0.49	8	0.8	Myo18b	
DMR12:49911001	12	49911001	49917000	6000	1	6.30E-17	-0.56	117	1.95	Myo18b	
DMR12:49938001	12	49938001	49939000	1000	1	3.50E-07	0.36	11	1.1	Myo18b	
DMR12:50121001	12	50121001	50123000	2000	1	3.30E-15	-0.83	13	0.65	Sez6l	
DMR12:50154001	12	50154001	50161000	7000	1	2.40E-09	0.48	75	1.07	Sez6l	
DMR12:50176001	12	50176001	50178000	2000	1	4.70E-07	0.39	29	1.45	Sez6l	
DMR12:50190001	12	50190001	50191000	1000	1	3.50E-11	0.66	24	2.4	Sez6l	
DMR12:50233001	12	50233001	50234000	1000	1	4.90E-10	0.52	26	2.6	Sez6l	
DMR12:50271001	12	50271001	50273000	2000	1	2.30E-08	0.6	40	2	Asphd2	Metabolism
DMR12:50375001	12	50375001	50376000	1000	1	6.10E-09	0.55	18	1.8	Tpst2	Transport

DMR12:50411001	12	50411001	50412000	1000	1	9.80E-07	-0.44	19	1.9	Crybb1;Cryba4	
DMR12:51222001	12	51222001	51225000	3000	1	7.30E-07	0.33	33	1.1	Mn1	
DMR12:51307001	12	51307001	51308000	1000	1	1.80E-07	-0.5	15	1.5	Pitpnb	Transport
DMR12:51375001	12	51375001	51379000	4000	1	1.30E-09	0.47	48	1.2	LOC102554233;Ttc28	Cytoskeleton
DMR12:51395001	12	51395001	51399000	4000	1	8.40E-10	0.44	94	2.35	Ttc28	Cytoskeleton
DMR12:51430001	12	51430001	51437000	7000	1	1.50E-08	0.31	107	1.53	Ttc28	Cytoskeleton
DMR12:51453001	12	51453001	51454000	1000	1	8.30E-09	0.65	34	3.4	Ttc28	Cytoskeleton
DMR12:51456001	12	51456001	51460000	4000	1	4.80E-07	0.45	96	2.4	Ttc28	Cytoskeleton
DMR12:51477001	12	51477001	51478000	1000	1	2.20E-09	0.5	17	1.7	Ttc28;LOC102553561	Cytoskeleton
DMR12:51583001	12	51583001	51584000	1000	1	6.40E-07	0.39	12	1.2	Ttc28	Cytoskeleton
DMR12:51597001	12	51597001	51602000	5000	1	9.90E-07	-0.39	87	1.74	Ttc28	Cytoskeleton
DMR12:51637001	12	51637001	51640000	3000	1	3.00E-07	-0.36	38	1.27	Ttc28	Cytoskeleton
DMR12:51643001	12	51643001	51650000	7000	1	3.70E-11	0.61	104	1.49	Ttc28	Cytoskeleton
DMR12:51695001	12	51695001	51698000	3000	1	5.10E-07	-0.43	56	1.87	Ttc28;LOC108352475	Cytoskeleton
DMR12:51756001	12	51756001	51757000	1000	1	4.50E-07	0.62	0	0	Ttc28	Cytoskeleton
DMR12:51806001	12	51806001	51807000	1000	1	7.90E-12	-0.42	11	1.1	Ttc28	Cytoskeleton
DMR12:51855001	12	51855001	51858000	3000	1	1.00E-07	-0.31	33	1.1	Chek2	Signaling
DMR12:52063001	12	52063001	52067000	4000	2	1.50E-07	-0.43	71	1.77	Ep400;Ddx51;Noc4l	Metabolism
DMR12:52125001	12	52125001	52126000	1000	1	2.20E-07	0.58	14	1.4	Galnt9	Golgi
DMR12:52359001	12	52359001	52361000	2000	1	9.60E-16	0.85	72	3.6	Fbrsl1	
DMR12:52367001	12	52367001	52368000	1000	1	2.30E-08	0.62	21	2.1	Fbrsl1	
DMR13:109001	13	109001	112000	3000	1	8.80E-08	0.42	61	2.03	Cntnap5a	
DMR13:267001	13	267001	269000	2000	1	1.00E-10	-0.55	5	0.25	Cntnap5a	
DMR13:6420001	13	6420001	6426000	6000	1	1.90E-10	0.33	41	0.68	Cntnap5c	
DMR13:6437001	13	6437001	6438000	1000	1	4.50E-20	0.52	9	0.9	Cntnap5c	
DMR13:6660001	13	6660001	6661000	1000	1	2.10E-07	-0.39	14	1.4	Cntnap5c	
DMR13:6738001	13	6738001	6743000	5000	1	1.60E-15	0.55	39	0.78	Cntnap5c	
DMR13:6779001	13	6779001	6781000	2000	1	1.50E-08	-0.4	56	2.8	Cntnap5c	
DMR13:6865001	13	6865001	6866000	1000	1	8.70E-08	-0.63	3	0.3	Cntnap5c	
DMR13:7061001	13	7061001	7067000	6000	2	7.80E-09	-0.32	60	1	Cntnap5c	
DMR13:7068001	13	7068001	7071000	3000	1	5.80E-15	0.7	24	0.8	Cntnap5c	
DMR13:7166001	13	7166001	7168000	2000	1	4.50E-08	0.5	32	1.6	Cntnap5c	
DMR13:7169001	13	7169001	7171000	2000	1	1.30E-07	-0.38	72	3.6	Cntnap5c	
DMR13:7274001	13	7274001	7279000	5000	1	1.90E-08	0.51	42	0.84	Cntnap5c	
DMR13:7402001	13	7402001	7404000	2000	1	3.50E-07	-0.57	10	0.5	Cntnap5c	
DMR13:7517001	13	7517001	7518000	1000	1	1.70E-32	0.58	7	0.7	Cntnap5c	
DMR13:19438001	13	19438001	19439000	1000	1	1.10E-07	-0.6	7	0.7	Olr1760-ps	
DMR13:21872001	13	21872001	21875000	3000	1	1.10E-07	0.38	31	1.03	Cntnap5b	
DMR13:22064001	13	22064001	22067000	3000	1	4.00E-09	-0.37	29	0.97	Cntnap5b	
DMR13:22100001	13	22100001	22103000	3000	1	3.60E-07	-0.29	23	0.77	Cntnap5b	
DMR13:22213001	13	22213001	22216000	3000	1	5.20E-08	-0.48	66	2.2	Cntnap5b	
DMR13:22526001	13	22526001	22532000	6000	1	1.40E-09	-0.42	207	3.45	Cntnap5b;LOC108352515	
DMR13:24708001	13	24708001	24709000	1000	1	5.80E-07	-0.31	5	0.5	Cdh20	Cytoskeleton
DMR13:24751001	13	24751001	24754000	3000	1	1.70E-07	-0.38	17	0.57	Cdh20	Cytoskeleton
DMR13:24855001	13	24855001	24857000	2000	1	2.10E-14	0.32	17	0.85	Cdh20	Cytoskeleton
DMR13:24902001	13	24902001	24903000	1000	1	7.20E-07	-0.32	24	2.4	Cdh20;LOC102547165	Cytoskeleton
DMR13:25248001	13	25248001	25250000	2000	1	9.90E-07	0.37	23	1.15	Rnf152	
DMR13:25254001	13	25254001	25256000	2000	1	1.70E-10	-0.46	12	0.6	Rnf152	
DMR13:25555001	13	25555001	25561000	6000	1	7.50E-14	0.38	51	0.85	Pign	Extracellular Matrix
DMR13:25659001	13	25659001	25661000	2000	1	7.40E-10	-0.51	10	0.5	Pign;RGD1307235	Extracellular Matrix
DMR13:25703001	13	25703001	25704000	1000	1	4.20E-07	-0.46	8	0.8	RGD1307235	
DMR13:25786001	13	25786001	25787000	1000	1	3.00E-07	0.26	8	0.8	Tnfrsf11a	Receptor
DMR13:25788001	13	25788001	25789000	1000	1	9.70E-07	0.39	10	1	Tnfrsf11a	Receptor
DMR13:25834001	13	25834001	25838000	4000	1	3.00E-09	0.58	64	1.6	Tnfrsf11a	Receptor
DMR13:25960001	13	25960001	25962000	2000	1	1.20E-14	-0.76	35	1.75	LOC108352517;Zcchc2	
DMR13:26180001	13	26180001	26181000	1000	1	9.00E-07	0.24	11	1.1	Phlpp1	Cytoskeleton
DMR13:26312001	13	26312001	26314000	2000	1	3.90E-09	-0.41	36	1.8	Phlpp1	Cytoskeleton
DMR13:26636001	13	26636001	26637000	1000	1	8.70E-11	-0.48	16	1.6	Bcl2;LOC102552849	
DMR13:26646001	13	26646001	26648000	2000	1	4.10E-09	-0.56	35	1.75	Bcl2;LOC102552849	
DMR13:26712001	13	26712001	26717000	5000	1	7.30E-09	-0.49	72	1.44	Bcl2	
DMR13:26798001	13	26798001	26799000	1000	1	5.60E-08	0.27	2	0.2	Kdsr	Metabolism
DMR13:26903001	13	26903001	26905000	2000	1	1.70E-09	0.75	23	1.15	Serpinb5	Protease; Proteolysis

DMR13:26926001	13	26926001	26929000	3000	1	2.10E-08	-0.28	25	0.83	Serpinb5	Protease; Proteolysis
DMR13:27039001	13	27039001	27041000	2000	1	5.90E-09	-0.48	13	0.65	Serpinb13	Protease; Proteolysis
DMR13:27070001	13	27070001	27071000	1000	1	9.40E-07	-0.57	2	0.2	Serpinb13	Protease; Proteolysis
DMR13:27280001	13	27280001	27282000	2000	1	2.40E-07	-0.48	14	0.7	LOC680414;Serpinb7	Protease; Proteolysis
DMR13:27863001	13	27863001	27868000	5000	2	9.00E-08	-0.4	48	0.96	Serpinb8	Protease; Proteolysis
DMR13:27890001	13	27890001	27891000	1000	1	1.10E-09	0.69	18	1.8	Serpinb8	Protease; Proteolysis
DMR13:31215001	13	31215001	31217000	2000	1	6.70E-07	-0.46	12	0.6	Cdh7	Cytoskeleton
DMR13:31750001	13	31750001	31751000	1000	1	9.10E-12	-0.59	3	0.3	Hnrnpa1-ps1	
DMR13:34271001	13	34271001	34273000	2000	2	3.90E-13	-0.49	20	1	Nifk	
DMR13:34491001	13	34491001	34493000	2000	1	2.50E-11	-0.46	31	1.55	Clasp1	Cytoskeleton
DMR13:34621001	13	34621001	34623000	2000	1	3.10E-07	-0.55	12	0.6	Tfcp2l1	Transcription
DMR13:34632001	13	34632001	34633000	1000	1	8.60E-07	0.52	9	0.9	Tfcp2l1	Transcription
DMR13:34638001	13	34638001	34641000	3000	1	9.80E-11	0.6	46	1.53	Tfcp2l1	Transcription
DMR13:34644001	13	34644001	34645000	1000	1	1.10E-13	0.57	14	1.4	Tfcp2l1	Transcription
DMR13:35020001	13	35020001	35022000	2000	1	8.10E-08	0.41	18	0.9	Gli2	Transcription
DMR13:35055001	13	35055001	35056000	1000	1	3.60E-07	0.31	11	1.1	Gli2	Transcription
DMR13:35437001	13	35437001	35438000	1000	1	2.40E-08	-0.38	27	2.7	Inhbb	Growth Factors
DMR13:35448001	13	35448001	35451000	3000	1	8.30E-11	0.5	48	1.6	Inhbb	Growth Factors
DMR13:35540001	13	35540001	35544000	4000	1	5.70E-07	-0.46	61	1.52	Ralb	Signaling
DMR13:35709001	13	35709001	35710000	1000	1	3.30E-07	-0.46	7	0.7	Ptpn4	Signaling
DMR13:35843001	13	35843001	35845000	2000	1	1.80E-07	-0.49	8	0.4	Ptpn4	Signaling
DMR13:36022001	13	36022001	36023000	1000	1	1.90E-08	-0.51	14	1.4	Cfap221	Development
DMR13:36086001	13	36086001	36091000	5000	1	1.00E-08	0.4	66	1.32	Sctr;Tmem37	Receptor;Transport
DMR13:36266001	13	36266001	36270000	4000	1	4.60E-08	-0.38	78	1.95	Steap3	
DMR13:36527001	13	36527001	36529000	2000	2	9.10E-08	-0.44	39	1.95	En1	Development
DMR13:37476001	13	37476001	37477000	1000	1	2.30E-07	-0.31	22	2.2	Ccdc93;Htr5b	Signaling
DMR13:37510001	13	37510001	37513000	3000	1	8.10E-09	-0.32	27	0.9	Ddx18	
DMR13:39771001	13	39771001	39773000	2000	2	3.20E-10	-0.54	23	1.15	Dpp10	Protease
DMR13:39788001	13	39788001	39790000	2000	1	2.10E-07	-0.59	6	0.3	Dpp10	Protease
DMR13:40071001	13	40071001	40072000	1000	1	3.20E-07	-0.44	3	0.3	Dpp10	Protease
DMR13:40123001	13	40123001	40125000	2000	1	3.00E-07	-0.61	4	0.2	Dpp10	Protease
DMR13:40187001	13	40187001	40189000	2000	1	6.70E-10	0.45	14	0.7	Dpp10	Protease
DMR13:40208001	13	40208001	40213000	5000	1	9.30E-16	-0.45	49	0.98	Dpp10	Protease
DMR13:40322001	13	40322001	40327000	5000	1	3.70E-08	-0.28	68	1.36	Dpp10	Protease
DMR13:40839001	13	40839001	40840000	1000	1	7.30E-10	0.73	19	1.9	Dpp10	Protease
DMR13:40952001	13	40952001	40954000	2000	1	6.80E-07	0.49	12	0.6	Dpp10	Protease
DMR13:40965001	13	40965001	40966000	1000	1	8.70E-08	-0.54	5	0.5	Dpp10	Protease
DMR13:41020001	13	41020001	41022000	2000	1	4.20E-09	0.57	10	0.5	Dpp10	Protease
DMR13:41103001	13	41103001	41104000	1000	1	5.60E-07	0.43	10	1	Dpp10	Protease
DMR13:41120001	13	41120001	41125000	5000	1	7.20E-08	-0.37	41	0.82	Dpp10	Protease
DMR13:41138001	13	41138001	41139000	1000	1	8.60E-17	0.83	22	2.2	Dpp10	Protease
DMR13:41734001	13	41734001	41735000	1000	1	1.20E-07	-0.42	12	1.2	Actr3;LOC102554198	Cytoskeleton
DMR13:41873001	13	41873001	41874000	1000	1	1.10E-08	-0.47	8	0.8	Slc35f5	Transport
DMR13:41898001	13	41898001	41899000	1000	1	7.30E-15	-0.64	8	0.8	Slc35f5	Transport
DMR13:42045001	13	42045001	42047000	2000	1	3.10E-08	0.47	11	0.55	Gpr39	Signaling
DMR13:42064001	13	42064001	42066000	2000	1	2.70E-09	0.57	36	1.8	Gpr39	Signaling
DMR13:42098001	13	42098001	42099000	1000	1	1.60E-07	-0.64	4	0.4	Gpr39	Signaling
DMR13:42100001	13	42100001	42101000	1000	1	7.40E-08	0.39	3	0.3	Gpr39	Signaling
DMR13:42132001	13	42132001	42135000	3000	1	8.60E-08	-0.34	27	0.9	Gpr39	Signaling
DMR13:42136001	13	42136001	42137000	1000	1	3.30E-07	-0.23	10	1	Gpr39	Signaling
DMR13:42187001	13	42187001	42190000	3000	2	1.50E-09	-0.58	35	1.17	Gpr39	Signaling
DMR13:42192001	13	42192001	42194000	2000	1	1.60E-07	-0.38	25	1.25	Gpr39	Signaling
DMR13:42385001	13	42385001	42387000	2000	1	6.40E-09	0.46	22	1.1	Nckap5	
DMR13:42423001	13	42423001	42425000	2000	1	7.90E-07	0.36	23	1.15	Nckap5	
DMR13:42426001	13	42426001	42432000	6000	1	1.90E-07	-0.35	59	0.98	Nckap5	
DMR13:42966001	13	42966001	42967000	1000	1	1.50E-19	0.76	4	0.4	Nckap5	
DMR13:43384001	13	43384001	43388000	4000	1	9.10E-08	0.53	55	1.38	RGD1560333	
DMR13:43394001	13	43394001	43395000	1000	1	3.80E-11	-0.58	2	0.2	RGD1560333;RGD1563709	
DMR13:43409001	13	43409001	43413000	4000	1	1.10E-11	-0.63	9	0.22	RGD1563709	
DMR13:44095001	13	44095001	44097000	2000	1	2.50E-10	0.62	41	2.05	Mgat5	Golgi
DMR13:44266001	13	44266001	44267000	1000	1	5.70E-07	0.4	11	1.1	Tmem163	
DMR13:44337001	13	44337001	44339000	2000	1	2.40E-08	-0.58	9	0.45	Tmem163	

DMR13:44429001	13	44429001	44434000	5000	1	4.10E-07	0.44	41	0.82	Acmsd	Metabolism
DMR13:44438001	13	44438001	44441000	3000	1	1.00E-13	-0.78	28	0.93	Acmsd	Metabolism
DMR13:44538001	13	44538001	44541000	3000	1	4.50E-10	0.52	40	1.33	Map3k19	
DMR13:44554001	13	44554001	44555000	1000	1	5.70E-07	-0.44	8	0.8	Map3k19	
DMR13:44628001	13	44628001	44629000	1000	1	1.30E-07	-0.48	4	0.4	Rab3gap1	Signaling
DMR13:44722001	13	44722001	44726000	4000	1	2.80E-09	0.55	56	1.4	Zranb3;Mir3473;LOC102548555	Transcription
DMR13:44773001	13	44773001	44775000	2000	1	2.00E-07	0.54	25	1.25	Zranb3	Transcription
DMR13:44894001	13	44894001	44896000	2000	1	3.50E-09	0.55	21	1.05	R3hdm1	
DMR13:45131001	13	45131001	45134000	3000	1	2.60E-07	-0.35	42	1.4	Dars	
DMR13:46225001	13	46225001	46227000	2000	1	5.20E-08	-0.51	14	0.7	Thsd7b	Cytoskeleton
DMR13:46300001	13	46300001	46302000	2000	1	3.20E-07	-0.71	12	0.6	Thsd7b	Cytoskeleton
DMR13:46666001	13	46666001	46667000	1000	1	6.00E-07	-0.29	11	1.1	Thsd7b	Cytoskeleton
DMR13:46729001	13	46729001	46730000	1000	1	1.30E-09	-0.73	1	0.1	Thsd7b	Cytoskeleton
DMR13:46827001	13	46827001	46832000	5000	1	3.40E-07	-0.34	56	1.12	Thsd7b	Cytoskeleton
DMR13:46872001	13	46872001	46874000	2000	1	2.50E-08	0.43	16	0.8	Thsd7b	Cytoskeleton
DMR13:46879001	13	46879001	46880000	1000	1	5.20E-07	-0.38	8	0.8	Thsd7b	Cytoskeleton
DMR13:46881001	13	46881001	46882000	1000	1	5.80E-08	-0.53	2	0.2	Thsd7b	Cytoskeleton
DMR13:47116001	13	47116001	47118000	2000	1	8.20E-07	-0.36	36	1.8	Cd55	
DMR13:47151001	13	47151001	47152000	1000	1	2.70E-08	0.47	13	1.3	Cd55	
DMR13:47271001	13	47271001	47277000	6000	1	6.20E-10	-0.3	60	1	Zp3r	
DMR13:47385001	13	47385001	47387000	2000	1	1.80E-07	0.57	18	0.9	C4bpa;LOC100360750;C4bpb	
DMR13:47670001	13	47670001	47673000	3000	1	2.40E-12	0.99	61	2.03	Il19	
DMR13:47730001	13	47730001	47734000	4000	1	6.60E-07	0.5	60	1.5	Il10	
DMR13:47751001	13	47751001	47753000	2000	1	2.20E-11	-0.65	12	0.6	Il10	
DMR13:47777001	13	47777001	47780000	3000	1	7.90E-10	0.59	55	1.83	Mapkapk2	Signaling
DMR13:47894001	13	47894001	47900000	6000	2	7.30E-10	0.77	135	2.25	Dyrk3	
DMR13:47937001	13	47937001	47940000	3000	1	5.20E-10	-0.47	45	1.5	Eif2d	Translation
DMR13:47972001	13	47972001	47976000	4000	1	3.00E-07	0.45	78	1.95	Rassf5	Cytoskeleton
DMR13:48019001	13	48019001	48022000	3000	1	6.50E-08	-0.37	92	3.07	Rassf5;LOC103691876;Ikbke	Cytoskeleton;Signaling
DMR13:48039001	13	48039001	48041000	2000	2	9.20E-10	0.68	26	1.3	LOC103691876;Ikbke	Signaling
DMR13:48461001	13	48461001	48463000	2000	1	9.40E-08	0.58	26	1.3	Rab7b	
DMR13:48470001	13	48470001	48472000	2000	1	3.20E-09	0.65	31	1.55	Rab7b	
DMR13:48483001	13	48483001	48484000	1000	1	3.30E-08	0.55	27	2.7	Rab7b	
DMR13:48598001	13	48598001	48599000	1000	1	3.20E-09	-0.48	5	0.5	Pm20d1;Slc41a1	Metabolism;Transport
DMR13:48812001	13	48812001	48813000	1000	1	8.20E-15	0.78	21	2.1	Elk4;Mfsd4	Transcription
DMR13:48925001	13	48925001	48931000	6000	1	3.50E-07	0.46	90	1.5	Cdk18	Signaling
DMR13:49108001	13	49108001	49112000	4000	2	2.10E-07	-0.49	68	1.7	Nuak2	Signaling
DMR13:49114001	13	49114001	49117000	3000	1	3.90E-07	0.35	48	1.6	Nuak2	Signaling
DMR13:49125001	13	49125001	49126000	1000	1	1.10E-07	0.57	13	1.3	Tmcc2	
DMR13:49171001	13	49171001	49174000	3000	1	1.50E-10	0.6	53	1.77	Tmcc2	
DMR13:49235001	13	49235001	49238000	3000	1	9.30E-07	-0.5	20	0.67	Dstyk	Signaling
DMR13:49336001	13	49336001	49338000	2000	1	4.90E-08	0.61	34	1.7	Nfasc	
DMR13:49684001	13	49684001	49688000	4000	1	1.10E-16	1	73	1.82	Lrrn2	Receptor
DMR13:49728001	13	49728001	49729000	1000	1	8.50E-10	0.66	34	3.4	Lrrn2	Receptor
DMR13:49928001	13	49928001	49930000	2000	1	1.10E-08	-0.39	28	1.4	Ppp1r15b	
DMR13:50182001	13	50182001	50183000	1000	1	7.60E-07	-0.57	7	0.7	Lax1;LOC108352533;LOC363987	Immune
DMR13:50449001	13	50449001	50453000	4000	1	4.50E-18	-0.58	94	2.35	Sox13	
DMR13:50551001	13	50551001	50554000	3000	1	2.50E-07	-0.41	61	2.03	Golt1a;Plekha6	Metabolism
DMR13:50953001	13	50953001	50954000	1000	1	2.40E-07	0.46	6	0.6	Chit1	Metabolism
DMR13:50988001	13	50988001	50989000	1000	1	7.70E-08	0.38	9	0.9	Chit1	Metabolism
DMR13:51082001	13	51082001	51084000	2000	1	2.60E-12	-0.53	26	1.3	Adora1	Signaling
DMR13:51152001	13	51152001	51153000	1000	1	1.10E-08	0.43	21	2.1	Ppfia4	
DMR13:51203001	13	51203001	51205000	2000	1	4.90E-09	-0.46	24	1.2	Tmem183a	
DMR13:51232001	13	51232001	51234000	2000	1	8.70E-07	-0.39	34	1.7	LOC289035;Cyb5r1;Adipor1	Transport;Metabolism;Signaling
DMR13:51243001	13	51243001	51244000	1000	1	8.30E-09	-0.73	6	0.6	Cyb5r1;Adipor1	Metabolism;Signaling
DMR13:51354001	13	51354001	51356000	2000	1	1.40E-11	0.87	48	2.4	LOC108352535;Mgat4e	Transport
DMR13:51365001	13	51365001	51366000	1000	1	1.40E-08	-0.41	24	2.4	LOC108352535;Mgat4e;LOC103691910	Transport
DMR13:51430001	13	51430001	51431000	1000	1	2.20E-09	-0.49	17	1.7	Kdm5b	Epigenetic

DMR13:51453001	13	51453001	51454000	1000	1	1.70E-11	-0.45	22	2.2	Kdm5b	Epigenetic
DMR13:51582001	13	51582001	51583000	1000	1	7.70E-07	0.55	13	1.3	Syt2;Ppp1r12b	Transport;Signaling
DMR13:51610001	13	51610001	51612000	2000	1	2.50E-07	-0.46	22	1.1	Ppp1r12b	Signaling
DMR13:51671001	13	51671001	51673000	2000	1	9.90E-09	-0.91	21	1.05	Ppp1r12b	Signaling
DMR13:51814001	13	51814001	51815000	1000	1	1.30E-08	0.73	25	2.5	Lgr6	
DMR13:51881001	13	51881001	51886000	5000	1	1.10E-08	0.64	56	1.12	Lgr6	
DMR13:51891001	13	51891001	51893000	2000	1	8.60E-07	0.43	35	1.75	Lgr6	
DMR13:51912001	13	51912001	51919000	7000	1	2.20E-08	-0.42	91	1.3	Lgr6	
DMR13:51938001	13	51938001	51939000	1000	1	7.10E-07	0.44	16	1.6	Lgr6;Ptprv	Signaling
DMR13:51970001	13	51970001	51971000	1000	1	9.60E-07	0.58	13	1.3	Ptpn7;Arl8a	Signaling
DMR13:52182001	13	52182001	52184000	2000	1	9.40E-08	-0.53	16	0.8	Lmod1;Shisa4	Cytoskeleton;Cytoskeleton
DMR13:52199001	13	52199001	52201000	2000	1	1.50E-12	0.8	45	2.25	Lmod1;Shisa4;Ipo9	Cytoskeleton;Cytoskeleton;Transport
DMR13:52220001	13	52220001	52223000	3000	1	5.00E-09	0.52	30	1	Ipo9	Transport
DMR13:52318001	13	52318001	52320000	2000	1	2.00E-08	-0.39	30	1.5	Nav1	
DMR13:52339001	13	52339001	52345000	6000	1	1.40E-08	0.72	87	1.45	Nav1	
DMR13:52356001	13	52356001	52359000	3000	1	3.50E-07	0.47	47	1.57	Nav1	
DMR13:52457001	13	52457001	52460000	3000	1	1.40E-07	0.51	37	1.23	Nav1;LOC108352536	
DMR13:52507001	13	52507001	52511000	4000	1	8.20E-07	-0.4	77	1.93	Nav1;LOC102553722	
DMR13:52593001	13	52593001	52596000	3000	1	1.60E-08	-0.4	47	1.57	LOC103691921;LOC108352537;Phlda3;LOC685812	
DMR13:52608001	13	52608001	52615000	7000	1	3.80E-08	0.49	140	2	LOC685812;Tnni1	Cytoskeleton
DMR13:52625001	13	52625001	52629000	4000	1	8.50E-07	-0.44	42	1.05	Tnni1	Cytoskeleton
DMR13:52634001	13	52634001	52635000	1000	1	6.00E-11	0.43	18	1.8	Tnni1	Cytoskeleton
DMR13:52667001	13	52667001	52670000	3000	1	1.40E-09	0.66	61	2.03	Lad1;Tnnt2	Development;Cytoskeleton
DMR13:52798001	13	52798001	52801000	3000	1	1.10E-07	0.5	30	1	Igfn1	
DMR13:52802001	13	52802001	52805000	3000	2	2.50E-13	0.66	43	1.43	Igfn1	
DMR13:52812001	13	52812001	52816000	4000	1	1.40E-10	0.75	62	1.55	Igfn1	
DMR13:52831001	13	52831001	52833000	2000	1	6.00E-09	0.58	36	1.8	Igfn1	
DMR13:52880001	13	52880001	52884000	4000	2	1.70E-09	-0.6	51	1.27	Tmem9;Ascl5;Cacna1s	Transcription;Transport
DMR13:53021001	13	53021001	53023000	2000	1	2.20E-08	-0.42	47	2.35	Kif21b;LOC498236	Cytoskeleton
DMR13:53105001	13	53105001	53107000	2000	1	2.80E-08	-0.39	36	1.8	RGD1311892	
DMR13:53158001	13	53158001	53161000	3000	1	2.70E-12	-0.5	57	1.9	Camsap2	
DMR13:53283001	13	53283001	53284000	1000	1	1.70E-07	0.65	20	2	RGD1564839	
DMR13:53469001	13	53469001	53471000	2000	1	8.60E-09	0.64	35	1.75	RGD1562613	
DMR13:53746001	13	53746001	53748000	2000	1	9.80E-08	0.44	28	1.4	Nr5a2	Transcription
DMR13:53786001	13	53786001	53787000	1000	1	6.00E-09	0.44	10	1	Nr5a2	Transcription
DMR13:53843001	13	53843001	53845000	2000	1	1.40E-09	0.78	68	3.4	Nr5a2;LOC108352538	Transcription
DMR13:53860001	13	53860001	53861000	1000	1	6.20E-08	-0.51	21	2.1	Nr5a2;LOC108352538	Transcription
DMR13:53927001	13	53927001	53929000	2000	1	1.20E-09	0.77	31	1.55	RGD1562134	
DMR13:54953001	13	54953001	54954000	1000	1	5.70E-07	0.56	21	2.1	Mir3570;Mir181a-1;Mir181b1	
DMR13:55124001	13	55124001	55125000	1000	1	2.10E-07	0.64	21	2.1	Ptprc	Signaling
DMR13:55449001	13	55449001	55450000	1000	1	6.50E-07	-0.65	1	0.1	Cct3-ps2	
DMR13:55507001	13	55507001	55508000	1000	1	7.10E-07	-0.51	22	2.2	Nek7	Signaling
DMR13:55558001	13	55558001	55559000	1000	1	4.70E-07	-0.3	17	1.7	Nek7	Signaling
DMR13:55586001	13	55586001	55587000	1000	1	4.90E-07	-0.6	5	0.5	Nek7	Signaling
DMR13:55607001	13	55607001	55609000	2000	1	4.30E-07	-0.37	20	1	Nek7;LOC103691924	Signaling
DMR13:55695001	13	55695001	55698000	3000	1	4.80E-07	-0.45	24	0.8	RGD1565063	
DMR13:55845001	13	55845001	55847000	2000	1	2.50E-09	-0.41	30	1.5	Lhx9	Development
DMR13:56009001	13	56009001	56010000	1000	1	1.40E-07	-0.44	6	0.6	Dennd1b	
DMR13:56084001	13	56084001	56085000	1000	1	2.40E-09	-0.46	7	0.7	Dennd1b	
DMR13:56372001	13	56372001	56373000	1000	1	9.00E-07	0.44	7	0.7	Crb1	Cytoskeleton
DMR13:56394001	13	56394001	56395000	1000	1	3.40E-10	0.57	21	2.1	Crb1	Cytoskeleton
DMR13:56514001	13	56514001	56516000	2000	1	5.50E-07	-0.38	44	2.2	Zbtb41	Transcription
DMR13:57325001	13	57325001	57326000	1000	1	6.70E-08	-0.33	8	0.8	Kcnt2	Transport
DMR13:57527001	13	57527001	57528000	1000	1	5.30E-12	0.68	16	1.6	Kcnt2	Transport
DMR13:63544001	13	63544001	63545000	1000	1	5.60E-08	0.73	12	1.2	Brinp3	
DMR13:63748001	13	63748001	63752000	4000	1	1.00E-07	-0.36	40	1	Brinp3	
DMR13:63929001	13	63929001	63930000	1000	1	2.10E-10	-0.55	5	0.5	Brinp3	

DMR13:67169001	13	67169001	67170000	1000	1	6.50E-07	-0.57	3	0.3	Pla2g4a	Metabolism
DMR13:67197001	13	67197001	67198000	1000	1	1.50E-10	-0.7	7	0.7	Pla2g4a	Metabolism
DMR13:67557001	13	67557001	67558000	1000	1	8.00E-13	0.64	20	2	Pdc	
DMR13:67559001	13	67559001	67561000	2000	2	3.30E-09	0.52	31	1.55	Pdc	
DMR13:67640001	13	67640001	67644000	4000	2	4.80E-11	-0.34	43	1.07	Tpr	Transport
DMR13:67662001	13	67662001	67664000	2000	1	1.80E-09	-0.43	16	0.8	Tpr;Prg4	Transport
DMR13:68033001	13	68033001	68034000	1000	1	8.90E-11	0.64	21	2.1	Hmcn1	
DMR13:68109001	13	68109001	68110000	1000	1	6.40E-08	0.49	18	1.8	Hmcn1	
DMR13:68240001	13	68240001	68245000	5000	1	3.30E-15	-0.58	52	1.04	Hmcn1	
DMR13:68251001	13	68251001	68252000	1000	1	2.40E-07	-0.5	9	0.9	Hmcn1	
DMR13:68320001	13	68320001	68321000	1000	1	9.30E-07	-0.37	12	1.2	Hmcn1	
DMR13:68708001	13	68708001	68710000	2000	1	4.80E-10	-0.54	24	1.2	Ivns1abp	Cytoskeleton
DMR13:68775001	13	68775001	68776000	1000	1	4.40E-09	-0.57	6	0.6	Swt1;Trmt1l	Epigenetic
DMR13:68870001	13	68870001	68871000	1000	1	6.90E-07	0.56	24	2.4	RGD1565977	
DMR13:68945001	13	68945001	68949000	4000	1	8.70E-10	0.68	51	1.27	Fam129a	
DMR13:68997001	13	68997001	69001000	4000	2	8.40E-08	-0.5	53	1.32	Fam129a	
DMR13:69008001	13	69008001	69009000	1000	1	3.90E-08	-0.35	23	2.3	Fam129a	
DMR13:69016001	13	69016001	69017000	1000	1	4.30E-08	-0.39	19	1.9	Fam129a	
DMR13:69028001	13	69028001	69029000	1000	1	1.20E-18	-0.68	16	1.6	Fam129a	
DMR13:69065001	13	69065001	69069000	4000	2	1.90E-07	-0.47	68	1.7	Fam129a	
DMR13:69122001	13	69122001	69126000	4000	1	4.50E-08	-0.48	74	1.85	Edem3	
DMR13:69298001	13	69298001	69300000	2000	1	4.60E-07	-0.38	51	2.55	RGD1309104	
DMR13:69336001	13	69336001	69338000	2000	1	4.20E-09	0.71	38	1.9	RGD1309104	
DMR13:69786001	13	69786001	69789000	3000	1	4.10E-08	-0.32	37	1.23	Tsen15;Colgalt2	Golgi
DMR13:69854001	13	69854001	69857000	3000	1	4.70E-09	-0.44	20	0.67	Colgalt2	Golgi
DMR13:69994001	13	69994001	69995000	1000	1	6.10E-07	-0.58	5	0.5	Rgl1	Transcription
DMR13:70013001	13	70013001	70015000	2000	1	7.20E-07	-0.41	25	1.25	Rgl1	Transcription
DMR13:70088001	13	70088001	70089000	1000	1	1.80E-08	0.42	13	1.3	Rgl1	Transcription
DMR13:70222001	13	70222001	70224000	2000	1	4.50E-13	0.74	40	2	LOC689881;Ncf2	Signaling
DMR13:70232001	13	70232001	70234000	2000	2	8.20E-11	-0.64	16	0.8	LOC689881;Ncf2	Signaling
DMR13:70247001	13	70247001	70250000	3000	1	4.60E-15	1.04	74	2.47	Ncf2;Smg7	Signaling;Metabolism
DMR13:70382001	13	70382001	70386000	4000	1	1.10E-07	-0.35	57	1.43	Nmnat2	Metabolism
DMR13:70394001	13	70394001	70395000	1000	1	7.90E-18	1.01	32	3.2	Nmnat2	Metabolism
DMR13:70396001	13	70396001	70398000	2000	1	4.30E-09	-0.38	32	1.6	Nmnat2	Metabolism
DMR13:70411001	13	70411001	70417000	6000	1	1.30E-07	-0.34	78	1.3	Nmnat2	Metabolism
DMR13:70466001	13	70466001	70472000	6000	1	9.60E-08	-0.38	75	1.25	Nmnat2;LOC102551684	Metabolism
DMR13:70488001	13	70488001	70498000	10000	1	2.80E-10	-0.41	96	0.96	Nmnat2;LOC102551684	Metabolism
DMR13:70572001	13	70572001	70576000	4000	1	6.20E-08	-0.67	48	1.2	Lamc2;LOC102552142	Extracellular Matrix
DMR13:70658001	13	70658001	70660000	2000	1	8.50E-09	0.61	32	1.6	Lamc1	Extracellular Matrix
DMR13:70674001	13	70674001	70676000	2000	1	4.10E-09	-0.5	43	2.15	Lamc1	Extracellular Matrix
DMR13:70683001	13	70683001	70686000	3000	1	1.30E-08	-0.38	50	1.67	Lamc1	Extracellular Matrix
DMR13:70733001	13	70733001	70735000	2000	1	2.40E-13	-0.48	22	1.1	Lamc1;LOC102551797	Extracellular Matrix
DMR13:70752001	13	70752001	70754000	2000	1	8.70E-07	-0.36	21	1.05	Lamc1;LOC102551797	Extracellular Matrix
DMR13:70771001	13	70771001	70775000	4000	1	2.70E-09	-0.54	47	1.18	Lamc1	Extracellular Matrix
DMR13:70851001	13	70851001	70852000	1000	1	4.80E-09	0.65	33	3.3	Shcbp1l	
DMR13:70854001	13	70854001	70855000	1000	1	2.30E-07	-0.59	9	0.9	Shcbp1l	
DMR13:70965001	13	70965001	70969000	4000	1	1.10E-08	0.56	64	1.6	Npl	Metabolism
DMR13:71080001	13	71080001	71081000	1000	1	5.80E-07	-0.43	12	1.2	Rgs8	Signaling
DMR13:71103001	13	71103001	71105000	2000	1	2.60E-09	0.54	41	2.05	Rgs8	Signaling
DMR13:71110001	13	71110001	71113000	3000	1	4.10E-07	-0.4	49	1.63	Rgs8	Signaling
DMR13:71161001	13	71161001	71164000	3000	1	3.70E-09	-0.42	44	1.47	LOC102552085;Rgs16	Signaling
DMR13:71186001	13	71186001	71189000	3000	1	7.60E-11	-0.76	56	1.87	Rgs16;Rnasel	Signaling
DMR13:71324001	13	71324001	71325000	1000	1	1.20E-08	-0.37	17	1.7	Teddm1;Glul	Metabolism
DMR13:71328001	13	71328001	71330000	2000	1	3.40E-09	-0.49	36	1.8	Teddm1;Glul	Metabolism
DMR13:71333001	13	71333001	71339000	6000	3	8.00E-10	-0.52	162	2.7	Teddm1;Glul	Metabolism
DMR13:71521001	13	71521001	71522000	1000	1	8.90E-09	0.57	10	1	Trnaw-cca	
DMR13:71669001	13	71669001	71670000	1000	1	2.50E-08	0.61	26	2.6	Zfp648	Transcription
DMR13:71907001	13	71907001	71908000	1000	1	9.90E-12	0.89	24	2.4	Cacna1e	Transport
DMR13:71991001	13	71991001	71993000	2000	1	2.00E-08	0.49	23	1.15	Cacna1e	Transport
DMR13:72424001	13	72424001	72426000	2000	1	5.30E-11	1.06	45	2.25	Cacna1e	Transport
DMR13:72435001	13	72435001	72439000	4000	2	1.90E-08	0.51	36	0.9	Cacna1e	Transport
DMR13:72504001	13	72504001	72505000	1000	1	1.40E-08	-0.45	3	0.3	Cacna1e	Transport

DMR13:72510001	13	72510001	72514000	4000	1	2.00E-07	-0.5	48	1.2	Cacna1e	Transport
DMR13:72522001	13	72522001	72525000	3000	1	3.60E-08	-0.68	22	0.73	Cacna1e	Transport
DMR13:72912001	13	72912001	72915000	3000	1	3.30E-08	-0.52	32	1.07	LOC102553617;Xpr1	Transport
DMR13:72943001	13	72943001	72944000	1000	1	2.40E-08	-0.43	3	0.3	Xpr1;LOC108352558	Transport
DMR13:73348001	13	73348001	73352000	4000	1	2.80E-11	0.86	76	1.9	Lhx4	Development
DMR13:73495001	13	73495001	73496000	1000	1	7.90E-12	1.01	24	2.4	Cep350	Cytoskeleton
DMR13:73498001	13	73498001	73499000	1000	1	8.80E-07	-0.39	23	2.3	Cep350	Cytoskeleton
DMR13:73530001	13	73530001	73532000	2000	1	7.70E-08	0.36	9	0.45	Cep350	Cytoskeleton
DMR13:73592001	13	73592001	73594000	2000	2	1.70E-11	-0.5	17	0.85	Cep350	Cytoskeleton
DMR13:73599001	13	73599001	73600000	1000	1	4.60E-09	-0.39	9	0.9	Cep350	Cytoskeleton
DMR13:73712001	13	73712001	73713000	1000	1	7.00E-13	-0.59	8	0.8	Tor1aip1;Tor1aip2	
DMR13:73772001	13	73772001	73773000	1000	1	4.10E-07	-0.38	15	1.5	Fam163a	
DMR13:73937001	13	73937001	73939000	2000	1	4.20E-08	0.37	24	1.2	Nphs2	Cytoskeleton
DMR13:74031001	13	74031001	74033000	2000	1	6.80E-07	-0.35	26	1.3	Axdnd1;Soat1	Metabolism
DMR13:74147001	13	74147001	74148000	1000	1	3.90E-12	-0.57	9	0.9	Abl2	
DMR13:74218001	13	74218001	74220000	2000	2	4.30E-15	-0.71	15	0.75	Abl2	
DMR13:74527001	13	74527001	74529000	2000	2	1.20E-08	-0.63	21	1.05	Ralgps2	Transcription
DMR13:74803001	13	74803001	74804000	1000	1	1.40E-10	0.62	23	2.3	Rasal2;LOC100912060	Signaling
DMR13:74963001	13	74963001	74964000	1000	1	5.00E-08	-0.36	5	0.5	Rasal2;LOC108352559	Signaling
DMR13:75091001	13	75091001	75092000	1000	1	9.20E-10	-0.53	10	1	Tp53i3	
DMR13:75120001	13	75120001	75122000	2000	1	8.00E-09	-0.45	31	1.55	Tp53i3	
DMR13:75180001	13	75180001	75182000	2000	1	2.00E-07	0.43	26	1.3	Sec16b	
DMR13:75203001	13	75203001	75204000	1000	1	6.30E-11	0.85	23	2.3	Sec16b	
DMR13:75974001	13	75974001	75975000	1000	1	6.50E-07	-0.4	10	1	Brinp2	
DMR13:76140001	13	76140001	76141000	1000	1	2.80E-07	-0.34	10	1	Astn1	
DMR13:76235001	13	76235001	76239000	4000	2	5.10E-10	-0.5	36	0.9	Astn1	
DMR13:76546001	13	76546001	76548000	2000	1	4.70E-09	0.69	25	1.25	Pappa2	
DMR13:76650001	13	76650001	76651000	1000	1	1.00E-07	-0.63	6	0.6	Pappa2	
DMR13:77629001	13	77629001	77630000	1000	1	8.90E-08	0.45	9	0.9	Tnr	Signaling
DMR13:77821001	13	77821001	77822000	1000	1	8.70E-09	0.5	15	1.5	LOC100302372;Tnn	Signaling
DMR13:77839001	13	77839001	77842000	3000	1	9.60E-09	0.51	25	0.83	Tnn	Signaling
DMR13:77853001	13	77853001	77858000	5000	2	3.00E-09	-0.44	60	1.2	Tnn	Signaling
DMR13:77930001	13	77930001	77931000	1000	1	1.70E-08	0.59	12	1.2	Mrps14	Translation
DMR13:77965001	13	77965001	77970000	5000	1	4.30E-07	-0.4	146	2.92	Cacybp;LOC103693675;Rabgap1l	Proteolysis;Signaling
DMR13:78424001	13	78424001	78426000	2000	1	1.10E-18	0.51	16	0.8	Rabgap1l	Signaling
DMR13:78457001	13	78457001	78460000	3000	1	8.50E-08	-0.4	29	0.97	Rabgap1l	Signaling
DMR13:78548001	13	78548001	78550000	2000	1	1.40E-08	-0.53	17	0.85	Rabgap1l	Signaling
DMR13:78551001	13	78551001	78553000	2000	1	5.40E-09	-0.54	11	0.55	Rabgap1l	Signaling
DMR13:78556001	13	78556001	78561000	5000	2	7.20E-12	-0.44	50	1	Rabgap1l	Signaling
DMR13:78813001	13	78813001	78817000	4000	1	7.90E-08	-0.3	56	1.4	Serpinc1;LOC108352561	Protease; Proteolysis
DMR13:78966001	13	78966001	78969000	3000	1	7.40E-07	0.5	23	0.77	Klhl20;Ankrd45	Cytoskeleton;Signaling
DMR13:78974001	13	78974001	78975000	1000	1	2.30E-10	0.75	25	2.5	Ankrd45;LOC100363459;LOC102551559	Signaling
DMR13:79039001	13	79039001	79040000	1000	1	2.30E-08	0.67	26	2.6	Slc9c2	
DMR13:79043001	13	79043001	79046000	3000	1	4.30E-09	-0.54	28	0.93	Slc9c2	
DMR13:79053001	13	79053001	79056000	3000	1	3.50E-07	-0.26	23	0.77	Slc9c2	
DMR13:79081001	13	79081001	79083000	2000	1	8.20E-07	-0.42	30	1.5	Slc9c2;Prdx6;LOC102552207	Metabolism
DMR13:79295001	13	79295001	79296000	1000	1	1.80E-09	-0.64	6	0.6	Tnfsf4	
DMR13:79717001	13	79717001	79719000	2000	1	3.30E-07	-0.38	19	0.95	Faslg	
DMR13:79847001	13	79847001	79849000	2000	1	8.90E-15	-0.79	18	0.9	LOC102554604;LOC103692015;RGD1309106	
DMR13:79895001	13	79895001	79897000	2000	2	9.10E-09	0.66	45	2.25	RGD1309106;Pigc;Dnm3	Golgi;Transport
DMR13:79920001	13	79920001	79922000	2000	1	2.10E-07	-0.48	50	2.5	Dnm3;LOC102554129	Transport
DMR13:80004001	13	80004001	80007000	3000	1	9.60E-10	-0.39	22	0.73	Dnm3	Transport
DMR13:80174001	13	80174001	80177000	3000	1	1.90E-07	-0.35	39	1.3	Dnm3	Transport
DMR13:80226001	13	80226001	80229000	3000	1	4.20E-10	-0.59	42	1.4	Dnm3	Transport
DMR13:80233001	13	80233001	80235000	2000	1	5.10E-07	-0.37	25	1.25	Dnm3	Transport
DMR13:80383001	13	80383001	80386000	3000	1	8.10E-09	-0.34	18	0.6	Dnm3;LOC102554073;LOC102555077	Transport
DMR13:80511001	13	80511001	80513000	2000	1	8.40E-08	-0.56	10	0.5	Myoc	Development

DMR13:80756001	13	80756001	80762000	6000	3	1.10E-09	0.51	106	1.77	Fmo2;LOC103692017;LOC103692016	Metabolism
DMR13:80795001	13	80795001	80800000	5000	1	4.80E-07	-0.37	45	0.9	LOC498264;Fmo6	Metabolism
DMR13:80823001	13	80823001	80825000	2000	1	4.40E-09	0.36	18	0.9	Fmo6	Metabolism
DMR13:80957001	13	80957001	80959000	2000	1	2.20E-10	0.87	28	1.4	Mroh9	
DMR13:81139001	13	81139001	81141000	2000	1	2.70E-08	-0.72	9	0.45	LOC103692020;Prrx1	
DMR13:81177001	13	81177001	81182000	5000	1	7.00E-14	0.8	72	1.44	Prrx1	
DMR13:81989001	13	81989001	81991000	2000	1	3.20E-09	0.42	48	2.4	Mettl11b	Epigenetic
DMR13:82125001	13	82125001	82132000	7000	1	5.60E-08	-0.3	66	0.94	Kifap3	Cytoskeleton
DMR13:82138001	13	82138001	82139000	1000	1	2.80E-07	-0.42	8	0.8	Kifap3	Cytoskeleton
DMR13:82202001	13	82202001	82203000	1000	1	1.10E-08	-0.44	8	0.8	Kifap3	Cytoskeleton
DMR13:82527001	13	82527001	82529000	2000	1	3.80E-10	-0.43	30	1.5	F5	Metabolism
DMR13:82578001	13	82578001	82581000	3000	1	1.80E-10	-0.44	60	2	Ccdc181	
DMR13:82608001	13	82608001	82610000	2000	1	4.90E-10	-0.52	39	1.95	Blzf1;Nme7	Transcription;Signaling
DMR13:82655001	13	82655001	82659000	4000	1	2.10E-07	0.52	44	1.1	Nme7	Signaling
DMR13:82723001	13	82723001	82724000	1000	1	1.90E-10	-0.39	16	1.6	Nme7	Signaling
DMR13:82763001	13	82763001	82765000	2000	1	4.00E-08	-0.41	17	0.85	Atp1b1	Transport
DMR13:82767001	13	82767001	82768000	1000	1	8.20E-08	-0.4	6	0.6	Atp1b1	Transport
DMR13:83073001	13	83073001	83074000	1000	1	1.80E-13	0.83	26	2.6	Dpt	Cytoskeleton
DMR13:83085001	13	83085001	83088000	3000	1	3.80E-07	0.4	34	1.13	Dpt;LOC102546371	Cytoskeleton
DMR13:83551001	13	83551001	83554000	3000	1	3.70E-07	-0.45	17	0.57	Gpr161	Signaling
DMR13:83739001	13	83739001	83740000	1000	1	2.20E-07	0.46	3	0.3	Adcy10	
DMR13:83771001	13	83771001	83772000	1000	1	8.40E-07	-0.39	21	2.1	Adcy10	
DMR13:83843001	13	83843001	83844000	1000	1	4.40E-07	-0.36	18	1.8	Mpzl1;Trnap-agg;Trnap-cgg	Cytoskeleton
DMR13:83855001	13	83855001	83859000	4000	1	5.80E-08	0.52	63	1.57	Trnap-agg;Trnap-cgg;Rcsd1	Cytoskeleton
DMR13:83878001	13	83878001	83879000	1000	1	1.90E-08	0.44	12	1.2	Rcsd1	Cytoskeleton
DMR13:83992001	13	83992001	83993000	1000	1	1.70E-10	0.96	42	4.2	Creg1;Cd247	Immune
DMR13:84005001	13	84005001	84006000	1000	1	6.00E-08	0.4	15	1.5	Cd247	Immune
DMR13:84018001	13	84018001	84022000	4000	1	3.70E-15	0.76	69	1.73	Cd247	Immune
DMR13:84041001	13	84041001	84042000	1000	1	7.90E-09	0.78	23	2.3	Cd247	Immune
DMR13:84208001	13	84208001	84211000	3000	1	1.50E-08	-0.47	21	0.7	Pou2f1	
DMR13:84305001	13	84305001	84306000	1000	1	1.70E-09	0.66	36	3.6	Dusp27	
DMR13:84323001	13	84323001	84324000	1000	1	3.60E-08	-0.36	16	1.6	Dusp27	
DMR13:84349001	13	84349001	84352000	3000	1	1.70E-07	0.54	59	1.97	Gpa33	
DMR13:84364001	13	84364001	84369000	5000	1	7.50E-08	0.8	76	1.52	Gpa33	
DMR13:84452001	13	84452001	84454000	2000	2	1.20E-12	1.03	61	3.05	Mael	
DMR13:84472001	13	84472001	84473000	1000	1	1.60E-10	0.56	18	1.8	Ildr2	Immune
DMR13:84486001	13	84486001	84491000	5000	1	5.20E-12	-0.46	104	2.08	Ildr2	Immune
DMR13:84529001	13	84529001	84535000	6000	1	1.60E-11	0.93	90	1.5	Ildr2;LOC103693676	Immune
DMR13:84763001	13	84763001	84765000	2000	1	7.10E-08	0.55	19	0.95	LOC685351;Fmo9	Metabolism
DMR13:85373001	13	85373001	85374000	1000	1	7.50E-07	0.31	18	1.8	Uck2	Signaling
DMR13:85400001	13	85400001	85401000	1000	1	7.70E-07	0.41	25	2.5	Uck2	Signaling
DMR13:85429001	13	85429001	85431000	2000	1	1.80E-07	-0.4	48	2.4	Uck2	Signaling
DMR13:85664001	13	85664001	85666000	2000	1	2.20E-07	0.36	26	1.3	Lrrc52	
DMR13:85805001	13	85805001	85810000	5000	1	2.40E-08	0.49	95	1.9	LOC103692054;Rxrg	Transcription
DMR13:85826001	13	85826001	85827000	1000	1	1.70E-14	0.77	24	2.4	LOC103692054;Rxrg	Transcription
DMR13:86032001	13	86032001	86033000	1000	1	5.90E-07	-0.45	11	1.1	Lmx1a	Development
DMR13:86051001	13	86051001	86054000	3000	1	2.20E-08	0.65	35	1.17	Lmx1a	Development
DMR13:86583001	13	86583001	86585000	2000	1	3.40E-07	-0.34	29	1.45	Pbx1	Development
DMR13:86645001	13	86645001	86646000	1000	1	2.70E-07	-0.35	14	1.4	Pbx1	Development
DMR13:86678001	13	86678001	86680000	2000	1	4.40E-10	-0.68	21	1.05	Pbx1	Development
DMR13:88005001	13	88005001	88006000	1000	1	4.30E-13	0.87	18	1.8	Rgs5	Signaling
DMR13:88261001	13	88261001	88264000	3000	1	3.80E-11	0.55	46	1.53	Ccdc190	
DMR13:88269001	13	88269001	88273000	4000	1	5.80E-15	0.84	97	2.42	Ccdc190	
DMR13:88356001	13	88356001	88358000	2000	2	1.30E-09	0.39	18	0.9	Ddr2	Receptor
DMR13:88458001	13	88458001	88463000	5000	1	4.20E-08	-0.54	62	1.24	LOC100911901;Uap1	
DMR13:88631001	13	88631001	88632000	1000	1	1.00E-11	0.72	19	1.9	Sh2d1b;LOC100361087	Cytoskeleton
DMR13:88743001	13	88743001	88744000	1000	1	1.10E-07	0.45	24	2.4	Nos1ap	Cytoskeleton
DMR13:88793001	13	88793001	88795000	2000	1	5.10E-08	-0.41	26	1.3	Nos1ap	Cytoskeleton
DMR13:88858001	13	88858001	88860000	2000	1	6.30E-07	0.43	27	1.35	Nos1ap	Cytoskeleton
DMR13:88865001	13	88865001	88869000	4000	2	2.90E-11	0.97	90	2.25	Nos1ap	Cytoskeleton
DMR13:88902001	13	88902001	88903000	1000	1	2.50E-09	-0.45	27	2.7	Nos1ap	Cytoskeleton

DMR13:88905001	13	88905001	88908000	3000	1	8.70E-08	0.54	42	1.4	Nos1ap	Cytoskeleton
DMR13:89006001	13	89006001	89007000	1000	1	6.30E-08	0.48	13	1.3	Olfml2b	Development
DMR13:89066001	13	89066001	89067000	1000	1	1.90E-09	-0.42	15	1.5	Atf6	
DMR13:89083001	13	89083001	89089000	6000	1	1.80E-11	0.4	67	1.12	Atf6	
DMR13:89094001	13	89094001	89096000	2000	1	2.10E-07	-0.35	40	2	Atf6	
DMR13:89110001	13	89110001	89115000	5000	1	2.70E-07	-0.27	46	0.92	Atf6	
DMR13:89145001	13	89145001	89148000	3000	2	1.10E-14	0.83	79	2.63	Atf6	
DMR13:89163001	13	89163001	89166000	3000	1	1.30E-10	-0.57	39	1.3	Atf6	
DMR13:89209001	13	89209001	89211000	2000	1	1.40E-08	-0.48	28	1.4	Atf6;LOC100911728	
DMR13:89236001	13	89236001	89238000	2000	1	1.80E-09	-0.4	32	1.6	Atf6	
DMR13:89240001	13	89240001	89242000	2000	1	5.00E-07	-0.39	32	1.6	Atf6	
DMR13:89252001	13	89252001	89255000	3000	1	6.20E-07	0.42	99	3.3	Atf6;LOC100362384;Dnmt3b-ps2;Dusp12	Transport;Signaling
DMR13:89283001	13	89283001	89286000	3000	1	1.80E-14	-0.61	37	1.23	Fcrlb	Immune
DMR13:89295001	13	89295001	89296000	1000	1	8.00E-07	0.62	13	1.3	Fcrlb;Fcrla	Immune
DMR13:89298001	13	89298001	89302000	4000	1	2.00E-14	0.81	84	2.1	Fcrlb;Fcrla	Immune
DMR13:89436001	13	89436001	89442000	6000	2	1.50E-13	0.83	316	5.27	LOC108348047;Trnae-cuc;Trnag-ucc;Trnad-guc;Trnal-cag;Trnag-gcc;LOC102557067	
DMR13:89443001	13	89443001	89449000	6000	2	8.70E-14	0.53	177	2.95	LOC108348047;Trnae-cuc;Trnag-ucc;Trnad-guc;Trnal-cag;Trnag-gcc;LOC102557067	
DMR13:89525001	13	89525001	89530000	5000	2	1.10E-08	-0.55	80	1.6	Sdhc;Mpz	Metabolism;Cytoskeleton
DMR13:89558001	13	89558001	89560000	2000	1	8.90E-09	0.4	32	1.6	Pcp4l1	
DMR13:89647001	13	89647001	89651000	4000	1	1.50E-09	0.46	63	1.57	B4galt3;Ppox;Usp21	Golgi;Metabolism;Protease
DMR13:89669001	13	89669001	89670000	1000	1	8.60E-07	-0.37	13	1.3	Usp21;Ufc1;LOC102547168	Protease
DMR13:89698001	13	89698001	89700000	2000	1	5.20E-07	0.56	43	2.15	Dedd	
DMR13:89955001	13	89955001	89956000	1000	1	1.60E-07	0.39	14	1.4	Cd244	Immune
DMR13:89981001	13	89981001	89984000	3000	1	2.60E-08	0.3	34	1.13	Cd244;LOC108352572	Immune
DMR13:89985001	13	89985001	89990000	5000	1	1.40E-09	-0.33	68	1.36	Cd244;LOC108352572;LOC103692282	Immune
DMR13:90048001	13	90048001	90049000	1000	1	1.00E-15	1.06	18	1.8	LOC102549964;Slamf7	Immune
DMR13:90236001	13	90236001	90237000	1000	1	3.50E-12	0.85	21	2.1	LOC103692288;Cd84	Immune
DMR13:90240001	13	90240001	90241000	1000	1	6.00E-07	0.43	5	0.5	LOC103692288;Cd84	Immune
DMR13:90282001	13	90282001	90284000	2000	1	1.80E-08	-0.46	16	0.8	Cd84;LOC103692287	Immune
DMR13:90385001	13	90385001	90390000	5000	2	4.00E-08	0.59	138	2.76	Vangl2;LOC108352574	
DMR13:90622001	13	90622001	90625000	3000	1	3.10E-07	-0.39	55	1.83	Atp1a4	Transport
DMR13:90674001	13	90674001	90678000	4000	1	4.20E-07	-0.34	49	1.23	Atp1a2	Transport
DMR13:90698001	13	90698001	90700000	2000	1	3.20E-16	1	72	3.6	Igsf8;Kcnj9	Immune;Transport
DMR13:90708001	13	90708001	90710000	2000	1	3.50E-12	0.63	36	1.8	Igsf8;Kcnj9	Immune;Transport
DMR13:90737001	13	90737001	90740000	3000	1	2.30E-07	0.37	47	1.57	Kcnj10	Transport
DMR13:90797001	13	90797001	90802000	5000	1	9.10E-07	0.35	77	1.54	LOC103692291;LOC102555223;Tagln2;Igsf9	Cytoskeleton
DMR13:90852001	13	90852001	90855000	3000	1	1.70E-08	0.49	25	0.83	Slamf9;LOC108352575	Immune
DMR13:90901001	13	90901001	90902000	1000	1	1.60E-08	-0.4	21	2.1	LOC102555374;Cfap45	
DMR13:90913001	13	90913001	90915000	2000	1	1.90E-10	-0.5	59	2.95	LOC102555374;Cfap45	
DMR13:90959001	13	90959001	90961000	2000	2	4.70E-09	0.42	29	1.45	Vsig8;Slamf8	Immune
DMR13:90970001	13	90970001	90971000	1000	1	4.00E-08	0.37	12	1.2	Slamf8	Immune
DMR13:91011001	13	91011001	91014000	3000	1	5.10E-08	-0.44	40	1.33	Dusp23	Signaling
DMR13:91016001	13	91016001	91017000	1000	1	8.10E-08	0.6	19	1.9	Dusp23	Signaling
DMR13:91077001	13	91077001	91082000	5000	1	1.20E-08	-0.43	51	1.02	Crp	
DMR13:91159001	13	91159001	91165000	6000	3	2.50E-12	0.77	331	5.52	Fcgr2a;Trnad-guc;Trnal-cag;LOC498276	
DMR13:91181001	13	91181001	91182000	1000	1	1.80E-07	0.51	32	3.2	LOC498276;Trnad-guc;Trnal-cag;LOC103693683	
DMR13:91183001	13	91183001	91184000	1000	1	8.40E-08	0.66	53	5.3	LOC498276;Trnad-guc;Trnal-cag;LOC103693683	
DMR13:91204001	13	91204001	91206000	2000	1	1.70E-07	0.56	60	3	LOC103693683;Trnag-ucc;LOC108352576;Trnag-gcc	

DMR13:91214001	13	91214001	91216000	2000	1	5.10E-14	0.84	72	3.6	Trnag-ucc;LOC108352576;Trnag-gcc;LOC100911825	
DMR13:91234001	13	91234001	91235000	1000	1	4.00E-09	0.67	49	4.9	LOC100911825;Trnad-guc;Trnal-cag;Trnag-ucc	
DMR13:91645001	13	91645001	91646000	1000	1	4.30E-08	-0.43	12	1.2	Olr1579;Olr1580-ps	
DMR13:91985001	13	91985001	91990000	5000	1	1.40E-07	-0.52	55	1.1	LOC102556096;RGD1562462	
DMR13:92137001	13	92137001	92139000	2000	1	1.60E-08	-0.45	16	0.8	Olr1587;Olr1588	
DMR13:92158001	13	92158001	92162000	4000	1	3.70E-08	-0.37	39	0.98	Olr1589	Receptor
DMR13:92174001	13	92174001	92175000	1000	1	4.50E-07	0.6	6	0.6	Olr1589;Olr1590	Receptor
DMR13:92230001	13	92230001	92232000	2000	1	1.80E-07	-0.44	14	0.7	Olr1593;Olr1594-ps;Olr1595	
DMR13:92313001	13	92313001	92319000	6000	2	2.20E-08	-0.39	72	1.2	Spta1	
DMR13:92382001	13	92382001	92387000	5000	1	3.40E-07	-0.34	43	0.86	Olr1600	Signaling
DMR13:92620001	13	92620001	92622000	2000	1	1.50E-07	0.44	20	1	Fmn2	
DMR13:92641001	13	92641001	92643000	2000	1	5.20E-09	-0.59	20	1	Fmn2	
DMR13:92680001	13	92680001	92682000	2000	1	1.90E-07	-0.42	12	0.6	Fmn2	
DMR13:92845001	13	92845001	92846000	1000	1	9.30E-09	0.56	4	0.4	Fmn2	
DMR13:92903001	13	92903001	92905000	2000	1	7.30E-07	-0.42	31	1.55	Grem2	
DMR13:92953001	13	92953001	92956000	3000	2	1.60E-10	-0.62	18	0.6	Grem2	
DMR13:93116001	13	93116001	93117000	1000	1	2.60E-15	0.82	29	2.9	Rgs7	
DMR13:93270001	13	93270001	93271000	1000	1	1.30E-08	0.42	8	0.8	Rgs7;LOC102547622	
DMR13:93375001	13	93375001	93379000	4000	1	5.30E-07	-0.47	10	0.25	Rgs7	
DMR13:93668001	13	93668001	93672000	4000	1	5.70E-07	0.54	61	1.52	Fh	Metabolism
DMR13:93788001	13	93788001	93789000	1000	1	9.10E-07	-0.37	8	0.8	LOC103692323;Wdr64	
DMR13:93974001	13	93974001	93975000	1000	1	2.40E-08	0.42	15	1.5	Becn2	Protease; Proteolysis
DMR13:94076001	13	94076001	94078000	2000	1	3.70E-07	-0.31	32	1.6	Pld5	Metabolism
DMR13:94103001	13	94103001	94105000	2000	1	4.30E-08	0.48	36	1.8	Pld5	Metabolism
DMR13:94118001	13	94118001	94120000	2000	1	1.30E-08	0.3	16	0.8	Pld5	Metabolism
DMR13:94139001	13	94139001	94141000	2000	1	1.90E-08	-0.29	9	0.45	Pld5	Metabolism
DMR13:94291001	13	94291001	94292000	1000	1	2.70E-13	1.03	23	2.3	Pld5	Metabolism
DMR13:94953001	13	94953001	94954000	1000	1	1.50E-20	0.96	5	0.5	Sdccag8	
DMR13:95145001	13	95145001	95146000	1000	1	1.70E-07	-0.33	7	0.7	Akt3	Signaling
DMR13:95190001	13	95190001	95192000	2000	1	4.00E-07	-0.37	13	0.65	Akt3	Signaling
DMR13:95305001	13	95305001	95308000	3000	1	3.40E-08	-0.37	25	0.83	Akt3	Signaling
DMR13:95409001	13	95409001	95415000	6000	1	8.20E-07	-0.33	55	0.92	Rpl30l1	Translation
DMR13:95950001	13	95950001	95951000	1000	1	2.20E-07	-0.37	5	0.5	Adss	Metabolism
DMR13:95980001	13	95980001	95982000	2000	1	7.40E-10	-0.45	16	0.8	RGD1565309	
DMR13:96019001	13	96019001	96020000	1000	1	3.30E-10	0.54	26	2.6	RGD1565309	
DMR13:96059001	13	96059001	96060000	1000	1	8.30E-07	-0.74	7	0.7	RGD1565309	
DMR13:96079001	13	96079001	96080000	1000	1	5.00E-07	-0.37	5	0.5	RGD1565309	
DMR13:96110001	13	96110001	96111000	1000	1	6.60E-08	0.51	14	1.4	RGD1565309;Desi2	Protease
DMR13:96124001	13	96124001	96126000	2000	1	5.70E-08	-0.41	29	1.45	Desi2	Protease
DMR13:96163001	13	96163001	96166000	3000	1	1.20E-08	0.72	60	2	Desi2;RGD1566217	Protease
DMR13:96190001	13	96190001	96193000	3000	1	4.00E-10	-0.5	28	0.93	RGD1563812	
DMR13:96296001	13	96296001	96297000	1000	1	2.60E-10	0.52	21	2.1	Efcab2	Cytoskeleton
DMR13:96357001	13	96357001	96359000	2000	1	1.70E-10	-0.38	19	0.95	Efcab2	Cytoskeleton
DMR13:96791001	13	96791001	96795000	4000	1	8.00E-14	0.88	140	3.5	Kif26b	Cytoskeleton
DMR13:96805001	13	96805001	96806000	1000	1	6.80E-08	0.59	5	0.5	Kif26b	Cytoskeleton
DMR13:96898001	13	96898001	96900000	2000	1	6.80E-09	0.58	40	2	Kif26b	Cytoskeleton
DMR13:96951001	13	96951001	96952000	1000	1	1.50E-09	0.46	10	1	Kif26b;LOC102553287	Cytoskeleton
DMR13:97120001	13	97120001	97124000	4000	1	2.40E-07	0.46	58	1.45	Kif26b;LOC102553199	Cytoskeleton
DMR13:97146001	13	97146001	97148000	2000	1	1.90E-08	0.43	40	2	Kif26b	Cytoskeleton
DMR13:97325001	13	97325001	97330000	5000	1	9.60E-08	-0.37	102	2.04	Smyd3	
DMR13:97340001	13	97340001	97341000	1000	1	4.10E-07	-0.3	8	0.8	Smyd3	
DMR13:97348001	13	97348001	97352000	4000	1	3.90E-09	-0.44	86	2.15	Smyd3	
DMR13:97414001	13	97414001	97420000	6000	1	3.00E-07	-0.33	84	1.4	Smyd3;LOC108352580	
DMR13:97427001	13	97427001	97431000	4000	1	2.40E-07	0.48	64	1.6	Smyd3;LOC108352580;LOC108352579	
DMR13:97473001	13	97473001	97475000	2000	1	5.10E-07	-0.55	22	1.1	Smyd3	
DMR13:97663001	13	97663001	97664000	1000	1	9.00E-08	-0.45	21	2.1	Smyd3;LOC100912475	
DMR13:97671001	13	97671001	97672000	1000	1	1.80E-08	-0.65	8	0.8	Smyd3	
DMR13:97770001	13	97770001	97773000	3000	1	1.50E-07	-0.36	41	1.37	Smyd3	

DMR13:97788001	13	97788001	97790000	2000	1	9.50E-07	0.43	21	1.05	Smyd3;LOC102552208	
DMR13:97873001	13	97873001	97874000	1000	1	2.00E-08	-0.46	18	1.8	Cnst	
DMR13:97881001	13	97881001	97882000	1000	1	9.30E-10	-0.66	8	0.8	Cnst;LOC102552409	
DMR13:98006001	13	98006001	98011000	5000	2	7.40E-08	0.57	81	1.62	Kif28p	Cytoskeleton
DMR13:98031001	13	98031001	98033000	2000	2	4.10E-09	-0.45	33	1.65	Kif28p;Ahctf1	Cytoskeleton;Cytoskeleton
DMR13:98223001	13	98223001	98224000	1000	1	3.50E-07	0.53	19	1.9	Cdc42bpa	Signaling
DMR13:98267001	13	98267001	98269000	2000	1	9.60E-08	-0.54	14	0.7	Cdc42bpa	Signaling
DMR13:98455001	13	98455001	98457000	2000	1	3.60E-07	0.34	22	1.1	Cdc42bpa;Adck3	Signaling
DMR13:98522001	13	98522001	98524000	2000	1	5.30E-08	0.6	34	1.7	Psen2	Proteolysis
DMR13:98613001	13	98613001	98615000	2000	1	1.90E-07	0.6	28	1.4	Itpkb	Signaling
DMR13:98678001	13	98678001	98680000	2000	1	2.80E-08	0.38	29	1.45	Itpkb	Signaling
DMR13:98704001	13	98704001	98708000	4000	2	2.40E-12	0.59	47	1.18	Itpkb	Signaling
DMR13:98709001	13	98709001	98710000	1000	1	2.80E-07	0.52	15	1.5	Itpkb	Signaling
DMR13:98733001	13	98733001	98736000	3000	1	1.60E-08	0.38	55	1.83	Stum	
DMR13:99017001	13	99017001	99018000	1000	1	1.30E-12	-0.52	24	2.4	Acbd3	
DMR13:99284001	13	99284001	99288000	4000	2	1.10E-15	-0.6	37	0.92	Ephx1	Metabolism
DMR13:99381001	13	99381001	99386000	5000	1	8.40E-09	-0.47	60	1.2	LOC498300;RGD1564463	Metabolism
DMR13:99690001	13	99690001	99691000	1000	1	6.10E-08	0.4	13	1.3	Cnih3	Transport
DMR13:99735001	13	99735001	99742000	7000	1	1.40E-08	0.38	103	1.47	Cnih3	Transport
DMR13:99811001	13	99811001	99812000	1000	1	8.40E-07	-0.52	8	0.8	Ccdc121	
DMR13:99813001	13	99813001	99814000	1000	1	5.70E-10	0.51	16	1.6	Ccdc121	
DMR13:100316001	13	100316001	100320000	4000	1	2.50E-13	0.73	93	2.33	Enah	Cytoskeleton
DMR13:100393001	13	100393001	100396000	3000	1	3.50E-08	-0.43	33	1.1	Enah	Cytoskeleton
DMR13:100459001	13	100459001	100461000	2000	1	2.10E-09	-0.39	31	1.55	Lbr;LOC103693700	Metabolism
DMR13:100750001	13	100750001	100752000	2000	1	1.60E-09	0.51	38	1.9	Fbxo28	
DMR13:100847001	13	100847001	100853000	6000	1	3.90E-09	-0.34	118	1.97	Tp53bp2	Signaling
DMR13:100876001	13	100876001	100878000	2000	1	6.60E-08	-0.35	27	1.35	Tp53bp2;Capn2	Signaling;Protease
DMR13:100910001	13	100910001	100914000	4000	1	1.50E-09	0.62	49	1.23	Capn2;LOC102555308	Protease
DMR13:100993001	13	100993001	100996000	3000	1	5.30E-07	0.34	6	0.2	Capn8	Protease
DMR13:100998001	13	100998001	1.01E+08	2000	1	4.50E-07	0.51	24	1.2	Capn8	Protease
DMR13:101033001	13	101033001	101037000	4000	1	7.70E-08	0.38	52	1.3	Capn8	Protease
DMR13:101160001	13	101160001	101162000	2000	1	5.10E-22	1.2	42	2.1	Ccdc185	
DMR13:101190001	13	101190001	101194000	4000	1	2.70E-09	0.75	73	1.82	Susd4	
DMR13:101208001	13	101208001	101210000	2000	1	3.60E-09	-0.5	46	2.3	Susd4	
DMR13:101229001	13	101229001	101232000	3000	2	2.20E-10	0.51	41	1.37	Susd4	
DMR13:101583001	13	101583001	101586000	3000	1	5.50E-08	-0.58	51	1.7	Disp1	
DMR13:101675001	13	101675001	101677000	2000	1	8.20E-08	-0.42	32	1.6	Fam177b;LOC108352623;Brox	
DMR13:101733001	13	101733001	101734000	1000	1	6.20E-09	-0.48	12	1.2	Aida;Mia3	Transport
DMR13:101768001	13	101768001	101769000	1000	1	1.10E-09	0.65	20	2	Mia3;Taf1a	Transport
DMR13:101778001	13	101778001	101780000	2000	1	1.20E-07	-0.43	29	1.45	Mia3;Taf1a	Transport
DMR13:101790001	13	101790001	101792000	2000	1	1.70E-07	-0.36	31	1.55	Taf1a	
DMR13:101795001	13	101795001	101797000	2000	1	1.30E-09	-0.42	19	0.95	Taf1a	
DMR13:101818001	13	101818001	101819000	1000	1	9.10E-18	1.15	29	2.9	Hhipl2	Signaling
DMR13:101825001	13	101825001	101828000	3000	1	3.30E-11	0.64	50	1.67	Hhipl2	Signaling
DMR13:101831001	13	101831001	101832000	1000	1	1.80E-07	0.49	15	1.5	Hhipl2	Signaling
DMR13:102638001	13	102638001	102641000	3000	1	1.80E-07	-0.43	101	3.37	Hlx	Development
DMR13:102652001	13	102652001	102660000	8000	2	1.80E-09	0.48	115	1.44	Hlx	Development
DMR13:102682001	13	102682001	102685000	3000	1	3.20E-08	0.51	58	1.93	1-Mar	
DMR13:102705001	13	102705001	102706000	1000	1	4.00E-09	-0.59	8	0.8	1-Mar	
DMR13:102807001	13	102807001	102809000	2000	2	7.50E-08	-0.42	53	2.65	Mark1	Signaling
DMR13:103246001	13	103246001	103247000	1000	1	7.70E-11	0.62	16	1.6	Iars2;Mir194-1	Translation
DMR13:103297001	13	103297001	103298000	1000	1	1.70E-09	-0.54	18	1.8	Bpnt1;Eprs	Signaling;Translation
DMR13:104156001	13	104156001	104159000	3000	1	4.20E-07	0.52	43	1.43	LOC103693693;RGD1561704	
DMR13:104312001	13	104312001	104313000	1000	1	6.50E-07	0.38	10	1	Dusp10	Signaling
DMR13:104319001	13	104319001	104320000	1000	1	9.10E-07	0.36	12	1.2	Dusp10	Signaling
DMR13:105094001	13	105094001	105099000	5000	1	5.90E-07	-0.35	47	0.94	Tgfb2	Growth Factors
DMR13:105109001	13	105109001	105112000	3000	1	6.40E-09	0.46	40	1.33	Tgfb2	Growth Factors
DMR13:105118001	13	105118001	105119000	1000	1	8.20E-07	-0.42	21	2.1	Tgfb2	Growth Factors
DMR13:105132001	13	105132001	105134000	2000	2	1.30E-07	0.61	31	1.55	Tgfb2	Growth Factors
DMR13:105142001	13	105142001	105143000	1000	1	1.10E-08	0.68	7	0.7	Tgfb2	Growth Factors
DMR13:105406001	13	105406001	105410000	4000	3	5.10E-21	1.37	128	3.2	Ddx3y	
DMR13:105611001	13	105611001	105613000	2000	1	1.30E-08	-0.4	26	1.3	Spata17	

DMR13:105793001	13	105793001	105794000	1000	1	3.80E-08	0.52	9	0.9	Gpatch2	Metabolism
DMR13:106060001	13	106060001	106062000	2000	2	6.30E-09	0.59	69	3.45	Esrrg	
DMR13:106191001	13	106191001	106195000	4000	2	2.50E-09	0.56	45	1.12	Esrrg	
DMR13:106214001	13	106214001	106218000	4000	1	2.50E-10	0.75	66	1.65	Esrrg	
DMR13:106221001	13	106221001	106222000	1000	1	2.80E-08	-0.36	23	2.3	Esrrg	
DMR13:106247001	13	106247001	106249000	2000	1	1.60E-08	0.41	27	1.35	Esrrg	
DMR13:106252001	13	106252001	106253000	1000	1	3.20E-11	-0.51	14	1.4	Esrrg	
DMR13:106254001	13	106254001	106256000	2000	1	1.00E-09	0.6	20	1	Esrrg	
DMR13:106290001	13	106290001	106291000	1000	1	4.20E-08	0.32	20	2	Esrrg	
DMR13:106298001	13	106298001	106304000	6000	2	5.10E-16	0.84	97	1.62	Esrrg	
DMR13:106310001	13	106310001	106311000	1000	1	4.10E-08	-0.41	20	2	Esrrg	
DMR13:106318001	13	106318001	106320000	2000	1	5.30E-14	0.79	50	2.5	Esrrg	
DMR13:106378001	13	106378001	106379000	1000	1	2.80E-17	0.83	23	2.3	Esrrg	
DMR13:106426001	13	106426001	106429000	3000	2	6.20E-10	0.67	45	1.5	Esrrg	
DMR13:106486001	13	106486001	106487000	1000	1	2.10E-07	0.54	10	1	Esrrg	
DMR13:106653001	13	106653001	106661000	8000	1	7.30E-07	-0.39	149	1.86	Esrrg	
DMR13:106751001	13	106751001	106752000	1000	1	1.10E-08	0.7	18	1.8	Ush2a	Extracellular Matrix
DMR13:106753001	13	106753001	106754000	1000	1	2.70E-15	1.03	33	3.3	Ush2a	Extracellular Matrix
DMR13:106793001	13	106793001	106794000	1000	1	5.00E-07	0.28	9	0.9	Ush2a	Extracellular Matrix
DMR13:106819001	13	106819001	106823000	4000	2	8.00E-09	0.58	57	1.43	Ush2a	Extracellular Matrix
DMR13:106939001	13	106939001	106940000	1000	1	1.50E-07	0.55	15	1.5	Ush2a	Extracellular Matrix
DMR13:106979001	13	106979001	106980000	1000	1	5.60E-08	0.53	15	1.5	Ush2a	Extracellular Matrix
DMR13:107019001	13	107019001	107025000	6000	1	3.70E-08	-0.45	124	2.07	Ush2a	Extracellular Matrix
DMR13:107032001	13	107032001	107034000	2000	1	4.40E-11	0.47	24	1.2	Ush2a	Extracellular Matrix
DMR13:107074001	13	107074001	107075000	1000	1	8.30E-07	0.48	8	0.8	Ush2a	Extracellular Matrix
DMR13:107152001	13	107152001	107158000	6000	1	3.20E-07	0.27	82	1.37	Ush2a	Extracellular Matrix
DMR13:107181001	13	107181001	107185000	4000	1	2.30E-07	0.5	57	1.43	Ush2a	Extracellular Matrix
DMR13:107245001	13	107245001	107250000	5000	2	1.60E-10	0.62	83	1.66	Ush2a	Extracellular Matrix
DMR13:107384001	13	107384001	107385000	1000	1	1.70E-07	0.39	5	0.5	Ush2a;LOC103692406	Extracellular Matrix
DMR13:107465001	13	107465001	107467000	2000	1	4.00E-08	-0.38	34	1.7	Kctd3	Cytoskeleton
DMR13:107696001	13	107696001	107698000	2000	1	7.80E-11	-0.52	16	0.8	Kcnk2	Transport
DMR13:107724001	13	107724001	107728000	4000	1	6.80E-07	0.31	49	1.23	Kcnk2	Transport
DMR13:107751001	13	107751001	107752000	1000	1	1.20E-10	0.68	17	1.7	Kcnk2	Transport
DMR13:107759001	13	107759001	107760000	1000	1	2.30E-07	-0.46	13	1.3	Kcnk2	Transport
DMR13:107783001	13	107783001	107786000	3000	1	2.00E-08	-0.51	36	1.2	Kcnk2	Transport
DMR13:107789001	13	107789001	107791000	2000	1	5.90E-10	0.88	44	2.2	Kcnk2	Transport
DMR13:107832001	13	107832001	107834000	2000	1	2.80E-08	-0.58	18	0.9	Kcnk2	Transport
DMR13:108153001	13	108153001	108154000	1000	1	4.10E-07	0.34	12	1.2	Cenpf	
DMR13:108409001	13	108409001	108410000	1000	1	6.60E-07	0.29	10	1	Prox1	Development
DMR13:108418001	13	108418001	108419000	1000	1	7.20E-09	-0.68	3	0.3	Prox1	Development
DMR13:108426001	13	108426001	108427000	1000	1	4.30E-08	-0.62	8	0.8	Prox1	Development
DMR13:108431001	13	108431001	108434000	3000	1	3.00E-08	0.5	49	1.63	Prox1	Development
DMR13:108660001	13	108660001	108661000	1000	1	4.10E-08	-0.47	20	2	Smyd2	Epigenetic
DMR13:108675001	13	108675001	108678000	3000	2	1.80E-12	-0.76	79	2.63	Smyd2	Epigenetic
DMR13:108726001	13	108726001	108727000	1000	1	9.10E-09	0.29	7	0.7	Ptpn14	Signaling
DMR13:108758001	13	108758001	108761000	3000	1	4.30E-09	0.58	38	1.27	Ptpn14	Signaling
DMR13:108815001	13	108815001	108818000	3000	1	2.80E-07	-0.39	60	2	Ptpn14	Signaling
DMR13:108819001	13	108819001	108820000	1000	1	2.90E-07	0.26	10	1	Ptpn14	Signaling
DMR13:109413001	13	109413001	109415000	2000	1	1.30E-10	-0.63	20	1	Rps6kc1	Signaling
DMR13:109542001	13	109542001	109545000	3000	1	2.80E-08	-0.37	58	1.93	Rps6kc1;Vash2	Signaling
DMR13:109572001	13	109572001	109576000	4000	2	2.80E-16	-0.59	45	1.12	Rps6kc1;LOC108348225	Signaling
DMR13:109583001	13	109583001	109584000	1000	1	8.00E-07	0.33	11	1.1	Rps6kc1;LOC108348225	Signaling
DMR13:109641001	13	109641001	109646000	5000	1	4.00E-08	-0.36	147	2.94	LOC498316;Tatdn3	
DMR13:109648001	13	109648001	109650000	2000	1	2.70E-07	-0.45	32	1.6	LOC498316;Tatdn3	
DMR13:109702001	13	109702001	109704000	2000	1	2.20E-07	0.49	44	2.2	Nsl1;Batf3	Transcription
DMR13:109979001	13	109979001	109984000	5000	1	1.50E-08	0.39	64	1.28	Nenf	Receptor
DMR13:110027001	13	110027001	110030000	3000	1	8.50E-07	0.37	64	2.13	Tmem206;Ppp2r5a	Signaling
DMR13:110251001	13	110251001	110252000	1000	1	3.60E-09	0.66	9	0.9	Dtl;Ints7	
DMR13:110275001	13	110275001	110280000	5000	2	3.20E-11	0.42	70	1.4	Ints7;LOC108352606	
DMR13:110417001	13	110417001	110420000	3000	1	2.30E-08	0.45	34	1.13	Lpgat1	Metabolism
DMR13:110691001	13	110691001	110694000	3000	1	9.40E-07	0.46	63	2.1	Slc30a1;LOC108352598	
DMR13:110794001	13	110794001	110798000	4000	1	5.30E-07	-0.37	50	1.25	Traf5	Cytoskeleton

DMR13:110924001	13	110924001	110927000	3000	1	1.90E-07	0.33	37	1.23	Kcnh1	Transport
DMR13:110939001	13	110939001	110941000	2000	1	1.70E-07	0.35	18	0.9	Kcnh1	Transport
DMR13:110994001	13	110994001	110996000	2000	2	6.50E-22	1.24	50	2.5	Kcnh1	Transport
DMR13:111050001	13	111050001	111053000	3000	1	4.10E-11	0.64	39	1.3	Kcnh1	Transport
DMR13:111057001	13	111057001	111059000	2000	1	1.30E-07	0.38	27	1.35	Kcnh1	Transport
DMR13:111063001	13	111063001	111067000	4000	1	1.10E-07	0.42	48	1.2	Kcnh1	Transport
DMR13:111071001	13	111071001	111072000	1000	1	4.60E-07	0.59	16	1.6	Kcnh1	Transport
DMR13:111157001	13	111157001	111159000	2000	1	7.50E-07	0.54	23	1.15	Kcnh1	Transport
DMR13:111212001	13	111212001	111214000	2000	1	2.90E-07	-0.34	26	1.3	Kcnh1	Transport
DMR13:111238001	13	111238001	111240000	2000	1	1.60E-07	0.57	25	1.25	Kcnh1;Hhat	Transport;Metabolism
DMR13:111268001	13	111268001	111269000	1000	1	4.00E-09	0.41	13	1.3	Hhat	Metabolism
DMR13:111282001	13	111282001	111284000	2000	1	1.70E-07	0.54	27	1.35	Hhat	Metabolism
DMR13:111286001	13	111286001	111290000	4000	1	6.70E-09	-0.56	50	1.25	Hhat	Metabolism
DMR13:111322001	13	111322001	111323000	1000	1	3.10E-07	-0.34	7	0.7	Hhat	Metabolism
DMR13:111362001	13	111362001	111364000	2000	1	1.10E-09	0.28	25	1.25	Hhat	Metabolism
DMR13:111426001	13	111426001	111428000	2000	1	2.10E-10	-0.73	8	0.4	Hhat	Metabolism
DMR13:111430001	13	111430001	111431000	1000	1	4.70E-15	0.78	18	1.8	Hhat	Metabolism
DMR13:111450001	13	111450001	111452000	2000	1	1.10E-08	0.39	24	1.2	Hhat	Metabolism
DMR13:111575001	13	111575001	111577000	2000	1	3.80E-07	-0.38	61	3.05	Sertad4;LOC108352599	
DMR13:111588001	13	111588001	111592000	4000	1	1.20E-07	-0.39	55	1.38	Sertad4;LOC108352599	
DMR13:111643001	13	111643001	111646000	3000	1	7.70E-09	0.69	33	1.1	Syt14	
DMR13:111712001	13	111712001	111714000	2000	1	2.60E-07	-0.47	22	1.1	Syt14	
DMR13:111970001	13	111970001	111971000	1000	1	1.30E-09	0.5	12	1.2	Hsd11b1	Metabolism
DMR13:111972001	13	111972001	111978000	6000	1	3.70E-07	0.35	49	0.82	Hsd11b1	Metabolism
DMR13:112074001	13	112074001	112080000	6000	1	2.40E-12	0.61	71	1.18	Lamb3;Camk1g	Extracellular Matrix;Signaling
DMR13:112103001	13	112103001	112106000	3000	1	7.50E-10	0.48	15	0.5	Camk1g	Signaling
DMR13:113516001	13	113516001	113518000	2000	1	2.60E-10	0.63	19	0.95	Plxna2	
DMR13:113579001	13	113579001	113583000	4000	1	4.80E-09	-0.32	31	0.78	Plxna2	
DMR13:113710001	13	113710001	113712000	2000	1	9.60E-10	0.5	19	0.95	Cd34	Cytoskeleton
DMR14:445001	14	445001	449000	4000	2	1.30E-15	0.99	82	2.05	RGD1564814;LOC100912107	
DMR14:1023001	14	1023001	1024000	1000	1	1.90E-12	0.75	12	1.2	Vom2r69	Signaling
DMR14:1098001	14	1098001	1100000	2000	1	2.90E-07	-0.3	11	0.55	Vom2r68	Signaling
DMR14:1456001	14	1456001	1462000	6000	3	2.60E-16	1.2	216	3.6	Crlf2;Csf2ra;LOC100365289;Ppp2r3b	Receptor;Signaling
DMR14:1465001	14	1465001	1466000	1000	1	5.00E-13	0.84	31	3.1	Crlf2;Csf2ra;LOC100365289;Ppp2r3b	Receptor;Signaling
DMR14:1472001	14	1472001	1476000	4000	4	7.40E-18	1.24	140	3.5	Csf2ra;LOC100365289;Ppp2r3b	Receptor;Signaling
DMR14:1657001	14	1657001	1661000	4000	1	7.70E-07	-0.33	30	0.75	Ccdc18	Cytoskeleton
DMR14:1666001	14	1666001	1667000	1000	1	4.30E-07	-0.52	11	1.1	Ccdc18	Cytoskeleton
DMR14:2059001	14	2059001	2062000	3000	1	8.20E-11	0.55	53	1.77	Idua;Slc26a1;Dgkq	Metabolism;Transport;Signaling
DMR14:2080001	14	2080001	2082000	2000	1	5.70E-07	0.44	34	1.7	Dgkq;Tmem175	Signaling;Transport
DMR14:2157001	14	2157001	2159000	2000	1	2.60E-07	-0.32	15	0.75	Gak	Transport
DMR14:2227001	14	2227001	2233000	6000	1	8.10E-10	0.63	78	1.3	Cplx1;LOC103692489	
DMR14:2662001	14	2662001	2665000	3000	1	2.70E-07	-0.32	32	1.07	Mtf2	Epigenetic
DMR14:2809001	14	2809001	2810000	1000	1	2.00E-09	0.46	13	1.3	Fam69a	
DMR14:2874001	14	2874001	2875000	1000	1	8.30E-07	0.48	23	2.3	Rpl5;LOC689892	Translation
DMR14:2892001	14	2892001	2896000	4000	2	4.70E-12	0.84	94	2.35	Evi5;LOC108352688	Signaling
DMR14:2980001	14	2980001	2983000	3000	1	3.00E-07	-0.45	42	1.4	Evi5	Signaling
DMR14:3229001	14	3229001	3232000	3000	2	8.10E-13	0.77	30	1	Glmn	Proteolysis
DMR14:3352001	14	3352001	3356000	4000	1	3.00E-07	-0.34	54	1.35	Btbd8;Lpcat2b;LOC108352691;Ephx4	Metabolism;Metabolism
DMR14:3378001	14	3378001	3379000	1000	1	1.10E-08	0.48	17	1.7	LOC108352691;Ephx4	Metabolism
DMR14:3383001	14	3383001	3385000	2000	1	4.20E-07	-0.35	32	1.6	Ephx4;LOC108352690	Metabolism
DMR14:3388001	14	3388001	3389000	1000	1	5.20E-07	-0.51	13	1.3	Ephx4;LOC108352690	Metabolism
DMR14:3430001	14	3430001	3432000	2000	1	2.50E-07	-0.51	33	1.65	Brdt	
DMR14:3528001	14	3528001	3529000	1000	1	4.10E-07	-0.32	26	2.6	Tgfbr3	Receptor
DMR14:3571001	14	3571001	3573000	2000	1	3.00E-07	-0.38	38	1.9	Tgfbr3	Receptor
DMR14:3652001	14	3652001	3653000	1000	1	3.90E-07	0.52	16	1.6	Tgfbr3	Receptor
DMR14:3655001	14	3655001	3659000	4000	1	2.60E-09	-0.5	83	2.08	Tgfbr3	Receptor

DMR14:3818001	14	3818001	3819000	1000	1	3.40E-08	-0.48	12	1.2	Cdc7	Signaling
DMR14:3881001	14	3881001	3886000	5000	1	4.30E-08	-0.55	46	0.92	Hfm1	Transcription
DMR14:4179001	14	4179001	4184000	5000	1	4.70E-07	-0.37	135	2.7	Zfp644	Transcription
DMR14:5101001	14	5101001	5102000	1000	1	4.40E-08	-0.51	31	3.1	Lrrc8d	Cytoskeleton
DMR14:5180001	14	5180001	5182000	2000	1	4.80E-07	0.42	34	1.7	Lrrc8d	Cytoskeleton
DMR14:5315001	14	5315001	5316000	1000	1	1.20E-10	0.83	38	3.8	Lrrc8c	Cytoskeleton
DMR14:5323001	14	5323001	5325000	2000	1	6.00E-07	0.32	31	1.55	Lrrc8c	Cytoskeleton
DMR14:5326001	14	5326001	5331000	5000	1	5.10E-07	-0.53	83	1.66	Lrrc8c	Cytoskeleton
DMR14:5380001	14	5380001	5386000	6000	1	9.30E-08	-0.28	72	1.2	Lrrc8c;LOC108352696	Cytoskeleton
DMR14:5471001	14	5471001	5474000	3000	1	1.40E-07	0.31	42	1.4	Lrrc8b	Cytoskeleton
DMR14:5490001	14	5490001	5491000	1000	1	4.70E-09	-0.49	18	1.8	Lrrc8b;LOC108352838	Cytoskeleton
DMR14:5605001	14	5605001	5610000	5000	1	8.10E-07	0.25	51	1.02	Abcg3l4	
DMR14:5623001	14	5623001	5626000	3000	1	6.70E-12	-0.46	47	1.57	Abcg3l4	
DMR14:5813001	14	5813001	5816000	3000	2	4.80E-08	0.64	30	1	Abcg3l3	Transport
DMR14:5820001	14	5820001	5823000	3000	1	1.10E-07	-0.27	32	1.07	Abcg3l3	Transport
DMR14:5845001	14	5845001	5847000	2000	1	3.80E-07	-0.49	10	0.5	Abcg3l3;LOC102554737;LOC679539;LOC100359633	Transport
DMR14:5988001	14	5988001	5989000	1000	1	2.80E-08	0.25	10	1	Abcg3l3;Abcg3	Transport
DMR14:6054001	14	6054001	6056000	2000	1	9.00E-10	0.76	25	1.25	Abcg3l3;Abcg3	Transport
DMR14:6504001	14	6504001	6505000	1000	1	2.30E-10	0.46	14	1.4	Abcg3l1	Transport
DMR14:6606001	14	6606001	6607000	1000	1	5.60E-08	-0.42	20	2	LOC679894;Pkd2	Transcription;Transport
DMR14:6646001	14	6646001	6647000	1000	1	1.80E-09	0.8	17	1.7	Pkd2	Transport
DMR14:6777001	14	6777001	6778000	1000	1	1.60E-14	0.69	23	2.3	LOC100910198;Mepe	
DMR14:6783001	14	6783001	6786000	3000	1	6.20E-15	0.75	73	2.43	LOC100910198;Mepe	
DMR14:6788001	14	6788001	6789000	1000	1	1.00E-10	0.61	12	1.2	LOC100910198;Mepe	
DMR14:6802001	14	6802001	6803000	1000	1	9.30E-23	1.31	41	4.1	LOC100910198;Mepe;Ibsp	Extracellular Matrix
DMR14:6805001	14	6805001	6806000	1000	1	1.30E-15	1.07	29	2.9	Ibsp	Extracellular Matrix
DMR14:6811001	14	6811001	6818000	7000	3	3.90E-09	-0.51	93	1.33	Ibsp	Extracellular Matrix
DMR14:6880001	14	6880001	6882000	2000	1	2.00E-07	0.48	43	2.15	Dmp1	
DMR14:6890001	14	6890001	6895000	5000	1	1.50E-13	0.86	91	1.82	Dmp1	
DMR14:6929001	14	6929001	6932000	3000	1	4.50E-16	0.93	85	2.83	Dmp1;LOC108352697;Dspp	
DMR14:7046001	14	7046001	7047000	1000	1	8.90E-08	-0.3	25	2.5	Nudt9;Hsd17b11	Metabolism
DMR14:7102001	14	7102001	7109000	7000	1	9.00E-09	-0.43	138	1.97	Hsd17b11;Hsd17b13	
DMR14:7127001	14	7127001	7131000	4000	1	1.90E-11	-0.43	93	2.33	Hsd17b13	
DMR14:7218001	14	7218001	7220000	2000	2	7.20E-16	-0.68	29	1.45	Klhl8;Aff1	Cytoskeleton;Transcription
DMR14:7224001	14	7224001	7225000	1000	1	9.00E-07	-0.44	28	2.8	Klhl8;Aff1	Cytoskeleton;Transcription
DMR14:7249001	14	7249001	7252000	3000	1	1.10E-07	-0.43	55	1.83	Aff1	Transcription
DMR14:7261001	14	7261001	7270000	9000	1	2.40E-07	0.37	190	2.11	Aff1	Transcription
DMR14:7379001	14	7379001	7380000	1000	1	3.90E-15	1.12	30	3	Aff1	Transcription
DMR14:7654001	14	7654001	7656000	2000	1	1.40E-07	-0.42	51	2.55	Slc10a6;LOC102546454;LOC102546534	Transport
DMR14:7658001	14	7658001	7659000	1000	1	6.00E-09	0.63	28	2.8	Slc10a6;LOC102546454;LOC102546534	Transport
DMR14:7682001	14	7682001	7684000	2000	1	4.20E-09	-0.53	45	2.25	Slc10a6;Ptpn13	Transport;Signaling
DMR14:7766001	14	7766001	7769000	3000	2	5.00E-09	-0.47	54	1.8	Ptpn13	Signaling
DMR14:7771001	14	7771001	7772000	1000	1	6.50E-08	-0.38	14	1.4	Ptpn13	Signaling
DMR14:7783001	14	7783001	7786000	3000	1	3.00E-11	-0.52	41	1.37	Ptpn13	Signaling
DMR14:7807001	14	7807001	7810000	3000	1	4.20E-07	-0.43	53	1.77	Ptpn13	Signaling
DMR14:7811001	14	7811001	7817000	6000	1	6.80E-12	-0.5	141	2.35	Ptpn13	Signaling
DMR14:7820001	14	7820001	7824000	4000	2	7.80E-09	-0.53	108	2.7	Ptpn13	Signaling
DMR14:7836001	14	7836001	7842000	6000	1	6.90E-08	-0.4	111	1.85	Ptpn13	Signaling
DMR14:7857001	14	7857001	7859000	2000	1	6.40E-08	-0.41	31	1.55	Ptpn13	Signaling
DMR14:8084001	14	8084001	8088000	4000	1	4.40E-10	0.72	93	2.33	Mapk10;LOC108352700	Signaling
DMR14:8133001	14	8133001	8136000	3000	1	3.00E-09	-0.51	65	2.17	Mapk10	Signaling
DMR14:8145001	14	8145001	8149000	4000	2	1.40E-15	-0.51	62	1.55	Mapk10;LOC103692565	Signaling
DMR14:8153001	14	8153001	8157000	4000	1	8.00E-12	1	81	2.02	Mapk10;LOC103692565	Signaling
DMR14:8182001	14	8182001	8183000	1000	1	1.50E-18	1.04	22	2.2	Mapk10	Signaling
DMR14:8212001	14	8212001	8214000	2000	1	1.50E-07	-0.44	20	1	Mapk10	Signaling
DMR14:8231001	14	8231001	8233000	2000	1	1.30E-16	1.1	61	3.05	Mapk10	Signaling

DMR14:8250001	14	8250001	8252000	2000	2	2.30E-11	-0.46	55	2.75	Mapk10	Signaling
DMR14:8259001	14	8259001	8262000	3000	1	3.50E-09	-0.45	67	2.23	Mapk10	Signaling
DMR14:8301001	14	8301001	8307000	6000	1	2.80E-09	0.44	71	1.18	Mapk10	Signaling
DMR14:8329001	14	8329001	8331000	2000	1	2.20E-07	-0.35	32	1.6	Mapk10	Signaling
DMR14:8434001	14	8434001	8436000	2000	1	5.20E-07	0.42	29	1.45	Arhgap24;LOC108352702	
DMR14:8523001	14	8523001	8527000	4000	1	9.60E-08	-0.69	52	1.3	Arhgap24;LOC108352701	
DMR14:8545001	14	8545001	8547000	2000	1	9.50E-10	-0.48	29	1.45	Arhgap24	
DMR14:8554001	14	8554001	8562000	8000	1	7.40E-08	-0.49	126	1.57	Arhgap24	
DMR14:8564001	14	8564001	8565000	1000	1	1.50E-07	0.6	26	2.6	Arhgap24	
DMR14:8610001	14	8610001	8613000	3000	1	2.00E-11	-0.53	43	1.43	Arhgap24	
DMR14:9017001	14	9017001	9018000	1000	1	2.20E-11	0.78	29	2.9	RGD1560931	
DMR14:9023001	14	9023001	9025000	2000	2	2.00E-15	1.18	59	2.95	RGD1560931	
DMR14:9161001	14	9161001	9162000	1000	1	5.00E-08	-0.43	22	2.2	Wdfy3	
DMR14:9163001	14	9163001	9165000	2000	1	5.70E-07	-0.34	53	2.65	Wdfy3	
DMR14:9228001	14	9228001	9230000	2000	1	4.80E-08	-0.48	36	1.8	Wdfy3;LOC103693724	
DMR14:9240001	14	9240001	9243000	3000	1	4.20E-09	-0.47	54	1.8	Wdfy3;LOC103693724	
DMR14:9275001	14	9275001	9278000	3000	1	4.40E-08	-0.37	79	2.63	Wdfy3	
DMR14:9307001	14	9307001	9308000	1000	1	3.20E-08	-0.4	32	3.2	Wdfy3;LOC103693723	
DMR14:9333001	14	9333001	9334000	1000	1	1.40E-08	0.58	25	2.5	Wdfy3	
DMR14:9465001	14	9465001	9466000	1000	1	1.20E-08	-0.44	13	1.3	Cds1;LOC108352705	Transport
DMR14:10525001	14	10525001	10526000	1000	1	4.60E-10	0.78	22	2.2	LOC108352710;Hpse	Metabolism
DMR14:10555001	14	10555001	10559000	4000	2	2.70E-12	-0.5	50	1.25	Hpse	Metabolism
DMR14:10568001	14	10568001	10574000	6000	1	6.10E-12	0.87	129	2.15	Hpse;Coq2	Metabolism;Metabolism
DMR14:10596001	14	10596001	10598000	2000	1	2.10E-08	0.48	40	2	Coq2	Metabolism
DMR14:10747001	14	10747001	10748000	1000	1	2.80E-07	-0.41	19	1.9	Cops4;LOC102550950	Protease
DMR14:10816001	14	10816001	10817000	1000	1	5.10E-07	0.47	24	2.4	Lin54	
DMR14:10859001	14	10859001	10862000	3000	1	6.50E-07	-0.43	33	1.1	LOC100911553;Sec31a	Transport
DMR14:11119001	14	11119001	11123000	4000	1	3.00E-07	0.71	62	1.55	Tmem150c	
DMR14:11138001	14	11138001	11143000	5000	2	2.30E-12	-0.58	80	1.6	Tmem150c	
DMR14:11210001	14	11210001	11212000	2000	1	8.50E-07	0.44	25	1.25	Hnrnpdl	
DMR14:11281001	14	11281001	11285000	4000	1	4.80E-10	-0.4	74	1.85	Hnrnpd;LOC102557303;LOC681250	
DMR14:11345001	14	11345001	11346000	1000	1	3.20E-09	-0.52	15	1.5	RGD1562073	
DMR14:11356001	14	11356001	11357000	1000	1	4.80E-08	0.45	31	3.1	RGD1562073	
DMR14:11491001	14	11491001	11492000	1000	1	1.70E-12	0.88	21	2.1	Rasgef1b	Transcription
DMR14:11509001	14	11509001	11511000	2000	1	3.40E-08	0.56	49	2.45	Rasgef1b	Transcription
DMR14:11576001	14	11576001	11580000	4000	1	1.50E-24	1.11	68	1.7	Rasgef1b	Transcription
DMR14:11584001	14	11584001	11585000	1000	1	5.00E-07	-0.34	12	1.2	Rasgef1b	Transcription
DMR14:11614001	14	11614001	11615000	1000	1	1.30E-11	-0.42	26	2.6	Rasgef1b	Transcription
DMR14:11671001	14	11671001	11672000	1000	1	8.10E-14	-0.58	15	1.5	Rasgef1b;LOC305181	Transcription
DMR14:11789001	14	11789001	11790000	1000	1	2.60E-07	0.42	9	0.9	Rasgef1b;LOC102552280	Transcription
DMR14:11840001	14	11840001	11841000	1000	1	5.60E-10	-0.68	7	0.7	Rasgef1b	Transcription
DMR14:11881001	14	11881001	11882000	1000	1	3.80E-09	-0.54	14	1.4	Rasgef1b	Transcription
DMR14:11888001	14	11888001	11890000	2000	2	3.90E-09	-0.42	46	2.3	Rasgef1b	Transcription
DMR14:11954001	14	11954001	11957000	3000	1	2.90E-12	-0.64	57	1.9	Rasgef1b	Transcription
DMR14:12001001	14	12001001	12014000	13000	2	3.60E-12	-0.42	315	2.42	Rasgef1b	Transcription
DMR14:12016001	14	12016001	12020000	4000	1	1.10E-11	-0.74	78	1.95	Rasgef1b	Transcription
DMR14:12237001	14	12237001	12242000	5000	1	1.50E-08	-0.5	63	1.26	Prkg2	Signaling
DMR14:12253001	14	12253001	12255000	2000	1	1.10E-08	-0.46	79	3.95	Prkg2	Signaling
DMR14:12265001	14	12265001	12267000	2000	1	9.20E-07	0.45	26	1.3	Prkg2	Signaling
DMR14:12975001	14	12975001	12976000	1000	1	3.80E-10	0.61	17	1.7	Fgf5	Growth Factors
DMR14:13193001	14	13193001	13195000	2000	1	4.20E-07	-0.48	26	1.3	Antxr2	Cytoskeleton
DMR14:13270001	14	13270001	13271000	1000	1	1.90E-07	-0.37	5	0.5	Antxr2	Cytoskeleton
DMR14:13272001	14	13272001	13275000	3000	1	7.80E-07	0.58	47	1.57	Antxr2	Cytoskeleton
DMR14:13297001	14	13297001	13298000	1000	1	1.30E-11	0.66	17	1.7	Antxr2	Cytoskeleton
DMR14:13311001	14	13311001	13312000	1000	1	7.90E-07	0.4	8	0.8	Antxr2	Cytoskeleton
DMR14:14137001	14	14137001	14140000	3000	1	1.50E-11	-0.47	44	1.47	Paqr3;Bmp2k	Signaling;Signaling
DMR14:14142001	14	14142001	14146000	4000	1	1.30E-09	-0.41	61	1.52	Bmp2k	Signaling
DMR14:14437001	14	14437001	14438000	1000	1	1.40E-09	0.58	28	2.8	Fras1	
DMR14:14447001	14	14447001	14448000	1000	1	1.20E-07	-0.52	12	1.2	Fras1	
DMR14:14502001	14	14502001	14506000	4000	1	1.00E-07	0.34	55	1.38	Fras1	

DMR14:14509001	14	14509001	14512000	3000	1	2.60E-07	0.39	48	1.6	Fras1	
DMR14:14524001	14	14524001	14525000	1000	1	1.90E-07	-0.49	19	1.9	Fras1	
DMR14:14564001	14	14564001	14566000	2000	1	6.80E-07	-0.51	24	1.2	Fras1	
DMR14:14695001	14	14695001	14696000	1000	1	1.40E-07	0.61	15	1.5	Fras1	
DMR14:14720001	14	14720001	14724000	4000	1	7.00E-10	-0.47	54	1.35	Fras1	
DMR14:14835001	14	14835001	14836000	1000	1	4.40E-07	0.49	67	6.7	Fras1	
DMR14:15097001	14	15097001	15101000	4000	1	2.20E-10	-0.51	29	0.72	Cnot6l	Translation
DMR14:16277001	14	16277001	16279000	2000	1	5.70E-08	-0.67	29	1.45	Ccng2	Signaling
DMR14:16619001	14	16619001	16621000	2000	1	1.40E-07	0.51	44	2.2	Shroom3	Cytoskeleton
DMR14:16631001	14	16631001	16632000	1000	1	3.10E-07	-0.35	26	2.6	Shroom3;LOC103693731	Cytoskeleton
DMR14:16645001	14	16645001	16646000	1000	1	6.20E-07	0.63	47	4.7	Shroom3;LOC103693731	Cytoskeleton
DMR14:16651001	14	16651001	16657000	6000	1	1.60E-10	-0.57	124	2.07	Shroom3	Cytoskeleton
DMR14:16668001	14	16668001	16673000	5000	1	8.00E-13	-0.56	111	2.22	Shroom3	Cytoskeleton
DMR14:16716001	14	16716001	16719000	3000	1	1.80E-07	-0.39	48	1.6	Shroom3	Cytoskeleton
DMR14:16771001	14	16771001	16773000	2000	1	4.20E-08	-0.47	37	1.85	Shroom3;LOC108352720	Cytoskeleton
DMR14:16813001	14	16813001	16814000	1000	1	3.40E-16	0.94	25	2.5	Shroom3	Cytoskeleton
DMR14:16818001	14	16818001	16820000	2000	1	5.90E-11	-0.57	50	2.5	Shroom3	Cytoskeleton
DMR14:16846001	14	16846001	16847000	1000	1	3.20E-09	0.78	33	3.3	Shroom3	Cytoskeleton
DMR14:16987001	14	16987001	16989000	2000	1	4.00E-12	-0.54	23	1.15	Stbd1;Fam47e	
DMR14:17010001	14	17010001	17012000	2000	1	6.30E-08	0.65	54	2.7	Fam47e;LOC103692672	
DMR14:17209001	14	17209001	17211000	2000	2	6.50E-09	-0.59	22	1.1	Art3;Cxcl10	Transport;Growth Factors
DMR14:17238001	14	17238001	17239000	1000	1	5.00E-08	0.39	21	2.1	Cxcl9;Sdad1	Growth Factors
DMR14:17266001	14	17266001	17271000	5000	1	1.60E-08	-0.54	102	2.04	Sdad1	
DMR14:17284001	14	17284001	17285000	1000	1	3.80E-10	0.83	31	3.1	Sdad1;Naaa	Protease
DMR14:17307001	14	17307001	17308000	1000	1	2.20E-07	0.41	18	1.8	Naaa;Ppef2	Protease;Signaling
DMR14:17343001	14	17343001	17346000	3000	1	6.10E-10	0.92	76	2.53	Ppef2	Signaling
DMR14:17361001	14	17361001	17363000	2000	1	1.40E-09	-0.52	30	1.5	Uso1	Transport
DMR14:17419001	14	17419001	17421000	2000	2	2.60E-11	-0.61	9	0.45	Uso1	Transport
DMR14:17438001	14	17438001	17440000	2000	2	4.10E-07	-0.43	39	1.95	Uso1;G3bp2	Transport;Metabolism
DMR14:17461001	14	17461001	17462000	1000	1	3.90E-09	0.62	18	1.8	G3bp2;LOC108352721	Metabolism
DMR14:17470001	14	17470001	17472000	2000	1	4.10E-10	-0.43	48	2.4	G3bp2;LOC108352721	Metabolism
DMR14:17575001	14	17575001	17578000	3000	2	4.60E-11	-0.44	59	1.97	Cdkl2;LOC100911949	Signaling
DMR14:17974001	14	17974001	17977000	3000	1	9.30E-08	-0.39	27	0.9	Parm1	
DMR14:18044001	14	18044001	18045000	1000	1	9.30E-07	0.55	12	1.2	Parm1	
DMR14:18082001	14	18082001	18083000	1000	1	6.00E-08	-0.42	9	0.9	Parm1	
DMR14:18233001	14	18233001	18235000	2000	1	3.60E-07	0.53	24	1.2	Btc	Growth Factors
DMR14:18514001	14	18514001	18515000	1000	1	2.90E-07	-0.62	3	0.3	Areg	Growth Factors
DMR14:18539001	14	18539001	18540000	1000	1	8.30E-23	1.12	9	0.9	Areg	Growth Factors
DMR14:18642001	14	18642001	18645000	3000	1	2.50E-07	0.31	24	0.8	Epgn;Mthfd2l	Growth Factors
DMR14:18706001	14	18706001	18707000	1000	1	9.60E-11	0.68	14	1.4	Mthfd2l	
DMR14:18708001	14	18708001	18709000	1000	1	3.50E-07	-0.36	17	1.7	Mthfd2l	
DMR14:18868001	14	18868001	18870000	2000	1	3.20E-08	0.72	42	2.1	Cxcl6;LOC103692658	
DMR14:18895001	14	18895001	18897000	2000	1	8.60E-09	0.74	39	1.95	Cxcl6	
DMR14:18919001	14	18919001	18922000	3000	2	2.70E-10	0.84	65	2.17	Cxcl6	
DMR14:18981001	14	18981001	18982000	1000	1	2.40E-07	0.5	23	2.3	Rassf6	Cytoskeleton
DMR14:18991001	14	18991001	18995000	4000	2	6.60E-08	-0.43	68	1.7	Rassf6	Cytoskeleton
DMR14:19000001	14	19000001	19003000	3000	2	1.80E-13	0.95	53	1.77	Rassf6	Cytoskeleton
DMR14:19088001	14	19088001	19091000	3000	1	3.50E-08	-0.33	46	1.53	Afm	Transport
DMR14:19134001	14	19134001	19136000	2000	1	4.40E-10	0.72	46	2.3	Afm;Afp	Transport
DMR14:19404001	14	19404001	19407000	3000	1	1.30E-07	-0.47	34	1.13	Ankrd17	Immune
DMR14:20168001	14	20168001	20169000	1000	1	8.60E-07	0.44	20	2	Npffr2	Signaling
DMR14:20263001	14	20263001	20265000	2000	1	3.20E-07	-0.52	19	0.95	Gc	Transport
DMR14:20273001	14	20273001	20277000	4000	1	2.70E-09	-0.57	40	1	Gc	Transport
DMR14:20292001	14	20292001	20295000	3000	1	4.30E-09	0.69	40	1.33	Gc	Transport
DMR14:20518001	14	20518001	20519000	1000	1	1.50E-10	-0.54	6	0.6	Slc4a4	Transport
DMR14:20584001	14	20584001	20585000	1000	1	4.70E-09	0.38	6	0.6	Slc4a4;LOC103692660	Transport
DMR14:20657001	14	20657001	20660000	3000	1	3.40E-07	-0.45	47	1.57	Slc4a4	Transport
DMR14:20721001	14	20721001	20722000	1000	1	3.40E-07	-0.43	7	0.7	Slc4a4	Transport
DMR14:20733001	14	20733001	20741000	8000	1	5.90E-11	-0.42	90	1.12	Slc4a4	Transport
DMR14:20825001	14	20825001	20830000	5000	1	2.00E-08	-0.47	54	1.08	Slc4a4	Transport
DMR14:21032001	14	21032001	21033000	1000	1	6.70E-08	-0.38	18	1.8	Grsf1	Translation

DMR14:21051001	14	21051001	21052000	1000	1	2.20E-08	-0.38	26	2.6	Grsf1;Rufy3	Translation
DMR14:21153001	14	21153001	21155000	2000	1	3.00E-09	-0.48	31	1.55	Rufy3;Utp3;RGD1561381	Metabolism
DMR14:21349001	14	21349001	21350000	1000	1	4.80E-13	0.86	20	2	Prol1	
DMR14:21571001	14	21571001	21574000	3000	2	1.80E-07	-0.37	29	0.97	Smr3b;Vcsa2	Protease; Proteolysis
DMR14:21676001	14	21676001	21679000	3000	1	4.00E-07	-0.24	17	0.57	Smr3b	
DMR14:21686001	14	21686001	21687000	1000	1	5.40E-07	0.26	6	0.6	Smr3b	
DMR14:21717001	14	21717001	21719000	2000	1	8.80E-08	0.56	18	0.9	Smr3b;Smr3a	Protease; Proteolysis
DMR14:21740001	14	21740001	21742000	2000	1	8.40E-07	0.45	33	1.65	LOC364127;Cabs1	
DMR14:21884001	14	21884001	21886000	2000	1	2.40E-08	0.71	31	1.55	Dzip1-ps1;Prr27	
DMR14:21897001	14	21897001	21899000	2000	2	2.30E-14	1.18	36	1.8	Prr27;Csn1s2b	
DMR14:21902001	14	21902001	21904000	2000	1	6.90E-07	-0.39	16	0.8	Prr27;Csn1s2b	
DMR14:21922001	14	21922001	21923000	1000	1	6.00E-07	-0.36	2	0.2	Csn1s2b;Csn1s2a	
DMR14:22032001	14	22032001	22033000	1000	1	1.10E-12	-0.4	3	0.3	RGD1562394	
DMR14:22034001	14	22034001	22035000	1000	1	1.70E-07	0.36	9	0.9	RGD1562394	
DMR14:22067001	14	22067001	22069000	2000	1	5.70E-07	-0.29	70	3.5	ste2	
DMR14:22764001	14	22764001	22767000	3000	1	2.00E-09	-0.39	23	0.77	RGD1565664	
DMR14:22831001	14	22831001	22836000	5000	1	6.00E-07	0.43	47	0.94	Ugt2b10	
DMR14:23156001	14	23156001	23157000	1000	1	1.20E-13	0.71	15	1.5	Tmprss11g	Protease
DMR14:23187001	14	23187001	23188000	1000	1	8.10E-08	-0.64	8	0.8	Tmprss11g	Protease
DMR14:23215001	14	23215001	23217000	2000	1	2.30E-07	-0.49	25	1.25	Tmprss11g;Tmprss11a	Protease
DMR14:23266001	14	23266001	23267000	1000	1	3.80E-11	-0.52	13	1.3	Tmprss11a	Protease
DMR14:23403001	14	23403001	23406000	3000	1	3.70E-21	0.95	51	1.7	Tmprss11c	Protease
DMR14:23488001	14	23488001	23489000	1000	1	1.20E-09	0.59	18	1.8	LOC103692776;Gnrhr	Signaling
DMR14:23977001	14	23977001	23978000	1000	1	4.10E-07	-0.53	3	0.3	Tmprss11f	Protease
DMR14:24057001	14	24057001	24060000	3000	1	8.10E-07	-0.32	24	0.8	Tmprss11f;LOC100364381	Protease
DMR14:24105001	14	24105001	24106000	1000	1	2.30E-07	0.48	15	1.5	Tmprss11b	
DMR14:25617001	14	25617001	25618000	1000	1	1.40E-08	-0.56	3	0.3	Epha5	Receptor
DMR14:25634001	14	25634001	25635000	1000	1	1.60E-07	-0.45	9	0.9	Epha5	Receptor
DMR14:25776001	14	25776001	25777000	1000	1	3.40E-07	0.52	17	1.7	Epha5	Receptor
DMR14:25841001	14	25841001	25842000	1000	1	7.10E-07	0.65	11	1.1	Epha5	Receptor
DMR14:28394001	14	28394001	28397000	3000	2	1.50E-16	0.99	50	1.67	Adgrl3	Signaling
DMR14:28440001	14	28440001	28442000	2000	2	4.50E-12	0.45	17	0.85	Adgrl3	Signaling
DMR14:28811001	14	28811001	28812000	1000	1	8.40E-10	-0.6	11	1.1	Adgrl3	Signaling
DMR14:28839001	14	28839001	28840000	1000	1	2.80E-07	0.48	13	1.3	Adgrl3	Signaling
DMR14:28904001	14	28904001	28906000	2000	1	7.50E-10	0.82	28	1.4	Adgrl3	Signaling
DMR14:30343001	14	30343001	30345000	2000	1	8.80E-08	0.45	28	1.4	Trnat-cgu	
DMR14:33039001	14	33039001	33040000	1000	1	4.00E-07	-0.49	26	2.6	Igfbp7;LOC103692786;LOC103692785;Trnac-gca	
DMR14:33057001	14	33057001	33058000	1000	1	4.40E-14	-0.49	21	2.1	Igfbp7	
DMR14:33131001	14	33131001	33133000	2000	1	5.10E-07	-0.4	40	2	Noa1;Rest	Transcription
DMR14:33136001	14	33136001	33140000	4000	1	1.50E-10	-0.45	86	2.15	Rest	Transcription
DMR14:33386001	14	33386001	33387000	1000	1	3.30E-07	0.37	4	0.4	Thegl	
DMR14:33396001	14	33396001	33397000	1000	1	3.30E-07	0.38	19	1.9	Thegl	
DMR14:33415001	14	33415001	33416000	1000	1	1.70E-10	0.75	31	3.1	Thegl	
DMR14:33661001	14	33661001	33664000	3000	3	5.70E-17	1.3	165	5.5	RGD1311575	
DMR14:33680001	14	33680001	33682000	2000	1	5.00E-09	-0.42	28	1.4	RGD1311575	
DMR14:33684001	14	33684001	33690000	6000	1	3.60E-09	-0.37	85	1.42	RGD1311575	
DMR14:33693001	14	33693001	33695000	2000	1	5.80E-07	-0.36	40	2	RGD1311575	
DMR14:33705001	14	33705001	33706000	1000	1	1.60E-07	-0.5	19	1.9	RGD1311575;LOC108352738	
DMR14:33767001	14	33767001	33768000	1000	1	1.10E-12	-0.66	9	0.9	RGD1311575	
DMR14:34184001	14	34184001	34186000	2000	1	8.50E-07	-0.41	18	0.9	Exoc1;LOC686911	Transcription
DMR14:34374001	14	34374001	34375000	1000	1	3.40E-07	-0.32	16	1.6	Nmu;Pdcl2	Epigenetic
DMR14:34384001	14	34384001	34385000	1000	1	2.50E-12	1.06	26	2.6	Nmu;Pdcl2	Epigenetic
DMR14:34403001	14	34403001	34405000	2000	1	1.90E-08	-0.39	21	1.05	Pdcl2	Epigenetic
DMR14:34415001	14	34415001	34417000	2000	1	5.80E-13	-0.58	26	1.3	Clock	
DMR14:34446001	14	34446001	34448000	2000	1	7.90E-07	-0.43	24	1.2	Clock	
DMR14:34457001	14	34457001	34458000	1000	1	1.20E-08	-0.45	13	1.3	Clock	
DMR14:34466001	14	34466001	34467000	1000	1	4.60E-13	-0.7	10	1	Clock	
DMR14:34576001	14	34576001	34582000	6000	1	2.10E-10	-0.4	61	1.02	Srd5a3	
DMR14:35066001	14	35066001	35069000	3000	1	2.30E-07	-0.37	48	1.6	Kit	Receptor
DMR14:35119001	14	35119001	35121000	2000	1	5.40E-07	0.38	37	1.85	Kit	Receptor
DMR14:35549001	14	35549001	35551000	2000	1	3.00E-07	-0.41	39	1.95	Pdgfra;LOC102554024	Receptor

DMR14:35692001	14	35692001	35694000	2000	2	3.50E-09	-0.52	16	0.8	LOC102556465;Chic2	
DMR14:36039001	14	36039001	36041000	2000	1	1.10E-07	-0.38	45	2.25	Lnx1	
DMR14:36084001	14	36084001	36086000	2000	1	4.30E-07	-0.33	30	1.5	Lnx1	
DMR14:36087001	14	36087001	36089000	2000	1	1.60E-10	-0.52	35	1.75	Lnx1	
DMR14:36116001	14	36116001	36117000	1000	1	1.70E-08	-0.52	15	1.5	Lnx1	
DMR14:36302001	14	36302001	36305000	3000	1	2.00E-11	-0.6	23	0.77	Scfd2	Transport
DMR14:36314001	14	36314001	36317000	3000	1	8.60E-08	-0.35	29	0.97	Scfd2	Transport
DMR14:36318001	14	36318001	36321000	3000	1	2.50E-08	0.29	43	1.43	Scfd2	Transport
DMR14:36340001	14	36340001	36343000	3000	1	2.00E-09	-0.79	31	1.03	Scfd2	Transport
DMR14:36482001	14	36482001	36484000	2000	1	6.30E-07	-0.38	27	1.35	Scfd2	Transport
DMR14:36506001	14	36506001	36511000	5000	1	4.40E-07	-0.28	58	1.16	Scfd2	Transport
DMR14:36545001	14	36545001	36548000	3000	1	8.20E-07	-0.38	53	1.77	Scfd2;Rasl11b	Transport
DMR14:36675001	14	36675001	36679000	4000	1	3.60E-07	0.63	77	1.93	Dancr;Usp46	Protease
DMR14:36714001	14	36714001	36718000	4000	1	4.30E-07	0.49	54	1.35	Usp46;LOC102554514	Protease
DMR14:36722001	14	36722001	36723000	1000	1	7.70E-08	0.67	26	2.6	Usp46	Protease
DMR14:36725001	14	36725001	36726000	1000	1	1.90E-09	0.58	16	1.6	Usp46	Protease
DMR14:37071001	14	37071001	37074000	3000	1	8.60E-07	-0.41	23	0.77	Spata18	
DMR14:37202001	14	37202001	37206000	4000	1	1.80E-07	-0.42	70	1.75	Dcun1d4	
DMR14:37326001	14	37326001	37328000	2000	1	2.50E-07	-0.36	24	1.2	Cwh43	
DMR14:37347001	14	37347001	37349000	2000	1	1.50E-08	-0.71	9	0.45	Cwh43	
DMR14:37628001	14	37628001	37629000	1000	1	2.00E-11	-0.46	15	1.5	Fryl	Cytoskeleton
DMR14:37645001	14	37645001	37647000	2000	1	2.10E-07	-0.53	44	2.2	Fryl	Cytoskeleton
DMR14:37700001	14	37700001	37704000	4000	1	3.00E-07	0.37	115	2.88	Fryl	Cytoskeleton
DMR14:37765001	14	37765001	37767000	2000	1	4.20E-07	0.59	44	2.2	Fryl;Zar1;Slc10a4	Cytoskeleton;Transport
DMR14:37778001	14	37778001	37781000	3000	1	4.50E-09	0.78	34	1.13	Slc10a4	Transport
DMR14:37934001	14	37934001	37938000	4000	1	1.80E-10	-0.56	56	1.4	Tec	
DMR14:37950001	14	37950001	37952000	2000	1	1.50E-11	-0.48	22	1.1	Tec	
DMR14:38039001	14	38039001	38040000	1000	1	5.80E-11	0.8	33	3.3	Txk	
DMR14:38094001	14	38094001	38097000	3000	1	6.60E-09	-0.79	41	1.37	Txk;Nipal1	
DMR14:38113001	14	38113001	38115000	2000	2	4.00E-10	0.86	40	2	Nipal1	
DMR14:38289001	14	38289001	38290000	1000	1	3.10E-07	-0.36	15	1.5	Corin	Binding Proteins
DMR14:38363001	14	38363001	38365000	2000	1	1.60E-08	-0.39	36	1.8	Corin;LOC680377;LOC1025502 73	Binding Proteins
DMR14:38384001	14	38384001	38385000	1000	1	2.00E-08	-0.37	16	1.6	Corin	Binding Proteins
DMR14:38444001	14	38444001	38445000	1000	1	1.90E-07	-0.34	12	1.2	Corin	Binding Proteins
DMR14:38530001	14	38530001	38531000	1000	1	2.70E-07	0.33	18	1.8	Atp10d	Transport
DMR14:38816001	14	38816001	38817000	1000	1	6.90E-08	0.52	18	1.8	Gabrb1	Ion Channel
DMR14:38839001	14	38839001	38840000	1000	1	1.40E-08	-0.38	3	0.3	Gabrb1;Olr1604-ps	Ion Channel
DMR14:38852001	14	38852001	38858000	6000	1	1.40E-08	-0.3	70	1.17	Gabrb1	Ion Channel
DMR14:38983001	14	38983001	38984000	1000	1	6.30E-11	0.49	16	1.6	Gabrb1	Ion Channel
DMR14:39015001	14	39015001	39016000	1000	1	1.20E-10	0.5	22	2.2	Gabrb1	Ion Channel
DMR14:39291001	14	39291001	39293000	2000	1	5.10E-08	0.52	20	1	Cox7b2	Metabolism
DMR14:39298001	14	39298001	39299000	1000	1	9.20E-07	0.43	8	0.8	Cox7b2	Metabolism
DMR14:39353001	14	39353001	39355000	2000	1	1.50E-07	-0.48	14	0.7	Cox7b2	Metabolism
DMR14:41711001	14	41711001	41712000	1000	1	1.40E-07	-0.47	9	0.9	Grxcr1	Metabolism
DMR14:42161001	14	42161001	42163000	2000	1	8.30E-13	0.75	40	2	Atp8a1	Transport
DMR14:42446001	14	42446001	42448000	2000	1	3.50E-10	-0.42	34	1.7	Bend4;LOC108352852	
DMR14:42494001	14	42494001	42497000	3000	1	3.30E-07	-0.34	25	0.83	Slc30a9	Transport
DMR14:42560001	14	42560001	42562000	2000	1	2.60E-09	-0.39	20	1	Tmem33	
DMR14:42709001	14	42709001	42711000	2000	1	1.10E-09	-0.61	35	1.75	Phox2b	Development
DMR14:42755001	14	42755001	42757000	2000	1	3.70E-10	0.77	40	2	Limch1	Cytoskeleton
DMR14:42863001	14	42863001	42864000	1000	1	4.20E-07	-0.56	6	0.6	Limch1	Cytoskeleton
DMR14:42933001	14	42933001	42934000	1000	1	2.70E-07	0.63	11	1.1	Limch1	Cytoskeleton
DMR14:42961001	14	42961001	42963000	2000	1	6.40E-08	-0.32	35	1.75	Limch1	Cytoskeleton
DMR14:43284001	14	43284001	43286000	2000	1	2.80E-08	-0.45	29	1.45	Apbb2	
DMR14:43345001	14	43345001	43346000	1000	1	3.00E-07	-0.43	11	1.1	Apbb2	
DMR14:43349001	14	43349001	43352000	3000	1	9.30E-08	-0.35	45	1.5	Apbb2	
DMR14:43379001	14	43379001	43380000	1000	1	6.10E-10	0.6	12	1.2	Apbb2	
DMR14:43688001	14	43688001	43689000	1000	1	4.80E-10	-0.57	8	0.8	RGD1563570	
DMR14:43708001	14	43708001	43709000	1000	1	2.60E-11	-0.47	13	1.3	Rbm47	Metabolism
DMR14:43718001	14	43718001	43720000	2000	1	5.70E-11	-0.71	25	1.25	Rbm47	Metabolism
DMR14:43728001	14	43728001	43729000	1000	1	4.20E-07	-0.53	3	0.3	Rbm47	Metabolism

DMR14:43756001	14	43756001	43759000	3000	1	1.90E-08	-0.41	31	1.03	Rbm47	Metabolism
DMR14:43777001	14	43777001	43779000	2000	1	3.70E-07	-0.42	36	1.8	Rbm47	Metabolism
DMR14:43786001	14	43786001	43787000	1000	1	1.50E-07	-0.37	10	1	Rbm47	Metabolism
DMR14:43830001	14	43830001	43832000	2000	1	4.50E-09	0.74	71	3.55	Rbm47	Metabolism
DMR14:43849001	14	43849001	43856000	7000	1	5.40E-07	-0.35	68	0.97	Rbm47	Metabolism
DMR14:44091001	14	44091001	44092000	1000	1	1.10E-07	-0.34	13	1.3	N4bp2	
DMR14:44429001	14	44429001	44430000	1000	1	2.60E-09	-0.52	8	0.8	Smim14	
DMR14:44458001	14	44458001	44460000	2000	1	1.00E-07	-0.47	22	1.1	Smim14	
DMR14:44487001	14	44487001	44488000	1000	1	2.90E-07	-0.62	13	1.3	Ugdh	Metabolism
DMR14:44556001	14	44556001	44559000	3000	1	2.30E-08	-0.41	118	3.93	Klb;LOC108352748	
DMR14:44911001	14	44911001	44914000	3000	1	3.40E-07	0.57	38	1.27	Tmem156	
DMR14:44967001	14	44967001	44972000	5000	1	5.80E-08	-0.49	59	1.18	Fam114a1;LOC108352749	
DMR14:45010001	14	45010001	45011000	1000	1	1.90E-07	0.57	15	1.5	Fam114a1	
DMR14:45033001	14	45033001	45035000	2000	1	2.20E-10	-0.53	30	1.5	Tlr6;LOC108352750	
DMR14:45333001	14	45333001	45334000	1000	1	9.10E-10	-0.5	5	0.5	LOC103692863;Mir328b	
DMR14:45747001	14	45747001	45749000	2000	1	3.10E-07	-0.35	26	1.3	Tbc1d1	Signaling
DMR14:45856001	14	45856001	45859000	3000	1	4.60E-08	0.43	31	1.03	Pgm2	Metabolism
DMR14:45866001	14	45866001	45867000	1000	1	1.40E-07	-0.36	4	0.4	Pgm2	Metabolism
DMR14:46630001	14	46630001	46640000	10000	8	2.90E-13	1.02	831	8.31	LOC102554740;Rn45s;Rn18s;Rn5-8s;Rn28s	
DMR14:46656001	14	46656001	46662000	6000	2	2.90E-13	0.47	144	2.4	Rn45s;Rn5-8s;Rn28s	
DMR14:46664001	14	46664001	46666000	2000	2	1.70E-14	-0.53	29	1.45	Rn45s;Rn28s	
DMR14:47203001	14	47203001	47205000	2000	1	3.00E-08	0.49	20	1	LOC108352753;Slc25a52;LOC108352830	
DMR14:48618001	14	48618001	48623000	5000	1	3.20E-11	-0.46	45	0.9	LOC685935;Dthd1	
DMR14:48748001	14	48748001	48752000	4000	1	3.60E-07	-0.28	39	0.98	Arap2	Signaling
DMR14:53153001	14	53153001	53154000	1000	1	9.10E-07	0.54	7	0.7	RGD1566292	
DMR14:53860001	14	53860001	53862000	2000	1	7.60E-08	0.36	22	1.1	Vom1r-ps103	
DMR14:53870001	14	53870001	53874000	4000	1	1.40E-10	-0.61	26	0.65	Vom1r-ps103	
DMR14:54671001	14	54671001	54672000	1000	1	3.10E-12	0.79	12	1.2	Pcdh7	Cytoskeleton
DMR14:54732001	14	54732001	54734000	2000	1	4.50E-10	-0.8	76	3.8	Pcdh7	Cytoskeleton
DMR14:54760001	14	54760001	54761000	1000	1	7.10E-09	0.42	14	1.4	Pcdh7	Cytoskeleton
DMR14:54777001	14	54777001	54778000	1000	1	5.40E-09	-0.65	12	1.2	Pcdh7	Cytoskeleton
DMR14:54814001	14	54814001	54815000	1000	1	1.30E-12	0.52	13	1.3	Pcdh7	Cytoskeleton
DMR14:55022001	14	55022001	55024000	2000	1	6.50E-07	-0.51	10	0.5	Pcdh7	Cytoskeleton
DMR14:55044001	14	55044001	55048000	4000	1	5.00E-07	0.55	57	1.43	Pcdh7	Cytoskeleton
DMR14:58872001	14	58872001	58875000	3000	1	1.10E-07	-0.28	29	0.97	RGD1562755	
DMR14:59216001	14	59216001	59218000	2000	1	6.90E-08	-0.37	32	1.6	Stim2	
DMR14:59241001	14	59241001	59243000	2000	1	5.80E-07	-0.33	24	1.2	Stim2	
DMR14:59288001	14	59288001	59289000	1000	1	5.90E-08	-0.47	13	1.3	Stim2	
DMR14:59789001	14	59789001	59792000	3000	1	1.80E-13	-0.54	29	0.97	Rbpj;LOC108352754	Transcription
DMR14:59845001	14	59845001	59848000	3000	1	8.10E-07	0.32	31	1.03	Rbpj	Transcription
DMR14:59849001	14	59849001	59850000	1000	1	1.50E-07	0.41	12	1.2	Rbpj	Transcription
DMR14:60046001	14	60046001	60048000	2000	1	5.00E-08	0.44	21	1.05	Smim20	
DMR14:60076001	14	60076001	60080000	4000	1	8.10E-16	0.9	113	2.83	Smim20	
DMR14:60133001	14	60133001	60134000	1000	1	1.10E-10	-0.42	17	1.7	LOC108352755;Sel1l3	
DMR14:60161001	14	60161001	60162000	1000	1	2.10E-09	-0.5	19	1.9	Sel1l3	
DMR14:60210001	14	60210001	60211000	1000	1	2.50E-07	-0.65	2	0.2	Sel1l3	
DMR14:60788001	14	60788001	60789000	1000	1	4.00E-08	0.4	17	1.7	Lgi2	
DMR14:60791001	14	60791001	60797000	6000	1	7.60E-07	0.43	120	2	Lgi2	
DMR14:60906001	14	60906001	60908000	2000	1	1.40E-13	0.31	16	0.8	Ccdc149	
DMR14:60921001	14	60921001	60923000	2000	1	6.70E-07	0.46	33	1.65	Ccdc149	
DMR14:60958001	14	60958001	60960000	2000	1	9.30E-28	1.28	71	3.55	Ccdc149;Sod3	Metabolism
DMR14:60963001	14	60963001	60965000	2000	1	2.50E-07	-0.57	26	1.3	Ccdc149;Sod3	Metabolism
DMR14:61968001	14	61968001	61970000	2000	1	8.60E-07	-0.39	10	0.5	Kctd8	
DMR14:62297001	14	62297001	62298000	1000	1	8.00E-14	0.78	22	2.2	Vom1r-ps31	
DMR14:62654001	14	62654001	62657000	3000	1	3.20E-11	-0.42	26	0.87	Gnpda2	Metabolism
DMR14:64691001	14	64691001	64693000	2000	1	6.30E-07	-0.4	24	1.2	Adgra3	Signaling
DMR14:65566001	14	65566001	65567000	1000	1	1.30E-07	-0.5	4	0.4	Kcnip4	
DMR14:65659001	14	65659001	65660000	1000	1	9.80E-14	0.67	18	1.8	Kcnip4	
DMR14:65694001	14	65694001	65696000	2000	1	8.60E-09	0.51	29	1.45	Kcnip4	
DMR14:65769001	14	65769001	65770000	1000	1	3.60E-09	0.43	4	0.4	Kcnip4	

DMR14:66193001	14	66193001	66195000	2000	1	1.40E-07	0.32	18	0.9	Kcnip4	
DMR14:66205001	14	66205001	66206000	1000	1	1.00E-07	-0.5	7	0.7	Kcnip4	
DMR14:66322001	14	66322001	66323000	1000	1	2.10E-07	0.42	7	0.7	Kcnip4;LOC103693056	
DMR14:66568001	14	66568001	66571000	3000	1	4.60E-07	0.51	28	0.93	Kcnip4	
DMR14:66589001	14	66589001	66591000	2000	1	4.90E-09	-0.54	22	1.1	Kcnip4	
DMR14:66666001	14	66666001	66667000	1000	1	2.30E-07	-0.55	5	0.5	Kcnip4	
DMR14:66760001	14	66760001	66762000	2000	1	8.70E-08	-0.45	19	0.95	Pacrgl	
DMR14:67121001	14	67121001	67123000	2000	1	3.30E-07	0.46	18	0.9	Slit2	
DMR14:69755001	14	69755001	69762000	7000	1	1.00E-06	-0.24	64	0.91	Lcorl	Transcription
DMR14:70099001	14	70099001	70100000	1000	1	3.20E-08	-0.45	7	0.7	Med28;LOC103693066;Lap3	Transcription;Protease
DMR14:70818001	14	70818001	70820000	2000	1	3.60E-07	-0.31	20	1	Ldb2	Transcription
DMR14:71052001	14	71052001	71054000	2000	1	2.60E-08	0.46	5	0.25	Ldb2	Transcription
DMR14:71059001	14	71059001	71062000	3000	1	2.40E-07	-0.47	22	0.73	Ldb2	Transcription
DMR14:71538001	14	71538001	71541000	3000	1	6.10E-08	-0.44	23	0.77	Prom1	
DMR14:71567001	14	71567001	71569000	2000	1	1.90E-07	-0.55	28	1.4	Prom1	
DMR14:71579001	14	71579001	71586000	7000	2	6.60E-11	-0.6	61	0.87	Prom1	
DMR14:71610001	14	71610001	71612000	2000	2	1.50E-10	-0.47	14	0.7	Prom1	
DMR14:71637001	14	71637001	71638000	1000	1	5.10E-07	-0.44	20	2	Prom1;Fgfbp1	
DMR14:71711001	14	71711001	71713000	2000	1	9.00E-07	0.45	21	1.05	Apex2l1;Cd38	Metabolism
DMR14:71904001	14	71904001	71906000	2000	1	7.60E-07	-0.55	21	1.05	Cc2d2a	
DMR14:71946001	14	71946001	71948000	2000	1	5.40E-07	-0.33	15	0.75	Cc2d2a	
DMR14:71975001	14	71975001	71977000	2000	1	3.60E-11	-0.51	37	1.85	Cc2d2a	
DMR14:72385001	14	72385001	72386000	1000	1	3.00E-09	-0.36	9	0.9	Cpeb2	Translation
DMR14:72954001	14	72954001	72956000	2000	1	2.00E-08	-0.37	14	0.7	Pp2d1	Signaling
DMR14:73760001	14	73760001	73764000	4000	1	3.70E-09	-0.4	53	1.32	Bod1l1	
DMR14:73801001	14	73801001	73802000	1000	1	1.30E-08	-0.39	18	1.8	Bod1l1	
DMR14:73884001	14	73884001	73886000	2000	1	1.00E-12	0.78	24	1.2	LOC100361803;Rab28	
DMR14:73918001	14	73918001	73919000	1000	1	7.80E-14	-0.8	7	0.7	Rab28	
DMR14:76674001	14	76674001	76676000	2000	1	2.50E-07	0.42	23	1.15	Clnk	Cytoskeleton
DMR14:76685001	14	76685001	76687000	2000	1	5.30E-07	0.6	30	1.5	Clnk	Cytoskeleton
DMR14:76695001	14	76695001	76697000	2000	1	9.90E-12	0.56	28	1.4	Clnk	Cytoskeleton
DMR14:76798001	14	76798001	76799000	1000	1	2.50E-08	0.58	8	0.8	Clnk	Cytoskeleton
DMR14:76823001	14	76823001	76824000	1000	1	1.50E-08	-0.42	9	0.9	Clnk	Cytoskeleton
DMR14:76875001	14	76875001	76877000	2000	1	1.10E-07	-0.37	22	1.1	Zfp518b;LOC498391	Transcription
DMR14:76878001	14	76878001	76880000	2000	1	1.40E-19	1.1	32	1.6	Zfp518b;LOC498391	Transcription
DMR14:76914001	14	76914001	76915000	1000	1	3.50E-12	0.96	29	2.9	Rrm1-ps1;LOC108352833	
DMR14:77033001	14	77033001	77034000	1000	1	3.30E-07	0.61	17	1.7	Wdr1	Cytoskeleton
DMR14:77070001	14	77070001	77071000	1000	1	2.70E-08	-0.41	16	1.6	Slc2a9	
DMR14:77180001	14	77180001	77185000	5000	1	5.50E-09	0.59	50	1	Slc2a9	
DMR14:77333001	14	77333001	77334000	1000	1	3.50E-10	0.62	13	1.3	Zbtb49	Transcription
DMR14:77348001	14	77348001	77349000	1000	1	4.50E-07	0.61	18	1.8	Zbtb49	Transcription
DMR14:77424001	14	77424001	77426000	2000	1	5.80E-09	-0.57	16	0.8	Stx18	Transcription
DMR14:77890001	14	77890001	77892000	2000	1	3.40E-07	-0.43	8	0.4	Stk32b	Signaling
DMR14:78103001	14	78103001	78105000	2000	1	5.50E-08	-0.61	25	1.25	Stk32b;LOC102554915	Signaling
DMR14:78128001	14	78128001	78130000	2000	1	1.70E-16	0.45	19	0.95	Evc2;LOC102553293	
DMR14:78133001	14	78133001	78137000	4000	1	1.50E-09	0.6	52	1.3	Evc2;LOC102553293	
DMR14:78149001	14	78149001	78150000	1000	1	3.70E-10	-0.44	16	1.6	Evc2	
DMR14:78172001	14	78172001	78174000	2000	1	5.10E-07	0.44	20	1	Evc2	
DMR14:78175001	14	78175001	78176000	1000	1	2.20E-07	0.57	16	1.6	Evc2	
DMR14:78193001	14	78193001	78195000	2000	1	1.50E-07	0.48	23	1.15	Evc2	
DMR14:78215001	14	78215001	78219000	4000	1	1.20E-07	0.41	46	1.15	Evc2;Evc	
DMR14:78424001	14	78424001	78426000	2000	1	4.20E-07	-0.44	51	2.55	Jakmip1	
DMR14:78476001	14	78476001	78477000	1000	1	3.10E-08	-0.39	15	1.5	Jakmip1	
DMR14:78654001	14	78654001	78655000	1000	1	8.40E-10	0.48	18	1.8	Wfs1	
DMR14:79003001	14	79003001	79004000	1000	1	6.50E-07	-0.59	2	0.2	Bloc1s4	
DMR14:79454001	14	79454001	79455000	1000	1	3.50E-08	-0.34	32	3.2	Ccdc96;Tada2b;Grpel1	Transcription;Transport
DMR14:79542001	14	79542001	79546000	4000	1	1.70E-07	0.34	80	2	Sorcs2	Transport
DMR14:79577001	14	79577001	79580000	3000	1	9.10E-09	-0.48	41	1.37	Sorcs2	Transport
DMR14:79610001	14	79610001	79612000	2000	1	6.00E-07	-0.6	27	1.35	Sorcs2	Transport
DMR14:79769001	14	79769001	79773000	4000	1	9.30E-09	0.35	78	1.95	Sorcs2	Transport
DMR14:79818001	14	79818001	79819000	1000	1	1.50E-07	0.62	15	1.5	Sorcs2	Transport

DMR14:79904001	14	79904001	79905000	1000	1	2.60E-09	0.55	15	1.5	Sorcs2	Transport
DMR14:79906001	14	79906001	79908000	2000	2	7.00E-07	0.5	25	1.25	Sorcs2	Transport
DMR14:79925001	14	79925001	79927000	2000	1	1.90E-08	0.57	40	2	Afap1	
DMR14:79940001	14	79940001	79946000	6000	2	1.40E-10	-0.57	106	1.77	Afap1	
DMR14:80047001	14	80047001	80050000	3000	1	3.50E-08	0.49	36	1.2	Ablim2	Cytoskeleton
DMR14:80101001	14	80101001	80102000	1000	1	2.90E-11	0.74	12	1.2	Ablim2	Cytoskeleton
DMR14:80106001	14	80106001	80108000	2000	1	4.60E-07	-0.28	28	1.4	Ablim2	Cytoskeleton
DMR14:80217001	14	80217001	80218000	1000	1	2.90E-12	0.39	7	0.7	Sh3tc1	
DMR14:80219001	14	80219001	80221000	2000	1	1.70E-08	0.57	34	1.7	Sh3tc1	
DMR14:80228001	14	80228001	80229000	1000	1	1.80E-07	0.45	13	1.3	Sh3tc1	
DMR14:80304001	14	80304001	80307000	3000	1	8.10E-08	0.52	27	0.9	Acox3	Metabolism
DMR14:80345001	14	80345001	80350000	5000	2	6.00E-09	-0.4	39	0.78	Acox3	Metabolism
DMR14:80386001	14	80386001	80387000	1000	1	9.50E-07	0.39	19	1.9	Trmt44	
DMR14:80422001	14	80422001	80424000	2000	1	3.10E-09	0.5	32	1.6	Cpz	Protease
DMR14:80426001	14	80426001	80427000	1000	1	7.00E-07	0.4	13	1.3	Cpz	Protease
DMR14:80531001	14	80531001	80533000	2000	1	5.00E-07	-0.38	33	1.65	Hmx1	Development
DMR14:80940001	14	80940001	80947000	7000	1	1.20E-10	0.56	101	1.44	Dok7	
DMR14:80966001	14	80966001	80968000	2000	1	2.10E-08	0.38	26	1.3	Dok7;Hgfac;Rgs12	Protease
DMR14:81033001	14	81033001	81035000	2000	1	7.60E-08	0.55	20	1	Rgs12;LOC108352780	
DMR14:81185001	14	81185001	81186000	1000	1	8.50E-07	-0.39	14	1.4	Htt	
DMR14:81397001	14	81397001	81399000	2000	1	1.40E-10	-0.46	27	1.35	Add1	Cytoskeleton
DMR14:81423001	14	81423001	81426000	3000	1	9.40E-07	-0.48	37	1.23	Add1;Sh3bp2	Cytoskeleton
DMR14:81785001	14	81785001	81786000	1000	1	4.30E-07	-0.3	7	0.7	Zfyve28	
DMR14:82041001	14	82041001	82042000	1000	1	1.20E-12	0.66	13	1.3	Poln;Nat8l	Transcription;Metabolism
DMR14:82263001	14	82263001	82266000	3000	1	1.40E-07	0.4	42	1.4	Letm1;Fgfr3	Transport;Receptor
DMR14:82283001	14	82283001	82285000	2000	1	1.30E-08	0.6	29	1.45	Fgfr3	Receptor
DMR14:82690001	14	82690001	82691000	1000	1	6.40E-08	-0.42	10	1	Maea	
DMR14:82702001	14	82702001	82704000	2000	1	9.20E-08	-0.37	18	0.9	Maea	
DMR14:82952001	14	82952001	82954000	2000	1	3.40E-13	-0.51	27	1.35	Slc5a1	Transport
DMR14:82973001	14	82973001	82974000	1000	1	1.10E-07	-0.33	22	2.2	Slc5a1	Transport
DMR14:83267001	14	83267001	83268000	1000	1	5.40E-08	-0.34	12	1.2	Prr14l	
DMR14:83280001	14	83280001	83281000	1000	1	2.60E-09	-0.36	15	1.5	Prr14l	
DMR14:83608001	14	83608001	83609000	1000	1	2.30E-11	-0.48	25	2.5	Limk2;LOC102546870	
DMR14:83736001	14	83736001	83737000	1000	1	8.50E-09	0.4	21	2.1	Pla2g3;Inpp5j	Signaling
DMR14:83739001	14	83739001	83741000	2000	2	8.30E-19	1.08	52	2.6	Pla2g3;Inpp5j	Signaling
DMR14:83752001	14	83752001	83754000	2000	1	8.70E-09	0.56	54	2.7	Inpp5j;Selm;Smtn	Signaling
DMR14:83762001	14	83762001	83765000	3000	1	2.50E-10	0.72	95	3.17	Selm;Smtn	
DMR14:83778001	14	83778001	83779000	1000	1	4.90E-19	1.2	27	2.7	Smtn	
DMR14:83899001	14	83899001	83901000	2000	1	2.00E-07	0.62	15	0.75	Morc2	
DMR14:83912001	14	83912001	83915000	3000	2	3.70E-14	0.85	25	0.83	Morc2	
DMR14:83916001	14	83916001	83917000	1000	1	2.40E-12	0.79	10	1	Morc2	
DMR14:83927001	14	83927001	83929000	2000	1	3.30E-08	0.64	26	1.3	Morc2	
DMR14:83930001	14	83930001	83931000	1000	1	1.70E-07	0.6	11	1.1	Morc2	
DMR14:83943001	14	83943001	83944000	1000	1	1.30E-08	0.7	12	1.2	Osbp2	
DMR14:83946001	14	83946001	83947000	1000	1	1.70E-08	0.62	27	2.7	Osbp2	
DMR14:83953001	14	83953001	83956000	3000	1	4.70E-07	0.42	53	1.77	Osbp2	
DMR14:83982001	14	83982001	83983000	1000	1	2.10E-09	-0.45	14	1.4	Osbp2	
DMR14:84028001	14	84028001	84029000	1000	1	2.90E-07	0.58	3	0.3	Osbp2;LOC108352785	
DMR14:84063001	14	84063001	84065000	2000	1	6.10E-08	0.4	25	1.25	Osbp2;LOC108352785	
DMR14:84067001	14	84067001	84070000	3000	1	4.40E-07	0.3	51	1.7	Osbp2	
DMR14:84098001	14	84098001	84101000	3000	1	2.30E-07	0.52	24	0.8	Osbp2	
DMR14:84251001	14	84251001	84258000	7000	1	2.10E-07	-0.32	84	1.2	Gal3st1	Transport
DMR14:84347001	14	84347001	84351000	4000	1	1.80E-08	-0.53	65	1.62	LOC102549281;Sec14l2	
DMR14:84582001	14	84582001	84584000	2000	1	8.10E-13	-0.49	28	1.4	Hormad2	
DMR14:84601001	14	84601001	84603000	2000	1	6.60E-07	-0.34	31	1.55	Hormad2;LOC103693146	
DMR14:84605001	14	84605001	84607000	2000	1	7.60E-08	-0.36	31	1.55	Hormad2;LOC103693146	
DMR14:84645001	14	84645001	84647000	2000	1	7.70E-11	-0.46	26	1.3	Hormad2	
DMR14:84670001	14	84670001	84672000	2000	1	9.80E-18	1.07	52	2.6	Hormad2	
DMR14:84757001	14	84757001	84759000	2000	1	4.30E-08	-0.45	16	0.8	Mtmr3	Signaling
DMR14:84803001	14	84803001	84804000	1000	1	2.70E-10	-0.51	9	0.9	Mtmr3	Signaling
DMR14:84879001	14	84879001	84880000	1000	1	1.30E-11	0.55	8	0.8	Ascc2	

DMR14:84935001	14	84935001	84936000	1000	1	9.50E-08	0.42	18	1.8	Uqcr10;Zmat5	Metabolism
DMR14:84958001	14	84958001	84959000	1000	1	3.50E-08	0.5	10	1	Zmat5;Cabp7	
DMR14:85094001	14	85094001	85095000	1000	1	5.00E-07	-0.47	5	0.5	Nf2	Cytoskeleton
DMR14:85119001	14	85119001	85122000	3000	1	6.10E-07	0.38	38	1.27	Nipsnap1	
DMR14:85128001	14	85128001	85129000	1000	1	3.30E-10	0.64	21	2.1	Nipsnap1;Thoc5	
DMR14:85163001	14	85163001	85164000	1000	1	4.50E-07	0.49	15	1.5	Thoc5	
DMR14:85168001	14	85168001	85175000	7000	1	4.30E-07	0.39	100	1.43	Thoc5;Nefh	
DMR14:85196001	14	85196001	85198000	2000	1	2.50E-12	-0.46	39	1.95	Nefh	
DMR14:85258001	14	85258001	85260000	2000	1	4.80E-09	0.52	24	1.2	Ap1b1	Transport
DMR14:85376001	14	85376001	85378000	2000	1	1.90E-08	-0.43	59	2.95	Emid1	
DMR14:85406001	14	85406001	85408000	2000	2	4.20E-11	0.76	41	2.05	Emid1	
DMR14:85410001	14	85410001	85412000	2000	1	5.40E-07	0.46	26	1.3	Emid1	
DMR14:85479001	14	85479001	85480000	1000	1	1.90E-07	-0.38	17	1.7	Kremen1	Receptor
DMR14:85485001	14	85485001	85488000	3000	1	3.90E-15	-0.5	55	1.83	Kremen1	Receptor
DMR14:85570001	14	85570001	85573000	3000	1	2.00E-08	-0.33	33	1.1	Znrf3	
DMR14:85781001	14	85781001	85783000	2000	1	9.20E-07	-0.33	31	1.55	Ccdc117;Frg2	
DMR14:85817001	14	85817001	85821000	4000	1	1.40E-07	0.47	60	1.5	Ankrd36	
DMR14:86054001	14	86054001	86055000	1000	1	3.30E-09	-0.4	12	1.2	Dbnl;Pgam2	Cytoskeleton;Metabolism
DMR14:86082001	14	86082001	86083000	1000	1	5.80E-08	0.48	10	1	Polm	Transcription
DMR14:86141001	14	86141001	86143000	2000	1	4.70E-10	-0.41	30	1.5	LOC108352788;Myl7;Gck	Cytoskeleton;Signaling
DMR14:86218001	14	86218001	86220000	2000	1	1.10E-11	0.65	52	2.6	Camk2b;LOC108352789	Signaling
DMR14:86237001	14	86237001	86238000	1000	1	9.00E-08	0.33	11	1.1	Camk2b	Signaling
DMR14:86330001	14	86330001	86331000	1000	1	8.90E-09	-0.53	7	0.7	Nudcd3	Cytoskeleton
DMR14:86352001	14	86352001	86353000	1000	1	2.30E-07	0.41	9	0.9	Npc1l1;LOC364192	
DMR14:86361001	14	86361001	86362000	1000	1	3.10E-08	-0.59	2	0.2	Npc1l1;LOC364192	
DMR14:86462001	14	86462001	86466000	4000	1	9.10E-09	-0.35	33	0.82	Npc1l1;Ogdh	Metabolism
DMR14:86486001	14	86486001	86488000	2000	1	9.30E-08	-0.58	30	1.5	Npc1l1;Ogdh	Metabolism
DMR14:86512001	14	86512001	86513000	1000	1	2.90E-12	-0.66	9	0.9	Npc1l1;LOC103693776	
DMR14:86558001	14	86558001	86560000	2000	1	3.00E-12	0.84	64	3.2	Npc1l1	
DMR14:86644001	14	86644001	86645000	1000	1	4.80E-12	-0.67	6	0.6	Zmiz2	
DMR14:86695001	14	86695001	86696000	1000	1	4.30E-07	-0.35	18	1.8	H2afv	Epigenetic
DMR14:86778001	14	86778001	86784000	6000	1	2.50E-10	0.64	107	1.78	LOC103693157;Myo1g	Cytoskeleton
DMR14:86902001	14	86902001	86903000	1000	1	1.00E-07	0.43	12	1.2	Wap	Protease; Proteolysis
DMR14:86908001	14	86908001	86911000	3000	1	1.20E-08	0.67	64	2.13	Wap	Protease; Proteolysis
DMR14:86922001	14	86922001	86923000	1000	1	6.70E-11	-0.49	36	3.6	Ramp3	Receptor
DMR14:86932001	14	86932001	86933000	1000	1	3.30E-07	-0.41	7	0.7	Ramp3	Receptor
DMR14:87302001	14	87302001	87304000	2000	1	2.40E-07	0.42	53	2.65	Adcy1	
DMR14:87324001	14	87324001	87326000	2000	1	1.70E-07	-0.54	16	0.8	Adcy1	
DMR14:87344001	14	87344001	87346000	2000	1	2.10E-11	-0.56	51	2.55	Adcy1	
DMR14:87348001	14	87348001	87355000	7000	2	1.00E-09	-0.49	107	1.53	Adcy1	
DMR14:87367001	14	87367001	87369000	2000	1	5.10E-08	-0.42	32	1.6	Adcy1	
DMR14:87399001	14	87399001	87400000	1000	1	4.20E-07	0.45	14	1.4	Adcy1	
DMR14:87402001	14	87402001	87406000	4000	1	7.80E-07	0.46	91	2.28	Adcy1	
DMR14:87416001	14	87416001	87418000	2000	1	7.30E-10	0.62	44	2.2	Adcy1	
DMR14:87438001	14	87438001	87439000	1000	1	3.40E-08	0.54	14	1.4	Adcy1;LOC102553858;Igfbp1	Protease; Proteolysis
DMR14:88696001	14	88696001	88697000	1000	1	1.70E-07	-0.34	13	1.3	Tns3	Cytoskeleton
DMR14:88783001	14	88783001	88785000	2000	1	2.50E-08	-0.41	42	2.1	Tns3	Cytoskeleton
DMR14:88904001	14	88904001	88906000	2000	1	4.80E-08	0.57	31	1.55	Tns3;LOC102557304;LOC103693171	Cytoskeleton
DMR14:89047001	14	89047001	89049000	2000	2	2.00E-09	-0.38	34	1.7	Pkd1l1	Transport
DMR14:89061001	14	89061001	89064000	3000	1	1.90E-10	0.63	33	1.1	Pkd1l1	Transport
DMR14:89073001	14	89073001	89075000	2000	1	1.70E-10	0.57	23	1.15	Pkd1l1	Transport
DMR14:89079001	14	89079001	89080000	1000	1	4.50E-12	-0.68	7	0.7	Pkd1l1	Transport
DMR14:89218001	14	89218001	89220000	2000	1	3.90E-09	-0.42	34	1.7	Hus1;Sun3	Transcription;Cytoskeleton
DMR14:89326001	14	89326001	89328000	2000	1	7.90E-07	-0.33	29	1.45	Upp1	Signaling
DMR14:89437001	14	89437001	89439000	2000	1	5.30E-07	0.44	41	2.05	Abca13	Transport
DMR14:89543001	14	89543001	89544000	1000	1	5.10E-07	0.4	9	0.9	Abca13	Transport
DMR14:89548001	14	89548001	89554000	6000	2	9.30E-08	0.41	65	1.08	Abca13	Transport
DMR14:89716001	14	89716001	89719000	3000	2	4.70E-09	-0.38	30	1	Abca13	Transport
DMR14:89843001	14	89843001	89844000	1000	1	4.20E-12	-0.77	3	0.3	Abca13	Transport

DMR14:89857001	14	89857001	89859000	2000	2	2.00E-11	0.41	24	1.2	Abca13	Transport
DMR14:91230001	14	91230001	91231000	1000	1	1.60E-08	0.49	11	1.1	Vwc2	
DMR14:91266001	14	91266001	91269000	3000	1	1.40E-07	-0.39	27	0.9	Vwc2	
DMR14:91367001	14	91367001	91368000	1000	1	8.70E-10	-0.42	6	0.6	Zpbp	
DMR14:91412001	14	91412001	91417000	5000	1	9.50E-07	-0.34	37	0.74	Zpbp	
DMR14:91434001	14	91434001	91436000	2000	1	1.80E-12	-0.61	44	2.2	Zpbp	
DMR14:91563001	14	91563001	91564000	1000	1	6.40E-10	-0.67	3	0.3	Zpbp;RGD1309870	
DMR14:91606001	14	91606001	91609000	3000	2	1.40E-15	0.95	52	1.73	RGD1309870	
DMR14:91779001	14	91779001	91781000	2000	1	2.30E-13	-0.92	10	0.5	Ikzf1	Transcription
DMR14:91805001	14	91805001	91812000	7000	1	1.20E-07	-0.48	93	1.33	Ikzf1	Transcription
DMR14:91836001	14	91836001	91839000	3000	1	8.00E-07	-0.62	44	1.47	Ikzf1	Transcription
DMR14:91867001	14	91867001	91868000	1000	1	2.50E-07	0.68	15	1.5	Ikzf1	Transcription
DMR14:91980001	14	91980001	91985000	5000	1	1.30E-08	0.72	127	2.54	Ddc	Metabolism
DMR14:92006001	14	92006001	92007000	1000	1	1.80E-09	-0.47	18	1.8	Ddc;LOC103693175	Metabolism
DMR14:99909001	14	99909001	99910000	1000	1	4.50E-07	-0.39	7	0.7	LOC108352804;Egfr	Receptor
DMR14:100130001	14	100130001	100133000	3000	1	8.20E-07	-0.41	40	1.33	Fbxo48	
DMR14:100162001	14	100162001	100165000	3000	1	7.20E-08	0.56	34	1.13	Plek	Cytoskeleton
DMR14:100320001	14	100320001	100321000	1000	1	5.30E-07	-0.66	11	1.1	Ppp3r1	
DMR14:100408001	14	100408001	100409000	1000	1	8.40E-07	-0.39	11	1.1	LOC100363290;C1d	Transcription
DMR14:100434001	14	100434001	100435000	1000	1	5.10E-09	0.53	18	1.8	C1d	Transcription
DMR14:102108001	14	102108001	102111000	3000	1	1.80E-07	-0.21	53	1.77	Olr1806-ps	
DMR14:102116001	14	102116001	102124000	8000	5	3.00E-11	-0.66	72	0.9	Olr1806-ps	
DMR14:103185001	14	103185001	103186000	1000	1	2.50E-07	-0.37	11	1.1	Meis1	Development
DMR14:103197001	14	103197001	103200000	3000	1	5.60E-07	-0.37	52	1.73	Meis1	Development
DMR14:104288001	14	104288001	104289000	1000	1	8.40E-09	0.54	24	2.4	Spred2	Cytoskeleton
DMR14:104290001	14	104290001	104291000	1000	1	7.20E-20	1.34	23	2.3	Spred2	Cytoskeleton
DMR14:104297001	14	104297001	104298000	1000	1	9.30E-09	0.72	29	2.9	Spred2	Cytoskeleton
DMR14:104455001	14	104455001	104458000	3000	1	9.10E-09	0.73	35	1.17	LOC100359517;Rpl38-ps2	
DMR14:104586001	14	104586001	104588000	2000	2	1.00E-17	1.16	60	3	Slc1a4	Transport
DMR14:104620001	14	104620001	104621000	1000	1	1.90E-07	0.44	12	1.2	Slc1a4;LOC108352807;LOC108352808	Transport
DMR14:104777001	14	104777001	104778000	1000	1	1.10E-08	0.63	24	2.4	Sertad2	Transcription
DMR14:104877001	14	104877001	104878000	1000	1	3.00E-08	-0.65	12	1.2	Sertad2	Transcription
DMR14:104898001	14	104898001	104900000	2000	1	4.60E-08	0.52	35	1.75	Sertad2	Transcription
DMR14:104917001	14	104917001	104919000	2000	1	2.20E-10	0.64	37	1.85	Aftph	
DMR14:105045001	14	105045001	105046000	1000	1	1.70E-09	-0.62	26	2.6	Lgalsl;LOC108352836	Extracellular Matrix
DMR14:106072001	14	106072001	106075000	3000	1	1.90E-16	0.96	58	1.93	Peli1	Proteolysis
DMR14:106157001	14	106157001	106163000	6000	1	2.60E-07	-0.38	68	1.13	Vps54	Transport
DMR14:106387001	14	106387001	106388000	1000	1	5.50E-08	-0.54	22	2.2	Mdh1;Wdpcp	Metabolism
DMR14:106881001	14	106881001	106882000	1000	1	2.90E-09	-0.55	18	1.8	Ehbp1	
DMR14:106906001	14	106906001	106907000	1000	1	9.40E-11	0.43	18	1.8	Ehbp1;LOC102551536	
DMR14:107041001	14	107041001	107045000	4000	1	3.40E-07	-0.39	64	1.6	Ehbp1;LOC102550584	
DMR14:107064001	14	107064001	107067000	3000	2	4.00E-17	0.83	168	5.6	Ehbp1;LOC102550584;LOC108352821	
DMR14:107098001	14	107098001	107100000	2000	1	8.00E-07	0.31	47	2.35	Ehbp1	
DMR14:107122001	14	107122001	107123000	1000	1	8.20E-11	0.67	27	2.7	Ehbp1	
DMR14:107274001	14	107274001	107276000	2000	1	2.30E-07	-0.31	35	1.75	Tmem17	
DMR14:107295001	14	107295001	107297000	2000	1	4.90E-08	0.62	47	2.35	Tmem17	
DMR14:107580001	14	107580001	107586000	6000	1	6.00E-08	0.44	123	2.05	LOC103693217;B3gnt2	Golgi
DMR14:107592001	14	107592001	107594000	2000	1	3.20E-07	-0.39	53	2.65	LOC103693217;B3gnt2	Golgi
DMR14:107733001	14	107733001	107734000	1000	1	4.20E-08	0.47	10	1	Commd1	
DMR14:107821001	14	107821001	107822000	1000	1	6.60E-07	-0.34	25	2.5	RGD1563743	
DMR14:108107001	14	108107001	108109000	2000	1	2.70E-09	-0.52	32	1.6	Usp34	Protease
DMR14:108122001	14	108122001	108125000	3000	1	5.00E-13	-0.58	29	0.97	Usp34	Protease
DMR14:108172001	14	108172001	108173000	1000	1	4.80E-09	-0.54	18	1.8	Usp34	Protease
DMR14:108197001	14	108197001	108199000	2000	1	6.40E-11	-0.58	21	1.05	Usp34	Protease
DMR14:108256001	14	108256001	108258000	2000	1	5.50E-12	-0.67	28	1.4	Usp34;Ahsa2	Protease;Transcription
DMR14:108295001	14	108295001	108296000	1000	1	2.90E-07	0.5	23	2.3	LOC690352;LOC108352815;RGD1305110;LOC364218	
DMR14:108383001	14	108383001	108384000	1000	1	2.10E-07	-0.44	6	0.6	RGD1305110	
DMR14:108428001	14	108428001	108430000	2000	1	3.10E-08	-0.39	69	3.45	Pus10	
DMR14:108496001	14	108496001	108497000	1000	1	7.20E-07	-0.61	7	0.7	Rel	Transcription

DMR14:108650001	14	108650001	108655000	5000	3	6.10E-08	-0.57	6	0.12	Papolg	Translation
DMR14:108831001	14	108831001	108834000	3000	1	1.80E-11	0.87	42	1.4	Bcl11a	Transcription
DMR14:108911001	14	108911001	108913000	2000	2	1.70E-19	1.32	109	5.45	Bcl11a	Transcription
DMR14:108921001	14	108921001	108923000	2000	1	2.20E-08	-0.47	35	1.75	Bcl11a;LOC102557119	Transcription
DMR14:110838001	14	110838001	110842000	4000	1	6.50E-07	-0.34	33	0.82	Vrk2	Signaling
DMR14:112739001	14	112739001	112740000	1000	1	4.40E-08	0.52	6	0.6	Ccdc85a	
DMR14:112771001	14	112771001	112773000	2000	1	5.50E-09	-0.64	8	0.4	Ccdc85a	
DMR14:113765001	14	113765001	113766000	1000	1	2.10E-07	-0.68	4	0.4	Ccdc88a	Transport
DMR14:113887001	14	113887001	113888000	1000	1	6.50E-08	-0.37	15	1.5	Ccdc88a	Transport
DMR14:113998001	14	113998001	114004000	6000	3	4.30E-09	0.38	64	1.07	Clhc1	Transport
DMR14:114019001	14	114019001	114021000	2000	1	2.40E-09	-0.5	2	0.1	Ndufs5-ps1	
DMR14:114142001	14	114142001	114145000	3000	1	2.30E-07	0.47	24	0.8	Rtn4	
DMR14:114231001	14	114231001	114232000	1000	1	1.50E-10	0.75	18	1.8	Eml6	
DMR14:114867001	14	114867001	114868000	1000	1	7.10E-10	0.57	15	1.5	RGD1562229	
DMR14:114956001	14	114956001	114957000	1000	1	1.20E-07	0.36	12	1.2	Acyp2;LOC100912100	Signaling
DMR14:115056001	14	115056001	115057000	1000	1	8.70E-22	1.34	20	2	Acyp2	Signaling
DMR14:115342001	14	115342001	115344000	2000	1	8.50E-08	-0.42	9	0.45	Erlec1;Asb3	
DMR15:142001	15	142001	145000	3000	1	7.10E-10	-0.69	20	0.67	Dlg5	Cytoskeleton
DMR15:187001	15	187001	190000	3000	1	4.70E-07	-0.51	36	1.2	Dlg5	Cytoskeleton
DMR15:220001	15	220001	223000	3000	1	8.10E-10	-0.44	37	1.23	Dlg5	Cytoskeleton
DMR15:399001	15	399001	401000	2000	1	1.70E-11	-0.8	9	0.45	Kcnma1	Transport
DMR15:472001	15	472001	473000	1000	1	7.70E-14	0.5	1	0.1	Kcnma1	Transport
DMR15:484001	15	484001	486000	2000	1	9.40E-10	0.46	29	1.45	Kcnma1	Transport
DMR15:832001	15	832001	833000	1000	1	2.80E-08	0.56	6	0.6	Kcnma1	Transport
DMR15:923001	15	923001	925000	2000	1	3.80E-07	-0.43	22	1.1	Kcnma1	Transport
DMR15:1014001	15	1014001	1017000	3000	1	7.20E-07	-0.33	23	0.77	Kcnma1	Transport
DMR15:2627001	15	2627001	2629000	2000	1	1.60E-08	0.42	41	2.05	Comtd1;Vdac2	Epigenetic;Transport
DMR15:2630001	15	2630001	2631000	1000	1	1.90E-07	0.68	15	1.5	Comtd1;Vdac2	Epigenetic;Transport
DMR15:2654001	15	2654001	2656000	2000	1	7.80E-10	0.66	34	1.7	Vdac2	Transport
DMR15:2798001	15	2798001	2802000	4000	1	6.30E-11	0.87	103	2.58	Dupd1;LOC108352871;Kat6b	Epigenetic
DMR15:2804001	15	2804001	2806000	2000	1	1.00E-10	0.47	38	1.9	Dupd1;LOC108352871;Kat6b	Epigenetic
DMR15:2857001	15	2857001	2858000	1000	1	2.90E-09	-0.48	27	2.7	Kat6b	Epigenetic
DMR15:2876001	15	2876001	2878000	2000	1	5.10E-09	-0.59	37	1.85	Kat6b	Epigenetic
DMR15:3039001	15	3039001	3040000	1000	1	8.30E-07	-0.36	27	2.7	Adk	
DMR15:3073001	15	3073001	3076000	3000	1	4.30E-07	-0.36	57	1.9	Adk	
DMR15:3102001	15	3102001	3103000	1000	1	1.20E-10	-0.55	28	2.8	Adk	
DMR15:3153001	15	3153001	3156000	3000	2	1.50E-09	-0.48	21	0.7	Adk	
DMR15:3170001	15	3170001	3173000	3000	1	8.00E-08	-0.32	57	1.9	Adk	
DMR15:3241001	15	3241001	3242000	1000	1	7.60E-07	-0.42	10	1	Adk	
DMR15:3243001	15	3243001	3247000	4000	1	2.00E-07	-0.42	92	2.3	Adk	
DMR15:3271001	15	3271001	3272000	1000	1	7.00E-07	0.31	6	0.6	Adk	
DMR15:3307001	15	3307001	3312000	5000	1	4.60E-09	-0.4	109	2.18	Adk	
DMR15:3324001	15	3324001	3325000	1000	1	3.70E-10	0.75	39	3.9	Adk	
DMR15:3408001	15	3408001	3409000	1000	1	6.50E-11	-0.6	14	1.4	Adk	
DMR15:3499001	15	3499001	3502000	3000	1	6.90E-10	-0.65	53	1.77	Vcl	
DMR15:3633001	15	3633001	3640000	7000	3	1.80E-12	-0.57	92	1.31	Plau	Protease
DMR15:3740001	15	3740001	3742000	2000	1	2.30E-09	0.59	18	0.9	NEWGENE_621802;NEWGENE_1304700	
DMR15:4024001	15	4024001	4026000	2000	1	6.70E-07	-0.47	16	0.8	Zswim8;Chchd1;Fut11;Sec24c	Golgi;Transport
DMR15:4124001	15	4124001	4125000	1000	1	1.10E-09	-0.43	13	1.3	Usp54;Ncl-ps1	Protease
DMR15:4441001	15	4441001	4442000	1000	1	3.40E-20	1.15	30	3	Ecd;Nudt13	
DMR15:4456001	15	4456001	4460000	4000	1	4.20E-09	0.33	54	1.35	Nudt13	
DMR15:4478001	15	4478001	4482000	4000	2	1.20E-11	0.8	84	2.1	Kcnk16;LOC108352873	Transport
DMR15:4483001	15	4483001	4485000	2000	1	3.20E-13	0.65	53	2.65	Kcnk16;LOC108352873	Transport
DMR15:4487001	15	4487001	4489000	2000	1	3.70E-09	0.86	51	2.55	Kcnk16;LOC108352873	Transport
DMR15:4585001	15	4585001	4587000	2000	1	1.50E-13	-0.58	62	3.1	Kcnk5	Transport
DMR15:4738001	15	4738001	4742000	4000	2	7.50E-09	-0.39	65	1.62	Gng2	Signaling
DMR15:4753001	15	4753001	4754000	1000	1	6.50E-07	-0.33	19	1.9	Gng2	Signaling
DMR15:4761001	15	4761001	4763000	2000	1	2.20E-09	-0.47	34	1.7	Gng2	Signaling
DMR15:4781001	15	4781001	4782000	1000	1	1.80E-07	-0.29	30	3	Gng2	Signaling
DMR15:4815001	15	4815001	4817000	2000	1	2.70E-08	-0.44	33	1.65	Gng2	Signaling
DMR15:4847001	15	4847001	4850000	3000	1	2.10E-07	0.39	89	2.97	Gng2;LOC102554918	Signaling

DMR15:4942001	15	4942001	4944000	2000	1	6.90E-07	0.39	22	1.1	Nid2	Signaling
DMR15:4945001	15	4945001	4948000	3000	1	5.00E-11	0.27	27	0.9	Nid2	Signaling
DMR15:5488001	15	5488001	5493000	5000	1	5.80E-09	-0.44	48	0.96	Spetex-2F;LOC102547093;LOC108352879	
DMR15:5544001	15	5544001	5546000	2000	1	5.10E-13	-0.52	22	1.1	Spetex-2C;LOC102549465	
DMR15:6241001	15	6241001	6242000	1000	1	7.80E-12	0.62	10	1	Zfp385d	
DMR15:6260001	15	6260001	6262000	2000	1	9.40E-08	-0.56	19	0.95	Zfp385d	
DMR15:6362001	15	6362001	6363000	1000	1	2.10E-08	-0.63	1	0.1	Zfp385d	
DMR15:6381001	15	6381001	6384000	3000	1	2.10E-08	-0.57	11	0.37	Zfp385d	
DMR15:6462001	15	6462001	6463000	1000	1	8.60E-07	-0.5	15	1.5	Zfp385d	
DMR15:6640001	15	6640001	6642000	2000	1	2.60E-11	-0.43	18	0.9	Zfp385d	
DMR15:6850001	15	6850001	6853000	3000	1	1.10E-07	-0.36	23	0.77	Zfp385d	
DMR15:6898001	15	6898001	6899000	1000	1	4.40E-08	-0.47	9	0.9	Zfp385d	
DMR15:7251001	15	7251001	7252000	1000	1	8.60E-07	-0.41	7	0.7	Zfp385d	
DMR15:7510001	15	7510001	7517000	7000	1	7.60E-11	-0.35	71	1.01	Ube2e2	Proteolysis
DMR15:7671001	15	7671001	7672000	1000	1	2.90E-08	-0.42	22	2.2	Ube2e2	Proteolysis
DMR15:7678001	15	7678001	7680000	2000	1	1.70E-07	-0.37	40	2	Ube2e2;LOC103693837	Proteolysis
DMR15:7687001	15	7687001	7688000	1000	1	7.60E-07	-0.4	8	0.8	Ube2e2;LOC103693837	Proteolysis
DMR15:7728001	15	7728001	7731000	3000	1	1.20E-10	0.58	50	1.67	Ube2e2	Proteolysis
DMR15:8152001	15	8152001	8156000	4000	3	2.40E-33	-0.78	51	1.27	Ube2e1;LOC102552559	Proteolysis
DMR15:8180001	15	8180001	8185000	5000	1	2.80E-08	-0.45	94	1.88	Ube2e1;Nkiras1;Rpl15	Proteolysis;Translation
DMR15:8232001	15	8232001	8235000	3000	1	2.80E-07	-0.58	21	0.7	Hspd1-ps20	
DMR15:8756001	15	8756001	8757000	1000	1	7.00E-07	0.42	10	1	Nr1d2	Transcription
DMR15:8894001	15	8894001	8896000	2000	2	6.00E-10	0.53	43	2.15	Thrb	Transcription
DMR15:8919001	15	8919001	8921000	2000	1	2.80E-09	-0.47	28	1.4	Thrb	Transcription
DMR15:8953001	15	8953001	8955000	2000	1	7.00E-11	0.74	33	1.65	Thrb;LOC103693275	Transcription
DMR15:8966001	15	8966001	8970000	4000	1	2.90E-07	-0.53	39	0.98	Thrb	Transcription
DMR15:9036001	15	9036001	9038000	2000	1	1.90E-12	-0.57	37	1.85	Thrb	Transcription
DMR15:9042001	15	9042001	9044000	2000	1	1.40E-07	-0.37	38	1.9	Thrb	Transcription
DMR15:9059001	15	9059001	9063000	4000	1	5.20E-07	0.47	50	1.25	Thrb	Transcription
DMR15:9078001	15	9078001	9081000	3000	1	6.20E-19	1.13	60	2	Thrb	Transcription
DMR15:9980001	15	9980001	9981000	1000	1	6.20E-07	0.46	16	1.6	Rarb	Transcription
DMR15:10033001	15	10033001	10034000	1000	1	5.50E-09	-0.48	13	1.3	Rarb	Transcription
DMR15:10092001	15	10092001	10094000	2000	1	3.80E-16	0.7	10	0.5	Rarb	Transcription
DMR15:10136001	15	10136001	10138000	2000	1	6.60E-09	-0.4	36	1.8	Rarb	Transcription
DMR15:10185001	15	10185001	10187000	2000	1	6.60E-07	0.49	34	1.7	Rarb	Transcription
DMR15:10226001	15	10226001	10230000	4000	2	2.50E-17	0.44	35	0.88	Rarb	Transcription
DMR15:10248001	15	10248001	10251000	3000	1	8.50E-07	0.55	51	1.7	Rarb	Transcription
DMR15:10306001	15	10306001	10307000	1000	1	2.90E-08	-0.43	2	0.2	Top2b	
DMR15:10466001	15	10466001	10467000	1000	1	1.90E-08	-0.77	4	0.4	LOC100909559;Oxsm	
DMR15:10634001	15	10634001	10635000	1000	1	1.60E-08	-0.44	15	1.5	RGD1559461	
DMR15:11659001	15	11659001	11662000	3000	1	1.50E-17	1.08	67	2.23	Nek10;LOC498453	Signaling
DMR15:11759001	15	11759001	11764000	5000	1	1.80E-08	-0.43	87	1.74	Nek10	Signaling
DMR15:11916001	15	11916001	11918000	2000	1	5.40E-07	-0.32	24	1.2	Slc4a7	Transport
DMR15:12478001	15	12478001	12482000	4000	2	6.10E-09	-0.4	69	1.73	Atxn7	
DMR15:12604001	15	12604001	12607000	3000	1	4.60E-17	-0.53	31	1.03	LOC102550375;RGD1565725	
DMR15:12621001	15	12621001	12624000	3000	1	2.40E-08	-0.41	19	0.63	RGD1565725	
DMR15:12671001	15	12671001	12672000	1000	1	2.20E-07	0.44	22	2.2	RGD1565725	
DMR15:12817001	15	12817001	12819000	2000	1	5.00E-08	-0.54	25	1.25	Fezf2	Transcription
DMR15:12825001	15	12825001	12826000	1000	1	4.70E-08	-0.39	15	1.5	Fezf2	Transcription
DMR15:12859001	15	12859001	12862000	3000	1	1.10E-15	-0.91	21	0.7	RGD1306063	
DMR15:12894001	15	12894001	12905000	11000	1	8.90E-09	-0.29	107	0.97	RGD1306063;LOC103693281	
DMR15:12940001	15	12940001	12944000	4000	1	4.10E-07	-0.25	35	0.88	Ptprg	Signaling
DMR15:13010001	15	13010001	13012000	2000	1	7.90E-07	-0.34	40	2	Ptprg	Signaling
DMR15:13083001	15	13083001	13084000	1000	1	2.70E-07	0.47	14	1.4	Ptprg	Signaling
DMR15:13202001	15	13202001	13205000	3000	1	6.50E-07	-0.52	44	1.47	Ptprg	Signaling
DMR15:13268001	15	13268001	13272000	4000	1	4.60E-07	0.52	54	1.35	Ptprg	Signaling
DMR15:13277001	15	13277001	13280000	3000	1	2.60E-09	-0.44	49	1.63	Ptprg	Signaling
DMR15:13281001	15	13281001	13282000	1000	1	2.30E-08	-0.45	10	1	Ptprg	Signaling
DMR15:13350001	15	13350001	13352000	2000	1	1.90E-07	0.54	43	2.15	Ptprg	Signaling
DMR15:13366001	15	13366001	13367000	1000	1	2.50E-08	0.59	14	1.4	Ptprg	Signaling

DMR15:13415001	15	13415001	13417000	2000	1	8.30E-10	0.75	34	1.7	Ptprg	Signaling
DMR15:13431001	15	13431001	13434000	3000	1	7.30E-09	0.54	25	0.83	Ptprg	Signaling
DMR15:13514001	15	13514001	13515000	1000	1	6.50E-07	0.61	23	2.3	Ptprg	Signaling
DMR15:13561001	15	13561001	13565000	4000	2	8.70E-10	-0.43	50	1.25	Ptprg	Signaling
DMR15:13566001	15	13566001	13568000	2000	1	8.70E-08	0.41	14	0.7	Ptprg	Signaling
DMR15:13596001	15	13596001	13600000	4000	2	6.80E-09	0.6	94	2.35	Ptprg	Signaling
DMR15:14845001	15	14845001	14848000	3000	1	7.00E-07	0.46	36	1.2	Synpr	Transport
DMR15:14849001	15	14849001	14850000	1000	1	5.70E-10	-0.65	3	0.3	Synpr	Transport
DMR15:14867001	15	14867001	14868000	1000	1	5.40E-08	0.4	11	1.1	Synpr	Transport
DMR15:14891001	15	14891001	14892000	1000	1	5.20E-07	0.37	7	0.7	Synpr;LOC102552767	Transport
DMR15:15510001	15	15510001	15512000	2000	1	3.10E-08	-0.65	18	0.9	Cadps;LOC108352890	Transport
DMR15:15582001	15	15582001	15584000	2000	1	7.10E-10	0.36	40	2	Cadps	Transport
DMR15:15612001	15	15612001	15614000	2000	1	4.90E-07	-0.33	28	1.4	Cadps	Transport
DMR15:15703001	15	15703001	15704000	1000	1	4.60E-07	0.43	9	0.9	Fhit	Signaling
DMR15:15867001	15	15867001	15868000	1000	1	2.70E-08	0.53	8	0.8	Fhit	Signaling
DMR15:16195001	15	16195001	16197000	2000	1	2.50E-07	0.52	22	1.1	Fhit	Signaling
DMR15:16252001	15	16252001	16254000	2000	1	1.80E-07	0.46	14	0.7	Fhit	Signaling
DMR15:16337001	15	16337001	16338000	1000	1	2.60E-10	-0.54	7	0.7	Fhit	Signaling
DMR15:16434001	15	16434001	16435000	1000	1	1.70E-37	1.32	34	3.4	Fhit	Signaling
DMR15:16478001	15	16478001	16480000	2000	1	1.50E-10	-0.56	19	0.95	Fhit	Signaling
DMR15:16491001	15	16491001	16492000	1000	1	1.40E-10	0.57	17	1.7	Fhit	Signaling
DMR15:16509001	15	16509001	16510000	1000	1	3.40E-08	0.5	5	0.5	Fhit	Signaling
DMR15:16684001	15	16684001	16685000	1000	1	3.90E-07	0.4	5	0.5	Fhit;LOC103693285	Signaling
DMR15:18324001	15	18324001	18326000	2000	2	1.90E-14	-0.71	36	1.8	Fam3d	Signaling
DMR15:18349001	15	18349001	18350000	1000	1	6.80E-08	-0.4	21	2.1	Fam3d	Signaling
DMR15:18402001	15	18402001	18405000	3000	1	3.10E-07	0.4	37	1.23	Fam107a	
DMR15:18424001	15	18424001	18425000	1000	1	4.90E-07	0.46	23	2.3	Fam107a	
DMR15:18487001	15	18487001	18492000	5000	1	3.70E-07	-0.42	99	1.98	Acox2;Kctd6	Metabolism;Cytoskeleton
DMR15:18589001	15	18589001	18592000	3000	1	1.70E-20	1.09	87	2.9	Pxk	
DMR15:18613001	15	18613001	18614000	1000	1	2.50E-09	0.68	24	2.4	Pxk	
DMR15:18679001	15	18679001	18680000	1000	1	1.00E-09	-0.5	22	2.2	Abhd6	Metabolism
DMR15:18700001	15	18700001	18704000	4000	1	2.50E-07	0.6	72	1.8	Abhd6;Dnase1l3	Metabolism
DMR15:18802001	15	18802001	18804000	2000	1	1.80E-07	-0.44	27	1.35	Flnb	
DMR15:18819001	15	18819001	18827000	8000	2	4.40E-07	-0.37	122	1.52	Flnb	
DMR15:19187001	15	19187001	19192000	5000	1	9.70E-13	-0.43	52	1.04	Ptgdrl	Signaling
DMR15:19353001	15	19353001	19354000	1000	1	3.30E-07	0.6	22	2.2	Ptger2	Signaling
DMR15:19478001	15	19478001	19480000	2000	1	5.00E-07	-0.45	28	1.4	Txndc16	Metabolism
DMR15:19527001	15	19527001	19532000	5000	1	1.70E-07	-0.28	45	0.9	Txndc16	Metabolism
DMR15:19578001	15	19578001	19583000	5000	1	1.30E-14	0.7	77	1.54	Gpr137c	
DMR15:19595001	15	19595001	19596000	1000	1	5.00E-09	-0.44	6	0.6	Gpr137c;LOC102552640	
DMR15:19614001	15	19614001	19615000	1000	1	1.10E-14	0.95	40	4	Gpr137c;LOC102552640;Ero1a	Metabolism
DMR15:19618001	15	19618001	19619000	1000	1	6.70E-11	-0.51	14	1.4	Gpr137c;LOC102552640;Ero1a	Metabolism
DMR15:19633001	15	19633001	19639000	6000	2	7.80E-10	0.56	106	1.77	Ero1a	Metabolism
DMR15:19796001	15	19796001	19798000	2000	1	1.20E-10	0.78	49	2.45	Fermt2	
DMR15:19814001	15	19814001	19816000	2000	1	1.90E-07	-0.32	44	2.2	Fermt2	
DMR15:19825001	15	19825001	19826000	1000	1	9.50E-11	-0.44	21	2.1	Fermt2	
DMR15:19976001	15	19976001	19981000	5000	1	4.50E-07	0.44	106	2.12	Ddhd1	Metabolism
DMR15:20801001	15	20801001	20805000	4000	2	7.40E-10	0.5	58	1.45	Bmp4;LOC102548186	Growth Factors
DMR15:21213001	15	21213001	21221000	8000	5	1.30E-12	-0.81	87	1.09	Olr1850-ps	
DMR15:23023001	15	23023001	23025000	2000	2	1.90E-13	0.68	58	2.9	Olr1809-ps	
DMR15:23562001	15	23562001	23563000	1000	1	7.10E-07	-0.39	16	1.6	Cdkn3;Cnih1	Signaling;Transport
DMR15:23685001	15	23685001	23690000	5000	1	4.20E-14	0.96	89	1.78	Samd4a	
DMR15:23710001	15	23710001	23713000	3000	1	1.60E-07	0.38	63	2.1	Samd4a	
DMR15:23757001	15	23757001	23760000	3000	1	6.10E-07	0.32	55	1.83	Samd4a	
DMR15:23762001	15	23762001	23764000	2000	1	2.00E-08	-0.47	30	1.5	Samd4a	
DMR15:23803001	15	23803001	23804000	1000	1	4.00E-08	0.39	18	1.8	Samd4a	
DMR15:23858001	15	23858001	23859000	1000	1	1.00E-07	0.64	7	0.7	Samd4a	
DMR15:23860001	15	23860001	23861000	1000	1	3.40E-10	0.46	24	2.4	Samd4a	
DMR15:23880001	15	23880001	23881000	1000	1	3.70E-09	-0.43	26	2.6	Samd4a	
DMR15:23950001	15	23950001	23953000	3000	1	1.00E-06	0.49	44	1.47	Gch1	Metabolism
DMR15:24051001	15	24051001	24052000	1000	1	6.60E-10	-0.54	15	1.5	Wdhd1;Socs4	Epigenetic;Signaling

DMR15:24136001	15	24136001	24138000	2000	1	3.70E-08	0.27	20	1	RGD1564783	
DMR15:24265001	15	24265001	24266000	1000	1	1.70E-07	0.54	18	1.8	Fbxo34	
DMR15:24279001	15	24279001	24280000	1000	1	2.60E-07	0.47	13	1.3	Fbxo34	
DMR15:24318001	15	24318001	24320000	2000	1	8.00E-08	-0.57	67	3.35	Fbxo34	
DMR15:24493001	15	24493001	24497000	4000	1	4.50E-07	-0.4	57	1.43	Ktn1	
DMR15:24510001	15	24510001	24516000	6000	1	2.30E-08	-0.38	79	1.32	Ktn1	
DMR15:24551001	15	24551001	24552000	1000	1	2.30E-08	-0.49	13	1.3	Ktn1	
DMR15:24935001	15	24935001	24939000	4000	1	3.00E-11	-0.5	78	1.95	Peli2	Proteolysis
DMR15:25061001	15	25061001	25062000	1000	1	2.00E-07	0.43	22	2.2	Peli2	Proteolysis
DMR15:25299001	15	25299001	25300000	1000	1	1.30E-11	0.88	29	2.9	Tmem260	
DMR15:25306001	15	25306001	25307000	1000	1	5.30E-07	-0.36	9	0.9	Tmem260	
DMR15:25308001	15	25308001	25310000	2000	1	2.90E-09	0.52	22	1.1	Tmem260	
DMR15:26175001	15	26175001	26177000	2000	1	4.00E-09	0.69	46	2.3	Slc35f4	
DMR15:26214001	15	26214001	26220000	6000	1	3.30E-07	-0.27	68	1.13	Slc35f4	
DMR15:26253001	15	26253001	26254000	1000	1	5.60E-13	1	24	2.4	Slc35f4;LOC102556222	
DMR15:26300001	15	26300001	26302000	2000	1	6.10E-09	-0.46	15	0.75	Slc35f4	
DMR15:26780001	15	26780001	26782000	2000	1	1.30E-08	0.74	24	1.2	Olr1624	Receptor
DMR15:27164001	15	27164001	27165000	1000	1	1.40E-08	0.54	22	2.2	Olr1878	
DMR15:27284001	15	27284001	27285000	1000	1	1.20E-07	-0.35	18	1.8	Olr1617	Signaling
DMR15:27474001	15	27474001	27475000	1000	1	1.10E-07	0.56	11	1.1	Olr1631	Signaling
DMR15:27607001	15	27607001	27613000	6000	1	8.50E-38	0.73	61	1.02	Olr1634-ps	
DMR15:27628001	15	27628001	27633000	5000	1	8.50E-09	-0.41	48	0.96	Olr1634-ps	
DMR15:27773001	15	27773001	27775000	2000	1	4.50E-07	0.5	45	2.25	Tep1	Transcription
DMR15:27857001	15	27857001	27859000	2000	1	6.80E-10	0.73	28	1.4	Osgep;Apex1;Tmem55b	Translation
DMR15:27869001	15	27869001	27870000	1000	1	1.10E-08	0.57	17	1.7	Pnp	Signaling
DMR15:27887001	15	27887001	27889000	2000	1	1.10E-11	-0.5	24	1.2	Pnp	Signaling
DMR15:28092001	15	28092001	28095000	3000	1	4.40E-12	-0.78	27	0.9	Rnase6;LOC103690270;Eddm3b;LOC103693840;Ang2	
DMR15:28104001	15	28104001	28107000	3000	1	7.70E-11	0.73	29	0.97	Eddm3b;LOC103693840;Ang2	
DMR15:28218001	15	28218001	28221000	3000	1	1.30E-08	-0.32	65	2.17	Ear11	
DMR15:28269001	15	28269001	28270000	1000	1	4.80E-10	0.69	38	3.8	Ear1	
DMR15:28304001	15	28304001	28306000	2000	1	2.90E-09	0.63	22	1.1	Slc39a2;Ndrg2	Transport;Protease
DMR15:28384001	15	28384001	28385000	1000	1	7.70E-09	0.51	15	1.5	Arhgef40	Transcription
DMR15:28389001	15	28389001	28393000	4000	1	3.00E-17	1.19	104	2.6	Arhgef40;Zfp219	Transcription;Transcription
DMR15:28394001	15	28394001	28395000	1000	1	9.20E-10	0.56	27	2.7	Arhgef40;Zfp219	Transcription;Transcription
DMR15:28557001	15	28557001	28559000	2000	1	2.90E-09	-0.63	27	1.35	Rpgrip1	
DMR15:28586001	15	28586001	28587000	1000	1	4.70E-08	0.45	14	1.4	Supt16h	Epigenetic
DMR15:28660001	15	28660001	28661000	1000	1	8.10E-12	-0.55	8	0.8	Chd8	
DMR15:28730001	15	28730001	28731000	1000	1	1.20E-32	1.32	28	2.8	Mettl3;Trnas-aga;Sall2	Epigenetic;Transcription
DMR15:28827001	15	28827001	28832000	5000	1	9.60E-07	-0.25	49	0.98	Olr1641	Receptor
DMR15:28844001	15	28844001	28848000	4000	1	1.50E-07	0.49	52	1.3	Olr1641;Olr1642	Receptor
DMR15:28863001	15	28863001	28864000	1000	1	1.10E-09	0.45	21	2.1	Olr1642;LOC100363123	Receptor
DMR15:30545001	15	30545001	30548000	3000	1	6.60E-09	-0.44	53	1.77	LOC102552674;Tcrva8;Trav14s1;LOC100360242	
DMR15:31572001	15	31572001	31574000	2000	1	1.10E-07	-0.54	10	0.5	LOC103693857;RGD1563477	
DMR15:31580001	15	31580001	31583000	3000	2	1.50E-07	0.53	34	1.13	LOC103693857;RGD1563477	
DMR15:32898001	15	32898001	32901000	3000	1	3.10E-07	0.57	36	1.2	Abhd4	
DMR15:32915001	15	32915001	32919000	4000	1	4.30E-08	-0.39	38	0.95	Abhd4;RGD1566241;Olr1646	Receptor
DMR15:33041001	15	33041001	33044000	3000	1	1.00E-08	-0.44	41	1.37	Slc7a7	Transport
DMR15:33067001	15	33067001	33069000	2000	1	6.20E-11	-0.73	19	0.95	Slc7a7;Mrpl52;Mmp14	Transport;Translation;Protease
DMR15:33128001	15	33128001	33129000	1000	1	3.00E-16	0.84	5	0.5	Rem2	
DMR15:33166001	15	33166001	33168000	2000	1	5.10E-09	-0.6	47	2.35	Prmt5;Trnar-acg	Golgi
DMR15:33239001	15	33239001	33241000	2000	1	4.00E-08	0.51	56	2.8	RGD1565222	
DMR15:33357001	15	33357001	33358000	1000	1	2.40E-08	0.65	29	2.9	RGD1560492;Cebpe	Transcription
DMR15:33382001	15	33382001	33385000	3000	1	1.20E-09	0.41	43	1.43	Slc7a8	Transport
DMR15:33522001	15	33522001	33525000	3000	1	1.90E-07	-0.34	38	1.27	Homez	Development

DMR15:33615001	15	33615001	33617000	2000	1	3.40E-09	0.71	45	2.25	Myh6;Myh7;LOC108352922;LOC108352923;Mir3546;Mir208a	
DMR15:33626001	15	33626001	33633000	7000	1	5.80E-07	0.36	117	1.67	Myh6;Myh7;Mir208b	
DMR15:33647001	15	33647001	33648000	1000	1	7.20E-08	-0.43	7	0.7	Myh7;Mir208b	
DMR15:33659001	15	33659001	33660000	1000	1	1.60E-17	0.83	31	3.1	Myh7	
DMR15:33673001	15	33673001	33674000	1000	1	2.00E-07	-0.63	5	0.5	Ngdn	Metabolism
DMR15:33721001	15	33721001	33723000	2000	2	2.40E-11	0.81	49	2.45	Zfhx2	Transcription
DMR15:33725001	15	33725001	33726000	1000	1	5.40E-11	0.47	31	3.1	Zfhx2	Transcription
DMR15:33730001	15	33730001	33732000	2000	1	1.10E-07	0.39	64	3.2	Zfhx2	Transcription
DMR15:33733001	15	33733001	33737000	4000	1	3.70E-07	0.42	70	1.75	Zfhx2	Transcription
DMR15:33749001	15	33749001	33752000	3000	1	7.10E-08	-0.4	66	2.2	Zfhx2;Thtpa;Ap1g2	Transcription;Signaling;Transport
DMR15:33814001	15	33814001	33817000	3000	1	1.20E-13	0.65	52	1.73	RGD1564324	Metabolism
DMR15:33818001	15	33818001	33821000	3000	1	2.90E-07	0.47	43	1.43	RGD1564324	Metabolism
DMR15:33875001	15	33875001	33878000	3000	1	2.80E-23	1.2	73	2.43	Dhrs2	Metabolism
DMR15:34046001	15	34046001	34049000	3000	1	3.60E-13	0.91	53	1.77	LOC102551831;RGD1565258	
DMR15:34198001	15	34198001	34200000	2000	2	7.30E-08	0.49	42	2.1	LOC102552093;Cpne6;Nrl	Transcription
DMR15:34206001	15	34206001	34211000	5000	1	7.60E-08	0.38	74	1.48	Nrl;Pck2	Transcription;Signaling
DMR15:34219001	15	34219001	34222000	3000	1	1.90E-08	0.51	69	2.3	Pck2	Signaling
DMR15:34239001	15	34239001	34242000	3000	1	4.70E-08	0.39	49	1.63	Dcaf11;LOC102552219;Fitm1	Proteolysis
DMR15:34283001	15	34283001	34286000	3000	2	1.30E-16	-0.65	58	1.93	Rnf31;Irf9;LOC103693570	Proteolysis;Transcription
DMR15:34358001	15	34358001	34361000	3000	1	6.30E-08	0.49	28	0.93	Nedd8;Gmpr2;Tinf2	Metabolism
DMR15:34387001	15	34387001	34388000	1000	1	2.30E-15	0.87	28	2.8	Tgm1;Rabggta	Transport;Metabolism
DMR15:34457001	15	34457001	34458000	1000	1	8.60E-08	-0.49	22	2.2	Ltb4r2;Ltb4r;Adcy4	Signaling
DMR15:34512001	15	34512001	34516000	4000	1	5.70E-07	0.23	43	1.07	Nfatc4;LOC102552484;Nynrin	Transcription;Translation
DMR15:34532001	15	34532001	34534000	2000	1	8.40E-09	0.88	52	2.6	Nynrin	Translation
DMR15:34672001	15	34672001	34675000	3000	1	7.40E-09	-0.6	24	0.8	Mcpt8l3;LOC498518	
DMR15:34748001	15	34748001	34749000	1000	1	2.70E-07	0.54	10	1	Mcpt4	Protease
DMR15:34751001	15	34751001	34753000	2000	1	2.30E-08	0.5	17	0.85	Mcpt4	Protease
DMR15:35102001	15	35102001	35103000	1000	1	6.40E-15	0.77	17	1.7	Ctsg	
DMR15:35127001	15	35127001	35128000	1000	1	1.10E-07	-0.61	6	0.6	Gzmn	Protease
DMR15:36100001	15	36100001	36102000	2000	1	8.00E-08	0.5	38	1.9	Gzmbl3	Protease
DMR15:36551001	15	36551001	36554000	3000	2	2.60E-17	0.93	64	2.13	Atp12a	Transport
DMR15:36629001	15	36629001	36634000	5000	1	9.90E-07	-0.36	40	0.8	Rnf17	
DMR15:36767001	15	36767001	36773000	6000	1	2.90E-07	-0.29	49	0.82	Cenpj	Cytoskeleton
DMR15:36824001	15	36824001	36827000	3000	1	7.90E-08	-0.57	46	1.53	Parp4	
DMR15:36996001	15	36996001	36998000	2000	1	1.20E-08	-0.51	15	0.75	Pspc1	Metabolism
DMR15:37144001	15	37144001	37145000	1000	1	2.50E-09	-0.36	5	0.5	RGD1563527	
DMR15:37403001	15	37403001	37406000	3000	2	4.10E-07	0.58	30	1	Gjb2;Gjb6	Cytoskeleton
DMR15:37416001	15	37416001	37419000	3000	1	7.00E-13	0.79	38	1.27	Gjb6;RGD1565170	Cytoskeleton
DMR15:37553001	15	37553001	37554000	1000	1	1.40E-07	0.35	17	1.7	Cryl1	Metabolism
DMR15:37638001	15	37638001	37640000	2000	1	4.30E-08	0.36	31	1.55	Cryl1	Metabolism
DMR15:37645001	15	37645001	37646000	1000	1	2.80E-12	0.7	14	1.4	Cryl1	Metabolism
DMR15:37775001	15	37775001	37776000	1000	1	1.80E-11	0.59	9	0.9	Ift88	
DMR15:37825001	15	37825001	37827000	2000	1	2.50E-12	0.65	50	2.5	Eef1akmt1;Xpo4	Transport
DMR15:38001001	15	38001001	38003000	2000	1	4.00E-08	0.58	22	1.1	Lats2;LOC108352928	Signaling
DMR15:38060001	15	38060001	38066000	6000	1	9.70E-10	0.59	87	1.45	LOC103693597;Sap18	
DMR15:38169001	15	38169001	38172000	3000	1	8.40E-14	0.83	72	2.4	Zdhhc20	
DMR15:38175001	15	38175001	38177000	2000	1	1.30E-07	0.49	21	1.05	Zdhhc20	
DMR15:38239001	15	38239001	38242000	3000	1	5.20E-07	-0.38	28	0.93	Micu2	Signaling
DMR15:38367001	15	38367001	38369000	2000	1	8.00E-10	-0.6	12	0.6	Fgf9	Growth Factors
DMR15:38388001	15	38388001	38389000	1000	1	4.60E-10	0.84	29	2.9	Fgf9	Growth Factors
DMR15:38790001	15	38790001	38793000	3000	1	2.30E-09	-0.67	23	0.77	Phf11;Setdb2	Transcription;Epigenetic
DMR15:38820001	15	38820001	38822000	2000	1	7.80E-08	-0.42	10	0.5	Phf11;Setdb2	Transcription;Epigenetic
DMR15:38827001	15	38827001	38828000	1000	1	2.10E-07	0.52	10	1	Phf11;Setdb2	Transcription;Epigenetic

DMR15:38843001	15	38843001	38844000	1000	1	1.50E-07	0.45	5	0.5	Phf11;Setdb2	Transcription;Epigenetic
DMR15:38929001	15	38929001	38930000	1000	1	1.30E-07	-0.38	14	1.4	Phf11;Setdb2;LOC102551654	Transcription;Epigenetic
DMR15:38973001	15	38973001	38975000	2000	1	2.40E-07	-0.42	15	0.75	Phf11;Setdb2	Transcription;Epigenetic
DMR15:39054001	15	39054001	39056000	2000	1	5.70E-11	0.52	25	1.25	Phf11;Setdb2;LOC102551719	Transcription;Epigenetic
DMR15:39118001	15	39118001	39120000	2000	1	1.20E-08	-0.57	22	1.1	Phf11;Setdb2	Transcription;Epigenetic
DMR15:39182001	15	39182001	39185000	3000	1	1.50E-18	1.02	44	1.47	Phf11;Setdb2	Transcription;Epigenetic
DMR15:39213001	15	39213001	39214000	1000	1	6.40E-07	0.64	22	2.2	Phf11;Setdb2	Transcription;Epigenetic
DMR15:39247001	15	39247001	39249000	2000	1	7.40E-07	0.42	14	0.7	Phf11;Setdb2	Transcription;Epigenetic
DMR15:39378001	15	39378001	39379000	1000	1	4.40E-09	-0.49	6	0.6	Phf11;Setdb2	Transcription;Epigenetic
DMR15:39447001	15	39447001	39448000	1000	1	1.20E-11	-0.53	2	0.2	Phf11;Setdb2	Transcription;Epigenetic
DMR15:39609001	15	39609001	39612000	3000	1	3.10E-07	0.35	1	0.03	Phf11;Setdb2;RGD1563302;LOC102552417	Transcription;Epigenetic
DMR15:39803001	15	39803001	39805000	2000	1	1.00E-07	-0.35	20	1	Cab39l	
DMR15:39873001	15	39873001	39874000	1000	1	6.40E-08	-0.54	19	1.9	Cdadc1	Metabolism
DMR15:39937001	15	39937001	39942000	5000	1	1.20E-12	0.7	66	1.32	Shisa2	Cytoskeleton
DMR15:39947001	15	39947001	39950000	3000	2	2.00E-07	0.55	78	2.6	Shisa2;Atp8a2	Cytoskeleton;Transport
DMR15:40032001	15	40032001	40035000	3000	1	4.00E-10	-0.42	39	1.3	Atp8a2	Transport
DMR15:40077001	15	40077001	40078000	1000	1	3.30E-09	-0.55	18	1.8	Atp8a2	Transport
DMR15:40094001	15	40094001	40095000	1000	1	5.20E-07	-0.42	26	2.6	Atp8a2	Transport
DMR15:40101001	15	40101001	40103000	2000	1	3.00E-07	0.43	23	1.15	Atp8a2;LOC108352984	Transport
DMR15:40132001	15	40132001	40133000	1000	1	3.30E-08	0.41	17	1.7	Atp8a2	Transport
DMR15:40194001	15	40194001	40195000	1000	1	6.80E-08	-0.47	15	1.5	Atp8a2	Transport
DMR15:40258001	15	40258001	40261000	3000	1	1.90E-07	-0.48	29	0.97	Atp8a2	Transport
DMR15:40351001	15	40351001	40355000	4000	1	5.30E-10	-0.36	85	2.12	Atp8a2;LOC103693865	Transport
DMR15:40587001	15	40587001	40589000	2000	1	1.20E-07	0.54	18	0.9	Mtmr6	Signaling
DMR15:40602001	15	40602001	40603000	1000	1	3.30E-07	0.31	17	1.7	Mtmr6	Signaling
DMR15:40963001	15	40963001	40964000	1000	1	2.10E-07	0.42	18	1.8	Spata13	Transcription
DMR15:41148001	15	41148001	41154000	6000	1	3.10E-07	0.58	91	1.52	Mipep	Protease
DMR15:41462001	15	41462001	41463000	1000	1	2.60E-07	0.39	13	1.3	Sacs	
DMR15:41480001	15	41480001	41486000	6000	3	7.50E-09	-0.7	35	0.58	Sacs	
DMR15:41492001	15	41492001	41494000	2000	1	8.40E-07	-0.33	25	1.25	Sacs	
DMR15:41560001	15	41560001	41561000	1000	1	1.50E-08	0.7	29	2.9	Sgcg	Cytoskeleton
DMR15:41730001	15	41730001	41731000	1000	1	7.70E-16	-0.61	24	2.4	Kpna3;LOC100911441	Transport
DMR15:41757001	15	41757001	41758000	1000	1	2.70E-08	-0.46	4	0.4	Kpna3	Transport
DMR15:41905001	15	41905001	41906000	1000	1	8.50E-12	0.7	61	6.1	Trim13	Proteolysis
DMR15:42544001	15	42544001	42548000	4000	1	7.40E-07	-0.31	44	1.1	Ccdc25;LOC102557042	
DMR15:42744001	15	42744001	42746000	2000	1	4.80E-07	-0.43	27	1.35	Adam2	Protease
DMR15:42756001	15	42756001	42760000	4000	1	1.20E-08	0.52	38	0.95	Adam2;Ephx2	Protease;Metabolism
DMR15:42978001	15	42978001	42979000	1000	1	5.60E-07	-0.37	10	1	Trim35	Proteolysis
DMR15:43347001	15	43347001	43349000	2000	1	7.20E-07	-0.6	16	0.8	Adra1a	Signaling
DMR15:43478001	15	43478001	43481000	3000	2	3.10E-08	0.5	59	1.97	Dpysl2	Metabolism
DMR15:43736001	15	43736001	43737000	1000	1	1.30E-07	0.38	11	1.1	Ppp2r2a;LOC102555410	Signaling
DMR15:43934001	15	43934001	43938000	4000	1	5.90E-07	0.44	55	1.38	Ebf2	Transcription
DMR15:44809001	15	44809001	44812000	3000	1	1.50E-11	-0.44	15	0.5	LOC108352935;Nefl	
DMR15:44867001	15	44867001	44868000	1000	1	3.30E-08	-0.69	4	0.4	Nefm	
DMR15:44869001	15	44869001	44871000	2000	1	4.50E-11	0.69	8	0.4	Nefm	
DMR15:45368001	15	45368001	45369000	1000	1	4.00E-07	-0.37	17	1.7	Dleu7	
DMR15:45461001	15	45461001	45463000	2000	1	6.00E-16	0.96	46	2.3	Rnaseh2b	
DMR15:45479001	15	45479001	45482000	3000	1	1.30E-07	-0.35	36	1.2	Rnaseh2b;Gucy1b2	Signaling
DMR15:45553001	15	45553001	45555000	2000	1	4.40E-11	0.59	50	2.5	Gucy1b2;LOC102554746	Signaling
DMR15:45732001	15	45732001	45734000	2000	1	4.80E-12	0.69	46	2.3	Fam124a	
DMR15:45735001	15	45735001	45737000	2000	1	7.30E-10	0.5	32	1.6	Fam124a	

DMR15:45778001	15	45778001	45779000	1000	1	8.20E-09	0.63	16	1.6	Fam124a	
DMR15:45830001	15	45830001	45832000	2000	1	3.70E-11	-0.55	16	0.8	Serpine3	Protease; Proteolysis
DMR15:46049001	15	46049001	46050000	1000	1	3.20E-07	-0.48	17	1.7	Wdfy2	
DMR15:46209001	15	46209001	46210000	1000	1	2.40E-17	0.7	35	3.5	Defb43	Signaling
DMR15:46305001	15	46305001	46307000	2000	1	9.40E-13	-0.49	68	3.4	Ctsb	Protease
DMR15:46407001	15	46407001	46409000	2000	1	3.10E-07	0.36	15	0.75	Gata4	Transcription
DMR15:46462001	15	46462001	46466000	4000	3	4.90E-09	0.5	38	0.95	Gata4	Transcription
DMR15:46549001	15	46549001	46552000	3000	1	6.60E-08	0.49	46	1.53	Blk	
DMR15:46591001	15	46591001	46594000	3000	1	1.10E-08	0.63	35	1.17	Blk	
DMR15:46610001	15	46610001	46612000	2000	2	1.30E-07	0.37	21	1.05	Fam167a	
DMR15:46692001	15	46692001	46694000	2000	1	1.20E-10	0.59	11	0.55	Tdh;Mtmr9	Metabolism;Signaling
DMR15:46895001	15	46895001	46896000	1000	1	1.50E-07	-0.49	13	1.3	Xkr6	
DMR15:47143001	15	47143001	47145000	2000	1	7.30E-09	0.49	27	1.35	Xkr6	
DMR15:47157001	15	47157001	47159000	2000	1	9.70E-07	-0.64	41	2.05	Xkr6	
DMR15:47172001	15	47172001	47174000	2000	1	3.10E-08	0.57	33	1.65	Xkr6	
DMR15:47201001	15	47201001	47202000	1000	1	1.60E-07	-0.5	8	0.8	LOC102553493;Pinx1	Metabolism
DMR15:47536001	15	47536001	47539000	3000	1	4.90E-07	-0.3	41	1.37	Msra	Metabolism
DMR15:47661001	15	47661001	47664000	3000	1	1.40E-09	-0.49	47	1.57	Msra	Metabolism
DMR15:47762001	15	47762001	47765000	3000	1	4.40E-10	-0.46	39	1.3	Msra	Metabolism
DMR15:47783001	15	47783001	47785000	2000	1	1.80E-07	0.47	8	0.4	Msra	Metabolism
DMR15:48186001	15	48186001	48188000	2000	1	5.90E-08	0.62	28	1.4	Kif13b;Hmbox1	Cytoskeleton
DMR15:48272001	15	48272001	48273000	1000	1	2.70E-07	-0.45	12	1.2	Hmbox1	
DMR15:48364001	15	48364001	48366000	2000	1	1.90E-07	0.58	24	1.2	Ints9	
DMR15:48446001	15	48446001	48450000	4000	1	4.40E-07	0.42	68	1.7	Extl3	Golgi
DMR15:48730001	15	48730001	48734000	4000	1	3.00E-12	0.71	56	1.4	Fbxo16	
DMR15:48829001	15	48829001	48830000	1000	1	6.20E-08	-0.64	13	1.3	Pnoc	Signaling
DMR15:51061001	15	51061001	51062000	1000	1	6.60E-07	0.44	6	0.6	Nkx3-1	Development
DMR15:51484001	15	51484001	51485000	1000	1	3.70E-07	-0.51	15	1.5	Rhobtb2;LOC103693695	Signaling
DMR15:51570001	15	51570001	51571000	1000	1	4.60E-07	-0.34	19	1.9	Pebp4	
DMR15:51668001	15	51668001	51673000	5000	1	2.70E-08	0.4	54	1.08	Pebp4	
DMR15:51732001	15	51732001	51736000	4000	1	1.50E-12	0.69	37	0.92	Pebp4	
DMR15:51743001	15	51743001	51750000	7000	1	3.10E-07	0.37	90	1.29	Pebp4;Egr3	Transcription
DMR15:51828001	15	51828001	51829000	1000	1	1.20E-09	-0.49	8	0.8	Bin3;Ccar2;RGD1308117	Epigenetic
DMR15:51888001	15	51888001	51889000	1000	1	4.90E-07	-0.66	2	0.2	Sorbs3;Ppp3cc	Signaling
DMR15:52009001	15	52009001	52011000	2000	1	7.80E-08	0.43	13	0.65	Slc39a14	Transport
DMR15:52115001	15	52115001	52116000	1000	1	4.40E-08	0.54	18	1.8	Piwil2	Translation
DMR15:52122001	15	52122001	52123000	1000	1	4.50E-08	-0.42	7	0.7	Piwil2;Polr3d	Translation;Transcription
DMR15:52236001	15	52236001	52237000	1000	1	8.70E-07	-0.34	18	1.8	Lgi3;Reep4;Hr	Transport;Epigenetic
DMR15:52434001	15	52434001	52435000	1000	1	1.00E-09	-0.57	7	0.7	Xpo7	Transport
DMR15:52846001	15	52846001	52851000	5000	1	3.10E-08	-0.24	53	1.06	Dpm3-ps1	
DMR15:54371001	15	54371001	54377000	6000	2	4.90E-09	-0.37	63	1.05	Fndc3a	Proteolysis
DMR15:54490001	15	54490001	54491000	1000	1	4.20E-07	0.54	9	0.9	Fndc3a	Proteolysis
DMR15:54502001	15	54502001	54503000	1000	1	1.20E-14	0.28	12	1.2	Fndc3a	Proteolysis
DMR15:54507001	15	54507001	54511000	4000	1	4.90E-07	-0.26	47	1.18	Fndc3a	Proteolysis
DMR15:55398001	15	55398001	55401000	3000	1	7.90E-10	-0.39	37	1.23	LOC103693716;Med4	Transcription
DMR15:55420001	15	55420001	55422000	2000	1	6.10E-09	0.52	25	1.25	Med4;Nudt15	Transcription
DMR15:56682001	15	56682001	56686000	4000	2	3.40E-62	1.5	42	1.05	Htr2a	Signaling
DMR15:56731001	15	56731001	56733000	2000	1	2.60E-07	0.54	37	1.85	Htr2a	Signaling
DMR15:56800001	15	56800001	56802000	2000	1	9.90E-07	0.43	31	1.55	Lrch1	
DMR15:56813001	15	56813001	56814000	1000	1	4.10E-08	-0.44	23	2.3	Lrch1	
DMR15:56900001	15	56900001	56901000	1000	1	3.50E-07	0.43	13	1.3	Lrch1	
DMR15:56907001	15	56907001	56909000	2000	1	2.30E-08	-0.4	29	1.45	Lrch1	
DMR15:57200001	15	57200001	57201000	1000	1	1.20E-08	-0.58	14	1.4	Lcp1	Cytoskeleton
DMR15:57206001	15	57206001	57210000	4000	1	1.20E-09	-0.52	49	1.23	Lcp1	Cytoskeleton
DMR15:57234001	15	57234001	57239000	5000	1	2.60E-08	-0.43	69	1.38	Lcp1	Cytoskeleton
DMR15:57281001	15	57281001	57282000	1000	1	1.30E-09	-0.44	5	0.5	Lcp1;Cpb2	Cytoskeleton;Protease
DMR15:57307001	15	57307001	57308000	1000	1	5.70E-07	0.61	9	0.9	Cpb2;LOC691812	Protease
DMR15:57567001	15	57567001	57569000	2000	1	7.70E-24	1.2	48	2.4	Siah3	Proteolysis
DMR15:57641001	15	57641001	57643000	2000	1	6.30E-10	0.69	39	1.95	Spert	
DMR15:57697001	15	57697001	57699000	2000	1	4.00E-10	-0.5	41	2.05	Spert;Erich6b	
DMR15:57709001	15	57709001	57710000	1000	1	4.50E-08	0.52	4	0.4	Spert;Erich6b;LOC692005	

DMR15:57713001	15	57713001	57714000	1000	1	1.80E-07	0.65	8	0.8	Spert;Erich6b;LOC692005	
DMR15:57738001	15	57738001	57739000	1000	1	8.80E-28	1.4	62	6.2	Spert;Erich6b	
DMR15:57786001	15	57786001	57787000	1000	1	3.00E-09	-0.45	10	1	Cog3	
DMR15:57818001	15	57818001	57821000	3000	1	9.40E-07	-0.38	52	1.73	Slc25a30	Transport
DMR15:57883001	15	57883001	57884000	1000	1	3.00E-07	0.63	25	2.5	LOC102552389;Tpt1	Cytoskeleton
DMR15:58045001	15	58045001	58048000	3000	1	1.60E-07	-0.62	28	0.93	Gtf2f2	Transcription
DMR15:58049001	15	58049001	58050000	1000	1	5.70E-07	-0.64	8	0.8	Gtf2f2	Transcription
DMR15:58068001	15	58068001	58069000	1000	1	5.60E-07	-0.54	53	5.3	Gtf2f2	Transcription
DMR15:58182001	15	58182001	58184000	2000	1	3.50E-10	-0.51	19	0.95	Nufip1	Metabolism
DMR15:58228001	15	58228001	58229000	1000	1	7.60E-07	0.56	13	1.3	Trnae-uuc	
DMR15:58231001	15	58231001	58234000	3000	1	1.00E-07	0.57	26	0.87	Trnae-uuc	
DMR15:58561001	15	58561001	58563000	2000	1	1.90E-07	-0.51	20	1	Tsc22d1	
DMR15:58567001	15	58567001	58570000	3000	1	2.60E-08	-0.51	44	1.47	Tsc22d1	
DMR15:58615001	15	58615001	58619000	4000	1	7.20E-09	-0.52	71	1.77	Tsc22d1	
DMR15:58629001	15	58629001	58636000	7000	3	5.00E-11	-0.54	101	1.44	Tsc22d1	
DMR15:58641001	15	58641001	58645000	4000	1	5.60E-08	-0.43	72	1.8	Tsc22d1	
DMR15:59164001	15	59164001	59166000	2000	1	7.20E-19	0.74	38	1.9	LOC108353005;Ccdc122	
DMR15:59230001	15	59230001	59232000	2000	1	6.70E-07	-0.34	25	1.25	Ccdc122	
DMR15:59287001	15	59287001	59291000	4000	1	1.40E-07	-0.33	32	0.8	Ccdc122	
DMR15:59335001	15	59335001	59337000	2000	1	8.80E-11	0.87	35	1.75	Enox1	Metabolism
DMR15:59350001	15	59350001	59353000	3000	1	7.00E-07	-0.46	46	1.53	Enox1	Metabolism
DMR15:59396001	15	59396001	59400000	4000	1	2.90E-13	-0.58	40	1	Enox1	Metabolism
DMR15:59549001	15	59549001	59551000	2000	1	1.70E-09	0.53	22	1.1	Enox1	Metabolism
DMR15:59583001	15	59583001	59586000	3000	1	1.30E-07	-0.29	171	5.7	Enox1	Metabolism
DMR15:59640001	15	59640001	59642000	2000	1	1.20E-07	0.53	19	0.95	Enox1	Metabolism
DMR15:59681001	15	59681001	59683000	2000	1	2.60E-13	-0.57	28	1.4	Enox1	Metabolism
DMR15:59739001	15	59739001	59747000	8000	1	1.40E-09	0.33	141	1.76	Enox1	Metabolism
DMR15:59796001	15	59796001	59798000	2000	1	1.60E-09	0.54	34	1.7	Enox1	Metabolism
DMR15:60005001	15	60005001	60006000	1000	1	8.10E-07	-0.34	15	1.5	Dnajc15	
DMR15:60077001	15	60077001	60080000	3000	2	6.80E-10	-0.75	30	1	Epsti1	
DMR15:60164001	15	60164001	60166000	2000	2	1.40E-09	-0.41	34	1.7	Epsti1	
DMR15:60833001	15	60833001	60834000	1000	1	7.60E-07	-0.37	13	1.3	Dgkh	Signaling
DMR15:60872001	15	60872001	60875000	3000	1	4.90E-07	0.54	39	1.3	Dgkh	Signaling
DMR15:60890001	15	60890001	60894000	4000	1	2.40E-07	-0.34	67	1.68	Dgkh	Signaling
DMR15:60901001	15	60901001	60905000	4000	1	1.40E-07	0.43	46	1.15	Dgkh	Signaling
DMR15:61139001	15	61139001	61141000	2000	1	2.60E-09	-0.6	24	1.2	Vwa8	
DMR15:61171001	15	61171001	61172000	1000	1	3.60E-07	-0.67	10	1	Vwa8	
DMR15:61247001	15	61247001	61249000	2000	1	7.80E-07	0.54	24	1.2	Vwa8	
DMR15:61352001	15	61352001	61357000	5000	1	1.70E-08	0.34	70	1.4	Vwa8	
DMR15:61588001	15	61588001	61590000	2000	1	6.00E-10	0.79	44	2.2	LOC100909790;Naa16	Metabolism
DMR15:61593001	15	61593001	61596000	3000	1	5.30E-20	1.14	60	2	LOC100909790;Naa16	Metabolism
DMR15:61814001	15	61814001	61816000	2000	1	1.50E-08	-0.38	30	1.5	Elf1;LOC102552221	Transcription
DMR15:61858001	15	61858001	61859000	1000	1	2.70E-09	-0.7	4	0.4	Elf1	Transcription
DMR15:61926001	15	61926001	61930000	4000	1	1.70E-07	-0.31	69	1.73	Lect1	
DMR15:61947001	15	61947001	61950000	3000	1	2.80E-10	0.71	48	1.6	Lect1	
DMR15:62197001	15	62197001	62198000	1000	1	6.70E-08	0.55	20	2	Pcdh8;LOC108352948	Cytoskeleton
DMR15:69959001	15	69959001	69961000	2000	1	9.10E-07	-0.36	21	1.05	Diaph3	
DMR15:70043001	15	70043001	70045000	2000	1	2.10E-07	-0.27	37	1.85	Diaph3	
DMR15:70122001	15	70122001	70123000	1000	1	3.10E-08	0.47	10	1	Diaph3	
DMR15:70155001	15	70155001	70158000	3000	1	2.00E-08	-0.25	29	0.97	Diaph3	
DMR15:70211001	15	70211001	70213000	2000	1	1.40E-08	-0.61	18	0.9	Diaph3	
DMR15:70292001	15	70292001	70296000	4000	1	5.90E-10	-0.39	37	0.92	Diaph3	
DMR15:70298001	15	70298001	70299000	1000	1	3.10E-07	0.39	13	1.3	Diaph3	
DMR15:70404001	15	70404001	70406000	2000	1	5.20E-07	-0.37	19	0.95	Diaph3	
DMR15:70802001	15	70802001	70803000	1000	1	1.20E-07	-0.44	14	1.4	Tdrd3	Translation
DMR15:70868001	15	70868001	70870000	2000	1	4.00E-09	-0.48	18	0.9	Tdrd3	Translation
DMR15:76779001	15	76779001	76780000	1000	1	4.20E-08	-0.41	2	0.2	Pcdh9	Cytoskeleton
DMR15:76892001	15	76892001	76893000	1000	1	1.60E-07	-0.37	14	1.4	Pcdh9	Cytoskeleton
DMR15:76934001	15	76934001	76935000	1000	1	7.10E-08	-0.53	13	1.3	Pcdh9	Cytoskeleton
DMR15:77051001	15	77051001	77052000	1000	1	8.30E-11	0.79	19	1.9	Pcdh9	Cytoskeleton
DMR15:77074001	15	77074001	77075000	1000	1	3.50E-15	-0.51	13	1.3	Pcdh9	Cytoskeleton
DMR15:77213001	15	77213001	77215000	2000	1	9.70E-09	0.61	26	1.3	Pcdh9	Cytoskeleton

DMR15:77286001	15	77286001	77288000	2000	1	6.60E-07	-0.48	20	1	Pcdh9	Cytoskeleton
DMR15:77336001	15	77336001	77338000	2000	2	2.20E-12	-0.61	12	0.6	Pcdh9	Cytoskeleton
DMR15:77382001	15	77382001	77383000	1000	1	4.10E-07	0.49	16	1.6	Pcdh9	Cytoskeleton
DMR15:77425001	15	77425001	77428000	3000	1	1.20E-07	0.49	23	0.77	Pcdh9	Cytoskeleton
DMR15:77464001	15	77464001	77465000	1000	1	5.50E-07	0.43	12	1.2	Pcdh9	Cytoskeleton
DMR15:77605001	15	77605001	77607000	2000	1	7.60E-09	0.54	26	1.3	Pcdh9	Cytoskeleton
DMR15:77685001	15	77685001	77686000	1000	1	9.80E-07	-0.35	16	1.6	Pcdh9	Cytoskeleton
DMR15:77717001	15	77717001	77718000	1000	1	3.40E-07	0.57	10	1	Pcdh9	Cytoskeleton
DMR15:77734001	15	77734001	77735000	1000	1	8.80E-13	0.98	36	3.6	Pcdh9	Cytoskeleton
DMR15:80298001	15	80298001	80299000	1000	1	1.90E-07	-0.39	12	1.2	Klhl1	Cytoskeleton
DMR15:80471001	15	80471001	80472000	1000	1	5.70E-11	-0.6	2	0.2	Klhl1	Cytoskeleton
DMR15:80566001	15	80566001	80567000	1000	1	1.30E-08	0.45	9	0.9	Klhl1	Cytoskeleton
DMR15:80578001	15	80578001	80581000	3000	1	5.00E-10	-0.3	27	0.9	Klhl1	Cytoskeleton
DMR15:80616001	15	80616001	80617000	1000	1	1.70E-07	-0.62	3	0.3	Klhl1	Cytoskeleton
DMR15:80664001	15	80664001	80666000	2000	1	4.40E-07	-0.56	6	0.3	Klhl1	Cytoskeleton
DMR15:82080001	15	82080001	82082000	2000	2	3.70E-13	0.96	47	2.35	Setsip	
DMR15:82149001	15	82149001	82152000	3000	1	1.70E-09	0.49	24	0.8	Dach1	Transcription
DMR15:82397001	15	82397001	82401000	4000	1	5.90E-07	-0.41	54	1.35	Dach1	Transcription
DMR15:82406001	15	82406001	82407000	1000	1	2.10E-08	0.66	21	2.1	Dach1	Transcription
DMR15:82447001	15	82447001	82449000	2000	1	3.30E-07	-0.45	21	1.05	Dach1	Transcription
DMR15:82454001	15	82454001	82455000	1000	1	1.70E-09	-0.7	3	0.3	Dach1	Transcription
DMR15:83418001	15	83418001	83420000	2000	1	1.00E-08	0.55	40	2	Mzt1	
DMR15:83479001	15	83479001	83480000	1000	1	1.80E-09	-0.47	12	1.2	Dis3;LOC100361028	Transcription
DMR15:83517001	15	83517001	83519000	2000	1	1.70E-11	-0.46	10	0.5	Pibf1	
DMR15:83597001	15	83597001	83598000	1000	1	1.00E-07	-0.29	10	1	Pibf1	
DMR15:83702001	15	83702001	83706000	4000	1	5.40E-09	-0.49	70	1.75	Klf5	Transcription
DMR15:83709001	15	83709001	83710000	1000	1	1.10E-09	0.72	31	3.1	Klf5	Transcription
DMR15:83714001	15	83714001	83715000	1000	1	3.20E-08	-0.51	13	1.3	Klf5	Transcription
DMR15:84328001	15	84328001	84329000	1000	1	7.20E-08	-0.64	2	0.2	Klf12;LOC103693766	Transcription
DMR15:84333001	15	84333001	84334000	1000	1	3.40E-09	0.47	8	0.8	Klf12;LOC103693766	Transcription
DMR15:84662001	15	84662001	84663000	1000	1	2.10E-17	-0.56	10	1	Klf12	Transcription
DMR15:85111001	15	85111001	85112000	1000	1	8.30E-09	0.55	10	1	RGD1560797	
DMR15:85121001	15	85121001	85124000	3000	1	1.10E-08	-0.4	24	0.8	RGD1560797;LOC108352953;LOC108352954	
DMR15:85928001	15	85928001	85929000	1000	1	7.50E-08	0.36	20	2	Tbc1d4	Signaling
DMR15:85949001	15	85949001	85951000	2000	1	2.50E-07	-0.46	25	1.25	Tbc1d4	Signaling
DMR15:86018001	15	86018001	86022000	4000	1	6.20E-07	-0.32	60	1.5	Tbc1d4	Signaling
DMR15:86133001	15	86133001	86134000	1000	1	2.60E-10	-0.4	15	1.5	RGD2320734;Commd6	
DMR15:86236001	15	86236001	86240000	4000	1	8.50E-07	-0.39	49	1.23	Lmo7	
DMR15:86274001	15	86274001	86275000	1000	1	2.10E-11	0.82	21	2.1	Lmo7	
DMR15:86304001	15	86304001	86305000	1000	1	2.30E-09	0.6	29	2.9	Lmo7	
DMR15:86309001	15	86309001	86311000	2000	1	3.40E-07	-0.34	44	2.2	Lmo7	
DMR15:86341001	15	86341001	86342000	1000	1	9.80E-07	0.51	12	1.2	Lmo7	
DMR15:86368001	15	86368001	86369000	1000	1	5.70E-09	-0.46	7	0.7	Lmo7	
DMR15:86381001	15	86381001	86382000	1000	1	6.10E-08	-0.49	7	0.7	Lmo7	
DMR15:86400001	15	86400001	86402000	2000	2	5.30E-08	-0.4	38	1.9	Lmo7	
DMR15:86416001	15	86416001	86418000	2000	1	2.90E-14	-0.49	35	1.75	Lmo7	
DMR15:86609001	15	86609001	86610000	1000	1	1.30E-09	0.57	15	1.5	LOC290444;RGD1562819	
DMR15:87407001	15	87407001	87408000	1000	1	1.30E-08	-0.53	8	0.8	Mycbp2	Proteolysis
DMR15:87687001	15	87687001	87689000	2000	1	1.20E-07	0.44	26	1.3	LOC108352960;Scel	
DMR15:87708001	15	87708001	87709000	1000	1	2.20E-14	0.84	16	1.6	Scel	
DMR15:87733001	15	87733001	87735000	2000	1	1.40E-09	0.45	36	1.8	Scel	
DMR15:87751001	15	87751001	87757000	6000	1	2.40E-09	0.76	66	1.1	Scel	
DMR15:87890001	15	87890001	87896000	6000	1	2.80E-07	-0.38	98	1.63	Slain1	
DMR15:87899001	15	87899001	87901000	2000	1	7.90E-07	0.35	19	0.95	Slain1	
DMR15:88037001	15	88037001	88039000	2000	1	1.10E-08	0.47	23	1.15	Ednrb;LOC102550182	
DMR15:88625001	15	88625001	88628000	3000	1	6.10E-18	-0.77	66	2.2	Pou4f1;Rnf219	
DMR15:89309001	15	89309001	89311000	2000	1	5.80E-08	-0.38	26	1.3	Rbm26	Metabolism
DMR15:89331001	15	89331001	89332000	1000	1	2.20E-08	-0.39	4	0.4	Rbm26	Metabolism
DMR15:89423001	15	89423001	89424000	1000	1	1.00E-07	-0.39	17	1.7	Ndfip2	
DMR15:89430001	15	89430001	89431000	1000	1	3.60E-12	0.62	11	1.1	Ndfip2	
DMR15:89445001	15	89445001	89447000	2000	1	1.30E-20	0.89	51	2.55	Ndfip2	

DMR15:90171001	15	90171001	90174000	3000	1	1.20E-08	-0.45	50	1.67	Spry2	Cytoskeleton
DMR15:90361001	15	90361001	90363000	2000	1	1.40E-11	0.78	27	1.35	LOC103693771;Trim52	Proteolysis
DMR15:93804001	15	93804001	93807000	3000	2	5.70E-10	-0.48	16	0.53	Mycbp2	Proteolysis
DMR15:93884001	15	93884001	93886000	2000	2	9.20E-13	-0.7	17	0.85	Mycbp2	Proteolysis
DMR15:95510001	15	95510001	95511000	1000	1	2.80E-08	0.72	23	2.3	Slitrk6	
DMR15:100284001	15	100284001	100285000	1000	1	3.80E-07	0.56	8	0.8	LOC102548462;Gpc5	
DMR15:100398001	15	100398001	100400000	2000	1	2.90E-08	-0.47	35	1.75	Gpc5	
DMR15:100402001	15	100402001	100404000	2000	1	5.60E-07	-0.48	14	0.7	Gpc5	
DMR15:100472001	15	100472001	100479000	7000	1	1.70E-07	0.35	66	0.94	Gpc5	
DMR15:100545001	15	100545001	100546000	1000	1	1.00E-07	0.41	9	0.9	Gpc5	
DMR15:100744001	15	100744001	100746000	2000	1	6.00E-07	0.62	31	1.55	Gpc5	
DMR15:100810001	15	100810001	100813000	3000	1	1.80E-10	0.72	18	0.6	Gpc5	
DMR15:100898001	15	100898001	100900000	2000	1	1.60E-08	0.41	17	0.85	Gpc5	
DMR15:100910001	15	100910001	100912000	2000	1	3.60E-07	-0.36	25	1.25	Gpc5	
DMR15:100913001	15	100913001	100914000	1000	1	3.90E-07	-0.34	3	0.3	Gpc5	
DMR15:100999001	15	100999001	1.01E+08	1000	1	3.90E-08	0.61	22	2.2	Gpc5	
DMR15:101138001	15	101138001	101139000	1000	1	7.30E-08	0.35	12	1.2	Gpc5	
DMR15:101237001	15	101237001	101239000	2000	2	2.90E-10	-0.61	3	0.15	Gpc5	
DMR15:101335001	15	101335001	101337000	2000	1	8.30E-08	0.53	12	0.6	Gpc5	
DMR15:101475001	15	101475001	101476000	1000	1	8.60E-07	0.37	12	1.2	Gpc5	
DMR15:101688001	15	101688001	101690000	2000	1	7.80E-08	-0.51	28	1.4	Gpc5	
DMR15:101757001	15	101757001	101758000	1000	1	3.20E-11	0.54	22	2.2	Gpc5	
DMR15:102156001	15	102156001	102158000	2000	2	2.10E-18	1.17	48	2.4	Gpc6	
DMR15:102428001	15	102428001	102429000	1000	1	9.40E-09	0.81	27	2.7	Gpc6	
DMR15:102449001	15	102449001	102450000	1000	1	7.10E-11	0.76	18	1.8	Gpc6	
DMR15:102457001	15	102457001	102458000	1000	1	5.00E-09	0.61	8	0.8	Gpc6	
DMR15:102499001	15	102499001	102502000	3000	1	1.20E-09	0.59	40	1.33	Gpc6;LOC103693784	
DMR15:102507001	15	102507001	102509000	2000	1	4.00E-22	0.96	35	1.75	Gpc6;LOC103693784	
DMR15:102514001	15	102514001	102516000	2000	1	1.10E-07	0.45	27	1.35	Gpc6	
DMR15:102524001	15	102524001	102525000	1000	1	5.20E-18	0.39	13	1.3	Gpc6	
DMR15:102540001	15	102540001	102541000	1000	1	4.60E-08	-0.42	5	0.5	Gpc6	
DMR15:102568001	15	102568001	102570000	2000	1	2.10E-08	0.36	21	1.05	Gpc6;LOC103693783	
DMR15:102582001	15	102582001	102584000	2000	1	2.00E-09	0.75	25	1.25	Gpc6;LOC103693783	
DMR15:102696001	15	102696001	102698000	2000	1	7.00E-09	0.45	24	1.2	Gpc6	
DMR15:102701001	15	102701001	102702000	1000	1	5.70E-09	-0.46	23	2.3	Gpc6	
DMR15:102749001	15	102749001	102750000	1000	1	9.50E-09	0.53	8	0.8	Gpc6	
DMR15:102808001	15	102808001	102809000	1000	1	1.30E-11	1.02	33	3.3	Gpc6	
DMR15:102875001	15	102875001	102877000	2000	1	2.20E-09	-0.43	38	1.9	Gpc6	
DMR15:102962001	15	102962001	102964000	2000	1	7.40E-07	0.5	27	1.35	Gpc6	
DMR15:102966001	15	102966001	102967000	1000	1	6.50E-07	-0.37	17	1.7	Gpc6	
DMR15:102987001	15	102987001	102988000	1000	1	5.80E-09	0.51	25	2.5	Gpc6	
DMR15:103073001	15	103073001	103074000	1000	1	3.70E-07	0.48	16	1.6	Gpc6	
DMR15:103095001	15	103095001	103099000	4000	1	1.20E-08	0.66	76	1.9	Gpc6	
DMR15:103116001	15	103116001	103120000	4000	1	1.40E-09	-0.43	70	1.75	Gpc6	
DMR15:103122001	15	103122001	103123000	1000	1	2.00E-14	0.87	45	4.5	Gpc6	
DMR15:103124001	15	103124001	103125000	1000	1	6.20E-17	0.65	21	2.1	Gpc6	
DMR15:103168001	15	103168001	103171000	3000	1	3.60E-10	0.8	64	2.13	Gpc6	
DMR15:103214001	15	103214001	103215000	1000	1	5.80E-07	-0.31	24	2.4	Dct	
DMR15:103236001	15	103236001	103241000	5000	1	9.20E-07	0.63	83	1.66	Dct	
DMR15:103726001	15	103726001	103730000	4000	1	9.80E-09	-0.42	104	2.6	Abcc4	Transport
DMR15:103739001	15	103739001	103741000	2000	2	5.60E-07	-0.4	73	3.65	Abcc4	Transport
DMR15:103776001	15	103776001	103777000	1000	1	2.80E-08	-0.6	9	0.9	Abcc4	Transport
DMR15:103779001	15	103779001	103787000	8000	3	3.00E-09	-0.38	180	2.25	Abcc4	Transport
DMR15:103830001	15	103830001	103832000	2000	1	8.80E-07	-0.43	51	2.55	Abcc4	Transport
DMR15:103848001	15	103848001	103850000	2000	1	6.80E-08	-0.41	61	3.05	Abcc4	Transport
DMR15:103861001	15	103861001	103864000	3000	1	1.80E-07	-0.36	71	2.37	Abcc4	Transport
DMR15:103881001	15	103881001	103883000	2000	1	6.50E-07	0.42	33	1.65	Abcc4	Transport
DMR15:103904001	15	103904001	103910000	6000	1	4.10E-09	-0.47	133	2.22	Abcc4	Transport
DMR15:104038001	15	104038001	104039000	1000	1	4.90E-07	-0.43	34	3.4	Cldn10	Cell Junction
DMR15:104049001	15	104049001	104054000	5000	2	2.30E-08	0.55	66	1.32	Cldn10	Cell Junction
DMR15:104055001	15	104055001	104056000	1000	1	4.00E-08	0.68	22	2.2	Cldn10	Cell Junction
DMR15:104110001	15	104110001	104112000	2000	1	4.80E-07	-0.51	34	1.7	Cldn10;Dzip1	Cell Junction

DMR15:104147001	15	104147001	104149000	2000	1	6.90E-08	-0.42	60	3	Dzip1	
DMR15:104156001	15	104156001	104160000	4000	2	2.50E-08	-0.44	101	2.52	Dzip1	
DMR15:104220001	15	104220001	104224000	4000	1	6.50E-07	-0.49	64	1.6	Dnajc3	
DMR15:104280001	15	104280001	104281000	1000	1	1.70E-07	-0.32	14	1.4	Uggt2	
DMR15:104290001	15	104290001	104291000	1000	1	2.10E-08	-0.38	14	1.4	Uggt2	
DMR15:104342001	15	104342001	104343000	1000	1	5.80E-07	0.41	7	0.7	Uggt2	
DMR15:104442001	15	104442001	104446000	4000	2	2.00E-18	1.04	91	2.28	Hs6st3	
DMR15:104463001	15	104463001	104467000	4000	1	9.20E-07	-0.27	64	1.6	Hs6st3	
DMR15:104494001	15	104494001	104496000	2000	1	4.70E-07	-0.38	33	1.65	Hs6st3	
DMR15:104571001	15	104571001	104576000	5000	1	2.20E-09	-0.43	105	2.1	Hs6st3	
DMR15:104622001	15	104622001	104625000	3000	1	2.70E-10	-0.47	28	0.93	Hs6st3	
DMR15:104629001	15	104629001	104631000	2000	1	7.00E-07	-0.32	52	2.6	Hs6st3	
DMR15:104641001	15	104641001	104645000	4000	1	3.10E-08	-0.28	40	1	Hs6st3	
DMR15:104728001	15	104728001	104731000	3000	1	1.10E-11	-0.54	46	1.53	Hs6st3	
DMR15:104776001	15	104776001	104777000	1000	1	4.90E-10	0.6	19	1.9	Hs6st3	
DMR15:104900001	15	104900001	104901000	1000	1	2.40E-14	-0.67	15	1.5	Hs6st3	
DMR15:104937001	15	104937001	104939000	2000	1	2.00E-08	-0.31	21	1.05	Hs6st3	
DMR15:105152001	15	105152001	105154000	2000	1	1.00E-07	-0.48	16	0.8	Hs6st3	
DMR15:105182001	15	105182001	105183000	1000	1	9.70E-11	0.92	31	3.1	Hs6st3	
DMR15:105205001	15	105205001	105206000	1000	1	6.60E-07	-0.34	25	2.5	Hs6st3	
DMR15:105216001	15	105216001	105218000	2000	1	3.80E-09	-0.5	38	1.9	Hs6st3;LOC102556639	
DMR15:105229001	15	105229001	105232000	3000	1	1.50E-08	0.78	57	1.9	Hs6st3;LOC102556639	
DMR15:105395001	15	105395001	105398000	3000	1	2.00E-19	1.37	64	2.13	LOC290492;LOC680256;Oxgr1;<br>LOC108352979	Signaling
DMR15:105662001	15	105662001	105665000	3000	1	7.00E-08	-0.45	57	1.9	Mbnl2	Translation
DMR15:105671001	15	105671001	105679000	8000	2	1.40E-09	-0.58	186	2.33	Mbnl2	Translation
DMR15:105692001	15	105692001	105696000	4000	1	4.10E-07	0.36	64	1.6	Mbnl2	Translation
DMR15:105706001	15	105706001	105707000	1000	1	6.60E-09	-0.45	13	1.3	Mbnl2	Translation
DMR15:105711001	15	105711001	105714000	3000	1	3.40E-10	-0.47	66	2.2	Mbnl2	Translation
DMR15:105778001	15	105778001	105780000	2000	1	5.30E-10	0.5	31	1.55	Mbnl2	Translation
DMR15:106250001	15	106250001	106254000	4000	1	2.60E-07	0.7	64	1.6	Ipo5	Transport
DMR15:106263001	15	106263001	106264000	1000	1	2.00E-07	-0.38	25	2.5	Ipo5	Transport
DMR15:106482001	15	106482001	106483000	1000	1	9.90E-14	-0.67	25	2.5	Farp1	
DMR15:106547001	15	106547001	106548000	1000	1	4.30E-08	0.41	16	1.6	Farp1	
DMR15:106585001	15	106585001	106586000	1000	1	1.10E-17	1.03	37	3.7	Farp1	
DMR15:106627001	15	106627001	106631000	4000	1	3.50E-11	-0.44	65	1.62	Farp1;Stk24	
DMR15:106639001	15	106639001	106641000	2000	1	8.20E-09	-0.54	29	1.45	Stk24	
DMR15:106675001	15	106675001	106679000	4000	1	4.50E-08	-0.45	76	1.9	Stk24	
DMR15:106826001	15	106826001	106827000	1000	1	2.80E-09	0.57	16	1.6	Slc15a1;Lnc004	Transport
DMR15:106845001	15	106845001	106846000	1000	1	4.80E-10	0.91	29	2.9	Slc15a1	Transport
DMR15:107170001	15	107170001	107172000	2000	1	6.60E-14	0.81	32	1.6	Olr1865-ps	
DMR15:107186001	15	107186001	107187000	1000	1	1.30E-07	-0.53	9	0.9	Olr1865-ps	
DMR15:107386001	15	107386001	107388000	2000	2	1.00E-07	0.41	60	3	Olr1775-ps	
DMR15:107402001	15	107402001	107403000	1000	1	3.90E-11	-0.59	3	0.3	Olr1775-ps	
DMR15:107948001	15	107948001	107955000	7000	1	4.40E-09	0.77	141	2.01	Dock9	Transcription
DMR15:108084001	15	108084001	108085000	1000	1	9.70E-07	0.37	14	1.4	Dock9	Transcription
DMR15:108148001	15	108148001	108150000	2000	1	1.20E-07	0.59	29	1.45	Dock9	Transcription
DMR15:108281001	15	108281001	108282000	1000	1	6.50E-07	-0.51	10	1	LOC103693804;Ubac2	
DMR15:108379001	15	108379001	108380000	1000	1	9.70E-07	0.4	3	0.3	Ubac2;Gpr183	Signaling
DMR15:108453001	15	108453001	108454000	1000	1	1.00E-08	0.77	27	2.7	LOC108352982;Timm8a2	
DMR15:108529001	15	108529001	108531000	2000	2	1.80E-09	-0.53	21	1.05	LOC103693805;Tm9sf2	Transport
DMR15:108714001	15	108714001	108715000	1000	1	7.90E-09	-0.36	25	2.5	Clybl	Metabolism
DMR15:108755001	15	108755001	108756000	1000	1	9.80E-09	-0.58	7	0.7	Clybl	Metabolism
DMR15:108794001	15	108794001	108795000	1000	1	5.20E-10	0.73	21	2.1	Clybl	Metabolism
DMR15:108947001	15	108947001	108952000	5000	1	1.30E-08	-0.39	41	0.82	Pcca	Metabolism
DMR15:108953001	15	108953001	108954000	1000	1	2.70E-07	-0.42	7	0.7	Pcca	Metabolism
DMR15:109043001	15	109043001	109044000	1000	1	6.60E-15	0.43	10	1	Pcca	Metabolism
DMR15:109060001	15	109060001	109061000	1000	1	3.10E-14	0.46	24	2.4	Pcca	Metabolism
DMR15:109928001	15	109928001	109930000	2000	1	8.30E-07	-0.49	13	0.65	Nalcn	Transport
DMR15:110055001	15	110055001	110056000	1000	1	1.10E-08	-0.27	9	0.9	Nalcn	Transport
DMR15:110160001	15	110160001	110161000	1000	1	2.20E-10	0.51	12	1.2	Itgbl1	Extracellular Matrix
DMR15:110211001	15	110211001	110215000	4000	1	4.80E-07	-0.37	33	0.82	Itgbl1	Extracellular Matrix

DMR15:110228001	15	110228001	110229000	1000	1	2.40E-07	-0.56	6	0.6	Itgbl1	Extracellular Matrix
DMR15:110268001	15	110268001	110269000	1000	1	1.90E-07	-0.35	4	0.4	Itgbl1	Extracellular Matrix
DMR15:110431001	15	110431001	110436000	5000	1	2.20E-07	-0.36	45	0.9	Fgf14	Growth Factors
DMR15:110473001	15	110473001	110475000	2000	2	4.60E-10	0.62	21	1.05	Fgf14	Growth Factors
DMR15:110507001	15	110507001	110513000	6000	1	6.40E-09	-0.28	62	1.03	Fgf14	Growth Factors
DMR15:110663001	15	110663001	110665000	2000	1	2.20E-08	-0.57	14	0.7	Fgf14	Growth Factors
DMR15:110715001	15	110715001	110716000	1000	1	2.60E-10	0.45	4	0.4	Fgf14	Growth Factors
DMR16:1784001	16	1784001	1787000	3000	1	2.80E-08	0.52	40	1.33	Zmiz1	
DMR16:1843001	16	1843001	1847000	4000	2	1.60E-09	-0.7	24	0.6	Zmiz1	
DMR16:1892001	16	1892001	1893000	1000	1	3.30E-11	0.78	23	2.3	Zmiz1;LOC108353066	
DMR16:1930001	16	1930001	1932000	2000	1	7.20E-09	0.66	26	1.3	Zmiz1	
DMR16:1950001	16	1950001	1955000	5000	1	3.90E-08	0.51	70	1.4	Zmiz1;LOC108353088	
DMR16:1988001	16	1988001	1990000	2000	1	2.40E-07	0.49	22	1.1	Ppif;Zcchc24	Transcription
DMR16:1994001	16	1994001	1996000	2000	1	1.10E-07	0.37	22	1.1	Ppif;Zcchc24	Transcription
DMR16:2037001	16	2037001	2039000	2000	1	5.50E-07	0.44	26	1.3	Zcchc24	
DMR16:2185001	16	2185001	2187000	2000	2	1.00E-08	-0.56	11	0.55	Slmap	Cytoskeleton
DMR16:2232001	16	2232001	2233000	1000	1	1.10E-11	0.9	26	2.6	Slmap	Cytoskeleton
DMR16:2376001	16	2376001	2377000	1000	1	1.30E-07	-0.44	2	0.2	Pde12;LOC108353069;Dnah12;LOC102547893	Translation;Cytoskeleton
DMR16:2394001	16	2394001	2395000	1000	1	6.40E-13	0.65	23	2.3	Dnah12;LOC102547893	Cytoskeleton
DMR16:2399001	16	2399001	2403000	4000	2	1.70E-08	-0.4	46	1.15	Dnah12	Cytoskeleton
DMR16:2405001	16	2405001	2406000	1000	1	8.60E-07	0.5	8	0.8	Dnah12	Cytoskeleton
DMR16:2465001	16	2465001	2467000	2000	1	1.10E-13	0.7	38	1.9	Dnah12;LOC102547798	Cytoskeleton
DMR16:2536001	16	2536001	2537000	1000	1	7.40E-10	0.53	17	1.7	Dnah12;Asb14	Cytoskeleton;Transport
DMR16:2618001	16	2618001	2619000	1000	1	2.50E-09	0.55	24	2.4	Hesx1	Development
DMR16:2620001	16	2620001	2621000	1000	1	1.40E-11	0.84	28	2.8	Hesx1	Development
DMR16:2628001	16	2628001	2629000	1000	1	4.40E-19	0.92	29	2.9	Hesx1	Development
DMR16:2697001	16	2697001	2701000	4000	2	9.70E-09	-0.45	61	1.52	Il17rd	Receptor
DMR16:2737001	16	2737001	2741000	4000	1	2.50E-09	0.44	62	1.55	Il17rd;LOC102548056;Arhgef3	Receptor;Transcription
DMR16:2782001	16	2782001	2783000	1000	1	2.20E-08	0.56	17	1.7	Arhgef3	Transcription
DMR16:2803001	16	2803001	2806000	3000	1	7.40E-11	-0.51	50	1.67	Arhgef3	Transcription
DMR16:2821001	16	2821001	2823000	2000	2	3.10E-13	0.43	21	1.05	Arhgef3;LOC108353070	Transcription
DMR16:2852001	16	2852001	2853000	1000	1	2.30E-07	0.35	13	1.3	Arhgef3	Transcription
DMR16:2864001	16	2864001	2869000	5000	1	1.00E-07	0.36	46	0.92	Arhgef3	Transcription
DMR16:2946001	16	2946001	2948000	2000	1	3.50E-09	-0.5	31	1.55	Arhgef3	Transcription
DMR16:3271001	16	3271001	3273000	2000	1	1.30E-08	0.43	32	1.6	Erc2	Transport
DMR16:3380001	16	3380001	3383000	3000	1	1.60E-19	1.14	57	1.9	Erc2;LOC108348452	Transport
DMR16:3398001	16	3398001	3401000	3000	2	3.30E-09	-0.57	14	0.47	Erc2;LOC103693877	Transport
DMR16:3422001	16	3422001	3423000	1000	1	1.10E-08	0.81	20	2	Erc2	Transport
DMR16:3465001	16	3465001	3466000	1000	1	5.00E-11	0.58	11	1.1	Erc2	Transport
DMR16:3511001	16	3511001	3513000	2000	1	8.60E-10	-0.46	21	1.05	Erc2	Transport
DMR16:3709001	16	3709001	3710000	1000	1	9.20E-08	0.44	29	2.9	Erc2;LOC100911661	Transport
DMR16:3808001	16	3808001	3811000	3000	1	6.40E-07	-0.42	63	2.1	Erc2;Fam213a	Transport
DMR16:3882001	16	3882001	3883000	1000	1	3.40E-10	0.52	13	1.3	Erc2;Anxa11	Transport;Signaling
DMR16:3897001	16	3897001	3900000	3000	1	5.90E-07	-0.35	24	0.8	Erc2;Anxa11	Transport;Signaling
DMR16:3913001	16	3913001	3915000	2000	2	2.70E-12	0.89	35	1.75	Erc2;Anxa11;LOC108353089	Transport;Signaling
DMR16:3956001	16	3956001	3958000	2000	1	4.60E-07	0.36	19	0.95	Erc2	Transport
DMR16:3967001	16	3967001	3970000	3000	1	6.40E-08	0.66	43	1.43	Erc2	Transport
DMR16:4221001	16	4221001	4224000	3000	2	5.00E-07	-0.4	50	1.67	Erc2	Transport
DMR16:4479001	16	4479001	4482000	3000	1	1.90E-09	0.81	54	1.8	Wnt5a	Signaling
DMR16:4895001	16	4895001	4897000	2000	1	2.30E-07	0.49	23	1.15	Cacna2d3	Transport
DMR16:5197001	16	5197001	5201000	4000	1	1.30E-08	-0.35	38	0.95	Cacna2d3	Transport
DMR16:5299001	16	5299001	5300000	1000	1	6.70E-10	-0.48	17	1.7	Cacna2d3	Transport
DMR16:5441001	16	5441001	5442000	1000	1	4.30E-08	-0.41	10	1	Cacna2d3	Transport
DMR16:5505001	16	5505001	5506000	1000	1	6.10E-07	0.34	16	1.6	Cacna2d3	Transport
DMR16:5586001	16	5586001	5587000	1000	1	7.70E-08	0.69	24	2.4	Cacna2d3	Transport
DMR16:5673001	16	5673001	5674000	1000	1	5.80E-10	0.31	18	1.8	Cacna2d3	Transport
DMR16:5676001	16	5676001	5677000	1000	1	2.80E-09	0.29	7	0.7	Cacna2d3	Transport
DMR16:5679001	16	5679001	5681000	2000	1	8.20E-23	1.21	38	1.9	Cacna2d3	Transport
DMR16:5715001	16	5715001	5716000	1000	1	5.30E-11	0.85	24	2.4	Cacna2d3	Transport
DMR16:5773001	16	5773001	5774000	1000	1	1.60E-08	0.56	9	0.9	Cacna2d3	Transport
DMR16:6056001	16	6056001	6059000	3000	1	5.30E-09	-0.48	89	2.97	Actr8;Il17rb	Cytoskeleton;Receptor

DMR16:6074001	16	6074001	6076000	2000	1	4.80E-08	0.55	34	1.7	Il17rb;Chdh	Receptor;Metabolism
DMR16:6103001	16	6103001	6106000	3000	1	1.60E-07	0.49	64	2.13	Chdh;Cacna1d	Metabolism;Transport
DMR16:6107001	16	6107001	6108000	1000	1	3.90E-08	0.65	23	2.3	Chdh;Cacna1d	Metabolism;Transport
DMR16:6116001	16	6116001	6118000	2000	1	2.90E-12	0.68	28	1.4	Chdh;Cacna1d	Metabolism;Transport
DMR16:6146001	16	6146001	6153000	7000	1	6.50E-07	0.35	128	1.83	Cacna1d	Transport
DMR16:6171001	16	6171001	6174000	3000	1	4.30E-07	-0.4	56	1.87	Cacna1d;LOC108348454	Transport
DMR16:6300001	16	6300001	6306000	6000	1	6.20E-07	0.35	115	1.92	Cacna1d;LOC103693892	Transport
DMR16:6361001	16	6361001	6365000	4000	1	1.20E-07	-0.35	80	2	Cacna1d	Transport
DMR16:6605001	16	6605001	6607000	2000	1	5.40E-14	0.71	26	1.3	LOC102555502;Tkt	Metabolism
DMR16:6768001	16	6768001	6769000	1000	1	7.50E-10	0.62	19	1.9	Sfmbt1	Epigenetic
DMR16:6812001	16	6812001	6813000	1000	1	3.70E-08	-0.47	13	1.3	Sfmbt1	Epigenetic
DMR16:6906001	16	6906001	6909000	3000	1	4.00E-08	-0.49	50	1.67	Sfmbt1;LOC103693895;Tmem110;LOC102555619	Epigenetic
DMR16:6981001	16	6981001	6983000	2000	1	2.20E-10	-0.51	28	1.4	Itih4;Itih3	Protease; Proteolysis
DMR16:7168001	16	7168001	7170000	2000	1	2.70E-10	-0.43	27	1.35	Pbrm1	Epigenetic
DMR16:7204001	16	7204001	7206000	2000	2	3.80E-07	0.62	19	0.95	Pbrm1;Smim4;Nt5dc2	Epigenetic;Signaling
DMR16:7219001	16	7219001	7222000	3000	1	5.00E-09	0.61	96	3.2	Smim4;Nt5dc2;Stab1	Signaling;Transport
DMR16:7228001	16	7228001	7236000	8000	1	1.80E-10	0.56	167	2.09	Nt5dc2;Stab1	Signaling;Transport
DMR16:7245001	16	7245001	7246000	1000	1	3.50E-07	0.38	32	3.2	Stab1;Nisch	Transport;Cytoskeleton
DMR16:7247001	16	7247001	7249000	2000	1	2.40E-08	-0.42	48	2.4	Stab1;Nisch	Transport;Cytoskeleton
DMR16:7307001	16	7307001	7311000	4000	2	5.60E-08	0.56	98	2.45	Sema3g	Signaling
DMR16:7322001	16	7322001	7323000	1000	1	4.30E-07	0.48	8	0.8	Sema3g;Phf7	Signaling;Transcription
DMR16:7357001	16	7357001	7361000	4000	2	1.00E-09	0.49	90	2.25	Dnah1	Cytoskeleton
DMR16:7393001	16	7393001	7396000	3000	1	9.50E-08	0.6	50	1.67	Dnah1	Cytoskeleton
DMR16:7519001	16	7519001	7521000	2000	1	7.30E-10	-0.51	35	1.75	Sh3bp5	Cytoskeleton
DMR16:7715001	16	7715001	7716000	1000	1	1.30E-07	0.54	19	1.9	Hacl1	Metabolism
DMR16:7722001	16	7722001	7724000	2000	1	1.40E-11	0.61	32	1.6	Hacl1	Metabolism
DMR16:7774001	16	7774001	7776000	2000	1	5.40E-12	0.89	58	2.9	Btd	Metabolism
DMR16:7789001	16	7789001	7790000	1000	1	3.40E-09	0.73	32	3.2	Btd	Metabolism
DMR16:7793001	16	7793001	7796000	3000	1	1.60E-10	0.51	58	1.93	Btd	Metabolism
DMR16:8126001	16	8126001	8132000	6000	1	6.00E-16	0.97	120	2	Galnt15	Golgi
DMR16:8209001	16	8209001	8212000	3000	1	4.70E-08	-0.37	43	1.43	Dph3;Oxnad1	Metabolism
DMR16:8229001	16	8229001	8231000	2000	1	5.00E-07	-0.35	80	4	Oxnad1	Metabolism
DMR16:8269001	16	8269001	8271000	2000	1	2.00E-08	0.54	39	1.95	Msmb	Signaling
DMR16:8287001	16	8287001	8288000	1000	1	2.30E-12	0.76	17	1.7	Msmb	Signaling
DMR16:8514001	16	8514001	8520000	6000	1	1.80E-08	0.53	126	2.1	Ogdhl	Metabolism
DMR16:8532001	16	8532001	8533000	1000	1	1.10E-08	-0.43	6	0.6	Ogdhl	Metabolism
DMR16:8566001	16	8566001	8568000	2000	1	4.50E-10	0.61	12	0.6	LOC680885;Chat	Metabolism
DMR16:8571001	16	8571001	8573000	2000	1	1.80E-08	0.55	44	2.2	LOC680885;Chat	Metabolism
DMR16:8657001	16	8657001	8658000	1000	1	1.60E-08	0.61	20	2	Chat	Metabolism
DMR16:8664001	16	8664001	8665000	1000	1	2.80E-17	1	29	2.9	Chat	Metabolism
DMR16:8748001	16	8748001	8750000	2000	1	2.00E-09	-0.47	34	1.7	Ercc6	
DMR16:8880001	16	8880001	8882000	2000	1	9.10E-09	0.55	34	1.7	RGD1564899	
DMR16:8885001	16	8885001	8891000	6000	1	5.90E-08	-0.45	108	1.8	RGD1564899	
DMR16:9018001	16	9018001	9019000	1000	1	5.10E-07	-0.62	5	0.5	RGD1561145	
DMR16:9042001	16	9042001	9044000	2000	1	9.30E-08	0.36	23	1.15	RGD1561145	
DMR16:9051001	16	9051001	9052000	1000	1	3.20E-09	-0.45	24	2.4	RGD1561145	
DMR16:9067001	16	9067001	9069000	2000	1	9.40E-08	-0.4	40	2	Fam170b	
DMR16:9166001	16	9166001	9168000	2000	1	5.30E-13	-0.52	42	2.1	Vstm4	Immune
DMR16:9225001	16	9225001	9227000	2000	1	1.70E-14	0.71	34	1.7	Wdfy4;Lrrc18	Cytoskeleton
DMR16:9335001	16	9335001	9336000	1000	1	2.10E-08	0.4	14	1.4	Wdfy4	
DMR16:9425001	16	9425001	9427000	2000	1	2.00E-08	-0.51	40	2	Wdfy4	
DMR16:9435001	16	9435001	9437000	2000	1	7.60E-07	0.45	40	2	Wdfy4	
DMR16:9567001	16	9567001	9572000	5000	1	1.10E-10	0.63	98	1.96	Arhgap22	
DMR16:9585001	16	9585001	9587000	2000	1	5.20E-07	0.44	41	2.05	Arhgap22	
DMR16:9596001	16	9596001	9599000	3000	1	2.20E-08	0.58	40	1.33	Arhgap22	
DMR16:9632001	16	9632001	9633000	1000	1	1.70E-09	-0.65	9	0.9	Mapk8	Signaling
DMR16:9741001	16	9741001	9742000	1000	1	1.50E-09	0.67	21	2.1	Frmpd2	
DMR16:9771001	16	9771001	9773000	2000	1	1.70E-09	-0.39	20	1	Frmpd2	
DMR16:9848001	16	9848001	9849000	1000	1	7.10E-09	0.49	10	1	Frmpd2	
DMR16:9937001	16	9937001	9942000	5000	1	1.60E-07	-0.34	31	0.62	Ptpn20	Signaling
DMR16:10272001	16	10272001	10274000	2000	1	3.90E-08	0.41	19	0.95	Gdf10;Gdf2;Rbp3	Growth Factors

DMR16:10383001	16	10383001	10384000	1000	1	4.10E-08	0.52	12	1.2	LOC108353091;Antxrl	Cytoskeleton
DMR16:10425001	16	10425001	10427000	2000	1	3.10E-07	0.46	27	1.35	Anxa8	Signaling
DMR16:10555001	16	10555001	10559000	4000	1	1.70E-07	0.47	62	1.55	Syt15	Transport
DMR16:10562001	16	10562001	10563000	1000	1	1.20E-07	0.61	32	3.2	Syt15;Fam35a	Transport
DMR16:10647001	16	10647001	10652000	5000	1	4.70E-11	-0.44	52	1.04	Fam35a;Glud1	Metabolism
DMR16:10727001	16	10727001	10728000	1000	1	4.30E-08	0.54	24	2.4	Sncg;Mmrn2	Transport
DMR16:10729001	16	10729001	10730000	1000	1	2.30E-07	0.64	14	1.4	Sncg;Mmrn2	Transport
DMR16:10828001	16	10828001	10830000	2000	1	8.50E-08	-0.39	17	0.85	Bmpr1a	Signaling
DMR16:10906001	16	10906001	10909000	3000	1	6.10E-13	0.65	47	1.57	Ldb3	Cytoskeleton
DMR16:10912001	16	10912001	10914000	2000	1	1.70E-08	0.35	23	1.15	Ldb3	Cytoskeleton
DMR16:10951001	16	10951001	10953000	2000	1	1.00E-07	0.56	25	1.25	Ldb3;Opn4;LOC100360810	Cytoskeleton;Signaling
DMR16:10973001	16	10973001	10975000	2000	2	9.00E-15	0.77	46	2.3	Opn4	Signaling
DMR16:11114001	16	11114001	11120000	6000	1	9.40E-09	-0.49	81	1.35	Wapl;Isca1-ps1;LOC108353076	
DMR16:11175001	16	11175001	11178000	3000	1	7.60E-07	0.48	41	1.37	Grid1	Receptor
DMR16:11211001	16	11211001	11213000	2000	1	1.20E-08	0.63	31	1.55	Grid1;Rpl38-ps3	Receptor
DMR16:11221001	16	11221001	11223000	2000	1	7.50E-08	0.33	32	1.6	Grid1;Rpl38-ps3	Receptor
DMR16:11243001	16	11243001	11246000	3000	1	2.50E-12	-0.52	34	1.13	Grid1;Mir346	Receptor
DMR16:11372001	16	11372001	11373000	1000	1	6.00E-08	0.23	9	0.9	Grid1	Receptor
DMR16:11573001	16	11573001	11575000	2000	1	1.40E-07	0.39	11	0.55	Grid1	Receptor
DMR16:11598001	16	11598001	11599000	1000	1	2.20E-08	-0.55	15	1.5	Grid1	Receptor
DMR16:11607001	16	11607001	11609000	2000	1	1.80E-07	-0.57	4	0.2	Grid1	Receptor
DMR16:11613001	16	11613001	11614000	1000	1	2.50E-07	-0.4	6	0.6	Grid1	Receptor
DMR16:11715001	16	11715001	11717000	2000	1	5.60E-07	0.37	21	1.05	Grid1	Receptor
DMR16:12708001	16	12708001	12710000	2000	1	9.90E-07	-0.41	10	0.5	RGD1562508;RGD1564225	
DMR16:14331001	16	14331001	14333000	2000	1	3.40E-11	0.46	15	0.75	Lrit2;Cdhr1	Cytoskeleton
DMR16:14359001	16	14359001	14361000	2000	1	3.80E-08	-0.39	33	1.65	LOC103693908;LOC290595;LOC108348380;LOC108353094;Ghitm	
DMR16:15774001	16	15774001	15775000	1000	1	1.80E-07	0.5	8	0.8	Nrg3	Growth Factors
DMR16:16132001	16	16132001	16134000	2000	1	7.20E-16	0.83	24	1.2	Nrg3	Growth Factors
DMR16:16164001	16	16164001	16167000	3000	1	2.50E-07	-0.4	22	0.73	Nrg3	Growth Factors
DMR16:16178001	16	16178001	16180000	2000	1	2.40E-07	0.32	18	0.9	Nrg3	Growth Factors
DMR16:16419001	16	16419001	16421000	2000	1	1.60E-08	-0.66	16	0.8	Nrg3	Growth Factors
DMR16:16558001	16	16558001	16559000	1000	1	8.50E-07	0.37	7	0.7	Nrg3	Growth Factors
DMR16:16718001	16	16718001	16719000	1000	1	1.60E-09	0.6	11	1.1	Nrg3	Growth Factors
DMR16:16753001	16	16753001	16754000	1000	1	2.80E-07	0.55	8	0.8	Nrg3	Growth Factors
DMR16:16763001	16	16763001	16765000	2000	1	8.70E-10	0.5	21	1.05	Nrg3	Growth Factors
DMR16:16950001	16	16950001	16952000	2000	1	3.00E-12	0.7	24	1.2	LOC108348383;RGD1564958	
DMR16:17535001	16	17535001	17538000	3000	1	2.80E-07	-0.32	38	1.27	Tspan14	
DMR16:17573001	16	17573001	17574000	1000	1	1.10E-07	-0.55	11	1.1	Tspan14	
DMR16:19215001	16	19215001	19221000	6000	1	5.80E-07	0.4	93	1.55	Klf2	Transcription
DMR16:19222001	16	19222001	19223000	1000	1	9.60E-09	0.61	32	3.2	Klf2	Transcription
DMR16:19227001	16	19227001	19233000	6000	1	1.10E-07	0.51	101	1.68	Klf2	Transcription
DMR16:19295001	16	19295001	19296000	1000	1	3.40E-07	0.61	14	1.4	Ap1m1;Fam32a	Transport
DMR16:19343001	16	19343001	19346000	3000	1	2.50E-09	0.47	33	1.1	Hsh2d;Rab8a	Immune
DMR16:19513001	16	19513001	19516000	3000	1	7.90E-08	0.64	46	1.53	Cyp4f18	
DMR16:19524001	16	19524001	19529000	5000	2	5.80E-09	0.72	101	2.02	Cyp4f18	
DMR16:19551001	16	19551001	19553000	2000	1	9.40E-07	-0.34	15	0.75	Zfp617;LOC102548752	Transcription
DMR16:19779001	16	19779001	19783000	4000	1	2.50E-07	0.63	59	1.48	Ocel1;Nr2f6;Ushbp1;Babam1	Transcription;Transcription
DMR16:19888001	16	19888001	19890000	2000	2	1.30E-18	1.17	85	4.25	Dda1;Ano8;Gtpbp3	Epigenetic
DMR16:19891001	16	19891001	19892000	1000	1	1.40E-07	0.5	17	1.7	Dda1;Ano8;Gtpbp3	Epigenetic
DMR16:19904001	16	19904001	19905000	1000	1	4.20E-09	0.48	13	1.3	Ano8;Gtpbp3;Plvap	Epigenetic
DMR16:20046001	16	20046001	20051000	5000	1	6.10E-07	-0.51	65	1.3	Fam129c;Colgalt1;Unc13a	Golgi
DMR16:20063001	16	20063001	20066000	3000	1	7.70E-08	0.36	31	1.03	Unc13a	
DMR16:20104001	16	20104001	20107000	3000	1	2.80E-08	0.46	46	1.53	Unc13a;Jak3	
DMR16:20113001	16	20113001	20115000	2000	1	3.00E-09	0.51	30	1.5	Unc13a;Jak3;Insl3;B3gnt3	Golgi
DMR16:20330001	16	20330001	20332000	2000	1	2.30E-07	0.44	22	1.1	Ccdc124;Kcnn1	Transport
DMR16:20378001	16	20378001	20381000	3000	1	6.20E-08	0.68	61	2.03	Il12rb1;Mast3	Receptor;Signaling
DMR16:20389001	16	20389001	20390000	1000	1	9.70E-10	0.55	20	2	Il12rb1;Mast3	Receptor;Signaling
DMR16:20403001	16	20403001	20406000	3000	1	9.20E-09	0.43	87	2.9	Mast3;Pik3r2	Signaling;Signaling

DMR16:20407001	16	20407001	20410000	3000	1	1.20E-07	0.51	59	1.97	Mast3;Pik3r2;LOC108348390	Signaling;Signaling
DMR16:20443001	16	20443001	20444000	1000	1	5.30E-08	0.56	25	2.5	Mpv17l2;Rab3a;Pde4c	Transport;Signaling
DMR16:20536001	16	20536001	20538000	2000	1	4.00E-10	-0.45	33	1.65	Pgpep1;Mk1	Protease;Cytoskeleton
DMR16:20673001	16	20673001	20675000	2000	1	2.10E-07	0.5	60	3	Kxd1;LOC102549939;Uba52;RGD1566239;Crlf1	Receptor
DMR16:20695001	16	20695001	20699000	4000	1	2.20E-07	0.44	52	1.3	Crlf1;Tmem59l;Klhl26	Receptor
DMR16:20817001	16	20817001	20819000	2000	1	1.10E-08	0.49	27	1.35	Comp;Upf1	
DMR16:20834001	16	20834001	20835000	1000	1	2.60E-07	-0.51	19	1.9	Upf1	
DMR16:20851001	16	20851001	20854000	3000	2	8.20E-07	-0.56	36	1.2	Upf1;Cers1;Gdf1;Cope	Growth Factors;Transport
DMR16:20928001	16	20928001	20933000	5000	1	3.90E-07	-0.48	61	1.22	Sugp2;LOC100361056;Armc6	Translation
DMR16:20983001	16	20983001	20984000	1000	1	2.20E-07	0.5	34	3.4	Slc25a42;Tmem161a	Transport
DMR16:20987001	16	20987001	20988000	1000	1	1.10E-07	0.6	23	2.3	Slc25a42;Tmem161a	Transport
DMR16:20992001	16	20992001	20993000	1000	1	6.20E-16	0.62	19	1.9	Slc25a42;Tmem161a;Mef2b	Transport;Transcription
DMR16:21020001	16	21020001	21022000	2000	1	4.40E-07	0.49	29	1.45	Mef2b;Borcs8;Rfxank	Transcription;Signaling
DMR16:21047001	16	21047001	21049000	2000	1	1.00E-08	-0.61	10	0.5	Rfxank;Nr2c2ap;Ncan	Signaling;Extracellular Matrix
DMR16:21061001	16	21061001	21062000	1000	1	1.00E-08	0.53	12	1.2	Ncan	Extracellular Matrix
DMR16:21099001	16	21099001	21103000	4000	1	2.90E-10	0.73	71	1.77	Hapln4;Tm6sf2;Sugp1	Extracellular Matrix;Translation
DMR16:21165001	16	21165001	21169000	4000	1	4.50E-10	-0.58	55	1.38	Mau2;Gatad2a	Transcription
DMR16:21298001	16	21298001	21299000	1000	1	1.40E-10	0.63	10	1	Yjefn3;Cilp2;Pbx4	Metabolism;Development
DMR16:21308001	16	21308001	21313000	5000	1	2.00E-13	0.67	87	1.74	Pbx4	Development
DMR16:21355001	16	21355001	21356000	1000	1	2.00E-07	-0.37	20	2	Lpar2;Gmip;Atp13a1	Signaling
DMR16:21357001	16	21357001	21358000	1000	1	2.40E-19	1.1	47	4.7	Lpar2;Gmip;Atp13a1	Signaling
DMR16:21462001	16	21462001	21463000	1000	1	8.60E-09	-0.38	9	0.9	Zfp964	
DMR16:21474001	16	21474001	21476000	2000	1	2.30E-08	0.55	27	1.35	Zfp964	
DMR16:21935001	16	21935001	21938000	3000	1	1.30E-07	0.35	16	0.53	RGD1563748	
DMR16:22006001	16	22006001	22020000	14000	4	3.90E-30	0.68	148	1.06	RGD1563748;RGD1564941	
DMR16:22022001	16	22022001	22025000	3000	1	2.10E-12	0.36	34	1.13	RGD1563748;RGD1564941	
DMR16:22029001	16	22029001	22032000	3000	1	2.60E-09	0.27	25	0.83	RGD1563748;RGD1564941	
DMR16:22036001	16	22036001	22037000	1000	1	3.40E-15	0.36	11	1.1	RGD1563748;RGD1564941	
DMR16:22044001	16	22044001	22047000	3000	1	3.20E-07	0.22	9	0.3	RGD1563748;RGD1564941	
DMR16:22048001	16	22048001	22050000	2000	1	8.90E-10	-0.27	9	0.45	RGD1563748;RGD1564941	
DMR16:22053001	16	22053001	22060000	7000	3	2.50E-10	0.47	90	1.29	RGD1563748;RGD1564941	
DMR16:22068001	16	22068001	22070000	2000	1	8.50E-13	0.28	18	0.9	RGD1563748;RGD1564941	
DMR16:22073001	16	22073001	22075000	2000	2	1.90E-13	0.29	20	1	RGD1563748;RGD1564941	
DMR16:22077001	16	22077001	22079000	2000	1	7.40E-07	0.18	17	0.85	RGD1563748;RGD1564941	
DMR16:22098001	16	22098001	22102000	4000	1	1.40E-10	-0.21	19	0.48	RGD1563748;RGD1564941;LOC103693919	
DMR16:22116001	16	22116001	22120000	4000	1	2.40E-14	0.37	42	1.05	RGD1563748;RGD1564941;LOC103693919;LOC108353083	
DMR16:22128001	16	22128001	22129000	1000	1	9.80E-07	0.21	8	0.8	RGD1563748;RGD1564941;LOC108353083;LOC108353082	
DMR16:22162001	16	22162001	22164000	2000	1	2.40E-07	-0.42	17	0.85	RGD1564941	
DMR16:22247001	16	22247001	22248000	1000	1	5.60E-11	0.49	7	0.7	Lzts1	
DMR16:22297001	16	22297001	22298000	1000	1	9.90E-09	-0.41	22	2.2	Lzts1	
DMR16:22364001	16	22364001	22366000	2000	1	4.60E-12	0.62	26	1.3	LOC102557578;Slc18a1	Transport
DMR16:22382001	16	22382001	22384000	2000	1	2.40E-09	-0.74	17	0.85	Slc18a1	Transport
DMR16:22554001	16	22554001	22555000	1000	1	9.60E-21	-0.87	20	2	Lpl	Metabolism
DMR16:22566001	16	22566001	22567000	1000	1	4.00E-15	0.82	18	1.8	Lpl	Metabolism
DMR16:22568001	16	22568001	22570000	2000	2	1.60E-10	-0.72	15	0.75	Lpl	Metabolism
DMR16:22614001	16	22614001	22615000	1000	1	6.50E-07	-0.43	9	0.9	Ints10	
DMR16:22792001	16	22792001	22793000	1000	1	3.90E-14	-0.82	22	2.2	Csgalnact1;LOC680966	Golgi
DMR16:22838001	16	22838001	22840000	2000	1	9.60E-07	-0.57	16	0.8	Csgalnact1	Golgi
DMR16:22882001	16	22882001	22883000	1000	1	2.30E-08	-0.43	15	1.5	Csgalnact1	Golgi
DMR16:22960001	16	22960001	22961000	1000	1	3.40E-07	-0.59	5	0.5	Csgalnact1	Golgi
DMR16:22972001	16	22972001	22973000	1000	1	3.70E-08	-0.58	4	0.4	Csgalnact1	Golgi
DMR16:23103001	16	23103001	23104000	1000	1	9.30E-07	0.65	19	1.9	Sh2d4a	Immune
DMR16:23111001	16	23111001	23112000	1000	1	7.10E-07	0.4	16	1.6	Sh2d4a	Immune

DMR16:23420001	16	23420001	23421000	1000	1	7.20E-07	-0.42	8	0.8	Psd3	Transcription
DMR16:23427001	16	23427001	23430000	3000	1	2.40E-09	-0.42	24	0.8	Psd3	Transcription
DMR16:23495001	16	23495001	23499000	4000	1	5.10E-07	-0.33	59	1.48	Psd3	Transcription
DMR16:23550001	16	23550001	23554000	4000	1	8.80E-08	-0.4	32	0.8	Psd3;LOC103693922	Transcription
DMR16:24950001	16	24950001	24951000	1000	1	6.10E-23	1.25	40	4	Tktl2	Metabolism
DMR16:25103001	16	25103001	25110000	7000	2	2.80E-12	-0.4	96	1.37	March1;LOC103693923	
DMR16:25207001	16	25207001	25214000	7000	1	2.00E-07	-0.37	80	1.14	1-Mar	
DMR16:25816001	16	25816001	25817000	1000	1	3.60E-07	-0.58	2	0.2	1-Mar	
DMR16:26601001	16	26601001	26603000	2000	1	6.60E-08	0.58	39	1.95	LOC100910468;Trim60	
DMR16:26743001	16	26743001	26747000	4000	2	5.00E-09	-0.54	55	1.38	Klhl2	Cytoskeleton
DMR16:26791001	16	26791001	26794000	3000	1	1.70E-07	-0.35	47	1.57	Klhl2	Cytoskeleton
DMR16:26940001	16	26940001	26942000	2000	1	1.00E-07	-0.49	20	1	Cpe	Protease
DMR16:26998001	16	26998001	26999000	1000	1	3.10E-10	0.75	22	2.2	Cpe	Protease
DMR16:27441001	16	27441001	27443000	2000	1	3.00E-08	-0.39	9	0.45	Tll1;LOC108348393	Protease
DMR16:28419001	16	28419001	28421000	2000	1	1.20E-08	-0.48	9	0.45	Spock3	Extracellular Matrix
DMR16:28425001	16	28425001	28426000	1000	1	2.10E-09	0.54	5	0.5	Spock3	Extracellular Matrix
DMR16:28461001	16	28461001	28463000	2000	1	3.30E-07	-0.32	17	0.85	Spock3	Extracellular Matrix
DMR16:28496001	16	28496001	28498000	2000	1	4.00E-16	-0.47	19	0.95	Spock3	Extracellular Matrix
DMR16:28596001	16	28596001	28597000	1000	1	4.40E-07	-0.53	2	0.2	Spock3	Extracellular Matrix
DMR16:28621001	16	28621001	28622000	1000	1	4.00E-07	-0.42	6	0.6	Spock3	Extracellular Matrix
DMR16:29843001	16	29843001	29846000	3000	1	2.70E-08	-0.28	27	0.9	Ddx60	
DMR16:29850001	16	29850001	29854000	4000	1	3.60E-10	-0.38	32	0.8	Ddx60	
DMR16:29892001	16	29892001	29895000	3000	1	9.80E-08	0.38	20	0.67	Ddx60	
DMR16:31366001	16	31366001	31368000	2000	1	8.20E-12	0.98	38	1.9	Palld	Cytoskeleton
DMR16:32067001	16	32067001	32073000	6000	2	2.20E-10	-0.4	119	1.98	Sh3rf1	
DMR16:32079001	16	32079001	32085000	6000	1	2.60E-12	-0.46	89	1.48	Sh3rf1	
DMR16:32111001	16	32111001	32115000	4000	3	6.70E-13	-0.47	70	1.75	Sh3rf1	
DMR16:32125001	16	32125001	32129000	4000	2	1.70E-10	-0.59	79	1.98	Sh3rf1	
DMR16:32141001	16	32141001	32142000	1000	1	5.00E-07	-0.53	10	1	Sh3rf1	
DMR16:32187001	16	32187001	32196000	9000	3	3.50E-14	-0.72	187	2.08	Sh3rf1	
DMR16:32223001	16	32223001	32226000	3000	1	4.10E-07	-0.38	45	1.5	Sh3rf1	
DMR16:32457001	16	32457001	32458000	1000	1	5.30E-08	-0.61	15	1.5	Clcn3	Transport
DMR16:32782001	16	32782001	32783000	1000	1	2.50E-08	-0.34	14	1.4	Mfap3l	
DMR16:34581001	16	34581001	34584000	3000	1	1.90E-08	-0.38	59	1.97	Galntl6	Golgi
DMR16:34614001	16	34614001	34620000	6000	2	1.70E-08	-0.32	78	1.3	Galntl6	Golgi
DMR16:34669001	16	34669001	34673000	4000	2	2.30E-10	-0.52	45	1.12	Galntl6;LOC103693941	Golgi
DMR16:34699001	16	34699001	34701000	2000	1	1.20E-08	-0.46	9	0.45	Galntl6	Golgi
DMR16:34795001	16	34795001	34797000	2000	2	3.70E-10	0.71	38	1.9	Galntl6;LOC100912321	Golgi
DMR16:34920001	16	34920001	34925000	5000	1	2.60E-07	-0.34	42	0.84	Galntl6	Golgi
DMR16:35055001	16	35055001	35056000	1000	1	5.50E-07	-0.48	5	0.5	Galntl6	Golgi
DMR16:35075001	16	35075001	35076000	1000	1	4.10E-08	0.51	8	0.8	Galntl6	Golgi
DMR16:35104001	16	35104001	35106000	2000	1	5.90E-07	-0.24	29	1.45	Galntl6	Golgi
DMR16:35109001	16	35109001	35110000	1000	1	4.30E-18	0.64	10	1	Galntl6	Golgi
DMR16:35199001	16	35199001	35201000	2000	1	4.90E-12	0.99	34	1.7	Galntl6;Rps27a-ps14	Golgi
DMR16:35565001	16	35565001	35566000	1000	1	2.40E-08	-0.49	6	0.6	Galntl6	Golgi
DMR16:35655001	16	35655001	35661000	6000	1	1.70E-07	-0.32	58	0.97	Galntl6	Golgi
DMR16:35717001	16	35717001	35721000	4000	1	8.10E-07	-0.38	36	0.9	Galntl6	Golgi
DMR16:35756001	16	35756001	35757000	1000	1	5.80E-07	-0.39	14	1.4	Galntl6	Golgi
DMR16:35773001	16	35773001	35775000	2000	1	1.40E-11	1.03	41	2.05	Galntl6	Golgi
DMR16:36004001	16	36004001	36007000	3000	1	4.20E-07	-0.39	43	1.43	Galnt7	Golgi
DMR16:36060001	16	36060001	36062000	2000	1	7.60E-08	-0.42	43	2.15	Galnt7	Golgi
DMR16:36670001	16	36670001	36671000	1000	1	2.90E-07	0.59	14	1.4	Pebp1-ps1	
DMR16:37748001	16	37748001	37785000	37000	27	9.60E-16	0.32	362	0.98	Glra3	Ion Channel
DMR16:39395001	16	39395001	39397000	2000	1	3.40E-07	-0.69	9	0.45	Gpm6a	Cytoskeleton
DMR16:39611001	16	39611001	39612000	1000	1	5.40E-07	-0.29	16	1.6	Gpm6a;LOC498628	Cytoskeleton
DMR16:41223001	16	41223001	41224000	1000	1	3.70E-09	-0.52	10	1	Aga	Protease
DMR16:46421001	16	46421001	46424000	3000	2	1.10E-21	1.16	82	2.73	Tenm3	
DMR16:46426001	16	46426001	46428000	2000	1	6.00E-07	-0.46	25	1.25	Tenm3	
DMR16:46449001	16	46449001	46450000	1000	1	2.60E-07	-0.34	17	1.7	Tenm3	
DMR16:46474001	16	46474001	46478000	4000	1	4.70E-08	-0.41	53	1.32	Tenm3;LOC102552351	
DMR16:46483001	16	46483001	46485000	2000	1	4.40E-08	0.5	37	1.85	Tenm3;LOC102552351	
DMR16:46578001	16	46578001	46582000	4000	1	1.60E-07	0.43	58	1.45	Tenm3	
DMR16:46808001	16	46808001	46811000	3000	1	4.00E-07	-0.37	58	1.93	Tenm3	

DMR16:46816001	16	46816001	46818000	2000	1	3.50E-08	-0.46	44	2.2	Tenm3	
DMR16:47219001	16	47219001	47220000	1000	1	5.00E-07	0.35	14	1.4	Dctd	Metabolism
DMR16:47385001	16	47385001	47387000	2000	1	3.60E-08	-0.43	26	1.3	Wwc2	
DMR16:47487001	16	47487001	47490000	3000	1	5.60E-09	-0.46	60	2	Wwc2	
DMR16:47498001	16	47498001	47500000	2000	1	1.50E-14	0.4	38	1.9	Wwc2	
DMR16:47658001	16	47658001	47662000	4000	1	6.40E-07	-0.57	39	0.98	Cdkn2aip	Transcription
DMR16:48001001	16	48001001	48004000	3000	1	1.70E-07	-0.55	34	1.13	Stox2	
DMR16:48014001	16	48014001	48017000	3000	2	4.40E-08	-0.56	40	1.33	Stox2	
DMR16:48057001	16	48057001	48058000	1000	1	7.40E-08	-0.31	33	3.3	Stox2	
DMR16:48067001	16	48067001	48069000	2000	1	6.60E-08	-0.37	36	1.8	Stox2	
DMR16:48085001	16	48085001	48088000	3000	1	6.70E-07	-0.49	53	1.77	Stox2	
DMR16:48130001	16	48130001	48132000	2000	1	1.50E-11	-0.48	29	1.45	Stox2	
DMR16:48145001	16	48145001	48147000	2000	1	3.50E-08	-0.31	37	1.85	Stox2	
DMR16:48169001	16	48169001	48171000	2000	1	1.70E-07	-0.46	25	1.25	Stox2	
DMR16:48182001	16	48182001	48183000	1000	1	1.50E-08	-0.36	19	1.9	Stox2	
DMR16:48195001	16	48195001	48197000	2000	1	1.30E-12	-0.48	40	2	Stox2	
DMR16:48324001	16	48324001	48328000	4000	1	3.10E-09	-0.47	29	0.72	LOC103693959;Enpp6	
DMR16:48397001	16	48397001	48398000	1000	1	4.30E-07	0.51	13	1.3	Enpp6	
DMR16:48431001	16	48431001	48432000	1000	1	6.20E-10	0.74	23	2.3	Enpp6	
DMR16:48628001	16	48628001	48631000	3000	1	8.00E-10	-0.59	28	0.93	Irf2	Transcription
DMR16:48636001	16	48636001	48639000	3000	1	1.00E-07	-0.31	41	1.37	Irf2	Transcription
DMR16:48644001	16	48644001	48645000	1000	1	1.40E-10	-0.45	20	2	Irf2	Transcription
DMR16:48875001	16	48875001	48876000	1000	1	6.10E-09	-0.57	6	0.6	Primpol	Transcription
DMR16:48928001	16	48928001	48931000	3000	1	7.20E-08	0.33	41	1.37	Cenpu;Acsl1	Metabolism
DMR16:49259001	16	49259001	49261000	2000	1	3.30E-07	0.41	27	1.35	Slc25a4	Transport
DMR16:49291001	16	49291001	49297000	6000	1	5.70E-10	-0.39	59	0.98	Cfap97	
DMR16:49337001	16	49337001	49341000	4000	1	1.30E-07	-0.51	41	1.02	Cfap97;Snx25	Cytoskeleton
DMR16:49362001	16	49362001	49364000	2000	1	8.20E-08	-0.45	27	1.35	Snx25	Cytoskeleton
DMR16:49397001	16	49397001	49403000	6000	1	1.50E-07	-0.33	51	0.85	Snx25	Cytoskeleton
DMR16:49424001	16	49424001	49426000	2000	1	4.70E-11	-0.57	31	1.55	Snx25	Cytoskeleton
DMR16:49542001	16	49542001	49543000	1000	1	2.90E-13	0.88	21	2.1	Pdlim3	Cytoskeleton
DMR16:49657001	16	49657001	49659000	2000	1	5.20E-22	1.23	65	3.25	Sorbs2	
DMR16:50046001	16	50046001	50047000	1000	1	3.80E-11	-0.47	11	1.1	Fam149a	
DMR16:50113001	16	50113001	50116000	3000	1	9.20E-09	-0.57	46	1.53	Fam149a;Cyp4v3	
DMR16:50166001	16	50166001	50168000	2000	1	3.00E-07	-0.39	23	1.15	Klkb1	Protease
DMR16:50189001	16	50189001	50190000	1000	1	5.30E-08	0.57	16	1.6	F11	Protease
DMR16:50348001	16	50348001	50350000	2000	1	7.40E-09	0.54	22	1.1	Mtnr1a	Signaling
DMR16:50384001	16	50384001	50388000	4000	1	1.30E-09	-0.39	95	2.38	Fat1	Cytoskeleton
DMR16:50408001	16	50408001	50414000	6000	1	6.90E-09	-0.36	119	1.98	Fat1	Cytoskeleton
DMR16:50433001	16	50433001	50435000	2000	1	3.30E-11	-0.44	31	1.55	Fat1	Cytoskeleton
DMR16:50463001	16	50463001	50464000	1000	1	1.40E-07	-0.52	18	1.8	Fat1	Cytoskeleton
DMR16:50486001	16	50486001	50489000	3000	1	5.10E-09	-0.48	75	2.5	Fat1	Cytoskeleton
DMR16:50493001	16	50493001	50497000	4000	1	9.90E-07	-0.3	67	1.68	Fat1	Cytoskeleton
DMR16:52016001	16	52016001	52017000	1000	1	7.30E-15	1.22	30	3	Zfp42	
DMR16:54133001	16	54133001	54135000	2000	1	3.30E-09	-0.56	9	0.45	Pcm1	
DMR16:54146001	16	54146001	54149000	3000	1	1.70E-09	0.87	37	1.23	Pcm1;Fgl1	Signaling
DMR16:54198001	16	54198001	54201000	3000	1	2.10E-10	-0.48	37	1.23	Fgl1	Signaling
DMR16:54308001	16	54308001	54317000	9000	1	6.90E-08	-0.32	153	1.7	Mtus1	
DMR16:54323001	16	54323001	54326000	3000	1	1.30E-18	-0.68	33	1.1	Mtus1	
DMR16:54423001	16	54423001	54425000	2000	1	1.30E-08	0.5	18	0.9	Pdgfrl;LOC690241;LOC100910853	Receptor
DMR16:54516001	16	54516001	54517000	1000	1	1.90E-14	0.84	19	1.9	Slc7a2	Transport
DMR16:54810001	16	54810001	54813000	3000	1	1.20E-09	-0.44	35	1.17	Mtmr7	Signaling
DMR16:54872001	16	54872001	54875000	3000	1	3.70E-07	-0.54	18	0.6	Vps37a	
DMR16:54895001	16	54895001	54896000	1000	1	6.40E-09	-0.48	9	0.9	Vps37a;Cnot7	Translation
DMR16:55007001	16	55007001	55009000	2000	1	3.60E-07	0.29	27	1.35	Zdhhc2	
DMR16:55010001	16	55010001	55012000	2000	1	8.30E-09	-0.71	11	0.55	Zdhhc2	
DMR16:56254001	16	56254001	56256000	2000	1	5.70E-08	0.35	22	1.1	Tusc3	Golgi
DMR16:56323001	16	56323001	56325000	2000	1	1.90E-07	-0.41	65	3.25	Tusc3	Golgi
DMR16:56327001	16	56327001	56328000	1000	1	8.00E-07	0.54	11	1.1	Tusc3	Golgi
DMR16:56353001	16	56353001	56355000	2000	1	2.80E-08	0.46	25	1.25	Tusc3	Golgi
DMR16:56865001	16	56865001	56866000	1000	1	8.40E-08	0.5	10	1	Msr1	Protease

DMR16:56878001	16	56878001	56879000	1000	1	5.90E-07	-0.55	3	0.3	Msr1	Protease
DMR16:56887001	16	56887001	56892000	5000	1	4.90E-07	-0.28	52	1.04	Msr1	Protease
DMR16:57203001	16	57203001	57206000	3000	1	9.20E-09	-0.46	17	0.57	Sgcz	
DMR16:57565001	16	57565001	57567000	2000	1	5.40E-07	-0.31	18	0.9	Sgcz	
DMR16:57681001	16	57681001	57682000	1000	1	1.90E-08	-0.33	12	1.2	Sgcz	
DMR16:57688001	16	57688001	57689000	1000	1	2.90E-09	0.43	9	0.9	Sgcz	
DMR16:57754001	16	57754001	57755000	1000	1	2.00E-07	-0.52	5	0.5	Sgcz	
DMR16:57802001	16	57802001	57804000	2000	1	2.00E-07	-0.31	20	1	Sgcz	
DMR16:57917001	16	57917001	57920000	3000	1	6.20E-07	-0.33	24	0.8	Sgcz	
DMR16:57947001	16	57947001	57949000	2000	2	1.20E-20	-0.67	5	0.25	Sgcz	
DMR16:57995001	16	57995001	57996000	1000	1	5.20E-07	-0.37	5	0.5	Sgcz	
DMR16:58080001	16	58080001	58081000	1000	1	1.00E-07	-0.66	6	0.6	Sgcz	
DMR16:58111001	16	58111001	58112000	1000	1	1.30E-08	0.56	12	1.2	Sgcz	
DMR16:58119001	16	58119001	58120000	1000	1	2.50E-07	-0.66	1	0.1	Sgcz	
DMR16:58837001	16	58837001	58840000	3000	1	2.80E-07	-0.48	42	1.4	Dlc1	Signaling
DMR16:58889001	16	58889001	58890000	1000	1	2.50E-13	-0.65	23	2.3	Dlc1	Signaling
DMR16:59062001	16	59062001	59063000	1000	1	2.20E-07	0.41	21	2.1	Dlc1;LOC108348402	Signaling
DMR16:59236001	16	59236001	59239000	3000	1	3.30E-10	-0.4	79	2.63	Dlc1	Signaling
DMR16:59332001	16	59332001	59334000	2000	2	3.00E-20	1.22	58	2.9	RGD1304810;LOC108348403;LOC103693971	
DMR16:59569001	16	59569001	59573000	4000	1	1.00E-08	-0.53	58	1.45	Lonrf1	Proteolysis
DMR16:59712001	16	59712001	59714000	2000	1	8.00E-16	0.79	48	2.4	Pragmin	
DMR16:59730001	16	59730001	59732000	2000	2	1.00E-09	0.49	30	1.5	Pragmin	
DMR16:60180001	16	60180001	60181000	1000	1	4.20E-07	-0.5	17	1.7	Mfhas1	Cytoskeleton
DMR16:60182001	16	60182001	60184000	2000	1	6.40E-08	-0.49	34	1.7	Mfhas1	Cytoskeleton
DMR16:60191001	16	60191001	60193000	2000	1	1.70E-08	-0.46	43	2.15	Mfhas1	Cytoskeleton
DMR16:60211001	16	60211001	60217000	6000	3	8.40E-13	-0.66	81	1.35	Mfhas1	Cytoskeleton
DMR16:60944001	16	60944001	60946000	2000	1	1.50E-07	-0.43	51	2.55	Tnks	Signaling
DMR16:61079001	16	61079001	61081000	2000	1	2.60E-12	0.45	51	2.55	Dusp4	Signaling
DMR16:61095001	16	61095001	61096000	1000	1	1.60E-13	0.77	22	2.2	Dusp4	Signaling
DMR16:61771001	16	61771001	61776000	5000	1	1.70E-08	-0.46	78	1.56	Leprotl1;Mboat4	Receptor;Metabolism
DMR16:61782001	16	61782001	61783000	1000	1	5.00E-08	0.53	18	1.8	Mboat4	Metabolism
DMR16:61784001	16	61784001	61786000	2000	1	6.10E-07	-0.39	26	1.3	Mboat4;Dctn6	Metabolism;Cytoskeleton
DMR16:61803001	16	61803001	61804000	1000	1	1.70E-08	0.69	8	0.8	Mboat4;Dctn6	Metabolism;Cytoskeleton
DMR16:61824001	16	61824001	61826000	2000	2	4.00E-09	0.84	34	1.7	Dctn6	Cytoskeleton
DMR16:61943001	16	61943001	61945000	2000	1	2.10E-08	0.48	36	1.8	Rbpms	Translation
DMR16:61958001	16	61958001	61964000	6000	1	1.70E-08	-0.42	107	1.78	Rbpms	Translation
DMR16:61976001	16	61976001	61977000	1000	1	3.70E-09	-0.47	17	1.7	Rbpms	Translation
DMR16:62001001	16	62001001	62004000	3000	1	4.00E-16	-0.79	55	1.83	Rbpms	Translation
DMR16:62075001	16	62075001	62077000	2000	1	2.40E-07	-0.35	32	1.6	Rbpms	Translation
DMR16:62170001	16	62170001	62173000	3000	1	2.00E-08	0.66	72	2.4	Gtf2e2	Transcription
DMR16:62191001	16	62191001	62192000	1000	1	3.50E-08	-0.42	17	1.7	Gsr	Metabolism
DMR16:62196001	16	62196001	62199000	3000	1	7.40E-07	-0.4	51	1.7	Gsr	Metabolism
DMR16:62223001	16	62223001	62225000	2000	1	4.70E-07	-0.5	31	1.55	Gsr	Metabolism
DMR16:62289001	16	62289001	62290000	1000	1	5.90E-14	-0.56	15	1.5	Ppp2cb	Signaling
DMR16:62300001	16	62300001	62301000	1000	1	1.20E-08	0.76	23	2.3	Ppp2cb	Signaling
DMR16:62426001	16	62426001	62428000	2000	2	1.30E-12	0.76	37	1.85	LOC108348448;Purg	Transcription
DMR16:62482001	16	62482001	62483000	1000	1	1.80E-09	-0.58	38	3.8	Purg;Wrn	Transcription;Epigenetic
DMR16:62500001	16	62500001	62502000	2000	1	2.20E-15	1.02	36	1.8	Wrn	Epigenetic
DMR16:62635001	16	62635001	62636000	1000	1	8.30E-07	0.61	19	1.9	RGD1563097	
DMR16:63036001	16	63036001	63037000	1000	1	1.10E-08	-0.54	14	1.4	Nrg1	Growth Factors
DMR16:63223001	16	63223001	63226000	3000	1	3.20E-10	0.38	26	0.87	Nrg1	Growth Factors
DMR16:63389001	16	63389001	63390000	1000	1	3.10E-10	0.48	18	1.8	Nrg1	Growth Factors
DMR16:63481001	16	63481001	63482000	1000	1	2.40E-07	0.4	6	0.6	Nrg1	Growth Factors
DMR16:63560001	16	63560001	63562000	2000	1	2.00E-09	-0.44	14	0.7	Nrg1	Growth Factors
DMR16:63647001	16	63647001	63650000	3000	1	8.60E-08	-0.46	33	1.1	Nrg1	Growth Factors
DMR16:63672001	16	63672001	63674000	2000	1	2.40E-07	-0.48	20	1	Nrg1	Growth Factors
DMR16:63703001	16	63703001	63705000	2000	1	1.40E-13	-0.59	21	1.05	Nrg1	Growth Factors
DMR16:63790001	16	63790001	63791000	1000	1	4.40E-15	-0.61	26	2.6	Nrg1	Growth Factors

DMR16:63830001	16	63830001	63831000	1000	1	2.10E-08	-0.78	7	0.7	Nrg1	Growth Factors
DMR16:63873001	16	63873001	63874000	1000	1	3.00E-19	-1.16	11	1.1	Nrg1	Growth Factors
DMR16:63888001	16	63888001	63889000	1000	1	8.10E-07	-0.57	15	1.5	Nrg1	Growth Factors
DMR16:63894001	16	63894001	63895000	1000	1	1.10E-07	0.5	13	1.3	Nrg1	Growth Factors
DMR16:63899001	16	63899001	63902000	3000	1	5.50E-08	-0.44	40	1.33	Nrg1	Growth Factors
DMR16:63914001	16	63914001	63916000	2000	1	9.50E-09	-0.43	33	1.65	Nrg1	Growth Factors
DMR16:64009001	16	64009001	64012000	3000	1	6.70E-07	-0.31	46	1.53	Nrg1;LOC103693977	Growth Factors
DMR16:64053001	16	64053001	64054000	1000	1	1.20E-10	0.69	26	2.6	Nrg1	Growth Factors
DMR16:64799001	16	64799001	64800000	1000	1	2.90E-09	0.58	16	1.6	LOC102555712;Dusp26	
DMR16:67127001	16	67127001	67129000	2000	1	1.30E-08	0.43	25	1.25	Unc5d	Receptor
DMR16:67138001	16	67138001	67142000	4000	2	8.70E-16	-0.62	44	1.1	Unc5d	Receptor
DMR16:67195001	16	67195001	67196000	1000	1	5.00E-08	-0.52	6	0.6	Unc5d	Receptor
DMR16:67277001	16	67277001	67278000	1000	1	1.50E-09	0.54	8	0.8	Unc5d	Receptor
DMR16:67308001	16	67308001	67311000	3000	1	7.00E-09	-0.4	32	1.07	Unc5d	Receptor
DMR16:67368001	16	67368001	67369000	1000	1	4.40E-10	-0.48	16	1.6	Unc5d	Receptor
DMR16:68469001	16	68469001	68471000	2000	1	5.90E-07	-0.67	14	0.7	RGD1562638	
DMR16:68498001	16	68498001	68499000	1000	1	5.40E-09	-0.67	2	0.2	RGD1562638	
DMR16:68586001	16	68586001	68589000	3000	1	7.80E-07	-0.32	28	0.93	RGD1562638	
DMR16:68820001	16	68820001	68822000	2000	1	3.90E-19	1.1	51	2.55	Poteg	Transport
DMR16:68829001	16	68829001	68831000	2000	1	6.50E-09	-0.48	31	1.55	Poteg	Transport
DMR16:68926001	16	68926001	68927000	1000	1	1.30E-08	0.65	24	2.4	RGD1562020;LOC100911312	
DMR16:68934001	16	68934001	68935000	1000	1	4.60E-10	0.71	23	2.3	RGD1562020;LOC100911312	
DMR16:68977001	16	68977001	68985000	8000	1	2.90E-07	-0.32	95	1.19	Eif4ebp1	Translation
DMR16:69053001	16	69053001	69057000	4000	1	1.60E-07	-0.34	70	1.75	Rab11fip1	
DMR16:69133001	16	69133001	69134000	1000	1	1.30E-08	0.51	25	2.5	Adgra2	Signaling
DMR16:69170001	16	69170001	69174000	4000	1	2.50E-09	-0.42	52	1.3	Prosc;Erlin2	
DMR16:69250001	16	69250001	69254000	4000	1	6.40E-08	0.59	70	1.75	Zfp703;LOC108348410	
DMR16:70006001	16	70006001	70007000	1000	1	3.40E-11	0.63	22	2.2	Pasd1	
DMR16:70071001	16	70071001	70074000	3000	1	3.10E-07	-0.3	30	1	RGD1308750;RGD1561481	Protease
DMR16:70821001	16	70821001	70822000	1000	1	1.00E-08	-0.48	8	0.8	Hook3	
DMR16:70914001	16	70914001	70917000	3000	1	1.20E-07	-0.46	28	0.93	Hgsnat;LOC102552292;Kcnu1	Transport
DMR16:70978001	16	70978001	70980000	2000	1	7.60E-10	0.69	38	1.9	Kcnu1	Transport
DMR16:71000001	16	71000001	71001000	1000	1	2.50E-10	0.81	28	2.8	Kcnu1	Transport
DMR16:71091001	16	71091001	71093000	2000	1	7.40E-12	-0.49	28	1.4	Ddhd2	Metabolism
DMR16:71130001	16	71130001	71132000	2000	1	1.50E-12	-0.57	33	1.65	Ddhd2;Plpp5;Whsc1l1	Metabolism;Signaling
DMR16:71135001	16	71135001	71138000	3000	1	6.50E-07	-0.34	58	1.93	Plpp5;Whsc1l1	Signaling
DMR16:71156001	16	71156001	71158000	2000	1	1.90E-09	-0.48	33	1.65	Whsc1l1	
DMR16:71264001	16	71264001	71265000	1000	1	9.80E-09	-0.43	28	2.8	Letm2;Fgfr1	Transport;Receptor
DMR16:71269001	16	71269001	71271000	2000	1	3.70E-08	0.54	45	2.25	Letm2;Fgfr1	Transport;Receptor
DMR16:71283001	16	71283001	71285000	2000	1	9.90E-07	-0.3	28	1.4	Fgfr1	Receptor
DMR16:71317001	16	71317001	71318000	1000	1	8.60E-08	0.77	32	3.2	Fgfr1	Receptor
DMR16:71653001	16	71653001	71657000	4000	2	1.30E-08	-0.48	98	2.45	Tacc1	
DMR16:71825001	16	71825001	71828000	3000	1	2.60E-07	-0.53	35	1.17	Adam9	Protease
DMR16:71863001	16	71863001	71865000	2000	1	1.80E-07	-0.43	20	1	Adam9	Protease
DMR16:71877001	16	71877001	71880000	3000	1	2.60E-07	-0.21	31	1.03	Adam9;Adam32	Protease
DMR16:71898001	16	71898001	71900000	2000	1	9.00E-08	-0.37	36	1.8	Adam32	Protease
DMR16:71906001	16	71906001	71907000	1000	1	2.30E-09	-0.54	12	1.2	Adam32	Protease
DMR16:71940001	16	71940001	71947000	7000	1	9.30E-08	-0.33	72	1.03	Adam32	Protease
DMR16:71981001	16	71981001	71983000	2000	1	8.10E-09	-0.39	14	0.7	Adam32	Protease
DMR16:71984001	16	71984001	71986000	2000	1	1.30E-07	-0.25	16	0.8	Adam32	Protease
DMR16:71996001	16	71996001	71997000	1000	1	7.90E-08	-0.29	11	1.1	Adam32	Protease
DMR16:72013001	16	72013001	72015000	2000	1	1.70E-08	-0.47	36	1.8	Adam5	Protease
DMR16:72101001	16	72101001	72109000	8000	2	1.40E-09	0.6	135	1.69	Adam3a	Protease
DMR16:72152001	16	72152001	72154000	2000	2	1.30E-09	-0.51	17	0.85	Adam18	Protease
DMR16:72157001	16	72157001	72158000	1000	1	8.40E-07	-0.47	10	1	Adam18	Protease
DMR16:72168001	16	72168001	72170000	2000	1	2.40E-11	0.78	50	2.5	Adam18	Protease
DMR16:72210001	16	72210001	72212000	2000	1	4.10E-08	-0.46	37	1.85	Adam18;Ido1	Protease;Metabolism
DMR16:72222001	16	72222001	72223000	1000	1	9.50E-09	0.69	22	2.2	Ido1;LOC108348416;Ido2	Metabolism
DMR16:72242001	16	72242001	72243000	1000	1	2.20E-10	-0.8	4	0.4	Ido2	Metabolism
DMR16:72270001	16	72270001	72272000	2000	1	4.10E-09	-0.53	7	0.35	Ido2	Metabolism
DMR16:72755001	16	72755001	72760000	5000	1	1.50E-07	0.34	65	1.3	Zmat4	
DMR16:72809001	16	72809001	72811000	2000	2	7.20E-12	-0.6	12	0.6	Zmat4	

DMR16:72812001	16	72812001	72813000	1000	1	5.20E-07	0.38	11	1.1	Zmat4	
DMR16:72954001	16	72954001	72958000	4000	1	4.40E-07	-0.43	59	1.48	Zmat4	
DMR16:73072001	16	73072001	73073000	1000	1	3.40E-08	0.52	11	1.1	Zmat4	
DMR16:73113001	16	73113001	73115000	2000	1	3.40E-07	0.53	25	1.25	Zmat4	
DMR16:73128001	16	73128001	73130000	2000	1	1.20E-07	0.51	38	1.9	Zmat4	
DMR16:73379001	16	73379001	73381000	2000	1	2.70E-09	0.53	31	1.55	Sfrp1	Receptor
DMR16:73382001	16	73382001	73385000	3000	2	3.70E-15	-0.62	70	2.33	Sfrp1	Receptor
DMR16:73413001	16	73413001	73414000	1000	1	4.90E-11	0.68	21	2.1	Sfrp1	Receptor
DMR16:73577001	16	73577001	73579000	2000	1	3.70E-07	-0.53	32	1.6	Golga7;Gins4	
DMR16:73587001	16	73587001	73589000	2000	1	8.20E-09	-0.44	29	1.45	Golga7;Gins4	
DMR16:73590001	16	73590001	73592000	2000	1	5.80E-07	0.39	34	1.7	Gins4	
DMR16:73605001	16	73605001	73607000	2000	1	9.20E-09	0.56	18	0.9	Gins4;Gpat4	Metabolism
DMR16:73667001	16	73667001	73671000	4000	2	6.80E-08	0.64	79	1.98	Nkx6-3	Development
DMR16:73730001	16	73730001	73733000	3000	1	7.20E-08	-0.68	36	1.2	Ank1;LOC102555020	
DMR16:73776001	16	73776001	73779000	3000	1	9.40E-07	-0.37	75	2.5	Ank1	
DMR16:73846001	16	73846001	73848000	2000	1	3.10E-09	-0.69	8	0.4	Ank1;LOC103693993	
DMR16:73914001	16	73914001	73915000	1000	1	5.80E-09	0.71	29	2.9	Ank1	
DMR16:74083001	16	74083001	74085000	2000	1	9.70E-12	0.83	63	3.15	LOC102556951;Ap3m2	Transport
DMR16:74092001	16	74092001	74096000	4000	1	7.70E-16	1.07	83	2.08	Ap3m2;Plat	Transport;Protease
DMR16:74258001	16	74258001	74262000	4000	1	2.10E-07	0.65	55	1.38	Polb;Dkk4	Transcription
DMR16:74271001	16	74271001	74273000	2000	1	5.50E-09	-0.56	39	1.95	Dkk4	
DMR16:74338001	16	74338001	74342000	4000	1	8.70E-07	0.37	54	1.35	Slc20a2	Transport
DMR16:74516001	16	74516001	74517000	1000	1	6.40E-09	-0.53	20	2	Slc25a15	Transport
DMR16:74621001	16	74621001	74622000	1000	1	1.30E-09	-0.46	16	1.6	Tpte2	Signaling
DMR16:74646001	16	74646001	74647000	1000	1	6.60E-10	-0.44	21	2.1	Tpte2;LOC102550098	Signaling
DMR16:74720001	16	74720001	74721000	1000	1	2.80E-08	-0.41	14	1.4	Thsd1;Vps36	Transport
DMR16:74786001	16	74786001	74787000	1000	1	1.30E-07	0.56	34	3.4	Ckap2;Nek3	Cytoskeleton;Signaling
DMR16:74923001	16	74923001	74926000	3000	1	4.50E-08	0.52	33	1.1	Atp7b	Transport
DMR16:75060001	16	75060001	75061000	1000	1	5.80E-07	-0.45	4	0.4	LOC685740;Defb38	Signaling
DMR16:75102001	16	75102001	75105000	3000	1	2.70E-12	-0.53	40	1.33	Defb37;Defb15	Signaling
DMR16:75129001	16	75129001	75132000	3000	1	3.40E-12	-0.63	35	1.17	Defb12	
DMR16:75296001	16	75296001	75298000	2000	1	3.40E-07	-0.57	9	0.45	Defb1	Signaling
DMR16:75349001	16	75349001	75352000	3000	1	5.10E-09	-0.3	48	1.6	Defa5;Defa24	Signaling
DMR16:75375001	16	75375001	75377000	2000	1	1.30E-09	-0.63	29	1.45	Defal1	Signaling
DMR16:75433001	16	75433001	75439000	6000	1	9.80E-09	-0.46	85	1.42	Defa8	Signaling
DMR16:75468001	16	75468001	75470000	2000	2	1.50E-07	-0.56	2	0.1	Defa9;Np4	Signaling
DMR16:75497001	16	75497001	75500000	3000	1	3.10E-09	-0.58	20	0.67	Defa10	Signaling
DMR16:75549001	16	75549001	75552000	3000	1	7.20E-08	-0.54	26	0.87	Defb51	
DMR16:75553001	16	75553001	75557000	4000	1	3.90E-07	-0.43	42	1.05	Defb51	
DMR16:75604001	16	75604001	75607000	3000	1	8.00E-07	-0.26	24	0.8	Defb5	Signaling
DMR16:75750001	16	75750001	75752000	2000	1	2.60E-21	1.14	47	2.35	Xkr5	
DMR16:75924001	16	75924001	75927000	3000	1	2.00E-13	-0.59	66	2.2	Mcph1	DNA Repair
DMR16:75937001	16	75937001	75941000	4000	1	2.10E-07	-0.47	79	1.98	Mcph1	DNA Repair
DMR16:75966001	16	75966001	75970000	4000	2	6.20E-07	-0.48	94	2.35	Mcph1;Angpt2	DNA Repair;Signaling
DMR16:76061001	16	76061001	76063000	2000	1	4.90E-17	-0.55	45	2.25	Mcph1	DNA Repair
DMR16:76082001	16	76082001	76085000	3000	1	1.50E-07	0.36	36	1.2	Mcph1	DNA Repair
DMR16:77259001	16	77259001	77262000	3000	1	7.90E-07	-0.46	29	0.97	Csmd1	
DMR16:77560001	16	77560001	77561000	1000	1	8.80E-08	-0.28	6	0.6	Csmd1	
DMR16:77570001	16	77570001	77571000	1000	1	1.30E-08	-0.66	4	0.4	Csmd1	
DMR16:77876001	16	77876001	77877000	1000	1	3.40E-07	0.48	12	1.2	Csmd1	
DMR16:77892001	16	77892001	77893000	1000	1	1.70E-08	0.67	8	0.8	Csmd1	
DMR16:77986001	16	77986001	77988000	2000	1	7.30E-11	-0.55	8	0.4	Csmd1	
DMR16:78120001	16	78120001	78122000	2000	2	4.20E-07	0.58	14	0.7	Csmd1	
DMR16:78380001	16	78380001	78385000	5000	1	3.00E-08	-0.41	40	0.8	Csmd1	
DMR16:78580001	16	78580001	78581000	1000	1	2.00E-07	-0.57	3	0.3	Csmd1	
DMR16:78697001	16	78697001	78700000	3000	1	5.10E-09	-0.43	47	1.57	Csmd1	
DMR16:78736001	16	78736001	78737000	1000	1	2.20E-07	0.53	7	0.7	Csmd1	
DMR16:78843001	16	78843001	78844000	1000	1	4.80E-08	-0.6	5	0.5	Csmd1	
DMR16:79587001	16	79587001	79590000	3000	2	1.30E-13	0.85	67	2.23	Myom2;LOC102551089	
DMR16:79611001	16	79611001	79613000	2000	2	5.40E-22	1.15	34	1.7	Myom2;LOC108348427	
DMR16:79615001	16	79615001	79619000	4000	1	7.70E-09	0.59	70	1.75	Myom2;LOC108348427	
DMR16:79644001	16	79644001	79646000	2000	1	2.20E-09	-0.42	37	1.85	Myom2	

DMR16:79758001	16	79758001	79767000	9000	1	3.10E-08	-0.48	308	3.42	Arhgef10	Transcription
DMR16:79868001	16	79868001	79871000	3000	1	2.60E-07	0.58	46	1.53	Dlgap2	Cytoskeleton
DMR16:79928001	16	79928001	79932000	4000	1	4.00E-07	0.36	35	0.88	Dlgap2	Cytoskeleton
DMR16:79964001	16	79964001	79966000	2000	1	2.80E-07	-0.47	19	0.95	Dlgap2	Cytoskeleton
DMR16:79969001	16	79969001	79970000	1000	1	2.30E-12	0.84	22	2.2	Dlgap2	Cytoskeleton
DMR16:79993001	16	79993001	79994000	1000	1	3.30E-10	0.56	13	1.3	Dlgap2	Cytoskeleton
DMR16:80045001	16	80045001	80046000	1000	1	2.00E-09	-0.38	16	1.6	Dlgap2	Cytoskeleton
DMR16:80171001	16	80171001	80172000	1000	1	1.80E-09	0.39	8	0.8	Dlgap2	Cytoskeleton
DMR16:80205001	16	80205001	80208000	3000	1	2.70E-11	-0.46	46	1.53	Dlgap2	Cytoskeleton
DMR16:80295001	16	80295001	80296000	1000	1	2.90E-07	0.5	11	1.1	Dlgap2	Cytoskeleton
DMR16:80339001	16	80339001	80342000	3000	1	1.00E-11	-0.46	57	1.9	Dlgap2	Cytoskeleton
DMR16:80368001	16	80368001	80370000	2000	1	8.80E-08	-0.38	49	2.45	Dlgap2	Cytoskeleton
DMR16:80516001	16	80516001	80519000	3000	1	3.40E-07	0.35	52	1.73	Dlgap2	Cytoskeleton
DMR16:80533001	16	80533001	80534000	1000	1	1.10E-09	-0.59	8	0.8	Dlgap2	Cytoskeleton
DMR16:80558001	16	80558001	80560000	2000	1	4.40E-08	0.39	29	1.45	Dlgap2;LOC108348428	Cytoskeleton
DMR16:80568001	16	80568001	80569000	1000	1	3.10E-11	0.82	14	1.4	Dlgap2;LOC108348428	Cytoskeleton
DMR16:80571001	16	80571001	80572000	1000	1	6.30E-13	-0.54	4	0.4	Dlgap2;LOC108348428	Cytoskeleton
DMR16:80579001	16	80579001	80582000	3000	1	7.90E-19	1.1	93	3.1	Dlgap2;LOC108348428	Cytoskeleton
DMR16:80627001	16	80627001	80628000	1000	1	2.80E-09	0.34	17	1.7	Erich1	
DMR16:80629001	16	80629001	80632000	3000	1	3.30E-07	-0.36	75	2.5	Erich1	
DMR16:80642001	16	80642001	80646000	4000	1	1.40E-10	-0.5	105	2.62	Erich1;LOC103693997	
DMR16:80662001	16	80662001	80664000	2000	1	2.10E-07	-0.35	65	3.25	Erich1	
DMR16:80786001	16	80786001	80788000	2000	1	3.30E-07	-0.36	43	2.15	Fbxo25;LOC108348429	
DMR16:80794001	16	80794001	80802000	8000	3	6.60E-11	-0.5	194	2.42	Fbxo25;LOC108348429	
DMR16:80845001	16	80845001	80846000	1000	1	5.30E-08	0.51	12	1.2	Champ1	
DMR16:81024001	16	81024001	81028000	4000	1	3.30E-07	-0.46	82	2.05	Dcun1d2;Tmco3	
DMR16:81029001	16	81029001	81031000	2000	1	6.90E-08	-0.44	49	2.45	Dcun1d2;Tmco3	
DMR16:81079001	16	81079001	81080000	1000	1	9.20E-08	-0.45	22	2.2	Tmco3;LOC102548465;Tfdp1	Transcription
DMR16:81084001	16	81084001	81087000	3000	2	1.60E-09	-0.65	44	1.47	LOC102548465;Tfdp1	Transcription
DMR16:81156001	16	81156001	81158000	2000	1	3.30E-07	0.3	50	2.5	Grk1	Signaling
DMR16:81246001	16	81246001	81248000	2000	1	8.70E-13	1.03	57	2.85	Gas6;LOC108349416;LOC103693998	Extracellular Matrix
DMR16:81328001	16	81328001	81330000	2000	1	4.70E-08	0.47	29	1.45	Rasa3	Signaling
DMR16:81408001	16	81408001	81410000	2000	2	2.30E-07	0.48	39	1.95	Rasa3	Signaling
DMR16:81519001	16	81519001	81520000	1000	1	4.50E-09	-0.36	15	1.5	Cdc16;Upf3a	Proteolysis
DMR16:81522001	16	81522001	81523000	1000	1	3.00E-08	-0.51	7	0.7	Cdc16;Upf3a	Proteolysis
DMR16:81535001	16	81535001	81536000	1000	1	8.40E-14	0.96	29	2.9	Upf3a	
DMR16:81595001	16	81595001	81598000	3000	1	7.50E-08	-0.68	57	1.9	NEWGENE_1582994	
DMR16:81639001	16	81639001	81648000	9000	2	3.70E-11	-0.54	204	2.27	Adprhl1;LOC102548530	Signaling
DMR16:81662001	16	81662001	81664000	2000	1	3.70E-08	0.49	33	1.65	LOC102548586;Grtp1	Signaling
DMR16:81666001	16	81666001	81667000	1000	1	3.00E-11	0.67	27	2.7	LOC102548586;Grtp1	Signaling
DMR16:81791001	16	81791001	81792000	1000	1	4.30E-13	0.82	21	2.1	Pcid2;Proz	Protease
DMR16:81795001	16	81795001	81796000	1000	1	5.90E-10	-0.56	27	2.7	Proz;F10;LOC108348431	Protease
DMR16:81826001	16	81826001	81828000	2000	1	6.50E-09	0.55	30	1.5	F10;F7	Protease
DMR16:81849001	16	81849001	81856000	7000	1	7.20E-08	0.53	158	2.26	Mcf2l	Transcription
DMR16:81921001	16	81921001	81923000	2000	1	6.10E-07	0.5	30	1.5	Mcf2l	Transcription
DMR16:83096001	16	83096001	83097000	1000	1	2.30E-11	-0.48	15	1.5	Arhgef7	Transcription
DMR16:83103001	16	83103001	83107000	4000	1	1.00E-08	-0.42	80	2	Arhgef7	Transcription
DMR16:83114001	16	83114001	83115000	1000	1	1.70E-15	-0.55	20	2	Arhgef7	Transcription
DMR16:83276001	16	83276001	83279000	3000	1	5.20E-08	-0.46	76	2.53	Ing1;Cars2	Epigenetic;Translation
DMR16:83311001	16	83311001	83313000	2000	1	3.60E-09	0.64	30	1.5	Cars2	Translation
DMR16:83364001	16	83364001	83366000	2000	1	4.10E-07	-0.44	37	1.85	LOC102553902;Rab20	
DMR16:83377001	16	83377001	83378000	1000	1	2.70E-07	-0.5	20	2	Rab20;Col4a2	Extracellular Matrix
DMR16:83392001	16	83392001	83393000	1000	1	5.70E-11	0.47	20	2	Rab20;Col4a2	Extracellular Matrix
DMR16:83395001	16	83395001	83396000	1000	1	2.70E-12	0.61	10	1	Col4a2	Extracellular Matrix
DMR16:83397001	16	83397001	83402000	5000	1	5.30E-11	0.68	81	1.62	Col4a2	Extracellular Matrix
DMR16:83405001	16	83405001	83406000	1000	1	3.60E-07	0.55	18	1.8	Col4a2	Extracellular Matrix
DMR16:83414001	16	83414001	83415000	1000	1	1.40E-19	1.02	31	3.1	Col4a2	Extracellular Matrix
DMR16:83421001	16	83421001	83427000	6000	1	7.30E-07	0.51	81	1.35	Col4a2	Extracellular Matrix
DMR16:83443001	16	83443001	83444000	1000	1	7.10E-08	0.3	24	2.4	Col4a2	Extracellular Matrix
DMR16:83452001	16	83452001	83453000	1000	1	2.40E-08	-0.47	28	2.8	Col4a2	Extracellular Matrix
DMR16:83481001	16	83481001	83484000	3000	1	1.80E-11	0.53	37	1.23	Col4a2	Extracellular Matrix

DMR16:83571001	16	83571001	83572000	1000	1	9.00E-08	0.6	15	1.5	Col4a1	Extracellular Matrix
DMR16:83592001	16	83592001	83593000	1000	1	1.30E-12	0.57	4	0.4	Col4a1	Extracellular Matrix
DMR16:83834001	16	83834001	83839000	5000	1	1.90E-07	-0.55	58	1.16	Irs2	
DMR16:84577001	16	84577001	84578000	1000	1	1.60E-12	-0.61	7	0.7	Myo16	
DMR16:84591001	16	84591001	84594000	3000	2	3.70E-11	0.31	40	1.33	Myo16	
DMR16:84613001	16	84613001	84617000	4000	1	4.60E-09	-0.58	50	1.25	Myo16;LOC103694009	
DMR16:84654001	16	84654001	84656000	2000	2	5.00E-08	-0.59	9	0.45	Myo16	
DMR16:84658001	16	84658001	84659000	1000	1	3.20E-14	0.53	15	1.5	Myo16	
DMR16:84664001	16	84664001	84665000	1000	1	1.70E-07	0.4	11	1.1	Myo16	
DMR16:84690001	16	84690001	84692000	2000	1	3.60E-08	-0.45	42	2.1	Myo16	
DMR16:84695001	16	84695001	84696000	1000	1	7.40E-08	-0.4	14	1.4	Myo16	
DMR16:84752001	16	84752001	84754000	2000	2	2.10E-08	-0.74	10	0.5	Myo16	
DMR16:84771001	16	84771001	84773000	2000	1	8.70E-07	-0.39	45	2.25	Myo16	
DMR16:84863001	16	84863001	84865000	2000	1	5.30E-07	-0.37	34	1.7	Myo16	
DMR16:85162001	16	85162001	85163000	1000	1	3.20E-09	-0.82	9	0.9	Myo16	
DMR16:85454001	16	85454001	85456000	2000	1	1.90E-07	-0.38	33	1.65	RGD1565902	
DMR16:85665001	16	85665001	85668000	3000	1	1.70E-12	0.78	47	1.57	Fam155a	
DMR16:85760001	16	85760001	85762000	2000	1	4.50E-07	0.42	23	1.15	Fam155a	
DMR16:85855001	16	85855001	85856000	1000	1	2.40E-11	0.74	21	2.1	Fam155a	
DMR16:85857001	16	85857001	85858000	1000	1	8.10E-08	-0.58	4	0.4	Fam155a	
DMR16:85885001	16	85885001	85889000	4000	1	8.40E-07	0.46	36	0.9	Fam155a	
DMR16:85899001	16	85899001	85901000	2000	1	3.90E-11	0.78	42	2.1	Fam155a	
DMR16:85903001	16	85903001	85904000	1000	1	3.60E-13	0.98	24	2.4	Fam155a	
DMR16:85957001	16	85957001	85961000	4000	1	1.00E-09	0.67	59	1.48	Fam155a	
DMR16:86583001	16	86583001	86584000	1000	1	5.10E-10	0.66	26	2.6	RGD1564807	Transcription
DMR16:86587001	16	86587001	86589000	2000	1	5.20E-09	-0.35	36	1.8	RGD1564807	Transcription
DMR16:86607001	16	86607001	86608000	1000	1	7.90E-16	-0.59	20	2	Arglu1	
DMR16:86634001	16	86634001	86635000	1000	1	1.90E-09	0.64	14	1.4	Arglu1;Efnb2	Signaling
DMR17:397001	17	397001	398000	1000	1	1.90E-07	-0.37	22	2.2	Fbp2;Fbp1	Metabolism
DMR17:530001	17	530001	533000	3000	1	5.20E-10	-0.28	27	0.9	Npepo	
DMR17:636001	17	636001	637000	1000	1	2.00E-08	0.45	13	1.3	Npepo	
DMR17:768001	17	768001	770000	2000	2	7.30E-08	-0.5	27	1.35	Npepo	
DMR17:1089001	17	1089001	1091000	2000	1	3.70E-09	0.68	33	1.65	Ptch1	
DMR17:1364001	17	1364001	1369000	5000	1	7.90E-08	-0.28	59	1.18	Ercc6l2	
DMR17:1403001	17	1403001	1404000	1000	1	2.20E-07	-0.38	11	1.1	Ercc6l2	
DMR17:1619001	17	1619001	1621000	2000	1	1.80E-09	0.7	46	2.3	Hsd17b3;Slc35d2	Transport
DMR17:1728001	17	1728001	1729000	1000	1	6.60E-14	-0.57	6	0.6	Cdc14b	Signaling
DMR17:1839001	17	1839001	1840000	1000	1	1.30E-13	0.88	18	1.8	Aaed1	
DMR17:2163001	17	2163001	2167000	4000	1	4.00E-08	0.55	40	1	RGD1563581	
DMR17:2425001	17	2425001	2427000	2000	1	8.60E-10	-0.53	8	0.4	Spata31a5	
DMR17:2663001	17	2663001	2665000	2000	1	5.30E-07	-0.39	13	0.65	Mfsd14b;LOC680346	
DMR17:3875001	17	3875001	3876000	1000	1	4.70E-07	0.35	11	1.1	Ctsr	Protease
DMR17:3993001	17	3993001	3994000	1000	1	2.20E-07	-0.42	6	0.6	Ctsq	Protease
DMR17:4210001	17	4210001	4211000	1000	1	2.70E-07	-0.65	6	0.6	Testin;LOC100912413	Protease
DMR17:4255001	17	4255001	4256000	1000	1	9.40E-08	0.48	12	1.2	Ctsll3	
DMR17:4296001	17	4296001	4301000	5000	1	2.60E-10	0.69	100	2	Dapk1	Signaling
DMR17:4449001	17	4449001	4450000	1000	1	3.20E-09	-0.45	33	3.3	Dapk1	Signaling
DMR17:4853001	17	4853001	4855000	2000	1	4.40E-09	-0.51	15	0.75	Gas1	
DMR17:5227001	17	5227001	5230000	3000	1	9.10E-07	-0.39	42	1.4	Zcchc6	
DMR17:5315001	17	5315001	5317000	2000	1	6.90E-08	0.42	37	1.85	LOC102547665;LOC102547829;Spata31d1d	
DMR17:5353001	17	5353001	5356000	3000	2	7.80E-09	-0.69	20	0.67	Spata31d3;LOC100912252	
DMR17:5457001	17	5457001	5460000	3000	1	6.40E-08	-0.35	31	1.03	Naa35	Metabolism
DMR17:5511001	17	5511001	5512000	1000	1	1.10E-07	-0.33	23	2.3	Agtpbp1	Protease
DMR17:5554001	17	5554001	5556000	2000	1	1.70E-11	-0.62	36	1.8	Agtpbp1	Protease
DMR17:5562001	17	5562001	5566000	4000	1	3.00E-07	-0.4	77	1.93	Agtpbp1	Protease
DMR17:5602001	17	5602001	5603000	1000	1	4.10E-10	0.54	17	1.7	Agtpbp1	Protease
DMR17:5935001	17	5935001	5937000	2000	1	3.20E-07	-0.35	39	1.95	Ntrk2	Receptor
DMR17:5953001	17	5953001	5954000	1000	1	1.40E-08	0.46	5	0.5	Ntrk2	Receptor
DMR17:5957001	17	5957001	5959000	2000	1	2.30E-11	0.6	42	2.1	Ntrk2	Receptor
DMR17:5975001	17	5975001	5977000	2000	1	2.30E-22	0.93	50	2.5	Ntrk2	Receptor
DMR17:5979001	17	5979001	5983000	4000	1	5.50E-07	-0.34	84	2.1	Ntrk2	Receptor

DMR17:6018001	17	6018001	6024000	6000	2	1.50E-14	1.04	121	2.02	Ntrk2	Receptor
DMR17:6048001	17	6048001	6051000	3000	1	3.70E-12	0.68	65	2.17	Ntrk2;LOC102548918	Receptor
DMR17:6052001	17	6052001	6054000	2000	1	2.50E-10	0.84	47	2.35	Ntrk2;LOC102548918	Receptor
DMR17:6076001	17	6076001	6077000	1000	1	3.90E-12	1.02	25	2.5	Ntrk2;LOC108353117	Receptor
DMR17:6078001	17	6078001	6079000	1000	1	1.70E-08	-0.41	20	2	Ntrk2;LOC108353117	Receptor
DMR17:6093001	17	6093001	6095000	2000	2	5.80E-11	0.72	48	2.4	Ntrk2;LOC108353117	Receptor
DMR17:6101001	17	6101001	6104000	3000	1	2.80E-10	0.38	40	1.33	Ntrk2	Receptor
DMR17:6114001	17	6114001	6118000	4000	1	4.00E-07	-0.39	64	1.6	Ntrk2	Receptor
DMR17:6147001	17	6147001	6149000	2000	1	1.70E-07	0.4	30	1.5	Ntrk2	Receptor
DMR17:6241001	17	6241001	6243000	2000	1	1.50E-08	0.55	41	2.05	Ntrk2	Receptor
DMR17:6463001	17	6463001	6465000	2000	1	2.90E-09	-0.42	33	1.65	Slc28a3	Transport
DMR17:6496001	17	6496001	6500000	4000	2	2.40E-07	0.47	102	2.55	Slc28a3	Transport
DMR17:6697001	17	6697001	6700000	3000	1	7.10E-07	0.55	54	1.8	RGD1311345;LOC103694036;Kif27	Cytoskeleton
DMR17:6705001	17	6705001	6708000	3000	1	9.70E-07	0.39	38	1.27	LOC103694036;Kif27	Cytoskeleton
DMR17:6813001	17	6813001	6815000	2000	1	7.60E-14	-0.53	47	2.35	Gkap1	Cytoskeleton
DMR17:6871001	17	6871001	6875000	4000	1	7.50E-12	-0.43	85	2.12	Ubqln1	
DMR17:6887001	17	6887001	6888000	1000	1	1.30E-07	0.76	21	2.1	Ubqln1;Idnk	Signaling
DMR17:6899001	17	6899001	6902000	3000	2	4.70E-10	0.81	55	1.83	Idnk;LOC681410	Signaling
DMR17:6926001	17	6926001	6928000	2000	1	1.10E-08	-0.4	48	2.4	LOC108348619;Klhl3	Cytoskeleton
DMR17:6933001	17	6933001	6938000	5000	2	4.70E-13	0.91	119	2.38	LOC108348619;Klhl3	Cytoskeleton
DMR17:6956001	17	6956001	6957000	1000	1	1.00E-18	0.75	40	4	Klhl3	Cytoskeleton
DMR17:7670001	17	7670001	7671000	1000	1	1.80E-12	0.48	10	1	Spock1;LOC108348496	Signaling
DMR17:7704001	17	7704001	7706000	2000	1	5.20E-07	-0.31	51	2.55	Spock1	Signaling
DMR17:7710001	17	7710001	7713000	3000	1	1.10E-09	-0.39	66	2.2	Spock1	Signaling
DMR17:7727001	17	7727001	7731000	4000	1	4.70E-07	0.31	56	1.4	Spock1	Signaling
DMR17:7771001	17	7771001	7774000	3000	1	1.40E-10	-0.45	39	1.3	Spock1	Signaling
DMR17:7797001	17	7797001	7800000	3000	2	1.60E-11	0.73	64	2.13	Spock1	Signaling
DMR17:7802001	17	7802001	7806000	4000	1	6.10E-11	0.7	75	1.88	Spock1	Signaling
DMR17:8138001	17	8138001	8143000	5000	1	1.10E-07	0.53	109	2.18	Trpc7	Transport
DMR17:8165001	17	8165001	8167000	2000	1	5.10E-08	-0.37	44	2.2	Trpc7	Transport
DMR17:8170001	17	8170001	8171000	1000	1	9.20E-09	0.44	13	1.3	Trpc7;LOC108348499	Transport
DMR17:8201001	17	8201001	8204000	3000	1	1.20E-08	0.37	45	1.5	Trpc7;LOC108348499;LOC102555284	Transport
DMR17:8228001	17	8228001	8229000	1000	1	1.80E-07	0.54	30	3	Trpc7;LOC102555284	Transport
DMR17:8252001	17	8252001	8255000	3000	1	1.20E-11	0.72	77	2.57	Trpc7	Transport
DMR17:8335001	17	8335001	8336000	1000	1	4.60E-09	-0.48	19	1.9	Smad5	Transcription
DMR17:8395001	17	8395001	8397000	2000	1	9.80E-08	0.42	29	1.45	Tgfbi	Cytoskeleton
DMR17:8435001	17	8435001	8436000	1000	1	9.50E-07	0.44	20	2	Tgfbi;LOC102555503	Cytoskeleton
DMR17:8521001	17	8521001	8524000	3000	2	1.80E-12	0.94	68	2.27	Fbxl21;LOC108348500	
DMR17:8571001	17	8571001	8572000	1000	1	5.40E-07	-0.37	7	0.7	Il9;Slc25a48	Cytokine;Transport
DMR17:8621001	17	8621001	8623000	2000	1	9.70E-09	0.74	40	2	RGD1562024	
DMR17:8631001	17	8631001	8634000	3000	1	2.00E-08	-0.47	49	1.63	RGD1562024	
DMR17:8896001	17	8896001	8899000	3000	2	4.90E-13	0.95	56	1.87	Catsper3	Transport
DMR17:9113001	17	9113001	9114000	1000	1	1.50E-09	0.68	25	2.5	Cxcl14	Growth Factors
DMR17:9221001	17	9221001	9225000	4000	1	9.50E-08	0.61	67	1.68	LOC108348503;LOC100360522;Tifab	
DMR17:9246001	17	9246001	9247000	1000	1	8.00E-07	0.49	22	2.2	Tifab	
DMR17:9341001	17	9341001	9343000	2000	1	2.00E-10	0.62	51	2.55	H2afy	
DMR17:9469001	17	9469001	9472000	3000	1	2.50E-09	0.61	51	1.7	RGD1566359;Ddx46	
DMR17:9503001	17	9503001	9507000	4000	1	2.90E-08	-0.38	84	2.1	Ddx46	
DMR17:9575001	17	9575001	9579000	4000	2	9.00E-13	0.71	63	1.57	Tmed9	Transport
DMR17:9647001	17	9647001	9650000	3000	1	2.70E-08	-0.37	44	1.47	Ddx41;LOC108348506;Dok3;Pdlim7	Cytoskeleton
DMR17:9703001	17	9703001	9708000	5000	1	6.80E-08	0.5	99	1.98	Dbn1;Prr7;Grk6	Cytoskeleton;Signaling
DMR17:9749001	17	9749001	9750000	1000	1	1.40E-08	0.55	22	2.2	F12;Pfn3;Slc34a1	Protease;Cytoskeleton;Transport
DMR17:9784001	17	9784001	9785000	1000	1	3.60E-07	0.56	20	2	Rgs14	
DMR17:9799001	17	9799001	9801000	2000	1	5.40E-15	-0.64	28	1.4	Rgs14;Lman2	Transport
DMR17:9850001	17	9850001	9851000	1000	1	9.30E-07	-0.32	10	1	Nsd1	
DMR17:9990001	17	9990001	9992000	2000	2	2.20E-18	1.03	60	3	LOC498705;Fgfr4	Receptor
DMR17:9993001	17	9993001	9995000	2000	1	6.00E-07	0.38	25	1.25	Fgfr4	Receptor

DMR17:10010001	17	10010001	10012000	2000	1	4.20E-09	0.63	45	2.25	Fgfr4;Zfp346	Receptor
DMR17:10014001	17	10014001	10016000	2000	1	2.10E-12	0.96	49	2.45	Fgfr4;Zfp346	Receptor
DMR17:10147001	17	10147001	10150000	3000	2	4.80E-09	0.6	66	2.2	Hk3;Unc5a	Signaling;Receptor
DMR17:10162001	17	10162001	10164000	2000	1	3.60E-09	0.51	43	2.15	Hk3;Unc5a	Signaling;Receptor
DMR17:10344001	17	10344001	10347000	3000	1	4.30E-14	0.91	86	2.87	Tspan17	
DMR17:10351001	17	10351001	10352000	1000	1	4.10E-08	0.75	25	2.5	Tspan17	
DMR17:10355001	17	10355001	10359000	4000	1	2.70E-07	0.41	77	1.93	Tspan17	
DMR17:10415001	17	10415001	10417000	2000	1	5.80E-07	0.38	24	1.2	Gprin1;Cdhr2	Cytoskeleton
DMR17:10423001	17	10423001	10424000	1000	1	5.20E-08	0.49	13	1.3	Gprin1;Cdhr2	Cytoskeleton
DMR17:10425001	17	10425001	10426000	1000	1	5.10E-12	0.68	21	2.1	Gprin1;Cdhr2	Cytoskeleton
DMR17:10577001	17	10577001	10579000	2000	1	6.70E-09	0.56	32	1.6	Arl10;LOC306766	
DMR17:10655001	17	10655001	10656000	1000	1	4.30E-07	0.52	25	2.5	LOC102551420;RGD1566033	
DMR17:10677001	17	10677001	10681000	4000	1	8.90E-07	-0.36	94	2.35	Thoc3	Metabolism
DMR17:10741001	17	10741001	10742000	1000	1	2.50E-11	0.71	14	1.4	Lnc012	
DMR17:10756001	17	10756001	10760000	4000	2	1.40E-13	1.06	91	2.28	Lnc012;Cplx2	
DMR17:10799001	17	10799001	10804000	5000	1	4.60E-08	0.34	79	1.58	Cplx2;LOC103694917	
DMR17:10808001	17	10808001	10810000	2000	1	4.30E-14	0.8	30	1.5	Cplx2;LOC103694917	
DMR17:11082001	17	11082001	11083000	1000	1	4.00E-08	0.61	45	4.5	Sfxn1	Transport
DMR17:11674001	17	11674001	11676000	2000	1	6.60E-08	-0.47	29	1.45	LOC108348511;Msx2	Development
DMR17:11906001	17	11906001	11911000	5000	2	4.20E-09	-0.42	40	0.8	RGD1561671	Transcription
DMR17:11914001	17	11914001	11916000	2000	1	5.70E-13	0.81	64	3.2	RGD1561671	Transcription
DMR17:11955001	17	11955001	11956000	1000	1	5.30E-07	0.62	19	1.9	Ror2	Receptor
DMR17:12001001	17	12001001	12004000	3000	2	1.80E-08	-0.42	45	1.5	Ror2	Receptor
DMR17:12029001	17	12029001	12032000	3000	1	1.40E-14	0.93	60	2	Ror2	Receptor
DMR17:12034001	17	12034001	12036000	2000	1	7.60E-07	0.32	37	1.85	Ror2	Receptor
DMR17:12047001	17	12047001	12048000	1000	1	1.50E-09	0.39	18	1.8	Ror2	Receptor
DMR17:12066001	17	12066001	12069000	3000	2	9.70E-10	-0.46	57	1.9	Ror2	Receptor
DMR17:12092001	17	12092001	12095000	3000	1	2.30E-07	-0.35	66	2.2	Ror2	Receptor
DMR17:12107001	17	12107001	12113000	6000	2	1.10E-13	-0.58	123	2.05	Ror2	Receptor
DMR17:12125001	17	12125001	12130000	5000	1	2.30E-12	0.67	113	2.26	Ror2	Receptor
DMR17:12276001	17	12276001	12279000	3000	1	1.10E-10	0.76	67	2.23	Nfil3	Transcription
DMR17:12332001	17	12332001	12333000	1000	1	2.00E-09	-0.42	23	2.3	Auh	Metabolism
DMR17:12377001	17	12377001	12379000	2000	1	1.30E-10	-0.42	43	2.15	Auh	Metabolism
DMR17:12637001	17	12637001	12639000	2000	1	7.00E-07	0.41	29	1.45	Syk	
DMR17:12640001	17	12640001	12642000	2000	1	4.50E-08	0.57	34	1.7	Syk	
DMR17:12647001	17	12647001	12649000	2000	1	5.70E-08	-0.31	36	1.8	Syk	
DMR17:12659001	17	12659001	12663000	4000	1	3.10E-07	-0.4	45	1.12	Syk	
DMR17:12726001	17	12726001	12729000	3000	1	3.50E-07	0.4	38	1.27	LOC103694041;Diras2	Signaling
DMR17:12735001	17	12735001	12736000	1000	1	1.10E-11	0.84	28	2.8	LOC103694041;Diras2	Signaling
DMR17:12763001	17	12763001	12767000	4000	1	1.00E-10	0.72	103	2.58	Diras2	Signaling
DMR17:13396001	17	13396001	13397000	1000	1	1.00E-09	0.72	35	3.5	Gadd45g	
DMR17:13556001	17	13556001	13557000	1000	1	9.70E-08	0.66	14	1.4	LOC103694042;Secisbp2	
DMR17:13751001	17	13751001	13753000	2000	1	1.80E-07	0.33	23	1.15	Shc3	Cytoskeleton
DMR17:13771001	17	13771001	13772000	1000	1	3.30E-16	0.99	18	1.8	Shc3	Cytoskeleton
DMR17:14602001	17	14602001	14603000	1000	1	7.20E-10	-0.43	7	0.7	LOC108348068;Ogn	
DMR17:14838001	17	14838001	14839000	1000	1	1.30E-08	0.51	13	1.3	Barx1	Development
DMR17:15366001	17	15366001	15367000	1000	1	3.90E-10	-0.53	21	2.1	Iars	Translation
DMR17:15749001	17	15749001	15750000	1000	1	1.00E-09	-0.47	22	2.2	Fgd3	Transcription
DMR17:15764001	17	15764001	15767000	3000	2	3.50E-16	-0.5	56	1.87	Fgd3	Transcription
DMR17:15872001	17	15872001	15874000	2000	1	3.70E-08	0.53	34	1.7	Ninj1	Cytoskeleton
DMR17:15929001	17	15929001	15933000	4000	1	9.70E-08	0.33	72	1.8	Wnk2	Signaling
DMR17:15939001	17	15939001	15941000	2000	1	7.40E-11	0.67	24	1.2	Wnk2	Signaling
DMR17:15983001	17	15983001	15988000	5000	1	1.20E-08	0.45	83	1.66	Wnk2	Signaling
DMR17:16007001	17	16007001	16010000	3000	1	1.10E-08	0.48	44	1.47	Wnk2	Signaling
DMR17:16113001	17	16113001	16114000	1000	1	8.80E-08	-0.36	8	0.8	Fam120a	
DMR17:16254001	17	16254001	16257000	3000	1	4.90E-07	0.55	47	1.57	Phf2	
DMR17:16354001	17	16354001	16355000	1000	1	7.30E-09	-0.49	25	2.5	Ptpdc1	Signaling
DMR17:16377001	17	16377001	16382000	5000	2	3.20E-08	0.56	69	1.38	Ptpdc1	Signaling
DMR17:16444001	17	16444001	16446000	2000	1	4.00E-10	0.66	35	1.75	LOC108348525;Zfp169;LOC108348526	Transcription
DMR17:16458001	17	16458001	16463000	5000	1	1.50E-13	0.91	77	1.54	Zfp169;LOC108348526	Transcription
DMR17:16682001	17	16682001	16683000	1000	1	5.80E-07	0.34	14	1.4	Id4	Transcription

DMR17:17807001	17	17807001	17808000	1000	1	2.70E-07	0.62	22	2.2	Rnf144b	Proteolysis
DMR17:17826001	17	17826001	17828000	2000	1	1.60E-09	-0.43	60	3	Rnf144b	Proteolysis
DMR17:17863001	17	17863001	17866000	3000	2	6.20E-10	-0.68	52	1.73	Rnf144b;LOC102555870	Proteolysis
DMR17:17869001	17	17869001	17872000	3000	1	3.50E-10	-0.42	51	1.7	Rnf144b;LOC102555870	Proteolysis
DMR17:17879001	17	17879001	17886000	7000	2	4.20E-09	-0.39	122	1.74	Rnf144b	Proteolysis
DMR17:18191001	17	18191001	18193000	2000	1	1.90E-07	-0.35	27	1.35	Kif13a	Cytoskeleton
DMR17:18198001	17	18198001	18200000	2000	1	1.50E-10	0.79	53	2.65	Kif13a	Cytoskeleton
DMR17:18238001	17	18238001	18242000	4000	2	6.50E-07	-0.35	109	2.72	Kif13a	Cytoskeleton
DMR17:18281001	17	18281001	18282000	1000	1	8.70E-09	-0.56	10	1	Kif13a	Cytoskeleton
DMR17:18285001	17	18285001	18286000	1000	1	2.10E-08	0.7	27	2.7	Kif13a	Cytoskeleton
DMR17:18299001	17	18299001	18305000	6000	1	4.30E-08	-0.45	104	1.73	Kif13a	Cytoskeleton
DMR17:18310001	17	18310001	18313000	3000	1	8.30E-08	-0.38	75	2.5	Kif13a	Cytoskeleton
DMR17:18327001	17	18327001	18328000	1000	1	3.70E-09	0.54	10	1	Kif13a	Cytoskeleton
DMR17:18355001	17	18355001	18357000	2000	1	2.20E-07	-0.47	20	1	LOC100362185;Nup153	Transport
DMR17:18365001	17	18365001	18366000	1000	1	2.20E-07	-0.4	7	0.7	Nup153	Transport
DMR17:18425001	17	18425001	18426000	1000	1	5.10E-17	0.86	28	2.8	Fam8a1	
DMR17:18450001	17	18450001	18453000	3000	1	1.90E-07	-0.33	83	2.77	Cap2	Cytoskeleton
DMR17:18502001	17	18502001	18511000	9000	1	2.00E-13	0.71	145	1.61	Cap2;LOC108353127	Cytoskeleton
DMR17:18556001	17	18556001	18558000	2000	1	2.00E-07	-0.4	29	1.45	Cap2	Cytoskeleton
DMR17:18587001	17	18587001	18590000	3000	1	2.70E-08	0.61	53	1.77	Cap2	Cytoskeleton
DMR17:18673001	17	18673001	18675000	2000	1	2.10E-07	0.73	65	3.25	Rbm24	
DMR17:19203001	17	19203001	19204000	1000	1	3.40E-08	-0.45	14	1.4	Atxn1	
DMR17:19265001	17	19265001	19266000	1000	1	1.50E-07	0.43	13	1.3	Atxn1	
DMR17:19315001	17	19315001	19318000	3000	1	1.90E-11	0.78	59	1.97	Atxn1	
DMR17:19414001	17	19414001	19418000	4000	2	6.80E-10	-0.62	37	0.92	Atxn1	
DMR17:19446001	17	19446001	19449000	3000	1	7.70E-15	-0.51	59	1.97	Atxn1	
DMR17:19465001	17	19465001	19467000	2000	1	2.10E-07	0.53	16	0.8	Atxn1	
DMR17:19500001	17	19500001	19502000	2000	1	4.00E-08	-0.33	23	1.15	Atxn1	
DMR17:19522001	17	19522001	19528000	6000	1	1.20E-08	-0.44	85	1.42	Atxn1	
DMR17:19560001	17	19560001	19566000	6000	1	3.80E-07	-0.37	90	1.5	Gmpr	Metabolism
DMR17:19573001	17	19573001	19574000	1000	1	1.80E-08	-0.41	27	2.7	Gmpr	Metabolism
DMR17:19584001	17	19584001	19588000	4000	1	1.90E-07	0.48	84	2.1	Gmpr	Metabolism
DMR17:19677001	17	19677001	19679000	2000	1	3.90E-10	0.68	41	2.05	Mylip	
DMR17:19681001	17	19681001	19683000	2000	1	4.50E-07	-0.4	31	1.55	Mylip	
DMR17:19698001	17	19698001	19702000	4000	1	2.00E-09	-0.39	59	1.48	Mylip	
DMR17:20220001	17	20220001	20225000	5000	1	7.00E-07	-0.36	45	0.9	Jarid2	Epigenetic
DMR17:21360001	17	21360001	21362000	2000	1	1.10E-10	-0.54	15	0.75	Smim13;LOC108353128;Mir3566;LOC102548659	
DMR17:21402001	17	21402001	21403000	1000	1	6.70E-07	0.4	17	1.7	Elovl2	Metabolism
DMR17:21429001	17	21429001	21432000	3000	1	1.10E-07	-0.41	38	1.27	Elovl2;LOC108348546;Sycp2l	Metabolism
DMR17:21438001	17	21438001	21440000	2000	1	9.20E-07	-0.36	41	2.05	LOC108348546;Sycp2l	
DMR17:21466001	17	21466001	21468000	2000	1	1.20E-07	-0.46	32	1.6	Sycp2l	
DMR17:21494001	17	21494001	21495000	1000	1	2.90E-12	0.77	42	4.2	Sycp2l;Gcm2	
DMR17:21503001	17	21503001	21505000	2000	1	1.90E-08	-0.42	32	1.6	Gcm2	
DMR17:21651001	17	21651001	21654000	3000	2	6.50E-09	0.58	57	1.9	Gcnt2	Golgi
DMR17:21662001	17	21662001	21663000	1000	1	9.00E-11	-0.44	14	1.4	Gcnt2	Golgi
DMR17:21671001	17	21671001	21672000	1000	1	6.00E-08	0.53	33	3.3	Gcnt2	Golgi
DMR17:21723001	17	21723001	21726000	3000	1	1.00E-10	0.81	51	1.7	Gcnt6	
DMR17:22315001	17	22315001	22317000	2000	1	4.00E-10	-0.44	35	1.75	Hivep1	
DMR17:22326001	17	22326001	22329000	3000	1	3.40E-07	-0.39	40	1.33	Hivep1	
DMR17:22356001	17	22356001	22357000	1000	1	7.40E-07	-0.49	15	1.5	Hivep1	
DMR17:22663001	17	22663001	22665000	2000	1	7.70E-07	-0.4	27	1.35	Adtrp;LOC100362172	Metabolism
DMR17:22693001	17	22693001	22695000	2000	1	3.00E-13	0.91	31	1.55	Adtrp;LOC102548261	
DMR17:22843001	17	22843001	22846000	3000	1	8.00E-07	-0.39	37	1.23	Tmem170b;LOC108353129	
DMR17:23165001	17	23165001	23167000	2000	2	5.30E-08	0.34	21	1.05	Nedd9;LOC108353140	
DMR17:23244001	17	23244001	23246000	2000	1	2.80E-07	-0.47	39	1.95	Phactr1	Signaling
DMR17:23280001	17	23280001	23282000	2000	1	3.50E-11	0.81	60	3	Phactr1	Signaling
DMR17:23345001	17	23345001	23347000	2000	1	3.70E-07	0.64	27	1.35	Phactr1	Signaling
DMR17:23408001	17	23408001	23409000	1000	1	6.00E-07	0.46	20	2	Phactr1	Signaling
DMR17:23426001	17	23426001	23427000	1000	1	2.40E-07	0.47	10	1	Phactr1	Signaling
DMR17:23480001	17	23480001	23481000	1000	1	1.20E-10	-0.48	17	1.7	Phactr1	Signaling
DMR17:23527001	17	23527001	23529000	2000	1	5.50E-08	-0.44	34	1.7	Phactr1	Signaling

DMR17:23594001	17	23594001	23595000	1000	1	1.30E-08	0.76	17	1.7	Phactr1	Signaling
DMR17:23789001	17	23789001	23790000	1000	1	1.10E-07	0.52	19	1.9	Tbc1d7;LOC102547830	Signaling
DMR17:23822001	17	23822001	23824000	2000	1	1.50E-08	0.85	40	2	Gfod1	Metabolism
DMR17:23830001	17	23830001	23831000	1000	1	3.60E-09	0.68	18	1.8	Gfod1	Metabolism
DMR17:23993001	17	23993001	23997000	4000	1	7.60E-08	0.5	57	1.43	Sirt5	
DMR17:24173001	17	24173001	24174000	1000	1	8.00E-08	-0.37	31	3.1	Mcur1	
DMR17:24274001	17	24274001	24276000	2000	2	5.70E-15	-0.55	31	1.55	Rnf182	
DMR17:24401001	17	24401001	24407000	6000	1	9.50E-09	0.63	103	1.72	Cd83	
DMR17:24659001	17	24659001	24660000	1000	1	2.40E-08	-0.44	12	1.2	Tfap2a	Transcription
DMR17:24663001	17	24663001	24665000	2000	2	6.30E-15	0.71	70	3.5	Tfap2a	Transcription
DMR17:24672001	17	24672001	24674000	2000	1	4.20E-07	-0.43	22	1.1	Tfap2a	Transcription
DMR17:24678001	17	24678001	24681000	3000	2	6.30E-08	0.64	47	1.57	Tfap2a	Transcription
DMR17:25082001	17	25082001	25083000	1000	1	1.50E-09	0.76	22	2.2	Ofcc1	
DMR17:25141001	17	25141001	25144000	3000	1	1.50E-09	-0.53	53	1.77	Ofcc1	
DMR17:25212001	17	25212001	25213000	1000	1	3.00E-08	-0.36	17	1.7	Ofcc1	
DMR17:25261001	17	25261001	25262000	1000	1	1.40E-07	0.5	15	1.5	Ofcc1	
DMR17:25312001	17	25312001	25314000	2000	1	2.20E-15	0.76	33	1.65	Ofcc1	
DMR17:25328001	17	25328001	25330000	2000	1	9.40E-07	-0.5	15	0.75	Ofcc1	
DMR17:25412001	17	25412001	25414000	2000	1	2.30E-09	0.54	28	1.4	Ofcc1	
DMR17:26473001	17	26473001	26475000	2000	1	1.50E-07	0.52	23	1.15	LOC100360173;Slc35b3	Transport
DMR17:26514001	17	26514001	26515000	1000	1	5.20E-07	0.62	24	2.4	Slc35b3	Transport
DMR17:26996001	17	26996001	27006000	10000	1	5.00E-10	-0.44	183	1.83	Bmp6	Growth Factors
DMR17:27248001	17	27248001	27252000	4000	1	2.50E-07	-0.48	52	1.3	Snrnp48	
DMR17:27288001	17	27288001	27289000	1000	1	7.70E-07	0.52	39	3.9	Snrnp48;Dsp	Cytoskeleton
DMR17:27290001	17	27290001	27295000	5000	1	2.50E-16	-0.6	100	2	Dsp	Cytoskeleton
DMR17:27326001	17	27326001	27328000	2000	1	4.80E-10	-0.54	34	1.7	Dsp	Cytoskeleton
DMR17:27478001	17	27478001	27479000	1000	1	3.80E-07	0.48	11	1.1	Cage1	
DMR17:27637001	17	27637001	27638000	1000	1	8.10E-08	-0.42	20	2	Rreb1	
DMR17:27642001	17	27642001	27644000	2000	1	1.20E-08	-0.56	52	2.6	Rreb1	
DMR17:27657001	17	27657001	27659000	2000	1	3.80E-07	-0.43	32	1.6	Rreb1	
DMR17:27675001	17	27675001	27676000	1000	1	1.30E-10	-0.43	10	1	Rreb1	
DMR17:27728001	17	27728001	27730000	2000	1	1.20E-07	0.4	36	1.8	Rreb1;LOC102546544	
DMR17:27929001	17	27929001	27931000	2000	1	2.70E-09	-0.51	66	3.3	Nrn1;LOC683105	
DMR17:27974001	17	27974001	27975000	1000	1	4.00E-08	-0.46	9	0.9	Nrn1	
DMR17:28165001	17	28165001	28167000	2000	1	3.80E-07	0.48	31	1.55	Ly86	
DMR17:28548001	17	28548001	28553000	5000	1	2.20E-07	0.48	44	0.88	F13a1	Transport
DMR17:29049001	17	29049001	29051000	2000	1	7.80E-11	0.56	25	1.25	Fars2	Translation
DMR17:29099001	17	29099001	29101000	2000	1	4.50E-07	-0.34	23	1.15	Fars2	Translation
DMR17:29270001	17	29270001	29272000	2000	1	5.30E-11	-0.39	17	0.85	Fars2	Translation
DMR17:29384001	17	29384001	29387000	3000	1	3.10E-13	0.68	18	0.6	Fars2	Translation
DMR17:29708001	17	29708001	29713000	5000	1	2.10E-12	-0.55	87	1.74	Cdyl	
DMR17:29837001	17	29837001	29839000	2000	1	5.10E-07	-0.48	6	0.3	Cdyl	
DMR17:30654001	17	30654001	30655000	1000	1	3.10E-11	-0.61	2	0.2	RGD1307537	
DMR17:30705001	17	30705001	30707000	2000	2	2.20E-14	-0.52	31	1.55	Prpf4b	Signaling
DMR17:30736001	17	30736001	30737000	1000	1	9.20E-07	0.6	26	2.6	Hmgb1-ps2	
DMR17:30969001	17	30969001	30972000	3000	1	5.50E-09	0.53	54	1.8	Pxdc1	
DMR17:30978001	17	30978001	30983000	5000	2	3.60E-12	0.48	37	0.74	Pxdc1	
DMR17:30989001	17	30989001	30991000	2000	1	2.20E-08	0.53	21	1.05	Pxdc1	
DMR17:31000001	17	31000001	31001000	1000	1	4.70E-10	-0.39	12	1.2	Pxdc1	
DMR17:31251001	17	31251001	31252000	1000	1	8.50E-07	0.32	12	1.2	Slc22a23	Transport
DMR17:31522001	17	31522001	31524000	2000	1	4.00E-08	0.51	31	1.55	Bphl	Metabolism
DMR17:31577001	17	31577001	31578000	1000	1	7.10E-07	-0.4	5	0.5	Ripk1	Signaling
DMR17:32186001	17	32186001	32189000	3000	1	7.50E-08	-0.44	25	0.83	Serpinb6;LOC102554685	
DMR17:32242001	17	32242001	32243000	1000	1	3.40E-10	0.41	7	0.7	RGD1564786	
DMR17:32267001	17	32267001	32269000	2000	1	9.50E-07	0.53	23	1.15	RGD1564786	
DMR17:32893001	17	32893001	32897000	4000	1	9.20E-09	-0.35	38	0.95	LOC108348553;Serpinb1a	Protease; Proteolysis
DMR17:33050001	17	33050001	33052000	2000	1	1.80E-07	0.39	13	0.65	Mylk4	Signaling
DMR17:33589001	17	33589001	33597000	8000	2	1.60E-21	0.64	79	0.99	Gmds	Metabolism
DMR17:34213001	17	34213001	34216000	3000	1	4.20E-09	-0.41	46	1.53	Foxq1	Transcription
DMR17:34773001	17	34773001	34774000	1000	1	1.40E-07	-0.65	3	0.3	Exoc2	
DMR17:34782001	17	34782001	34785000	3000	1	6.90E-08	-0.7	20	0.67	Exoc2	
DMR17:34882001	17	34882001	34883000	1000	1	3.40E-08	0.39	4	0.4	Irf4	Transcription

DMR17:34986001	17	34986001	34988000	2000	2	4.00E-08	-0.72	23	1.15	Dusp22	Signaling
DMR17:35918001	17	35918001	35919000	1000	1	2.30E-07	0.49	16	1.6	Agtr1a	
DMR17:35958001	17	35958001	35959000	1000	1	1.60E-10	-0.55	20	2	Agtr1a	
DMR17:36067001	17	36067001	36071000	4000	1	3.20E-08	-0.42	71	1.77	Mboat1	Metabolism
DMR17:36087001	17	36087001	36089000	2000	1	1.70E-07	0.43	38	1.9	Mboat1	Metabolism
DMR17:36446001	17	36446001	36447000	1000	1	1.10E-10	-0.79	4	0.4	RGD1561310;Cdkal1	Translation
DMR17:36486001	17	36486001	36488000	2000	1	3.60E-07	-0.43	13	0.65	Cdkal1	
DMR17:36979001	17	36979001	36980000	1000	1	6.30E-12	-0.49	13	1.3	Cdkal1	
DMR17:38466001	17	38466001	38469000	3000	1	1.50E-08	-0.37	47	1.57	Prl5a2	Hormone
DMR17:38983001	17	38983001	38984000	1000	1	2.20E-11	0.7	15	1.5	Prl7d1	Hormone
DMR17:39233001	17	39233001	39234000	1000	1	5.20E-07	-0.3	5	0.5	Prl8a9	Hormone
DMR17:39235001	17	39235001	39237000	2000	1	9.90E-09	0.25	17	0.85	Prl8a9	Hormone
DMR17:39276001	17	39276001	39277000	1000	1	2.50E-13	0.83	6	0.6	Prl3d4	Hormone
DMR17:39424001	17	39424001	39426000	2000	1	9.00E-07	-0.21	38	1.9	Prl8a5	Hormone
DMR17:39556001	17	39556001	39557000	1000	1	5.60E-07	-0.56	2	0.2	Prl2b1;Prl8a2	Hormone
DMR17:41942001	17	41942001	41946000	4000	1	1.10E-08	-0.42	51	1.27	Dcdc2	
DMR17:41991001	17	41991001	41992000	1000	1	3.10E-07	-0.42	12	1.2	Dcdc2	
DMR17:42172001	17	42172001	42178000	6000	1	9.30E-09	-0.25	69	1.15	LOC103694089;RGD1307443;Rpl37-ps1	
DMR17:42218001	17	42218001	42220000	2000	1	1.50E-08	0.36	20	1	RGD1307443;Tdp2	
DMR17:42324001	17	42324001	42326000	2000	1	2.40E-08	0.52	46	2.3	LOC102548126;Fam65b	
DMR17:42550001	17	42550001	42553000	3000	1	3.40E-07	-0.44	42	1.4	Fam65b;LOC102552189	
DMR17:42606001	17	42606001	42609000	3000	1	3.70E-14	0.49	48	1.6	Cmah	
DMR17:42613001	17	42613001	42614000	1000	1	1.20E-09	-0.52	11	1.1	Cmah	
DMR17:42730001	17	42730001	42733000	3000	1	3.30E-07	-0.26	30	1	Prl3a1	Hormone
DMR17:42819001	17	42819001	42821000	2000	1	3.60E-14	0.59	20	1	Prl3c1;LOC691541	Hormone
DMR17:42845001	17	42845001	42846000	1000	1	4.00E-07	0.38	22	2.2	Prl3d2	Hormone
DMR17:43235001	17	43235001	43236000	1000	1	2.20E-08	-0.34	12	1.2	Carmil1	
DMR17:43240001	17	43240001	43241000	1000	1	7.90E-07	0.6	13	1.3	Carmil1	
DMR17:43286001	17	43286001	43288000	2000	1	1.70E-08	-0.69	11	0.55	Carmil1	
DMR17:43452001	17	43452001	43454000	2000	1	1.30E-10	-0.52	18	0.9	LOC100909739;Slc17a4	Transport
DMR17:43481001	17	43481001	43482000	1000	1	3.90E-11	0.67	10	1	Slc17a4;Slc17a1	Transport
DMR17:43533001	17	43533001	43534000	1000	1	1.50E-08	-0.57	5	0.5	Slc17a3	Transport
DMR17:43553001	17	43553001	43558000	5000	1	5.20E-08	-0.33	39	0.78	Slc17a3;Slc17a2	Transport
DMR17:43838001	17	43838001	43839000	1000	1	2.80E-07	0.39	12	1.2	Trnas-gcu;Trnaq-uug;Trnas-uga;Trnam-cau;Trnaw-cca;Trnar-ucg	
DMR17:43852001	17	43852001	43854000	2000	1	6.10E-08	0.64	40	2	Trnaw-cca;Trnar-ucg;Trnas-aga;Trnar-acg	
DMR17:44403001	17	44403001	44409000	6000	6	4.90E-37	1.02	123	2.05	RGD1561897	
DMR17:44410001	17	44410001	44412000	2000	2	1.70E-31	0.99	26	1.3	RGD1561897	
DMR17:44416001	17	44416001	44419000	3000	2	2.30E-20	1.23	37	1.23	RGD1561897	
DMR17:44420001	17	44420001	44427000	7000	6	1.50E-27	0.95	193	2.76	RGD1561897	
DMR17:44534001	17	44534001	44535000	1000	1	2.00E-10	-0.54	14	1.4	Hist1h4b;Hist1h2bk;LOC680322;Trnat-agu;Trnai-aau;LOC102556078;Trnas-cga;Trnar-acg	Epigenetic
DMR17:44589001	17	44589001	44590000	1000	1	1.60E-08	-0.58	4	0.4	Trnas-gcu;Pom121l2;Trnam-cau	Transport
DMR17:44612001	17	44612001	44618000	6000	2	1.80E-24	0.49	59	0.98	Zfp184	
DMR17:44903001	17	44903001	44904000	1000	1	1.70E-07	-0.57	2	0.2	LOC102557552;Olr1656-ps	
DMR17:45008001	17	45008001	45015000	7000	1	9.90E-07	0.38	70	1	Olr1658	Receptor
DMR17:45176001	17	45176001	45178000	2000	1	2.10E-16	0.88	21	1.05	Nkapl	
DMR17:45562001	17	45562001	45563000	1000	1	8.20E-11	0.68	20	2	Trnaa-cgc;Trnat-agu;Trnaa-agc;Trnaa-ugc	
DMR17:45648001	17	45648001	45650000	2000	1	7.80E-08	-0.48	18	0.9	LOC102547106;Trnaf-gaa;Trnae-cuc	
DMR17:45735001	17	45735001	45738000	3000	1	1.80E-08	-0.35	24	0.8	LOC100912958;RGD1564243	Receptor
DMR17:45745001	17	45745001	45749000	4000	2	3.40E-09	-0.43	36	0.9	RGD1564243	
DMR17:45806001	17	45806001	45810000	4000	1	2.20E-09	0.31	41	1.02	Olr1664	Receptor
DMR17:46656001	17	46656001	46657000	1000	1	6.50E-13	-0.56	5	0.5	Elmo1	Cytoskeleton
DMR17:46713001	17	46713001	46722000	9000	1	6.60E-07	-0.37	102	1.13	Elmo1	Cytoskeleton
DMR17:46786001	17	46786001	46787000	1000	1	4.10E-07	0.39	9	0.9	Elmo1	Cytoskeleton

DMR17:46809001	17	46809001	46810000	1000	1	1.30E-07	-0.57	4	0.4	Elmo1	Cytoskeleton
DMR17:46833001	17	46833001	46834000	1000	1	9.40E-08	-0.43	11	1.1	Elmo1	Cytoskeleton
DMR17:46862001	17	46862001	46863000	1000	1	3.50E-07	-0.55	6	0.6	Elmo1	Cytoskeleton
DMR17:47261001	17	47261001	47262000	1000	1	1.10E-09	0.56	17	1.7	Gpr141;LOC102546579	Signaling
DMR17:48404001	17	48404001	48409000	5000	1	2.50E-07	-0.37	50	1	Amph;LOC102555260	
DMR17:48632001	17	48632001	48634000	2000	1	7.70E-13	-0.58	9	0.45	Vps41	Transport
DMR17:49108001	17	49108001	49110000	2000	1	2.90E-14	0.71	26	1.3	Pou6f2	
DMR17:49256001	17	49256001	49257000	1000	1	7.70E-09	0.37	15	1.5	Pou6f2	
DMR17:49357001	17	49357001	49358000	1000	1	5.40E-07	0.27	35	3.5	Pou6f2	
DMR17:49469001	17	49469001	49470000	1000	1	2.20E-08	0.62	19	1.9	Pou6f2	
DMR17:49516001	17	49516001	49517000	1000	1	3.60E-11	0.66	31	3.1	Pou6f2;Tgif2-ps1	
DMR17:50336001	17	50336001	50338000	2000	1	8.60E-12	0.44	12	0.6	Sugct	Transport
DMR17:50398001	17	50398001	50400000	2000	1	5.60E-07	-0.27	31	1.55	Sugct	Transport
DMR17:50542001	17	50542001	50543000	1000	1	1.80E-08	0.4	6	0.6	Sugct	Transport
DMR17:50615001	17	50615001	50619000	4000	1	1.00E-10	-0.38	36	0.9	Sugct	Transport
DMR17:50943001	17	50943001	50944000	1000	1	2.90E-10	0.65	20	2	Sugct;LOC108348620	Transport
DMR17:52296001	17	52296001	52298000	2000	1	3.60E-08	0.53	43	2.15	LOC103694103;Gli3	Transcription
DMR17:52612001	17	52612001	52613000	1000	1	1.30E-08	-0.62	3	0.3	RGD1563690	
DMR17:52762001	17	52762001	52763000	1000	1	4.20E-07	-0.35	9	0.9	Psme1-ps1	
DMR17:53400001	17	53400001	53402000	2000	1	4.70E-12	-0.6	7	0.35	Hecw1;LOC100912102	Proteolysis
DMR17:53433001	17	53433001	53440000	7000	1	4.00E-08	0.46	67	0.96	Hecw1	Proteolysis
DMR17:53446001	17	53446001	53448000	2000	1	2.00E-07	0.36	34	1.7	Hecw1	Proteolysis
DMR17:53607001	17	53607001	53614000	7000	1	7.00E-07	-0.3	71	1.01	Hecw1;LOC103694104	Proteolysis
DMR17:53700001	17	53700001	53701000	1000	1	2.00E-07	-0.57	7	0.7	Hecw1;LOC100912163	Proteolysis
DMR17:53745001	17	53745001	53748000	3000	1	6.40E-11	-0.51	11	0.37	Hecw1;LOC100912163	Proteolysis
DMR17:53946001	17	53946001	53950000	4000	1	2.80E-07	-0.42	26	0.65	Arid4b;Ggps1	Transcription
DMR17:53973001	17	53973001	53974000	1000	1	1.10E-08	0.74	32	3.2	Ggps1;Tbce	Transcription
DMR17:54037001	17	54037001	54038000	1000	1	1.60E-07	0.43	11	1.1	Tbce;B3galnt2	Transcription;Golgi
DMR17:54293001	17	54293001	54294000	1000	1	5.00E-09	-0.64	3	0.3	Arhgap12	Signaling
DMR17:54316001	17	54316001	54318000	2000	1	5.80E-08	-0.46	13	0.65	Arhgap12	Signaling
DMR17:54678001	17	54678001	54681000	3000	1	1.30E-07	-0.35	25	0.83	Zeb1	Transcription
DMR17:55292001	17	55292001	55293000	1000	1	8.30E-07	-0.39	16	1.6	Svil	Cytoskeleton
DMR17:55376001	17	55376001	55378000	2000	1	4.10E-08	-0.41	36	1.8	Svil	Cytoskeleton
DMR17:56226001	17	56226001	56227000	1000	1	7.00E-07	0.4	10	1	RGD1564062	
DMR17:56919001	17	56919001	56920000	1000	1	3.50E-11	0.72	16	1.6	Bambi	
DMR17:57338001	17	57338001	57339000	1000	1	4.10E-08	-0.49	12	1.2	Epc1	Epigenetic
DMR17:57375001	17	57375001	57376000	1000	1	3.10E-07	-0.43	12	1.2	Epc1	Epigenetic
DMR17:57399001	17	57399001	57402000	3000	1	2.40E-10	-0.44	17	0.57	Epc1	Epigenetic
DMR17:57403001	17	57403001	57404000	1000	1	6.10E-09	-0.57	7	0.7	Epc1	Epigenetic
DMR17:57958001	17	57958001	57959000	1000	1	1.80E-16	-0.85	8	0.8	RGD1564999;LOC102552003	
DMR17:57990001	17	57990001	57991000	1000	1	9.30E-07	-0.39	12	1.2	Idi1;Wdr37	Metabolism
DMR17:58090001	17	58090001	58091000	1000	1	5.00E-07	-0.43	24	2.4	Adarb2	Metabolism
DMR17:58193001	17	58193001	58194000	1000	1	1.40E-08	0.56	16	1.6	Adarb2	Metabolism
DMR17:58210001	17	58210001	58213000	3000	1	8.10E-08	0.49	54	1.8	Adarb2	Metabolism
DMR17:58215001	17	58215001	58217000	2000	1	3.50E-07	0.4	30	1.5	Adarb2	Metabolism
DMR17:58274001	17	58274001	58279000	5000	1	9.10E-08	-0.61	56	1.12	Adarb2	Metabolism
DMR17:58288001	17	58288001	58289000	1000	1	3.10E-07	-0.33	11	1.1	Adarb2	Metabolism
DMR17:60101001	17	60101001	60102000	1000	1	1.50E-07	-0.64	11	1.1	Mpp7	Cytoskeleton
DMR17:60145001	17	60145001	60147000	2000	1	6.90E-07	-0.38	16	0.8	Mpp7	Cytoskeleton
DMR17:60371001	17	60371001	60374000	3000	1	1.70E-07	-0.42	8	0.27	Armc4	
DMR17:60411001	17	60411001	60412000	1000	1	6.00E-09	-0.56	13	1.3	Armc4	
DMR17:60458001	17	60458001	60459000	1000	1	7.50E-14	1.02	53	5.3	Armc4	
DMR17:60529001	17	60529001	60531000	2000	2	4.10E-09	-0.77	29	1.45	LOC103694121;Mkx	Development
DMR17:60949001	17	60949001	60950000	1000	1	3.70E-07	-0.48	8	0.8	Wac	Cytoskeleton
DMR17:62300001	17	62300001	62302000	2000	1	2.50E-07	0.54	10	0.5	Gjd4	Cytoskeleton
DMR17:62331001	17	62331001	62333000	2000	1	1.30E-07	-0.42	30	1.5	Ccny	
DMR17:62361001	17	62361001	62364000	3000	1	2.90E-09	-0.45	17	0.57	Ccny	
DMR17:62471001	17	62471001	62473000	2000	1	1.90E-07	-0.4	23	1.15	Ccny;LOC100909729	
DMR17:63269001	17	63269001	63271000	2000	1	2.50E-10	-0.47	19	0.95	Larp4b	Metabolism
DMR17:63714001	17	63714001	63715000	1000	1	9.70E-13	0.57	10	1	Dip2c	
DMR17:63767001	17	63767001	63769000	2000	1	2.70E-07	-0.44	8	0.4	Dip2c	
DMR17:64000001	17	64000001	64001000	1000	1	4.30E-07	0.4	12	1.2	Chrm3	Signaling

DMR17:64032001	17	64032001	64037000	5000	1	5.80E-07	-0.3	52	1.04	Chrm3	Signaling
DMR17:64095001	17	64095001	64099000	4000	2	4.80E-11	-0.56	11	0.28	Chrm3	Signaling
DMR17:64104001	17	64104001	64106000	2000	1	3.40E-07	-0.56	13	0.65	Chrm3	Signaling
DMR17:64247001	17	64247001	64253000	6000	1	3.70E-08	0.5	76	1.27	Chrm3	Signaling
DMR17:64417001	17	64417001	64418000	1000	1	1.60E-08	-0.5	6	0.6	Chrm3	Signaling
DMR17:65717001	17	65717001	65723000	6000	1	2.30E-07	-0.36	97	1.62	Ryr2	Ion Channel
DMR17:65742001	17	65742001	65743000	1000	1	5.20E-08	-0.4	14	1.4	Ryr2	Ion Channel
DMR17:65828001	17	65828001	65830000	2000	1	1.10E-09	0.49	37	1.85	Ryr2	Ion Channel
DMR17:65882001	17	65882001	65884000	2000	1	3.10E-07	-0.34	18	0.9	Ryr2	Ion Channel
DMR17:65907001	17	65907001	65909000	2000	1	1.20E-09	-0.61	11	0.55	Ryr2	Ion Channel
DMR17:66303001	17	66303001	66304000	1000	1	8.80E-07	0.44	7	0.7	Mtr;Actn2	
DMR17:66330001	17	66330001	66332000	2000	1	2.40E-07	-0.34	38	1.9	Actn2	
DMR17:66333001	17	66333001	66339000	6000	1	3.80E-07	0.51	76	1.27	Actn2	
DMR17:66492001	17	66492001	66494000	2000	1	3.60E-07	-0.35	28	1.4	Heatr1;Lgals8	Metabolism;Extracellular Matrix
DMR17:66537001	17	66537001	66539000	2000	1	6.00E-07	-0.32	20	1	Anlnl1	Cytoskeleton
DMR17:66549001	17	66549001	66552000	3000	1	7.00E-07	-0.41	55	1.83	Anlnl1	Cytoskeleton
DMR17:67924001	17	67924001	67925000	1000	1	5.40E-08	-0.57	5	0.5	Klf6	Transcription
DMR17:69385001	17	69385001	69386000	1000	1	1.30E-07	-0.52	7	0.7	Akr1e2;Akr1c2	Metabolism
DMR17:69583001	17	69583001	69588000	5000	1	3.00E-07	-0.31	85	1.7	Akr1c12l1	Metabolism
DMR17:69621001	17	69621001	69626000	5000	1	6.00E-07	-0.26	34	0.68	Akr1c12	Metabolism
DMR17:69651001	17	69651001	69652000	1000	1	1.40E-07	-0.56	3	0.3	Akr1c12;Akr1c19	Metabolism
DMR17:69654001	17	69654001	69655000	1000	1	1.80E-08	-0.56	1	0.1	Akr1c12;Akr1c19	Metabolism
DMR17:69839001	17	69839001	69841000	2000	1	4.00E-08	0.32	9	0.45	Akr1cl	
DMR17:69953001	17	69953001	69954000	1000	1	2.60E-08	-0.54	9	0.9	Ucn3	
DMR17:70001001	17	70001001	70004000	3000	1	7.30E-07	-0.42	34	1.13	Net1	Transcription
DMR17:70039001	17	70039001	70043000	4000	1	8.90E-07	0.51	49	1.23	LOC103694125;Calml5	
DMR17:70211001	17	70211001	70213000	2000	1	4.50E-10	0.49	37	1.85	Rpl29-ps3;Asb13	
DMR17:70231001	17	70231001	70232000	1000	1	1.40E-10	0.79	17	1.7	Asb13	
DMR17:70335001	17	70335001	70337000	2000	1	1.70E-12	0.82	30	1.5	Gdi2	Signaling
DMR17:70376001	17	70376001	70378000	2000	1	1.80E-15	1.21	58	2.9	Ankrd16	
DMR17:70381001	17	70381001	70384000	3000	1	6.90E-07	-0.43	50	1.67	Ankrd16	
DMR17:70417001	17	70417001	70419000	2000	1	1.50E-07	-0.3	22	1.1	Fbxo18	
DMR17:70461001	17	70461001	70462000	1000	1	1.70E-13	0.82	26	2.6	Il15ra	Receptor
DMR17:70463001	17	70463001	70465000	2000	1	8.20E-08	-0.44	25	1.25	Il15ra	Receptor
DMR17:70580001	17	70580001	70584000	4000	1	1.60E-13	0.94	63	1.57	LOC102553333;Rbm17	Translation
DMR17:70653001	17	70653001	70654000	1000	1	9.70E-07	-0.31	15	1.5	Pfkfb3	Metabolism
DMR17:70965001	17	70965001	70971000	6000	2	6.40E-10	-0.59	86	1.43	Prkcq	Signaling
DMR17:71003001	17	71003001	71005000	2000	2	3.40E-20	1.3	49	2.45	Prkcq	Signaling
DMR17:71022001	17	71022001	71023000	1000	1	3.10E-09	-0.43	9	0.9	Prkcq	Signaling
DMR17:71026001	17	71026001	71028000	2000	1	1.80E-07	0.36	28	1.4	Prkcq	Signaling
DMR17:71081001	17	71081001	71084000	3000	1	7.70E-13	0.81	70	2.33	Prkcq	Signaling
DMR17:71106001	17	71106001	71107000	1000	1	1.80E-08	0.63	11	1.1	Prkcq	Signaling
DMR17:71113001	17	71113001	71118000	5000	2	9.30E-08	0.51	88	1.76	Prkcq	Signaling
DMR17:71176001	17	71176001	71179000	3000	1	2.20E-13	-0.57	23	0.77	RGD1564416	
DMR17:71182001	17	71182001	71186000	4000	1	9.10E-07	-0.55	27	0.68	RGD1564416	
DMR17:71792001	17	71792001	71794000	2000	1	1.30E-07	0.32	24	1.2	Sfmbt2	Epigenetic
DMR17:71845001	17	71845001	71853000	8000	2	3.70E-08	-0.45	202	2.52	Sfmbt2	Epigenetic
DMR17:71854001	17	71854001	71856000	2000	1	1.10E-08	-0.46	50	2.5	Sfmbt2	Epigenetic
DMR17:71907001	17	71907001	71911000	4000	1	1.50E-11	-0.69	57	1.43	Sfmbt2	Epigenetic
DMR17:72156001	17	72156001	72160000	4000	1	9.10E-15	0.65	45	1.12	Itih2	Protease; Proteolysis
DMR17:72199001	17	72199001	72200000	1000	1	2.60E-10	-0.46	25	2.5	Itih2;Kin;Atp5c1	Protease; Proteolysis
DMR17:72278001	17	72278001	72283000	5000	2	1.90E-09	-0.54	67	1.34	Taf3	
DMR17:72389001	17	72389001	72391000	2000	1	1.30E-15	-0.61	36	1.8	Taf3;LOC102546306	
DMR17:72435001	17	72435001	72438000	3000	2	4.30E-16	-0.76	38	1.27	Gata3	Transcription
DMR17:72439001	17	72439001	72444000	5000	2	3.50E-10	-0.53	86	1.72	Gata3	Transcription
DMR17:75354001	17	75354001	75355000	1000	1	9.00E-07	0.37	14	1.4	Celf2	
DMR17:75458001	17	75458001	75464000	6000	2	7.20E-11	-0.42	56	0.93	Celf2	
DMR17:75582001	17	75582001	75583000	1000	1	1.00E-07	0.35	16	1.6	Celf2	
DMR17:75656001	17	75656001	75658000	2000	1	6.70E-11	-0.53	21	1.05	Celf2	
DMR17:75664001	17	75664001	75666000	2000	1	1.40E-07	0.51	31	1.55	Celf2	
DMR17:75749001	17	75749001	75751000	2000	2	5.50E-12	-0.58	25	1.25	Usp6nl	Signaling

DMR17:75812001	17	75812001	75813000	1000	1	8.70E-07	-0.49	8	0.8	Usp6nl	Signaling
DMR17:75838001	17	75838001	75839000	1000	1	2.60E-11	-0.41	17	1.7	Usp6nl	Signaling
DMR17:76190001	17	76190001	76191000	1000	1	3.10E-07	0.46	11	1.1	Upf2	Metabolism
DMR17:76205001	17	76205001	76208000	3000	1	1.10E-08	-0.42	48	1.6	Upf2	Metabolism
DMR17:76294001	17	76294001	76298000	4000	1	7.60E-12	0.84	76	1.9	Upf2;LOC103694151;Dhtkd1	Metabolism;Metabolism
DMR17:76329001	17	76329001	76330000	1000	1	9.90E-07	-0.26	24	2.4	Dhtkd1;LOC108353134	Metabolism
DMR17:76401001	17	76401001	76405000	4000	1	2.30E-16	-0.67	73	1.82	Nudt5;Cdc123	Signaling
DMR17:76406001	17	76406001	76409000	3000	1	5.50E-14	-0.6	42	1.4	Nudt5;Cdc123	Signaling
DMR17:76588001	17	76588001	76590000	2000	1	5.80E-09	-0.44	41	2.05	Camk1d	Signaling
DMR17:76626001	17	76626001	76630000	4000	1	1.80E-09	-0.5	31	0.78	Camk1d;LOC108348588	Signaling
DMR17:76668001	17	76668001	76673000	5000	1	3.10E-07	-0.46	63	1.26	Camk1d	Signaling
DMR17:76881001	17	76881001	76886000	5000	2	3.60E-14	1.03	67	1.34	Camk1d	Signaling
DMR17:76990001	17	76990001	76991000	1000	1	9.10E-10	0.41	17	1.7	Ccdc3	
DMR17:77081001	17	77081001	77088000	7000	1	2.30E-09	-0.38	75	1.07	Ccdc3	
DMR17:77102001	17	77102001	77103000	1000	1	2.70E-10	0.76	30	3	Ccdc3	
DMR17:77191001	17	77191001	77192000	1000	1	2.50E-08	-0.51	27	2.7	Optn	
DMR17:77280001	17	77280001	77282000	2000	1	1.50E-08	-0.42	46	2.3	Phyh	Metabolism
DMR17:77288001	17	77288001	77289000	1000	1	1.50E-07	0.48	28	2.8	Phyh	Metabolism
DMR17:77494001	17	77494001	77498000	4000	1	2.70E-08	-0.42	96	2.4	Bend7;LOC103694130	
DMR17:77611001	17	77611001	77614000	3000	1	5.60E-08	-0.52	29	0.97	Prpf18	Translation
DMR17:77635001	17	77635001	77637000	2000	1	3.60E-07	-0.37	46	2.3	Prpf18;LOC108348591;Frmd4a	Translation
DMR17:77673001	17	77673001	77674000	1000	1	6.10E-12	-0.53	33	3.3	Frmd4a	
DMR17:77737001	17	77737001	77741000	4000	1	3.00E-11	-0.47	71	1.77	Frmd4a	
DMR17:77790001	17	77790001	77792000	2000	1	4.80E-11	-0.4	29	1.45	Frmd4a	
DMR17:78477001	17	78477001	78480000	3000	1	9.00E-08	0.44	48	1.6	Fam107b	
DMR17:78517001	17	78517001	78520000	3000	1	8.70E-08	-0.36	59	1.97	Fam107b	
DMR17:78531001	17	78531001	78533000	2000	1	1.00E-07	-0.47	23	1.15	Fam107b	
DMR17:78549001	17	78549001	78551000	2000	1	1.10E-07	0.43	26	1.3	Fam107b	
DMR17:78585001	17	78585001	78587000	2000	1	3.00E-08	-0.5	14	0.7	Fam107b;LOC108348592	
DMR17:78603001	17	78603001	78605000	2000	1	3.40E-09	0.66	35	1.75	Fam107b;LOC108348592	
DMR17:78643001	17	78643001	78645000	2000	1	2.30E-07	-0.41	41	2.05	Fam107b	
DMR17:78658001	17	78658001	78659000	1000	1	1.80E-07	0.42	19	1.9	Fam107b	
DMR17:78663001	17	78663001	78666000	3000	1	1.30E-10	-0.44	42	1.4	Fam107b	
DMR17:78774001	17	78774001	78775000	1000	1	6.60E-08	0.55	9	0.9	Suv39h2;Dclre1c	Epigenetic;Transcription
DMR17:78827001	17	78827001	78830000	3000	2	5.80E-09	0.62	57	1.9	Meig1	
DMR17:78873001	17	78873001	78876000	3000	1	1.40E-08	0.56	52	1.73	Rpl34-ps1;Olah	Metabolism
DMR17:78884001	17	78884001	78885000	1000	1	1.40E-11	0.88	25	2.5	Rpl34-ps1;Olah	Metabolism
DMR17:78906001	17	78906001	78909000	3000	1	1.30E-07	0.42	53	1.77	Olah;Acbd7;Rpp38	Metabolism;Transport
DMR17:78912001	17	78912001	78913000	1000	1	7.60E-07	0.45	19	1.9	Olah;Acbd7;Rpp38;Nmt2	Metabolism;Transport;Transport
DMR17:78969001	17	78969001	78977000	8000	1	6.30E-07	-0.43	96	1.2	Nmt2	Transport
DMR17:79029001	17	79029001	79032000	3000	1	6.10E-10	0.96	91	3.03	Fam171a1	
DMR17:79037001	17	79037001	79039000	2000	1	9.80E-15	0.81	58	2.9	Fam171a1	
DMR17:79146001	17	79146001	79148000	2000	1	1.10E-08	-0.45	43	2.15	Fam171a1	
DMR17:79362001	17	79362001	79364000	2000	1	1.70E-09	0.44	23	1.15	Itga8	Extracellular Matrix
DMR17:79374001	17	79374001	79376000	2000	1	9.60E-13	0.69	39	1.95	Itga8	Extracellular Matrix
DMR17:79380001	17	79380001	79381000	1000	1	1.50E-07	-0.44	13	1.3	Itga8	Extracellular Matrix
DMR17:79667001	17	79667001	79669000	2000	1	1.60E-11	0.78	44	2.2	Itga8	Extracellular Matrix
DMR17:79711001	17	79711001	79714000	3000	1	3.60E-09	0.69	37	1.23	Fam188a;LOC103694135	
DMR17:79772001	17	79772001	79774000	2000	2	9.60E-09	-0.46	31	1.55	Fam188a	
DMR17:80253001	17	80253001	80255000	2000	1	3.00E-08	0.72	29	1.45	LOC108348618;Pter	Metabolism
DMR17:80425001	17	80425001	80427000	2000	1	1.80E-07	0.31	28	1.4	Rsu1	Cytoskeleton
DMR17:80437001	17	80437001	80438000	1000	1	2.20E-07	-0.47	17	1.7	Rsu1	Cytoskeleton
DMR17:80546001	17	80546001	80547000	1000	1	7.60E-11	-0.46	55	5.5	Rsu1	Cytoskeleton
DMR17:80560001	17	80560001	80565000	5000	2	8.30E-08	0.7	102	2.04	Rsu1	Cytoskeleton
DMR17:80572001	17	80572001	80574000	2000	1	2.30E-11	0.8	46	2.3	Rsu1	Cytoskeleton
DMR17:80795001	17	80795001	80799000	4000	1	2.00E-07	-0.39	64	1.6	Cubn;LOC102550536	
DMR17:80800001	17	80800001	80803000	3000	1	1.20E-08	-0.49	65	2.17	Cubn;LOC102550536	
DMR17:80805001	17	80805001	80811000	6000	1	3.70E-09	0.7	105	1.75	Cubn;LOC102550536	

DMR17:80873001	17	80873001	80874000	1000	1	1.90E-10	-0.54	8	0.8	Vim	
DMR17:80879001	17	80879001	80880000	1000	1	4.50E-07	-0.32	18	1.8	Vim	
DMR17:80887001	17	80887001	80890000	3000	1	2.80E-12	0.58	55	1.83	Vim	
DMR17:80951001	17	80951001	80952000	1000	1	7.80E-12	-0.6	5	0.5	St8sia6	Transport
DMR17:80959001	17	80959001	80962000	3000	1	1.80E-18	1.08	74	2.47	St8sia6	Transport
DMR17:81003001	17	81003001	81007000	4000	2	1.70E-09	-0.43	67	1.68	St8sia6	Transport
DMR17:81173001	17	81173001	81175000	2000	1	1.00E-15	0.56	17	0.85	Hacd1	
DMR17:81202001	17	81202001	81203000	1000	1	6.80E-11	0.66	21	2.1	Hacd1;Stam	Cytoskeleton
DMR17:81275001	17	81275001	81283000	8000	1	7.70E-07	-0.49	117	1.46	Tmem236	
DMR17:81349001	17	81349001	81350000	1000	1	3.00E-11	-0.56	4	0.4	Tmem236;Mrc1	
DMR17:81403001	17	81403001	81406000	3000	1	3.60E-09	-0.44	43	1.43	Mrc1	
DMR17:81413001	17	81413001	81415000	2000	1	1.40E-07	-0.4	14	0.7	Mrc1	
DMR17:81421001	17	81421001	81422000	1000	1	1.40E-08	-0.54	2	0.2	Mrc1	
DMR17:81493001	17	81493001	81495000	2000	1	1.70E-08	0.25	22	1.1	Slc39a12	Transport
DMR17:81522001	17	81522001	81525000	3000	1	1.80E-07	-0.34	27	0.9	Slc39a12	Transport
DMR17:81745001	17	81745001	81747000	2000	1	3.50E-07	0.51	26	1.3	Cacnb2	Transport
DMR17:81839001	17	81839001	81840000	1000	1	1.20E-09	-0.44	7	0.7	Cacnb2	Transport
DMR17:82067001	17	82067001	82072000	5000	1	6.50E-07	-0.58	50	1	Nsun6;Arl5b	Signaling
DMR17:82084001	17	82084001	82086000	2000	1	3.40E-07	-0.37	27	1.35	Arl5b	Signaling
DMR17:82438001	17	82438001	82439000	1000	1	1.10E-07	-0.54	1	0.1	Malrd1	
DMR17:82444001	17	82444001	82447000	3000	1	9.60E-07	-0.46	15	0.5	Malrd1	
DMR17:82571001	17	82571001	82575000	4000	2	9.40E-15	0.61	44	1.1	Malrd1	
DMR17:82653001	17	82653001	82655000	2000	1	6.90E-07	-0.63	11	0.55	Malrd1	
DMR17:82941001	17	82941001	82943000	2000	1	2.10E-07	0.49	9	0.45	Malrd1	
DMR17:83109001	17	83109001	83110000	1000	1	2.20E-07	0.33	9	0.9	Malrd1	
DMR17:83325001	17	83325001	83327000	2000	1	1.60E-07	-0.53	10	0.5	Plxdc2	
DMR17:83377001	17	83377001	83381000	4000	1	6.50E-07	-0.3	38	0.95	Plxdc2	
DMR17:83427001	17	83427001	83428000	1000	1	3.20E-08	-0.41	6	0.6	Plxdc2	
DMR17:83534001	17	83534001	83535000	1000	1	8.40E-09	0.29	28	2.8	Plxdc2	
DMR17:84253001	17	84253001	84257000	4000	1	2.80E-09	0.44	44	1.1	Nebl	
DMR17:84874001	17	84874001	84877000	3000	1	7.80E-08	-0.47	27	0.9	Mllt10	Transcription
DMR17:85019001	17	85019001	85020000	1000	1	1.30E-08	-0.45	4	0.4	Dnajc1	Transcription
DMR17:85098001	17	85098001	85100000	2000	1	7.60E-09	-0.59	10	0.5	Dnajc1	Transcription
DMR17:85652001	17	85652001	85658000	6000	1	1.70E-09	-0.37	60	1	Pip4k2a	Signaling
DMR17:85704001	17	85704001	85707000	3000	1	1.70E-08	-0.38	57	1.9	Pip4k2a	Signaling
DMR17:85749001	17	85749001	85750000	1000	1	2.10E-13	0.99	52	5.2	Pip4k2a	Signaling
DMR17:85756001	17	85756001	85757000	1000	1	1.40E-09	0.73	22	2.2	Pip4k2a	Signaling
DMR17:85894001	17	85894001	85897000	3000	1	1.40E-10	-0.49	50	1.67	LOC102556022;Armc3;LOC100361451	
DMR17:85909001	17	85909001	85912000	3000	1	1.70E-08	0.55	67	2.23	Armc3;LOC100361451;LOC103694139	
DMR17:85949001	17	85949001	85952000	3000	1	2.50E-17	1.03	68	2.27	Armc3;LOC680565	
DMR17:85957001	17	85957001	85959000	2000	1	2.10E-07	0.39	39	1.95	Armc3;LOC680565	
DMR17:87220001	17	87220001	87224000	4000	1	3.70E-14	-0.66	54	1.35	Etl4	
DMR17:87355001	17	87355001	87356000	1000	1	7.10E-07	0.55	13	1.3	Etl4	
DMR17:87358001	17	87358001	87361000	3000	1	1.70E-14	0.74	51	1.7	Etl4	
DMR17:87369001	17	87369001	87370000	1000	1	2.40E-07	-0.36	13	1.3	Etl4	
DMR17:87411001	17	87411001	87415000	4000	2	9.00E-09	-0.6	80	2	Etl4	
DMR17:87538001	17	87538001	87540000	2000	1	3.60E-07	-0.43	36	1.8	Etl4	
DMR17:87557001	17	87557001	87560000	3000	1	6.10E-07	-0.3	74	2.47	Etl4	
DMR17:87563001	17	87563001	87564000	1000	1	6.00E-08	-0.44	9	0.9	Etl4	
DMR17:87680001	17	87680001	87683000	3000	1	5.90E-07	0.5	33	1.1	Etl4	
DMR17:87689001	17	87689001	87692000	3000	1	1.40E-08	0.52	53	1.77	Etl4	
DMR17:87724001	17	87724001	87730000	6000	2	2.50E-11	-0.53	135	2.25	Arhgap21	
DMR17:87731001	17	87731001	87733000	2000	1	2.30E-07	-0.36	38	1.9	Arhgap21	
DMR17:87799001	17	87799001	87802000	3000	1	6.00E-09	-0.42	41	1.37	Arhgap21	
DMR17:87915001	17	87915001	87918000	3000	1	5.10E-07	0.52	35	1.17	Prtfdc1	Golgi
DMR17:87928001	17	87928001	87931000	3000	1	7.10E-07	-0.43	47	1.57	Prtfdc1	Golgi
DMR17:87960001	17	87960001	87962000	2000	1	3.00E-07	-0.4	23	1.15	Prtfdc1	Golgi
DMR17:88010001	17	88010001	88011000	1000	1	1.10E-07	0.57	16	1.6	Prtfdc1	Golgi
DMR17:88034001	17	88034001	88036000	2000	1	8.40E-07	0.51	41	2.05	Prtfdc1;LOC102550221	Golgi
DMR17:88139001	17	88139001	88141000	2000	1	3.70E-09	0.68	43	2.15	RGD1566369	Translation

DMR17:88367001	17	88367001	88369000	2000	2	3.30E-11	-0.41	18	0.9	Gpr158	Signaling
DMR17:88437001	17	88437001	88440000	3000	1	2.60E-11	-0.65	31	1.03	Gpr158;LOC102549062	Signaling
DMR17:88487001	17	88487001	88489000	2000	2	9.80E-09	-0.5	16	0.8	Gpr158	Signaling
DMR17:89617001	17	89617001	89620000	3000	1	1.50E-12	-0.42	39	1.3	Potec	Transport
DMR17:89886001	17	89886001	89887000	1000	1	2.00E-09	0.67	27	2.7	LOC100910957;Acbd5	Transport
DMR17:89894001	17	89894001	89895000	1000	1	7.00E-07	-0.4	7	0.7	Acbd5	Transport
DMR17:90103001	17	90103001	90104000	1000	1	6.20E-09	-0.45	12	1.2	Apbb1ip	Cytoskeleton
DMR17:90134001	17	90134001	90135000	1000	1	2.80E-15	1.3	21	2.1	Apbb1ip	Cytoskeleton
DMR17:90157001	17	90157001	90159000	2000	1	5.20E-07	0.34	15	0.75	Apbb1ip;LOC685062	Cytoskeleton
DMR17:90432001	17	90432001	90433000	1000	1	4.10E-07	-0.36	7	0.7	Lyst	
DMR17:90774001	17	90774001	90779000	5000	1	3.60E-08	-0.63	42	0.84	Ero1b	Metabolism
DMR18:289001	18	289001	292000	3000	3	4.70E-21	1.25	92	3.07	Mtmr1	Signaling
DMR18:294001	18	294001	299000	5000	2	4.70E-15	1	152	3.04	Mtmr1	Signaling
DMR18:306001	18	306001	309000	3000	1	2.00E-11	-0.54	65	2.17	Mtmr1	Signaling
DMR18:331001	18	331001	333000	2000	1	3.50E-08	-0.37	17	0.85	Mtmr1	Signaling
DMR18:364001	18	364001	365000	1000	1	2.30E-26	1.32	41	4.1	F8	Metabolism
DMR18:372001	18	372001	375000	3000	3	4.10E-22	1.2	145	4.83	F8	Metabolism
DMR18:377001	18	377001	379000	2000	1	2.40E-22	1.24	111	5.55	F8	Metabolism
DMR18:382001	18	382001	385000	3000	1	1.50E-13	0.96	120	4	F8	Metabolism
DMR18:386001	18	386001	392000	6000	2	1.40E-10	0.72	229	3.82	F8;Fundc2	Metabolism
DMR18:394001	18	394001	397000	3000	2	2.20E-22	1.36	156	5.2	F8;Fundc2	Metabolism
DMR18:408001	18	408001	411000	3000	2	5.30E-10	0.7	116	3.87	F8;Fundc2;LOC102546764;Mtcp1;LOC102546433	Metabolism
DMR18:913001	18	913001	914000	1000	1	3.60E-07	-0.46	3	0.3	Colec12	
DMR18:1412001	18	1412001	1413000	1000	1	4.30E-08	0.63	20	2	RGD1565904	
DMR18:1765001	18	1765001	1770000	5000	2	1.00E-10	0.65	91	1.82	Greb1l;LOC684764	
DMR18:2075001	18	2075001	2076000	1000	1	2.80E-08	0.71	17	1.7	Mib1;LOC103694162	Proteolysis
DMR18:3367001	18	3367001	3369000	2000	1	4.70E-08	0.53	22	1.1	Cables1	
DMR18:3439001	18	3439001	3440000	1000	1	2.10E-08	0.61	10	1	Tmem241;LOC103694167	Transport
DMR18:3631001	18	3631001	3633000	2000	1	5.40E-08	0.55	28	1.4	Npc1	
DMR18:3753001	18	3753001	3755000	2000	1	7.40E-07	0.29	25	1.25	Lama3	Extracellular Matrix
DMR18:3771001	18	3771001	3772000	1000	1	3.40E-09	0.35	8	0.8	Lama3	Extracellular Matrix
DMR18:3968001	18	3968001	3970000	2000	1	3.50E-09	0.38	24	1.2	Ttc39c	
DMR18:4276001	18	4276001	4278000	2000	1	1.10E-07	0.32	30	1.5	Osbpl1a	
DMR18:4303001	18	4303001	4305000	2000	2	5.90E-10	-0.6	17	0.85	Osbpl1a	
DMR18:4358001	18	4358001	4359000	1000	1	8.10E-07	-0.3	22	2.2	Hrh4	Signaling
DMR18:4382001	18	4382001	4383000	1000	1	2.70E-07	0.3	16	1.6	Hrh4	Signaling
DMR18:5089001	18	5089001	5091000	2000	1	2.90E-07	0.78	50	2.5	Zfp521;LOC108348745;LOC102549314	Transcription
DMR18:5194001	18	5194001	5197000	3000	1	7.50E-07	-0.36	75	2.5	Zfp521	Transcription
DMR18:5238001	18	5238001	5239000	1000	1	7.30E-07	-0.33	11	1.1	Zfp521	Transcription
DMR18:5277001	18	5277001	5280000	3000	1	2.10E-09	0.55	54	1.8	Zfp521	Transcription
DMR18:5322001	18	5322001	5325000	3000	1	1.50E-12	0.53	36	1.2	Zfp521	Transcription
DMR18:6014001	18	6014001	6016000	2000	1	2.30E-07	0.64	37	1.85	Ss18	Transcription
DMR18:6033001	18	6033001	6035000	2000	1	1.40E-07	-0.61	17	0.85	Ss18	Transcription
DMR18:6239001	18	6239001	6240000	1000	1	2.40E-07	-0.43	8	0.8	Taf4b	Transcription
DMR18:6306001	18	6306001	6310000	4000	1	1.10E-09	0.42	61	1.52	Taf4b	Transcription
DMR18:6454001	18	6454001	6457000	3000	1	6.10E-07	-0.34	42	1.4	Kctd1	Cytoskeleton
DMR18:6815001	18	6815001	6825000	10000	1	7.20E-08	-0.33	131	1.31	LOC102551834;Chst9	Transport
DMR18:6987001	18	6987001	6989000	2000	1	1.20E-07	-0.57	15	0.75	Chst9	Transport
DMR18:7108001	18	7108001	7110000	2000	1	6.70E-08	-0.4	30	1.5	Chst9;LOC102552005;LOC108348751	Transport
DMR18:7125001	18	7125001	7128000	3000	1	9.00E-11	0.84	44	1.47	Chst9;LOC102552005;LOC108348751	Transport
DMR18:8194001	18	8194001	8195000	1000	1	6.50E-08	-0.34	13	1.3	Cdh2	Cytoskeleton
DMR18:8334001	18	8334001	8336000	2000	1	6.20E-09	-0.53	21	1.05	Cdh2	Cytoskeleton
DMR18:8349001	18	8349001	8352000	3000	1	4.60E-07	-0.42	32	1.07	Cdh2	Cytoskeleton
DMR18:11789001	18	11789001	11790000	1000	1	1.00E-13	0.8	33	3.3	Dsc3	Cytoskeleton
DMR18:11865001	18	11865001	11867000	2000	1	4.20E-08	-0.43	18	0.9	Dsc2	Cytoskeleton
DMR18:12075001	18	12075001	12076000	1000	1	5.30E-08	-0.58	5	0.5	Dsg4	Cytoskeleton
DMR18:12079001	18	12079001	12084000	5000	1	7.10E-07	0.32	61	1.22	Dsg4	Cytoskeleton
DMR18:12219001	18	12219001	12220000	1000	1	2.40E-11	-0.68	28	2.8	RGD1562080	

DMR18:12279001	18	12279001	12283000	4000	1	2.10E-08	-0.36	63	1.57	RGD1562080;LOC102554807	
DMR18:12286001	18	12286001	12288000	2000	1	1.70E-09	0.69	51	2.55	RGD1562080;LOC102554807	
DMR18:12293001	18	12293001	12294000	1000	1	1.30E-15	1.12	23	2.3	RGD1562080	
DMR18:12841001	18	12841001	12842000	1000	1	2.70E-08	0.66	13	1.3	Ccdc178	
DMR18:13081001	18	13081001	13082000	1000	1	7.50E-07	0.41	17	1.7	Ccdc178	
DMR18:13197001	18	13197001	13200000	3000	1	5.10E-20	1.14	43	1.43	Ccdc178	
DMR18:13334001	18	13334001	13335000	1000	1	2.50E-08	-0.44	14	1.4	Asxl3	
DMR18:13368001	18	13368001	13370000	2000	1	1.20E-07	0.53	20	1	Asxl3	
DMR18:13685001	18	13685001	13686000	1000	1	4.60E-07	-0.65	29	2.9	Nol4	
DMR18:13757001	18	13757001	13758000	1000	1	2.40E-09	-0.36	16	1.6	Nol4	
DMR18:13833001	18	13833001	13835000	2000	1	5.90E-07	0.45	26	1.3	Nol4	
DMR18:13849001	18	13849001	13850000	1000	1	1.80E-09	0.73	11	1.1	Nol4	
DMR18:13961001	18	13961001	13964000	3000	1	8.10E-07	-0.5	33	1.1	Nol4	
DMR18:13967001	18	13967001	13971000	4000	1	8.50E-07	-0.23	30	0.75	Nol4	
DMR18:14658001	18	14658001	14659000	1000	1	1.60E-08	0.56	22	2.2	Dtna	Proteolysis
DMR18:15023001	18	15023001	15028000	5000	2	2.30E-12	0.71	72	1.44	Mapre2	Cytoskeleton
DMR18:15086001	18	15086001	15088000	2000	1	1.50E-08	0.63	44	2.2	Mapre2;Mep1b	Cytoskeleton;Protease
DMR18:15107001	18	15107001	15108000	1000	1	1.00E-08	0.62	14	1.4	Mapre2	Cytoskeleton
DMR18:15242001	18	15242001	15244000	2000	1	1.10E-19	0.95	41	2.05	Mapre2;LOC291746	Cytoskeleton
DMR18:15272001	18	15272001	15273000	1000	1	4.60E-09	-0.59	4	0.4	Mapre2;LOC498826	Cytoskeleton;Epigenetic
DMR18:15408001	18	15408001	15413000	5000	2	1.90E-11	0.88	87	1.74	Mapre2	Cytoskeleton
DMR18:15450001	18	15450001	15452000	2000	2	5.70E-11	-0.53	27	1.35	Mapre2;LOC100909691;LOC102551486	Cytoskeleton
DMR18:15499001	18	15499001	15508000	9000	1	2.30E-09	0.68	152	1.69	Mapre2;B4galt6;LOC103694173	Cytoskeleton;Golgi
DMR18:15552001	18	15552001	15554000	2000	1	2.50E-07	0.51	18	0.9	Mapre2;Ttr;LOC102551809	Cytoskeleton;Metabolism
DMR18:15582001	18	15582001	15584000	2000	1	2.10E-08	-0.45	32	1.6	Mapre2;Dsg2	Cytoskeleton;Cytoskeleton
DMR18:15591001	18	15591001	15593000	2000	1	3.30E-14	0.88	49	2.45	Mapre2;Dsg2	Cytoskeleton;Cytoskeleton
DMR18:15601001	18	15601001	15606000	5000	1	9.50E-08	-0.35	82	1.64	Mapre2;Dsg2	Cytoskeleton;Cytoskeleton
DMR18:15662001	18	15662001	15667000	5000	1	3.60E-08	0.5	34	0.68	Mapre2;Dsg3	Cytoskeleton;Cytoskeleton
DMR18:15674001	18	15674001	15675000	1000	1	8.70E-07	-0.4	10	1	Mapre2;Dsg3	Cytoskeleton;Cytoskeleton
DMR18:15690001	18	15690001	15692000	2000	1	5.80E-10	0.62	42	2.1	Mapre2;Dsg3;LOC108353181;LOC100362510	Cytoskeleton;Cytoskeleton
DMR18:16139001	18	16139001	16140000	1000	1	1.10E-08	-0.46	6	0.6	Galnt1	Golgi
DMR18:16192001	18	16192001	16194000	2000	1	6.10E-08	-0.29	50	2.5	Galnt1;LOC498829	Golgi
DMR18:16197001	18	16197001	16198000	1000	1	8.00E-10	0.83	27	2.7	Galnt1;LOC498829	Golgi
DMR18:16499001	18	16499001	16502000	3000	1	1.40E-09	-0.38	43	1.43	Rprd1a;LOC102553572	Signaling
DMR18:16674001	18	16674001	16676000	2000	1	2.30E-08	-0.46	43	2.15	Fhod3	
DMR18:16685001	18	16685001	16686000	1000	1	2.70E-07	-0.38	15	1.5	Fhod3	
DMR18:16918001	18	16918001	16919000	1000	1	1.00E-07	-0.64	2	0.2	Fhod3	
DMR18:17039001	18	17039001	17041000	2000	1	1.80E-07	0.55	25	1.25	Fhod3;LOC103694179	
DMR18:17180001	18	17180001	17182000	2000	1	9.50E-09	0.46	31	1.55	Fhod3;LOC108348756	
DMR18:17217001	18	17217001	17220000	3000	1	1.60E-08	0.47	61	2.03	Fhod3	
DMR18:17320001	18	17320001	17321000	1000	1	1.90E-08	-0.44	24	2.4	Fhod3	
DMR18:17371001	18	17371001	17372000	1000	1	6.40E-12	0.56	13	1.3	Tpgs2	Cytoskeleton
DMR18:17463001	18	17463001	17468000	5000	1	6.00E-07	-0.29	33	0.66	RGD1562608	
DMR18:17685001	18	17685001	17691000	6000	1	7.50E-12	0.51	74	1.23	RGD1562608;Celf4	
DMR18:17715001	18	17715001	17716000	1000	1	8.80E-07	0.63	11	1.1	Celf4	
DMR18:17762001	18	17762001	17763000	1000	1	7.90E-08	0.4	12	1.2	Celf4	
DMR18:17918001	18	17918001	17922000	4000	1	2.30E-13	0.83	71	1.77	Celf4;LOC102555477	
DMR18:17935001	18	17935001	17937000	2000	1	1.60E-10	0.62	37	1.85	Celf4;LOC291758	
DMR18:18031001	18	18031001	18032000	1000	1	5.50E-07	0.45	10	1	Celf4	
DMR18:18060001	18	18060001	18066000	6000	1	7.10E-08	0.37	94	1.57	Celf4	
DMR18:19156001	18	19156001	19157000	1000	1	1.40E-08	-0.53	4	0.4	Spata45	
DMR18:22851001	18	22851001	22853000	2000	1	4.40E-07	-0.29	12	0.6	RGD1564026	

DMR18:23698001	18	23698001	23699000	1000	1	2.50E-07	-0.6	2	0.2	Rit2	Signaling
DMR18:23718001	18	23718001	23720000	2000	1	3.90E-07	-0.42	17	0.85	Rit2	Signaling
DMR18:23795001	18	23795001	23797000	2000	1	8.60E-07	-0.33	15	0.75	Rit2	Signaling
DMR18:23809001	18	23809001	23810000	1000	1	5.70E-07	-0.62	11	1.1	Rit2	Signaling
DMR18:23967001	18	23967001	23969000	2000	1	2.40E-08	-0.36	18	0.9	Rit2	Signaling
DMR18:24499001	18	24499001	24501000	2000	1	4.10E-07	0.43	27	1.35	Sap130	Epigenetic
DMR18:24745001	18	24745001	24750000	5000	1	1.10E-12	0.61	89	1.78	Lims2;Gpr17;Myo7b	Cytoskeleton;Signaling;Cytoskeleton
DMR18:24753001	18	24753001	24754000	1000	1	3.60E-13	0.72	21	2.1	Lims2;Myo7b	Cytoskeleton;Cytoskeleton
DMR18:24824001	18	24824001	24827000	3000	1	2.30E-09	0.65	49	1.63	Myo7b	Cytoskeleton
DMR18:24828001	18	24828001	24830000	2000	1	8.20E-07	0.52	30	1.5	Myo7b;LOC102546309	Cytoskeleton
DMR18:24916001	18	24916001	24919000	3000	1	2.80E-08	0.51	55	1.83	Proc	Protease
DMR18:24963001	18	24963001	24964000	1000	1	4.90E-07	-0.44	16	1.6	Map3k2	
DMR18:24984001	18	24984001	24986000	2000	1	1.60E-08	0.3	24	1.2	Map3k2	
DMR18:25159001	18	25159001	25161000	2000	1	9.20E-07	0.36	22	1.1	Bin1	
DMR18:25195001	18	25195001	25197000	2000	1	1.30E-13	0.71	40	2	Bin1	
DMR18:25221001	18	25221001	25226000	5000	2	4.50E-09	0.66	103	2.06	Bin1	
DMR18:25346001	18	25346001	25350000	4000	1	8.40E-09	-0.34	50	1.25	Gypc	
DMR18:25608001	18	25608001	25611000	3000	1	5.00E-07	-0.34	44	1.47	Tslp	
DMR18:25624001	18	25624001	25626000	2000	1	5.80E-07	0.36	24	1.2	Tslp	
DMR18:25738001	18	25738001	25739000	1000	1	4.40E-07	-0.36	20	2	Camk4	Signaling
DMR18:26174001	18	26174001	26176000	2000	1	4.70E-07	0.46	33	1.65	Nrep	
DMR18:26179001	18	26179001	26183000	4000	4	2.00E-39	1.26	132	3.3	Nrep	
DMR18:26190001	18	26190001	26195000	5000	1	7.40E-07	-0.39	88	1.76	Nrep	
DMR18:26196001	18	26196001	26198000	2000	1	7.30E-09	-0.46	55	2.75	Nrep	
DMR18:26559001	18	26559001	26561000	2000	1	8.30E-08	0.48	41	2.05	Epb41l4a	
DMR18:26574001	18	26574001	26577000	3000	1	5.10E-08	-0.37	31	1.03	Epb41l4a	
DMR18:26674001	18	26674001	26675000	1000	1	6.20E-09	-0.35	15	1.5	Epb41l4a	
DMR18:26713001	18	26713001	26715000	2000	1	7.90E-07	-0.36	27	1.35	Epb41l4a	
DMR18:26773001	18	26773001	26774000	1000	1	1.90E-09	-0.58	12	1.2	Epb41l4a	
DMR18:27180001	18	27180001	27185000	5000	1	1.50E-08	-0.48	79	1.58	Pkd2l2	Transport
DMR18:27274001	18	27274001	27275000	1000	1	6.80E-07	-0.37	9	0.9	Fam13b	
DMR18:27336001	18	27336001	27337000	1000	1	3.00E-08	0.51	25	2.5	Wnt8a	Signaling
DMR18:27387001	18	27387001	27389000	2000	1	6.50E-07	0.41	24	1.2	Brd8	
DMR18:27497001	18	27497001	27500000	3000	1	4.00E-13	0.52	62	2.07	Gfra3	Receptor
DMR18:27509001	18	27509001	27511000	2000	1	9.60E-08	0.39	19	0.95	Gfra3	Receptor
DMR18:28048001	18	28048001	28051000	3000	1	1.60E-07	0.54	47	1.57	Ctnna1	Cytoskeleton
DMR18:28083001	18	28083001	28084000	1000	1	2.50E-07	-0.5	7	0.7	Sil1	
DMR18:28114001	18	28114001	28117000	3000	2	4.30E-09	-0.6	29	0.97	Sil1	
DMR18:28126001	18	28126001	28128000	2000	2	5.30E-08	-0.34	19	0.95	Sil1	
DMR18:28156001	18	28156001	28160000	4000	2	1.50E-08	-0.4	41	1.02	Sil1	
DMR18:28163001	18	28163001	28164000	1000	1	7.80E-07	-0.37	10	1	Sil1	
DMR18:28222001	18	28222001	28223000	1000	1	2.00E-08	0.56	12	1.2	Sil1	
DMR18:28374001	18	28374001	28375000	1000	1	5.40E-07	-0.44	5	0.5	Matr3	
DMR18:28422001	18	28422001	28424000	2000	1	4.40E-08	0.56	48	2.4	Paip2;Slc23a1	Transport
DMR18:28440001	18	28440001	28442000	2000	1	4.90E-07	0.38	27	1.35	Mzb1;Prob1;Spata24	
DMR18:28446001	18	28446001	28448000	2000	2	9.10E-10	0.6	19	0.95	Mzb1;Prob1;Spata24	
DMR18:28804001	18	28804001	28805000	1000	1	1.60E-07	0.62	13	1.3	Psd2	Transcription
DMR18:28816001	18	28816001	28818000	2000	1	3.80E-09	-0.55	9	0.45	Psd2	Transcription
DMR18:28856001	18	28856001	28859000	3000	1	2.10E-07	0.5	42	1.4	Nrg2	Growth Factors
DMR18:28871001	18	28871001	28875000	4000	1	8.20E-08	-0.45	51	1.27	Nrg2	Growth Factors
DMR18:28898001	18	28898001	28901000	3000	1	2.30E-08	0.55	48	1.6	Nrg2	Growth Factors
DMR18:28945001	18	28945001	28947000	2000	1	4.40E-09	-0.44	29	1.45	Nrg2	Growth Factors
DMR18:29209001	18	29209001	29210000	1000	1	5.80E-07	-0.54	2	0.2	Cystm1	
DMR18:29343001	18	29343001	29345000	2000	1	4.70E-07	0.44	43	2.15	Hbegf;Slc4a9	Growth Factors;Transport
DMR18:30045001	18	30045001	30046000	1000	1	2.30E-07	-0.6	0	0	Pcdha4	Cytoskeleton
DMR18:30095001	18	30095001	30099000	4000	1	6.80E-14	-0.39	33	0.82	Pcdha4	Cytoskeleton
DMR18:30308001	18	30308001	30311000	3000	1	6.10E-08	-0.38	62	2.07	Pcdhb1	Cytoskeleton
DMR18:30516001	18	30516001	30518000	2000	1	8.00E-11	0.62	47	2.35	LOC100360378;Pcdhb12;LOC108348233;Pcdhb14	Cytoskeleton

DMR18:30821001	18	30821001	30822000	1000	1	1.90E-07	0.62	21	2.1	Pcdhga1;Pcdhga2;Pcdhga3;RGD1560482;Pcdhga4	Cytoskeleton
DMR18:30832001	18	30832001	30833000	1000	1	7.00E-11	0.82	22	2.2	Pcdhga1;Pcdhga2;Pcdhga3;RGD1560482;Pcdhga4;LOC108353166;Pcdhga5	Cytoskeleton
DMR18:30842001	18	30842001	30843000	1000	1	1.50E-10	0.8	25	2.5	Pcdhga1;Pcdhga2;Pcdhga3;Pcdhga4;LOC108353166;Pcdhga5;LOC103694196	Cytoskeleton
DMR18:30844001	18	30844001	30845000	1000	1	1.30E-07	0.53	9	0.9	Pcdhga1;Pcdhga2;Pcdhga3;Pcdhga4;LOC108353166;Pcdhga5;LOC103694196	Cytoskeleton
DMR18:30974001	18	30974001	30975000	1000	1	1.40E-09	0.62	12	1.2	Pcdhga1;Pcdhga2;Pcdhga3;Pcdhga5;Pcdhga7;Pcdhga8;Pcdhga9;Pcdhga10;Pcdhgb7;Pcdhga11;Pcdhgb8;Pcdhga12;Pcdhgc3;Pcdhgc5;Diaph1	Cytoskeleton
DMR18:31093001	18	31093001	31094000	1000	1	8.90E-07	-0.42	9	0.9	Hdac3;Rell2;Fchsd1	
DMR18:31125001	18	31125001	31126000	1000	1	8.70E-09	0.62	18	1.8	Arap3	Signaling
DMR18:31129001	18	31129001	31130000	1000	1	1.10E-10	0.63	16	1.6	Arap3	Signaling
DMR18:31336001	18	31336001	31338000	2000	1	2.90E-07	0.44	37	1.85	Pcdh1	Cytoskeleton
DMR18:31402001	18	31402001	31403000	1000	1	1.50E-07	-0.52	10	1	RGD735029	
DMR18:31416001	18	31416001	31417000	1000	1	5.80E-07	0.6	23	2.3	RGD735029;Pcdh12	Cytoskeleton
DMR18:31837001	18	31837001	31838000	1000	1	2.30E-07	-0.47	6	0.6	Nr3c1;Arhgap26	Signaling
DMR18:31895001	18	31895001	31899000	4000	1	1.00E-12	0.41	39	0.98	Nr3c1;Arhgap26	Signaling
DMR18:31914001	18	31914001	31915000	1000	1	2.30E-10	0.59	7	0.7	Nr3c1;Arhgap26	Signaling
DMR18:31953001	18	31953001	31957000	4000	1	5.80E-10	-0.59	58	1.45	Nr3c1;Arhgap26	Signaling
DMR18:31960001	18	31960001	31961000	1000	1	9.30E-07	0.26	6	0.6	Nr3c1;Arhgap26	Signaling
DMR18:32004001	18	32004001	32005000	1000	1	5.20E-08	0.49	7	0.7	Nr3c1;Arhgap26	Signaling
DMR18:32059001	18	32059001	32064000	5000	1	1.20E-07	-0.4	62	1.24	Nr3c1;Arhgap26	Signaling
DMR18:32093001	18	32093001	32096000	3000	1	5.70E-17	0.69	36	1.2	Nr3c1;Arhgap26	Signaling
DMR18:32105001	18	32105001	32108000	3000	1	7.40E-07	0.32	30	1	Nr3c1;Arhgap26	Signaling
DMR18:32176001	18	32176001	32181000	5000	1	1.60E-07	0.37	45	0.9	Nr3c1;Arhgap26	Signaling
DMR18:32281001	18	32281001	32286000	5000	1	3.40E-08	0.52	64	1.28	Nr3c1;Arhgap26;Fgf1	Signaling;Growth Factors
DMR18:32294001	18	32294001	32297000	3000	1	1.80E-07	0.42	45	1.5	Nr3c1;Arhgap26;Fgf1	Signaling;Growth Factors
DMR18:32363001	18	32363001	32364000	1000	1	3.30E-07	0.39	21	2.1	Nr3c1;Arhgap26;Fgf1	Signaling;Growth Factors
DMR18:32392001	18	32392001	32394000	2000	1	6.20E-15	0.98	29	1.45	Nr3c1;Arhgap26	Signaling
DMR18:32491001	18	32491001	32493000	2000	1	1.90E-07	0.37	35	1.75	Nr3c1;Arhgap26	Signaling
DMR18:32613001	18	32613001	32616000	3000	1	5.30E-13	0.88	49	1.63	Nr3c1;Spry4	Cytoskeleton
DMR18:32625001	18	32625001	32626000	1000	1	6.90E-09	0.63	13	1.3	Nr3c1	
DMR18:32703001	18	32703001	32704000	1000	1	1.90E-07	0.45	23	2.3	Nr3c1	
DMR18:32965001	18	32965001	32966000	1000	1	1.70E-16	1.36	38	3.8	Pabpc2	
DMR18:33442001	18	33442001	33443000	1000	1	9.00E-11	-0.73	7	0.7	Yipf5;Kctd16	
DMR18:33472001	18	33472001	33476000	4000	1	1.80E-07	-0.33	39	0.98	Kctd16	
DMR18:35731001	18	35731001	35733000	2000	1	8.40E-07	0.4	20	1	Mcc;LOC103694203	
DMR18:36592001	18	36592001	36593000	1000	1	7.20E-08	0.58	10	1	Rbm27	Metabolism
DMR18:36807001	18	36807001	36811000	4000	1	6.10E-07	-0.31	27	0.68	Tcerg1;LOC103694264	Transcription
DMR18:36812001	18	36812001	36813000	1000	1	3.00E-07	-0.27	9	0.9	Tcerg1	Transcription
DMR18:36822001	18	36822001	36823000	1000	1	3.00E-13	-0.55	3	0.3	Tcerg1	Transcription
DMR18:37066001	18	37066001	37072000	6000	1	5.20E-07	-0.24	68	1.13	Ppp2r2b	Signaling
DMR18:37078001	18	37078001	37081000	3000	1	5.90E-07	0.35	47	1.57	Ppp2r2b	Signaling
DMR18:37139001	18	37139001	37141000	2000	1	2.10E-09	-0.38	12	0.6	Ppp2r2b;LOC108348777	Signaling
DMR18:37208001	18	37208001	37209000	1000	1	6.20E-07	0.54	46	4.6	Ppp2r2b	Signaling
DMR18:37354001	18	37354001	37358000	4000	1	9.20E-07	-0.35	41	1.02	Ppp2r2b	Signaling
DMR18:37413001	18	37413001	37415000	2000	1	9.40E-07	0.39	13	0.65	Ppp2r2b	Signaling
DMR18:37880001	18	37880001	37881000	1000	1	6.10E-09	-0.3	8	0.8	Jakmip2	
DMR18:37917001	18	37917001	37918000	1000	1	4.10E-09	-0.56	7	0.7	Jakmip2	
DMR18:37988001	18	37988001	37992000	4000	2	2.60E-08	-0.34	41	1.02	Jakmip2	
DMR18:38098001	18	38098001	38099000	1000	1	1.30E-19	0.91	13	1.3	Jakmip2	
DMR18:38291001	18	38291001	38292000	1000	1	1.20E-08	-0.61	2	0.2	Scgb3a2	
DMR18:40594001	18	40594001	40597000	3000	1	5.30E-07	-0.35	34	1.13	Tmed7;Eif1a	Transport;Translation

DMR18:40781001	18	40781001	40782000	1000	1	3.70E-11	0.72	17	1.7	Ap3s1;LOC103694206	Transport
DMR18:40971001	18	40971001	40975000	4000	1	2.70E-07	-0.43	30	0.75	Arl14epl	Signaling
DMR18:41332001	18	41332001	41335000	3000	3	1.40E-27	1.54	85	2.83	Sema6a	Signaling
DMR18:41899001	18	41899001	41900000	1000	1	3.90E-08	0.54	4	0.4	RGD1561114;LOC108348788	
DMR18:41908001	18	41908001	41909000	1000	1	8.90E-09	0.22	8	0.8	RGD1561114;LOC108348788;RGD1561627	
DMR18:41930001	18	41930001	41934000	4000	1	2.70E-08	-0.36	56	1.4	RGD1561627;LOC108348787;RGD1565083	
DMR18:43952001	18	43952001	43953000	1000	1	9.40E-11	-0.47	22	2.2	Dtwd2	
DMR18:44513001	18	44513001	44515000	2000	1	2.40E-13	-0.41	14	0.7	Dmxl1	
DMR18:44759001	18	44759001	44762000	3000	1	2.70E-08	-0.37	23	0.77	Tnfaip8	
DMR18:44842001	18	44842001	44845000	3000	2	6.20E-13	-0.59	38	1.27	Hsd17b4	
DMR18:45024001	18	45024001	45025000	1000	1	2.60E-16	0.78	19	1.9	Fam170a	
DMR18:45026001	18	45026001	45028000	2000	1	3.10E-10	0.62	31	1.55	Fam170a	
DMR18:46037001	18	46037001	46040000	3000	1	2.60E-07	-0.35	34	1.13	Prr16	
DMR18:46085001	18	46085001	46094000	9000	1	1.70E-07	-0.34	109	1.21	Prr16	
DMR18:47516001	18	47516001	47520000	4000	1	9.50E-09	-0.37	42	1.05	Lox;LOC681584	Metabolism
DMR18:47551001	18	47551001	47554000	3000	1	2.00E-09	0.56	40	1.33	Lox	Metabolism
DMR18:48124001	18	48124001	48125000	1000	1	9.70E-07	0.4	10	1	LOC100362233;Snx2	Cytoskeleton
DMR18:48136001	18	48136001	48139000	3000	1	6.70E-11	-0.59	21	0.7	LOC100362233;Snx2	Cytoskeleton
DMR18:48168001	18	48168001	48169000	1000	1	4.00E-12	-0.61	9	0.9	Snx2	Cytoskeleton
DMR18:48206001	18	48206001	48207000	1000	1	9.60E-08	-0.51	12	1.2	Snx24	Cytoskeleton
DMR18:48239001	18	48239001	48245000	6000	2	7.40E-11	-0.42	97	1.62	Snx24;LOC103694215	Cytoskeleton
DMR18:48288001	18	48288001	48289000	1000	1	3.20E-08	-0.37	14	1.4	Snx24	Cytoskeleton
DMR18:48523001	18	48523001	48527000	4000	1	9.10E-07	-0.45	57	1.43	Prdm6	Transcription
DMR18:48545001	18	48545001	48548000	3000	1	1.20E-07	-0.59	28	0.93	Prdm6	Transcription
DMR18:48877001	18	48877001	48878000	1000	1	5.90E-07	-0.5	8	0.8	Csnk1g3	Signaling
DMR18:49895001	18	49895001	49897000	2000	1	5.80E-08	0.44	35	1.75	Zfp608	
DMR18:49913001	18	49913001	49921000	8000	1	9.50E-09	-0.42	124	1.55	Zfp608	
DMR18:51514001	18	51514001	51516000	2000	1	1.10E-09	-0.41	22	1.1	Gramd3	
DMR18:51524001	18	51524001	51525000	1000	1	1.10E-08	-0.64	15	1.5	Gramd3	
DMR18:51542001	18	51542001	51543000	1000	1	6.90E-07	-0.34	12	1.2	Gramd3	
DMR18:51667001	18	51667001	51668000	1000	1	1.30E-08	-0.42	24	2.4	Phax	Metabolism
DMR18:51794001	18	51794001	51796000	2000	1	3.40E-08	-0.44	26	1.3	Lmnb1	
DMR18:51892001	18	51892001	51894000	2000	1	1.20E-07	0.38	24	1.2	3-Mar	
DMR18:51895001	18	51895001	51897000	2000	1	3.70E-07	-0.49	25	1.25	3-Mar	
DMR18:51995001	18	51995001	51996000	1000	1	2.10E-08	0.67	16	1.6	March3;LOC102547345	
DMR18:52219001	18	52219001	52221000	2000	1	4.50E-07	-0.32	36	1.8	LOC108348736;Megf10	Extracellular Matrix
DMR18:52245001	18	52245001	52248000	3000	1	3.60E-07	-0.63	17	0.57	Megf10	Extracellular Matrix
DMR18:52303001	18	52303001	52306000	3000	1	6.50E-08	-0.46	64	2.13	Megf10	Extracellular Matrix
DMR18:52353001	18	52353001	52356000	3000	1	3.30E-12	0.67	69	2.3	Megf10;LOC103694223	Extracellular Matrix
DMR18:52540001	18	52540001	52542000	2000	1	9.70E-09	0.34	28	1.4	Ctxn3	
DMR18:52961001	18	52961001	52962000	1000	1	5.10E-07	-0.41	20	2	Slc12a2	Transport
DMR18:53094001	18	53094001	53097000	3000	1	1.20E-09	-0.48	62	2.07	Fbn2	Extracellular Matrix
DMR18:53104001	18	53104001	53108000	4000	1	2.20E-11	-0.48	63	1.57	Fbn2	Extracellular Matrix
DMR18:53120001	18	53120001	53123000	3000	2	3.30E-13	-0.52	47	1.57	Fbn2	Extracellular Matrix
DMR18:53148001	18	53148001	53151000	3000	1	3.90E-09	-0.49	48	1.6	Fbn2	Extracellular Matrix
DMR18:53644001	18	53644001	53649000	5000	2	5.20E-07	-0.31	43	0.86	Slc27a6	Transport
DMR18:53912001	18	53912001	53913000	1000	1	4.70E-07	0.45	6	0.6	LOC100359716;Adamts19	Protease
DMR18:53971001	18	53971001	53977000	6000	2	6.80E-11	-0.57	57	0.95	Adamts19	Protease
DMR18:53978001	18	53978001	53980000	2000	1	2.10E-07	-0.57	9	0.45	Adamts19	Protease
DMR18:54067001	18	54067001	54069000	2000	1	5.90E-07	-0.49	17	0.85	Adamts19	Protease
DMR18:54078001	18	54078001	54079000	1000	1	2.40E-07	0.44	12	1.2	Adamts19	Protease
DMR18:54386001	18	54386001	54387000	1000	1	5.30E-07	0.38	11	1.1	Chsy3	Golgi
DMR18:55678001	18	55678001	55683000	5000	1	7.80E-08	-0.33	52	1.04	RGD1305184;MGC105567	
DMR18:55699001	18	55699001	55704000	5000	1	2.30E-07	-0.35	47	0.94	MGC105567	
DMR18:55708001	18	55708001	55715000	7000	1	5.20E-11	-0.34	64	0.91	MGC105567;LOC102550835	
DMR18:55756001	18	55756001	55758000	2000	1	3.80E-07	-0.53	19	0.95	Smim3	
DMR18:55848001	18	55848001	55849000	1000	1	3.10E-13	0.91	28	2.8	Rbm22;Myoz3	Translation;Cytoskeleton
DMR18:55853001	18	55853001	55855000	2000	1	1.90E-07	-0.41	34	1.7	Myoz3;Synpo	Cytoskeleton;Cytoskeleton

DMR18:55870001	18	55870001	55876000	6000	3	6.40E-18	1.09	141	2.35	Myoz3;Synpo	Cytoskeleton;Cytoskeleton
DMR18:55913001	18	55913001	55915000	2000	1	5.90E-07	0.45	30	1.5	Synpo	Cytoskeleton
DMR18:55938001	18	55938001	55943000	5000	1	6.10E-09	0.61	82	1.64	LOC103694228;Ndst1	Transport
DMR18:55959001	18	55959001	55960000	1000	1	9.80E-08	0.53	10	1	Ndst1	Transport
DMR18:55964001	18	55964001	55966000	2000	1	8.30E-11	0.63	31	1.55	Ndst1	Transport
DMR18:56087001	18	56087001	56089000	2000	1	3.10E-14	0.75	38	1.9	Cd74;Tcof1	Cytoskeleton;Metabolism
DMR18:56090001	18	56090001	56092000	2000	1	1.10E-07	0.47	29	1.45	Cd74;Tcof1	Cytoskeleton;Metabolism
DMR18:56285001	18	56285001	56288000	3000	1	7.00E-09	0.51	47	1.57	Camk2a	Signaling
DMR18:56310001	18	56310001	56314000	4000	1	5.00E-07	0.5	57	1.43	Slc6a7	Transport
DMR18:56317001	18	56317001	56318000	1000	1	1.20E-07	0.37	7	0.7	Slc6a7;LOC103690061	Transport
DMR18:56334001	18	56334001	56340000	6000	1	1.70E-10	-0.48	87	1.45	Slc6a7;LOC103690061;Cdx1	Transport;Development
DMR18:56360001	18	56360001	56363000	3000	1	2.60E-09	0.59	45	1.5	Cdx1;Pdgfrb;LOC102555480	Development;Receptor
DMR18:56386001	18	56386001	56389000	3000	2	8.40E-13	0.72	66	2.2	Pdgfrb	Receptor
DMR18:56456001	18	56456001	56457000	1000	1	2.10E-07	0.63	13	1.3	Csf1r;Hmgxb3	Receptor
DMR18:56532001	18	56532001	56533000	1000	1	8.80E-10	-0.46	9	0.9	Slc26a2	Transport
DMR18:56571001	18	56571001	56572000	1000	1	9.00E-07	0.48	10	1	Pde6a	Signaling
DMR18:56573001	18	56573001	56575000	2000	1	3.90E-07	0.59	31	1.55	Pde6a	Signaling
DMR18:56588001	18	56588001	56589000	1000	1	5.70E-09	0.47	11	1.1	Pde6a	Signaling
DMR18:56634001	18	56634001	56636000	2000	1	9.30E-08	-0.54	34	1.7	Ppargc1b	Transcription
DMR18:56723001	18	56723001	56724000	1000	1	3.10E-08	0.65	14	1.4	Mir3557;Mir378	
DMR18:56735001	18	56735001	56737000	2000	1	4.90E-08	-0.32	15	0.75	Mir3557;Mir378	
DMR18:56808001	18	56808001	56814000	6000	1	4.00E-11	0.68	70	1.17	Arhgef37	
DMR18:56881001	18	56881001	56884000	3000	1	1.50E-08	-0.45	50	1.67	Arhgef37;Csnk1a1	Signaling
DMR18:57090001	18	57090001	57091000	1000	1	1.60E-08	0.54	19	1.9	Afap1l1	
DMR18:57098001	18	57098001	57100000	2000	1	5.40E-07	0.57	25	1.25	Afap1l1	
DMR18:57109001	18	57109001	57110000	1000	1	3.90E-07	-0.35	9	0.9	Afap1l1	
DMR18:57176001	18	57176001	57178000	2000	1	7.80E-07	-0.44	4	0.2	Ablim3	Cytoskeleton
DMR18:57189001	18	57189001	57191000	2000	1	8.60E-11	0.49	18	0.9	Ablim3	Cytoskeleton
DMR18:57229001	18	57229001	57233000	4000	1	5.10E-09	-0.59	57	1.43	Ablim3	Cytoskeleton
DMR18:57240001	18	57240001	57242000	2000	1	2.00E-10	0.63	37	1.85	Ablim3	Cytoskeleton
DMR18:57289001	18	57289001	57292000	3000	1	3.00E-09	-0.44	55	1.83	Sh3tc2	
DMR18:57381001	18	57381001	57382000	1000	1	2.50E-07	0.45	13	1.3	Sh3tc2	
DMR18:57525001	18	57525001	57527000	2000	2	5.40E-17	1.01	39	1.95	Adrb2;LOC102556293	Signaling
DMR18:57645001	18	57645001	57651000	6000	1	1.50E-08	0.4	71	1.18	Htr4;LOC108348797	Signaling
DMR18:57689001	18	57689001	57692000	3000	1	1.50E-07	-0.35	9	0.3	Htr4	Signaling
DMR18:57715001	18	57715001	57718000	3000	1	2.70E-07	-0.53	32	1.07	Htr4	Signaling
DMR18:57835001	18	57835001	57837000	2000	1	2.40E-09	-0.46	35	1.75	Fbxo38	
DMR18:57923001	18	57923001	57926000	3000	1	8.00E-14	-0.52	29	0.97	Spink7;Spink13	Protease; Proteolysis
DMR18:57940001	18	57940001	57943000	3000	1	5.20E-11	-0.46	25	0.83	Spink13	Protease; Proteolysis
DMR18:57982001	18	57982001	57986000	4000	1	8.10E-07	-0.24	39	0.98	Spink13;Spink10	Protease; Proteolysis
DMR18:58024001	18	58024001	58025000	1000	1	8.80E-07	-0.51	11	1.1	Spink13	Protease; Proteolysis
DMR18:58050001	18	58050001	58053000	3000	1	5.90E-07	-0.35	31	1.03	Spink13	Protease; Proteolysis
DMR18:59099001	18	59099001	59101000	2000	1	3.80E-10	-0.43	44	2.2	Wdr7	
DMR18:59103001	18	59103001	59106000	3000	1	4.80E-08	-0.48	50	1.67	Wdr7	
DMR18:59126001	18	59126001	59127000	1000	1	1.40E-07	0.4	19	1.9	Wdr7	
DMR18:59818001	18	59818001	59821000	3000	1	9.10E-07	-0.4	52	1.73	LOC102553013;RGD1562699;Onecut2	Development
DMR18:59867001	18	59867001	59870000	3000	1	3.20E-07	0.4	32	1.07	Onecut2	Development
DMR18:60022001	18	60022001	60023000	1000	1	1.20E-08	0.37	11	1.1	Atp8b1	Transport
DMR18:60028001	18	60028001	60030000	2000	1	6.00E-07	-0.28	35	1.75	Atp8b1	Transport
DMR18:60055001	18	60055001	60057000	2000	1	3.30E-07	0.51	35	1.75	Atp8b1	Transport
DMR18:60067001	18	60067001	60068000	1000	1	6.70E-08	0.56	14	1.4	Atp8b1	Transport
DMR18:60419001	18	60419001	60420000	1000	1	3.50E-07	-0.28	18	1.8	Nedd4l	Proteolysis
DMR18:60422001	18	60422001	60424000	2000	1	1.70E-07	0.48	27	1.35	Nedd4l	Proteolysis
DMR18:60471001	18	60471001	60473000	2000	1	3.20E-09	0.51	34	1.7	Nedd4l	Proteolysis
DMR18:60499001	18	60499001	60501000	2000	1	2.30E-08	-0.44	28	1.4	Nedd4l	Proteolysis
DMR18:60575001	18	60575001	60576000	1000	1	1.50E-07	-0.37	13	1.3	Nedd4l	Proteolysis
DMR18:60596001	18	60596001	60597000	1000	1	4.50E-09	-0.42	15	1.5	Nedd4l	Proteolysis

DMR18:60601001	18	60601001	60603000	2000	1	1.40E-08	-0.64	22	1.1	Nedd4l	Proteolysis
DMR18:60625001	18	60625001	60627000	2000	1	1.80E-09	0.51	43	2.15	Nedd4l;LOC108353172	Proteolysis
DMR18:60979001	18	60979001	60980000	1000	1	6.10E-08	0.71	24	2.4	Alpk2	Signaling
DMR18:61010001	18	61010001	61013000	3000	1	3.30E-10	-0.54	28	0.93	Alpk2;LOC108348802	Signaling
DMR18:61167001	18	61167001	61168000	1000	1	7.10E-10	-0.5	4	0.4	Malt1	Protease
DMR18:61231001	18	61231001	61234000	3000	1	8.90E-08	0.5	52	1.73	LOC100363296;RGD1560815	
DMR18:61321001	18	61321001	61322000	1000	1	1.30E-08	-0.5	28	2.8	Zfp532	
DMR18:61323001	18	61323001	61325000	2000	1	1.30E-09	-0.41	55	2.75	Zfp532	
DMR18:61335001	18	61335001	61336000	1000	1	2.20E-07	-0.38	24	2.4	Zfp532	
DMR18:61386001	18	61386001	61388000	2000	1	3.80E-07	-0.36	34	1.7	Oacyl	Metabolism
DMR18:61394001	18	61394001	61395000	1000	1	5.90E-07	0.41	4	0.4	Oacyl	Metabolism
DMR18:61422001	18	61422001	61424000	2000	1	8.10E-07	0.46	43	2.15	Oacyl	Metabolism
DMR18:61689001	18	61689001	61691000	2000	1	5.00E-09	-0.35	45	2.25	Lman1	Transport
DMR18:61714001	18	61714001	61715000	1000	1	9.80E-09	-0.38	5	0.5	Lman1	Transport
DMR18:61748001	18	61748001	61750000	2000	1	2.50E-09	-0.45	36	1.8	Ccbe1	Extracellular Matrix
DMR18:61783001	18	61783001	61786000	3000	1	2.00E-07	0.45	30	1	Ccbe1	Extracellular Matrix
DMR18:61811001	18	61811001	61813000	2000	1	4.80E-07	-0.42	19	0.95	Ccbe1	Extracellular Matrix
DMR18:61822001	18	61822001	61824000	2000	1	2.80E-07	-0.4	13	0.65	Ccbe1	Extracellular Matrix
DMR18:61999001	18	61999001	62001000	2000	1	2.00E-07	0.37	22	1.1	Ccbe1	Extracellular Matrix
DMR18:62021001	18	62021001	62022000	1000	1	3.20E-08	0.46	22	2.2	Ccbe1;LOC108348805;LOC108348806	Extracellular Matrix
DMR18:62149001	18	62149001	62150000	1000	1	4.70E-07	0.46	18	1.8	LOC291547;Pmaip1	
DMR18:62459001	18	62459001	62464000	5000	1	5.60E-09	0.56	64	1.28	RGD1564795	
DMR18:62821001	18	62821001	62823000	2000	1	3.50E-08	-0.52	26	1.3	Gnal;LOC102551933	Signaling
DMR18:62954001	18	62954001	62956000	2000	1	5.10E-08	0.54	28	1.4	Gnal;Mppe1	Signaling;Metabolism
DMR18:63006001	18	63006001	63007000	1000	1	1.10E-07	0.57	21	2.1	Impa2	Signaling
DMR18:63036001	18	63036001	63041000	5000	1	1.30E-07	0.42	95	1.9	Impa2	Signaling
DMR18:63074001	18	63074001	63075000	1000	1	1.70E-07	-0.42	10	1	LOC103694232;Cidea	
DMR18:63095001	18	63095001	63096000	1000	1	1.50E-09	0.62	7	0.7	Cidea	
DMR18:63368001	18	63368001	63369000	1000	1	6.20E-07	-0.39	12	1.2	Cep76	
DMR18:63381001	18	63381001	63382000	1000	1	9.10E-09	-0.37	13	1.3	Cep76	
DMR18:63620001	18	63620001	63621000	1000	1	4.80E-08	0.54	5	0.5	Seh1l	
DMR18:63761001	18	63761001	63763000	2000	1	6.30E-16	0.94	36	1.8	Ldlrad4	
DMR18:63772001	18	63772001	63774000	2000	1	1.90E-08	-0.42	36	1.8	Ldlrad4	
DMR18:63805001	18	63805001	63808000	3000	1	7.10E-08	-0.38	57	1.9	Ldlrad4	
DMR18:63821001	18	63821001	63829000	8000	1	4.10E-07	-0.27	157	1.96	Ldlrad4;RGD1562758	
DMR18:63837001	18	63837001	63840000	3000	2	1.50E-13	-0.62	73	2.43	Ldlrad4	
DMR18:63903001	18	63903001	63904000	1000	1	4.30E-18	-0.73	2	0.2	Ldlrad4	
DMR18:63958001	18	63958001	63961000	3000	1	5.70E-07	-0.44	43	1.43	Ldlrad4	
DMR18:63982001	18	63982001	63983000	1000	1	1.00E-11	0.7	22	2.2	Ldlrad4	
DMR18:64049001	18	64049001	64051000	2000	1	4.40E-07	-0.41	32	1.6	Ldlrad4;Fam210a	
DMR18:65302001	18	65302001	65303000	1000	1	5.30E-07	-0.45	9	0.9	Tcf4	Transcription
DMR18:65340001	18	65340001	65343000	3000	1	1.50E-07	-0.36	51	1.7	Tcf4	Transcription
DMR18:65349001	18	65349001	65350000	1000	1	1.40E-07	0.27	15	1.5	Tcf4	Transcription
DMR18:65359001	18	65359001	65360000	1000	1	2.00E-10	0.33	6	0.6	Tcf4	Transcription
DMR18:65413001	18	65413001	65419000	6000	1	7.20E-08	-0.41	143	2.38	Tcf4	Transcription
DMR18:65487001	18	65487001	65489000	2000	1	5.70E-08	-0.35	35	1.75	Tcf4	Transcription
DMR18:65821001	18	65821001	65825000	4000	1	8.30E-08	-0.33	61	1.52	Mbd2	
DMR18:65886001	18	65886001	65887000	1000	1	5.20E-09	0.72	27	2.7	Mbd2	
DMR18:66528001	18	66528001	66529000	1000	1	7.00E-08	0.39	5	0.5	LOC100360803;Dcc	
DMR18:66536001	18	66536001	66538000	2000	1	9.00E-10	0.52	17	0.85	Dcc	
DMR18:66594001	18	66594001	66595000	1000	1	8.90E-07	-0.41	14	1.4	Dcc	
DMR18:66738001	18	66738001	66739000	1000	1	8.50E-08	0.5	14	1.4	Dcc	
DMR18:66816001	18	66816001	66818000	2000	1	6.80E-07	0.4	20	1	Dcc	
DMR18:67144001	18	67144001	67145000	1000	1	9.10E-09	0.53	13	1.3	Dcc	
DMR18:67433001	18	67433001	67434000	1000	1	4.90E-09	-0.42	11	1.1	Dcc	
DMR18:67458001	18	67458001	67460000	2000	1	4.60E-09	-0.26	19	0.95	Dcc	
DMR18:68399001	18	68399001	68401000	2000	1	4.30E-13	0.7	31	1.55	Ccdc68	
DMR18:68414001	18	68414001	68418000	4000	1	1.10E-07	0.35	49	1.23	Ccdc68	
DMR18:68475001	18	68475001	68476000	1000	1	3.10E-11	-0.51	15	1.5	Ccdc68	
DMR18:68522001	18	68522001	68524000	2000	1	1.00E-07	-0.46	31	1.55	Rab27b	
DMR18:68617001	18	68617001	68620000	3000	1	7.90E-07	-0.26	38	1.27	Rab27b	

DMR18:68637001	18	68637001	68640000	3000	2	8.30E-08	-0.56	32	1.07	Rab27b;LOC108348810	
DMR18:69776001	18	69776001	69782000	6000	3	5.40E-11	-0.63	74	1.23	Me2	Metabolism
DMR18:69860001	18	69860001	69862000	2000	1	1.10E-09	0.73	44	2.2	Mro;LOC102551811	
DMR18:69896001	18	69896001	69897000	1000	1	8.40E-09	0.52	36	3.6	Mapk4	Signaling
DMR18:69954001	18	69954001	69957000	3000	1	3.60E-07	-0.34	74	2.47	Mapk4	Signaling
DMR18:70003001	18	70003001	70007000	4000	2	5.20E-11	-0.53	86	2.15	Mapk4	Signaling
DMR18:70014001	18	70014001	70015000	1000	1	1.20E-07	-0.38	16	1.6	Mapk4	Signaling
DMR18:70176001	18	70176001	70178000	2000	1	8.60E-08	-0.38	29	1.45	LOC108348861;Ska1	
DMR18:70249001	18	70249001	70250000	1000	1	1.80E-10	-0.39	13	1.3	LOC108348812;Mbd1	
DMR18:70317001	18	70317001	70319000	2000	1	7.30E-10	0.57	29	1.45	Cfap53;LOC108348813	
DMR18:70424001	18	70424001	70426000	2000	1	4.50E-09	0.67	29	1.45	LOC678956;Myo5b	Cytoskeleton
DMR18:70440001	18	70440001	70441000	1000	1	6.20E-07	-0.31	21	2.1	Myo5b	Cytoskeleton
DMR18:70530001	18	70530001	70537000	7000	1	2.10E-10	-0.45	122	1.74	Myo5b	Cytoskeleton
DMR18:70753001	18	70753001	70756000	3000	1	5.30E-09	-0.43	65	2.17	Acaa2	Metabolism
DMR18:70916001	18	70916001	70920000	4000	1	3.00E-07	-0.37	56	1.4	Lipg	Metabolism
DMR18:71005001	18	71005001	71007000	2000	1	7.90E-08	-0.44	26	1.3	Dym	
DMR18:71205001	18	71205001	71210000	5000	1	2.60E-08	-0.4	59	1.18	Dym	
DMR18:71211001	18	71211001	71213000	2000	1	2.50E-07	-0.37	27	1.35	Dym	
DMR18:71250001	18	71250001	71251000	1000	1	9.60E-07	0.45	21	2.1	Dym	
DMR18:71307001	18	71307001	71308000	1000	1	5.40E-08	0.37	5	0.5	Dym	
DMR18:71422001	18	71422001	71424000	2000	1	8.00E-10	-0.39	59	2.95	Smad7	Transcription
DMR18:71534001	18	71534001	71536000	2000	2	6.70E-09	-0.55	7	0.35	Ctif	Metabolism
DMR18:71558001	18	71558001	71559000	1000	1	3.30E-08	-0.6	22	2.2	Ctif	Metabolism
DMR18:71658001	18	71658001	71660000	2000	1	1.20E-10	-0.6	12	0.6	Ctif	Metabolism
DMR18:72204001	18	72204001	72208000	4000	1	3.30E-07	0.41	57	1.43	Zbtb7c	
DMR18:72387001	18	72387001	72390000	3000	1	9.80E-13	0.47	31	1.03	Zbtb7c	
DMR18:72428001	18	72428001	72432000	4000	1	3.70E-16	-0.63	63	1.57	Zbtb7c	
DMR18:72439001	18	72439001	72445000	6000	2	1.50E-15	1.33	114	1.9	Zbtb7c	
DMR18:72453001	18	72453001	72456000	3000	1	3.30E-07	0.44	56	1.87	Zbtb7c	
DMR18:72461001	18	72461001	72462000	1000	1	6.90E-08	0.6	10	1	Zbtb7c	
DMR18:72472001	18	72472001	72474000	2000	1	6.00E-07	0.44	62	3.1	Zbtb7c	
DMR18:73149001	18	73149001	73151000	2000	1	4.20E-09	-0.6	33	1.65	Skor2	
DMR18:73157001	18	73157001	73158000	1000	1	1.20E-09	-0.68	22	2.2	Skor2	
DMR18:73218001	18	73218001	73219000	1000	1	3.70E-09	0.58	27	2.7	Hdhd2	Signaling
DMR18:73247001	18	73247001	73251000	4000	1	2.50E-08	-0.36	99	2.48	Hdhd2	Signaling
DMR18:73294001	18	73294001	73298000	4000	1	3.00E-07	0.58	82	2.05	Katnal2	Cytoskeleton
DMR18:73314001	18	73314001	73315000	1000	1	1.70E-07	-0.38	6	0.6	Katnal2	Cytoskeleton
DMR18:73324001	18	73324001	73326000	2000	1	7.20E-07	-0.36	29	1.45	Katnal2	Cytoskeleton
DMR18:73554001	18	73554001	73557000	3000	1	2.10E-07	0.53	41	1.37	LOC102552068;LOC108348820;St8sia5	Transport
DMR18:73616001	18	73616001	73619000	3000	1	5.40E-07	-0.33	49	1.63	St8sia5	Transport
DMR18:73624001	18	73624001	73627000	3000	1	6.90E-14	0.81	70	2.33	St8sia5	Transport
DMR18:73645001	18	73645001	73647000	2000	1	4.30E-12	0.74	55	2.75	LOC102551787;Loxhd1	
DMR18:73686001	18	73686001	73688000	2000	2	3.60E-19	-0.74	21	1.05	Loxhd1	
DMR18:73725001	18	73725001	73728000	3000	1	1.20E-13	-0.57	47	1.57	Loxhd1	
DMR18:73733001	18	73733001	73735000	2000	1	3.20E-07	-0.52	29	1.45	Loxhd1	
DMR18:73805001	18	73805001	73809000	4000	2	2.50E-09	-0.52	59	1.48	Loxhd1	
DMR18:73827001	18	73827001	73829000	2000	1	1.90E-07	0.55	43	2.15	Rnf165	Proteolysis
DMR18:74030001	18	74030001	74033000	3000	1	1.40E-07	-0.38	45	1.5	RGD1308601	
DMR18:74159001	18	74159001	74162000	3000	1	4.70E-07	-0.35	64	2.13	Haus1;Atp5a1	
DMR18:74163001	18	74163001	74165000	2000	1	1.20E-10	-0.44	53	2.65	Atp5a1;Pstpip2	Cytoskeleton
DMR18:74173001	18	74173001	74174000	1000	1	2.20E-07	0.63	19	1.9	Atp5a1;Pstpip2	Cytoskeleton
DMR18:74281001	18	74281001	74286000	5000	1	6.50E-08	0.42	79	1.58	Pstpip2	Cytoskeleton
DMR18:74344001	18	74344001	74345000	1000	1	1.80E-07	-0.32	7	0.7	Epg5	
DMR18:74423001	18	74423001	74425000	2000	1	4.10E-08	0.41	21	1.05	Siglec15	
DMR18:74492001	18	74492001	74493000	1000	1	5.90E-07	0.43	21	2.1	Slc14a1;LOC102553274	Transport
DMR18:74518001	18	74518001	74519000	1000	1	2.80E-07	0.52	22	2.2	Slc14a2	Transport
DMR18:74540001	18	74540001	74542000	2000	1	5.00E-09	0.42	34	1.7	Slc14a2	Transport
DMR18:74555001	18	74555001	74558000	3000	1	1.70E-09	-0.48	61	2.03	Slc14a2	Transport
DMR18:74593001	18	74593001	74595000	2000	2	1.80E-12	-0.58	16	0.8	Slc14a2	Transport
DMR18:74604001	18	74604001	74607000	3000	1	5.20E-07	-0.42	34	1.13	Slc14a2	Transport
DMR18:74698001	18	74698001	74700000	2000	1	3.20E-07	-0.39	34	1.7	Slc14a2	Transport

DMR18:74750001	18	74750001	74751000	1000	1	1.30E-10	0.46	5	0.5	Slc14a2;LOC102553338	Transport
DMR18:74801001	18	74801001	74803000	2000	1	2.00E-09	0.72	33	1.65	Slc14a2	Transport
DMR18:74806001	18	74806001	74807000	1000	1	3.80E-07	-0.39	6	0.6	Slc14a2	Transport
DMR18:74832001	18	74832001	74833000	1000	1	4.40E-12	0.6	12	1.2	Slc14a2	Transport
DMR18:74844001	18	74844001	74846000	2000	2	1.40E-15	-0.86	11	0.55	Slc14a2	Transport
DMR18:74883001	18	74883001	74884000	1000	1	2.40E-09	-0.54	9	0.9	Slc14a2	Transport
DMR18:74932001	18	74932001	74933000	1000	1	9.90E-08	0.43	11	1.1	Slc14a2	Transport
DMR18:75205001	18	75205001	75206000	1000	1	1.90E-16	0.81	35	3.5	Setbp1	Epigenetic
DMR18:75300001	18	75300001	75301000	1000	1	9.30E-11	-0.57	14	1.4	Setbp1	Epigenetic
DMR18:75352001	18	75352001	75353000	1000	1	4.00E-07	-0.56	11	1.1	Setbp1	Epigenetic
DMR18:76553001	18	76553001	76555000	2000	1	7.30E-09	0.57	44	2.2	Pard6g	Cell Junction
DMR18:76650001	18	76650001	76654000	4000	1	8.90E-08	-0.42	62	1.55	Adnp2;LOC108348823	
DMR18:76700001	18	76700001	76701000	1000	1	1.90E-15	0.85	30	3	LOC108348825;LOC108348824;LOC108348740;Rbfa	
DMR18:76702001	18	76702001	76705000	3000	1	2.40E-12	0.72	74	2.47	LOC108348825;LOC108348824;LOC108348740;Rbfa	
DMR18:76742001	18	76742001	76743000	1000	1	3.30E-08	0.67	25	2.5	Txnl4a;Hsbp1l1	
DMR18:76759001	18	76759001	76762000	3000	1	5.10E-11	0.63	30	1	Hsbp1l1;Pqlc1	
DMR18:76776001	18	76776001	76777000	1000	1	5.30E-09	0.51	47	4.7	Pqlc1	
DMR18:76784001	18	76784001	76788000	4000	1	1.70E-14	-0.56	76	1.9	Pqlc1	
DMR18:76795001	18	76795001	76799000	4000	1	2.10E-07	-0.36	111	2.78	Pqlc1;Kcng2	Transport
DMR18:76840001	18	76840001	76841000	1000	1	1.70E-07	-0.36	21	2.1	Kcng2	Transport
DMR18:76850001	18	76850001	76853000	3000	1	1.20E-15	0.7	65	2.17	Kcng2	Transport
DMR18:76912001	18	76912001	76915000	3000	1	5.30E-10	0.74	56	1.87	LOC103694249;Ctdp1	Signaling
DMR18:77199001	18	77199001	77202000	3000	2	5.50E-10	0.41	45	1.5	Nfatc1	Transcription
DMR18:77217001	18	77217001	77218000	1000	1	1.10E-07	0.57	13	1.3	Nfatc1	Transcription
DMR18:77271001	18	77271001	77275000	4000	1	2.40E-09	0.64	74	1.85	Nfatc1	Transcription
DMR18:77281001	18	77281001	77284000	3000	1	2.60E-11	0.74	67	2.23	Nfatc1	Transcription
DMR18:77326001	18	77326001	77327000	1000	1	8.60E-07	0.46	12	1.2	Nfatc1	Transcription
DMR18:77333001	18	77333001	77336000	3000	1	4.00E-07	0.41	51	1.7	Atp9b	Transport
DMR18:77350001	18	77350001	77351000	1000	1	6.80E-15	0.83	8	0.8	Atp9b	Transport
DMR18:77575001	18	77575001	77579000	4000	1	4.80E-08	0.43	122	3.05	Sall3	Transcription
DMR18:77583001	18	77583001	77589000	6000	1	8.00E-09	-0.53	106	1.77	Sall3;LOC108348827	Transcription
DMR18:79263001	18	79263001	79264000	1000	1	2.70E-07	0.68	16	1.6	Galr1	Signaling
DMR18:79383001	18	79383001	79386000	3000	1	4.60E-07	0.36	50	1.67	Mbp	Cytoskeleton
DMR18:79389001	18	79389001	79393000	4000	1	7.30E-07	-0.53	52	1.3	Mbp	Cytoskeleton
DMR18:79422001	18	79422001	79427000	5000	1	1.70E-28	1	98	1.96	Mbp;LOC108348833	Cytoskeleton
DMR18:79438001	18	79438001	79442000	4000	1	9.00E-07	0.47	43	1.07	Mbp;Zfp236	Cytoskeleton;Transcription
DMR18:79490001	18	79490001	79491000	1000	1	6.40E-08	0.42	13	1.3	Zfp236	Transcription
DMR18:79513001	18	79513001	79517000	4000	1	4.20E-07	-0.34	29	0.72	Zfp236	Transcription
DMR18:79757001	18	79757001	79759000	2000	2	1.10E-13	1.05	74	3.7	LOC102552572;Zfp516	Transcription
DMR18:79785001	18	79785001	79786000	1000	1	3.90E-09	-0.54	23	2.3	Zfp516	Transcription
DMR18:79788001	18	79788001	79791000	3000	1	4.10E-07	-0.4	50	1.67	Zfp516	Transcription
DMR18:79828001	18	79828001	79838000	10000	2	3.30E-09	-0.58	189	1.89	Zfp516	Transcription
DMR18:81135001	18	81135001	81138000	3000	2	2.80E-20	0.43	40	1.33	Zfp407	Transcription
DMR18:81139001	18	81139001	81143000	4000	1	4.80E-07	-0.35	45	1.12	Zfp407	Transcription
DMR18:81148001	18	81148001	81149000	1000	1	4.70E-08	-0.46	13	1.3	Zfp407	Transcription
DMR18:81185001	18	81185001	81187000	2000	1	1.60E-16	0.61	33	1.65	Zfp407	Transcription
DMR18:81303001	18	81303001	81306000	3000	1	2.10E-08	-0.52	24	0.8	Zfp407	Transcription
DMR18:81318001	18	81318001	81319000	1000	1	2.40E-08	-0.66	4	0.4	Zfp407	Transcription
DMR18:81341001	18	81341001	81343000	2000	1	2.70E-10	0.48	38	1.9	Zfp407	Transcription
DMR18:81366001	18	81366001	81367000	1000	1	5.30E-07	-0.38	14	1.4	Zfp407	Transcription
DMR18:81400001	18	81400001	81401000	1000	1	4.70E-15	-0.89	4	0.4	Zfp407	Transcription
DMR18:81405001	18	81405001	81406000	1000	1	5.70E-07	-0.38	11	1.1	Zfp407	Transcription
DMR18:81428001	18	81428001	81430000	2000	1	6.40E-07	-0.41	38	1.9	Zfp407	Transcription
DMR18:81433001	18	81433001	81435000	2000	1	2.30E-07	-0.44	23	1.15	Zfp407	Transcription
DMR18:81520001	18	81520001	81526000	6000	1	1.00E-08	0.73	124	2.07	Cndp1;Cndp2	Protease
DMR18:81581001	18	81581001	81583000	2000	1	3.30E-09	0.7	33	1.65	Fam69c	
DMR18:81591001	18	81591001	81594000	3000	1	3.70E-07	0.5	60	2	Fam69c	
DMR18:81803001	18	81803001	81806000	3000	1	1.10E-08	0.59	57	1.9	Fbxo15;Timm21	
DMR18:81818001	18	81818001	81819000	1000	1	7.20E-07	0.48	27	2.7	Fbxo15;LOC103694257	

DMR18:86355001	18	86355001	86357000	2000	1	3.40E-08	-0.39	14	0.7	Cd226;LOC102546621	
DMR18:86431001	18	86431001	86432000	1000	1	1.40E-07	-0.38	6	0.6	Dok6	
DMR18:86667001	18	86667001	86668000	1000	1	1.10E-08	0.46	4	0.4	Dok6	
DMR19:543001	19	543001	545000	2000	1	2.10E-07	0.45	20	1	LOC685670;Fam96b	
DMR19:637001	19	637001	640000	3000	1	2.60E-09	0.61	42	1.4	Car7	
DMR19:681001	19	681001	682000	1000	1	1.50E-17	1.4	36	3.6	Nae1;Terb1	Proteolysis
DMR19:868001	19	868001	870000	2000	1	1.20E-20	0.98	40	2	Cmtm1;LOC102554842	Transport
DMR19:891001	19	891001	892000	1000	1	3.80E-10	0.46	2	0.2	Cmtm2a;LOC102555510	Transport
DMR19:948001	19	948001	950000	2000	1	3.60E-07	0.51	22	1.1	Tk2;LOC108348885;LOC108348886;Bean1	Signaling
DMR19:951001	19	951001	952000	1000	1	4.70E-10	0.51	13	1.3	LOC108348885;LOC108348886;Bean1	
DMR19:975001	19	975001	977000	2000	2	4.30E-10	-0.78	7	0.35	Bean1	
DMR19:1000001	19	1000001	1001000	1000	1	7.70E-07	-0.32	17	1.7	Bean1	
DMR19:1024001	19	1024001	1027000	3000	1	1.60E-07	0.52	47	1.57	Cdh5	Cytoskeleton
DMR19:1058001	19	1058001	1059000	1000	1	9.10E-08	0.56	10	1	Cdh5	Cytoskeleton
DMR19:1077001	19	1077001	1078000	1000	1	2.10E-07	0.49	46	4.6	Cdh5	Cytoskeleton
DMR19:2493001	19	2493001	2494000	1000	1	9.20E-08	0.59	8	0.8	Cdh11	Cytoskeleton
DMR19:6082001	19	6082001	6083000	1000	1	2.20E-10	-0.59	6	0.6	Cdh8;LOC102547668	Cytoskeleton
DMR19:6118001	19	6118001	6119000	1000	1	1.80E-09	-0.4	12	1.2	Cdh8	Cytoskeleton
DMR19:8951001	19	8951001	8952000	1000	1	1.80E-08	-0.72	4	0.4	Polr2l-ps1	
DMR19:9603001	19	9603001	9606000	3000	1	2.70E-11	-0.46	36	1.2	Got2	Metabolism
DMR19:9618001	19	9618001	9621000	3000	1	2.50E-09	-0.35	34	1.13	Got2;Slc38a7	Metabolism;Transport
DMR19:9687001	19	9687001	9688000	1000	1	1.50E-07	-0.48	8	0.8	Cnot1	Translation
DMR19:9730001	19	9730001	9732000	2000	1	2.10E-08	-0.37	26	1.3	Cnot1	Translation
DMR19:9811001	19	9811001	9814000	3000	1	1.90E-11	-0.49	63	2.1	Ndrg4;LOC688942	Protease
DMR19:9891001	19	9891001	9895000	4000	1	5.30E-07	-0.43	67	1.68	Prss54	Protease
DMR19:9924001	19	9924001	9925000	1000	1	1.30E-18	0.99	39	3.9	Ccdc113	
DMR19:9965001	19	9965001	9967000	2000	1	3.90E-09	-0.45	41	2.05	Csnk2a2	
DMR19:10003001	19	10003001	10005000	2000	1	6.30E-07	-0.36	25	1.25	Csnk2a2	
DMR19:10078001	19	10078001	10080000	2000	1	3.40E-07	0.53	38	1.9	Mmp15	Protease
DMR19:10102001	19	10102001	10103000	1000	1	1.90E-11	0.8	23	2.3	Mmp15;Usb1	Protease
DMR19:10197001	19	10197001	10200000	3000	2	2.50E-09	-0.51	66	2.2	Cngb1	Ion Channel
DMR19:10320001	19	10320001	10322000	2000	1	1.40E-08	0.59	28	1.4	Kifc3;LOC103694284	Cytoskeleton
DMR19:10339001	19	10339001	10341000	2000	1	3.30E-07	0.59	29	1.45	Kifc3;Katnb1	Cytoskeleton
DMR19:10346001	19	10346001	10349000	3000	1	5.70E-07	-0.46	34	1.13	Kifc3;Katnb1	Cytoskeleton
DMR19:10401001	19	10401001	10402000	1000	1	1.30E-08	0.49	14	1.4	Adgrg3	Signaling
DMR19:10485001	19	10485001	10487000	2000	1	1.70E-07	-0.36	65	3.25	Adgrg5	Signaling
DMR19:10505001	19	10505001	10506000	1000	1	6.00E-11	-0.51	6	0.6	Adgrg5	Signaling
DMR19:10517001	19	10517001	10518000	1000	1	5.30E-09	0.37	19	1.9	Adgrg5;Ccdc102a	Signaling
DMR19:10626001	19	10626001	10628000	2000	1	1.40E-07	0.7	42	2.1	Ccl17;LOC307650	Growth Factors
DMR19:10685001	19	10685001	10692000	7000	2	3.40E-08	-0.57	87	1.24	Ccl22;LOC102550889	Growth Factors
DMR19:10739001	19	10739001	10745000	6000	1	6.40E-14	0.82	87	1.45	LOC102550889;Pllp;Arl2bp	Transport
DMR19:10747001	19	10747001	10748000	1000	1	1.30E-07	0.46	11	1.1	Pllp;Arl2bp	Transport
DMR19:10943001	19	10943001	10944000	1000	1	9.30E-08	0.45	15	1.5	Nlrc5	Cytoskeleton
DMR19:10951001	19	10951001	10953000	2000	1	1.00E-07	0.57	23	1.15	Nlrc5	Cytoskeleton
DMR19:10982001	19	10982001	10984000	2000	1	3.20E-11	-0.54	20	1	Nlrc5;LOC108348891	Cytoskeleton
DMR19:10986001	19	10986001	10987000	1000	1	9.70E-12	-0.55	15	1.5	Nlrc5;LOC108348891	Cytoskeleton
DMR19:11006001	19	11006001	11011000	5000	1	3.90E-09	-0.42	58	1.16	Nlrc5;LOC102553150	Cytoskeleton
DMR19:11189001	19	11189001	11191000	2000	1	5.00E-09	-0.44	21	1.05	Nup93	Transport
DMR19:11238001	19	11238001	11239000	1000	1	3.00E-07	0.55	5	0.5	Nup93	Transport
DMR19:11555001	19	11555001	11560000	5000	1	1.10E-08	-0.41	83	1.66	Gnao1	Signaling
DMR19:11612001	19	11612001	11613000	1000	1	3.60E-07	-0.54	11	1.1	Gnao1	Signaling
DMR19:12102001	19	12102001	12104000	2000	1	9.90E-08	-0.53	15	0.75	Ces5a	Metabolism
DMR19:12546001	19	12546001	12547000	1000	1	2.00E-08	-0.4	18	1.8	Large1	Golgi
DMR19:12589001	19	12589001	12591000	2000	1	1.80E-07	-0.48	15	0.75	Large1	Golgi
DMR19:12609001	19	12609001	12610000	1000	1	3.80E-10	0.64	21	2.1	Large1	Golgi
DMR19:12692001	19	12692001	12694000	2000	1	5.90E-07	-0.44	16	0.8	Large1	Golgi
DMR19:12753001	19	12753001	12755000	2000	1	1.30E-07	-0.35	35	1.75	Large1	Golgi
DMR19:12772001	19	12772001	12775000	3000	2	1.80E-09	-0.43	56	1.87	Large1	Golgi
DMR19:12798001	19	12798001	12799000	1000	1	1.80E-08	-0.49	23	2.3	Large1	Golgi
DMR19:12832001	19	12832001	12835000	3000	2	9.60E-11	-0.59	59	1.97	Large1;LOC108348896	Golgi

DMR19:12857001	19	12857001	12859000	2000	1	6.70E-13	-0.5	24	1.2	Large1;LOC108348896	Golgi
DMR19:14569001	19	14569001	14570000	1000	1	3.00E-08	0.53	10	1	Mcm5	Transcription
DMR19:15214001	19	15214001	15216000	2000	1	6.40E-09	0.67	16	0.8	Ces1d	Metabolism
DMR19:15226001	19	15226001	15227000	1000	1	1.20E-15	-0.56	10	1	Ces1d	Metabolism
DMR19:15427001	19	15427001	15428000	1000	1	1.60E-08	-0.38	10	1	Slc6a2	Transport
DMR19:15462001	19	15462001	15466000	4000	1	6.00E-07	-0.4	55	1.38	Lpcat2	Metabolism
DMR19:15554001	19	15554001	15555000	1000	1	8.80E-08	-0.39	12	1.2	Mmp2	Protease
DMR19:15558001	19	15558001	15562000	4000	1	1.20E-07	0.61	59	1.48	Mmp2	Protease
DMR19:15718001	19	15718001	15720000	2000	1	1.70E-07	-0.39	27	1.35	Irx6	Development
DMR19:15724001	19	15724001	15725000	1000	1	4.40E-08	0.34	10	1	Irx6	Development
DMR19:15839001	19	15839001	15840000	1000	1	8.70E-09	0.79	35	3.5	LOC102554898;Irx3	Development
DMR19:16357001	19	16357001	16358000	1000	1	5.00E-07	0.5	26	2.6	LOC103694293;Trnas-aga	
DMR19:16775001	19	16775001	16779000	4000	2	8.10E-11	0.39	73	1.82	Fto	Metabolism
DMR19:16885001	19	16885001	16886000	1000	1	1.60E-10	-0.63	20	2	Fto	Metabolism
DMR19:16976001	19	16976001	16978000	2000	1	7.50E-08	-0.42	39	1.95	Fto	Metabolism
DMR19:17087001	19	17087001	17090000	3000	1	1.40E-10	-0.38	48	1.6	Fto	Metabolism
DMR19:17314001	19	17314001	17316000	2000	1	1.10E-08	0.5	29	1.45	Rbl2	Epigenetic
DMR19:17465001	19	17465001	17469000	4000	1	2.10E-16	-0.62	63	1.57	Chd9	
DMR19:18110001	19	18110001	18113000	3000	1	1.20E-07	-0.45	33	1.1	Tox3	
DMR19:18156001	19	18156001	18157000	1000	1	6.80E-08	0.31	8	0.8	Tox3	
DMR19:18166001	19	18166001	18168000	2000	1	1.00E-07	-0.29	39	1.95	Tox3	
DMR19:19392001	19	19392001	19394000	2000	1	5.30E-08	0.34	37	1.85	Nod2;Snx20	Cytoskeleton
DMR19:19437001	19	19437001	19438000	1000	1	8.80E-07	0.41	19	1.9	Nkd1	
DMR19:19463001	19	19463001	19466000	3000	1	1.10E-07	-0.41	45	1.5	Nkd1	
DMR19:19490001	19	19490001	19493000	3000	1	9.20E-11	0.4	41	1.37	Nkd1	
DMR19:19712001	19	19712001	19715000	3000	1	2.60E-13	0.63	50	1.67	Adcy7	
DMR19:19721001	19	19721001	19724000	3000	1	4.50E-18	0.64	65	2.17	Adcy7	
DMR19:19748001	19	19748001	19750000	2000	1	1.30E-07	-0.37	35	1.75	Adcy7	
DMR19:19866001	19	19866001	19867000	1000	1	8.90E-08	-0.33	10	1	Heatr3	
DMR19:19904001	19	19904001	19906000	2000	1	8.00E-11	-0.57	23	1.15	Heatr3;Cnep1r1;LOC108348906	
DMR19:20230001	19	20230001	20231000	1000	1	8.90E-07	-0.41	16	1.6	Zfp423	
DMR19:20332001	19	20332001	20333000	1000	1	9.80E-08	-0.37	25	2.5	Zfp423	
DMR19:20336001	19	20336001	20337000	1000	1	2.70E-10	0.47	16	1.6	Zfp423	
DMR19:21245001	19	21245001	21246000	1000	1	6.20E-08	-0.44	7	0.7	N4bp1	Translation
DMR19:21493001	19	21493001	21494000	1000	1	1.00E-12	0.61	11	1.1	Lonp2	Protease
DMR19:21501001	19	21501001	21504000	3000	1	8.90E-11	-0.71	32	1.07	Lonp2	Protease
DMR19:21561001	19	21561001	21562000	1000	1	9.30E-07	0.46	10	1	Abcc12	Transport
DMR19:21563001	19	21563001	21566000	3000	1	5.30E-07	0.41	44	1.47	Abcc12	Transport
DMR19:21574001	19	21574001	21579000	5000	1	3.70E-07	0.39	67	1.34	Abcc12	Transport
DMR19:22044001	19	22044001	22045000	1000	1	7.60E-07	-0.38	15	1.5	Phkb	Signaling
DMR19:22137001	19	22137001	22140000	3000	1	3.00E-08	-0.43	23	0.77	Phkb	Signaling
DMR19:22397001	19	22397001	22398000	1000	1	6.80E-07	0.26	9	0.9	Itfg1	
DMR19:22439001	19	22439001	22441000	2000	1	2.50E-08	-0.34	55	2.75	Neto2	Protease
DMR19:22461001	19	22461001	22464000	3000	1	1.80E-12	0.35	30	1	Neto2	Protease
DMR19:22496001	19	22496001	22497000	1000	1	5.80E-07	-0.34	26	2.6	Neto2	Protease
DMR19:22566001	19	22566001	22568000	2000	1	9.20E-07	-0.41	21	1.05	Dnaja2	Transcription
DMR19:22705001	19	22705001	22708000	3000	1	1.70E-07	-0.44	41	1.37	RGD1308706;LOC100363129	
DMR19:23397001	19	23397001	23400000	3000	1	1.00E-06	0.36	70	2.33	LOC102553576;Sall1;LOC103694299	Transcription
DMR19:23610001	19	23610001	23611000	1000	1	1.60E-08	0.4	72	7.2	Il15	Cytokine
DMR19:23945001	19	23945001	23946000	1000	1	4.80E-09	-0.48	19	1.9	Zfp330	
DMR19:24054001	19	24054001	24057000	3000	3	2.90E-25	0.81	157	5.23	Rnf150	
DMR19:24214001	19	24214001	24216000	2000	1	1.20E-08	0.57	26	1.3	Rnf150	
DMR19:24245001	19	24245001	24247000	2000	1	3.90E-07	0.29	19	0.95	Rnf150	
DMR19:24418001	19	24418001	24420000	2000	1	1.50E-08	0.54	44	2.2	Tbc1d9	Signaling
DMR19:24425001	19	24425001	24427000	2000	1	1.20E-08	0.43	30	1.5	Tbc1d9	Signaling
DMR19:24429001	19	24429001	24431000	2000	1	8.30E-09	-0.43	34	1.7	Tbc1d9	Signaling
DMR19:24509001	19	24509001	24512000	3000	1	4.40E-09	0.43	44	1.47	Mgat4d	Transport
DMR19:24574001	19	24574001	24576000	2000	1	1.30E-09	-0.45	43	2.15	Clgn;Scoc	Transcription
DMR19:24606001	19	24606001	24607000	1000	1	3.90E-10	-0.5	9	0.9	Scoc	
DMR19:24666001	19	24666001	24672000	6000	1	3.40E-12	0.79	100	1.67	Olr1667;LOC688497	

DMR19:24859001	19	24859001	24861000	2000	1	2.20E-15	-0.59	38	1.9	Ddx39a;Adgre5	Signaling
DMR19:25125001	19	25125001	25126000	1000	1	6.20E-12	0.56	17	1.7	Prkaca;Samd1;LOC686013;LOC100912606;RGD1310262;Mir1199	Signaling;Epigenetic
DMR19:25146001	19	25146001	25148000	2000	1	2.20E-07	0.44	55	2.75	Palm3;Il27ra	Receptor
DMR19:25166001	19	25166001	25168000	2000	1	7.00E-14	0.62	50	2.5	Il27ra;Rln3;LOC102555451	Receptor
DMR19:25233001	19	25233001	25236000	3000	1	2.40E-10	-0.49	31	1.03	Podnl1	
DMR19:25241001	19	25241001	25245000	4000	1	1.10E-07	0.65	80	2	Podnl1;Cc2d1a	
DMR19:25333001	19	25333001	25335000	2000	1	8.00E-07	0.48	49	2.45	Zswim4	
DMR19:25468001	19	25468001	25470000	2000	1	3.00E-07	0.42	32	1.6	Cacna1a	Transport
DMR19:25477001	19	25477001	25481000	4000	1	2.50E-07	-0.45	68	1.7	Cacna1a	Transport
DMR19:25517001	19	25517001	25520000	3000	1	3.60E-08	0.45	32	1.07	Cacna1a	Transport
DMR19:25542001	19	25542001	25544000	2000	1	7.60E-07	0.4	32	1.6	Cacna1a	Transport
DMR19:25627001	19	25627001	25630000	3000	1	7.30E-07	0.49	65	2.17	Cacna1a	Transport
DMR19:25707001	19	25707001	25710000	3000	1	2.00E-11	0.62	56	1.87	Cacna1a	Transport
DMR19:25726001	19	25726001	25729000	3000	1	1.60E-07	0.37	62	2.07	Cacna1a	Transport
DMR19:25757001	19	25757001	25759000	2000	1	9.40E-14	0.61	32	1.6	Cacna1a	Transport
DMR19:25815001	19	25815001	25816000	1000	1	3.30E-09	0.6	28	2.8	Trmt1;Lyl1;Nf1x	Epigenetic;Transcription;Transcription
DMR19:25819001	19	25819001	25820000	1000	1	2.10E-11	0.88	46	4.6	Trmt1;Lyl1;Nf1x	Epigenetic;Transcription;Transcription
DMR19:25821001	19	25821001	25825000	4000	1	1.00E-08	0.56	90	2.25	Trmt1;Lyl1;Nf1x	Epigenetic;Transcription;Transcription
DMR19:25863001	19	25863001	25866000	3000	1	5.30E-09	-0.37	60	2	Nf1x	Transcription
DMR19:25888001	19	25888001	25891000	3000	1	8.40E-13	-0.52	71	2.37	Nf1x	Transcription
DMR19:25923001	19	25923001	25925000	2000	1	1.10E-10	0.76	35	1.75	Nf1x;Dand5	Transcription
DMR19:25945001	19	25945001	25946000	1000	1	7.90E-07	0.47	15	1.5	Dand5;Gadd45gip1;Rad23a	DNA Repair
DMR19:26023001	19	26023001	26025000	2000	2	6.70E-09	0.62	50	2.5	Klf1;Dnase2;MAST1	Transcription;Transcription
DMR19:26029001	19	26029001	26032000	3000	2	7.30E-10	0.75	70	2.33	Klf1;Dnase2;MAST1;Mir3550	Transcription;Transcription
DMR19:26111001	19	26111001	26116000	5000	3	1.20E-10	0.6	118	2.36	Hook2;Best2	Transport;Transport
DMR19:26124001	19	26124001	26126000	2000	1	5.00E-16	1	54	2.7	Hook2;Best2;Asna1	Transport;Transport
DMR19:26144001	19	26144001	26145000	1000	1	8.40E-07	-0.38	10	1	Asna1;RGD1564093;Tnpo2	Transport
DMR19:26155001	19	26155001	26158000	3000	2	4.10E-07	-0.45	36	1.2	RGD1564093;Tnpo2	Transport
DMR19:26248001	19	26248001	26249000	1000	1	3.50E-09	-0.38	22	2.2	Zfp791	Transcription
DMR19:26259001	19	26259001	26260000	1000	1	8.00E-07	0.28	8	0.8	Zfp791;LOC108353207;LOC108353208	Transcription
DMR19:27429001	19	27429001	27430000	1000	1	5.90E-07	-0.33	14	1.4	Mylk3	Signaling
DMR19:27442001	19	27442001	27445000	3000	1	7.10E-08	0.3	26	0.87	Mylk3	Signaling
DMR19:28368001	19	28368001	28374000	6000	2	2.00E-18	1.02	106	1.77	LOC501317;LOC103694306;LOC102557368;Vom1r21	Receptor
DMR19:28654001	19	28654001	28659000	5000	1	1.50E-15	0.51	44	0.88	LOC501317;LOC691712;RGD1562660	
DMR19:28667001	19	28667001	28671000	4000	1	3.40E-08	0.37	28	0.7	LOC501317;LOC691712;RGD1562660	
DMR19:28746001	19	28746001	28750000	4000	1	1.50E-08	0.45	44	1.1	LOC501317;LOC691712;RGD1562660;LOC102550448	
DMR19:28815001	19	28815001	28816000	1000	1	1.40E-07	0.42	8	0.8	LOC501317;RGD1562660	
DMR19:28856001	19	28856001	28859000	3000	3	2.90E-21	0.57	20	0.67	LOC501317;RGD1562660	
DMR19:28860001	19	28860001	28861000	1000	1	2.60E-08	0.24	10	1	LOC501317;RGD1562660	
DMR19:28864001	19	28864001	28869000	5000	1	4.50E-07	0.7	40	0.8	LOC501317;RGD1562660	
DMR19:28874001	19	28874001	28881000	7000	2	5.10E-24	0.92	67	0.96	LOC501317;RGD1562660	
DMR19:28885001	19	28885001	28890000	5000	2	3.50E-09	0.55	36	0.72	LOC501317;RGD1562660	
DMR19:29605001	19	29605001	29606000	1000	1	6.00E-07	0.52	10	1	Inpp4b	
DMR19:29692001	19	29692001	29693000	1000	1	1.20E-10	0.54	15	1.5	Inpp4b	
DMR19:29769001	19	29769001	29771000	2000	1	3.70E-12	0.35	11	0.55	Inpp4b	
DMR19:29800001	19	29800001	29802000	2000	1	1.90E-09	0.7	43	2.15	Inpp4b;LOC103694314	Epigenetic
DMR19:30023001	19	30023001	30027000	4000	1	5.20E-14	0.46	39	0.98	Inpp4b	
DMR19:30039001	19	30039001	30041000	2000	1	1.50E-32	0.54	24	1.2	Inpp4b	
DMR19:30241001	19	30241001	30242000	1000	1	8.80E-08	0.34	13	1.3	Inpp4b	
DMR19:30269001	19	30269001	30270000	1000	1	6.50E-07	-0.34	20	2	Inpp4b	

DMR19:30311001	19	30311001	30313000	2000	1	1.30E-13	0.89	30	1.5	Inpp4b;RGD1565102	
DMR19:30347001	19	30347001	30349000	2000	1	5.50E-08	-0.41	8	0.4	Inpp4b	
DMR19:30792001	19	30792001	30793000	1000	1	1.30E-07	0.51	20	2	LOC688904;Gab1	Cytoskeleton
DMR19:30796001	19	30796001	30799000	3000	1	9.40E-07	-0.39	46	1.53	Gab1	Cytoskeleton
DMR19:30810001	19	30810001	30816000	6000	1	2.10E-11	0.92	100	1.67	Gab1	Cytoskeleton
DMR19:30839001	19	30839001	30842000	3000	1	1.90E-07	-0.44	44	1.47	Gab1	Cytoskeleton
DMR19:30850001	19	30850001	30853000	3000	2	5.80E-08	-0.54	52	1.73	Gab1	Cytoskeleton
DMR19:30882001	19	30882001	30883000	1000	1	1.10E-08	-0.34	17	1.7	Gab1	Cytoskeleton
DMR19:31035001	19	31035001	31036000	1000	1	1.40E-07	0.42	13	1.3	Frem3	
DMR19:31518001	19	31518001	31521000	3000	1	9.60E-07	-0.35	34	1.13	Hhip	Signaling
DMR19:31553001	19	31553001	31557000	4000	1	5.20E-08	0.35	60	1.5	Hhip	Signaling
DMR19:32180001	19	32180001	32183000	3000	1	4.90E-07	-0.38	38	1.27	LOC100360036;Smad1	Transcription
DMR19:32199001	19	32199001	32203000	4000	1	3.10E-09	-0.45	69	1.73	Smad1	Transcription
DMR19:32213001	19	32213001	32216000	3000	1	7.60E-07	-0.39	75	2.5	Smad1	Transcription
DMR19:32217001	19	32217001	32220000	3000	2	2.90E-12	-0.49	74	2.47	Smad1	Transcription
DMR19:32330001	19	32330001	32335000	5000	1	7.50E-07	-0.37	80	1.6	Mmaa;LOC102550010;LOC498933	Metabolism
DMR19:32387001	19	32387001	32388000	1000	1	3.30E-07	-0.57	5	0.5	LOC102550010;LOC498933;Zfp827	Transcription
DMR19:32451001	19	32451001	32454000	3000	1	3.30E-07	0.54	41	1.37	Zfp827	Transcription
DMR19:32458001	19	32458001	32461000	3000	1	8.10E-09	0.5	39	1.3	Zfp827	Transcription
DMR19:32462001	19	32462001	32463000	1000	1	2.00E-10	0.41	11	1.1	Zfp827	Transcription
DMR19:32545001	19	32545001	32546000	1000	1	2.90E-07	0.51	35	3.5	Zfp827;LOC103694317	Transcription
DMR19:32599001	19	32599001	32600000	1000	1	2.70E-07	0.53	19	1.9	Zfp827	Transcription
DMR19:32775001	19	32775001	32776000	1000	1	5.40E-09	0.61	7	0.7	Lsm6	Translation
DMR19:32791001	19	32791001	32792000	1000	1	6.40E-09	0.45	12	1.2	Lsm6	Translation
DMR19:32816001	19	32816001	32817000	1000	1	1.10E-08	0.46	4	0.4	RGD1564764	
DMR19:32821001	19	32821001	32822000	1000	1	8.40E-10	0.71	13	1.3	RGD1564764	
DMR19:32950001	19	32950001	32953000	3000	1	1.00E-09	-0.49	50	1.67	Slc10a7	Transport
DMR19:32976001	19	32976001	32978000	2000	1	1.70E-07	-0.38	34	1.7	Slc10a7;LOC103694318	Transport
DMR19:33196001	19	33196001	33197000	1000	1	9.00E-07	0.57	13	1.3	Ttc29	
DMR19:33372001	19	33372001	33378000	6000	1	2.30E-07	-0.31	69	1.15	Ttc29	
DMR19:33392001	19	33392001	33394000	2000	1	1.10E-08	0.64	29	1.45	Ttc29	
DMR19:33953001	19	33953001	33955000	2000	1	1.80E-09	0.49	16	0.8	Ednra	
DMR19:34103001	19	34103001	34105000	2000	1	3.60E-08	-0.39	27	1.35	Prmt9	Golgi
DMR19:34366001	19	34366001	34369000	3000	1	1.60E-09	0.61	49	1.63	Arhgap10	Signaling
DMR19:34423001	19	34423001	34424000	1000	1	1.20E-07	-0.5	7	0.7	Nr3c2	
DMR19:34432001	19	34432001	34433000	1000	1	8.60E-07	-0.47	6	0.6	Nr3c2	
DMR19:37004001	19	37004001	37005000	1000	1	9.00E-10	0.66	12	1.2	LOC108348882;RGD1564100	
DMR19:37007001	19	37007001	37010000	3000	3	1.10E-21	1	67	2.23	LOC108348882;RGD1564100;Slc25a36l1	
DMR19:37011001	19	37011001	37015000	4000	4	7.70E-23	0.79	93	2.33	RGD1564100;Slc25a36l1	
DMR19:37042001	19	37042001	37043000	1000	1	6.80E-07	-0.48	2	0.2	Ces2g;LOC102548129;Ces2h	Metabolism
DMR19:37084001	19	37084001	37086000	2000	2	5.30E-08	-0.46	10	0.5	Ces3a;Ces4a	Metabolism
DMR19:37099001	19	37099001	37102000	3000	1	1.30E-11	0.86	55	1.83	Ces4a	Metabolism
DMR19:37103001	19	37103001	37109000	6000	1	3.90E-08	0.53	74	1.23	Ces4a	Metabolism
DMR19:37220001	19	37220001	37221000	1000	1	5.30E-11	-0.79	25	2.5	B3gnt9;Tradd;Fbxl8;Hsf4	Golgi;Transcription
DMR19:37228001	19	37228001	37231000	3000	2	3.60E-07	0.48	30	1	Tradd;Fbxl8;Hsf4;Nol3;Kiaa0895l	Transcription
DMR19:37237001	19	37237001	37240000	3000	1	1.40E-07	0.54	60	2	Hsf4;Nol3;Kiaa0895l;Exoc3l1	Transcription;Transport
DMR19:37341001	19	37341001	37343000	2000	1	4.10E-08	0.55	18	0.9	Plekhg4;Kctd19	Transcription;Cytoskeleton
DMR19:37479001	19	37479001	37481000	2000	1	5.40E-07	0.51	32	1.6	Hsd11b2;Atp6v0d1	Metabolism;Metabolism
DMR19:37548001	19	37548001	37549000	1000	1	9.40E-20	0.75	22	2.2	Agrp;Fam65a	Signaling
DMR19:37669001	19	37669001	37672000	3000	1	3.00E-07	0.49	65	2.17	Carmil2;Acd;Pard6a;Enkd1;RGD1561415;Gfod2	Cell Junction;Metabolism
DMR19:37778001	19	37778001	37779000	1000	1	1.40E-08	-0.5	14	1.4	Ranbp10	Cytoskeleton
DMR19:37918001	19	37918001	37923000	5000	1	1.80E-12	1.03	129	2.58	Psmb10;Lcat;Slc12a4	Protease;Metabolism;Transport
DMR19:37924001	19	37924001	37927000	3000	1	2.70E-07	-0.34	40	1.33	Lcat;Slc12a4	Metabolism;Transport

DMR19:37950001	19	37950001	37953000	3000	1	3.10E-12	0.74	70	2.33	Dpep3	Protease
DMR19:37975001	19	37975001	37976000	1000	1	9.30E-10	0.6	14	1.4	Dpep2	Protease
DMR19:38025001	19	38025001	38028000	3000	1	1.30E-09	0.57	32	1.07	Dus2	Translation
DMR19:38158001	19	38158001	38159000	1000	1	8.40E-10	-0.61	3	0.3	Slc7a6	Transport
DMR19:38335001	19	38335001	38337000	2000	1	1.40E-07	0.46	29	1.45	Eif5b-ps1	
DMR19:38429001	19	38429001	38431000	2000	1	7.60E-07	0.32	34	1.7	Nqo1	Metabolism
DMR19:38469001	19	38469001	38471000	2000	1	1.90E-08	-0.48	13	0.65	Nfat5	Transcription
DMR19:38590001	19	38590001	38592000	2000	1	9.60E-07	-0.54	31	1.55	Zfp90	Transcription
DMR19:38781001	19	38781001	38783000	2000	1	3.80E-13	0.55	29	1.45	Cdh1	Cytoskeleton
DMR19:38784001	19	38784001	38786000	2000	1	1.40E-08	-0.54	18	0.9	Cdh1	Cytoskeleton
DMR19:38815001	19	38815001	38817000	2000	1	4.60E-07	-0.59	23	1.15	Cdh1	Cytoskeleton
DMR19:38831001	19	38831001	38834000	3000	1	6.70E-08	-0.31	48	1.6	Cdh1	Cytoskeleton
DMR19:38892001	19	38892001	38893000	1000	1	1.20E-07	0.39	10	1	Tango6	
DMR19:39037001	19	39037001	39041000	4000	1	4.10E-08	0.52	38	0.95	Tango6	
DMR19:39118001	19	39118001	39119000	1000	1	1.50E-09	-0.38	18	1.8	Utp4;LOC102548090;Sntb2	
DMR19:39136001	19	39136001	39137000	1000	1	9.20E-07	-0.3	19	1.9	Sntb2	
DMR19:39265001	19	39265001	39266000	1000	1	8.20E-07	0.5	18	1.8	Cog8;Nip7;Tmed6;Terf2	Cytoskeleton;Transport
DMR19:39273001	19	39273001	39279000	6000	1	2.30E-08	-0.48	68	1.13	Tmed6;Terf2	Transport
DMR19:39628001	19	39628001	39629000	1000	1	9.70E-16	0.79	24	2.4	RGD1562833	
DMR19:40904001	19	40904001	40908000	4000	1	4.30E-10	0.47	99	2.48	Il34;Mtss1l	Cytokine
DMR19:40917001	19	40917001	40920000	3000	1	5.80E-07	0.53	59	1.97	Mtss1l;Vac14	Cytoskeleton
DMR19:41131001	19	41131001	41134000	3000	1	2.30E-08	0.54	40	1.33	Hydin	
DMR19:41138001	19	41138001	41140000	2000	1	3.30E-07	0.54	42	2.1	Hydin	
DMR19:41188001	19	41188001	41189000	1000	1	5.00E-07	-0.36	14	1.4	Hydin	
DMR19:41241001	19	41241001	41242000	1000	1	1.50E-12	0.4	6	0.6	Hydin	
DMR19:41289001	19	41289001	41290000	1000	1	2.70E-07	0.3	11	1.1	Hydin	
DMR19:41322001	19	41322001	41327000	5000	1	3.00E-10	-0.6	78	1.56	Hydin	
DMR19:41328001	19	41328001	41331000	3000	1	3.20E-07	0.41	44	1.47	Hydin	
DMR19:41377001	19	41377001	41378000	1000	1	2.70E-12	0.56	16	1.6	Hydin	
DMR19:41435001	19	41435001	41436000	1000	1	4.30E-08	0.65	14	1.4	Cmtr2;LOC108348934	
DMR19:41492001	19	41492001	41494000	2000	1	1.00E-08	-0.45	14	0.7	Calb2	Signaling
DMR19:41669001	19	41669001	41674000	5000	1	1.50E-10	-0.35	105	2.1	Tat	Metabolism
DMR19:41675001	19	41675001	41678000	3000	1	2.20E-14	1.24	66	2.2	Tat	Metabolism
DMR19:41686001	19	41686001	41687000	1000	1	5.80E-07	0.71	18	1.8	Tat	Metabolism
DMR19:41712001	19	41712001	41713000	1000	1	6.40E-07	-0.35	21	2.1	Marveld3	Cytoskeleton
DMR19:41736001	19	41736001	41738000	2000	1	3.40E-08	0.34	15	0.75	Phlpp2	Cytoskeleton
DMR19:41803001	19	41803001	41804000	1000	1	3.30E-08	-0.45	18	1.8	Phlpp2;Ap1g1	Cytoskeleton
DMR19:41830001	19	41830001	41831000	1000	1	1.10E-08	-0.48	13	1.3	Ap1g1	
DMR19:42156001	19	42156001	42158000	2000	1	4.30E-07	-0.46	36	1.8	Pmfbp1	
DMR19:42159001	19	42159001	42163000	4000	1	1.20E-07	0.47	58	1.45	Pmfbp1	
DMR19:42182001	19	42182001	42183000	1000	1	1.10E-09	-0.45	24	2.4	Pmfbp1	
DMR19:42757001	19	42757001	42760000	3000	1	1.00E-09	0.71	95	3.17	Zfhx3	Transcription
DMR19:42849001	19	42849001	42851000	2000	2	4.10E-12	-0.49	52	2.6	Zfhx3;LOC108348931	Transcription
DMR19:42868001	19	42868001	42871000	3000	1	1.90E-08	-0.52	55	1.83	Zfhx3	Transcription
DMR19:42872001	19	42872001	42875000	3000	1	3.70E-08	-0.39	74	2.47	Zfhx3	Transcription
DMR19:42879001	19	42879001	42881000	2000	1	1.90E-07	-0.35	39	1.95	Zfhx3	Transcription
DMR19:42923001	19	42923001	42925000	2000	1	4.20E-08	-0.35	43	2.15	Zfhx3;LOC108353195	Transcription
DMR19:43157001	19	43157001	43159000	2000	1	3.30E-08	0.48	27	1.35	Pdpr;LOC690718;Clec18a	Metabolism;Immune
DMR19:43291001	19	43291001	43292000	1000	1	2.50E-07	-0.38	16	1.6	St3gal2	Transport
DMR19:43344001	19	43344001	43346000	2000	1	4.20E-12	0.65	42	2.1	Fuk	
DMR19:43347001	19	43347001	43351000	4000	1	5.60E-07	0.32	75	1.88	Fuk;Cog4	Transport
DMR19:43553001	19	43553001	43554000	1000	1	2.40E-08	0.56	17	1.7	Fa2h	Metabolism
DMR19:43790001	19	43790001	43791000	1000	1	1.90E-07	-0.51	10	1	Znrf1	Proteolysis
DMR19:43880001	19	43880001	43882000	2000	1	6.10E-08	0.49	35	1.75	Zfp1	
DMR19:43893001	19	43893001	43897000	4000	1	1.10E-18	0.8	72	1.8	Zfp1;Ctrb1	Protease
DMR19:43905001	19	43905001	43910000	5000	3	7.30E-20	1.4	100	2	Ctrb1	Protease
DMR19:43915001	19	43915001	43916000	1000	1	5.70E-10	-0.37	22	2.2	Ctrb1	Protease
DMR19:43918001	19	43918001	43919000	1000	1	7.40E-15	0.68	19	1.9	Ctrb1	Protease
DMR19:43958001	19	43958001	43962000	4000	1	1.90E-07	-0.33	56	1.4	Bcar1;LOC103694331	
DMR19:44062001	19	44062001	44063000	1000	1	9.70E-11	0.63	11	1.1	Cfdp1	
DMR19:44155001	19	44155001	44157000	2000	1	2.10E-13	0.73	31	1.55	Tmem231;Gabarapl2	Cytoskeleton
DMR19:44267001	19	44267001	44268000	1000	1	7.30E-07	0.52	24	2.4	RGD1563834	

DMR19:44829001	19	44829001	44834000	5000	1	6.20E-07	-0.45	61	1.22	Cntnap4	
DMR19:44844001	19	44844001	44846000	2000	1	5.40E-09	0.65	40	2	Cntnap4	
DMR19:44908001	19	44908001	44909000	1000	1	3.00E-11	-0.5	14	1.4	Cntnap4	
DMR19:44922001	19	44922001	44924000	2000	1	2.40E-08	-0.38	23	1.15	Cntnap4	
DMR19:44938001	19	44938001	44942000	4000	1	1.60E-09	-0.53	66	1.65	Cntnap4	
DMR19:45057001	19	45057001	45059000	2000	1	4.10E-08	0.41	15	0.75	Cntnap4	
DMR19:45114001	19	45114001	45116000	2000	1	7.60E-07	0.46	24	1.2	Cntnap4	
DMR19:45898001	19	45898001	45899000	1000	1	9.40E-07	0.43	8	0.8	RGD1565047	
DMR19:46076001	19	46076001	46077000	1000	1	5.60E-08	-0.48	12	1.2	Adamts18	Protease
DMR19:46089001	19	46089001	46091000	2000	1	5.10E-09	-0.48	27	1.35	Adamts18	Protease
DMR19:46117001	19	46117001	46119000	2000	1	2.10E-09	-0.5	24	1.2	Adamts18	Protease
DMR19:46135001	19	46135001	46137000	2000	1	5.90E-08	-0.69	24	1.2	Adamts18;LOC690181	Protease
DMR19:46138001	19	46138001	46142000	4000	1	1.10E-10	0.91	52	1.3	Adamts18;LOC690181	Protease
DMR19:46164001	19	46164001	46167000	3000	1	1.90E-08	-0.48	54	1.8	Adamts18	Protease
DMR19:46625001	19	46625001	46626000	1000	1	3.90E-07	0.52	12	1.2	Vat1l	
DMR19:46662001	19	46662001	46663000	1000	1	4.50E-09	0.54	23	2.3	Vat1l	
DMR19:46671001	19	46671001	46672000	1000	1	1.00E-09	0.71	19	1.9	Vat1l	
DMR19:46673001	19	46673001	46674000	1000	1	2.40E-08	0.46	5	0.5	Vat1l	
DMR19:46756001	19	46756001	46759000	3000	1	1.60E-14	0.41	26	0.87	Wwox	
DMR19:46780001	19	46780001	46783000	3000	1	7.40E-08	0.35	47	1.57	Wwox;LOC108348988	
DMR19:46917001	19	46917001	46919000	2000	1	4.40E-07	0.39	16	0.8	Wwox	
DMR19:46995001	19	46995001	46996000	1000	1	1.70E-09	0.5	12	1.2	Wwox	
DMR19:47000001	19	47000001	47001000	1000	1	2.20E-08	0.58	7	0.7	Wwox	
DMR19:47018001	19	47018001	47021000	3000	1	3.90E-07	0.41	58	1.93	Wwox	
DMR19:47048001	19	47048001	47051000	3000	2	1.60E-08	-0.42	62	2.07	Wwox	
DMR19:47116001	19	47116001	47118000	2000	1	1.90E-07	0.42	29	1.45	Wwox	
DMR19:47123001	19	47123001	47125000	2000	1	2.00E-07	0.32	24	1.2	Wwox	
DMR19:47221001	19	47221001	47222000	1000	1	2.00E-08	0.42	17	1.7	Wwox	
DMR19:47236001	19	47236001	47237000	1000	1	1.20E-09	0.56	4	0.4	Wwox	
DMR19:47293001	19	47293001	47295000	2000	1	9.00E-08	0.35	21	1.05	Wwox	
DMR19:47340001	19	47340001	47344000	4000	1	4.70E-07	0.42	56	1.4	Wwox;LOC108348987	
DMR19:47361001	19	47361001	47362000	1000	1	4.80E-07	0.51	9	0.9	Wwox	
DMR19:47418001	19	47418001	47420000	2000	1	4.70E-07	0.4	12	0.6	Wwox	
DMR19:47439001	19	47439001	47440000	1000	1	7.00E-07	-0.3	29	2.9	Wwox	
DMR19:47450001	19	47450001	47453000	3000	1	4.20E-07	-0.37	35	1.17	Wwox	
DMR19:47499001	19	47499001	47500000	1000	1	8.80E-12	0.78	29	2.9	Wwox	
DMR19:47532001	19	47532001	47533000	1000	1	8.30E-07	0.54	11	1.1	Wwox	
DMR19:47541001	19	47541001	47542000	1000	1	3.10E-07	0.33	18	1.8	Wwox	
DMR19:47582001	19	47582001	47584000	2000	1	2.70E-07	0.3	41	2.05	Wwox	
DMR19:47608001	19	47608001	47609000	1000	1	1.20E-15	0.87	23	2.3	Wwox;LOC108348984;LOC108348986	
DMR19:47685001	19	47685001	47686000	1000	1	9.70E-07	0.38	17	1.7	Wwox;LOC108348985	
DMR19:47687001	19	47687001	47690000	3000	1	2.10E-07	-0.51	40	1.33	Wwox;LOC108348985	
DMR19:47702001	19	47702001	47705000	3000	1	2.50E-17	-0.63	32	1.07	Wwox;LOC108348985	
DMR19:49009001	19	49009001	49011000	2000	1	1.70E-12	0.8	36	1.8	Dynlrb2	Cytoskeleton
DMR19:49101001	19	49101001	49103000	2000	1	3.80E-08	0.36	19	0.95	Cdyl2	
DMR19:49104001	19	49104001	49105000	1000	1	1.20E-09	0.57	17	1.7	Cdyl2	
DMR19:49107001	19	49107001	49111000	4000	1	2.80E-17	0.93	96	2.4	Cdyl2	
DMR19:49128001	19	49128001	49129000	1000	1	2.60E-08	0.52	7	0.7	Cdyl2	
DMR19:49171001	19	49171001	49172000	1000	1	1.10E-08	-0.45	25	2.5	Cdyl2	
DMR19:49231001	19	49231001	49232000	1000	1	5.30E-10	0.74	11	1.1	Cdyl2	
DMR19:49494001	19	49494001	49497000	3000	1	1.40E-07	0.41	57	1.9	Atmin;LOC103694344	
DMR19:49553001	19	49553001	49554000	1000	1	1.20E-08	0.59	20	2	Pkd1l2	Transport
DMR19:49609001	19	49609001	49610000	1000	1	2.20E-20	0.98	22	2.2	Pkd1l2	Transport
DMR19:49632001	19	49632001	49635000	3000	1	1.70E-07	0.46	44	1.47	Pkd1l2;Bco1	Transport;Metabolism
DMR19:49741001	19	49741001	49743000	2000	1	5.00E-07	-0.35	43	2.15	Gan	Cytoskeleton
DMR19:49858001	19	49858001	49861000	3000	1	6.20E-07	-0.39	45	1.5	Cmip	
DMR19:49926001	19	49926001	49927000	1000	1	1.60E-07	0.58	14	1.4	Cmip	
DMR19:50009001	19	50009001	50012000	3000	2	2.80E-12	0.84	51	1.7	Cmip	
DMR19:50044001	19	50044001	50045000	1000	1	2.20E-07	-0.45	27	2.7	Plcg2	Metabolism
DMR19:50203001	19	50203001	50205000	2000	2	1.40E-11	0.85	54	2.7	Sdr42e1	Metabolism
DMR19:50214001	19	50214001	50215000	1000	1	4.50E-15	0.73	29	2.9	Sdr42e1	Metabolism

DMR19:50243001	19	50243001	50250000	7000	2	5.70E-18	-0.59	107	1.53	Hsd17b2	Metabolism
DMR19:50375001	19	50375001	50378000	3000	1	6.40E-08	-0.46	48	1.6	Mphosph6	
DMR19:50894001	19	50894001	50896000	2000	1	3.70E-08	0.54	20	1	Cdh13	Cytoskeleton
DMR19:50913001	19	50913001	50914000	1000	1	1.90E-07	0.31	22	2.2	Cdh13	Cytoskeleton
DMR19:50933001	19	50933001	50935000	2000	1	3.20E-07	-0.36	29	1.45	Cdh13	Cytoskeleton
DMR19:50948001	19	50948001	50949000	1000	1	9.00E-07	0.53	11	1.1	Cdh13	Cytoskeleton
DMR19:50998001	19	50998001	51004000	6000	2	1.20E-10	0.7	84	1.4	Cdh13	Cytoskeleton
DMR19:51019001	19	51019001	51020000	1000	1	2.60E-07	0.4	18	1.8	Cdh13	Cytoskeleton
DMR19:51058001	19	51058001	51063000	5000	1	1.70E-08	-0.34	91	1.82	Cdh13	Cytoskeleton
DMR19:51097001	19	51097001	51098000	1000	1	3.40E-16	1.19	21	2.1	Cdh13	Cytoskeleton
DMR19:51128001	19	51128001	51129000	1000	1	9.50E-13	0.88	23	2.3	Cdh13	Cytoskeleton
DMR19:51154001	19	51154001	51155000	1000	1	2.30E-07	0.68	27	2.7	Cdh13	Cytoskeleton
DMR19:51189001	19	51189001	51191000	2000	1	5.70E-08	-0.41	17	0.85	Cdh13	Cytoskeleton
DMR19:51206001	19	51206001	51209000	3000	1	6.50E-09	-0.44	44	1.47	Cdh13;LOC102552714	Cytoskeleton
DMR19:51233001	19	51233001	51234000	1000	1	3.70E-09	0.7	24	2.4	Cdh13;LOC102552714	Cytoskeleton
DMR19:51252001	19	51252001	51254000	2000	1	2.00E-12	-0.55	15	0.75	Cdh13	Cytoskeleton
DMR19:51271001	19	51271001	51272000	1000	1	5.00E-07	0.6	19	1.9	Cdh13	Cytoskeleton
DMR19:51291001	19	51291001	51292000	1000	1	1.20E-08	0.28	6	0.6	Cdh13	Cytoskeleton
DMR19:51340001	19	51340001	51343000	3000	1	8.30E-07	-0.31	51	1.7	Cdh13	Cytoskeleton
DMR19:51346001	19	51346001	51349000	3000	1	1.10E-09	0.51	50	1.67	Cdh13	Cytoskeleton
DMR19:51396001	19	51396001	51400000	4000	2	1.80E-17	1.09	89	2.22	Cdh13	Cytoskeleton
DMR19:51432001	19	51432001	51436000	4000	1	4.30E-10	0.62	80	2	Cdh13	Cytoskeleton
DMR19:51437001	19	51437001	51438000	1000	1	1.80E-18	1.09	27	2.7	Cdh13	Cytoskeleton
DMR19:51447001	19	51447001	51452000	5000	1	2.00E-14	0.67	58	1.16	Cdh13	Cytoskeleton
DMR19:51460001	19	51460001	51463000	3000	1	2.70E-07	0.57	52	1.73	Cdh13	Cytoskeleton
DMR19:51594001	19	51594001	51596000	2000	1	9.60E-08	-0.43	64	3.2	Cdh13	Cytoskeleton
DMR19:51650001	19	51650001	51651000	1000	1	2.00E-17	-0.56	16	1.6	Cdh13	Cytoskeleton
DMR19:51699001	19	51699001	51701000	2000	1	3.70E-07	0.34	24	1.2	Cdh13	Cytoskeleton
DMR19:51703001	19	51703001	51704000	1000	1	3.80E-08	0.65	22	2.2	Cdh13	Cytoskeleton
DMR19:51748001	19	51748001	51750000	2000	1	8.80E-22	0.71	20	1	Cdh13	Cytoskeleton
DMR19:51764001	19	51764001	51766000	2000	2	1.30E-09	0.45	21	1.05	Cdh13	Cytoskeleton
DMR19:51767001	19	51767001	51768000	1000	1	1.40E-08	0.48	13	1.3	Cdh13	Cytoskeleton
DMR19:51769001	19	51769001	51770000	1000	1	6.50E-09	0.53	15	1.5	Cdh13	Cytoskeleton
DMR19:51839001	19	51839001	51840000	1000	1	3.40E-08	0.38	11	1.1	Cdh13	Cytoskeleton
DMR19:51964001	19	51964001	51967000	3000	1	3.10E-12	0.57	50	1.67	Cdh13	Cytoskeleton
DMR19:51983001	19	51983001	51984000	1000	1	7.90E-13	0.8	14	1.4	Hsbp1	
DMR19:52086001	19	52086001	52088000	2000	1	3.30E-11	0.85	140	7	Osgin1;Necab2	
DMR19:52091001	19	52091001	52093000	2000	1	1.00E-07	0.61	27	1.35	Osgin1;Necab2	
DMR19:52097001	19	52097001	52099000	2000	1	3.50E-08	-0.43	41	2.05	Necab2	
DMR19:52110001	19	52110001	52118000	8000	1	1.80E-08	0.46	116	1.45	Necab2;Slc38a8	Transport
DMR19:52197001	19	52197001	52198000	1000	1	2.20E-08	-0.52	8	0.8	Mbtps1	Protease
DMR19:52258001	19	52258001	52259000	1000	1	9.20E-08	0.53	33	3.3	Taf1c;Adad2	Transcription;Metabolism
DMR19:52260001	19	52260001	52262000	2000	1	1.30E-07	0.6	36	1.8	Taf1c;Adad2;Kcng4	Transcription;Metabolism;Transport
DMR19:52273001	19	52273001	52274000	1000	1	2.60E-13	0.77	45	4.5	Adad2;Kcng4	Metabolism;Transport
DMR19:52349001	19	52349001	52352000	3000	1	8.90E-07	-0.37	53	1.77	Atp2c2	Transport
DMR19:52368001	19	52368001	52370000	2000	1	4.90E-07	0.55	33	1.65	Atp2c2;LOC102549202	Transport
DMR19:52382001	19	52382001	52383000	1000	1	6.30E-09	0.65	32	3.2	Atp2c2	Transport
DMR19:52476001	19	52476001	52479000	3000	1	4.90E-09	0.31	25	0.83	Cotl1	Cytoskeleton
DMR19:52560001	19	52560001	52562000	2000	1	2.70E-07	0.52	39	1.95	Usp10	Protease
DMR19:52598001	19	52598001	52599000	1000	1	3.20E-08	-0.45	19	1.9	Usp10	Protease
DMR19:52635001	19	52635001	52639000	4000	1	8.60E-07	0.56	60	1.5	Crispld2	Immune
DMR19:52679001	19	52679001	52685000	6000	2	1.30E-11	0.57	90	1.5	Crispld2	Immune
DMR19:52687001	19	52687001	52688000	1000	1	4.00E-07	-0.5	8	0.8	Crispld2	Immune
DMR19:52768001	19	52768001	52772000	4000	1	2.20E-07	-0.39	50	1.25	RGD1304884	
DMR19:52784001	19	52784001	52786000	2000	1	4.40E-07	0.56	22	1.1	RGD1304884	
DMR19:52792001	19	52792001	52796000	4000	1	6.10E-08	-0.31	61	1.52	RGD1304884	
DMR19:52799001	19	52799001	52802000	3000	1	1.90E-08	-0.48	85	2.83	RGD1304884	
DMR19:52804001	19	52804001	52805000	1000	1	1.40E-07	0.55	15	1.5	RGD1304884	
DMR19:52835001	19	52835001	52840000	5000	1	4.30E-09	0.47	77	1.54	RGD1304884	
DMR19:52900001	19	52900001	52901000	1000	1	3.70E-08	0.27	16	1.6	RGD1304884	

DMR19:52924001	19	52924001	52925000	1000	1	1.20E-09	0.54	25	2.5	RGD1304884	
DMR19:52945001	19	52945001	52946000	1000	1	1.20E-12	0.83	40	4	RGD1304884	
DMR19:53001001	19	53001001	53008000	7000	2	1.90E-12	-0.65	174	2.49	RGD1304884;Fendrr;Foxf1	
DMR19:53015001	19	53015001	53016000	1000	1	3.40E-09	-0.55	33	3.3	RGD1304884;Fendrr;Foxf1;Mthfsd	Metabolism
DMR19:53099001	19	53099001	53100000	1000	1	1.30E-12	0.92	24	2.4	RGD1304884	
DMR19:53147001	19	53147001	53149000	2000	1	5.20E-09	0.77	42	2.1	RGD1304884	
DMR19:53226001	19	53226001	53228000	2000	2	1.30E-09	0.29	23	1.15	RGD1304884	
DMR19:53229001	19	53229001	53232000	3000	1	3.00E-08	0.6	33	1.1	RGD1304884	
DMR19:53236001	19	53236001	53237000	1000	1	1.10E-07	0.56	15	1.5	RGD1304884	
DMR19:53259001	19	53259001	53261000	2000	2	6.50E-10	0.6	20	1	RGD1304884	
DMR19:53265001	19	53265001	53273000	8000	3	1.50E-10	0.55	154	1.93	RGD1304884;LOC367563	
DMR19:53275001	19	53275001	53277000	2000	1	3.30E-07	0.44	28	1.4	RGD1304884;LOC367563	
DMR19:53293001	19	53293001	53294000	1000	1	7.90E-15	0.59	20	2	RGD1304884	
DMR19:53318001	19	53318001	53320000	2000	1	6.70E-08	0.42	27	1.35	RGD1304884	
DMR19:53334001	19	53334001	53336000	2000	1	2.10E-08	-0.4	38	1.9	RGD1304884	
DMR19:53346001	19	53346001	53352000	6000	2	2.20E-10	0.71	151	2.52	RGD1304884;LOC108348955	
DMR19:53391001	19	53391001	53393000	2000	1	8.50E-17	0.79	65	3.25	RGD1304884;LOC108348955	
DMR19:53416001	19	53416001	53421000	5000	1	8.00E-10	0.67	108	2.16	RGD1304884	
DMR19:53425001	19	53425001	53430000	5000	1	3.70E-14	0.9	114	2.28	RGD1304884	
DMR19:53432001	19	53432001	53435000	3000	1	1.50E-18	0.84	56	1.87	RGD1304884	
DMR19:53724001	19	53724001	53725000	1000	1	9.90E-08	0.49	26	2.6	RGD1304884	
DMR19:53764001	19	53764001	53767000	3000	1	3.40E-07	0.33	36	1.2	Fam92b	
DMR19:53797001	19	53797001	53798000	1000	1	7.60E-08	0.48	15	1.5	LOC102551361;Gse1	
DMR19:53806001	19	53806001	53809000	3000	1	1.20E-07	0.61	39	1.3	LOC102551361;Gse1	
DMR19:53812001	19	53812001	53813000	1000	1	4.50E-09	-0.4	24	2.4	Gse1	
DMR19:53821001	19	53821001	53824000	3000	1	6.10E-07	0.34	64	2.13	Gse1	
DMR19:53847001	19	53847001	53848000	1000	1	5.80E-09	0.45	17	1.7	Gse1	
DMR19:53854001	19	53854001	53857000	3000	1	2.20E-10	0.64	41	1.37	Gse1;LOC103694351	
DMR19:53886001	19	53886001	53893000	7000	1	2.20E-10	-0.58	103	1.47	Gse1	
DMR19:53913001	19	53913001	53915000	2000	1	1.30E-08	0.48	27	1.35	Gse1;LOC102551937	
DMR19:53927001	19	53927001	53933000	6000	1	2.60E-08	0.57	85	1.42	Gse1;LOC102551937	
DMR19:53938001	19	53938001	53940000	2000	1	5.40E-09	0.58	27	1.35	Gse1	
DMR19:53953001	19	53953001	53954000	1000	1	1.50E-09	0.57	14	1.4	Gse1	
DMR19:53955001	19	53955001	53959000	4000	1	2.60E-08	0.5	49	1.23	Gse1	
DMR19:53967001	19	53967001	53971000	4000	1	6.80E-09	0.45	53	1.32	Gse1	
DMR19:53985001	19	53985001	53987000	2000	1	9.60E-09	0.4	43	2.15	Gse1	
DMR19:54003001	19	54003001	54004000	1000	1	1.60E-08	0.4	9	0.9	Gse1;LOC108348949	
DMR19:54018001	19	54018001	54020000	2000	2	4.30E-08	0.51	39	1.95	Gse1;LOC102551873	
DMR19:54046001	19	54046001	54048000	2000	1	1.40E-07	0.43	26	1.3	Gse1	
DMR19:54050001	19	54050001	54054000	4000	3	1.30E-09	0.54	45	1.12	Gse1;RGD1565689	
DMR19:54082001	19	54082001	54083000	1000	1	7.90E-08	0.49	19	1.9	Gse1	
DMR19:54103001	19	54103001	54106000	3000	1	6.20E-09	0.58	61	2.03	Gse1	
DMR19:54166001	19	54166001	54170000	4000	1	1.50E-09	0.75	64	1.6	Gins2	Transcription
DMR19:54184001	19	54184001	54187000	3000	1	5.60E-09	0.59	47	1.57	RGD1309651	
DMR19:54322001	19	54322001	54324000	2000	1	8.70E-07	0.41	44	2.2	Irf8	Transcription
DMR19:54333001	19	54333001	54335000	2000	1	2.40E-10	0.58	32	1.6	Irf8;LOC108348951	Transcription
DMR19:54344001	19	54344001	54345000	1000	1	3.00E-08	0.49	19	1.9	Irf8;LOC108348951	Transcription
DMR19:54578001	19	54578001	54579000	1000	1	5.20E-07	0.5	16	1.6	Jph3	
DMR19:54623001	19	54623001	54624000	1000	1	1.00E-07	0.4	11	1.1	Jph3;Mir3564	
DMR19:54829001	19	54829001	54833000	4000	2	5.20E-30	0.54	41	1.02	Banp	
DMR19:54888001	19	54888001	54895000	7000	2	7.20E-11	0.45	97	1.39	Zfp469	
DMR19:54928001	19	54928001	54933000	5000	2	8.00E-08	0.69	79	1.58	Zfp469	
DMR19:54969001	19	54969001	54974000	5000	1	3.60E-12	0.57	103	2.06	Zfp469	
DMR19:54985001	19	54985001	54990000	5000	1	3.00E-08	0.46	79	1.58	Zfp469	
DMR19:55058001	19	55058001	55060000	2000	1	2.30E-08	0.5	29	1.45	Zfp469	
DMR19:55076001	19	55076001	55078000	2000	1	9.50E-09	0.42	44	2.2	Zfp469	
DMR19:55079001	19	55079001	55080000	1000	1	1.50E-08	0.49	29	2.9	Zfp469	
DMR19:55119001	19	55119001	55128000	9000	1	8.30E-11	0.61	170	1.89	Zfpm1	Transcription
DMR19:55151001	19	55151001	55152000	1000	1	7.70E-07	0.33	19	1.9	Zfpm1	Transcription
DMR19:55184001	19	55184001	55186000	2000	1	9.10E-07	-0.35	41	2.05	Mlnr	Transcription
DMR19:55238001	19	55238001	55239000	1000	1	1.40E-07	0.57	14	1.4	Zc3h18;Il17c	

DMR19:55294001	19	55294001	55295000	1000	1	4.60E-07	0.5	23	2.3	Snai3;Rnf166;Ctu2	Transcription;Translation
DMR19:55338001	19	55338001	55341000	3000	1	9.90E-09	0.62	44	1.47	Piezo1	
DMR19:55390001	19	55390001	55395000	5000	1	8.10E-07	0.48	102	2.04	Cdt1;Aprt;Galns	Transcription;Transport;Metabolism
DMR19:55432001	19	55432001	55437000	5000	3	1.30E-09	0.7	114	2.28	Galns;Trappc2l;Pabpn1l;Cbfa2t3	Metabolism;Transport;Translation;Transcription
DMR19:55458001	19	55458001	55460000	2000	1	2.90E-10	0.6	40	2	Cbfa2t3;LOC108348958	Transcription
DMR19:55512001	19	55512001	55517000	5000	1	3.00E-08	0.36	79	1.58	Cbfa2t3	Transcription
DMR19:55518001	19	55518001	55523000	5000	1	3.00E-07	0.32	94	1.88	Cbfa2t3	Transcription
DMR19:55598001	19	55598001	55601000	3000	1	9.10E-09	0.43	59	1.97	Acsf3;LOC102551324	Metabolism
DMR19:55608001	19	55608001	55609000	1000	1	3.70E-07	0.46	17	1.7	Acsf3;LOC102551324;LOC108348960	Metabolism
DMR19:55610001	19	55610001	55611000	1000	1	1.70E-07	0.48	7	0.7	Acsf3;LOC102551324;LOC108348960	Metabolism
DMR19:55635001	19	55635001	55638000	3000	1	2.20E-07	0.5	50	1.67	Acsf3	Metabolism
DMR19:55677001	19	55677001	55680000	3000	1	2.20E-10	0.5	48	1.6	Cdh15	Cytoskeleton
DMR19:55698001	19	55698001	55703000	5000	2	1.20E-07	-0.39	82	1.64	Cdh15;Ankrd11	Cytoskeleton
DMR19:55710001	19	55710001	55721000	11000	2	1.40E-09	-0.51	248	2.25	Ankrd11	
DMR19:55795001	19	55795001	55796000	1000	1	4.50E-09	-0.44	14	1.4	Ankrd11	
DMR19:55802001	19	55802001	55804000	2000	1	3.90E-10	-0.44	30	1.5	Ankrd11	
DMR19:55837001	19	55837001	55838000	1000	1	1.70E-10	-0.53	7	0.7	Ankrd11	
DMR19:55850001	19	55850001	55853000	3000	1	4.80E-08	-0.43	40	1.33	Ankrd11	
DMR19:55875001	19	55875001	55877000	2000	1	5.00E-07	0.61	47	2.35	LOC108348981;LOC102551789;Spg7	Protease
DMR19:55894001	19	55894001	55898000	4000	1	1.40E-09	-0.45	71	1.77	Spg7	Protease
DMR19:55901001	19	55901001	55903000	2000	1	5.00E-07	-0.38	28	1.4	Spg7	Protease
DMR19:55931001	19	55931001	55932000	1000	1	7.30E-07	-0.38	17	1.7	Cpne7	
DMR19:55935001	19	55935001	55937000	2000	1	9.90E-07	0.35	29	1.45	Cpne7	
DMR19:56082001	19	56082001	56083000	1000	1	1.20E-07	0.58	20	2	Fanca	
DMR19:56093001	19	56093001	56095000	2000	1	6.70E-07	-0.35	45	2.25	Fanca	
DMR19:56150001	19	56150001	56153000	3000	1	4.20E-09	-0.44	55	1.83	Spire2	Cytoskeleton
DMR19:56293001	19	56293001	56295000	2000	1	8.00E-07	-0.36	40	2	Afg3l1;Dbndd1	Protease
DMR19:56336001	19	56336001	56340000	4000	4	4.20E-25	0.68	109	2.72	Gas8	Cytoskeleton
DMR19:56639001	19	56639001	56643000	4000	1	1.70E-07	-0.48	50	1.25	Ccsap;LOC108348965	
DMR19:56841001	19	56841001	56842000	1000	1	2.00E-08	0.37	15	1.5	LOC108348967;RGD1563459	
DMR19:56975001	19	56975001	56978000	3000	1	2.70E-07	0.44	44	1.47	Cox6c-ps1	
DMR19:56986001	19	56986001	56988000	2000	1	3.00E-08	0.44	26	1.3	Cox6c-ps1	
DMR19:57067001	19	57067001	57069000	2000	1	7.00E-07	-0.33	51	2.55	Galnt2	Golgi
DMR19:57083001	19	57083001	57086000	3000	1	2.50E-08	-0.64	85	2.83	Galnt2	Golgi
DMR19:57106001	19	57106001	57108000	2000	1	1.00E-07	0.55	47	2.35	Galnt2	Golgi
DMR19:57176001	19	57176001	57183000	7000	1	3.70E-07	-0.32	153	2.19	Pgbd5	Epigenetic
DMR19:57184001	19	57184001	57185000	1000	1	2.80E-07	-0.4	28	2.8	Pgbd5	Epigenetic
DMR19:57284001	19	57284001	57286000	2000	1	5.30E-14	-0.53	36	1.8	Cog2	
DMR19:57337001	19	57337001	57339000	2000	1	6.20E-11	0.44	22	1.1	Agt;Capn9	Protease; Proteolysis;Protease
DMR19:57348001	19	57348001	57351000	3000	2	1.20E-18	0.92	47	1.57	Capn9	Protease
DMR19:57358001	19	57358001	57360000	2000	1	7.10E-08	0.55	18	0.9	Capn9	Protease
DMR19:57368001	19	57368001	57376000	8000	1	1.40E-07	-0.54	114	1.43	Capn9	Protease
DMR19:57412001	19	57412001	57415000	3000	1	2.00E-14	0.76	53	1.77	RGD1559896	
DMR19:57522001	19	57522001	57528000	6000	1	4.20E-12	0.83	102	1.7	Fam89a	Cytoskeleton
DMR19:57544001	19	57544001	57545000	1000	1	1.10E-08	0.46	21	2.1	LOC102549725;Trim67	Proteolysis
DMR19:57546001	19	57546001	57547000	1000	1	6.80E-07	0.45	11	1.1	LOC102549725;Trim67	Proteolysis
DMR19:57561001	19	57561001	57567000	6000	1	2.50E-07	0.52	79	1.32	Trim67	Proteolysis
DMR19:57613001	19	57613001	57614000	1000	1	5.20E-10	-0.62	15	1.5	RGD1562218;Gnpat	Metabolism
DMR19:57677001	19	57677001	57680000	3000	1	9.70E-09	-0.48	82	2.73	Egln1	
DMR19:57884001	19	57884001	57885000	1000	1	1.40E-10	0.7	31	3.1	Disc1	
DMR19:57887001	19	57887001	57888000	1000	1	1.70E-07	0.48	16	1.6	Disc1	
DMR19:57956001	19	57956001	57957000	1000	1	3.70E-07	0.34	13	1.3	Disc1;LOC108348971	
DMR19:57975001	19	57975001	57976000	1000	1	6.30E-30	1.04	18	1.8	Disc1	
DMR19:58000001	19	58000001	58002000	2000	1	2.10E-12	0.55	24	1.2	Disc1;LOC103694360	

DMR19:58003001	19	58003001	58007000	4000	1	5.80E-08	0.5	45	1.12	Disc1;LOC103694360	
DMR19:58008001	19	58008001	58009000	1000	1	1.50E-07	0.46	26	2.6	Disc1	
DMR19:58065001	19	58065001	58068000	3000	1	4.40E-08	-0.58	72	2.4	Disc1	
DMR19:58262001	19	58262001	58264000	2000	1	9.60E-07	0.61	34	1.7	Sipa1l2	Signaling
DMR19:58358001	19	58358001	58360000	2000	1	1.90E-09	0.59	23	1.15	Sipa1l2	Signaling
DMR19:58409001	19	58409001	58410000	1000	1	7.30E-16	0.77	13	1.3	Sipa1l2	Signaling
DMR19:58431001	19	58431001	58437000	6000	1	3.90E-08	-0.38	97	1.62	Set	Epigenetic
DMR19:58596001	19	58596001	58597000	1000	1	2.30E-07	0.54	7	0.7	Pcnx2	
DMR19:58598001	19	58598001	58600000	2000	1	2.70E-18	1.19	40	2	Pcnx2	
DMR19:58605001	19	58605001	58608000	3000	1	5.50E-08	0.43	56	1.87	Pcnx2;LOC108353198	
DMR19:58663001	19	58663001	58666000	3000	1	5.90E-07	0.51	33	1.1	Pcnx2;LOC108353197	
DMR19:58719001	19	58719001	58721000	2000	1	1.90E-09	-0.54	13	0.65	Pcnx2	
DMR19:58722001	19	58722001	58723000	1000	1	5.30E-08	0.38	7	0.7	Pcnx2	
DMR19:58724001	19	58724001	58726000	2000	1	1.10E-09	0.49	25	1.25	Pcnx2	
DMR19:58836001	19	58836001	58837000	1000	1	5.10E-08	-0.46	8	0.8	Kcnk1	Transport
DMR19:58864001	19	58864001	58865000	1000	1	9.20E-07	0.36	8	0.8	Kcnk1	Transport
DMR19:58997001	19	58997001	5.90E+07	3000	1	6.20E-07	0.31	46	1.53	Slc35f3	
DMR19:59046001	19	59046001	59051000	5000	1	2.50E-07	0.36	82	1.64	Slc35f3	
DMR19:59053001	19	59053001	59054000	1000	1	2.70E-08	-0.46	15	1.5	Slc35f3	
DMR19:59153001	19	59153001	59155000	2000	1	1.00E-09	0.59	24	1.2	Slc35f3	
DMR19:59214001	19	59214001	59219000	5000	2	4.20E-12	0.45	53	1.06	Slc35f3	
DMR19:59241001	19	59241001	59242000	1000	1	6.60E-07	0.28	10	1	Slc35f3	
DMR19:59274001	19	59274001	59275000	1000	1	4.00E-12	-0.58	25	2.5	Slc35f3	
DMR19:59319001	19	59319001	59320000	1000	1	1.90E-08	-0.47	14	1.4	Coa6	Transcription
DMR19:59890001	19	59890001	59894000	4000	1	2.10E-13	-0.45	55	1.38	Rbm34	Translation
DMR19:59904001	19	59904001	59909000	5000	1	8.60E-07	-0.29	77	1.54	Rbm34;LOC100912076	Translation
DMR19:59979001	19	59979001	59980000	1000	1	1.70E-07	0.35	12	1.2	LOC365026;Hmg1l1	
DMR19:59984001	19	59984001	59986000	2000	1	4.10E-08	-0.4	27	1.35	LOC365026;Hmg1l1;LOC679596	
DMR19:59988001	19	59988001	59990000	2000	1	6.30E-08	0.53	33	1.65	LOC365026;Hmg1l1;LOC679596	
DMR19:60015001	19	60015001	60017000	2000	1	7.70E-07	0.46	27	1.35	Pard3	
DMR19:60048001	19	60048001	60050000	2000	1	7.40E-07	-0.49	31	1.55	Pard3	
DMR19:60111001	19	60111001	60116000	5000	1	2.60E-07	-0.39	87	1.74	Pard3	
DMR19:60135001	19	60135001	60136000	1000	1	3.50E-09	-0.61	0	0	Pard3;LOC102548266	
DMR19:60150001	19	60150001	60155000	5000	3	7.90E-12	-0.45	91	1.82	Pard3	
DMR19:60278001	19	60278001	60284000	6000	1	7.20E-08	-0.41	80	1.33	Pard3	
DMR19:60326001	19	60326001	60328000	2000	1	1.80E-07	-0.45	35	1.75	Pard3	
DMR19:60329001	19	60329001	60331000	2000	1	2.60E-15	-0.63	31	1.55	Pard3	
DMR19:60460001	19	60460001	60461000	1000	1	1.30E-07	-0.54	8	0.8	Pard3	
DMR19:60572001	19	60572001	60573000	1000	1	3.80E-07	0.43	16	1.6	Pard3	
DMR19:60580001	19	60580001	60583000	3000	1	7.50E-09	0.38	41	1.37	Pard3	
DMR19:61483001	19	61483001	61485000	2000	1	9.80E-07	0.57	34	1.7	Nrp1	
DMR19:61900001	19	61900001	61902000	2000	1	6.90E-08	0.64	16	0.8	Ccdc7	
DMR19:61932001	19	61932001	61935000	3000	1	5.80E-09	-0.22	27	0.9	Ccdc7	
DMR19:61946001	19	61946001	61947000	1000	1	1.30E-07	0.36	11	1.1	Ccdc7	
DMR19:61974001	19	61974001	61975000	1000	1	2.90E-07	0.51	8	0.8	Ccdc7;LOC307960	
DMR19:62054001	19	62054001	62056000	2000	1	5.10E-08	-0.37	22	1.1	Ccdc7	
DMR19:62109001	19	62109001	62110000	1000	1	5.90E-08	-0.35	5	0.5	Ccdc7	
DMR19:62131001	19	62131001	62136000	5000	1	8.40E-08	-0.25	53	1.06	Ccdc7	
DMR20:54001	20	54001	55000	1000	1	4.60E-08	0.51	15	1.5	2600a1a2	
DMR20:57001	20	57001	62000	5000	1	4.10E-13	0.63	142	2.84	2600a1a2	
DMR20:63001	20	63001	66000	3000	1	6.30E-07	0.24	73	2.43	2600a1a2	
DMR20:161001	20	161001	162000	1000	1	4.80E-08	0.29	15	1.5	RT1-CE10;LOC108353254;LOC103694368;LOC108349020	Immune
DMR20:163001	20	163001	164000	1000	1	5.20E-11	0.4	13	1.3	RT1-CE10;LOC108353254;LOC103694368;LOC108349020	Immune
DMR20:228001	20	228001	231000	3000	1	4.50E-09	0.44	73	2.43	Tmlhe	Metabolism
DMR20:260001	20	260001	263000	3000	1	7.20E-18	0.62	58	1.93	Tmlhe	Metabolism
DMR20:264001	20	264001	265000	1000	1	6.70E-08	0.72	22	2.2	Tmlhe	Metabolism

DMR20:326001	20	326001	330000	4000	1	6.30E-08	-0.35	41	1.02	LOC100362712;Olr1668;LOC108353249	Receptor
DMR20:504001	20	504001	505000	1000	1	5.00E-12	0.47	7	0.7	Olr1680;Olr1681	
DMR20:1060001	20	1060001	1062000	2000	1	6.80E-07	-0.42	12	0.6	Olr1697;Olr1698-ps	
DMR20:1206001	20	1206001	1207000	1000	1	2.50E-07	-0.25	9	0.9	Olr1710;Olr1711-ps	Signaling
DMR20:1229001	20	1229001	1237000	8000	1	3.60E-09	-0.25	99	1.24	Olr1712-ps	
DMR20:1347001	20	1347001	1348000	1000	1	2.70E-07	-0.54	3	0.3	RT1-M2	Immune
DMR20:1526001	20	1526001	1527000	1000	1	9.30E-08	-0.44	2	0.2	Olr1718;Olr1719-ps;Olr1720	
DMR20:1594001	20	1594001	1599000	5000	1	7.50E-07	-0.62	31	0.62	Olr1723-ps;Olr1724	
DMR20:1677001	20	1677001	1679000	2000	1	3.50E-14	0.88	34	1.7	Olr1731;LOC680336	
DMR20:1798001	20	1798001	1801000	3000	1	7.30E-08	-0.38	21	0.7	Olr1738;Olr1739	Receptor
DMR20:1979001	20	1979001	1982000	3000	2	7.10E-10	0.73	104	3.47	Gabbr1	Signaling
DMR20:2000001	20	2000001	2001000	1000	1	5.30E-07	0.51	10	1	Mog	Immune
DMR20:2058001	20	2058001	2059000	1000	1	2.70E-11	0.72	29	2.9	RT1-M4;LOC102554759;Rps15-ps1;RT1-M6-1	Immune
DMR20:2062001	20	2062001	2064000	2000	1	3.80E-11	-0.68	9	0.45	LOC102554759;Rps15-ps1;RT1-M6-1	Immune
DMR20:2144001	20	2144001	2145000	1000	1	1.70E-07	-0.44	9	0.9	Trim31	Proteolysis
DMR20:2152001	20	2152001	2157000	5000	1	3.60E-07	-0.57	42	0.84	Trim31	Proteolysis
DMR20:2190001	20	2190001	2191000	1000	1	6.10E-07	0.43	32	3.2	Rpl39-ps;Pbp-ps;Trim10;Trim15;Trim15-ps1	Proteolysis
DMR20:2232001	20	2232001	2233000	1000	1	5.70E-09	0.55	21	2.1	Trim26	Proteolysis
DMR20:2346001	20	2346001	2350000	4000	1	3.20E-08	0.6	30	0.75	RT1-M1-5	
DMR20:2542001	20	2542001	2543000	1000	1	1.50E-14	0.89	25	2.5	RT1-P2-ps1	
DMR20:2549001	20	2549001	2550000	1000	1	3.80E-09	-0.37	4	0.4	RT1-P2-ps1;Rcrg1-ps40;Nerg-ps15	
DMR20:2604001	20	2604001	2607000	3000	1	4.00E-07	-0.25	21	0.7	2824a1;2661a1a2;2795a1;2886a1-a1a2	
DMR20:2985001	20	2985001	2987000	2000	1	4.10E-08	0.34	13	0.65	2920a1a2;2846a1a2;2619a1_ps1;2927a1;Nerg-ps12;Rcrg1-ps31	
DMR20:3024001	20	3024001	3026000	2000	1	2.20E-07	0.27	19	0.95	LOC108353242;2812a1a2;Nerg-ps5	
DMR20:3053001	20	3053001	3055000	2000	1	4.00E-08	-0.47	14	0.7	LOC102555141;Rcrg2-ps3;Nerg-ps3;2509a1;Rcrg1-ps7;LOC102552510	
DMR20:3071001	20	3071001	3072000	1000	1	4.80E-07	-0.43	9	0.9	2509a1;Rcrg1-ps7;LOC102552510;Rcrg2-ps6;Rcrg1-ps6;Rcrg1-ps4;LOC102555235;Rcrg2-ps2	
DMR20:3352001	20	3352001	3354000	2000	1	1.80E-08	0.42	30	1.5	Ppp1r10;Mrps18b;Atat1;LOC108353245	Signaling;Translation
DMR20:3382001	20	3382001	3385000	3000	1	1.80E-11	-0.47	109	3.63	Dhx16;Ppp1r18;Mir6328	Transcription
DMR20:3424001	20	3424001	3427000	3000	1	5.10E-09	-0.49	115	3.83	Mdc1;Tubb5;Flot1	Cytoskeleton
DMR20:3428001	20	3428001	3430000	2000	1	1.10E-11	0.65	43	2.15	Mdc1;Tubb5;Flot1;Ier3	Cytoskeleton
DMR20:3450001	20	3450001	3452000	2000	1	2.30E-08	-0.5	33	1.65	Ier3	
DMR20:3532001	20	3532001	3534000	2000	1	5.50E-07	0.53	35	1.75	Cb707485	
DMR20:3565001	20	3565001	3569000	4000	3	6.90E-12	0.75	96	2.4	Ddr1	Receptor
DMR20:3601001	20	3601001	3604000	3000	2	1.00E-08	0.68	58	1.93	Vars2;Sfta2;Dpcr1	Translation;Transport
DMR20:3622001	20	3622001	3624000	2000	1	2.10E-13	-0.5	35	1.75	Dpcr1;CB741658	
DMR20:3627001	20	3627001	3629000	2000	1	3.00E-07	-0.37	46	2.3	Dpcr1;CB741658	
DMR20:3666001	20	3666001	3669000	3000	1	6.00E-07	0.31	39	1.3	Mt1m	
DMR20:3670001	20	3670001	3671000	1000	1	7.30E-27	1.24	30	3	Mt1m	
DMR20:3702001	20	3702001	3703000	1000	1	1.30E-09	0.6	18	1.8	Cdsn	
DMR20:3736001	20	3736001	3740000	4000	2	1.80E-08	0.56	90	2.25	Psors1c2;Cchcr1;Tcf19;Pou5f1	Transcription
DMR20:3813001	20	3813001	3815000	2000	1	3.00E-14	0.87	75	3.75	Ring1;Mir219-1;Hsd17b8;Slc39a7;Rxrb	Proteolysis;Metabolism;Transport;Transcription
DMR20:3891001	20	3891001	3892000	1000	1	2.20E-07	0.64	38	3.8	RT1-Ha;RT1-DOa	Immune
DMR20:3970001	20	3970001	3975000	5000	2	2.90E-16	1.2	95	1.9	Psmb9;Tap1	Protease;Transport
DMR20:3980001	20	3980001	3982000	2000	1	8.60E-07	0.5	45	2.25	Psmb9;Tap1;Psmb8	Protease;Transport
DMR20:3988001	20	3988001	3989000	1000	1	3.60E-09	0.68	21	2.1	Psmb9;Tap1;Psmb8;Tap2	Protease;Transport
DMR20:4039001	20	4039001	4041000	2000	1	2.00E-07	0.57	50	2.5	LOC102554184;RT1-Bb	Immune

DMR20:4047001	20	4047001	4048000	1000	1	1.00E-07	0.64	21	2.1	LOC102554184;RT1-Bb	Immune
DMR20:4129001	20	4129001	4131000	2000	1	7.30E-08	0.53	48	2.4	RT1-Db2;Arrb2-ps;RT1-Da;Btnl2	Immune;Immune
DMR20:4135001	20	4135001	4136000	1000	1	7.50E-09	0.57	19	1.9	RT1-Db2;RT1-Da;Btnl2	Immune;Immune
DMR20:4291001	20	4291001	4293000	2000	1	4.50E-12	-0.5	93	4.65	RGD1624210;Cyp21a1-ps;C4b	Immune;Protease; Proteolysis
DMR20:4312001	20	4312001	4315000	3000	1	5.90E-07	0.38	57	1.9	C4b;Stk19;Stk19-ps	Protease; Proteolysis
DMR20:4319001	20	4319001	4321000	2000	1	1.40E-07	0.5	36	1.8	C4b;Stk19;Stk19-ps;Notch4	Protease; Proteolysis
DMR20:4322001	20	4322001	4324000	2000	1	6.80E-07	-0.41	43	2.15	C4b;Stk19;Stk19-ps;Notch4	Protease; Proteolysis
DMR20:4347001	20	4347001	4348000	1000	1	3.20E-11	-0.52	24	2.4	Stk19;Notch4;Gpsm3;Pbx2	Development
DMR20:4413001	20	4413001	4414000	1000	1	1.70E-11	0.79	32	3.2	Stk19;Fkbpl;Atf6b	Transcription
DMR20:4423001	20	4423001	4425000	2000	1	3.00E-07	-0.39	37	1.85	Stk19;Fkbpl;Atf6b	Transcription
DMR20:4431001	20	4431001	4435000	4000	1	6.40E-08	-0.37	80	2	Stk19;Atf6b	
DMR20:4448001	20	4448001	4452000	4000	1	8.80E-14	0.76	102	2.55	Stk19	
DMR20:4552001	20	4552001	4556000	4000	1	4.20E-08	0.65	92	2.3	Cfb;C2	
DMR20:4607001	20	4607001	4608000	1000	1	1.70E-07	0.5	33	3.3	Slc44a4;Neu1	Transport;Metabolism
DMR20:4612001	20	4612001	4615000	3000	1	9.60E-13	0.91	98	3.27	Slc44a4;Neu1	Transport;Metabolism
DMR20:4623001	20	4623001	4625000	2000	1	4.30E-07	-0.41	37	1.85	Neu1	Metabolism
DMR20:4670001	20	4670001	4673000	3000	1	1.50E-09	0.44	46	1.53	RT1-CE6;LOC103694379	
DMR20:4685001	20	4685001	4686000	1000	1	2.90E-12	0.31	15	1.5	RT1-CE6;LOC103694379;LOC108353247;RT1-CE7	Immune
DMR20:4688001	20	4688001	4690000	2000	1	1.10E-12	0.55	28	1.4	LOC103694379;LOC108353247;RT1-CE7	Immune
DMR20:4695001	20	4695001	4696000	1000	1	4.10E-10	0.29	9	0.9	LOC108353247;RT1-CE7	Immune
DMR20:4751001	20	4751001	4758000	7000	1	3.80E-09	0.49	89	1.27	LOC102555972;Sacm2l-ps4	
DMR20:4777001	20	4777001	4781000	4000	1	1.80E-08	0.43	46	1.15	RT1-CE2;Sacm2l-ps3	
DMR20:4801001	20	4801001	4804000	3000	1	2.20E-08	0.28	54	1.8	Ddx39b	
DMR20:4805001	20	4805001	4807000	2000	1	5.00E-08	0.53	36	1.8	Ddx39b	
DMR20:4816001	20	4816001	4818000	2000	1	2.00E-08	-0.47	66	3.3	Ddx39b;Atp6v1g2;Nfkbil1	Metabolism;Signaling
DMR20:4822001	20	4822001	4823000	1000	1	1.80E-07	0.32	21	2.1	Ddx39b;Atp6v1g2;Nfkbil1	Metabolism;Signaling
DMR20:4848001	20	4848001	4851000	3000	1	5.30E-09	-0.38	48	1.6	Nfkbil1;Lta;LOC103694380	Signaling
DMR20:4928001	20	4928001	4933000	5000	1	1.20E-07	0.34	65	1.3	Hspa1l;RT1-CE3;RT1-CE4;Sacm2l-ps2;LOC103694382	Immune
DMR20:4939001	20	4939001	4941000	2000	2	1.50E-08	0.75	5	0.25	Hspa1l;RT1-CE3;LOC103694382	Immune
DMR20:4978001	20	4978001	4979000	1000	1	3.40E-07	-0.46	20	2	Lsm2;Rpl13-ps1;LOC108349032	Translation
DMR20:5025001	20	5025001	5027000	2000	1	6.90E-07	-0.37	25	1.25	Vwa7;Sapcd1;Msh5	Transcription
DMR20:5038001	20	5038001	5042000	4000	1	1.20E-07	-0.49	98	2.45	Msh5;Clic1;Ddah2	Transcription;Transport;Metabolism
DMR20:5067001	20	5067001	5072000	5000	1	7.20E-07	-0.33	82	1.64	Ly6g6c;Ly6g6d;Ly6g6e;Ly6g6f;Rps25-ps2;Abhd16a	Protease
DMR20:5152001	20	5152001	5155000	3000	2	2.70E-11	-0.63	84	2.8	Prrc2a;E230034O05Rik;Aif1	Metabolism;Signaling
DMR20:5157001	20	5157001	5158000	1000	1	8.90E-09	-0.46	7	0.7	Prrc2a;E230034O05Rik;Aif1	Metabolism;Signaling
DMR20:5163001	20	5163001	5167000	4000	1	5.20E-07	-0.39	53	1.32	Prrc2a;E230034O05Rik;Aif1;Ncr3;Lst1	Metabolism;Signaling
DMR20:5190001	20	5190001	5194000	4000	1	1.60E-07	-0.53	100	2.5	Ltb;Tnf;LOC103690381;LOC103689996	
DMR20:5329001	20	5329001	5330000	1000	1	2.20E-09	0.55	21	2.1	LOC100911886;Sacm2l-ps5;Sacm2l-ps7;Cdk2ap1-ps6	
DMR20:5372001	20	5372001	5374000	2000	1	1.60E-07	0.6	14	0.7	Sacm2l-ps1;LOC224733l-2;RT1-A2;Cdk2ap1-ps1	Immune
DMR20:5562001	20	5562001	5564000	2000	1	1.50E-15	0.85	42	2.1	Syngap1;Zbtb9	Signaling;Cytoskeleton
DMR20:5595001	20	5595001	5600000	5000	1	1.90E-07	0.41	69	1.38	Bak1	
DMR20:5602001	20	5602001	5604000	2000	1	5.50E-10	0.75	42	2.1	Bak1	
DMR20:5609001	20	5609001	5614000	5000	1	3.10E-21	1.05	110	2.2	Bak1;Ggnbp1	Protease
DMR20:5679001	20	5679001	5682000	3000	1	2.80E-07	0.4	87	2.9	Itpr3	Ion Channel
DMR20:5703001	20	5703001	5704000	1000	1	6.30E-08	0.63	39	3.9	Itpr3;Uqcc2	Ion Channel;Transcription
DMR20:5731001	20	5731001	5737000	6000	2	7.90E-11	0.65	140	2.33	Uqcc2;Ip6k3	Transcription;Signaling
DMR20:5790001	20	5790001	5791000	1000	1	1.90E-10	0.67	22	2.2	Lemd2;Clpsl2	Signaling

DMR20:5794001	20	5794001	5796000	2000	1	5.70E-07	0.55	66	3.3	Lemd2;Clpsl2;Clps	Signaling
DMR20:5797001	20	5797001	5798000	1000	1	9.80E-12	0.53	12	1.2	Clpsl2;Clps	Signaling
DMR20:5809001	20	5809001	5810000	1000	1	2.10E-07	0.48	8	0.8	Clps;Lhfpl5	Signaling
DMR20:5817001	20	5817001	5820000	3000	1	2.10E-07	0.42	64	2.13	Lhfpl5;Srpk1	Signaling
DMR20:5884001	20	5884001	5886000	2000	1	1.10E-10	0.55	30	1.5	Slc26a8	Transport
DMR20:5957001	20	5957001	5961000	4000	2	8.70E-08	-0.41	72	1.8	Mapk14	Signaling
DMR20:5983001	20	5983001	5984000	1000	1	3.40E-07	-0.34	21	2.1	Mapk14	Signaling
DMR20:6013001	20	6013001	6016000	3000	1	3.60E-07	0.46	78	2.6	Mapk13	Signaling
DMR20:6027001	20	6027001	6029000	2000	1	9.50E-17	0.69	50	2.5	Mapk13	Signaling
DMR20:6043001	20	6043001	6045000	2000	1	8.70E-12	0.6	33	1.65	Brpf3	Transcription
DMR20:6114001	20	6114001	6116000	2000	1	2.60E-17	1.28	53	2.65	Pnpla1	Metabolism
DMR20:6130001	20	6130001	6131000	1000	1	5.80E-12	0.87	18	1.8	Pnpla1	Metabolism
DMR20:6146001	20	6146001	6147000	1000	1	2.30E-07	0.51	15	1.5	Pnpla1;LOC689561	Metabolism
DMR20:6182001	20	6182001	6183000	1000	1	3.80E-10	0.58	23	2.3	Pxt1	
DMR20:6190001	20	6190001	6191000	1000	1	2.10E-07	0.54	24	2.4	Pxt1	
DMR20:6192001	20	6192001	6194000	2000	1	1.30E-07	0.57	38	1.9	Pxt1	
DMR20:6195001	20	6195001	6197000	2000	1	1.80E-08	0.64	37	1.85	Pxt1;Kctd20	
DMR20:6207001	20	6207001	6209000	2000	1	5.90E-07	-0.37	40	2	Pxt1;Kctd20	
DMR20:6244001	20	6244001	6246000	2000	1	8.90E-07	-0.37	40	2	Stk38	Signaling
DMR20:6264001	20	6264001	6266000	2000	1	4.80E-11	0.57	28	1.4	Stk38	Signaling
DMR20:6291001	20	6291001	6296000	5000	2	1.30E-09	-0.49	73	1.46	Srsf3	Translation
DMR20:6349001	20	6349001	6350000	1000	1	1.40E-08	0.49	9	0.9	Cdkn1a	Signaling
DMR20:6356001	20	6356001	6357000	1000	1	1.20E-07	0.53	44	4.4	Cdkn1a;Rab44	Signaling
DMR20:6389001	20	6389001	6394000	5000	1	4.60E-08	0.42	91	1.82	Rab44	
DMR20:6494001	20	6494001	6498000	4000	2	1.90E-10	-0.46	92	2.3	Cpne5;LOC102547155	
DMR20:6515001	20	6515001	6518000	3000	1	2.50E-08	-0.45	80	2.67	LOC102547155;Ppil1	Transcription
DMR20:6555001	20	6555001	6558000	3000	1	9.20E-08	0.5	91	3.03	Ppil1;LOC108348120;Mln	Transcription
DMR20:6682001	20	6682001	6685000	3000	1	7.00E-07	0.6	42	1.4	Ppil1	Transcription
DMR20:6728001	20	6728001	6732000	4000	1	2.30E-07	0.5	78	1.95	Ppil1	Transcription
DMR20:6734001	20	6734001	6739000	5000	2	1.20E-10	0.72	101	2.02	Ppil1;Grm4	Transcription;Signaling
DMR20:6741001	20	6741001	6742000	1000	1	2.30E-08	0.5	14	1.4	Ppil1;Grm4	Transcription;Signaling
DMR20:6750001	20	6750001	6756000	6000	1	6.20E-10	0.58	165	2.75	Ppil1;Grm4	Transcription;Signaling
DMR20:6873001	20	6873001	6876000	3000	1	3.70E-09	-0.57	49	1.63	Ppil1;RGD735065	Transcription
DMR20:6901001	20	6901001	6902000	1000	1	8.70E-11	0.77	16	1.6	RGD735065	
DMR20:6911001	20	6911001	6913000	2000	1	1.90E-07	-0.32	41	2.05	RGD735065	
DMR20:6958001	20	6958001	6961000	3000	1	5.10E-07	-0.31	105	3.5	Mtch1;LOC102552169	Transport
DMR20:6990001	20	6990001	6992000	2000	1	8.60E-07	0.34	34	1.7	Fgd2	Transcription
DMR20:7124001	20	7124001	7126000	2000	1	6.80E-07	0.43	33	1.65	Hmga1	Transcription
DMR20:7196001	20	7196001	7197000	1000	1	6.40E-08	0.46	24	2.4	Nudt3	Signaling
DMR20:7200001	20	7200001	7202000	2000	1	7.40E-11	-0.43	31	1.55	Nudt3	Signaling
DMR20:7238001	20	7238001	7240000	2000	2	5.80E-09	0.68	59	2.95	Pacsin1	Cytoskeleton
DMR20:7246001	20	7246001	7247000	1000	1	1.90E-07	0.64	28	2.8	Pacsin1	Cytoskeleton
DMR20:7250001	20	7250001	7252000	2000	1	6.00E-07	0.46	33	1.65	Pacsin1	Cytoskeleton
DMR20:7257001	20	7257001	7260000	3000	1	2.80E-07	0.55	51	1.7	Pacsin1	Cytoskeleton
DMR20:7434001	20	7434001	7435000	1000	1	7.40E-07	-0.47	28	2.8	Snrpc;Uhrf1bp1	
DMR20:7444001	20	7444001	7445000	1000	1	2.20E-07	-0.51	6	0.6	Uhrf1bp1	
DMR20:7500001	20	7500001	7503000	3000	1	3.60E-08	-0.54	58	1.93	Anks1a	Cytoskeleton
DMR20:7512001	20	7512001	7513000	1000	1	5.50E-07	0.59	17	1.7	Anks1a	Cytoskeleton
DMR20:7518001	20	7518001	7521000	3000	1	4.30E-07	-0.38	79	2.63	Anks1a	Cytoskeleton
DMR20:7583001	20	7583001	7585000	2000	1	2.70E-07	-0.35	62	3.1	Anks1a	Cytoskeleton
DMR20:7615001	20	7615001	7617000	2000	1	5.50E-07	0.48	58	2.9	Anks1a	Cytoskeleton
DMR20:7744001	20	7744001	7745000	1000	1	4.80E-09	0.39	17	1.7	Scube3;LOC108349040;Zfp523	Extracellular Matrix
DMR20:7760001	20	7760001	7761000	1000	1	5.00E-08	-0.36	13	1.3	LOC108349040;Zfp523	
DMR20:7792001	20	7792001	7803000	11000	1	5.30E-09	0.61	208	1.89	Zfp523;Def6	Cytoskeleton
DMR20:7876001	20	7876001	7878000	2000	1	5.90E-09	0.55	49	2.45	Ppard	Transcription
DMR20:7897001	20	7897001	7898000	1000	1	4.50E-11	0.59	29	2.9	Fance	
DMR20:7906001	20	7906001	7907000	1000	1	8.90E-07	0.42	20	2	Fance;Rpl10a;Tead3	Translation;Transcription
DMR20:8026001	20	8026001	8027000	1000	1	4.10E-10	-0.51	13	1.3	Fkbp5	Transcription
DMR20:8043001	20	8043001	8048000	5000	1	2.30E-15	-0.87	56	1.12	Fkbp5;LOC103694392	Transcription
DMR20:8093001	20	8093001	8096000	3000	1	2.10E-07	-0.37	35	1.17	Fkbp5	Transcription
DMR20:8106001	20	8106001	8107000	1000	1	3.60E-07	-0.43	16	1.6	Fkbp5;Armc12	Transcription

DMR20:8124001	20	8124001	8125000	1000	1	2.10E-09	-0.37	20	2	Armc12	
DMR20:8162001	20	8162001	8163000	1000	1	9.10E-08	-0.4	26	2.6	Pim1	Signaling
DMR20:8193001	20	8193001	8195000	2000	1	8.70E-12	0.72	62	3.1	LOC687478;LOC102547645;Tmem217	
DMR20:8236001	20	8236001	8238000	2000	1	5.70E-08	-0.49	58	2.9	Tbc1d22b	Signaling
DMR20:8259001	20	8259001	8265000	6000	1	6.40E-10	0.58	105	1.75	Tbc1d22b	Signaling
DMR20:8345001	20	8345001	8348000	3000	1	8.20E-08	-0.38	63	2.1	Cmtr1	
DMR20:8390001	20	8390001	8391000	1000	1	4.40E-07	0.49	19	1.9	Cmtr1;Ccdc167	
DMR20:8505001	20	8505001	8510000	5000	1	1.90E-07	0.46	72	1.44	LOC288777;Mdga1	
DMR20:8518001	20	8518001	8522000	4000	1	4.30E-07	0.44	81	2.02	Mdga1	
DMR20:8528001	20	8528001	8532000	4000	2	3.30E-11	0.64	105	2.62	Mdga1	
DMR20:8533001	20	8533001	8535000	2000	2	3.00E-13	0.84	56	2.8	Mdga1	
DMR20:8538001	20	8538001	8540000	2000	1	1.50E-13	0.84	38	1.9	Mdga1	
DMR20:8561001	20	8561001	8563000	2000	1	3.10E-09	0.65	39	1.95	Mdga1	
DMR20:8681001	20	8681001	8686000	5000	1	8.80E-07	0.58	93	1.86	Zfand3	
DMR20:8687001	20	8687001	8688000	1000	1	2.00E-10	0.76	29	2.9	Zfand3	
DMR20:8703001	20	8703001	8706000	3000	1	1.10E-07	-0.46	41	1.37	Zfand3	
DMR20:8717001	20	8717001	8718000	1000	1	2.90E-10	-0.56	14	1.4	Zfand3	
DMR20:8727001	20	8727001	8730000	3000	1	2.00E-07	-0.41	37	1.23	Zfand3	
DMR20:8752001	20	8752001	8755000	3000	1	4.80E-10	-0.43	47	1.57	Zfand3	
DMR20:8774001	20	8774001	8777000	3000	1	5.70E-13	-0.54	53	1.77	Zfand3	
DMR20:8782001	20	8782001	8784000	2000	1	3.70E-13	-0.57	32	1.6	Zfand3	
DMR20:8836001	20	8836001	8838000	2000	1	2.40E-07	-0.49	33	1.65	Zfand3	
DMR20:8853001	20	8853001	8859000	6000	1	2.80E-08	-0.41	77	1.28	Zfand3	
DMR20:8874001	20	8874001	8879000	5000	1	9.60E-09	-0.44	102	2.04	Zfand3	
DMR20:8900001	20	8900001	8901000	1000	1	1.40E-11	0.56	18	1.8	Zfand3;Btbd9	
DMR20:9004001	20	9004001	9006000	2000	1	1.30E-08	-0.41	28	1.4	Btbd9	
DMR20:9049001	20	9049001	9051000	2000	1	6.50E-07	-0.36	46	2.3	Btbd9	
DMR20:9101001	20	9101001	9106000	5000	1	1.90E-08	-0.38	91	1.82	Btbd9	
DMR20:9117001	20	9117001	9120000	3000	1	3.10E-08	-0.44	43	1.43	Btbd9	
DMR20:9138001	20	9138001	9139000	1000	1	7.00E-07	-0.33	23	2.3	Btbd9	
DMR20:9148001	20	9148001	9151000	3000	1	1.20E-08	-0.4	61	2.03	Btbd9	
DMR20:9159001	20	9159001	9160000	1000	1	7.30E-07	-0.39	19	1.9	Btbd9	
DMR20:9232001	20	9232001	9235000	3000	1	4.50E-07	-0.29	64	2.13	Btbd9	
DMR20:9283001	20	9283001	9284000	1000	1	5.50E-08	0.43	26	2.6	Glo1	Metabolism
DMR20:9285001	20	9285001	9290000	5000	1	6.00E-09	0.62	106	2.12	Glo1	Metabolism
DMR20:9303001	20	9303001	9304000	1000	1	3.80E-07	-0.43	20	2	Dnah8	Cytoskeleton
DMR20:9325001	20	9325001	9329000	4000	1	1.30E-08	0.76	73	1.82	Dnah8	Cytoskeleton
DMR20:9350001	20	9350001	9351000	1000	1	5.40E-07	-0.56	15	1.5	Dnah8	Cytoskeleton
DMR20:9354001	20	9354001	9356000	2000	2	2.60E-12	0.99	50	2.5	Dnah8	Cytoskeleton
DMR20:9366001	20	9366001	9368000	2000	1	1.10E-08	-0.32	40	2	Dnah8	Cytoskeleton
DMR20:9385001	20	9385001	9386000	1000	1	4.70E-09	0.55	20	2	Dnah8;LOC103694393	Cytoskeleton
DMR20:9392001	20	9392001	9394000	2000	1	5.70E-12	0.91	68	3.4	Dnah8;LOC103694393	Cytoskeleton
DMR20:9400001	20	9400001	9407000	7000	2	4.90E-12	0.76	136	1.94	Dnah8	Cytoskeleton
DMR20:9416001	20	9416001	9417000	1000	1	5.90E-09	0.5	32	3.2	Dnah8	Cytoskeleton
DMR20:9418001	20	9418001	9423000	5000	2	7.30E-16	0.86	109	2.18	Dnah8	Cytoskeleton
DMR20:9426001	20	9426001	9429000	3000	2	4.50E-21	1.03	85	2.83	Dnah8	Cytoskeleton
DMR20:9431001	20	9431001	9433000	2000	2	9.90E-16	1.15	76	3.8	Dnah8	Cytoskeleton
DMR20:9460001	20	9460001	9461000	1000	1	2.20E-11	0.91	19	1.9	Dnah8;LOC100909773	Cytoskeleton
DMR20:9468001	20	9468001	9472000	4000	1	1.10E-07	0.47	79	1.98	Dnah8;LOC100909773	Cytoskeleton
DMR20:9477001	20	9477001	9480000	3000	1	2.80E-09	-0.36	69	2.3	Dnah8	Cytoskeleton
DMR20:9483001	20	9483001	9487000	4000	1	1.00E-18	1.12	76	1.9	Dnah8	Cytoskeleton
DMR20:9511001	20	9511001	9512000	1000	1	9.10E-07	0.41	18	1.8	Dnah8	Cytoskeleton
DMR20:9525001	20	9525001	9534000	9000	1	8.40E-11	-0.47	175	1.94	Dnah8	Cytoskeleton
DMR20:9561001	20	9561001	9564000	3000	1	3.90E-07	0.31	40	1.33	Dnah8	Cytoskeleton
DMR20:9567001	20	9567001	9572000	5000	2	4.10E-17	1	131	2.62	Dnah8	Cytoskeleton
DMR20:9576001	20	9576001	9577000	1000	1	6.80E-10	-0.73	4	0.4	Glp1r	Receptor
DMR20:9627001	20	9627001	9628000	1000	1	2.80E-07	0.52	20	2	Glp1r	Receptor
DMR20:9636001	20	9636001	9637000	1000	1	3.30E-08	0.51	22	2.2	Glp1r;Umodl1	Receptor
DMR20:9642001	20	9642001	9644000	2000	2	3.70E-12	0.98	56	2.8	Umodl1	Receptor
DMR20:9649001	20	9649001	9653000	4000	1	5.70E-07	0.41	50	1.25	Umodl1	Receptor
DMR20:9661001	20	9661001	9666000	5000	2	1.10E-10	-0.72	54	1.08	Umodl1	Receptor

DMR20:9667001	20	9667001	9668000	1000	1	1.30E-11	0.34	19	1.9	Umodl1	Receptor
DMR20:9732001	20	9732001	9734000	2000	1	6.10E-07	0.4	26	1.3	Abcg1	Transport
DMR20:9842001	20	9842001	9843000	1000	1	1.90E-07	0.69	26	2.6	Abcg1;Tff3	Transport;Signaling
DMR20:9869001	20	9869001	9874000	5000	2	2.90E-19	0.98	123	2.46	Tff2	Signaling
DMR20:9877001	20	9877001	9878000	1000	1	5.50E-09	0.52	13	1.3	Tff2	Signaling
DMR20:9881001	20	9881001	9885000	4000	1	1.90E-07	-0.33	45	1.12	Tff2;Tff1	Signaling
DMR20:9900001	20	9900001	9902000	2000	1	3.20E-09	0.48	42	2.1	Tff1;Tmprss3	Signaling;Protease
DMR20:9962001	20	9962001	9967000	5000	2	6.00E-09	-0.6	65	1.3	Ubash3a	
DMR20:9991001	20	9991001	9992000	1000	1	1.10E-14	0.65	19	1.9	Ubash3a;Rsph1	
DMR20:10037001	20	10037001	10041000	4000	1	1.00E-09	-0.49	80	2	Slc37a1	Transport
DMR20:10045001	20	10045001	10046000	1000	1	4.90E-07	0.52	8	0.8	Slc37a1	Transport
DMR20:10054001	20	10054001	10056000	2000	1	1.30E-09	-0.55	57	2.85	Slc37a1	Transport
DMR20:10125001	20	10125001	10130000	5000	1	2.70E-07	0.43	82	1.64	Pde9a	Signaling
DMR20:10192001	20	10192001	10195000	3000	1	2.10E-08	0.48	54	1.8	Pde9a	Signaling
DMR20:10196001	20	10196001	10198000	2000	1	3.30E-08	0.51	42	2.1	Pde9a	Signaling
DMR20:10247001	20	10247001	10249000	2000	1	1.10E-09	0.57	34	1.7	LOC103694394;Wdr4	Translation
DMR20:10278001	20	10278001	10280000	2000	1	8.00E-11	-0.54	12	0.6	Ndufv3	Metabolism
DMR20:10283001	20	10283001	10287000	4000	1	8.70E-12	0.51	64	1.6	Ndufv3	Metabolism
DMR20:10335001	20	10335001	10336000	1000	1	7.50E-08	0.56	21	2.1	Pknox1	Development
DMR20:10374001	20	10374001	10375000	1000	1	1.70E-12	0.92	27	2.7	Cbs	Metabolism
DMR20:10378001	20	10378001	10380000	2000	2	4.40E-10	-0.47	24	1.2	Cbs	Metabolism
DMR20:10389001	20	10389001	10390000	1000	1	1.30E-07	-0.53	17	1.7	Cbs;U2af1	Metabolism;Translatio n
DMR20:10439001	20	10439001	10440000	1000	1	1.30E-10	0.57	18	1.8	Cryaa	
DMR20:10659001	20	10659001	10662000	3000	1	1.40E-08	0.43	57	1.9	Sik1	Signaling
DMR20:10667001	20	10667001	10673000	6000	2	8.60E-10	0.72	173	2.88	Sik1;LOC108349047	Signaling
DMR20:10938001	20	10938001	10940000	2000	1	1.30E-12	-0.56	39	1.95	Pdxk	Signaling
DMR20:10965001	20	10965001	10966000	1000	1	1.50E-13	0.77	35	3.5	Cstb	Protease; Proteolysis
DMR20:11091001	20	11091001	11093000	2000	1	4.20E-08	-0.33	29	1.45	Agpat3	Metabolism
DMR20:11132001	20	11132001	11133000	1000	1	1.60E-10	-0.48	38	3.8	Agpat3	Metabolism
DMR20:11136001	20	11136001	11141000	5000	1	3.20E-08	-0.36	121	2.42	Agpat3	Metabolism
DMR20:11321001	20	11321001	11324000	3000	3	7.90E-14	0.97	69	2.3	Icoslg	Immune
DMR20:11330001	20	11330001	11331000	1000	1	9.40E-07	0.38	20	2	Icoslg	Immune
DMR20:11332001	20	11332001	11338000	6000	1	1.20E-11	0.8	111	1.85	Icoslg;Dnmt3l	Immune;Epigenetic
DMR20:11359001	20	11359001	11362000	3000	2	1.60E-12	0.81	56	1.87	Dnmt3l;Aire	Epigenetic
DMR20:11412001	20	11412001	11413000	1000	1	2.80E-07	0.61	24	2.4	Pfkl;RGD1309594	Metabolism
DMR20:11468001	20	11468001	11471000	3000	1	8.30E-07	-0.42	70	2.33	Trpm2	Transport
DMR20:11472001	20	11472001	11476000	4000	2	2.80E-07	0.55	75	1.88	Trpm2	Transport
DMR20:11477001	20	11477001	11481000	4000	1	3.10E-10	0.67	79	1.98	Trpm2;Lrrc3	Transport
DMR20:11482001	20	11482001	11484000	2000	1	8.20E-07	0.52	25	1.25	Trpm2;Lrrc3	Transport
DMR20:11494001	20	11494001	11499000	5000	1	3.90E-07	-0.4	80	1.6	Lrrc3;Tspear	Signaling
DMR20:11561001	20	11561001	11563000	2000	1	2.10E-11	0.78	31	1.55	RGD1561557;LOC690415;LOC6 90425	
DMR20:11689001	20	11689001	11695000	6000	2	1.50E-08	0.49	95	1.58	Ube2g2;LOC108349048	
DMR20:11759001	20	11759001	11763000	4000	1	1.20E-08	-0.45	93	2.33	Pttg1ip	
DMR20:11802001	20	11802001	11806000	4000	1	4.20E-08	0.5	62	1.55	Itgb2	Extracellular Matrix
DMR20:11880001	20	11880001	11886000	6000	1	1.40E-10	0.33	137	2.28	Fam207a	
DMR20:11902001	20	11902001	11905000	3000	1	1.30E-07	0.67	51	1.7	Fam207a	
DMR20:11996001	20	11996001	12002000	6000	1	1.00E-07	0.55	78	1.3	Adarb1	Metabolism
DMR20:12024001	20	12024001	12026000	2000	1	5.60E-10	-0.57	35	1.75	Adarb1	Metabolism
DMR20:12081001	20	12081001	12083000	2000	1	2.20E-07	0.42	22	1.1	Adarb1	Metabolism
DMR20:12092001	20	12092001	12093000	1000	1	7.90E-09	-0.51	11	1.1	Adarb1	Metabolism
DMR20:12097001	20	12097001	12100000	3000	1	6.00E-07	0.42	68	2.27	Adarb1	Metabolism
DMR20:12276001	20	12276001	12282000	6000	3	2.40E-16	-0.6	110	1.83	Col18a1	Extracellular Matrix
DMR20:12320001	20	12320001	12323000	3000	1	1.50E-09	0.45	42	1.4	Col18a1	Extracellular Matrix
DMR20:12510001	20	12510001	12514000	4000	1	8.10E-08	-0.32	65	1.62	Pcbp3	Metabolism
DMR20:12530001	20	12530001	12531000	1000	1	5.50E-08	-0.39	14	1.4	Pcbp3	Metabolism
DMR20:12555001	20	12555001	12558000	3000	1	5.00E-08	0.48	28	0.93	Pcbp3	Metabolism
DMR20:12602001	20	12602001	12604000	2000	1	4.30E-07	0.4	30	1.5	Pcbp3	Metabolism
DMR20:12658001	20	12658001	12660000	2000	1	1.80E-08	0.56	37	1.85	Col6a1	Extracellular Matrix
DMR20:12672001	20	12672001	12673000	1000	1	3.00E-12	0.67	12	1.2	Col6a1	Extracellular Matrix
DMR20:12674001	20	12674001	12678000	4000	1	9.60E-08	0.69	91	2.28	Col6a1	Extracellular Matrix

DMR20:12776001	20	12776001	12778000	2000	1	1.40E-10	-0.4	26	1.3	Col6a2	Extracellular Matrix
DMR20:12786001	20	12786001	12787000	1000	1	3.20E-07	0.55	14	1.4	Col6a2	Extracellular Matrix
DMR20:12795001	20	12795001	12798000	3000	1	4.30E-10	-0.54	38	1.27	Col6a2;Ftcd	Extracellular Matrix
DMR20:12807001	20	12807001	12808000	1000	1	1.90E-07	0.42	14	1.4	Col6a2;Ftcd	Extracellular Matrix
DMR20:12816001	20	12816001	12818000	2000	1	5.00E-19	0.98	45	2.25	Ftcd;Spatc1l	
DMR20:12846001	20	12846001	12853000	7000	2	1.30E-07	0.47	115	1.64	Lss	Metabolism
DMR20:12860001	20	12860001	12863000	3000	1	3.20E-09	0.45	48	1.6	Lss	Metabolism
DMR20:12893001	20	12893001	12894000	1000	1	1.40E-09	-0.52	15	1.5	Mcm3ap	Cytoskeleton
DMR20:12934001	20	12934001	12935000	1000	1	4.50E-07	0.53	16	1.6	Ybey;RGD1564149;Pcnt	
DMR20:13022001	20	13022001	13023000	1000	1	1.50E-08	-0.34	21	2.1	Pcnt	
DMR20:13041001	20	13041001	13042000	1000	1	1.00E-09	-0.41	27	2.7	Pcnt;Dip2a	
DMR20:13051001	20	13051001	13066000	15000	1	2.90E-07	0.29	266	1.77	Dip2a	
DMR20:13111001	20	13111001	13114000	3000	3	2.60E-13	0.6	70	2.33	Dip2a	
DMR20:13133001	20	13133001	13136000	3000	1	3.20E-09	0.74	73	2.43	Dip2a;S100b	Signaling
DMR20:13177001	20	13177001	13179000	2000	1	6.20E-07	-0.38	36	1.8	Prmt2;LOC108348157;LOC103690153	Golgi
DMR20:13198001	20	13198001	13201000	3000	1	2.90E-07	-0.39	77	2.57	Prmt2	Golgi
DMR20:13205001	20	13205001	13208000	3000	1	1.70E-23	1.13	62	2.07	Prmt2;Mir678;LOC103694402	Golgi
DMR20:13215001	20	13215001	13216000	1000	1	1.60E-10	0.55	26	2.6	Prmt2;Mir678;LOC103694402	Golgi
DMR20:13415001	20	13415001	13418000	3000	1	5.60E-07	-0.48	53	1.77	Slc5a4	Transport
DMR20:13446001	20	13446001	13449000	3000	2	3.10E-10	-0.73	31	1.03	Slc5a4	Transport
DMR20:13452001	20	13452001	13459000	7000	1	2.20E-07	-0.38	129	1.84	Slc5a4	Transport
DMR20:13522001	20	13522001	13529000	7000	2	1.70E-22	1.18	95	1.36	Slc5a4b	Transport
DMR20:13562001	20	13562001	13564000	2000	1	2.30E-07	0.41	26	1.3	Zfp280b	Transcription
DMR20:13586001	20	13586001	13590000	4000	1	5.80E-11	0.82	82	2.05	Zfp280b;LOC103690331	Transcription
DMR20:13627001	20	13627001	13630000	3000	1	4.70E-07	-0.34	34	1.13	RGD1563815	
DMR20:13640001	20	13640001	13643000	3000	1	8.10E-07	-0.44	53	1.77	RGD1563815	
DMR20:13649001	20	13649001	13653000	4000	1	1.40E-08	0.83	91	2.28	RGD1563815;LOC102552984;Vpreb3;RGD1564162	Immune
DMR20:13712001	20	13712001	13713000	1000	1	6.60E-09	0.7	24	2.4	Smarcb1;Derl3;Mif	Transcription;Transport;Metabolism
DMR20:13716001	20	13716001	13717000	1000	1	8.10E-09	-0.42	22	2.2	Derl3;Mif	Transport;Metabolism
DMR20:13757001	20	13757001	13759000	2000	1	6.60E-11	0.56	48	2.4	Gstt2;LOC108349022	
DMR20:13781001	20	13781001	13784000	3000	2	5.80E-11	0.85	66	2.2	Gstt4	
DMR20:13820001	20	13820001	13822000	2000	1	4.10E-08	0.44	46	2.3	Gstt1;Gstt3;Ddt	Metabolism
DMR20:13880001	20	13880001	13883000	3000	1	6.50E-07	0.4	58	1.93	Cabin1	Signaling
DMR20:13895001	20	13895001	13896000	1000	1	1.70E-08	0.57	9	0.9	Cabin1	Signaling
DMR20:13950001	20	13950001	13952000	2000	1	3.30E-07	0.56	46	2.3	Cabin1;LOC102556116	Signaling
DMR20:13958001	20	13958001	13963000	5000	1	3.20E-07	0.58	90	1.8	Cabin1;Susd2	Signaling
DMR20:14076001	20	14076001	14077000	1000	1	8.60E-09	0.59	18	1.8	Snrpd3	Translation
DMR20:14182001	20	14182001	14188000	6000	1	1.70E-14	0.72	84	1.4	LOC102556333;Upb1	Metabolism
DMR20:14191001	20	14191001	14192000	1000	1	9.20E-07	0.31	15	1.5	Upb1	Metabolism
DMR20:14253001	20	14253001	14256000	3000	1	4.40E-08	0.77	55	1.83	Adora2a	Signaling
DMR20:14257001	20	14257001	14258000	1000	1	5.00E-20	0.74	28	2.8	Adora2a	Signaling
DMR20:14403001	20	14403001	14405000	2000	1	3.00E-07	-0.54	29	1.45	Specc1l	
DMR20:14419001	20	14419001	14420000	1000	1	1.00E-13	0.88	28	2.8	Bcr	Signaling
DMR20:14432001	20	14432001	14433000	1000	1	4.20E-07	0.49	30	3	Bcr	Signaling
DMR20:14459001	20	14459001	14461000	2000	2	3.70E-11	0.65	39	1.95	Bcr	Signaling
DMR20:14501001	20	14501001	14502000	1000	1	2.80E-08	-0.47	13	1.3	Bcr	Signaling
DMR20:14556001	20	14556001	14558000	2000	1	1.60E-14	0.61	36	1.8	Bcr;Rab36	Signaling
DMR20:14574001	20	14574001	14577000	3000	1	6.00E-09	0.58	49	1.63	Rab36;Rtdr1	
DMR20:14583001	20	14583001	14584000	1000	1	3.40E-13	0.72	28	2.8	Rab36;Rtdr1;Gnaz	Signaling
DMR20:14590001	20	14590001	14592000	2000	1	3.30E-23	1.08	48	2.4	Rtdr1;Gnaz	Signaling
DMR20:14621001	20	14621001	14623000	2000	1	2.50E-07	0.55	46	2.3	Rtdr1;Gnaz	Signaling
DMR20:14634001	20	14634001	14637000	3000	1	5.50E-07	-0.3	88	2.93	Rtdr1;Gnaz	Signaling
DMR20:14640001	20	14640001	14642000	2000	1	1.40E-08	0.43	29	1.45	Rtdr1;Gnaz	Signaling
DMR20:14700001	20	14700001	14701000	1000	1	5.90E-16	0.66	12	1.2	RGD1561987	
DMR20:14820001	20	14820001	14826000	6000	2	9.00E-09	-0.38	56	0.93	Polr3d-ps1	
DMR20:14952001	20	14952001	14953000	1000	1	5.70E-16	0.87	40	4	Pcdh15	Cytoskeleton
DMR20:14969001	20	14969001	14970000	1000	1	1.20E-07	-0.37	25	2.5	Pcdh15;LOC103694424	Cytoskeleton
DMR20:14971001	20	14971001	14975000	4000	2	3.80E-13	0.83	58	1.45	Pcdh15;LOC103694424	Cytoskeleton
DMR20:14984001	20	14984001	14991000	7000	1	1.60E-13	0.35	81	1.16	Pcdh15	Cytoskeleton

DMR20:15016001	20	15016001	15019000	3000	1	1.10E-07	0.6	50	1.67	Pcdh15	Cytoskeleton
DMR20:15107001	20	15107001	15108000	1000	1	2.00E-08	-0.35	13	1.3	Pcdh15	Cytoskeleton
DMR20:15212001	20	15212001	15213000	1000	1	1.10E-09	0.75	23	2.3	Pcdh15	Cytoskeleton
DMR20:15243001	20	15243001	15246000	3000	1	8.50E-08	0.41	54	1.8	Pcdh15	Cytoskeleton
DMR20:15760001	20	15760001	15761000	1000	1	2.00E-08	0.6	22	2.2	NEWGENE_1590969	
DMR20:15779001	20	15779001	15782000	3000	1	4.90E-09	-0.62	39	1.3	NEWGENE_1590969	
DMR20:15797001	20	15797001	15800000	3000	1	8.60E-13	-0.46	28	0.93	NEWGENE_1590969	
DMR20:15847001	20	15847001	15849000	2000	1	1.50E-07	-0.6	14	0.7	NEWGENE_1590969	
DMR20:15866001	20	15866001	15873000	7000	1	3.60E-07	-0.29	77	1.1	NEWGENE_1590969	
DMR20:15919001	20	15919001	15921000	2000	1	2.40E-07	-0.37	17	0.85	NEWGENE_1590969	
DMR20:15957001	20	15957001	15958000	1000	1	5.00E-18	0.95	24	2.4	NEWGENE_1590969	
DMR20:16136001	20	16136001	16138000	2000	1	2.10E-08	-0.38	16	0.8	NEWGENE_1590969	
DMR20:16172001	20	16172001	16176000	4000	1	1.10E-10	0.72	55	1.38	NEWGENE_1590969	
DMR20:16179001	20	16179001	16180000	1000	1	1.10E-07	-0.73	0	0	NEWGENE_1590969	
DMR20:16302001	20	16302001	16304000	2000	1	2.80E-07	0.65	37	1.85	NEWGENE_1590969	
DMR20:16306001	20	16306001	16308000	2000	1	1.20E-10	0.69	49	2.45	NEWGENE_1590969	
DMR20:16356001	20	16356001	16358000	2000	1	1.10E-07	0.61	23	1.15	NEWGENE_1590969	
DMR20:16379001	20	16379001	16380000	1000	1	2.60E-10	0.46	13	1.3	NEWGENE_1590969	
DMR20:16435001	20	16435001	16436000	1000	1	1.30E-07	0.35	7	0.7	NEWGENE_1590969	
DMR20:16588001	20	16588001	16594000	6000	1	4.90E-09	0.56	77	1.28	NEWGENE_1590969	
DMR20:16649001	20	16649001	16650000	1000	1	1.00E-14	1.06	21	2.1	NEWGENE_1590969;LOC108349009	
DMR20:16672001	20	16672001	16676000	4000	2	3.20E-11	-0.68	25	0.62	NEWGENE_1590969	
DMR20:16715001	20	16715001	16716000	1000	1	4.40E-07	0.54	14	1.4	NEWGENE_1590969	
DMR20:17083001	20	17083001	17084000	1000	1	6.10E-07	-0.34	14	1.4	Zwint;LOC108349051	
DMR20:17748001	20	17748001	17749000	1000	1	1.30E-07	-0.46	23	2.3	RGD1559903	
DMR20:18465001	20	18465001	18469000	4000	1	1.00E-13	-0.57	83	2.08	Ipmk	Signaling
DMR20:18470001	20	18470001	18473000	3000	2	1.70E-08	-0.46	78	2.6	Ipmk	Signaling
DMR20:18481001	20	18481001	18482000	1000	1	9.90E-12	-0.6	31	3.1	Ipmk	Signaling
DMR20:18483001	20	18483001	18487000	4000	1	1.10E-07	-0.38	95	2.38	Ipmk;LOC103694404;Cisd1	Signaling
DMR20:18504001	20	18504001	18506000	2000	2	1.60E-07	-0.45	41	2.05	Cisd1;LOC108349023	
DMR20:18535001	20	18535001	18538000	3000	1	3.70E-10	0.58	58	1.93	Ube2d1	Proteolysis
DMR20:18548001	20	18548001	18551000	3000	1	6.30E-10	0.76	64	2.13	Ube2d1	Proteolysis
DMR20:18574001	20	18574001	18576000	2000	1	4.90E-08	0.43	49	2.45	Ube2d1	Proteolysis
DMR20:18584001	20	18584001	18586000	2000	1	2.70E-09	0.39	34	1.7	Ube2d1;Tfam	Proteolysis
DMR20:18615001	20	18615001	18618000	3000	1	2.30E-09	0.6	51	1.7	Tfam	
DMR20:18835001	20	18835001	18837000	2000	1	1.00E-10	-0.45	63	3.15	Bicc1	Metabolism
DMR20:18904001	20	18904001	18905000	1000	1	9.50E-23	1	38	3.8	Bicc1	Metabolism
DMR20:18937001	20	18937001	18938000	1000	1	1.70E-07	0.61	20	2	Bicc1	Metabolism
DMR20:19369001	20	19369001	19373000	4000	2	1.40E-09	0.59	81	2.02	Phyhipl;Fam13c	
DMR20:19380001	20	19380001	19382000	2000	1	4.10E-08	-0.47	37	1.85	Fam13c	
DMR20:19406001	20	19406001	19407000	1000	1	1.30E-16	1.13	26	2.6	Fam13c	
DMR20:19420001	20	19420001	19421000	1000	1	3.40E-07	0.52	10	1	Fam13c	
DMR20:19434001	20	19434001	19435000	1000	1	3.60E-08	-0.36	13	1.3	Fam13c	
DMR20:19444001	20	19444001	19446000	2000	1	5.80E-12	0.75	39	1.95	Fam13c	
DMR20:19467001	20	19467001	19470000	3000	1	2.60E-07	0.66	86	2.87	Fam13c	
DMR20:19624001	20	19624001	19627000	3000	1	2.30E-08	0.65	51	1.7	LOC102552716;Slc16a9	
DMR20:19656001	20	19656001	19659000	3000	1	4.60E-07	0.42	53	1.77	Slc16a9	
DMR20:19665001	20	19665001	19666000	1000	1	5.90E-24	1.26	27	2.7	Slc16a9	
DMR20:19729001	20	19729001	19735000	6000	3	1.60E-08	-0.51	192	3.2	Mrln;Ccdc6	
DMR20:19814001	20	19814001	19815000	1000	1	5.40E-07	-0.33	29	2.9	Ccdc6	
DMR20:19821001	20	19821001	19822000	1000	1	1.40E-07	-0.38	28	2.8	Ccdc6	
DMR20:19936001	20	19936001	19939000	3000	1	2.70E-07	-0.41	33	1.1	Ank3	
DMR20:19948001	20	19948001	19951000	3000	1	1.30E-07	-0.43	65	2.17	Ank3	
DMR20:19954001	20	19954001	19955000	1000	1	5.80E-07	0.44	25	2.5	Ank3	
DMR20:19960001	20	19960001	19962000	2000	1	9.20E-08	-0.36	42	2.1	Ank3	
DMR20:19975001	20	19975001	19976000	1000	1	6.20E-07	0.61	26	2.6	Ank3	
DMR20:20031001	20	20031001	20037000	6000	2	2.00E-07	0.49	124	2.07	Ank3	
DMR20:20038001	20	20038001	20039000	1000	1	1.30E-10	-0.52	19	1.9	Ank3	
DMR20:20109001	20	20109001	20113000	4000	1	4.00E-10	0.56	72	1.8	Ank3	
DMR20:20117001	20	20117001	20122000	5000	1	2.20E-09	-0.52	79	1.58	Ank3	
DMR20:20192001	20	20192001	20193000	1000	1	1.80E-09	-0.4	21	2.1	Ank3	

DMR20:20205001	20	20205001	20207000	2000	1	2.10E-07	0.36	22	1.1	Ank3;LOC102554153	
DMR20:20211001	20	20211001	20212000	1000	1	6.20E-07	-0.39	22	2.2	Ank3;LOC102554153	
DMR20:20236001	20	20236001	20241000	5000	1	3.70E-07	-0.32	120	2.4	Ank3;LOC108353248	
DMR20:20260001	20	20260001	20261000	1000	1	4.60E-08	-0.39	17	1.7	Ank3	
DMR20:20375001	20	20375001	20384000	9000	1	6.80E-09	-0.46	181	2.01	Ank3;LOC102554005;LOC102553944	
DMR20:20408001	20	20408001	20411000	3000	1	5.80E-08	-0.4	69	2.3	Ank3	
DMR20:20435001	20	20435001	20444000	9000	2	2.40E-07	-0.36	290	3.22	Ank3	
DMR20:20459001	20	20459001	20460000	1000	1	1.20E-07	-0.25	6	0.6	Ank3	
DMR20:20540001	20	20540001	20543000	3000	1	7.60E-07	-0.43	59	1.97	Rpl29-ps2	
DMR20:20553001	20	20553001	20555000	2000	2	2.00E-10	0.56	66	3.3	Rpl29-ps2	
DMR20:20571001	20	20571001	20572000	1000	1	6.70E-08	0.68	18	1.8	Cdk1	Signaling
DMR20:20601001	20	20601001	20602000	1000	1	1.50E-14	1.17	16	1.6	Cdk1	Signaling
DMR20:20658001	20	20658001	20659000	1000	1	4.90E-10	0.9	49	4.9	Rhobtb1	Signaling
DMR20:20665001	20	20665001	20666000	1000	1	4.30E-08	-0.46	18	1.8	Rhobtb1	Signaling
DMR20:20677001	20	20677001	20680000	3000	1	4.20E-07	0.49	68	2.27	Rhobtb1	Signaling
DMR20:20681001	20	20681001	20682000	1000	1	5.60E-08	-0.3	21	2.1	Rhobtb1	Signaling
DMR20:21246001	20	21246001	21247000	1000	1	4.30E-08	-0.45	9	0.9	LOC102546397;RGD1564320	
DMR20:21248001	20	21248001	21250000	2000	1	3.90E-07	0.46	24	1.2	LOC102546397;RGD1564320	
DMR20:21266001	20	21266001	21267000	1000	1	1.30E-11	0.9	23	2.3	RGD1564320	
DMR20:21273001	20	21273001	21275000	2000	1	2.90E-10	-0.46	36	1.8	RGD1564320	
DMR20:21331001	20	21331001	21335000	4000	1	6.60E-08	-0.32	69	1.73	RGD1306739	
DMR20:21367001	20	21367001	21375000	8000	1	6.40E-08	0.49	125	1.56	RGD1306739	
DMR20:21420001	20	21420001	21421000	1000	1	1.60E-07	-0.38	18	1.8	RGD1306739	
DMR20:21643001	20	21643001	21644000	1000	1	5.70E-08	0.41	20	2	Tmem26	
DMR20:21687001	20	21687001	21688000	1000	1	8.30E-07	0.4	17	1.7	Tmem26	
DMR20:21727001	20	21727001	21729000	2000	2	5.30E-09	-0.56	48	2.4	Arid5b	
DMR20:21761001	20	21761001	21763000	2000	1	6.70E-09	-0.51	25	1.25	Arid5b	
DMR20:21764001	20	21764001	21765000	1000	1	1.40E-07	0.6	28	2.8	Arid5b	
DMR20:21770001	20	21770001	21771000	1000	1	2.10E-08	0.62	27	2.7	Arid5b	
DMR20:21809001	20	21809001	21810000	1000	1	4.70E-07	-0.45	14	1.4	Arid5b	
DMR20:21825001	20	21825001	21828000	3000	3	5.40E-09	-0.5	70	2.33	Arid5b	
DMR20:21829001	20	21829001	21831000	2000	1	2.40E-08	-0.44	52	2.6	Arid5b	
DMR20:21854001	20	21854001	21855000	1000	1	6.50E-09	0.6	26	2.6	Arid5b;LOC103694408	
DMR20:21870001	20	21870001	21874000	4000	2	6.50E-10	-0.42	68	1.7	Arid5b;LOC103694408	
DMR20:21879001	20	21879001	21880000	1000	1	1.10E-12	1.05	36	3.6	Arid5b	
DMR20:21886001	20	21886001	21889000	3000	1	8.00E-07	-0.37	77	2.57	Arid5b;LOC108349057	
DMR20:21890001	20	21890001	21896000	6000	1	6.40E-08	0.36	64	1.07	Arid5b;LOC108349057;LOC102550601	
DMR20:21910001	20	21910001	21915000	5000	1	8.30E-18	0.81	92	1.84	LOC108349057;LOC102550601;Rtkn2	Cytoskeleton
DMR20:21950001	20	21950001	21951000	1000	1	2.20E-08	0.62	23	2.3	Rtkn2	Cytoskeleton
DMR20:21960001	20	21960001	21962000	2000	1	8.90E-14	0.87	52	2.6	Rtkn2	Cytoskeleton
DMR20:21994001	20	21994001	21995000	1000	1	1.20E-12	0.71	36	3.6	Rtkn2	Cytoskeleton
DMR20:22009001	20	22009001	22010000	1000	1	4.40E-17	0.97	25	2.5	Rtkn2	Cytoskeleton
DMR20:22077001	20	22077001	22079000	2000	1	4.70E-09	-0.58	17	0.85	Zfp365	
DMR20:22084001	20	22084001	22087000	3000	1	7.10E-10	0.62	76	2.53	Zfp365	
DMR20:22456001	20	22456001	22457000	1000	1	4.50E-11	0.94	38	3.8	Ado;Egr2	Transcription
DMR20:22804001	20	22804001	22806000	2000	1	2.60E-08	-0.47	27	1.35	Jmjd1c	Epigenetic
DMR20:22824001	20	22824001	22829000	5000	2	4.50E-11	-0.68	77	1.54	Jmjd1c	Epigenetic
DMR20:22837001	20	22837001	22839000	2000	1	6.30E-10	-0.68	17	0.85	Jmjd1c	Epigenetic
DMR20:22859001	20	22859001	22862000	3000	1	9.90E-07	-0.37	30	1	Jmjd1c	Epigenetic
DMR20:22899001	20	22899001	22906000	7000	1	3.10E-07	-0.44	154	2.2	Jmjd1c;Reep3	Epigenetic;Transport
DMR20:22969001	20	22969001	22971000	2000	1	5.10E-23	1.26	46	2.3	Reep3	Transport
DMR20:25351001	20	25351001	25352000	1000	1	1.50E-09	-0.5	7	0.7	Ctnna3	
DMR20:25387001	20	25387001	25390000	3000	1	3.50E-07	-0.44	33	1.1	Ctnna3	
DMR20:25393001	20	25393001	25395000	2000	2	5.00E-12	-0.59	5	0.25	Ctnna3	
DMR20:25661001	20	25661001	25662000	1000	1	1.10E-07	-0.59	6	0.6	Ctnna3	
DMR20:25813001	20	25813001	25814000	1000	1	1.60E-07	0.38	10	1	Ctnna3	
DMR20:25854001	20	25854001	25859000	5000	1	7.40E-09	-0.36	28	0.56	Ctnna3	
DMR20:25899001	20	25899001	25901000	2000	1	8.00E-09	-0.58	8	0.4	Ctnna3	
DMR20:25934001	20	25934001	25936000	2000	1	1.80E-07	-0.46	21	1.05	Ctnna3	

DMR20:25958001	20	25958001	25960000	2000	1	6.80E-10	0.77	25	1.25	Ctnna3	
DMR20:26016001	20	26016001	26017000	1000	1	9.50E-08	-0.4	4	0.4	Ctnna3;Lrrtm3	Receptor
DMR20:26075001	20	26075001	26076000	1000	1	1.90E-14	0.49	18	1.8	Ctnna3;Lrrtm3	Receptor
DMR20:26179001	20	26179001	26180000	1000	1	2.30E-10	-0.53	4	0.4	Ctnna3	
DMR20:26235001	20	26235001	26236000	1000	1	4.30E-07	0.44	9	0.9	Ctnna3	
DMR20:26452001	20	26452001	26457000	5000	1	9.90E-12	-0.43	39	0.78	Ctnna3	
DMR20:26460001	20	26460001	26464000	4000	1	3.70E-08	0.48	52	1.3	Ctnna3	
DMR20:26543001	20	26543001	26544000	1000	1	1.00E-11	0.73	14	1.4	Ctnna3;LOC108349010	
DMR20:26547001	20	26547001	26549000	2000	1	1.10E-08	-0.46	24	1.2	Ctnna3;LOC108349010	
DMR20:26588001	20	26588001	26589000	1000	1	6.10E-07	-0.6	3	0.3	Ctnna3	
DMR20:26919001	20	26919001	26922000	3000	1	4.40E-07	-0.71	25	0.83	Dnajc12	Transcription
DMR20:27158001	20	27158001	27161000	3000	2	4.60E-09	-0.51	23	0.77	Pbld2;Hnrnph3	Translation
DMR20:27615001	20	27615001	27617000	2000	1	7.40E-08	-0.71	13	0.65	Trappc3l	
DMR20:27830001	20	27830001	27831000	1000	1	3.00E-07	-0.44	5	0.5	Gcc2	Cytoskeleton
DMR20:27843001	20	27843001	27846000	3000	1	1.00E-12	-0.71	26	0.87	Gcc2	Cytoskeleton
DMR20:27928001	20	27928001	27929000	1000	1	6.70E-07	-0.42	12	1.2	Lims1	Cytoskeleton
DMR20:27936001	20	27936001	27938000	2000	1	3.40E-10	-0.72	9	0.45	Lims1	Cytoskeleton
DMR20:28169001	20	28169001	28171000	2000	1	6.60E-09	0.41	9	0.45	Edar	
DMR20:28176001	20	28176001	28179000	3000	1	4.70E-10	0.37	15	0.5	Edar	
DMR20:28244001	20	28244001	28249000	5000	2	2.90E-11	0.67	79	1.58	Edar	
DMR20:28401001	20	28401001	28403000	2000	1	9.60E-10	-0.43	77	3.85	Sh3rf3	
DMR20:28531001	20	28531001	28538000	7000	1	5.40E-07	0.36	74	1.06	Sh3rf3	
DMR20:28596001	20	28596001	28597000	1000	1	3.90E-07	-0.39	14	1.4	Sh3rf3	
DMR20:28704001	20	28704001	28706000	2000	1	2.60E-08	0.41	22	1.1	Sh3rf3	
DMR20:28809001	20	28809001	28812000	3000	2	9.30E-36	-1.24	27	0.9	Sept10;Sowahc	
DMR20:28974001	20	28974001	28975000	1000	1	9.50E-08	-0.41	18	1.8	P4ha1	Golgi
DMR20:28976001	20	28976001	28977000	1000	1	3.70E-07	-0.37	24	2.4	P4ha1	Golgi
DMR20:28985001	20	28985001	28986000	1000	1	6.90E-07	-0.32	14	1.4	Pla2g12b	Metabolism
DMR20:28990001	20	28990001	28991000	1000	1	1.70E-08	0.58	14	1.4	Pla2g12b	Metabolism
DMR20:28996001	20	28996001	28998000	2000	1	4.20E-07	-0.32	40	2	Pla2g12b	Metabolism
DMR20:29020001	20	29020001	29021000	1000	1	2.70E-16	1.09	33	3.3	Oit3	Receptor
DMR20:29066001	20	29066001	29067000	1000	1	1.50E-16	0.54	4	0.4	Mcu	
DMR20:29107001	20	29107001	29109000	2000	1	5.30E-07	0.56	23	1.15	Mcu	
DMR20:29174001	20	29174001	29176000	2000	1	8.40E-07	-0.3	33	1.65	Mcu	
DMR20:29279001	20	29279001	29281000	2000	1	6.70E-09	0.6	40	2	LOC503340;Micu1	Signaling
DMR20:29298001	20	29298001	29299000	1000	1	2.30E-09	-0.48	6	0.6	Micu1	Signaling
DMR20:29309001	20	29309001	29312000	3000	1	7.60E-08	0.49	44	1.47	Micu1	Signaling
DMR20:29592001	20	29592001	29594000	2000	1	9.40E-08	-0.66	27	1.35	Ascc1	
DMR20:29629001	20	29629001	29630000	1000	1	3.10E-07	0.45	6	0.6	Ascc1	
DMR20:29672001	20	29672001	29675000	3000	2	1.80E-13	0.72	49	1.63	Spock2	Extracellular Matrix
DMR20:29767001	20	29767001	29768000	1000	1	6.20E-08	0.42	16	1.6	Chst3	Transport
DMR20:29822001	20	29822001	29824000	2000	1	2.30E-09	0.53	28	1.4	Psap	Cytoskeleton
DMR20:29865001	20	29865001	29873000	8000	1	1.50E-12	0.69	123	1.54	Psap;Cdh23	Cytoskeleton;Cytoskeleton
DMR20:29894001	20	29894001	29897000	3000	1	2.50E-08	0.38	31	1.03	Cdh23;Vsir	Cytoskeleton;Immune
DMR20:29961001	20	29961001	29967000	6000	1	3.80E-08	0.43	113	1.88	Cdh23;LOC100361018	Cytoskeleton
DMR20:30089001	20	30089001	30094000	5000	1	1.60E-07	0.47	66	1.32	Cdh23	Cytoskeleton
DMR20:30104001	20	30104001	30106000	2000	1	1.30E-07	0.53	35	1.75	Cdh23	Cytoskeleton
DMR20:30116001	20	30116001	30117000	1000	1	1.30E-08	0.63	22	2.2	Cdh23	Cytoskeleton
DMR20:30131001	20	30131001	30133000	2000	1	8.70E-07	0.45	32	1.6	Cdh23	Cytoskeleton
DMR20:30134001	20	30134001	30136000	2000	1	7.50E-08	0.48	34	1.7	Cdh23	Cytoskeleton
DMR20:30154001	20	30154001	30156000	2000	2	1.20E-08	0.62	30	1.5	Cdh23	Cytoskeleton
DMR20:30158001	20	30158001	30162000	4000	1	4.10E-07	0.52	67	1.68	Cdh23	Cytoskeleton
DMR20:30202001	20	30202001	30204000	2000	1	4.00E-07	-0.41	38	1.9	Cdh23	Cytoskeleton
DMR20:30212001	20	30212001	30215000	3000	1	1.80E-09	0.44	32	1.07	Cdh23	Cytoskeleton
DMR20:30227001	20	30227001	30230000	3000	1	7.00E-07	0.49	55	1.83	Cdh23	Cytoskeleton
DMR20:30257001	20	30257001	30263000	6000	1	4.40E-08	0.49	112	1.87	Cdh23	Cytoskeleton
DMR20:30289001	20	30289001	30290000	1000	1	8.50E-09	0.4	13	1.3	Slc29a3	Transport
DMR20:30318001	20	30318001	30321000	3000	1	8.70E-07	0.34	25	0.83	Slc29a3	Transport
DMR20:30342001	20	30342001	30347000	5000	1	5.00E-09	0.59	79	1.58	Unc5b	Receptor
DMR20:30348001	20	30348001	30350000	2000	1	2.70E-07	0.38	57	2.85	Unc5b	Receptor
DMR20:30714001	20	30714001	30718000	4000	1	7.50E-07	0.39	47	1.18	Sgpl1	

DMR20:30791001	20	30791001	30794000	3000	1	2.60E-16	0.83	52	1.73	Tbata;LOC108349065	
DMR20:30803001	20	30803001	30805000	2000	2	4.60E-07	0.38	35	1.75	Tbata;LOC108349065;Adamts14	Protease
DMR20:30853001	20	30853001	30857000	4000	1	2.90E-08	0.58	55	1.38	Adamts14	Protease
DMR20:30869001	20	30869001	30871000	2000	1	5.10E-07	-0.37	43	2.15	Adamts14	Protease
DMR20:30890001	20	30890001	30892000	2000	2	2.90E-07	0.48	25	1.25	Adamts14	Protease
DMR20:30915001	20	30915001	30919000	4000	1	1.20E-11	0.73	82	2.05	Prf1	
DMR20:30933001	20	30933001	30937000	4000	1	1.50E-09	0.69	55	1.38	Pald1	Signaling
DMR20:30957001	20	30957001	30965000	8000	1	3.60E-09	0.56	152	1.9	Pald1	Signaling
DMR20:31035001	20	31035001	31036000	1000	1	1.80E-07	0.62	24	2.4	Nodal	Growth Factors
DMR20:31038001	20	31038001	31040000	2000	1	7.60E-08	0.47	23	1.15	Nodal	Growth Factors
DMR20:31154001	20	31154001	31156000	2000	1	6.70E-07	0.53	31	1.55	Lrrc20;LOC108349066	
DMR20:31199001	20	31199001	31201000	2000	1	2.60E-08	0.43	33	1.65	Lrrc20	
DMR20:31235001	20	31235001	31236000	1000	1	4.40E-11	0.8	13	1.3	Npffr1	Signaling
DMR20:31316001	20	31316001	31320000	4000	1	6.00E-08	0.37	67	1.68	Sar1a;Tysnd1;Aifm2	Signaling;Protease;Metabolism
DMR20:31348001	20	31348001	31352000	4000	1	9.50E-07	0.4	52	1.3	Aifm2;H2afy2	Metabolism;Epigenetic
DMR20:31353001	20	31353001	31356000	3000	1	4.60E-08	0.62	41	1.37	Aifm2;H2afy2	Metabolism;Epigenetic
DMR20:31363001	20	31363001	31365000	2000	1	3.60E-08	0.47	60	3	Aifm2;H2afy2	Metabolism;Epigenetic
DMR20:31450001	20	31450001	31452000	2000	1	5.30E-07	-0.38	44	2.2	Col13a1	Extracellular Matrix
DMR20:31559001	20	31559001	31560000	1000	1	5.00E-07	0.52	24	2.4	Col13a1	Extracellular Matrix
DMR20:31802001	20	31802001	31805000	3000	1	3.00E-09	0.49	53	1.77	Tspan15	
DMR20:31809001	20	31809001	31811000	2000	1	3.00E-09	-0.42	28	1.4	Tspan15	
DMR20:31846001	20	31846001	31848000	2000	1	2.30E-07	0.53	27	1.35	Tspan15	
DMR20:31849001	20	31849001	31851000	2000	1	4.50E-12	0.73	37	1.85	Tspan15	
DMR20:31890001	20	31890001	31893000	3000	1	2.00E-07	0.46	68	2.27	Tacr2	Signaling
DMR20:31911001	20	31911001	31914000	3000	2	1.30E-13	0.87	74	2.47	Tacr2;Hk1	Signaling;Signaling
DMR20:31924001	20	31924001	31926000	2000	2	8.10E-09	-0.37	26	1.3	Hk1	Signaling
DMR20:31937001	20	31937001	31939000	2000	2	9.60E-10	0.68	52	2.6	Hk1	Signaling
DMR20:32151001	20	32151001	32154000	3000	1	3.70E-07	0.64	73	2.43	Srgn;LOC108353232;LOC108349067	
DMR20:32217001	20	32217001	32218000	1000	1	4.70E-07	0.61	16	1.6	Kif1bp;Ddx21	
DMR20:32322001	20	32322001	32332000	10000	3	4.70E-09	-0.54	137	1.37	Stox1	
DMR20:32349001	20	32349001	32351000	2000	1	3.20E-10	0.77	32	1.6	Stox1;LOC108349068	
DMR20:32352001	20	32352001	32354000	2000	2	8.70E-08	-0.73	93	4.65	Stox1;LOC108349068	
DMR20:32355001	20	32355001	32358000	3000	1	3.30E-09	0.7	51	1.7	Stox1;LOC108349068	
DMR20:32419001	20	32419001	32421000	2000	2	3.10E-11	-0.37	83	4.15	Ccar1	Epigenetic
DMR20:32422001	20	32422001	32427000	5000	1	7.00E-10	-0.54	62	1.24	Ccar1	Epigenetic
DMR20:32597001	20	32597001	32599000	2000	1	6.90E-08	-0.41	21	1.05	LOC108349016;Gprc6a	Signaling
DMR20:32704001	20	32704001	32706000	2000	1	1.20E-11	-0.45	16	0.8	LOC365574;Rfx6	Transcription
DMR20:33129001	20	33129001	33130000	1000	1	2.20E-18	1.2	31	3.1	Ros1	Receptor
DMR20:33386001	20	33386001	33387000	1000	1	7.80E-07	-0.42	22	2.2	Dcbld1	Protease
DMR20:33429001	20	33429001	33430000	1000	1	1.70E-11	-0.51	31	3.1	Dcbld1	Protease
DMR20:33440001	20	33440001	33441000	1000	1	1.30E-09	0.59	10	1	Dcbld1	Protease
DMR20:33476001	20	33476001	33477000	1000	1	5.80E-07	-0.39	15	1.5	Gopc	Transport
DMR20:33569001	20	33569001	33570000	1000	1	9.60E-07	-0.36	12	1.2	Nus1;LOC102546398	
DMR20:33855001	20	33855001	33857000	2000	1	6.80E-07	0.37	13	0.65	Slc35f1	
DMR20:33912001	20	33912001	33915000	3000	1	9.60E-07	-0.32	99	3.3	Slc35f1;LOC100911233	
DMR20:33920001	20	33920001	33922000	2000	1	1.70E-12	-0.65	22	1.1	Slc35f1	
DMR20:33949001	20	33949001	33950000	1000	1	1.40E-07	-0.56	14	1.4	Slc35f1;LOC103689994;LOC102547010	
DMR20:34044001	20	34044001	34046000	2000	1	1.50E-09	0.47	20	1	Slc35f1	
DMR20:34061001	20	34061001	34062000	1000	1	3.10E-07	-0.46	17	1.7	Slc35f1	
DMR20:34198001	20	34198001	34199000	1000	1	6.10E-10	-0.53	2	0.2	Slc35f1;LOC108349070	
DMR20:34239001	20	34239001	34241000	2000	1	9.50E-08	-0.34	34	1.7	Slc35f1	
DMR20:34254001	20	34254001	34255000	1000	1	4.20E-09	-0.44	3	0.3	Slc35f1	
DMR20:34271001	20	34271001	34272000	1000	1	9.30E-07	-0.46	8	0.8	Slc35f1	
DMR20:34360001	20	34360001	34363000	3000	1	3.20E-08	0.68	31	1.03	Slc35f1	
DMR20:34381001	20	34381001	34382000	1000	1	5.00E-07	-0.45	0	0	Slc35f1	

DMR20:34565001	20	34565001	34568000	3000	1	5.50E-07	-0.32	18	0.6	Cep85l	
DMR20:34614001	20	34614001	34616000	2000	1	2.80E-08	-0.65	5	0.25	Cep85l	
DMR20:34686001	20	34686001	34689000	3000	1	2.00E-15	0.8	47	1.57	Cep85l	
DMR20:34696001	20	34696001	34698000	2000	1	7.70E-12	0.76	40	2	Cep85l	
DMR20:34731001	20	34731001	34733000	2000	1	1.80E-07	-0.42	9	0.45	Cep85l	
DMR20:34744001	20	34744001	34745000	1000	1	8.50E-15	1.01	30	3	Cep85l;LOC691736	
DMR20:34940001	20	34940001	34942000	2000	1	1.50E-07	0.51	27	1.35	Mcm9;Fam184a	Transcription
DMR20:34947001	20	34947001	34949000	2000	1	2.40E-07	-0.39	17	0.85	Fam184a	
DMR20:34954001	20	34954001	34958000	4000	1	9.90E-13	-0.58	98	2.45	Fam184a	
DMR20:34984001	20	34984001	34988000	4000	1	6.10E-07	-0.26	47	1.18	Fam184a	
DMR20:34999001	20	34999001	3.50E+07	1000	1	7.70E-17	1.14	28	2.8	Fam184a	
DMR20:35007001	20	35007001	35010000	3000	1	1.70E-07	-0.36	23	0.77	Fam184a	
DMR20:35035001	20	35035001	35039000	4000	1	2.00E-07	-0.33	61	1.52	Fam184a	
DMR20:35248001	20	35248001	35249000	1000	1	7.60E-07	0.4	12	1.2	LOC108353234;Man1a1	Golgi
DMR20:35259001	20	35259001	35260000	1000	1	4.00E-07	-0.41	13	1.3	LOC108353234;Man1a1	Golgi
DMR20:35360001	20	35360001	35361000	1000	1	8.10E-11	0.5	23	2.3	Man1a1	Golgi
DMR20:35429001	20	35429001	35431000	2000	1	3.10E-07	-0.36	28	1.4	Man1a1	Golgi
DMR20:37558001	20	37558001	37559000	1000	1	5.50E-11	0.73	7	0.7	Tbc1d32	
DMR20:37881001	20	37881001	37882000	1000	1	1.90E-10	-0.49	14	1.4	Gja1	Cytoskeleton
DMR20:37887001	20	37887001	37889000	2000	1	2.10E-09	0.67	48	2.4	Gja1	Cytoskeleton
DMR20:38931001	20	38931001	38933000	2000	1	2.90E-07	0.67	38	1.9	LOC108349075;Hsf2	Transcription
DMR20:40354001	20	40354001	40356000	2000	1	2.00E-11	0.41	29	1.45	Hs3st5;LOC102553345	Transport
DMR20:40368001	20	40368001	40369000	1000	1	3.60E-07	0.55	18	1.8	Hs3st5	Transport
DMR20:40417001	20	40417001	40420000	3000	1	5.90E-16	0.55	31	1.03	Hs3st5	Transport
DMR20:40526001	20	40526001	40527000	1000	1	3.50E-13	0.86	21	2.1	Hs3st5	Transport
DMR20:40528001	20	40528001	40529000	1000	1	8.50E-07	-0.55	1	0.1	Hs3st5	Transport
DMR20:40615001	20	40615001	40616000	1000	1	1.10E-07	-0.39	10	1	Pkib	Signaling
DMR20:41391001	20	41391001	41392000	1000	1	7.20E-07	-0.49	5	0.5	Frk	
DMR20:42961001	20	42961001	42963000	2000	1	1.00E-08	0.79	25	1.25	Marcks	
DMR20:43087001	20	43087001	43090000	3000	1	7.90E-08	-0.46	53	1.77	Hdac2	
DMR20:44099001	20	44099001	44102000	3000	1	4.90E-07	0.46	65	2.17	Lama4	
DMR20:44165001	20	44165001	44167000	2000	1	2.90E-11	-0.5	24	1.2	Lama4;LOC102550544	
DMR20:44204001	20	44204001	44206000	2000	2	2.10E-30	1.12	165	8.25	Lama4;Fam229b	
DMR20:44215001	20	44215001	44217000	2000	1	1.90E-07	-0.34	24	1.2	Fam229b;Tube1	Cytoskeleton
DMR20:44251001	20	44251001	44252000	1000	1	8.90E-07	-0.46	5	0.5	Wisp3	
DMR20:44255001	20	44255001	44256000	1000	1	3.80E-12	0.81	24	2.4	Wisp3	
DMR20:44508001	20	44508001	44510000	2000	1	4.20E-08	-0.71	14	0.7	Fyn	
DMR20:44521001	20	44521001	44523000	2000	1	2.90E-07	-0.38	44	2.2	Fyn	
DMR20:44552001	20	44552001	44556000	4000	1	1.50E-09	-0.54	88	2.2	Fyn	
DMR20:44562001	20	44562001	44566000	4000	1	3.80E-09	-0.48	88	2.2	Fyn	
DMR20:44594001	20	44594001	44596000	2000	2	4.80E-07	-0.47	47	2.35	Fyn;LOC108349083	
DMR20:44600001	20	44600001	44604000	4000	1	3.40E-07	-0.46	78	1.95	Fyn;LOC108349083	
DMR20:44615001	20	44615001	44619000	4000	2	2.10E-09	-0.49	89	2.22	Fyn;LOC108349083	
DMR20:44636001	20	44636001	44639000	3000	1	7.90E-15	0.93	96	3.2	Fyn	
DMR20:44705001	20	44705001	44710000	5000	1	5.00E-09	-0.54	85	1.7	Traf3ip2;LOC102551236	Cytoskeleton
DMR20:44724001	20	44724001	44729000	5000	2	5.00E-10	-0.59	99	1.98	Traf3ip2	Cytoskeleton
DMR20:44732001	20	44732001	44736000	4000	1	1.00E-07	0.37	67	1.68	Traf3ip2	Cytoskeleton
DMR20:44833001	20	44833001	44838000	5000	1	5.10E-10	-0.44	32	0.64	Rev3l	Transcription
DMR20:44863001	20	44863001	44865000	2000	1	1.70E-08	-0.38	32	1.6	Rev3l	Transcription
DMR20:44899001	20	44899001	44902000	3000	1	6.70E-17	-0.6	56	1.87	Rev3l	Transcription
DMR20:44964001	20	44964001	44966000	2000	1	9.30E-08	-0.37	8	0.4	Rev3l;LOC100359898	Transcription
DMR20:45023001	20	45023001	45024000	1000	1	6.80E-07	0.57	18	1.8	RGD1304770	Transport
DMR20:45030001	20	45030001	45032000	2000	1	6.60E-08	-0.44	23	1.15	RGD1304770;RGD1561777	Transport
DMR20:45037001	20	45037001	45040000	3000	1	1.20E-08	0.73	30	1	RGD1561777	Transport
DMR20:45045001	20	45045001	45050000	5000	1	1.90E-09	-0.36	38	0.76	RGD1561777	Transport
DMR20:45093001	20	45093001	45094000	1000	1	8.10E-07	0.44	10	1	Naglt1	
DMR20:45107001	20	45107001	45109000	2000	2	4.20E-16	0.95	43	2.15	Naglt1	
DMR20:45113001	20	45113001	45115000	2000	1	4.20E-17	0.86	42	2.1	Naglt1;RGD1310495	Transport
DMR20:45137001	20	45137001	45138000	1000	1	1.90E-11	-0.52	17	1.7	RGD1310495;Slc16a10	Transport
DMR20:45162001	20	45162001	45165000	3000	1	2.80E-08	0.53	40	1.33	Slc16a10	Transport
DMR20:45169001	20	45169001	45174000	5000	1	5.20E-10	-0.38	48	0.96	Slc16a10	Transport

DMR20:45313001	20	45313001	45315000	2000	1	1.10E-10	0.81	30	1.5	LOC103694442;LOC108349085;Rpf2	
DMR20:45316001	20	45316001	45317000	1000	1	7.20E-14	-0.6	34	3.4	LOC103694442;LOC108349085;Rpf2	
DMR20:45341001	20	45341001	45342000	1000	1	7.70E-10	-0.5	18	1.8	Rpf2;Gtf3c6	
DMR20:45408001	20	45408001	45411000	3000	2	4.30E-16	-0.63	26	0.87	Amd1	Metabolism
DMR20:45450001	20	45450001	45455000	5000	1	8.60E-07	-0.33	38	0.76	LOC100361424;LOC100361476;Cdk19	Signaling
DMR20:45466001	20	45466001	45470000	4000	1	6.90E-13	0.55	19	0.48	Cdk19	Signaling
DMR20:45536001	20	45536001	45537000	1000	1	2.90E-08	0.66	22	2.2	Cdk19	Signaling
DMR20:45591001	20	45591001	45593000	2000	1	1.50E-07	0.59	33	1.65	Cdk19	Signaling
DMR20:45650001	20	45650001	45651000	1000	1	1.40E-07	0.69	25	2.5	Slc22a16	
DMR20:45652001	20	45652001	45654000	2000	1	4.10E-08	-0.54	45	2.25	Slc22a16	
DMR20:46002001	20	46002001	46004000	2000	1	2.40E-08	0.58	38	1.9	Fig4	Signaling
DMR20:46101001	20	46101001	46103000	2000	2	2.70E-07	-0.44	44	2.2	Ak9	Signaling
DMR20:46124001	20	46124001	46126000	2000	1	1.80E-11	0.99	37	1.85	Ak9	Signaling
DMR20:46135001	20	46135001	46137000	2000	2	6.70E-15	0.95	71	3.55	Ak9	Signaling
DMR20:46238001	20	46238001	46240000	2000	1	7.60E-08	-0.36	38	1.9	Ppil6	Transcription
DMR20:46454001	20	46454001	46456000	2000	1	2.00E-12	-0.54	49	2.45	Foxo3	
DMR20:46457001	20	46457001	46460000	3000	1	7.60E-10	-0.46	69	2.3	Foxo3	
DMR20:46476001	20	46476001	46480000	4000	1	3.40E-11	-0.48	84	2.1	Foxo3	
DMR20:46481001	20	46481001	46483000	2000	1	2.70E-08	-0.47	45	2.25	Foxo3	
DMR20:46498001	20	46498001	46499000	1000	1	4.10E-08	-0.44	15	1.5	Foxo3	
DMR20:46505001	20	46505001	46507000	2000	1	3.80E-07	-0.41	99	4.95	Foxo3	
DMR20:46508001	20	46508001	46513000	5000	2	5.10E-08	-0.48	94	1.88	Foxo3	
DMR20:46525001	20	46525001	46527000	2000	1	8.30E-07	0.38	39	1.95	Foxo3;LOC102555657	
DMR20:46571001	20	46571001	46573000	2000	1	2.10E-10	-0.38	43	2.15	RGD1561147	
DMR20:46620001	20	46620001	46628000	8000	1	8.90E-08	-0.36	180	2.25	Cep57l1	
DMR20:46744001	20	46744001	46747000	3000	1	4.20E-07	-0.56	54	1.8	Sesn1	Metabolism
DMR20:46760001	20	46760001	46762000	2000	1	3.40E-08	-0.36	29	1.45	Sesn1;Armc2	Metabolism
DMR20:46800001	20	46800001	46801000	1000	1	6.00E-11	0.44	4	0.4	Armc2	
DMR20:46802001	20	46802001	46805000	3000	1	2.80E-07	0.55	62	2.07	Armc2	
DMR20:46836001	20	46836001	46838000	2000	1	3.70E-21	1	39	1.95	Armc2	
DMR20:46986001	20	46986001	46990000	4000	2	8.10E-15	0.88	80	2	RGD1560975	
DMR20:46992001	20	46992001	46995000	3000	1	8.60E-11	0.69	64	2.13	RGD1560975	
DMR20:47076001	20	47076001	47078000	2000	1	3.60E-16	-0.64	50	2.5	Lace1	
DMR20:47086001	20	47086001	47090000	4000	1	1.80E-07	-0.36	56	1.4	Lace1	
DMR20:47098001	20	47098001	47100000	2000	1	1.80E-11	-0.65	52	2.6	Lace1	
DMR20:47173001	20	47173001	47176000	3000	1	2.20E-12	0.71	79	2.63	Lace1	
DMR20:47195001	20	47195001	47197000	2000	2	3.90E-12	0.76	50	2.5	Lace1	
DMR20:47201001	20	47201001	47204000	3000	1	3.20E-07	0.42	62	2.07	Lace1	
DMR20:47270001	20	47270001	47271000	1000	1	1.70E-10	0.58	20	2	Snx3	
DMR20:47272001	20	47272001	47273000	1000	1	3.60E-08	0.54	23	2.3	Snx3	
DMR20:47559001	20	47559001	47563000	4000	1	5.50E-09	-0.35	55	1.38	Sec63	Cytoskeleton
DMR20:47592001	20	47592001	47593000	1000	1	5.00E-08	0.39	13	1.3	LOC365595;Scml4	Epigenetic
DMR20:47605001	20	47605001	47608000	3000	1	6.90E-10	0.56	54	1.8	LOC365595;Scml4	Epigenetic
DMR20:47613001	20	47613001	47614000	1000	1	1.30E-07	0.32	5	0.5	Scml4	Epigenetic
DMR20:47628001	20	47628001	47632000	4000	1	7.10E-07	0.38	52	1.3	Scml4	Epigenetic
DMR20:47644001	20	47644001	47645000	1000	1	3.20E-08	-0.61	6	0.6	Scml4	Epigenetic
DMR20:47752001	20	47752001	47755000	3000	1	4.00E-09	0.76	80	2.67	Sobp	
DMR20:47791001	20	47791001	47794000	3000	1	4.20E-08	-0.33	57	1.9	Sobp	
DMR20:47801001	20	47801001	47804000	3000	2	1.60E-15	0.64	64	2.13	Sobp	
DMR20:47806001	20	47806001	47812000	6000	1	7.10E-10	-0.45	114	1.9	Sobp	
DMR20:47825001	20	47825001	47830000	5000	1	2.60E-15	1.01	132	2.64	Sobp;LOC108349095	
DMR20:47846001	20	47846001	47847000	1000	1	8.80E-12	-0.56	28	2.8	Sobp;LOC108349095	
DMR20:47868001	20	47868001	47873000	5000	1	4.80E-07	-0.42	106	2.12	Sobp	
DMR20:47955001	20	47955001	47960000	5000	2	1.60E-12	0.93	79	1.58	Pdss2	
DMR20:47999001	20	47999001	48003000	4000	2	9.90E-10	-0.62	124	3.1	Pdss2	
DMR20:48020001	20	48020001	48023000	3000	1	6.30E-07	-0.41	47	1.57	Pdss2	
DMR20:48049001	20	48049001	48052000	3000	1	5.10E-07	-0.43	64	2.13	Pdss2	
DMR20:48074001	20	48074001	48077000	3000	1	5.30E-09	-0.35	68	2.27	Pdss2;LOC102551730	
DMR20:48078001	20	48078001	48080000	2000	1	2.80E-08	-0.45	44	2.2	Pdss2;LOC102551730	

DMR20:48099001	20	48099001	48100000	1000	1	9.80E-12	0.36	56	5.6	Pdss2	
DMR20:48167001	20	48167001	48169000	2000	1	1.90E-07	-0.34	41	2.05	Pdss2	
DMR20:48176001	20	48176001	48178000	2000	1	4.70E-08	0.28	33	1.65	Pdss2	
DMR20:48187001	20	48187001	48193000	6000	1	1.80E-07	-0.39	137	2.28	Pdss2	
DMR20:48212001	20	48212001	48214000	2000	1	4.40E-07	0.38	48	2.4	RGD1564849	
DMR20:48457001	20	48457001	48458000	1000	1	6.00E-19	1.12	31	3.1	Wasf1	Cytoskeleton
DMR20:48464001	20	48464001	48467000	3000	2	1.60E-09	-0.4	60	2	Wasf1	Cytoskeleton
DMR20:48482001	20	48482001	48485000	3000	1	9.80E-15	-0.46	60	2	Wasf1	Cytoskeleton
DMR20:48493001	20	48493001	48494000	1000	1	1.60E-07	-0.31	15	1.5	Wasf1;Cdc40	Cytoskeleton;Translation
DMR20:48505001	20	48505001	48507000	2000	1	3.30E-07	-0.29	36	1.8	Wasf1;Cdc40	Cytoskeleton;Translation
DMR20:48560001	20	48560001	48561000	1000	1	1.80E-08	-0.43	39	3.9	Cdc40	Translation
DMR20:48576001	20	48576001	48580000	4000	1	9.20E-08	-0.46	72	1.8	Cdc40;Mettl24	Translation;Epigenetic
DMR20:48648001	20	48648001	48651000	3000	1	1.70E-07	-0.4	54	1.8	Mettl24	Epigenetic
DMR20:48657001	20	48657001	48658000	1000	1	1.70E-09	-0.39	26	2.6	Mettl24	Epigenetic
DMR20:48680001	20	48680001	48684000	4000	2	1.10E-14	0.99	63	1.57	Mettl24	Epigenetic
DMR20:48695001	20	48695001	48696000	1000	1	5.90E-08	-0.49	12	1.2	Mettl24	Epigenetic
DMR20:48740001	20	48740001	48741000	1000	1	6.00E-07	0.49	32	3.2	Ddo	Metabolism
DMR20:48743001	20	48743001	48744000	1000	1	1.30E-09	0.76	35	3.5	Ddo	Metabolism
DMR20:48844001	20	48844001	48847000	3000	1	7.90E-09	-0.49	50	1.67	Qrsl1	Metabolism
DMR20:48849001	20	48849001	48850000	1000	1	7.60E-07	-0.41	25	2.5	Qrsl1	Metabolism
DMR20:48886001	20	48886001	48887000	1000	1	2.40E-08	-0.47	10	1	Qrsl1;Rtn4ip1	Metabolism;Metabolism
DMR20:48994001	20	48994001	48998000	4000	2	3.10E-19	-0.58	57	1.43	Aim1	
DMR20:49083001	20	49083001	49086000	3000	3	2.80E-12	-0.58	76	2.53	Aim1	
DMR20:49094001	20	49094001	49099000	5000	1	5.80E-07	-0.29	46	0.92	Aim1	
DMR20:49106001	20	49106001	49107000	1000	1	3.10E-14	-0.63	18	1.8	Aim1	
DMR20:49343001	20	49343001	49344000	1000	1	1.40E-07	-0.37	16	1.6	Atg5	Transport
DMR20:49355001	20	49355001	49358000	3000	1	8.60E-12	-0.59	42	1.4	Atg5	Transport
DMR20:49379001	20	49379001	49380000	1000	1	8.50E-07	-0.44	3	0.3	Atg5	Transport
DMR20:49381001	20	49381001	49382000	1000	1	1.30E-07	-0.43	27	2.7	Atg5	Transport
DMR20:49396001	20	49396001	49404000	8000	2	2.00E-12	0.66	160	2	Atg5;LOC103694450	Transport
DMR20:49468001	20	49468001	49472000	4000	1	1.40E-07	0.51	94	2.35	Prdm1	Transcription
DMR20:49473001	20	49473001	49477000	4000	1	2.70E-13	-0.72	88	2.2	Prdm1	Transcription
DMR20:49481001	20	49481001	49482000	1000	1	7.20E-07	-0.39	27	2.7	Prdm1	Transcription
DMR20:49489001	20	49489001	49491000	2000	1	4.90E-08	0.55	46	2.3	Prdm1	Transcription
DMR20:49526001	20	49526001	49529000	3000	1	1.90E-11	0.8	58	1.93	Prdm1	Transcription
DMR20:50204001	20	50204001	50210000	6000	2	1.70E-13	-0.57	122	2.03	Prep	Protease
DMR20:50236001	20	50236001	50240000	4000	1	6.20E-12	-0.41	114	2.85	Prep	Protease
DMR20:50262001	20	50262001	50265000	3000	1	8.30E-08	-0.48	53	1.77	Prep	Protease
DMR20:50387001	20	50387001	50388000	1000	1	6.80E-09	0.49	18	1.8	Popdc3	
DMR20:50393001	20	50393001	50394000	1000	1	3.90E-09	0.61	17	1.7	Popdc3;LOC108353235	
DMR20:50404001	20	50404001	50405000	1000	1	7.70E-07	-0.34	18	1.8	Popdc3;LOC108353235	
DMR20:50406001	20	50406001	50409000	3000	1	5.70E-07	0.45	41	1.37	Popdc3;LOC108353235	
DMR20:50462001	20	50462001	50463000	1000	1	4.30E-17	0.98	28	2.8	Bves	
DMR20:50480001	20	50480001	50482000	2000	1	1.40E-10	0.67	33	1.65	Bves;Lin28b	Metabolism
DMR20:50565001	20	50565001	50567000	2000	2	4.60E-10	-0.62	42	2.1	Lin28b	Metabolism
DMR20:50646001	20	50646001	50650000	4000	1	3.80E-07	-0.45	42	1.05	Hace1	Proteolysis
DMR20:50666001	20	50666001	50669000	3000	1	5.00E-07	0.27	60	2	Hace1;LOC103694456	Proteolysis
DMR20:50696001	20	50696001	50700000	4000	1	6.70E-11	-0.41	69	1.73	Hace1	Proteolysis
DMR20:53840001	20	53840001	53841000	1000	1	7.70E-09	0.38	10	1	Grik2	Receptor
DMR20:53857001	20	53857001	53859000	2000	1	1.50E-08	-0.58	6	0.3	Grik2	Receptor
DMR20:53875001	20	53875001	53878000	3000	1	2.80E-09	-0.59	11	0.37	Grik2	Receptor
DMR20:53970001	20	53970001	53971000	1000	1	5.20E-07	-0.44	9	0.9	Grik2	Receptor
DMR20:54102001	20	54102001	54103000	1000	1	2.60E-07	-0.43	5	0.5	Grik2	Receptor
DMR20:54378001	20	54378001	54379000	1000	1	1.90E-07	0.38	2	0.2	Grik2	Receptor
DMR20:54474001	20	54474001	54475000	1000	1	5.70E-07	0.68	17	1.7	Grik2	Receptor
DMR20:55513001	20	55513001	55514000	1000	1	2.20E-08	-0.36	10	1	Ascc3	Cytoskeleton
DMR20:55552001	20	55552001	55555000	3000	1	1.50E-07	-0.35	23	0.77	Ascc3	Cytoskeleton
DMR20:55566001	20	55566001	55567000	1000	1	1.20E-08	0.65	23	2.3	Ascc3	Cytoskeleton
DMR20:56199001	20	56199001	56201000	2000	1	9.00E-10	0.66	66	3.3	LOC103694460;Cd99	

DMRX:290001	X	290001	293000	3000	1	2.30E-17	0.7	43	1.43	Olr1756-ps	
DMRX:713001	X	713001	714000	1000	1	5.70E-10	-0.78	6	0.6	Ssx2	
DMRX:2031001	X	2031001	2032000	1000	1	1.40E-11	-0.84	5	0.5	Jade3	Transcription
DMRX:2075001	X	2075001	2076000	1000	1	3.40E-07	-0.69	4	0.4	Rp2	Signaling
DMRX:2593001	X	2593001	2595000	2000	1	3.10E-07	-0.25	19	0.95	Slc9a7	Transport
DMRX:3593001	X	3593001	3594000	1000	1	2.50E-07	-0.8	2	0.2	RGD1561575	
DMRX:4715001	X	4715001	4716000	1000	1	8.40E-07	-0.52	1	0.1	RGD1559731	
DMRX:5226001	X	5226001	5229000	3000	2	1.60E-11	-0.89	10	0.33	RGD1564748	
DMRX:5805001	X	5805001	5817000	12000	2	4.50E-08	-0.36	110	0.92	LOC691655;Efhc2	Signaling
DMRX:5922001	X	5922001	5923000	1000	1	1.90E-16	0.81	9	0.9	Efhc2	Signaling
DMRX:6036001	X	6036001	6038000	2000	1	8.60E-08	-0.25	16	0.8	Efhc2	Signaling
DMRX:10202001	X	10202001	10203000	1000	1	5.10E-08	-0.47	16	1.6	Nyx	Receptor
DMRX:10399001	X	10399001	10401000	2000	1	6.10E-07	-0.61	27	1.35	Ddx3x	
DMRX:10537001	X	10537001	10539000	2000	1	1.20E-07	-0.41	20	1	Usp9x	Protease
DMRX:11069001	X	11069001	11073000	4000	1	2.10E-17	0.55	41	1.02	Med14;RGD1565685	Transcription
DMRX:11138001	X	11138001	11140000	2000	2	3.20E-10	-0.65	22	1.1	Atp6ap2	Receptor
DMRX:13884001	X	13884001	13890000	6000	1	3.20E-08	-0.35	50	0.83	Sytl5	
DMRX:14098001	X	14098001	14099000	1000	1	6.40E-11	-0.45	5	0.5	Sytl5	
DMRX:14287001	X	14287001	14290000	3000	1	3.90E-08	-0.52	44	1.47	Rpgr	Proteolysis
DMRX:14305001	X	14305001	14306000	1000	1	1.70E-08	-0.52	7	0.7	Rpgr	Proteolysis
DMRX:14396001	X	14396001	14401000	5000	1	1.90E-08	-0.44	34	0.68	Lancl3	
DMRX:14452001	X	14452001	14453000	1000	1	7.40E-08	-0.49	4	0.4	Lancl3	
DMRX:14951001	X	14951001	14956000	5000	5	1.10E-09	-0.61	30	0.6	Slc38a5	Transport
DMRX:15055001	X	15055001	15059000	4000	2	6.40E-07	-0.46	65	1.62	Porcn;Ebp;Tbc1d25	Metabolism;Metabolism;Signaling
DMRX:15452001	X	15452001	15453000	1000	1	1.30E-09	-0.47	31	3.1	Timm17b;Pqbp1;Slc35a2;Pim2	Cytoskeleton;Transport;Signaling
DMRX:15505001	X	15505001	15507000	2000	2	3.70E-09	-0.59	21	1.05	Otud5;LOC102555178;Kcnd1	Protease;Transport
DMRX:15757001	X	15757001	15758000	1000	1	3.30E-07	-0.5	8	0.8	Ccdc22;Foxp3;LOC108349208	
DMRX:15765001	X	15765001	15766000	1000	1	4.50E-08	-0.6	8	0.8	Foxp3;LOC108349208;Ppp1r3f	
DMRX:15783001	X	15783001	15785000	2000	1	6.80E-07	-0.4	26	1.3	Ppp1r3f	
DMRX:15789001	X	15789001	15790000	1000	1	1.10E-08	-0.34	7	0.7	Ppp1r3f	
DMRX:15948001	X	15948001	15950000	2000	1	2.70E-21	1.08	47	2.35	RGD1566093	
DMRX:16125001	X	16125001	16126000	1000	1	3.80E-10	-0.34	9	0.9	Mir362;Mir3560;Mir501;Mir500	
DMRX:16303001	X	16303001	16305000	2000	1	3.50E-07	-0.64	22	1.1	Akap4;Ccnb3	Cytoskeleton;Signaling
DMRX:16468001	X	16468001	16470000	2000	1	1.50E-07	-0.54	18	0.9	Dgkk	Signaling
DMRX:16806001	X	16806001	16808000	2000	1	1.80E-11	-0.59	17	0.85	Shroom4	Cytoskeleton
DMRX:16810001	X	16810001	16811000	1000	1	9.40E-07	0.45	10	1	Shroom4	Cytoskeleton
DMRX:16950001	X	16950001	16952000	2000	1	5.30E-07	-0.34	17	0.85	RGD1563606	
DMRX:17165001	X	17165001	17167000	2000	1	3.80E-10	-0.42	10	0.5	LOC103694470;Nudt11	
DMRX:17224001	X	17224001	17225000	1000	1	4.80E-08	-0.47	5	0.5	Nudt11	
DMRX:18874001	X	18874001	18875000	1000	1	4.90E-14	-0.77	15	1.5	Klf8	Transcription
DMRX:20477001	X	20477001	20478000	1000	1	2.30E-09	-0.58	8	0.8	FAM120C	
DMRX:20784001	X	20784001	20786000	2000	1	6.30E-09	-0.53	18	0.9	FAM120C	
DMRX:20968001	X	20968001	20969000	1000	1	3.00E-14	-0.63	3	0.3	FAM120C	
DMRX:20997001	X	20997001	2.10E+07	3000	1	1.30E-08	-0.29	25	0.83	FAM120C	
DMRX:21136001	X	21136001	21138000	2000	1	5.60E-08	-0.81	6	0.3	Phf8	
DMRX:21575001	X	21575001	21577000	2000	1	2.80E-08	-0.55	37	1.85	Huwe1;Mirlet7f-2;Mir98	Proteolysis
DMRX:23576001	X	23576001	23577000	1000	1	1.00E-09	-0.61	9	0.9	Shroom2	Cytoskeleton
DMRX:25795001	X	25795001	25797000	2000	1	2.40E-10	-0.43	21	1.05	Mid1	Proteolysis
DMRX:26381001	X	26381001	26386000	5000	1	5.60E-09	-0.37	48	0.96	Arhgap6	Signaling
DMRX:26495001	X	26495001	26497000	2000	1	1.50E-07	-0.42	16	0.8	Arhgap6	Signaling
DMRX:26739001	X	26739001	26740000	1000	1	5.60E-13	-0.68	3	0.3	Arhgap6	Signaling
DMRX:27350001	X	27350001	27351000	1000	1	1.80E-08	0.58	19	1.9	Frmpd4	
DMRX:27944001	X	27944001	27946000	2000	1	3.60E-09	-0.42	14	0.7	Frmpd4	
DMRX:28117001	X	28117001	28119000	2000	1	6.50E-07	-0.36	16	0.8	Frmpd4	
DMRX:28560001	X	28560001	28562000	2000	1	2.20E-07	-0.47	31	1.55	Tlr8	Receptor
DMRX:30603001	X	30603001	30604000	1000	1	3.70E-08	-0.79	5	0.5	Glra2	Ion Channel
DMRX:30704001	X	30704001	30705000	1000	1	4.60E-07	0.39	6	0.6	Glra2	Ion Channel
DMRX:31752001	X	31752001	31758000	6000	1	1.10E-09	-0.43	58	0.97	Asb9;Asb11	
DMRX:31847001	X	31847001	31848000	1000	1	2.10E-11	0.81	24	2.4	Figf;LOC108349213;Pir	Transcription

DMRX:32079001	X	32079001	32080000	1000	1	3.90E-12	0.45	7	0.7	Ace2	Protease
DMRX:32151001	X	32151001	32152000	1000	1	8.40E-07	-0.47	6	0.6	Tmem27	
DMRX:33817001	X	33817001	33818000	1000	1	4.80E-07	-0.54	5	0.5	Reps2;LOC108349215	Transport
DMRX:33829001	X	33829001	33831000	2000	1	1.90E-09	-0.76	17	0.85	Reps2;LOC108349215	Transport
DMRX:33889001	X	33889001	33890000	1000	1	6.90E-07	-0.37	6	0.6	Reps2	Transport
DMRX:34717001	X	34717001	34718000	1000	1	7.50E-10	-0.65	2	0.2	RGD1566197	
DMRX:34724001	X	34724001	34725000	1000	1	6.20E-10	-0.59	10	1	RGD1566197;Rai2	
DMRX:35576001	X	35576001	35578000	2000	1	2.90E-07	-0.65	19	0.95	Cdkl5	Signaling
DMRX:35632001	X	35632001	35635000	3000	1	1.50E-12	-0.49	33	1.1	Cdkl5;Gja6	Signaling;Cytoskeleton
DMRX:35831001	X	35831001	35832000	1000	1	2.40E-10	-0.58	8	0.8	Ppef1	Signaling
DMRX:35873001	X	35873001	35876000	3000	2	1.10E-09	-0.36	29	0.97	Ppef1;LOC108349217	Signaling
DMRX:36443001	X	36443001	36445000	2000	1	1.70E-08	0.28	17	0.85	Phka2	
DMRX:36446001	X	36446001	36449000	3000	3	6.00E-21	1.01	46	1.53	Phka2	
DMRX:36450001	X	36450001	36458000	8000	5	6.20E-31	1.05	144	1.8	Phka2;LOC108353287	
DMRX:36461001	X	36461001	36469000	8000	2	2.90E-20	0.86	186	2.33	Phka2;LOC108353287	
DMRX:36470001	X	36470001	36473000	3000	3	3.40E-18	0.72	60	2	Phka2;LOC108353287	
DMRX:36846001	X	36846001	36847000	1000	1	6.50E-09	-0.42	22	2.2	Phka2	
DMRX:37825001	X	37825001	37827000	2000	2	7.30E-09	-0.85	8	0.4	Sh3kbp1	
DMRX:37921001	X	37921001	37922000	1000	1	5.40E-07	-0.5	5	0.5	Sh3kbp1	
DMRX:38126001	X	38126001	38127000	1000	1	2.10E-10	-0.67	12	1.2	Sh3kbp1	
DMRX:38340001	X	38340001	38341000	1000	1	2.30E-08	-0.53	12	1.2	RGD1562161	
DMRX:42947001	X	42947001	42952000	5000	1	1.10E-07	-0.54	14	0.28	RGD1559536	Protease; Proteolysis
DMRX:43298001	X	43298001	43300000	2000	2	6.80E-14	1.27	58	2.9	LOC680190;RGD1566060	Cell Junction
DMRX:43301001	X	43301001	43303000	2000	2	6.70E-16	1.28	58	2.9	LOC680190;RGD1566060	Cell Junction
DMRX:45111001	X	45111001	45112000	1000	1	2.90E-07	-0.72	8	0.8	Prkx	Signaling
DMRX:45589001	X	45589001	45590000	1000	1	9.20E-07	-0.49	3	0.3	RGD1563234	
DMRX:46636001	X	46636001	46637000	1000	1	2.10E-07	-0.37	6	0.6	Cfap47	
DMRX:48189001	X	48189001	48190000	1000	1	1.40E-07	-0.74	1	0.1	Da2-19	
DMRX:49638001	X	49638001	49642000	4000	2	3.70E-09	0.32	52	1.3	Srsf8	Translation
DMRX:51189001	X	51189001	51195000	6000	2	1.10E-09	-0.37	82	1.37	Dmd	
DMRX:51414001	X	51414001	51417000	3000	1	9.00E-15	0.58	27	0.9	Dmd;LOC103690859	
DMRX:51787001	X	51787001	51792000	5000	4	2.10E-10	0.57	277	5.54	Dmd;LOC102549011	
DMRX:52351001	X	52351001	52352000	1000	1	1.00E-07	-0.32	8	0.8	Dmd	
DMRX:52449001	X	52449001	52450000	1000	1	3.00E-07	-0.68	5	0.5	Dmd	
DMRX:52505001	X	52505001	52508000	3000	1	7.30E-09	-0.39	26	0.87	Dmd	
DMRX:52555001	X	52555001	52557000	2000	1	3.00E-11	0.3	15	0.75	Dmd	
DMRX:53039001	X	53039001	53047000	8000	2	1.50E-07	-0.48	85	1.06	Dmd	
DMRX:53128001	X	53128001	53134000	6000	1	6.20E-07	-0.31	60	1	Dmd	
DMRX:53309001	X	53309001	53313000	4000	1	4.60E-08	0.22	56	1.4	Dmd	
DMRX:54083001	X	54083001	54084000	1000	1	8.00E-07	-0.82	12	1.2	Tab3	
DMRX:54271001	X	54271001	54272000	1000	1	6.40E-08	-0.58	13	1.3	Gk;LOC100363193	Metabolism
DMRX:55550001	X	55550001	55554000	4000	1	6.20E-07	-0.27	37	0.92	Il1rapl1	Receptor
DMRX:56325001	X	56325001	56333000	8000	1	5.20E-19	0.74	70	0.88	Il1rapl1	Receptor
DMRX:56387001	X	56387001	56389000	2000	1	8.70E-11	-0.4	23	1.15	Il1rapl1	Receptor
DMRX:56427001	X	56427001	56428000	1000	1	1.70E-11	0.68	20	2	Il1rapl1	Receptor
DMRX:56782001	X	56782001	56786000	4000	1	1.10E-08	-0.32	39	0.98	Il1rapl1	Receptor
DMRX:56790001	X	56790001	56793000	3000	1	9.20E-07	-0.31	27	0.9	Il1rapl1	Receptor
DMRX:57708001	X	57708001	57715000	7000	1	1.10E-07	0.28	75	1.07	RGD1565286	
DMRX:61107001	X	61107001	61108000	1000	1	1.10E-24	1.05	19	1.9	MGC114483;Mageb4	Cytoskeleton
DMRX:61149001	X	61149001	61150000	1000	1	9.10E-29	1.08	14	1.4	Mageb4	Cytoskeleton
DMRX:62387001	X	62387001	62388000	1000	1	1.90E-07	-0.5	13	1.3	Pola1	Transcription
DMRX:62895001	X	62895001	62896000	1000	1	1.20E-07	-0.73	1	0.1	Pdk3	Signaling
DMRX:63165001	X	63165001	63166000	1000	1	1.70E-17	-0.8	19	1.9	Zfx	Transcription
DMRX:64223001	X	64223001	64224000	1000	1	3.60E-11	0.84	12	1.2	Spin4	
DMRX:64336001	X	64336001	64339000	3000	1	1.10E-08	-0.31	29	0.97	Arhgef9	Transcription
DMRX:64366001	X	64366001	64368000	2000	1	3.60E-07	-0.47	21	1.05	Arhgef9	Transcription
DMRX:65291001	X	65291001	65292000	1000	1	1.60E-09	-0.53	16	1.6	Msn	Cytoskeleton
DMRX:65578001	X	65578001	65582000	4000	1	2.30E-11	-0.52	41	1.02	Heph	Metabolism
DMRX:68210001	X	68210001	68213000	3000	1	1.30E-07	-0.24	33	1.1	Ophn1	Signaling
DMRX:68214001	X	68214001	68218000	4000	1	1.20E-07	-0.37	41	1.02	Ophn1	Signaling
DMRX:68228001	X	68228001	68230000	2000	1	1.00E-07	-0.42	16	0.8	Ophn1	Signaling
DMRX:68294001	X	68294001	68296000	2000	1	5.40E-07	-0.5	17	0.85	Ophn1	Signaling

DMRX:68424001	X	68424001	68425000	1000	1	1.50E-07	0.47	9	0.9	Ophn1	Signaling
DMRX:69214001	X	69214001	69218000	4000	3	4.20E-10	-0.73	92	2.3	LOC108349229;Pja1	Proteolysis
DMRX:69836001	X	69836001	69841000	5000	1	1.20E-09	-0.48	46	0.92	Eda	
DMRX:70678001	X	70678001	70680000	2000	1	1.20E-11	-0.43	19	0.95	Tex11	
DMRX:70762001	X	70762001	70764000	2000	1	6.70E-18	1	13	0.65	Tex11	
DMRX:70772001	X	70772001	70773000	1000	1	7.80E-07	-0.49	12	1.2	Tex11	
DMRX:70934001	X	70934001	70937000	3000	2	1.80E-08	-0.38	24	0.8	Tex11;LOC100362309	
DMRX:70999001	X	70999001	71001000	2000	1	1.80E-07	0.43	19	0.95	Snx12	
DMRX:71130001	X	71130001	71132000	2000	2	5.00E-08	-0.62	11	0.55	Snx12;LOC102548326	
DMRX:71548001	X	71548001	71549000	1000	1	2.30E-13	-0.69	9	0.9	Ogt	Golgi
DMRX:71590001	X	71590001	71593000	3000	2	1.50E-07	0.66	268	8.93	Ogt;LOC102548506;LOC103690864	Golgi
DMRX:71970001	X	71970001	71972000	2000	1	9.90E-07	-0.48	27	1.35	Nhsl2;LOC100911832	
DMRX:72485001	X	72485001	72486000	1000	1	7.50E-08	-0.45	22	2.2	Phka1	Signaling
DMRX:72682001	X	72682001	72690000	8000	2	6.50E-18	1.12	149	1.86	LOC680024;Dmrtc1c1	Transcription
DMRX:73002001	X	73002001	73003000	1000	1	2.20E-10	0.79	19	1.9	LOC100360246;RGD1563270	
DMRX:73399001	X	73399001	73402000	3000	3	2.60E-22	1.05	16	0.53	Zfp449	Transcription
DMRX:73496001	X	73496001	73501000	5000	2	9.70E-08	0.71	78	1.56	Fam9b	
DMRX:74410001	X	74410001	74412000	2000	1	6.20E-07	-0.52	17	0.85	Jpx	
DMRX:74679001	X	74679001	74680000	1000	1	3.20E-10	-0.48	14	1.4	Slc16a2	
DMRX:74977001	X	74977001	74978000	1000	1	2.30E-09	-0.67	13	1.3	RGD1561931;Mir672	
DMRX:76039001	X	76039001	76042000	3000	1	3.20E-09	-0.54	24	0.8	LOC100912991;LOC102557587;Pbdc1	
DMRX:76899001	X	76899001	76901000	2000	1	1.80E-11	-0.61	34	1.7	Atrx	Transcription
DMRX:77255001	X	77255001	77258000	3000	1	2.40E-07	-0.41	29	0.97	LOC680814;Pgk1	Metabolism
DMRX:77294001	X	77294001	77296000	2000	2	2.00E-13	0.92	88	4.4	Taf9b;LOC100359505	
DMRX:77485001	X	77485001	77487000	2000	1	9.70E-08	-0.44	66	3.3	Fndc3c1	Proteolysis
DMRX:77514001	X	77514001	77516000	2000	1	5.20E-13	-0.39	21	1.05	Fndc3c1	Proteolysis
DMRX:77547001	X	77547001	77549000	2000	1	1.70E-08	-0.53	18	0.9	Fndc3c1	Proteolysis
DMRX:77879001	X	77879001	77880000	1000	1	1.20E-08	-0.71	7	0.7	RGD1560821	Translation
DMRX:78176001	X	78176001	78181000	5000	1	7.00E-07	-0.28	43	0.86	P2ry10	Signaling
DMRX:78204001	X	78204001	78205000	1000	1	1.10E-07	-0.7	6	0.6	P2ry10	Signaling
DMRX:78217001	X	78217001	78218000	1000	1	3.80E-07	-0.39	9	0.9	P2ry10;LOC108353297	Signaling
DMRX:78384001	X	78384001	78387000	3000	2	3.60E-11	-0.43	27	0.9	Gpr174;LOC102552443	Signaling
DMRX:79066001	X	79066001	79068000	2000	1	1.20E-08	-0.63	13	0.65	RGD1561552	Cytoskeleton
DMRX:80135001	X	80135001	80136000	1000	1	8.80E-16	-0.75	11	1.1	Hmgn5	Epigenetic
DMRX:80280001	X	80280001	80281000	1000	1	7.70E-08	0.57	10	1	Sh3bgrl	
DMRX:82277001	X	82277001	82278000	1000	1	4.00E-07	-0.55	3	0.3	Cylc1	Development
DMRX:84690001	X	84690001	84695000	5000	1	6.40E-07	-0.4	45	0.9	Chm	Signaling
DMRX:85272001	X	85272001	85276000	4000	1	3.50E-07	-0.26	31	0.78	Dach2	Transcription
DMRX:92639001	X	92639001	92640000	1000	1	1.30E-16	0.99	2	0.2	Pcdh11x	
DMRX:92919001	X	92919001	92920000	1000	1	2.60E-09	0.55	5	0.5	Pcdh11x	
DMRX:93090001	X	93090001	93093000	3000	1	8.20E-07	-0.34	63	2.1	Pcdh11x	
DMRX:93139001	X	93139001	93140000	1000	1	6.00E-13	0.56	15	1.5	Pcdh11x	
DMRX:97267001	X	97267001	97270000	3000	1	3.10E-09	-0.64	7	0.23	Cldn34c4	Cell Junction
DMRX:99763001	X	99763001	99766000	3000	1	2.20E-20	0.79	29	0.97	Diaph2	
DMRX:104245001	X	104245001	104247000	2000	1	6.00E-07	0.32	35	1.75	Rps27a-ps4	
DMRX:104973001	X	104973001	104974000	1000	1	1.40E-08	-0.5	6	0.6	Xkrx	
DMRX:104986001	X	104986001	104987000	1000	1	2.80E-07	-0.48	9	0.9	Xkrx;LOC102554368	
DMRX:105002001	X	105002001	105003000	1000	1	7.70E-08	0.34	1	0.1	LOC102554368;Arl13a	
DMRX:105006001	X	105006001	105007000	1000	1	2.00E-07	-0.66	2	0.2	LOC102554368;Arl13a	
DMRX:105093001	X	105093001	105095000	2000	1	1.10E-07	-0.53	18	0.9	RGD1565132	
DMRX:105489001	X	105489001	105493000	4000	1	1.80E-07	-0.3	35	0.88	Armcx4	
DMRX:105658001	X	105658001	105659000	1000	1	2.90E-07	-0.58	13	1.3	RGD1564480	
DMRX:106182001	X	106182001	106185000	3000	2	1.90E-08	-0.44	9	0.3	Tcp11x2	Cytoskeleton
DMRX:106302001	X	106302001	106303000	1000	1	7.00E-11	-0.65	11	1.1	LOC102553815;Gprasp1	Transcription
DMRX:106499001	X	106499001	106500000	1000	1	1.40E-08	-0.72	5	0.5	MGC109340	
DMRX:107046001	X	107046001	107050000	4000	1	2.80E-07	0.38	47	1.18	LOC100361246;Trnaa-ugc	
DMRX:107052001	X	107052001	107057000	5000	2	1.80E-07	0.62	96	1.92	LOC100361246;Trnaa-ugc	
DMRX:107250001	X	107250001	107251000	1000	1	7.00E-07	0.47	17	1.7	LOC100361093;Trnaa-ugc	
DMRX:107387001	X	107387001	107388000	1000	1	1.00E-07	-0.53	8	0.8	Morf4l2	Epigenetic
DMRX:108824001	X	108824001	108825000	1000	1	7.70E-08	-0.53	3	0.3	Il1rapl2	Receptor

DMRX:109160001	X	109160001	109167000	7000	2	4.40E-12	-0.41	62	0.89	Il1rapl2	Receptor
DMRX:109431001	X	109431001	109433000	2000	1	2.80E-09	0.27	41	2.05	Il1rapl2	Receptor
DMRX:109468001	X	109468001	109470000	2000	2	4.60E-09	-0.71	17	0.85	Il1rapl2	Receptor
DMRX:110973001	X	110973001	110978000	5000	3	8.20E-08	-0.33	43	0.86	Tbc1d8b	Signaling
DMRX:111877001	X	111877001	111880000	3000	2	1.40E-07	-0.61	29	0.97	Tsc22d3	
DMRX:113230001	X	113230001	113231000	1000	1	4.60E-07	-0.61	13	1.3	RGD1566136	
DMRX:113408001	X	113408001	113411000	3000	2	6.50E-08	-0.36	21	0.7	Gucy2f	Signaling
DMRX:114084001	X	114084001	114085000	1000	1	2.90E-13	-0.72	16	1.6	Tmem164	
DMRX:114087001	X	114087001	114088000	1000	1	8.10E-07	-0.41	18	1.8	Tmem164	
DMRX:114096001	X	114096001	114100000	4000	1	7.30E-12	-0.58	48	1.2	Tmem164	
DMRX:114225001	X	114225001	114227000	2000	1	6.10E-07	-0.56	17	0.85	Ammecr1	
DMRX:114557001	X	114557001	114559000	2000	1	3.90E-09	-0.53	29	1.45	Chrdl1	
DMRX:114627001	X	114627001	114628000	1000	1	8.30E-07	-0.62	3	0.3	Chrdl1	
DMRX:114815001	X	114815001	114817000	2000	1	1.40E-10	-0.44	16	0.8	Pak3	Signaling
DMRX:115173001	X	115173001	115174000	1000	1	1.80E-08	-0.56	15	1.5	Dcx	
DMRX:115269001	X	115269001	115270000	1000	1	1.80E-08	0.62	26	2.6	RGD1566355	
DMRX:115655001	X	115655001	115658000	3000	1	3.20E-07	-0.25	31	1.03	Trpc5	Transport
DMRX:115703001	X	115703001	115705000	2000	1	9.00E-09	-0.6	22	1.1	Trpc5;Trpc5os	Transport
DMRX:115779001	X	115779001	115780000	1000	1	7.50E-07	-0.67	14	1.4	Trpc5	Transport
DMRX:117248001	X	117248001	117250000	2000	2	2.20E-09	-0.58	9	0.45	Olr1758-ps	
DMRX:117541001	X	117541001	117543000	2000	1	4.30E-08	-0.59	12	0.6	Olr1762-ps	
DMRX:118544001	X	118544001	118545000	1000	1	3.10E-11	-0.57	4	0.4	Lrch2	
DMRX:118727001	X	118727001	118729000	2000	1	1.90E-09	-0.46	20	1	Ott	
DMRX:119071001	X	119071001	119073000	2000	1	4.40E-09	-0.58	16	0.8	Pls3	Cytoskeleton
DMRX:119075001	X	119075001	119078000	3000	1	1.80E-12	-0.57	35	1.17	Pls3	Cytoskeleton
DMRX:123073001	X	123073001	123076000	3000	1	6.60E-07	-0.27	29	0.97	RGD1561445	
DMRX:123317001	X	123317001	123320000	3000	1	1.20E-20	0.37	28	0.93	RGD1565032;LOC100361883	
DMRX:123737001	X	123737001	123738000	1000	1	8.60E-11	-0.62	14	1.4	Upf3b	
DMRX:123990001	X	123990001	123991000	1000	1	9.10E-07	0.58	18	1.8	Rhox2;Rhox8;LOC100364002	Development
DMRX:123998001	X	123998001	123999000	1000	1	3.60E-13	0.98	31	3.1	Rhox2;LOC100364002	Development
DMRX:124621001	X	124621001	124622000	1000	1	6.40E-07	-0.53	5	0.5	Atp1b4	Transport
DMRX:128178001	X	128178001	128184000	6000	2	1.10E-07	-0.33	65	1.08	Thoc2	Transcription
DMRX:129220001	X	129220001	129221000	1000	1	8.40E-07	-0.59	3	0.3	Tenm1	
DMRX:134756001	X	134756001	134758000	2000	1	4.50E-07	-0.51	18	0.9	Ocrl	Signaling
DMRX:135180001	X	135180001	135182000	2000	1	1.10E-13	-0.73	20	1	Bcorl1	
DMRX:135184001	X	135184001	135186000	2000	1	9.90E-07	-0.36	21	1.05	Bcorl1	
DMRX:135251001	X	135251001	135253000	2000	1	9.80E-08	-0.52	31	1.55	Elf4	Transcription
DMRX:135750001	X	135750001	135755000	5000	2	8.90E-11	-0.76	26	0.52	RGD1564955	
DMRX:136145001	X	136145001	136147000	2000	1	4.40E-16	0.51	32	1.6	Enox2;LOC103690897	Metabolism
DMRX:136746001	X	136746001	136747000	1000	1	3.20E-14	0.76	4	0.4	Olr1765	Signaling
DMRX:136894001	X	136894001	136895000	1000	1	2.00E-11	-0.69	3	0.3	Olr1766	Receptor
DMRX:138917001	X	138917001	138919000	2000	1	6.00E-09	-0.62	8	0.4	Hs6st2	Transport
DMRX:139457001	X	139457001	139458000	1000	1	2.80E-10	-0.62	11	1.1	Gpc4	
DMRX:139935001	X	139935001	139936000	1000	1	8.30E-09	-0.57	8	0.8	Gpc3	
DMRX:143369001	X	143369001	143370000	1000	1	7.00E-08	-0.76	5	0.5	Atp11c	Transport
DMRX:143445001	X	143445001	143446000	1000	1	1.90E-09	-0.39	13	1.3	Atp11c	Transport
DMRX:144352001	X	144352001	144353000	1000	1	3.80E-07	-0.77	2	0.2	RGD1561327	Cytoskeleton
DMRX:144706001	X	144706001	144708000	2000	1	8.90E-07	-0.41	8	0.4	Ldoc1	
DMRX:149826001	X	149826001	149828000	2000	1	2.10E-10	-0.55	9	0.45	Olr1759-ps	
DMRX:150476001	X	150476001	150479000	3000	1	1.70E-22	1.18	39	1.3	Olr1761-ps	
DMRX:150480001	X	150480001	150482000	2000	1	1.70E-10	-0.34	8	0.4	Olr1761-ps	
DMRX:150492001	X	150492001	150494000	2000	1	3.10E-13	0.62	37	1.85	Olr1761-ps	
DMRX:150542001	X	150542001	150550000	8000	3	1.30E-09	-0.47	44	0.55	Olr1770-ps;LOC103694521	
DMRX:151301001	X	151301001	151306000	5000	1	2.00E-07	-0.43	51	1.02	LOC108349259;Mir3580;Mir465	
DMRX:153047001	X	153047001	153048000	1000	1	2.00E-08	-0.58	4	0.4	Pnma5	
DMRX:153951001	X	153951001	153954000	3000	1	6.00E-07	-0.35	28	0.93	Aff2	Transcription
DMRX:155833001	X	155833001	155839000	6000	3	4.00E-09	0.34	170	2.83	Dkc1	Cell Cycle
DMRX:155840001	X	155840001	155845000	5000	2	5.60E-25	0.72	128	2.56	Dkc1	Cell Cycle
DMRX:155850001	X	155850001	155852000	2000	1	5.60E-15	0.89	124	6.2	Dkc1	Cell Cycle
DMRX:155853001	X	155853001	155856000	3000	1	8.30E-07	-0.33	112	3.73	Dkc1	Cell Cycle
DMRX:155865001	X	155865001	155866000	1000	1	3.70E-07	0.57	26	2.6	Dkc1	Cell Cycle

DMRX:155868001	X	155868001	155869000	1000	1	8.40E-08	0.54	26	2.6	Dkc1	Cell Cycle
DMRX:156687001	X	156687001	156689000	2000	1	7.30E-08	-0.63	7	0.35	Mecp2;LOC103694527	Transcription
DMRX:156934001	X	156934001	156935000	1000	1	3.90E-08	-0.61	4	0.4	L1cam	
DMRX:157282001	X	157282001	157283000	1000	1	2.70E-13	0.48	6	0.6	Atp2b3	Transport
DMRX:157424001	X	157424001	157425000	1000	1	1.80E-16	0.97	11	1.1	Zfp92;LOC103694532	
DMRX:157886001	X	157886001	157888000	2000	1	4.40E-07	-0.56	11	0.55	Fam122b	
DMRX:158830001	X	158830001	158831000	1000	1	8.60E-11	-0.49	11	1.1	Ints6l	Transcription
DMRX:159894001	X	159894001	159899000	5000	1	3.60E-07	-0.24	45	0.9	Rbmx	
DMRY:413001	Y	413001	415000	2000	2	3.10E-10	-0.61	16	0.8	LOC103694545;LOC103694546;<br>Uba1y	Proteolysis
DMRY:1122001	Y	1122001	1125000	3000	1	6.30E-07	-0.4	23	0.77	Kdm6a	Epigenetic
DMRY:1135001	Y	1135001	1136000	1000	1	3.40E-08	-0.46	4	0.4	Kdm6a	Epigenetic
DMRY:1157001	Y	1157001	1160000	3000	1	2.00E-07	-0.47	26	0.87	Kdm6a	Epigenetic
DMRY:1173001	Y	1173001	1177000	4000	1	2.90E-08	-0.64	24	0.6	Kdm6a	Epigenetic
DMRY:1179001	Y	1179001	1180000	1000	1	2.30E-08	-0.54	4	0.4	Kdm6a	Epigenetic
DMRY:1208001	Y	1208001	1209000	1000	1	7.10E-07	-0.71	1	0.1	Kdm6a;Ddx3	Epigenetic
DMRY:1316001	Y	1316001	1320000	4000	1	1.60E-07	-0.36	29	0.72	Usp9y	Protease
DMRY:1355001	Y	1355001	1358000	3000	1	3.90E-07	-0.39	32	1.07	Usp9y	Protease
DMRY:1392001	Y	1392001	1395000	3000	1	3.40E-07	-0.25	32	1.07	Usp9y	Protease
DMRY:1409001	Y	1409001	1415000	6000	1	1.00E-08	-0.36	68	1.13	Usp9y	Protease
