# Supplementary material for: Environmental induced transgenerational inheritance impacts systems epigenetics in disease etiology
Source: Sci Rep. 2022 Apr 19;12:5452. doi: 10.1038/s41598-022-09336-0 (PMC9018793; doi:10.1038/s41598-022-09336-0)
Supplement: Supplementary file 21 — Supplementary Table S13. [file 41598_2022_9336_MOESM21_ESM.pdf]

Supplemental Table S13
Methoxychlor DMR p<1e-06

DMR Name	Chr	Start	Stop	Length	# Sig Win	minP	maxLFC	CpG #	CpG Density	Gene Annotation	Gene Category
DMR1:537001	1	537001	540000	3000	1	8.20E-07	-0.53	13	0.43	Vom2r-ps5;LOC102556463	
DMR1:626001	1	626001	628000	2000	1	2.90E-09	0.62	75	3.75	Vom2r-ps5	
DMR1:1104001	1	1104001	1108000	4000	1	9.00E-08	-1.09	68	1.7	Raet1e	Immune
DMR1:1672001	1	1672001	1673000	1000	1	4.80E-07	-0.52	15	1.5	LOC100910848;LOC108348623;RGD1561843	
DMR1:4490001	1	4490001	4492000	2000	1	3.40E-07	-0.66	17	0.85	Adgb	
DMR1:6589001	1	6589001	6591000	2000	1	9.50E-10	0.47	27	1.35	Utrn	
DMR1:7212001	1	7212001	7214000	2000	1	6.80E-07	0.5	27	1.35	LOC108349485;Plagl1	Transcription
DMR1:7233001	1	7233001	7234000	1000	1	5.70E-10	0.48	18	1.8	Plagl1	Transcription
DMR1:7249001	1	7249001	7252000	3000	1	9.50E-07	0.49	61	2.03	Plagl1;LOC108349486	Transcription
DMR1:7950001	1	7950001	7955000	5000	1	5.80E-08	0.45	74	1.48	Aig1	
DMR1:8006001	1	8006001	8007000	1000	1	8.70E-07	0.41	13	1.3	Aig1	
DMR1:8311001	1	8311001	8316000	5000	1	1.50E-07	0.44	142	2.84	Hivep2	
DMR1:13571001	1	13571001	13574000	3000	1	6.90E-07	0.65	73	2.43	Nhsl1	
DMR1:15810001	1	15810001	15811000	1000	1	2.00E-07	0.44	20	2	Bclaf1	
DMR1:15965001	1	15965001	15968000	3000	1	9.60E-09	0.48	61	2.03	Pde7b	Signaling
DMR1:16841001	1	16841001	16844000	3000	1	1.30E-08	0.47	44	1.47	Hbs1l	Translation
DMR1:16868001	1	16868001	16870000	2000	1	6.30E-08	0.47	32	1.6	Hbs1l;LOC108348873	Translation
DMR1:17642001	1	17642001	17648000	6000	1	4.70E-07	0.42	196	3.27	Ptprk	Signaling
DMR1:17998001	1	17998001	1.80E+07	2000	1	3.20E-07	0.4	34	1.7	Ptprk	Signaling
DMR1:19310001	1	19310001	19313000	3000	1	3.40E-09	0.44	23	0.77	Arhgap18	Signaling
DMR1:20122001	1	20122001	20123000	1000	1	2.70E-18	0.63	11	1.1	Samd3;LOC100360791	
DMR1:20929001	1	20929001	20930000	1000	1	4.60E-09	0.57	3	0.3	Epb41l2	
DMR1:21021001	1	21021001	21022000	1000	1	2.50E-07	0.59	3	0.3	Epb41l2	
DMR1:21138001	1	21138001	21144000	6000	1	2.90E-07	0.51	107	1.78	LOC102552728;Akap7;LOC100364016	Translation
DMR1:21540001	1	21540001	21545000	5000	1	3.90E-07	0.5	130	2.6	Arg1;Med23	Metabolism;Transcription
DMR1:21614001	1	21614001	21615000	1000	1	3.20E-07	0.51	17	1.7	Enpp3	
DMR1:22262001	1	22262001	22264000	2000	1	4.20E-07	0.42	44	2.2	Stx7	Transcription
DMR1:22671001	1	22671001	22675000	4000	1	1.10E-07	-0.49	34	0.85	Vnn3	Metabolism
DMR1:23394001	1	23394001	23396000	2000	1	2.10E-10	0.54	17	0.85	Eya4	
DMR1:24019001	1	24019001	24020000	1000	1	7.70E-08	0.65	21	2.1	Slc2a12	
DMR1:24224001	1	24224001	24225000	1000	1	2.80E-08	0.61	8	0.8	Sgk1	Signaling
DMR1:24247001	1	24247001	24249000	2000	1	2.70E-07	0.47	23	1.15	Sgk1	Signaling
DMR1:31894001	1	31894001	31895000	1000	1	9.90E-07	0.53	14	1.4	Tppp	Cytoskeleton
DMR1:32067001	1	32067001	32068000	1000	1	9.10E-09	0.48	22	2.2	Nkd2	
DMR1:32079001	1	32079001	32081000	2000	1	7.80E-08	0.42	38	1.9	Nkd2;Slc12a7	Transport
DMR1:32093001	1	32093001	32100000	7000	1	1.10E-08	0.51	137	1.96	Nkd2;Slc12a7	Transport
DMR1:32263001	1	32263001	32264000	1000	1	7.50E-08	0.51	16	1.6	Tert	Transcription
DMR1:37254001	1	37254001	37257000	3000	1	4.70E-08	-0.74	18	0.6	Adcy2	
DMR1:37258001	1	37258001	37259000	1000	1	6.00E-07	-0.78	10	1	Adcy2	
DMR1:37270001	1	37270001	37278000	8000	1	2.10E-10	-0.42	60	0.75	Adcy2	
DMR1:37301001	1	37301001	37306000	5000	1	1.20E-07	-0.52	27	0.54	Adcy2	
DMR1:37323001	1	37323001	37331000	8000	1	1.50E-08	-0.64	52	0.65	Adcy2	
DMR1:37465001	1	37465001	37470000	5000	1	1.30E-08	-1.38	50	1	Adcy2	
DMR1:37483001	1	37483001	37488000	5000	1	6.80E-08	-0.48	34	0.68	Adcy2	
DMR1:37675001	1	37675001	37680000	5000	1	2.10E-07	-0.35	40	0.8	Adcy2	
DMR1:41429001	1	41429001	41431000	2000	1	8.90E-18	0.47	26	1.3	Esr1	
DMR1:43538001	1	43538001	43539000	1000	1	3.30E-07	-0.57	11	1.1	Oprm1	Signaling
DMR1:45993001	1	45993001	45994000	1000	1	5.70E-08	0.49	13	1.3	Arid1b	
DMR1:46067001	1	46067001	46071000	4000	1	5.90E-07	0.54	55	1.38	Arid1b	
DMR1:46916001	1	46916001	46918000	2000	1	7.10E-09	0.53	28	1.4	Synj2;LOC103690968	Signaling
DMR1:47236001	1	47236001	47238000	2000	1	2.60E-07	0.56	20	1	Sytl3	
DMR1:48022001	1	48022001	48023000	1000	1	2.20E-07	0.49	25	2.5	Acat2l1;LOC108349718;Tcp1	Metabolism;Translation
DMR1:48715001	1	48715001	48724000	9000	1	4.00E-09	0.6	178	1.98	Map3k4;Agpat4	Signaling;Metabolism
DMR1:49314001	1	49314001	49316000	2000	1	1.20E-08	0.51	18	0.9	Park2	
DMR1:49416001	1	49416001	49417000	1000	1	1.30E-07	0.48	10	1	Park2	
DMR1:50212001	1	50212001	50213000	1000	1	1.50E-07	0.55	14	1.4	Pacrg	
DMR1:52997001	1	52997001	52999000	2000	1	7.70E-07	0.48	27	1.35	Prr18;Sft2d1	
DMR1:53239001	1	53239001	53240000	1000	1	2.50E-07	0.43	22	2.2	Rps6ka2	Golgi
DMR1:53293001	1	53293001	53295000	2000	1	2.70E-07	0.52	50	2.5	Rps6ka2	Golgi
DMR1:53308001	1	53308001	53310000	2000	1	9.60E-07	0.43	15	0.75	Rps6ka2	Golgi

DMR1:55899001	1	55899001	55901000	2000	1	2.90E-13	0.62	12	0.6	Vom2r9	Signaling
DMR1:56319001	1	56319001	56321000	2000	1	6.20E-07	0.43	26	1.3	Smoc2	Signaling
DMR1:56925001	1	56925001	56927000	2000	1	4.40E-07	0.57	20	1	Wdr27	
DMR1:57797001	1	57797001	57799000	2000	1	1.70E-07	0.55	36	1.8	Rgmb	
DMR1:61125001	1	61125001	61126000	1000	1	7.30E-07	-0.46	9	0.9	Vom1r-ps24;Vom1r20	Receptor
DMR1:63560001	1	63560001	63561000	1000	1	1.80E-09	0.4	23	2.3	Vom2r26	Signaling
DMR1:66027001	1	66027001	66030000	3000	1	8.10E-07	-0.45	30	1	Vom2r36;Vom2r35;LOC108348434	Signaling
DMR1:67196001	1	67196001	67199000	3000	1	3.90E-08	-0.49	27	0.9	Vom1r43	Receptor
DMR1:69481001	1	69481001	69485000	4000	1	8.60E-07	-0.44	37	0.92	Vom2r-ps44	
DMR1:69642001	1	69642001	69645000	3000	1	6.20E-07	-0.87	18	0.6	Vom2r-ps46;LOC103691032	
DMR1:72435001	1	72435001	72439000	4000	1	3.50E-07	0.44	71	1.77	Sbk2;Ssc5d	Signaling;Protease
DMR1:72451001	1	72451001	72453000	2000	1	3.90E-09	0.48	41	2.05	Ssc5d;Nat14	Protease;Metabolism
DMR1:76337001	1	76337001	76340000	3000	1	7.50E-08	-0.99	16	0.53	Sult2a2;LOC100363218	
DMR1:76411001	1	76411001	76418000	7000	1	3.80E-08	-0.66	30	0.43	Sult2a2	
DMR1:76443001	1	76443001	76447000	4000	1	1.10E-07	-0.72	24	0.6	Sult2a2	
DMR1:76581001	1	76581001	76590000	9000	2	5.20E-07	-0.61	34	0.38	Sult2a6;Sult2a1	
DMR1:78097001	1	78097001	78107000	10000	2	1.90E-07	0.48	236	2.36	Dhx34	Transcription
DMR1:78157001	1	78157001	78159000	2000	1	7.20E-10	0.5	36	1.8	C5ar2	Signaling
DMR1:78779001	1	78779001	78781000	2000	1	2.00E-07	0.47	50	2.5	Prkd2	Signaling
DMR1:79805001	1	79805001	79806000	1000	1	1.30E-07	0.5	18	1.8	Pglyrp1;Ccdc61	Epigenetic
DMR1:80260001	1	80260001	80262000	2000	1	6.40E-07	0.42	19	0.95	Ercc1;Cd3eap;Ppp1r13l	Transcription;Transcription
DMR1:80355001	1	80355001	80357000	2000	1	4.80E-07	0.72	12	0.6	Mark4	Signaling
DMR1:80505001	1	80505001	80507000	2000	1	7.10E-07	0.42	27	1.35	Clasrp;LOC108348935	Translation
DMR1:80645001	1	80645001	80648000	3000	1	4.60E-08	0.52	26	0.87	Nectin2	
DMR1:80838001	1	80838001	80840000	2000	1	3.80E-07	0.42	39	1.95	PVR	
DMR1:80917001	1	80917001	80918000	1000	1	9.80E-07	0.46	5	0.5	Ceacam20;Zfp180	Transcription
DMR1:81290001	1	81290001	81292000	2000	1	3.80E-10	0.48	39	1.95	Smg9;Irgc	
DMR1:81351001	1	81351001	81354000	3000	1	4.80E-07	0.49	53	1.77	Plaur	Receptor
DMR1:81458001	1	81458001	81460000	2000	1	1.60E-07	0.51	35	1.75	Zfp575;Ethe1	Transcription;Metabolism
DMR1:81862001	1	81862001	81863000	1000	1	4.20E-11	0.59	11	1.1	Atp1a3	Transport
DMR1:81895001	1	81895001	81896000	1000	1	2.00E-07	0.47	13	1.3	Grik5	Receptor
DMR1:81933001	1	81933001	81936000	3000	2	4.80E-10	0.51	54	1.8	Grik5	Receptor
DMR1:81978001	1	81978001	81980000	2000	1	2.30E-07	0.45	43	2.15	Pou2f2	
DMR1:82005001	1	82005001	82007000	2000	1	4.00E-07	0.45	55	2.75	Pou2f2;LOC103691054	
DMR1:82076001	1	82076001	82079000	3000	1	5.70E-08	0.55	54	1.8	Dedd2;Zfp526	
DMR1:82113001	1	82113001	82116000	3000	1	1.50E-11	0.57	85	2.83	Gsk3a;Erf	Signaling;Transcription
DMR1:82139001	1	82139001	82145000	6000	1	2.20E-07	0.5	142	2.37	Cic	Transcription
DMR1:82159001	1	82159001	82162000	3000	1	2.40E-07	0.48	50	1.67	Cic;Pafah1b3;Prr19	Transcription;Golgi
DMR1:82199001	1	82199001	82202000	3000	1	2.00E-07	0.45	57	1.9	Megf8	Extracellular Matrix
DMR1:82204001	1	82204001	82211000	7000	2	1.10E-09	0.63	143	2.04	Megf8	Extracellular Matrix
DMR1:82215001	1	82215001	82220000	5000	2	1.70E-08	0.5	86	1.72	Megf8	Extracellular Matrix
DMR1:82257001	1	82257001	82261000	4000	1	6.80E-07	0.47	73	1.82	LOC102549342;Lipe;Cxcl17	Metabolism;Growth Factors
DMR1:82277001	1	82277001	82280000	3000	1	3.80E-09	0.53	67	2.23	LOC102549342;Cxcl17	Growth Factors
DMR1:82288001	1	82288001	82291000	3000	1	9.20E-07	0.46	70	2.33	LOC102549342;Cxcl17	Growth Factors
DMR1:82506001	1	82506001	82510000	4000	1	7.60E-07	0.43	82	2.05	Tgfb1;Ccdc97;Hnrnpul1	Growth Factors
DMR1:83651001	1	83651001	83653000	2000	1	1.00E-09	0.58	19	0.95	LOC103691073;Cyp2a3	Metabolism
DMR1:84053001	1	84053001	84055000	2000	1	5.80E-08	0.49	28	1.4	Adck4	
DMR1:84061001	1	84061001	84063000	2000	1	1.20E-07	0.49	27	1.35	Adck4;Numbl	Cytoskeleton
DMR1:84171001	1	84171001	84173000	2000	1	8.50E-08	0.44	26	1.3	Shkbp1;Sptbn4	Cytoskeleton
DMR1:84297001	1	84297001	84300000	3000	1	6.20E-08	0.53	58	1.93	Sertad1;Prx	Transcription
DMR1:84323001	1	84323001	84327000	4000	1	1.80E-07	0.48	62	1.55	Prx;LOC102552059;Hipk4	
DMR1:84338001	1	84338001	84341000	3000	1	9.40E-08	0.52	60	2	Hipk4;Pld3	Metabolism
DMR1:84418001	1	84418001	84421000	3000	1	4.20E-10	0.55	43	1.43	Akt2	Signaling
DMR1:84443001	1	84443001	84457000	14000	1	1.20E-07	0.46	244	1.74	Akt2;LOC103691074	Signaling
DMR1:85016001	1	85016001	85022000	6000	2	4.20E-09	0.74	153	2.55	Fcgbp	Extracellular Matrix
DMR1:85027001	1	85027001	85028000	1000	1	3.70E-08	0.51	24	2.4	Fcgbp	Extracellular Matrix
DMR1:85194001	1	85194001	85197000	3000	1	8.80E-07	0.53	60	2	Pak4	Signaling
DMR1:86332001	1	86332001	86339000	7000	1	2.40E-07	-0.46	36	0.51	Vom2r-ps22	
DMR1:86656001	1	86656001	86659000	3000	1	3.40E-09	-0.59	23	0.77	Vom1r-ps8	
DMR1:86936001	1	86936001	86943000	7000	2	4.70E-08	0.45	140	2	Sars2;LOC102550248;Ccer2;Nfkbib;Sirt2	Translation
DMR1:87014001	1	87014001	87017000	3000	1	3.20E-07	0.47	63	2.1	Hnrnpl;Ech1;Lgals4	Metabolism;Extracellular Matrix
DMR1:87066001	1	87066001	87067000	1000	1	3.40E-08	0.54	18	1.8	Capn12;LOC102550499	Protease

DMR1:87104001	1	87104001	87108000	4000	1	4.40E-07	0.57	49	1.23	Actn4	
DMR1:87137001	1	87137001	87139000	2000	1	1.40E-07	0.44	25	1.25	Actn4;LOC102550585	
DMR1:87157001	1	87157001	87159000	2000	2	1.60E-07	0.56	47	2.35	Actn4;LOC102550585;LOC100909725	
DMR1:87269001	1	87269001	87272000	3000	1	8.80E-09	0.5	71	2.37	Sipa1l3	Signaling
DMR1:87369001	1	87369001	87371000	2000	1	4.00E-13	0.61	43	2.15	Sipa1l3	Signaling
DMR1:87391001	1	87391001	87392000	1000	1	4.00E-08	0.63	13	1.3	Sipa1l3;LOC108348907	Signaling
DMR1:87446001	1	87446001	87451000	5000	1	2.30E-08	0.47	90	1.8	Sipa1l3	Signaling
DMR1:87458001	1	87458001	87460000	2000	1	1.60E-09	0.48	40	2	Sipa1l3	Signaling
DMR1:87949001	1	87949001	87955000	6000	1	2.80E-10	0.51	81	1.35	Map4k1;Ryr1	Ion Channel
DMR1:88003001	1	88003001	88006000	3000	1	5.10E-09	0.52	79	2.63	Ryr1	Ion Channel
DMR1:88009001	1	88009001	88010000	1000	1	5.70E-09	0.53	38	3.8	Ryr1	Ion Channel
DMR1:88085001	1	88085001	88086000	1000	1	2.00E-08	0.49	16	1.6	Rasgrp4;Fam98c	Transcription;Translation
DMR1:88159001	1	88159001	88161000	2000	1	6.10E-08	0.55	46	2.3	Catsperg1;Kcnk6	Transport
DMR1:88748001	1	88748001	88750000	2000	1	1.30E-07	0.5	36	1.8	Thap8;Clip3	Transcription
DMR1:89128001	1	89128001	89133000	5000	1	4.10E-07	0.44	68	1.36	Haus5;LOC100912333;LOC688924	
DMR1:89340001	1	89340001	89343000	3000	1	3.10E-08	0.47	51	1.7	LOC102554355;Mag;LOC108349549	Immune
DMR1:89495001	1	89495001	89496000	1000	1	1.80E-07	0.48	21	2.1	Fxyd1;Lgi4;Fxyd3	Transport
DMR1:89955001	1	89955001	89958000	3000	1	5.30E-07	0.6	40	1.33	Scgb1b30;Wtip	
DMR1:89963001	1	89963001	89968000	5000	1	8.10E-07	0.39	87	1.74	Scgb1b30;Wtip	
DMR1:89973001	1	89973001	89976000	3000	1	5.60E-09	0.58	55	1.83	Wtip	
DMR1:90068001	1	90068001	90071000	3000	1	1.70E-08	0.55	57	1.9	Gpi	Metabolism
DMR1:90073001	1	90073001	90077000	4000	2	2.90E-08	0.44	75	1.88	Gpi	Metabolism
DMR1:90511001	1	90511001	90517000	6000	1	4.10E-08	0.54	149	2.48	Kctd15	Cytoskeleton
DMR1:90560001	1	90560001	90563000	3000	1	4.80E-07	0.46	68	2.27	Chst8;LOC108349467;LOC108349466	Transport
DMR1:90679001	1	90679001	90682000	3000	1	6.90E-07	0.48	39	1.3	Chst8	Transport
DMR1:91223001	1	91223001	91229000	6000	1	1.30E-07	0.47	80	1.33	Pepd	Protease
DMR1:91357001	1	91357001	91358000	1000	1	8.10E-07	0.38	19	1.9	Cebpa	Transcription
DMR1:91443001	1	91443001	91447000	4000	2	2.30E-08	0.51	70	1.75	Slc7a10;Lrp3	Transport;Binding Proteins
DMR1:92705001	1	92705001	92708000	3000	1	9.90E-07	0.39	123	4.1	Tshz3	Transcription
DMR1:92722001	1	92722001	92724000	2000	1	9.90E-07	0.42	38	1.9	Tshz3	Transcription
DMR1:93756001	1	93756001	93757000	1000	1	5.60E-07	-0.71	16	1.6	Zfp536	Transcription
DMR1:94044001	1	94044001	94048000	4000	1	8.10E-07	0.44	58	1.45	Zfp536	Transcription
DMR1:94075001	1	94075001	94077000	2000	1	5.10E-08	0.52	38	1.9	Zfp536	Transcription
DMR1:94651001	1	94651001	94655000	4000	1	8.70E-07	0.78	72	1.8	LOC108349756;Pop4	
DMR1:94820001	1	94820001	94821000	1000	1	5.10E-08	0.43	13	1.3	LOC108348090;RGD1584023	
DMR1:95414001	1	95414001	95415000	1000	1	1.20E-10	0.62	9	0.9	LOC102548133;LOC102548014;Zfp939	
DMR1:99079001	1	99079001	99080000	1000	1	1.60E-12	0.35	19	1.9	Vom2r38;Vom2r37;LOC108349760;LOC103691104	Signaling
DMR1:100314001	1	100314001	100315000	1000	1	5.40E-08	0.52	15	1.5	Shank1	
DMR1:100328001	1	100328001	100331000	3000	1	1.40E-08	0.51	62	2.07	Shank1	
DMR1:100463001	1	100463001	100464000	1000	1	6.00E-08	0.43	11	1.1	Lrrc4b;Aspdh;Josd2	Metabolism;Protease
DMR1:100475001	1	100475001	100478000	3000	1	1.90E-08	0.45	37	1.23	Lrrc4b;Aspdh;Josd2;LOC100909904	Metabolism;Protease
DMR1:100490001	1	100490001	100493000	3000	1	3.10E-07	0.45	45	1.5	LOC100909904;Emc10;Fam71e1	Epigenetic
DMR1:100542001	1	100542001	100551000	9000	1	1.30E-07	0.54	232	2.58	Spib;Pold1;Nr1h2	Transcription;Transcription;Transcription
DMR1:100581001	1	100581001	100582000	1000	1	8.40E-07	0.46	10	1	Napsa	
DMR1:100774001	1	100774001	100777000	3000	1	4.60E-07	0.46	44	1.47	Vrk3	Signaling
DMR1:100897001	1	100897001	100899000	2000	1	8.30E-08	0.46	38	1.9	Med25;Fuz;Ap2a1	Transcription;Transport
DMR1:100904001	1	100904001	100907000	3000	1	4.40E-07	0.66	51	1.7	Fuz;Ap2a1	Transport
DMR1:101005001	1	101005001	101007000	2000	1	1.10E-10	0.65	20	1	Irf3;Scaf1;Rras	Transcription;Translation;Signaling
DMR1:101008001	1	101008001	101010000	2000	1	3.90E-08	0.49	25	1.25	Scaf1;Rras	Translation;Signaling
DMR1:101030001	1	101030001	101032000	2000	1	2.90E-09	0.51	33	1.65	Prr12	
DMR1:101218001	1	101218001	101221000	3000	2	1.10E-08	0.46	37	1.23	Dkkl1;Tead2;Cd37	Transcription
DMR1:101275001	1	101275001	101279000	4000	1	2.50E-07	0.46	76	1.9	RGD1562492;LOC108349572	Transport
DMR1:101314001	1	101314001	101318000	4000	1	5.30E-07	0.58	70	1.75	Trpm4;Hrc	Transport
DMR1:101319001	1	101319001	101321000	2000	1	1.60E-08	0.65	38	1.9	Trpm4;Hrc;Ppfia3	Transport
DMR1:101359001	1	101359001	101360000	1000	1	1.20E-09	0.51	16	1.6	Ppfia3;Lin7b;Snrnp70	Cytoskeleton;Translation
DMR1:101367001	1	101367001	101369000	2000	1	9.90E-07	0.46	73	3.65	Ppfia3;Lin7b;Snrnp70	Cytoskeleton;Translation
DMR1:101413001	1	101413001	101415000	2000	1	2.40E-08	0.47	49	2.45	Kcna7;Ntf4;Lhb;Ruvbl2	Transport;Growth Factors;Hormone
DMR1:101637001	1	101637001	101643000	6000	1	7.30E-07	0.46	88	1.47	Mamstr;Fut2	Golgi
DMR1:101692001	1	101692001	101699000	7000	2	3.90E-09	0.49	159	2.27	Car11;Dbp;Sphk2;Rpl18;Fam83e;Spaca4	Transcription;Signaling;Translation

DMR1:101709001	1	101709001	101710000	1000	1	2.80E-08	0.56	19	1.9	Sphk2;Rpl18;Fam83e;Spaca4;Sult2b1	Signaling;Translation;Transport
DMR1:101873001	1	101873001	101874000	1000	1	1.30E-07	0.51	15	1.5	Kdelr1;Syngr4	Transport
DMR1:102231001	1	102231001	102232000	1000	1	8.40E-08	0.54	25	2.5	Ush1c	Cytoskeleton
DMR1:102505001	1	102505001	102508000	3000	1	3.00E-08	0.5	67	2.23	Sergef	Proteolysis
DMR1:102551001	1	102551001	102556000	5000	1	2.50E-07	0.46	79	1.58	Sergef	Proteolysis
DMR1:102635001	1	102635001	102640000	5000	1	7.90E-09	0.59	93	1.86	Sergef	Proteolysis
DMR1:102653001	1	102653001	102655000	2000	1	4.00E-12	0.59	27	1.35	Sergef	Proteolysis
DMR1:102681001	1	102681001	102682000	1000	1	1.30E-07	0.51	12	1.2	Tph1	
DMR1:102824001	1	102824001	102828000	4000	1	4.20E-07	0.57	45	1.12	Hps5	
DMR1:104187001	1	104187001	104188000	1000	1	2.30E-07	0.47	18	1.8	E2f8	Transcription
DMR1:104738001	1	104738001	104742000	4000	1	3.60E-07	0.4	73	1.82	Nav2	
DMR1:104886001	1	104886001	104888000	2000	1	5.40E-07	0.4	40	2	Nav2	
DMR1:104907001	1	104907001	104910000	3000	1	1.10E-09	0.57	51	1.7	Nav2	
DMR1:105411001	1	105411001	105414000	3000	1	5.80E-09	0.48	28	0.93	Nell1	Signaling
DMR1:105458001	1	105458001	105461000	3000	1	3.70E-07	0.46	33	1.1	Nell1	Signaling
DMR1:107389001	1	107389001	107392000	3000	1	1.20E-07	-0.47	9	0.3	Ccdc179	
DMR1:114139001	1	114139001	114141000	2000	1	2.20E-07	-0.44	21	1.05	Siglech;LOC108349722	
DMR1:114429001	1	114429001	114433000	4000	1	8.10E-11	0.54	39	0.98	Nipa1;LOC108349578	
DMR1:116630001	1	116630001	116634000	4000	1	7.60E-15	0.83	30	0.75	Ube3a	Proteolysis
DMR1:124634001	1	124634001	124636000	2000	1	4.80E-11	0.59	34	1.7	Otud7a	Protease
DMR1:125054001	1	125054001	125056000	2000	1	3.90E-07	0.5	27	1.35	Trpm1	Transport
DMR1:125190001	1	125190001	125195000	5000	3	2.70E-11	0.73	91	1.82	Fan1	
DMR1:125426001	1	125426001	125430000	4000	1	9.30E-08	0.46	48	1.2	Apba2	Transport
DMR1:125457001	1	125457001	125463000	6000	1	8.00E-10	0.51	120	2	Apba2	Transport
DMR1:125690001	1	125690001	125692000	2000	1	2.40E-10	0.75	39	1.95	Fam189a1	
DMR1:126165001	1	126165001	126166000	1000	1	2.50E-07	0.48	15	1.5	Tjp1	Cell Junction
DMR1:126912001	1	126912001	126915000	3000	1	4.60E-07	0.46	48	1.6	Pcsk6	Protease
DMR1:127151001	1	127151001	127157000	6000	1	4.30E-07	0.84	76	1.27	LOC102549616;LOC108349001;Lrrk1	Signaling
DMR1:128937001	1	128937001	128939000	2000	1	8.40E-08	0.45	25	1.25	Igf1r	Receptor
DMR1:129086001	1	129086001	129088000	2000	1	5.50E-07	0.44	39	1.95	Igf1r	Receptor
DMR1:129119001	1	129119001	129122000	3000	1	4.60E-09	0.59	52	1.73	Igf1r	Receptor
DMR1:129166001	1	129166001	129169000	3000	1	6.60E-07	0.51	83	2.77	Igf1r	Receptor
DMR1:134727001	1	134727001	134734000	7000	1	2.10E-08	0.49	119	1.7	Rgma	
DMR1:136058001	1	136058001	136060000	2000	1	2.70E-08	0.47	28	1.4	Slco3a1	Transport
DMR1:137308001	1	137308001	137310000	2000	1	8.40E-07	0.47	32	1.6	Akap13	
DMR1:138080001	1	138080001	138081000	1000	1	1.40E-08	0.44	3	0.3	Agbl1	Protease
DMR1:138351001	1	138351001	138352000	1000	1	3.30E-08	-0.77	5	0.5	Agbl1	Protease
DMR1:140486001	1	140486001	140488000	2000	1	3.30E-08	0.63	21	1.05	Mrpl46;Mrps11	Translation
DMR1:140597001	1	140597001	140600000	3000	1	2.50E-08	0.49	54	1.8	Aen;Isg20	Transcription
DMR1:140816001	1	140816001	140823000	7000	1	1.70E-08	0.55	87	1.24	Acan;Hapln3	Extracellular Matrix
DMR1:141057001	1	141057001	141061000	4000	1	2.50E-10	0.54	67	1.68	Abhd2	Protease
DMR1:141122001	1	141122001	141123000	1000	1	5.70E-09	0.5	19	1.9	Fanci	
DMR1:141778001	1	141778001	141780000	2000	1	2.10E-08	0.5	31	1.55	Zfp710	Transcription
DMR1:141841001	1	141841001	141843000	2000	1	9.40E-07	0.47	45	2.25	Zfp710;LOC100911225	Transcription
DMR1:141860001	1	141860001	141862000	2000	1	4.30E-11	0.55	64	3.2	Zfp710	Transcription
DMR1:141864001	1	141864001	141867000	3000	1	3.70E-07	0.44	55	1.83	Zfp710;Idh2	Transcription;Metabolism
DMR1:142178001	1	142178001	142179000	1000	1	5.30E-07	0.39	12	1.2	Fes;LOC102553801;Furin	Protease
DMR1:142730001	1	142730001	142733000	3000	1	5.20E-07	0.44	49	1.63	Wdr73;Nmb;LOC108349599;Sec11a	Protease
DMR1:142851001	1	142851001	142852000	1000	1	1.40E-07	0.49	21	2.1	Zfp592	
DMR1:142902001	1	142902001	142903000	1000	1	4.80E-07	0.42	17	1.7	Alpk3;LOC102555867	Signaling
DMR1:142906001	1	142906001	142910000	4000	1	1.00E-06	0.59	80	2	Alpk3;LOC102555867	Signaling
DMR1:143392001	1	143392001	143393000	1000	1	2.20E-09	0.51	23	2.3	Fsd2;Whamm	Proteolysis
DMR1:143655001	1	143655001	143657000	2000	1	2.60E-09	0.46	37	1.85	Btbd1;Tm6sf1	Proteolysis
DMR1:143679001	1	143679001	143682000	3000	1	4.60E-07	0.61	49	1.63	Tm6sf1;LOC102556283	
DMR1:144162001	1	144162001	144166000	4000	1	1.50E-07	0.45	75	1.88	Sh3gl3	
DMR1:146430001	1	146430001	146431000	1000	1	8.90E-07	0.56	9	0.9	Arnt2	Transcription
DMR1:147609001	1	147609001	147612000	3000	1	4.70E-15	0.69	24	0.8	Cyp2c7	Metabolism
DMR1:148978001	1	148978001	148982000	4000	1	5.80E-07	-0.56	23	0.58	Vom2r40	
DMR1:150345001	1	150345001	150348000	3000	1	7.40E-08	-0.62	6	0.2	Folh1	Protease
DMR1:153608001	1	153608001	153609000	1000	1	4.40E-08	-0.65	11	1.1	Fzd4	Receptor
DMR1:153933001	1	153933001	153934000	1000	1	4.30E-07	0.52	2	0.2	Me3	Metabolism
DMR1:154119001	1	154119001	154127000	8000	1	2.30E-08	0.69	248	3.1	Ccdc81;LOC108349851;LOC102549852	
DMR1:156969001	1	156969001	156974000	5000	1	6.00E-07	-0.4	46	0.92	Dlg2	Cytoskeleton
DMR1:157588001	1	157588001	157591000	3000	1	3.90E-07	0.51	27	0.9	Rab30	

DMR1:161668001	1	161668001	161669000	1000	1	3.90E-08	0.45	17	1.7	Tenm4;LOC108349818	
DMR1:161732001	1	161732001	161735000	3000	1	2.70E-08	0.48	41	1.37	Tenm4	
DMR1:163024001	1	163024001	163026000	2000	1	9.00E-07	0.49	35	1.75	Myo7a	Cytoskeleton
DMR1:163137001	1	163137001	163141000	4000	1	2.80E-08	0.42	42	1.05	Capn5;B3gnt6	Protease;Golgi
DMR1:163230001	1	163230001	163234000	4000	1	3.00E-07	-0.48	17	0.42	Acer3	
DMR1:163319001	1	163319001	163320000	1000	1	4.50E-09	0.54	25	2.5	Tsku	Receptor
DMR1:163876001	1	163876001	163878000	2000	1	3.70E-07	0.55	23	1.15	Uvrag	
DMR1:164115001	1	164115001	164117000	2000	1	1.10E-08	0.51	23	1.15	Dgat2	Metabolism
DMR1:164248001	1	164248001	164252000	4000	1	2.10E-09	0.53	56	1.4	Map6	
DMR1:164378001	1	164378001	164383000	5000	1	2.40E-10	0.53	88	1.76	Gdpd5	Signaling
DMR1:164883001	1	164883001	164884000	1000	1	1.00E-07	0.55	17	1.7	Xrra1;LOC102547456	
DMR1:164908001	1	164908001	164911000	3000	1	2.00E-08	0.55	68	2.27	Xrra1;LOC102547456;Rnf169	
DMR1:164923001	1	164923001	164925000	2000	1	4.50E-08	0.45	27	1.35	Xrra1;Rnf169	
DMR1:165199001	1	165199001	165201000	2000	1	3.00E-08	0.45	26	1.3	LOC100912071;Kcne3	Transport
DMR1:165885001	1	165885001	165888000	3000	1	1.40E-08	0.52	51	1.7	Fam168a;Relt	Cytoskeleton;Receptor
DMR1:165907001	1	165907001	165909000	2000	1	2.60E-10	0.58	23	1.15	Relt	Receptor
DMR1:166437001	1	166437001	166441000	4000	1	1.40E-09	0.58	43	1.07	Stard10	
DMR1:166534001	1	166534001	166536000	2000	1	3.80E-07	0.44	40	2	Arap1;Pde2a	Signaling;Signaling
DMR1:166598001	1	166598001	166602000	4000	1	9.30E-09	0.49	55	1.38	Pde2a;Mir139	Signaling
DMR1:167073001	1	167073001	167077000	4000	2	8.30E-09	0.62	100	2.5	Numa1	Cytoskeleton
DMR1:168896001	1	168896001	168897000	1000	1	2.80E-08	-0.54	8	0.8	Olr127	Receptor
DMR1:169057001	1	169057001	169062000	5000	1	1.90E-07	-0.37	56	1.12	Olr131	Receptor
DMR1:169173001	1	169173001	169180000	7000	2	6.90E-10	0.46	237	3.39	LOC102555557;Olr139;LOC689243	Receptor
DMR1:169562001	1	169562001	169564000	2000	1	6.10E-09	0.5	24	1.2	Olr152;Olr153;Olr155	Receptor
DMR1:170595001	1	170595001	170596000	1000	1	7.10E-10	0.53	21	2.1	Ilk;Taf10;Tpp1;Dchs1	Signaling;Transcription;Protease;Cytoskeleton
DMR1:170650001	1	170650001	170652000	2000	1	2.10E-07	-1.17	17	0.85	Mrpl17	Translation
DMR1:172272001	1	172272001	172278000	6000	1	9.30E-07	-0.34	57	0.95	Olr246;Olr247	Signaling
DMR1:172331001	1	172331001	172335000	4000	1	3.00E-07	-0.5	31	0.78	RGD1562400	Signaling
DMR1:173599001	1	173599001	173601000	2000	1	3.30E-07	0.67	23	1.15	Tub	
DMR1:174648001	1	174648001	174649000	1000	1	9.30E-07	0.49	22	2.2	LOC365348;Ipo7	Transport
DMR1:175608001	1	175608001	175616000	8000	1	3.50E-08	0.61	120	1.5	Ampd3	Metabolism
DMR1:177079001	1	177079001	177082000	3000	1	1.50E-07	0.44	34	1.13	Mical2	
DMR1:177379001	1	177379001	177380000	1000	1	3.40E-07	0.48	22	2.2	Parva	Cytoskeleton
DMR1:177519001	1	177519001	177523000	4000	1	6.90E-07	0.41	46	1.15	Tead1	Transcription
DMR1:178810001	1	178810001	178812000	2000	1	2.30E-07	0.45	16	0.8	Spon1	Cytoskeleton
DMR1:182843001	1	182843001	182844000	1000	1	5.50E-07	0.33	18	1.8	Ssty1	
DMR1:182847001	1	182847001	182849000	2000	1	3.60E-07	0.29	33	1.65	Ssty1	
DMR1:185290001	1	185290001	185292000	2000	1	8.50E-07	0.49	22	1.1	Pik3c2a	Signaling
DMR1:185779001	1	185779001	185781000	2000	1	1.30E-08	0.49	23	1.15	Sox6	
DMR1:189958001	1	189958001	189959000	1000	1	1.30E-12	0.71	14	1.4	Thumpd1;Crym	Metabolism
DMR1:191725001	1	191725001	191727000	2000	2	1.50E-08	0.71	17	0.85	Scnn1g	Transport
DMR1:191737001	1	191737001	191738000	1000	1	7.00E-08	0.67	15	1.5	Scnn1g	Transport
DMR1:191923001	1	191923001	191924000	1000	1	3.30E-07	0.46	21	2.1	Cog7	
DMR1:191960001	1	191960001	191961000	1000	1	5.10E-07	0.51	22	2.2	Gga2	
DMR1:192516001	1	192516001	192521000	5000	1	4.80E-11	0.69	66	1.32	Prkcb	Signaling
DMR1:194105001	1	194105001	194106000	1000	1	5.70E-07	0.67	8	0.8	Hs3st4	
DMR1:196886001	1	196886001	196888000	2000	1	1.00E-08	0.5	36	1.8	Nsmce1;LOC103691233	
DMR1:197030001	1	197030001	197031000	1000	1	1.90E-07	0.45	15	1.5	Il21r;Gtf3c1	Receptor;Transcription
DMR1:197675001	1	197675001	197678000	3000	1	4.10E-08	0.59	76	2.53	Sbk1	Signaling
DMR1:198302001	1	198302001	198304000	2000	1	8.10E-08	0.59	29	1.45	Ino80e;Hirip3;Taok2	Signaling
DMR1:198421001	1	198421001	198423000	2000	1	6.00E-07	0.44	37	1.85	Cdipt;LOC103691236;Mvp	Transport;Cytoskeleton
DMR1:198683001	1	198683001	198684000	1000	1	4.00E-07	0.46	32	3.2	Znf48;Zfp771	Transcription
DMR1:199103001	1	199103001	199107000	4000	1	6.80E-07	0.45	89	2.22	Bcl7c	
DMR1:199417001	1	199417001	199419000	2000	1	2.10E-09	0.54	21	1.05	Fus	Metabolism
DMR1:199453001	1	199453001	199456000	3000	1	4.70E-07	0.49	41	1.37	Trim72	Proteolysis
DMR1:199617001	1	199617001	199618000	1000	1	3.90E-07	0.44	21	2.1	Itgad;Cox6a2	Extracellular Matrix;Metabolism
DMR1:199660001	1	199660001	199662000	2000	1	3.80E-08	0.5	51	2.55	LOC103691238;Armc5;Tgfb1i1	Transcription;Cytoskeleton
DMR1:200102001	1	200102001	200103000	1000	1	2.60E-09	0.66	22	2.2	Inpp5f	Signaling
DMR1:200691001	1	200691001	200693000	2000	1	9.70E-07	0.44	59	2.95	Fgfr2	Receptor
DMR1:201290001	1	201290001	201294000	4000	1	9.60E-07	0.5	71	1.77	Tacc2	
DMR1:201345001	1	201345001	201347000	2000	1	1.60E-07	0.47	30	1.5	Btbd16	
DMR1:201527001	1	201527001	201530000	3000	1	8.40E-07	0.41	42	1.4	Htra1	Protease
DMR1:201927001	1	201927001	201928000	1000	1	2.40E-07	0.47	10	1	RGD1305014	

DMR1:205783001	1	205783001	205785000	2000	1	2.30E-10	0.51	32	1.6	Uros;Bccip;Dhx32	Metabolism;Transcription
DMR1:206217001	1	206217001	206219000	2000	1	4.90E-10	0.61	12	0.6	Adam12	Protease
DMR1:207123001	1	207123001	207126000	3000	1	6.00E-08	0.54	33	1.1	Dock1;Fam196a	Transcription
DMR1:207152001	1	207152001	207155000	3000	2	1.20E-07	0.5	65	2.17	Dock1;Fam196a	Transcription
DMR1:207183001	1	207183001	207184000	1000	1	8.30E-08	0.48	12	1.2	Dock1	Transcription
DMR1:207407001	1	207407001	207410000	3000	1	2.40E-07	0.53	27	0.9	Dock1	Transcription
DMR1:209239001	1	209239001	209241000	2000	1	6.80E-07	0.45	27	1.35	Mgmt	
DMR1:209283001	1	209283001	209286000	3000	1	2.50E-08	0.48	46	1.53	Mgmt	
DMR1:212186001	1	212186001	212187000	1000	1	5.80E-07	0.39	19	1.9	Adgra1	Signaling
DMR1:212678001	1	212678001	212680000	2000	1	1.40E-09	0.58	32	1.6	Olr288	Receptor
DMR1:213614001	1	213614001	213621000	7000	1	1.10E-07	0.56	75	1.07	Bet1l;Ric8a;Sirt3	Transcription
DMR1:213729001	1	213729001	213732000	3000	2	1.30E-13	0.71	140	4.67	LOC102555474;Pgghg	Metabolism
DMR1:214245001	1	214245001	214247000	2000	1	2.00E-07	0.48	40	2	Phrf1;Irf7;Cdhr5	Transcription;Cytoskeleton
DMR1:214743001	1	214743001	214745000	2000	1	6.10E-08	0.45	22	1.1	Muc5ac	
DMR1:214777001	1	214777001	214779000	2000	1	2.40E-07	0.57	20	1	Muc5b	Extracellular Matrix
DMR1:214793001	1	214793001	214796000	3000	1	3.40E-07	0.45	45	1.5	Muc5b	Extracellular Matrix
DMR1:215545001	1	215545001	215550000	5000	1	7.70E-08	0.77	61	1.22	Ifitm10;Ctsd	
DMR1:215597001	1	215597001	215603000	6000	1	1.60E-07	0.4	113	1.88	LOC102546801;Syt8;Tnni2	Transport;Cytoskeleton
DMR1:215650001	1	215650001	215654000	4000	1	1.70E-08	0.55	42	1.05	Lsp1;Prr33	Cytoskeleton
DMR1:215655001	1	215655001	215657000	2000	1	2.40E-08	0.5	27	1.35	Lsp1;Prr33;Tnnt3	Cytoskeleton;Cytoskeleton
DMR1:215738001	1	215738001	215742000	4000	1	4.70E-07	0.48	55	1.38	H19;Mir675;LOC102547221	
DMR1:216658001	1	216658001	216660000	2000	1	1.70E-07	0.48	21	1.05	Cdkn1c	Signaling
DMR1:217001001	1	217001001	217003000	2000	1	2.60E-07	0.47	20	1	Nadsyn1	Metabolism
DMR1:217357001	1	217357001	217358000	1000	1	5.20E-07	0.45	25	2.5	Shank2	
DMR1:217763001	1	217763001	217764000	1000	1	1.10E-11	0.69	19	1.9	Ano1	
DMR1:218413001	1	218413001	218417000	4000	1	4.00E-13	0.58	37	0.92	Tpcn2	Transport
DMR1:218559001	1	218559001	218561000	2000	1	1.80E-08	0.46	24	1.2	Cpt1a	Metabolism
DMR1:218620001	1	218620001	218622000	2000	1	4.70E-09	0.5	28	1.4	Cpt1a	Metabolism
DMR1:218952001	1	218952001	218954000	2000	2	3.20E-08	0.65	31	1.55	RGD1311946	
DMR1:219353001	1	219353001	219360000	7000	3	1.30E-08	0.6	125	1.79	Cdk2ap2;Pitpnm1;Aip;LOC108349683	Signaling;Transport;Transcription
DMR1:220133001	1	220133001	220136000	3000	1	5.60E-07	0.42	36	1.2	Actn3;Zdhhc24	
DMR1:220493001	1	220493001	220495000	2000	1	2.60E-11	0.55	27	1.35	LOC102555354;LOC102555167;Rab1b;Klc2	Cytoskeleton
DMR1:220719001	1	220719001	220722000	3000	1	2.00E-07	0.52	53	1.77	Catsper1;Cst6	Transport
DMR1:220763001	1	220763001	220768000	5000	1	6.60E-07	0.39	108	2.16	LOC103691278;Sart1;LOC102555676;Tsga10ip	Extracellular Matrix
DMR1:221053001	1	221053001	221059000	6000	1	4.00E-07	0.43	80	1.33	Map3k11;LOC102546344;Kcnk7;Ehbp1l1	Signaling;Transport
DMR1:221223001	1	221223001	221224000	1000	1	1.10E-08	0.52	18	1.8	Frmd8	Cytoskeleton
DMR1:221283001	1	221283001	221285000	2000	1	4.70E-07	0.51	19	0.95	Cdc42ep2;LOC108348875	
DMR1:221487001	1	221487001	221489000	2000	1	2.80E-08	0.47	34	1.7	Naaladl1;Sac3d1;Snx15	Protease;Cytoskeleton;Signaling
DMR1:221812001	1	221812001	221819000	7000	1	1.40E-07	0.41	97	1.39	Nrxn2	
DMR1:221863001	1	221863001	221865000	2000	1	1.00E-08	0.56	58	2.9	Nrxn2	
DMR1:221898001	1	221898001	221904000	6000	2	7.70E-07	0.58	119	1.98	Nrxn2;Slc22a12	Transport
DMR1:224594001	1	224594001	224597000	3000	1	2.70E-07	-0.5	12	0.4	Slc22a25	Transport
DMR1:224703001	1	224703001	224707000	4000	1	5.40E-07	0.49	85	2.12	Slc22a25	Transport
DMR1:225161001	1	225161001	225163000	2000	1	9.70E-11	0.6	36	1.8	Tut1;Eef1g	Metabolism
DMR1:225180001	1	225180001	225181000	1000	1	4.90E-09	0.53	11	1.1	Eef1g;Ahnak	
DMR1:225930001	1	225930001	225932000	2000	1	2.40E-07	0.47	61	3.05	Fads2;Incenp	
DMR1:226049001	1	226049001	226052000	3000	1	6.00E-07	0.45	33	1.1	Fads2;Best1;LOC102554361	Transport
DMR1:226287001	1	226287001	226291000	4000	1	4.80E-07	0.51	41	1.02	Myrf;Dagla	Metabolism
DMR1:226297001	1	226297001	226298000	1000	1	1.10E-10	0.66	9	0.9	Myrf;Dagla	Metabolism
DMR1:226314001	1	226314001	226320000	6000	1	1.10E-07	0.56	101	1.68	Dagla	Metabolism
DMR1:226338001	1	226338001	226339000	1000	1	4.00E-07	0.58	19	1.9	Dagla	Metabolism
DMR1:226439001	1	226439001	226443000	4000	1	7.30E-08	0.6	56	1.4	Syt7	Transport
DMR1:226756001	1	226756001	226758000	2000	1	1.50E-09	0.55	24	1.2	Vps37c	
DMR1:226777001	1	226777001	226780000	3000	1	8.30E-07	0.4	47	1.57	Vps37c;Cd5	
DMR1:226917001	1	226917001	226922000	5000	1	7.70E-07	0.75	62	1.24	Slc15a3;Tmem132a;Tmem109	Transport
DMR1:226967001	1	226967001	226970000	3000	1	1.20E-08	0.52	39	1.3	Prpf19;Zp1;Ptgdr2	Translation;Signaling
DMR1:230087001	1	230087001	230093000	6000	1	5.80E-07	-0.49	50	0.83	Olr358;Olr359-ps	Receptor
DMR1:230429001	1	230429001	230431000	2000	1	1.10E-07	-0.71	4	0.2	Olr370-ps	
DMR1:231541001	1	231541001	231547000	6000	1	3.60E-09	-0.56	68	1.13	Tle4	Transcription
DMR1:233188001	1	233188001	233191000	3000	1	1.70E-08	0.51	41	1.37	Cep78	

DMR1:234365001	1	234365001	234366000	1000	1	1.00E-08	0.53	11	1.1	Rorb;LOC102554891	Transcription
DMR1:234757001	1	234757001	234759000	2000	1	1.30E-08	0.6	29	1.45	Nmrk1;Ostf1	Signaling
DMR1:239298001	1	239298001	239299000	1000	1	8.40E-11	0.57	11	1.1	Abhd17b;LOC108348948	Protease
DMR1:239916001	1	239916001	239918000	2000	1	5.00E-11	0.53	32	1.6	Trpm3	Transport
DMR1:240058001	1	240058001	240059000	1000	1	6.70E-07	1.2	16	1.6	Trpm3;LOC100912018	Transport
DMR1:241819001	1	241819001	241821000	2000	1	5.90E-08	0.52	25	1.25	Fam189a2	
DMR1:242675001	1	242675001	242679000	4000	2	5.20E-12	0.53	20	0.5	Pgm5	Metabolism
DMR1:245473001	1	245473001	245477000	4000	1	6.60E-07	0.39	46	1.15	Kcnv2;Pum3	Transport
DMR1:247568001	1	247568001	247570000	2000	1	7.00E-07	0.38	35	1.75	Pdcd1lg2;LOC102547032	Immune
DMR1:251479001	1	251479001	251480000	1000	1	4.40E-07	0.43	21	2.1	Pten	Signaling
DMR1:252936001	1	252936001	252938000	2000	1	5.90E-07	0.38	51	2.55	Lipa;Ifit1bl;Ifit1	Metabolism
DMR1:256265001	1	256265001	256266000	1000	1	5.60E-07	0.47	11	1.1	Exoc6	Transport
DMR1:257394001	1	257394001	257395000	1000	1	8.10E-07	0.43	17	1.7	Plce1	Metabolism
DMR1:259942001	1	259942001	259944000	2000	1	2.20E-07	0.55	36	1.8	Ccnj	Signaling
DMR1:260109001	1	260109001	260111000	2000	1	1.80E-10	0.62	34	1.7	Ccnj	Signaling
DMR1:261508001	1	261508001	261509000	1000	1	7.80E-08	0.47	17	1.7	Golga7b	
DMR1:261528001	1	261528001	261531000	3000	2	3.60E-07	0.49	50	1.67	Mir3085;Crtac1	
DMR1:261662001	1	261662001	261663000	1000	1	1.10E-07	0.43	9	0.9	Crtac1	
DMR1:262001001	1	262001001	262003000	2000	1	4.60E-07	0.53	49	2.45	Hps1	
DMR1:262056001	1	262056001	262057000	1000	1	2.90E-08	0.52	7	0.7	LOC108349471;Hpse2;LOC102554961	
DMR1:262361001	1	262361001	262362000	1000	1	6.00E-08	0.59	10	1	Hpse2	
DMR1:263928001	1	263928001	263930000	2000	1	9.30E-09	0.51	56	2.8	Bloc1s2;Pkd2l1	Transport
DMR1:264520001	1	264520001	264523000	3000	1	5.10E-07	0.38	72	2.4	Pax2	
DMR1:264525001	1	264525001	264530000	5000	1	6.00E-07	0.45	92	1.84	Pax2	
DMR1:264563001	1	264563001	264568000	5000	1	2.50E-07	0.42	94	1.88	Pax2	
DMR1:264788001	1	264788001	264790000	2000	1	7.60E-12	0.55	33	1.65	Pdzd7;Sfxn3	Cytoskeleton;Transport
DMR1:265255001	1	265255001	265256000	1000	1	2.40E-07	0.65	10	1	Btrc	Cytoskeleton
DMR1:265785001	1	265785001	265791000	6000	1	3.60E-07	0.43	122	2.03	Ldb1	Transcription
DMR1:266080001	1	266080001	266083000	3000	1	1.10E-10	0.73	27	0.9	Psd;Fbxl15;Cuedc2;Mir146b	Transcription
DMR1:266302001	1	266302001	266304000	2000	1	2.00E-07	0.37	17	0.85	Arl3;LOC108349707	
DMR1:266375001	1	266375001	266377000	2000	1	1.60E-08	0.46	37	1.85	Wbp1l	
DMR1:266469001	1	266469001	266475000	6000	1	2.80E-07	0.47	169	2.82	Borcs7;As3mt	Epigenetic
DMR1:266957001	1	266957001	266963000	6000	1	4.10E-08	0.5	78	1.3	Neurl1	Proteolysis
DMR1:266965001	1	266965001	266966000	1000	1	1.60E-07	0.53	20	2	Neurl1	Proteolysis
DMR1:267004001	1	267004001	267011000	7000	1	7.00E-09	0.47	118	1.69	Neurl1;LOC108349530	Proteolysis
DMR1:267151001	1	267151001	267153000	2000	1	6.90E-07	0.45	36	1.8	Sh3pxd2a	
DMR1:267162001	1	267162001	267164000	2000	1	3.80E-07	0.81	27	1.35	Sh3pxd2a	
DMR1:267424001	1	267424001	267425000	1000	1	1.10E-10	0.57	19	1.9	Slk;Col17a1	Extracellular Matrix
DMR1:267538001	1	267538001	267539000	1000	1	2.80E-08	0.68	6	0.6	Cfap43	
DMR1:268240001	1	268240001	268252000	12000	1	1.10E-08	0.51	144	1.2	Sorcs3	Transport
DMR1:271352001	1	271352001	271354000	2000	1	9.10E-07	0.7	14	0.7	Ccdc147	
DMR1:272805001	1	272805001	272807000	2000	1	1.60E-07	-0.88	15	0.75	Ins1	Growth Factors
DMR1:273734001	1	273734001	273736000	2000	1	9.40E-07	0.48	40	2	Xpnpep1	Protease
DMR1:273740001	1	273740001	273743000	3000	1	9.30E-08	0.5	48	1.6	Xpnpep1;LOC102550358	Protease
DMR1:274257001	1	274257001	274258000	1000	1	2.30E-07	0.54	30	3	Dusp5	Signaling
DMR1:274584001	1	274584001	274585000	1000	1	1.40E-07	0.6	16	1.6	Rbm20	
DMR1:275891001	1	275891001	275893000	2000	2	6.30E-10	0.72	115	5.75	Gpam	Metabolism
DMR1:276303001	1	276303001	276304000	1000	1	5.70E-07	0.44	14	1.4	Zdhhc6;Vti1a	Transcription
DMR1:276447001	1	276447001	276448000	1000	1	4.00E-10	0.52	19	1.9	Vti1a;LOC103691376	Transcription
DMR1:277721001	1	277721001	277725000	4000	1	1.00E-06	0.55	88	2.2	Vwa2;Afap1l2	Extracellular Matrix
DMR1:277728001	1	277728001	277729000	1000	1	3.90E-07	0.55	15	1.5	Vwa2;Afap1l2	Extracellular Matrix
DMR1:277865001	1	277865001	277866000	1000	1	4.30E-07	0.39	19	1.9	Ablim1	
DMR1:278028001	1	278028001	278030000	2000	1	3.40E-07	0.44	47	2.35	Ablim1;LOC102550100	
DMR1:278320001	1	278320001	278322000	2000	1	4.60E-08	0.48	52	2.6	Trub1	Metabolism
DMR1:279083001	1	279083001	279089000	6000	1	7.60E-07	0.52	85	1.42	Atrnl1	Extracellular Matrix
DMR1:279394001	1	279394001	279395000	1000	1	2.00E-07	0.54	16	1.6	Gfra1	Receptor
DMR1:279480001	1	279480001	279483000	3000	1	9.10E-07	0.72	45	1.5	Gfra1	Receptor
DMR1:279961001	1	279961001	279964000	3000	1	7.50E-08	0.63	47	1.57	Hspa12a	
DMR1:280033001	1	280033001	280035000	2000	1	5.30E-07	0.4	39	1.95	Hspa12a;LOC100363557	
DMR1:280149001	1	280149001	280150000	1000	1	1.30E-07	0.53	6	0.6	Shtn1	
DMR1:280171001	1	280171001	280172000	1000	1	7.30E-08	0.41	21	2.1	Shtn1	
DMR1:280409001	1	280409001	280412000	3000	1	7.20E-08	0.51	52	1.73	Slc18a2	Transport
DMR1:282152001	1	282152001	282153000	1000	1	9.30E-07	0.45	15	1.5	Eif3a	Translation
DMR2:5452001	2	5452001	5457000	5000	1	2.70E-08	0.38	54	1.08	Fam172a	
DMR2:9028001	2	9028001	9033000	5000	1	7.50E-07	0.28	33	0.66	Adgrv1	Signaling

DMR2:9390001	2	9390001	9392000	2000	1	4.10E-07	0.41	34	1.7	Adgrv1	Signaling
DMR2:20125001	2	20125001	20126000	1000	1	2.20E-07	0.52	11	1.1	Atg10	Proteolysis
DMR2:20700001	2	20700001	20702000	2000	1	6.40E-09	0.55	36	1.8	Ssbp2	Transcription
DMR2:21797001	2	21797001	21798000	1000	1	1.10E-07	0.43	21	2.1	Msh3	Transcription
DMR2:22037001	2	22037001	22039000	2000	1	5.90E-07	0.42	29	1.45	Fam151b	
DMR2:22259001	2	22259001	22262000	3000	1	1.30E-07	0.51	36	1.2	Serinc5	Signaling
DMR2:22648001	2	22648001	22653000	5000	1	8.10E-08	0.63	92	1.84	Cmya5	Proteolysis
DMR2:23802001	2	23802001	23803000	1000	1	1.00E-07	0.5	26	2.6	Lhfpl2	
DMR2:23810001	2	23810001	23812000	2000	1	2.40E-07	0.5	41	2.05	Lhfpl2	
DMR2:24057001	2	24057001	24060000	3000	1	3.70E-07	0.48	34	1.13	Ap3b1	Transport
DMR2:24155001	2	24155001	24159000	4000	2	8.20E-08	0.61	57	1.43	Ap3b1	Transport
DMR2:25072001	2	25072001	25074000	2000	1	1.10E-07	0.48	42	2.1	Aggf1	
DMR2:26307001	2	26307001	26312000	5000	1	2.90E-07	0.58	75	1.5	Iqgap2	Signaling
DMR2:26495001	2	26495001	26496000	1000	1	5.20E-07	0.47	20	2	Sv2c	
DMR2:27290001	2	27290001	27291000	1000	1	2.50E-08	0.57	22	2.2	Ankdd1b	
DMR2:29613001	2	29613001	29615000	2000	1	2.40E-07	0.46	31	1.55	Mrps27	Translation
DMR2:29641001	2	29641001	29644000	3000	1	5.50E-07	0.44	30	1	Mrps27	Translation
DMR2:30375001	2	30375001	30376000	1000	1	3.80E-10	0.55	11	1.1	Smn1;Naip6	Translation
DMR2:30699001	2	30699001	30701000	2000	1	6.10E-07	0.42	41	2.05	Ccdc125;Cdk7	Signaling
DMR2:31760001	2	31760001	31763000	3000	1	8.20E-07	0.45	54	1.8	Pik3r1	Signaling
DMR2:33060001	2	33060001	33061000	1000	1	2.30E-07	0.52	13	1.3	NEWGENE_1310139	
DMR2:34298001	2	34298001	34302000	4000	1	2.00E-12	0.52	35	0.88	Trim23;Ppwd1	Signaling;Transcription
DMR2:37982001	2	37982001	37984000	2000	1	6.70E-09	0.6	23	1.15	Ipo11	Transport
DMR2:39448001	2	39448001	39451000	3000	1	9.70E-08	0.49	28	0.93	Ercc8	Transcription
DMR2:40510001	2	40510001	40511000	1000	1	4.00E-07	-0.61	7	0.7	Pde4d	Signaling
DMR2:40954001	2	40954001	40959000	5000	1	1.60E-08	0.46	66	1.32	Pde4d;LOC108350210	Signaling
DMR2:41424001	2	41424001	41425000	1000	1	9.70E-08	0.54	9	0.9	Pde4d	Signaling
DMR2:44493001	2	44493001	44494000	1000	1	6.30E-07	-0.47	11	1.1	Ddx4	
DMR2:44585001	2	44585001	44589000	4000	3	1.30E-09	0.52	40	1	Slc38a9	Transport
DMR2:44696001	2	44696001	44698000	2000	1	3.50E-08	0.55	22	1.1	Plpp1	Signaling
DMR2:44713001	2	44713001	44715000	2000	1	9.50E-09	0.59	19	0.95	Plpp1	Signaling
DMR2:44755001	2	44755001	44756000	1000	1	3.10E-07	0.52	12	1.2	Skiv2l2	
DMR2:44828001	2	44828001	44835000	7000	1	1.10E-07	0.42	110	1.57	Dhx29	Transcription
DMR2:45058001	2	45058001	45061000	3000	1	5.20E-08	0.56	34	1.13	Gzmk	Protease
DMR2:45460001	2	45460001	45464000	4000	1	6.70E-07	0.43	69	1.73	Snx18	Cytoskeleton
DMR2:46362001	2	46362001	46366000	4000	1	5.40E-08	0.5	63	1.57	Ndufs4	Metabolism
DMR2:49404001	2	49404001	49406000	2000	1	9.10E-07	0.53	19	0.95	Parp8	
DMR2:53864001	2	53864001	53865000	1000	1	3.30E-10	0.57	18	1.8	Oxct1	Transport
DMR2:54673001	2	54673001	54675000	2000	1	3.50E-07	0.94	28	1.4	Mroh2b;LOC102553573;LOC108349954	
DMR2:57088001	2	57088001	57090000	2000	1	2.80E-07	0.7	27	1.35	Wdr70	
DMR2:60423001	2	60423001	60426000	3000	1	2.00E-08	-0.45	28	0.93	Dnajc21	Transcription
DMR2:60444001	2	60444001	60446000	2000	1	3.30E-08	0.45	34	1.7	Dnajc21;Brix1	Transcription;Metabolism
DMR2:60488001	2	60488001	60492000	4000	1	6.10E-09	0.51	41	1.02	LOC108349965;Ttc23l	
DMR2:60918001	2	60918001	60919000	1000	1	2.10E-08	0.61	7	0.7	C1qtnf3	
DMR2:61100001	2	61100001	61103000	3000	1	6.50E-07	0.42	20	0.67	Adamts12	Protease
DMR2:62256001	2	62256001	62257000	1000	1	6.30E-07	0.43	10	1	Mtmr12	Signaling
DMR2:62524001	2	62524001	62526000	2000	1	1.80E-07	0.58	19	0.95	Pdzd2	Cytokine
DMR2:69579001	2	69579001	69581000	2000	1	1.50E-09	0.63	12	0.6	Cdh10	Cytoskeleton
DMR2:74501001	2	74501001	74503000	2000	1	7.70E-09	-0.67	11	0.55	Cdh18	Cytoskeleton
DMR2:74637001	2	74637001	74641000	4000	1	9.00E-07	-0.84	20	0.5	Cdh18	Cytoskeleton
DMR2:79406001	2	79406001	79408000	2000	1	5.80E-07	-0.69	18	0.9	Fbxl7	
DMR2:80157001	2	80157001	80158000	1000	1	5.20E-10	0.5	12	1.2	Ankh	
DMR2:80607001	2	80607001	80609000	2000	1	9.20E-09	0.47	25	1.25	Trio	Transcription
DMR2:80734001	2	80734001	80735000	1000	1	4.50E-07	0.43	13	1.3	Trio	Transcription
DMR2:81292001	2	81292001	81293000	1000	1	6.90E-07	-0.46	10	1	Dnah5	Cytoskeleton
DMR2:83774001	2	83774001	83776000	2000	1	1.90E-08	0.6	22	1.1	Ctnnd2	Cytoskeleton
DMR2:84012001	2	84012001	84014000	2000	1	1.70E-07	0.44	21	1.05	Ctnnd2	Cytoskeleton
DMR2:84092001	2	84092001	84094000	2000	1	9.30E-08	0.45	39	1.95	Ctnnd2	Cytoskeleton
DMR2:84376001	2	84376001	84377000	1000	1	4.40E-07	0.51	12	1.2	Ankrd33b	
DMR2:84393001	2	84393001	84395000	2000	1	2.00E-08	0.54	26	1.3	Ankrd33b	
DMR2:84398001	2	84398001	84401000	3000	1	6.00E-07	0.4	52	1.73	Ankrd33b	
DMR2:86984001	2	86984001	86986000	2000	1	2.60E-08	-0.5	18	0.9	Zfp458	Transcription
DMR2:88374001	2	88374001	88378000	4000	1	8.70E-12	0.36	26	0.65	E2f5;Lrrcc1	Transcription;Cytoskeleton
DMR2:91475001	2	91475001	91477000	2000	1	2.60E-07	-0.69	7	0.35	Pcsk1	Protease

DMR2:91498001	2	91498001	91499000	1000	1	9.80E-08	-0.97	8	0.8	Pcsk1;LOC102548697	Protease
DMR2:93577001	2	93577001	93580000	3000	1	3.30E-08	0.5	29	0.97	Chmp4c	Transport
DMR2:93645001	2	93645001	93646000	1000	1	9.80E-08	0.51	9	0.9	Chmp4c	Transport
DMR2:100668001	2	100668001	100669000	1000	1	1.60E-08	0.43	11	1.1	Ythdf3	
DMR2:104082001	2	104082001	104086000	4000	1	2.60E-07	-0.47	35	0.88	Pde7a	Signaling
DMR2:104921001	2	104921001	104923000	2000	1	9.60E-12	0.78	16	0.8	Hltf;Gyg1	Golgi
DMR2:111753001	2	111753001	111755000	2000	1	9.40E-07	0.43	16	0.8	Nlgn1	Cytoskeleton
DMR2:113017001	2	113017001	113018000	1000	1	8.60E-08	0.55	9	0.9	Tnfsf10	
DMR2:113169001	2	113169001	113171000	2000	1	1.90E-07	0.58	27	1.35	Fndc3b	Proteolysis
DMR2:113330001	2	113330001	113333000	3000	1	2.00E-07	0.43	34	1.13	Fndc3b	Proteolysis
DMR2:114327001	2	114327001	114329000	2000	1	6.30E-08	0.55	20	1	Tnik	Signaling
DMR2:116572001	2	116572001	116573000	1000	1	2.70E-10	0.53	11	1.1	Egfem1	
DMR2:118277001	2	118277001	118281000	4000	1	7.20E-09	0.58	72	1.8	Kcnmb2;LOC102549015	Transport
DMR2:119005001	2	119005001	119007000	2000	1	1.20E-07	0.5	21	1.05	Gnb4;LOC103691541	Signaling
DMR2:119211001	2	119211001	119212000	1000	1	3.30E-11	0.58	12	1.2	Usp13	Protease
DMR2:122405001	2	122405001	122406000	1000	1	7.70E-08	0.49	10	1	Atp11b	Transport
DMR2:122881001	2	122881001	122883000	2000	1	1.50E-08	-0.69	61	3.05	Rslcan18;Arse;Qrfpr	Metabolism;Signaling
DMR2:123359001	2	123359001	123360000	1000	1	3.20E-09	0.59	13	1.3	Trpc3	Transport
DMR2:127760001	2	127760001	127764000	4000	1	4.50E-09	-0.48	28	0.7	Mfsd8;Abhd18	
DMR2:128006001	2	128006001	128007000	1000	1	1.00E-07	0.72	15	1.5	Pgrmc2	Receptor
DMR2:141441001	2	141441001	141443000	2000	1	3.80E-08	0.53	17	0.85	LOC103691555;Foxo1	
DMR2:142806001	2	142806001	142809000	3000	1	3.80E-09	0.58	49	1.63	Frem2	
DMR2:144296001	2	144296001	144298000	2000	1	2.40E-08	0.46	21	1.05	Sertm1	
DMR2:144451001	2	144451001	144455000	4000	1	1.20E-07	0.51	52	1.3	Ccna1	Signaling
DMR2:145065001	2	145065001	145069000	4000	1	5.40E-07	0.84	39	0.98	Nbea	
DMR2:145222001	2	145222001	145223000	1000	1	1.10E-07	0.66	8	0.8	Nbea;LOC102552778	
DMR2:145292001	2	145292001	145294000	2000	1	3.80E-07	0.46	23	1.15	Nbea	
DMR2:149232001	2	149232001	149234000	2000	1	1.70E-08	0.54	20	1	Med12l	Transcription
DMR2:155483001	2	155483001	155486000	3000	1	5.70E-07	-0.79	15	0.5	Vom2r-ps74	
DMR2:166656001	2	166656001	166658000	2000	1	4.30E-07	0.5	20	1	Sptssb	Golgi
DMR2:173766001	2	173766001	173767000	1000	1	1.70E-09	0.44	17	1.7	Wdr49;LOC102556029	
DMR2:174388001	2	174388001	174390000	2000	1	4.50E-07	-0.35	26	1.3	Golim4	
DMR2:174889001	2	174889001	174890000	1000	1	1.20E-07	-0.59	6	0.6	Fstl5	Protease; Proteolysis
DMR2:178023001	2	178023001	178025000	2000	1	2.70E-09	0.58	23	1.15	Rapgef2	Transcription
DMR2:183439001	2	183439001	183444000	5000	1	2.00E-09	0.33	41	0.82	Fhdc1	
DMR2:184865001	2	184865001	184866000	1000	1	2.10E-07	0.57	15	1.5	Fam160a1	
DMR2:185345001	2	185345001	185348000	3000	1	3.30E-09	0.68	50	1.67	Sh3d19	
DMR2:185351001	2	185351001	185352000	1000	1	5.30E-08	0.45	25	2.5	Sh3d19	
DMR2:186105001	2	186105001	186108000	3000	1	3.10E-07	0.46	35	1.17	Lrba;Dclk2	Signaling
DMR2:186430001	2	186430001	186432000	2000	1	3.00E-09	0.45	43	2.15	Kirrel	
DMR2:186452001	2	186452001	186454000	2000	1	7.10E-07	0.43	29	1.45	Kirrel	
DMR2:187751001	2	187751001	187754000	3000	2	2.90E-10	0.8	37	1.23	Paqr6;Bglap;Pmf1	Signaling
DMR2:188122001	2	188122001	188125000	3000	1	6.60E-09	0.54	38	1.27	Syt11	Transport
DMR2:188213001	2	188213001	188214000	1000	1	4.20E-08	0.5	19	1.9	Gon4l;Msto1	Transcription
DMR2:188331001	2	188331001	188333000	2000	1	4.90E-11	0.58	16	0.8	Ash1l	Epigenetic
DMR2:188549001	2	188549001	188553000	4000	1	1.90E-11	0.56	98	2.45	Thbs3;Mir92b;Muc1;Trim46;Krtcap2	Cytoskeleton;Proteolysis
DMR2:188733001	2	188733001	188735000	2000	1	4.30E-07	0.47	31	1.55	Flad1;Lenep;Cks1b	Transport;Cytoskeleton
DMR2:188771001	2	188771001	188776000	5000	1	9.60E-07	0.39	85	1.7	Pygo2;Pbxip1;Pmvk	Signaling
DMR2:188998001	2	188998001	189003000	5000	1	2.00E-08	0.6	62	1.24	Kcnn3	Transport
DMR2:189088001	2	189088001	189090000	2000	1	8.50E-07	0.38	37	1.85	Adar;Chrnb2	Metabolism;Ion Channel
DMR2:189501001	2	189501001	189505000	4000	1	1.10E-08	-0.6	24	0.6	Nup210l	Transport
DMR2:189725001	2	189725001	189727000	2000	1	2.00E-08	0.43	21	1.05	Gatad2b	Transcription
DMR2:192159001	2	192159001	192161000	2000	1	3.20E-07	-0.62	8	0.4	Pglyrp3	
DMR2:195641001	2	195641001	195644000	3000	2	1.30E-08	0.65	57	1.9	Rorc;Lingo4;Tdrkh	Transcription;Receptor;Cytoskeleton
DMR2:196043001	2	196043001	196045000	2000	1	2.40E-08	0.56	24	1.2	Pogz;Psmb4;LOC108350065	Transcription;Protease
DMR2:198174001	2	198174001	198175000	1000	1	2.50E-07	0.43	14	1.4	Vps45	Transport
DMR2:199340001	2	199340001	199342000	2000	1	5.50E-08	0.51	36	1.8	Bcl9	
DMR2:199396001	2	199396001	199400000	4000	1	9.50E-07	0.45	84	2.1	Bcl9	
DMR2:199925001	2	199925001	199927000	2000	1	7.50E-07	0.47	26	1.3	Pde4dip	
DMR2:200238001	2	200238001	200239000	1000	1	5.20E-07	0.45	17	1.7	Notch2	Extracellular Matrix
DMR2:200259001	2	200259001	200262000	3000	1	6.00E-09	0.52	55	1.83	Notch2	Extracellular Matrix
DMR2:200309001	2	200309001	200315000	6000	1	9.80E-07	0.53	124	2.07	Notch2	Extracellular Matrix
DMR2:202284001	2	202284001	202286000	2000	1	2.90E-07	0.56	22	1.1	Spag17	
DMR2:202431001	2	202431001	202434000	3000	1	4.10E-09	0.47	32	1.07	Spag17	

DMR2:202536001	2	202536001	202537000	1000	1	7.70E-07	0.52	11	1.1	Gdap2	
DMR2:202812001	2	202812001	202813000	1000	1	2.60E-08	0.51	12	1.2	Fam46c	
DMR2:202982001	2	202982001	202985000	3000	1	7.30E-10	0.84	76	2.53	Man1a2	Golgi
DMR2:203013001	2	203013001	203015000	2000	1	2.90E-08	0.53	14	0.7	Man1a2	Golgi
DMR2:203826001	2	203826001	203829000	3000	2	4.30E-08	0.51	68	2.27	Igsf3	Immune
DMR2:203839001	2	203839001	203841000	2000	1	2.50E-09	0.53	31	1.55	Igsf3	Immune
DMR2:205604001	2	205604001	205605000	1000	1	3.70E-07	0.53	14	1.4	Dennd2c	
DMR2:207126001	2	207126001	207127000	1000	1	5.60E-08	0.44	9	0.9	Slc16a1	Transport
DMR2:207283001	2	207283001	207289000	6000	1	1.60E-07	0.45	115	1.92	Rhoc;Mov10	Signaling
DMR2:207941001	2	207941001	207943000	2000	1	1.30E-07	0.54	23	1.15	Kcnd3	Transport
DMR2:208335001	2	208335001	208336000	1000	1	6.00E-09	0.59	15	1.5	LOC100911347;Adora3	Signaling
DMR2:209455001	2	209455001	209457000	2000	1	3.60E-08	-1.06	7	0.35	Lrif1	
DMR2:210171001	2	210171001	210175000	4000	1	4.10E-10	0.5	20	0.5	Slc16a4	Transport
DMR2:210882001	2	210882001	210889000	7000	3	4.00E-08	0.6	117	1.67	Ampd2;Gnat2;Gnai3	Metabolism;Signaling
DMR2:211119001	2	211119001	211120000	1000	1	3.90E-14	0.65	11	1.1	Sort1	Transport
DMR2:211758001	2	211758001	211759000	1000	1	3.80E-07	0.38	14	1.4	Henmt1;Fam102b	
DMR2:211792001	2	211792001	211795000	3000	1	2.70E-08	0.63	46	1.53	Fam102b	
DMR2:211938001	2	211938001	211939000	1000	1	1.60E-08	0.59	7	0.7	Slc25a24	Transport
DMR2:217846001	2	217846001	217848000	2000	1	5.50E-09	0.5	14	0.7	Olfm3	Development
DMR2:218647001	2	218647001	218648000	1000	1	1.10E-08	0.57	11	1.1	S1pr1	Signaling
DMR2:220305001	2	220305001	220308000	3000	1	9.50E-07	0.52	32	1.07	Plppr4	Signaling
DMR2:222191001	2	222191001	222192000	1000	1	9.30E-07	0.41	11	1.1	Dpyd	Metabolism
DMR2:224915001	2	224915001	224917000	2000	1	5.40E-07	0.53	41	2.05	Alg14	Transport
DMR2:225725001	2	225725001	225728000	3000	1	2.50E-08	0.88	52	1.73	Abca4	Transport
DMR2:230341001	2	230341001	230343000	2000	1	1.50E-10	0.55	52	2.6	Arsj;Sec24b	Transport
DMR2:231119001	2	231119001	231122000	3000	1	6.30E-10	-0.98	11	0.37	Camk2d	Signaling
DMR2:231973001	2	231973001	231975000	2000	1	8.80E-07	0.4	25	1.25	Neurog2	Transcription
DMR2:235332001	2	235332001	235333000	1000	1	7.10E-07	0.48	7	0.7	Pla2g12a;Casp6	Metabolism;Protease
DMR2:235555001	2	235555001	235559000	4000	1	8.50E-07	0.43	54	1.35	Col25a1	Extracellular Matrix
DMR2:235816001	2	235816001	235820000	4000	2	1.50E-07	0.48	54	1.35	Ostc	Transport
DMR2:236265001	2	236265001	236268000	3000	1	2.20E-07	0.74	51	1.7	Lef1;LOC103691708	Transcription
DMR2:236341001	2	236341001	236343000	2000	1	1.90E-07	0.47	36	1.8	Lef1	Transcription
DMR2:237893001	2	237893001	237894000	1000	1	8.20E-09	0.63	15	1.5	Tbck	Signaling
DMR2:240425001	2	240425001	240429000	4000	1	6.60E-07	0.4	62	1.55	Cenpe	
DMR2:240559001	2	240559001	240561000	2000	1	1.20E-08	0.27	16	0.8	Slc9b1	
DMR2:241962001	2	241962001	241963000	1000	1	1.10E-08	0.5	6	0.6	Ppp3ca	Signaling
DMR2:242164001	2	242164001	242166000	2000	1	4.30E-09	0.51	38	1.9	Ppp3ca	Signaling
DMR2:243430001	2	243430001	243432000	2000	1	7.50E-11	0.58	20	1	Trmt10a	Epigenetic
DMR2:244082001	2	244082001	244086000	4000	1	1.30E-07	0.45	86	2.15	Tspan5	
DMR2:244826001	2	244826001	244828000	2000	1	6.60E-07	-0.45	15	0.75	Stpg2;LOC100910816	Development
DMR2:248428001	2	248428001	248432000	4000	1	8.60E-07	-0.87	24	0.6	Gbp1	Signaling
DMR2:248684001	2	248684001	248689000	5000	1	3.90E-07	0.52	71	1.42	Kyat3	Metabolism
DMR2:251292001	2	251292001	251293000	1000	1	4.80E-07	0.44	16	1.6	Col24a1	Extracellular Matrix
DMR2:252340001	2	252340001	252345000	5000	1	1.30E-10	0.59	43	0.86	Spata1	
DMR2:256709001	2	256709001	256710000	1000	1	9.70E-07	-0.66	14	1.4	Adgrl4	Signaling
DMR2:257378001	2	257378001	257380000	2000	1	3.60E-08	0.54	22	1.1	Gipc2	Cytoskeleton
DMR2:257441001	2	257441001	257442000	1000	1	8.10E-08	0.48	11	1.1	Fubp1	Metabolism
DMR2:257538001	2	257538001	257540000	2000	1	3.70E-08	0.5	44	2.2	Miga1;LOC108350144	
DMR2:257913001	2	257913001	257915000	2000	1	2.10E-07	0.46	22	1.1	Pigk	
DMR2:259017001	2	259017001	259019000	2000	1	3.50E-08	0.47	29	1.45	St6galnac5	
DMR2:259025001	2	259025001	259027000	2000	1	4.90E-09	0.58	43	2.15	St6galnac5	
DMR2:259486001	2	259486001	259493000	7000	3	4.20E-08	0.45	118	1.69	St6galnac3	
DMR2:259922001	2	259922001	259923000	1000	1	4.40E-07	0.51	17	1.7	St6galnac3;LOC103691740	
DMR2:260276001	2	260276001	260277000	1000	1	3.00E-07	0.38	15	1.5	Slc44a5	Transport
DMR2:260323001	2	260323001	260325000	2000	2	3.50E-15	0.7	32	1.6	Slc44a5;LOC102553583	Transport
DMR2:260386001	2	260386001	260387000	1000	1	5.90E-13	1.13	20	2	Slc44a5	Transport
DMR2:260522001	2	260522001	260524000	2000	1	4.10E-08	0.5	24	1.2	Slc44a5	Transport
DMR2:260582001	2	260582001	260585000	3000	1	1.90E-08	0.71	29	0.97	Lhx8	Development
DMR2:261154001	2	261154001	261157000	3000	1	7.00E-07	-0.57	8	0.27	Tnni3k	
DMR2:261359001	2	261359001	261382000	23000	19	3.40E-12	1.21	645	2.8	Fpgt	Transport
DMR3:1888001	3	1888001	1893000	5000	1	1.20E-07	0.45	73	1.46	Cacna1b	Transport
DMR3:1921001	3	1921001	1922000	1000	1	1.20E-09	0.62	13	1.3	Cacna1b	Transport
DMR3:2030001	3	2030001	2033000	3000	1	1.40E-07	0.43	30	1	Ehmt1	
DMR3:2279001	3	2279001	2280000	1000	1	5.60E-07	0.46	16	1.6	Nsmf;Entpd8	Signaling
DMR3:2569001	3	2569001	2570000	1000	1	3.30E-07	0.58	18	1.8	Man1b1;LOC103691747;Dpp7;Uap1l1	Golgi;Protease

DMR3:2627001	3	2627001	2630000	3000	1	7.90E-08	0.71	44	1.47	Entpd2;Npdc1;LOC366006;Fut7	Signaling;Golgi
DMR3:2650001	3	2650001	2654000	4000	1	3.50E-07	0.51	69	1.73	Fut7;Abca2	Golgi;Transport
DMR3:2655001	3	2655001	2670000	15000	2	6.10E-07	0.5	449	2.99	Fut7;Abca2;Clic3;RGD1306215	Golgi;Transport;Transport
DMR3:2785001	3	2785001	2786000	1000	1	1.70E-08	0.5	20	2	Edf1;Mamdc4	
DMR3:2943001	3	2943001	2948000	5000	2	5.80E-08	0.48	67	1.34	LOC100360027;Lcn11;Lcn3	Transport
DMR3:3270001	3	3270001	3276000	6000	1	5.80E-09	0.69	128	2.13	Glt6d1;Lcn9	Golgi;Transport
DMR3:3388001	3	3388001	3392000	4000	1	1.60E-08	0.52	120	3	Camsap1;LOC100360117	
DMR3:3540001	3	3540001	3541000	1000	1	3.20E-07	0.56	19	1.9	Nacc2	
DMR3:3567001	3	3567001	3568000	1000	1	2.10E-09	0.55	14	1.4	Nacc2	
DMR3:3733001	3	3733001	3738000	5000	1	3.20E-07	0.46	84	1.68	Ccdc187	Cytoskeleton
DMR3:3757001	3	3757001	3760000	3000	1	4.50E-07	0.43	44	1.47	Ccdc187;Gpsm1	Cytoskeleton
DMR3:3775001	3	3775001	3778000	3000	1	9.90E-09	0.48	60	2	Gpsm1	
DMR3:3943001	3	3943001	3945000	2000	1	7.60E-12	0.62	31	1.55	Notch1	Extracellular Matrix
DMR3:5553001	3	5553001	5557000	4000	1	1.10E-08	0.57	74	1.85	Adamts13;LOC102548484;Cacfd1	Protease
DMR3:5765001	3	5765001	5767000	2000	1	2.50E-07	0.43	25	1.25	Sardh;LOC102548789	Metabolism
DMR3:5815001	3	5815001	5818000	3000	1	8.90E-07	0.44	62	2.07	Vav2	
DMR3:5865001	3	5865001	5871000	6000	1	2.50E-08	0.49	88	1.47	Vav2	
DMR3:5885001	3	5885001	5889000	4000	1	1.60E-07	0.42	60	1.5	Vav2	
DMR3:5927001	3	5927001	5929000	2000	1	9.10E-07	0.39	39	1.95	Vav2	
DMR3:6034001	3	6034001	6036000	2000	1	9.80E-07	0.56	29	1.45	Brd3	
DMR3:6286001	3	6286001	6300000	14000	1	2.50E-08	0.45	324	2.31	Rxra	Transcription
DMR3:6609001	3	6609001	6613000	4000	1	2.00E-09	0.5	73	1.82	Fcnb	
DMR3:6797001	3	6797001	6799000	2000	1	3.30E-07	0.48	74	3.7	Olfm1;LOC102549127	Development
DMR3:7619001	3	7619001	7620000	1000	1	8.40E-09	0.6	16	1.6	Cfap77	Development
DMR3:7647001	3	7647001	7648000	1000	1	5.20E-09	0.61	10	1	Ttf1	
DMR3:7671001	3	7671001	7672000	1000	1	7.70E-07	0.68	11	1.1	LOC108350345;Setx	
DMR3:8605001	3	8605001	8608000	3000	1	2.10E-08	0.56	70	2.33	Sptan1;Wdr34	Cytoskeleton
DMR3:8672001	3	8672001	8674000	2000	1	1.20E-11	0.7	33	1.65	Zer1	
DMR3:8767001	3	8767001	8769000	2000	2	9.70E-19	0.92	57	2.85	Kyat1	Metabolism
DMR3:8827001	3	8827001	8829000	2000	1	9.00E-07	0.44	36	1.8	Lrrc8a;Phyhd1	Cytoskeleton;Metabolism
DMR3:8906001	3	8906001	8907000	1000	1	8.70E-09	0.61	17	1.7	Nup188;Sh3glb2	
DMR3:8965001	3	8965001	8967000	2000	1	4.10E-08	0.75	32	1.6	LOC686066;Dolpp1;Crat	Golgi;Metabolism
DMR3:9233001	3	9233001	9234000	1000	1	1.00E-07	0.48	9	0.9	Nup214;Aif1l	Transport;Signaling
DMR3:9279001	3	9279001	9284000	5000	2	7.80E-08	0.56	75	1.5	Lamc3	Extracellular Matrix
DMR3:9371001	3	9371001	9373000	2000	1	4.30E-07	0.54	38	1.9	Fibcd1	Signaling
DMR3:9829001	3	9829001	9832000	3000	1	8.20E-07	0.54	46	1.53	RGD1305178;Usp20	Protease
DMR3:9872001	3	9872001	9876000	4000	1	5.00E-07	0.46	105	2.62	Fnbp1	
DMR3:10027001	3	10027001	10030000	3000	1	6.30E-07	0.65	79	2.63	Fibcd1l1;Qrfp	
DMR3:10487001	3	10487001	10489000	2000	1	5.80E-07	0.39	42	2.1	Hmcn2	
DMR3:10633001	3	10633001	10636000	3000	1	6.10E-09	0.68	46	1.53	Gpr107	Signaling
DMR3:11021001	3	11021001	11023000	2000	1	2.40E-09	0.64	26	1.3	Nup214	Transport
DMR3:11045001	3	11045001	11047000	2000	1	2.60E-10	0.68	38	1.9	Nup214	Transport
DMR3:11088001	3	11088001	11092000	4000	1	7.10E-08	0.47	83	2.08	LOC102555629;Fam78a	
DMR3:11250001	3	11250001	11251000	1000	1	2.70E-07	0.5	7	0.7	Prrc2b;LOC103691765;Pomt1	Metabolism;Transport
DMR3:11365001	3	11365001	11367000	2000	1	2.10E-08	0.5	47	2.35	Dnm1	Transport
DMR3:11571001	3	11571001	11574000	3000	1	5.10E-07	0.4	33	1.1	Fam102a	
DMR3:11716001	3	11716001	11719000	3000	1	2.20E-07	0.63	54	1.8	Eng;Fpgs	Receptor;Metabolism
DMR3:11824001	3	11824001	11827000	3000	1	3.00E-07	0.53	44	1.47	Ptrh1;Cfap157;Stxbp1	Metabolism;Development;Transport
DMR3:11910001	3	11910001	11916000	6000	1	5.90E-07	0.48	83	1.38	Fam129b	
DMR3:12024001	3	12024001	12025000	1000	1	3.90E-08	0.68	19	1.9	Slc2a8;Garnl3	Signaling
DMR3:12026001	3	12026001	12029000	3000	1	1.30E-07	0.59	59	1.97	Slc2a8;Garnl3	Signaling
DMR3:12081001	3	12081001	12084000	3000	1	4.40E-08	0.67	54	1.8	Garnl3	Signaling
DMR3:12269001	3	12269001	12272000	3000	1	1.70E-07	0.59	35	1.17	Ralgps1;Angptl2	Transcription;Signaling
DMR3:12777001	3	12777001	12786000	9000	1	5.80E-07	0.43	158	1.76	Mvb12b	
DMR3:12790001	3	12790001	12791000	1000	1	1.30E-07	0.59	12	1.2	Mvb12b	
DMR3:12804001	3	12804001	12811000	7000	3	1.30E-07	0.63	130	1.86	Mvb12b	
DMR3:12879001	3	12879001	12884000	5000	1	1.20E-08	0.6	68	1.36	Mvb12b	
DMR3:12905001	3	12905001	12908000	3000	1	4.70E-12	0.79	37	1.23	Mvb12b	
DMR3:13696001	3	13696001	13700000	4000	1	3.20E-07	0.69	84	2.1	Mapkap1	Cytoskeleton
DMR3:13709001	3	13709001	13711000	2000	1	5.00E-08	0.5	33	1.65	Mapkap1	Cytoskeleton
DMR3:14231001	3	14231001	14233000	2000	1	3.40E-07	-0.39	14	0.7	C5	
DMR3:14659001	3	14659001	14663000	4000	1	7.20E-08	0.66	56	1.4	Ggta1	Golgi
DMR3:14876001	3	14876001	14881000	5000	1	2.40E-07	0.53	105	2.1	RGD1564343;Dab2ip	Signaling
DMR3:14934001	3	14934001	14938000	4000	1	8.60E-08	0.45	70	1.75	Dab2ip	Signaling

DMR3:14939001	3	14939001	14944000	5000	2	1.10E-08	0.52	82	1.64	Dab2ip	Signaling
DMR3:14957001	3	14957001	14959000	2000	1	3.80E-09	0.51	51	2.55	Dab2ip	Signaling
DMR3:14971001	3	14971001	14984000	13000	2	5.40E-10	0.58	240	1.85	Dab2ip	Signaling
DMR3:15101001	3	15101001	15103000	2000	1	9.20E-07	0.48	36	1.8	Ttll11	Cytoskeleton
DMR3:15269001	3	15269001	15272000	3000	1	1.70E-07	0.41	45	1.5	Ttll11	Cytoskeleton
DMR3:15292001	3	15292001	15298000	6000	1	2.70E-08	0.56	78	1.3	Ttll11	Cytoskeleton
DMR3:15351001	3	15351001	15355000	4000	1	5.50E-07	0.51	70	1.75	LOC108350358;Ndufa8	Metabolism
DMR3:15552001	3	15552001	15554000	2000	1	8.20E-07	0.52	17	0.85	Ptgs1	Metabolism
DMR3:21353001	3	21353001	21357000	4000	1	3.20E-07	-0.58	7	0.17	Olr434	Signaling
DMR3:21891001	3	21891001	21893000	2000	1	9.40E-07	-0.6	6	0.3	Strbp	Metabolism
DMR3:22886001	3	22886001	22889000	3000	1	9.70E-07	0.55	30	1	Nek6;Psmb7	Protease
DMR3:25592001	3	25592001	25595000	3000	1	7.10E-07	-0.43	29	0.97	Lrp1b	
DMR3:25843001	3	25843001	25844000	1000	1	1.80E-08	-1.08	5	0.5	Lrp1b	
DMR3:27012001	3	27012001	27015000	3000	1	8.90E-07	-0.91	17	0.57	Lrp1b	
DMR3:28997001	3	28997001	28999000	2000	1	5.50E-07	-1.13	11	0.55	Arhgap15	Signaling
DMR3:29968001	3	29968001	29970000	2000	2	5.20E-09	0.48	37	1.85	Zeb2	Transcription
DMR3:35129001	3	35129001	35131000	2000	1	1.50E-08	0.5	20	1	Kif5c	
DMR3:35170001	3	35170001	35172000	2000	1	5.30E-07	0.47	28	1.4	Kif5c	
DMR3:35603001	3	35603001	35605000	2000	1	6.30E-09	0.51	29	1.45	Lypd6	
DMR3:35774001	3	35774001	35776000	2000	1	3.70E-08	0.44	24	1.2	Mmadhc	
DMR3:35808001	3	35808001	35809000	1000	1	2.80E-07	0.5	12	1.2	Mmadhc;LOC102555425	
DMR3:37569001	3	37569001	37571000	2000	1	7.50E-09	0.48	25	1.25	Tnfaip6	
DMR3:38373001	3	38373001	38376000	3000	1	9.40E-07	0.43	24	0.8	RGD1564306	
DMR3:38414001	3	38414001	38419000	5000	1	1.40E-07	0.4	89	1.78	RGD1560248	
DMR3:38436001	3	38436001	38440000	4000	1	4.80E-07	0.43	66	1.65	RGD1560248	
DMR3:41177001	3	41177001	41179000	2000	1	3.10E-07	-1.05	8	0.4	Kcnj3	Transport
DMR3:44191001	3	44191001	44193000	2000	1	5.50E-07	0.43	40	2	Cytip;LOC102555754	
DMR3:45264001	3	45264001	45266000	2000	1	1.00E-10	0.53	26	1.3	Pkp4	Cytoskeleton
DMR3:45289001	3	45289001	45291000	2000	1	1.20E-08	0.67	20	1	Pkp4	Cytoskeleton
DMR3:45346001	3	45346001	45351000	5000	1	4.00E-07	0.39	81	1.62	Pkp4	Cytoskeleton
DMR3:45717001	3	45717001	45720000	3000	2	8.20E-10	0.52	49	1.63	Tanc1	
DMR3:45723001	3	45723001	45725000	2000	1	6.50E-07	0.48	30	1.5	Tanc1	
DMR3:45745001	3	45745001	45747000	2000	1	4.00E-09	0.54	23	1.15	Tanc1	
DMR3:45755001	3	45755001	45757000	2000	1	1.70E-08	0.46	50	2.5	Tanc1	
DMR3:45824001	3	45824001	45826000	2000	1	9.20E-07	0.39	35	1.75	Tanc1	
DMR3:45887001	3	45887001	45892000	5000	1	4.30E-09	0.44	109	2.18	Tanc1	
DMR3:45912001	3	45912001	45917000	5000	1	5.10E-07	0.58	119	2.38	Tanc1;Wdsub1	
DMR3:45924001	3	45924001	45926000	2000	1	7.90E-07	0.62	38	1.9	Tanc1;Wdsub1	
DMR3:46038001	3	46038001	46039000	1000	1	3.20E-11	0.52	22	2.2	Baz2b	Epigenetic
DMR3:46442001	3	46442001	46444000	2000	1	7.40E-10	0.5	37	1.85	Ly75	
DMR3:48456001	3	48456001	48458000	2000	1	3.50E-10	0.52	29	1.45	Gcg;Fap	Protease
DMR3:51012001	3	51012001	51018000	6000	1	9.30E-08	0.5	63	1.05	Grb14;LOC102552585	Cytoskeleton
DMR3:51534001	3	51534001	51536000	2000	1	8.90E-07	-0.63	25	1.25	LOC102553383;Scn3a	Transport
DMR3:51537001	3	51537001	51538000	1000	1	5.80E-09	0.49	14	1.4	LOC102553383;Scn3a	Transport
DMR3:52200001	3	52200001	52203000	3000	2	1.60E-08	0.48	46	1.53	Galnt3	Golgi
DMR3:55436001	3	55436001	55438000	2000	1	7.90E-07	0.57	23	1.15	Nostrin;Spc25	
DMR3:56362001	3	56362001	56364000	2000	1	3.20E-08	0.53	20	1	Myo3b	
DMR3:56826001	3	56826001	56828000	2000	1	6.90E-07	-0.43	19	0.95	Erich2	
DMR3:57628001	3	57628001	57630000	2000	1	1.80E-07	0.42	39	1.95	Mettl8	
DMR3:57634001	3	57634001	57636000	2000	1	5.40E-07	0.7	15	0.75	Mettl8	
DMR3:58145001	3	58145001	58147000	2000	1	9.40E-17	0.75	39	1.95	Metap1d;LOC108350393	Protease
DMR3:58914001	3	58914001	58918000	4000	1	2.20E-07	0.5	58	1.45	Rapgef4	Transcription
DMR3:60501001	3	60501001	60503000	2000	1	1.00E-11	0.58	27	1.35	LOC102552170;Chn1	
DMR3:60625001	3	60625001	60628000	3000	1	3.80E-07	0.4	23	0.77	Chn1	
DMR3:61423001	3	61423001	61424000	1000	1	4.60E-10	0.71	10	1	LOC102553552;Lnp	
DMR3:61607001	3	61607001	61608000	1000	1	3.50E-08	-1.12	11	1.1	Hoxd12;Hoxd11;Hoxd10	Development
DMR3:62861001	3	62861001	62863000	2000	1	4.90E-08	0.58	18	0.9	Pde11a	Signaling
DMR3:62876001	3	62876001	62880000	4000	1	1.60E-08	0.51	45	1.12	Pde11a	Signaling
DMR3:63493001	3	63493001	63495000	2000	1	2.90E-08	0.53	17	0.85	Prkra	
DMR3:64043001	3	64043001	64044000	1000	1	5.70E-08	0.48	10	1	Sestd1	
DMR3:64263001	3	64263001	64265000	2000	1	5.70E-07	0.45	22	1.1	Zfp385b	
DMR3:66832001	3	66832001	66833000	1000	1	3.40E-08	0.48	13	1.3	Pde1a	Signaling
DMR3:67677001	3	67677001	67679000	2000	1	8.00E-09	0.4	36	1.8	Frzb	Receptor
DMR3:69449001	3	69449001	69451000	2000	1	3.00E-07	-1.12	18	0.9	Zfp804a	
DMR3:71035001	3	71035001	71039000	4000	1	7.00E-07	0.48	36	0.9	Zc3h15	Transcription

DMR3:75496001	3	75496001	75499000	3000	1	2.30E-13	0.39	27	0.9	Olr564-ps;LOC108350559;Olr565-ps	
DMR3:75900001	3	75900001	75902000	2000	1	9.80E-07	-0.39	14	0.7	Olr585-ps;Olr586	Signaling
DMR3:76239001	3	76239001	76240000	1000	1	9.30E-07	-0.89	7	0.7	Olr606;Olr607	Receptor
DMR3:78416001	3	78416001	78422000	6000	2	2.70E-10	0.44	69	1.15	Olr704;Olr705	Receptor
DMR3:78646001	3	78646001	78647000	1000	1	8.30E-07	0.46	9	0.9	Olr717	Receptor
DMR3:79256001	3	79256001	79259000	3000	1	5.80E-09	0.53	49	1.63	Ptprj	Signaling
DMR3:79276001	3	79276001	79280000	4000	1	1.10E-07	0.56	49	1.23	Ptprj	Signaling
DMR3:79299001	3	79299001	79302000	3000	1	3.00E-08	0.55	29	0.97	Ptprj	Signaling
DMR3:79643001	3	79643001	79644000	1000	1	4.70E-10	0.54	24	2.4	Agbl2	Protease
DMR3:79670001	3	79670001	79671000	1000	1	4.40E-07	0.75	6	0.6	Mtch2	Transport
DMR3:80550001	3	80550001	80552000	2000	1	3.10E-10	0.58	57	2.85	F2;Znf408;Arhgap1	Protease;Signaling
DMR3:80675001	3	80675001	80677000	2000	1	1.00E-09	0.58	32	1.6	Ambra1	
DMR3:81510001	3	81510001	81512000	2000	1	3.00E-08	0.57	51	2.55	Chst1	Transport
DMR3:82117001	3	82117001	82120000	3000	1	8.40E-08	0.54	33	1.1	Tspan18	
DMR3:82579001	3	82579001	82581000	2000	1	2.80E-08	0.58	30	1.5	Alx4	Development
DMR3:86015001	3	86015001	86018000	3000	1	5.90E-07	-0.92	17	0.57	Lrrc4c	
DMR3:91243001	3	91243001	91245000	2000	1	7.10E-12	-1.03	25	1.25	LOC103691850;Traf6	Cytoskeleton
DMR3:91343001	3	91343001	91347000	4000	1	3.20E-11	0.55	52	1.3	Prr5l	
DMR3:91765001	3	91765001	91769000	4000	1	5.10E-07	0.39	50	1.25	Ldlrad3	Binding Proteins
DMR3:93196001	3	93196001	93197000	1000	1	4.00E-08	0.46	15	1.5	Ehf	Transcription
DMR3:93998001	3	93998001	93999000	1000	1	8.70E-07	0.48	12	1.2	Fbxo3	
DMR3:94921001	3	94921001	94926000	5000	1	5.60E-07	-0.42	34	0.68	Ccdc73	
DMR3:103729001	3	103729001	103732000	3000	1	2.30E-07	0.41	55	1.83	Lpcat4;Nutm1	Metabolism
DMR3:104083001	3	104083001	104084000	1000	1	3.50E-07	0.47	12	1.2	Aven;LOC108350584	
DMR3:105277001	3	105277001	105278000	1000	1	2.10E-07	0.57	11	1.1	Scg5;Arhgap11a	Transcription;Signaling
DMR3:108937001	3	108937001	108943000	6000	1	8.80E-07	-0.45	58	0.97	Fam98b	
DMR3:109034001	3	109034001	109037000	3000	1	6.50E-07	0.58	59	1.97	Rasgrp1	Transcription
DMR3:110224001	3	110224001	110226000	2000	1	8.80E-07	0.4	24	1.2	Eif2ak4	Signaling
DMR3:110482001	3	110482001	110484000	2000	1	2.30E-09	0.47	28	1.4	Pak6;Ankrd63	Signaling
DMR3:111008001	3	111008001	111009000	1000	1	3.90E-07	-0.64	15	1.5	Dnajc17;Zfyve19	Transcription
DMR3:111568001	3	111568001	111572000	4000	1	9.70E-07	0.45	54	1.35	Ltk;Rpap1	Receptor
DMR3:113037001	3	113037001	113039000	2000	1	8.30E-08	0.51	20	1	Tgm7l1;Tgm7	Transport
DMR3:113314001	3	113314001	113315000	1000	1	7.00E-07	0.44	14	1.4	Ppip5k1;Ckmt1b;Strc	Signaling;Cytoskeleton
DMR3:113894001	3	113894001	113895000	1000	1	6.50E-13	0.57	10	1	Casc4	
DMR3:114012001	3	114012001	114017000	5000	1	3.50E-07	0.58	59	1.18	Spg11	
DMR3:116903001	3	116903001	116906000	3000	1	1.10E-07	0.44	39	1.3	Sema6d	Signaling
DMR3:117498001	3	117498001	117499000	1000	1	6.50E-07	0.4	11	1.1	Slc12a1;LOC102555270	Transport
DMR3:117984001	3	117984001	117985000	1000	1	6.00E-08	0.46	19	1.9	Shc4	Cytoskeleton
DMR3:118027001	3	118027001	118028000	1000	1	5.00E-07	0.43	20	2	Secisbp2l	
DMR3:118118001	3	118118001	118119000	1000	1	1.30E-08	0.59	12	1.2	Cops2	
DMR3:118149001	3	118149001	118152000	3000	1	7.00E-08	-0.7	16	0.53	Galk2	Metabolism
DMR3:118167001	3	118167001	118171000	4000	1	9.30E-08	-0.46	40	1	Galk2	Metabolism
DMR3:118341001	3	118341001	118343000	2000	1	1.20E-07	0.5	26	1.3	Fam227b;Fgf7	Growth Factors
DMR3:118413001	3	118413001	118416000	3000	1	7.60E-08	-0.53	25	0.83	Fam227b	
DMR3:118786001	3	118786001	118787000	1000	1	8.20E-09	0.58	6	0.6	Atp8b4	Transport
DMR3:118959001	3	118959001	118960000	1000	1	1.90E-08	0.48	15	1.5	Atp8b4	Transport
DMR3:119076001	3	119076001	119077000	1000	1	2.00E-07	0.52	14	1.4	Hdc	Metabolism
DMR3:119575001	3	119575001	119576000	1000	1	8.30E-08	0.49	22	2.2	Blvra;Ncaph	Metabolism
DMR3:119605001	3	119605001	119609000	4000	1	3.20E-07	0.47	61	1.52	Ncaph;Itpripl1	
DMR3:120424001	3	120424001	120425000	1000	1	1.60E-08	0.55	13	1.3	Acoxl	Metabolism
DMR3:120764001	3	120764001	120765000	1000	1	5.30E-08	0.46	9	0.9	Bcl2l11	
DMR3:121231001	3	121231001	121232000	1000	1	3.90E-07	0.45	16	1.6	Anapc1;Mertk	Proteolysis;Receptor
DMR3:121620001	3	121620001	121623000	3000	1	4.90E-08	0.44	53	1.77	Ttl;Polr1b	Transcription
DMR3:121673001	3	121673001	121677000	4000	1	8.10E-08	0.51	47	1.18	Chchd5	
DMR3:121970001	3	121970001	121972000	2000	1	4.80E-07	-0.51	11	0.55	RGD1566226	
DMR3:122141001	3	122141001	122145000	4000	1	6.30E-08	0.5	68	1.7	Sirpa	Receptor
DMR3:122195001	3	122195001	122197000	2000	1	1.60E-07	0.51	45	2.25	Pdyn	Signaling
DMR3:122836001	3	122836001	122841000	5000	1	1.10E-07	0.5	90	1.8	Ebf4	Transcription
DMR3:123129001	3	123129001	123130000	1000	1	1.50E-08	0.7	15	1.5	Avp;LOC103691888;Ubox5	Signaling
DMR3:124913001	3	124913001	124916000	3000	1	3.70E-11	0.56	49	1.63	Cds2	Transport
DMR3:125523001	3	125523001	125525000	2000	1	2.30E-08	0.52	34	1.7	Crls1;Lrrn4	Transport;Immune
DMR3:128719001	3	128719001	128721000	2000	1	4.80E-08	0.43	40	2	Plcb4	Metabolism
DMR3:129753001	3	129753001	129754000	1000	1	6.10E-07	0.68	13	1.3	Snap25	Transcription
DMR3:136083001	3	136083001	136085000	2000	1	3.00E-07	0.45	16	0.8	Macrod2	
DMR3:136217001	3	136217001	136219000	2000	1	1.80E-07	0.48	16	0.8	Macrod2	

DMR3:137960001	3	137960001	137962000	2000	1	5.90E-07	0.4	41	2.05	Bfsp1	
DMR3:140651001	3	140651001	140654000	3000	1	3.20E-08	0.44	42	1.4	Ralgapa2	Signaling
DMR3:146478001	3	146478001	146480000	2000	1	3.60E-07	0.62	20	1	Acss1;Vsx1;LOC102547396	Metabolism;Development
DMR3:147104001	3	147104001	147111000	7000	1	7.90E-07	0.48	135	1.93	Sdcbp2;Snph	Transport;Transcription
DMR3:147127001	3	147127001	147129000	2000	1	8.10E-08	0.51	29	1.45	Snph	Transcription
DMR3:147248001	3	147248001	147250000	2000	1	1.90E-08	0.82	29	1.45	Psmf1	Protease; Proteolysis
DMR3:147802001	3	147802001	147804000	2000	1	1.40E-07	0.52	23	1.15	Rbck1	Proteolysis
DMR3:148350001	3	148350001	148353000	3000	2	8.20E-08	0.53	38	1.27	Tpx2	Cytoskeleton
DMR3:148525001	3	148525001	148527000	2000	2	3.20E-10	0.59	24	1.2	Xkr7;LOC108350503	
DMR3:148537001	3	148537001	148538000	1000	1	2.20E-07	0.54	15	1.5	Xkr7;LOC108350503;Ccm2l	
DMR3:148789001	3	148789001	148791000	2000	1	1.10E-07	0.51	26	1.3	Kif3b	Cytoskeleton
DMR3:148835001	3	148835001	148837000	2000	1	3.20E-07	0.43	18	0.9	LOC102554727;Asxl1	
DMR3:149281001	3	149281001	149283000	2000	1	2.00E-08	0.49	27	1.35	Efcab8	
DMR3:149681001	3	149681001	149684000	3000	1	1.50E-07	0.55	41	1.37	Bpifb1	
DMR3:150836001	3	150836001	150837000	1000	1	6.30E-07	0.42	17	1.7	Pigu	
DMR3:151282001	3	151282001	151284000	2000	2	4.20E-11	0.48	43	2.15	Procr	Signaling
DMR3:151342001	3	151342001	151345000	3000	1	2.10E-08	0.46	62	2.07	Mmp24	Protease
DMR3:151384001	3	151384001	151385000	1000	1	4.30E-08	0.45	18	1.8	Uqcc1	Transcription
DMR3:151390001	3	151390001	151393000	3000	1	1.50E-11	0.56	61	2.03	Uqcc1	Transcription
DMR3:151433001	3	151433001	151442000	9000	1	7.50E-08	0.54	155	1.72	Uqcc1	Transcription
DMR3:151536001	3	151536001	151538000	2000	1	5.40E-07	0.55	56	2.8	Cep250;LOC102550306	Epigenetic
DMR3:151563001	3	151563001	151564000	1000	1	3.80E-08	0.54	14	1.4	LOC102550306;Ergic3;Fer1l4	Transport
DMR3:151565001	3	151565001	151570000	5000	1	5.10E-07	0.48	86	1.72	Ergic3;Fer1l4	Transport
DMR3:152348001	3	152348001	152349000	1000	1	7.00E-08	0.51	9	0.9	Phf20	
DMR3:152867001	3	152867001	152868000	1000	1	5.40E-07	0.44	12	1.2	Myl9;LOC102551821	Cytoskeleton
DMR3:153103001	3	153103001	153106000	3000	1	1.30E-07	0.51	36	1.2	Ndrg3;LOC102547986;Dsn1	Protease
DMR3:153195001	3	153195001	153196000	1000	1	2.60E-07	0.45	17	1.7	Soga1;Tldc2	
DMR3:153454001	3	153454001	153457000	3000	1	1.90E-07	0.58	56	1.87	Rpn2;Ghrh	Golgi;Signaling
DMR3:153509001	3	153509001	153510000	1000	1	9.00E-07	0.41	13	1.3	Manbal	
DMR3:153529001	3	153529001	153530000	1000	1	7.70E-07	0.53	18	1.8	Manbal	
DMR3:153585001	3	153585001	153595000	10000	1	4.40E-07	0.46	202	2.02	Src	
DMR3:154360001	3	154360001	154361000	1000	1	1.60E-09	0.58	10	1	Ctnnbl1	
DMR3:154593001	3	154593001	154596000	3000	1	6.70E-10	0.54	32	1.07	Tgm2	Transport
DMR3:154682001	3	154682001	154684000	2000	1	1.20E-15	0.74	44	2.2	RGD1563354	Transcription
DMR3:155164001	3	155164001	155165000	1000	1	2.80E-07	0.55	18	1.8	Ppp1r16b	Signaling
DMR3:157027001	3	157027001	157029000	2000	1	1.10E-09	0.53	23	1.15	Chd6	
DMR3:157836001	3	157836001	157844000	8000	1	1.60E-07	0.56	82	1.02	Ptprt	Signaling
DMR3:158045001	3	158045001	158046000	1000	1	7.20E-07	0.43	15	1.5	Ptprt	Signaling
DMR3:159623001	3	159623001	159631000	8000	1	2.20E-07	0.47	126	1.57	Tox2	
DMR3:159675001	3	159675001	159676000	1000	1	5.30E-07	0.53	15	1.5	Tox2	
DMR3:159688001	3	159688001	159690000	2000	1	1.70E-07	0.51	19	0.95	Tox2	
DMR3:159691001	3	159691001	159693000	2000	1	7.10E-07	0.47	46	2.3	Tox2	
DMR3:160005001	3	160005001	160009000	4000	1	5.20E-08	0.45	76	1.9	Ttpal;Serinc3	Transport;Signaling
DMR3:160121001	3	160121001	160125000	4000	1	1.20E-07	0.46	68	1.7	Pkig;Ada	Signaling;Metabolism
DMR3:160412001	3	160412001	160413000	1000	1	8.10E-08	0.62	19	1.9	Pabpc1l	
DMR3:160477001	3	160477001	160480000	3000	1	8.80E-07	0.46	62	2.07	Stk4	Signaling
DMR3:160516001	3	160516001	160517000	1000	1	7.40E-07	0.46	12	1.2	Stk4	Signaling
DMR3:160865001	3	160865001	160866000	1000	1	8.50E-08	0.47	20	2	Rbpjl;Sdc4	Transcription;Receptor
DMR3:160910001	3	160910001	160916000	6000	1	2.20E-08	0.48	93	1.55	Sys1;Tp53tg5	Transport
DMR3:160954001	3	160954001	160958000	4000	1	7.20E-07	0.44	63	1.57	Pigt	
DMR3:161092001	3	161092001	161097000	5000	1	1.90E-07	0.52	75	1.5	Wfdc16	Protease; Proteolysis
DMR3:161452001	3	161452001	161456000	4000	1	5.50E-07	0.41	90	2.25	Slc12a5;Ncoa5	Transport;Transcription
DMR3:161459001	3	161459001	161463000	4000	3	2.70E-08	0.51	125	3.12	Slc12a5;Ncoa5	Transport;Transcription
DMR3:161504001	3	161504001	161508000	4000	1	1.60E-09	0.51	70	1.75	Ncoa5	Transcription
DMR3:161515001	3	161515001	161517000	2000	1	8.10E-08	0.39	31	1.55	Cd40	Receptor
DMR3:161901001	3	161901001	161909000	8000	1	5.40E-09	0.54	179	2.24	Cdh22	Cytoskeleton
DMR3:161911001	3	161911001	161912000	1000	1	5.20E-08	0.53	24	2.4	Cdh22	Cytoskeleton
DMR3:162426001	3	162426001	162429000	3000	1	4.00E-08	0.49	51	1.7	Eya2	
DMR3:162432001	3	162432001	162434000	2000	1	6.20E-09	0.7	61	3.05	Eya2	
DMR3:163407001	3	163407001	163410000	3000	1	1.00E-08	0.61	56	1.87	Prex1	Transcription
DMR3:163438001	3	163438001	163439000	1000	1	8.50E-08	0.52	19	1.9	Prex1	Transcription
DMR3:163447001	3	163447001	163453000	6000	1	1.90E-08	0.52	104	1.73	Prex1	Transcription
DMR3:163851001	3	163851001	163858000	7000	1	6.90E-07	0.47	196	2.8	Znfx1;Kcnb1	Transport
DMR3:163953001	3	163953001	163955000	2000	1	1.30E-08	0.53	47	2.35	LOC102552303;Ptgis	
DMR3:163981001	3	163981001	163982000	1000	1	1.70E-09	0.52	14	1.4	Ptgis	

DMR3:164031001	3	164031001	164033000	2000	1	8.20E-07	0.48	43	2.15	B4galt5	Golgi
DMR3:164042001	3	164042001	164043000	1000	1	5.30E-10	0.55	25	2.5	B4galt5	Golgi
DMR3:164181001	3	164181001	164183000	2000	1	2.70E-07	0.41	23	1.15	Slc9a8	Transport
DMR3:164199001	3	164199001	164202000	3000	1	6.60E-08	0.59	39	1.3	Slc9a8	Transport
DMR3:164278001	3	164278001	164280000	2000	1	7.60E-07	0.52	38	1.9	Snai1	Transcription
DMR3:164945001	3	164945001	164948000	3000	1	7.10E-09	0.55	43	1.43	Adnp	
DMR3:165268001	3	165268001	165272000	4000	1	9.10E-08	0.45	83	2.08	Nfatc2	Transcription
DMR3:165413001	3	165413001	165417000	4000	1	4.60E-13	0.68	115	2.88	Atp9a	Transport
DMR3:165424001	3	165424001	165427000	3000	1	1.40E-08	0.52	107	3.57	Atp9a	Transport
DMR3:165493001	3	165493001	165496000	3000	1	3.40E-08	0.66	30	1	Atp9a	Transport
DMR3:165731001	3	165731001	165733000	2000	1	1.80E-07	0.44	49	2.45	Zfp64	Transcription
DMR3:167960001	3	167960001	167961000	1000	1	8.90E-10	0.57	15	1.5	Bcas1	
DMR3:168486001	3	168486001	168488000	2000	1	1.30E-09	0.47	55	2.75	Dok5;LOC102552784	
DMR3:170398001	3	170398001	170405000	7000	1	5.70E-09	0.51	130	1.86	Cstf1;Cass4	
DMR3:170901001	3	170901001	170906000	5000	1	9.60E-08	0.42	114	2.28	Bmp7	Growth Factors
DMR3:171149001	3	171149001	171154000	5000	1	8.40E-08	0.46	135	2.7	LOC102550488;Ctcfl	Transcription
DMR3:171866001	3	171866001	171872000	6000	1	7.20E-07	0.47	142	2.37	Vapb;Apcdd1l	Transport
DMR3:171895001	3	171895001	171897000	2000	1	2.10E-07	0.49	41	2.05	Apcdd1l	
DMR3:172196001	3	172196001	172201000	5000	1	3.30E-07	0.53	106	2.12	Npepl1	Protease
DMR3:172408001	3	172408001	172410000	2000	1	9.50E-08	0.44	35	1.75	Gnas;LOC103692002	Signaling
DMR3:174002001	3	174002001	174005000	3000	1	2.10E-07	0.45	59	1.97	Cdh26	Cytoskeleton
DMR3:175490001	3	175490001	175491000	1000	1	5.50E-07	0.42	21	2.1	Osbpl2	
DMR3:175551001	3	175551001	175565000	14000	2	1.70E-10	0.56	370	2.64	Adrm1;Lama5	Extracellular Matrix
DMR3:175651001	3	175651001	175654000	3000	1	1.40E-08	0.46	59	1.97	Cables2;LOC103691993;Rbbp8nl	Transcription
DMR3:175769001	3	175769001	175773000	4000	1	8.00E-07	0.5	62	1.55	LOC102552888;Mir1b	
DMR3:175859001	3	175859001	175862000	3000	1	3.60E-07	0.48	59	1.97	Slco4a1;LOC102552621;LOC102552550	Transport
DMR3:176236001	3	176236001	176238000	2000	1	2.40E-07	0.63	21	1.05	Slc17a9	Transport
DMR3:176457001	3	176457001	176459000	2000	1	7.60E-07	0.57	20	1	Nkain4	
DMR3:176591001	3	176591001	176593000	2000	1	4.80E-08	0.74	49	2.45	Kcnq2	Transport
DMR3:176733001	3	176733001	176734000	1000	1	1.70E-07	0.49	23	2.3	Fndc11;Helz2	Transcription
DMR3:177066001	3	177066001	177068000	2000	1	6.50E-07	0.43	36	1.8	Uckl1	Signaling
DMR4:468001	4	468001	470000	2000	2	3.00E-18	0.93	38	1.9	Cnpy1	
DMR4:2142001	4	2142001	2144000	2000	2	1.10E-12	0.56	5	0.25	Lmbr1	Receptor
DMR4:4042001	4	4042001	4044000	2000	1	4.80E-08	0.53	19	0.95	Dpp6	Protease
DMR4:4058001	4	4058001	4064000	6000	1	7.60E-09	0.53	91	1.52	Dpp6	Protease
DMR4:4888001	4	4888001	4891000	3000	1	5.00E-08	-0.55	21	0.7	Dpp6	Protease
DMR4:6573001	4	6573001	6577000	4000	1	1.20E-07	0.46	75	1.88	Prkag2	Signaling
DMR4:6595001	4	6595001	6603000	8000	1	1.00E-07	0.48	151	1.89	Prkag2	Signaling
DMR4:6634001	4	6634001	6635000	1000	1	1.80E-07	0.44	29	2.9	Prkag2	Signaling
DMR4:6667001	4	6667001	6670000	3000	1	2.00E-07	0.46	49	1.63	Prkag2;LOC102554253	Signaling
DMR4:6701001	4	6701001	6703000	2000	1	2.80E-07	0.47	33	1.65	Prkag2;LOC103692022;LOC102554193	Signaling
DMR4:6761001	4	6761001	6764000	3000	1	3.00E-07	0.47	56	1.87	Prkag2	Signaling
DMR4:6870001	4	6870001	6875000	5000	1	6.10E-07	0.68	84	1.68	Rheb	Signaling
DMR4:6877001	4	6877001	6880000	3000	2	6.10E-08	0.38	58	1.93	Rheb	Signaling
DMR4:7078001	4	7078001	7082000	4000	1	4.90E-11	0.6	70	1.75	Smarcd3	Epigenetic
DMR4:7206001	4	7206001	7212000	6000	1	9.10E-07	0.49	130	2.17	Gbx1;Agap3;LOC102554667	Development
DMR4:7369001	4	7369001	7370000	1000	1	8.80E-12	0.83	12	1.2	Kcnh2	Transport
DMR4:7759001	4	7759001	7761000	2000	1	9.40E-12	0.6	43	2.15	Fam126a;LOC103692024	
DMR4:8112001	4	8112001	8117000	5000	1	1.60E-13	0.73	57	1.14	Srpk2	Signaling
DMR4:9198001	4	9198001	9201000	3000	1	5.60E-10	0.62	49	1.63	Orc5	Cell Cycle
DMR4:9368001	4	9368001	9371000	3000	1	2.60E-07	0.42	41	1.37	Reln	Extracellular Matrix
DMR4:9408001	4	9408001	9413000	5000	1	1.70E-08	0.42	103	2.06	Reln	Extracellular Matrix
DMR4:9689001	4	9689001	9690000	1000	1	6.20E-07	0.41	22	2.2	Reln	Extracellular Matrix
DMR4:9865001	4	9865001	9868000	3000	1	1.00E-10	0.55	54	1.8	Slc26a5;Psmc2	Transport;Protease
DMR4:9900001	4	9900001	9901000	1000	1	3.70E-08	0.79	17	1.7	Dnajc2;Pmpcb	Transcription;Protease
DMR4:10146001	4	10146001	10147000	1000	1	1.20E-08	0.59	16	1.6	Fbxl13;Lrrc17	Receptor
DMR4:11378001	4	11378001	11379000	1000	1	2.60E-08	0.61	8	0.8	Magi2	
DMR4:14337001	4	14337001	14341000	4000	1	1.50E-07	-0.44	39	0.98	Sema3c	Signaling
DMR4:21427001	4	21427001	21429000	2000	1	2.80E-09	0.46	19	0.95	Grm3	Signaling
DMR4:25450001	4	25450001	25454000	4000	1	8.60E-07	-0.41	33	0.82	Steap1	
DMR4:25519001	4	25519001	25520000	1000	1	2.80E-07	0.55	11	1.1	Steap2	
DMR4:26035001	4	26035001	26038000	3000	1	8.40E-09	-0.39	33	1.1	Cdk14	Signaling
DMR4:26191001	4	26191001	26194000	3000	1	8.90E-07	0.68	36	1.2	Cdk14	Signaling
DMR4:26216001	4	26216001	26217000	1000	1	8.40E-07	0.52	13	1.3	Cdk14;LOC102549548	Signaling

DMR4:27359001	4	27359001	27360000	1000	1	4.40E-09	0.54	26	2.6	Mterf1	
DMR4:27392001	4	27392001	27394000	2000	1	1.10E-08	-0.77	15	0.75	Lrrd1	Cytoskeleton
DMR4:27866001	4	27866001	27869000	3000	1	1.10E-08	0.47	56	1.87	Cdk6	Signaling
DMR4:28449001	4	28449001	28453000	4000	1	1.10E-08	0.49	39	0.98	Vps50	
DMR4:28975001	4	28975001	28979000	4000	1	1.20E-07	-0.59	37	0.92	Gngt1	Signaling
DMR4:29978001	4	29978001	29982000	4000	1	5.50E-07	0.39	77	1.93	Ppp1r9a	
DMR4:30080001	4	30080001	30084000	4000	1	2.50E-07	0.42	42	1.05	Ppp1r9a	
DMR4:34041001	4	34041001	34043000	2000	1	1.40E-09	0.55	27	1.35	Col28a1	Extracellular Matrix
DMR4:38810001	4	38810001	38815000	5000	1	1.90E-07	-0.61	44	0.88	Thsd7a	Cytoskeleton
DMR4:39521001	4	39521001	39522000	1000	1	6.70E-08	0.48	17	1.7	Tmem106b	
DMR4:42213001	4	42213001	42214000	1000	1	7.10E-07	0.45	8	0.8	Mdfic	
DMR4:43093001	4	43093001	43094000	1000	1	4.20E-07	-0.8	11	1.1	Cttnbp2	
DMR4:45301001	4	45301001	45302000	1000	1	1.40E-11	0.68	10	1	Wnt2	Signaling
DMR4:48439001	4	48439001	48442000	3000	2	3.40E-18	0.65	40	1.33	Kcnd2	Transport
DMR4:50434001	4	50434001	50436000	2000	1	3.90E-08	0.42	24	1.2	Cadps2	Transport
DMR4:54677001	4	54677001	54683000	6000	1	3.00E-09	-0.38	60	1	Grm8	Signaling
DMR4:54938001	4	54938001	54945000	7000	4	3.40E-12	-2.11	27	0.39	Grm8	Signaling
DMR4:54958001	4	54958001	54960000	2000	1	2.90E-07	-0.6	15	0.75	Grm8	Signaling
DMR4:55010001	4	55010001	55016000	6000	1	9.50E-08	0.67	34	0.57	Grm8	Signaling
DMR4:58833001	4	58833001	58838000	5000	1	3.00E-07	0.46	86	1.72	Podxl;LOC102548336	Cytoskeleton
DMR4:58856001	4	58856001	58857000	1000	1	6.50E-07	0.44	13	1.3	Podxl	Cytoskeleton
DMR4:61175001	4	61175001	61176000	1000	1	1.60E-08	-0.58	12	1.2	Exoc4	Transport
DMR4:62092001	4	62092001	62095000	3000	1	3.60E-07	0.47	26	0.87	Cald1	Cytoskeleton
DMR4:62414001	4	62414001	62418000	4000	1	9.50E-07	-0.36	59	1.48	Wdr91	
DMR4:62457001	4	62457001	62459000	2000	1	3.60E-08	0.45	34	1.7	Stra8	
DMR4:62523001	4	62523001	62525000	2000	1	5.10E-07	0.5	27	1.35	RGD1565367	Transport
DMR4:62552001	4	62552001	62556000	4000	1	3.50E-08	0.52	80	2	Cnot4	Proteolysis
DMR4:62859001	4	62859001	62861000	2000	1	5.10E-07	0.34	42	2.1	Fam180a	
DMR4:64529001	4	64529001	64532000	3000	1	1.80E-07	0.44	64	2.13	Dgki	Signaling
DMR4:64907001	4	64907001	64909000	2000	1	5.50E-07	0.51	41	2.05	Creb3l2	
DMR4:65759001	4	65759001	65760000	1000	1	3.10E-07	0.47	19	1.9	Atp6v0a4	Metabolism
DMR4:65899001	4	65899001	65904000	5000	1	2.20E-09	0.52	108	2.16	RGD1306271	
DMR4:66677001	4	66677001	66684000	7000	1	3.00E-07	0.39	74	1.06	Tbxas1	Metabolism
DMR4:67185001	4	67185001	67186000	1000	1	9.40E-09	0.5	16	1.6	Rab19;Mkrn1	Proteolysis
DMR4:67518001	4	67518001	67520000	2000	1	1.60E-07	-0.54	33	1.65	Braf;LOC102554766	Signaling
DMR4:71079001	4	71079001	71080000	1000	1	8.10E-10	0.65	14	1.4	RGD1562066	Signaling
DMR4:72539001	4	72539001	72541000	2000	1	1.70E-08	-0.68	19	0.95	Olr815-ps	
DMR4:76233001	4	76233001	76234000	1000	1	1.70E-07	0.57	8	0.8	Cntnap2	
DMR4:76749001	4	76749001	76751000	2000	1	2.60E-07	0.63	20	1	Cntnap2	
DMR4:77904001	4	77904001	77906000	2000	1	2.50E-07	0.53	16	0.8	LOC108350703;Trnac-gca	
DMR4:77949001	4	77949001	77952000	3000	1	1.90E-10	-0.64	19	0.63	LOC108350703;Trnac-gca	
DMR4:77961001	4	77961001	77963000	2000	1	7.70E-08	0.45	25	1.25	LOC108350703;Trnac-gca;Fgfr1-ps1	
DMR4:78032001	4	78032001	78033000	1000	1	5.00E-07	0.47	17	1.7	LOC108350703;Krba1	
DMR4:78054001	4	78054001	78055000	1000	1	4.70E-08	0.41	15	1.5	Krba1	
DMR4:78089001	4	78089001	78092000	3000	1	4.20E-08	0.69	44	1.47	Sspo	Extracellular Matrix
DMR4:78120001	4	78120001	78121000	1000	1	3.50E-07	0.45	14	1.4	Sspo	Extracellular Matrix
DMR4:78209001	4	78209001	78210000	1000	1	1.10E-07	0.46	17	1.7	Lrrc61;Rarres2	Cytoskeleton
DMR4:78495001	4	78495001	78496000	1000	1	3.00E-08	0.57	6	0.6	Aoc1	Metabolism
DMR4:80051001	4	80051001	80053000	2000	1	1.80E-09	0.61	30	1.5	Osbpl3	
DMR4:81893001	4	81893001	81895000	2000	1	8.30E-09	0.51	22	1.1	Skap2	Cytoskeleton
DMR4:85424001	4	85424001	85426000	2000	1	3.80E-10	0.6	12	0.6	Fam188b	
DMR4:85517001	4	85517001	85519000	2000	1	4.70E-07	0.36	31	1.55	Fam188b;LOC108350810	
DMR4:87030001	4	87030001	87031000	1000	1	6.70E-07	-0.54	7	0.7	Avl9	
DMR4:87227001	4	87227001	87229000	2000	1	5.60E-12	0.59	27	1.35	Fkbp9;Nt5c3a	Metabolism
DMR4:89094001	4	89094001	89095000	1000	1	1.50E-07	0.42	10	1	Herc3	Proteolysis
DMR4:91298001	4	91298001	91301000	3000	1	6.60E-26	0.84	21	0.7	Ccser1	
DMR4:91945001	4	91945001	91947000	2000	1	7.90E-07	-0.72	11	0.55	Ccser1	
DMR4:92338001	4	92338001	92340000	2000	1	4.00E-07	-0.74	22	1.1	Ccser1	
DMR4:94209001	4	94209001	94211000	2000	1	8.40E-07	0.4	15	0.75	Grid2	Receptor
DMR4:97323001	4	97323001	97325000	2000	1	4.90E-10	0.56	27	1.35	RGD1564699	
DMR4:97687001	4	97687001	97688000	1000	1	8.60E-07	0.36	13	1.3	Gng12;LOC103692153	Signaling
DMR4:99308001	4	99308001	99309000	1000	1	8.90E-07	0.61	9	0.9	Rmnd5a	
DMR4:99721001	4	99721001	99722000	1000	1	9.70E-08	0.54	17	1.7	Reep1	Transport
DMR4:100062001	4	100062001	100064000	2000	1	7.10E-09	0.56	19	0.95	Atoh8	Transcription
DMR4:100415001	4	100415001	100417000	2000	1	9.10E-07	0.4	31	1.55	Capg;Elmod3	Cytoskeleton;Cytoskeleton

DMR4:100975001	4	100975001	100977000	2000	1	8.10E-07	0.48	32	1.6	Dnah6	Cytoskeleton
DMR4:101190001	4	101190001	101193000	3000	1	1.20E-08	0.48	20	0.67	Suclg1	Metabolism
DMR4:108889001	4	108889001	108892000	3000	1	3.20E-07	-0.54	27	0.9	Ctnna2	Cytoskeleton
DMR4:108933001	4	108933001	108940000	7000	1	1.30E-08	-0.41	60	0.86	Ctnna2	Cytoskeleton
DMR4:110724001	4	110724001	110726000	2000	1	2.30E-07	0.8	5	0.25	Lrrtm4	Receptor
DMR4:112694001	4	112694001	112695000	1000	1	1.60E-08	0.47	12	1.2	Gcfc2;Mrpl19	Epigenetic;Translation
DMR4:113730001	4	113730001	113732000	2000	1	4.50E-09	0.46	34	1.7	Sema4f	Signaling
DMR4:113830001	4	113830001	113832000	2000	1	2.90E-07	0.46	26	1.3	M1ap	
DMR4:113905001	4	113905001	113907000	2000	1	8.80E-07	0.47	25	1.25	Dqx1;NEWGENE_1595506;Pcgf1	Transcription
DMR4:115091001	4	115091001	115092000	1000	1	4.30E-08	0.5	13	1.3	Tet3	
DMR4:115104001	4	115104001	115107000	3000	1	7.80E-10	0.5	62	2.07	Tet3	
DMR4:115183001	4	115183001	115184000	1000	1	1.90E-08	0.43	17	1.7	Dguok	Signaling
DMR4:115758001	4	115758001	115759000	1000	1	4.70E-07	0.48	16	1.6	Dysf	Transport
DMR4:115830001	4	115830001	115833000	3000	1	7.80E-09	0.72	48	1.6	Dysf	Transport
DMR4:116916001	4	116916001	116918000	2000	1	9.10E-09	0.49	59	2.95	Spr	Metabolism
DMR4:117058001	4	117058001	117060000	2000	1	3.30E-14	0.61	25	1.25	Sfxn5	Transport
DMR4:117537001	4	117537001	117539000	2000	1	3.10E-10	0.52	26	1.3	Nat8f5;Nat8	Metabolism
DMR4:117687001	4	117687001	117690000	3000	1	3.30E-07	0.77	33	1.1	Figla;Add2	Transcription;Cytoskeleton
DMR4:118195001	4	118195001	118196000	1000	1	5.00E-07	0.48	15	1.5	Pcyox1	Metabolism
DMR4:118320001	4	118320001	118322000	2000	1	8.10E-07	0.51	27	1.35	LOC102554318;Pcbp1;LOC102554481	
DMR4:118424001	4	118424001	118427000	3000	1	5.20E-09	0.47	18	0.6	Asprv1	
DMR4:118565001	4	118565001	118567000	2000	1	2.40E-08	0.61	28	1.4	Anxa4	Signaling
DMR4:118977001	4	118977001	118980000	3000	1	4.80E-08	0.52	47	1.57	Antxr1;LOC108350726	Cytoskeleton
DMR4:119032001	4	119032001	119035000	3000	1	4.80E-07	0.41	36	1.2	Antxr1	Cytoskeleton
DMR4:119703001	4	119703001	119704000	1000	1	3.50E-09	0.49	13	1.3	Rab43	
DMR4:119970001	4	119970001	119973000	3000	1	3.50E-07	0.49	34	1.13	Rab7a	
DMR4:120705001	4	120705001	120711000	6000	1	1.50E-09	0.54	92	1.53	Mgll	Metabolism
DMR4:120778001	4	120778001	120780000	2000	1	6.40E-08	0.61	26	1.3	Mgll;Abtb1;Podxl2	Metabolism
DMR4:120785001	4	120785001	120790000	5000	1	2.50E-07	0.49	135	2.7	Abtb1;Podxl2	
DMR4:121233001	4	121233001	121234000	1000	1	2.40E-09	0.49	28	2.8	Plxna1	
DMR4:121333001	4	121333001	121334000	1000	1	4.80E-08	0.54	10	1	Chchd6	
DMR4:121618001	4	121618001	121620000	2000	1	1.10E-08	0.57	23	1.15	Txnrd3	Metabolism
DMR4:122112001	4	122112001	122115000	3000	1	3.50E-11	0.4	80	2.67	Vom1r103	
DMR4:122654001	4	122654001	122656000	2000	1	8.60E-07	0.49	17	0.85	Nup210	Transport
DMR4:122869001	4	122869001	122870000	1000	1	8.00E-07	0.53	15	1.5	Fbln2	Extracellular Matrix
DMR4:123393001	4	123393001	123394000	1000	1	6.40E-08	0.48	17	1.7	Iqsec1	Transcription
DMR4:123448001	4	123448001	123456000	8000	2	5.00E-10	0.48	129	1.61	Iqsec1	Transcription
DMR4:123485001	4	123485001	123491000	6000	1	3.30E-07	0.49	96	1.6	Slc41a3	Transport
DMR4:123914001	4	123914001	123917000	3000	1	8.60E-07	0.44	48	1.6	Fgd5	Transcription
DMR4:124319001	4	124319001	124322000	3000	1	1.10E-07	-0.73	22	0.73	Prickle2	Cytoskeleton
DMR4:125718001	4	125718001	125721000	3000	1	2.00E-07	0.78	38	1.27	Magi1	
DMR4:126619001	4	126619001	126623000	4000	2	1.40E-07	0.56	88	2.2	Slc25a26;Lrig1	Transport;Receptor
DMR4:129384001	4	129384001	129386000	2000	1	3.20E-08	0.54	20	1	Fam19a4	
DMR4:129405001	4	129405001	129406000	1000	1	3.90E-08	0.65	14	1.4	Fam19a4	
DMR4:130353001	4	130353001	130354000	1000	1	8.00E-07	0.44	10	1	Mitf	
DMR4:131862001	4	131862001	131865000	3000	1	2.60E-07	0.44	44	1.47	Foxp1	
DMR4:133758001	4	133758001	133759000	1000	1	3.30E-08	0.49	18	1.8	Pdzrn3	
DMR4:133929001	4	133929001	133930000	1000	1	1.80E-09	0.56	14	1.4	Pdzrn3	
DMR4:138421001	4	138421001	138422000	1000	1	8.00E-07	-0.85	8	0.8	Cntn4	Cytoskeleton
DMR4:140401001	4	140401001	140403000	2000	1	3.30E-09	0.63	17	0.85	Itpr1	Ion Channel
DMR4:145113001	4	145113001	145114000	1000	1	4.80E-09	0.65	6	0.6	LOC108350744;Lhfpl4	
DMR4:145137001	4	145137001	145138000	1000	1	2.60E-09	0.77	18	1.8	Lhfpl4	
DMR4:145243001	4	145243001	145245000	2000	1	4.90E-08	0.42	20	1	Mtmr14;Cpne9	Signaling
DMR4:145776001	4	145776001	145778000	2000	1	7.60E-07	0.63	20	1	Atp2b2	Transport
DMR4:145829001	4	145829001	145830000	1000	1	3.80E-07	0.42	22	2.2	Atp2b2	Transport
DMR4:146114001	4	146114001	146118000	4000	1	1.50E-07	0.57	46	1.15	Slc6a11	Transport
DMR4:146255001	4	146255001	146256000	1000	1	2.20E-08	0.5	12	1.2	Slc6a1	Transport
DMR4:146276001	4	146276001	146280000	4000	1	1.50E-07	0.62	75	1.88	Slc6a1	Transport
DMR4:146283001	4	146283001	146284000	1000	1	1.40E-08	0.51	29	2.9	Slc6a1;LOC102548867	Transport
DMR4:146782001	4	146782001	146783000	1000	1	4.00E-07	0.43	12	1.2	Atg7;Vgll4	Proteolysis;Transcription
DMR4:146789001	4	146789001	146791000	2000	1	3.10E-08	0.48	29	1.45	Vgll4	Transcription
DMR4:146808001	4	146808001	146813000	5000	1	5.40E-08	0.48	88	1.76	Vgll4	Transcription
DMR4:146937001	4	146937001	146938000	1000	1	2.40E-07	0.55	15	1.5	Tamm41	
DMR4:146941001	4	146941001	146945000	4000	1	7.50E-12	0.57	79	1.98	Tamm41	

DMR4:147173001	4	147173001	147175000	2000	1	9.00E-07	0.51	25	1.25	Syn2;Timp4	Transport;Protease; Proteolysis
DMR4:147864001	4	147864001	147868000	4000	1	5.00E-09	0.58	93	2.33	Plxnd1	
DMR4:147872001	4	147872001	147875000	3000	1	3.50E-08	0.63	30	1	Plxnd1	
DMR4:147876001	4	147876001	147880000	4000	1	3.70E-07	0.46	80	2	Plxnd1	
DMR4:148189001	4	148189001	148195000	6000	2	3.00E-09	0.7	72	1.2	Fam21c;LOC108350750;Zfand4	
DMR4:148780001	4	148780001	148782000	2000	1	5.30E-07	0.5	38	1.9	Rassf4;LOC500300	Cytoskeleton
DMR4:149804001	4	149804001	149806000	2000	1	5.10E-07	0.66	17	0.85	RGD1563294	
DMR4:150432001	4	150432001	150434000	2000	1	5.10E-08	0.52	32	1.6	Bms1	
DMR4:150594001	4	150594001	150598000	4000	1	1.90E-07	0.44	101	2.52	Ankrd26	
DMR4:150718001	4	150718001	150721000	3000	1	2.00E-09	0.57	47	1.57	Cacna1c	Transport
DMR4:150747001	4	150747001	150751000	4000	1	5.60E-07	0.47	52	1.3	Cacna1c	Transport
DMR4:150825001	4	150825001	150831000	6000	1	8.50E-08	0.46	97	1.62	Cacna1c	Transport
DMR4:150855001	4	150855001	150861000	6000	2	3.10E-07	0.49	122	2.03	Cacna1c	Transport
DMR4:150962001	4	150962001	150963000	1000	1	3.90E-07	0.53	17	1.7	Cacna1c	Transport
DMR4:151039001	4	151039001	151041000	2000	1	2.10E-07	0.48	27	1.35	Cacna1c	Transport
DMR4:151064001	4	151064001	151067000	3000	1	2.80E-07	0.53	39	1.3	Cacna1c	Transport
DMR4:151978001	4	151978001	151981000	3000	1	6.60E-07	0.41	52	1.73	Fbxl14	
DMR4:152186001	4	152186001	152187000	1000	1	9.60E-07	-0.39	8	0.8	Erc1	Transport
DMR4:153484001	4	153484001	153487000	3000	1	9.60E-09	0.53	89	2.97	Mical3	
DMR4:153494001	4	153494001	153500000	6000	1	8.10E-09	0.6	111	1.85	Mical3	
DMR4:153501001	4	153501001	153505000	4000	1	8.50E-08	0.47	88	2.2	Mical3	
DMR4:153634001	4	153634001	153636000	2000	1	6.10E-07	0.51	39	1.95	Mical3	
DMR4:153934001	4	153934001	153936000	2000	1	6.20E-09	0.56	43	2.15	Slc6a12	Transport
DMR4:153954001	4	153954001	153957000	3000	1	3.70E-07	0.49	53	1.77	Iqsec3	Transcription
DMR4:154230001	4	154230001	154231000	1000	1	1.80E-08	0.42	7	0.7	Mug2	Protease; Proteolysis
DMR4:155687001	4	155687001	155692000	5000	1	5.60E-11	0.53	74	1.48	Foxj2;C3ar1	Signaling
DMR4:156133001	4	156133001	156139000	6000	1	6.10E-07	-0.44	67	1.12	Clec4b2	Transport
DMR4:157086001	4	157086001	157088000	2000	1	5.20E-11	0.71	23	1.15	Clstn3;LOC102553636	Transport
DMR4:157354001	4	157354001	157356000	2000	1	9.80E-08	0.49	40	2	Usp5;Cdca3;Gnb3;P3h3	Protease;Signaling;Extracellular Matrix
DMR4:157472001	4	157472001	157475000	3000	1	4.00E-08	0.49	46	1.53	Cops7a	
DMR4:157663001	4	157663001	157664000	1000	1	3.50E-08	0.49	22	2.2	Nop2;Iffo1	Metabolism
DMR4:157971001	4	157971001	157977000	6000	3	4.60E-08	0.48	101	1.68	Cd9	
DMR4:158143001	4	158143001	158146000	3000	1	4.60E-09	0.51	52	1.73	Vwf	
DMR4:158338001	4	158338001	158339000	1000	1	7.00E-07	0.49	11	1.1	Ano2	
DMR4:159263001	4	159263001	159265000	2000	1	2.70E-07	0.42	36	1.8	Kcna6	Transport
DMR4:160054001	4	160054001	160057000	3000	2	6.00E-10	0.69	33	1.1	Parp11	
DMR4:160322001	4	160322001	160323000	1000	1	6.50E-09	0.44	20	2	Prmt8	Golgi
DMR4:160642001	4	160642001	160644000	2000	1	5.70E-07	0.49	28	1.4	Tspan9	
DMR4:161636001	4	161636001	161639000	3000	1	4.20E-10	0.58	76	2.53	LOC100362138;Tulp3	
DMR4:161711001	4	161711001	161714000	3000	1	1.90E-07	0.51	29	0.97	LOC108350769;LOC100362909;Nrip2	Proteolysis
DMR4:163376001	4	163376001	163381000	5000	1	5.80E-09	-0.52	35	0.7	Klrd1	
DMR4:163484001	4	163484001	163487000	3000	2	1.40E-07	-0.66	22	0.73	LOC100359471;Klri1	
DMR4:167941001	4	167941001	167945000	4000	2	1.50E-09	0.5	58	1.45	Etv6	Transcription
DMR4:168130001	4	168130001	168131000	1000	1	4.90E-08	0.48	18	1.8	Bcl2l14	
DMR4:168219001	4	168219001	168223000	4000	1	2.10E-09	0.56	64	1.6	Lrp6	Binding Proteins
DMR4:168226001	4	168226001	168229000	3000	1	2.90E-08	0.51	37	1.23	Lrp6	Binding Proteins
DMR4:168358001	4	168358001	168359000	1000	1	2.20E-07	0.44	21	2.1	Mansc1	
DMR4:169012001	4	169012001	169013000	1000	1	6.40E-11	0.67	12	1.2	Fam234b;Gsg1	Cytoskeleton
DMR4:170647001	4	170647001	170651000	4000	1	2.90E-08	0.61	76	1.9	Gucy2c	Signaling
DMR4:171826001	4	171826001	171827000	1000	1	7.30E-08	0.49	18	1.8	Dera	Metabolism
DMR4:173933001	4	173933001	173936000	3000	1	8.80E-09	0.51	23	0.77	Pik3c2g	Signaling
DMR4:174158001	4	174158001	174163000	5000	1	4.50E-07	0.48	67	1.34	Plcz1	Metabolism
DMR4:174682001	4	174682001	174684000	2000	2	9.10E-10	0.59	28	1.4	Plekha5	
DMR4:174699001	4	174699001	174701000	2000	1	4.50E-07	0.38	22	1.1	Plekha5	
DMR4:175672001	4	175672001	175674000	2000	1	1.70E-07	-0.64	18	0.9	Pde3a	Signaling
DMR4:176998001	4	176998001	177001000	3000	1	2.20E-07	0.53	30	1	Cmas	Metabolism
DMR4:177162001	4	177162001	177168000	6000	1	1.50E-07	0.43	123	2.05	St8sia1	Transport
DMR4:178092001	4	178092001	178094000	2000	1	9.80E-07	0.4	60	3	Sox5	
DMR4:178169001	4	178169001	178174000	5000	1	9.00E-10	0.54	77	1.54	Sox5	
DMR4:178865001	4	178865001	178866000	1000	1	4.60E-07	0.56	11	1.1	Sox5	
DMR4:178944001	4	178944001	178947000	3000	1	1.30E-07	0.64	34	1.13	Sox5	
DMR4:179404001	4	179404001	179406000	2000	1	1.60E-09	0.59	25	1.25	Lrmp	
DMR4:180333001	4	180333001	180335000	2000	1	5.50E-08	0.52	39	1.95	Sspn;LOC102550089;LOC108350848	

DMR4:180545001	4	180545001	180547000	2000	1	8.70E-08	0.45	26	1.3	Itpr2	Ion Channel
DMR4:180666001	4	180666001	180668000	2000	1	5.20E-09	0.53	25	1.25	Itpr2	Ion Channel
DMR4:180740001	4	180740001	180742000	2000	1	6.80E-08	0.58	37	1.85	Itpr2	Ion Channel
DMR4:181296001	4	181296001	181299000	3000	1	9.60E-10	0.66	85	2.83	Ppfibp1	
DMR4:181339001	4	181339001	181343000	4000	1	3.00E-07	0.55	61	1.52	Ppfibp1;LOC690784	
DMR4:181666001	4	181666001	181668000	2000	1	6.70E-10	0.53	22	1.1	Pthlh	Hormone
DMR4:181886001	4	181886001	181888000	2000	1	1.80E-07	0.48	52	2.6	Ccdc91	
DMR4:182009001	4	182009001	182013000	4000	1	7.90E-07	0.52	53	1.32	Ccdc91	
DMR5:2407001	5	2407001	2410000	3000	1	1.20E-10	0.61	23	0.77	Stau2	
DMR5:2430001	5	2430001	2433000	3000	1	7.80E-07	0.56	42	1.4	Stau2	
DMR5:3132001	5	3132001	3134000	2000	1	1.90E-07	0.47	26	1.3	Kcnb2	Transport
DMR5:5561001	5	5561001	5562000	1000	1	2.00E-08	0.46	18	1.8	Ncoa2	Epigenetic
DMR5:8494001	5	8494001	8497000	3000	1	3.00E-09	-0.57	21	0.7	Cpa6	Protease
DMR5:8788001	5	8788001	8789000	1000	1	2.50E-07	0.44	18	1.8	Cspp1	Cell Cycle
DMR5:16360001	5	16360001	16361000	1000	1	8.60E-09	1.31	8	0.8	Tmem68	
DMR5:16504001	5	16504001	16506000	2000	1	3.40E-07	-0.62	17	0.85	RGD1564981	
DMR5:18886001	5	18886001	18889000	3000	1	1.50E-07	0.45	40	1.33	Fam110b	
DMR5:22513001	5	22513001	22514000	1000	1	4.60E-07	-0.33	14	1.4	Clvs1	Transport
DMR5:22721001	5	22721001	22724000	3000	1	1.50E-10	0.46	44	1.47	Asph	Golgi
DMR5:24300001	5	24300001	24303000	3000	1	2.00E-07	0.53	56	1.87	LOC108351119;Ndufaf6	
DMR5:24605001	5	24605001	24606000	1000	1	3.60E-07	0.4	17	1.7	Esrp1	Translation
DMR5:25672001	5	25672001	25676000	4000	1	3.40E-07	0.42	77	1.93	Tmem67	
DMR5:28252001	5	28252001	28253000	1000	1	9.20E-07	0.45	6	0.6	Lrrc69	Cytoskeleton
DMR5:28532001	5	28532001	28534000	2000	1	9.80E-07	-0.51	19	0.95	Necab1	
DMR5:32596001	5	32596001	32603000	7000	1	6.90E-09	-0.52	58	0.83	Cnbd1	
DMR5:33438001	5	33438001	33440000	2000	1	3.50E-07	-0.89	19	0.95	Cngb3	Ion Channel
DMR5:33556001	5	33556001	33559000	3000	1	4.90E-10	0.63	22	0.73	Cpne3;LOC108351084	
DMR5:40870001	5	40870001	40871000	1000	1	1.50E-07	-0.61	6	0.6	Manea	
DMR5:43689001	5	43689001	43692000	3000	1	5.20E-07	0.49	36	1.2	Epha7	Receptor
DMR5:47739001	5	47739001	47740000	1000	1	1.00E-07	0.43	9	0.9	Bach2	
DMR5:47766001	5	47766001	47767000	1000	1	1.90E-07	0.46	22	2.2	Bach2	
DMR5:47803001	5	47803001	47804000	1000	1	2.30E-10	0.59	13	1.3	Bach2	
DMR5:48094001	5	48094001	48098000	4000	2	2.40E-09	0.56	72	1.8	Ankrd6	
DMR5:48111001	5	48111001	48113000	2000	1	2.90E-09	0.49	47	2.35	Ankrd6;LOC102552310	
DMR5:48128001	5	48128001	48130000	2000	1	2.80E-07	0.51	29	1.45	Ankrd6	
DMR5:48134001	5	48134001	48137000	3000	1	3.20E-07	-1.27	39	1.3	Ankrd6	
DMR5:49324001	5	49324001	49329000	5000	1	1.70E-07	0.43	87	1.74	Cnr1	Signaling
DMR5:51542001	5	51542001	51545000	3000	1	2.00E-07	-0.3	38	1.27	Lingo2	
DMR5:56486001	5	56486001	56489000	3000	1	6.30E-13	0.65	46	1.53	Aco1;Ddx58	Metabolism
DMR5:57116001	5	57116001	57118000	2000	1	1.00E-08	0.53	31	1.55	B4galt1	
DMR5:57346001	5	57346001	57348000	2000	1	2.40E-08	0.5	37	1.85	Nfx1	Transcription
DMR5:57452001	5	57452001	57454000	2000	1	6.50E-07	0.47	35	1.75	Nol6	Metabolism
DMR5:58044001	5	58044001	58050000	6000	1	2.60E-07	0.46	155	2.58	Cntfr	Receptor
DMR5:58069001	5	58069001	58071000	2000	1	2.00E-07	0.45	29	1.45	Cntfr	Receptor
DMR5:58177001	5	58177001	58179000	2000	1	3.00E-08	0.47	40	2	LOC102547621;Ccl19;LOC689481	Growth Factors
DMR5:58284001	5	58284001	58287000	3000	1	1.00E-08	0.57	49	1.63	Fam205a	
DMR5:58375001	5	58375001	58376000	1000	1	3.00E-07	0.44	19	1.9	Phf24	Signaling
DMR5:58381001	5	58381001	58385000	4000	1	1.70E-08	0.53	88	2.2	Phf24;Dnajb5	Signaling;Transcription
DMR5:58398001	5	58398001	58401000	3000	1	5.60E-07	0.56	71	2.37	Dnajb5	Transcription
DMR5:58869001	5	58869001	58875000	6000	2	1.50E-07	0.52	163	2.72	Rusc2;Fam166b	
DMR5:58940001	5	58940001	58944000	4000	1	1.10E-07	0.45	79	1.98	LOC103692345;Tesk1;Cd72	
DMR5:59155001	5	59155001	59157000	2000	1	6.70E-08	0.47	40	2	Npr2;Spag8;Hint2;Fam221b;Tmem8b	Signaling;Signaling;Cytoskeleton
DMR5:59176001	5	59176001	59180000	4000	1	1.20E-07	0.53	62	1.55	Tmem8b	Cytoskeleton
DMR5:59228001	5	59228001	59230000	2000	1	2.80E-10	0.65	32	1.6	Hrct1	
DMR5:59413001	5	59413001	59416000	3000	2	2.70E-07	0.62	60	2	Reck;Glipr2	Protease; Proteolysis;Immune
DMR5:59501001	5	59501001	59508000	7000	1	1.90E-07	0.67	122	1.74	Clta;Gne	Transport;Transcription
DMR5:59809001	5	59809001	59811000	2000	1	6.90E-07	0.47	20	1	Melk	Signaling
DMR5:60154001	5	60154001	60155000	1000	1	1.40E-08	0.55	16	1.6	Pax5	
DMR5:60609001	5	60609001	60612000	3000	1	1.30E-10	0.52	53	1.77	Fbxo10	Proteolysis
DMR5:60983001	5	60983001	60984000	1000	1	9.10E-07	0.51	23	2.3	Shb	
DMR5:60992001	5	60992001	61005000	13000	1	5.10E-07	0.51	266	2.05	Shb	
DMR5:61010001	5	61010001	61012000	2000	1	1.30E-07	0.38	35	1.75	Shb	
DMR5:61042001	5	61042001	61044000	2000	1	1.60E-08	0.55	38	1.9	Shb	

DMR5:61053001	5	61053001	61058000	5000	1	1.80E-08	0.53	61	1.22	Shb	
DMR5:61707001	5	61707001	61709000	2000	1	1.50E-07	0.47	23	1.15	Tmod1;Tstd2	Cytoskeleton;Transport
DMR5:62163001	5	62163001	62170000	7000	1	1.30E-08	0.52	109	1.56	Trim14;Coro2a	Proteolysis;Cytoskeleton
DMR5:62281001	5	62281001	62282000	1000	1	8.70E-07	0.43	20	2	Gabbr2	Signaling
DMR5:62323001	5	62323001	62327000	4000	1	3.90E-08	0.48	65	1.62	Gabbr2	Signaling
DMR5:62340001	5	62340001	62343000	3000	1	9.30E-07	0.44	47	1.57	Gabbr2	Signaling
DMR5:62583001	5	62583001	62585000	2000	1	3.60E-10	0.57	31	1.55	Gabbr2	Signaling
DMR5:62740001	5	62740001	62744000	4000	1	3.60E-08	0.49	76	1.9	Galnt12	Golgi
DMR5:62754001	5	62754001	62757000	3000	1	6.90E-07	0.48	40	1.33	Galnt12	Golgi
DMR5:64074001	5	64074001	64075000	1000	1	4.90E-08	0.51	12	1.2	Invs	
DMR5:64391001	5	64391001	64393000	2000	1	5.10E-08	0.45	16	0.8	Tmeff1	
DMR5:64394001	5	64394001	64397000	3000	1	9.40E-07	0.46	61	2.03	Tmeff1	
DMR5:70290001	5	70290001	70292000	2000	1	6.10E-08	0.44	22	1.1	Slc44a1;LOC102551474	Transport
DMR5:70321001	5	70321001	70323000	2000	1	6.40E-09	0.47	62	3.1	Slc44a1;LOC102551474	Transport
DMR5:70383001	5	70383001	70384000	1000	1	3.80E-09	0.51	14	1.4	Slc44a1	Transport
DMR5:70473001	5	70473001	70475000	2000	1	1.00E-08	0.51	19	0.95	Fsd1l;LOC100360453	Proteolysis
DMR5:70634001	5	70634001	70637000	3000	1	2.70E-08	0.59	26	0.87	Tmem38b	Transport
DMR5:73494001	5	73494001	73497000	3000	1	7.80E-07	0.65	48	1.6	Actl7b;Actl7a;Ikbkap	Cytoskeleton
DMR5:74128001	5	74128001	74131000	3000	1	3.30E-07	0.45	57	1.9	Epb41l4b	
DMR5:74297001	5	74297001	74303000	6000	1	4.30E-11	0.56	104	1.73	Ptpn3;LOC108350978	Signaling
DMR5:75211001	5	75211001	75213000	2000	1	3.00E-07	0.54	32	1.6	Svep1	
DMR5:75655001	5	75655001	75660000	5000	2	1.40E-10	0.52	80	1.6	Lpar1	Signaling
DMR5:76396001	5	76396001	76398000	2000	1	1.60E-07	0.44	50	2.5	Ugcg	Golgi
DMR5:77005001	5	77005001	77009000	4000	1	7.60E-10	0.57	68	1.7	RGD1310951;Inip	
DMR5:77132001	5	77132001	77133000	1000	1	7.90E-07	0.58	12	1.2	Snx30	Cytoskeleton
DMR5:78312001	5	78312001	78315000	3000	1	3.90E-07	0.44	40	1.33	Wdr31;LOC102547534	
DMR5:78911001	5	78911001	78914000	3000	1	5.00E-08	0.54	41	1.37	Zfp618	Transcription
DMR5:78970001	5	78970001	78974000	4000	1	1.20E-07	0.43	49	1.23	Zfp618;Ambp	Transcription
DMR5:79223001	5	79223001	79226000	3000	1	4.20E-10	0.59	30	1	Akna;LOC102547858;Whrn	Cytoskeleton
DMR5:79277001	5	79277001	79279000	2000	1	3.40E-07	0.62	27	1.35	Whrn	Cytoskeleton
DMR5:81431001	5	81431001	81433000	2000	1	6.50E-07	-0.94	47	2.35	Astn2;Trim32	
DMR5:100601001	5	100601001	100602000	1000	1	7.30E-08	0.56	9	0.9	Nfib	Transcription
DMR5:104504001	5	104504001	104507000	3000	1	9.40E-07	0.39	32	1.07	Adamtsl1	
DMR5:104814001	5	104814001	104816000	2000	1	2.30E-25	1.14	21	1.05	Fam154a	
DMR5:105285001	5	105285001	105288000	3000	1	6.30E-07	0.4	42	1.4	Acer2;LOC108351102	
DMR5:105358001	5	105358001	105360000	2000	2	1.30E-07	0.41	18	0.9	Slc24a2	Transport
DMR5:106950001	5	106950001	106951000	1000	1	7.80E-09	0.62	27	2.7	Ifna5;LOC690903	
DMR5:113622001	5	113622001	113625000	3000	1	7.20E-07	-0.62	17	0.57	Ift74;Lrrc19	
DMR5:113785001	5	113785001	113786000	1000	1	2.90E-07	0.53	11	1.1	Tek	Receptor
DMR5:114949001	5	114949001	114950000	1000	1	1.50E-07	0.6	6	0.6	Hook1	Transport
DMR5:116740001	5	116740001	116742000	2000	1	4.00E-08	0.53	33	1.65	Nfia	Transcription
DMR5:117097001	5	117097001	117099000	2000	1	2.50E-07	0.54	21	1.05	Patj	
DMR5:118737001	5	118737001	118740000	3000	1	3.20E-07	0.43	33	1.1	Pgm1	Metabolism
DMR5:118809001	5	118809001	118812000	3000	1	5.20E-07	0.89	25	0.83	Pgm1	Metabolism
DMR5:119768001	5	119768001	119770000	2000	1	4.80E-07	0.56	33	1.65	Cachd1	Transport
DMR5:120255001	5	120255001	120257000	2000	1	1.60E-08	0.55	22	1.1	Ak4	Signaling
DMR5:122909001	5	122909001	122911000	2000	1	9.40E-07	0.54	15	0.75	Oma1	Protease
DMR5:124367001	5	124367001	124368000	1000	1	7.40E-07	0.81	4	0.4	C8a	
DMR5:126059001	5	126059001	126067000	8000	2	6.60E-08	0.44	102	1.27	Pcsk9;Bsnd	Protease
DMR5:126182001	5	126182001	126184000	2000	1	5.20E-09	0.48	36	1.8	Dhcr24	Metabolism
DMR5:126343001	5	126343001	126346000	3000	1	9.40E-07	0.47	40	1.33	Fam151a;Acot11	Metabolism
DMR5:126380001	5	126380001	126381000	1000	1	9.40E-08	0.46	20	2	Acot11	Metabolism
DMR5:126512001	5	126512001	126517000	5000	1	5.10E-07	0.38	109	2.18	LOC689013;Ssbp3	Transcription
DMR5:126918001	5	126918001	126919000	1000	1	1.60E-08	0.45	6	0.6	Dio1	
DMR5:127101001	5	127101001	127102000	1000	1	1.90E-09	0.64	17	1.7	Glis1	Transcription
DMR5:127112001	5	127112001	127115000	3000	1	4.50E-07	0.52	34	1.13	Glis1;LOC102551042	Transcription
DMR5:127130001	5	127130001	127134000	4000	1	9.80E-08	0.41	86	2.15	Glis1;LOC102551042	Transcription
DMR5:127442001	5	127442001	127444000	2000	1	1.40E-07	0.5	53	2.65	Lrp8	Binding Proteins
DMR5:127657001	5	127657001	127660000	3000	1	8.20E-12	0.55	33	1.1	Scp2	Transport
DMR5:127765001	5	127765001	127766000	1000	1	6.90E-07	0.45	12	1.2	Echdc2	Metabolism
DMR5:128830001	5	128830001	128831000	1000	1	2.70E-07	0.47	10	1	Osbpl9	
DMR5:129011001	5	129011001	129013000	2000	1	7.80E-10	0.61	16	0.8	Eps15	Transport
DMR5:129052001	5	129052001	129053000	1000	1	2.90E-07	0.69	17	1.7	Ttc39a	
DMR5:133318001	5	133318001	133321000	3000	1	3.00E-07	0.45	22	0.73	Trabd2b	Protease
DMR5:133880001	5	133880001	133883000	3000	1	8.70E-09	0.51	40	1.33	Tal1	Transcription

DMR5:134726001	5	134726001	134731000	5000	2	1.20E-10	0.52	83	1.66	Mknk1;Kncn	Signaling
DMR5:134776001	5	134776001	134782000	6000	1	1.70E-08	0.42	104	1.73	Dmbx1	Development
DMR5:135380001	5	135380001	135382000	2000	1	4.00E-07	-0.8	14	0.7	Ipp	Cytoskeleton
DMR5:135596001	5	135596001	135597000	1000	1	1.70E-09	0.52	22	2.2	Tesk2	
DMR5:136133001	5	136133001	136137000	4000	1	4.10E-08	0.51	67	1.68	Tmem53;Rnf220	
DMR5:136230001	5	136230001	136232000	2000	1	2.80E-07	0.43	51	2.55	Rnf220	
DMR5:136592001	5	136592001	136593000	1000	1	5.10E-09	0.52	23	2.3	Klf17	Transcription
DMR5:136984001	5	136984001	136985000	1000	1	1.20E-07	0.47	21	2.1	Kdm4a	Epigenetic
DMR5:137044001	5	137044001	137046000	2000	1	2.10E-09	0.6	35	1.75	Ptprf	Signaling
DMR5:137047001	5	137047001	137062000	15000	1	6.30E-07	0.46	318	2.12	Ptprf	Signaling
DMR5:137095001	5	137095001	137100000	5000	1	1.70E-11	0.54	95	1.9	Ptprf;LOC102553423	Signaling
DMR5:137112001	5	137112001	137117000	5000	1	8.70E-07	0.45	88	1.76	Ptprf;LOC102553423	Signaling
DMR5:137257001	5	137257001	137260000	3000	1	7.50E-07	0.39	37	1.23	Elovl1;Cdc20;LOC103692460;Mpl	Metabolism;Proteolysis;Receptor
DMR5:137401001	5	137401001	137407000	6000	1	9.70E-07	0.39	127	2.12	Cfap57	
DMR5:137454001	5	137454001	137455000	1000	1	3.60E-08	0.65	0	0	Cfap57;Ebna1bp2	
DMR5:137930001	5	137930001	137931000	1000	1	7.40E-11	0.59	12	1.2	Olr869;LOC679953	Receptor
DMR5:138477001	5	138477001	138479000	2000	1	2.30E-08	0.45	29	1.45	Ppcs;Zmynd12	Transport
DMR5:138488001	5	138488001	138493000	5000	1	3.10E-10	0.52	64	1.28	Zmynd12	
DMR5:138934001	5	138934001	138936000	2000	1	6.30E-08	0.6	31	1.55	Hivep3	
DMR5:139020001	5	139020001	139022000	2000	1	5.70E-07	0.51	21	1.05	Hivep3	
DMR5:139084001	5	139084001	139086000	2000	1	3.30E-07	0.53	34	1.7	Hivep3	
DMR5:139821001	5	139821001	139826000	5000	1	9.50E-07	0.42	78	1.56	Rims3	Transport
DMR5:140824001	5	140824001	140826000	2000	1	5.90E-09	0.59	28	1.4	Bmp8b;Oxct2a;Ppie	Growth Factors;Transport;Transcription
DMR5:140986001	5	140986001	140989000	3000	1	4.00E-08	0.44	58	1.93	Pabpc4	
DMR5:141438001	5	141438001	141439000	1000	1	2.90E-07	-0.53	8	0.8	Akirin1	
DMR5:141487001	5	141487001	141488000	1000	1	6.90E-07	0.4	14	1.4	Rhbdl2;LOC103692469	
DMR5:142690001	5	142690001	142692000	2000	1	2.40E-07	0.51	25	1.25	Utp11;Fhl3	Metabolism;Transcription
DMR5:143709001	5	143709001	143710000	1000	1	2.50E-08	0.47	17	1.7	Grik3	Receptor
DMR5:144147001	5	144147001	144151000	4000	1	9.40E-07	0.49	71	1.77	Stk40;LOC108351032;Eva1b	Signaling
DMR5:144250001	5	144250001	144253000	3000	1	2.60E-07	0.46	80	2.67	Map7d1	Cytoskeleton
DMR5:144733001	5	144733001	144735000	2000	1	6.20E-08	0.67	19	0.95	Psmb2;Tfap2e	Protease;Transcription
DMR5:144775001	5	144775001	144778000	3000	1	4.40E-08	0.46	62	2.07	Ncdn;LOC100294508	Cytoskeleton
DMR5:144866001	5	144866001	144870000	4000	1	3.70E-07	0.43	53	1.32	LOC100294508;Zmym4	Transcription
DMR5:144878001	5	144878001	144881000	3000	1	5.50E-07	0.54	56	1.87	LOC100294508;Zmym4	Transcription
DMR5:145250001	5	145250001	145255000	5000	1	6.90E-07	0.54	93	1.86	Dlgap3	Cytoskeleton
DMR5:145260001	5	145260001	145261000	1000	1	2.00E-08	0.65	12	1.2	Dlgap3	Cytoskeleton
DMR5:146247001	5	146247001	146249000	2000	1	3.40E-11	0.56	35	1.75	Csmd2	
DMR5:146270001	5	146270001	146271000	1000	1	4.40E-08	0.56	5	0.5	Csmd2;LOC108351071	
DMR5:146361001	5	146361001	146362000	1000	1	2.10E-12	0.64	10	1	Csmd2	
DMR5:146368001	5	146368001	146369000	1000	1	3.10E-07	0.54	25	2.5	Csmd2	
DMR5:146533001	5	146533001	146538000	5000	1	2.90E-09	0.47	79	1.58	Csmd2	
DMR5:146540001	5	146540001	146542000	2000	1	9.90E-09	0.51	41	2.05	Csmd2	
DMR5:146573001	5	146573001	146575000	2000	1	6.80E-10	0.55	37	1.85	Csmd2	
DMR5:146706001	5	146706001	146715000	9000	1	2.00E-07	0.46	131	1.46	Csmd2	
DMR5:146825001	5	146825001	146827000	2000	1	2.10E-07	0.56	16	0.8	Phc2	Epigenetic
DMR5:146854001	5	146854001	146861000	7000	1	3.00E-11	0.58	120	1.71	Phc2	Epigenetic
DMR5:146963001	5	146963001	146967000	4000	1	8.80E-08	0.54	104	2.6	Zfp362	Transcription
DMR5:147120001	5	147120001	147123000	3000	1	3.00E-08	0.52	60	2	Azin2	Metabolism
DMR5:147141001	5	147141001	147144000	3000	1	2.40E-07	0.43	67	2.23	Azin2	Metabolism
DMR5:147316001	5	147316001	147317000	1000	1	2.80E-08	0.49	19	1.9	Fndc5	
DMR5:147327001	5	147327001	147332000	5000	1	5.20E-07	0.45	95	1.9	Fndc5;S100pbp	
DMR5:147572001	5	147572001	147573000	1000	1	1.60E-07	0.42	30	3	Zbtb8a;LOC108351038	Cytoskeleton
DMR5:148161001	5	148161001	148165000	4000	1	4.60E-07	0.53	80	2	Spocd1;Adgrb2	Signaling
DMR5:148206001	5	148206001	148213000	7000	1	6.60E-07	0.45	124	1.77	Adgrb2	Signaling
DMR5:148364001	5	148364001	148369000	5000	1	7.30E-07	0.5	116	2.32	Hcrtr1	Signaling
DMR5:148436001	5	148436001	148439000	3000	1	3.90E-07	0.43	52	1.73	RGD1562036	Metabolism
DMR5:148953001	5	148953001	148954000	1000	1	8.90E-07	0.47	22	2.2	Sdc3	Receptor
DMR5:148962001	5	148962001	148964000	2000	1	6.50E-13	0.81	23	1.15	Sdc3;LOC108351040	Receptor
DMR5:150088001	5	150088001	150089000	1000	1	7.50E-07	0.56	20	2	Tmem200b;Epb41	
DMR5:150426001	5	150426001	150428000	2000	1	5.40E-07	0.39	26	1.3	Gmeb1	
DMR5:151162001	5	151162001	151163000	1000	1	4.90E-08	0.51	17	1.7	LOC108351145;Fgr	
DMR5:151230001	5	151230001	151233000	3000	1	9.70E-08	0.53	61	2.03	Ahdc1	

DMR5:151260001	5	151260001	151267000	7000	1	1.50E-07	0.5	208	2.97	Ahdc1	
DMR5:151502001	5	151502001	151506000	4000	1	3.80E-09	0.62	69	1.73	Wdtc1	Proteolysis
DMR5:152341001	5	152341001	152345000	4000	1	3.60E-09	0.59	74	1.85	Ubxn11	Signaling
DMR5:152372001	5	152372001	152377000	5000	1	4.10E-07	0.45	53	1.06	Cep85	
DMR5:152476001	5	152476001	152478000	2000	1	1.10E-08	0.5	27	1.35	LOC108351043;Fam110d	
DMR5:152854001	5	152854001	152860000	6000	1	1.90E-07	0.52	155	2.58	Man1c1	Golgi
DMR5:152897001	5	152897001	152902000	5000	1	3.50E-08	0.53	117	2.34	Man1c1	Golgi
DMR5:152956001	5	152956001	152957000	1000	1	1.80E-07	0.49	22	2.2	Ldlrap1	Cytoskeleton
DMR5:153680001	5	153680001	153681000	1000	1	1.10E-09	0.55	24	2.4	Srrm1	Translation
DMR5:154084001	5	154084001	154085000	1000	1	4.00E-07	0.53	13	1.3	Il22ra1	Receptor
DMR5:155118001	5	155118001	155119000	1000	1	3.00E-07	0.51	28	2.8	Ephb2	Receptor
DMR5:155333001	5	155333001	155338000	5000	1	1.30E-07	0.46	93	1.86	Zbtb40	Transcription
DMR5:155829001	5	155829001	155834000	5000	1	1.60E-08	0.6	94	1.88	Hspg2	
DMR5:155857001	5	155857001	155866000	9000	1	1.50E-07	0.42	191	2.12	Hspg2	
DMR5:155869001	5	155869001	155873000	4000	1	4.10E-07	0.42	55	1.38	Hspg2	
DMR5:155874001	5	155874001	155879000	5000	1	1.70E-07	0.48	97	1.94	Hspg2	
DMR5:155905001	5	155905001	155906000	1000	1	8.10E-09	0.57	16	1.6	Hspg2;Ldlrad2	
DMR5:155964001	5	155964001	155967000	3000	1	1.80E-07	0.58	36	1.2	Usp48	Protease
DMR5:156242001	5	156242001	156244000	2000	1	7.40E-08	0.5	39	1.95	Ece1	Protease
DMR5:156488001	5	156488001	156489000	1000	1	5.80E-07	0.44	12	1.2	Eif4g3	Translation
DMR5:156540001	5	156540001	156543000	3000	1	2.20E-09	0.49	71	2.37	Eif4g3	Translation
DMR5:157163001	5	157163001	157165000	2000	1	8.10E-07	0.44	37	1.85	Ubxn10;Pla2g2c	Metabolism
DMR5:157346001	5	157346001	157351000	5000	1	3.50E-07	0.52	117	2.34	Otud3	Protease
DMR5:157581001	5	157581001	157582000	1000	1	5.90E-09	0.5	7	0.7	Minos1	
DMR5:157719001	5	157719001	157720000	1000	1	6.30E-10	0.66	14	1.4	Capzb	Cytoskeleton
DMR5:157765001	5	157765001	157769000	4000	1	7.80E-07	0.49	82	2.05	Pqlc2;Akr7a2;LOC108351049	Metabolism
DMR5:157815001	5	157815001	157816000	1000	1	2.90E-07	0.55	21	2.1	Akr7a3;Mrto4;Emc1	Metabolism
DMR5:157867001	5	157867001	157870000	3000	1	9.00E-07	0.43	48	1.6	Ubr4	Proteolysis
DMR5:157890001	5	157890001	157891000	1000	1	1.40E-07	0.58	13	1.3	Ubr4;Trnav-aac	Proteolysis
DMR5:157926001	5	157926001	157930000	4000	2	2.40E-09	0.61	68	1.7	Ubr4	Proteolysis
DMR5:157951001	5	157951001	157953000	2000	1	6.50E-08	0.48	38	1.9	Ubr4	Proteolysis
DMR5:158077001	5	158077001	158081000	4000	1	2.40E-07	-0.75	60	1.5	Iffo2;Aldh4a1	Metabolism
DMR5:158500001	5	158500001	158501000	1000	1	5.30E-09	0.51	17	1.7	LOC108351052;Igsf21	
DMR5:158542001	5	158542001	158547000	5000	1	2.80E-08	0.49	89	1.78	Igsf21;LOC102547437	
DMR5:158552001	5	158552001	158556000	4000	1	5.30E-08	0.53	82	2.05	Igsf21;LOC102547437	
DMR5:158584001	5	158584001	158585000	1000	1	4.80E-07	0.46	16	1.6	Igsf21	
DMR5:158629001	5	158629001	158632000	3000	1	1.70E-07	0.52	45	1.5	Igsf21	
DMR5:159280001	5	159280001	159282000	2000	1	1.10E-07	0.43	53	2.65	Padi4	
DMR5:159287001	5	159287001	159296000	9000	2	3.70E-07	0.56	154	1.71	Padi4	
DMR5:159330001	5	159330001	159334000	4000	1	1.40E-07	0.56	70	1.75	Padi3;Padi1	
DMR5:159345001	5	159345001	159346000	1000	1	1.20E-08	0.5	11	1.1	Padi3;Padi1	
DMR5:159442001	5	159442001	159444000	2000	1	1.80E-08	0.53	49	2.45	Padi2	
DMR5:159480001	5	159480001	159481000	1000	1	2.60E-13	0.7	13	1.3	Padi2;Sdhb	Metabolism
DMR5:159523001	5	159523001	159525000	2000	1	6.70E-07	0.57	29	1.45	Atp13a2;Mfap2	
DMR5:159542001	5	159542001	159546000	4000	1	1.20E-07	0.61	95	2.38	Mfap2;Crocc	Epigenetic
DMR5:159555001	5	159555001	159562000	7000	1	4.10E-07	0.5	148	2.11	Crocc	Epigenetic
DMR5:159677001	5	159677001	159680000	3000	1	3.60E-10	0.62	35	1.17	Fbxo42	
DMR5:159757001	5	159757001	159761000	4000	1	7.10E-07	0.59	74	1.85	Trnai-aau;LOC108351054;Arhgef19	Transcription
DMR5:159849001	5	159849001	159856000	7000	2	4.90E-09	0.8	143	2.04	Epha2	Receptor
DMR5:160008001	5	160008001	160024000	16000	1	5.00E-08	0.43	478	2.99	Zbtb17;Spen	Transcription;Metabolism
DMR5:160171001	5	160171001	160173000	2000	1	3.80E-08	0.52	51	2.55	Tmem82;Slc25a34;Plekhm2	
DMR5:160198001	5	160198001	160202000	4000	1	9.00E-07	0.43	82	2.05	Plekhm2	
DMR5:160211001	5	160211001	160214000	3000	1	3.70E-07	0.46	52	1.73	Plekhm2	
DMR5:160435001	5	160435001	160437000	2000	1	2.20E-07	0.54	27	1.35	Fhad1	
DMR5:160534001	5	160534001	160538000	4000	1	2.40E-08	0.47	75	1.88	Fhad1;LOC102553488	
DMR5:160600001	5	160600001	160603000	3000	1	1.10E-08	0.49	51	1.7	Tmem51	
DMR5:160758001	5	160758001	160763000	5000	1	5.50E-12	0.61	87	1.74	Kazn	
DMR5:160834001	5	160834001	160838000	4000	1	5.40E-07	0.45	93	2.33	Kazn	
DMR5:161464001	5	161464001	161470000	6000	1	7.70E-08	0.44	87	1.45	Kazn	
DMR5:161533001	5	161533001	161536000	3000	1	1.20E-07	0.84	50	1.67	Kazn	
DMR5:164798001	5	164798001	164799000	1000	1	1.70E-08	0.51	12	1.2	Nppb;Nppa	
DMR5:164927001	5	164927001	164928000	1000	1	2.50E-08	0.51	17	1.7	Draxin;LOC108351058	
DMR5:164938001	5	164938001	164942000	4000	1	8.60E-09	0.47	37	0.92	Draxin;Mad2l2	
DMR5:165062001	5	165062001	165065000	3000	1	8.90E-09	0.59	65	2.17	Disp3	
DMR5:165239001	5	165239001	165244000	5000	1	5.50E-10	0.59	89	1.78	Ubiad1	Transport

DMR5:165271001	5	165271001	165274000	3000	1	2.90E-07	0.53	51	1.7	Mtor	Signaling
DMR5:165738001	5	165738001	165743000	5000	1	3.10E-08	0.57	99	1.98	Casz1;LOC103692511	Transcription
DMR5:165896001	5	165896001	165899000	3000	1	4.10E-07	0.39	51	1.7	Pex14	Transport
DMR5:165971001	5	165971001	165978000	7000	1	1.90E-07	0.61	140	2	Pgd	Metabolism
DMR5:166157001	5	166157001	166161000	4000	1	6.70E-07	0.51	84	2.1	Ube4b	Proteolysis
DMR5:166684001	5	166684001	166685000	1000	1	1.80E-08	0.53	23	2.3	Tmem201	
DMR5:166705001	5	166705001	166706000	1000	1	3.50E-07	0.42	11	1.1	Tmem201;Slc25a33	Transport
DMR5:166913001	5	166913001	166917000	4000	1	4.50E-07	0.46	95	2.38	Spsb1	
DMR5:167000001	5	167000001	167004000	4000	1	4.80E-07	0.61	133	3.33	H6pd	Metabolism
DMR5:167031001	5	167031001	167032000	1000	1	4.20E-09	0.56	10	1	H6pd;LOC108351062	Metabolism
DMR5:167209001	5	167209001	167210000	1000	1	4.30E-07	0.44	26	2.6	Slc2a7	
DMR5:167231001	5	167231001	167233000	2000	1	2.50E-09	0.44	41	2.05	Car6	
DMR5:167591001	5	167591001	167595000	4000	1	6.60E-07	0.4	56	1.4	Rere	
DMR5:168197001	5	168197001	168200000	3000	1	1.50E-08	0.51	38	1.27	Camta1	Transcription
DMR5:168666001	5	168666001	168668000	2000	2	2.20E-08	0.65	55	2.75	Camta1	Transcription
DMR5:168675001	5	168675001	168677000	2000	1	6.50E-07	0.5	47	2.35	Camta1	Transcription
DMR5:168778001	5	168778001	168785000	7000	1	6.60E-10	0.61	120	1.71	Camta1	Transcription
DMR5:168854001	5	168854001	168856000	2000	1	1.70E-07	0.42	54	2.7	Camta1;LOC102546752	Transcription
DMR5:168959001	5	168959001	168966000	7000	1	3.40E-07	0.4	106	1.51	Camta1;LOC103692523	Transcription
DMR5:169152001	5	169152001	169159000	7000	1	4.90E-09	0.49	164	2.34	Dnajc11;Thap3;Phf13	Transcription
DMR5:169206001	5	169206001	169208000	2000	1	9.00E-08	0.5	38	1.9	Zbtb48;Tas1r1;Nol9	Transcription;Signaling;Translation
DMR5:169215001	5	169215001	169216000	1000	1	4.30E-07	0.5	14	1.4	Tas1r1;Nol9	Signaling;Translation
DMR5:169260001	5	169260001	169266000	6000	1	2.40E-07	0.46	117	1.95	Plekhg5	
DMR5:169563001	5	169563001	169566000	3000	1	2.00E-07	0.54	95	3.17	Chd5;Kcnab2	
DMR5:169572001	5	169572001	169580000	8000	2	2.80E-09	0.55	177	2.21	Chd5;Kcnab2	
DMR5:169607001	5	169607001	169609000	2000	1	5.50E-07	0.5	67	3.35	Kcnab2	
DMR5:169647001	5	169647001	169652000	5000	1	5.00E-07	0.42	106	2.12	Kcnab2;LOC500594;Nphp4	Translation
DMR5:169728001	5	169728001	169732000	4000	1	5.30E-07	0.47	125	3.12	Nphp4	
DMR5:170628001	5	170628001	170633000	5000	1	1.90E-07	0.46	105	2.1	Ajap1	
DMR5:170644001	5	170644001	170645000	1000	1	4.10E-09	0.57	22	2.2	Ajap1	
DMR5:171380001	5	171380001	171384000	4000	1	3.00E-07	0.49	82	2.05	Tp73	Transcription
DMR5:171408001	5	171408001	171415000	7000	1	1.90E-08	0.5	123	1.76	Tp73	Transcription
DMR5:172069001	5	172069001	172070000	1000	1	1.20E-10	0.53	15	1.5	Actrt2	Cytoskeleton
DMR5:172298001	5	172298001	172302000	4000	1	9.80E-07	0.51	77	1.93	Mmel1;Fam213b	Protease
DMR5:172389001	5	172389001	172397000	8000	1	4.30E-07	0.5	179	2.24	Pank4;Plch2	Signaling;Metabolism
DMR5:172399001	5	172399001	172402000	3000	1	8.10E-08	0.64	43	1.43	Plch2	Metabolism
DMR5:172417001	5	172417001	172425000	8000	1	6.90E-08	0.5	120	1.5	Plch2	Metabolism
DMR5:172434001	5	172434001	172435000	1000	1	6.40E-07	0.5	16	1.6	Plch2	Metabolism
DMR5:172446001	5	172446001	172447000	1000	1	7.20E-08	0.48	16	1.6	Plch2	Metabolism
DMR5:172510001	5	172510001	172514000	4000	1	6.70E-07	0.51	56	1.4	Morn1;LOC108351067	Signaling
DMR5:172579001	5	172579001	172581000	2000	1	2.90E-07	0.44	15	0.75	Ski	
DMR5:172703001	5	172703001	172704000	1000	1	5.70E-07	0.39	13	1.3	Prkcz	Signaling
DMR6:829001	6	829001	835000	6000	1	8.30E-08	0.41	123	2.05	Crim1	
DMR6:883001	6	883001	886000	3000	1	1.50E-08	0.48	55	1.83	Crim1	
DMR6:1164001	6	1164001	1167000	3000	1	5.60E-07	0.47	73	2.43	Vit	Extracellular Matrix
DMR6:1296001	6	1296001	1298000	2000	1	7.80E-08	0.51	29	1.45	Strn	
DMR6:2926001	6	2926001	2930000	4000	1	1.30E-07	0.5	47	1.18	Dhx57	Transcription
DMR6:3043001	6	3043001	3045000	2000	1	3.20E-07	0.4	67	3.35	Arhgef33	Transcription
DMR6:3698001	6	3698001	3700000	2000	1	5.00E-08	0.45	22	1.1	Tmem178a	
DMR6:6672001	6	6672001	6673000	1000	1	2.10E-08	0.5	33	3.3	Eml4;LOC102554883	
DMR6:6913001	6	6913001	6914000	1000	1	4.90E-10	0.49	13	1.3	Mta3	Development
DMR6:8525001	6	8525001	8528000	3000	1	2.80E-09	0.46	40	1.33	Camkmt;LOC103692549	Golgi
DMR6:8591001	6	8591001	8593000	2000	1	1.20E-08	0.5	30	1.5	Camkmt	Golgi
DMR6:8628001	6	8628001	8629000	1000	1	9.60E-08	0.43	22	2.2	Camkmt	Golgi
DMR6:9650001	6	9650001	9651000	1000	1	8.20E-07	0.39	13	1.3	Prkce	Signaling
DMR6:9736001	6	9736001	9738000	2000	1	7.80E-08	0.64	29	1.45	Prkce	Signaling
DMR6:9743001	6	9743001	9744000	1000	1	4.30E-09	0.58	15	1.5	Prkce	Signaling
DMR6:9811001	6	9811001	9813000	2000	1	7.90E-08	0.45	36	1.8	Prkce	Signaling
DMR6:10331001	6	10331001	10333000	2000	1	1.20E-08	0.47	41	2.05	Epas1	Transcription
DMR6:10551001	6	10551001	10553000	2000	1	6.90E-07	0.45	36	1.8	Rhoq	Signaling
DMR6:10952001	6	10952001	10955000	3000	1	5.70E-08	0.46	67	2.23	Ttc7a	
DMR6:10960001	6	10960001	10964000	4000	1	1.50E-09	0.6	75	1.88	Ttc7a	
DMR6:10969001	6	10969001	10972000	3000	1	4.80E-07	0.54	62	2.07	Ttc7a	
DMR6:11473001	6	11473001	11476000	3000	1	1.20E-08	0.48	53	1.77	Kcnk12	Transport

DMR6:11642001	6	11642001	11644000	2000	1	7.90E-09	0.54	47	2.35	Msh6	Transcription
DMR6:11649001	6	11649001	11652000	3000	1	3.70E-07	0.42	41	1.37	Msh6	Transcription
DMR6:12372001	6	12372001	12374000	2000	1	3.30E-07	0.48	49	2.45	Ston1	Transport
DMR6:12408001	6	12408001	12413000	5000	1	2.70E-08	0.5	33	0.66	Gtf2a1l	Transcription
DMR6:12510001	6	12510001	12512000	2000	1	2.70E-07	0.43	15	0.75	Lhcgr	Signaling
DMR6:12982001	6	12982001	12984000	2000	1	1.30E-12	0.51	25	1.25	Fshr	Signaling
DMR6:13933001	6	13933001	13934000	1000	1	1.90E-07	0.57	12	1.2	Nrxn1	
DMR6:13962001	6	13962001	13963000	1000	1	1.90E-07	0.47	25	2.5	Nrxn1;LOC102550619	
DMR6:18998001	6	18998001	19001000	3000	1	1.90E-07	-0.49	24	0.8	Cwf19l2	
DMR6:21057001	6	21057001	21059000	2000	1	3.40E-11	0.72	19	0.95	Fam98a	Translation
DMR6:21379001	6	21379001	21381000	2000	1	1.50E-08	0.5	29	1.45	Ltbp1	Extracellular Matrix
DMR6:22960001	6	22960001	22961000	1000	1	7.30E-09	0.52	17	1.7	Alk	Receptor
DMR6:23012001	6	23012001	23013000	1000	1	2.80E-07	-0.47	13	1.3	Alk;LOC108351180	Receptor
DMR6:23325001	6	23325001	23327000	2000	1	7.00E-16	0.79	11	0.55	Clip4	Transcription
DMR6:24507001	6	24507001	24509000	2000	1	7.60E-10	0.66	13	0.65	Lclat1	Metabolism
DMR6:25297001	6	25297001	25299000	2000	1	2.30E-07	0.44	31	1.55	Srd5a2	Metabolism
DMR6:25884001	6	25884001	25887000	3000	1	8.90E-08	0.56	45	1.5	Bre	
DMR6:26476001	6	26476001	26479000	3000	2	2.90E-11	0.65	35	1.17	Ift172;Krtcap3;Nrbp1	Development;Signaling
DMR6:26538001	6	26538001	26542000	4000	1	2.50E-07	0.44	100	2.5	Ppm1g;Zfp513;Snx17;Eif2b4	Signaling;Cytoskeleton;Translation
DMR6:26876001	6	26876001	26878000	2000	1	9.30E-07	0.53	19	0.95	Tmem214;Mapre3	Cytoskeleton
DMR6:27170001	6	27170001	27173000	3000	1	2.70E-07	0.47	38	1.27	Kcnk3	Transport
DMR6:27396001	6	27396001	27402000	6000	1	4.10E-10	0.57	96	1.6	Otof	Transport
DMR6:27406001	6	27406001	27409000	3000	1	6.20E-07	0.47	65	2.17	Otof	Transport
DMR6:27437001	6	27437001	27438000	1000	1	3.60E-10	0.58	20	2	Drc1	
DMR6:27523001	6	27523001	27526000	3000	1	4.10E-07	0.44	37	1.23	Adgrf3	
DMR6:28057001	6	28057001	28058000	1000	1	1.20E-07	0.44	15	1.5	Dtnb	Proteolysis
DMR6:28169001	6	28169001	28171000	2000	1	2.10E-11	0.59	39	1.95	Dtnb	Proteolysis
DMR6:28214001	6	28214001	28217000	3000	1	2.20E-07	0.46	45	1.5	Dnmt3a	Epigenetic
DMR6:28449001	6	28449001	28452000	3000	1	4.80E-08	0.43	44	1.47	Efr3b	
DMR6:28681001	6	28681001	28684000	3000	1	3.90E-07	0.45	38	1.27	Ncoa1	Epigenetic
DMR6:29186001	6	29186001	29189000	3000	1	4.50E-07	0.47	45	1.5	Klhl29	Cytoskeleton
DMR6:29239001	6	29239001	29240000	1000	1	4.80E-11	0.67	20	2	Klhl29	Cytoskeleton
DMR6:29353001	6	29353001	29354000	1000	1	7.90E-08	0.52	26	2.6	Klhl29;LOC102554916	Cytoskeleton
DMR6:29371001	6	29371001	29373000	2000	1	4.40E-09	0.62	36	1.8	Klhl29;LOC102554916	Cytoskeleton
DMR6:30103001	6	30103001	30108000	5000	2	1.70E-10	0.61	87	1.74	Fam228a;Itsn2	Transport
DMR6:42784001	6	42784001	42785000	1000	1	5.80E-07	0.42	16	1.6	RGD1561890;LOC690346	
DMR6:43041001	6	43041001	43045000	4000	1	7.60E-07	0.48	79	1.98	Hpcal1	
DMR6:43085001	6	43085001	43087000	2000	2	3.50E-08	0.49	42	2.1	Hpcal1	
DMR6:43399001	6	43399001	43402000	3000	1	8.90E-07	0.54	41	1.37	Cpsf3;Iah1;Adam17	Translation;Metabolism;Protease
DMR6:43833001	6	43833001	43836000	3000	1	1.30E-08	0.59	42	1.4	Klf11;Cys1	Transcription
DMR6:44126001	6	44126001	44128000	2000	1	2.30E-07	0.55	28	1.4	Mboat2	Metabolism
DMR6:44330001	6	44330001	44331000	1000	1	8.70E-07	0.5	15	1.5	Kidins220	
DMR6:48563001	6	48563001	48564000	1000	1	2.90E-07	0.71	3	0.3	Myt1l;LOC103692607	Transcription
DMR6:49858001	6	49858001	49860000	2000	2	9.90E-08	0.54	25	1.25	Acp1;Sh3yl1	Signaling;Cytoskeleton
DMR6:50519001	6	50519001	50527000	8000	1	8.80E-07	0.5	161	2.01	Lamb1	Extracellular Matrix
DMR6:52635001	6	52635001	52637000	2000	1	2.70E-11	0.56	28	1.4	Atxn7l1	
DMR6:52670001	6	52670001	52673000	3000	1	1.20E-09	0.54	63	2.1	Atxn7l1	
DMR6:60459001	6	60459001	60461000	2000	1	5.90E-08	0.51	15	0.75	Dock4	Transcription
DMR6:69507001	6	69507001	69508000	1000	1	8.50E-07	-0.84	6	0.6	Olr873-ps	
DMR6:71211001	6	71211001	71212000	1000	1	1.40E-16	0.43	5	0.5	Prkd1	Signaling
DMR6:72137001	6	72137001	72139000	2000	1	7.90E-09	0.52	23	1.15	Scfd1;LOC680823	Transport
DMR6:73115001	6	73115001	73116000	1000	1	1.60E-07	0.5	6	0.6	Nubpl	
DMR6:73644001	6	73644001	73647000	3000	1	3.00E-08	0.55	57	1.9	Akap6	
DMR6:73791001	6	73791001	73795000	4000	1	8.00E-07	0.44	51	1.27	Akap6	
DMR6:73853001	6	73853001	73859000	6000	1	9.40E-07	0.6	93	1.55	Akap6	
DMR6:76280001	6	76280001	76281000	1000	1	1.90E-08	0.42	14	1.4	Nfkbia	
DMR6:76471001	6	76471001	76474000	3000	1	8.90E-07	-0.4	26	0.87	Ralgapa1	Signaling
DMR6:76499001	6	76499001	76500000	1000	1	3.70E-09	0.55	14	1.4	Ralgapa1	Signaling
DMR6:77623001	6	77623001	77626000	3000	1	5.10E-07	0.69	29	0.97	Pax9;Slc25a21	Transport
DMR6:77808001	6	77808001	77810000	2000	1	3.90E-07	0.52	19	0.95	Slc25a21	Transport
DMR6:78619001	6	78619001	78623000	4000	1	3.20E-07	0.42	52	1.3	RGD1560556	
DMR6:78700001	6	78700001	78701000	1000	1	1.70E-11	0.64	5	0.5	RGD1560556;LOC366632	
DMR6:88773001	6	88773001	88776000	3000	1	1.30E-08	-0.5	33	1.1	Mdga2;LOC103692647	

DMR6:91919001	6	91919001	91920000	1000	1	3.60E-07	0.43	17	1.7	Sos2	Transcription
DMR6:92309001	6	92309001	92312000	3000	3	9.80E-10	0.79	139	4.63	Atl1	Signaling
DMR6:93797001	6	93797001	93799000	2000	1	1.20E-09	0.56	21	1.05	RGD1559545	
DMR6:95419001	6	95419001	95423000	4000	1	7.50E-07	0.5	112	2.8	Pcnx4;Dhrs7	Metabolism
DMR6:96553001	6	96553001	96554000	1000	1	8.30E-07	0.43	23	2.3	Prkch	Signaling
DMR6:98328001	6	98328001	98335000	7000	1	1.50E-10	0.68	102	1.46	Rhoj	Signaling
DMR6:98425001	6	98425001	98426000	1000	1	7.70E-08	0.43	12	1.2	Ppp2r5e	Signaling
DMR6:99041001	6	99041001	99044000	3000	1	1.90E-07	0.59	76	2.53	Syne2	
DMR6:99140001	6	99140001	99145000	5000	1	4.50E-07	0.48	100	2	Syne2	
DMR6:99164001	6	99164001	99167000	3000	1	5.90E-08	0.46	47	1.57	Esr2	
DMR6:99650001	6	99650001	99655000	5000	1	2.70E-07	0.46	114	2.28	Plekhg3;Sptb	
DMR6:99676001	6	99676001	99677000	1000	1	2.70E-08	0.48	27	2.7	Sptb	
DMR6:99690001	6	99690001	99692000	2000	1	2.70E-07	0.45	40	2	Sptb	
DMR6:99746001	6	99746001	99751000	5000	1	1.30E-07	0.57	97	1.94	Sptb	
DMR6:99865001	6	99865001	99868000	3000	1	9.50E-09	0.51	46	1.53	Rab15;LOC102546646	
DMR6:100460001	6	100460001	100461000	1000	1	9.40E-07	-0.84	7	0.7	Fut8	Golgi
DMR6:101312001	6	101312001	101314000	2000	1	1.10E-07	-0.65	13	0.65	RGD1562540	
DMR6:102245001	6	102245001	102248000	3000	1	4.40E-09	0.53	43	1.43	Plekhh1;LOC102547892	
DMR6:103019001	6	103019001	103021000	2000	1	8.30E-12	0.69	23	1.15	Rad51b	Transcription
DMR6:103387001	6	103387001	103389000	2000	1	2.00E-08	0.59	32	1.6	Actn1	
DMR6:104733001	6	104733001	104735000	2000	1	4.00E-07	0.46	27	1.35	Smoc1	Signaling
DMR6:104787001	6	104787001	104788000	1000	1	2.10E-07	0.46	18	1.8	Smoc1	Signaling
DMR6:104831001	6	104831001	104838000	7000	1	3.90E-08	0.66	96	1.37	Smoc1	Signaling
DMR6:104839001	6	104839001	104842000	3000	1	9.40E-07	0.48	25	0.83	Smoc1	Signaling
DMR6:105163001	6	105163001	105165000	2000	1	3.10E-07	-0.56	9	0.45	Adam4	Protease
DMR6:105459001	6	105459001	105461000	2000	1	5.20E-07	0.48	41	2.05	Map3k9	Signaling
DMR6:105462001	6	105462001	105463000	1000	1	2.40E-08	0.47	28	2.8	Map3k9	Signaling
DMR6:106389001	6	106389001	106390000	1000	1	2.10E-07	-0.58	20	2	Rgs6	
DMR6:106395001	6	106395001	106396000	1000	1	9.70E-07	0.45	22	2.2	Rgs6	
DMR6:106440001	6	106440001	106442000	2000	1	8.90E-16	0.98	11	0.55	Rgs6	
DMR6:106527001	6	106527001	106529000	2000	1	1.30E-10	0.4	21	1.05	Rgs6	
DMR6:106538001	6	106538001	106540000	2000	1	6.80E-07	0.44	30	1.5	Rgs6	
DMR6:107004001	6	107004001	107006000	2000	1	1.80E-07	0.4	42	2.1	Dcaf4	
DMR6:107643001	6	107643001	107648000	5000	1	7.80E-07	0.43	105	2.1	Pnma1;Elmsan1	
DMR6:108306001	6	108306001	108308000	2000	1	5.60E-07	0.53	42	2.1	Vsx2;Abcd4	Development;Transport
DMR6:108524001	6	108524001	108526000	2000	1	3.60E-08	0.49	14	0.7	Ltbp2	Extracellular Matrix
DMR6:108604001	6	108604001	108608000	4000	1	2.80E-08	0.45	60	1.5	Ltbp2	Extracellular Matrix
DMR6:109682001	6	109682001	109685000	3000	1	3.10E-07	0.49	43	1.43	Flvcr2;RGD1310769;Ttll5	Transport;Cytoskeleton
DMR6:109718001	6	109718001	109719000	1000	1	7.60E-09	0.45	13	1.3	Ttll5;LOC103692679	Cytoskeleton
DMR6:109844001	6	109844001	109845000	1000	1	5.30E-07	0.49	11	1.1	Ttll5	Cytoskeleton
DMR6:109962001	6	109962001	109963000	1000	1	3.50E-08	0.7	14	1.4	Ift43	
DMR6:110095001	6	110095001	110097000	2000	1	6.10E-07	0.48	47	2.35	Gpatch2l	Metabolism
DMR6:110447001	6	110447001	110451000	4000	1	1.20E-07	0.48	66	1.65	Esrrb	
DMR6:110616001	6	110616001	110619000	3000	1	3.50E-07	0.36	34	1.13	Vash1	
DMR6:110626001	6	110626001	110629000	3000	1	3.70E-07	0.56	52	1.73	Vash1	
DMR6:111385001	6	111385001	111387000	2000	1	1.60E-07	0.58	13	0.65	Sptlc2	Metabolism
DMR6:111683001	6	111683001	111686000	3000	1	5.20E-10	0.52	45	1.5	Adck1	Transport
DMR6:111706001	6	111706001	111711000	5000	1	2.40E-09	0.55	66	1.32	Adck1	Transport
DMR6:115587001	6	115587001	115588000	1000	1	5.40E-07	0.46	21	2.1	Sel1l	
DMR6:122519001	6	122519001	122520000	1000	1	1.50E-08	0.53	18	1.8	Kcnk10	Transport
DMR6:123803001	6	123803001	123805000	2000	1	6.20E-08	0.48	30	1.5	Efcab11	Signaling
DMR6:124209001	6	124209001	124210000	1000	1	9.70E-07	-0.47	12	1.2	LOC108351372;Calm1	Signaling
DMR6:124311001	6	124311001	124313000	2000	1	5.20E-10	0.58	27	1.35	Ttc7b	
DMR6:124567001	6	124567001	124570000	3000	1	2.80E-07	0.67	38	1.27	Rps6ka5	Golgi
DMR6:124918001	6	124918001	124922000	4000	1	1.40E-09	0.55	92	2.3	Ccdc88c	Transport
DMR6:124978001	6	124978001	124983000	5000	1	8.80E-09	0.47	107	2.14	Ccdc88c	Transport
DMR6:125019001	6	125019001	125020000	1000	1	6.70E-08	0.54	13	1.3	Ccdc88c	Transport
DMR6:125888001	6	125888001	125891000	3000	1	9.20E-08	0.55	59	1.97	Cpsf2	
DMR6:126505001	6	126505001	126508000	3000	1	2.30E-09	0.52	49	1.63	Itpk1	Signaling
DMR6:126569001	6	126569001	126573000	4000	1	8.90E-08	0.57	72	1.8	Itpk1;LOC103692695	Signaling
DMR6:127287001	6	127287001	127293000	6000	2	4.20E-09	0.53	93	1.55	Otub2;Ddx24	Protease
DMR6:128764001	6	128764001	128766000	2000	1	4.60E-07	0.49	37	1.85	Glrx5;LOC100912332	Metabolism
DMR6:129570001	6	129570001	129573000	3000	1	4.50E-07	0.51	58	1.93	Ak7	Signaling
DMR6:132105001	6	132105001	132110000	5000	2	3.60E-07	0.6	84	1.68	Ccnk;Ccdc85c	Signaling
DMR6:132112001	6	132112001	132119000	7000	1	4.70E-07	0.5	147	2.1	Ccnk;Ccdc85c	Signaling

DMR6:132222001	6	132222001	132223000	1000	1	1.50E-07	0.5	20	2	Hhipl1	Signaling
DMR6:132870001	6	132870001	132871000	1000	1	2.30E-09	0.51	15	1.5	Wdr25	Cytoskeleton
DMR6:133662001	6	133662001	133665000	3000	1	2.10E-09	0.52	56	1.87	LOC102557203;RGD1566401	
DMR6:133700001	6	133700001	133705000	5000	2	3.60E-11	0.54	58	1.16	RGD1566401;Mir673;Mir493;Mir337;Mir3544;Mir540;Mir665;Rtl1;Mir431;Mir433;Mir127	
DMR6:133724001	6	133724001	133728000	4000	1	1.20E-10	0.77	68	1.7	Rtl1;Mir3543;Mir434;Mir136;Mir341;Mir1188	
DMR6:133734001	6	133734001	133741000	7000	2	1.90E-08	0.55	147	2.1	Mir341;Mir1188;Mir370	
DMR6:133904001	6	133904001	133905000	1000	1	5.30E-07	0.47	10	1	Mir410;Mir3072	
DMR6:134818001	6	134818001	134823000	5000	1	1.50E-07	0.49	82	1.64	Ppp2r5c	Signaling
DMR6:134845001	6	134845001	134850000	5000	2	4.50E-08	0.57	84	1.68	Ppp2r5c;LOC102547242;LOC102547175	Signaling
DMR6:134869001	6	134869001	134870000	1000	1	8.20E-08	0.54	19	1.9	Ppp2r5c	Signaling
DMR6:135613001	6	135613001	135618000	5000	1	4.30E-07	0.44	84	1.68	Traf3	Cytoskeleton
DMR6:135717001	6	135717001	135719000	2000	1	4.70E-07	0.47	49	2.45	Traf3;Amn	Cytoskeleton
DMR6:135747001	6	135747001	135754000	7000	1	1.80E-07	0.48	206	2.94	Cdc42bpb	Signaling
DMR6:135787001	6	135787001	135789000	2000	1	3.10E-08	0.49	42	2.1	Cdc42bpb	Signaling
DMR6:135791001	6	135791001	135792000	1000	1	3.60E-07	0.46	17	1.7	Cdc42bpb	Signaling
DMR6:135808001	6	135808001	135809000	1000	1	6.90E-08	0.4	26	2.6	Cdc42bpb	Signaling
DMR6:136387001	6	136387001	136388000	1000	1	3.00E-09	0.55	13	1.3	Xrcc3;Zfyve21;Ppp1r13b	Transcription;Signaling
DMR6:136742001	6	136742001	136756000	14000	2	1.60E-07	0.56	389	2.78	Kif26a	Cytoskeleton
DMR6:137176001	6	137176001	137179000	3000	1	1.50E-07	0.55	53	1.77	Inf2;Adssl1	Metabolism
DMR6:137180001	6	137180001	137184000	4000	1	1.20E-07	0.54	58	1.45	Inf2;Adssl1	Metabolism
DMR6:137293001	6	137293001	137296000	3000	1	5.90E-07	0.45	53	1.77	Cep170b	Cytoskeleton
DMR6:137297001	6	137297001	137307000	10000	1	1.30E-07	0.52	257	2.57	Cep170b;LOC102552635	Cytoskeleton
DMR6:137387001	6	137387001	137389000	2000	1	1.90E-07	0.57	50	2.5	Ahnak2;RGD1307315;Cdca4	Transcription
DMR6:137673001	6	137673001	137675000	2000	1	2.50E-08	0.55	40	2	Gpr132	Signaling
DMR6:137712001	6	137712001	137725000	13000	1	1.80E-07	0.45	380	2.92	Jag2	
DMR6:137741001	6	137741001	137743000	2000	1	8.50E-07	0.54	43	2.15	Jag2;Nudt14	Signaling
DMR6:137757001	6	137757001	137759000	2000	1	8.20E-07	0.54	20	1	Brf1	Transcription
DMR6:137838001	6	137838001	137842000	4000	1	1.20E-07	0.58	58	1.45	Pacs2	
DMR6:137883001	6	137883001	137885000	2000	1	3.30E-07	0.5	32	1.6	Pacs2;Tex22	
DMR6:137917001	6	137917001	137919000	2000	1	1.20E-07	0.45	37	1.85	Mta1	Development
DMR6:144306001	6	144306001	144308000	2000	1	1.10E-07	0.42	29	1.45	Ncapg2	
DMR6:144380001	6	144380001	144381000	1000	1	1.00E-07	0.43	13	1.3	LOC102548847;Ptprn2	Signaling
DMR6:145052001	6	145052001	145054000	2000	1	1.00E-11	0.6	40	2	Ptprn2	Signaling
DMR6:145747001	6	145747001	145749000	2000	1	2.90E-07	0.46	34	1.7	Cdca7l	
DMR7:2466001	7	2466001	2468000	2000	1	5.20E-14	0.52	19	0.95	Naca	Transcription
DMR7:2542001	7	2542001	2546000	4000	1	1.60E-08	0.51	65	1.62	Baz2a;Rbms2	
DMR7:2924001	7	2924001	2927000	3000	1	8.10E-08	0.51	74	2.47	Esyt1;LOC102552826	
DMR7:2935001	7	2935001	2940000	5000	1	2.70E-08	0.63	95	1.9	Esyt1;LOC102552826	
DMR7:5070001	7	5070001	5074000	4000	2	1.60E-10	-0.84	23	0.58	Olr890-ps	
DMR7:5755001	7	5755001	5756000	1000	1	4.70E-09	0.66	5	0.5	Olr1024	
DMR7:10939001	7	10939001	10947000	8000	2	1.80E-09	0.67	152	1.9	Ankrd24;Sirt6;LOC102551996	
DMR7:11127001	7	11127001	11129000	2000	1	1.50E-07	0.42	31	1.55	Celf5;LOC102552420	
DMR7:11154001	7	11154001	11162000	8000	1	2.20E-07	0.46	193	2.41	LOC102552420;Nfic	Transcription
DMR7:11208001	7	11208001	11213000	5000	2	2.10E-09	0.54	122	2.44	LOC102552770;Fzr1;Mfsd12;LOC690617	Proteolysis
DMR7:11251001	7	11251001	11255000	4000	1	3.20E-09	0.61	99	2.48	Hmg20b;LOC102552827;Gipc3;Tbxa2r;Cactin	Cytoskeleton;Signaling
DMR7:11432001	7	11432001	11440000	8000	1	4.80E-09	0.64	234	2.92	Pias4;Zbtb7a	
DMR7:11487001	7	11487001	11490000	3000	1	1.00E-07	0.42	44	1.47	Map2k2;Creb3l3	Signaling
DMR7:11530001	7	11530001	11535000	5000	1	2.30E-07	0.52	103	2.06	Sgta	
DMR7:11536001	7	11536001	11539000	3000	1	4.90E-07	0.49	63	2.1	Sgta;Slc39a3	Transport
DMR7:11778001	7	11778001	11779000	1000	1	6.70E-07	0.57	14	1.4	Jsrp1;Amh;Sf3a2;Plekhj1;Dot1l	Translation;Transport;Epigenetic
DMR7:11862001	7	11862001	11867000	5000	1	5.80E-09	0.64	82	1.64	Ap3d1;Izumo4	Transport
DMR7:11891001	7	11891001	11895000	4000	1	1.40E-07	0.52	87	2.17	Mob3a;LOC108351611	Signaling
DMR7:11915001	7	11915001	11921000	6000	1	9.80E-07	0.51	112	1.87	Mknk2;Btbd2	Signaling;Proteolysis
DMR7:12036001	7	12036001	12042000	6000	3	8.20E-11	0.65	173	2.88	Rexo1;Atp8b3	Transcription;Transport
DMR7:12505001	7	12505001	12508000	3000	1	8.60E-07	0.46	74	2.47	Sbno2;Gpx4	Metabolism
DMR7:12591001	7	12591001	12599000	8000	1	6.60E-07	0.48	196	2.45	Arhgap45;Arid3a;Kiss1r	Transcription;Signaling

DMR7:12841001	7	12841001	12850000	9000	2	7.00E-08	0.52	288	3.2	Fgf22;Polrmt;Hcn2	Growth Factors;Transcription;Transport
DMR7:12855001	7	12855001	12863000	8000	1	4.50E-07	0.57	204	2.55	Polrmt;Hcn2;LOC108351407	Transcription;Transport
DMR7:12961001	7	12961001	12964000	3000	1	1.60E-08	0.62	62	2.07	Shc2;C2cd4c	Cytoskeleton
DMR7:12966001	7	12966001	12967000	1000	1	5.00E-07	0.51	17	1.7	Shc2;C2cd4c	Cytoskeleton
DMR7:13064001	7	13064001	13068000	4000	1	1.50E-07	0.46	92	2.3	Mier2;Plpp2	Development;Signaling
DMR7:13597001	7	13597001	13601000	4000	1	4.30E-07	0.46	50	1.25	Olr1083	Receptor
DMR7:14222001	7	14222001	14231000	9000	1	7.30E-07	0.52	145	1.61	Ephx3;Brd4	Metabolism
DMR7:14350001	7	14350001	14357000	7000	1	3.50E-07	0.52	140	2	Akap8l	Cytoskeleton
DMR7:14378001	7	14378001	14384000	6000	1	8.70E-07	0.49	114	1.9	Akap8l;Wiz	Cytoskeleton;Transcription
DMR7:14496001	7	14496001	14499000	3000	1	5.80E-09	0.65	61	2.03	Cyp4f39;LOC102555438	Metabolism
DMR7:15706001	7	15706001	15708000	2000	1	4.00E-08	0.37	14	0.7	Olr1094-ps;Olr1092	
DMR7:16489001	7	16489001	16492000	3000	1	8.50E-09	-0.64	24	0.8	Olr934-ps	
DMR7:17388001	7	17388001	17390000	2000	1	8.00E-09	-0.83	12	0.6	Vom1r-ps90	
DMR7:20341001	7	20341001	20342000	1000	1	5.10E-13	0.8	18	1.8	RGD1564409;LOC100912315;LOC103690230	
DMR7:20386001	7	20386001	20388000	2000	1	8.60E-08	0.52	17	0.85	RGD1564409;LOC102552467;LOC103692792	
DMR7:20461001	7	20461001	20464000	3000	1	1.00E-08	0.52	33	1.1	LOC103692793;LOC687602;RGD1566251	
DMR7:23497001	7	23497001	23501000	4000	1	6.50E-07	0.36	46	1.15	Syn3	Transport
DMR7:23865001	7	23865001	23867000	2000	1	9.10E-07	0.46	15	0.75	Bpifc	
DMR7:24001001	7	24001001	24004000	3000	1	1.10E-11	0.62	54	1.8	Pwp1	Epigenetic
DMR7:24045001	7	24045001	24048000	3000	1	1.00E-10	0.52	65	2.17	Btbd11	Cytoskeleton
DMR7:24092001	7	24092001	24100000	8000	1	6.60E-10	0.52	145	1.81	Btbd11	Cytoskeleton
DMR7:24863001	7	24863001	24864000	1000	1	9.30E-08	0.47	12	1.2	Tcp11l2	Cytoskeleton
DMR7:25977001	7	25977001	25978000	1000	1	2.00E-08	0.52	11	1.1	LOC100910996;Ric8b	
DMR7:26277001	7	26277001	26284000	7000	1	2.10E-07	0.42	167	2.39	Appl2	Cytoskeleton
DMR7:26813001	7	26813001	26815000	2000	1	2.60E-07	0.48	43	2.15	Chst11	Transport
DMR7:26885001	7	26885001	26887000	2000	1	3.50E-07	0.51	34	1.7	Chst11	Transport
DMR7:27311001	7	27311001	27312000	1000	1	8.20E-07	-0.7	10	1	LOC362863;LOC102550216;Nt5dc3	Signaling
DMR7:27326001	7	27326001	27330000	4000	1	2.00E-09	0.56	79	1.98	Nt5dc3	Signaling
DMR7:27336001	7	27336001	27339000	3000	1	5.30E-10	0.68	39	1.3	Nt5dc3	Signaling
DMR7:28910001	7	28910001	28913000	3000	1	3.10E-07	0.53	39	1.3	Dram1	
DMR7:29306001	7	29306001	29309000	3000	1	4.40E-07	0.56	60	2	Utp20	
DMR7:30254001	7	30254001	30257000	3000	1	3.70E-08	0.5	65	2.17	Slc17a8	Transport
DMR7:31085001	7	31085001	31086000	1000	1	1.70E-10	-0.66	3	0.3	Anks1b;LOC108351597	Cytoskeleton
DMR7:31271001	7	31271001	31273000	2000	1	5.00E-08	0.44	19	0.95	Anks1b	Cytoskeleton
DMR7:33768001	7	33768001	33769000	1000	1	4.40E-07	0.47	8	0.8	RGD1565866	
DMR7:34113001	7	34113001	34116000	3000	1	9.90E-08	0.41	73	2.43	Elk3	Transcription
DMR7:34477001	7	34477001	34480000	3000	1	6.90E-08	0.52	24	0.8	Ccdc38;Snrpf	Translation
DMR7:34547001	7	34547001	34548000	1000	1	5.70E-08	0.43	16	1.6	Ntn4	Extracellular Matrix
DMR7:34608001	7	34608001	34609000	1000	1	4.50E-07	0.5	11	1.1	Ntn4;LOC100362560	Extracellular Matrix
DMR7:35026001	7	35026001	35028000	2000	1	4.40E-10	0.61	36	1.8	Fgd6	Transcription
DMR7:35172001	7	35172001	35173000	1000	1	5.30E-07	0.53	7	0.7	Ndufa12	Metabolism
DMR7:35920001	7	35920001	35921000	1000	1	4.60E-08	0.39	27	2.7	Plxnc1	
DMR7:35968001	7	35968001	35970000	2000	1	1.90E-08	0.45	32	1.6	Plxnc1	
DMR7:35987001	7	35987001	35990000	3000	1	2.30E-08	0.51	40	1.33	Plxnc1	
DMR7:41382001	7	41382001	41384000	2000	1	4.70E-07	0.46	31	1.55	Poc1b	
DMR7:47433001	7	47433001	47434000	1000	1	2.80E-08	0.49	17	1.7	Tmtc2	Golgi
DMR7:49026001	7	49026001	49027000	1000	1	3.50E-08	-0.84	9	0.9	Ppfia2	
DMR7:51891001	7	51891001	51892000	1000	1	6.80E-07	-0.64	12	1.2	Otogl	Extracellular Matrix
DMR7:53277001	7	53277001	53282000	5000	1	6.60E-09	0.56	87	1.74	E2f7	Transcription
DMR7:53308001	7	53308001	53310000	2000	1	6.80E-07	0.45	50	2.5	E2f7	Transcription
DMR7:53956001	7	53956001	53958000	2000	1	3.30E-09	0.42	20	1	Osbpl8	
DMR7:54774001	7	54774001	54776000	2000	1	7.90E-11	0.51	28	1.4	Krr1;Glipr1	Metabolism;Immune
DMR7:58050001	7	58050001	58051000	1000	1	1.10E-09	0.46	11	1.1	Tph2	
DMR7:59936001	7	59936001	59938000	2000	1	9.70E-07	0.5	27	1.35	Rab3ip	Transcription
DMR7:60402001	7	60402001	60403000	1000	1	2.50E-09	0.59	15	1.5	Cpsf6	Translation
DMR7:60814001	7	60814001	60817000	3000	1	1.30E-09	0.69	26	0.87	Nup107	Transport
DMR7:63540001	7	63540001	63545000	5000	1	1.20E-07	0.47	100	2	Rassf3	Cytoskeleton
DMR7:64031001	7	64031001	64034000	3000	1	2.10E-08	0.48	52	1.73	Srgap1	Signaling
DMR7:64863001	7	64863001	64867000	4000	1	4.50E-10	0.51	72	1.8	Grip1;Helb	Transcription
DMR7:65194001	7	65194001	65195000	1000	1	8.00E-08	0.5	19	1.9	Hmga2;LOC102549373	Transcription

DMR7:66311001	7	66311001	66314000	3000	1	4.40E-07	0.42	30	1	Fam19a2	
DMR7:70314001	7	70314001	70317000	3000	2	2.60E-12	0.76	49	1.63	Avil;Tsfm;Mettl21b	Cytoskeleton;Translation
DMR7:70360001	7	70360001	70361000	1000	1	1.00E-07	0.5	25	2.5	Cdk4;Tspan31;LOC103692874;Agap2	Signaling
DMR7:70394001	7	70394001	70395000	1000	1	3.60E-12	0.54	16	1.6	Os9	
DMR7:70527001	7	70527001	70531000	4000	1	6.00E-07	0.5	81	2.02	Kif5a	Cytoskeleton
DMR7:70846001	7	70846001	70853000	7000	1	3.10E-09	0.46	162	2.31	Nxph4;Lrp1	Signaling;Binding Proteins
DMR7:70854001	7	70854001	70861000	7000	1	6.10E-08	0.41	138	1.97	Lrp1	Binding Proteins
DMR7:70899001	7	70899001	70901000	2000	1	1.10E-09	0.47	43	2.15	Lrp1	Binding Proteins
DMR7:71023001	7	71023001	71026000	3000	2	8.30E-09	0.52	58	1.93	Myo1a;Tac3	Cytoskeleton
DMR7:71126001	7	71126001	71135000	9000	2	2.10E-10	0.68	223	2.48	Rdh7	Metabolism
DMR7:71283001	7	71283001	71284000	1000	1	2.10E-14	0.68	15	1.5	LOC102549197;Mterf3	
DMR7:71796001	7	71796001	71797000	1000	1	7.80E-10	0.4	20	2	Cpq	Protease
DMR7:72937001	7	72937001	72939000	2000	1	2.70E-08	0.45	27	1.35	Laptm4b	Transport
DMR7:73275001	7	73275001	73277000	2000	1	5.30E-11	0.6	22	1.1	Rida;Pop1	Metabolism
DMR7:73409001	7	73409001	73412000	3000	1	1.20E-07	0.49	10	0.33	Nipal2	
DMR7:76421001	7	76421001	76422000	1000	1	5.40E-09	0.51	15	1.5	Ncald	
DMR7:76872001	7	76872001	76874000	2000	1	8.00E-07	0.49	31	1.55	Ubr5	Proteolysis
DMR7:80528001	7	80528001	80530000	2000	1	1.00E-06	0.53	25	1.25	Oxr1	
DMR7:87532001	7	87532001	87536000	4000	1	1.20E-07	-0.36	40	1	Csmd3	
DMR7:94928001	7	94928001	94929000	1000	1	1.80E-08	0.53	14	1.4	Deptor	Transcription
DMR7:95245001	7	95245001	95246000	1000	1	1.20E-07	0.47	6	0.6	Col14a1	Extracellular Matrix
DMR7:95283001	7	95283001	95284000	1000	1	6.00E-07	0.39	13	1.3	Col14a1;Mrpl13	Extracellular Matrix;Translation
DMR7:95614001	7	95614001	95615000	1000	1	2.40E-07	0.48	13	1.3	Sntb1	
DMR7:97612001	7	97612001	97614000	2000	1	8.00E-09	0.64	28	1.4	Zhx2;LOC102553568	Development
DMR7:97678001	7	97678001	97682000	4000	1	1.70E-07	0.44	68	1.7	Zhx2	Development
DMR7:97784001	7	97784001	97793000	9000	2	4.20E-07	0.48	109	1.21	LOC683899;Tbc1d31;Rps27a-ps10	
DMR7:97914001	7	97914001	97915000	1000	1	5.20E-07	0.46	10	1	Zhx1	Development
DMR7:98066001	7	98066001	98067000	1000	1	4.20E-07	0.59	9	0.9	Fbxo32	
DMR7:98682001	7	98682001	98683000	1000	1	1.40E-09	0.62	8	0.8	Tmem65	
DMR7:98829001	7	98829001	98832000	3000	1	3.60E-07	0.46	44	1.47	Ndufb9;Mtss1	Metabolism;Cytoskeleton
DMR7:98845001	7	98845001	98849000	4000	1	9.50E-07	0.45	45	1.12	Mtss1	Cytoskeleton
DMR7:98908001	7	98908001	98910000	2000	1	6.70E-08	0.49	31	1.55	Mtss1	Cytoskeleton
DMR7:107025001	7	107025001	107027000	2000	1	8.30E-07	0.76	19	0.95	Trnak-cuu	
DMR7:111729001	7	111729001	111730000	1000	1	1.20E-07	0.45	7	0.7	Rps19l1	Translation
DMR7:112932001	7	112932001	112933000	1000	1	5.40E-07	-0.61	6	0.6	Fam135b	
DMR7:113895001	7	113895001	113898000	3000	1	2.50E-07	0.53	29	0.97	Kcnk9;LOC103692936	Transport
DMR7:114116001	7	114116001	114117000	1000	1	1.40E-07	0.52	16	1.6	Trappc9	
DMR7:114823001	7	114823001	114824000	1000	1	5.30E-08	0.62	10	1	Slc45a4	Transport
DMR7:114991001	7	114991001	114994000	3000	1	6.90E-07	0.48	50	1.67	Ptp4a3;Mroh5	Signaling
DMR7:115864001	7	115864001	115867000	3000	1	3.60E-07	0.61	30	1	LOC102550355;Mroh4	
DMR7:116480001	7	116480001	116484000	4000	1	5.50E-08	0.59	83	2.08	LOC108351510;RGD1565410	
DMR7:116554001	7	116554001	116556000	2000	1	4.90E-07	0.5	21	1.05	Ly6al	
DMR7:116614001	7	116614001	116616000	2000	1	7.50E-07	0.62	16	0.8	Ly6h	
DMR7:116879001	7	116879001	116881000	2000	1	8.80E-07	0.45	34	1.7	Zc3h3;Gsdmd	
DMR7:117082001	7	117082001	117086000	4000	1	1.50E-08	0.52	76	1.9	RGD1563870	
DMR7:117231001	7	117231001	117245000	14000	1	2.40E-08	0.52	644	4.6	Plec	Cytoskeleton
DMR7:117360001	7	117360001	117365000	5000	1	4.10E-08	0.53	124	2.48	Smpd5;Oplah	Metabolism
DMR7:117366001	7	117366001	117368000	2000	1	8.50E-09	0.57	29	1.45	Oplah	Metabolism
DMR7:117428001	7	117428001	117430000	2000	2	3.50E-07	0.46	39	1.95	Maf1;Wdr97;Hgh1;Tssk5	Transcription;Signaling
DMR7:117658001	7	117658001	117664000	6000	2	2.00E-09	0.58	149	2.48	Adck5;Cpsf1	Transport;Translation
DMR7:118234001	7	118234001	118236000	2000	1	4.70E-08	0.47	18	0.9	Rbfox2	Translation
DMR7:119340001	7	119340001	119343000	3000	1	1.50E-07	0.46	40	1.33	Cacng2	Transport
DMR7:119391001	7	119391001	119392000	1000	1	1.50E-07	0.91	12	1.2	Ift27	
DMR7:119419001	7	119419001	119428000	9000	1	6.40E-07	0.67	148	1.64	Ift27;Pvalb	Signaling
DMR7:119597001	7	119597001	119599000	2000	1	1.70E-08	0.48	33	1.65	Tex33	
DMR7:119659001	7	119659001	119663000	4000	1	7.00E-08	0.52	55	1.38	Kctd17;Tmprss6	Protease
DMR7:120055001	7	120055001	120058000	3000	1	2.90E-07	0.58	32	1.07	LOC108351511;Cdc42ep1	
DMR7:120363001	7	120363001	120366000	3000	1	4.00E-10	0.51	42	1.4	Micall1;RGD1359634	
DMR7:120484001	7	120484001	120485000	1000	1	5.00E-07	0.54	29	2.9	Pick1;Slc16a8;Baiap2l2	Transport;Transport;Cytoskeleton
DMR7:120516001	7	120516001	120521000	5000	1	9.20E-07	0.42	89	1.78	Baiap2l2;Pla2g6	Cytoskeleton;Metabolism
DMR7:120548001	7	120548001	120549000	1000	1	8.10E-08	0.46	14	1.4	Pla2g6	Metabolism
DMR7:120572001	7	120572001	120573000	1000	1	5.60E-07	0.44	11	1.1	Maff	Transcription
DMR7:120609001	7	120609001	120612000	3000	1	1.40E-08	0.49	41	1.37	Tmem184b	Transport

DMR7:120621001	7	120621001	120622000	1000	1	1.80E-08	0.52	22	2.2	Tmem184b	Transport
DMR7:120718001	7	120718001	120725000	7000	2	1.70E-07	0.56	161	2.3	Kcnj4	Transport
DMR7:121020001	7	121020001	121023000	3000	1	5.10E-10	0.53	47	1.57	Nptxr	
DMR7:121052001	7	121052001	121054000	2000	1	4.30E-07	0.48	76	3.8	Cbx6;LOC102547313	
DMR7:121136001	7	121136001	121139000	3000	1	9.40E-07	0.56	53	1.77	Apobec3b;Cbx7	Proteolysis
DMR7:121234001	7	121234001	121236000	2000	1	1.00E-08	0.6	31	1.55	Pdgfb;LOC108351514	Growth Factors
DMR7:121332001	7	121332001	121335000	3000	1	5.00E-10	0.53	54	1.8	Syngr1;LOC103692946	Transport
DMR7:121367001	7	121367001	121368000	1000	1	8.20E-07	0.45	20	2	Tab1	Signaling
DMR7:121383001	7	121383001	121385000	2000	1	9.70E-09	0.56	27	1.35	Tab1;Mgat3	Signaling;Golgi
DMR7:121824001	7	121824001	121825000	1000	1	6.80E-08	0.48	25	2.5	Grap2	
DMR7:121864001	7	121864001	121867000	3000	1	2.80E-09	0.51	53	1.77	Grap2;LOC108351516	
DMR7:121987001	7	121987001	121990000	3000	1	9.60E-08	0.45	33	1.1	Tnrc6b	Metabolism
DMR7:122164001	7	122164001	122165000	1000	1	7.60E-09	0.42	15	1.5	Adsl;LOC100362980	Metabolism
DMR7:122186001	7	122186001	122189000	3000	1	1.30E-07	0.47	54	1.8	Adsl	Metabolism
DMR7:122293001	7	122293001	122294000	1000	1	3.50E-07	0.44	19	1.9	Mkl1	
DMR7:122992001	7	122992001	122996000	4000	1	4.70E-07	0.4	83	2.08	Zc3h7b	Metabolism
DMR7:123022001	7	123022001	123026000	4000	1	8.20E-08	0.59	77	1.93	Zc3h7b;Tef	Metabolism;Transcription
DMR7:123465001	7	123465001	123468000	3000	1	3.90E-10	0.52	58	1.93	Tnfrsf13c;Cenpm	Receptor
DMR7:124081001	7	124081001	124083000	2000	1	5.20E-07	0.46	28	1.4	A4galt	Transport
DMR7:124118001	7	124118001	124120000	2000	1	7.90E-07	0.52	52	2.6	A4galt	Transport
DMR7:124152001	7	124152001	124153000	1000	1	7.60E-07	0.6	13	1.3	Arfgap3	Signaling
DMR7:124538001	7	124538001	124545000	7000	1	5.70E-07	0.43	116	1.66	Scube1	Extracellular Matrix
DMR7:124579001	7	124579001	124580000	1000	1	1.70E-07	0.57	8	0.8	Scube1	Extracellular Matrix
DMR7:124582001	7	124582001	124583000	1000	1	2.70E-08	0.54	13	1.3	Scube1	Extracellular Matrix
DMR7:124589001	7	124589001	124591000	2000	1	1.00E-07	0.53	21	1.05	Scube1	Extracellular Matrix
DMR7:124733001	7	124733001	124736000	3000	1	4.60E-08	0.57	49	1.63	Mpped1	Metabolism
DMR7:124778001	7	124778001	124782000	4000	1	1.50E-08	0.53	66	1.65	Efcab6;LOC102549885	Signaling
DMR7:125082001	7	125082001	125085000	3000	1	1.50E-07	0.51	39	1.3	Samm50	
DMR7:125342001	7	125342001	125348000	6000	1	6.70E-07	0.57	97	1.62	LOC108351521;RGD1566029	
DMR7:125363001	7	125363001	125366000	3000	1	1.20E-09	0.55	45	1.5	RGD1566029	
DMR7:125577001	7	125577001	125581000	4000	1	6.30E-07	0.49	74	1.85	Prr5	
DMR7:125667001	7	125667001	125670000	3000	1	1.40E-09	0.52	43	1.43	Phf21b	
DMR7:125698001	7	125698001	125699000	1000	1	4.20E-07	0.56	26	2.6	Phf21b	
DMR7:125786001	7	125786001	125791000	5000	1	6.40E-10	0.55	90	1.8	Arhgap8;LOC102550959	Signaling
DMR7:126598001	7	126598001	126600000	2000	1	3.30E-08	0.63	36	1.8	Mirlet7c-2;Mirlet7b	
DMR7:126839001	7	126839001	126845000	6000	1	3.30E-07	0.45	98	1.63	Celsr1	Cytoskeleton
DMR7:126891001	7	126891001	126898000	7000	1	3.30E-07	0.41	123	1.76	Celsr1	Cytoskeleton
DMR7:126908001	7	126908001	126909000	1000	1	3.30E-07	0.39	23	2.3	Celsr1	Cytoskeleton
DMR7:127030001	7	127030001	127032000	2000	1	4.20E-07	0.47	40	2	Cerk	Signaling
DMR7:127171001	7	127171001	127172000	1000	1	9.50E-07	0.7	5	0.5	Tbc1d22a	Signaling
DMR7:127175001	7	127175001	127176000	1000	1	2.40E-07	0.53	11	1.1	Tbc1d22a	Signaling
DMR7:128691001	7	128691001	128692000	1000	1	9.20E-08	0.62	7	0.7	Fam19a5	
DMR7:130015001	7	130015001	130017000	2000	1	9.20E-07	0.43	33	1.65	Mov10l1	
DMR7:130066001	7	130066001	130070000	4000	1	8.00E-09	0.59	71	1.77	Trabd;Selo	
DMR7:130166001	7	130166001	130168000	2000	1	1.60E-09	0.47	38	1.9	Plxnb2;LOC102549176;Dennd6b	
DMR7:130352001	7	130352001	130357000	5000	1	9.60E-08	0.54	69	1.38	Ncaph2;Tymp;Odf3b;Klhdc7b;Syce3	Golgi;Development
DMR7:130448001	7	130448001	130451000	3000	1	2.10E-08	0.51	68	2.27	Mapk8ip2;Arsa	Cytoskeleton;Metabolism
DMR7:130500001	7	130500001	130502000	2000	1	2.30E-09	0.52	30	1.5	Shank3	
DMR7:132687001	7	132687001	132689000	2000	1	4.30E-07	0.44	27	1.35	Slc2a13	Transport
DMR7:132923001	7	132923001	132924000	1000	1	1.10E-09	0.51	15	1.5	Lrrk2	Cytoskeleton
DMR7:133347001	7	133347001	133348000	1000	1	3.20E-07	0.56	22	2.2	Cntn1	
DMR7:133400001	7	133400001	133403000	3000	1	3.40E-07	0.42	59	1.97	Cntn1	
DMR7:134609001	7	134609001	134611000	2000	1	3.30E-07	0.61	20	1	Zcrb1;Pphln1;LOC108351532	
DMR7:134645001	7	134645001	134646000	1000	1	1.20E-07	0.43	11	1.1	Pphln1;LOC108351532	
DMR7:134763001	7	134763001	134770000	7000	1	5.90E-07	0.44	129	1.84	Prickle1	Cytoskeleton
DMR7:134807001	7	134807001	134811000	4000	1	1.90E-07	0.46	59	1.48	Prickle1	Cytoskeleton
DMR7:135806001	7	135806001	135811000	5000	1	3.70E-07	0.4	107	2.14	Pus7l;Irak4;LOC689928	
DMR7:135891001	7	135891001	135892000	1000	1	1.10E-07	0.41	15	1.5	Tmem117	
DMR7:137755001	7	137755001	137758000	3000	1	2.60E-09	0.8	52	1.73	Arid2	
DMR7:137974001	7	137974001	137980000	6000	1	3.40E-07	0.47	163	2.72	Slc38a1	Transport
DMR7:138726001	7	138726001	138730000	4000	1	6.50E-07	-0.66	48	1.2	LOC102551484;Pced1b	
DMR7:139198001	7	139198001	139205000	7000	1	2.20E-07	0.5	87	1.24	Rpap3;Endou	
DMR7:139245001	7	139245001	139246000	1000	1	7.20E-07	0.43	22	2.2	Rapgef3;LOC102548155	Transcription
DMR7:139711001	7	139711001	139715000	4000	1	1.40E-07	0.48	66	1.65	Pfkm;Asb8	Metabolism
DMR7:139835001	7	139835001	139836000	1000	1	3.20E-07	0.43	22	2.2	Olr1877	

DMR7:140097001	7	140097001	140098000	1000	1	3.80E-09	0.51	15	1.5	Lalba;Olr1111	Receptor
DMR7:140322001	7	140322001	140324000	2000	1	1.40E-07	0.67	49	2.45	Cacnb3;Ddx23	Transport
DMR7:140528001	7	140528001	140535000	7000	1	8.70E-08	0.5	160	2.29	Kmt2d	
DMR7:140585001	7	140585001	140588000	3000	1	4.30E-08	0.46	42	1.4	Dhh;Lmbr1l	Receptor
DMR7:140869001	7	140869001	140875000	6000	1	3.00E-08	0.63	92	1.53	Kcnh3;Spats2	Transport
DMR7:140993001	7	140993001	1.41E+08	7000	1	5.60E-08	0.6	77	1.1	Fmnl3	
DMR7:141749001	7	141749001	141751000	2000	2	6.70E-08	0.61	52	2.6	Dip2b	
DMR7:141801001	7	141801001	141805000	4000	1	2.50E-07	0.51	85	2.12	Dip2b;LOC102550811	
DMR7:141846001	7	141846001	141849000	3000	1	6.50E-08	0.47	68	2.27	Dip2b	
DMR7:141884001	7	141884001	141885000	1000	1	1.40E-07	0.57	12	1.2	Dip2b;Atf1	
DMR7:142037001	7	142037001	142039000	2000	1	3.80E-07	0.57	31	1.55	Slc11a2	Transport
DMR7:142457001	7	142457001	142460000	3000	1	4.60E-07	0.43	77	2.57	Slc4a8	Transport
DMR7:142566001	7	142566001	142569000	3000	1	1.60E-07	0.5	65	2.17	Scn8a	Transport
DMR7:142699001	7	142699001	142701000	2000	1	1.70E-07	0.65	21	1.05	Fignl2	
DMR7:142715001	7	142715001	142717000	2000	1	3.20E-08	0.5	32	1.6	Fignl2	
DMR7:142994001	7	142994001	142997000	3000	1	9.90E-07	0.39	67	2.23	Krt80	
DMR7:143069001	7	143069001	143071000	2000	1	4.90E-07	0.41	45	2.25	Krt7;Krt83	
DMR7:143550001	7	143550001	143552000	2000	1	6.20E-09	0.54	30	1.5	Krt78	
DMR7:143715001	7	143715001	143718000	3000	1	1.70E-09	0.56	60	2	Tns2;Spryd3	Cytoskeleton;Cytoskeleton
DMR7:143721001	7	143721001	143723000	2000	1	1.80E-09	0.71	31	1.55	Tns2;Spryd3	Cytoskeleton;Cytoskeleton
DMR7:143857001	7	143857001	143859000	2000	1	2.80E-07	0.4	39	1.95	Rarg	Transcription
DMR7:143860001	7	143860001	143863000	3000	1	8.00E-07	0.42	65	2.17	Rarg	Transcription
DMR7:145140001	7	145140001	145145000	5000	1	4.00E-07	0.48	56	1.12	Pde1b;Ppp1r1a;LOC102547969	Signaling;Signaling
DMR8:5779001	8	5779001	5780000	1000	1	7.00E-09	0.45	19	1.9	Mmp8	Protease
DMR8:5836001	8	5836001	5837000	1000	1	2.60E-07	0.48	11	1.1	Mmp20	Protease
DMR8:6878001	8	6878001	6879000	1000	1	2.30E-07	-0.59	13	1.3	Trpc6	Transport
DMR8:7160001	8	7160001	7162000	2000	1	1.10E-07	0.5	19	0.95	Pgr	
DMR8:12161001	8	12161001	12162000	1000	1	2.70E-07	0.48	18	1.8	Maml2	Transcription
DMR8:13039001	8	13039001	13041000	2000	1	7.60E-08	0.55	29	1.45	Amotl1	
DMR8:13080001	8	13080001	13083000	3000	1	2.90E-08	0.49	66	2.2	Amotl1;LOC108351653	
DMR8:13821001	8	13821001	13823000	2000	1	1.40E-07	0.47	48	2.4	Med17;Vstm5	Transcription;Immune
DMR8:14142001	8	14142001	14144000	2000	1	2.50E-13	0.68	24	1.2	Deup1	Cytoskeleton
DMR8:14247001	8	14247001	14250000	3000	1	4.20E-07	0.5	62	2.07	Slc36a4	Transport
DMR8:19320001	8	19320001	19323000	3000	1	9.70E-07	-0.76	5	0.17	Olr1144	Receptor
DMR8:21892001	8	21892001	21896000	4000	1	3.60E-08	0.46	84	2.1	LOC500956;Angptl6;Ppan	Signaling;Metabolism
DMR8:21985001	8	21985001	21987000	2000	1	2.70E-07	0.4	43	2.15	LOC367036;S1pr2	Signaling
DMR8:22366001	8	22366001	22367000	1000	1	5.40E-08	0.51	12	1.2	Slc44a2	Transport
DMR8:22538001	8	22538001	22542000	4000	2	1.10E-09	0.68	66	1.65	Dnm2;Tmed1;LOC100360348	Transport;Transport
DMR8:22620001	8	22620001	22623000	3000	1	4.00E-08	0.75	62	2.07	Carm1;Yipf2;RGD1309188	Golgi
DMR8:22674001	8	22674001	22675000	1000	1	2.70E-07	0.39	15	1.5	Smarca4	Epigenetic
DMR8:22852001	8	22852001	22855000	3000	1	2.60E-09	0.53	63	2.1	Dock6;Angptl8	Transcription
DMR8:22884001	8	22884001	22886000	2000	1	9.90E-07	0.45	24	1.2	Dock6	Transcription
DMR8:22968001	8	22968001	22971000	3000	1	1.80E-07	0.65	36	1.2	Plppr2;Swsap1;Epor;Rgl3	Signaling;Receptor;Transcription
DMR8:23929001	8	23929001	23930000	1000	1	1.10E-08	0.6	18	1.8	Bbs9	
DMR8:24031001	8	24031001	24032000	1000	1	4.10E-07	0.46	29	2.9	Bbs9	
DMR8:25585001	8	25585001	25587000	2000	1	2.50E-07	0.48	29	1.45	Dpy19l1	
DMR8:26736001	8	26736001	26738000	2000	1	6.70E-07	0.71	15	0.75	Eepd1	
DMR8:27782001	8	27782001	27784000	2000	1	1.30E-13	0.65	26	1.3	B3gat1	Golgi
DMR8:29615001	8	29615001	29616000	1000	1	7.00E-10	0.51	8	0.8	Opcml	Immune
DMR8:30502001	8	30502001	30503000	1000	1	5.70E-08	0.56	14	1.4	Ntm	Immune
DMR8:30678001	8	30678001	30682000	4000	1	1.10E-07	-0.35	44	1.1	Ntm	Immune
DMR8:30711001	8	30711001	30713000	2000	1	6.10E-07	0.38	26	1.3	Ntm	Immune
DMR8:31515001	8	31515001	31517000	2000	1	4.50E-08	0.68	17	0.85	Snx19	Cytoskeleton
DMR8:33630001	8	33630001	33632000	2000	1	1.60E-10	0.55	33	1.65	Fli1	Transcription
DMR8:35796001	8	35796001	35798000	2000	1	5.10E-07	0.47	32	1.6	Kirrel3	
DMR8:36226001	8	36226001	36228000	2000	1	1.80E-08	0.58	36	1.8	Kirrel3	
DMR8:36653001	8	36653001	36654000	1000	1	8.30E-09	0.4	18	1.8	Cdon	
DMR8:36696001	8	36696001	36699000	3000	1	2.80E-08	0.62	28	0.93	Cdon;LOC102548010	
DMR8:37816001	8	37816001	37819000	3000	1	1.50E-08	-0.75	22	0.73	RGD1560348	
DMR8:37881001	8	37881001	37882000	1000	1	1.70E-07	-0.37	12	1.2	RGD1560348	
DMR8:39737001	8	39737001	39739000	2000	1	3.20E-08	0.8	97	4.85	Slc37a2	Transport
DMR8:39892001	8	39892001	39894000	2000	1	7.30E-09	0.62	15	0.75	LOC100911068;Robo3	
DMR8:44211001	8	44211001	44217000	6000	1	3.20E-07	0.42	96	1.6	Gramd1b	
DMR8:44246001	8	44246001	44247000	1000	1	5.20E-08	0.49	17	1.7	Gramd1b	

DMR8:44904001	8	44904001	44905000	1000	1	2.40E-08	0.58	11	1.1	Clmp	
DMR8:45696001	8	45696001	45698000	2000	1	7.30E-07	0.43	31	1.55	Lnc215	
DMR8:46227001	8	46227001	46231000	4000	1	1.40E-07	0.42	67	1.68	Sorl1	Transport
DMR8:46258001	8	46258001	46262000	4000	1	6.80E-09	0.48	53	1.32	Sorl1	Transport
DMR8:46274001	8	46274001	46278000	4000	1	2.00E-07	0.53	64	1.6	Sorl1	Transport
DMR8:46617001	8	46617001	46619000	2000	1	1.70E-07	0.62	35	1.75	Tecta	
DMR8:46922001	8	46922001	46927000	5000	1	5.20E-08	0.45	71	1.42	Grik4	Receptor
DMR8:47149001	8	47149001	47154000	5000	1	6.10E-07	0.47	61	1.22	Grik4	Receptor
DMR8:47458001	8	47458001	47464000	6000	1	6.00E-07	0.44	84	1.4	Pou2f3	
DMR8:47482001	8	47482001	47487000	5000	1	7.00E-08	0.53	51	1.02	Pou2f3	
DMR8:48130001	8	48130001	48132000	2000	1	9.40E-10	0.52	25	1.25	Nectin1	
DMR8:48387001	8	48387001	48389000	2000	1	9.70E-08	0.41	28	1.4	LOC103693068;Thy1	Cytoskeleton
DMR8:48412001	8	48412001	48413000	1000	1	7.00E-10	0.61	24	2.4	Usp2	Protease
DMR8:48664001	8	48664001	48665000	1000	1	3.90E-09	0.54	15	1.5	Dpagt1;H2afx;Hmbs	Golgi;Epigenetic;Metabolism
DMR8:48703001	8	48703001	48707000	4000	2	3.60E-09	0.59	55	1.38	Hyou1;Slc37a4	Transport
DMR8:48718001	8	48718001	48720000	2000	1	5.40E-07	0.47	39	1.95	Hyou1;Slc37a4;Trappc4;Rps25;Ccdc84	Transport;Transport;Translation
DMR8:48831001	8	48831001	48834000	3000	1	3.30E-08	0.53	61	2.03	Bcl9l;Cxcr5	
DMR8:48968001	8	48968001	48971000	3000	1	1.00E-07	0.54	43	1.43	Ddx6	
DMR8:49852001	8	49852001	49856000	4000	1	1.90E-08	0.44	61	1.52	Dscaml1	Cytoskeleton
DMR8:49971001	8	49971001	49980000	9000	3	1.90E-09	0.6	160	1.78	Dscaml1	Cytoskeleton
DMR8:50377001	8	50377001	50378000	1000	1	6.10E-08	0.61	8	0.8	Sik3	Signaling
DMR8:50498001	8	50498001	50501000	3000	1	4.00E-08	0.53	58	1.93	Sik3	Signaling
DMR8:50530001	8	50530001	50532000	2000	1	1.00E-07	0.59	32	1.6	Sik3;Apoa1;Apoc3;Apoa4	Signaling;Binding Proteins
DMR8:52032001	8	52032001	52033000	1000	1	7.80E-07	0.44	15	1.5	Cadm1	
DMR8:52738001	8	52738001	52740000	2000	1	8.30E-09	0.45	22	1.1	Nxpe4;Nxpe1	
DMR8:53041001	8	53041001	53043000	2000	1	3.00E-12	0.58	35	1.75	Zbtb16	Transcription
DMR8:53258001	8	53258001	53259000	1000	1	3.70E-07	0.48	17	1.7	Htr3b	Ion Channel
DMR8:53832001	8	53832001	53833000	1000	1	7.40E-07	0.6	5	0.5	LOC102556026;Ncam1	
DMR8:53839001	8	53839001	53844000	5000	1	2.60E-08	0.47	79	1.58	Ncam1	
DMR8:55128001	8	55128001	55131000	3000	1	6.40E-07	0.43	36	1.2	Dixdc1	Cytoskeleton
DMR8:56390001	8	56390001	56391000	1000	1	5.80E-08	0.44	9	0.9	Fdx1	Metabolism
DMR8:57898001	8	57898001	57901000	3000	1	1.50E-09	0.53	51	1.7	Exph5;LOC108351708	
DMR8:59244001	8	59244001	59245000	1000	1	1.00E-08	0.63	13	1.3	Acsbg1	Metabolism
DMR8:59610001	8	59610001	59614000	4000	1	2.50E-10	0.58	65	1.62	Chrna3;Chrnb4	Ion Channel
DMR8:59615001	8	59615001	59616000	1000	1	3.90E-07	0.5	23	2.3	Chrna3;Chrnb4	Ion Channel
DMR8:59814001	8	59814001	59817000	3000	1	5.80E-13	0.64	32	1.07	Nrg4	Growth Factors
DMR8:60625001	8	60625001	60628000	3000	1	2.80E-07	-0.59	19	0.63	RGD1563578;LOC691004	
DMR8:60663001	8	60663001	60665000	2000	1	7.30E-09	-1.39	25	1.25	RGD1563578	
DMR8:60909001	8	60909001	60912000	3000	1	1.40E-07	0.46	33	1.1	Peak1	Signaling
DMR8:61275001	8	61275001	61277000	2000	1	1.40E-07	0.51	76	3.8	Lingo1	Receptor
DMR8:61325001	8	61325001	61328000	3000	1	6.50E-08	0.42	39	1.3	Lingo1	Receptor
DMR8:61450001	8	61450001	61452000	2000	2	1.40E-07	0.45	25	1.25	Lingo1	Receptor
DMR8:61592001	8	61592001	61594000	2000	1	4.00E-07	0.45	39	1.95	Snx33;LOC103693101	Cytoskeleton
DMR8:61915001	8	61915001	61920000	5000	1	5.10E-10	0.52	90	1.8	LOC691110;RGD1305464	
DMR8:62235001	8	62235001	62239000	4000	1	2.10E-08	0.51	38	0.95	Scamp5	Transport
DMR8:62376001	8	62376001	62377000	1000	1	2.20E-07	0.5	17	1.7	Scamp2;Ulk3;Cplx3;LOC103693104;Lman1l	Transport;Signaling;Transport
DMR8:62378001	8	62378001	62379000	1000	1	1.30E-08	0.5	16	1.6	Scamp2;Ulk3;Cplx3;LOC103693104;Lman1l	Transport;Signaling;Transport
DMR8:62625001	8	62625001	62627000	2000	1	1.10E-09	0.36	11	0.55	Arid3b	Transcription
DMR8:62741001	8	62741001	62742000	1000	1	7.60E-07	0.45	20	2	Sema7a	Signaling
DMR8:62807001	8	62807001	62812000	5000	1	1.10E-07	0.47	55	1.1	Cyp11a1;Ccdc33	
DMR8:63018001	8	63018001	63020000	2000	1	1.30E-07	0.45	27	1.35	Pml	
DMR8:63466001	8	63466001	63468000	2000	1	1.80E-07	0.48	40	2	Rec114	
DMR8:63613001	8	63613001	63618000	5000	2	1.70E-07	0.51	74	1.48	Hcn4	Transport
DMR8:64197001	8	64197001	64198000	1000	1	5.70E-09	0.28	8	0.8	Arih1	Proteolysis
DMR8:64376001	8	64376001	64378000	2000	1	3.10E-09	0.54	28	1.4	Celf6	
DMR8:64447001	8	64447001	64449000	2000	1	7.10E-10	0.56	20	1	Parp6	
DMR8:64486001	8	64486001	64487000	1000	1	8.80E-12	0.66	11	1.1	Pkm	Signaling
DMR8:65053001	8	65053001	65056000	3000	1	3.80E-08	0.53	39	1.3	Thsd4	
DMR8:65401001	8	65401001	65403000	2000	1	7.60E-08	0.4	32	1.6	Thsd4	
DMR8:66948001	8	66948001	66949000	1000	1	1.40E-08	0.5	23	2.3	Paqr5	Signaling
DMR8:68583001	8	68583001	68585000	2000	1	4.60E-07	0.44	19	0.95	Smad3	Transcription

DMR8:68598001	8	68598001	68599000	1000	1	2.00E-07	0.44	12	1.2	Smad3	Transcription
DMR8:68896001	8	68896001	68898000	2000	1	2.70E-10	0.56	27	1.35	Smad6	Transcription
DMR8:69563001	8	69563001	69564000	1000	1	5.60E-07	-0.46	6	0.6	Map2k1;Tmem185a	Signaling
DMR8:69875001	8	69875001	69880000	5000	1	7.10E-07	0.47	63	1.26	Megf11	Extracellular Matrix
DMR8:69992001	8	69992001	69997000	5000	1	5.70E-10	0.54	93	1.86	Megf11;LOC102548470	Extracellular Matrix
DMR8:70142001	8	70142001	70144000	2000	1	2.80E-07	0.51	29	1.45	Megf11	Extracellular Matrix
DMR8:71336001	8	71336001	71340000	4000	1	1.30E-07	0.47	41	1.02	Zfp609	
DMR8:71870001	8	71870001	71872000	2000	1	4.10E-09	0.61	17	0.85	Dapk2	Signaling
DMR8:71909001	8	71909001	71910000	1000	1	6.20E-07	0.4	18	1.8	Dapk2	Signaling
DMR8:73028001	8	73028001	73029000	1000	1	7.90E-08	0.44	12	1.2	Tln2;Mir190	
DMR8:73080001	8	73080001	73081000	1000	1	3.90E-07	0.46	12	1.2	Tln2	
DMR8:73827001	8	73827001	73829000	2000	1	1.40E-09	0.47	35	1.75	Vps13c	Transport
DMR8:75571001	8	75571001	75575000	4000	2	1.70E-07	0.59	73	1.82	Rora	Transcription
DMR8:76989001	8	76989001	76992000	3000	1	5.70E-08	0.57	25	0.83	Sltm	
DMR8:77176001	8	77176001	77179000	3000	1	3.30E-07	0.5	16	0.53	Adam10	Protease
DMR8:77285001	8	77285001	77289000	4000	1	9.90E-09	0.53	52	1.3	Lipc	Metabolism
DMR8:77711001	8	77711001	77713000	2000	1	1.70E-08	0.5	20	1	Aldh1a2	Metabolism
DMR8:78873001	8	78873001	78875000	2000	1	9.60E-07	-0.45	18	0.9	Zfp280d	Transcription
DMR8:80383001	8	80383001	80386000	3000	1	6.00E-11	0.48	22	0.73	Unc13c	
DMR8:81121001	8	81121001	81123000	2000	1	1.40E-07	0.48	41	2.05	Wdr72	
DMR8:82288001	8	82288001	82289000	1000	1	8.00E-07	0.94	22	2.2	Gnb5;Bcl2l10	Signaling
DMR8:82398001	8	82398001	82400000	2000	1	9.30E-08	0.59	38	1.9	Leo1;LOC100912347	Transcription
DMR8:82493001	8	82493001	82495000	2000	1	3.30E-08	0.51	23	1.15	Tmod3	Cytoskeleton
DMR8:82774001	8	82774001	82779000	5000	1	2.10E-08	-0.82	22	0.44	Bmp5	Growth Factors
DMR8:82831001	8	82831001	82839000	8000	1	2.60E-09	-0.72	51	0.64	Bmp5	Growth Factors
DMR8:82846001	8	82846001	82850000	4000	1	1.00E-09	-0.45	21	0.52	Bmp5	Growth Factors
DMR8:84772001	8	84772001	84773000	1000	1	3.70E-07	0.47	21	2.1	Lrrc1	
DMR8:85219001	8	85219001	85220000	1000	1	2.10E-07	0.59	14	1.4	Elovl5	Metabolism
DMR8:85907001	8	85907001	85908000	1000	1	6.30E-07	0.55	13	1.3	Slc17a5	Transport
DMR8:87620001	8	87620001	87622000	2000	1	1.50E-10	0.47	26	1.3	Myo6	Cytoskeleton
DMR8:87763001	8	87763001	87766000	3000	1	6.60E-08	0.48	53	1.77	Impg1	Extracellular Matrix
DMR8:87767001	8	87767001	87768000	1000	1	9.70E-08	0.43	10	1	Impg1	Extracellular Matrix
DMR8:90369001	8	90369001	90374000	5000	1	6.20E-08	-0.53	37	0.74	Irak1bp1	
DMR8:91135001	8	91135001	91137000	2000	1	1.10E-07	0.42	24	1.2	Sh3bgrl2	
DMR8:91373001	8	91373001	91374000	1000	1	1.30E-08	0.57	14	1.4	Ttk	Signaling
DMR8:94164001	8	94164001	94166000	2000	1	4.10E-07	0.47	28	1.4	Dopey1	
DMR8:94325001	8	94325001	94328000	3000	1	7.50E-07	0.43	36	1.2	Me1	Metabolism
DMR8:94865001	8	94865001	94867000	2000	1	2.00E-07	0.53	51	2.55	Mrap2;Cep162	
DMR8:94892001	8	94892001	94895000	3000	1	7.10E-08	0.49	40	1.33	Cep162;LOC103690444	
DMR8:96584001	8	96584001	96587000	3000	1	2.10E-07	0.4	94	3.13	Mthfs	Metabolism
DMR8:103477001	8	103477001	103480000	3000	1	7.80E-07	-0.62	39	1.3	PCOLCE2	Protease
DMR8:103598001	8	103598001	103599000	1000	1	2.30E-07	0.45	16	1.6	Pls1	Cytoskeleton
DMR8:104735001	8	104735001	104736000	1000	1	8.70E-10	0.7	7	0.7	Pxylp1	Signaling
DMR8:104843001	8	104843001	104845000	2000	1	5.80E-08	0.44	41	2.05	Spsb4	
DMR8:104883001	8	104883001	104886000	3000	1	1.50E-09	0.58	43	1.43	Spsb4	
DMR8:105446001	8	105446001	105448000	2000	1	6.40E-07	0.49	22	1.1	Clstn2	Transport
DMR8:105700001	8	105700001	105701000	1000	1	7.90E-07	0.41	9	0.9	Clstn2	Transport
DMR8:106343001	8	106343001	106344000	1000	1	5.70E-10	0.64	15	1.5	Nmnat3	Metabolism
DMR8:106544001	8	106544001	106545000	1000	1	1.80E-08	0.44	15	1.5	RGD1565403	
DMR8:107624001	8	107624001	107625000	1000	1	7.90E-07	0.44	23	2.3	Mras	Signaling
DMR8:107629001	8	107629001	107633000	4000	1	5.70E-08	0.47	71	1.77	Mras	Signaling
DMR8:107708001	8	107708001	107710000	2000	1	3.10E-07	0.53	56	2.8	Nme9	
DMR8:108738001	8	108738001	108739000	1000	1	2.80E-07	0.44	13	1.3	Il20rb	Receptor
DMR8:108771001	8	108771001	108775000	4000	2	4.00E-10	-0.97	88	2.2	Il20rb	Receptor
DMR8:109428001	8	109428001	109430000	2000	1	7.60E-08	0.43	26	1.3	Pccb	Metabolism
DMR8:110627001	8	110627001	110630000	3000	1	6.40E-07	0.51	34	1.13	Ephb1	Receptor
DMR8:110701001	8	110701001	110704000	3000	1	2.60E-07	0.45	40	1.33	Ephb1	Receptor
DMR8:111557001	8	111557001	111560000	3000	1	1.30E-07	0.52	39	1.3	Slco2a1	Transport
DMR8:111635001	8	111635001	111639000	4000	1	5.60E-07	0.48	64	1.6	Rab6b	
DMR8:111673001	8	111673001	111677000	4000	1	8.00E-08	0.46	50	1.25	Rab6b;Srprb	Transport
DMR8:111972001	8	111972001	111974000	2000	1	1.30E-08	0.5	28	1.4	Bfsp2;Tmem108	
DMR8:112841001	8	112841001	112845000	4000	1	1.20E-07	0.42	46	1.15	Acpp	
DMR8:113117001	8	113117001	113118000	1000	1	2.30E-07	0.38	14	1.4	Cpne4	
DMR8:113318001	8	113318001	113322000	4000	1	7.70E-07	0.39	63	1.57	Cpne4	
DMR8:114757001	8	114757001	114759000	2000	1	6.70E-08	0.58	39	1.95	Col6a4	

DMR8:114917001	8	114917001	114919000	2000	1	6.60E-07	0.46	79	3.95	Twf2;Tlr9;Alas1	Cytoskeleton;Receptor;Metabolism
DMR8:115135001	8	115135001	115136000	1000	1	1.10E-11	0.56	16	1.6	Rpl29;Acy1;Abhd14a	Translation
DMR8:115270001	8	115270001	115272000	2000	1	3.70E-07	0.44	25	1.25	Iqcf3;LOC102550160	
DMR8:115393001	8	115393001	115397000	4000	1	6.70E-08	0.52	35	0.88	Tex264;Rad54l2	
DMR8:116043001	8	116043001	116045000	2000	1	7.50E-07	0.59	29	1.45	Mapkapk3;LOC102557197;Cish	Signaling;Signaling
DMR8:116321001	8	116321001	116322000	1000	1	3.20E-07	0.51	13	1.3	Rassf1;Tusc2;Hyal2	Cytoskeleton;Metabolism
DMR8:116443001	8	116443001	116455000	12000	1	6.90E-08	0.45	258	2.15	Gnat1;Sema3f	Signaling;Signaling
DMR8:116836001	8	116836001	116841000	5000	2	2.30E-08	0.57	78	1.56	Ip6k1;Gmppb;Rnf123;Amigo3	Signaling;Transport
DMR8:116882001	8	116882001	116892000	10000	3	4.90E-08	0.5	310	3.1	Bsn	
DMR8:116940001	8	116940001	116942000	2000	1	1.80E-07	0.52	31	1.55	Bsn	
DMR8:117640001	8	117640001	117645000	5000	2	4.70E-08	0.47	121	2.42	Celsr3;Slc26a6	Cytoskeleton;Transport
DMR8:117848001	8	117848001	117854000	6000	2	1.80E-07	0.51	138	2.3	Plxnb1;LOC102553408	
DMR8:117926001	8	117926001	117928000	2000	1	4.10E-07	0.44	29	1.45	Spink8;Nme6;Camp	Protease; Proteolysis;Signaling;Signaling
DMR8:117970001	8	117970001	117973000	3000	1	2.90E-09	0.53	26	0.87	Cdc25a	Signaling
DMR8:118629001	8	118629001	118633000	4000	1	5.30E-08	0.61	99	2.48	Scap;Ptpn23	Transport
DMR8:118939001	8	118939001	118940000	1000	1	1.60E-07	0.46	26	2.6	Ccdc12	Translation
DMR8:118996001	8	118996001	118998000	2000	1	3.30E-07	0.51	27	1.35	Pth1r	Receptor
DMR8:119208001	8	119208001	119211000	3000	1	3.40E-07	0.42	66	2.2	RGD1564138;Tdgf1	Signaling
DMR8:119707001	8	119707001	119709000	2000	1	3.90E-07	0.38	46	2.3	Dclk3	Signaling
DMR8:122544001	8	122544001	122548000	4000	1	6.50E-07	0.45	72	1.8	Ccr4;Trim71	Proteolysis
DMR8:123231001	8	123231001	123232000	1000	1	5.00E-08	0.53	18	1.8	Osbpl10	
DMR8:123238001	8	123238001	123245000	7000	1	5.10E-09	0.59	158	2.26	Osbpl10	
DMR8:123283001	8	123283001	123284000	1000	1	5.20E-11	0.69	8	0.8	Osbpl10	
DMR8:123364001	8	123364001	123365000	1000	1	1.90E-08	0.63	4	0.4	Stt3b	Golgi
DMR8:124034001	8	124034001	124035000	1000	1	7.80E-07	0.49	19	1.9	Gadl1	
DMR8:124313001	8	124313001	124315000	2000	1	1.40E-07	0.58	41	2.05	Tgfbr2	Signaling
DMR8:127156001	8	127156001	127158000	2000	1	7.70E-07	0.43	48	2.4	Ctdspl;Eomes	Transcription
DMR8:127476001	8	127476001	127478000	2000	2	2.50E-07	0.49	32	1.6	Ctdspl;Itga9	Extracellular Matrix
DMR8:127735001	8	127735001	127737000	2000	1	2.10E-11	0.73	42	2.1	Ctdspl;Vill	Cytoskeleton
DMR8:127758001	8	127758001	127760000	2000	1	3.90E-07	0.4	49	2.45	Vill;Plcd1	Cytoskeleton;Metabolism
DMR8:128066001	8	128066001	128069000	3000	1	6.70E-08	0.5	51	1.7	Xylb	Metabolism
DMR8:128122001	8	128122001	128125000	3000	2	1.60E-07	0.49	69	2.3	Acvr2b;LOC108351831;Exog	Signaling
DMR8:128138001	8	128138001	128139000	1000	1	1.40E-07	0.49	26	2.6	LOC108351831;Exog	
DMR8:128152001	8	128152001	128154000	2000	1	5.50E-08	0.51	29	1.45	Exog	
DMR8:128228001	8	128228001	128234000	6000	1	4.60E-07	0.41	139	2.32	Scn5a	Transport
DMR8:128308001	8	128308001	128313000	5000	1	8.00E-09	0.48	126	2.52	Scn10a	Transport
DMR8:128487001	8	128487001	128492000	5000	1	3.00E-09	0.5	91	1.82	Scn11a	Transport
DMR8:128640001	8	128640001	128644000	4000	1	4.40E-07	0.48	61	1.52	Ttc21a	
DMR8:128703001	8	128703001	128709000	6000	1	6.70E-08	0.58	124	2.07	Xirp1	Cytoskeleton
DMR8:129058001	8	129058001	129062000	4000	1	2.70E-07	0.47	49	1.23	Myrip	Cytoskeleton
DMR8:129116001	8	129116001	129117000	1000	1	6.30E-07	0.45	16	1.6	Myrip	Cytoskeleton
DMR8:129241001	8	129241001	129243000	2000	1	1.60E-11	0.5	37	1.85	Entpd3;Rpl14	Signaling;Translation
DMR8:129800001	8	129800001	129803000	3000	1	8.50E-07	0.45	52	1.73	Ulk4	Signaling
DMR8:129877001	8	129877001	129879000	2000	1	1.40E-07	0.47	27	1.35	Ulk4	Signaling
DMR8:130021001	8	130021001	130023000	2000	1	7.20E-07	0.44	37	1.85	Trak1	Transport
DMR8:130055001	8	130055001	130058000	3000	1	3.90E-08	0.55	56	1.87	Trak1	Transport
DMR8:130107001	8	130107001	130108000	1000	1	2.40E-07	0.53	16	1.6	Trak1	Transport
DMR8:130403001	8	130403001	130408000	5000	1	2.50E-07	0.55	101	2.02	Nktr;Zbtb47;Klhl40	Transcription;Transcription;Cytoskeleton
DMR8:130591001	8	130591001	130595000	4000	1	2.40E-08	0.56	23	0.58	Fam198a	
DMR8:130828001	8	130828001	130829000	1000	1	8.90E-07	0.45	13	1.3	Ano10;LOC103690476	
DMR8:131941001	8	131941001	131946000	5000	1	1.60E-07	0.48	62	1.24	Zfp167;Znf660	Transcription
DMR8:132191001	8	132191001	132194000	3000	1	5.00E-07	0.46	21	0.7	Zdhhc3	
DMR8:132707001	8	132707001	132709000	2000	1	2.80E-07	0.52	23	1.15	RGD1566368;Slc6a20	Transport
DMR9:108001	9	108001	109000	1000	1	1.10E-08	0.49	11	1.1	Efhb;LOC103690615	Signaling
DMR9:1432001	9	1432001	1434000	2000	1	6.70E-11	-0.37	30	1.5	Tbc1d5;LOC108348193	Signaling
DMR9:2112001	9	2112001	2115000	3000	1	4.20E-08	0.54	31	1.03	RGD1562851	
DMR9:3394001	9	3394001	3395000	1000	1	6.40E-08	0.98	8	0.8	Kcnh8	Transport
DMR9:3481001	9	3481001	3483000	2000	1	1.00E-10	0.35	19	0.95	Kcnh8	Transport
DMR9:3703001	9	3703001	3704000	1000	1	5.90E-07	0.4	12	1.2	Kcnh8	Transport
DMR9:4171001	9	4171001	4179000	8000	1	2.40E-10	0.37	80	1	Sult1c2a	Transport
DMR9:4591001	9	4591001	4593000	2000	1	9.50E-08	0.25	28	1.4	RGD1562392;LOC108351870	Transport

DMR9:7068001	9	7068001	7070000	2000	1	1.80E-07	0.62	10	0.5	St6gal2	Transport
DMR9:9619001	9	9619001	9621000	2000	1	4.50E-07	0.49	21	1.05	Vav1	
DMR9:9658001	9	9658001	9661000	3000	1	3.00E-09	0.66	45	1.5	Vav1	
DMR9:10414001	9	10414001	10415000	1000	1	3.70E-07	0.42	18	1.8	Catsperd	
DMR9:10450001	9	10450001	10453000	3000	1	3.90E-07	0.47	74	2.47	Lonp1;Rpl36;LOC301124;Safb	Protease;Translation
DMR9:10487001	9	10487001	10490000	3000	1	5.60E-08	0.59	60	2	Safb2;Tincr	
DMR9:10610001	9	10610001	10612000	2000	1	2.60E-07	0.42	30	1.5	Ptprs	Signaling
DMR9:10656001	9	10656001	10658000	2000	1	5.90E-07	0.65	37	1.85	Ptprs;Kdm4b	Signaling;Epigenetic
DMR9:12883001	9	12883001	12892000	9000	2	1.40E-07	0.53	166	1.84	Rftn1	
DMR9:13118001	9	13118001	13122000	4000	1	1.20E-07	0.44	67	1.68	Kif6	Cytoskeleton
DMR9:13921001	9	13921001	13926000	5000	1	1.00E-09	0.47	78	1.56	Lrfn2;LOC108351889	
DMR9:13954001	9	13954001	13956000	2000	1	1.40E-07	0.44	21	1.05	Lrfn2	
DMR9:14000001	9	14000001	14003000	3000	1	5.90E-08	0.45	48	1.6	Lrfn2	
DMR9:14582001	9	14582001	14584000	2000	1	3.20E-10	0.61	18	0.9	Nfya	Transcription
DMR9:14585001	9	14585001	14587000	2000	1	1.20E-13	0.7	15	0.75	Nfya	Transcription
DMR9:14666001	9	14666001	14668000	2000	1	5.30E-07	0.44	37	1.85	Treml2;Treml4	
DMR9:14721001	9	14721001	14722000	1000	1	2.70E-08	0.45	18	1.8	Trem3;Trem1	
DMR9:14785001	9	14785001	14787000	2000	1	7.60E-08	0.46	23	1.15	Ncr2	
DMR9:15093001	9	15093001	15097000	4000	1	2.80E-07	0.5	65	1.62	Foxp4	
DMR9:15560001	9	15560001	15562000	2000	1	6.50E-07	0.4	44	2.2	RGD1561662	
DMR9:15665001	9	15665001	15666000	1000	1	2.20E-07	0.41	18	1.8	Trerf1	
DMR9:15698001	9	15698001	15703000	5000	1	9.50E-07	0.43	124	2.48	Trerf1	
DMR9:15716001	9	15716001	15719000	3000	1	5.30E-07	0.47	60	2	Trerf1	
DMR9:15747001	9	15747001	15750000	3000	1	8.60E-08	0.48	75	2.5	Trerf1	
DMR9:16623001	9	16623001	16626000	3000	1	3.60E-07	0.47	54	1.8	Mea1;Klhdc3;Rrp36;LOC680835	
DMR9:16728001	9	16728001	16730000	2000	1	7.10E-08	0.62	53	2.65	Ptk7;Srf	Receptor
DMR9:16891001	9	16891001	16895000	4000	1	3.50E-07	0.56	84	2.1	Ttbk1	Signaling
DMR9:17128001	9	17128001	17129000	1000	1	1.30E-07	0.45	23	2.3	Yipf3;Polr1c;Xpo5	Transcription;Transport
DMR9:17240001	9	17240001	17241000	1000	1	9.80E-08	0.86	11	1.1	Rsph9;Mrps18a	Translation
DMR9:17796001	9	17796001	17800000	4000	1	1.70E-07	0.5	54	1.35	Slc29a1	Transport
DMR9:17821001	9	17821001	17823000	2000	1	1.50E-07	0.42	35	1.75	Hsp90ab1;Slc35b2;Nfkbie	Signaling;Transport;Transpor t
DMR9:18691001	9	18691001	18694000	3000	1	6.90E-07	0.42	44	1.47	Runx2	Transcription
DMR9:18766001	9	18766001	18769000	3000	1	3.10E-09	0.54	47	1.57	Runx2	Transcription
DMR9:19835001	9	19835001	19837000	2000	1	5.30E-09	0.47	29	1.45	Cyp39a1	Metabolism
DMR9:19865001	9	19865001	19866000	1000	1	4.70E-07	-0.7	7	0.7	Cyp39a1	Metabolism
DMR9:20000001	9	20000001	20002000	2000	1	3.70E-07	0.42	22	1.1	Ankrd66	Signaling
DMR9:21899001	9	21899001	21903000	4000	1	1.00E-08	-0.73	14	0.35	Olr1828-ps	
DMR9:21904001	9	21904001	21912000	8000	4	2.80E-09	-0.59	64	0.8	Olr1828-ps	
DMR9:27119001	9	27119001	27120000	1000	1	7.90E-07	0.48	27	2.7	Tram2	
DMR9:27163001	9	27163001	27166000	3000	1	1.00E-07	0.5	60	2	Tram2	
DMR9:30707001	9	30707001	30709000	2000	1	9.80E-08	0.49	17	0.85	Col19a1	Extracellular Matrix
DMR9:30838001	9	30838001	30842000	4000	1	5.30E-07	-0.47	54	1.35	Col19a1	Extracellular Matrix
DMR9:37787001	9	37787001	37788000	1000	1	1.30E-07	0.52	15	1.5	Ccdc115;Trnae-uuc;LOC102551518	
DMR9:37960001	9	37960001	37965000	5000	1	5.70E-07	0.46	91	1.82	Dst	Cytoskeleton
DMR9:38200001	9	38200001	38203000	3000	1	4.80E-08	0.47	63	2.1	Dst;LOC102551615	Cytoskeleton
DMR9:38244001	9	38244001	38247000	3000	1	2.10E-08	0.47	50	1.67	Dst	Cytoskeleton
DMR9:38465001	9	38465001	38466000	1000	1	1.00E-08	0.5	23	2.3	Bag2;Rab23	Transcription
DMR9:41065001	9	41065001	41066000	1000	1	2.20E-08	0.58	16	1.6	Prss40	Protease
DMR9:42881001	9	42881001	42885000	4000	1	5.50E-07	0.5	76	1.9	Arid5a	Transcription
DMR9:43086001	9	43086001	43088000	2000	1	2.10E-08	0.53	31	1.55	Cnnm4;Cnnm3	
DMR9:43358001	9	43358001	43361000	3000	1	6.40E-07	0.4	75	2.5	Zap70;Tmem131	
DMR9:43504001	9	43504001	43505000	1000	1	6.80E-10	0.58	11	1.1	Tmem131;LOC102554557	
DMR9:43682001	9	43682001	43688000	6000	2	8.90E-11	0.56	117	1.95	Vwa3b;LOC102554996	
DMR9:43702001	9	43702001	43706000	4000	1	4.00E-07	0.4	67	1.68	Vwa3b	
DMR9:43764001	9	43764001	43771000	7000	1	8.90E-07	0.63	91	1.3	Vwa3b	
DMR9:43984001	9	43984001	43987000	3000	1	9.50E-08	0.48	64	2.13	Inpp4a	
DMR9:44102001	9	44102001	44107000	5000	1	3.50E-07	0.37	109	2.18	Mgat4a	Transport
DMR9:44275001	9	44275001	44277000	2000	1	4.90E-07	0.42	28	1.4	RGD1310819	
DMR9:44985001	9	44985001	44987000	2000	1	2.70E-07	0.5	25	1.25	Aff3	Transcription
DMR9:45027001	9	45027001	45028000	1000	1	3.20E-09	0.45	17	1.7	Aff3	Transcription
DMR9:45083001	9	45083001	45088000	5000	1	4.00E-07	0.52	83	1.66	Aff3	Transcription
DMR9:45122001	9	45122001	45123000	1000	1	1.20E-07	0.47	10	1	Aff3	Transcription
DMR9:45256001	9	45256001	45261000	5000	1	8.20E-08	0.45	85	1.7	Aff3	Transcription
DMR9:46461001	9	46461001	46464000	3000	1	4.40E-07	0.52	61	2.03	Rfx8	Transcription

DMR9:46993001	9	46993001	46994000	1000	1	2.70E-07	0.41	20	2	Il1r1	Receptor
DMR9:47252001	9	47252001	47253000	1000	1	3.20E-07	0.41	13	1.3	Il18rap	Receptor
DMR9:49852001	9	49852001	49854000	2000	1	4.90E-10	0.56	28	1.4	Tgfbrap1	
DMR9:53168001	9	53168001	53170000	2000	1	3.30E-10	0.55	12	0.6	Pms1	Transcription
DMR9:53728001	9	53728001	53729000	1000	1	2.80E-07	0.41	20	2	Nemp2;LOC100912307	
DMR9:55527001	9	55527001	55531000	4000	1	2.50E-11	-0.47	42	1.05	Tmeff2	
DMR9:60049001	9	60049001	60053000	4000	1	1.70E-07	0.46	68	1.7	Slc39a10	Transport
DMR9:60230001	9	60230001	60233000	3000	1	4.80E-07	-0.34	51	1.7	Dnah7	Cytoskeleton
DMR9:60515001	9	60515001	60517000	2000	1	7.40E-10	0.59	24	1.2	Hecw2	Proteolysis
DMR9:61427001	9	61427001	61429000	2000	1	3.90E-08	0.4	37	1.85	Ankrd44;LOC108351932	Cytoskeleton
DMR9:63569001	9	63569001	63570000	1000	1	2.20E-12	0.68	15	1.5	Satb2	Epigenetic
DMR9:64740001	9	64740001	64742000	2000	1	4.70E-07	0.51	35	1.75	Spats2l	
DMR9:64812001	9	64812001	64813000	1000	1	1.50E-09	0.51	27	2.7	Spats2l	
DMR9:66339001	9	66339001	66341000	2000	1	1.60E-08	0.53	37	1.85	RGD1562029	
DMR9:67138001	9	67138001	67139000	1000	1	1.60E-07	0.44	26	2.6	Cyp20a1	Metabolism
DMR9:69386001	9	69386001	69387000	1000	1	1.50E-07	0.42	18	1.8	Pard3b	
DMR9:69425001	9	69425001	69428000	3000	1	4.20E-08	0.47	47	1.57	Pard3b	
DMR9:70170001	9	70170001	70171000	1000	1	8.80E-10	0.5	6	0.6	Adam23	Protease
DMR9:73446001	9	73446001	73447000	1000	1	1.20E-08	0.5	11	1.1	Map2	
DMR9:73624001	9	73624001	73625000	1000	1	2.30E-07	0.4	23	2.3	Unc80	
DMR9:74056001	9	74056001	74057000	1000	1	2.10E-11	0.67	6	0.6	Lancl1	
DMR9:74156001	9	74156001	74162000	6000	1	7.70E-07	0.65	83	1.38	Cps1	Metabolism
DMR9:75398001	9	75398001	75404000	6000	1	3.50E-08	-0.53	58	0.97	Erbb4	Receptor
DMR9:78932001	9	78932001	78937000	5000	1	2.80E-07	0.47	74	1.48	Fn1	Signaling
DMR9:79793001	9	79793001	79794000	1000	1	3.00E-08	0.57	10	1	LOC108351949;March4;LOC102555358	Proteolysis
DMR9:79827001	9	79827001	79829000	2000	1	2.10E-07	-0.43	24	1.2	March4;LOC102555358;LOC108351948	Proteolysis
DMR9:81204001	9	81204001	81209000	5000	1	3.70E-07	0.43	95	1.9	Tns1	Cytoskeleton
DMR9:81372001	9	81372001	81373000	1000	1	6.20E-07	0.47	15	1.5	Tns1	Cytoskeleton
DMR9:81633001	9	81633001	81635000	2000	1	2.30E-08	0.54	31	1.55	Pnkd;Catip	
DMR9:81843001	9	81843001	81845000	2000	1	9.00E-08	0.49	19	0.95	Plcd4;Zfp142	Metabolism;Transcription
DMR9:84316001	9	84316001	84317000	1000	1	3.60E-08	0.48	25	2.5	Sgpp2;Farsb	Signaling;Translation
DMR9:86317001	9	86317001	86320000	3000	1	5.70E-07	0.54	39	1.3	Dock10	
DMR9:88116001	9	88116001	88119000	3000	1	6.60E-11	0.55	36	1.2	Rhbdd1	Protease
DMR9:91978001	9	91978001	91980000	2000	2	2.20E-08	0.5	22	1.1	Dner	
DMR9:92511001	9	92511001	92512000	1000	1	4.30E-09	0.59	35	3.5	Slc16a14;LOC102550706	Transport
DMR9:93730001	9	93730001	93732000	2000	1	2.60E-08	0.77	27	1.35	Nppc	Hormone
DMR9:94101001	9	94101001	94102000	1000	1	2.80E-09	0.5	6	0.6	Dis3l2	Transcription
DMR9:94191001	9	94191001	94193000	2000	1	6.00E-07	0.5	48	2.4	Alppl2;Alpp;Alpi	Signaling
DMR9:94268001	9	94268001	94269000	1000	1	3.60E-08	0.57	15	1.5	Prss56	Protease
DMR9:96277001	9	96277001	96280000	3000	1	9.10E-08	0.55	109	3.63	Sh3bp4	
DMR9:96767001	9	96767001	96770000	3000	1	3.30E-07	0.67	72	2.4	Agap1	
DMR9:96793001	9	96793001	96797000	4000	1	8.60E-07	0.43	63	1.57	Agap1	
DMR9:96857001	9	96857001	96860000	3000	1	4.40E-09	0.61	42	1.4	Agap1	
DMR9:96897001	9	96897001	96898000	1000	1	3.90E-07	0.46	30	3	Agap1	
DMR9:97002001	9	97002001	97004000	2000	1	8.80E-07	0.48	27	1.35	Agap1	
DMR9:98113001	9	98113001	98115000	2000	1	2.90E-07	0.46	29	1.45	Mlph;Prlh;Rab17	Cytoskeleton
DMR9:98182001	9	98182001	98185000	3000	1	2.90E-08	0.48	38	1.27	Lrrfip1	Transcription
DMR9:98471001	9	98471001	98472000	1000	1	3.40E-09	0.5	18	1.8	Espnl	Cytoskeleton
DMR9:98576001	9	98576001	98579000	3000	1	2.70E-09	0.57	52	1.73	Per2	Transcription
DMR9:99057001	9	99057001	99059000	2000	1	1.30E-08	0.52	33	1.65	Hdac4	
DMR9:99157001	9	99157001	99159000	2000	1	1.90E-08	0.5	13	0.65	Hdac4	
DMR9:99590001	9	99590001	99593000	3000	1	4.20E-08	0.73	44	1.47	Ppp1r7	Signaling
DMR9:100085001	9	100085001	100087000	2000	1	2.80E-07	0.63	40	2	Ppp1r7;Dusp28;Rnpepl1	Signaling
DMR9:100169001	9	100169001	100177000	8000	2	1.10E-08	0.58	143	1.79	Ppp1r7;Aqp12a;Kif1a	Signaling;Cytoskeleton
DMR9:100220001	9	100220001	100224000	4000	1	1.70E-07	0.42	77	1.93	Ppp1r7;Kif1a	Signaling;Cytoskeleton
DMR9:100302001	9	100302001	100303000	1000	1	5.30E-11	0.61	13	1.3	Ppp1r7;RGD1563692	Signaling
DMR9:100354001	9	100354001	100357000	3000	2	2.50E-07	0.52	50	1.67	Ppp1r7;Crocc2	Signaling
DMR9:100853001	9	100853001	100858000	5000	1	3.30E-08	0.54	73	1.46	Thap4	
DMR9:105661001	9	105661001	105662000	1000	1	1.50E-07	0.46	16	1.6	RGD1560925	
DMR9:110188001	9	110188001	110190000	2000	1	6.20E-07	0.47	27	1.35	Efna5	Signaling
DMR9:110265001	9	110265001	110268000	3000	1	5.10E-07	0.37	52	1.73	Efna5	Signaling
DMR9:110760001	9	110760001	110762000	2000	1	4.90E-07	0.4	39	1.95	Fbxl17	Metabolism
DMR9:113002001	9	113002001	113003000	1000	1	1.60E-08	0.54	18	1.8	Tmem232	

DMR9:113005001	9	113005001	113007000	2000	1	2.00E-08	0.44	33	1.65	Tmem232	
DMR9:113078001	9	113078001	113081000	3000	1	6.00E-07	0.42	53	1.77	Tmem232	
DMR9:113267001	9	113267001	113269000	2000	1	2.60E-09	0.61	37	1.85	Tmem232;LOC103690585	
DMR9:113440001	9	113440001	113445000	5000	1	2.70E-07	0.4	154	3.08	Rab31	
DMR9:113658001	9	113658001	113660000	2000	1	2.10E-08	0.54	83	4.15	Ankrd12	
DMR9:113701001	9	113701001	113703000	2000	1	1.30E-07	0.75	35	1.75	Twsg1	
DMR9:114119001	9	114119001	114123000	4000	1	5.40E-07	0.44	47	1.18	LOC103693248;Ddx11	Epigenetic
DMR9:115046001	9	115046001	115048000	2000	1	9.00E-07	0.51	38	1.9	Ptprm	Signaling
DMR9:115314001	9	115314001	115316000	2000	1	1.40E-07	0.49	40	2	Ptprm	Signaling
DMR9:115351001	9	115351001	115353000	2000	1	3.80E-07	0.38	36	1.8	Ptprm	Signaling
DMR9:116066001	9	116066001	116067000	1000	1	2.60E-08	0.45	6	0.6	Arhgap28	Signaling
DMR9:116138001	9	116138001	116140000	2000	1	4.40E-08	0.52	32	1.6	Arhgap28	Signaling
DMR9:117335001	9	117335001	117337000	2000	1	3.10E-07	0.48	33	1.65	Epb41l3	
DMR9:118530001	9	118530001	118531000	1000	1	5.10E-10	0.55	6	0.6	Dlgap1	Cytoskeleton
DMR9:118904001	9	118904001	118907000	3000	1	1.80E-07	0.41	55	1.83	Dlgap1	Cytoskeleton
DMR9:121978001	9	121978001	121979000	1000	1	2.80E-08	-1.09	3	0.3	Clul1;LOC102550127	
DMR10:3018001	10	3018001	3024000	6000	1	4.80E-07	0.4	82	1.37	Shisa9	
DMR10:3204001	10	3204001	3205000	1000	1	6.50E-07	0.39	11	1.1	Rrn3	Transcription
DMR10:3900001	10	3900001	3903000	3000	1	7.50E-07	0.37	34	1.13	Snx29	Cytoskeleton
DMR10:4010001	10	4010001	4016000	6000	1	3.30E-07	0.47	62	1.03	Snx29;LOC108352047	Cytoskeleton
DMR10:4088001	10	4088001	4091000	3000	1	6.40E-10	0.8	28	0.93	Snx29;LOC102554761	Cytoskeleton
DMR10:5087001	10	5087001	5089000	2000	1	9.90E-07	0.55	20	1	Clec16a	
DMR10:5246001	10	5246001	5247000	1000	1	5.90E-09	0.71	14	1.4	Ciita	
DMR10:7300001	10	7300001	7302000	2000	1	4.60E-08	0.42	23	1.15	Tmem114;LOC102546970	
DMR10:8996001	10	8996001	8997000	1000	1	3.50E-08	0.54	9	0.9	Rbfox1	Translation
DMR10:9241001	10	9241001	9242000	1000	1	9.70E-07	0.53	9	0.9	Rbfox1;LOC108352195;LOC108352196	Translation
DMR10:9959001	10	9959001	9962000	3000	1	1.60E-07	0.5	43	1.43	Rbfox1	Translation
DMR10:10256001	10	10256001	10258000	2000	1	8.20E-08	0.46	24	1.2	Rbfox1	Translation
DMR10:10640001	10	10640001	10642000	2000	1	5.90E-08	0.64	29	1.45	Ppl	Cytoskeleton
DMR10:10669001	10	10669001	10677000	8000	1	6.00E-07	0.47	178	2.22	Ppl	Cytoskeleton
DMR10:10703001	10	10703001	10707000	4000	1	1.60E-07	0.43	39	0.98	Ubn1	Cytoskeleton
DMR10:10763001	10	10763001	10766000	3000	1	2.90E-07	0.49	49	1.63	Glyr1;Rogdi;Smim22;Sept12	Metabolism;Transcription
DMR10:11122001	10	11122001	11124000	2000	1	3.00E-08	0.52	63	3.15	Coro7;Vasn	Cytoskeleton;Receptor
DMR10:11156001	10	11156001	11158000	2000	1	4.10E-07	0.54	35	1.75	Pam16;Glis2	Transport;Transcription
DMR10:11159001	10	11159001	11164000	5000	1	6.50E-07	0.41	81	1.62	Pam16;Glis2	Transport;Transcription
DMR10:11493001	10	11493001	11498000	5000	1	1.20E-07	0.46	89	1.78	Adcy9	
DMR10:11798001	10	11798001	11803000	5000	1	4.00E-09	0.46	94	1.88	Slx4;LOC108352058;Nlrc3	Cytoskeleton
DMR10:11810001	10	11810001	11813000	3000	1	1.90E-09	0.5	65	2.17	Slx4;LOC108352058;Nlrc3	Cytoskeleton
DMR10:11840001	10	11840001	11842000	2000	1	4.10E-10	0.68	26	1.3	Nlrc3;Cluap1	Cytoskeleton
DMR10:11848001	10	11848001	11852000	4000	1	3.70E-08	0.59	51	1.27	Nlrc3;Cluap1	Cytoskeleton
DMR10:12031001	10	12031001	12033000	2000	2	3.90E-09	0.66	29	1.45	Zfp263	Transcription
DMR10:12637001	10	12637001	12638000	1000	1	1.70E-07	-0.67	6	0.6	Olr1374;Olr1375	Receptor
DMR10:13157001	10	13157001	13160000	3000	1	4.40E-07	0.49	111	3.7	Srrm2;Tceb2	
DMR10:13593001	10	13593001	13596000	3000	1	5.30E-10	0.51	59	1.97	LOC100158225;LOC102551327;Ccnf	Signaling
DMR10:13597001	10	13597001	13599000	2000	1	4.20E-07	0.55	50	2.5	LOC100158225;LOC102551327;Ccnf	Signaling
DMR10:13641001	10	13641001	13644000	3000	1	5.50E-07	0.48	45	1.5	Abca17	Transport
DMR10:13812001	10	13812001	13817000	5000	2	2.60E-08	0.58	125	2.5	Eci1;E4f1;Dnase1l2	Metabolism;Transcription
DMR10:13833001	10	13833001	13835000	2000	1	5.10E-07	-0.67	21	1.05	E4f1;Pgp;Mlst8;Bricd5	Transcription;Signaling
DMR10:13841001	10	13841001	13844000	3000	1	5.30E-08	0.46	69	2.3	E4f1;Pgp;Mlst8;Bricd5;Caskin1	Transcription;Signaling;Cytoskeleton
DMR10:13864001	10	13864001	13871000	7000	1	6.80E-07	0.51	171	2.44	Caskin1;Traf7	Cytoskeleton
DMR10:13879001	10	13879001	13889000	10000	1	2.70E-07	0.55	134	1.34	Caskin1;Traf7;LOC102551976;Rab26	Cytoskeleton
DMR10:14083001	10	14083001	14085000	2000	1	8.40E-08	0.46	42	2.1	Rnf151;Rps2;Ndufb10;Rpl3l	Translation;Metabolism
DMR10:14467001	10	14467001	14469000	2000	1	5.90E-09	0.49	41	2.05	Ift140;Telo2	Development
DMR10:14525001	10	14525001	14529000	4000	2	5.20E-08	0.56	51	1.27	Clcn7;Ccdc154;LOC103693290;LOC685273	Transport
DMR10:14732001	10	14732001	14739000	7000	1	8.00E-08	0.46	179	2.56	Tpsb2;Tpsg1;Cacna1h	Protease;Transport
DMR10:14762001	10	14762001	14766000	4000	1	8.40E-15	0.55	55	1.38	Cacna1h	Transport
DMR10:14849001	10	14849001	14851000	2000	2	4.10E-07	0.53	27	1.35	Sstr5	Signaling
DMR10:15170001	10	15170001	15173000	3000	1	5.20E-08	0.45	70	2.33	Ccdc78;Fam173a;Metrn;LOC108352064;Fbxl16	
DMR10:15207001	10	15207001	15209000	2000	2	1.60E-10	0.6	46	2.3	Jmjd8;Stub1;Rhbdl1;Rhot2;Wdr90	Golgi;Proteolysis;Signaling
DMR10:15259001	10	15259001	15264000	5000	1	6.60E-13	0.65	44	0.88	Mettl26;Rab40c	
DMR10:15918001	10	15918001	15919000	1000	1	2.00E-10	0.54	24	2.4	Nsg2	
DMR10:16863001	10	16863001	16866000	3000	1	5.30E-09	0.55	54	1.8	Ergic1;LOC108352069	

DMR10:16922001	10	16922001	16929000	7000	1	6.30E-07	0.5	115	1.64	Ergic1;LOC102552476	
DMR10:17085001	10	17085001	17090000	5000	1	1.00E-07	0.47	84	1.68	Neurl1b;LOC102555877	Proteolysis
DMR10:17238001	10	17238001	17240000	2000	1	1.50E-07	0.54	38	1.9	Sh3pxd2b	
DMR10:17266001	10	17266001	17271000	5000	1	3.30E-08	0.52	91	1.82	Sh3pxd2b	
DMR10:17426001	10	17426001	17429000	3000	1	9.00E-07	0.45	46	1.53	Stk10	
DMR10:17458001	10	17458001	17467000	9000	1	6.30E-08	0.65	128	1.42	Stk10	
DMR10:18653001	10	18653001	18655000	2000	1	3.30E-08	0.56	35	1.75	Kcnip1	
DMR10:18753001	10	18753001	18755000	2000	1	4.00E-08	0.64	34	1.7	Kcnip1	
DMR10:18797001	10	18797001	18800000	3000	1	5.60E-08	0.8	55	1.83	Kcnip1	
DMR10:19515001	10	19515001	19519000	4000	1	3.80E-07	0.41	59	1.48	Dock2	
DMR10:19541001	10	19541001	19543000	2000	1	5.40E-10	0.77	24	1.2	Dock2	
DMR10:19700001	10	19700001	19701000	1000	1	5.10E-07	0.41	20	2	RGD1564301	
DMR10:20005001	10	20005001	20006000	1000	1	8.40E-07	0.47	9	0.9	Slit3	
DMR10:20257001	10	20257001	20259000	2000	1	3.20E-08	0.63	31	1.55	Slit3	
DMR10:20679001	10	20679001	20681000	2000	1	7.70E-07	0.45	48	2.4	Wwc1	
DMR10:20688001	10	20688001	20689000	1000	1	6.90E-07	0.53	32	3.2	Wwc1	
DMR10:20870001	10	20870001	20873000	3000	1	4.60E-09	0.46	45	1.5	Tenm2	
DMR10:21057001	10	21057001	21059000	2000	1	4.30E-07	0.45	48	2.4	Tenm2	
DMR10:21134001	10	21134001	21138000	4000	1	9.80E-07	0.42	52	1.3	Tenm2	
DMR10:21202001	10	21202001	21205000	3000	1	3.50E-07	0.44	50	1.67	Tenm2	
DMR10:21757001	10	21757001	21759000	2000	1	1.00E-07	0.44	28	1.4	Tenm2	
DMR10:28852001	10	28852001	28853000	1000	1	3.70E-07	0.74	45	4.5	Atp10b	Transport
DMR10:29137001	10	29137001	29143000	6000	1	5.10E-07	0.39	118	1.97	Ccnjl	Signaling
DMR10:29150001	10	29150001	29151000	1000	1	8.00E-10	0.58	17	1.7	Ccnjl	Signaling
DMR10:29170001	10	29170001	29172000	2000	1	4.40E-07	0.48	26	1.3	Ccnjl	Signaling
DMR10:29333001	10	29333001	29334000	1000	1	3.90E-07	0.47	13	1.3	Pwwp2a;Ttc1	Epigenetic
DMR10:29376001	10	29376001	29377000	1000	1	5.00E-08	0.54	15	1.5	Ttc1	
DMR10:30052001	10	30052001	30053000	1000	1	6.60E-08	0.44	19	1.9	Il12b	Receptor
DMR10:31317001	10	31317001	31320000	3000	1	1.10E-07	0.53	60	2	Cyfip2;Fndc9	Cytoskeleton
DMR10:31334001	10	31334001	31337000	3000	1	5.70E-07	0.42	51	1.7	Cyfip2;Fndc9	Cytoskeleton
DMR10:31392001	10	31392001	31394000	2000	1	2.30E-08	0.58	89	4.45	Cyfip2;LOC108352178	Cytoskeleton
DMR10:35452001	10	35452001	35455000	3000	1	2.80E-07	0.48	44	1.47	Rasgef1c	Transcription
DMR10:35630001	10	35630001	35631000	1000	1	1.90E-07	0.46	11	1.1	Tbc1d9b	Signaling
DMR10:37221001	10	37221001	37222000	1000	1	4.80E-07	0.42	11	1.1	Sec24a;Sar1b	Transport
DMR10:37255001	10	37255001	37257000	2000	1	1.20E-10	0.58	26	1.3	Sar1b	
DMR10:37280001	10	37280001	37281000	1000	1	1.90E-11	0.72	27	2.7	Jade2	Transcription
DMR10:37634001	10	37634001	37642000	8000	1	3.20E-07	0.51	123	1.54	Tcf7	Transcription
DMR10:38529001	10	38529001	38532000	3000	1	8.80E-07	0.41	54	1.8	Fstl4;LOC108352083	Protease; Proteolysis
DMR10:39238001	10	39238001	39239000	1000	1	5.00E-07	0.39	34	3.4	Slc22a5	Transport
DMR10:39402001	10	39402001	39403000	1000	1	5.40E-08	0.48	12	1.2	Pdlim4	Cytoskeleton
DMR10:39714001	10	39714001	39717000	3000	1	1.80E-07	0.44	48	1.6	Acsl6;Meikin	Metabolism
DMR10:40292001	10	40292001	40293000	1000	1	9.40E-08	0.69	7	0.7	Tnip1	
DMR10:42894001	10	42894001	42897000	3000	1	1.30E-07	0.5	47	1.57	Fam114a2	
DMR10:43202001	10	43202001	43204000	2000	1	1.30E-12	0.68	37	1.85	Galnt10	Golgi
DMR10:43812001	10	43812001	43813000	1000	1	8.30E-07	0.4	32	3.2	Lypd8	Cytoskeleton
DMR10:44661001	10	44661001	44668000	7000	1	3.20E-08	-0.56	30	0.43	Olr1449;RGD1559534	Receptor
DMR10:45382001	10	45382001	45389000	7000	1	2.20E-07	0.51	149	2.13	Obscn	
DMR10:45949001	10	45949001	45951000	2000	1	6.70E-07	0.54	34	1.7	Olr1463;LOC691571	Receptor
DMR10:46023001	10	46023001	46026000	3000	1	1.90E-07	0.48	15	0.5	Mprip	Cytoskeleton
DMR10:46510001	10	46510001	46514000	4000	1	5.80E-08	0.45	162	4.05	Rai1	Transcription
DMR10:46536001	10	46536001	46541000	5000	1	8.40E-07	0.43	57	1.14	Rai1	Transcription
DMR10:46871001	10	46871001	46875000	4000	1	6.20E-08	0.54	62	1.55	Myo15a	Cytoskeleton
DMR10:47645001	10	47645001	47646000	1000	1	5.00E-07	0.5	21	2.1	Slc47a1	Transport
DMR10:47977001	10	47977001	47978000	1000	1	7.50E-09	0.61	15	1.5	Slc5a10;Fam83g	Transport
DMR10:48558001	10	48558001	48565000	7000	1	2.70E-13	0.59	102	1.46	Adora2b	Signaling
DMR10:49312001	10	49312001	49316000	4000	1	7.10E-08	0.52	63	1.57	Tvp23b	
DMR10:49388001	10	49388001	49389000	1000	1	6.00E-09	0.49	14	1.4	Cdrt4	
DMR10:50441001	10	50441001	50443000	2000	1	1.90E-07	0.53	29	1.45	Cox10	Metabolism
DMR10:50483001	10	50483001	50485000	2000	1	3.10E-07	0.51	29	1.45	Cox10	Metabolism
DMR10:52470001	10	52470001	52472000	2000	1	2.40E-07	0.39	22	1.1	Dnah9	Cytoskeleton
DMR10:53015001	10	53015001	53016000	1000	1	3.60E-07	-0.59	16	1.6	Shisa6	
DMR10:53495001	10	53495001	53498000	3000	1	8.40E-08	0.54	34	1.13	Pirt;LOC102554819	
DMR10:53619001	10	53619001	53621000	2000	1	5.50E-08	0.57	25	1.25	Myh3	
DMR10:53626001	10	53626001	53627000	1000	1	2.60E-07	0.5	19	1.9	Myh3	
DMR10:54154001	10	54154001	54157000	3000	2	2.30E-12	0.66	36	1.2	Gas7	Cytoskeleton

DMR10:54500001	10	54500001	54502000	2000	1	6.90E-09	0.51	29	1.45	Cfap52	
DMR10:54937001	10	54937001	54939000	2000	1	1.20E-07	0.54	24	1.2	Ntn1	Extracellular Matrix
DMR10:54972001	10	54972001	54973000	1000	1	7.30E-07	0.44	25	2.5	Ntn1	Extracellular Matrix
DMR10:55370001	10	55370001	55374000	4000	1	6.90E-08	0.49	63	1.57	Myh10	
DMR10:55544001	10	55544001	55546000	2000	1	1.50E-07	0.41	35	1.75	Arhgef15;Slc25a35	Transcription
DMR10:56006001	10	56006001	56010000	4000	1	8.30E-07	0.49	108	2.7	Cyb5d1;Naa38;Tmem88;Kdm6b	Translation;Epigenetic
DMR10:56177001	10	56177001	56181000	4000	1	3.30E-07	0.44	54	1.35	Efnb3;Wrap53;Tp53	Signaling;Transcription
DMR10:56354001	10	56354001	56357000	3000	1	3.70E-07	0.51	41	1.37	Polr2a;Slc35g3	Transcription;Transport
DMR10:56553001	10	56553001	56557000	4000	1	3.90E-08	0.6	61	1.52	Ybx2;Slc2a4;LOC102555366	
DMR10:58809001	10	58809001	58810000	1000	1	9.70E-09	0.56	15	1.5	Slc13a5	Transport
DMR10:59012001	10	59012001	59015000	3000	2	6.70E-09	0.57	36	1.2	Mybbp1a;Spns2	Epigenetic;Transport
DMR10:59600001	10	59600001	59601000	1000	1	3.70E-08	0.48	15	1.5	Camkk1	Signaling
DMR10:61278001	10	61278001	61280000	2000	1	1.90E-08	0.49	30	1.5	Rap1gap2	Signaling
DMR10:61304001	10	61304001	61306000	2000	1	3.10E-09	0.54	24	1.2	Rap1gap2	Signaling
DMR10:61342001	10	61342001	61346000	4000	1	2.10E-09	0.72	60	1.5	Rap1gap2	Signaling
DMR10:61718001	10	61718001	61724000	6000	1	1.20E-07	0.5	95	1.58	Sgsm2	Signaling
DMR10:61755001	10	61755001	61757000	2000	1	8.50E-09	0.54	30	1.5	Sgsm2;Tsr1;Srr	Signaling;Metabolism
DMR10:62269001	10	62269001	62271000	2000	1	4.70E-07	0.61	29	1.45	Serpinf2;Wdr81	Protease; Proteolysis
DMR10:62272001	10	62272001	62273000	1000	1	1.80E-09	0.58	14	1.4	Serpinf2;Wdr81	Protease; Proteolysis
DMR10:62780001	10	62780001	62781000	1000	1	5.20E-13	0.59	16	1.6	Ssh2	Signaling
DMR10:63212001	10	63212001	63216000	4000	1	8.80E-07	0.45	46	1.15	Blmh	Protease
DMR10:63804001	10	63804001	63811000	7000	1	3.60E-07	0.47	99	1.41	Inpp5k;Myo1c	Signaling;Cytoskeleton
DMR10:64060001	10	64060001	64064000	4000	1	3.00E-07	0.45	65	1.62	Rph3al;LOC102549419	
DMR10:64107001	10	64107001	64112000	5000	1	1.50E-07	0.64	64	1.28	Rph3al	
DMR10:64433001	10	64433001	64435000	2000	1	2.50E-08	0.49	26	1.3	Nxn	Metabolism
DMR10:64461001	10	64461001	64465000	4000	1	1.10E-07	0.5	51	1.27	Nxn	Metabolism
DMR10:64599001	10	64599001	64606000	7000	1	1.70E-13	0.62	122	1.74	Abr	Signaling
DMR10:64656001	10	64656001	64658000	2000	1	1.90E-07	0.5	44	2.2	Abr	Signaling
DMR10:64979001	10	64979001	64980000	1000	1	5.90E-07	0.51	17	1.7	LOC103693392;Myo18a	
DMR10:65412001	10	65412001	65415000	3000	1	3.00E-10	0.53	63	2.1	Fam222b;Nek8;Traf4	Signaling;Cytoskeleton
DMR10:66778001	10	66778001	66779000	1000	1	4.40E-08	0.62	13	1.3	Nf1	Signaling
DMR10:67023001	10	67023001	67025000	2000	1	7.40E-07	0.45	24	1.2	Rab11fip4	
DMR10:67714001	10	67714001	67715000	1000	1	1.30E-09	0.52	12	1.2	Rhbdl3	
DMR10:68347001	10	68347001	68348000	1000	1	3.50E-08	0.48	18	1.8	Asic2	Transport
DMR10:68379001	10	68379001	68382000	3000	1	7.40E-10	0.51	39	1.3	Asic2	Transport
DMR10:68565001	10	68565001	68569000	4000	1	3.30E-11	0.63	33	0.82	Asic2;LOC688465	Transport
DMR10:68571001	10	68571001	68572000	1000	1	1.00E-08	0.58	15	1.5	Asic2	Transport
DMR10:68779001	10	68779001	68780000	1000	1	5.10E-08	0.47	18	1.8	Asic2	Transport
DMR10:69785001	10	69785001	69787000	2000	1	4.30E-08	0.51	36	1.8	Tmem132e	
DMR10:70221001	10	70221001	70223000	2000	2	6.20E-08	0.53	36	1.8	Rffl;Rad51d	Proteolysis;Transcription
DMR10:70449001	10	70449001	70451000	2000	1	1.60E-07	0.45	27	1.35	Slfn3	
DMR10:70747001	10	70747001	70749000	2000	1	5.00E-07	0.54	38	1.9	Ccl5	Growth Factors
DMR10:71175001	10	71175001	71177000	2000	1	2.10E-09	0.57	26	1.3	Hnf1b	Transcription
DMR10:71578001	10	71578001	71581000	3000	1	4.40E-07	0.49	27	0.9	Acaca	
DMR10:74553001	10	74553001	74554000	1000	1	3.40E-08	0.47	22	2.2	Trim37;Ppm1e	Proteolysis;Signaling
DMR10:74792001	10	74792001	74793000	1000	1	5.20E-09	0.55	14	1.4	Tex14	
DMR10:75004001	10	75004001	75007000	3000	1	4.30E-07	0.47	54	1.8	Rnf43	
DMR10:75063001	10	75063001	75066000	3000	1	3.90E-07	0.5	49	1.63	Tspoap1	
DMR10:75186001	10	75186001	75188000	2000	2	1.30E-09	0.58	23	1.15	Olr1521;Olr1522	Receptor
DMR10:75618001	10	75618001	75620000	2000	1	4.40E-07	0.49	24	1.2	Ccdc182	
DMR10:75815001	10	75815001	75817000	2000	1	4.30E-07	0.61	33	1.65	Msi2	
DMR10:76152001	10	76152001	76154000	2000	1	9.80E-08	0.66	38	1.9	Akap1	Cytoskeleton
DMR10:77738001	10	77738001	77739000	1000	1	2.90E-08	0.46	14	1.4	Mmd	Signaling
DMR10:77759001	10	77759001	77760000	1000	1	5.70E-07	0.37	21	2.1	Mmd	Signaling
DMR10:80823001	10	80823001	80824000	1000	1	7.70E-07	0.44	5	0.5	Car10	
DMR10:81129001	10	81129001	81132000	3000	1	4.00E-10	0.6	33	1.1	Car10	
DMR10:82224001	10	82224001	82225000	1000	1	1.20E-07	0.51	14	1.4	Spata20;LOC102555637;Epn3;Mycbpap	
DMR10:82262001	10	82262001	82263000	1000	1	2.10E-07	0.45	13	1.3	Mycbpap;LOC108352211;Rsad1	
DMR10:82743001	10	82743001	82744000	1000	1	6.60E-08	0.52	17	1.7	LOC102556395;Col1a1	Extracellular Matrix
DMR10:82846001	10	82846001	82847000	1000	1	8.30E-07	0.47	15	1.5	Samd14;Pdk2;Itga3	Signaling;Extracellular Matrix
DMR10:83074001	10	83074001	83076000	2000	1	2.10E-08	0.51	33	1.65	LOC103693422;Tac4	
DMR10:83634001	10	83634001	83635000	1000	1	1.10E-08	0.63	13	1.3	LOC102547047;Phospho1;Abi3	Signaling;Cytoskeleton
DMR10:84433001	10	84433001	84436000	3000	1	3.30E-08	0.6	64	2.13	Skap1	Cytoskeleton

DMR10:84484001	10	84484001	84486000	2000	1	7.70E-07	0.41	35	1.75	Skap1	Cytoskeleton
DMR10:85134001	10	85134001	85136000	2000	1	5.90E-07	0.5	28	1.4	Kpnb1;Npepps	Transport;Protease
DMR10:85457001	10	85457001	85469000	12000	2	3.00E-07	0.5	235	1.96	Srcin1	
DMR10:85649001	10	85649001	85652000	3000	1	1.80E-07	0.46	45	1.5	Pcgf2;Trnan-guu;Psmb3;Pip4k2b	Protease;Signaling
DMR10:85883001	10	85883001	85887000	4000	1	1.60E-08	0.46	53	1.32	Plxdc1	
DMR10:85902001	10	85902001	85906000	4000	1	8.10E-07	0.43	77	1.93	Plxdc1	
DMR10:86351001	10	86351001	86352000	1000	1	2.30E-10	0.66	14	1.4	Pnmt;Pgap3	Epigenetic
DMR10:86797001	10	86797001	86800000	3000	1	1.00E-08	0.6	23	0.77	Wipf2	Cytoskeleton
DMR10:86863001	10	86863001	86865000	2000	1	2.20E-08	0.49	35	1.75	Rara	Transcription
DMR10:86958001	10	86958001	86961000	3000	1	7.00E-08	0.44	67	2.23	Igfbp4	Protease; Proteolysis
DMR10:87283001	10	87283001	87285000	2000	1	1.80E-07	0.43	31	1.55	Krt28;LOC108352132	
DMR10:87299001	10	87299001	87301000	2000	1	7.30E-07	0.44	45	2.25	LOC108352132;Krt10	
DMR10:88091001	10	88091001	88095000	4000	1	1.10E-07	0.44	76	1.9	LOC108352134;Krt9	
DMR10:88157001	10	88157001	88166000	9000	1	7.10E-08	0.53	154	1.71	Krt16;Krt17;Krt42	
DMR10:88187001	10	88187001	88188000	1000	1	1.60E-07	0.44	25	2.5	Krt42	
DMR10:88477001	10	88477001	88478000	1000	1	5.70E-07	0.49	17	1.7	Ttc25	
DMR10:88556001	10	88556001	88558000	2000	1	8.90E-07	0.41	37	1.85	Zfp385c	
DMR10:88591001	10	88591001	88592000	1000	1	3.40E-09	0.53	18	1.8	Zfp385c;Dhx58	
DMR10:88598001	10	88598001	88600000	2000	1	1.10E-10	0.56	24	1.2	Zfp385c;Dhx58	
DMR10:88611001	10	88611001	88618000	7000	1	2.50E-07	0.47	157	2.24	Dhx58;Kat2a;Hspb9;Rab5c	
DMR10:88952001	10	88952001	88957000	5000	1	8.20E-10	0.56	82	1.64	Atp6v0a1	Metabolism
DMR10:89035001	10	89035001	89038000	3000	1	8.50E-07	0.45	72	2.4	Fam134c;Tubg1	Cytoskeleton
DMR10:89190001	10	89190001	89191000	1000	1	2.40E-10	0.57	33	3.3	Vps25;Wnk4;LOC103693430;Coa3;Cntd1	Transport;Signaling;Transcription
DMR10:90739001	10	90739001	90741000	2000	1	6.20E-07	0.56	36	1.8	Adam11;LOC102547852	Protease
DMR10:90769001	10	90769001	90772000	3000	1	3.00E-08	0.46	64	2.13	LOC102550942;Gjc1	Cytoskeleton
DMR10:91018001	10	91018001	91020000	2000	1	7.00E-11	0.53	42	2.1	Kif18b	Cytoskeleton
DMR10:91161001	10	91161001	91165000	4000	1	5.60E-07	0.61	61	1.52	Nmt1;Plcd3	Transport;Metabolism
DMR10:91184001	10	91184001	91185000	1000	1	1.10E-08	0.45	16	1.6	Plcd3;Acbd4	Metabolism;Transport
DMR10:91416001	10	91416001	91419000	3000	1	2.30E-07	0.45	60	2	Arhgap27	Signaling
DMR10:91787001	10	91787001	91791000	4000	1	1.20E-11	0.62	77	1.93	Wnt9b	Signaling
DMR10:91928001	10	91928001	91932000	4000	1	7.60E-08	0.42	67	1.68	Nsf	Transport
DMR10:91956001	10	91956001	91958000	2000	1	3.20E-07	0.48	44	2.2	Nsf	Transport
DMR10:94225001	10	94225001	94228000	3000	1	1.40E-09	0.54	46	1.53	Kcnh6;Dcaf7	Transport
DMR10:94521001	10	94521001	94524000	3000	1	1.30E-07	0.59	54	1.8	Scn4a;LOC108352146	Transport
DMR10:94580001	10	94580001	94582000	2000	1	4.40E-07	0.37	49	2.45	Icam2;Ern1	Translation
DMR10:94706001	10	94706001	94710000	4000	1	2.70E-07	0.46	84	2.1	LOC102546698;Tex2	
DMR10:94732001	10	94732001	94739000	7000	1	1.60E-09	0.6	102	1.46	Tex2	
DMR10:94897001	10	94897001	94901000	4000	1	2.70E-11	0.5	63	1.57	Pecam1	Immune
DMR10:95489001	10	95489001	95491000	2000	1	1.50E-07	0.43	42	2.1	Pitpnc1	
DMR10:95504001	10	95504001	95505000	1000	1	5.10E-09	0.52	27	2.7	Pitpnc1;LOC102549520	
DMR10:95613001	10	95613001	95614000	1000	1	8.50E-07	0.41	19	1.9	Pitpnc1	
DMR10:95952001	10	95952001	95955000	3000	1	4.00E-07	0.48	78	2.6	Cacng4	Transport
DMR10:95993001	10	95993001	95997000	4000	1	1.60E-07	0.47	70	1.75	Cacng4;LOC108352150	Transport
DMR10:96472001	10	96472001	96474000	2000	1	1.10E-07	0.45	23	1.15	Prkca	Signaling
DMR10:96515001	10	96515001	96516000	1000	1	2.80E-11	0.61	15	1.5	Prkca	Signaling
DMR10:96733001	10	96733001	96735000	2000	1	2.00E-07	0.39	37	1.85	Cep112	
DMR10:97218001	10	97218001	97237000	19000	2	4.80E-07	0.46	444	2.34	Axin2	Cytoskeleton
DMR10:98004001	10	98004001	98007000	3000	1	9.20E-07	0.43	33	1.1	Fam20a	
DMR10:98500001	10	98500001	98502000	2000	1	9.60E-07	0.72	16	0.8	Abca6	Transport
DMR10:101688001	10	101688001	101691000	3000	1	1.20E-07	0.45	55	1.83	LOC102549836;Mir297	
DMR10:101803001	10	101803001	101806000	3000	1	3.30E-09	0.64	54	1.8	Slc39a11	Transport
DMR10:101888001	10	101888001	101889000	1000	1	4.10E-07	0.45	25	2.5	Slc39a11;LOC108352158	Transport
DMR10:101917001	10	101917001	101923000	6000	1	5.30E-08	0.58	110	1.83	Slc39a11	Transport
DMR10:101955001	10	101955001	101958000	3000	1	5.70E-09	0.48	41	1.37	Slc39a11	Transport
DMR10:102110001	10	102110001	102112000	2000	1	7.50E-07	0.5	41	2.05	Slc39a11	Transport
DMR10:102321001	10	102321001	102323000	2000	1	1.30E-07	0.46	32	1.6	Sdk2	
DMR10:102530001	10	102530001	102532000	2000	1	5.80E-07	0.52	39	1.95	Sdk2;LOC102547687	
DMR10:102542001	10	102542001	102544000	2000	1	6.80E-07	0.46	43	2.15	Sdk2;LOC102547687	
DMR10:102567001	10	102567001	102568000	1000	1	3.00E-07	0.6	12	1.2	Sdk2	
DMR10:103277001	10	103277001	103280000	3000	1	2.60E-08	0.56	66	2.2	Dnai2	Cytoskeleton
DMR10:103715001	10	103715001	103719000	4000	1	2.20E-07	0.5	61	1.52	Rab37;Slc9a3r1;LOC103693478	
DMR10:103732001	10	103732001	103734000	2000	1	9.90E-09	0.5	31	1.55	Slc9a3r1;LOC103693478;Nat9;Tmem104	
DMR10:103787001	10	103787001	103789000	2000	1	4.20E-07	0.49	48	2.4	Tmem104;LOC102554289;Grin2c	Receptor

DMR10:103792001	10	103792001	103794000	2000	1	3.60E-07	0.53	67	3.35	Tmem104;LOC102554289;Grin2c	Receptor
DMR10:104138001	10	104138001	104142000	4000	1	6.60E-07	0.55	69	1.73	Nup85;Gga3	Development
DMR10:104338001	10	104338001	104343000	5000	1	9.60E-07	0.45	111	2.22	Mir3577;Caskin2	Cytoskeleton
DMR10:104349001	10	104349001	104352000	3000	1	3.30E-08	0.5	58	1.93	Mir3577;Caskin2;Tsen54	Cytoskeleton;Translation
DMR10:104506001	10	104506001	104508000	2000	1	7.00E-07	0.6	26	1.3	Sap30bp	Transcription
DMR10:104557001	10	104557001	104561000	4000	1	2.50E-07	0.52	111	2.78	Itgb4;Galk1	Extracellular Matrix;Metabolism
DMR10:104629001	10	104629001	104632000	3000	2	6.70E-13	0.78	56	1.87	Unc13d;Wbp2;LOC102552044	
DMR10:104648001	10	104648001	104653000	5000	1	6.00E-07	0.44	82	1.64	LOC102552044;Trim47	
DMR10:105100001	10	105100001	105105000	5000	1	2.40E-07	0.68	161	3.22	Ten1;Evpl	Cytoskeleton
DMR10:105106001	10	105106001	105110000	4000	1	7.40E-07	0.64	91	2.28	Evpl	Cytoskeleton
DMR10:105111001	10	105111001	105115000	4000	1	1.20E-07	0.43	88	2.2	Evpl;Srp68	Cytoskeleton;Metabolism
DMR10:105163001	10	105163001	105166000	3000	1	1.50E-07	0.62	80	2.67	Galr2;LOC100911734	Signaling
DMR10:105281001	10	105281001	105284000	3000	1	1.50E-07	0.65	69	2.3	Foxj1;LOC108352160;Rnf157	Proteolysis
DMR10:105337001	10	105337001	105339000	2000	1	8.40E-08	0.55	34	1.7	Rnf157	Proteolysis
DMR10:105401001	10	105401001	105408000	7000	1	7.60E-08	0.49	198	2.83	Ubald2;Qrich2	
DMR10:105577001	10	105577001	105584000	7000	1	1.20E-07	0.51	190	2.71	Aanat;Rhbdf2	Metabolism;Protease
DMR10:105622001	10	105622001	105628000	6000	2	5.20E-08	0.45	155	2.58	Cygb;Prcd	
DMR10:105755001	10	105755001	105757000	2000	1	1.20E-09	0.65	45	2.25	Mxra7;LOC103693479	
DMR10:105906001	10	105906001	105909000	3000	1	4.20E-09	0.94	23	0.77	Mgat5b	Golgi
DMR10:106101001	10	106101001	106108000	7000	1	3.60E-08	0.5	147	2.1	Sec14l1;LOC102547461	
DMR10:106707001	10	106707001	106710000	3000	1	1.80E-11	0.64	64	2.13	Tnrc6c	Metabolism
DMR10:106787001	10	106787001	106789000	2000	1	1.50E-07	0.61	53	2.65	Tmc6;Tmc8	
DMR10:106792001	10	106792001	106795000	3000	1	8.10E-07	0.5	87	2.9	Tmc6;Tmc8;LOC688282	Immune
DMR10:107012001	10	107012001	107014000	2000	1	9.40E-08	0.64	34	1.7	Pgs1	Transport
DMR10:107023001	10	107023001	107027000	4000	1	2.90E-07	0.45	58	1.45	Pgs1;Dnah17	Transport;Cytoskeleton
DMR10:107053001	10	107053001	107054000	1000	1	9.20E-09	0.54	35	3.5	Dnah17	Cytoskeleton
DMR10:107077001	10	107077001	107082000	5000	1	3.40E-08	0.45	138	2.76	Dnah17	Cytoskeleton
DMR10:107096001	10	107096001	107098000	2000	1	1.80E-08	0.47	41	2.05	Dnah17	Cytoskeleton
DMR10:107119001	10	107119001	107121000	2000	2	1.50E-07	0.54	43	2.15	Dnah17	Cytoskeleton
DMR10:107302001	10	107302001	107305000	3000	1	1.50E-09	0.53	49	1.63	Usp36	Protease
DMR10:107368001	10	107368001	107374000	6000	1	6.70E-07	0.52	124	2.07	Timp2;Cep295nl	Protease; Proteolysis
DMR10:107533001	10	107533001	107540000	7000	1	5.70E-09	0.53	159	2.27	Rbfox3	Translation
DMR10:107541001	10	107541001	107545000	4000	1	8.60E-07	0.43	63	1.57	Rbfox3	Translation
DMR10:107790001	10	107790001	107794000	4000	1	3.20E-08	0.51	82	2.05	Rbfox3	Translation
DMR10:108385001	10	108385001	108386000	1000	1	1.90E-07	0.51	14	1.4	Ccdc40;Gaa	Metabolism
DMR10:108387001	10	108387001	108393000	6000	1	5.00E-07	0.53	114	1.9	Ccdc40;Gaa	Metabolism
DMR10:108397001	10	108397001	108399000	2000	1	1.30E-07	0.46	44	2.2	Ccdc40;Gaa	Metabolism
DMR10:108648001	10	108648001	108649000	1000	1	1.10E-07	0.46	18	1.8	Endov	
DMR10:108864001	10	108864001	108866000	2000	1	6.40E-08	0.53	37	1.85	Rptor	
DMR10:108952001	10	108952001	108954000	2000	1	3.50E-07	0.5	35	1.75	Rptor	
DMR10:109075001	10	109075001	109079000	4000	1	9.50E-07	0.42	80	2	LOC108352229;Chmp6	Transport
DMR10:109196001	10	109196001	109200000	4000	1	1.80E-07	0.45	89	2.22	Baiap2;Aatk;Mir3065;Mir338	Cytoskeleton;Signaling
DMR10:109415001	10	109415001	109416000	1000	1	6.90E-08	0.49	12	1.2	Bahcc1	Transcription
DMR10:109477001	10	109477001	109483000	6000	1	7.20E-08	0.55	170	2.83	Bahcc1	Transcription
DMR10:109632001	10	109632001	109635000	3000	1	1.20E-07	0.47	54	1.8	Pde6g;Oxld1;Ccdc137;Arl16;Hgs	Signaling;Metabolism
DMR10:109754001	10	109754001	109755000	1000	1	9.80E-07	0.51	14	1.4	P4hb;LOC108352167;Arhgdia	Transcription;Signaling
DMR10:109819001	10	109819001	109822000	3000	1	2.70E-07	0.62	61	2.03	Mafg;Pycr1;Myadml2	Transcription;Metabolism
DMR10:110146001	10	110146001	110149000	3000	1	3.70E-08	0.54	80	2.67	Slc16a3;Csnk1d	Transport;Signaling
DMR10:111429001	10	111429001	111436000	7000	1	8.80E-07	-0.46	79	1.13	Vom2r-ps3	
DMR11:3142001	11	3142001	3144000	2000	1	9.60E-07	0.39	21	1.05	Vgll3	Transcription
DMR11:9713001	11	9713001	9715000	2000	1	2.10E-08	0.59	24	1.2	Robo1	
DMR11:10089001	11	10089001	10094000	5000	1	2.00E-07	0.52	77	1.54	Robo1	
DMR11:10107001	11	10107001	10112000	5000	1	7.50E-09	0.51	97	1.94	Robo1	
DMR11:10133001	11	10133001	10136000	3000	1	4.60E-07	0.46	36	1.2	Robo1	
DMR11:11330001	11	11330001	11331000	1000	1	1.30E-07	0.66	11	1.1	Robo2	
DMR11:16843001	11	16843001	16845000	2000	1	6.50E-08	0.57	31	1.55	Cxadr	
DMR11:20021001	11	20021001	20022000	1000	1	9.40E-09	-0.81	6	0.6	Ncam2	
DMR11:24440001	11	24440001	24442000	2000	1	1.60E-07	0.45	34	1.7	App	Protease; Proteolysis
DMR11:27020001	11	27020001	27022000	2000	1	7.00E-07	0.4	34	1.7	N6amt1;Ltn1	Epigenetic;Proteolysis
DMR11:30596001	11	30596001	30601000	5000	1	1.90E-07	0.45	83	1.66	Hunk	
DMR11:30658001	11	30658001	30664000	6000	1	3.20E-07	0.45	120	2	Hunk	
DMR11:30954001	11	30954001	30957000	3000	1	6.20E-07	0.54	33	1.1	Urb1	
DMR11:31817001	11	31817001	31822000	5000	1	1.20E-07	0.49	75	1.5	Son	Translation
DMR11:31884001	11	31884001	31886000	2000	1	1.50E-11	0.7	27	1.35	Cryzl1	Metabolism

DMR11:32001001	11	32001001	32006000	5000	1	2.50E-07	0.44	71	1.42	Itsn1	Transport
DMR11:32258001	11	32258001	32261000	3000	1	3.30E-08	0.59	36	1.2	Mrps6	Translation
DMR11:32500001	11	32500001	32501000	1000	1	1.80E-08	0.73	25	2.5	Kcne1;LOC108352307	Transport
DMR11:32673001	11	32673001	32678000	5000	1	2.10E-08	0.5	88	1.76	Clic6	Transport
DMR11:33569001	11	33569001	33574000	5000	1	2.80E-07	0.55	110	2.2	LOC102549086;Mir802	
DMR11:33940001	11	33940001	33943000	3000	1	2.20E-09	0.47	33	1.1	Dopey2;LOC103693520	
DMR11:33945001	11	33945001	33949000	4000	1	8.10E-07	0.53	63	1.57	Dopey2;LOC103693520	
DMR11:35592001	11	35592001	35593000	1000	1	7.30E-07	0.42	8	0.8	Kcnj15	Transport
DMR11:35639001	11	35639001	35641000	2000	1	3.10E-07	0.46	37	1.85	Erg	Transcription
DMR11:36560001	11	36560001	36561000	1000	1	1.60E-07	0.52	14	1.4	Sh3bgr	
DMR11:37513001	11	37513001	37515000	2000	1	6.00E-08	0.52	25	1.25	Dscam	Cytoskeleton
DMR11:37790001	11	37790001	37791000	1000	1	1.40E-10	0.53	15	1.5	Bace2	Protease
DMR11:42843001	11	42843001	42845000	2000	2	1.90E-07	0.6	30	1.5	Epha6	Receptor
DMR11:43131001	11	43131001	43137000	6000	1	6.10E-07	-0.41	60	1	Olr1528	Signaling
DMR11:44996001	11	44996001	44999000	3000	1	5.50E-08	-0.61	43	1.43	Col8a1	Extracellular Matrix
DMR11:45907001	11	45907001	45915000	8000	1	1.40E-07	0.39	241	3.01	Lnp1;LOC103693553	
DMR11:46237001	11	46237001	46238000	1000	1	5.70E-07	0.45	10	1	Abi3bp	
DMR11:47043001	11	47043001	47045000	2000	1	7.40E-08	0.48	22	1.1	Senp7;LOC102552047;Trmt10c	Protease;Epigenetic
DMR11:50993001	11	50993001	50996000	3000	1	2.90E-07	-0.73	29	0.97	Alcam	Immune
DMR11:53007001	11	53007001	53008000	1000	1	2.10E-08	0.57	14	1.4	Bbx	Transcription
DMR11:54381001	11	54381001	54382000	1000	1	3.50E-07	0.49	13	1.3	RGD1310335	
DMR11:61136001	11	61136001	61137000	1000	1	5.90E-07	0.45	15	1.5	Boc	
DMR11:61450001	11	61450001	61452000	2000	1	1.20E-07	0.57	9	0.45	Usf3	
DMR11:62085001	11	62085001	62086000	1000	1	2.20E-07	0.48	11	1.1	Zbtb20	Transcription
DMR11:62123001	11	62123001	62124000	1000	1	1.40E-07	0.46	13	1.3	Zbtb20	Transcription
DMR11:62483001	11	62483001	62485000	2000	1	5.50E-15	0.39	19	0.95	Zbtb20	Transcription
DMR11:62486001	11	62486001	62488000	2000	1	7.10E-13	0.33	25	1.25	Zbtb20	Transcription
DMR11:64618001	11	64618001	64620000	2000	1	8.90E-08	0.42	26	1.3	Arhgap31	Signaling
DMR11:64682001	11	64682001	64684000	2000	1	1.10E-07	0.48	24	1.2	Arhgap31	Signaling
DMR11:64804001	11	64804001	64807000	3000	1	8.00E-08	0.65	32	1.07	Timmdc1;Cd80	Immune
DMR11:64862001	11	64862001	64866000	4000	1	1.20E-08	0.53	79	1.98	Cd80;LOC108352341;Adprh	Immune;Metabolism
DMR11:64961001	11	64961001	64964000	3000	1	2.90E-21	1.99	26	0.87	Popdc2;Cox17	Transcription
DMR11:65017001	11	65017001	65019000	2000	1	1.00E-08	0.56	26	1.3	Maats1;Nr1i2	Transcription
DMR11:65020001	11	65020001	65023000	3000	1	2.70E-09	0.64	41	1.37	Maats1;Nr1i2	Transcription
DMR11:65984001	11	65984001	65985000	1000	1	4.10E-07	0.48	16	1.6	Hgd	Metabolism
DMR11:66509001	11	66509001	66510000	1000	1	1.10E-07	-0.62	3	0.3	Stxbp5l	Transport
DMR11:66605001	11	66605001	66607000	2000	1	6.00E-07	0.47	30	1.5	Polq	Transcription
DMR11:66954001	11	66954001	66956000	2000	1	3.10E-07	0.49	26	1.3	Slc15a2	Transport
DMR11:67082001	11	67082001	67085000	3000	1	7.70E-09	0.51	47	1.57	Cd86	Immune
DMR11:68412001	11	68412001	68413000	1000	1	2.20E-09	0.51	14	1.4	Sema5b	Signaling
DMR11:68569001	11	68569001	68573000	4000	1	1.20E-07	0.47	84	2.1	Pdia5	Transcription
DMR11:69127001	11	69127001	69128000	1000	1	1.80E-09	0.5	33	3.3	Mylk	
DMR11:69483001	11	69483001	69486000	3000	1	1.80E-10	0.56	53	1.77	Kalrn	Transcription
DMR11:69828001	11	69828001	69831000	3000	1	1.70E-07	0.52	44	1.47	Kalrn	Transcription
DMR11:69953001	11	69953001	69959000	6000	1	1.30E-10	0.54	104	1.73	Kalrn	Transcription
DMR11:70059001	11	70059001	70062000	3000	1	5.40E-07	0.42	56	1.87	LOC108352345;Itgb5	
DMR11:70074001	11	70074001	70075000	1000	1	5.60E-07	0.55	10	1	Itgb5;LOC108352346	
DMR11:70089001	11	70089001	70090000	1000	1	5.40E-07	0.4	18	1.8	Itgb5;LOC108352346	
DMR11:70256001	11	70256001	70260000	4000	1	4.50E-07	0.62	54	1.35	Heg1	
DMR11:70535001	11	70535001	70536000	1000	1	3.80E-07	0.47	9	0.9	Zfp148	
DMR11:71034001	11	71034001	71037000	3000	1	2.20E-07	0.44	52	1.73	Lrch3	
DMR11:71171001	11	71171001	71175000	4000	1	8.80E-07	0.4	85	2.12	Rubcn	
DMR11:71351001	11	71351001	71356000	5000	1	1.20E-07	0.54	106	2.12	Tnk2	
DMR11:71532001	11	71532001	71533000	1000	1	7.80E-07	0.38	11	1.1	Zdhhc19;Slc51a	Transport
DMR11:72580001	11	72580001	72586000	6000	2	9.70E-09	0.68	92	1.53	Bdh1	Metabolism
DMR11:72602001	11	72602001	72605000	3000	1	7.70E-07	0.37	96	3.2	Bdh1	Metabolism
DMR11:73097001	11	73097001	73098000	1000	1	6.60E-13	0.76	8	0.8	Acap2	
DMR11:73754001	11	73754001	73755000	1000	1	1.10E-13	0.65	16	1.6	Tmem44;LOC102548792	
DMR11:74755001	11	74755001	74757000	2000	1	7.60E-08	0.52	39	1.95	Opa1	Transport
DMR11:75500001	11	75500001	75502000	2000	1	5.50E-07	0.47	30	1.5	Mb21d2	
DMR11:75597001	11	75597001	75598000	1000	1	8.20E-07	0.5	13	1.3	Fgf12	Growth Factors
DMR11:75661001	11	75661001	75663000	2000	1	1.90E-07	0.46	21	1.05	Fgf12	Growth Factors
DMR11:79509001	11	79509001	79511000	2000	1	5.10E-11	0.74	30	1.5	Lpp	Signaling
DMR11:81386001	11	81386001	81388000	2000	1	9.00E-07	0.59	29	1.45	Eif4a2	
DMR11:81447001	11	81447001	81449000	2000	1	6.50E-09	0.34	21	1.05	Kng1l1	

DMR11:82162001	11	82162001	82163000	1000	1	3.80E-07	0.42	25	2.5	Dgkg	Signaling
DMR11:82251001	11	82251001	82254000	3000	1	1.10E-07	0.43	62	2.07	Etv5;Trnam-cau	Transcription
DMR11:83128001	11	83128001	83131000	3000	1	3.50E-07	0.39	37	1.23	Vps8;LOC108352385	Cytoskeleton
DMR11:83528001	11	83528001	83535000	7000	1	1.90E-08	0.45	214	3.06	Ephb3	Receptor
DMR11:83947001	11	83947001	83952000	5000	2	7.40E-09	0.61	77	1.54	Psmd2;LOC102551589;Ece2	Protease;Protease
DMR11:84332001	11	84332001	84333000	1000	1	6.10E-09	0.58	19	1.9	Map6d1;Yeats2	Transcription
DMR11:85161001	11	85161001	85163000	2000	1	7.10E-08	-0.69	12	0.6	Olr1565;LOC363825	Receptor
DMR11:86909001	11	86909001	86916000	7000	1	2.30E-08	0.61	166	2.37	Zdhhc8;Ccdc188;LOC102555338	
DMR11:86985001	11	86985001	86995000	10000	1	2.50E-07	0.61	215	2.15	Rtn4r	
DMR11:87556001	11	87556001	87560000	4000	2	1.10E-09	0.55	73	1.82	Smpd4;Ccdc74a;Med15	Signaling
DMR11:87563001	11	87563001	87566000	3000	1	7.70E-10	0.59	52	1.73	Ccdc74a;Med15	
DMR11:87880001	11	87880001	87882000	2000	1	1.70E-07	0.47	27	1.35	Pi4ka	Signaling
DMR11:87984001	11	87984001	87986000	2000	1	5.60E-08	0.52	24	1.2	Pi4ka;Tmem191c;Hic2	Signaling;Transcription
DMR11:88162001	11	88162001	88164000	2000	1	2.50E-08	0.58	30	1.5	Ppil2;Ypel1	Transcription
DMR11:88332001	11	88332001	88333000	1000	1	2.40E-07	0.41	10	1	Ppm1f	Signaling
DMR12:1070001	12	1070001	1074000	4000	1	2.10E-07	0.47	66	1.65	Stard13	Signaling
DMR12:1502001	12	1502001	1503000	1000	1	1.40E-07	0.46	31	3.1	LOC103693612;LOC102554571;Rn5s	
DMR12:1993001	12	1993001	1999000	6000	2	4.70E-09	0.56	149	2.48	Arhgef18;Pex11g	
DMR12:2132001	12	2132001	2133000	1000	1	5.90E-07	0.45	12	1.2	Camsap3	
DMR12:2155001	12	2155001	2158000	3000	1	9.90E-10	0.61	61	2.03	Camsap3;Xab2	Translation
DMR12:2660001	12	2660001	2661000	1000	1	4.20E-13	0.78	12	1.2	LOC103690865;Cd209f	Transport
DMR12:5033001	12	5033001	5034000	1000	1	7.60E-07	-0.76	9	0.9	Vom2r-ps91	
DMR12:5484001	12	5484001	5486000	2000	1	4.00E-07	0.43	39	1.95	Zfp958	Transcription
DMR12:5659001	12	5659001	5662000	3000	1	6.90E-07	0.45	62	2.07	Fry	Cytoskeleton
DMR12:5669001	12	5669001	5670000	1000	1	8.00E-07	0.6	21	2.1	Fry	Cytoskeleton
DMR12:6887001	12	6887001	6888000	1000	1	5.80E-08	0.58	18	1.8	Alox5ap	Transport
DMR12:7546001	12	7546001	7547000	1000	1	2.70E-10	0.7	37	3.7	Katnal1;LOC102555153	Cytoskeleton
DMR12:8117001	12	8117001	8119000	2000	1	1.80E-07	0.57	30	1.5	Slc7a1;Mtus2	Transport
DMR12:9768001	12	9768001	9777000	9000	2	6.80E-09	0.79	197	2.19	Lnx2	
DMR12:10337001	12	10337001	10338000	1000	1	8.00E-07	0.46	19	1.9	Wasf3	Cytoskeleton
DMR12:11278001	12	11278001	11280000	2000	1	9.70E-08	0.5	23	1.15	Arpc1a	Cytoskeleton
DMR12:11493001	12	11493001	11498000	5000	1	2.70E-07	0.51	90	1.8	Smurf1	Proteolysis
DMR12:12711001	12	12711001	12719000	8000	2	3.80E-09	0.6	109	1.36	Rsph10b;Pms2	Transcription
DMR12:12886001	12	12886001	12889000	3000	1	6.20E-09	0.6	61	2.03	Usp42;Cyth3	Protease;Transcription
DMR12:12890001	12	12890001	12891000	1000	1	5.80E-09	0.59	25	2.5	Cyth3	Transcription
DMR12:13311001	12	13311001	13316000	5000	1	6.80E-07	0.43	100	2	LOC683674;LOC108352423;Zfp853	Transcription
DMR12:13394001	12	13394001	13395000	1000	1	4.40E-08	0.4	13	1.3	Zfp12;Spdye4	
DMR12:13859001	12	13859001	13864000	5000	1	9.50E-09	0.65	108	2.16	Tnrc18	Transcription
DMR12:13894001	12	13894001	13901000	7000	1	9.80E-07	0.47	133	1.9	Tnrc18	Transcription
DMR12:13905001	12	13905001	13907000	2000	1	3.90E-10	0.59	33	1.65	Tnrc18;Slc29a4	Transcription;Transport
DMR12:13921001	12	13921001	13924000	3000	1	1.30E-07	0.45	52	1.73	Tnrc18;Slc29a4	Transcription;Transport
DMR12:14217001	12	14217001	14219000	2000	1	7.90E-09	0.77	53	2.65	Foxk1;LOC103693620	
DMR12:14547001	12	14547001	14551000	4000	1	2.30E-07	0.42	76	1.9	Sdk1	
DMR12:15021001	12	15021001	15025000	4000	1	1.40E-07	0.42	55	1.38	Sdk1	
DMR12:15150001	12	15150001	15155000	5000	1	1.90E-08	0.52	94	1.88	Sdk1	
DMR12:15337001	12	15337001	15339000	2000	1	3.60E-08	0.51	21	1.05	Sdk1;LOC108352493	
DMR12:16006001	12	16006001	16013000	7000	1	3.00E-08	0.55	118	1.69	Amz1;Brat1	
DMR12:16108001	12	16108001	16111000	3000	1	3.70E-07	0.44	67	2.23	Ttyh3;Lfng	Transport;Golgi
DMR12:16729001	12	16729001	16731000	2000	1	6.00E-07	0.57	41	2.05	Mad1l1	
DMR12:17318001	12	17318001	17319000	1000	1	3.20E-09	0.71	13	1.3	LOC498154;Gper1;LOC102546864	Signaling
DMR12:17443001	12	17443001	17445000	2000	1	1.10E-09	0.53	46	2.3	Adap1	
DMR12:17617001	12	17617001	17621000	4000	1	2.30E-07	0.48	54	1.35	Prkar1b	Signaling
DMR12:17696001	12	17696001	17699000	3000	1	6.20E-10	0.7	52	1.73	Prkar1b	Signaling
DMR12:18100001	12	18100001	18102000	2000	1	1.40E-07	0.66	17	0.85	RGD1566386	Transcription
DMR12:18532001	12	18532001	18534000	2000	2	3.40E-08	-0.88	92	4.6	Asmt;Akap17a;Asmtl;Il3ra	Epigenetic
DMR12:21426001	12	21426001	21429000	3000	1	9.80E-07	0.64	27	0.9	Zcwpw1;LOC102549330	
DMR12:22241001	12	22241001	22244000	3000	1	1.80E-07	0.5	66	2.2	Gnb2;Gigyf1	Signaling
DMR12:22467001	12	22467001	22471000	4000	1	1.20E-07	0.48	81	2.02	Srrt;Ufsp1;Ache	Metabolism
DMR12:22535001	12	22535001	22537000	2000	1	6.20E-14	0.75	18	0.9	Muc3	
DMR12:23448001	12	23448001	23449000	1000	1	4.00E-07	0.41	14	1.4	Cux1	Development
DMR12:23879001	12	23879001	23881000	2000	1	2.40E-07	0.46	30	1.5	Srrm3	
DMR12:24290001	12	24290001	24293000	3000	1	4.40E-07	0.97	68	2.27	Hip1	Cytoskeleton
DMR12:24677001	12	24677001	24681000	4000	1	5.10E-10	0.53	87	2.17	Dnajc30;Wbscr22;Stx1a	Transcription;Transcription
DMR12:24809001	12	24809001	24813000	4000	1	3.50E-08	0.52	76	1.9	Wbscr28	
DMR12:24971001	12	24971001	24972000	1000	1	4.80E-07	0.5	20	2	LOC102553484;Eln	Development

DMR12:25021001	12	25021001	25022000	1000	1	2.20E-08	0.48	22	2.2	Eln	Development
DMR12:25025001	12	25025001	25028000	3000	1	2.50E-11	0.61	33	1.1	Eln;Limk1	Development
DMR12:25039001	12	25039001	25041000	2000	1	8.80E-08	0.56	31	1.55	Limk1	
DMR12:25046001	12	25046001	25050000	4000	1	4.60E-07	0.51	62	1.55	Limk1	
DMR12:25497001	12	25497001	25499000	2000	1	1.20E-08	0.5	37	1.85	Gtf2i;LOC103691302;Ncf1	Transcription
DMR12:29623001	12	29623001	29624000	1000	1	4.40E-07	0.46	15	1.5	Caln1;LOC108352439	
DMR12:29896001	12	29896001	29898000	2000	1	4.30E-08	0.47	38	1.9	Tyw1;LOC103691321	Metabolism
DMR12:30095001	12	30095001	30098000	3000	1	4.80E-07	0.42	74	2.47	Tpst1	Transport
DMR12:30132001	12	30132001	30137000	5000	1	9.30E-10	0.55	89	1.78	Crcp	Signaling
DMR12:30138001	12	30138001	30140000	2000	1	1.30E-07	0.38	31	1.55	Crcp	Signaling
DMR12:31184001	12	31184001	31189000	5000	1	4.20E-07	0.42	102	2.04	Adgrd1	Signaling
DMR12:31207001	12	31207001	31209000	2000	1	1.30E-08	0.51	44	2.2	Adgrd1	Signaling
DMR12:31935001	12	31935001	31938000	3000	1	7.70E-08	0.43	53	1.77	Tmem132d	
DMR12:31942001	12	31942001	31943000	1000	1	8.80E-07	0.41	21	2.1	Tmem132d	
DMR12:36398001	12	36398001	36399000	1000	1	3.50E-07	0.46	23	2.3	Tmem132b	
DMR12:36984001	12	36984001	36995000	11000	1	3.40E-07	0.48	268	2.44	Ncor2	Epigenetic
DMR12:37018001	12	37018001	37022000	4000	2	5.30E-07	0.45	111	2.78	Ncor2	Epigenetic
DMR12:37030001	12	37030001	37035000	5000	1	8.00E-08	0.61	143	2.86	Ncor2;Fam101a	Epigenetic
DMR12:37039001	12	37039001	37041000	2000	2	4.30E-10	0.58	61	3.05	Ncor2;Fam101a	Epigenetic
DMR12:37751001	12	37751001	37754000	3000	1	5.30E-08	0.51	55	1.83	Mphosph9	
DMR12:37807001	12	37807001	37810000	3000	1	4.90E-10	0.51	59	1.97	Pitpnm2	Transport
DMR12:37873001	12	37873001	37884000	11000	1	1.00E-08	0.58	177	1.61	Pitpnm2	Transport
DMR12:37893001	12	37893001	37905000	12000	1	4.60E-08	0.57	300	2.5	Pitpnm2;Arl6ip4;Ogfod2;LOC103691351	Transport;Metabolism
DMR12:38012001	12	38012001	38017000	5000	1	1.10E-08	0.45	112	2.24	Vps37b;Hip1r	Cytoskeleton
DMR12:38486001	12	38486001	38488000	2000	1	1.00E-07	0.44	22	1.1	Vps33a;Diablo	Transport
DMR12:38718001	12	38718001	38719000	1000	1	7.50E-07	0.45	18	1.8	Wdr66	
DMR12:38769001	12	38769001	38771000	2000	1	1.50E-09	0.59	33	1.65	Wdr66	
DMR12:38835001	12	38835001	38837000	2000	1	3.80E-07	0.38	33	1.65	LOC102547495;Hpd;LOC100359816	Metabolism
DMR12:38844001	12	38844001	38848000	4000	1	6.00E-07	0.48	107	2.67	Hpd;LOC100359816	Metabolism
DMR12:38890001	12	38890001	38894000	4000	1	2.50E-07	0.52	98	2.45	Rhof;Tmem120b	Signaling
DMR12:39134001	12	39134001	39137000	3000	1	3.30E-09	0.51	35	1.17	Kdm2b	
DMR12:39222001	12	39222001	39224000	2000	1	5.30E-07	0.48	44	2.2	Anapc5	
DMR12:39262001	12	39262001	39264000	2000	1	9.10E-07	0.44	29	1.45	Camkk2	
DMR12:39303001	12	39303001	39306000	3000	1	4.30E-07	0.39	68	2.27	Camkk2;P2rx4	Ion Channel
DMR12:39387001	12	39387001	39388000	1000	1	7.50E-08	0.48	16	1.6	P2rx7	Ion Channel
DMR12:39652001	12	39652001	39655000	3000	1	1.60E-07	0.5	43	1.43	Arpc3;LOC102548903;LOC108352447	Cytoskeleton
DMR12:39869001	12	39869001	39871000	2000	1	5.90E-07	0.45	42	2.1	Ppp1cc	Signaling
DMR12:40110001	12	40110001	40111000	1000	1	7.90E-10	0.51	16	1.6	Cux2	Development
DMR12:40115001	12	40115001	40116000	1000	1	7.10E-07	0.46	28	2.8	Cux2	Development
DMR12:40193001	12	40193001	40196000	3000	1	2.90E-08	0.52	53	1.77	Cux2	Development
DMR12:40199001	12	40199001	40205000	6000	1	5.60E-07	0.51	113	1.88	Cux2	Development
DMR12:40705001	12	40705001	40706000	1000	1	5.10E-07	0.54	14	1.4	Trafd1;Hectd4	Proteolysis
DMR12:40950001	12	40950001	40955000	5000	1	1.50E-08	0.68	75	1.5	Ptpn11	Signaling
DMR12:41119001	12	41119001	41122000	3000	1	1.60E-09	0.5	61	2.03	Rph3a	
DMR12:41363001	12	41363001	41364000	1000	1	1.30E-07	0.58	12	1.2	Oas3;Oas2	Metabolism
DMR12:41401001	12	41401001	41404000	3000	1	4.70E-10	0.51	42	1.4	Dtx1	Proteolysis
DMR12:41474001	12	41474001	41480000	6000	1	1.80E-07	0.52	130	2.17	Cfap73;Ddx54;Rita1	
DMR12:41518001	12	41518001	41523000	5000	2	9.70E-09	0.57	83	1.66	Tpcn1	Transport
DMR12:42085001	12	42085001	42088000	3000	1	1.90E-07	0.46	56	1.87	Tbx5;LOC102549962	Transcription
DMR12:43424001	12	43424001	43430000	6000	1	1.30E-08	0.68	104	1.73	Med13l	
DMR12:43432001	12	43432001	43436000	4000	1	8.60E-07	0.35	92	2.3	Med13l	
DMR12:43438001	12	43438001	43441000	3000	1	4.10E-07	0.47	49	1.63	Med13l	
DMR12:43560001	12	43560001	43561000	1000	1	4.90E-11	0.65	21	2.1	Med13l	
DMR12:43967001	12	43967001	43972000	5000	1	3.80E-07	0.47	99	1.98	Rnft2;LOC102553921	
DMR12:44073001	12	44073001	44076000	3000	1	3.60E-08	0.48	47	1.57	Fbxw8	
DMR12:44129001	12	44129001	44132000	3000	1	1.90E-07	0.5	43	1.43	Fbxw8;Tesc	
DMR12:44225001	12	44225001	44229000	4000	2	1.10E-08	0.61	63	1.57	Nos1	Metabolism
DMR12:44256001	12	44256001	44263000	7000	1	1.80E-07	0.67	108	1.54	Nos1;Fbxo21	Metabolism
DMR12:44643001	12	44643001	44644000	1000	1	9.10E-07	0.41	21	2.1	Ksr2	Signaling
DMR12:44762001	12	44762001	44763000	1000	1	3.70E-10	0.56	21	2.1	Ksr2	Signaling
DMR12:44983001	12	44983001	44987000	4000	1	4.20E-07	0.48	80	2	Vsig10	
DMR12:45030001	12	45030001	45033000	3000	1	3.10E-07	0.4	69	2.3	Pebp1;Taok3	Protease; Proteolysis;Signaling
DMR12:45043001	12	45043001	45044000	1000	1	3.30E-09	0.5	19	1.9	Taok3	Signaling

DMR12:45081001	12	45081001	45084000	3000	1	3.10E-07	0.54	55	1.83	Taok3	Signaling
DMR12:45215001	12	45215001	45216000	1000	1	8.50E-08	0.48	33	3.3	Suds3	
DMR12:46093001	12	46093001	46094000	1000	1	6.70E-09	0.51	23	2.3	Ccdc60	
DMR12:46103001	12	46103001	46106000	3000	1	9.90E-07	0.46	61	2.03	Ccdc60	
DMR12:46171001	12	46171001	46173000	2000	1	6.00E-11	0.69	32	1.6	Ccdc60	
DMR12:46321001	12	46321001	46327000	6000	1	3.00E-07	0.5	141	2.35	Prkab1;LOC108352463;Cit	Signaling;Signaling
DMR12:46379001	12	46379001	46381000	2000	1	8.10E-11	0.62	32	1.6	Cit	Signaling
DMR12:46438001	12	46438001	46441000	3000	1	1.90E-07	0.47	53	1.77	Cit	Signaling
DMR12:46659001	12	46659001	46660000	1000	1	8.30E-09	0.51	18	1.8	Bicdl1	Transport
DMR12:46705001	12	46705001	46712000	7000	1	9.10E-07	0.51	159	2.27	Bicdl1;Rab35	Transport
DMR12:46730001	12	46730001	46741000	11000	1	2.90E-07	0.49	294	2.67	Gcn1l1	
DMR12:46769001	12	46769001	46776000	7000	1	5.10E-07	0.43	176	2.51	Gcn1l1	
DMR12:46777001	12	46777001	46779000	2000	1	1.50E-07	0.5	38	1.9	Gcn1l1	
DMR12:46782001	12	46782001	46784000	2000	1	8.00E-09	0.5	45	2.25	Gcn1l1;Rplp0	Translation
DMR12:47052001	12	47052001	47055000	3000	1	6.60E-07	0.4	51	1.7	Srsf9	Translation
DMR12:47130001	12	47130001	47132000	2000	1	3.10E-07	0.56	41	2.05	Rnf10;Pop5	Transport
DMR12:47299001	12	47299001	47300000	1000	1	5.10E-08	0.51	25	2.5	Sppl3	Proteolysis
DMR12:47349001	12	47349001	47351000	2000	1	3.40E-08	0.65	29	1.45	Sppl3	Proteolysis
DMR12:47637001	12	47637001	47642000	5000	1	5.20E-08	0.43	96	1.92	Git2;Tchp	
DMR12:47743001	12	47743001	47753000	10000	2	1.00E-09	0.52	248	2.48	Trpv4;Fam222a	Transport
DMR12:47937001	12	47937001	47940000	3000	1	6.00E-07	0.39	71	2.37	Mmab;Ube3b	Metabolism;Proteolysis
DMR12:47956001	12	47956001	47960000	4000	1	8.00E-07	0.48	104	2.6	Ube3b	Proteolysis
DMR12:48011001	12	48011001	48014000	3000	2	2.10E-08	0.45	88	2.93	Kctd10;LOC108352465	Cytoskeleton
DMR12:48127001	12	48127001	48136000	9000	2	3.50E-09	0.7	190	2.11	Foxn4;Acacb	
DMR12:48148001	12	48148001	48158000	10000	1	1.80E-09	0.56	207	2.07	Acacb	
DMR12:48164001	12	48164001	48167000	3000	1	9.90E-07	0.56	49	1.63	Acacb	
DMR12:48440001	12	48440001	48444000	4000	1	5.10E-09	0.54	79	1.98	Ssh1	Signaling
DMR12:48552001	12	48552001	48557000	5000	2	6.40E-09	0.52	109	2.18	Coro1c;LOC100361749	Cytoskeleton
DMR12:48839001	12	48839001	48846000	7000	1	2.90E-09	0.66	116	1.66	Wscd2	
DMR12:48849001	12	48849001	48851000	2000	1	2.10E-07	0.52	29	1.45	Wscd2	
DMR12:49333001	12	49333001	49334000	1000	1	5.00E-08	0.64	34	3.4	Sgsm1	Signaling
DMR12:49484001	12	49484001	49486000	2000	1	2.50E-07	0.7	48	2.4	RGD1306556	
DMR12:49487001	12	49487001	49489000	2000	1	8.50E-08	0.52	28	1.4	RGD1306556	
DMR12:49499001	12	49499001	49502000	3000	1	4.00E-11	0.5	64	2.13	RGD1306556;LOC102552141	
DMR12:49655001	12	49655001	49658000	3000	1	1.70E-08	0.86	38	1.27	Grk3	Signaling
DMR12:49812001	12	49812001	49814000	2000	1	3.40E-08	0.57	12	0.6	Myo18b	
DMR12:50369001	12	50369001	50372000	3000	1	9.10E-08	0.52	39	1.3	Tpst2	Transport
DMR12:51212001	12	51212001	51215000	3000	1	2.10E-07	0.46	69	2.3	Mn1	
DMR12:51395001	12	51395001	51403000	8000	1	5.30E-09	0.57	178	2.22	Ttc28	Cytoskeleton
DMR12:51442001	12	51442001	51446000	4000	1	3.80E-08	0.57	91	2.28	Ttc28	Cytoskeleton
DMR12:51571001	12	51571001	51575000	4000	1	3.10E-08	0.54	64	1.6	Ttc28;LOC108352474	Cytoskeleton
DMR12:51655001	12	51655001	51657000	2000	1	2.00E-07	0.39	37	1.85	Ttc28	Cytoskeleton
DMR12:51751001	12	51751001	51752000	1000	1	7.70E-20	0.73	20	2	Ttc28	Cytoskeleton
DMR12:52018001	12	52018001	52019000	1000	1	1.30E-07	0.5	20	2	Ep400	
DMR12:52305001	12	52305001	52313000	8000	1	2.20E-08	0.57	125	1.56	Fbrsl1	
DMR12:52361001	12	52361001	52363000	2000	1	9.80E-07	0.5	28	1.4	Fbrsl1	
DMR13:540001	13	540001	541000	1000	1	2.00E-08	-0.65	9	0.9	Cntnap5a;LOC102546561	
DMR13:6774001	13	6774001	6780000	6000	1	1.10E-07	0.48	200	3.33	Cntnap5c	
DMR13:7517001	13	7517001	7519000	2000	1	4.40E-21	0.52	17	0.85	Cntnap5c	
DMR13:25203001	13	25203001	25204000	1000	1	2.40E-08	0.5	13	1.3	Rnf152	
DMR13:25521001	13	25521001	25523000	2000	1	1.10E-08	0.46	12	0.6	Pign	Extracellular Matrix
DMR13:25608001	13	25608001	25609000	1000	1	2.30E-07	0.47	14	1.4	Pign	Extracellular Matrix
DMR13:26223001	13	26223001	26224000	1000	1	5.60E-07	0.45	8	0.8	Phlpp1	Cytoskeleton
DMR13:26740001	13	26740001	26743000	3000	3	2.90E-09	0.68	96	3.2	Bcl2	
DMR13:34830001	13	34830001	34834000	4000	1	6.90E-08	0.59	121	3.02	LOC679673;Gli2	Transcription
DMR13:34942001	13	34942001	34943000	1000	1	7.80E-07	0.62	12	1.2	Gli2	Transcription
DMR13:35010001	13	35010001	35013000	3000	1	2.60E-07	0.44	76	2.53	Gli2	Transcription
DMR13:35938001	13	35938001	35940000	2000	1	3.60E-07	0.46	25	1.25	Tmem177	
DMR13:35974001	13	35974001	35979000	5000	1	1.60E-07	0.47	96	1.92	Cfap221	Development
DMR13:40288001	13	40288001	40289000	1000	1	3.20E-08	-1.02	13	1.3	Dpp10	Protease
DMR13:40474001	13	40474001	40476000	2000	1	4.20E-07	-0.36	15	0.75	Dpp10	Protease
DMR13:42127001	13	42127001	42129000	2000	1	3.50E-10	0.78	30	1.5	Gpr39	Signaling
DMR13:42966001	13	42966001	42967000	1000	1	1.60E-09	0.65	4	0.4	Nckap5	
DMR13:45054001	13	45054001	45056000	2000	1	1.70E-07	0.54	26	1.3	Mcm6	Transcription
DMR13:46601001	13	46601001	46602000	1000	1	1.20E-08	-0.7	9	0.9	Thsd7b	Cytoskeleton

DMR13:47446001	13	47446001	47451000	5000	1	8.60E-07	0.53	44	0.88	Pfkfb2;Yod1;LOC498222	Metabolism;Protease
DMR13:47547001	13	47547001	47552000	5000	1	6.50E-07	0.55	88	1.76	Fcamr	Immune
DMR13:48068001	13	48068001	48069000	1000	1	7.60E-08	0.53	9	0.9	Srgap2	Signaling
DMR13:49105001	13	49105001	49107000	2000	1	1.00E-07	0.63	35	1.75	Nuak2	Signaling
DMR13:49286001	13	49286001	49295000	9000	1	7.10E-09	0.53	194	2.16	Rbbp5;Tmem81;Cntn2	
DMR13:49331001	13	49331001	49338000	7000	1	1.80E-07	0.46	120	1.71	Nfasc	
DMR13:49374001	13	49374001	49375000	1000	1	1.20E-07	0.47	25	2.5	Nfasc	
DMR13:49409001	13	49409001	49411000	2000	1	4.50E-11	0.67	26	1.3	Nfasc	
DMR13:49422001	13	49422001	49423000	1000	1	1.20E-07	0.47	15	1.5	Nfasc	
DMR13:49684001	13	49684001	49693000	9000	2	3.70E-09	0.52	141	1.57	Lrrn2	Receptor
DMR13:49863001	13	49863001	49865000	2000	1	2.30E-09	0.51	27	1.35	Pik3c2b	Signaling
DMR13:49903001	13	49903001	49906000	3000	1	6.40E-10	0.56	44	1.47	Pik3c2b	Signaling
DMR13:50102001	13	50102001	50103000	1000	1	2.90E-07	0.42	14	1.4	Atp2b4	Transport
DMR13:50249001	13	50249001	50251000	2000	1	2.00E-07	0.4	36	1.8	LOC498231;Snrpe	Translation
DMR13:50488001	13	50488001	50491000	3000	1	2.50E-07	0.47	49	1.63	Sox13;Etnk2	Signaling
DMR13:50556001	13	50556001	50561000	5000	1	7.70E-08	0.48	106	2.12	Golt1a;Plekha6	Metabolism
DMR13:50723001	13	50723001	50726000	3000	1	2.60E-07	0.42	61	2.03	Optc	Extracellular Matrix
DMR13:50877001	13	50877001	50879000	2000	1	8.20E-07	0.47	43	2.15	Fmod	
DMR13:51039001	13	51039001	51044000	5000	1	9.00E-07	0.44	97	1.94	Chi3l1;Mybph;Adora1	Metabolism;Signaling
DMR13:51047001	13	51047001	51049000	2000	1	7.80E-07	0.49	27	1.35	Mybph;Adora1	Signaling
DMR13:51141001	13	51141001	51146000	5000	1	3.80E-07	0.44	46	0.92	Ppfia4	
DMR13:51155001	13	51155001	51161000	6000	1	2.80E-07	0.44	120	2	Ppfia4	
DMR13:51309001	13	51309001	51311000	2000	1	4.30E-07	0.64	29	1.45	Klhl12;Rabif	Cytoskeleton;Transcription
DMR13:51594001	13	51594001	51595000	1000	1	4.80E-07	0.47	18	1.8	Ppp1r12b	Signaling
DMR13:51714001	13	51714001	51716000	2000	1	3.40E-07	0.51	32	1.6	Ppp1r12b	Signaling
DMR13:51831001	13	51831001	51832000	1000	1	2.70E-07	0.47	11	1.1	Lgr6	
DMR13:51936001	13	51936001	51939000	3000	1	9.20E-08	0.56	50	1.67	Lgr6;Ptprv	Signaling
DMR13:52159001	13	52159001	52160000	1000	1	5.70E-07	0.39	16	1.6	Lmod1	Cytoskeleton
DMR13:52396001	13	52396001	52399000	3000	1	4.80E-07	0.39	59	1.97	Nav1	
DMR13:52685001	13	52685001	52686000	1000	1	2.10E-09	0.59	10	1	Tnnt2	Cytoskeleton
DMR13:52808001	13	52808001	52811000	3000	1	4.40E-07	0.45	35	1.17	Igfn1	
DMR13:52859001	13	52859001	52867000	8000	1	8.20E-08	0.42	139	1.74	Tmem9	
DMR13:52939001	13	52939001	52946000	7000	1	7.50E-07	0.45	146	2.09	Cacna1s	Transport
DMR13:52972001	13	52972001	52975000	3000	1	2.20E-07	0.41	57	1.9	Cacna1s;Kif21b	Transport;Cytoskeleton
DMR13:53030001	13	53030001	53034000	4000	1	1.20E-07	0.58	62	1.55	Kif21b;LOC498236	Cytoskeleton
DMR13:53154001	13	53154001	53156000	2000	1	1.70E-08	0.54	40	2	Camsap2	
DMR13:53323001	13	53323001	53324000	1000	1	2.30E-07	0.75	9	0.9	Ddx59;LOC102547307	
DMR13:55509001	13	55509001	55511000	2000	1	1.60E-09	0.63	26	1.3	Nek7	Signaling
DMR13:55551001	13	55551001	55552000	1000	1	6.80E-08	0.57	19	1.9	Nek7	Signaling
DMR13:57235001	13	57235001	57236000	1000	1	2.90E-07	-0.7	8	0.8	Kcnt2	Transport
DMR13:63690001	13	63690001	63692000	2000	1	5.80E-07	-0.55	12	0.6	Brinp3	
DMR13:68027001	13	68027001	68029000	2000	1	4.50E-07	0.49	37	1.85	Hmcn1	
DMR13:68137001	13	68137001	68138000	1000	1	3.50E-07	0.42	5	0.5	Hmcn1	
DMR13:68974001	13	68974001	68979000	5000	1	5.10E-10	0.53	66	1.32	Fam129a	
DMR13:69958001	13	69958001	69962000	4000	2	8.30E-12	0.57	58	1.45	Rgl1;LOC102551528	Transcription
DMR13:69984001	13	69984001	69985000	1000	1	1.30E-07	0.52	14	1.4	Rgl1	Transcription
DMR13:70064001	13	70064001	70066000	2000	1	3.50E-08	0.49	31	1.55	Rgl1	Transcription
DMR13:70179001	13	70179001	70181000	2000	1	1.70E-08	0.55	29	1.45	Rgl1;Arpc5	Transcription;Cytoskeleton
DMR13:70192001	13	70192001	70193000	1000	1	3.70E-08	0.51	26	2.6	Arpc5;LOC100362914	Cytoskeleton
DMR13:71336001	13	71336001	71340000	4000	1	8.60E-08	0.47	104	2.6	Glul	Metabolism
DMR13:71977001	13	71977001	71980000	3000	1	9.20E-07	0.52	53	1.77	Cacna1e	Transport
DMR13:72837001	13	72837001	72839000	2000	1	3.10E-10	0.52	24	1.2	Stx6	Transcription
DMR13:72853001	13	72853001	72856000	3000	1	6.10E-07	0.49	44	1.47	Stx6;RGD1304622	Transcription;Cell Junction
DMR13:73423001	13	73423001	73424000	1000	1	4.30E-07	0.43	17	1.7	LOC102554128;Qsox1	Metabolism
DMR13:73580001	13	73580001	73583000	3000	1	5.70E-07	0.49	46	1.53	Cep350	Cytoskeleton
DMR13:73661001	13	73661001	73662000	1000	1	5.20E-08	0.5	16	1.6	RGD1561738;Tor1aip1	
DMR13:74027001	13	74027001	74030000	3000	1	5.50E-07	0.47	38	1.27	Axdnd1;Soat1	Metabolism
DMR13:74254001	13	74254001	74256000	2000	1	5.20E-07	0.52	24	1.2	Abl2;Tor3a	Transcription
DMR13:74486001	13	74486001	74487000	1000	1	1.20E-08	0.66	9	0.9	Ralgps2	Transcription
DMR13:74794001	13	74794001	74798000	4000	1	1.10E-07	0.47	54	1.35	Rasal2;LOC100912060	Signaling
DMR13:75189001	13	75189001	75192000	3000	1	9.00E-07	0.48	50	1.67	Sec16b	
DMR13:76036001	13	76036001	76037000	1000	1	5.40E-09	0.56	17	1.7	Brinp2	
DMR13:78424001	13	78424001	78426000	2000	1	4.80E-08	0.4	16	0.8	Rabgap1l	Signaling
DMR13:79371001	13	79371001	79373000	2000	1	5.50E-07	-0.52	13	0.65	LOC103692000;Tnfsf18	
DMR13:79941001	13	79941001	79943000	2000	1	1.30E-11	0.68	41	2.05	Dnm3;LOC102554129	Transport

DMR13:80124001	13	80124001	80126000	2000	1	6.00E-08	0.57	30	1.5	Dnm3;Mir199a2;Mir214;Mir3120	Transport
DMR13:80246001	13	80246001	80248000	2000	1	2.30E-07	0.5	22	1.1	Dnm3	Transport
DMR13:81691001	13	81691001	81693000	2000	1	1.60E-07	0.45	16	0.8	Gorab	
DMR13:82751001	13	82751001	82753000	2000	1	2.30E-07	0.43	27	1.35	Atp1b1	Transport
DMR13:83509001	13	83509001	83511000	2000	1	6.70E-07	0.43	22	1.1	Tiprl	
DMR13:83557001	13	83557001	83560000	3000	1	7.30E-07	0.45	55	1.83	Gpr161	Signaling
DMR13:83870001	13	83870001	83871000	1000	1	7.10E-08	-0.54	14	1.4	Rcsd1	Cytoskeleton
DMR13:84325001	13	84325001	84326000	1000	1	3.70E-07	0.56	9	0.9	Dusp27;Gpa33	
DMR13:84611001	13	84611001	84615000	4000	1	5.60E-07	0.45	46	1.15	Tada1;Pogk	Epigenetic
DMR13:85257001	13	85257001	85258000	1000	1	5.40E-07	0.49	11	1.1	RGD1563749	
DMR13:85335001	13	85335001	85338000	3000	1	3.50E-08	0.52	62	2.07	RGD1563679	
DMR13:85422001	13	85422001	85424000	2000	1	9.70E-07	0.4	36	1.8	Uck2	Signaling
DMR13:85822001	13	85822001	85823000	1000	1	8.40E-08	0.47	14	1.4	LOC103692054;Rxrg	Transcription
DMR13:86568001	13	86568001	86571000	3000	1	4.80E-07	0.66	34	1.13	Pbx1	Development
DMR13:88380001	13	88380001	88381000	1000	1	9.70E-07	0.53	6	0.6	Ddr2	Receptor
DMR13:88630001	13	88630001	88633000	3000	1	9.70E-08	0.64	52	1.73	Sh2d1b;LOC100361087	Cytoskeleton
DMR13:89007001	13	89007001	89010000	3000	2	6.00E-08	0.48	57	1.9	Olfml2b	Development
DMR13:89052001	13	89052001	89055000	3000	1	7.40E-07	0.43	29	0.97	Atf6	
DMR13:90600001	13	90600001	90601000	1000	1	2.40E-11	0.69	9	0.9	LOC103692289;Casq1;Atp1a4	Signaling;Transport
DMR13:91013001	13	91013001	91014000	1000	1	8.00E-08	0.55	21	2.1	Dusp23	Signaling
DMR13:93352001	13	93352001	93354000	2000	1	6.60E-07	0.43	13	0.65	Rgs7	
DMR13:93680001	13	93680001	93681000	1000	1	7.40E-08	0.46	14	1.4	Fh;Kmo	Metabolism;Metabolism
DMR13:94103001	13	94103001	94105000	2000	1	5.70E-07	0.55	36	1.8	Pld5	Metabolism
DMR13:94962001	13	94962001	94964000	2000	1	7.20E-10	0.57	27	1.35	Sdccag8	
DMR13:95952001	13	95952001	95953000	1000	1	2.90E-07	0.47	16	1.6	Adss	Metabolism
DMR13:96844001	13	96844001	96845000	1000	1	9.30E-09	0.48	16	1.6	Kif26b	Cytoskeleton
DMR13:96866001	13	96866001	96867000	1000	1	2.70E-07	0.37	9	0.9	Kif26b	Cytoskeleton
DMR13:98524001	13	98524001	98526000	2000	1	9.80E-07	0.46	39	1.95	Psen2	Proteolysis
DMR13:100353001	13	100353001	100354000	1000	1	6.60E-12	0.59	10	1	Enah	Cytoskeleton
DMR13:100663001	13	100663001	100665000	2000	1	2.40E-07	0.5	34	1.7	LOC108352583;Degs1;LOC108352584	Metabolism
DMR13:100923001	13	100923001	100924000	1000	1	9.50E-10	0.52	12	1.2	Capn2	Protease
DMR13:101310001	13	101310001	101319000	9000	1	1.70E-07	0.56	140	1.56	Susd4	
DMR13:101472001	13	101472001	101475000	3000	1	4.10E-07	0.53	30	1	Disp1	
DMR13:101552001	13	101552001	101553000	1000	1	3.50E-07	0.51	4	0.4	Disp1	
DMR13:101793001	13	101793001	101794000	1000	1	1.60E-07	0.42	25	2.5	Taf1a	
DMR13:105182001	13	105182001	105185000	3000	1	7.40E-08	0.66	35	1.17	Rrp15	
DMR13:105480001	13	105480001	105487000	7000	1	4.90E-07	-0.45	92	1.31	Spata17	
DMR13:105738001	13	105738001	105740000	2000	1	7.40E-07	0.4	27	1.35	Gpatch2	Metabolism
DMR13:105788001	13	105788001	105789000	1000	1	2.30E-07	0.47	19	1.9	Gpatch2	Metabolism
DMR13:106492001	13	106492001	106493000	1000	1	1.90E-10	0.69	9	0.9	Esrrg	
DMR13:107059001	13	107059001	107060000	1000	1	4.00E-07	0.53	9	0.9	Ush2a	Extracellular Matrix
DMR13:107070001	13	107070001	107072000	2000	1	1.10E-08	0.64	31	1.55	Ush2a	Extracellular Matrix
DMR13:107295001	13	107295001	107297000	2000	1	2.20E-07	0.42	43	2.15	Ush2a	Extracellular Matrix
DMR13:107370001	13	107370001	107374000	4000	2	7.70E-08	0.44	62	1.55	Ush2a;LOC103692406	Extracellular Matrix
DMR13:108660001	13	108660001	108661000	1000	1	4.10E-07	0.51	20	2	Smyd2	Epigenetic
DMR13:109451001	13	109451001	109454000	3000	1	9.10E-09	0.61	61	2.03	Rps6kc1	Signaling
DMR13:109458001	13	109458001	109459000	1000	1	1.50E-07	0.44	25	2.5	Rps6kc1	Signaling
DMR13:109577001	13	109577001	109578000	1000	1	1.20E-08	0.62	22	2.2	Rps6kc1;LOC108348225	Signaling
DMR13:110278001	13	110278001	110281000	3000	1	8.60E-07	0.48	43	1.43	Ints7;LOC108352606	
DMR13:110384001	13	110384001	110388000	4000	1	1.70E-09	0.6	35	0.88	Lpgat1	Metabolism
DMR13:110662001	13	110662001	110668000	6000	1	1.20E-07	0.38	123	2.05	Slc30a1	
DMR13:111025001	13	111025001	111027000	2000	1	1.00E-08	0.45	37	1.85	Kcnh1	Transport
DMR13:111121001	13	111121001	111122000	1000	1	3.60E-08	0.52	17	1.7	Kcnh1	Transport
DMR14:2043001	14	2043001	2046000	3000	1	3.40E-07	0.44	54	1.8	Idua;Slc26a1	Metabolism;Transport
DMR14:2072001	14	2072001	2076000	4000	1	4.50E-07	0.41	77	1.93	Dgkq;Tmem175	Signaling;Transport
DMR14:2535001	14	2535001	2536000	1000	1	5.10E-07	-0.34	8	0.8	Ccdc18	Cytoskeleton
DMR14:3031001	14	3031001	3032000	1000	1	9.50E-17	0.77	18	1.8	Evi5	Signaling
DMR14:5005001	14	5005001	5006000	1000	1	3.90E-07	0.48	18	1.8	Zfp326	Cytoskeleton
DMR14:5190001	14	5190001	5193000	3000	1	8.90E-07	0.42	56	1.87	Lrrc8d	Cytoskeleton
DMR14:7023001	14	7023001	7027000	4000	1	5.70E-07	0.43	54	1.35	Sparcl1;LOC100359907;Nudt9	Extracellular Matrix;Metabolism
DMR14:7037001	14	7037001	7041000	4000	1	1.90E-08	0.49	70	1.75	LOC100359907;Nudt9	Metabolism
DMR14:7074001	14	7074001	7076000	2000	1	9.10E-07	0.43	27	1.35	Hsd17b11;LOC108352698	
DMR14:7389001	14	7389001	7392000	3000	1	1.00E-07	0.37	43	1.43	Aff1;LOC100910240	Transcription
DMR14:7695001	14	7695001	7699000	4000	1	6.60E-08	0.59	70	1.75	Ptpn13	Signaling

DMR14:7815001	14	7815001	7817000	2000	1	1.80E-08	0.61	36	1.8	Ptpn13	Signaling
DMR14:8130001	14	8130001	8135000	5000	1	9.00E-08	0.51	97	1.94	Mapk10	Signaling
DMR14:8289001	14	8289001	8290000	1000	1	9.10E-09	0.5	13	1.3	Mapk10	Signaling
DMR14:8374001	14	8374001	8377000	3000	1	2.10E-07	0.44	38	1.27	Mapk10;Arhgap24	Signaling
DMR14:9431001	14	9431001	9432000	1000	1	4.50E-10	0.62	33	3.3	Cds1	Transport
DMR14:10484001	14	10484001	10492000	8000	1	4.00E-08	0.58	207	2.59	Helq	
DMR14:10602001	14	10602001	10604000	2000	1	6.50E-07	0.37	42	2.1	Coq2	Metabolism
DMR14:10783001	14	10783001	10785000	2000	1	5.50E-07	0.39	22	1.1	Lin54	
DMR14:11685001	14	11685001	11689000	4000	1	2.10E-11	0.61	94	2.35	Rasgef1b;LOC305181	Transcription
DMR14:11758001	14	11758001	11759000	1000	1	3.90E-07	0.42	25	2.5	Rasgef1b	Transcription
DMR14:13215001	14	13215001	13218000	3000	1	7.40E-07	0.51	50	1.67	Antxr2	Cytoskeleton
DMR14:14202001	14	14202001	14206000	4000	1	8.00E-07	0.43	68	1.7	Bmp2k	Signaling
DMR14:14454001	14	14454001	14456000	2000	1	4.00E-09	0.49	46	2.3	Fras1	
DMR14:14634001	14	14634001	14635000	1000	1	4.50E-08	0.4	16	1.6	Fras1	
DMR14:15436001	14	15436001	15437000	1000	1	8.20E-09	-0.51	7	0.7	RGD1561699;LOC108348100	
DMR14:16800001	14	16800001	16801000	1000	1	1.10E-08	0.5	15	1.5	Shroom3	Cytoskeleton
DMR14:16943001	14	16943001	16944000	1000	1	3.80E-07	0.47	22	2.2	Ccdc158	
DMR14:17058001	14	17058001	17063000	5000	1	7.30E-07	0.38	66	1.32	Scarb2	Transport
DMR14:17084001	14	17084001	17087000	3000	1	4.30E-07	0.45	43	1.43	Scarb2	Transport
DMR14:17257001	14	17257001	17260000	3000	1	3.30E-09	0.48	54	1.8	Sdad1	
DMR14:18539001	14	18539001	18540000	1000	1	2.50E-07	0.75	9	0.9	Areg	Growth Factors
DMR14:20022001	14	20022001	20024000	2000	1	2.50E-08	0.68	21	1.05	Adamts3	Protease
DMR14:21049001	14	21049001	21053000	4000	1	7.40E-07	0.5	79	1.98	Grsf1;Rufy3	Translation
DMR14:26707001	14	26707001	26710000	3000	1	1.70E-07	-0.5	8	0.27	Tecrl	Metabolism
DMR14:33642001	14	33642001	33643000	1000	1	9.00E-08	0.51	20	2	Aasdh;LOC108352737;RGD1311575	Metabolism
DMR14:33692001	14	33692001	33694000	2000	1	3.20E-07	0.76	32	1.6	RGD1311575	
DMR14:34050001	14	34050001	34052000	2000	1	7.90E-07	0.41	30	1.5	Cep135	Epigenetic
DMR14:35101001	14	35101001	35102000	1000	1	1.10E-07	0.46	14	1.4	Kit	Receptor
DMR14:35140001	14	35140001	35145000	5000	1	2.00E-09	0.68	85	1.7	Kit	Receptor
DMR14:36082001	14	36082001	36087000	5000	1	6.10E-07	0.39	73	1.46	Lnx1	
DMR14:36184001	14	36184001	36186000	2000	1	1.00E-07	0.43	25	1.25	Fip1l1	
DMR14:36329001	14	36329001	36331000	2000	1	8.50E-07	0.46	11	0.55	Scfd2	Transport
DMR14:37147001	14	37147001	37148000	1000	1	1.10E-15	0.72	20	2	Lrrc66	Receptor
DMR14:37879001	14	37879001	37881000	2000	1	3.40E-07	0.47	29	1.45	Slain2;LOC102556376	
DMR14:38837001	14	38837001	38840000	3000	1	1.20E-07	-0.68	20	0.67	Gabrb1;Olr1604-ps	Ion Channel
DMR14:41988001	14	41988001	41990000	2000	1	8.90E-10	0.48	41	2.05	Atp8a1	Transport
DMR14:42150001	14	42150001	42151000	1000	1	9.20E-09	0.54	14	1.4	Atp8a1	Transport
DMR14:42416001	14	42416001	42419000	3000	1	3.90E-07	0.51	33	1.1	Bend4	
DMR14:42774001	14	42774001	42775000	1000	1	3.70E-07	0.45	15	1.5	Limch1	Cytoskeleton
DMR14:43782001	14	43782001	43783000	1000	1	5.20E-07	0.47	15	1.5	Rbm47	Metabolism
DMR14:44564001	14	44564001	44565000	1000	1	8.40E-07	0.39	22	2.2	Klb;LOC108352748	
DMR14:44815001	14	44815001	44817000	2000	1	4.20E-08	0.53	28	1.4	LOC100910864;Klhl5	Cytoskeleton
DMR14:45693001	14	45693001	45694000	1000	1	8.60E-07	0.54	12	1.2	Tbc1d1	Signaling
DMR14:60571001	14	60571001	60573000	2000	1	3.80E-08	0.48	16	0.8	Pi4k2b	Signaling
DMR14:62297001	14	62297001	62298000	1000	1	5.60E-07	0.64	22	2.2	Vom1r-ps31	
DMR14:64691001	14	64691001	64693000	2000	1	2.40E-07	0.43	24	1.2	Adgra3	Signaling
DMR14:69776001	14	69776001	69777000	1000	1	8.80E-08	-0.84	6	0.6	Lcorl	Transcription
DMR14:69993001	14	69993001	69994000	1000	1	7.60E-07	0.38	9	0.9	Fam184b	
DMR14:71548001	14	71548001	71550000	2000	1	6.00E-08	0.43	26	1.3	Prom1	
DMR14:71556001	14	71556001	71560000	4000	1	3.10E-07	0.4	45	1.12	Prom1	
DMR14:77121001	14	77121001	77123000	2000	1	3.40E-07	0.49	26	1.3	Slc2a9	
DMR14:77153001	14	77153001	77154000	1000	1	5.10E-07	0.53	9	0.9	Slc2a9	
DMR14:77275001	14	77275001	77277000	2000	1	9.50E-08	0.44	42	2.1	Tmem128	
DMR14:77954001	14	77954001	77957000	3000	1	8.50E-07	-0.47	27	0.9	Stk32b	Signaling
DMR14:78040001	14	78040001	78041000	1000	1	7.00E-08	0.52	10	1	Stk32b	Signaling
DMR14:78499001	14	78499001	78501000	2000	1	1.10E-08	0.63	25	1.25	Jakmip1	
DMR14:78721001	14	78721001	78723000	2000	1	8.70E-07	0.55	31	1.55	Ppp2r2c	Signaling
DMR14:79299001	14	79299001	79301000	2000	2	4.70E-09	0.58	16	0.8	Tbc1d14;LOC680039	Signaling
DMR14:79692001	14	79692001	79695000	3000	1	3.20E-07	0.56	35	1.17	Sorcs2	Transport
DMR14:79698001	14	79698001	79699000	1000	1	1.20E-07	0.51	28	2.8	Sorcs2	Transport
DMR14:79719001	14	79719001	79721000	2000	1	4.70E-07	0.45	41	2.05	Sorcs2;Psapl1	Transport;Cytoskeleton
DMR14:79961001	14	79961001	79963000	2000	1	2.40E-08	0.48	33	1.65	Afap1	
DMR14:80113001	14	80113001	80115000	2000	1	4.00E-09	0.51	39	1.95	Ablim2	Cytoskeleton
DMR14:80408001	14	80408001	80412000	4000	1	3.50E-10	0.66	49	1.23	Cpz	Protease
DMR14:80943001	14	80943001	80944000	1000	1	9.70E-09	0.47	14	1.4	Dok7	

DMR14:80969001	14	80969001	80974000	5000	1	5.20E-09	0.57	92	1.84	Dok7;Hgfac;Rgs12	Protease
DMR14:81343001	14	81343001	81346000	3000	1	7.70E-07	0.43	46	1.53	Grk4;Nop14	Signaling;Metabolism
DMR14:81459001	14	81459001	81461000	2000	1	2.20E-10	0.55	34	1.7	Sh3bp2	
DMR14:81736001	14	81736001	81737000	1000	1	5.30E-09	0.46	9	0.9	Zfyve28	
DMR14:81788001	14	81788001	81790000	2000	1	3.30E-07	0.5	39	1.95	Zfyve28	
DMR14:83132001	14	83132001	83133000	1000	1	3.10E-07	0.63	6	0.6	Depdc5	Signaling
DMR14:83582001	14	83582001	83585000	3000	1	7.20E-07	0.46	52	1.73	Pik3ip1;Limk2	Protease
DMR14:83761001	14	83761001	83768000	7000	3	1.80E-08	0.52	174	2.49	Selm;Smtn	
DMR14:83769001	14	83769001	83771000	2000	1	3.80E-10	0.57	46	2.3	Smtn	
DMR14:83923001	14	83923001	83927000	4000	1	1.20E-09	0.48	66	1.65	Morc2	
DMR14:84218001	14	84218001	84223000	5000	2	1.40E-08	0.59	64	1.28	Pes1;Gal3st1	Metabolism;Transport
DMR14:84286001	14	84286001	84289000	3000	2	2.40E-07	0.56	48	1.6	Sec14l4;LOC108352786;LOC103693135	
DMR14:84365001	14	84365001	84366000	1000	1	2.50E-07	0.51	17	1.7	Sec14l2;Rnf215	
DMR14:84443001	14	84443001	84446000	3000	1	2.10E-07	0.53	52	1.73	Tbc1d10a;Gatsl3	Signaling
DMR14:84693001	14	84693001	84696000	3000	1	6.20E-08	0.39	71	2.37	Mtmr3	Signaling
DMR14:84863001	14	84863001	84866000	3000	1	1.50E-07	0.41	49	1.63	Ascc2	
DMR14:84968001	14	84968001	84971000	3000	1	5.20E-08	0.56	73	2.43	Zmat5;Cabp7	
DMR14:84991001	14	84991001	84995000	4000	1	3.20E-07	0.57	55	1.38	Nf2	Cytoskeleton
DMR14:85360001	14	85360001	85361000	1000	1	6.40E-09	0.64	19	1.9	Ewsr1;Rhbdd3;Emid1	Metabolism;Protease
DMR14:85388001	14	85388001	85390000	2000	1	1.30E-07	0.56	40	2	Emid1	
DMR14:85529001	14	85529001	85531000	2000	1	4.60E-07	0.64	52	2.6	LOC102551828;Znrf3	
DMR14:86010001	14	86010001	86011000	1000	1	1.70E-07	0.53	14	1.4	Urgcp;LOC108352787	
DMR14:86106001	14	86106001	86114000	8000	1	1.40E-10	0.55	239	2.99	Aebp1;Pold2	Protease;Transcription
DMR14:86121001	14	86121001	86122000	1000	1	6.50E-09	0.58	10	1	Aebp1;Pold2;LOC108352788	Protease;Transcription
DMR14:86209001	14	86209001	86211000	2000	1	3.70E-08	0.45	45	2.25	Ykt6;Camk2b;LOC108352789	Signaling
DMR14:86378001	14	86378001	86382000	4000	1	1.10E-08	0.74	73	1.82	Npc1l1;LOC103693777;Ddx56;Tmed4	Transport
DMR14:86916001	14	86916001	86920000	4000	1	2.90E-07	0.44	69	1.73	Ramp3	Receptor
DMR14:87374001	14	87374001	87382000	8000	1	1.50E-07	0.57	94	1.18	Adcy1	
DMR14:88726001	14	88726001	88729000	3000	1	5.90E-08	0.49	45	1.5	Tns3	Cytoskeleton
DMR14:89201001	14	89201001	89202000	1000	1	2.20E-08	0.45	14	1.4	Pkd1l1;Hus1	Transport;Transcription
DMR14:91850001	14	91850001	91854000	4000	1	3.70E-07	0.51	77	1.93	Ikzf1	Transcription
DMR14:92013001	14	92013001	92019000	6000	2	4.40E-10	0.59	77	1.28	LOC103693175;Grb10	Cytoskeleton
DMR14:101746001	14	101746001	101761000	15000	2	7.10E-08	-0.65	96	0.64	Olr1827-ps	
DMR14:103189001	14	103189001	103190000	1000	1	8.00E-07	0.49	13	1.3	Meis1	Development
DMR14:104596001	14	104596001	104599000	3000	1	5.60E-07	0.53	58	1.93	Slc1a4	Transport
DMR14:106143001	14	106143001	106144000	1000	1	6.50E-07	0.65	11	1.1	Vps54	Transport
DMR14:106608001	14	106608001	106610000	2000	1	1.40E-07	0.49	24	1.2	Wdpcp	
DMR14:106956001	14	106956001	106960000	4000	1	4.40E-07	0.69	68	1.7	Ehbp1	
DMR14:107589001	14	107589001	107592000	3000	1	5.20E-07	0.54	56	1.87	LOC103693217;B3gnt2	Golgi
DMR14:108837001	14	108837001	108838000	1000	1	1.60E-08	0.49	18	1.8	Bcl11a	Transcription
DMR14:108858001	14	108858001	108859000	1000	1	3.90E-09	0.64	11	1.1	Bcl11a	Transcription
DMR14:110730001	14	110730001	110731000	1000	1	4.60E-07	0.42	7	0.7	Fancl;Vrk2	Proteolysis;Signaling
DMR14:112712001	14	112712001	112713000	1000	1	5.00E-07	-1.07	6	0.6	Ccdc85a	
DMR14:114230001	14	114230001	114232000	2000	1	6.70E-07	0.67	32	1.6	Eml6	
DMR14:114646001	14	114646001	114648000	2000	1	1.20E-07	0.55	17	0.85	Sptbn1	
DMR15:621001	15	621001	622000	1000	1	5.10E-09	-0.64	2	0.2	Kcnma1	Transport
DMR15:2696001	15	2696001	2698000	2000	1	4.50E-08	0.45	18	0.9	Samd8	
DMR15:2860001	15	2860001	2862000	2000	1	3.40E-07	0.4	43	2.15	Kat6b	Epigenetic
DMR15:2934001	15	2934001	2938000	4000	1	2.30E-12	0.6	66	1.65	Kat6b	Epigenetic
DMR15:3522001	15	3522001	3526000	4000	1	2.10E-08	0.52	39	0.98	Vcl	
DMR15:3738001	15	3738001	3739000	1000	1	2.50E-07	0.65	18	1.8	NEWGENE_621802	
DMR15:4383001	15	4383001	4386000	3000	1	1.30E-07	0.53	25	0.83	Fam149b1	
DMR15:4584001	15	4584001	4586000	2000	1	9.50E-09	0.54	54	2.7	Kcnk5	Transport
DMR15:4605001	15	4605001	4606000	1000	1	1.60E-07	0.41	26	2.6	Kcnk5	Transport
DMR15:5640001	15	5640001	5641000	1000	1	7.00E-07	0.29	5	0.5	Spetex-2C;LOC108349412	
DMR15:7205001	15	7205001	7207000	2000	1	2.90E-07	-0.47	13	0.65	Zfp385d	
DMR15:7624001	15	7624001	7627000	3000	1	1.10E-07	-0.44	32	1.07	Ube2e2;LOC100366184	Proteolysis
DMR15:7686001	15	7686001	7687000	1000	1	6.70E-11	0.58	21	2.1	Ube2e2;LOC103693837	Proteolysis
DMR15:11712001	15	11712001	11715000	3000	1	3.00E-07	-0.64	29	0.97	Nek10	Signaling
DMR15:12427001	15	12427001	12429000	2000	1	3.70E-07	0.47	26	1.3	Atxn7;LOC108352888	
DMR15:12966001	15	12966001	12968000	2000	1	2.50E-08	0.52	18	0.9	Ptprg	Signaling
DMR15:13412001	15	13412001	13413000	1000	1	2.60E-08	0.45	17	1.7	Ptprg	Signaling
DMR15:13544001	15	13544001	13545000	1000	1	1.20E-10	0.52	17	1.7	Ptprg	Signaling
DMR15:15731001	15	15731001	15732000	1000	1	4.80E-07	0.46	7	0.7	Fhit	Signaling

DMR15:16434001	15	16434001	16435000	1000	1	3.50E-11	0.89	34	3.4	Fhit	Signaling
DMR15:18349001	15	18349001	18351000	2000	1	4.90E-07	0.47	33	1.65	Fam3d	Signaling
DMR15:18454001	15	18454001	18461000	7000	1	2.10E-08	0.5	140	2	Acox2	Metabolism
DMR15:18768001	15	18768001	18771000	3000	1	4.60E-08	0.69	74	2.47	Flnb	
DMR15:18784001	15	18784001	18785000	1000	1	2.80E-07	0.54	12	1.2	Flnb	
DMR15:19489001	15	19489001	19491000	2000	1	4.70E-07	0.47	30	1.5	Txndc16	Metabolism
DMR15:19800001	15	19800001	19805000	5000	1	1.00E-06	0.4	101	2.02	Fermt2	
DMR15:23581001	15	23581001	23582000	1000	1	1.30E-07	0.74	15	1.5	Cnih1	Transport
DMR15:23755001	15	23755001	23756000	1000	1	1.30E-07	0.48	31	3.1	Samd4a	
DMR15:23781001	15	23781001	23783000	2000	1	3.30E-10	0.54	40	2	Samd4a	
DMR15:23866001	15	23866001	23870000	4000	1	1.50E-09	0.5	91	2.28	Samd4a	
DMR15:24318001	15	24318001	24320000	2000	1	8.90E-08	0.66	67	3.35	Fbxo34	
DMR15:25351001	15	25351001	25352000	1000	1	7.60E-10	0.83	18	1.8	Tmem260;LOC102554488	
DMR15:25354001	15	25354001	25356000	2000	1	1.60E-08	0.52	32	1.6	Tmem260;LOC102554488	
DMR15:25963001	15	25963001	25966000	3000	1	3.50E-07	0.46	44	1.47	Ap5m1	
DMR15:26598001	15	26598001	26600000	2000	1	7.80E-07	0.51	17	0.85	RGD1310110	
DMR15:27607001	15	27607001	27613000	6000	1	1.80E-13	0.5	61	1.02	Olr1634-ps	
DMR15:33042001	15	33042001	33047000	5000	2	4.80E-08	0.63	58	1.16	Slc7a7	Transport
DMR15:33099001	15	33099001	33103000	4000	2	1.80E-09	0.56	64	1.6	LOC102550180;Lrp10	Binding Proteins
DMR15:33225001	15	33225001	33227000	2000	1	2.00E-07	0.47	57	2.85	Ajuba;RGD1565222	Transcription
DMR15:33367001	15	33367001	33371000	4000	1	7.30E-13	0.63	46	1.15	Cebpe;Slc7a8	Transcription;Transport
DMR15:33586001	15	33586001	33588000	2000	1	1.30E-07	0.56	26	1.3	Slc22a17;Efs;Il25	Transport
DMR15:33610001	15	33610001	33615000	5000	1	2.70E-07	0.41	124	2.48	Cmtm5;Myh6;Myh7;LOC108352922;LOC108352923;Mir3546;Mir208a	Transport
DMR15:33706001	15	33706001	33715000	9000	1	2.80E-07	0.52	164	1.82	Zfhx2	Transcription
DMR15:33822001	15	33822001	33827000	5000	2	2.00E-16	0.72	68	1.36	RGD1564324	Metabolism
DMR15:33843001	15	33843001	33845000	2000	1	3.40E-08	0.67	24	1.2	RGD1564324	Metabolism
DMR15:34189001	15	34189001	34194000	5000	1	6.00E-07	0.4	118	2.36	Carmil3;LOC102552093;Cpne6;Nrl	Transcription
DMR15:34484001	15	34484001	34485000	1000	1	9.10E-07	0.48	12	1.2	Ripk3;LOC102552386;Nfatc4	Signaling;Transcription
DMR15:34746001	15	34746001	34749000	3000	1	2.10E-08	-0.86	26	0.87	Mcpt4	Protease
DMR15:34752001	15	34752001	34755000	3000	1	4.30E-08	-0.76	26	0.87	Mcpt4	Protease
DMR15:34761001	15	34761001	34763000	2000	1	1.30E-07	-0.49	11	0.55	Mcpt4;RGD1562290	Protease
DMR15:35407001	15	35407001	35411000	4000	1	1.00E-06	-0.41	35	0.88	Gzmb	Protease
DMR15:36748001	15	36748001	36750000	2000	1	3.80E-07	0.52	20	1	Rnf17;Cenpj	Cytoskeleton
DMR15:37332001	15	37332001	37336000	4000	1	1.80E-07	0.4	132	3.3	Gja3;LOC102549747	Cytoskeleton
DMR15:37575001	15	37575001	37577000	2000	1	2.50E-10	0.5	27	1.35	Cryl1	Metabolism
DMR15:37952001	15	37952001	37955000	3000	1	1.90E-08	0.59	74	2.47	Lats2	Signaling
DMR15:37986001	15	37986001	37988000	2000	1	2.50E-08	0.46	26	1.3	Lats2;LOC108352928	Signaling
DMR15:38234001	15	38234001	38242000	8000	1	1.20E-10	0.54	80	1	Micu2	Signaling
DMR15:38907001	15	38907001	38909000	2000	1	3.30E-07	0.44	18	0.9	Phf11;Setdb2	Transcription;Epigenetic
DMR15:40071001	15	40071001	40073000	2000	1	6.40E-07	0.4	25	1.25	Atp8a2	Transport
DMR15:40323001	15	40323001	40327000	4000	1	3.00E-07	0.47	57	1.43	Atp8a2	Transport
DMR15:40402001	15	40402001	40404000	2000	1	2.10E-07	0.44	24	1.2	Atp8a2	Transport
DMR15:41054001	15	41054001	41057000	3000	1	4.30E-07	0.44	63	2.1	Spata13	Transcription
DMR15:41544001	15	41544001	41545000	1000	1	4.20E-07	0.54	12	1.2	Sgcg	Cytoskeleton
DMR15:42927001	15	42927001	42930000	3000	1	7.80E-07	0.6	24	0.8	Ptk2b	
DMR15:44604001	15	44604001	44609000	5000	1	1.70E-12	0.59	68	1.36	Dock5	Transcription
DMR15:45367001	15	45367001	45369000	2000	1	3.00E-07	0.49	26	1.3	Dleu7	
DMR15:45533001	15	45533001	45534000	1000	1	4.50E-07	0.45	13	1.3	Gucy1b2	Signaling
DMR15:47144001	15	47144001	47145000	1000	1	6.20E-07	0.49	7	0.7	Xkr6	
DMR15:47546001	15	47546001	47550000	4000	1	8.40E-07	0.5	46	1.15	Msra	Metabolism
DMR15:47650001	15	47650001	47651000	1000	1	8.60E-09	0.58	5	0.5	Msra	Metabolism
DMR15:48790001	15	48790001	48792000	2000	1	3.60E-08	0.5	25	1.25	Zfp395	Transcription
DMR15:48800001	15	48800001	48804000	4000	1	3.00E-08	0.54	66	1.65	Zfp395;Pnoc	Transcription;Signaling
DMR15:51862001	15	51862001	51864000	2000	1	8.60E-07	0.48	33	1.65	Pdlim2;Sorbs3	Cytoskeleton
DMR15:55087001	15	55087001	55089000	2000	1	8.90E-09	0.5	26	1.3	Rb1	Epigenetic
DMR15:55406001	15	55406001	55409000	3000	1	9.20E-07	0.53	69	2.3	LOC103693716;Med4;Nudt15	Transcription
DMR15:57392001	15	57392001	57394000	2000	2	9.50E-08	0.48	52	2.6	Zc3h13	
DMR15:57935001	15	57935001	57938000	3000	1	2.50E-07	0.46	55	1.83	LOC108353038;Gtf2f2	Transcription
DMR15:58012001	15	58012001	58013000	1000	1	2.20E-07	0.57	10	1	Gtf2f2;Kctd4	Transcription;Cytoskeleton
DMR15:60197001	15	60197001	60198000	1000	1	3.40E-07	0.43	16	1.6	Epsti1	
DMR15:60888001	15	60888001	60892000	4000	2	1.40E-07	0.46	75	1.88	Dgkh	Signaling
DMR15:60929001	15	60929001	60931000	2000	1	6.30E-08	0.48	32	1.6	Dgkh	Signaling
DMR15:61352001	15	61352001	61355000	3000	1	2.90E-08	0.44	46	1.53	Vwa8	
DMR15:70121001	15	70121001	70124000	3000	1	4.70E-07	0.42	36	1.2	Diaph3	

DMR15:70779001	15	70779001	70786000	7000	1	1.20E-07	0.46	71	1.01	Tdrd3	Translation
DMR15:80274001	15	80274001	80275000	1000	1	9.70E-09	-0.98	5	0.5	Klhl1	Cytoskeleton
DMR15:83587001	15	83587001	83588000	1000	1	9.50E-07	0.45	20	2	Pibf1	
DMR15:84329001	15	84329001	84331000	2000	1	9.90E-08	0.47	19	0.95	Klf12;LOC103693766	Transcription
DMR15:84332001	15	84332001	84334000	2000	1	3.10E-08	0.53	17	0.85	Klf12;LOC103693766	Transcription
DMR15:86332001	15	86332001	86334000	2000	1	3.90E-07	0.79	19	0.95	Lmo7	
DMR15:86399001	15	86399001	86401000	2000	1	9.00E-07	0.37	34	1.7	Lmo7	
DMR15:86468001	15	86468001	86470000	2000	1	3.30E-07	0.52	41	2.05	Lmo7	
DMR15:100484001	15	100484001	100485000	1000	1	9.80E-08	0.54	10	1	Gpc5	
DMR15:103334001	15	103334001	103336000	2000	1	1.90E-13	0.78	25	1.25	Tgds;Gpr180	Metabolism
DMR15:103703001	15	103703001	103707000	4000	1	9.10E-08	0.42	96	2.4	Abcc4	Transport
DMR15:103769001	15	103769001	103772000	3000	1	2.70E-07	0.5	70	2.33	Abcc4	Transport
DMR15:103773001	15	103773001	103782000	9000	1	9.10E-07	0.41	140	1.56	Abcc4	Transport
DMR15:103901001	15	103901001	103911000	10000	1	3.60E-07	0.45	215	2.15	Abcc4	Transport
DMR15:104022001	15	104022001	104025000	3000	1	3.00E-08	0.57	32	1.07	Cldn10	Cell Junction
DMR15:104035001	15	104035001	104039000	4000	1	3.70E-09	0.63	80	2	Cldn10	Cell Junction
DMR15:104988001	15	104988001	104991000	3000	1	2.40E-07	0.43	57	1.9	Hs6st3;LOC102546571	
DMR15:105656001	15	105656001	105657000	1000	1	9.40E-08	0.43	17	1.7	Mbnl2	Translation
DMR15:105742001	15	105742001	105746000	4000	1	1.30E-10	0.8	88	2.2	Mbnl2	Translation
DMR15:105773001	15	105773001	105775000	2000	1	4.40E-07	0.7	40	2	Mbnl2	Translation
DMR15:106442001	15	106442001	106444000	2000	1	2.60E-07	0.5	55	2.75	Farp1	
DMR15:106617001	15	106617001	106621000	4000	1	1.20E-07	0.43	98	2.45	Farp1;Stk24	
DMR15:106622001	15	106622001	106624000	2000	1	5.20E-08	0.55	42	2.1	Farp1;Stk24	
DMR15:106643001	15	106643001	106645000	2000	1	4.60E-09	0.47	28	1.4	Stk24	
DMR15:106652001	15	106652001	106654000	2000	1	7.50E-08	0.51	26	1.3	Stk24	
DMR15:106656001	15	106656001	106660000	4000	1	7.10E-09	0.53	93	2.33	Stk24	
DMR15:106664001	15	106664001	106669000	5000	1	3.20E-10	0.59	72	1.44	Stk24	
DMR15:107955001	15	107955001	107957000	2000	1	7.30E-11	0.57	42	2.1	Dock9	Transcription
DMR15:108016001	15	108016001	108019000	3000	1	4.50E-07	0.45	49	1.63	Dock9	Transcription
DMR15:108684001	15	108684001	108689000	5000	1	1.10E-07	0.81	57	1.14	Clybl	Metabolism
DMR15:108716001	15	108716001	108719000	3000	1	8.60E-08	0.53	42	1.4	Clybl	Metabolism
DMR15:108802001	15	108802001	108803000	1000	1	1.20E-08	0.8	35	3.5	Clybl	Metabolism
DMR15:108810001	15	108810001	108814000	4000	1	2.50E-07	0.42	60	1.5	Clybl	Metabolism
DMR15:110211001	15	110211001	110214000	3000	1	4.90E-08	-0.54	22	0.73	Itgbl1	Extracellular Matrix
DMR16:1737001	16	1737001	1743000	6000	1	2.80E-07	0.63	85	1.42	Zmiz1;LOC102553140	
DMR16:1762001	16	1762001	1763000	1000	1	8.00E-08	0.74	7	0.7	Zmiz1;LOC102553140	
DMR16:1941001	16	1941001	1946000	5000	1	1.00E-09	0.63	63	1.26	Zmiz1	
DMR16:1964001	16	1964001	1965000	1000	1	3.00E-07	0.41	23	2.3	Zmiz1;LOC108353088	
DMR16:2541001	16	2541001	2543000	2000	1	9.10E-07	0.44	30	1.5	Dnah12;Asb14	Cytoskeleton;Transport
DMR16:2860001	16	2860001	2864000	4000	1	8.90E-07	0.48	60	1.5	Arhgef3	Transcription
DMR16:2945001	16	2945001	2948000	3000	1	7.60E-07	0.44	47	1.57	Arhgef3	Transcription
DMR16:3268001	16	3268001	3270000	2000	1	3.60E-07	0.46	22	1.1	Erc2	Transport
DMR16:3934001	16	3934001	3936000	2000	1	6.80E-07	0.5	31	1.55	Erc2;RGD1565017	Transport
DMR16:6326001	16	6326001	6327000	1000	1	1.70E-10	0.57	23	2.3	Cacna1d	Transport
DMR16:6393001	16	6393001	6395000	2000	1	8.50E-10	0.57	42	2.1	Cacna1d	Transport
DMR16:6933001	16	6933001	6938000	5000	1	1.20E-07	0.44	96	1.92	Tmem110	
DMR16:6955001	16	6955001	6964000	9000	1	7.90E-07	0.44	174	1.93	Tmem110;Mustn1;Itih4	Protease; Proteolysis
DMR16:7216001	16	7216001	7226000	10000	1	4.80E-08	0.49	294	2.94	Smim4;Nt5dc2;Stab1	Signaling;Transport
DMR16:7240001	16	7240001	7246000	6000	2	2.10E-15	0.73	150	2.5	Stab1;Nisch	Transport;Cytoskeleton
DMR16:7259001	16	7259001	7261000	2000	1	9.80E-07	0.51	86	4.3	Stab1;Nisch	Transport;Cytoskeleton
DMR16:7318001	16	7318001	7324000	6000	1	9.70E-08	0.64	82	1.37	Sema3g;Phf7	Signaling;Transcription
DMR16:7374001	16	7374001	7380000	6000	1	1.70E-07	0.54	127	2.12	Dnah1	Cytoskeleton
DMR16:8210001	16	8210001	8214000	4000	1	5.60E-09	0.49	53	1.32	Dph3;Oxnad1	Metabolism
DMR16:8359001	16	8359001	8360000	1000	1	8.10E-07	0.49	18	1.8	Timm23;Parg	Transport;Metabolism
DMR16:8752001	16	8752001	8754000	2000	1	3.80E-08	0.51	29	1.45	Ercc6	
DMR16:9032001	16	9032001	9035000	3000	1	8.60E-07	0.46	39	1.3	RGD1561145	
DMR16:9770001	16	9770001	9775000	5000	1	5.00E-07	0.56	60	1.2	Frmpd2	
DMR16:10489001	16	10489001	10491000	2000	1	9.10E-08	0.5	38	1.9	Npy4r	Signaling
DMR16:10519001	16	10519001	10533000	14000	1	5.10E-09	0.56	254	1.81	Gprin2	
DMR16:10711001	16	10711001	10713000	2000	1	1.70E-08	0.46	23	1.15	Fam25a;Sncg	Transport
DMR16:10877001	16	10877001	10879000	2000	1	8.60E-07	0.65	21	1.05	Ldb3	Cytoskeleton
DMR16:10926001	16	10926001	10927000	1000	1	7.30E-07	0.48	24	2.4	Ldb3	Cytoskeleton
DMR16:10941001	16	10941001	10948000	7000	1	6.60E-07	0.46	119	1.7	Ldb3;Opn4;LOC100360810	Cytoskeleton;Signaling
DMR16:11931001	16	11931001	11932000	1000	1	8.10E-07	0.54	15	1.5	Grid1	Receptor

DMR16:14351001	16	14351001	14353000	2000	1	2.80E-07	0.48	19	0.95	Cdhr1;LOC103693908;LOC290595;LOC108348380	Cytoskeleton
DMR16:17592001	16	17592001	17594000	2000	1	5.50E-07	0.59	37	1.85	Tspan14	
DMR16:17603001	16	17603001	17606000	3000	1	1.00E-08	0.57	45	1.5	Tspan14;Sh2d4b	Immune
DMR16:18832001	16	18832001	18834000	2000	1	1.50E-07	0.55	58	2.9	LOC103693913;Sin3b	Epigenetic
DMR16:19186001	16	19186001	19187000	1000	1	3.50E-10	0.6	24	2.4	Eps15l1	Transport
DMR16:19217001	16	19217001	19220000	3000	1	8.20E-08	0.43	49	1.63	Klf2	Transcription
DMR16:19288001	16	19288001	19291000	3000	1	2.00E-07	0.69	49	1.63	Ap1m1	Transport
DMR16:19883001	16	19883001	19885000	2000	1	2.00E-11	0.64	42	2.1	Mrpl34;LOC103693916;Dda1;Ano8	Translation
DMR16:19975001	16	19975001	19977000	2000	1	5.30E-08	0.52	43	2.15	Mvb12a;Tmem221	
DMR16:20160001	16	20160001	20165000	5000	1	1.80E-10	0.56	71	1.42	Fcho1;LOC108348388;LOC108348387	Cytoskeleton
DMR16:20471001	16	20471001	20477000	6000	1	4.50E-08	0.51	151	2.52	Pde4c;LOC102547294;Jund	Signaling;Transcription
DMR16:20504001	16	20504001	20511000	7000	1	7.20E-09	0.52	101	1.44	Lsm4	Translation
DMR16:20697001	16	20697001	20699000	2000	1	1.30E-07	0.47	30	1.5	Tmem59l;Klhl26	
DMR16:20725001	16	20725001	20728000	3000	1	6.90E-07	0.54	122	4.07	Klhl26	
DMR16:20879001	16	20879001	20885000	6000	2	6.90E-09	0.5	117	1.95	Cope;Ddx49;Homer3	Transport
DMR16:20887001	16	20887001	20890000	3000	1	2.50E-07	0.5	54	1.8	Ddx49;Homer3	
DMR16:20991001	16	20991001	20993000	2000	1	5.60E-12	0.69	34	1.7	Slc25a42;Tmem161a;Mef2b	Transport;Transcription
DMR16:21114001	16	21114001	21116000	2000	1	1.10E-11	0.58	21	1.05	Sugp1	Translation
DMR16:21216001	16	21216001	21218000	2000	1	5.60E-13	0.67	28	1.4	Gatad2a	Transcription
DMR16:21248001	16	21248001	21250000	2000	1	9.50E-07	0.57	24	1.2	Gatad2a	Transcription
DMR16:22029001	16	22029001	22032000	3000	1	8.30E-08	0.3	25	0.83	RGD1563748;RGD1564941	
DMR16:22115001	16	22115001	22118000	3000	2	5.30E-08	0.27	36	1.2	RGD1563748;RGD1564941;LOC103693919;LOC108353083	
DMR16:22119001	16	22119001	22123000	4000	1	1.00E-07	0.34	30	0.75	RGD1563748;RGD1564941;LOC108353083	
DMR16:22183001	16	22183001	22184000	1000	1	7.40E-07	0.41	39	3.9	RGD1564941	
DMR16:23314001	16	23314001	23317000	3000	3	7.90E-10	0.64	68	2.27	Psd3	Transcription
DMR16:25565001	16	25565001	25568000	3000	1	4.90E-07	-0.45	27	0.9	1-Mar	
DMR16:35013001	16	35013001	35014000	1000	1	1.40E-08	0.62	8	0.8	Galntl6	Golgi
DMR16:35109001	16	35109001	35110000	1000	1	1.00E-12	0.6	10	1	Galntl6	Golgi
DMR16:35564001	16	35564001	35566000	2000	1	6.80E-07	-0.53	13	0.65	Galntl6	Golgi
DMR16:35632001	16	35632001	35635000	3000	1	2.50E-07	-0.44	30	1	Galntl6	Golgi
DMR16:35719001	16	35719001	35721000	2000	1	4.30E-07	-0.42	16	0.8	Galntl6	Golgi
DMR16:36096001	16	36096001	36107000	11000	2	6.20E-08	0.44	361	3.28	LOC102555039;Sap30	Epigenetic
DMR16:46897001	16	46897001	46902000	5000	1	2.60E-07	0.47	66	1.32	Tenm3	
DMR16:47381001	16	47381001	47386000	5000	1	1.70E-08	0.52	76	1.52	Wwc2	
DMR16:47479001	16	47479001	47490000	11000	1	4.70E-09	0.56	191	1.74	Wwc2	
DMR16:47726001	16	47726001	47730000	4000	1	3.50E-08	0.54	41	1.02	Ing2	Epigenetic
DMR16:48182001	16	48182001	48183000	1000	1	5.80E-07	0.4	19	1.9	Stox2	
DMR16:48578001	16	48578001	48580000	2000	1	3.10E-08	0.44	30	1.5	Irf2	Transcription
DMR16:49291001	16	49291001	49297000	6000	1	2.10E-07	-0.44	59	0.98	Cfap97	
DMR16:50027001	16	50027001	50028000	1000	1	1.60E-07	0.5	15	1.5	Tlr3	
DMR16:50347001	16	50347001	50349000	2000	1	5.40E-09	0.54	42	2.1	Mtnr1a	Signaling
DMR16:52163001	16	52163001	52165000	2000	1	7.80E-07	-0.77	10	0.5	LOC682860;Triml1	Proteolysis
DMR16:54025001	16	54025001	54026000	1000	1	4.90E-07	0.52	14	1.4	Asah1	Protease
DMR16:54781001	16	54781001	54783000	2000	1	5.50E-07	0.38	22	1.1	Mtmr7	Signaling
DMR16:54808001	16	54808001	54812000	4000	1	1.00E-08	-0.57	38	0.95	Mtmr7	Signaling
DMR16:55049001	16	55049001	55052000	3000	1	6.50E-08	0.5	39	1.3	Micu3	Signaling
DMR16:57455001	16	57455001	57457000	2000	1	7.20E-07	-0.53	23	1.15	Sgcz	
DMR16:57565001	16	57565001	57567000	2000	1	3.30E-07	-0.41	18	0.9	Sgcz	
DMR16:57795001	16	57795001	57797000	2000	1	7.70E-07	0.56	6	0.3	Sgcz	
DMR16:59672001	16	59672001	59674000	2000	1	9.40E-07	0.6	32	1.6	Pragmin	
DMR16:59675001	16	59675001	59676000	1000	1	2.00E-07	0.5	27	2.7	Pragmin	
DMR16:61052001	16	61052001	61055000	3000	1	5.80E-07	0.43	32	1.07	Tnks	Signaling
DMR16:61803001	16	61803001	61804000	1000	1	5.80E-07	0.72	8	0.8	Mboat4;Dctn6	Metabolism;Cytoskeleton
DMR16:62000001	16	62000001	62004000	4000	1	3.70E-11	0.54	68	1.7	Rbpms	Translation
DMR16:62068001	16	62068001	62070000	2000	1	9.60E-07	0.47	28	1.4	Rbpms	Translation
DMR16:62086001	16	62086001	62089000	3000	1	1.40E-07	0.46	77	2.57	Rbpms	Translation
DMR16:62531001	16	62531001	62533000	2000	1	2.90E-09	0.68	21	1.05	Wrn	Epigenetic
DMR16:62552001	16	62552001	62553000	1000	1	4.30E-07	0.6	12	1.2	Wrn	Epigenetic
DMR16:63223001	16	63223001	63226000	3000	1	9.40E-07	0.35	26	0.87	Nrg1	Growth Factors
DMR16:63642001	16	63642001	63646000	4000	1	1.60E-08	0.46	64	1.6	Nrg1	Growth Factors
DMR16:67062001	16	67062001	67064000	2000	1	3.00E-07	0.4	35	1.75	Unc5d	Receptor
DMR16:67286001	16	67286001	67289000	3000	1	2.80E-07	0.71	26	0.87	Unc5d	Receptor

DMR16:69171001	16	69171001	69173000	2000	1	1.20E-07	0.61	22	1.1	Prosc;Erlin2	
DMR16:69203001	16	69203001	69205000	2000	1	9.90E-07	0.58	12	0.6	Erlin2	
DMR16:70006001	16	70006001	70007000	1000	1	2.10E-07	0.62	22	2.2	Pasd1	
DMR16:71290001	16	71290001	71291000	1000	1	7.50E-07	0.47	21	2.1	Fgfr1	Receptor
DMR16:71864001	16	71864001	71867000	3000	1	3.70E-07	0.52	42	1.4	Adam9	Protease
DMR16:71883001	16	71883001	71886000	3000	2	8.40E-11	0.88	29	0.97	Adam9;Adam32	Protease
DMR16:72918001	16	72918001	72925000	7000	1	5.20E-07	0.43	103	1.47	Zmat4	
DMR16:73368001	16	73368001	73371000	3000	2	3.20E-07	0.4	60	2	Sfrp1	Receptor
DMR16:73587001	16	73587001	73590000	3000	2	3.90E-09	0.51	48	1.6	Golga7;Gins4	
DMR16:73634001	16	73634001	73636000	2000	1	1.50E-08	0.55	51	2.55	Gpat4;LOC108348420	Metabolism
DMR16:73765001	16	73765001	73771000	6000	1	6.20E-07	0.57	168	2.8	Ank1;LOC100910418	
DMR16:73773001	16	73773001	73775000	2000	1	2.40E-07	0.44	54	2.7	Ank1;LOC100910418	
DMR16:74203001	16	74203001	74205000	2000	1	9.60E-08	0.5	51	2.55	Ikbkb	Signaling
DMR16:74346001	16	74346001	74349000	3000	1	1.90E-07	0.47	70	2.33	Slc20a2	Transport
DMR16:74730001	16	74730001	74732000	2000	1	6.40E-08	0.45	41	2.05	Vps36	Transport
DMR16:75821001	16	75821001	75822000	1000	1	2.00E-10	0.58	13	1.3	Agpat5	Metabolism
DMR16:75998001	16	75998001	76001000	3000	1	6.60E-09	0.51	46	1.53	Mcph1;Angpt2	DNA Repair;Signaling
DMR16:76097001	16	76097001	76102000	5000	1	2.00E-08	0.41	41	0.82	Mcph1	DNA Repair
DMR16:79780001	16	79780001	79785000	5000	1	1.70E-07	0.44	153	3.06	Arhgef10	Transcription
DMR16:79794001	16	79794001	79800000	6000	1	1.00E-08	0.66	150	2.5	Arhgef10	Transcription
DMR16:80406001	16	80406001	80410000	4000	1	9.80E-07	0.42	59	1.48	Dlgap2	Cytoskeleton
DMR16:80435001	16	80435001	80437000	2000	1	6.50E-07	0.47	37	1.85	Dlgap2	Cytoskeleton
DMR16:80727001	16	80727001	80729000	2000	1	4.40E-08	0.5	45	2.25	Tdrp	
DMR16:80769001	16	80769001	80770000	1000	1	2.30E-07	0.49	13	1.3	Fbxo25	
DMR16:81156001	16	81156001	81157000	1000	1	9.20E-07	0.36	29	2.9	Grk1	Signaling
DMR16:81174001	16	81174001	81176000	2000	2	6.70E-11	0.64	25	1.25	Grk1;Tmem255b	Signaling
DMR16:81211001	16	81211001	81220000	9000	1	2.50E-07	0.56	198	2.2	Tmem255b;Gas6	Extracellular Matrix
DMR16:81239001	16	81239001	81242000	3000	1	8.20E-08	0.48	57	1.9	Gas6;LOC108349416	Extracellular Matrix
DMR16:81317001	16	81317001	81322000	5000	1	1.60E-07	0.54	101	2.02	LOC290876;Rasa3	Signaling
DMR16:81640001	16	81640001	81646000	6000	1	7.70E-07	0.46	150	2.5	Adprhl1;LOC102548530	Signaling
DMR16:81674001	16	81674001	81675000	1000	1	1.10E-07	0.49	26	2.6	Grtp1	Signaling
DMR16:81715001	16	81715001	81717000	2000	1	1.30E-08	0.55	35	1.75	Lamp1;Cul4a	Transport;Proteolysis
DMR16:81737001	16	81737001	81740000	3000	2	2.80E-18	0.87	41	1.37	Cul4a	Proteolysis
DMR16:81845001	16	81845001	81848000	3000	1	8.40E-07	0.39	70	2.33	Mcf2l	Transcription
DMR16:81923001	16	81923001	81924000	1000	1	9.70E-07	0.42	17	1.7	Mcf2l	Transcription
DMR16:81931001	16	81931001	81935000	4000	1	4.90E-08	0.55	81	2.02	Mcf2l	Transcription
DMR16:82048001	16	82048001	82053000	5000	1	1.00E-07	0.64	92	1.84	Atp11a	Transport
DMR16:82102001	16	82102001	82104000	2000	1	1.20E-08	0.49	25	1.25	Atp11a;LOC102548949	Transport
DMR16:82218001	16	82218001	82222000	4000	1	1.80E-07	0.5	72	1.8	Tubgcp3	Cytoskeleton
DMR16:83004001	16	83004001	83008000	4000	1	3.30E-07	0.47	82	2.05	Tex29;Arhgef7	Transcription
DMR16:83037001	16	83037001	83039000	2000	1	3.30E-07	0.59	38	1.9	Arhgef7	Transcription
DMR16:83104001	16	83104001	83105000	1000	1	2.80E-11	0.66	18	1.8	Arhgef7	Transcription
DMR16:83111001	16	83111001	83112000	1000	1	9.40E-07	0.47	28	2.8	Arhgef7	Transcription
DMR16:83551001	16	83551001	83553000	2000	1	1.10E-07	0.49	50	2.5	Col4a1	Extracellular Matrix
DMR16:84667001	16	84667001	84668000	1000	1	3.70E-07	0.46	12	1.2	Myo16	
DMR16:85775001	16	85775001	85777000	2000	1	5.90E-08	0.52	13	0.65	Fam155a	
DMR17:674001	17	674001	675000	1000	1	2.80E-09	0.55	14	1.4	Npepo	
DMR17:768001	17	768001	770000	2000	1	2.80E-09	0.6	27	1.35	Npepo	
DMR17:1051001	17	1051001	1054000	3000	1	5.30E-09	0.46	57	1.9	Ptch1	
DMR17:1750001	17	1750001	1751000	1000	1	1.80E-07	0.39	14	1.4	Cdc14b	Signaling
DMR17:5318001	17	5318001	5324000	6000	2	1.30E-07	0.44	127	2.12	LOC102547665;LOC102547829;Spata31d1d;Spata31d1b	
DMR17:5334001	17	5334001	5337000	3000	1	4.10E-07	0.48	60	2	Spata31d1d;Spata31d1b;Spata31d3	
DMR17:6184001	17	6184001	6188000	4000	1	5.20E-07	0.56	99	2.48	Ntrk2	Receptor
DMR17:6779001	17	6779001	6781000	2000	1	1.40E-07	0.48	33	1.65	Kif27;LOC102551320;LOC102551121;Gkap1	Cytoskeleton;Cytoskeleton
DMR17:7021001	17	7021001	7026000	5000	1	1.20E-11	0.67	114	2.28	Klhl3;Mir874	Cytoskeleton
DMR17:7709001	17	7709001	7714000	5000	1	2.40E-07	0.4	99	1.98	Spock1	Signaling
DMR17:8276001	17	8276001	8280000	4000	1	1.90E-08	0.62	64	1.6	Trpc7	Transport
DMR17:8342001	17	8342001	8345000	3000	1	1.30E-07	0.45	49	1.63	Smad5	Transcription
DMR17:9329001	17	9329001	9334000	5000	1	1.60E-07	0.52	98	1.96	H2afy	
DMR17:9548001	17	9548001	9551000	3000	1	7.30E-07	0.37	57	1.9	B4galt7;Tmed9	Golgi;Transport
DMR17:9617001	17	9617001	9623000	6000	1	1.40E-08	0.45	114	1.9	Fam193b;LOC108348505;Ddx41	
DMR17:9655001	17	9655001	9663000	8000	2	4.30E-08	0.5	140	1.75	Pdlim7	Cytoskeleton
DMR17:9688001	17	9688001	9692000	4000	1	6.50E-09	0.52	84	2.1	Dbn1;Prr7	Cytoskeleton

DMR17:9811001	17	9811001	9818000	7000	1	2.60E-09	0.55	173	2.47	Lman2;LOC102550438	Transport
DMR17:9833001	17	9833001	9836000	3000	1	5.40E-11	0.57	68	2.27	LOC102550438;Mxd3;Prelid1;Rab24;Nsd1	Transcription
DMR17:10141001	17	10141001	10157000	16000	1	5.90E-07	0.47	366	2.29	Hk3;Unc5a	Signaling;Receptor
DMR17:10180001	17	10180001	10184000	4000	1	1.20E-07	0.53	103	2.58	Unc5a	Receptor
DMR17:10346001	17	10346001	10349000	3000	1	1.30E-07	0.43	50	1.67	Tspan17	
DMR17:10355001	17	10355001	10359000	4000	1	5.30E-11	0.65	77	1.93	Tspan17	
DMR17:10460001	17	10460001	10461000	1000	1	1.10E-07	-1.03	10	1	Cdhr2;Rnf44	Cytoskeleton
DMR17:10482001	17	10482001	10483000	1000	1	2.90E-07	0.42	12	1.2	Rnf44;Faf2	
DMR17:10608001	17	10608001	10612000	4000	1	5.50E-10	0.71	59	1.48	LOC306766;Simc1	
DMR17:10775001	17	10775001	10777000	2000	1	1.80E-11	0.64	43	2.15	Lnc012;Cplx2;LOC103694917	
DMR17:11673001	17	11673001	11675000	2000	1	2.90E-08	0.48	29	1.45	LOC108348511;Msx2	Development
DMR17:11924001	17	11924001	11927000	3000	1	7.50E-10	0.56	59	1.97	RGD1561671	Transcription
DMR17:11987001	17	11987001	11989000	2000	2	8.80E-07	0.66	37	1.85	Ror2	Receptor
DMR17:12003001	17	12003001	12005000	2000	1	1.20E-07	0.47	22	1.1	Ror2	Receptor
DMR17:12065001	17	12065001	12066000	1000	1	3.20E-11	0.6	26	2.6	Ror2	Receptor
DMR17:14470001	17	14470001	14472000	2000	1	1.70E-08	0.49	16	0.8	Trnak-cuu;LOC689448;LOC689458;LOC108353119	
DMR17:14616001	17	14616001	14617000	1000	1	6.10E-08	-0.71	12	1.2	LOC108348068;Ogn	
DMR17:15362001	17	15362001	15364000	2000	1	1.80E-07	0.47	51	2.55	Iars	Translation
DMR17:15382001	17	15382001	15384000	2000	1	9.10E-10	0.68	12	0.6	Iars	Translation
DMR17:15676001	17	15676001	15683000	7000	1	3.00E-08	0.6	151	2.16	Bicd2	
DMR17:15794001	17	15794001	15795000	1000	1	6.70E-07	0.45	9	0.9	Fgd3	Transcription
DMR17:15864001	17	15864001	15871000	7000	1	3.00E-09	0.66	141	2.01	Card19;Ninj1	Cytoskeleton
DMR17:15928001	17	15928001	15932000	4000	1	2.00E-08	0.63	61	1.52	Wnk2	Signaling
DMR17:16025001	17	16025001	16026000	1000	1	8.60E-08	0.44	16	1.6	Wnk2	Signaling
DMR17:16279001	17	16279001	16281000	2000	1	1.90E-07	0.42	36	1.8	Phf2	
DMR17:17996001	17	17996001	1.80E+07	4000	1	5.00E-07	0.58	91	2.28	Dek;Kdm1b	Epigenetic;Metabolism
DMR17:18020001	17	18020001	18022000	2000	1	1.60E-07	0.44	48	2.4	Kdm1b;Tpmt	Metabolism;Epigenetic
DMR17:18142001	17	18142001	18145000	3000	1	2.20E-11	0.58	50	1.67	LOC108348531;Kif13a	Cytoskeleton
DMR17:18226001	17	18226001	18231000	5000	1	2.20E-10	0.62	86	1.72	Kif13a	Cytoskeleton
DMR17:18288001	17	18288001	18290000	2000	1	1.20E-08	0.45	42	2.1	Kif13a	Cytoskeleton
DMR17:18459001	17	18459001	18461000	2000	1	2.50E-08	0.5	34	1.7	Cap2	Cytoskeleton
DMR17:18508001	17	18508001	18513000	5000	1	1.30E-11	0.64	77	1.54	Cap2;LOC108353127	Cytoskeleton
DMR17:19329001	17	19329001	19334000	5000	1	2.80E-07	0.42	83	1.66	Atxn1	
DMR17:20251001	17	20251001	20252000	1000	1	6.80E-07	-0.54	11	1.1	Jarid2	Epigenetic
DMR17:20280001	17	20280001	20281000	1000	1	7.80E-07	0.39	13	1.3	Jarid2	Epigenetic
DMR17:21433001	17	21433001	21434000	1000	1	3.60E-07	0.59	10	1	LOC108348546;Sycp2l	
DMR17:23552001	17	23552001	23554000	2000	1	6.90E-08	0.42	18	0.9	Phactr1	Signaling
DMR17:23783001	17	23783001	23785000	2000	1	4.90E-08	0.62	29	1.45	Tbc1d7	Signaling
DMR17:23996001	17	23996001	2.40E+07	4000	1	1.60E-07	0.51	58	1.45	Sirt5	
DMR17:26947001	17	26947001	26953000	6000	1	3.80E-07	0.41	115	1.92	Txndc5;Bmp6	Transcription;Growth Factors
DMR17:27442001	17	27442001	27444000	2000	1	8.30E-08	0.47	25	1.25	Riok1;Cage1	Signaling
DMR17:27543001	17	27543001	27545000	2000	1	9.80E-09	0.51	44	2.2	Rreb1	
DMR17:27554001	17	27554001	27560000	6000	1	1.30E-07	0.5	151	2.52	Rreb1	
DMR17:27641001	17	27641001	27644000	3000	1	3.90E-08	0.58	60	2	Rreb1	
DMR17:27951001	17	27951001	27954000	3000	1	9.40E-08	0.69	54	1.8	Nrn1	
DMR17:27974001	17	27974001	27976000	2000	1	3.90E-07	0.49	36	1.8	Nrn1	
DMR17:29729001	17	29729001	29732000	3000	1	6.80E-08	0.49	62	2.07	Cdyl	
DMR17:31250001	17	31250001	31252000	2000	2	1.20E-10	0.56	20	1	Slc22a23	Transport
DMR17:32625001	17	32625001	32626000	1000	1	7.70E-07	0.46	12	1.2	RGD1562844	
DMR17:33589001	17	33589001	33593000	4000	1	5.80E-11	0.52	35	0.88	Gmds	Metabolism
DMR17:36358001	17	36358001	36359000	1000	1	6.80E-07	0.45	18	1.8	E2f3	Transcription
DMR17:36486001	17	36486001	36488000	2000	1	1.30E-08	0.55	13	0.65	Cdkal1	
DMR17:42311001	17	42311001	42312000	1000	1	9.40E-08	0.47	11	1.1	Gmnn;LOC102548126	
DMR17:44570001	17	44570001	44571000	1000	1	4.90E-08	0.38	13	1.3	Prss16;Trnaq-cug;Trnas-gcu	Protease
DMR17:44823001	17	44823001	44824000	1000	1	6.40E-09	0.51	17	1.7	LOC100360950;Hist1h2bd	Epigenetic
DMR17:50542001	17	50542001	50546000	4000	1	7.20E-07	0.42	31	0.78	Sugct	Transport
DMR17:50606001	17	50606001	50611000	5000	1	6.20E-12	0.78	40	0.8	Sugct	Transport
DMR17:54013001	17	54013001	54015000	2000	1	9.90E-10	0.66	58	2.9	Tbce	Transcription
DMR17:54656001	17	54656001	54658000	2000	1	1.10E-08	0.54	36	1.8	Zeb1	Transcription
DMR17:55718001	17	55718001	55721000	3000	1	3.00E-07	0.41	46	1.53	RGD1562037;LOC102557539	
DMR17:56939001	17	56939001	56941000	2000	1	5.70E-08	0.46	27	1.35	RGD1564129	

DMR17:60428001	17	60428001	60431000	3000	1	3.50E-10	0.58	48	1.6	Armc4	
DMR17:62410001	17	62410001	62411000	1000	1	7.20E-07	-0.48	9	0.9	Ccny	
DMR17:68584001	17	68584001	68586000	2000	1	1.90E-08	0.44	34	1.7	Pfkp;LOC100360669	Metabolism
DMR17:69830001	17	69830001	69832000	2000	1	6.00E-07	0.48	21	1.05	Akr1c14;Akr1cl	
DMR17:72159001	17	72159001	72162000	3000	1	6.10E-07	0.4	48	1.6	Itih2	Protease; Proteolysis
DMR17:72185001	17	72185001	72187000	2000	1	1.00E-07	0.45	36	1.8	Itih2	Protease; Proteolysis
DMR17:72386001	17	72386001	72388000	2000	1	7.10E-08	0.52	40	2	Taf3;LOC102546306	
DMR17:75799001	17	75799001	75805000	6000	1	1.80E-07	0.4	89	1.48	Usp6nl	Signaling
DMR17:75813001	17	75813001	75815000	2000	1	9.10E-07	0.45	34	1.7	Usp6nl	Signaling
DMR17:76175001	17	76175001	76181000	6000	1	4.50E-09	0.5	78	1.3	Upf2	Metabolism
DMR17:76182001	17	76182001	76184000	2000	1	2.70E-08	0.47	25	1.25	Upf2	Metabolism
DMR17:76415001	17	76415001	76420000	5000	2	4.50E-09	0.54	76	1.52	Nudt5;Cdc123	Signaling
DMR17:76451001	17	76451001	76456000	5000	1	1.80E-07	0.44	91	1.82	Cdc123	
DMR17:76666001	17	76666001	76669000	3000	1	3.90E-08	0.49	60	2	Camk1d	Signaling
DMR17:76892001	17	76892001	76895000	3000	1	1.70E-09	0.57	34	1.13	Camk1d	Signaling
DMR17:77170001	17	77170001	77172000	2000	1	5.90E-07	0.41	35	1.75	Optn	
DMR17:78790001	17	78790001	78797000	7000	1	1.10E-07	0.43	137	1.96	Suv39h2;Dclre1c;LOC100125598;LOC108353135	Epigenetic;Transcription
DMR17:79082001	17	79082001	79083000	1000	1	9.00E-08	0.45	24	2.4	Fam171a1	
DMR17:79091001	17	79091001	79098000	7000	1	3.00E-09	0.62	167	2.39	Fam171a1	
DMR17:79122001	17	79122001	79123000	1000	1	8.40E-07	0.45	11	1.1	Fam171a1;LOC103694133	
DMR17:80800001	17	80800001	80802000	2000	1	9.50E-07	0.49	41	2.05	Cubn;LOC102550536	
DMR17:80844001	17	80844001	80846000	2000	1	5.90E-07	0.42	32	1.6	Trdmt1	Translation
DMR17:80979001	17	80979001	80982000	3000	1	1.90E-07	-0.55	22	0.73	St8sia6	Transport
DMR17:86075001	17	86075001	86077000	2000	1	1.20E-09	0.56	33	1.65	Msrb2	Metabolism
DMR17:87467001	17	87467001	87469000	2000	1	5.70E-09	0.53	27	1.35	Etl4	
DMR17:87731001	17	87731001	87733000	2000	1	2.10E-08	0.57	38	1.9	Arhgap21	
DMR17:87744001	17	87744001	87746000	2000	1	1.20E-07	0.5	48	2.4	Arhgap21	
DMR17:88537001	17	88537001	88539000	2000	1	3.10E-08	0.49	35	1.75	Gpr158	Signaling
DMR17:90107001	17	90107001	90109000	2000	1	2.30E-07	0.5	23	1.15	Apbb1ip	Cytoskeleton
DMR18:3155001	18	3155001	3157000	2000	1	5.70E-07	0.77	14	0.7	Rbbp8	Transcription
DMR18:3344001	18	3344001	3346000	2000	1	7.10E-07	0.47	20	1	Cables1;LOC102555446	
DMR18:3616001	18	3616001	3617000	1000	1	3.10E-07	0.49	22	2.2	RGD1311805;Npc1	
DMR18:3676001	18	3676001	3679000	3000	1	2.00E-08	0.54	43	1.43	Ankrd29	
DMR18:3917001	18	3917001	3921000	4000	2	1.60E-11	0.83	43	1.07	Lama3	Extracellular Matrix
DMR18:3966001	18	3966001	3973000	7000	1	3.70E-09	0.56	90	1.29	Ttc39c	
DMR18:3997001	18	3997001	3999000	2000	1	1.30E-07	0.65	29	1.45	Ttc39c	
DMR18:4131001	18	4131001	4135000	4000	1	2.80E-11	0.74	46	1.15	Osbpl1a	
DMR18:4276001	18	4276001	4278000	2000	1	1.60E-08	0.47	30	1.5	Osbpl1a	
DMR18:4281001	18	4281001	4283000	2000	1	8.70E-13	0.64	28	1.4	Osbpl1a	
DMR18:5324001	18	5324001	5325000	1000	1	3.30E-09	0.52	4	0.4	Zfp521	Transcription
DMR18:6029001	18	6029001	6032000	3000	1	3.40E-07	0.43	61	2.03	Ss18	Transcription
DMR18:8312001	18	8312001	8315000	3000	1	2.50E-07	0.55	40	1.33	Cdh2	Cytoskeleton
DMR18:11757001	18	11757001	11758000	1000	1	7.90E-07	0.71	9	0.9	Dsc3	Cytoskeleton
DMR18:12264001	18	12264001	12266000	2000	1	7.80E-11	0.65	20	1	RGD1562080;LOC102554807	
DMR18:15256001	18	15256001	15257000	1000	1	4.00E-09	0.54	13	1.3	Mapre2	Cytoskeleton
DMR18:15467001	18	15467001	15468000	1000	1	2.00E-09	0.55	11	1.1	Mapre2;LOC102551486;B4galt6	Cytoskeleton;Golgi
DMR18:15595001	18	15595001	15597000	2000	1	3.80E-07	0.48	53	2.65	Mapre2;Dsg2	Cytoskeleton;Cytoskeleton
DMR18:15630001	18	15630001	15632000	2000	1	1.80E-07	0.48	41	2.05	Mapre2;Dsg2;LOC102551904	Cytoskeleton;Cytoskeleton
DMR18:15745001	18	15745001	15746000	1000	1	5.90E-08	0.52	13	1.3	Mapre2	Cytoskeleton
DMR18:15885001	18	15885001	15888000	3000	1	1.40E-07	0.5	36	1.2	Zscan30;Zfp35	Transcription
DMR18:16541001	18	16541001	16542000	1000	1	3.40E-07	0.44	25	2.5	Slc39a6;Elp2	Transport
DMR18:17770001	18	17770001	17772000	2000	1	6.20E-07	0.53	35	1.75	Celf4	
DMR18:17815001	18	17815001	17816000	1000	1	1.80E-08	0.55	10	1	Celf4	
DMR18:17828001	18	17828001	17834000	6000	1	2.00E-10	0.53	85	1.42	Celf4	
DMR18:17920001	18	17920001	17921000	1000	1	2.10E-08	0.46	13	1.3	Celf4;LOC102555477	
DMR18:23987001	18	23987001	23988000	1000	1	5.20E-07	0.74	6	0.6	Rit2	Signaling
DMR18:24455001	18	24455001	24456000	1000	1	4.20E-14	0.61	9	0.9	Sap130	Epigenetic
DMR18:24687001	18	24687001	24688000	1000	1	2.00E-07	0.52	22	2.2	Wdr33;Sft2d3	Translation
DMR18:25007001	18	25007001	25010000	3000	1	8.50E-07	0.52	24	0.8	Map3k2	
DMR18:25047001	18	25047001	25049000	2000	1	2.30E-08	0.5	46	2.3	Ercc3	Epigenetic
DMR18:25210001	18	25210001	25215000	5000	1	4.60E-14	0.7	103	2.06	Bin1	
DMR18:26181001	18	26181001	26183000	2000	1	6.80E-07	0.52	64	3.2	Nrep	
DMR18:27077001	18	27077001	27078000	1000	1	1.90E-07	0.62	12	1.2	Apc	
DMR18:27127001	18	27127001	27134000	7000	3	1.60E-08	0.5	146	2.09	Srp19;Reep5;LOC102549915	Transport;Transport

DMR18:28056001	18	28056001	28058000	2000	1	2.10E-07	0.46	35	1.75	Ctnna1;Sil1	Cytoskeleton
DMR18:28067001	18	28067001	28068000	1000	1	4.10E-08	0.57	28	2.8	Sil1	
DMR18:28635001	18	28635001	28643000	8000	1	3.00E-09	0.65	161	2.01	Cxxc5;LOC103694188	
DMR18:28650001	18	28650001	28652000	2000	1	2.40E-07	0.5	49	2.45	Cxxc5	
DMR18:28924001	18	28924001	28926000	2000	1	8.40E-07	0.53	22	1.1	Nrg2;LOC103694189	Growth Factors
DMR18:30795001	18	30795001	30797000	2000	1	1.90E-10	0.6	23	1.15	RGD1563159	
DMR18:31032001	18	31032001	31035000	3000	1	5.60E-07	0.55	26	0.87	Diaph1	
DMR18:31565001	18	31565001	31567000	2000	1	3.00E-07	0.55	34	1.7	Ndfip1	
DMR18:31887001	18	31887001	31889000	2000	1	6.60E-07	0.41	34	1.7	Nr3c1;Arhgap26	Signaling
DMR18:32404001	18	32404001	32406000	2000	1	6.80E-13	0.66	28	1.4	Nr3c1;Arhgap26	Signaling
DMR18:32574001	18	32574001	32577000	3000	1	3.20E-12	0.63	38	1.27	Nr3c1;LOC102547511	
DMR18:36460001	18	36460001	36462000	2000	1	8.00E-09	0.79	11	0.55	Sh3rf2	
DMR18:37175001	18	37175001	37176000	1000	1	2.30E-07	0.43	6	0.6	Ppp2r2b	Signaling
DMR18:37891001	18	37891001	37892000	1000	1	8.10E-07	-0.43	8	0.8	Jakmip2	
DMR18:41067001	18	41067001	41068000	1000	1	9.30E-07	0.43	13	1.3	Commd10	
DMR18:44691001	18	44691001	44692000	1000	1	2.20E-08	0.52	14	1.4	Tnfaip8	
DMR18:44814001	18	44814001	44816000	2000	1	7.80E-07	0.48	19	0.95	Hsd17b4	
DMR18:48664001	18	48664001	48668000	4000	1	8.30E-10	0.59	62	1.55	Cep120	
DMR18:51843001	18	51843001	51847000	4000	1	1.10E-08	0.62	69	1.73	3-Mar	
DMR18:52431001	18	52431001	52433000	2000	1	1.40E-07	0.41	65	3.25	Prrc1	
DMR18:53732001	18	53732001	53734000	2000	1	6.70E-07	0.45	30	1.5	Isoc1	Metabolism
DMR18:55872001	18	55872001	55874000	2000	1	4.20E-07	0.39	48	2.4	Synpo	Cytoskeleton
DMR18:56061001	18	56061001	56062000	1000	1	2.20E-07	0.47	25	2.5	Cd74	Cytoskeleton
DMR18:56390001	18	56390001	56392000	2000	1	6.50E-09	0.51	45	2.25	Pdgfrb	Receptor
DMR18:56559001	18	56559001	56561000	2000	1	3.00E-08	0.5	26	1.3	Pde6a	Signaling
DMR18:57073001	18	57073001	57074000	1000	1	8.60E-07	0.41	11	1.1	Afap1l1	
DMR18:57142001	18	57142001	57146000	4000	1	3.20E-07	0.42	68	1.7	Ablim3	Cytoskeleton
DMR18:57402001	18	57402001	57403000	1000	1	8.00E-07	0.47	22	2.2	Sh3tc2	
DMR18:57838001	18	57838001	57841000	3000	1	4.10E-07	0.4	71	2.37	Fbxo38	
DMR18:58290001	18	58290001	58296000	6000	1	9.40E-09	0.52	91	1.52	Apcdd1	
DMR18:59334001	18	59334001	59336000	2000	1	8.30E-07	0.38	37	1.85	Wdr7	
DMR18:59364001	18	59364001	59365000	1000	1	8.10E-08	-0.63	3	0.3	Wdr7	
DMR18:59942001	18	59942001	59948000	6000	1	5.60E-09	0.67	87	1.45	Fech	Metabolism
DMR18:60428001	18	60428001	60429000	1000	1	4.00E-07	0.47	7	0.7	Nedd4l	Proteolysis
DMR18:60682001	18	60682001	60684000	2000	1	4.10E-09	0.53	34	1.7	Nedd4l	Proteolysis
DMR18:60702001	18	60702001	60707000	5000	1	1.60E-07	0.41	92	1.84	Nedd4l	Proteolysis
DMR18:61001001	18	61001001	61004000	3000	1	2.50E-07	0.41	56	1.87	Alpk2;LOC108348802	Signaling
DMR18:61250001	18	61250001	61252000	2000	1	3.50E-07	0.46	30	1.5	Zfp532	
DMR18:61409001	18	61409001	61410000	1000	1	5.70E-07	0.45	9	0.9	Oacyl	Metabolism
DMR18:61429001	18	61429001	61431000	2000	2	4.30E-07	0.43	40	2	Oacyl	Metabolism
DMR18:61499001	18	61499001	61500000	1000	1	7.60E-10	0.52	9	0.9	Sec11c	Protease
DMR18:63907001	18	63907001	63909000	2000	1	5.50E-07	0.55	37	1.85	Ldlrad4	
DMR18:63957001	18	63957001	63959000	2000	1	1.80E-08	0.54	36	1.8	Ldlrad4	
DMR18:65296001	18	65296001	65298000	2000	1	8.20E-07	0.51	32	1.6	Tcf4	Transcription
DMR18:65367001	18	65367001	65369000	2000	1	3.30E-08	0.47	26	1.3	Tcf4	Transcription
DMR18:69941001	18	69941001	69944000	3000	1	5.80E-07	0.53	70	2.33	Mapk4	Signaling
DMR18:70194001	18	70194001	70196000	2000	1	1.40E-07	0.43	57	2.85	Ska1;Cxxc1	
DMR18:70255001	18	70255001	70260000	5000	1	1.00E-08	0.5	101	2.02	Mbd1;Cfap53	
DMR18:70310001	18	70310001	70313000	3000	1	3.10E-07	0.4	44	1.47	Cfap53;LOC108348813	
DMR18:70631001	18	70631001	70636000	5000	2	4.20E-08	0.49	97	1.94	Myo5b	Cytoskeleton
DMR18:70751001	18	70751001	70754000	3000	1	6.50E-08	0.45	49	1.63	Acaa2	Metabolism
DMR18:71234001	18	71234001	71235000	1000	1	2.70E-08	0.53	18	1.8	Dym	
DMR18:71401001	18	71401001	71404000	3000	1	9.40E-07	0.53	36	1.2	LOC102556053;Smad7	Transcription
DMR18:71405001	18	71405001	71409000	4000	1	1.20E-07	0.55	60	1.5	Smad7	Transcription
DMR18:73156001	18	73156001	73158000	2000	1	2.50E-07	-0.75	59	2.95	Skor2	
DMR18:73248001	18	73248001	73250000	2000	1	7.50E-08	0.48	81	4.05	Hdhd2	Signaling
DMR18:73328001	18	73328001	73330000	2000	1	3.30E-08	0.45	25	1.25	Katnal2	Cytoskeleton
DMR18:74336001	18	74336001	74338000	2000	2	6.50E-14	0.79	35	1.75	Epg5	
DMR18:74347001	18	74347001	74348000	1000	1	1.50E-09	0.57	13	1.3	Epg5	
DMR18:74494001	18	74494001	74497000	3000	1	2.30E-10	0.59	68	2.27	Slc14a1;LOC102553274	Transport
DMR18:74663001	18	74663001	74665000	2000	1	3.20E-09	0.61	15	0.75	Slc14a2	Transport
DMR18:76624001	18	76624001	76629000	5000	1	3.70E-07	0.49	128	2.56	Pard6g;Adnp2	Cell Junction
DMR18:76651001	18	76651001	76653000	2000	1	1.30E-10	0.61	42	2.1	Adnp2;LOC108348823	
DMR18:76795001	18	76795001	76798000	3000	2	2.40E-08	0.51	96	3.2	Pqlc1	
DMR18:76890001	18	76890001	76893000	3000	2	1.80E-08	0.8	44	1.47	Kcng2	Transport

DMR18:77234001	18	77234001	77235000	1000	1	1.40E-07	0.5	29	2.9	Nfatc1	Transcription
DMR18:77275001	18	77275001	77276000	1000	1	4.60E-07	0.5	36	3.6	Nfatc1	Transcription
DMR18:77284001	18	77284001	77286000	2000	1	8.70E-10	0.63	34	1.7	Nfatc1	Transcription
DMR18:77289001	18	77289001	77291000	2000	1	1.70E-08	0.42	59	2.95	Nfatc1	Transcription
DMR18:77368001	18	77368001	77371000	3000	1	5.60E-09	0.54	39	1.3	Atp9b	Transport
DMR18:77389001	18	77389001	77390000	1000	1	6.40E-07	0.45	14	1.4	Atp9b	Transport
DMR18:79828001	18	79828001	79831000	3000	1	6.40E-07	0.43	68	2.27	Zfp516	Transcription
DMR18:79846001	18	79846001	79849000	3000	1	4.10E-08	0.73	62	2.07	Zfp516	Transcription
DMR18:79858001	18	79858001	79863000	5000	1	8.20E-08	0.52	89	1.78	Zfp516	Transcription
DMR18:81282001	18	81282001	81284000	2000	1	2.00E-07	0.68	30	1.5	Zfp407;LOC102552911	Transcription
DMR18:81601001	18	81601001	81605000	4000	1	6.30E-09	0.5	48	1.2	Fam69c	
DMR18:86588001	18	86588001	86589000	1000	1	3.00E-09	-0.93	4	0.4	Dok6	
DMR18:87614001	18	87614001	87616000	2000	1	7.20E-10	0.55	18	0.9	Tmx3	Transcription
DMR19:686001	19	686001	687000	1000	1	4.50E-08	-0.62	13	1.3	Nae1;Terb1	Proteolysis
DMR19:9768001	19	9768001	9772000	4000	1	7.90E-07	0.45	92	2.3	Cnot1;Setd6;Ndrg4	Translation;Epigenetic;Protease
DMR19:9781001	19	9781001	9785000	4000	1	5.40E-08	0.5	62	1.55	Ndrg4	Protease
DMR19:10084001	19	10084001	10089000	5000	1	8.80E-08	0.52	101	2.02	Mmp15	Protease
DMR19:10183001	19	10183001	10185000	2000	1	6.20E-07	0.51	21	1.05	Cngb1	Ion Channel
DMR19:10339001	19	10339001	10340000	1000	1	9.40E-10	0.79	12	1.2	Kifc3;Katnb1	Cytoskeleton
DMR19:10349001	19	10349001	10352000	3000	1	9.40E-07	0.46	50	1.67	Kifc3;Katnb1	Cytoskeleton
DMR19:10541001	19	10541001	10543000	2000	1	1.50E-07	0.45	31	1.55	Ccdc102a	
DMR19:10624001	19	10624001	10626000	2000	1	1.30E-07	0.38	38	1.9	Ccl17;LOC307650	Growth Factors
DMR19:10645001	19	10645001	10647000	2000	1	5.00E-07	0.46	35	1.75	Cx3cl1	Growth Factors
DMR19:10854001	19	10854001	10857000	3000	1	3.30E-07	0.47	43	1.43	Fam192a;Cpne2	
DMR19:10861001	19	10861001	10867000	6000	1	3.80E-07	0.45	109	1.82	Fam192a;Cpne2	
DMR19:11422001	19	11422001	11423000	1000	1	2.10E-11	0.5	22	2.2	Bbs2;Ogfod1	Epigenetic
DMR19:11431001	19	11431001	11432000	1000	1	1.10E-07	0.41	24	2.4	Bbs2;Ogfod1	Epigenetic
DMR19:11487001	19	11487001	11489000	2000	2	7.40E-10	0.69	61	3.05	Amfr	Proteolysis
DMR19:11619001	19	11619001	11620000	1000	1	1.40E-07	0.69	25	2.5	Gnao1	Signaling
DMR19:11636001	19	11636001	11641000	5000	1	1.10E-08	0.5	84	1.68	Gnao1	Signaling
DMR19:12674001	19	12674001	12675000	1000	1	9.50E-07	0.47	17	1.7	Large1	Golgi
DMR19:12921001	19	12921001	12927000	6000	1	2.80E-07	0.49	95	1.58	Large1	Golgi
DMR19:14224001	19	14224001	14226000	2000	1	7.50E-07	0.51	41	2.05	Isx	Development
DMR19:14660001	19	14660001	14663000	3000	1	6.00E-08	0.54	88	2.93	Rasd2	
DMR19:16792001	19	16792001	16796000	4000	1	8.20E-07	0.44	63	1.57	Fto	Metabolism
DMR19:16863001	19	16863001	16866000	3000	1	1.20E-07	0.47	40	1.33	Fto	Metabolism
DMR19:17083001	19	17083001	17087000	4000	2	7.30E-09	0.49	53	1.32	Fto	Metabolism
DMR19:20176001	19	20176001	20178000	2000	1	8.30E-09	0.53	33	1.65	Zfp423	
DMR19:20332001	19	20332001	20334000	2000	2	5.70E-12	0.6	43	2.15	Zfp423	
DMR19:21450001	19	21450001	21454000	4000	3	4.20E-11	1.01	82	2.05	Lonp2	Protease
DMR19:21577001	19	21577001	21582000	5000	1	7.70E-07	0.54	67	1.34	Abcc12	Transport
DMR19:22572001	19	22572001	22573000	1000	1	9.40E-10	0.47	12	1.2	Dnaja2	Transcription
DMR19:22612001	19	22612001	22621000	9000	1	1.40E-07	0.49	194	2.16	Gpt2;LOC102548349	Metabolism
DMR19:23399001	19	23399001	23402000	3000	1	7.90E-07	0.43	100	3.33	Sall1;LOC103694299	Transcription
DMR19:24322001	19	24322001	24324000	2000	1	8.70E-07	0.47	21	1.05	LOC108348920;LOC102554553;Tbc1d9;LOC108348919	Signaling
DMR19:24704001	19	24704001	24707000	3000	1	7.50E-07	0.58	60	2	LOC102556660;Ndufb7;Tecr	Metabolism;Metabolism
DMR19:25074001	19	25074001	25076000	2000	1	1.40E-07	-0.5	26	1.3	Adgrl1;Asf1b	Signaling;Epigenetic
DMR19:25559001	19	25559001	25562000	3000	1	8.70E-07	0.43	50	1.67	Cacna1a	Transport
DMR19:25833001	19	25833001	25835000	2000	1	8.60E-07	0.42	34	1.7	Nf1x;LOC102546551	Transcription
DMR19:25865001	19	25865001	25868000	3000	1	2.70E-08	0.58	53	1.77	Nf1x	Transcription
DMR19:28660001	19	28660001	28661000	1000	1	5.20E-09	0.55	36	3.6	LOC501317;LOC691712;RGD1562660	
DMR19:29611001	19	29611001	29612000	1000	1	7.80E-08	0.38	12	1.2	Inpp4b	
DMR19:29810001	19	29810001	29811000	1000	1	1.60E-07	0.43	15	1.5	Inpp4b;LOC103694314	Epigenetic
DMR19:30039001	19	30039001	30040000	1000	1	4.70E-19	0.51	13	1.3	Inpp4b	
DMR19:30212001	19	30212001	30213000	1000	1	7.50E-07	0.53	13	1.3	Inpp4b	
DMR19:32398001	19	32398001	32401000	3000	1	1.80E-08	0.54	70	2.33	Zfp827	Transcription
DMR19:32466001	19	32466001	32469000	3000	1	1.00E-08	0.59	38	1.27	Zfp827	Transcription
DMR19:32476001	19	32476001	32481000	5000	1	7.60E-08	0.43	74	1.48	Zfp827	Transcription
DMR19:32549001	19	32549001	32551000	2000	1	5.60E-09	0.4	31	1.55	Zfp827	Transcription
DMR19:32588001	19	32588001	32589000	1000	1	8.00E-08	0.5	21	2.1	Zfp827	Transcription
DMR19:32924001	19	32924001	32927000	3000	1	8.00E-09	0.62	25	0.83	Slc10a7	Transport
DMR19:33012001	19	33012001	33014000	2000	1	2.40E-07	0.51	31	1.55	Slc10a7	Transport
DMR19:34390001	19	34390001	34391000	1000	1	6.50E-07	0.6	13	1.3	Arhgap10	Signaling

DMR19:37424001	19	37424001	37425000	1000	1	1.30E-07	0.51	17	1.7	Lrrc36;Tppp3;Zdhhc1	Cytoskeleton
DMR19:37480001	19	37480001	37481000	1000	1	9.80E-07	0.4	14	1.4	Hsd11b2;Atp6v0d1	Metabolism;Metabolism
DMR19:37646001	19	37646001	37648000	2000	1	4.30E-07	0.51	17	0.85	Ctcf;Carmil2	Transcription
DMR19:38115001	19	38115001	38117000	2000	1	6.50E-09	0.56	33	1.65	Nfatc3;Esrp2	Transcription;Translation
DMR19:38469001	19	38469001	38470000	1000	1	2.90E-07	0.55	7	0.7	Nfat5	Transcription
DMR19:38785001	19	38785001	38786000	1000	1	5.40E-08	0.6	11	1.1	Cdh1	Cytoskeleton
DMR19:38934001	19	38934001	38937000	3000	1	3.40E-08	-0.56	22	0.73	Tango6	
DMR19:41971001	19	41971001	41972000	1000	1	3.10E-07	-0.67	8	0.8	Ist1	
DMR19:42763001	19	42763001	42772000	9000	1	1.00E-11	0.67	313	3.48	Zfhx3	Transcription
DMR19:42874001	19	42874001	42876000	2000	1	4.80E-07	0.41	39	1.95	Zfhx3	Transcription
DMR19:43907001	19	43907001	43914000	7000	1	6.40E-09	0.43	102	1.46	Ctrb1	Protease
DMR19:46098001	19	46098001	46100000	2000	1	2.80E-07	0.54	38	1.9	Adamts18	Protease
DMR19:46837001	19	46837001	46841000	4000	1	1.70E-07	0.52	54	1.35	Wwox	
DMR19:47400001	19	47400001	47401000	1000	1	8.30E-08	0.44	25	2.5	Wwox	
DMR19:49181001	19	49181001	49182000	1000	1	3.30E-07	0.52	15	1.5	Cdyl2	
DMR19:49722001	19	49722001	49724000	2000	1	3.10E-07	0.46	32	1.6	Gan	Cytoskeleton
DMR19:49807001	19	49807001	49808000	1000	1	4.30E-07	0.47	13	1.3	Cmip;LOC690350	
DMR19:49937001	19	49937001	49938000	1000	1	1.70E-08	0.5	16	1.6	Cmip	
DMR19:49941001	19	49941001	49946000	5000	1	1.70E-08	0.45	118	2.36	Cmip	
DMR19:50069001	19	50069001	50070000	1000	1	6.40E-07	0.47	28	2.8	Plcg2	Metabolism
DMR19:50141001	19	50141001	50148000	7000	1	1.60E-07	0.48	158	2.26	Plcg2	Metabolism
DMR19:50222001	19	50222001	50226000	4000	1	3.70E-07	0.47	72	1.8	Sdr42e1	Metabolism
DMR19:51053001	19	51053001	51054000	1000	1	3.70E-08	0.48	15	1.5	Cdh13	Cytoskeleton
DMR19:51080001	19	51080001	51082000	2000	1	7.30E-07	0.46	27	1.35	Cdh13	Cytoskeleton
DMR19:51545001	19	51545001	51547000	2000	1	5.30E-08	0.49	50	2.5	Cdh13	Cytoskeleton
DMR19:51642001	19	51642001	51643000	1000	1	9.20E-09	0.47	9	0.9	Cdh13	Cytoskeleton
DMR19:51785001	19	51785001	51787000	2000	1	2.70E-07	0.51	43	2.15	Cdh13	Cytoskeleton
DMR19:52111001	19	52111001	52113000	2000	1	6.10E-09	0.73	33	1.65	Necab2;Slc38a8	Transport
DMR19:52246001	19	52246001	52251000	5000	1	6.60E-10	0.59	109	2.18	Dnaaf1;Taf1c;Adad2	Signaling;Transcription;Metabolism
DMR19:52388001	19	52388001	52395000	7000	1	9.20E-07	0.73	116	1.66	Atp2c2;LOC103694346	Transport
DMR19:52432001	19	52432001	52433000	1000	1	4.10E-07	0.58	8	0.8	Tldc1	
DMR19:52598001	19	52598001	52600000	2000	1	2.60E-08	0.56	49	2.45	Usp10	Protease
DMR19:52642001	19	52642001	52644000	2000	1	3.80E-08	0.86	29	1.45	Crispld2	Immune
DMR19:52715001	19	52715001	52717000	2000	1	4.60E-07	0.57	30	1.5	Crispld2	Immune
DMR19:53181001	19	53181001	53183000	2000	1	1.90E-09	0.49	29	1.45	RGD1304884;LOC103694348	
DMR19:53283001	19	53283001	53285000	2000	1	3.60E-07	0.53	33	1.65	RGD1304884;LOC367563	
DMR19:53318001	19	53318001	53323000	5000	1	5.10E-07	0.51	84	1.68	RGD1304884	
DMR19:53376001	19	53376001	53377000	1000	1	1.70E-07	0.61	20	2	RGD1304884;LOC108348955	
DMR19:53395001	19	53395001	53397000	2000	1	3.50E-07	0.48	45	2.25	RGD1304884;LOC108348955	
DMR19:53648001	19	53648001	53652000	4000	1	1.40E-10	0.61	101	2.52	RGD1304884;Map1lc3b;Zcchc14	Cytoskeleton
DMR19:53734001	19	53734001	53735000	1000	1	1.40E-07	0.52	14	1.4	RGD1304884;Fam92b	
DMR19:53827001	19	53827001	53828000	1000	1	1.30E-08	0.57	20	2	Gse1	
DMR19:54021001	19	54021001	54026000	5000	1	2.70E-11	0.49	96	1.92	Gse1;LOC102551873	
DMR19:54126001	19	54126001	54128000	2000	1	9.90E-07	0.4	46	2.3	Gse1	
DMR19:54135001	19	54135001	54141000	6000	1	1.50E-07	0.53	166	2.77	Gse1	
DMR19:54881001	19	54881001	54888000	7000	1	2.40E-07	0.54	129	1.84	Zfp469	
DMR19:54919001	19	54919001	54920000	1000	1	8.20E-09	0.44	14	1.4	Zfp469	
DMR19:54934001	19	54934001	54940000	6000	1	6.10E-09	0.46	144	2.4	Zfp469	
DMR19:54984001	19	54984001	54989000	5000	1	2.80E-09	0.58	88	1.76	Zfp469	
DMR19:54996001	19	54996001	55002000	6000	1	8.10E-07	0.46	118	1.97	Zfp469	
DMR19:55071001	19	55071001	55074000	3000	1	3.50E-07	0.53	76	2.53	Zfp469	
DMR19:55230001	19	55230001	55237000	7000	1	2.60E-07	0.43	109	1.56	Zc3h18;Il17c	
DMR19:55258001	19	55258001	55267000	9000	1	7.90E-08	0.46	245	2.72	Il17c;Cyba;Mvd;Snai3	Metabolism;Transcription
DMR19:55324001	19	55324001	55326000	2000	1	4.10E-08	0.59	63	3.15	Piezo1	
DMR19:55450001	19	55450001	55458000	8000	1	8.00E-07	0.45	203	2.54	Cbfa2t3;LOC108348958	Transcription
DMR19:55507001	19	55507001	55511000	4000	1	1.60E-08	0.44	68	1.7	Cbfa2t3	Transcription
DMR19:55709001	19	55709001	55715000	6000	1	2.90E-07	0.42	137	2.28	Ankrd11	
DMR19:55762001	19	55762001	55765000	3000	1	3.50E-07	0.45	43	1.43	Ankrd11	
DMR19:55850001	19	55850001	55852000	2000	1	1.40E-10	0.67	33	1.65	Ankrd11	
DMR19:55900001	19	55900001	55904000	4000	1	1.60E-07	0.49	67	1.68	Spg7	Protease
DMR19:55931001	19	55931001	55933000	2000	1	3.60E-07	0.49	33	1.65	Cpne7	
DMR19:55934001	19	55934001	55939000	5000	1	6.40E-08	0.48	97	1.94	Cpne7	
DMR19:56189001	19	56189001	56191000	2000	1	6.30E-08	0.58	31	1.55	Tcf25	
DMR19:56336001	19	56336001	56340000	4000	1	2.00E-08	0.48	109	2.72	Gas8	Cytoskeleton

DMR19:56600001	19	56600001	56603000	3000	1	2.40E-10	0.57	46	1.53	Rab4a	
DMR19:56703001	19	56703001	56704000	1000	1	2.60E-11	0.65	4	0.4	Nup133	
DMR19:57087001	19	57087001	57094000	7000	1	6.10E-07	0.57	164	2.34	Galnt2	Golgi
DMR19:57472001	19	57472001	57475000	3000	1	2.00E-09	0.52	69	2.3	Ttc13;Arv1	
DMR19:57477001	19	57477001	57478000	1000	1	1.50E-07	0.46	21	2.1	Ttc13;Arv1	
DMR19:57616001	19	57616001	57623000	7000	1	2.60E-07	0.64	85	1.21	RGD1562218;Gnpat	Metabolism
DMR19:58752001	19	58752001	58754000	2000	1	1.90E-07	0.49	46	2.3	RGD1306091	
DMR19:61995001	19	61995001	61996000	1000	1	5.70E-07	-0.57	6	0.6	Ccdc7	
DMR19:62054001	19	62054001	62056000	2000	1	2.00E-07	0.41	22	1.1	Ccdc7	
DMR20:375001	20	375001	377000	2000	1	5.80E-07	-0.41	23	1.15	Olr1669-ps;Olr1670	Receptor
DMR20:2203001	20	2203001	2204000	1000	1	5.60E-11	0.68	16	1.6	Trim15;Trim15-ps1;Trim26	Proteolysis
DMR20:2856001	20	2856001	2858000	2000	1	2.10E-08	0.78	20	1	Btnl8	Immune
DMR20:2886001	20	2886001	2888000	2000	1	2.60E-08	0.96	27	1.35	Btnl6-ps1	
DMR20:3327001	20	3327001	3328000	1000	1	7.50E-07	0.46	20	2	Abcf1;Mir877;Ppp1r10	Translation;Signaling
DMR20:3408001	20	3408001	3411000	3000	1	7.30E-07	0.42	50	1.67	Nrm;Mdc1	
DMR20:3589001	20	3589001	3595000	6000	1	7.70E-07	0.43	130	2.17	Gtf2h4;LOC108349028;Vars2;Sfta2	Transcription;Translation;Transport
DMR20:3747001	20	3747001	3749000	2000	1	6.30E-07	0.45	53	2.65	Cchcr1;Tcf19;Pou5f1	Transcription
DMR20:4153001	20	4153001	4157000	4000	1	7.30E-09	0.48	71	1.77	Btnl2;Btnl3	Immune
DMR20:4476001	20	4476001	4478000	2000	1	2.80E-07	0.68	44	2.2	Stk19;Tnxa-ps1;Cyp21a1	
DMR20:4495001	20	4495001	4497000	2000	1	1.00E-07	0.54	45	2.25	Stk19;Tnxa-ps1;Cyp21a1;LOC103689965	Protease; Proteolysis
DMR20:4583001	20	4583001	4588000	5000	2	1.20E-09	0.55	201	4.02	Zbtb12;Ehmt2;Slc44a4	Transcription;Transport
DMR20:4841001	20	4841001	4845000	4000	1	3.80E-07	0.45	82	2.05	Nfkbil1;Lta	Signaling
DMR20:5024001	20	5024001	5025000	1000	1	2.80E-07	0.48	16	1.6	Vwa7;Sapcd1;Msh5	Transcription
DMR20:5087001	20	5087001	5094000	7000	1	3.90E-09	0.53	132	1.89	Rps25-ps2;Abhd16a;Ly6g5c	Protease
DMR20:5162001	20	5162001	5164000	2000	1	6.30E-07	0.49	27	1.35	Prrc2a;E230034O05Rik;Aif1;Ncr3	Metabolism;Signaling
DMR20:5459001	20	5459001	5461000	2000	1	1.10E-09	0.55	59	2.95	Wdr46;Pfdn6;Rgl2;Tapbp	Transcription;Immune
DMR20:5470001	20	5470001	5472000	2000	1	3.20E-07	0.55	39	1.95	Rgl2;Tapbp;Zbtb22;Daxx	Transcription;Immune;Cytoskeleton
DMR20:5656001	20	5656001	5658000	2000	1	1.10E-07	0.52	39	1.95	Itpr3	Ion Channel
DMR20:5686001	20	5686001	5690000	4000	1	1.90E-09	0.52	86	2.15	Itpr3	Ion Channel
DMR20:5695001	20	5695001	5700000	5000	1	7.70E-07	0.48	119	2.38	Itpr3	Ion Channel
DMR20:5797001	20	5797001	5798000	1000	1	1.50E-07	0.5	12	1.2	Clpsl2;Clps	Signaling
DMR20:5905001	20	5905001	5906000	1000	1	7.30E-08	0.44	20	2	Slc26a8	Transport
DMR20:6418001	20	6418001	6422000	4000	1	4.00E-07	0.49	98	2.45	Rab44;LOC100910615;Cpne5	
DMR20:6432001	20	6432001	6433000	1000	1	1.10E-07	0.52	18	1.8	Cpne5	
DMR20:6521001	20	6521001	6524000	3000	1	6.50E-07	0.43	38	1.27	Ppil1	Transcription
DMR20:6624001	20	6624001	6627000	3000	1	3.70E-11	0.58	77	2.57	Ppil1	Transcription
DMR20:6642001	20	6642001	6644000	2000	1	6.50E-07	0.44	38	1.9	Ppil1	Transcription
DMR20:6678001	20	6678001	6684000	6000	1	7.40E-07	0.44	99	1.65	Ppil1	Transcription
DMR20:6739001	20	6739001	6742000	3000	1	2.50E-09	0.69	53	1.77	Ppil1;Grm4	Transcription;Signaling
DMR20:6795001	20	6795001	6796000	1000	1	1.00E-09	0.71	19	1.9	Ppil1;Grm4	Transcription;Signaling
DMR20:6901001	20	6901001	6903000	2000	1	6.40E-10	0.57	36	1.8	RGD735065	
DMR20:6906001	20	6906001	6911000	5000	1	1.20E-07	0.42	92	1.84	RGD735065	
DMR20:6977001	20	6977001	6980000	3000	1	3.30E-07	0.49	76	2.53	Fgd2	Transcription
DMR20:7118001	20	7118001	7123000	5000	1	1.10E-07	0.5	114	2.28	Hmga1	Transcription
DMR20:7549001	20	7549001	7552000	3000	1	5.00E-07	0.47	67	2.23	Anks1a	Cytoskeleton
DMR20:7583001	20	7583001	7586000	3000	1	2.90E-08	0.46	96	3.2	Anks1a	Cytoskeleton
DMR20:7587001	20	7587001	7590000	3000	1	9.10E-09	0.46	74	2.47	Anks1a	Cytoskeleton
DMR20:7627001	20	7627001	7637000	10000	2	3.40E-08	0.5	294	2.94	Anks1a;Tcp11	Cytoskeleton;Cytoskeleton
DMR20:7778001	20	7778001	7783000	5000	2	2.60E-07	0.72	169	3.38	Zfp523;Def6	Cytoskeleton
DMR20:7812001	20	7812001	7814000	2000	1	3.60E-08	0.51	45	2.25	Def6;Ppard	Cytoskeleton;Transcription
DMR20:7897001	20	7897001	7902000	5000	1	3.30E-08	0.47	122	2.44	Fance;Rpl10a;Tead3	Translation;Transcription
DMR20:7940001	20	7940001	7943000	3000	1	3.50E-07	0.43	64	2.13	Tead3;Tulp1	Transcription
DMR20:8110001	20	8110001	8113000	3000	1	1.40E-09	0.47	73	2.43	Armc12	
DMR20:8229001	20	8229001	8232000	3000	1	2.50E-09	0.61	67	2.23	Tbc1d22b	Signaling
DMR20:8269001	20	8269001	8273000	4000	1	1.10E-08	0.51	101	2.52	Tbc1d22b	Signaling
DMR20:8294001	20	8294001	8300000	6000	1	1.20E-07	0.55	115	1.92	Rnf8	Proteolysis
DMR20:8310001	20	8310001	8311000	1000	1	5.80E-07	0.45	14	1.4	Rnf8	Proteolysis
DMR20:8569001	20	8569001	8572000	3000	1	2.80E-07	0.48	62	2.07	Mdga1	
DMR20:8890001	20	8890001	8901000	11000	2	1.10E-07	0.56	235	2.14	Zfand3;Btbd9	
DMR20:9101001	20	9101001	9103000	2000	1	4.40E-08	0.48	31	1.55	Btbd9	
DMR20:9180001	20	9180001	9183000	3000	1	7.10E-08	0.49	50	1.67	Btbd9	
DMR20:9434001	20	9434001	9437000	3000	1	3.90E-07	0.41	90	3	Dnah8	Cytoskeleton

DMR20:9519001	20	9519001	9521000	2000	1	7.70E-07	0.41	31	1.55	Dnah8	Cytoskeleton
DMR20:9825001	20	9825001	9827000	2000	1	3.70E-07	0.49	34	1.7	Abcg1	Transport
DMR20:9830001	20	9830001	9838000	8000	1	4.30E-09	0.51	166	2.08	Abcg1	Transport
DMR20:9978001	20	9978001	9979000	1000	1	1.00E-08	0.54	23	2.3	Ubash3a	
DMR20:10138001	20	10138001	10141000	3000	1	9.70E-08	0.53	57	1.9	Pde9a	Signaling
DMR20:10218001	20	10218001	10219000	1000	1	3.10E-07	0.55	8	0.8	Pde9a	Signaling
DMR20:10395001	20	10395001	10403000	8000	1	5.50E-08	0.49	165	2.06	Cbs;U2af1	Metabolism;Translation
DMR20:10739001	20	10739001	10749000	10000	1	6.90E-07	0.68	188	1.88	Hsf2bp	
DMR20:11128001	20	11128001	11133000	5000	1	1.60E-07	0.5	124	2.48	Agpat3	Metabolism
DMR20:11418001	20	11418001	11420000	2000	1	7.50E-07	0.48	42	2.1	Pfkl;RGD1309594	Metabolism
DMR20:11554001	20	11554001	11558000	4000	1	3.70E-07	0.54	45	1.12	LOC690386;RGD1561557;LOC690415	
DMR20:11751001	20	11751001	11753000	2000	1	1.80E-11	0.57	37	1.85	Pttg1ip	
DMR20:11788001	20	11788001	11793000	5000	1	5.90E-07	0.49	95	1.9	Itgb2	Extracellular Matrix
DMR20:11868001	20	11868001	11872000	4000	1	9.80E-08	0.51	83	2.08	Fam207a	
DMR20:12313001	20	12313001	12329000	16000	1	4.80E-08	0.52	262	1.64	Col18a1;Slc19a1	Extracellular Matrix;Transport
DMR20:12341001	20	12341001	12343000	2000	1	1.60E-07	0.49	71	3.55	Col18a1;Slc19a1	Extracellular Matrix;Transport
DMR20:12621001	20	12621001	12624000	3000	1	1.10E-08	0.53	53	1.77	Pcbp3	Metabolism
DMR20:12881001	20	12881001	12885000	4000	1	3.20E-07	0.48	67	1.68	Mcm3ap	Cytoskeleton
DMR20:12978001	20	12978001	12979000	1000	1	9.90E-07	0.66	20	2	Pcnt	
DMR20:13692001	20	13692001	13694000	2000	1	2.70E-08	0.57	33	1.65	Smarcb1;Derl3	Transcription;Transport
DMR20:13858001	20	13858001	13860000	2000	1	1.10E-07	0.45	37	1.85	Cabin1	Signaling
DMR20:13948001	20	13948001	13955000	7000	1	2.20E-08	0.51	123	1.76	Cabin1;LOC102556116	Signaling
DMR20:14046001	20	14046001	14047000	1000	1	1.80E-07	0.42	14	1.4	Ggt1;Lrrc75b	Protease
DMR20:14273001	20	14273001	14277000	4000	1	2.60E-09	0.6	89	2.22	Adora2a	Signaling
DMR20:14278001	20	14278001	14282000	4000	1	5.50E-07	0.42	82	2.05	Adora2a;Specc1l	Signaling
DMR20:14424001	20	14424001	14425000	1000	1	1.10E-07	0.52	8	0.8	Bcr	Signaling
DMR20:14538001	20	14538001	14540000	2000	1	7.60E-07	0.43	28	1.4	Bcr	Signaling
DMR20:14594001	20	14594001	14600000	6000	1	3.00E-07	0.55	157	2.62	Rtdr1;Gnaz	Signaling
DMR20:14601001	20	14601001	14605000	4000	1	3.30E-09	0.51	136	3.4	Rtdr1;Gnaz	Signaling
DMR20:14618001	20	14618001	14632000	14000	1	3.50E-08	0.44	386	2.76	Rtdr1;Gnaz	Signaling
DMR20:14634001	20	14634001	14639000	5000	1	7.20E-09	0.57	146	2.92	Rtdr1;Gnaz	Signaling
DMR20:14984001	20	14984001	14989000	5000	1	4.00E-12	0.39	46	0.92	Pcdh15	Cytoskeleton
DMR20:15146001	20	15146001	15147000	1000	1	1.00E-07	0.54	6	0.6	Pcdh15	Cytoskeleton
DMR20:16022001	20	16022001	16023000	1000	1	1.50E-07	-0.69	17	1.7	NEWGENE_1590969	
DMR20:16344001	20	16344001	16346000	2000	1	2.10E-07	-0.68	9	0.45	NEWGENE_1590969	
DMR20:18823001	20	18823001	18825000	2000	1	6.30E-07	0.49	24	1.2	Bicc1	Metabolism
DMR20:19682001	20	19682001	19683000	1000	1	2.40E-07	0.61	5	0.5	Slc16a9	
DMR20:20104001	20	20104001	20106000	2000	1	1.90E-07	0.5	62	3.1	Ank3	
DMR20:21951001	20	21951001	21952000	1000	1	2.10E-07	0.46	24	2.4	Rtkn2	Cytoskeleton
DMR20:22904001	20	22904001	22910000	6000	1	4.50E-08	0.41	139	2.32	Jmjd1c;Reep3	Epigenetic;Transport
DMR20:25310001	20	25310001	25312000	2000	1	2.60E-07	0.43	34	1.7	Ctnna3	
DMR20:26445001	20	26445001	26446000	1000	1	3.90E-07	0.58	19	1.9	Ctnna3	
DMR20:26904001	20	26904001	26905000	1000	1	4.00E-07	0.42	14	1.4	Dnajc12	Transcription
DMR20:28513001	20	28513001	28514000	1000	1	5.80E-09	0.68	8	0.8	Sh3rf3	
DMR20:29421001	20	29421001	29423000	2000	1	3.00E-07	0.48	35	1.75	Micu1	Signaling
DMR20:29860001	20	29860001	29863000	3000	1	8.20E-07	0.43	47	1.57	Psap;Cdh23	Cytoskeleton;Cytoskeleton
DMR20:29866001	20	29866001	29868000	2000	1	2.90E-07	0.61	28	1.4	Psap;Cdh23	Cytoskeleton;Cytoskeleton
DMR20:30169001	20	30169001	30175000	6000	1	2.00E-07	0.5	111	1.85	Cdh23	Cytoskeleton
DMR20:30194001	20	30194001	30197000	3000	1	7.00E-08	0.51	61	2.03	Cdh23	Cytoskeleton
DMR20:30241001	20	30241001	30243000	2000	1	1.20E-09	0.62	23	1.15	Cdh23	Cytoskeleton
DMR20:30342001	20	30342001	30345000	3000	2	1.60E-07	0.56	51	1.7	Unc5b	Receptor
DMR20:30360001	20	30360001	30365000	5000	1	2.20E-12	0.61	71	1.42	Unc5b	Receptor
DMR20:30834001	20	30834001	30836000	2000	1	3.20E-09	0.46	39	1.95	Adamts14	Protease
DMR20:30846001	20	30846001	30847000	1000	1	5.00E-07	0.44	11	1.1	Adamts14	Protease
DMR20:30958001	20	30958001	30966000	8000	1	7.70E-07	0.45	149	1.86	Pald1	Signaling
DMR20:31497001	20	31497001	31501000	4000	1	9.10E-08	0.53	80	2	Col13a1	Extracellular Matrix
DMR20:31505001	20	31505001	31510000	5000	1	2.50E-07	0.46	80	1.6	Col13a1;LOC102554788	Extracellular Matrix
DMR20:31603001	20	31603001	31605000	2000	1	4.80E-07	0.45	15	0.75	Col13a1	Extracellular Matrix
DMR20:31827001	20	31827001	31830000	3000	1	3.90E-07	0.43	63	2.1	Tspan15	
DMR20:31987001	20	31987001	31988000	1000	1	7.70E-07	0.43	26	2.6	Hk1	Signaling
DMR20:32093001	20	32093001	32094000	1000	1	1.70E-07	0.48	17	1.7	Vps26a	Transport
DMR20:32274001	20	32274001	32275000	1000	1	9.20E-08	0.41	18	1.8	Ddx50;Stox1;LOC108353233	
DMR20:32314001	20	32314001	32316000	2000	1	5.60E-07	0.44	40	2	Stox1	

DMR20:34250001	20	34250001	34252000	2000	1	5.60E-08	0.54	23	1.15	Slc35f1	
DMR20:34599001	20	34599001	34602000	3000	1	9.00E-07	-0.46	31	1.03	Cep85l	
DMR20:34993001	20	34993001	34995000	2000	1	9.50E-07	0.51	27	1.35	Fam184a	
DMR20:44546001	20	44546001	44550000	4000	2	8.20E-08	0.46	87	2.17	Fyn	
DMR20:44687001	20	44687001	44692000	5000	1	1.40E-08	0.66	112	2.24	LOC108349084;Traf3ip2;LOC102551236	Cytoskeleton
DMR20:45152001	20	45152001	45157000	5000	1	4.50E-07	0.65	58	1.16	Slc16a10	Transport
DMR20:45515001	20	45515001	45517000	2000	1	5.60E-09	0.7	35	1.75	Cdk19	Signaling
DMR20:46785001	20	46785001	46786000	1000	1	3.60E-08	0.47	26	2.6	Armc2	
DMR20:46801001	20	46801001	46803000	2000	1	5.60E-07	0.42	30	1.5	Armc2	
DMR20:46849001	20	46849001	46855000	6000	1	7.60E-08	0.51	104	1.73	Armc2	
DMR20:46866001	20	46866001	46868000	2000	1	9.50E-07	0.48	22	1.1	Armc2;LOC103694446	
DMR20:47102001	20	47102001	47104000	2000	1	1.30E-08	0.58	30	1.5	Lace1	
DMR20:47169001	20	47169001	47171000	2000	1	1.40E-07	0.36	44	2.2	Lace1	
DMR20:47630001	20	47630001	47632000	2000	1	4.10E-08	0.5	34	1.7	Scml4	Epigenetic
DMR20:47764001	20	47764001	47767000	3000	1	5.60E-08	0.46	64	2.13	Sobp	
DMR20:47811001	20	47811001	47815000	4000	1	3.20E-08	0.49	83	2.08	Sobp;LOC108349095	
DMR20:48257001	20	48257001	48258000	1000	1	9.40E-07	0.4	16	1.6	Bend3	
DMR20:48735001	20	48735001	48737000	2000	1	8.30E-07	0.49	59	2.95	Ddo	Metabolism
DMR20:48932001	20	48932001	48937000	5000	1	1.30E-07	0.57	112	2.24	Rtn4ip1;Aim1	Metabolism
DMR20:48954001	20	48954001	48956000	2000	1	2.30E-10	0.6	41	2.05	Aim1	
DMR20:48986001	20	48986001	48989000	3000	1	1.00E-07	0.9	39	1.3	Aim1	
DMR20:49019001	20	49019001	49022000	3000	1	6.50E-13	0.57	58	1.93	Aim1	
DMR20:49065001	20	49065001	49067000	2000	1	2.90E-18	1.27	24	1.2	Aim1	
DMR20:49073001	20	49073001	49077000	4000	1	5.90E-07	0.42	62	1.55	Aim1	
DMR20:49295001	20	49295001	49298000	3000	1	1.50E-08	-0.75	31	1.03	LOC102554815;LOC103694449;Atg5	Transport
DMR20:49330001	20	49330001	49331000	1000	1	9.70E-08	0.45	15	1.5	Atg5	Transport
DMR20:49463001	20	49463001	49465000	2000	1	2.60E-07	0.47	23	1.15	LOC102554933;Prdm1	Transcription
DMR20:50228001	20	50228001	50237000	9000	4	2.60E-17	1.21	238	2.64	Prep	Protease
DMR20:50775001	20	50775001	50779000	4000	1	2.50E-07	0.59	42	1.05	Hace1	Proteolysis
DMRX:290001	X	290001	302000	12000	1	3.20E-07	-0.59	99	0.82	Olr1756-ps	
DMRX:969001	X	969001	975000	6000	1	3.00E-12	0.53	65	1.08	Zfp182	
DMRX:20577001	X	20577001	20578000	1000	1	3.70E-07	-1.37	7	0.7	FAM120C	
DMRX:35979001	X	35979001	35980000	1000	1	5.40E-07	0.62	11	1.1	Phka2	
DMRX:36385001	X	36385001	36389000	4000	1	8.60E-07	-0.39	30	0.75	Phka2	
DMRX:53087001	X	53087001	53088000	1000	1	8.70E-08	-0.46	13	1.3	Dmd	
DMRX:54868001	X	54868001	54874000	6000	1	2.30E-07	0.57	56	0.93	mageb1l1	
DMRX:61241001	X	61241001	61242000	1000	1	6.40E-07	-0.38	11	1.1	Mageb18	Cytoskeleton
DMRX:75172001	X	75172001	75174000	2000	1	4.60E-07	-0.5	18	0.9	Abcb7	Transport
DMRX:99762001	X	99762001	99765000	3000	2	1.50E-07	0.54	49	1.63	Diaph2	
DMRX:107395001	X	107395001	107396000	1000	1	6.50E-12	0.84	19	1.9	Morf4l2;LOC102555540	Epigenetic
DMRX:117248001	X	117248001	117253000	5000	1	3.50E-07	-0.63	24	0.48	Olr1758-ps	
DMRX:123317001	X	123317001	123320000	3000	1	8.20E-09	0.3	28	0.93	RGD1565032;LOC100361883	
DMRX:135252001	X	135252001	135253000	1000	1	5.40E-07	0.51	14	1.4	Elf4	Transcription
DMRX:138459001	X	138459001	138460000	1000	1	3.00E-07	0.6	6	0.6	Mbnl3;LOC108353302	Translation
DMRX:143388001	X	143388001	143390000	2000	1	2.40E-09	0.65	48	2.4	Atp11c	Transport
DMRX:153008001	X	153008001	153010000	2000	1	1.10E-07	0.59	34	1.7	Zfp185	
DMRX:153065001	X	153065001	153068000	3000	1	6.90E-07	0.55	37	1.23	Pnma3;Xlr4a	
DMRX:155833001	X	155833001	155839000	6000	2	2.20E-07	0.37	170	2.83	Dkc1	Cell Cycle
DMRX:155840001	X	155840001	155848000	8000	3	2.00E-08	0.46	279	3.49	Dkc1	Cell Cycle
