# Supplementary material for: Environmental induced transgenerational inheritance impacts systems epigenetics in disease etiology
Source: Sci Rep. 2022 Apr 19;12:5452. doi: 10.1038/s41598-022-09336-0 (PMC9018793; doi:10.1038/s41598-022-09336-0)
Supplement: Supplementary file 22 — Supplementary Table S14. [file 41598_2022_9336_MOESM22_ESM.pdf]

Supplemental Table S14
Atrazine DMR p<1e-06

DMR Name	Chr	Start	Stop	Length	# Sig Win	minP	maxLFC	CpG #	CpG Density	Gene Annotation	Gene Category
DMR1:2407001	1	2407001	2409000	2000	1	2.40E-07	0.37	16	0.8	Ust	Transport
DMR1:15917001	1	15917001	15918000	1000	1	5.00E-08	0.33	8	0.8	Pde7b	Signaling
DMR1:16526001	1	16526001	16527000	1000	1	4.00E-07	-0.35	19	1.9	Ahi1	
DMR1:17366001	1	17366001	17367000	1000	1	3.60E-08	0.53	5	0.5	Themis	
DMR1:18708001	1	18708001	18709000	1000	1	5.70E-07	0.42	5	0.5	Lama2	Extracellular Matrix
DMR1:20011001	1	20011001	20013000	2000	1	4.50E-08	0.75	15	0.75	L3mbtl3	Epigenetic
DMR1:21157001	1	21157001	21158000	1000	1	2.20E-08	0.34	2	0.2	Akap7	Translation
DMR1:21597001	1	21597001	21598000	1000	1	2.10E-07	0.33	4	0.4	Med23	Transcription
DMR1:21772001	1	21772001	21775000	3000	1	3.20E-07	0.32	17	0.57	Enpp1	
DMR1:23425001	1	23425001	23426000	1000	1	8.80E-07	-0.44	23	2.3	Eya4	
DMR1:28312001	1	28312001	28314000	2000	1	8.60E-07	-0.56	26	1.3	Rnf217	Proteolysis
DMR1:28387001	1	28387001	28389000	2000	1	1.00E-07	0.28	13	0.65	Rnf217	Proteolysis
DMR1:32105001	1	32105001	32108000	3000	1	2.80E-07	-0.47	71	2.37	Slc12a7	Transport
DMR1:32126001	1	32126001	32128000	2000	1	5.60E-07	-0.65	30	1.5	Slc12a7	Transport
DMR1:32209001	1	32209001	32211000	2000	1	2.50E-08	-0.43	30	1.5	Slc6a19	Transport
DMR1:32649001	1	32649001	32652000	3000	1	7.30E-07	-0.56	36	1.2	Irx4	Development
DMR1:37127001	1	37127001	37129000	2000	1	4.10E-07	0.44	4	0.2	Adcy2	
DMR1:39912001	1	39912001	39915000	3000	1	2.50E-08	0.3	25	0.83	Ppp1r14c	Signaling
DMR1:40378001	1	40378001	40379000	1000	1	5.80E-07	-0.52	9	0.9	Plekhg1;LOC108349517	
DMR1:41646001	1	41646001	41647000	1000	1	4.40E-07	0.36	9	0.9	Syne1	
DMR1:41982001	1	41982001	41985000	3000	1	1.30E-09	0.51	23	0.77	Syne1	
DMR1:42134001	1	42134001	42136000	2000	1	5.10E-07	0.71	10	0.5	Myct1	
DMR1:43545001	1	43545001	43546000	1000	1	4.20E-08	-0.52	6	0.6	Oprm1	Signaling
DMR1:46923001	1	46923001	46924000	1000	1	9.00E-08	-0.3	15	1.5	Synj2;LOC103690968	Signaling
DMR1:47167001	1	47167001	47168000	1000	1	9.90E-07	-0.3	14	1.4	Tmem181	
DMR1:47464001	1	47464001	47465000	1000	1	8.70E-07	-0.51	9	0.9	Rsph3	Development
DMR1:48560001	1	48560001	48562000	2000	1	9.50E-12	0.48	13	0.65	Plg;Dut-ps	Protease
DMR1:48709001	1	48709001	48711000	2000	1	2.50E-07	-0.38	39	1.95	Map3k4;Agpat4	Signaling;Metabolism
DMR1:50484001	1	50484001	50486000	2000	1	1.20E-08	0.39	11	0.55	Pacrg	
DMR1:52916001	1	52916001	52918000	2000	1	5.00E-09	-0.77	28	1.4	T2	
DMR1:55658001	1	55658001	55659000	1000	1	7.20E-08	-0.36	7	0.7	LOC102556502;LOC103690993;LOC102546375;LOC108349807;RGD1561185	
DMR1:56807001	1	56807001	56808000	1000	1	6.20E-07	-0.56	9	0.9	Wdr27	
DMR1:64131001	1	64131001	64132000	1000	1	9.50E-09	-0.58	12	1.2	Tmc4;Leng1;Cnot3;Mir3572	Transcription
DMR1:70239001	1	70239001	70242000	3000	1	1.80E-07	0.31	22	0.73	Peg3;Apeg3;Zim1	Transcription
DMR1:72952001	1	72952001	72956000	4000	1	6.80E-07	0.66	34	0.85	Eps8l1;Rdh13	Cytoskeleton;Golgi
DMR1:75748001	1	75748001	75749000	1000	1	1.20E-07	0.5	4	0.4	Bsph1	
DMR1:78103001	1	78103001	78107000	4000	1	7.90E-07	-0.46	89	2.22	Dhx34	Transcription
DMR1:78155001	1	78155001	78156000	1000	1	7.00E-07	0.61	23	2.3	C5ar2	Signaling
DMR1:78459001	1	78459001	78461000	2000	1	5.90E-07	-0.37	41	2.05	Arhgap35	Signaling
DMR1:81203001	1	81203001	81204000	1000	1	6.00E-14	0.45	11	1.1	Tescl;Lypd5	Receptor
DMR1:81381001	1	81381001	81383000	2000	1	7.30E-10	-0.54	61	3.05	Zfp428;Srrm5	
DMR1:81784001	1	81784001	81788000	4000	1	8.90E-10	-0.51	94	2.35	Arhgef1;RGD1563034	Transcription
DMR1:81887001	1	81887001	81890000	3000	1	4.10E-09	-0.54	70	2.33	Atp1a3;Grik5	Transport;Receptor
DMR1:82460001	1	82460001	82463000	3000	1	8.20E-12	-0.63	61	2.03	Bckdha;Exosc5;Tmem91	Metabolism;Transcription
DMR1:82965001	1	82965001	82968000	3000	1	4.90E-08	-0.75	42	1.4	LOC100911309;Cd177	
DMR1:83201001	1	83201001	83203000	2000	1	7.90E-07	0.38	15	0.75	Cyp2b3	Metabolism
DMR1:84839001	1	84839001	84842000	3000	1	2.30E-07	0.37	37	1.23	Wdr87	
DMR1:85195001	1	85195001	85197000	2000	1	7.00E-08	-0.39	53	2.65	Pak4	Signaling
DMR1:87202001	1	87202001	87204000	2000	1	1.10E-08	-0.49	28	1.4	LOC103690068;Spint2	Protease; Proteolysis
DMR1:87949001	1	87949001	87953000	4000	1	2.50E-08	-0.38	60	1.5	Map4k1;Ryr1	Ion Channel
DMR1:88014001	1	88014001	88015000	1000	1	1.90E-07	0.44	8	0.8	Ryr1	Ion Channel
DMR1:88362001	1	88362001	88365000	3000	1	5.10E-07	-0.71	43	1.43	Zfp14	
DMR1:89128001	1	89128001	89129000	1000	1	9.40E-07	-0.45	26	2.6	Haus5;LOC100912333;LOC688924	
DMR1:89308001	1	89308001	89310000	2000	1	9.40E-07	-0.41	39	1.95	LOC102554169;Ffar3;Ffar1;Cd22	
DMR1:89526001	1	89526001	89529000	3000	1	6.90E-07	-0.39	48	1.6	Hpn	Protease
DMR1:92731001	1	92731001	92733000	2000	1	3.20E-08	-0.73	44	2.2	Tshz3	Transcription
DMR1:93844001	1	93844001	93846000	2000	1	4.10E-07	-0.37	55	2.75	Zfp536;LOC103691087	Transcription
DMR1:93960001	1	93960001	93965000	5000	1	5.30E-08	-0.47	55	1.1	Zfp536	Transcription
DMR1:94001001	1	94001001	94002000	1000	1	2.80E-07	-0.55	21	2.1	Zfp536	Transcription
DMR1:98572001	1	98572001	98573000	1000	1	4.80E-08	0.39	10	1	Siglec5	Immune
DMR1:99114001	1	99114001	99115000	1000	1	2.90E-09	0.37	4	0.4	Vom2r38;Vom2r37;LOC100912942;LOC108349568	Signaling
DMR1:100314001	1	100314001	100316000	2000	1	4.40E-07	-0.42	27	1.35	Shank1	
DMR1:100328001	1	100328001	100331000	3000	1	1.00E-12	-0.65	62	2.07	Shank1	

DMR1:101176001	1	101176001	101177000	1000	1	7.30E-09	-0.33	10	1	Slc17a7;Gfy;Pth2;Ccdc155	Transport
DMR1:101607001	1	101607001	101608000	1000	1	7.80E-07	-0.49	19	1.9	Fut1;Izumo1;LOC103691112;Rasip1	Golgi
DMR1:101931001	1	101931001	101932000	1000	1	7.00E-07	-0.4	15	1.5	Ccdc114	
DMR1:102332001	1	102332001	102333000	1000	1	1.00E-06	-0.73	14	1.4	Otog	Extracellular Matrix
DMR1:102491001	1	102491001	102494000	3000	1	4.60E-07	-0.4	44	1.47	Sergef	Proteolysis
DMR1:102892001	1	102892001	102894000	2000	1	4.20E-10	-0.45	30	1.5	LOC102552634;Ldha	Metabolism
DMR1:104198001	1	104198001	104201000	3000	1	2.30E-08	-0.4	56	1.87	E2f8	Transcription
DMR1:105207001	1	105207001	105209000	2000	1	5.70E-07	-0.4	30	1.5	Prmt3	Golgi
DMR1:106159001	1	106159001	106160000	1000	1	3.80E-07	-0.53	6	0.6	Nell1	Signaling
DMR1:116047001	1	116047001	116051000	4000	1	3.80E-07	0.49	21	0.52	Atp10a	Transport
DMR1:124459001	1	124459001	124460000	1000	1	9.40E-08	0.41	4	0.4	Otud7a	Protease
DMR1:125599001	1	125599001	125604000	5000	1	7.10E-07	0.39	64	1.28	Fam189a1	
DMR1:126800001	1	126800001	126804000	4000	1	6.00E-07	-0.5	71	1.77	Pcsk6	Protease
DMR1:137824001	1	137824001	137826000	2000	2	1.60E-10	0.48	10	0.5	Agbl1	Protease
DMR1:138693001	1	138693001	138695000	2000	1	2.10E-07	0.47	42	2.1	Agbl1	Protease
DMR1:140025001	1	140025001	140026000	1000	1	1.30E-09	0.47	6	0.6	Ntrk3	Receptor
DMR1:140125001	1	140125001	140127000	2000	1	3.00E-08	-0.4	16	0.8	Ntrk3	Receptor
DMR1:140783001	1	140783001	140784000	1000	1	6.50E-07	-0.55	13	1.3	Acan	Extracellular Matrix
DMR1:141026001	1	141026001	141028000	2000	1	3.50E-09	-0.47	44	2.2	Abhd2	Protease
DMR1:141030001	1	141030001	141032000	2000	1	6.80E-09	-0.56	31	1.55	Abhd2	Protease
DMR1:141841001	1	141841001	141843000	2000	1	1.20E-09	-0.45	45	2.25	Zfp710;LOC100911225	Transcription
DMR1:142311001	1	142311001	142312000	1000	1	8.40E-07	-0.44	17	1.7	Blm	Epigenetic
DMR1:143455001	1	143455001	143456000	1000	1	7.30E-08	-0.49	17	1.7	Homer2	
DMR1:145815001	1	145815001	145818000	3000	1	4.00E-07	-0.5	31	1.03	Il16;LOC103691182	Cytokine
DMR1:148976001	1	148976001	148977000	1000	1	9.70E-07	0.3	2	0.2	Vom2r40	
DMR1:155379001	1	155379001	155380000	1000	1	1.90E-07	0.36	6	0.6	Dlg2	Cytoskeleton
DMR1:156026001	1	156026001	156028000	2000	1	8.70E-08	0.46	8	0.4	Dlg2;LOC108349816	Cytoskeleton
DMR1:156571001	1	156571001	156572000	1000	1	2.90E-07	-0.63	7	0.7	Dlg2	Cytoskeleton
DMR1:161732001	1	161732001	161733000	1000	1	4.90E-07	-0.3	13	1.3	Tenm4	
DMR1:161748001	1	161748001	161749000	1000	1	1.90E-07	-0.41	22	2.2	Tenm4	
DMR1:162246001	1	162246001	162249000	3000	2	7.20E-10	0.34	5	0.17	Gab2	Cytoskeleton
DMR1:162474001	1	162474001	162477000	3000	2	4.50E-08	-0.42	42	1.4	Ints4	
DMR1:166617001	1	166617001	166618000	1000	1	7.30E-10	-0.52	15	1.5	Pde2a	Signaling
DMR1:167334001	1	167334001	167338000	4000	1	2.00E-07	-0.4	70	1.75	Pgap2;Rhog	Golgi;Signaling
DMR1:167680001	1	167680001	167681000	1000	1	7.10E-07	-0.41	11	1.1	Olr42-ps;Trim21	Proteolysis
DMR1:169015001	1	169015001	169017000	2000	1	8.10E-08	0.51	9	0.45	Hbe1	
DMR1:170002001	1	170002001	170003000	1000	1	1.60E-08	0.46	5	0.5	Olr193	Signaling
DMR1:170850001	1	170850001	170852000	2000	1	3.20E-07	0.41	9	0.45	Olr213	Receptor
DMR1:171140001	1	171140001	171141000	1000	1	1.00E-07	0.47	3	0.3	Olr228-ps;LOC100362585	
DMR1:174351001	1	174351001	174352000	1000	1	4.00E-07	-0.41	29	2.9	LOC499240;Ascl3;Tmem9b	
DMR1:178912001	1	178912001	178914000	2000	1	1.40E-07	0.42	4	0.2	Spon1	Cytoskeleton
DMR1:188701001	1	188701001	188702000	1000	1	8.70E-08	-0.53	35	3.5	Gprc5b;LOC102550985	Signaling
DMR1:189911001	1	189911001	189912000	1000	1	6.40E-07	-0.4	7	0.7	Thumpd1;Zp2;LOC103691218	
DMR1:190007001	1	190007001	190008000	1000	1	1.70E-07	0.45	4	0.4	Thumpd1;Abca14	Transport
DMR1:190699001	1	190699001	190704000	5000	1	7.40E-08	-0.52	44	0.88	Vwa3a	
DMR1:191092001	1	191092001	191094000	2000	1	1.20E-09	0.27	17	0.85	Otoa	Cytoskeleton
DMR1:191737001	1	191737001	191738000	1000	1	9.50E-07	-0.56	15	1.5	Scnn1g	Transport
DMR1:192264001	1	192264001	192268000	4000	1	6.80E-08	0.33	52	1.3	Prkcb	Signaling
DMR1:192438001	1	192438001	192441000	3000	1	7.90E-07	0.5	45	1.5	Prkcb	Signaling
DMR1:192517001	1	192517001	192520000	3000	1	6.20E-07	-0.39	35	1.17	Prkcb	Signaling
DMR1:193065001	1	193065001	193068000	3000	1	2.70E-07	0.46	38	1.27	Tnrc6a	Metabolism
DMR1:195098001	1	195098001	195101000	3000	1	1.00E-06	0.41	13	0.43	Snurf;Snrpn;LOC103690242	Translation
DMR1:197908001	1	197908001	197910000	2000	1	8.10E-07	-0.47	48	2.4	Tufm;Atxn2l	Translation;Metabolism
DMR1:198392001	1	198392001	198394000	2000	1	9.90E-07	-0.44	44	2.2	Asphd1;Sez6l2	Metabolism
DMR1:199513001	1	199513001	199515000	2000	1	4.50E-07	0.28	31	1.55	Itgam	
DMR1:199658001	1	199658001	199662000	4000	1	1.70E-07	-0.44	107	2.67	LOC103691238;Armc5;Tgfb1i1	Transcription;Cytoskeleton
DMR1:200102001	1	200102001	200103000	1000	1	1.90E-09	-0.63	22	2.2	Inpp5f	Signaling
DMR1:207124001	1	207124001	207126000	2000	1	1.80E-09	-0.49	21	1.05	Dock1;Fam196a	Transcription
DMR1:209536001	1	209536001	209537000	1000	1	8.50E-08	-0.49	35	3.5	Ebf3	Transcription
DMR1:212651001	1	212651001	212652000	1000	1	5.80E-07	0.31	11	1.1	Olr287	Receptor
DMR1:213598001	1	213598001	213600000	2000	1	1.90E-09	-0.63	38	1.9	Scgb1c1;Odf3;Bet1l;Ric8a	Growth Factors;Development;Transcription
DMR1:213607001	1	213607001	213609000	2000	1	4.20E-07	-0.35	52	2.6	Odf3;Bet1l;Ric8a;Sirt3	Development;Transcription
DMR1:213745001	1	213745001	213746000	1000	1	5.00E-07	-0.39	25	2.5	Pgghg;Ifitm5;LOC108349669;Ifitm2	Metabolism
DMR1:214809001	1	214809001	214810000	1000	1	2.20E-07	-0.43	25	2.5	Muc5b	Extracellular Matrix
DMR1:215030001	1	215030001	215033000	3000	1	5.30E-07	-0.51	101	3.37	Dusp8	Signaling
DMR1:216581001	1	216581001	216584000	3000	1	1.90E-07	-0.41	49	1.63	Kcnq1	Transport
DMR1:217430001	1	217430001	217432000	2000	1	5.60E-07	0.33	14	0.7	Shank2	
DMR1:217821001	1	217821001	217822000	1000	1	1.00E-07	-0.37	19	1.9	Ano1	

DMR1:219535001	1	219535001	219542000	7000	1	7.10E-07	-0.46	103	1.47	Ankrd13d;Grk2	Signaling
DMR1:220493001	1	220493001	220494000	1000	1	2.40E-07	-0.4	13	1.3	LOC102555354;LOC102555167;Rab1b;Klc2	Cytoskeleton
DMR1:221804001	1	221804001	221807000	3000	1	2.60E-07	0.27	17	0.57	Nrxn2	
DMR1:221895001	1	221895001	221896000	1000	1	3.00E-07	0.31	4	0.4	Nrxn2	
DMR1:226878001	1	226878001	226880000	2000	1	1.20E-07	-0.35	26	1.3	Cd6	Protease
DMR1:228065001	1	228065001	228066000	1000	1	9.30E-08	0.43	7	0.7	Oosp1	
DMR1:228476001	1	228476001	228478000	2000	1	2.10E-08	0.51	11	0.55	Olr321	Receptor
DMR1:233488001	1	233488001	233489000	1000	1	2.00E-07	-0.57	10	1	Gnaq	Signaling
DMR1:235261001	1	235261001	235262000	1000	1	2.50E-07	-0.48	12	1.2	Gna14	Signaling
DMR1:240121001	1	240121001	240123000	2000	1	3.50E-08	0.6	6	0.3	Trpm3	Transport
DMR1:241614001	1	241614001	241615000	1000	1	1.10E-07	-0.44	17	1.7	Apba1	Transport
DMR1:241641001	1	241641001	241644000	3000	1	4.80E-07	0.26	8	0.27	Apba1	Transport
DMR1:242747001	1	242747001	242751000	4000	1	3.70E-07	0.26	31	0.78	Pgm5	Metabolism
DMR1:249043001	1	249043001	249045000	2000	1	3.10E-07	0.33	12	0.6	Prkg1	
DMR1:249637001	1	249637001	249638000	1000	1	4.20E-07	-0.52	17	1.7	Prkg1	
DMR1:251342001	1	251342001	251344000	2000	1	1.80E-08	0.45	16	0.8	Atad1;Cyp2b15;LOC100910127	Metabolism
DMR1:251388001	1	251388001	251391000	3000	1	6.30E-09	0.3	31	1.03	Atad1	
DMR1:252449001	1	252449001	252451000	2000	1	5.60E-08	0.26	11	0.55	Ankrd22	
DMR1:252546001	1	252546001	252548000	2000	1	5.90E-07	-0.57	27	1.35	Stambpl1;Acta2;LOC687722	Protease;Cytoskeleton
DMR1:255036001	1	255036001	255038000	2000	1	9.80E-07	-0.38	16	0.8	Pcgf5	Epigenetic
DMR1:256726001	1	256726001	256727000	1000	1	1.10E-07	-0.4	11	1.1	Myof;LOC108349463	Transport
DMR1:260323001	1	260323001	260325000	2000	1	6.60E-07	-0.51	20	1	Dntt;Opalin	Transcription
DMR1:260953001	1	260953001	260956000	3000	1	7.60E-08	-0.35	57	1.9	Slit1;LOC102553433	
DMR1:261964001	1	261964001	261970000	6000	1	5.80E-07	0.24	96	1.6	Pyroxd2	Metabolism
DMR1:262013001	1	262013001	262014000	1000	1	2.80E-07	-0.5	21	2.1	Hps1;LOC108349471;Hpse2	
DMR1:263934001	1	263934001	263935000	1000	1	9.00E-07	-0.33	17	1.7	Pkd2l1	Transport
DMR1:263954001	1	263954001	263958000	4000	1	2.90E-07	-0.43	42	1.05	Pkd2l1	Transport
DMR1:264053001	1	264053001	264054000	1000	1	2.60E-07	0.34	18	1.8	LOC108349475;Scd2	
DMR1:264113001	1	264113001	264115000	2000	1	3.20E-07	-0.37	27	1.35	Scd4	
DMR1:264511001	1	264511001	264513000	2000	1	1.40E-07	-0.47	41	2.05	Pax2	
DMR1:265627001	1	265627001	265629000	2000	1	4.60E-07	0.41	16	0.8	C1H10orf76	
DMR1:265821001	1	265821001	265823000	2000	1	2.00E-08	-0.39	36	1.8	Pprc1;Nolc1	Transcription
DMR1:267046001	1	267046001	267051000	5000	1	1.50E-07	-0.47	141	2.82	Neurl1;Sh3pxd2a	Proteolysis
DMR1:267460001	1	267460001	267462000	2000	1	2.80E-07	-0.38	40	2	Col17a1;LOC103691371	Extracellular Matrix
DMR1:267542001	1	267542001	267543000	1000	1	3.10E-08	-0.35	22	2.2	Cfap43	
DMR1:268164001	1	268164001	268165000	1000	1	3.30E-08	0.29	5	0.5	Sorcs3	Transport
DMR1:268478001	1	268478001	268479000	1000	1	4.80E-07	-0.77	3	0.3	Sorcs3	Transport
DMR1:274588001	1	274588001	274591000	3000	1	7.30E-07	0.34	33	1.1	Rbm20	
DMR1:277918001	1	277918001	277920000	2000	1	4.70E-07	-0.49	57	2.85	Ablim1;LOC103691383	
DMR1:277981001	1	277981001	277982000	1000	1	1.20E-07	-0.54	17	1.7	Ablim1	
DMR1:278685001	1	278685001	278687000	2000	1	2.70E-07	0.28	16	0.8	Atrnl1	Extracellular Matrix
DMR1:279294001	1	279294001	279295000	1000	1	2.20E-08	0.29	10	1	Gfra1	Receptor
DMR1:279339001	1	279339001	279341000	2000	1	3.40E-07	0.37	7	0.35	Gfra1	Receptor
DMR1:280046001	1	280046001	280048000	2000	1	3.90E-08	-0.51	6	0.3	Hspa12a;LOC100363557	
DMR1:280213001	1	280213001	280215000	2000	1	2.20E-07	0.31	22	1.1	Shtn1	
DMR2:2533001	2	2533001	2535000	2000	1	7.60E-07	-0.56	12	0.6	Ell2;LOC102549076	Transcription
DMR2:2913001	2	2913001	2914000	1000	1	4.30E-07	0.28	4	0.4	Ttc37	
DMR2:9245001	2	9245001	9246000	1000	1	1.90E-07	0.29	9	0.9	Adgrv1	Signaling
DMR2:12392001	2	12392001	12393000	1000	1	2.20E-07	0.39	4	0.4	Tmem161b;LOC100909625	
DMR2:17726001	2	17726001	17727000	1000	1	1.30E-08	0.43	4	0.4	Edil3	Metabolism
DMR2:18068001	2	18068001	18069000	1000	1	6.50E-07	0.37	3	0.3	Edil3	Metabolism
DMR2:18558001	2	18558001	18559000	1000	1	1.40E-07	0.4	7	0.7	Vcan	Extracellular Matrix
DMR2:18804001	2	18804001	18806000	2000	1	2.80E-08	0.41	18	0.9	Xrcc4	Transcription
DMR2:20700001	2	20700001	20703000	3000	1	6.90E-09	-0.56	55	1.83	Ssbp2	Transcription
DMR2:22339001	2	22339001	22342000	3000	1	5.90E-07	-0.56	40	1.33	Thbs4	
DMR2:24736001	2	24736001	24740000	4000	3	1.70E-08	0.3	10	0.25	Pde8b	Signaling
DMR2:25776001	2	25776001	25778000	2000	1	4.70E-09	-0.47	61	3.05	Arhgef28	
DMR2:27025001	2	27025001	27027000	2000	1	4.70E-07	-0.41	32	1.6	Poc5;Ankdd1b	
DMR2:27290001	2	27290001	27292000	2000	1	5.50E-09	-0.42	29	1.45	Ankdd1b	
DMR2:28011001	2	28011001	28013000	2000	1	9.70E-07	0.32	19	0.95	Hexb	Metabolism
DMR2:28053001	2	28053001	28056000	3000	1	4.90E-07	-0.41	88	2.93	Enc1	
DMR2:28367001	2	28367001	28368000	1000	1	1.30E-07	-0.7	8	0.8	Utp15;LOC100910438	Translation;Signaling
DMR2:28793001	2	28793001	28794000	1000	1	3.90E-08	-0.68	6	0.6	LOC108349927;Tmem171;LOC102546725	
DMR2:33295001	2	33295001	33297000	2000	1	2.20E-08	0.34	13	0.65	NEWGENE_1310139	
DMR2:33321001	2	33321001	33324000	3000	1	4.60E-07	0.34	26	0.87	NEWGENE_1310139	
DMR2:34528001	2	34528001	34529000	1000	1	3.10E-07	0.37	6	0.6	Adamts6	Protease
DMR2:38116001	2	38116001	38117000	1000	1	2.20E-07	0.45	7	0.7	Ipo11;Dimt1	Transport;Epigenetic
DMR2:45772001	2	45772001	45775000	3000	1	8.70E-07	-0.45	42	1.4	Arl15;LOC100362624	

DMR2:45805001	2	45805001	45807000	2000	1	4.90E-07	0.39	10	0.5	Arl15	
DMR2:50103001	2	50103001	50104000	1000	1	1.50E-08	0.28	5	0.5	Hcn1	Transport
DMR2:54787001	2	54787001	54790000	3000	1	4.10E-07	0.39	17	0.57	C7	
DMR2:56459001	2	56459001	56461000	2000	1	1.10E-07	-0.41	52	2.6	Lifr	Receptor
DMR2:56594001	2	56594001	56595000	1000	1	9.20E-08	0.31	8	0.8	Egflam	Extracellular Matrix
DMR2:58251001	2	58251001	58252000	1000	1	8.60E-08	0.39	4	0.4	Ranbp3l	Cytoskeleton
DMR2:61130001	2	61130001	61135000	5000	1	1.50E-07	0.41	31	0.62	Adamts12	Protease
DMR2:61294001	2	61294001	61295000	1000	1	7.40E-07	0.43	3	0.3	Adamts12	Protease
DMR2:62454001	2	62454001	62455000	1000	1	2.80E-07	-0.38	19	1.9	Pdzd2	Cytokine
DMR2:62503001	2	62503001	62509000	6000	1	8.30E-07	-0.32	134	2.23	Pdzd2	Cytokine
DMR2:72284001	2	72284001	72285000	1000	1	2.00E-07	0.47	4	0.4	Cdh12	Cytoskeleton
DMR2:74463001	2	74463001	74464000	1000	1	6.10E-07	0.3	2	0.2	Cdh18	Cytoskeleton
DMR2:81327001	2	81327001	81329000	2000	1	2.50E-08	0.44	9	0.45	Dnah5;LOC108349969	Cytoskeleton
DMR2:89585001	2	89585001	89586000	1000	1	4.00E-08	0.45	6	0.6	Ralyl	
DMR2:100382001	2	100382001	100383000	1000	1	4.50E-10	0.71	4	0.4	Hnf4g	Transcription
DMR2:109617001	2	109617001	109621000	4000	1	5.30E-07	0.48	21	0.52	Naaladl2	
DMR2:109653001	2	109653001	109655000	2000	1	4.10E-08	0.39	7	0.35	Naaladl2	
DMR2:111542001	2	111542001	111543000	1000	1	4.60E-08	0.46	5	0.5	Nlgn1	Cytoskeleton
DMR2:114418001	2	114418001	114421000	3000	1	1.20E-08	0.55	30	1	Slc2a2	
DMR2:116907001	2	116907001	116909000	2000	1	5.30E-09	0.45	11	0.55	Egfem1	
DMR2:116991001	2	116991001	116993000	2000	1	1.80E-08	0.46	12	0.6	Egfem1	
DMR2:117805001	2	117805001	117808000	3000	1	2.00E-08	-0.46	24	0.8	Mecom;LOC103691544	Transcription
DMR2:123375001	2	123375001	123380000	5000	1	4.40E-08	-0.52	128	2.56	Trpc3	Transport
DMR2:123462001	2	123462001	123464000	2000	1	1.60E-07	0.48	17	0.85	Trpc3	Transport
DMR2:140937001	2	140937001	140939000	2000	1	1.30E-09	0.43	19	0.95	Maml3	
DMR2:142325001	2	142325001	142327000	2000	1	9.70E-08	-0.37	47	2.35	Lhfp	
DMR2:145211001	2	145211001	145212000	1000	1	6.20E-08	0.43	6	0.6	Nbea;LOC102552778	
DMR2:155567001	2	155567001	155569000	2000	1	4.20E-07	0.3	4	0.2	Kcnab1	
DMR2:166685001	2	166685001	166686000	1000	1	7.90E-08	0.27	4	0.4	Sptssb	Golgi
DMR2:174565001	2	174565001	174566000	1000	1	7.30E-07	0.32	8	0.8	Fstl5	Protease; Proteolysis
DMR2:178221001	2	178221001	178223000	2000	1	3.10E-07	-0.36	22	1.1	LOC499643;LOC108350042;LOC365827;Fnip2	
DMR2:178513001	2	178513001	178515000	2000	1	4.00E-08	0.29	19	0.95	Rxfp1;LOC108350043	Signaling
DMR2:184864001	2	184864001	184866000	2000	1	3.40E-08	-0.37	31	1.55	Fam160a1	
DMR2:185285001	2	185285001	185289000	4000	1	2.80E-07	-0.6	74	1.85	Sh3d19;Prss48	Protease
DMR2:185758001	2	185758001	185759000	1000	1	1.30E-08	-0.51	12	1.2	Lrba	
DMR2:186334001	2	186334001	186336000	2000	1	4.30E-07	0.4	23	1.15	Cd1d1	
DMR2:186430001	2	186430001	186432000	2000	1	2.10E-08	-0.4	43	2.15	Kirrel	
DMR2:187189001	2	187189001	187190000	1000	1	2.90E-09	-0.44	13	1.3	Insrr	Receptor
DMR2:187488001	2	187488001	187489000	1000	1	9.60E-09	-0.37	17	1.7	Iqgap3	Signaling
DMR2:187941001	2	187941001	187944000	3000	1	2.40E-07	-0.42	28	0.93	Ubqln4;Ssr2	Transport
DMR2:188211001	2	188211001	188215000	4000	1	2.50E-07	-0.33	82	2.05	Gon4l;Msto1	Transcription
DMR2:189908001	2	189908001	189910000	2000	1	6.60E-07	0.46	10	0.5	LOC361990;S100a1;S100a13	Signaling
DMR2:190094001	2	190094001	190095000	1000	1	7.90E-07	0.33	6	0.6	S100a9	Signaling
DMR2:194429001	2	194429001	194432000	3000	1	5.50E-07	0.45	8	0.27	RGD1564313	Proteolysis
DMR2:194505001	2	194505001	194507000	2000	1	1.20E-07	0.42	14	0.7	LOC365852;LOC100363449;RGD1560510	Proteolysis
DMR2:194554001	2	194554001	194555000	1000	1	4.60E-07	0.35	3	0.3	RGD1565369	
DMR2:195155001	2	195155001	195156000	1000	1	8.10E-07	0.31	5	0.5	RGD1563667	Proteolysis
DMR2:195333001	2	195333001	195334000	1000	1	6.60E-07	0.29	3	0.3	RGD1563667	Proteolysis
DMR2:195710001	2	195710001	195711000	1000	1	4.90E-07	-0.63	11	1.1	Riiad1;Celf3	
DMR2:196043001	2	196043001	196045000	2000	1	4.80E-08	-0.48	24	1.2	Pogz;Psmb4;LOC108350065	Transcription;Protease
DMR2:198778001	2	198778001	198780000	2000	1	3.90E-07	-0.39	55	2.75	Pex11b;Itga10	Extracellular Matrix
DMR2:200387001	2	200387001	200389000	2000	1	6.20E-07	0.53	21	1.05	Reg4	
DMR2:203251001	2	203251001	203253000	2000	1	5.00E-07	0.35	9	0.45	Vtcn1	Immune
DMR2:209364001	2	209364001	209366000	2000	1	9.70E-07	0.33	9	0.45	Lrif1	
DMR2:217888001	2	217888001	217890000	2000	1	3.80E-07	0.42	8	0.4	Olfm3	Development
DMR2:220306001	2	220306001	220308000	2000	1	2.40E-07	-0.5	23	1.15	Plppr4	Signaling
DMR2:222364001	2	222364001	222365000	1000	1	6.30E-08	0.36	5	0.5	Dpyd	Metabolism
DMR2:227787001	2	227787001	227788000	1000	1	2.90E-11	0.44	4	0.4	Ndst3	Transport
DMR2:235327001	2	235327001	235329000	2000	1	7.70E-08	-0.53	49	2.45	Pla2g12a	Metabolism
DMR2:235533001	2	235533001	235536000	3000	1	9.80E-07	-0.53	43	1.43	Col25a1;LOC103691705	Extracellular Matrix
DMR2:235610001	2	235610001	235612000	2000	1	2.00E-07	-0.37	25	1.25	Col25a1	Extracellular Matrix
DMR2:235814001	2	235814001	235815000	1000	1	2.30E-07	-0.53	10	1	Ostc	Transport
DMR2:236314001	2	236314001	236317000	3000	1	4.90E-08	-0.48	47	1.57	Lef1	Transcription
DMR2:238766001	2	238766001	238767000	1000	1	8.20E-08	-0.44	19	1.9	Tet2	
DMR2:242873001	2	242873001	242874000	1000	1	5.50E-07	-0.44	20	2	Ddit4l	
DMR2:243526001	2	243526001	243528000	2000	1	6.20E-08	0.38	19	0.95	Adh7;LOC102556058	Metabolism
DMR2:243833001	2	243833001	243835000	2000	1	6.90E-08	0.32	12	0.6	Eif4e	Translation
DMR2:244707001	2	244707001	244710000	3000	1	1.90E-07	0.33	20	0.67	Stpg2	Development

DMR2:246740001	2	246740001	246741000	1000	1	1.30E-07	0.44	7	0.7	LOC103691720;Pdha2	Metabolism
DMR2:247254001	2	247254001	247255000	1000	1	9.20E-08	0.5	4	0.4	Unc5c	Receptor
DMR2:250777001	2	250777001	250778000	1000	1	6.50E-07	0.29	9	0.9	Clca2;Clca4l;LOC108350129	Transport
DMR2:252145001	2	252145001	252146000	1000	1	9.50E-07	-0.51	12	1.2	Lpar3	Signaling
DMR2:258513001	2	258513001	258515000	2000	1	4.20E-07	0.27	12	0.6	St6galnac5	
DMR2:258828001	2	258828001	258829000	1000	1	1.40E-07	-0.41	28	2.8	St6galnac5;Adgrl2	Signaling
DMR2:259491001	2	259491001	259493000	2000	1	1.90E-08	-0.44	31	1.55	St6galnac3	
DMR2:259568001	2	259568001	259571000	3000	1	2.20E-07	-0.4	46	1.53	St6galnac3	
DMR2:259592001	2	259592001	259595000	3000	1	7.70E-07	-0.38	61	2.03	St6galnac3	
DMR2:259721001	2	259721001	259723000	2000	1	1.50E-07	0.28	12	0.6	St6galnac3;LOC108350218	
DMR2:260238001	2	260238001	260240000	2000	1	5.80E-07	-0.42	26	1.3	Slc44a5	Transport
DMR2:260322001	2	260322001	260329000	7000	1	2.80E-07	-0.36	121	1.73	Slc44a5;LOC102553583	Transport
DMR2:260492001	2	260492001	260493000	1000	1	1.10E-07	0.36	10	1	Slc44a5	Transport
DMR2:261000001	2	261000001	261002000	2000	1	1.80E-07	0.54	23	1.15	Erich3;LOC102555516;LOC108350146	
DMR2:261236001	2	261236001	261238000	2000	1	3.10E-07	0.47	10	0.5	Tnni3k	
DMR2:261369001	2	261369001	261384000	15000	3	1.00E-09	-0.87	385	2.57	Fpgt	Transport
DMR2:261431001	2	261431001	261432000	1000	1	3.40E-07	0.38	0	0	Lrriq3	
DMR2:262933001	2	262933001	262935000	2000	1	2.10E-07	0.31	16	0.8	Negr1	Immune
DMR2:263557001	2	263557001	263558000	1000	1	3.30E-07	0.37	4	0.4	Negr1	Immune
DMR3:2279001	3	2279001	2280000	1000	1	4.20E-07	-0.4	16	1.6	Nsmf;Entpd8	Signaling
DMR3:2629001	3	2629001	2630000	1000	1	2.80E-07	-0.43	21	2.1	Entpd2;Npdc1;LOC366006;Fut7	Signaling;Golgi
DMR3:3272001	3	3272001	3275000	3000	1	4.50E-07	-0.59	76	2.53	Glt6d1;Lcn9	Golgi;Transport
DMR3:3327001	3	3327001	3330000	3000	1	2.20E-09	-0.95	32	1.07	Kcnt1	Transport
DMR3:3348001	3	3348001	3354000	6000	1	1.40E-07	-0.48	101	1.68	Kcnt1	Transport
DMR3:3363001	3	3363001	3364000	1000	1	2.80E-07	-0.64	21	2.1	Kcnt1;Camsap1	Transport
DMR3:5554001	3	5554001	5557000	3000	1	9.10E-07	-0.42	61	2.03	Adamts13;LOC102548484;Cacfd1	Protease
DMR3:5633001	3	5633001	5634000	1000	1	3.60E-08	-0.56	22	2.2	Adamtsl2	Protease
DMR3:5702001	3	5702001	5704000	2000	1	2.70E-07	-0.41	37	1.85	Fam163b;Dbh	Metabolism
DMR3:7227001	3	7227001	7228000	1000	1	4.10E-07	-0.64	8	0.8	Tsc1	
DMR3:7616001	3	7616001	7617000	1000	1	4.80E-07	-0.41	20	2	Cfap77	Development
DMR3:7727001	3	7727001	7728000	1000	1	3.10E-07	-0.42	10	1	Setx	
DMR3:8355001	3	8355001	8358000	3000	1	2.20E-07	-0.59	44	1.47	Trub2;Coq4;Slc27a4	Metabolism;Transport
DMR3:8414001	3	8414001	8416000	2000	1	9.80E-07	0.25	10	0.5	Urm1;Mir2964;Mir219-2;Cercam	Golgi
DMR3:8683001	3	8683001	8688000	5000	1	7.90E-09	-0.43	65	1.3	Zer1	
DMR3:8946001	3	8946001	8947000	1000	1	1.70E-09	-0.69	19	1.9	Miga2;LOC686066	
DMR3:10153001	3	10153001	10156000	3000	1	2.30E-07	-0.46	44	1.47	Abl1;Exosc2	Translation
DMR3:10337001	3	10337001	10338000	1000	1	5.60E-07	-0.39	17	1.7	Ass1	Metabolism
DMR3:10452001	3	10452001	10453000	1000	1	2.90E-07	-0.49	22	2.2	Hmcn2	
DMR3:10457001	3	10457001	10459000	2000	1	6.30E-07	0.33	16	0.8	Hmcn2	
DMR3:11981001	3	11981001	11983000	2000	1	6.60E-07	-0.41	43	2.15	Fam129b;Lrsam1	
DMR3:11999001	3	11999001	1.20E+07	1000	1	2.60E-10	-0.57	21	2.1	Lrsam1;Rpl12	Translation
DMR3:12026001	3	12026001	12027000	1000	1	2.30E-07	-0.61	24	2.4	Slc2a8;Garnl3	Signaling
DMR3:12630001	3	12630001	12632000	2000	1	8.30E-09	-0.53	41	2.05	Lmx1b	Development
DMR3:12768001	3	12768001	12772000	4000	1	6.90E-08	-0.42	69	1.73	Mvb12b	
DMR3:15056001	3	15056001	15058000	2000	1	1.40E-12	-0.5	40	2	Dab2ip	Signaling
DMR3:15134001	3	15134001	15136000	2000	1	8.30E-07	-0.46	49	2.45	Ttll11	Cytoskeleton
DMR3:15155001	3	15155001	15157000	2000	1	3.50E-07	0.41	31	1.55	Ttll11	Cytoskeleton
DMR3:15303001	3	15303001	15309000	6000	1	1.60E-08	-0.41	89	1.48	Ttll11	Cytoskeleton
DMR3:21555001	3	21555001	21559000	4000	1	2.60E-08	0.58	29	0.72	LOC100911251;Klf5-ps2	
DMR3:22052001	3	22052001	22055000	3000	1	6.50E-08	-0.59	57	1.9	Crb2;Dennd1a	Cytoskeleton
DMR3:25612001	3	25612001	25614000	2000	1	7.50E-07	0.26	10	0.5	Lrp1b	
DMR3:25631001	3	25631001	25633000	2000	1	3.70E-08	0.67	9	0.45	Lrp1b	
DMR3:26599001	3	26599001	26600000	1000	1	2.80E-07	0.34	1	0.1	Lrp1b	
DMR3:28957001	3	28957001	28958000	1000	1	3.30E-07	-0.53	4	0.4	Arhgap15	Signaling
DMR3:29681001	3	29681001	29682000	1000	1	5.30E-07	0.5	13	1.3	Gtdc1	Golgi
DMR3:35680001	3	35680001	35681000	1000	1	7.80E-07	-0.38	15	1.5	Lypd6	
DMR3:37674001	3	37674001	37680000	6000	1	4.20E-07	-0.44	100	1.67	Neb	
DMR3:37691001	3	37691001	37693000	2000	1	5.20E-07	-0.47	43	2.15	Neb	
DMR3:37740001	3	37740001	37741000	1000	1	1.80E-07	-0.39	12	1.2	Neb	
DMR3:44029001	3	44029001	44030000	1000	1	1.50E-07	-0.85	8	0.8	Galnt5	Golgi
DMR3:45173001	3	45173001	45175000	2000	1	7.90E-07	-0.83	18	0.9	Ccdc148	
DMR3:45915001	3	45915001	45916000	1000	1	9.50E-08	-0.65	38	3.8	Tanc1;Wdsub1	
DMR3:46426001	3	46426001	46430000	4000	1	3.40E-08	-0.41	102	2.55	Ly75	
DMR3:46560001	3	46560001	46561000	1000	1	6.00E-07	0.26	6	0.6	Pla2r1	
DMR3:46586001	3	46586001	46588000	2000	1	1.70E-15	-7.65	24	1.2	Pla2r1	
DMR3:48221001	3	48221001	48222000	1000	1	5.40E-08	-0.64	13	1.3	Slc4a10	Transport
DMR3:51160001	3	51160001	51161000	1000	1	5.70E-07	0.4	6	0.6	Cobll1	
DMR3:55498001	3	55498001	55501000	3000	1	7.70E-07	-0.49	35	1.17	LOC108350389;Abcb11	Transport
DMR3:57722001	3	57722001	57723000	1000	1	9.10E-07	-0.32	23	2.3	Cybrd1	Metabolism

DMR3:58103001	3	58103001	58106000	3000	1	3.00E-07	0.37	31	1.03	Metap1d	Protease
DMR3:58145001	3	58145001	58147000	2000	1	5.50E-07	-0.34	39	1.95	Metap1d;LOC108350393	Protease
DMR3:63144001	3	63144001	63146000	2000	1	6.90E-07	0.29	12	0.6	Pde11a	Signaling
DMR3:63791001	3	63791001	63792000	1000	1	9.00E-08	-0.71	9	0.9	Ttn	
DMR3:64029001	3	64029001	64031000	2000	1	1.80E-08	0.32	18	0.9	Ccdc141;Sestd1	
DMR3:75598001	3	75598001	75599000	1000	1	2.20E-07	0.69	5	0.5	Olr570-ps;Olr571-ps	
DMR3:82585001	3	82585001	82587000	2000	1	1.70E-07	-0.54	30	1.5	Alx4	Development
DMR3:86529001	3	86529001	86531000	2000	1	1.70E-09	0.59	4	0.2	Lrrc4c	
DMR3:97970001	3	97970001	97972000	2000	1	6.90E-07	-0.48	33	1.65	LOC103691854;Arl14ep	
DMR3:109045001	3	109045001	109046000	1000	1	6.80E-07	-0.37	23	2.3	Rasgrp1	Transcription
DMR3:110453001	3	110453001	110456000	3000	1	6.40E-09	-0.61	55	1.83	Pak6	Signaling
DMR3:110566001	3	110566001	110567000	1000	1	2.70E-07	0.27	2	0.2	RGD1565536;Phgr1;Disp2	
DMR3:111850001	3	111850001	111851000	1000	1	5.80E-09	-0.44	23	2.3	Sptbn5	
DMR3:113190001	3	113190001	113191000	1000	1	4.40E-08	-0.43	10	1	Tp53bp1	Transcription
DMR3:113612001	3	113612001	113613000	1000	1	8.30E-07	-0.46	8	0.8	Frmd5	
DMR3:114205001	3	114205001	114207000	2000	1	6.70E-07	-0.36	28	1.4	Sord	Metabolism
DMR3:114299001	3	114299001	114300000	1000	1	2.40E-07	-0.39	17	1.7	Shf	
DMR3:114705001	3	114705001	114707000	2000	2	1.80E-07	0.42	17	0.85	Gatm	Transport
DMR3:117832001	3	117832001	117834000	2000	1	1.60E-07	-0.37	33	1.65	Cep152	
DMR3:118118001	3	118118001	118119000	1000	1	7.70E-07	-0.42	12	1.2	Cops2	
DMR3:120587001	3	120587001	120589000	2000	1	9.90E-07	-0.4	21	1.05	Acoxl	Metabolism
DMR3:120667001	3	120667001	120668000	1000	1	2.30E-08	-0.62	14	1.4	Acoxl	Metabolism
DMR3:121965001	3	121965001	121969000	4000	1	2.40E-07	0.36	31	0.78	RGD1566226	
DMR3:123614001	3	123614001	123618000	4000	1	1.20E-08	-0.43	85	2.12	Adam33;Siglec1	Protease
DMR3:129986001	3	129986001	129987000	1000	1	1.20E-08	0.45	28	2.8	Slx4ip	
DMR3:130050001	3	130050001	130054000	4000	1	2.10E-07	0.46	27	0.68	Slx4ip	
DMR3:130085001	3	130085001	130087000	2000	1	4.80E-07	-0.5	39	1.95	Jag1	
DMR3:131356001	3	131356001	131357000	1000	1	6.40E-07	-0.62	16	1.6	LOC108350568;Btbd3	Proteolysis
DMR3:136084001	3	136084001	136085000	1000	1	1.10E-07	-0.39	8	0.8	Macrod2	
DMR3:140265001	3	140265001	140267000	2000	1	8.20E-07	-0.67	18	0.9	Cfap61	
DMR3:143883001	3	143883001	143885000	2000	1	1.20E-08	-0.67	21	1.05	Andpro	
DMR3:147421001	3	147421001	147427000	6000	1	3.80E-07	-0.42	102	1.7	Angpt4	Signaling
DMR3:148558001	3	148558001	148559000	1000	1	2.50E-07	-0.42	14	1.4	Ccm2l	
DMR3:150225001	3	150225001	150226000	1000	1	8.70E-07	-0.66	12	1.2	Chmp4b	Transport
DMR3:150557001	3	150557001	150560000	3000	1	2.80E-07	-0.43	52	1.73	Asip	Signaling
DMR3:152500001	3	152500001	152501000	1000	1	4.00E-07	-0.63	14	1.4	Epb41l1	
DMR3:152518001	3	152518001	152519000	1000	1	1.80E-07	-0.47	16	1.6	Epb41l1	
DMR3:154633001	3	154633001	154636000	3000	1	2.60E-07	-0.95	55	1.83	Tgm2	Transport
DMR3:159328001	3	159328001	159333000	5000	1	2.60E-07	-0.36	78	1.56	L3mbtl1	Epigenetic
DMR3:159636001	3	159636001	159638000	2000	1	3.70E-07	-0.4	32	1.6	Tox2	
DMR3:159985001	3	159985001	159986000	1000	1	3.80E-08	-0.39	24	2.4	LOC502692;Ttpal	Transport
DMR3:160110001	3	160110001	160111000	1000	1	9.20E-07	-0.46	19	1.9	Pkig;Ada	Signaling;Metabolism
DMR3:163470001	3	163470001	163471000	1000	1	3.10E-08	-0.4	19	1.9	Prex1	Transcription
DMR3:163933001	3	163933001	163935000	2000	1	2.80E-07	-0.46	43	2.15	Kcnb1;LOC102552303	Transport
DMR3:164654001	3	164654001	164656000	2000	1	1.10E-07	-0.54	52	2.6	Ptpn1	Signaling
DMR3:165435001	3	165435001	165439000	4000	1	5.60E-07	-0.46	96	2.4	Atp9a	Transport
DMR3:166657001	3	166657001	166659000	2000	1	8.80E-09	0.37	27	1.35	Tshz2	Transcription
DMR3:166748001	3	166748001	166750000	2000	1	2.40E-07	-0.37	54	2.7	Tshz2	Transcription
DMR3:168006001	3	168006001	168009000	3000	1	1.70E-11	-0.47	46	1.53	Bcas1	
DMR3:168485001	3	168485001	168490000	5000	1	5.40E-07	-0.37	137	2.74	Dok5;LOC102552784	
DMR3:170455001	3	170455001	170458000	3000	1	3.30E-08	-0.67	44	1.47	Rtfdc1;Gcnt7	Golgi
DMR3:170460001	3	170460001	170461000	1000	1	5.20E-07	-0.46	20	2	Rtfdc1;Gcnt7	Golgi
DMR3:171935001	3	171935001	171936000	1000	1	1.20E-08	0.32	3	0.3	Apcdd1l	
DMR3:172715001	3	172715001	172720000	5000	1	1.10E-08	0.27	66	1.32	Zfp831	
DMR3:172759001	3	172759001	172760000	1000	1	2.50E-07	0.35	2	0.2	Zfp831	
DMR3:173895001	3	173895001	173896000	1000	1	2.90E-07	0.41	5	0.5	Sycp2	Epigenetic
DMR3:175253001	3	175253001	175255000	2000	1	7.40E-07	0.36	31	1.55	Cdh4	Cytoskeleton
DMR3:175541001	3	175541001	175543000	2000	1	2.00E-07	-0.53	19	0.95	Osbpl2;Adrm1	
DMR3:175555001	3	175555001	175569000	14000	2	5.90E-08	-0.45	396	2.83	Adrm1;Lama5	Extracellular Matrix
DMR3:176937001	3	176937001	176939000	2000	1	6.80E-07	0.39	9	0.45	Zbtb46	Cytoskeleton
DMR3:177066001	3	177066001	177068000	2000	1	1.30E-08	-0.58	36	1.8	Uckl1	Signaling
DMR3:177327001	3	177327001	177329000	2000	1	4.00E-07	-0.48	20	1	Myt1	Transcription
DMR3:177377001	3	177377001	177380000	3000	1	6.70E-08	-0.47	38	1.27	Pcmtd2;Polr3k;LOC102549636	Epigenetic;Transcription
DMR4:2712001	4	2712001	2714000	2000	1	7.50E-08	-0.49	20	1	Dnajb6	
DMR4:4320001	4	4320001	4322000	2000	1	3.20E-07	-0.36	29	1.45	Dpp6	Protease
DMR4:4827001	4	4827001	4830000	3000	1	5.80E-08	-0.61	19	0.63	Dpp6	Protease
DMR4:7133001	4	7133001	7137000	4000	1	6.10E-07	-0.57	50	1.25	Abcf2;Iqca1l	Translation
DMR4:7276001	4	7276001	7279000	3000	1	4.50E-07	-0.39	71	2.37	Slc4a2;LOC103695197;Cdk5;Asic3	Transport;Signaling;Transport
DMR4:9175001	4	9175001	9178000	3000	1	6.60E-07	0.39	31	1.03	Orc5;LOC103692027	Cell Cycle

DMR4:9407001	4	9407001	9408000	1000	1	1.70E-07	0.38	6	0.6	Reln	Extracellular Matrix
DMR4:10428001	4	10428001	10430000	2000	1	2.50E-07	-0.5	22	1.1	Ccdc146;Gsap	Development;Signaling
DMR4:17555001	4	17555001	17561000	6000	1	1.70E-07	-0.35	185	3.08	Sema3e	Signaling
DMR4:18278001	4	18278001	18279000	1000	1	2.30E-07	0.35	4	0.4	Sema3a	Signaling
DMR4:25522001	4	25522001	25523000	1000	1	1.80E-09	-0.44	12	1.2	Steap2	
DMR4:27825001	4	27825001	27829000	4000	1	4.60E-07	-0.31	83	2.08	Cdk6	Signaling
DMR4:31580001	4	31580001	31582000	2000	1	4.50E-08	0.42	15	0.75	Slc25a13	Transport
DMR4:34047001	4	34047001	34049000	2000	1	7.50E-07	-0.51	34	1.7	Col28a1	Extracellular Matrix
DMR4:34064001	4	34064001	34065000	1000	1	2.70E-08	-0.78	5	0.5	Col28a1	Extracellular Matrix
DMR4:34451001	4	34451001	34452000	1000	1	2.30E-07	-0.42	18	1.8	Glcci1	
DMR4:38691001	4	38691001	38693000	2000	1	2.00E-07	-0.47	25	1.25	Thsd7a	Cytoskeleton
DMR4:44340001	4	44340001	44341000	1000	1	9.10E-07	-0.39	41	4.1	Tes	Cytoskeleton
DMR4:44809001	4	44809001	44810000	1000	1	1.70E-07	-0.58	10	1	Met	Receptor
DMR4:48871001	4	48871001	48873000	2000	1	8.60E-08	-0.34	32	1.6	Tspan12	
DMR4:54544001	4	54544001	54546000	2000	1	3.90E-07	0.43	17	0.85	Grm8	Signaling
DMR4:54720001	4	54720001	54721000	1000	1	5.90E-08	0.36	2	0.2	Grm8	Signaling
DMR4:54966001	4	54966001	54968000	2000	1	5.70E-07	0.54	10	0.5	Grm8	Signaling
DMR4:55012001	4	55012001	55016000	4000	1	5.60E-07	0.41	13	0.32	Grm8	Signaling
DMR4:57893001	4	57893001	57894000	1000	1	1.40E-07	-0.67	6	0.6	Cpa4	Protease
DMR4:59478001	4	59478001	59479000	1000	1	7.10E-07	-0.33	13	1.3	Plxna4;LOC108350688	
DMR4:64841001	4	64841001	64842000	1000	1	1.00E-07	-0.49	21	2.1	Dgki	Signaling
DMR4:67622001	4	67622001	67623000	1000	1	2.90E-10	0.34	2	0.2	Mrps33	Translation
DMR4:70964001	4	70964001	70966000	2000	1	8.20E-07	0.36	16	0.8	Trpv5	Transport
DMR4:74794001	4	74794001	74799000	5000	1	3.50E-08	0.4	21	0.42	Cntnap2	
DMR4:79565001	4	79565001	79566000	1000	1	1.60E-07	0.56	2	0.2	Npy;LOC100912228	Signaling
DMR4:80037001	4	80037001	80039000	2000	1	6.80E-07	-0.45	33	1.65	Osbpl3	
DMR4:80080001	4	80080001	80084000	4000	1	1.80E-07	-0.47	59	1.48	Osbpl3	
DMR4:82881001	4	82881001	82883000	2000	1	2.00E-07	-0.38	29	1.45	Jazf1	
DMR4:85691001	4	85691001	85692000	1000	1	1.00E-07	-0.54	16	1.6	Adcyap1r1	Receptor
DMR4:85707001	4	85707001	85708000	1000	1	3.60E-07	-0.48	14	1.4	Adcyap1r1	Receptor
DMR4:87175001	4	87175001	87176000	1000	1	9.80E-07	0.48	3	0.3	Fkbp9	
DMR4:88270001	4	88270001	88271000	1000	1	3.70E-07	0.52	2	0.2	Vom1r86	Receptor
DMR4:89264001	4	89264001	89265000	1000	1	8.30E-07	0.32	8	0.8	Fam13a	
DMR4:92492001	4	92492001	92493000	1000	1	3.50E-08	0.46	5	0.5	Ccser1	
DMR4:94302001	4	94302001	94304000	2000	1	4.20E-07	0.26	15	0.75	Grid2	Receptor
DMR4:111189001	4	111189001	111191000	2000	1	5.80E-07	0.39	8	0.4	Lrrtm4	Receptor
DMR4:112713001	4	112713001	112714000	1000	1	7.20E-07	-0.39	19	1.9	Mrpl19;Eva1a	Translation
DMR4:113382001	4	113382001	113383000	1000	1	2.90E-07	0.34	2	0.2	Tacr1	Signaling
DMR4:115758001	4	115758001	115761000	3000	1	1.90E-08	-0.46	36	1.2	Dysf	Transport
DMR4:116984001	4	116984001	116987000	3000	2	1.10E-07	-0.47	43	1.43	Emx1;Sfxn5	Development;Transport
DMR4:117711001	4	117711001	117713000	2000	1	1.10E-07	0.38	25	1.25	Add2	Cytoskeleton
DMR4:119145001	4	119145001	119146000	1000	1	6.60E-07	0.26	9	0.9	Gkn2;Gkn1	
DMR4:121233001	4	121233001	121238000	5000	1	3.10E-10	-0.46	100	2	Plxna1	
DMR4:121606001	4	121606001	121607000	1000	1	3.90E-07	-0.42	17	1.7	Txnrd3	Metabolism
DMR4:122319001	4	122319001	122321000	2000	1	3.60E-07	-0.56	19	0.95	Zxdc;Cfap100	Transcription
DMR4:123423001	4	123423001	123424000	1000	1	2.70E-07	-0.37	11	1.1	Iqsec1	Transcription
DMR4:124483001	4	124483001	124484000	1000	1	3.00E-08	-0.59	14	1.4	Prickle2	Cytoskeleton
DMR4:129957001	4	129957001	129959000	2000	1	4.20E-07	-0.38	27	1.35	Frmd4b	
DMR4:136597001	4	136597001	136598000	1000	1	3.40E-07	0.47	6	0.6	Cntn6	
DMR4:145722001	4	145722001	145723000	1000	1	5.90E-07	-0.49	18	1.8	Atp2b2	Transport
DMR4:145853001	4	145853001	145856000	3000	1	4.90E-07	-0.52	48	1.6	Atp2b2	Transport
DMR4:146221001	4	146221001	146222000	1000	1	3.70E-07	-0.59	7	0.7	Slc6a11	Transport
DMR4:146822001	4	146822001	146823000	1000	1	2.10E-07	-0.45	23	2.3	Vgll4	Transcription
DMR4:147711001	4	147711001	147712000	1000	1	6.50E-07	-0.7	15	1.5	Cand2;Rpl32;Efcab12	Proteolysis;Translation;Signaling
DMR4:147831001	4	147831001	147833000	2000	1	8.30E-08	-0.44	34	1.7	Ift122;Rho	Signaling
DMR4:150227001	4	150227001	150234000	7000	1	1.90E-08	-0.41	155	2.21	Ret	Receptor
DMR4:150719001	4	150719001	150721000	2000	1	1.10E-07	-0.38	34	1.7	Cacna1c	Transport
DMR4:150882001	4	150882001	150885000	3000	1	9.50E-07	-0.33	34	1.13	Cacna1c	Transport
DMR4:151279001	4	151279001	151281000	2000	1	1.50E-07	-0.42	30	1.5	Cacna1c;Dcp1b	Transport;Translation
DMR4:153494001	4	153494001	153496000	2000	1	1.20E-07	-0.48	41	2.05	Mical3	
DMR4:156113001	4	156113001	156114000	1000	1	3.50E-07	0.41	2	0.2	Clec4b2	Transport
DMR4:157081001	4	157081001	157082000	1000	1	6.70E-07	0.24	9	0.9	Clstn3;LOC102553636	Transport
DMR4:157662001	4	157662001	157665000	3000	1	4.20E-07	-0.43	56	1.87	Nop2;Iffo1	Metabolism
DMR4:158516001	4	158516001	158517000	1000	1	1.10E-07	0.32	10	1	Ano2	
DMR4:159242001	4	159242001	159244000	2000	1	5.30E-09	0.45	13	0.65	LOC103692224;Kcna6	Transport
DMR4:160161001	4	160161001	160164000	3000	1	7.40E-07	0.54	28	0.93	Cracr2a	
DMR4:160229001	4	160229001	160233000	4000	1	2.10E-08	-0.42	64	1.6	Cracr2a	
DMR4:160863001	4	160863001	160865000	2000	1	1.20E-08	0.31	14	0.7	LOC102552479;Senp17	Protease
DMR4:167862001	4	167862001	167864000	2000	1	3.30E-08	-0.52	41	2.05	Etv6	Transcription

DMR4:168841001	4	168841001	168842000	1000	1	4.00E-10	-0.54	20	2	Gprc5a	Signaling
DMR4:170591001	4	170591001	170593000	2000	1	1.90E-07	-0.5	34	1.7	Plbd1	Metabolism
DMR4:170945001	4	170945001	170947000	2000	1	3.80E-08	-0.52	26	1.3	Pde6h	Signaling
DMR4:171086001	4	171086001	171088000	2000	1	1.70E-07	-0.71	51	2.55	Rerg	Signaling
DMR4:171525001	4	171525001	171528000	3000	1	2.20E-09	0.34	49	1.63	Eps8	Cytoskeleton
DMR4:171568001	4	171568001	171570000	2000	1	6.50E-07	0.31	19	0.95	Eps8	Cytoskeleton
DMR4:171585001	4	171585001	171589000	4000	1	3.00E-07	0.4	66	1.65	Eps8	Cytoskeleton
DMR4:171772001	4	171772001	171773000	1000	1	7.10E-08	-0.53	18	1.8	Dera	Metabolism
DMR4:175424001	4	175424001	175426000	2000	1	1.10E-08	0.3	12	0.6	Pde3a	Signaling
DMR4:176170001	4	176170001	176172000	2000	1	3.00E-10	0.47	11	0.55	Slco1a1	Transport
DMR4:176667001	4	176667001	176668000	1000	1	1.60E-08	0.3	6	0.6	Gys2	
DMR4:176916001	4	176916001	176918000	2000	1	3.80E-08	-0.53	42	2.1	Abcc9	Transport
DMR4:178752001	4	178752001	178753000	1000	1	5.10E-07	0.28	6	0.6	Sox5	
DMR4:180755001	4	180755001	180757000	2000	1	4.40E-07	0.33	19	0.95	Itpr2	Ion Channel
DMR4:180806001	4	180806001	180807000	1000	1	8.00E-07	0.31	15	1.5	Itpr2	Ion Channel
DMR4:182050001	4	182050001	182052000	2000	1	7.00E-07	-0.36	32	1.6	Ccdc91	
DMR4:182515001	4	182515001	182517000	2000	1	4.90E-07	0.34	18	0.9	Far2	
DMR5:713001	5	713001	714000	1000	1	6.30E-07	-0.43	8	0.8	Crispld1	Immune
DMR5:16585001	5	16585001	16586000	1000	1	1.50E-07	0.28	9	0.9	Lyn	
DMR5:21836001	5	21836001	21839000	3000	1	5.10E-07	-0.52	54	1.8	Chd7	
DMR5:21929001	5	21929001	21932000	3000	1	6.90E-07	-0.47	96	3.2	Chd7	
DMR5:24426001	5	24426001	24427000	1000	1	3.30E-07	-0.46	24	2.4	Tp53inp1;LOC102547431;Ccne2	Signaling
DMR5:28534001	5	28534001	28535000	1000	1	7.40E-07	-0.55	7	0.7	Necab1	
DMR5:32806001	5	32806001	32807000	1000	1	1.10E-07	0.51	7	0.7	Cnbd1	
DMR5:47493001	5	47493001	47497000	4000	1	7.20E-07	-0.43	59	1.48	Bach2	
DMR5:47608001	5	47608001	47613000	5000	1	1.90E-07	-0.3	87	1.74	Bach2	
DMR5:47741001	5	47741001	47743000	2000	1	2.60E-07	-0.29	39	1.95	Bach2	
DMR5:58088001	5	58088001	58091000	3000	1	1.50E-09	-0.4	55	1.83	Cntfr;Rpp25l;Dctn3	Receptor;Translation;Cytoskeleton
DMR5:58176001	5	58176001	58178000	2000	1	2.50E-09	-0.41	35	1.75	Ccl27;LOC102547621;Ccl19;LOC689481	Growth Factors;Growth Factors
DMR5:58694001	5	58694001	58697000	3000	1	1.30E-09	0.57	24	0.8	Unc13b	
DMR5:59216001	5	59216001	59217000	1000	1	6.60E-07	-0.47	15	1.5	Olr834	Receptor
DMR5:59438001	5	59438001	59440000	2000	1	3.90E-07	-0.42	24	1.2	Glipr2	Immune
DMR5:61662001	5	61662001	61664000	2000	1	3.40E-07	0.3	25	1.25	Tmod1	Cytoskeleton
DMR5:62233001	5	62233001	62235000	2000	1	1.60E-07	-0.43	49	2.45	Tbc1d2	Signaling
DMR5:62331001	5	62331001	62335000	4000	1	4.00E-08	-0.53	69	1.73	Gabbr2	Signaling
DMR5:62384001	5	62384001	62385000	1000	1	1.60E-07	0.25	3	0.3	Gabbr2	Signaling
DMR5:62500001	5	62500001	62504000	4000	1	1.00E-07	0.43	66	1.65	Gabbr2	Signaling
DMR5:62627001	5	62627001	62630000	3000	1	2.20E-07	-0.35	54	1.8	Gabbr2	Signaling
DMR5:65075001	5	65075001	65076000	1000	1	1.20E-08	0.44	4	0.4	Grin3a	Receptor
DMR5:65078001	5	65078001	65079000	1000	1	6.90E-07	0.47	4	0.4	Grin3a	Receptor
DMR5:69760001	5	69760001	69761000	1000	1	9.50E-08	0.33	10	1	Olr852	Receptor
DMR5:69850001	5	69850001	69852000	2000	1	1.60E-07	-0.47	37	1.85	Nipsnap3b;Abca1	Transport
DMR5:70301001	5	70301001	70303000	2000	1	7.30E-07	0.55	26	1.3	Slc44a1;LOC102551474	Transport
DMR5:70577001	5	70577001	70578000	1000	1	9.50E-07	-0.46	11	1.1	Fktn	
DMR5:74014001	5	74014001	74017000	3000	1	2.30E-08	0.42	29	0.97	Frrs1l	
DMR5:74294001	5	74294001	74296000	2000	1	2.30E-07	-0.44	41	2.05	Ptpn3;LOC108350978	Signaling
DMR5:75276001	5	75276001	75280000	4000	1	4.30E-07	0.25	31	0.78	Svep1	
DMR5:77014001	5	77014001	77015000	1000	1	1.10E-07	-0.49	12	1.2	Inip	
DMR5:77288001	5	77288001	77292000	4000	1	9.50E-10	0.35	25	0.62	LOC100363405;Mup4	Transcription;Transport
DMR5:77306001	5	77306001	77309000	3000	1	1.10E-07	0.31	20	0.67	Mup4;Obp3	Transport
DMR5:77935001	5	77935001	77940000	5000	1	8.20E-07	0.25	35	0.7	Zfp37	Transcription
DMR5:78245001	5	78245001	78246000	1000	1	1.90E-07	-0.5	11	1.1	Slc31a1;Cdc26	Transport
DMR5:79309001	5	79309001	79310000	1000	1	5.60E-07	-0.54	10	1	Whrn	Cytoskeleton
DMR5:81734001	5	81734001	81736000	2000	1	8.40E-07	0.33	8	0.4	Astn2	
DMR5:100533001	5	100533001	100534000	1000	1	7.10E-07	-0.31	11	1.1	Nfib;LOC103692390	Transcription
DMR5:102767001	5	102767001	102769000	2000	1	3.90E-07	-0.4	18	0.9	Bnc2	Transcription
DMR5:103249001	5	103249001	103251000	2000	1	8.30E-08	0.43	6	0.3	Cntln	
DMR5:104950001	5	104950001	104952000	2000	1	3.80E-07	-0.42	24	1.2	Rraga;Fam29a	Signaling
DMR5:105219001	5	105219001	105222000	3000	1	8.60E-08	0.49	23	0.77	LOC100911372;Acer2	
DMR5:106960001	5	106960001	106962000	2000	2	4.30E-12	0.47	13	0.65	Ifna5;LOC690903	
DMR5:110327001	5	110327001	110328000	1000	1	2.50E-07	0.56	7	0.7	Zfp352	Transcription
DMR5:118770001	5	118770001	118771000	1000	1	1.60E-08	-0.32	25	2.5	Pgm1	Metabolism
DMR5:119244001	5	119244001	119247000	3000	1	9.50E-07	-0.36	74	2.47	Ror1	Receptor
DMR5:123388001	5	123388001	123391000	3000	1	1.90E-08	0.42	18	0.6	Dab1	Cytoskeleton
DMR5:128287001	5	128287001	128289000	2000	1	3.70E-08	0.37	13	0.65	Zfyve9	
DMR5:136133001	5	136133001	136136000	3000	1	3.40E-07	-0.49	57	1.9	Tmem53;Rnf220	
DMR5:136361001	5	136361001	136364000	3000	1	9.30E-07	0.31	29	0.97	Rnf220;LOC690462	
DMR5:136531001	5	136531001	136536000	5000	1	1.10E-08	-0.47	98	1.96	Eri3;Dmap1;LOC108351139	Transcription;Epigenetic
DMR5:137045001	5	137045001	137046000	1000	1	2.30E-07	-0.51	18	1.8	Ptprf	Signaling

DMR5:137095001	5	137095001	137098000	3000	1	1.60E-07	-0.38	68	2.27	Ptprf;LOC102553423	Signaling
DMR5:139039001	5	139039001	139041000	2000	1	1.70E-07	-0.41	56	2.8	Hivep3	
DMR5:139806001	5	139806001	139808000	2000	1	2.10E-08	-0.44	25	1.25	Rims3	Transport
DMR5:142159001	5	142159001	142160000	1000	1	3.40E-07	-0.49	11	1.1	RGD1563049	
DMR5:143628001	5	143628001	143629000	1000	1	2.40E-07	-0.41	22	2.2	Grik3	Receptor
DMR5:143711001	5	143711001	143716000	5000	1	9.00E-08	-0.52	118	2.36	Grik3	Receptor
DMR5:144305001	5	144305001	144308000	3000	1	7.40E-07	-0.47	52	1.73	Trappc3;Col8a2	Extracellular Matrix
DMR5:144337001	5	144337001	144338000	1000	1	2.00E-08	-0.56	26	2.6	Col8a2;Adprhl2;Tekt2	Extracellular Matrix;Metabolism;Cytoskeleton
DMR5:146526001	5	146526001	146527000	1000	1	1.70E-08	-0.39	25	2.5	Csmd2	
DMR5:148223001	5	148223001	148224000	1000	1	1.90E-07	-0.46	29	2.9	Adgrb2	Signaling
DMR5:150068001	5	150068001	150070000	2000	1	2.60E-07	-0.49	40	2	Srsf4;Tmem200b	Translation
DMR5:151230001	5	151230001	151233000	3000	1	1.90E-07	-0.38	61	2.03	Ahdc1	
DMR5:152755001	5	152755001	152757000	2000	1	2.30E-07	-0.42	53	2.65	Mtfr1l;Sepn1	
DMR5:152855001	5	152855001	152860000	5000	1	1.70E-07	-0.34	139	2.78	Man1c1	Golgi
DMR5:153121001	5	153121001	153122000	1000	1	6.30E-07	-0.55	13	1.3	Tmem57	
DMR5:153225001	5	153225001	153228000	3000	1	5.60E-07	-0.41	60	2	Rhd;Tmem50a	Transport
DMR5:154051001	5	154051001	154054000	3000	1	8.40E-07	0.3	48	1.6	Ifnlr1	Receptor
DMR5:154151001	5	154151001	154152000	1000	1	5.70E-07	0.27	9	0.9	Myom3	
DMR5:157815001	5	157815001	157820000	5000	1	6.00E-07	-0.67	79	1.58	Akr7a3;Mrto4;Emc1	Metabolism
DMR5:158740001	5	158740001	158741000	1000	1	9.10E-07	0.33	4	0.4	Igsf21	
DMR5:159480001	5	159480001	159481000	1000	1	5.10E-07	-0.41	13	1.3	Padi2;Sdhb	Metabolism
DMR5:160011001	5	160011001	160027000	16000	2	1.40E-07	-0.6	546	3.41	Zbtb17;Spen	Transcription;Metabolism
DMR5:161009001	5	161009001	161011000	2000	1	1.90E-07	-0.46	38	1.9	Kazn	
DMR5:161267001	5	161267001	161268000	1000	1	1.50E-07	0.31	17	1.7	Kazn;LOC102554076	
DMR5:165689001	5	165689001	165690000	1000	1	2.10E-10	-0.54	21	2.1	Casz1	Transcription
DMR5:165740001	5	165740001	165743000	3000	1	1.00E-07	-0.48	64	2.13	Casz1;LOC103692511	Transcription
DMR5:165759001	5	165759001	165766000	7000	1	2.60E-07	-0.56	182	2.6	Casz1	Transcription
DMR5:165804001	5	165804001	165806000	2000	1	6.70E-11	-0.6	36	1.8	Pex14	Transport
DMR5:166679001	5	166679001	166680000	1000	1	2.30E-07	-0.43	26	2.6	Tmem201	
DMR5:166684001	5	166684001	166685000	1000	1	9.00E-07	-0.53	23	2.3	Tmem201	
DMR5:166935001	5	166935001	166936000	1000	1	2.70E-07	-0.54	21	2.1	Spsb1	
DMR5:167591001	5	167591001	167595000	4000	1	3.30E-07	-0.36	56	1.4	Rere	
DMR5:167655001	5	167655001	167656000	1000	1	5.50E-07	-0.47	24	2.4	Rere	
DMR5:168619001	5	168619001	168621000	2000	1	1.20E-08	0.32	29	1.45	Camta1	Transcription
DMR5:169572001	5	169572001	169575000	3000	1	4.50E-09	-0.48	81	2.7	Chd5;Kcnab2	
DMR5:169647001	5	169647001	169652000	5000	1	7.50E-07	-0.42	106	2.12	Kcnab2;LOC500594;Nphp4	Translation
DMR5:169741001	5	169741001	169748000	7000	1	3.00E-07	-0.46	161	2.3	Nphp4	
DMR5:170667001	5	170667001	170672000	5000	1	3.90E-07	-0.37	123	2.46	Ajap1	
DMR5:171244001	5	171244001	171247000	3000	1	5.30E-08	-0.5	62	2.07	LOC103692524;RGD1304567;Dffb	Transcription
DMR5:171323001	5	171323001	171325000	2000	1	9.80E-07	-0.37	39	1.95	Ccdc27;LOC102551562	
DMR5:171332001	5	171332001	171335000	3000	1	6.60E-07	-0.54	94	3.13	Ccdc27;LOC102551562	
DMR5:171396001	5	171396001	171397000	1000	1	9.30E-09	-0.47	26	2.6	Tp73	Transcription
DMR5:172698001	5	172698001	172704000	6000	1	1.20E-07	-0.44	133	2.22	Prkcz	Signaling
DMR5:173595001	5	173595001	173600000	5000	1	3.40E-07	-0.47	109	2.18	LOC100362942;Agrn	Extracellular Matrix
DMR5:173654001	5	173654001	173658000	4000	1	7.10E-07	-0.62	84	2.1	Perm1;Plekhn1;Klhl17;Noc2l	Cytoskeleton
DMR6:899001	6	899001	901000	2000	1	3.80E-07	-0.34	41	2.05	Crim1	
DMR6:955001	6	955001	956000	1000	1	1.40E-07	-0.37	17	1.7	Crim1;Fez2	
DMR6:6989001	6	6989001	6994000	5000	1	7.30E-08	0.28	78	1.56	Mta3	Development
DMR6:9644001	6	9644001	9646000	2000	1	9.80E-07	-0.35	39	1.95	Prkce	Signaling
DMR6:10536001	6	10536001	10537000	1000	1	8.80E-07	-0.32	28	2.8	Rhoq	Signaling
DMR6:10930001	6	10930001	10935000	5000	1	1.30E-08	-0.46	82	1.64	Ttc7a	
DMR6:10953001	6	10953001	10954000	1000	1	1.30E-07	-0.44	25	2.5	Ttc7a	
DMR6:10960001	6	10960001	10967000	7000	1	7.90E-09	-0.49	124	1.77	Ttc7a	
DMR6:23269001	6	23269001	23270000	1000	1	2.20E-07	-0.42	12	1.2	Clip4	Transcription
DMR6:24570001	6	24570001	24571000	1000	1	2.50E-07	-0.52	18	1.8	LOC685881;Capn13	Protease
DMR6:25656001	6	25656001	25658000	2000	1	4.20E-09	-0.51	31	1.55	Bre	
DMR6:26773001	6	26773001	26774000	1000	1	7.90E-07	0.36	11	1.1	Tcf23;Prr30	Transcription
DMR6:26782001	6	26782001	26783000	1000	1	7.40E-07	-0.55	22	2.2	Tcf23;Prr30;Preb;Abhd1	Transcription;Transcription;Protease
DMR6:28407001	6	28407001	28409000	2000	1	8.30E-07	-0.35	39	1.95	Efr3b	
DMR6:28641001	6	28641001	28642000	1000	1	2.00E-08	-0.5	25	2.5	Adcy3;Cenpo	
DMR6:43834001	6	43834001	43836000	2000	1	1.90E-08	-0.55	19	0.95	Klf11;Cys1	Transcription
DMR6:55606001	6	55606001	55607000	1000	1	4.40E-07	-0.45	24	2.4	Bzw2	Transcription
DMR6:55667001	6	55667001	55668000	1000	1	1.50E-07	-0.4	10	1	Ankmy2;LOC102556505	
DMR6:55688001	6	55688001	55689000	1000	1	8.40E-07	-0.3	18	1.8	Ankmy2;Lrrc72	
DMR6:56743001	6	56743001	56745000	2000	1	1.80E-11	0.56	3	0.15	Vom2r49	Signaling
DMR6:57918001	6	57918001	57922000	4000	1	4.30E-07	0.3	21	0.52	Dgkb	Signaling
DMR6:58150001	6	58150001	58151000	1000	1	2.40E-09	-0.71	6	0.6	Dgkb	Signaling
DMR6:60285001	6	60285001	60287000	2000	1	7.30E-08	-0.36	25	1.25	Zfp277	Transcription

DMR6:61280001	6	61280001	61283000	3000	1	6.40E-08	-0.55	29	0.97	Immp2l	
DMR6:61479001	6	61479001	61480000	1000	1	6.80E-08	0.35	5	0.5	Immp2l	
DMR6:71243001	6	71243001	71244000	1000	1	5.40E-07	-0.52	9	0.9	Prkd1	Signaling
DMR6:72656001	6	72656001	72657000	1000	1	7.70E-07	-0.42	19	1.9	Heatr5a	
DMR6:73633001	6	73633001	73634000	1000	1	5.30E-07	0.34	5	0.5	Akap6	
DMR6:73890001	6	73890001	73892000	2000	1	7.20E-11	-0.54	17	0.85	Akap6	
DMR6:74839001	6	74839001	74842000	3000	1	7.20E-09	-0.66	24	0.8	Npas3	
DMR6:80313001	6	80313001	80314000	1000	1	2.90E-07	0.43	2	0.2	Fbxo33	
DMR6:91548001	6	91548001	91549000	1000	1	2.40E-07	-0.56	11	1.1	Klhdc1	
DMR6:98345001	6	98345001	98347000	2000	1	4.20E-09	0.38	20	1	Rhoj	Signaling
DMR6:102522001	6	102522001	102524000	2000	1	9.80E-08	0.48	13	0.65	Rad51b	Transcription
DMR6:103432001	6	103432001	103434000	2000	1	7.70E-07	-0.4	20	1	Actn1	
DMR6:103565001	6	103565001	103566000	1000	1	4.40E-09	0.55	9	0.9	Dcaf5;Scarna3	Proteolysis
DMR6:104073001	6	104073001	104075000	2000	1	5.90E-07	-0.54	27	1.35	Galnt16	Golgi
DMR6:105358001	6	105358001	105359000	1000	1	9.90E-07	0.46	10	1	Ttc9	Transcription
DMR6:105378001	6	105378001	105381000	3000	1	2.50E-07	-0.35	60	2	Ttc9	Transcription
DMR6:105736001	6	105736001	105738000	2000	1	5.50E-10	-0.46	53	2.65	Pcnx1;LOC108351268	
DMR6:106539001	6	106539001	106540000	1000	1	3.80E-07	-0.43	15	1.5	Rgs6	
DMR6:107258001	6	107258001	107261000	3000	1	2.40E-07	-0.49	78	2.6	Papln	Protease
DMR6:108117001	6	108117001	108119000	2000	1	2.50E-08	0.32	15	0.75	Entpd5;Bbof1	Signaling
DMR6:108293001	6	108293001	108296000	3000	1	1.00E-09	-0.46	44	1.47	Vsx2	Development
DMR6:108928001	6	108928001	108929000	1000	1	9.90E-14	-0.55	11	1.1	Prox2;Dlst	Development;Transport
DMR6:110231001	6	110231001	110232000	1000	1	2.90E-07	-0.45	13	1.3	Gpatch2l;LOC108351277	Metabolism
DMR6:113322001	6	113322001	113323000	1000	1	3.00E-08	-0.62	2	0.2	Nrxn3	
DMR6:115402001	6	115402001	115404000	2000	1	2.20E-07	0.34	21	1.05	Ston2	Transport
DMR6:115406001	6	115406001	115411000	5000	1	3.30E-07	0.31	112	2.24	Ston2	Transport
DMR6:124920001	6	124920001	124922000	2000	1	5.50E-07	-0.44	51	2.55	Ccdc88c	Transport
DMR6:124986001	6	124986001	124988000	2000	1	2.00E-08	-0.46	54	2.7	Ccdc88c	Transport
DMR6:126195001	6	126195001	126199000	4000	1	5.90E-07	-0.48	49	1.23	Rin3	Transcription
DMR6:126262001	6	126262001	126263000	1000	1	6.30E-08	-0.48	20	2	Rin3;LOC103692692	Transcription
DMR6:127119001	6	127119001	127125000	6000	1	8.60E-07	-0.45	115	1.92	Prima1	
DMR6:128515001	6	128515001	128519000	4000	1	4.00E-08	-0.43	81	2.02	Clmn	
DMR6:129576001	6	129576001	129579000	3000	1	5.30E-07	-0.42	47	1.57	Ak7	Signaling
DMR6:131883001	6	131883001	131889000	6000	1	3.80E-07	-0.37	152	2.53	Bcl11b	Transcription
DMR6:132152001	6	132152001	132154000	2000	1	7.20E-07	-0.51	48	2.4	Ccdc85c	
DMR6:132422001	6	132422001	132431000	9000	1	5.00E-07	-0.4	230	2.56	Eml1	
DMR6:132915001	6	132915001	132917000	2000	1	8.30E-10	-0.52	37	1.85	Wdr25	Cytoskeleton
DMR6:133745001	6	133745001	133748000	3000	1	3.50E-07	-0.47	28	0.93	Mir370	
DMR6:135689001	6	135689001	135691000	2000	1	5.80E-08	-0.55	53	2.65	Traf3	Cytoskeleton
DMR6:135743001	6	135743001	135749000	6000	1	9.30E-09	-0.55	150	2.5	Cdc42bpb	Signaling
DMR6:136740001	6	136740001	136744000	4000	1	3.90E-07	-0.41	80	2	Kif26a	Cytoskeleton
DMR6:137712001	6	137712001	137726000	14000	2	1.10E-08	-0.57	392	2.8	Jag2	
DMR6:137883001	6	137883001	137887000	4000	2	5.80E-07	-0.58	77	1.93	Pacs2;Tex22	
DMR6:142644001	6	142644001	142645000	1000	1	8.00E-07	0.59	2	0.2	LOC102552859;Olr874-ps	
DMR6:143068001	6	143068001	143069000	1000	1	6.50E-07	0.34	5	0.5	RGD1561166	
DMR7:3499001	7	3499001	3501000	2000	1	1.90E-07	0.51	16	0.8	Olr878	Receptor
DMR7:5405001	7	5405001	5406000	1000	1	4.70E-07	0.44	3	0.3	Olr903	Receptor
DMR7:7322001	7	7322001	7324000	2000	1	4.10E-07	0.43	13	0.65	LOC108351564;Olr953-ps	
DMR7:11208001	7	11208001	11213000	5000	1	3.30E-09	-0.49	122	2.44	LOC102552770;Fzr1;Mfsd12;LOC690617	Proteolysis
DMR7:11586001	7	11586001	11587000	1000	1	4.30E-07	-0.55	10	1	Gng7;LOC102553301	Signaling
DMR7:11777001	7	11777001	11780000	3000	1	8.70E-07	-0.57	52	1.73	Jsrp1;Amh;Sf3a2;Plekhj1;Dot1l	Translation;Transport;Epigenetic
DMR7:11915001	7	11915001	11919000	4000	1	3.80E-07	-0.47	83	2.08	Mknk2	Signaling
DMR7:12194001	7	12194001	12199000	5000	2	3.70E-08	-0.52	184	3.68	Mbd3;Mex3d	Metabolism
DMR7:12649001	7	12649001	12650000	1000	1	1.40E-09	-0.66	13	1.3	Arhgap45;Elane;Prtn3;Plppr3	Protease;Signaling
DMR7:12843001	7	12843001	12850000	7000	1	7.90E-07	-0.5	260	3.71	Polrmt;Hcn2	Transcription;Transport
DMR7:13007001	7	13007001	13009000	2000	1	3.30E-08	-0.35	31	1.55	Theg	
DMR7:13045001	7	13045001	13058000	13000	1	3.10E-07	-0.37	306	2.35	Mier2;Plpp2	Development;Signaling
DMR7:15638001	7	15638001	15640000	2000	1	1.40E-07	0.37	4	0.2	Olr1091	Signaling
DMR7:18444001	7	18444001	18446000	2000	1	7.90E-07	0.29	12	0.6	Adamts10;Myo1f	Protease;Cytoskeleton
DMR7:23498001	7	23498001	23500000	2000	1	6.00E-07	0.25	25	1.25	Syn3	Transport
DMR7:23521001	7	23521001	23523000	2000	1	2.40E-08	-0.48	29	1.45	Syn3	Transport
DMR7:23865001	7	23865001	23867000	2000	1	5.00E-07	-0.37	15	0.75	Bpifc	
DMR7:24053001	7	24053001	24060000	7000	1	1.10E-07	-0.47	133	1.9	Btbd11	Cytoskeleton
DMR7:24928001	7	24928001	24929000	1000	1	5.30E-10	-0.36	18	1.8	Ckap4	
DMR7:25109001	7	25109001	25113000	4000	1	6.40E-07	-0.49	126	3.15	Nuak1	Signaling
DMR7:25114001	7	25114001	25115000	1000	1	2.60E-07	0.26	7	0.7	Nuak1	Signaling
DMR7:26286001	7	26286001	26289000	3000	1	1.40E-08	0.39	13	0.43	Appl2	Cytoskeleton
DMR7:26815001	7	26815001	26824000	9000	1	6.60E-07	-0.47	165	1.83	Chst11	Transport
DMR7:29030001	7	29030001	29033000	3000	1	4.50E-08	0.3	38	1.27	Gnptab;Chpt1;Sycp3	Transport

DMR7:29929001	7	29929001	29931000	2000	1	5.30E-08	-0.6	36	1.8	Ano4	
DMR7:30011001	7	30011001	30012000	1000	1	3.40E-07	0.43	4	0.4	Ano4;Nr1h4	Transcription
DMR7:31228001	7	31228001	31230000	2000	1	2.30E-07	0.29	19	0.95	Anks1b	Cytoskeleton
DMR7:33661001	7	33661001	33662000	1000	1	9.50E-10	0.48	43	4.3	RGD1565866	
DMR7:35709001	7	35709001	35711000	2000	1	5.40E-08	0.58	2	0.1	LOC100912201;Cep83os	
DMR7:35974001	7	35974001	35976000	2000	1	4.20E-07	0.24	30	1.5	Plxnc1	
DMR7:38743001	7	38743001	38745000	2000	1	6.80E-08	0.43	16	0.8	Dcn	
DMR7:38912001	7	38912001	38915000	3000	1	2.10E-07	0.36	7	0.23	Epyc	
DMR7:40160001	7	40160001	40162000	2000	1	2.00E-09	0.91	13	0.65	Tmtc3	
DMR7:40166001	7	40166001	40167000	1000	1	2.00E-08	0.5	11	1.1	Tmtc3	
DMR7:43606001	7	43606001	43608000	2000	1	1.90E-09	0.44	7	0.35	Mgat4c;LOC685339	Transport
DMR7:50300001	7	50300001	50302000	2000	1	4.60E-07	0.36	3	0.15	Syt1;LOC103692848	Transport
DMR7:51859001	7	51859001	51861000	2000	1	5.00E-12	0.49	13	0.65	Otogl	Extracellular Matrix
DMR7:53291001	7	53291001	53295000	4000	1	2.10E-08	-0.52	90	2.25	E2f7	Transcription
DMR7:57382001	7	57382001	57383000	1000	1	2.00E-08	0.52	7	0.7	Trhde	Protease
DMR7:58149001	7	58149001	58151000	2000	1	4.70E-07	0.32	25	1.25	Tph2	
DMR7:59421001	7	59421001	59422000	1000	1	5.30E-11	-0.88	10	1	Ptprb	Receptor
DMR7:60716001	7	60716001	60717000	1000	1	8.50E-08	-0.42	7	0.7	Cpm;Mdm2	Protease;Epigenetic
DMR7:60737001	7	60737001	60738000	1000	1	3.00E-08	0.62	3	0.3	Mdm2	Epigenetic
DMR7:61205001	7	61205001	61206000	1000	1	2.10E-07	0.33	21	2.1	Mdm1	
DMR7:64994001	7	64994001	64996000	2000	1	8.50E-10	-0.47	39	1.95	Tmbim4;Llph	
DMR7:66354001	7	66354001	66356000	2000	1	5.70E-08	0.46	17	0.85	Fam19a2	
DMR7:71011001	7	71011001	71012000	1000	1	1.30E-07	-0.57	19	1.9	Myo1a	Cytoskeleton
DMR7:71287001	7	71287001	71289000	2000	2	6.80E-09	-0.46	22	1.1	Mterf3;Ptdss1	Transport
DMR7:73003001	7	73003001	73004000	1000	1	7.80E-08	-0.52	14	1.4	Matn2	Extracellular Matrix
DMR7:74609001	7	74609001	74610000	1000	1	2.50E-07	0.48	7	0.7	Vps13b	
DMR7:75324001	7	75324001	75326000	2000	1	6.00E-09	-0.41	31	1.55	Snx31	Cytoskeleton
DMR7:76154001	7	76154001	76155000	1000	1	5.60E-08	0.46	11	1.1	Grhl2	Transcription
DMR7:81360001	7	81360001	81361000	1000	1	1.70E-08	-0.55	11	1.1	Angpt1	Signaling
DMR7:95666001	7	95666001	95668000	2000	1	2.40E-08	0.39	18	0.9	Sntb1;LOC102552118	
DMR7:97693001	7	97693001	97699000	6000	1	5.00E-07	-0.43	155	2.58	Zhx2;LOC102553429	Development
DMR7:97783001	7	97783001	97787000	4000	1	1.30E-07	-0.39	42	1.05	LOC683899;Tbc1d31;Rps27a-ps10	
DMR7:98822001	7	98822001	98824000	2000	1	4.00E-07	-0.38	38	1.9	Tatdn1;Ndufb9;Mtss1	Metabolism;Cytoskeleton
DMR7:99172001	7	99172001	99174000	2000	1	1.10E-08	0.43	9	0.45	Cyp2b1	Metabolism
DMR7:102709001	7	102709001	102711000	2000	1	1.20E-07	0.38	19	0.95	Pvt1	
DMR7:107782001	7	107782001	107784000	2000	1	3.60E-08	0.48	8	0.4	Ndrg1;LOC108351506	Protease
DMR7:108608001	7	108608001	108609000	1000	1	5.00E-07	0.36	3	0.3	Phf20l1	
DMR7:114121001	7	114121001	114123000	2000	1	2.30E-07	0.4	11	0.55	Trappc9	
DMR7:114601001	7	114601001	114603000	2000	1	1.20E-09	0.34	8	0.4	Ptk2	
DMR7:114719001	7	114719001	114721000	2000	1	3.70E-08	0.48	19	0.95	Dennd3	
DMR7:114959001	7	114959001	114960000	1000	1	4.30E-07	0.43	7	0.7	Gpr20;Ptp4a3	Signaling
DMR7:116412001	7	116412001	116413000	1000	1	2.80E-07	0.31	12	1.2	Ly6i	
DMR7:116961001	7	116961001	116963000	2000	1	4.60E-07	-0.49	34	1.7	Pycrl;Tsta3	Metabolism
DMR7:117170001	7	117170001	117173000	3000	1	2.60E-07	-0.53	90	3	Eppk1;LOC680875	Cytoskeleton
DMR7:117231001	7	117231001	117253000	22000	3	2.40E-08	-0.58	841	3.82	Plec	Cytoskeleton
DMR7:117422001	7	117422001	117426000	4000	1	3.50E-07	-0.59	100	2.5	Sharpin;Maf1;Wdr97;Hgh1	Proteolysis;Transcription
DMR7:119395001	7	119395001	119403000	8000	1	3.10E-07	-0.47	178	2.22	Ift27	
DMR7:119572001	7	119572001	119575000	3000	1	7.60E-08	-0.37	52	1.73	Csf2rb	Receptor
DMR7:120065001	7	120065001	120068000	3000	1	1.60E-07	-0.48	81	2.7	Cdc42ep1;Lgals2	Extracellular Matrix
DMR7:120458001	7	120458001	120460000	2000	1	5.20E-08	0.28	12	0.6	Pick1	Transport
DMR7:120718001	7	120718001	120720000	2000	1	6.90E-10	-0.64	87	4.35	Kcnj4	Transport
DMR7:122544001	7	122544001	122545000	1000	1	8.40E-08	-0.46	12	1.2	Slc25a17	
DMR7:123004001	7	123004001	123006000	2000	1	3.30E-07	-0.5	42	2.1	Zc3h7b	Metabolism
DMR7:124356001	7	124356001	124358000	2000	1	8.90E-07	-0.44	40	2	Ttll1	Cytoskeleton
DMR7:124428001	7	124428001	124430000	2000	1	9.60E-07	-0.33	24	1.2	Mcat	
DMR7:124546001	7	124546001	124550000	4000	1	1.90E-09	-0.42	103	2.58	Scube1	Extracellular Matrix
DMR7:124581001	7	124581001	124583000	2000	1	3.30E-07	-0.57	32	1.6	Scube1	Extracellular Matrix
DMR7:125086001	7	125086001	125090000	4000	1	1.30E-07	0.29	46	1.15	Samm50	
DMR7:126051001	7	126051001	126053000	2000	1	7.10E-08	-0.36	20	1	Ribc2	Cytoskeleton
DMR7:126416001	7	126416001	126418000	2000	1	5.00E-10	-0.39	25	1.25	Wnt7b	Signaling
DMR7:126792001	7	126792001	126795000	3000	1	2.30E-07	-0.54	62	2.07	Celsr1	Cytoskeleton
DMR7:126836001	7	126836001	126842000	6000	1	2.10E-09	-0.42	110	1.83	Celsr1	Cytoskeleton
DMR7:126955001	7	126955001	126957000	2000	1	1.20E-08	-0.53	53	2.65	Gramd4	
DMR7:127012001	7	127012001	127013000	1000	1	2.80E-08	-0.57	25	2.5	Gramd4;Cerk	Signaling
DMR7:127175001	7	127175001	127176000	1000	1	9.90E-07	-0.48	11	1.1	Tbc1d22a	Signaling
DMR7:128663001	7	128663001	128665000	2000	1	5.60E-08	-0.46	47	2.35	Fam19a5	
DMR7:128697001	7	128697001	128698000	1000	1	4.20E-07	-0.37	33	3.3	Fam19a5	
DMR7:130260001	7	130260001	130271000	11000	1	8.40E-07	-0.48	171	1.55	Ppp6r2;Sbf1	Signaling;Signaling
DMR7:134643001	7	134643001	134646000	3000	1	9.70E-12	-0.45	40	1.33	Pphln1;LOC108351532	

DMR7:136018001	7	136018001	136019000	1000	1	1.70E-07	-0.49	16	1.6	Tmem117	
DMR7:136191001	7	136191001	136192000	1000	1	3.10E-07	0.26	8	0.8	Tmem117	
DMR7:136272001	7	136272001	136273000	1000	1	1.80E-07	-0.39	15	1.5	Tmem117	
DMR7:136522001	7	136522001	136523000	1000	1	1.80E-07	-0.44	24	2.4	Nell2	Signaling
DMR7:136589001	7	136589001	136590000	1000	1	4.70E-08	0.42	5	0.5	Nell2	Signaling
DMR7:136641001	7	136641001	136644000	3000	1	1.20E-07	0.37	26	0.87	Nell2;LOC102546804	Signaling
DMR7:137002001	7	137002001	137004000	2000	1	2.70E-07	0.41	9	0.45	Dbx2	
DMR7:137978001	7	137978001	137981000	3000	1	4.30E-07	-0.39	89	2.97	Slc38a1	Transport
DMR7:139313001	7	139313001	139318000	5000	1	2.20E-07	-0.4	102	2.04	Hdac7;LOC108351548	Epigenetic
DMR7:139345001	7	139345001	139346000	1000	1	2.90E-11	-0.58	29	2.9	LOC108351548;Vdr	Transcription
DMR7:139347001	7	139347001	139350000	3000	1	2.00E-08	-0.42	46	1.53	Vdr	Transcription
DMR7:139473001	7	139473001	139476000	3000	1	1.80E-07	-0.35	66	2.2	Col2a1	Extracellular Matrix
DMR7:140526001	7	140526001	140533000	7000	1	7.10E-07	-0.51	174	2.49	Kmt2d	
DMR7:140909001	7	140909001	140914000	5000	1	2.60E-08	-0.45	107	2.14	Kcnh3;Mcrs1;LOC103692979	Transport
DMR7:141707001	7	141707001	141708000	1000	1	3.80E-07	0.33	10	1	Dip2b	
DMR7:142413001	7	142413001	142414000	1000	1	7.40E-08	0.39	1	0.1	Slc4a8	Transport
DMR7:143444001	7	143444001	143447000	3000	1	4.20E-07	0.41	24	0.8	LOC300249;Krt1	
DMR7:143845001	7	143845001	143848000	3000	1	2.20E-07	-0.4	67	2.23	Itgb7;Rarg	Extracellular Matrix;Transcription
DMR7:144332001	7	144332001	144334000	2000	1	6.50E-08	-0.42	43	2.15	Calcoco1	
DMR7:144777001	7	144777001	144780000	3000	1	1.10E-07	-0.49	54	1.8	Smug1	Epigenetic
DMR8:527001	8	527001	529000	2000	1	3.30E-07	0.33	43	2.15	Gucy1a2	Signaling
DMR8:2644001	8	2644001	2646000	2000	1	8.10E-08	0.32	8	0.4	Casp4;LOC102557277	Protease
DMR8:5300001	8	5300001	5301000	1000	1	8.20E-07	0.44	4	0.4	Dync2h1	Cytoskeleton
DMR8:7161001	8	7161001	7162000	1000	1	1.40E-09	-0.52	14	1.4	Pgr	
DMR8:14522001	8	14522001	14523000	1000	1	7.20E-07	-0.29	16	1.6	Fat3	Cytoskeleton
DMR8:20533001	8	20533001	20535000	2000	1	1.90E-07	0.45	4	0.2	Olr1174	Receptor
DMR8:21705001	8	21705001	21707000	2000	1	2.20E-07	0.46	10	0.5	Olfm2	Development
DMR8:22452001	8	22452001	22454000	2000	1	1.60E-07	-0.38	37	1.85	Qtrt1;Dnm2	Translation;Transport
DMR8:22620001	8	22620001	22624000	4000	1	9.60E-07	-0.41	77	1.93	Carm1;Yipf2;RGD1309188	Golgi
DMR8:22781001	8	22781001	22782000	1000	1	7.60E-08	-0.42	23	2.3	Ldlr;Spc24;Kank2	Binding Proteins;Cytoskeleton
DMR8:22837001	8	22837001	22840000	3000	1	3.20E-07	-0.47	77	2.57	Dock6	Transcription
DMR8:23843001	8	23843001	23846000	3000	1	9.70E-07	0.3	15	0.5	Bbs9	
DMR8:23880001	8	23880001	23881000	1000	1	9.00E-07	-0.43	19	1.9	Bbs9	
DMR8:24448001	8	24448001	24449000	1000	1	5.10E-08	0.35	7	0.7	Bmper	Extracellular Matrix
DMR8:26670001	8	26670001	26672000	2000	1	6.70E-07	-0.34	25	1.25	Eepd1	
DMR8:28406001	8	28406001	28408000	2000	1	2.90E-07	-0.42	32	1.6	Igsf9b	
DMR8:30728001	8	30728001	30729000	1000	1	1.60E-07	0.29	12	1.2	Ntm	Immune
DMR8:31518001	8	31518001	31520000	2000	1	4.60E-07	-0.55	14	0.7	Snx19	Cytoskeleton
DMR8:33543001	8	33543001	33547000	4000	1	8.40E-07	-0.49	64	1.6	Fli1	Transcription
DMR8:43990001	8	43990001	43993000	3000	1	1.30E-07	0.54	14	0.47	Tmem225	
DMR8:44349001	8	44349001	44350000	1000	1	1.00E-07	0.42	8	0.8	Gramd1b	
DMR8:45696001	8	45696001	45697000	1000	1	3.40E-08	-0.39	18	1.8	Lnc215	
DMR8:46921001	8	46921001	46923000	2000	1	6.30E-07	-0.39	25	1.25	Grik4	Receptor
DMR8:47224001	8	47224001	47225000	1000	1	4.70E-07	-0.46	21	2.1	Grik4	Receptor
DMR8:47241001	8	47241001	47246000	5000	1	7.20E-07	-0.53	74	1.48	Grik4;LOC102549942	Receptor
DMR8:48463001	8	48463001	48465000	2000	1	1.80E-09	-0.41	31	1.55	LOC103693069;Mcam	Immune
DMR8:48703001	8	48703001	48704000	1000	1	9.20E-07	-0.47	10	1	Hyou1	
DMR8:49496001	8	49496001	49497000	1000	1	2.10E-08	-0.51	11	1.1	Tmprss4;LOC102546809	Protease
DMR8:49848001	8	49848001	49851000	3000	1	8.20E-07	-0.42	40	1.33	Dscaml1	Cytoskeleton
DMR8:49956001	8	49956001	49958000	2000	1	2.20E-08	-0.43	26	1.3	Dscaml1	Cytoskeleton
DMR8:49976001	8	49976001	49980000	4000	1	6.40E-09	-0.51	77	1.93	Dscaml1	Cytoskeleton
DMR8:50496001	8	50496001	50502000	6000	1	4.60E-10	-0.43	119	1.98	Sik3	Signaling
DMR8:53785001	8	53785001	53786000	1000	1	9.40E-07	-0.5	12	1.2	Ttc12	
DMR8:57800001	8	57800001	57801000	1000	1	4.70E-07	0.35	11	1.1	Ddx10	
DMR8:58598001	8	58598001	58599000	1000	1	5.60E-08	0.52	6	0.6	Tnfaip8l3	
DMR8:59001001	8	59001001	59007000	6000	3	8.50E-08	0.41	16	0.27	Dmxl2	
DMR8:61287001	8	61287001	61289000	2000	1	3.70E-07	-0.43	34	1.7	Lingo1	Receptor
DMR8:62452001	8	62452001	62453000	1000	1	7.20E-07	0.31	9	0.9	Cyp1a2	Metabolism
DMR8:63379001	8	63379001	63381000	2000	1	3.60E-07	0.36	94	4.7	Nptn	Cytoskeleton
DMR8:65401001	8	65401001	65403000	2000	1	1.90E-07	-0.33	32	1.6	Thsd4	
DMR8:66331001	8	66331001	66337000	6000	1	1.70E-09	-0.57	119	1.98	Tle3;LOC102551666	Transcription
DMR8:67325001	8	67325001	67326000	1000	1	4.50E-08	-0.39	14	1.4	Anp32a	Epigenetic
DMR8:67386001	8	67386001	67390000	4000	1	1.50E-07	0.29	60	1.5	Coro2b	Cytoskeleton
DMR8:67612001	8	67612001	67613000	1000	1	3.10E-07	-0.49	13	1.3	Itga11;LOC102546312;LOC103693115	Extracellular Matrix
DMR8:67669001	8	67669001	67671000	2000	1	5.00E-07	-0.67	23	1.15	Itga11	Extracellular Matrix
DMR8:68580001	8	68580001	68581000	1000	1	2.50E-07	-0.64	13	1.3	Smad3	Transcription
DMR8:68644001	8	68644001	68646000	2000	1	8.60E-07	-0.3	28	1.4	Smad3	Transcription
DMR8:69986001	8	69986001	69989000	3000	1	5.80E-07	-0.52	37	1.23	Megf11	Extracellular Matrix
DMR8:70134001	8	70134001	70135000	1000	1	9.30E-07	-0.39	15	1.5	Megf11	Extracellular Matrix

DMR8:70418001	8	70418001	70419000	1000	1	2.20E-07	-0.64	15	1.5	Slc24a1;LOC103693120	Transport
DMR8:73019001	8	73019001	73020000	1000	1	9.90E-08	-0.45	24	2.4	Tln2	
DMR8:73093001	8	73093001	73094000	1000	1	6.10E-07	0.18	10	1	Tln2	
DMR8:73437001	8	73437001	73438000	1000	1	2.30E-08	-0.52	22	2.2	Tln2	
DMR8:73799001	8	73799001	73802000	3000	1	1.30E-07	-0.49	54	1.8	Vps13c	Transport
DMR8:75731001	8	75731001	75732000	1000	1	1.70E-08	0.29	9	0.9	Anxa2	Signaling
DMR8:82176001	8	82176001	82177000	1000	1	1.50E-07	-0.43	18	1.8	Myo5c	Cytoskeleton
DMR8:82949001	8	82949001	82950000	1000	1	7.60E-07	0.28	6	0.6	Bmp5	Growth Factors
DMR8:92932001	8	92932001	92935000	3000	1	7.20E-07	0.34	29	0.97	Fam46a	
DMR8:102664001	8	102664001	102666000	2000	1	3.70E-07	0.46	7	0.35	Slc9a9	Transport
DMR8:104731001	8	104731001	104732000	1000	1	2.20E-09	-0.36	22	2.2	Pxylp1	Signaling
DMR8:105946001	8	105946001	105947000	1000	1	9.60E-07	0.3	8	0.8	Clstn2	Transport
DMR8:106417001	8	106417001	106418000	1000	1	1.10E-07	-0.44	26	2.6	Nmnat3	Metabolism
DMR8:106420001	8	106420001	106423000	3000	1	6.30E-07	-0.52	49	1.63	Nmnat3	Metabolism
DMR8:111517001	8	111517001	111518000	1000	1	2.70E-07	-0.38	24	2.4	Slco2a1	Transport
DMR8:111558001	8	111558001	111561000	3000	2	3.90E-10	-0.66	49	1.63	Slco2a1	Transport
DMR8:111564001	8	111564001	111568000	4000	2	1.40E-07	-0.55	87	2.17	Slco2a1	Transport
DMR8:113536001	8	113536001	113538000	2000	1	4.70E-09	-0.5	20	1	Cpne4	
DMR8:113624001	8	113624001	113627000	3000	1	3.70E-07	-0.45	57	1.9	Mrpl3	Translation
DMR8:115381001	8	115381001	115385000	4000	1	4.70E-07	-0.54	61	1.52	Tex264;LOC108351777;Rad54l2	
DMR8:115908001	8	115908001	115910000	2000	1	7.20E-07	0.48	10	0.5	Dock3	Transcription
DMR8:116227001	8	116227001	116229000	2000	1	5.70E-07	-0.5	32	1.6	Cacna2d2	Transport
DMR8:116266001	8	116266001	116271000	5000	1	2.00E-07	-0.35	114	2.28	Cacna2d2	Transport
DMR8:116320001	8	116320001	116322000	2000	1	9.40E-07	-0.43	31	1.55	Rassf1;Tusc2;Hyal2	Cytoskeleton;Metabolism
DMR8:116415001	8	116415001	116418000	3000	1	9.00E-07	-0.44	39	1.3	Slc38a3	Transport
DMR8:116423001	8	116423001	116425000	2000	1	5.50E-09	-0.73	34	1.7	Slc38a3;Gnat1	Transport;Signaling
DMR8:116839001	8	116839001	116843000	4000	1	3.40E-07	-0.57	74	1.85	Gmppb;Rnf123;Amigo3	Transport
DMR8:117790001	8	117790001	117795000	5000	1	3.90E-08	-0.34	101	2.02	LOC100911077;LOC108351779;Shisa5;Trex1;Atrip	Cytoskeleton
DMR8:120099001	8	120099001	120100000	1000	1	3.50E-08	0.41	6	0.6	Trnap-agg	
DMR8:122442001	8	122442001	122447000	5000	1	5.90E-07	-0.41	117	2.34	Glb1;Tmppe	Metabolism
DMR8:122550001	8	122550001	122555000	5000	1	2.60E-07	-0.39	143	2.86	Trim71	Proteolysis
DMR8:123283001	8	123283001	123284000	1000	1	2.60E-07	-0.6	8	0.8	Osbpl10	
DMR8:124034001	8	124034001	124035000	1000	1	1.00E-07	-0.39	19	1.9	Gadl1	
DMR8:124987001	8	124987001	124991000	4000	1	5.60E-07	0.26	50	1.25	Rbms3	
DMR8:125179001	8	125179001	125181000	2000	1	6.20E-07	-0.37	42	2.1	Rbms3	
DMR8:125575001	8	125575001	125577000	2000	1	2.10E-07	0.27	23	1.15	Rbms3;LOC102550774	
DMR8:127813001	8	127813001	127816000	3000	1	4.50E-09	-0.5	70	2.33	Dlec1	
DMR8:128409001	8	128409001	128411000	2000	1	9.00E-07	0.25	23	1.15	Scn10a	Transport
DMR8:128613001	8	128613001	128617000	4000	1	1.50E-07	-0.39	104	2.6	Wdr48;Gorasp1;Ttc21a	
DMR8:129829001	8	129829001	129833000	4000	1	2.30E-07	-0.39	55	1.38	Ulk4	Signaling
DMR8:130009001	8	130009001	130012000	3000	1	6.10E-07	-0.36	79	2.63	Trak1	Transport
DMR8:130079001	8	130079001	130084000	5000	1	4.00E-07	-0.42	104	2.08	Trak1	Transport
DMR8:132555001	8	132555001	132556000	1000	1	5.30E-07	-0.56	8	0.8	Limd1	Transcription
DMR9:8045001	9	8045001	8047000	2000	1	6.70E-07	0.37	3	0.15	LOC100911278;Adgre4	Signaling
DMR9:10286001	9	10286001	10290000	4000	1	1.90E-07	-0.44	82	2.05	Rfx2;Prr22;Dus3l;Nrtn	Transcription;Translation;Growth Factors
DMR9:10344001	9	10344001	10346000	2000	1	5.90E-07	-0.52	60	3	Ndufa11;Vmac;Ranbp3	Metabolism;Cytoskeleton
DMR9:15162001	9	15162001	15164000	2000	1	3.20E-08	-0.44	28	1.4	Mdfi	
DMR9:16624001	9	16624001	16626000	2000	1	7.50E-07	-0.37	49	2.45	Mea1;Klhdc3;Rrp36;LOC680835	
DMR9:16673001	9	16673001	16674000	1000	1	1.10E-08	-0.37	26	2.6	Ptk7	Receptor
DMR9:16891001	9	16891001	16892000	1000	1	6.30E-07	-0.61	20	2	Ttbk1	Signaling
DMR9:20956001	9	20956001	20958000	2000	1	1.60E-07	0.42	21	1.05	Adgrf2;Adgrf4	
DMR9:37925001	9	37925001	37928000	3000	1	6.40E-07	-0.4	69	2.3	Dst	Cytoskeleton
DMR9:38134001	9	38134001	38135000	1000	1	3.30E-09	-0.4	26	2.6	Dst	Cytoskeleton
DMR9:38340001	9	38340001	38345000	5000	1	7.50E-08	-0.67	73	1.46	Bend6	
DMR9:41455001	9	41455001	41457000	2000	1	1.10E-07	-0.6	33	1.65	RGD1560533	
DMR9:42880001	9	42880001	42885000	5000	1	4.20E-07	-0.46	89	1.78	Arid5a	Transcription
DMR9:43358001	9	43358001	43362000	4000	1	7.30E-07	-0.41	95	2.38	Zap70;Tmem131	
DMR9:43682001	9	43682001	43685000	3000	1	4.70E-07	-0.44	55	1.83	Vwa3b;LOC102554996	
DMR9:43711001	9	43711001	43712000	1000	1	2.20E-07	0.3	4	0.4	Vwa3b	
DMR9:43729001	9	43729001	43733000	4000	1	4.10E-08	-0.43	59	1.48	Vwa3b	
DMR9:43955001	9	43955001	43956000	1000	1	3.40E-08	0.31	1	0.1	Inpp4a	
DMR9:45186001	9	45186001	45188000	2000	1	8.40E-07	-0.49	49	2.45	Aff3	Transcription
DMR9:45548001	9	45548001	45551000	3000	1	1.60E-08	-0.41	68	2.27	Chst10	Transport
DMR9:46030001	9	46030001	46032000	2000	1	6.10E-07	-0.35	56	2.8	Npas2	
DMR9:47172001	9	47172001	47173000	1000	1	7.10E-09	0.45	2	0.2	Il1rl1	Receptor
DMR9:49936001	9	49936001	49938000	2000	1	2.10E-08	0.38	23	1.15	Fhl2	Transcription
DMR9:50282001	9	50282001	50284000	2000	1	7.70E-07	-0.53	31	1.55	Nck2	Cytoskeleton

DMR9:51531001	9	51531001	51532000	1000	1	3.60E-07	0.4	5	0.5	Gulp1	Cytoskeleton
DMR9:51656001	9	51656001	51657000	1000	1	3.30E-08	0.47	2	0.2	Hspd1-ps13	
DMR9:55373001	9	55373001	55375000	2000	1	6.00E-07	-0.47	20	1	Tmeff2	
DMR9:55646001	9	55646001	55648000	2000	1	8.20E-08	0.34	8	0.4	Tmeff2	
DMR9:60301001	9	60301001	60303000	2000	1	1.10E-07	0.37	14	0.7	Dnah7	Cytoskeleton
DMR9:62281001	9	62281001	62283000	2000	1	9.10E-07	0.44	10	0.5	Plcl1;LOC103690536	Metabolism
DMR9:66339001	9	66339001	66342000	3000	1	1.60E-08	-0.49	46	1.53	RGD1562029	
DMR9:66869001	9	66869001	66873000	4000	1	1.30E-07	0.31	35	0.88	Wdr12;Carf	Transcription
DMR9:68594001	9	68594001	68596000	2000	1	8.70E-07	0.26	15	0.75	Pard3b	
DMR9:68821001	9	68821001	68823000	2000	1	5.30E-07	0.33	10	0.5	Pard3b	
DMR9:69122001	9	69122001	69123000	1000	1	9.10E-07	-0.33	8	0.8	Pard3b	
DMR9:71302001	9	71302001	71304000	2000	1	3.00E-07	0.52	11	0.55	Creb1;Mettl21a	
DMR9:72081001	9	72081001	72083000	2000	1	6.40E-07	-0.68	12	0.6	Pth2r	Receptor
DMR9:79937001	9	79937001	79942000	5000	1	3.20E-08	-0.46	166	3.32	Smarcal1	Transcription
DMR9:81799001	9	81799001	81801000	2000	1	3.80E-07	0.29	26	1.3	Usp37;Cnot9	Protease
DMR9:82160001	9	82160001	82161000	1000	1	2.80E-08	-0.64	15	1.5	Cryba2;Mir375;Cfap65	Development
DMR9:82611001	9	82611001	82615000	4000	1	3.30E-08	-0.42	85	2.12	Speg	
DMR9:82692001	9	82692001	82694000	2000	1	1.90E-07	-0.36	39	1.95	Obsl1;Inha	Growth Factors
DMR9:83408001	9	83408001	83413000	5000	1	2.70E-07	-0.43	168	3.36	LOC290508;Spetex-2E	
DMR9:84316001	9	84316001	84317000	1000	1	1.80E-07	-0.43	25	2.5	Sgpp2;Farsb	Signaling;Translation
DMR9:84702001	9	84702001	84703000	1000	1	2.80E-08	0.44	3	0.3	Kcne4	Transport
DMR9:85548001	9	85548001	85549000	1000	1	5.50E-08	-0.5	6	0.6	Mrpl44;LOC108352030	Translation
DMR9:88167001	9	88167001	88168000	1000	1	9.50E-07	-0.44	11	1.1	Rhbdd1	Protease
DMR9:93599001	9	93599001	93600000	1000	1	9.40E-08	-0.59	7	0.7	Pde6d;Cops7b	Signaling
DMR9:93727001	9	93727001	93728000	1000	1	3.20E-08	-0.41	20	2	Nppc	Hormone
DMR9:93833001	9	93833001	93835000	2000	1	3.10E-09	0.48	19	0.95	Dis3l2	Transcription
DMR9:94935001	9	94935001	94938000	3000	1	6.70E-08	-0.5	57	1.9	Sag	Cytoskeleton
DMR9:95015001	9	95015001	95018000	3000	1	4.80E-08	0.3	18	0.6	Dgkd	Signaling
DMR9:95322001	9	95322001	95323000	1000	1	2.40E-07	-0.52	12	1.2	Mroh2a	
DMR9:96274001	9	96274001	96281000	7000	1	4.80E-08	-0.48	171	2.44	Sh3bp4	
DMR9:96826001	9	96826001	96829000	3000	1	1.50E-07	-0.46	44	1.47	Agap1	
DMR9:96857001	9	96857001	96858000	1000	1	2.40E-07	-0.57	13	1.3	Agap1	
DMR9:96864001	9	96864001	96865000	1000	1	2.10E-07	-0.53	18	1.8	Agap1	
DMR9:97002001	9	97002001	97006000	4000	1	3.90E-08	-0.44	64	1.6	Agap1	
DMR9:98384001	9	98384001	98385000	1000	1	5.90E-07	0.36	12	1.2	LOC301604;LOC102547878;Ube2f	Proteolysis
DMR9:98580001	9	98580001	98582000	2000	1	1.30E-08	-0.42	30	1.5	Per2	Transcription
DMR9:99753001	9	99753001	99754000	1000	1	1.20E-07	-0.47	16	1.6	Ppp1r7;Olr1349;Olr1350;Olr1351	Signaling;Signaling
DMR9:99939001	9	99939001	99940000	1000	1	3.90E-07	-0.37	16	1.6	Ppp1r7	Signaling
DMR9:100201001	9	100201001	100206000	5000	1	2.90E-11	-0.48	98	1.96	Ppp1r7;Kif1a	Signaling;Cytoskeleton
DMR9:111194001	9	111194001	111196000	2000	1	4.70E-07	0.5	10	0.5	Gin1	
DMR9:113021001	9	113021001	113022000	1000	1	1.10E-09	-0.38	29	2.9	Tmem232	
DMR9:115851001	9	115851001	115852000	1000	1	4.20E-08	0.31	9	0.9	Lrrc30	Cytoskeleton
DMR9:117395001	9	117395001	117398000	3000	1	1.10E-07	0.25	4	0.13	Epb41l3;LOC100361186	
DMR9:117471001	9	117471001	117472000	1000	1	4.40E-08	0.32	3	0.3	Epb41l3;RGD1563667	Proteolysis
DMR10:848001	10	848001	850000	2000	1	1.30E-07	-0.4	33	1.65	LOC103693254;Myh11	
DMR10:2820001	10	2820001	2822000	2000	1	4.40E-07	0.3	10	0.5	Shisa9	
DMR10:3715001	10	3715001	3716000	1000	1	5.30E-07	-0.42	20	2	Cpped1	
DMR10:3840001	10	3840001	3845000	5000	1	9.90E-07	-0.58	83	1.66	Snx29	Cytoskeleton
DMR10:4198001	10	4198001	4199000	1000	1	4.60E-07	0.4	9	0.9	Snx29	Cytoskeleton
DMR10:7036001	10	7036001	7040000	4000	1	2.40E-08	0.32	22	0.55	RGD1564086;Carhsp1	Metabolism
DMR10:9614001	10	9614001	9615000	1000	1	3.30E-07	0.29	8	0.8	Rbfox1	Translation
DMR10:10401001	10	10401001	10403000	2000	1	8.60E-07	0.3	17	0.85	Rbfox1	Translation
DMR10:11873001	10	11873001	11877000	4000	1	9.70E-07	-0.5	65	1.62	Cluap1;RGD1561796;Naa60;LOC102551430	Metabolism
DMR10:13530001	10	13530001	13533000	3000	1	6.40E-07	-0.62	50	1.67	Pdpk1;Amdhd2;Atp6v0c	Signaling;Metabolism;Metabolism
DMR10:13601001	10	13601001	13603000	2000	1	7.20E-07	-0.35	35	1.75	LOC100158225;LOC102551327;Ccnf	Signaling
DMR10:14774001	10	14774001	14778000	4000	1	1.10E-08	-0.49	90	2.25	Cacna1h	Transport
DMR10:15503001	10	15503001	15505000	2000	1	6.70E-08	-0.43	40	2	Tmem8a;Mrpl28	Cytoskeleton;Translation
DMR10:15678001	10	15678001	15681000	3000	1	9.10E-07	-0.47	97	3.23	Rhbdf1;Snrnp25	Protease
DMR10:16865001	10	16865001	16866000	1000	1	1.90E-10	-0.49	18	1.8	Ergic1;LOC108352069	
DMR10:20121001	10	20121001	20122000	1000	1	4.00E-08	-0.68	16	1.6	Slit3	
DMR10:20863001	10	20863001	20864000	1000	1	7.80E-07	-0.36	35	3.5	Tenm2	
DMR10:21513001	10	21513001	21514000	1000	1	6.20E-07	0.32	7	0.7	Tenm2	
DMR10:23695001	10	23695001	23696000	1000	1	1.20E-07	0.37	8	0.8	Ebf1	Transcription
DMR10:29395001	10	29395001	29397000	2000	1	3.40E-07	-0.35	38	1.9	LOC108352077;Adra1b	Signaling
DMR10:38492001	10	38492001	38493000	1000	1	6.10E-07	-0.66	13	1.3	Fstl4	Protease; Proteolysis
DMR10:38830001	10	38830001	38833000	3000	1	7.00E-09	-1.21	38	1.27	Shroom1;Sowaha	Cytoskeleton
DMR10:38882001	10	38882001	38884000	2000	1	6.80E-09	-0.77	29	1.45	8-Sep	
DMR10:39340001	10	39340001	39341000	1000	1	1.10E-07	-0.62	17	1.7	Slc22a4	Transport
DMR10:39450001	10	39450001	39454000	4000	1	6.50E-08	-0.48	87	2.17	P4ha2	Golgi

DMR10:40311001	10	40311001	40312000	1000	1	4.20E-07	-0.79	9	0.9	Tnip1;Anxa6	Signaling
DMR10:40772001	10	40772001	40774000	2000	1	8.50E-07	-0.42	41	2.05	Sparc;LOC108352218	Extracellular Matrix
DMR10:40879001	10	40879001	40880000	1000	1	3.60E-07	0.38	25	2.5	Glra1	Ion Channel
DMR10:42467001	10	42467001	42470000	3000	1	5.60E-07	0.25	25	0.83	Gria1	Receptor
DMR10:42599001	10	42599001	42600000	1000	1	7.80E-10	0.56	3	0.3	Gria1	Receptor
DMR10:42665001	10	42665001	42667000	2000	1	4.90E-08	0.42	9	0.45	Gria1	Receptor
DMR10:43614001	10	43614001	43615000	1000	1	7.40E-09	-0.49	15	1.5	Mrpl22;LOC102555634	Translation
DMR10:44227001	10	44227001	44230000	3000	1	8.60E-07	0.38	26	0.87	Olr1431;LOC691352	Receptor;Cytoskeleton
DMR10:45280001	10	45280001	45281000	1000	1	1.50E-08	0.41	8	0.8	Rnf187;Hist3h2ba	Proteolysis;Epigenetic
DMR10:45399001	10	45399001	45400000	1000	1	1.10E-09	0.47	10	1	Obscn	
DMR10:45435001	10	45435001	45437000	2000	1	4.30E-09	-0.59	29	1.45	Obscn	
DMR10:45455001	10	45455001	45456000	1000	1	7.10E-10	1.25	13	1.3	Obscn	
DMR10:45474001	10	45474001	45475000	1000	1	1.40E-08	-0.52	19	1.9	Obscn	
DMR10:45590001	10	45590001	45591000	1000	1	1.60E-09	-0.57	20	2	Wnt3a	Signaling
DMR10:46602001	10	46602001	46604000	2000	1	3.20E-07	-0.62	31	1.55	Srebf1;Tom1l2	
DMR10:46951001	10	46951001	46955000	4000	1	4.50E-08	-0.45	82	2.05	Llgl1;Flii	Transport;Cytoskeleton
DMR10:47297001	10	47297001	47299000	2000	1	1.30E-08	0.54	16	0.8	Kcnj12	Transport
DMR10:47555001	10	47555001	47557000	2000	1	1.20E-07	-0.41	17	0.85	Aldh3a2	Metabolism
DMR10:47710001	10	47710001	47711000	1000	1	5.00E-11	0.51	9	0.9	LOC108352219;Rnf112	Signaling
DMR10:47760001	10	47760001	47761000	1000	1	3.60E-07	0.38	7	0.7	Mfap4;Mapk7	Signaling
DMR10:50492001	10	50492001	50493000	1000	1	1.60E-08	-0.58	16	1.6	Cox10	Metabolism
DMR10:51517001	10	51517001	51519000	2000	1	7.60E-07	-0.45	22	1.1	Arhgap44	
DMR10:52352001	10	52352001	52354000	2000	1	2.00E-09	-0.6	19	0.95	Zfp18;Dnah9	Cytoskeleton
DMR10:52369001	10	52369001	52370000	1000	1	8.30E-07	-0.49	10	1	Dnah9	Cytoskeleton
DMR10:54248001	10	54248001	54249000	1000	1	6.60E-11	-0.53	10	1	Gas7;Rcvrn	Cytoskeleton
DMR10:55372001	10	55372001	55373000	1000	1	9.40E-09	-0.43	25	2.5	Myh10	
DMR10:55688001	10	55688001	55690000	2000	1	2.10E-07	-0.52	44	2.2	Vamp2;Per1;Trnat-agu;Trnas-cga	Transcription
DMR10:55929001	10	55929001	55933000	4000	1	7.10E-07	-0.79	57	1.43	Cntrob;Trappc1;Kcnab3;RGD1563441	Transport
DMR10:55960001	10	55960001	55962000	2000	1	6.30E-09	-0.41	50	2.5	Chd3;LOC102557195	
DMR10:56170001	10	56170001	56171000	1000	1	3.20E-08	-0.48	19	1.9	Efnb3;Wrap53	Signaling
DMR10:56647001	10	56647001	56650000	3000	2	3.80E-07	-0.43	65	2.17	Dlg4	Cytoskeleton
DMR10:57049001	10	57049001	57050000	1000	1	7.90E-08	-0.39	16	1.6	Arrb2;Med11	Cytoskeleton
DMR10:57156001	10	57156001	57157000	1000	1	4.80E-07	0.34	10	1	LOC100361777;Pld2	Metabolism
DMR10:57422001	10	57422001	57426000	4000	1	6.80E-10	-0.79	56	1.4	LOC108352100;Scimp	
DMR10:59164001	10	59164001	59165000	1000	1	3.20E-07	0.36	6	0.6	Ube2g1	
DMR10:59324001	10	59324001	59326000	2000	1	5.30E-08	-0.61	22	1.1	Ankfy1	Transport
DMR10:60438001	10	60438001	60439000	1000	1	3.40E-07	0.4	1	0.1	Olr1492	Receptor
DMR10:63054001	10	63054001	63055000	1000	1	3.50E-07	0.34	7	0.7	Efcab5	Signaling
DMR10:63670001	10	63670001	63674000	4000	1	2.10E-07	-0.35	75	1.88	Rilp;Scarf1;Slc43a2	Transport
DMR10:63804001	10	63804001	63806000	2000	1	3.00E-07	-0.45	40	2	Inpp5k;Myo1c	Signaling;Cytoskeleton
DMR10:64616001	10	64616001	64617000	1000	1	2.40E-07	-0.45	15	1.5	Abr	Signaling
DMR10:64940001	10	64940001	64943000	3000	1	2.80E-08	0.42	38	1.27	LOC102551552;Pipox	Metabolism
DMR10:65025001	10	65025001	65030000	5000	1	9.80E-08	-0.44	73	1.46	Myo18a;LOC103693393	
DMR10:65412001	10	65412001	65414000	2000	1	4.60E-08	-0.36	40	2	Fam222b;Nek8;Traf4	Signaling;Cytoskeleton
DMR10:65631001	10	65631001	65635000	4000	1	8.50E-07	-0.41	50	1.25	Foxn1	
DMR10:68470001	10	68470001	68473000	3000	1	1.80E-07	0.46	32	1.07	Asic2	Transport
DMR10:68574001	10	68574001	68575000	1000	1	2.80E-08	0.38	5	0.5	Asic2	Transport
DMR10:68616001	10	68616001	68618000	2000	1	1.00E-09	1.05	47	2.35	Asic2	Transport
DMR10:69248001	10	69248001	69250000	2000	1	6.80E-07	0.35	6	0.3	Asic2	Transport
DMR10:70122001	10	70122001	70123000	1000	1	4.90E-08	-0.59	18	1.8	Cct6b;Zfp830;LOC103693403	Translation
DMR10:73243001	10	73243001	73245000	2000	1	1.90E-08	0.57	14	0.7	Bcas3	
DMR10:74230001	10	74230001	74232000	2000	1	7.80E-08	0.38	16	0.8	Ypel2	
DMR10:74613001	10	74613001	74617000	4000	1	2.10E-08	0.31	20	0.5	Ppm1e;LOC102557246	Signaling
DMR10:74980001	10	74980001	74981000	1000	1	2.10E-07	0.32	10	1	Rnf43	
DMR10:77861001	10	77861001	77862000	1000	1	8.10E-12	-0.51	28	2.8	Hlf;LOC103693416	Transcription
DMR10:77912001	10	77912001	77914000	2000	1	2.50E-07	-0.38	24	1.2	Hlf	Transcription
DMR10:82257001	10	82257001	82260000	3000	1	2.40E-07	-0.38	42	1.4	Mycbpap;LOC108352211;Rsad1	
DMR10:83969001	10	83969001	83970000	1000	1	3.60E-13	-0.54	21	2.1	Calcoco2;Ttll6	Cytoskeleton
DMR10:85162001	10	85162001	85163000	1000	1	5.30E-09	-0.8	11	1.1	Npepps	Protease
DMR10:85390001	10	85390001	85393000	3000	1	2.10E-07	-0.47	71	2.37	LOC102557607;Arhgap23	
DMR10:85464001	10	85464001	85467000	3000	1	3.50E-07	-0.57	84	2.8	Srcin1	
DMR10:86754001	10	86754001	86755000	1000	1	4.80E-10	-0.59	17	1.7	Casc3;Rapgefl1	
DMR10:86936001	10	86936001	86937000	1000	1	5.30E-09	-0.32	11	1.1	Top2a	Transcription
DMR10:86968001	10	86968001	86971000	3000	2	3.80E-08	-0.86	67	2.23	Igfbp4;Tns4	Protease; Proteolysis;Cytoskeleton
DMR10:87002001	10	87002001	87004000	2000	1	4.20E-07	-0.46	35	1.75	Tns4	Cytoskeleton
DMR10:87382001	10	87382001	87386000	4000	1	4.90E-07	-0.47	94	2.35	Krt23	
DMR10:87465001	10	87465001	87468000	3000	1	2.30E-07	0.4	24	0.8	Krt40;LOC108352226;Krtap3-3;LOC680043;Krtap3-2	
DMR10:88871001	10	88871001	88872000	1000	1	7.70E-07	-0.42	17	1.7	Ptrf	

DMR10:90324001	10	90324001	90328000	4000	1	3.10E-09	0.34	29	0.72	Pno1-ps1	
DMR10:90998001	10	90998001	90999000	1000	1	7.50E-07	-0.67	20	2	FAM187A;Gfap;Kif18b	Cytoskeleton
DMR10:91569001	10	91569001	91570000	1000	1	7.40E-08	0.22	10	1	Lrrc37a	Receptor
DMR10:91700001	10	91700001	91701000	1000	1	8.80E-07	-0.6	19	1.9	Prp2l1;LOC108352186;Lyzl6;LOC103693460;Rprml	Receptor
DMR10:93523001	10	93523001	93525000	2000	1	9.10E-07	-0.43	37	1.85	Mrc2	
DMR10:93608001	10	93608001	93610000	2000	1	2.80E-07	-0.41	33	1.65	10-Mar	
DMR10:94504001	10	94504001	94508000	4000	1	5.00E-08	-0.48	108	2.7	Cd79b;Scn4a	Immune;Transport
DMR10:94585001	10	94585001	94586000	1000	1	1.20E-07	-0.35	19	1.9	Icam2;Ern1	Translation
DMR10:95930001	10	95930001	95934000	4000	1	1.90E-07	-0.53	68	1.7	Cacng1	Transport
DMR10:97642001	10	97642001	97644000	2000	1	7.20E-07	-0.6	31	1.55	Gna13	Signaling
DMR10:101799001	10	101799001	101806000	7000	1	8.80E-07	-0.5	123	1.76	Slc39a11	Transport
DMR10:101830001	10	101830001	101836000	6000	1	2.20E-07	-0.53	127	2.12	Slc39a11	Transport
DMR10:101888001	10	101888001	101889000	1000	1	5.10E-10	-0.46	25	2.5	Slc39a11;LOC108352158	Transport
DMR10:102111001	10	102111001	102113000	2000	1	4.40E-08	-0.44	33	1.65	Slc39a11	Transport
DMR10:103190001	10	103190001	103192000	2000	1	9.50E-07	-0.59	36	1.8	Rpl38	Translation
DMR10:103326001	10	103326001	103328000	2000	1	6.50E-07	-0.47	32	1.6	Kif19;LOC102548333;LOC103693476;Btbd17	Cytoskeleton
DMR10:103635001	10	103635001	103638000	3000	1	9.60E-07	0.42	54	1.8	LOC103693477;Rab37	
DMR10:103745001	10	103745001	103748000	3000	1	2.00E-07	-0.43	50	1.67	Nat9;Tmem104	
DMR10:103788001	10	103788001	103789000	1000	1	1.80E-07	-0.44	23	2.3	Tmem104;LOC102554289;Grin2c	Receptor
DMR10:103930001	10	103930001	103931000	1000	1	6.40E-09	-0.56	23	2.3	Cdr2l	
DMR10:104436001	10	104436001	104440000	4000	1	2.60E-07	-0.52	121	3.02	LOC690323;Recql5	Epigenetic
DMR10:104603001	10	104603001	104605000	2000	1	5.50E-07	-0.38	44	2.2	Unk;Unc13d	
DMR10:104617001	10	104617001	104622000	5000	1	2.20E-08	-0.49	125	2.5	Unk;Unc13d;Wbp2	
DMR10:105106001	10	105106001	105108000	2000	1	6.50E-07	-0.63	54	2.7	Evpl	Cytoskeleton
DMR10:105716001	10	105716001	105718000	2000	1	7.20E-07	-0.33	52	2.6	St6galnac1	
DMR10:106707001	10	106707001	106708000	1000	1	3.30E-07	-0.52	28	2.8	Tnrc6c	Metabolism
DMR10:107053001	10	107053001	107057000	4000	1	8.30E-08	0.34	86	2.15	Dnah17	Cytoskeleton
DMR10:107150001	10	107150001	107152000	2000	1	8.90E-07	-0.38	48	2.4	Dnah17;LOC103693482	Cytoskeleton
DMR10:107337001	10	107337001	107339000	2000	1	3.50E-07	-0.45	56	2.8	Timp2	Protease; Proteolysis
DMR10:107448001	10	107448001	107450000	2000	1	7.20E-09	0.41	28	1.4	Cant1;LOC102548791;C1qtnf1	Signaling
DMR10:107745001	10	107745001	107747000	2000	1	1.20E-07	-0.36	38	1.9	Rbfox3	Translation
DMR10:107797001	10	107797001	107800000	3000	1	9.80E-09	-0.44	53	1.77	Rbfox3	Translation
DMR10:109632001	10	109632001	109634000	2000	1	1.00E-06	-0.38	44	2.2	Pde6g;Oxld1;Ccdc137;Arl16;Hgs	Signaling;Metabolism
DMR10:110015001	10	110015001	110016000	1000	1	1.90E-07	0.4	6	0.6	Fasn	
DMR10:110671001	10	110671001	110672000	1000	1	9.90E-07	-0.39	15	1.5	Tbcd	Transcription
DMR11:643001	11	643001	644000	1000	1	1.10E-08	0.56	4	0.4	Epha3	Receptor
DMR11:9413001	11	9413001	9414000	1000	1	5.90E-07	0.43	9	0.9	Robo1	
DMR11:9983001	11	9983001	9984000	1000	1	9.40E-07	-0.44	23	2.3	Robo1	
DMR11:10086001	11	10086001	10091000	5000	1	5.70E-07	-0.38	105	2.1	Robo1	
DMR11:27988001	11	27988001	27990000	2000	1	7.60E-07	0.5	9	0.45	Grik1	Receptor
DMR11:31057001	11	31057001	31059000	2000	1	1.70E-10	-0.53	46	2.3	Eva1c	
DMR11:31817001	11	31817001	31819000	2000	1	1.30E-09	-0.5	42	2.1	Son	Translation
DMR11:35664001	11	35664001	35669000	5000	1	4.40E-07	-0.39	81	1.62	Erg	Transcription
DMR11:37464001	11	37464001	37465000	1000	1	4.30E-08	0.36	4	0.4	Dscam	Cytoskeleton
DMR11:38025001	11	38025001	38029000	4000	1	1.10E-07	-0.7	60	1.5	Mx2	Transport
DMR11:43614001	11	43614001	43616000	2000	1	2.90E-09	0.45	5	0.25	Olr1556-ps;Olr1557	Signaling
DMR11:50827001	11	50827001	50829000	2000	1	9.90E-08	-0.66	10	0.5	Alcam	Immune
DMR11:52838001	11	52838001	52842000	4000	1	6.40E-08	0.49	34	0.85	LOC102548829;Ccdc54	
DMR11:54309001	11	54309001	54311000	2000	1	9.50E-13	0.5	8	0.4	Myh15	
DMR11:54502001	11	54502001	54505000	3000	1	1.40E-08	0.53	15	0.5	Retnlg	
DMR11:54521001	11	54521001	54523000	2000	2	1.20E-09	0.93	14	0.7	Retnlg	
DMR11:54668001	11	54668001	54671000	3000	1	2.70E-08	0.56	16	0.53	Trat1	Immune
DMR11:54847001	11	54847001	54848000	1000	1	4.80E-08	0.49	4	0.4	Morc1	
DMR11:57361001	11	57361001	57362000	1000	1	8.70E-10	-0.6	11	1.1	Phldb2	
DMR11:60121001	11	60121001	60122000	1000	1	3.50E-07	-0.8	7	0.7	Tmprss7	Protease
DMR11:60168001	11	60168001	60169000	1000	1	5.40E-16	-1.16	9	0.9	LOC685680;Gcsam	
DMR11:61135001	11	61135001	61137000	2000	1	5.00E-10	-0.41	29	1.45	Boc	
DMR11:64481001	11	64481001	64482000	1000	1	1.10E-07	-3.99	8	0.8	RGD1563835;LOC363788;RGD1306995	
DMR11:64933001	11	64933001	64940000	7000	1	4.90E-07	0.59	154	2.2	Popdc2	
DMR11:65059001	11	65059001	65064000	5000	1	9.50E-11	-0.59	79	1.58	Nr1i2;Gsk3b	Transcription;Signaling
DMR11:66017001	11	66017001	66019000	2000	1	8.50E-07	0.29	21	1.05	Hgd	Metabolism
DMR11:69484001	11	69484001	69487000	3000	1	5.80E-07	-0.33	58	1.93	Kalrn	Transcription
DMR11:69762001	11	69762001	69763000	1000	1	3.50E-07	-0.35	30	3	Kalrn	Transcription
DMR11:71172001	11	71172001	71175000	3000	1	8.00E-07	-0.3	68	2.27	Rubcn	
DMR11:71528001	11	71528001	71531000	3000	1	1.10E-08	0.3	40	1.33	Zdhhc19;Slc51a	Transport
DMR11:72600001	11	72600001	72607000	7000	1	8.60E-07	-0.33	193	2.76	Bdh1	Metabolism
DMR11:72734001	11	72734001	72736000	2000	1	2.30E-07	0.5	32	1.6	Apod	Binding Proteins
DMR11:73666001	11	73666001	73668000	2000	1	2.60E-07	-0.5	32	1.6	Fam43a	

DMR11:76806001	11	76806001	76808000	2000	1	1.60E-07	-0.42	29	1.45	Ccdc50;LOC102548576;LOC102557186	
DMR11:77602001	11	77602001	77603000	1000	1	5.00E-10	-0.47	13	1.3	Il1rap	Receptor
DMR11:78071001	11	78071001	78072000	1000	1	4.20E-07	0.47	4	0.4	P3h2;LOC103690757	Extracellular Matrix
DMR11:78446001	11	78446001	78447000	1000	1	4.90E-07	-0.44	8	0.8	Tp63	Transcription
DMR11:79337001	11	79337001	79338000	1000	1	1.80E-09	0.49	9	0.9	Lpp	Signaling
DMR11:82541001	11	82541001	82544000	3000	1	3.50E-07	-0.68	71	2.37	Igf2bp2	Metabolism
DMR11:83528001	11	83528001	83535000	7000	1	8.00E-08	-0.44	214	3.06	Ephb3	Receptor
DMR11:83948001	11	83948001	83952000	4000	1	2.40E-07	-0.38	72	1.8	Psmd2;LOC102551589;Ece2	Protease;Protease
DMR11:86909001	11	86909001	86914000	5000	1	1.10E-08	-0.53	136	2.72	Zdhhc8;Ccdc188;LOC102555338	
DMR11:87019001	11	87019001	87020000	1000	1	7.50E-08	-0.62	14	1.4	Rtn4r	
DMR11:87068001	11	87068001	87069000	1000	1	5.20E-07	-0.4	18	1.8	Prodh1;Dgcr6	Metabolism
DMR11:87556001	11	87556001	87559000	3000	1	4.00E-07	-0.48	59	1.97	Smpd4;Ccdc74a;Med15	Signaling
DMR11:88398001	11	88398001	88400000	2000	1	3.80E-07	0.31	15	0.75	Igll1	Immune
DMR11:88448001	11	88448001	88451000	3000	1	4.80E-07	0.37	19	0.63	Spag6	Cytoskeleton
DMR12:1265001	12	1265001	1266000	1000	1	3.20E-09	0.33	3	0.3	Stard13	Signaling
DMR12:2089001	12	2089001	2093000	4000	1	1.00E-07	-0.43	122	3.05	Pnpla6	Metabolism
DMR12:4164001	12	4164001	4168000	4000	1	8.70E-07	0.39	28	0.7	Vom2r60;RGD1559710	Signaling
DMR12:5658001	12	5658001	5663000	5000	1	4.70E-07	-0.36	90	1.8	Fry	Cytoskeleton
DMR12:8291001	12	8291001	8292000	1000	1	3.50E-09	-0.5	24	2.4	Mtus2	
DMR12:9060001	12	9060001	9061000	1000	1	4.90E-07	-0.37	14	1.4	Flt1;LOC102547693	Receptor
DMR12:9716001	12	9716001	9717000	1000	1	4.60E-08	-0.38	26	2.6	Polr1d	
DMR12:10246001	12	10246001	10249000	3000	1	8.80E-08	0.32	53	1.77	Gpr12	Signaling
DMR12:11845001	12	11845001	11847000	2000	1	1.30E-07	-0.52	28	1.4	Tmem130	Signaling
DMR12:13910001	12	13910001	13912000	2000	1	5.80E-07	-0.39	64	3.2	Tnrc18;Slc29a4	Transcription;Transport
DMR12:14004001	12	14004001	14006000	2000	1	3.40E-07	0.48	13	0.65	Wipi2	
DMR12:15696001	12	15696001	15698000	2000	1	6.90E-07	-0.52	31	1.55	Card11	
DMR12:16180001	12	16180001	16182000	2000	1	1.20E-07	0.39	12	0.6	Grifin	Extracellular Matrix
DMR12:17978001	12	17978001	17979000	1000	1	1.00E-08	-0.44	19	1.9	Fam20c	
DMR12:18200001	12	18200001	18203000	3000	1	3.60E-07	0.3	9	0.3	Vom2r-ps98	
DMR12:19689001	12	19689001	19692000	3000	1	6.30E-07	0.66	16	0.53	Pvrig;LOC108352428	
DMR12:20131001	12	20131001	20135000	4000	1	3.40E-09	0.49	23	0.58	Pilrb;LOC108352496	
DMR12:20136001	12	20136001	20142000	6000	1	1.40E-07	0.53	34	0.57	Pilrb;LOC108352496	
DMR12:20284001	12	20284001	20286000	2000	2	2.80E-07	0.57	7	0.35	LOC685157;RGD1560262	Immune
DMR12:20386001	12	20386001	20388000	2000	1	2.60E-08	0.59	8	0.4	Vom2r-ps103	
DMR12:20460001	12	20460001	20468000	8000	1	7.40E-08	0.62	39	0.49	Vom2r-ps100	
DMR12:20476001	12	20476001	20478000	2000	1	4.80E-10	0.57	6	0.3	Vom2r-ps100;LOC680910	Immune
DMR12:20530001	12	20530001	20531000	1000	1	3.30E-07	0.82	11	1.1	Vom2r-ps100;LOC102554946	
DMR12:20534001	12	20534001	20535000	1000	1	1.70E-07	0.83	13	1.3	Vom2r-ps100;LOC102554946	
DMR12:20642001	12	20642001	20646000	4000	2	2.70E-08	0.48	23	0.58	Vom2r-ps100;RGD1561730	Immune
DMR12:20658001	12	20658001	20661000	3000	1	3.50E-09	0.87	28	0.93	Vom2r-ps100;RGD1561730	Immune
DMR12:20663001	12	20663001	20671000	8000	1	1.60E-07	0.7	35	0.44	Vom2r-ps100;RGD1561730	Immune
DMR12:20672001	12	20672001	20675000	3000	1	5.70E-07	0.94	20	0.67	Vom2r-ps100	
DMR12:20680001	12	20680001	20682000	2000	1	8.30E-07	0.93	19	0.95	Vom2r-ps100	
DMR12:21928001	12	21928001	21930000	2000	2	5.40E-11	0.79	12	0.6	RGD1561143;LOC100910497	Immune
DMR12:21932001	12	21932001	21937000	5000	2	2.50E-10	0.53	66	1.32	RGD1561143;LOC100910497	Immune
DMR12:22540001	12	22540001	22542000	2000	1	1.20E-07	0.44	21	1.05	Muc3	
DMR12:23646001	12	23646001	23651000	5000	1	2.00E-08	-0.45	175	3.5	Upk3bl	
DMR12:23900001	12	23900001	23905000	5000	1	3.40E-07	0.38	59	1.18	Srrm3	
DMR12:23996001	12	23996001	23997000	1000	1	5.20E-07	-0.37	27	2.7	Styxl1;Tmem120a;Por	Metabolism
DMR12:24717001	12	24717001	24719000	2000	2	7.80E-08	0.48	12	0.6	Stx1a;Abhd11os;Abhd11	Transcription
DMR12:25326001	12	25326001	25327000	1000	1	2.20E-08	0.5	6	0.6	Gtf2ird1	Transcription
DMR12:25376001	12	25376001	25377000	1000	1	2.10E-11	0.41	15	1.5	Gtf2ird1;LOC108352437	Transcription
DMR12:25402001	12	25402001	25404000	2000	1	4.20E-07	0.26	25	1.25	Gtf2i	Transcription
DMR12:28996001	12	28996001	28998000	2000	1	3.50E-08	0.33	21	1.05	Wbscr17	
DMR12:29033001	12	29033001	29034000	1000	1	2.20E-11	0.56	7	0.7	Wbscr17;LOC100912262	
DMR12:30538001	12	30538001	30541000	3000	1	7.90E-07	-0.48	49	1.63	Gbas	
DMR12:31387001	12	31387001	31388000	1000	1	6.40E-07	0.44	12	1.2	Rimbp2	
DMR12:31557001	12	31557001	31558000	1000	1	5.40E-07	-0.26	22	2.2	Rimbp2	
DMR12:31986001	12	31986001	31996000	10000	1	7.40E-07	-0.4	130	1.3	Tmem132d	
DMR12:32007001	12	32007001	32011000	4000	1	5.80E-08	0.37	45	1.12	Tmem132d	
DMR12:32808001	12	32808001	32809000	1000	1	5.40E-07	-0.47	30	3	Tmem132c	
DMR12:32955001	12	32955001	32956000	1000	1	1.70E-07	0.28	7	0.7	Tmem132c	
DMR12:36208001	12	36208001	36209000	1000	1	6.00E-08	0.4	4	0.4	Tmem132b;LOC100911699	
DMR12:36309001	12	36309001	36312000	3000	1	2.20E-08	0.29	24	0.8	Tmem132b	
DMR12:36889001	12	36889001	36891000	2000	1	8.50E-08	-0.66	36	1.8	Ncor2	Epigenetic
DMR12:36983001	12	36983001	36985000	2000	1	6.30E-07	-0.5	55	2.75	Ncor2	Epigenetic
DMR12:37236001	12	37236001	37239000	3000	1	7.40E-07	-0.32	73	2.43	Ccdc92;Dnah10	
DMR12:37715001	12	37715001	37718000	3000	1	1.30E-10	0.58	19	0.63	Mphosph9	
DMR12:37877001	12	37877001	37879000	2000	1	8.00E-07	-0.63	32	1.6	Pitpnm2	Transport

DMR12:37896001	12	37896001	37897000	1000	1	4.20E-07	-0.54	13	1.3	Pitpnm2;Arl6ip4	Transport
DMR12:38205001	12	38205001	38209000	4000	1	5.20E-07	-0.52	81	2.02	Kntc1	
DMR12:38417001	12	38417001	38419000	2000	1	6.10E-08	-0.36	39	1.95	Clip1	Transcription
DMR12:38891001	12	38891001	38894000	3000	1	4.50E-08	-0.64	76	2.53	Rhof;Tmem120b	Signaling
DMR12:39348001	12	39348001	39349000	1000	1	1.90E-07	-0.41	28	2.8	P2rx7	Ion Channel
DMR12:40023001	12	40023001	40024000	1000	1	7.70E-07	-0.44	32	3.2	Cux2	Development
DMR12:40081001	12	40081001	40085000	4000	1	7.90E-07	-0.35	84	2.1	Cux2	Development
DMR12:40213001	12	40213001	40218000	5000	1	3.90E-07	-0.5	106	2.12	Cux2;Fam109a	Development
DMR12:40262001	12	40262001	40264000	2000	1	8.10E-08	-0.67	30	1.5	Sh2b3;Atxn2	Cytoskeleton;Metabolism
DMR12:42131001	12	42131001	42134000	3000	1	3.70E-09	0.4	29	0.97	Tbx5	Transcription
DMR12:44243001	12	44243001	44249000	6000	1	2.80E-07	-0.38	133	2.22	Nos1;Fbxo21	Metabolism
DMR12:44382001	12	44382001	44384000	2000	1	3.30E-07	-0.31	45	2.25	Nos1	Metabolism
DMR12:44655001	12	44655001	44660000	5000	1	8.30E-07	-0.43	69	1.38	Ksr2	Signaling
DMR12:44967001	12	44967001	44968000	1000	1	3.50E-07	-0.39	18	1.8	Wsb2;Vsig10	
DMR12:44976001	12	44976001	44977000	1000	1	4.70E-08	-0.4	23	2.3	Vsig10	
DMR12:46170001	12	46170001	46173000	3000	1	2.10E-07	-0.7	48	1.6	Ccdc60	
DMR12:46325001	12	46325001	46327000	2000	1	5.10E-07	-0.48	58	2.9	Prkab1;LOC108352463;Cit	Signaling;Signaling
DMR12:47224001	12	47224001	47228000	4000	1	6.10E-07	-0.38	66	1.65	LOC102548245;Mlec	
DMR12:47684001	12	47684001	47685000	1000	1	4.80E-07	-0.61	30	3	Gltp	
DMR12:47746001	12	47746001	47750000	4000	1	4.80E-08	-0.47	141	3.52	Trpv4;Fam222a	Transport
DMR12:47969001	12	47969001	47975000	6000	1	1.80E-08	-0.51	147	2.45	Ube3b	Proteolysis
DMR12:48651001	12	48651001	48656000	5000	1	5.20E-07	-0.42	114	2.28	Sart3;Ficd	Translation
DMR12:49375001	12	49375001	49377000	2000	1	2.70E-07	-0.46	38	1.9	Sgsm1	Signaling
DMR12:49481001	12	49481001	49487000	6000	1	4.50E-10	-0.75	155	2.58	RGD1306556	
DMR12:49548001	12	49548001	49558000	10000	1	2.60E-08	0.32	223	2.23	RGD1306556;Crybb3	
DMR12:50293001	12	50293001	50294000	1000	1	4.80E-07	-0.48	20	2	Hps4;LOC102556781	
DMR12:50466001	12	50466001	50468000	2000	1	7.90E-08	-0.54	24	1.2	Miat	
DMR12:51221001	12	51221001	51223000	2000	1	8.90E-08	0.29	18	0.9	Mn1	
DMR12:51977001	12	51977001	51978000	1000	1	4.70E-08	-0.47	7	0.7	Ep400	
DMR12:52305001	12	52305001	52307000	2000	1	1.30E-07	-0.51	57	2.85	Fbrsl1	
DMR13:7403001	13	7403001	7404000	1000	1	2.30E-07	0.36	1	0.1	Cntnap5c	
DMR13:13668001	13	13668001	13669000	1000	1	9.70E-07	0.33	6	0.6	Znf658	
DMR13:22528001	13	22528001	22530000	2000	1	2.20E-07	-0.41	87	4.35	Cntnap5b;LOC108352515	
DMR13:22583001	13	22583001	22585000	2000	1	2.20E-07	0.37	6	0.3	Cntnap5b	
DMR13:27362001	13	27362001	27363000	1000	1	1.70E-07	0.37	3	0.3	Serpinb7	Protease; Proteolysis
DMR13:27915001	13	27915001	27916000	1000	1	2.10E-07	-0.37	38	3.8	Serpinb8	Protease; Proteolysis
DMR13:35540001	13	35540001	35544000	4000	1	2.40E-09	0.25	61	1.52	Ralb	Signaling
DMR13:37415001	13	37415001	37420000	5000	1	4.70E-07	-0.36	121	2.42	Ccdc93	
DMR13:39788001	13	39788001	39790000	2000	1	9.10E-08	0.47	6	0.3	Dpp10	Protease
DMR13:42510001	13	42510001	42511000	1000	1	2.50E-08	0.36	7	0.7	Nckap5	
DMR13:42664001	13	42664001	42666000	2000	1	9.00E-07	0.47	12	0.6	Nckap5	
DMR13:43926001	13	43926001	43927000	1000	1	9.70E-08	-0.57	16	1.6	Mgat5	Golgi
DMR13:44183001	13	44183001	44186000	3000	1	9.00E-07	0.29	31	1.03	Tmem163	
DMR13:44323001	13	44323001	44325000	2000	1	3.80E-07	0.28	10	0.5	Tmem163	
DMR13:45026001	13	45026001	45027000	1000	1	2.30E-09	-0.46	27	2.7	Lct	Metabolism
DMR13:46023001	13	46023001	46026000	3000	1	1.10E-07	-0.47	38	1.27	Thsd7b	Cytoskeleton
DMR13:46729001	13	46729001	46730000	1000	1	9.30E-09	0.6	1	0.1	Thsd7b	Cytoskeleton
DMR13:47448001	13	47448001	47450000	2000	1	1.40E-08	-0.55	17	0.85	Pfkfb2;Yod1;LOC498222	Metabolism;Protease
DMR13:48531001	13	48531001	48534000	3000	1	7.50E-07	-0.61	44	1.47	LOC103691878;Slc26a9	Transport
DMR13:49392001	13	49392001	49394000	2000	1	3.80E-07	-0.56	26	1.3	Nfasc	
DMR13:50243001	13	50243001	50245000	2000	1	4.10E-07	-0.52	25	1.25	Zc3h11a;LOC498231;Snrpe	Translation;Translation
DMR13:50482001	13	50482001	50484000	2000	1	3.70E-07	-0.4	21	1.05	Sox13;Etnk2	Signaling
DMR13:50496001	13	50496001	50498000	2000	1	9.90E-07	-0.42	34	1.7	Etnk2;Ren;LOC102550525	Signaling
DMR13:50990001	13	50990001	50991000	1000	1	7.70E-08	0.32	8	0.8	Chit1	Metabolism
DMR13:52429001	13	52429001	52431000	2000	1	7.00E-07	-0.54	30	1.5	Nav1	
DMR13:52698001	13	52698001	52700000	2000	1	4.10E-08	0.33	11	0.55	Pkp1	Cytoskeleton
DMR13:53169001	13	53169001	53171000	2000	1	4.70E-07	0.49	11	0.55	Camsap2	
DMR13:57173001	13	57173001	57174000	1000	1	3.20E-07	0.31	7	0.7	Kcnt2	Transport
DMR13:69834001	13	69834001	69839000	5000	1	6.40E-08	0.39	69	1.38	Colgalt2	Golgi
DMR13:69929001	13	69929001	69932000	3000	1	6.30E-07	-0.49	47	1.57	Rgl1	Transcription
DMR13:69958001	13	69958001	69962000	4000	1	3.30E-07	-0.45	58	1.45	Rgl1;LOC102551528	Transcription
DMR13:70663001	13	70663001	70667000	4000	1	1.30E-08	0.32	69	1.73	Lamc1	Extracellular Matrix
DMR13:71112001	13	71112001	71114000	2000	1	2.20E-07	-0.5	34	1.7	Rgs8	Signaling
DMR13:71337001	13	71337001	71339000	2000	1	2.60E-07	-0.4	65	3.25	Glul	Metabolism
DMR13:73422001	13	73422001	73424000	2000	1	8.70E-07	-0.39	29	1.45	LOC102554128;Qsox1	Metabolism
DMR13:74250001	13	74250001	74253000	3000	1	6.00E-07	-0.4	31	1.03	Abl2;Tor3a	Transcription
DMR13:74263001	13	74263001	74264000	1000	1	5.50E-08	-0.39	7	0.7	Abl2;Tor3a	Transcription
DMR13:74988001	13	74988001	74990000	2000	1	8.20E-07	0.32	1	0.05	Rasal2	Signaling
DMR13:77891001	13	77891001	77892000	1000	1	4.40E-11	-0.51	21	2.1	Tnn	Signaling

DMR13:83859001	13	83859001	83860000	1000	1	2.20E-07	-0.5	10	1	Rcsd1	Cytoskeleton
DMR13:85226001	13	85226001	85228000	2000	1	1.90E-07	0.35	11	0.55	Fam78b	
DMR13:86531001	13	86531001	86532000	1000	1	6.00E-07	-0.45	12	1.2	Pbx1	Development
DMR13:87831001	13	87831001	87833000	2000	2	1.20E-08	-1.09	56	2.8	Nuf2	Cytoskeleton
DMR13:90068001	13	90068001	90069000	1000	1	7.10E-07	-0.45	12	1.2	Slamf7	Immune
DMR13:90822001	13	90822001	90824000	2000	1	8.30E-08	-0.41	48	2.4	Tagln2;Igsf9	Cytoskeleton
DMR13:91883001	13	91883001	91884000	1000	1	3.00E-07	-0.49	15	1.5	Cadm3	
DMR13:95141001	13	95141001	95142000	1000	1	1.50E-09	0.55	12	1.2	Akt3	Signaling
DMR13:98226001	13	98226001	98228000	2000	1	2.00E-07	0.31	19	0.95	Cdc42bpa	Signaling
DMR13:98727001	13	98727001	98730000	3000	1	2.90E-07	-0.46	53	1.77	Stum	
DMR13:98731001	13	98731001	98733000	2000	1	2.70E-07	-0.34	49	2.45	Stum	
DMR13:99419001	13	99419001	99420000	1000	1	3.80E-07	-0.3	19	1.9	Nvl	
DMR13:100872001	13	100872001	100875000	3000	1	1.70E-07	-0.44	45	1.5	Tp53bp2;Capn2	Signaling;Protease
DMR13:101232001	13	101232001	101233000	1000	1	4.20E-07	-0.4	24	2.4	Susd4	
DMR13:101307001	13	101307001	101309000	2000	1	1.30E-07	-0.4	56	2.8	Susd4	
DMR13:105534001	13	105534001	105536000	2000	1	4.00E-07	-0.83	23	1.15	Spata17	
DMR13:105597001	13	105597001	105599000	2000	1	6.80E-08	-0.48	20	1	Spata17	
DMR13:106087001	13	106087001	106089000	2000	1	1.60E-07	0.27	19	0.95	Esrrg	
DMR13:108675001	13	108675001	108679000	4000	1	2.00E-09	-0.7	105	2.62	Smyd2	Epigenetic
DMR13:108724001	13	108724001	108725000	1000	1	5.20E-07	-0.42	36	3.6	Ptpn14	Signaling
DMR13:109452001	13	109452001	109453000	1000	1	2.40E-07	-0.46	22	2.2	Rps6kc1	Signaling
DMR13:109546001	13	109546001	109548000	2000	1	1.30E-07	-0.42	34	1.7	Rps6kc1;Vash2	Signaling
DMR13:111191001	13	111191001	111192000	1000	1	5.10E-09	0.33	7	0.7	Kcnh1	Transport
DMR14:3068001	14	3068001	3070000	2000	1	3.30E-07	0.35	9	0.45	Gfi1	Transcription
DMR14:3209001	14	3209001	3211000	2000	1	3.60E-07	0.29	19	0.95	Rpap2;Glmn	Proteolysis
DMR14:3599001	14	3599001	3601000	2000	1	3.90E-07	0.28	18	0.9	Tgfbr3	Receptor
DMR14:5305001	14	5305001	5306000	1000	1	9.20E-07	-0.39	19	1.9	Lrrc8c	Cytoskeleton
DMR14:6601001	14	6601001	6602000	1000	1	1.30E-08	-0.45	22	2.2	LOC679894;Pkd2	Transcription;Transport
DMR14:7123001	14	7123001	7124000	1000	1	1.20E-07	0.36	4	0.4	Hsd17b13	
DMR14:7286001	14	7286001	7287000	1000	1	7.00E-11	-0.5	20	2	Aff1	Transcription
DMR14:7813001	14	7813001	7814000	1000	1	7.60E-10	-0.41	35	3.5	Ptpn13	Signaling
DMR14:11100001	14	11100001	11102000	2000	1	5.20E-07	-0.5	44	2.2	LOC108352712;Tmem150c	
DMR14:11169001	14	11169001	11171000	2000	1	8.50E-07	-0.44	48	2.4	Tmem150c;Enoph1	Signaling
DMR14:13329001	14	13329001	13331000	2000	1	2.10E-10	-0.51	57	2.85	Antxr2	Cytoskeleton
DMR14:14665001	14	14665001	14666000	1000	1	3.60E-08	0.29	7	0.7	Fras1	
DMR14:14720001	14	14720001	14724000	4000	1	6.40E-08	0.38	54	1.35	Fras1	
DMR14:14787001	14	14787001	14789000	2000	1	5.10E-07	0.35	31	1.55	Fras1	
DMR14:17632001	14	17632001	17633000	1000	1	7.50E-08	-0.44	16	1.6	Rchy1;LOC102546535	Proteolysis
DMR14:22966001	14	22966001	22968000	2000	1	2.70E-07	0.52	21	1.05	Ythdc1	
DMR14:33386001	14	33386001	33388000	2000	1	3.90E-07	0.29	10	0.5	Thegl	
DMR14:33696001	14	33696001	33700000	4000	1	5.50E-09	0.49	78	1.95	RGD1311575	
DMR14:37642001	14	37642001	37643000	1000	1	4.20E-08	0.37	8	0.8	Fryl	Cytoskeleton
DMR14:37819001	14	37819001	37821000	2000	1	2.40E-07	-0.43	34	1.7	Slain2	
DMR14:39767001	14	39767001	39768000	1000	1	4.00E-07	0.41	6	0.6	Gabra2	Ion Channel
DMR14:43494001	14	43494001	43495000	1000	1	7.50E-09	-0.4	19	1.9	Apbb2	
DMR14:43756001	14	43756001	43758000	2000	1	7.40E-07	0.3	19	0.95	Rbm47	Metabolism
DMR14:44033001	14	44033001	44036000	3000	1	3.80E-07	0.3	30	1	N4bp2	
DMR14:44225001	14	44225001	44226000	1000	1	1.20E-07	-0.38	15	1.5	Pds5a;LOC680579	Epigenetic
DMR14:78670001	14	78670001	78672000	2000	1	6.40E-07	-0.55	33	1.65	Wfs1;Ppp2r2c	Signaling
DMR14:78695001	14	78695001	78698000	3000	1	6.10E-07	-0.67	41	1.37	Ppp2r2c	Signaling
DMR14:79711001	14	79711001	79718000	7000	1	5.70E-08	0.31	93	1.33	Sorcs2;Psapl1	Transport;Cytoskeleton
DMR14:80424001	14	80424001	80426000	2000	1	2.90E-07	-0.53	33	1.65	Cpz	Protease
DMR14:80950001	14	80950001	80953000	3000	1	4.30E-07	-0.54	36	1.2	Dok7	
DMR14:81358001	14	81358001	81362000	4000	1	5.60E-09	-0.38	63	1.57	Nop14;Mfsd10;Add1	Metabolism;Cytoskeleton
DMR14:81812001	14	81812001	81813000	1000	1	1.30E-08	0.3	4	0.4	Zfyve28;Mxd4;LOC108352782	Transcription
DMR14:82058001	14	82058001	82059000	1000	1	1.70E-07	-0.63	10	1	Nat8l;RGD1560394	Metabolism
DMR14:84351001	14	84351001	84355000	4000	1	2.20E-07	-0.42	66	1.65	LOC102549281;Sec14l2	
DMR14:84443001	14	84443001	84446000	3000	1	4.50E-07	-0.47	52	1.73	Tbc1d10a;Gatsl3	Signaling
DMR14:85234001	14	85234001	85238000	4000	1	4.70E-08	0.39	60	1.5	Ap1b1	Transport
DMR14:85796001	14	85796001	85798000	2000	1	2.40E-07	0.36	12	0.6	Frg2	
DMR14:86157001	14	86157001	86159000	2000	1	1.10E-07	0.34	32	1.6	Myl7;Gck	Cytoskeleton;Signaling
DMR14:86178001	14	86178001	86182000	4000	1	6.30E-07	-0.32	69	1.73	Gck	Signaling
DMR14:86219001	14	86219001	86222000	3000	1	6.20E-09	-0.43	59	1.97	Camk2b;LOC108352789	Signaling
DMR14:86917001	14	86917001	86918000	1000	1	7.80E-08	-0.39	19	1.9	Ramp3	Receptor
DMR14:89167001	14	89167001	89169000	2000	1	8.00E-07	0.38	25	1.25	Pkd1l1	Transport
DMR14:89740001	14	89740001	89741000	1000	1	3.20E-07	-0.83	5	0.5	Abca13	Transport
DMR14:91916001	14	91916001	91919000	3000	1	6.10E-08	-0.35	61	2.03	Ddc	Metabolism
DMR14:99517001	14	99517001	99520000	3000	1	4.10E-08	0.38	12	0.4	Vstm2a	Immune
DMR14:100386001	14	100386001	100389000	3000	1	3.90E-07	-0.79	39	1.3	Wdr92	Metabolism

DMR14:104286001	14	104286001	104289000	3000	1	2.50E-07	-0.33	56	1.87	Spred2	Cytoskeleton
DMR14:106953001	14	106953001	106955000	2000	1	1.50E-07	-0.4	27	1.35	Ehbp1	
DMR14:108507001	14	108507001	108508000	1000	1	9.40E-07	0.41	8	0.8	Rel	Transcription
DMR14:108650001	14	108650001	108655000	5000	1	5.50E-07	0.47	6	0.12	Papolg	Translation
DMR14:114019001	14	114019001	114023000	4000	1	5.90E-07	0.36	11	0.28	Ndufs5-ps1	
DMR15:2906001	15	2906001	2908000	2000	2	2.30E-09	-0.48	55	2.75	Kat6b	Epigenetic
DMR15:2933001	15	2933001	2935000	2000	1	1.20E-07	-0.41	25	1.25	Kat6b	Epigenetic
DMR15:3169001	15	3169001	3171000	2000	1	2.80E-07	-0.52	35	1.75	Adk	
DMR15:4474001	15	4474001	4477000	3000	1	7.90E-07	-0.35	68	2.27	Kcnk16;LOC108352873	Transport
DMR15:4755001	15	4755001	4757000	2000	1	5.60E-11	-0.63	28	1.4	Gng2	Signaling
DMR15:6457001	15	6457001	6458000	1000	1	5.10E-10	0.46	5	0.5	Zfp385d	
DMR15:7789001	15	7789001	7790000	1000	1	3.40E-07	-0.61	25	2.5	Ube2e2	Proteolysis
DMR15:8135001	15	8135001	8137000	2000	1	1.00E-07	-0.34	29	1.45	Ube2e1	Proteolysis
DMR15:11815001	15	11815001	11816000	1000	1	2.90E-07	-0.43	12	1.2	Nek10	Signaling
DMR15:15529001	15	15529001	15530000	1000	1	2.40E-07	-0.36	42	4.2	Cadps	Transport
DMR15:15552001	15	15552001	15553000	1000	1	4.10E-08	0.43	3	0.3	Cadps	Transport
DMR15:15586001	15	15586001	15590000	4000	1	9.20E-07	-0.36	71	1.77	Cadps	Transport
DMR15:18339001	15	18339001	18343000	4000	1	7.00E-08	0.47	27	0.68	Fam3d	Signaling
DMR15:18359001	15	18359001	18360000	1000	1	3.00E-08	0.27	9	0.9	Fam3d	Signaling
DMR15:23751001	15	23751001	23753000	2000	1	3.40E-07	0.33	29	1.45	Samd4a	
DMR15:24318001	15	24318001	24320000	2000	1	6.80E-09	-0.67	67	3.35	Fbxo34	
DMR15:24495001	15	24495001	24500000	5000	1	1.70E-07	-0.41	76	1.52	Ktn1	
DMR15:25074001	15	25074001	25079000	5000	1	3.10E-08	-0.44	149	2.98	Peli2	Proteolysis
DMR15:28630001	15	28630001	28632000	2000	1	3.20E-07	-0.55	32	1.6	Chd8	
DMR15:28659001	15	28659001	28661000	2000	1	7.00E-09	0.41	20	1	Chd8	
DMR15:30463001	15	30463001	30464000	1000	1	1.90E-07	0.42	4	0.4	LOC691067;RGD1564212	
DMR15:36555001	15	36555001	36556000	1000	1	2.00E-08	-0.41	16	1.6	Atp12a	Transport
DMR15:37616001	15	37616001	37619000	3000	1	1.50E-07	-0.45	35	1.17	Cryl1	Metabolism
DMR15:39985001	15	39985001	39986000	1000	1	4.70E-07	-0.35	22	2.2	Atp8a2	Transport
DMR15:40397001	15	40397001	40398000	1000	1	4.90E-07	0.36	10	1	Atp8a2	Transport
DMR15:46393001	15	46393001	46396000	3000	1	1.10E-09	-0.66	43	1.43	Gata4	Transcription
DMR15:47693001	15	47693001	47694000	1000	1	6.50E-07	-0.36	11	1.1	Msra	Metabolism
DMR15:51537001	15	51537001	51541000	4000	1	1.80E-07	-0.67	53	1.32	Pebp4	
DMR15:52284001	15	52284001	52286000	2000	1	5.40E-07	0.26	9	0.45	Fam160b2;Dmtn	Cytoskeleton
DMR15:57824001	15	57824001	57827000	3000	1	8.10E-08	-0.61	35	1.17	Slc25a30	Transport
DMR15:61271001	15	61271001	61273000	2000	1	3.60E-09	-0.52	37	1.85	Vwa8	
DMR15:61756001	15	61756001	61759000	3000	1	9.20E-10	0.3	17	0.57	Wbp4	
DMR15:83703001	15	83703001	83705000	2000	1	8.20E-08	-0.4	47	2.35	Klf5	Transcription
DMR15:100671001	15	100671001	100673000	2000	1	7.80E-07	0.28	17	0.85	Gpc5	
DMR15:100714001	15	100714001	100715000	1000	1	1.40E-07	0.47	2	0.2	Gpc5	
DMR15:103694001	15	103694001	103699000	5000	1	1.90E-07	-0.34	128	2.56	Abcc4	Transport
DMR15:103781001	15	103781001	103782000	1000	1	3.80E-07	0.3	0	0	Abcc4	Transport
DMR15:104017001	15	104017001	104018000	1000	1	9.50E-08	0.28	3	0.3	Cldn10	Cell Junction
DMR15:105230001	15	105230001	105232000	2000	1	5.10E-09	-0.93	50	2.5	Hs6st3;LOC102556639	
DMR15:105259001	15	105259001	105263000	4000	1	2.20E-07	0.46	59	1.48	Hs6st3	
DMR15:106511001	15	106511001	106512000	1000	1	2.00E-08	0.89	12	1.2	Farp1	
DMR15:106552001	15	106552001	106555000	3000	1	4.30E-08	-0.5	67	2.23	Farp1	
DMR15:108411001	15	108411001	108413000	2000	1	5.20E-07	-0.44	38	1.9	Ubac2	
DMR15:109815001	15	109815001	109816000	1000	1	7.70E-07	0.32	2	0.2	Nalcn	Transport
DMR16:1975001	16	1975001	1978000	3000	1	4.70E-08	0.34	19	0.63	Ppif	Transcription
DMR16:6665001	16	6665001	6668000	3000	1	2.00E-07	-0.47	68	2.27	Prkcd	Signaling
DMR16:7237001	16	7237001	7242000	5000	1	6.20E-07	-0.42	126	2.52	Stab1	Transport
DMR16:8748001	16	8748001	8749000	1000	1	3.80E-09	-0.49	24	2.4	Ercc6	
DMR16:8759001	16	8759001	8761000	2000	1	4.00E-07	-0.36	50	2.5	Ercc6	
DMR16:9302001	16	9302001	9304000	2000	1	1.20E-07	-0.4	28	1.4	Wdfy4	
DMR16:9568001	16	9568001	9571000	3000	1	3.80E-09	-0.4	49	1.63	Arhgap22	
DMR16:10396001	16	10396001	10401000	5000	1	1.70E-08	0.51	81	1.62	Antxrl	Cytoskeleton
DMR16:10748001	16	10748001	10750000	2000	1	5.90E-07	-0.46	45	2.25	Mmrn2;Bmpr1a	Signaling
DMR16:12460001	16	12460001	12461000	1000	1	6.70E-07	0.46	6	0.6	LOC108353079;RGD1566353	
DMR16:18696001	16	18696001	18697000	1000	1	1.30E-08	0.36	10	1	Mat1a	Metabolism
DMR16:18855001	16	18855001	18858000	3000	1	2.80E-07	-0.53	42	1.4	Sin3b	Epigenetic
DMR16:19115001	16	19115001	19116000	1000	1	9.10E-07	0.24	9	0.9	Calr3	Transcription
DMR16:19796001	16	19796001	19797000	1000	1	4.20E-08	-0.59	14	1.4	Ushbp1;Babam1;Ankle1;RGD1565495	
DMR16:19906001	16	19906001	19908000	2000	1	1.40E-08	-0.43	31	1.55	Gtpbp3;Plvap	Epigenetic
DMR16:20162001	16	20162001	20164000	2000	1	4.50E-07	-0.37	26	1.3	Fcho1;LOC108348388;LOC108348387	Cytoskeleton
DMR16:22723001	16	22723001	22724000	1000	1	1.10E-08	0.32	11	1.1	Csgalnact1	Golgi
DMR16:22987001	16	22987001	22989000	2000	1	8.70E-07	0.37	16	0.8	Csgalnact1	Golgi
DMR16:23037001	16	23037001	23038000	1000	1	2.50E-09	0.5	5	0.5	Csgalnact1	Golgi
DMR16:25645001	16	25645001	25648000	3000	1	1.40E-07	0.28	20	0.67	1-Mar	
DMR16:26636001	16	26636001	26637000	1000	1	3.90E-08	0.31	7	0.7	Tmem192	

DMR16:27441001	16	27441001	27443000	2000	2	1.20E-07	0.52	9	0.45	Tll1;LOC108348393	Protease
DMR16:31363001	16	31363001	31364000	1000	1	7.40E-11	-0.79	7	0.7	Palld	Cytoskeleton
DMR16:37183001	16	37183001	37184000	1000	1	5.70E-08	-0.43	5	0.5	Fbxo8;Cep44	Transcription
DMR16:37748001	16	37748001	37784000	36000	1	4.30E-07	0.2	350	0.97	Glra3	Ion Channel
DMR16:46437001	16	46437001	46439000	2000	1	6.60E-07	0.46	16	0.8	Tenm3	
DMR16:50408001	16	50408001	50411000	3000	1	4.30E-07	-0.35	53	1.77	Fat1	Cytoskeleton
DMR16:54302001	16	54302001	54305000	3000	1	7.20E-07	-0.52	39	1.3	Mtus1	
DMR16:54629001	16	54629001	54631000	2000	1	2.10E-07	0.45	5	0.25	Adam24	Protease
DMR16:68997001	16	68997001	68998000	1000	1	1.10E-07	0.31	5	0.5	Adrb3	Signaling
DMR16:73019001	16	73019001	73020000	1000	1	7.30E-07	0.41	9	0.9	Zmat4	
DMR16:73368001	16	73368001	73370000	2000	1	9.10E-07	-0.33	40	2	Sfrp1	Receptor
DMR16:73767001	16	73767001	73778000	11000	1	4.00E-08	-0.74	291	2.65	Ank1;LOC100910418	
DMR16:74190001	16	74190001	74191000	1000	1	4.30E-07	-0.36	16	1.6	Ikbkb;LOC108348421	Signaling
DMR16:74550001	16	74550001	74553000	3000	1	1.30E-07	-0.4	73	2.43	Slc25a15;Tpte2	Transport;Signaling
DMR16:74749001	16	74749001	74751000	2000	1	3.10E-08	-0.51	21	1.05	Vps36;Ckap2	Transport;Cytoskeleton
DMR16:75108001	16	75108001	75109000	1000	1	2.60E-07	-0.74	9	0.9	Defb15;Defb12	
DMR16:75176001	16	75176001	75178000	2000	1	1.50E-07	0.45	6	0.3	Defb9	Signaling
DMR16:75756001	16	75756001	75758000	2000	1	4.90E-07	0.26	19	0.95	Xkr5	
DMR16:77475001	16	77475001	77476000	1000	1	9.10E-07	0.31	3	0.3	Csmd1	
DMR16:77880001	16	77880001	77883000	3000	1	5.20E-08	0.31	22	0.73	Csmd1	
DMR16:78795001	16	78795001	78797000	2000	1	1.50E-09	0.43	18	0.9	Csmd1	
DMR16:79634001	16	79634001	79635000	1000	1	2.50E-08	0.31	4	0.4	Myom2	
DMR16:79796001	16	79796001	79801000	5000	1	4.80E-07	-0.7	141	2.82	Arhgef10	Transcription
DMR16:80022001	16	80022001	80025000	3000	1	9.60E-07	-0.51	50	1.67	Dlgap2	Cytoskeleton
DMR16:80570001	16	80570001	80572000	2000	1	5.10E-07	0.38	10	0.5	Dlgap2;LOC108348428	Cytoskeleton
DMR16:80750001	16	80750001	80752000	2000	1	4.60E-07	-0.48	41	2.05	Tdrp	
DMR16:81139001	16	81139001	81142000	3000	1	1.40E-08	-0.37	79	2.63	Atp4b	Transport
DMR16:81213001	16	81213001	81218000	5000	1	4.20E-07	-0.49	135	2.7	Tmem255b;Gas6	Extracellular Matrix
DMR16:81219001	16	81219001	81220000	1000	1	1.70E-07	-0.71	23	2.3	Tmem255b;Gas6	Extracellular Matrix
DMR16:81420001	16	81420001	81427000	7000	1	3.70E-07	-0.38	154	2.2	Rasa3	Signaling
DMR16:81532001	16	81532001	81534000	2000	1	1.80E-07	-0.43	31	1.55	Upf3a	
DMR16:81970001	16	81970001	81976000	6000	1	7.90E-07	-0.42	116	1.93	Mcf2l	Transcription
DMR16:83006001	16	83006001	83009000	3000	1	2.10E-08	-0.44	68	2.27	Tex29;Arhgef7	Transcription
DMR16:85157001	16	85157001	85159000	2000	1	6.70E-14	0.6	12	0.6	Myo16;Marco	Extracellular Matrix
DMR16:85161001	16	85161001	85163000	2000	1	9.50E-08	0.86	13	0.65	Myo16	
DMR16:85181001	16	85181001	85182000	1000	1	5.90E-08	-0.46	14	1.4	Myo16	
DMR16:85539001	16	85539001	85540000	1000	1	5.20E-08	0.34	5	0.5	Fam155a	
DMR16:90645001	16	90645001	90647000	2000	1	1.40E-07	-0.39	16	0.8	Shcbp1	
DMR17:1349001	17	1349001	1350000	1000	1	5.20E-07	-0.53	7	0.7	Ercc6l2	
DMR17:2380001	17	2380001	2381000	1000	1	7.70E-09	-0.84	12	1.2	Cntnap3b	
DMR17:4363001	17	4363001	4365000	2000	1	9.50E-07	-0.36	49	2.45	Dapk1	Signaling
DMR17:5262001	17	5262001	5263000	1000	1	2.30E-07	-0.54	15	1.5	Zcchc6	
DMR17:5575001	17	5575001	5578000	3000	1	7.10E-07	-0.35	74	2.47	Agtpbp1	Protease
DMR17:6016001	17	6016001	6018000	2000	1	5.20E-08	0.29	35	1.75	Ntrk2	Receptor
DMR17:6481001	17	6481001	6485000	4000	1	4.30E-08	-0.31	78	1.95	Slc28a3	Transport
DMR17:6515001	17	6515001	6517000	2000	1	3.90E-07	-0.4	42	2.1	Slc28a3	Transport
DMR17:8201001	17	8201001	8205000	4000	1	8.50E-07	-0.42	63	1.57	Trpc7;LOC108348499;LOC102555284	Transport
DMR17:9332001	17	9332001	9335000	3000	1	8.60E-08	-0.37	70	2.33	H2afy	
DMR17:9656001	17	9656001	9659000	3000	1	3.80E-07	-0.33	64	2.13	Pdlim7	Cytoskeleton
DMR17:10166001	17	10166001	10168000	2000	1	3.40E-08	-0.5	42	2.1	Unc5a	Receptor
DMR17:10739001	17	10739001	10740000	1000	1	1.10E-07	-0.44	24	2.4	Lnc012	
DMR17:12077001	17	12077001	12081000	4000	1	2.20E-07	-0.38	88	2.2	Ror2	Receptor
DMR17:12109001	17	12109001	12113000	4000	1	5.20E-07	-0.37	89	2.22	Ror2	Receptor
DMR17:12764001	17	12764001	12765000	1000	1	1.70E-07	-0.39	32	3.2	Diras2	Signaling
DMR17:18021001	17	18021001	18023000	2000	1	2.70E-10	-0.37	70	3.5	Kdm1b;Tpmt	Metabolism;Epigenetic
DMR17:21388001	17	21388001	21392000	4000	1	6.60E-08	-0.41	62	1.55	Elovl2	Metabolism
DMR17:22295001	17	22295001	22296000	1000	1	2.50E-07	-0.33	12	1.2	Hivep1	
DMR17:23163001	17	23163001	23164000	1000	1	1.90E-07	-0.47	24	2.4	Nedd9;LOC108353140	
DMR17:25161001	17	25161001	25163000	2000	1	4.40E-07	0.32	31	1.55	Ofcc1	
DMR17:27555001	17	27555001	27559000	4000	1	6.10E-07	-0.49	122	3.05	Rreb1	
DMR17:28496001	17	28496001	28498000	2000	1	2.30E-07	-0.68	24	1.2	F13a1	Transport
DMR17:28579001	17	28579001	28580000	1000	1	2.60E-07	-0.77	10	1	F13a1	Transport
DMR17:31344001	17	31344001	31345000	1000	1	1.40E-08	-0.38	13	1.3	Slc22a23	Transport
DMR17:38445001	17	38445001	38448000	3000	1	2.80E-07	0.37	32	1.07	Prl5a2	Hormone
DMR17:39834001	17	39834001	39835000	1000	1	7.50E-07	0.5	4	0.4	Prl	Hormone
DMR17:44498001	17	44498001	44499000	1000	1	4.90E-07	-0.42	13	1.3	Trnas-gcu	
DMR17:53744001	17	53744001	53745000	1000	1	5.50E-07	0.36	9	0.9	Hecw1;LOC100912163	Proteolysis
DMR17:55288001	17	55288001	55291000	3000	1	7.70E-07	-0.39	54	1.8	Svil	Cytoskeleton
DMR17:55400001	17	55400001	55402000	2000	1	1.60E-07	-0.47	34	1.7	Svil	Cytoskeleton

DMR17:56044001	17	56044001	56045000	1000	1	6.00E-07	-0.39	17	1.7	Mtpap	Metabolism
DMR17:64096001	17	64096001	64098000	2000	1	9.20E-10	0.39	2	0.1	Chrm3	Signaling
DMR17:75772001	17	75772001	75775000	3000	1	9.90E-08	-0.38	70	2.33	Usp6nl	Signaling
DMR17:76678001	17	76678001	76680000	2000	1	8.60E-07	0.22	36	1.8	Camk1d	Signaling
DMR17:77746001	17	77746001	77748000	2000	1	1.00E-07	0.45	23	1.15	Frmd4a	
DMR17:79081001	17	79081001	79084000	3000	1	1.80E-07	-0.36	55	1.83	Fam171a1	
DMR17:79363001	17	79363001	79365000	2000	1	1.40E-08	0.36	14	0.7	Itga8	Extracellular Matrix
DMR17:83510001	17	83510001	83514000	4000	1	2.80E-07	0.89	35	0.88	Plxdc2	
DMR17:84894001	17	84894001	84897000	3000	1	5.60E-10	0.33	28	0.93	Mllt10	Transcription
DMR17:84989001	17	84989001	84990000	1000	1	5.20E-10	-0.51	27	2.7	Mllt10;Dnajc1	Transcription;Transcription
DMR17:87207001	17	87207001	87208000	1000	1	7.60E-08	0.37	4	0.4	Etl4	
DMR17:89108001	17	89108001	89109000	1000	1	5.70E-09	0.32	5	0.5	Myo3a	
DMR17:90274001	17	90274001	90275000	1000	1	6.70E-07	-0.66	10	1	Gng4	Signaling
DMR17:90427001	17	90427001	90428000	1000	1	6.90E-07	0.46	4	0.4	Lyst	
DMR18:3471001	18	3471001	3475000	4000	1	6.30E-07	-0.38	68	1.7	Tmem241	Transport
DMR18:5227001	18	5227001	5228000	1000	1	7.60E-08	-0.4	23	2.3	Zfp521	Transcription
DMR18:6427001	18	6427001	6429000	2000	1	5.30E-09	0.31	29	1.45	Kctd1	Cytoskeleton
DMR18:6891001	18	6891001	6892000	1000	1	8.30E-08	0.37	11	1.1	Chst9	Transport
DMR18:8268001	18	8268001	8270000	2000	1	9.30E-07	0.28	21	1.05	Cdh2;LOC102552679	Cytoskeleton
DMR18:11871001	18	11871001	11873000	2000	1	2.10E-08	0.3	13	0.65	Dsc1	Cytoskeleton
DMR18:15662001	18	15662001	15664000	2000	1	3.40E-07	0.46	18	0.9	Mapre2;Dsg3	Cytoskeleton;Cytoskeleton
DMR18:17815001	18	17815001	17816000	1000	1	5.90E-08	-0.44	10	1	Celf4	
DMR18:23808001	18	23808001	23810000	2000	1	3.70E-09	0.47	17	0.85	Rit2	Signaling
DMR18:25338001	18	25338001	25341000	3000	1	1.60E-07	0.26	41	1.37	Gypc	
DMR18:28816001	18	28816001	28818000	2000	1	1.00E-07	0.35	9	0.45	Psd2	Transcription
DMR18:32353001	18	32353001	32354000	1000	1	1.70E-08	-0.44	20	2	Nr3c1;Arhgap26;Fgf1	Signaling;Growth Factors
DMR18:36552001	18	36552001	36554000	2000	1	8.30E-07	-0.51	19	0.95	Lars	Translation
DMR18:37052001	18	37052001	37054000	2000	1	4.20E-07	0.37	12	0.6	Ppp2r2b	Signaling
DMR18:37285001	18	37285001	37286000	1000	1	7.50E-08	-0.63	3	0.3	Ppp2r2b	Signaling
DMR18:37863001	18	37863001	37865000	2000	1	4.40E-09	-0.44	16	0.8	LOC108348779;Jakmip2	
DMR18:37953001	18	37953001	37955000	2000	1	1.60E-07	0.44	7	0.35	Jakmip2	
DMR18:48450001	18	48450001	48453000	3000	1	8.90E-08	-0.46	63	2.1	Prdm6	Transcription
DMR18:49900001	18	49900001	49901000	1000	1	7.50E-08	-0.67	11	1.1	Zfp608	
DMR18:51213001	18	51213001	51215000	2000	1	2.80E-08	0.39	9	0.45	RGD1560341	
DMR18:51842001	18	51842001	51845000	3000	1	2.70E-08	-0.5	61	2.03	3-Mar	
DMR18:52227001	18	52227001	52229000	2000	1	1.00E-09	0.52	18	0.9	Megf10	Extracellular Matrix
DMR18:52303001	18	52303001	52305000	2000	1	1.70E-08	-0.4	43	2.15	Megf10	Extracellular Matrix
DMR18:52326001	18	52326001	52329000	3000	1	7.80E-08	-1.2	54	1.8	Megf10;LOC103694223	Extracellular Matrix
DMR18:55872001	18	55872001	55873000	1000	1	3.70E-09	-0.41	21	2.1	Synpo	Cytoskeleton
DMR18:56001001	18	56001001	56005000	4000	1	8.40E-07	-0.36	61	1.52	Ndst1	Transport
DMR18:60575001	18	60575001	60576000	1000	1	2.60E-08	0.31	13	1.3	Nedd4l	Proteolysis
DMR18:63788001	18	63788001	63790000	2000	1	1.40E-08	-0.44	55	2.75	Ldlrad4	
DMR18:63960001	18	63960001	63961000	1000	1	7.00E-07	-0.35	13	1.3	Ldlrad4	
DMR18:63991001	18	63991001	63992000	1000	1	3.40E-09	0.42	4	0.4	Ldlrad4	
DMR18:67610001	18	67610001	67611000	1000	1	3.70E-07	-0.51	11	1.1	Dcc	
DMR18:68972001	18	68972001	68974000	2000	1	5.80E-07	0.39	16	0.8	LOC361346;Stard6	
DMR18:70693001	18	70693001	70694000	1000	1	3.20E-07	0.27	24	2.4	Myo5b	Cytoskeleton
DMR18:71420001	18	71420001	71422000	2000	1	8.40E-07	-0.37	38	1.9	Smad7	Transcription
DMR18:71658001	18	71658001	71660000	2000	1	4.30E-08	0.37	12	0.6	Ctif	Metabolism
DMR18:71704001	18	71704001	71707000	3000	1	6.20E-07	-0.68	39	1.3	Ctif	Metabolism
DMR18:73815001	18	73815001	73819000	4000	1	4.60E-07	0.27	63	1.57	Loxhd1	
DMR18:73841001	18	73841001	73843000	2000	1	8.20E-09	0.38	13	0.65	Rnf165	Proteolysis
DMR18:77211001	18	77211001	77216000	5000	1	7.20E-07	-0.48	123	2.46	Nfatc1	Transcription
DMR18:77234001	18	77234001	77235000	1000	1	2.10E-10	-0.5	29	2.9	Nfatc1	Transcription
DMR18:77241001	18	77241001	77244000	3000	1	4.50E-07	-0.51	66	2.2	Nfatc1	Transcription
DMR18:79241001	18	79241001	79242000	1000	1	2.90E-07	0.26	10	1	Galr1	Signaling
DMR18:79244001	18	79244001	79245000	1000	1	7.40E-07	-0.56	17	1.7	Galr1	Signaling
DMR18:79794001	18	79794001	79796000	2000	1	9.20E-09	-0.54	48	2.4	Zfp516	Transcription
DMR18:79804001	18	79804001	79805000	1000	1	7.00E-12	0.49	7	0.7	Zfp516	Transcription
DMR18:80869001	18	80869001	80870000	1000	1	1.50E-07	0.33	12	1.2	Tshz1	Transcription
DMR18:81283001	18	81283001	81285000	2000	1	2.70E-07	-0.7	28	1.4	Zfp407;LOC102552911	Transcription
DMR18:81440001	18	81440001	81442000	2000	1	1.50E-09	0.63	18	0.9	Zfp407	Transcription
DMR18:81497001	18	81497001	81498000	1000	1	3.70E-08	0.39	9	0.9	Cndp1	Protease
DMR18:81531001	18	81531001	81534000	3000	1	1.10E-08	-0.5	55	1.83	Cndp2	Protease
DMR18:81819001	18	81819001	81822000	3000	1	7.50E-12	0.48	26	0.87	Fbxo15;LOC103694257	
DMR19:2617001	19	2617001	2618000	1000	1	1.50E-07	0.3	6	0.6	RGD1564126	
DMR19:6323001	19	6323001	6324000	1000	1	7.00E-09	0.41	2	0.2	Cdh8	Cytoskeleton
DMR19:10037001	19	10037001	10041000	4000	1	3.20E-07	-0.43	76	1.9	Cfap20	
DMR19:15828001	19	15828001	15830000	2000	1	2.90E-10	0.37	10	0.5	LOC102554898;Irx3	Development
DMR19:19448001	19	19448001	19450000	2000	1	2.80E-07	-0.39	34	1.7	Nkd1	

DMR19:19486001	19	19486001	19489000	3000	1	1.80E-08	-0.51	55	1.83	Nkd1	
DMR19:20205001	19	20205001	20207000	2000	1	6.10E-07	-0.3	50	2.5	Zfp423	
DMR19:21586001	19	21586001	21587000	1000	1	8.70E-07	0.32	8	0.8	Abcc12	Transport
DMR19:22116001	19	22116001	22117000	1000	1	1.90E-07	-0.42	25	2.5	Phkb	Signaling
DMR19:24322001	19	24322001	24324000	2000	1	8.70E-08	-0.46	21	1.05	LOC108348920;LOC102554553;Tbc1d9;LOC108348919	Signaling
DMR19:24502001	19	24502001	24503000	1000	1	6.70E-08	-0.42	15	1.5	Mgat4d	Transport
DMR19:30817001	19	30817001	30820000	3000	1	2.30E-08	-0.42	72	2.4	Gab1	Cytoskeleton
DMR19:31854001	19	31854001	31855000	1000	1	5.30E-08	-0.39	12	1.2	Anapc10	Proteolysis
DMR19:32550001	19	32550001	32551000	1000	1	2.40E-11	-0.43	17	1.7	Zfp827	Transcription
DMR19:33226001	19	33226001	33227000	1000	1	2.70E-09	-0.5	11	1.1	Ttc29	
DMR19:33336001	19	33336001	33341000	5000	1	8.30E-07	0.44	49	0.98	Ttc29	
DMR19:34323001	19	34323001	34324000	1000	1	9.20E-07	0.4	4	0.4	Arhgap10	Signaling
DMR19:34664001	19	34664001	34665000	1000	1	6.40E-07	0.39	9	0.9	Nr3c2	
DMR19:38469001	19	38469001	38471000	2000	1	1.80E-09	-0.54	13	0.65	Nfat5	Transcription
DMR19:38784001	19	38784001	38786000	2000	1	6.90E-07	-0.51	18	0.9	Cdh1	Cytoskeleton
DMR19:42119001	19	42119001	42125000	6000	1	4.20E-07	-0.45	128	2.13	Txnl4b;Dhx38;Pmfbp1	Metabolism;Transcription
DMR19:43212001	19	43212001	43213000	1000	1	1.30E-08	-0.45	26	2.6	Aars;Ddx19b	
DMR19:43964001	19	43964001	43966000	2000	1	1.30E-08	-0.59	32	1.6	Bcar1;LOC103694331	
DMR19:44930001	19	44930001	44933000	3000	1	8.90E-07	0.7	28	0.93	Cntnap4	
DMR19:46155001	19	46155001	46156000	1000	1	5.80E-07	0.31	7	0.7	Adamts18;LOC690181	Protease
DMR19:46985001	19	46985001	46986000	1000	1	6.90E-07	-0.33	32	3.2	Wwox	
DMR19:49450001	19	49450001	49451000	1000	1	1.00E-07	-0.39	24	2.4	Cmc2;Cenpn	
DMR19:50222001	19	50222001	50224000	2000	1	2.80E-07	-0.51	44	2.2	Sdr42e1	Metabolism
DMR19:50282001	19	50282001	50284000	2000	1	3.50E-08	0.26	19	0.95	Hsd17b2	Metabolism
DMR19:51667001	19	51667001	51668000	1000	1	3.20E-07	0.3	1	0.1	Cdh13	Cytoskeleton
DMR19:52860001	19	52860001	52863000	3000	1	8.60E-08	-0.46	53	1.77	RGD1304884	
DMR19:53376001	19	53376001	53377000	1000	1	4.00E-07	-0.59	20	2	RGD1304884;LOC108348955	
DMR19:53801001	19	53801001	53802000	1000	1	7.60E-07	-0.44	30	3	LOC102551361;Gse1	
DMR19:54021001	19	54021001	54026000	5000	1	2.10E-07	-0.45	96	1.92	Gse1;LOC102551873	
DMR19:54117001	19	54117001	54119000	2000	1	7.10E-07	-0.34	40	2	Gse1	
DMR19:54120001	19	54120001	54124000	4000	1	6.60E-08	-0.53	87	2.17	Gse1	
DMR19:54134001	19	54134001	54142000	8000	1	1.10E-07	-0.52	222	2.78	Gse1;Gins2	Transcription
DMR19:54322001	19	54322001	54326000	4000	1	1.00E-06	-0.51	104	2.6	Irf8	Transcription
DMR19:54981001	19	54981001	54982000	1000	1	5.10E-07	-0.4	22	2.2	Zfp469	
DMR19:54984001	19	54984001	54989000	5000	2	7.90E-09	-0.6	88	1.76	Zfp469	
DMR19:54999001	19	54999001	5.50E+07	1000	1	1.10E-12	-0.64	26	2.6	Zfp469	
DMR19:56129001	19	56129001	56130000	1000	1	1.50E-07	-0.76	12	1.2	Fanca;Spire2	Cytoskeleton
DMR19:56602001	19	56602001	56603000	1000	1	1.60E-07	-0.45	18	1.8	Rab4a	
DMR19:56668001	19	56668001	56669000	1000	1	8.10E-07	-0.35	14	1.4	Acta1	Cytoskeleton
DMR19:57083001	19	57083001	57087000	4000	1	1.90E-07	-0.6	102	2.55	Galnt2	Golgi
DMR19:57561001	19	57561001	57563000	2000	1	2.50E-07	0.37	20	1	Trim67	Proteolysis
DMR19:58319001	19	58319001	58323000	4000	1	9.80E-07	-0.77	54	1.35	Sipa1l2	Signaling
DMR19:58825001	19	58825001	58826000	1000	1	5.90E-08	-0.41	17	1.7	Kcnk1	Transport
DMR19:60247001	19	60247001	60248000	1000	1	1.90E-07	0.31	7	0.7	Pard3	
DMR19:60278001	19	60278001	60279000	1000	1	9.00E-08	0.35	4	0.4	Pard3	
DMR20:145001	20	145001	154000	9000	4	8.10E-09	-0.77	152	1.69	RT1-CE10;LOC108353253;LOC224733l-9;Cdk2ap1-ps3;LOC108353254;LOC103694368;LOC108349020	Immune
DMR20:366001	20	366001	367000	1000	1	1.50E-07	-0.38	21	2.1	Olr1669-ps	
DMR20:831001	20	831001	832000	1000	1	2.40E-08	0.41	11	1.1	Olr1692;Olr1693	Signaling
DMR20:2586001	20	2586001	2587000	1000	1	3.10E-07	0.23	9	0.9	2994a1;Nerg-ps10;Nerg-ps13;Rcrg1-ps34;Rcrg1-ps24	
DMR20:2757001	20	2757001	2760000	3000	1	1.80E-08	0.58	23	0.77	Btnl7	Immune
DMR20:2987001	20	2987001	2989000	2000	1	3.70E-10	0.39	11	0.55	2619a1_ps1;2927a1;Nerg-ps12;Rcrg1-ps31;2869a1a2	
DMR20:3147001	20	3147001	3152000	5000	3	1.40E-14	-1.14	62	1.24	RT1-O2-ps;RGD1562652;RT1-N2;RT1-S2;Rps2-ps1;RT1-O1	Immune
DMR20:3257001	20	3257001	3258000	1000	1	1.40E-09	0.79	17	1.7	LOC360231;RT1-T24-3;LOC108353244;2310ex4-5;LOC103694375	Immune
DMR20:3259001	20	3259001	3261000	2000	1	1.70E-07	0.84	27	1.35	LOC360231;RT1-T24-3;LOC108353244;2310ex4-5;LOC103694375;RT1-T24-1	Immune
DMR20:3612001	20	3612001	3613000	1000	1	6.90E-08	-0.43	19	1.9	Sfta2;Dpcr1	Transport
DMR20:3747001	20	3747001	3749000	2000	2	1.00E-08	-0.46	53	2.65	Cchcr1;Tcf19;Pou5f1	Transcription
DMR20:4452001	20	4452001	4459000	7000	1	3.30E-07	-0.43	124	1.77	Stk19	
DMR20:4580001	20	4580001	4588000	8000	1	2.80E-07	-0.59	285	3.56	LOC102553878;Zbtb12;Ehmt2;Slc44a4	Transcription;Transport
DMR20:4665001	20	4665001	4675000	10000	1	1.30E-07	-0.61	142	1.42	RT1-CE6;LOC103694379	

DMR20:4678001	20	4678001	4682000	4000	1	1.80E-08	-0.4	38	0.95	RT1-CE6;LOC103694379;LOC108353247	
DMR20:4776001	20	4776001	4778000	2000	1	4.90E-10	-0.43	21	1.05	RT1-CE2;Sacm2l-ps3	
DMR20:4914001	20	4914001	4919000	5000	2	1.10E-07	-0.5	74	1.48	Hspa1l;Cdk2ap1-ps5;RT1-CE3;RT1-CE4;Sacm2l-ps2	Immune
DMR20:4922001	20	4922001	4923000	1000	1	5.10E-09	-0.56	6	0.6	Hspa1l;RT1-CE3;RT1-CE4;Sacm2l-ps2	Immune
DMR20:4926001	20	4926001	4930000	4000	1	4.10E-08	-0.51	63	1.57	Hspa1l;RT1-CE3;RT1-CE4;Sacm2l-ps2;LOC103694382	Immune
DMR20:4947001	20	4947001	4950000	3000	1	6.20E-08	-0.71	44	1.47	Hspa1l;RT1-CE3;LOC103694382;LOC103694387	Immune
DMR20:5107001	20	5107001	5112000	5000	1	7.00E-07	-0.42	100	2	Ly6g5c;Ly6g5b;Csnk2b;Gpank1;G4;Apom	Signaling;Binding Proteins
DMR20:5145001	20	5145001	5150000	5000	1	1.60E-07	-0.56	139	2.78	Bag6;Prrc2a;E230034O05Rik	Transcription;Metabolism
DMR20:5188001	20	5188001	5194000	6000	1	3.50E-07	-0.46	143	2.38	Lst1;Ltb;Tnf;LOC103690381;LOC103689996	
DMR20:5444001	20	5444001	5446000	2000	1	5.10E-07	-0.39	49	2.45	Vps52;Rps18;B3galt4;Wdr46;Pfdn6	Transport;Translation;Golgi
DMR20:5695001	20	5695001	5701000	6000	1	5.40E-07	-0.53	161	2.68	Itpr3	Ion Channel
DMR20:5819001	20	5819001	5820000	1000	1	2.10E-08	-0.57	26	2.6	Lhfpl5;Srpk1	Signaling
DMR20:6818001	20	6818001	6820000	2000	1	2.30E-08	-0.46	50	2.5	Ppil1	Transcription
DMR20:7709001	20	7709001	7715000	6000	2	1.10E-07	-0.5	125	2.08	Scube3;LOC108349040	Extracellular Matrix
DMR20:7830001	20	7830001	7833000	3000	1	8.60E-09	-0.48	59	1.97	Ppard	Transcription
DMR20:7935001	20	7935001	7936000	1000	1	1.70E-07	-0.49	22	2.2	Tead3;Tulp1	Transcription
DMR20:8303001	20	8303001	8304000	1000	1	4.90E-08	-0.43	37	3.7	Rnf8	Proteolysis
DMR20:8532001	20	8532001	8535000	3000	1	7.00E-07	-0.52	67	2.23	Mdga1	
DMR20:8804001	20	8804001	8805000	1000	1	1.20E-07	-0.54	17	1.7	Zfand3	
DMR20:9994001	20	9994001	9999000	5000	1	7.00E-07	-0.33	98	1.96	Ubash3a;Rsph1	
DMR20:11715001	20	11715001	11717000	2000	1	1.10E-07	-0.54	53	2.65	Ube2g2;LOC108349048;Sumo3	
DMR20:11779001	20	11779001	11781000	2000	1	1.80E-07	-0.48	35	1.75	Itgb2	Extracellular Matrix
DMR20:12313001	20	12313001	12315000	2000	1	6.20E-09	-0.51	26	1.3	Col18a1	Extracellular Matrix
DMR20:12362001	20	12362001	12365000	3000	1	1.60E-07	-0.36	81	2.7	Slc19a1;LOC100912209	Transport
DMR20:12629001	20	12629001	12632000	3000	1	1.90E-07	-0.4	56	1.87	Pcbp3	Metabolism
DMR20:13951001	20	13951001	13955000	4000	1	4.00E-07	-0.67	77	1.93	Cabin1;LOC102556116	Signaling
DMR20:14596001	20	14596001	14605000	9000	2	3.20E-08	-0.51	253	2.81	Rtdr1;Gnaz	Signaling
DMR20:14657001	20	14657001	14662000	5000	1	2.10E-07	-0.44	95	1.9	Rtdr1	
DMR20:15138001	20	15138001	15139000	1000	1	3.20E-07	-0.48	15	1.5	Pcdh15	Cytoskeleton
DMR20:15162001	20	15162001	15163000	1000	1	1.10E-07	0.25	8	0.8	Pcdh15	Cytoskeleton
DMR20:16479001	20	16479001	16481000	2000	1	1.70E-09	0.37	11	0.55	NEWGENE_1590969	
DMR20:18590001	20	18590001	18593000	3000	1	1.70E-07	0.68	22	0.73	Ube2d1;Tfam	Proteolysis
DMR20:18897001	20	18897001	18902000	5000	1	1.90E-09	-0.49	92	1.84	Bicc1	Metabolism
DMR20:18913001	20	18913001	18914000	1000	1	8.00E-11	-0.54	22	2.2	Bicc1	Metabolism
DMR20:19382001	20	19382001	19384000	2000	1	9.20E-07	-0.73	76	3.8	Fam13c	
DMR20:25393001	20	25393001	25395000	2000	1	8.80E-07	0.42	5	0.25	Ctnna3	
DMR20:26268001	20	26268001	26270000	2000	1	8.00E-07	0.32	12	0.6	Ctnna3	
DMR20:29955001	20	29955001	29967000	12000	1	3.40E-08	-0.49	217	1.81	Cdh23;LOC100361018	Cytoskeleton
DMR20:31104001	20	31104001	31107000	3000	1	6.10E-07	-0.43	40	1.33	Lrrc20	
DMR20:31464001	20	31464001	31468000	4000	1	3.30E-07	-0.37	83	2.08	Col13a1	Extracellular Matrix
DMR20:31498001	20	31498001	31500000	2000	1	6.90E-10	-0.49	44	2.2	Col13a1	Extracellular Matrix
DMR20:31564001	20	31564001	31565000	1000	1	9.00E-07	-0.46	14	1.4	Col13a1	Extracellular Matrix
DMR20:31816001	20	31816001	31817000	1000	1	9.80E-08	0.3	5	0.5	Tspan15	
DMR20:32292001	20	32292001	32294000	2000	1	2.80E-08	-0.51	48	2.4	Stox1;LOC108353233	
DMR20:32304001	20	32304001	32305000	1000	1	1.40E-08	-0.56	8	0.8	Stox1;LOC108353233	
DMR20:34686001	20	34686001	34689000	3000	1	9.40E-07	0.37	47	1.57	Cep85l	
DMR20:34730001	20	34730001	34733000	3000	1	7.70E-07	0.29	20	0.67	Cep85l	
DMR20:45409001	20	45409001	45411000	2000	1	4.40E-07	0.36	14	0.7	Amd1	Metabolism
DMR20:46129001	20	46129001	46130000	1000	1	4.60E-07	0.3	8	0.8	Ak9	Signaling
DMR20:47041001	20	47041001	47043000	2000	1	3.10E-07	-0.53	46	2.3	Lace1	
DMR20:48115001	20	48115001	48116000	1000	1	3.80E-07	0.44	11	1.1	Pdss2	
DMR20:48567001	20	48567001	48569000	2000	1	6.30E-07	-0.31	48	2.4	Cdc40	Translation
DMR20:50229001	20	50229001	50235000	6000	1	2.30E-10	-0.52	153	2.55	Prep	Protease
DMR20:55404001	20	55404001	55406000	2000	1	6.20E-09	-0.87	14	0.7	Ascc3;LOC108349089	Cytoskeleton
DMRX:16364001	X	16364001	16365000	1000	1	5.60E-07	0.56	3	0.3	Ccnb3	Signaling
DMRX:17165001	X	17165001	17167000	2000	1	7.80E-09	0.32	10	0.5	LOC103694470;Nudt11	
DMRX:24910001	X	24910001	24911000	1000	1	9.30E-08	0.39	6	0.6	Wwc3	
DMRX:25465001	X	25465001	25467000	2000	1	1.30E-07	0.4	4	0.2	Mid1	Proteolysis
DMRX:35901001	X	35901001	35903000	2000	1	8.90E-07	0.42	16	0.8	Ppef1	Signaling
DMRX:37825001	X	37825001	37827000	2000	1	3.20E-09	0.55	8	0.4	Sh3kbp1	
DMRX:45131001	X	45131001	45134000	3000	2	1.10E-08	0.39	11	0.37	Prkx	Signaling
DMRX:52030001	X	52030001	52031000	1000	1	8.80E-07	0.53	6	0.6	Dmd	
DMRX:63597001	X	63597001	63603000	6000	3	1.90E-08	0.37	21	0.35	Apoo	
DMRX:71962001	X	71962001	71965000	3000	1	2.10E-07	-0.53	39	1.3	Nhsl2;LOC100911832	
DMRX:78932001	X	78932001	78937000	5000	1	7.10E-09	-0.53	165	3.3	Fam46d	

DMRX:107611001	X	107611001	107612000	1000	1	2.30E-08	0.34	10	1	Tmsbl1	
DMRX:109375001	X	109375001	109376000	1000	1	7.20E-08	0.4	5	0.5	Il1rapl2	Receptor
DMRX:116313001	X	116313001	116314000	1000	1	1.20E-07	0.55	0	0	Zcchc16	
DMRX:117248001	X	117248001	117250000	2000	1	7.20E-09	0.48	9	0.45	Olr1758-ps	
DMRX:124468001	X	124468001	124469000	1000	1	5.00E-07	0.45	8	0.8	Tmem255a	
DMRX:135742001	X	135742001	135748000	6000	1	4.90E-07	0.4	32	0.53	RGD1564955	
DMRX:140302001	X	140302001	140304000	2000	1	3.90E-07	0.51	58	2.9	Gpr101	Signaling
DMRX:156645001	X	156645001	156646000	1000	1	1.50E-07	-0.58	13	1.3	Mecp2	Transcription
DMRY:413001	Y	413001	415000	2000	1	4.00E-07	0.39	16	0.8	LOC103694545;LOC103694546;Uba1y	Proteolysis
