# Supplementary material for: Environmental induced transgenerational inheritance impacts systems epigenetics in disease etiology
Source: Sci Rep. 2022 Apr 19;12:5452. doi: 10.1038/s41598-022-09336-0 (PMC9018793; doi:10.1038/s41598-022-09336-0)
Supplement: Supplementary file 23 — Supplementary Table S15. [file 41598_2022_9336_MOESM23_ESM.pdf]

Supplemental Table S15
Glyphosate DMR p<1e-04

DMR Name	Chr	Start	Stop	Length	# Sig Win	minP	maxLFC	CpG #	CpG Density	Gene Annotation	Gene Category
DMR1:2588001	1	2588001	2589000	1000	1	2.30E-05	0.33	15	1.5	Ust	Transport
DMR1:3855001	1	3855001	3856000	1000	1	1.40E-05	-0.42	26	2.6	RGD1560633;Stxbp5	Translation;Transport
DMR1:7057001	1	7057001	7058000	1000	1	6.30E-05	0.31	13	1.3	Stx11	Transcription
DMR1:7264001	1	7264001	7266000	2000	1	1.70E-05	-0.28	42	2.1	Plagl1;LOC108349486	Transcription
DMR1:7326001	1	7326001	7327000	1000	1	2.20E-05	0.27	18	1.8	Zc2hc1b;Ltv1	Transcription
DMR1:16917001	1	16917001	16919000	2000	1	6.10E-06	0.24	32	1.6	Aldh8a1	Metabolism
DMR1:22116001	1	22116001	22117000	1000	1	5.70E-05	0.3	12	1.2	LOC502223;Moxd1	Metabolism
DMR1:31519001	1	31519001	31520000	1000	1	1.60E-06	0.78	9	0.9	LOC100911237;LOC689660;Rpl26-ps2	
DMR1:31521001	1	31521001	31524000	3000	3	2.10E-05	0.67	19	0.63	LOC100911237;Rpl26-ps2;Lrrc14b	
DMR1:40665001	1	40665001	40666000	1000	1	5.90E-05	0.47	11	1.1	Mthfd1l	
DMR1:46223001	1	46223001	46225000	2000	1	1.80E-05	0.39	38	1.9	Arid1b	
DMR1:46654001	1	46654001	46656000	2000	1	8.20E-05	0.42	43	2.15	Zdhhc14	
DMR1:48678001	1	48678001	48679000	1000	1	4.60E-05	0.27	17	1.7	Map3k4	Signaling
DMR1:53133001	1	53133001	53134000	1000	1	2.10E-05	-0.38	7	0.7	Fgfr1op	
DMR1:57735001	1	57735001	57737000	2000	1	7.50E-05	0.24	23	1.15	Chd1	
DMR1:61420001	1	61420001	61421000	1000	1	3.70E-05	0.27	13	1.3	Zfp52	Transcription
DMR1:63469001	1	63469001	63471000	2000	1	7.60E-05	-0.37	21	1.05	Vom2r-ps41	
DMR1:63712001	1	63712001	63713000	1000	1	5.30E-06	-0.76	5	0.5	RGD1562625	
DMR1:63715001	1	63715001	63716000	1000	1	1.30E-06	-0.63	5	0.5	RGD1562625	
DMR1:63748001	1	63748001	63752000	4000	1	2.90E-05	-0.25	42	1.05	Pirb;Lilrb3l	Immune
DMR1:63753001	1	63753001	63758000	5000	1	7.10E-06	-0.44	56	1.12	Lilrb3l	Immune
DMR1:63761001	1	63761001	63762000	1000	1	5.10E-05	-0.41	7	0.7	Lilrb3l	Immune
DMR1:63775001	1	63775001	63776000	1000	1	5.10E-05	-0.88	8	0.8	Lilrb3l	Immune
DMR1:63782001	1	63782001	63783000	1000	1	3.10E-05	-0.67	8	0.8	Lilrb3l;LOC690938	Immune
DMR1:63785001	1	63785001	63790000	5000	5	2.20E-08	-0.87	46	0.92	Lilrb3l;LOC690938	Immune
DMR1:63998001	1	63998001	64002000	4000	1	5.50E-05	-0.64	22	0.55	Lilrb3a	Immune
DMR1:64004001	1	64004001	64005000	1000	1	2.70E-06	-0.66	5	0.5	Lilrb3a	Immune
DMR1:64071001	1	64071001	64073000	2000	1	2.90E-05	-0.64	9	0.45	Lilrb3b	Immune
DMR1:64117001	1	64117001	64119000	2000	1	4.10E-05	-0.38	16	0.8	Mboat7;Tmc4;Leng1	
DMR1:64483001	1	64483001	64485000	2000	1	1.80E-06	0.37	26	1.3	Olr1l;LOC103689958;Olr386	Receptor
DMR1:64489001	1	64489001	64490000	1000	1	2.80E-05	0.27	18	1.8	Olr1l;LOC103689958;Olr386	Receptor
DMR1:69371001	1	69371001	69372000	1000	1	7.60E-05	-0.26	7	0.7	Nlrp4a	
DMR1:69486001	1	69486001	69487000	1000	1	1.30E-06	-0.93	6	0.6	Vom2r-ps44;Vom2r-ps45	
DMR1:70429001	1	70429001	70431000	2000	1	6.40E-05	-0.29	30	1.5	Olr3	Receptor
DMR1:71453001	1	71453001	71454000	1000	1	1.40E-05	0.43	12	1.2	Zfp787;Nlrp5	Transcription
DMR1:72360001	1	72360001	72361000	1000	1	9.70E-05	-0.33	6	0.6	Zfp865;LOC100911196	Transcription
DMR1:75143001	1	75143001	75144000	1000	1	3.90E-05	0.33	28	2.8	Vom1r-ps66;RGD1564801	Signaling
DMR1:79129001	1	79129001	79130000	1000	1	3.10E-05	0.4	19	1.9	Ceacam3	Immune
DMR1:79637001	1	79637001	79638000	1000	1	2.20E-05	-0.61	10	1	LOC102550882;LOC102552765;Mill1;LOC102552856	Immune
DMR1:81407001	1	81407001	81409000	2000	1	2.30E-05	0.26	54	2.7	Irgq;Pinlyp;Xrcc1	Cytoskeleton;Transcription
DMR1:82578001	1	82578001	82581000	3000	1	8.20E-05	0.24	57	1.9	Axl	Receptor
DMR1:82600001	1	82600001	82601000	1000	1	4.80E-08	-1.06	21	2.1	Cyp2s1	Metabolism
DMR1:85046001	1	85046001	85049000	3000	1	5.60E-05	0.21	59	1.97	Fcgbp;Fcgbpl1	Extracellular Matrix
DMR1:85207001	1	85207001	85209000	2000	1	2.00E-05	0.6	21	1.05	Pak4;Nccrp1	Signaling
DMR1:87288001	1	87288001	87291000	3000	1	9.70E-05	0.34	35	1.17	Sipa1l3	Signaling
DMR1:89281001	1	89281001	89282000	1000	1	1.00E-05	-0.45	34	3.4	Ffar2;LOC102554169	
DMR1:94403001	1	94403001	94404000	1000	1	7.60E-05	-0.57	46	4.6	Uri1;LOC108349566	Epigenetic
DMR1:98392001	1	98392001	98393000	1000	1	1.40E-05	-0.42	5	0.5	Cd33	Immune
DMR1:101318001	1	101318001	101319000	1000	1	9.50E-05	0.23	17	1.7	Trpm4;Hrc;Ppfia3	Transport
DMR1:101517001	1	101517001	101519000	2000	1	2.70E-07	0.23	26	1.3	Tulp2;Nucb1;Ppp1r15a;Plekha4	Signaling
DMR1:101797001	1	101797001	101798000	1000	1	1.70E-06	0.3	18	1.8	Lmtk3;Cyth2;Kcnj14	Transcription;Transport
DMR1:101946001	1	101946001	101947000	1000	1	3.30E-06	0.35	40	4	Abcc6	Transport
DMR1:124455001	1	124455001	124456000	1000	1	8.20E-05	-0.53	5	0.5	Otud7a	Protease
DMR1:124484001	1	124484001	124486000	2000	1	2.80E-05	0.3	13	0.65	Otud7a;Hmgn5b	Protease
DMR1:125033001	1	125033001	125034000	1000	1	8.20E-05	0.32	15	1.5	Trpm1;Mir211	Transport
DMR1:125458001	1	125458001	125460000	2000	1	3.10E-05	-0.56	60	3	Apba2	Transport
DMR1:125658001	1	125658001	125660000	2000	1	7.50E-05	0.33	19	0.95	Fam189a1;Ndnl2	
DMR1:126767001	1	126767001	126769000	2000	1	1.30E-06	0.43	44	2.2	Pcsk6;LOC108349587	Protease
DMR1:140854001	1	140854001	140855000	1000	1	2.20E-05	0.28	22	2.2	Hapln3;Mfge8	Extracellular Matrix;Metabolism
DMR1:142982001	1	142982001	142983000	1000	1	8.10E-05	0.42	11	1.1	Slc28a1	Transport
DMR1:144143001	1	144143001	144147000	4000	1	3.00E-06	0.27	39	0.98	Sh3gl3	
DMR1:157615001	1	157615001	157616000	1000	1	4.30E-06	0.49	14	1.4	Rab30	
DMR1:161654001	1	161654001	161656000	2000	1	2.40E-05	0.32	25	1.25	Tenm4;LOC108349818	
DMR1:164359001	1	164359001	164360000	1000	1	4.20E-05	0.37	9	0.9	Gdpd5	Signaling

DMR1:166574001	1	166574001	166576000	2000	1	6.50E-05	0.19	41	2.05	Pde2a	Signaling
DMR1:176459001	1	176459001	176460000	1000	1	6.60E-05	-0.44	8	0.8	Galnt18	Golgi
DMR1:187178001	1	187178001	187179000	1000	1	2.70E-05	0.26	9	0.9	Xylt1	Transport
DMR1:221872001	1	221872001	221875000	3000	1	4.00E-05	0.16	130	4.33	Nrxn2	
DMR1:224871001	1	224871001	224872000	1000	1	1.90E-05	0.23	12	1.2	Chrm1	Signaling
DMR1:226107001	1	226107001	226108000	1000	1	2.50E-05	0.2	16	1.6	Fads2;Fads3	
DMR1:237892001	1	237892001	237894000	2000	1	7.80E-05	-0.6	14	0.7	Anxa1	Signaling
DMR1:240592001	1	240592001	240594000	2000	1	3.90E-05	0.32	26	1.3	Trpm3;Aldh1a7	Transport;Metabolism
DMR1:250937001	1	250937001	250941000	4000	1	9.70E-06	0.45	65	1.62	Sgms1	
DMR1:251958001	1	251958001	251959000	1000	1	1.30E-05	-0.31	5	0.5	Rnls	
DMR1:254703001	1	254703001	254704000	1000	1	4.20E-05	-0.54	4	0.4	Rpp30	Translation
DMR1:260394001	1	260394001	260395000	1000	1	6.30E-05	-0.4	9	0.9	Tll2	Protease
DMR1:260878001	1	260878001	260879000	1000	1	4.80E-05	0.33	14	1.4	Slit1	
DMR1:261995001	1	261995001	261999000	4000	1	2.10E-05	0.36	66	1.65	Pyroxd2;Hps1	Metabolism
DMR1:262002001	1	262002001	262004000	2000	1	3.00E-05	0.3	59	2.95	Hps1	
DMR1:262007001	1	262007001	262010000	3000	1	1.80E-06	0.25	45	1.5	Hps1;LOC108349471	
DMR1:268598001	1	268598001	268599000	1000	1	1.90E-06	0.44	8	0.8	Sorcs3	Transport
DMR1:279966001	1	279966001	279967000	1000	1	3.60E-05	0.43	18	1.8	Hspa12a	
DMR2:4001001	2	4001001	4002000	1000	1	1.60E-05	-0.23	10	1	Mctp1	
DMR2:5553001	2	5553001	5554000	1000	1	1.20E-05	-0.5	25	2.5	Fam172a	
DMR2:12526001	2	12526001	12528000	2000	1	4.10E-05	-0.42	9	0.45	Tmem161b;LOC103691414	
DMR2:21964001	2	21964001	21966000	2000	1	7.20E-05	0.41	36	1.8	Dhfr	Metabolism
DMR2:22652001	2	22652001	22653000	1000	1	7.60E-05	0.31	27	2.7	Cmya5	Proteolysis
DMR2:24165001	2	24165001	24166000	1000	1	7.90E-05	0.47	13	1.3	Ap3b1	Transport
DMR2:34308001	2	34308001	34309000	1000	1	5.30E-05	-0.4	2	0.2	Ppwd1;Cenpk	Transcription
DMR2:44176001	2	44176001	44177000	1000	1	5.80E-05	-0.33	17	1.7	Ankrd55;LOC103691456	Cytoskeleton
DMR2:49352001	2	49352001	49363000	11000	4	9.50E-08	0.22	335	3.05	Parp8;LOC100910954	
DMR2:49366001	2	49366001	49367000	1000	1	4.60E-05	0.18	16	1.6	Parp8;LOC100910954	
DMR2:49391001	2	49391001	49396000	5000	1	7.20E-06	0.36	114	2.28	Parp8;LOC100910954	
DMR2:67060001	2	67060001	67061000	1000	1	1.70E-06	-0.32	2	0.2	Cdh9	Cytoskeleton
DMR2:76684001	2	76684001	76685000	1000	1	7.00E-05	0.26	27	2.7	Sirpd	Receptor
DMR2:115858001	2	115858001	115859000	1000	1	4.00E-05	0.21	16	1.6	Skil	
DMR2:118272001	2	118272001	118274000	2000	2	2.80E-06	0.32	22	1.1	Kcnmb2;LOC102549015	Transport
DMR2:118984001	2	118984001	118985000	1000	1	3.80E-07	-0.71	6	0.6	Gnb4	Signaling
DMR2:119410001	2	119410001	119411000	1000	1	2.90E-06	0.32	9	0.9	Pex5l	Transport
DMR2:124399001	2	124399001	124400000	1000	1	7.50E-05	-0.42	9	0.9	Spry1	Cytoskeleton
DMR2:128567001	2	128567001	128568000	1000	1	4.90E-05	-0.56	4	0.4	Sclt1	
DMR2:150764001	2	150764001	150765000	1000	1	5.50E-05	-0.37	10	1	Mbnl1	Translation
DMR2:154598001	2	154598001	154600000	2000	1	3.10E-06	-0.36	8	0.4	Gmps;Vom2r44	Metabolism;Signaling
DMR2:166057001	2	166057001	166058000	1000	1	8.10E-06	-0.44	8	0.8	Ppm1l;LOC102548099	Signaling
DMR2:168235001	2	168235001	168237000	2000	1	3.40E-08	-0.54	6	0.3	Vom1r56	
DMR2:182253001	2	182253001	182254000	1000	1	4.60E-05	0.4	22	2.2	Dchs2	
DMR2:192661001	2	192661001	192662000	1000	1	2.50E-05	-0.54	1	0.1	LOC108350274;Sprr1a	
DMR2:196804001	2	196804001	196806000	2000	1	5.60E-05	-0.46	17	0.85	Olr1864-ps	
DMR2:198003001	2	198003001	198004000	1000	1	4.80E-05	0.25	9	0.9	LOC108348218;Aph1a;Car14	Protease
DMR2:199821001	2	199821001	199823000	2000	1	8.50E-05	-0.42	29	1.45	Fmo5;Prkab2	Metabolism;Signaling
DMR2:210309001	2	210309001	210310000	1000	1	2.90E-06	-0.35	10	1	Slc6a17	Transport
DMR2:211276001	2	211276001	211280000	4000	3	1.60E-06	0.26	14	0.35	RGD1310209	
DMR2:212286001	2	212286001	212287000	1000	1	9.20E-05	0.38	10	1	Vav3	
DMR2:225081001	2	225081001	225082000	1000	1	9.90E-06	-0.42	5	0.5	Slc44a3	Transport
DMR2:227353001	2	227353001	227354000	1000	1	2.80E-05	0.34	18	1.8	Synpo2	Cytoskeleton
DMR2:231897001	2	231897001	231898000	1000	1	4.40E-05	-0.42	8	0.8	Zgrf1	
DMR2:235669001	2	235669001	235670000	1000	1	4.90E-05	-0.45	12	1.2	Col25a1	Extracellular Matrix
DMR2:244096001	2	244096001	244097000	1000	1	5.40E-07	0.23	13	1.3	Tspan5	
DMR2:247159001	2	247159001	247161000	2000	1	3.40E-05	0.47	25	1.25	Unc5c	Receptor
DMR2:252629001	2	252629001	252630000	1000	1	2.00E-05	0.27	3	0.3	Prkacb	Signaling
DMR2:258196001	2	258196001	258197000	1000	1	1.70E-05	0.4	9	0.9	St6galnac5	
DMR2:260378001	2	260378001	260381000	3000	1	7.50E-08	0.38	58	1.93	Slc44a5	Transport
DMR3:11963001	3	11963001	11966000	3000	1	6.10E-05	0.33	73	2.43	Fam129b;Lrsam1	
DMR3:12084001	3	12084001	12085000	1000	1	5.70E-05	0.24	13	1.3	Garnl3	Signaling
DMR3:12879001	3	12879001	12881000	2000	1	9.10E-05	0.44	30	1.5	Mvb12b	
DMR3:16993001	3	16993001	16994000	1000	1	2.30E-05	-0.43	4	0.4	RGD1563231	Immune
DMR3:25278001	3	25278001	25279000	1000	1	4.10E-06	0.28	8	0.8	Lrp1b	
DMR3:44465001	3	44465001	44468000	3000	1	7.00E-05	0.39	42	1.4	Acvr1	Signaling
DMR3:45188001	3	45188001	45190000	2000	1	1.10E-05	0.29	26	1.3	Ccdc148	
DMR3:45716001	3	45716001	45717000	1000	1	5.20E-05	0.38	12	1.2	Tanc1	
DMR3:51360001	3	51360001	51362000	2000	1	4.10E-05	0.29	23	1.15	Slc38a11	Transport
DMR3:53804001	3	53804001	53805000	1000	1	3.80E-05	-0.45	9	0.9	B3galt1	Golgi
DMR3:54181001	3	54181001	54182000	1000	1	4.80E-05	-0.4	9	0.9	B3galt1	Golgi

DMR3:57681001	3	57681001	57682000	1000	1	4.20E-05	-0.3	6	0.6	Dcaf17	
DMR3:60152001	3	60152001	60155000	3000	1	2.60E-05	-0.45	46	1.53	Wipf1	Cytoskeleton
DMR3:63604001	3	63604001	63605000	1000	1	6.60E-05	0.29	29	2.9	Ttn	
DMR3:64078001	3	64078001	64079000	1000	1	3.10E-05	0.32	8	0.8	Sestd1	
DMR3:72177001	3	72177001	72178000	1000	1	7.70E-05	-0.45	13	1.3	Serping1	Protease; Proteolysis
DMR3:72764001	3	72764001	72766000	2000	1	1.90E-05	-0.29	4	0.2	Olr438-ps;Olr439	Receptor
DMR3:81431001	3	81431001	81432000	1000	1	3.60E-05	-0.45	17	1.7	LOC108350417;Chst1	Transport
DMR3:86381001	3	86381001	86382000	1000	1	5.90E-05	-0.35	8	0.8	Lrrc4c	
DMR3:86790001	3	86790001	86791000	1000	1	9.20E-05	-0.32	4	0.4	Lrrc4c	
DMR3:93001001	3	93001001	93002000	1000	1	4.90E-05	-0.32	5	0.5	Apip	Metabolism
DMR3:93225001	3	93225001	93226000	1000	1	2.00E-05	0.27	7	0.7	Ehf	Transcription
DMR3:112364001	3	112364001	112365000	1000	1	1.50E-05	-0.49	7	0.7	Snap23;Lrrc57;LOC691918	Transcription
DMR3:112707001	3	112707001	112708000	1000	1	9.50E-06	-0.46	8	0.8	Ttbk2	Signaling
DMR3:113144001	3	113144001	113147000	3000	2	2.80E-06	0.35	12	0.4	Tubgcp4	Cytoskeleton
DMR3:114404001	3	114404001	114405000	1000	1	2.80E-05	-0.39	7	0.7	Slc28a2	Transport
DMR3:119661001	3	119661001	119664000	3000	1	7.40E-05	0.41	33	1.1	Snrnp200;Ciao1	Cytoskeleton
DMR3:121673001	3	121673001	121674000	1000	1	8.20E-05	0.41	14	1.4	Chchd5	
DMR3:142738001	3	142738001	142739000	1000	1	5.00E-05	0.35	2	0.2	Sstr4;Thbd	Signaling;Receptor
DMR3:151372001	3	151372001	151373000	1000	1	4.70E-05	0.34	13	1.3	Eif6;Fam83c	Translation
DMR3:152965001	3	152965001	152970000	5000	2	2.00E-05	0.75	61	1.22	Sla2;LOC103691946;Ndrg3	Signaling;Protease
DMR3:152972001	3	152972001	152975000	3000	2	3.20E-05	0.68	48	1.6	Sla2;LOC103691946;Ndrg3	Signaling;Protease
DMR3:153030001	3	153030001	153034000	4000	4	3.60E-07	0.89	52	1.3	Ndrg3;LOC102554288	Protease
DMR3:153035001	3	153035001	153042000	7000	1	2.60E-05	0.51	75	1.07	Ndrg3;LOC102554288	Protease
DMR3:153043001	3	153043001	153048000	5000	3	2.80E-06	0.73	53	1.06	Ndrg3;LOC102554288	Protease
DMR3:153059001	3	153059001	153068000	9000	4	1.20E-06	0.91	121	1.34	Ndrg3	Protease
DMR3:160270001	3	160270001	160272000	2000	1	5.10E-08	0.34	48	2.4	Rims4	Transport
DMR3:161290001	3	161290001	161292000	2000	1	7.10E-05	0.3	41	2.05	Zswim3;Zswim1;Spata25;Neurl2;Ctsa	Proteolysis;Protease
DMR3:161582001	3	161582001	161585000	3000	1	5.50E-06	0.3	52	1.73	Cdh22	Cytoskeleton
DMR3:161920001	3	161920001	161922000	2000	1	2.60E-05	0.29	28	1.4	Cdh22	Cytoskeleton
DMR3:162290001	3	162290001	162291000	1000	1	2.00E-05	0.27	30	3	Eya2	
DMR3:163366001	3	163366001	163368000	2000	1	3.70E-05	0.17	20	1	Prex1	Transcription
DMR3:163781001	3	163781001	163782000	1000	1	5.40E-05	0.26	21	2.1	Ddx27;Znfx1	
DMR4:14557001	4	14557001	14558000	1000	1	1.30E-06	-0.45	3	0.3	RGD1563537	
DMR4:24035001	4	24035001	24037000	2000	1	1.80E-06	-0.55	10	0.5	Zfp804b	
DMR4:26241001	4	26241001	26242000	1000	1	8.70E-05	0.27	5	0.5	Cdk14;LOC102549548	Signaling
DMR4:26266001	4	26266001	26268000	2000	1	1.90E-05	-0.42	6	0.3	Cdk14;LOC102549548	Signaling
DMR4:33145001	4	33145001	33147000	2000	1	7.20E-05	-0.23	11	0.55	Olr1757-ps	
DMR4:38947001	4	38947001	38949000	2000	1	4.10E-05	-0.49	21	1.05	Thsd7a;LOC103692074	Cytoskeleton
DMR4:48579001	4	48579001	48580000	1000	1	8.60E-05	0.29	22	2.2	Kcnd2	Transport
DMR4:49441001	4	49441001	49442000	1000	1	1.90E-06	-0.56	10	1	Fam3c	Signaling
DMR4:50400001	4	50400001	50401000	1000	1	8.50E-06	0.36	12	1.2	Cadps2	Transport
DMR4:51885001	4	51885001	51886000	1000	1	3.50E-05	-0.47	7	0.7	Pot1	Transcription
DMR4:58337001	4	58337001	58338000	1000	1	6.60E-05	0.29	22	2.2	Mir3556a;Mir29a;Mir3587;Mir29b1	
DMR4:60616001	4	60616001	60617000	1000	1	9.90E-05	-0.45	2	0.2	Exoc4	Transport
DMR4:70898001	4	70898001	70899000	1000	1	1.10E-05	-0.32	5	0.5	Ephb6	Receptor
DMR4:79326001	4	79326001	79327000	1000	1	7.70E-05	-0.4	10	1	RGD1564712	
DMR4:82793001	4	82793001	82794000	1000	1	3.20E-05	-0.33	10	1	Tax1bp1	
DMR4:84133001	4	84133001	84134000	1000	1	5.00E-05	0.35	14	1.4	Cpvl	
DMR4:88209001	4	88209001	88210000	1000	1	7.10E-05	-0.39	3	0.3	Vom1r84	Receptor
DMR4:88488001	4	88488001	88489000	1000	1	8.90E-05	-0.26	2	0.2	Vopp1	
DMR4:95202001	4	95202001	95203000	1000	1	3.50E-05	-0.37	8	0.8	Grid2	Receptor
DMR4:95297001	4	95297001	95299000	2000	1	5.60E-05	-0.41	18	0.9	Grid2	Receptor
DMR4:98693001	4	98693001	98695000	2000	1	4.10E-05	0.27	23	1.15	Eif2ak3	Signaling
DMR4:107984001	4	107984001	107985000	1000	1	3.60E-05	-0.49	12	1.2	Ctnna2	Cytoskeleton
DMR4:115216001	4	115216001	115217000	1000	1	1.60E-05	0.34	10	1	Dguok;Actg2	Signaling;Cytoskeleton
DMR4:119181001	4	119181001	119182000	1000	1	1.50E-07	0.52	1	0.1	Gkn2;Gkn3;LOC102554941	
DMR4:120178001	4	120178001	120180000	2000	1	9.20E-05	0.41	29	1.45	Tcp1-ps1	
DMR4:121677001	4	121677001	121680000	3000	1	9.70E-06	-0.86	64	2.13	Vom1r90;LOC100363221	Receptor
DMR4:128812001	4	128812001	128813000	1000	1	2.90E-05	0.37	15	1.5	Fam19a1	
DMR4:136832001	4	136832001	136833000	1000	1	1.80E-05	-0.52	6	0.6	Cntn6	
DMR4:151324001	4	151324001	151325000	1000	1	2.00E-06	0.19	12	1.2	Cacna2d4	Transport
DMR4:152957001	4	152957001	152959000	2000	1	1.80E-06	-0.31	17	0.85	Kdm5a	Epigenetic
DMR4:158420001	4	158420001	158421000	1000	1	8.40E-05	0.35	18	1.8	Ano2	
DMR4:158488001	4	158488001	158489000	1000	1	9.40E-05	-0.42	12	1.2	Ano2	
DMR4:160810001	4	160810001	160811000	1000	1	1.80E-05	0.43	8	0.8	Senp18	Protease
DMR4:162779001	4	162779001	162780000	1000	1	9.50E-08	-0.4	4	0.4	Klrb1	Receptor
DMR4:163217001	4	163217001	163218000	1000	1	4.70E-06	-0.46	7	0.7	Clec1a;Clec7a	
DMR4:174839001	4	174839001	174840000	1000	1	2.90E-05	-0.41	12	1.2	Aebp2	
DMR4:179009001	4	179009001	179010000	1000	1	5.20E-05	0.28	28	2.8	Sox5	

DMR4:181454001	4	181454001	181455000	1000	1	4.40E-05	0.33	17	1.7	Mrps35	Translation
DMR5:8491001	5	8491001	8492000	1000	1	3.00E-05	0.53	15	1.5	Cpa6	Protease
DMR5:8702001	5	8702001	8703000	1000	1	9.10E-05	0.55	6	0.6	Arfgef1	Transcription
DMR5:16532001	5	16532001	16533000	1000	1	8.60E-05	-0.45	13	1.3	Lyn	
DMR5:18765001	5	18765001	18766000	1000	1	8.30E-06	-0.52	6	0.6	Fam110b	
DMR5:18769001	5	18769001	18770000	1000	1	2.90E-05	0.3	17	1.7	Fam110b	
DMR5:18796001	5	18796001	18797000	1000	1	3.70E-05	0.52	12	1.2	Fam110b	
DMR5:18859001	5	18859001	18860000	1000	1	1.70E-05	-0.55	2	0.2	Fam110b	
DMR5:21663001	5	21663001	21664000	1000	1	1.40E-06	-0.37	15	1.5	Rab2a	
DMR5:50165001	5	50165001	50167000	2000	1	3.00E-06	-0.31	22	1.1	Cfap206	
DMR5:56985001	5	56985001	56986000	1000	1	3.60E-06	-0.7	14	1.4	Aptx	DNA Repair
DMR5:57745001	5	57745001	57746000	1000	1	7.80E-06	-0.45	7	0.7	Ubap1	
DMR5:57901001	5	57901001	57902000	1000	1	4.40E-05	0.34	13	1.3	RGD1561916;LOC102553821;Fam219a	
DMR5:58201001	5	58201001	58203000	2000	1	3.10E-06	0.43	74	3.7	Ccl21	Growth Factors
DMR5:60966001	5	60966001	60967000	1000	1	5.10E-06	0.27	22	2.2	Mcart1;Shb	
DMR5:60996001	5	60996001	60997000	1000	1	7.00E-05	0.48	12	1.2	Shb	
DMR5:74194001	5	74194001	74195000	1000	1	2.80E-05	0.31	25	2.5	Epb41l4b	
DMR5:74350001	5	74350001	74352000	2000	1	1.70E-05	-0.38	22	1.1	Ptpn3	Signaling
DMR5:136333001	5	136333001	136334000	1000	1	4.60E-06	0.45	14	1.4	Rnf220	
DMR5:136841001	5	136841001	136842000	1000	1	6.30E-05	0.28	13	1.3	St3gal3;LOC102553361	Transport
DMR5:137046001	5	137046001	137047000	1000	1	1.20E-05	0.19	18	1.8	Ptprf	Signaling
DMR5:137219001	5	137219001	137220000	1000	1	4.40E-05	0.37	25	2.5	Szt2	
DMR5:138698001	5	138698001	138703000	5000	2	4.80E-05	0.38	54	1.08	Guca2b	Signaling
DMR5:139788001	5	139788001	139790000	2000	1	6.80E-05	0.26	26	1.3	Rims3	Transport
DMR5:141082001	5	141082001	141084000	2000	1	3.60E-09	0.35	34	1.7	Macf1	Cytoskeleton
DMR5:142914001	5	142914001	142915000	1000	1	2.10E-05	0.39	11	1.1	Epha10;Cdca8	Receptor
DMR5:146945001	5	146945001	146946000	1000	1	4.30E-05	0.31	21	2.1	A3galt2;Zfp362	Golgi;Transcription
DMR5:148208001	5	148208001	148209000	1000	1	5.60E-05	0.23	32	3.2	Adgrb2	Signaling
DMR5:150069001	5	150069001	150070000	1000	1	6.00E-05	0.26	16	1.6	Srsf4;Tmem200b	Translation
DMR5:150875001	5	150875001	150876000	1000	1	6.70E-05	-0.39	5	0.5	Eya3	
DMR5:151771001	5	151771001	151773000	2000	1	2.90E-06	-0.49	25	1.25	Nudc;Nr0b2	Cytoskeleton;Transcription
DMR5:152831001	5	152831001	152832000	1000	1	1.30E-05	0.3	27	2.7	Man1c1	Golgi
DMR5:152965001	5	152965001	152966000	1000	1	1.40E-05	0.3	32	3.2	Ldlrap1	Cytoskeleton
DMR5:153207001	5	153207001	153208000	1000	1	7.50E-05	0.24	14	1.4	Rhd	Transport
DMR5:155242001	5	155242001	155243000	1000	1	2.20E-05	0.2	6	0.6	C1qb	
DMR5:155875001	5	155875001	155876000	1000	1	3.20E-05	0.29	30	3	Hspg2	
DMR5:156058001	5	156058001	156062000	4000	1	4.70E-05	0.25	95	2.38	Rap1gap	Signaling
DMR5:156620001	5	156620001	156621000	1000	1	4.70E-05	0.34	18	1.8	Sh2d5;Kif17	Cytoskeleton;Cytoskeleton
DMR5:157925001	5	157925001	157927000	2000	1	5.70E-05	0.36	38	1.9	Ubr4	Proteolysis
DMR5:159049001	5	159049001	159050000	1000	1	3.00E-06	0.24	13	1.3	Arhgef10l	Transcription
DMR5:167025001	5	167025001	167028000	3000	1	7.00E-06	0.23	60	2	H6pd;LOC108351062	Metabolism
DMR5:171273001	5	171273001	171274000	1000	1	7.90E-05	0.4	17	1.7	Cep104	
DMR5:173169001	5	173169001	173170000	1000	1	3.40E-06	0.47	8	0.8	Ssu72	Signaling
DMR5:173591001	5	173591001	173592000	1000	1	9.40E-05	0.29	20	2	LOC100362942;Agrn	Extracellular Matrix
DMR5:173610001	5	173610001	173613000	3000	1	3.20E-05	0.25	61	2.03	Agrn	Extracellular Matrix
DMR6:6809001	6	6809001	6810000	1000	1	7.60E-05	-0.36	5	0.5	Kcng3	Transport
DMR6:11005001	6	11005001	11006000	1000	1	6.80E-05	0.44	16	1.6	Ttc7a;LOC681766	
DMR6:11846001	6	11846001	11851000	5000	1	2.90E-05	0.29	194	3.88	RGD1562306	
DMR6:21809001	6	21809001	21810000	1000	1	3.00E-05	0.44	9	0.9	Ttc27;LOC108351178	
DMR6:24677001	6	24677001	24678000	1000	1	6.00E-05	-0.41	8	0.8	Capn13;LOC102553955	Protease
DMR6:26759001	6	26759001	26760000	1000	1	9.60E-05	0.45	8	0.8	Tcf23	Transcription
DMR6:50524001	6	50524001	50526000	2000	1	2.20E-05	0.39	22	1.1	Lamb1	Extracellular Matrix
DMR6:51047001	6	51047001	51048000	1000	1	6.10E-05	0.5	18	1.8	Cog5	
DMR6:52356001	6	52356001	52358000	2000	1	9.70E-05	0.27	33	1.65	Cdhr3	Cytoskeleton
DMR6:54171001	6	54171001	54172000	1000	1	1.20E-05	0.34	20	2	Hdac9	
DMR6:78312001	6	78312001	78313000	1000	1	3.50E-05	-0.19	7	0.7	Mipol1	
DMR6:78719001	6	78719001	78721000	2000	1	3.80E-05	-0.35	13	0.65	RGD1560556	
DMR6:92382001	6	92382001	92383000	1000	1	4.00E-05	-0.5	8	0.8	Sav1;LOC102548804	
DMR6:99154001	6	99154001	99155000	1000	1	2.20E-05	-0.48	8	0.8	Syne2;Esr2	
DMR6:100416001	6	100416001	100418000	2000	1	6.20E-05	-0.25	18	0.9	Fut8	Golgi
DMR6:100443001	6	100443001	100445000	2000	1	2.60E-06	-0.51	15	0.75	Fut8	Golgi
DMR6:100470001	6	100470001	100474000	4000	2	8.30E-06	-0.6	25	0.62	Fut8	Golgi
DMR6:100495001	6	100495001	100496000	1000	1	6.10E-05	-0.5	9	0.9	Fut8	Golgi
DMR6:100533001	6	100533001	100534000	1000	1	1.30E-06	-0.45	2	0.2	Fut8	Golgi
DMR6:109056001	6	109056001	109058000	2000	1	1.40E-06	0.33	28	1.4	Eif2b2;Mlh3	Translation;Transcription
DMR6:109093001	6	109093001	109094000	1000	1	9.20E-05	0.27	24	2.4	Mlh3;Acyp1	Transcription;Signaling
DMR6:109700001	6	109700001	109701000	1000	1	5.50E-05	0.47	11	1.1	RGD1310769;Ttll5	Cytoskeleton
DMR6:110007001	6	110007001	110008000	1000	1	2.70E-05	0.31	25	2.5	Ift43	
DMR6:125529001	6	125529001	125530000	1000	1	3.40E-05	-0.45	3	0.3	Tc2n	

DMR6:126047001	6	126047001	126048000	1000	1	5.60E-05	0.29	23	2.3	Slc24a4	Transport
DMR6:126868001	6	126868001	126871000	3000	1	2.10E-05	0.23	69	2.3	Unc79	
DMR6:126923001	6	126923001	126924000	1000	1	3.40E-05	0.35	15	1.5	Unc79	
DMR6:126928001	6	126928001	126933000	5000	3	1.50E-05	0.23	38	0.76	Unc79	
DMR6:132279001	6	132279001	132280000	1000	1	4.00E-05	0.24	19	1.9	Cyp46a1	Metabolism
DMR6:136008001	6	136008001	136010000	2000	1	9.30E-05	0.41	13	0.65	Eif5	Translation
DMR6:137702001	6	137702001	137703000	1000	1	7.00E-05	0.46	18	1.8	LOC103692726;Jag2	
DMR6:144392001	6	144392001	144394000	2000	1	1.30E-06	0.25	18	0.9	LOC102548847;Ptprn2	Signaling
DMR6:145407001	6	145407001	145408000	1000	1	9.40E-05	-0.41	10	1	Rapgef5	Transcription
DMR6:146802001	6	146802001	146803000	1000	1	6.20E-05	-0.39	9	0.9	Abcb5	Transport
DMR7:9470001	7	9470001	9472000	2000	1	6.00E-05	-0.43	6	0.3	Olr1069	Signaling
DMR7:11191001	7	11191001	11195000	4000	1	8.00E-05	0.33	98	2.45	Smim24;LOC102552675;Dohh;LOC102552770;Fzr1	Metabolism;Proteolysis
DMR7:11508001	7	11508001	11509000	1000	1	4.10E-05	0.26	25	2.5	Creb3l3;Thop1	Protease
DMR7:12409001	7	12409001	12410000	1000	1	3.90E-05	0.35	20	2	RGD1562114;Cirbp;Midn	
DMR7:17963001	7	17963001	17965000	2000	1	6.00E-05	0.49	48	2.4	Vom1r106;Vom1r-ps96	Receptor
DMR7:27711001	7	27711001	27712000	1000	1	3.80E-06	0.32	28	2.8	RGD1560034	
DMR7:28794001	7	28794001	28795000	1000	1	7.40E-05	-0.48	11	1.1	Ccdc53;LOC102551862	
DMR7:29066001	7	29066001	29068000	2000	1	3.60E-05	-0.36	30	1.5	Chpt1;LOC102554140	Transport
DMR7:29373001	7	29373001	29374000	1000	1	2.40E-05	0.26	21	2.1	Utp20	
DMR7:31773001	7	31773001	31774000	1000	1	6.40E-05	-0.35	8	0.8	Apaf1	
DMR7:33746001	7	33746001	33748000	2000	1	7.00E-05	0.19	23	1.15	RGD1565866	
DMR7:58454001	7	58454001	58456000	2000	1	2.80E-05	0.42	37	1.85	Lgr5	Signaling
DMR7:64383001	7	64383001	64384000	1000	1	5.30E-06	0.56	12	1.2	Vps54-ps1	
DMR7:71026001	7	71026001	71028000	2000	1	4.90E-07	0.28	36	1.8	Myo1a;Tac3;Zbtb39	Cytoskeleton;Transcription
DMR7:71126001	7	71126001	71135000	9000	2	5.90E-06	0.47	223	2.48	Rdh7	Metabolism
DMR7:71961001	7	71961001	71963000	2000	1	6.80E-05	-0.53	4	0.2	Cpq	Protease
DMR7:73275001	7	73275001	73277000	2000	1	2.20E-05	0.63	22	1.1	Rida;Pop1	Metabolism
DMR7:74278001	7	74278001	74279000	1000	1	9.10E-05	-0.3	8	0.8	Vps13b	
DMR7:74633001	7	74633001	74634000	1000	1	8.50E-05	-0.29	3	0.3	Vps13b	
DMR7:76865001	7	76865001	76866000	1000	1	4.70E-05	0.27	34	3.4	Ubr5	Proteolysis
DMR7:78860001	7	78860001	78861000	1000	1	1.10E-06	0.38	17	1.7	LOC103692892;Lrp12	Binding Proteins
DMR7:95496001	7	95496001	95497000	1000	1	7.80E-05	0.23	7	0.7	Sntb1	
DMR7:109134001	7	109134001	109137000	3000	1	6.50E-05	0.48	18	0.6	Zfat	Transcription
DMR7:109148001	7	109148001	109150000	2000	1	6.70E-06	0.42	18	0.9	Zfat	Transcription
DMR7:114953001	7	114953001	114954000	1000	1	5.50E-07	0.53	19	1.9	Gpr20	
DMR7:116667001	7	116667001	116668000	1000	1	7.10E-05	0.31	11	1.1	Zfp41;Gli4	Transcription
DMR7:117694001	7	117694001	117698000	4000	1	2.80E-05	0.33	87	2.17	Vps28;Tonsl;LOC108348051	Transport
DMR7:119135001	7	119135001	119136000	1000	1	3.30E-11	-1.6	16	1.6	LOC102553868;Txn2	Metabolism
DMR7:119269001	7	119269001	119271000	2000	1	5.00E-05	-0.33	33	1.65	Cacng2	Transport
DMR7:120254001	7	120254001	120256000	2000	1	7.70E-05	0.33	33	1.65	H1f0;Gcat	Metabolism
DMR7:123248001	7	123248001	123249000	1000	1	9.70E-05	-0.4	6	0.6	Desi1;LOC103692949	Protease
DMR7:126474001	7	126474001	126475000	1000	1	7.60E-05	0.36	9	0.9	Wnt7b;LOC102554082	Signaling
DMR7:127025001	7	127025001	127027000	2000	1	8.60E-05	0.24	36	1.8	Cerk	Signaling
DMR7:127129001	7	127129001	127130000	1000	1	4.70E-05	0.34	13	1.3	Tbc1d22a	Signaling
DMR7:135866001	7	135866001	135868000	2000	1	1.40E-06	0.27	16	0.8	LOC108351539;Ndufb4-ps1	
DMR7:136697001	7	136697001	136699000	2000	1	3.80E-05	-0.39	18	0.9	Nell2	Signaling
DMR7:139447001	7	139447001	139449000	2000	1	5.50E-05	0.29	66	3.3	Tmem106c;Col2a1	Extracellular Matrix
DMR7:139737001	7	139737001	139738000	1000	1	8.90E-05	0.41	15	1.5	Asb8	
DMR7:140292001	7	140292001	140293000	1000	1	2.70E-05	0.44	22	2.2	Adcy6;LOC102555399	
DMR7:142028001	7	142028001	142029000	1000	1	4.50E-05	0.46	16	1.6	Mettl7a;Higd1c;Slc11a2	Epigenetic;Transport
DMR7:142305001	7	142305001	142306000	1000	1	6.40E-05	0.39	22	2.2	Bin2;Cela1	Protease
DMR7:143457001	7	143457001	143458000	1000	1	1.00E-04	0.24	19	1.9	Krt1;Krt77	
DMR8:8971001	8	8971001	8972000	1000	1	9.70E-05	-0.55	6	0.6	Cntn5	
DMR8:29539001	8	29539001	29540000	1000	1	3.80E-05	0.32	10	1	Opcml	Immune
DMR8:30059001	8	30059001	30060000	1000	1	9.50E-05	-0.32	10	1	Ntm	Immune
DMR8:40492001	8	40492001	40493000	1000	1	2.80E-05	-0.47	4	0.4	Olr1204	Receptor
DMR8:47185001	8	47185001	47186000	1000	1	2.00E-06	-0.49	14	1.4	Grik4	Receptor
DMR8:49166001	8	49166001	49168000	2000	1	6.60E-05	0.27	31	1.55	Kmt2a	Epigenetic
DMR8:50430001	8	50430001	50431000	1000	1	4.70E-05	0.37	12	1.2	Sik3;LOC103693072	Signaling
DMR8:53434001	8	53434001	53435000	1000	1	6.30E-05	-0.37	8	0.8	Tmprss5	Protease
DMR8:60250001	8	60250001	60251000	1000	1	1.60E-05	-0.4	15	1.5	Scaper	
DMR8:61457001	8	61457001	61458000	1000	1	1.10E-05	0.22	16	1.6	Lingo1	Receptor
DMR8:62520001	8	62520001	62521000	1000	1	9.40E-06	0.39	12	1.2	Edc3;Clk3	Signaling
DMR8:67716001	8	67716001	67717000	1000	1	7.50E-05	-0.27	15	1.5	Fem1b	
DMR8:82804001	8	82804001	82808000	4000	1	2.20E-05	-0.29	32	0.8	Bmp5	Growth Factors
DMR8:83342001	8	83342001	83343000	1000	1	1.00E-04	-0.44	8	0.8	Hcrtr2	Signaling
DMR8:87621001	8	87621001	87622000	1000	1	9.70E-05	0.18	7	0.7	Myo6	Cytoskeleton
DMR8:91539001	8	91539001	91541000	2000	1	2.40E-05	0.2	17	0.85	Bckdhb	Metabolism

DMR8:102496001	8	102496001	102497000	1000	1	1.20E-07	-0.47	17	1.7	Slc9a9;LOC103693163	Transport
DMR8:105349001	8	105349001	105350000	1000	1	9.90E-05	-0.4	8	0.8	Clstn2	Transport
DMR8:107729001	8	107729001	107730000	1000	1	9.50E-06	0.31	21	2.1	Nme9;Armc8	
DMR8:112834001	8	112834001	112836000	2000	1	6.20E-05	0.41	31	1.55	Dnajc13;Acpp	Transcription
DMR8:113220001	8	113220001	113221000	1000	1	1.60E-05	-0.33	10	1	Cpne4	
DMR8:114410001	8	114410001	114411000	1000	1	7.30E-05	-0.45	16	1.6	Col6a6	
DMR8:116006001	8	116006001	116008000	2000	1	7.90E-05	0.36	30	1.5	Mapkapk3	Signaling
DMR8:117268001	8	117268001	117271000	3000	1	1.60E-05	0.3	80	2.67	Lamb2;Usp19	Extracellular Matrix;Protease
DMR8:118815001	8	118815001	118816000	1000	1	8.20E-05	-0.34	12	1.2	Setd2	Epigenetic
DMR8:122529001	8	122529001	122530000	1000	1	7.20E-06	-0.71	9	0.9	Ccr4	
DMR8:123207001	8	123207001	123209000	2000	1	7.70E-05	0.23	43	2.15	Osbpl10	
DMR8:124305001	8	124305001	124306000	1000	1	8.70E-06	-0.48	26	2.6	Gadl1;Tgfbr2	Signaling
DMR8:125382001	8	125382001	125384000	2000	1	3.20E-05	0.31	27	1.35	Rbms3	
DMR8:125543001	8	125543001	125544000	1000	1	8.30E-07	0.45	16	1.6	Rbms3;LOC102550774	
DMR8:127650001	8	127650001	127653000	3000	1	2.60E-05	0.35	53	1.77	Ctdspl;LOC108351797	
DMR8:127671001	8	127671001	127672000	1000	1	4.30E-05	0.25	26	2.6	Ctdspl	
DMR8:127714001	8	127714001	127716000	2000	1	5.60E-05	0.22	56	2.8	Ctdspl;Mir26a	
DMR8:128341001	8	128341001	128342000	1000	1	2.70E-05	0.27	26	2.6	Scn10a;LOC108351811	Transport
DMR9:6975001	9	6975001	6976000	1000	1	4.10E-05	-0.28	3	0.3	St6gal2	Transport
DMR9:14790001	9	14790001	14794000	4000	1	2.00E-05	0.22	45	1.12	Ncr2	
DMR9:14796001	9	14796001	14800000	4000	1	3.50E-05	0.2	44	1.1	Ncr2	
DMR9:16697001	9	16697001	16698000	1000	1	2.20E-05	0.33	19	1.9	Ptk7	Receptor
DMR9:28717001	9	28717001	28718000	1000	1	7.30E-05	-0.26	6	0.6	Rims1	Transport
DMR9:41236001	9	41236001	41237000	1000	1	2.00E-05	0.3	16	1.6	Arhgef4	Transcription
DMR9:42798001	9	42798001	42799000	1000	1	8.30E-05	-0.28	7	0.7	Uggt1;LOC102553975	Golgi
DMR9:49641001	9	49641001	49643000	2000	1	4.90E-05	-0.22	12	0.6	Mrps9	Translation
DMR9:52137001	9	52137001	52138000	1000	1	4.10E-05	-0.5	8	0.8	Col5a2	Extracellular Matrix
DMR9:60558001	9	60558001	60559000	1000	1	5.30E-05	0.29	9	0.9	Hecw2	Proteolysis
DMR9:62198001	9	62198001	62199000	1000	1	6.70E-05	0.49	8	0.8	Plcl1;LOC108351933	Metabolism
DMR9:67324001	9	67324001	67326000	2000	1	5.30E-06	0.3	28	1.4	Raph1	Cytoskeleton
DMR9:70197001	9	70197001	70198000	1000	1	1.40E-05	0.3	5	0.5	Adam23	Protease
DMR9:82159001	9	82159001	82160000	1000	1	2.10E-05	0.24	11	1.1	Cryba2;Mir375;Cfap65	Development
DMR9:85443001	9	85443001	85444000	1000	1	2.50E-05	0.27	18	1.8	Ap1s3;LOC108351958	Transport
DMR9:88140001	9	88140001	88145000	5000	1	3.00E-05	0.45	34	0.68	Rhbdd1;LOC108351962	Protease
DMR9:88220001	9	88220001	88221000	1000	1	4.10E-05	-0.33	14	1.4	Rhbdd1	Protease
DMR9:88399001	9	88399001	88401000	2000	1	3.80E-05	0.36	32	1.6	Col4a3;LOC102554007;LOC103693235	Extracellular Matrix
DMR9:92073001	9	92073001	92074000	1000	1	7.80E-05	-0.48	7	0.7	Dner	
DMR9:97010001	9	97010001	97011000	1000	1	2.60E-07	0.42	16	1.6	Agap1	
DMR9:99966001	9	99966001	99969000	3000	1	1.20E-05	0.4	45	1.5	Ppp1r7	Signaling
DMR9:100835001	9	100835001	100836000	1000	1	2.40E-05	0.3	12	1.2	Bok	
DMR9:110076001	9	110076001	110077000	1000	1	1.10E-05	0.28	31	3.1	Efna5	Signaling
DMR9:110104001	9	110104001	110105000	1000	1	1.00E-05	0.39	18	1.8	Efna5	Signaling
DMR9:110665001	9	110665001	110666000	1000	1	4.60E-05	0.36	20	2	Fbxl17	Metabolism
DMR9:110724001	9	110724001	110725000	1000	1	2.00E-05	0.3	17	1.7	Fbxl17	Metabolism
DMR9:111860001	9	111860001	111861000	1000	1	2.70E-05	0.25	11	1.1	Fer	
DMR9:113444001	9	113444001	113445000	1000	1	1.10E-07	0.35	27	2.7	Rab31	
DMR9:114504001	9	114504001	114509000	5000	1	1.70E-05	0.26	130	2.6	Mtcl1;LOC108351984	
DMR9:114605001	9	114605001	114607000	2000	1	4.30E-05	0.27	56	2.8	Mtcl1	
DMR10:9207001	10	9207001	9208000	1000	1	8.00E-05	-0.31	4	0.4	Rbfox1	Translation
DMR10:10077001	10	10077001	10079000	2000	1	1.20E-05	0.25	40	2	Rbfox1	Translation
DMR10:13895001	10	13895001	13896000	1000	1	6.80E-05	0.33	14	1.4	Traf7;LOC102551976;Rab26	
DMR10:14128001	10	14128001	14130000	2000	1	2.80E-06	0.38	42	2.1	Hs3st6	Transport
DMR10:14602001	10	14602001	14606000	4000	1	1.30E-09	0.99	114	2.85	Unkl;Gnptg;Tsr3;Baiap3;Mir3547	Signaling
DMR10:15875001	10	15875001	15876000	1000	1	3.00E-05	0.25	10	1	Nsg2	
DMR10:20650001	10	20650001	20651000	1000	1	1.30E-05	0.33	22	2.2	Rars	
DMR10:28614001	10	28614001	28615000	1000	1	2.70E-07	0.39	16	1.6	Atp10b	Transport
DMR10:35385001	10	35385001	35386000	1000	1	7.00E-05	0.3	23	2.3	Rasgef1c	Transcription
DMR10:38210001	10	38210001	38211000	1000	1	9.40E-05	-0.38	7	0.7	Fstl4	Protease; Proteolysis
DMR10:38220001	10	38220001	38221000	1000	1	9.90E-07	0.48	18	1.8	Fstl4	Protease; Proteolysis
DMR10:43827001	10	43827001	43831000	4000	2	2.30E-07	0.23	39	0.98	Lypd8;LOC108352180	Cytoskeleton
DMR10:48433001	10	48433001	48434000	1000	1	2.20E-05	0.31	11	1.1	Specc1	
DMR10:55168001	10	55168001	55169000	1000	1	4.40E-05	-0.24	11	1.1	Ccdc42	
DMR10:55689001	10	55689001	55690000	1000	1	4.90E-06	0.26	22	2.2	Vamp2;Per1;Trnat-agu;Trnas-cga	Transcription
DMR10:57109001	10	57109001	57110000	1000	1	3.70E-06	-0.4	20	2	Tm4sf5	
DMR10:59876001	10	59876001	59877000	1000	1	3.70E-06	-0.36	5	0.5	Aspa	
DMR10:62753001	10	62753001	62754000	1000	1	2.20E-05	-0.53	7	0.7	Ssh2	Signaling
DMR10:65458001	10	65458001	65459000	1000	1	5.80E-05	-0.29	11	1.1	Rab34;Proca1	
DMR10:68446001	10	68446001	68448000	2000	1	9.70E-06	-0.32	27	1.35	Asic2	Transport
DMR10:68751001	10	68751001	68752000	1000	1	6.60E-05	0.25	6	0.6	Asic2	Transport

DMR10:81064001	10	81064001	81066000	2000	1	8.00E-05	-0.24	10	0.5	Car10	
DMR10:84123001	10	84123001	84125000	2000	1	7.20E-06	0.33	21	1.05	LOC100911410;Mir196c;Hoxb9;Hoxb8	
DMR10:86329001	10	86329001	86330000	1000	1	8.30E-06	0.33	29	2.9	Stard3;Tcap	
DMR10:86358001	10	86358001	86359000	1000	1	4.40E-05	-0.46	16	1.6	Pgap3;Erbb2	Receptor
DMR10:87269001	10	87269001	87270000	1000	1	5.50E-05	0.22	22	2.2	Krt27;Krt28	
DMR10:88221001	10	88221001	88224000	3000	1	3.20E-07	0.48	33	1.1	Krt42;Eif1	Translation
DMR10:90642001	10	90642001	90643000	1000	1	2.40E-05	-0.38	6	0.6	Meioc;Ccdc43	
DMR10:91039001	10	91039001	91040000	1000	1	4.40E-05	-0.42	18	1.8	LOC103693459;C1ql1	
DMR10:91793001	10	91793001	91794000	1000	1	3.80E-05	0.36	11	1.1	Wnt9b	Signaling
DMR10:92224001	10	92224001	92225000	1000	1	1.50E-06	0.55	18	1.8	Crhr1	Receptor
DMR10:94078001	10	94078001	94079000	1000	1	3.50E-05	0.32	7	0.7	Tanc2;LOC103693484;LOC102556346	
DMR10:94091001	10	94091001	94092000	1000	1	4.80E-06	-0.36	16	1.6	Tanc2;LOC102556346	
DMR10:101761001	10	101761001	101762000	1000	1	2.20E-05	0.38	16	1.6	Slc39a11	Transport
DMR10:104602001	10	104602001	104605000	3000	1	3.60E-06	0.34	63	2.1	Unk;Unc13d	
DMR10:105135001	10	105135001	105136000	1000	1	6.10E-05	-0.32	11	1.1	Srp68	Metabolism
DMR10:106323001	10	106323001	106324000	1000	1	9.90E-06	0.29	16	1.6	9-Sep	
DMR10:109017001	10	109017001	109019000	2000	1	6.80E-05	0.31	35	1.75	Rptor	
DMR10:110851001	10	110851001	110852000	1000	1	2.00E-07	-0.47	9	0.9	B3gntl1	Golgi
DMR11:7353001	11	7353001	7354000	1000	1	3.90E-05	-0.41	8	0.8	Gbe1	Metabolism
DMR11:10033001	11	10033001	10034000	1000	1	1.20E-05	0.44	19	1.9	Robo1	
DMR11:11902001	11	11902001	11905000	3000	1	5.10E-07	-0.69	44	1.47	Robo2	
DMR11:12414001	11	12414001	12415000	1000	1	1.50E-05	-0.43	1	0.1	Robo2	
DMR11:12519001	11	12519001	12520000	1000	1	7.30E-06	-0.38	7	0.7	Robo2	
DMR11:12598001	11	12598001	12600000	2000	1	4.20E-11	1.51	16	0.8	Robo2	
DMR11:12620001	11	12620001	12621000	1000	1	4.90E-06	0.69	15	1.5	Robo2	
DMR11:13977001	11	13977001	13978000	1000	1	2.60E-06	-0.39	7	0.7	Lipi	Metabolism
DMR11:25356001	11	25356001	25357000	1000	1	1.80E-05	-0.42	18	1.8	Adamts1	Protease
DMR11:34467001	11	34467001	34468000	1000	1	7.90E-05	0.45	10	1	Hlcs	Metabolism
DMR11:42618001	11	42618001	42619000	1000	1	7.80E-05	0.39	13	1.3	Epha6	Receptor
DMR11:42771001	11	42771001	42772000	1000	1	1.10E-05	-0.68	8	0.8	Epha6	Receptor
DMR11:43074001	11	43074001	43075000	1000	1	1.90E-05	-0.34	4	0.4	Gabrr3;LOC108352321	Ion Channel
DMR11:55246001	11	55246001	55248000	2000	1	2.50E-05	-0.49	11	0.55	Dppa4	
DMR11:61183001	11	61183001	61184000	1000	1	2.30E-07	0.39	19	1.9	Cfap44	
DMR11:66603001	11	66603001	66604000	1000	1	3.40E-07	-0.92	2	0.2	Polq	Transcription
DMR11:67753001	11	67753001	67754000	1000	1	3.00E-05	-0.41	19	1.9	Parp9;Dtx3l	Proteolysis
DMR11:69770001	11	69770001	69773000	3000	1	8.70E-05	0.27	66	2.2	Kalrn	Transcription
DMR11:83934001	11	83934001	83935000	1000	1	1.30E-05	0.34	17	1.7	Eif4g1;Psmd2;LOC102551589	Translation;Protease
DMR11:89687001	11	89687001	89689000	2000	1	2.20E-06	-0.57	32	1.6	RGD1560337	
DMR12:2352001	12	2352001	2354000	2000	1	2.90E-06	-0.43	9	0.45	Cd209a	Transport
DMR12:5102001	12	5102001	5104000	2000	1	6.40E-05	-0.38	6	0.3	Vom2r58	Signaling
DMR12:12390001	12	12390001	12392000	2000	1	4.70E-06	0.32	49	2.45	Tecpr1	
DMR12:12899001	12	12899001	12900000	1000	1	2.40E-05	0.27	22	2.2	Cyth3	Transcription
DMR12:15797001	12	15797001	15798000	1000	1	1.10E-06	-0.35	11	1.1	Card11	
DMR12:19343001	12	19343001	19344000	1000	1	6.90E-05	-0.4	15	1.5	Cnpy4;Mblac1	
DMR12:20140001	12	20140001	20141000	1000	1	6.40E-05	-0.44	7	0.7	Pilrb;LOC108352496	
DMR12:21928001	12	21928001	21930000	2000	1	5.00E-05	-0.38	12	0.6	RGD1561143;LOC100910497	Immune
DMR12:23631001	12	23631001	23633000	2000	1	3.60E-05	-0.53	51	2.55	Rasa4	Signaling
DMR12:23958001	12	23958001	23959000	1000	1	4.30E-05	-0.31	11	1.1	Mdh2;Styxl1	Metabolism
DMR12:23984001	12	23984001	23985000	1000	1	6.20E-05	-0.36	9	0.9	Styxl1;Tmem120a	
DMR12:25199001	12	25199001	25200000	1000	1	3.30E-05	0.33	17	1.7	Clip2	Transcription
DMR12:25397001	12	25397001	25401000	4000	1	1.80E-05	0.26	88	2.2	Gtf2i	Transcription
DMR12:29054001	12	29054001	29055000	1000	1	1.80E-05	0.41	16	1.6	Wbscr17;LOC100912262	
DMR12:30615001	12	30615001	30616000	1000	1	1.90E-06	0.25	23	2.3	Sept14;LOC108352487	Cytoskeleton
DMR12:36903001	12	36903001	36907000	4000	1	8.20E-07	0.29	83	2.08	Ncor2	Epigenetic
DMR12:37542001	12	37542001	37543000	1000	1	4.30E-05	-0.34	9	0.9	Snrnp35;Rilpl2	Translation
DMR12:37801001	12	37801001	37802000	1000	1	8.40E-05	0.28	15	1.5	Pitpnm2	Transport
DMR12:38519001	12	38519001	38520000	1000	1	2.60E-05	0.35	16	1.6	Lrrc43;Il31	Signaling
DMR12:39155001	12	39155001	39156000	1000	1	1.30E-05	0.21	20	2	Kdm2b;Rnf34	Proteolysis
DMR12:39587001	12	39587001	39588000	1000	1	5.00E-05	0.39	21	2.1	Atp2a2	Transport
DMR12:40200001	12	40200001	40203000	3000	1	3.40E-05	0.42	59	1.97	Cux2	Development
DMR12:40912001	12	40912001	40913000	1000	1	2.00E-05	0.26	16	1.6	Ptpn11	Signaling
DMR12:41346001	12	41346001	41347000	1000	1	4.80E-05	0.38	10	1	Oas3;Oas2	Metabolism
DMR12:41617001	12	41617001	41619000	2000	1	6.20E-05	0.37	43	2.15	Plbd2;Sds	Metabolism;Metabolism
DMR12:44052001	12	44052001	44053000	1000	1	6.10E-05	0.24	6	0.6	Fbxw8	
DMR12:44872001	12	44872001	44873000	1000	1	4.00E-06	0.36	24	2.4	Ksr2	Signaling
DMR12:45753001	12	45753001	45754000	1000	1	7.20E-05	0.46	16	1.6	Srrm4	
DMR12:45766001	12	45766001	45768000	2000	1	4.20E-05	0.52	22	1.1	Srrm4	
DMR12:45836001	12	45836001	45838000	2000	1	8.10E-05	0.44	41	2.05	Srrm4	
DMR12:45867001	12	45867001	45871000	4000	2	3.80E-07	0.99	70	1.75	Srrm4	
DMR12:45888001	12	45888001	45890000	2000	1	5.00E-05	-0.65	23	1.15	Srrm4	

DMR12:47564001	12	47564001	47565000	1000	1	6.20E-05	0.19	29	2.9	RGD1560398;Ankrd13a	
DMR12:47645001	12	47645001	47647000	2000	1	4.80E-05	0.32	60	3	Tchp;LOC102547088	
DMR12:50312001	12	50312001	50313000	1000	1	8.10E-05	0.39	18	1.8	Hps4;LOC102556781;Srrd;Tfip11	Translation
DMR12:52020001	12	52020001	52021000	1000	1	1.30E-05	-0.31	9	0.9	Ep400	
DMR13:25661001	13	25661001	25663000	2000	1	5.80E-05	-0.41	14	0.7	Pign;RGD1307235	Extracellular Matrix
DMR13:40070001	13	40070001	40072000	2000	1	6.30E-05	0.32	5	0.25	Dpp10	Protease
DMR13:42127001	13	42127001	42129000	2000	2	2.90E-05	0.43	30	1.5	Gpr39	Signaling
DMR13:42347001	13	42347001	42348000	1000	1	5.70E-06	0.31	10	1	Nckap5	
DMR13:44852001	13	44852001	44853000	1000	1	4.30E-05	0.3	4	0.4	R3hdm1	
DMR13:47425001	13	47425001	47426000	1000	1	1.70E-05	-0.52	3	0.3	Pfkfb2	Metabolism
DMR13:50519001	13	50519001	50520000	1000	1	4.10E-05	0.22	24	2.4	Ren;LOC102550525;Kiss1	
DMR13:50547001	13	50547001	50548000	1000	1	4.90E-05	0.24	23	2.3	Kiss1;Golt1a;Plekha6	Metabolism
DMR13:55608001	13	55608001	55609000	1000	1	3.40E-05	0.36	11	1.1	Nek7;LOC103691924	Signaling
DMR13:75094001	13	75094001	75095000	1000	1	8.10E-05	-0.43	15	1.5	Tp53i3	
DMR13:80728001	13	80728001	80729000	1000	1	3.80E-05	-0.47	6	0.6	Fmo1	Metabolism
DMR13:82440001	13	82440001	82442000	2000	1	9.40E-05	0.49	33	1.65	Selp	
DMR13:89578001	13	89578001	89579000	1000	1	3.00E-05	0.32	8	0.8	Nr1i3	Transcription
DMR13:90918001	13	90918001	90919000	1000	1	2.90E-05	0.3	19	1.9	Cfap45	
DMR13:102928001	13	102928001	102929000	1000	1	3.30E-05	-0.44	27	2.7	Mark1	Signaling
DMR13:107401001	13	107401001	107402000	1000	1	3.70E-05	0.34	20	2	Ush2a	Extracellular Matrix
DMR13:109367001	13	109367001	109368000	1000	1	8.90E-06	-0.45	5	0.5	Rps6kc1	Signaling
DMR14:8578001	14	8578001	8579000	1000	1	5.50E-06	0.3	23	2.3	Arhgap24	
DMR14:9299001	14	9299001	9301000	2000	1	6.50E-05	0.28	34	1.7	Wdfy3	
DMR14:12307001	14	12307001	12311000	4000	3	1.30E-05	0.42	86	2.15	Prkg2	Signaling
DMR14:12355001	14	12355001	12357000	2000	1	2.50E-05	0.44	23	1.15	Bmp3	Growth Factors
DMR14:12396001	14	12396001	12397000	1000	1	2.70E-05	0.34	16	1.6	Bmp3	Growth Factors
DMR14:15444001	14	15444001	15445000	1000	1	7.00E-05	-0.3	2	0.2	LOC108348100;RGD1565660	
DMR14:28752001	14	28752001	28753000	1000	1	8.50E-05	-0.42	8	0.8	Adgrl3	Signaling
DMR14:34118001	14	34118001	34119000	1000	1	1.30E-05	0.28	1	0.1	Cep135	Epigenetic
DMR14:39374001	14	39374001	39375000	1000	1	3.50E-05	0.33	19	1.9	Cox7b2	Metabolism
DMR14:44952001	14	44952001	44953000	1000	1	3.20E-05	0.34	12	1.2	Fam114a1	
DMR14:46626001	14	46626001	46641000	15000	2	9.00E-06	-0.42	998	6.65	LOC102554740;Rn45s;Rn18s;Rn5-8s;Rn28s	
DMR14:62256001	14	62256001	62257000	1000	1	6.70E-05	-0.3	6	0.6	LOC100363278;Vom1r-ps33;Vom1r-ps32	
DMR14:76874001	14	76874001	76875000	1000	1	5.50E-05	0.3	13	1.3	Zfp518b;LOC498391	Transcription
DMR14:79699001	14	79699001	79700000	1000	1	1.00E-04	0.17	15	1.5	Sorcs2	Transport
DMR14:81789001	14	81789001	81790000	1000	1	2.10E-05	0.31	19	1.9	Zfyve28	
DMR14:85475001	14	85475001	85477000	2000	1	4.40E-06	0.25	30	1.5	Kremen1	Receptor
DMR14:86286001	14	86286001	86287000	1000	1	2.70E-05	0.27	15	1.5	Camk2b	Signaling
DMR14:86792001	14	86792001	86793000	1000	1	7.50E-06	0.31	17	1.7	Myo1g;LOC102547700	Cytoskeleton
DMR14:100190001	14	100190001	100191000	1000	1	4.50E-05	0.36	10	1	Plek	Cytoskeleton
DMR14:100192001	14	100192001	100193000	1000	1	4.10E-06	0.33	10	1	Plek	Cytoskeleton
DMR14:100249001	14	100249001	100250000	1000	1	1.20E-07	0.76	25	2.5	Cnrip1	
DMR14:100257001	14	100257001	100258000	1000	1	3.30E-06	0.57	13	1.3	Cnrip1	
DMR14:100400001	14	100400001	100401000	1000	1	8.20E-07	0.47	27	2.7	Wdr92;LOC100363290	Metabolism
DMR14:100402001	14	100402001	100404000	2000	1	2.10E-05	0.27	32	1.6	Wdr92;LOC100363290	Metabolism
DMR14:107671001	14	107671001	107673000	2000	1	1.70E-06	0.38	40	2	Commd1;LOC108352812	
DMR14:114339001	14	114339001	114340000	1000	1	8.80E-05	-0.37	6	0.6	Eml6	
DMR14:114697001	14	114697001	114699000	2000	1	7.70E-05	0.42	15	0.75	Sptbn1;LOC108352820	
DMR15:4455001	15	4455001	4459000	4000	1	2.50E-07	0.55	57	1.43	Nudt13	
DMR15:6859001	15	6859001	6860000	1000	1	9.10E-08	-0.39	8	0.8	Zfp385d	
DMR15:7834001	15	7834001	7836000	2000	1	1.50E-06	0.22	8	0.4	Ube2e2	Proteolysis
DMR15:10096001	15	10096001	10097000	1000	1	9.10E-05	0.34	11	1.1	Rarb	Transcription
DMR15:38142001	15	38142001	38143000	1000	1	8.40E-06	0.33	8	0.8	Zdhhc20	
DMR15:40370001	15	40370001	40371000	1000	1	5.00E-05	0.25	10	1	Atp8a2;LOC103693865	Transport
DMR15:59495001	15	59495001	59496000	1000	1	3.20E-05	0.36	7	0.7	Enox1	Metabolism
DMR15:59733001	15	59733001	59734000	1000	1	3.70E-05	0.35	20	2	Enox1	Metabolism
DMR15:83636001	15	83636001	83637000	1000	1	4.20E-05	0.34	15	1.5	Pibf1	
DMR15:83718001	15	83718001	83720000	2000	1	3.90E-05	0.4	30	1.5	Klf5	Transcription
DMR16:7674001	16	7674001	7676000	2000	1	1.50E-05	0.4	21	1.05	Colq;LOC108353071	Extracellular Matrix
DMR16:7875001	16	7875001	7876000	1000	1	4.70E-05	0.27	4	0.4	Ankrd28	Cytoskeleton
DMR16:9517001	16	9517001	9518000	1000	1	6.40E-05	0.32	22	2.2	Arhgap22	
DMR16:10525001	16	10525001	10529000	4000	1	3.20E-05	0.38	84	2.1	Gprin2	
DMR16:14383001	16	14383001	14384000	1000	1	1.00E-05	0.49	26	2.6	Ghitm	
DMR16:16173001	16	16173001	16175000	2000	1	5.60E-05	0.55	13	0.65	Nrg3	Growth Factors
DMR16:16393001	16	16393001	16394000	1000	1	6.90E-06	-0.37	6	0.6	Nrg3	Growth Factors
DMR16:17533001	16	17533001	17534000	1000	1	7.40E-05	0.29	19	1.9	Tspan14	
DMR16:19108001	16	19108001	19113000	5000	4	2.80E-08	0.25	14	0.28	Calr3	Transcription
DMR16:19208001	16	19208001	19209000	1000	1	5.40E-05	0.36	20	2	Eps15l1	Transport
DMR16:20009001	16	20009001	20011000	2000	1	1.10E-05	0.31	48	2.4	LOC108353096;Slc27a1;Pgls	Transport;Metabolism

DMR16:20562001	16	20562001	20563000	1000	1	2.30E-05	0.28	24	2.4	Gdf15;Lrrc25	Growth Factors
DMR16:23797001	16	23797001	23798000	1000	1	2.40E-05	-0.45	3	0.3	Psd3	Transcription
DMR16:29727001	16	29727001	29728000	1000	1	3.30E-07	0.59	13	1.3	Anxa10	Signaling
DMR16:46733001	16	46733001	46734000	1000	1	1.20E-05	0.33	14	1.4	Tenm3	
DMR16:46832001	16	46832001	46833000	1000	1	6.00E-05	0.31	31	3.1	Tenm3	
DMR16:47919001	16	47919001	47920000	1000	1	2.10E-05	0.26	7	0.7	Trappc11	
DMR16:48241001	16	48241001	48242000	1000	1	8.30E-05	-0.25	16	1.6	Stox2	
DMR16:50418001	16	50418001	50428000	10000	1	3.40E-05	0.52	174	1.74	Fat1	Cytoskeleton
DMR16:50482001	16	50482001	50485000	3000	1	4.10E-05	0.35	70	2.33	Fat1	Cytoskeleton
DMR16:51067001	16	51067001	51070000	3000	1	9.10E-08	-0.59	22	0.73	RGD1563604	
DMR16:55003001	16	55003001	55004000	1000	1	2.80E-05	0.39	17	1.7	Zdhhc2	
DMR16:56261001	16	56261001	56262000	1000	1	6.60E-05	0.3	4	0.4	Tusc3	Golgi
DMR16:61988001	16	61988001	61990000	2000	1	6.60E-05	0.26	17	0.85	Rbpms	Translation
DMR16:62363001	16	62363001	62364000	1000	1	1.80E-06	-0.34	15	1.5	Tex15	
DMR16:71744001	16	71744001	71745000	1000	1	9.20E-05	0.57	5	0.5	Plekha2	
DMR16:71993001	16	71993001	71994000	1000	1	4.30E-05	-0.29	11	1.1	Adam32	Protease
DMR16:81624001	16	81624001	81626000	2000	1	7.60E-06	0.29	43	2.15	NEWGENE_1582994;Adprhl1	Signaling
DMR16:81642001	16	81642001	81645000	3000	1	4.80E-05	0.31	76	2.53	Adprhl1;LOC102548530	Signaling
DMR17:17918001	17	17918001	17919000	1000	1	5.80E-05	0.27	25	2.5	Rnf144b	Proteolysis
DMR17:20095001	17	20095001	20096000	1000	1	4.20E-06	0.35	22	2.2	Dtnbp1	
DMR17:23518001	17	23518001	23520000	2000	1	6.10E-06	-0.32	15	0.75	Phactr1	Signaling
DMR17:39834001	17	39834001	39835000	1000	1	1.50E-07	-0.51	4	0.4	Prl	Hormone
DMR17:45174001	17	45174001	45175000	1000	1	3.30E-05	-0.37	17	1.7	Nkapl	
DMR17:48422001	17	48422001	48423000	1000	1	2.70E-05	-0.31	5	0.5	Amph	
DMR17:50259001	17	50259001	50260000	1000	1	6.00E-05	0.39	2	0.2	Sugct	Transport
DMR17:53509001	17	53509001	53510000	1000	1	7.50E-05	-0.35	4	0.4	Hecw1	Proteolysis
DMR17:76698001	17	76698001	76699000	1000	1	6.40E-06	-0.54	4	0.4	Camk1d	Signaling
DMR17:77191001	17	77191001	77192000	1000	1	8.10E-05	0.29	27	2.7	Optn	
DMR17:80432001	17	80432001	80433000	1000	1	3.50E-05	0.39	14	1.4	Rsu1	Cytoskeleton
DMR17:83105001	17	83105001	83106000	1000	1	2.40E-05	-0.32	5	0.5	Malrd1	
DMR17:87521001	17	87521001	87522000	1000	1	4.20E-05	0.28	14	1.4	Etl4	
DMR17:88648001	17	88648001	88649000	1000	1	4.00E-05	0.35	14	1.4	Gpr158	Signaling
DMR18:3836001	18	3836001	3838000	2000	1	7.10E-05	0.33	22	1.1	Lama3	Extracellular Matrix
DMR18:4154001	18	4154001	4155000	1000	1	2.60E-05	0.46	14	1.4	Osbpl1a	
DMR18:13426001	18	13426001	13427000	1000	1	2.50E-05	0.25	19	1.9	Asxl3	
DMR18:16733001	18	16733001	16734000	1000	1	7.40E-06	-0.53	10	1	Fhod3	
DMR18:17286001	18	17286001	17289000	3000	1	6.90E-05	0.2	48	1.6	Fhod3	
DMR18:24929001	18	24929001	24931000	2000	1	9.10E-05	0.28	49	2.45	Proc	Protease
DMR18:29175001	18	29175001	29176000	1000	1	4.50E-05	0.27	3	0.3	Cystm1	
DMR18:32163001	18	32163001	32164000	1000	1	8.00E-05	-0.39	11	1.1	Nr3c1;Arhgap26	Signaling
DMR18:40203001	18	40203001	40204000	1000	1	3.30E-05	-0.53	8	0.8	Pggt1b	Metabolism
DMR18:47641001	18	47641001	47642000	1000	1	3.00E-05	-0.4	9	0.9	RGD1564428	
DMR18:51215001	18	51215001	51216000	1000	1	8.70E-05	-0.46	13	1.3	RGD1560341	
DMR18:51601001	18	51601001	51603000	2000	1	2.30E-05	0.42	23	1.15	Gramd3	
DMR18:51802001	18	51802001	51803000	1000	1	3.80E-05	-0.32	8	0.8	Lmnb1	
DMR18:51812001	18	51812001	51813000	1000	1	1.00E-06	0.56	15	1.5	Lmnb1	
DMR18:51927001	18	51927001	51928000	1000	1	4.40E-06	0.79	9	0.9	3-Mar	
DMR18:51969001	18	51969001	51970000	1000	1	3.40E-05	0.33	9	0.9	3-Mar	
DMR18:52435001	18	52435001	52436000	1000	1	3.10E-05	0.31	18	1.8	Prrc1	
DMR18:56350001	18	56350001	56352000	2000	1	4.70E-05	0.33	18	0.9	Cdx1	Development
DMR18:57797001	18	57797001	57798000	1000	1	9.10E-05	0.3	34	3.4	Htr4	Signaling
DMR18:64114001	18	64114001	64117000	3000	3	7.10E-08	-1.37	89	2.97	Rnmt;Mc5r	Epigenetic;Signaling
DMR18:73284001	18	73284001	73287000	3000	1	2.90E-05	0.26	61	2.03	Hdhd2;Katnal2	Signaling;Cytoskeleton
DMR18:79348001	18	79348001	79349000	1000	1	3.30E-05	0.26	21	2.1	Mbp	Cytoskeleton
DMR18:79808001	18	79808001	79810000	2000	1	2.00E-05	0.29	15	0.75	Zfp516	Transcription
DMR19:12474001	19	12474001	12475000	1000	1	1.70E-05	-0.52	5	0.5	Large1	Golgi
DMR19:25508001	19	25508001	25509000	1000	1	4.70E-06	0.38	15	1.5	Cacna1a	Transport
DMR19:26034001	19	26034001	26035000	1000	1	9.00E-05	0.31	14	1.4	Dnase2;MAST1;Mir3550	Transcription
DMR19:38155001	19	38155001	38156000	1000	1	8.50E-05	-0.37	12	1.2	Pla2g15;Slc7a6	Metabolism;Transport
DMR19:39217001	19	39217001	39218000	1000	1	1.40E-05	-0.42	13	1.3	Sntb2	
DMR19:43668001	19	43668001	43669000	1000	1	2.30E-05	0.47	15	1.5	Wdr59	
DMR19:51478001	19	51478001	51479000	1000	1	2.70E-05	0.42	11	1.1	Cdh13	Cytoskeleton
DMR19:62046001	19	62046001	62047000	1000	1	1.90E-05	-0.34	5	0.5	Ccdc7	
DMR20:232001	20	232001	233000	1000	1	1.20E-05	0.39	17	1.7	Tmlhe	Metabolism
DMR20:458001	20	458001	459000	1000	1	6.10E-06	-0.53	2	0.2	Olr1675	
DMR20:475001	20	475001	476000	1000	1	4.90E-05	-0.44	5	0.5	Olr1678	
DMR20:488001	20	488001	490000	2000	1	1.50E-05	-0.49	18	0.9	LOC100910263;Olr1680	
DMR20:501001	20	501001	503000	2000	1	2.50E-06	-0.53	9	0.45	Olr1680	
DMR20:512001	20	512001	513000	1000	1	7.80E-05	-0.61	8	0.8	Olr1681	
DMR20:515001	20	515001	516000	1000	1	6.80E-06	-0.59	2	0.2	Olr1681	

DMR20:1060001	20	1060001	1063000	3000	1	2.10E-06	0.43	21	0.7	Olr1697;Olr1698-ps	
DMR20:3845001	20	3845001	3848000	3000	2	5.90E-09	0.37	55	1.83	Col11a2	Extracellular Matrix
DMR20:4021001	20	4021001	4027000	6000	1	2.50E-05	0.56	104	1.73	RT1-DOb;LOC102554184	Immune
DMR20:4051001	20	4051001	4052000	1000	1	1.40E-05	1.1	2	0.2	LOC102554184;RT1-Bb	Immune
DMR20:4056001	20	4056001	4057000	1000	1	3.80E-06	1.78	10	1	RT1-Bb;RT1-Ba	Immune
DMR20:4058001	20	4058001	4059000	1000	1	2.10E-05	1.8	10	1	RT1-Bb;RT1-Ba	Immune
DMR20:5560001	20	5560001	5562000	2000	1	3.50E-05	0.26	42	2.1	Syngap1;Zbtb9	Signaling;Cytoskeleton
DMR20:5790001	20	5790001	5792000	2000	1	5.80E-05	-0.29	44	2.2	Lemd2;Clpsl2	Signaling
DMR20:8890001	20	8890001	8893000	3000	1	7.80E-05	0.31	88	2.93	Zfand3;Btbd9	
DMR20:9684001	20	9684001	9685000	1000	1	3.20E-05	0.36	27	2.7	Umodl1	Receptor
DMR20:10178001	20	10178001	10180000	2000	1	1.70E-05	-0.28	34	1.7	Pde9a	Signaling
DMR20:27087001	20	27087001	27088000	1000	1	6.10E-05	0.22	9	0.9	Atoh7;LOC100362906	Transcription
DMR20:30288001	20	30288001	30290000	2000	1	5.50E-05	-0.29	26	1.3	Slc29a3	Transport
DMR20:47099001	20	47099001	47100000	1000	1	9.40E-05	0.28	25	2.5	Lace1	
DMR20:47133001	20	47133001	47135000	2000	1	5.30E-05	-0.44	31	1.55	Lace1	
DMR20:54228001	20	54228001	54229000	1000	1	5.30E-05	-0.34	9	0.9	Grik2	Receptor
DMRX:295001	X	295001	297000	2000	1	6.80E-05	-0.35	12	0.6	Olr1756-ps	
DMRX:13935001	X	13935001	13936000	1000	1	8.50E-05	-0.42	6	0.6	Sytl5	
DMRX:20100001	X	20100001	20101000	1000	1	6.20E-05	-0.42	5	0.5	LOC685282;Fgd1	Transcription
DMRX:20468001	X	20468001	20469000	1000	1	9.00E-05	-0.46	5	0.5	Wnk3	Signaling
DMRX:20638001	X	20638001	20641000	3000	1	1.80E-06	0.31	86	2.87	FAM120C	
DMRX:32078001	X	32078001	32079000	1000	1	1.30E-06	-0.45	8	0.8	Ace2	Protease
DMRX:73496001	X	73496001	73497000	1000	1	3.70E-05	-0.42	26	2.6	Fam9b	
DMRX:80135001	X	80135001	80136000	1000	1	3.10E-06	0.33	11	1.1	Hmgn5	Epigenetic
DMRX:140295001	X	140295001	140296000	1000	1	1.40E-05	-0.39	6	0.6	Gpr101	Signaling
DMRX:144757001	X	144757001	144758000	1000	1	4.80E-06	-0.47	3	0.3	Ldoc1	
DMRX:149839001	X	149839001	149841000	2000	1	1.10E-05	-0.29	9	0.45	Olr1759-ps	
DMRX:155840001	X	155840001	155842000	2000	1	6.00E-06	0.19	46	2.3	Dkc1	Cell Cycle
DMRX:159538001	X	159538001	159539000	1000	1	3.00E-05	-0.42	8	0.8	Htatsf1	Translation
