# Supplementary material for: Environmental induced transgenerational inheritance impacts systems epigenetics in disease etiology
Source: Sci Rep. 2022 Apr 19;12:5452. doi: 10.1038/s41598-022-09336-0 (PMC9018793; doi:10.1038/s41598-022-09336-0)
Supplement: Supplementary file 24 — Supplementary Table S16. [file 41598_2022_9336_MOESM24_ESM.pdf]

Supplemental Table S16

Control Disease Specific DMR List Kidney Disease p<1e-04

DMR Name	Chr	start	Length	# Sig Win	minP	maxLFC	CpG #	CpG Density	Gene Annotation	Gene Category
DMR1:23273001	1	23273001	1000	1	6.28E-05	-0.7312807	9	0.9	Eya4	
DMR1:112340001	1	112340001	2000	1	5.06E-05	0.8574235	17	0.85	Gabrg3	Ion Channel
DMR1:147868001	1	147868001	1000	1	2.07E-05	0.7744235	5	0.5	Cyp2c7	Metabolism
DMR1:148050001	1	148050001	4000	1	6.69E-05	0.7483809	17	0.425	Cyp2c7	Metabolism
DMR1:177759001	1	177759001	1000	1	1.89E-05	-0.7583758	15	1.5	Rassf10	Cytoskeleton
DMR1:207330001	1	207330001	1000	1	2.20E-05	-1.08022	11	1.1	Dock1	Transcription
DMR1:226841001	1	226841001	1000	1	8.98E-05	-0.686229	17	1.7	Cd6	Protease
DMR1:236869001	1	236869001	1000	1	4.36E-06	-0.7190888	12	1.2	Prune2	
DMR2:141482001	2	141482001	1000	1	3.82E-05	0.6201602	10	1	Foxo1	
DMR3:12153001	3	12153001	2000	1	8.18E-05	0.8702456	15	0.75	Garnl3	Signaling
DMR3:75676001	3	75676001	1000	1	3.61E-05	0.8093305	5	0.5	Olr574-ps	
DMR3:80695001	3	80695001	1000	1	1.01E-05	0.7693405	7	0.7	Ambra1	
DMR3:105144001	3	105144001	1000	1	3.29E-05	-0.7037919	13	1.3	Fmn1	
DMR3:146691001	3	146691001	1000	1	8.65E-05	-0.8516025	11	1.1	Abhd12;Gins1	Protease;Transcription
DMR4:34660001	4	34660001	1000	1	8.12E-05	-0.7518886	23	2.3	Ica1	
DMR4:65930001	4	65930001	2000	1	9.67E-05	-0.6567059	24	1.2	RGD1306271	
DMR4:91729001	4	91729001	1000	1	3.21E-05	-1.1577226	9	0.9	Ccser1	
DMR4:131635001	4	131635001	3000	1	4.69E-05	-0.5686922	52	1.733333333	Foxp1	
DMR6:73763001	6	73763001	1000	1	8.09E-05	-0.7137271	6	0.6	Akap6	
DMR7:135881001	7	135881001	1000	1	1.61E-05	0.6826721	11	1.1	LOC108351539;Tmem117	
DMR8:1729001	8	1729001	1000	1	9.67E-05	1.0480164	4	0.4	Gria4;LOC108351645	Receptor
DMR8:127594001	8	127594001	1000	1	3.01E-05	-0.5842882	13	1.3	Ctdspl	
DMR9:55261001	9	55261001	1000	1	1.36E-05	-0.7553721	16	1.6	Sdpr	
DMR9:75766001	9	75766001	1000	1	1.09E-05	-1.1260551	8	0.8	Erbb4	Receptor
DMR10:5917001	10	5917001	1000	1	8.79E-05	0.8494384	10	1	Grin2a	Receptor
DMR10:74379001	10	74379001	1000	1	7.81E-05	0.6670201	10	1	Gdpd1;Smg8	Metabolism
DMR11:59175001	11	59175001	1000	1	6.51E-05	-0.6432228	14	1.4	Lsamp	Immune
DMR11:88878001	11	88878001	1000	1	4.90E-05	0.8778215	6	0.6	Dnm1l;Yars2	Transport;Translation
DMR12:12888001	12	12888001	1000	1	8.34E-06	0.6708001	16	1.6	Usp42;Cyth3	Protease;Transcription
DMR12:30175001	12	30175001	1000	1	1.58E-05	-0.9355604	19	1.9	Asl	Metabolism
DMR13:95328001	13	95328001	1000	1	6.20E-05	0.8072515	6	0.6	Akt3	Signaling
DMR14:81637001	14	81637001	1000	1	8.89E-05	0.8565192	6	0.6	Fam193a	
DMR16:11557001	16	11557001	1000	1	5.35E-05	-0.8430885	15	1.5	Grid1	Receptor
DMR17:34828001	17	34828001	2000	1	6.05E-05	0.8166612	24	1.2	Exoc2	
DMR18:28114001	18	28114001	1000	1	4.99E-05	0.6980537	9	0.9	Sil1	
DMR18:40243001	18	40243001	1000	1	7.79E-05	0.7785961	4	0.4	Ccdc112	
DMR20:45250001	20	45250001	1000	1	3.27E-05	-0.6712674	10	1	Slc16a10	Transport
DMRX:2489001	X	2489001	1000	1	8.76E-05	0.4572671	8	0.8	Slc9a7;LOC102547012	Transport
DMRX:13985001	X	13985001	1000	1	6.38E-05	-0.6914888	7	0.7	Sytl5;Hypm;LOC100363125	Epigenetic
DMRX:39778001	X	39778001	1000	1	5.85E-05	-0.7620438	9	0.9	Cnksr2	
DMRX:105500001	X	105500001	1000	1	2.60E-05	-1.1944128	12	1.2	Armcx4	
