# Supplementary material for: Environmental induced transgenerational inheritance impacts systems epigenetics in disease etiology
Source: Sci Rep. 2022 Apr 19;12:5452. doi: 10.1038/s41598-022-09336-0 (PMC9018793; doi:10.1038/s41598-022-09336-0)
Supplement: Supplementary file 25 — Supplementary Table S17. [file 41598_2022_9336_MOESM25_ESM.pdf]

Supplemental Table S17

Control Disease Specific DMR List Prostate Disease p<1e-04

DMR Name	Chr	start	Length	# Sig Win	minP	maxLFC	CpG #	CpG Density	Gene Annotation	Gene Category
DMR1:18741001	1	18741001	1000	1	5.53E-05	0.854816	10	1	Lama2	Extracellular Matrix
DMR1:66401001	1	66401001	1000	1	3.62E-05	0.9164668	3	0.3	Vom1r53;Vom1r-ps61	Receptor
DMR1:78373001	1	78373001	2000	1	3.07E-05	-0.800062	37	1.85	Zc3h4	Transcription
DMR1:78445001	1	78445001	2000	1	5.29E-06	-1.0087544	22	1.1	Npas1;Arhgap35	Transcription;Signaling
DMR1:81908001	1	81908001	1000	1	1.29E-05	-0.9615648	25	2.5	Grik5	Receptor
DMR1:113108001	1	113108001	1000	1	4.48E-05	0.9334706	8	0.8	Gabrb3	Ion Channel
DMR1:135840001	1	135840001	1000	1	2.14E-05	-0.8391799	16	1.6	Slco3a1	Transport
DMR1:142058001	1	142058001	1000	1	7.49E-05	0.7516636	16	1.6	LOC102549117;Ttll13;Ngrn;Vps33b	Cytoskeleton;Transport
DMR1:157398001	1	157398001	1000	1	3.54E-06	-1.1497272	13	1.3	LOC100361001;Ccdc90b	
DMR1:157444001	1	157444001	1000	1	8.73E-05	0.7540154	9	0.9	Ankrd42	
DMR1:178659001	1	178659001	1000	1	9.06E-06	-0.9941003	8	0.8	Spon1	Cytoskeleton
DMR1:193167001	1	193167001	1000	1	5.34E-05	-0.8865461	11	1.1	Slc5a11	Transport
DMR1:213628001	1	213628001	1000	1	1.07E-06	1.1535319	11	1.1	Sirt3;Psmd13	Protease
DMR1:219817001	1	219817001	1000	1	4.08E-06	-1.7735678	10	1	Pc	Metabolism
DMR1:251383001	1	251383001	1000	1	2.08E-05	-0.994783	9	0.9	Atad1	
DMR1:260989001	1	260989001	1000	1	6.25E-05	-1.0481511	7	0.7	Slit1	
DMR1:279401001	1	279401001	2000	1	5.79E-05	-1.5171704	24	1.2	Gfra1	Receptor
DMR2:26263001	2	26263001	1000	1	4.53E-07	-1.3924634	29	2.9	Iqgap2	Signaling
DMR2:27998001	2	27998001	1000	1	1.51E-05	-0.956572	17	1.7	Hexb	Metabolism
DMR2:29424001	2	29424001	2000	1	3.78E-05	1.0140238	18	0.9	Zfp366	Transcription
DMR2:113856001	2	113856001	1000	1	2.49E-05	0.9646342	12	1.2	Pld1	Metabolism
DMR2:116019001	2	116019001	3000	1	4.04E-05	-0.9249182	40	1.333333333	LOC100910976;Phc3	Epigenetic
DMR2:140789001	2	140789001	1000	1	1.05E-05	-1.7758574	12	1.2	Maml3	
DMR2:157936001	2	157936001	2000	1	9.75E-05	0.7832285	14	0.7	Veph1	
DMR2:165748001	2	165748001	1000	1	2.41E-05	0.9339002	12	1.2	Kpna4	Transport
DMR2:166010001	2	166010001	1000	1	9.79E-05	1.1094604	3	0.3	Ppm1l	Signaling
DMR2:170470001	2	170470001	1000	1	7.96E-06	-1.1592696	6	0.6	Slitrk3	
DMR2:194317001	2	194317001	1000	1	8.15E-05	-1.0386252	24	2.4	RGD1560096;LOC100911153	
DMR2:196447001	2	196447001	1000	1	3.14E-05	0.9633438	7	0.7	Prune	
DMR2:206114001	2	206114001	3000	1	7.26E-06	-1.2649103	35	1.166666667	Syt6	Transport
DMR2:211122001	2	211122001	1000	1	4.89E-05	1.1443368	8	0.8	Sort1	Transport
DMR2:237780001	2	237780001	1000	1	5.91E-06	1.03288	6	0.6	Tbck	Signaling
DMR2:244157001	2	244157001	2000	1	3.45E-06	-1.0465624	26	1.3	Tspan5	
DMR2:259420001	2	259420001	1000	1	4.23E-05	1.0296125	28	2.8	St6galnac3	
DMR3:9413001	3	9413001	1000	1	2.38E-05	-1.7620784	14	1.4	Qrfp;LOC100909750	
DMR3:14970001	3	14970001	1000	1	6.55E-05	-0.8845103	15	1.5	Dab2ip	Signaling
DMR3:15928001	3	15928001	2000	1	8.08E-05	0.9970325	7	0.35	Olr401	Receptor
DMR3:29201001	3	29201001	1000	1	1.16E-06	-1.4204697	13	1.3	Arhgap15	Signaling
DMR3:71270001	3	71270001	1000	1	3.27E-05	0.9660949	11	1.1	Fam171b;LOC108350410;Zswim2	Proteolysis
DMR3:80054001	3	80054001	1000	1	8.04E-05	-1.3528144	11	1.1	Ddb2	DNA Repair
DMR3:97863001	3	97863001	1000	1	1.50E-05	0.7886955	11	1.1	Mpped2	Metabolism
DMR3:107606001	3	107606001	1000	1	4.53E-06	-1.3394878	5	0.5	Meis2	Development
DMR3:123354001	3	123354001	1000	1	7.25E-06	0.894589	13	1.3	RGD1565616	
DMR3:140642001	3	140642001	1000	1	3.64E-05	-1.2391323	8	0.8	Ralgapa2	Signaling
DMR3:146382001	3	146382001	1000	1	5.27E-05	0.9329821	18	1.8	Cst7;Apmap	
DMR3:147497001	3	147497001	2000	1	6.68E-05	-0.9173585	37	1.85	Fam110a;LOC108350501;Slc52a3	Transport

DMR4:10135001	4	10135001	1000	1	2.21E-05	0.8201648	17	1.7	Fbxl13;Lrrc17	Receptor
DMR4:13463001	4	13463001	1000	1	3.30E-05	-0.797894	7	0.7	Gnai1	Signaling
DMR4:13493001	4	13493001	1000	1	8.41E-05	0.7210264	14	1.4	Gnai1	Signaling
DMR4:34952001	4	34952001	1000	1	4.41E-06	-1.747268	4	0.4	Nxph1	Signaling
DMR4:38689001	4	38689001	1000	1	7.79E-05	-0.9698153	10	1	Thsd7a	Cytoskeleton
DMR4:41954001	4	41954001	1000	1	2.46E-05	1.0113264	5	0.5	Foxp2	
DMR4:66701001	4	66701001	1000	1	6.54E-07	-1.3911975	10	1	Tbxas1	Metabolism
DMR4:88128001	4	88128001	1000	1	3.76E-05	-0.9362946	9	0.9	Vom1r82;Vom1r-ps73	Receptor
DMR4:92320001	4	92320001	2000	1	7.01E-05	0.7509574	20	1	Ccser1	
DMR4:100782001	4	100782001	1000	1	6.30E-05	1.0655125	11	1.1	Kcmf1	Proteolysis
DMR4:109506001	4	109506001	1000	1	7.22E-06	0.945609	7	0.7	Reg1a	
DMR4:136128001	4	136128001	1000	1	9.67E-05	0.9490067	4	0.4	RGD1560353	
DMR4:150053001	4	150053001	1000	1	5.26E-06	-1.0672269	16	1.6	Rasgef1a	Transcription
DMR4:153471001	4	153471001	1000	1	2.75E-05	-1.367647	10	1	Bid	
DMR4:155036001	4	155036001	1000	1	5.76E-05	-0.8972337	8	0.8	Klrg1	Receptor
DMR4:178268001	4	178268001	1000	1	3.76E-05	-0.7768582	11	1.1	Sox5	
DMR5:1351001	5	1351001	1000	1	5.17E-05	0.7320135	7	0.7	Gdap1	
DMR5:100635001	5	100635001	2000	1	8.66E-05	0.8713185	27	1.35	Nfib	Transcription
DMR5:121898001	5	121898001	1000	1	6.58E-05	0.6742562	6	0.6	Pde4b	Signaling
DMR5:124501001	5	124501001	1000	1	4.12E-05	-0.9338555	12	1.2	RGD1564074;LOC102549337;LOC108351070	
DMR5:136934001	5	136934001	1000	1	4.80E-05	-1.2535084	0	0	St3gal3	Transport
DMR6:1361001	6	1361001	1000	1	2.04E-05	-1.4468619	0	0	Heatr5b;LOC103692530	
DMR6:23078001	6	23078001	1000	1	1.70E-05	-1.1580728	11	1.1	Alk	Receptor
DMR6:42744001	6	42744001	1000	1	9.47E-05	0.8957485	16	1.6	Nol10	
DMR6:60268001	6	60268001	2000	1	7.58E-05	0.9254667	11	0.55	Zfp277	Transcription
DMR6:74423001	6	74423001	1000	1	3.20E-06	1.0901933	11	1.1	Npas3	
DMR6:94953001	6	94953001	1000	1	5.46E-05	-0.765357	12	1.2	Rtn1	
DMR6:98505001	6	98505001	1000	1	4.91E-05	-1.2985809	10	1	Ppp2r5e	Signaling
DMR6:99733001	6	99733001	1000	1	7.21E-05	-1.3733168	11	1.1	Sptb	
DMR6:105625001	6	105625001	1000	1	4.30E-05	0.9442599	12	1.2	Pcnx1	
DMR6:111286001	6	111286001	1000	1	8.98E-05	-1.457754	7	0.7	Vipas39;Ahsa1	Transcription
DMR6:139187001	6	139187001	1000	1	2.72E-06	-0.9152298	8	0.8	Ighg	
DMR7:24873001	7	24873001	1000	1	6.03E-05	0.8174427	15	1.5	Tcp11l2	Cytoskeleton
DMR7:59424001	7	59424001	1000	1	8.62E-07	1.2318012	11	1.1	Ptprb	Receptor
DMR7:61237001	7	61237001	1000	1	1.93E-05	-1.0455508	20	2	Il22	
DMR7:72987001	7	72987001	1000	1	5.13E-05	-1.0165246	8	0.8	Matn2	Extracellular Matrix
DMR7:74189001	7	74189001	1000	1	3.14E-05	0.7341953	8	0.8	Vps13b;LOC103692884	
DMR7:75013001	7	75013001	1000	1	2.23E-06	-1.5368609	10	1	Spag1	
DMR7:95325001	7	95325001	1000	1	1.33E-05	-0.820115	17	1.7	Mtbp	
DMR7:104774001	7	104774001	1000	1	8.18E-05	0.6201044	8	0.8	Asap1	
DMR7:126249001	7	126249001	1000	1	7.76E-05	-1.1037051	13	1.3	Atxn10	
DMR7:131788001	7	131788001	1000	1	4.94E-05	0.9182703	16	1.6	Cpne8	
DMR7:140430001	7	140430001	1000	1	6.21E-06	-0.9185492	9	0.9	Arf3	Signaling
DMR8:52135001	8	52135001	1000	1	5.59E-05	0.9430185	13	1.3	Cadm1	
DMR8:70814001	8	70814001	2000	1	7.16E-06	1.0840201	12	0.6	Clpx	Protease
DMR8:71394001	8	71394001	1000	1	3.84E-05	-0.9120015	15	1.5	Trip4	Transcription
DMR8:72423001	8	72423001	1000	1	9.17E-05	-1.5937364	9	0.9	Car12	
DMR8:73171001	8	73171001	2000	1	9.40E-05	-0.7001694	30	1.5	Tln2	
DMR8:94042001	8	94042001	1000	1	4.12E-05	1.0880531	20	2	Ube3d;LOC102551813	Proteolysis
DMR8:112557001	8	112557001	1000	1	8.01E-05	-1.1819191	25	2.5	Nphp3	
DMR8:113370001	8	113370001	1000	1	9.56E-05	0.8470525	15	1.5	Cpne4	
DMR8:118276001	8	118276001	1000	1	7.43E-05	-1.1137037	8	0.8	Smarcc1	Epigenetic
DMR8:125472001	8	125472001	2000	1	5.53E-05	1.074522	22	1.1	Rbms3	
DMR8:130117001	8	130117001	1000	1	5.89E-05	-0.737371	14	1.4	Cck	
DMR8:132137001	8	132137001	1000	1	3.44E-05	-1.157877	9	0.9	Tgm4	Transport

DMR9:9983001	9	9983001	1000	1	6.23E-05	-1.0556914	16	1.6	Dennd1c;Crb3;Slc25a23	Transport
DMR9:30048001	9	30048001	2000	1	5.91E-05	0.785702	51	2.55	B3gat2	Golgi
DMR9:43626001	9	43626001	1000	1	8.93E-05	-1.2600906	9	0.9	Vwa3b	
DMR9:43687001	9	43687001	2000	1	2.19E-05	-1.3313964	26	1.3	Vwa3b;LOC102554996	
DMR9:92685001	9	92685001	1000	1	9.82E-05	0.775366	8	0.8	Sp100	
DMR9:93853001	9	93853001	1000	1	5.78E-05	1.0003755	4	0.4	Dis3l2	Transcription
DMR10:10420001	10	10420001	2000	1	6.73E-05	-1.2603501	20	1	Rbfox1	Translation
DMR10:45518001	10	45518001	1000	1	1.65E-05	-1.3987207	9	0.9	Iba57;Gjc2	Cytoskeleton
DMR10:48350001	10	48350001	1000	1	1.52E-06	-2.0068375	8	0.8	Specc1;LOC108352092	
DMR10:61555001	10	61555001	1000	1	3.03E-06	-2.2288952	5	0.5	Pafah1b1	
DMR10:74281001	10	74281001	2000	1	3.69E-05	-1.2460552	28	1.4	Ypel2	
DMR10:75503001	10	75503001	1000	1	4.75E-06	-1.4359235	13	1.3	Cuedc1	
DMR10:84396001	10	84396001	1000	1	2.97E-05	-1.4155052	23	2.3	Skap1	Cytoskeleton
DMR10:90369001	10	90369001	1000	1	9.25E-05	-0.8355743	14	1.4	Grn	
DMR10:94179001	10	94179001	1000	1	3.96E-05	-1.1338278	16	1.6	Ace	Protease
DMR10:103907001	10	103907001	1000	1	1.60E-05	-0.9529153	12	1.2	Otop3;Hid1	Transport
DMR11:11310001	11	11310001	1000	1	1.21E-06	-1.5036524	9	0.9	Robo2	
DMR11:35133001	11	35133001	1000	1	4.55E-05	-1.4943286	8	0.8	Kcnj6	Transport
DMR11:42272001	11	42272001	2000	1	5.11E-05	-1.5836122	10	0.5	Epha6	Receptor
DMR11:42854001	11	42854001	2000	1	8.32E-05	1.1495119	13	0.65	Arl6	Signaling
DMR11:43609001	11	43609001	1000	1	1.00E-05	-1.0090548	23	2.3	Olr1556-ps;Olr1557	Signaling
DMR11:46288001	11	46288001	1000	1	1.90E-05	1.0329015	9	0.9	Abi3bp	
DMR11:67065001	11	67065001	2000	1	5.37E-05	-0.9284323	12	0.6	Cd86	Immune
DMR11:68748001	11	68748001	1000	1	9.13E-05	-0.8460416	10	1	Adcy5	
DMR11:70420001	11	70420001	3000	1	5.79E-05	-1.1162565	46	1.533333333	Slc12a8	Transport
DMR11:70789001	11	70789001	1000	1	4.77E-07	1.1598299	13	1.3	Osbpl11	
DMR11:78441001	11	78441001	1000	1	9.83E-05	0.927396	7	0.7	Tp63	Transcription
DMR11:80940001	11	80940001	1000	1	6.62E-05	0.6284591	19	1.9	St6gal1	Transport
DMR11:88161001	11	88161001	1000	1	7.91E-05	-1.0345288	22	2.2	Ppil2;Ypel1	Transcription
DMR12:52147001	12	52147001	2000	1	7.00E-05	-1.5493706	28	1.4	Galnt9	Golgi
DMR13:42269001	13	42269001	1000	1	6.27E-05	0.9637845	15	1.5	Lypd1;Nckap5	
DMR13:51643001	13	51643001	1000	1	7.63E-05	0.7475067	12	1.2	Ppp1r12b	Signaling
DMR13:68730001	13	68730001	1000	1	5.10E-05	-1.653853	5	0.5	Ivns1abp;Swt1	Cytoskeleton
DMR13:80097001	13	80097001	1000	1	6.37E-05	-0.9964099	11	1.1	Dnm3	Transport
DMR13:89026001	13	89026001	1000	1	1.68E-05	-0.9169685	11	1.1	Olfml2b	Development
DMR14:3374001	14	3374001	2000	1	6.50E-05	-1.0877938	19	0.95	LOC108352691;Ephx4	Metabolism
DMR14:28542001	14	28542001	1000	1	8.07E-05	1.100244	6	0.6	Adgrl3	Signaling
DMR14:39798001	14	39798001	1000	1	7.46E-05	1.1510019	10	1	Gabra2	Ion Channel
DMR14:42058001	14	42058001	1000	1	9.26E-05	1.0843085	20	2	Atp8a1	Transport
DMR14:81650001	14	81650001	1000	1	9.32E-05	0.7664825	8	0.8	Rnf4	
DMR14:83716001	14	83716001	1000	1	5.17E-05	0.797182	11	1.1	Pla2g3	
DMR14:86943001	14	86943001	1000	1	9.27E-05	-1.1026988	14	1.4	Ramp3	Receptor
DMR14:110845001	14	110845001	1000	1	7.70E-05	-1.424126	5	0.5	Vrk2	Signaling
DMR15:12647001	15	12647001	1000	1	9.59E-05	0.8776811	3	0.3	RGD1565725	
DMR15:28583001	15	28583001	1000	1	3.62E-05	1.0979691	4	0.4	Rpgrip1;Supt16h	Epigenetic
DMR15:42741001	15	42741001	1000	1	9.68E-05	1.0302388	7	0.7	Adam2	Protease
DMR15:49090001	15	49090001	1000	1	5.36E-05	-0.9052391	12	1.2	Scara5	Protease
DMR15:83587001	15	83587001	1000	1	9.04E-05	0.6783149	20	2	Pibf1	
DMR15:102433001	15	102433001	1000	1	2.91E-05	-1.3137333	6	0.6	Gpc6	
DMR16:10916001	16	10916001	1000	1	6.16E-05	-0.9579719	11	1.1	Ldb3	Cytoskeleton
DMR16:25109001	16	25109001	1000	1	4.72E-05	-0.7755225	22	2.2	March1;LOC103693923	
DMR16:74313001	16	74313001	2000	1	9.89E-05	0.8738379	28	1.4	Vdac3;Slc20a2	Transport;Transport
DMR17:9984001	17	9984001	2000	1	2.59E-07	-1.2944254	33	1.65	LOC498705;Fgfr4	Receptor
DMR17:12267001	17	12267001	1000	1	1.58E-05	0.6528051	23	2.3	Nfil3	Transcription
DMR17:52452001	17	52452001	1000	1	7.27E-05	-1.3811232	12	1.2	Gli3	Transcription
DMR17:55385001	17	55385001	2000	1	5.10E-05	-1.2033716	22	1.1	Svil	Cytoskeleton

DMR17:78629001	17	78629001	1000	1	7.59E-05	0.8059408	10	1	Fam107b	
DMR17:89038001	17	89038001	1000	1	2.62E-05	-1.5904602	8	0.8	Myo3a	
DMR18:6058001	18	6058001	1000	1	1.00E-04	-0.7476919	11	1.1	Ss18	Transcription
DMR18:15475001	18	15475001	1000	1	1.47E-05	-0.9728362	9	0.9	Mapre2;B4galt6	Cytoskeleton;Golgi
DMR18:15585001	18	15585001	1000	1	8.19E-05	0.7490375	8	0.8	Mapre2;Dsg2	Cytoskeleton;Cytoskeleton
DMR18:24607001	18	24607001	1000	1	8.77E-05	-1.013537	10	1	Wdr33	Translation
DMR18:29465001	18	29465001	1000	1	5.06E-06	1.0826924	6	0.6	Ankhd1	Immune
DMR18:36458001	18	36458001	1000	1	2.09E-05	-1.0401608	9	0.9	Sh3rf2	
DMR18:72251001	18	72251001	3000	1	5.39E-05	-1.3100605	50	1.666666667	Zbtb7c	
DMR18:80894001	18	80894001	1000	1	3.13E-06	-0.8775452	20	2	Tshz1	Transcription
DMR19:10844001	19	10844001	1000	1	1.24E-05	-1.7070181	12	1.2	Fam192a	
DMR19:22357001	19	22357001	1000	1	1.98E-05	-1.5686771	7	0.7	Itfg1	
DMR19:25350001	19	25350001	1000	1	1.46E-05	-1.0560732	7	0.7	Zswim4;LOC288913;Mri1	Metabolism
DMR19:38132001	19	38132001	1000	1	3.93E-05	-0.9182287	8	0.8	Pla2g15	Metabolism
DMR19:55064001	19	55064001	1000	1	3.32E-05	-1.0122056	14	1.4	Zfp469	
DMR19:57194001	19	57194001	1000	1	6.54E-05	-1.2570157	11	1.1	Pgbd5;LOC108348970	Epigenetic
DMR20:855001	20	855001	1000	1	2.74E-06	1.1302513	9	0.9	Dstnl1;Olr1694;Olr1695	Receptor
DMR20:3746001	20	3746001	1000	1	5.57E-05	-0.7623111	13	1.3	Cchcr1;Tcf19;Pou5f1	Transcription
DMR20:7477001	20	7477001	1000	1	6.05E-05	0.9497646	18	1.8	Uhrf1bp1;Taf11;Anks1a	Transcription;Cytoskeleton
DMR20:9469001	20	9469001	1000	1	5.68E-05	-1.2993096	20	2	Dnah8;LOC100909773	Cytoskeleton
DMR20:14593001	20	14593001	1000	1	1.65E-05	0.9397435	27	2.7	Rtdr1;Gnaz	Signaling
DMR20:15169001	20	15169001	1000	1	6.92E-05	-0.9610051	14	1.4	Pcdh15	Cytoskeleton
DMR20:27045001	20	27045001	1000	1	4.47E-05	-1.1271657	7	0.7	Mypn	
DMR20:41093001	20	41093001	1000	1	3.88E-05	1.1403495	12	1.2	Tspyl1;Tspyl4	Epigenetic
DMRX:16793001	X	16793001	2000	1	7.17E-05	-1.1362849	26	1.3	Shroom4	Cytoskeleton
DMRX:75388001	X	75388001	1000	1	9.16E-06	-1.4896734	7	0.7	Uprt	Signaling
DMRX:123470001	X	123470001	1000	1	1.07E-05	-1.9945293	4	0.4	RGD1564541	
DMRX:124935001	X	124935001	1000	1	2.27E-05	-1.3722726	7	0.7	C1galt1c1	Transport
