# Supplementary material for: Environmental induced transgenerational inheritance impacts systems epigenetics in disease etiology
Source: Sci Rep. 2022 Apr 19;12:5452. doi: 10.1038/s41598-022-09336-0 (PMC9018793; doi:10.1038/s41598-022-09336-0)
Supplement: Supplementary file 26 — Supplementary Table S18. [file 41598_2022_9336_MOESM26_ESM.pdf]

Supplemental Table S18

Control Disease Specific DMR List Testis Disease p<1e-04

DMR Name	Chr	start	Length	# Sig Win	minP	maxLFC	CpG #	CpG Density	Gene Annotation	Gene Category
DMR1:50172001	1	50172001	1000	1	2.23E-05	1.0932597	14	1.4	Pacrg;LOC102552213	
DMR1:105445001	1	105445001	3000	1	4.83E-07	1.1668185	31	1.033333333	Nell1	Signaling
DMR1:125800001	1	125800001	1000	1	2.33E-05	1.0392594	11	1.1	Fam189a1	
DMR1:167598001	1	167598001	1000	1	2.43E-05	-1.0887562	7	0.7	Olr39;Olr40	Receptor
DMR1:169968001	1	169968001	2000	1	3.17E-05	-1.2102138	10	0.5	Olr190;Olr191-ps	Receptor
DMR1:170789001	1	170789001	1000	1	3.05E-06	-1.4272057	6	0.6	LOC103691201;Olr210	Receptor
DMR1:206047001	1	206047001	2000	1	1.87E-05	0.8249494	29	1.45	Adam12	Protease
DMR1:220284001	1	220284001	1000	1	2.83E-05	-1.2734559	8	0.8	Npas4;LOC108349788	Transcription
DMR1:226529001	1	226529001	1000	1	5.26E-05	-1.142635	6	0.6	Ppp1r32	Signaling
DMR1:260340001	1	260340001	1000	1	2.03E-05	-1.0243682	8	0.8	Opalin;Tll2	Protease
DMR1:266869001	1	266869001	1000	1	3.36E-05	0.7392738	12	1.2	Taf5;Usmg5;Pdcd11	Metabolism
DMR2:26332001	2	26332001	1000	1	6.24E-05	-1.3233459	8	0.8	Iqgap2	Signaling
DMR2:56447001	2	56447001	1000	1	2.90E-05	-0.8998846	18	1.8	Lifr	Receptor
DMR2:109755001	2	109755001	2000	1	2.88E-05	-1.0243643	14	0.7	Naaladl2	
DMR2:118307001	2	118307001	1000	1	2.69E-05	-0.9525995	11	1.1	Kcnmb2	Transport
DMR2:123620001	2	123620001	1000	1	5.41E-05	-1.0563026	8	0.8	RGD1307100	
DMR2:187466001	2	187466001	1000	1	4.17E-05	-1.062288	9	0.9	Iqgap3	Signaling
DMR2:210888001	2	210888001	1000	1	3.92E-05	0.9000301	13	1.3	Gnat2;Gnai3	Signaling
DMR2:217932001	2	217932001	2000	1	8.34E-05	0.5542757	22	1.1	Olfm3	Development
DMR2:231488001	2	231488001	2000	1	8.55E-05	0.7822113	23	1.15	Ank2	
DMR2:240734001	2	240734001	1000	1	2.66E-05	0.6676652	10	1	Manba	Golgi
DMR2:257907001	2	257907001	1000	1	2.75E-05	-1.8735186	11	1.1	Pigk	
DMR2:260088001	2	260088001	1000	1	1.20E-06	-1.451855	4	0.4	Msh4	Transcription
DMR3:10524001	3	10524001	1000	1	9.92E-06	0.7067297	17	1.7	Hmcn2	
DMR3:23086001	3	23086001	1000	1	8.27E-05	1.1059351	6	0.6	Nr6a1	
DMR3:28445001	3	28445001	1000	1	6.20E-05	1.1463541	19	1.9	Kynu;LOC102547849	Metabolism
DMR3:89746001	3	89746001	1000	1	1.16E-05	-1.49961	3	0.3	RGD1560961	
DMR3:132894001	3	132894001	1000	1	2.20E-05	-1.1468049	7	0.7	Tasp1	Protease
DMR3:149911001	3	149911001	1000	1	9.91E-05	-1.1076675	5	0.5	Snta1	
DMR3:153509001	3	153509001	1000	1	7.23E-05	-0.7031376	13	1.3	Manbal	
DMR3:159757001	3	159757001	1000	1	2.58E-05	-0.8229721	16	1.6	Jph2	
DMR4:7848001	4	7848001	2000	1	5.05E-05	0.9341973	30	1.5	Tomm7;Rint1	Transport
DMR4:16654001	4	16654001	1000	1	1.12E-05	1.0911422	20	2	Pclo	
DMR4:35237001	4	35237001	1000	1	7.40E-05	0.8539285	14	1.4	Nxph1	Signaling
DMR4:55718001	4	55718001	1000	1	9.96E-05	-0.867968	16	1.6	Gcc1;Arf5;Fscn3	Signaling;Cytoskeleton
DMR4:76001001	4	76001001	1000	1	6.22E-05	-1.5683956	4	0.4	Cntnap2	
DMR4:95221001	4	95221001	1000	1	7.00E-05	-1.105648	3	0.3	Grid2	Receptor
DMR4:115210001	4	115210001	2000	1	1.10E-06	-1.175012	21	1.05	Dguok;Actg2	Signaling;Cytoskeleton
DMR4:117159001	4	117159001	1000	1	2.90E-05	0.7481354	10	1	Rab11fip5;LOC681041	
DMR4:144571001	4	144571001	1000	1	3.63E-05	0.717137	9	0.9	Rad18	Proteolysis
DMR4:157379001	4	157379001	1000	1	8.13E-05	-0.8165458	19	1.9	P3h3;Gpr162;Cd4	Extracellular Matrix;Signaling
DMR5:5674001	5	5674001	1000	1	6.27E-06	0.8359909	15	1.5	Ncoa2	Epigenetic
DMR5:25352001	5	25352001	2000	1	8.46E-05	0.6591164	43	2.15	Gem;Cdh17	Cytoskeleton
DMR5:39303001	5	39303001	1000	1	2.86E-05	-1.598929	1	0.1	Gpr63	Signaling
DMR5:59614001	5	59614001	1000	1	7.37E-05	0.8416933	10	1	Rnf38	
DMR5:65058001	5	65058001	1000	1	2.33E-05	-1.1970684	10	1	Grin3a	Receptor
DMR5:117702001	5	117702001	1000	1	3.87E-06	0.6770351	9	0.9	Dock7;Angptl3	Transcription;Signaling
DMR5:120548001	5	120548001	1000	1	2.72E-05	-1.2010026	7	0.7	Lepr	Receptor
DMR6:25941001	6	25941001	1000	1	8.57E-05	-0.8557548	8	0.8	Bre	

DMR6:55759001	6	55759001	1000	1	5.01E-05	-1.2520514	11	1.1	Lrrc72;LOC108351332	
DMR6:98924001	6	98924001	2000	1	8.64E-05	0.8026595	35	1.75	Syne2	
DMR6:102229001	6	102229001	1000	1	5.56E-05	0.9992065	7	0.7	Plekhh1	
DMR6:106528001	6	106528001	2000	1	2.32E-05	1.0882572	18	0.9	Rgs6	
DMR6:142655001	6	142655001	2000	1	3.27E-05	-1.507383	10	0.5	Olr874-ps	
DMR7:107289001	7	107289001	1000	1	2.30E-05	-2.103047	4	0.4	Tmem71	
DMR7:133056001	7	133056001	2000	1	1.24E-05	0.85179	22	1.1	Muc19;Smgc	
DMR8:22949001	8	22949001	1000	1	7.16E-05	-1.234167	10	1	Ccdc159;Plppr2	Signaling
DMR8:102781001	8	102781001	1000	1	7.06E-05	1.1084139	12	1.2	Slc9a9	Transport
DMR8:109373001	8	109373001	1000	1	8.92E-05	-1.186721	9	0.9	LOC686039;Pccb	Metabolism
DMR8:112798001	8	112798001	1000	1	1.69E-05	-0.9245756	12	1.2	Dnajc13;LOC108351774	Transcription
DMR8:115690001	8	115690001	1000	1	1.11E-05	-1.5180889	6	0.6	Dock3	Transcription
DMR8:130987001	8	130987001	1000	1	4.81E-05	0.9058536	6	0.6	Abhd5	
DMR9:18730001	9	18730001	1000	1	2.58E-05	0.6425946	16	1.6	Runx2	Transcription
DMR9:110732001	9	110732001	2000	1	2.35E-05	0.9068017	25	1.25	Fbxl17	Metabolism
DMR10:4105001	10	4105001	1000	1	6.95E-05	-1.0256769	10	1	Snx29;LOC102554761	Cytoskeleton
DMR10:4257001	10	4257001	1000	1	1.94E-06	0.9627367	16	1.6	Snx29;Tnfrsf17	Cytoskeleton;Receptor
DMR10:16700001	10	16700001	1000	1	2.04E-06	-1.1773133	13	1.3	Crebrf	
DMR10:19424001	10	19424001	1000	1	9.03E-05	1.0795031	14	1.4	Dock2	
DMR10:39006001	10	39006001	1000	1	1.68E-05	0.8920687	14	1.4	Rad50	
DMR10:39615001	10	39615001	1000	1	6.33E-05	0.9165452	12	1.2	Il3	
DMR10:70223001	10	70223001	1000	1	5.19E-05	-1.4071759	4	0.4	Rffl;Rad51d	Proteolysis;Transcription
DMR10:70413001	10	70413001	1000	1	3.87E-05	0.8509195	12	1.2	Slfn4	
DMR11:32766001	11	32766001	2000	1	7.80E-06	1.0030439	34	1.7	Runx1	Transcription
DMR11:46372001	11	46372001	2000	1	2.90E-05	0.6774885	15	0.75	Abi3bp	
DMR11:65348001	11	65348001	3000	1	1.90E-05	-1.3458588	29	0.966666667	Gpr156	Signaling
DMR11:77474001	11	77474001	1000	1	5.89E-05	0.7357429	15	1.5	Il1rap	Receptor
DMR12:19223001	12	19223001	1000	1	4.75E-05	-1.2724393	4	0.4	LOC103691085;Zkscan1	Transcription
DMR12:25617001	12	25617001	2000	1	3.04E-06	-0.7959906	35	1.75	Gatsl2	
DMR12:29272001	12	29272001	1000	1	2.81E-05	-1.2999287	5	0.5	Wbscr17	
DMR12:36280001	12	36280001	1000	1	2.74E-05	-1.2900828	6	0.6	Tmem132b	
DMR13:43971001	13	43971001	1000	1	2.56E-05	-1.2583088	16	1.6	Mgat5	Golgi
DMR13:46907001	13	46907001	1000	1	7.91E-05	-1.0424288	7	0.7	Thsd7b	Cytoskeleton
DMR13:50567001	13	50567001	1000	1	2.04E-06	-0.9083485	10	1	Plekha6	
DMR13:67847001	13	67847001	1000	1	1.44E-05	-1.655772	6	0.6	Hmcn1	
DMR13:70678001	13	70678001	1000	1	6.10E-05	0.7140943	6	0.6	Lamc1	Extracellular Matrix
DMR13:80352001	13	80352001	2000	1	1.10E-05	-1.0132854	29	1.45	Dnm3	Transport
DMR13:84620001	13	84620001	2000	1	5.58E-05	0.9378591	13	0.65	Pogk	Epigenetic
DMR13:96937001	13	96937001	2000	1	7.79E-05	-0.6274298	33	1.65	Kif26b;LOC102553287	Cytoskeleton
DMR13:97913001	13	97913001	2000	1	2.34E-06	1.0543574	33	1.65	Cnst;LOC108352581	
DMR13:104155001	13	104155001	1000	1	4.88E-05	1.1785554	27	2.7	LOC108352591;LOC103693693;RGD1561704	
DMR14:9402001	14	9402001	2000	1	1.35E-05	0.6847818	46	2.3	Cds1	Transport
DMR14:30323001	14	30323001	2000	1	2.12E-05	0.896648	21	1.05	LOC102547286;Mir193b	
DMR14:39764001	14	39764001	1000	1	7.63E-05	-1.2512733	4	0.4	Gabra2	Ion Channel
DMR14:54795001	14	54795001	1000	1	8.30E-05	-0.8735223	4	0.4	Pcdh7	Cytoskeleton
DMR14:81195001	14	81195001	1000	1	7.34E-05	0.8145542	13	1.3	Htt	
DMR15:854001	15	854001	2000	1	1.63E-05	1.0430129	18	0.9	Kcnma1	Transport
DMR15:16462001	15	16462001	1000	1	5.97E-05	0.6205196	6	0.6	Fhit	Signaling
DMR15:36856001	15	36856001	1000	1	3.66E-06	0.9970962	9	0.9	Parp4	
DMR15:45553001	15	45553001	1000	1	9.42E-05	0.8575624	23	2.3	Gucy1b2;LOC102554746	Signaling
DMR15:51247001	15	51247001	1000	1	8.32E-05	-0.9066323	15	1.5	Entpd4	Signaling
DMR16:18666001	16	18666001	1000	1	2.17E-05	-0.8765654	12	1.2	Dydc1	
DMR16:81964001	16	81964001	3000	1	1.01E-06	-0.9989191	50	1.666666667	Mcf2l	Transcription
DMR16:84870001	16	84870001	2000	1	4.41E-06	0.9257533	25	1.25	Myo16	

DMR17:1638001	17	1638001	2000	1	5.10E-05	0.9427902	24	1.2	Slc35d2	Transport
DMR17:25116001	17	25116001	1000	1	8.92E-05	-0.9210783	6	0.6	Ofcc1	
DMR17:33617001	17	33617001	2000	1	4.21E-05	0.8088542	21	1.05	Gmds	Metabolism
DMR17:44693001	17	44693001	1000	1	1.06E-05	-1.3921242	1	0.1	Trnak-uuu;Trnam-cau;Trnai-uau	
DMR17:56981001	17	56981001	1000	1	4.42E-05	0.6726882	11	1.1	Cul2	Proteolysis
DMR17:63717001	17	63717001	1000	1	5.28E-05	-1.5193084	4	0.4	Dip2c	
DMR18:17769001	18	17769001	1000	1	7.84E-05	-0.8698488	19	1.9	Celf4	
DMR18:37104001	18	37104001	1000	1	6.60E-05	-0.8595384	4	0.4	Ppp2r2b	Signaling
DMR18:39352001	18	39352001	1000	1	8.79E-06	0.9783874	9	0.9	Kcnn2	Transport
DMR18:44608001	18	44608001	1000	1	9.77E-05	-0.741983	14	1.4	Dmxl1	
DMR18:86604001	18	86604001	1000	1	9.90E-06	1.0365602	7	0.7	Dok6	
DMR19:33304001	19	33304001	1000	1	1.44E-05	-0.8057259	8	0.8	Ttc29	
DMR19:52918001	19	52918001	1000	1	8.75E-06	-1.5983857	10	1	RGD1304884	
DMR19:54958001	19	54958001	1000	1	3.53E-05	-0.8609873	15	1.5	Zfp469	
DMR20:5467001	20	5467001	1000	1	6.51E-05	-1.5414968	27	2.7	Pfdn6;Rgl2;Tapbp;Zbtb22	Transcription;Immune;Cytoskeleton
DMR20:6394001	20	6394001	1000	1	3.77E-05	0.7320208	25	2.5	Rab44	
DMR20:16135001	20	16135001	1000	1	6.84E-05	0.9857205	7	0.7	NEWGENE_1590969	
DMRX:124201001	X	124201001	1000	1	4.25E-06	1.2952713	11	1.1	Rhox10	Development
