# Supplementary material for: Environmental induced transgenerational inheritance impacts systems epigenetics in disease etiology
Source: Sci Rep. 2022 Apr 19;12:5452. doi: 10.1038/s41598-022-09336-0 (PMC9018793; doi:10.1038/s41598-022-09336-0)
Supplement: Supplementary file 27 — Supplementary Table S19. [file 41598_2022_9336_MOESM27_ESM.pdf]

Supplemental Table S19

Control Disease Specific DMR List Obesity p<1e-04

DMR Name	Chr	start	Length	# Sig Win	minP	maxLFC	CpG #	CpG Density	Gene Annotation	Gene Category
DMR1:47092001	1	47092001	1000	1	6.77E-05	-0.6705156	13	1.3	Tulp4	
DMR1:185926001	1	185926001	1000	1	8.79E-06	-0.5973816	6	0.6	Sox6	
DMR1:192990001	1	192990001	1000	1	6.38E-05	0.7619292	24	2.4	Tnrc6a	Metabolism
DMR2:54285001	2	54285001	1000	1	5.60E-06	0.796869	4	0.4	Plcxd3	
DMR2:140845001	2	140845001	2000	1	4.45E-06	0.8898087	13	0.65	Maml3;Dusp14l1	
DMR2:173810001	2	173810001	1000	1	7.72E-05	0.6982523	4	0.4	Wdr49	
DMR2:204588001	2	204588001	1000	1	7.70E-05	0.5695057	9	0.9	Vangl1	
DMR2:238607001	2	238607001	1000	1	4.48E-07	-0.6907805	9	0.9	Ppa2	Signaling
DMR2:259705001	2	259705001	1000	1	3.70E-05	0.6870502	9	0.9	St6galnac3	
DMR3:154984001	3	154984001	1000	1	9.40E-05	-0.8248921	13	1.3	Ralgapb;LOC100911217	
DMR3:159350001	3	159350001	1000	1	6.22E-05	-0.8302046	17	1.7	L3mbtl1	Epigenetic
DMR3:161963001	3	161963001	1000	1	3.08E-05	-0.8536292	15	1.5	Cdh22;LOC108350574;LOC102555457	Cytoskeleton
DMR5:1455001	5	1455001	1000	1	8.91E-05	0.7974307	5	0.5	Jph1	
DMR5:34737001	5	34737001	2000	1	4.62E-05	0.6615696	15	0.75	Nkain3	
DMR5:69223001	5	69223001	1000	1	6.31E-05	0.8081726	7	0.7	Olr844	Receptor
DMR5:148283001	5	148283001	1000	1	7.58E-05	-0.6924392	24	2.4	Col16a1	Extracellular Matrix
DMR5:157329001	5	157329001	1000	1	9.43E-05	-0.581789	11	1.1	Pla2g2e	
DMR5:166596001	5	166596001	1000	1	5.57E-05	-0.7484603	29	2.9	Clstn1;Pik3cd	Transport;Signaling
DMR5:168840001	5	168840001	1000	1	1.97E-05	-0.8165504	7	0.7	Camta1;LOC102546752	Transcription
DMR6:7595001	6	7595001	1000	1	4.31E-05	0.8190873	6	0.6	Thada	Cytoskeleton
DMR6:28774001	6	28774001	2000	1	3.20E-05	0.8433196	17	0.85	Ncoa1	Epigenetic
DMR6:124373001	6	124373001	1000	1	5.98E-05	0.6781132	4	0.4	Ttc7b	
DMR6:132385001	6	132385001	1000	1	7.34E-05	-0.7375671	27	2.7	Eml1	
DMR7:130475001	7	130475001	3000	1	4.99E-05	-0.7492488	56	1.866666667	Shank3	
DMR9:20943001	9	20943001	1000	1	7.01E-05	-0.5074172	10	1	Adgrf2;Adgrf4	
DMR9:95452001	9	95452001	1000	1	2.47E-05	0.7943162	12	1.2	Trpm8	Transport
DMR9:113077001	9	113077001	1000	1	5.45E-05	-0.6915921	12	1.2	Tmem232	
DMR11:53214001	11	53214001	2000	1	6.41E-05	-0.5917816	29	1.45	Bbx	Transcription
DMR11:83170001	11	83170001	1000	1	7.24E-05	-0.5933829	13	1.3	Vps8	Cytoskeleton
DMR11:86638001	11	86638001	1000	1	8.56E-05	-0.5235692	17	1.7	Gnb1l	Cytoskeleton
DMR13:51808001	13	51808001	1000	1	6.15E-05	-0.6015422	11	1.1	Ube2t;Lgr6	Proteolysis
DMR14:23009001	14	23009001	1000	1	7.67E-05	0.5914459	7	0.7	Ythdc1;LOC108352729	
DMR15:109341001	15	109341001	1000	1	7.51E-05	0.8156664	9	0.9	Ggact;Tmtc4	
DMR16:21360001	16	21360001	1000	1	6.36E-05	0.5908184	16	1.6	Gmip;Atp13a1	
DMR17:43221001	17	43221001	2000	1	7.06E-06	-0.5731665	16	0.8	Carmil1	
DMR18:31587001	18	31587001	1000	1	2.91E-06	0.8010367	2	0.2	Ndfip1;LOC108348773	
DMR18:35517001	18	35517001	1000	1	1.60E-05	-0.9452615	11	1.1	Dcp2	
DMR19:11228001	19	11228001	1000	1	8.54E-06	0.9955328	9	0.9	Nup93	Transport
