# Supplementary material for: Environmental induced transgenerational inheritance impacts systems epigenetics in disease etiology
Source: Sci Rep. 2022 Apr 19;12:5452. doi: 10.1038/s41598-022-09336-0 (PMC9018793; doi:10.1038/s41598-022-09336-0)
Supplement: Supplementary file 28 — Supplementary Table S20. [file 41598_2022_9336_MOESM28_ESM.pdf]

Supplemental Table S20

Control Disease Specific DMR List Multiple Disease p<1e-04

DMR Name	Chr	start	Length	# Sig Win	minP	maxLFC	CpG #	CpG Density	Gene Annotation	Gene Category
DMR1:4794001	1	4794001	1000	1	2.29E-05	0.8321222	5	0.5	Grm1	Signaling
DMR1:22632001	1	22632001	2000	1	4.71E-05	0.7893283	25	1.25	Vnn1	Metabolism
DMR1:25724001	1	25724001	3000	1	4.78E-05	0.6452931	17	0.566666667	Trdn	
DMR1:37325001	1	37325001	6000	1	1.27E-05	0.7417191	43	0.716666667	Adcy2	
DMR1:37466001	1	37466001	2000	1	3.59E-05	0.72453	9	0.45	Adcy2	
DMR1:37552001	1	37552001	4000	1	7.50E-05	0.4090331	17	0.425	Adcy2;LOC100362709	
DMR1:40548001	1	40548001	1000	1	5.29E-05	-0.4102053	10	1	Mthfd1l	
DMR1:41379001	1	41379001	1000	1	9.25E-05	0.8582724	6	0.6	Esr1	
DMR1:60285001	1	60285001	3000	1	8.80E-05	0.6762065	8	0.266666667	Vom1r10	Receptor
DMR1:64331001	1	64331001	1000	1	1.54E-05	-0.5784422	9	0.9	Cacng6;Cacng8;Mir935	Transport
DMR1:78686001	1	78686001	1000	1	7.65E-05	0.6690524	45	4.5	Ap2s1;LOC679748	Transport
DMR1:80046001	1	80046001	1000	1	5.19E-05	0.5953872	9	0.9	Fbxo46;Qpctl	Transport
DMR1:82335001	1	82335001	1000	1	8.41E-05	-0.4589535	15	1.5	LOC102549342;Ceacam1	
DMR1:87260001	1	87260001	1000	1	3.09E-05	0.6372939	12	1.2	Dpf1;Sipa1l3	Epigenetic;Signaling
DMR1:91796001	1	91796001	1000	1	2.23E-05	0.7305144	4	0.4	Tdrd12	Cytoskeleton
DMR1:105592001	1	105592001	1000	1	5.95E-05	-0.4389166	16	1.6	Nell1	Signaling
DMR1:113218001	1	113218001	1000	1	3.60E-05	0.7983014	8	0.8	Gabrb3	Ion Channel
DMR1:129233001	1	129233001	1000	1	5.41E-05	-0.5589916	10	1	Pgpep1l	Protease
DMR1:141506001	1	141506001	1000	1	6.60E-05	0.5433529	5	0.5	Wdr93;Snrpep2	Metabolism
DMR1:147933001	1	147933001	1000	1	9.18E-05	0.7372606	5	0.5	Cyp2c7	Metabolism
DMR1:165421001	1	165421001	1000	1	4.60E-05	-0.4466356	19	1.9	C2cd3	
DMR1:166450001	1	166450001	1000	1	6.88E-05	0.7302119	14	1.4	Stard10;Arap1	Signaling
DMR1:168511001	1	168511001	1000	1	5.02E-05	-0.5419899	9	0.9	Olr97;Olr98	Receptor
DMR1:169218001	1	169218001	2000	1	5.22E-06	-0.4027623	24	1.2	Olr140;Olr141;Olr142	Receptor
DMR1:198124001	1	198124001	1000	1	9.73E-05	0.5857809	35	3.5	Slx1b;RGD1565363;Coro1a	Transcription;Cytoskeleton
DMR1:211204001	1	211204001	1000	1	9.78E-05	-0.5365102	14	1.4	Mapk1ip1;Ppp2r2d	Signaling
DMR1:211461001	1	211461001	1000	1	4.01E-06	0.794344	9	0.9	Stk32c	Signaling
DMR1:214129001	1	214129001	1000	1	8.87E-06	0.7766056	8	0.8	Ptdss2	Transport
DMR1:215704001	1	215704001	1000	1	3.63E-05	0.6822935	15	1.5	Mrpl23	Translation
DMR1:220164001	1	220164001	1000	1	4.77E-05	0.684664	13	1.3	Bbs1;Dpp3	Protease
DMR1:227648001	1	227648001	1000	1	2.93E-05	0.8304276	4	0.4	Ms4a4c	Transport
DMR1:238403001	1	238403001	1000	1	2.75E-05	0.9374462	21	2.1	Tmc1	
DMR1:238443001	1	238443001	1000	1	7.03E-05	0.7591778	2	0.2	Tmc1	
DMR1:254615001	1	254615001	2000	1	3.66E-05	0.6215998	7	0.35	Htr7;LOC108349452	Signaling
DMR1:256032001	1	256032001	1000	1	1.13E-05	0.7181824	8	0.8	Kif11	Cytoskeleton
DMR1:256683001	1	256683001	1000	1	9.04E-06	-0.4877304	8	0.8	Myof	Transport
DMR1:270382001	1	270382001	1000	1	4.04E-05	0.6304558	6	0.6	Sorcs1	Transport
DMR2:8969001	2	8969001	2000	1	1.05E-05	-0.6972448	12	0.6	Adgrv1	Signaling
DMR2:20092001	2	20092001	6000	1	6.40E-05	0.5820561	81	1.35	Atg10	Proteolysis
DMR2:27106001	2	27106001	1000	1	3.08E-05	0.5518589	17	1.7	Ankdd1b;Arhgef26	Transcription
DMR2:56533001	2	56533001	1000	1	9.26E-06	0.5714792	13	1.3	Egflam;LOC108349957	Extracellular Matrix
DMR2:66936001	2	66936001	1000	1	6.33E-05	-0.8757172	5	0.5	Cdh9	Cytoskeleton
DMR2:92569001	2	92569001	1000	1	3.66E-05	0.7852594	1	0.1	tGap1;LOC102553273;LOC100363165	Signaling
DMR2:105086001	2	105086001	2000	1	7.27E-05	-0.4393141	14	0.7	Cpb1	Protease
DMR2:112331001	2	112331001	2000	1	5.87E-05	0.8578393	16	0.8	Spata16	
DMR2:116658001	2	116658001	1000	1	1.78E-06	0.8742525	7	0.7	Egfem1	
DMR2:117413001	2	117413001	1000	1	7.11E-06	0.8749458	26	2.6	Mecom	Transcription
DMR2:157506001	2	157506001	1000	1	5.90E-05	0.5235314	12	1.2	Lekr1	
DMR2:164299001	2	164299001	1000	1	5.67E-06	0.6885532	4	0.4	Rsrc1	

DMR2:185960001	2	185960001	1000	1	2.97E-05	0.5679984	8	0.8	Lrba	
DMR2:191294001	2	191294001	2000	1	1.08E-05	0.7445041	6	0.3	RGD1562234	
DMR2:198776001	2	198776001	1000	1	1.54E-05	-0.5160305	15	1.5	Pex11b;Itga10	Extracellular Matrix
DMR2:206297001	2	206297001	1000	1	2.38E-05	0.7874272	5	0.5	Dclre1b;Ap4b1;Bcl2l15	Transcription;Transport
DMR2:208018001	2	208018001	2000	1	8.04E-05	-0.4538176	21	1.05	Kcnd3	Transport
DMR2:210891001	2	210891001	1000	1	2.98E-05	-0.5670618	17	1.7	Gnat2;Gnai3	Signaling
DMR2:212420001	2	212420001	1000	1	5.77E-05	-0.4416258	11	1.1	Vav3	
DMR2:216369001	2	216369001	1000	1	1.96E-05	0.4840825	4	0.4	Amy2a3	
DMR2:220472001	2	220472001	1000	1	6.35E-05	0.829696	21	2.1	Frrs1;Palmd	Metabolism
DMR2:259914001	2	259914001	1000	1	1.02E-05	0.7606793	33	3.3	St6galnac3;LOC103691740	
DMR3:2203001	3	2203001	1000	1	9.21E-05	-0.667932	6	0.6	Pnpla7	Metabolism
DMR3:25596001	3	25596001	1000	1	3.71E-05	-0.4517498	10	1	Lrp1b	
DMR3:27172001	3	27172001	1000	1	6.92E-05	0.7197664	3	0.3	Lrp1b	
DMR3:35158001	3	35158001	1000	1	5.99E-05	-0.7425338	13	1.3	Kif5c	
DMR3:44144001	3	44144001	1000	1	8.25E-05	0.6780003	9	0.9	Cytip	
DMR3:44298001	3	44298001	1000	1	5.87E-05	0.5676765	20	2	Acvr1c	Signaling
DMR3:44507001	3	44507001	1000	1	2.73E-05	-0.5703598	10	1	Acvr1	Signaling
DMR3:53842001	3	53842001	1000	1	8.76E-05	-0.4520487	16	1.6	B3galt1	Golgi
DMR3:55916001	3	55916001	1000	1	2.52E-05	0.7981129	10	1	Bbs5;Klhl41;Fastkd1	Cytoskeleton
DMR3:61806001	3	61806001	1000	1	2.64E-05	0.9119824	5	0.5	Mtx2	
DMR3:73369001	3	73369001	1000	1	9.34E-05	0.6590103	7	0.7	Olr473	Receptor
DMR3:103192001	3	103192001	2000	1	2.92E-05	0.7791043	32	1.6	LOC100363452;Olr783	Translation;Receptor
DMR3:103985001	3	103985001	2000	1	8.52E-05	0.7534972	24	1.2	Chrm5;Aven	Signaling
DMR3:113198001	3	113198001	1000	1	8.81E-05	-0.5131495	12	1.2	Tp53bp1	Transcription
DMR3:114241001	3	114241001	2000	1	9.66E-05	-0.5119763	26	1.3	Duox2;Duoxa2;Duoxa1;Duox1	Metabolism
DMR3:118194001	3	118194001	2000	1	2.36E-05	-0.6471189	21	1.05	Galk2	Metabolism
DMR3:118287001	3	118287001	2000	1	8.13E-05	-0.4427833	31	1.55	Fam227b	
DMR3:125434001	3	125434001	1000	1	1.45E-05	-0.5083482	7	0.7	Chgb	
DMR3:128424001	3	128424001	1000	1	2.81E-05	0.7596704	5	0.5	Plcb1	Metabolism
DMR3:136632001	3	136632001	1000	1	4.16E-05	0.8357859	5	0.5	Kif16b	Cytoskeleton
DMR3:151376001	3	151376001	2000	1	9.97E-05	-0.4535071	34	1.7	Fam83c;Uqcc1	Transcription
DMR3:175351001	3	175351001	1000	1	5.52E-05	-0.5712951	13	1.3	Taf4	Transcription
DMR4:6213001	4	6213001	1000	1	7.84E-06	0.9045318	4	0.4	Kmt2c	
DMR4:7382001	4	7382001	1000	1	8.75E-05	0.5418838	29	2.9	Kcnh2	Transport
DMR4:10917001	4	10917001	1000	1	2.56E-06	-0.6488804	9	0.9	Phtf2	Development
DMR4:23888001	4	23888001	1000	1	5.20E-05	0.717274	4	0.4	Zfp804b	
DMR4:67782001	4	67782001	2000	1	9.48E-05	0.6188973	18	0.9	Tmem178b	
DMR4:75892001	4	75892001	1000	1	5.71E-05	-0.5959389	6	0.6	Cntnap2	
DMR4:118420001	4	118420001	3000	1	6.04E-05	-0.4319985	34	1.133333333	Asprv1	
DMR4:126057001	4	126057001	2000	1	1.56E-07	-0.6089826	34	1.7	Magi1	
DMR4:148353001	4	148353001	1000	1	8.36E-05	0.9260003	9	0.9	March8;LOC680441	
DMR4:152222001	4	152222001	1000	1	2.55E-05	0.6902249	10	1	Erc1	Transport
DMR4:152943001	4	152943001	1000	1	1.97E-05	0.7478482	3	0.3	Kdm5a	Epigenetic
DMR4:157371001	4	157371001	1000	1	2.24E-05	0.8270452	38	3.8	P3h3;Gpr162;Cd4	Extracellular Matrix;Signaling
DMR5:29581001	5	29581001	1000	1	8.78E-05	0.510626	11	1.1	Decr1	Metabolism
DMR5:50645001	5	50645001	1000	1	1.49E-05	-0.5174625	10	1	Mob3b;LOC102549230	Signaling
DMR5:68820001	5	68820001	1000	1	2.43E-06	0.7915333	18	1.8	Olr848	Receptor
DMR5:70650001	5	70650001	1000	1	6.64E-05	0.7135846	10	1	Tmem38b;LOC102547886	Transport
DMR5:76809001	5	76809001	2000	1	3.97E-05	0.6816549	13	0.65	Hsdl2	Metabolism
DMR5:93395001	5	93395001	1000	1	8.62E-06	0.7772635	7	0.7	Ptprd;LOC108351111	Signaling
DMR5:128507001	5	128507001	1000	1	3.97E-05	-0.4733821	12	1.2	Rab3b	
DMR5:131831001	5	131831001	1000	1	6.84E-05	0.7546465	12	1.2	Slc5a9	Transport
DMR5:136803001	5	136803001	1000	1	5.72E-05	-0.4101622	11	1.1	St3gal3	Transport

DMR5:139223001	5	139223001	1000	1	5.14E-05	-0.620054	28	2.8	Foxo6	
DMR5:145241001	5	145241001	1000	1	6.42E-05	-0.6314847	7	0.7	Dlgap3	Cytoskeleton
DMR5:154705001	5	154705001	1000	1	1.54E-05	0.7422456	11	1.1	Hnrnpr	Metabolism
DMR5:157521001	5	157521001	1000	1	1.33E-05	-0.6474673	14	1.4	Htr6;Nbl1	Signaling
DMR5:161720001	5	161720001	3000	1	1.69E-05	-0.5241704	38	1.266666667	Kazn;RGD1560231	
DMR5:168557001	5	168557001	1000	1	6.19E-05	0.6749609	37	3.7	Camta1;LOC102546564	Transcription
DMR6:1664001	6	1664001	1000	1	7.53E-05	0.7201184	19	1.9	Qpct	Transport
DMR6:4451001	6	4451001	1000	1	4.12E-06	-0.5161387	9	0.9	Slc8a1	Transport
DMR6:8056001	6	8056001	2000	1	8.92E-05	0.5718973	24	1.2	Lrpprc	
DMR6:9637001	6	9637001	1000	1	3.61E-05	-0.5293751	13	1.3	Prkce	Signaling
DMR6:23239001	6	23239001	1000	1	6.71E-05	-0.5889771	16	1.6	Clip4	Transcription
DMR6:24379001	6	24379001	1000	1	8.32E-05	0.6298056	11	1.1	Lclat1	Metabolism
DMR6:24898001	6	24898001	1000	1	5.15E-05	-0.4262389	7	0.7	Galnt14	Golgi
DMR6:27392001	6	27392001	1000	1	1.04E-05	-0.510819	13	1.3	Otof	Transport
DMR6:28303001	6	28303001	1000	1	8.01E-05	-0.8036023	16	1.6	Dnmt3a;LOC100911610	Epigenetic
DMR6:37091001	6	37091001	1000	1	2.78E-05	0.7078414	2	0.2	Vsnl1	
DMR6:42171001	6	42171001	1000	1	2.20E-05	-0.4296248	9	0.9	Rock2	Signaling
DMR6:43053001	6	43053001	1000	1	4.39E-05	-0.4515876	17	1.7	Hpcal1	
DMR6:48574001	6	48574001	1000	1	3.40E-05	0.7120572	5	0.5	Myt1l;LOC103692607	Transcription
DMR6:48666001	6	48666001	1000	1	8.07E-05	-0.507078	6	0.6	Myt1l;LOC108351218	Transcription
DMR6:73619001	6	73619001	1000	1	8.00E-05	0.7375251	27	2.7	Akap6	
DMR6:94909001	6	94909001	2000	1	7.62E-05	0.6534184	1	0.05	Ccdc175	
DMR6:97195001	6	97195001	4000	1	4.60E-05	0.7195394	49	1.225	Syt16;LOC108351259	
DMR6:100009001	6	100009001	1000	1	1.20E-05	-0.4528709	12	1.2	Max	Transcription
DMR6:106469001	6	106469001	1000	1	1.92E-05	-0.5581839	12	1.2	Rgs6;LOC100912402;LOC108351269	
DMR6:115411001	6	115411001	1000	1	4.03E-05	-0.4701396	12	1.2	Ston2	Transport
DMR6:127678001	6	127678001	2000	1	3.50E-05	-0.4125806	21	1.05	Serpina9	Protease; Proteolysis
DMR6:131844001	6	131844001	1000	1	9.98E-05	-0.5120348	26	2.6	Bcl11b	Transcription
DMR7:3817001	7	3817001	1000	1	6.08E-05	-0.5112376	25	2.5	Olr882-ps	
DMR7:5626001	7	5626001	2000	1	2.57E-05	-0.6367885	34	1.7	Olr1845	
DMR7:11523001	7	11523001	3000	1	1.66E-05	-0.4612555	43	1.433333333	Thop1;Sgta	Protease
DMR7:24142001	7	24142001	1000	1	9.96E-05	0.6365111	23	2.3	Btbd11	Cytoskeleton
DMR7:49610001	7	49610001	1000	1	3.49E-05	-0.8349975	8	0.8	Lin7a	Cytoskeleton
DMR7:87936001	7	87936001	2000	1	7.76E-05	0.585535	42	2.1	Csmd3	
DMR7:90324001	7	90324001	1000	1	4.21E-05	0.7989499	24	2.4	Trps1	Transcription
DMR7:113065001	7	113065001	1000	1	3.26E-05	0.7244519	7	0.7	Col22a1	Extracellular Matrix
DMR7:114836001	7	114836001	1000	1	2.07E-05	0.6888036	3	0.3	Slc45a4	Transport
DMR7:122576001	7	122576001	1000	1	5.44E-05	0.7010512	11	1.1	Slc25a17;St13	
DMR7:127206001	7	127206001	1000	1	2.19E-06	-0.5723411	12	1.2	Tbc1d22a	Signaling
DMR7:137250001	7	137250001	1000	1	9.70E-05	-0.5881601	10	1	Ano6	
DMR8:19723001	8	19723001	2000	1	1.26E-05	0.8200296	12	0.6	Olr1153-ps	
DMR8:22384001	8	22384001	1000	1	4.66E-06	0.7077448	32	3.2	Slc44a2	Transport
DMR8:22922001	8	22922001	2000	1	4.94E-06	-0.7189269	18	0.9	Rab3d;Tmem205	
DMR8:25741001	8	25741001	1000	1	8.44E-06	0.8586909	3	0.3	Dpy19l2	
DMR8:55133001	8	55133001	1000	1	3.28E-05	-0.4439904	9	0.9	Dixdc1	Cytoskeleton
DMR8:59563001	8	59563001	1000	1	4.47E-05	0.6039516	10	1	Chrna5	Ion Channel
DMR8:71836001	8	71836001	1000	1	8.64E-06	-0.5414252	12	1.2	Dapk2	Signaling
DMR8:72509001	8	72509001	1000	1	8.76E-05	0.6476604	5	0.5	Aph1b	Protease
DMR8:103678001	8	103678001	1000	1	3.93E-05	0.7690014	9	0.9	Atr	Signaling
DMR8:105973001	8	105973001	1000	1	8.26E-05	0.3908693	8	0.8	Clstn2	Transport
DMR8:115932001	8	115932001	1000	1	5.92E-05	0.6662783	8	0.8	Dock3;LOC102550698	Transcription
DMR8:116499001	8	116499001	1000	1	6.40E-05	0.9266104	12	1.2	Rbm5	
DMR8:116806001	8	116806001	2000	1	3.88E-05	0.5898284	18	0.9	Ip6k1	Signaling
DMR8:116980001	8	116980001	1000	1	1.81E-05	0.6444469	7	0.7	Dag1	Cytoskeleton

DMR8:117617001	8	117617001	1000	1	9.83E-05	-0.4572397	15	1.5	Nckipsd;Celsr3	Cytoskeleton;Cytoskeleton
DMR8:117767001	8	117767001	1000	1	9.03E-05	-0.4504239	22	2.2	Pfkfb4;LOC100911077	Metabolism
DMR8:118157001	8	118157001	2000	1	4.99E-05	-0.5282389	27	1.35	Map4;Dhx30	Transcription
DMR8:118999001	8	118999001	1000	1	8.44E-05	-0.4723915	20	2	Pth1r	Receptor
DMR8:124882001	8	124882001	2000	1	4.33E-05	0.7375086	17	0.85	Rbms3	
DMR8:130275001	8	130275001	1000	1	5.27E-05	-0.6059201	11	1.1	Vipr1	Receptor
DMR8:132664001	8	132664001	1000	1	1.00E-05	0.8576849	12	1.2	Sacm1l;RGD1566368	Signaling
DMR9:14669001	9	14669001	2000	1	5.97E-05	-0.4576098	13	0.65	Treml2;Treml4	
DMR9:15617001	9	15617001	1000	1	5.05E-05	0.7487699	24	2.4	Guca1a;Guca1b	
DMR9:27067001	9	27067001	1000	1	2.12E-06	0.9038938	15	1.5	LOC102549795;Efhc1	Signaling
DMR9:61542001	9	61542001	1000	1	8.59E-05	0.5731267	5	0.5	Tmem258b	
DMR9:88140001	9	88140001	4000	1	4.77E-05	-0.8093889	23	0.575	Rhbdd1;LOC108351962	Protease
DMR9:93146001	9	93146001	2000	1	7.85E-05	0.4829176	22	1.1	Psmd1	Protease
DMR9:95386001	9	95386001	1000	1	7.35E-05	-0.6436119	9	0.9	LOC680352;Trpm8	Transport
DMR9:110087001	9	110087001	1000	1	3.91E-05	-0.6278456	14	1.4	Efna5	Signaling
DMR10:1424001	10	1424001	1000	1	3.01E-05	0.6582385	9	0.9	Parn	Translation
DMR10:5744001	10	5744001	2000	1	2.98E-05	-0.544027	17	0.85	Grin2a	Receptor
DMR10:5914001	10	5914001	2000	1	3.63E-05	-0.5433186	20	1	Grin2a	Receptor
DMR10:20806001	10	20806001	2000	1	1.37E-05	-0.6333812	42	2.1	Wwc1	
DMR10:31172001	10	31172001	1000	1	4.20E-06	-0.5593429	10	1	Adam19	Protease
DMR10:40542001	10	40542001	1000	1	4.17E-06	-0.4521971	7	0.7	Slc36a1	Transport
DMR10:45723001	10	45723001	3000	1	7.94E-05	0.617807	40	1.333333333	Prss38;LOC102546363	Protease
DMR10:46375001	10	46375001	1000	1	3.02E-05	-0.5235923	13	1.3	Pemt	Epigenetic
DMR10:56063001	10	56063001	1000	1	4.13E-05	-0.4383434	11	1.1	Dnah2	Cytoskeleton
DMR10:57253001	10	57253001	2000	1	9.17E-05	0.693944	16	0.8	Chrne;Rpl36a-ps2;Gp1ba	Ion Channel;Receptor
DMR10:68249001	10	68249001	1000	1	1.35E-05	0.6883847	7	0.7	Asic2	Transport
DMR10:72482001	10	72482001	1000	1	1.28E-05	0.7410202	5	0.5	Usp32;RGD1310166	Protease
DMR10:83835001	10	83835001	1000	1	9.07E-06	-0.5248265	16	1.6	LOC102550169;Gip	
DMR10:92311001	10	92311001	2000	1	4.41E-05	-0.5730629	21	1.05	Mapt;LOC100912629	
DMR10:94772001	10	94772001	2000	1	2.17E-05	0.667	19	0.95	Tex2	
DMR10:98717001	10	98717001	1000	1	8.13E-06	0.8030074	35	3.5	Map2k6	Signaling
DMR10:105931001	10	105931001	1000	1	8.18E-05	-0.6160539	17	1.7	Mgat5b	Golgi
DMR11:9786001	11	9786001	1000	1	7.61E-05	0.6072892	30	3	Robo1	
DMR11:11267001	11	11267001	1000	1	1.16E-05	0.5147006	8	0.8	Robo2	
DMR11:40503001	11	40503001	1000	1	2.29E-05	-0.6525725	10	1	RGD1559516	
DMR11:54898001	11	54898001	1000	1	5.97E-05	0.7722158	10	1	Morc1	
DMR11:59443001	11	59443001	1000	1	3.22E-05	0.6777652	10	1	Lsamp	Immune
DMR11:64597001	11	64597001	1000	1	9.72E-05	0.8005265	13	1.3	Arhgap31	Signaling
DMR11:70283001	11	70283001	1000	1	1.40E-05	-0.5800666	17	1.7	Heg1	
DMR11:79267001	11	79267001	2000	1	5.85E-05	0.5675329	22	1.1	Lpp	Signaling
DMR11:85277001	11	85277001	3000	1	8.35E-05	-0.4140686	32	1.066666667	Olr1568	
DMR12:8349001	12	8349001	1000	1	3.57E-07	0.9842046	24	2.4	Mtus2	
DMR12:10586001	12	10586001	1000	1	3.20E-05	0.4576596	10	1	Rnf6	
DMR12:28694001	12	28694001	2000	1	2.07E-05	-0.6404385	16	0.8	Wbscr17	
DMR12:43948001	12	43948001	2000	1	1.00E-05	0.5894592	50	2.5	RGD1562310;Rnft2	
DMR12:48087001	12	48087001	1000	1	7.76E-05	0.760466	24	2.4	Myo1h	
DMR12:51788001	12	51788001	4000	1	3.91E-05	-0.4537759	44	1.1	Ttc28	Cytoskeleton
DMR13:25814001	13	25814001	2000	1	5.49E-05	-0.4987873	35	1.75	Tnfrsf11a	Receptor
DMR13:26917001	13	26917001	1000	1	7.68E-05	0.701531	5	0.5	Serpinb5	Protease; Proteolysis
DMR13:32371001	13	32371001	1000	1	1.52E-06	0.8436015	5	0.5	Cdh19	Cytoskeleton
DMR13:47411001	13	47411001	1000	1	7.32E-05	-0.5742908	21	2.1	Pfkfb2	Metabolism
DMR13:85225001	13	85225001	1000	1	1.93E-06	-0.4354695	20	2	Fam78b	
DMR13:89001001	13	89001001	1000	1	3.61E-05	-0.4635353	16	1.6	Olfml2b	Development
DMR13:98477001	13	98477001	2000	1	8.99E-05	-0.4491669	34	1.7	Adck3	
DMR13:107385001	13	107385001	2000	1	7.63E-05	0.5472605	32	1.6	Ush2a;LOC103692406	Extracellular Matrix

DMR13:107891001	13	107891001	1000	1	1.50E-06	-0.569634	4	0.4	Kcnk2	Transport
DMR14:8301001	14	8301001	1000	1	9.05E-05	-0.4683347	11	1.1	Mapk10	Signaling
DMR14:11562001	14	11562001	1000	1	3.81E-06	-0.5051718	10	1	Rasgef1b	Transcription
DMR14:14811001	14	14811001	1000	1	1.05E-05	-0.5520419	12	1.2	Fras1	
DMR14:16408001	14	16408001	2000	1	7.23E-06	-0.5849745	20	1	11-Sep	
DMR14:38644001	14	38644001	1000	1	2.30E-05	-0.4669825	12	1.2	Gabrb1	Ion Channel
DMR14:45331001	14	45331001	1000	1	7.76E-05	0.9943543	25	2.5	LOC103692863;Mir328b	
DMR14:45787001	14	45787001	1000	1	7.40E-06	-0.5079373	12	1.2	Tbc1d1	Signaling
DMR14:80151001	14	80151001	2000	1	4.29E-05	-0.3741372	27	1.35	Ablim2	Cytoskeleton
DMR14:86821001	14	86821001	1000	1	9.19E-05	-0.4333428	14	1.4	Ccm2;LOC108352791	
DMR14:99909001	14	99909001	1000	1	2.90E-05	-0.4813506	7	0.7	LOC108352804;Egfr	Receptor
DMR14:108159001	14	108159001	1000	1	3.53E-06	0.6141111	10	1	Usp34	Protease
DMR15:4053001	15	4053001	1000	1	9.84E-05	-0.5268169	9	0.9	Sec24c	Transport
DMR15:4811001	15	4811001	1000	1	8.94E-05	-0.5943212	19	1.9	Gng2	Signaling
DMR15:11305001	15	11305001	1000	1	1.60E-05	-0.5584456	6	0.6	Lrrc3b	Receptor
DMR15:28267001	15	28267001	2000	1	2.32E-05	0.7103886	19	0.95	Ear1	
DMR15:33047001	15	33047001	2000	1	3.33E-05	-0.5076111	28	1.4	Slc7a7	Transport
DMR15:35828001	15	35828001	1000	1	8.74E-05	0.4878	4	0.4	Olr1282-ps	
DMR15:40270001	15	40270001	1000	1	1.84E-05	0.6816511	8	0.8	Atp8a2	Transport
DMR15:45889001	15	45889001	2000	1	8.87E-05	-0.4874561	10	0.5	Ints6	Transcription
DMR15:58689001	15	58689001	1000	1	3.77E-05	-0.551727	17	1.7	Serp2	Transcription
DMR15:108747001	15	108747001	1000	1	5.52E-05	-0.656146	7	0.7	Clybl;LOC102555437	Metabolism
DMR16:16905001	16	16905001	1000	1	1.27E-05	0.7545601	5	0.5	Nrg3	Growth Factors
DMR16:77220001	16	77220001	1000	1	7.13E-05	0.713552	3	0.3	Csmd1	
DMR17:13706001	17	13706001	1000	1	3.14E-07	0.7724906	7	0.7	Shc3	Cytoskeleton
DMR17:33525001	17	33525001	1000	1	9.35E-05	0.5912981	11	1.1	Gmds	Metabolism
DMR17:70499001	17	70499001	1000	1	4.91E-05	0.6791659	10	1	Il2ra	Receptor
DMR17:78806001	17	78806001	1000	1	4.40E-05	-0.4932276	5	0.5	Dclre1c;LOC108353135;Meig1	Transcription
DMR17:87667001	17	87667001	1000	1	3.90E-06	-0.5356888	6	0.6	Etl4	
DMR17:89014001	17	89014001	1000	1	1.16E-06	1.1184314	25	2.5	Myo3a	
DMR18:15168001	18	15168001	1000	1	7.07E-05	0.7398884	33	3.3	Mapre2;Rnf138	Cytoskeleton;Proteolysis
DMR18:15924001	18	15924001	1000	1	1.49E-05	0.7012881	8	0.8	Zfp24	
DMR18:28199001	18	28199001	1000	1	8.49E-05	-0.5014114	4	0.4	Sil1	
DMR18:44822001	18	44822001	1000	1	7.87E-05	-0.4394688	12	1.2	Hsd17b4	
DMR18:74664001	18	74664001	1000	1	7.01E-06	-0.6977451	5	0.5	Slc14a2	Transport
DMR18:79570001	18	79570001	1000	1	5.01E-05	-0.495215	7	0.7	RGD1562171	
DMR18:84171001	18	84171001	1000	1	8.43E-06	-0.5685034	6	0.6	RGD1559726	Cytoskeleton
DMR19:681001	19	681001	2000	1	8.62E-05	0.9804419	56	2.8	Nae1;Terb1	Proteolysis
DMR19:19779001	19	19779001	1000	1	4.50E-06	-0.4822127	19	1.9	Papd5	
DMR19:30860001	19	30860001	2000	1	9.06E-05	-0.4668513	33	1.65	Gab1	Cytoskeleton
DMR19:53148001	19	53148001	1000	1	3.70E-05	0.7966136	31	3.1	RGD1304884	
DMR19:57866001	19	57866001	2000	1	6.32E-05	-0.4483516	30	1.5	Disc1	
DMR19:59217001	19	59217001	1000	1	6.00E-05	-0.437994	3	0.3	Slc35f3	
DMR20:6393001	20	6393001	1000	1	1.98E-05	-0.5286853	18	1.8	Rab44	
DMR20:14064001	20	14064001	2000	1	2.21E-05	-0.5706479	20	1	Lrrc75b	
DMR20:18455001	20	18455001	1000	1	7.26E-05	0.8445721	23	2.3	Ipmk	Signaling
DMR20:33490001	20	33490001	1000	1	2.37E-07	-0.6051189	10	1	Gopc	Transport
DMR20:35033001	20	35033001	1000	1	6.48E-05	-0.5487309	7	0.7	Fam184a	
DMR20:46135001	20	46135001	2000	1	7.93E-06	0.7161798	71	3.55	Ak9	Signaling
DMRX:11172001	X	11172001	1000	1	5.58E-05	-0.8068151	9	0.9	Atp6ap2	Receptor
DMRX:14481001	X	14481001	4000	1	9.62E-05	-0.4666906	42	1.05	Lancl3	
DMRX:111828001	X	111828001	1000	1	6.33E-05	0.6122087	11	1.1	Prps1	Signaling
