# Supplementary material for: Environmental induced transgenerational inheritance impacts systems epigenetics in disease etiology
Source: Sci Rep. 2022 Apr 19;12:5452. doi: 10.1038/s41598-022-09336-0 (PMC9018793; doi:10.1038/s41598-022-09336-0)
Supplement: Supplementary file 29 — Supplementary Table S21. [file 41598_2022_9336_MOESM29_ESM.pdf]

Supplemental Table S21

Exposure Correlated Kidney Disease DMR Associated Genes

**Control**	
RASSF10	Ras association domain family member 10
FOXO1	forkhead box O1
CYP2C8	cytochrome P450 family 2 subfamily C member 8
ERBB4	erb-b2 receptor tyrosine kinase 4
GRIN2A	glutamate ionotropic receptor NMDA type subunit 2A
**Dioxin**	
CCR1	C-C motif chemokine receptor 1
COQ8B	coenzyme Q8B
DDR1	discoidin domain receptor tyrosine kinase 1
SDCCAG8	SHH signaling and ciliogenesis regulator SDCCAG8
TCF7L2	transcription factor 7 like 2
**Plastics**	
ACTN1	actinin alpha 1
AOC3	amine oxidase copper containing 3
EGFR	epidermal growth factor receptor
IFT140	intraflagellar transport 140
ITGB3	integrin subunit beta 3
MIR26A1	microRNA 26a-1
NLRP1	NLR family pyrin domain containing 1
REL	REL proto-oncogene, NF-kB subunit
STK4	serine/threonine kinase 4
TRPC6	transient receptor potential cation channel subfamily C member 6
**Pesticides**	
SGK1	serum/glucocorticoid regulated kinase 1
TFF3	trefoil factor 3
IFT140	intraflagellar transport 140
NFATC2	nuclear factor of activated T cells 2
MAGI2	membrane associated guanylate kinase, WW and PDZ domain containing 2
NEDD4L	NEDD4 like E3 ubiquitin protein ligase
NOS1	nitric oxide synthase 1
NRG1	neuregulin 1
PREP	prolyl endopeptidase
BICC1	BicC family RNA binding protein 1
SLC15A2	solute carrier family 15 member 2
SDC4	syndecan 4
COL4A2	collagen type IV alpha 2 chain
GNB3	G protein subunit beta 3
CRIM1	cysteine rich transmembrane BMP regulator 1
BAK1	BCL2 antagonist/killer 1
PODXL	podocalyxin like

Glyphosate

LOXL2	lysyl oxidase like 2
RELA	RELA proto-oncogene, NF-kB subunit
ATG5	autophagy related 5
TNFAIP8	TNF alpha induced protein 8
CDH2	cadherin 2
VWF	von Willebrand factor
BCHE	butyrylcholinesterase

Methoxychlor

APP	amyloid beta precursor protein
RET	ret proto-oncogene
BBS10	Bardet-Biedl syndrome 10
GSK3B	glycogen synthase kinase 3 beta
FGF21	fibroblast growth factor 21
CYBB	cytochrome b-245 beta chain
NEDD4L	NEDD4 like E3 ubiquitin protein ligase
TNFSF13B	TNF superfamily member 13b
NOS1AP	nitric oxide synthase 1 adaptor protein
OAT	ornithine aminotransferase
SCARB2	scavenger receptor class B member 2

Atrazine

RAC1	Rac family small GTPase 1
SEMA3A	semaphorin 3A
AOX1	aldehyde oxidase 1
ZEB2	zinc finger E-box binding homeobox 2
NR1H4	nuclear receptor subfamily 1 group H member 4
KLF2	Kruppel like factor 2
HPD	4-hydroxyphenylpyruvate dioxygenase
MAPK8	mitogen-activated protein kinase 8

Jet Fuel

COQ8B	coenzyme Q8B
HLA-DQB1	major histocompatibility complex, class II, DQ beta 1
CD274	CD274 molecule
CNR2	cannabinoid receptor 2
TGFA	transforming growth factor alpha
CNDP2	carnosine dipeptidase 2
