# Supplementary material for: Environmental induced transgenerational inheritance impacts systems epigenetics in disease etiology
Source: Sci Rep. 2022 Apr 19;12:5452. doi: 10.1038/s41598-022-09336-0 (PMC9018793; doi:10.1038/s41598-022-09336-0)
Supplement: Supplementary file 30 — Supplementary Table S22. [file 41598_2022_9336_MOESM30_ESM.pdf]

Supplemental Table S22

Exposure Correlated Prostate Disease DMR Associated Genes

**Control**	
MAML3	mastermind like transcriptional coactivator 3
ABL1	ABL proto-oncogene 1, non-receptor tyrosine kinase
CD86	CD86 molecule
SIRT3	sirtuin 3
FOXP2	forkhead box P2
TSPYL1	TSPY like 1
HEATR5B	HEAT repeat containing 5B
APMAP	adipocyte plasma membrane associated protein
FAM110A	family with sequence similarity 110 member A
SLCO3A1	solute carrier organic anion transporter family member 3A1
EPHA6	EPH receptor A6
NFIB	nuclear factor I B
SMARCC1	SWI/SNF related, matrix associated, actin dependent regulator of chromatin subfamily c member 1
BID	BH3 interacting domain death agonist
SORT1	sortilin 1
DSG2	desmoglein 2
KPNA4	karyopherin subunit alpha 4
ALK	ALK receptor tyrosine kinase
PDE4B	phosphodiesterase 4B
PLD1	phospholipase D1
RPL37	ribosomal protein L37
TBXAS1	thromboxane A synthase 1
MEIS2	Meis homeobox 2
IQGAP2	IQ motif containing GTPase activating protein 2
GRN	granulin precursor
GLI3	GLI family zinc finger 3
UPRT	uracil phosphoribosyltransferase homolog
DAB2IP	DAB2 interacting protein
SOX5	SRY-box transcription factor 5
ZNF366	zinc finger protein 366
CBX2	chromobox 2
TGM4	transglutaminase 4
ROBO2	roundabout guidance receptor 2
ST6GAL1	ST6 beta-galactoside alpha-2,6-sialyltransferase 1
FGFR4	fibroblast growth factor receptor 4
SLIT1	slit guidance ligand 1
PODXL	podocalyxin like
ASAP1	ArfGAP with SH3 domain, ankyrin repeat and PH domain 1
ST3GAL3	ST3 beta-galactoside alpha-2,3-sialyltransferase 3
SPAG1	sperm associated antigen 1
ACE	angiotensin I converting enzyme
POU5F1	POU class 5 homeobox 1
CADM1	cell adhesion molecule 1
CCK	cholecystokinin

Dioxin

DLG4	discs large MAGUK scaffold protein 4
MITF	melanocyte inducing transcription factor
HFE	homeostatic iron regulator
PRKCZ	protein kinase C zeta
NFIC	nuclear factor I C
PTPRA	protein tyrosine phosphatase receptor type A
FBXW8	F-box and WD repeat domain containing 8
MACROH2A1	macroH2A.1 histone
SMAD3	SMAD family member 3
NOTCH3	notch receptor 3
TFF3	trefoil factor 3
IQGAP2	IQ motif containing GTPase activating protein 2
PTPRK	protein tyrosine phosphatase receptor type K
HCRTR2	hypocretin receptor 2
TACC2	transforming acidic coiled-coil containing protein 2
HLA-A	major histocompatibility complex, class I, A
ORAI3	ORAI calcium release-activated calcium modulator 3
SEC31A	SEC31 homolog A, COPII coat complex component
CD200R1	CD200 receptor 1

Pesticides

SHANK2	SH3 and multiple ankyrin repeat domains 2
LIMA1	LIM domain and actin binding 1
PRKCZ	protein kinase C zeta
TBXT	T-box transcription factor T
GSTO1	glutathione S-transferase omega 1
CCDC181	coiled-coil domain containing 181
KLK3	kallikrein related peptidase 3
GSTO2	glutathione S-transferase omega 2
MYCN	MYCN proto-oncogene, bHLH transcription factor
SALL4	spalt like transcription factor 4
SOX5	SRY-box transcription factor 5
ZNF366	zinc finger protein 366
ACAT1	acetyl-CoA acetyltransferase 1
RNF126	ring finger protein 126
PRKAG2	protein kinase AMP-activated non-catalytic subunit gamma 2
KLK1	kallikrein 1
PHLPP1	PH domain and leucine rich repeat protein phosphatase 1
ETV6	ETS variant transcription factor 6
QSOX1	quiescin sulfhydryl oxidase 1
RORA	RAR related orphan receptor A
SLIT1	slit guidance ligand 1
KLK5	kallikrein related peptidase 5
CDK5	cyclin dependent kinase 5
PRKCB	protein kinase C beta
FAM13C	family with sequence similarity 13 member C

Glyphosate

MTHFR	methylenetetrahydrofolate reductase
MYCBP2	MYC binding protein 2
MAP2K1	mitogen-activated protein kinase kinase 1
KAT5	lysine acetyltransferase 5
RNU1-1	RNA, U1 small nuclear 1
NUAK1	NUAK family kinase 1
FN1	fibronectin 1
SFMBT2	Scm like with four mbt domains 2
SLIT2	slit guidance ligand 2
AHR	aryl hydrocarbon receptor
CTNND2	catenin delta 2
CDON	cell adhesion associated, oncogene regulated
RUNX1	RUNX family transcription factor 1
SUCLA2	succinate-CoA ligase ADP-forming subunit beta
ARHGAP10	Rho GTPase activating protein 10
RELA	RELA proto-oncogene, NF-kB subunit
PDGFRA	platelet derived growth factor receptor alpha
AXL	AXL receptor tyrosine kinase
RABEP1	rabaptin, RAB GTPase binding effector protein 1
PLA2G7	phospholipase A2 group VII
RNF40	ring finger protein 40
SCGN	secretagogin, EF-hand calcium binding protein

Methoxychlor

RNU1-1	RNA, U1 small nuclear 1
MCPH1	microcephalin 1
STAT1	signal transducer and activator of transcription 1
HNF1B	HNF1 homeobox B
RUNX1	RUNX family transcription factor 1
DACH1	dachshund family transcription factor 1
MIR30D	microRNA 30d
NDRG3	NDRG family member 3
HIP1	huntingtin interacting protein 1
TBC1D4	TBC1 domain family member 4
TNIK	TRAF2 and NCK interacting kinase
THADA	THADA armadillo repeat containing
DDAH1	dimethylarginine dimethylaminohydrolase 1
ELAVL1	ELAV like RNA binding protein 1
