# Supplementary material for: Environmental induced transgenerational inheritance impacts systems epigenetics in disease etiology
Source: Sci Rep. 2022 Apr 19;12:5452. doi: 10.1038/s41598-022-09336-0 (PMC9018793; doi:10.1038/s41598-022-09336-0)
Supplement: Supplementary file 31 — Supplementary Table S23. [file 41598_2022_9336_MOESM31_ESM.pdf]

Supplemental Table S23

Disease Correlated Puberty Abnormalities DMR Associated Genes

**Atrazine**	
INSR	insulin receptor
DLG2	discs large MAGUK scaffold protein 2
**Jet Fuel**	
FGFR1	fibroblast growth factor receptor 1
PPP3CA	protein phosphatase 3 catalytic subunit alpha
CYP1A1	cytochrome P450 family 1 subfamily A member 1
