# Supplementary material for: Environmental induced transgenerational inheritance impacts systems epigenetics in disease etiology
Source: Sci Rep. 2022 Apr 19;12:5452. doi: 10.1038/s41598-022-09336-0 (PMC9018793; doi:10.1038/s41598-022-09336-0)
Supplement: Supplementary file 32 — Supplementary Table S24. [file 41598_2022_9336_MOESM32_ESM.pdf]

Supplemental Table S24

Disease Correlated Testis Disease DMR Associated Genes

**Control**	
HTT	huntingtin
TASP1	taspase 1
CHEK2	checkpoint kinase 2
**Dioxin**	
FGFR1	fibroblast growth factor receptor 1
EGFR	epidermal growth factor receptor
ENTPD6	ectonucleoside triphosphate diphosphohydrolase 6
PDE4D	phosphodiesterase 4D
**Pesticides**	
DUSP6	dual specificity phosphatase 6
RARB	retinoic acid receptor beta
BCL2	BCL2 apoptosis regulator
**Atrazine**	
KITLG	KIT ligand
SMAD2	SMAD family member 2
GPC6	glypican 6
PTEN	phosphatase and tensin homolog
TERT	telomerase reverse transcriptase
PTPN11	protein tyrosine phosphatase non-receptor type 11
TGFBR2	transforming growth factor beta receptor 2
TASP1	taspase 1
PDE4D	phosphodiesterase 4D
PPARG	peroxisome proliferator activated receptor gamma
CIT	citron rho-interacting serine/threonine kinase
NR5A2	nuclear receptor subfamily 5 group A member 2
