# Supplementary material for: Environmental induced transgenerational inheritance impacts systems epigenetics in disease etiology
Source: Sci Rep. 2022 Apr 19;12:5452. doi: 10.1038/s41598-022-09336-0 (PMC9018793; doi:10.1038/s41598-022-09336-0)
Supplement: Supplementary file 33 — Supplementary Table S25. [file 41598_2022_9336_MOESM33_ESM.pdf]

# Supplemental Table S25

## Exposure Correlated Obesity DMR Associated Genes

| | |
|---|---|
| **Control** | |
| TRPM8 | transient receptor potential cation channel subfamily M member 8 |
| PIK3CD | phosphatidylinositol-4,5-bisphosphate 3-kinase catalytic subunit delta |
| DUSP14 | dual specificity phosphatase 14 |
| NCOA1 | nuclear receptor coactivator 1 |
| PLA2G2E | phospholipase A2 group IIE |
| THADA | THADA armadillo repeat containing |
| SOX6 | SRY-box transcription factor 6 |
| CDH22 | cadherin 22 |
| | |
| **Dioxin** | |
| FTO | FTO alpha-ketoglutarate dependent dioxygenase |
| TP53 | tumor protein p53 |
| SATB2 | SATB homeobox 2 |
| GRIA1 | glutamate ionotropic receptor AMPA type subunit 1 |
| MTR | 5-methyltetrahydrofolate-homocysteine methyltransferase |
| TRHDE | thyrotropin releasing hormone degrading enzyme |
| NEGR1 | neuronal growth regulator 1 |
| FOXP1 | forkhead box P1 |
| SLC51B | solute carrier family 51 subunit beta |
| FXN | frataxin |
| ARHGAP21 | Rho GTPase activating protein 21 |
| PAFAH1B1 | platelet activating factor acetylhydrolase 1b regulatory subunit 1 |
| DOC2B | double C2 domain beta |
| LRP1 | LDL receptor related protein 1 |
| SLC5A1 | solute carrier family 5 member 1 |
| PRLH | prolactin releasing hormone |
| | |
| **Glyphosate** | |
| IDH2 | isocitrate dehydrogenase (NADP(+)) 2 |
| AACS | acetoacetyl-CoA synthetase |
| JAK2 | Janus kinase 2 |
| NTRK2 | neurotrophic receptor tyrosine kinase 2 |
| ANK2 | ankyrin 2 |
| PTPRD | protein tyrosine phosphatase receptor type D |
| GRIA1 | glutamate ionotropic receptor AMPA type subunit 1 |
| RPL3 | ribosomal protein L3 |
| CERS2 | ceramide synthase 2 |
| F2 | coagulation factor II, thrombin |
| GUCA2B | guanylate cyclase activator 2B |
| PTPRS | protein tyrosine phosphatase receptor type S |

| | |
|---|---|
| ZFHX3 | zinc finger homeobox 3 |
| VIP | vasoactive intestinal peptide |
| MAT2B | methionine adenosyltransferase 2B |
| F8 | coagulation factor VIII |
| SEC16B | SEC16 homolog B, endoplasmic reticulum export factor |
| NRXN3 | neurexin 3 |
| G3BP2 | G3BP stress granule assembly factor 2 |

**Methoxychlor**

| | |
|---|---|
| NFATC2 | nuclear factor of activated T cells 2 |
| HHAT | hedgehog acyltransferase |
| EPHX2 | epoxide hydrolase 2 |
| ARNT2 | aryl hydrocarbon receptor nuclear translocator 2 |
| CHRNA3 | cholinergic receptor nicotinic alpha 3 subunit |
| PDGFC | platelet derived growth factor C |
| ATP1A1 | ATPase Na+/K+ transporting subunit alpha 1 |
| APP | amyloid beta precursor protein |
| ANK2 | ankyrin 2 |
| CHRNB4 | cholinergic receptor nicotinic beta 4 subunit |
| KLK3 | kallikrein related peptidase 3 |
| MAPK6 | mitogen-activated protein kinase 6 |
| SPTLC1 | serine palmitoyltransferase long chain base subunit 1 |
| DPP4 | dipeptidyl peptidase 4 |
| TRPM6 | transient receptor potential cation channel subfamily M member 6 |
| MIR30D | microRNA 30d |
| SESN3 | sestrin 3 |
| CD24 | CD24 molecule |
| GALNT2 | polypeptide N-acetylgalactosaminyltransferase 2 |
| ITPR1 | inositol 1,4,5-trisphosphate receptor type 1 |

**Atrazine**

| | |
|---|---|
| POLG | DNA polymerase gamma, catalytic subunit |
| STK39 | serine/threonine kinase 39 |
| PTPRT | protein tyrosine phosphatase receptor type T |
| THRAP3 | thyroid hormone receptor associated protein 3 |
| CNOT6L | CCR4-NOT transcription complex subunit 6 like |
| LILRB3 | leukocyte immunoglobulin like receptor B3 |
| TBC1D4 | TBC1 domain family member 4 |
| FAT3 | FAT atypical cadherin 3 |
| MYOD1 | myogenic differentiation 1 |
| ACSS3 | acyl-CoA synthetase short chain family member 3 |
| TCF4 | transcription factor 4 |
| DKK3 | dickkopf WNT signaling pathway inhibitor 3 |
| CRHR2 | corticotropin releasing hormone receptor 2 |
| SP1 | Sp1 transcription factor |
| SDCCAG8 | SHH signaling and ciliogenesis regulator SDCCAG8 |

| | |
|---|---|
| TCF7L2 | transcription factor 7 like 2 |
| CTSB | cathepsin B |
| MEST | mesoderm specific transcript |
| LRP2 | LDL receptor related protein 2 |
| NRXN3 | neurexin 3 |
| SOX6 | SRY-box transcription factor 6 |

**Jet Fuel**

| | |
|---|---|
| ALDH9A1 | aldehyde dehydrogenase 9 family member A1 |
| PROC | protein C, inactivator of coagulation factors Va and VIIIa |
| CCNA2 | cyclin A2 |
| CCN1 | cellular communication network factor 1 |
| GALNT10 | polypeptide N-acetylgalactosaminyltransferase 10 |
| PINK1 | PTEN induced kinase 1 |
| NHLRC1 | NHL repeat containing E3 ubiquitin protein ligase 1 |
| BDKRB1 | bradykinin receptor B1 |
| ATP1A1 | ATPase Na+/K+ transporting subunit alpha 1 |
| NTN1 | netrin 1 |
| SCN5A | sodium voltage-gated channel alpha subunit 5 |
| NEGR1 | neuronal growth regulator 1 |
| PRKCB | protein kinase C beta |
| GALNT2 | polypeptide N-acetylgalactosaminyltransferase 2 |
| DDOST | dolichyl-diphosphooligosaccharide--protein glycosyltransferase non-catalytic subunit |
| CASR | calcium sensing receptor |
