# Supplementary material for: Environmental induced transgenerational inheritance impacts systems epigenetics in disease etiology
Source: Sci Rep. 2022 Apr 19;12:5452. doi: 10.1038/s41598-022-09336-0 (PMC9018793; doi:10.1038/s41598-022-09336-0)
Supplement: Supplementary file 34 — Supplementary Table S26. [file 41598_2022_9336_MOESM34_ESM.pdf]

Supplemental Table S26

Exposure Correlated Multiple Disease DMR Associated Genes

**Control**	
ATP6AP2	ATPase H+ transporting accessory protein 2
DLGAP3	DLG associated protein 3
NBL1	NBL1, DAN family BMP antagonist
PFKFB2	6-phosphofructo-2-kinase/fructose-2,6-biphosphatase 2
PFKFB4	6-phosphofructo-2-kinase/fructose-2,6-biphosphatase 4
ADCY2	adenylate cyclase 2
DNMT3A	DNA methyltransferase 3 alpha
UBASH3A	ubiquitin associated and SH3 domain containing A
FOXO6	forkhead box O6
RAB3D	RAB3D, member RAS oncogene family
EGFR	epidermal growth factor receptor
IPMK	inositol polyphosphate multikinase
LRP1B	LDL receptor related protein 1B
CHGB	chromogranin B
ESR1	estrogen receptor 1
KDM5A	lysine demethylase 5A
KCNK2	potassium two pore domain channel subfamily K member 2
GPR162	G protein-coupled receptor 162
MAPT	microtubule associated protein tau
GNAI2	G protein subunit alpha i2
PTH1R	parathyroid hormone 1 receptor
RBM5	RNA binding motif protein 5
MMAB	metabolism of cobalamin associated B
MOB3B	MOB kinase activator 3B
MECOM	MDS1 and EVI1 complex locus
ABLIM2	actin binding LIM protein family member 2
IP6K1	inositol hexakisphosphate kinase 1
ZNF24	zinc finger protein 24
RGS6	regulator of G protein signaling 6
PEMT	phosphatidylethanolamine N-methyltransferase
PRKCE	protein kinase C epsilon
LCLAT1	lysocardiolipin acyltransferase 1
CEACAM1	CEA cell adhesion molecule 1
HSD17B4	hydroxysteroid 17-beta dehydrogenase 4
CYP2C8	cytochrome P450 family 2 subfamily C member 8
SLC45A4	solute carrier family 45 member 4
CHRNA5	cholinergic receptor nicotinic alpha 5 subunit
PRPS1	phosphoribosyl pyrophosphate synthetase 1
SLC14A2	solute carrier family 14 member 2
TNFRSF11A	TNF receptor superfamily member 11a
CHRNE	cholinergic receptor nicotinic epsilon subunit
PTPRD	protein tyrosine phosphatase receptor type D
DIXDC1	DIX domain containing 1
SERPINB5	serpin family B member 5

MAPK10	mitogen-activated protein kinase 10
TRPS1	transcriptional repressor GATA binding 1
MAGI1	membrane associated guanylate kinase, WW and PDZ domain containing 1
SLC36A1	solute carrier family 36 member 1
MAP2K6	mitogen-activated protein kinase kinase 6
PTPRS	protein tyrosine phosphatase receptor type S
ASIC2	acid sensing ion channel subunit 2
AKAP6	A-kinase anchoring protein 6
VIPR1	vasoactive intestinal peptide receptor 1
PTDSS2	phosphatidylserine synthase 2
SORCS1	sortilin related VPS10 domain containing receptor 1
ROCK2	Rho associated coiled-coil containing protein kinase 2
AMPH	amphiphysin
ATR	ATR serine/threonine kinase
VAV3	vav guanine nucleotide exchange factor 3
CSMD1	CUB and Sushi multiple domains 1
CNTNAP2	contactin associated protein 2
DAG1	dystroglycan 1
CD4	CD4 molecule
MUC1	mucin 1, cell surface associated
DAPK2	death associated protein kinase 2
ACVR1C	activin A receptor type 1C
BBS5	Bardet-Biedl syndrome 5
RAB3B	RAB3B, member RAS oncogene family
SLC7A7	solute carrier family 7 member 7
HTR6	5-hydroxytryptamine receptor 6
GIP	gastric inhibitory polypeptide
QPCTL	glutaminyl-peptide cyclotransferase like
RNF6	ring finger protein 6
ARHGEF26	Rho guanine nucleotide exchange factor 26
TRPM8	transient receptor potential cation channel subfamily M member 8
SGTA	small glutamine rich tetratricopeptide repeat containing alpha
LSAMP	limbic system associated membrane protein
VNN1	vanin 1
GABRB3	gamma-aminobutyric acid type A receptor subunit beta3
LRPPRC	leucine rich pentatricopeptide repeat containing
TBC1D1	TBC1 domain family member 1
USP34	ubiquitin specific peptidase 34
INTS6	integrator complex subunit 6
MYOF	myoferlin
BBS1	Bardet-Biedl syndrome 1
FRAS1	Fraser extracellular matrix complex subunit 1
MYT1L	myelin transcription factor 1 like
C1QTNF1	C1q and TNF related 1
NR1D1	nuclear receptor subfamily 1 group D member 1
ADAM19	ADAM metallopeptidase domain 19
GRIN2A	glutamate ionotropic receptor NMDA type subunit 2A
ROBO1	roundabout guidance receptor 1
ROBO2	roundabout guidance receptor 2
GRM1	glutamate metabotropic receptor 1

IL2RA	interleukin 2 receptor subunit alpha
ST3GAL3	ST3 beta-galactoside alpha-2,3-sialyltransferase 3
KMT2C	lysine methyltransferase 2C
KCNH2	potassium voltage-gated channel subfamily H member 2

Dioxin

None

Plastics

MAGI2	membrane associated guanylate kinase, WW and PDZ domain containing 2
TCP1	t-complex 1
TP63	tumor protein p63
COL12A1	collagen type XII alpha 1 chain
FBXO11	F-box protein 11
PDE4B	phosphodiesterase 4B
INTS6	integrator complex subunit 6
MAPK10	mitogen-activated protein kinase 10
PGF	placental growth factor
GSTA3	glutathione S-transferase alpha 3
TGFA	transforming growth factor alpha
HS6ST3	heparan sulfate 6-O-sulfotransferase 3
PPP2R5C	protein phosphatase 2 regulatory subunit B'gamma
SRGAP1	SLIT-ROBO Rho GTPase activating protein 1
SOX5	SRY-box transcription factor 5
FAIM	Fas apoptotic inhibitory molecule
XAF1	XIAP associated factor 1
KCNC2	potassium voltage-gated channel subfamily C member 2
TOLLIP	toll interacting protein
KRT16	keratin 16
MSH6	mutS homolog 6
MTREX	Mtr4 exosome RNA helicase
BMPR2	bone morphogenetic protein receptor type 2

Pesticides

HORMAD2	HORMA domain containing 2
ADARB1	adenosine deaminase RNA specific B1
FDXR	ferredoxin reductase
PTCH1	patched 1
CACNA1G	calcium voltage-gated channel subunit alpha1 G
SACS	sacsin molecular chaperone
CAV2	caveolin 2
CPT1B	carnitine palmitoyltransferase 1B
PIEZO1	piezo type mechanosensitive ion channel component 1
APOH	apolipoprotein H
RUNX1	RUNX family transcription factor 1
TP73	tumor protein p73
APP	amyloid beta precursor protein
FAS	Fas cell surface death receptor
ING1	inhibitor of growth family member 1
FGF1	fibroblast growth factor 1

KITLG	KIT ligand
SERPINH1	serpin family H member 1
SULT2A1	sulfotransferase family 2A member 1
MYCBP2	MYC binding protein 2
FGF2	fibroblast growth factor 2
CDKAL1	CDK5 regulatory subunit associated protein 1 like 1
CHD7	chromodomain helicase DNA binding protein 7
PMS2	PMS1 homolog 2, mismatch repair system component
HS6ST3	heparan sulfate 6-O-sulfotransferase 3
AQP3	aquaporin 3 (Gill blood group)
KYNU	kynureninase
FGF13	fibroblast growth factor 13
GAS6	growth arrest specific 6
RNLS	renalase, FAD dependent amine oxidase
ABLIM2	actin binding LIM protein family member 2
FGFR2	fibroblast growth factor receptor 2
MTREX	Mtr4 exosome RNA helicase
LGALS3	galectin 3
LGALS4	galectin 4
MITF	melanocyte inducing transcription factor
TRPM3	transient receptor potential cation channel subfamily M member 3
TRIO	trio Rho guanine nucleotide exchange factor
PLPP2	phospholipid phosphatase 2
CFD	complement factor D
COX7B2	cytochrome c oxidase subunit 7B2
ATAD2	ATPase family AAA domain containing 2
PTPN13	protein tyrosine phosphatase non-receptor type 13
MLF1	myeloid leukemia factor 1
PTPN14	protein tyrosine phosphatase non-receptor type 14
DYSF	dysferlin
MMD	monocyte to macrophage differentiation associated
SLC6A2	solute carrier family 6 member 2
P3H4	prolyl 3-hydroxylase family member 4 (inactive)
CADPS	calcium dependent secretion activator
ANLN	anillin actin binding protein
BID	BH3 interacting domain death agonist
SLC12A8	solute carrier family 12 member 8
ARHGEF11	Rho guanine nucleotide exchange factor 11
ZDHHC14	zinc finger DHHC-type palmitoyltransferase 14
ELANE	elastase, neutrophil expressed
ADAM17	ADAM metallopeptidase domain 17
CIDEA	cell death inducing DFFA like effector a
BLVRA	biliverdin reductase A
PTPRK	protein tyrosine phosphatase receptor type K
TMEM26	transmembrane protein 26
CERK	ceramide kinase
TRIM16	tripartite motif containing 16
NCF1	neutrophil cytosolic factor 1
NELL2	neural EGFL like 2
SNAPIN	SNAP associated protein

PTPRR	protein tyrosine phosphatase receptor type R
KIF13A	kinesin family member 13A
GUCY2C	guanylate cyclase 2C
PRDM16	PR/SET domain 16
AKAP6	A-kinase anchoring protein 6
POU2F2	POU class 2 homeobox 2
TALDO1	transaldolase 1
FADS1	fatty acid desaturase 1
GPAT4	glycerol-3-phosphate acyltransferase 4
PCP4	Purkinje cell protein 4
PCSK1	proprotein convertase subtilisin/kexin type 1
RPS6	ribosomal protein S6
PCSK2	proprotein convertase subtilisin/kexin type 2
ARRB2	arrestin beta 2
STK39	serine/threonine kinase 39
PLEKHA7	pleckstrin homology domain containing A7
NFIA	nuclear factor I A
ARSB	arylsulfatase B
EIF3D	eukaryotic translation initiation factor 3 subunit D
SMARCAL1	SWI/SNF related, matrix associated, actin dependent regulator of chromatin, subfamily a like 1
HEATR5B	HEAT repeat containing 5B
PROK2	prokineticin 2
SRRM4	serine/arginine repetitive matrix 4
NFE2L2	nuclear factor, erythroid 2 like 2
LMO7	LIM domain 7
RAPGEF4	Rap guanine nucleotide exchange factor 4
PCYT2	phosphate cytidylyltransferase 2, ethanolamine
ABCC4	ATP binding cassette subfamily C member 4
USP9X	ubiquitin specific peptidase 9 X-linked
STAB1	stabilin 1
ARVCF	ARVCF delta catenin family member
GPAM	glycerol-3-phosphate acyltransferase, mitochondrial
TBX15	T-box transcription factor 15
PDE4B	phosphodiesterase 4B
SLURP1	secreted LY6/PLAUR domain containing 1
LOX	lysyl oxidase
CSPG4	chondroitin sulfate proteoglycan 4
MSRB3	methionine sulfoxide reductase B3
GHR	growth hormone receptor
SLC38A1	solute carrier family 38 member 1
EPAS1	endothelial PAS domain protein 1
AGTR1	angiotensin II receptor type 1
TCF4	transcription factor 4
BICC1	BicC family RNA binding protein 1
SLCO2A1	solute carrier organic anion transporter family member 2A1
ABCB1	ATP binding cassette subfamily B member 1
ZFP36L1	ZFP36 ring finger protein like 1
ITK	IL2 inducible T cell kinase
TNFSF4	TNF superfamily member 4
DKC1	dyskerin pseudouridine synthase 1

KLHL9	kelch like family member 9
TCF7L2	transcription factor 7 like 2
KCTD15	potassium channel tetramerization domain containing 15
MRC1	mannose receptor C-type 1
CLDN2	claudin 2
EPHA3	EPH receptor A3
PRDM6	PR/SET domain 6
SLIT1	slit guidance ligand 1
CIT	citron rho-interacting serine/threonine kinase
LRP5	LDL receptor related protein 5
EPHB1	EPH receptor B1
m_Svs2	semenogelin 1
NKAIN2	sodium/potassium transporting ATPase interacting 2
TRPM8	transient receptor potential cation channel subfamily M member 8
RABGAP1	RAB GTPase activating protein 1
LSAMP	limbic system associated membrane protein
JAK1	Janus kinase 1
SMARCA4	SWI/SNF related, matrix associated, actin dependent regulator of chromatin, subfamily a, member 4
EPOR	erythropoietin receptor
PYCARD	PYD and CARD domain containing
IFNL1	interferon lambda 1
UNC5B	unc-5 netrin receptor B
GLDC	glycine decarboxylase
ARID5B	AT-rich interaction domain 5B
SCARB2	scavenger receptor class B member 2
TMEFF2	transmembrane protein with EGF like and two follistatin like domains 2
SIX2	SIX homeobox 2
AKT2	AKT serine/threonine kinase 2
IDO1	indoleamine 2,3-dioxygenase 1
NOS1	nitric oxide synthase 1
TFAP2D	transcription factor AP-2 delta
ALAS1	5'-aminolevulinate synthase 1
SLC30A3	solute carrier family 30 member 3
GLP1R	glucagon like peptide 1 receptor
EPB41L3	erythrocyte membrane protein band 4.1 like 3
ALDH1A1	aldehyde dehydrogenase 1 family member A1
UCP1	uncoupling protein 1
ALDH2	aldehyde dehydrogenase 2 family member
SCN5A	sodium voltage-gated channel alpha subunit 5
m_Scgb1b2	secretoglobin, family 1B, member 2
PRDM2	PR/SET domain 2
CAPN9	calpain 9
B3GAT1	beta-1,3-glucuronyltransferase 1
MIR200B	microRNA 200b
IDE	insulin degrading enzyme
MEX3D	mex-3 RNA binding family member D
RLN3	relaxin 3
SOX6	SRY-box transcription factor 6
MIR429	microRNA 429
C4A	complement C4A (Rodgers blood group)

C4B	complement C4B (Chido blood group)
COBLL1	cordon-bleu WH2 repeat protein like 1
MSRA	methionine sulfoxide reductase A
RARB	retinoic acid receptor beta
KCNJ5	potassium inwardly rectifying channel subfamily J member 5
NRG1	neuregulin 1
SNAP47	synaptosome associated protein 47
TDO2	tryptophan 2,3-dioxygenase
PRF1	perforin 1
SIRT7	sirtuin 7
ANKS1B	ankyrin repeat and sterile alpha motif domain containing 1B
TEAD4	TEA domain transcription factor 4
BTG2	BTG anti-proliferation factor 2
MT1A	metallothionein 1A
MT1B	metallothionein 1B
CMIP	c-Maf inducing protein
NR1H4	nuclear receptor subfamily 1 group H member 4
MAD1L1	mitotic arrest deficient 1 like 1
TECTA	tectorin alpha
NEGR1	neuronal growth regulator 1
MT1G	metallothionein 1G
NISCH	nischarin
KCNK2	potassium two pore domain channel subfamily K member 2
TUBA1A	tubulin alpha 1a
IQGAP2	IQ motif containing GTPase activating protein 2
MT1X	metallothionein 1X
RNU1-1	RNA, U1 small nuclear 1
POC5	POC5 centriolar protein
m_Gm26504	predicted gene, 26504
NCOR2	nuclear receptor corepressor 2
DPT	dermatopontin
DPYD	dihydropyrimidine dehydrogenase
CHEK2	checkpoint kinase 2
HLA-A	major histocompatibility complex, class I, A
SOX10	SRY-box transcription factor 10
SEC31A	SEC31 homolog A, COPII coat complex component
ALDH7A1	aldehyde dehydrogenase 7 family member A1
ABCA1	ATP binding cassette subfamily A member 1
PRKCA	protein kinase C alpha
ABCB4	ATP binding cassette subfamily B member 4
PRKCB	protein kinase C beta
CLMP	CXADR like membrane protein
NAALAD2	N-acetylated alpha-linked acidic dipeptidase 2
CYP2B6	cytochrome P450 family 2 subfamily B member 6
PRKCH	protein kinase C eta
CYP2C19	cytochrome P450 family 2 subfamily C member 19
MBOAT7	membrane bound O-acyltransferase domain containing 7
PHF2	PHD finger protein 2
PRKCQ	protein kinase C theta
PRKCZ	protein kinase C zeta

ABL2	ABL proto-oncogene 2, non-receptor tyrosine kinase
CRIM1	cysteine rich transmembrane BMP regulator 1
CDH2	cadherin 2
IGF1R	insulin like growth factor 1 receptor
FSHR	follicle stimulating hormone receptor
VAV2	vav guanine nucleotide exchange factor 2
CSPP1	centrosome and spindle pole associated protein 1
CXCL16	C-X-C motif chemokine ligand 16
IGF2R	insulin like growth factor 2 receptor
CCL22	C-C motif chemokine ligand 22
TFF3	trefoil factor 3
MTRR	5-methyltetrahydrofolate-homocysteine methyltransferase reductase
RALY	RALY heterogeneous nuclear ribonucleoprotein
NEDD4L	NEDD4 like E3 ubiquitin protein ligase
AMFR	autocrine motility factor receptor
CDH13	cadherin 13
MAP2K6	mitogen-activated protein kinase kinase 6
TGFA	transforming growth factor alpha
SRGAP1	SLIT-ROBO Rho GTPase activating protein 1
TGFB1	transforming growth factor beta 1
MKX	mohawk homeobox
SDC4	syndecan 4
RFX4	regulatory factor X4
SPTB	spectrin beta, erythrocytic
TNFAIP8	TNF alpha induced protein 8
COL5A1	collagen type V alpha 1 chain
KLRB1	killer cell lectin like receptor B1
LRRK2	leucine rich repeat kinase 2
PRNP	prion protein
SREBF2	sterol regulatory element binding transcription factor 2
FAM20A	FAM20A golgi associated secretory pathway pseudokinase
RGS12	regulator of G protein signaling 12
KLC1	kinesin light chain 1
PIK3CB	phosphatidylinositol-4,5-bisphosphate 3-kinase catalytic subunit beta
KDM4A	lysine demethylase 4A
KLF12	Kruppel like factor 12
SEMA3F	semaphorin 3F
PDGFC	platelet derived growth factor C
SEMG1	semenogelin 1
PSD3	pleckstrin and Sec7 domain containing 3
F13A1	coagulation factor XIII A chain
PDLIM7	PDZ and LIM domain 7
ARHGEF12	Rho guanine nucleotide exchange factor 12
MAPKAPK2	MAPK activated protein kinase 2
ANK2	ankyrin 2
TLR9	toll like receptor 9
AXL	AXL receptor tyrosine kinase
PARD3	par-3 family cell polarity regulator
COL18A1	collagen type XVIII alpha 1 chain
CRTC1	CREB regulated transcription coactivator 1

NUAK2	NUAK family kinase 2
GHRL	ghrelin and obestatin prepropeptide
FANCA	FA complementation group A
GPC6	glypican 6
HNRNPL	heterogeneous nuclear ribonucleoprotein L
CERT1	ceramide transporter 1
THRB	thyroid hormone receptor beta
HASPIN	histone H3 associated protein kinase
SLCO5A1	solute carrier organic anion transporter family member 5A1
SIK3	SIK family kinase 3
PRTN3	proteinase 3
ARHGAP21	Rho GTPase activating protein 21
MED13L	mediator complex subunit 13L
ALDH1A2	aldehyde dehydrogenase 1 family member A2
PKP1	plakophilin 1
PSEN1	presenilin 1
IL1RAP	interleukin 1 receptor accessory protein
ANXA6	annexin A6
PLA2G2A	phospholipase A2 group IIA
ANXA11	annexin A11
CD200R1	CD200 receptor 1
PER3	period circadian regulator 3
ECH1	enoyl-CoA hydratase 1
SPRED2	sprouty related EVH1 domain containing 2
FBLN1	fibulin 1
TRPM7	transient receptor potential cation channel subfamily M member 7
PLA2G5	phospholipase A2 group V
DKK2	dickkopf WNT signaling pathway inhibitor 2
FASN	fatty acid synthase
KSR2	kinase suppressor of ras 2
ORM1	orosomucoid 1
HHAT	hedgehog acyltransferase
UNC13B	unc-13 homolog B
CARM1	coactivator associated arginine methyltransferase 1
ADAM12	ADAM metallopeptidase domain 12
MYO6	myosin VI
TSHZ3	teashirt zinc finger homeobox 3
CDCA5	cell division cycle associated 5
XBP1	X-box binding protein 1
MLYCD	malonyl-CoA decarboxylase
MSI2	musashi RNA binding protein 2
SHROOM3	shroom family member 3
IL20RA	interleukin 20 receptor subunit alpha
CLEC3B	C-type lectin domain family 3 member B
PPP1R12A	protein phosphatase 1 regulatory subunit 12A
IL20	interleukin 20
NPC1L1	NPC1 like intracellular cholesterol transporter 1
TARDBP	TAR DNA binding protein
MYT1L	myelin transcription factor 1 like
P2RX3	purinergic receptor P2X 3

G3BP1	G3BP stress granule assembly factor 1
ROBO1	roundabout guidance receptor 1
EXOC2	exocyst complex component 2
PSMD9	proteasome 26S subunit, non-ATPase 9
SF3B1	splicing factor 3b subunit 1
PDZD2	PDZ domain containing 2
RORA	RAR related orphan receptor A
TNS1	tensin 1
IL15RA	interleukin 15 receptor subunit alpha
NUP107	nucleoporin 107
RHBDF2	rhomboid 5 homolog 2
SLC9A3R1	SLC9A3 regulator 1
NRXN3	neurexin 3
ADAM10	ADAM metallopeptidase domain 10
TPH2	tryptophan hydroxylase 2
TNFAIP8L2	TNF alpha induced protein 8 like 2
**Glyphosate**	
MIR21	microRNA 21
SREBF2	sterol regulatory element binding transcription factor 2
PPARGC1B	PPARG coactivator 1 beta
FLNB	filamin B
ZFP1	ZFP1 zinc finger protein
PRMT7	protein arginine methyltransferase 7
DNMT3A	DNA methyltransferase 3 alpha
m_Mir21a	microRNA 21a
SLC15A3	solute carrier family 15 member 3
ANK2	ankyrin 2
IPMK	inositol polyphosphate multikinase
PDE4A	phosphodiesterase 4A
PARD3	par-3 family cell polarity regulator
RELN	reelin
CERS6	ceramide synthase 6
MYCBP2	MYC binding protein 2
ITGB3	integrin subunit beta 3
VWA8	von Willebrand factor A domain containing 8
ZBTB16	zinc finger and BTB domain containing 16
SORBS1	sorbin and SH3 domain containing 1
DMTN	dematin actin binding protein
AQP7	aquaporin 7
MIR33A	microRNA 33a
FAM71F1	family with sequence similarity 71 member F1
AQP9	aquaporin 9
TRAF3	TNF receptor associated factor 3
MRTFA	myocardin related transcription factor A
SLIT1	slit guidance ligand 1
G3BP2	G3BP stress granule assembly factor 2
EPHB1	EPH receptor B1
ARHGEF39	Rho guanine nucleotide exchange factor 39
HELZ2	helicase with zinc finger 2

TDRD1	tudor domain containing 1
ATP10D	ATPase phospholipid transporting 10D (putative)
SLC2A9	solute carrier family 2 member 9
FBXW8	F-box and WD repeat domain containing 8
MACROH2A1	macroH2A.1 histone
RELA	RELA proto-oncogene, NF-kB subunit
PYCARD	PYD and CARD domain containing
RBFOX1	RNA binding fox-1 homolog 1
DMD	dystrophin
F11R	F11 receptor
SLC12A8	solute carrier family 12 member 8
GRIA1	glutamate ionotropic receptor AMPA type subunit 1
TRPM2	transient receptor potential cation channel subfamily M member 2
CDC37	cell division cycle 37, HSP90 cochaperone
SPOCK1	SPARC (osteonectin), cwcv and kazal like domains proteoglycan 1
KAT5	lysine acetyltransferase 5
NELL2	neural EGFL like 2
TXNIP	thioredoxin interacting protein
LGR4	leucine rich repeat containing G protein-coupled receptor 4
KIF13A	kinesin family member 13A
FGF17	fibroblast growth factor 17
AKAP6	A-kinase anchoring protein 6
UCHL3	ubiquitin C-terminal hydrolase L3
PHLPP2	PH domain and leucine rich repeat protein phosphatase 2
CD274	CD274 molecule
TNS1	tensin 1

Methoxychlor

PEX19	peroxisomal biogenesis factor 19
CD1D	CD1d molecule
MSRA	methionine sulfoxide reductase A
TOR1A	torsin family 1 member A
SCLY	selenocysteine lyase
CDON	cell adhesion associated, oncogene regulated
PDE11A	phosphodiesterase 11A
TM6SF2	transmembrane 6 superfamily member 2
RAPGEF4	Rap guanine nucleotide exchange factor 4
TNFSF15	TNF superfamily member 15
SLC25A15	solute carrier family 25 member 15
TEAD4	TEA domain transcription factor 4
DHODH	dihydroorotate dehydrogenase (quinone)
MXI1	MAX interactor 1, dimerization protein
UBE3C	ubiquitin protein ligase E3C
MAD1L1	mitotic arrest deficient 1 like 1
PARD3	par-3 family cell polarity regulator
COL18A1	collagen type XVIII alpha 1 chain
RELN	reelin
TENT5B	terminal nucleotidyltransferase 5B
SULT2A1	sulfotransferase family 2A member 1
SLCO2B1	solute carrier organic anion transporter family member 2B1

MYCBP2	MYC binding protein 2
RNU1-1	RNA, U1 small nuclear 1
GSTA2	glutathione S-transferase alpha 2
PIP5K1A	phosphatidylinositol-4-phosphate 5-kinase type 1 alpha
GLCCI1	glucocorticoid induced 1
MIR30D	microRNA 30d
GABPA	GA binding protein transcription factor subunit alpha
FBXL7	F-box and leucine rich repeat protein 7
ITPR1	inositol 1,4,5-trisphosphate receptor type 1
NKAIN2	sodium/potassium transporting ATPase interacting 2
FASN	fatty acid synthase
PAWR	pro-apoptotic WT1 regulator
RDH16	retinol dehydrogenase 16
PARP1	poly(ADP-ribose) polymerase 1
LSAMP	limbic system associated membrane protein
PTPN13	protein tyrosine phosphatase non-receptor type 13
KIRREL1	kirre like nephrin family adhesion molecule 1
SLC14A2	solute carrier family 14 member 2
LRPPRC	leucine rich pentatricopeptide repeat containing
MAPK4	mitogen-activated protein kinase 4
GOT1	glutamic-oxaloacetic transaminase 1
MTTP	microsomal triglyceride transfer protein
MAPK6	mitogen-activated protein kinase 6
PLG	plasminogen
IRF6	interferon regulatory factor 6
NR6A1	nuclear receptor subfamily 6 group A member 1
NELL2	neural EGFL like 2
PARP2	poly(ADP-ribose) polymerase 2
KIF13A	kinesin family member 13A
ASIC2	acid sensing ion channel subunit 2
STK4	serine/threonine kinase 4
COL4A3	collagen type IV alpha 3 chain
CPE	carboxypeptidase E
NOTCH1	notch receptor 1
ROS1	ROS proto-oncogene 1, receptor tyrosine kinase
NRP2	neuropilin 2
NRXN3	neurexin 3
IDE	insulin degrading enzyme
IGF2BP2	insulin like growth factor 2 mRNA binding protein 2

Atrazine

CDK9	cyclin dependent kinase 9
STK39	serine/threonine kinase 39
SHANK2	SH3 and multiple ankyrin repeat domains 2
SMARCA5	SWI/SNF related, matrix associated, actin dependent regulator of chromatin, subfamily a, member 5
KLF8	Kruppel like factor 8
DNMT3B	DNA methyltransferase 3 beta
FLT3	fms related receptor tyrosine kinase 3
DYRK1B	dual specificity tyrosine phosphorylation regulated kinase 1B
ANKS1B	ankyrin repeat and sterile alpha motif domain containing 1B

GKN1	gastrokine 1
TTK	TTK protein kinase
SULT2A1	sulfotransferase family 2A member 1
MYCBP2	MYC binding protein 2
KCNMA1	potassium calcium-activated channel subfamily M alpha 1
CBX7	chromobox 7
RNU1-1	RNA, U1 small nuclear 1
GNAQ	G protein subunit alpha q
THRB	thyroid hormone receptor beta
HTR5A	5-hydroxytryptamine receptor 5A
HSD3B1	hydroxy-delta-5-steroid dehydrogenase, 3 beta- and steroid delta-isomerase 1
SEC31A	SEC31 homolog A, COPII coat complex component
GJA5	gap junction protein alpha 5
PTPN11	protein tyrosine phosphatase non-receptor type 11
ABCA1	ATP binding cassette subfamily A member 1
KRT16	keratin 16
AKT3	AKT serine/threonine kinase 3
BMPER	BMP binding endothelial regulator
FRS2	fibroblast growth factor receptor substrate 2
AMBRA1	autophagy and beclin 1 regulator 1
SH3PXD2B	SH3 and PX domains 2B
PRKD1	protein kinase D1
JARID2	jumonji and AT-rich interaction domain containing 2
PLEKHA2	pleckstrin homology domain containing A2
FBN1	fibrillin 1
NXN	nucleoredoxin
HDAC9	histone deacetylase 9
GIT1	GIT ArfGAP 1
SLC25A24	solute carrier family 25 member 24
GPR158	G protein-coupled receptor 158
CDH10	cadherin 10
PPM1L	protein phosphatase, Mg2+/Mn2+ dependent 1L
RPTOR	regulatory associated protein of MTOR complex 1
ARID1B	AT-rich interaction domain 1B
CACNA1C	calcium voltage-gated channel subunit alpha1 C
FAIM	Fas apoptotic inhibitory molecule
AKR1C3	aldo-keto reductase family 1 member C3
MSH3	mutS homolog 3
UBD	ubiquitin D
SORCS1	sortilin related VPS10 domain containing receptor 1
PDZD2	PDZ domain containing 2
MBD1	methyl-CpG binding domain protein 1
NRXN3	neurexin 3
APOBEC3B	apolipoprotein B mRNA editing enzyme catalytic subunit 3B
OSBPL3	oxysterol binding protein like 3
MBL2	mannose binding lectin 2

Jet Fuel

PROC	protein C, inactivator of coagulation factors Va and VIIIa
ERN1	endoplasmic reticulum to nucleus signaling 1

CFHR1	complement factor H related 1
MYO18A	myosin XVIIIA
PDGFD	platelet derived growth factor D
NCEH1	neutral cholesterol ester hydrolase 1
ANKS1B	ankyrin repeat and sterile alpha motif domain containing 1B
RAPGEF4	Rap guanine nucleotide exchange factor 4
SMIM31	small integral membrane protein 31
MCF2L	MCF.2 cell line derived transforming sequence like
PMS1	PMS1 homolog 1, mismatch repair system component
NR1H4	nuclear receptor subfamily 1 group H member 4
MGAT5	alpha-1,6-mannosylglycoprotein 6-beta-N-acetylglucosaminyltransferase
DNER	delta/notch like EGF repeat containing
HINT1	histidine triad nucleotide binding protein 1
MYCBP2	MYC binding protein 2
PELI1	pellino E3 ubiquitin protein ligase 1
RNU1-1	RNA, U1 small nuclear 1
CDKAL1	CDK5 regulatory subunit associated protein 1 like 1
MCPH1	microcephalin 1
HTR6	5-hydroxytryptamine receptor 6
KANK1	KN motif and ankyrin repeat domains 1
PITX3	paired like homeodomain 3
MAGEA4	MAGE family member A4
IL2RB	interleukin 2 receptor subunit beta
EPHB1	EPH receptor B1
CRY1	cryptochrome circadian regulator 1
DKK2	dickkopf WNT signaling pathway inhibitor 2
EPHB2	EPH receptor B2
ERN2	endoplasmic reticulum to nucleus signaling 2
PRKCI	protein kinase C iota
ENPP1	ectonucleotide pyrophosphatase/phosphodiesterase 1
TRIP13	thyroid hormone receptor interactor 13
HBB	hemoglobin subunit beta
TRPA1	transient receptor potential cation channel subfamily A member 1
SUCLG2	succinate-CoA ligase GDP-forming subunit beta
ADAM12	ADAM metallopeptidase domain 12
PBX3	PBX homeobox 3
ICAM2	intercellular adhesion molecule 2
SLC6A6	solute carrier family 6 member 6
TPX2	TPX2 microtubule nucleation factor
APC	APC regulator of WNT signaling pathway
GSTP1	glutathione S-transferase pi 1
SATB2	SATB homeobox 2
EIF2AK3	eukaryotic translation initiation factor 2 alpha kinase 3
SNAP29	synaptosome associated protein 29
IFIH1	interferon induced with helicase C domain 1
VCL	vinculin
SLC9A3R2	SLC9A3 regulator 2
MAGI1	membrane associated guanylate kinase, WW and PDZ domain containing 1
DAB2IP	DAB2 interacting protein
DUSP1	dual specificity phosphatase 1

KIF13A	kinesin family member 13A
PTX3	pentraxin 3
TGFBR3	transforming growth factor beta receptor 3
LRIG3	leucine rich repeats and immunoglobulin like domains 3
CUL3	cullin 3
TAFA5	TAFA chemokine like family member 5
UBE2S	ubiquitin conjugating enzyme E2 S
TRIM28	tripartite motif containing 28
TNIK	TRAF2 and NCK interacting kinase
ATRN	attractin
NRXN3	neurexin 3
STX8	syntaxin 8
