# Supplementary material for: Environmental induced transgenerational inheritance impacts systems epigenetics in disease etiology
Source: Sci Rep. 2022 Apr 19;12:5452. doi: 10.1038/s41598-022-09336-0 (PMC9018793; doi:10.1038/s41598-022-09336-0)
Supplement: Supplementary file 35 — Supplementary Table S27. [file 41598_2022_9336_MOESM35_ESM.pdf]

## Supplemental Table S27

### Kidney Disease Module Associated Gene

**Blue Module DMR**

| | |
|---|---|
| CAV1 | caveolin 1 |
| RICTOR | RPTOR independent companion of MTOR complex 2 |
| MCTP2 | multiple C2 and transmembrane domain containing 2 |
| CR1L | complement C3b/C4b receptor 1 like |
| APP | amyloid beta precursor protein |
| CR2 | complement C3d receptor 2 |
| ADCYAP1 | adenylate cyclase activating polypeptide 1 |
| EGFR | epidermal growth factor receptor |
| NCK1 | NCK adaptor protein 1 |
| PAH | phenylalanine hydroxylase |
| BBS9 | Bardet-Biedl syndrome 9 |
| HIPK2 | homeodomain interacting protein kinase 2 |
| CHIT1 | chitinase 1 |
| CDKAL1 | CDK5 regulatory subunit associated protein 1 like 1 |
| MMAB | metabolism of cobalamin associated B |
| CXCL10 | C-X-C motif chemokine ligand 10 |
| LIN7C | lin-7 homolog C, crumbs cell polarity complex component |
| REG3A | regenerating family member 3 alpha |
| RNLS | renalase, FAD dependent amine oxidase |
| GPC5 | glypican 5 |
| CCDC171 | coiled-coil domain containing 171 |
| MIF | macrophage migration inhibitory factor |
| HSD11B1 | hydroxysteroid 11-beta dehydrogenase 1 |
| ABCG2 | ATP binding cassette subfamily G member 2 (Junior blood group) |
| ABCC6 | ATP binding cassette subfamily C member 6 |
| CALCRL | calcitonin receptor like receptor |
| FHIT | fragile histidine triad diadenosine triphosphatase |
| TINAG | tubulointerstitial nephritis antigen |
| ADRA1B | adrenoceptor alpha 1B |
| TRPC1 | transient receptor potential cation channel subfamily C member 1 |
| TRPC3 | transient receptor potential cation channel subfamily C member 3 |
| ADAM17 | ADAM metallopeptidase domain 17 |
| RHOA | ras homolog family member A |
| TRPC5 | transient receptor potential cation channel subfamily C member 5 |
| NR3C2 | nuclear receptor subfamily 3 group C member 2 |
| MMP1 | matrix metallopeptidase 1 |
| PTPRN2 | protein tyrosine phosphatase receptor type N2 |
| PON2 | paraoxonase 2 |
| ELMO1 | engulfment and cell motility 1 |
| ITGA2 | integrin subunit alpha 2 |

| | |
|---|---|
| SORCS1 | sortilin related VPS10 domain containing receptor 1 |
| HMCN1 | hemicentin 1 |
| MMP10 | matrix metallopeptidase 10 |
| TSG101 | tumor susceptibility 101 |
| MMP13 | matrix metallopeptidase 13 |
| PVT1 | Pvt1 oncogene |
| PDE5A | phosphodiesterase 5A |
| SCLT1 | sodium channel and clathrin linker 1 |
| PCSK2 | proprotein convertase subtilisin/kexin type 2 |
| CASP12 | caspase 12 (gene/pseudogene) |
| MAGI2 | membrane associated guanylate kinase, WW and PDZ domain containing 2 |
| GZMB | granzyme B |
| SLC4A4 | solute carrier family 4 member 4 |
| SLCO1B3 | solute carrier organic anion transporter family member 1B3 |
| PDE1A | phosphodiesterase 1A |
| NFKB1 | nuclear factor kappa B subunit 1 |
| ABCC4 | ATP binding cassette subfamily C member 4 |
| ARL13A | ADP ribosylation factor like GTPase 13A |
| SLC34A1 | solute carrier family 34 member 1 |
| EFEMP1 | EGF containing fibulin extracellular matrix protein 1 |
| TBXAS1 | thromboxane A synthase 1 |
| LOX | lysyl oxidase |
| CD226 | CD226 molecule |
| m_Slco1a1 | solute carrier organic anion transporter family, member 1a1 |
| PPIA | peptidylprolyl isomerase A |
| m_Slco1a4 | solute carrier organic anion transporter family, member 1a4 |
| HLA-B | major histocompatibility complex, class I, B |
| ITGB3 | integrin subunit beta 3 |
| GHR | growth hormone receptor |
| NARS2 | asparaginyl-tRNA synthetase 2, mitochondrial |
| BRAF | B-Raf proto-oncogene, serine/threonine kinase |
| AGTR1 | angiotensin II receptor type 1 |
| ABCB1 | ATP binding cassette subfamily B member 1 |
| SLCO1A2 | solute carrier organic anion transporter family member 1A2 |
| SV2B | synaptic vesicle glycoprotein 2B |
| MUC16 | mucin 16, cell surface associated |
| CORIN | corin, serine peptidase |
| AHRR | aryl-hydrocarbon receptor repressor |
| ENPP1 | ectonucleotide pyrophosphatase/phosphodiesterase 1 |
| SERPINB7 | serpin family B member 7 |
| SLC39A8 | solute carrier family 39 member 8 |
| DMD | dystrophin |
| LTBP2 | latent transforming growth factor beta binding protein 2 |
| NLRP3 | NLR family pyrin domain containing 3 |
| PEPD | peptidase D |

| | |
|---|---|
| FRMD3 | FERM domain containing 3 |
| CMA1 | chymase 1 |
| m_Abcb1b | ATP-binding cassette, subfamily B (MDR/TAP), member 1B |
| NOS2 | nitric oxide synthase 2 |
| ERBB4 | erb-b2 receptor tyrosine kinase 4 |
| CLOCK | clock circadian regulator |
| IDE | insulin degrading enzyme |
| CTSL | cathepsin L |
| CFH | complement factor H |
| THSD7A | thrombospondin type 1 domain containing 7A |
| MSR1 | macrophage scavenger receptor 1 |
| CD72 | CD72 molecule |
| C5 | complement C5 |
| CFI | complement factor I |
| UGT2B17 | UDP glucuronosyltransferase family 2 member B17 |
| GSTK1 | glutathione S-transferase kappa 1 |
| ALMS1 | ALMS1 centrosome and basal body associated protein |
| ESR1 | estrogen receptor 1 |
| NPR3 | natriuretic peptide receptor 3 |
| ALOX5 | arachidonate 5-lipoxygenase |
| SLC17A3 | solute carrier family 17 member 3 |
| NLRP1 | NLR family pyrin domain containing 1 |
| SERPINA3 | serpin family A member 3 |
| IFNA1 | interferon alpha 1 |
| PIP5K1A | phosphatidylinositol-4-phosphate 5-kinase type 1 alpha |
| ABCC2 | ATP binding cassette subfamily C member 2 |
| NUP160 | nucleoporin 160 |
| CYP1A1 | cytochrome P450 family 1 subfamily A member 1 |
| IFNAR1 | interferon alpha and beta receptor subunit 1 |
| IFNG | interferon gamma |
| CYP2C19 | cytochrome P450 family 2 subfamily C member 19 |
| PRKD1 | protein kinase D1 |
| CYP2C8 | cytochrome P450 family 2 subfamily C member 8 |
| CYP2C9 | cytochrome P450 family 2 subfamily C member 9 |
| PRKDC | protein kinase, DNA-activated, catalytic subunit |
| PRKG1 | protein kinase cGMP-dependent 1 |
| RCAN1 | regulator of calcineurin 1 |
| CYP2E1 | cytochrome P450 family 2 subfamily E member 1 |
| CNR1 | cannabinoid receptor 1 |
| IGF2 | insulin like growth factor 2 |
| TFF3 | trefoil factor 3 |
| CYP4A11 | cytochrome P450 family 4 subfamily A member 11 |
| KIR3DL1 | killer cell immunoglobulin like receptor, three Ig domains and long cytoplasmic tail 1 |
| IQCB1 | IQ motif containing B1 |
| CYP7A1 | cytochrome P450 family 7 subfamily A member 1 |

| | |
|---|---|
| TG | thyroglobulin |
| IGH | immunoglobulin heavy locus |
| ATP6V0A4 | ATPase H+ transporting V0 subunit a4 |
| CACNA1C | calcium voltage-gated channel subunit alpha1 C |
| F2RL1 | F2R like trypsin receptor 1 |
| TGFB2 | transforming growth factor beta 2 |
| PLA2R1 | phospholipase A2 receptor 1 |
| COL4A6 | collagen type IV alpha 6 chain |
| KLRB1 | killer cell lectin like receptor B1 |
| CC2D2A | coiled-coil and C2 domain containing 2A |
| FAM20A | FAM20A golgi associated secretory pathway pseudokinase |
| PROS1 | protein S |
| DKK1 | dickkopf WNT signaling pathway inhibitor 1 |
| THPO | thrombopoietin |
| PDGFC | platelet derived growth factor C |
| AVP | arginine vasopressin |
| ANGPT1 | angiopoietin 1 |
| PRSS1 | serine protease 1 |
| TJP1 | tight junction protein 1 |
| UFL1 | UFM1 specific ligase 1 |
| TMPRSS15 | transmembrane serine protease 15 |
| GHRL | ghrelin and obestatin prepropeptide |
| G6PD | glucose-6-phosphate dehydrogenase |
| MYC | MYC proto-oncogene, bHLH transcription factor |
| NOX4 | NADPH oxidase 4 |
| PKHD1 | PKHD1 ciliary IPT domain containing fibrocystin/polyductin |
| GRK4 | G protein-coupled receptor kinase 4 |
| OPRM1 | opioid receptor mu 1 |
| CEP290 | centrosomal protein 290 |
| INVS | inversin |
| TLR2 | toll like receptor 2 |
| PLA2G4A | phospholipase A2 group IVA |
| TRPM7 | transient receptor potential cation channel subfamily M member 7 |
| SGCD | sarcoglycan delta |
| CES1 | carboxylesterase 1 |
| CAMK2A | calcium/calmodulin dependent protein kinase II alpha |
| UNC13B | unc-13 homolog B |
| IL23R | interleukin 23 receptor |
| TMOD1 | tropomodulin 1 |
| SYTL2 | synaptotagmin like 2 |
| FCAR | Fc fragment of IgA receptor |
| MYO9A | myosin IXA |
| SEMA3C | semaphorin 3C |
| CFTR | CF transmembrane conductance regulator |
| HDAC9 | histone deacetylase 9 |

| | |
|---|---|
| CCL4 | C-C motif chemokine ligand 4 |
| CP | ceruloplasmin |
| SLIT2 | slit guidance ligand 2 |
| GRIN2A | glutamate ionotropic receptor NMDA type subunit 2A |
| STK3 | serine/threonine kinase 3 |
| ROBO2 | roundabout guidance receptor 2 |
| KCNQ5 | potassium voltage-gated channel subfamily Q member 5 |
| NR3C1 | nuclear receptor subfamily 3 group C member 1 |
| ROCK1 | Rho associated coiled-coil containing protein kinase 1 |
| HPGD | 15-hydroxyprostaglandin dehydrogenase |
| CDKL5 | cyclin dependent kinase like 5 |
| CLDN7 | claudin 7 |
| IL16 | interleukin 16 |
