# Supplementary material for: Environmental induced transgenerational inheritance impacts systems epigenetics in disease etiology
Source: Sci Rep. 2022 Apr 19;12:5452. doi: 10.1038/s41598-022-09336-0 (PMC9018793; doi:10.1038/s41598-022-09336-0)
Supplement: Supplementary file 36 — Supplementary Table S28. [file 41598_2022_9336_MOESM36_ESM.pdf]

Supplemental Table S28

Prostate Disease Module Associated Gene

Light Green Module DMR

(No prostate disease-associated genes in light green module)
