# Supplementary material for: Environmental induced transgenerational inheritance impacts systems epigenetics in disease etiology
Source: Sci Rep. 2022 Apr 19;12:5452. doi: 10.1038/s41598-022-09336-0 (PMC9018793; doi:10.1038/s41598-022-09336-0)
Supplement: Supplementary file 37 — Supplementary Table S29. [file 41598_2022_9336_MOESM37_ESM.pdf]

Supplemental Table S29

Puberty Disease Module Associated Gene

**Yellow Module DMR**	
PPARG	peroxisome proliferator activated receptor gamma
LHCGR	luteinizing hormone/choriogonadotropin receptor
CCDC141	coiled-coil domain containing 141
ESR1	estrogen receptor 1
GH1	growth hormone 1
ESRRG	estrogen related receptor gamma
LIN28A	lin-28 homolog A
PAPSS2	3'-phosphoadenosine 5'-phosphosulfate synthase 2
GPER1	G protein-coupled estrogen receptor 1
CHD7	chromodomain helicase DNA binding protein 7
KLB	klotho beta
KISS1R	KISS1 receptor
FNDC5	fibronectin type III domain containing 5
SORBS1	sorbin and SH3 domain containing 1
CYP1A1	cytochrome P450 family 1 subfamily A member 1
WDR11	WD repeat domain 11
CYP1B1	cytochrome P450 family 1 subfamily B member 1
LEP	leptin
NTN1	netrin 1
HSD11B1	hydroxysteroid 11-beta dehydrogenase 1
DLG2	discs large MAGUK scaffold protein 2
INSR	insulin receptor
GNRHR	gonadotropin releasing hormone receptor
PDYN	prodynorphin
IGF1R	insulin like growth factor 1 receptor
SHBG	sex hormone binding globulin
STAT5B	signal transducer and activator of transcription 5B
PROKR2	prokineticin receptor 2
TACR3	tachykinin receptor 3
PTEN	phosphatase and tensin homolog
GLI2	GLI family zinc finger 2
ERBB2	erb-b2 receptor tyrosine kinase 2
TGFA	transforming growth factor alpha
ERBB4	erb-b2 receptor tyrosine kinase 4
PPP3CA	protein phosphatase 3 catalytic subunit alpha
CYP11B1	cytochrome P450 family 11 subfamily B member 1
ZNF462	zinc finger protein 462
POU2F2	POU class 2 homeobox 2
NR3C1	nuclear receptor subfamily 3 group C member 1
CYP19A1	cytochrome P450 family 19 subfamily A member 1

CYP21A2	cytochrome P450 family 21 subfamily A member 2
TSHR	thyroid stimulating hormone receptor

Green Module DMR

SYCP1	synaptonemal complex protein 1
WDPCP	WD repeat containing planar cell polarity effector
HHAT	hedgehog acyltransferase
PDE11A	phosphodiesterase 11A
LHCGR	luteinizing hormone/choriogonadotropin receptor
IGF2	insulin like growth factor 2
CFTR	CF transmembrane conductance regulator
ESR1	estrogen receptor 1
PDE4B	phosphodiesterase 4B
OPHN1	oligophrenin 1
MAP2K1	mitogen-activated protein kinase kinase 1
GHR	growth hormone receptor
ADAMTS16	ADAM metallopeptidase with thrombospondin type 1 motif 16
NOS2	nitric oxide synthase 2
MAGEA4	MAGE family member A4
TASP1	taspase 1
PDE5A	phosphodiesterase 5A
KLK13	kallikrein related peptidase 13

Blue Module DMR

IGSF1	immunoglobulin superfamily member 1
AVP	arginine vasopressin
NOS2	nitric oxide synthase 2
ERBB4	erb-b2 receptor tyrosine kinase 4
XKR4	XK related 4
KIFAP3	kinesin associated protein 3
PAPPA2	pappalysin 2
CYP1A1	cytochrome P450 family 1 subfamily A member 1
NR3C1	nuclear receptor subfamily 3 group C member 1
CDKL5	cyclin dependent kinase like 5
ESR1	estrogen receptor 1
HSD11B1	hydroxysteroid 11-beta dehydrogenase 1
DLG2	discs large MAGUK scaffold protein 2
TSHR	thyroid stimulating hormone receptor
