# Supplementary material for: Environmental induced transgenerational inheritance impacts systems epigenetics in disease etiology
Source: Sci Rep. 2022 Apr 19;12:5452. doi: 10.1038/s41598-022-09336-0 (PMC9018793; doi:10.1038/s41598-022-09336-0)
Supplement: Supplementary file 38 — Supplementary Table S30. [file 41598_2022_9336_MOESM38_ESM.pdf]

Supplemental Table S30

Testis Disease Module Associated Gene

Green Module DMR

SYCP1	synaptonemal complex protein 1
WDPCP	WD repeat containing planar cell polarity effector
HHAT	hedgehog acyltransferase
PDE11A	phosphodiesterase 11A
LHCGR	luteinizing hormone/choriogonadotropin receptor
IGF2	insulin like growth factor 2
CFTR	CF transmembrane conductance regulator
ESR1	estrogen receptor 1
PDE4B	phosphodiesterase 4B
OPHN1	oligophrenin 1
MAP2K1	mitogen-activated protein kinase kinase 1
GHR	growth hormone receptor
ADAMTS16	ADAM metallopeptidase with thrombospondin type 1 motif 16
NOS2	nitric oxide synthase 2
MAGEA4	MAGE family member A4
TASP1	taspase 1
PDE5A	phosphodiesterase 5A
KLK13	kallikrein related peptidase 13
