# Supplementary material for: Environmental induced transgenerational inheritance impacts systems epigenetics in disease etiology
Source: Sci Rep. 2022 Apr 19;12:5452. doi: 10.1038/s41598-022-09336-0 (PMC9018793; doi:10.1038/s41598-022-09336-0)
Supplement: Supplementary file 40 — Supplementary Table S32. [file 41598_2022_9336_MOESM40_ESM.pdf]

Supplemental Table S32

Multiple Disease Module Associated Gene

Salmon Module DMR

DKC1	dyskerin pseudouridine synthase 1
AMPH	amphiphysin
TSHZ2	teashirt zinc finger homeobox 2
RHEB	Ras homolog, mTORC1 binding

Purple Module DMR

(No multiple disease-associated genes in purple module)

Black Module DMR

PHOSPHO1	phosphoethanolamine/phosphocholine phosphatase 1
CFH	complement factor H
CPT1A	carnitine palmitoyltransferase 1A
AACS	acetoacetyl-CoA synthetase
KMO	kynurenine 3-monooxygenase
CSDE1	cold shock domain containing E1
ADCY2	adenylate cyclase 2
NRG1	neuregulin 1
GRHL2	grainyhead like transcription factor 2
RUNX1	RUNX family transcription factor 1
C5	complement C5
PXDN	peroxidasin
IPMK	inositol polyphosphate multikinase
LRP1B	LDL receptor related protein 1B
ANKRD26	ankyrin repeat domain 26
SULT1C2	sulfotransferase family 1C member 2
INHA	inhibin subunit alpha
TPSAB1	tryptase alpha/beta 1
LTB4R	leukotriene B4 receptor
PIP5K1A	phosphatidylinositol-4-phosphate 5-kinase type 1 alpha
CHEK2	checkpoint kinase 2
SOX9	SRY-box transcription factor 9
LDLR	low density lipoprotein receptor
FGFR1	fibroblast growth factor receptor 1
GAS8	growth arrest specific 8
ARHGAP10	Rho GTPase activating protein 10
ETV6	ETS variant transcription factor 6
OSMR	oncostatin M receptor
WWOX	WW domain containing oxidoreductase
NTN1	netrin 1
PRKN	parkin RBR E3 ubiquitin protein ligase
CLIC5	chloride intracellular channel 5
FH	fumarate hydratase

INSR	insulin receptor
PKD1L2	polycystin 1 like 2 (gene/pseudogene)
PAX2	paired box 2
BTBD9	BTB domain containing 9
SMYD3	SET and MYND domain containing 3
LGALS9	galectin 9
PRKD1	protein kinase D1
r_Peg3	paternally expressed 3
SUCLG2	succinate-CoA ligase GDP-forming subunit beta
NBEA	neurobeachin
PRKG1	protein kinase cGMP-dependent 1
RAPGEF3	Rap guanine nucleotide exchange factor 3
PBX1	PBX homeobox 1
POLR2D	RNA polymerase II subunit D
MAP3K11	mitogen-activated protein kinase kinase kinase 11
PTPRF	protein tyrosine phosphatase receptor type F
TRPM1	transient receptor potential cation channel subfamily M member 1
ARID3B	AT-rich interaction domain 3B
RIPK3	receptor interacting serine/threonine kinase 3
COL1A1	collagen type I alpha 1 chain
CSF1R	colony stimulating factor 1 receptor
KIF13A	kinesin family member 13A
CACNA1D	calcium voltage-gated channel subunit alpha1 D
TGFBR3	transforming growth factor beta receptor 3
AMPH	amphiphysin
IGSF3	immunoglobulin superfamily member 3
SRC	SRC proto-oncogene, non-receptor tyrosine kinase
BMP7	bone morphogenetic protein 7
FLG	filaggrin
FADS2	fatty acid desaturase 2
PCSK2	proprotein convertase subtilisin/kexin type 2
PKD2L2	polycystin 2 like 2, transient receptor potential cation channel
TRIM66	tripartite motif containing 66
PXN	paxillin
NCEH1	neutral cholesterol ester hydrolase 1
SRRM4	serine/arginine repetitive matrix 4
PPARD	peroxisome proliferator activated receptor delta
NFKB1	nuclear factor kappa B subunit 1
ANK2	ankyrin 2
CD4	CD4 molecule
MIEN1	migration and invasion enhancer 1
TPSB2	tryptase beta 2
E2F5	E2F transcription factor 5
CSRP1	cysteine and glycine rich protein 1
ZMYND8	zinc finger MYND-type containing 8
OPCML	opioid binding protein/cell adhesion molecule like
EBF1	EBF transcription factor 1

NUDT3	nudix hydrolase 3
ARHGAP21	Rho GTPase activating protein 21
MED13L	mediator complex subunit 13L
PKP1	plakophilin 1
SULF2	sulfatase 2
SLIT1	slit guidance ligand 1
CIT	citron rho-interacting serine/threonine kinase
G3BP2	G3BP stress granule assembly factor 2
EPHB1	EPH receptor B1
OSBPL8	oxysterol binding protein like 8
SERPINB7	serpin family B member 7
MYLK	myosin light chain kinase
IL1RAPL1	interleukin 1 receptor accessory protein like 1
LPAR1	lysophosphatidic acid receptor 1
FAT1	FAT atypical cadherin 1
LIMA1	LIM domain and actin binding 1
PPP1R15A	protein phosphatase 1 regulatory subunit 15A
TSHZ3	teashirt zinc finger homeobox 3
HOXC8	homeobox C8
CSRP3	cysteine and glycine rich protein 3
PEG3	paternally expressed 3
ARID5B	AT-rich interaction domain 5B
NPC1L1	NPC1 like intracellular cholesterol transporter 1
CNOT7	CCR4-NOT transcription complex subunit 7
PCTP	phosphatidylcholine transfer protein
LMO4	LIM domain only 4
ERBB2	erb-b2 receptor tyrosine kinase 2
GTF2IRD1	GTF2I repeat domain containing 1
FCGR2B	Fc fragment of IgG receptor IIb
GLP1R	glucagon like peptide 1 receptor
LAMA4	laminin subunit alpha 4
G3BP1	G3BP stress granule assembly factor 1
ATG7	autophagy related 7
GRIN2B	glutamate ionotropic receptor NMDA type subunit 2B
ZFHX3	zinc finger homeobox 3
CPD	carboxypeptidase D
TYROBP	transmembrane immune signaling adaptor TYROBP
GALR1	galanin receptor 1
ZBTB38	zinc finger and BTB domain containing 38
XRCC1	X-ray repair cross complementing 1
PIAS1	protein inhibitor of activated STAT 1
ALPK1	alpha kinase 1
PA2G4	proliferation-associated 2G4
TNIK	TRAF2 and NCK interacting kinase
NRXN3	neurexin 3
