# Supplementary material for: Environmental induced transgenerational inheritance impacts systems epigenetics in disease etiology
Source: Sci Rep. 2022 Apr 19;12:5452. doi: 10.1038/s41598-022-09336-0 (PMC9018793; doi:10.1038/s41598-022-09336-0)
Supplement: Supplementary file 19 — Supplementary Table S11. [file 41598_2022_9336_MOESM19_ESM.pdf]

Supplemental Table S11
Plastics DMR p<1e-06

DMR Name	Chr	Start	Stop	Length	# Sig Win	minP	maxLFC	CpG #	CpG Density	Gene Annotation	Gene Category
DMR1:444001	1	444001	445000	1000	1	5.60E-07	0.49	5	0.5	Vom2r-ps5	
DMR1:474001	1	474001	475000	1000	1	5.60E-17	0.74	16	1.6	Vom2r-ps5	
DMR1:476001	1	476001	477000	1000	1	1.40E-15	0.94	21	2.1	Vom2r-ps5	
DMR1:495001	1	495001	497000	2000	1	6.00E-07	-0.36	30	1.5	Vom2r-ps5	
DMR1:512001	1	512001	516000	4000	2	2.40E-26	0.85	16	0.4	Vom2r-ps5;LOC103690914	
DMR1:522001	1	522001	523000	1000	1	1.00E-13	0.67	6	0.6	Vom2r-ps5;LOC103690914	
DMR1:539001	1	539001	542000	3000	1	2.10E-07	0.46	27	0.9	Vom2r-ps5;LOC102556463	
DMR1:548001	1	548001	550000	2000	1	7.20E-12	0.58	15	0.75	Vom2r-ps5;LOC102556463	
DMR1:562001	1	562001	563000	1000	1	1.30E-23	0.76	7	0.7	Vom2r-ps5	
DMR1:565001	1	565001	566000	1000	1	4.80E-11	-0.61	7	0.7	Vom2r-ps5	
DMR1:590001	1	590001	593000	3000	1	2.50E-14	-0.44	55	1.83	Vom2r-ps5	
DMR1:601001	1	601001	602000	1000	1	2.30E-08	0.42	22	2.2	Vom2r-ps5	
DMR1:613001	1	613001	616000	3000	3	7.10E-20	-0.59	35	1.17	Vom2r-ps5	
DMR1:618001	1	618001	622000	4000	3	5.00E-17	-0.47	45	1.12	Vom2r-ps5	
DMR1:626001	1	626001	627000	1000	1	1.30E-10	0.59	53	5.3	Vom2r-ps5	
DMR1:651001	1	651001	656000	5000	2	9.80E-09	-0.63	86	1.72	Vom2r-ps5	
DMR1:663001	1	663001	670000	7000	1	9.50E-07	0.22	48	0.69	Vom2r-ps5	
DMR1:671001	1	671001	674000	3000	1	9.90E-09	-0.43	30	1	Vom2r-ps5	
DMR1:680001	1	680001	686000	6000	2	1.60E-15	-0.5	67	1.12	Vom2r-ps5	
DMR1:695001	1	695001	696000	1000	1	8.20E-10	-0.51	12	1.2	Vom2r-ps5	
DMR1:721001	1	721001	730000	9000	5	5.10E-16	0.56	62	0.69	Vom2r4	Signaling
DMR1:739001	1	739001	741000	2000	2	4.10E-13	-0.49	25	1.25	Vom2r6	Signaling
DMR1:760001	1	760001	761000	1000	1	3.10E-10	0.65	3	0.3	Vom2r6	Signaling
DMR1:795001	1	795001	796000	1000	1	9.70E-13	0.67	8	0.8	Vom2r5	Signaling
DMR1:798001	1	798001	799000	1000	1	1.10E-14	0.69	6	0.6	Vom2r5	Signaling
DMR1:1106001	1	1106001	1108000	2000	2	1.70E-08	0.41	41	2.05	Raet1e	Immune
DMR1:1130001	1	1130001	1131000	1000	1	3.00E-07	0.52	3	0.3	Raet1e	Immune
DMR1:1426001	1	1426001	1430000	4000	2	1.50E-18	0.68	41	1.02	Rrlt	
DMR1:1433001	1	1433001	1434000	1000	1	3.90E-09	0.57	5	0.5	Rrlt	
DMR1:1443001	1	1443001	1444000	1000	1	1.90E-08	0.67	2	0.2	Rrlt	
DMR1:1445001	1	1445001	1446000	1000	1	1.70E-14	0.74	9	0.9	Rrlt;Raet1c	
DMR1:1459001	1	1459001	1462000	3000	2	6.40E-22	0.76	18	0.6	Raet1c;Raet1d	
DMR1:1464001	1	1464001	1465000	1000	1	4.10E-16	0.66	11	1.1	Raet1c;Raet1d	
DMR1:1477001	1	1477001	1479000	2000	2	3.00E-18	0.38	5	0.25	Raet1d	
DMR1:1483001	1	1483001	1487000	4000	1	9.20E-07	-0.44	31	0.78	Raet1d	
DMR1:1613001	1	1613001	1614000	1000	1	1.80E-09	-0.44	8	0.8	Vom2r-ps10	
DMR1:1665001	1	1665001	1669000	4000	3	1.60E-15	0.72	31	0.78	LOC100910848;LOC108348623;RGD1561843	
DMR1:1672001	1	1672001	1674000	2000	1	2.40E-22	0.87	24	1.2	LOC100910848;LOC108348623;RGD1561843	
DMR1:1675001	1	1675001	1677000	2000	1	8.90E-23	0.96	13	0.65	LOC100910848;LOC108348623;RGD1561843	
DMR1:1683001	1	1683001	1685000	2000	1	1.20E-09	-0.62	19	0.95	LOC108348623;RGD1561843	
DMR1:1693001	1	1693001	1696000	3000	1	2.20E-14	0.65	43	1.43	LOC108348623;Lrp11	
DMR1:1699001	1	1699001	1701000	2000	2	7.50E-19	0.66	12	0.6	Lrp11	
DMR1:1710001	1	1710001	1714000	4000	1	2.00E-14	0.54	51	1.27	Lrp11	
DMR1:1720001	1	1720001	1724000	4000	1	1.80E-16	0.68	60	1.5	Lrp11	
DMR1:1765001	1	1765001	1766000	1000	1	3.10E-07	0.49	4	0.4	Pcmt1;Nup43	Epigenetic
DMR1:1817001	1	1817001	1819000	2000	1	2.70E-10	0.57	19	0.95	Lats1;LOC684011;Katna1	Signaling;Cytoskeleton
DMR1:1823001	1	1823001	1824000	1000	1	2.80E-25	0.76	2	0.2	Lats1;LOC684011;Katna1	Signaling;Cytoskeleton
DMR1:1835001	1	1835001	1838000	3000	2	2.90E-16	-0.69	39	1.3	Katna1	Cytoskeleton
DMR1:1851001	1	1851001	1852000	1000	1	9.30E-10	0.57	1	0.1	Katna1;LOC493584	Cytoskeleton
DMR1:1881001	1	1881001	1882000	1000	1	8.60E-20	0.78	8	0.8	Ginm1;RGD1563636	
DMR1:1893001	1	1893001	1895000	2000	1	5.70E-07	0.45	46	2.3	Ginm1;RGD1563636;Ppil4	Transcription
DMR1:1904001	1	1904001	1905000	1000	1	1.80E-07	-0.66	10	1	Ppil4	Transcription
DMR1:1919001	1	1919001	1921000	2000	1	1.50E-10	0.55	16	0.8	Ppil4	Transcription
DMR1:1953001	1	1953001	1955000	2000	1	2.70E-09	-0.41	18	0.9	Zc3h12d	Translation
DMR1:1975001	1	1975001	1976000	1000	1	3.30E-13	0.51	22	2.2	Zc3h12d	Translation
DMR1:1977001	1	1977001	1978000	1000	1	2.30E-09	-0.49	17	1.7	Zc3h12d	Translation

DMR1:1990001	1	1990001	1993000	3000	1	6.80E-08	-0.48	50	1.67	Tab2	
DMR1:1999001	1	1999001	2003000	4000	1	7.60E-07	-0.5	75	1.88	Tab2	
DMR1:2009001	1	2009001	2010000	1000	1	3.20E-07	-0.48	13	1.3	Tab2	
DMR1:2013001	1	2013001	2014000	1000	1	1.60E-09	0.41	18	1.8	Tab2	
DMR1:2366001	1	2366001	2369000	3000	2	2.00E-17	0.85	36	1.2	Ust;LOC108348767	Transport
DMR1:2372001	1	2372001	2373000	1000	1	2.20E-11	0.41	24	2.4	Ust	Transport
DMR1:2378001	1	2378001	2380000	2000	1	3.00E-10	0.49	39	1.95	Ust	Transport
DMR1:2384001	1	2384001	2386000	2000	1	8.10E-07	-0.49	19	0.95	Ust	Transport
DMR1:2397001	1	2397001	2398000	1000	1	9.90E-15	0.71	5	0.5	Ust	Transport
DMR1:2408001	1	2408001	2409000	1000	1	4.40E-13	0.7	4	0.4	Ust	Transport
DMR1:2434001	1	2434001	2435000	1000	1	2.80E-11	0.43	12	1.2	Ust	Transport
DMR1:2439001	1	2439001	2444000	5000	1	7.40E-15	0.55	57	1.14	Ust	Transport
DMR1:2459001	1	2459001	2462000	3000	1	5.40E-21	0.68	49	1.63	Ust	Transport
DMR1:2466001	1	2466001	2474000	8000	2	5.60E-12	0.53	138	1.73	Ust	Transport
DMR1:2491001	1	2491001	2493000	2000	1	4.70E-08	0.48	17	0.85	Ust	Transport
DMR1:2494001	1	2494001	2497000	3000	2	6.00E-07	-0.4	41	1.37	Ust	Transport
DMR1:2504001	1	2504001	2506000	2000	1	2.20E-21	0.87	27	1.35	Ust	Transport
DMR1:2525001	1	2525001	2530000	5000	3	8.50E-23	0.75	72	1.44	Ust	Transport
DMR1:2532001	1	2532001	2533000	1000	1	2.80E-09	0.46	13	1.3	Ust	Transport
DMR1:2548001	1	2548001	2550000	2000	1	3.00E-07	-0.44	14	0.7	Ust	Transport
DMR1:2552001	1	2552001	2559000	7000	2	3.20E-13	0.78	68	0.97	Ust	Transport
DMR1:2565001	1	2565001	2570000	5000	3	1.10E-17	0.57	70	1.4	Ust	Transport
DMR1:2594001	1	2594001	2595000	1000	1	4.50E-08	0.35	13	1.3	Ust	Transport
DMR1:2605001	1	2605001	2608000	3000	1	6.30E-12	0.67	56	1.87	Ust	Transport
DMR1:2618001	1	2618001	2619000	1000	1	6.30E-10	0.45	16	1.6	Ust	Transport
DMR1:2780001	1	2780001	2781000	1000	1	2.10E-08	0.64	11	1.1	Sash1	
DMR1:2782001	1	2782001	2790000	8000	3	4.80E-12	-0.51	162	2.02	Sash1	
DMR1:2817001	1	2817001	2820000	3000	1	1.70E-11	0.46	45	1.5	Sash1	
DMR1:2826001	1	2826001	2828000	2000	1	6.40E-10	0.45	36	1.8	Sash1	
DMR1:2832001	1	2832001	2843000	11000	1	1.30E-07	-0.44	217	1.97	Sash1	
DMR1:2845001	1	2845001	2847000	2000	1	1.80E-12	0.64	24	1.2	Sash1	
DMR1:2848001	1	2848001	2851000	3000	1	7.90E-07	0.3	50	1.67	Sash1	
DMR1:2852001	1	2852001	2855000	3000	1	3.20E-10	0.42	63	2.1	Sash1	
DMR1:2856001	1	2856001	2858000	2000	1	3.20E-09	-0.47	39	1.95	Sash1	
DMR1:2859001	1	2859001	2861000	2000	1	8.80E-16	0.55	25	1.25	Sash1	
DMR1:2868001	1	2868001	2871000	3000	1	1.70E-12	0.4	62	2.07	Sash1	
DMR1:2888001	1	2888001	2893000	5000	1	5.80E-07	-0.56	89	1.78	Sash1	
DMR1:2895001	1	2895001	2896000	1000	1	9.80E-08	0.35	18	1.8	Sash1	
DMR1:2900001	1	2900001	2901000	1000	1	1.50E-08	0.64	6	0.6	Sash1	
DMR1:2906001	1	2906001	2909000	3000	1	2.80E-07	0.38	48	1.6	Sash1	
DMR1:2910001	1	2910001	2911000	1000	1	3.00E-17	0.75	11	1.1	Sash1	
DMR1:2929001	1	2929001	2931000	2000	1	5.20E-18	0.52	42	2.1	Sash1	
DMR1:2953001	1	2953001	2958000	5000	2	1.80E-12	-0.43	69	1.38	Sash1	
DMR1:3720001	1	3720001	3721000	1000	1	4.00E-12	0.52	11	1.1	Samd5	
DMR1:3731001	1	3731001	3735000	4000	1	8.70E-11	-0.43	60	1.5	Samd5	
DMR1:3741001	1	3741001	3742000	1000	1	3.40E-07	0.31	14	1.4	Samd5	
DMR1:3846001	1	3846001	3848000	2000	2	4.60E-23	0.91	20	1	RGD1560633	Translation
DMR1:3861001	1	3861001	3865000	4000	1	3.70E-08	-0.61	84	2.1	Stxbp5	Transport
DMR1:3892001	1	3892001	3899000	7000	1	2.00E-08	0.43	75	1.07	Stxbp5	Transport
DMR1:3902001	1	3902001	3906000	4000	2	1.20E-15	0.64	55	1.38	Stxbp5	Transport
DMR1:3985001	1	3985001	3989000	4000	3	3.50E-11	-0.62	17	0.42	Stxbp5	Transport
DMR1:3997001	1	3997001	3999000	2000	1	9.80E-07	-0.51	33	1.65	Stxbp5	Transport
DMR1:4004001	1	4004001	4007000	3000	1	4.10E-08	-0.59	44	1.47	Stxbp5	Transport
DMR1:4382001	1	4382001	4389000	7000	1	2.90E-09	0.63	57	0.81	Vom1r-ps1	
DMR1:4390001	1	4390001	4393000	3000	1	6.00E-07	-0.58	29	0.97	Vom1r-ps1	
DMR1:4441001	1	4441001	4442000	1000	1	1.50E-11	-0.56	4	0.4	Adgb;LOC102555130	
DMR1:4448001	1	4448001	4449000	1000	1	3.30E-08	-0.65	24	2.4	Adgb;LOC102555130	
DMR1:4469001	1	4469001	4473000	4000	2	2.10E-10	-0.42	28	0.7	Adgb	
DMR1:4524001	1	4524001	4530000	6000	5	6.30E-24	-0.52	65	1.08	Adgb	
DMR1:4536001	1	4536001	4537000	1000	1	1.50E-14	0.51	14	1.4	Adgb	
DMR1:4546001	1	4546001	4549000	3000	1	4.50E-08	0.34	33	1.1	Adgb	
DMR1:4559001	1	4559001	4566000	7000	5	7.30E-19	-0.5	82	1.17	Adgb	
DMR1:4575001	1	4575001	4576000	1000	1	7.10E-12	0.5	15	1.5	Adgb	
DMR1:4592001	1	4592001	4594000	2000	1	1.80E-07	0.36	23	1.15	Adgb	
DMR1:4627001	1	4627001	4629000	2000	1	3.00E-13	0.89	8	0.4	Rab32	

DMR1:4741001	1	4741001	4745000	4000	1	1.20E-11	-0.62	19	0.48	Grm1	Signaling
DMR1:4754001	1	4754001	4755000	1000	1	6.00E-08	-0.68	12	1.2	Grm1	Signaling
DMR1:4791001	1	4791001	4792000	1000	1	3.00E-07	-0.65	6	0.6	Grm1	Signaling
DMR1:4822001	1	4822001	4824000	2000	1	7.20E-12	0.45	21	1.05	Grm1	Signaling
DMR1:4849001	1	4849001	4850000	1000	1	2.40E-08	-0.63	13	1.3	Grm1	Signaling
DMR1:4875001	1	4875001	4876000	1000	1	3.60E-16	0.8	1	0.1	Grm1	Signaling
DMR1:4888001	1	4888001	4890000	2000	1	7.80E-07	-0.5	8	0.4	Grm1	Signaling
DMR1:4902001	1	4902001	4903000	1000	1	2.40E-07	0.42	8	0.8	Grm1	Signaling
DMR1:4949001	1	4949001	4951000	2000	1	2.30E-09	0.44	10	0.5	Grm1	Signaling
DMR1:4969001	1	4969001	4970000	1000	1	2.70E-10	0.55	9	0.9	Grm1	Signaling
DMR1:4975001	1	4975001	4977000	2000	1	1.60E-13	0.58	16	0.8	Grm1	Signaling
DMR1:5015001	1	5015001	5016000	1000	1	2.50E-07	0.38	9	0.9	Grm1	Signaling
DMR1:5057001	1	5057001	5062000	5000	1	5.70E-08	0.39	24	0.48	Grm1	Signaling
DMR1:5065001	1	5065001	5067000	2000	1	4.40E-09	0.43	16	0.8	Grm1	Signaling
DMR1:5073001	1	5073001	5076000	3000	1	8.50E-09	0.46	22	0.73	Grm1	Signaling
DMR1:5079001	1	5079001	5081000	2000	2	1.30E-13	0.67	12	0.6	Grm1	Signaling
DMR1:5082001	1	5082001	5084000	2000	2	3.30E-20	0.82	29	1.45	Grm1	Signaling
DMR1:5101001	1	5101001	5107000	6000	1	5.80E-16	0.7	62	1.03	Grm1	Signaling
DMR1:5114001	1	5114001	5116000	2000	1	3.30E-07	0.42	29	1.45	Grm1	Signaling
DMR1:5132001	1	5132001	5134000	2000	1	2.20E-16	0.61	39	1.95	Grm1	Signaling
DMR1:5136001	1	5136001	5138000	2000	1	2.50E-10	0.46	32	1.6	Grm1	Signaling
DMR1:5150001	1	5150001	5153000	3000	1	3.40E-13	0.77	41	1.37	Grm1	Signaling
DMR1:5155001	1	5155001	5157000	2000	2	7.50E-17	0.97	22	1.1	Grm1	Signaling
DMR1:5236001	1	5236001	5238000	2000	1	6.60E-07	0.41	22	1.1	Dctn3l1;Shprh	Proteolysis
DMR1:5245001	1	5245001	5246000	1000	1	4.30E-09	0.43	11	1.1	Dctn3l1;Shprh	Proteolysis
DMR1:5248001	1	5248001	5252000	4000	2	4.30E-09	-0.63	50	1.25	Dctn3l1;Shprh	Proteolysis
DMR1:5256001	1	5256001	5257000	1000	1	8.10E-07	-0.45	15	1.5	Shprh	Proteolysis
DMR1:5263001	1	5263001	5264000	1000	1	8.70E-15	0.58	11	1.1	Shprh	Proteolysis
DMR1:5270001	1	5270001	5274000	4000	1	3.60E-09	0.54	40	1	Shprh	Proteolysis
DMR1:5282001	1	5282001	5284000	2000	1	1.70E-09	-0.42	41	2.05	Shprh	Proteolysis
DMR1:5378001	1	5378001	5385000	7000	2	2.90E-13	0.54	77	1.1	Fbxo30	
DMR1:5440001	1	5440001	5443000	3000	1	9.50E-24	0.76	34	1.13	Epm2a	
DMR1:5447001	1	5447001	5448000	1000	1	9.90E-07	-0.47	3	0.3	Epm2a	
DMR1:5450001	1	5450001	5451000	1000	1	2.00E-08	0.42	14	1.4	Epm2a	
DMR1:5452001	1	5452001	5455000	3000	1	4.70E-08	-0.45	22	0.73	Epm2a	
DMR1:5456001	1	5456001	5463000	7000	2	4.40E-19	-0.51	76	1.09	Epm2a	
DMR1:5495001	1	5495001	5496000	1000	1	4.50E-12	0.6	5	0.5	Epm2a;LOC102547170	
DMR1:5501001	1	5501001	5504000	3000	1	2.60E-09	0.54	32	1.07	Epm2a	
DMR1:5532001	1	5532001	5534000	2000	2	1.90E-11	0.56	26	1.3	Epm2a	
DMR1:5544001	1	5544001	5545000	1000	1	4.30E-08	-0.61	22	2.2	Epm2a	
DMR1:5556001	1	5556001	5562000	6000	3	2.70E-13	0.53	70	1.17	Epm2a	
DMR1:5574001	1	5574001	5577000	3000	1	8.10E-08	-0.63	8	0.27	Epm2a	
DMR1:6459001	1	6459001	6460000	1000	1	6.40E-15	0.63	6	0.6	Utrn	
DMR1:6507001	1	6507001	6508000	1000	1	3.90E-10	0.57	8	0.8	Utrn	
DMR1:6511001	1	6511001	6512000	1000	1	1.60E-11	-0.53	4	0.4	Utrn	
DMR1:6515001	1	6515001	6519000	4000	1	1.90E-10	0.42	64	1.6	Utrn	
DMR1:6521001	1	6521001	6525000	4000	1	1.10E-07	-0.65	51	1.27	Utrn	
DMR1:6528001	1	6528001	6529000	1000	1	1.20E-12	0.46	18	1.8	Utrn	
DMR1:6530001	1	6530001	6533000	3000	1	8.70E-20	0.75	38	1.27	Utrn	
DMR1:6536001	1	6536001	6537000	1000	1	2.90E-18	0.88	6	0.6	Utrn	
DMR1:6545001	1	6545001	6548000	3000	1	1.00E-13	0.47	66	2.2	Utrn	
DMR1:6560001	1	6560001	6563000	3000	1	6.20E-13	0.6	38	1.27	Utrn	
DMR1:6565001	1	6565001	6567000	2000	1	1.30E-09	-0.44	13	0.65	Utrn	
DMR1:6569001	1	6569001	6571000	2000	1	2.20E-08	0.38	28	1.4	Utrn	
DMR1:6572001	1	6572001	6576000	4000	1	4.30E-09	0.49	48	1.2	Utrn	
DMR1:6583001	1	6583001	6584000	1000	1	8.10E-08	-0.66	16	1.6	Utrn	
DMR1:6587001	1	6587001	6589000	2000	1	2.30E-07	-0.45	28	1.4	Utrn	
DMR1:6590001	1	6590001	6595000	5000	1	7.20E-17	0.65	63	1.26	Utrn	
DMR1:6596001	1	6596001	6598000	2000	1	9.50E-07	0.37	30	1.5	Utrn	
DMR1:6599001	1	6599001	6601000	2000	1	3.60E-07	0.4	20	1	Utrn	
DMR1:6625001	1	6625001	6630000	5000	1	8.90E-10	0.66	95	1.9	Utrn	
DMR1:6638001	1	6638001	6641000	3000	1	9.30E-18	0.68	32	1.07	Utrn	
DMR1:6645001	1	6645001	6646000	1000	1	1.20E-15	0.96	7	0.7	Utrn	
DMR1:6649001	1	6649001	6653000	4000	2	1.30E-18	0.68	50	1.25	Utrn	
DMR1:6654001	1	6654001	6655000	1000	1	8.80E-11	0.49	15	1.5	Utrn	

DMR1:6659001	1	6659001	6661000	2000	1	1.50E-14	0.73	11	0.55	Utrn	
DMR1:6675001	1	6675001	6678000	3000	1	3.40E-09	0.42	37	1.23	Utrn	
DMR1:6685001	1	6685001	6688000	3000	1	3.60E-10	0.62	25	0.83	Utrn	
DMR1:6711001	1	6711001	6713000	2000	2	1.80E-14	0.52	29	1.45	Utrn	
DMR1:6715001	1	6715001	6718000	3000	3	1.20E-13	-0.64	11	0.37	Utrn	
DMR1:6724001	1	6724001	6726000	2000	1	2.00E-08	0.38	32	1.6	Utrn	
DMR1:6733001	1	6733001	6735000	2000	1	1.10E-15	0.66	29	1.45	Utrn	
DMR1:6738001	1	6738001	6745000	7000	1	4.00E-09	0.55	72	1.03	Utrn	
DMR1:6753001	1	6753001	6756000	3000	2	1.60E-13	0.52	46	1.53	Utrn	
DMR1:6759001	1	6759001	6772000	13000	3	2.70E-16	0.64	203	1.56	Utrn	
DMR1:6773001	1	6773001	6776000	3000	1	9.00E-10	0.62	53	1.77	Utrn	
DMR1:6777001	1	6777001	6782000	5000	2	8.40E-11	0.52	68	1.36	Utrn	
DMR1:6785001	1	6785001	6787000	2000	1	2.50E-11	0.48	42	2.1	Utrn	
DMR1:6795001	1	6795001	6798000	3000	1	9.70E-10	0.47	25	0.83	Utrn	
DMR1:6822001	1	6822001	6834000	12000	1	8.50E-14	0.56	168	1.4	Utrn	
DMR1:6845001	1	6845001	6849000	4000	1	9.90E-09	-0.51	46	1.15	Utrn	
DMR1:6863001	1	6863001	6865000	2000	2	6.20E-35	1.11	16	0.8	Utrn	
DMR1:6870001	1	6870001	6874000	4000	1	6.70E-10	-0.45	43	1.07	Utrn	
DMR1:6892001	1	6892001	6893000	1000	1	3.30E-13	0.7	4	0.4	Utrn	
DMR1:6924001	1	6924001	6927000	3000	1	6.90E-12	0.44	37	1.23	Utrn;LOC103690918	
DMR1:6940001	1	6940001	6941000	1000	1	3.20E-12	0.62	30	3	Utrn	
DMR1:6943001	1	6943001	6946000	3000	1	2.70E-07	-0.57	50	1.67	Utrn	
DMR1:6948001	1	6948001	6952000	4000	1	2.00E-08	0.31	74	1.85	Utrn	
DMR1:6953001	1	6953001	6959000	6000	1	1.20E-08	0.32	98	1.63	Utrn	
DMR1:6971001	1	6971001	6976000	5000	1	6.60E-11	0.42	62	1.24	Utrn	
DMR1:7010001	1	7010001	7011000	1000	1	1.60E-11	0.47	14	1.4	Trnal-uaa	
DMR1:7023001	1	7023001	7029000	6000	1	2.60E-11	0.46	56	0.93	Trnal-uaa	
DMR1:7058001	1	7058001	7060000	2000	1	4.80E-16	0.52	30	1.5	Stx11	Transcription
DMR1:7061001	1	7061001	7062000	1000	1	5.90E-14	0.48	21	2.1	Stx11	Transcription
DMR1:7063001	1	7063001	7064000	1000	1	3.20E-13	0.73	4	0.4	Stx11	Transcription
DMR1:7066001	1	7066001	7071000	5000	2	2.60E-08	0.39	65	1.3	Stx11	Transcription
DMR1:7074001	1	7074001	7076000	2000	2	4.40E-09	0.36	32	1.6	Stx11	Transcription
DMR1:7093001	1	7093001	7096000	3000	1	2.40E-17	0.52	43	1.43	Sf3b5	Translation
DMR1:7109001	1	7109001	7110000	1000	1	5.10E-15	0.51	15	1.5	Sf3b5	Translation
DMR1:7231001	1	7231001	7232000	1000	1	2.50E-19	0.61	22	2.2	Plagl1	Transcription
DMR1:7233001	1	7233001	7241000	8000	1	1.60E-07	-0.43	144	1.8	Plagl1	Transcription
DMR1:7243001	1	7243001	7246000	3000	1	7.10E-08	-0.54	41	1.37	Plagl1	Transcription
DMR1:7247001	1	7247001	7248000	1000	1	1.10E-32	0.82	19	1.9	Plagl1	Transcription
DMR1:7256001	1	7256001	7260000	4000	3	8.80E-15	0.62	60	1.5	Plagl1;LOC108349486	Transcription
DMR1:7269001	1	7269001	7271000	2000	1	3.40E-13	0.77	22	1.1	Plagl1;LOC108349486	Transcription
DMR1:7283001	1	7283001	7285000	2000	1	1.70E-07	0.47	27	1.35	Zc2hc1b	Transcription
DMR1:7304001	1	7304001	7306000	2000	2	1.40E-08	-0.52	14	0.7	Zc2hc1b	Transcription
DMR1:7312001	1	7312001	7315000	3000	2	1.60E-19	0.87	35	1.17	Zc2hc1b;Ltv1	Transcription
DMR1:7316001	1	7316001	7319000	3000	2	7.30E-14	0.45	47	1.57	Zc2hc1b;Ltv1	Transcription
DMR1:7337001	1	7337001	7341000	4000	1	1.90E-14	0.56	41	1.02	Ltv1	
DMR1:7357001	1	7357001	7362000	5000	2	4.70E-08	-0.5	64	1.28	Phactr2	Signaling
DMR1:7363001	1	7363001	7368000	5000	1	7.20E-07	-0.46	116	2.32	Phactr2	Signaling
DMR1:7384001	1	7384001	7389000	5000	1	9.10E-08	-0.47	78	1.56	Phactr2	Signaling
DMR1:7415001	1	7415001	7426000	11000	3	2.10E-19	-0.62	146	1.33	Phactr2	Signaling
DMR1:7427001	1	7427001	7431000	4000	3	6.50E-36	-0.63	47	1.18	Phactr2	Signaling
DMR1:7433001	1	7433001	7436000	3000	1	1.80E-09	-0.33	65	2.17	Phactr2	Signaling
DMR1:7448001	1	7448001	7449000	1000	1	3.90E-07	-0.47	6	0.6	Phactr2	Signaling
DMR1:7462001	1	7462001	7465000	3000	1	6.60E-19	0.74	52	1.73	Phactr2	Signaling
DMR1:7473001	1	7473001	7474000	1000	1	4.30E-11	0.57	10	1	Phactr2	Signaling
DMR1:7476001	1	7476001	7480000	4000	1	3.80E-08	-0.49	36	0.9	Phactr2	Signaling
DMR1:7500001	1	7500001	7503000	3000	1	3.30E-07	0.35	28	0.93	Phactr2	Signaling
DMR1:7538001	1	7538001	7542000	4000	2	1.60E-13	-0.69	37	0.92	Phactr2	Signaling
DMR1:7545001	1	7545001	7546000	1000	1	5.00E-07	0.44	24	2.4	Phactr2	Signaling
DMR1:7559001	1	7559001	7561000	2000	2	4.30E-21	0.92	14	0.7	Phactr2	Signaling
DMR1:7575001	1	7575001	7581000	6000	2	3.40E-07	-0.38	132	2.2	Phactr2	Signaling
DMR1:7582001	1	7582001	7588000	6000	4	5.60E-14	0.54	86	1.43	Phactr2	Signaling
DMR1:7597001	1	7597001	7601000	4000	2	2.80E-14	-0.79	49	1.23	Phactr2	Signaling
DMR1:7618001	1	7618001	7620000	2000	1	1.20E-08	-0.59	38	1.9	Phactr2	Signaling
DMR1:7635001	1	7635001	7641000	6000	4	6.00E-12	0.5	94	1.57	Phactr2;LOC103690919	Signaling
DMR1:7665001	1	7665001	7670000	5000	1	9.90E-13	0.49	90	1.8	Fuca2	Metabolism

DMR1:7681001	1	7681001	7685000	4000	2	8.30E-09	-0.56	44	1.1	Fuca2;Pex3	Metabolism
DMR1:7692001	1	7692001	7695000	3000	1	5.10E-08	-0.44	13	0.43	Pex3	
DMR1:7699001	1	7699001	7704000	5000	2	4.30E-08	-0.45	56	1.12	Pex3	
DMR1:7736001	1	7736001	7737000	1000	1	4.80E-12	-0.62	12	1.2	Pex3;Adat2	Metabolism
DMR1:7747001	1	7747001	7751000	4000	2	1.90E-20	1.16	62	1.55	Adat2	Metabolism
DMR1:7795001	1	7795001	7797000	2000	1	1.50E-12	0.47	23	1.15	RGD1563591	
DMR1:7802001	1	7802001	7803000	1000	1	4.70E-11	0.47	9	0.9	RGD1563591	
DMR1:7824001	1	7824001	7826000	2000	1	5.00E-07	-0.36	43	2.15	Aig1	
DMR1:7832001	1	7832001	7834000	2000	1	2.00E-08	-0.58	41	2.05	Aig1	
DMR1:7838001	1	7838001	7840000	2000	1	2.50E-13	0.48	21	1.05	Aig1	
DMR1:7845001	1	7845001	7848000	3000	1	9.50E-19	0.95	35	1.17	Aig1	
DMR1:7853001	1	7853001	7855000	2000	2	7.50E-19	0.65	43	2.15	Aig1	
DMR1:7869001	1	7869001	7872000	3000	1	1.10E-13	0.48	58	1.93	Aig1	
DMR1:7879001	1	7879001	7885000	6000	1	3.70E-09	0.37	94	1.57	Aig1	
DMR1:7893001	1	7893001	7897000	4000	1	1.90E-20	0.97	39	0.98	Aig1	
DMR1:7901001	1	7901001	7902000	1000	1	1.70E-08	0.45	11	1.1	Aig1	
DMR1:7909001	1	7909001	7915000	6000	2	1.20E-16	0.73	103	1.72	Aig1	
DMR1:7918001	1	7918001	7919000	1000	1	6.10E-09	0.42	17	1.7	Aig1	
DMR1:7921001	1	7921001	7922000	1000	1	6.10E-08	0.48	12	1.2	Aig1	
DMR1:7923001	1	7923001	7926000	3000	1	8.60E-16	0.66	36	1.2	Aig1	
DMR1:7942001	1	7942001	7944000	2000	1	7.90E-07	-0.39	42	2.1	Aig1	
DMR1:7946001	1	7946001	7949000	3000	2	2.10E-08	-0.43	54	1.8	Aig1	
DMR1:7952001	1	7952001	7954000	2000	1	2.60E-09	0.38	34	1.7	Aig1	
DMR1:7955001	1	7955001	7956000	1000	1	5.60E-09	0.44	14	1.4	Aig1	
DMR1:7962001	1	7962001	7963000	1000	1	4.20E-11	0.4	15	1.5	Aig1	
DMR1:7975001	1	7975001	7976000	1000	1	9.50E-09	-0.58	14	1.4	Aig1	
DMR1:7979001	1	7979001	7984000	5000	2	9.90E-08	-0.4	58	1.16	Aig1	
DMR1:7997001	1	7997001	8.00E+06	3000	1	1.40E-09	0.52	55	1.83	Aig1	
DMR1:8007001	1	8007001	8008000	1000	1	7.00E-12	0.58	7	0.7	Aig1	
DMR1:8019001	1	8019001	8020000	1000	1	1.70E-08	0.43	10	1	Aig1	
DMR1:8021001	1	8021001	8024000	3000	1	1.20E-09	0.38	40	1.33	Aig1	
DMR1:8148001	1	8148001	8154000	6000	4	2.60E-40	1.2	86	1.43	Hivep2	
DMR1:8155001	1	8155001	8157000	2000	2	1.80E-12	0.49	25	1.25	Hivep2	
DMR1:8158001	1	8158001	8163000	5000	2	1.90E-14	0.49	66	1.32	Hivep2	
DMR1:8169001	1	8169001	8171000	2000	1	2.50E-08	0.43	36	1.8	Hivep2	
DMR1:8186001	1	8186001	8189000	3000	1	3.20E-11	0.49	48	1.6	Hivep2	
DMR1:8190001	1	8190001	8193000	3000	1	4.80E-11	0.46	49	1.63	Hivep2;LOC102551141	
DMR1:8204001	1	8204001	8208000	4000	1	1.00E-08	0.39	58	1.45	Hivep2;LOC102551141;LOC108348466	
DMR1:8211001	1	8211001	8214000	3000	1	2.40E-07	-0.42	56	1.87	Hivep2;LOC102551141;LOC108348466	
DMR1:8267001	1	8267001	8270000	3000	1	8.20E-08	-0.69	33	1.1	Hivep2	
DMR1:8273001	1	8273001	8276000	3000	3	7.10E-20	0.59	55	1.83	Hivep2	
DMR1:8279001	1	8279001	8280000	1000	1	8.00E-09	-0.62	9	0.9	Hivep2	
DMR1:8300001	1	8300001	8304000	4000	1	8.60E-08	-0.46	65	1.62	Hivep2	
DMR1:8308001	1	8308001	8311000	3000	1	2.80E-08	-0.61	35	1.17	Hivep2	
DMR1:8333001	1	8333001	8337000	4000	1	5.10E-15	0.55	50	1.25	Hivep2	
DMR1:8586001	1	8586001	8587000	1000	1	4.70E-19	0.64	10	1	Adgrg6	Signaling
DMR1:8592001	1	8592001	8594000	2000	1	3.90E-08	0.55	16	0.8	Adgrg6	Signaling
DMR1:8598001	1	8598001	8602000	4000	1	9.30E-07	-0.59	42	1.05	Adgrg6	Signaling
DMR1:8641001	1	8641001	8645000	4000	1	7.00E-09	0.47	48	1.2	Adgrg6	Signaling
DMR1:8658001	1	8658001	8661000	3000	1	4.40E-08	0.55	35	1.17	Adgrg6	Signaling
DMR1:8664001	1	8664001	8668000	4000	1	5.80E-07	0.34	50	1.25	Adgrg6	Signaling
DMR1:8669001	1	8669001	8671000	2000	1	1.00E-07	0.38	27	1.35	Adgrg6	Signaling
DMR1:8697001	1	8697001	8701000	4000	2	3.50E-16	-0.72	55	1.38	Adgrg6	Signaling
DMR1:8705001	1	8705001	8707000	2000	2	2.20E-09	0.5	21	1.05	Adgrg6	Signaling
DMR1:8727001	1	8727001	8728000	1000	1	3.30E-19	1.08	1	0.1	Adgrg6	Signaling
DMR1:8760001	1	8760001	8762000	2000	1	2.20E-12	0.79	9	0.45	Adgrg6;LOC103690920	Signaling
DMR1:8829001	1	8829001	8832000	3000	1	5.70E-08	-0.51	52	1.73	Vta1	Transport
DMR1:8866001	1	8866001	8867000	1000	1	3.40E-09	0.38	7	0.7	Vta1	Transport
DMR1:8869001	1	8869001	8871000	2000	1	2.70E-11	0.66	19	0.95	Vta1;LOC103690921	Transport
DMR1:8897001	1	8897001	8899000	2000	1	5.70E-10	0.58	38	1.9	LOC103690921;Gje1	Cytoskeleton
DMR1:8945001	1	8945001	8948000	3000	1	2.00E-08	0.47	33	1.1	Nmbr	Receptor
DMR1:8955001	1	8955001	8957000	2000	1	1.70E-11	0.38	16	0.8	Nmbr	Receptor

DMR1:11766001	1	11766001	11768000	2000	2	8.70E-18	0.59	31	1.55	LOC108349829;LOC100909392;Ect2l	
DMR1:11792001	1	11792001	11793000	1000	1	1.90E-07	0.55	7	0.7	Ect2l	
DMR1:11795001	1	11795001	11799000	4000	3	6.10E-12	0.64	61	1.52	Ect2l	
DMR1:11801001	1	11801001	11802000	1000	1	2.60E-17	0.71	15	1.5	Ect2l	
DMR1:11999001	1	11999001	12001000	2000	1	2.90E-15	0.72	8	0.4	LOC100909599;Mcc	
DMR1:12009001	1	12009001	12013000	4000	2	8.80E-09	-0.38	33	0.82	Mcc	
DMR1:12014001	1	12014001	12015000	1000	1	3.80E-14	0.6	7	0.7	Mcc	
DMR1:12102001	1	12102001	12105000	3000	2	3.60E-07	-0.63	23	0.77	Mcc	
DMR1:12834001	1	12834001	12835000	1000	1	2.80E-09	0.35	20	2	Cited2	Transcription
DMR1:12907001	1	12907001	12912000	5000	2	8.60E-09	-0.41	167	3.34	LOC102554594;Txlnb	Transport
DMR1:12919001	1	12919001	12920000	1000	1	2.70E-14	0.7	15	1.5	LOC102554594;Txlnb	Transport
DMR1:12921001	1	12921001	12922000	1000	1	1.90E-07	-0.44	12	1.2	LOC102554594;Txlnb	Transport
DMR1:12927001	1	12927001	12931000	4000	1	5.40E-13	0.58	49	1.23	Txlnb	Transport
DMR1:12945001	1	12945001	12947000	2000	1	5.70E-09	0.41	44	2.2	Txlnb;LOC100360362	Transport
DMR1:12954001	1	12954001	12955000	1000	1	2.30E-07	0.51	9	0.9	Txlnb;LOC100360362	Transport
DMR1:12964001	1	12964001	12974000	10000	5	2.30E-13	-0.57	137	1.37	Txlnb	Transport
DMR1:13023001	1	13023001	13028000	5000	3	4.80E-12	-0.52	32	0.64	Heca	
DMR1:13031001	1	13031001	13034000	3000	1	2.20E-13	-0.59	8	0.27	Heca	
DMR1:13035001	1	13035001	13036000	1000	1	3.30E-10	0.55	12	1.2	Heca	
DMR1:13053001	1	13053001	13055000	2000	1	7.00E-07	-0.35	15	0.75	Heca	
DMR1:13152001	1	13152001	13156000	4000	2	2.40E-14	0.55	33	0.82	LOC108349444;Abracl	
DMR1:13164001	1	13164001	13167000	3000	2	3.60E-09	0.38	59	1.97	LOC108349444;Abracl	
DMR1:13168001	1	13168001	13169000	1000	1	4.30E-29	0.81	16	1.6	LOC108349444;Abracl	
DMR1:13176001	1	13176001	13180000	4000	2	1.40E-18	0.68	43	1.07	Abracl	
DMR1:13181001	1	13181001	13185000	4000	1	2.70E-16	0.56	75	1.88	Abracl	
DMR1:13255001	1	13255001	13257000	2000	1	1.00E-11	0.57	25	1.25	Reps1	Transport
DMR1:13268001	1	13268001	13271000	3000	3	7.40E-10	-0.62	27	0.9	Reps1	Transport
DMR1:13272001	1	13272001	13276000	4000	1	7.00E-10	0.5	39	0.98	Reps1	Transport
DMR1:13279001	1	13279001	13281000	2000	1	7.00E-07	-0.52	29	1.45	Reps1	Transport
DMR1:13282001	1	13282001	13285000	3000	2	1.60E-11	0.49	33	1.1	Reps1	Transport
DMR1:13292001	1	13292001	13293000	1000	1	2.10E-19	0.68	17	1.7	Reps1;NEWGENE_2319083	Transport
DMR1:13318001	1	13318001	13319000	1000	1	4.00E-07	0.53	7	0.7	NEWGENE_2319083	
DMR1:13321001	1	13321001	13325000	4000	3	5.40E-11	0.69	71	1.77	NEWGENE_2319083	
DMR1:13326001	1	13326001	13329000	3000	1	1.20E-09	0.49	32	1.07	NEWGENE_2319083	
DMR1:13341001	1	13341001	13342000	1000	1	3.30E-07	0.3	15	1.5	NEWGENE_2319083	
DMR1:13351001	1	13351001	13356000	5000	1	8.00E-30	0.93	81	1.62	NEWGENE_2319083	
DMR1:13358001	1	13358001	13361000	3000	1	9.70E-14	0.53	32	1.07	NEWGENE_2319083;LOC103690924	
DMR1:13385001	1	13385001	13386000	1000	1	1.00E-13	0.44	22	2.2	Ccdc28a;LOC102549233	Receptor
DMR1:13390001	1	13390001	13392000	2000	1	8.30E-14	0.57	51	2.55	Ccdc28a;LOC102549233	Receptor
DMR1:13461001	1	13461001	13466000	5000	2	2.20E-12	0.86	67	1.34	LOC102549339;Nhsl1	
DMR1:13478001	1	13478001	13479000	1000	1	1.30E-09	-0.63	11	1.1	Nhsl1	
DMR1:13480001	1	13480001	13482000	2000	1	4.10E-07	0.28	30	1.5	Nhsl1	
DMR1:13493001	1	13493001	13495000	2000	1	1.40E-07	-0.46	42	2.1	Nhsl1	
DMR1:13500001	1	13500001	13504000	4000	1	1.70E-14	0.52	67	1.68	Nhsl1	
DMR1:13513001	1	13513001	13518000	5000	3	8.70E-13	0.51	97	1.94	Nhsl1	
DMR1:13520001	1	13520001	13522000	2000	1	1.80E-07	-0.54	36	1.8	Nhsl1	
DMR1:13525001	1	13525001	13527000	2000	1	3.40E-25	0.65	44	2.2	Nhsl1	
DMR1:13529001	1	13529001	13532000	3000	2	6.30E-22	0.76	42	1.4	Nhsl1	
DMR1:13534001	1	13534001	13535000	1000	1	2.70E-11	0.53	6	0.6	Nhsl1	
DMR1:13549001	1	13549001	13553000	4000	2	2.00E-14	0.77	79	1.98	Nhsl1	
DMR1:13563001	1	13563001	13568000	5000	1	1.70E-19	0.67	65	1.3	Nhsl1	
DMR1:13582001	1	13582001	13584000	2000	1	4.80E-10	0.42	32	1.6	Nhsl1	
DMR1:13591001	1	13591001	13592000	1000	1	7.30E-11	0.44	12	1.2	Nhsl1	
DMR1:13598001	1	13598001	13606000	8000	1	4.90E-08	-0.44	153	1.91	Nhsl1	
DMR1:13607001	1	13607001	13608000	1000	1	4.10E-07	0.37	17	1.7	Nhsl1	
DMR1:13610001	1	13610001	13614000	4000	1	6.10E-10	0.37	82	2.05	Nhsl1	
DMR1:13634001	1	13634001	13636000	2000	2	3.30E-13	0.48	21	1.05	Nhsl1	
DMR1:13643001	1	13643001	13653000	10000	3	2.70E-17	-0.65	187	1.87	Nhsl1	
DMR1:13662001	1	13662001	13666000	4000	1	9.20E-17	0.7	73	1.82	Nhsl1	
DMR1:13669001	1	13669001	13674000	5000	4	7.20E-21	0.64	81	1.62	Nhsl1	
DMR1:13688001	1	13688001	13689000	1000	1	3.60E-09	-0.47	12	1.2	Nhsl1	
DMR1:13690001	1	13690001	13697000	7000	2	3.20E-16	0.5	99	1.41	Nhsl1	
DMR1:13698001	1	13698001	13699000	1000	1	1.90E-07	0.31	31	3.1	Nhsl1	

DMR1:13700001	1	13700001	13704000	4000	1	4.70E-09	0.38	95	2.38	Nhsl1;Hebp2	
DMR1:13709001	1	13709001	13713000	4000	1	2.50E-14	0.5	59	1.48	Nhsl1;Hebp2	
DMR1:13724001	1	13724001	13727000	3000	1	1.30E-08	0.56	22	0.73	Hebp2	
DMR1:13826001	1	13826001	13828000	2000	1	1.70E-07	-0.39	12	0.6	Arfgef3	Transcription
DMR1:13834001	1	13834001	13837000	3000	1	3.80E-09	-0.75	43	1.43	Arfgef3	Transcription
DMR1:13862001	1	13862001	13865000	3000	1	5.00E-08	0.41	13	0.43	Arfgef3	Transcription
DMR1:13897001	1	13897001	13900000	3000	1	1.40E-09	0.43	52	1.73	Arfgef3	Transcription
DMR1:13903001	1	13903001	13905000	2000	1	6.60E-11	0.36	17	0.85	Arfgef3	Transcription
DMR1:14115001	1	14115001	14121000	6000	4	1.10E-16	0.66	87	1.45	RGD1560303	Signaling
DMR1:14122001	1	14122001	14125000	3000	1	4.80E-10	0.38	38	1.27	RGD1560303	Signaling
DMR1:14126001	1	14126001	14128000	2000	1	2.40E-07	-0.64	24	1.2	RGD1560303	Signaling
DMR1:14218001	1	14218001	14222000	4000	1	2.90E-10	0.66	34	0.85	Perp	
DMR1:14233001	1	14233001	14235000	2000	1	5.50E-07	0.36	26	1.3	Perp	
DMR1:14397001	1	14397001	14399000	2000	2	4.90E-14	0.49	28	1.4	Tnfaip3	Protease
DMR1:14410001	1	14410001	14413000	3000	1	1.50E-08	-0.48	54	1.8	Tnfaip3	Protease
DMR1:14421001	1	14421001	14422000	1000	1	4.40E-08	0.59	4	0.4	Tnfaip3	Protease
DMR1:15055001	1	15055001	15059000	4000	3	7.30E-15	0.59	35	0.88	Ifngr1	Receptor
DMR1:15082001	1	15082001	15090000	8000	4	5.40E-20	0.67	121	1.51	Ifngr1;Il22ra2	Receptor
DMR1:15091001	1	15091001	15094000	3000	1	2.60E-10	-0.65	30	1	Il22ra2	Receptor
DMR1:15096001	1	15096001	15099000	3000	1	1.60E-08	-0.68	53	1.77	Il22ra2	Receptor
DMR1:15100001	1	15100001	15102000	2000	1	1.60E-16	0.75	40	2	Il22ra2	Receptor
DMR1:15106001	1	15106001	15107000	1000	1	9.10E-08	-0.38	13	1.3	Il22ra2	Receptor
DMR1:15108001	1	15108001	15110000	2000	2	4.20E-13	0.5	27	1.35	Il22ra2	Receptor
DMR1:15112001	1	15112001	15117000	5000	1	6.60E-22	0.81	66	1.32	Il22ra2	Receptor
DMR1:15171001	1	15171001	15172000	1000	1	2.20E-20	0.73	9	0.9	Il20ra	Receptor
DMR1:15173001	1	15173001	15176000	3000	1	2.00E-20	0.7	35	1.17	Il20ra	Receptor
DMR1:15182001	1	15182001	15184000	2000	1	6.00E-20	0.88	28	1.4	Il20ra	Receptor
DMR1:15197001	1	15197001	15198000	1000	1	3.70E-11	0.68	4	0.4	Il20ra	Receptor
DMR1:15204001	1	15204001	15206000	2000	1	3.00E-11	-0.49	47	2.35	Il20ra	Receptor
DMR1:15207001	1	15207001	15208000	1000	1	1.30E-09	0.41	18	1.8	Il20ra	Receptor
DMR1:15216001	1	15216001	15220000	4000	1	4.70E-14	0.61	45	1.12	Il20ra	Receptor
DMR1:15229001	1	15229001	15230000	1000	1	8.30E-10	0.37	6	0.6	Il20ra	Receptor
DMR1:15274001	1	15274001	15289000	15000	5	2.00E-20	-0.48	207	1.38	Slc35d3	Transport
DMR1:15314001	1	15314001	15319000	5000	1	4.40E-08	0.29	19	0.38	Pex7	
DMR1:15320001	1	15320001	15326000	6000	2	3.30E-23	1.04	53	0.88	Pex7	
DMR1:15328001	1	15328001	15329000	1000	1	1.10E-09	0.52	10	1	Pex7	
DMR1:15332001	1	15332001	15333000	1000	1	3.60E-08	-0.52	13	1.3	Pex7	
DMR1:15367001	1	15367001	15368000	1000	1	5.90E-09	-0.48	13	1.3	Pex7	
DMR1:15369001	1	15369001	15373000	4000	3	7.00E-15	0.48	87	2.17	Pex7	
DMR1:15375001	1	15375001	15378000	3000	1	2.50E-10	0.47	39	1.3	Pex7	
DMR1:15381001	1	15381001	15382000	1000	1	1.60E-15	0.7	7	0.7	Pex7;LOC102547604	
DMR1:15402001	1	15402001	15403000	1000	1	2.70E-07	0.5	5	0.5	LOC102547411;Map3k5	Signaling
DMR1:15409001	1	15409001	15410000	1000	1	7.10E-08	-0.48	14	1.4	LOC102547411;Map3k5	Signaling
DMR1:15418001	1	15418001	15421000	3000	1	4.60E-10	0.38	46	1.53	LOC102547411;Map3k5	Signaling
DMR1:15424001	1	15424001	15425000	1000	1	8.50E-09	0.33	21	2.1	Map3k5	Signaling
DMR1:15451001	1	15451001	15452000	1000	1	8.00E-10	-0.44	21	2.1	Map3k5	Signaling
DMR1:15459001	1	15459001	15461000	2000	2	8.00E-12	0.56	33	1.65	Map3k5	Signaling
DMR1:15462001	1	15462001	15465000	3000	1	2.30E-12	0.51	44	1.47	Map3k5	Signaling
DMR1:15470001	1	15470001	15472000	2000	2	1.20E-09	0.69	16	0.8	Map3k5	Signaling
DMR1:15473001	1	15473001	15478000	5000	3	8.40E-16	0.82	77	1.54	Map3k5	Signaling
DMR1:15486001	1	15486001	15490000	4000	2	6.10E-29	0.72	88	2.2	Map3k5	Signaling
DMR1:15499001	1	15499001	15500000	1000	1	6.40E-08	0.35	13	1.3	Map3k5	Signaling
DMR1:15502001	1	15502001	15505000	3000	1	1.70E-14	0.87	67	2.23	Map3k5	Signaling
DMR1:15515001	1	15515001	15516000	1000	1	7.90E-08	-0.4	18	1.8	Map3k5	Signaling
DMR1:15523001	1	15523001	15525000	2000	1	1.40E-08	0.38	55	2.75	Map3k5	Signaling
DMR1:15529001	1	15529001	15536000	7000	1	1.20E-22	0.62	112	1.6	Map3k5;LOC102547350	Signaling
DMR1:15539001	1	15539001	15543000	4000	1	7.10E-15	0.79	55	1.38	Map3k5;LOC102547350	Signaling
DMR1:15547001	1	15547001	15552000	5000	2	5.10E-09	0.39	105	2.1	Map3k5;LOC102547350	Signaling
DMR1:15554001	1	15554001	15559000	5000	1	9.40E-08	-0.59	68	1.36	Map3k5;LOC102547350	Signaling
DMR1:15565001	1	15565001	15570000	5000	1	2.10E-18	0.58	89	1.78	Map3k5;LOC102547350	Signaling
DMR1:15571001	1	15571001	15572000	1000	1	4.40E-09	-0.58	6	0.6	Map3k5	Signaling
DMR1:15579001	1	15579001	15580000	1000	1	1.70E-23	0.64	23	2.3	Map3k5	Signaling
DMR1:15590001	1	15590001	15593000	3000	1	1.10E-17	0.65	71	2.37	Map3k5	Signaling
DMR1:15637001	1	15637001	15640000	3000	1	1.30E-08	0.38	48	1.6	Map7;LOC108349472	Cytoskeleton
DMR1:15644001	1	15644001	15648000	4000	3	2.50E-23	0.82	57	1.43	Map7;LOC108349472	Cytoskeleton

DMR1:15650001	1	15650001	15651000	1000	1	8.80E-08	-0.65	12	1.2	Map7	Cytoskeleton
DMR1:15664001	1	15664001	15666000	2000	1	7.80E-12	0.42	33	1.65	Map7	Cytoskeleton
DMR1:15672001	1	15672001	15674000	2000	1	3.90E-07	0.35	40	2	Map7	Cytoskeleton
DMR1:15678001	1	15678001	15679000	1000	1	6.60E-09	0.37	17	1.7	Map7	Cytoskeleton
DMR1:15689001	1	15689001	15701000	12000	4	1.40E-18	0.77	225	1.88	Map7	Cytoskeleton
DMR1:15702001	1	15702001	15703000	1000	1	1.80E-14	0.51	44	4.4	Map7	Cytoskeleton
DMR1:15725001	1	15725001	15729000	4000	1	3.60E-11	0.44	70	1.75	Map7;LOC102551251	Cytoskeleton
DMR1:15730001	1	15730001	15734000	4000	2	9.50E-23	0.67	77	1.93	Map7;LOC102551251	Cytoskeleton
DMR1:15735001	1	15735001	15737000	2000	1	5.20E-11	0.46	38	1.9	Map7;LOC102551251	Cytoskeleton
DMR1:15752001	1	15752001	15755000	3000	1	4.00E-26	0.95	47	1.57	Map7	Cytoskeleton
DMR1:15791001	1	15791001	15795000	4000	1	1.70E-22	0.69	79	1.98	Bclaf1	
DMR1:15805001	1	15805001	15806000	1000	1	2.80E-10	-0.75	7	0.7	Bclaf1	
DMR1:15822001	1	15822001	15823000	1000	1	1.30E-07	-0.54	6	0.6	Bclaf1	
DMR1:15877001	1	15877001	15882000	5000	2	2.00E-11	-0.78	58	1.16	Pde7b	Signaling
DMR1:15902001	1	15902001	15903000	1000	1	1.10E-09	0.49	5	0.5	Pde7b	Signaling
DMR1:15923001	1	15923001	15928000	5000	3	2.50E-31	0.88	77	1.54	Pde7b	Signaling
DMR1:15929001	1	15929001	15931000	2000	1	1.90E-08	-0.47	30	1.5	Pde7b	Signaling
DMR1:15934001	1	15934001	15938000	4000	3	4.80E-13	0.59	54	1.35	Pde7b	Signaling
DMR1:15942001	1	15942001	15943000	1000	1	2.50E-10	0.39	14	1.4	Pde7b	Signaling
DMR1:15944001	1	15944001	15945000	1000	1	4.50E-14	0.62	13	1.3	Pde7b	Signaling
DMR1:15947001	1	15947001	15949000	2000	2	1.30E-11	0.43	31	1.55	Pde7b	Signaling
DMR1:15953001	1	15953001	15954000	1000	1	8.40E-07	-0.55	0	0	Pde7b	Signaling
DMR1:15959001	1	15959001	15962000	3000	1	1.50E-13	0.56	36	1.2	Pde7b	Signaling
DMR1:15964001	1	15964001	15966000	2000	1	1.30E-19	0.58	34	1.7	Pde7b	Signaling
DMR1:15970001	1	15970001	15974000	4000	1	7.40E-07	-0.43	31	0.78	Pde7b	Signaling
DMR1:15988001	1	15988001	15992000	4000	1	5.60E-11	0.65	52	1.3	Pde7b	Signaling
DMR1:15994001	1	15994001	15996000	2000	1	5.30E-10	0.45	20	1	Pde7b	Signaling
DMR1:16005001	1	16005001	16009000	4000	2	5.80E-08	0.38	62	1.55	Pde7b	Signaling
DMR1:16021001	1	16021001	16023000	2000	1	7.30E-07	0.27	54	2.7	Pde7b	Signaling
DMR1:16027001	1	16027001	16032000	5000	1	4.30E-07	0.45	66	1.32	Pde7b	Signaling
DMR1:16033001	1	16033001	16034000	1000	1	2.80E-12	0.41	19	1.9	Pde7b	Signaling
DMR1:16063001	1	16063001	16068000	5000	1	3.40E-20	0.71	60	1.2	Pde7b	Signaling
DMR1:16091001	1	16091001	16094000	3000	1	2.50E-08	-0.47	39	1.3	Pde7b	Signaling
DMR1:16115001	1	16115001	16117000	2000	1	7.30E-07	-0.61	30	1.5	Pde7b	Signaling
DMR1:16118001	1	16118001	16121000	3000	1	1.30E-21	0.89	26	0.87	Pde7b	Signaling
DMR1:16141001	1	16141001	16143000	2000	1	1.30E-13	0.46	28	1.4	Pde7b	Signaling
DMR1:16176001	1	16176001	16180000	4000	1	1.20E-08	0.33	54	1.35	Pde7b	Signaling
DMR1:16183001	1	16183001	16185000	2000	1	1.10E-07	0.46	10	0.5	Pde7b	Signaling
DMR1:16186001	1	16186001	16190000	4000	2	8.30E-15	0.63	38	0.95	Pde7b	Signaling
DMR1:16198001	1	16198001	16201000	3000	1	3.20E-07	0.42	35	1.17	Pde7b	Signaling
DMR1:16205001	1	16205001	16207000	2000	1	1.30E-08	0.36	31	1.55	Pde7b	Signaling
DMR1:16209001	1	16209001	16210000	1000	1	5.60E-17	0.82	1	0.1	Pde7b	Signaling
DMR1:16498001	1	16498001	16500000	2000	2	7.60E-07	-0.51	24	1.2	Ahi1	
DMR1:16521001	1	16521001	16522000	1000	1	3.60E-10	0.49	17	1.7	Ahi1	
DMR1:16551001	1	16551001	16552000	1000	1	2.50E-13	0.77	11	1.1	Ahi1	
DMR1:16557001	1	16557001	16558000	1000	1	9.00E-10	-0.58	12	1.2	Ahi1	
DMR1:16653001	1	16653001	16657000	4000	1	6.40E-09	0.3	71	1.77	RGD1560020_predicted	
DMR1:16658001	1	16658001	16660000	2000	1	5.80E-07	0.46	41	2.05	RGD1560020_predicted	
DMR1:16665001	1	16665001	16666000	1000	1	1.60E-17	1.02	8	0.8	RGD1560020_predicted	
DMR1:16668001	1	16668001	16670000	2000	1	1.20E-24	0.75	33	1.65	RGD1560020_predicted	
DMR1:16814001	1	16814001	16817000	3000	1	9.70E-15	0.53	51	1.7	Hbs1l	Translation
DMR1:16843001	1	16843001	16844000	1000	1	3.00E-10	0.38	11	1.1	Hbs1l	Translation
DMR1:16845001	1	16845001	16847000	2000	1	1.40E-17	0.58	25	1.25	Hbs1l	Translation
DMR1:16871001	1	16871001	16874000	3000	1	3.40E-07	-0.51	46	1.53	Hbs1l;LOC108348873	Translation
DMR1:16880001	1	16880001	16881000	1000	1	8.30E-09	0.42	9	0.9	Hbs1l;LOC108349717	Translation
DMR1:16882001	1	16882001	16890000	8000	4	2.20E-12	-0.74	75	0.94	Hbs1l;LOC108349717	Translation
DMR1:16896001	1	16896001	16899000	3000	2	1.10E-07	-0.55	28	0.93	Hbs1l;LOC108349717	Translation
DMR1:16904001	1	16904001	16906000	2000	1	3.30E-07	0.41	27	1.35	Hbs1l;Aldh8a1	Translation;Metabolism
DMR1:16929001	1	16929001	16930000	1000	1	3.70E-09	0.36	15	1.5	Aldh8a1	Metabolism
DMR1:16932001	1	16932001	16935000	3000	1	5.00E-19	0.66	43	1.43	Aldh8a1	Metabolism
DMR1:17155001	1	17155001	17158000	3000	1	4.60E-08	0.48	51	1.7	Themis	
DMR1:17176001	1	17176001	17179000	3000	2	4.50E-14	-0.66	10	0.33	Themis	
DMR1:17180001	1	17180001	17181000	1000	1	6.60E-14	0.55	11	1.1	Themis	
DMR1:17192001	1	17192001	17195000	3000	1	3.00E-10	-0.6	12	0.4	Themis	

DMR1:17222001	1	17222001	17224000	2000	1	7.50E-10	-0.59	13	0.65	Themis	
DMR1:17228001	1	17228001	17233000	5000	4	2.80E-26	-0.52	44	0.88	Themis	
DMR1:17253001	1	17253001	17255000	2000	1	1.70E-11	0.67	17	0.85	Themis;LOC108349008;LOC102552380	
DMR1:17258001	1	17258001	17259000	1000	1	9.20E-07	0.49	4	0.4	Themis;LOC108349008;LOC102552380;LOC103690941	
DMR1:17263001	1	17263001	17264000	1000	1	6.20E-07	0.34	11	1.1	Themis;LOC102552380;LOC103690941	
DMR1:17269001	1	17269001	17271000	2000	1	1.00E-08	-0.48	24	1.2	Themis;LOC103690941	
DMR1:17277001	1	17277001	17278000	1000	1	7.80E-12	0.6	12	1.2	Themis;LOC103690941	
DMR1:17283001	1	17283001	17284000	1000	1	1.20E-10	-0.61	8	0.8	Themis	
DMR1:17287001	1	17287001	17288000	1000	1	1.50E-10	0.61	4	0.4	Themis	
DMR1:17294001	1	17294001	17296000	2000	1	6.90E-09	-0.43	9	0.45	Themis	
DMR1:17303001	1	17303001	17306000	3000	2	5.80E-07	-0.4	21	0.7	Themis	
DMR1:17325001	1	17325001	17326000	1000	1	1.30E-09	-0.51	7	0.7	Themis	
DMR1:17327001	1	17327001	17328000	1000	1	2.70E-14	-0.76	3	0.3	Themis	
DMR1:17353001	1	17353001	17356000	3000	1	7.70E-23	0.93	47	1.57	Themis	
DMR1:17382001	1	17382001	17384000	2000	2	4.20E-11	-0.72	5	0.25	Themis	
DMR1:17458001	1	17458001	17462000	4000	1	8.00E-07	-0.56	73	1.82	Ptprk	Signaling
DMR1:17470001	1	17470001	17473000	3000	3	1.40E-07	-0.49	53	1.77	Ptprk	Signaling
DMR1:17486001	1	17486001	17489000	3000	1	7.60E-07	-0.51	51	1.7	Ptprk	Signaling
DMR1:17522001	1	17522001	17523000	1000	1	1.20E-09	-0.6	9	0.9	Ptprk	Signaling
DMR1:17527001	1	17527001	17529000	2000	1	6.10E-09	0.52	13	0.65	Ptprk	Signaling
DMR1:17535001	1	17535001	17536000	1000	1	2.30E-14	0.65	12	1.2	Ptprk	Signaling
DMR1:17539001	1	17539001	17546000	7000	2	7.70E-08	-0.52	100	1.43	Ptprk;LOC102552760	Signaling
DMR1:17566001	1	17566001	17568000	2000	1	9.50E-19	-0.45	22	1.1	Ptprk;LOC102552760;LOC100911400	Signaling
DMR1:17590001	1	17590001	17591000	1000	1	2.50E-07	0.51	7	0.7	Ptprk;LOC103690149	Signaling
DMR1:17622001	1	17622001	17623000	1000	1	6.80E-08	0.41	19	1.9	Ptprk;LOC103690149	Signaling
DMR1:17638001	1	17638001	17639000	1000	1	9.30E-07	0.5	13	1.3	Ptprk;LOC103690149	Signaling
DMR1:17643001	1	17643001	17644000	1000	1	4.60E-14	0.41	45	4.5	Ptprk	Signaling
DMR1:17648001	1	17648001	17649000	1000	1	9.50E-07	-0.34	18	1.8	Ptprk	Signaling
DMR1:17670001	1	17670001	17673000	3000	2	4.60E-08	-0.62	32	1.07	Ptprk	Signaling
DMR1:17698001	1	17698001	17700000	2000	1	8.20E-11	0.51	13	0.65	Ptprk	Signaling
DMR1:17725001	1	17725001	17726000	1000	1	3.70E-07	0.34	21	2.1	Ptprk	Signaling
DMR1:17748001	1	17748001	17750000	2000	1	1.50E-09	0.51	29	1.45	Ptprk	Signaling
DMR1:17753001	1	17753001	17758000	5000	2	6.30E-12	0.69	82	1.64	Ptprk	Signaling
DMR1:17817001	1	17817001	17820000	3000	1	1.40E-08	-0.57	45	1.5	Ptprk	Signaling
DMR1:17826001	1	17826001	17829000	3000	1	1.10E-08	0.51	28	0.93	Ptprk	Signaling
DMR1:17834001	1	17834001	17838000	4000	1	5.50E-07	0.37	58	1.45	Ptprk	Signaling
DMR1:17852001	1	17852001	17855000	3000	1	7.40E-08	-0.56	49	1.63	Ptprk	Signaling
DMR1:17864001	1	17864001	17865000	1000	1	1.50E-11	0.45	15	1.5	Ptprk	Signaling
DMR1:17904001	1	17904001	17906000	2000	1	4.40E-16	0.67	27	1.35	Ptprk	Signaling
DMR1:17916001	1	17916001	17917000	1000	1	8.50E-13	0.61	11	1.1	Ptprk	Signaling
DMR1:17927001	1	17927001	17928000	1000	1	8.10E-08	-0.57	9	0.9	Ptprk	Signaling
DMR1:17957001	1	17957001	17959000	2000	1	6.10E-12	0.63	23	1.15	Ptprk	Signaling
DMR1:17981001	1	17981001	17987000	6000	1	3.00E-12	0.77	69	1.15	Ptprk	Signaling
DMR1:18022001	1	18022001	18027000	5000	2	7.60E-10	-0.72	51	1.02	Ptprk	Signaling
DMR1:18028001	1	18028001	18034000	6000	3	2.00E-08	-0.49	87	1.45	Ptprk	Signaling
DMR1:18036001	1	18036001	18038000	2000	1	4.70E-17	0.67	20	1	Ptprk	Signaling
DMR1:18050001	1	18050001	18056000	6000	1	1.10E-08	-0.52	70	1.17	Ptprk	Signaling
DMR1:18494001	1	18494001	18495000	1000	1	2.50E-10	0.8	6	0.6	Lama2	Extracellular Matrix
DMR1:18513001	1	18513001	18517000	4000	2	2.20E-21	-0.55	29	0.72	Lama2	Extracellular Matrix
DMR1:18531001	1	18531001	18533000	2000	1	1.40E-07	0.52	20	1	Lama2	Extracellular Matrix
DMR1:18555001	1	18555001	18556000	1000	1	1.50E-08	0.49	10	1	Lama2	Extracellular Matrix
DMR1:18559001	1	18559001	18562000	3000	2	5.50E-12	-0.46	33	1.1	Lama2	Extracellular Matrix
DMR1:18567001	1	18567001	18568000	1000	1	1.30E-10	-0.84	12	1.2	Lama2	Extracellular Matrix
DMR1:18583001	1	18583001	18585000	2000	1	4.40E-12	0.62	17	0.85	Lama2	Extracellular Matrix
DMR1:18589001	1	18589001	18591000	2000	1	1.20E-10	-0.64	9	0.45	Lama2	Extracellular Matrix
DMR1:18596001	1	18596001	18597000	1000	1	1.90E-09	0.66	3	0.3	Lama2	Extracellular Matrix
DMR1:18599001	1	18599001	18603000	4000	3	3.40E-11	-0.56	16	0.4	Lama2	Extracellular Matrix
DMR1:18615001	1	18615001	18618000	3000	1	9.50E-10	0.44	30	1	Lama2	Extracellular Matrix
DMR1:18629001	1	18629001	18630000	1000	1	5.10E-09	0.51	11	1.1	Lama2	Extracellular Matrix
DMR1:18637001	1	18637001	18639000	2000	1	6.10E-07	-0.54	11	0.55	Lama2	Extracellular Matrix
DMR1:18647001	1	18647001	18648000	1000	1	4.60E-10	0.59	6	0.6	Lama2	Extracellular Matrix

DMR1:18668001	1	18668001	18670000	2000	1	8.30E-07	-0.53	19	0.95	Lama2	Extracellular Matrix
DMR1:18683001	1	18683001	18684000	1000	1	1.40E-07	-0.56	6	0.6	Lama2	Extracellular Matrix
DMR1:18691001	1	18691001	18693000	2000	1	2.70E-10	0.54	10	0.5	Lama2	Extracellular Matrix
DMR1:18694001	1	18694001	18695000	1000	1	9.70E-08	-0.47	12	1.2	Lama2	Extracellular Matrix
DMR1:18700001	1	18700001	18704000	4000	3	2.00E-21	-0.51	48	1.2	Lama2	Extracellular Matrix
DMR1:18705001	1	18705001	18709000	4000	2	5.70E-17	0.88	26	0.65	Lama2	Extracellular Matrix
DMR1:18737001	1	18737001	18740000	3000	3	6.20E-16	0.67	29	0.97	Lama2	Extracellular Matrix
DMR1:18765001	1	18765001	18766000	1000	1	6.70E-09	-0.52	3	0.3	Lama2	Extracellular Matrix
DMR1:18781001	1	18781001	18786000	5000	1	3.10E-08	-0.45	52	1.04	Lama2	Extracellular Matrix
DMR1:18787001	1	18787001	18790000	3000	1	1.60E-07	-0.47	20	0.67	Lama2	Extracellular Matrix
DMR1:18802001	1	18802001	18804000	2000	1	9.20E-11	0.59	13	0.65	Lama2	Extracellular Matrix
DMR1:18805001	1	18805001	18807000	2000	1	5.90E-09	0.67	20	1	Lama2	Extracellular Matrix
DMR1:18816001	1	18816001	18817000	1000	1	4.60E-07	-0.48	14	1.4	Lama2	Extracellular Matrix
DMR1:18853001	1	18853001	18855000	2000	2	1.20E-17	0.65	9	0.45	Lama2	Extracellular Matrix
DMR1:18880001	1	18880001	18881000	1000	1	2.40E-16	0.51	12	1.2	Lama2	Extracellular Matrix
DMR1:18891001	1	18891001	18896000	5000	4	1.00E-10	-0.77	17	0.34	Lama2	Extracellular Matrix
DMR1:18926001	1	18926001	18931000	5000	2	2.50E-07	0.44	56	1.12	Lama2	Extracellular Matrix
DMR1:18936001	1	18936001	18938000	2000	2	1.10E-16	-0.86	6	0.3	Lama2	Extracellular Matrix
DMR1:18955001	1	18955001	18956000	1000	1	1.10E-11	0.44	15	1.5	Lama2	Extracellular Matrix
DMR1:18960001	1	18960001	18965000	5000	1	9.20E-11	0.51	64	1.28	Lama2	Extracellular Matrix
DMR1:18967001	1	18967001	18970000	3000	2	1.00E-09	0.53	26	0.87	Lama2	Extracellular Matrix
DMR1:18985001	1	18985001	18986000	1000	1	7.90E-13	0.83	9	0.9	Lama2	Extracellular Matrix
DMR1:18993001	1	18993001	18996000	3000	2	5.10E-10	0.55	23	0.77	Lama2	Extracellular Matrix
DMR1:19001001	1	19001001	19010000	9000	4	7.10E-29	-0.55	102	1.13	Lama2	Extracellular Matrix
DMR1:19015001	1	19015001	19021000	6000	2	2.90E-18	-0.6	37	0.62	Lama2	Extracellular Matrix
DMR1:19031001	1	19031001	19032000	1000	1	7.70E-09	0.43	3	0.3	Lama2	Extracellular Matrix
DMR1:19038001	1	19038001	19039000	1000	1	7.10E-08	0.5	7	0.7	Lama2	Extracellular Matrix
DMR1:19059001	1	19059001	19060000	1000	1	3.80E-13	0.64	7	0.7	Lama2	Extracellular Matrix
DMR1:19068001	1	19068001	19069000	1000	1	1.30E-18	0.66	11	1.1	Lama2	Extracellular Matrix
DMR1:19073001	1	19073001	19082000	9000	5	4.10E-25	-0.59	102	1.13	Lama2	Extracellular Matrix
DMR1:19097001	1	19097001	19098000	1000	1	2.00E-16	0.59	8	0.8	Lama2	Extracellular Matrix
DMR1:19100001	1	19100001	19101000	1000	1	6.10E-13	0.57	11	1.1	Lama2	Extracellular Matrix
DMR1:19103001	1	19103001	19107000	4000	2	6.00E-08	-0.54	19	0.48	Lama2	Extracellular Matrix
DMR1:19135001	1	19135001	19137000	2000	1	2.40E-08	-0.57	9	0.45	Lama2	Extracellular Matrix
DMR1:19148001	1	19148001	19149000	1000	1	1.40E-08	0.4	13	1.3	Lama2	Extracellular Matrix
DMR1:19153001	1	19153001	19155000	2000	1	5.10E-12	-0.54	8	0.4	Lama2	Extracellular Matrix
DMR1:19221001	1	19221001	19222000	1000	1	2.50E-14	0.65	12	1.2	LOC108349500;Arhgap18	Signaling
DMR1:19227001	1	19227001	19228000	1000	1	6.10E-08	-0.61	10	1	LOC108349500;Arhgap18	Signaling
DMR1:19229001	1	19229001	19233000	4000	4	1.10E-14	-0.95	21	0.52	Arhgap18	Signaling
DMR1:19238001	1	19238001	19240000	2000	1	2.20E-11	0.52	34	1.7	Arhgap18	Signaling
DMR1:19241001	1	19241001	19242000	1000	1	7.10E-08	-0.53	4	0.4	Arhgap18	Signaling
DMR1:19255001	1	19255001	19259000	4000	2	9.60E-09	0.38	100	2.5	Arhgap18	Signaling
DMR1:19260001	1	19260001	19264000	4000	2	6.20E-08	0.44	44	1.1	Arhgap18	Signaling
DMR1:19285001	1	19285001	19290000	5000	1	4.20E-09	0.46	61	1.22	Arhgap18;LOC103690928	Signaling
DMR1:19291001	1	19291001	19294000	3000	1	6.80E-09	-0.61	35	1.17	Arhgap18;LOC103690928	Signaling
DMR1:19306001	1	19306001	19308000	2000	1	1.70E-13	0.61	19	0.95	Arhgap18	Signaling
DMR1:19310001	1	19310001	19314000	4000	2	1.60E-11	0.58	31	0.78	Arhgap18	Signaling
DMR1:19319001	1	19319001	19320000	1000	1	7.80E-10	0.41	30	3	Arhgap18	Signaling
DMR1:19341001	1	19341001	19342000	1000	1	9.20E-07	0.32	15	1.5	Arhgap18	Signaling
DMR1:19364001	1	19364001	19367000	3000	1	2.00E-07	0.4	39	1.3	Arhgap18	Signaling
DMR1:19369001	1	19369001	19370000	1000	1	3.80E-10	-0.55	16	1.6	Arhgap18	Signaling
DMR1:19378001	1	19378001	19381000	3000	1	1.10E-12	0.44	45	1.5	Arhgap18	Signaling
DMR1:19389001	1	19389001	19392000	3000	1	5.40E-08	-0.5	41	1.37	Arhgap18	Signaling
DMR1:19411001	1	19411001	19415000	4000	2	7.40E-09	-0.49	22	0.55	Arhgap18	Signaling
DMR1:19430001	1	19430001	19434000	4000	1	4.30E-09	0.45	47	1.18	Arhgap18	Signaling
DMR1:19446001	1	19446001	19449000	3000	1	3.00E-10	0.42	34	1.13	Arhgap18	Signaling
DMR1:19450001	1	19450001	19453000	3000	1	6.90E-12	0.64	40	1.33	Arhgap18	Signaling
DMR1:19455001	1	19455001	19458000	3000	2	2.10E-17	0.6	14	0.47	Arhgap18	Signaling
DMR1:19466001	1	19466001	19467000	1000	1	4.20E-15	0.74	10	1	Arhgap18	Signaling
DMR1:19470001	1	19470001	19471000	1000	1	1.20E-07	0.39	11	1.1	Arhgap18	Signaling
DMR1:20010001	1	20010001	20015000	5000	3	6.70E-21	0.98	46	0.92	L3mbtl3	Epigenetic
DMR1:20020001	1	20020001	20023000	3000	1	3.20E-20	0.6	49	1.63	L3mbtl3	Epigenetic
DMR1:20025001	1	20025001	20030000	5000	2	3.20E-14	0.66	50	1	L3mbtl3	Epigenetic
DMR1:20031001	1	20031001	20033000	2000	1	1.30E-14	0.55	31	1.55	L3mbtl3	Epigenetic
DMR1:20034001	1	20034001	20037000	3000	1	8.00E-11	0.42	31	1.03	L3mbtl3	Epigenetic

DMR1:20055001	1	20055001	20057000	2000	2	8.00E-13	0.52	17	0.85	L3mbtl3	Epigenetic
DMR1:20071001	1	20071001	20073000	2000	1	1.10E-12	0.44	21	1.05	L3mbtl3	Epigenetic
DMR1:20084001	1	20084001	20086000	2000	1	5.50E-07	-0.47	25	1.25	L3mbtl3	Epigenetic
DMR1:20114001	1	20114001	20116000	2000	1	2.70E-09	-0.32	50	2.5	Samd3;LOC100360791	
DMR1:20117001	1	20117001	20122000	5000	3	4.80E-20	-0.5	44	0.88	Samd3;LOC100360791	
DMR1:20129001	1	20129001	20130000	1000	1	3.10E-07	-0.54	9	0.9	Samd3	
DMR1:20132001	1	20132001	20133000	1000	1	1.50E-07	0.4	3	0.3	Samd3	
DMR1:20326001	1	20326001	20328000	2000	1	3.00E-08	0.4	15	0.75	Tmem200a	
DMR1:20335001	1	20335001	20337000	2000	2	2.70E-12	0.54	25	1.25	Tmem200a	
DMR1:20369001	1	20369001	20373000	4000	1	2.10E-17	0.66	49	1.23	Tmem200a	
DMR1:20377001	1	20377001	20379000	2000	2	4.80E-17	0.98	7	0.35	Tmem200a	
DMR1:20393001	1	20393001	20394000	1000	1	2.30E-15	0.87	5	0.5	Tmem200a	
DMR1:20398001	1	20398001	20399000	1000	1	7.60E-11	0.58	9	0.9	Tmem200a	
DMR1:20424001	1	20424001	20427000	3000	2	2.00E-08	-0.55	13	0.43	RGD1559962	
DMR1:20856001	1	20856001	20858000	2000	1	6.00E-07	0.53	26	1.3	Smlr1	
DMR1:20862001	1	20862001	20864000	2000	1	9.90E-08	-0.63	13	0.65	Smlr1	
DMR1:20872001	1	20872001	20873000	1000	1	7.90E-08	-0.38	3	0.3	Smlr1;Epb41l2	
DMR1:20876001	1	20876001	20877000	1000	1	1.60E-10	0.4	23	2.3	Smlr1;Epb41l2;LOC102552668	
DMR1:20887001	1	20887001	20892000	5000	1	1.00E-10	0.39	67	1.34	Smlr1;Epb41l2;LOC102552668	
DMR1:20903001	1	20903001	20904000	1000	1	1.90E-11	0.4	28	2.8	Epb41l2	
DMR1:20916001	1	20916001	20920000	4000	1	1.50E-07	0.35	46	1.15	Epb41l2	
DMR1:20923001	1	20923001	20927000	4000	2	5.80E-10	0.45	54	1.35	Epb41l2	
DMR1:20929001	1	20929001	20933000	4000	1	1.00E-14	0.52	46	1.15	Epb41l2	
DMR1:20944001	1	20944001	20947000	3000	1	2.60E-11	0.41	39	1.3	Epb41l2	
DMR1:20961001	1	20961001	20966000	5000	1	1.40E-19	0.64	63	1.26	Epb41l2	
DMR1:20982001	1	20982001	20984000	2000	1	3.40E-29	0.87	29	1.45	Epb41l2	
DMR1:20985001	1	20985001	20987000	2000	2	1.00E-19	0.5	58	2.9	Epb41l2	
DMR1:21001001	1	21001001	21002000	1000	1	8.70E-11	0.44	12	1.2	Epb41l2	
DMR1:21003001	1	21003001	21007000	4000	1	7.40E-08	-0.36	57	1.43	Epb41l2	
DMR1:21021001	1	21021001	21022000	1000	1	3.60E-11	0.55	3	0.3	Epb41l2	
DMR1:21029001	1	21029001	21034000	5000	2	1.00E-14	-0.74	25	0.5	Epb41l2	
DMR1:21035001	1	21035001	21045000	10000	1	3.80E-08	-0.62	160	1.6	Epb41l2	
DMR1:21071001	1	21071001	21074000	3000	1	9.30E-13	0.4	59	1.97	Epb41l2;LOC108349502	
DMR1:21133001	1	21133001	21134000	1000	1	3.20E-16	0.54	26	2.6	LOC102552728;Akap7	Translation
DMR1:21138001	1	21138001	21140000	2000	1	8.10E-11	0.48	48	2.4	LOC102552728;Akap7;LOC100364016	Translation
DMR1:21144001	1	21144001	21151000	7000	1	7.00E-09	-0.49	136	1.94	Akap7;LOC100364016	Translation
DMR1:21201001	1	21201001	21208000	7000	2	9.80E-22	0.7	121	1.73	Akap7	Translation
DMR1:21216001	1	21216001	21219000	3000	3	2.60E-15	0.7	30	1	Akap7	Translation
DMR1:21224001	1	21224001	21229000	5000	3	1.20E-19	0.62	81	1.62	Akap7	Translation
DMR1:21231001	1	21231001	21234000	3000	2	4.30E-15	-0.65	56	1.87	Akap7	Translation
DMR1:21237001	1	21237001	21241000	4000	1	1.50E-11	0.46	55	1.38	Akap7	Translation
DMR1:21242001	1	21242001	21243000	1000	1	1.90E-08	0.44	7	0.7	Akap7	Translation
DMR1:21247001	1	21247001	21248000	1000	1	4.50E-17	0.71	3	0.3	Akap7	Translation
DMR1:21265001	1	21265001	21266000	1000	1	4.90E-08	-0.52	17	1.7	Akap7	Translation
DMR1:21531001	1	21531001	21532000	1000	1	3.00E-11	0.5	16	1.6	Arg1;Med23	Metabolism;Transcription
DMR1:21552001	1	21552001	21555000	3000	1	4.80E-17	0.66	44	1.47	Med23	Transcription
DMR1:21561001	1	21561001	21565000	4000	1	3.70E-08	-0.67	64	1.6	Med23	Transcription
DMR1:21573001	1	21573001	21577000	4000	2	7.80E-11	-0.64	22	0.55	Med23	Transcription
DMR1:21591001	1	21591001	21594000	3000	1	1.30E-10	0.44	46	1.53	Med23	Transcription
DMR1:21597001	1	21597001	21598000	1000	1	7.70E-09	0.59	4	0.4	Med23	Transcription
DMR1:21603001	1	21603001	21605000	2000	1	8.10E-15	0.61	26	1.3	Enpp3	
DMR1:21606001	1	21606001	21610000	4000	1	2.10E-08	0.61	57	1.43	Enpp3	
DMR1:21619001	1	21619001	21621000	2000	1	1.10E-17	0.6	34	1.7	Enpp3	
DMR1:21627001	1	21627001	21633000	6000	3	6.90E-14	0.64	57	0.95	Enpp3	
DMR1:21650001	1	21650001	21653000	3000	1	4.20E-16	0.65	35	1.17	Enpp3	
DMR1:21670001	1	21670001	21677000	7000	1	6.60E-09	0.47	102	1.46	Enpp3	
DMR1:21680001	1	21680001	21682000	2000	2	6.20E-12	-0.66	30	1.5	Enpp3	
DMR1:21683001	1	21683001	21688000	5000	2	1.10E-11	0.51	83	1.66	Enpp3;LOC102553363	
DMR1:21689001	1	21689001	21693000	4000	1	1.50E-07	-0.48	52	1.3	Enpp3;LOC102553363	
DMR1:21739001	1	21739001	21741000	2000	2	2.20E-14	-0.54	21	1.05	Enpp1	
DMR1:21755001	1	21755001	21756000	1000	1	3.80E-23	0.89	5	0.5	Enpp1	
DMR1:21768001	1	21768001	21783000	15000	2	8.30E-16	0.73	214	1.43	Enpp1	
DMR1:21786001	1	21786001	21789000	3000	1	5.50E-15	0.58	46	1.53	Enpp1	

DMR1:21790001	1	21790001	21803000	13000	1	5.20E-09	0.55	191	1.47	Enpp1	
DMR1:21808001	1	21808001	21811000	3000	1	5.30E-07	0.32	39	1.3	Enpp1	
DMR1:21812001	1	21812001	21816000	4000	1	2.00E-11	0.45	53	1.32	Enpp1	
DMR1:21860001	1	21860001	21863000	3000	1	5.70E-10	0.5	39	1.3	Ctgf;LOC108349248;LOC103690930	
DMR1:21864001	1	21864001	21866000	2000	1	6.10E-11	0.43	24	1.2	Ctgf;LOC108349248;LOC103690930	
DMR1:22116001	1	22116001	22118000	2000	1	1.10E-07	0.32	27	1.35	LOC502223;Moxd1	Metabolism
DMR1:22119001	1	22119001	22120000	1000	1	3.40E-08	0.5	14	1.4	LOC502223;Moxd1	Metabolism
DMR1:22126001	1	22126001	22128000	2000	2	1.60E-13	0.48	25	1.25	LOC502223;Moxd1	Metabolism
DMR1:22131001	1	22131001	22135000	4000	1	1.90E-07	-0.58	44	1.1	Moxd1	Metabolism
DMR1:22146001	1	22146001	22148000	2000	1	3.80E-07	-0.37	37	1.85	Moxd1	Metabolism
DMR1:22158001	1	22158001	22160000	2000	1	1.00E-16	0.52	45	2.25	Moxd1;LOC108349454	Metabolism
DMR1:22187001	1	22187001	22193000	6000	4	2.00E-19	-0.51	59	0.98	Moxd1	Metabolism
DMR1:22235001	1	22235001	22236000	1000	1	4.70E-07	0.47	10	1	Stx7	Transcription
DMR1:22239001	1	22239001	22241000	2000	2	3.20E-13	0.89	4	0.2	Stx7	Transcription
DMR1:22251001	1	22251001	22254000	3000	2	6.70E-12	0.57	46	1.53	Stx7	Transcription
DMR1:22257001	1	22257001	22265000	8000	3	1.00E-10	0.65	126	1.57	Stx7	Transcription
DMR1:22274001	1	22274001	22276000	2000	1	2.60E-14	0.65	26	1.3	Stx7;LOC108349187	Transcription
DMR1:22336001	1	22336001	22340000	4000	3	1.30E-22	-0.59	31	0.78	Taar8c	Signaling
DMR1:22359001	1	22359001	22361000	2000	1	1.30E-07	0.61	20	1	Taar8a	Signaling
DMR1:22419001	1	22419001	22422000	3000	2	9.50E-18	-0.7	11	0.37	LOC108349728;Taar7i-ps	
DMR1:22436001	1	22436001	22441000	5000	2	4.30E-15	-0.57	23	0.46	Taar7i-ps	
DMR1:22481001	1	22481001	22482000	1000	1	5.20E-10	0.54	6	0.6	Taar7f-ps;Taar7e;Taar7d	Signaling
DMR1:22489001	1	22489001	22497000	8000	4	1.30E-21	-0.44	84	1.05	Taar7e;Taar7d	Signaling
DMR1:22509001	1	22509001	22512000	3000	2	7.30E-18	0.64	20	0.67	Taar7c;Taar7b	Signaling
DMR1:22525001	1	22525001	22528000	3000	1	2.10E-16	0.72	38	1.27	Taar7b;Taar7a;Taar6	Signaling
DMR1:22533001	1	22533001	22534000	1000	1	4.10E-08	0.32	12	1.2	Taar7a;Taar6	Signaling
DMR1:22541001	1	22541001	22543000	2000	1	1.00E-07	0.45	22	1.1	Taar6;Taar5	Signaling
DMR1:22545001	1	22545001	22547000	2000	2	2.40E-17	1	20	1	Taar6;Taar5	Signaling
DMR1:22575001	1	22575001	22577000	2000	1	8.50E-07	-0.42	34	1.7	LOC102554023;Taar3;Taar2	Signaling
DMR1:22578001	1	22578001	22580000	2000	1	9.90E-07	-0.46	42	2.1	LOC102554023;Taar3;Taar2	Signaling
DMR1:22609001	1	22609001	22611000	2000	2	1.90E-12	0.53	38	1.9	LOC103690932;Vnn1	Metabolism
DMR1:22625001	1	22625001	22626000	1000	1	1.40E-07	0.5	9	0.9	Vnn1	Metabolism
DMR1:22631001	1	22631001	22633000	2000	2	5.20E-11	0.54	28	1.4	Vnn1	Metabolism
DMR1:22654001	1	22654001	22657000	3000	1	2.50E-09	-0.7	54	1.8	Vnn3	Metabolism
DMR1:22710001	1	22710001	22723000	13000	5	8.20E-21	0.82	148	1.14	Slc18b1	Transport
DMR1:22728001	1	22728001	22735000	7000	3	2.00E-11	0.56	46	0.66	Slc18b1	Transport
DMR1:22736001	1	22736001	22737000	1000	1	2.60E-15	0.85	6	0.6	Slc18b1	Transport
DMR1:22738001	1	22738001	22740000	2000	2	3.00E-14	-0.53	32	1.6	Slc18b1	Transport
DMR1:22741001	1	22741001	22742000	1000	1	9.70E-11	0.37	17	1.7	Slc18b1	Transport
DMR1:22744001	1	22744001	22747000	3000	1	1.40E-12	0.49	57	1.9	Slc18b1	Transport
DMR1:22766001	1	22766001	22772000	6000	3	5.10E-13	-0.6	51	0.85	LOC100910237;Rps12	Translation
DMR1:23248001	1	23248001	23250000	2000	1	5.40E-18	1	17	0.85	Eya4	
DMR1:23256001	1	23256001	23258000	2000	1	1.50E-10	0.55	18	0.9	Eya4	
DMR1:23259001	1	23259001	23262000	3000	2	2.00E-24	1.04	30	1	Eya4	
DMR1:23278001	1	23278001	23281000	3000	1	1.40E-11	0.44	39	1.3	Eya4	
DMR1:23301001	1	23301001	23302000	1000	1	1.40E-09	0.51	9	0.9	Eya4	
DMR1:23313001	1	23313001	23317000	4000	1	2.20E-08	0.5	72	1.8	Eya4	
DMR1:23320001	1	23320001	23322000	2000	1	2.40E-08	0.4	25	1.25	Eya4	
DMR1:23331001	1	23331001	23334000	3000	1	4.90E-07	0.48	17	0.57	Eya4	
DMR1:23354001	1	23354001	23356000	2000	1	3.70E-07	-0.44	25	1.25	Eya4	
DMR1:23363001	1	23363001	23364000	1000	1	2.80E-08	0.43	8	0.8	Eya4	
DMR1:23369001	1	23369001	23374000	5000	1	2.20E-07	0.33	61	1.22	Eya4	
DMR1:23386001	1	23386001	23389000	3000	1	1.70E-16	0.59	29	0.97	Eya4	
DMR1:23393001	1	23393001	23397000	4000	1	6.30E-11	0.48	35	0.88	Eya4	
DMR1:23398001	1	23398001	23399000	1000	1	3.70E-10	0.4	13	1.3	Eya4	
DMR1:23421001	1	23421001	23422000	1000	1	4.60E-09	-0.65	12	1.2	Eya4	
DMR1:23439001	1	23439001	23440000	1000	1	1.60E-07	0.4	7	0.7	Eya4	
DMR1:23453001	1	23453001	23454000	1000	1	2.10E-07	0.4	8	0.8	Eya4	
DMR1:23490001	1	23490001	23494000	4000	1	1.20E-09	0.47	29	0.72	Eya4	
DMR1:23519001	1	23519001	23520000	1000	1	1.10E-08	0.49	12	1.2	Eya4	
DMR1:23535001	1	23535001	23536000	1000	1	3.60E-08	0.54	8	0.8	Eya4;LOC100910446	Transcription
DMR1:23539001	1	23539001	23542000	3000	3	2.30E-17	1	15	0.5	Eya4;LOC100910446	Transcription
DMR1:23552001	1	23552001	23554000	2000	1	1.80E-14	0.66	33	1.65	Eya4;LOC100910446	Transcription

DMR1:23557001	1	23557001	23559000	2000	1	8.60E-09	0.56	19	0.95	Eya4;LOC100910446;Taar8b	Transcription;Signaling
DMR1:23561001	1	23561001	23564000	3000	1	2.60E-08	0.64	25	0.83	Eya4;LOC100910446;Taar8b	Transcription;Signaling
DMR1:23581001	1	23581001	23582000	1000	1	2.40E-07	-0.53	18	1.8	Eya4	
DMR1:23593001	1	23593001	23596000	3000	1	1.00E-13	-0.77	17	0.57	Eya4	
DMR1:23616001	1	23616001	23620000	4000	2	1.50E-20	0.71	52	1.3	Eya4	
DMR1:23892001	1	23892001	23897000	5000	2	3.20E-08	-0.59	59	1.18	Tcf21	Transcription
DMR1:23903001	1	23903001	23905000	2000	1	9.80E-07	0.35	28	1.4	Tcf21	Transcription
DMR1:23911001	1	23911001	23913000	2000	1	1.20E-12	0.8	39	1.95	Tcf21	Transcription
DMR1:23918001	1	23918001	23920000	2000	1	2.20E-09	0.43	18	0.9	Tcf21	Transcription
DMR1:23972001	1	23972001	23974000	2000	1	3.10E-17	0.82	32	1.6	Tbpl1	Transcription
DMR1:23989001	1	23989001	23999000	10000	2	1.70E-18	0.77	125	1.25	Tbpl1;Slc2a12	Transcription
DMR1:24008001	1	24008001	24016000	8000	5	4.20E-15	0.54	117	1.46	Tbpl1;Slc2a12	Transcription
DMR1:24027001	1	24027001	24035000	8000	3	5.20E-17	0.61	105	1.31	Slc2a12	
DMR1:24036001	1	24036001	24041000	5000	1	5.70E-12	0.69	73	1.46	Slc2a12	
DMR1:24043001	1	24043001	24049000	6000	2	3.00E-08	0.44	82	1.37	Slc2a12	
DMR1:24180001	1	24180001	24182000	2000	1	1.80E-17	0.71	16	0.8	Sgk1	Signaling
DMR1:24209001	1	24209001	24210000	1000	1	5.50E-10	0.38	18	1.8	Sgk1	Signaling
DMR1:24213001	1	24213001	24214000	1000	1	6.60E-11	0.52	13	1.3	Sgk1	Signaling
DMR1:24219001	1	24219001	24225000	6000	2	6.00E-20	0.71	80	1.33	Sgk1	Signaling
DMR1:24228001	1	24228001	24232000	4000	2	8.80E-26	1.01	47	1.18	Sgk1	Signaling
DMR1:24233001	1	24233001	24235000	2000	1	6.80E-08	0.44	17	0.85	Sgk1	Signaling
DMR1:24255001	1	24255001	24256000	1000	1	4.00E-12	0.65	11	1.1	Sgk1	Signaling
DMR1:24262001	1	24262001	24263000	1000	1	7.60E-08	-0.45	15	1.5	Sgk1	Signaling
DMR1:24266001	1	24266001	24269000	3000	3	3.70E-07	-0.39	36	1.2	Sgk1	Signaling
DMR1:24282001	1	24282001	24285000	3000	1	1.80E-10	0.46	31	1.03	Sgk1	Signaling
DMR1:24291001	1	24291001	24293000	2000	1	3.20E-07	0.3	33	1.65	Sgk1;LOC108349236	Signaling
DMR1:24312001	1	24312001	24313000	1000	1	6.90E-12	0.76	8	0.8	Sgk1;LOC108349236;LOC108349238	Signaling
DMR1:25165001	1	25165001	25166000	1000	1	1.90E-08	-0.58	5	0.5	Clvs2	Transport
DMR1:25179001	1	25179001	25182000	3000	2	1.10E-13	0.63	39	1.3	Clvs2	Transport
DMR1:25183001	1	25183001	25184000	1000	1	1.60E-12	0.62	4	0.4	Clvs2	Transport
DMR1:25222001	1	25222001	25226000	4000	2	2.40E-13	-0.68	19	0.48	Clvs2	Transport
DMR1:25399001	1	25399001	25401000	2000	1	1.50E-08	-0.57	4	0.2	Trdn	
DMR1:25425001	1	25425001	25426000	1000	1	1.20E-09	0.72	7	0.7	Trdn	
DMR1:25443001	1	25443001	25445000	2000	1	2.90E-09	0.61	6	0.3	Trdn	
DMR1:25456001	1	25456001	25457000	1000	1	3.80E-12	0.64	7	0.7	Trdn	
DMR1:25462001	1	25462001	25465000	3000	3	1.00E-12	-0.84	12	0.4	Trdn	
DMR1:25468001	1	25468001	25469000	1000	1	7.80E-16	0.87	4	0.4	Trdn	
DMR1:25472001	1	25472001	25473000	1000	1	8.40E-19	0.76	8	0.8	Trdn	
DMR1:25478001	1	25478001	25482000	4000	2	4.10E-28	0.91	33	0.82	Trdn	
DMR1:25490001	1	25490001	25493000	3000	2	1.30E-08	0.6	21	0.7	Trdn	
DMR1:25513001	1	25513001	25514000	1000	1	6.10E-10	0.73	6	0.6	Trdn	
DMR1:25527001	1	25527001	25529000	2000	1	2.20E-07	-0.54	9	0.45	Trdn	
DMR1:25537001	1	25537001	25539000	2000	1	1.80E-09	-0.43	16	0.8	Trdn	
DMR1:25543001	1	25543001	25544000	1000	1	9.70E-09	0.58	5	0.5	Trdn	
DMR1:25563001	1	25563001	25567000	4000	2	2.70E-07	0.54	25	0.62	Trdn	
DMR1:25569001	1	25569001	25573000	4000	2	1.20E-10	-0.61	10	0.25	Trdn	
DMR1:25591001	1	25591001	25594000	3000	2	3.30E-18	-0.75	11	0.37	Trdn	
DMR1:25613001	1	25613001	25615000	2000	1	2.40E-23	0.98	3	0.15	Trdn	
DMR1:25622001	1	25622001	25625000	3000	1	1.10E-17	-0.79	8	0.27	Trdn	
DMR1:25653001	1	25653001	25654000	1000	1	5.40E-08	0.49	7	0.7	Trdn	
DMR1:25668001	1	25668001	25670000	2000	1	7.40E-18	0.6	16	0.8	Trdn	
DMR1:25699001	1	25699001	25708000	9000	5	2.30E-19	-0.38	107	1.19	Trdn	
DMR1:25730001	1	25730001	25732000	2000	1	1.80E-14	-0.8	11	0.55	Trdn	
DMR1:25742001	1	25742001	25750000	8000	5	7.30E-19	-0.44	95	1.19	Trdn	
DMR1:25760001	1	25760001	25763000	3000	1	9.80E-10	0.7	15	0.5	Trdn	
DMR1:28311001	1	28311001	28314000	3000	1	3.80E-10	0.4	41	1.37	Rnf217	Proteolysis
DMR1:28319001	1	28319001	28324000	5000	1	4.40E-08	0.57	29	0.58	Rnf217	Proteolysis
DMR1:28327001	1	28327001	28330000	3000	1	1.30E-19	0.94	32	1.07	Rnf217	Proteolysis
DMR1:28332001	1	28332001	28336000	4000	1	4.40E-07	0.34	42	1.05	Rnf217	Proteolysis
DMR1:28349001	1	28349001	28352000	3000	2	5.40E-11	-0.77	37	1.23	Rnf217	Proteolysis
DMR1:28361001	1	28361001	28366000	5000	1	3.20E-22	1	60	1.2	Rnf217	Proteolysis
DMR1:28452001	1	28452001	28453000	1000	1	2.60E-12	0.65	6	0.6	Tpd52l1	

DMR1:28465001	1	28465001	28467000	2000	2	5.50E-21	-0.79	7	0.35	Tpd52l1	
DMR1:28483001	1	28483001	28484000	1000	1	1.40E-07	-0.67	10	1	Tpd52l1	
DMR1:28485001	1	28485001	28486000	1000	1	8.20E-07	-0.46	8	0.8	Tpd52l1	
DMR1:28498001	1	28498001	28499000	1000	1	8.50E-18	0.69	12	1.2	Tpd52l1	
DMR1:28509001	1	28509001	28511000	2000	1	1.00E-25	0.97	17	0.85	Tpd52l1	
DMR1:28543001	1	28543001	28544000	1000	1	3.90E-08	0.31	18	1.8	Tpd52l1	
DMR1:28546001	1	28546001	28548000	2000	1	8.20E-23	0.74	30	1.5	Tpd52l1	
DMR1:28554001	1	28554001	28557000	3000	3	2.00E-21	0.78	23	0.77	Tpd52l1	
DMR1:28559001	1	28559001	28563000	4000	3	5.20E-11	0.53	38	0.95	Tpd52l1;Hddc2	
DMR1:28570001	1	28570001	28571000	1000	1	5.40E-07	-0.49	15	1.5	Tpd52l1;Hddc2	
DMR1:28587001	1	28587001	28591000	4000	2	1.20E-13	0.64	32	0.8	Tpd52l1;Hddc2	
DMR1:29208001	1	29208001	29210000	2000	1	1.10E-15	0.76	11	0.55	Hey2;Trnae-cuc	Transcription
DMR1:29211001	1	29211001	29213000	2000	1	8.90E-07	0.38	17	0.85	Hey2;Trnae-cuc	Transcription
DMR1:29241001	1	29241001	29244000	3000	1	2.60E-09	0.4	29	0.97	Ncoa7	
DMR1:29247001	1	29247001	29250000	3000	1	1.40E-07	-0.61	31	1.03	Ncoa7	
DMR1:29251001	1	29251001	29257000	6000	1	1.30E-10	0.61	72	1.2	Ncoa7	
DMR1:29264001	1	29264001	29266000	2000	1	2.80E-07	0.29	37	1.85	Ncoa7	
DMR1:29276001	1	29276001	29279000	3000	1	8.40E-08	0.44	26	0.87	Ncoa7	
DMR1:29280001	1	29280001	29283000	3000	1	3.80E-14	0.48	40	1.33	Ncoa7	
DMR1:29285001	1	29285001	29286000	1000	1	6.10E-11	0.38	16	1.6	Ncoa7	
DMR1:29295001	1	29295001	29298000	3000	1	8.10E-08	0.33	35	1.17	Ncoa7	
DMR1:29313001	1	29313001	29317000	4000	2	6.10E-10	-0.49	20	0.5	Ncoa7	
DMR1:29345001	1	29345001	29349000	4000	3	2.80E-22	0.73	35	0.88	Ncoa7;LOC108348877	
DMR1:29350001	1	29350001	29351000	1000	1	5.00E-08	0.36	14	1.4	Ncoa7;LOC108348877	
DMR1:29358001	1	29358001	29362000	4000	4	4.90E-16	-0.42	56	1.4	Ncoa7;LOC108348877	
DMR1:29364001	1	29364001	29366000	2000	1	3.40E-14	0.53	23	1.15	Ncoa7	
DMR1:29367001	1	29367001	29368000	1000	1	3.10E-08	-0.57	10	1	Ncoa7	
DMR1:29377001	1	29377001	29380000	3000	1	1.90E-07	-0.42	45	1.5	Ncoa7	
DMR1:29408001	1	29408001	29410000	2000	2	1.60E-16	0.75	16	0.8	Hint3	DNA Repair
DMR1:29427001	1	29427001	29431000	4000	1	9.70E-08	-0.55	12	0.3	Hint3;Trmt11	DNA Repair;Epigenetic
DMR1:29433001	1	29433001	29434000	1000	1	5.50E-09	0.37	12	1.2	Hint3;Trmt11	DNA Repair;Epigenetic
DMR1:29440001	1	29440001	29442000	2000	1	5.10E-18	0.62	28	1.4	Trmt11	Epigenetic
DMR1:29445001	1	29445001	29447000	2000	1	4.10E-11	-0.49	30	1.5	Trmt11	Epigenetic
DMR1:29448001	1	29448001	29450000	2000	1	4.20E-12	0.58	23	1.15	Trmt11	Epigenetic
DMR1:29451001	1	29451001	29455000	4000	1	3.90E-07	-0.51	36	0.9	Trmt11	Epigenetic
DMR1:29467001	1	29467001	29469000	2000	1	6.10E-08	0.43	26	1.3	Trmt11	Epigenetic
DMR1:29479001	1	29479001	29482000	3000	1	2.10E-09	0.38	44	1.47	Trmt11	Epigenetic
DMR1:29495001	1	29495001	29500000	5000	1	5.30E-07	0.3	71	1.42	Trmt11	Epigenetic
DMR1:29538001	1	29538001	29540000	2000	1	8.50E-07	0.41	25	1.25	Trmt11	Epigenetic
DMR1:29557001	1	29557001	29558000	1000	1	1.10E-08	0.4	8	0.8	Trmt11	Epigenetic
DMR1:29565001	1	29565001	29568000	3000	2	1.30E-10	0.57	14	0.47	Trmt11	Epigenetic
DMR1:29569001	1	29569001	29571000	2000	2	1.00E-23	0.94	19	0.95	Trmt11	Epigenetic
DMR1:29922001	1	29922001	29923000	1000	1	3.30E-09	0.49	14	1.4	Cenpw	
DMR1:29925001	1	29925001	29927000	2000	1	4.90E-09	-0.46	7	0.35	Cenpw	
DMR1:30745001	1	30745001	30746000	1000	1	1.50E-15	0.58	18	1.8	Rspo3	
DMR1:30768001	1	30768001	30769000	1000	1	3.90E-10	0.58	4	0.4	Rspo3	
DMR1:30893001	1	30893001	30895000	2000	1	2.60E-08	-0.5	7	0.35	Echdc1	Metabolism
DMR1:30897001	1	30897001	30899000	2000	1	3.70E-12	0.46	21	1.05	Echdc1	Metabolism
DMR1:30912001	1	30912001	30915000	3000	1	5.40E-13	0.53	16	0.53	Echdc1	Metabolism
DMR1:31034001	1	31034001	31037000	3000	3	3.00E-13	-0.59	6	0.2	LOC102554862;Soga3	
DMR1:31078001	1	31078001	31080000	2000	1	1.20E-07	0.45	9	0.45	Soga3	
DMR1:31104001	1	31104001	31105000	1000	1	1.70E-09	-0.42	5	0.5	Soga3	
DMR1:31126001	1	31126001	31128000	2000	1	1.50E-07	0.36	25	1.25	Soga3	
DMR1:31257001	1	31257001	31258000	1000	1	3.40E-22	0.94	8	0.8	Mrpl36;LOC100912599	Translation;Metabolism
DMR1:31260001	1	31260001	31261000	1000	1	6.00E-10	0.67	6	0.6	Mrpl36;LOC100912599	Translation;Metabolism
DMR1:31411001	1	31411001	31412000	1000	1	5.50E-13	0.59	7	0.7	RGD1311933	
DMR1:31419001	1	31419001	31422000	3000	1	7.50E-15	0.76	30	1	RGD1311933	
DMR1:31522001	1	31522001	31524000	2000	1	7.00E-09	0.64	10	0.5	LOC100911237;Rpl26-ps2;Lrrc14b	
DMR1:31526001	1	31526001	31527000	1000	1	7.50E-08	0.54	5	0.5	LOC100911237;Rpl26-ps2;Lrrc14b	

DMR1:31537001	1	31537001	31544000	7000	1	6.40E-11	0.39	68	0.97	Rpl26-ps2;Lrrc14b;Ccdc127;Sdha;LOC103694986	Metabolism
DMR1:31597001	1	31597001	31600000	3000	2	3.10E-09	0.56	17	0.57	Pdcd6;Ahrr	Cytoskeleton;Transcription
DMR1:31601001	1	31601001	31602000	1000	1	1.20E-07	-0.45	17	1.7	Pdcd6;Ahrr	Cytoskeleton;Transcription
DMR1:31622001	1	31622001	31628000	6000	3	2.00E-19	-0.56	40	0.67	Ahrr	Transcription
DMR1:31665001	1	31665001	31666000	1000	1	1.10E-12	0.74	5	0.5	Ahrr;LOC102548594	Transcription
DMR1:31667001	1	31667001	31670000	3000	1	3.20E-09	0.52	23	0.77	Ahrr;LOC102548594	Transcription
DMR1:31681001	1	31681001	31682000	1000	1	2.50E-15	0.82	6	0.6	Ahrr;LOC102548594;LOC108349507	Transcription
DMR1:31683001	1	31683001	31684000	1000	1	1.10E-12	0.48	23	2.3	Ahrr;LOC108349507	Transcription
DMR1:31715001	1	31715001	31722000	7000	2	2.50E-11	0.52	89	1.27	Exoc3;Slc9a3	Transport;Transport
DMR1:31725001	1	31725001	31729000	4000	1	2.40E-08	0.4	35	0.88	Exoc3;Slc9a3	Transport;Transport
DMR1:31764001	1	31764001	31767000	3000	1	2.80E-12	0.63	22	0.73	Slc9a3	Transport
DMR1:31768001	1	31768001	31769000	1000	1	1.60E-15	0.48	13	1.3	Slc9a3	Transport
DMR1:31773001	1	31773001	31776000	3000	2	1.10E-17	0.59	43	1.43	Slc9a3	Transport
DMR1:31838001	1	31838001	31839000	1000	1	6.10E-07	-0.53	9	0.9	Cep72	
DMR1:31843001	1	31843001	31847000	4000	2	1.40E-14	-0.49	53	1.32	Cep72	
DMR1:31857001	1	31857001	31859000	2000	1	6.20E-07	-0.6	29	1.45	Cep72;Tppp	Cytoskeleton
DMR1:31906001	1	31906001	31907000	1000	1	2.70E-12	0.47	20	2	Zdhhc11	
DMR1:31920001	1	31920001	31921000	1000	1	4.70E-16	0.77	6	0.6	Zdhhc11	
DMR1:31923001	1	31923001	31924000	1000	1	1.20E-26	0.97	9	0.9	Zdhhc11	
DMR1:31929001	1	31929001	31934000	5000	1	7.00E-28	0.9	63	1.26	Zdhhc11;LOC108348816;Brd9	Epigenetic
DMR1:31935001	1	31935001	31939000	4000	2	6.20E-10	0.49	48	1.2	Zdhhc11;LOC108348816;Brd9	Epigenetic
DMR1:31940001	1	31940001	31943000	3000	1	2.50E-15	0.63	19	0.63	Zdhhc11;LOC108348816;Brd9	Epigenetic
DMR1:32011001	1	32011001	32014000	3000	3	1.50E-13	0.87	23	0.77	Trip13	Epigenetic
DMR1:32079001	1	32079001	32081000	2000	1	4.00E-21	0.55	38	1.9	Nkd2;Slc12a7	Transport
DMR1:32098001	1	32098001	32100000	2000	1	3.60E-08	0.39	58	2.9	Slc12a7	Transport
DMR1:32102001	1	32102001	32105000	3000	1	3.50E-20	1.02	32	1.07	Slc12a7	Transport
DMR1:32114001	1	32114001	32115000	1000	1	2.30E-07	-0.48	12	1.2	Slc12a7	Transport
DMR1:32130001	1	32130001	32135000	5000	1	9.90E-07	-0.51	78	1.56	Slc12a7	Transport
DMR1:32136001	1	32136001	32138000	2000	1	6.60E-12	0.52	24	1.2	Slc12a7	Transport
DMR1:32161001	1	32161001	32165000	4000	2	3.10E-09	-0.55	53	1.32	Slc12a7	Transport
DMR1:32168001	1	32168001	32169000	1000	1	1.10E-12	0.83	24	2.4	Slc12a7	Transport
DMR1:32176001	1	32176001	32178000	2000	1	1.10E-10	0.43	29	1.45	Slc12a7	Transport
DMR1:32202001	1	32202001	32206000	4000	2	2.80E-14	0.64	51	1.27	Slc6a19	Transport
DMR1:32209001	1	32209001	32212000	3000	2	1.80E-09	0.4	39	1.3	Slc6a19;Slc6a18	Transport
DMR1:32226001	1	32226001	32227000	1000	1	1.90E-16	0.82	10	1	Slc6a19;Slc6a18	Transport
DMR1:32249001	1	32249001	32251000	2000	1	2.10E-08	0.44	11	0.55	LOC103690946;Tert	Transcription
DMR1:32269001	1	32269001	32270000	1000	1	5.20E-11	0.53	10	1	Tert	Transcription
DMR1:32303001	1	32303001	32304000	1000	1	1.60E-08	-0.66	11	1.1	Clptm1l;LOC102548924	
DMR1:32313001	1	32313001	32314000	1000	1	1.20E-12	0.81	10	1	LOC102548924;Slc6a3	Transport
DMR1:32317001	1	32317001	32319000	2000	2	8.00E-13	0.82	12	0.6	LOC102548924;Slc6a3	Transport
DMR1:32329001	1	32329001	32334000	5000	1	7.70E-07	0.32	63	1.26	LOC102548924;Slc6a3	Transport
DMR1:32374001	1	32374001	32375000	1000	1	7.50E-08	0.47	2	0.2	Lpcat1	Metabolism
DMR1:32378001	1	32378001	32379000	1000	1	3.50E-08	-0.62	13	1.3	Lpcat1	Metabolism
DMR1:32404001	1	32404001	32407000	3000	1	9.40E-21	0.86	68	2.27	Lpcat1	Metabolism
DMR1:32424001	1	32424001	32426000	2000	1	1.70E-09	-0.56	32	1.6	Lpcat1	Metabolism
DMR1:32624001	1	32624001	32627000	3000	2	1.50E-09	0.63	26	0.87	Irx4	Development
DMR1:32638001	1	32638001	32639000	1000	1	6.40E-16	0.91	39	3.9	Irx4	Development
DMR1:32648001	1	32648001	32651000	3000	1	1.10E-08	-0.54	32	1.07	Irx4	Development
DMR1:33921001	1	33921001	33923000	2000	1	8.30E-12	-0.67	30	1.5	Irx1	Development
DMR1:33925001	1	33925001	33926000	1000	1	1.80E-07	0.44	7	0.7	Irx1	Development
DMR1:35081001	1	35081001	35082000	1000	1	1.10E-09	-0.39	6	0.6	Adamts16	Protease
DMR1:35095001	1	35095001	35096000	1000	1	8.50E-10	0.5	7	0.7	Adamts16;LOC102554097	Protease
DMR1:35125001	1	35125001	35127000	2000	1	7.40E-11	0.65	7	0.35	Adamts16	Protease
DMR1:35128001	1	35128001	35129000	1000	1	6.20E-07	0.37	14	1.4	Adamts16	Protease
DMR1:35131001	1	35131001	35134000	3000	1	4.10E-11	0.57	25	0.83	Adamts16	Protease
DMR1:35135001	1	35135001	35137000	2000	1	1.70E-13	0.57	24	1.2	Adamts16	Protease
DMR1:35144001	1	35144001	35147000	3000	1	3.50E-08	-0.4	32	1.07	Adamts16	Protease
DMR1:35150001	1	35150001	35158000	8000	5	1.70E-24	-0.54	94	1.18	Adamts16	Protease
DMR1:35180001	1	35180001	35181000	1000	1	4.50E-20	1.06	4	0.4	Adamts16	Protease
DMR1:35210001	1	35210001	35211000	1000	1	1.10E-08	0.58	10	1	Adamts16	Protease

DMR1:35276001	1	35276001	35277000	1000	1	8.10E-08	0.45	9	0.9	Ice1	Golgi
DMR1:35299001	1	35299001	35303000	4000	1	9.20E-08	0.38	50	1.25	Ice1	Golgi
DMR1:35311001	1	35311001	35315000	4000	2	1.60E-08	-0.58	48	1.2	Ice1	Golgi
DMR1:35332001	1	35332001	35334000	2000	1	2.10E-17	0.81	19	0.95	Ice1	Golgi
DMR1:36104001	1	36104001	36106000	2000	1	3.20E-16	0.63	24	1.2	Med10	Transcription
DMR1:36119001	1	36119001	36120000	1000	1	5.30E-07	0.44	7	0.7	Med10	Transcription
DMR1:36121001	1	36121001	36125000	4000	2	7.50E-09	0.45	45	1.12	Med10	Transcription
DMR1:36175001	1	36175001	36178000	3000	1	3.40E-07	0.42	14	0.47	Ube2ql1	Proteolysis
DMR1:36183001	1	36183001	36185000	2000	2	4.40E-16	0.82	23	1.15	Ube2ql1	Proteolysis
DMR1:36212001	1	36212001	36213000	1000	1	4.90E-07	0.32	16	1.6	Ube2ql1	Proteolysis
DMR1:36285001	1	36285001	36289000	4000	1	9.20E-12	0.41	42	1.05	LOC108349442;Nsun2	Epigenetic
DMR1:36304001	1	36304001	36308000	4000	1	6.70E-07	-0.59	63	1.57	Nsun2	Epigenetic
DMR1:36314001	1	36314001	36316000	2000	1	5.00E-07	-0.51	20	1	Nsun2;Srd5a1	Epigenetic;Metabolism
DMR1:36340001	1	36340001	36347000	7000	3	2.60E-16	0.62	71	1.01	Srd5a1	Metabolism
DMR1:36352001	1	36352001	36354000	2000	1	3.10E-16	0.54	34	1.7	Srd5a1	Metabolism
DMR1:36363001	1	36363001	36367000	4000	2	2.10E-08	0.5	30	0.75	Srd5a1	Metabolism
DMR1:36413001	1	36413001	36415000	2000	1	1.50E-08	-0.47	14	0.7	Papd7	
DMR1:36430001	1	36430001	36434000	4000	1	3.50E-07	-0.56	70	1.75	Papd7	
DMR1:36440001	1	36440001	36448000	8000	4	1.30E-15	0.55	83	1.04	Papd7	
DMR1:37035001	1	37035001	37036000	1000	1	3.70E-08	0.36	15	1.5	Adcy2	
DMR1:37061001	1	37061001	37063000	2000	1	1.50E-07	0.41	8	0.4	Adcy2	
DMR1:37064001	1	37064001	37065000	1000	1	3.20E-15	0.74	7	0.7	Adcy2	
DMR1:37090001	1	37090001	37092000	2000	1	1.40E-14	0.65	12	0.6	Adcy2	
DMR1:37109001	1	37109001	37114000	5000	2	9.20E-15	-0.55	53	1.06	Adcy2	
DMR1:37194001	1	37194001	37195000	1000	1	1.90E-12	0.62	9	0.9	Adcy2	
DMR1:37201001	1	37201001	37202000	1000	1	4.90E-07	-0.53	11	1.1	Adcy2	
DMR1:37249001	1	37249001	37250000	1000	1	1.80E-34	1.18	8	0.8	Adcy2	
DMR1:37280001	1	37280001	37281000	1000	1	1.20E-11	0.5	15	1.5	Adcy2	
DMR1:37353001	1	37353001	37357000	4000	3	1.60E-20	-0.5	51	1.27	Adcy2	
DMR1:37400001	1	37400001	37407000	7000	3	9.90E-17	-0.57	52	0.74	Adcy2	
DMR1:37418001	1	37418001	37419000	1000	1	5.00E-08	-0.49	16	1.6	Adcy2	
DMR1:37422001	1	37422001	37423000	1000	1	1.90E-07	0.47	11	1.1	Adcy2	
DMR1:37457001	1	37457001	37459000	2000	1	2.20E-11	0.64	7	0.35	Adcy2	
DMR1:37465001	1	37465001	37468000	3000	1	8.50E-11	-0.62	14	0.47	Adcy2	
DMR1:37483001	1	37483001	37488000	5000	3	1.30E-16	-0.71	34	0.68	Adcy2	
DMR1:37537001	1	37537001	37539000	2000	1	3.30E-07	0.54	16	0.8	Adcy2	
DMR1:37543001	1	37543001	37544000	1000	1	5.90E-09	-0.51	2	0.2	Adcy2	
DMR1:37549001	1	37549001	37551000	2000	1	1.80E-08	0.33	14	0.7	Adcy2;LOC100362709	
DMR1:37559001	1	37559001	37560000	1000	1	3.60E-07	-0.52	14	1.4	Adcy2;LOC100362709	
DMR1:37573001	1	37573001	37576000	3000	1	2.10E-13	0.55	21	0.7	Adcy2	
DMR1:37578001	1	37578001	37585000	7000	5	2.30E-24	-0.53	79	1.13	Adcy2	
DMR1:37628001	1	37628001	37630000	2000	2	6.60E-11	0.53	16	0.8	Adcy2	
DMR1:37650001	1	37650001	37656000	6000	1	7.80E-11	-0.54	28	0.47	Adcy2	
DMR1:37661001	1	37661001	37664000	3000	2	2.50E-12	-0.46	28	0.93	Adcy2	
DMR1:37672001	1	37672001	37685000	13000	10	2.00E-28	0.98	81	0.62	Adcy2	
DMR1:37709001	1	37709001	37714000	5000	4	1.80E-19	1.02	35	0.7	RGD1308544	
DMR1:37718001	1	37718001	37728000	10000	4	2.60E-15	0.64	98	0.98	RGD1308544;Fastkd3	
DMR1:37754001	1	37754001	37757000	3000	1	1.30E-11	-0.61	27	0.9	Mtrr	Metabolism
DMR1:37759001	1	37759001	37768000	9000	6	6.10E-12	-0.52	95	1.06	Mtrr	Metabolism
DMR1:37772001	1	37772001	37774000	2000	1	7.50E-09	0.41	22	1.1	Mtrr	Metabolism
DMR1:37777001	1	37777001	37779000	2000	1	6.20E-17	0.61	21	1.05	Mtrr	Metabolism
DMR1:37866001	1	37866001	37872000	6000	3	1.70E-26	-0.66	66	1.1	RGD1566325;LOC103690951	
DMR1:37890001	1	37890001	37894000	4000	1	1.80E-18	0.65	20	0.5	RGD1566325;LOC103690951	
DMR1:37902001	1	37902001	37903000	1000	1	3.40E-14	0.72	2	0.2	RGD1566325	
DMR1:37973001	1	37973001	37976000	3000	1	1.70E-07	-0.41	17	0.57	LOC102549842;Zfp748	Transcription
DMR1:37987001	1	37987001	37990000	3000	2	5.20E-17	0.75	24	0.8	Zfp748	
DMR1:37996001	1	37996001	38001000	5000	3	2.40E-14	-0.53	45	0.9	Zfp748	
DMR1:38519001	1	38519001	38521000	2000	2	1.90E-11	-0.72	11	0.55	RGD1564382;LOC102551340	Transcription
DMR1:38524001	1	38524001	38526000	2000	1	2.10E-09	0.46	11	0.55	RGD1564382;LOC102551340	Transcription
DMR1:38529001	1	38529001	38534000	5000	4	1.50E-16	-0.66	30	0.6	RGD1564382;LOC102551340	Transcription
DMR1:38535001	1	38535001	38538000	3000	1	8.90E-16	0.62	25	0.83	RGD1564382;LOC102551340	Transcription
DMR1:39815001	1	39815001	39817000	2000	1	7.60E-09	0.51	4	0.2	Ppp1r14c	Signaling
DMR1:39818001	1	39818001	39820000	2000	1	3.40E-14	0.54	7	0.35	Ppp1r14c	Signaling
DMR1:39830001	1	39830001	39837000	7000	4	2.70E-19	-0.49	70	1	Ppp1r14c	Signaling

DMR1:39841001	1	39841001	39844000	3000	2	1.60E-12	0.68	20	0.67	Ppp1r14c	Signaling
DMR1:39847001	1	39847001	39851000	4000	2	2.10E-13	-0.5	25	0.62	Ppp1r14c	Signaling
DMR1:39877001	1	39877001	39879000	2000	1	1.60E-07	0.45	14	0.7	Ppp1r14c	Signaling
DMR1:39891001	1	39891001	39894000	3000	2	1.30E-08	0.64	13	0.43	Ppp1r14c	Signaling
DMR1:39897001	1	39897001	39899000	2000	1	1.20E-10	0.49	13	0.65	Ppp1r14c	Signaling
DMR1:39903001	1	39903001	39909000	6000	4	2.20E-08	-0.53	34	0.57	Ppp1r14c	Signaling
DMR1:39912001	1	39912001	39915000	3000	1	1.70E-08	0.38	25	0.83	Ppp1r14c	Signaling
DMR1:39921001	1	39921001	39923000	2000	1	7.60E-08	-0.6	27	1.35	Ppp1r14c	Signaling
DMR1:39925001	1	39925001	39927000	2000	1	8.60E-11	0.69	10	0.5	Ppp1r14c	Signaling
DMR1:40080001	1	40080001	40084000	4000	2	7.50E-07	-0.44	17	0.42	Iyd	Metabolism
DMR1:40104001	1	40104001	40110000	6000	5	2.80E-28	-0.67	56	0.93	Iyd	Metabolism
DMR1:40290001	1	40290001	40296000	6000	3	1.70E-20	0.76	39	0.65	LOC103690956;Plekhg1	
DMR1:40313001	1	40313001	40314000	1000	1	1.80E-12	0.46	0	0	Plekhg1	
DMR1:40320001	1	40320001	40321000	1000	1	6.90E-10	0.54	3	0.3	Plekhg1	
DMR1:40340001	1	40340001	40341000	1000	1	7.30E-09	-0.64	9	0.9	Plekhg1	
DMR1:40383001	1	40383001	40385000	2000	1	6.90E-07	0.38	19	0.95	Plekhg1;LOC108349517	
DMR1:40429001	1	40429001	40430000	1000	1	5.00E-12	0.69	2	0.2	Plekhg1	
DMR1:40437001	1	40437001	40439000	2000	1	1.40E-23	0.95	16	0.8	Plekhg1	
DMR1:40443001	1	40443001	40446000	3000	2	4.10E-11	0.54	27	0.9	Plekhg1	
DMR1:40452001	1	40452001	40453000	1000	1	2.70E-14	0.62	7	0.7	Plekhg1	
DMR1:40468001	1	40468001	40471000	3000	2	8.20E-09	0.43	15	0.5	Plekhg1;LOC679794;LOC679776	
DMR1:40482001	1	40482001	40484000	2000	1	3.40E-17	0.64	33	1.65	Plekhg1;LOC679794;LOC679776	
DMR1:40495001	1	40495001	40498000	3000	2	1.20E-10	0.49	41	1.37	Plekhg1	
DMR1:40501001	1	40501001	40502000	1000	1	1.30E-08	-0.63	9	0.9	Plekhg1	
DMR1:40538001	1	40538001	40539000	1000	1	1.80E-07	-0.45	18	1.8	Mthfd1l	
DMR1:40555001	1	40555001	40557000	2000	1	2.50E-15	0.72	17	0.85	Mthfd1l	
DMR1:40562001	1	40562001	40564000	2000	1	3.50E-17	0.62	14	0.7	Mthfd1l	
DMR1:40571001	1	40571001	40573000	2000	2	9.50E-09	-0.55	24	1.2	Mthfd1l	
DMR1:40598001	1	40598001	40599000	1000	1	1.90E-07	-0.64	10	1	Mthfd1l	
DMR1:40611001	1	40611001	40618000	7000	1	1.10E-08	-0.46	59	0.84	Mthfd1l	
DMR1:40619001	1	40619001	40623000	4000	1	1.70E-14	0.82	53	1.32	Mthfd1l;LOC102546677	
DMR1:40637001	1	40637001	40639000	2000	1	1.70E-09	0.53	17	0.85	Mthfd1l;LOC102546677	
DMR1:40654001	1	40654001	40655000	1000	1	5.00E-12	0.48	14	1.4	Mthfd1l;LOC102546677	
DMR1:40670001	1	40670001	40671000	1000	1	3.60E-12	0.4	18	1.8	Mthfd1l	
DMR1:40711001	1	40711001	40712000	1000	1	1.70E-09	0.48	11	1.1	Mthfd1l	
DMR1:40713001	1	40713001	40715000	2000	1	2.90E-08	-0.68	20	1	Mthfd1l	
DMR1:40723001	1	40723001	40724000	1000	1	5.80E-08	0.35	17	1.7	Mthfd1l	
DMR1:40857001	1	40857001	40859000	2000	1	7.50E-07	0.34	25	1.25	Akap12	Cytoskeleton
DMR1:40860001	1	40860001	40861000	1000	1	1.80E-07	0.43	8	0.8	Akap12	Cytoskeleton
DMR1:40919001	1	40919001	40924000	5000	1	1.40E-18	0.95	67	1.34	Zbtb2	Transcription
DMR1:40963001	1	40963001	40965000	2000	1	7.00E-09	0.43	12	0.6	Rmnd1	
DMR1:40972001	1	40972001	40978000	6000	1	3.70E-09	-0.41	60	1	Rmnd1;Armt1	
DMR1:41001001	1	41001001	41005000	4000	1	2.00E-07	-0.55	45	1.12	Armt1	
DMR1:41006001	1	41006001	41007000	1000	1	3.70E-15	0.67	8	0.8	Armt1	
DMR1:41008001	1	41008001	41009000	1000	1	1.10E-07	-0.65	6	0.6	Armt1	
DMR1:41029001	1	41029001	41033000	4000	1	6.80E-15	0.54	34	0.85	Ccdc170	
DMR1:41045001	1	41045001	41048000	3000	2	4.00E-10	-0.42	21	0.7	Ccdc170	
DMR1:41049001	1	41049001	41052000	3000	3	2.20E-14	-0.45	31	1.03	Ccdc170	
DMR1:41054001	1	41054001	41055000	1000	1	4.70E-07	0.4	14	1.4	Ccdc170	
DMR1:41057001	1	41057001	41059000	2000	2	1.30E-15	0.79	16	0.8	Ccdc170	
DMR1:41088001	1	41088001	41091000	3000	2	6.70E-11	0.68	23	0.77	Ccdc170	
DMR1:41127001	1	41127001	41128000	1000	1	9.00E-07	0.58	6	0.6	Ccdc170	
DMR1:41205001	1	41205001	41210000	5000	2	1.60E-20	0.92	42	0.84	Esr1	
DMR1:41211001	1	41211001	41212000	1000	1	4.30E-12	0.69	13	1.3	Esr1	
DMR1:41232001	1	41232001	41234000	2000	1	2.20E-19	0.85	23	1.15	Esr1	
DMR1:41236001	1	41236001	41239000	3000	1	4.40E-11	-0.53	11	0.37	Esr1	
DMR1:41241001	1	41241001	41244000	3000	1	6.80E-11	-0.54	22	0.73	Esr1	
DMR1:41255001	1	41255001	41263000	8000	6	6.70E-16	-0.49	75	0.94	Esr1	
DMR1:41268001	1	41268001	41269000	1000	1	2.00E-12	0.58	2	0.2	Esr1	
DMR1:41277001	1	41277001	41279000	2000	1	1.60E-12	0.73	11	0.55	Esr1	
DMR1:41289001	1	41289001	41290000	1000	1	1.10E-24	0.88	8	0.8	Esr1	
DMR1:41305001	1	41305001	41307000	2000	2	4.60E-07	-0.59	10	0.5	Esr1	
DMR1:41310001	1	41310001	41313000	3000	1	1.30E-07	-0.59	23	0.77	Esr1	

DMR1:41321001	1	41321001	41322000	1000	1	4.00E-07	0.39	14	1.4	Esr1	
DMR1:41347001	1	41347001	41348000	1000	1	2.20E-09	0.47	8	0.8	Esr1	
DMR1:41371001	1	41371001	41376000	5000	3	8.50E-12	-0.8	48	0.96	Esr1	
DMR1:41377001	1	41377001	41379000	2000	2	2.30E-15	0.95	10	0.5	Esr1	
DMR1:41386001	1	41386001	41387000	1000	1	9.30E-09	0.54	2	0.2	Esr1	
DMR1:41389001	1	41389001	41391000	2000	2	2.10E-21	0.85	24	1.2	Esr1	
DMR1:41422001	1	41422001	41425000	3000	2	1.10E-12	-0.88	24	0.8	Esr1	
DMR1:41427001	1	41427001	41432000	5000	3	2.30E-25	-0.57	57	1.14	Esr1	
DMR1:41433001	1	41433001	41436000	3000	1	5.80E-08	-0.3	35	1.17	Esr1	
DMR1:41459001	1	41459001	41460000	1000	1	1.20E-14	0.64	9	0.9	Esr1	
DMR1:41497001	1	41497001	41499000	2000	1	3.60E-07	0.44	16	0.8	Esr1	
DMR1:41506001	1	41506001	41510000	4000	1	1.40E-11	0.56	39	0.98	Esr1	
DMR1:41517001	1	41517001	41519000	2000	2	2.50E-31	0.92	11	0.55	Esr1	
DMR1:41522001	1	41522001	41523000	1000	1	1.70E-11	0.56	6	0.6	Esr1	
DMR1:41546001	1	41546001	41548000	2000	1	2.10E-08	-0.43	16	0.8	Esr1	
DMR1:41630001	1	41630001	41637000	7000	2	3.20E-08	0.37	108	1.54	Syne1	
DMR1:41646001	1	41646001	41648000	2000	2	9.80E-13	0.58	20	1	Syne1	
DMR1:41663001	1	41663001	41665000	2000	1	1.70E-07	-0.49	34	1.7	Syne1	
DMR1:41679001	1	41679001	41683000	4000	1	6.70E-08	0.52	48	1.2	Syne1	
DMR1:41684001	1	41684001	41687000	3000	1	8.50E-08	0.49	27	0.9	Syne1	
DMR1:41688001	1	41688001	41690000	2000	1	1.20E-09	0.76	24	1.2	Syne1	
DMR1:41697001	1	41697001	41698000	1000	1	3.70E-07	0.49	7	0.7	Syne1	
DMR1:41720001	1	41720001	41721000	1000	1	4.90E-09	-0.5	13	1.3	Syne1	
DMR1:41736001	1	41736001	41738000	2000	1	6.00E-17	0.81	22	1.1	Syne1	
DMR1:41744001	1	41744001	41746000	2000	1	1.10E-09	-0.7	23	1.15	Syne1	
DMR1:41747001	1	41747001	41749000	2000	1	1.60E-09	0.43	17	0.85	Syne1	
DMR1:41767001	1	41767001	41769000	2000	1	4.80E-10	0.49	19	0.95	Syne1	
DMR1:41873001	1	41873001	41875000	2000	1	1.30E-08	0.6	18	0.9	Syne1	
DMR1:41880001	1	41880001	41884000	4000	3	4.70E-16	-0.68	56	1.4	Syne1	
DMR1:41888001	1	41888001	41890000	2000	1	5.50E-14	0.77	23	1.15	Syne1	
DMR1:41903001	1	41903001	41905000	2000	1	1.40E-08	-0.67	21	1.05	Syne1	
DMR1:41915001	1	41915001	41916000	1000	1	2.90E-10	0.57	4	0.4	Syne1	
DMR1:41918001	1	41918001	41921000	3000	1	9.20E-15	-0.96	45	1.5	Syne1	
DMR1:41946001	1	41946001	41953000	7000	1	3.20E-11	0.45	71	1.01	Syne1	
DMR1:41954001	1	41954001	41955000	1000	1	3.30E-08	0.54	4	0.4	Syne1	
DMR1:41961001	1	41961001	41963000	2000	1	2.40E-12	0.64	11	0.55	Syne1	
DMR1:42007001	1	42007001	42011000	4000	1	5.70E-07	0.35	41	1.02	Syne1	
DMR1:42033001	1	42033001	42034000	1000	1	6.20E-07	0.37	3	0.3	Syne1	
DMR1:42048001	1	42048001	42050000	2000	1	5.20E-20	0.68	21	1.05	Syne1	
DMR1:42061001	1	42061001	42063000	2000	1	6.20E-07	-0.42	26	1.3	Syne1	
DMR1:42065001	1	42065001	42066000	1000	1	8.80E-09	0.35	17	1.7	Syne1	
DMR1:42074001	1	42074001	42075000	1000	1	3.60E-10	0.5	11	1.1	Syne1	
DMR1:42111001	1	42111001	42113000	2000	1	4.40E-17	0.79	17	0.85	LOC102546605;Myct1	
DMR1:42138001	1	42138001	42139000	1000	1	3.00E-08	0.61	14	1.4	Myct1	
DMR1:42180001	1	42180001	42182000	2000	1	1.70E-11	0.77	19	0.95	Vip	Signaling
DMR1:42184001	1	42184001	42185000	1000	1	1.70E-13	0.72	7	0.7	Vip	Signaling
DMR1:42209001	1	42209001	42211000	2000	1	1.40E-10	0.74	14	0.7	Ccdc170	
DMR1:42250001	1	42250001	42254000	4000	1	1.70E-07	-0.38	25	0.62	Ccdc170	
DMR1:42457001	1	42457001	42458000	1000	1	5.40E-07	0.35	7	0.7	Fbxo5	
DMR1:42461001	1	42461001	42462000	1000	1	3.60E-07	-0.56	11	1.1	Fbxo5	
DMR1:42477001	1	42477001	42478000	1000	1	6.10E-11	0.44	10	1	Fbxo5;Mtrf1l	Translation
DMR1:42479001	1	42479001	42480000	1000	1	3.40E-08	0.41	17	1.7	Mtrf1l	Translation
DMR1:42501001	1	42501001	42503000	2000	2	2.70E-09	0.54	7	0.35	Rgs17	Signaling
DMR1:42506001	1	42506001	42508000	2000	2	4.10E-19	0.7	16	0.8	Rgs17	Signaling
DMR1:42539001	1	42539001	42541000	2000	1	1.00E-07	0.43	11	0.55	Rgs17	Signaling
DMR1:42542001	1	42542001	42545000	3000	1	5.10E-09	-0.63	43	1.43	Rgs17	Signaling
DMR1:42549001	1	42549001	42551000	2000	1	6.90E-08	-0.33	23	1.15	Rgs17	Signaling
DMR1:42567001	1	42567001	42568000	1000	1	2.40E-08	0.45	9	0.9	Rgs17	Signaling
DMR1:42783001	1	42783001	42785000	2000	2	2.10E-10	0.73	5	0.25	RGD1562524	
DMR1:42787001	1	42787001	42788000	1000	1	5.80E-08	0.49	4	0.4	RGD1562524	
DMR1:43447001	1	43447001	43456000	9000	6	6.40E-12	-0.44	95	1.06	Oprm1	Signaling
DMR1:43461001	1	43461001	43462000	1000	1	1.00E-07	0.51	4	0.4	Oprm1	Signaling
DMR1:43464001	1	43464001	43468000	4000	1	1.50E-09	0.54	45	1.12	Oprm1	Signaling
DMR1:43485001	1	43485001	43491000	6000	1	1.70E-10	0.67	72	1.2	Oprm1	Signaling
DMR1:43503001	1	43503001	43505000	2000	1	2.60E-07	0.39	19	0.95	Oprm1	Signaling

DMR1:43520001	1	43520001	43521000	1000	1	5.50E-16	0.59	6	0.6	Oprm1	Signaling
DMR1:43538001	1	43538001	43540000	2000	1	1.40E-12	0.61	22	1.1	Oprm1	Signaling
DMR1:43568001	1	43568001	43576000	8000	3	2.20E-10	0.59	65	0.81	Oprm1;Ipcef1	Signaling
DMR1:43579001	1	43579001	43581000	2000	1	3.70E-07	0.49	9	0.45	Oprm1;Ipcef1	Signaling
DMR1:43622001	1	43622001	43623000	1000	1	1.70E-15	0.52	9	0.9	Oprm1;Ipcef1	Signaling
DMR1:43648001	1	43648001	43651000	3000	1	1.80E-19	0.7	29	0.97	Oprm1;Ipcef1;LOC102553925	Signaling
DMR1:43657001	1	43657001	43659000	2000	1	6.80E-08	0.49	12	0.6	Oprm1;Ipcef1;LOC102553925	Signaling
DMR1:43671001	1	43671001	43672000	1000	1	2.90E-13	0.67	8	0.8	Oprm1;Ipcef1;LOC102553925	Signaling
DMR1:43678001	1	43678001	43681000	3000	1	1.60E-14	0.54	28	0.93	Oprm1;Ipcef1	Signaling
DMR1:43689001	1	43689001	43690000	1000	1	6.50E-08	0.46	7	0.7	Oprm1;Ipcef1	Signaling
DMR1:43694001	1	43694001	43696000	2000	1	2.90E-10	-0.56	28	1.4	Oprm1;Ipcef1	Signaling
DMR1:43702001	1	43702001	43709000	7000	1	2.00E-07	0.39	102	1.46	Oprm1;Ipcef1	Signaling
DMR1:43730001	1	43730001	43734000	4000	2	2.80E-15	-0.6	32	0.8	Ipcef1;LOC680118	
DMR1:43740001	1	43740001	43741000	1000	1	5.50E-27	0.85	16	1.6	Ipcef1	
DMR1:43793001	1	43793001	43794000	1000	1	9.70E-08	0.34	7	0.7	Cnksr3	
DMR1:43815001	1	43815001	43818000	3000	1	3.90E-10	0.45	30	1	Cnksr3	
DMR1:43819001	1	43819001	43823000	4000	1	6.20E-13	0.47	52	1.3	Cnksr3	
DMR1:43831001	1	43831001	43832000	1000	1	1.30E-14	0.48	25	2.5	Cnksr3	
DMR1:43840001	1	43840001	43841000	1000	1	3.30E-08	0.45	6	0.6	Cnksr3	
DMR1:43853001	1	43853001	43856000	3000	2	3.00E-30	0.97	40	1.33	Cnksr3	
DMR1:43874001	1	43874001	43882000	8000	1	1.00E-09	0.37	130	1.62	Cnksr3	
DMR1:43922001	1	43922001	43927000	5000	2	1.10E-09	-0.57	54	1.08	RGD1560234	
DMR1:44010001	1	44010001	44013000	3000	1	1.30E-07	0.32	59	1.97	Scaf8	Translation
DMR1:44066001	1	44066001	44069000	3000	1	1.20E-09	0.54	18	0.6	Scaf8	Translation
DMR1:44071001	1	44071001	44073000	2000	1	6.50E-10	0.52	10	0.5	Scaf8	Translation
DMR1:44101001	1	44101001	44102000	1000	1	1.10E-08	-0.6	16	1.6	Scaf8	Translation
DMR1:44113001	1	44113001	44115000	2000	1	1.00E-07	0.31	29	1.45	Scaf8	Translation
DMR1:44229001	1	44229001	44231000	2000	1	9.90E-12	0.65	16	0.8	Tiam2	
DMR1:44251001	1	44251001	44252000	1000	1	8.70E-10	0.41	8	0.8	Tiam2	
DMR1:44268001	1	44268001	44270000	2000	1	1.60E-07	-0.57	25	1.25	Tiam2	
DMR1:44291001	1	44291001	44293000	2000	1	8.50E-08	-0.51	24	1.2	Tiam2	
DMR1:44302001	1	44302001	44304000	2000	1	6.00E-09	0.41	17	0.85	Tiam2	
DMR1:44351001	1	44351001	44352000	1000	1	8.70E-08	0.35	12	1.2	Tiam2	
DMR1:44359001	1	44359001	44361000	2000	1	1.80E-08	0.32	17	0.85	Tiam2	
DMR1:44362001	1	44362001	44366000	4000	2	3.30E-14	0.49	53	1.32	Tiam2	
DMR1:44380001	1	44380001	44383000	3000	2	9.60E-16	-0.73	31	1.03	Tiam2	
DMR1:44388001	1	44388001	44389000	1000	1	4.20E-07	-0.6	18	1.8	Tiam2	
DMR1:44390001	1	44390001	44393000	3000	1	3.40E-14	0.84	17	0.57	Tiam2	
DMR1:44414001	1	44414001	44419000	5000	1	4.10E-08	-0.71	110	2.2	Tiam2	
DMR1:44421001	1	44421001	44423000	2000	1	1.10E-08	-0.51	32	1.6	Tiam2	
DMR1:44439001	1	44439001	44441000	2000	1	9.10E-19	-0.59	19	0.95	Tiam2;Tfb1m;Cldn20;LOC102547288	Epigenetic;Cell Junction
DMR1:44466001	1	44466001	44467000	1000	1	2.40E-08	0.34	15	1.5	Tfb1m	Epigenetic
DMR1:44537001	1	44537001	44544000	7000	6	4.00E-17	-0.45	88	1.26	LOC103690965;Nox3	Metabolism
DMR1:44548001	1	44548001	44549000	1000	1	9.10E-08	0.51	9	0.9	Nox3	Metabolism
DMR1:44550001	1	44550001	44551000	1000	1	4.80E-07	0.52	7	0.7	Nox3	Metabolism
DMR1:44560001	1	44560001	44565000	5000	4	1.60E-12	0.54	28	0.56	Nox3	Metabolism
DMR1:44575001	1	44575001	44577000	2000	1	6.00E-07	-0.45	27	1.35	Nox3	Metabolism
DMR1:44579001	1	44579001	44582000	3000	1	2.70E-14	0.61	35	1.17	Nox3	Metabolism
DMR1:44590001	1	44590001	44594000	4000	1	4.40E-09	0.58	42	1.05	Nox3	Metabolism
DMR1:44598001	1	44598001	44599000	1000	1	1.60E-21	1.03	15	1.5	Nox3	Metabolism
DMR1:44606001	1	44606001	44607000	1000	1	2.00E-11	0.56	10	1	Nox3	Metabolism
DMR1:44616001	1	44616001	44618000	2000	1	2.00E-09	-0.6	26	1.3	Nox3	Metabolism
DMR1:45911001	1	45911001	45915000	4000	1	1.20E-09	0.45	59	1.48	Arid1b	
DMR1:45918001	1	45918001	45920000	2000	1	2.90E-07	-0.35	34	1.7	Arid1b	
DMR1:45921001	1	45921001	45922000	1000	1	1.70E-10	0.45	10	1	Arid1b	
DMR1:45923001	1	45923001	45924000	1000	1	4.50E-10	0.51	10	1	Arid1b	
DMR1:45929001	1	45929001	45932000	3000	1	4.20E-07	-0.57	32	1.07	Arid1b	
DMR1:45933001	1	45933001	45934000	1000	1	2.20E-10	0.47	4	0.4	Arid1b	
DMR1:45935001	1	45935001	45937000	2000	1	1.60E-13	0.58	25	1.25	Arid1b	
DMR1:45948001	1	45948001	45952000	4000	2	1.90E-12	-0.51	39	0.98	Arid1b	
DMR1:45953001	1	45953001	45959000	6000	1	1.10E-15	0.53	91	1.52	Arid1b	
DMR1:45960001	1	45960001	45963000	3000	2	5.20E-13	0.52	49	1.63	Arid1b	
DMR1:45974001	1	45974001	45977000	3000	1	7.80E-15	0.61	37	1.23	Arid1b	
DMR1:45985001	1	45985001	45987000	2000	1	1.90E-07	0.34	24	1.2	Arid1b	

DMR1:45999001	1	45999001	46002000	3000	1	7.10E-16	0.72	25	0.83	Arid1b	
DMR1:46009001	1	46009001	46017000	8000	1	2.90E-18	0.86	98	1.23	Arid1b	
DMR1:46049001	1	46049001	46056000	7000	1	6.50E-10	-0.45	102	1.46	Arid1b	
DMR1:46064001	1	46064001	46065000	1000	1	8.70E-14	0.48	15	1.5	Arid1b	
DMR1:46126001	1	46126001	46128000	2000	1	1.70E-07	-0.6	23	1.15	Arid1b	
DMR1:46132001	1	46132001	46137000	5000	3	2.00E-08	-0.53	64	1.28	Arid1b	
DMR1:46145001	1	46145001	46146000	1000	1	6.30E-08	0.36	16	1.6	Arid1b	
DMR1:46180001	1	46180001	46181000	1000	1	1.80E-07	-0.59	9	0.9	Arid1b	
DMR1:46202001	1	46202001	46203000	1000	1	2.50E-07	-0.5	12	1.2	Arid1b	
DMR1:46239001	1	46239001	46244000	5000	2	1.50E-14	0.49	102	2.04	Arid1b	
DMR1:46295001	1	46295001	46296000	1000	1	1.50E-09	0.38	25	2.5	Tmem242	
DMR1:46301001	1	46301001	46302000	1000	1	6.90E-12	0.51	6	0.6	Tmem242;Ldhal6b	
DMR1:46324001	1	46324001	46325000	1000	1	1.50E-07	0.49	7	0.7	Tmem242	
DMR1:46335001	1	46335001	46336000	1000	1	8.10E-13	0.78	9	0.9	Tmem242	
DMR1:46366001	1	46366001	46369000	3000	1	7.10E-07	-0.45	37	1.23	LOC108348585;Zdhhc14	
DMR1:46383001	1	46383001	46384000	1000	1	1.10E-07	-0.49	12	1.2	LOC108348585;Zdhhc14	
DMR1:46402001	1	46402001	46405000	3000	1	6.00E-26	0.95	53	1.77	Zdhhc14	
DMR1:46422001	1	46422001	46426000	4000	1	1.60E-10	0.47	56	1.4	Zdhhc14	
DMR1:46427001	1	46427001	46431000	4000	1	1.20E-07	0.43	49	1.23	Zdhhc14	
DMR1:46436001	1	46436001	46437000	1000	1	1.70E-07	0.43	8	0.8	Zdhhc14	
DMR1:46451001	1	46451001	46452000	1000	1	2.70E-07	0.33	19	1.9	Zdhhc14	
DMR1:46468001	1	46468001	46469000	1000	1	3.10E-10	0.39	8	0.8	Zdhhc14	
DMR1:46480001	1	46480001	46483000	3000	1	9.10E-20	0.64	38	1.27	Zdhhc14	
DMR1:46485001	1	46485001	46487000	2000	1	1.50E-08	0.38	28	1.4	Zdhhc14	
DMR1:46491001	1	46491001	46494000	3000	1	4.80E-07	0.29	55	1.83	Zdhhc14	
DMR1:46495001	1	46495001	46507000	12000	4	2.30E-14	-0.51	209	1.74	Zdhhc14	
DMR1:46523001	1	46523001	46530000	7000	1	1.90E-09	0.41	99	1.41	Zdhhc14	
DMR1:46550001	1	46550001	46555000	5000	1	5.40E-16	0.7	71	1.42	Zdhhc14	
DMR1:46556001	1	46556001	46557000	1000	1	2.80E-13	0.49	19	1.9	Zdhhc14	
DMR1:46574001	1	46574001	46578000	4000	2	1.80E-21	-0.54	51	1.27	Zdhhc14	
DMR1:46592001	1	46592001	46593000	1000	1	1.90E-10	0.53	13	1.3	Zdhhc14	
DMR1:46601001	1	46601001	46602000	1000	1	3.60E-16	0.74	8	0.8	Zdhhc14	
DMR1:46605001	1	46605001	46610000	5000	2	4.80E-20	0.57	87	1.74	Zdhhc14	
DMR1:46623001	1	46623001	46626000	3000	1	1.00E-08	0.63	33	1.1	Zdhhc14	
DMR1:46638001	1	46638001	46643000	5000	3	1.30E-25	-0.74	64	1.28	Zdhhc14	
DMR1:46654001	1	46654001	46661000	7000	2	2.70E-13	-0.49	102	1.46	Zdhhc14	
DMR1:46665001	1	46665001	46668000	3000	1	2.30E-13	0.64	40	1.33	Zdhhc14	
DMR1:46799001	1	46799001	46804000	5000	3	3.60E-16	-0.68	59	1.18	Snx9	Cytoskeleton
DMR1:46808001	1	46808001	46811000	3000	1	5.90E-09	-0.58	36	1.2	Snx9	Cytoskeleton
DMR1:46823001	1	46823001	46827000	4000	2	1.40E-07	0.4	73	1.82	Snx9;LOC100365065;Synj2	Cytoskeleton;Signalin g
DMR1:46849001	1	46849001	46850000	1000	1	1.10E-08	0.41	17	1.7	Synj2;LOC102551310	Signaling
DMR1:46854001	1	46854001	46860000	6000	1	1.00E-24	0.81	93	1.55	Synj2;LOC102551310	Signaling
DMR1:46902001	1	46902001	46903000	1000	1	2.80E-08	-0.52	5	0.5	Synj2;LOC103690968	Signaling
DMR1:46906001	1	46906001	46909000	3000	2	1.60E-12	0.43	41	1.37	Synj2;LOC103690968	Signaling
DMR1:46923001	1	46923001	46927000	4000	2	2.10E-11	0.43	67	1.68	Synj2;LOC103690968;Serac1	Signaling
DMR1:46944001	1	46944001	46945000	1000	1	8.00E-23	0.82	8	0.8	Serac1	
DMR1:46963001	1	46963001	46968000	5000	1	4.10E-07	-0.43	79	1.58	Serac1	
DMR1:46980001	1	46980001	46982000	2000	1	2.40E-07	0.39	18	0.9	Serac1;Gtf2h5	Transcription
DMR1:46995001	1	46995001	46997000	2000	1	2.10E-07	0.37	25	1.25	Gtf2h5;LOC102551377;Tulp4	Transcription
DMR1:47013001	1	47013001	47015000	2000	1	2.30E-08	-0.55	18	0.9	LOC102551377;Tulp4	
DMR1:47031001	1	47031001	47034000	3000	1	1.80E-07	-0.54	28	0.93	Tulp4	
DMR1:47039001	1	47039001	47042000	3000	2	6.90E-08	0.41	45	1.5	Tulp4	
DMR1:47057001	1	47057001	47059000	2000	1	1.90E-13	0.5	18	0.9	Tulp4	
DMR1:47065001	1	47065001	47066000	1000	1	5.50E-07	-0.62	16	1.6	Tulp4	
DMR1:47077001	1	47077001	47079000	2000	2	2.30E-08	-0.6	35	1.75	Tulp4	
DMR1:47081001	1	47081001	47083000	2000	1	8.30E-07	-0.46	18	0.9	Tulp4	
DMR1:47104001	1	47104001	47105000	1000	1	9.80E-19	0.67	14	1.4	Tulp4	
DMR1:47123001	1	47123001	47129000	6000	1	2.70E-07	0.32	103	1.72	Tulp4	
DMR1:47137001	1	47137001	47138000	1000	1	3.80E-07	0.32	35	3.5	Tulp4	
DMR1:47176001	1	47176001	47177000	1000	1	5.20E-08	-0.64	16	1.6	Tmem181	
DMR1:47181001	1	47181001	47185000	4000	1	1.10E-09	0.42	46	1.15	Tmem181	
DMR1:47214001	1	47214001	47218000	4000	1	6.10E-08	-0.61	45	1.12	Dynlt1;Sytl3	Cytoskeleton
DMR1:47228001	1	47228001	47230000	2000	1	8.80E-07	-0.48	39	1.95	Sytl3	
DMR1:47240001	1	47240001	47241000	1000	1	6.80E-07	-0.51	14	1.4	Sytl3	

DMR1:47251001	1	47251001	47252000	1000	1	3.90E-22	0.78	6	0.6	Sytl3	
DMR1:47254001	1	47254001	47255000	1000	1	2.60E-07	0.5	9	0.9	Sytl3	
DMR1:47256001	1	47256001	47259000	3000	1	3.50E-14	0.72	42	1.4	Sytl3	
DMR1:47264001	1	47264001	47265000	1000	1	3.30E-09	-0.71	10	1	Sytl3	
DMR1:47272001	1	47272001	47277000	5000	1	7.10E-18	0.9	55	1.1	Sytl3	
DMR1:47327001	1	47327001	47331000	4000	1	6.00E-10	0.45	101	2.52	Ezr	Cytoskeleton
DMR1:47341001	1	47341001	47343000	2000	1	4.80E-09	0.54	16	0.8	Ezr	Cytoskeleton
DMR1:47412001	1	47412001	47416000	4000	2	7.60E-12	0.8	41	1.02	Rsph3	Development
DMR1:47417001	1	47417001	47423000	6000	3	1.30E-14	0.87	46	0.77	Rsph3	Development
DMR1:47433001	1	47433001	47437000	4000	1	3.00E-25	0.77	54	1.35	Rsph3	Development
DMR1:47457001	1	47457001	47458000	1000	1	1.70E-09	-0.68	3	0.3	Rsph3	Development
DMR1:47480001	1	47480001	47483000	3000	1	8.60E-10	0.47	30	1	Rsph3	Development
DMR1:47487001	1	47487001	47488000	1000	1	1.50E-13	0.56	18	1.8	Rsph3;Tagap	Development;Signaling
DMR1:47495001	1	47495001	47496000	1000	1	2.50E-08	-0.68	26	2.6	Tagap	Signaling
DMR1:47580001	1	47580001	47585000	5000	1	1.00E-13	0.56	88	1.76	LOC102551987;RGD1560015	
DMR1:47594001	1	47594001	47596000	2000	1	2.80E-11	0.43	22	1.1	RGD1560015;Fndc1	
DMR1:47616001	1	47616001	47619000	3000	1	7.90E-13	0.46	48	1.6	Fndc1	
DMR1:47660001	1	47660001	47666000	6000	2	1.60E-09	0.44	88	1.47	Fndc1	
DMR1:47672001	1	47672001	47676000	4000	1	8.10E-16	0.69	64	1.6	Fndc1	
DMR1:47684001	1	47684001	47686000	2000	1	2.10E-07	0.42	27	1.35	Fndc1	
DMR1:47906001	1	47906001	47907000	1000	1	1.60E-14	0.79	7	0.7	Sod2	Metabolism
DMR1:47908001	1	47908001	47909000	1000	1	4.20E-10	0.4	16	1.6	Sod2	Metabolism
DMR1:47913001	1	47913001	47915000	2000	1	2.10E-07	-0.58	26	1.3	Sod2	Metabolism
DMR1:47984001	1	47984001	47986000	2000	1	2.80E-08	0.34	23	1.15	Acat2	Metabolism
DMR1:47989001	1	47989001	47994000	5000	2	1.20E-13	-0.57	44	0.88	Acat2	Metabolism
DMR1:47996001	1	47996001	47997000	1000	1	5.40E-07	0.7	38	3.8	Acat2	Metabolism
DMR1:47998001	1	47998001	47999000	1000	1	5.80E-07	-0.34	17	1.7	Acat2;Acat2l1	Metabolism
DMR1:48006001	1	48006001	48008000	2000	1	2.00E-08	-0.61	19	0.95	Acat2l1;LOC108349718	Metabolism
DMR1:48009001	1	48009001	48014000	5000	2	2.80E-09	-0.43	65	1.3	Acat2l1;LOC108349718	Metabolism
DMR1:48015001	1	48015001	48016000	1000	1	4.80E-09	0.43	32	3.2	Acat2l1;LOC108349718;Tcp1	Metabolism;Translation
DMR1:48034001	1	48034001	48038000	4000	2	3.00E-10	0.56	67	1.68	Acat2l1;Tcp1;Mrpl18;Pnldc1	Metabolism;Translation;Translation;Translation
DMR1:48042001	1	48042001	48044000	2000	1	1.00E-07	-0.52	25	1.25	Tcp1;Mrpl18;Pnldc1	Translation;Translation;Translation
DMR1:48049001	1	48049001	48054000	5000	2	1.60E-12	0.51	65	1.3	Pnldc1	Translation
DMR1:48055001	1	48055001	48058000	3000	1	3.10E-09	0.44	53	1.77	Pnldc1	Translation
DMR1:48066001	1	48066001	48070000	4000	1	4.70E-07	0.33	73	1.82	Pnldc1;Mas1l;Mas1	Translation;Signaling
DMR1:48077001	1	48077001	48079000	2000	2	1.70E-14	0.88	30	1.5	Mas1l;Mas1	Signaling
DMR1:48080001	1	48080001	48081000	1000	1	3.50E-07	0.33	18	1.8	Mas1l;Mas1	Signaling
DMR1:48082001	1	48082001	48086000	4000	1	9.00E-17	0.44	66	1.65	Mas1l;Mas1	Signaling
DMR1:48087001	1	48087001	48088000	1000	1	1.50E-10	0.46	7	0.7	Mas1l;Mas1	Signaling
DMR1:48089001	1	48089001	48091000	2000	1	6.30E-07	-0.57	30	1.5	Mas1l;Mas1	Signaling
DMR1:48099001	1	48099001	48100000	1000	1	1.10E-07	-0.57	15	1.5	Mas1l;Mas1	Signaling
DMR1:48177001	1	48177001	48182000	5000	1	5.70E-08	-0.49	64	1.28	Mas1l;Igf2r	Transport
DMR1:48187001	1	48187001	48189000	2000	1	1.60E-12	0.5	22	1.1	Mas1l;Igf2r	Transport
DMR1:48207001	1	48207001	48211000	4000	2	9.70E-10	0.49	44	1.1	Mas1l;Igf2r	Transport
DMR1:48224001	1	48224001	48226000	2000	1	1.60E-07	-0.46	26	1.3	Igf2r	Transport
DMR1:48270001	1	48270001	48271000	1000	1	9.30E-07	0.32	13	1.3	Igf2r;Slc22a1	Transport;Transport
DMR1:48280001	1	48280001	48285000	5000	3	5.60E-15	0.45	54	1.08	Slc22a1	Transport
DMR1:48286001	1	48286001	48287000	1000	1	3.20E-07	-0.5	20	2	Slc22a1	Transport
DMR1:48296001	1	48296001	48302000	6000	1	8.70E-07	0.39	63	1.05	Slc22a1	Transport
DMR1:48310001	1	48310001	48312000	2000	2	5.90E-18	1.02	31	1.55	Slc22a1;Slc22a2	Transport
DMR1:48313001	1	48313001	48314000	1000	1	4.00E-08	0.4	14	1.4	Slc22a2	Transport
DMR1:48327001	1	48327001	48328000	1000	1	7.50E-07	-0.4	12	1.2	Slc22a2	Transport
DMR1:48331001	1	48331001	48334000	3000	2	1.80E-22	0.94	18	0.6	Slc22a2	Transport
DMR1:48340001	1	48340001	48347000	7000	3	3.00E-23	1.07	75	1.07	Slc22a2;LOC103690972	Transport
DMR1:48364001	1	48364001	48367000	3000	1	2.00E-12	0.61	36	1.2	Slc22a2	Transport
DMR1:48423001	1	48423001	48425000	2000	1	2.40E-20	0.67	32	1.6	LOC102553224;Slc22a3	Transport
DMR1:48432001	1	48432001	48433000	1000	1	5.90E-14	0.6	13	1.3	Slc22a3	Transport
DMR1:48461001	1	48461001	48463000	2000	1	3.40E-13	0.54	24	1.2	Slc22a3	Transport
DMR1:48484001	1	48484001	48487000	3000	1	7.70E-22	0.7	48	1.6	Slc22a3	Transport
DMR1:48488001	1	48488001	48492000	4000	1	1.20E-07	-0.4	69	1.73	Slc22a3	Transport

DMR1:48503001	1	48503001	48505000	2000	1	4.80E-10	0.47	27	1.35	Slc22a3	Transport
DMR1:48531001	1	48531001	48535000	4000	1	4.60E-17	0.64	48	1.2	Slc22a3;Plg	Transport;Protease
DMR1:48542001	1	48542001	48545000	3000	1	1.10E-14	0.61	46	1.53	Plg	Protease
DMR1:48568001	1	48568001	48570000	2000	1	2.10E-10	-0.61	20	1	Plg;Dut-ps	Protease
DMR1:48574001	1	48574001	48576000	2000	2	3.30E-07	0.56	17	0.85	Dut-ps;LOC108349106	
DMR1:48639001	1	48639001	48642000	3000	1	3.80E-08	0.39	52	1.73	Map3k4	Signaling
DMR1:48656001	1	48656001	48657000	1000	1	8.40E-14	0.6	11	1.1	Map3k4	Signaling
DMR1:48665001	1	48665001	48666000	1000	1	1.60E-07	-0.42	11	1.1	Map3k4	Signaling
DMR1:48670001	1	48670001	48672000	2000	1	8.50E-07	-0.52	33	1.65	Map3k4	Signaling
DMR1:48680001	1	48680001	48683000	3000	1	4.20E-43	1.02	57	1.9	Map3k4	Signaling
DMR1:48701001	1	48701001	48714000	13000	3	1.70E-12	0.51	227	1.75	Map3k4;Agpat4	Signaling;Metabolism
DMR1:48733001	1	48733001	48734000	1000	1	5.50E-09	0.54	13	1.3	Agpat4	Metabolism
DMR1:48744001	1	48744001	48745000	1000	1	4.80E-09	0.32	14	1.4	Agpat4	Metabolism
DMR1:48758001	1	48758001	48760000	2000	2	6.10E-23	0.69	30	1.5	Agpat4;LOC108349099	Metabolism
DMR1:48783001	1	48783001	48784000	1000	1	4.60E-23	0.9	13	1.3	Agpat4;LOC108349099	Metabolism
DMR1:48796001	1	48796001	48799000	3000	1	2.80E-10	0.54	28	0.93	Agpat4	Metabolism
DMR1:48800001	1	48800001	48801000	1000	1	1.50E-21	0.7	12	1.2	Agpat4	Metabolism
DMR1:48807001	1	48807001	48808000	1000	1	1.80E-07	0.47	6	0.6	Agpat4	Metabolism
DMR1:48870001	1	48870001	48871000	1000	1	8.20E-11	0.41	19	1.9	Park2	
DMR1:48875001	1	48875001	48877000	2000	2	2.50E-12	0.5	24	1.2	Park2	
DMR1:48880001	1	48880001	48882000	2000	1	1.50E-09	0.38	34	1.7	Park2	
DMR1:48885001	1	48885001	48887000	2000	1	8.30E-21	0.84	19	0.95	Park2	
DMR1:48913001	1	48913001	48914000	1000	1	1.00E-12	0.52	9	0.9	Park2;LOC103690973	
DMR1:48915001	1	48915001	48916000	1000	1	1.40E-07	0.37	5	0.5	Park2;LOC103690973	
DMR1:48927001	1	48927001	48929000	2000	1	6.50E-10	0.57	48	2.4	Park2	
DMR1:48930001	1	48930001	48931000	1000	1	2.80E-08	0.57	12	1.2	Park2	
DMR1:48935001	1	48935001	48936000	1000	1	3.30E-10	0.64	6	0.6	Park2	
DMR1:48946001	1	48946001	48947000	1000	1	3.30E-17	0.65	13	1.3	Park2	
DMR1:48949001	1	48949001	48950000	1000	1	3.50E-09	0.65	7	0.7	Park2	
DMR1:48969001	1	48969001	48970000	1000	1	8.30E-07	-0.47	16	1.6	Park2	
DMR1:48980001	1	48980001	48981000	1000	1	8.40E-07	0.39	14	1.4	Park2	
DMR1:48999001	1	48999001	49004000	5000	2	1.30E-12	-0.6	94	1.88	Park2	
DMR1:49039001	1	49039001	49044000	5000	1	3.90E-12	-0.66	68	1.36	Park2	
DMR1:49052001	1	49052001	49056000	4000	1	1.20E-07	-0.41	45	1.12	Park2	
DMR1:49079001	1	49079001	49081000	2000	2	4.80E-15	0.62	11	0.55	Park2	
DMR1:49095001	1	49095001	49101000	6000	1	6.00E-17	0.69	87	1.45	Park2	
DMR1:49102001	1	49102001	49105000	3000	2	2.40E-08	-0.5	57	1.9	Park2	
DMR1:49163001	1	49163001	49164000	1000	1	7.40E-10	-0.54	23	2.3	Park2	
DMR1:49175001	1	49175001	49177000	2000	2	1.30E-11	-0.48	21	1.05	Park2	
DMR1:49178001	1	49178001	49180000	2000	1	2.20E-09	0.4	35	1.75	Park2	
DMR1:49197001	1	49197001	49198000	1000	1	8.20E-10	0.41	25	2.5	Park2	
DMR1:49213001	1	49213001	49215000	2000	1	3.00E-08	0.34	35	1.75	Park2	
DMR1:49247001	1	49247001	49248000	1000	1	5.50E-11	-0.53	9	0.9	Park2	
DMR1:49252001	1	49252001	49253000	1000	1	8.40E-09	-0.68	13	1.3	Park2	
DMR1:49261001	1	49261001	49263000	2000	1	6.70E-08	-0.37	29	1.45	Park2	
DMR1:49267001	1	49267001	49270000	3000	1	7.10E-09	0.36	30	1	Park2	
DMR1:49287001	1	49287001	49292000	5000	1	2.90E-07	0.32	78	1.56	Park2	
DMR1:49331001	1	49331001	49332000	1000	1	8.30E-10	0.4	24	2.4	Park2	
DMR1:49339001	1	49339001	49340000	1000	1	5.10E-09	0.39	16	1.6	Park2	
DMR1:49352001	1	49352001	49356000	4000	1	6.80E-10	0.37	58	1.45	Park2	
DMR1:49377001	1	49377001	49379000	2000	2	1.70E-11	-0.56	38	1.9	Park2	
DMR1:49397001	1	49397001	49398000	1000	1	1.20E-10	0.4	11	1.1	Park2	
DMR1:49415001	1	49415001	49417000	2000	1	7.80E-09	0.57	14	0.7	Park2	
DMR1:49419001	1	49419001	49426000	7000	1	1.30E-07	-0.66	79	1.13	Park2	
DMR1:49438001	1	49438001	49442000	4000	4	1.90E-13	-0.63	18	0.45	Park2	
DMR1:49451001	1	49451001	49454000	3000	1	1.70E-11	0.54	31	1.03	Park2	
DMR1:49529001	1	49529001	49532000	3000	2	4.60E-12	0.74	23	0.77	Park2	
DMR1:49564001	1	49564001	49572000	8000	4	3.70E-18	-0.59	102	1.27	Park2	
DMR1:49595001	1	49595001	49596000	1000	1	5.00E-07	0.4	6	0.6	Park2	
DMR1:49602001	1	49602001	49604000	2000	1	1.90E-09	0.37	24	1.2	Park2	
DMR1:49613001	1	49613001	49615000	2000	1	2.10E-08	-0.6	30	1.5	Park2	
DMR1:49622001	1	49622001	49625000	3000	1	8.50E-10	0.39	47	1.57	Park2	
DMR1:49657001	1	49657001	49658000	1000	1	7.20E-09	-0.75	11	1.1	Park2	
DMR1:49700001	1	49700001	49702000	2000	1	4.50E-07	-0.48	29	1.45	Park2	

DMR1:49731001	1	49731001	49733000	2000	1	2.90E-07	-0.56	15	0.75	Park2	
DMR1:49735001	1	49735001	49737000	2000	1	1.70E-07	-0.52	16	0.8	Park2	
DMR1:49757001	1	49757001	49763000	6000	1	1.50E-08	-0.61	97	1.62	Park2	
DMR1:49778001	1	49778001	49779000	1000	1	6.60E-10	0.65	1	0.1	Park2	
DMR1:49802001	1	49802001	49805000	3000	2	1.30E-16	0.82	21	0.7	Park2	
DMR1:49807001	1	49807001	49810000	3000	3	5.60E-17	0.57	39	1.3	Park2	
DMR1:49811001	1	49811001	49812000	1000	1	4.30E-12	0.67	7	0.7	Park2	
DMR1:49817001	1	49817001	49818000	1000	1	5.40E-07	-0.61	13	1.3	Park2	
DMR1:49826001	1	49826001	49828000	2000	1	7.90E-12	0.43	20	1	Park2	
DMR1:49847001	1	49847001	49849000	2000	1	2.70E-17	0.65	17	0.85	Park2	
DMR1:49853001	1	49853001	49857000	4000	3	2.30E-23	1.03	30	0.75	Park2	
DMR1:49889001	1	49889001	49890000	1000	1	4.80E-07	0.43	15	1.5	Park2	
DMR1:49908001	1	49908001	49911000	3000	1	5.00E-14	0.58	32	1.07	Park2	
DMR1:49915001	1	49915001	49922000	7000	4	1.10E-28	-0.61	88	1.26	Park2	
DMR1:49951001	1	49951001	49959000	8000	2	2.00E-11	-0.88	111	1.39	Park2;LOC108349802	
DMR1:50008001	1	50008001	50011000	3000	2	1.70E-10	0.49	23	0.77	Park2	
DMR1:50021001	1	50021001	50022000	1000	1	5.40E-08	0.55	4	0.4	Park2;LOC103694996	
DMR1:50028001	1	50028001	50029000	1000	1	1.10E-09	0.43	9	0.9	Park2;LOC103694996	
DMR1:50041001	1	50041001	50042000	1000	1	1.80E-07	-0.37	5	0.5	Park2	
DMR1:50063001	1	50063001	50065000	2000	2	1.90E-08	0.62	15	0.75	Park2;Pacrg	
DMR1:50081001	1	50081001	50086000	5000	1	1.70E-09	0.41	48	0.96	Pacrg	
DMR1:50110001	1	50110001	50113000	3000	1	3.00E-20	0.63	28	0.93	Pacrg	
DMR1:50121001	1	50121001	50123000	2000	1	8.90E-08	-0.58	21	1.05	Pacrg	
DMR1:50130001	1	50130001	50132000	2000	1	1.60E-11	-0.79	22	1.1	Pacrg;LOC102552213	
DMR1:50141001	1	50141001	50142000	1000	1	7.50E-21	0.75	10	1	Pacrg;LOC102552213	
DMR1:50144001	1	50144001	50145000	1000	1	1.30E-18	0.7	3	0.3	Pacrg;LOC102552213	
DMR1:50146001	1	50146001	50148000	2000	1	3.70E-07	-0.46	31	1.55	Pacrg;LOC102552213	
DMR1:50158001	1	50158001	50160000	2000	1	5.90E-08	-0.6	18	0.9	Pacrg;LOC102552213	
DMR1:50171001	1	50171001	50175000	4000	2	1.20E-08	-0.64	37	0.92	Pacrg;LOC102552213	
DMR1:50222001	1	50222001	50223000	1000	1	4.60E-07	-0.48	17	1.7	Pacrg	
DMR1:50227001	1	50227001	50231000	4000	1	5.70E-07	-0.46	43	1.07	Pacrg	
DMR1:50233001	1	50233001	50236000	3000	2	1.40E-08	0.52	37	1.23	Pacrg	
DMR1:50243001	1	50243001	50245000	2000	1	7.40E-08	0.46	16	0.8	Pacrg	
DMR1:50256001	1	50256001	50258000	2000	1	1.70E-07	0.53	21	1.05	Pacrg	
DMR1:50276001	1	50276001	50279000	3000	2	3.80E-15	0.67	32	1.07	Pacrg	
DMR1:50287001	1	50287001	50290000	3000	1	2.90E-08	0.39	37	1.23	Pacrg	
DMR1:50293001	1	50293001	50295000	2000	1	7.40E-11	0.46	25	1.25	Pacrg	
DMR1:50313001	1	50313001	50314000	1000	1	8.40E-09	0.56	6	0.6	Pacrg	
DMR1:50347001	1	50347001	50348000	1000	1	5.90E-16	0.84	5	0.5	Pacrg	
DMR1:50351001	1	50351001	50362000	11000	2	1.80E-12	0.47	147	1.34	Pacrg	
DMR1:50367001	1	50367001	50369000	2000	1	1.50E-08	0.39	30	1.5	Pacrg	
DMR1:50371001	1	50371001	50372000	1000	1	3.10E-09	0.45	13	1.3	Pacrg	
DMR1:50387001	1	50387001	50388000	1000	1	6.80E-08	-0.62	10	1	Pacrg	
DMR1:50390001	1	50390001	50393000	3000	1	2.50E-08	0.45	23	0.77	Pacrg	
DMR1:50397001	1	50397001	50399000	2000	1	4.30E-07	0.34	30	1.5	Pacrg	
DMR1:50402001	1	50402001	50406000	4000	1	8.30E-09	0.4	40	1	Pacrg	
DMR1:50448001	1	50448001	50449000	1000	1	4.80E-17	0.6	6	0.6	Pacrg	
DMR1:50455001	1	50455001	50456000	1000	1	2.90E-09	0.59	12	1.2	Pacrg	
DMR1:50483001	1	50483001	50487000	4000	2	8.60E-16	0.72	35	0.88	Pacrg	
DMR1:50491001	1	50491001	50494000	3000	2	2.40E-09	-0.7	42	1.4	Pacrg	
DMR1:50988001	1	50988001	50990000	2000	1	1.60E-07	0.45	24	1.2	Qk	
DMR1:51612001	1	51612001	51615000	3000	1	6.00E-10	0.57	26	0.87	LOC108348950;Pabpc6	
DMR1:52323001	1	52323001	52324000	1000	1	3.20E-11	0.55	12	1.2	MGC94891	
DMR1:52337001	1	52337001	52339000	2000	2	3.40E-14	0.71	17	0.85	MGC94891	
DMR1:52341001	1	52341001	52344000	3000	2	8.00E-23	0.75	32	1.07	MGC94891	
DMR1:52357001	1	52357001	52361000	4000	1	8.50E-09	-0.45	90	2.25	MGC94891;Pde10a	Signaling
DMR1:52373001	1	52373001	52376000	3000	3	1.20E-33	0.89	36	1.2	Pde10a	Signaling
DMR1:52388001	1	52388001	52390000	2000	1	1.30E-07	0.34	36	1.8	Pde10a	Signaling
DMR1:52391001	1	52391001	52393000	2000	1	6.80E-08	-0.58	44	2.2	Pde10a	Signaling
DMR1:52394001	1	52394001	52399000	5000	1	9.00E-12	0.45	104	2.08	Pde10a	Signaling
DMR1:52408001	1	52408001	52416000	8000	3	1.20E-16	0.47	158	1.98	Pde10a	Signaling
DMR1:52418001	1	52418001	52421000	3000	1	1.30E-09	0.45	66	2.2	Pde10a	Signaling
DMR1:52424001	1	52424001	52428000	4000	2	1.90E-09	-0.64	45	1.12	Pde10a	Signaling
DMR1:52441001	1	52441001	52443000	2000	1	2.50E-13	0.5	22	1.1	Pde10a	Signaling
DMR1:52453001	1	52453001	52455000	2000	1	4.40E-11	0.38	32	1.6	Pde10a	Signaling

DMR1:52472001	1	52472001	52475000	3000	3	2.90E-15	0.58	47	1.57	Pde10a	Signaling
DMR1:52476001	1	52476001	52477000	1000	1	6.90E-14	0.49	21	2.1	Pde10a	Signaling
DMR1:52505001	1	52505001	52507000	2000	1	6.40E-19	0.61	30	1.5	Pde10a	Signaling
DMR1:52514001	1	52514001	52515000	1000	1	1.30E-07	0.41	6	0.6	Pde10a	Signaling
DMR1:52550001	1	52550001	52554000	4000	1	1.20E-24	1.02	49	1.23	Pde10a	Signaling
DMR1:52877001	1	52877001	52879000	2000	1	6.90E-11	0.37	34	1.7	T	
DMR1:52880001	1	52880001	52882000	2000	1	2.50E-13	-0.85	13	0.65	T	
DMR1:52902001	1	52902001	52905000	3000	2	3.10E-19	0.79	30	1	T;T2	
DMR1:52906001	1	52906001	52908000	2000	1	3.90E-08	0.4	28	1.4	T;T2	
DMR1:52917001	1	52917001	52919000	2000	1	2.60E-08	0.38	31	1.55	T2	
DMR1:52920001	1	52920001	52922000	2000	1	7.00E-14	0.73	14	0.7	T2	
DMR1:52927001	1	52927001	52931000	4000	4	8.20E-35	0.99	47	1.18	T2	
DMR1:52932001	1	52932001	52934000	2000	1	2.80E-10	0.68	27	1.35	T2	
DMR1:52936001	1	52936001	52940000	4000	1	8.40E-09	0.72	50	1.25	T2	
DMR1:52948001	1	52948001	52951000	3000	1	2.50E-21	0.87	29	0.97	T2	
DMR1:52957001	1	52957001	52959000	2000	1	2.70E-10	0.43	28	1.4	T2	
DMR1:52961001	1	52961001	52962000	1000	1	1.50E-10	0.65	9	0.9	T2	
DMR1:52977001	1	52977001	52981000	4000	1	9.50E-07	-0.38	52	1.3	T2;LOC684284;Prr18	
DMR1:52985001	1	52985001	52991000	6000	1	1.20E-10	0.44	91	1.52	LOC684284;Prr18;Sft2d1	
DMR1:52998001	1	52998001	53001000	3000	2	6.00E-09	-0.58	52	1.73	Prr18;Sft2d1	
DMR1:53008001	1	53008001	53010000	2000	1	4.40E-10	0.48	10	0.5	Sft2d1	
DMR1:53017001	1	53017001	53018000	1000	1	4.90E-16	0.63	8	0.8	Sft2d1;Mpc1	
DMR1:53045001	1	53045001	53048000	3000	1	1.20E-13	0.8	36	1.2	Mpc1	
DMR1:53061001	1	53061001	53062000	1000	1	2.50E-20	0.75	11	1.1	Ccr6	
DMR1:53066001	1	53066001	53071000	5000	1	1.20E-14	0.63	63	1.26	Ccr6	
DMR1:53072001	1	53072001	53073000	1000	1	1.30E-07	0.32	20	2	Ccr6	
DMR1:53081001	1	53081001	53083000	2000	2	2.50E-09	0.63	19	0.95	Ccr6	
DMR1:53095001	1	53095001	53096000	1000	1	9.10E-18	1.06	1	0.1	Ccr6	
DMR1:53144001	1	53144001	53146000	2000	1	4.70E-08	0.5	12	0.6	Fgfr1op	
DMR1:53155001	1	53155001	53157000	2000	1	2.90E-07	0.34	18	0.9	Fgfr1op	
DMR1:53181001	1	53181001	53187000	6000	2	5.10E-10	-0.73	82	1.37	Rnaset2	
DMR1:53202001	1	53202001	53205000	3000	1	2.40E-18	0.86	28	0.93	Rnaset2	
DMR1:53212001	1	53212001	53213000	1000	1	1.20E-11	0.64	2	0.2	Rps6ka2	Golgi
DMR1:53234001	1	53234001	53236000	2000	2	6.00E-10	-0.57	27	1.35	Rps6ka2	Golgi
DMR1:53237001	1	53237001	53241000	4000	1	1.30E-08	0.39	74	1.85	Rps6ka2	Golgi
DMR1:53273001	1	53273001	53278000	5000	1	1.30E-17	0.95	52	1.04	Rps6ka2	Golgi
DMR1:53282001	1	53282001	53283000	1000	1	2.20E-08	0.38	18	1.8	Rps6ka2	Golgi
DMR1:53286001	1	53286001	53287000	1000	1	3.70E-09	0.35	10	1	Rps6ka2	Golgi
DMR1:53303001	1	53303001	53311000	8000	2	6.40E-12	0.49	93	1.16	Rps6ka2	Golgi
DMR1:53314001	1	53314001	53315000	1000	1	8.40E-12	0.55	12	1.2	Rps6ka2	Golgi
DMR1:53316001	1	53316001	53320000	4000	1	3.40E-10	0.4	68	1.7	Rps6ka2	Golgi
DMR1:53325001	1	53325001	53327000	2000	1	9.20E-10	-0.8	23	1.15	Rps6ka2	Golgi
DMR1:53336001	1	53336001	53341000	5000	1	6.40E-23	0.65	81	1.62	Rps6ka2	Golgi
DMR1:53342001	1	53342001	53354000	12000	2	2.70E-17	0.96	138	1.15	Rps6ka2	Golgi
DMR1:53356001	1	53356001	53358000	2000	1	7.50E-07	0.38	24	1.2	Rps6ka2	Golgi
DMR1:53373001	1	53373001	53376000	3000	2	7.80E-15	0.46	48	1.6	Rps6ka2	Golgi
DMR1:53406001	1	53406001	53407000	1000	1	3.90E-12	0.48	20	2	Rps6ka2	Golgi
DMR1:53413001	1	53413001	53414000	1000	1	2.20E-07	0.37	11	1.1	Rps6ka2	Golgi
DMR1:53415001	1	53415001	53418000	3000	1	1.70E-08	-0.57	51	1.7	Rps6ka2	Golgi
DMR1:53420001	1	53420001	53422000	2000	1	1.90E-10	0.6	29	1.45	Rps6ka2	Golgi
DMR1:53442001	1	53442001	53444000	2000	1	2.40E-08	0.42	20	1	Rps6ka2	Golgi
DMR1:53472001	1	53472001	53474000	2000	1	8.30E-07	-0.57	35	1.75	Rps6ka2	Golgi
DMR1:53488001	1	53488001	53489000	1000	1	2.10E-15	0.55	14	1.4	Rps6ka2	Golgi
DMR1:53492001	1	53492001	53495000	3000	2	2.10E-21	0.64	56	1.87	Rps6ka2	Golgi
DMR1:53533001	1	53533001	53539000	6000	2	2.10E-14	0.63	67	1.12	Tcp10b	Cytoskeleton
DMR1:53543001	1	53543001	53545000	2000	1	6.20E-18	0.77	26	1.3	Tcp10b	Cytoskeleton
DMR1:53551001	1	53551001	53553000	2000	1	2.10E-13	0.46	27	1.35	Tcp10b;Ttll2	Cytoskeleton
DMR1:53563001	1	53563001	53569000	6000	2	2.00E-16	0.57	65	1.08	Tcp10b;Ttll2;Unc93a	Cytoskeleton;Signaling
DMR1:53600001	1	53600001	53602000	2000	1	6.80E-12	0.56	16	0.8	Unc93a;LOC102548318	Signaling
DMR1:53709001	1	53709001	53710000	1000	1	1.30E-10	0.55	4	0.4	Afdn	Cytoskeleton
DMR1:53716001	1	53716001	53718000	2000	1	3.90E-09	-0.72	17	0.85	Afdn	Cytoskeleton
DMR1:53722001	1	53722001	53724000	2000	1	1.00E-13	0.56	14	0.7	Afdn	Cytoskeleton
DMR1:53755001	1	53755001	53756000	1000	1	5.60E-09	-0.59	13	1.3	Afdn	Cytoskeleton
DMR1:53790001	1	53790001	53791000	1000	1	1.70E-13	0.67	7	0.7	Afdn	Cytoskeleton

DMR1:53822001	1	53822001	53825000	3000	2	3.20E-18	0.95	13	0.43	Afdn	Cytoskeleton
DMR1:53846001	1	53846001	53848000	2000	2	3.20E-17	0.64	18	0.9	Afdn;LOC103690979	Cytoskeleton
DMR1:54023001	1	54023001	54025000	2000	1	1.20E-13	0.61	22	1.1	RGD1560718	Signaling
DMR1:54027001	1	54027001	54030000	3000	3	2.50E-16	0.86	28	0.93	RGD1560718	Signaling
DMR1:54034001	1	54034001	54036000	2000	1	2.10E-08	-0.25	39	1.95	RGD1560718	Signaling
DMR1:54037001	1	54037001	54042000	5000	3	3.80E-14	-0.37	32	0.64	RGD1560718	Signaling
DMR1:54043001	1	54043001	54044000	1000	1	1.80E-13	0.26	5	0.5	RGD1560718	Signaling
DMR1:54046001	1	54046001	54048000	2000	1	9.60E-19	0.6	14	0.7	RGD1560718;LOC102552087;LOC108349470	Signaling
DMR1:54312001	1	54312001	54313000	1000	1	6.40E-08	-0.39	8	0.8	LOC103691009;Frmd1	
DMR1:54314001	1	54314001	54315000	1000	1	4.70E-10	0.52	9	0.9	LOC103691009;Frmd1;LOC102550907	
DMR1:54321001	1	54321001	54325000	4000	3	1.30E-17	0.9	33	0.82	Frmd1;LOC102550907	
DMR1:54336001	1	54336001	54338000	2000	1	3.10E-08	0.43	10	0.5	Frmd1;LOC102550907	
DMR1:54604001	1	54604001	54607000	3000	1	1.50E-07	-0.57	9	0.3	Vom2r7	
DMR1:54621001	1	54621001	54628000	7000	2	1.90E-17	-0.54	54	0.77	Vom2r7	
DMR1:54840001	1	54840001	54842000	2000	1	2.70E-18	0.8	16	0.8	Smok2a	Signaling
DMR1:54846001	1	54846001	54848000	2000	2	2.40E-08	-0.4	34	1.7	Smok2a	Signaling
DMR1:54849001	1	54849001	54850000	1000	1	3.40E-13	0.52	6	0.6	Smok2a	Signaling
DMR1:54856001	1	54856001	54860000	4000	1	1.40E-10	-0.29	33	0.82	Smok2a	Signaling
DMR1:55220001	1	55220001	55226000	6000	1	1.20E-12	0.28	56	0.93	RGD1561667	Signaling
DMR1:55227001	1	55227001	55231000	4000	1	5.20E-07	0.28	21	0.52	RGD1561667	Signaling
DMR1:55232001	1	55232001	55234000	2000	1	9.40E-08	0.19	22	1.1	RGD1561667	Signaling
DMR1:55654001	1	55654001	55660000	6000	1	1.70E-16	0.45	51	0.85	LOC102556502;LOC103690993;LOC102546375;LOC108349807;RGD1561185	
DMR1:55671001	1	55671001	55673000	2000	1	1.10E-13	0.3	36	1.8	LOC108349807;RGD1561185	
DMR1:55853001	1	55853001	55855000	2000	1	6.50E-07	0.44	14	0.7	Vom2r9	Signaling
DMR1:55858001	1	55858001	55859000	1000	1	5.30E-08	0.44	8	0.8	Vom2r9	Signaling
DMR1:55891001	1	55891001	55892000	1000	1	1.70E-08	0.68	8	0.8	Vom2r9	Signaling
DMR1:55898001	1	55898001	55899000	1000	1	7.50E-13	0.63	8	0.8	Vom2r9	Signaling
DMR1:56151001	1	56151001	56152000	1000	1	7.00E-07	0.57	13	1.3	Dact2	
DMR1:56153001	1	56153001	56157000	4000	1	4.90E-11	0.74	44	1.1	Dact2	
DMR1:56158001	1	56158001	56159000	1000	1	1.20E-08	-0.55	11	1.1	Dact2	
DMR1:56170001	1	56170001	56172000	2000	1	5.90E-19	0.63	28	1.4	Dact2	
DMR1:56245001	1	56245001	56250000	5000	1	5.60E-08	0.61	46	0.92	LOC108349078;Smoc2	Signaling
DMR1:56252001	1	56252001	56257000	5000	4	1.70E-17	0.8	37	0.74	Smoc2	Signaling
DMR1:56265001	1	56265001	56266000	1000	1	1.60E-07	0.51	8	0.8	Smoc2	Signaling
DMR1:56269001	1	56269001	56270000	1000	1	1.40E-10	0.62	5	0.5	Smoc2	Signaling
DMR1:56275001	1	56275001	56276000	1000	1	1.70E-12	0.6	4	0.4	Smoc2	Signaling
DMR1:56285001	1	56285001	56287000	2000	1	4.50E-10	0.39	25	1.25	Smoc2	Signaling
DMR1:56288001	1	56288001	56290000	2000	1	2.50E-08	-0.58	32	1.6	Smoc2	Signaling
DMR1:56304001	1	56304001	56306000	2000	1	3.90E-08	-0.74	21	1.05	Smoc2	Signaling
DMR1:56329001	1	56329001	56330000	1000	1	5.50E-07	-0.5	15	1.5	Smoc2	Signaling
DMR1:56344001	1	56344001	56346000	2000	1	2.10E-16	0.62	13	0.65	Smoc2	Signaling
DMR1:56375001	1	56375001	56377000	2000	1	1.10E-11	0.56	11	0.55	Smoc2	Signaling
DMR1:56673001	1	56673001	56676000	3000	1	5.00E-09	-0.59	46	1.53	Thbs2	
DMR1:56680001	1	56680001	56681000	1000	1	2.80E-07	-0.54	19	1.9	Thbs2	
DMR1:56682001	1	56682001	56683000	1000	1	5.20E-10	0.42	18	1.8	Thbs2	
DMR1:56814001	1	56814001	56816000	2000	1	4.30E-12	0.57	9	0.45	Wdr27	
DMR1:56842001	1	56842001	56848000	6000	2	1.90E-11	-0.64	39	0.65	Wdr27	
DMR1:56866001	1	56866001	56868000	2000	1	8.10E-14	0.5	40	2	Wdr27;LOC108349525	
DMR1:56869001	1	56869001	56872000	3000	1	2.20E-09	-0.54	48	1.6	Wdr27;LOC108349525	
DMR1:56884001	1	56884001	56885000	1000	1	2.20E-07	-0.4	22	2.2	Wdr27;LOC108349525	
DMR1:56895001	1	56895001	56898000	3000	1	1.40E-13	-0.68	19	0.63	Wdr27	
DMR1:56903001	1	56903001	56906000	3000	1	9.50E-16	0.82	41	1.37	Wdr27	
DMR1:56907001	1	56907001	56909000	2000	1	2.50E-09	0.54	8	0.4	Wdr27	
DMR1:56936001	1	56936001	56940000	4000	1	5.30E-09	-0.52	36	0.9	Wdr27;LOC100125364	
DMR1:56991001	1	56991001	56994000	3000	2	1.20E-11	-0.73	36	1.2	Tcte3;Ermard	Cytoskeleton
DMR1:57308001	1	57308001	57313000	5000	3	7.50E-26	1.18	63	1.26	Dll1	
DMR1:57318001	1	57318001	57320000	2000	1	6.70E-09	-0.68	44	2.2	Dll1	
DMR1:57330001	1	57330001	57331000	1000	1	2.10E-08	0.36	21	2.1	Dll1	
DMR1:57341001	1	57341001	57342000	1000	1	7.70E-11	0.49	9	0.9	Fam120b	
DMR1:57351001	1	57351001	57356000	5000	1	2.60E-07	-0.5	57	1.14	Fam120b	
DMR1:57369001	1	57369001	57374000	5000	1	1.10E-07	-0.73	86	1.72	Fam120b	

DMR1:57376001	1	57376001	57378000	2000	1	2.40E-07	-0.38	12	0.6	Fam120b	
DMR1:57392001	1	57392001	57394000	2000	1	2.40E-08	-0.6	17	0.85	Fam120b	
DMR1:57465001	1	57465001	57466000	1000	1	1.40E-14	0.6	11	1.1	Psmb1	Protease
DMR1:57470001	1	57470001	57473000	3000	1	1.20E-10	0.49	46	1.53	Psmb1	Protease
DMR1:57484001	1	57484001	57485000	1000	1	1.70E-14	0.54	12	1.2	Psmb1;Tbp	Protease;Transcription
DMR1:57535001	1	57535001	57538000	3000	1	1.10E-07	0.4	24	0.8	Prdm9	Transcription
DMR1:57562001	1	57562001	57565000	3000	1	2.50E-08	0.41	19	0.63	Prdm9;LOC102551889	Transcription
DMR1:57682001	1	57682001	57683000	1000	1	4.40E-30	0.88	18	1.8	LOC108349526;Chd1	
DMR1:57700001	1	57700001	57703000	3000	1	5.20E-11	-0.69	33	1.1	Chd1	
DMR1:57704001	1	57704001	57706000	2000	1	9.30E-09	0.51	24	1.2	Chd1	
DMR1:57712001	1	57712001	57714000	2000	1	4.50E-08	0.38	27	1.35	Chd1	
DMR1:57718001	1	57718001	57719000	1000	1	2.60E-09	-0.58	15	1.5	Chd1	
DMR1:57731001	1	57731001	57734000	3000	1	1.80E-13	0.45	36	1.2	Chd1	
DMR1:57741001	1	57741001	57742000	1000	1	6.60E-13	0.69	7	0.7	Chd1	
DMR1:57760001	1	57760001	57762000	2000	1	2.80E-10	0.51	24	1.2	Chd1	
DMR1:59131001	1	59131001	59132000	1000	1	4.00E-10	0.5	10	1	LOC108349836;Riok2	Signaling
DMR1:59161001	1	59161001	59163000	2000	1	5.50E-08	-0.62	25	1.25	Lix1	
DMR1:59171001	1	59171001	59172000	1000	1	3.20E-16	0.79	5	0.5	Lix1;LOC102552556	
DMR1:59177001	1	59177001	59178000	1000	1	3.50E-07	-0.58	11	1.1	Lix1;LOC102552556	
DMR1:59179001	1	59179001	59180000	1000	1	5.80E-15	0.72	6	0.6	Lix1;LOC102552556	
DMR1:59181001	1	59181001	59182000	1000	1	7.50E-10	0.52	9	0.9	Lix1;LOC102552556	
DMR1:59185001	1	59185001	59188000	3000	1	7.00E-07	0.42	26	0.87	Lix1;LOC102552556	
DMR1:59285001	1	59285001	59286000	1000	1	2.70E-07	0.54	8	0.8	Lnpep	Protease
DMR1:59304001	1	59304001	59305000	1000	1	1.50E-09	0.7	6	0.6	Lnpep	Protease
DMR1:59309001	1	59309001	59311000	2000	1	5.70E-07	0.43	27	1.35	Lnpep	Protease
DMR1:59318001	1	59318001	59320000	2000	1	8.30E-15	0.99	10	0.5	Lnpep	Protease
DMR1:59342001	1	59342001	59344000	2000	1	1.90E-18	1.03	8	0.4	Lnpep	Protease
DMR1:59351001	1	59351001	59352000	1000	1	1.70E-08	0.5	3	0.3	Lnpep	Protease
DMR1:59357001	1	59357001	59358000	1000	1	6.10E-11	0.68	2	0.2	Lnpep	Protease
DMR1:59537001	1	59537001	59543000	6000	1	7.70E-08	0.44	37	0.62	Vom2r-ps19	
DMR1:59555001	1	59555001	59556000	1000	1	4.80E-13	0.47	11	1.1	Vom2r-ps19	
DMR1:59580001	1	59580001	59584000	4000	1	2.40E-10	-0.54	24	0.6	Vom2r-ps20;LOC102553133	
DMR1:59700001	1	59700001	59702000	2000	1	8.20E-07	0.53	66	3.3	LOC688452;Mir99b;Mir3596c;Mirlet7e;Mir125a	
DMR1:59703001	1	59703001	59704000	1000	1	4.90E-14	0.83	14	1.4	LOC688452;Mir99b;Mir3596c;Mirlet7e;Mir125a	
DMR1:59705001	1	59705001	59706000	1000	1	4.20E-21	0.79	9	0.9	LOC688452;Mir99b;Mir3596c;Mirlet7e;Mir125a	
DMR1:59707001	1	59707001	59708000	1000	1	2.10E-14	0.8	5	0.5	LOC688452;Mir99b;Mir3596c;Mirlet7e;Mir125a	
DMR1:59716001	1	59716001	59718000	2000	1	2.80E-07	0.4	18	0.9	LOC688452;Has1	Golgi
DMR1:59724001	1	59724001	59726000	2000	1	3.20E-17	0.75	14	0.7	LOC688452;Has1	Golgi
DMR1:59732001	1	59732001	59734000	2000	2	9.90E-15	0.74	10	0.5	Has1	Golgi
DMR1:59735001	1	59735001	59738000	3000	1	1.40E-11	0.5	15	0.5	Has1	Golgi
DMR1:59752001	1	59752001	59758000	6000	4	1.10E-20	0.78	57	0.95	LOC108349528;Fpr1	Signaling
DMR1:59773001	1	59773001	59774000	1000	1	5.80E-07	-0.36	5	0.5	LOC108349528;Fpr1	Signaling
DMR1:59777001	1	59777001	59780000	3000	1	4.20E-09	-0.6	31	1.03	Fpr1	Signaling
DMR1:59789001	1	59789001	59791000	2000	2	2.80E-08	-0.5	8	0.4	Fpr2	Signaling
DMR1:59818001	1	59818001	59822000	4000	2	1.00E-11	-0.55	32	0.8	Fpr2	Signaling
DMR1:59887001	1	59887001	59893000	6000	1	1.30E-14	-0.44	64	1.07	Fpr-rs3	Signaling
DMR1:59898001	1	59898001	59899000	1000	1	4.40E-26	1.14	21	2.1	Fpr-rs3	Signaling
DMR1:59947001	1	59947001	59951000	4000	1	5.20E-13	0.66	18	0.45	Vom2r-ps23	
DMR1:59958001	1	59958001	59960000	2000	1	9.70E-07	-0.35	12	0.6	Vom2r-ps23	
DMR1:59974001	1	59974001	59977000	3000	1	1.10E-11	-0.55	11	0.37	Vom2r-ps23	
DMR1:60047001	1	60047001	60049000	2000	1	9.70E-15	0.82	5	0.25	LOC108349105;Vom1r6	Receptor
DMR1:60094001	1	60094001	60095000	1000	1	8.60E-07	0.51	6	0.6	Vom1r7	Receptor
DMR1:60111001	1	60111001	60113000	2000	1	4.30E-08	0.52	8	0.4	LOC690283;Vom1r8	Receptor
DMR1:60115001	1	60115001	60116000	1000	1	1.40E-07	0.61	2	0.2	LOC690283;Vom1r8	Receptor
DMR1:60123001	1	60123001	60128000	5000	2	2.10E-08	0.57	39	0.78	Vom1r8;LOC102556252	Receptor
DMR1:60254001	1	60254001	60256000	2000	1	5.20E-14	0.78	7	0.35	Vom1r9	Receptor
DMR1:60264001	1	60264001	60276000	12000	3	6.60E-09	-0.55	74	0.62	Vom1r10	Receptor
DMR1:60284001	1	60284001	60287000	3000	1	7.10E-08	-0.52	8	0.27	Vom1r10	Receptor
DMR1:60394001	1	60394001	60399000	5000	4	1.70E-11	-0.68	24	0.48	Vom1r12	Receptor
DMR1:60499001	1	60499001	60501000	2000	1	2.50E-11	0.54	11	0.55	Vom1r-ps10	

DMR1:60546001	1	60546001	60551000	5000	2	1.50E-08	0.44	113	2.26	Vom1r-ps11	
DMR1:60624001	1	60624001	60627000	3000	2	1.80E-09	-0.55	6	0.2	Vom1r15	Receptor
DMR1:60643001	1	60643001	60644000	1000	1	2.30E-09	-0.65	2	0.2	Vom1r15	Receptor
DMR1:60681001	1	60681001	60683000	2000	1	2.60E-12	0.49	11	0.55	Vom1r-ps14	
DMR1:60686001	1	60686001	60689000	3000	3	5.40E-11	-0.48	28	0.93	Vom1r-ps15	
DMR1:60694001	1	60694001	60699000	5000	2	3.20E-13	-0.45	41	0.82	Vom1r-ps15;Vom1r-ps16	
DMR1:60747001	1	60747001	60751000	4000	1	8.10E-14	-0.4	48	1.2	Vom1r-ps17	
DMR1:60771001	1	60771001	60773000	2000	2	8.20E-10	0.65	5	0.25	Vom1r-ps18	
DMR1:60840001	1	60840001	60844000	4000	1	1.60E-22	0.74	14	0.35	Vom1r-ps22;Vom1r-ps21	
DMR1:61063001	1	61063001	61066000	3000	1	2.20E-08	-0.39	19	0.63	Vom1r-ps23	
DMR1:61074001	1	61074001	61075000	1000	1	2.60E-07	0.57	6	0.6	Vom1r-ps23	
DMR1:61076001	1	61076001	61079000	3000	2	5.50E-21	-0.43	25	0.83	Vom1r-ps23	
DMR1:61089001	1	61089001	61091000	2000	1	3.80E-10	-0.66	8	0.4	Vom1r19	Receptor
DMR1:61097001	1	61097001	61101000	4000	2	5.50E-20	0.74	61	1.52	Vom1r19	Receptor
DMR1:61119001	1	61119001	61121000	2000	1	2.50E-11	-0.59	14	0.7	Vom1r-ps24	
DMR1:61122001	1	61122001	61128000	6000	2	1.90E-13	-0.41	60	1	Vom1r-ps24;Vom1r20	Receptor
DMR1:61210001	1	61210001	61213000	3000	2	1.00E-12	-0.45	26	0.87	Vom1r17	
DMR1:61224001	1	61224001	61227000	3000	1	1.20E-10	-0.42	22	0.73	Vom1r17;LOC103691016;Trnar-ccu	
DMR1:61286001	1	61286001	61291000	5000	1	7.10E-16	0.57	31	0.62	LOC102552527;Zfp51	Transcription
DMR1:61301001	1	61301001	61304000	3000	1	2.90E-07	0.38	22	0.73	Zfp51	Transcription
DMR1:61332001	1	61332001	61334000	2000	2	4.80E-09	0.48	8	0.4	Zfp51	Transcription
DMR1:61351001	1	61351001	61353000	2000	1	4.10E-10	0.4	22	1.1	Zfp53;LOC690453	
DMR1:61354001	1	61354001	61359000	5000	3	2.90E-09	-0.61	28	0.56	Zfp53;LOC690453	
DMR1:61360001	1	61360001	61364000	4000	1	1.30E-07	0.47	31	0.78	Zfp53;LOC690453	
DMR1:61410001	1	61410001	61413000	3000	1	2.40E-08	-0.32	19	0.63	Zfp52	Transcription
DMR1:61420001	1	61420001	61421000	1000	1	2.50E-10	0.54	13	1.3	Zfp52	Transcription
DMR1:61433001	1	61433001	61435000	2000	1	4.30E-10	0.52	13	0.65	Zfp52;LOC102550851	Transcription
DMR1:61436001	1	61436001	61439000	3000	1	1.90E-08	0.54	21	0.7	Zfp52;LOC102550851	Transcription
DMR1:61511001	1	61511001	61518000	7000	6	4.80E-20	-0.61	77	1.1	LOC108348215;Znf761	Transcription
DMR1:61536001	1	61536001	61539000	3000	1	2.10E-07	0.34	7	0.23	Znf761	
DMR1:61549001	1	61549001	61554000	5000	1	1.20E-09	0.55	56	1.12	RGD1565566	
DMR1:61563001	1	61563001	61571000	8000	5	4.80E-25	-0.53	85	1.06	RGD1565566	
DMR1:61609001	1	61609001	61618000	9000	3	8.70E-19	-0.52	74	0.82	Vom1r-ps26;Vom1r22	Receptor
DMR1:61636001	1	61636001	61643000	7000	2	6.10E-13	-0.51	47	0.67	Vom1r22	Receptor
DMR1:61672001	1	61672001	61676000	4000	2	1.50E-14	-0.42	35	0.88	Vom1r23	Receptor
DMR1:61687001	1	61687001	61689000	2000	1	9.00E-18	0.71	17	0.85	Vom1r23	Receptor
DMR1:61690001	1	61690001	61696000	6000	5	2.10E-12	-0.61	52	0.87	Vom1r23;LOC102553001	Receptor
DMR1:61807001	1	61807001	61808000	1000	1	3.60E-08	0.47	7	0.7	Vom2r-ps24	
DMR1:61844001	1	61844001	61846000	2000	1	9.00E-12	-0.48	21	1.05	Vom2r-ps24	
DMR1:62286001	1	62286001	62290000	4000	1	3.20E-21	0.76	21	0.52	RGD1566248	
DMR1:62292001	1	62292001	62293000	1000	1	4.30E-14	0.44	16	1.6	RGD1566248	
DMR1:62297001	1	62297001	62300000	3000	1	1.10E-12	-0.45	17	0.57	RGD1566248	
DMR1:62307001	1	62307001	62310000	3000	1	9.80E-10	-0.74	15	0.5	Zfp945	
DMR1:62494001	1	62494001	62495000	1000	1	3.10E-11	0.61	5	0.5	Vom2r80	Signaling
DMR1:62514001	1	62514001	62515000	1000	1	7.60E-12	0.69	9	0.9	Vom2r80	Signaling
DMR1:62542001	1	62542001	62547000	5000	1	2.00E-08	0.56	31	0.62	Zfp40	Transcription
DMR1:62550001	1	62550001	62552000	2000	1	9.10E-08	0.49	9	0.45	Zfp40	Transcription
DMR1:62559001	1	62559001	62561000	2000	1	3.40E-19	0.79	5	0.25	Zfp40	Transcription
DMR1:62571001	1	62571001	62572000	1000	1	1.70E-07	-0.48	5	0.5	Zfp40	Transcription
DMR1:62605001	1	62605001	62607000	2000	2	1.70E-12	-0.48	22	1.1	Vom2r-ps30	
DMR1:62630001	1	62630001	62631000	1000	1	4.90E-07	0.42	8	0.8	Vom2r-ps30	
DMR1:62632001	1	62632001	62640000	8000	4	1.20E-11	-0.54	59	0.74	Vom2r-ps30	
DMR1:62659001	1	62659001	62662000	3000	1	1.70E-09	-0.6	13	0.43	Vom2r-ps31;Vom2r15	Signaling
DMR1:62827001	1	62827001	62829000	2000	1	1.40E-13	0.6	11	0.55	LOC690686;Vom2r16	Signaling
DMR1:62857001	1	62857001	62859000	2000	1	6.40E-09	-0.56	13	0.65	Vom2r16	Signaling
DMR1:62884001	1	62884001	62887000	3000	1	1.60E-07	-0.38	26	0.87	Vom2r-ps32	
DMR1:62888001	1	62888001	62889000	1000	1	1.10E-08	-0.39	8	0.8	Vom2r-ps32	
DMR1:62891001	1	62891001	62893000	2000	1	8.10E-11	-0.56	10	0.5	Vom2r-ps32	
DMR1:63172001	1	63172001	63175000	3000	1	1.80E-08	-0.54	14	0.47	Vom1r110	Receptor
DMR1:63205001	1	63205001	63206000	1000	1	9.00E-08	0.6	6	0.6	Vom1r28;LOC365167	
DMR1:63233001	1	63233001	63237000	4000	3	1.30E-21	-0.56	48	1.2	Vom1r29	Receptor
DMR1:63258001	1	63258001	63261000	3000	2	1.90E-12	-0.44	25	0.83	Vom1r109	
DMR1:63303001	1	63303001	63304000	1000	1	2.80E-08	0.63	8	0.8	Zik1;LOC102554457	
DMR1:63343001	1	63343001	63348000	5000	3	4.60E-14	-0.45	71	1.42	Vom1r30	Receptor

DMR1:63437001	1	63437001	63442000	5000	3	7.90E-15	-0.58	43	0.86	Vom2r-ps41	
DMR1:63468001	1	63468001	63474000	6000	1	1.30E-12	-0.69	51	0.85	Vom2r-ps41	
DMR1:63542001	1	63542001	63556000	14000	12	7.40E-23	-0.5	176	1.26	Vom2r-ps42;Vom2r26	Signaling
DMR1:63560001	1	63560001	63563000	3000	2	2.30E-28	-0.69	38	1.27	Vom2r26	Signaling
DMR1:63564001	1	63564001	63567000	3000	1	4.00E-07	-0.36	31	1.03	Vom2r26	Signaling
DMR1:63572001	1	63572001	63577000	5000	4	6.80E-13	-0.65	66	1.32	Vom2r26	Signaling
DMR1:63579001	1	63579001	63582000	3000	1	1.30E-10	0.6	15	0.5	Vom2r26	Signaling
DMR1:63667001	1	63667001	63678000	11000	7	2.80E-21	-0.47	161	1.46	Lilrc2	Immune
DMR1:63687001	1	63687001	63688000	1000	1	3.50E-09	-0.57	3	0.3	Lilrc2	Immune
DMR1:63689001	1	63689001	63693000	4000	2	5.30E-11	-0.5	33	0.82	Lilrc2	Immune
DMR1:63696001	1	63696001	63710000	14000	8	4.00E-29	-0.6	144	1.03	RGD1562625	
DMR1:63723001	1	63723001	63732000	9000	4	3.50E-24	-0.59	109	1.21	RGD1562625;Pirb	
DMR1:63735001	1	63735001	63736000	1000	1	1.90E-08	0.67	1	0.1	RGD1562625;Pirb	
DMR1:63748001	1	63748001	63754000	6000	4	4.10E-11	-0.61	57	0.95	Pirb;Lilrb3l	Immune
DMR1:63755001	1	63755001	63758000	3000	1	2.10E-07	-0.69	36	1.2	Lilrb3l	Immune
DMR1:63761001	1	63761001	63762000	1000	1	4.30E-07	-0.53	7	0.7	Lilrb3l	Immune
DMR1:63767001	1	63767001	63774000	7000	3	1.50E-12	-0.84	40	0.57	Lilrb3l	Immune
DMR1:63786001	1	63786001	63787000	1000	1	3.10E-12	-0.88	5	0.5	Lilrb3l;LOC690938	Immune
DMR1:63788001	1	63788001	63790000	2000	1	1.90E-07	-0.9	24	1.2	Lilrb3l;LOC690938	Immune
DMR1:63791001	1	63791001	63792000	1000	1	7.30E-07	-0.36	18	1.8	Lilrb3l;LOC690938	Immune
DMR1:63814001	1	63814001	63815000	1000	1	1.10E-07	0.48	8	0.8	Lilrb3l;LOC683420	Immune
DMR1:63822001	1	63822001	63827000	5000	3	1.60E-11	-0.5	41	0.82	Lilrb3l;LOC683420	Immune
DMR1:63836001	1	63836001	63838000	2000	1	1.10E-11	-0.64	18	0.9	Lilrb3l;LOC683420	Immune
DMR1:63854001	1	63854001	63869000	15000	11	3.70E-25	-0.55	177	1.18	Lilrb3l	Immune
DMR1:63892001	1	63892001	63906000	14000	10	1.00E-21	-0.53	153	1.09	Lilrb1	
DMR1:63913001	1	63913001	63915000	2000	1	1.60E-07	-0.32	12	0.6	Lilra3	
DMR1:63935001	1	63935001	63953000	18000	13	1.20E-24	-0.57	208	1.16	Lilra3	
DMR1:63998001	1	63998001	6.40E+07	2000	1	1.90E-10	-0.79	12	0.6	Lilrb3a	Immune
DMR1:64038001	1	64038001	64040000	2000	2	3.70E-17	0.89	34	1.7	Tsen34;Mboat7l1;Tmc4b	Translation;Metabolism
DMR1:64050001	1	64050001	64051000	1000	1	8.20E-07	0.47	1	0.1	Mboat7l1;Tmc4b	Metabolism
DMR1:64067001	1	64067001	64068000	1000	1	2.90E-08	-0.6	6	0.6	Lilrb3b	Immune
DMR1:64106001	1	64106001	64108000	2000	1	4.20E-22	0.94	35	1.75	Tsen34l1;Mboat7;Tmc4	
DMR1:64123001	1	64123001	64124000	1000	1	2.60E-17	0.88	3	0.3	Mboat7;Tmc4;Leng1;Cnot3	Transcription
DMR1:64125001	1	64125001	64126000	1000	1	5.90E-07	0.37	13	1.3	Tmc4;Leng1;Cnot3;Mir3572	Transcription
DMR1:64130001	1	64130001	64132000	2000	1	5.60E-13	0.53	28	1.4	Tmc4;Leng1;Cnot3;Mir3572	Transcription
DMR1:64175001	1	64175001	64185000	10000	6	2.30E-21	0.9	101	1.01	Tfpt;Ndufa3;Oscar	Metabolism;Immune
DMR1:64193001	1	64193001	64194000	1000	1	5.20E-11	0.52	21	2.1	Oscar	Immune
DMR1:64261001	1	64261001	64264000	3000	1	4.30E-13	0.53	32	1.07	Tarm1;Vstm1	Immune
DMR1:64268001	1	64268001	64272000	4000	1	1.50E-07	0.49	37	0.92	Vstm1	
DMR1:64310001	1	64310001	64311000	1000	1	1.00E-26	0.9	14	1.4	Cacng6	Transport
DMR1:64314001	1	64314001	64315000	1000	1	4.00E-09	0.43	7	0.7	Cacng6	Transport
DMR1:64324001	1	64324001	64329000	5000	1	2.00E-14	0.51	55	1.1	Cacng6;Cacng8;Mir935	Transport
DMR1:64331001	1	64331001	64336000	5000	1	2.70E-11	0.85	120	2.4	Cacng6;Cacng8;Mir935	Transport
DMR1:64339001	1	64339001	64343000	4000	2	7.10E-12	0.66	61	1.52	Cacng8;Mir935	Transport
DMR1:64349001	1	64349001	64356000	7000	6	1.10E-23	0.97	111	1.59	Cacng8;LOC103691003	Transport
DMR1:64360001	1	64360001	64361000	1000	1	4.10E-18	0.67	7	0.7	Cacng8;LOC103691003	Transport
DMR1:64379001	1	64379001	64381000	2000	1	7.30E-09	0.52	22	1.1	LOC103691003;Cacng7	Transport
DMR1:64384001	1	64384001	64388000	4000	1	9.10E-07	0.34	48	1.2	LOC103691003;Cacng7	Transport
DMR1:64398001	1	64398001	64401000	3000	2	7.60E-22	0.62	39	1.3	LOC103691003;Cacng7;Prkcg	Transport;Signaling
DMR1:64414001	1	64414001	64415000	1000	1	5.70E-12	0.67	17	1.7	Cacng7;Prkcg	Transport;Signaling
DMR1:64418001	1	64418001	64421000	3000	1	7.80E-17	0.69	21	0.7	Prkcg	Signaling
DMR1:64422001	1	64422001	64425000	3000	1	2.50E-13	0.56	25	0.83	Prkcg	Signaling
DMR1:64428001	1	64428001	64429000	1000	1	1.20E-17	0.81	9	0.9	Prkcg;Myadm	Signaling
DMR1:64430001	1	64430001	64432000	2000	2	3.90E-23	0.93	34	1.7	Prkcg;Myadm	Signaling
DMR1:64436001	1	64436001	64437000	1000	1	7.10E-07	0.41	5	0.5	Prkcg;Myadm	Signaling
DMR1:64439001	1	64439001	64441000	2000	1	4.30E-10	0.43	44	2.2	Prkcg;Myadm	Signaling
DMR1:64476001	1	64476001	64478000	2000	2	2.00E-13	0.52	21	1.05	Olr1l;LOC103689958	Receptor
DMR1:64484001	1	64484001	64486000	2000	1	6.00E-22	0.68	25	1.25	Olr1l;LOC103689958;Olr386	Receptor
DMR1:64489001	1	64489001	64491000	2000	1	5.00E-13	0.48	28	1.4	Olr1l;LOC103689958;Olr386	Receptor
DMR1:64494001	1	64494001	64495000	1000	1	4.10E-10	0.59	8	0.8	LOC103689958;Olr386	Receptor
DMR1:64516001	1	64516001	64524000	8000	1	9.80E-07	0.39	94	1.18	Nlrp12	
DMR1:64525001	1	64525001	64527000	2000	1	1.10E-07	-0.4	22	1.1	Nlrp12;Mir295-1;Mir294	
DMR1:64551001	1	64551001	64552000	1000	1	3.90E-09	0.36	13	1.3	Nlrp12	
DMR1:64553001	1	64553001	64556000	3000	1	5.40E-07	0.46	36	1.2	Nlrp12	

DMR1:64658001	1	64658001	64660000	2000	2	8.20E-17	0.73	17	0.85	LOC108348628;Vom2r27	Signaling
DMR1:64669001	1	64669001	64673000	4000	2	3.50E-14	-0.55	28	0.7	LOC108348628;Vom2r27	Signaling
DMR1:64688001	1	64688001	64692000	4000	3	2.90E-19	0.73	20	0.5	Vom2r27	Signaling
DMR1:64704001	1	64704001	64707000	3000	1	6.80E-10	0.66	12	0.4	Vom2r27;LOC100365524	Signaling
DMR1:64839001	1	64839001	64843000	4000	3	5.00E-08	0.46	36	0.9	LOC103691004;Vom2r12	
DMR1:64856001	1	64856001	64858000	2000	1	1.90E-07	-0.35	18	0.9	Vom2r12	
DMR1:64861001	1	64861001	64866000	5000	2	7.50E-14	-0.52	66	1.32	Vom2r12	
DMR1:64867001	1	64867001	64868000	1000	1	1.20E-08	-0.41	7	0.7	Vom2r12	
DMR1:64875001	1	64875001	64880000	5000	3	5.50E-13	-0.57	54	1.08	Vom2r12	
DMR1:64883001	1	64883001	64887000	4000	1	2.30E-10	0.6	32	0.8	Vom2r12	
DMR1:64909001	1	64909001	64910000	1000	1	1.80E-09	-0.61	2	0.2	Vom2r12	
DMR1:64935001	1	64935001	64939000	4000	2	1.50E-11	-0.53	26	0.65	Vom2r12;Vom2r13	Signaling
DMR1:64953001	1	64953001	64958000	5000	1	3.90E-09	-0.49	36	0.72	Vom2r12;Vom2r13	Signaling
DMR1:65009001	1	65009001	65013000	4000	2	1.00E-13	-0.5	51	1.27	LOC108349538;Vom2r-ps26	
DMR1:65020001	1	65020001	65021000	1000	1	2.70E-07	0.69	6	0.6	Vom2r-ps26	
DMR1:65026001	1	65026001	65029000	3000	1	1.40E-14	0.57	21	0.7	Vom2r-ps26	
DMR1:65538001	1	65538001	65541000	3000	1	2.00E-14	0.59	96	3.2	Mzf1;Ube2m;Chmp2a;Trim28	Transcription;Proteolysis;Transport
DMR1:65553001	1	65553001	65554000	1000	1	2.20E-33	1.06	7	0.7	Chmp2a;Trim28;Zbtb45	Transport;Transcription
DMR1:65577001	1	65577001	65580000	3000	1	8.00E-07	0.47	30	1	Zbtb45;LOC108348450;LOC108348449;Slc27a5;LOC108348441	Transcription;Transport
DMR1:65655001	1	65655001	65656000	1000	1	7.60E-09	0.39	11	1.1	LOC102552901;Rnf225;Rps5	Translation
DMR1:65671001	1	65671001	65672000	1000	1	8.50E-10	0.54	13	1.3	Rps5;Zfp128	Translation
DMR1:65684001	1	65684001	65685000	1000	1	2.50E-07	0.51	8	0.8	Zfp128	
DMR1:65697001	1	65697001	65698000	1000	1	1.00E-21	0.9	3	0.3	Zfp128	
DMR1:65748001	1	65748001	65749000	1000	1	5.80E-20	0.99	1	0.1	Zfp110	
DMR1:65759001	1	65759001	65767000	8000	3	6.90E-16	-0.55	80	1	Zfp110;LOC108348438	
DMR1:65783001	1	65783001	65785000	2000	1	6.70E-12	0.51	16	0.8	Zfp329	Transcription
DMR1:65836001	1	65836001	65838000	2000	1	7.90E-14	1.02	29	1.45	Zscan18	Transcription
DMR1:65853001	1	65853001	65854000	1000	1	8.40E-15	0.51	11	1.1	Zscan18;LOC108349536	Transcription
DMR1:65861001	1	65861001	65872000	11000	5	1.10E-17	-0.5	103	0.94	Zscan18;LOC108349536	Transcription
DMR1:65897001	1	65897001	65898000	1000	1	2.70E-08	0.44	8	0.8	LOC108349536;Vom2r36	Signaling
DMR1:65924001	1	65924001	65933000	9000	2	1.50E-09	0.4	91	1.01	LOC108349536;Vom2r36;Vom2r33	Signaling
DMR1:65946001	1	65946001	65947000	1000	1	6.30E-15	0.95	0	0	Vom2r36;Vom2r33	Signaling
DMR1:65954001	1	65954001	65955000	1000	1	2.80E-07	0.44	10	1	Vom2r36;Vom2r33	Signaling
DMR1:65976001	1	65976001	65992000	16000	8	5.30E-28	-0.51	182	1.14	Vom2r36	Signaling
DMR1:65996001	1	65996001	65999000	3000	2	2.00E-11	-0.44	27	0.9	Vom2r36;Vom2r35	Signaling
DMR1:66009001	1	66009001	66013000	4000	4	1.70E-10	-0.44	33	0.82	Vom2r36;Vom2r35	Signaling
DMR1:66027001	1	66027001	66033000	6000	1	1.20E-14	-0.52	77	1.28	Vom2r36;Vom2r35;LOC108348434	Signaling
DMR1:66034001	1	66034001	66039000	5000	2	2.00E-07	-0.62	89	1.78	Vom2r36;LOC108348434	Signaling
DMR1:66070001	1	66070001	66071000	1000	1	1.00E-08	-0.33	6	0.6	Vom2r36	Signaling
DMR1:66080001	1	66080001	66082000	2000	2	1.00E-10	0.52	22	1.1	Vom2r36	Signaling
DMR1:66091001	1	66091001	66093000	2000	1	6.10E-07	-0.53	7	0.35	Vom2r36	Signaling
DMR1:66115001	1	66115001	66118000	3000	1	1.10E-08	-0.4	55	1.83	Vom2r36;LOC108349537;LOC108349534	Signaling
DMR1:66119001	1	66119001	66125000	6000	2	3.80E-08	-0.5	142	2.37	Vom2r36;LOC108349537;LOC108349534;Mir295-2	Signaling
DMR1:66145001	1	66145001	66147000	2000	1	3.90E-11	-0.44	9	0.45	Vom2r36	Signaling
DMR1:66224001	1	66224001	66225000	1000	1	1.10E-08	-0.49	0	0	LOC691722;Zfp606	Transcription
DMR1:66247001	1	66247001	66254000	7000	2	3.50E-21	0.82	52	0.74	Zfp606	Transcription
DMR1:66263001	1	66263001	66266000	3000	1	1.20E-15	-0.64	17	0.57	Zfp606	Transcription
DMR1:66279001	1	66279001	66281000	2000	1	3.70E-07	-0.41	7	0.35	Vom2r-ps59	
DMR1:66284001	1	66284001	66289000	5000	2	1.50E-19	-0.47	53	1.06	Vom2r-ps59	
DMR1:66344001	1	66344001	66349000	5000	2	8.30E-14	0.58	50	1	Vom1r-ps64	
DMR1:66362001	1	66362001	66363000	1000	1	1.60E-07	0.58	4	0.4	Vom1r-ps64;Vom1r-ps63	
DMR1:66373001	1	66373001	66377000	4000	1	1.10E-08	-0.57	19	0.48	Vom1r-ps63;Vom1r-ps60	
DMR1:66393001	1	66393001	66394000	1000	1	1.50E-07	-0.5	20	2	Vom1r54;Vom1r53	Receptor
DMR1:66399001	1	66399001	66403000	4000	2	2.50E-09	0.52	23	0.58	Vom1r54;Vom1r53;Vom1r-ps61	Receptor
DMR1:66677001	1	66677001	66678000	1000	1	8.70E-18	0.6	13	1.3	Vom1r57	Receptor
DMR1:66680001	1	66680001	66682000	2000	2	5.10E-17	0.96	26	1.3	Vom1r57	Receptor
DMR1:66684001	1	66684001	66686000	2000	2	8.50E-22	0.89	7	0.35	Vom1r57	Receptor

DMR1:66691001	1	66691001	66695000	4000	1	2.20E-10	-0.45	18	0.45	Vom1r57	Receptor
DMR1:66701001	1	66701001	66706000	5000	5	3.40E-18	-0.56	40	0.8	Vom1r-ps65	
DMR1:66719001	1	66719001	66723000	4000	1	7.80E-08	-0.61	29	0.72	Vom1r-ps65;LOC108349838	
DMR1:66750001	1	66750001	66758000	8000	1	4.30E-14	0.52	80	1	Vom1r-ps55	
DMR1:66801001	1	66801001	66804000	3000	1	5.00E-17	0.7	13	0.43	Vom1r-ps57	
DMR1:66808001	1	66808001	66809000	1000	1	5.50E-19	0.99	6	0.6	Vom1r-ps57	
DMR1:66832001	1	66832001	66836000	4000	3	9.90E-10	-0.53	19	0.48	Vom1r-ps56	
DMR1:66838001	1	66838001	66839000	1000	1	6.10E-13	-0.52	7	0.7	Vom1r-ps56	
DMR1:66844001	1	66844001	66845000	1000	1	7.40E-09	0.51	8	0.8	Vom1r-ps56	
DMR1:66855001	1	66855001	66870000	15000	7	3.00E-16	0.58	135	0.9	Vom1r51	Receptor
DMR1:66891001	1	66891001	66893000	2000	1	1.70E-09	0.57	15	0.75	Zfp551	
DMR1:66901001	1	66901001	66902000	1000	1	9.20E-07	-0.57	18	1.8	Zfp551	
DMR1:66976001	1	66976001	66977000	1000	1	2.50E-09	0.61	8	0.8	Vom1r49;LOC108348146	
DMR1:66978001	1	66978001	66981000	3000	1	5.00E-08	-0.66	10	0.33	Vom1r49;LOC108348146	
DMR1:66982001	1	66982001	66987000	5000	1	4.30E-12	-0.57	31	0.62	LOC108346146;Vom1r-ps53;Vom1r-ps52	
DMR1:66998001	1	66998001	6.70E+07	2000	2	7.10E-11	-0.49	18	0.9	Vom1r-ps53;Vom1r-ps52	
DMR1:67020001	1	67020001	67025000	5000	2	1.00E-17	-0.62	37	0.74	Vom1r47	Receptor
DMR1:67027001	1	67027001	67028000	1000	1	4.70E-09	0.48	7	0.7	Vom1r47	Receptor
DMR1:67030001	1	67030001	67031000	1000	1	9.00E-12	-0.62	7	0.7	Vom1r47	Receptor
DMR1:67053001	1	67053001	67056000	3000	1	7.70E-12	-0.5	23	0.77	Vom1r46	Receptor
DMR1:67057001	1	67057001	67058000	1000	1	7.90E-07	-0.55	19	1.9	Vom1r46	Receptor
DMR1:67086001	1	67086001	67089000	3000	1	7.70E-08	-0.51	12	0.4	Vom1r-ps51	
DMR1:67098001	1	67098001	67103000	5000	2	2.70E-07	-0.42	38	0.76	Vom1r-ps51	
DMR1:67119001	1	67119001	67129000	10000	4	1.10E-13	-0.62	70	0.7	Vom1r45	Receptor
DMR1:67143001	1	67143001	67146000	3000	2	5.60E-09	0.6	14	0.47	Vom1r45	Receptor
DMR1:67170001	1	67170001	67172000	2000	1	1.10E-11	-0.63	8	0.4	Vom1r44	Receptor
DMR1:67177001	1	67177001	67178000	1000	1	4.50E-09	0.66	6	0.6	Vom1r44	Receptor
DMR1:67180001	1	67180001	67181000	1000	1	6.90E-11	0.61	4	0.4	Vom1r44	Receptor
DMR1:67188001	1	67188001	67190000	2000	1	2.00E-12	-0.75	5	0.25	Vom1r44	Receptor
DMR1:67192001	1	67192001	67200000	8000	3	3.80E-15	-0.47	90	1.12	Vom1r43	Receptor
DMR1:67203001	1	67203001	67205000	2000	1	9.50E-07	0.42	9	0.45	Vom1r43	Receptor
DMR1:67211001	1	67211001	67214000	3000	1	6.20E-09	0.63	6	0.2	Vom1r43	Receptor
DMR1:67216001	1	67216001	67217000	1000	1	3.50E-07	-0.4	6	0.6	Vom1r43	Receptor
DMR1:67264001	1	67264001	67271000	7000	4	4.10E-14	-0.73	41	0.59	LOC103691020;Vom1r42	
DMR1:67276001	1	67276001	67281000	5000	1	2.70E-08	-0.42	32	0.64	LOC103691020;Vom1r42	
DMR1:67285001	1	67285001	67286000	1000	1	1.80E-10	0.65	1	0.1	LOC103691020;Vom1r42	
DMR1:67297001	1	67297001	67300000	3000	3	1.40E-14	0.89	9	0.3	LOC103691020;Vom1r42	
DMR1:67309001	1	67309001	67310000	1000	1	1.00E-16	0.64	17	1.7	Vom1r42;LOC691645	
DMR1:67342001	1	67342001	67349000	7000	1	3.90E-11	0.57	42	0.6	LOC100362054;Vom1r-ps108	Transcription
DMR1:67504001	1	67504001	67507000	3000	1	8.40E-09	-0.63	15	0.5	Vom2r78	
DMR1:67513001	1	67513001	67516000	3000	2	7.10E-11	-0.6	17	0.57	Vom2r78	
DMR1:67523001	1	67523001	67527000	4000	1	2.30E-11	0.63	28	0.7	Vom2r78	
DMR1:67532001	1	67532001	67535000	3000	2	1.80E-24	0.76	18	0.6	Vom2r78	
DMR1:67667001	1	67667001	67668000	1000	1	5.30E-10	0.68	13	1.3	Vom1r40	
DMR1:67692001	1	67692001	67693000	1000	1	9.70E-07	-0.44	6	0.6	Vom1r-ps48	
DMR1:67736001	1	67736001	67738000	2000	1	7.80E-12	0.79	5	0.25	Vom1r41	Receptor
DMR1:67747001	1	67747001	67753000	6000	2	7.70E-11	0.58	39	0.65	Vom1r41	Receptor
DMR1:68099001	1	68099001	68108000	9000	1	3.60E-08	-0.29	72	0.8	LOC683242;Vom2r-ps57	
DMR1:68113001	1	68113001	68114000	1000	1	3.00E-10	0.56	6	0.6	Vom2r-ps57	
DMR1:68126001	1	68126001	68127000	1000	1	1.60E-09	-0.38	9	0.9	Vom2r-ps57	
DMR1:68132001	1	68132001	68135000	3000	2	3.60E-15	0.81	16	0.53	Vom2r-ps57	
DMR1:68206001	1	68206001	68213000	7000	2	1.90E-21	-0.58	74	1.06	Vom1r-ps38;Vom1r29	Receptor
DMR1:68517001	1	68517001	68519000	2000	1	4.60E-07	-0.44	9	0.45	Vom1r27	Receptor
DMR1:68522001	1	68522001	68523000	1000	1	3.30E-10	0.59	6	0.6	Vom1r27	Receptor
DMR1:68583001	1	68583001	68586000	3000	2	8.20E-11	-0.8	13	0.43	Vom1r-ps34	
DMR1:68637001	1	68637001	68640000	3000	1	5.50E-09	-0.52	13	0.43	Vom1r-ps35;LOC100911644	
DMR1:68662001	1	68662001	68663000	1000	1	3.60E-14	0.72	7	0.7	Vom1r-ps36;Vom1r63	Receptor
DMR1:68668001	1	68668001	68669000	1000	1	5.20E-11	0.67	1	0.1	Vom1r-ps36;Vom1r63	Receptor
DMR1:68997001	1	68997001	68998000	1000	1	2.00E-11	-0.41	6	0.6	Vom2r3	
DMR1:69326001	1	69326001	69327000	1000	1	1.40E-15	0.64	4	0.4	Nlrp4a	
DMR1:69344001	1	69344001	69346000	2000	1	9.60E-07	-0.32	14	0.7	Nlrp4a	
DMR1:69363001	1	69363001	69369000	6000	2	1.00E-25	-0.58	63	1.05	Nlrp4a	
DMR1:69370001	1	69370001	69373000	3000	1	9.80E-07	0.39	28	0.93	Nlrp4a	
DMR1:69380001	1	69380001	69385000	5000	3	1.60E-17	-0.65	74	1.48	Nlrp4a	

DMR1:69390001	1	69390001	69397000	7000	6	7.30E-17	-0.49	70	1	Nlrp4a	
DMR1:69401001	1	69401001	69403000	2000	2	1.30E-11	-0.78	8	0.4	Nlrp4a	
DMR1:69404001	1	69404001	69409000	5000	1	1.40E-12	-0.51	32	0.64	Nlrp4a	
DMR1:69413001	1	69413001	69414000	1000	1	7.60E-07	-0.42	7	0.7	Nlrp4a	
DMR1:69417001	1	69417001	69420000	3000	2	1.40E-12	-0.5	21	0.7	Nlrp4a	
DMR1:69434001	1	69434001	69435000	1000	1	2.90E-09	0.68	5	0.5	Nlrp4a	
DMR1:69437001	1	69437001	69442000	5000	2	6.10E-15	0.56	38	0.76	Nlrp4a	
DMR1:69447001	1	69447001	69448000	1000	1	4.60E-08	-0.44	3	0.3	Nlrp4a	
DMR1:69482001	1	69482001	69484000	2000	1	6.30E-13	-0.47	19	0.95	Vom2r-ps44	
DMR1:69493001	1	69493001	69494000	1000	1	9.00E-13	-0.56	12	1.2	Vom2r-ps44;Vom2r-ps45;LOC100362711	
DMR1:69495001	1	69495001	69496000	1000	1	1.60E-19	0.74	7	0.7	Vom2r-ps44;Vom2r-ps45;LOC100362711	
DMR1:69509001	1	69509001	69516000	7000	4	4.90E-19	-0.55	80	1.14	Vom2r-ps45;LOC100362711	
DMR1:69533001	1	69533001	69534000	1000	1	3.90E-08	-0.61	1	0.1	Vom2r-ps45;LOC100362757	
DMR1:69548001	1	69548001	69550000	2000	1	3.90E-07	-0.3	18	0.9	Vom2r28	Signaling
DMR1:69567001	1	69567001	69574000	7000	3	3.50E-19	-0.65	49	0.7	Vom2r28;LOC103691055	Signaling
DMR1:69593001	1	69593001	69597000	4000	2	1.50E-12	-0.39	30	0.75	Vom2r28	Signaling
DMR1:69605001	1	69605001	69607000	2000	2	3.10E-22	0.82	12	0.6	Zfp418	Transcription
DMR1:69608001	1	69608001	69609000	1000	1	3.50E-24	0.65	14	1.4	Zfp418	Transcription
DMR1:69613001	1	69613001	69615000	2000	1	2.80E-10	0.59	12	0.6	Zfp418	Transcription
DMR1:69624001	1	69624001	69625000	1000	1	2.50E-07	-0.58	6	0.6	Zfp418	Transcription
DMR1:69633001	1	69633001	69634000	1000	1	9.10E-11	0.61	6	0.6	Zfp418	Transcription
DMR1:69638001	1	69638001	69639000	1000	1	2.50E-10	0.52	8	0.8	Zfp418;Vom2r-ps46;LOC103691032	Transcription
DMR1:69731001	1	69731001	69736000	5000	2	1.60E-12	-0.53	41	0.82	Zfp978	
DMR1:69745001	1	69745001	69746000	1000	1	1.10E-07	0.49	14	1.4	Zfp978	
DMR1:69749001	1	69749001	69753000	4000	1	3.60E-08	-0.48	8	0.2	Zfp978	
DMR1:69779001	1	69779001	69782000	3000	2	3.20E-08	-0.42	9	0.3	Mfsd14a;Hiat1-ps1	
DMR1:69783001	1	69783001	69785000	2000	2	6.00E-12	-0.65	12	0.6	Mfsd14a;Hiat1-ps1	
DMR1:69786001	1	69786001	69789000	3000	3	8.30E-12	-0.46	76	2.53	Mfsd14a;Hiat1-ps1	
DMR1:69808001	1	69808001	69809000	1000	1	4.00E-11	0.64	7	0.7	Mfsd14a;Hiat1-ps1	
DMR1:69837001	1	69837001	69840000	3000	1	3.20E-07	-0.36	23	0.77	LOC108349745;Zfp773-ps1;LOC108349540	Transcription
DMR1:69845001	1	69845001	69846000	1000	1	1.50E-09	-0.57	17	1.7	LOC108349745;Zfp773-ps1;LOC108349540;Zfp772	Transcription;Transcription
DMR1:69861001	1	69861001	69862000	1000	1	5.00E-12	0.76	2	0.2	LOC108349540;Zfp772;LOC102547517;LOC102547412	Transcription
DMR1:69869001	1	69869001	69875000	6000	2	5.60E-13	-0.5	41	0.68	Zfp772;LOC102547517;LOC102547412	Transcription
DMR1:69928001	1	69928001	69930000	2000	1	1.10E-07	-0.45	14	0.7	Vom1r-ps42	
DMR1:69946001	1	69946001	69950000	4000	1	1.30E-08	-0.44	53	1.32	LOC103695010;Aurkc	Signaling
DMR1:69962001	1	69962001	69964000	2000	1	1.20E-07	0.35	29	1.45	LOC103695010;Aurkc;LOC108349541;LOC103691033	Signaling
DMR1:69970001	1	69970001	69974000	4000	1	2.30E-15	0.56	47	1.18	Aurkc;LOC108349541;LOC103691033;LOC103690204;LOC100910305	Signaling
DMR1:69997001	1	69997001	7.00E+07	3000	2	1.10E-07	-0.62	26	0.87	LOC103690204;Usp29	Protease
DMR1:70010001	1	70010001	70012000	2000	1	1.40E-10	0.61	34	1.7	Usp29;LOC102547903	Protease
DMR1:70020001	1	70020001	70026000	6000	2	5.10E-17	0.59	64	1.07	Usp29;LOC102547903	Protease
DMR1:70039001	1	70039001	70042000	3000	1	1.30E-07	-0.41	27	0.9	Usp29;LOC102547903	Protease
DMR1:70135001	1	70135001	70137000	2000	2	4.60E-14	0.81	18	0.9	LOC102547903;Mir3099	
DMR1:70139001	1	70139001	70142000	3000	2	8.80E-14	0.47	55	1.83	LOC102547903;Mir3099	
DMR1:70231001	1	70231001	70233000	2000	1	5.20E-08	-0.47	45	2.25	Peg3;Apeg3	Transcription
DMR1:70251001	1	70251001	70255000	4000	2	3.80E-10	0.4	57	1.43	Zim1	
DMR1:70262001	1	70262001	70263000	1000	1	9.90E-15	0.88	8	0.8	Zim1	
DMR1:70428001	1	70428001	70430000	2000	1	6.40E-10	0.64	28	1.4	Olr3	Receptor
DMR1:70432001	1	70432001	70434000	2000	2	1.00E-08	0.61	13	0.65	Olr3	Receptor
DMR1:70435001	1	70435001	70436000	1000	1	6.30E-08	0.49	2	0.2	Olr3	Receptor
DMR1:70451001	1	70451001	70453000	2000	1	2.50E-26	1	14	0.7	Olr4	Signaling
DMR1:70504001	1	70504001	70508000	4000	1	6.70E-09	-0.48	27	0.68	Olr8;LOC103691061	Signaling
DMR1:71117001	1	71117001	71121000	4000	1	6.30E-11	0.63	62	1.55	Smim17	
DMR1:71126001	1	71126001	71132000	6000	4	4.50E-15	-0.58	92	1.53	Smim17	
DMR1:71142001	1	71142001	71143000	1000	1	2.10E-13	0.71	8	0.8	Smim17	
DMR1:71145001	1	71145001	71148000	3000	1	3.40E-07	-0.44	8	0.27	Smim17	

DMR1:71174001	1	71174001	71175000	1000	1	7.60E-11	0.45	7	0.7	Smim17;Zfp28;Zfp78	Transcription
DMR1:71197001	1	71197001	71198000	1000	1	1.30E-09	0.69	33	3.3	Zfp78;LOC102548162	Transcription
DMR1:71231001	1	71231001	71234000	3000	2	4.40E-21	0.9	23	0.77	LOC102548162;Zfp583	
DMR1:71244001	1	71244001	71246000	2000	1	2.60E-09	0.43	61	3.05	Zfp583	
DMR1:71248001	1	71248001	71249000	1000	1	7.90E-16	0.58	14	1.4	Zfp583	
DMR1:71250001	1	71250001	71251000	1000	1	2.70E-12	0.54	8	0.8	Zfp583	
DMR1:71263001	1	71263001	71266000	3000	1	2.30E-07	-0.49	43	1.43	Zfp583;Zfp667	
DMR1:71277001	1	71277001	71282000	5000	2	6.50E-11	0.55	54	1.08	Zfp667	
DMR1:71298001	1	71298001	71300000	2000	1	1.20E-10	0.45	26	1.3	Zfp667;LOC108349542;LOC102548473;LOC103691035	
DMR1:71320001	1	71320001	71325000	5000	1	2.40E-18	0.91	48	0.96	LOC102548473;LOC103691035;Zscan5b	Transcription
DMR1:71326001	1	71326001	71328000	2000	2	3.60E-18	1.01	20	1	LOC103691035;Zscan5b	Transcription
DMR1:71345001	1	71345001	71349000	4000	1	1.70E-07	-0.43	58	1.45	Zscan5b;Galp	Transcription
DMR1:71350001	1	71350001	71357000	7000	1	4.50E-14	0.48	123	1.76	Zscan5b;Galp	Transcription
DMR1:71360001	1	71360001	71361000	1000	1	5.50E-13	0.74	6	0.6	Galp	
DMR1:71362001	1	71362001	71363000	1000	1	1.50E-13	0.66	8	0.8	Galp	
DMR1:71398001	1	71398001	71400000	2000	1	4.40E-14	0.7	63	3.15	Zfp444	Transcription
DMR1:71420001	1	71420001	71421000	1000	1	2.60E-08	-0.65	21	2.1	Zfp787	Transcription
DMR1:71428001	1	71428001	71430000	2000	2	7.00E-12	0.54	14	0.7	Zfp787	Transcription
DMR1:71449001	1	71449001	71451000	2000	1	6.60E-16	0.51	23	1.15	Zfp787;Nlrp5	Transcription
DMR1:71457001	1	71457001	71458000	1000	1	3.90E-23	0.89	15	1.5	Zfp787;Nlrp5	Transcription
DMR1:71466001	1	71466001	71469000	3000	1	1.00E-18	0.77	53	1.77	Nlrp5	
DMR1:71560001	1	71560001	71563000	3000	1	6.70E-07	-0.42	21	0.7	Vom2r-ps118	
DMR1:71564001	1	71564001	71565000	1000	1	7.50E-07	-0.55	3	0.3	Vom2r-ps118	
DMR1:71675001	1	71675001	71678000	3000	2	6.00E-18	0.8	34	1.13	Nlrp4	
DMR1:71721001	1	71721001	71724000	3000	1	7.70E-18	-0.69	18	0.6	Nlrp4;RGD1564651	
DMR1:71725001	1	71725001	71732000	7000	3	1.50E-15	-0.49	76	1.09	Nlrp4;RGD1564651	
DMR1:71773001	1	71773001	71778000	5000	3	1.50E-14	0.82	24	0.48	LOC691325;Obox2	Development
DMR1:71780001	1	71780001	71782000	2000	1	5.10E-10	0.41	17	0.85	Obox2	Development
DMR1:71860001	1	71860001	71861000	1000	1	3.80E-09	0.66	3	0.3	Nlrp9	
DMR1:71864001	1	71864001	71870000	6000	5	1.90E-22	-0.53	67	1.12	Nlrp9	
DMR1:71873001	1	71873001	71875000	2000	2	3.90E-21	0.97	19	0.95	Nlrp9	
DMR1:71882001	1	71882001	71886000	4000	2	3.60E-13	0.59	17	0.42	Nlrp9	
DMR1:71889001	1	71889001	71890000	1000	1	2.50E-13	0.86	5	0.5	Nlrp9	
DMR1:71899001	1	71899001	71903000	4000	3	2.20E-18	-0.68	49	1.23	Nlrp9	
DMR1:71952001	1	71952001	71953000	1000	1	2.00E-09	0.63	5	0.5	Vom1r34	Receptor
DMR1:72122001	1	72122001	72126000	4000	2	1.90E-10	-0.68	14	0.35	Vom1r35	Receptor
DMR1:72171001	1	72171001	72175000	4000	3	1.20E-10	-0.59	47	1.18	Vom1r36	Receptor
DMR1:72181001	1	72181001	72182000	1000	1	1.20E-07	0.39	8	0.8	Vom1r36	Receptor
DMR1:72187001	1	72187001	72193000	6000	6	1.00E-14	0.57	44	0.73	Vom1r36;LOC108349748	Receptor
DMR1:72199001	1	72199001	72201000	2000	1	2.80E-09	0.39	41	2.05	Vom1r36;LOC108349748;Vom1r37	Receptor
DMR1:72225001	1	72225001	72226000	1000	1	4.70E-17	0.62	10	1	Vom1r-ps45;Vom1r38	Receptor
DMR1:72236001	1	72236001	72241000	5000	1	1.50E-17	-0.43	44	0.88	Vom1r38;Vom2r-ps47	Receptor
DMR1:72263001	1	72263001	72264000	1000	1	1.30E-15	0.67	15	1.5	Vom2r-ps47;LOC108349544;Rasl2-9	Signaling
DMR1:72265001	1	72265001	72272000	7000	4	1.10E-13	-0.62	47	0.67	Vom2r-ps47;LOC108349544;Rasl2-9;Rfpl4a	Signaling
DMR1:72275001	1	72275001	72279000	4000	1	2.80E-08	0.44	47	1.18	LOC108349544;Rasl2-9;Rfpl4a	Signaling
DMR1:72281001	1	72281001	72282000	1000	1	5.20E-07	-0.55	11	1.1	LOC108349544;Rasl2-9;Rfpl4a	Signaling
DMR1:72284001	1	72284001	72285000	1000	1	4.00E-07	-0.53	9	0.9	Rfpl4a;Epn1	
DMR1:72298001	1	72298001	72302000	4000	1	9.10E-15	0.48	60	1.5	Epn1	
DMR1:72322001	1	72322001	72324000	2000	2	6.70E-33	0.97	38	1.9	U2af2;Ccdc106	
DMR1:72339001	1	72339001	72341000	2000	2	2.60E-12	0.76	110	5.5	U2af2;Ccdc106;Zfp580	Transcription
DMR1:72354001	1	72354001	72358000	4000	1	1.20E-15	0.72	87	2.17	Zfp865;LOC100911196	Transcription
DMR1:72380001	1	72380001	72381000	1000	1	7.80E-07	0.47	40	4	LOC100911196;Zfp524;Fiz1	Transcription
DMR1:72388001	1	72388001	72393000	5000	1	1.80E-10	0.42	56	1.12	Zfp524;Fiz1;Zfp579	Transcription
DMR1:72399001	1	72399001	72401000	2000	2	2.90E-08	0.6	86	4.3	Zfp579	Transcription
DMR1:72403001	1	72403001	72404000	1000	1	9.30E-15	0.72	6	0.6	Zfp579	Transcription
DMR1:72406001	1	72406001	72409000	3000	1	4.10E-08	0.55	21	0.7	Zfp579	Transcription
DMR1:72419001	1	72419001	72420000	1000	1	3.80E-16	0.65	8	0.8	Sbk3;Sbk2	Signaling
DMR1:72421001	1	72421001	72427000	6000	3	2.60E-18	0.6	90	1.5	Sbk3;Sbk2	Signaling
DMR1:72435001	1	72435001	72438000	3000	2	1.30E-29	0.67	53	1.77	Sbk2;Ssc5d	Signaling;Protease

DMR1:72441001	1	72441001	72445000	4000	2	3.90E-23	0.78	61	1.52	Sbk2;Ssc5d	Signaling;Protease
DMR1:72447001	1	72447001	72452000	5000	3	1.80E-12	0.46	79	1.58	Ssc5d	Protease
DMR1:72461001	1	72461001	72462000	1000	1	8.20E-23	0.77	12	1.2	Ssc5d;Nat14;Zfp628	Protease;Metabolism;Transcription
DMR1:72467001	1	72467001	72472000	5000	2	3.00E-15	0.57	105	2.1	Ssc5d;Nat14;Zfp628;LOC684792	Protease;Metabolism;Transcription
DMR1:72479001	1	72479001	72481000	2000	2	1.90E-21	0.71	22	1.1	Zfp628;LOC684792	Transcription
DMR1:72518001	1	72518001	72522000	4000	2	1.60E-12	0.49	40	1	LOC684270;LOC292586;Isoc2b	Metabolism
DMR1:72533001	1	72533001	72535000	2000	1	3.20E-16	0.7	13	0.65	Isoc2b	
DMR1:72536001	1	72536001	72540000	4000	1	1.00E-15	0.74	66	1.65	Isoc2b;Shisa7	
DMR1:72546001	1	72546001	72548000	2000	2	1.10E-08	0.56	67	3.35	Isoc2b;Shisa7	
DMR1:72550001	1	72550001	72554000	4000	2	3.70E-15	0.89	114	2.85	Isoc2b;Shisa7	
DMR1:72591001	1	72591001	72595000	4000	2	1.50E-16	-0.63	35	0.88	Ube2s	Proteolysis
DMR1:72617001	1	72617001	72618000	1000	1	1.80E-07	0.48	4	0.4	Rpl28;Tmem238	Translation
DMR1:72620001	1	72620001	72621000	1000	1	8.40E-07	-0.42	19	1.9	Rpl28;Tmem238;Tmem190	Translation
DMR1:72626001	1	72626001	72628000	2000	2	8.00E-20	0.67	20	1	Rpl28;Tmem238;Tmem190;Il11	Translation;Cytokine
DMR1:72645001	1	72645001	72654000	9000	3	1.70E-09	-0.77	89	0.99	Il11;Fam71e2;Cox6b2	Cytokine;Metabolism
DMR1:72666001	1	72666001	72669000	3000	1	4.80E-07	0.41	49	1.63	Fam71e2;Cox6b2;Kmt5c	Metabolism
DMR1:72682001	1	72682001	72687000	5000	2	1.30E-12	0.5	41	0.82	Kmt5c;Tmem150b	
DMR1:72724001	1	72724001	72726000	2000	2	3.70E-17	0.86	25	1.25	Brsk1;LOC108349434;Hspbp1	Signaling
DMR1:72727001	1	72727001	72730000	3000	2	8.20E-20	0.88	68	2.27	Brsk1;LOC108349434;Hspbp1	Signaling
DMR1:72750001	1	72750001	72753000	3000	1	7.90E-14	0.55	27	0.9	Hspbp1;Ppp6r1	Signaling
DMR1:72758001	1	72758001	72761000	3000	1	5.60E-08	0.56	83	2.77	Hspbp1;Ppp6r1	Signaling
DMR1:72776001	1	72776001	72779000	3000	2	4.40E-17	0.85	20	0.67	Ppp6r1;Tmem86b	Signaling
DMR1:72793001	1	72793001	72795000	2000	1	2.50E-12	0.44	27	1.35	Ppp6r1;Tmem86b;LOC102547606	Signaling
DMR1:72804001	1	72804001	72806000	2000	1	2.80E-13	0.65	20	1	Tmem86b;LOC102547606;Ptprh	Signaling
DMR1:72815001	1	72815001	72816000	1000	1	5.70E-07	0.44	17	1.7	Tmem86b;Ptprh	Signaling
DMR1:72818001	1	72818001	72819000	1000	1	1.70E-14	0.56	15	1.5	Ptprh	Signaling
DMR1:72826001	1	72826001	72832000	6000	1	2.50E-07	0.43	59	0.98	Ptprh	Signaling
DMR1:72833001	1	72833001	72834000	1000	1	1.70E-08	0.41	13	1.3	Ptprh	Signaling
DMR1:72837001	1	72837001	72843000	6000	1	2.70E-09	0.34	51	0.85	Ptprh	Signaling
DMR1:72849001	1	72849001	72855000	6000	1	9.50E-07	-0.37	68	1.13	Ptprh;Syt5;LOC108349515	Signaling;Transport
DMR1:72884001	1	72884001	72887000	3000	2	2.50E-17	0.7	58	1.93	Dnaaf3;Tnni3;Tnnt1	Transcription;Cytoskeleton;Cytoskeleton
DMR1:72897001	1	72897001	72899000	2000	1	7.80E-08	0.37	20	1	Tnnt1;Ppp1r12c	Cytoskeleton;Signaling
DMR1:72932001	1	72932001	72933000	1000	1	3.70E-08	0.45	14	1.4	Ppp1r12c;Eps8l1	Signaling;Cytoskeleton
DMR1:72934001	1	72934001	72935000	1000	1	3.30E-10	0.51	24	2.4	Ppp1r12c;Eps8l1	Signaling;Cytoskeleton
DMR1:72938001	1	72938001	72941000	3000	1	5.50E-07	0.46	15	0.5	Eps8l1	Cytoskeleton
DMR1:72944001	1	72944001	72946000	2000	1	8.50E-13	0.53	23	1.15	Eps8l1	Cytoskeleton
DMR1:72950001	1	72950001	72953000	3000	3	2.00E-23	0.87	31	1.03	Eps8l1;Rdh13	Cytoskeleton;Golgi
DMR1:72965001	1	72965001	72972000	7000	5	6.00E-31	-0.67	92	1.31	Rdh13	Golgi
DMR1:72976001	1	72976001	72977000	1000	1	1.30E-13	0.57	11	1.1	Rdh13	Golgi
DMR1:72982001	1	72982001	72990000	8000	1	1.40E-15	-0.59	81	1.01	Rdh13;Gp6	Golgi;Immune
DMR1:72997001	1	72997001	7.30E+07	3000	1	6.80E-07	0.43	18	0.6	Gp6	Immune
DMR1:73014001	1	73014001	73018000	4000	2	4.10E-13	0.6	24	0.6	Gp6	Immune
DMR1:73032001	1	73032001	73033000	1000	1	5.10E-16	0.72	11	1.1	Gp6	Immune
DMR1:73235001	1	73235001	73236000	1000	1	4.40E-10	0.53	8	0.8	Ncr1;Fcar	Immune
DMR1:73238001	1	73238001	73244000	6000	3	3.80E-17	0.64	46	0.77	Fcar;LOC108349720	Immune
DMR1:73252001	1	73252001	73256000	4000	2	1.20E-11	-0.43	34	0.85	Fcar;LOC108349720	Immune
DMR1:73296001	1	73296001	73297000	1000	1	1.00E-07	0.62	2	0.2	LOC100361171;Kir3dl1	Immune
DMR1:73308001	1	73308001	73309000	1000	1	8.90E-11	0.51	6	0.6	Kir3dl1	Immune
DMR1:73321001	1	73321001	73324000	3000	2	1.40E-10	-0.62	23	0.77	Kir3dl1	Immune
DMR1:73329001	1	73329001	73333000	4000	1	7.70E-09	-0.3	49	1.23	Kir3dl1	Immune
DMR1:73334001	1	73334001	73338000	4000	3	1.70E-16	0.63	33	0.82	Kir3dl1	Immune
DMR1:73339001	1	73339001	73341000	2000	1	1.10E-07	-0.47	9	0.45	Kir3dl1	Immune
DMR1:73602001	1	73602001	73605000	3000	2	4.50E-10	-0.51	20	0.67	Lilrb3	Immune
DMR1:73621001	1	73621001	73622000	1000	1	1.60E-10	0.62	5	0.5	Lilrb3	Immune
DMR1:73655001	1	73655001	73658000	3000	1	3.90E-18	0.78	11	0.37	Lilra5	Immune
DMR1:73667001	1	73667001	73668000	1000	1	7.50E-10	-0.65	7	0.7	Lilra5	Immune

DMR1:73677001	1	73677001	73679000	2000	1	6.70E-10	0.48	12	0.6	Lilra5;LOC691536	Immune
DMR1:73688001	1	73688001	73689000	1000	1	5.90E-10	0.67	4	0.4	Lilra5;LOC691536	Immune
DMR1:73713001	1	73713001	73715000	2000	1	4.90E-10	0.54	3	0.15	Cdc42ep5;Leng9;Leng8	Cytoskeleton
DMR1:73716001	1	73716001	73717000	1000	1	1.70E-08	0.59	2	0.2	Cdc42ep5;Leng9;Leng8	Cytoskeleton
DMR1:73722001	1	73722001	73724000	2000	2	4.20E-20	0.72	47	2.35	Cdc42ep5;Leng9;Leng8	Cytoskeleton
DMR1:73740001	1	73740001	73743000	3000	1	7.10E-14	0.75	26	0.87	Leng8;Ttyh1;Lair1	Cytoskeleton;Transport;Immune
DMR1:73750001	1	73750001	73751000	1000	1	7.50E-10	0.58	1	0.1	Ttyh1;Lair1	Transport;Immune
DMR1:73754001	1	73754001	73755000	1000	1	3.90E-10	0.54	13	1.3	Ttyh1;Lair1	Transport;Immune
DMR1:73757001	1	73757001	73758000	1000	1	3.90E-09	0.56	5	0.5	Ttyh1;Lair1	Transport;Immune
DMR1:73759001	1	73759001	73760000	1000	1	1.00E-15	0.63	9	0.9	Ttyh1;Lair1	Transport;Immune
DMR1:73793001	1	73793001	73794000	1000	1	2.40E-10	0.45	10	1	Lair1	Immune
DMR1:73795001	1	73795001	73796000	1000	1	1.40E-26	0.9	8	0.8	Lair1	Immune
DMR1:73798001	1	73798001	73802000	4000	1	4.50E-10	-0.45	17	0.42	Lair1	Immune
DMR1:73813001	1	73813001	73815000	2000	2	4.90E-12	0.77	9	0.45	Lair1	Immune
DMR1:73835001	1	73835001	73836000	1000	1	2.00E-15	0.62	7	0.7	Lair1	Immune
DMR1:73843001	1	73843001	73846000	3000	1	4.00E-08	-0.44	13	0.43	Lair1	Immune
DMR1:73924001	1	73924001	73925000	1000	1	1.80E-09	0.63	7	0.7	Nlrp4b	
DMR1:73967001	1	73967001	73970000	3000	2	1.70E-10	-0.49	15	0.5	Nlrp4b	
DMR1:73971001	1	73971001	73973000	2000	1	7.80E-16	0.68	15	0.75	Nlrp4b	
DMR1:74214001	1	74214001	74215000	1000	1	1.70E-08	0.56	2	0.2	Vom2r-ps48	
DMR1:74258001	1	74258001	74260000	2000	1	1.00E-07	-0.52	7	0.35	Vom2r29	Signaling
DMR1:74263001	1	74263001	74266000	3000	1	6.30E-11	-0.39	25	0.83	Vom2r29	Signaling
DMR1:74282001	1	74282001	74285000	3000	2	1.10E-10	-0.55	18	0.6	Vom2r29	Signaling
DMR1:74428001	1	74428001	74436000	8000	1	1.20E-07	-0.25	96	1.2	Vom2r-ps51	
DMR1:74447001	1	74447001	74453000	6000	5	9.70E-13	-0.51	70	1.17	Vom2r-ps51	
DMR1:74475001	1	74475001	74478000	3000	2	9.20E-09	-0.47	17	0.57	Vom2r-ps52	
DMR1:74494001	1	74494001	74495000	1000	1	3.70E-07	0.48	6	0.6	Vom2r-ps52	
DMR1:74519001	1	74519001	74522000	3000	1	7.90E-07	-0.58	11	0.37	Vom1r-ps47	
DMR1:74588001	1	74588001	74591000	3000	1	1.30E-11	-0.51	15	0.5	Vom2r-ps53	
DMR1:74593001	1	74593001	74594000	1000	1	4.40E-08	0.52	8	0.8	Vom2r-ps53;LOC108349750	
DMR1:74692001	1	74692001	74693000	1000	1	1.40E-11	0.59	5	0.5	Vom2r-ps54	
DMR1:74774001	1	74774001	74777000	3000	1	2.10E-12	-0.42	16	0.53	Vom2r30	
DMR1:74794001	1	74794001	74795000	1000	1	1.90E-07	0.49	11	1.1	Vom2r30;LOC100361673	
DMR1:74796001	1	74796001	74799000	3000	1	1.90E-07	-0.49	15	0.5	Vom2r30;LOC100361673	
DMR1:74801001	1	74801001	74804000	3000	1	7.00E-09	0.46	21	0.7	Vom2r30;LOC100361673	
DMR1:74882001	1	74882001	74883000	1000	1	2.10E-07	0.63	1	0.1	Vom2r-ps55	
DMR1:74895001	1	74895001	74896000	1000	1	1.60E-13	-0.59	9	0.9	Vom2r-ps55	
DMR1:74919001	1	74919001	74920000	1000	1	1.20E-07	0.56	8	0.8	Vom2r-ps55	
DMR1:74979001	1	74979001	74985000	6000	3	1.60E-10	-0.68	34	0.57	LOC100911630;LOC100911587;Vom2r31	Signaling
DMR1:75122001	1	75122001	75128000	6000	1	1.20E-08	-0.4	62	1.03	Vom1r58;Vom1r-ps66	Receptor
DMR1:75136001	1	75136001	75141000	5000	1	5.40E-14	-0.52	38	0.76	Vom1r58;Vom1r-ps66	Receptor
DMR1:75142001	1	75142001	75145000	3000	1	5.10E-07	-0.42	42	1.4	Vom1r58;Vom1r-ps66;RGD1564801	Receptor;Signaling
DMR1:75154001	1	75154001	75157000	3000	1	7.10E-09	-0.4	67	2.23	RGD1564801;Vom1r59	Signaling;Receptor
DMR1:75158001	1	75158001	75161000	3000	1	5.70E-07	-0.46	19	0.63	RGD1564801;Vom1r59	Signaling;Receptor
DMR1:75200001	1	75200001	75207000	7000	3	1.70E-16	0.57	41	0.59	Vom1r60	Receptor
DMR1:75289001	1	75289001	75290000	1000	1	2.20E-07	0.63	3	0.3	Vom1r61	Receptor
DMR1:75291001	1	75291001	75296000	5000	1	1.20E-10	-0.41	55	1.1	Vom1r61	Receptor
DMR1:75300001	1	75300001	75309000	9000	5	3.10E-28	-0.62	101	1.12	Vom1r61	Receptor
DMR1:75322001	1	75322001	75323000	1000	1	1.20E-16	0.69	9	0.9	Vom1r62	Receptor
DMR1:75325001	1	75325001	75327000	2000	1	1.60E-08	-0.57	15	0.75	Vom1r62;RGD1310257	Receptor
DMR1:75335001	1	75335001	75337000	2000	1	2.20E-19	0.94	23	1.15	Vom1r62;RGD1310257	Receptor
DMR1:75355001	1	75355001	75358000	3000	1	5.10E-09	0.6	87	2.9	RGD1310257;Lig1	Methylation
DMR1:75387001	1	75387001	75388000	1000	1	2.30E-11	0.62	5	0.5	Lig1	Methylation
DMR1:75392001	1	75392001	75393000	1000	1	3.30E-07	0.57	3	0.3	Lig1	Methylation
DMR1:75430001	1	75430001	75434000	4000	1	1.20E-07	-0.38	34	0.85	Pla2g4c	Metabolism
DMR1:75435001	1	75435001	75440000	5000	3	1.10E-14	-0.49	46	0.92	Pla2g4c	Metabolism
DMR1:75453001	1	75453001	75456000	3000	2	2.60E-16	0.56	30	1	Pla2g4c	Metabolism
DMR1:75457001	1	75457001	75464000	7000	4	3.00E-28	-0.62	96	1.37	Pla2g4c	Metabolism
DMR1:75479001	1	75479001	75480000	1000	1	9.40E-08	0.72	5	0.5	Pla2g4c	Metabolism
DMR1:75628001	1	75628001	75635000	7000	2	1.90E-20	-0.49	80	1.14	LOC691813;Peli1-ps1	
DMR1:75646001	1	75646001	75654000	8000	4	2.60E-13	-0.55	87	1.09	Peli1-ps1	
DMR1:75692001	1	75692001	75695000	3000	3	4.90E-15	0.75	12	0.4	Cabp5	

DMR1:75718001	1	75718001	75723000	5000	1	1.10E-08	-0.47	33	0.66	Bsph1	
DMR1:75748001	1	75748001	75749000	1000	1	1.50E-13	0.96	4	0.4	Bsph1	
DMR1:75752001	1	75752001	75754000	2000	2	1.00E-12	0.85	11	0.55	Bsph1	
DMR1:75758001	1	75758001	75766000	8000	5	5.50E-15	-0.42	109	1.36	Bsph1	
DMR1:75777001	1	75777001	75784000	7000	1	6.70E-11	0.64	74	1.06	Bsph1	
DMR1:75799001	1	75799001	75801000	2000	1	5.00E-18	0.58	20	1	Bsph1	
DMR1:75832001	1	75832001	75834000	2000	1	9.70E-08	0.47	22	1.1	Bsph2	
DMR1:76302001	1	76302001	76303000	1000	1	1.10E-10	0.52	7	0.7	Sult2a2	
DMR1:76353001	1	76353001	76356000	3000	1	1.60E-07	-0.55	29	0.97	Sult2a2;LOC100363218	
DMR1:76358001	1	76358001	76359000	1000	1	3.00E-07	0.42	9	0.9	Sult2a2	
DMR1:76390001	1	76390001	76392000	2000	1	3.10E-07	0.49	7	0.35	Sult2a2	
DMR1:76393001	1	76393001	76398000	5000	2	1.20E-12	-0.57	43	0.86	Sult2a2	
DMR1:76412001	1	76412001	76413000	1000	1	1.20E-07	-0.53	4	0.4	Sult2a2	
DMR1:76422001	1	76422001	76425000	3000	1	9.50E-13	-0.69	84	2.8	Sult2a2	
DMR1:76428001	1	76428001	76431000	3000	1	1.20E-18	-0.57	43	1.43	Sult2a2	
DMR1:76437001	1	76437001	76440000	3000	2	1.00E-07	-0.54	7	0.23	Sult2a2	
DMR1:76450001	1	76450001	76457000	7000	3	1.50E-10	-0.44	55	0.79	Sult2a2	
DMR1:76458001	1	76458001	76460000	2000	1	3.10E-11	-0.39	26	1.3	Sult2a2	
DMR1:76498001	1	76498001	76499000	1000	1	6.60E-07	-0.72	10	1	Sult2a2	
DMR1:76520001	1	76520001	76521000	1000	1	6.80E-07	0.5	3	0.3	Sult2a2;Sult2a6	
DMR1:76562001	1	76562001	76567000	5000	2	5.50E-11	-0.45	43	0.86	Sult2a6;Sult2a1	
DMR1:76568001	1	76568001	76569000	1000	1	1.10E-07	0.47	8	0.8	Sult2a6;Sult2a1	
DMR1:76581001	1	76581001	76583000	2000	1	4.30E-07	-0.45	13	0.65	Sult2a6;Sult2a1	
DMR1:76592001	1	76592001	76595000	3000	1	1.50E-07	-0.44	13	0.43	Sult2a6;Sult2a1	
DMR1:76616001	1	76616001	76619000	3000	1	8.30E-10	0.47	44	1.47	Sult2a6;Sult2a1	
DMR1:76653001	1	76653001	76656000	3000	1	6.10E-08	-0.66	22	0.73	Sult2a6;Srp72-ps1	
DMR1:76660001	1	76660001	76662000	2000	1	1.10E-09	0.58	9	0.45	Sult2a6;Srp72-ps1	
DMR1:76680001	1	76680001	76685000	5000	3	7.30E-21	-0.51	61	1.22	Sult2a6	
DMR1:76701001	1	76701001	76704000	3000	1	1.20E-08	-0.64	11	0.37	Sult2a6	
DMR1:76706001	1	76706001	76709000	3000	2	1.60E-11	-0.55	81	2.7	Sult2a6	
DMR1:77104001	1	77104001	77105000	1000	1	2.20E-10	0.67	9	0.9	RGD1565590	
DMR1:77523001	1	77523001	77524000	1000	1	1.90E-19	0.78	10	1	LOC102551565;Crxos1;Sepw1	
DMR1:77539001	1	77539001	77541000	2000	1	1.80E-11	-0.47	16	0.8	Sepw1;LOC103690015	Translation
DMR1:77625001	1	77625001	77630000	5000	1	3.20E-08	-0.42	23	0.46	Obox1;LOC102554917	
DMR1:77758001	1	77758001	77762000	4000	2	3.70E-14	0.5	43	1.07	Crx	Development
DMR1:77763001	1	77763001	77765000	2000	2	3.10E-15	0.57	33	1.65	Crx	Development
DMR1:77858001	1	77858001	77861000	3000	1	2.30E-22	0.62	55	1.83	Ehd2;Gltscr1	Transport
DMR1:77865001	1	77865001	77866000	1000	1	8.10E-07	0.48	18	1.8	Ehd2;Gltscr1;LOC103691043	Transport
DMR1:77876001	1	77876001	77880000	4000	2	6.10E-30	0.97	50	1.25	Gltscr1;LOC103691043	
DMR1:77881001	1	77881001	77885000	4000	1	4.60E-07	0.4	53	1.32	Gltscr1;LOC103691043	
DMR1:77894001	1	77894001	77899000	5000	1	5.10E-12	0.4	66	1.32	Gltscr1	
DMR1:77906001	1	77906001	77907000	1000	1	7.10E-09	0.36	15	1.5	Gltscr1	
DMR1:77989001	1	77989001	77992000	3000	1	8.30E-10	0.46	24	0.8	Zfp541;Napa	Transport
DMR1:78019001	1	78019001	78021000	2000	1	1.50E-09	0.36	29	1.45	Napa;Kptn;Slc8a2	Transport;Transport
DMR1:78025001	1	78025001	78026000	1000	1	6.90E-18	1.02	28	2.8	Kptn;Slc8a2	Transport
DMR1:78034001	1	78034001	78036000	2000	2	1.20E-23	0.86	63	3.15	Slc8a2	Transport
DMR1:78039001	1	78039001	78042000	3000	1	5.60E-08	0.54	82	2.73	Slc8a2	Transport
DMR1:78043001	1	78043001	78046000	3000	2	5.50E-18	0.54	38	1.27	Slc8a2	Transport
DMR1:78054001	1	78054001	78057000	3000	2	1.30E-08	0.33	50	1.67	Slc8a2;Meis3	Transport;Development
DMR1:78063001	1	78063001	78068000	5000	3	2.40E-17	0.8	50	1	Slc8a2;Meis3;Dhx34	Transport;Development;Transcription
DMR1:78078001	1	78078001	78080000	2000	2	7.30E-20	0.83	5	0.25	Meis3;Dhx34	Development;Transcription
DMR1:78089001	1	78089001	78094000	5000	3	3.80E-25	0.96	114	2.28	Meis3;Dhx34	Development;Transcription
DMR1:78105001	1	78105001	78107000	2000	1	3.60E-12	0.49	47	2.35	Dhx34	Transcription
DMR1:78108001	1	78108001	78111000	3000	1	6.80E-16	0.53	67	2.23	Dhx34	Transcription
DMR1:78172001	1	78172001	78173000	1000	1	5.60E-13	0.54	5	0.5	C5ar2	Signaling
DMR1:78177001	1	78177001	78181000	4000	2	2.10E-19	0.67	68	1.7	C5ar2;LOC684925;C5ar1	Signaling
DMR1:78209001	1	78209001	78211000	2000	1	3.20E-17	0.61	24	1.2	Inafm1;Ccdc9	
DMR1:78224001	1	78224001	78226000	2000	1	1.90E-14	0.72	52	2.6	Ccdc9	
DMR1:78229001	1	78229001	78230000	1000	1	3.80E-12	0.77	4	0.4	Ccdc9	
DMR1:78282001	1	78282001	78287000	5000	1	4.50E-07	0.4	75	1.5	Sae1	Proteolysis
DMR1:78289001	1	78289001	78291000	2000	1	1.40E-23	0.88	29	1.45	Sae1	Proteolysis

DMR1:78337001	1	78337001	78338000	1000	1	6.30E-09	0.47	13	1.3	Sae1	Proteolysis
DMR1:78346001	1	78346001	78348000	2000	1	4.30E-08	0.51	16	0.8	Zc3h4	Transcription
DMR1:78369001	1	78369001	78370000	1000	1	7.10E-11	0.64	9	0.9	Zc3h4	Transcription
DMR1:78375001	1	78375001	78376000	1000	1	3.00E-08	0.37	16	1.6	Zc3h4	Transcription
DMR1:78406001	1	78406001	78408000	2000	1	1.40E-16	0.74	17	0.85	Tmem160	
DMR1:78432001	1	78432001	78437000	5000	1	1.40E-08	0.41	61	1.22	Npas1	Transcription
DMR1:78446001	1	78446001	78450000	4000	1	1.60E-16	0.6	52	1.3	Npas1;Arhgap35	Transcription;Signaling
DMR1:78458001	1	78458001	78461000	3000	1	8.50E-10	0.42	51	1.7	Arhgap35	Signaling
DMR1:78488001	1	78488001	78491000	3000	2	2.40E-13	0.63	27	0.9	Arhgap35	Signaling
DMR1:78504001	1	78504001	78505000	1000	1	2.30E-08	0.54	6	0.6	Arhgap35	Signaling
DMR1:78514001	1	78514001	78516000	2000	1	3.60E-07	-0.52	23	1.15	Arhgap35	Signaling
DMR1:78518001	1	78518001	78523000	5000	1	8.90E-07	-0.46	75	1.5	Arhgap35	Signaling
DMR1:78525001	1	78525001	78526000	1000	1	5.30E-17	0.85	2	0.2	Arhgap35	Signaling
DMR1:78534001	1	78534001	78535000	1000	1	4.70E-08	0.48	9	0.9	Arhgap35	Signaling
DMR1:78549001	1	78549001	78551000	2000	1	1.60E-08	-0.63	10	0.5	Arhgap35;LOC108349548	Signaling
DMR1:78569001	1	78569001	78570000	1000	1	1.30E-08	-0.64	5	0.5	Arhgap35	Signaling
DMR1:78571001	1	78571001	78573000	2000	2	3.50E-20	0.79	81	4.05	Arhgap35	Signaling
DMR1:78618001	1	78618001	78622000	4000	2	7.20E-12	0.62	54	1.35	LOC102550403;LOC108349752;Ceacam9	
DMR1:78635001	1	78635001	78637000	2000	1	5.90E-09	0.53	25	1.25	LOC108349752;Ceacam9	
DMR1:78644001	1	78644001	78649000	5000	2	1.70E-17	0.54	70	1.4	LOC108349752;Ceacam9	
DMR1:78672001	1	78672001	78673000	1000	1	4.10E-09	0.44	11	1.1	Ceacam9;Ap2s1	Transport
DMR1:78676001	1	78676001	78679000	3000	1	3.50E-07	0.41	48	1.6	Ap2s1;LOC679748	Transport
DMR1:78681001	1	78681001	78682000	1000	1	6.50E-07	0.38	19	1.9	Ap2s1;LOC679748	Transport
DMR1:78688001	1	78688001	78689000	1000	1	2.50E-08	-0.63	13	1.3	Ap2s1;LOC679748	Transport
DMR1:78701001	1	78701001	78702000	1000	1	1.10E-22	0.88	8	0.8	Slc1a5	Transport
DMR1:78756001	1	78756001	78757000	1000	1	1.20E-22	0.67	16	1.6	Strn4	
DMR1:78779001	1	78779001	78785000	6000	1	1.20E-07	0.39	110	1.83	Prkd2	Signaling
DMR1:78818001	1	78818001	78822000	4000	1	3.70E-12	0.75	97	2.42	Dact3;Gng8	Signaling
DMR1:78831001	1	78831001	78835000	4000	1	4.00E-24	0.72	85	2.12	Gng8;Ptgir;Calm3	Signaling;Signaling;Signaling
DMR1:78843001	1	78843001	78851000	8000	3	1.80E-14	0.64	152	1.9	Ptgir;Calm3	Signaling;Signaling
DMR1:78867001	1	78867001	78872000	5000	3	8.50E-22	0.68	63	1.26	Pnmal2	
DMR1:78899001	1	78899001	78906000	7000	2	2.50E-16	0.78	60	0.86	Pnmal1	
DMR1:78924001	1	78924001	78927000	3000	1	7.40E-20	0.5	40	1.33	Ccdc8	
DMR1:78965001	1	78965001	78968000	3000	1	1.30E-12	0.49	39	1.3	Ppp5c;Hif3a	Signaling;Transcription
DMR1:78970001	1	78970001	78971000	1000	1	2.20E-07	0.4	10	1	Ppp5c;Hif3a	Signaling;Transcription
DMR1:78976001	1	78976001	78988000	12000	3	1.30E-11	0.59	158	1.32	Ppp5c;Hif3a	Signaling;Transcription
DMR1:78990001	1	78990001	78994000	4000	3	4.90E-16	0.69	48	1.2	Hif3a	Transcription
DMR1:78995001	1	78995001	78997000	2000	2	3.80E-11	0.44	16	0.8	Hif3a	Transcription
DMR1:78999001	1	78999001	79001000	2000	1	5.20E-11	0.66	25	1.25	Hif3a	Transcription
DMR1:79019001	1	79019001	79024000	5000	3	2.30E-28	-0.66	54	1.08	Cgm4	
DMR1:79040001	1	79040001	79042000	2000	1	9.00E-14	0.57	20	1	Cgm4;LOC103691046	
DMR1:79054001	1	79054001	79056000	2000	1	1.10E-22	1.01	7	0.35	LOC103691046;Psg16	
DMR1:79066001	1	79066001	79067000	1000	1	4.70E-12	0.5	10	1	Psg16	
DMR1:79086001	1	79086001	79089000	3000	2	1.20E-10	-0.42	30	1	Psg16	
DMR1:79113001	1	79113001	79120000	7000	5	4.00E-25	-0.56	70	1	Ceacam3	Immune
DMR1:79138001	1	79138001	79140000	2000	1	1.70E-07	0.52	17	0.85	Ceacam3	Immune
DMR1:79143001	1	79143001	79144000	1000	1	4.10E-21	0.67	10	1	Ceacam3	Immune
DMR1:79170001	1	79170001	79176000	6000	4	2.10E-22	-0.56	67	1.12	Psgb1	
DMR1:79178001	1	79178001	79180000	2000	2	2.30E-09	-0.68	4	0.2	Psgb1	
DMR1:79191001	1	79191001	79195000	4000	1	2.60E-08	0.4	33	0.82	Psgb1	
DMR1:79196001	1	79196001	79197000	1000	1	2.70E-09	0.32	5	0.5	Psgb1	
DMR1:79240001	1	79240001	79246000	6000	4	1.60E-29	-0.62	80	1.33	Psg29	
DMR1:79248001	1	79248001	79249000	1000	1	1.20E-11	-0.53	4	0.4	Psg29	
DMR1:79253001	1	79253001	79256000	3000	1	2.00E-09	0.42	34	1.13	Psg29	
DMR1:79389001	1	79389001	79392000	3000	1	7.10E-07	0.48	22	0.73	Ceacam11	Immune
DMR1:79471001	1	79471001	79472000	1000	1	5.70E-09	0.49	14	1.4	Igfl3	
DMR1:79635001	1	79635001	79636000	1000	1	6.80E-11	0.54	13	1.3	LOC102550882;LOC102552765;Mill1;LOC102552856	Immune

DMR1:79639001	1	79639001	79643000	4000	1	4.40E-09	0.65	29	0.72	LOC102552765;Mill1;LOC102552856	Immune
DMR1:79644001	1	79644001	79645000	1000	1	2.90E-13	0.54	9	0.9	Mill1;LOC102552856	Immune
DMR1:79658001	1	79658001	79659000	1000	1	2.50E-11	0.53	6	0.6	Mill1	Immune
DMR1:79660001	1	79660001	79665000	5000	3	6.10E-17	0.81	62	1.24	Mill1	Immune
DMR1:79671001	1	79671001	79673000	2000	1	2.80E-11	0.61	11	0.55	LOC292672;Psg19	Immune
DMR1:79691001	1	79691001	79692000	1000	1	5.70E-13	0.54	10	1	Psg19;LOC102557319	Immune
DMR1:79742001	1	79742001	79745000	3000	1	1.10E-07	0.34	44	1.47	LOC100362871;Micb	Immune
DMR1:79746001	1	79746001	79750000	4000	2	5.90E-09	0.53	58	1.45	LOC100362871;Micb	Immune
DMR1:79763001	1	79763001	79770000	7000	3	3.20E-21	1.24	79	1.13	Micb	Immune
DMR1:79777001	1	79777001	79779000	2000	1	1.40E-07	0.39	23	1.15	Micb	Immune
DMR1:79786001	1	79786001	79789000	3000	1	8.40E-08	0.37	48	1.6	Pglyrp1;Ccdc61	Epigenetic
DMR1:79793001	1	79793001	79795000	2000	1	3.10E-32	1	23	1.15	Pglyrp1;Ccdc61	Epigenetic
DMR1:79797001	1	79797001	79800000	3000	2	8.40E-13	0.66	118	3.93	Pglyrp1;Ccdc61	Epigenetic
DMR1:79811001	1	79811001	79813000	2000	2	4.80E-25	0.71	22	1.1	Ccdc61	Epigenetic
DMR1:79819001	1	79819001	79822000	3000	2	1.40E-22	0.86	33	1.1	Ccdc61;Nova2	Epigenetic;Metabolism
DMR1:79823001	1	79823001	79826000	3000	1	3.40E-14	0.93	38	1.27	Ccdc61;Nova2	Epigenetic;Metabolism
DMR1:79828001	1	79828001	79830000	2000	2	5.00E-13	0.44	15	0.75	Nova2	Metabolism
DMR1:79832001	1	79832001	79834000	2000	2	7.80E-14	0.47	40	2	Nova2	Metabolism
DMR1:79840001	1	79840001	79842000	2000	1	1.50E-09	0.43	23	1.15	Nova2	Metabolism
DMR1:79849001	1	79849001	79851000	2000	2	1.30E-22	1.01	21	1.05	Nova2	Metabolism
DMR1:79859001	1	79859001	79861000	2000	1	7.40E-17	0.58	29	1.45	Nova2	Metabolism
DMR1:79863001	1	79863001	79864000	1000	1	9.40E-13	0.64	12	1.2	Nova2	Metabolism
DMR1:79871001	1	79871001	79872000	1000	1	2.50E-08	0.7	29	2.9	Nova2	Metabolism
DMR1:79881001	1	79881001	79885000	4000	2	1.30E-19	0.9	41	1.02	Nanos2	Metabolism
DMR1:79944001	1	79944001	79945000	1000	1	8.00E-10	-0.6	12	1.2	Sympk	
DMR1:79960001	1	79960001	79963000	3000	1	4.40E-17	0.52	44	1.47	Sympk;Rsph6a;LOC103691057;LOC108349062	Cytoskeleton
DMR1:79974001	1	79974001	79977000	3000	1	5.10E-13	0.56	42	1.4	Rsph6a;LOC103691057;LOC108349062;Dmwd	Cytoskeleton
DMR1:80013001	1	80013001	80014000	1000	1	1.00E-07	0.48	10	1	Six5;LOC102553424	Development
DMR1:80017001	1	80017001	80019000	2000	1	5.10E-11	0.42	21	1.05	LOC102553424;Fbxo46	
DMR1:80021001	1	80021001	80022000	1000	1	1.40E-07	0.56	4	0.4	LOC102553424;Fbxo46	
DMR1:80047001	1	80047001	80050000	3000	1	2.50E-08	-0.44	41	1.37	Fbxo46;Qpctl;Snrpd2	Transport;Translation
DMR1:80052001	1	80052001	80055000	3000	1	3.50E-08	0.31	40	1.33	Fbxo46;Qpctl;Snrpd2;Gipr	Transport;Translation;Receptor
DMR1:80073001	1	80073001	80075000	2000	1	1.90E-07	-0.58	37	1.85	Gipr	Receptor
DMR1:80076001	1	80076001	80078000	2000	2	1.30E-16	0.7	31	1.55	Gipr;Eml2	Receptor
DMR1:80079001	1	80079001	80081000	2000	1	1.20E-08	0.49	38	1.9	Gipr;Eml2	Receptor
DMR1:80107001	1	80107001	80112000	5000	3	2.00E-26	0.89	84	1.68	Eml2	
DMR1:80114001	1	80114001	80115000	1000	1	3.00E-07	0.57	33	3.3	Eml2	
DMR1:80118001	1	80118001	80120000	2000	1	3.80E-08	0.34	26	1.3	Eml2;Gpr4	Signaling
DMR1:80125001	1	80125001	80127000	2000	2	1.00E-10	0.57	48	2.4	Eml2;Gpr4	Signaling
DMR1:80176001	1	80176001	80179000	3000	1	5.00E-09	0.49	58	1.93	Opa3;Vasp	Cytoskeleton
DMR1:80180001	1	80180001	80181000	1000	1	6.30E-21	0.92	16	1.6	Opa3;Vasp;Ppm1n	Cytoskeleton
DMR1:80204001	1	80204001	80207000	3000	3	2.30E-12	0.64	50	1.67	Rtn2;Fosb;LOC103691049	Transcription
DMR1:80231001	1	80231001	80232000	1000	1	2.30E-07	0.37	38	3.8	Fosb;LOC108349552;LOC108349753	Transcription
DMR1:80251001	1	80251001	80253000	2000	2	2.70E-17	0.86	29	1.45	LOC108349753;Ercc1	Transcription
DMR1:80261001	1	80261001	80262000	1000	1	1.10E-15	0.47	7	0.7	Ercc1;Cd3eap;Ppp1r13l	Transcription;Transcription
DMR1:80264001	1	80264001	80268000	4000	1	4.80E-11	0.41	57	1.43	Ercc1;Cd3eap;Ppp1r13l	Transcription;Transcription
DMR1:80296001	1	80296001	80298000	2000	1	9.00E-09	0.38	32	1.6	Ppp1r13l;Ercc2;Mir343;Klc3	Epigenetic;Cytoskeleton
DMR1:80300001	1	80300001	80301000	1000	1	2.50E-10	0.43	18	1.8	Ppp1r13l;Ercc2;Mir343;Klc3	Epigenetic;Cytoskeleton
DMR1:80307001	1	80307001	80308000	1000	1	2.60E-13	0.49	22	2.2	Ercc2;Mir343;Klc3	Epigenetic;Cytoskeleton
DMR1:80329001	1	80329001	80331000	2000	2	2.40E-17	0.86	46	2.3	Ckm;Mark4	Signaling;Signaling
DMR1:80335001	1	80335001	80337000	2000	1	1.90E-22	0.66	59	2.95	Ckm;Mark4	Signaling;Signaling
DMR1:80354001	1	80354001	80360000	6000	3	5.60E-25	1.53	75	1.25	Mark4	Signaling

DMR1:80366001	1	80366001	80369000	3000	2	3.00E-15	0.62	99	3.3	Mark4;Exoc3l2	Signaling;Transport
DMR1:80374001	1	80374001	80375000	1000	1	9.70E-23	0.88	33	3.3	Mark4;Exoc3l2	Signaling;Transport
DMR1:80379001	1	80379001	80380000	1000	1	2.50E-13	0.52	13	1.3	Exoc3l2	Transport
DMR1:80381001	1	80381001	80382000	1000	1	1.10E-18	0.61	23	2.3	Exoc3l2	Transport
DMR1:80391001	1	80391001	80392000	1000	1	3.50E-15	0.44	22	2.2	Exoc3l2	Transport
DMR1:80400001	1	80400001	80402000	2000	1	1.70E-07	0.36	31	1.55	Exoc3l2	Transport
DMR1:80407001	1	80407001	80409000	2000	1	3.80E-30	0.9	38	1.9	Exoc3l2;Bloc1s3;Trappc6a	Transport
DMR1:80412001	1	80412001	80414000	2000	1	5.60E-09	0.4	35	1.75	Exoc3l2;Bloc1s3;Trappc6a	Transport
DMR1:80421001	1	80421001	80423000	2000	1	8.30E-11	0.46	30	1.5	Bloc1s3;Trappc6a;Nkpd1	
DMR1:80501001	1	80501001	80503000	2000	1	1.20E-29	0.86	29	1.45	Zfp296;Clasrp	Transcription;Translation
DMR1:80504001	1	80504001	80506000	2000	1	8.60E-09	0.38	37	1.85	Clasrp;LOC108348935	Translation
DMR1:80507001	1	80507001	80509000	2000	1	5.10E-09	0.53	24	1.2	Clasrp;LOC108348935;Relb	Translation;Transcription
DMR1:80543001	1	80543001	80545000	2000	1	1.10E-11	0.81	87	4.35	Relb;Clptm1	Transcription
DMR1:80568001	1	80568001	80571000	3000	1	2.00E-08	-0.47	49	1.63	Clptm1;LOC108348938	
DMR1:80589001	1	80589001	80591000	2000	1	5.50E-11	0.4	33	1.65	Clptm1;Apoc2;Apoc4	Binding Proteins
DMR1:80592001	1	80592001	80594000	2000	1	9.80E-08	0.29	24	1.2	Apoc2;Apoc4	Binding Proteins
DMR1:80596001	1	80596001	80597000	1000	1	1.00E-08	0.39	9	0.9	Apoc2;Apoc4;Apoc1	Binding Proteins
DMR1:80636001	1	80636001	80638000	2000	1	7.20E-11	0.45	30	1.5	Tomm40;Nectin2	Transport
DMR1:80639001	1	80639001	80641000	2000	1	6.90E-09	0.4	29	1.45	Tomm40;Nectin2	Transport
DMR1:80662001	1	80662001	80665000	3000	2	1.40E-23	0.86	47	1.57	Nectin2;Bcam	Immune
DMR1:80675001	1	80675001	80679000	4000	1	6.00E-08	0.43	93	2.33	Nectin2;Bcam	Immune
DMR1:80680001	1	80680001	80682000	2000	1	3.40E-08	0.39	39	1.95	Bcam	Immune
DMR1:80686001	1	80686001	80687000	1000	1	4.90E-08	0.37	15	1.5	Bcam	Immune
DMR1:80695001	1	80695001	80698000	3000	1	4.60E-17	0.57	28	0.93	Bcam;Cblc	Immune;Metabolism
DMR1:80703001	1	80703001	80705000	2000	1	2.20E-08	0.42	25	1.25	Cblc	Metabolism
DMR1:80706001	1	80706001	80707000	1000	1	3.50E-07	-0.57	11	1.1	Cblc	Metabolism
DMR1:80737001	1	80737001	80738000	1000	1	2.40E-09	0.46	22	2.2	Bcl3	Transport
DMR1:80748001	1	80748001	80749000	1000	1	2.50E-21	0.9	9	0.9	Bcl3	Transport
DMR1:80753001	1	80753001	80755000	2000	1	2.50E-13	0.46	27	1.35	Bcl3	Transport
DMR1:80787001	1	80787001	80791000	4000	2	3.20E-10	-0.56	55	1.38	Ceacam16;LOC102547975;Ceacam19	
DMR1:80796001	1	80796001	80797000	1000	1	3.50E-07	0.41	22	2.2	LOC102547975;Ceacam19	
DMR1:80798001	1	80798001	80799000	1000	1	5.20E-07	-0.51	15	1.5	LOC102547975;Ceacam19	
DMR1:80802001	1	80802001	80805000	3000	1	3.00E-24	0.82	41	1.37	LOC102547975;Ceacam19	
DMR1:80809001	1	80809001	80810000	1000	1	8.70E-07	0.47	13	1.3	LOC102547975;Ceacam19	
DMR1:80823001	1	80823001	80824000	1000	1	4.30E-07	-0.59	14	1.4	PVR	
DMR1:80832001	1	80832001	80834000	2000	1	1.20E-30	0.73	23	1.15	PVR	
DMR1:80836001	1	80836001	80841000	5000	4	5.30E-23	0.65	77	1.54	PVR;Igsf23	
DMR1:80851001	1	80851001	80853000	2000	1	2.20E-15	0.87	44	2.2	Igsf23;LOC108349553	
DMR1:80856001	1	80856001	80858000	2000	1	6.70E-07	0.3	39	1.95	Igsf23;LOC108349553	
DMR1:80859001	1	80859001	80864000	5000	3	3.50E-13	0.55	91	1.82	Igsf23;LOC108349553	
DMR1:80865001	1	80865001	80867000	2000	1	1.90E-09	0.38	28	1.4	Igsf23	
DMR1:80872001	1	80872001	80874000	2000	1	7.00E-07	-0.56	17	0.85	Igsf23;Ceacam20	
DMR1:80875001	1	80875001	80877000	2000	1	3.50E-16	0.82	59	2.95	Igsf23;Ceacam20	
DMR1:80881001	1	80881001	80883000	2000	1	5.80E-21	0.82	21	1.05	Ceacam20;LOC102548132	
DMR1:80899001	1	80899001	80902000	3000	2	3.00E-12	0.53	35	1.17	Ceacam20;LOC102548132	
DMR1:80909001	1	80909001	80911000	2000	1	1.70E-07	0.57	31	1.55	Ceacam20;Zfp180	Transcription
DMR1:80912001	1	80912001	80916000	4000	2	1.10E-11	0.56	40	1	Ceacam20;Zfp180	Transcription
DMR1:80925001	1	80925001	80926000	1000	1	2.20E-12	0.52	14	1.4	Zfp180;LOC108348994	Transcription
DMR1:80954001	1	80954001	80955000	1000	1	7.00E-12	0.48	10	1	Zfp112	Transcription
DMR1:80969001	1	80969001	80973000	4000	1	6.20E-18	0.57	72	1.8	Zfp112;Znf235;LOC102551479	Transcription
DMR1:81034001	1	81034001	81040000	6000	3	4.30E-12	0.62	108	1.8	Zfp111	Transcription
DMR1:81129001	1	81129001	81131000	2000	1	1.20E-07	0.67	61	3.05	Zfp61;Zfp94	Transcription
DMR1:81137001	1	81137001	81140000	3000	1	1.90E-07	0.32	89	2.97	Zfp61;Zfp94	Transcription
DMR1:81141001	1	81141001	81143000	2000	1	9.60E-07	0.29	30	1.5	Zfp94;LOC103691053	
DMR1:81158001	1	81158001	81159000	1000	1	2.10E-08	0.49	15	1.5	Zfp94;LOC103691053;LOC102548695	
DMR1:81177001	1	81177001	81184000	7000	1	1.20E-07	-0.4	152	2.17	LOC102548695;Tescl	
DMR1:81203001	1	81203001	81208000	5000	3	6.30E-16	0.84	67	1.34	Tescl;Lypd5	Receptor
DMR1:81209001	1	81209001	81212000	3000	1	1.20E-08	0.42	53	1.77	Lypd5	Receptor
DMR1:81222001	1	81222001	81228000	6000	2	8.60E-10	0.65	79	1.32	Lypd5;Kcnn4	Receptor;Transport
DMR1:81229001	1	81229001	81238000	9000	4	4.80E-16	0.65	154	1.71	Kcnn4	Transport
DMR1:81240001	1	81240001	81241000	1000	1	5.70E-07	0.35	13	1.3	Kcnn4	Transport

DMR1:81250001	1	81250001	81251000	1000	1	9.00E-21	0.54	24	2.4	Kcnn4;Smg9	Transport
DMR1:81254001	1	81254001	81256000	2000	1	6.30E-13	0.4	48	2.4	Kcnn4;Smg9	Transport
DMR1:81273001	1	81273001	81278000	5000	3	1.40E-15	0.58	99	1.98	Smg9	
DMR1:81290001	1	81290001	81292000	2000	2	2.90E-13	0.49	39	1.95	Smg9;Irgc	
DMR1:81300001	1	81300001	81301000	1000	1	2.80E-14	0.69	11	1.1	Irgc	
DMR1:81321001	1	81321001	81323000	2000	2	2.70E-17	0.98	29	1.45	Plaur	Receptor
DMR1:81328001	1	81328001	81331000	3000	1	2.40E-07	0.41	65	2.17	Plaur	Receptor
DMR1:81332001	1	81332001	81334000	2000	1	7.70E-13	0.47	32	1.6	Plaur	Receptor
DMR1:81351001	1	81351001	81355000	4000	2	2.40E-14	0.55	70	1.75	Plaur	Receptor
DMR1:81359001	1	81359001	81362000	3000	1	6.90E-08	0.39	32	1.07	Cadm4;Zfp428	
DMR1:81368001	1	81368001	81370000	2000	1	9.50E-16	0.44	45	2.25	Cadm4;Zfp428;Srrm5	
DMR1:81379001	1	81379001	81386000	7000	3	3.40E-15	0.61	139	1.99	Cadm4;Zfp428;Srrm5;Irgq	
DMR1:81409001	1	81409001	81411000	2000	2	1.90E-19	0.85	40	2	Irgq;Pinlyp;Xrcc1	Cytoskeleton;Transcription
DMR1:81427001	1	81427001	81431000	4000	2	2.20E-36	1.23	72	1.8	Xrcc1	Transcription
DMR1:81434001	1	81434001	81436000	2000	1	2.10E-19	0.59	60	3	Xrcc1	Transcription
DMR1:81438001	1	81438001	81439000	1000	1	1.40E-08	0.4	13	1.3	Xrcc1;Zfp575	Transcription;Transcription
DMR1:81475001	1	81475001	81482000	7000	1	6.10E-13	0.63	113	1.61	Ethe1;Phldb3;LOC108349554	Metabolism
DMR1:81490001	1	81490001	81492000	2000	1	2.20E-08	0.4	25	1.25	Phldb3;LOC108349554;Lypd3	Receptor
DMR1:81523001	1	81523001	81524000	1000	1	9.90E-16	1.01	12	1.2	Tex101	
DMR1:81525001	1	81525001	81528000	3000	2	7.20E-20	0.67	30	1	Tex101	
DMR1:81536001	1	81536001	81543000	7000	2	3.50E-09	-0.5	50	0.71	Tex101;LOC100910565	
DMR1:81599001	1	81599001	81604000	5000	1	4.00E-07	0.31	102	2.04	RGD1565655;LOC499098;LOC100909700	
DMR1:81635001	1	81635001	81638000	3000	1	2.60E-12	0.56	39	1.3	LOC100909700;LOC108349555;LOC100910734;Ceacam4	
DMR1:81649001	1	81649001	81653000	4000	2	2.30E-11	0.34	78	1.95	LOC100910734;Ceacam4	
DMR1:81675001	1	81675001	81677000	2000	1	2.20E-11	0.61	21	1.05	LOC108349556;Ceacam6	
DMR1:81684001	1	81684001	81686000	2000	1	8.20E-08	0.32	26	1.3	LOC108349556;Ceacam6	
DMR1:81720001	1	81720001	81722000	2000	1	3.80E-18	0.65	37	1.85	Lypd4	
DMR1:81761001	1	81761001	81763000	2000	1	2.60E-08	-0.53	31	1.55	Rps19;Cd79a;Arhgef1	Translation;Immune;Transcription
DMR1:81783001	1	81783001	81788000	5000	1	2.50E-15	0.47	105	2.1	Arhgef1;RGD1563034	Transcription
DMR1:81789001	1	81789001	81792000	3000	1	2.10E-07	0.42	33	1.1	Arhgef1;RGD1563034	Transcription
DMR1:81793001	1	81793001	81801000	8000	2	7.00E-18	0.57	182	2.28	Arhgef1;RGD1563034	Transcription
DMR1:81802001	1	81802001	81806000	4000	3	1.30E-30	1.08	103	2.58	Arhgef1;RGD1563034	Transcription
DMR1:81808001	1	81808001	81817000	9000	5	7.50E-20	0.71	194	2.16	RGD1563034	
DMR1:81848001	1	81848001	81849000	1000	1	5.20E-16	0.59	7	0.7	Rabac1;Atp1a3	Transport;Transport
DMR1:81852001	1	81852001	81854000	2000	2	4.00E-18	0.57	57	2.85	Rabac1;Atp1a3	Transport;Transport
DMR1:81860001	1	81860001	81865000	5000	2	1.90E-10	0.42	82	1.64	Atp1a3	Transport
DMR1:81875001	1	81875001	81876000	1000	1	5.20E-14	0.54	22	2.2	Atp1a3;Grik5	Transport;Receptor
DMR1:81877001	1	81877001	81881000	4000	2	4.10E-29	1.05	56	1.4	Atp1a3;Grik5	Transport;Receptor
DMR1:81882001	1	81882001	81885000	3000	1	1.10E-11	0.72	78	2.6	Atp1a3;Grik5	Transport;Receptor
DMR1:81889001	1	81889001	81893000	4000	2	1.30E-21	0.58	118	2.95	Atp1a3;Grik5	Transport;Receptor
DMR1:81897001	1	81897001	81901000	4000	1	1.20E-25	0.8	74	1.85	Grik5	Receptor
DMR1:81906001	1	81906001	81907000	1000	1	2.60E-09	0.47	13	1.3	Grik5	Receptor
DMR1:81911001	1	81911001	81914000	3000	1	4.90E-11	0.44	27	0.9	Grik5	Receptor
DMR1:81924001	1	81924001	81926000	2000	1	7.80E-16	0.55	40	2	Grik5	Receptor
DMR1:81927001	1	81927001	81929000	2000	1	5.10E-08	0.4	27	1.35	Grik5	Receptor
DMR1:81933001	1	81933001	81939000	6000	4	8.80E-29	0.71	107	1.78	Grik5	Receptor
DMR1:81941001	1	81941001	81950000	9000	6	4.60E-22	0.85	124	1.38	Grik5;Zfp574	Receptor
DMR1:81967001	1	81967001	81968000	1000	1	9.30E-08	0.41	34	3.4	Zfp574;Pou2f2	
DMR1:81974001	1	81974001	81980000	6000	5	5.00E-17	0.64	125	2.08	Pou2f2	
DMR1:81981001	1	81981001	81982000	1000	1	3.00E-22	0.63	11	1.1	Pou2f2	
DMR1:81986001	1	81986001	81990000	4000	1	6.00E-11	0.48	92	2.3	Pou2f2	
DMR1:81993001	1	81993001	81999000	6000	3	1.10E-15	0.55	118	1.97	Pou2f2;LOC103691054	
DMR1:82005001	1	82005001	82007000	2000	2	2.50E-15	0.54	55	2.75	Pou2f2;LOC103691054	
DMR1:82008001	1	82008001	82011000	3000	1	5.60E-07	0.32	34	1.13	Pou2f2;LOC103691054	
DMR1:82014001	1	82014001	82016000	2000	1	7.40E-09	0.42	9	0.45	Pou2f2;LOC103691054	
DMR1:82019001	1	82019001	82027000	8000	5	3.70E-33	0.97	134	1.68	Pou2f2;LOC103691054	
DMR1:82032001	1	82032001	82034000	2000	1	3.10E-09	0.41	45	2.25	Pou2f2;LOC103691054	
DMR1:82038001	1	82038001	82045000	7000	3	3.40E-19	-0.59	148	2.11	Pou2f2	
DMR1:82047001	1	82047001	82051000	4000	4	3.20E-24	0.89	172	4.3	Pou2f2	
DMR1:82072001	1	82072001	82075000	3000	1	4.00E-14	0.55	72	2.4	Dedd2	

DMR1:82082001	1	82082001	82087000	5000	1	6.40E-07	-0.58	73	1.46	Dedd2;Zfp526	
DMR1:82102001	1	82102001	82104000	2000	1	9.30E-13	0.56	23	1.15	Zfp526;Gsk3a;Erf	Signaling;Transcription
DMR1:82114001	1	82114001	82116000	2000	2	1.10E-16	0.55	75	3.75	Gsk3a;Erf	Signaling;Transcription
DMR1:82131001	1	82131001	82133000	2000	1	1.10E-08	0.5	14	0.7	Cic	Transcription
DMR1:82137001	1	82137001	82138000	1000	1	1.20E-18	0.88	29	2.9	Cic	Transcription
DMR1:82141001	1	82141001	82142000	1000	1	1.30E-18	0.68	47	4.7	Cic	Transcription
DMR1:82160001	1	82160001	82161000	1000	1	3.90E-16	0.55	13	1.3	Cic;Pafah1b3;Prr19	Transcription;Golgi
DMR1:82177001	1	82177001	82182000	5000	1	2.00E-12	0.38	81	1.62	Prr19;Tmem145;Megf8	Extracellular Matrix
DMR1:82186001	1	82186001	82189000	3000	1	1.50E-08	0.42	36	1.2	Tmem145;Megf8	Extracellular Matrix
DMR1:82190001	1	82190001	82191000	1000	1	3.50E-12	0.61	4	0.4	Tmem145;Megf8	Extracellular Matrix
DMR1:82193001	1	82193001	82199000	6000	3	4.80E-31	0.84	107	1.78	Tmem145;Megf8	Extracellular Matrix
DMR1:82202001	1	82202001	82207000	5000	1	1.20E-12	0.44	87	1.74	Megf8	Extracellular Matrix
DMR1:82208001	1	82208001	82210000	2000	1	3.00E-09	0.43	50	2.5	Megf8	Extracellular Matrix
DMR1:82215001	1	82215001	82217000	2000	2	2.00E-16	0.52	38	1.9	Megf8	Extracellular Matrix
DMR1:82222001	1	82222001	82226000	4000	1	4.20E-27	0.99	68	1.7	Megf8;Cnfn	Extracellular Matrix
DMR1:82229001	1	82229001	82232000	3000	1	7.30E-13	0.5	68	2.27	Megf8;Cnfn	Extracellular Matrix
DMR1:82236001	1	82236001	82244000	8000	2	1.70E-11	0.52	105	1.31	Megf8;Cnfn;LOC102549342;Lipe	Extracellular Matrix;Metabolism
DMR1:82250001	1	82250001	82255000	5000	3	4.20E-20	0.67	88	1.76	LOC102549342;Lipe	Metabolism
DMR1:82271001	1	82271001	82273000	2000	1	2.60E-07	-0.46	35	1.75	LOC102549342;Lipe;Cxcl17	Metabolism;Growth Factors
DMR1:82277001	1	82277001	82281000	4000	1	1.60E-17	0.63	75	1.88	LOC102549342;Cxcl17	Growth Factors
DMR1:82314001	1	82314001	82318000	4000	3	1.30E-08	-0.64	46	1.15	LOC102549342;Ceacam1	
DMR1:82319001	1	82319001	82321000	2000	1	5.40E-11	0.52	37	1.85	LOC102549342;Ceacam1	
DMR1:82322001	1	82322001	82326000	4000	1	7.50E-08	0.33	98	2.45	LOC102549342;Ceacam1	
DMR1:82327001	1	82327001	82330000	3000	1	8.40E-16	0.54	72	2.4	LOC102549342;Ceacam1	
DMR1:82331001	1	82331001	82337000	6000	1	4.10E-17	0.59	97	1.62	LOC102549342;Ceacam1	
DMR1:82345001	1	82345001	82349000	4000	1	1.40E-07	-0.39	69	1.73	LOC102549342;Ceacam1	
DMR1:82351001	1	82351001	82354000	3000	1	1.20E-13	0.42	57	1.9	LOC102549342;Ceacam1	
DMR1:82398001	1	82398001	82399000	1000	1	1.60E-18	0.93	8	0.8	LOC102549342;Erich4	
DMR1:82403001	1	82403001	82404000	1000	1	1.80E-07	0.3	8	0.8	LOC102549342;Erich4;Atp5sl	
DMR1:82407001	1	82407001	82412000	5000	3	2.70E-26	0.9	69	1.38	LOC102549342;Erich4;Atp5sl;B3gnt8	Golgi
DMR1:82420001	1	82420001	82421000	1000	1	7.40E-07	0.61	44	4.4	Atp5sl;B3gnt8;Bckdha	Golgi;Metabolism
DMR1:82432001	1	82432001	82435000	3000	2	1.30E-14	0.47	53	1.77	B3gnt8;Bckdha	Golgi;Metabolism
DMR1:82444001	1	82444001	82445000	1000	1	6.60E-08	0.47	5	0.5	Bckdha;Exosc5	Metabolism;Transcription
DMR1:82446001	1	82446001	82447000	1000	1	4.20E-08	0.68	4	0.4	Bckdha;Exosc5	Metabolism;Transcription
DMR1:82471001	1	82471001	82472000	1000	1	7.70E-09	0.71	14	1.4	Exosc5;Tmem91;B9d2;Tgfb1	Transcription;Development;Growth Factors
DMR1:82475001	1	82475001	82479000	4000	1	5.50E-07	0.32	56	1.4	Tmem91;B9d2;Tgfb1	Development;Growth Factors
DMR1:82482001	1	82482001	82485000	3000	1	2.40E-09	0.61	99	3.3	B9d2;Tgfb1	Development;Growth Factors
DMR1:82495001	1	82495001	82501000	6000	1	2.90E-13	0.64	118	1.97	Tgfb1;Ccdc97	Growth Factors
DMR1:82502001	1	82502001	82506000	4000	4	1.50E-22	0.8	71	1.77	Tgfb1;Ccdc97;Hnrnpul1	Growth Factors
DMR1:82525001	1	82525001	82526000	1000	1	6.10E-16	0.62	12	1.2	Hnrnpul1	
DMR1:82554001	1	82554001	82556000	2000	2	7.90E-13	-0.57	27	1.35	Hnrnpul1;Axl	Receptor
DMR1:82562001	1	82562001	82567000	5000	3	8.50E-27	0.81	81	1.62	Axl	Receptor
DMR1:82575001	1	82575001	82579000	4000	2	1.30E-20	0.76	56	1.4	Axl	Receptor
DMR1:82580001	1	82580001	82581000	1000	1	7.80E-10	0.59	21	2.1	Axl	Receptor
DMR1:82582001	1	82582001	82586000	4000	3	1.80E-15	0.54	84	2.1	Axl;Cyp2s1	Receptor;Metabolism
DMR1:82591001	1	82591001	82596000	5000	2	2.20E-24	0.62	80	1.6	Cyp2s1	Metabolism
DMR1:82606001	1	82606001	82608000	2000	1	2.40E-08	0.4	21	1.05	Cyp2s1	Metabolism
DMR1:82890001	1	82890001	82892000	2000	1	2.30E-07	-0.42	15	0.75	RGD1564380;LOC100911185	
DMR1:82956001	1	82956001	82960000	4000	1	5.40E-07	0.39	81	2.02	LOC102553892;LOC100911309;Cd177	
DMR1:82963001	1	82963001	82964000	1000	1	9.20E-07	0.41	4	0.4	LOC100911309;Cd177	
DMR1:82994001	1	82994001	82998000	4000	3	1.90E-09	0.65	58	1.45	Cd177;LOC108349464;RGD1563502;LOC103689942	

DMR1:83109001	1	83109001	83118000	9000	4	1.90E-23	0.8	71	0.79	Cyp2b2	Metabolism
DMR1:83155001	1	83155001	83161000	6000	3	8.60E-12	0.58	42	0.7	Cyp2b3	Metabolism
DMR1:83200001	1	83200001	83202000	2000	1	3.10E-16	-0.59	8	0.4	Cyp2b3	Metabolism
DMR1:83204001	1	83204001	83210000	6000	4	3.90E-17	0.93	45	0.75	Cyp2b3	Metabolism
DMR1:83225001	1	83225001	83233000	8000	5	4.60E-14	-0.55	78	0.98	Cyp2b3	Metabolism
DMR1:83234001	1	83234001	83235000	1000	1	2.40E-18	0.84	2	0.2	Cyp2b3	Metabolism
DMR1:83250001	1	83250001	83253000	3000	2	1.80E-09	-0.45	35	1.17	Eif4g2-ps2	
DMR1:83258001	1	83258001	83260000	2000	1	1.50E-19	0.71	10	0.5	Eif4g2-ps2	
DMR1:83391001	1	83391001	83392000	1000	1	9.60E-17	0.79	2	0.2	Cyp2b21	Metabolism
DMR1:83394001	1	83394001	83398000	4000	2	1.50E-11	0.54	17	0.42	Cyp2b21	Metabolism
DMR1:83401001	1	83401001	83411000	10000	6	3.30E-15	0.59	76	0.76	Cyp2b21	Metabolism
DMR1:83424001	1	83424001	83425000	1000	1	1.60E-10	0.6	2	0.2	Cyp2b21	Metabolism
DMR1:83433001	1	83433001	83434000	1000	1	3.40E-10	0.51	4	0.4	Epc2l1	
DMR1:83441001	1	83441001	83445000	4000	1	1.50E-09	-0.65	10	0.25	Epc2l1	
DMR1:83513001	1	83513001	83517000	4000	4	1.20E-26	0.96	34	0.85	Cyp2b12	Metabolism
DMR1:83519001	1	83519001	83523000	4000	2	4.50E-23	1.14	19	0.48	Cyp2b12	Metabolism
DMR1:83525001	1	83525001	83527000	2000	1	1.70E-12	0.57	18	0.9	Cyp2b12	Metabolism
DMR1:83624001	1	83624001	83626000	2000	1	5.30E-08	0.36	48	2.4	Cyp2g1	Metabolism
DMR1:83628001	1	83628001	83630000	2000	1	7.90E-10	0.56	15	0.75	Cyp2g1	Metabolism
DMR1:83645001	1	83645001	83647000	2000	1	1.70E-23	0.76	28	1.4	Cyp2g1;LOC103691073;Cyp2a3	Metabolism
DMR1:83651001	1	83651001	83652000	1000	1	3.60E-12	0.51	9	0.9	LOC103691073;Cyp2a3	Metabolism
DMR1:83656001	1	83656001	83657000	1000	1	4.30E-07	0.4	15	1.5	LOC103691073;Cyp2a3	Metabolism
DMR1:83668001	1	83668001	83672000	4000	1	5.70E-08	-0.4	20	0.5	Cyp2a3	Metabolism
DMR1:83730001	1	83730001	83733000	3000	2	2.40E-09	0.63	16	0.53	Cyp2a1	Metabolism
DMR1:83737001	1	83737001	83742000	5000	2	6.30E-15	0.76	40	0.8	Cyp2a2	Metabolism
DMR1:83746001	1	83746001	83748000	2000	1	2.10E-14	0.61	12	0.6	Cyp2a2	Metabolism
DMR1:83750001	1	83750001	83751000	1000	1	8.50E-08	0.36	14	1.4	Cyp2a2	Metabolism
DMR1:83770001	1	83770001	83771000	1000	1	1.30E-08	0.4	9	0.9	Cyp2a2	Metabolism
DMR1:83813001	1	83813001	83814000	1000	1	6.00E-07	-0.52	13	1.3	LOC108349422;Sdccag1-ps1;LOC365218	
DMR1:83822001	1	83822001	83824000	2000	2	3.80E-15	0.71	14	0.7	Sdccag1-ps1;LOC365218	
DMR1:83828001	1	83828001	83832000	4000	2	2.50E-07	0.36	48	1.2	Sdccag1-ps1;LOC365218	
DMR1:83923001	1	83923001	83928000	5000	3	1.60E-17	-0.57	49	0.98	Cyp2f4	
DMR1:83929001	1	83929001	83930000	1000	1	1.80E-12	0.53	15	1.5	Cyp2f4	
DMR1:83931001	1	83931001	83935000	4000	2	4.20E-15	0.62	43	1.07	Cyp2f4	
DMR1:83940001	1	83940001	83943000	3000	2	8.40E-22	0.85	50	1.67	Cyp2f4	
DMR1:83949001	1	83949001	83951000	2000	1	1.10E-15	0.78	31	1.55	Cyp2f4	
DMR1:83970001	1	83970001	83971000	1000	1	1.20E-10	0.44	21	2.1	Cyp2t1;Egln2;Rab4b	Metabolism
DMR1:83975001	1	83975001	83977000	2000	1	2.20E-16	0.71	60	3	Cyp2t1;Egln2;Rab4b	Metabolism
DMR1:83982001	1	83982001	83989000	7000	2	9.80E-29	0.93	98	1.4	Egln2;Rab4b;Mia	
DMR1:84002001	1	84002001	84004000	2000	1	6.00E-08	-0.53	28	1.4	Mia;Snrpa;RGD1560854	Translation
DMR1:84009001	1	84009001	84011000	2000	1	1.40E-12	0.63	20	1	Snrpa;RGD1560854;Itpkc	Translation;Signaling
DMR1:84023001	1	84023001	84025000	2000	1	3.40E-08	-0.5	29	1.45	RGD1560854;Itpkc	Signaling
DMR1:84039001	1	84039001	84040000	1000	1	1.40E-15	0.62	25	2.5	Itpkc;Adck4	Signaling
DMR1:84043001	1	84043001	84047000	4000	1	1.80E-11	0.45	96	2.4	Itpkc;Adck4	Signaling
DMR1:84052001	1	84052001	84055000	3000	2	5.40E-20	0.68	37	1.23	Adck4	
DMR1:84056001	1	84056001	84057000	1000	1	4.00E-08	0.33	14	1.4	Adck4	
DMR1:84073001	1	84073001	84074000	1000	1	1.20E-19	0.59	22	2.2	Adck4;Numbl;LOC100909621	Cytoskeleton
DMR1:84079001	1	84079001	84083000	4000	2	1.80E-23	1.02	54	1.35	Numbl;LOC100909621	Cytoskeleton
DMR1:84112001	1	84112001	84113000	1000	1	3.10E-14	0.53	13	1.3	LOC108348928;Ltbp4	Extracellular Matrix
DMR1:84130001	1	84130001	84131000	1000	1	1.10E-14	0.68	16	1.6	Ltbp4	Extracellular Matrix
DMR1:84134001	1	84134001	84136000	2000	1	7.00E-19	0.51	38	1.9	Ltbp4	Extracellular Matrix
DMR1:84137001	1	84137001	84139000	2000	1	3.70E-07	0.58	73	3.65	Ltbp4	Extracellular Matrix
DMR1:84153001	1	84153001	84157000	4000	2	3.60E-21	0.89	64	1.6	Ltbp4;Shkbp1	Extracellular Matrix;Cytoskeleton
DMR1:84160001	1	84160001	84161000	1000	1	4.80E-15	0.52	17	1.7	Ltbp4;Shkbp1;Sptbn4	Extracellular Matrix;Cytoskeleton
DMR1:84170001	1	84170001	84173000	3000	1	3.10E-21	0.58	44	1.47	Shkbp1;Sptbn4	Cytoskeleton
DMR1:84188001	1	84188001	84191000	3000	1	3.90E-13	0.52	27	0.9	Sptbn4	
DMR1:84195001	1	84195001	84196000	1000	1	8.10E-07	0.49	14	1.4	Sptbn4	
DMR1:84202001	1	84202001	84206000	4000	2	3.40E-21	0.85	54	1.35	Sptbn4	
DMR1:84224001	1	84224001	84228000	4000	2	1.20E-24	1.11	48	1.2	Sptbn4	
DMR1:84232001	1	84232001	84238000	6000	3	2.00E-34	0.88	92	1.53	Sptbn4	
DMR1:84240001	1	84240001	84242000	2000	1	8.80E-17	0.64	27	1.35	Sptbn4	
DMR1:84243001	1	84243001	84245000	2000	1	3.40E-09	0.4	36	1.8	Sptbn4	

DMR1:84261001	1	84261001	84262000	1000	1	2.90E-18	0.63	17	1.7	Sptbn4;Blvrb	Metabolism
DMR1:84265001	1	84265001	84268000	3000	2	1.30E-17	0.8	44	1.47	Blvrb	Metabolism
DMR1:84279001	1	84279001	84280000	1000	1	8.00E-08	-0.63	18	1.8	Blvrb;Sertad3	Metabolism
DMR1:84314001	1	84314001	84315000	1000	1	1.40E-14	0.57	11	1.1	Prx;LOC102552059	
DMR1:84321001	1	84321001	84328000	7000	3	2.30E-14	-0.76	132	1.89	Prx;LOC102552059;Hipk4	
DMR1:84332001	1	84332001	84333000	1000	1	3.80E-08	0.46	15	1.5	Prx;Hipk4;Pld3	Metabolism
DMR1:84345001	1	84345001	84347000	2000	1	1.20E-17	0.67	26	1.3	Hipk4;Pld3	Metabolism
DMR1:84348001	1	84348001	84350000	2000	1	1.40E-07	0.42	33	1.65	Hipk4;Pld3	Metabolism
DMR1:84368001	1	84368001	84370000	2000	1	1.10E-07	-0.66	23	1.15	Pld3;RGD1307554	Metabolism
DMR1:84371001	1	84371001	84376000	5000	1	4.80E-12	0.52	58	1.16	Pld3;RGD1307554	Metabolism
DMR1:84384001	1	84384001	84391000	7000	2	4.00E-15	0.51	90	1.29	RGD1307554;Akt2	Signaling
DMR1:84393001	1	84393001	84395000	2000	2	1.80E-10	0.49	17	0.85	RGD1307554;Akt2	Signaling
DMR1:84415001	1	84415001	84418000	3000	1	7.90E-17	0.54	50	1.67	Akt2	Signaling
DMR1:84424001	1	84424001	84427000	3000	1	3.40E-07	-0.49	42	1.4	Akt2	Signaling
DMR1:84428001	1	84428001	84431000	3000	2	1.70E-19	0.57	37	1.23	Akt2	Signaling
DMR1:84434001	1	84434001	84436000	2000	1	6.30E-21	0.69	29	1.45	Akt2	Signaling
DMR1:84449001	1	84449001	84451000	2000	1	3.50E-08	0.34	38	1.9	Akt2	Signaling
DMR1:84455001	1	84455001	84458000	3000	1	1.30E-09	0.51	48	1.6	Akt2;LOC103691074	Signaling
DMR1:84493001	1	84493001	84496000	3000	1	1.50E-13	0.71	35	1.17	Map3k10	Signaling
DMR1:84666001	1	84666001	84670000	4000	2	3.10E-07	-0.45	21	0.52	LOC100910046;Zfp780b	Transcription
DMR1:84677001	1	84677001	84679000	2000	1	1.80E-10	0.41	26	1.3	Zfp780b	Transcription
DMR1:84687001	1	84687001	84691000	4000	1	5.40E-07	-0.45	27	0.68	Zfp780b;LOC102553565	Transcription
DMR1:84827001	1	84827001	84830000	3000	1	3.30E-07	0.33	42	1.4	Wdr87	
DMR1:84833001	1	84833001	84837000	4000	4	2.00E-18	0.89	23	0.58	Wdr87	
DMR1:84839001	1	84839001	84845000	6000	3	2.10E-14	0.91	54	0.9	Wdr87	
DMR1:84900001	1	84900001	84901000	1000	1	1.70E-13	0.5	11	1.1	Zfp780b-ps1;LOC102549611;LOC108349329	Transcription
DMR1:84936001	1	84936001	84937000	1000	1	8.60E-07	-0.6	9	0.9	Zfp59	
DMR1:84938001	1	84938001	84940000	2000	1	5.30E-07	-0.52	22	1.1	Zfp59	
DMR1:84941001	1	84941001	84945000	4000	1	1.40E-13	0.62	22	0.55	Zfp59	
DMR1:84953001	1	84953001	84955000	2000	2	1.70E-10	0.54	17	0.85	Zfp59	
DMR1:84970001	1	84970001	84971000	1000	1	4.70E-10	0.45	9	0.9	Zfp59;Psmc4	Protease
DMR1:84974001	1	84974001	84975000	1000	1	1.00E-13	0.55	8	0.8	Psmc4	Protease
DMR1:84981001	1	84981001	84986000	5000	1	1.20E-17	0.59	101	2.02	Psmc4;LOC102549879	Protease
DMR1:84995001	1	84995001	8.50E+07	5000	1	6.20E-11	0.51	62	1.24	Psmc4;LOC102549879;Fcgbp	Protease;Extracellular Matrix
DMR1:85002001	1	85002001	85004000	2000	2	4.90E-32	0.92	18	0.9	LOC102549879;Fcgbp	Extracellular Matrix
DMR1:85014001	1	85014001	85015000	1000	1	2.60E-14	0.47	20	2	Fcgbp	Extracellular Matrix
DMR1:85020001	1	85020001	85023000	3000	2	5.30E-21	0.57	69	2.3	Fcgbp	Extracellular Matrix
DMR1:85026001	1	85026001	85029000	3000	2	2.10E-16	0.57	63	2.1	Fcgbp	Extracellular Matrix
DMR1:85030001	1	85030001	85035000	5000	5	5.90E-22	0.79	74	1.48	Fcgbp	Extracellular Matrix
DMR1:85045001	1	85045001	85052000	7000	5	2.80E-11	0.43	110	1.57	Fcgbp;Fcgbpl1	Extracellular Matrix
DMR1:85088001	1	85088001	85092000	4000	2	8.70E-17	0.55	72	1.8	Fcgbpl1	Extracellular Matrix
DMR1:85093001	1	85093001	85102000	9000	3	2.60E-13	0.59	137	1.52	Fcgbpl1;Fbl	Extracellular Matrix;Translation
DMR1:85107001	1	85107001	85109000	2000	2	1.10E-24	0.75	41	2.05	Fbl;Dyrk1b	Translation
DMR1:85114001	1	85114001	85117000	3000	1	2.10E-16	0.61	53	1.77	Fbl;Dyrk1b	Translation
DMR1:85118001	1	85118001	85121000	3000	2	5.80E-29	0.76	76	2.53	Fbl;Dyrk1b	Translation
DMR1:85132001	1	85132001	85133000	1000	1	6.20E-19	0.62	12	1.2	Acp7	Signaling
DMR1:85139001	1	85139001	85141000	2000	1	1.10E-09	0.49	20	1	Acp7	Signaling
DMR1:85147001	1	85147001	85150000	3000	1	7.20E-09	0.42	50	1.67	Acp7	Signaling
DMR1:85165001	1	85165001	85166000	1000	1	4.20E-10	0.39	13	1.3	Acp7;Pak4	Signaling;Signaling
DMR1:85179001	1	85179001	85181000	2000	1	3.60E-07	0.38	30	1.5	Pak4	Signaling
DMR1:85186001	1	85186001	85190000	4000	1	1.30E-12	0.46	45	1.12	Pak4	Signaling
DMR1:85209001	1	85209001	85211000	2000	2	1.20E-09	0.5	3	0.15	Pak4;Nccrp1;Sycn	Signaling
DMR1:85215001	1	85215001	85218000	3000	3	4.10E-27	1.05	58	1.93	Nccrp1;Sycn	
DMR1:85221001	1	85221001	85223000	2000	2	4.20E-18	0.9	24	1.2	Nccrp1;Sycn	
DMR1:85250001	1	85250001	85254000	4000	1	2.70E-14	0.5	46	1.15	Ifnl1	Cytokine
DMR1:85259001	1	85259001	85261000	2000	1	1.90E-21	0.71	12	0.6	Ifnl1	Cytokine
DMR1:85281001	1	85281001	85283000	2000	2	2.00E-10	0.49	19	0.95	Lrfn1	
DMR1:85290001	1	85290001	85292000	2000	1	1.60E-10	0.73	107	5.35	Lrfn1	
DMR1:85300001	1	85300001	85306000	6000	1	2.10E-07	0.4	105	1.75	Lrfn1;Gmfg	Signaling
DMR1:85319001	1	85319001	85320000	1000	1	8.60E-08	-0.5	14	1.4	Gmfg;Samd4b	Signaling
DMR1:85324001	1	85324001	85327000	3000	1	5.10E-15	0.61	75	2.5	Gmfg;Samd4b	Signaling

DMR1:85332001	1	85332001	85333000	1000	1	6.20E-07	0.55	7	0.7	Gmfg;Samd4b	Signaling
DMR1:85354001	1	85354001	85360000	6000	2	1.80E-17	0.75	67	1.12	Samd4b;Paf1;Med29	Transcription
DMR1:85362001	1	85362001	85365000	3000	1	1.20E-14	0.55	42	1.4	Samd4b;Paf1;Med29	Transcription
DMR1:85388001	1	85388001	85389000	1000	1	4.20E-07	0.4	13	1.3	Zfp36;Trnai-uau;Plekhg2	Metabolism
DMR1:85390001	1	85390001	85391000	1000	1	1.20E-07	0.34	19	1.9	Zfp36;Trnai-uau;Plekhg2	Metabolism
DMR1:85399001	1	85399001	85401000	2000	1	8.40E-19	0.8	25	1.25	Plekhg2;Rps16;LOC102550060	Translation
DMR1:85402001	1	85402001	85407000	5000	1	6.00E-07	-0.62	70	1.4	Plekhg2;Rps16;LOC102550060	Translation
DMR1:85411001	1	85411001	85414000	3000	2	1.90E-19	0.92	38	1.27	Rps16;LOC102550060	Translation
DMR1:85432001	1	85432001	85435000	3000	2	7.30E-11	0.48	52	1.73	LOC685751;LOC108349843;Supt5h	
DMR1:85446001	1	85446001	85449000	3000	2	6.50E-09	0.44	35	1.17	Supt5h	
DMR1:85450001	1	85450001	85452000	2000	2	2.10E-08	-0.47	22	1.1	Supt5h	
DMR1:85453001	1	85453001	85459000	6000	2	5.70E-22	1.16	59	0.98	Supt5h	
DMR1:85462001	1	85462001	85465000	3000	1	1.80E-13	0.47	48	1.6	Supt5h;Timm50	
DMR1:85522001	1	85522001	85523000	1000	1	2.10E-17	0.67	9	0.9	Eid2	
DMR1:85524001	1	85524001	85526000	2000	1	1.10E-20	0.62	32	1.6	Eid2	
DMR1:85549001	1	85549001	85550000	1000	1	1.40E-07	0.54	10	1	Nccrp1	
DMR1:85557001	1	85557001	85558000	1000	1	8.30E-16	0.97	12	1.2	Nccrp1;LOC100911458	
DMR1:86013001	1	86013001	86018000	5000	2	2.20E-10	-0.63	39	0.78	LOC690191;Vom1r-ps4	
DMR1:86048001	1	86048001	86050000	2000	2	5.10E-09	-0.46	16	0.8	Vom1r-ps5	
DMR1:86051001	1	86051001	86055000	4000	2	6.90E-22	-0.58	54	1.35	Vom1r-ps5	
DMR1:86057001	1	86057001	86058000	1000	1	2.40E-09	0.6	4	0.4	Vom1r-ps5	
DMR1:86079001	1	86079001	86084000	5000	2	6.50E-10	0.55	37	0.74	Fpr-rs4	Signaling
DMR1:86095001	1	86095001	86097000	2000	2	1.30E-13	-0.6	9	0.45	Vom2r10	Signaling
DMR1:86110001	1	86110001	86111000	1000	1	5.00E-16	0.83	6	0.6	Vom2r10	Signaling
DMR1:86117001	1	86117001	86120000	3000	1	8.30E-08	-0.34	20	0.67	Vom2r10	Signaling
DMR1:86121001	1	86121001	86122000	1000	1	1.30E-14	0.68	6	0.6	Vom2r10	Signaling
DMR1:86125001	1	86125001	86126000	1000	1	1.70E-07	0.5	4	0.4	Vom2r10	Signaling
DMR1:86132001	1	86132001	86142000	10000	4	4.30E-13	-0.46	92	0.92	Vom2r10	Signaling
DMR1:86275001	1	86275001	86276000	1000	1	2.30E-07	0.58	2	0.2	Vom1r-ps6	
DMR1:86281001	1	86281001	86282000	1000	1	7.80E-07	-0.58	6	0.6	Vom1r-ps6	
DMR1:86299001	1	86299001	86300000	1000	1	4.60E-09	0.71	5	0.5	Vom2r-ps22	
DMR1:86316001	1	86316001	86321000	5000	2	4.30E-11	-0.61	25	0.5	Vom2r-ps22	
DMR1:86335001	1	86335001	86339000	4000	2	2.50E-13	0.63	21	0.52	Vom2r-ps22	
DMR1:86352001	1	86352001	86355000	3000	2	1.70E-08	0.46	7	0.23	Vom1r2	Receptor
DMR1:86369001	1	86369001	86370000	1000	1	3.80E-07	0.42	8	0.8	Vom1r2	Receptor
DMR1:86522001	1	86522001	86524000	2000	1	7.40E-10	0.53	5	0.25	Vom1r4	Receptor
DMR1:86615001	1	86615001	86616000	1000	1	7.60E-11	0.62	3	0.3	Vom2r11	Signaling
DMR1:86617001	1	86617001	86618000	1000	1	5.00E-13	0.77	5	0.5	Vom2r11	Signaling
DMR1:86628001	1	86628001	86633000	5000	1	8.50E-07	-0.41	33	0.66	Vom2r11	Signaling
DMR1:86645001	1	86645001	86646000	1000	1	1.00E-11	0.55	15	1.5	LOC681853;Vom1r-ps8	
DMR1:86648001	1	86648001	86650000	2000	2	1.10E-07	0.58	11	0.55	LOC681853;Vom1r-ps8	
DMR1:86656001	1	86656001	86659000	3000	1	4.00E-08	0.44	23	0.77	Vom1r-ps8	
DMR1:86683001	1	86683001	86687000	4000	2	2.60E-20	-0.56	40	1	Vom1r5	
DMR1:86774001	1	86774001	86775000	1000	1	1.30E-07	-0.41	18	1.8	Vom2r7;LOC102549906	
DMR1:86863001	1	86863001	86869000	6000	3	4.10E-26	1.01	67	1.12	LOC108349512;Fbxo27	
DMR1:86886001	1	86886001	86887000	1000	1	1.50E-08	0.58	7	0.7	Fbxo27	
DMR1:86892001	1	86892001	86894000	2000	1	1.60E-08	0.43	28	1.4	Fbxo17	
DMR1:86901001	1	86901001	86902000	1000	1	1.30E-11	0.43	16	1.6	Fbxo17	
DMR1:86911001	1	86911001	86913000	2000	1	8.80E-15	0.58	21	1.05	Fbxo17;Mrps12	Translation
DMR1:86916001	1	86916001	86917000	1000	1	2.30E-07	-0.59	14	1.4	Fbxo17;Mrps12;Sars2;LOC102550248	Translation;Translation
DMR1:86918001	1	86918001	86919000	1000	1	5.20E-09	0.45	17	1.7	Fbxo17;Mrps12;Sars2;LOC102550248	Translation;Translation
DMR1:86925001	1	86925001	86931000	6000	1	2.60E-28	0.85	108	1.8	Fbxo17;Mrps12;Sars2;LOC102550248;Ccer2	Translation;Translation
DMR1:86933001	1	86933001	86937000	4000	2	3.30E-30	1.16	52	1.3	Mrps12;Sars2;LOC102550248;Ccer2;Nfkbib	Translation;Translation
DMR1:86938001	1	86938001	86942000	4000	2	1.10E-14	0.46	94	2.35	Sars2;LOC102550248;Ccer2;Nfkbib;Sirt2	Translation
DMR1:86943001	1	86943001	86947000	4000	1	1.80E-15	0.57	45	1.12	Sars2;Ccer2;Nfkbib;Sirt2	Translation
DMR1:86956001	1	86956001	86959000	3000	1	9.90E-10	0.47	43	1.43	Nfkbib;Sirt2	
DMR1:86973001	1	86973001	86975000	2000	1	3.90E-09	0.51	22	1.1	Sirt2;Rinl	Transcription
DMR1:86982001	1	86982001	86984000	2000	1	3.90E-10	0.36	29	1.45	Rinl	Transcription
DMR1:86999001	1	86999001	8.70E+07	1000	1	7.50E-08	-0.48	12	1.2	Hnrnpl;Ech1	Metabolism

DMR1:87001001	1	87001001	87004000	3000	1	1.60E-08	0.43	74	2.47	Hnrnpl;Ech1	Metabolism
DMR1:87010001	1	87010001	87011000	1000	1	2.10E-07	0.36	18	1.8	Hnrnpl;Ech1;Lgals4	Metabolism;Extracellular Matrix
DMR1:87018001	1	87018001	87019000	1000	1	1.30E-09	0.42	18	1.8	Ech1;Lgals4	Metabolism;Extracellular Matrix
DMR1:87021001	1	87021001	87023000	2000	2	5.90E-18	0.51	26	1.3	Ech1;Lgals4	Metabolism;Extracellular Matrix
DMR1:87026001	1	87026001	87030000	4000	1	1.60E-11	0.54	53	1.32	Lgals4	Extracellular Matrix
DMR1:87033001	1	87033001	87036000	3000	2	9.50E-14	0.48	51	1.7	Lgals4;Lgals7	Extracellular Matrix
DMR1:87042001	1	87042001	87044000	2000	2	3.30E-07	-0.38	32	1.6	Lgals7	Extracellular Matrix
DMR1:87050001	1	87050001	87052000	2000	1	1.40E-07	-0.47	31	1.55	Lgals7	Extracellular Matrix
DMR1:87054001	1	87054001	87057000	3000	1	3.60E-08	0.46	30	1	Lgals7;Capn12;LOC102550499	Extracellular Matrix;Protease
DMR1:87061001	1	87061001	87063000	2000	2	1.80E-13	0.54	17	0.85	Capn12;LOC102550499	Protease
DMR1:87064001	1	87064001	87066000	2000	1	1.30E-17	0.64	36	1.8	Capn12;LOC102550499	Protease
DMR1:87078001	1	87078001	87083000	5000	1	1.10E-12	0.47	97	1.94	Capn12;LOC102550499;Actn4	Protease
DMR1:87108001	1	87108001	87109000	1000	1	3.20E-13	0.54	8	0.8	Actn4	
DMR1:87122001	1	87122001	87123000	1000	1	6.40E-15	0.72	10	1	Actn4	
DMR1:87137001	1	87137001	87139000	2000	1	3.80E-07	0.35	25	1.25	Actn4;LOC102550585	
DMR1:87144001	1	87144001	87147000	3000	2	9.80E-31	0.83	55	1.83	Actn4;LOC102550585	
DMR1:87148001	1	87148001	87151000	3000	1	1.00E-21	1	54	1.8	Actn4;LOC102550585	
DMR1:87155001	1	87155001	87156000	1000	1	1.10E-24	1.23	40	4	Actn4;LOC102550585	
DMR1:87157001	1	87157001	87160000	3000	2	1.90E-12	0.66	71	2.37	Actn4;LOC102550585;LOC100909725	
DMR1:87211001	1	87211001	87213000	2000	1	4.00E-19	0.63	22	1.1	Spint2	Protease; Proteolysis
DMR1:87229001	1	87229001	87230000	1000	1	8.70E-13	0.46	20	2	Spint2;Ppp1r14a	Protease; Proteolysis;Signaling
DMR1:87231001	1	87231001	87234000	3000	1	4.70E-10	0.41	66	2.2	Spint2;Ppp1r14a	Protease; Proteolysis;Signaling
DMR1:87247001	1	87247001	87248000	1000	1	3.90E-12	0.49	15	1.5	Dpf1	Epigenetic
DMR1:87251001	1	87251001	87252000	1000	1	2.00E-07	-0.43	17	1.7	Dpf1;Sipa1l3	Epigenetic;Signaling
DMR1:87264001	1	87264001	87267000	3000	1	6.40E-16	0.7	54	1.8	Dpf1;Sipa1l3	Epigenetic;Signaling
DMR1:87272001	1	87272001	87277000	5000	2	1.70E-08	0.44	57	1.14	Sipa1l3	Signaling
DMR1:87301001	1	87301001	87303000	2000	2	2.20E-23	0.73	34	1.7	Sipa1l3	Signaling
DMR1:87308001	1	87308001	87311000	3000	1	5.60E-12	0.6	43	1.43	Sipa1l3	Signaling
DMR1:87312001	1	87312001	87320000	8000	2	4.50E-08	-0.49	162	2.02	Sipa1l3	Signaling
DMR1:87360001	1	87360001	87362000	2000	1	8.70E-13	0.41	35	1.75	Sipa1l3	Signaling
DMR1:87442001	1	87442001	87443000	1000	1	9.30E-12	0.43	13	1.3	Sipa1l3	Signaling
DMR1:87450001	1	87450001	87451000	1000	1	1.20E-11	0.56	25	2.5	Sipa1l3	Signaling
DMR1:87458001	1	87458001	87459000	1000	1	1.10E-16	0.51	21	2.1	Sipa1l3	Signaling
DMR1:87460001	1	87460001	87464000	4000	1	6.20E-24	0.69	72	1.8	Sipa1l3	Signaling
DMR1:87466001	1	87466001	87467000	1000	1	1.40E-08	0.39	18	1.8	Sipa1l3	Signaling
DMR1:87644001	1	87644001	87645000	1000	1	1.60E-09	0.52	27	2.7	Zfp84	Transcription
DMR1:87649001	1	87649001	87651000	2000	1	1.60E-07	-0.6	28	1.4	Zfp84	Transcription
DMR1:87654001	1	87654001	87660000	6000	2	5.90E-20	0.64	127	2.12	Zfp84;LOC102547413	Transcription
DMR1:87893001	1	87893001	87894000	1000	1	4.20E-13	0.79	21	2.1	LOC102547059;Zfp569	
DMR1:87917001	1	87917001	87920000	3000	1	7.80E-10	0.46	37	1.23	LOC102547059;Eif3k	Translation
DMR1:87938001	1	87938001	87944000	6000	2	8.50E-13	0.48	73	1.22	Eif3k;Map4k1	Translation
DMR1:87947001	1	87947001	87948000	1000	1	1.60E-08	0.62	13	1.3	Eif3k;Map4k1	Translation
DMR1:87951001	1	87951001	87954000	3000	2	8.60E-11	0.44	35	1.17	Map4k1;Ryr1	Ion Channel
DMR1:87963001	1	87963001	87969000	6000	1	1.50E-27	0.86	97	1.62	Map4k1;Ryr1	Ion Channel
DMR1:87970001	1	87970001	87972000	2000	2	3.40E-12	0.68	21	1.05	Ryr1	Ion Channel
DMR1:87977001	1	87977001	87980000	3000	1	8.50E-07	0.32	84	2.8	Ryr1	Ion Channel
DMR1:87984001	1	87984001	87989000	5000	2	4.00E-34	1.33	101	2.02	Ryr1	Ion Channel
DMR1:87999001	1	87999001	88001000	2000	2	5.70E-16	0.6	36	1.8	Ryr1	Ion Channel
DMR1:88002001	1	88002001	88006000	4000	2	3.90E-19	0.71	106	2.65	Ryr1	Ion Channel
DMR1:88007001	1	88007001	88010000	3000	1	4.10E-08	0.4	79	2.63	Ryr1	Ion Channel
DMR1:88017001	1	88017001	88018000	1000	1	3.60E-07	-0.41	8	0.8	Ryr1	Ion Channel
DMR1:88020001	1	88020001	88021000	1000	1	2.60E-19	0.9	22	2.2	Ryr1	Ion Channel
DMR1:88023001	1	88023001	88025000	2000	1	2.10E-14	0.74	28	1.4	Ryr1	Ion Channel
DMR1:88028001	1	88028001	88033000	5000	1	1.80E-15	0.61	92	1.84	Ryr1	Ion Channel
DMR1:88034001	1	88034001	88036000	2000	1	3.90E-21	0.75	24	1.2	Ryr1	Ion Channel
DMR1:88041001	1	88041001	88044000	3000	1	8.40E-07	0.33	58	1.93	Ryr1	Ion Channel
DMR1:88048001	1	88048001	88051000	3000	1	5.00E-09	0.48	70	2.33	Ryr1	Ion Channel

DMR1:88057001	1	88057001	88064000	7000	5	4.30E-21	0.76	115	1.64	Ryr1	Ion Channel
DMR1:88076001	1	88076001	88079000	3000	1	2.00E-09	0.33	53	1.77	Ryr1;Rasgrp4	Ion Channel;Transcription
DMR1:88080001	1	88080001	88082000	2000	1	8.50E-26	0.62	35	1.75	Rasgrp4	Transcription
DMR1:88085001	1	88085001	88086000	1000	1	6.20E-20	0.58	16	1.6	Rasgrp4;Fam98c	Transcription;Translation
DMR1:88091001	1	88091001	88098000	7000	1	5.70E-22	0.89	107	1.53	Rasgrp4;Fam98c;Spred3	Transcription;Translation;Cytoskeleton
DMR1:88109001	1	88109001	88111000	2000	1	8.30E-15	0.74	36	1.8	Spred3;Ggn;Psmd8	Cytoskeleton;Protease
DMR1:88134001	1	88134001	88136000	2000	1	3.10E-17	0.63	31	1.55	LOC100910426;Catsperg1	
DMR1:88150001	1	88150001	88152000	2000	1	1.30E-24	1.02	44	2.2	Catsperg1	
DMR1:88157001	1	88157001	88158000	1000	1	1.50E-25	1.24	43	4.3	Catsperg1	
DMR1:88159001	1	88159001	88162000	3000	1	1.30E-11	0.62	70	2.33	Catsperg1;Kcnk6	Transport
DMR1:88183001	1	88183001	88187000	4000	2	5.80E-12	0.55	69	1.73	Kcnk6;Yif1b;LOC100361913;LOC103691097	Transport
DMR1:88188001	1	88188001	88191000	3000	2	3.90E-14	0.82	47	1.57	Yif1b;LOC100361913;LOC103691097	
DMR1:88198001	1	88198001	88200000	2000	1	1.90E-08	0.4	60	3	Yif1b;LOC100361913;LOC103691097;LOC103690163	Transcription
DMR1:88229001	1	88229001	88230000	1000	1	6.00E-07	-0.52	15	1.5	Zfp74	
DMR1:88251001	1	88251001	88255000	4000	2	1.00E-12	0.66	47	1.18	Zfp74;LOC100359523	
DMR1:88339001	1	88339001	88341000	2000	1	9.20E-12	0.54	25	1.25	LOC108348102;Zfp14	
DMR1:88416001	1	88416001	88417000	1000	1	2.70E-07	-0.5	26	2.6	Zfp566	Transcription
DMR1:88422001	1	88422001	88423000	1000	1	1.90E-26	0.91	11	1.1	Zfp566	Transcription
DMR1:88468001	1	88468001	88470000	2000	2	6.90E-21	0.84	31	1.55	Zfp260	
DMR1:88521001	1	88521001	88522000	1000	1	2.60E-17	0.7	9	0.9	LOC100912787;Zfp420	
DMR1:88531001	1	88531001	88532000	1000	1	8.50E-13	1.03	8	0.8	LOC100912787;Zfp420	
DMR1:88549001	1	88549001	88550000	1000	1	5.40E-10	0.47	16	1.6	Zfp420;LOC102550277	
DMR1:88582001	1	88582001	88583000	1000	1	4.00E-07	-0.48	16	1.6	Zfp382	
DMR1:88588001	1	88588001	88591000	3000	2	1.20E-09	0.42	61	2.03	Zfp382	
DMR1:88593001	1	88593001	88595000	2000	2	6.70E-17	0.61	34	1.7	Zfp382;LOC100912964	
DMR1:88602001	1	88602001	88604000	2000	1	2.40E-08	-0.47	36	1.8	Zfp382;LOC100912964	
DMR1:88609001	1	88609001	88612000	3000	1	6.80E-10	0.54	50	1.67	Zfp382;LOC100912964;Cox7a1	
DMR1:88622001	1	88622001	88623000	1000	1	1.80E-10	0.42	23	2.3	Cox7a1;LOC100912380;LOC108349558	
DMR1:88682001	1	88682001	88683000	1000	1	8.20E-08	0.46	23	2.3	Tbcb;Polr2i;LOC100912425	Transcription
DMR1:88700001	1	88700001	88703000	3000	2	1.50E-13	0.61	37	1.23	LOC100912425;Wdr62	
DMR1:88711001	1	88711001	88714000	3000	1	6.40E-07	0.39	42	1.4	Wdr62	
DMR1:88748001	1	88748001	88750000	2000	2	2.70E-11	0.53	36	1.8	Thap8;Clip3	Transcription
DMR1:88751001	1	88751001	88752000	1000	1	1.90E-11	0.53	18	1.8	Thap8;Clip3	Transcription
DMR1:88753001	1	88753001	88755000	2000	1	2.70E-08	0.43	45	2.25	Thap8;Clip3	Transcription
DMR1:88771001	1	88771001	88772000	1000	1	1.10E-14	0.56	23	2.3	Clip3;Alkbh6;Syne4;RGD1562079	Transcription;Metabolism
DMR1:88775001	1	88775001	88777000	2000	1	2.30E-13	0.55	37	1.85	Clip3;Alkbh6;Syne4;RGD1562079	Transcription;Metabolism
DMR1:88782001	1	88782001	88784000	2000	1	3.40E-12	0.61	19	0.95	Alkbh6;Syne4;RGD1562079	Metabolism
DMR1:88787001	1	88787001	88788000	1000	1	7.40E-08	0.42	22	2.2	Syne4;RGD1562079	
DMR1:88789001	1	88789001	88793000	4000	2	1.30E-10	0.62	53	1.32	RGD1562079	
DMR1:88813001	1	88813001	88819000	6000	4	1.70E-16	0.71	93	1.55	Lrfn3	
DMR1:88820001	1	88820001	88824000	4000	4	1.80E-18	0.58	87	2.17	Lrfn3	
DMR1:88825001	1	88825001	88826000	1000	1	1.40E-26	0.9	9	0.9	Lrfn3	
DMR1:88872001	1	88872001	88876000	4000	1	1.60E-08	0.38	65	1.62	LOC688666;Tyrobp;Hcst;Nfkbid	Cytoskeleton;Signaling
DMR1:88877001	1	88877001	88879000	2000	1	2.50E-08	0.49	29	1.45	Tyrobp;Hcst;Nfkbid	Cytoskeleton;Signaling
DMR1:88880001	1	88880001	88882000	2000	1	6.30E-14	0.5	33	1.65	Tyrobp;Hcst;Nfkbid	Cytoskeleton;Signaling
DMR1:88889001	1	88889001	88893000	4000	1	5.30E-07	0.34	70	1.75	Tyrobp;Hcst;Nfkbid;Aplp1	Cytoskeleton;Signaling;Protease; Proteolysis
DMR1:88900001	1	88900001	88902000	2000	1	1.40E-12	0.53	28	1.4	Nfkbid;Aplp1;Kirrel2	Signaling;Protease; Proteolysis

DMR1:88911001	1	88911001	88915000	4000	2	3.10E-28	0.83	61	1.52	Aplp1;Kirrel2;Nphs1	Protease; Proteolysis
DMR1:88921001	1	88921001	88923000	2000	2	2.00E-19	0.68	34	1.7	Kirrel2;Nphs1	
DMR1:88924001	1	88924001	88928000	4000	2	1.80E-10	0.39	55	1.38	Kirrel2;Nphs1	
DMR1:88930001	1	88930001	88934000	4000	3	2.80E-21	0.67	78	1.95	Kirrel2;Nphs1	
DMR1:88941001	1	88941001	88944000	3000	1	7.10E-14	0.61	54	1.8	Nphs1	
DMR1:88966001	1	88966001	88967000	1000	1	6.30E-07	0.43	6	0.6	Prodh2;RGD1560986	Metabolism
DMR1:88971001	1	88971001	88972000	1000	1	3.70E-09	0.32	17	1.7	Prodh2;RGD1560986;Arhgap33	Metabolism;Signaling
DMR1:88978001	1	88978001	88981000	3000	2	8.00E-31	0.88	62	2.07	Prodh2;RGD1560986;Arhgap33	Metabolism;Signaling
DMR1:88986001	1	88986001	88991000	5000	1	9.40E-11	0.55	92	1.84	RGD1560986;Arhgap33;Proser3	Signaling
DMR1:89026001	1	89026001	89027000	1000	1	1.20E-07	0.44	6	0.6	Psenen;U2af1l4;Igflr1;Kmt2b	Translation;Receptor;Epigenetic
DMR1:89038001	1	89038001	89040000	2000	1	1.10E-25	0.7	44	2.2	Kmt2b;Mir3569;Zbtb32	Epigenetic;Transcription
DMR1:89053001	1	89053001	89054000	1000	1	4.80E-22	1.06	21	2.1	Zbtb32;Upk1a	Transcription
DMR1:89058001	1	89058001	89061000	3000	1	2.60E-11	0.53	42	1.4	Zbtb32;Upk1a	Transcription
DMR1:89107001	1	89107001	89108000	1000	1	3.90E-07	0.44	15	1.5	Rbm42;Haus5	
DMR1:89122001	1	89122001	89125000	3000	2	4.00E-11	0.59	57	1.9	Haus5;LOC100912333;LOC688924	
DMR1:89126001	1	89126001	89133000	7000	2	5.60E-17	0.65	84	1.2	Haus5;LOC100912333;LOC688924	
DMR1:89161001	1	89161001	89165000	4000	2	5.00E-21	0.78	60	1.5	LOC688924;Atp4a	Transport
DMR1:89184001	1	89184001	89189000	5000	1	1.10E-13	0.67	76	1.52	Atp4a;Tmem147;Gapdhs	Transport;Metabolism
DMR1:89201001	1	89201001	89206000	5000	3	6.80E-14	0.53	68	1.36	Gapdhs;Sbsn;Dmkn	Metabolism
DMR1:89210001	1	89210001	89216000	6000	2	3.90E-16	0.67	89	1.48	Sbsn;Dmkn	
DMR1:89224001	1	89224001	89226000	2000	1	6.30E-08	0.37	32	1.6	Dmkn;Krtdap	
DMR1:89261001	1	89261001	89264000	3000	2	1.80E-20	0.84	45	1.5	LOC100361079;Ffar2	Translation
DMR1:89265001	1	89265001	89267000	2000	1	9.00E-09	0.59	37	1.85	LOC100361079;Ffar2	Translation
DMR1:89271001	1	89271001	89272000	1000	1	1.60E-22	0.75	20	2	Ffar2;LOC102554169	
DMR1:89276001	1	89276001	89278000	2000	1	9.00E-17	0.67	23	1.15	Ffar2;LOC102554169	
DMR1:89294001	1	89294001	89297000	3000	1	2.80E-15	0.7	43	1.43	LOC102554169;Ffar3	
DMR1:89299001	1	89299001	89302000	3000	1	2.10E-11	0.48	47	1.57	LOC102554169;Ffar3;Ffar1	
DMR1:89310001	1	89310001	89314000	4000	1	1.20E-09	0.35	72	1.8	LOC102554169;Ffar3;Ffar1;Cd22	
DMR1:89320001	1	89320001	89323000	3000	1	1.80E-24	0.81	43	1.43	Cd22	
DMR1:89330001	1	89330001	89335000	5000	1	5.60E-11	0.45	64	1.28	Cd22;LOC102554355	
DMR1:89336001	1	89336001	89343000	7000	1	9.40E-18	0.76	97	1.39	Cd22;LOC102554355;Mag;LOC108349549	Immune
DMR1:89344001	1	89344001	89345000	1000	1	5.60E-10	0.6	6	0.6	LOC102554355;Mag;LOC108349549	Immune
DMR1:89375001	1	89375001	89381000	6000	2	4.50E-13	0.68	73	1.22	Hamp;Usf2;Lsr	Immune
DMR1:89426001	1	89426001	89429000	3000	3	7.40E-17	0.66	34	1.13	Fam187b	Immune
DMR1:89467001	1	89467001	89468000	1000	1	8.70E-07	0.31	15	1.5	Fxyd5;Fxyd7	Transport
DMR1:89474001	1	89474001	89481000	7000	2	5.10E-16	0.56	118	1.69	Fxyd5;Fxyd7;Fxyd1	Transport
DMR1:89485001	1	89485001	89487000	2000	1	9.80E-07	0.51	58	2.9	Fxyd7;Fxyd1;Lgi4	Transport
DMR1:89488001	1	89488001	89493000	5000	1	5.00E-07	0.31	83	1.66	Fxyd7;Fxyd1;Lgi4;Fxyd3	Transport
DMR1:89494001	1	89494001	89498000	4000	1	9.70E-20	0.6	55	1.38	Fxyd7;Fxyd1;Lgi4;Fxyd3	Transport
DMR1:89502001	1	89502001	89504000	2000	1	1.90E-08	0.39	35	1.75	Lgi4;Fxyd3	Transport
DMR1:89505001	1	89505001	89507000	2000	1	2.00E-21	0.79	24	1.2	Lgi4;Fxyd3	Transport
DMR1:89508001	1	89508001	89510000	2000	1	4.50E-18	0.59	25	1.25	Lgi4;Fxyd3	Transport
DMR1:89515001	1	89515001	89517000	2000	1	4.20E-07	0.36	20	1	Fxyd3	Transport
DMR1:89520001	1	89520001	89522000	2000	2	5.00E-29	0.95	20	1	Fxyd3	Transport
DMR1:89539001	1	89539001	89543000	4000	3	4.30E-13	0.52	78	1.95	Hpn;Scn1b	Protease;Transport
DMR1:89572001	1	89572001	89574000	2000	1	9.40E-15	0.53	54	2.7	Gramd1a	
DMR1:89578001	1	89578001	89582000	4000	4	2.40E-20	0.76	59	1.48	Gramd1a	
DMR1:89624001	1	89624001	89626000	2000	2	5.40E-13	0.56	19	0.95	LOC108349560;RGD1561430	
DMR1:89630001	1	89630001	89631000	1000	1	2.80E-09	0.41	19	1.9	LOC108349560;RGD1561430	
DMR1:89633001	1	89633001	89634000	1000	1	4.10E-09	0.42	13	1.3	LOC108349560;RGD1561430	
DMR1:89638001	1	89638001	89643000	5000	2	3.40E-09	-0.61	52	1.04	LOC108349560;RGD1561430;RGD1563307	Epigenetic
DMR1:89651001	1	89651001	89653000	2000	1	1.40E-07	0.49	41	2.05	RGD1563307	Epigenetic

DMR1:89711001	1	89711001	89717000	6000	2	6.40E-14	0.6	56	0.93	Scgb1b24;Scgb2b2	
DMR1:89728001	1	89728001	89730000	2000	1	4.10E-13	0.47	31	1.55	Scgb1b24;Scgb2b2	
DMR1:89733001	1	89733001	89735000	2000	1	2.20E-08	-0.4	22	1.1	Scgb2b2	
DMR1:89783001	1	89783001	89784000	1000	1	4.10E-10	0.52	8	0.8	Scgb2b24;Apbh	
DMR1:89788001	1	89788001	89791000	3000	1	5.70E-11	-0.4	32	1.07	Scgb2b24;Apbh	
DMR1:89795001	1	89795001	89796000	1000	1	1.10E-07	-0.65	6	0.6	Apbh	
DMR1:89958001	1	89958001	89967000	9000	2	6.80E-13	-0.75	119	1.32	Scgb1b30;Wtip	
DMR1:89974001	1	89974001	89979000	5000	1	2.90E-10	-0.45	56	1.12	Wtip	
DMR1:89982001	1	89982001	89985000	3000	1	4.60E-08	-0.44	35	1.17	Wtip	
DMR1:89986001	1	89986001	89987000	1000	1	2.70E-08	-0.37	5	0.5	Wtip	
DMR1:89989001	1	89989001	89991000	2000	2	3.90E-21	0.79	23	1.15	Wtip	
DMR1:89996001	1	89996001	89998000	2000	1	2.40E-07	0.38	14	0.7	Wtip	
DMR1:90003001	1	90003001	90005000	2000	1	1.40E-17	0.88	17	0.85	Wtip;Uba2	Proteolysis
DMR1:90006001	1	90006001	90008000	2000	1	6.00E-07	0.57	18	0.9	Wtip;Uba2	Proteolysis
DMR1:90019001	1	90019001	90020000	1000	1	3.80E-18	0.69	10	1	Uba2	Proteolysis
DMR1:90043001	1	90043001	90047000	4000	1	3.90E-07	0.35	41	1.02	Uba2;Pdcd2l	Proteolysis
DMR1:90074001	1	90074001	90076000	2000	1	1.50E-12	0.41	41	2.05	Gpi	Metabolism
DMR1:90086001	1	90086001	90089000	3000	1	7.30E-08	0.35	59	1.97	Gpi;RGD1308428	Metabolism
DMR1:90111001	1	90111001	90113000	2000	1	1.20E-07	0.52	12	0.6	RGD1308428	
DMR1:90124001	1	90124001	90126000	2000	1	1.20E-07	0.44	20	1	RGD1308428;LOC108349721	
DMR1:90150001	1	90150001	90151000	1000	1	4.60E-08	0.48	7	0.7	RGD1308428	
DMR1:90166001	1	90166001	90167000	1000	1	1.90E-11	0.77	7	0.7	RGD1308428	
DMR1:90185001	1	90185001	90187000	2000	1	4.60E-13	0.73	27	1.35	RGD1308428	
DMR1:90207001	1	90207001	90208000	1000	1	2.80E-10	0.37	11	1.1	Lsm14a	Metabolism
DMR1:90210001	1	90210001	90211000	1000	1	2.80E-07	0.4	18	1.8	Lsm14a	Metabolism
DMR1:90253001	1	90253001	90259000	6000	2	1.10E-14	0.7	71	1.18	Lsm14a	Metabolism
DMR1:90536001	1	90536001	90539000	3000	2	1.90E-22	0.69	54	1.8	Chst8	Transport
DMR1:90558001	1	90558001	90559000	1000	1	3.50E-25	0.82	28	2.8	Chst8;LOC108349467	Transport
DMR1:90561001	1	90561001	90563000	2000	1	3.30E-25	0.71	47	2.35	Chst8;LOC108349467;LOC108349466	Transport
DMR1:90564001	1	90564001	90566000	2000	1	1.40E-07	-0.49	35	1.75	Chst8;LOC108349467;LOC108349466	Transport
DMR1:90568001	1	90568001	90574000	6000	2	2.50E-12	0.65	83	1.38	Chst8;LOC108349467;LOC108349466	Transport
DMR1:90579001	1	90579001	90580000	1000	1	1.90E-13	0.45	20	2	Chst8;LOC108349467;LOC108349466	Transport
DMR1:90581001	1	90581001	90584000	3000	1	2.10E-09	0.52	35	1.17	Chst8;LOC108349467;LOC108349466	Transport
DMR1:90594001	1	90594001	90595000	1000	1	6.50E-14	0.79	11	1.1	Chst8	Transport
DMR1:90609001	1	90609001	90611000	2000	1	3.50E-08	0.38	32	1.6	Chst8	Transport
DMR1:90641001	1	90641001	90646000	5000	1	3.00E-08	-0.57	97	1.94	Chst8	Transport
DMR1:90650001	1	90650001	90657000	7000	3	2.10E-09	-0.67	92	1.31	Chst8	Transport
DMR1:90671001	1	90671001	90673000	2000	2	1.20E-10	0.48	40	2	Chst8	Transport
DMR1:90694001	1	90694001	90701000	7000	1	2.80E-07	-0.5	109	1.56	Chst8	Transport
DMR1:90716001	1	90716001	90720000	4000	2	3.60E-16	0.55	49	1.23	RGD1560088	
DMR1:90721001	1	90721001	90722000	1000	1	5.40E-08	-0.66	16	1.6	RGD1560088	
DMR1:90732001	1	90732001	90734000	2000	1	8.30E-09	0.38	25	1.25	RGD1560088	
DMR1:90815001	1	90815001	90817000	2000	2	2.70E-20	0.76	19	0.95	LOC103690062;Pepd	Protease
DMR1:90836001	1	90836001	90843000	7000	4	4.00E-21	-0.58	63	0.9	Pepd	Protease
DMR1:90856001	1	90856001	90858000	2000	1	4.70E-08	0.42	24	1.2	Pepd	Protease
DMR1:90877001	1	90877001	90880000	3000	1	8.60E-28	0.75	31	1.03	Pepd	Protease
DMR1:90893001	1	90893001	90896000	3000	1	1.90E-13	0.64	35	1.17	Pepd	Protease
DMR1:90908001	1	90908001	90911000	3000	1	2.30E-08	0.57	23	0.77	Pepd;LOC108348197	Protease
DMR1:90937001	1	90937001	90940000	3000	2	2.50E-13	0.61	44	1.47	Pepd;LOC108348111	Protease
DMR1:90941001	1	90941001	90943000	2000	1	3.40E-10	0.53	56	2.8	Pepd;LOC108348111;LOC108348128	Protease
DMR1:90945001	1	90945001	90947000	2000	1	3.40E-08	0.61	17	0.85	Pepd;LOC108348111;LOC108348128	Protease
DMR1:90953001	1	90953001	90955000	2000	2	1.70E-08	0.43	33	1.65	Pepd;LOC108348111;LOC108348128;LOC108348106;LOC108348122	Protease
DMR1:90957001	1	90957001	90960000	3000	1	1.40E-09	0.47	69	2.3	Pepd;LOC108348111;LOC108348128;LOC108348106;LOC108348122	Protease

DMR1:90964001	1	90964001	90965000	1000	1	2.00E-11	0.43	30	3	Pepd;LOC108348128;LOC108348106;LOC108348122	Protease
DMR1:90975001	1	90975001	90978000	3000	1	2.40E-20	0.63	56	1.87	Pepd;LOC108348122;LOC102548322	Protease
DMR1:90980001	1	90980001	90981000	1000	1	3.70E-16	0.56	25	2.5	Pepd;LOC108348122;LOC102548322	Protease
DMR1:91027001	1	91027001	91030000	3000	1	7.30E-10	0.5	31	1.03	Pepd;Wdr62;Ovol3	Protease
DMR1:91046001	1	91046001	91047000	1000	1	6.00E-07	0.44	27	2.7	Pepd;Ovol3;LOC103690054;LOC103690005	Protease;Transcription
DMR1:91071001	1	91071001	91072000	1000	1	1.90E-10	0.46	24	2.4	Pepd;Capns1;LOC108349561;LOC687508	Protease;Metabolism
DMR1:91152001	1	91152001	91153000	1000	1	1.30E-09	0.46	19	1.9	Pepd;LOC100912070	Protease
DMR1:91160001	1	91160001	91161000	1000	1	6.10E-08	0.43	12	1.2	Pepd;LOC100912070;LOC100912309	Protease
DMR1:91180001	1	91180001	91182000	2000	1	1.10E-11	0.59	29	1.45	Pepd;LOC100912309;Pdcd5	Protease;Signaling
DMR1:91187001	1	91187001	91188000	1000	1	7.60E-21	0.64	15	1.5	Pepd;Pdcd5	Protease;Signaling
DMR1:91189001	1	91189001	91191000	2000	2	1.10E-24	0.98	24	1.2	Pepd	Protease
DMR1:91193001	1	91193001	91196000	3000	3	8.40E-21	0.91	32	1.07	Pepd;LOC100912350	Protease
DMR1:91216001	1	91216001	91223000	7000	1	1.50E-07	-0.51	93	1.33	Pepd;LOC100912350	Protease
DMR1:91224001	1	91224001	91229000	5000	1	3.90E-08	0.35	72	1.44	Pepd	Protease
DMR1:91235001	1	91235001	91237000	2000	1	9.20E-08	-0.46	34	1.7	Pepd	Protease
DMR1:91243001	1	91243001	91244000	1000	1	1.60E-09	0.36	13	1.3	Pepd	Protease
DMR1:91248001	1	91248001	91252000	4000	1	3.30E-07	0.48	78	1.95	Pepd	Protease
DMR1:91253001	1	91253001	91255000	2000	1	6.30E-07	0.31	62	3.1	Pepd	Protease
DMR1:91260001	1	91260001	91261000	1000	1	9.50E-08	-0.52	16	1.6	Pepd	Protease
DMR1:91285001	1	91285001	91288000	3000	1	3.30E-10	0.67	29	0.97	Pepd;Cebpg	Protease;Transcription
DMR1:91301001	1	91301001	91303000	2000	1	2.90E-09	0.67	9	0.45	Cebpg	Transcription
DMR1:91304001	1	91304001	91307000	3000	1	2.50E-07	0.3	43	1.43	Cebpg	Transcription
DMR1:91359001	1	91359001	91360000	1000	1	1.30E-07	-0.42	17	1.7	Cebpa	Transcription
DMR1:91368001	1	91368001	91369000	1000	1	4.20E-15	0.8	18	1.8	Cebpa;LOC108349562	Transcription
DMR1:91426001	1	91426001	91431000	5000	1	6.30E-08	-0.34	70	1.4	Slc7a10	Transport
DMR1:91436001	1	91436001	91440000	4000	3	1.00E-14	0.78	54	1.35	Slc7a10;Lrp3	Transport;Binding Proteins
DMR1:91469001	1	91469001	91471000	2000	1	8.40E-16	-0.81	40	2	Lrp3	Binding Proteins
DMR1:91506001	1	91506001	91508000	2000	1	1.50E-11	0.53	31	1.55	Wdr88	
DMR1:91509001	1	91509001	91511000	2000	1	9.10E-16	0.67	14	0.7	Wdr88	
DMR1:91521001	1	91521001	91522000	1000	1	4.30E-07	-0.49	9	0.9	Wdr88;Gpatch1	
DMR1:91526001	1	91526001	91528000	2000	1	3.90E-11	0.72	50	2.5	Wdr88;Gpatch1	
DMR1:91532001	1	91532001	91535000	3000	2	3.00E-15	-0.7	23	0.77	Wdr88;Gpatch1	
DMR1:91543001	1	91543001	91546000	3000	1	4.50E-07	-0.5	30	1	Gpatch1	
DMR1:91553001	1	91553001	91557000	4000	1	1.60E-10	0.45	44	1.1	Gpatch1	
DMR1:91558001	1	91558001	91561000	3000	1	1.50E-07	0.38	30	1	Gpatch1	
DMR1:91578001	1	91578001	91580000	2000	1	9.10E-21	0.77	33	1.65	Gpatch1;LOC687679	
DMR1:91581001	1	91581001	91585000	4000	1	3.90E-10	0.56	50	1.25	Gpatch1;LOC687679	
DMR1:91604001	1	91604001	91606000	2000	2	2.10E-09	-0.44	28	1.4	Rhpn2	Cytoskeleton
DMR1:91612001	1	91612001	91617000	5000	1	8.10E-10	0.54	58	1.16	Rhpn2	Cytoskeleton
DMR1:91634001	1	91634001	91636000	2000	1	8.10E-09	-0.55	30	1.5	Rhpn2	Cytoskeleton
DMR1:91640001	1	91640001	91642000	2000	1	1.20E-09	0.38	38	1.9	Rhpn2	Cytoskeleton
DMR1:91643001	1	91643001	91645000	2000	1	1.80E-22	0.77	38	1.9	Rhpn2	Cytoskeleton
DMR1:91646001	1	91646001	91647000	1000	1	7.40E-09	0.48	5	0.5	Rhpn2	Cytoskeleton
DMR1:91648001	1	91648001	91649000	1000	1	1.90E-10	0.52	11	1.1	Rhpn2;Faap24	Cytoskeleton
DMR1:91653001	1	91653001	91655000	2000	2	2.60E-12	0.47	40	2	Rhpn2;Faap24;Cep89	Cytoskeleton
DMR1:91658001	1	91658001	91663000	5000	1	1.30E-07	-0.44	72	1.44	Rhpn2;Faap24;Cep89	Cytoskeleton
DMR1:91664001	1	91664001	91669000	5000	3	4.70E-15	0.64	57	1.14	Rhpn2;Faap24;Cep89	Cytoskeleton
DMR1:91677001	1	91677001	91679000	2000	1	4.30E-10	0.39	28	1.4	Cep89	
DMR1:91686001	1	91686001	91687000	1000	1	1.30E-07	0.36	13	1.3	Cep89	
DMR1:91699001	1	91699001	91708000	9000	3	3.40E-11	0.43	155	1.72	Cep89;Slc7a9	Transport
DMR1:91720001	1	91720001	91722000	2000	1	4.00E-08	-0.49	36	1.8	Slc7a9	Transport
DMR1:91724001	1	91724001	91728000	4000	1	5.10E-08	-0.49	45	1.12	Slc7a9	Transport
DMR1:91744001	1	91744001	91747000	3000	1	6.50E-09	0.44	53	1.77	Slc7a9;Tdrd12;LOC108349436	Transport;Cytoskeleton
DMR1:91758001	1	91758001	91760000	2000	1	8.50E-08	0.34	27	1.35	Tdrd12	Cytoskeleton
DMR1:91768001	1	91768001	91771000	3000	1	7.20E-09	-0.51	29	0.97	Tdrd12	Cytoskeleton
DMR1:91775001	1	91775001	91777000	2000	1	6.90E-08	-0.44	12	0.6	Tdrd12	Cytoskeleton

DMR1:91788001	1	91788001	91790000	2000	1	8.00E-07	0.32	16	0.8	Tdrd12	Cytoskeleton
DMR1:91797001	1	91797001	91799000	2000	1	3.10E-07	0.5	20	1	Tdrd12	Cytoskeleton
DMR1:91804001	1	91804001	91805000	1000	1	9.20E-13	0.48	11	1.1	Tdrd12	Cytoskeleton
DMR1:91818001	1	91818001	91819000	1000	1	8.80E-09	0.47	7	0.7	Tdrd12;Nudt19	Cytoskeleton
DMR1:91843001	1	91843001	91845000	2000	1	3.60E-24	1.03	18	0.9	Nudt19	
DMR1:91854001	1	91854001	91855000	1000	1	5.90E-10	-0.52	20	2	Nudt19;Rgs9bp;Ankrd27	
DMR1:91866001	1	91866001	91870000	4000	1	1.10E-10	0.54	69	1.73	Ankrd27	
DMR1:91890001	1	91890001	91892000	2000	1	4.20E-11	0.51	36	1.8	Ankrd27	
DMR1:91899001	1	91899001	91902000	3000	1	1.50E-10	-0.63	46	1.53	Ankrd27	
DMR1:91968001	1	91968001	91969000	1000	1	5.70E-13	0.64	16	1.6	Ankrd27;Pdcd5	Signaling
DMR1:92025001	1	92025001	92031000	6000	1	7.70E-07	-0.42	102	1.7	Dpy19l3	
DMR1:92032001	1	92032001	92033000	1000	1	3.50E-07	-0.58	12	1.2	Dpy19l3	
DMR1:92037001	1	92037001	92043000	6000	4	9.50E-09	-0.64	101	1.68	Dpy19l3	
DMR1:92060001	1	92060001	92067000	7000	3	1.40E-13	0.79	118	1.69	Dpy19l3	
DMR1:92074001	1	92074001	92078000	4000	1	4.60E-08	0.49	51	1.27	Dpy19l3	
DMR1:92093001	1	92093001	92096000	3000	1	9.20E-11	-0.65	16	0.53	Zfp507	Transcription
DMR1:92128001	1	92128001	92129000	1000	1	2.10E-20	0.88	14	1.4	Zfp507	Transcription
DMR1:92694001	1	92694001	92696000	2000	1	6.10E-07	-0.37	20	1	Tshz3	Transcription
DMR1:92717001	1	92717001	92719000	2000	1	6.80E-26	0.7	19	0.95	Tshz3	Transcription
DMR1:92724001	1	92724001	92729000	5000	1	3.60E-10	0.47	84	1.68	Tshz3	Transcription
DMR1:92732001	1	92732001	92734000	2000	1	9.50E-08	0.37	40	2	Tshz3	Transcription
DMR1:92735001	1	92735001	92736000	1000	1	1.40E-09	0.43	20	2	Tshz3	Transcription
DMR1:92738001	1	92738001	92743000	5000	1	1.40E-07	0.4	90	1.8	Tshz3	Transcription
DMR1:92744001	1	92744001	92751000	7000	1	2.30E-21	0.72	144	2.06	Tshz3	Transcription
DMR1:92763001	1	92763001	92771000	8000	2	4.10E-18	0.75	137	1.71	Tshz3;LOC108349564	Transcription
DMR1:93242001	1	93242001	93243000	1000	1	6.70E-07	-0.49	25	2.5	RGD1562402	Translation
DMR1:93251001	1	93251001	93252000	1000	1	4.70E-08	0.42	7	0.7	RGD1562402	Translation
DMR1:93684001	1	93684001	93688000	4000	1	9.10E-07	-0.37	82	2.05	Zfp536	Transcription
DMR1:93694001	1	93694001	93695000	1000	1	1.80E-09	0.46	7	0.7	Zfp536	Transcription
DMR1:93719001	1	93719001	93722000	3000	2	4.80E-17	0.77	51	1.7	Zfp536	Transcription
DMR1:93747001	1	93747001	93748000	1000	1	1.30E-08	0.36	17	1.7	Zfp536	Transcription
DMR1:93749001	1	93749001	93751000	2000	2	6.10E-09	0.38	21	1.05	Zfp536	Transcription
DMR1:93757001	1	93757001	93760000	3000	1	1.90E-15	0.94	39	1.3	Zfp536	Transcription
DMR1:93771001	1	93771001	93772000	1000	1	3.70E-10	0.4	15	1.5	Zfp536;LOC108349103	Transcription
DMR1:93783001	1	93783001	93786000	3000	1	7.00E-07	0.37	46	1.53	Zfp536;LOC108349103	Transcription
DMR1:93787001	1	93787001	93788000	1000	1	3.80E-07	0.6	6	0.6	Zfp536;LOC108349103	Transcription
DMR1:93796001	1	93796001	93798000	2000	1	1.10E-17	0.63	15	0.75	Zfp536;LOC108349103	Transcription
DMR1:93808001	1	93808001	93810000	2000	1	7.50E-14	0.66	22	1.1	Zfp536;LOC108349103	Transcription
DMR1:93811001	1	93811001	93816000	5000	1	1.60E-16	0.59	71	1.42	Zfp536;LOC108349103	Transcription
DMR1:93831001	1	93831001	93835000	4000	1	5.40E-07	0.3	60	1.5	Zfp536;LOC103691087	Transcription
DMR1:93838001	1	93838001	93841000	3000	1	4.70E-14	-0.76	45	1.5	Zfp536;LOC103691087	Transcription
DMR1:93854001	1	93854001	93856000	2000	1	2.40E-15	0.58	27	1.35	Zfp536	Transcription
DMR1:93898001	1	93898001	93911000	13000	2	1.70E-09	0.47	227	1.75	Zfp536	Transcription
DMR1:93957001	1	93957001	93959000	2000	1	1.60E-16	1.03	29	1.45	Zfp536	Transcription
DMR1:93960001	1	93960001	93962000	2000	2	6.60E-21	0.85	15	0.75	Zfp536	Transcription
DMR1:93963001	1	93963001	93964000	1000	1	7.00E-08	0.38	19	1.9	Zfp536	Transcription
DMR1:93965001	1	93965001	93966000	1000	1	1.50E-24	0.89	14	1.4	Zfp536	Transcription
DMR1:93977001	1	93977001	93981000	4000	3	1.10E-15	0.5	59	1.48	Zfp536	Transcription
DMR1:93982001	1	93982001	93987000	5000	2	1.30E-15	0.62	45	0.9	Zfp536	Transcription
DMR1:93989001	1	93989001	93991000	2000	2	3.00E-12	0.71	23	1.15	Zfp536	Transcription
DMR1:94014001	1	94014001	94015000	1000	1	9.80E-16	0.59	14	1.4	Zfp536	Transcription
DMR1:94021001	1	94021001	94022000	1000	1	1.00E-11	0.39	9	0.9	Zfp536	Transcription
DMR1:94025001	1	94025001	94027000	2000	2	8.40E-11	-0.57	22	1.1	Zfp536	Transcription
DMR1:94046001	1	94046001	94047000	1000	1	6.00E-07	0.33	17	1.7	Zfp536	Transcription
DMR1:94051001	1	94051001	94056000	5000	2	8.30E-23	1.04	58	1.16	Zfp536	Transcription
DMR1:94062001	1	94062001	94063000	1000	1	1.70E-09	0.42	17	1.7	Zfp536	Transcription
DMR1:94066001	1	94066001	94071000	5000	1	1.50E-08	0.42	105	2.1	Zfp536	Transcription
DMR1:94072001	1	94072001	94073000	1000	1	1.40E-10	0.46	18	1.8	Zfp536	Transcription
DMR1:94079001	1	94079001	94085000	6000	2	1.20E-09	-0.53	79	1.32	Zfp536	Transcription
DMR1:94089001	1	94089001	94090000	1000	1	3.90E-12	0.7	5	0.5	Zfp536	Transcription
DMR1:94111001	1	94111001	94114000	3000	1	6.70E-07	0.42	55	1.83	Zfp536	Transcription
DMR1:94115001	1	94115001	94117000	2000	1	9.40E-10	0.44	34	1.7	Zfp536	Transcription
DMR1:94120001	1	94120001	94125000	5000	1	7.00E-09	0.38	76	1.52	Zfp536	Transcription
DMR1:94141001	1	94141001	94147000	6000	2	5.80E-19	0.92	71	1.18	Zfp536	Transcription
DMR1:94162001	1	94162001	94165000	3000	1	1.10E-11	0.56	32	1.07	Zfp536	Transcription

DMR1:94337001	1	94337001	94340000	3000	2	1.70E-12	-0.69	29	0.97	LOC108349449;Uri1	Epigenetic
DMR1:94341001	1	94341001	94344000	3000	1	2.40E-07	-0.55	44	1.47	Uri1	Epigenetic
DMR1:94345001	1	94345001	94346000	1000	1	4.00E-09	0.46	15	1.5	Uri1	Epigenetic
DMR1:94360001	1	94360001	94362000	2000	1	9.90E-09	-0.4	17	0.85	Uri1	Epigenetic
DMR1:94478001	1	94478001	94480000	2000	1	1.40E-11	0.49	26	1.3	Ccne1	Signaling
DMR1:94483001	1	94483001	94484000	1000	1	1.60E-10	-0.58	14	1.4	Ccne1	Signaling
DMR1:94503001	1	94503001	94504000	1000	1	1.80E-19	0.87	16	1.6	Ccne1;LOC502323	Signaling
DMR1:94505001	1	94505001	94507000	2000	1	3.30E-19	0.58	27	1.35	Ccne1;LOC502323	Signaling
DMR1:94604001	1	94604001	94607000	3000	1	5.50E-09	0.4	47	1.57	Plekhf1;LOC108349450	
DMR1:94612001	1	94612001	94614000	2000	1	6.50E-08	-0.48	22	1.1	Plekhf1;LOC108349450	
DMR1:94618001	1	94618001	94623000	5000	1	6.40E-15	0.56	69	1.38	Plekhf1;LOC108349450	
DMR1:94656001	1	94656001	94659000	3000	1	3.50E-07	-0.6	30	1	Pop4	
DMR1:94666001	1	94666001	94668000	2000	1	4.40E-07	-0.42	27	1.35	Pop4;LOC361559	
DMR1:94670001	1	94670001	94673000	3000	1	7.70E-16	0.9	13	0.43	Pop4;LOC361559	
DMR1:94678001	1	94678001	94681000	3000	1	1.40E-09	0.51	28	0.93	Pop4;LOC361559	
DMR1:94714001	1	94714001	94718000	4000	1	5.10E-17	0.61	56	1.4	LOC361559;Uqcrb-ps1	
DMR1:94719001	1	94719001	94723000	4000	1	1.90E-07	-0.49	54	1.35	LOC361559;Uqcrb-ps1	
DMR1:94834001	1	94834001	94835000	1000	1	4.60E-08	0.48	9	0.9	RGD1584023	
DMR1:94845001	1	94845001	94847000	2000	1	9.20E-12	0.5	22	1.1	RGD1584023;LOC102548231	
DMR1:95075001	1	95075001	95078000	3000	1	1.60E-11	0.56	54	1.8	Rps27a-ps30	
DMR1:95430001	1	95430001	95432000	2000	1	1.70E-12	0.51	26	1.3	LOC102548014;Zfp939	
DMR1:97646001	1	97646001	97649000	3000	1	1.40E-12	0.59	20	0.67	Vstm2b	Immune
DMR1:97654001	1	97654001	97656000	2000	1	7.20E-11	0.4	21	1.05	Vstm2b	Immune
DMR1:97668001	1	97668001	97669000	1000	1	9.80E-08	0.52	7	0.7	Vstm2b	Immune
DMR1:97705001	1	97705001	97706000	1000	1	1.40E-12	0.56	5	0.5	LOC690265;MGC114499	
DMR1:97725001	1	97725001	97726000	1000	1	1.10E-07	-0.55	4	0.4	MGC114499	
DMR1:98177001	1	98177001	98180000	3000	1	1.90E-07	0.34	67	2.23	Zfp141	
DMR1:98182001	1	98182001	98183000	1000	1	4.70E-12	0.44	8	0.8	Zfp141	
DMR1:98205001	1	98205001	98209000	4000	2	7.00E-09	0.51	41	1.02	Zfp141;LOC102555672	
DMR1:98382001	1	98382001	98385000	3000	1	5.80E-12	0.55	28	0.93	Cd33	Immune
DMR1:98386001	1	98386001	98388000	2000	1	2.40E-15	0.84	18	0.9	Cd33	Immune
DMR1:98406001	1	98406001	98410000	4000	2	1.70E-08	-0.52	28	0.7	Cd33;Siglecl1	Immune
DMR1:98411001	1	98411001	98413000	2000	2	3.30E-16	0.84	11	0.55	Cd33;Siglecl1	Immune
DMR1:98423001	1	98423001	98425000	2000	1	4.10E-22	0.61	25	1.25	Siglecl1;LOC690483	Immune
DMR1:98428001	1	98428001	98430000	2000	1	1.60E-11	0.54	28	1.4	Siglecl1;LOC690483	Immune
DMR1:98436001	1	98436001	98438000	2000	1	4.10E-16	0.79	14	0.7	LOC690483;Iglon5	Immune;Immune
DMR1:98443001	1	98443001	98445000	2000	1	3.80E-08	-0.44	25	1.25	LOC690483;Iglon5	Immune;Immune
DMR1:98450001	1	98450001	98452000	2000	2	1.70E-19	0.74	26	1.3	Iglon5;Vsig10l	Immune
DMR1:98467001	1	98467001	98469000	2000	2	1.50E-21	0.8	9	0.45	Iglon5;Vsig10l;Etfb	Immune;Metabolism
DMR1:98483001	1	98483001	98485000	2000	1	3.50E-20	0.64	17	0.85	Etfb;Cldnd2;Nkg7	Metabolism;Cytoskeleton
DMR1:98487001	1	98487001	98490000	3000	1	7.40E-11	0.79	79	2.63	Etfb;Cldnd2;Nkg7;Lim2	Metabolism;Cytoskeleton
DMR1:98495001	1	98495001	98498000	3000	1	6.50E-13	0.51	45	1.5	Etfb;Cldnd2;Nkg7;Lim2;C1H19orf84;LOC102550061	Metabolism;Cytoskeleton
DMR1:98499001	1	98499001	98500000	1000	1	4.80E-09	0.61	4	0.4	Cldnd2;Nkg7;Lim2;C1H19orf84;LOC102550061	Cytoskeleton
DMR1:98502001	1	98502001	98503000	1000	1	1.70E-20	0.59	17	1.7	Nkg7;Lim2;C1H19orf84;LOC102550061;Siglec10	Cytoskeleton;Immune
DMR1:98505001	1	98505001	98507000	2000	2	1.20E-26	1	21	1.05	Lim2;C1H19orf84;LOC102550061;Siglec10	Cytoskeleton;Immune
DMR1:98524001	1	98524001	98525000	1000	1	2.00E-27	1.19	6	0.6	Siglec10	Immune
DMR1:98560001	1	98560001	98563000	3000	2	9.60E-16	1	11	0.37	Siglec5	Immune
DMR1:98570001	1	98570001	98575000	5000	2	2.10E-12	0.56	65	1.3	Siglec5	Immune
DMR1:98613001	1	98613001	98614000	1000	1	1.50E-08	0.48	8	0.8	Dzf17	
DMR1:98646001	1	98646001	98648000	2000	2	1.80E-19	1	8	0.4	RGD1565346	
DMR1:98650001	1	98650001	98651000	1000	1	1.20E-08	0.5	4	0.4	RGD1565346	
DMR1:98764001	1	98764001	98765000	1000	1	8.40E-14	0.71	3	0.3	RGD1564800	
DMR1:98901001	1	98901001	98910000	9000	1	1.00E-07	-0.45	52	0.58	Vom2r38	
DMR1:98912001	1	98912001	98915000	3000	1	1.80E-13	0.69	13	0.43	Vom2r38	
DMR1:98924001	1	98924001	98925000	1000	1	3.10E-16	0.59	9	0.9	Vom2r38	
DMR1:98927001	1	98927001	98932000	5000	2	6.10E-11	-0.54	27	0.54	Vom2r38	
DMR1:98941001	1	98941001	98946000	5000	3	2.30E-19	-0.67	56	1.12	Vom2r38	
DMR1:98979001	1	98979001	98986000	7000	4	3.70E-10	-0.36	81	1.16	Vom2r38;Vom2r37	Signaling
DMR1:99018001	1	99018001	99021000	3000	1	2.10E-12	-0.53	21	0.7	Vom2r38;Vom2r37	Signaling

DMR1:99030001	1	99030001	99038000	8000	5	6.60E-29	-0.47	63	0.79	Vom2r38;Vom2r37	Signaling
DMR1:99041001	1	99041001	99045000	4000	2	1.30E-08	0.28	19	0.48	Vom2r38;Vom2r37;LOC100911353;LOC108349760	Signaling
DMR1:99046001	1	99046001	99048000	2000	1	1.40E-07	-0.27	29	1.45	Vom2r38;Vom2r37;LOC100911353;LOC108349760	Signaling
DMR1:99050001	1	99050001	99054000	4000	1	4.80E-07	0.22	52	1.3	Vom2r38;Vom2r37;LOC100911353;LOC108349760	Signaling
DMR1:99056001	1	99056001	99063000	7000	5	2.50E-31	-0.44	61	0.87	Vom2r38;Vom2r37;LOC100911353;LOC108349760	Signaling
DMR1:99078001	1	99078001	99080000	2000	1	2.30E-12	0.29	24	1.2	Vom2r38;Vom2r37;LOC108349760;LOC103691104	Signaling
DMR1:99084001	1	99084001	99091000	7000	6	3.30E-13	0.59	57	0.81	Vom2r38;Vom2r37;LOC108349760;LOC103691104	Signaling
DMR1:99096001	1	99096001	99098000	2000	1	1.70E-07	0.31	8	0.4	Vom2r38;Vom2r37;LOC103691104;LOC100912942	Signaling
DMR1:99099001	1	99099001	99100000	1000	1	1.70E-11	-0.35	22	2.2	Vom2r38;Vom2r37;LOC103691104;LOC100912942	Signaling
DMR1:99102001	1	99102001	99104000	2000	1	1.90E-07	-0.4	20	1	Vom2r38;Vom2r37;LOC103691104;LOC100912942	Signaling
DMR1:99108001	1	99108001	99111000	3000	1	5.30E-08	-0.72	16	0.53	Vom2r38;Vom2r37;LOC100912942	Signaling
DMR1:99114001	1	99114001	99120000	6000	3	3.30E-13	0.64	83	1.38	Vom2r38;Vom2r37;LOC100912942;LOC108349568	Signaling
DMR1:99122001	1	99122001	99127000	5000	2	8.00E-09	-0.55	78	1.56	Vom2r38;Vom2r37;LOC108349568	Signaling
DMR1:99155001	1	99155001	99156000	1000	1	4.20E-07	0.57	5	0.5	Vom2r38	
DMR1:99287001	1	99287001	99292000	5000	2	1.90E-09	0.57	33	0.66	Klk1c12	
DMR1:99313001	1	99313001	99319000	6000	2	1.60E-11	0.93	47	0.78	Klk1c12;LOC108349761;LOC100911689	
DMR1:99352001	1	99352001	99355000	3000	2	2.40E-08	0.47	18	0.6	LOC100911838;Klk1c8	
DMR1:99364001	1	99364001	99368000	4000	2	5.10E-09	-0.49	42	1.05	Klk1c8	
DMR1:99381001	1	99381001	99384000	3000	2	1.50E-12	0.71	29	0.97	Klk15	Protease
DMR1:99388001	1	99388001	99391000	3000	1	3.20E-13	0.7	25	0.83	Klk15	Protease
DMR1:99393001	1	99393001	99395000	2000	1	1.10E-14	0.53	14	0.7	Klk15	Protease
DMR1:99400001	1	99400001	99401000	1000	1	7.40E-11	0.61	8	0.8	Klk15	Protease
DMR1:99404001	1	99404001	99406000	2000	1	5.40E-09	0.47	28	1.4	Klk15;LOC100911930	Protease
DMR1:99515001	1	99515001	99516000	1000	1	1.50E-10	0.47	8	0.8	LOC102556967;Zfp819	Transcription;Transcription
DMR1:99537001	1	99537001	99541000	4000	1	1.30E-07	-0.63	85	2.12	Zfp819	Transcription
DMR1:99552001	1	99552001	99554000	2000	1	5.00E-08	0.42	27	1.35	Ceacam18	
DMR1:99561001	1	99561001	99563000	2000	1	7.90E-17	0.53	26	1.3	Ceacam18	
DMR1:99566001	1	99566001	99569000	3000	1	2.50E-10	0.49	37	1.23	Ceacam18;Siglec8	Immune
DMR1:99573001	1	99573001	99576000	3000	1	2.80E-08	0.39	41	1.37	Ceacam18;Siglec8	Immune
DMR1:99580001	1	99580001	99582000	2000	1	2.00E-11	0.49	26	1.3	Ceacam18;Siglec8	Immune
DMR1:99599001	1	99599001	99600000	1000	1	2.70E-08	0.59	22	2.2	Ctu1	
DMR1:99609001	1	99609001	99611000	2000	1	5.50E-10	0.52	42	2.1	Ctu1;Klk14	Protease
DMR1:99622001	1	99622001	99624000	2000	1	1.10E-07	0.6	19	0.95	Klk14;LOC103691107	Protease
DMR1:99639001	1	99639001	99641000	2000	2	2.20E-17	-0.48	25	1.25	LOC103691107;Klk13	Protease
DMR1:99648001	1	99648001	99650000	2000	1	7.50E-20	0.89	39	1.95	Klk13	Protease
DMR1:99652001	1	99652001	99661000	9000	5	1.50E-26	0.84	87	0.97	Klk13	Protease
DMR1:99664001	1	99664001	99665000	1000	1	1.40E-07	0.51	7	0.7	Klk13	Protease
DMR1:99697001	1	99697001	99700000	3000	2	1.70E-15	-0.85	26	0.87	Klk12	Protease
DMR1:99705001	1	99705001	99711000	6000	3	2.60E-14	0.7	81	1.35	Klk12;Klk11;Klk10	Protease
DMR1:99714001	1	99714001	99717000	3000	3	4.00E-17	0.64	53	1.77	Klk12;Klk11;Klk10	Protease
DMR1:99719001	1	99719001	99720000	1000	1	1.80E-17	0.77	24	2.4	Klk12;Klk11;Klk10;Klk9	Protease
DMR1:99721001	1	99721001	99724000	3000	2	2.10E-22	0.68	40	1.33	Klk12;Klk11;Klk10;Klk9	Protease
DMR1:99741001	1	99741001	99743000	2000	1	1.50E-07	0.52	25	1.25	Klk9;Klk8;Klk7	Protease
DMR1:99751001	1	99751001	99754000	3000	3	2.50E-21	1.01	24	0.8	Klk8;Klk7;Klk6	Protease
DMR1:99757001	1	99757001	99765000	8000	5	2.30E-33	1.1	107	1.34	Klk7;Klk6	Protease
DMR1:99766001	1	99766001	99767000	1000	1	1.10E-19	0.72	17	1.7	Klk6	Protease
DMR1:99769001	1	99769001	99772000	3000	2	6.80E-13	0.53	26	0.87	Klk6;Klk5	Protease
DMR1:99776001	1	99776001	99778000	2000	2	1.30E-19	0.81	22	1.1	Klk6;Klk5	Protease
DMR1:99780001	1	99780001	99782000	2000	1	2.60E-20	0.89	22	1.1	Klk5	Protease
DMR1:99783001	1	99783001	99786000	3000	2	1.70E-21	0.87	30	1	Klk5	Protease
DMR1:99799001	1	99799001	99801000	2000	1	3.10E-10	0.42	26	1.3	Klk5	Protease

DMR1:99803001	1	99803001	99804000	1000	1	2.40E-10	0.54	10	1	Klk5	Protease
DMR1:99826001	1	99826001	99829000	3000	2	1.70E-12	0.53	42	1.4	Klk4	Protease
DMR1:99840001	1	99840001	99844000	4000	2	9.50E-12	0.51	53	1.32	Klk4	Protease
DMR1:99880001	1	99880001	99883000	3000	2	1.10E-10	0.82	21	0.7	LOC687880;Klk1c10	
DMR1:99884001	1	99884001	99885000	1000	1	6.70E-07	0.46	10	1	LOC687880;Klk1c10	
DMR1:99894001	1	99894001	99898000	4000	2	1.20E-19	0.77	40	1	LOC687880;Klk1c10	
DMR1:99946001	1	99946001	99947000	1000	1	4.20E-12	0.54	11	1.1	LOC102546874;Klk1c6	Protease
DMR1:99948001	1	99948001	99953000	5000	2	3.20E-14	0.85	52	1.04	LOC102546874;Klk1c6;LOC102546987	Protease
DMR1:99958001	1	99958001	99959000	1000	1	1.00E-15	0.74	8	0.8	Klk1c6;LOC102546987	Protease
DMR1:99961001	1	99961001	99962000	1000	1	5.40E-08	-0.5	6	0.6	Klk1c6;LOC102546987	Protease
DMR1:99963001	1	99963001	99965000	2000	1	8.20E-07	0.35	19	0.95	Klk1c6;LOC102546987	Protease
DMR1:99968001	1	99968001	99969000	1000	1	5.30E-14	0.45	16	1.6	Klk1c6;LOC102546987	Protease
DMR1:99976001	1	99976001	99986000	10000	5	1.10E-15	0.82	105	1.05	LOC102546987;Klk1c2;LOC690674	
DMR1:99988001	1	99988001	99992000	4000	2	5.30E-14	-0.61	43	1.07	Klk1c2;LOC690674	
DMR1:100049001	1	100049001	100053000	4000	1	5.00E-07	-0.41	37	0.92	LOC103690048;Klk5l;LOC102549238	
DMR1:100057001	1	100057001	100058000	1000	1	1.20E-09	0.56	11	1.1	Klk5l;LOC102549238	
DMR1:100061001	1	100061001	100062000	1000	1	2.60E-09	0.55	3	0.3	Klk5l;LOC102549238	
DMR1:100063001	1	100063001	100066000	3000	1	3.10E-07	0.43	25	0.83	Klk5l;LOC102549238	
DMR1:100086001	1	100086001	100090000	4000	2	1.60E-11	0.44	42	1.05	LOC102549343;Klk1c9	
DMR1:100096001	1	100096001	100101000	5000	1	2.80E-08	0.43	47	0.94	Klk1c9	
DMR1:100128001	1	100128001	100130000	2000	2	3.30E-11	0.43	17	0.85	LOC690730;Klk1	
DMR1:100131001	1	100131001	100135000	4000	3	5.90E-18	0.54	35	0.88	LOC690730;Klk1;LOC108349762;Klk1c3	Protease
DMR1:100139001	1	100139001	100146000	7000	4	5.70E-18	0.56	72	1.03	LOC690730;Klk1;LOC108349762;Klk1c3	Protease
DMR1:100149001	1	100149001	100151000	2000	1	2.90E-13	0.76	13	0.65	LOC108349762;Klk1c3	Protease
DMR1:100155001	1	100155001	100158000	3000	1	2.00E-13	0.71	24	0.8	Klk1c3;LOC108348116;LOC108349569	Protease
DMR1:100160001	1	100160001	100163000	3000	2	1.20E-14	0.59	25	0.83	Klk1c3;LOC108348116;LOC108349569;LOC103691133	Protease
DMR1:100170001	1	100170001	100175000	5000	1	1.50E-11	0.51	53	1.06	Klk1c3;LOC108348116;LOC108349569;LOC103691133	Protease
DMR1:100195001	1	100195001	100196000	1000	1	2.00E-09	0.57	6	0.6	LOC102553035;Klk1b3;LOC102552731	
DMR1:100200001	1	100200001	100204000	4000	1	1.80E-11	0.43	38	0.95	LOC102553035;Klk1b3;LOC102552731;LOC102552485	
DMR1:100212001	1	100212001	100213000	1000	1	8.00E-10	0.43	14	1.4	Klk1b3;LOC102552731;LOC102552485;Fv1	
DMR1:100230001	1	100230001	100234000	4000	1	1.60E-08	-0.6	50	1.25	LOC102552731;Fv1;Acpt;LOC103691109;LOC102552286	
DMR1:100238001	1	100238001	100241000	3000	2	2.00E-18	0.72	31	1.03	Acpt;LOC103691109;LOC102552286;Gpr32	
DMR1:100253001	1	100253001	100256000	3000	1	4.30E-15	0.54	53	1.77	Gpr32	
DMR1:100291001	1	100291001	100294000	3000	2	4.40E-10	0.71	107	3.57	Clec11a;Shank1	Signaling
DMR1:100296001	1	100296001	100301000	5000	2	5.00E-15	0.63	195	3.9	Clec11a;Shank1	Signaling
DMR1:100303001	1	100303001	100305000	2000	1	2.30E-08	0.36	31	1.55	Clec11a;Shank1	Signaling
DMR1:100306001	1	100306001	100311000	5000	3	9.80E-31	1.08	63	1.26	Shank1	
DMR1:100318001	1	100318001	100321000	3000	1	7.20E-07	-0.47	44	1.47	Shank1	
DMR1:100322001	1	100322001	100327000	5000	3	5.80E-11	0.75	105	2.1	Shank1	
DMR1:100328001	1	100328001	100333000	5000	3	7.10E-24	0.69	84	1.68	Shank1	
DMR1:100334001	1	100334001	100337000	3000	3	9.60E-22	0.91	34	1.13	Shank1;RGD1309036	
DMR1:100342001	1	100342001	100345000	3000	3	5.50E-23	0.71	65	2.17	Shank1;RGD1309036	
DMR1:100346001	1	100346001	100347000	1000	1	5.10E-12	0.7	62	6.2	Shank1;RGD1309036	
DMR1:100349001	1	100349001	100350000	1000	1	4.80E-10	0.45	17	1.7	Shank1;RGD1309036	
DMR1:100351001	1	100351001	100357000	6000	3	1.10E-13	0.41	81	1.35	Shank1;RGD1309036	
DMR1:100359001	1	100359001	100361000	2000	1	2.30E-15	0.97	60	3	RGD1309036	
DMR1:100366001	1	100366001	100368000	2000	1	1.30E-08	0.43	24	1.2	RGD1309036	
DMR1:100391001	1	100391001	100392000	1000	1	4.80E-08	0.45	11	1.1	Syt3	Transport
DMR1:100394001	1	100394001	100396000	2000	1	5.50E-25	0.8	35	1.75	Syt3	Transport
DMR1:100407001	1	100407001	100412000	5000	1	5.10E-12	0.52	87	1.74	Syt3	Transport
DMR1:100414001	1	100414001	100415000	1000	1	3.30E-23	0.77	14	1.4	Syt3	Transport
DMR1:100422001	1	100422001	100435000	13000	5	8.30E-33	1.12	256	1.97	LOC108349570;Lrrc4b	

DMR1:100437001	1	100437001	100439000	2000	2	8.60E-14	0.48	29	1.45	LOC108349570;Lrrc4b	
DMR1:100441001	1	100441001	100444000	3000	1	2.80E-13	0.67	27	0.9	LOC108349570;Lrrc4b	
DMR1:100446001	1	100446001	100449000	3000	3	2.30E-21	0.99	52	1.73	Lrrc4b	
DMR1:100456001	1	100456001	100457000	1000	1	3.50E-07	-0.53	15	1.5	Lrrc4b	
DMR1:100462001	1	100462001	100466000	4000	1	4.60E-10	0.4	49	1.23	Lrrc4b;Aspdh;Josd2	Metabolism;Protease
DMR1:100471001	1	100471001	100472000	1000	1	2.80E-07	0.37	12	1.2	Lrrc4b;Aspdh;Josd2	Metabolism;Protease
DMR1:100475001	1	100475001	100477000	2000	2	7.50E-28	0.6	24	1.2	Lrrc4b;Aspdh;Josd2	Metabolism;Protease
DMR1:100478001	1	100478001	100490000	12000	10	1.40E-25	1.02	118	0.98	Lrrc4b;Aspdh;Josd2;LOC100909904;Emc10	Metabolism;Protease;Epigenetic
DMR1:100491001	1	100491001	100493000	2000	1	6.70E-14	0.48	28	1.4	LOC100909904;Emc10;Fam71e1	Epigenetic
DMR1:100495001	1	100495001	100497000	2000	1	6.80E-09	0.42	38	1.9	LOC100909904;Emc10;Fam71e1;Mybpc2	Epigenetic
DMR1:100511001	1	100511001	100517000	6000	3	3.20E-21	0.93	74	1.23	Emc10;Fam71e1;Mybpc2	Epigenetic
DMR1:100520001	1	100520001	100526000	6000	5	4.40E-26	0.93	57	0.95	Mybpc2;Spib	Transcription
DMR1:100544001	1	100544001	100548000	4000	2	1.50E-18	0.68	133	3.33	Spib;Pold1;Nr1h2	Transcription;Transcription;Transcription
DMR1:100550001	1	100550001	100551000	1000	1	1.40E-15	0.51	17	1.7	Pold1;Nr1h2	Transcription;Transcription
DMR1:100555001	1	100555001	100560000	5000	2	8.60E-15	0.75	159	3.18	Pold1;Nr1h2	Transcription;Transcription
DMR1:100565001	1	100565001	100568000	3000	1	2.10E-11	0.61	41	1.37	Nr1h2;Napsa	Transcription
DMR1:100569001	1	100569001	100573000	4000	3	6.00E-21	0.72	46	1.15	Nr1h2;Napsa	Transcription
DMR1:100575001	1	100575001	100582000	7000	1	9.40E-12	0.43	85	1.21	Napsa	
DMR1:100588001	1	100588001	100594000	6000	3	4.30E-23	1.15	142	2.37	Napsa;Kcnc3	Transport
DMR1:100595001	1	100595001	100596000	1000	1	7.10E-11	0.59	15	1.5	Napsa;Kcnc3	Transport
DMR1:100598001	1	100598001	100605000	7000	6	3.30E-23	0.71	137	1.96	Napsa;Kcnc3;Myh14	Transport
DMR1:100622001	1	100622001	100625000	3000	2	2.80E-08	0.34	60	2	Myh14	
DMR1:100629001	1	100629001	100632000	3000	2	2.60E-24	0.79	66	2.2	Myh14	
DMR1:100637001	1	100637001	100645000	8000	4	1.30E-45	1.07	165	2.06	Myh14	
DMR1:100668001	1	100668001	100670000	2000	1	3.60E-10	0.45	38	1.9	Myh14;RGD1563905	
DMR1:100710001	1	100710001	100711000	1000	1	7.40E-08	0.59	6	0.6	Izumo2	
DMR1:100730001	1	100730001	100734000	4000	2	1.80E-13	-0.48	20	0.5	Izumo2;Zfp473	Transcription
DMR1:100750001	1	100750001	100755000	5000	2	4.70E-24	0.71	58	1.16	Zfp473;Vrk3	Transcription;Signaling
DMR1:100781001	1	100781001	100784000	3000	1	8.40E-29	1.05	50	1.67	Vrk3	Signaling
DMR1:100789001	1	100789001	100792000	3000	2	1.70E-20	0.67	48	1.6	Vrk3	Signaling
DMR1:100807001	1	100807001	100811000	4000	2	6.30E-22	0.68	50	1.25	Atf5;Nup62;Il4i1	Transcription;Transport;Metabolism
DMR1:100874001	1	100874001	100875000	1000	1	1.30E-08	0.43	20	2	Ptov1;Med25	Transcription
DMR1:100913001	1	100913001	100915000	2000	1	1.80E-12	0.42	31	1.55	Ap2a1	Transport
DMR1:100925001	1	100925001	100926000	1000	1	3.30E-19	0.92	9	0.9	Ap2a1;Tsks	Transport
DMR1:100935001	1	100935001	100936000	1000	1	2.60E-12	0.81	1	0.1	Ap2a1;Tsks	Transport
DMR1:100958001	1	100958001	100959000	1000	1	2.10E-17	0.51	14	1.4	Tsks;Cpt1c	Metabolism
DMR1:100962001	1	100962001	100966000	4000	2	7.90E-27	1.04	93	2.33	Tsks;Cpt1c;Prmt1	Metabolism;Golgi
DMR1:100978001	1	100978001	100979000	1000	1	3.90E-10	0.41	14	1.4	Cpt1c;Prmt1;Bcl2l12	Metabolism;Golgi
DMR1:100984001	1	100984001	100986000	2000	1	7.20E-07	0.29	23	1.15	Prmt1;Bcl2l12;Irf3	Golgi;Transcription
DMR1:101002001	1	101002001	101004000	2000	1	2.40E-29	0.91	100	5	Bcl2l12;Irf3;Scaf1;Rras	Transcription;Translation;Signaling
DMR1:101006001	1	101006001	101008000	2000	1	3.60E-07	-0.61	27	1.35	Irf3;Scaf1;Rras	Transcription;Translation;Signaling
DMR1:101014001	1	101014001	101019000	5000	3	1.10E-19	0.69	73	1.46	Scaf1;Rras;Prr12	Translation;Signaling
DMR1:101021001	1	101021001	101024000	3000	2	6.70E-11	-0.54	61	2.03	Rras;Prr12	Signaling
DMR1:101031001	1	101031001	101032000	1000	1	1.40E-07	0.35	21	2.1	Prr12	
DMR1:101037001	1	101037001	101038000	1000	1	2.60E-13	0.45	15	1.5	Prr12;Prrg2	Protease
DMR1:101039001	1	101039001	101045000	6000	5	1.90E-19	0.69	196	3.27	Prr12;Prrg2	Protease
DMR1:101058001	1	101058001	101060000	2000	1	9.40E-12	0.48	17	0.85	Prrg2;Nosip	Protease
DMR1:101070001	1	101070001	101073000	3000	1	1.20E-18	0.62	84	2.8	Nosip;Rcn3	Signaling
DMR1:101075001	1	101075001	101079000	4000	2	3.20E-11	0.43	85	2.12	Nosip;Rcn3;Fcgrt	Signaling;Immune
DMR1:101095001	1	101095001	101100000	5000	2	2.50E-17	0.74	72	1.44	Rcn3;Fcgrt	Signaling;Immune
DMR1:101129001	1	101129001	101131000	2000	2	5.80E-08	0.43	26	1.3	Rpl13a;Flt3lg;Aldh16a1	Translation;Growth Factors;Metabolism

DMR1:101153001	1	101153001	101156000	3000	1	2.60E-14	0.69	47	1.57	Aldh16a1;Pih1d1;LOC108349571;Slc17a7	Metabolism;Transport
DMR1:101164001	1	101164001	101166000	2000	2	2.50E-37	0.92	46	2.3	Pih1d1;LOC108349571;Slc17a7;Gfy;Pth2	Transport
DMR1:101177001	1	101177001	101178000	1000	1	1.20E-17	0.73	12	1.2	Slc17a7;Gfy;Pth2;Ccdc155	Transport
DMR1:101185001	1	101185001	101187000	2000	2	6.80E-17	0.76	36	1.8	Gfy;Pth2;Ccdc155	
DMR1:101192001	1	101192001	101193000	1000	1	4.50E-13	0.49	10	1	Ccdc155	
DMR1:101194001	1	101194001	101198000	4000	2	2.00E-23	0.74	29	0.72	Ccdc155;Dkkl1	
DMR1:101201001	1	101201001	101202000	1000	1	7.30E-08	0.52	8	0.8	Ccdc155;Dkkl1	
DMR1:101217001	1	101217001	101221000	4000	3	2.90E-20	0.67	52	1.3	Dkkl1;Tead2;Cd37	Transcription
DMR1:101232001	1	101232001	101233000	1000	1	3.00E-10	0.43	38	3.8	Tead2;Cd37;Slc6a16	Transcription;Transport
DMR1:101238001	1	101238001	101239000	1000	1	2.30E-09	0.54	9	0.9	Tead2;Cd37;Slc6a16	Transcription;Transport
DMR1:101248001	1	101248001	101250000	2000	1	3.00E-12	0.52	18	0.9	Slc6a16	Transport
DMR1:101266001	1	101266001	101285000	19000	3	4.20E-12	0.58	254	1.34	Slc6a16;RGD1562492;LOC108349572;Trpm4	Transport;Transport
DMR1:101286001	1	101286001	101288000	2000	1	9.70E-07	0.39	27	1.35	RGD1562492;LOC108349572;Trpm4	Transport;Transport
DMR1:101296001	1	101296001	101299000	3000	1	1.10E-10	0.5	59	1.97	LOC108349572;Trpm4	Transport
DMR1:101308001	1	101308001	101312000	4000	3	7.40E-29	0.94	80	2	Trpm4	Transport
DMR1:101316001	1	101316001	101318000	2000	1	2.00E-09	0.41	46	2.3	Trpm4;Hrc	Transport
DMR1:101323001	1	101323001	101327000	4000	1	7.40E-09	0.43	91	2.28	Trpm4;Hrc;Ppfia3	Transport
DMR1:101335001	1	101335001	101337000	2000	2	1.70E-16	0.55	36	1.8	Hrc;Ppfia3	
DMR1:101339001	1	101339001	101341000	2000	1	2.20E-08	0.45	39	1.95	Ppfia3	
DMR1:101342001	1	101342001	101347000	5000	1	1.90E-15	0.8	84	1.68	Ppfia3	
DMR1:101355001	1	101355001	101356000	1000	1	8.20E-21	0.85	7	0.7	Ppfia3;Lin7b	Cytoskeleton
DMR1:101359001	1	101359001	101360000	1000	1	1.80E-21	0.62	16	1.6	Ppfia3;Lin7b;Snrnp70	Cytoskeleton;Translation
DMR1:101366001	1	101366001	101369000	3000	1	1.50E-28	0.81	79	2.63	Ppfia3;Lin7b;Snrnp70	Cytoskeleton;Translation
DMR1:101373001	1	101373001	101376000	3000	1	6.80E-17	0.62	29	0.97	Snrnp70	Translation
DMR1:101389001	1	101389001	101391000	2000	1	8.90E-14	0.7	16	0.8	Snrnp70;Kcna7	Translation;Transport
DMR1:101394001	1	101394001	101395000	1000	1	5.60E-09	0.37	15	1.5	Snrnp70;Kcna7	Translation;Transport
DMR1:101399001	1	101399001	101401000	2000	1	5.70E-08	0.51	32	1.6	Kcna7;Ntf4;Lhb	Transport;Growth Factors;Hormone
DMR1:101402001	1	101402001	101405000	3000	2	3.70E-10	0.57	36	1.2	Kcna7;Ntf4;Lhb;Ruvbl2	Transport;Growth Factors;Hormone
DMR1:101412001	1	101412001	101414000	2000	1	1.20E-08	0.38	39	1.95	Kcna7;Ntf4;Lhb;Ruvbl2	Transport;Growth Factors;Hormone
DMR1:101416001	1	101416001	101419000	3000	1	7.00E-12	0.48	45	1.5	Ntf4;Lhb;Ruvbl2;Gys1	Growth Factors;Hormone
DMR1:101453001	1	101453001	101456000	3000	1	5.70E-07	-0.55	37	1.23	Gys1;Ftl1;Bax;Dhdh	Transport;Metabolism
DMR1:101457001	1	101457001	101461000	4000	1	2.60E-09	0.62	76	1.9	Gys1;Ftl1;Bax;Dhdh	Transport;Metabolism
DMR1:101463001	1	101463001	101465000	2000	1	1.70E-11	0.56	26	1.3	Bax;Dhdh;Tulp2	Metabolism
DMR1:101516001	1	101516001	101522000	6000	3	8.80E-25	0.92	102	1.7	Tulp2;Nucb1;Ppp1r15a;Plekha4	Signaling
DMR1:101533001	1	101533001	101536000	3000	1	3.60E-11	0.39	42	1.4	Plekha4;Hsd17b14	Metabolism
DMR1:101544001	1	101544001	101547000	3000	1	9.40E-11	0.49	44	1.47	Plekha4;Hsd17b14;LOC102556190;Bcat2	Metabolism;Metabolism
DMR1:101574001	1	101574001	101577000	3000	1	8.10E-07	-0.52	41	1.37	Bcat2	Metabolism
DMR1:101581001	1	101581001	101582000	1000	1	1.70E-10	0.77	16	1.6	Bcat2	Metabolism
DMR1:101589001	1	101589001	101592000	3000	2	9.50E-25	0.94	25	0.83	Fgf21;Fut1	Growth Factors;Golgi
DMR1:101594001	1	101594001	101595000	1000	1	1.60E-17	0.68	11	1.1	Fgf21;Fut1;Izumo1;LOC103691112	Growth Factors;Golgi
DMR1:101608001	1	101608001	101610000	2000	1	3.90E-22	0.7	38	1.9	Fut1;Izumo1;LOC103691112;Rasip1	Golgi
DMR1:101617001	1	101617001	101620000	3000	1	1.50E-07	0.37	39	1.3	Izumo1;LOC103691112;Rasip1;Mamstr	
DMR1:101638001	1	101638001	101641000	3000	3	5.50E-13	0.52	47	1.57	Mamstr;Fut2	Golgi
DMR1:101643001	1	101643001	101645000	2000	1	7.00E-07	0.35	42	2.1	Fut2	Golgi

DMR1:101647001	1	101647001	101651000	4000	3	6.20E-31	1.26	50	1.25	Fut2	Golgi
DMR1:101670001	1	101670001	101672000	2000	1	6.40E-16	0.52	43	2.15	Sec1;Ntn5	Golgi;Extracellular Matrix
DMR1:101676001	1	101676001	101677000	1000	1	7.20E-07	0.39	12	1.2	Sec1;Ntn5;Car11	Golgi;Extracellular Matrix
DMR1:101680001	1	101680001	101683000	3000	3	1.50E-19	0.8	71	2.37	Sec1;Ntn5;Car11;Dbp;Sphk2	Golgi;Extracellular Matrix;Transcription;Signaling
DMR1:101710001	1	101710001	101711000	1000	1	2.10E-17	0.88	9	0.9	Sphk2;Rpl18;Fam83e;Spaca4;Sult2b1	Signaling;Translation;Transport
DMR1:101712001	1	101712001	101713000	1000	1	1.50E-17	0.5	22	2.2	Rpl18;Fam83e;Spaca4;Sult2b1	Translation;Transport
DMR1:101731001	1	101731001	101732000	1000	1	2.50E-11	0.75	7	0.7	Sult2b1	Transport
DMR1:101740001	1	101740001	101751000	11000	9	1.40E-37	1.13	134	1.22	Sult2b1	Transport
DMR1:101753001	1	101753001	101760000	7000	3	8.80E-27	1.03	117	1.67	Sult2b1	Transport
DMR1:101764001	1	101764001	101765000	1000	1	1.10E-07	-0.65	10	1	Sult2b1;Lmtk3	Transport
DMR1:101767001	1	101767001	101768000	1000	1	3.50E-07	0.34	14	1.4	Sult2b1;Lmtk3	Transport
DMR1:101777001	1	101777001	101780000	3000	1	3.00E-14	0.53	55	1.83	Sult2b1;Lmtk3	Transport
DMR1:101781001	1	101781001	101782000	1000	1	1.20E-13	0.74	34	3.4	Sult2b1;Lmtk3	Transport
DMR1:101796001	1	101796001	101798000	2000	1	1.20E-10	0.49	37	1.85	Lmtk3;Cyth2;Kcnj14	Transcription;Transport
DMR1:101812001	1	101812001	101815000	3000	1	5.90E-18	0.57	42	1.4	Cyth2;Kcnj14;Grwd1;Grin2d	Transcription;Transport;Receptor
DMR1:101828001	1	101828001	101832000	4000	1	6.50E-07	0.32	61	1.52	Grwd1;Grin2d	Receptor
DMR1:101843001	1	101843001	101849000	6000	1	7.40E-10	0.47	126	2.1	Grin2d	Receptor
DMR1:101850001	1	101850001	101857000	7000	5	5.20E-29	1.16	191	2.73	Grin2d;Kdelr1	Receptor;Transport
DMR1:101875001	1	101875001	101879000	4000	2	9.20E-14	0.73	32	0.8	Kdelr1;Syngr4;Tmem143	Transport
DMR1:101897001	1	101897001	101899000	2000	1	2.40E-12	0.61	21	1.05	Tmem143;Emp3;Ccdc114	Cytoskeleton
DMR1:101900001	1	101900001	101901000	1000	1	6.20E-08	0.33	21	2.1	Tmem143;Emp3;Ccdc114	Cytoskeleton
DMR1:101902001	1	101902001	101905000	3000	3	2.30E-20	0.74	41	1.37	Tmem143;Emp3;Ccdc114	Cytoskeleton
DMR1:101922001	1	101922001	101925000	3000	1	4.60E-07	0.4	31	1.03	Ccdc114	
DMR1:101929001	1	101929001	101934000	5000	3	2.00E-16	0.58	96	1.92	Ccdc114	
DMR1:101936001	1	101936001	101937000	1000	1	3.50E-08	0.32	8	0.8	Ccdc114	
DMR1:101951001	1	101951001	101955000	4000	2	1.20E-12	0.51	85	2.12	Abcc6	Transport
DMR1:101963001	1	101963001	101965000	2000	2	6.60E-13	0.58	24	1.2	Abcc6	Transport
DMR1:101966001	1	101966001	101968000	2000	1	1.40E-07	0.45	21	1.05	Abcc6	Transport
DMR1:101972001	1	101972001	101978000	6000	5	8.60E-28	0.83	86	1.43	Abcc6	Transport
DMR1:101980001	1	101980001	101981000	1000	1	2.70E-08	0.38	15	1.5	Abcc6	Transport
DMR1:101987001	1	101987001	101991000	4000	2	2.30E-33	1.47	60	1.5	Abcc6	Transport
DMR1:101994001	1	101994001	101996000	2000	1	2.80E-18	0.61	28	1.4	Abcc6	Transport
DMR1:102001001	1	102001001	102005000	4000	2	2.40E-18	0.57	73	1.82	Abcc6	Transport
DMR1:102008001	1	102008001	102012000	4000	1	4.30E-14	0.5	66	1.65	Abcc6;Nomo1	Transport
DMR1:102031001	1	102031001	102032000	1000	1	9.30E-07	-0.52	23	2.3	Nomo1	
DMR1:102037001	1	102037001	102042000	5000	1	3.40E-08	0.37	72	1.44	Nomo1	
DMR1:102065001	1	102065001	102066000	1000	1	5.20E-08	-0.41	20	2	Nomo1;LOC103691113	
DMR1:102068001	1	102068001	102070000	2000	1	4.60E-18	0.46	47	2.35	Nomo1;LOC103691113;Ncr3lg1	
DMR1:102071001	1	102071001	102079000	8000	3	1.10E-11	-0.51	126	1.57	Nomo1;Ncr3lg1	
DMR1:102093001	1	102093001	102095000	2000	1	2.20E-07	0.39	29	1.45	Ncr3lg1;LOC102550828;Kcnj11	Transport
DMR1:102096001	1	102096001	102102000	6000	3	2.20E-08	-0.57	84	1.4	Ncr3lg1;LOC102550828;Kcnj11;Abcc8	Transport;Transport
DMR1:102106001	1	102106001	102108000	2000	1	8.20E-07	0.46	76	3.8	LOC102550828;Kcnj11;Abcc8	Transport;Transport
DMR1:102116001	1	102116001	102117000	1000	1	2.40E-07	0.29	17	1.7	Kcnj11;Abcc8	Transport;Transport
DMR1:102122001	1	102122001	102125000	3000	1	6.00E-15	0.6	67	2.23	Abcc8	Transport
DMR1:102136001	1	102136001	102140000	4000	1	1.10E-08	0.44	64	1.6	Abcc8	Transport
DMR1:102147001	1	102147001	102149000	2000	1	6.30E-16	0.47	46	2.3	Abcc8	Transport
DMR1:102154001	1	102154001	102155000	1000	1	1.20E-12	0.46	8	0.8	Abcc8	Transport
DMR1:102156001	1	102156001	102160000	4000	3	6.20E-23	0.83	51	1.27	Abcc8	Transport
DMR1:102169001	1	102169001	102173000	4000	2	2.50E-22	0.62	46	1.15	Abcc8	Transport
DMR1:102174001	1	102174001	102179000	5000	1	6.70E-10	0.45	56	1.12	Abcc8	Transport
DMR1:102187001	1	102187001	102190000	3000	1	4.20E-13	0.42	67	2.23	Abcc8	Transport
DMR1:102221001	1	102221001	102222000	1000	1	8.40E-26	0.76	19	1.9	Ush1c	Cytoskeleton
DMR1:102228001	1	102228001	102229000	1000	1	8.20E-09	-0.58	11	1.1	Ush1c	Cytoskeleton
DMR1:102230001	1	102230001	102232000	2000	1	4.00E-07	0.41	40	2	Ush1c	Cytoskeleton
DMR1:102238001	1	102238001	102239000	1000	1	1.80E-07	-0.52	24	2.4	Ush1c	Cytoskeleton

DMR1:102247001	1	102247001	102254000	7000	3	5.30E-17	-0.81	81	1.16	Ush1c;Otog	Cytoskeleton;Extracellular Matrix
DMR1:102258001	1	102258001	102260000	2000	1	1.70E-15	0.59	33	1.65	Ush1c;Otog	Cytoskeleton;Extracellular Matrix
DMR1:102261001	1	102261001	102264000	3000	3	7.80E-16	0.61	32	1.07	Ush1c;Otog	Cytoskeleton;Extracellular Matrix
DMR1:102279001	1	102279001	102280000	1000	1	3.80E-12	0.47	23	2.3	Otog;LOC103691114	Extracellular Matrix
DMR1:102285001	1	102285001	102287000	2000	1	2.00E-07	-0.56	39	1.95	Otog;LOC103691114	Extracellular Matrix
DMR1:102288001	1	102288001	102290000	2000	1	7.60E-08	0.38	33	1.65	Otog;LOC103691114	Extracellular Matrix
DMR1:102303001	1	102303001	102307000	4000	2	8.70E-27	1.04	70	1.75	Otog;LOC103691114	Extracellular Matrix
DMR1:102308001	1	102308001	102310000	2000	1	2.70E-10	0.47	25	1.25	Otog;LOC103691114	Extracellular Matrix
DMR1:102311001	1	102311001	102314000	3000	1	2.00E-10	0.5	38	1.27	Otog	Extracellular Matrix
DMR1:102318001	1	102318001	102319000	1000	1	3.30E-09	0.53	6	0.6	Otog	Extracellular Matrix
DMR1:102320001	1	102320001	102322000	2000	1	5.30E-07	0.32	51	2.55	Otog	Extracellular Matrix
DMR1:102337001	1	102337001	102341000	4000	1	5.90E-09	0.58	51	1.27	Otog	Extracellular Matrix
DMR1:102388001	1	102388001	102391000	3000	2	9.20E-26	0.69	70	2.33	Myod1	Transcription
DMR1:102403001	1	102403001	102404000	1000	1	3.60E-11	0.43	14	1.4	Myod1	Transcription
DMR1:102407001	1	102407001	102410000	3000	2	6.10E-20	0.82	46	1.53	Myod1;Kcnc1	Transcription;Transport
DMR1:102424001	1	102424001	102425000	1000	1	4.50E-11	0.45	21	2.1	Kcnc1	Transport
DMR1:102426001	1	102426001	102431000	5000	2	5.80E-21	0.69	78	1.56	Kcnc1	Transport
DMR1:102433001	1	102433001	102436000	3000	2	7.10E-17	0.52	60	2	Kcnc1	Transport
DMR1:102441001	1	102441001	102445000	4000	2	6.90E-13	0.77	60	1.5	Kcnc1	Transport
DMR1:102452001	1	102452001	102456000	4000	2	4.80E-13	0.45	101	2.52	Kcnc1;Sergef	Transport;Proteolysis
DMR1:102461001	1	102461001	102463000	2000	2	2.00E-18	0.52	40	2	Kcnc1;Sergef	Transport;Proteolysis
DMR1:102471001	1	102471001	102473000	2000	1	6.80E-07	0.44	21	1.05	Sergef	Proteolysis
DMR1:102479001	1	102479001	102483000	4000	1	4.30E-12	0.49	68	1.7	Sergef	Proteolysis
DMR1:102541001	1	102541001	102549000	8000	4	2.40E-27	-0.56	96	1.2	Sergef	Proteolysis
DMR1:102550001	1	102550001	102554000	4000	1	7.60E-08	0.39	68	1.7	Sergef	Proteolysis
DMR1:102557001	1	102557001	102564000	7000	4	2.60E-29	0.98	88	1.26	Sergef	Proteolysis
DMR1:102570001	1	102570001	102572000	2000	1	1.20E-23	0.74	29	1.45	Sergef	Proteolysis
DMR1:102579001	1	102579001	102580000	1000	1	3.60E-10	0.54	8	0.8	Sergef	Proteolysis
DMR1:102581001	1	102581001	102582000	1000	1	4.80E-07	-0.45	14	1.4	Sergef	Proteolysis
DMR1:102583001	1	102583001	102589000	6000	1	2.00E-07	-0.44	100	1.67	Sergef	Proteolysis
DMR1:102592001	1	102592001	102593000	1000	1	1.10E-08	0.41	16	1.6	Sergef	Proteolysis
DMR1:102595001	1	102595001	102597000	2000	1	4.30E-17	0.74	28	1.4	Sergef	Proteolysis
DMR1:102605001	1	102605001	102608000	3000	3	1.40E-18	0.59	23	0.77	Sergef	Proteolysis
DMR1:102618001	1	102618001	102623000	5000	2	7.00E-14	0.72	61	1.22	Sergef	Proteolysis
DMR1:102630001	1	102630001	102632000	2000	1	2.90E-11	0.48	38	1.9	Sergef	Proteolysis
DMR1:102642001	1	102642001	102646000	4000	2	4.70E-21	0.92	60	1.5	Sergef	Proteolysis
DMR1:102656001	1	102656001	102658000	2000	1	1.60E-07	-0.41	34	1.7	Sergef	Proteolysis
DMR1:102673001	1	102673001	102676000	3000	2	8.30E-19	0.68	39	1.3	Sergef;Tph1	Proteolysis
DMR1:102688001	1	102688001	102691000	3000	1	8.30E-08	0.36	45	1.5	Tph1	
DMR1:102703001	1	102703001	102706000	3000	1	2.90E-21	0.5	43	1.43	Tph1	
DMR1:102727001	1	102727001	102729000	2000	1	2.20E-07	0.29	19	0.95	LOC691143;Saal1	Binding Proteins
DMR1:102766001	1	102766001	102768000	2000	1	6.70E-24	0.76	47	2.35	LOC108349573;Saa4	
DMR1:102820001	1	102820001	102821000	1000	1	2.60E-07	-0.38	13	1.3	Hps5	
DMR1:102833001	1	102833001	102834000	1000	1	2.10E-07	-0.58	14	1.4	Hps5	
DMR1:102838001	1	102838001	102840000	2000	1	1.50E-13	0.46	28	1.4	Hps5;Gtf2h1	Transcription
DMR1:102842001	1	102842001	102845000	3000	2	4.40E-27	0.69	39	1.3	Hps5;Gtf2h1	Transcription
DMR1:102887001	1	102887001	102889000	2000	2	1.20E-12	0.47	37	1.85	Gtf2h1;LOC102552634	Transcription
DMR1:102916001	1	102916001	102917000	1000	1	1.00E-14	0.65	5	0.5	Ldha;Ldhc	Metabolism
DMR1:102928001	1	102928001	102931000	3000	1	1.40E-16	0.57	30	1	Ldhc	Metabolism
DMR1:102941001	1	102941001	102943000	2000	2	2.50E-09	-0.56	28	1.4	Ldhc;Tsg101	Metabolism;Proteolysis
DMR1:102951001	1	102951001	102956000	5000	1	8.20E-14	0.81	43	0.86	Tsg101	Proteolysis
DMR1:102977001	1	102977001	102984000	7000	4	1.90E-17	-0.65	95	1.36	Tsg101;Uevld	Proteolysis
DMR1:102999001	1	102999001	103001000	2000	1	2.20E-11	0.59	32	1.6	Uevld	Proteolysis
DMR1:103004001	1	103004001	103006000	2000	1	8.40E-16	0.54	27	1.35	Uevld	Proteolysis
DMR1:103020001	1	103020001	103021000	1000	1	3.10E-08	0.55	12	1.2	Uevld	Proteolysis
DMR1:103070001	1	103070001	103071000	1000	1	4.30E-07	-0.48	4	0.4	Vom1r70;LOC108349847	Receptor
DMR1:103163001	1	103163001	103165000	2000	1	1.60E-07	0.46	27	1.35	LOC108349285;Tmem86a	
DMR1:103183001	1	103183001	103187000	4000	2	4.50E-08	0.58	40	1	Tmem86a;Igsf22	
DMR1:103195001	1	103195001	103198000	3000	2	2.30E-18	0.64	39	1.3	Igsf22;Ptpn5	
DMR1:103199001	1	103199001	103202000	3000	1	3.40E-08	0.39	48	1.6	Igsf22;Ptpn5	

DMR1:103223001	1	103223001	103226000	3000	2	1.70E-15	-0.49	40	1.33	Ptpn5	
DMR1:103227001	1	103227001	103231000	4000	1	4.70E-08	0.46	51	1.27	Ptpn5	
DMR1:103232001	1	103232001	103233000	1000	1	3.20E-10	0.36	19	1.9	Ptpn5	
DMR1:103267001	1	103267001	103268000	1000	1	1.30E-13	0.74	2	0.2	Ptpn5	
DMR1:103300001	1	103300001	103304000	4000	3	3.70E-10	-0.61	23	0.58	Mrgprx3	Signaling
DMR1:103322001	1	103322001	103326000	4000	1	3.60E-08	0.49	45	1.12	Mrgprx3	Signaling
DMR1:103328001	1	103328001	103329000	1000	1	7.20E-17	0.57	12	1.2	Mrgprx3	Signaling
DMR1:103372001	1	103372001	103373000	1000	1	2.50E-07	-0.53	11	1.1	LOC100364836;LOC108349574;Mrgprx1	Signaling
DMR1:103375001	1	103375001	103377000	2000	1	5.40E-10	0.65	13	0.65	LOC100364836;LOC108349574;Mrgprx1	Signaling
DMR1:103381001	1	103381001	103382000	1000	1	2.80E-14	0.65	4	0.4	LOC108349574;Mrgprx1	Signaling
DMR1:103383001	1	103383001	103385000	2000	1	2.90E-09	0.47	10	0.5	Mrgprx1	Signaling
DMR1:103390001	1	103390001	103392000	2000	2	2.80E-18	0.76	20	1	Mrgprx1	Signaling
DMR1:103420001	1	103420001	103424000	4000	3	6.50E-19	-0.48	58	1.45	Mrgprb4	Signaling
DMR1:103431001	1	103431001	103433000	2000	1	3.10E-07	0.39	14	0.7	Mrgprb4	Signaling
DMR1:103541001	1	103541001	103543000	2000	1	4.40E-08	0.38	19	0.95	Mrgprb5	Signaling
DMR1:103730001	1	103730001	103737000	7000	2	7.00E-18	-0.84	56	0.8	Mrgprx2	Signaling
DMR1:103738001	1	103738001	103739000	1000	1	2.40E-09	-0.54	8	0.8	Mrgprx2	Signaling
DMR1:103749001	1	103749001	103751000	2000	2	6.70E-14	-0.74	9	0.45	Mrgprx2	Signaling
DMR1:103756001	1	103756001	103759000	3000	2	2.90E-10	0.61	11	0.37	Mrgprx2	Signaling
DMR1:103812001	1	103812001	103814000	2000	1	1.00E-09	0.65	10	0.5	Mrgprx2l;LOC365251	Signaling
DMR1:103816001	1	103816001	103819000	3000	2	1.60E-10	-0.68	11	0.37	Mrgprx2l;LOC365251	Signaling
DMR1:103891001	1	103891001	103894000	3000	2	1.70E-08	-0.73	15	0.5	Mrgprb3	Signaling
DMR1:103895001	1	103895001	103896000	1000	1	2.20E-07	0.36	5	0.5	Mrgprb3	Signaling
DMR1:103901001	1	103901001	103906000	5000	3	2.70E-13	-0.65	38	0.76	Mrgprb3	Signaling
DMR1:103911001	1	103911001	103912000	1000	1	1.80E-07	-0.44	5	0.5	Mrgprb3	Signaling
DMR1:103993001	1	103993001	103995000	2000	2	7.60E-27	0.85	19	0.95	RGD1560730	Signaling
DMR1:104002001	1	104002001	104003000	1000	1	1.90E-08	0.48	4	0.4	RGD1560730	Signaling
DMR1:104004001	1	104004001	104006000	2000	1	2.40E-07	-0.88	12	0.6	RGD1560730	Signaling
DMR1:104010001	1	104010001	104018000	8000	5	7.70E-15	-0.5	49	0.61	RGD1560730;Mrgprb2	Signaling
DMR1:104022001	1	104022001	104023000	1000	1	2.00E-09	0.49	2	0.2	RGD1560730;Mrgprb2	Signaling
DMR1:104044001	1	104044001	104047000	3000	1	9.20E-11	-0.39	31	1.03	Mrgprb2	
DMR1:104052001	1	104052001	104054000	2000	2	7.40E-16	0.85	6	0.3	Mrgprb2	
DMR1:104055001	1	104055001	104059000	4000	4	2.20E-08	-0.51	15	0.38	Mrgprb2	
DMR1:104114001	1	104114001	104115000	1000	1	9.20E-09	0.45	11	1.1	Zdhhc13	
DMR1:104116001	1	104116001	104119000	3000	1	6.60E-09	0.31	17	0.57	Zdhhc13	
DMR1:104136001	1	104136001	104140000	4000	1	3.10E-10	0.5	44	1.1	Zdhhc13;Csrp3	Cytoskeleton
DMR1:104141001	1	104141001	104142000	1000	1	9.40E-08	-0.67	10	1	Zdhhc13;Csrp3	Cytoskeleton
DMR1:104168001	1	104168001	104172000	4000	1	5.70E-07	-0.59	44	1.1	Csrp3	Cytoskeleton
DMR1:104173001	1	104173001	104178000	5000	1	9.80E-07	-0.55	58	1.16	Csrp3;E2f8	Cytoskeleton;Transcription
DMR1:104193001	1	104193001	104199000	6000	2	7.50E-20	0.46	143	2.38	E2f8	Transcription
DMR1:104585001	1	104585001	104586000	1000	1	2.30E-15	0.65	15	1.5	Nav2	
DMR1:104612001	1	104612001	104616000	4000	2	3.20E-09	-0.53	55	1.38	Nav2	
DMR1:104622001	1	104622001	104624000	2000	1	7.20E-07	0.34	26	1.3	Nav2	
DMR1:104631001	1	104631001	104635000	4000	1	4.40E-10	0.61	59	1.48	Nav2	
DMR1:104654001	1	104654001	104656000	2000	1	4.10E-10	0.56	24	1.2	Nav2	
DMR1:104677001	1	104677001	104679000	2000	1	1.50E-07	-0.48	34	1.7	Nav2	
DMR1:104681001	1	104681001	104682000	1000	1	7.00E-10	0.44	7	0.7	Nav2	
DMR1:104683001	1	104683001	104685000	2000	1	9.60E-07	-0.58	26	1.3	Nav2	
DMR1:104693001	1	104693001	104700000	7000	1	6.50E-08	-0.73	140	2	Nav2	
DMR1:104718001	1	104718001	104721000	3000	2	1.90E-15	0.78	28	0.93	Nav2	
DMR1:104724001	1	104724001	104725000	1000	1	1.20E-09	0.41	16	1.6	Nav2	
DMR1:104739001	1	104739001	104744000	5000	1	1.60E-07	-0.42	92	1.84	Nav2	
DMR1:104755001	1	104755001	104758000	3000	2	2.70E-14	0.51	42	1.4	Nav2	
DMR1:104766001	1	104766001	104767000	1000	1	1.30E-09	0.72	19	1.9	Nav2	
DMR1:104775001	1	104775001	104778000	3000	2	1.20E-10	-0.55	49	1.63	Nav2	
DMR1:104782001	1	104782001	104784000	2000	1	6.00E-07	0.33	37	1.85	Nav2	
DMR1:104792001	1	104792001	104798000	6000	1	8.40E-11	0.56	133	2.22	Nav2	
DMR1:104800001	1	104800001	104801000	1000	1	7.00E-11	0.42	17	1.7	Nav2	
DMR1:104832001	1	104832001	104834000	2000	1	8.30E-11	0.65	26	1.3	Nav2	
DMR1:104836001	1	104836001	104838000	2000	2	5.70E-13	0.49	24	1.2	Nav2	
DMR1:104853001	1	104853001	104856000	3000	1	1.90E-15	0.6	52	1.73	Nav2	
DMR1:104857001	1	104857001	104858000	1000	1	4.00E-21	0.65	18	1.8	Nav2	

DMR1:104860001	1	104860001	104861000	1000	1	2.70E-19	0.68	12	1.2	Nav2	
DMR1:104871001	1	104871001	104873000	2000	1	1.90E-07	-0.61	25	1.25	Nav2	
DMR1:104885001	1	104885001	104893000	8000	1	1.60E-10	0.45	153	1.91	Nav2	
DMR1:104904001	1	104904001	104907000	3000	1	5.20E-07	0.32	47	1.57	Nav2	
DMR1:104916001	1	104916001	104921000	5000	1	7.80E-13	0.5	81	1.62	Nav2	
DMR1:104922001	1	104922001	104924000	2000	1	5.00E-09	-0.5	32	1.6	Nav2	
DMR1:104945001	1	104945001	104949000	4000	1	9.50E-09	0.42	59	1.48	Nav2	
DMR1:105105001	1	105105001	105107000	2000	1	3.10E-12	0.43	40	2	Htatip2;Prmt3	Metabolism;Golgi
DMR1:105117001	1	105117001	105118000	1000	1	1.20E-07	-0.6	7	0.7	Htatip2;Prmt3	Metabolism;Golgi
DMR1:105124001	1	105124001	105126000	2000	1	1.80E-08	-0.48	19	0.95	Prmt3	Golgi
DMR1:105131001	1	105131001	105132000	1000	1	2.50E-25	1.03	1	0.1	Prmt3	Golgi
DMR1:105152001	1	105152001	105153000	1000	1	3.70E-10	0.6	7	0.7	Prmt3	Golgi
DMR1:105173001	1	105173001	105175000	2000	1	6.50E-09	-0.48	14	0.7	Prmt3	Golgi
DMR1:105192001	1	105192001	105195000	3000	1	2.20E-08	0.58	21	0.7	Prmt3	Golgi
DMR1:105288001	1	105288001	105290000	2000	1	2.60E-07	0.36	36	1.8	Slc6a5	Transport
DMR1:105298001	1	105298001	105303000	5000	1	5.10E-07	-0.46	68	1.36	Slc6a5	Transport
DMR1:105308001	1	105308001	105312000	4000	1	1.10E-07	0.42	69	1.73	Slc6a5	Transport
DMR1:105346001	1	105346001	105348000	2000	1	6.40E-11	0.49	27	1.35	Slc6a5;Nell1	Transport;Signaling
DMR1:105351001	1	105351001	105355000	4000	1	4.80E-12	0.56	53	1.32	Nell1	Signaling
DMR1:105365001	1	105365001	105368000	3000	1	2.20E-11	0.49	34	1.13	Nell1	Signaling
DMR1:105379001	1	105379001	105381000	2000	1	1.20E-09	0.59	21	1.05	Nell1	Signaling
DMR1:105382001	1	105382001	105384000	2000	2	2.70E-21	0.78	22	1.1	Nell1	Signaling
DMR1:105389001	1	105389001	105390000	1000	1	3.30E-19	0.72	10	1	Nell1	Signaling
DMR1:105394001	1	105394001	105396000	2000	2	6.90E-29	1.02	13	0.65	Nell1	Signaling
DMR1:105397001	1	105397001	105398000	1000	1	2.10E-13	0.68	4	0.4	Nell1	Signaling
DMR1:105399001	1	105399001	105401000	2000	1	8.00E-08	0.31	38	1.9	Nell1	Signaling
DMR1:105403001	1	105403001	105406000	3000	1	8.70E-21	1	34	1.13	Nell1	Signaling
DMR1:105414001	1	105414001	105416000	2000	1	1.10E-15	0.83	9	0.45	Nell1	Signaling
DMR1:105417001	1	105417001	105419000	2000	1	3.10E-09	0.38	27	1.35	Nell1	Signaling
DMR1:105425001	1	105425001	105426000	1000	1	2.90E-14	0.65	14	1.4	Nell1	Signaling
DMR1:105435001	1	105435001	105438000	3000	1	1.80E-07	0.45	26	0.87	Nell1	Signaling
DMR1:105448001	1	105448001	105452000	4000	2	1.20E-12	-0.51	27	0.68	Nell1	Signaling
DMR1:105457001	1	105457001	105460000	3000	2	7.10E-13	0.57	26	0.87	Nell1	Signaling
DMR1:105461001	1	105461001	105469000	8000	3	1.90E-20	0.76	101	1.26	Nell1	Signaling
DMR1:105478001	1	105478001	105480000	2000	1	8.10E-08	0.32	21	1.05	Nell1	Signaling
DMR1:105488001	1	105488001	105489000	1000	1	1.50E-14	0.6	6	0.6	Nell1	Signaling
DMR1:105496001	1	105496001	105499000	3000	1	3.70E-18	0.55	38	1.27	Nell1	Signaling
DMR1:105500001	1	105500001	105502000	2000	1	1.30E-07	0.4	20	1	Nell1	Signaling
DMR1:105504001	1	105504001	105506000	2000	1	5.70E-08	0.47	22	1.1	Nell1	Signaling
DMR1:105507001	1	105507001	105508000	1000	1	9.20E-07	0.32	13	1.3	Nell1	Signaling
DMR1:105515001	1	105515001	105517000	2000	1	5.40E-07	-0.31	22	1.1	Nell1	Signaling
DMR1:105530001	1	105530001	105543000	13000	5	7.20E-20	0.49	133	1.02	Nell1	Signaling
DMR1:105554001	1	105554001	105559000	5000	1	2.20E-09	-0.72	74	1.48	Nell1	Signaling
DMR1:105565001	1	105565001	105571000	6000	3	2.50E-25	-0.51	69	1.15	Nell1	Signaling
DMR1:105575001	1	105575001	105576000	1000	1	2.20E-07	0.58	3	0.3	Nell1	Signaling
DMR1:105581001	1	105581001	105582000	1000	1	2.60E-18	0.98	4	0.4	Nell1	Signaling
DMR1:105591001	1	105591001	105593000	2000	1	1.20E-07	0.34	24	1.2	Nell1	Signaling
DMR1:105594001	1	105594001	105597000	3000	1	1.40E-08	0.45	17	0.57	Nell1	Signaling
DMR1:105608001	1	105608001	105609000	1000	1	1.30E-07	-0.63	14	1.4	Nell1	Signaling
DMR1:105611001	1	105611001	105615000	4000	1	1.60E-09	0.47	40	1	Nell1;LOC102557182	Signaling
DMR1:105643001	1	105643001	105644000	1000	1	3.00E-09	0.55	4	0.4	Nell1	Signaling
DMR1:105656001	1	105656001	105658000	2000	1	9.00E-13	0.71	13	0.65	Nell1	Signaling
DMR1:105665001	1	105665001	105668000	3000	1	1.70E-15	0.84	33	1.1	Nell1	Signaling
DMR1:105680001	1	105680001	105681000	1000	1	3.00E-14	0.48	15	1.5	Nell1	Signaling
DMR1:105682001	1	105682001	105684000	2000	1	1.10E-13	0.7	14	0.7	Nell1	Signaling
DMR1:105698001	1	105698001	105699000	1000	1	1.30E-13	0.56	11	1.1	Nell1	Signaling
DMR1:105700001	1	105700001	105701000	1000	1	2.00E-16	0.64	17	1.7	Nell1	Signaling
DMR1:105704001	1	105704001	105709000	5000	1	4.10E-09	0.49	48	0.96	Nell1	Signaling
DMR1:105711001	1	105711001	105713000	2000	1	9.80E-14	0.83	15	0.75	Nell1	Signaling
DMR1:105756001	1	105756001	105757000	1000	1	1.50E-10	0.42	13	1.3	Nell1	Signaling
DMR1:105769001	1	105769001	105770000	1000	1	4.10E-14	0.61	8	0.8	Nell1	Signaling
DMR1:105794001	1	105794001	105796000	2000	1	2.40E-14	0.57	45	2.25	Nell1	Signaling
DMR1:105801001	1	105801001	105803000	2000	1	1.40E-12	0.65	24	1.2	Nell1	Signaling
DMR1:105808001	1	105808001	105809000	1000	1	4.70E-13	0.54	7	0.7	Nell1	Signaling
DMR1:105816001	1	105816001	105818000	2000	1	1.50E-15	0.63	19	0.95	Nell1	Signaling

DMR1:105819001	1	105819001	105820000	1000	1	8.00E-08	0.43	5	0.5	Nell1	Signaling
DMR1:105832001	1	105832001	105837000	5000	2	3.20E-18	0.64	52	1.04	Nell1	Signaling
DMR1:105846001	1	105846001	105849000	3000	1	1.30E-17	0.65	20	0.67	Nell1	Signaling
DMR1:105858001	1	105858001	105859000	1000	1	4.60E-21	0.97	5	0.5	Nell1	Signaling
DMR1:105873001	1	105873001	105879000	6000	2	1.30E-26	-0.71	50	0.83	Nell1	Signaling
DMR1:105897001	1	105897001	105898000	1000	1	3.70E-16	0.57	12	1.2	Nell1	Signaling
DMR1:105899001	1	105899001	105900000	1000	1	3.10E-08	0.38	8	0.8	Nell1	Signaling
DMR1:105903001	1	105903001	105905000	2000	1	1.10E-07	0.38	28	1.4	Nell1	Signaling
DMR1:105923001	1	105923001	105924000	1000	1	4.00E-12	0.79	7	0.7	Nell1	Signaling
DMR1:105928001	1	105928001	105931000	3000	1	6.10E-10	0.57	29	0.97	Nell1;LOC100125384	Signaling
DMR1:105965001	1	105965001	105967000	2000	1	6.20E-09	0.64	12	0.6	Nell1	Signaling
DMR1:105977001	1	105977001	105980000	3000	1	3.10E-09	0.46	23	0.77	Nell1	Signaling
DMR1:106002001	1	106002001	106005000	3000	1	4.50E-08	0.52	12	0.4	Nell1	Signaling
DMR1:106006001	1	106006001	106009000	3000	2	2.20E-11	-0.4	32	1.07	Nell1	Signaling
DMR1:106021001	1	106021001	106022000	1000	1	7.00E-07	0.42	5	0.5	Nell1	Signaling
DMR1:106032001	1	106032001	106033000	1000	1	3.10E-15	0.76	3	0.3	Nell1	Signaling
DMR1:106042001	1	106042001	106044000	2000	1	1.40E-09	0.46	33	1.65	Nell1	Signaling
DMR1:106077001	1	106077001	106078000	1000	1	2.30E-22	0.96	9	0.9	Nell1	Signaling
DMR1:106097001	1	106097001	106100000	3000	2	1.20E-07	-0.56	17	0.57	Nell1	Signaling
DMR1:106111001	1	106111001	106112000	1000	1	8.20E-10	0.49	6	0.6	Nell1;LOC103691116	Signaling
DMR1:106128001	1	106128001	106131000	3000	1	1.10E-18	0.67	20	0.67	Nell1	Signaling
DMR1:106133001	1	106133001	106137000	4000	1	1.00E-09	0.49	38	0.95	Nell1	Signaling
DMR1:106150001	1	106150001	106154000	4000	3	1.10E-15	0.72	28	0.7	Nell1	Signaling
DMR1:106175001	1	106175001	106176000	1000	1	1.70E-07	0.57	5	0.5	Nell1	Signaling
DMR1:106202001	1	106202001	106204000	2000	1	1.40E-10	-0.76	23	1.15	Nell1	Signaling
DMR1:106208001	1	106208001	106210000	2000	2	2.20E-11	-0.54	28	1.4	Nell1	Signaling
DMR1:106222001	1	106222001	106224000	2000	2	3.40E-13	0.72	18	0.9	Nell1	Signaling
DMR1:106868001	1	106868001	106869000	1000	1	5.60E-09	-0.66	9	0.9	Ano5	
DMR1:106893001	1	106893001	106894000	1000	1	2.80E-10	0.5	6	0.6	Ano5	
DMR1:106904001	1	106904001	106905000	1000	1	5.20E-09	0.46	7	0.7	Ano5	
DMR1:106906001	1	106906001	106909000	3000	2	6.20E-15	0.63	28	0.93	Ano5;LOC102550279	
DMR1:106944001	1	106944001	106947000	3000	2	1.60E-10	0.5	36	1.2	Ano5	
DMR1:106978001	1	106978001	106979000	1000	1	6.20E-09	0.69	2	0.2	Ano5;Slc17a6	Transport
DMR1:106982001	1	106982001	106983000	1000	1	5.80E-09	0.44	12	1.2	Slc17a6	Transport
DMR1:106994001	1	106994001	106995000	1000	1	4.40E-07	0.4	5	0.5	Slc17a6	Transport
DMR1:107047001	1	107047001	107051000	4000	3	1.80E-13	0.67	24	0.6	Slc17a6	Transport
DMR1:107248001	1	107248001	107249000	1000	1	4.00E-07	-0.57	6	0.6	Gas2	
DMR1:107252001	1	107252001	107254000	2000	1	6.10E-16	0.79	9	0.45	Gas2	
DMR1:107260001	1	107260001	107261000	1000	1	9.40E-08	0.71	2	0.2	Gas2	
DMR1:107287001	1	107287001	107288000	1000	1	1.20E-08	0.4	7	0.7	Gas2	
DMR1:107387001	1	107387001	107392000	5000	2	4.80E-10	-0.71	15	0.3	Ccdc179	
DMR1:107393001	1	107393001	107394000	1000	1	2.10E-09	-0.55	4	0.4	Ccdc179	
DMR1:109631001	1	109631001	109633000	2000	1	2.60E-15	0.94	5	0.25	Hspd1-ps7;LOC103691117	
DMR1:109634001	1	109634001	109636000	2000	1	6.60E-09	0.74	13	0.65	Hspd1-ps7;LOC103691117	
DMR1:111839001	1	111839001	111841000	2000	1	6.50E-09	0.53	11	0.55	Luzp2	
DMR1:111858001	1	111858001	111859000	1000	1	1.40E-07	0.56	7	0.7	Luzp2	
DMR1:111867001	1	111867001	111869000	2000	1	6.00E-29	0.95	6	0.3	Luzp2	
DMR1:111904001	1	111904001	111905000	1000	1	3.00E-07	0.47	4	0.4	Luzp2	
DMR1:111944001	1	111944001	111945000	1000	1	1.10E-09	0.53	8	0.8	Luzp2	
DMR1:111973001	1	111973001	111974000	1000	1	9.80E-08	0.5	10	1	Luzp2	
DMR1:112004001	1	112004001	112007000	3000	1	5.70E-09	0.43	19	0.63	Luzp2	
DMR1:112011001	1	112011001	112012000	1000	1	3.10E-07	0.5	9	0.9	Luzp2	
DMR1:112051001	1	112051001	112052000	1000	1	1.80E-12	0.52	10	1	Luzp2	
DMR1:112199001	1	112199001	112200000	1000	1	8.80E-10	0.43	15	1.5	Gabrg3	Ion Channel
DMR1:112233001	1	112233001	112234000	1000	1	9.30E-08	0.45	5	0.5	Gabrg3	Ion Channel
DMR1:112241001	1	112241001	112242000	1000	1	1.60E-07	0.65	6	0.6	Gabrg3	Ion Channel
DMR1:112280001	1	112280001	112281000	1000	1	2.20E-13	0.67	4	0.4	Gabrg3;LOC103691121	Ion Channel
DMR1:112294001	1	112294001	112295000	1000	1	5.60E-14	0.66	4	0.4	Gabrg3	Ion Channel
DMR1:112319001	1	112319001	112320000	1000	1	3.70E-09	0.47	5	0.5	Gabrg3	Ion Channel
DMR1:112325001	1	112325001	112326000	1000	1	2.50E-13	0.74	6	0.6	Gabrg3	Ion Channel
DMR1:112330001	1	112330001	112332000	2000	2	5.70E-14	0.93	11	0.55	Gabrg3	Ion Channel
DMR1:112334001	1	112334001	112336000	2000	1	1.30E-10	0.53	15	0.75	Gabrg3	Ion Channel
DMR1:112339001	1	112339001	112340000	1000	1	6.50E-07	-0.34	8	0.8	Gabrg3	Ion Channel
DMR1:112346001	1	112346001	112347000	1000	1	1.30E-08	0.51	3	0.3	Gabrg3	Ion Channel
DMR1:112354001	1	112354001	112356000	2000	1	7.30E-11	0.49	12	0.6	Gabrg3	Ion Channel

DMR1:112362001	1	112362001	112363000	1000	1	1.30E-07	0.6	6	0.6	Gabrg3	Ion Channel
DMR1:112364001	1	112364001	112365000	1000	1	9.40E-07	-0.57	2	0.2	Gabrg3	Ion Channel
DMR1:112372001	1	112372001	112374000	2000	1	1.00E-12	0.85	11	0.55	Gabrg3	Ion Channel
DMR1:112375001	1	112375001	112378000	3000	2	4.00E-16	0.72	18	0.6	Gabrg3	Ion Channel
DMR1:112437001	1	112437001	112439000	2000	1	1.60E-22	0.89	5	0.25	Gabrg3	Ion Channel
DMR1:112447001	1	112447001	112452000	5000	3	1.30E-25	-0.57	55	1.1	Gabrg3	Ion Channel
DMR1:112453001	1	112453001	112456000	3000	1	3.90E-09	0.52	25	0.83	Gabrg3	Ion Channel
DMR1:112463001	1	112463001	112466000	3000	2	5.00E-13	0.68	12	0.4	Gabrg3	Ion Channel
DMR1:112479001	1	112479001	112480000	1000	1	2.40E-11	0.66	2	0.2	Gabrg3	Ion Channel
DMR1:112501001	1	112501001	112503000	2000	1	5.70E-07	0.4	16	0.8	Gabrg3	Ion Channel
DMR1:112522001	1	112522001	112523000	1000	1	1.00E-08	0.59	5	0.5	Gabrg3	Ion Channel
DMR1:112596001	1	112596001	112597000	1000	1	5.50E-08	0.41	10	1	Gabrg3	Ion Channel
DMR1:112599001	1	112599001	112602000	3000	1	3.10E-07	0.59	18	0.6	Gabrg3	Ion Channel
DMR1:112633001	1	112633001	112635000	2000	2	9.90E-11	0.57	10	0.5	Gabrg3	Ion Channel
DMR1:112659001	1	112659001	112660000	1000	1	9.40E-10	-0.42	11	1.1	Gabrg3	Ion Channel
DMR1:112711001	1	112711001	112712000	1000	1	4.30E-08	0.5	4	0.4	Gabrg3	Ion Channel
DMR1:112721001	1	112721001	112729000	8000	3	2.40E-15	-0.51	85	1.06	Gabrg3	Ion Channel
DMR1:112758001	1	112758001	112763000	5000	1	6.90E-07	-0.58	22	0.44	Gabrg3	Ion Channel
DMR1:112770001	1	112770001	112775000	5000	1	2.30E-07	0.51	28	0.56	Gabrg3	Ion Channel
DMR1:112783001	1	112783001	112791000	8000	3	6.70E-24	-0.55	70	0.88	Gabrg3	Ion Channel
DMR1:112803001	1	112803001	112804000	1000	1	3.30E-11	0.73	8	0.8	Gabrg3	Ion Channel
DMR1:112824001	1	112824001	112827000	3000	1	1.10E-09	-0.74	19	0.63	Gabra5	Ion Channel
DMR1:112836001	1	112836001	112838000	2000	2	9.90E-14	-0.6	5	0.25	Gabra5	Ion Channel
DMR1:112849001	1	112849001	112853000	4000	3	9.80E-19	-0.56	45	1.12	Gabra5	Ion Channel
DMR1:112888001	1	112888001	112890000	2000	1	7.90E-08	-0.6	15	0.75	Gabra5	Ion Channel
DMR1:112911001	1	112911001	112913000	2000	1	2.70E-16	0.66	17	0.85	Gabra5	Ion Channel
DMR1:112919001	1	112919001	112922000	3000	1	1.20E-10	0.49	21	0.7	Gabra5	Ion Channel
DMR1:112937001	1	112937001	112938000	1000	1	1.60E-09	0.41	7	0.7	Gabra5	Ion Channel
DMR1:112952001	1	112952001	112954000	2000	1	1.30E-07	0.49	11	0.55	Gabra5	Ion Channel
DMR1:113036001	1	113036001	113039000	3000	1	1.10E-10	0.49	34	1.13	Gabrb3	Ion Channel
DMR1:113093001	1	113093001	113095000	2000	1	1.60E-07	0.52	8	0.4	Gabrb3	Ion Channel
DMR1:113103001	1	113103001	113104000	1000	1	3.20E-09	0.74	5	0.5	Gabrb3	Ion Channel
DMR1:113107001	1	113107001	113108000	1000	1	9.00E-07	0.48	3	0.3	Gabrb3	Ion Channel
DMR1:113115001	1	113115001	113118000	3000	2	6.50E-09	-0.5	19	0.63	Gabrb3	Ion Channel
DMR1:113129001	1	113129001	113130000	1000	1	3.40E-14	0.77	2	0.2	Gabrb3	Ion Channel
DMR1:113141001	1	113141001	113143000	2000	2	5.40E-11	0.85	7	0.35	Gabrb3	Ion Channel
DMR1:113147001	1	113147001	113149000	2000	2	1.90E-09	0.51	19	0.95	Gabrb3	Ion Channel
DMR1:113164001	1	113164001	113165000	1000	1	2.50E-09	0.6	7	0.7	Gabrb3	Ion Channel
DMR1:113170001	1	113170001	113172000	2000	1	3.50E-09	0.38	18	0.9	Gabrb3	Ion Channel
DMR1:113179001	1	113179001	113181000	2000	1	1.60E-10	0.57	27	1.35	Gabrb3	Ion Channel
DMR1:113194001	1	113194001	113196000	2000	1	1.90E-09	0.68	5	0.25	Gabrb3	Ion Channel
DMR1:113223001	1	113223001	113225000	2000	2	4.70E-09	0.51	9	0.45	Gabrb3	Ion Channel
DMR1:113228001	1	113228001	113230000	2000	1	1.20E-11	0.73	15	0.75	Gabrb3	Ion Channel
DMR1:113414001	1	113414001	113417000	3000	1	4.50E-09	0.55	14	0.47	Luzp2	
DMR1:113421001	1	113421001	113423000	2000	1	4.30E-07	-0.46	19	0.95	Luzp2	
DMR1:113427001	1	113427001	113429000	2000	1	1.00E-08	0.49	18	0.9	Luzp2	
DMR1:113436001	1	113436001	113438000	2000	1	1.00E-07	-0.61	5	0.25	Luzp2	
DMR1:113448001	1	113448001	113450000	2000	1	1.10E-07	-0.35	14	0.7	Luzp2	
DMR1:113484001	1	113484001	113485000	1000	1	6.90E-07	0.54	3	0.3	Luzp2;LOC100361285	
DMR1:113486001	1	113486001	113491000	5000	1	1.20E-10	0.76	38	0.76	Luzp2;LOC100361285;LOC103691130	
DMR1:113497001	1	113497001	113498000	1000	1	3.60E-08	0.6	12	1.2	Luzp2;LOC100361285;LOC103691130	
DMR1:113520001	1	113520001	113522000	2000	2	2.90E-17	1.08	6	0.3	Luzp2	
DMR1:113535001	1	113535001	113536000	1000	1	5.50E-17	0.87	6	0.6	Luzp2	
DMR1:113538001	1	113538001	113539000	1000	1	7.20E-09	0.55	4	0.4	Luzp2	
DMR1:114040001	1	114040001	114044000	4000	1	2.70E-09	0.53	48	1.2	Siglech	
DMR1:114048001	1	114048001	114049000	1000	1	1.60E-12	0.61	13	1.3	Siglech	
DMR1:114069001	1	114069001	114071000	2000	2	1.90E-15	0.5	19	0.95	Siglech	
DMR1:114078001	1	114078001	114079000	1000	1	1.50E-08	0.33	14	1.4	Siglech	
DMR1:114087001	1	114087001	114093000	6000	3	2.70E-15	0.54	52	0.87	Siglech	
DMR1:114132001	1	114132001	114134000	2000	1	1.00E-15	-0.79	8	0.4	Siglech	
DMR1:114135001	1	114135001	114141000	6000	4	1.40E-16	-0.56	71	1.18	Siglech;LOC108349722	
DMR1:114156001	1	114156001	114157000	1000	1	2.80E-19	0.87	12	1.2	Siglech	
DMR1:114162001	1	114162001	114165000	3000	2	6.40E-12	-0.51	12	0.4	Siglech;LOC100911716	

DMR1:114180001	1	114180001	114184000	4000	2	2.00E-09	0.55	28	0.7	LOC100911716;Tubgcp5	Cytoskeleton
DMR1:114260001	1	114260001	114263000	3000	1	3.50E-11	0.53	30	1	Cyfip1	Cytoskeleton
DMR1:114274001	1	114274001	114275000	1000	1	1.50E-07	0.31	12	1.2	Cyfip1	Cytoskeleton
DMR1:114299001	1	114299001	114302000	3000	2	1.10E-07	-0.59	16	0.53	Cyfip1	Cytoskeleton
DMR1:114303001	1	114303001	114308000	5000	3	2.80E-14	0.48	49	0.98	Cyfip1	Cytoskeleton
DMR1:114364001	1	114364001	114366000	2000	1	1.90E-16	0.69	17	0.85	Nipa2;LOC108348727	
DMR1:114379001	1	114379001	114381000	2000	1	3.40E-15	0.76	8	0.4	Nipa2;LOC108348727;Nipa1	
DMR1:114406001	1	114406001	114407000	1000	1	7.40E-10	0.5	17	1.7	LOC108348727;Nipa1	
DMR1:114429001	1	114429001	114433000	4000	1	2.30E-08	0.35	39	0.98	Nipa1;LOC108349578	
DMR1:114445001	1	114445001	114446000	1000	1	1.30E-09	0.59	4	0.4	LOC108349578;Herc2	Transcription
DMR1:114450001	1	114450001	114452000	2000	1	4.00E-07	0.34	13	0.65	LOC108349578;Herc2	Transcription
DMR1:114458001	1	114458001	114460000	2000	1	9.70E-07	0.45	15	0.75	LOC108349578;Herc2	Transcription
DMR1:114467001	1	114467001	114468000	1000	1	7.10E-07	0.4	8	0.8	Herc2	Transcription
DMR1:114485001	1	114485001	114486000	1000	1	9.90E-11	0.57	8	0.8	Herc2	Transcription
DMR1:114495001	1	114495001	114499000	4000	1	9.40E-11	0.48	31	0.78	Herc2	Transcription
DMR1:114501001	1	114501001	114502000	1000	1	1.00E-08	0.5	3	0.3	Herc2	Transcription
DMR1:114508001	1	114508001	114510000	2000	1	3.70E-09	-0.67	11	0.55	Herc2	Transcription
DMR1:114519001	1	114519001	114526000	7000	3	8.70E-29	-0.54	76	1.09	Herc2	Transcription
DMR1:114531001	1	114531001	114534000	3000	1	2.10E-13	0.82	23	0.77	Herc2	Transcription
DMR1:114536001	1	114536001	114542000	6000	1	2.90E-19	0.75	40	0.67	Herc2	Transcription
DMR1:114547001	1	114547001	114549000	2000	1	9.00E-10	0.4	10	0.5	Herc2	Transcription
DMR1:114563001	1	114563001	114565000	2000	1	8.60E-13	0.56	16	0.8	Herc2	Transcription
DMR1:114585001	1	114585001	114588000	3000	3	1.40E-14	0.7	16	0.53	Herc2	Transcription
DMR1:114590001	1	114590001	114591000	1000	1	1.00E-08	-0.43	8	0.8	Herc2	Transcription
DMR1:114593001	1	114593001	114595000	2000	2	9.90E-18	-0.53	19	0.95	Herc2	Transcription
DMR1:114609001	1	114609001	114613000	4000	1	2.90E-11	-0.52	43	1.07	Herc2	Transcription
DMR1:114620001	1	114620001	114622000	2000	1	1.50E-09	-0.61	7	0.35	Herc2	Transcription
DMR1:114631001	1	114631001	114634000	3000	1	1.10E-27	0.98	23	0.77	Herc2	Transcription
DMR1:114660001	1	114660001	114661000	1000	1	2.80E-07	0.46	10	1	Herc2;LOC100365773	Transcription
DMR1:115962001	1	115962001	115964000	2000	1	1.20E-09	0.59	8	0.4	Atp10a	Transport
DMR1:115985001	1	115985001	115986000	1000	1	3.20E-08	0.52	10	1	Atp10a	Transport
DMR1:115993001	1	115993001	115995000	2000	1	1.90E-12	0.68	21	1.05	Atp10a	Transport
DMR1:116006001	1	116006001	116010000	4000	1	1.70E-13	0.68	33	0.82	Atp10a	Transport
DMR1:116015001	1	116015001	116017000	2000	1	1.50E-23	0.76	14	0.7	Atp10a	Transport
DMR1:116029001	1	116029001	116031000	2000	2	1.10E-08	-0.48	21	1.05	Atp10a	Transport
DMR1:116050001	1	116050001	116051000	1000	1	2.20E-18	0.81	11	1.1	Atp10a	Transport
DMR1:116074001	1	116074001	116076000	2000	2	2.00E-14	-0.48	16	0.8	Atp10a	Transport
DMR1:116082001	1	116082001	116089000	7000	4	2.70E-28	-0.56	62	0.89	Atp10a	Transport
DMR1:116097001	1	116097001	116098000	1000	1	3.10E-12	-0.93	8	0.8	Atp10a	Transport
DMR1:116109001	1	116109001	116111000	2000	1	7.70E-16	0.74	4	0.2	Atp10a	Transport
DMR1:116124001	1	116124001	116126000	2000	1	1.50E-07	0.45	16	0.8	Atp10a	Transport
DMR1:116147001	1	116147001	116152000	5000	1	1.70E-07	0.4	39	0.78	Atp10a;Fpr3;LOC108349580	Transport;Signaling
DMR1:116583001	1	116583001	116585000	2000	1	1.60E-11	0.63	8	0.4	Ube3a	Proteolysis
DMR1:116620001	1	116620001	116624000	4000	2	1.20E-07	-0.58	23	0.58	Ube3a	Proteolysis
DMR1:116629001	1	116629001	116634000	5000	1	3.20E-07	0.46	35	0.7	Ube3a	Proteolysis
DMR1:116666001	1	116666001	116669000	3000	3	1.80E-12	0.81	10	0.33	Ube3a;LOC103691125	Proteolysis
DMR1:116670001	1	116670001	116673000	3000	2	1.60E-16	0.6	23	0.77	Ube3a;LOC103691125;LOC103691124	Proteolysis
DMR1:120218001	1	120218001	120219000	1000	1	3.70E-08	-0.58	10	1	RGD1560408	
DMR1:120234001	1	120234001	120235000	1000	1	6.50E-22	0.96	2	0.2	RGD1560408	
DMR1:122301001	1	122301001	122302000	1000	1	7.40E-09	0.44	7	0.7	Mir344a-2	
DMR1:122307001	1	122307001	122308000	1000	1	1.40E-08	-0.36	13	1.3	Mir344a-2	
DMR1:122404001	1	122404001	122405000	1000	1	3.90E-17	0.85	6	0.6	Mir344g	
DMR1:122407001	1	122407001	122409000	2000	2	1.90E-24	0.86	11	0.55	Mir344g	
DMR1:122983001	1	122983001	122986000	3000	1	5.80E-08	0.48	29	0.97	Ndn	Cytoskeleton
DMR1:123027001	1	123027001	123028000	1000	1	5.00E-09	0.54	12	1.2	Magel2	Cytoskeleton
DMR1:123054001	1	123054001	123055000	1000	1	8.00E-08	0.5	7	0.7	Mkrn3	Proteolysis
DMR1:123103001	1	123103001	123109000	6000	3	1.00E-18	0.64	72	1.2	Peg12;LOC108349584;LOC679961	
DMR1:123298001	1	123298001	123300000	2000	1	3.70E-09	0.6	10	0.5	Vom2r-ps62	
DMR1:123304001	1	123304001	123306000	2000	2	2.10E-08	0.6	6	0.3	Vom2r-ps62	
DMR1:123309001	1	123309001	123313000	4000	1	1.40E-08	-0.78	10	0.25	Vom2r-ps62	
DMR1:123325001	1	123325001	123331000	6000	2	8.90E-14	-0.46	60	1	Vom2r-ps62	
DMR1:123335001	1	123335001	123343000	8000	5	1.20E-23	-0.55	86	1.07	Vom2r-ps62	
DMR1:123903001	1	123903001	123904000	1000	1	1.40E-14	0.53	24	2.4	Chrna7	Ion Channel

DMR1:123980001	1	123980001	123985000	5000	1	5.70E-07	-0.49	49	0.98	Chrna7	Ion Channel
DMR1:124010001	1	124010001	124011000	1000	1	2.80E-07	-0.49	8	0.8	Chrna7	Ion Channel
DMR1:124022001	1	124022001	124023000	1000	1	9.40E-07	-0.55	8	0.8	Chrna7	Ion Channel
DMR1:124323001	1	124323001	124327000	4000	1	7.50E-08	0.37	37	0.92	Otud7a	Protease
DMR1:124328001	1	124328001	124329000	1000	1	1.40E-09	0.6	3	0.3	Otud7a	Protease
DMR1:124359001	1	124359001	124360000	1000	1	8.30E-10	0.76	5	0.5	Otud7a	Protease
DMR1:124361001	1	124361001	124362000	1000	1	1.70E-09	-0.77	12	1.2	Otud7a	Protease
DMR1:124400001	1	124400001	124401000	1000	1	1.20E-07	0.45	7	0.7	Otud7a;LOC680151	Protease
DMR1:124411001	1	124411001	124414000	3000	1	1.70E-11	-0.38	60	2	Otud7a	Protease
DMR1:124426001	1	124426001	124428000	2000	1	4.40E-13	0.71	15	0.75	Otud7a	Protease
DMR1:124436001	1	124436001	124437000	1000	1	7.50E-15	0.66	3	0.3	Otud7a	Protease
DMR1:124438001	1	124438001	124439000	1000	1	4.90E-08	0.43	7	0.7	Otud7a	Protease
DMR1:124442001	1	124442001	124443000	1000	1	9.30E-12	0.44	8	0.8	Otud7a	Protease
DMR1:124456001	1	124456001	124458000	2000	1	6.50E-07	0.36	12	0.6	Otud7a	Protease
DMR1:124459001	1	124459001	124460000	1000	1	8.20E-07	0.41	4	0.4	Otud7a	Protease
DMR1:124468001	1	124468001	124471000	3000	3	6.80E-19	0.75	41	1.37	Otud7a;Hmgn5b	Protease
DMR1:124474001	1	124474001	124477000	3000	3	7.40E-26	0.76	22	0.73	Otud7a;Hmgn5b	Protease
DMR1:124484001	1	124484001	124487000	3000	1	5.40E-12	0.67	20	0.67	Otud7a;Hmgn5b	Protease
DMR1:124490001	1	124490001	124491000	1000	1	5.90E-09	0.48	7	0.7	Otud7a;Hmgn5b	Protease
DMR1:124505001	1	124505001	124508000	3000	1	4.00E-15	0.59	25	0.83	Otud7a	Protease
DMR1:124525001	1	124525001	124528000	3000	1	2.60E-14	0.58	25	0.83	Otud7a	Protease
DMR1:124534001	1	124534001	124535000	1000	1	6.60E-08	0.5	9	0.9	Otud7a	Protease
DMR1:124545001	1	124545001	124550000	5000	1	2.90E-08	-0.62	25	0.5	Otud7a	Protease
DMR1:124558001	1	124558001	124559000	1000	1	1.20E-09	-0.66	4	0.4	Otud7a	Protease
DMR1:124562001	1	124562001	124563000	1000	1	4.50E-08	0.38	8	0.8	Otud7a	Protease
DMR1:124587001	1	124587001	124589000	2000	1	1.80E-14	0.63	15	0.75	Otud7a	Protease
DMR1:124616001	1	124616001	124617000	1000	1	5.50E-09	0.53	10	1	Otud7a	Protease
DMR1:124622001	1	124622001	124624000	2000	1	4.50E-10	0.57	22	1.1	Otud7a	Protease
DMR1:124625001	1	124625001	124626000	1000	1	1.30E-07	0.39	16	1.6	Otud7a	Protease
DMR1:124628001	1	124628001	124629000	1000	1	1.30E-12	0.85	4	0.4	Otud7a	Protease
DMR1:124634001	1	124634001	124637000	3000	2	3.70E-20	0.45	49	1.63	Otud7a	Protease
DMR1:124641001	1	124641001	124644000	3000	1	6.20E-08	-0.49	35	1.17	Otud7a	Protease
DMR1:124656001	1	124656001	124662000	6000	2	1.60E-25	0.91	79	1.32	Otud7a	Protease
DMR1:124664001	1	124664001	124665000	1000	1	1.90E-12	0.57	6	0.6	Otud7a	Protease
DMR1:124795001	1	124795001	124802000	7000	1	8.30E-16	0.71	93	1.33	Klf13	Transcription
DMR1:124972001	1	124972001	124977000	5000	2	9.10E-13	-0.56	78	1.56	Trpm1	Transport
DMR1:124985001	1	124985001	124986000	1000	1	7.60E-12	0.52	17	1.7	Trpm1	Transport
DMR1:125035001	1	125035001	125038000	3000	2	4.00E-16	0.7	30	1	Trpm1;Mir211	Transport
DMR1:125040001	1	125040001	125041000	1000	1	1.60E-11	-0.69	16	1.6	Trpm1;Mir211	Transport
DMR1:125054001	1	125054001	125058000	4000	2	1.40E-20	0.69	52	1.3	Trpm1	Transport
DMR1:125064001	1	125064001	125065000	1000	1	2.20E-10	-0.63	21	2.1	Trpm1	Transport
DMR1:125079001	1	125079001	125083000	4000	2	2.10E-07	0.43	67	1.68	Trpm1	Transport
DMR1:125106001	1	125106001	125110000	4000	2	9.40E-21	0.59	67	1.68	Trpm1	Transport
DMR1:125114001	1	125114001	125119000	5000	3	3.10E-15	-0.61	25	0.5	Mtmr10	Signaling
DMR1:125131001	1	125131001	125132000	1000	1	3.60E-20	1.05	7	0.7	Mtmr10	Signaling
DMR1:125133001	1	125133001	125134000	1000	1	1.60E-29	0.89	13	1.3	Mtmr10	Signaling
DMR1:125150001	1	125150001	125154000	4000	2	6.20E-12	0.56	42	1.05	Mtmr10	Signaling
DMR1:125165001	1	125165001	125166000	1000	1	3.70E-07	0.4	7	0.7	Mtmr10	Signaling
DMR1:125176001	1	125176001	125177000	1000	1	3.30E-08	0.49	5	0.5	Mtmr10;Fan1	Signaling
DMR1:125202001	1	125202001	125207000	5000	1	7.40E-13	0.4	75	1.5	Fan1;Mphosph10	Metabolism
DMR1:125232001	1	125232001	125236000	4000	2	2.10E-13	-0.62	50	1.25	Mphosph10;Mcee	Metabolism;Metabolism
DMR1:125252001	1	125252001	125253000	1000	1	1.20E-07	0.39	7	0.7	Mcee	Metabolism
DMR1:125256001	1	125256001	125257000	1000	1	9.50E-25	0.89	3	0.3	Mcee	Metabolism
DMR1:125357001	1	125357001	125362000	5000	1	1.10E-12	0.67	57	1.14	LOC108349006;Apba2	Transport
DMR1:125397001	1	125397001	125398000	1000	1	3.60E-24	0.81	14	1.4	Apba2	Transport
DMR1:125400001	1	125400001	125402000	2000	1	1.50E-08	-0.5	15	0.75	Apba2	Transport
DMR1:125403001	1	125403001	125405000	2000	1	1.10E-12	0.55	22	1.1	Apba2	Transport
DMR1:125416001	1	125416001	125418000	2000	1	5.10E-07	0.35	40	2	Apba2	Transport
DMR1:125434001	1	125434001	125435000	1000	1	1.90E-08	-0.48	16	1.6	Apba2	Transport
DMR1:125436001	1	125436001	125437000	1000	1	6.50E-08	-0.64	12	1.2	Apba2	Transport
DMR1:125447001	1	125447001	125450000	3000	1	5.20E-23	0.89	48	1.6	Apba2	Transport
DMR1:125451001	1	125451001	125455000	4000	1	9.90E-12	0.54	58	1.45	Apba2	Transport
DMR1:125457001	1	125457001	125463000	6000	4	1.70E-10	0.82	120	2	Apba2	Transport
DMR1:125474001	1	125474001	125475000	1000	1	2.70E-11	0.72	6	0.6	Apba2	Transport

DMR1:125485001	1	125485001	125486000	1000	1	3.30E-07	-0.38	17	1.7	Apba2	Transport
DMR1:125499001	1	125499001	125501000	2000	1	3.60E-26	0.93	9	0.45	Apba2	Transport
DMR1:125517001	1	125517001	125521000	4000	1	2.80E-07	0.38	45	1.12	Apba2	Transport
DMR1:125533001	1	125533001	125542000	9000	2	2.00E-19	0.62	131	1.46	Apba2	Transport
DMR1:125583001	1	125583001	125586000	3000	1	2.20E-07	0.34	51	1.7	Fam189a1	
DMR1:125594001	1	125594001	125596000	2000	1	1.80E-07	0.33	38	1.9	Fam189a1	
DMR1:125602001	1	125602001	125604000	2000	2	2.60E-20	0.93	16	0.8	Fam189a1	
DMR1:125608001	1	125608001	125614000	6000	2	5.20E-09	-0.51	94	1.57	Fam189a1	
DMR1:125621001	1	125621001	125623000	2000	1	1.10E-07	-0.57	28	1.4	Fam189a1	
DMR1:125624001	1	125624001	125627000	3000	1	2.90E-07	-0.52	35	1.17	Fam189a1	
DMR1:125630001	1	125630001	125631000	1000	1	5.30E-08	0.37	10	1	Fam189a1	
DMR1:125634001	1	125634001	125637000	3000	2	5.70E-11	-0.51	30	1	Fam189a1	
DMR1:125683001	1	125683001	125684000	1000	1	3.20E-08	-0.57	14	1.4	Fam189a1	
DMR1:125726001	1	125726001	125729000	3000	2	1.30E-16	0.83	89	2.97	Fam189a1	
DMR1:125754001	1	125754001	125756000	2000	1	1.60E-07	-0.4	44	2.2	Fam189a1	
DMR1:125780001	1	125780001	125786000	6000	2	1.50E-07	0.41	60	1	Fam189a1	
DMR1:125795001	1	125795001	125797000	2000	1	5.00E-12	0.41	27	1.35	Fam189a1	
DMR1:125798001	1	125798001	125800000	2000	1	1.00E-07	0.33	33	1.65	Fam189a1	
DMR1:125870001	1	125870001	125876000	6000	2	3.70E-07	-0.49	95	1.58	Fam189a1;Matr3-ps1	
DMR1:125880001	1	125880001	125882000	2000	2	4.40E-26	0.97	16	0.8	Fam189a1;Matr3-ps1	
DMR1:125893001	1	125893001	125895000	2000	1	8.30E-11	0.48	30	1.5	Fam189a1	
DMR1:125896001	1	125896001	125897000	1000	1	2.30E-12	0.58	13	1.3	Fam189a1	
DMR1:125905001	1	125905001	125912000	7000	2	2.30E-17	0.63	108	1.54	Fam189a1	
DMR1:125920001	1	125920001	125923000	3000	1	1.20E-07	0.32	47	1.57	Fam189a1	
DMR1:125927001	1	125927001	125930000	3000	1	2.10E-10	-0.43	29	0.97	Fam189a1	
DMR1:125934001	1	125934001	125935000	1000	1	6.30E-10	0.44	11	1.1	Fam189a1	
DMR1:125936001	1	125936001	125937000	1000	1	8.40E-07	0.57	11	1.1	Fam189a1	
DMR1:125948001	1	125948001	125950000	2000	1	4.20E-07	0.45	18	0.9	Fam189a1	
DMR1:125951001	1	125951001	125952000	1000	1	9.10E-08	-0.45	20	2	Fam189a1	
DMR1:125957001	1	125957001	125961000	4000	1	1.20E-10	0.44	76	1.9	Fam189a1	
DMR1:125970001	1	125970001	125971000	1000	1	4.50E-08	0.68	19	1.9	Fam189a1	
DMR1:125977001	1	125977001	125978000	1000	1	4.30E-16	0.55	13	1.3	Fam189a1;LOC103691154	
DMR1:126135001	1	126135001	126138000	3000	1	2.50E-19	0.7	35	1.17	Tjp1	Cell Junction
DMR1:126139001	1	126139001	126141000	2000	1	1.30E-10	0.59	27	1.35	Tjp1	Cell Junction
DMR1:126154001	1	126154001	126158000	4000	2	4.30E-15	0.54	41	1.02	Tjp1	Cell Junction
DMR1:126168001	1	126168001	126169000	1000	1	8.70E-07	-0.45	16	1.6	Tjp1	Cell Junction
DMR1:126176001	1	126176001	126178000	2000	2	7.60E-11	0.49	23	1.15	Tjp1	Cell Junction
DMR1:126258001	1	126258001	126261000	3000	1	1.90E-10	0.67	31	1.03	Tjp1	Cell Junction
DMR1:126270001	1	126270001	126277000	7000	1	6.80E-11	0.57	87	1.24	Tjp1	Cell Junction
DMR1:126289001	1	126289001	126292000	3000	1	7.90E-09	-0.55	42	1.4	Tjp1	Cell Junction
DMR1:126311001	1	126311001	126312000	1000	1	8.00E-16	0.57	12	1.2	Tjp1	Cell Junction
DMR1:126320001	1	126320001	126324000	4000	3	3.80E-21	0.69	38	0.95	Tjp1	Cell Junction
DMR1:126327001	1	126327001	126336000	9000	1	3.30E-07	0.31	118	1.31	Tjp1	Cell Junction
DMR1:126339001	1	126339001	126342000	3000	1	9.00E-20	0.66	34	1.13	Tjp1	Cell Junction
DMR1:126360001	1	126360001	126363000	3000	3	8.00E-09	-0.43	27	0.9	Tjp1	Cell Junction
DMR1:126380001	1	126380001	126382000	2000	2	5.70E-29	0.92	38	1.9	Tjp1	Cell Junction
DMR1:126386001	1	126386001	126390000	4000	1	7.30E-09	0.38	88	2.2	Tjp1	Cell Junction
DMR1:126413001	1	126413001	126418000	5000	3	4.30E-17	0.86	52	1.04	Tjp1	Cell Junction
DMR1:126436001	1	126436001	126440000	4000	4	2.20E-18	-0.52	58	1.45	Tjp1	Cell Junction
DMR1:126441001	1	126441001	126447000	6000	4	8.10E-17	0.83	63	1.05	Tjp1	Cell Junction
DMR1:126469001	1	126469001	126475000	6000	4	3.60E-29	-0.56	68	1.13	Tjp1;LOC102546680	Cell Junction
DMR1:126509001	1	126509001	126511000	2000	1	7.60E-08	0.5	22	1.1	Tjp1;LOC102546538	Cell Junction
DMR1:126535001	1	126535001	126538000	3000	1	3.70E-07	-0.47	38	1.27	Tarsl2	Translation
DMR1:126576001	1	126576001	126577000	1000	1	9.80E-10	0.7	16	1.6	Tm2d3;LOC108349769	
DMR1:126597001	1	126597001	126598000	1000	1	8.50E-08	0.56	5	0.5	Tm2d3;LOC108349586	
DMR1:126601001	1	126601001	126603000	2000	1	5.40E-16	0.53	43	2.15	Tm2d3;LOC108349586	
DMR1:126766001	1	126766001	126771000	5000	2	1.20E-12	0.58	88	1.76	Pcsk6;LOC108349587	Protease
DMR1:126779001	1	126779001	126780000	1000	1	3.90E-07	0.3	10	1	Pcsk6;LOC108349587	Protease
DMR1:126798001	1	126798001	126802000	4000	1	5.30E-10	0.44	55	1.38	Pcsk6	Protease
DMR1:126832001	1	126832001	126837000	5000	1	4.00E-08	-0.37	86	1.72	Pcsk6	Protease
DMR1:126857001	1	126857001	126861000	4000	1	9.60E-14	0.49	51	1.27	Pcsk6	Protease
DMR1:126864001	1	126864001	126867000	3000	2	5.10E-13	0.62	42	1.4	Pcsk6	Protease
DMR1:126888001	1	126888001	126896000	8000	4	7.50E-29	-0.53	83	1.04	Pcsk6	Protease
DMR1:126904001	1	126904001	126908000	4000	3	5.80E-08	0.45	78	1.95	Pcsk6	Protease
DMR1:126981001	1	126981001	126983000	2000	1	2.00E-09	0.52	16	0.8	Snrpa1;Vimp	Translation

DMR1:126984001	1	126984001	126986000	2000	1	1.30E-10	-0.62	21	1.05	Snrpa1;Vimp	Translation
DMR1:127016001	1	127016001	127019000	3000	1	2.00E-09	0.4	41	1.37	LOC108349588;Chsy1	Golgi
DMR1:127069001	1	127069001	127073000	4000	2	4.40E-09	-0.6	77	1.93	Chsy1	Golgi
DMR1:127077001	1	127077001	127079000	2000	1	8.00E-08	0.38	36	1.8	Chsy1	Golgi
DMR1:127163001	1	127163001	127164000	1000	1	8.10E-07	0.34	6	0.6	LOC102549616;LOC108349001;Lrrk1;LOC108349000	Signaling
DMR1:127184001	1	127184001	127185000	1000	1	2.90E-14	0.73	9	0.9	Lrrk1;LOC108349000	Signaling
DMR1:127199001	1	127199001	127201000	2000	1	1.70E-09	0.43	35	1.75	Lrrk1	Signaling
DMR1:127225001	1	127225001	127226000	1000	1	1.70E-21	0.82	9	0.9	Lrrk1	Signaling
DMR1:127242001	1	127242001	127243000	1000	1	1.00E-19	0.84	6	0.6	Lrrk1	Signaling
DMR1:127249001	1	127249001	127257000	8000	2	5.00E-24	1.01	132	1.65	Lrrk1	Signaling
DMR1:127278001	1	127278001	127279000	1000	1	8.60E-09	0.51	5	0.5	Lrrk1	Signaling
DMR1:127284001	1	127284001	127286000	2000	1	6.90E-10	0.4	27	1.35	Lrrk1	Signaling
DMR1:127302001	1	127302001	127307000	5000	1	1.20E-08	0.58	61	1.22	Lrrk1;Aldh1a3	Signaling;Metabolism
DMR1:127308001	1	127308001	127311000	3000	1	3.20E-09	0.4	48	1.6	Lrrk1;Aldh1a3	Signaling;Metabolism
DMR1:127319001	1	127319001	127320000	1000	1	4.10E-07	0.34	16	1.6	Aldh1a3	Metabolism
DMR1:127331001	1	127331001	127335000	4000	2	2.90E-15	0.52	66	1.65	Aldh1a3	Metabolism
DMR1:127342001	1	127342001	127343000	1000	1	2.60E-08	-0.57	13	1.3	Aldh1a3	Metabolism
DMR1:127526001	1	127526001	127536000	10000	3	1.60E-18	-0.66	123	1.23	Asb7	Transport
DMR1:127538001	1	127538001	127542000	4000	1	1.10E-19	0.65	72	1.8	Asb7	Transport
DMR1:127550001	1	127550001	127551000	1000	1	1.40E-10	0.47	12	1.2	Asb7	Transport
DMR1:127577001	1	127577001	127578000	1000	1	5.70E-07	0.6	7	0.7	Asb7	Transport
DMR1:127580001	1	127580001	127583000	3000	2	1.40E-13	0.65	13	0.43	Asb7	Transport
DMR1:127611001	1	127611001	127612000	1000	1	8.80E-12	0.44	22	2.2	Lins1	
DMR1:127630001	1	127630001	127632000	2000	1	5.20E-09	-0.46	5	0.25	Lins1	
DMR1:127705001	1	127705001	127707000	2000	1	6.00E-15	0.68	26	1.3	Cers3	
DMR1:127734001	1	127734001	127735000	1000	1	6.30E-08	0.62	15	1.5	Cers3	
DMR1:127741001	1	127741001	127742000	1000	1	6.50E-07	0.6	7	0.7	Cers3	
DMR1:127747001	1	127747001	127749000	2000	1	6.60E-07	-0.46	21	1.05	Cers3	
DMR1:127752001	1	127752001	127753000	1000	1	2.00E-16	0.71	6	0.6	Cers3	
DMR1:127766001	1	127766001	127767000	1000	1	1.20E-07	0.57	3	0.3	Cers3	
DMR1:127768001	1	127768001	127772000	4000	2	2.50E-08	-0.54	53	1.32	Cers3	
DMR1:127776001	1	127776001	127777000	1000	1	7.20E-08	-0.6	14	1.4	Cers3	
DMR1:127786001	1	127786001	127794000	8000	3	8.40E-16	0.7	72	0.9	Cers3;Adamts17	Protease
DMR1:127796001	1	127796001	127802000	6000	1	5.20E-08	-0.59	69	1.15	Adamts17	Protease
DMR1:127811001	1	127811001	127815000	4000	1	1.60E-11	-0.64	73	1.82	Adamts17	Protease
DMR1:127825001	1	127825001	127828000	3000	2	6.10E-13	0.52	25	0.83	Adamts17	Protease
DMR1:127855001	1	127855001	127857000	2000	1	2.40E-09	0.52	18	0.9	Adamts17	Protease
DMR1:127858001	1	127858001	127862000	4000	1	1.10E-10	0.47	88	2.2	Adamts17	Protease
DMR1:127894001	1	127894001	127898000	4000	1	3.80E-07	-0.33	42	1.05	Adamts17	Protease
DMR1:127899001	1	127899001	127900000	1000	1	1.40E-07	-0.56	17	1.7	Adamts17	Protease
DMR1:127913001	1	127913001	127922000	9000	5	3.80E-14	0.62	91	1.01	Adamts17	Protease
DMR1:127945001	1	127945001	127949000	4000	2	4.80E-24	0.61	52	1.3	Adamts17	Protease
DMR1:127976001	1	127976001	127978000	2000	1	3.40E-08	-0.59	33	1.65	Adamts17	Protease
DMR1:127990001	1	127990001	127991000	1000	1	9.30E-11	0.45	13	1.3	Adamts17	Protease
DMR1:127992001	1	127992001	127993000	1000	1	3.80E-15	0.84	7	0.7	Adamts17	Protease
DMR1:128041001	1	128041001	128044000	3000	2	1.30E-08	0.45	31	1.03	Adamts17	Protease
DMR1:128057001	1	128057001	128058000	1000	1	6.60E-07	-0.47	14	1.4	Adamts17	Protease
DMR1:128067001	1	128067001	128071000	4000	1	5.80E-07	0.4	42	1.05	Adamts17	Protease
DMR1:128085001	1	128085001	128086000	1000	1	1.30E-10	0.69	6	0.6	Adamts17	Protease
DMR1:128087001	1	128087001	128091000	4000	1	4.10E-07	-0.7	56	1.4	Adamts17	Protease
DMR1:128118001	1	128118001	128121000	3000	3	1.60E-15	-0.68	10	0.33	Adamts17	Protease
DMR1:128131001	1	128131001	128132000	1000	1	3.30E-10	-0.56	14	1.4	Adamts17	Protease
DMR1:128235001	1	128235001	128237000	2000	1	2.30E-10	0.39	14	0.7	Mef2a	Transcription
DMR1:128252001	1	128252001	128254000	2000	1	1.90E-08	0.51	15	0.75	Mef2a	Transcription
DMR1:128307001	1	128307001	128310000	3000	1	3.00E-10	-0.55	16	0.53	Mef2a	Transcription
DMR1:128311001	1	128311001	128313000	2000	1	9.80E-08	0.48	7	0.35	Mef2a	Transcription
DMR1:128315001	1	128315001	128319000	4000	2	6.50E-18	-0.47	41	1.02	Mef2a	Transcription
DMR1:128321001	1	128321001	128322000	1000	1	4.40E-09	0.5	13	1.3	Mef2a	Transcription
DMR1:128329001	1	128329001	128331000	2000	2	8.30E-14	0.61	20	1	Mef2a	Transcription
DMR1:128332001	1	128332001	128333000	1000	1	2.20E-08	-0.7	5	0.5	Mef2a	Transcription
DMR1:128350001	1	128350001	128351000	1000	1	2.40E-07	-0.47	11	1.1	Mef2a	Transcription
DMR1:128503001	1	128503001	128507000	4000	1	6.50E-15	0.5	55	1.38	Lrrc28	

DMR1:128508001	1	128508001	128511000	3000	1	3.60E-08	0.66	17	0.57	Lrrc28	
DMR1:128512001	1	128512001	128514000	2000	1	3.60E-08	-0.62	27	1.35	Lrrc28	
DMR1:128517001	1	128517001	128518000	1000	1	5.40E-10	0.75	10	1	Lrrc28	
DMR1:128522001	1	128522001	128523000	1000	1	9.70E-10	-0.61	15	1.5	Lrrc28	
DMR1:128524001	1	128524001	128527000	3000	1	2.50E-07	-0.55	49	1.63	Lrrc28	
DMR1:128542001	1	128542001	128544000	2000	2	3.90E-10	-0.48	14	0.7	Lrrc28	
DMR1:128554001	1	128554001	128559000	5000	2	6.50E-08	0.42	53	1.06	Lrrc28	
DMR1:128574001	1	128574001	128576000	2000	1	5.90E-08	-0.55	27	1.35	Lrrc28	
DMR1:128579001	1	128579001	128581000	2000	1	1.10E-10	-0.58	16	0.8	Lrrc28	
DMR1:128597001	1	128597001	128602000	5000	1	1.80E-15	0.6	86	1.72	Lrrc28;Ttc23;LOC108349234	
DMR1:128607001	1	128607001	128609000	2000	1	4.30E-10	0.37	28	1.4	Lrrc28;Ttc23;LOC108349234	
DMR1:128611001	1	128611001	128616000	5000	1	3.60E-07	0.49	73	1.46	Lrrc28;Ttc23;LOC108349234	
DMR1:128617001	1	128617001	128621000	4000	1	5.10E-10	0.54	42	1.05	Ttc23;LOC108349234	
DMR1:128645001	1	128645001	128647000	2000	1	1.70E-07	-0.5	18	0.9	Ttc23	
DMR1:128660001	1	128660001	128664000	4000	1	4.10E-10	0.42	63	1.57	Ttc23	
DMR1:128682001	1	128682001	128683000	1000	1	9.10E-12	0.74	9	0.9	Ttc23;Synm	
DMR1:128696001	1	128696001	128698000	2000	1	9.20E-11	0.43	56	2.8	Ttc23;Synm	
DMR1:128708001	1	128708001	128709000	1000	1	1.10E-11	0.44	8	0.8	Synm	
DMR1:128725001	1	128725001	128727000	2000	2	3.20E-20	0.64	31	1.55	Synm	
DMR1:128728001	1	128728001	128729000	1000	1	9.60E-17	0.58	23	2.3	Synm	
DMR1:128730001	1	128730001	128732000	2000	1	9.30E-07	0.33	24	1.2	Synm	
DMR1:128919001	1	128919001	128921000	2000	1	7.90E-07	-0.45	26	1.3	Igf1r	Receptor
DMR1:128938001	1	128938001	128939000	1000	1	1.60E-23	0.66	10	1	Igf1r	Receptor
DMR1:128959001	1	128959001	128961000	2000	1	1.70E-10	-0.67	39	1.95	Igf1r	Receptor
DMR1:128963001	1	128963001	128969000	6000	3	1.20E-12	0.53	65	1.08	Igf1r	Receptor
DMR1:128971001	1	128971001	128976000	5000	2	5.30E-15	0.53	86	1.72	Igf1r	Receptor
DMR1:128983001	1	128983001	128984000	1000	1	1.80E-17	0.65	17	1.7	Igf1r	Receptor
DMR1:128994001	1	128994001	128996000	2000	1	4.30E-09	0.37	32	1.6	Igf1r	Receptor
DMR1:128999001	1	128999001	129006000	7000	2	2.50E-14	0.54	125	1.79	Igf1r	Receptor
DMR1:129011001	1	129011001	129016000	5000	1	5.30E-16	0.54	64	1.28	Igf1r	Receptor
DMR1:129021001	1	129021001	129027000	6000	1	1.30E-07	-0.44	110	1.83	Igf1r	Receptor
DMR1:129028001	1	129028001	129029000	1000	1	8.50E-12	0.57	17	1.7	Igf1r	Receptor
DMR1:129057001	1	129057001	129060000	3000	1	3.60E-07	0.35	52	1.73	Igf1r	Receptor
DMR1:129061001	1	129061001	129062000	1000	1	1.30E-08	0.41	11	1.1	Igf1r	Receptor
DMR1:129075001	1	129075001	129078000	3000	2	2.80E-07	-0.46	37	1.23	Igf1r	Receptor
DMR1:129081001	1	129081001	129083000	2000	1	2.00E-09	-0.62	33	1.65	Igf1r	Receptor
DMR1:129086001	1	129086001	129089000	3000	2	2.80E-14	0.55	62	2.07	Igf1r	Receptor
DMR1:129090001	1	129090001	129093000	3000	1	1.20E-07	0.34	57	1.9	Igf1r	Receptor
DMR1:129097001	1	129097001	129105000	8000	1	6.50E-07	0.33	118	1.48	Igf1r	Receptor
DMR1:129116001	1	129116001	129118000	2000	1	7.50E-08	0.34	29	1.45	Igf1r	Receptor
DMR1:129152001	1	129152001	129154000	2000	1	7.20E-07	-0.61	34	1.7	Igf1r	Receptor
DMR1:129163001	1	129163001	129171000	8000	1	1.50E-11	0.53	191	2.39	Igf1r	Receptor
DMR1:129181001	1	129181001	129186000	5000	1	1.50E-07	-0.44	97	1.94	Igf1r	Receptor
DMR1:129187001	1	129187001	129188000	1000	1	7.70E-07	0.31	23	2.3	Igf1r	Receptor
DMR1:129219001	1	129219001	129222000	3000	2	5.80E-11	0.52	34	1.13	Igf1r;Pgpep1l	Receptor;Protease
DMR1:129250001	1	129250001	129253000	3000	1	4.20E-07	0.39	40	1.33	Pgpep1l;Fam169b	Protease
DMR1:129263001	1	129263001	129266000	3000	2	2.70E-08	-0.62	41	1.37	Fam169b	
DMR1:129267001	1	129267001	129270000	3000	1	6.50E-08	0.43	36	1.2	Fam169b	
DMR1:129280001	1	129280001	129284000	4000	1	9.60E-10	0.44	53	1.32	Fam169b	
DMR1:129285001	1	129285001	129286000	1000	1	3.10E-08	0.51	9	0.9	Fam169b	
DMR1:129287001	1	129287001	129288000	1000	1	8.60E-08	-0.57	18	1.8	Fam169b	
DMR1:129291001	1	129291001	129293000	2000	1	7.70E-08	0.47	25	1.25	Fam169b	
DMR1:129323001	1	129323001	129327000	4000	2	1.70E-28	0.76	72	1.8	Fam169b	
DMR1:129330001	1	129330001	129332000	2000	1	9.40E-21	0.73	24	1.2	Fam169b	
DMR1:129754001	1	129754001	129757000	3000	1	8.30E-10	-0.6	45	1.5	Arrdc4	
DMR1:129762001	1	129762001	129763000	1000	1	2.30E-08	0.53	10	1	Arrdc4	
DMR1:129764001	1	129764001	129765000	1000	1	2.30E-09	0.41	14	1.4	Arrdc4	
DMR1:129767001	1	129767001	129768000	1000	1	4.80E-09	-0.65	13	1.3	Arrdc4	
DMR1:129777001	1	129777001	129778000	1000	1	1.10E-14	0.76	5	0.5	Arrdc4	
DMR1:130525001	1	130525001	130531000	6000	3	9.20E-12	0.63	68	1.13	Olr9-ps	
DMR1:130534001	1	130534001	130536000	2000	1	1.80E-08	-0.73	25	1.25	Olr9-ps	
DMR1:131436001	1	131436001	131439000	3000	1	7.60E-16	0.63	35	1.17	LOC102553173;Nr2f2	Transcription
DMR1:132188001	1	132188001	132189000	1000	1	1.80E-14	0.62	13	1.3	RGD1562781	
DMR1:132196001	1	132196001	132202000	6000	3	9.30E-12	0.5	73	1.22	RGD1562781	
DMR1:132203001	1	132203001	132210000	7000	2	4.20E-12	-0.38	53	0.76	RGD1562781	

DMR1:133349001	1	133349001	133351000	2000	1	9.80E-10	0.6	18	0.9	Mctp2	
DMR1:133384001	1	133384001	133386000	2000	1	5.10E-08	-0.59	12	0.6	Mctp2	
DMR1:133395001	1	133395001	133398000	3000	1	1.20E-11	-0.72	47	1.57	Mctp2	
DMR1:133418001	1	133418001	133419000	1000	1	3.70E-09	-0.65	5	0.5	Mctp2	
DMR1:133444001	1	133444001	133446000	2000	1	9.90E-15	0.77	20	1	Mctp2	
DMR1:133463001	1	133463001	133464000	1000	1	2.80E-07	0.5	8	0.8	Mctp2	
DMR1:133468001	1	133468001	133470000	2000	1	3.00E-07	-0.44	22	1.1	Mctp2	
DMR1:133486001	1	133486001	133488000	2000	1	3.50E-17	0.66	29	1.45	Mctp2	
DMR1:133497001	1	133497001	133498000	1000	1	6.70E-07	-0.49	11	1.1	Mctp2	
DMR1:133529001	1	133529001	133536000	7000	5	1.30E-21	-0.59	57	0.81	Mctp2	
DMR1:133550001	1	133550001	133551000	1000	1	7.40E-14	0.8	3	0.3	Mctp2	
DMR1:133565001	1	133565001	133567000	2000	1	8.20E-10	-0.56	17	0.85	Mctp2	
DMR1:134688001	1	134688001	134692000	4000	2	2.70E-13	-0.66	53	1.32	Rgma	
DMR1:134694001	1	134694001	134696000	2000	1	1.70E-08	0.6	29	1.45	Rgma	
DMR1:134719001	1	134719001	134721000	2000	1	1.20E-10	0.57	17	0.85	Rgma	
DMR1:134736001	1	134736001	134737000	1000	1	8.00E-08	0.41	14	1.4	Rgma	
DMR1:134765001	1	134765001	134770000	5000	1	4.70E-10	0.52	49	0.98	Chd2	
DMR1:134801001	1	134801001	134802000	1000	1	9.30E-18	0.71	6	0.6	Chd2	
DMR1:134806001	1	134806001	134807000	1000	1	5.30E-19	0.6	46	4.6	Chd2	
DMR1:134834001	1	134834001	134835000	1000	1	1.70E-07	-0.47	8	0.8	Chd2	
DMR1:134852001	1	134852001	134856000	4000	2	1.00E-09	0.54	22	0.55	Chd2	
DMR1:134871001	1	134871001	134873000	2000	1	1.00E-08	0.41	19	0.95	Chd2;LOC108349594	
DMR1:135086001	1	135086001	135088000	2000	1	5.10E-07	0.34	21	1.05	LOC103691177;Fam174b	
DMR1:135093001	1	135093001	135098000	5000	1	2.10E-22	0.73	53	1.06	LOC103691177;Fam174b	
DMR1:135102001	1	135102001	135107000	5000	4	2.40E-20	-0.65	64	1.28	Fam174b	
DMR1:135114001	1	135114001	135120000	6000	3	6.40E-10	0.43	83	1.38	Fam174b	
DMR1:135121001	1	135121001	135122000	1000	1	4.50E-10	0.5	14	1.4	Fam174b	
DMR1:135134001	1	135134001	135137000	3000	2	1.10E-15	0.89	32	1.07	Fam174b	
DMR1:135142001	1	135142001	135146000	4000	2	2.80E-12	0.37	74	1.85	Fam174b	
DMR1:135147001	1	135147001	135149000	2000	1	3.00E-07	0.38	25	1.25	Fam174b	
DMR1:135169001	1	135169001	135173000	4000	1	3.30E-13	0.48	56	1.4	Fam174b	
DMR1:135177001	1	135177001	135179000	2000	2	1.60E-17	0.58	37	1.85	Fam174b;LOC103691163	
DMR1:135185001	1	135185001	135190000	5000	1	8.30E-18	0.66	45	0.9	Fam174b;LOC103691163	
DMR1:135327001	1	135327001	135328000	1000	1	1.50E-10	0.5	7	0.7	LOC108349596;St8sia2	Transport
DMR1:135361001	1	135361001	135363000	2000	1	5.80E-13	0.6	16	0.8	St8sia2	Transport
DMR1:135374001	1	135374001	135376000	2000	1	1.80E-08	0.53	14	0.7	St8sia2	Transport
DMR1:135401001	1	135401001	135402000	1000	1	9.10E-17	0.74	13	1.3	St8sia2	Transport
DMR1:135410001	1	135410001	135412000	2000	2	4.00E-08	-0.46	20	1	St8sia2	Transport
DMR1:135413001	1	135413001	135414000	1000	1	1.90E-07	0.42	10	1	St8sia2	Transport
DMR1:135786001	1	135786001	135787000	1000	1	6.00E-10	0.68	9	0.9	Slco3a1	Transport
DMR1:135815001	1	135815001	135818000	3000	1	8.20E-09	-0.45	38	1.27	Slco3a1	Transport
DMR1:135820001	1	135820001	135822000	2000	1	2.30E-11	0.48	18	0.9	Slco3a1	Transport
DMR1:135824001	1	135824001	135827000	3000	1	1.30E-07	0.49	24	0.8	Slco3a1	Transport
DMR1:135831001	1	135831001	135832000	1000	1	2.40E-09	0.36	8	0.8	Slco3a1	Transport
DMR1:135870001	1	135870001	135871000	1000	1	7.70E-10	0.49	9	0.9	Slco3a1	Transport
DMR1:135872001	1	135872001	135873000	1000	1	2.10E-17	0.57	8	0.8	Slco3a1	Transport
DMR1:135882001	1	135882001	135884000	2000	1	2.50E-08	0.53	28	1.4	Slco3a1	Transport
DMR1:135931001	1	135931001	135932000	1000	1	4.40E-09	0.63	2	0.2	Slco3a1	Transport
DMR1:135954001	1	135954001	135963000	9000	1	3.40E-07	0.36	100	1.11	Slco3a1;LOC108348469	Transport
DMR1:135965001	1	135965001	135966000	1000	1	5.50E-07	0.52	6	0.6	Slco3a1;LOC108348469	Transport
DMR1:135976001	1	135976001	135980000	4000	1	6.80E-07	0.52	30	0.75	Slco3a1;LOC108348469	Transport
DMR1:135992001	1	135992001	135993000	1000	1	2.30E-09	0.45	9	0.9	Slco3a1	Transport
DMR1:136004001	1	136004001	136006000	2000	1	1.70E-08	0.42	23	1.15	Slco3a1	Transport
DMR1:136034001	1	136034001	136035000	1000	1	6.50E-10	0.46	10	1	Slco3a1	Transport
DMR1:136044001	1	136044001	136045000	1000	1	8.10E-10	-0.5	10	1	Slco3a1	Transport
DMR1:136049001	1	136049001	136051000	2000	1	8.70E-11	0.46	22	1.1	Slco3a1	Transport
DMR1:136070001	1	136070001	136071000	1000	1	8.60E-12	0.52	13	1.3	Slco3a1	Transport
DMR1:136079001	1	136079001	136085000	6000	1	6.90E-07	0.47	57	0.95	Slco3a1	Transport
DMR1:136653001	1	136653001	136657000	4000	1	3.00E-14	0.66	32	0.8	Sv2b	
DMR1:136677001	1	136677001	136681000	4000	2	3.10E-17	0.71	44	1.1	Sv2b	
DMR1:136682001	1	136682001	136683000	1000	1	6.70E-07	0.31	12	1.2	Sv2b	
DMR1:136685001	1	136685001	136686000	1000	1	5.70E-10	-0.8	15	1.5	Sv2b	
DMR1:136688001	1	136688001	136690000	2000	1	8.50E-10	0.72	9	0.45	Sv2b	
DMR1:136692001	1	136692001	136694000	2000	2	6.80E-25	1.11	29	1.45	Sv2b	
DMR1:136696001	1	136696001	136697000	1000	1	4.10E-08	0.39	8	0.8	Sv2b	

DMR1:136702001	1	136702001	136704000	2000	2	4.80E-17	0.98	14	0.7	Sv2b	
DMR1:136719001	1	136719001	136721000	2000	1	4.30E-10	-0.61	6	0.3	Sv2b	
DMR1:136724001	1	136724001	136727000	3000	2	5.60E-09	0.54	13	0.43	Sv2b	
DMR1:136733001	1	136733001	136734000	1000	1	9.40E-08	0.59	10	1	Sv2b	
DMR1:136761001	1	136761001	136762000	1000	1	2.00E-10	0.62	3	0.3	Sv2b	
DMR1:136765001	1	136765001	136766000	1000	1	3.90E-12	0.57	4	0.4	Sv2b	
DMR1:136783001	1	136783001	136784000	1000	1	1.30E-09	0.56	4	0.4	Sv2b	
DMR1:136785001	1	136785001	136786000	1000	1	3.30E-08	0.4	8	0.8	Sv2b	
DMR1:136788001	1	136788001	136792000	4000	4	1.50E-10	0.51	34	0.85	Sv2b	
DMR1:136811001	1	136811001	136813000	2000	1	1.10E-15	0.72	12	0.6	Sv2b	
DMR1:136828001	1	136828001	136830000	2000	1	1.50E-12	0.54	13	0.65	Sv2b	
DMR1:136834001	1	136834001	136837000	3000	1	1.00E-11	0.68	24	0.8	Sv2b;LOC108349288	
DMR1:136840001	1	136840001	136841000	1000	1	9.90E-11	0.63	6	0.6	Sv2b;LOC108349288	
DMR1:137080001	1	137080001	137081000	1000	1	7.90E-07	0.44	10	1	Akap13	
DMR1:137092001	1	137092001	137095000	3000	3	4.10E-25	0.99	22	0.73	Akap13	
DMR1:137127001	1	137127001	137129000	2000	2	1.20E-23	0.92	20	1	Akap13	
DMR1:137136001	1	137136001	137140000	4000	4	3.40E-13	-0.65	21	0.52	Akap13	
DMR1:137148001	1	137148001	137149000	1000	1	7.30E-09	-0.55	15	1.5	Akap13	
DMR1:137169001	1	137169001	137170000	1000	1	1.00E-10	0.48	5	0.5	Akap13	
DMR1:137196001	1	137196001	137198000	2000	1	4.60E-12	0.63	14	0.7	Akap13	
DMR1:137203001	1	137203001	137204000	1000	1	3.70E-07	0.5	2	0.2	Akap13	
DMR1:137237001	1	137237001	137240000	3000	1	1.00E-13	0.55	51	1.7	Akap13	
DMR1:137279001	1	137279001	137285000	6000	1	1.10E-07	0.59	51	0.85	Akap13	
DMR1:137296001	1	137296001	137297000	1000	1	3.20E-07	-0.58	10	1	Akap13	
DMR1:137302001	1	137302001	137303000	1000	1	2.00E-08	-0.41	7	0.7	Akap13	
DMR1:137316001	1	137316001	137318000	2000	2	5.60E-12	-0.78	10	0.5	Akap13	
DMR1:137322001	1	137322001	137324000	2000	1	9.30E-08	0.35	20	1	Akap13;Klhl25	
DMR1:137328001	1	137328001	137329000	1000	1	3.50E-18	1.03	8	0.8	Klhl25	
DMR1:137335001	1	137335001	137337000	2000	1	1.80E-14	0.57	30	1.5	Klhl25	
DMR1:137347001	1	137347001	137350000	3000	1	2.40E-07	0.46	40	1.33	Klhl25;LOC103691164	
DMR1:137363001	1	137363001	137365000	2000	1	7.10E-15	0.69	25	1.25	Klhl25;LOC103691164	
DMR1:137799001	1	137799001	137801000	2000	1	1.10E-10	0.63	22	1.1	LOC108349597;Agbl1	Protease
DMR1:137805001	1	137805001	137806000	1000	1	3.10E-10	0.46	7	0.7	LOC108349597;Agbl1	Protease
DMR1:137824001	1	137824001	137825000	1000	1	3.20E-10	0.57	2	0.2	Agbl1	Protease
DMR1:137828001	1	137828001	137833000	5000	2	1.90E-12	0.46	49	0.98	Agbl1	Protease
DMR1:137843001	1	137843001	137848000	5000	3	8.40E-11	0.63	25	0.5	Agbl1	Protease
DMR1:137850001	1	137850001	137851000	1000	1	3.40E-17	0.99	4	0.4	Agbl1	Protease
DMR1:137858001	1	137858001	137862000	4000	1	1.10E-16	0.54	33	0.82	Agbl1	Protease
DMR1:137882001	1	137882001	137889000	7000	2	1.70E-07	-0.58	57	0.81	Agbl1	Protease
DMR1:137909001	1	137909001	137913000	4000	1	7.20E-07	-0.47	26	0.65	Agbl1;LOC108349814	Protease
DMR1:137914001	1	137914001	137915000	1000	1	9.50E-13	0.49	13	1.3	Agbl1;LOC108349814	Protease
DMR1:137927001	1	137927001	137929000	2000	1	4.40E-07	0.45	25	1.25	Agbl1	Protease
DMR1:137979001	1	137979001	137980000	1000	1	2.50E-13	0.63	8	0.8	Agbl1	Protease
DMR1:138018001	1	138018001	138020000	2000	1	2.50E-07	-0.48	12	0.6	Agbl1	Protease
DMR1:138055001	1	138055001	138057000	2000	1	1.30E-11	-0.66	5	0.25	Agbl1	Protease
DMR1:138063001	1	138063001	138069000	6000	4	2.50E-14	-0.38	53	0.88	Agbl1	Protease
DMR1:138074001	1	138074001	138075000	1000	1	3.80E-08	0.59	8	0.8	Agbl1	Protease
DMR1:138082001	1	138082001	138086000	4000	1	2.40E-15	-0.72	8	0.2	Agbl1	Protease
DMR1:138115001	1	138115001	138117000	2000	1	1.50E-09	0.54	13	0.65	Agbl1	Protease
DMR1:138119001	1	138119001	138121000	2000	1	5.70E-08	0.55	19	0.95	Agbl1	Protease
DMR1:138134001	1	138134001	138136000	2000	1	3.70E-07	0.51	19	0.95	Agbl1	Protease
DMR1:138144001	1	138144001	138146000	2000	1	1.20E-09	0.61	16	0.8	Agbl1	Protease
DMR1:138161001	1	138161001	138162000	1000	1	1.30E-08	0.61	2	0.2	Agbl1	Protease
DMR1:138164001	1	138164001	138165000	1000	1	5.60E-16	-0.42	9	0.9	Agbl1	Protease
DMR1:138194001	1	138194001	138195000	1000	1	4.30E-10	-0.52	10	1	Agbl1	Protease
DMR1:138196001	1	138196001	138197000	1000	1	1.20E-08	0.48	3	0.3	Agbl1	Protease
DMR1:138216001	1	138216001	138217000	1000	1	2.50E-14	0.81	5	0.5	Agbl1	Protease
DMR1:138226001	1	138226001	138227000	1000	1	6.20E-11	0.62	5	0.5	Agbl1	Protease
DMR1:138231001	1	138231001	138232000	1000	1	4.10E-07	0.52	1	0.1	Agbl1	Protease
DMR1:138263001	1	138263001	138268000	5000	1	9.50E-10	-0.41	35	0.7	Agbl1	Protease
DMR1:138319001	1	138319001	138326000	7000	5	2.20E-24	-0.55	79	1.13	Agbl1	Protease
DMR1:138327001	1	138327001	138329000	2000	1	3.00E-14	0.65	8	0.4	Agbl1	Protease
DMR1:138330001	1	138330001	138332000	2000	1	3.20E-11	-0.75	12	0.6	Agbl1	Protease
DMR1:138363001	1	138363001	138365000	2000	1	6.50E-15	0.66	9	0.45	Agbl1	Protease
DMR1:138381001	1	138381001	138385000	4000	2	7.10E-08	-0.57	19	0.48	Agbl1	Protease

DMR1:138402001	1	138402001	138403000	1000	1	9.80E-15	0.58	12	1.2	Agbl1	Protease
DMR1:138404001	1	138404001	138405000	1000	1	2.80E-14	0.72	3	0.3	Agbl1	Protease
DMR1:138422001	1	138422001	138423000	1000	1	2.30E-07	0.46	4	0.4	Agbl1	Protease
DMR1:138492001	1	138492001	138493000	1000	1	8.20E-10	0.57	2	0.2	Agbl1	Protease
DMR1:138495001	1	138495001	138496000	1000	1	2.10E-10	0.55	6	0.6	Agbl1	Protease
DMR1:138519001	1	138519001	138520000	1000	1	2.10E-08	0.55	10	1	Agbl1	Protease
DMR1:138553001	1	138553001	138554000	1000	1	6.10E-10	0.45	8	0.8	Agbl1	Protease
DMR1:138600001	1	138600001	138601000	1000	1	1.70E-08	0.61	4	0.4	Agbl1	Protease
DMR1:138683001	1	138683001	138684000	1000	1	3.20E-09	0.62	6	0.6	Agbl1	Protease
DMR1:138693001	1	138693001	138695000	2000	2	6.70E-12	0.95	42	2.1	Agbl1	Protease
DMR1:138699001	1	138699001	138700000	1000	1	4.30E-12	0.72	6	0.6	Agbl1	Protease
DMR1:138703001	1	138703001	138704000	1000	1	2.80E-20	0.88	7	0.7	Agbl1	Protease
DMR1:138716001	1	138716001	138717000	1000	1	8.20E-12	0.64	2	0.2	Agbl1	Protease
DMR1:138718001	1	138718001	138720000	2000	1	7.40E-07	0.51	7	0.35	Agbl1	Protease
DMR1:138721001	1	138721001	138722000	1000	1	1.80E-11	-0.73	12	1.2	Agbl1	Protease
DMR1:138829001	1	138829001	138831000	2000	2	7.00E-10	-0.6	10	0.5	Vom1r-ps67	
DMR1:138840001	1	138840001	138847000	7000	3	7.10E-17	-0.51	56	0.8	Vom1r-ps67	
DMR1:139890001	1	139890001	139893000	3000	1	6.70E-21	0.85	38	1.27	Ntrk3	Receptor
DMR1:139895001	1	139895001	139898000	3000	2	4.50E-09	0.66	15	0.5	Ntrk3	Receptor
DMR1:139948001	1	139948001	139950000	2000	1	2.70E-07	-0.41	21	1.05	Ntrk3	Receptor
DMR1:139972001	1	139972001	139974000	2000	1	1.70E-12	0.58	13	0.65	Ntrk3	Receptor
DMR1:140025001	1	140025001	140027000	2000	2	4.20E-18	0.64	17	0.85	Ntrk3	Receptor
DMR1:140068001	1	140068001	140070000	2000	1	5.80E-14	0.53	27	1.35	Ntrk3	Receptor
DMR1:140079001	1	140079001	140080000	1000	1	9.00E-10	0.53	10	1	Ntrk3	Receptor
DMR1:140087001	1	140087001	140088000	1000	1	6.00E-09	0.5	7	0.7	Ntrk3	Receptor
DMR1:140150001	1	140150001	140151000	1000	1	3.70E-14	0.74	1	0.1	Ntrk3	Receptor
DMR1:140159001	1	140159001	140160000	1000	1	1.00E-09	0.53	2	0.2	Ntrk3	Receptor
DMR1:140180001	1	140180001	140184000	4000	1	1.70E-13	0.58	30	0.75	Ntrk3	Receptor
DMR1:140185001	1	140185001	140186000	1000	1	1.50E-07	0.38	8	0.8	Ntrk3	Receptor
DMR1:140192001	1	140192001	140193000	1000	1	1.20E-09	0.58	7	0.7	Ntrk3	Receptor
DMR1:140196001	1	140196001	140199000	3000	1	6.00E-16	0.93	26	0.87	Ntrk3	Receptor
DMR1:140228001	1	140228001	140229000	1000	1	7.00E-10	0.55	8	0.8	Ntrk3	Receptor
DMR1:140241001	1	140241001	140245000	4000	1	9.80E-08	0.47	32	0.8	Ntrk3	Receptor
DMR1:140250001	1	140250001	140251000	1000	1	4.20E-07	0.44	11	1.1	Ntrk3	Receptor
DMR1:140461001	1	140461001	140462000	1000	1	1.90E-17	0.54	22	2.2	Mrpl46	Translation
DMR1:140473001	1	140473001	140477000	4000	2	1.10E-10	-0.46	53	1.32	Mrpl46;Mrps11	Translation
DMR1:140491001	1	140491001	140492000	1000	1	5.20E-09	-0.48	22	2.2	Mrps11	Translation
DMR1:140495001	1	140495001	140497000	2000	2	2.90E-35	1.04	23	1.15	Mrps11	Translation
DMR1:140509001	1	140509001	140514000	5000	1	3.20E-19	0.57	74	1.48	Det1	Proteolysis
DMR1:140515001	1	140515001	140516000	1000	1	2.80E-19	1	3	0.3	Det1	Proteolysis
DMR1:140523001	1	140523001	140526000	3000	1	5.90E-13	0.59	37	1.23	Det1	Proteolysis
DMR1:140543001	1	140543001	140545000	2000	1	1.60E-12	0.61	16	0.8	Det1	Proteolysis
DMR1:140564001	1	140564001	140569000	5000	2	1.70E-07	-0.55	62	1.24	LOC102547416;Mir7a-2	
DMR1:140575001	1	140575001	140578000	3000	1	3.00E-07	0.28	55	1.83	LOC102547416;Mir7a-2;Aen	Transcription
DMR1:140587001	1	140587001	140588000	1000	1	8.20E-10	0.35	21	2.1	Aen	Transcription
DMR1:140598001	1	140598001	140600000	2000	2	3.20E-13	0.59	48	2.4	Aen;Isg20	Transcription
DMR1:140608001	1	140608001	140610000	2000	1	1.30E-12	0.52	38	1.9	Isg20	Transcription
DMR1:140752001	1	140752001	140753000	1000	1	2.70E-07	-0.45	16	1.6	Acan	Extracellular Matrix
DMR1:140757001	1	140757001	140759000	2000	1	5.50E-12	0.51	30	1.5	Acan	Extracellular Matrix
DMR1:140776001	1	140776001	140778000	2000	1	8.10E-11	0.59	23	1.15	Acan	Extracellular Matrix
DMR1:140787001	1	140787001	140791000	4000	1	8.40E-15	0.84	43	1.07	Acan	Extracellular Matrix
DMR1:140817001	1	140817001	140819000	2000	1	8.70E-07	-0.44	31	1.55	Acan;Hapln3	Extracellular Matrix
DMR1:140833001	1	140833001	140834000	1000	1	2.10E-12	0.6	12	1.2	Acan;Hapln3	Extracellular Matrix
DMR1:140849001	1	140849001	140851000	2000	1	1.20E-07	0.38	26	1.3	Hapln3;Mfge8	Extracellular Matrix;Metabolism
DMR1:140855001	1	140855001	140859000	4000	1	1.70E-17	0.62	63	1.57	Hapln3;Mfge8	Extracellular Matrix;Metabolism
DMR1:140862001	1	140862001	140865000	3000	1	5.70E-10	0.37	40	1.33	Mfge8	Metabolism
DMR1:140989001	1	140989001	140991000	2000	1	1.80E-09	0.44	22	1.1	LOC108348916;Abhd2	Protease
DMR1:140999001	1	140999001	141001000	2000	1	1.10E-17	0.59	38	1.9	LOC108348916;Abhd2	Protease
DMR1:141013001	1	141013001	141018000	5000	1	5.80E-07	-0.39	40	0.8	Abhd2	Protease
DMR1:141020001	1	141020001	141026000	6000	1	1.30E-16	0.65	77	1.28	Abhd2	Protease
DMR1:141036001	1	141036001	141038000	2000	1	2.90E-23	0.89	27	1.35	Abhd2	Protease
DMR1:141039001	1	141039001	141040000	1000	1	4.50E-17	0.7	12	1.2	Abhd2	Protease
DMR1:141060001	1	141060001	141065000	5000	1	1.00E-27	0.77	67	1.34	Abhd2	Protease

DMR1:141104001	1	141104001	141107000	3000	1	2.20E-16	0.74	41	1.37	Rlbp1;Fanci	Transport
DMR1:141113001	1	141113001	141116000	3000	2	2.10E-16	0.61	28	0.93	Rlbp1;Fanci	Transport
DMR1:141117001	1	141117001	141121000	4000	1	7.30E-15	0.52	56	1.4	Rlbp1;Fanci	Transport
DMR1:141122001	1	141122001	141124000	2000	1	7.10E-08	0.36	36	1.8	Fanci	
DMR1:141128001	1	141128001	141129000	1000	1	8.30E-13	0.45	18	1.8	Fanci	
DMR1:141131001	1	141131001	141132000	1000	1	8.10E-07	-0.56	17	1.7	Fanci	
DMR1:141133001	1	141133001	141134000	1000	1	5.00E-09	0.36	15	1.5	Fanci	
DMR1:141135001	1	141135001	141137000	2000	1	7.10E-07	-0.35	39	1.95	Fanci	
DMR1:141150001	1	141150001	141154000	4000	1	7.00E-09	-0.57	58	1.45	Fanci	
DMR1:141168001	1	141168001	141170000	2000	1	8.20E-08	-0.54	17	0.85	Fanci;Polg	Transcription
DMR1:141191001	1	141191001	141194000	3000	1	8.40E-11	-0.44	31	1.03	Polg;Trnar-ucg;LOC102547943	Transcription
DMR1:141196001	1	141196001	141197000	1000	1	1.50E-19	0.76	10	1	Polg;Trnar-ucg;LOC102547943	Transcription
DMR1:141222001	1	141222001	141223000	1000	1	1.80E-08	0.46	18	1.8	LOC685596;LOC102555346;Mir3597-3;Mir9-3	
DMR1:141327001	1	141327001	141328000	1000	1	6.20E-11	0.45	16	1.6	Rhcg	Transport
DMR1:141331001	1	141331001	141333000	2000	1	3.40E-14	0.54	44	2.2	Rhcg	Transport
DMR1:141352001	1	141352001	141355000	3000	1	7.60E-11	0.43	35	1.17	Rhcg	Transport
DMR1:141393001	1	141393001	141396000	3000	1	1.20E-11	0.47	28	0.93	Ticrr	
DMR1:141405001	1	141405001	141406000	1000	1	1.20E-09	0.43	6	0.6	Ticrr	
DMR1:141445001	1	141445001	141446000	1000	1	5.40E-07	0.32	21	2.1	Kif7	Cytoskeleton
DMR1:141450001	1	141450001	141452000	2000	1	3.40E-09	0.42	36	1.8	Kif7;Plin1	Cytoskeleton
DMR1:141458001	1	141458001	141462000	4000	2	4.80E-22	0.89	95	2.38	Kif7;Plin1	Cytoskeleton
DMR1:141478001	1	141478001	141481000	3000	2	3.10E-10	0.6	52	1.73	Plin1;Pex11a;Wdr93	Metabolism
DMR1:141500001	1	141500001	141502000	2000	2	5.80E-15	0.85	12	0.6	Wdr93;Snrpep2	Metabolism
DMR1:141507001	1	141507001	141510000	3000	1	2.10E-09	0.38	21	0.7	Wdr93;Snrpep2	Metabolism
DMR1:141530001	1	141530001	141532000	2000	1	6.30E-07	-0.53	31	1.55	Wdr93;Mesp1	Metabolism
DMR1:141555001	1	141555001	141559000	4000	1	9.10E-11	-0.58	43	1.07	Mesp2;Anpep	Protease
DMR1:141574001	1	141574001	141577000	3000	2	4.20E-18	0.62	43	1.43	Anpep	Protease
DMR1:141579001	1	141579001	141582000	3000	1	2.60E-13	0.48	51	1.7	Anpep	Protease
DMR1:141583001	1	141583001	141590000	7000	2	1.60E-09	0.59	144	2.06	Anpep	Protease
DMR1:141597001	1	141597001	141601000	4000	1	5.60E-13	0.62	64	1.6	Anpep	Protease
DMR1:141602001	1	141602001	141605000	3000	1	5.50E-07	-0.49	54	1.8	Anpep;Ap3s2	Protease;Transport
DMR1:141619001	1	141619001	141625000	6000	3	3.10E-12	-0.56	73	1.22	Ap3s2	Transport
DMR1:141635001	1	141635001	141637000	2000	1	2.50E-09	0.52	16	0.8	Ap3s2	Transport
DMR1:141646001	1	141646001	141647000	1000	1	3.40E-16	0.55	14	1.4	Ap3s2	Transport
DMR1:141674001	1	141674001	141676000	2000	2	1.60E-07	-0.4	34	1.7	Arpin	
DMR1:141679001	1	141679001	141682000	3000	1	2.60E-09	0.45	39	1.3	Arpin	
DMR1:141776001	1	141776001	141780000	4000	1	2.70E-12	0.44	61	1.52	Zfp710	Transcription
DMR1:141798001	1	141798001	141803000	5000	2	1.30E-16	0.97	74	1.48	Zfp710	Transcription
DMR1:141805001	1	141805001	141809000	4000	3	1.60E-22	-0.66	54	1.35	Zfp710	Transcription
DMR1:141813001	1	141813001	141814000	1000	1	3.10E-08	0.61	13	1.3	Zfp710;LOC100911186	Transcription
DMR1:141816001	1	141816001	141817000	1000	1	6.40E-10	0.42	17	1.7	Zfp710;LOC100911186	Transcription
DMR1:141838001	1	141838001	141843000	5000	3	4.20E-23	1	64	1.28	Zfp710;LOC100911186;LOC100911225	Transcription
DMR1:141850001	1	141850001	141853000	3000	1	9.50E-20	0.77	43	1.43	Zfp710	Transcription
DMR1:141854001	1	141854001	141856000	2000	2	1.80E-11	0.55	26	1.3	Zfp710	Transcription
DMR1:141865001	1	141865001	141867000	2000	1	2.40E-12	0.47	45	2.25	Zfp710;Idh2	Transcription;Metabolism
DMR1:141898001	1	141898001	141902000	4000	1	1.90E-07	-0.5	50	1.25	Idh2	Metabolism
DMR1:141980001	1	141980001	141982000	2000	2	1.20E-08	0.5	22	1.1	Sema4b	Signaling
DMR1:142062001	1	142062001	142064000	2000	1	3.10E-20	0.86	15	0.75	Ngrn;Vps33b	Transport
DMR1:142071001	1	142071001	142072000	1000	1	8.20E-08	-0.56	9	0.9	Vps33b	Transport
DMR1:142076001	1	142076001	142080000	4000	1	5.00E-07	-0.46	49	1.23	Vps33b;Prc1	Transport;Cytoskeleton
DMR1:142089001	1	142089001	142091000	2000	1	1.50E-32	0.96	39	1.95	Vps33b;Prc1	Transport;Cytoskeleton
DMR1:142103001	1	142103001	142107000	4000	1	1.00E-07	0.38	53	1.32	Prc1;Rccd1	Cytoskeleton
DMR1:142144001	1	142144001	142147000	3000	1	1.20E-07	0.32	61	2.03	Unc45a;Hddc3;LOC108349446;Man2a2	Metabolism
DMR1:142160001	1	142160001	142163000	3000	2	5.80E-18	0.85	66	2.2	Man2a2	
DMR1:142168001	1	142168001	142169000	1000	1	2.10E-10	0.42	16	1.6	Man2a2;Fes	
DMR1:142172001	1	142172001	142174000	2000	1	5.30E-09	0.39	40	2	Man2a2;Fes	
DMR1:142205001	1	142205001	142206000	1000	1	1.30E-17	0.61	12	1.2	Furin	Protease
DMR1:142239001	1	142239001	142242000	3000	1	1.60E-10	0.37	49	1.63	LOC108349447;Blm	Epigenetic
DMR1:142278001	1	142278001	142279000	1000	1	3.10E-13	0.62	11	1.1	Blm	Epigenetic

DMR1:142283001	1	142283001	142286000	3000	2	3.70E-23	0.77	23	0.77	Blm	Epigenetic
DMR1:142291001	1	142291001	142294000	3000	1	2.60E-21	0.73	47	1.57	Blm	Epigenetic
DMR1:142304001	1	142304001	142306000	2000	1	9.40E-07	-0.53	16	0.8	Blm	Epigenetic
DMR1:142327001	1	142327001	142329000	2000	2	4.80E-12	-0.79	25	1.25	Blm	Epigenetic
DMR1:142394001	1	142394001	142395000	1000	1	2.20E-21	0.89	13	1.3	LOC100909455;Crtc3	Transcription
DMR1:142397001	1	142397001	142398000	1000	1	2.30E-09	0.58	6	0.6	LOC100909455;Crtc3	Transcription
DMR1:142403001	1	142403001	142405000	2000	1	2.20E-21	0.86	22	1.1	LOC100909455;Crtc3	Transcription
DMR1:142407001	1	142407001	142408000	1000	1	1.40E-15	0.51	23	2.3	Crtc3	Transcription
DMR1:142412001	1	142412001	142416000	4000	2	7.10E-20	0.65	49	1.23	Crtc3	Transcription
DMR1:142440001	1	142440001	142444000	4000	1	2.20E-14	0.69	44	1.1	Crtc3	Transcription
DMR1:142459001	1	142459001	142461000	2000	1	8.60E-08	0.46	33	1.65	Crtc3	Transcription
DMR1:142490001	1	142490001	142491000	1000	1	3.30E-11	0.44	8	0.8	Crtc3	Transcription
DMR1:142492001	1	142492001	142494000	2000	1	7.20E-07	-0.55	19	0.95	Crtc3	Transcription
DMR1:142507001	1	142507001	142511000	4000	2	6.30E-11	-0.59	42	1.05	Crtc3;LOC103691178	Transcription
DMR1:142512001	1	142512001	142517000	5000	1	5.30E-07	0.32	68	1.36	LOC103691178;Iqgap1	Signaling
DMR1:142543001	1	142543001	142546000	3000	1	2.20E-10	0.38	50	1.67	Iqgap1	Signaling
DMR1:142549001	1	142549001	142555000	6000	1	5.20E-08	-0.56	93	1.55	Iqgap1	Signaling
DMR1:142562001	1	142562001	142563000	1000	1	3.60E-19	0.6	16	1.6	Iqgap1	Signaling
DMR1:142572001	1	142572001	142575000	3000	1	5.20E-09	-0.67	39	1.3	Iqgap1	Signaling
DMR1:142578001	1	142578001	142579000	1000	1	4.00E-17	0.48	15	1.5	Iqgap1	Signaling
DMR1:142592001	1	142592001	142597000	5000	2	5.70E-15	0.57	75	1.5	Iqgap1	Signaling
DMR1:142614001	1	142614001	142615000	1000	1	9.60E-07	0.39	17	1.7	Iqgap1	Signaling
DMR1:142618001	1	142618001	142619000	1000	1	1.50E-07	-0.41	8	0.8	Iqgap1	Signaling
DMR1:142677001	1	142677001	142678000	1000	1	3.40E-11	0.53	20	2	Zscan2	Transcription
DMR1:142685001	1	142685001	142687000	2000	1	4.20E-12	0.49	32	1.6	Zscan2	Transcription
DMR1:142751001	1	142751001	142752000	1000	1	9.80E-07	-0.41	10	1	Sec11a	Protease
DMR1:142833001	1	142833001	142835000	2000	1	9.60E-09	0.42	25	1.25	Zfp592;LOC108349600	
DMR1:142852001	1	142852001	142855000	3000	1	1.80E-11	0.51	30	1	Zfp592	
DMR1:142878001	1	142878001	142879000	1000	1	9.90E-21	0.69	12	1.2	Zfp592;Alpk3	Signaling
DMR1:142884001	1	142884001	142893000	9000	3	1.30E-09	0.38	151	1.68	Alpk3;LOC102555867	Signaling
DMR1:142895001	1	142895001	142897000	2000	1	1.10E-14	0.5	30	1.5	Alpk3;LOC102555867	Signaling
DMR1:142898001	1	142898001	142900000	2000	1	1.80E-23	1.05	17	0.85	Alpk3;LOC102555867	Signaling
DMR1:142902001	1	142902001	142906000	4000	3	7.90E-38	1.17	50	1.25	Alpk3;LOC102555867	Signaling
DMR1:142908001	1	142908001	142910000	2000	1	3.60E-09	0.35	29	1.45	Alpk3;LOC102555867	Signaling
DMR1:142918001	1	142918001	142920000	2000	1	2.50E-08	0.44	43	2.15	Alpk3;LOC102555804	Signaling
DMR1:142922001	1	142922001	142926000	4000	1	6.40E-07	-0.45	57	1.43	Alpk3;LOC102555804	Signaling
DMR1:142927001	1	142927001	142929000	2000	1	1.10E-18	0.57	41	2.05	Alpk3;LOC102555804	Signaling
DMR1:142952001	1	142952001	142959000	7000	1	3.00E-17	0.68	81	1.16	Slc28a1	Transport
DMR1:142963001	1	142963001	142970000	7000	3	6.40E-21	0.72	86	1.23	Slc28a1	Transport
DMR1:142971001	1	142971001	142974000	3000	2	3.70E-10	0.45	35	1.17	Slc28a1	Transport
DMR1:142979001	1	142979001	142980000	1000	1	1.70E-08	0.38	16	1.6	Slc28a1	Transport
DMR1:142992001	1	142992001	142995000	3000	2	6.70E-23	0.73	35	1.17	Slc28a1	Transport
DMR1:143000001	1	143000001	143008000	8000	1	2.50E-07	0.32	119	1.49	Slc28a1	Transport
DMR1:143030001	1	143030001	143034000	4000	2	2.00E-10	0.58	36	0.9	Pde8a	Signaling
DMR1:143055001	1	143055001	143057000	2000	1	6.40E-07	0.39	20	1	Pde8a	Signaling
DMR1:143084001	1	143084001	143089000	5000	2	3.40E-18	0.71	52	1.04	Pde8a	Signaling
DMR1:143109001	1	143109001	143111000	2000	1	1.20E-08	-0.56	30	1.5	Pde8a	Signaling
DMR1:143144001	1	143144001	143147000	3000	1	1.40E-09	0.46	60	2	Pde8a	Signaling
DMR1:143148001	1	143148001	143151000	3000	2	8.20E-09	-0.57	42	1.4	Pde8a	Signaling
DMR1:143179001	1	143179001	143180000	1000	1	4.70E-11	-0.54	12	1.2	Rps17;Cpeb1	Translation;Translati on
DMR1:143227001	1	143227001	143231000	4000	1	2.30E-07	-0.4	9	0.22	Cpeb1	Translation
DMR1:143236001	1	143236001	143237000	1000	1	8.50E-07	-0.44	6	0.6	Cpeb1	Translation
DMR1:143258001	1	143258001	143261000	3000	2	1.20E-15	-0.46	34	1.13	Cpeb1	Translation
DMR1:143284001	1	143284001	143286000	2000	1	6.50E-08	0.47	16	0.8	Cpeb1;Ap3b2	Translation;Transport
DMR1:143323001	1	143323001	143327000	4000	2	2.80E-08	-0.49	44	1.1	Ap3b2;LOC102555928;LOC1003 60933;LOC102556224	Transport
DMR1:143365001	1	143365001	143367000	2000	1	3.40E-08	0.4	33	1.65	LOC102556224;Scarna15;Fsd2	Proteolysis
DMR1:143377001	1	143377001	143381000	4000	2	4.00E-16	0.52	58	1.45	Fsd2	Proteolysis
DMR1:143391001	1	143391001	143394000	3000	1	5.90E-08	0.36	51	1.7	Fsd2;Whamm	Proteolysis
DMR1:143396001	1	143396001	143398000	2000	1	1.80E-11	0.53	24	1.2	Fsd2;Whamm	Proteolysis
DMR1:143413001	1	143413001	143414000	1000	1	6.40E-08	-0.6	7	0.7	Whamm	
DMR1:143427001	1	143427001	143428000	1000	1	5.00E-08	0.28	17	1.7	Whamm	
DMR1:143450001	1	143450001	143456000	6000	1	9.50E-08	0.35	92	1.53	Homer2	

DMR1:143486001	1	143486001	143489000	3000	1	3.20E-08	0.48	42	1.4	Homer2	
DMR1:143491001	1	143491001	143494000	3000	3	9.80E-27	0.71	29	0.97	Homer2	
DMR1:143518001	1	143518001	143523000	5000	5	3.90E-23	0.95	46	0.92	Homer2	
DMR1:143524001	1	143524001	143525000	1000	1	2.10E-13	0.48	17	1.7	Homer2	
DMR1:143530001	1	143530001	143534000	4000	1	5.70E-15	0.6	74	1.85	Homer2	
DMR1:143538001	1	143538001	143539000	1000	1	2.00E-10	0.39	11	1.1	Homer2	
DMR1:143609001	1	143609001	143611000	2000	1	1.10E-14	0.61	24	1.2	RGD1305713;Btbd1	Proteolysis
DMR1:143619001	1	143619001	143621000	2000	1	4.40E-12	0.48	33	1.65	Btbd1	Proteolysis
DMR1:143627001	1	143627001	143631000	4000	2	1.40E-08	0.42	28	0.7	Btbd1	Proteolysis
DMR1:143642001	1	143642001	143644000	2000	1	1.40E-07	0.37	25	1.25	Btbd1	Proteolysis
DMR1:143649001	1	143649001	143657000	8000	3	1.50E-19	0.6	74	0.92	Btbd1;Tm6sf1	Proteolysis
DMR1:143659001	1	143659001	143670000	11000	4	2.20E-17	0.67	133	1.21	Tm6sf1;LOC102556283	
DMR1:143677001	1	143677001	143678000	1000	1	8.70E-21	0.75	14	1.4	Tm6sf1;LOC102556283	
DMR1:143688001	1	143688001	143689000	1000	1	6.30E-07	0.37	7	0.7	Tm6sf1;LOC102556283	
DMR1:143702001	1	143702001	143704000	2000	1	4.20E-08	-0.45	27	1.35	Tm6sf1;Hdgfrp3	
DMR1:143709001	1	143709001	143710000	1000	1	9.00E-18	0.65	9	0.9	Tm6sf1;Hdgfrp3	
DMR1:143728001	1	143728001	143729000	1000	1	1.30E-15	0.82	3	0.3	Hdgfrp3	
DMR1:143802001	1	143802001	143804000	2000	1	1.30E-13	0.51	31	1.55	Bnc1	Transcription
DMR1:143811001	1	143811001	143813000	2000	1	2.00E-08	-0.41	26	1.3	Bnc1	Transcription
DMR1:143827001	1	143827001	143830000	3000	2	6.40E-19	0.56	34	1.13	Bnc1	Transcription
DMR1:143858001	1	143858001	143860000	2000	1	1.80E-07	0.41	30	1.5	LOC102556513;Bnc1	Transcription
DMR1:143886001	1	143886001	143889000	3000	1	2.70E-08	-0.64	31	1.03	Bnc1	Transcription
DMR1:144091001	1	144091001	144092000	1000	1	2.10E-11	0.5	7	0.7	Sh3gl3	
DMR1:144094001	1	144094001	144096000	2000	2	4.00E-09	0.43	48	2.4	Sh3gl3	
DMR1:144102001	1	144102001	144103000	1000	1	2.00E-10	0.37	9	0.9	Sh3gl3	
DMR1:144107001	1	144107001	144110000	3000	2	1.20E-10	-0.44	35	1.17	Sh3gl3	
DMR1:144123001	1	144123001	144124000	1000	1	1.80E-16	0.68	7	0.7	Sh3gl3	
DMR1:144126001	1	144126001	144133000	7000	4	3.30E-24	0.88	55	0.79	Sh3gl3	
DMR1:144144001	1	144144001	144150000	6000	4	1.30E-20	0.86	61	1.02	Sh3gl3	
DMR1:144158001	1	144158001	144160000	2000	1	4.10E-09	0.58	14	0.7	Sh3gl3	
DMR1:144163001	1	144163001	144167000	4000	1	1.90E-17	0.52	62	1.55	Sh3gl3	
DMR1:144198001	1	144198001	144200000	2000	2	1.50E-31	1.24	18	0.9	Sh3gl3	
DMR1:144201001	1	144201001	144202000	1000	1	4.00E-10	0.41	20	2	Sh3gl3	
DMR1:144254001	1	144254001	144255000	1000	1	1.10E-07	0.51	9	0.9	Adamtsl3	Protease
DMR1:144267001	1	144267001	144269000	2000	1	4.80E-10	0.48	15	0.75	Adamtsl3	Protease
DMR1:144271001	1	144271001	144273000	2000	1	4.30E-08	0.62	28	1.4	Adamtsl3	Protease
DMR1:144276001	1	144276001	144280000	4000	1	2.70E-07	0.34	47	1.18	Adamtsl3	Protease
DMR1:144310001	1	144310001	144312000	2000	1	3.70E-09	0.41	17	0.85	Adamtsl3	Protease
DMR1:144370001	1	144370001	144371000	1000	1	3.90E-07	-0.47	13	1.3	Adamtsl3	Protease
DMR1:144377001	1	144377001	144379000	2000	2	8.70E-13	0.55	16	0.8	Adamtsl3	Protease
DMR1:144381001	1	144381001	144386000	5000	3	3.50E-10	0.66	36	0.72	Adamtsl3	Protease
DMR1:144413001	1	144413001	144419000	6000	2	2.40E-12	-0.44	60	1	Adamtsl3	Protease
DMR1:144426001	1	144426001	144429000	3000	1	5.40E-08	0.43	32	1.07	Adamtsl3	Protease
DMR1:144437001	1	144437001	144441000	4000	3	2.40E-08	-0.63	8	0.2	Adamtsl3	Protease
DMR1:144443001	1	144443001	144445000	2000	2	1.30E-11	0.69	12	0.6	Adamtsl3	Protease
DMR1:144456001	1	144456001	144459000	3000	1	1.00E-12	0.67	23	0.77	Adamtsl3	Protease
DMR1:144470001	1	144470001	144471000	1000	1	1.50E-11	0.77	9	0.9	Adamtsl3	Protease
DMR1:144529001	1	144529001	144530000	1000	1	2.80E-10	0.49	9	0.9	Adamtsl3	Protease
DMR1:144547001	1	144547001	144550000	3000	2	6.50E-11	0.45	41	1.37	Adamtsl3	Protease
DMR1:144571001	1	144571001	144573000	2000	1	1.90E-13	0.75	10	0.5	Adamtsl3	Protease
DMR1:144627001	1	144627001	144628000	1000	1	2.70E-07	-0.63	15	1.5	Efl1	Translation
DMR1:144632001	1	144632001	144633000	1000	1	4.60E-09	0.62	9	0.9	Efl1	Translation
DMR1:144637001	1	144637001	144639000	2000	1	5.70E-20	0.81	11	0.55	Efl1	Translation
DMR1:144640001	1	144640001	144643000	3000	2	7.80E-21	0.75	20	0.67	Efl1	Translation
DMR1:144660001	1	144660001	144661000	1000	1	5.00E-08	0.4	10	1	Efl1	Translation
DMR1:144682001	1	144682001	144683000	1000	1	9.70E-10	0.65	6	0.6	Efl1	Translation
DMR1:144694001	1	144694001	144695000	1000	1	4.10E-07	0.54	7	0.7	Efl1	Translation
DMR1:144718001	1	144718001	144720000	2000	1	4.20E-21	0.82	24	1.2	Efl1	Translation
DMR1:144721001	1	144721001	144722000	1000	1	9.60E-22	1.2	17	1.7	Efl1	Translation
DMR1:144744001	1	144744001	144748000	4000	1	7.90E-08	0.39	35	0.88	Efl1	Translation
DMR1:144839001	1	144839001	144841000	2000	1	6.30E-08	0.31	27	1.35	Mex3b	Metabolism
DMR1:145125001	1	145125001	145126000	1000	1	9.50E-07	0.46	12	1.2	RGD1566271	
DMR1:145705001	1	145705001	145707000	2000	2	1.00E-17	0.85	12	0.6	Tmc3	
DMR1:145709001	1	145709001	145714000	5000	1	1.40E-07	0.5	58	1.16	Tmc3	
DMR1:145717001	1	145717001	145720000	3000	1	4.30E-08	0.34	30	1	Tmc3	

DMR1:145738001	1	145738001	145741000	3000	2	9.50E-19	0.91	20	0.67	Tmc3	
DMR1:145743001	1	145743001	145745000	2000	1	7.30E-07	-0.46	26	1.3	Tmc3	
DMR1:145748001	1	145748001	145755000	7000	3	3.00E-19	-0.47	81	1.16	Tmc3	
DMR1:145775001	1	145775001	145776000	1000	1	3.10E-10	0.62	8	0.8	Stard5;Il16	Cytokine
DMR1:145800001	1	145800001	145803000	3000	1	5.20E-09	-0.54	37	1.23	Il16;LOC103691182	Cytokine
DMR1:145814001	1	145814001	145816000	2000	2	8.60E-09	0.49	16	0.8	Il16;LOC103691182	Cytokine
DMR1:145817001	1	145817001	145821000	4000	1	1.00E-12	0.65	35	0.88	Il16;LOC103691182	Cytokine
DMR1:145853001	1	145853001	145856000	3000	1	1.10E-08	0.44	22	0.73	Il16	Cytokine
DMR1:145857001	1	145857001	145864000	7000	2	4.20E-20	-0.52	81	1.16	Il16	Cytokine
DMR1:145876001	1	145876001	145877000	1000	1	5.30E-07	0.39	12	1.2	Il16	Cytokine
DMR1:145909001	1	145909001	145912000	3000	2	1.30E-10	0.76	19	0.63	Cfap161	Development
DMR1:145914001	1	145914001	145917000	3000	2	5.70E-11	-0.62	12	0.4	Cfap161	Development
DMR1:145919001	1	145919001	145926000	7000	2	6.50E-10	0.47	49	0.7	Cfap161	Development
DMR1:145931001	1	145931001	145933000	2000	2	2.70E-19	0.84	28	1.4	Cfap161	Development
DMR1:145937001	1	145937001	145945000	8000	5	1.30E-19	-0.58	91	1.14	Cfap161	Development
DMR1:146019001	1	146019001	146020000	1000	1	2.40E-08	0.5	6	0.6	Mesdc1	
DMR1:146060001	1	146060001	146061000	1000	1	2.30E-10	0.5	10	1	Cemip	
DMR1:146077001	1	146077001	146079000	2000	1	1.50E-08	0.48	20	1	Cemip	
DMR1:146086001	1	146086001	146087000	1000	1	2.90E-09	0.45	14	1.4	Cemip	
DMR1:146088001	1	146088001	146091000	3000	1	9.90E-07	0.36	28	0.93	Cemip	
DMR1:146098001	1	146098001	146099000	1000	1	1.00E-08	-0.59	12	1.2	Cemip	
DMR1:146110001	1	146110001	146111000	1000	1	1.80E-10	0.48	10	1	Cemip	
DMR1:146144001	1	146144001	146146000	2000	1	5.90E-16	0.5	32	1.6	Cemip	
DMR1:146173001	1	146173001	146176000	3000	1	7.20E-11	0.59	20	0.67	Cemip	
DMR1:146195001	1	146195001	146196000	1000	1	3.70E-09	0.59	5	0.5	Cemip	
DMR1:146198001	1	146198001	146202000	4000	3	1.40E-12	-0.61	28	0.7	Cemip	
DMR1:146204001	1	146204001	146206000	2000	1	3.60E-09	0.59	7	0.35	Cemip	
DMR1:146244001	1	146244001	146245000	1000	1	7.30E-08	0.28	12	1.2	Abhd17c	Protease
DMR1:146249001	1	146249001	146251000	2000	1	5.50E-07	-0.53	31	1.55	Abhd17c	Protease
DMR1:146278001	1	146278001	146279000	1000	1	3.30E-09	0.46	5	0.5	Abhd17c	Protease
DMR1:146280001	1	146280001	146281000	1000	1	1.50E-23	0.92	8	0.8	Abhd17c;LOC108349171	Protease
DMR1:146293001	1	146293001	146299000	6000	3	2.00E-21	-0.55	57	0.95	Abhd17c;LOC108349171	Protease
DMR1:146391001	1	146391001	146393000	2000	1	1.70E-16	0.68	16	0.8	Arnt2	Transcription
DMR1:146405001	1	146405001	146407000	2000	1	3.40E-15	0.58	28	1.4	Arnt2	Transcription
DMR1:146425001	1	146425001	146426000	1000	1	6.50E-07	0.38	11	1.1	Arnt2	Transcription
DMR1:146431001	1	146431001	146432000	1000	1	8.40E-09	0.37	22	2.2	Arnt2	Transcription
DMR1:146454001	1	146454001	146456000	2000	1	3.60E-08	-0.65	23	1.15	Arnt2	Transcription
DMR1:146497001	1	146497001	146499000	2000	1	9.10E-09	0.42	26	1.3	Arnt2	Transcription
DMR1:146518001	1	146518001	146519000	1000	1	1.50E-07	-0.45	18	1.8	Arnt2	Transcription
DMR1:146528001	1	146528001	146530000	2000	2	1.90E-11	0.68	8	0.4	Arnt2	Transcription
DMR1:146540001	1	146540001	146542000	2000	1	9.80E-12	0.73	17	0.85	Arnt2	Transcription
DMR1:146547001	1	146547001	146548000	1000	1	3.50E-11	0.43	14	1.4	Arnt2	Transcription
DMR1:146562001	1	146562001	146564000	2000	1	4.60E-09	0.55	10	0.5	Arnt2	Transcription
DMR1:146723001	1	146723001	146725000	2000	1	1.20E-07	0.46	22	1.1	Fah	
DMR1:146783001	1	146783001	146785000	2000	1	1.50E-13	0.79	10	0.5	Zfand6	
DMR1:147020001	1	147020001	147021000	1000	1	1.10E-08	0.55	11	1.1	LOC100911047;F8a1	
DMR1:147025001	1	147025001	147026000	1000	1	3.90E-09	0.52	9	0.9	F8a1;Xlr3a	
DMR1:147030001	1	147030001	147036000	6000	1	2.50E-09	0.35	91	1.52	F8a1;Xlr3a	
DMR1:147038001	1	147038001	147039000	1000	1	6.20E-07	0.42	11	1.1	Xlr3a	
DMR1:147046001	1	147046001	147049000	3000	1	4.30E-08	0.48	47	1.57	Xlr3a	
DMR1:147057001	1	147057001	147058000	1000	1	2.00E-08	0.39	16	1.6	Xlr3a;LOC100366231	
DMR1:147059001	1	147059001	147061000	2000	2	2.60E-15	0.54	36	1.8	Xlr3a;LOC100366231	
DMR1:147408001	1	147408001	147413000	5000	1	1.20E-07	-0.54	23	0.46	Cyp2c7	Metabolism
DMR1:147420001	1	147420001	147422000	2000	1	2.70E-17	-0.82	6	0.3	Cyp2c7	Metabolism
DMR1:147440001	1	147440001	147444000	4000	2	3.60E-10	-0.47	25	0.62	Cyp2c7	Metabolism
DMR1:147464001	1	147464001	147472000	8000	2	7.80E-09	-0.53	175	2.19	Cyp2c7	Metabolism
DMR1:147483001	1	147483001	147485000	2000	1	6.50E-09	0.5	10	0.5	Cyp2c7	Metabolism
DMR1:147493001	1	147493001	147495000	2000	1	4.80E-08	0.59	11	0.55	Cyp2c7	Metabolism
DMR1:147496001	1	147496001	147497000	1000	1	1.00E-10	0.6	7	0.7	Cyp2c7	Metabolism
DMR1:147502001	1	147502001	147504000	2000	2	3.10E-18	0.84	8	0.4	Cyp2c7	Metabolism
DMR1:147508001	1	147508001	147509000	1000	1	1.50E-10	0.52	10	1	Cyp2c7	Metabolism
DMR1:147518001	1	147518001	147520000	2000	2	5.30E-08	-0.37	14	0.7	Cyp2c7;LOC100361434	Metabolism
DMR1:147523001	1	147523001	147525000	2000	2	4.10E-13	0.59	14	0.7	Cyp2c7;LOC100361434	Metabolism
DMR1:147535001	1	147535001	147538000	3000	1	5.40E-17	-0.45	32	1.07	Cyp2c7;LOC100361434	Metabolism
DMR1:147549001	1	147549001	147556000	7000	4	5.00E-19	0.49	63	0.9	Cyp2c7;LOC100361434	Metabolism

DMR1:147560001	1	147560001	147563000	3000	2	7.60E-11	-0.52	18	0.6	Cyp2c7;LOC100361434	Metabolism
DMR1:147564001	1	147564001	147565000	1000	1	2.10E-09	0.47	10	1	Cyp2c7;LOC100361434	Metabolism
DMR1:147566001	1	147566001	147567000	1000	1	1.50E-10	0.57	8	0.8	Cyp2c7;LOC100361434	Metabolism
DMR1:147568001	1	147568001	147572000	4000	2	1.20E-11	-0.49	21	0.52	Cyp2c7;LOC100361434	Metabolism
DMR1:147587001	1	147587001	147592000	5000	1	5.10E-07	-0.44	30	0.6	Cyp2c7;LOC100361434	Metabolism
DMR1:147602001	1	147602001	147604000	2000	2	1.20E-09	-0.51	6	0.3	Cyp2c7;LOC100361434	Metabolism
DMR1:147607001	1	147607001	147608000	1000	1	8.60E-17	0.68	3	0.3	Cyp2c7	Metabolism
DMR1:147609001	1	147609001	147612000	3000	2	5.40E-14	0.57	24	0.8	Cyp2c7	Metabolism
DMR1:147653001	1	147653001	147655000	2000	1	3.80E-07	0.31	19	0.95	Cyp2c7	Metabolism
DMR1:147663001	1	147663001	147666000	3000	1	3.10E-14	-0.61	12	0.4	Cyp2c7	Metabolism
DMR1:147683001	1	147683001	147686000	3000	2	2.90E-18	0.68	10	0.33	Cyp2c7;LOC100359554	Metabolism
DMR1:147691001	1	147691001	147692000	1000	1	1.90E-10	0.74	5	0.5	Cyp2c7;LOC100359554	Metabolism
DMR1:147693001	1	147693001	147694000	1000	1	3.60E-09	0.4	5	0.5	Cyp2c7;LOC100359554	Metabolism
DMR1:147701001	1	147701001	147703000	2000	1	1.20E-17	0.86	7	0.35	Cyp2c7;LOC100359554	Metabolism
DMR1:147715001	1	147715001	147724000	9000	5	2.10E-17	-0.47	117	1.3	Cyp2c7;Cyp2c6v1	Metabolism
DMR1:147737001	1	147737001	147738000	1000	1	1.00E-11	0.64	12	1.2	Cyp2c7;Cyp2c6v1	Metabolism
DMR1:147742001	1	147742001	147746000	4000	2	1.50E-14	0.57	30	0.75	Cyp2c7;Cyp2c6v1	Metabolism
DMR1:147747001	1	147747001	147748000	1000	1	1.30E-11	0.62	7	0.7	Cyp2c7;Cyp2c6v1	Metabolism
DMR1:147774001	1	147774001	147775000	1000	1	3.30E-07	0.41	5	0.5	Cyp2c7;Cyp2c6v1	Metabolism
DMR1:147783001	1	147783001	147790000	7000	3	1.50E-13	0.54	49	0.7	Cyp2c7;Cyp2c6v1	Metabolism
DMR1:147791001	1	147791001	147796000	5000	1	9.20E-09	0.46	34	0.68	Cyp2c7;Cyp2c6v1	Metabolism
DMR1:147797001	1	147797001	147800000	3000	1	7.40E-17	0.53	12	0.4	Cyp2c7;Cyp2c6v1	Metabolism
DMR1:147802001	1	147802001	147803000	1000	1	4.40E-10	0.5	6	0.6	Cyp2c7;Cyp2c6v1	Metabolism
DMR1:147810001	1	147810001	147812000	2000	1	3.00E-16	0.58	9	0.45	Cyp2c7;Cyp2c6v1	Metabolism
DMR1:147816001	1	147816001	147818000	2000	1	2.30E-12	0.54	13	0.65	Cyp2c7;Cyp2c6v1	Metabolism
DMR1:147819001	1	147819001	147822000	3000	1	1.20E-23	0.62	18	0.6	Cyp2c7;Cyp2c6v1	Metabolism
DMR1:147823001	1	147823001	147830000	7000	1	1.20E-07	-0.49	52	0.74	Cyp2c7;Cyp2c6v1	Metabolism
DMR1:147831001	1	147831001	147834000	3000	3	1.40E-18	0.5	24	0.8	Cyp2c7	Metabolism
DMR1:147835001	1	147835001	147843000	8000	3	3.60E-15	-0.58	37	0.46	Cyp2c7	Metabolism
DMR1:147848001	1	147848001	147853000	5000	2	5.90E-12	-0.52	25	0.5	Cyp2c7	Metabolism
DMR1:147857001	1	147857001	147861000	4000	2	2.40E-30	0.79	31	0.78	Cyp2c7	Metabolism
DMR1:147866001	1	147866001	147867000	1000	1	5.40E-15	0.64	2	0.2	Cyp2c7	Metabolism
DMR1:147869001	1	147869001	147872000	3000	1	3.10E-07	-0.32	26	0.87	Cyp2c7	Metabolism
DMR1:147884001	1	147884001	147885000	1000	1	4.50E-07	0.41	5	0.5	Cyp2c7;LOC100911718	Metabolism
DMR1:147892001	1	147892001	147902000	10000	2	4.00E-15	-0.53	71	0.71	Cyp2c7;LOC100911718	Metabolism
DMR1:147903001	1	147903001	147906000	3000	1	9.60E-12	0.47	21	0.7	Cyp2c7	Metabolism
DMR1:147913001	1	147913001	147915000	2000	2	9.00E-10	-0.63	53	2.65	Cyp2c7	Metabolism
DMR1:147922001	1	147922001	147925000	3000	1	2.90E-10	-0.59	8	0.27	Cyp2c7	Metabolism
DMR1:147936001	1	147936001	147944000	8000	1	6.00E-12	-0.37	65	0.81	Cyp2c7	Metabolism
DMR1:147986001	1	147986001	147988000	2000	1	9.00E-08	-0.51	13	0.65	Cyp2c7	Metabolism
DMR1:148034001	1	148034001	148036000	2000	1	1.50E-09	-0.68	4	0.2	Cyp2c7	Metabolism
DMR1:148061001	1	148061001	148069000	8000	3	1.40E-22	0.85	62	0.78	Cyp2c7	Metabolism
DMR1:148075001	1	148075001	148081000	6000	2	1.50E-18	-0.65	32	0.53	Cyp2c7	Metabolism
DMR1:148085001	1	148085001	148088000	3000	2	8.20E-15	-0.56	8	0.27	Cyp2c7	Metabolism
DMR1:148092001	1	148092001	148095000	3000	2	1.30E-15	-0.62	14	0.47	Cyp2c7	Metabolism
DMR1:148114001	1	148114001	148115000	1000	1	8.70E-13	0.54	18	1.8	Cyp2c7	Metabolism
DMR1:148117001	1	148117001	148120000	3000	2	1.70E-14	0.65	19	0.63	Cyp2c7	Metabolism
DMR1:148125001	1	148125001	148127000	2000	2	2.00E-23	0.83	14	0.7	Cyp2c7	Metabolism
DMR1:148425001	1	148425001	148426000	1000	1	4.60E-09	0.41	32	3.2	Vbp1	Transcription
DMR1:148427001	1	148427001	148430000	3000	3	1.60E-18	-0.99	179	5.97	Vbp1	Transcription
DMR1:148455001	1	148455001	148457000	2000	1	1.00E-13	0.56	53	2.65	LOC102546990;Mpp1	
DMR1:148461001	1	148461001	148464000	3000	2	1.40E-17	0.49	72	2.4	Mpp1	
DMR1:148835001	1	148835001	148837000	2000	1	1.90E-16	0.61	12	0.6	LOC100912014;Olr225-ps;Olr226	Receptor
DMR1:148846001	1	148846001	148847000	1000	1	5.80E-07	0.47	11	1.1	Olr225-ps;Olr226	Receptor
DMR1:148852001	1	148852001	148855000	3000	2	6.30E-10	0.7	11	0.37	Olr225-ps;Olr226;LOC100912097	Receptor
DMR1:148911001	1	148911001	148927000	16000	5	6.70E-25	-0.69	112	0.7	Vom2r40	
DMR1:148931001	1	148931001	148934000	3000	1	5.50E-08	-0.6	6	0.2	Vom2r40	
DMR1:148941001	1	148941001	148945000	4000	1	6.30E-07	0.48	15	0.38	Vom2r40	
DMR1:148946001	1	148946001	148947000	1000	1	4.60E-13	0.52	12	1.2	Vom2r40	
DMR1:148950001	1	148950001	148954000	4000	1	6.60E-07	0.42	16	0.4	Vom2r40	
DMR1:148961001	1	148961001	148964000	3000	1	5.50E-07	0.38	16	0.53	Vom2r40	
DMR1:148966001	1	148966001	148971000	5000	1	5.30E-09	0.38	28	0.56	Vom2r40	
DMR1:148976001	1	148976001	148977000	1000	1	1.60E-12	0.56	2	0.2	Vom2r40	

DMR1:148978001	1	148978001	148985000	7000	1	3.60E-11	0.51	46	0.66	Vom2r40	
DMR1:149160001	1	149160001	149162000	2000	1	3.10E-18	-0.44	16	0.8	LOC108349603;Vom2r42	Signaling
DMR1:149163001	1	149163001	149168000	5000	2	3.30E-07	-0.41	28	0.56	LOC108349603;Vom2r42	Signaling
DMR1:149179001	1	149179001	149182000	3000	1	9.30E-15	0.69	22	0.73	LOC108349603;Vom2r42	Signaling
DMR1:149184001	1	149184001	149185000	1000	1	9.40E-08	0.54	9	0.9	LOC108349603;Vom2r42	Signaling
DMR1:149300001	1	149300001	149304000	4000	2	1.20E-13	-0.46	39	0.98	Vom2r-ps63	
DMR1:149494001	1	149494001	149499000	5000	1	4.40E-08	-0.35	34	0.68	Vom2r43	Signaling
DMR1:149500001	1	149500001	149501000	1000	1	2.50E-07	-0.56	5	0.5	Vom2r43	Signaling
DMR1:149527001	1	149527001	149529000	2000	1	2.60E-12	0.82	9	0.45	Vom2r43	Signaling
DMR1:149784001	1	149784001	149785000	1000	1	3.00E-08	0.6	8	0.8	LOC100909935;Olr13	Receptor
DMR1:149828001	1	149828001	149832000	4000	3	9.40E-11	-0.39	43	1.07	Olr15-ps	
DMR1:149968001	1	149968001	149969000	1000	1	3.70E-17	0.99	5	0.5	Olr20	Signaling
DMR1:149972001	1	149972001	149973000	1000	1	9.00E-07	-0.46	5	0.5	Olr20	Signaling
DMR1:150011001	1	150011001	150013000	2000	1	3.00E-08	-0.74	15	0.75	Olr21-ps	
DMR1:150014001	1	150014001	150017000	3000	1	5.30E-08	-0.52	13	0.43	Olr21-ps	
DMR1:150019001	1	150019001	150026000	7000	5	7.70E-21	-0.45	76	1.09	Olr22-ps	
DMR1:150032001	1	150032001	150035000	3000	1	5.80E-10	-0.39	45	1.5	Olr22-ps	
DMR1:150077001	1	150077001	150080000	3000	1	7.40E-08	-0.5	24	0.8	Olr23;LOC100360319	Signaling
DMR1:150102001	1	150102001	150104000	2000	2	2.90E-07	0.55	10	0.5	Olr24	Signaling
DMR1:150132001	1	150132001	150134000	2000	1	1.10E-09	0.56	10	0.5	Olr25	Signaling
DMR1:150137001	1	150137001	150138000	1000	1	5.00E-07	0.63	5	0.5	Olr25	Signaling
DMR1:150161001	1	150161001	150168000	7000	4	1.70E-19	-0.57	99	1.41	Olr26-ps;Olr27	Signaling
DMR1:150215001	1	150215001	150216000	1000	1	1.00E-06	-0.64	8	0.8	Olr29;Olr30	Signaling
DMR1:150279001	1	150279001	150280000	1000	1	5.10E-09	-0.4	7	0.7	Olr33-ps	
DMR1:150305001	1	150305001	150306000	1000	1	3.30E-08	0.46	4	0.4	Olr34	Signaling
DMR1:150334001	1	150334001	150335000	1000	1	7.80E-09	0.53	6	0.6	Folh1	Protease
DMR1:150355001	1	150355001	150356000	1000	1	2.00E-10	0.65	3	0.3	Folh1	Protease
DMR1:150360001	1	150360001	150361000	1000	1	1.80E-15	0.8	5	0.5	Folh1	Protease
DMR1:150370001	1	150370001	150375000	5000	2	1.00E-07	-0.4	36	0.72	Folh1;LOC103691192	Protease
DMR1:150400001	1	150400001	150401000	1000	1	3.50E-07	0.37	7	0.7	Folh1	Protease
DMR1:150403001	1	150403001	150405000	2000	2	4.40E-19	0.74	11	0.55	Folh1;Vom2r-ps66	Protease
DMR1:150499001	1	150499001	150501000	2000	2	3.30E-15	-0.7	3	0.15	Vom2r-ps67	
DMR1:150512001	1	150512001	150515000	3000	1	7.80E-07	-0.43	13	0.43	Vom2r-ps67	
DMR1:150789001	1	150789001	150792000	3000	2	5.60E-16	-0.86	6	0.2	LOC100360510;LOC100910339;<br>Nox4	Metabolism
DMR1:150796001	1	150796001	150797000	1000	1	1.20E-11	0.68	16	1.6	LOC100910339;Nox4	Metabolism
DMR1:150809001	1	150809001	150810000	1000	1	1.20E-08	0.52	4	0.4	Nox4	Metabolism
DMR1:150822001	1	150822001	150825000	3000	2	9.40E-13	-0.63	9	0.3	Nox4	Metabolism
DMR1:150837001	1	150837001	150838000	1000	1	9.10E-08	-0.51	5	0.5	Nox4	Metabolism
DMR1:150857001	1	150857001	150859000	2000	2	2.40E-14	0.79	9	0.45	Nox4	Metabolism
DMR1:150877001	1	150877001	150879000	2000	1	1.00E-12	0.76	12	0.6	Nox4	Metabolism
DMR1:150885001	1	150885001	150886000	1000	1	3.30E-09	-0.55	5	0.5	Nox4	Metabolism
DMR1:150912001	1	150912001	150913000	1000	1	3.20E-09	-0.41	14	1.4	Nox4	Metabolism
DMR1:150954001	1	150954001	150955000	1000	1	1.60E-15	0.93	1	0.1	Nox4;LOC103691187	Metabolism
DMR1:150962001	1	150962001	150964000	2000	2	2.50E-16	0.73	20	1	Nox4;LOC103691187	Metabolism
DMR1:150986001	1	150986001	150992000	6000	3	3.30E-15	-0.44	50	0.83	Nox4	Metabolism
DMR1:151001001	1	151001001	151004000	3000	1	1.20E-12	-0.71	13	0.43	Tyr	Metabolism
DMR1:151005001	1	151005001	151008000	3000	1	6.40E-09	0.6	55	1.83	Tyr	Metabolism
DMR1:151011001	1	151011001	151012000	1000	1	5.90E-08	0.45	8	0.8	Tyr	Metabolism
DMR1:151026001	1	151026001	151029000	3000	1	8.20E-11	-0.66	6	0.2	Tyr	Metabolism
DMR1:151034001	1	151034001	151037000	3000	1	2.00E-09	-0.46	11	0.37	Tyr	Metabolism
DMR1:151050001	1	151050001	151052000	2000	1	1.60E-18	0.95	11	0.55	Tyr	Metabolism
DMR1:151067001	1	151067001	151070000	3000	1	3.00E-11	-0.44	18	0.6	Tyr	Metabolism
DMR1:151115001	1	151115001	151117000	2000	1	1.30E-07	0.59	10	0.5	Tyr	Metabolism
DMR1:151221001	1	151221001	151222000	1000	1	5.80E-18	0.92	6	0.6	Grm5	Signaling
DMR1:151223001	1	151223001	151224000	1000	1	3.90E-10	0.53	7	0.7	Grm5	Signaling
DMR1:151238001	1	151238001	151239000	1000	1	3.10E-17	0.93	10	1	Grm5	Signaling
DMR1:151253001	1	151253001	151255000	2000	1	3.20E-18	0.68	7	0.35	Grm5	Signaling
DMR1:151260001	1	151260001	151267000	7000	3	1.00E-25	-0.59	71	1.01	Grm5	Signaling
DMR1:151278001	1	151278001	151280000	2000	2	1.30E-08	0.48	10	0.5	Grm5	Signaling
DMR1:151290001	1	151290001	151292000	2000	1	9.00E-10	-0.62	14	0.7	Grm5	Signaling
DMR1:151293001	1	151293001	151295000	2000	1	3.10E-22	-0.63	10	0.5	Grm5	Signaling
DMR1:151323001	1	151323001	151324000	1000	1	2.00E-13	0.76	7	0.7	Grm5;LOC102555017	Signaling
DMR1:151366001	1	151366001	151368000	2000	1	4.20E-14	0.81	9	0.45	Grm5	Signaling
DMR1:151377001	1	151377001	151378000	1000	1	1.20E-16	0.8	9	0.9	Grm5	Signaling

DMR1:151383001	1	151383001	151385000	2000	2	7.90E-14	0.82	13	0.65	Grm5	Signaling
DMR1:151388001	1	151388001	151390000	2000	1	1.40E-08	0.48	23	1.15	Grm5;LOC102555105	Signaling
DMR1:151452001	1	151452001	151454000	2000	1	8.60E-15	0.87	8	0.4	Grm5	Signaling
DMR1:151475001	1	151475001	151476000	1000	1	1.70E-18	0.85	11	1.1	Grm5	Signaling
DMR1:151491001	1	151491001	151492000	1000	1	5.30E-12	0.73	4	0.4	Grm5	Signaling
DMR1:151494001	1	151494001	151502000	8000	5	1.40E-21	-0.55	83	1.04	Grm5	Signaling
DMR1:151510001	1	151510001	151511000	1000	1	2.50E-13	0.59	7	0.7	Grm5	Signaling
DMR1:151545001	1	151545001	151547000	2000	1	4.70E-08	-0.59	7	0.35	Grm5	Signaling
DMR1:151556001	1	151556001	151562000	6000	1	4.40E-09	0.45	24	0.4	Grm5	Signaling
DMR1:151584001	1	151584001	151585000	1000	1	9.50E-08	0.58	5	0.5	Grm5	Signaling
DMR1:151594001	1	151594001	151597000	3000	3	5.50E-21	-0.47	35	1.17	Grm5	Signaling
DMR1:151600001	1	151600001	151606000	6000	1	9.80E-08	-0.35	54	0.9	Grm5	Signaling
DMR1:151611001	1	151611001	151612000	1000	1	5.20E-12	-0.82	6	0.6	Grm5	Signaling
DMR1:151613001	1	151613001	151614000	1000	1	8.40E-08	0.35	13	1.3	Grm5	Signaling
DMR1:151632001	1	151632001	151634000	2000	1	3.10E-15	0.55	12	0.6	Grm5	Signaling
DMR1:151657001	1	151657001	151660000	3000	3	5.30E-22	1.02	12	0.4	Grm5	Signaling
DMR1:151697001	1	151697001	151699000	2000	1	7.50E-12	-0.44	16	0.8	Grm5	Signaling
DMR1:151703001	1	151703001	151705000	2000	1	4.90E-09	-0.53	6	0.3	Grm5	Signaling
DMR1:151713001	1	151713001	151715000	2000	2	4.10E-14	0.75	14	0.7	Grm5	Signaling
DMR1:151727001	1	151727001	151729000	2000	1	1.40E-10	0.66	10	0.5	Grm5	Signaling
DMR1:151736001	1	151736001	151737000	1000	1	4.00E-07	0.51	11	1.1	Grm5	Signaling
DMR1:151740001	1	151740001	151742000	2000	1	1.50E-10	0.62	14	0.7	Grm5	Signaling
DMR1:151757001	1	151757001	151759000	2000	1	5.30E-10	-0.48	18	0.9	Grm5	Signaling
DMR1:151760001	1	151760001	151761000	1000	1	6.20E-24	1.01	5	0.5	Grm5	Signaling
DMR1:151765001	1	151765001	151766000	1000	1	1.40E-10	0.63	2	0.2	Grm5	Signaling
DMR1:151774001	1	151774001	151777000	3000	2	6.40E-22	0.92	14	0.47	Grm5	Signaling
DMR1:151792001	1	151792001	151796000	4000	2	1.80E-10	0.65	23	0.58	Grm5	Signaling
DMR1:151910001	1	151910001	151912000	2000	1	9.10E-07	-0.49	12	0.6	LOC102554680;Ctsc	Protease
DMR1:151952001	1	151952001	151953000	1000	1	1.90E-07	-0.56	9	0.9	Ctsc	Protease
DMR1:151954001	1	151954001	151958000	4000	3	9.80E-12	-0.46	28	0.7	Ctsc	Protease
DMR1:152085001	1	152085001	152086000	1000	1	8.90E-08	0.54	6	0.6	Rab38	
DMR1:152093001	1	152093001	152101000	8000	3	6.70E-10	-0.58	49	0.61	Rab38	
DMR1:152102001	1	152102001	152105000	3000	3	4.20E-20	-0.57	35	1.17	Rab38	
DMR1:152109001	1	152109001	152111000	2000	1	1.00E-11	0.55	14	0.7	Rab38	
DMR1:152114001	1	152114001	152115000	1000	1	5.80E-07	-0.51	13	1.3	Rab38	
DMR1:152126001	1	152126001	152127000	1000	1	6.40E-12	0.65	6	0.6	Rab38	
DMR1:152134001	1	152134001	152136000	2000	1	1.30E-07	-0.41	18	0.9	Rab38	
DMR1:152146001	1	152146001	152148000	2000	1	2.70E-14	-0.69	7	0.35	Rab38	
DMR1:152151001	1	152151001	152152000	1000	1	1.60E-09	0.6	2	0.2	Rab38	
DMR1:152876001	1	152876001	152879000	3000	1	1.90E-16	0.65	19	0.63	Tmem135	
DMR1:152886001	1	152886001	152887000	1000	1	8.00E-07	0.44	12	1.2	Tmem135	
DMR1:152900001	1	152900001	152902000	2000	1	2.40E-12	-0.52	18	0.9	Tmem135	
DMR1:152924001	1	152924001	152933000	9000	6	1.70E-24	-0.55	99	1.1	Tmem135	
DMR1:152941001	1	152941001	152945000	4000	2	1.10E-07	0.41	36	0.9	Tmem135	
DMR1:152954001	1	152954001	152955000	1000	1	2.00E-09	0.5	7	0.7	Tmem135	
DMR1:152974001	1	152974001	152976000	2000	2	1.10E-10	0.63	12	0.6	Tmem135	
DMR1:152980001	1	152980001	152982000	2000	1	8.30E-09	0.51	11	0.55	Tmem135	
DMR1:152986001	1	152986001	152989000	3000	2	1.80E-14	0.64	21	0.7	Tmem135	
DMR1:153005001	1	153005001	153008000	3000	1	5.60E-07	-0.35	28	0.93	Tmem135	
DMR1:153020001	1	153020001	153022000	2000	1	9.20E-08	0.55	12	0.6	Tmem135	
DMR1:153035001	1	153035001	153037000	2000	2	1.10E-08	-0.74	3	0.15	Tmem135	
DMR1:153038001	1	153038001	153039000	1000	1	5.90E-13	0.51	8	0.8	Tmem135	
DMR1:153042001	1	153042001	153047000	5000	1	1.70E-11	0.51	35	0.7	Tmem135	
DMR1:153050001	1	153050001	153052000	2000	1	8.50E-08	-0.41	12	0.6	Tmem135	
DMR1:153057001	1	153057001	153063000	6000	3	2.40E-13	-0.55	50	0.83	Tmem135	
DMR1:153085001	1	153085001	153093000	8000	4	3.70E-21	-0.54	88	1.1	Tmem135	
DMR1:153583001	1	153583001	153586000	3000	2	1.70E-25	0.9	29	0.97	Fzd4	Receptor
DMR1:153592001	1	153592001	153593000	1000	1	4.10E-09	-0.54	29	2.9	Fzd4	Receptor
DMR1:153604001	1	153604001	153606000	2000	1	1.20E-08	0.34	23	1.15	Fzd4	Receptor
DMR1:153607001	1	153607001	153609000	2000	1	4.20E-07	-0.43	22	1.1	Fzd4	Receptor
DMR1:153689001	1	153689001	153692000	3000	1	1.80E-07	-0.62	44	1.47	RGD1562517	
DMR1:153698001	1	153698001	153702000	4000	2	9.80E-25	-0.6	75	1.88	RGD1562517	
DMR1:153750001	1	153750001	153751000	1000	1	8.40E-10	0.45	12	1.2	Prss23;LOC103691188	Protease
DMR1:153857001	1	153857001	153859000	2000	1	5.50E-09	-0.38	31	1.55	LOC102549783;Me3	Metabolism
DMR1:153869001	1	153869001	153870000	1000	1	6.60E-09	0.5	9	0.9	LOC102549783;Me3	Metabolism

DMR1:153887001	1	153887001	153889000	2000	2	2.80E-09	0.66	19	0.95	Me3	Metabolism
DMR1:153902001	1	153902001	153903000	1000	1	7.40E-10	-0.64	20	2	Me3	Metabolism
DMR1:153906001	1	153906001	153907000	1000	1	1.20E-08	0.53	2	0.2	Me3	Metabolism
DMR1:153908001	1	153908001	153912000	4000	2	1.20E-18	-0.6	41	1.02	Me3	Metabolism
DMR1:153937001	1	153937001	153939000	2000	2	3.10E-12	-0.54	16	0.8	Me3	Metabolism
DMR1:153940001	1	153940001	153941000	1000	1	5.80E-16	-0.49	5	0.5	Me3	Metabolism
DMR1:153957001	1	153957001	153959000	2000	1	4.00E-13	0.65	23	1.15	Me3	Metabolism
DMR1:153962001	1	153962001	153964000	2000	1	4.70E-07	0.52	12	0.6	Me3	Metabolism
DMR1:153985001	1	153985001	153986000	1000	1	2.40E-07	-0.35	9	0.9	Me3	Metabolism
DMR1:153987001	1	153987001	153990000	3000	1	7.10E-27	0.89	26	0.87	Me3	Metabolism
DMR1:153993001	1	153993001	153996000	3000	1	5.10E-08	0.48	19	0.63	Me3	Metabolism
DMR1:154009001	1	154009001	154010000	1000	1	4.70E-08	0.46	8	0.8	Me3	Metabolism
DMR1:154019001	1	154019001	154024000	5000	2	8.60E-13	0.54	34	0.68	Me3	Metabolism
DMR1:154040001	1	154040001	154044000	4000	1	3.20E-12	-0.51	14	0.35	Me3	Metabolism
DMR1:154054001	1	154054001	154055000	1000	1	2.80E-09	0.72	7	0.7	Me3;Ccdc81	Metabolism
DMR1:154085001	1	154085001	154088000	3000	1	2.90E-17	0.91	30	1	Ccdc81	
DMR1:154092001	1	154092001	154097000	5000	4	2.00E-10	-0.46	69	1.38	Ccdc81	
DMR1:154136001	1	154136001	154139000	3000	1	3.20E-07	0.32	38	1.27	LOC108349851;LOC102549852;<br>Hikeshi	
DMR1:154150001	1	154150001	154151000	1000	1	3.60E-12	0.54	13	1.3	Hikeshi	
DMR1:154175001	1	154175001	154176000	1000	1	3.20E-13	0.61	12	1.2	Hikeshi	
DMR1:154211001	1	154211001	154213000	2000	1	1.30E-11	0.49	28	1.4	Eed	Epigenetic
DMR1:154393001	1	154393001	154394000	1000	1	6.50E-14	0.97	5	0.5	Picalm	Transport
DMR1:154411001	1	154411001	154412000	1000	1	1.30E-10	0.71	9	0.9	Picalm	Transport
DMR1:154435001	1	154435001	154437000	2000	1	4.40E-07	0.36	18	0.9	Picalm	Transport
DMR1:154452001	1	154452001	154453000	1000	1	1.80E-08	-0.62	7	0.7	Picalm	Transport
DMR1:154470001	1	154470001	154471000	1000	1	1.40E-08	0.43	12	1.2	Ccdc83	
DMR1:154488001	1	154488001	154494000	6000	5	2.20E-31	-0.64	64	1.07	Ccdc83	
DMR1:154499001	1	154499001	154500000	1000	1	1.70E-09	-0.52	8	0.8	Ccdc83	
DMR1:154509001	1	154509001	154510000	1000	1	3.60E-11	0.77	5	0.5	Ccdc83	
DMR1:154528001	1	154528001	154529000	1000	1	2.20E-09	0.52	10	1	Sytl2	
DMR1:154548001	1	154548001	154549000	1000	1	7.80E-07	0.43	4	0.4	Sytl2	
DMR1:154586001	1	154586001	154587000	1000	1	1.70E-19	0.95	6	0.6	Sytl2	
DMR1:154820001	1	154820001	154821000	1000	1	2.30E-10	0.59	15	1.5	Rps27a-ps26	
DMR1:154828001	1	154828001	154830000	2000	1	1.00E-12	0.81	11	0.55	Rps27a-ps26	
DMR1:154910001	1	154910001	154911000	1000	1	8.70E-14	0.76	10	1	Rps27a-ps27;Dlg2	Cytoskeleton
DMR1:154918001	1	154918001	154920000	2000	1	1.90E-28	1.18	9	0.45	Rps27a-ps27;Dlg2	Cytoskeleton
DMR1:154948001	1	154948001	154949000	1000	1	1.60E-17	0.87	11	1.1	Dlg2	Cytoskeleton
DMR1:154966001	1	154966001	154967000	1000	1	4.20E-07	-0.61	15	1.5	Dlg2	Cytoskeleton
DMR1:155040001	1	155040001	155041000	1000	1	1.40E-09	0.6	1	0.1	Dlg2	Cytoskeleton
DMR1:155043001	1	155043001	155045000	2000	1	1.00E-13	0.72	13	0.65	Dlg2	Cytoskeleton
DMR1:155090001	1	155090001	155091000	1000	1	1.50E-09	0.5	7	0.7	Dlg2	Cytoskeleton
DMR1:155093001	1	155093001	155096000	3000	1	2.70E-11	0.61	16	0.53	Dlg2	Cytoskeleton
DMR1:155099001	1	155099001	155101000	2000	1	1.40E-10	0.39	11	0.55	Dlg2	Cytoskeleton
DMR1:155340001	1	155340001	155343000	3000	1	1.10E-19	0.88	25	0.83	Dlg2	Cytoskeleton
DMR1:155454001	1	155454001	155455000	1000	1	1.10E-10	0.53	12	1.2	Dlg2	Cytoskeleton
DMR1:155469001	1	155469001	155470000	1000	1	9.10E-07	-0.67	17	1.7	Dlg2	Cytoskeleton
DMR1:155508001	1	155508001	155510000	2000	1	6.70E-09	0.43	11	0.55	Dlg2	Cytoskeleton
DMR1:155511001	1	155511001	155512000	1000	1	3.60E-07	0.46	6	0.6	Dlg2	Cytoskeleton
DMR1:155518001	1	155518001	155521000	3000	1	2.90E-07	-0.47	35	1.17	Dlg2	Cytoskeleton
DMR1:155525001	1	155525001	155526000	1000	1	2.50E-12	0.61	9	0.9	Dlg2	Cytoskeleton
DMR1:155532001	1	155532001	155534000	2000	1	9.30E-08	0.48	2	0.1	Dlg2	Cytoskeleton
DMR1:155565001	1	155565001	155572000	7000	3	6.80E-23	-0.5	87	1.24	Dlg2	Cytoskeleton
DMR1:155580001	1	155580001	155581000	1000	1	1.20E-07	0.41	11	1.1	Dlg2	Cytoskeleton
DMR1:155660001	1	155660001	155661000	1000	1	2.10E-09	0.59	10	1	Dlg2	Cytoskeleton
DMR1:155663001	1	155663001	155665000	2000	1	1.30E-09	0.54	15	0.75	Dlg2	Cytoskeleton
DMR1:155679001	1	155679001	155680000	1000	1	1.30E-07	0.42	9	0.9	Dlg2	Cytoskeleton
DMR1:155710001	1	155710001	155716000	6000	4	3.40E-18	-0.58	57	0.95	Dlg2	Cytoskeleton
DMR1:155726001	1	155726001	155728000	2000	1	1.50E-24	1.05	14	0.7	Dlg2	Cytoskeleton
DMR1:155731001	1	155731001	155732000	1000	1	4.60E-13	0.58	7	0.7	Dlg2	Cytoskeleton
DMR1:155781001	1	155781001	155782000	1000	1	1.10E-09	0.57	15	1.5	Dlg2	Cytoskeleton
DMR1:155861001	1	155861001	155863000	2000	1	2.50E-10	0.68	18	0.9	Dlg2	Cytoskeleton
DMR1:155866001	1	155866001	155867000	1000	1	5.20E-07	0.74	5	0.5	Dlg2	Cytoskeleton
DMR1:155872001	1	155872001	155877000	5000	1	6.30E-14	-0.63	43	0.86	Dlg2;LOC293116	Cytoskeleton
DMR1:155891001	1	155891001	155893000	2000	1	3.00E-08	-0.37	21	1.05	Dlg2;LOC293116	Cytoskeleton

DMR1:155897001	1	155897001	155898000	1000	1	2.30E-15	0.86	6	0.6	Dlg2;LOC293116	Cytoskeleton
DMR1:155940001	1	155940001	155944000	4000	1	6.90E-08	-0.48	36	0.9	Dlg2	Cytoskeleton
DMR1:155985001	1	155985001	155989000	4000	2	1.30E-17	-0.53	40	1	Dlg2;LOC108349816	Cytoskeleton
DMR1:156008001	1	156008001	156011000	3000	1	2.40E-15	0.75	22	0.73	Dlg2;LOC108349816	Cytoskeleton
DMR1:156017001	1	156017001	156019000	2000	2	1.50E-14	0.7	13	0.65	Dlg2;LOC108349816	Cytoskeleton
DMR1:156025001	1	156025001	156026000	1000	1	6.10E-07	0.56	3	0.3	Dlg2;LOC108349816	Cytoskeleton
DMR1:156031001	1	156031001	156032000	1000	1	2.00E-15	0.88	4	0.4	Dlg2;LOC108349816	Cytoskeleton
DMR1:156036001	1	156036001	156042000	6000	4	1.60E-11	0.51	38	0.63	Dlg2;LOC108349816	Cytoskeleton
DMR1:156043001	1	156043001	156044000	1000	1	2.00E-09	0.56	9	0.9	Dlg2;LOC108349816	Cytoskeleton
DMR1:156067001	1	156067001	156068000	1000	1	3.80E-21	0.94	8	0.8	Dlg2;LOC108349816	Cytoskeleton
DMR1:156071001	1	156071001	156072000	1000	1	3.40E-11	0.71	5	0.5	Dlg2;LOC108349816	Cytoskeleton
DMR1:156086001	1	156086001	156088000	2000	1	4.20E-07	-0.53	21	1.05	Dlg2;LOC108349816	Cytoskeleton
DMR1:156106001	1	156106001	156108000	2000	1	3.00E-07	0.38	17	0.85	Dlg2;LOC108349816	Cytoskeleton
DMR1:156110001	1	156110001	156118000	8000	4	1.30E-15	-0.49	70	0.88	Dlg2;LOC108349816	Cytoskeleton
DMR1:156189001	1	156189001	156193000	4000	2	3.20E-09	-0.55	25	0.62	Dlg2;LOC108349816	Cytoskeleton
DMR1:156206001	1	156206001	156207000	1000	1	5.40E-07	0.54	7	0.7	Dlg2;LOC108349816	Cytoskeleton
DMR1:156211001	1	156211001	156213000	2000	1	2.00E-07	0.55	12	0.6	Dlg2;LOC108349816	Cytoskeleton
DMR1:156238001	1	156238001	156239000	1000	1	3.70E-17	0.97	5	0.5	Dlg2;LOC108349816	Cytoskeleton
DMR1:156240001	1	156240001	156243000	3000	3	5.50E-12	-0.8	9	0.3	Dlg2;LOC108349816	Cytoskeleton
DMR1:156245001	1	156245001	156247000	2000	1	5.90E-09	0.55	5	0.25	Dlg2;LOC108349816	Cytoskeleton
DMR1:156271001	1	156271001	156273000	2000	2	1.20E-11	-0.77	3	0.15	Dlg2;LOC108349816;Tmem126b	Cytoskeleton
DMR1:156287001	1	156287001	156288000	1000	1	2.50E-08	-0.62	9	0.9	Dlg2;Tmem126a;Crebzf	Cytoskeleton;Transcription
DMR1:156301001	1	156301001	156304000	3000	2	1.60E-11	-0.61	14	0.47	Dlg2;Tmem126a;Crebzf	Cytoskeleton;Transcription
DMR1:156306001	1	156306001	156309000	3000	1	3.40E-18	0.76	20	0.67	Dlg2;Crebzf	Cytoskeleton;Transcription
DMR1:156311001	1	156311001	156316000	5000	2	1.80E-07	0.52	27	0.54	Dlg2;LOC100911776	Cytoskeleton
DMR1:156347001	1	156347001	156348000	1000	1	1.70E-20	1.01	4	0.4	Dlg2;Sytl2	Cytoskeleton
DMR1:156380001	1	156380001	156381000	1000	1	9.70E-07	0.49	4	0.4	Dlg2;Sytl2	Cytoskeleton
DMR1:156403001	1	156403001	156404000	1000	1	2.00E-07	0.45	9	0.9	Dlg2;Sytl2	Cytoskeleton
DMR1:156406001	1	156406001	156407000	1000	1	1.00E-08	0.43	14	1.4	Dlg2;Sytl2	Cytoskeleton
DMR1:156449001	1	156449001	156452000	3000	2	2.30E-22	-0.49	45	1.5	Dlg2;Sytl2	Cytoskeleton
DMR1:156454001	1	156454001	156455000	1000	1	8.40E-11	0.59	10	1	Dlg2	Cytoskeleton
DMR1:156474001	1	156474001	156484000	10000	4	4.50E-23	-0.56	82	0.82	Dlg2;Ccdc83	Cytoskeleton
DMR1:156493001	1	156493001	156496000	3000	1	1.50E-10	0.64	22	0.73	Dlg2;Ccdc83	Cytoskeleton
DMR1:156500001	1	156500001	156505000	5000	3	1.30E-13	-0.45	47	0.94	Dlg2;Ccdc83	Cytoskeleton
DMR1:156509001	1	156509001	156512000	3000	1	2.40E-15	0.78	21	0.7	Dlg2;Ccdc83	Cytoskeleton
DMR1:156524001	1	156524001	156525000	1000	1	5.90E-11	0.69	5	0.5	Dlg2	Cytoskeleton
DMR1:156546001	1	156546001	156547000	1000	1	1.60E-09	0.67	3	0.3	Dlg2	Cytoskeleton
DMR1:156559001	1	156559001	156560000	1000	1	2.20E-18	0.72	8	0.8	Dlg2	Cytoskeleton
DMR1:156566001	1	156566001	156569000	3000	1	2.70E-17	0.85	4	0.13	Dlg2	Cytoskeleton
DMR1:156605001	1	156605001	156606000	1000	1	6.40E-09	0.49	14	1.4	Dlg2	Cytoskeleton
DMR1:156618001	1	156618001	156619000	1000	1	5.40E-11	0.59	4	0.4	Dlg2	Cytoskeleton
DMR1:156624001	1	156624001	156625000	1000	1	3.80E-08	0.6	10	1	Dlg2	Cytoskeleton
DMR1:156649001	1	156649001	156650000	1000	1	1.10E-15	0.68	6	0.6	Dlg2	Cytoskeleton
DMR1:156665001	1	156665001	156667000	2000	1	5.10E-09	-0.45	21	1.05	Dlg2	Cytoskeleton
DMR1:156688001	1	156688001	156689000	1000	1	1.10E-07	0.56	5	0.5	Dlg2	Cytoskeleton
DMR1:156699001	1	156699001	156700000	1000	1	2.50E-14	0.77	8	0.8	Dlg2	Cytoskeleton
DMR1:156701001	1	156701001	156703000	2000	1	4.80E-08	-0.84	12	0.6	Dlg2	Cytoskeleton
DMR1:156732001	1	156732001	156733000	1000	1	8.80E-07	0.43	6	0.6	Dlg2	Cytoskeleton
DMR1:156738001	1	156738001	156739000	1000	1	6.10E-09	0.54	6	0.6	Dlg2	Cytoskeleton
DMR1:156750001	1	156750001	156752000	2000	2	2.10E-08	0.56	14	0.7	Dlg2	Cytoskeleton
DMR1:156763001	1	156763001	156765000	2000	2	4.50E-08	-0.59	12	0.6	Dlg2	Cytoskeleton
DMR1:156767001	1	156767001	156768000	1000	1	2.30E-07	-0.58	2	0.2	Dlg2;LOC108349817	Cytoskeleton
DMR1:156775001	1	156775001	156777000	2000	2	1.30E-10	0.67	11	0.55	Dlg2;LOC108349817	Cytoskeleton
DMR1:156790001	1	156790001	156791000	1000	1	7.80E-11	0.61	3	0.3	Dlg2	Cytoskeleton
DMR1:156796001	1	156796001	156798000	2000	1	5.30E-07	-0.46	8	0.4	Dlg2	Cytoskeleton
DMR1:156832001	1	156832001	156839000	7000	4	1.20E-17	-0.54	63	0.9	Dlg2	Cytoskeleton
DMR1:156851001	1	156851001	156852000	1000	1	8.20E-07	0.38	6	0.6	Dlg2	Cytoskeleton
DMR1:156858001	1	156858001	156863000	5000	4	1.50E-12	-0.5	50	1	Dlg2	Cytoskeleton
DMR1:156867001	1	156867001	156868000	1000	1	3.00E-07	0.38	9	0.9	Dlg2	Cytoskeleton
DMR1:156869001	1	156869001	156870000	1000	1	3.70E-09	0.51	5	0.5	Dlg2	Cytoskeleton
DMR1:156879001	1	156879001	156880000	1000	1	4.40E-17	0.72	5	0.5	Dlg2	Cytoskeleton

DMR1:156959001	1	156959001	156961000	2000	2	1.40E-20	0.75	18	0.9	Dlg2	Cytoskeleton
DMR1:156969001	1	156969001	156977000	8000	5	7.70E-24	-0.54	76	0.95	Dlg2	Cytoskeleton
DMR1:156978001	1	156978001	156979000	1000	1	2.90E-13	0.66	8	0.8	Dlg2	Cytoskeleton
DMR1:156985001	1	156985001	156986000	1000	1	1.60E-08	0.56	5	0.5	Dlg2	Cytoskeleton
DMR1:156991001	1	156991001	156992000	1000	1	2.10E-10	-0.66	10	1	Dlg2	Cytoskeleton
DMR1:156994001	1	156994001	156996000	2000	1	1.40E-16	0.73	11	0.55	Dlg2	Cytoskeleton
DMR1:156997001	1	156997001	156999000	2000	2	1.10E-14	0.76	10	0.5	Dlg2	Cytoskeleton
DMR1:157003001	1	157003001	157004000	1000	1	4.40E-10	0.52	10	1	Dlg2	Cytoskeleton
DMR1:157027001	1	157027001	157031000	4000	2	1.00E-08	0.41	48	1.2	Dlg2	Cytoskeleton
DMR1:157032001	1	157032001	157034000	2000	1	1.50E-11	0.6	27	1.35	Dlg2	Cytoskeleton
DMR1:157035001	1	157035001	157039000	4000	1	1.20E-13	0.57	44	1.1	Dlg2	Cytoskeleton
DMR1:157062001	1	157062001	157064000	2000	1	1.80E-15	0.95	7	0.35	Dlg2	Cytoskeleton
DMR1:157080001	1	157080001	157083000	3000	2	1.10E-16	0.76	24	0.8	Dlg2	Cytoskeleton
DMR1:157113001	1	157113001	157114000	1000	1	6.90E-12	0.61	7	0.7	Dlg2	Cytoskeleton
DMR1:157144001	1	157144001	157146000	2000	1	4.90E-08	0.41	20	1	Dlg2	Cytoskeleton
DMR1:157151001	1	157151001	157152000	1000	1	1.20E-09	-0.55	17	1.7	Dlg2	Cytoskeleton
DMR1:157153001	1	157153001	157154000	1000	1	3.20E-07	-0.5	14	1.4	Dlg2	Cytoskeleton
DMR1:157188001	1	157188001	157189000	1000	1	9.10E-12	0.78	7	0.7	Dlg2	Cytoskeleton
DMR1:157252001	1	157252001	157254000	2000	1	5.80E-08	-0.49	36	1.8	Dlg2	Cytoskeleton
DMR1:157272001	1	157272001	157273000	1000	1	1.20E-10	0.63	10	1	Dlg2	Cytoskeleton
DMR1:157390001	1	157390001	157399000	9000	2	3.90E-08	-0.5	96	1.07	LOC100361001;Ccdc90b	
DMR1:157436001	1	157436001	157439000	3000	1	5.20E-07	0.45	18	0.6	Ankrd42	
DMR1:157443001	1	157443001	157444000	1000	1	1.00E-06	0.43	5	0.5	Ankrd42	
DMR1:157495001	1	157495001	157497000	2000	1	1.10E-13	0.55	20	1	Pcf11;LOC680479	Translation
DMR1:157504001	1	157504001	157510000	6000	1	9.60E-09	0.42	60	1	Pcf11;LOC680479;LOC100912320	Translation
DMR1:157586001	1	157586001	157587000	1000	1	9.60E-12	0.8	6	0.6	Rab30	
DMR1:157599001	1	157599001	157607000	8000	3	2.00E-23	-0.55	98	1.23	Rab30	
DMR1:157615001	1	157615001	157618000	3000	1	1.10E-09	0.57	32	1.07	Rab30	
DMR1:157638001	1	157638001	157641000	3000	1	1.90E-07	0.46	27	0.9	Rab30;LOC103691196	
DMR1:157682001	1	157682001	157684000	2000	2	2.30E-10	-0.55	11	0.55	Ddias	
DMR1:157685001	1	157685001	157686000	1000	1	2.10E-08	-0.61	5	0.5	Ddias	
DMR1:157696001	1	157696001	157697000	1000	1	1.60E-08	0.41	9	0.9	Ddias;Prcp	Protease
DMR1:157698001	1	157698001	157699000	1000	1	4.60E-09	0.46	5	0.5	Ddias;Prcp	Protease
DMR1:157707001	1	157707001	157711000	4000	1	4.10E-16	0.54	25	0.62	Ddias;Prcp	Protease
DMR1:157723001	1	157723001	157726000	3000	3	2.40E-20	0.64	21	0.7	Prcp	Protease
DMR1:157734001	1	157734001	157736000	2000	1	2.90E-09	0.45	18	0.9	Prcp	Protease
DMR1:157742001	1	157742001	157744000	2000	1	3.00E-07	0.43	16	0.8	Prcp	Protease
DMR1:157756001	1	157756001	157757000	1000	1	2.00E-10	0.71	9	0.9	Prcp	Protease
DMR1:157910001	1	157910001	157913000	3000	1	6.20E-07	-0.37	24	0.8	Fam181b	
DMR1:161174001	1	161174001	161175000	1000	1	2.70E-07	0.43	25	2.5	Tenm4	
DMR1:161179001	1	161179001	161182000	3000	1	6.40E-15	0.69	23	0.77	Tenm4	
DMR1:161183001	1	161183001	161184000	1000	1	1.60E-10	0.76	75	7.5	Tenm4	
DMR1:161195001	1	161195001	161196000	1000	1	3.10E-09	0.56	13	1.3	Tenm4	
DMR1:161214001	1	161214001	161216000	2000	2	1.50E-08	-0.61	18	0.9	Tenm4;Mir708	
DMR1:161238001	1	161238001	161239000	1000	1	1.20E-16	0.85	4	0.4	Tenm4	
DMR1:161247001	1	161247001	161250000	3000	1	7.20E-08	-0.57	23	0.77	Tenm4	
DMR1:161254001	1	161254001	161255000	1000	1	1.10E-15	0.55	9	0.9	Tenm4	
DMR1:161286001	1	161286001	161288000	2000	1	1.20E-08	0.38	17	0.85	Tenm4	
DMR1:161325001	1	161325001	161328000	3000	2	1.80E-15	0.87	43	1.43	Tenm4	
DMR1:161330001	1	161330001	161331000	1000	1	1.40E-09	0.35	14	1.4	Tenm4	
DMR1:161333001	1	161333001	161336000	3000	1	6.00E-10	0.46	31	1.03	Tenm4	
DMR1:161361001	1	161361001	161363000	2000	2	3.10E-13	0.84	8	0.4	Tenm4	
DMR1:161379001	1	161379001	161380000	1000	1	7.30E-07	0.44	4	0.4	Tenm4	
DMR1:161390001	1	161390001	161391000	1000	1	3.90E-10	0.59	8	0.8	Tenm4	
DMR1:161392001	1	161392001	161394000	2000	1	7.60E-07	0.44	19	0.95	Tenm4	
DMR1:161428001	1	161428001	161429000	1000	1	6.60E-08	-0.56	17	1.7	Tenm4	
DMR1:161430001	1	161430001	161431000	1000	1	4.50E-09	0.52	10	1	Tenm4	
DMR1:161457001	1	161457001	161460000	3000	1	2.60E-16	0.81	18	0.6	Tenm4	
DMR1:161473001	1	161473001	161481000	8000	3	3.60E-11	0.57	87	1.09	Tenm4	
DMR1:161490001	1	161490001	161491000	1000	1	9.20E-07	-0.59	11	1.1	Tenm4	
DMR1:161493001	1	161493001	161495000	2000	2	3.40E-12	-0.64	24	1.2	Tenm4	
DMR1:161508001	1	161508001	161512000	4000	1	7.90E-07	-0.64	38	0.95	Tenm4	
DMR1:161554001	1	161554001	161557000	3000	2	1.20E-11	0.61	29	0.97	Tenm4	
DMR1:161577001	1	161577001	161580000	3000	1	7.30E-08	0.46	24	0.8	Tenm4	

DMR1:161619001	1	161619001	161625000	6000	1	4.20E-09	-0.75	80	1.33	Tenm4	
DMR1:161637001	1	161637001	161640000	3000	2	3.70E-08	-0.52	50	1.67	Tenm4	
DMR1:161655001	1	161655001	161656000	1000	1	9.50E-09	0.41	12	1.2	Tenm4;LOC108349818	
DMR1:161668001	1	161668001	161670000	2000	1	1.20E-11	0.42	26	1.3	Tenm4;LOC108349818	
DMR1:161679001	1	161679001	161682000	3000	1	8.30E-20	0.68	61	2.03	Tenm4;LOC108349818	
DMR1:161708001	1	161708001	161709000	1000	1	4.20E-07	-0.41	15	1.5	Tenm4	
DMR1:161726001	1	161726001	161727000	1000	1	2.50E-21	0.77	14	1.4	Tenm4	
DMR1:161732001	1	161732001	161733000	1000	1	7.50E-15	0.55	13	1.3	Tenm4	
DMR1:161737001	1	161737001	161741000	4000	1	4.40E-08	0.31	46	1.15	Tenm4	
DMR1:161753001	1	161753001	161754000	1000	1	4.60E-15	0.77	9	0.9	Tenm4	
DMR1:161756001	1	161756001	161757000	1000	1	2.00E-08	-0.6	10	1	Tenm4	
DMR1:161764001	1	161764001	161768000	4000	1	1.40E-16	0.72	58	1.45	Tenm4	
DMR1:161771001	1	161771001	161772000	1000	1	4.60E-07	0.42	5	0.5	Tenm4	
DMR1:161776001	1	161776001	161777000	1000	1	6.50E-08	-0.48	14	1.4	Tenm4	
DMR1:161784001	1	161784001	161785000	1000	1	4.20E-07	-0.49	11	1.1	Tenm4	
DMR1:161798001	1	161798001	161802000	4000	1	8.60E-07	-0.36	54	1.35	Tenm4	
DMR1:161804001	1	161804001	161806000	2000	1	1.60E-09	-0.49	36	1.8	Tenm4	
DMR1:161808001	1	161808001	161811000	3000	1	6.10E-07	-0.41	35	1.17	Tenm4	
DMR1:161812001	1	161812001	161814000	2000	1	8.60E-07	-0.49	41	2.05	Tenm4	
DMR1:161831001	1	161831001	161835000	4000	1	2.00E-07	0.35	53	1.32	Tenm4	
DMR1:161841001	1	161841001	161845000	4000	1	8.90E-07	-0.63	62	1.55	Tenm4	
DMR1:161850001	1	161850001	161852000	2000	2	2.30E-13	0.56	25	1.25	Tenm4	
DMR1:161862001	1	161862001	161864000	2000	1	5.20E-08	-0.55	40	2	Tenm4	
DMR1:161870001	1	161870001	161874000	4000	1	2.10E-11	-0.71	60	1.5	Tenm4	
DMR1:161878001	1	161878001	161882000	4000	1	9.60E-08	0.38	75	1.88	Tenm4	
DMR1:161890001	1	161890001	161892000	2000	2	9.00E-17	0.81	26	1.3	Tenm4	
DMR1:161933001	1	161933001	161935000	2000	1	8.30E-11	-0.55	20	1	Nars2	Translation
DMR1:161937001	1	161937001	161938000	1000	1	6.50E-07	-0.48	7	0.7	Nars2	Translation
DMR1:161939001	1	161939001	161942000	3000	1	6.90E-10	-0.43	20	0.67	Nars2	Translation
DMR1:161949001	1	161949001	161950000	1000	1	1.80E-08	-0.52	13	1.3	Nars2	Translation
DMR1:161956001	1	161956001	161957000	1000	1	4.50E-07	-0.51	12	1.2	Nars2	Translation
DMR1:161960001	1	161960001	161963000	3000	1	1.40E-09	-0.49	41	1.37	Nars2	Translation
DMR1:161983001	1	161983001	161984000	1000	1	8.70E-07	-0.49	7	0.7	Nars2;LOC108349194	Translation
DMR1:161988001	1	161988001	161989000	1000	1	2.50E-07	-0.46	14	1.4	Nars2;LOC108349194	Translation
DMR1:162000001	1	162000001	162001000	1000	1	7.90E-11	0.49	6	0.6	Nars2;LOC108349194	Translation
DMR1:162018001	1	162018001	162024000	6000	4	6.50E-25	-0.53	69	1.15	Nars2	Translation
DMR1:162084001	1	162084001	162089000	5000	2	4.10E-20	0.87	29	0.58	Gab2	Cytoskeleton
DMR1:162096001	1	162096001	162098000	2000	2	7.70E-12	0.46	10	0.5	Gab2	Cytoskeleton
DMR1:162114001	1	162114001	162121000	7000	1	7.20E-07	0.35	40	0.57	Gab2	Cytoskeleton
DMR1:162154001	1	162154001	162160000	6000	4	7.40E-23	-0.47	60	1	Gab2	Cytoskeleton
DMR1:162199001	1	162199001	162200000	1000	1	3.30E-09	0.65	2	0.2	Gab2	Cytoskeleton
DMR1:162201001	1	162201001	162202000	1000	1	9.60E-08	0.32	7	0.7	Gab2	Cytoskeleton
DMR1:162225001	1	162225001	162226000	1000	1	1.70E-16	0.61	10	1	Gab2	Cytoskeleton
DMR1:162250001	1	162250001	162251000	1000	1	3.60E-10	-0.53	8	0.8	Gab2	Cytoskeleton
DMR1:162264001	1	162264001	162271000	7000	4	4.30E-14	-0.67	47	0.67	Gab2	Cytoskeleton
DMR1:162286001	1	162286001	162287000	1000	1	9.70E-14	0.56	33	3.3	Gab2;Usp35	Cytoskeleton;Protease
DMR1:162318001	1	162318001	162320000	2000	1	1.80E-14	0.8	32	1.6	Kctd21	Cytoskeleton
DMR1:162324001	1	162324001	162326000	2000	1	2.50E-09	0.47	25	1.25	Kctd21;LOC102550214	Cytoskeleton
DMR1:162329001	1	162329001	162331000	2000	1	2.10E-08	0.35	39	1.95	Kctd21;LOC102550214	Cytoskeleton
DMR1:162335001	1	162335001	162342000	7000	3	2.00E-14	0.72	93	1.33	Kctd21;LOC102550214;Alg8	Cytoskeleton;Golgi
DMR1:162387001	1	162387001	162390000	3000	1	2.20E-08	0.34	39	1.3	Thrsp;RGD1562118	
DMR1:162399001	1	162399001	162401000	2000	2	8.70E-21	0.64	17	0.85	RGD1562118;LOC102557251	
DMR1:162404001	1	162404001	162406000	2000	1	6.70E-07	0.39	17	0.85	RGD1562118;LOC102557251	
DMR1:162408001	1	162408001	162413000	5000	1	5.70E-12	0.7	54	1.08	LOC102557251;Kctd14	Cytoskeleton
DMR1:162415001	1	162415001	162420000	5000	1	5.10E-07	0.42	74	1.48	LOC102557251;Kctd14	Cytoskeleton
DMR1:162438001	1	162438001	162439000	1000	1	1.70E-09	0.44	14	1.4	Ints4	
DMR1:162456001	1	162456001	162459000	3000	1	2.10E-18	0.65	26	0.87	Ints4	
DMR1:162472001	1	162472001	162473000	1000	1	3.80E-11	0.48	3	0.3	Ints4	
DMR1:162474001	1	162474001	162477000	3000	1	3.80E-10	0.43	42	1.4	Ints4	
DMR1:162478001	1	162478001	162482000	4000	1	6.90E-07	0.3	29	0.72	Ints4	
DMR1:162486001	1	162486001	162489000	3000	3	3.60E-08	-0.65	22	0.73	Ints4	
DMR1:162490001	1	162490001	162491000	1000	1	7.90E-08	-0.61	10	1	Ints4	
DMR1:162517001	1	162517001	162518000	1000	1	6.90E-18	0.78	18	1.8	Ints4;Aamdc	
DMR1:162570001	1	162570001	162571000	1000	1	2.20E-09	-0.76	11	1.1	Rsf1	Epigenetic

DMR1:162574001	1	162574001	162576000	2000	1	3.70E-07	0.36	22	1.1	Rsf1	Epigenetic
DMR1:162583001	1	162583001	162584000	1000	1	2.10E-14	0.68	20	2	Rsf1	Epigenetic
DMR1:162590001	1	162590001	162592000	2000	1	4.60E-08	-0.47	16	0.8	Rsf1	Epigenetic
DMR1:162597001	1	162597001	162601000	4000	3	4.80E-12	0.66	19	0.48	Rsf1	Epigenetic
DMR1:162619001	1	162619001	162622000	3000	2	3.70E-15	0.67	22	0.73	Rsf1	Epigenetic
DMR1:162623001	1	162623001	162626000	3000	1	1.70E-08	-0.6	19	0.63	Rsf1	Epigenetic
DMR1:162648001	1	162648001	162653000	5000	2	9.90E-16	0.55	42	0.84	Rsf1	Epigenetic
DMR1:162656001	1	162656001	162659000	3000	1	1.10E-08	0.54	35	1.17	Rsf1	Epigenetic
DMR1:162660001	1	162660001	162662000	2000	1	2.00E-18	0.6	29	1.45	Rsf1	Epigenetic
DMR1:162695001	1	162695001	162696000	1000	1	5.40E-07	-0.55	7	0.7	Clns1a;LOC102557309;Aqp11	Transport
DMR1:162702001	1	162702001	162704000	2000	1	1.80E-08	-0.62	41	2.05	Clns1a;Aqp11	Transport
DMR1:162716001	1	162716001	162717000	1000	1	6.50E-07	0.57	11	1.1	Aqp11	
DMR1:162719001	1	162719001	162723000	4000	2	6.10E-16	0.59	70	1.75	Aqp11	
DMR1:162754001	1	162754001	162761000	7000	3	2.10E-23	-0.53	73	1.04	LOC102550562;Pak1	Signaling
DMR1:162802001	1	162802001	162803000	1000	1	8.50E-12	-0.72	6	0.6	Pak1	Signaling
DMR1:162809001	1	162809001	162811000	2000	1	3.20E-08	0.31	13	0.65	Pak1	Signaling
DMR1:162818001	1	162818001	162819000	1000	1	3.40E-07	0.44	2	0.2	Pak1	Signaling
DMR1:162822001	1	162822001	162825000	3000	1	5.60E-12	0.52	17	0.57	Pak1	Signaling
DMR1:162847001	1	162847001	162849000	2000	2	2.20E-15	0.6	17	0.85	Pak1	Signaling
DMR1:162889001	1	162889001	162890000	1000	1	6.20E-10	0.53	15	1.5	Pak1;Gdpd4	Signaling;Signaling
DMR1:162893001	1	162893001	162896000	3000	2	1.30E-21	0.72	21	0.7	Pak1;Gdpd4	Signaling;Signaling
DMR1:162921001	1	162921001	162923000	2000	1	1.10E-18	0.54	37	1.85	Gdpd4;LOC293133	Signaling
DMR1:162924001	1	162924001	162928000	4000	2	3.10E-12	0.71	30	0.75	Gdpd4;LOC293133	Signaling
DMR1:162933001	1	162933001	162936000	3000	1	1.70E-19	0.86	52	1.73	Gdpd4;LOC293133	Signaling
DMR1:162941001	1	162941001	162943000	2000	1	2.10E-08	0.43	25	1.25	Gdpd4;LOC293133	Signaling
DMR1:162944001	1	162944001	162949000	5000	1	4.10E-12	-0.65	46	0.92	Gdpd4;LOC293133	Signaling
DMR1:162961001	1	162961001	162965000	4000	2	3.70E-15	-0.53	37	0.92	Gdpd4	Signaling
DMR1:162970001	1	162970001	162972000	2000	1	2.80E-10	0.54	22	1.1	Gdpd4	Signaling
DMR1:162976001	1	162976001	162980000	4000	1	3.50E-11	0.43	43	1.07	Gdpd4	Signaling
DMR1:162985001	1	162985001	162987000	2000	1	1.20E-23	0.7	44	2.2	Gdpd4	Signaling
DMR1:162988001	1	162988001	162992000	4000	1	1.10E-15	0.68	42	1.05	Gdpd4;Myo7a	Signaling;Cytoskeleton
DMR1:163004001	1	163004001	163005000	1000	1	1.50E-07	-0.52	17	1.7	Gdpd4;Myo7a	Signaling;Cytoskeleton
DMR1:163007001	1	163007001	163008000	1000	1	3.90E-08	0.4	17	1.7	Gdpd4;Myo7a	Signaling;Cytoskeleton
DMR1:163017001	1	163017001	163018000	1000	1	4.70E-07	-0.37	11	1.1	Myo7a	Cytoskeleton
DMR1:163030001	1	163030001	163034000	4000	1	1.90E-13	0.49	73	1.82	Myo7a	Cytoskeleton
DMR1:163037001	1	163037001	163039000	2000	1	3.80E-07	-0.46	16	0.8	Myo7a	Cytoskeleton
DMR1:163061001	1	163061001	163062000	1000	1	8.90E-09	0.58	7	0.7	Myo7a	Cytoskeleton
DMR1:163082001	1	163082001	163083000	1000	1	1.80E-25	0.9	17	1.7	Capn5	Protease
DMR1:163109001	1	163109001	163110000	1000	1	6.40E-07	0.25	23	2.3	Capn5	Protease
DMR1:163112001	1	163112001	163113000	1000	1	1.80E-20	0.71	13	1.3	Capn5	Protease
DMR1:163118001	1	163118001	163122000	4000	2	1.80E-08	-0.42	45	1.12	Capn5	Protease
DMR1:163131001	1	163131001	163133000	2000	1	1.60E-09	0.55	23	1.15	Capn5	Protease
DMR1:163146001	1	163146001	163147000	1000	1	9.50E-15	0.59	13	1.3	B3gnt6	Golgi
DMR1:163189001	1	163189001	163199000	10000	3	4.50E-19	-0.52	103	1.03	Acer3	
DMR1:163201001	1	163201001	163203000	2000	2	1.40E-07	0.5	11	0.55	Acer3	
DMR1:163208001	1	163208001	163210000	2000	1	7.50E-12	0.71	9	0.45	Acer3	
DMR1:163219001	1	163219001	163220000	1000	1	3.30E-09	0.54	4	0.4	Acer3	
DMR1:163223001	1	163223001	163224000	1000	1	4.40E-08	0.57	8	0.8	Acer3	
DMR1:163228001	1	163228001	163234000	6000	3	1.40E-21	-0.71	34	0.57	Acer3	
DMR1:163236001	1	163236001	163237000	1000	1	2.10E-19	0.74	7	0.7	Acer3	
DMR1:163238001	1	163238001	163245000	7000	5	1.40E-19	-0.55	79	1.13	Acer3	
DMR1:163246001	1	163246001	163247000	1000	1	4.00E-10	0.58	5	0.5	Acer3	
DMR1:163251001	1	163251001	163252000	1000	1	5.70E-10	0.45	16	1.6	Acer3	
DMR1:163269001	1	163269001	163276000	7000	1	3.90E-14	0.58	46	0.66	Acer3	
DMR1:163315001	1	163315001	163316000	1000	1	9.50E-07	-0.52	12	1.2	Tsku	Receptor
DMR1:163329001	1	163329001	163332000	3000	1	4.50E-14	0.63	24	0.8	Tsku;LOC108349448	Receptor
DMR1:163334001	1	163334001	163335000	1000	1	1.60E-07	0.46	7	0.7	Tsku;LOC108349448	Receptor
DMR1:163338001	1	163338001	163340000	2000	1	4.30E-14	0.52	19	0.95	Tsku;LOC108349448	Receptor
DMR1:163385001	1	163385001	163387000	2000	1	5.10E-25	0.88	21	1.05	RGD1559902	
DMR1:163388001	1	163388001	163390000	2000	1	4.60E-26	0.79	22	1.1	RGD1559902;Gucy2d	Signaling
DMR1:163398001	1	163398001	163399000	1000	1	3.00E-17	0.78	20	2	RGD1559902;Gucy2d	Signaling
DMR1:163410001	1	163410001	163411000	1000	1	1.00E-18	0.83	18	1.8	Gucy2d	Signaling

DMR1:163415001	1	163415001	163419000	4000	2	3.40E-19	0.97	58	1.45	Gucy2d	Signaling
DMR1:163438001	1	163438001	163442000	4000	1	1.50E-14	0.68	42	1.05	Gucy2d;Lrrc32	Signaling
DMR1:163450001	1	163450001	163451000	1000	1	6.50E-07	-0.56	11	1.1	Lrrc32	
DMR1:163460001	1	163460001	163465000	5000	2	8.10E-11	0.54	61	1.22	Lrrc32	
DMR1:163592001	1	163592001	163596000	4000	1	3.30E-08	0.31	41	1.02	Emsy	Transcription
DMR1:163627001	1	163627001	163628000	1000	1	7.50E-08	0.35	22	2.2	Emsy	Transcription
DMR1:163767001	1	163767001	163770000	3000	1	5.80E-09	0.5	33	1.1	Trnap-ugg;Trnap-agg	
DMR1:163774001	1	163774001	163775000	1000	1	1.70E-08	0.84	69	6.9	Trnap-ugg;Trnap-agg	
DMR1:163798001	1	163798001	163801000	3000	1	2.10E-07	0.41	49	1.63	Wnt11	Signaling
DMR1:163835001	1	163835001	163840000	5000	2	5.80E-25	0.77	73	1.46	Uvrag	
DMR1:163863001	1	163863001	163864000	1000	1	5.60E-13	0.46	9	0.9	Uvrag	
DMR1:163903001	1	163903001	163904000	1000	1	3.40E-09	0.45	13	1.3	Uvrag	
DMR1:163919001	1	163919001	163920000	1000	1	4.60E-07	0.36	11	1.1	Uvrag	
DMR1:163929001	1	163929001	163931000	2000	1	2.00E-10	-0.78	24	1.2	Uvrag	
DMR1:163971001	1	163971001	163977000	6000	1	2.30E-10	0.47	63	1.05	Uvrag	
DMR1:164003001	1	164003001	164004000	1000	1	2.10E-10	0.46	14	1.4	Uvrag	
DMR1:164032001	1	164032001	164033000	1000	1	1.60E-11	0.57	5	0.5	Uvrag	
DMR1:164043001	1	164043001	164045000	2000	1	5.90E-08	0.33	23	1.15	Uvrag	
DMR1:164056001	1	164056001	164059000	3000	2	4.70E-33	1.25	25	0.83	Uvrag	
DMR1:164073001	1	164073001	164084000	11000	5	8.90E-19	0.66	105	0.95	Uvrag	
DMR1:164098001	1	164098001	164101000	3000	1	3.20E-07	0.36	34	1.13	Uvrag	
DMR1:164129001	1	164129001	164130000	1000	1	7.80E-07	-0.59	8	0.8	Dgat2	Metabolism
DMR1:164135001	1	164135001	164137000	2000	1	1.30E-18	0.54	38	1.9	Dgat2	Metabolism
DMR1:164173001	1	164173001	164176000	3000	2	6.90E-17	0.87	32	1.07	Mogat2	Metabolism
DMR1:164183001	1	164183001	164184000	1000	1	1.10E-07	0.5	9	0.9	Mogat2	Metabolism
DMR1:164194001	1	164194001	164195000	1000	1	3.60E-08	0.49	6	0.6	Mogat2	Metabolism
DMR1:164196001	1	164196001	164204000	8000	1	4.30E-14	0.68	112	1.4	Mogat2	Metabolism
DMR1:164208001	1	164208001	164210000	2000	1	2.10E-17	0.66	22	1.1	Mogat2	Metabolism
DMR1:164216001	1	164216001	164219000	3000	1	1.20E-12	0.53	50	1.67	Map6	
DMR1:164220001	1	164220001	164221000	1000	1	4.30E-07	-0.53	8	0.8	Map6	
DMR1:164243001	1	164243001	164244000	1000	1	2.80E-09	0.47	6	0.6	Map6	
DMR1:164251001	1	164251001	164253000	2000	1	1.00E-12	0.49	34	1.7	Map6	
DMR1:164270001	1	164270001	164278000	8000	1	2.70E-15	0.58	161	2.01	Map6	
DMR1:164281001	1	164281001	164284000	3000	1	2.00E-07	-0.54	32	1.07	Map6	
DMR1:164285001	1	164285001	164289000	4000	2	8.10E-28	0.97	44	1.1	Map6	
DMR1:164294001	1	164294001	164296000	2000	1	8.80E-27	0.85	11	0.55	Map6;Serpinh1	Protease; Proteolysis
DMR1:164309001	1	164309001	164311000	2000	2	1.20E-15	0.84	15	0.75	Serpinh1	Protease; Proteolysis
DMR1:164326001	1	164326001	164328000	2000	1	1.10E-12	0.49	28	1.4	Gdpd5	Signaling
DMR1:164340001	1	164340001	164341000	1000	1	4.20E-10	0.43	9	0.9	Gdpd5	Signaling
DMR1:164374001	1	164374001	164376000	2000	1	1.10E-12	0.42	20	1	Gdpd5	Signaling
DMR1:164380001	1	164380001	164384000	4000	1	1.40E-08	0.37	76	1.9	Gdpd5	Signaling
DMR1:164387001	1	164387001	164392000	5000	2	2.40E-12	0.4	86	1.72	Gdpd5	Signaling
DMR1:164399001	1	164399001	164403000	4000	1	3.00E-10	-0.59	48	1.2	Gdpd5	Signaling
DMR1:164413001	1	164413001	164415000	2000	1	4.60E-12	0.48	19	0.95	Gdpd5;Klhl35	Signaling;Cytoskeleton
DMR1:164447001	1	164447001	164450000	3000	2	1.20E-20	0.67	63	2.1	Rps3;LOC108349777	Translation
DMR1:164491001	1	164491001	164493000	2000	1	3.00E-14	0.67	17	0.85	LOC100912335;Arrb1	Cytoskeleton
DMR1:164497001	1	164497001	164498000	1000	1	1.40E-11	0.56	10	1	LOC100912335;Arrb1	Cytoskeleton
DMR1:164521001	1	164521001	164522000	1000	1	3.30E-09	0.53	6	0.6	Arrb1;Mir326	Cytoskeleton
DMR1:164543001	1	164543001	164546000	3000	1	4.70E-07	-0.34	44	1.47	Arrb1	Cytoskeleton
DMR1:164548001	1	164548001	164552000	4000	1	2.40E-16	0.58	65	1.62	Arrb1	Cytoskeleton
DMR1:164561001	1	164561001	164563000	2000	1	4.50E-08	0.38	32	1.6	Arrb1	Cytoskeleton
DMR1:164611001	1	164611001	164614000	3000	2	2.30E-18	0.74	20	0.67	LOC108349134;Slco2b1	Transport
DMR1:164644001	1	164644001	164647000	3000	2	8.60E-15	0.63	34	1.13	Slco2b1	Transport
DMR1:164655001	1	164655001	164656000	1000	1	2.00E-13	0.84	5	0.5	Slco2b1	Transport
DMR1:164661001	1	164661001	164666000	5000	2	2.10E-16	0.61	62	1.24	Slco2b1	Transport
DMR1:164674001	1	164674001	164680000	6000	1	8.30E-21	0.66	76	1.27	Slco2b1	Transport
DMR1:164726001	1	164726001	164727000	1000	1	2.70E-23	0.91	7	0.7	Olr36	Receptor
DMR1:164755001	1	164755001	164756000	1000	1	2.20E-07	0.32	17	1.7	Olr37	
DMR1:164791001	1	164791001	164797000	6000	3	9.30E-20	-0.59	64	1.07	Neu3	Metabolism
DMR1:164798001	1	164798001	164800000	2000	2	2.40E-21	0.9	16	0.8	Neu3;LOC102553078	Metabolism
DMR1:164809001	1	164809001	164810000	1000	1	5.10E-07	-0.68	16	1.6	Neu3;LOC102553078	Metabolism
DMR1:164831001	1	164831001	164836000	5000	1	3.70E-07	-0.53	59	1.18	Spcs2	Protease

DMR1:164863001	1	164863001	164865000	2000	1	3.40E-11	0.42	31	1.55	Xrra1;LOC102547456	
DMR1:164958001	1	164958001	164963000	5000	1	1.30E-11	0.52	51	1.02	Rnf169	
DMR1:164964001	1	164964001	164970000	6000	1	3.20E-08	0.4	68	1.13	Rnf169	
DMR1:164978001	1	164978001	164979000	1000	1	3.80E-12	0.59	7	0.7	Rnf169	
DMR1:164984001	1	164984001	164986000	2000	2	2.10E-27	1.04	27	1.35	Rnf169	
DMR1:165006001	1	165006001	165009000	3000	2	6.70E-36	1.01	78	2.6	Chrdl2	
DMR1:165014001	1	165014001	165015000	1000	1	2.60E-11	0.36	12	1.2	Chrdl2	
DMR1:165019001	1	165019001	165022000	3000	2	1.50E-07	0.37	47	1.57	Chrdl2	
DMR1:165033001	1	165033001	165036000	3000	2	2.30E-20	1.02	27	0.9	Chrdl2	
DMR1:165085001	1	165085001	165089000	4000	1	4.40E-07	-0.47	40	1	Pold3	Transcription
DMR1:165110001	1	165110001	165111000	1000	1	5.90E-18	0.63	25	2.5	Pold3	Transcription
DMR1:165112001	1	165112001	165113000	1000	1	7.70E-10	-0.59	13	1.3	Pold3	Transcription
DMR1:165129001	1	165129001	165132000	3000	1	5.70E-11	0.43	37	1.23	Pold3	Transcription
DMR1:165162001	1	165162001	165165000	3000	1	2.90E-20	0.51	37	1.23	Lipt2	Transport
DMR1:165167001	1	165167001	165170000	3000	1	8.10E-13	0.42	48	1.6	Lipt2	Transport
DMR1:165194001	1	165194001	165199000	5000	1	3.20E-09	0.45	82	1.64	LOC100912071;Kcne3	Transport
DMR1:165200001	1	165200001	165208000	8000	1	3.30E-09	0.36	83	1.04	LOC100912071;Kcne3	Transport
DMR1:165264001	1	165264001	165266000	2000	1	1.30E-07	-0.44	30	1.5	Pgm2l1	Metabolism
DMR1:165296001	1	165296001	165298000	2000	2	1.10E-10	0.39	45	2.25	Pgm2l1;P4ha3	Metabolism;Golgi
DMR1:165306001	1	165306001	165311000	5000	1	1.10E-18	0.75	72	1.44	P4ha3	Golgi
DMR1:165317001	1	165317001	165320000	3000	1	3.60E-12	0.47	32	1.07	P4ha3	Golgi
DMR1:165322001	1	165322001	165323000	1000	1	4.20E-08	-0.44	17	1.7	P4ha3	Golgi
DMR1:165324001	1	165324001	165325000	1000	1	3.30E-15	0.55	18	1.8	P4ha3	Golgi
DMR1:165333001	1	165333001	165335000	2000	1	1.10E-10	0.51	22	1.1	P4ha3;Ppme1	Golgi
DMR1:165343001	1	165343001	165344000	1000	1	3.00E-07	0.38	10	1	Ppme1	Golgi
DMR1:165351001	1	165351001	165353000	2000	1	7.40E-13	0.68	15	0.75	Ppme1	Golgi
DMR1:165393001	1	165393001	165395000	2000	1	2.10E-18	0.67	17	0.85	C2cd3	
DMR1:165396001	1	165396001	165397000	1000	1	2.50E-08	0.47	10	1	C2cd3	
DMR1:165442001	1	165442001	165443000	1000	1	2.70E-23	0.9	8	0.8	C2cd3	
DMR1:165445001	1	165445001	165446000	1000	1	2.80E-07	0.44	7	0.7	C2cd3	
DMR1:165466001	1	165466001	165469000	3000	2	1.70E-14	0.49	30	1	C2cd3	
DMR1:165499001	1	165499001	165502000	3000	1	1.60E-09	0.63	25	0.83	Ucp3;Ucp2	Transport
DMR1:165504001	1	165504001	165505000	1000	1	1.10E-08	0.62	2	0.2	Ucp3;Ucp2	Transport
DMR1:165542001	1	165542001	165545000	3000	1	5.70E-10	-0.56	32	1.07	LOC103691200;Coa4	Transcription
DMR1:165554001	1	165554001	165557000	3000	1	1.10E-07	-0.39	49	1.63	Coa4;Mrpl48	Transcription;Translation
DMR1:165571001	1	165571001	165573000	2000	1	4.00E-14	0.56	25	1.25	Mrpl48	Translation
DMR1:165576001	1	165576001	165580000	4000	2	5.00E-12	0.59	46	1.15	Mrpl48	Translation
DMR1:165614001	1	165614001	165615000	1000	1	1.20E-11	0.64	3	0.3	Mrpl48;Rab6a	Translation
DMR1:165653001	1	165653001	165654000	1000	1	1.30E-16	0.61	7	0.7	Rab6a	
DMR1:165678001	1	165678001	165679000	1000	1	1.90E-07	0.39	18	1.8	Plekhb1;LOC108349605	
DMR1:165686001	1	165686001	165688000	2000	1	9.70E-14	0.59	24	1.2	Plekhb1;LOC108349605	
DMR1:165692001	1	165692001	165694000	2000	2	1.50E-15	0.77	24	1.2	Plekhb1;LOC108349605	
DMR1:165721001	1	165721001	165723000	2000	1	4.70E-13	0.62	15	0.75	Fam168a	Cytoskeleton
DMR1:165724001	1	165724001	165726000	2000	2	3.60E-13	0.68	78	3.9	Fam168a	Cytoskeleton
DMR1:165749001	1	165749001	165750000	1000	1	6.60E-11	-0.64	12	1.2	Fam168a	Cytoskeleton
DMR1:165753001	1	165753001	165754000	1000	1	1.50E-07	0.45	10	1	Fam168a	Cytoskeleton
DMR1:165781001	1	165781001	165783000	2000	1	1.50E-10	0.6	15	0.75	Fam168a	Cytoskeleton
DMR1:165823001	1	165823001	165825000	2000	1	5.80E-11	0.47	16	0.8	Fam168a	Cytoskeleton
DMR1:165841001	1	165841001	165848000	7000	4	2.00E-13	-0.51	72	1.03	Fam168a	Cytoskeleton
DMR1:165857001	1	165857001	165858000	1000	1	2.00E-13	0.59	9	0.9	Fam168a	Cytoskeleton
DMR1:165863001	1	165863001	165865000	2000	1	9.00E-21	0.83	16	0.8	Fam168a	Cytoskeleton
DMR1:165873001	1	165873001	165874000	1000	1	1.10E-09	0.47	8	0.8	Fam168a	Cytoskeleton
DMR1:165908001	1	165908001	165909000	1000	1	6.30E-15	0.59	16	1.6	Relt	Receptor
DMR1:165912001	1	165912001	165915000	3000	1	4.30E-10	0.43	39	1.3	Relt;Mir3102	Receptor
DMR1:165970001	1	165970001	165974000	4000	1	4.90E-16	0.74	55	1.38	P2ry6	Signaling
DMR1:165976001	1	165976001	165978000	2000	1	1.20E-17	0.72	20	1	P2ry6	Signaling
DMR1:165985001	1	165985001	165987000	2000	1	2.70E-10	0.5	30	1.5	P2ry6	Signaling
DMR1:166011001	1	166011001	166014000	3000	1	8.70E-11	0.61	25	0.83	P2ry6	Signaling
DMR1:166019001	1	166019001	166022000	3000	1	3.30E-13	0.41	44	1.47	P2ry2	Signaling
DMR1:166042001	1	166042001	166043000	1000	1	4.80E-08	0.44	10	1	P2ry2;LOC108349453	Signaling
DMR1:166047001	1	166047001	166054000	7000	1	2.80E-09	0.41	92	1.31	P2ry2;LOC108349453	Signaling
DMR1:166147001	1	166147001	166148000	1000	1	8.30E-07	0.42	7	0.7	Fchsd2	
DMR1:166164001	1	166164001	166165000	1000	1	5.10E-13	0.59	13	1.3	Fchsd2	
DMR1:166167001	1	166167001	166168000	1000	1	1.10E-13	0.74	15	1.5	Fchsd2	

DMR1:166178001	1	166178001	166180000	2000	2	3.30E-10	0.56	14	0.7	Fchsd2	
DMR1:166186001	1	166186001	166188000	2000	2	1.40E-17	0.87	6	0.3	Fchsd2	
DMR1:166189001	1	166189001	166190000	1000	1	7.40E-12	-0.57	6	0.6	Fchsd2	
DMR1:166195001	1	166195001	166197000	2000	1	1.00E-10	0.57	10	0.5	Fchsd2	
DMR1:166200001	1	166200001	166207000	7000	4	2.30E-18	-0.52	69	0.99	Fchsd2	
DMR1:166229001	1	166229001	166231000	2000	1	2.70E-07	-0.46	14	0.7	Fchsd2	
DMR1:166237001	1	166237001	166239000	2000	1	9.40E-08	0.45	16	0.8	Fchsd2	
DMR1:166241001	1	166241001	166248000	7000	5	1.70E-21	-0.55	68	0.97	Fchsd2	
DMR1:166269001	1	166269001	166271000	2000	1	1.70E-07	-0.5	16	0.8	Fchsd2	
DMR1:166276001	1	166276001	166279000	3000	1	1.40E-09	-0.46	26	0.87	Fchsd2;LOC108349606	Translation
DMR1:166280001	1	166280001	166285000	5000	4	1.40E-16	-0.51	74	1.48	Fchsd2;LOC108349606	Translation
DMR1:166290001	1	166290001	166292000	2000	1	4.60E-07	0.57	18	0.9	Fchsd2;LOC108349606	Translation
DMR1:166310001	1	166310001	166313000	3000	2	1.00E-08	-0.48	24	0.8	Fchsd2;LOC100912515	Receptor
DMR1:166315001	1	166315001	166317000	2000	1	1.90E-14	0.63	13	0.65	Fchsd2	
DMR1:166319001	1	166319001	166321000	2000	2	2.60E-11	0.59	9	0.45	Fchsd2	
DMR1:166331001	1	166331001	166333000	2000	2	1.20E-10	0.69	13	0.65	Fchsd2	
DMR1:166335001	1	166335001	166336000	1000	1	1.20E-09	0.57	9	0.9	Fchsd2	
DMR1:166351001	1	166351001	166361000	10000	7	6.50E-20	-0.56	124	1.24	Fchsd2	
DMR1:166371001	1	166371001	166372000	1000	1	4.20E-10	0.64	4	0.4	Fchsd2	
DMR1:166389001	1	166389001	166392000	3000	1	1.60E-12	-0.53	11	0.37	Fchsd2;Atg16l2	
DMR1:166415001	1	166415001	166418000	3000	1	4.50E-09	0.39	40	1.33	Atg16l2;LOC102546541	
DMR1:166424001	1	166424001	166425000	1000	1	1.80E-12	0.49	10	1	Atg16l2;LOC102546541;Stard10	
DMR1:166438001	1	166438001	166439000	1000	1	3.40E-07	0.36	17	1.7	Stard10	
DMR1:166442001	1	166442001	166446000	4000	2	2.20E-16	0.74	33	0.82	Stard10	
DMR1:166467001	1	166467001	166469000	2000	1	7.10E-09	0.33	41	2.05	Stard10;Arap1	Signaling
DMR1:166471001	1	166471001	166474000	3000	2	1.20E-08	0.41	44	1.47	Arap1	Signaling
DMR1:166477001	1	166477001	166478000	1000	1	2.40E-12	0.68	3	0.3	Arap1	Signaling
DMR1:166479001	1	166479001	166493000	14000	2	2.50E-13	-0.54	190	1.36	Arap1	Signaling
DMR1:166498001	1	166498001	166503000	5000	4	4.10E-22	0.71	57	1.14	Arap1	Signaling
DMR1:166514001	1	166514001	166517000	3000	2	1.50E-18	0.59	47	1.57	Arap1	Signaling
DMR1:166528001	1	166528001	166529000	1000	1	3.80E-19	0.68	19	1.9	Arap1;Pde2a	Signaling;Signaling
DMR1:166534001	1	166534001	166537000	3000	3	1.40E-10	0.48	50	1.67	Arap1;Pde2a	Signaling;Signaling
DMR1:166547001	1	166547001	166551000	4000	1	1.40E-19	0.97	30	0.75	Pde2a	Signaling
DMR1:166565001	1	166565001	166571000	6000	3	3.40E-16	0.49	73	1.22	Pde2a	Signaling
DMR1:166574001	1	166574001	166576000	2000	2	2.60E-11	0.4	41	2.05	Pde2a	Signaling
DMR1:166589001	1	166589001	166594000	5000	2	1.50E-40	1.24	56	1.12	Pde2a;Mir139	Signaling
DMR1:166602001	1	166602001	166604000	2000	2	2.90E-17	0.71	25	1.25	Pde2a	Signaling
DMR1:166605001	1	166605001	166609000	4000	1	2.60E-08	-0.48	67	1.68	Pde2a	Signaling
DMR1:166611001	1	166611001	166612000	1000	1	1.80E-07	0.43	17	1.7	Pde2a	Signaling
DMR1:166628001	1	166628001	166629000	1000	1	1.00E-17	0.6	21	2.1	Pde2a	Signaling
DMR1:166634001	1	166634001	166639000	5000	4	1.20E-14	-0.87	61	1.22	Pde2a	Signaling
DMR1:166655001	1	166655001	166656000	1000	1	4.30E-07	0.35	8	0.8	Art2b	
DMR1:166661001	1	166661001	166662000	1000	1	5.60E-23	0.87	7	0.7	Art2b	
DMR1:166673001	1	166673001	166674000	1000	1	1.80E-07	0.46	12	1.2	Art2b	
DMR1:166675001	1	166675001	166676000	1000	1	3.80E-07	-0.55	7	0.7	Art2b	
DMR1:166681001	1	166681001	166682000	1000	1	1.40E-07	0.56	3	0.3	Art2b	
DMR1:166692001	1	166692001	166694000	2000	1	1.80E-16	0.77	13	0.65	Art2b	
DMR1:166727001	1	166727001	166732000	5000	1	8.00E-10	0.53	80	1.6	LOC365317;Clpb	Protease
DMR1:166737001	1	166737001	166738000	1000	1	3.10E-07	0.5	3	0.3	LOC365317;Clpb	Protease
DMR1:166780001	1	166780001	166782000	2000	2	1.80E-28	0.94	20	1	Clpb	Protease
DMR1:166797001	1	166797001	166800000	3000	2	1.80E-16	0.53	37	1.23	Clpb	Protease
DMR1:166801001	1	166801001	166802000	1000	1	6.00E-12	0.48	11	1.1	Clpb	Protease
DMR1:166821001	1	166821001	166822000	1000	1	8.70E-10	0.53	10	1	Clpb	Protease
DMR1:166862001	1	166862001	166863000	1000	1	7.10E-13	0.78	5	0.5	Clpb	Protease
DMR1:166876001	1	166876001	166878000	2000	1	3.10E-13	0.5	16	0.8	Clpb;LOC102547177	Protease
DMR1:166881001	1	166881001	166884000	3000	1	1.40E-18	0.82	30	1	LOC102547177;Phox2a	Development
DMR1:166892001	1	166892001	166894000	2000	1	2.90E-10	0.6	81	4.05	LOC102547177;Phox2a;Inppl1	Development
DMR1:166895001	1	166895001	166896000	1000	1	2.20E-19	0.82	13	1.3	Phox2a;Inppl1	Development
DMR1:166926001	1	166926001	166927000	1000	1	2.10E-11	0.73	5	0.5	Folr2;LOC102547245;Folr1	
DMR1:166937001	1	166937001	166940000	3000	1	1.60E-11	-0.59	41	1.37	Folr2;Folr1	
DMR1:166941001	1	166941001	166945000	4000	2	8.20E-16	0.6	51	1.27	Folr2;Folr1	
DMR1:166950001	1	166950001	166952000	2000	1	7.40E-16	0.54	32	1.6	Folr1	
DMR1:166953001	1	166953001	166956000	3000	1	6.20E-09	0.36	32	1.07	Folr1	
DMR1:166972001	1	166972001	166973000	1000	1	1.00E-07	0.34	27	2.7	Anapc15	

DMR1:166977001	1	166977001	166979000	2000	1	1.60E-15	0.6	25	1.25	Anapc15;Lrtomt	
DMR1:167037001	1	167037001	167038000	1000	1	2.10E-09	0.48	12	1.2	Numa1	Cytoskeleton
DMR1:167050001	1	167050001	167054000	4000	2	1.00E-19	1.04	46	1.15	Numa1	Cytoskeleton
DMR1:167058001	1	167058001	167061000	3000	1	4.90E-16	0.93	24	0.8	Numa1	Cytoskeleton
DMR1:167068001	1	167068001	167070000	2000	1	8.10E-07	-0.49	30	1.5	Numa1	Cytoskeleton
DMR1:167074001	1	167074001	167079000	5000	2	5.30E-08	0.42	108	2.16	Numa1	Cytoskeleton
DMR1:167103001	1	167103001	167104000	1000	1	2.20E-11	0.61	13	1.3	Il18bp;Rnf121	Proteolysis
DMR1:167148001	1	167148001	167149000	1000	1	2.30E-08	0.47	5	0.5	Rnf121;LOC102549471	Proteolysis
DMR1:167169001	1	167169001	167172000	3000	1	4.30E-21	0.78	33	1.1	Rnf121;LOC102549471;LOC108348605;Trpc2	Proteolysis;Transport
DMR1:167173001	1	167173001	167176000	3000	1	3.40E-18	1.01	27	0.9	Rnf121;LOC102549471;Trpc2	Proteolysis;Transport
DMR1:167187001	1	167187001	167192000	5000	2	2.10E-18	0.77	100	2	LOC102549471;Trpc2;Art5	Transport;Transport
DMR1:167228001	1	167228001	167229000	1000	1	1.30E-07	-0.32	12	1.2	Nup98	Transport
DMR1:167230001	1	167230001	167234000	4000	1	2.80E-11	-0.46	44	1.1	Nup98	Transport
DMR1:167252001	1	167252001	167253000	1000	1	7.30E-07	-0.49	9	0.9	Nup98	Transport
DMR1:167257001	1	167257001	167260000	3000	1	2.40E-10	0.46	25	0.83	Nup98	Transport
DMR1:167261001	1	167261001	167262000	1000	1	1.20E-10	0.48	10	1	Nup98	Transport
DMR1:167273001	1	167273001	167275000	2000	1	1.60E-08	0.48	12	0.6	Nup98	Transport
DMR1:167290001	1	167290001	167292000	2000	1	3.10E-11	0.7	11	0.55	Nup98;LOC108348799	Transport
DMR1:167294001	1	167294001	167296000	2000	1	2.40E-26	0.9	15	0.75	Nup98;LOC108348799	Transport
DMR1:167328001	1	167328001	167330000	2000	2	1.80E-21	0.73	24	1.2	Pgap2;Rhog	Golgi;Signaling
DMR1:167337001	1	167337001	167339000	2000	1	7.20E-09	0.41	34	1.7	Pgap2;Rhog	Golgi;Signaling
DMR1:167344001	1	167344001	167347000	3000	1	1.20E-07	0.3	38	1.27	Pgap2;Rhog;LOC108348579	Golgi;Signaling
DMR1:167360001	1	167360001	167368000	8000	5	1.00E-12	-0.45	92	1.15	LOC108348579;Stim1	
DMR1:167417001	1	167417001	167420000	3000	1	6.40E-16	0.55	9	0.3	Stim1	
DMR1:167441001	1	167441001	167443000	2000	1	8.10E-15	0.63	13	0.65	Stim1	
DMR1:167453001	1	167453001	167455000	2000	1	8.20E-11	0.68	11	0.55	Stim1	
DMR1:167461001	1	167461001	167462000	1000	1	1.50E-07	-0.47	8	0.8	Stim1;LOC103691224	
DMR1:167481001	1	167481001	167484000	3000	1	6.10E-16	0.58	19	0.63	Stim1;LOC103691224	
DMR1:167502001	1	167502001	167503000	1000	1	1.40E-07	-0.64	7	0.7	Stim1	
DMR1:167517001	1	167517001	167520000	3000	3	7.10E-17	0.64	22	0.73	Stim1	
DMR1:167523001	1	167523001	167524000	1000	1	4.70E-11	0.53	6	0.6	Stim1	
DMR1:167526001	1	167526001	167528000	2000	2	2.90E-16	0.65	18	0.9	Stim1	
DMR1:167531001	1	167531001	167532000	1000	1	4.40E-18	0.69	15	1.5	Stim1;Rrm1	Metabolism
DMR1:167538001	1	167538001	167539000	1000	1	1.60E-07	0.63	50	5	Stim1;Rrm1	Metabolism
DMR1:167564001	1	167564001	167566000	2000	1	2.80E-07	-0.46	24	1.2	Rrm1	Metabolism
DMR1:167590001	1	167590001	167594000	4000	1	9.10E-08	-0.36	27	0.68	Olr38-ps;Olr39	Receptor
DMR1:167598001	1	167598001	167600000	2000	2	5.40E-14	0.76	16	0.8	Olr39;Olr40	Receptor
DMR1:167603001	1	167603001	167605000	2000	1	3.00E-07	0.42	34	1.7	Olr40	Receptor
DMR1:167615001	1	167615001	167617000	2000	1	2.90E-07	-0.48	18	0.9	Olr40	Receptor
DMR1:167649001	1	167649001	167654000	5000	1	5.50E-13	-0.62	25	0.5	Olr41	Receptor
DMR1:167670001	1	167670001	167672000	2000	1	1.50E-07	-0.29	37	1.85	Olr41	Receptor
DMR1:167682001	1	167682001	167685000	3000	2	8.30E-21	0.99	21	0.7	Olr42-ps;Trim21	Proteolysis
DMR1:167689001	1	167689001	167697000	8000	3	8.00E-17	-0.49	87	1.09	Olr42-ps;Trim21	Proteolysis
DMR1:167698001	1	167698001	167699000	1000	1	3.50E-12	0.45	13	1.3	Trim21	Proteolysis
DMR1:167700001	1	167700001	167703000	3000	1	6.80E-10	-0.53	29	0.97	Trim21	Proteolysis
DMR1:167710001	1	167710001	167712000	2000	1	2.20E-16	0.78	14	0.7	Trim21;Olr43	Proteolysis;Receptor
DMR1:167727001	1	167727001	167729000	2000	1	1.80E-11	-0.48	8	0.4	Olr43;Olr44	Receptor
DMR1:167738001	1	167738001	167740000	2000	1	1.10E-11	0.6	12	0.6	Olr44;Olr45	Receptor
DMR1:167746001	1	167746001	167747000	1000	1	5.50E-10	0.48	5	0.5	Olr45	Receptor
DMR1:167762001	1	167762001	167763000	1000	1	7.30E-20	0.96	4	0.4	Olr46;Bcl2l1-ps1;Olr47	Receptor
DMR1:167769001	1	167769001	167770000	1000	1	3.80E-09	0.37	19	1.9	Olr46;Bcl2l1-ps1;Olr47	Receptor
DMR1:167779001	1	167779001	167780000	1000	1	3.90E-08	0.43	2	0.2	Olr47;Olr48	Receptor
DMR1:167793001	1	167793001	167796000	3000	1	2.80E-09	-0.73	53	1.77	Olr48	Receptor
DMR1:167824001	1	167824001	167828000	4000	2	1.60E-13	0.75	34	0.85	LOC684179;LOC103690775;Trim68	Receptor;Proteolysis
DMR1:167844001	1	167844001	167847000	3000	1	1.20E-08	-0.43	11	0.37	LOC103690775;Trim68	Proteolysis
DMR1:167851001	1	167851001	167853000	2000	1	3.10E-08	0.48	13	0.65	Trim68;LOC684208	Proteolysis;Receptor
DMR1:167857001	1	167857001	167861000	4000	1	1.30E-10	0.55	37	0.92	Trim68;LOC684208;Olr63	Proteolysis;Receptor
DMR1:167881001	1	167881001	167882000	1000	1	1.30E-08	0.43	6	0.6	Olr63;Olr62;Olr61	Receptor
DMR1:167925001	1	167925001	167928000	3000	1	1.90E-08	0.31	27	0.9	Olr59;Olr58-ps;Olr57	Receptor
DMR1:167932001	1	167932001	167935000	3000	1	3.20E-13	0.8	11	0.37	Olr59;Olr57;Olr56	Receptor
DMR1:167943001	1	167943001	167945000	2000	1	1.10E-07	0.46	10	0.5	Olr59;Olr57;Olr56;Olr55-ps	Receptor
DMR1:167953001	1	167953001	167955000	2000	2	9.20E-22	0.91	4	0.2	Olr56;Olr55-ps;Olr54-ps	Receptor
DMR1:167967001	1	167967001	167969000	2000	1	8.10E-08	0.35	18	0.9	Olr54-ps;Olr53	Receptor

DMR1:168010001	1	168010001	168012000	2000	1	2.10E-16	0.72	9	0.45	Olr51;Olr50	Receptor
DMR1:168075001	1	168075001	168078000	3000	1	8.10E-10	0.62	20	0.67	LOC103690133;LOC684365;LOC684376;Or51t1	Receptor
DMR1:168079001	1	168079001	168080000	1000	1	2.00E-15	0.9	6	0.6	LOC103690133;LOC684365;LOC684376;Or51t1	Receptor
DMR1:168090001	1	168090001	168091000	1000	1	1.90E-07	0.48	2	0.2	LOC684376;Or51t1;Olr66-ps	Receptor
DMR1:168102001	1	168102001	168109000	7000	4	3.30E-25	-0.6	70	1	Or51t1;Olr66-ps;Olr67	Receptor
DMR1:168130001	1	168130001	168131000	1000	1	3.70E-08	1.23	6	0.6	Nras-ps1;Olr68	Receptor
DMR1:168150001	1	168150001	168154000	4000	2	6.00E-14	-0.47	45	1.12	Olr69;Olr70	Receptor
DMR1:168155001	1	168155001	168161000	6000	1	8.10E-16	0.76	64	1.07	Olr70;Olr71-ps	Receptor
DMR1:168188001	1	168188001	168190000	2000	2	2.50E-13	-0.71	8	0.4	Olr73-ps;Olr74	Receptor
DMR1:168194001	1	168194001	168195000	1000	1	2.70E-10	0.44	12	1.2	Olr73-ps;Olr74;Olr75	Receptor
DMR1:168198001	1	168198001	168200000	2000	1	1.30E-07	0.67	13	0.65	Olr74;Olr75	Receptor
DMR1:168218001	1	168218001	168220000	2000	1	1.70E-07	0.43	17	0.85	Olr76-ps;Olr77	Receptor
DMR1:168250001	1	168250001	168255000	5000	2	2.30E-15	-0.53	40	0.8	Olr79	Receptor
DMR1:168272001	1	168272001	168273000	1000	1	3.80E-08	0.56	4	0.4	Olr80	Receptor
DMR1:168274001	1	168274001	168279000	5000	2	3.90E-18	-0.65	39	0.78	Olr80	Receptor
DMR1:168305001	1	168305001	168306000	1000	1	2.80E-09	0.57	4	0.4	Olr82	Receptor
DMR1:168330001	1	168330001	168331000	1000	1	3.20E-12	0.68	4	0.4	Olr83	Receptor
DMR1:168344001	1	168344001	168345000	1000	1	4.60E-12	0.71	8	0.8	Olr84;Olr85	Receptor
DMR1:168376001	1	168376001	168378000	2000	2	1.30E-11	0.64	8	0.4	Olr86	Receptor
DMR1:168380001	1	168380001	168381000	1000	1	6.60E-14	0.82	34	3.4	Olr86;Olr87	Receptor
DMR1:168382001	1	168382001	168385000	3000	1	7.40E-12	0.53	13	0.43	Olr86;Olr87	Receptor
DMR1:168396001	1	168396001	168397000	1000	1	2.10E-07	0.49	4	0.4	Olr87;Olr88	Receptor
DMR1:168416001	1	168416001	168418000	2000	1	2.10E-07	0.39	11	0.55	Olr89;Olr90-ps	Receptor
DMR1:168420001	1	168420001	168421000	1000	1	2.20E-09	0.62	1	0.1	Olr89;Olr90-ps	Receptor
DMR1:168429001	1	168429001	168438000	9000	5	2.00E-20	-0.59	86	0.96	Olr90-ps;Olr91	Receptor
DMR1:168454001	1	168454001	168455000	1000	1	6.00E-08	0.48	5	0.5	Olr91;Olr92;Olr93	Receptor
DMR1:168495001	1	168495001	168496000	1000	1	9.50E-07	-0.51	2	0.2	Olr96;Olr97	Receptor
DMR1:168505001	1	168505001	168512000	7000	4	1.20E-16	-0.46	82	1.17	Olr97;Olr98	Receptor
DMR1:168525001	1	168525001	168526000	1000	1	9.40E-07	-0.47	3	0.3	Olr98;Olr99-ps;Olr100-ps	Receptor
DMR1:168537001	1	168537001	168540000	3000	2	7.60E-10	-0.47	33	1.1	Olr99-ps;Olr100-ps;Olr101	Receptor
DMR1:168541001	1	168541001	168543000	2000	1	1.10E-09	-0.4	15	0.75	Olr100-ps;Olr101	Receptor
DMR1:168548001	1	168548001	168549000	1000	1	1.10E-08	0.49	4	0.4	Olr101;Olr102-ps	Receptor
DMR1:168558001	1	168558001	168559000	1000	1	1.90E-08	0.54	2	0.2	Olr102-ps	
DMR1:168561001	1	168561001	168568000	7000	3	1.80E-14	-0.48	82	1.17	Olr102-ps;Olr103	Receptor
DMR1:168570001	1	168570001	168573000	3000	1	7.80E-12	-0.78	57	1.9	Olr103	Receptor
DMR1:168580001	1	168580001	168582000	2000	2	7.60E-10	-0.54	10	0.5	Olr103;Olr104	Receptor
DMR1:168588001	1	168588001	168592000	4000	3	2.90E-11	-0.54	27	0.68	Olr104;Olr105	Receptor
DMR1:168626001	1	168626001	168634000	8000	5	1.10E-20	-0.63	127	1.59	Olr107;Olr108;Olr109	Receptor
DMR1:168664001	1	168664001	168671000	7000	4	7.70E-26	-0.55	79	1.13	Olr110;Olr111	Receptor
DMR1:168674001	1	168674001	168676000	2000	1	4.50E-16	0.55	20	1	Olr111	
DMR1:168695001	1	168695001	168696000	1000	1	1.20E-11	0.49	6	0.6	Olr112;Olr113	Receptor
DMR1:168714001	1	168714001	168721000	7000	5	1.90E-23	-0.75	90	1.29	Olr114;Olr115	Receptor
DMR1:168731001	1	168731001	168738000	7000	3	2.30E-25	-0.46	61	0.87	Olr115;LOC499215;Olr116-ps	Receptor
DMR1:168756001	1	168756001	168758000	2000	1	1.90E-07	0.58	26	1.3	Olr116-ps;Olr117-ps;LOC108349608	
DMR1:168763001	1	168763001	168766000	3000	1	1.50E-10	-0.62	66	2.2	Olr117-ps;LOC108349608	
DMR1:168804001	1	168804001	168811000	7000	5	2.40E-19	-0.49	72	1.03	Olr119;Olr120;Olr121	Receptor
DMR1:168826001	1	168826001	168827000	1000	1	1.40E-07	0.56	3	0.3	Olr121;Olr122	Receptor
DMR1:168836001	1	168836001	168839000	3000	1	2.40E-10	-0.45	11	0.37	Olr122;LOC100361642	Receptor
DMR1:168840001	1	168840001	168843000	3000	1	5.70E-07	0.51	12	0.4	Olr122;LOC100361642;Olr124	Receptor
DMR1:168858001	1	168858001	168859000	1000	1	1.40E-08	-0.59	7	0.7	Olr124;Olr125	Receptor
DMR1:168864001	1	168864001	168866000	2000	1	1.10E-15	0.82	13	0.65	Olr125	Receptor
DMR1:168868001	1	168868001	168870000	2000	1	4.80E-11	0.52	18	0.9	Olr125	Receptor
DMR1:168873001	1	168873001	168875000	2000	2	5.20E-12	0.72	7	0.35	Olr125;Olr126	Receptor
DMR1:168876001	1	168876001	168877000	1000	1	5.10E-12	0.54	9	0.9	Olr125;Olr126	Receptor
DMR1:168893001	1	168893001	168895000	2000	2	1.80E-20	-0.57	14	0.7	Olr126;Olr127	Receptor
DMR1:168896001	1	168896001	168898000	2000	2	2.40E-09	-0.42	19	0.95	Olr127	Receptor
DMR1:168910001	1	168910001	168920000	10000	6	8.80E-19	0.56	141	1.41	Olr127;Olr128	Receptor
DMR1:168921001	1	168921001	168925000	4000	2	8.10E-11	0.57	40	1	Olr128	Receptor
DMR1:168938001	1	168938001	168941000	3000	2	6.70E-11	-0.42	45	1.5	Olr128;Hbb-b1	Receptor
DMR1:168944001	1	168944001	168945000	1000	1	3.50E-09	0.56	3	0.3	Hbb-b1	
DMR1:168946001	1	168946001	168953000	7000	5	3.10E-19	-0.55	66	0.94	Hbb-b1;LOC100134871	

DMR1:168962001	1	168962001	168963000	1000	1	5.90E-11	0.65	4	0.4	Hbb-b1;LOC100134871;LOC689064;Hbb	
DMR1:168966001	1	168966001	168967000	1000	1	3.30E-09	-0.73	6	0.6	LOC100134871;LOC689064;Hbb	
DMR1:168977001	1	168977001	168978000	1000	1	2.80E-08	0.57	6	0.6	Hbb;LOC502358	
DMR1:168979001	1	168979001	168982000	3000	1	3.30E-08	0.55	10	0.33	Hbb;LOC502358	
DMR1:168995001	1	168995001	168996000	1000	1	3.60E-12	0.76	3	0.3	LOC502358;Hbg1;Hbe2;Hbe1	
DMR1:168998001	1	168998001	1.69E+08	2000	1	1.30E-17	0.83	15	0.75	LOC502358;Hbg1;Hbe2;Hbe1	
DMR1:169012001	1	169012001	169014000	2000	1	3.70E-13	0.83	13	0.65	Hbe1	
DMR1:169015001	1	169015001	169017000	2000	1	1.30E-08	0.53	9	0.45	Hbe1	
DMR1:169044001	1	169044001	169047000	3000	2	1.20E-12	0.71	18	0.6	Olr130;Olr131	Receptor
DMR1:169057001	1	169057001	169062000	5000	4	2.30E-31	-0.62	56	1.12	Olr131	Receptor
DMR1:169064001	1	169064001	169068000	4000	2	2.90E-14	-0.46	35	0.88	Olr132	Receptor
DMR1:169082001	1	169082001	169086000	4000	2	2.20E-21	-0.5	42	1.05	Olr132;Olr133-ps	Receptor
DMR1:169090001	1	169090001	169095000	5000	1	3.80E-11	0.76	24	0.48	Olr133-ps	
DMR1:169117001	1	169117001	169118000	1000	1	6.30E-07	0.48	6	0.6	Olr134-ps;Olr135	Receptor
DMR1:169131001	1	169131001	169139000	8000	7	2.30E-24	-0.54	75	0.94	Olr136;LOC499219	Receptor;Transcription
DMR1:169176001	1	169176001	169180000	4000	1	2.50E-07	0.31	128	3.2	LOC102555557;Olr139;LOC689243	Receptor
DMR1:169183001	1	169183001	169186000	3000	2	1.50E-08	0.48	25	0.83	LOC102555557;Olr139;LOC689243	Receptor
DMR1:169188001	1	169188001	169196000	8000	3	1.00E-14	-0.37	108	1.35	LOC102555557;Olr139;LOC689243	Receptor
DMR1:169198001	1	169198001	169204000	6000	1	1.20E-07	-0.38	42	0.7	Olr140;Olr141	Receptor
DMR1:169209001	1	169209001	169210000	1000	1	1.10E-10	0.48	12	1.2	Olr140;Olr141	Receptor
DMR1:169216001	1	169216001	169219000	3000	3	9.10E-26	-0.56	32	1.07	Olr140;Olr141	Receptor
DMR1:169220001	1	169220001	169223000	3000	1	5.00E-10	-0.42	23	0.77	Olr141;Olr142	Receptor
DMR1:169238001	1	169238001	169239000	1000	1	2.60E-08	0.64	6	0.6	Olr142;Olr143;Olr144	Receptor
DMR1:169244001	1	169244001	169245000	1000	1	3.50E-17	0.79	7	0.7	Olr143;Olr144	Receptor
DMR1:169256001	1	169256001	169259000	3000	2	2.50E-12	-0.45	32	1.07	Olr144	Receptor
DMR1:169260001	1	169260001	169265000	5000	2	6.60E-25	-0.54	60	1.2	Olr145	Receptor
DMR1:169286001	1	169286001	169287000	1000	1	5.70E-18	0.62	9	0.9	Olr146-ps;Olr147-ps	
DMR1:169291001	1	169291001	169294000	3000	1	1.20E-08	0.51	20	0.67	Olr147-ps	
DMR1:169295001	1	169295001	169297000	2000	1	1.90E-19	0.7	16	0.8	Olr147-ps;Olr148	Receptor
DMR1:169311001	1	169311001	169314000	3000	2	8.90E-19	0.73	15	0.5	Olr148;RGD1562433	Receptor
DMR1:169322001	1	169322001	169325000	3000	1	3.30E-08	0.54	18	0.6	RGD1562433;Ubqln3	
DMR1:169337001	1	169337001	169339000	2000	1	9.60E-10	0.4	14	0.7	Ubqln3;Ubqlnl;RGD1310717	Development
DMR1:169363001	1	169363001	169370000	7000	5	4.10E-21	-0.6	91	1.3	RGD1310717;Olr149	Development;Receptor
DMR1:169377001	1	169377001	169383000	6000	5	1.80E-17	-0.72	76	1.27	Olr149;Olr150;LOC108348458	Receptor
DMR1:169386001	1	169386001	169387000	1000	1	2.00E-10	0.46	24	2.4	Olr150;LOC108348458	Receptor
DMR1:169389001	1	169389001	169390000	1000	1	5.10E-11	0.54	9	0.9	Olr150;LOC108348458	Receptor
DMR1:169396001	1	169396001	169400000	4000	3	4.00E-08	0.42	42	1.05	Olr150;LOC108348458;Olr151-ps	Receptor
DMR1:169411001	1	169411001	169414000	3000	1	1.90E-11	0.53	35	1.17	Olr151-ps;Trim6	Proteolysis
DMR1:169416001	1	169416001	169418000	2000	2	2.60E-10	-0.55	28	1.4	Trim6	Proteolysis
DMR1:169484001	1	169484001	169486000	2000	1	2.70E-09	0.59	8	0.4	LOC102555738;Trim30c;LOC102554029	Proteolysis
DMR1:169487001	1	169487001	169488000	1000	1	7.30E-09	0.45	8	0.8	LOC102555738;Trim30c;LOC102554029	Proteolysis
DMR1:169489001	1	169489001	169490000	1000	1	4.10E-14	0.62	12	1.2	Trim30c;LOC102554029	Proteolysis
DMR1:169492001	1	169492001	169493000	1000	1	1.80E-09	-0.52	11	1.1	Trim30c;LOC102554029	Proteolysis
DMR1:169515001	1	169515001	169520000	5000	3	1.80E-10	-0.43	29	0.58	Trim30c;Trim30	Proteolysis
DMR1:169521001	1	169521001	169522000	1000	1	1.60E-11	0.67	6	0.6	Trim30c;Trim30	Proteolysis
DMR1:169525001	1	169525001	169526000	1000	1	2.40E-10	0.64	1	0.1	Trim30	
DMR1:169535001	1	169535001	169537000	2000	2	4.10E-21	0.91	10	0.5	Trim30;LOC499224	
DMR1:169546001	1	169546001	169548000	2000	1	5.40E-07	0.51	22	1.1	Trim30;LOC499224;Olr152	Receptor
DMR1:169565001	1	169565001	169567000	2000	2	3.90E-13	0.77	8	0.4	Olr153;Olr155;Olr154	Receptor
DMR1:169579001	1	169579001	169587000	8000	6	2.00E-25	-0.57	97	1.21	Olr155;Olr154	Receptor
DMR1:169600001	1	169600001	169601000	1000	1	1.00E-11	0.67	5	0.5	Olr155;Olr156	Receptor
DMR1:169612001	1	169612001	169614000	2000	1	7.10E-11	0.7	11	0.55	Olr157	
DMR1:169619001	1	169619001	169620000	1000	1	1.90E-14	0.7	4	0.4	Olr157	
DMR1:169621001	1	169621001	169625000	4000	1	3.80E-11	-0.48	29	0.72	Olr157	
DMR1:169648001	1	169648001	169650000	2000	1	1.00E-10	0.49	8	0.4	Olr159	Receptor

DMR1:169651001	1	169651001	169653000	2000	1	5.80E-08	-0.6	6	0.3	Olr159;Olr160	Receptor
DMR1:169664001	1	169664001	169665000	1000	1	8.50E-07	0.51	3	0.3	Olr160;Olr161	Receptor
DMR1:169671001	1	169671001	169672000	1000	1	1.90E-09	0.84	6	0.6	Olr160;Olr161	Receptor
DMR1:169687001	1	169687001	169689000	2000	1	4.90E-08	-0.34	16	0.8	Olr162;Olr163	Receptor
DMR1:169690001	1	169690001	169695000	5000	3	1.50E-16	-0.44	88	1.76	Olr162;Olr163	Receptor
DMR1:169704001	1	169704001	169706000	2000	1	2.30E-10	-0.44	15	0.75	Olr163	Receptor
DMR1:169727001	1	169727001	169738000	11000	4	3.90E-09	-0.61	64	0.58	LOC100910107;Olr164	Protease;Receptor
DMR1:169748001	1	169748001	169751000	3000	2	6.50E-13	0.47	53	1.77	Olr164	Receptor
DMR1:169752001	1	169752001	169759000	7000	3	4.50E-10	-0.43	54	0.77	Olr164	Receptor
DMR1:169760001	1	169760001	169761000	1000	1	1.20E-07	0.43	5	0.5	Olr165	Receptor
DMR1:169762001	1	169762001	169764000	2000	1	5.60E-15	0.59	8	0.4	Olr165	Receptor
DMR1:169791001	1	169791001	169794000	3000	1	2.30E-14	0.8	9	0.3	Olr166-ps;Olr167	Receptor
DMR1:169805001	1	169805001	169806000	1000	1	4.20E-14	0.91	1	0.1	Olr166-ps;Olr167	Receptor
DMR1:169826001	1	169826001	169838000	12000	7	4.80E-31	-0.63	146	1.22	Olr179	Receptor
DMR1:169841001	1	169841001	169844000	3000	1	1.00E-13	0.7	21	0.7	Olr179	Receptor
DMR1:169854001	1	169854001	169855000	1000	1	3.50E-10	0.64	4	0.4	Olr179;Olr180	Receptor
DMR1:169862001	1	169862001	169864000	2000	1	5.40E-13	0.6	6	0.3	Olr180	Receptor
DMR1:169865001	1	169865001	169873000	8000	5	2.20E-19	-0.45	78	0.98	Olr180;Olr181	Receptor
DMR1:169876001	1	169876001	169879000	3000	3	2.20E-10	-0.67	17	0.57	Olr181;Olr182-ps	Receptor
DMR1:169890001	1	169890001	169893000	3000	1	2.10E-11	0.73	19	0.63	Olr181;Olr182-ps;Olr183;Olr184	Receptor
DMR1:169929001	1	169929001	169931000	2000	1	1.40E-13	-0.55	21	1.05	Olr186;Olr187-ps	Receptor
DMR1:169932001	1	169932001	169934000	2000	1	5.50E-09	0.53	9	0.45	Olr187-ps	
DMR1:169936001	1	169936001	169938000	2000	1	2.80E-21	0.7	17	0.85	Olr187-ps;Olr188	Receptor
DMR1:169952001	1	169952001	169957000	5000	1	5.20E-09	0.59	38	0.76	Olr188;Olr189;Olr190	Receptor
DMR1:169968001	1	169968001	169970000	2000	2	4.80E-09	0.56	10	0.5	Olr190;Olr191-ps	Receptor
DMR1:169975001	1	169975001	169978000	3000	3	1.30E-09	0.65	17	0.57	Olr190;Olr191-ps;RGD1559839	Receptor
DMR1:169984001	1	169984001	169985000	1000	1	2.70E-10	0.61	4	0.4	RGD1559839;Olr192	
DMR1:170051001	1	170051001	170053000	2000	2	6.80E-15	0.75	11	0.55	Olr195-ps	
DMR1:170054001	1	170054001	170057000	3000	2	2.70E-12	-0.55	14	0.47	Olr195-ps	
DMR1:170069001	1	170069001	170072000	3000	1	2.90E-08	-0.45	28	0.93	Olr196	Receptor
DMR1:170075001	1	170075001	170082000	7000	5	5.90E-25	-0.57	71	1.01	Olr196;Olr197	Receptor
DMR1:170099001	1	170099001	170101000	2000	1	9.70E-11	0.58	14	0.7	Olr197;Olr198	Receptor
DMR1:170123001	1	170123001	170124000	1000	1	1.80E-10	0.6	4	0.4	Olr199	Receptor
DMR1:170128001	1	170128001	170135000	7000	4	8.00E-13	-0.43	56	0.8	Olr199	Receptor
DMR1:170138001	1	170138001	170141000	3000	2	9.10E-21	1	15	0.5	Olr200	Receptor
DMR1:170153001	1	170153001	170159000	6000	2	5.00E-17	-0.67	36	0.6	Olr200	Receptor
DMR1:170162001	1	170162001	170166000	4000	3	2.80E-12	-0.42	34	0.85	Olr201	Receptor
DMR1:170181001	1	170181001	170183000	2000	2	1.00E-14	0.6	15	0.75	Olr201;Olr202	Receptor
DMR1:170184001	1	170184001	170185000	1000	1	1.10E-10	0.58	3	0.3	Olr201;Olr202	Receptor
DMR1:170195001	1	170195001	170198000	3000	1	2.10E-08	0.38	38	1.27	Olr202;LOC686660	Receptor
DMR1:170201001	1	170201001	170203000	2000	1	4.20E-07	0.46	15	0.75	LOC686660;RGD1561034	Receptor
DMR1:170251001	1	170251001	170253000	2000	1	3.30E-10	0.6	15	0.75	Cnga4;LOC108349007;Cckbr	Ion Channel;Signaling
DMR1:170267001	1	170267001	170270000	3000	3	1.10E-14	0.67	44	1.47	LOC108349007;Cckbr	Signaling
DMR1:170281001	1	170281001	170285000	4000	2	3.90E-17	0.98	41	1.02	Cckbr	Signaling
DMR1:170373001	1	170373001	170376000	3000	1	2.30E-11	0.66	29	0.97	Smpd1	Signaling
DMR1:170395001	1	170395001	170397000	2000	1	1.10E-13	0.46	31	1.55	Smpd1;Apbb1	Signaling
DMR1:170398001	1	170398001	170401000	3000	1	7.80E-08	0.34	32	1.07	Apbb1	
DMR1:170405001	1	170405001	170406000	1000	1	2.00E-17	0.49	11	1.1	Apbb1	
DMR1:170407001	1	170407001	170410000	3000	1	1.80E-17	0.67	29	0.97	Apbb1	
DMR1:170416001	1	170416001	170418000	2000	1	4.00E-07	0.44	15	0.75	Apbb1;Hpx	Protease
DMR1:170419001	1	170419001	170421000	2000	1	1.70E-15	0.61	24	1.2	Apbb1;Hpx	Protease
DMR1:170426001	1	170426001	170428000	2000	1	1.50E-09	0.46	19	0.95	Hpx	Protease
DMR1:170438001	1	170438001	170440000	2000	2	5.10E-21	0.74	14	0.7	Hpx;Trim3	Protease;Proteolysis
DMR1:170449001	1	170449001	170454000	5000	4	3.10E-21	0.76	106	2.12	Trim3	Proteolysis
DMR1:170455001	1	170455001	170461000	6000	4	5.00E-14	0.53	66	1.1	Trim3;Arfip2	Proteolysis;Transport
DMR1:170484001	1	170484001	170485000	1000	1	8.20E-24	0.91	3	0.3	Timm10b;Dnhd1	Transport;Cytoskeleton
DMR1:170486001	1	170486001	170493000	7000	6	6.80E-16	-0.56	76	1.09	Dnhd1	Cytoskeleton
DMR1:170506001	1	170506001	170510000	4000	1	2.50E-08	-0.54	14	0.35	Dnhd1	Cytoskeleton
DMR1:170521001	1	170521001	170522000	1000	1	6.70E-19	0.66	13	1.3	Dnhd1	Cytoskeleton
DMR1:170523001	1	170523001	170526000	3000	1	8.40E-13	0.62	22	0.73	Dnhd1	Cytoskeleton
DMR1:170528001	1	170528001	170531000	3000	1	4.20E-08	-0.36	25	0.83	Dnhd1	Cytoskeleton
DMR1:170540001	1	170540001	170543000	3000	1	3.00E-13	-0.81	9	0.3	Dnhd1	Cytoskeleton

DMR1:170545001	1	170545001	170547000	2000	2	1.30E-14	1.02	3	0.15	Dnhd1	Cytoskeleton
DMR1:170553001	1	170553001	170555000	2000	2	1.60E-14	0.67	6	0.3	Dnhd1	Cytoskeleton
DMR1:170559001	1	170559001	170561000	2000	1	5.30E-07	0.47	14	0.7	Dnhd1	Cytoskeleton
DMR1:170562001	1	170562001	170563000	1000	1	1.50E-09	0.49	7	0.7	Dnhd1	Cytoskeleton
DMR1:170595001	1	170595001	170598000	3000	1	1.00E-13	0.54	82	2.73	Ilk;Taf10;Tpp1;Dchs1	Signaling;Transcription;Protease;Cytoskeleton
DMR1:170608001	1	170608001	170611000	3000	1	7.50E-11	0.53	33	1.1	Dchs1	Cytoskeleton
DMR1:170612001	1	170612001	170614000	2000	1	6.80E-07	-0.52	35	1.75	Dchs1	Cytoskeleton
DMR1:170619001	1	170619001	170622000	3000	3	1.10E-18	0.55	40	1.33	Dchs1	Cytoskeleton
DMR1:170623001	1	170623001	170630000	7000	2	5.90E-19	1.14	165	2.36	Dchs1	Cytoskeleton
DMR1:170634001	1	170634001	170636000	2000	1	1.40E-07	0.3	20	1	Dchs1	Cytoskeleton
DMR1:170684001	1	170684001	170688000	4000	2	3.60E-09	-0.49	29	0.72	Olr229	
DMR1:170702001	1	170702001	170706000	4000	1	8.80E-07	-0.57	24	0.6	Olr229	
DMR1:170716001	1	170716001	170717000	1000	1	6.80E-11	0.51	15	1.5	Olr207-ps	
DMR1:170718001	1	170718001	170722000	4000	1	1.80E-12	-0.56	33	0.82	Olr207-ps	
DMR1:170727001	1	170727001	170732000	5000	2	2.40E-12	-0.33	57	1.14	Olr207-ps;LOC103691201	
DMR1:170753001	1	170753001	170756000	3000	1	1.60E-10	-0.57	7	0.23	LOC103691201;Olr208;Olr209	Receptor
DMR1:170805001	1	170805001	170811000	6000	2	5.00E-10	-0.56	28	0.47	Olr210;Olr211	Receptor
DMR1:170820001	1	170820001	170827000	7000	3	2.20E-14	-0.66	46	0.66	Olr212-ps	
DMR1:170830001	1	170830001	170831000	1000	1	5.20E-07	0.5	3	0.3	Olr212-ps;Olr213	Receptor
DMR1:170835001	1	170835001	170836000	1000	1	9.00E-13	0.61	6	0.6	Olr212-ps;Olr213	Receptor
DMR1:170850001	1	170850001	170855000	5000	1	4.60E-08	0.43	24	0.48	Olr213;Olr214	Receptor
DMR1:170870001	1	170870001	170871000	1000	1	4.30E-08	-0.47	5	0.5	Olr214	Receptor
DMR1:170874001	1	170874001	170879000	5000	3	4.60E-10	-0.61	17	0.34	Olr214;Olr215	Receptor
DMR1:170894001	1	170894001	170897000	3000	2	1.80E-09	-0.66	11	0.37	Olr215;Olr216-ps;LOC100911720	Receptor
DMR1:170905001	1	170905001	170912000	7000	3	4.20E-21	-0.61	97	1.39	Olr216-ps;LOC100911720;Olr217	Receptor
DMR1:170936001	1	170936001	170937000	1000	1	8.60E-07	0.47	7	0.7	Olr218;Olr219	Receptor
DMR1:170941001	1	170941001	170944000	3000	1	9.40E-08	-0.44	11	0.37	Olr219	Receptor
DMR1:170957001	1	170957001	170959000	2000	1	1.50E-07	-0.33	19	0.95	Olr220	
DMR1:170960001	1	170960001	170962000	2000	1	3.20E-09	0.51	7	0.35	Olr220	
DMR1:170971001	1	170971001	170973000	2000	1	6.60E-09	-0.44	16	0.8	Olr220	
DMR1:170990001	1	170990001	170997000	7000	4	1.80E-23	-0.51	64	0.91	Olr221	Receptor
DMR1:171000001	1	171000001	171001000	1000	1	3.60E-11	0.77	5	0.5	Olr222	Receptor
DMR1:171005001	1	171005001	171006000	1000	1	3.60E-07	0.47	5	0.5	Olr222	Receptor
DMR1:171014001	1	171014001	171015000	1000	1	2.30E-13	0.55	8	0.8	Olr222	Receptor
DMR1:171042001	1	171042001	171044000	2000	1	5.70E-09	0.6	11	0.55	Olr224	Signaling
DMR1:171116001	1	171116001	171121000	5000	3	2.00E-18	-0.9	16	0.32	Olr228-ps	
DMR1:171124001	1	171124001	171126000	2000	2	5.50E-15	0.71	11	0.55	Olr228-ps;LOC100362585	
DMR1:171140001	1	171140001	171143000	3000	1	4.20E-12	-0.55	19	0.63	Olr228-ps;LOC100362585	
DMR1:171177001	1	171177001	171179000	2000	1	1.10E-22	0.83	14	0.7	Olr230;Olr231	Receptor
DMR1:171193001	1	171193001	171194000	1000	1	2.20E-12	0.6	3	0.3	Olr231;Olr232	Receptor
DMR1:171200001	1	171200001	171201000	1000	1	7.60E-09	0.53	9	0.9	Olr232;Olr233	Receptor
DMR1:171202001	1	171202001	171205000	3000	2	9.40E-17	-0.51	20	0.67	Olr232;Olr233	Receptor
DMR1:171271001	1	171271001	171272000	1000	1	6.60E-16	0.7	10	1	Olr234	Receptor
DMR1:171294001	1	171294001	171299000	5000	4	1.60E-18	-0.62	60	1.2	Nlrp14	
DMR1:171316001	1	171316001	171317000	1000	1	6.70E-08	-0.64	5	0.5	Nlrp14	
DMR1:171318001	1	171318001	171320000	2000	1	2.20E-09	0.53	26	1.3	Nlrp14	
DMR1:171335001	1	171335001	171336000	1000	1	2.70E-10	0.63	16	1.6	Nlrp14	
DMR1:171347001	1	171347001	171348000	1000	1	3.70E-11	0.47	13	1.3	Nlrp14	
DMR1:171408001	1	171408001	171409000	1000	1	3.20E-13	0.72	19	1.9	Rbmxl2	
DMR1:171413001	1	171413001	171415000	2000	2	5.50E-15	0.72	13	0.65	Rbmxl2	
DMR1:171432001	1	171432001	171437000	5000	1	3.30E-16	-0.57	31	0.62	Rbmxl2	
DMR1:171588001	1	171588001	171591000	3000	1	3.20E-07	0.48	32	1.07	LOC100912015;Syt9	Transport
DMR1:171615001	1	171615001	171618000	3000	1	2.00E-07	0.42	23	0.77	Syt9	Transport
DMR1:171620001	1	171620001	171624000	4000	2	3.00E-12	-0.45	32	0.8	Syt9	Transport
DMR1:171630001	1	171630001	171631000	1000	1	5.60E-14	0.66	8	0.8	Syt9	Transport
DMR1:171637001	1	171637001	171638000	1000	1	1.20E-07	-0.47	13	1.3	Syt9	Transport
DMR1:171650001	1	171650001	171652000	2000	1	9.00E-11	0.48	14	0.7	Syt9	Transport
DMR1:171671001	1	171671001	171672000	1000	1	1.80E-09	0.54	12	1.2	Syt9	Transport
DMR1:171685001	1	171685001	171687000	2000	1	5.30E-07	0.33	12	0.6	Syt9	Transport
DMR1:171688001	1	171688001	171689000	1000	1	8.30E-12	0.67	7	0.7	Syt9	Transport
DMR1:171698001	1	171698001	171700000	2000	1	6.80E-08	0.45	21	1.05	Syt9	Transport

DMR1:171705001	1	171705001	171706000	1000	1	6.00E-14	0.48	10	1	Syt9	Transport
DMR1:171722001	1	171722001	171723000	1000	1	7.30E-07	-0.52	12	1.2	Syt9	Transport
DMR1:171735001	1	171735001	171739000	4000	1	1.00E-12	0.54	28	0.7	Syt9	Transport
DMR1:171750001	1	171750001	171756000	6000	3	6.10E-14	0.71	47	0.78	Syt9	Transport
DMR1:171759001	1	171759001	171760000	1000	1	3.10E-12	0.59	7	0.7	Syt9	Transport
DMR1:171763001	1	171763001	171764000	1000	1	1.40E-09	0.33	14	1.4	Syt9	Transport
DMR1:171766001	1	171766001	171767000	1000	1	2.40E-08	0.35	9	0.9	Syt9	Transport
DMR1:171778001	1	171778001	171779000	1000	1	2.20E-07	0.49	9	0.9	Syt9	Transport
DMR1:171784001	1	171784001	171785000	1000	1	1.20E-07	0.42	6	0.6	Olfml1	Development
DMR1:171813001	1	171813001	171814000	1000	1	5.00E-09	0.41	8	0.8	Olfml1;Ppfibp2	Development
DMR1:171844001	1	171844001	171846000	2000	1	1.10E-12	-0.75	21	1.05	Ppfibp2	
DMR1:171861001	1	171861001	171862000	1000	1	1.90E-09	-0.53	21	2.1	Ppfibp2	
DMR1:171870001	1	171870001	171871000	1000	1	1.20E-11	0.64	9	0.9	Ppfibp2	
DMR1:171899001	1	171899001	171900000	1000	1	2.70E-07	0.43	4	0.4	Ppfibp2	
DMR1:171906001	1	171906001	171907000	1000	1	9.90E-07	0.5	3	0.3	Ppfibp2	
DMR1:171915001	1	171915001	171919000	4000	1	1.60E-12	0.49	39	0.98	Ppfibp2	
DMR1:171920001	1	171920001	171921000	1000	1	1.80E-10	-0.75	9	0.9	Ppfibp2	
DMR1:171933001	1	171933001	171937000	4000	1	5.80E-08	-0.33	39	0.98	Ppfibp2	
DMR1:171950001	1	171950001	171951000	1000	1	1.80E-08	0.53	5	0.5	Ppfibp2	
DMR1:171977001	1	171977001	171978000	1000	1	1.00E-14	0.64	5	0.5	Ppfibp2;Cyb5r2	Metabolism
DMR1:172002001	1	172002001	172003000	1000	1	4.70E-07	0.56	3	0.3	LOC499235;Ovch2	Protease
DMR1:172032001	1	172032001	172036000	4000	2	8.40E-14	-0.66	18	0.45	Ovch2	Protease
DMR1:172038001	1	172038001	172040000	2000	2	4.30E-19	-0.73	5	0.25	Ovch2	Protease
DMR1:172063001	1	172063001	172065000	2000	2	8.30E-14	0.66	8	0.4	Olr236-ps;Olr237;Olr238-ps	Signaling
DMR1:172067001	1	172067001	172074000	7000	2	1.00E-10	-0.48	49	0.7	Olr237;Olr238-ps;Olr239	Signaling
DMR1:172076001	1	172076001	172086000	10000	6	1.30E-19	0.55	86	0.86	Olr238-ps;Olr239;Olr240	Signaling
DMR1:172088001	1	172088001	172091000	3000	2	9.60E-11	0.78	17	0.57	Olr240	Signaling
DMR1:172132001	1	172132001	172133000	1000	1	7.80E-11	0.64	6	0.6	Olr241;LOC103691202	Signaling
DMR1:172158001	1	172158001	172161000	3000	1	6.60E-12	0.58	18	0.6	Olr242	Signaling
DMR1:172205001	1	172205001	172206000	1000	1	1.60E-07	0.48	6	0.6	Olr244	Signaling
DMR1:172213001	1	172213001	172215000	2000	1	1.30E-14	0.5	16	0.8	Olr244	Signaling
DMR1:172235001	1	172235001	172236000	1000	1	3.80E-07	0.51	2	0.2	Olr244;Olr245	Signaling
DMR1:172248001	1	172248001	172252000	4000	1	1.40E-08	0.54	26	0.65	Olr244;Olr245	Signaling
DMR1:172274001	1	172274001	172278000	4000	3	6.60E-20	-0.48	42	1.05	Olr246;Olr247	Signaling
DMR1:172329001	1	172329001	172334000	5000	1	1.10E-12	-0.52	40	0.8	RGD1562400	Signaling
DMR1:172352001	1	172352001	172354000	2000	1	3.80E-18	0.71	12	0.6	Olr250	Signaling
DMR1:172374001	1	172374001	172375000	1000	1	4.50E-19	0.86	0	0	Olr251;LOC690646	Signaling
DMR1:172381001	1	172381001	172386000	5000	1	4.80E-07	-0.39	41	0.82	LOC690646;Olr252	Signaling
DMR1:172507001	1	172507001	172511000	4000	2	9.80E-13	0.63	9	0.22	Olr256-ps	
DMR1:172537001	1	172537001	172544000	7000	5	1.30E-22	-0.55	74	1.06	Olr257	Signaling
DMR1:172546001	1	172546001	172547000	1000	1	3.80E-15	0.73	6	0.6	Olr257	Signaling
DMR1:172557001	1	172557001	172558000	1000	1	1.20E-10	0.53	6	0.6	Olr257;Olr258-ps;Olr259	Signaling
DMR1:172560001	1	172560001	172562000	2000	2	9.20E-14	0.45	28	1.4	Olr257;Olr258-ps;Olr259	Signaling
DMR1:172571001	1	172571001	172578000	7000	4	2.10E-12	-0.58	46	0.66	Olr259	Signaling
DMR1:172592001	1	172592001	172593000	1000	1	5.10E-07	-0.43	4	0.4	Olr260	Signaling
DMR1:172626001	1	172626001	172629000	3000	1	1.40E-07	0.49	16	0.53	Olr262;Olr263	Signaling
DMR1:172679001	1	172679001	172684000	5000	3	7.30E-26	-0.52	71	1.42	Olr265-ps	
DMR1:172686001	1	172686001	172688000	2000	2	3.50E-18	0.84	11	0.55	Olr265-ps	
DMR1:172689001	1	172689001	172691000	2000	1	5.00E-10	0.53	10	0.5	Olr265-ps	
DMR1:172707001	1	172707001	172709000	2000	2	1.60E-14	0.64	7	0.35	Olr266-ps;LOC690723	
DMR1:172734001	1	172734001	172741000	7000	3	2.80E-10	-0.49	46	0.66	Olr267	Signaling
DMR1:172745001	1	172745001	172747000	2000	1	2.90E-08	-0.38	9	0.45	Olr268	Signaling
DMR1:172753001	1	172753001	172755000	2000	1	1.70E-10	0.71	9	0.45	Olr268	Signaling
DMR1:172761001	1	172761001	172763000	2000	1	1.50E-07	-0.61	6	0.3	Olr268;Olr269-ps	Signaling
DMR1:172796001	1	172796001	172799000	3000	3	2.40E-09	-0.6	17	0.57	Olr270;Olr271	Signaling
DMR1:172885001	1	172885001	172886000	1000	1	2.30E-07	0.56	1	0.1	Olr1875;Olr276	Signaling
DMR1:172903001	1	172903001	172904000	1000	1	1.10E-07	-0.67	3	0.3	Olr276	Signaling
DMR1:172947001	1	172947001	172950000	3000	2	1.50E-13	-0.45	39	1.3	Olr278	Signaling
DMR1:173113001	1	173113001	173116000	3000	1	5.20E-11	0.51	21	0.7	Olr279	
DMR1:173167001	1	173167001	173172000	5000	2	4.70E-10	-0.42	51	1.02	Olr203	Receptor
DMR1:173175001	1	173175001	173178000	3000	1	9.90E-09	0.43	24	0.8	Olr203	Receptor
DMR1:173183001	1	173183001	173186000	3000	1	7.40E-07	0.36	20	0.67	Olr203;LOC499230	Receptor
DMR1:173335001	1	173335001	173340000	5000	3	1.00E-11	0.54	19	0.38	Gvinp1	
DMR1:173348001	1	173348001	173350000	2000	1	1.70E-08	-0.72	13	0.65	Gvinp1	
DMR1:173379001	1	173379001	173391000	12000	4	2.10E-14	0.72	101	0.84	Olr285	Receptor

DMR1:173414001	1	173414001	173417000	3000	1	3.50E-07	-0.4	26	0.87	Olr284-ps	
DMR1:173418001	1	173418001	173422000	4000	1	1.20E-07	-0.29	44	1.1	Olr284-ps	
DMR1:173432001	1	173432001	173434000	2000	1	4.60E-11	0.49	22	1.1	Olr284-ps;LOC100363312;Olr283	Receptor
DMR1:173435001	1	173435001	173436000	1000	1	2.40E-11	-0.53	13	1.3	LOC100363312;Olr283	Receptor
DMR1:173451001	1	173451001	173454000	3000	1	7.10E-16	0.44	26	0.87	Olr283;Olr282	Receptor
DMR1:173466001	1	173466001	173468000	2000	1	1.80E-16	0.78	18	0.9	Olr282;Olr281	Receptor
DMR1:173475001	1	173475001	173477000	2000	1	8.00E-07	0.56	5	0.25	Olr281	
DMR1:173479001	1	173479001	173481000	2000	1	4.00E-07	0.44	9	0.45	Olr281	
DMR1:173517001	1	173517001	173520000	3000	1	8.40E-10	0.47	10	0.33	Nlrp10	
DMR1:173521001	1	173521001	173522000	1000	1	8.20E-16	0.74	6	0.6	Nlrp10	
DMR1:173569001	1	173569001	173571000	2000	1	2.00E-17	0.48	30	1.5	Tub	
DMR1:173590001	1	173590001	173592000	2000	1	4.10E-11	0.43	34	1.7	Tub	
DMR1:173593001	1	173593001	173594000	1000	1	2.80E-09	0.4	23	2.3	Tub	
DMR1:173595001	1	173595001	173596000	1000	1	2.40E-12	0.55	10	1	Tub	
DMR1:173604001	1	173604001	173606000	2000	1	3.50E-08	0.4	22	1.1	Tub	
DMR1:173609001	1	173609001	173610000	1000	1	1.60E-14	0.81	5	0.5	Tub	
DMR1:173616001	1	173616001	173619000	3000	3	6.50E-19	0.93	43	1.43	Tub	
DMR1:173621001	1	173621001	173623000	2000	1	2.50E-07	0.34	28	1.4	Tub;Ric3	
DMR1:173625001	1	173625001	173626000	1000	1	1.10E-09	0.45	19	1.9	Tub;Ric3	
DMR1:173636001	1	173636001	173638000	2000	1	3.50E-10	-0.6	12	0.6	Tub;Ric3	
DMR1:173645001	1	173645001	173646000	1000	1	1.00E-07	0.35	11	1.1	Ric3;LOC108349532	
DMR1:173648001	1	173648001	173652000	4000	2	1.10E-14	-0.68	37	0.92	Ric3;LOC108349532	
DMR1:173665001	1	173665001	173668000	3000	3	2.20E-18	-0.54	32	1.07	Ric3	
DMR1:173685001	1	173685001	173686000	1000	1	8.10E-09	0.37	10	1	Ric3	
DMR1:173741001	1	173741001	173744000	3000	1	4.80E-09	0.53	48	1.6	Lmo1	
DMR1:173768001	1	173768001	173770000	2000	2	3.10E-08	0.5	16	0.8	Lmo1	
DMR1:173883001	1	173883001	173885000	2000	1	2.90E-11	0.72	19	0.95	Stk33	Signaling
DMR1:173889001	1	173889001	173890000	1000	1	1.20E-15	0.6	4	0.4	Stk33	Signaling
DMR1:173891001	1	173891001	173897000	6000	4	1.30E-12	-0.58	37	0.62	Stk33	Signaling
DMR1:173899001	1	173899001	173900000	1000	1	9.90E-08	0.41	2	0.2	Stk33	Signaling
DMR1:173901001	1	173901001	173902000	1000	1	1.20E-07	0.52	4	0.4	Stk33	Signaling
DMR1:173904001	1	173904001	173908000	4000	2	8.90E-10	0.57	26	0.65	Stk33	Signaling
DMR1:173936001	1	173936001	173937000	1000	1	1.60E-08	0.51	6	0.6	Stk33	Signaling
DMR1:173949001	1	173949001	173950000	1000	1	1.00E-07	0.35	13	1.3	Stk33	Signaling
DMR1:173966001	1	173966001	173969000	3000	1	2.00E-30	1.16	18	0.6	Stk33	Signaling
DMR1:173978001	1	173978001	173979000	1000	1	7.60E-20	1.01	11	1.1	Stk33	Signaling
DMR1:173982001	1	173982001	173984000	2000	1	3.90E-07	-0.45	16	0.8	Stk33	Signaling
DMR1:173990001	1	173990001	173993000	3000	2	8.40E-09	0.46	25	0.83	Stk33	Signaling
DMR1:173994001	1	173994001	173996000	2000	1	2.50E-07	-0.52	9	0.45	Stk33	Signaling
DMR1:173998001	1	173998001	174004000	6000	2	4.30E-15	-0.45	57	0.95	Stk33	Signaling
DMR1:174013001	1	174013001	174016000	3000	1	1.50E-12	0.54	28	0.93	Stk33	Signaling
DMR1:174030001	1	174030001	174031000	1000	1	1.40E-11	0.51	5	0.5	Stk33	Signaling
DMR1:174032001	1	174032001	174035000	3000	1	7.30E-09	0.53	21	0.7	Stk33	Signaling
DMR1:174099001	1	174099001	174102000	3000	2	3.30E-13	0.63	30	1	Trim66	Epigenetic
DMR1:174106001	1	174106001	174108000	2000	1	2.00E-13	0.49	43	2.15	Trim66	Epigenetic
DMR1:174115001	1	174115001	174117000	2000	1	4.60E-15	0.68	16	0.8	Trim66	Epigenetic
DMR1:174123001	1	174123001	174126000	3000	1	1.90E-09	-0.44	32	1.07	Trim66;Rpl27a	Epigenetic;Translation
DMR1:174127001	1	174127001	174131000	4000	1	3.70E-07	0.36	51	1.27	Trim66;Rpl27a;St5	Epigenetic;Translation
DMR1:174171001	1	174171001	174172000	1000	1	7.00E-08	-0.6	9	0.9	St5	
DMR1:174190001	1	174190001	174196000	6000	1	3.70E-08	0.37	70	1.17	St5	
DMR1:174198001	1	174198001	174200000	2000	1	4.20E-08	0.38	17	0.85	St5	
DMR1:174208001	1	174208001	174209000	1000	1	3.50E-19	0.67	5	0.5	St5	
DMR1:174222001	1	174222001	174225000	3000	3	9.70E-12	0.54	16	0.53	St5	
DMR1:174294001	1	174294001	174298000	4000	2	1.10E-16	-0.5	47	1.18	St5	
DMR1:174321001	1	174321001	174327000	6000	3	2.10E-13	-0.48	43	0.72	Akip1	
DMR1:174337001	1	174337001	174338000	1000	1	1.20E-13	0.83	0	0	Akip1;LOC499240	
DMR1:174353001	1	174353001	174356000	3000	1	7.50E-20	0.63	51	1.7	LOC499240;Ascl3;Tmem9b	
DMR1:174365001	1	174365001	174367000	2000	2	2.00E-16	0.92	15	0.75	Ascl3;Tmem9b	
DMR1:174405001	1	174405001	174408000	3000	3	5.20E-11	0.49	16	0.53	Nrip3	Proteolysis
DMR1:174414001	1	174414001	174415000	1000	1	3.10E-11	0.39	18	1.8	Nrip3	Proteolysis
DMR1:174427001	1	174427001	174432000	5000	1	2.00E-18	0.56	78	1.56	Scube2	Extracellular Matrix
DMR1:174445001	1	174445001	174447000	2000	1	7.80E-08	0.51	7	0.35	Scube2	Extracellular Matrix

DMR1:174450001	1	174450001	174454000	4000	1	1.00E-10	0.47	50	1.25	Scube2	Extracellular Matrix
DMR1:174463001	1	174463001	174465000	2000	2	3.90E-12	0.62	13	0.65	Scube2	Extracellular Matrix
DMR1:174471001	1	174471001	174472000	1000	1	8.90E-08	0.29	16	1.6	Scube2	Extracellular Matrix
DMR1:174487001	1	174487001	174493000	6000	1	1.50E-07	0.38	70	1.17	Scube2	Extracellular Matrix
DMR1:174501001	1	174501001	174503000	2000	1	1.40E-07	0.43	31	1.55	Scube2	Extracellular Matrix
DMR1:174532001	1	174532001	174533000	1000	1	1.20E-10	0.44	10	1	Dennd5a;LOC108349590	
DMR1:174536001	1	174536001	174539000	3000	2	6.60E-10	0.48	20	0.67	Dennd5a;LOC108349590	
DMR1:174601001	1	174601001	174602000	1000	1	9.20E-08	0.32	35	3.5	Tmem41b	
DMR1:174603001	1	174603001	174604000	1000	1	6.50E-10	0.45	7	0.7	Tmem41b	
DMR1:174739001	1	174739001	174741000	2000	1	6.90E-08	0.5	33	1.65	Zfp143	
DMR1:174746001	1	174746001	174749000	3000	1	3.70E-07	0.41	33	1.1	Zfp143	
DMR1:174763001	1	174763001	174764000	1000	1	4.40E-08	0.49	12	1.2	Wee1	Signaling
DMR1:174779001	1	174779001	174782000	3000	1	1.30E-10	0.62	22	0.73	Wee1	Signaling
DMR1:174797001	1	174797001	174798000	1000	1	9.40E-17	0.62	6	0.6	Wee1	Signaling
DMR1:174800001	1	174800001	174802000	2000	1	3.40E-10	0.56	21	1.05	Wee1	Signaling
DMR1:174854001	1	174854001	174856000	2000	1	4.10E-07	-0.36	25	1.25	Swap70	Cytoskeleton
DMR1:174869001	1	174869001	174872000	3000	2	5.20E-23	0.85	23	0.77	Swap70	Cytoskeleton
DMR1:174892001	1	174892001	174893000	1000	1	1.70E-07	-0.58	8	0.8	Swap70	Cytoskeleton
DMR1:174896001	1	174896001	174899000	3000	1	1.10E-07	0.47	21	0.7	Swap70	Cytoskeleton
DMR1:174913001	1	174913001	174918000	5000	1	2.30E-08	-0.35	75	1.5	Swap70	Cytoskeleton
DMR1:174929001	1	174929001	174934000	5000	1	4.10E-16	0.58	54	1.08	Swap70	Cytoskeleton
DMR1:174942001	1	174942001	174943000	1000	1	1.30E-08	0.43	17	1.7	Sbf2	Signaling
DMR1:174953001	1	174953001	174955000	2000	1	4.60E-07	-0.72	22	1.1	Sbf2	Signaling
DMR1:174963001	1	174963001	174965000	2000	1	5.50E-12	0.43	25	1.25	Sbf2	Signaling
DMR1:174979001	1	174979001	174981000	2000	1	2.00E-11	0.61	12	0.6	Sbf2	Signaling
DMR1:174986001	1	174986001	174988000	2000	1	1.70E-14	0.47	28	1.4	Sbf2	Signaling
DMR1:174999001	1	174999001	175002000	3000	2	1.40E-07	-0.57	10	0.33	Sbf2	Signaling
DMR1:175053001	1	175053001	175054000	1000	1	3.90E-13	0.57	13	1.3	Sbf2	Signaling
DMR1:175106001	1	175106001	175113000	7000	5	1.60E-14	0.47	81	1.16	Sbf2	Signaling
DMR1:175129001	1	175129001	175134000	5000	3	1.80E-15	-0.54	57	1.14	Sbf2	Signaling
DMR1:175143001	1	175143001	175145000	2000	2	3.50E-16	0.52	30	1.5	Sbf2	Signaling
DMR1:175154001	1	175154001	175158000	4000	2	1.80E-14	0.62	46	1.15	Sbf2	Signaling
DMR1:175184001	1	175184001	175186000	2000	1	2.50E-14	0.59	9	0.45	Sbf2;LOC108349611	Signaling
DMR1:175190001	1	175190001	175192000	2000	1	1.60E-11	0.69	11	0.55	Sbf2;LOC108349611	Signaling
DMR1:175199001	1	175199001	175200000	1000	1	3.10E-11	0.64	5	0.5	Sbf2	Signaling
DMR1:175203001	1	175203001	175207000	4000	2	1.50E-12	0.61	28	0.7	Sbf2	Signaling
DMR1:175220001	1	175220001	175223000	3000	1	2.40E-12	0.63	19	0.63	Sbf2	Signaling
DMR1:175245001	1	175245001	175248000	3000	1	1.90E-09	0.6	12	0.4	Sbf2	Signaling
DMR1:175258001	1	175258001	175260000	2000	1	1.50E-10	0.61	7	0.35	Sbf2	Signaling
DMR1:175270001	1	175270001	175274000	4000	2	5.80E-10	-0.48	25	0.62	Sbf2	Signaling
DMR1:175278001	1	175278001	175283000	5000	3	1.80E-12	0.76	35	0.7	Sbf2	Signaling
DMR1:175284001	1	175284001	175286000	2000	2	8.00E-27	0.74	6	0.3	Sbf2	Signaling
DMR1:175287001	1	175287001	175295000	8000	6	3.40E-23	-0.54	103	1.29	Sbf2	Signaling
DMR1:175300001	1	175300001	175302000	2000	1	1.20E-15	0.8	6	0.3	Sbf2	Signaling
DMR1:175309001	1	175309001	175310000	1000	1	3.90E-11	-0.61	5	0.5	Sbf2	Signaling
DMR1:175372001	1	175372001	175375000	3000	1	6.60E-07	-0.39	52	1.73	Sbf2	Signaling
DMR1:175390001	1	175390001	175393000	3000	1	4.40E-10	-0.54	30	1	Sbf2	Signaling
DMR1:175403001	1	175403001	175404000	1000	1	7.00E-08	0.63	4	0.4	Sbf2	Signaling
DMR1:175430001	1	175430001	175435000	5000	4	3.80E-12	-0.76	28	0.56	Sbf2;Adm	Signaling;Hormone
DMR1:175436001	1	175436001	175438000	2000	1	6.50E-13	0.58	20	1	Adm	Hormone
DMR1:175441001	1	175441001	175443000	2000	2	6.30E-13	0.54	19	0.95	Adm	Hormone
DMR1:175575001	1	175575001	175578000	3000	1	6.10E-12	0.55	35	1.17	LOC103691204;Ampd3	Metabolism
DMR1:175615001	1	175615001	175616000	1000	1	1.10E-10	0.45	11	1.1	Ampd3	Metabolism
DMR1:175622001	1	175622001	175625000	3000	1	7.30E-18	0.63	60	2	Ampd3;rnf141	Metabolism
DMR1:175641001	1	175641001	175643000	2000	1	2.00E-17	0.6	18	0.9	rnf141	
DMR1:175666001	1	175666001	175669000	3000	1	9.60E-16	0.67	40	1.33	rnf141;Lyve1	Receptor
DMR1:175692001	1	175692001	175693000	1000	1	3.60E-09	0.48	10	1	Mrvi1	Cytoskeleton
DMR1:175710001	1	175710001	175713000	3000	2	2.10E-16	0.69	28	0.93	Mrvi1;LOC103691205	Cytoskeleton
DMR1:175723001	1	175723001	175726000	3000	1	3.30E-07	0.45	29	0.97	Mrvi1;LOC103691205	Cytoskeleton
DMR1:175735001	1	175735001	175736000	1000	1	1.50E-09	0.42	10	1	Mrvi1;LOC103691205	Cytoskeleton
DMR1:175747001	1	175747001	175749000	2000	1	1.70E-21	0.85	22	1.1	Mrvi1	Cytoskeleton
DMR1:175751001	1	175751001	175754000	3000	1	5.70E-13	0.7	20	0.67	Mrvi1	Cytoskeleton
DMR1:175769001	1	175769001	175771000	2000	1	3.20E-10	0.33	30	1.5	Mrvi1	Cytoskeleton
DMR1:175781001	1	175781001	175783000	2000	1	4.70E-07	0.35	34	1.7	Mrvi1	Cytoskeleton
DMR1:175795001	1	175795001	175797000	2000	1	6.50E-08	0.41	27	1.35	Mrvi1	Cytoskeleton

DMR1:175811001	1	175811001	175813000	2000	1	3.60E-08	0.42	24	1.2	Mrvi1	Cytoskeleton
DMR1:175830001	1	175830001	175832000	2000	1	8.70E-12	-0.84	28	1.4	Ctr9	Transcription
DMR1:175846001	1	175846001	175847000	1000	1	6.30E-10	0.47	9	0.9	Ctr9	Transcription
DMR1:175849001	1	175849001	175850000	1000	1	1.10E-10	0.5	8	0.8	Ctr9	Transcription
DMR1:175860001	1	175860001	175862000	2000	1	1.00E-10	-0.77	23	1.15	Ctr9;LOC103691206	Transcription
DMR1:175887001	1	175887001	175893000	6000	2	7.60E-10	-0.57	61	1.02	Eif4g2	Translation
DMR1:176068001	1	176068001	176070000	2000	1	8.80E-09	0.41	22	1.1	RGD1566189	
DMR1:176315001	1	176315001	176316000	1000	1	1.50E-07	0.59	5	0.5	Galnt18	Golgi
DMR1:176343001	1	176343001	176344000	1000	1	7.00E-07	0.34	9	0.9	Galnt18	Golgi
DMR1:176351001	1	176351001	176352000	1000	1	2.80E-11	0.62	2	0.2	Galnt18	Golgi
DMR1:176420001	1	176420001	176421000	1000	1	2.00E-08	0.45	10	1	Galnt18	Golgi
DMR1:176427001	1	176427001	176436000	9000	5	6.50E-25	-0.56	121	1.34	Galnt18	Golgi
DMR1:176509001	1	176509001	176511000	2000	1	8.60E-13	0.74	9	0.45	Galnt18	Golgi
DMR1:176530001	1	176530001	176532000	2000	1	9.80E-09	0.57	22	1.1	Galnt18	Golgi
DMR1:176533001	1	176533001	176535000	2000	1	9.60E-09	0.47	22	1.1	Galnt18	Golgi
DMR1:176549001	1	176549001	176550000	1000	1	3.40E-09	0.58	9	0.9	Galnt18	Golgi
DMR1:176571001	1	176571001	176574000	3000	1	3.10E-17	0.92	41	1.37	Galnt18	Golgi
DMR1:176576001	1	176576001	176583000	7000	1	7.50E-08	0.41	62	0.89	Galnt18	Golgi
DMR1:176612001	1	176612001	176615000	3000	2	8.90E-17	0.65	51	1.7	Galnt18	Golgi
DMR1:176849001	1	176849001	176850000	1000	1	4.10E-08	0.4	9	0.9	Usp47	Protease
DMR1:176851001	1	176851001	176852000	1000	1	3.80E-08	0.39	10	1	Usp47	Protease
DMR1:176911001	1	176911001	176912000	1000	1	1.10E-09	0.49	6	0.6	Usp47	Protease
DMR1:176954001	1	176954001	176955000	1000	1	1.90E-14	0.56	13	1.3	Dkk3	
DMR1:176991001	1	176991001	176995000	4000	2	1.30E-16	0.59	55	1.38	Dkk3	
DMR1:177055001	1	177055001	177057000	2000	2	3.30E-09	-0.62	23	1.15	Mical2	
DMR1:177061001	1	177061001	177066000	5000	2	4.10E-11	0.46	54	1.08	Mical2	
DMR1:177077001	1	177077001	177078000	1000	1	8.10E-07	0.48	3	0.3	Mical2	
DMR1:177080001	1	177080001	177082000	2000	2	2.90E-19	0.68	22	1.1	Mical2	
DMR1:177100001	1	177100001	177102000	2000	1	4.60E-08	0.38	30	1.5	Mical2	
DMR1:177105001	1	177105001	177106000	1000	1	2.60E-14	0.62	11	1.1	Mical2	
DMR1:177109001	1	177109001	177111000	2000	1	4.30E-07	0.43	20	1	Mical2	
DMR1:177121001	1	177121001	177132000	11000	1	2.40E-07	0.31	123	1.12	Mical2	
DMR1:177147001	1	177147001	177150000	3000	1	6.50E-17	0.9	38	1.27	Mical2	
DMR1:177152001	1	177152001	177154000	2000	1	5.40E-07	0.34	20	1	Mical2	
DMR1:177158001	1	177158001	177159000	1000	1	2.40E-16	0.59	19	1.9	Mical2	
DMR1:177180001	1	177180001	177183000	3000	1	4.70E-07	0.33	35	1.17	Mical2;Micalcl	
DMR1:177223001	1	177223001	177226000	3000	1	2.00E-09	0.42	35	1.17	Micalcl	
DMR1:177244001	1	177244001	177247000	3000	1	7.40E-13	0.52	27	0.9	LOC108349616;Parva	Cytoskeleton
DMR1:177253001	1	177253001	177255000	2000	1	2.10E-24	0.67	20	1	LOC108349616;Parva	Cytoskeleton
DMR1:177256001	1	177256001	177261000	5000	1	2.90E-12	0.56	45	0.9	LOC108349616;Parva	Cytoskeleton
DMR1:177262001	1	177262001	177264000	2000	1	3.30E-08	0.56	28	1.4	Parva	Cytoskeleton
DMR1:177291001	1	177291001	177295000	4000	2	7.20E-16	0.67	45	1.12	Parva	Cytoskeleton
DMR1:177296001	1	177296001	177298000	2000	1	1.00E-10	0.51	15	0.75	Parva	Cytoskeleton
DMR1:177301001	1	177301001	177302000	1000	1	1.70E-07	0.29	12	1.2	Parva	Cytoskeleton
DMR1:177313001	1	177313001	177318000	5000	2	3.30E-12	-0.71	66	1.32	Parva	Cytoskeleton
DMR1:177321001	1	177321001	177323000	2000	1	1.40E-10	0.38	14	0.7	Parva	Cytoskeleton
DMR1:177326001	1	177326001	177328000	2000	1	2.90E-12	0.48	35	1.75	Parva	Cytoskeleton
DMR1:177374001	1	177374001	177376000	2000	1	5.20E-12	0.73	15	0.75	Parva	Cytoskeleton
DMR1:177379001	1	177379001	177381000	2000	1	1.70E-12	0.59	38	1.9	Parva	Cytoskeleton
DMR1:177385001	1	177385001	177387000	2000	1	2.40E-07	0.31	27	1.35	Parva	Cytoskeleton
DMR1:177389001	1	177389001	177390000	1000	1	5.10E-08	0.39	20	2	Parva	Cytoskeleton
DMR1:177392001	1	177392001	177394000	2000	1	2.70E-22	0.77	28	1.4	Parva	Cytoskeleton
DMR1:177415001	1	177415001	177416000	1000	1	1.80E-20	0.81	11	1.1	Parva	Cytoskeleton
DMR1:177489001	1	177489001	177490000	1000	1	2.50E-10	0.58	8	0.8	Tead1	Transcription
DMR1:177498001	1	177498001	177501000	3000	1	5.00E-07	-0.52	41	1.37	Tead1	Transcription
DMR1:177509001	1	177509001	177511000	2000	2	8.30E-19	0.62	32	1.6	Tead1	Transcription
DMR1:177524001	1	177524001	177529000	5000	1	6.00E-11	-0.6	50	1	Tead1	Transcription
DMR1:177538001	1	177538001	177541000	3000	1	2.40E-19	0.68	29	0.97	Tead1	Transcription
DMR1:177544001	1	177544001	177547000	3000	1	1.50E-10	0.41	42	1.4	Tead1	Transcription
DMR1:177555001	1	177555001	177558000	3000	1	2.00E-14	0.78	30	1	Tead1	Transcription
DMR1:177575001	1	177575001	177577000	2000	1	5.30E-07	0.35	17	0.85	Tead1	Transcription
DMR1:177578001	1	177578001	177582000	4000	1	4.20E-07	-0.51	37	0.92	Tead1;LOC108349617	Transcription
DMR1:177615001	1	177615001	177616000	1000	1	3.60E-10	-0.49	12	1.2	Tead1	Transcription
DMR1:177634001	1	177634001	177643000	9000	2	5.40E-08	-0.59	134	1.49	Tead1	Transcription
DMR1:177648001	1	177648001	177654000	6000	1	3.90E-09	0.4	81	1.35	Tead1	Transcription

DMR1:177656001	1	177656001	177657000	1000	1	3.30E-15	0.65	5	0.5	Tead1	Transcription
DMR1:177664001	1	177664001	177665000	1000	1	3.50E-08	0.34	14	1.4	Tead1	Transcription
DMR1:177672001	1	177672001	177675000	3000	1	8.10E-15	0.64	39	1.3	Tead1	Transcription
DMR1:177710001	1	177710001	177711000	1000	1	1.10E-08	0.44	13	1.3	Tead1	Transcription
DMR1:177723001	1	177723001	177725000	2000	1	1.50E-22	0.86	27	1.35	Tead1	Transcription
DMR1:178033001	1	178033001	178038000	5000	1	4.10E-11	0.45	66	1.32	Arntl	Transcription
DMR1:178042001	1	178042001	178043000	1000	1	3.80E-11	0.52	10	1	Arntl	Transcription
DMR1:178044001	1	178044001	178049000	5000	1	2.90E-08	0.43	58	1.16	Arntl	Transcription
DMR1:178057001	1	178057001	178061000	4000	1	3.30E-13	0.49	103	2.58	Arntl	Transcription
DMR1:178067001	1	178067001	178070000	3000	1	9.90E-13	0.42	55	1.83	Arntl	Transcription
DMR1:178086001	1	178086001	178089000	3000	2	4.80E-09	0.54	28	0.93	Arntl	Transcription
DMR1:178100001	1	178100001	178101000	1000	1	7.80E-22	0.52	7	0.7	Arntl	Transcription
DMR1:178122001	1	178122001	178127000	5000	1	8.10E-07	0.39	62	1.24	Arntl	Transcription
DMR1:178206001	1	178206001	178211000	5000	3	2.80E-15	-0.7	17	0.34	Btbd10;Pth	Hormone
DMR1:178213001	1	178213001	178214000	1000	1	1.80E-07	0.62	4	0.4	Pth	Hormone
DMR1:178221001	1	178221001	178227000	6000	1	5.70E-07	0.44	51	0.85	Pth	Hormone
DMR1:178354001	1	178354001	178355000	1000	1	2.10E-08	0.57	6	0.6	Far1	
DMR1:178406001	1	178406001	178408000	2000	2	1.50E-08	0.52	16	0.8	Far1	
DMR1:178422001	1	178422001	178423000	1000	1	9.90E-07	-0.49	4	0.4	Far1;LOC102548137	
DMR1:178569001	1	178569001	178573000	4000	1	4.60E-09	-0.58	44	1.1	Spon1	Cytoskeleton
DMR1:178607001	1	178607001	178608000	1000	1	1.30E-13	0.59	19	1.9	Spon1;LOC102548271	Cytoskeleton
DMR1:178610001	1	178610001	178616000	6000	1	5.30E-15	0.49	80	1.33	Spon1;LOC102548271	Cytoskeleton
DMR1:178627001	1	178627001	178632000	5000	2	4.60E-16	0.6	60	1.2	Spon1	Cytoskeleton
DMR1:178647001	1	178647001	178648000	1000	1	1.10E-15	0.65	10	1	Spon1	Cytoskeleton
DMR1:178660001	1	178660001	178661000	1000	1	3.50E-28	0.77	16	1.6	Spon1	Cytoskeleton
DMR1:178666001	1	178666001	178667000	1000	1	2.00E-09	0.66	4	0.4	Spon1	Cytoskeleton
DMR1:178680001	1	178680001	178688000	8000	4	4.10E-21	-0.59	94	1.18	Spon1	Cytoskeleton
DMR1:178704001	1	178704001	178706000	2000	2	7.20E-11	0.46	23	1.15	Spon1	Cytoskeleton
DMR1:178721001	1	178721001	178724000	3000	2	2.40E-16	0.61	27	0.9	Spon1	Cytoskeleton
DMR1:178777001	1	178777001	178778000	1000	1	1.10E-14	0.61	14	1.4	Spon1	Cytoskeleton
DMR1:178781001	1	178781001	178782000	1000	1	1.90E-08	-0.73	12	1.2	Spon1	Cytoskeleton
DMR1:178786001	1	178786001	178791000	5000	1	7.10E-10	-0.49	61	1.22	Spon1	Cytoskeleton
DMR1:178800001	1	178800001	178803000	3000	1	1.40E-10	0.64	38	1.27	Spon1	Cytoskeleton
DMR1:178811001	1	178811001	178815000	4000	1	4.40E-15	0.73	50	1.25	Spon1	Cytoskeleton
DMR1:178816001	1	178816001	178821000	5000	2	5.00E-15	-0.56	77	1.54	Spon1	Cytoskeleton
DMR1:178841001	1	178841001	178843000	2000	1	4.30E-10	0.44	28	1.4	Spon1	Cytoskeleton
DMR1:178844001	1	178844001	178848000	4000	1	1.70E-09	0.38	51	1.27	Spon1;LOC108349620	Cytoskeleton
DMR1:178854001	1	178854001	178857000	3000	2	3.80E-17	0.63	47	1.57	Spon1;LOC108349620	Cytoskeleton
DMR1:178864001	1	178864001	178865000	1000	1	4.30E-23	0.87	8	0.8	Spon1;LOC108349620	Cytoskeleton
DMR1:178868001	1	178868001	178869000	1000	1	7.00E-14	0.67	11	1.1	Spon1	Cytoskeleton
DMR1:178887001	1	178887001	178892000	5000	4	1.00E-26	-0.64	39	0.78	Spon1	Cytoskeleton
DMR1:178912001	1	178912001	178914000	2000	1	2.40E-08	0.59	4	0.2	Spon1	Cytoskeleton
DMR1:178915001	1	178915001	178917000	2000	1	1.20E-08	0.36	23	1.15	Spon1	Cytoskeleton
DMR1:178918001	1	178918001	178923000	5000	1	6.10E-15	0.52	64	1.28	Spon1	Cytoskeleton
DMR1:178935001	1	178935001	178937000	2000	1	1.20E-07	-0.44	32	1.6	Spon1;Rras2	Cytoskeleton;Signaling
DMR1:178941001	1	178941001	178952000	11000	3	1.70E-10	-0.47	152	1.38	Spon1;Rras2	Cytoskeleton;Signaling
DMR1:178965001	1	178965001	178967000	2000	1	5.60E-10	0.36	20	1	Rras2	Signaling
DMR1:178971001	1	178971001	178974000	3000	3	3.10E-24	0.7	28	0.93	Rras2	Signaling
DMR1:178978001	1	178978001	178981000	3000	1	1.60E-08	0.48	41	1.37	Rras2	Signaling
DMR1:179007001	1	179007001	179008000	1000	1	1.20E-07	0.3	11	1.1	Rras2	Signaling
DMR1:179018001	1	179018001	179024000	6000	2	1.30E-10	0.52	79	1.32	Rras2	Signaling
DMR1:182084001	1	182084001	182085000	1000	1	3.60E-09	0.58	3	0.3	Ythdc2	Transcription
DMR1:182111001	1	182111001	182112000	1000	1	6.10E-10	0.46	7	0.7	Ythdc2	Transcription
DMR1:182119001	1	182119001	182121000	2000	1	1.10E-09	0.53	18	0.9	Ythdc2	Transcription
DMR1:182123001	1	182123001	182124000	1000	1	2.00E-08	0.52	3	0.3	Ythdc2	Transcription
DMR1:182833001	1	182833001	182836000	3000	1	7.00E-09	-0.78	19	0.63	Ssty1	
DMR1:182840001	1	182840001	182844000	4000	1	2.00E-24	0.59	63	1.57	Ssty1	
DMR1:182845001	1	182845001	182854000	9000	6	9.90E-15	0.76	101	1.12	Ssty1	
DMR1:183672001	1	183672001	183676000	4000	1	5.60E-10	0.45	52	1.3	RGD1565301	
DMR1:183678001	1	183678001	183679000	1000	1	1.10E-19	0.86	7	0.7	RGD1565301	
DMR1:183684001	1	183684001	183685000	1000	1	9.90E-23	0.87	13	1.3	RGD1565301	
DMR1:183724001	1	183724001	183725000	1000	1	2.80E-07	-0.38	13	1.3	Copb1;LOC102548665;Psma1	Transport;Protease
DMR1:183728001	1	183728001	183729000	1000	1	2.70E-10	0.64	12	1.2	Copb1;LOC102548665;Psma1	Transport;Protease

DMR1:183751001	1	183751001	183753000	2000	1	1.50E-09	0.4	28	1.4	Psma1	Protease
DMR1:183881001	1	183881001	183885000	4000	1	2.40E-14	0.62	27	0.68	Pde3b	Signaling
DMR1:183888001	1	183888001	183889000	1000	1	4.10E-10	0.52	6	0.6	Pde3b	Signaling
DMR1:183908001	1	183908001	183909000	1000	1	4.00E-08	-0.6	7	0.7	Pde3b;LOC102548890	Signaling
DMR1:183935001	1	183935001	183940000	5000	3	1.20E-17	0.69	53	1.06	Pde3b	Signaling
DMR1:183942001	1	183942001	183944000	2000	1	6.80E-07	0.34	11	0.55	Pde3b	Signaling
DMR1:183947001	1	183947001	183948000	1000	1	5.30E-15	0.77	9	0.9	Pde3b	Signaling
DMR1:183962001	1	183962001	183963000	1000	1	2.00E-13	0.59	3	0.3	Pde3b	Signaling
DMR1:183981001	1	183981001	183982000	1000	1	3.30E-11	0.52	5	0.5	Pde3b	Signaling
DMR1:183996001	1	183996001	183997000	1000	1	1.40E-08	-0.75	11	1.1	Pde3b	Signaling
DMR1:184015001	1	184015001	184016000	1000	1	4.60E-09	0.57	5	0.5	Pde3b	Signaling
DMR1:184018001	1	184018001	184022000	4000	3	1.70E-13	-0.54	34	0.85	Pde3b	Signaling
DMR1:184025001	1	184025001	184031000	6000	3	3.60E-20	-0.5	83	1.38	Pde3b	Signaling
DMR1:184034001	1	184034001	184039000	5000	1	1.00E-15	-0.59	60	1.2	Pde3b	Signaling
DMR1:184077001	1	184077001	184078000	1000	1	7.40E-12	0.44	9	0.9	Pde3b;Cyp2r1	Signaling;Metabolism
DMR1:184098001	1	184098001	184100000	2000	2	8.60E-09	-0.72	17	0.85	Cyp2r1	Metabolism
DMR1:184174001	1	184174001	184177000	3000	1	1.10E-16	-0.64	8	0.27	Calca	Hormone
DMR1:184196001	1	184196001	184197000	1000	1	6.30E-07	-0.6	8	0.8	Calca;LOC108349622	Hormone
DMR1:184198001	1	184198001	184199000	1000	1	5.40E-13	0.53	13	1.3	Calca;LOC108349622	Hormone
DMR1:184294001	1	184294001	184296000	2000	2	2.50E-18	0.96	9	0.45	Insc	
DMR1:184319001	1	184319001	184320000	1000	1	7.50E-08	-0.67	10	1	Insc	
DMR1:184324001	1	184324001	184328000	4000	1	4.80E-09	0.39	35	0.88	Insc	
DMR1:184331001	1	184331001	184334000	3000	2	4.70E-07	0.46	28	0.93	Insc	
DMR1:184336001	1	184336001	184337000	1000	1	2.00E-08	0.49	7	0.7	Insc	
DMR1:184370001	1	184370001	184372000	2000	1	6.50E-10	0.55	11	0.55	Insc	
DMR1:184412001	1	184412001	184413000	1000	1	2.10E-14	0.71	10	1	Insc	
DMR1:184416001	1	184416001	184419000	3000	1	1.60E-09	0.66	16	0.53	Insc	
DMR1:185111001	1	185111001	185112000	1000	1	1.10E-08	0.46	6	0.6	Nucb2	Signaling
DMR1:185124001	1	185124001	185126000	2000	2	2.40E-13	-0.66	26	1.3	Nucb2	Signaling
DMR1:185128001	1	185128001	185130000	2000	1	3.20E-12	0.49	20	1	Nucb2	Signaling
DMR1:185139001	1	185139001	185140000	1000	1	6.40E-11	0.66	9	0.9	Nucb2;LOC108349624	Signaling
DMR1:185151001	1	185151001	185154000	3000	1	8.00E-09	0.41	30	1	Nucb2;LOC108349624	Signaling
DMR1:185201001	1	185201001	185203000	2000	1	1.50E-10	0.42	14	0.7	Pik3c2a	Signaling
DMR1:185321001	1	185321001	185322000	1000	1	3.60E-07	-0.59	8	0.8	Pik3c2a;Rps13	Signaling;Translation
DMR1:185437001	1	185437001	185441000	4000	1	4.50E-08	-0.42	49	1.23	Plekha7	
DMR1:185467001	1	185467001	185468000	1000	1	1.20E-07	-0.55	10	1	Plekha7	
DMR1:185495001	1	185495001	185497000	2000	1	1.70E-10	0.47	31	1.55	Plekha7	
DMR1:185552001	1	185552001	185554000	2000	1	4.50E-21	0.88	21	1.05	RGD1311703	
DMR1:185575001	1	185575001	185576000	1000	1	9.50E-08	-0.58	9	0.9	RGD1311703;LOC102549121	
DMR1:185629001	1	185629001	185631000	2000	1	2.10E-22	0.96	29	1.45	Sox6	
DMR1:185632001	1	185632001	185634000	2000	2	9.90E-10	0.76	74	3.7	Sox6	
DMR1:185685001	1	185685001	185686000	1000	1	2.30E-10	0.56	6	0.6	Sox6	
DMR1:185697001	1	185697001	185700000	3000	1	6.00E-18	0.7	21	0.7	Sox6	
DMR1:185736001	1	185736001	185737000	1000	1	1.80E-35	1.19	1	0.1	Sox6	
DMR1:185741001	1	185741001	185743000	2000	1	1.80E-10	0.53	20	1	Sox6	
DMR1:185744001	1	185744001	185746000	2000	1	9.80E-09	0.42	11	0.55	Sox6	
DMR1:185755001	1	185755001	185760000	5000	3	7.10E-12	-0.44	43	0.86	Sox6	
DMR1:185765001	1	185765001	185766000	1000	1	9.20E-07	-0.43	9	0.9	Sox6	
DMR1:185772001	1	185772001	185774000	2000	1	2.20E-09	0.53	65	3.25	Sox6	
DMR1:185793001	1	185793001	185801000	8000	4	1.80E-13	0.6	61	0.76	Sox6	
DMR1:185813001	1	185813001	185815000	2000	1	9.00E-10	0.53	18	0.9	Sox6	
DMR1:185819001	1	185819001	185821000	2000	1	2.70E-07	0.33	14	0.7	Sox6	
DMR1:185849001	1	185849001	185850000	1000	1	1.60E-12	0.8	7	0.7	Sox6	
DMR1:185915001	1	185915001	185917000	2000	2	1.20E-12	0.49	9	0.45	Sox6	
DMR1:185926001	1	185926001	185927000	1000	1	1.90E-20	0.79	6	0.6	Sox6	
DMR1:185940001	1	185940001	185941000	1000	1	9.80E-14	0.54	8	0.8	Sox6	
DMR1:185954001	1	185954001	185956000	2000	1	4.50E-11	0.74	3	0.15	Sox6	
DMR1:185977001	1	185977001	185978000	1000	1	7.70E-07	-0.56	11	1.1	Sox6	
DMR1:186000001	1	186000001	186001000	1000	1	4.80E-09	0.55	11	1.1	Sox6	
DMR1:186026001	1	186026001	186030000	4000	1	3.30E-10	0.52	29	0.72	Sox6	
DMR1:186105001	1	186105001	186107000	2000	1	4.20E-09	-0.55	17	0.85	Sox6	
DMR1:186152001	1	186152001	186153000	1000	1	1.90E-08	0.64	2	0.2	Sox6	
DMR1:186162001	1	186162001	186163000	1000	1	4.20E-07	-0.47	10	1	Sox6	
DMR1:186185001	1	186185001	186186000	1000	1	1.80E-09	-0.66	13	1.3	Sox6	

DMR1:186189001	1	186189001	186190000	1000	1	1.20E-08	0.51	4	0.4	Sox6	
DMR1:186933001	1	186933001	186935000	2000	1	9.10E-13	0.59	19	0.95	Xylt1	Transport
DMR1:186943001	1	186943001	186945000	2000	2	1.60E-12	0.66	17	0.85	Xylt1	Transport
DMR1:186953001	1	186953001	186955000	2000	1	1.20E-11	0.54	8	0.4	Xylt1	Transport
DMR1:186965001	1	186965001	186966000	1000	1	5.30E-10	0.63	10	1	Xylt1	Transport
DMR1:186967001	1	186967001	186971000	4000	3	4.90E-12	0.52	42	1.05	Xylt1	Transport
DMR1:186982001	1	186982001	186983000	1000	1	7.90E-15	0.57	6	0.6	Xylt1	Transport
DMR1:186987001	1	186987001	186992000	5000	1	3.20E-13	0.74	48	0.96	Xylt1	Transport
DMR1:186997001	1	186997001	186998000	1000	1	1.30E-15	0.64	3	0.3	Xylt1	Transport
DMR1:187008001	1	187008001	187011000	3000	1	1.50E-20	0.94	40	1.33	Xylt1	Transport
DMR1:187027001	1	187027001	187029000	2000	1	2.00E-09	-0.55	24	1.2	Xylt1	Transport
DMR1:187030001	1	187030001	187032000	2000	1	3.20E-08	-0.65	38	1.9	Xylt1	Transport
DMR1:187042001	1	187042001	187043000	1000	1	7.80E-10	0.38	19	1.9	Xylt1	Transport
DMR1:187062001	1	187062001	187064000	2000	1	3.30E-07	-0.38	26	1.3	Xylt1	Transport
DMR1:187089001	1	187089001	187093000	4000	1	7.90E-15	0.77	31	0.78	Xylt1	Transport
DMR1:187120001	1	187120001	187124000	4000	3	5.80E-19	0.71	48	1.2	Xylt1	Transport
DMR1:187169001	1	187169001	187170000	1000	1	1.20E-21	0.82	13	1.3	Xylt1	Transport
DMR1:187176001	1	187176001	187177000	1000	1	5.50E-08	-0.42	13	1.3	Xylt1	Transport
DMR1:187245001	1	187245001	187246000	1000	1	5.80E-10	-0.66	14	1.4	Xylt1	Transport
DMR1:187251001	1	187251001	187252000	1000	1	4.00E-10	0.63	3	0.3	Xylt1	Transport
DMR1:187750001	1	187750001	187754000	4000	1	6.40E-17	0.65	37	0.92	Rps15a	Translation
DMR1:187755001	1	187755001	187756000	1000	1	1.40E-08	0.38	10	1	Rps15a	Translation
DMR1:187788001	1	187788001	187789000	1000	1	5.30E-10	0.4	11	1.1	Arl6ip1;Smg1	
DMR1:187793001	1	187793001	187794000	1000	1	3.20E-09	0.61	12	1.2	Smg1	
DMR1:187824001	1	187824001	187827000	3000	1	2.40E-08	-0.74	19	0.63	Smg1	
DMR1:187835001	1	187835001	187837000	2000	1	3.30E-23	0.73	9	0.45	Smg1	
DMR1:187839001	1	187839001	187840000	1000	1	1.20E-11	0.57	6	0.6	Smg1	
DMR1:187880001	1	187880001	187881000	1000	1	2.00E-13	0.66	9	0.9	Smg1	
DMR1:187889001	1	187889001	187895000	6000	1	2.70E-08	0.52	57	0.95	Smg1	
DMR1:188005001	1	188005001	188006000	1000	1	9.10E-12	-0.64	9	0.9	Clec19a	
DMR1:188007001	1	188007001	188008000	1000	1	1.60E-07	0.43	6	0.6	Clec19a	
DMR1:188054001	1	188054001	188057000	3000	1	5.90E-13	0.63	36	1.2	Syt17	Transport
DMR1:188058001	1	188058001	188059000	1000	1	2.70E-07	0.34	10	1	Syt17	Transport
DMR1:188061001	1	188061001	188066000	5000	2	1.60E-21	0.82	43	0.86	Syt17	Transport
DMR1:188067001	1	188067001	188070000	3000	1	5.30E-16	0.57	41	1.37	Syt17	Transport
DMR1:188080001	1	188080001	188081000	1000	1	5.40E-09	-0.64	9	0.9	Syt17	Transport
DMR1:188082001	1	188082001	188083000	1000	1	1.30E-11	0.65	5	0.5	Syt17	Transport
DMR1:188090001	1	188090001	188091000	1000	1	1.90E-17	0.69	17	1.7	Syt17	Transport
DMR1:188106001	1	188106001	188107000	1000	1	1.30E-09	0.48	7	0.7	Syt17	Transport
DMR1:188138001	1	188138001	188140000	2000	1	2.40E-15	0.68	14	0.7	Itpripl2	
DMR1:188165001	1	188165001	188168000	3000	2	2.20E-10	0.54	44	1.47	LOC108349625;Coq7	
DMR1:188175001	1	188175001	188181000	6000	1	1.40E-13	0.5	83	1.38	LOC108349625;Coq7	
DMR1:188183001	1	188183001	188191000	8000	3	7.40E-23	-0.53	123	1.54	Coq7;Tmc7;LOC102550534	
DMR1:188227001	1	188227001	188228000	1000	1	5.70E-12	0.5	16	1.6	Tmc7	
DMR1:188235001	1	188235001	188242000	7000	1	1.30E-14	0.62	83	1.19	Tmc7	
DMR1:188246001	1	188246001	188249000	3000	1	1.30E-15	0.7	32	1.07	Tmc7	
DMR1:188280001	1	188280001	188283000	3000	1	2.90E-07	0.29	28	0.93	Tmc5	
DMR1:188291001	1	188291001	188292000	1000	1	1.40E-12	0.5	13	1.3	Tmc5	
DMR1:188293001	1	188293001	188296000	3000	2	1.10E-12	0.45	34	1.13	Tmc5	
DMR1:188311001	1	188311001	188314000	3000	2	5.00E-09	0.45	35	1.17	Tmc5	
DMR1:188319001	1	188319001	188320000	1000	1	5.10E-11	0.87	8	0.8	Tmc5	
DMR1:188342001	1	188342001	188344000	2000	2	5.40E-14	0.71	18	0.9	Tmc5	
DMR1:188383001	1	188383001	188385000	2000	1	8.00E-10	-0.46	15	0.75	Gde1	Signaling
DMR1:188408001	1	188408001	188409000	1000	1	1.10E-14	0.83	2	0.2	Gde1;Ccp110	Signaling
DMR1:188418001	1	188418001	188420000	2000	1	5.70E-09	-0.52	30	1.5	Ccp110	
DMR1:188434001	1	188434001	188438000	4000	1	8.90E-10	0.55	41	1.02	Ccp110	
DMR1:188450001	1	188450001	188451000	1000	1	2.10E-08	0.54	7	0.7	Ccp110;LOC361635	
DMR1:188557001	1	188557001	188560000	3000	3	8.10E-15	-0.42	34	1.13	LOC361635;Knop1	
DMR1:188582001	1	188582001	188588000	6000	1	1.30E-07	-0.67	74	1.23	Iqck	
DMR1:188607001	1	188607001	188610000	3000	1	2.30E-14	0.63	33	1.1	Iqck	
DMR1:188614001	1	188614001	188617000	3000	2	8.00E-10	0.4	24	0.8	Iqck	
DMR1:188672001	1	188672001	188673000	1000	1	2.20E-10	0.49	9	0.9	Iqck	
DMR1:188685001	1	188685001	188689000	4000	1	1.10E-09	-0.5	64	1.6	Iqck;Gprc5b;LOC102550985	Signaling
DMR1:188854001	1	188854001	188855000	1000	1	2.70E-10	0.46	11	1.1	Gpr139	
DMR1:188884001	1	188884001	188885000	1000	1	3.30E-07	0.47	10	1	Gpr139	

DMR1:188890001	1	188890001	188893000	3000	1	6.90E-08	-0.52	39	1.3	Gpr139;LOC108349626	
DMR1:189158001	1	189158001	189159000	1000	1	7.90E-07	0.38	9	0.9	Gp2	Receptor
DMR1:189172001	1	189172001	189173000	1000	1	1.30E-08	0.51	4	0.4	Gp2	Receptor
DMR1:189188001	1	189188001	189192000	4000	2	2.60E-18	0.95	26	0.65	Gp2;Umod	Receptor
DMR1:189229001	1	189229001	189233000	4000	1	9.60E-08	0.45	53	1.32	Pdilt;Acsm5	Transcription;Metabolism
DMR1:189239001	1	189239001	189240000	1000	1	5.70E-13	0.74	4	0.4	Pdilt;Acsm5	Transcription;Metabolism
DMR1:189271001	1	189271001	189276000	5000	1	3.30E-07	-0.35	35	0.7	Acsm5	Metabolism
DMR1:189314001	1	189314001	189315000	1000	1	4.80E-16	0.83	8	0.8	Acsm2a	
DMR1:189319001	1	189319001	189321000	2000	1	5.80E-30	1.24	14	0.7	Acsm2a	
DMR1:189333001	1	189333001	189335000	2000	1	1.70E-11	0.72	14	0.7	Acsm2a	
DMR1:189346001	1	189346001	189347000	1000	1	1.60E-07	0.4	4	0.4	RGD1559600	
DMR1:189349001	1	189349001	189353000	4000	2	7.20E-11	0.78	20	0.5	RGD1559600;Acsm1	
DMR1:189366001	1	189366001	189368000	2000	1	1.60E-11	0.5	20	1	RGD1559600;Acsm1;LOC102551259	
DMR1:189379001	1	189379001	189382000	3000	1	8.70E-08	-0.45	20	0.67	Acsm1;LOC102551259	
DMR1:189402001	1	189402001	189403000	1000	1	3.50E-09	0.65	3	0.3	Acsm1	
DMR1:189429001	1	189429001	189430000	1000	1	1.50E-12	0.52	11	1.1	Acsm4	Metabolism
DMR1:189462001	1	189462001	189463000	1000	1	7.10E-09	0.56	18	1.8	Acsm4;Thumpd1	Metabolism
DMR1:189464001	1	189464001	189467000	3000	1	1.90E-09	-0.59	28	0.93	Acsm4;Thumpd1	Metabolism
DMR1:189481001	1	189481001	189482000	1000	1	8.80E-07	0.43	32	3.2	Thumpd1	
DMR1:189496001	1	189496001	189498000	2000	1	1.20E-10	-0.6	6	0.3	Thumpd1;LOC102551569	
DMR1:189499001	1	189499001	189504000	5000	1	1.50E-08	0.44	90	1.8	Thumpd1;LOC102551569	
DMR1:189505001	1	189505001	189511000	6000	2	7.80E-17	-0.51	93	1.55	Thumpd1;LOC102551569;Acsm3	Metabolism
DMR1:189516001	1	189516001	189517000	1000	1	3.50E-09	0.49	3	0.3	Thumpd1;LOC102551569;Acsm3	Metabolism
DMR1:189576001	1	189576001	189581000	5000	2	5.40E-09	0.45	36	0.72	Thumpd1;Exnef	
DMR1:189602001	1	189602001	189605000	3000	1	1.90E-07	0.47	7	0.23	Thumpd1;Exnef	
DMR1:189663001	1	189663001	189664000	1000	1	4.50E-07	0.36	7	0.7	Thumpd1;Dcun1d3;Lyrm1	
DMR1:189690001	1	189690001	189694000	4000	1	6.20E-12	0.52	31	0.78	Thumpd1;Lyrm1;LOC108349819	
DMR1:189716001	1	189716001	189719000	3000	1	6.70E-07	-0.53	39	1.3	Thumpd1;Dnah3	
DMR1:189720001	1	189720001	189722000	2000	2	1.50E-22	0.78	15	0.75	Thumpd1;Dnah3	
DMR1:189734001	1	189734001	189736000	2000	1	9.90E-13	0.53	19	0.95	Thumpd1;Dnah3	
DMR1:189766001	1	189766001	189768000	2000	1	6.60E-07	-0.43	17	0.85	Thumpd1;Dnah3	
DMR1:189776001	1	189776001	189777000	1000	1	2.60E-18	0.81	6	0.6	Thumpd1;Dnah3	
DMR1:189787001	1	189787001	189788000	1000	1	7.20E-13	0.6	13	1.3	Thumpd1;Dnah3	
DMR1:189799001	1	189799001	189800000	1000	1	1.20E-19	0.87	5	0.5	Thumpd1;Dnah3	
DMR1:189891001	1	189891001	189892000	1000	1	1.70E-29	0.97	6	0.6	Thumpd1;Tmem159;LOC108349820;Zp2	
DMR1:189907001	1	189907001	189908000	1000	1	7.30E-25	0.86	17	1.7	Thumpd1;Zp2	
DMR1:189918001	1	189918001	189919000	1000	1	3.90E-08	-0.47	11	1.1	Thumpd1;Zp2;LOC103691218	
DMR1:189977001	1	189977001	189978000	1000	1	7.80E-15	0.78	6	0.6	Thumpd1;Abca14	Transport
DMR1:190007001	1	190007001	190009000	2000	1	7.70E-11	0.74	5	0.25	Thumpd1;Abca14	Transport
DMR1:190018001	1	190018001	190024000	6000	1	2.00E-07	-0.6	62	1.03	Thumpd1;Abca14	Transport
DMR1:190036001	1	190036001	190039000	3000	1	2.10E-07	-0.39	10	0.33	Thumpd1;Abca14	Transport
DMR1:190086001	1	190086001	190089000	3000	3	7.50E-10	-0.58	10	0.33	Thumpd1;Abca15	Transport
DMR1:190095001	1	190095001	190097000	2000	2	2.80E-23	0.92	9	0.45	Thumpd1;Abca15	Transport
DMR1:190104001	1	190104001	190110000	6000	1	3.30E-10	0.44	47	0.78	Thumpd1;Abca15	Transport
DMR1:190114001	1	190114001	190120000	6000	2	9.10E-13	-0.48	43	0.72	Thumpd1;Abca15	Transport
DMR1:190126001	1	190126001	190132000	6000	4	1.90E-19	-0.49	66	1.1	Thumpd1;Abca15;Hspd1-ps5	Transport
DMR1:190154001	1	190154001	190162000	8000	4	5.30E-16	-0.94	62	0.78	Thumpd1;Abca15	Transport
DMR1:190185001	1	190185001	190187000	2000	1	1.70E-17	1.04	8	0.4	Thumpd1;Abca15	Transport
DMR1:190197001	1	190197001	190200000	3000	2	8.60E-12	-0.64	10	0.33	Thumpd1;Abca16	Transport
DMR1:190203001	1	190203001	190205000	2000	1	1.50E-07	0.49	9	0.45	Thumpd1;Abca16	Transport
DMR1:190231001	1	190231001	190234000	3000	1	8.30E-08	0.61	14	0.47	Thumpd1;Abca16	Transport
DMR1:190237001	1	190237001	190240000	3000	2	2.60E-11	-0.49	19	0.63	Thumpd1;Abca16	Transport
DMR1:190244001	1	190244001	190247000	3000	1	8.10E-12	-0.53	38	1.27	Thumpd1;Abca16	Transport
DMR1:190249001	1	190249001	190252000	3000	1	2.00E-15	0.72	16	0.53	Thumpd1;Abca16	Transport
DMR1:190259001	1	190259001	190263000	4000	3	1.80E-18	0.41	35	0.88	Thumpd1;Abca16	Transport
DMR1:190269001	1	190269001	190271000	2000	2	7.80E-11	-0.55	6	0.3	Thumpd1;Abca16	Transport
DMR1:190274001	1	190274001	190276000	2000	1	2.50E-10	0.7	9	0.45	Thumpd1;Abca16	Transport
DMR1:190294001	1	190294001	190295000	1000	1	9.80E-08	0.51	5	0.5	Thumpd1;Abca16	Transport

DMR1:190298001	1	190298001	190303000	5000	1	1.40E-07	-0.46	39	0.78	Thumpd1;Abca16	Transport
DMR1:190304001	1	190304001	190306000	2000	1	1.50E-11	0.57	20	1	Thumpd1;Abca16	Transport
DMR1:190309001	1	190309001	190313000	4000	2	4.10E-15	-0.61	22	0.55	Thumpd1;Abca16	Transport
DMR1:190331001	1	190331001	190334000	3000	2	1.50E-13	0.56	19	0.63	Thumpd1;Abca16	Transport
DMR1:190357001	1	190357001	190358000	1000	1	8.80E-07	0.52	6	0.6	Thumpd1;Abca16	Transport
DMR1:190363001	1	190363001	190365000	2000	1	2.30E-21	0.92	19	0.95	Thumpd1;Abca16	Transport
DMR1:190546001	1	190546001	190547000	1000	1	7.30E-08	0.36	19	1.9	LOC691519;LOC108349627;Uqcrc2	Protease
DMR1:190548001	1	190548001	190552000	4000	3	3.70E-12	-0.6	44	1.1	LOC691519;LOC108349627;Uqcrc2	Protease
DMR1:190560001	1	190560001	190561000	1000	1	7.90E-07	0.43	9	0.9	Uqcrc2	Protease
DMR1:190562001	1	190562001	190566000	4000	3	7.20E-18	-0.5	44	1.1	Uqcrc2	Protease
DMR1:190568001	1	190568001	190569000	1000	1	1.50E-12	0.53	16	1.6	Uqcrc2	Protease
DMR1:190579001	1	190579001	190581000	2000	2	7.10E-19	0.73	16	0.8	Uqcrc2;Pdzd9	Protease
DMR1:190676001	1	190676001	190677000	1000	1	5.60E-09	0.43	8	0.8	LOC102547219;LOC102555900;Vwa3a	
DMR1:190703001	1	190703001	190705000	2000	1	8.20E-09	-0.61	32	1.6	Vwa3a	
DMR1:190717001	1	190717001	190721000	4000	2	7.80E-21	0.78	37	0.92	Vwa3a	
DMR1:190722001	1	190722001	190728000	6000	2	1.10E-26	0.9	76	1.27	Vwa3a	
DMR1:190740001	1	190740001	190741000	1000	1	7.50E-18	0.79	7	0.7	Vwa3a	
DMR1:190742001	1	190742001	190744000	2000	1	6.70E-07	-0.55	20	1	Vwa3a	
DMR1:190746001	1	190746001	190747000	1000	1	2.10E-17	0.72	28	2.8	Vwa3a;Sdr42e2	
DMR1:190748001	1	190748001	190749000	1000	1	5.20E-10	0.73	6	0.6	Vwa3a;Sdr42e2	
DMR1:190759001	1	190759001	190760000	1000	1	6.70E-08	-0.64	18	1.8	Sdr42e2	
DMR1:190765001	1	190765001	190767000	2000	2	1.90E-08	0.36	35	1.75	Sdr42e2;Trnal-uag	
DMR1:190783001	1	190783001	190784000	1000	1	3.70E-07	0.49	9	0.9	Trnal-uag;LOC102556194;Eef2k	
DMR1:190785001	1	190785001	190788000	3000	2	4.20E-09	-0.42	37	1.23	LOC102556194;Eef2k	
DMR1:190793001	1	190793001	190794000	1000	1	7.10E-13	0.62	8	0.8	Eef2k	
DMR1:190814001	1	190814001	190815000	1000	1	4.90E-08	0.29	17	1.7	Eef2k	
DMR1:190816001	1	190816001	190818000	2000	1	1.20E-21	0.79	34	1.7	Eef2k	
DMR1:190819001	1	190819001	190823000	4000	1	5.10E-08	0.41	55	1.38	Eef2k	
DMR1:190828001	1	190828001	190834000	6000	1	1.50E-17	0.49	99	1.65	Eef2k	
DMR1:190842001	1	190842001	190846000	4000	1	2.50E-09	-0.6	44	1.1	Eef2k;LOC108348725;Trnal-aag;Polr3e	Transcription
DMR1:190894001	1	190894001	190896000	2000	1	9.50E-08	-0.52	27	1.35	LOC108349629;Cdr2	
DMR1:190909001	1	190909001	190910000	1000	1	1.90E-12	0.57	11	1.1	Cdr2;LOC691551	
DMR1:190918001	1	190918001	190920000	2000	1	4.40E-08	0.38	22	1.1	Cdr2;LOC691551	
DMR1:190957001	1	190957001	190958000	1000	1	1.20E-12	0.64	5	0.5	LOC691551;Mettl9	
DMR1:190979001	1	190979001	190980000	1000	1	7.10E-07	0.52	3	0.3	Mettl9	
DMR1:190986001	1	190986001	190989000	3000	2	6.10E-18	0.74	26	0.87	Mettl9;Igsf6	Immune
DMR1:191022001	1	191022001	191025000	3000	1	1.00E-11	-0.67	20	0.67	Otoa	Cytoskeleton
DMR1:191046001	1	191046001	191048000	2000	1	8.30E-08	-0.58	18	0.9	Otoa	Cytoskeleton
DMR1:191056001	1	191056001	191057000	1000	1	7.70E-09	0.71	11	1.1	Otoa	Cytoskeleton
DMR1:191067001	1	191067001	191071000	4000	1	5.40E-07	0.37	59	1.48	Otoa	Cytoskeleton
DMR1:191072001	1	191072001	191074000	2000	2	4.40E-21	0.87	20	1	Otoa	Cytoskeleton
DMR1:191089001	1	191089001	191090000	1000	1	4.80E-14	0.79	1	0.1	Otoa	Cytoskeleton
DMR1:191097001	1	191097001	191099000	2000	1	3.00E-07	0.41	25	1.25	Otoa	Cytoskeleton
DMR1:191335001	1	191335001	191337000	2000	2	3.70E-08	0.37	20	1	Hs3st2	Transport
DMR1:191338001	1	191338001	191339000	1000	1	1.50E-11	0.53	8	0.8	Hs3st2	Transport
DMR1:191342001	1	191342001	191344000	2000	1	4.20E-07	0.48	16	0.8	Hs3st2	Transport
DMR1:191356001	1	191356001	191361000	5000	1	1.40E-07	0.37	41	0.82	Hs3st2	Transport
DMR1:191383001	1	191383001	191384000	1000	1	7.20E-08	0.37	7	0.7	Hs3st2	Transport
DMR1:191386001	1	191386001	191396000	10000	5	5.20E-18	0.77	132	1.32	Hs3st2	Transport
DMR1:191397001	1	191397001	191398000	1000	1	5.50E-15	0.79	4	0.4	Hs3st2	Transport
DMR1:191402001	1	191402001	191403000	1000	1	1.60E-08	0.57	0	0	Hs3st2	Transport
DMR1:191404001	1	191404001	191406000	2000	1	8.10E-07	0.43	17	0.85	Hs3st2	Transport
DMR1:191422001	1	191422001	191424000	2000	2	7.70E-16	0.72	11	0.55	Hs3st2	Transport
DMR1:191432001	1	191432001	191434000	2000	1	5.40E-08	0.44	16	0.8	Hs3st2	Transport
DMR1:191435001	1	191435001	191440000	5000	1	1.20E-13	0.68	47	0.94	Hs3st2	Transport
DMR1:191443001	1	191443001	191444000	1000	1	9.20E-09	0.61	14	1.4	Hs3st2	Transport
DMR1:191456001	1	191456001	191462000	6000	3	2.70E-23	-0.58	85	1.42	Hs3st2	Transport
DMR1:191463001	1	191463001	191466000	3000	2	5.40E-10	-0.5	34	1.13	Hs3st2	Transport
DMR1:191475001	1	191475001	191476000	1000	1	9.20E-09	0.48	10	1	Hs3st2	Transport
DMR1:191601001	1	191601001	191606000	5000	4	2.10E-14	-0.52	39	0.78	Usp31	Protease
DMR1:191610001	1	191610001	191614000	4000	1	5.80E-07	-0.55	48	1.2	Usp31	Protease

DMR1:191697001	1	191697001	191699000	2000	2	5.20E-08	0.48	21	1.05	Scnn1g	Transport
DMR1:191716001	1	191716001	191717000	1000	1	5.80E-08	-0.63	10	1	Scnn1g	Transport
DMR1:191723001	1	191723001	191724000	1000	1	1.30E-19	0.65	9	0.9	Scnn1g	Transport
DMR1:191732001	1	191732001	191733000	1000	1	6.90E-07	-0.51	5	0.5	Scnn1g	Transport
DMR1:191817001	1	191817001	191823000	6000	1	3.20E-07	-0.38	55	0.92	LOC102549682;Scnn1b	Transport
DMR1:191832001	1	191832001	191834000	2000	1	8.20E-10	0.44	24	1.2	LOC102549682;Scnn1b	Transport
DMR1:191844001	1	191844001	191845000	1000	1	3.40E-10	0.47	11	1.1	Scnn1b	Transport
DMR1:191851001	1	191851001	191854000	3000	2	1.00E-19	0.81	27	0.9	Scnn1b	Transport
DMR1:191861001	1	191861001	191864000	3000	1	2.00E-20	0.92	34	1.13	Scnn1b	Transport
DMR1:191869001	1	191869001	191871000	2000	1	2.50E-09	0.56	26	1.3	Scnn1b	Transport
DMR1:191877001	1	191877001	191878000	1000	1	8.00E-14	0.52	12	1.2	Scnn1b	Transport
DMR1:191883001	1	191883001	191888000	5000	1	8.10E-13	0.46	84	1.68	Scnn1b;Cog7	Transport
DMR1:191906001	1	191906001	191908000	2000	1	1.60E-08	0.61	13	0.65	Cog7	
DMR1:191934001	1	191934001	191937000	3000	1	2.80E-09	0.44	36	1.2	Cog7	
DMR1:191940001	1	191940001	191944000	4000	2	2.30E-10	0.49	56	1.4	Cog7;Gga2	
DMR1:191952001	1	191952001	191956000	4000	1	2.40E-09	0.54	64	1.6	Cog7;Gga2	
DMR1:191964001	1	191964001	191965000	1000	1	1.20E-11	0.37	12	1.2	Gga2	
DMR1:191976001	1	191976001	191978000	2000	1	1.90E-07	-0.44	25	1.25	Gga2	
DMR1:191982001	1	191982001	191984000	2000	1	5.20E-19	0.58	23	1.15	Gga2	
DMR1:191992001	1	191992001	191993000	1000	1	1.80E-09	0.43	7	0.7	Gga2;Ears2	Translation
DMR1:192031001	1	192031001	192032000	1000	1	6.90E-09	0.41	12	1.2	Ears2;Ubfd1	Translation
DMR1:192036001	1	192036001	192038000	2000	2	4.00E-11	-0.5	20	1	Ubfd1;Ndufab1	Transport
DMR1:192081001	1	192081001	192082000	1000	1	4.40E-08	0.5	6	0.6	Palb2;Dctn5	Cytoskeleton
DMR1:192090001	1	192090001	192091000	1000	1	7.20E-09	0.43	13	1.3	Palb2;Dctn5	Cytoskeleton
DMR1:192119001	1	192119001	192121000	2000	1	1.50E-09	0.52	18	0.9	Plk1;Ern2	Signaling;Translation
DMR1:192152001	1	192152001	192156000	4000	1	2.10E-09	0.39	52	1.3	Ern2	Translation
DMR1:192162001	1	192162001	192165000	3000	1	4.70E-20	0.73	40	1.33	Chp2	
DMR1:192171001	1	192171001	192173000	2000	1	3.70E-13	0.41	33	1.65	Chp2	
DMR1:192240001	1	192240001	192242000	2000	1	2.70E-09	0.42	42	2.1	Prkcb	Signaling
DMR1:192247001	1	192247001	192248000	1000	1	3.70E-07	0.33	12	1.2	Prkcb	Signaling
DMR1:192257001	1	192257001	192260000	3000	2	6.40E-08	0.55	22	0.73	Prkcb	Signaling
DMR1:192262001	1	192262001	192264000	2000	1	2.50E-09	0.51	12	0.6	Prkcb	Signaling
DMR1:192265001	1	192265001	192269000	4000	2	1.40E-21	0.81	32	0.8	Prkcb	Signaling
DMR1:192271001	1	192271001	192274000	3000	1	6.10E-07	0.3	52	1.73	Prkcb	Signaling
DMR1:192288001	1	192288001	192290000	2000	2	4.30E-12	0.74	9	0.45	Prkcb	Signaling
DMR1:192292001	1	192292001	192295000	3000	1	4.90E-14	0.77	39	1.3	Prkcb	Signaling
DMR1:192307001	1	192307001	192309000	2000	1	2.40E-09	0.43	29	1.45	Prkcb	Signaling
DMR1:192312001	1	192312001	192313000	1000	1	1.00E-07	0.36	7	0.7	Prkcb	Signaling
DMR1:192314001	1	192314001	192315000	1000	1	1.10E-09	0.43	10	1	Prkcb	Signaling
DMR1:192350001	1	192350001	192353000	3000	2	1.10E-09	-0.66	38	1.27	Prkcb	Signaling
DMR1:192365001	1	192365001	192366000	1000	1	7.00E-14	0.53	18	1.8	Prkcb	Signaling
DMR1:192374001	1	192374001	192379000	5000	3	4.40E-14	0.57	51	1.02	Prkcb	Signaling
DMR1:192397001	1	192397001	192399000	2000	2	1.80E-15	0.5	36	1.8	Prkcb	Signaling
DMR1:192400001	1	192400001	192401000	1000	1	6.20E-08	0.57	12	1.2	Prkcb	Signaling
DMR1:192410001	1	192410001	192413000	3000	1	9.00E-07	0.31	45	1.5	Prkcb	Signaling
DMR1:192430001	1	192430001	192433000	3000	1	5.30E-09	0.4	39	1.3	Prkcb	Signaling
DMR1:192436001	1	192436001	192442000	6000	1	1.00E-08	0.42	73	1.22	Prkcb	Signaling
DMR1:192447001	1	192447001	192448000	1000	1	1.00E-13	0.56	11	1.1	Prkcb	Signaling
DMR1:192461001	1	192461001	192463000	2000	1	9.00E-09	0.37	26	1.3	Prkcb	Signaling
DMR1:192476001	1	192476001	192478000	2000	1	1.20E-08	-0.51	28	1.4	Prkcb	Signaling
DMR1:192494001	1	192494001	192496000	2000	2	2.60E-19	0.57	19	0.95	Prkcb	Signaling
DMR1:192509001	1	192509001	192510000	1000	1	3.40E-07	-0.6	14	1.4	Prkcb	Signaling
DMR1:192516001	1	192516001	192518000	2000	1	7.30E-08	-0.43	23	1.15	Prkcb	Signaling
DMR1:192547001	1	192547001	192554000	7000	1	6.40E-07	0.34	87	1.24	Prkcb	Signaling
DMR1:192557001	1	192557001	192559000	2000	2	3.50E-25	0.82	26	1.3	Prkcb	Signaling
DMR1:192560001	1	192560001	192564000	4000	1	3.80E-11	0.71	46	1.15	Prkcb	Signaling
DMR1:192624001	1	192624001	192626000	2000	1	2.00E-09	0.48	21	1.05	Cacng3	Transport
DMR1:192632001	1	192632001	192634000	2000	1	6.50E-17	0.97	25	1.25	Cacng3	Transport
DMR1:192651001	1	192651001	192652000	1000	1	4.60E-20	0.79	12	1.2	Cacng3	Transport
DMR1:192653001	1	192653001	192656000	3000	1	1.30E-07	0.46	32	1.07	Cacng3	Transport
DMR1:192667001	1	192667001	192668000	1000	1	1.30E-08	0.49	11	1.1	Cacng3	Transport
DMR1:192687001	1	192687001	192690000	3000	1	4.00E-09	-0.71	35	1.17	Cacng3	Transport
DMR1:192972001	1	192972001	192978000	6000	2	3.00E-20	0.8	77	1.28	Rbbp6	Proteolysis
DMR1:192979001	1	192979001	192983000	4000	1	3.00E-16	0.87	39	0.98	Tnrc6a	Metabolism
DMR1:192984001	1	192984001	192985000	1000	1	1.00E-17	0.56	18	1.8	Tnrc6a	Metabolism

DMR1:192996001	1	192996001	192997000	1000	1	5.20E-08	-0.64	16	1.6	Tnrc6a	Metabolism
DMR1:192998001	1	192998001	1.93E+08	2000	1	6.20E-07	-0.39	22	1.1	Tnrc6a	Metabolism
DMR1:193041001	1	193041001	193046000	5000	2	4.30E-16	0.62	87	1.74	Tnrc6a	Metabolism
DMR1:193066001	1	193066001	193067000	1000	1	9.10E-08	0.38	15	1.5	Tnrc6a	Metabolism
DMR1:193086001	1	193086001	193089000	3000	1	1.80E-07	-0.45	22	0.73	Tnrc6a	Metabolism
DMR1:193090001	1	193090001	193092000	2000	1	9.00E-15	0.69	18	0.9	Tnrc6a	Metabolism
DMR1:193106001	1	193106001	193109000	3000	1	5.70E-11	-0.61	22	0.73	Tnrc6a	Metabolism
DMR1:193122001	1	193122001	193125000	3000	1	2.00E-07	-0.53	33	1.1	Tnrc6a	Metabolism
DMR1:193143001	1	193143001	193144000	1000	1	4.20E-08	-0.56	24	2.4	Tnrc6a;Slc5a11	Metabolism;Transport
DMR1:193158001	1	193158001	193159000	1000	1	9.10E-07	-0.53	12	1.2	Slc5a11	Transport
DMR1:193164001	1	193164001	193166000	2000	1	1.90E-11	0.56	29	1.45	Slc5a11	Transport
DMR1:193195001	1	193195001	193197000	2000	1	1.30E-08	-0.53	18	0.9	Slc5a11	Transport
DMR1:193199001	1	193199001	193204000	5000	2	1.10E-14	0.67	53	1.06	Slc5a11	Transport
DMR1:193208001	1	193208001	193212000	4000	3	2.10E-11	0.49	51	1.27	Slc5a11	Transport
DMR1:193221001	1	193221001	193222000	1000	1	3.70E-07	-0.56	9	0.9	Slc5a11	Transport
DMR1:193230001	1	193230001	193233000	3000	2	4.40E-09	-0.49	9	0.3	Slc5a11;Arhgap17	Transport
DMR1:193234001	1	193234001	193235000	1000	1	2.80E-12	0.65	8	0.8	Slc5a11;Arhgap17	Transport
DMR1:193245001	1	193245001	193247000	2000	1	5.20E-11	0.43	37	1.85	Arhgap17	
DMR1:193255001	1	193255001	193260000	5000	1	8.90E-13	-0.73	56	1.12	Arhgap17	
DMR1:193264001	1	193264001	193266000	2000	1	4.80E-09	0.53	12	0.6	Arhgap17	
DMR1:193315001	1	193315001	193319000	4000	1	5.30E-09	0.38	40	1	Arhgap17	
DMR1:193320001	1	193320001	193321000	1000	1	1.60E-10	0.75	3	0.3	Arhgap17	
DMR1:193331001	1	193331001	193333000	2000	1	5.70E-09	0.39	17	0.85	Arhgap17;Lcmt1	Epigenetic
DMR1:193344001	1	193344001	193345000	1000	1	7.00E-11	0.66	9	0.9	Lcmt1	Epigenetic
DMR1:193358001	1	193358001	193360000	2000	1	2.70E-10	0.43	20	1	Lcmt1	Epigenetic
DMR1:193394001	1	193394001	193401000	7000	1	1.20E-07	-0.59	65	0.93	Lcmt1	Epigenetic
DMR1:193704001	1	193704001	193706000	2000	1	1.30E-13	-0.73	9	0.45	Paip2l1	
DMR1:193709001	1	193709001	193716000	7000	4	1.20E-21	-0.52	75	1.07	Paip2l1	
DMR1:193832001	1	193832001	193834000	2000	1	4.90E-08	0.64	7	0.35	Hs3st4	
DMR1:193853001	1	193853001	193856000	3000	1	6.90E-12	0.64	22	0.73	Hs3st4;LOC108349632	
DMR1:193859001	1	193859001	193862000	3000	1	8.30E-12	0.64	19	0.63	Hs3st4	
DMR1:193882001	1	193882001	193884000	2000	1	3.70E-20	0.79	19	0.95	Hs3st4	
DMR1:193904001	1	193904001	193905000	1000	1	9.80E-08	0.58	7	0.7	Hs3st4	
DMR1:194041001	1	194041001	194044000	3000	2	5.30E-15	0.53	24	0.8	Hs3st4	
DMR1:194057001	1	194057001	194058000	1000	1	2.40E-09	0.41	8	0.8	Hs3st4	
DMR1:194064001	1	194064001	194066000	2000	1	4.80E-10	-0.52	23	1.15	Hs3st4;LOC103691223	
DMR1:194111001	1	194111001	194116000	5000	2	4.60E-09	0.54	56	1.12	Hs3st4	
DMR1:194138001	1	194138001	194139000	1000	1	1.80E-17	0.83	8	0.8	Hs3st4	
DMR1:194161001	1	194161001	194163000	2000	1	2.20E-11	0.42	21	1.05	Hs3st4	
DMR1:194170001	1	194170001	194174000	4000	1	2.50E-07	0.63	46	1.15	Hs3st4	
DMR1:194176001	1	194176001	194177000	1000	1	3.10E-09	0.49	8	0.8	Hs3st4	
DMR1:194183001	1	194183001	194184000	1000	1	1.50E-21	0.85	13	1.3	Hs3st4	
DMR1:194194001	1	194194001	194195000	1000	1	5.80E-07	0.41	5	0.5	Hs3st4	
DMR1:194213001	1	194213001	194214000	1000	1	3.90E-07	0.38	15	1.5	Hs3st4	
DMR1:194224001	1	194224001	194226000	2000	1	1.20E-13	0.72	19	0.95	Hs3st4	
DMR1:194248001	1	194248001	194249000	1000	1	7.60E-13	0.73	57	5.7	Hs3st4	
DMR1:194757001	1	194757001	194758000	1000	1	1.10E-11	0.63	6	0.6	LOC100911676;Nupr1	
DMR1:194771001	1	194771001	194773000	2000	1	1.20E-12	0.61	24	1.2	Nupr1	
DMR1:195425001	1	195425001	195431000	6000	5	3.90E-11	-0.5	51	0.85	LOC365268;RGD1561206	
DMR1:196832001	1	196832001	196833000	1000	1	9.90E-14	0.62	31	3.1	Kdm8	
DMR1:196876001	1	196876001	196878000	2000	2	1.20E-09	0.42	16	0.8	Nsmce1;LOC103691233	
DMR1:196888001	1	196888001	196890000	2000	2	1.10E-12	0.49	33	1.65	Nsmce1;LOC103691233	
DMR1:196935001	1	196935001	196936000	1000	1	1.90E-07	-0.52	13	1.3	Il4r	Receptor
DMR1:196962001	1	196962001	196965000	3000	2	3.50E-15	0.86	39	1.3	Il4r	Receptor
DMR1:196972001	1	196972001	196978000	6000	1	3.20E-17	0.59	64	1.07	Il4r	Receptor
DMR1:197007001	1	197007001	197009000	2000	1	4.50E-08	0.34	32	1.6	Il21r	Receptor
DMR1:197024001	1	197024001	197028000	4000	1	4.80E-15	0.73	37	0.92	Il21r;Gtf3c1	Receptor;Transcription
DMR1:197030001	1	197030001	197032000	2000	1	5.50E-08	0.34	24	1.2	Il21r;Gtf3c1	Receptor;Transcription
DMR1:197044001	1	197044001	197045000	1000	1	2.10E-07	-0.65	19	1.9	Gtf3c1	Transcription
DMR1:197064001	1	197064001	197066000	2000	1	7.90E-07	-0.49	27	1.35	Gtf3c1	Transcription
DMR1:197075001	1	197075001	197078000	3000	1	5.80E-07	0.43	29	0.97	Gtf3c1	Transcription
DMR1:197086001	1	197086001	197087000	1000	1	2.10E-07	0.44	16	1.6	Gtf3c1	Transcription

DMR1:197095001	1	197095001	197097000	2000	1	6.60E-08	0.38	18	0.9	Gtf3c1;LOC361646	Transcription
DMR1:197108001	1	197108001	197109000	1000	1	1.50E-13	0.66	6	0.6	Gtf3c1;LOC361646	Transcription
DMR1:197270001	1	197270001	197273000	3000	3	1.10E-22	0.9	29	0.97	LOC361646;Gsg1l	Cytoskeleton
DMR1:197279001	1	197279001	197283000	4000	3	1.10E-16	0.74	44	1.1	Gsg1l	Cytoskeleton
DMR1:197288001	1	197288001	197290000	2000	1	2.80E-07	0.39	16	0.8	Gsg1l	Cytoskeleton
DMR1:197292001	1	197292001	197295000	3000	1	2.70E-20	0.86	38	1.27	Gsg1l	Cytoskeleton
DMR1:197310001	1	197310001	197313000	3000	1	5.20E-07	-0.52	33	1.1	Gsg1l	Cytoskeleton
DMR1:197314001	1	197314001	197317000	3000	1	5.80E-08	0.41	44	1.47	Gsg1l	Cytoskeleton
DMR1:197318001	1	197318001	197320000	2000	2	2.30E-20	0.83	25	1.25	Gsg1l	Cytoskeleton
DMR1:197325001	1	197325001	197328000	3000	1	3.20E-09	0.41	38	1.27	Gsg1l	Cytoskeleton
DMR1:197329001	1	197329001	197330000	1000	1	4.10E-10	0.59	4	0.4	Gsg1l	Cytoskeleton
DMR1:197334001	1	197334001	197337000	3000	1	2.30E-12	0.64	32	1.07	Gsg1l	Cytoskeleton
DMR1:197339001	1	197339001	197342000	3000	1	2.50E-18	0.96	48	1.6	Gsg1l	Cytoskeleton
DMR1:197343001	1	197343001	197347000	4000	1	8.50E-07	0.32	50	1.25	Gsg1l	Cytoskeleton
DMR1:197358001	1	197358001	197359000	1000	1	1.80E-08	0.41	9	0.9	Gsg1l	Cytoskeleton
DMR1:197397001	1	197397001	197399000	2000	1	2.20E-14	0.73	21	1.05	Gsg1l	Cytoskeleton
DMR1:197404001	1	197404001	197411000	7000	1	2.90E-09	0.52	98	1.4	Gsg1l	Cytoskeleton
DMR1:197412001	1	197412001	197415000	3000	2	8.50E-10	0.5	17	0.57	Gsg1l	Cytoskeleton
DMR1:197425001	1	197425001	197429000	4000	1	4.30E-10	-0.53	82	2.05	Gsg1l	Cytoskeleton
DMR1:197441001	1	197441001	197442000	1000	1	1.80E-08	0.36	10	1	Gsg1l	Cytoskeleton
DMR1:197458001	1	197458001	197461000	3000	1	8.40E-14	0.47	40	1.33	Gsg1l	Cytoskeleton
DMR1:197465001	1	197465001	197468000	3000	1	1.00E-09	0.54	36	1.2	Gsg1l	Cytoskeleton
DMR1:197469001	1	197469001	197471000	2000	1	1.80E-17	0.77	26	1.3	Gsg1l	Cytoskeleton
DMR1:197529001	1	197529001	197530000	1000	1	1.80E-09	-0.59	4	0.4	Xpo6	
DMR1:197538001	1	197538001	197541000	3000	1	1.70E-08	0.44	21	0.7	Xpo6	
DMR1:197543001	1	197543001	197548000	5000	1	1.90E-09	0.49	32	0.64	Xpo6	
DMR1:197549001	1	197549001	197551000	2000	2	1.20E-07	-0.53	16	0.8	Xpo6	
DMR1:197592001	1	197592001	197594000	2000	1	2.40E-07	0.47	13	0.65	Xpo6;LOC102548734;LOC108349639	
DMR1:197624001	1	197624001	197628000	4000	1	1.60E-09	0.53	48	1.2	LOC108349640;Sbk1	Signaling
DMR1:197631001	1	197631001	197636000	5000	4	1.50E-22	0.72	62	1.24	LOC108349640;Sbk1	Signaling
DMR1:197637001	1	197637001	197639000	2000	1	3.90E-07	0.41	18	0.9	LOC108349640;Sbk1	Signaling
DMR1:197754001	1	197754001	197757000	3000	1	1.20E-12	0.45	48	1.6	LOC102548820;Lat	
DMR1:197759001	1	197759001	197761000	2000	1	9.50E-12	0.65	25	1.25	Lat	
DMR1:197768001	1	197768001	197770000	2000	2	3.10E-08	0.43	30	1.5	Lat;Spns1	Transport
DMR1:197775001	1	197775001	197777000	2000	1	4.90E-10	0.36	28	1.4	Lat;Spns1;Nfatc2ip	Transport
DMR1:197779001	1	197779001	197783000	4000	2	5.00E-07	-0.49	57	1.43	Lat;Spns1;Nfatc2ip	Transport
DMR1:197785001	1	197785001	197787000	2000	1	4.00E-10	0.61	26	1.3	Spns1;Nfatc2ip	Transport
DMR1:197800001	1	197800001	197801000	1000	1	1.60E-08	0.58	56	5.6	Nfatc2ip	
DMR1:197813001	1	197813001	197816000	3000	2	2.00E-10	0.59	61	2.03	LOC100361060;Cd19;LOC108349641	
DMR1:197835001	1	197835001	197836000	1000	1	3.80E-08	-0.42	6	0.6	LOC108349641;Rabep2	
DMR1:197840001	1	197840001	197842000	2000	2	1.30E-16	0.65	25	1.25	Rabep2	
DMR1:197849001	1	197849001	197859000	10000	3	1.40E-22	0.64	181	1.81	Rabep2;Atp2a1	Transport
DMR1:197863001	1	197863001	197865000	2000	2	7.00E-11	0.51	34	1.7	Rabep2;Atp2a1	Transport
DMR1:197867001	1	197867001	197869000	2000	1	8.30E-08	-0.66	34	1.7	Atp2a1;Sh2b1	Transport;Cytoskeleton
DMR1:197871001	1	197871001	197872000	1000	1	4.30E-22	0.91	20	2	Atp2a1;Sh2b1	Transport;Cytoskeleton
DMR1:197882001	1	197882001	197886000	4000	1	1.30E-09	0.39	68	1.7	Atp2a1;Sh2b1	Transport;Cytoskeleton
DMR1:197901001	1	197901001	197902000	1000	1	9.40E-14	0.69	5	0.5	Tufm;Atxn2l	Translation;Metabolism
DMR1:197908001	1	197908001	197910000	2000	1	3.00E-10	0.49	48	2.4	Tufm;Atxn2l	Translation;Metabolism
DMR1:197927001	1	197927001	197929000	2000	1	1.30E-07	-0.54	20	1	Atxn2l	Metabolism
DMR1:197958001	1	197958001	197959000	1000	1	5.20E-07	0.42	12	1.2	Mt1-ps1;Eif3c	Translation
DMR1:197975001	1	197975001	197977000	2000	2	8.20E-14	0.49	19	0.95	Eif3c;Cln3	Translation;Transport
DMR1:197994001	1	197994001	197996000	2000	1	1.50E-08	0.35	30	1.5	Cln3;Apobr;Il27	Transport;Cytokine
DMR1:197999001	1	197999001	198002000	3000	1	6.50E-10	0.43	41	1.37	Cln3;Apobr;Il27	Transport;Cytokine
DMR1:198006001	1	198006001	198007000	1000	1	2.50E-18	0.77	8	0.8	Cln3;Apobr;Il27	Transport;Cytokine
DMR1:198033001	1	198033001	198034000	1000	1	3.40E-07	0.39	11	1.1	LOC687726;NEWGENE_69363	
DMR1:198045001	1	198045001	198048000	3000	1	5.90E-07	0.42	33	1.1	NEWGENE_69363	
DMR1:198053001	1	198053001	198054000	1000	1	5.80E-10	0.48	10	1	NEWGENE_69363	

DMR1:198075001	1	198075001	198082000	7000	1	2.80E-10	0.59	61	0.87	Sgf29;LOC691797	
DMR1:198109001	1	198109001	198110000	1000	1	6.90E-16	0.76	6	0.6	Sgf29;Sult1a1;Slx1b	Transport;Transcription
DMR1:198135001	1	198135001	198137000	2000	1	2.40E-08	0.35	14	0.7	Coro1a;LOC108349642	Cytoskeleton
DMR1:198182001	1	198182001	198184000	2000	1	1.60E-18	0.83	52	2.6	LOC102551806;LOC103691234;Mapk3	Signaling
DMR1:198199001	1	198199001	198202000	3000	1	3.80E-09	0.33	56	1.87	Mapk3;Gdpd3;Ypel3	Signaling;Metabolism
DMR1:198243001	1	198243001	198246000	3000	2	5.30E-18	0.69	79	2.63	Aldoa;LOC103691235;Fam57b	Metabolism
DMR1:198258001	1	198258001	198260000	2000	2	9.30E-36	1.02	39	1.95	Fam57b;RGD1563217	
DMR1:198264001	1	198264001	198267000	3000	2	8.60E-13	0.57	55	1.83	Fam57b;RGD1563217	
DMR1:198268001	1	198268001	198270000	2000	1	4.60E-16	0.81	17	0.85	Fam57b;RGD1563217	
DMR1:198272001	1	198272001	198275000	3000	1	4.50E-14	0.6	33	1.1	Fam57b;RGD1563217;Doc2a	
DMR1:198278001	1	198278001	198281000	3000	3	2.40E-18	0.57	42	1.4	RGD1563217;Doc2a;Ino80e	
DMR1:198295001	1	198295001	198296000	1000	1	1.60E-08	-0.57	9	0.9	Doc2a;Ino80e;Hirip3;Taok2	Signaling
DMR1:198299001	1	198299001	198301000	2000	2	8.60E-17	0.68	35	1.75	Ino80e;Hirip3;Taok2	Signaling
DMR1:198304001	1	198304001	198305000	1000	1	2.20E-18	0.76	15	1.5	Ino80e;Hirip3;Taok2	Signaling
DMR1:198342001	1	198342001	198344000	2000	1	7.50E-17	0.79	20	1	Taok2;LOC108349643	Signaling
DMR1:198345001	1	198345001	198347000	2000	1	3.90E-08	-0.64	20	1	Taok2;LOC108349643	Signaling
DMR1:198348001	1	198348001	198350000	2000	1	6.40E-10	0.48	27	1.35	Taok2	Signaling
DMR1:198355001	1	198355001	198357000	2000	1	2.90E-20	0.91	21	1.05	Taok2;Kctd13	Signaling
DMR1:198369001	1	198369001	198371000	2000	1	2.40E-08	0.5	3	0.15	Kctd13;Asphd1	Metabolism
DMR1:198375001	1	198375001	198376000	1000	1	5.30E-18	0.52	23	2.3	Kctd13;Asphd1;Sez6l2	Metabolism
DMR1:198378001	1	198378001	198380000	2000	1	5.70E-10	0.37	29	1.45	Kctd13;Asphd1;Sez6l2	Metabolism
DMR1:198382001	1	198382001	198383000	1000	1	1.70E-11	0.78	37	3.7	Kctd13;Asphd1;Sez6l2	Metabolism
DMR1:198384001	1	198384001	198386000	2000	1	1.50E-07	0.44	35	1.75	Kctd13;Asphd1;Sez6l2	Metabolism
DMR1:198404001	1	198404001	198406000	2000	1	6.00E-07	0.43	15	0.75	Sez6l2;LOC102552638;Cdipt	Transport
DMR1:198418001	1	198418001	198420000	2000	2	2.00E-10	0.46	20	1	LOC102552638;Cdipt;LOC103691236;Mvp	Transport;Cytoskeleton
DMR1:198452001	1	198452001	198453000	1000	1	1.80E-17	0.8	15	1.5	Mvp;Pagr1;Prrt2;Maz;Kif22	Cytoskeleton;Transcription;Cytoskeleton
DMR1:198457001	1	198457001	198459000	2000	1	5.30E-11	0.4	39	1.95	Mvp;Pagr1;Prrt2;Maz;Kif22	Cytoskeleton;Transcription;Cytoskeleton
DMR1:198466001	1	198466001	198467000	1000	1	5.90E-07	-0.61	16	1.6	Maz;Kif22;LOC108349644	Transcription;Cytoskeleton
DMR1:198469001	1	198469001	198472000	3000	1	2.20E-07	-0.55	34	1.13	Maz;Kif22;LOC108349644	Transcription;Cytoskeleton
DMR1:198473001	1	198473001	198475000	2000	1	6.50E-15	0.63	24	1.2	Kif22;LOC108349644;Zg16	Cytoskeleton
DMR1:198550001	1	198550001	198553000	3000	1	6.50E-10	0.46	69	2.3	Qprt;LOC108349646	
DMR1:198560001	1	198560001	198563000	3000	2	7.30E-33	0.94	37	1.23	Qprt;LOC108349646;Spn	
DMR1:198580001	1	198580001	198582000	2000	2	1.30E-18	0.81	19	0.95	Spn	
DMR1:198583001	1	198583001	198585000	2000	1	3.00E-07	0.36	21	1.05	Spn	
DMR1:198591001	1	198591001	198592000	1000	1	9.20E-10	0.47	9	0.9	Spn	
DMR1:198630001	1	198630001	198632000	2000	1	9.60E-07	-0.48	26	1.3	Cd2bp2;Tbc1d10b	Translation;Signaling
DMR1:198641001	1	198641001	198644000	3000	2	3.10E-19	0.72	52	1.73	Cd2bp2;Tbc1d10b	Translation;Signaling
DMR1:198673001	1	198673001	198675000	2000	1	2.00E-20	0.64	31	1.55	Znf48	Transcription
DMR1:198682001	1	198682001	198684000	2000	1	1.40E-08	0.4	51	2.55	Znf48;Zfp771	Transcription
DMR1:198727001	1	198727001	198728000	1000	1	7.00E-32	1.06	9	0.9	Sephs2;LOC102553602;LOC365373	
DMR1:198731001	1	198731001	198735000	4000	2	1.80E-21	0.79	63	1.57	Sephs2;LOC102553602;LOC365373;LOC100361565;Itgal	Extracellular Matrix
DMR1:198736001	1	198736001	198739000	3000	2	8.20E-08	0.39	31	1.03	LOC102553602;LOC365373;LOC100361565;Itgal	Extracellular Matrix
DMR1:198743001	1	198743001	198745000	2000	1	1.20E-09	0.63	20	1	LOC102553602;LOC365373;LOC100361565;Itgal	Extracellular Matrix
DMR1:198750001	1	198750001	198752000	2000	1	3.30E-14	0.55	27	1.35	LOC100361565;Itgal	Extracellular Matrix
DMR1:198756001	1	198756001	198761000	5000	1	1.10E-12	0.67	67	1.34	Itgal	Extracellular Matrix
DMR1:198763001	1	198763001	198766000	3000	1	1.80E-07	-0.44	42	1.4	Itgal	Extracellular Matrix
DMR1:198773001	1	198773001	198776000	3000	1	1.60E-12	0.47	66	2.2	Itgal	Extracellular Matrix
DMR1:198782001	1	198782001	198784000	2000	1	2.70E-14	0.58	16	0.8	Itgal;Znf768	Extracellular Matrix;Transcription
DMR1:198796001	1	198796001	198797000	1000	1	4.40E-17	1.07	16	1.6	Znf768;LOC102547029	Transcription
DMR1:198805001	1	198805001	198808000	3000	1	3.60E-11	0.61	23	0.77	Znf768;LOC102547029	Transcription
DMR1:198903001	1	198903001	198905000	2000	1	1.90E-09	0.54	25	1.25	Zfp689;Prr14	
DMR1:198906001	1	198906001	198907000	1000	1	1.30E-07	0.32	17	1.7	Zfp689;Prr14	

DMR1:198932001	1	198932001	198934000	2000	1	1.60E-30	1.09	31	1.55	Fbrs	
DMR1:198965001	1	198965001	198966000	1000	1	4.30E-11	0.49	10	1	Srcap	
DMR1:198983001	1	198983001	198989000	6000	2	1.30E-22	0.66	71	1.18	Srcap	
DMR1:199004001	1	199004001	199005000	1000	1	2.20E-19	0.7	20	2	Srcap;Tmem265	
DMR1:199009001	1	199009001	199011000	2000	1	9.50E-13	0.47	17	0.85	Srcap;Tmem265;Phkg2	Signaling
DMR1:199020001	1	199020001	199025000	5000	2	7.00E-13	0.51	77	1.54	Tmem265;Phkg2;Ccdc189	Signaling
DMR1:199026001	1	199026001	199027000	1000	1	1.30E-11	0.44	21	2.1	Phkg2;Ccdc189	Signaling
DMR1:199038001	1	199038001	199040000	2000	1	8.40E-07	-0.52	44	2.2	Phkg2;Ccdc189;Rnf40	Signaling
DMR1:199048001	1	199048001	199050000	2000	1	1.30E-10	0.52	26	1.3	Rnf40;Zfp629	
DMR1:199065001	1	199065001	199066000	1000	1	4.30E-07	0.53	15	1.5	Zfp629	
DMR1:199074001	1	199074001	199075000	1000	1	1.20E-08	-0.54	15	1.5	Zfp629	
DMR1:199112001	1	199112001	199115000	3000	1	1.20E-12	0.69	50	1.67	Bcl7c	
DMR1:199117001	1	199117001	199118000	1000	1	5.60E-07	-0.4	11	1.1	Bcl7c	
DMR1:199178001	1	199178001	199180000	2000	1	1.70E-09	0.37	36	1.8	Ctf1;Ctf2	
DMR1:199184001	1	199184001	199185000	1000	1	5.70E-17	0.72	10	1	Ctf2;LOC102554987	
DMR1:199205001	1	199205001	199212000	7000	4	3.60E-12	-0.53	106	1.51	Fbxl19;Orai3	Transport
DMR1:199228001	1	199228001	199231000	3000	1	7.10E-09	-0.57	43	1.43	Orai3;Setd1b	Transport;Epigenetic
DMR1:199232001	1	199232001	199235000	3000	2	1.00E-24	0.74	61	2.03	Orai3;Setd1b	Transport;Epigenetic
DMR1:199242001	1	199242001	199244000	2000	1	4.70E-10	0.41	21	1.05	Setd1b;Hsd3b7;Stx1b	Epigenetic;Metabolism;Transcription
DMR1:199263001	1	199263001	199264000	1000	1	4.60E-07	0.29	20	2	Stx1b;LOC108349648	Transcription
DMR1:199312001	1	199312001	199317000	5000	1	3.50E-07	0.36	122	2.44	Zfp668;Zfp646	Transcription
DMR1:199374001	1	199374001	199376000	2000	1	5.90E-07	0.33	35	1.75	Kat8;Prss8;Prss36	Epigenetic;Protease
DMR1:199378001	1	199378001	199379000	1000	1	1.40E-15	0.46	17	1.7	Kat8;Prss8;Prss36	Epigenetic;Protease
DMR1:199387001	1	199387001	199390000	3000	1	3.20E-11	0.49	28	0.93	Prss8;Prss36	Protease
DMR1:199403001	1	199403001	199405000	2000	1	7.30E-13	0.69	14	0.7	Prss36;Fus	Protease;Metabolism
DMR1:199417001	1	199417001	199418000	1000	1	1.40E-22	0.69	15	1.5	Fus	Metabolism
DMR1:199424001	1	199424001	199427000	3000	2	4.40E-36	0.93	34	1.13	Fus	Metabolism
DMR1:199432001	1	199432001	199433000	1000	1	9.30E-07	-0.53	8	0.8	Fus;Pycard	Metabolism
DMR1:199455001	1	199455001	199456000	1000	1	2.40E-25	0.78	19	1.9	Trim72	Proteolysis
DMR1:199460001	1	199460001	199462000	2000	2	5.80E-15	0.55	8	0.4	Trim72;LOC102556076	Proteolysis
DMR1:199484001	1	199484001	199487000	3000	1	1.30E-11	0.53	27	0.9	LOC102556076;LOC102556135;Itgam	
DMR1:199491001	1	199491001	199497000	6000	2	5.60E-15	0.55	81	1.35	LOC102556076;LOC102556135;Itgam	
DMR1:199498001	1	199498001	199503000	5000	1	3.00E-11	0.75	56	1.12	LOC102556135;Itgam	
DMR1:199519001	1	199519001	199521000	2000	1	2.90E-11	0.44	34	1.7	Itgam	
DMR1:199537001	1	199537001	199543000	6000	2	6.40E-08	0.44	106	1.77	Itgam	
DMR1:199544001	1	199544001	199547000	3000	1	1.10E-08	0.42	42	1.4	Itgam;Itgax	Extracellular Matrix
DMR1:199549001	1	199549001	199552000	3000	2	1.90E-08	-0.47	42	1.4	Itgam;Itgax	Extracellular Matrix
DMR1:199559001	1	199559001	199561000	2000	1	1.10E-10	0.37	35	1.75	Itgax	Extracellular Matrix
DMR1:199568001	1	199568001	199571000	3000	1	6.50E-08	-0.63	40	1.33	Itgax	Extracellular Matrix
DMR1:199572001	1	199572001	199576000	4000	1	2.50E-08	0.39	65	1.62	Itgax	Extracellular Matrix
DMR1:199583001	1	199583001	199585000	2000	1	9.70E-10	-0.7	16	0.8	Itgax	Extracellular Matrix
DMR1:199596001	1	199596001	199598000	2000	1	3.30E-14	0.48	26	1.3	Itgad	Extracellular Matrix
DMR1:199601001	1	199601001	199603000	2000	2	2.20E-20	0.69	30	1.5	Itgad	Extracellular Matrix
DMR1:199613001	1	199613001	199616000	3000	1	3.40E-07	-0.54	39	1.3	Itgad;Cox6a2	Extracellular Matrix;Metabolism
DMR1:199617001	1	199617001	199619000	2000	1	9.40E-13	0.5	31	1.55	Itgad;Cox6a2	Extracellular Matrix;Metabolism
DMR1:199635001	1	199635001	199638000	3000	1	2.00E-07	0.34	35	1.17	Cox6a2;LOC103691238	Metabolism;Transcription
DMR1:199646001	1	199646001	199647000	1000	1	3.90E-09	-0.46	9	0.9	LOC103691238;Armc5	Transcription
DMR1:199659001	1	199659001	199662000	3000	3	9.20E-16	0.54	80	2.67	LOC103691238;Armc5;Tgfb1i1	Transcription;Cytoskeleton
DMR1:199679001	1	199679001	199683000	4000	2	8.60E-11	0.6	52	1.3	Tgfb1i1;Slc5a2;RGD1310127	Cytoskeleton;Transport
DMR1:199693001	1	199693001	199695000	2000	2	1.20E-27	1.11	43	2.15	Slc5a2;RGD1310127	Transport
DMR1:199702001	1	199702001	199703000	1000	1	1.30E-09	0.39	9	0.9	RGD1310127;LOC102556439	
DMR1:199705001	1	199705001	199706000	1000	1	4.60E-09	-0.62	10	1	RGD1310127;LOC102556439	
DMR1:199708001	1	199708001	199711000	3000	1	4.80E-16	-0.59	27	0.9	RGD1310127;LOC102556439;Ahsp	Transcription
DMR1:199716001	1	199716001	199717000	1000	1	4.10E-20	0.77	33	3.3	RGD1310127;LOC102556439;Ahsp	Transcription

DMR1:199727001	1	199727001	199729000	2000	1	6.00E-09	0.36	18	0.9	Ahsp	Transcription
DMR1:199773001	1	199773001	199775000	2000	2	1.10E-07	0.37	27	1.35	LOC102546301;LOC103691239; Rgs10	
DMR1:199792001	1	199792001	199796000	4000	1	1.30E-08	-0.52	59	1.48	Rgs10	
DMR1:199814001	1	199814001	199820000	6000	1	1.80E-07	-0.38	88	1.47	Rgs10	
DMR1:199826001	1	199826001	199832000	6000	1	1.40E-07	-0.5	102	1.7	Rgs10	
DMR1:199834001	1	199834001	199836000	2000	1	1.10E-16	0.56	30	1.5	Tial1	Translation
DMR1:199855001	1	199855001	199857000	2000	2	1.50E-11	0.52	11	0.55	Tial1	Translation
DMR1:199874001	1	199874001	199877000	3000	1	3.40E-08	0.43	32	1.07	Tial1;LOC687874;LOC108349783	Translation
DMR1:199951001	1	199951001	199955000	4000	1	7.30E-08	-0.49	62	1.55	Bag3	
DMR1:200040001	1	200040001	200041000	1000	1	1.90E-13	0.79	5	0.5	Inpp5f	Signaling
DMR1:200102001	1	200102001	200103000	1000	1	4.80E-19	0.9	22	2.2	Inpp5f	Signaling
DMR1:200107001	1	200107001	200108000	1000	1	4.60E-08	-0.57	23	2.3	Inpp5f	Signaling
DMR1:200135001	1	200135001	200137000	2000	1	1.60E-12	0.6	22	1.1	Mcmbp	
DMR1:200145001	1	200145001	200147000	2000	1	6.90E-08	0.29	16	0.8	Mcmbp	
DMR1:200169001	1	200169001	200170000	1000	1	3.50E-18	0.88	8	0.8	Mcmbp;Sec23ip	Metabolism
DMR1:200197001	1	200197001	200203000	6000	2	1.50E-08	-0.52	67	1.12	Sec23ip	Metabolism
DMR1:200204001	1	200204001	200208000	4000	1	7.50E-11	0.4	52	1.3	Sec23ip	Metabolism
DMR1:200209001	1	200209001	200211000	2000	1	5.30E-07	-0.55	34	1.7	Sec23ip	Metabolism
DMR1:200214001	1	200214001	200215000	1000	1	1.40E-07	-0.4	11	1.1	Sec23ip	Metabolism
DMR1:200217001	1	200217001	200219000	2000	1	1.40E-13	0.69	26	1.3	Sec23ip	Metabolism
DMR1:200583001	1	200583001	200586000	3000	1	4.20E-08	-0.54	32	1.07	Fgfr2	Receptor
DMR1:200596001	1	200596001	200598000	2000	1	8.80E-08	-0.53	29	1.45	Fgfr2	Receptor
DMR1:200599001	1	200599001	200605000	6000	1	6.20E-09	-0.6	78	1.3	Fgfr2	Receptor
DMR1:200610001	1	200610001	200612000	2000	1	2.10E-10	0.43	34	1.7	Fgfr2	Receptor
DMR1:200657001	1	200657001	200662000	5000	1	1.20E-09	0.65	88	1.76	Fgfr2	Receptor
DMR1:200663001	1	200663001	200669000	6000	1	2.50E-08	-0.44	83	1.38	Fgfr2	Receptor
DMR1:200689001	1	200689001	200694000	5000	1	8.60E-10	0.35	123	2.46	Fgfr2	Receptor
DMR1:200701001	1	200701001	200703000	2000	1	3.90E-19	0.68	32	1.6	Fgfr2	Receptor
DMR1:200835001	1	200835001	200837000	2000	1	1.60E-11	0.52	17	0.85	Ate1	Translation
DMR1:200863001	1	200863001	200864000	1000	1	1.20E-12	0.75	9	0.9	Ate1;LOC108349651	Translation
DMR1:200892001	1	200892001	200893000	1000	1	1.10E-07	0.45	7	0.7	Ate1	Translation
DMR1:200981001	1	200981001	200982000	1000	1	1.40E-13	0.56	5	0.5	Ate1	Translation
DMR1:201008001	1	201008001	201009000	1000	1	4.80E-07	0.63	10	1	Ate1;LOC108349649	Translation
DMR1:201091001	1	201091001	201093000	2000	1	5.60E-12	0.43	30	1.5	Ate1;RGD1563285	Translation
DMR1:201103001	1	201103001	201110000	7000	1	5.70E-15	0.43	103	1.47	RGD1563285;Nsmce4a	
DMR1:201115001	1	201115001	201116000	1000	1	2.50E-11	-0.85	16	1.6	Nsmce4a	
DMR1:201156001	1	201156001	201160000	4000	1	1.70E-08	-0.51	36	0.9	Tacc2	
DMR1:201176001	1	201176001	201181000	5000	1	1.00E-09	-0.58	68	1.36	Tacc2	
DMR1:201186001	1	201186001	201187000	1000	1	1.30E-09	-0.73	21	2.1	Tacc2	
DMR1:201191001	1	201191001	201192000	1000	1	1.60E-09	0.35	14	1.4	Tacc2	
DMR1:201232001	1	201232001	201236000	4000	1	1.10E-09	0.53	64	1.6	Tacc2;LOC103691241	
DMR1:201254001	1	201254001	201256000	2000	1	1.70E-07	-0.49	22	1.1	Tacc2;LOC103691241	
DMR1:201258001	1	201258001	201259000	1000	1	3.40E-07	-0.49	20	2	Tacc2	
DMR1:201263001	1	201263001	201264000	1000	1	4.40E-11	0.48	7	0.7	Tacc2	
DMR1:201291001	1	201291001	201293000	2000	2	2.00E-14	0.54	23	1.15	Tacc2	
DMR1:201318001	1	201318001	201320000	2000	1	5.20E-10	0.52	27	1.35	Tacc2	
DMR1:201323001	1	201323001	201324000	1000	1	5.60E-09	0.44	25	2.5	Tacc2	
DMR1:201333001	1	201333001	201336000	3000	2	1.50E-07	-0.49	47	1.57	Tacc2;Btbd16	
DMR1:201338001	1	201338001	201343000	5000	2	2.10E-14	0.51	69	1.38	Tacc2;Btbd16	
DMR1:201358001	1	201358001	201361000	3000	3	1.10E-13	0.5	72	2.4	Btbd16	
DMR1:201381001	1	201381001	201388000	7000	1	3.30E-14	0.52	72	1.03	Btbd16	
DMR1:201392001	1	201392001	201393000	1000	1	3.90E-08	-0.44	14	1.4	Btbd16	
DMR1:201423001	1	201423001	201424000	1000	1	1.40E-10	0.4	15	1.5	Plekha1	
DMR1:201427001	1	201427001	201428000	1000	1	9.80E-07	0.34	11	1.1	Plekha1	
DMR1:201435001	1	201435001	201436000	1000	1	9.30E-07	-0.52	10	1	Plekha1	
DMR1:201473001	1	201473001	201475000	2000	1	2.10E-08	0.43	25	1.25	Plekha1	
DMR1:201484001	1	201484001	201486000	2000	1	3.20E-08	-0.62	26	1.3	Plekha1	
DMR1:201487001	1	201487001	201488000	1000	1	2.40E-10	-0.53	8	0.8	Plekha1	
DMR1:201503001	1	201503001	201506000	3000	1	9.80E-07	-0.41	42	1.4	Htra1	Protease
DMR1:201514001	1	201514001	201517000	3000	1	1.80E-07	-0.55	37	1.23	Htra1	Protease
DMR1:201527001	1	201527001	201532000	5000	2	5.00E-14	-0.5	71	1.42	Htra1	Protease
DMR1:201621001	1	201621001	201622000	1000	1	3.70E-08	0.68	3	0.3	Dmbt1	Protease
DMR1:201631001	1	201631001	201635000	4000	1	8.10E-08	0.42	35	0.88	Dmbt1	Protease

DMR1:201648001	1	201648001	201649000	1000	1	1.40E-08	0.35	5	0.5	Dmbt1	Protease
DMR1:201675001	1	201675001	201676000	1000	1	8.30E-12	-0.7	12	1.2	Dmbt1	Protease
DMR1:201684001	1	201684001	201685000	1000	1	9.40E-11	0.66	8	0.8	Dmbt1	Protease
DMR1:201687001	1	201687001	201689000	2000	1	1.80E-07	-0.38	22	1.1	Dmbt1	Protease
DMR1:201890001	1	201890001	201892000	2000	2	1.80E-15	0.58	28	1.4	Cuzd1;RGD1559891	Receptor
DMR1:201900001	1	201900001	201902000	2000	1	1.50E-07	-0.48	27	1.35	Cuzd1;RGD1559891;RGD1560958	Receptor
DMR1:201909001	1	201909001	201910000	1000	1	3.50E-08	0.56	5	0.5	RGD1559891;RGD1560958;Fam24a	
DMR1:201981001	1	201981001	201985000	4000	1	2.20E-08	-0.4	88	2.2	Ikzf5;Acadsb	Transcription;Metabolism
DMR1:201986001	1	201986001	201987000	1000	1	2.90E-07	0.53	7	0.7	Ikzf5;Acadsb	Transcription;Metabolism
DMR1:202001001	1	202001001	202003000	2000	1	4.80E-08	0.41	9	0.45	Acadsb	Metabolism
DMR1:202013001	1	202013001	202019000	6000	2	4.20E-22	1.01	62	1.03	Acadsb	Metabolism
DMR1:202449001	1	202449001	202451000	2000	1	2.30E-08	-0.45	25	1.25	Plpp4	Signaling
DMR1:202454001	1	202454001	202455000	1000	1	1.40E-07	0.49	10	1	Plpp4	Signaling
DMR1:202467001	1	202467001	202471000	4000	1	2.90E-11	-0.48	52	1.3	Plpp4	Signaling
DMR1:202488001	1	202488001	202491000	3000	1	7.20E-22	0.74	24	0.8	Plpp4	Signaling
DMR1:202506001	1	202506001	202508000	2000	1	4.70E-07	-0.49	14	0.7	Plpp4	Signaling
DMR1:202513001	1	202513001	202514000	1000	1	7.90E-08	0.44	5	0.5	Plpp4	Signaling
DMR1:202522001	1	202522001	202524000	2000	2	7.30E-17	0.56	17	0.85	Plpp4	Signaling
DMR1:202567001	1	202567001	202569000	2000	2	1.20E-22	0.99	20	1	Plpp4;LOC691951	Signaling
DMR1:202570001	1	202570001	202575000	5000	1	1.10E-10	-0.71	80	1.6	Plpp4;LOC691951	Signaling
DMR1:202758001	1	202758001	202762000	4000	2	8.50E-07	-0.39	43	1.07	Wdr11	
DMR1:202764001	1	202764001	202766000	2000	2	1.00E-16	0.79	9	0.45	Wdr11	
DMR1:202768001	1	202768001	202769000	1000	1	1.20E-12	0.78	4	0.4	Wdr11	
DMR1:202779001	1	202779001	202781000	2000	1	1.10E-07	0.38	29	1.45	Wdr11	
DMR1:202783001	1	202783001	202785000	2000	1	9.20E-10	-0.67	29	1.45	Wdr11	
DMR1:202805001	1	202805001	202807000	2000	1	5.30E-07	-0.59	25	1.25	Wdr11	
DMR1:202823001	1	202823001	202826000	3000	1	1.10E-07	0.38	34	1.13	Wdr11;LOC102555739	
DMR1:203140001	1	203140001	203141000	1000	1	1.80E-07	0.57	8	0.8	Dmbt1	Protease
DMR1:203150001	1	203150001	203151000	1000	1	6.60E-10	0.76	2	0.2	Dmbt1	Protease
DMR1:203162001	1	203162001	203164000	2000	1	1.90E-10	0.61	15	0.75	Dmbt1	Protease
DMR1:203166001	1	203166001	203167000	1000	1	2.90E-08	0.71	7	0.7	Dmbt1	Protease
DMR1:203226001	1	203226001	203228000	2000	1	1.20E-10	-0.44	22	1.1	Dmbt1	Protease
DMR1:203230001	1	203230001	203232000	2000	1	1.20E-12	0.41	34	1.7	Dmbt1	Protease
DMR1:203503001	1	203503001	203504000	1000	1	5.80E-11	0.52	10	1	Hmx3	Development
DMR1:203532001	1	203532001	203537000	5000	1	1.30E-07	-0.49	78	1.56	Hmx2;Bub3	Development;Metabolism
DMR1:204045001	1	204045001	204046000	1000	1	2.40E-14	1.04	5	0.5	Cpxm2	Protease
DMR1:204049001	1	204049001	204052000	3000	1	1.30E-09	-0.68	34	1.13	Cpxm2	Protease
DMR1:204054001	1	204054001	204057000	3000	1	8.90E-07	-0.53	30	1	Cpxm2	Protease
DMR1:204062001	1	204062001	204064000	2000	2	4.70E-11	-0.47	35	1.75	Cpxm2	Protease
DMR1:204122001	1	204122001	204124000	2000	2	2.10E-07	-0.57	18	0.9	Cpxm2	Protease
DMR1:204126001	1	204126001	204127000	1000	1	3.50E-12	0.62	4	0.4	Cpxm2	Protease
DMR1:204142001	1	204142001	204144000	2000	1	4.00E-07	-0.52	28	1.4	Cpxm2	Protease
DMR1:204254001	1	204254001	204256000	2000	1	1.80E-10	0.33	50	2.5	Chst15	Transport
DMR1:204270001	1	204270001	204273000	3000	3	1.30E-09	-0.52	48	1.6	Chst15	Transport
DMR1:204300001	1	204300001	204301000	1000	1	4.40E-12	0.71	4	0.4	Chst15	Transport
DMR1:204329001	1	204329001	204330000	1000	1	1.40E-07	0.5	6	0.6	Chst15;LOC103691248	Transport
DMR1:204568001	1	204568001	204575000	7000	2	3.10E-08	-0.46	72	1.03	Oat	Metabolism
DMR1:204580001	1	204580001	204581000	1000	1	1.60E-20	0.81	11	1.1	Oat	Metabolism
DMR1:204583001	1	204583001	204586000	3000	1	3.20E-16	0.88	41	1.37	Oat	Metabolism
DMR1:204597001	1	204597001	204599000	2000	1	8.60E-07	-0.57	24	1.2	Nkx1-2	Development
DMR1:204607001	1	204607001	204610000	3000	1	4.20E-07	-0.43	37	1.23	Nkx1-2;Lhpp	Development;Signaling
DMR1:204620001	1	204620001	204623000	3000	1	7.10E-11	0.48	41	1.37	Lhpp	Signaling
DMR1:204627001	1	204627001	204628000	1000	1	9.70E-10	0.41	13	1.3	Lhpp	Signaling
DMR1:204661001	1	204661001	204664000	3000	1	2.10E-23	0.65	42	1.4	Lhpp	Signaling
DMR1:204665001	1	204665001	204666000	1000	1	1.40E-08	0.4	17	1.7	Lhpp	Signaling
DMR1:204692001	1	204692001	204694000	2000	2	4.40E-12	-0.6	42	2.1	Lhpp	Signaling
DMR1:204695001	1	204695001	204699000	4000	1	1.10E-09	0.48	72	1.8	Lhpp	Signaling
DMR1:204705001	1	204705001	204707000	2000	1	2.60E-19	0.76	22	1.1	Lhpp;Fam53b	Signaling
DMR1:204732001	1	204732001	204733000	1000	1	2.00E-11	0.6	6	0.6	Fam53b	

DMR1:204763001	1	204763001	204764000	1000	1	6.60E-11	0.67	10	1	Fam53b	
DMR1:204777001	1	204777001	204780000	3000	1	2.80E-10	0.46	32	1.07	Fam53b	
DMR1:204786001	1	204786001	204787000	1000	1	6.20E-07	0.31	19	1.9	Fam53b	
DMR1:204808001	1	204808001	204810000	2000	1	1.50E-07	-0.47	20	1	Fam53b;LOC102552031	
DMR1:204814001	1	204814001	204815000	1000	1	1.80E-12	0.73	4	0.4	Fam53b;LOC102552031	
DMR1:204854001	1	204854001	204856000	2000	1	3.10E-10	0.52	48	2.4	Mettl10;Fam175b	
DMR1:204864001	1	204864001	204865000	1000	1	4.60E-09	0.52	6	0.6	Mettl10;Fam175b	
DMR1:204966001	1	204966001	204967000	1000	1	2.10E-21	0.81	3	0.3	Zranb1	Protease
DMR1:204973001	1	204973001	204975000	2000	1	3.10E-07	-0.68	10	0.5	Zranb1	Protease
DMR1:205008001	1	205008001	205010000	2000	1	2.00E-28	0.93	35	1.75	Zranb1;Ctbp2	Protease;Transcription
DMR1:205024001	1	205024001	205028000	4000	1	1.60E-07	-0.48	59	1.48	Ctbp2	Transcription
DMR1:205608001	1	205608001	205610000	2000	2	8.30E-13	0.47	17	0.85	Tex36	
DMR1:205615001	1	205615001	205619000	4000	2	1.30E-24	0.8	36	0.9	Tex36	
DMR1:205628001	1	205628001	205630000	2000	1	7.80E-10	0.53	23	1.15	Tex36;LOC102552830	
DMR1:205731001	1	205731001	205734000	3000	1	1.30E-20	0.68	60	2	Edrf1	
DMR1:205754001	1	205754001	205760000	6000	1	3.00E-13	0.42	93	1.55	Mmp21;Uros	Protease;Metabolism
DMR1:205778001	1	205778001	205781000	3000	1	5.80E-07	0.51	27	0.9	Uros;Bccip;Dhx32	Metabolism;Transcription
DMR1:205793001	1	205793001	205795000	2000	1	4.60E-07	0.37	38	1.9	Bccip;Dhx32	Transcription
DMR1:205799001	1	205799001	205800000	1000	1	5.40E-14	0.5	10	1	Bccip;Dhx32	Transcription
DMR1:205801001	1	205801001	205809000	8000	1	5.20E-09	0.4	98	1.23	Dhx32	Transcription
DMR1:205812001	1	205812001	205813000	1000	1	1.50E-07	0.52	5	0.5	Dhx32	Transcription
DMR1:205846001	1	205846001	205848000	2000	1	6.80E-08	0.41	15	0.75	Dhx32;Fank1	Transcription
DMR1:205863001	1	205863001	205867000	4000	1	8.10E-14	-0.69	40	1	Fank1	
DMR1:205874001	1	205874001	205876000	2000	1	5.00E-07	0.33	17	0.85	Fank1;LOC108349724	
DMR1:205884001	1	205884001	205886000	2000	1	7.10E-08	0.49	8	0.4	Fank1;LOC108349724	
DMR1:205915001	1	205915001	205917000	2000	2	1.20E-08	0.44	13	0.65	Fank1	
DMR1:205920001	1	205920001	205923000	3000	1	2.50E-10	-0.54	25	0.83	Fank1	
DMR1:205926001	1	205926001	205928000	2000	2	2.30E-07	0.47	12	0.6	Fank1	
DMR1:205932001	1	205932001	205934000	2000	1	3.70E-07	0.35	16	0.8	Fank1	
DMR1:205942001	1	205942001	205950000	8000	4	1.20E-19	-0.49	94	1.18	Fank1;Adam12	Protease
DMR1:205999001	1	205999001	2.06E+08	1000	1	6.80E-13	0.5	12	1.2	Adam12	Protease
DMR1:206015001	1	206015001	206016000	1000	1	2.50E-19	0.84	7	0.7	Adam12	Protease
DMR1:206044001	1	206044001	206046000	2000	1	4.20E-08	0.48	18	0.9	Adam12	Protease
DMR1:206047001	1	206047001	206049000	2000	1	9.80E-08	-0.6	29	1.45	Adam12	Protease
DMR1:206053001	1	206053001	206054000	1000	1	3.00E-08	0.42	15	1.5	Adam12	Protease
DMR1:206055001	1	206055001	206056000	1000	1	1.60E-09	0.48	9	0.9	Adam12	Protease
DMR1:206069001	1	206069001	206072000	3000	3	9.00E-11	0.54	18	0.6	Adam12;LOC108349655	Protease
DMR1:206107001	1	206107001	206110000	3000	1	5.70E-07	0.39	32	1.07	Adam12;LOC108349655	Protease
DMR1:206111001	1	206111001	206116000	5000	2	7.70E-12	0.45	52	1.04	Adam12;LOC108349655	Protease
DMR1:206149001	1	206149001	206151000	2000	1	9.40E-21	0.78	23	1.15	Adam12	Protease
DMR1:206192001	1	206192001	206196000	4000	2	2.60E-17	-0.52	66	1.65	Adam12	Protease
DMR1:206220001	1	206220001	206222000	2000	2	7.20E-15	0.79	20	1	Adam12	Protease
DMR1:206233001	1	206233001	206242000	9000	5	1.40E-19	-0.46	103	1.14	Adam12	Protease
DMR1:206268001	1	206268001	206270000	2000	1	1.90E-09	0.87	41	2.05	Adam12	Protease
DMR1:206293001	1	206293001	206295000	2000	1	3.90E-08	-0.55	19	0.95	Adam12	Protease
DMR1:206906001	1	206906001	206908000	2000	1	1.10E-13	0.62	26	1.3	Dock1	Transcription
DMR1:206911001	1	206911001	206912000	1000	1	4.60E-11	0.56	9	0.9	Dock1	Transcription
DMR1:206936001	1	206936001	206940000	4000	2	3.90E-23	0.72	47	1.18	Dock1	Transcription
DMR1:206959001	1	206959001	206962000	3000	3	1.90E-23	0.84	22	0.73	Dock1	Transcription
DMR1:206978001	1	206978001	206979000	1000	1	3.20E-12	-0.79	19	1.9	Dock1	Transcription
DMR1:206987001	1	206987001	206990000	3000	1	3.70E-09	-0.53	38	1.27	Dock1	Transcription
DMR1:207007001	1	207007001	207012000	5000	1	1.00E-11	-0.47	66	1.32	Dock1	Transcription
DMR1:207035001	1	207035001	207037000	2000	1	1.10E-08	0.46	9	0.45	Dock1	Transcription
DMR1:207079001	1	207079001	207083000	4000	1	6.40E-09	0.4	74	1.85	Dock1	Transcription
DMR1:207094001	1	207094001	207099000	5000	1	1.90E-08	-0.62	60	1.2	Dock1	Transcription
DMR1:207102001	1	207102001	207105000	3000	2	2.80E-11	0.59	28	0.93	Dock1	Transcription
DMR1:207139001	1	207139001	207140000	1000	1	2.30E-12	0.72	4	0.4	Dock1;Fam196a	Transcription
DMR1:207150001	1	207150001	207152000	2000	2	5.90E-09	-0.7	21	1.05	Dock1;Fam196a	Transcription
DMR1:207180001	1	207180001	207182000	2000	2	9.00E-18	0.75	11	0.55	Dock1;Fam196a	Transcription
DMR1:207190001	1	207190001	207193000	3000	2	2.50E-09	0.36	33	1.1	Dock1	Transcription
DMR1:207204001	1	207204001	207205000	1000	1	8.90E-15	0.62	16	1.6	Dock1	Transcription
DMR1:207235001	1	207235001	207238000	3000	1	3.00E-11	0.63	32	1.07	Dock1	Transcription

DMR1:207256001	1	207256001	207258000	2000	1	4.20E-14	0.75	13	0.65	Dock1	Transcription
DMR1:207273001	1	207273001	207275000	2000	1	6.00E-07	-0.57	22	1.1	Dock1	Transcription
DMR1:207286001	1	207286001	207288000	2000	1	3.30E-15	0.81	11	0.55	Dock1	Transcription
DMR1:207304001	1	207304001	207305000	1000	1	2.60E-25	0.98	6	0.6	Dock1	Transcription
DMR1:207352001	1	207352001	207354000	2000	2	5.40E-14	0.98	20	1	Dock1	Transcription
DMR1:207358001	1	207358001	207362000	4000	1	9.00E-14	0.55	51	1.27	Dock1	Transcription
DMR1:207381001	1	207381001	207382000	1000	1	8.70E-07	-0.36	15	1.5	Dock1;LOC103691254	Transcription
DMR1:207402001	1	207402001	207406000	4000	1	5.90E-19	0.61	55	1.38	Dock1	Transcription
DMR1:207418001	1	207418001	207423000	5000	2	2.50E-15	0.86	54	1.08	Dock1	Transcription
DMR1:207493001	1	207493001	207499000	6000	4	7.40E-18	0.97	43	0.72	Nps	
DMR1:207501001	1	207501001	207503000	2000	1	6.90E-11	0.51	33	1.65	Nps	
DMR1:207520001	1	207520001	207522000	2000	2	7.20E-17	0.84	16	0.8	Nps	
DMR1:207650001	1	207650001	207654000	4000	1	7.80E-13	0.62	30	0.75	Foxi2	Transcription
DMR1:207665001	1	207665001	207668000	3000	1	7.20E-07	-0.39	25	0.83	Foxi2	Transcription
DMR1:207745001	1	207745001	207746000	1000	1	2.50E-09	0.57	7	0.7	Clrn3	
DMR1:207749001	1	207749001	207750000	1000	1	5.60E-09	0.36	11	1.1	Clrn3	
DMR1:207812001	1	207812001	207813000	1000	1	2.60E-07	0.36	20	2	Ptpre	Signaling
DMR1:207857001	1	207857001	207860000	3000	1	4.30E-10	-0.56	46	1.53	Ptpre	Signaling
DMR1:207863001	1	207863001	207865000	2000	2	4.00E-18	0.55	53	2.65	Ptpre	Signaling
DMR1:207940001	1	207940001	207943000	3000	2	1.10E-19	1.05	38	1.27	Ptpre;LOC108349658;LOC102551963	Signaling
DMR1:207948001	1	207948001	207953000	5000	1	2.70E-10	-0.62	64	1.28	Ptpre;LOC108349658;LOC102551963	Signaling
DMR1:207986001	1	207986001	207987000	1000	1	1.40E-08	0.47	14	1.4	Ptpre;Mki67	Signaling
DMR1:208014001	1	208014001	208015000	1000	1	5.40E-16	0.63	10	1	Mki67	
DMR1:209242001	1	209242001	209247000	5000	4	1.50E-13	0.75	33	0.66	Mgmt	
DMR1:209252001	1	209252001	209255000	3000	2	4.20E-11	-0.63	51	1.7	Mgmt	
DMR1:209262001	1	209262001	209281000	19000	6	6.30E-21	0.94	277	1.46	Mgmt	
DMR1:209316001	1	209316001	209317000	1000	1	3.60E-08	-0.65	15	1.5	Mgmt	
DMR1:209321001	1	209321001	209324000	3000	2	6.20E-09	-0.47	48	1.6	Mgmt	
DMR1:209353001	1	209353001	209355000	2000	1	1.40E-07	-0.49	36	1.8	Mgmt	
DMR1:209375001	1	209375001	209376000	1000	1	7.30E-08	0.39	11	1.1	Mgmt	
DMR1:209377001	1	209377001	209380000	3000	1	6.60E-22	0.71	50	1.67	Mgmt	
DMR1:209448001	1	209448001	209450000	2000	1	3.10E-07	-0.47	11	0.55	Mgmt	
DMR1:209534001	1	209534001	209537000	3000	1	8.80E-07	-0.44	69	2.3	Ebf3	Transcription
DMR1:209562001	1	209562001	209567000	5000	1	2.60E-08	-0.62	97	1.94	Ebf3	Transcription
DMR1:209578001	1	209578001	209581000	3000	1	7.90E-08	-0.56	34	1.13	Ebf3	Transcription
DMR1:209584001	1	209584001	209588000	4000	1	4.00E-14	0.74	53	1.32	Ebf3	Transcription
DMR1:209590001	1	209590001	209598000	8000	2	1.50E-10	-0.66	112	1.4	Ebf3	Transcription
DMR1:209602001	1	209602001	209603000	1000	1	1.30E-15	0.67	12	1.2	Ebf3	Transcription
DMR1:209607001	1	209607001	209610000	3000	1	4.00E-07	-0.47	43	1.43	Ebf3	Transcription
DMR1:209620001	1	209620001	209625000	5000	1	1.70E-08	-0.55	109	2.18	Ebf3	Transcription
DMR1:209761001	1	209761001	209764000	3000	1	3.90E-08	0.35	55	1.83	Glrx3	Metabolism
DMR1:209771001	1	209771001	209773000	2000	1	2.60E-22	0.85	13	0.65	Glrx3	Metabolism
DMR1:209786001	1	209786001	209788000	2000	1	6.50E-08	0.38	25	1.25	Glrx3;LOC103691257	Metabolism
DMR1:210552001	1	210552001	210557000	5000	1	1.20E-07	0.41	50	1	Tcerg1l	Transcription
DMR1:210612001	1	210612001	210625000	13000	3	2.50E-13	-0.58	135	1.04	Tcerg1l	Transcription
DMR1:210639001	1	210639001	210640000	1000	1	6.30E-09	0.46	5	0.5	Tcerg1l	Transcription
DMR1:210644001	1	210644001	210646000	2000	1	2.90E-12	0.68	20	1	Tcerg1l	Transcription
DMR1:210653001	1	210653001	210654000	1000	1	3.50E-13	0.61	10	1	Tcerg1l	Transcription
DMR1:210703001	1	210703001	210705000	2000	1	4.40E-08	-0.71	25	1.25	Tcerg1l	Transcription
DMR1:210737001	1	210737001	210738000	1000	1	4.80E-12	0.71	4	0.4	Tcerg1l	Transcription
DMR1:211252001	1	211252001	211253000	1000	1	3.40E-11	0.51	9	0.9	Bnip3	
DMR1:211272001	1	211272001	211274000	2000	1	3.40E-07	0.37	32	1.6	Bnip3;LOC102553541	
DMR1:211287001	1	211287001	211288000	1000	1	5.00E-15	0.66	12	1.2	LOC102553541;LOC108349665;Jakmip3	
DMR1:211289001	1	211289001	211291000	2000	2	4.80E-14	-0.6	44	2.2	LOC108349665;Jakmip3	
DMR1:211297001	1	211297001	211299000	2000	2	2.40E-09	-0.43	26	1.3	LOC108349665;Jakmip3	
DMR1:211300001	1	211300001	211306000	6000	3	1.00E-20	0.57	55	0.92	LOC108349665;Jakmip3	
DMR1:211318001	1	211318001	211319000	1000	1	3.30E-13	0.46	18	1.8	Jakmip3	
DMR1:211325001	1	211325001	211327000	2000	1	2.10E-28	1.23	25	1.25	Jakmip3	
DMR1:211328001	1	211328001	211333000	5000	2	2.60E-10	0.42	48	0.96	Jakmip3	
DMR1:211341001	1	211341001	211345000	4000	2	1.00E-16	0.53	38	0.95	Jakmip3	
DMR1:211368001	1	211368001	211372000	4000	1	5.10E-13	0.42	84	2.1	Jakmip3	
DMR1:211393001	1	211393001	211394000	1000	1	2.30E-17	0.9	3	0.3	Jakmip3	

DMR1:211434001	1	211434001	211436000	2000	1	4.50E-27	1.13	27	1.35	Dpysl4;Stk32c	Metabolism;Signaling
DMR1:211445001	1	211445001	211451000	6000	1	3.40E-08	0.4	87	1.45	Dpysl4;Stk32c	Metabolism;Signaling
DMR1:211453001	1	211453001	211454000	1000	1	4.80E-16	0.73	5	0.5	Stk32c	Signaling
DMR1:211505001	1	211505001	211507000	2000	1	9.00E-07	0.32	18	0.9	Stk32c	Signaling
DMR1:211515001	1	211515001	211517000	2000	1	7.20E-10	0.5	12	0.6	Stk32c	Signaling
DMR1:211524001	1	211524001	211526000	2000	1	1.50E-08	0.41	15	0.75	Stk32c	Signaling
DMR1:211535001	1	211535001	211538000	3000	1	2.00E-10	-0.62	25	0.83	Lrrc27	
DMR1:211540001	1	211540001	211542000	2000	1	7.80E-07	0.35	13	0.65	Lrrc27	
DMR1:211593001	1	211593001	211595000	2000	1	7.70E-07	-0.44	28	1.4	Pwwp2b	Epigenetic
DMR1:211603001	1	211603001	211604000	1000	1	1.70E-10	0.39	9	0.9	Pwwp2b	Epigenetic
DMR1:211606001	1	211606001	211613000	7000	1	8.70E-07	0.36	124	1.77	Pwwp2b;LOC103691258	Epigenetic
DMR1:211740001	1	211740001	211741000	1000	1	4.80E-07	0.38	8	0.8	Inpp5a	Signaling
DMR1:211763001	1	211763001	211764000	1000	1	7.80E-07	-0.55	12	1.2	Inpp5a	Signaling
DMR1:211777001	1	211777001	211778000	1000	1	8.40E-12	0.77	10	1	Inpp5a	Signaling
DMR1:211782001	1	211782001	211784000	2000	1	4.20E-13	0.72	23	1.15	Inpp5a	Signaling
DMR1:211786001	1	211786001	211789000	3000	2	3.10E-10	0.56	24	0.8	Inpp5a	Signaling
DMR1:211797001	1	211797001	211799000	2000	1	6.60E-07	-0.44	15	0.75	Inpp5a	Signaling
DMR1:211800001	1	211800001	211801000	1000	1	9.90E-08	-0.51	14	1.4	Inpp5a	Signaling
DMR1:211819001	1	211819001	211821000	2000	1	6.60E-07	-0.61	21	1.05	Inpp5a	Signaling
DMR1:211831001	1	211831001	211832000	1000	1	3.80E-08	0.44	22	2.2	Inpp5a	Signaling
DMR1:211933001	1	211933001	211937000	4000	2	2.40E-27	1.06	37	0.92	Nkx6-2;Cfap46	Development
DMR1:211942001	1	211942001	211945000	3000	1	1.10E-10	0.46	43	1.43	Cfap46	
DMR1:211959001	1	211959001	211961000	2000	1	4.00E-09	0.4	53	2.65	Cfap46	
DMR1:211980001	1	211980001	211985000	5000	1	7.20E-10	-0.49	74	1.48	Cfap46	
DMR1:212001001	1	212001001	212006000	5000	1	1.60E-11	0.63	76	1.52	Cfap46	
DMR1:212023001	1	212023001	212026000	3000	1	3.00E-09	0.44	32	1.07	Cfap46	
DMR1:212161001	1	212161001	212163000	2000	1	1.30E-16	0.6	30	1.5	Adgra1	Signaling
DMR1:212171001	1	212171001	212176000	5000	3	4.10E-22	0.75	70	1.4	Adgra1	Signaling
DMR1:212220001	1	212220001	212221000	1000	1	3.90E-11	0.6	10	1	Adgra1;Kndc1	Signaling;Transcription
DMR1:212246001	1	212246001	212248000	2000	2	2.30E-16	0.75	18	0.9	Kndc1	Transcription
DMR1:212260001	1	212260001	212262000	2000	1	5.80E-08	-0.58	21	1.05	Kndc1	Transcription
DMR1:212270001	1	212270001	212272000	2000	1	7.50E-10	0.42	13	0.65	Kndc1;Utf1	Transcription
DMR1:212302001	1	212302001	212308000	6000	3	3.60E-14	0.53	73	1.22	Mir202;LOC108349785;LOC103691261;Adam8	Protease
DMR1:212322001	1	212322001	212323000	1000	1	7.50E-15	0.88	22	2.2	LOC103691261;Adam8	Protease
DMR1:212505001	1	212505001	212509000	4000	3	1.70E-13	0.61	28	0.7	LOC680741;Msx3	Development
DMR1:212528001	1	212528001	212530000	2000	2	7.30E-14	0.86	22	1.1	LOC102551319;Caly	
DMR1:212554001	1	212554001	212555000	1000	1	3.50E-20	0.83	6	0.6	Caly;Prap1;Fuom	
DMR1:212587001	1	212587001	212588000	1000	1	2.20E-08	-0.62	7	0.7	Echs1;Paox	Metabolism;Metabolism
DMR1:212599001	1	212599001	212601000	2000	1	6.60E-08	0.47	23	1.15	Paox;Mtg1	Metabolism
DMR1:212636001	1	212636001	212637000	1000	1	6.40E-08	0.36	14	1.4	Olr287;Olr286	Receptor
DMR1:212643001	1	212643001	212647000	4000	1	1.50E-08	-0.4	33	0.82	Olr287;Olr286	Receptor
DMR1:212655001	1	212655001	212660000	5000	4	6.10E-14	0.71	37	0.74	Olr287	Receptor
DMR1:212668001	1	212668001	212669000	1000	1	1.50E-07	0.35	14	1.4	Olr288	Receptor
DMR1:212672001	1	212672001	212673000	1000	1	1.90E-07	-0.59	9	0.9	Olr288	Receptor
DMR1:212676001	1	212676001	212680000	4000	1	4.40E-07	0.39	49	1.23	Olr288	Receptor
DMR1:212687001	1	212687001	212688000	1000	1	1.10E-10	0.65	4	0.4	Olr288;Cd163l1	Receptor
DMR1:212723001	1	212723001	212729000	6000	5	5.60E-16	-0.44	64	1.07	Olr292	
DMR1:212737001	1	212737001	212740000	3000	2	3.40E-11	-0.46	24	0.8	Olr292;LOC103691266	
DMR1:212771001	1	212771001	212774000	3000	1	1.50E-10	0.54	36	1.2	Olr292	
DMR1:212784001	1	212784001	212791000	7000	2	3.30E-15	-0.53	63	0.9	Olr292	
DMR1:212798001	1	212798001	212802000	4000	2	1.70E-13	-0.5	22	0.55	Olr292	
DMR1:212822001	1	212822001	212825000	3000	1	5.20E-07	-0.39	13	0.43	Olr292	
DMR1:212831001	1	212831001	212832000	1000	1	2.50E-08	0.61	3	0.3	Olr292;Olr289	Signaling
DMR1:212844001	1	212844001	212847000	3000	1	6.10E-13	-0.74	10	0.33	Olr292;Olr289	Signaling
DMR1:212849001	1	212849001	212850000	1000	1	7.90E-16	0.57	12	1.2	Olr292	
DMR1:212852001	1	212852001	212858000	6000	5	1.60E-21	-0.52	64	1.07	Olr292;Olr290-ps	
DMR1:212866001	1	212866001	212867000	1000	1	1.50E-14	0.55	6	0.6	Olr292;Olr290-ps;LOC100360557	Signaling
DMR1:212878001	1	212878001	212879000	1000	1	5.10E-15	0.8	5	0.5	Olr292;LOC100360557	Signaling
DMR1:212880001	1	212880001	212883000	3000	1	1.10E-11	0.43	33	1.1	Olr292;LOC100360557	Signaling

DMR1:212884001	1	212884001	212885000	1000	1	3.10E-12	0.63	4	0.4	Olr292	
DMR1:212889001	1	212889001	212890000	1000	1	5.60E-09	0.58	9	0.9	Olr292;Olr293-ps	
DMR1:212891001	1	212891001	212905000	14000	8	2.00E-23	0.63	135	0.96	Olr292;Olr293-ps;Olr294-ps;Olr295	Signaling
DMR1:212906001	1	212906001	212907000	1000	1	1.60E-12	0.66	6	0.6	Olr293-ps;Olr294-ps;Olr295	Signaling
DMR1:212908001	1	212908001	212909000	1000	1	4.30E-07	-0.54	2	0.2	Olr294-ps;Olr295	Signaling
DMR1:212922001	1	212922001	212924000	2000	1	1.60E-08	0.64	10	0.5	Olr295	Signaling
DMR1:212932001	1	212932001	212934000	2000	2	9.10E-12	0.52	11	0.55	Olr296-ps;Olr297	
DMR1:212939001	1	212939001	212940000	1000	1	4.30E-09	0.57	5	0.5	Olr296-ps;Olr297	
DMR1:212978001	1	212978001	212986000	8000	5	6.40E-24	0.7	80	1	Olr298	
DMR1:212992001	1	212992001	212994000	2000	2	1.20E-11	0.55	11	0.55	Olr298	
DMR1:213002001	1	213002001	213003000	1000	1	3.40E-07	0.45	3	0.3	Olr300;Olr299	Signaling
DMR1:213019001	1	213019001	213020000	1000	1	2.10E-17	0.87	7	0.7	Olr300;Olr299	Signaling
DMR1:213025001	1	213025001	213027000	2000	1	3.30E-08	-0.45	7	0.35	Olr300	
DMR1:213045001	1	213045001	213052000	7000	1	2.90E-09	-0.35	38	0.54	Olr300;Olr301-ps;LOC108349862	
DMR1:213059001	1	213059001	213064000	5000	1	2.70E-14	0.59	17	0.34	LOC108349862;Olr302	Signaling
DMR1:213078001	1	213078001	213085000	7000	2	6.20E-13	-0.54	60	0.86	Olr302;Olr303	Signaling
DMR1:213104001	1	213104001	213105000	1000	1	2.90E-07	-0.55	3	0.3	Olr303	
DMR1:213135001	1	213135001	213142000	7000	6	2.60E-19	-0.51	84	1.2	Olr304	Signaling
DMR1:213158001	1	213158001	213161000	3000	1	3.40E-11	0.55	16	0.53	Olr305	Signaling
DMR1:213166001	1	213166001	213167000	1000	1	6.50E-07	0.4	5	0.5	Olr305	Signaling
DMR1:213187001	1	213187001	213188000	1000	1	6.50E-07	-0.5	11	1.1	Olr306	Signaling
DMR1:213198001	1	213198001	213199000	1000	1	2.00E-12	-0.63	5	0.5	Olr306	Signaling
DMR1:213200001	1	213200001	213201000	1000	1	1.30E-07	-0.45	4	0.4	Olr306	Signaling
DMR1:213202001	1	213202001	213204000	2000	1	1.50E-07	-0.37	16	0.8	Olr306	Signaling
DMR1:213282001	1	213282001	213287000	5000	1	1.60E-16	-0.47	74	1.48	Olr308	Signaling
DMR1:213338001	1	213338001	213339000	1000	1	3.60E-11	-0.57	11	1.1	Olr309;Olr310	Signaling
DMR1:213350001	1	213350001	213353000	3000	2	6.00E-10	0.56	23	0.77	Olr310	Signaling
DMR1:213366001	1	213366001	213371000	5000	4	1.50E-14	-0.47	60	1.2	Olr311	Signaling
DMR1:213380001	1	213380001	213385000	5000	3	5.40E-10	-0.53	19	0.38	Olr311	Signaling
DMR1:213396001	1	213396001	213402000	6000	2	1.40E-20	-0.57	68	1.13	Olr312	Signaling
DMR1:213410001	1	213410001	213414000	4000	2	9.60E-15	-0.52	37	0.92	Olr312	Signaling
DMR1:213419001	1	213419001	213420000	1000	1	8.90E-07	0.48	2	0.2	Olr313	Signaling
DMR1:213425001	1	213425001	213426000	1000	1	1.40E-12	0.61	6	0.6	Olr313	Signaling
DMR1:213432001	1	213432001	213437000	5000	5	7.60E-27	-0.59	55	1.1	Olr313;Olr314-ps	Signaling
DMR1:213438001	1	213438001	213444000	6000	1	9.10E-12	-0.49	57	0.95	Olr314-ps;Olr315	Signaling
DMR1:213462001	1	213462001	213463000	1000	1	6.30E-10	0.63	2	0.2	Olr315	Signaling
DMR1:213500001	1	213500001	213502000	2000	1	2.10E-07	0.41	6	0.3	Cyp2e1	Metabolism
DMR1:213503001	1	213503001	213508000	5000	4	1.70E-16	0.72	54	1.08	Cyp2e1	Metabolism
DMR1:213515001	1	213515001	213517000	2000	1	2.10E-18	0.77	17	0.85	Cyp2e1;Syce1	Metabolism
DMR1:213520001	1	213520001	213521000	1000	1	1.50E-18	0.73	6	0.6	Cyp2e1;Syce1;LOC103691262	Metabolism
DMR1:213530001	1	213530001	213532000	2000	2	1.30E-08	-0.64	12	0.6	Cyp2e1;Syce1;LOC103691262	Metabolism
DMR1:213547001	1	213547001	213554000	7000	3	6.50E-20	0.78	55	0.79	LOC681212;RGD1559948;LOC103691263	
DMR1:213557001	1	213557001	213561000	4000	1	3.70E-10	-0.81	34	0.85	RGD1559948;LOC103691263	
DMR1:213567001	1	213567001	213569000	2000	1	2.50E-09	-0.61	16	0.8	RGD1559948;LOC103691263;RGD1309350	Metabolism
DMR1:213583001	1	213583001	213592000	9000	7	1.90E-11	-0.68	93	1.03	RGD1309350;LOC108349668;Scgb1c1;Odf3	Metabolism;Growth Factors;Development
DMR1:213593001	1	213593001	213595000	2000	1	1.10E-13	0.6	23	1.15	LOC108349668;Scgb1c1;Odf3;Bet1l	Growth Factors;Development;Transcription
DMR1:213616001	1	213616001	213622000	6000	2	4.10E-16	0.63	58	0.97	Bet1l;Ric8a;Sirt3	Transcription
DMR1:213623001	1	213623001	213628000	5000	1	9.10E-15	0.66	66	1.32	Sirt3;Psmd13	Protease
DMR1:213650001	1	213650001	213652000	2000	1	2.00E-18	0.56	30	1.5	Psmd13;Cox8b	Protease;Metabolism
DMR1:213668001	1	213668001	213676000	8000	7	8.40E-24	-0.54	95	1.19	Nlrp6;LOC108348167	
DMR1:213724001	1	213724001	213725000	1000	1	3.00E-14	0.54	18	1.8	LOC102555474;Pgghg	Metabolism
DMR1:213726001	1	213726001	213732000	6000	2	3.40E-10	0.45	212	3.53	LOC102555474;Pgghg	Metabolism
DMR1:213734001	1	213734001	213735000	1000	1	4.50E-11	0.74	27	2.7	LOC102555474;Pgghg;Ifitm5	Metabolism
DMR1:213740001	1	213740001	213743000	3000	1	6.90E-09	0.36	47	1.57	LOC102555474;Pgghg;Ifitm5;LOC108349669;Ifitm2	Metabolism

DMR1:213747001	1	213747001	213751000	4000	2	9.70E-09	-0.56	45	1.12	Pgghg;Ifitm5;LOC108349669;Ifitm2	Metabolism
DMR1:213760001	1	213760001	213764000	4000	2	6.90E-17	0.59	74	1.85	LOC108349669;Ifitm2;Ifitm1	
DMR1:213774001	1	213774001	213780000	6000	3	3.30E-09	0.57	80	1.33	Ifitm1	
DMR1:213809001	1	213809001	213811000	2000	1	1.20E-12	0.59	32	1.6	Ifitm3;Ifitm6	
DMR1:213823001	1	213823001	213826000	3000	2	7.60E-23	0.98	72	2.4	Ifitm6	
DMR1:213834001	1	213834001	213835000	1000	1	7.10E-35	1.18	16	1.6	Ifitm6	
DMR1:213836001	1	213836001	213843000	7000	2	1.30E-19	0.8	73	1.04	Ifitm6	
DMR1:213856001	1	213856001	213862000	6000	1	1.20E-09	0.52	103	1.72	Ifitm6;LOC108348748;B4galnt4	Golgi
DMR1:213863001	1	213863001	213865000	2000	2	1.00E-20	0.76	24	1.2	Ifitm6;LOC108348748;B4galnt4	Golgi
DMR1:213903001	1	213903001	213905000	2000	2	3.80E-09	0.43	26	1.3	Pkp3;Sigirr;Ano9	Cytoskeleton;Receptor
DMR1:213914001	1	213914001	213916000	2000	1	9.80E-08	0.37	22	1.1	Sigirr;Ano9	Receptor
DMR1:214121001	1	214121001	214122000	1000	1	4.60E-07	0.45	5	0.5	Ptdss2	Transport
DMR1:214124001	1	214124001	214125000	1000	1	9.00E-19	0.71	13	1.3	Ptdss2	Transport
DMR1:214166001	1	214166001	214168000	2000	1	2.20E-20	0.78	26	1.3	Rnh1	
DMR1:214217001	1	214217001	214218000	1000	1	1.70E-07	0.52	4	0.4	LOC102553043;Mir3574;Mir210;Phrf1	
DMR1:214313001	1	214313001	214315000	2000	1	7.10E-08	0.41	20	1	Deaf1;Tmem80	Transcription
DMR1:214316001	1	214316001	214317000	1000	1	1.60E-19	0.94	8	0.8	Deaf1;Tmem80	Transcription
DMR1:214347001	1	214347001	214352000	5000	1	2.40E-13	0.46	88	1.76	Eps8l2	Cytoskeleton
DMR1:214510001	1	214510001	214512000	2000	1	1.90E-09	0.69	67	3.35	Chid1	
DMR1:214529001	1	214529001	214532000	3000	1	1.80E-09	0.51	31	1.03	Ap2a2	Transport
DMR1:214545001	1	214545001	214548000	3000	1	3.90E-09	0.65	20	0.67	Ap2a2	Transport
DMR1:214566001	1	214566001	214570000	4000	2	7.60E-10	0.44	52	1.3	Ap2a2	Transport
DMR1:214588001	1	214588001	214589000	1000	1	2.80E-21	0.83	14	1.4	Ap2a2	Transport
DMR1:214591001	1	214591001	214593000	2000	1	5.20E-11	0.59	19	0.95	Ap2a2	Transport
DMR1:214610001	1	214610001	214617000	7000	6	1.80E-31	0.8	147	2.1	Ap2a2;Muc6	Transport;Extracellular Matrix
DMR1:214630001	1	214630001	214633000	3000	2	9.90E-20	0.92	38	1.27	Muc6	Extracellular Matrix
DMR1:214651001	1	214651001	214654000	3000	1	2.20E-11	0.6	34	1.13	Muc2	Extracellular Matrix
DMR1:214660001	1	214660001	214661000	1000	1	2.00E-10	0.49	13	1.3	Muc2	Extracellular Matrix
DMR1:214681001	1	214681001	214684000	3000	2	1.80E-21	0.7	16	0.53	Muc2	Extracellular Matrix
DMR1:214724001	1	214724001	214725000	1000	1	1.20E-16	0.84	9	0.9	Muc5ac	
DMR1:214742001	1	214742001	214748000	6000	3	1.90E-12	0.47	93	1.55	Muc5ac	
DMR1:214757001	1	214757001	214758000	1000	1	3.20E-12	0.64	8	0.8	Muc5ac	
DMR1:214792001	1	214792001	214796000	4000	1	2.70E-10	0.43	61	1.52	Muc5b	Extracellular Matrix
DMR1:214802001	1	214802001	214804000	2000	2	1.70E-11	0.45	41	2.05	Muc5b	Extracellular Matrix
DMR1:214918001	1	214918001	214919000	1000	1	3.20E-11	0.46	13	1.3	Brsk2	Signaling
DMR1:214931001	1	214931001	214934000	3000	1	3.90E-07	0.4	52	1.73	Brsk2;LOC103691267	Signaling
DMR1:214938001	1	214938001	214940000	2000	1	1.10E-07	0.45	30	1.5	Brsk2;LOC103691267	Signaling
DMR1:214947001	1	214947001	214951000	4000	1	5.60E-20	0.69	74	1.85	Brsk2;LOC103691267;Mob2	Signaling;Signaling
DMR1:214961001	1	214961001	214963000	2000	1	1.20E-07	-0.47	21	1.05	Mob2	Signaling
DMR1:215024001	1	215024001	215026000	2000	1	2.40E-30	0.85	20	1	Dusp8	Signaling
DMR1:215045001	1	215045001	215047000	2000	1	2.00E-09	0.48	16	0.8	Dusp8	Signaling
DMR1:215132001	1	215132001	215133000	1000	1	1.80E-16	0.83	5	0.5	LOC108349786;Krtap5-1;LOC103691269	
DMR1:215134001	1	215134001	215135000	1000	1	8.10E-21	0.94	13	1.3	LOC108349786;Krtap5-1;LOC103691269	
DMR1:215512001	1	215512001	215515000	3000	1	6.90E-15	0.52	50	1.67	Ifitm10	
DMR1:215520001	1	215520001	215521000	1000	1	1.40E-13	0.64	8	0.8	Ifitm10	
DMR1:215522001	1	215522001	215528000	6000	6	5.50E-20	0.83	58	0.97	Ifitm10	
DMR1:215530001	1	215530001	215533000	3000	3	2.20E-21	0.89	22	0.73	Ifitm10;Ctsd	
DMR1:215597001	1	215597001	215600000	3000	1	6.50E-19	0.52	55	1.83	LOC102546801;Syt8;Tnni2	Transport;Cytoskeleton
DMR1:215610001	1	215610001	215612000	2000	1	5.60E-09	0.43	39	1.95	LOC102546801;Syt8;Tnni2	Transport;Cytoskeleton
DMR1:215633001	1	215633001	215638000	5000	5	5.40E-22	0.93	18	0.36	Lsp1	Cytoskeleton
DMR1:215639001	1	215639001	215641000	2000	2	1.50E-17	0.79	26	1.3	Lsp1	Cytoskeleton
DMR1:215650001	1	215650001	215652000	2000	2	4.00E-09	0.46	21	1.05	Lsp1;Prr33	Cytoskeleton
DMR1:215655001	1	215655001	215658000	3000	1	2.80E-15	0.65	39	1.3	Lsp1;Prr33;Tnnt3	Cytoskeleton;Cytoskeleton
DMR1:215659001	1	215659001	215661000	2000	1	2.80E-08	0.39	34	1.7	Lsp1;Prr33;Tnnt3	Cytoskeleton;Cytoskeleton

DMR1:215662001	1	215662001	215664000	2000	1	8.50E-21	0.94	23	1.15	Lsp1;Prr33;Tnnt3	Cytoskeleton;Cytoskeleton
DMR1:215685001	1	215685001	215686000	1000	1	7.90E-14	0.56	13	1.3	Tnnt3	Cytoskeleton
DMR1:215694001	1	215694001	215700000	6000	1	2.40E-13	0.48	69	1.15	Mrpl23	Translation
DMR1:215744001	1	215744001	215750000	6000	3	1.50E-34	1.04	168	2.8	H19;Mir675;LOC102547221;LOC102548505	
DMR1:215819001	1	215819001	215821000	2000	2	1.40E-20	0.83	12	0.6	Igf2	Growth Factors
DMR1:215824001	1	215824001	215827000	3000	2	7.50E-21	0.87	18	0.6	Igf2;Mir483	Growth Factors
DMR1:215832001	1	215832001	215837000	5000	3	1.80E-19	0.79	207	4.14	Igf2;Mir483;LOC100909737;LOC102548568	Growth Factors
DMR1:216081001	1	216081001	216082000	1000	1	2.50E-13	0.47	11	1.1	Th	
DMR1:216144001	1	216144001	216146000	2000	1	3.10E-23	0.81	15	0.75	Ascl2	Transcription
DMR1:216164001	1	216164001	216166000	2000	1	9.90E-08	0.38	25	1.25	Ascl2	Transcription
DMR1:216184001	1	216184001	216188000	4000	2	5.10E-15	0.52	63	1.57	Tspan32	
DMR1:216194001	1	216194001	216197000	3000	2	9.90E-15	0.63	37	1.23	Tspan32	
DMR1:216199001	1	216199001	216200000	1000	1	6.30E-12	0.7	5	0.5	Tspan32	
DMR1:216207001	1	216207001	216211000	4000	1	4.70E-09	0.64	35	0.88	Tspan32	
DMR1:216212001	1	216212001	216213000	1000	1	8.30E-29	1.13	2	0.2	Tspan32	
DMR1:216227001	1	216227001	216228000	1000	1	5.20E-10	0.44	9	0.9	Cd81	
DMR1:216242001	1	216242001	216243000	1000	1	1.20E-18	1.05	8	0.8	Cd81	
DMR1:216265001	1	216265001	216266000	1000	1	2.20E-13	0.57	13	1.3	Tssc4;Trpm5	Transport
DMR1:216283001	1	216283001	216285000	2000	1	3.00E-18	0.82	15	0.75	Trpm5;Kcnq1	Transport;Transport
DMR1:216302001	1	216302001	216306000	4000	1	1.20E-14	0.77	40	1	Kcnq1	Transport
DMR1:216307001	1	216307001	216309000	2000	2	2.10E-30	1.04	11	0.55	Kcnq1	Transport
DMR1:216316001	1	216316001	216320000	4000	2	1.40E-14	0.61	54	1.35	Kcnq1	Transport
DMR1:216337001	1	216337001	216338000	1000	1	7.40E-12	0.52	11	1.1	Kcnq1	Transport
DMR1:216339001	1	216339001	216340000	1000	1	4.30E-10	0.43	7	0.7	Kcnq1	Transport
DMR1:216345001	1	216345001	216348000	3000	1	4.70E-08	0.38	49	1.63	Kcnq1	Transport
DMR1:216367001	1	216367001	216370000	3000	1	3.00E-11	0.68	35	1.17	Kcnq1	Transport
DMR1:216386001	1	216386001	216388000	2000	2	1.20E-26	0.85	19	0.95	Kcnq1	Transport
DMR1:216443001	1	216443001	216444000	1000	1	8.20E-07	-0.61	4	0.4	Kcnq1	Transport
DMR1:216453001	1	216453001	216455000	2000	1	1.10E-09	0.53	16	0.8	Kcnq1	Transport
DMR1:216477001	1	216477001	216478000	1000	1	5.40E-08	-0.47	14	1.4	Kcnq1	Transport
DMR1:216519001	1	216519001	216520000	1000	1	3.20E-07	-0.58	13	1.3	Kcnq1	Transport
DMR1:216550001	1	216550001	216552000	2000	1	4.60E-07	0.36	25	1.25	Kcnq1	Transport
DMR1:216557001	1	216557001	216559000	2000	1	3.00E-08	0.39	21	1.05	Kcnq1	Transport
DMR1:216590001	1	216590001	216593000	3000	1	1.40E-15	0.51	46	1.53	Kcnq1	Transport
DMR1:216594001	1	216594001	216597000	3000	3	2.60E-17	0.86	13	0.43	Kcnq1	Transport
DMR1:216598001	1	216598001	216601000	3000	2	7.70E-19	0.93	19	0.63	Kcnq1	Transport
DMR1:216622001	1	216622001	216626000	4000	2	2.40E-10	0.46	46	1.15	Kcnq1	Transport
DMR1:216633001	1	216633001	216635000	2000	1	5.90E-12	0.63	19	0.95	Kcnq1	Transport
DMR1:216654001	1	216654001	216655000	1000	1	3.90E-07	-0.47	17	1.7	Cdkn1c	Signaling
DMR1:216679001	1	216679001	216681000	2000	2	9.50E-14	0.52	24	1.2	Slc22a18	Transport
DMR1:216682001	1	216682001	216686000	4000	1	1.60E-07	0.38	82	2.05	Slc22a18	Transport
DMR1:216689001	1	216689001	216691000	2000	1	6.10E-12	0.61	21	1.05	Slc22a18	Transport
DMR1:216729001	1	216729001	216730000	1000	1	9.40E-07	-0.64	7	0.7	Nap1l4	Epigenetic
DMR1:216737001	1	216737001	216738000	1000	1	4.60E-07	-0.44	9	0.9	Nap1l4	Epigenetic
DMR1:216805001	1	216805001	216806000	1000	1	7.60E-07	-0.33	16	1.6	Cars;Tnfrsf26	Translation;Receptor
DMR1:216830001	1	216830001	216833000	3000	1	1.10E-15	0.84	20	0.67	Tnfrsf26	Receptor
DMR1:216835001	1	216835001	216837000	2000	1	8.60E-09	0.62	18	0.9	Tnfrsf26;Tnfrsf22	Receptor
DMR1:216838001	1	216838001	216839000	1000	1	4.10E-10	0.63	5	0.5	Tnfrsf26;Tnfrsf22	Receptor
DMR1:216844001	1	216844001	216846000	2000	2	3.70E-15	0.69	17	0.85	Tnfrsf22	
DMR1:216851001	1	216851001	216852000	1000	1	9.10E-12	0.53	10	1	Tnfrsf22	
DMR1:216856001	1	216856001	216857000	1000	1	4.20E-15	0.91	4	0.4	Tnfrsf22;Osbpl5	
DMR1:216865001	1	216865001	216866000	1000	1	2.70E-08	0.45	6	0.6	Tnfrsf22;Osbpl5	
DMR1:216876001	1	216876001	216878000	2000	1	1.70E-18	0.73	25	1.25	Osbpl5	
DMR1:216892001	1	216892001	216893000	1000	1	4.20E-09	0.5	6	0.6	Osbpl5	
DMR1:216910001	1	216910001	216912000	2000	1	2.30E-13	-0.74	27	1.35	Osbpl5	
DMR1:216927001	1	216927001	216929000	2000	1	2.90E-08	0.36	30	1.5	Osbpl5;LOC103691270	
DMR1:216938001	1	216938001	216940000	2000	2	1.00E-19	0.77	30	1.5	LOC103691270;RGD1562011	Signaling
DMR1:216946001	1	216946001	216952000	6000	2	2.90E-14	0.76	50	0.83	LOC103691270;RGD1562011;Mrgprg	Signaling
DMR1:216959001	1	216959001	216960000	1000	1	8.10E-07	0.34	5	0.5	Mrgprg;Mrgpre	Signaling
DMR1:216964001	1	216964001	216966000	2000	1	8.10E-08	0.59	21	1.05	Mrgprg;Mrgpre	Signaling
DMR1:216991001	1	216991001	216993000	2000	1	1.20E-08	0.36	14	0.7	Nadsyn1	Metabolism

DMR1:217015001	1	217015001	217018000	3000	1	5.80E-13	0.43	36	1.2	Nadsyn1;Dhcr7	Metabolism;Metabolism
DMR1:217027001	1	217027001	217030000	3000	2	6.20E-21	0.83	40	1.33	Dhcr7;LOC102552318	Metabolism;Cytoskeleton
DMR1:217175001	1	217175001	217177000	2000	2	1.50E-15	0.75	12	0.6	Shank2	
DMR1:217200001	1	217200001	217201000	1000	1	1.30E-18	0.74	14	1.4	Shank2	
DMR1:217204001	1	217204001	217205000	1000	1	4.40E-15	0.72	10	1	Shank2	
DMR1:217208001	1	217208001	217210000	2000	1	6.20E-23	1.02	22	1.1	Shank2	
DMR1:217211001	1	217211001	217214000	3000	1	8.90E-18	0.67	34	1.13	Shank2	
DMR1:217215001	1	217215001	217220000	5000	3	8.40E-17	0.78	69	1.38	Shank2	
DMR1:217233001	1	217233001	217234000	1000	1	3.30E-10	0.64	6	0.6	Shank2	
DMR1:217239001	1	217239001	217244000	5000	1	1.20E-07	0.44	72	1.44	Shank2	
DMR1:217251001	1	217251001	217253000	2000	1	3.70E-14	0.83	19	0.95	Shank2	
DMR1:217257001	1	217257001	217258000	1000	1	1.60E-11	0.51	8	0.8	Shank2	
DMR1:217316001	1	217316001	217318000	2000	1	9.80E-10	0.39	26	1.3	Shank2	
DMR1:217321001	1	217321001	217324000	3000	2	1.10E-24	0.85	29	0.97	Shank2	
DMR1:217325001	1	217325001	217326000	1000	1	4.90E-10	0.62	6	0.6	Shank2	
DMR1:217344001	1	217344001	217345000	1000	1	2.00E-17	0.49	28	2.8	Shank2	
DMR1:217353001	1	217353001	217359000	6000	1	3.30E-11	0.47	86	1.43	Shank2	
DMR1:217372001	1	217372001	217374000	2000	1	1.50E-11	0.73	20	1	Shank2	
DMR1:217383001	1	217383001	217385000	2000	2	9.30E-15	0.71	26	1.3	Shank2	
DMR1:217387001	1	217387001	217389000	2000	1	3.40E-11	0.64	20	1	Shank2	
DMR1:217396001	1	217396001	217399000	3000	3	9.40E-21	0.81	28	0.93	Shank2	
DMR1:217401001	1	217401001	217402000	1000	1	1.80E-07	0.44	10	1	Shank2	
DMR1:217430001	1	217430001	217434000	4000	2	1.10E-17	0.82	39	0.98	Shank2	
DMR1:217435001	1	217435001	217439000	4000	3	3.30E-20	0.88	67	1.68	Shank2	
DMR1:217441001	1	217441001	217443000	2000	2	3.10E-15	0.81	10	0.5	Shank2	
DMR1:217444001	1	217444001	217448000	4000	1	1.40E-09	0.39	50	1.25	Shank2	
DMR1:217459001	1	217459001	217460000	1000	1	1.40E-13	0.57	46	4.6	Shank2	
DMR1:217466001	1	217466001	217468000	2000	1	4.60E-07	0.4	32	1.6	Shank2	
DMR1:217471001	1	217471001	217476000	5000	2	1.10E-29	1.11	58	1.16	Shank2	
DMR1:217493001	1	217493001	217495000	2000	1	2.40E-10	0.63	10	0.5	Shank2	
DMR1:217547001	1	217547001	217549000	2000	2	1.60E-11	0.71	28	1.4	Shank2	
DMR1:217645001	1	217645001	217646000	1000	1	3.40E-09	-0.69	12	1.2	Cttn;Ppfia1	Cytoskeleton
DMR1:217670001	1	217670001	217672000	2000	1	2.10E-08	0.37	24	1.2	Ppfia1	
DMR1:217682001	1	217682001	217687000	5000	1	4.10E-08	-0.45	59	1.18	Ppfia1;LOC108349676	
DMR1:217706001	1	217706001	217707000	1000	1	8.40E-07	0.58	7	0.7	Ppfia1;LOC100364769	
DMR1:217724001	1	217724001	217725000	1000	1	1.50E-08	0.52	6	0.6	Ppfia1;LOC100364769	
DMR1:217743001	1	217743001	217744000	1000	1	1.70E-07	-0.48	9	0.9	LOC100364769;Fadd	
DMR1:217750001	1	217750001	217752000	2000	2	6.50E-19	0.91	13	0.65	Fadd;Ano1	
DMR1:217756001	1	217756001	217757000	1000	1	1.10E-29	0.87	7	0.7	Fadd;Ano1	
DMR1:217763001	1	217763001	217764000	1000	1	9.60E-11	0.51	19	1.9	Ano1	
DMR1:217807001	1	217807001	217810000	3000	3	1.70E-23	0.82	19	0.63	Ano1	
DMR1:217816001	1	217816001	217818000	2000	1	1.50E-22	0.77	19	0.95	Ano1	
DMR1:217828001	1	217828001	217832000	4000	3	7.80E-22	0.93	34	0.85	Ano1	
DMR1:217855001	1	217855001	217856000	1000	1	3.70E-07	0.39	13	1.3	Ano1	
DMR1:217871001	1	217871001	217874000	3000	2	5.30E-14	0.61	38	1.27	Ano1	
DMR1:217879001	1	217879001	217880000	1000	1	3.80E-17	0.71	13	1.3	Ano1	
DMR1:217886001	1	217886001	217890000	4000	1	2.40E-20	0.94	40	1	Ano1	
DMR1:217893001	1	217893001	217899000	6000	4	1.70E-21	0.92	54	0.9	Ano1	
DMR1:217906001	1	217906001	217907000	1000	1	7.90E-07	0.32	10	1	Ano1	
DMR1:217996001	1	217996001	217999000	3000	2	5.20E-16	-0.47	48	1.6	Fgf3;LOC108349678	Growth Factors
DMR1:218005001	1	218005001	218007000	2000	2	3.30E-18	0.69	33	1.65	Fgf3;LOC108349678	Growth Factors
DMR1:218011001	1	218011001	218012000	1000	1	1.40E-09	0.4	13	1.3	Fgf3;LOC108349678	Growth Factors
DMR1:218036001	1	218036001	218039000	3000	1	1.40E-09	-0.8	44	1.47	Fgf4	Growth Factors
DMR1:218050001	1	218050001	218053000	3000	1	7.50E-08	0.5	42	1.4	Fgf19	Growth Factors
DMR1:218358001	1	218358001	218361000	3000	1	3.40E-09	0.62	34	1.13	Rmt1	
DMR1:218362001	1	218362001	218365000	3000	2	9.60E-23	0.72	40	1.33	Rmt1	
DMR1:218366001	1	218366001	218372000	6000	3	5.70E-22	0.95	58	0.97	Rmt1	
DMR1:218384001	1	218384001	218386000	2000	1	3.20E-16	0.72	16	0.8	Rmt1	
DMR1:218413001	1	218413001	218415000	2000	1	1.10E-08	0.54	7	0.35	Tpcn2	Transport
DMR1:218416001	1	218416001	218417000	1000	1	6.30E-17	0.48	12	1.2	Tpcn2	Transport
DMR1:218422001	1	218422001	218424000	2000	1	9.60E-10	0.43	27	1.35	Tpcn2	Transport
DMR1:218432001	1	218432001	218435000	3000	2	1.20E-18	0.76	47	1.57	Tpcn2	Transport
DMR1:218462001	1	218462001	218464000	2000	1	1.60E-16	0.85	20	1	Mrgprf	Signaling

DMR1:218522001	1	218522001	218525000	3000	2	5.80E-14	0.81	37	1.23	Ighmbp2;Mrpl21	Transcription;Translation
DMR1:218548001	1	218548001	218551000	3000	2	9.50E-11	0.6	37	1.23	Mrpl21	Translation
DMR1:218555001	1	218555001	218557000	2000	1	9.50E-17	0.84	15	0.75	Mrpl21	Translation
DMR1:218558001	1	218558001	218560000	2000	1	5.90E-07	0.32	21	1.05	Cpt1a	Metabolism
DMR1:218597001	1	218597001	218605000	8000	1	4.30E-08	0.46	114	1.43	Cpt1a	Metabolism
DMR1:218616001	1	218616001	218618000	2000	1	8.10E-07	-0.57	27	1.35	Cpt1a	Metabolism
DMR1:218619001	1	218619001	218623000	4000	2	1.50E-12	0.6	51	1.27	Cpt1a;Tesmin	Metabolism
DMR1:218627001	1	218627001	218628000	1000	1	1.80E-07	-0.63	14	1.4	Cpt1a;Tesmin	Metabolism
DMR1:218653001	1	218653001	218654000	1000	1	3.10E-11	0.5	13	1.3	Tesmin;Gal	
DMR1:218708001	1	218708001	218710000	2000	1	2.30E-11	0.56	13	0.65	Ppp6r3	Signaling
DMR1:218779001	1	218779001	218780000	1000	1	1.80E-09	0.6	3	0.3	Ppp6r3	Signaling
DMR1:218784001	1	218784001	218785000	1000	1	8.80E-07	-0.47	9	0.9	Ppp6r3	Signaling
DMR1:218798001	1	218798001	218799000	1000	1	2.20E-07	0.4	9	0.9	Ppp6r3;LOC102552426;LOC103691272	Signaling
DMR1:218812001	1	218812001	218814000	2000	1	9.20E-11	0.79	10	0.5	Ppp6r3;LOC102552426;LOC103691272;Trnaa-agc;Lrp5	Signaling;Receptor
DMR1:218817001	1	218817001	218818000	1000	1	9.90E-09	0.42	18	1.8	Ppp6r3;LOC103691272;Trnaa-agc;Lrp5	Signaling;Receptor
DMR1:218829001	1	218829001	218834000	5000	1	1.00E-08	-0.55	75	1.5	Lrp5	Receptor
DMR1:218850001	1	218850001	218856000	6000	1	2.00E-13	-0.83	84	1.4	Lrp5	Receptor
DMR1:218880001	1	218880001	218881000	1000	1	1.10E-10	0.48	12	1.2	Lrp5	Receptor
DMR1:218889001	1	218889001	218891000	2000	1	5.70E-08	0.39	30	1.5	Lrp5	Receptor
DMR1:218926001	1	218926001	218928000	2000	1	9.80E-07	-0.54	18	0.9	Lrp5;LOC108349725	Receptor
DMR1:218934001	1	218934001	218936000	2000	1	1.10E-11	0.43	28	1.4	RGD1311946	
DMR1:218959001	1	218959001	218960000	1000	1	1.30E-11	0.45	10	1	RGD1311946	
DMR1:218994001	1	218994001	218996000	2000	1	7.80E-09	0.47	15	0.75	LOC108349680;LOC108349681;Kmt5b	
DMR1:219061001	1	219061001	219063000	2000	2	1.80E-12	-0.55	19	0.95	Trnap-agg	
DMR1:219129001	1	219129001	219134000	5000	2	1.60E-11	0.42	77	1.54	Chka;Tcirg1;Ndufs8	Signaling;Metabolism;Metabolism
DMR1:219171001	1	219171001	219173000	2000	2	1.30E-10	0.43	27	1.35	Aldh3b1;Unc93b1	Metabolism
DMR1:219186001	1	219186001	219187000	1000	1	7.50E-08	0.52	5	0.5	Aldh3b1;Unc93b1;LOC688778	Metabolism
DMR1:219192001	1	219192001	219196000	4000	3	1.70E-16	0.67	34	0.85	Unc93b1;LOC688778	Metabolism
DMR1:219210001	1	219210001	219216000	6000	2	2.00E-15	0.49	79	1.32	LOC688778;Aldh3b2	Metabolism
DMR1:219219001	1	219219001	219224000	5000	3	8.70E-08	0.34	75	1.5	Aldh3b2;Acy3	
DMR1:219243001	1	219243001	219245000	2000	2	3.20E-11	0.5	46	2.3	Acy3;Tbx10;LOC103691273;Nudt8;Doc2g;Ndufv1	Transcription;Signaling;Metabolism
DMR1:219269001	1	219269001	219270000	1000	1	4.40E-10	0.41	20	2	Ndufv1	Metabolism
DMR1:219295001	1	219295001	219296000	1000	1	1.90E-10	0.64	5	0.5	Gstp1	Transport
DMR1:219306001	1	219306001	219308000	2000	1	1.70E-07	0.34	23	1.15	RGD1307603	Transport
DMR1:219309001	1	219309001	219312000	3000	1	5.80E-07	0.38	45	1.5	RGD1307603	Transport
DMR1:219317001	1	219317001	219318000	1000	1	7.40E-09	0.45	6	0.6	RGD1307603	Transport
DMR1:219319001	1	219319001	219322000	3000	2	6.40E-13	0.81	34	1.13	RGD1307603;Cabp2	Transport
DMR1:219324001	1	219324001	219335000	11000	4	2.70E-15	0.6	139	1.26	Cabp2	
DMR1:219359001	1	219359001	219360000	1000	1	1.80E-11	0.46	29	2.9	Pitpnm1;Aip;LOC108349683	Transport;Transcription
DMR1:219375001	1	219375001	219376000	1000	1	5.40E-15	0.59	12	1.2	Aip;LOC108349683;Tmem134;Cabp4	Transcription
DMR1:219394001	1	219394001	219397000	3000	1	3.30E-07	0.35	40	1.33	Cabp4;Gpr152;LOC108349682;Coro1b;Ptprcap;Rps6kb2	Signaling;Cytoskeleton;Golgi
DMR1:219409001	1	219409001	219412000	3000	2	2.80E-08	0.41	34	1.13	Coro1b;Ptprcap;Rps6kb2;Carns1	Cytoskeleton;Golgi
DMR1:219465001	1	219465001	219471000	6000	1	2.70E-24	0.71	67	1.12	Clcf1;Pold4	Transcription
DMR1:219473001	1	219473001	219475000	2000	1	1.40E-13	0.81	9	0.45	Clcf1;Pold4	Transcription
DMR1:219484001	1	219484001	219485000	1000	1	5.50E-14	0.54	9	0.9	Pold4	Transcription
DMR1:219502001	1	219502001	219503000	1000	1	7.80E-08	0.48	6	0.6	LOC103691274;Ssh3	Signaling
DMR1:219535001	1	219535001	219538000	3000	1	7.00E-28	0.69	56	1.87	Ankrd13d;Grk2	Signaling
DMR1:219574001	1	219574001	219577000	3000	1	6.10E-13	0.47	58	1.93	Kdm2a	
DMR1:219618001	1	219618001	219619000	1000	1	9.30E-08	0.42	11	1.1	Kdm2a	
DMR1:219629001	1	219629001	219631000	2000	1	1.80E-23	0.9	10	0.5	Kdm2a	
DMR1:219661001	1	219661001	219662000	1000	1	4.50E-08	0.53	16	1.6	Rhod	Signaling
DMR1:219693001	1	219693001	219696000	3000	1	9.80E-07	0.32	39	1.3	Syt12	Transport
DMR1:219700001	1	219700001	219701000	1000	1	9.50E-15	0.65	11	1.1	Syt12	Transport
DMR1:219702001	1	219702001	219704000	2000	1	1.90E-19	0.66	24	1.2	Syt12	Transport

DMR1:219724001	1	219724001	219727000	3000	1	8.50E-16	0.53	60	2	Syt12	Transport
DMR1:219761001	1	219761001	219762000	1000	1	1.40E-09	0.42	6	0.6	LOC689065;Pc	Metabolism
DMR1:219770001	1	219770001	219772000	2000	1	4.50E-20	0.92	8	0.4	Pc	Metabolism
DMR1:219802001	1	219802001	219805000	3000	1	3.10E-09	0.43	27	0.9	Pc	Metabolism
DMR1:219815001	1	219815001	219816000	1000	1	4.50E-13	0.55	8	0.8	Pc	Metabolism
DMR1:219871001	1	219871001	219872000	1000	1	3.50E-09	-0.5	12	1.2	Rce1;RGD1563323	Protease
DMR1:219896001	1	219896001	219898000	2000	1	1.20E-15	-0.62	17	0.85	RGD1563323	
DMR1:219902001	1	219902001	219910000	8000	4	4.40E-29	0.75	86	1.07	RGD1563323	
DMR1:219921001	1	219921001	219923000	2000	2	1.60E-23	-0.55	31	1.55	RGD1563323	
DMR1:219959001	1	219959001	219962000	3000	1	9.00E-16	0.53	35	1.17	RGD1563323;Sptbn2;LOC102553046	
DMR1:219969001	1	219969001	219973000	4000	2	3.90E-18	0.66	48	1.2	Sptbn2;LOC102553046	
DMR1:219976001	1	219976001	219978000	2000	1	2.90E-11	0.47	40	2	Sptbn2;LOC102553046	
DMR1:219981001	1	219981001	219982000	1000	1	1.10E-09	-0.57	19	1.9	Sptbn2	
DMR1:219988001	1	219988001	219992000	4000	2	2.80E-09	0.41	76	1.9	Sptbn2	
DMR1:219996001	1	219996001	220001000	5000	1	1.50E-18	0.54	95	1.9	Sptbn2;Rbm4b	Translation
DMR1:220063001	1	220063001	220066000	3000	1	5.30E-07	-0.48	22	0.73	Rbm14;Ccs	Translation;Metabolism
DMR1:220069001	1	220069001	220072000	3000	1	8.70E-13	0.55	37	1.23	Rbm14;Ccs;LOC108349684	Translation;Metabolism
DMR1:220098001	1	220098001	220101000	3000	1	1.20E-09	0.58	37	1.23	Ccs;Ccdc87	Metabolism
DMR1:220103001	1	220103001	220106000	3000	1	1.40E-08	0.43	33	1.1	Ccs;Ccdc87;Ctsf	Metabolism;Protease
DMR1:220108001	1	220108001	220111000	3000	2	3.20E-10	0.48	35	1.17	Ccdc87;Ctsf;Actn3	Protease
DMR1:220120001	1	220120001	220122000	2000	1	3.00E-07	-0.54	32	1.6	Ctsf;Actn3	Protease
DMR1:220128001	1	220128001	220129000	1000	1	1.20E-07	0.46	8	0.8	Ctsf;Actn3;Zdhhc24	Protease
DMR1:220133001	1	220133001	220136000	3000	1	5.90E-24	0.67	36	1.2	Actn3;Zdhhc24	
DMR1:220139001	1	220139001	220142000	3000	2	5.30E-19	0.64	32	1.07	Actn3;Zdhhc24;Bbs1	
DMR1:220165001	1	220165001	220166000	1000	1	1.80E-14	0.9	31	3.1	Bbs1;Dpp3	Protease
DMR1:220182001	1	220182001	220184000	2000	1	8.00E-09	0.38	28	1.4	Dpp3;Peli3	Protease;Proteolysis
DMR1:220211001	1	220211001	220217000	6000	2	3.70E-09	-0.52	67	1.12	Peli3	Proteolysis
DMR1:220239001	1	220239001	220240000	1000	1	2.20E-09	0.69	10	1	mrpl11	
DMR1:220251001	1	220251001	220254000	3000	1	1.60E-07	0.38	37	1.23	mrpl11;Npas4	Transcription
DMR1:220344001	1	220344001	220345000	1000	1	1.60E-12	0.62	16	1.6	Brms1;LOC103691276;Rin1;Cd248	Transcription
DMR1:220346001	1	220346001	220349000	3000	3	6.90E-23	0.84	49	1.63	LOC103691276;Rin1;Cd248;LOC102554272	Transcription
DMR1:220354001	1	220354001	220356000	2000	1	5.00E-09	0.51	32	1.6	Cd248;LOC102554272;LOC100911932;LOC102554421	
DMR1:220363001	1	220363001	220364000	1000	1	2.30E-08	0.5	11	1.1	Cd248;LOC102554272;LOC100911932;LOC102554421;Tmem151a	
DMR1:220373001	1	220373001	220374000	1000	1	8.60E-09	0.57	16	1.6	LOC100911932;LOC102554421;Tmem151a;LOC108349789	
DMR1:220460001	1	220460001	220463000	3000	1	1.10E-09	0.6	56	1.87	LOC102554466;Tmem151a;Yif1a	
DMR1:220476001	1	220476001	220480000	4000	3	4.70E-15	0.61	82	2.05	Tmem151a;Yif1a;Cnih2;LOC102555354;LOC102555167;Rab1b	Transport
DMR1:220529001	1	220529001	220530000	1000	1	9.70E-07	-0.69	6	0.6	Pacs1	
DMR1:220555001	1	220555001	220557000	2000	1	4.30E-07	0.41	13	0.65	Pacs1	
DMR1:220558001	1	220558001	220559000	1000	1	2.20E-10	0.57	9	0.9	Pacs1	
DMR1:220570001	1	220570001	220571000	1000	1	3.40E-07	-0.66	11	1.1	Pacs1	
DMR1:220600001	1	220600001	220602000	2000	1	7.20E-09	0.51	14	0.7	Pacs1;LOC102554619	
DMR1:220607001	1	220607001	220609000	2000	2	1.20E-08	-0.69	14	0.7	Pacs1;LOC102554619	
DMR1:220625001	1	220625001	220626000	1000	1	7.30E-07	0.43	8	0.8	Pacs1	
DMR1:220630001	1	220630001	220631000	1000	1	3.20E-15	0.84	6	0.6	Pacs1	
DMR1:220663001	1	220663001	220664000	1000	1	5.50E-11	0.4	11	1.1	Sf3b2;Gal3st3	Translation;Transport
DMR1:220678001	1	220678001	220679000	1000	1	3.50E-19	0.61	2	0.2	Gal3st3	Transport
DMR1:220687001	1	220687001	220688000	1000	1	9.00E-12	0.43	11	1.1	Gal3st3	Transport
DMR1:220718001	1	220718001	220720000	2000	2	2.70E-07	0.4	44	2.2	Catsper1;Cst6	Transport
DMR1:220722001	1	220722001	220724000	2000	1	8.20E-16	0.51	24	1.2	Catsper1;Cst6	Transport
DMR1:220735001	1	220735001	220738000	3000	1	5.10E-09	0.41	35	1.17	Cst6;Banf1;Eif1ad	Translation
DMR1:220767001	1	220767001	220769000	2000	1	1.70E-07	0.3	26	1.3	LOC103691278;Sart1;LOC102555676;Tsga10ip	Extracellular Matrix

DMR1:220802001	1	220802001	220803000	1000	1	4.30E-24	0.73	7	0.7	Drap1;Bles03	Transcription
DMR1:220816001	1	220816001	220819000	3000	1	2.60E-14	0.49	44	1.47	Drap1;Bles03;Fosl1	Transcription;Transcription
DMR1:220822001	1	220822001	220824000	2000	1	1.30E-10	-0.73	17	0.85	Fosl1	Transcription
DMR1:220873001	1	220873001	220875000	2000	1	6.40E-07	-0.45	21	1.05	Mus81;Cfl1;Snx32;LOC102551929	Cytoskeleton;Cytoskeleton
DMR1:220891001	1	220891001	220894000	3000	3	2.00E-30	0.79	66	2.2	Snx32;LOC102551929;LOC102556290	Cytoskeleton
DMR1:220919001	1	220919001	220921000	2000	1	2.70E-07	-0.45	39	1.95	LOC102556290;Ovol1	
DMR1:220926001	1	220926001	220928000	2000	1	5.80E-07	-0.53	36	1.8	LOC102556290;Ovol1	
DMR1:220944001	1	220944001	220946000	2000	1	1.50E-08	0.41	27	1.35	Ovol1;Ap5b1	
DMR1:220956001	1	220956001	220959000	3000	1	2.90E-07	0.35	30	1	Ap5b1;Rnaseh2c;Kat5	Epigenetic
DMR1:221047001	1	221047001	221049000	2000	1	2.20E-11	0.48	23	1.15	Pcnx3;Map3k11;LOC102546344;Kcnk7	Signaling;Transport
DMR1:221055001	1	221055001	221059000	4000	1	2.70E-09	0.41	51	1.27	Map3k11;LOC102546344;Kcnk7;Ehbp1l1	Signaling;Transport
DMR1:221068001	1	221068001	221069000	1000	1	6.50E-09	0.4	10	1	Kcnk7;Ehbp1l1	Transport
DMR1:221071001	1	221071001	221072000	1000	1	5.40E-15	0.61	14	1.4	Ehbp1l1	
DMR1:221078001	1	221078001	221084000	6000	1	7.90E-30	0.78	91	1.52	Ehbp1l1;Fam89b;Sssca1	Cytoskeleton
DMR1:221102001	1	221102001	221103000	1000	1	4.80E-10	0.46	10	1	Ltbp3	Extracellular Matrix
DMR1:221104001	1	221104001	221108000	4000	2	6.80E-13	0.49	67	1.68	Ltbp3;Scyl1	Extracellular Matrix;Signaling
DMR1:221122001	1	221122001	221124000	2000	2	8.20E-11	0.45	49	2.45	Ltbp3;Scyl1	Extracellular Matrix;Signaling
DMR1:221138001	1	221138001	221139000	1000	1	2.50E-12	0.61	12	1.2	Scyl1	Signaling
DMR1:221222001	1	221222001	221226000	4000	1	5.30E-10	-0.72	57	1.43	Frmd8	Cytoskeleton
DMR1:221227001	1	221227001	221230000	3000	1	1.20E-15	0.54	51	1.7	Frmd8;Slc25a45	Cytoskeleton;Transport
DMR1:221250001	1	221250001	221251000	1000	1	4.10E-08	0.49	16	1.6	Slc25a45;Tigd3;Dpf2	Transport;Epigenetic;Epigenetic
DMR1:221258001	1	221258001	221263000	5000	2	4.10E-07	-0.39	66	1.32	Tigd3;Dpf2;Cdc42ep2	Epigenetic;Epigenetic
DMR1:221283001	1	221283001	221286000	3000	1	5.10E-12	0.55	35	1.17	Cdc42ep2;LOC108348875	
DMR1:221287001	1	221287001	221290000	3000	2	2.30E-24	0.89	27	0.9	Cdc42ep2;LOC108348875;Pola2	Transcription
DMR1:221325001	1	221325001	221326000	1000	1	1.00E-10	0.45	12	1.2	Pola2;Slc22a20	Transcription;Transport
DMR1:221338001	1	221338001	221341000	3000	1	2.20E-13	0.56	50	1.67	Slc22a20;Capn1	Transport;Protease
DMR1:221346001	1	221346001	221348000	2000	2	2.80E-16	0.64	30	1.5	Slc22a20;Capn1	Transport;Protease
DMR1:221362001	1	221362001	221363000	1000	1	3.90E-18	0.58	13	1.3	Capn1;LOC108349685	Protease
DMR1:221365001	1	221365001	221367000	2000	2	2.20E-07	0.38	34	1.7	Capn1;LOC108349685	Protease
DMR1:221398001	1	221398001	221402000	4000	1	2.00E-07	-0.59	60	1.5	LOC108349685;Syvn1	
DMR1:221468001	1	221468001	221470000	2000	1	1.10E-21	1.03	16	0.8	Cdca5;Naaladl1	Protease
DMR1:221471001	1	221471001	221473000	2000	1	2.30E-19	0.99	34	1.7	Cdca5;Naaladl1	Protease
DMR1:221487001	1	221487001	221489000	2000	1	9.80E-07	0.32	34	1.7	Naaladl1;Sac3d1;Snx15	Protease;Cytoskeleton;Signaling
DMR1:221494001	1	221494001	221495000	1000	1	4.50E-17	0.84	7	0.7	Naaladl1;Sac3d1;Snx15;Arl2	Protease;Cytoskeleton;Signaling
DMR1:221523001	1	221523001	221525000	2000	1	1.10E-13	0.48	28	1.4	Arl2	
DMR1:221536001	1	221536001	221537000	1000	1	2.80E-20	0.73	12	1.2	Batf2;Majin	Transcription
DMR1:221548001	1	221548001	221553000	5000	1	5.80E-18	0.61	59	1.18	Batf2;Majin	Transcription
DMR1:221568001	1	221568001	221569000	1000	1	2.30E-08	-0.6	8	0.8	Majin;LOC108349686	
DMR1:221580001	1	221580001	221583000	3000	1	4.10E-09	0.69	26	0.87	Majin;LOC108349686	
DMR1:221591001	1	221591001	221593000	2000	1	9.00E-07	-0.56	24	1.2	Majin;Gpha2;Ppp2r5b	Hormone;Signaling
DMR1:221608001	1	221608001	221612000	4000	2	9.70E-20	0.91	31	0.78	Ppp2r5b;Atg2a	Signaling
DMR1:221630001	1	221630001	221632000	2000	1	4.30E-19	1	23	1.15	Atg2a;Mir194-2;Mir192	
DMR1:221681001	1	221681001	221683000	2000	1	8.30E-08	0.4	38	1.9	Cdc42bpg	Signaling
DMR1:221697001	1	221697001	221699000	2000	1	1.90E-09	0.46	14	0.7	Cdc42bpg;Men1	Signaling
DMR1:221705001	1	221705001	221707000	2000	1	8.00E-09	0.48	46	2.3	Men1;Map4k2	
DMR1:221746001	1	221746001	221749000	3000	1	1.90E-20	0.59	84	2.8	Sf1;Pygm	Translation;Golgi
DMR1:221761001	1	221761001	221764000	3000	1	8.70E-09	0.48	61	2.03	Pygm;Rasgrp2	Golgi;Transcription
DMR1:221778001	1	221778001	221785000	7000	2	1.90E-18	0.87	84	1.2	Pygm;Rasgrp2;Nrxn2	Golgi;Transcription
DMR1:221792001	1	221792001	221795000	3000	1	5.50E-13	0.75	91	3.03	Rasgrp2;Nrxn2	Transcription
DMR1:221798001	1	221798001	221801000	3000	2	6.70E-12	0.65	42	1.4	Rasgrp2;Nrxn2	Transcription
DMR1:221803001	1	221803001	221811000	8000	4	1.00E-19	0.63	89	1.11	Nrxn2	
DMR1:221814001	1	221814001	221816000	2000	1	3.30E-12	0.45	30	1.5	Nrxn2	

DMR1:221818001	1	221818001	221820000	2000	1	9.40E-16	0.54	32	1.6	Nrxn2	
DMR1:221821001	1	221821001	221823000	2000	1	2.40E-20	0.65	38	1.9	Nrxn2	
DMR1:221825001	1	221825001	221828000	3000	1	8.60E-08	0.44	41	1.37	Nrxn2	
DMR1:221829001	1	221829001	221831000	2000	1	6.10E-20	0.51	48	2.4	Nrxn2	
DMR1:221837001	1	221837001	221844000	7000	3	2.30E-13	0.57	107	1.53	Nrxn2	
DMR1:221845001	1	221845001	221847000	2000	1	7.90E-11	0.52	32	1.6	Nrxn2	
DMR1:221851001	1	221851001	221854000	3000	1	8.60E-09	0.4	30	1	Nrxn2	
DMR1:221860001	1	221860001	221862000	2000	2	3.20E-25	0.82	20	1	Nrxn2	
DMR1:221863001	1	221863001	221866000	3000	1	3.30E-13	0.44	79	2.63	Nrxn2	
DMR1:221867001	1	221867001	221868000	1000	1	3.30E-08	0.36	24	2.4	Nrxn2	
DMR1:221869001	1	221869001	221875000	6000	5	1.90E-23	0.79	188	3.13	Nrxn2	
DMR1:221878001	1	221878001	221882000	4000	2	6.90E-29	1.09	41	1.02	Nrxn2	
DMR1:221883001	1	221883001	221889000	6000	4	1.60E-22	0.67	83	1.38	Nrxn2	
DMR1:221890001	1	221890001	221892000	2000	1	1.10E-10	0.45	32	1.6	Nrxn2	
DMR1:221921001	1	221921001	221924000	3000	1	1.20E-17	0.7	38	1.27	Slc22a12	Transport
DMR1:221927001	1	221927001	221932000	5000	1	1.10E-14	0.53	87	1.74	Slc22a12	Transport
DMR1:221982001	1	221982001	221985000	3000	1	1.20E-10	0.63	34	1.13	RGD1566262	
DMR1:221996001	1	221996001	222001000	5000	2	3.20E-14	0.51	82	1.64	RGD1566262	
DMR1:222077001	1	222077001	222078000	1000	1	2.30E-07	0.39	14	1.4	Rps6ka4	Golgi
DMR1:222095001	1	222095001	222097000	2000	1	2.50E-11	-0.7	27	1.35	Rps6ka4;Ccdc88b	Golgi;Transport
DMR1:222102001	1	222102001	222104000	2000	1	8.70E-14	0.52	21	1.05	Rps6ka4;Ccdc88b	Golgi;Transport
DMR1:222107001	1	222107001	222108000	1000	1	1.80E-29	0.75	32	3.2	Rps6ka4;Ccdc88b	Golgi;Transport
DMR1:222110001	1	222110001	222112000	2000	2	7.30E-10	0.39	31	1.55	Ccdc88b	Transport
DMR1:222117001	1	222117001	222119000	2000	2	2.20E-15	0.71	28	1.4	Ccdc88b	Transport
DMR1:222216001	1	222216001	222219000	3000	1	1.40E-07	0.29	48	1.6	Bad;Plcb3	Metabolism
DMR1:222239001	1	222239001	222241000	2000	2	1.70E-14	-0.61	30	1.5	Ppp1r14b;Fkbp2;Vegfb;Dnajc4;Nudt22;Trpt1	Signaling;Growth Factors;Signaling;Transport
DMR1:222294001	1	222294001	222295000	1000	1	3.00E-14	0.84	10	1	Stip1	
DMR1:222321001	1	222321001	222326000	5000	3	1.20E-16	0.71	91	1.82	Macrod1	
DMR1:222332001	1	222332001	222334000	2000	1	1.90E-07	0.4	24	1.2	Macrod1;Flrt1	
DMR1:222342001	1	222342001	222343000	1000	1	6.10E-07	0.34	13	1.3	Macrod1;Flrt1	
DMR1:222361001	1	222361001	222365000	4000	1	2.30E-14	0.84	46	1.15	Macrod1;Flrt1	
DMR1:222372001	1	222372001	222375000	3000	1	1.20E-11	0.45	38	1.27	Macrod1;Flrt1	
DMR1:222378001	1	222378001	222381000	3000	1	1.10E-12	0.41	41	1.37	Macrod1;Flrt1	
DMR1:222387001	1	222387001	222388000	1000	1	9.00E-12	0.44	24	2.4	Macrod1;Flrt1	
DMR1:222405001	1	222405001	222411000	6000	1	1.20E-07	0.36	84	1.4	Macrod1;Flrt1	
DMR1:222412001	1	222412001	222414000	2000	1	5.40E-10	0.62	12	0.6	Macrod1;Flrt1	
DMR1:222418001	1	222418001	222420000	2000	2	1.60E-10	0.49	48	2.4	Macrod1;Flrt1	
DMR1:222432001	1	222432001	222435000	3000	1	2.30E-11	0.64	36	1.2	Macrod1;LOC108349688	
DMR1:222438001	1	222438001	222441000	3000	2	7.50E-14	0.54	59	1.97	Macrod1;LOC108349688	
DMR1:222442001	1	222442001	222445000	3000	2	1.60E-19	0.62	62	2.07	Macrod1;LOC108349688;Otub1	Protease
DMR1:222462001	1	222462001	222463000	1000	1	9.90E-07	0.3	11	1.1	Otub1;Cox8a	Protease;Metabolism
DMR1:222469001	1	222469001	222470000	1000	1	1.60E-07	0.43	14	1.4	Otub1;Cox8a;Naa40	Protease;Metabolism
DMR1:222474001	1	222474001	222475000	1000	1	8.10E-18	0.94	1	0.1	Cox8a;Naa40;LOC102551606	Metabolism
DMR1:222477001	1	222477001	222478000	1000	1	3.20E-08	0.42	11	1.1	Cox8a;Naa40;LOC102551606	Metabolism
DMR1:222509001	1	222509001	222510000	1000	1	2.80E-10	0.64	9	0.9	Rcor2	
DMR1:222512001	1	222512001	222515000	3000	2	6.90E-11	0.51	23	0.77	Rcor2	
DMR1:222522001	1	222522001	222525000	3000	1	2.00E-09	0.52	46	1.53	Rcor2;Mark2	Signaling
DMR1:222531001	1	222531001	222532000	1000	1	2.20E-08	0.38	15	1.5	Rcor2;Mark2	Signaling
DMR1:222534001	1	222534001	222536000	2000	1	2.20E-07	-0.48	25	1.25	Rcor2;Mark2	Signaling
DMR1:222560001	1	222560001	222561000	1000	1	4.00E-09	0.4	15	1.5	Mark2	Signaling
DMR1:222579001	1	222579001	222581000	2000	1	2.10E-12	0.51	15	0.75	Mark2	Signaling
DMR1:222586001	1	222586001	222587000	1000	1	7.10E-08	0.64	5	0.5	Mark2	Signaling
DMR1:222589001	1	222589001	222590000	1000	1	9.00E-09	0.54	33	3.3	Mark2	Signaling
DMR1:222592001	1	222592001	222593000	1000	1	1.90E-10	-0.6	12	1.2	Mark2	Signaling
DMR1:222599001	1	222599001	222601000	2000	1	6.40E-09	0.48	25	1.25	Mark2;RGD1308106	Signaling
DMR1:222660001	1	222660001	222662000	2000	1	1.70E-10	0.41	35	1.75	RGD1560108	
DMR1:222671001	1	222671001	222673000	2000	2	1.40E-09	0.42	44	2.2	RGD1560108;Rtn3	
DMR1:222679001	1	222679001	222680000	1000	1	5.10E-07	-0.66	8	0.8	RGD1560108;Rtn3	
DMR1:222698001	1	222698001	222699000	1000	1	1.40E-08	0.44	5	0.5	Rtn3	
DMR1:222759001	1	222759001	222760000	1000	1	4.90E-11	0.5	3	0.3	Atl3	Signaling
DMR1:222812001	1	222812001	222822000	10000	4	3.40E-30	-0.55	107	1.07	LOC686151;RGD1563402	

DMR1:222842001	1	222842001	222844000	2000	1	1.90E-07	-0.61	20	1	Pla2g16	
DMR1:222853001	1	222853001	222854000	1000	1	8.10E-11	0.37	16	1.6	Pla2g16	
DMR1:222855001	1	222855001	222857000	2000	1	7.00E-13	0.5	19	0.95	Pla2g16	
DMR1:222858001	1	222858001	222859000	1000	1	4.30E-15	0.54	10	1	Pla2g16	
DMR1:222860001	1	222860001	222862000	2000	1	6.70E-10	0.35	21	1.05	Pla2g16	
DMR1:222863001	1	222863001	222865000	2000	1	1.80E-09	0.33	19	0.95	Pla2g16	
DMR1:222866001	1	222866001	222868000	2000	1	5.10E-12	0.79	14	0.7	Pla2g16	
DMR1:222870001	1	222870001	222873000	3000	1	5.90E-08	0.41	27	0.9	Pla2g16	
DMR1:222875001	1	222875001	222876000	1000	1	1.60E-09	0.62	6	0.6	Pla2g16	
DMR1:222883001	1	222883001	222886000	3000	1	7.30E-09	-0.42	21	0.7	Pla2g16;Lgals12	Extracellular Matrix
DMR1:222890001	1	222890001	222892000	2000	2	6.80E-23	0.98	50	2.5	Lgals12	Extracellular Matrix
DMR1:222916001	1	222916001	222917000	1000	1	2.80E-07	0.44	6	0.6	Hrasls5	
DMR1:222919001	1	222919001	222920000	1000	1	4.10E-08	0.43	5	0.5	Hrasls5	
DMR1:222922001	1	222922001	222925000	3000	2	9.00E-11	-0.6	4	0.13	Hrasls5	
DMR1:222943001	1	222943001	222944000	1000	1	9.40E-13	0.74	3	0.3	Hrasls5	
DMR1:222968001	1	222968001	222969000	1000	1	6.20E-11	0.61	1	0.1	LOC293712;Slc22a24	
DMR1:222993001	1	222993001	222998000	5000	1	2.10E-13	-0.59	50	1	Slc22a24	
DMR1:223011001	1	223011001	223015000	4000	1	6.40E-08	0.37	52	1.3	Slc22a24;LOC100910252	
DMR1:223075001	1	223075001	223076000	1000	1	3.80E-07	0.4	29	2.9	Slc22a24	
DMR1:223255001	1	223255001	223259000	4000	1	2.40E-07	-0.38	29	0.72	Slc22a24;Sirpb2	
DMR1:223334001	1	223334001	223337000	3000	1	1.20E-17	-0.81	11	0.37	Slc22a24	
DMR1:223363001	1	223363001	223365000	2000	1	4.60E-12	-0.45	24	1.2	Slc22a24	
DMR1:223393001	1	223393001	223396000	3000	2	8.50E-23	-0.58	30	1	Slc22a24	
DMR1:223454001	1	223454001	223461000	7000	2	1.50E-25	-0.6	75	1.07	Slc22a24	
DMR1:223470001	1	223470001	223477000	7000	3	9.80E-16	-0.52	76	1.09	Slc22a24	
DMR1:223490001	1	223490001	223496000	6000	3	9.20E-11	-0.44	50	0.83	Slc22a24	
DMR1:223499001	1	223499001	223505000	6000	1	4.50E-12	0.52	44	0.73	Slc22a24	
DMR1:223508001	1	223508001	223515000	7000	2	1.50E-12	-0.52	60	0.86	Slc22a24	
DMR1:223529001	1	223529001	223532000	3000	1	1.00E-07	-0.45	12	0.4	Slc22a24	
DMR1:224312001	1	224312001	224318000	6000	2	1.50E-20	-0.57	64	1.07	UST4r	Transport
DMR1:224326001	1	224326001	224341000	15000	2	2.00E-13	-0.62	195	1.3	UST4r	Transport
DMR1:224348001	1	224348001	224356000	8000	4	6.20E-14	-0.49	53	0.66	UST4r	Transport
DMR1:224361001	1	224361001	224366000	5000	3	2.50E-13	0.71	16	0.32	UST4r	Transport
DMR1:224367001	1	224367001	224370000	3000	1	4.90E-19	0.66	28	0.93	UST4r	Transport
DMR1:224372001	1	224372001	224376000	4000	1	2.90E-13	-0.58	17	0.42	UST4r	Transport
DMR1:224384001	1	224384001	224387000	3000	2	1.20E-13	0.59	21	0.7	UST4r	Transport
DMR1:224399001	1	224399001	224404000	5000	3	3.20E-25	-0.52	43	0.86	UST4r	Transport
DMR1:224450001	1	224450001	224452000	2000	1	4.50E-14	-0.4	24	1.2	Ust5r	Transport
DMR1:224453001	1	224453001	224457000	4000	4	4.60E-23	-0.55	46	1.15	Ust5r	Transport
DMR1:224466001	1	224466001	224477000	11000	6	4.40E-25	-0.61	78	0.71	Ust5r	Transport
DMR1:224480001	1	224480001	224501000	21000	11	1.70E-25	-0.58	207	0.99	Ust5r	Transport
DMR1:224504001	1	224504001	224507000	3000	2	9.00E-11	0.67	11	0.37	Ust5r	Transport
DMR1:224508001	1	224508001	224515000	7000	3	5.30E-11	-0.54	56	0.8	Ust5r	Transport
DMR1:224517001	1	224517001	224521000	4000	3	9.30E-24	-0.59	52	1.3	Ust5r	Transport
DMR1:224534001	1	224534001	224543000	9000	4	1.70E-14	-0.53	89	0.99	Ust5r	Transport
DMR1:224602001	1	224602001	224603000	1000	1	6.20E-12	0.72	6	0.6	Slc22a25	Transport
DMR1:224659001	1	224659001	224664000	5000	3	1.80E-17	-0.55	49	0.98	Slc22a25	Transport
DMR1:224669001	1	224669001	224677000	8000	6	4.40E-25	-0.57	76	0.95	Slc22a25;LOC108349689	Transport
DMR1:224681001	1	224681001	224682000	1000	1	1.30E-13	0.58	11	1.1	Slc22a25;LOC108349689	Transport
DMR1:224683001	1	224683001	224689000	6000	2	8.10E-12	-0.5	39	0.65	Slc22a25;LOC108349689	Transport
DMR1:224699001	1	224699001	224700000	1000	1	1.20E-07	0.53	5	0.5	Slc22a25	Transport
DMR1:224793001	1	224793001	224794000	1000	1	3.80E-07	0.36	8	0.8	LOC103691288;Slc22a8	Transport
DMR1:224803001	1	224803001	224804000	1000	1	2.20E-07	0.34	13	1.3	Slc22a8	Transport
DMR1:224811001	1	224811001	224813000	2000	2	1.60E-13	0.66	21	1.05	Slc22a8;LOC102550957	Transport
DMR1:224835001	1	224835001	224843000	8000	2	3.50E-16	0.65	89	1.11	LOC102550957;Slc22a6	Transport
DMR1:224864001	1	224864001	224865000	1000	1	2.50E-19	0.72	7	0.7	Chrm1	Signaling
DMR1:224869001	1	224869001	224870000	1000	1	1.80E-13	0.59	13	1.3	Chrm1	Signaling
DMR1:224871001	1	224871001	224873000	2000	1	5.90E-09	0.5	23	1.15	Chrm1	Signaling
DMR1:224888001	1	224888001	224890000	2000	1	3.00E-09	0.4	23	1.15	Chrm1	Signaling
DMR1:224894001	1	224894001	224896000	2000	1	3.10E-08	0.43	13	0.65	Chrm1	Signaling
DMR1:224899001	1	224899001	224900000	1000	1	2.90E-16	0.57	8	0.8	Slc3a2	
DMR1:224902001	1	224902001	224903000	1000	1	8.30E-12	0.59	14	1.4	Slc3a2	
DMR1:224904001	1	224904001	224906000	2000	2	9.00E-15	0.52	22	1.1	Slc3a2	
DMR1:224936001	1	224936001	224938000	2000	2	1.70E-08	-0.64	21	1.05	LOC690251;Wdr74;Stx5	Translation;Transcription

DMR1:224946001	1	224946001	224947000	1000	1	2.00E-12	0.79	4	0.4	Wdr74;Stx5	Translation;Transcription
DMR1:224966001	1	224966001	224968000	2000	2	6.80E-07	-0.42	30	1.5	Nxf1;Tmem223;Tmem179b;Taf6l	Metabolism;Transcription
DMR1:224996001	1	224996001	224997000	1000	1	1.60E-07	-0.53	17	1.7	Taf6l;Polr2g;Zbtb3;Ttc9c	Transcription;Transcription;Transcription;Transcription
DMR1:225010001	1	225010001	225012000	2000	1	3.60E-07	-0.64	18	0.9	Zbtb3;Ttc9c;Hnrnpul2	Transcription;Transcription
DMR1:225105001	1	225105001	225109000	4000	1	1.70E-08	0.41	34	0.85	Ints5;Ganab	Metabolism
DMR1:225110001	1	225110001	225116000	6000	1	6.70E-10	0.4	95	1.58	Ganab;B3gat3	Metabolism;Golgi
DMR1:225142001	1	225142001	225145000	3000	2	3.90E-17	0.61	51	1.7	Eml3;Mta2;Tut1	Development;Metabolism
DMR1:225183001	1	225183001	225184000	1000	1	5.70E-08	0.52	10	1	Eef1g;Ahnak	
DMR1:225189001	1	225189001	225194000	5000	1	1.40E-07	-0.55	61	1.22	Ahnak	
DMR1:225216001	1	225216001	225222000	6000	2	9.10E-23	0.91	57	0.95	Ahnak	
DMR1:225246001	1	225246001	225249000	3000	1	9.00E-20	0.75	36	1.2	Ahnak;LOC108349690	
DMR1:225250001	1	225250001	225254000	4000	3	2.80E-11	0.82	24	0.6	Ahnak;LOC108349690	
DMR1:225255001	1	225255001	225257000	2000	1	1.00E-18	0.59	47	2.35	Ahnak	
DMR1:225258001	1	225258001	225259000	1000	1	8.20E-24	0.74	10	1	Ahnak	
DMR1:225261001	1	225261001	225263000	2000	1	4.10E-07	-0.43	25	1.25	Ahnak	
DMR1:225269001	1	225269001	225272000	3000	1	6.80E-10	0.44	36	1.2	Ahnak;Scgb1a1	Growth Factors
DMR1:225280001	1	225280001	225281000	1000	1	1.70E-09	0.42	5	0.5	Ahnak;Scgb1a1	Growth Factors
DMR1:225282001	1	225282001	225284000	2000	1	6.30E-10	0.36	30	1.5	Ahnak;Scgb1a1	Growth Factors
DMR1:225287001	1	225287001	225293000	6000	1	7.20E-14	0.69	77	1.28	Ahnak;Scgb1a1	Growth Factors
DMR1:225294001	1	225294001	225298000	4000	1	1.20E-12	0.51	61	1.52	Ahnak	
DMR1:225322001	1	225322001	225324000	2000	1	4.40E-10	0.49	10	0.5	Ahnak;Asrgl1	Protease
DMR1:225332001	1	225332001	225334000	2000	1	1.30E-08	-0.52	9	0.45	Ahnak;Asrgl1	Protease
DMR1:225336001	1	225336001	225337000	1000	1	1.30E-10	0.64	3	0.3	Ahnak;Asrgl1	Protease
DMR1:225338001	1	225338001	225345000	7000	5	1.20E-19	0.67	94	1.34	Ahnak;Asrgl1	Protease
DMR1:225347001	1	225347001	225348000	1000	1	7.00E-10	0.73	3	0.3	Ahnak	
DMR1:225349001	1	225349001	225352000	3000	3	6.50E-22	-0.53	37	1.23	Ahnak	
DMR1:225353001	1	225353001	225356000	3000	1	8.70E-10	-0.46	27	0.9	Ahnak	
DMR1:225362001	1	225362001	225369000	7000	2	4.50E-09	-0.49	83	1.19	Ahnak	
DMR1:225373001	1	225373001	225380000	7000	2	2.60E-11	-0.65	112	1.6	Ahnak	
DMR1:225394001	1	225394001	225396000	2000	1	2.00E-09	-0.38	34	1.7	Ahnak	
DMR1:225460001	1	225460001	225466000	6000	4	1.70E-22	-0.5	58	0.97	LOC108348288;RGD1566289	
DMR1:225469001	1	225469001	225471000	2000	1	3.80E-08	0.51	38	1.9	LOC108348288;RGD1566289	
DMR1:225478001	1	225478001	225481000	3000	2	2.80E-13	-0.55	29	0.97	RGD1566289	
DMR1:225564001	1	225564001	225566000	2000	1	2.00E-14	0.79	13	0.65	RGD1566289	
DMR1:225572001	1	225572001	225573000	1000	1	2.80E-07	0.36	19	1.9	RGD1566289;LOC690454	
DMR1:225574001	1	225574001	225581000	7000	6	4.00E-15	-0.48	69	0.99	RGD1566289;LOC690454	
DMR1:225584001	1	225584001	225588000	4000	1	5.90E-07	-0.52	10	0.25	RGD1566289;LOC690454;Scgb1d2	
DMR1:225601001	1	225601001	225603000	2000	2	3.90E-12	0.72	11	0.55	Scgb1d2	
DMR1:225604001	1	225604001	225605000	1000	1	2.90E-07	-0.34	5	0.5	Scgb1d2	
DMR1:225684001	1	225684001	225687000	3000	2	1.60E-12	-0.47	10	0.33	Psbpc2	Transport
DMR1:225694001	1	225694001	225698000	4000	2	3.00E-15	0.75	13	0.32	Psbpc2	Transport
DMR1:225699001	1	225699001	225701000	2000	1	1.10E-07	-0.47	11	0.55	Psbpc2	Transport
DMR1:225810001	1	225810001	225816000	6000	1	9.70E-13	0.48	43	0.72	Scgb2a1	
DMR1:225840001	1	225840001	225843000	3000	2	1.40E-09	-0.51	14	0.47	Scgb2a1	
DMR1:225894001	1	225894001	225895000	1000	1	4.40E-11	0.53	28	2.8	Scgb2a2	
DMR1:225898001	1	225898001	225899000	1000	1	1.00E-07	0.58	6	0.6	Scgb2a2;Fads2	
DMR1:225929001	1	225929001	225933000	4000	1	8.90E-18	0.69	82	2.05	Fads2;Incenp	
DMR1:225955001	1	225955001	225962000	7000	1	1.50E-09	0.58	53	0.76	Fads2;Incenp;LOC102552566	
DMR1:225964001	1	225964001	225969000	5000	1	8.20E-11	0.47	50	1	Fads2;LOC102552566	
DMR1:225971001	1	225971001	225973000	2000	1	8.50E-08	0.53	20	1	Fads2;LOC102552566	
DMR1:226009001	1	226009001	226017000	8000	2	5.00E-13	0.54	131	1.64	Fads2;LOC102552566;LOC102552394	
DMR1:226047001	1	226047001	226049000	2000	1	2.10E-08	0.43	27	1.35	Fads2;Best1;LOC102554361	Transport
DMR1:226051001	1	226051001	226054000	3000	1	1.90E-12	0.69	35	1.17	Fads2;Best1	Transport
DMR1:226072001	1	226072001	226074000	2000	1	4.40E-16	0.72	16	0.8	Fads2;Rab3il1	Transcription
DMR1:226081001	1	226081001	226083000	2000	1	5.30E-20	0.6	29	1.45	Fads2;Rab3il1;Fads3	Transcription
DMR1:226107001	1	226107001	226108000	1000	1	1.30E-14	0.54	16	1.6	Fads2;Fads3	
DMR1:226118001	1	226118001	226119000	1000	1	6.60E-08	0.34	14	1.4	Fads2;Fads3	

DMR1:226124001	1	226124001	226126000	2000	1	2.30E-12	0.56	19	0.95	Fads2	
DMR1:226134001	1	226134001	226137000	3000	2	1.10E-07	0.34	36	1.2	Fads2	
DMR1:226160001	1	226160001	226161000	1000	1	6.20E-11	0.44	9	0.9	Fads2	
DMR1:226223001	1	226223001	226226000	3000	1	3.50E-14	0.65	26	0.87	LOC690604;Fads1	
DMR1:226249001	1	226249001	226251000	2000	1	3.10E-08	-0.48	28	1.4	Fads1;Fen1;Tmem258;Myrf	DNA Repair
DMR1:226269001	1	226269001	226273000	4000	1	1.10E-11	0.48	69	1.73	Tmem258;Myrf	
DMR1:226275001	1	226275001	226278000	3000	2	2.10E-17	0.68	49	1.63	Myrf	
DMR1:226296001	1	226296001	226298000	2000	1	1.50E-07	0.44	25	1.25	Myrf;Dagla	Metabolism
DMR1:226308001	1	226308001	226312000	4000	2	1.20E-19	0.69	40	1	Dagla	Metabolism
DMR1:226338001	1	226338001	226340000	2000	1	6.90E-14	0.67	32	1.6	Dagla	Metabolism
DMR1:226357001	1	226357001	226360000	3000	1	1.20E-26	0.91	26	0.87	Dagla;LOC100911105;LOC102552972;LOC108349691	Metabolism
DMR1:226422001	1	226422001	226426000	4000	4	8.70E-27	1.07	38	0.95	LOC108349692;Syt7	Transport
DMR1:226427001	1	226427001	226428000	1000	1	1.20E-18	0.8	10	1	LOC108349692;Syt7	Transport
DMR1:226438001	1	226438001	226441000	3000	1	4.10E-09	0.52	41	1.37	LOC108349692;Syt7	Transport
DMR1:226447001	1	226447001	226449000	2000	1	2.50E-10	0.45	30	1.5	Syt7	Transport
DMR1:226464001	1	226464001	226468000	4000	1	1.10E-19	0.78	75	1.88	Syt7	Transport
DMR1:226472001	1	226472001	226476000	4000	3	5.30E-27	0.95	54	1.35	Syt7	Transport
DMR1:226477001	1	226477001	226479000	2000	1	7.00E-22	0.69	43	2.15	Syt7	Transport
DMR1:226494001	1	226494001	226495000	1000	1	1.50E-16	0.62	12	1.2	Syt7;Lrrc10b	Transport
DMR1:226513001	1	226513001	226515000	2000	1	2.40E-09	0.41	28	1.4	Ppp1r32	Signaling
DMR1:226524001	1	226524001	226526000	2000	1	7.50E-12	0.45	39	1.95	Ppp1r32	Signaling
DMR1:226529001	1	226529001	226531000	2000	1	2.30E-07	0.42	22	1.1	Ppp1r32	Signaling
DMR1:226559001	1	226559001	226561000	2000	2	1.40E-08	-0.44	7	0.35	Sdhaf2	
DMR1:226582001	1	226582001	226584000	2000	2	9.50E-26	0.84	13	0.65	Sdhaf2;Cpsf7	Translation
DMR1:226609001	1	226609001	226611000	2000	1	2.30E-07	-0.54	21	1.05	LOC103691286;LOC102554522;Tmem216;RGD1561525;Cyb561a3	Metabolism
DMR1:226626001	1	226626001	226628000	2000	1	1.60E-07	-0.46	36	1.8	RGD1561525;Cyb561a3;Tmem138	Metabolism
DMR1:226689001	1	226689001	226694000	5000	2	2.50E-14	0.59	57	1.14	Ddb1;Vwce	Translation
DMR1:226699001	1	226699001	226701000	2000	2	6.80E-17	1.03	16	0.8	Vwce	
DMR1:226710001	1	226710001	226712000	2000	1	2.50E-11	0.56	23	1.15	Vwce	
DMR1:226715001	1	226715001	226716000	1000	1	7.60E-11	0.44	17	1.7	Vwce;LOC108348129	
DMR1:226738001	1	226738001	226740000	2000	1	2.30E-07	-0.51	26	1.3	LOC108348129;Vps37c	
DMR1:226749001	1	226749001	226751000	2000	1	6.30E-21	0.7	32	1.6	Vps37c	
DMR1:226775001	1	226775001	226778000	3000	1	3.30E-28	1.05	37	1.23	Vps37c;Cd5	
DMR1:226781001	1	226781001	226783000	2000	1	1.00E-09	0.43	26	1.3	Vps37c;Cd5	
DMR1:226784001	1	226784001	226786000	2000	2	4.20E-18	0.65	37	1.85	Vps37c;Cd5	
DMR1:226787001	1	226787001	226790000	3000	1	4.00E-13	0.62	57	1.9	Cd5	
DMR1:226794001	1	226794001	226795000	1000	1	8.90E-07	0.39	15	1.5	Cd5	
DMR1:226799001	1	226799001	226802000	3000	1	6.20E-07	-0.46	42	1.4	Cd5	
DMR1:226845001	1	226845001	226847000	2000	1	3.40E-12	0.64	23	1.15	Cd6	Protease
DMR1:226848001	1	226848001	226850000	2000	1	4.30E-07	0.53	35	1.75	Cd6	Protease
DMR1:226852001	1	226852001	226853000	1000	1	1.60E-16	0.75	6	0.6	Cd6	Protease
DMR1:226869001	1	226869001	226872000	3000	2	9.10E-23	0.66	44	1.47	Cd6	Protease
DMR1:226873001	1	226873001	226875000	2000	1	1.60E-07	-0.35	27	1.35	Cd6	Protease
DMR1:226876001	1	226876001	226880000	4000	1	2.10E-07	0.36	58	1.45	Cd6	Protease
DMR1:226889001	1	226889001	226891000	2000	1	7.60E-18	0.68	16	0.8	Cd6;Slc15a3	Protease;Transport
DMR1:226896001	1	226896001	226898000	2000	1	5.00E-11	0.75	41	2.05	Cd6;Slc15a3	Protease;Transport
DMR1:226954001	1	226954001	226959000	5000	2	5.90E-10	-0.75	58	1.16	Prpf19;Zp1	Translation
DMR1:226981001	1	226981001	226983000	2000	1	3.90E-18	0.72	27	1.35	Zp1;Ptgdr2	Signaling
DMR1:226985001	1	226985001	226987000	2000	1	2.00E-14	0.47	22	1.1	Ptgdr2;Ccdc86	Signaling
DMR1:226988001	1	226988001	226989000	1000	1	7.80E-16	0.43	16	1.6	Ptgdr2;Ccdc86	Signaling
DMR1:227005001	1	227005001	227006000	1000	1	1.90E-08	0.33	11	1.1	Ptgdr2;Ccdc86	Signaling
DMR1:227021001	1	227021001	227022000	1000	1	1.30E-14	0.6	8	0.8	Ms4a10	Transport
DMR1:227028001	1	227028001	227029000	1000	1	4.10E-13	0.64	9	0.9	Ms4a10;Ms4a15	Transport
DMR1:227039001	1	227039001	227041000	2000	1	5.80E-07	0.43	31	1.55	Ms4a10;Ms4a15;Ms4a18	Transport
DMR1:227055001	1	227055001	227057000	2000	2	2.20E-11	0.46	17	0.85	Ms4a15;Ms4a18	Transport
DMR1:227071001	1	227071001	227075000	4000	2	4.40E-08	-0.39	37	0.92	Ms4a18	Transport
DMR1:227080001	1	227080001	227082000	2000	2	1.40E-09	0.6	8	0.4	Ms4a18	Transport
DMR1:227123001	1	227123001	227127000	4000	1	3.00E-13	-0.62	14	0.35	LOC100910973;LOC293740;Ms4a8	Transport
DMR1:227128001	1	227128001	227136000	8000	4	2.70E-17	-0.47	60	0.75	LOC293740;Ms4a8	Transport

DMR1:227241001	1	227241001	227243000	2000	1	1.30E-19	0.99	5	0.25	LOC102557108;LOC100911403;Ms4a6bl	
DMR1:227252001	1	227252001	227254000	2000	2	2.60E-10	0.78	7	0.35	LOC102557108;Ms4a6bl	
DMR1:227260001	1	227260001	227267000	7000	4	2.30E-20	-0.47	62	0.89	LOC102557108;Ms4a6bl	
DMR1:227364001	1	227364001	227366000	2000	1	5.10E-20	0.87	11	0.55	LOC100912336;Ms4a13-ps1	Transport
DMR1:227371001	1	227371001	227375000	4000	2	1.50E-15	0.71	30	0.75	LOC100912336;Ms4a13-ps1	Transport
DMR1:227377001	1	227377001	227378000	1000	1	6.00E-07	0.34	8	0.8	Ms4a13-ps1	
DMR1:227416001	1	227416001	227419000	3000	1	1.20E-08	0.37	46	1.53	Ms4a12;LOC103691292	Transport
DMR1:227420001	1	227420001	227422000	2000	2	1.30E-21	0.73	28	1.4	Ms4a12;LOC103691292;Ms4a1	Transport
DMR1:227426001	1	227426001	227427000	1000	1	2.60E-09	0.47	8	0.8	Ms4a12;Ms4a1	Transport
DMR1:227438001	1	227438001	227441000	3000	3	7.30E-09	0.42	23	0.77	Ms4a1;Ms4a5	Transport
DMR1:227442001	1	227442001	227443000	1000	1	2.70E-15	0.64	11	1.1	Ms4a1;Ms4a5	Transport
DMR1:227452001	1	227452001	227455000	3000	2	1.20E-16	0.76	18	0.6	Ms4a5	Transport
DMR1:227460001	1	227460001	227461000	1000	1	1.20E-23	0.81	9	0.9	Ms4a5	Transport
DMR1:227464001	1	227464001	227466000	2000	1	1.10E-07	0.61	12	0.6	Ms4a5;Ms4a14	Transport
DMR1:227471001	1	227471001	227474000	3000	2	5.10E-09	-0.55	16	0.53	Ms4a14	Transport
DMR1:227500001	1	227500001	227502000	2000	1	2.20E-15	0.71	6	0.3	Ms4a7	Transport
DMR1:227507001	1	227507001	227509000	2000	1	1.10E-09	0.48	13	0.65	Ms4a7	Transport
DMR1:227514001	1	227514001	227517000	3000	1	6.30E-16	-0.77	32	1.07	Ms4a7	Transport
DMR1:227566001	1	227566001	227567000	1000	1	6.10E-08	-0.37	8	0.8	Ms4a4a	Transport
DMR1:227568001	1	227568001	227571000	3000	3	1.40E-27	-0.57	31	1.03	Ms4a4a	Transport
DMR1:227592001	1	227592001	227593000	1000	1	3.40E-12	0.79	4	0.4	Ms4a4a	Transport
DMR1:227594001	1	227594001	227595000	1000	1	5.10E-16	0.9	5	0.5	Ms4a4a	Transport
DMR1:227630001	1	227630001	227636000	6000	4	1.90E-08	-0.72	46	0.77	Ms4a4c	Transport
DMR1:227644001	1	227644001	227645000	1000	1	1.10E-09	-0.72	4	0.4	Ms4a4c	Transport
DMR1:227665001	1	227665001	227666000	1000	1	7.90E-07	0.43	3	0.3	Ms4a4c;Ms4a6c	Transport
DMR1:227681001	1	227681001	227686000	5000	2	9.60E-14	-0.59	47	0.94	Ms4a6c;LOC690870	
DMR1:227814001	1	227814001	227815000	1000	1	2.10E-10	0.58	6	0.6	Ms4a6b	
DMR1:227819001	1	227819001	227824000	5000	2	2.90E-16	0.82	17	0.34	Ms4a6b	
DMR1:227910001	1	227910001	227912000	2000	2	8.40E-15	0.64	11	0.55	LOC690836;LOC100362826;Ms4a6a	Transport
DMR1:227913001	1	227913001	227914000	1000	1	2.60E-10	0.59	10	1	LOC690836;LOC100362826;Ms4a6a	Transport
DMR1:227937001	1	227937001	227939000	2000	1	6.60E-07	-0.52	5	0.25	Ms4a6a	Transport
DMR1:227940001	1	227940001	227944000	4000	2	3.90E-09	0.52	23	0.58	Ms4a6a	Transport
DMR1:227954001	1	227954001	227956000	2000	1	5.70E-10	0.5	13	0.65	Ms4a2	Transport
DMR1:227967001	1	227967001	227968000	1000	1	4.30E-09	0.51	8	0.8	Ms4a2	Transport
DMR1:227971001	1	227971001	227975000	4000	2	4.10E-14	-0.74	21	0.52	Ms4a2;Ms4a3	Transport
DMR1:228006001	1	228006001	228007000	1000	1	5.10E-10	-0.51	8	0.8	Oosp2	
DMR1:228009001	1	228009001	228011000	2000	1	7.60E-36	1.19	9	0.45	Oosp2	
DMR1:228024001	1	228024001	228028000	4000	3	4.50E-24	-0.54	43	1.07	Oosp2;Oosp1	
DMR1:228034001	1	228034001	228036000	2000	1	8.30E-20	0.71	18	0.9	Oosp1	
DMR1:228050001	1	228050001	228052000	2000	1	7.70E-07	-0.48	28	1.4	Oosp1	
DMR1:228060001	1	228060001	228062000	2000	1	7.00E-12	0.56	25	1.25	Oosp1	
DMR1:228065001	1	228065001	228068000	3000	2	9.60E-20	0.81	24	0.8	Oosp1	
DMR1:228136001	1	228136001	228137000	1000	1	3.80E-16	0.94	23	2.3	Gif;Stx3;Mrpl16	Transcription;Translation
DMR1:228138001	1	228138001	228142000	4000	1	4.00E-14	0.8	30	0.75	Gif;Stx3;Mrpl16	Transcription;Translation
DMR1:228188001	1	228188001	228189000	1000	1	3.50E-16	0.85	10	1	Stx3;LOC103691293	Transcription
DMR1:228224001	1	228224001	228225000	1000	1	5.60E-07	0.44	3	0.3	Olr1874;LOC309220	
DMR1:228322001	1	228322001	228326000	4000	3	2.40E-20	0.81	23	0.58	Olr319	Signaling
DMR1:228341001	1	228341001	228343000	2000	2	1.70E-13	0.57	16	0.8	Patl1	Translation
DMR1:228371001	1	228371001	228373000	2000	1	8.70E-09	-0.43	21	1.05	Patl1;LOC103691294	Translation
DMR1:228405001	1	228405001	228406000	1000	1	7.30E-07	-0.48	10	1	LOC103691294;Osbp	
DMR1:228419001	1	228419001	228424000	5000	2	7.20E-12	0.62	48	0.96	Osbp	
DMR1:228431001	1	228431001	228432000	1000	1	5.60E-08	0.43	6	0.6	Osbp;Trnaf-gaa;Trnak-uuu	
DMR1:228433001	1	228433001	228434000	1000	1	5.10E-10	0.59	6	0.6	Osbp;Trnaf-gaa;Trnak-uuu	
DMR1:228447001	1	228447001	228449000	2000	2	1.40E-14	0.67	15	0.75	Trnak-uuu;Trnaf-gaa;Trnal-uaa;Trnar-ucu;Trnav-uac;LOC100363176;Olr320-ps	
DMR1:228463001	1	228463001	228466000	3000	1	3.70E-10	0.61	28	0.93	Olr320-ps;Olr321	Receptor
DMR1:228471001	1	228471001	228472000	1000	1	6.80E-09	0.68	3	0.3	Olr321	Receptor
DMR1:228476001	1	228476001	228479000	3000	2	2.50E-16	0.86	15	0.5	Olr321	Receptor
DMR1:228495001	1	228495001	228501000	6000	4	1.30E-30	-0.54	61	1.02	Olr322	Receptor

DMR1:228527001	1	228527001	228528000	1000	1	3.60E-08	0.38	11	1.1	Olr322;Olr323;Olr324	Receptor
DMR1:228531001	1	228531001	228538000	7000	3	2.40E-18	0.89	47	0.67	Olr322;Olr323;Olr324;Olr325	Receptor
DMR1:228543001	1	228543001	228544000	1000	1	7.00E-23	0.84	8	0.8	Olr322;Olr324;Olr325	Receptor
DMR1:228613001	1	228613001	228621000	8000	5	4.10E-18	0.53	78	0.98	Olr330	Signaling
DMR1:228639001	1	228639001	228641000	2000	1	3.70E-12	-0.53	3	0.15	Olr330	Signaling
DMR1:228672001	1	228672001	228676000	4000	1	1.70E-07	0.31	36	0.9	Olr337	Signaling
DMR1:228689001	1	228689001	228692000	3000	1	4.70E-07	-0.46	38	1.27	Olr337;Pfpl	Signaling
DMR1:228700001	1	228700001	228702000	2000	1	2.00E-07	0.64	13	0.65	Pfpl	
DMR1:228748001	1	228748001	228751000	3000	1	6.10E-12	-0.76	24	0.8	Dtx4	Proteolysis
DMR1:228762001	1	228762001	228763000	1000	1	1.00E-12	0.55	9	0.9	Dtx4	Proteolysis
DMR1:228766001	1	228766001	228773000	7000	6	2.20E-18	0.93	43	0.61	Dtx4	Proteolysis
DMR1:228994001	1	228994001	228996000	2000	1	8.40E-09	-0.45	9	0.45	Fam111a	
DMR1:229004001	1	229004001	229007000	3000	2	2.30E-21	0.9	32	1.07	Fam111a	
DMR1:229062001	1	229062001	229063000	1000	1	2.20E-07	-0.4	12	1.2	Glyatl1;Glyat	Metabolism
DMR1:229068001	1	229068001	229069000	1000	1	1.40E-10	0.61	4	0.4	Glyat	Metabolism
DMR1:229072001	1	229072001	229073000	1000	1	1.70E-07	0.47	5	0.5	Glyat	Metabolism
DMR1:229083001	1	229083001	229084000	1000	1	3.90E-13	0.77	5	0.5	Glyat	Metabolism
DMR1:229198001	1	229198001	229205000	7000	2	1.90E-11	-0.58	59	0.84	RGD1305704	
DMR1:229391001	1	229391001	229394000	3000	1	8.30E-08	0.52	10	0.33	Olr335-ps	
DMR1:229445001	1	229445001	229450000	5000	3	4.60E-10	-0.49	70	1.4	Olr334;Olr333-ps	Signaling
DMR1:229451001	1	229451001	229453000	2000	2	5.70E-24	1.19	10	0.5	Olr334;Olr333-ps;Olr332	Signaling
DMR1:229465001	1	229465001	229467000	2000	1	8.50E-09	-0.41	14	0.7	Olr332	Signaling
DMR1:229468001	1	229468001	229471000	3000	1	6.60E-09	-0.54	20	0.67	Olr332;Olr387	Signaling
DMR1:229473001	1	229473001	229476000	3000	1	1.40E-16	0.94	16	0.53	Olr387	Signaling
DMR1:229490001	1	229490001	229491000	1000	1	9.70E-07	0.43	2	0.2	Olr387	Signaling
DMR1:229524001	1	229524001	229527000	3000	1	5.20E-15	-0.63	7	0.23	Glyatl2	
DMR1:229542001	1	229542001	229543000	1000	1	3.70E-08	0.53	6	0.6	Glyatl2	
DMR1:229552001	1	229552001	229555000	3000	3	3.40E-13	0.76	20	0.67	Glyatl2	
DMR1:229590001	1	229590001	229591000	1000	1	2.40E-14	0.8	9	0.9	Cntf	
DMR1:229616001	1	229616001	229617000	1000	1	2.80E-08	0.43	9	0.9	Zfp91	Transcription
DMR1:229619001	1	229619001	229620000	1000	1	1.60E-07	0.42	10	1	Zfp91	Transcription
DMR1:229631001	1	229631001	229633000	2000	1	1.90E-07	-0.51	16	0.8	Zfp91;Lpxn	Transcription;Cytoskeleton
DMR1:229651001	1	229651001	229652000	1000	1	2.10E-19	0.78	9	0.9	Lpxn	Cytoskeleton
DMR1:229656001	1	229656001	229660000	4000	2	8.80E-28	0.85	26	0.65	Lpxn	Cytoskeleton
DMR1:229662001	1	229662001	229663000	1000	1	3.00E-07	-0.35	11	1.1	Lpxn	Cytoskeleton
DMR1:229664001	1	229664001	229666000	2000	2	1.80E-26	0.87	22	1.1	Lpxn	Cytoskeleton
DMR1:229675001	1	229675001	229677000	2000	1	8.20E-12	-0.5	15	0.75	Lpxn	Cytoskeleton
DMR1:229736001	1	229736001	229746000	10000	4	2.60E-18	-0.57	127	1.27	Olr341;Olr340	Receptor
DMR1:229753001	1	229753001	229754000	1000	1	3.10E-09	0.48	9	0.9	Olr341;Olr340	Receptor
DMR1:229760001	1	229760001	229761000	1000	1	6.40E-10	-0.65	12	1.2	Olr341;Olr340	Receptor
DMR1:229763001	1	229763001	229764000	1000	1	2.50E-09	0.64	4	0.4	Olr341;Olr340	Receptor
DMR1:229796001	1	229796001	229799000	3000	2	5.40E-10	-0.51	10	0.33	Olr343	Receptor
DMR1:229801001	1	229801001	229802000	1000	1	1.90E-11	0.59	4	0.4	Olr343	Receptor
DMR1:229813001	1	229813001	229818000	5000	1	1.50E-10	0.42	37	0.74	Olr343;Olr344	Receptor
DMR1:229822001	1	229822001	229824000	2000	1	4.40E-10	0.65	7	0.35	Olr344	Receptor
DMR1:229847001	1	229847001	229848000	1000	1	4.80E-13	0.87	3	0.3	Olr346	Receptor
DMR1:229852001	1	229852001	229855000	3000	1	6.00E-18	0.68	21	0.7	Olr346	Receptor
DMR1:229856001	1	229856001	229857000	1000	1	9.60E-09	-0.75	9	0.9	Olr346	Receptor
DMR1:229867001	1	229867001	229870000	3000	1	2.50E-08	-0.42	15	0.5	Olr347	Receptor
DMR1:229871001	1	229871001	229873000	2000	2	3.70E-18	0.76	11	0.55	Olr347	Receptor
DMR1:229882001	1	229882001	229886000	4000	1	1.50E-11	-0.54	27	0.68	Olr347;Olr348	Receptor
DMR1:229897001	1	229897001	229898000	1000	1	9.90E-09	0.47	13	1.3	Olr348	Receptor
DMR1:229899001	1	229899001	229902000	3000	2	4.30E-12	0.65	9	0.3	Olr348	Receptor
DMR1:229921001	1	229921001	229925000	4000	3	6.40E-14	0.76	26	0.65	Olr349	Receptor
DMR1:229931001	1	229931001	229939000	8000	2	5.10E-15	0.52	77	0.96	Olr349;Cct7-ps1	Receptor
DMR1:229950001	1	229950001	229952000	2000	2	9.00E-13	-0.7	7	0.35	Olr350-ps	
DMR1:229956001	1	229956001	229957000	1000	1	2.90E-12	0.7	4	0.4	Olr350-ps	
DMR1:229963001	1	229963001	229964000	1000	1	2.00E-21	0.79	12	1.2	Olr350-ps	
DMR1:229965001	1	229965001	229977000	12000	9	1.40E-29	-0.58	131	1.09	Olr350-ps	
DMR1:229982001	1	229982001	229989000	7000	5	1.90E-20	-0.54	81	1.16	Olr351-ps	
DMR1:229992001	1	229992001	229993000	1000	1	1.90E-21	0.84	5	0.5	Olr351-ps	
DMR1:229999001	1	229999001	230003000	4000	2	5.10E-10	-0.65	17	0.42	Olr351-ps;Olr352	Receptor
DMR1:230019001	1	230019001	230020000	1000	1	4.70E-08	0.57	5	0.5	Olr352;Olr353-ps	Receptor
DMR1:230025001	1	230025001	230029000	4000	3	8.50E-21	-0.52	69	1.73	Olr353-ps;Olr354;Olr355-ps	Receptor

DMR1:230059001	1	230059001	230062000	3000	3	1.20E-13	0.75	9	0.3	Olr357	Receptor
DMR1:230063001	1	230063001	230064000	1000	1	2.90E-11	-0.59	3	0.3	Olr357	Receptor
DMR1:230065001	1	230065001	230066000	1000	1	5.80E-10	-0.51	3	0.3	Olr357	Receptor
DMR1:230068001	1	230068001	230071000	3000	1	6.70E-09	0.48	11	0.37	Olr358	Receptor
DMR1:230080001	1	230080001	230082000	2000	2	8.90E-16	0.83	11	0.55	Olr358	Receptor
DMR1:230087001	1	230087001	230092000	5000	2	8.70E-10	-0.47	46	0.92	Olr358;Olr359-ps	Receptor
DMR1:230105001	1	230105001	230106000	1000	1	3.20E-10	0.6	7	0.7	Olr360	Receptor
DMR1:230111001	1	230111001	230112000	1000	1	5.10E-07	0.48	5	0.5	Olr360;RGD1559854	Receptor
DMR1:230113001	1	230113001	230114000	1000	1	1.70E-11	0.61	10	1	Olr360;RGD1559854	Receptor
DMR1:230150001	1	230150001	230158000	8000	4	5.90E-32	-0.64	67	0.84	Olr361	Receptor
DMR1:230273001	1	230273001	230274000	1000	1	1.30E-11	0.54	5	0.5	Olr363	Receptor
DMR1:230276001	1	230276001	230279000	3000	2	1.00E-10	-0.73	7	0.23	Olr363	Receptor
DMR1:230311001	1	230311001	230313000	2000	1	6.60E-07	-0.37	7	0.35	Olr365	Receptor
DMR1:230346001	1	230346001	230347000	1000	1	2.40E-10	-0.5	3	0.3	Olr366-ps	
DMR1:230367001	1	230367001	230368000	1000	1	1.10E-19	0.76	10	1	Olr367	Receptor
DMR1:230369001	1	230369001	230372000	3000	2	3.00E-10	-0.45	26	0.87	Olr367	Receptor
DMR1:230379001	1	230379001	230382000	3000	1	1.60E-17	0.79	12	0.4	Olr367;Olr368-ps	Receptor
DMR1:230387001	1	230387001	230391000	4000	1	1.90E-07	-0.41	26	0.65	Olr367;Olr368-ps	Receptor
DMR1:230395001	1	230395001	230402000	7000	4	4.00E-24	-0.59	69	0.99	Olr368-ps	
DMR1:230436001	1	230436001	230443000	7000	3	4.20E-17	-0.51	60	0.86	Olr370-ps;Olr371	Receptor
DMR1:230448001	1	230448001	230449000	1000	1	5.80E-07	0.5	2	0.2	Olr371	Receptor
DMR1:230452001	1	230452001	230453000	1000	1	9.80E-07	0.53	7	0.7	Olr371	Receptor
DMR1:230458001	1	230458001	230459000	1000	1	1.30E-10	0.55	7	0.7	Olr371	Receptor
DMR1:230528001	1	230528001	230529000	1000	1	6.60E-07	-0.51	2	0.2	Olr372	Signaling
DMR1:230546001	1	230546001	230548000	2000	1	6.80E-08	-0.66	5	0.25	Olr373	Signaling
DMR1:230557001	1	230557001	230563000	6000	3	1.80E-18	-0.54	63	1.05	Olr373	Signaling
DMR1:230588001	1	230588001	230595000	7000	1	1.50E-13	-0.58	47	0.67	Olr375	Signaling
DMR1:230613001	1	230613001	230616000	3000	3	2.00E-11	-0.51	24	0.8	Olr375	Signaling
DMR1:230619001	1	230619001	230626000	7000	1	7.00E-09	-0.41	38	0.54	Olr376	Receptor
DMR1:230641001	1	230641001	230645000	4000	2	1.10E-14	-0.57	22	0.55	LOC100359718;Olr377	Receptor
DMR1:230655001	1	230655001	230658000	3000	2	9.50E-10	-0.63	8	0.27	LOC100359718;Olr377;Olr378	Receptor
DMR1:230662001	1	230662001	230663000	1000	1	1.60E-09	0.68	0	0	Olr378	Receptor
DMR1:230723001	1	230723001	230727000	4000	2	1.30E-19	-0.58	36	0.9	Olr382	Receptor
DMR1:230739001	1	230739001	230742000	3000	2	9.90E-10	-0.42	15	0.5	Olr382	Receptor
DMR1:230769001	1	230769001	230772000	3000	1	6.00E-07	-0.43	8	0.27	Olr383	Receptor
DMR1:230813001	1	230813001	230815000	2000	1	4.80E-07	0.44	13	0.65	Olr384	Receptor
DMR1:230818001	1	230818001	230820000	2000	1	1.00E-09	0.56	8	0.4	Olr384	Receptor
DMR1:230831001	1	230831001	230832000	1000	1	3.60E-09	-0.59	5	0.5	Vom2r-ps69	
DMR1:230836001	1	230836001	230837000	1000	1	3.40E-18	0.68	4	0.4	Vom2r-ps69	
DMR1:230838001	1	230838001	230841000	3000	1	6.80E-10	-0.52	15	0.5	Vom2r-ps69	
DMR1:230842001	1	230842001	230843000	1000	1	9.30E-08	-0.52	4	0.4	Vom2r-ps69	
DMR1:231372001	1	231372001	231373000	1000	1	8.60E-08	0.47	7	0.7	RGD1561986	
DMR1:231384001	1	231384001	231388000	4000	3	9.30E-24	-0.82	13	0.32	Tle4	Transcription
DMR1:231415001	1	231415001	231417000	2000	1	6.10E-11	0.47	21	1.05	Tle4	Transcription
DMR1:231469001	1	231469001	231470000	1000	1	1.20E-12	0.56	11	1.1	Tle4	Transcription
DMR1:231487001	1	231487001	231488000	1000	1	4.40E-08	0.47	5	0.5	Tle4	Transcription
DMR1:231540001	1	231540001	231544000	4000	4	9.40E-21	-0.55	56	1.4	Tle4	Transcription
DMR1:233127001	1	233127001	233128000	1000	1	6.50E-08	0.34	10	1	Psat1	Metabolism
DMR1:233133001	1	233133001	233137000	4000	1	5.30E-10	-0.55	44	1.1	Psat1	Metabolism
DMR1:233147001	1	233147001	233149000	2000	1	1.50E-07	0.55	17	0.85	Psat1	Metabolism
DMR1:233188001	1	233188001	233191000	3000	1	1.30E-11	0.55	41	1.37	Cep78	
DMR1:233205001	1	233205001	233207000	2000	1	1.30E-17	0.92	14	0.7	Cep78	
DMR1:233372001	1	233372001	233376000	4000	1	8.50E-07	0.38	47	1.18	Gnaq	Signaling
DMR1:233402001	1	233402001	233403000	1000	1	3.00E-23	0.62	9	0.9	Gnaq	Signaling
DMR1:233406001	1	233406001	233407000	1000	1	9.20E-13	0.56	9	0.9	Gnaq	Signaling
DMR1:233484001	1	233484001	233485000	1000	1	7.00E-07	0.53	8	0.8	Gnaq	Signaling
DMR1:233500001	1	233500001	233501000	1000	1	5.00E-07	0.42	8	0.8	Gnaq	Signaling
DMR1:233537001	1	233537001	233538000	1000	1	5.00E-08	0.36	12	1.2	Gnaq	Signaling
DMR1:233562001	1	233562001	233565000	3000	2	1.30E-13	0.79	24	0.8	Gnaq	Signaling
DMR1:233572001	1	233572001	233573000	1000	1	2.00E-07	-0.59	12	1.2	Gnaq	Signaling
DMR1:233597001	1	233597001	233601000	4000	3	1.10E-20	0.85	43	1.07	Gnaq	Signaling
DMR1:233610001	1	233610001	233616000	6000	3	3.70E-20	-0.53	78	1.3	Gnaq	Signaling
DMR1:233622001	1	233622001	233623000	1000	1	1.00E-15	0.55	22	2.2	Gnaq	Signaling
DMR1:234243001	1	234243001	234244000	1000	1	4.00E-07	-0.57	3	0.3	Rorb	Transcription
DMR1:234297001	1	234297001	234299000	2000	1	2.90E-07	0.5	20	1	Rorb;LOC108349695	Transcription

DMR1:234317001	1	234317001	234318000	1000	1	7.90E-13	-0.8	10	1	Rorb;LOC102554891	Transcription
DMR1:234325001	1	234325001	234327000	2000	2	2.80E-20	0.82	18	0.9	Rorb;LOC102554891	Transcription
DMR1:234359001	1	234359001	234361000	2000	1	1.40E-12	0.54	24	1.2	Rorb;LOC102554891	Transcription
DMR1:234365001	1	234365001	234368000	3000	1	8.60E-07	0.35	41	1.37	Rorb;LOC102554891	Transcription
DMR1:234378001	1	234378001	234384000	6000	3	4.80E-18	-0.54	67	1.12	Rorb	Transcription
DMR1:234385001	1	234385001	234387000	2000	1	1.30E-09	0.41	37	1.85	Rorb	Transcription
DMR1:234410001	1	234410001	234411000	1000	1	1.30E-10	-0.71	21	2.1	Rorb	Transcription
DMR1:234413001	1	234413001	234414000	1000	1	1.00E-09	0.59	20	2	Rorb	Transcription
DMR1:234434001	1	234434001	234436000	2000	1	1.90E-08	-0.54	28	1.4	Rorb	Transcription
DMR1:234468001	1	234468001	234471000	3000	1	4.40E-09	-0.52	22	0.73	Trpm6	Transport
DMR1:234496001	1	234496001	234498000	2000	1	2.70E-11	0.49	24	1.2	Trpm6	Transport
DMR1:234500001	1	234500001	234511000	11000	3	2.30E-20	-0.59	116	1.05	Trpm6	Transport
DMR1:234537001	1	234537001	234541000	4000	1	2.00E-18	0.81	47	1.18	Trpm6	Transport
DMR1:234543001	1	234543001	234545000	2000	2	1.30E-17	0.8	17	0.85	Trpm6	Transport
DMR1:234573001	1	234573001	234577000	4000	2	5.00E-23	0.81	36	0.9	Trpm6	Transport
DMR1:234582001	1	234582001	234583000	1000	1	7.40E-14	0.82	5	0.5	Trpm6	Transport
DMR1:234606001	1	234606001	234609000	3000	3	3.00E-18	-0.67	24	0.8	Trpm6;LOC108349696	Transport
DMR1:234624001	1	234624001	234625000	1000	1	3.50E-07	-0.44	12	1.2	Trpm6;LOC108349696;LOC102554711	Transport
DMR1:234633001	1	234633001	234635000	2000	1	3.60E-11	0.45	26	1.3	Trpm6;LOC102554711	Transport
DMR1:234639001	1	234639001	234641000	2000	1	4.00E-11	0.61	14	0.7	Trpm6;LOC102554711	Transport
DMR1:234674001	1	234674001	234675000	1000	1	3.90E-07	0.42	7	0.7	LOC499331;Carnmt1	
DMR1:234685001	1	234685001	234687000	2000	1	1.70E-11	0.42	17	0.85	Carnmt1	
DMR1:234706001	1	234706001	234712000	6000	1	6.50E-10	0.47	58	0.97	Carnmt1;Nmrk1	Signaling
DMR1:234734001	1	234734001	234735000	1000	1	4.80E-07	-0.53	5	0.5	Nmrk1;LOC108349697	Signaling
DMR1:234758001	1	234758001	234759000	1000	1	4.70E-13	0.49	16	1.6	Nmrk1;Ostf1	Signaling
DMR1:234784001	1	234784001	234786000	2000	1	1.90E-07	-0.61	22	1.1	Ostf1	
DMR1:234797001	1	234797001	234801000	4000	2	7.80E-23	0.85	51	1.27	Ostf1	
DMR1:234805001	1	234805001	234808000	3000	3	1.60E-17	0.78	13	0.43	Ostf1	
DMR1:235155001	1	235155001	235161000	6000	5	6.00E-27	-0.65	60	1	Gna14	Signaling
DMR1:235172001	1	235172001	235173000	1000	1	2.00E-07	0.42	7	0.7	Gna14	Signaling
DMR1:235187001	1	235187001	235191000	4000	2	1.20E-15	0.67	53	1.32	Gna14	Signaling
DMR1:235194001	1	235194001	235198000	4000	1	8.80E-12	-0.45	48	1.2	Gna14;LOC103691297	Signaling
DMR1:235201001	1	235201001	235202000	1000	1	1.70E-10	0.52	3	0.3	Gna14;LOC103691297	Signaling
DMR1:235235001	1	235235001	235239000	4000	1	7.80E-07	0.33	60	1.5	Gna14	Signaling
DMR1:235243001	1	235243001	235244000	1000	1	7.20E-09	0.6	7	0.7	Gna14	Signaling
DMR1:235258001	1	235258001	235260000	2000	1	1.10E-11	0.51	19	0.95	Gna14	Signaling
DMR1:235276001	1	235276001	235278000	2000	2	4.30E-14	0.73	10	0.5	Gna14	Signaling
DMR1:235297001	1	235297001	235299000	2000	1	5.00E-22	0.68	12	0.6	Gna14	Signaling
DMR1:235308001	1	235308001	235310000	2000	1	1.60E-18	1.03	6	0.3	Gna14	Signaling
DMR1:235318001	1	235318001	235320000	2000	1	1.50E-07	0.37	22	1.1	Gna14	Signaling
DMR1:235328001	1	235328001	235331000	3000	1	1.60E-12	0.53	19	0.63	Gna14	Signaling
DMR1:235361001	1	235361001	235365000	4000	1	1.70E-25	1.06	31	0.78	Vps13a	
DMR1:235406001	1	235406001	235408000	2000	1	8.50E-15	-0.57	11	0.55	Vps13a	
DMR1:235444001	1	235444001	235447000	3000	1	9.80E-15	-0.55	20	0.67	Vps13a	
DMR1:235455001	1	235455001	235459000	4000	1	1.50E-09	-0.56	17	0.42	Vps13a	
DMR1:235460001	1	235460001	235462000	2000	1	2.20E-12	0.7	8	0.4	Vps13a	
DMR1:235475001	1	235475001	235477000	2000	1	4.30E-17	0.86	9	0.45	Vps13a	
DMR1:235486001	1	235486001	235487000	1000	1	5.30E-09	0.64	4	0.4	Vps13a	
DMR1:235503001	1	235503001	235505000	2000	2	1.20E-21	-0.49	22	1.1	Vps13a	
DMR1:235506001	1	235506001	235508000	2000	2	1.30E-12	0.55	19	0.95	Vps13a	
DMR1:235510001	1	235510001	235512000	2000	1	4.00E-13	0.64	15	0.75	Vps13a	
DMR1:235517001	1	235517001	235518000	1000	1	5.00E-13	0.61	6	0.6	Vps13a	
DMR1:235520001	1	235520001	235523000	3000	2	2.90E-08	-0.49	29	0.97	Vps13a	
DMR1:235527001	1	235527001	235528000	1000	1	7.50E-08	-0.56	7	0.7	Vps13a	
DMR1:235548001	1	235548001	235549000	1000	1	5.60E-08	0.38	9	0.9	Vps13a	
DMR1:235562001	1	235562001	235563000	1000	1	4.30E-07	0.5	4	0.4	Vps13a	
DMR1:235571001	1	235571001	235573000	2000	1	2.30E-07	-0.41	16	0.8	Vps13a	
DMR1:235578001	1	235578001	235581000	3000	1	3.10E-07	0.4	31	1.03	Vps13a	
DMR1:235616001	1	235616001	235618000	2000	2	1.00E-13	0.52	17	0.85	Vps13a	
DMR1:235646001	1	235646001	235647000	1000	1	4.30E-12	0.81	3	0.3	Vps13a	
DMR1:235662001	1	235662001	235664000	2000	1	2.10E-09	-0.48	29	1.45	Vps13a	
DMR1:235672001	1	235672001	235673000	1000	1	1.40E-22	0.7	16	1.6	Vps13a	
DMR1:235678001	1	235678001	235679000	1000	1	1.60E-10	0.44	15	1.5	Vps13a	
DMR1:235694001	1	235694001	235699000	5000	3	8.10E-09	-0.52	54	1.08	Vps13a	

DMR1:235720001	1	235720001	235721000	1000	1	2.70E-07	0.4	7	0.7	Vps13a	
DMR1:235728001	1	235728001	235729000	1000	1	3.00E-16	0.76	4	0.4	Vps13a	
DMR1:235754001	1	235754001	235756000	2000	1	3.50E-12	0.59	15	0.75	Vps13a	
DMR1:235768001	1	235768001	235771000	3000	2	4.00E-10	-0.47	22	0.73	Vps13a	
DMR1:235772001	1	235772001	235779000	7000	2	1.40E-07	-0.58	33	0.47	Vps13a	
DMR1:235782001	1	235782001	235789000	7000	4	2.60E-29	-0.67	73	1.04	Vps13a	
DMR1:235791001	1	235791001	235792000	1000	1	2.20E-09	0.48	9	0.9	Vps13a	
DMR1:235809001	1	235809001	235811000	2000	1	3.10E-11	0.46	15	0.75	Vps13a	
DMR1:235823001	1	235823001	235824000	1000	1	5.60E-15	0.72	10	1	Vps13a	
DMR1:236029001	1	236029001	236030000	1000	1	2.30E-14	0.64	17	1.7	Pcsk5	Protease
DMR1:236046001	1	236046001	236049000	3000	1	2.90E-08	-0.41	12	0.4	Pcsk5	Protease
DMR1:236054001	1	236054001	236056000	2000	2	3.20E-13	0.61	19	0.95	Pcsk5	Protease
DMR1:236062001	1	236062001	236063000	1000	1	1.10E-12	-0.7	17	1.7	Pcsk5	Protease
DMR1:236082001	1	236082001	236086000	4000	3	1.30E-18	0.92	29	0.72	Pcsk5	Protease
DMR1:236135001	1	236135001	236139000	4000	1	3.90E-21	0.82	30	0.75	Pcsk5	Protease
DMR1:236142001	1	236142001	236144000	2000	2	6.10E-09	0.61	12	0.6	Pcsk5	Protease
DMR1:236153001	1	236153001	236154000	1000	1	1.80E-16	0.65	9	0.9	Pcsk5	Protease
DMR1:236159001	1	236159001	236162000	3000	1	1.80E-11	0.55	26	0.87	Pcsk5	Protease
DMR1:236196001	1	236196001	236197000	1000	1	4.70E-07	-0.63	11	1.1	Pcsk5	Protease
DMR1:236200001	1	236200001	236201000	1000	1	1.30E-14	0.64	8	0.8	Pcsk5	Protease
DMR1:236268001	1	236268001	236271000	3000	2	4.60E-10	0.71	21	0.7	Pcsk5	Protease
DMR1:236273001	1	236273001	236275000	2000	2	7.80E-09	0.52	13	0.65	Pcsk5	Protease
DMR1:236276001	1	236276001	236277000	1000	1	3.90E-08	-0.6	15	1.5	Pcsk5	Protease
DMR1:236299001	1	236299001	236301000	2000	1	1.70E-26	1.02	20	1	Pcsk5	Protease
DMR1:236318001	1	236318001	236320000	2000	1	3.60E-08	0.44	16	0.8	Pcsk5	Protease
DMR1:236482001	1	236482001	236485000	3000	1	4.00E-17	0.71	31	1.03	Rfk	
DMR1:236490001	1	236490001	236492000	2000	1	2.20E-11	0.52	12	0.6	Rfk	
DMR1:236494001	1	236494001	236496000	2000	1	1.30E-07	-0.52	21	1.05	Rfk	
DMR1:236552001	1	236552001	236553000	1000	1	1.70E-13	0.61	9	0.9	Gcnt1	Golgi
DMR1:236558001	1	236558001	236560000	2000	1	5.50E-16	0.59	19	0.95	Gcnt1	Golgi
DMR1:236564001	1	236564001	236565000	1000	1	2.80E-08	0.43	6	0.6	Gcnt1	Golgi
DMR1:236575001	1	236575001	236577000	2000	2	7.90E-10	-0.56	21	1.05	Gcnt1	Golgi
DMR1:236666001	1	236666001	236669000	3000	2	4.20E-10	-0.74	30	1	Prune2	
DMR1:236678001	1	236678001	236681000	3000	2	5.30E-07	0.4	50	1.67	Prune2	
DMR1:236715001	1	236715001	236717000	2000	1	9.00E-08	0.48	15	0.75	Prune2	
DMR1:236727001	1	236727001	236730000	3000	2	1.20E-08	0.35	33	1.1	Prune2	
DMR1:236731001	1	236731001	236733000	2000	1	1.60E-07	0.4	30	1.5	Prune2	
DMR1:236750001	1	236750001	236751000	1000	1	3.20E-15	0.82	6	0.6	Prune2	
DMR1:236755001	1	236755001	236757000	2000	1	3.20E-19	0.77	23	1.15	Prune2	
DMR1:236760001	1	236760001	236761000	1000	1	2.70E-08	0.57	6	0.6	Prune2	
DMR1:236763001	1	236763001	236764000	1000	1	3.10E-09	0.45	4	0.4	Prune2	
DMR1:236768001	1	236768001	236769000	1000	1	1.20E-10	-0.61	14	1.4	Prune2	
DMR1:236786001	1	236786001	236789000	3000	1	2.40E-10	-0.52	14	0.47	Prune2	
DMR1:236827001	1	236827001	236828000	1000	1	6.50E-07	0.34	9	0.9	Prune2	
DMR1:236829001	1	236829001	236830000	1000	1	1.40E-22	0.89	11	1.1	Prune2	
DMR1:236831001	1	236831001	236835000	4000	1	7.80E-07	0.36	46	1.15	Prune2	
DMR1:236853001	1	236853001	236855000	2000	1	1.20E-10	0.48	20	1	Prune2	
DMR1:236905001	1	236905001	236908000	3000	1	1.00E-23	0.77	27	0.9	Prune2	
DMR1:237579001	1	237579001	237585000	6000	3	1.40E-12	-0.45	31	0.52	RGD1563931	
DMR1:237897001	1	237897001	237898000	1000	1	1.00E-11	0.72	3	0.3	Anxa1	Signaling
DMR1:237908001	1	237908001	237911000	3000	1	4.60E-10	-0.63	30	1	Anxa1	Signaling
DMR1:237920001	1	237920001	237921000	1000	1	1.70E-10	0.5	11	1.1	Anxa1	Signaling
DMR1:238240001	1	238240001	238242000	2000	1	8.30E-24	0.88	20	1	Aldh1a1	Metabolism
DMR1:238259001	1	238259001	238260000	1000	1	1.20E-09	0.44	14	1.4	Aldh1a1	Metabolism
DMR1:238354001	1	238354001	238356000	2000	2	5.80E-11	-0.63	28	1.4	Tmc1	
DMR1:238370001	1	238370001	238371000	1000	1	1.40E-16	0.87	8	0.8	Tmc1	
DMR1:238379001	1	238379001	238380000	1000	1	9.00E-16	0.62	13	1.3	Tmc1	
DMR1:238382001	1	238382001	238384000	2000	1	5.70E-12	0.64	11	0.55	Tmc1	
DMR1:238385001	1	238385001	238388000	3000	1	1.10E-08	0.5	33	1.1	Tmc1	
DMR1:238404001	1	238404001	238405000	1000	1	1.40E-11	0.55	15	1.5	Tmc1	
DMR1:238417001	1	238417001	238420000	3000	1	2.20E-10	0.52	31	1.03	Tmc1	
DMR1:238440001	1	238440001	238442000	2000	1	2.30E-09	0.4	14	0.7	Tmc1	
DMR1:238458001	1	238458001	238460000	2000	1	1.30E-08	0.44	7	0.35	Tmc1	
DMR1:238463001	1	238463001	238465000	2000	1	2.20E-14	0.42	34	1.7	Tmc1	
DMR1:238505001	1	238505001	238512000	7000	5	2.70E-20	-0.54	67	0.96	Tmc1	

DMR1:238518001	1	238518001	238519000	1000	1	3.40E-07	0.47	9	0.9	Tmc1	
DMR1:238836001	1	238836001	238838000	2000	1	3.30E-11	0.73	8	0.4	Zfand5	
DMR1:238859001	1	238859001	238860000	1000	1	1.10E-08	0.64	3	0.3	Zfand5;LOC102551544	
DMR1:238862001	1	238862001	238869000	7000	6	1.50E-19	-0.52	94	1.34	Zfand5;LOC102551544;LOC103691306	
DMR1:238997001	1	238997001	238998000	1000	1	2.70E-07	-0.43	7	0.7	Gda	Metabolism
DMR1:239009001	1	239009001	239013000	4000	3	4.00E-12	0.76	36	0.9	Gda	Metabolism
DMR1:239046001	1	239046001	239047000	1000	1	6.90E-19	0.56	12	1.2	Gda	Metabolism
DMR1:239058001	1	239058001	239059000	1000	1	2.70E-07	0.49	10	1	Gda	Metabolism
DMR1:239065001	1	239065001	239071000	6000	1	1.80E-07	0.46	51	0.85	Gda	Metabolism
DMR1:239207001	1	239207001	239208000	1000	1	2.30E-09	-0.52	14	1.4	RGD1359158	
DMR1:239211001	1	239211001	239215000	4000	1	1.80E-12	0.6	39	0.98	RGD1359158	
DMR1:239225001	1	239225001	239229000	4000	1	2.60E-07	0.39	36	0.9	RGD1359158	
DMR1:239258001	1	239258001	239259000	1000	1	8.50E-11	0.51	10	1	RGD1359158;Abhd17b	Protease
DMR1:239274001	1	239274001	239276000	2000	2	2.60E-08	0.5	12	0.6	RGD1359158;Abhd17b	Protease
DMR1:239288001	1	239288001	239289000	1000	1	4.00E-08	0.44	8	0.8	Abhd17b	Protease
DMR1:239298001	1	239298001	239299000	1000	1	2.10E-09	0.42	11	1.1	Abhd17b;LOC108348948	Protease
DMR1:239427001	1	239427001	239428000	1000	1	3.90E-12	0.45	19	1.9	Tmem2	
DMR1:239432001	1	239432001	239435000	3000	1	1.60E-08	0.37	31	1.03	Tmem2	
DMR1:239446001	1	239446001	239452000	6000	1	6.80E-09	-0.53	80	1.33	Tmem2	
DMR1:239460001	1	239460001	239462000	2000	1	6.60E-08	-0.6	21	1.05	Tmem2	
DMR1:239765001	1	239765001	239766000	1000	1	6.70E-12	0.72	6	0.6	Trpm3	Transport
DMR1:239768001	1	239768001	239770000	2000	1	7.30E-07	-0.44	17	0.85	Trpm3	Transport
DMR1:239799001	1	239799001	239801000	2000	2	2.00E-08	0.44	11	0.55	Trpm3	Transport
DMR1:239809001	1	239809001	239813000	4000	1	7.40E-10	0.65	47	1.18	Trpm3	Transport
DMR1:239829001	1	239829001	239830000	1000	1	3.80E-16	-0.74	4	0.4	Trpm3	Transport
DMR1:239838001	1	239838001	239839000	1000	1	3.40E-16	0.97	12	1.2	Trpm3;LOC108349821	Transport
DMR1:239845001	1	239845001	239846000	1000	1	4.40E-08	-0.52	13	1.3	Trpm3;LOC108349821	Transport
DMR1:239850001	1	239850001	239851000	1000	1	1.20E-08	0.41	10	1	Trpm3;LOC108349821	Transport
DMR1:239858001	1	239858001	239860000	2000	1	1.80E-08	0.58	7	0.35	Trpm3;LOC108349821	Transport
DMR1:239861001	1	239861001	239863000	2000	1	4.10E-07	-0.5	4	0.2	Trpm3	Transport
DMR1:239890001	1	239890001	239892000	2000	2	5.20E-08	-0.44	23	1.15	Trpm3	Transport
DMR1:239893001	1	239893001	239895000	2000	2	5.50E-18	0.81	8	0.4	Trpm3	Transport
DMR1:239905001	1	239905001	239908000	3000	2	3.40E-12	0.63	27	0.9	Trpm3	Transport
DMR1:239909001	1	239909001	239912000	3000	2	2.70E-10	0.57	27	0.9	Trpm3	Transport
DMR1:239916001	1	239916001	239918000	2000	1	7.70E-20	0.65	32	1.6	Trpm3	Transport
DMR1:239921001	1	239921001	239923000	2000	2	8.70E-16	0.65	16	0.8	Trpm3	Transport
DMR1:239985001	1	239985001	239987000	2000	1	1.40E-14	0.53	22	1.1	Trpm3	Transport
DMR1:239994001	1	239994001	239995000	1000	1	1.60E-07	0.36	9	0.9	Trpm3	Transport
DMR1:240010001	1	240010001	240011000	1000	1	8.00E-10	0.45	10	1	Trpm3	Transport
DMR1:240020001	1	240020001	240021000	1000	1	3.80E-11	0.65	4	0.4	Trpm3	Transport
DMR1:240033001	1	240033001	240036000	3000	2	3.20E-19	0.67	21	0.7	Trpm3;LOC108349822	Transport
DMR1:240037001	1	240037001	240039000	2000	1	8.10E-13	0.68	18	0.9	Trpm3;LOC108349822	Transport
DMR1:240041001	1	240041001	240042000	1000	1	2.30E-11	0.7	8	0.8	Trpm3;LOC108349822	Transport
DMR1:240043001	1	240043001	240048000	5000	2	2.30E-14	0.78	27	0.54	Trpm3;LOC108349822;LOC100912018	Transport
DMR1:240128001	1	240128001	240129000	1000	1	4.80E-07	0.38	13	1.3	Trpm3	Transport
DMR1:240131001	1	240131001	240132000	1000	1	2.80E-07	0.4	7	0.7	Trpm3	Transport
DMR1:240139001	1	240139001	240140000	1000	1	1.40E-09	-0.69	9	0.9	Trpm3	Transport
DMR1:240141001	1	240141001	240142000	1000	1	4.60E-09	-0.52	4	0.4	Trpm3	Transport
DMR1:240152001	1	240152001	240156000	4000	2	1.40E-07	0.53	27	0.68	Trpm3	Transport
DMR1:240207001	1	240207001	240208000	1000	1	6.10E-07	-0.6	18	1.8	Trpm3	Transport
DMR1:240216001	1	240216001	240221000	5000	3	3.20E-15	0.63	49	0.98	Trpm3	Transport
DMR1:240223001	1	240223001	240226000	3000	1	7.10E-07	0.37	30	1	Trpm3	Transport
DMR1:240235001	1	240235001	240236000	1000	1	3.20E-07	0.42	6	0.6	Trpm3	Transport
DMR1:240283001	1	240283001	240284000	1000	1	2.50E-07	-0.66	6	0.6	Trpm3	Transport
DMR1:240296001	1	240296001	240302000	6000	2	5.10E-15	0.56	50	0.83	Trpm3	Transport
DMR1:240314001	1	240314001	240315000	1000	1	7.00E-16	0.82	6	0.6	Trpm3	Transport
DMR1:240322001	1	240322001	240326000	4000	3	9.20E-21	0.85	32	0.8	Trpm3	Transport
DMR1:240341001	1	240341001	240343000	2000	2	2.80E-11	0.63	9	0.45	Trpm3;LOC108349823	Transport
DMR1:240356001	1	240356001	240358000	2000	2	3.30E-11	0.66	27	1.35	Trpm3;LOC108349823	Transport
DMR1:240373001	1	240373001	240374000	1000	1	4.90E-08	0.45	9	0.9	Trpm3;LOC102552001	Transport
DMR1:240377001	1	240377001	240379000	2000	1	2.10E-14	0.51	27	1.35	Trpm3;LOC102552001	Transport
DMR1:240383001	1	240383001	240385000	2000	2	1.20E-07	-0.68	31	1.55	Trpm3;LOC102552001	Transport
DMR1:240429001	1	240429001	240430000	1000	1	7.10E-07	-0.65	11	1.1	Trpm3	Transport

DMR1:240432001	1	240432001	240433000	1000	1	6.70E-09	0.67	3	0.3	Trpm3	Transport
DMR1:240449001	1	240449001	240451000	2000	1	2.50E-08	0.58	11	0.55	Trpm3	Transport
DMR1:240452001	1	240452001	240456000	4000	1	1.30E-09	-0.53	13	0.32	Trpm3	Transport
DMR1:240462001	1	240462001	240463000	1000	1	1.40E-18	0.69	2	0.2	Trpm3	Transport
DMR1:240479001	1	240479001	240480000	1000	1	7.60E-12	0.47	10	1	Trpm3	Transport
DMR1:240508001	1	240508001	240509000	1000	1	4.80E-20	0.88	7	0.7	Trpm3	Transport
DMR1:240573001	1	240573001	240577000	4000	3	8.20E-19	-0.57	50	1.25	Trpm3;LOC108349824;Aldh1a7	Transport;Metabolism
DMR1:240592001	1	240592001	240595000	3000	1	1.20E-11	0.62	33	1.1	Trpm3;Aldh1a7	Transport;Metabolism
DMR1:240606001	1	240606001	240607000	1000	1	2.90E-09	0.66	7	0.7	Trpm3;Aldh1a7	Transport;Metabolism
DMR1:240614001	1	240614001	240616000	2000	1	3.20E-08	0.39	19	0.95	Trpm3	Transport
DMR1:240689001	1	240689001	240691000	2000	1	4.90E-09	-0.44	32	1.6	Trpm3	Transport
DMR1:240708001	1	240708001	240711000	3000	1	1.90E-07	0.44	37	1.23	Trpm3	Transport
DMR1:240717001	1	240717001	240722000	5000	2	5.00E-08	-0.64	58	1.16	Trpm3	Transport
DMR1:240732001	1	240732001	240736000	4000	2	2.30E-08	-0.65	53	1.32	Trpm3	Transport
DMR1:240782001	1	240782001	240784000	2000	1	1.40E-11	0.74	12	0.6	RGD1561453	
DMR1:240912001	1	240912001	240918000	6000	1	7.80E-08	-0.63	74	1.23	Klf9	Transcription
DMR1:240922001	1	240922001	240925000	3000	1	1.20E-09	0.38	40	1.33	Klf9	Transcription
DMR1:240975001	1	240975001	240977000	2000	1	8.30E-13	0.64	9	0.45	Smc5	
DMR1:240983001	1	240983001	240986000	3000	2	1.10E-14	-0.59	25	0.83	Smc5	
DMR1:240992001	1	240992001	240994000	2000	2	6.70E-13	0.52	19	0.95	Smc5	
DMR1:241004001	1	241004001	241005000	1000	1	1.10E-07	-0.57	9	0.9	Smc5	
DMR1:241015001	1	241015001	241020000	5000	2	2.10E-34	-0.6	55	1.1	Smc5	
DMR1:241022001	1	241022001	241024000	2000	1	6.90E-11	0.5	25	1.25	Smc5	
DMR1:241036001	1	241036001	241040000	4000	1	5.40E-08	0.46	36	0.9	Smc5	
DMR1:241055001	1	241055001	241061000	6000	2	6.70E-08	0.48	42	0.7	Smc5	
DMR1:241086001	1	241086001	241088000	2000	1	1.60E-23	0.82	22	1.1	Mamdc2	
DMR1:241110001	1	241110001	241112000	2000	1	1.20E-08	0.53	25	1.25	Mamdc2	
DMR1:241116001	1	241116001	241118000	2000	1	3.60E-08	0.52	14	0.7	Mamdc2	
DMR1:241120001	1	241120001	241122000	2000	1	1.50E-15	0.75	21	1.05	Mamdc2	
DMR1:241400001	1	241400001	241401000	1000	1	1.50E-25	0.7	10	1	RGD1560242	
DMR1:241408001	1	241408001	241413000	5000	1	1.20E-07	-0.4	48	0.96	RGD1560242	
DMR1:241442001	1	241442001	241444000	2000	1	8.10E-09	0.44	12	0.6	RGD1560242	
DMR1:241451001	1	241451001	241458000	7000	3	1.60E-14	0.71	85	1.21	RGD1560242;RGD1564433	
DMR1:241523001	1	241523001	241527000	4000	3	1.00E-12	-0.7	25	0.62	Ptar1	Metabolism
DMR1:241529001	1	241529001	241531000	2000	1	4.60E-08	0.37	10	0.5	Ptar1	Metabolism
DMR1:241560001	1	241560001	241561000	1000	1	3.70E-08	-0.58	14	1.4	Ptar1	Metabolism
DMR1:241588001	1	241588001	241592000	4000	1	3.90E-07	0.38	42	1.05	Apba1	Transport
DMR1:241596001	1	241596001	241599000	3000	1	7.00E-12	0.49	41	1.37	Apba1	Transport
DMR1:241608001	1	241608001	241609000	1000	1	4.70E-09	-0.79	13	1.3	Apba1	Transport
DMR1:241623001	1	241623001	241624000	1000	1	4.50E-08	0.44	10	1	Apba1	Transport
DMR1:241646001	1	241646001	241648000	2000	1	8.00E-08	0.47	13	0.65	Apba1	Transport
DMR1:241697001	1	241697001	241704000	7000	1	7.30E-07	-0.47	111	1.59	Apba1	Transport
DMR1:241712001	1	241712001	241713000	1000	1	1.80E-08	0.53	6	0.6	Apba1	Transport
DMR1:241721001	1	241721001	241722000	1000	1	1.10E-11	0.57	9	0.9	Apba1	Transport
DMR1:241739001	1	241739001	241746000	7000	2	1.40E-16	-0.74	84	1.2	Apba1	Transport
DMR1:241763001	1	241763001	241766000	3000	1	1.00E-07	-0.5	67	2.23	Apba1	Transport
DMR1:241780001	1	241780001	241784000	4000	1	2.40E-07	0.33	85	2.12	Apba1	Transport
DMR1:241793001	1	241793001	241795000	2000	1	1.60E-08	-0.54	42	2.1	Apba1	Transport
DMR1:241799001	1	241799001	241803000	4000	2	6.80E-13	0.64	54	1.35	Apba1	Transport
DMR1:241814001	1	241814001	241815000	1000	1	3.30E-11	0.44	10	1	Fam189a2	
DMR1:241816001	1	241816001	241818000	2000	1	1.20E-13	0.5	22	1.1	Fam189a2	
DMR1:241822001	1	241822001	241826000	4000	1	5.60E-08	0.37	68	1.7	Fam189a2	
DMR1:241849001	1	241849001	241850000	1000	1	7.20E-07	0.47	2	0.2	Fam189a2	
DMR1:241854001	1	241854001	241857000	3000	1	1.80E-07	-0.38	29	0.97	Fam189a2	
DMR1:241866001	1	241866001	241868000	2000	1	3.40E-07	-0.52	20	1	Fam189a2	
DMR1:241976001	1	241976001	241980000	4000	1	1.50E-14	0.67	49	1.23	Tjp2	Cell Junction
DMR1:241986001	1	241986001	241988000	2000	1	3.70E-09	0.48	22	1.1	Tjp2	Cell Junction
DMR1:242049001	1	242049001	242050000	1000	1	1.10E-15	0.77	3	0.3	Tjp2	Cell Junction
DMR1:242056001	1	242056001	242057000	1000	1	2.20E-09	-0.49	17	1.7	Tjp2	Cell Junction
DMR1:242074001	1	242074001	242076000	2000	1	4.90E-11	-0.61	33	1.65	Tjp2	Cell Junction
DMR1:242077001	1	242077001	242079000	2000	1	6.60E-11	0.46	31	1.55	Tjp2	Cell Junction
DMR1:242136001	1	242136001	242139000	3000	1	4.30E-12	0.69	22	0.73	Fxn	Transport

DMR1:242148001	1	242148001	242151000	3000	1	3.20E-07	0.34	49	1.63	Fxn	Transport
DMR1:242163001	1	242163001	242164000	1000	1	1.00E-11	0.48	6	0.6	Pip5k1b	Signaling
DMR1:242171001	1	242171001	242175000	4000	4	1.40E-23	0.66	57	1.43	Pip5k1b	Signaling
DMR1:242182001	1	242182001	242185000	3000	1	1.90E-15	0.67	28	0.93	Pip5k1b	Signaling
DMR1:242199001	1	242199001	242201000	2000	1	1.30E-19	0.72	24	1.2	Pip5k1b	Signaling
DMR1:242223001	1	242223001	242225000	2000	2	9.10E-15	0.63	11	0.55	Pip5k1b	Signaling
DMR1:242241001	1	242241001	242243000	2000	1	1.10E-10	0.41	28	1.4	Pip5k1b	Signaling
DMR1:242256001	1	242256001	242257000	1000	1	9.60E-20	0.84	5	0.5	Pip5k1b	Signaling
DMR1:242260001	1	242260001	242267000	7000	1	3.20E-13	0.5	105	1.5	Pip5k1b	Signaling
DMR1:242297001	1	242297001	242303000	6000	1	8.90E-07	0.36	78	1.3	Pip5k1b	Signaling
DMR1:242306001	1	242306001	242310000	4000	1	8.10E-11	-0.76	44	1.1	Pip5k1b	Signaling
DMR1:242331001	1	242331001	242342000	11000	4	7.00E-26	-0.58	99	0.9	Pip5k1b	Signaling
DMR1:242350001	1	242350001	242353000	3000	1	4.90E-08	0.35	48	1.6	Pip5k1b	Signaling
DMR1:242361001	1	242361001	242363000	2000	1	1.80E-09	0.34	29	1.45	Pip5k1b;Fam122a	Signaling
DMR1:242364001	1	242364001	242365000	1000	1	3.10E-10	0.43	17	1.7	Pip5k1b;Fam122a	Signaling
DMR1:242367001	1	242367001	242370000	3000	1	2.00E-10	0.44	36	1.2	Pip5k1b;Fam122a	Signaling
DMR1:242387001	1	242387001	242388000	1000	1	4.50E-07	0.27	7	0.7	Pip5k1b	Signaling
DMR1:242444001	1	242444001	242445000	1000	1	8.00E-12	0.52	6	0.6	Pip5k1b	Signaling
DMR1:242606001	1	242606001	242608000	2000	1	8.80E-09	-0.44	24	1.2	Pgm5	Metabolism
DMR1:242656001	1	242656001	242658000	2000	1	3.10E-14	0.68	29	1.45	Pgm5	Metabolism
DMR1:242666001	1	242666001	242668000	2000	1	3.00E-08	0.57	10	0.5	Pgm5	Metabolism
DMR1:242669001	1	242669001	242681000	12000	4	2.70E-18	0.71	118	0.98	Pgm5	Metabolism
DMR1:242687001	1	242687001	242689000	2000	1	9.50E-08	0.35	18	0.9	Pgm5	Metabolism
DMR1:242693001	1	242693001	242696000	3000	2	4.80E-10	-0.89	30	1	Pgm5	Metabolism
DMR1:242697001	1	242697001	242699000	2000	1	1.80E-08	-0.34	20	1	Pgm5	Metabolism
DMR1:242706001	1	242706001	242707000	1000	1	9.20E-12	0.68	6	0.6	Pgm5	Metabolism
DMR1:242721001	1	242721001	242722000	1000	1	4.70E-07	0.41	8	0.8	Pgm5	Metabolism
DMR1:242743001	1	242743001	242745000	2000	1	2.00E-12	0.57	19	0.95	Pgm5	Metabolism
DMR1:242750001	1	242750001	242751000	1000	1	4.20E-10	0.45	9	0.9	Pgm5	Metabolism
DMR1:242752001	1	242752001	242755000	3000	2	1.60E-12	0.59	33	1.1	Pgm5	Metabolism
DMR1:242759001	1	242759001	242761000	2000	1	4.90E-15	0.88	19	0.95	Pgm5	Metabolism
DMR1:242769001	1	242769001	242771000	2000	1	6.00E-16	0.91	15	0.75	Pgm5	Metabolism
DMR1:242772001	1	242772001	242774000	2000	1	1.90E-19	0.8	21	1.05	Pgm5	Metabolism
DMR1:242775001	1	242775001	242776000	1000	1	4.20E-14	0.6	8	0.8	Pgm5	Metabolism
DMR1:242801001	1	242801001	242803000	2000	1	1.70E-16	0.78	13	0.65	Foxd4	Transcription
DMR1:242839001	1	242839001	242840000	1000	1	5.40E-17	0.84	3	0.3	Cbwd1	
DMR1:242863001	1	242863001	242865000	2000	1	6.30E-10	0.55	16	0.8	Cbwd1;LOC102556915	
DMR1:242869001	1	242869001	242870000	1000	1	3.30E-07	0.37	8	0.8	Cbwd1;LOC102556915	
DMR1:242929001	1	242929001	242932000	3000	2	4.60E-09	-0.56	14	0.47	Dock8	Transcription
DMR1:242933001	1	242933001	242935000	2000	2	2.00E-17	0.87	12	0.6	Dock8	Transcription
DMR1:242937001	1	242937001	242938000	1000	1	5.40E-10	0.43	12	1.2	Dock8	Transcription
DMR1:242941001	1	242941001	242944000	3000	1	1.10E-07	0.33	42	1.4	Dock8	Transcription
DMR1:242945001	1	242945001	242947000	2000	2	4.40E-09	-0.53	34	1.7	Dock8	Transcription
DMR1:242961001	1	242961001	242962000	1000	1	5.00E-07	-0.5	16	1.6	Dock8;C1H9orf66	Transcription
DMR1:242970001	1	242970001	242973000	3000	2	2.10E-16	0.58	35	1.17	Dock8;C1H9orf66	Transcription
DMR1:242975001	1	242975001	242979000	4000	2	7.50E-10	0.46	53	1.32	Dock8	Transcription
DMR1:242988001	1	242988001	242989000	1000	1	6.70E-08	0.34	11	1.1	Dock8	Transcription
DMR1:242994001	1	242994001	242996000	2000	2	4.50E-28	0.92	11	0.55	Dock8	Transcription
DMR1:243011001	1	243011001	243013000	2000	1	4.40E-07	-0.53	16	0.8	Dock8	Transcription
DMR1:243015001	1	243015001	243018000	3000	1	2.50E-08	-0.7	34	1.13	Dock8	Transcription
DMR1:243023001	1	243023001	243025000	2000	1	2.80E-09	-0.45	29	1.45	Dock8	Transcription
DMR1:243067001	1	243067001	243068000	1000	1	1.40E-15	0.62	12	1.2	Dock8	Transcription
DMR1:243106001	1	243106001	243111000	5000	1	5.60E-20	0.71	76	1.52	Dock8;Hmgb1-ps4	Transcription
DMR1:243113001	1	243113001	243117000	4000	1	1.80E-07	-0.41	80	2	Dock8;Hmgb1-ps4	Transcription
DMR1:243127001	1	243127001	243132000	5000	1	2.50E-13	0.44	85	1.7	Dock8	Transcription
DMR1:243155001	1	243155001	243160000	5000	2	4.80E-15	0.64	74	1.48	Dock8	Transcription
DMR1:243216001	1	243216001	243218000	2000	1	9.00E-10	0.37	23	1.15	Kank1	Cytoskeleton
DMR1:243229001	1	243229001	243231000	2000	2	3.80E-09	0.69	27	1.35	Kank1	Cytoskeleton
DMR1:243232001	1	243232001	243233000	1000	1	1.50E-16	0.93	16	1.6	Kank1	Cytoskeleton
DMR1:243235001	1	243235001	243241000	6000	2	3.80E-14	0.49	82	1.37	Kank1	Cytoskeleton
DMR1:243274001	1	243274001	243277000	3000	1	3.10E-07	-0.38	40	1.33	Kank1	Cytoskeleton
DMR1:243283001	1	243283001	243284000	1000	1	1.80E-08	0.57	7	0.7	Kank1	Cytoskeleton
DMR1:243299001	1	243299001	243302000	3000	1	3.90E-08	-0.45	65	2.17	Kank1;LOC102554752	Cytoskeleton
DMR1:243306001	1	243306001	243310000	4000	1	3.70E-10	-0.73	57	1.43	Kank1;LOC102554752	Cytoskeleton
DMR1:243334001	1	243334001	243337000	3000	1	6.50E-07	0.31	43	1.43	Kank1	Cytoskeleton

DMR1:243341001	1	243341001	243344000	3000	1	2.50E-10	0.59	39	1.3	Kank1	Cytoskeleton
DMR1:243345001	1	243345001	243351000	6000	1	6.70E-07	0.35	69	1.15	Kank1	Cytoskeleton
DMR1:243366001	1	243366001	243371000	5000	1	1.30E-12	0.48	77	1.54	Kank1	Cytoskeleton
DMR1:243470001	1	243470001	243473000	3000	1	2.70E-07	-0.66	52	1.73	LOC100359573;Dmrt1	Transcription
DMR1:243485001	1	243485001	243487000	2000	2	2.70E-09	0.44	20	1	Dmrt1	Transcription
DMR1:243488001	1	243488001	243490000	2000	1	2.00E-08	0.37	30	1.5	Dmrt1	Transcription
DMR1:243493001	1	243493001	243494000	1000	1	2.70E-08	0.31	25	2.5	Dmrt1	Transcription
DMR1:243498001	1	243498001	243501000	3000	1	1.90E-08	0.41	63	2.1	Dmrt1	Transcription
DMR1:243503001	1	243503001	243505000	2000	1	8.20E-10	0.44	18	0.9	Dmrt1	Transcription
DMR1:243506001	1	243506001	243509000	3000	1	9.40E-07	0.34	33	1.1	Dmrt1	Transcription
DMR1:243515001	1	243515001	243518000	3000	2	1.90E-14	-0.56	51	1.7	Dmrt1	Transcription
DMR1:243545001	1	243545001	243549000	4000	2	7.10E-23	0.56	71	1.77	Dmrt1	Transcription
DMR1:243559001	1	243559001	243562000	3000	2	2.00E-12	0.48	20	0.67	Dmrt1	Transcription
DMR1:243565001	1	243565001	243570000	5000	1	1.60E-11	-0.76	80	1.6	Dmrt1	Transcription
DMR1:243571001	1	243571001	243573000	2000	1	5.20E-15	0.42	18	0.9	Dmrt1	Transcription
DMR1:243592001	1	243592001	243593000	1000	1	7.80E-18	0.68	15	1.5	Dmrt1;Dmrt3	Transcription
DMR1:243595001	1	243595001	243600000	5000	1	8.30E-08	-0.46	64	1.28	Dmrt3	Transcription
DMR1:243606001	1	243606001	243607000	1000	1	5.10E-09	0.44	13	1.3	Dmrt3	Transcription
DMR1:243609001	1	243609001	243610000	1000	1	1.90E-09	-0.56	19	1.9	Dmrt3	Transcription
DMR1:243611001	1	243611001	243613000	2000	2	5.20E-22	0.91	28	1.4	Dmrt3	Transcription
DMR1:244605001	1	244605001	244608000	3000	2	3.10E-18	-0.52	39	1.3	LOC108349293;Smarca2	Epigenetic
DMR1:244609001	1	244609001	244610000	1000	1	6.60E-08	0.36	8	0.8	LOC108349293;Smarca2	Epigenetic
DMR1:244612001	1	244612001	244614000	2000	1	3.40E-11	0.54	21	1.05	LOC108349293;Smarca2	Epigenetic
DMR1:244626001	1	244626001	244628000	2000	1	1.70E-07	0.44	20	1	LOC108349293;Smarca2	Epigenetic
DMR1:244640001	1	244640001	244642000	2000	1	1.60E-11	0.43	33	1.65	Smarca2	Epigenetic
DMR1:244655001	1	244655001	244656000	1000	1	1.10E-07	0.33	13	1.3	Smarca2	Epigenetic
DMR1:244667001	1	244667001	244670000	3000	1	2.80E-09	-0.59	50	1.67	Smarca2	Epigenetic
DMR1:244686001	1	244686001	244690000	4000	1	6.40E-07	0.34	67	1.68	Smarca2	Epigenetic
DMR1:244699001	1	244699001	244702000	3000	1	3.10E-12	0.69	40	1.33	Smarca2	Epigenetic
DMR1:244707001	1	244707001	244708000	1000	1	8.10E-08	0.38	9	0.9	Smarca2	Epigenetic
DMR1:244715001	1	244715001	244716000	1000	1	3.90E-11	-0.8	13	1.3	Smarca2	Epigenetic
DMR1:244727001	1	244727001	244728000	1000	1	6.30E-07	-0.39	18	1.8	Smarca2	Epigenetic
DMR1:244734001	1	244734001	244735000	1000	1	9.20E-10	0.51	14	1.4	Smarca2	Epigenetic
DMR1:244743001	1	244743001	244745000	2000	2	1.00E-10	-0.63	25	1.25	Smarca2	Epigenetic
DMR1:244765001	1	244765001	244769000	4000	3	7.00E-20	0.62	62	1.55	Smarca2	Epigenetic
DMR1:244780001	1	244780001	244782000	2000	1	8.80E-07	0.3	28	1.4	Smarca2	Epigenetic
DMR1:244792001	1	244792001	244797000	5000	4	3.80E-20	-0.54	80	1.6	Smarca2	Epigenetic
DMR1:245261001	1	245261001	245262000	1000	1	6.80E-12	0.66	5	0.5	Vldlr	Binding Proteins
DMR1:245274001	1	245274001	245276000	2000	2	1.70E-14	0.57	12	0.6	Vldlr	Binding Proteins
DMR1:245396001	1	245396001	245397000	1000	1	5.80E-07	0.42	10	1	Kcnv2	Transport
DMR1:245433001	1	245433001	245434000	1000	1	1.00E-18	0.73	13	1.3	Kcnv2	Transport
DMR1:245442001	1	245442001	245444000	2000	1	3.10E-12	0.58	29	1.45	Kcnv2	Transport
DMR1:245464001	1	245464001	245466000	2000	1	3.30E-07	-0.52	37	1.85	Kcnv2	Transport
DMR1:245474001	1	245474001	245476000	2000	2	1.40E-16	0.65	23	1.15	Kcnv2;Pum3	Transport
DMR1:245492001	1	245492001	245498000	6000	4	2.60E-17	-0.52	64	1.07	Pum3	
DMR1:245499001	1	245499001	245502000	3000	1	1.00E-10	-0.49	19	0.63	Pum3	
DMR1:245509001	1	245509001	245511000	2000	1	4.00E-13	0.42	33	1.65	Pum3	
DMR1:245853001	1	245853001	245855000	2000	1	9.90E-08	-0.63	24	1.2	Rfx3	Transcription
DMR1:245874001	1	245874001	245876000	2000	2	2.40E-09	0.5	14	0.7	Rfx3	Transcription
DMR1:245880001	1	245880001	245883000	3000	1	6.20E-10	-0.49	16	0.53	Rfx3	Transcription
DMR1:245891001	1	245891001	245893000	2000	2	4.20E-13	0.76	14	0.7	Rfx3	Transcription
DMR1:245916001	1	245916001	245917000	1000	1	3.10E-12	0.49	6	0.6	Rfx3	Transcription
DMR1:245946001	1	245946001	245947000	1000	1	7.60E-20	0.77	15	1.5	Rfx3	Transcription
DMR1:245955001	1	245955001	245959000	4000	3	3.20E-16	-0.44	43	1.07	Rfx3	Transcription
DMR1:245960001	1	245960001	245963000	3000	2	1.90E-12	-0.49	41	1.37	Rfx3	Transcription
DMR1:245976001	1	245976001	245978000	2000	2	1.10E-07	0.36	21	1.05	Rfx3	Transcription
DMR1:245983001	1	245983001	245984000	1000	1	4.20E-08	0.39	7	0.7	Rfx3	Transcription
DMR1:246041001	1	246041001	246043000	2000	1	6.30E-09	-0.63	25	1.25	Rfx3	Transcription
DMR1:246050001	1	246050001	246054000	4000	1	5.50E-11	0.64	34	0.85	Rfx3	Transcription
DMR1:246080001	1	246080001	246081000	1000	1	2.50E-07	0.41	8	0.8	Rfx3	Transcription
DMR1:246082001	1	246082001	246083000	1000	1	3.20E-08	0.46	5	0.5	Rfx3	Transcription
DMR1:246375001	1	246375001	246379000	4000	1	2.50E-11	0.4	74	1.85	Glis3	Transcription
DMR1:246408001	1	246408001	246409000	1000	1	4.90E-08	0.47	8	0.8	Glis3	Transcription
DMR1:246418001	1	246418001	246420000	2000	1	4.40E-07	-0.5	21	1.05	Glis3	Transcription
DMR1:246439001	1	246439001	246441000	2000	1	7.70E-07	0.33	27	1.35	Glis3	Transcription

DMR1:246449001	1	246449001	246452000	3000	1	1.10E-07	0.39	31	1.03	Glis3	Transcription
DMR1:246460001	1	246460001	246463000	3000	1	4.00E-07	-0.49	49	1.63	Glis3	Transcription
DMR1:246475001	1	246475001	246477000	2000	1	2.70E-07	0.36	28	1.4	Glis3	Transcription
DMR1:246478001	1	246478001	246480000	2000	1	5.00E-22	0.64	40	2	Glis3	Transcription
DMR1:246482001	1	246482001	246485000	3000	1	4.90E-13	0.84	35	1.17	Glis3	Transcription
DMR1:246495001	1	246495001	246498000	3000	1	6.00E-13	0.59	27	0.9	Glis3	Transcription
DMR1:246512001	1	246512001	246515000	3000	1	9.40E-08	-0.38	39	1.3	Glis3	Transcription
DMR1:246518001	1	246518001	246520000	2000	1	5.20E-07	0.31	20	1	Glis3	Transcription
DMR1:246535001	1	246535001	246538000	3000	2	1.50E-11	0.68	15	0.5	Glis3	Transcription
DMR1:246540001	1	246540001	246541000	1000	1	9.00E-15	-0.9	12	1.2	Glis3	Transcription
DMR1:246542001	1	246542001	246545000	3000	1	9.90E-08	-0.51	38	1.27	Glis3	Transcription
DMR1:246562001	1	246562001	246565000	3000	1	4.60E-10	0.54	39	1.3	Glis3;LOC102556263	Transcription
DMR1:246574001	1	246574001	246576000	2000	1	6.80E-11	-0.6	35	1.75	Glis3;LOC102556263	Transcription
DMR1:246655001	1	246655001	246656000	1000	1	2.80E-20	0.86	6	0.6	LOC103691327;NEWGENE_1565505	
DMR1:246675001	1	246675001	246676000	1000	1	3.20E-11	0.41	15	1.5	NEWGENE_1565505	
DMR1:246685001	1	246685001	246696000	11000	1	2.90E-07	0.34	143	1.3	NEWGENE_1565505;LOC102550766	
DMR1:246701001	1	246701001	246705000	4000	1	1.30E-10	0.53	39	0.98	NEWGENE_1565505;LOC102550766	
DMR1:246706001	1	246706001	246707000	1000	1	3.60E-19	0.73	10	1	NEWGENE_1565505;LOC102550766	
DMR1:246732001	1	246732001	246733000	1000	1	5.30E-10	-0.65	13	1.3	NEWGENE_1565505;LOC103691328	
DMR1:246737001	1	246737001	246738000	1000	1	1.00E-08	-0.69	14	1.4	NEWGENE_1565505;LOC103691328	
DMR1:246749001	1	246749001	246750000	1000	1	9.50E-07	-0.37	12	1.2	NEWGENE_1565505;LOC103691328	
DMR1:246766001	1	246766001	246767000	1000	1	9.20E-16	0.62	14	1.4	NEWGENE_1565505	
DMR1:246776001	1	246776001	246779000	3000	1	5.50E-08	-0.37	49	1.63	NEWGENE_1565505	
DMR1:246780001	1	246780001	246782000	2000	1	7.40E-07	-0.4	31	1.55	NEWGENE_1565505	
DMR1:246792001	1	246792001	246795000	3000	1	6.30E-12	-0.66	55	1.83	NEWGENE_1565505	
DMR1:246959001	1	246959001	246960000	1000	1	2.40E-09	0.4	14	1.4	LOC102546345;Slc1a1	Transport
DMR1:246965001	1	246965001	246967000	2000	1	2.80E-15	0.63	26	1.3	LOC102546345;Slc1a1	Transport
DMR1:246977001	1	246977001	246982000	5000	2	4.00E-13	-0.58	66	1.32	Slc1a1	Transport
DMR1:246984001	1	246984001	246987000	3000	1	6.50E-08	-0.47	47	1.57	Slc1a1	Transport
DMR1:246988001	1	246988001	246992000	4000	1	2.40E-10	0.48	63	1.57	Slc1a1	Transport
DMR1:247002001	1	247002001	247005000	3000	2	1.40E-10	0.41	16	0.53	Slc1a1	Transport
DMR1:247033001	1	247033001	247039000	6000	1	5.40E-07	-0.53	84	1.4	Slc1a1;Spata6l	Transport
DMR1:247048001	1	247048001	247050000	2000	1	1.60E-14	0.7	20	1	Spata6l	
DMR1:247070001	1	247070001	247075000	5000	1	5.30E-08	-0.54	78	1.56	Spata6l;Plpp6	Signaling
DMR1:247100001	1	247100001	247103000	3000	1	5.80E-09	-0.51	25	0.83	LOC680727;RGD1565057;Cdc37l1	Transcription
DMR1:247126001	1	247126001	247130000	4000	3	4.70E-15	-0.55	33	0.82	Cdc37l1	Transcription
DMR1:247136001	1	247136001	247139000	3000	1	7.90E-12	-0.7	37	1.23	Cdc37l1;Ak3	Transcription;Signaling
DMR1:247157001	1	247157001	247162000	5000	1	7.30E-10	0.39	59	1.18	Ak3	Signaling
DMR1:247163001	1	247163001	247166000	3000	1	5.20E-08	-0.55	32	1.07	Ak3	Signaling
DMR1:247173001	1	247173001	247175000	2000	1	7.40E-08	0.35	32	1.6	Ak3	Signaling
DMR1:247178001	1	247178001	247179000	1000	1	1.50E-10	0.47	8	0.8	Ak3	Signaling
DMR1:247218001	1	247218001	247224000	6000	2	2.50E-20	0.93	66	1.1	Rcl1	Metabolism
DMR1:247234001	1	247234001	247235000	1000	1	9.40E-08	0.51	6	0.6	Rcl1	Metabolism
DMR1:247237001	1	247237001	247238000	1000	1	1.40E-08	-0.45	9	0.9	Rcl1	Metabolism
DMR1:247239001	1	247239001	247241000	2000	1	1.50E-08	0.47	14	0.7	Rcl1	Metabolism
DMR1:247253001	1	247253001	247256000	3000	2	1.40E-16	0.55	35	1.17	Rcl1;Mir101-2	Metabolism
DMR1:247267001	1	247267001	247268000	1000	1	4.60E-11	0.54	15	1.5	Rcl1;Mir101-2	Metabolism
DMR1:247282001	1	247282001	247283000	1000	1	1.50E-07	0.36	16	1.6	Rcl1	Metabolism
DMR1:247388001	1	247388001	247396000	8000	4	2.30E-20	0.89	109	1.36	Jak2	
DMR1:247431001	1	247431001	247434000	3000	1	5.10E-13	0.71	29	0.97	Jak2	
DMR1:247443001	1	247443001	247446000	3000	1	1.80E-08	0.5	40	1.33	Jak2	
DMR1:247450001	1	247450001	247452000	2000	1	1.20E-15	0.58	25	1.25	Jak2	
DMR1:247456001	1	247456001	247460000	4000	2	5.50E-14	0.85	35	0.88	Jak2	
DMR1:247463001	1	247463001	247467000	4000	2	6.10E-18	-0.56	26	0.65	Jak2;Insl6;LOC102546806	Hormone
DMR1:247474001	1	247474001	247476000	2000	1	2.70E-11	0.55	25	1.25	Insl6;LOC102546806;Rln1	Hormone
DMR1:247490001	1	247490001	247498000	8000	1	3.90E-09	0.34	107	1.34	Rln1;Plgrkt	Hormone

DMR1:247532001	1	247532001	247536000	4000	1	6.70E-07	0.43	81	2.02	Cd274	Immune
DMR1:247543001	1	247543001	247547000	4000	1	6.60E-15	0.9	40	1	Cd274;LOC680908	Immune
DMR1:247557001	1	247557001	247560000	3000	1	5.40E-14	0.5	34	1.13	LOC680908;Pdcd1lg2;LOC102547032	Immune
DMR1:247568001	1	247568001	247570000	2000	1	1.90E-09	0.4	35	1.75	Pdcd1lg2;LOC102547032	Immune
DMR1:247573001	1	247573001	247575000	2000	2	1.80E-10	0.57	16	0.8	Pdcd1lg2;LOC102547032	Immune
DMR1:247576001	1	247576001	247578000	2000	1	1.30E-07	-0.46	6	0.3	Pdcd1lg2;LOC102547032	Immune
DMR1:247584001	1	247584001	247586000	2000	2	8.10E-16	0.51	31	1.55	Pdcd1lg2	Immune
DMR1:247598001	1	247598001	247602000	4000	1	3.50E-09	0.4	71	1.77	Pdcd1lg2	Immune
DMR1:247603001	1	247603001	247604000	1000	1	5.30E-07	-0.4	7	0.7	Pdcd1lg2	Immune
DMR1:247610001	1	247610001	247613000	3000	1	1.30E-17	0.55	42	1.4	Pdcd1lg2	Immune
DMR1:247614001	1	247614001	247617000	3000	3	6.50E-09	0.44	19	0.63	Pdcd1lg2	Immune
DMR1:247633001	1	247633001	247635000	2000	1	9.60E-17	0.68	16	0.8	Pdcd1lg2	Immune
DMR1:247679001	1	247679001	247681000	2000	1	2.60E-09	0.38	21	1.05	Ric1	
DMR1:247692001	1	247692001	247693000	1000	1	2.40E-08	0.42	8	0.8	Ric1	
DMR1:247706001	1	247706001	247707000	1000	1	3.00E-10	0.6	4	0.4	Ric1	
DMR1:247708001	1	247708001	247710000	2000	1	6.30E-07	-0.49	11	0.55	Ric1	
DMR1:247718001	1	247718001	247719000	1000	1	5.50E-07	-0.57	10	1	Ric1	
DMR1:247730001	1	247730001	247734000	4000	2	2.80E-12	0.69	31	0.78	Ric1	
DMR1:247744001	1	247744001	247748000	4000	3	4.60E-12	0.64	31	0.78	Ric1	
DMR1:247768001	1	247768001	247771000	3000	1	9.00E-11	-0.45	29	0.97	Ric1	
DMR1:247796001	1	247796001	247797000	1000	1	3.10E-10	0.66	10	1	Ermp1	Protease
DMR1:247798001	1	247798001	247800000	2000	2	7.80E-08	0.37	32	1.6	Ermp1	Protease
DMR1:247807001	1	247807001	247808000	1000	1	6.90E-16	0.49	12	1.2	Ermp1	Protease
DMR1:247863001	1	247863001	247864000	1000	1	1.70E-19	0.58	18	1.8	Mlana	
DMR1:247865001	1	247865001	247867000	2000	1	1.70E-23	0.95	7	0.35	Mlana	
DMR1:247878001	1	247878001	247881000	3000	2	5.20E-19	0.83	42	1.4	Mlana	
DMR1:247888001	1	247888001	247890000	2000	1	2.00E-12	0.52	39	1.95	Mlana;RGD1311595	
DMR1:247917001	1	247917001	247919000	2000	1	5.70E-11	0.58	16	0.8	RGD1311595	
DMR1:247943001	1	247943001	247946000	3000	1	4.90E-19	0.86	25	0.83	RGD1311595;LOC108349433	
DMR1:247965001	1	247965001	247967000	2000	1	3.40E-09	0.56	13	0.65	RGD1311595	
DMR1:247982001	1	247982001	247985000	3000	1	1.30E-07	-0.66	57	1.9	RGD1311595;Ranbp6	Transport
DMR1:247991001	1	247991001	247998000	7000	1	4.30E-08	-0.48	66	0.94	Ranbp6	Transport
DMR1:248105001	1	248105001	248107000	2000	1	9.60E-16	0.87	52	2.6	Il33	
DMR1:248116001	1	248116001	248118000	2000	1	8.40E-10	0.44	35	1.75	Il33	
DMR1:248147001	1	248147001	248150000	3000	2	2.90E-09	0.43	39	1.3	Il33	
DMR1:248182001	1	248182001	248190000	8000	4	4.20E-25	-0.61	107	1.34	Tpd52l3	
DMR1:248194001	1	248194001	248195000	1000	1	4.70E-08	0.53	18	1.8	Tpd52l3	
DMR1:248202001	1	248202001	248203000	1000	1	5.60E-14	0.68	10	1	Tpd52l3	
DMR1:248216001	1	248216001	248219000	3000	1	2.50E-12	0.4	50	1.67	LOC681078;Uhrf2	Proteolysis
DMR1:248220001	1	248220001	248221000	1000	1	3.00E-11	0.53	9	0.9	LOC681078;Uhrf2	Proteolysis
DMR1:248222001	1	248222001	248228000	6000	3	7.20E-11	0.41	78	1.3	LOC681078;Uhrf2	Proteolysis
DMR1:248274001	1	248274001	248278000	4000	2	2.90E-08	-0.62	37	0.92	Uhrf2	Proteolysis
DMR1:248287001	1	248287001	248290000	3000	1	2.80E-14	0.63	28	0.93	Uhrf2;Gldc	Proteolysis;Metabolism
DMR1:248291001	1	248291001	248293000	2000	1	1.90E-09	-0.56	29	1.45	Uhrf2;Gldc	Proteolysis;Metabolism
DMR1:248296001	1	248296001	248298000	2000	1	2.30E-07	-0.44	36	1.8	Uhrf2;Gldc	Proteolysis;Metabolism
DMR1:248312001	1	248312001	248315000	3000	1	1.30E-07	0.43	51	1.7	Gldc	Metabolism
DMR1:248321001	1	248321001	248327000	6000	2	2.40E-10	-0.57	75	1.25	Gldc	Metabolism
DMR1:248381001	1	248381001	248384000	3000	2	2.20E-19	0.9	17	0.57	Gldc	Metabolism
DMR1:248451001	1	248451001	248455000	4000	2	9.50E-10	0.74	42	1.05	Mbl2;LOC102550442	Extracellular Matrix
DMR1:248905001	1	248905001	248910000	5000	4	2.30E-19	-0.53	70	1.4	Dkk1;LOC100912084	
DMR1:249017001	1	249017001	249018000	1000	1	2.20E-07	0.35	8	0.8	Prkg1	
DMR1:249054001	1	249054001	249056000	2000	1	6.20E-08	0.44	10	0.5	Prkg1	
DMR1:249060001	1	249060001	249061000	1000	1	1.00E-09	-0.67	20	2	Prkg1	
DMR1:249062001	1	249062001	249063000	1000	1	1.20E-15	0.71	11	1.1	Prkg1	
DMR1:249066001	1	249066001	249068000	2000	1	2.20E-08	0.42	28	1.4	Prkg1	
DMR1:249071001	1	249071001	249075000	4000	1	2.30E-07	-0.48	48	1.2	Prkg1	
DMR1:249077001	1	249077001	249078000	1000	1	6.30E-09	-0.74	12	1.2	Prkg1	
DMR1:249086001	1	249086001	249087000	1000	1	4.80E-16	0.68	12	1.2	Prkg1	
DMR1:249202001	1	249202001	249203000	1000	1	1.20E-08	0.33	24	2.4	Prkg1	
DMR1:249204001	1	249204001	249211000	7000	1	9.80E-07	-0.56	74	1.06	Prkg1	
DMR1:249212001	1	249212001	249214000	2000	2	4.90E-11	0.58	9	0.45	Prkg1	

DMR1:249221001	1	249221001	249223000	2000	1	9.30E-09	0.43	16	0.8	Prkg1	
DMR1:249235001	1	249235001	249244000	9000	8	1.30E-16	0.5	106	1.18	Prkg1	
DMR1:249258001	1	249258001	249259000	1000	1	3.00E-11	0.59	17	1.7	Prkg1	
DMR1:249297001	1	249297001	249298000	1000	1	4.80E-08	0.56	5	0.5	Prkg1	
DMR1:249303001	1	249303001	249311000	8000	5	1.00E-16	-0.46	97	1.21	Prkg1	
DMR1:249317001	1	249317001	249319000	2000	2	3.50E-10	-0.51	15	0.75	Prkg1	
DMR1:249330001	1	249330001	249335000	5000	1	1.50E-07	-0.45	158	3.16	Prkg1	
DMR1:249344001	1	249344001	249353000	9000	3	7.80E-23	-0.57	105	1.17	Prkg1	
DMR1:249361001	1	249361001	249365000	4000	1	2.50E-09	0.46	44	1.1	Prkg1	
DMR1:249375001	1	249375001	249377000	2000	1	5.80E-07	0.39	16	0.8	Prkg1	
DMR1:249388001	1	249388001	249390000	2000	1	8.70E-09	0.47	17	0.85	Prkg1	
DMR1:249406001	1	249406001	249411000	5000	2	7.70E-13	0.52	65	1.3	Prkg1	
DMR1:249417001	1	249417001	249418000	1000	1	1.90E-13	0.73	7	0.7	Prkg1	
DMR1:249419001	1	249419001	249420000	1000	1	4.30E-10	0.49	20	2	Prkg1	
DMR1:249425001	1	249425001	249436000	11000	3	7.70E-11	-0.63	135	1.23	Prkg1	
DMR1:249440001	1	249440001	249443000	3000	1	7.20E-12	0.46	35	1.17	Prkg1	
DMR1:249449001	1	249449001	249456000	7000	1	2.80E-07	-0.54	78	1.11	Prkg1	
DMR1:249458001	1	249458001	249459000	1000	1	5.50E-10	0.44	8	0.8	Prkg1	
DMR1:249466001	1	249466001	249472000	6000	1	4.80E-11	0.42	81	1.35	Prkg1	
DMR1:249474001	1	249474001	249481000	7000	4	3.10E-31	-0.6	76	1.09	Prkg1	
DMR1:249500001	1	249500001	249503000	3000	2	1.90E-14	0.69	20	0.67	Prkg1	
DMR1:249504001	1	249504001	249505000	1000	1	3.00E-12	0.61	10	1	Prkg1	
DMR1:249517001	1	249517001	249519000	2000	2	1.70E-29	0.95	21	1.05	Prkg1	
DMR1:249522001	1	249522001	249523000	1000	1	7.20E-09	0.49	6	0.6	Prkg1	
DMR1:249524001	1	249524001	249526000	2000	1	7.80E-13	0.52	24	1.2	Prkg1	
DMR1:249528001	1	249528001	249529000	1000	1	3.90E-13	0.49	10	1	Prkg1	
DMR1:249530001	1	249530001	249531000	1000	1	6.70E-12	0.53	5	0.5	Prkg1	
DMR1:249548001	1	249548001	249550000	2000	1	2.40E-07	0.48	17	0.85	Prkg1	
DMR1:249576001	1	249576001	249577000	1000	1	6.60E-07	0.42	18	1.8	Prkg1;Cstf2t	Translation
DMR1:249585001	1	249585001	249591000	6000	3	1.00E-10	-0.64	65	1.08	Prkg1;Cstf2t	Translation
DMR1:249609001	1	249609001	249612000	3000	1	6.60E-12	0.44	39	1.3	Prkg1	
DMR1:249615001	1	249615001	249618000	3000	2	1.40E-15	0.59	24	0.8	Prkg1	
DMR1:249624001	1	249624001	249632000	8000	2	3.50E-09	0.53	90	1.12	Prkg1	
DMR1:249637001	1	249637001	249639000	2000	1	3.50E-25	0.99	26	1.3	Prkg1	
DMR1:249644001	1	249644001	249649000	5000	2	4.20E-10	0.53	46	0.92	Prkg1	
DMR1:249650001	1	249650001	249651000	1000	1	2.70E-10	0.71	6	0.6	Prkg1	
DMR1:249657001	1	249657001	249661000	4000	3	1.40E-10	-0.49	58	1.45	Prkg1	
DMR1:249665001	1	249665001	249666000	1000	1	1.10E-15	0.91	13	1.3	Prkg1	
DMR1:249688001	1	249688001	249689000	1000	1	3.90E-07	0.41	6	0.6	Prkg1	
DMR1:249692001	1	249692001	249697000	5000	1	2.40E-20	1.67	38	0.76	Prkg1	
DMR1:249698001	1	249698001	249700000	2000	2	3.80E-13	0.57	25	1.25	Prkg1	
DMR1:249708001	1	249708001	249715000	7000	4	2.50E-26	-0.53	85	1.21	Prkg1	
DMR1:249719001	1	249719001	249724000	5000	3	1.20E-21	-0.57	86	1.72	Prkg1	
DMR1:249733001	1	249733001	249737000	4000	3	4.10E-17	-0.57	38	0.95	Prkg1	
DMR1:249793001	1	249793001	249795000	2000	1	1.60E-11	0.54	25	1.25	Prkg1	
DMR1:249798001	1	249798001	249804000	6000	3	1.80E-12	0.86	50	0.83	Prkg1	
DMR1:249820001	1	249820001	249826000	6000	2	4.70E-11	0.59	46	0.77	Prkg1	
DMR1:249827001	1	249827001	249829000	2000	1	7.20E-16	0.6	28	1.4	Prkg1	
DMR1:249848001	1	249848001	249853000	5000	2	2.60E-11	0.45	57	1.14	Prkg1	
DMR1:249857001	1	249857001	249858000	1000	1	6.90E-14	0.74	5	0.5	Prkg1	
DMR1:249873001	1	249873001	249875000	2000	1	8.40E-08	0.41	5	0.25	Prkg1	
DMR1:249879001	1	249879001	249880000	1000	1	4.60E-09	0.46	8	0.8	Prkg1	
DMR1:249910001	1	249910001	249912000	2000	1	2.20E-10	0.55	12	0.6	Prkg1	
DMR1:249915001	1	249915001	249917000	2000	1	7.40E-10	0.55	10	0.5	Prkg1	
DMR1:249979001	1	249979001	249982000	3000	2	5.70E-09	-0.56	30	1	Prkg1	
DMR1:249987001	1	249987001	249988000	1000	1	9.10E-10	0.61	6	0.6	Prkg1	
DMR1:249999001	1	249999001	2.50E+08	1000	1	6.20E-07	0.41	8	0.8	Prkg1	
DMR1:250034001	1	250034001	250036000	2000	1	5.20E-14	-0.45	20	1	Prkg1	
DMR1:250098001	1	250098001	250100000	2000	1	2.40E-12	-0.72	22	1.1	Prkg1	
DMR1:250101001	1	250101001	250103000	2000	1	2.80E-07	0.48	17	0.85	Prkg1	
DMR1:250112001	1	250112001	250114000	2000	1	1.20E-13	0.67	25	1.25	Prkg1	
DMR1:250126001	1	250126001	250132000	6000	2	1.30E-08	0.49	46	0.77	Prkg1	
DMR1:250137001	1	250137001	250140000	3000	1	2.10E-13	-0.54	25	0.83	Prkg1	
DMR1:250184001	1	250184001	250186000	2000	1	6.80E-08	-0.36	36	1.8	Prkg1	
DMR1:250187001	1	250187001	250189000	2000	1	2.90E-07	0.34	32	1.6	Prkg1	

DMR1:250252001	1	250252001	250253000	1000	1	5.10E-10	0.47	9	0.9	Prkg1	
DMR1:250256001	1	250256001	250262000	6000	1	1.10E-09	0.57	71	1.18	Prkg1	
DMR1:250285001	1	250285001	250288000	3000	2	4.80E-07	-0.57	31	1.03	Prkg1	
DMR1:250431001	1	250431001	250432000	1000	1	4.00E-08	0.4	9	0.9	LOC103691334;A1cf	Metabolism
DMR1:250446001	1	250446001	250452000	6000	1	5.20E-11	0.54	57	0.95	A1cf	Metabolism
DMR1:250464001	1	250464001	250465000	1000	1	7.40E-21	0.81	5	0.5	A1cf	Metabolism
DMR1:250488001	1	250488001	250489000	1000	1	1.10E-10	0.57	8	0.8	A1cf	Metabolism
DMR1:250498001	1	250498001	250503000	5000	1	1.00E-11	0.63	58	1.16	A1cf	Metabolism
DMR1:250521001	1	250521001	250525000	4000	2	1.50E-19	0.94	28	0.7	A1cf	Metabolism
DMR1:250545001	1	250545001	250549000	4000	1	7.00E-08	0.47	33	0.82	Asah2	
DMR1:250552001	1	250552001	250555000	3000	2	1.30E-10	0.46	22	0.73	Asah2	
DMR1:250569001	1	250569001	250570000	1000	1	1.70E-07	0.39	7	0.7	Asah2	
DMR1:250579001	1	250579001	250583000	4000	1	5.20E-09	0.41	43	1.07	Asah2	
DMR1:250584001	1	250584001	250587000	3000	1	2.30E-07	-0.45	44	1.47	Asah2	
DMR1:250596001	1	250596001	250599000	3000	2	3.40E-07	0.41	29	0.97	Asah2	
DMR1:250600001	1	250600001	250604000	4000	2	4.40E-14	-0.6	10	0.25	Asah2	
DMR1:250605001	1	250605001	250607000	2000	1	8.80E-08	0.56	18	0.9	Asah2	
DMR1:250608001	1	250608001	250609000	1000	1	2.30E-11	0.56	13	1.3	Asah2	
DMR1:250617001	1	250617001	250627000	10000	2	1.70E-18	0.87	117	1.17	Asah2	
DMR1:250642001	1	250642001	250645000	3000	1	8.60E-07	-0.57	19	0.63	Asah2	
DMR1:250646001	1	250646001	250647000	1000	1	1.70E-08	0.51	4	0.4	Asah2	
DMR1:250655001	1	250655001	250656000	1000	1	5.70E-07	-0.47	10	1	Asah2	
DMR1:250663001	1	250663001	250665000	2000	1	2.10E-08	0.55	44	2.2	Asah2	
DMR1:250681001	1	250681001	250683000	2000	2	1.40E-11	0.64	26	1.3	Sgms1	
DMR1:250697001	1	250697001	250703000	6000	3	6.90E-28	0.76	51	0.85	Sgms1	
DMR1:250717001	1	250717001	250721000	4000	2	7.70E-09	0.39	47	1.18	Sgms1	
DMR1:250725001	1	250725001	250728000	3000	2	6.40E-11	-0.66	60	2	Sgms1	
DMR1:250738001	1	250738001	250742000	4000	1	3.40E-07	0.3	48	1.2	Sgms1	
DMR1:250751001	1	250751001	250753000	2000	1	2.20E-07	0.51	9	0.45	Sgms1	
DMR1:250776001	1	250776001	250781000	5000	1	5.90E-07	-0.47	76	1.52	Sgms1	
DMR1:250786001	1	250786001	250788000	2000	1	1.40E-11	0.52	23	1.15	Sgms1	
DMR1:250823001	1	250823001	250826000	3000	1	1.80E-35	0.88	50	1.67	Sgms1	
DMR1:250857001	1	250857001	250860000	3000	1	1.40E-14	0.72	41	1.37	Sgms1	
DMR1:250863001	1	250863001	250871000	8000	2	1.60E-12	0.67	93	1.16	Sgms1	
DMR1:250874001	1	250874001	250875000	1000	1	3.70E-09	0.68	4	0.4	Sgms1	
DMR1:250889001	1	250889001	250892000	3000	1	3.40E-07	-0.35	37	1.23	Sgms1	
DMR1:250895001	1	250895001	250898000	3000	2	1.50E-14	0.72	39	1.3	Sgms1	
DMR1:250927001	1	250927001	250928000	1000	1	7.10E-09	0.44	11	1.1	Sgms1	
DMR1:250942001	1	250942001	250945000	3000	1	6.50E-07	-0.58	46	1.53	Sgms1	
DMR1:250956001	1	250956001	250958000	2000	1	2.70E-08	-0.37	27	1.35	Sgms1	
DMR1:250961001	1	250961001	250962000	1000	1	1.80E-15	0.73	8	0.8	Sgms1	
DMR1:250989001	1	250989001	250990000	1000	1	4.00E-13	0.68	9	0.9	Nutf2-ps1	
DMR1:250993001	1	250993001	250994000	1000	1	3.60E-19	0.52	12	1.2	Nutf2-ps1	
DMR1:250995001	1	250995001	250997000	2000	1	1.10E-07	0.37	12	0.6	Nutf2-ps1;LOC681274	
DMR1:250999001	1	250999001	251001000	2000	1	6.10E-07	-0.47	28	1.4	Nutf2-ps1;LOC681274	
DMR1:251053001	1	251053001	251058000	5000	1	4.10E-10	0.43	69	1.38	Minpp1	Signaling
DMR1:251060001	1	251060001	251063000	3000	2	5.00E-10	-0.54	50	1.67	Minpp1	Signaling
DMR1:251075001	1	251075001	251077000	2000	1	1.10E-07	-0.57	35	1.75	Minpp1	Signaling
DMR1:251078001	1	251078001	251084000	6000	1	9.20E-20	0.93	69	1.15	Minpp1	Signaling
DMR1:251138001	1	251138001	251140000	2000	2	3.90E-14	-0.62	13	0.65	Papss2	
DMR1:251160001	1	251160001	251163000	3000	1	1.60E-09	0.5	35	1.17	Papss2	
DMR1:251164001	1	251164001	251165000	1000	1	1.70E-08	-0.45	22	2.2	Papss2	
DMR1:251170001	1	251170001	251171000	1000	1	8.30E-09	0.58	9	0.9	Papss2	
DMR1:251182001	1	251182001	251185000	3000	1	2.90E-18	0.6	63	2.1	Papss2	
DMR1:251190001	1	251190001	251195000	5000	2	1.50E-21	0.83	44	0.88	Papss2	
DMR1:251198001	1	251198001	251199000	1000	1	3.10E-09	0.43	15	1.5	Papss2	
DMR1:251202001	1	251202001	251205000	3000	1	1.30E-09	-0.56	47	1.57	Papss2	
DMR1:251243001	1	251243001	251245000	2000	1	5.10E-09	-0.59	20	1	Atad1	
DMR1:251254001	1	251254001	251261000	7000	3	5.50E-11	0.49	64	0.91	Atad1	
DMR1:251278001	1	251278001	251279000	1000	1	6.60E-09	0.68	12	1.2	Atad1	
DMR1:251286001	1	251286001	251288000	2000	1	3.80E-10	0.59	11	0.55	Atad1	
DMR1:251290001	1	251290001	251291000	1000	1	2.20E-11	0.66	1	0.1	Atad1	
DMR1:251305001	1	251305001	251306000	1000	1	3.50E-07	0.51	4	0.4	Atad1	
DMR1:251327001	1	251327001	251328000	1000	1	8.40E-13	0.9	3	0.3	Atad1;Cyp2b15	Metabolism
DMR1:251331001	1	251331001	251334000	3000	3	6.60E-13	0.83	22	0.73	Atad1;Cyp2b15	Metabolism

DMR1:251335001	1	251335001	251337000	2000	2	4.50E-11	0.64	13	0.65	Atad1;Cyp2b15	Metabolism
DMR1:251339001	1	251339001	251340000	1000	1	3.30E-09	0.51	1	0.1	Atad1;Cyp2b15	Metabolism
DMR1:251341001	1	251341001	251344000	3000	1	7.40E-08	0.49	29	0.97	Atad1;Cyp2b15;LOC100910127	Metabolism
DMR1:251353001	1	251353001	251355000	2000	1	3.60E-14	0.91	15	0.75	Atad1;LOC100910127	Metabolism
DMR1:251358001	1	251358001	251361000	3000	3	1.70E-14	0.88	22	0.73	Atad1;LOC100910127	Metabolism
DMR1:251362001	1	251362001	251364000	2000	2	8.00E-11	0.69	14	0.7	Atad1;LOC100910127	Metabolism
DMR1:251368001	1	251368001	251369000	1000	1	4.90E-14	0.64	13	1.3	Atad1;LOC100910127	Metabolism
DMR1:251382001	1	251382001	251383000	1000	1	3.70E-08	0.48	14	1.4	Atad1	
DMR1:251384001	1	251384001	251390000	6000	3	5.30E-10	0.6	53	0.88	Atad1	
DMR1:251437001	1	251437001	251438000	1000	1	6.60E-07	0.42	6	0.6	Pten	Signaling
DMR1:251458001	1	251458001	251459000	1000	1	1.90E-14	0.97	8	0.8	Pten	Signaling
DMR1:251479001	1	251479001	251480000	1000	1	1.70E-07	0.37	21	2.1	Pten	Signaling
DMR1:251486001	1	251486001	251487000	1000	1	9.00E-10	0.44	20	2	Pten	Signaling
DMR1:251587001	1	251587001	251592000	5000	3	9.50E-14	-0.61	57	1.14	RGD1565695	
DMR1:251594001	1	251594001	251597000	3000	1	3.70E-10	0.54	34	1.13	RGD1565695	
DMR1:251602001	1	251602001	251604000	2000	1	1.90E-17	0.55	37	1.85	RGD1565695	
DMR1:251605001	1	251605001	251607000	2000	1	2.20E-11	0.57	23	1.15	RGD1565695;LOC102547905	
DMR1:251610001	1	251610001	251611000	1000	1	1.20E-10	0.42	14	1.4	RGD1565695;LOC102547905	
DMR1:251827001	1	251827001	251832000	5000	3	1.10E-21	0.85	46	0.92	Rnls	
DMR1:251867001	1	251867001	251870000	3000	1	5.40E-09	0.53	25	0.83	Rnls	
DMR1:251872001	1	251872001	251873000	1000	1	2.60E-09	0.49	7	0.7	Rnls	
DMR1:251877001	1	251877001	251882000	5000	4	3.40E-13	-0.54	37	0.74	Rnls	
DMR1:251899001	1	251899001	251903000	4000	1	9.50E-09	0.4	43	1.07	Rnls	
DMR1:251920001	1	251920001	251925000	5000	2	5.70E-10	-0.58	63	1.26	Rnls	
DMR1:251954001	1	251954001	251963000	9000	6	4.70E-24	-0.55	81	0.9	Rnls	
DMR1:251965001	1	251965001	251967000	2000	2	8.90E-11	0.6	21	1.05	Rnls	
DMR1:251978001	1	251978001	251981000	3000	1	1.80E-08	0.58	38	1.27	Rnls	
DMR1:251999001	1	251999001	252001000	2000	1	4.50E-14	0.61	22	1.1	Rnls;Rps4x-ps1	
DMR1:252010001	1	252010001	252013000	3000	2	7.00E-20	0.68	25	0.83	Rnls;Rps4x-ps1	
DMR1:252040001	1	252040001	252041000	1000	1	1.00E-09	0.5	5	0.5	Rnls	
DMR1:252046001	1	252046001	252047000	1000	1	2.80E-14	0.67	5	0.5	Rnls	
DMR1:252055001	1	252055001	252062000	7000	4	4.70E-16	0.61	53	0.76	Rnls	
DMR1:252070001	1	252070001	252072000	2000	1	3.30E-12	-0.74	8	0.4	Rnls	
DMR1:252074001	1	252074001	252075000	1000	1	6.40E-15	0.47	9	0.9	Rnls	
DMR1:252077001	1	252077001	252084000	7000	5	3.20E-27	-0.62	75	1.07	Rnls	
DMR1:252089001	1	252089001	252093000	4000	2	2.30E-16	0.78	33	0.82	Rnls;Lipo1	Metabolism
DMR1:252124001	1	252124001	252127000	3000	1	4.30E-08	0.49	26	0.87	Lipo1	Metabolism
DMR1:252270001	1	252270001	252279000	9000	4	3.40E-23	-0.51	75	0.83	Lipf	Metabolism
DMR1:252290001	1	252290001	252297000	7000	3	1.30E-07	-0.5	61	0.87	Lipf	Metabolism
DMR1:252303001	1	252303001	252304000	1000	1	2.00E-15	0.51	14	1.4	Lipf	Metabolism
DMR1:252323001	1	252323001	252325000	2000	1	1.20E-12	0.59	12	0.6	Lipk	Metabolism
DMR1:252326001	1	252326001	252327000	1000	1	3.50E-10	0.42	14	1.4	Lipk	Metabolism
DMR1:252341001	1	252341001	252342000	1000	1	3.50E-14	0.74	8	0.8	Lipk	Metabolism
DMR1:252343001	1	252343001	252344000	1000	1	6.50E-14	0.83	9	0.9	Lipk	Metabolism
DMR1:252348001	1	252348001	252349000	1000	1	6.50E-08	0.49	2	0.2	Lipk	Metabolism
DMR1:252364001	1	252364001	252368000	4000	2	3.80E-07	0.43	29	0.72	Lipk;Lipn	Metabolism
DMR1:252398001	1	252398001	252401000	3000	1	2.00E-07	-0.59	31	1.03	Lipn;Lipm	Metabolism
DMR1:252402001	1	252402001	252403000	1000	1	6.80E-16	0.65	6	0.6	Lipn;Lipm	Metabolism
DMR1:252412001	1	252412001	252413000	1000	1	9.40E-15	0.78	7	0.7	Lipm	Metabolism
DMR1:252426001	1	252426001	252428000	2000	1	4.30E-07	-0.56	15	0.75	Lipm;Ankrd22	Metabolism
DMR1:252435001	1	252435001	252437000	2000	1	4.10E-07	-0.54	25	1.25	Lipm;Ankrd22	Metabolism
DMR1:252442001	1	252442001	252443000	1000	1	6.90E-20	0.78	9	0.9	Ankrd22	
DMR1:252444001	1	252444001	252446000	2000	1	7.50E-07	-0.56	25	1.25	Ankrd22	
DMR1:252447001	1	252447001	252451000	4000	2	1.20E-18	0.9	23	0.58	Ankrd22	
DMR1:252457001	1	252457001	252458000	1000	1	2.00E-07	-0.61	12	1.2	Ankrd22	
DMR1:252464001	1	252464001	252466000	2000	2	2.00E-20	0.77	15	0.75	Ankrd22	
DMR1:252480001	1	252480001	252484000	4000	1	7.20E-11	0.65	31	0.78	Stambpl1	Protease
DMR1:252493001	1	252493001	252494000	1000	1	4.60E-17	0.6	12	1.2	Stambpl1	Protease
DMR1:252495001	1	252495001	252497000	2000	1	6.30E-07	-0.45	30	1.5	Stambpl1	Protease
DMR1:252505001	1	252505001	252507000	2000	2	1.50E-11	0.57	14	0.7	Stambpl1	Protease
DMR1:252516001	1	252516001	252519000	3000	1	4.10E-12	0.73	22	0.73	Stambpl1	Protease
DMR1:252520001	1	252520001	252521000	1000	1	7.10E-09	0.47	6	0.6	Stambpl1	Protease
DMR1:252533001	1	252533001	252534000	1000	1	4.40E-17	0.64	14	1.4	Stambpl1;Acta2	Protease;Cytoskeleton

DMR1:252540001	1	252540001	252541000	1000	1	1.70E-08	0.36	15	1.5	Stambpl1;Acta2	Protease;Cytoskeleton
DMR1:252554001	1	252554001	252557000	3000	2	3.70E-13	0.54	46	1.53	Acta2;LOC687722	Cytoskeleton
DMR1:252579001	1	252579001	252583000	4000	1	8.40E-10	-0.4	61	1.52	Fas	Receptor
DMR1:252591001	1	252591001	252594000	3000	1	4.80E-10	0.45	25	0.83	Fas	Receptor
DMR1:252598001	1	252598001	252600000	2000	2	3.40E-21	1.07	11	0.55	Fas	Receptor
DMR1:252601001	1	252601001	252602000	1000	1	9.90E-15	0.7	13	1.3	Fas	Receptor
DMR1:252615001	1	252615001	252618000	3000	1	2.30E-14	0.68	28	0.93	Fas	Receptor
DMR1:252620001	1	252620001	252621000	1000	1	7.40E-07	-0.42	14	1.4	Fas	Receptor
DMR1:252630001	1	252630001	252631000	1000	1	2.60E-11	0.49	16	1.6	Fas	Receptor
DMR1:252800001	1	252800001	252802000	2000	1	5.30E-19	0.62	16	0.8	Ch25h	Metabolism
DMR1:252810001	1	252810001	252812000	2000	1	6.30E-19	0.62	30	1.5	Ch25h;Lipa	Metabolism;Metabolism
DMR1:252831001	1	252831001	252833000	2000	2	4.90E-13	0.58	20	1	Lipa	Metabolism
DMR1:252851001	1	252851001	252853000	2000	1	3.90E-10	0.7	18	0.9	Lipa	Metabolism
DMR1:252882001	1	252882001	252885000	3000	1	3.40E-24	0.97	34	1.13	Lipa;Ifit2	Metabolism
DMR1:252894001	1	252894001	252896000	2000	1	1.40E-12	0.51	30	1.5	Lipa;Ifit2	Metabolism
DMR1:252910001	1	252910001	252914000	4000	1	1.70E-08	0.36	56	1.4	Lipa;Ifit2;Ifit3;Ifit1bl	Metabolism
DMR1:252961001	1	252961001	252963000	2000	1	6.40E-08	0.43	18	0.9	Lipa;Ifit1bl	Metabolism
DMR1:252965001	1	252965001	252967000	2000	2	5.10E-14	0.62	16	0.8	Lipa;Ifit1bl;Slc16a12	Metabolism;Transport
DMR1:252968001	1	252968001	252970000	2000	1	2.40E-07	-0.48	12	0.6	Lipa;Ifit1bl;Slc16a12	Metabolism;Transport
DMR1:252993001	1	252993001	252997000	4000	1	4.00E-11	0.6	22	0.55	Slc16a12	Transport
DMR1:253004001	1	253004001	253008000	4000	1	1.90E-08	0.42	33	0.82	Slc16a12	Transport
DMR1:253026001	1	253026001	253029000	3000	1	2.40E-14	0.48	45	1.5	Slc16a12	Transport
DMR1:253030001	1	253030001	253031000	1000	1	1.60E-21	0.99	2	0.2	Slc16a12	Transport
DMR1:253041001	1	253041001	253044000	3000	2	8.60E-16	0.87	26	0.87	Slc16a12	Transport
DMR1:253059001	1	253059001	253061000	2000	2	7.60E-20	0.81	7	0.35	Slc16a12	Transport
DMR1:253105001	1	253105001	253107000	2000	2	4.00E-07	0.33	17	0.85	Pank1	Signaling
DMR1:253110001	1	253110001	253112000	2000	1	9.30E-16	0.6	25	1.25	Pank1	Signaling
DMR1:253115001	1	253115001	253121000	6000	1	1.50E-08	-0.59	90	1.5	Pank1;Mir107	Signaling
DMR1:253124001	1	253124001	253128000	4000	1	1.10E-11	0.48	49	1.23	Pank1;Mir107	Signaling
DMR1:253138001	1	253138001	253141000	3000	1	1.50E-09	0.56	50	1.67	Pank1;Mir107	Signaling
DMR1:253149001	1	253149001	253151000	2000	1	6.60E-17	0.6	22	1.1	Pank1	Signaling
DMR1:253165001	1	253165001	253169000	4000	1	2.20E-12	0.77	44	1.1	Pank1	Signaling
DMR1:253172001	1	253172001	253173000	1000	1	4.60E-09	0.68	3	0.3	Pank1	Signaling
DMR1:253178001	1	253178001	253181000	3000	1	4.70E-08	0.36	36	1.2	Pank1	Signaling
DMR1:253224001	1	253224001	253226000	2000	1	2.40E-29	1	12	0.6	LOC102549070;Kif20b	Cytoskeleton
DMR1:253227001	1	253227001	253228000	1000	1	3.50E-07	0.43	7	0.7	LOC102549070;Kif20b	Cytoskeleton
DMR1:253234001	1	253234001	253235000	1000	1	4.80E-14	0.49	19	1.9	Kif20b	Cytoskeleton
DMR1:253272001	1	253272001	253274000	2000	1	7.10E-10	0.64	14	0.7	Kif20b	Cytoskeleton
DMR1:253282001	1	253282001	253293000	11000	7	5.70E-19	0.58	128	1.16	Kif20b;Ears2l1	Cytoskeleton
DMR1:253311001	1	253311001	253312000	1000	1	3.30E-09	0.61	6	0.6	Ears2l1	
DMR1:254548001	1	254548001	254552000	4000	2	4.20E-10	0.45	37	0.92	Htr7	Signaling
DMR1:254556001	1	254556001	254558000	2000	2	4.80E-24	0.64	28	1.4	Htr7	Signaling
DMR1:254573001	1	254573001	254578000	5000	4	4.10E-20	-0.65	47	0.94	Htr7	Signaling
DMR1:254580001	1	254580001	254582000	2000	1	2.40E-11	-0.57	9	0.45	Htr7	Signaling
DMR1:254583001	1	254583001	254585000	2000	1	1.30E-11	0.57	11	0.55	Htr7	Signaling
DMR1:254586001	1	254586001	254589000	3000	2	2.60E-08	-0.55	11	0.37	Htr7	Signaling
DMR1:254612001	1	254612001	254616000	4000	3	6.20E-18	-0.45	23	0.58	Htr7;LOC108349452	Signaling
DMR1:254622001	1	254622001	254624000	2000	1	4.00E-08	0.59	16	0.8	Htr7;LOC108349452	Signaling
DMR1:254625001	1	254625001	254635000	10000	6	4.50E-27	-0.56	128	1.28	Htr7;LOC108349452	Signaling
DMR1:254653001	1	254653001	254656000	3000	1	3.40E-07	-0.42	29	0.97	Htr7	Signaling
DMR1:254680001	1	254680001	254683000	3000	2	1.80E-12	0.62	17	0.57	Htr7	Signaling
DMR1:254688001	1	254688001	254691000	3000	1	3.60E-20	0.81	22	0.73	Rpp30	Translation
DMR1:254692001	1	254692001	254695000	3000	1	1.10E-23	0.88	29	0.97	Rpp30	Translation
DMR1:254704001	1	254704001	254705000	1000	1	2.80E-15	0.96	8	0.8	Rpp30	Translation
DMR1:254706001	1	254706001	254707000	1000	1	1.50E-08	0.43	17	1.7	Rpp30	Translation
DMR1:254723001	1	254723001	254725000	2000	1	8.00E-10	0.35	34	1.7	Rpp30;Ankrd1	Translation
DMR1:254727001	1	254727001	254730000	3000	2	4.60E-16	0.61	60	2	Rpp30;Ankrd1	Translation
DMR1:254733001	1	254733001	254735000	2000	1	8.00E-09	0.56	19	0.95	Ankrd1	
DMR1:254736001	1	254736001	254738000	2000	1	1.90E-25	0.84	17	0.85	Ankrd1	
DMR1:254963001	1	254963001	254965000	2000	1	3.50E-10	0.44	52	2.6	Pcgf5	Epigenetic
DMR1:254996001	1	254996001	254999000	3000	1	6.60E-12	0.44	69	2.3	Pcgf5	Epigenetic

DMR1:255002001	1	255002001	255006000	4000	1	1.00E-09	0.42	73	1.82	Pcgf5	Epigenetic
DMR1:255008001	1	255008001	255012000	4000	1	7.80E-11	0.44	69	1.73	Pcgf5	Epigenetic
DMR1:255015001	1	255015001	255017000	2000	1	2.90E-16	0.54	32	1.6	Pcgf5	Epigenetic
DMR1:255028001	1	255028001	255029000	1000	1	1.00E-12	0.44	23	2.3	Pcgf5	Epigenetic
DMR1:255036001	1	255036001	255039000	3000	1	7.50E-16	0.64	25	0.83	Pcgf5	Epigenetic
DMR1:255045001	1	255045001	255048000	3000	1	8.40E-08	0.47	22	0.73	Pcgf5	Epigenetic
DMR1:255060001	1	255060001	255061000	1000	1	5.30E-10	-0.53	19	1.9	Pcgf5	Epigenetic
DMR1:255082001	1	255082001	255086000	4000	2	1.70E-14	-0.56	42	1.05	Pcgf5	Epigenetic
DMR1:255094001	1	255094001	255095000	1000	1	4.50E-08	0.39	16	1.6	Pcgf5	Epigenetic
DMR1:255100001	1	255100001	255101000	1000	1	3.60E-08	-0.46	16	1.6	Pcgf5	Epigenetic
DMR1:255175001	1	255175001	255177000	2000	1	4.90E-09	0.67	15	0.75	Hectd2	Proteolysis
DMR1:255181001	1	255181001	255185000	4000	2	5.90E-13	0.5	57	1.43	Hectd2	Proteolysis
DMR1:255190001	1	255190001	255193000	3000	2	6.40E-13	0.58	23	0.77	Hectd2	Proteolysis
DMR1:255202001	1	255202001	255203000	1000	1	7.90E-11	0.62	7	0.7	Hectd2	Proteolysis
DMR1:255208001	1	255208001	255211000	3000	1	5.90E-08	-0.42	20	0.67	Hectd2	Proteolysis
DMR1:255222001	1	255222001	255226000	4000	1	9.50E-09	-0.54	26	0.65	Hectd2	Proteolysis
DMR1:255248001	1	255248001	255250000	2000	1	1.10E-07	0.38	12	0.6	Hectd2	Proteolysis
DMR1:255382001	1	255382001	255388000	6000	4	1.40E-15	-0.49	39	0.65	Ppp1r3c	Signaling
DMR1:255558001	1	255558001	255561000	3000	1	3.30E-09	0.55	18	0.6	Fgfbp3;Btaf1	Epigenetic
DMR1:255603001	1	255603001	255604000	1000	1	1.70E-10	0.58	3	0.3	Btaf1	Epigenetic
DMR1:255615001	1	255615001	255617000	2000	1	3.40E-09	-0.42	20	1	Btaf1	Epigenetic
DMR1:255618001	1	255618001	255622000	4000	1	2.90E-07	-0.57	38	0.95	Btaf1	Epigenetic
DMR1:255639001	1	255639001	255641000	2000	1	7.40E-07	-0.41	13	0.65	Btaf1	Epigenetic
DMR1:255645001	1	255645001	255651000	6000	1	1.40E-08	0.48	86	1.43	Btaf1;Cpeb3	Epigenetic;Translation
DMR1:255687001	1	255687001	255689000	2000	1	1.20E-08	-0.6	27	1.35	Cpeb3	Translation
DMR1:255706001	1	255706001	255709000	3000	2	1.80E-11	0.68	36	1.2	Cpeb3	Translation
DMR1:255717001	1	255717001	255721000	4000	1	1.90E-07	0.31	61	1.52	Cpeb3	Translation
DMR1:255732001	1	255732001	255737000	5000	3	7.10E-28	1.42	50	1	Cpeb3	Translation
DMR1:255739001	1	255739001	255742000	3000	1	8.10E-08	0.32	31	1.03	Cpeb3	Translation
DMR1:255760001	1	255760001	255762000	2000	1	1.50E-07	-0.47	23	1.15	Cpeb3	Translation
DMR1:255780001	1	255780001	255784000	4000	1	7.20E-13	0.44	48	1.2	Cpeb3	Translation
DMR1:255787001	1	255787001	255790000	3000	1	2.80E-07	0.49	22	0.73	Cpeb3	Translation
DMR1:255793001	1	255793001	255797000	4000	1	1.50E-12	0.58	50	1.25	Cpeb3;LOC108349460	Translation
DMR1:255799001	1	255799001	255803000	4000	1	3.50E-08	0.41	66	1.65	Cpeb3;LOC108349460	Translation
DMR1:255903001	1	255903001	255905000	2000	2	2.80E-11	0.45	21	1.05	LOC102549247;Ide	Protease
DMR1:255906001	1	255906001	255910000	4000	2	3.70E-13	-0.47	41	1.02	LOC102549247;Ide	Protease
DMR1:255913001	1	255913001	255914000	1000	1	1.30E-09	0.38	17	1.7	LOC102549247;Ide	Protease
DMR1:255927001	1	255927001	255930000	3000	2	1.50E-15	0.62	70	2.33	Ide	Protease
DMR1:255941001	1	255941001	255942000	1000	1	8.10E-11	0.66	6	0.6	Ide;LOC103691353	Protease
DMR1:255976001	1	255976001	255977000	1000	1	9.10E-18	0.52	9	0.9	Ide	Protease
DMR1:255979001	1	255979001	255988000	9000	3	8.80E-14	-0.44	101	1.12	Ide	Protease
DMR1:256000001	1	256000001	256002000	2000	1	2.00E-12	0.56	27	1.35	Ide	Protease
DMR1:256003001	1	256003001	256006000	3000	2	4.60E-14	0.78	23	0.77	Ide	Protease
DMR1:256007001	1	256007001	256012000	5000	1	1.00E-17	0.61	53	1.06	Ide	Protease
DMR1:256017001	1	256017001	256022000	5000	1	1.40E-08	0.42	59	1.18	Ide	Protease
DMR1:256040001	1	256040001	256042000	2000	1	1.50E-17	0.76	15	0.75	Kif11	Cytoskeleton
DMR1:256051001	1	256051001	256058000	7000	5	1.60E-31	-0.61	84	1.2	Kif11	Cytoskeleton
DMR1:256063001	1	256063001	256067000	4000	1	1.70E-09	0.52	36	0.9	Kif11	Cytoskeleton
DMR1:256106001	1	256106001	256111000	5000	1	4.70E-07	0.29	77	1.54	Hhex	Development
DMR1:256112001	1	256112001	256114000	2000	1	3.60E-13	0.4	22	1.1	Hhex	Development
DMR1:256115001	1	256115001	256116000	1000	1	2.40E-17	0.9	9	0.9	Hhex	Development
DMR1:256205001	1	256205001	256210000	5000	2	7.10E-16	0.58	76	1.52	Exoc6	Transport
DMR1:256211001	1	256211001	256215000	4000	2	6.20E-14	0.66	60	1.5	Exoc6	Transport
DMR1:256233001	1	256233001	256239000	6000	1	1.60E-07	0.33	52	0.87	Exoc6	Transport
DMR1:256242001	1	256242001	256244000	2000	1	2.60E-13	0.65	15	0.75	Exoc6	Transport
DMR1:256245001	1	256245001	256249000	4000	2	2.10E-18	-0.53	60	1.5	Exoc6	Transport
DMR1:256264001	1	256264001	256265000	1000	1	1.10E-12	0.47	8	0.8	Exoc6	Transport
DMR1:256266001	1	256266001	256267000	1000	1	1.50E-09	0.42	10	1	Exoc6	Transport
DMR1:256268001	1	256268001	256270000	2000	1	8.90E-07	-0.43	23	1.15	Exoc6	Transport
DMR1:256271001	1	256271001	256273000	2000	1	7.30E-10	0.63	27	1.35	Exoc6	Transport
DMR1:256274001	1	256274001	256276000	2000	1	4.50E-13	0.47	19	0.95	Exoc6	Transport
DMR1:256280001	1	256280001	256282000	2000	1	2.40E-08	0.47	20	1	Exoc6	Transport
DMR1:256289001	1	256289001	256292000	3000	1	7.80E-12	-0.5	22	0.73	Exoc6	Transport
DMR1:256295001	1	256295001	256296000	1000	1	1.00E-17	0.59	10	1	Exoc6	Transport

DMR1:256299001	1	256299001	256302000	3000	2	1.80E-14	0.51	33	1.1	Exoc6	Transport
DMR1:256311001	1	256311001	256314000	3000	1	1.40E-07	0.3	34	1.13	Exoc6	Transport
DMR1:256315001	1	256315001	256322000	7000	5	1.00E-13	-0.64	64	0.91	Exoc6	Transport
DMR1:256324001	1	256324001	256327000	3000	2	1.80E-09	0.5	36	1.2	Exoc6	Transport
DMR1:256336001	1	256336001	256338000	2000	1	3.40E-11	0.46	22	1.1	Exoc6	Transport
DMR1:256345001	1	256345001	256350000	5000	1	5.20E-12	0.52	71	1.42	Exoc6	Transport
DMR1:256353001	1	256353001	256354000	1000	1	7.10E-08	0.47	9	0.9	Exoc6	Transport
DMR1:256359001	1	256359001	256363000	4000	2	2.10E-21	0.89	40	1	Exoc6;Cyp26c1	Transport;Metabolism
DMR1:256374001	1	256374001	256375000	1000	1	2.40E-14	0.5	19	1.9	Exoc6;Cyp26c1;LOC102549441;Cyp26a1	Transport;Metabolism
DMR1:256389001	1	256389001	256396000	7000	1	2.70E-08	0.48	103	1.47	Cyp26c1;LOC102549441;Cyp26a1	Metabolism
DMR1:256444001	1	256444001	256446000	2000	1	6.10E-19	0.9	22	1.1	RGD1562542	
DMR1:256447001	1	256447001	256452000	5000	1	1.80E-08	-0.61	61	1.22	RGD1562542	
DMR1:256588001	1	256588001	256594000	6000	3	2.60E-19	0.67	100	1.67	Myof	Transport
DMR1:256603001	1	256603001	256604000	1000	1	2.60E-09	0.35	21	2.1	Myof	Transport
DMR1:256605001	1	256605001	256609000	4000	2	3.10E-09	-0.57	73	1.82	Myof	Transport
DMR1:256611001	1	256611001	256619000	8000	2	4.80E-13	0.64	110	1.38	Myof	Transport
DMR1:256620001	1	256620001	256622000	2000	1	5.10E-07	-0.54	15	0.75	Myof	Transport
DMR1:256636001	1	256636001	256637000	1000	1	1.00E-07	0.39	14	1.4	Myof	Transport
DMR1:256638001	1	256638001	256642000	4000	1	2.80E-08	0.43	49	1.23	Myof	Transport
DMR1:256644001	1	256644001	256646000	2000	1	4.00E-07	0.37	35	1.75	Myof	Transport
DMR1:256654001	1	256654001	256656000	2000	2	9.40E-18	0.84	15	0.75	Myof	Transport
DMR1:256667001	1	256667001	256670000	3000	2	2.30E-12	0.57	26	0.87	Myof	Transport
DMR1:256671001	1	256671001	256678000	7000	2	1.20E-09	-0.51	86	1.23	Myof	Transport
DMR1:256713001	1	256713001	256717000	4000	1	9.20E-07	-0.56	44	1.1	Myof;LOC100911555	Transport
DMR1:256718001	1	256718001	256720000	2000	1	3.70E-10	0.49	22	1.1	Myof;LOC100911555	Transport
DMR1:256721001	1	256721001	256725000	4000	1	9.10E-09	0.45	48	1.2	Myof;LOC100911555;LOC108349463	Transport
DMR1:256726001	1	256726001	256727000	1000	1	7.70E-10	0.47	11	1.1	Myof;LOC108349463	Transport
DMR1:256728001	1	256728001	256731000	3000	2	2.60E-08	-0.45	37	1.23	Myof;LOC108349463	Transport
DMR1:256732001	1	256732001	256734000	2000	1	1.00E-10	0.59	29	1.45	Myof;LOC108349463	Transport
DMR1:256741001	1	256741001	256743000	2000	1	2.00E-07	0.36	25	1.25	Myof;LOC108349463;Cep55	Transport
DMR1:256751001	1	256751001	256753000	2000	1	5.20E-09	0.5	18	0.9	Cep55	
DMR1:256789001	1	256789001	256794000	5000	2	3.10E-15	0.61	46	0.92	Ffar4	Signaling
DMR1:256819001	1	256819001	256820000	1000	1	1.60E-17	0.94	6	0.6	Rbp4;Pde6c	Transport;Signaling
DMR1:256866001	1	256866001	256870000	4000	3	2.40E-16	-0.56	77	1.93	Pde6c	Signaling
DMR1:256872001	1	256872001	256878000	6000	2	8.40E-14	0.58	78	1.3	Pde6c;Fra10ac1	Signaling;Signaling
DMR1:256900001	1	256900001	256903000	3000	2	1.90E-14	0.58	102	3.4	Fra10ac1	Signaling
DMR1:256929001	1	256929001	256931000	2000	2	6.00E-13	0.59	29	1.45	RGD1561251	
DMR1:256940001	1	256940001	256943000	3000	1	4.40E-10	0.46	40	1.33	RGD1561251	
DMR1:256967001	1	256967001	256968000	1000	1	3.70E-18	0.69	13	1.3	Lgi1;LOC103691354	
DMR1:256969001	1	256969001	256972000	3000	1	1.60E-07	0.33	39	1.3	Lgi1;LOC103691354	
DMR1:256975001	1	256975001	256978000	3000	1	1.10E-07	-0.6	38	1.27	Lgi1	
DMR1:256983001	1	256983001	256984000	1000	1	2.60E-18	0.67	12	1.2	Lgi1	
DMR1:257081001	1	257081001	257082000	1000	1	8.50E-08	-0.59	10	1	Slc35g1	Transport
DMR1:257090001	1	257090001	257094000	4000	2	1.00E-13	0.57	48	1.2	Slc35g1	Transport
DMR1:257144001	1	257144001	257147000	3000	2	5.00E-12	0.48	33	1.1	Plce1	Metabolism
DMR1:257150001	1	257150001	257153000	3000	1	7.80E-09	0.49	39	1.3	Plce1	Metabolism
DMR1:257176001	1	257176001	257178000	2000	1	2.80E-17	0.56	26	1.3	Plce1	Metabolism
DMR1:257184001	1	257184001	257186000	2000	1	2.10E-07	0.42	18	0.9	Plce1	Metabolism
DMR1:257187001	1	257187001	257191000	4000	1	3.50E-17	0.68	42	1.05	Plce1	Metabolism
DMR1:257199001	1	257199001	257202000	3000	1	1.80E-12	0.55	45	1.5	Plce1	Metabolism
DMR1:257206001	1	257206001	257209000	3000	1	3.50E-07	-0.39	29	0.97	Plce1	Metabolism
DMR1:257213001	1	257213001	257218000	5000	3	1.10E-14	-0.6	68	1.36	Plce1;LOC102550070	Metabolism
DMR1:257221001	1	257221001	257222000	1000	1	6.30E-13	0.46	20	2	Plce1;LOC102550070	Metabolism
DMR1:257227001	1	257227001	257231000	4000	1	1.90E-13	0.45	43	1.07	Plce1;LOC102550070	Metabolism
DMR1:257232001	1	257232001	257233000	1000	1	4.60E-11	0.5	11	1.1	Plce1;LOC102550070	Metabolism
DMR1:257241001	1	257241001	257244000	3000	2	2.60E-24	1.07	27	0.9	Plce1	Metabolism
DMR1:257247001	1	257247001	257250000	3000	1	8.20E-13	-0.67	50	1.67	Plce1	Metabolism
DMR1:257253001	1	257253001	257255000	2000	1	2.20E-07	-0.6	35	1.75	Plce1	Metabolism
DMR1:257257001	1	257257001	257262000	5000	1	6.50E-09	0.38	74	1.48	Plce1	Metabolism
DMR1:257266001	1	257266001	257270000	4000	1	1.40E-10	0.43	49	1.23	Plce1	Metabolism
DMR1:257271001	1	257271001	257274000	3000	1	9.70E-07	0.36	53	1.77	Plce1	Metabolism

DMR1:257282001	1	257282001	257284000	2000	1	6.30E-08	0.47	11	0.55	Plce1	Metabolism
DMR1:257286001	1	257286001	257288000	2000	1	4.20E-08	-0.54	29	1.45	Plce1	Metabolism
DMR1:257303001	1	257303001	257304000	1000	1	3.10E-08	0.39	15	1.5	Plce1	Metabolism
DMR1:257325001	1	257325001	257326000	1000	1	4.90E-08	0.56	6	0.6	Plce1	Metabolism
DMR1:257333001	1	257333001	257335000	2000	1	8.60E-09	0.41	26	1.3	Plce1	Metabolism
DMR1:257336001	1	257336001	257337000	1000	1	5.10E-07	0.32	14	1.4	Plce1	Metabolism
DMR1:257364001	1	257364001	257366000	2000	1	2.60E-13	0.47	16	0.8	Plce1	Metabolism
DMR1:257368001	1	257368001	257369000	1000	1	2.20E-17	0.5	13	1.3	Plce1	Metabolism
DMR1:257372001	1	257372001	257386000	14000	6	1.10E-15	0.85	135	0.96	Plce1	Metabolism
DMR1:257388001	1	257388001	257395000	7000	1	1.40E-21	0.77	104	1.49	Plce1	Metabolism
DMR1:257408001	1	257408001	257413000	5000	2	3.00E-15	-0.79	27	0.54	Plce1	Metabolism
DMR1:257419001	1	257419001	257420000	1000	1	7.40E-09	0.44	5	0.5	Plce1	Metabolism
DMR1:257431001	1	257431001	257433000	2000	1	5.00E-16	0.87	21	1.05	Plce1	Metabolism
DMR1:257436001	1	257436001	257439000	3000	1	2.40E-19	0.79	50	1.67	Plce1	Metabolism
DMR1:257456001	1	257456001	257457000	1000	1	5.70E-08	0.56	4	0.4	Plce1	Metabolism
DMR1:257462001	1	257462001	257465000	3000	1	9.20E-07	-0.38	44	1.47	Plce1;Noc3l	Metabolism
DMR1:257467001	1	257467001	257469000	2000	1	7.10E-07	0.31	24	1.2	Plce1;Noc3l	Metabolism
DMR1:257492001	1	257492001	257496000	4000	1	2.70E-13	0.55	41	1.02	Noc3l	
DMR1:257500001	1	257500001	257503000	3000	1	5.90E-10	0.4	22	0.73	Noc3l	
DMR1:257506001	1	257506001	257507000	1000	1	2.70E-07	0.46	14	1.4	Noc3l	
DMR1:257526001	1	257526001	257527000	1000	1	1.90E-07	0.55	5	0.5	Tbc1d12	Signaling
DMR1:257539001	1	257539001	257540000	1000	1	3.40E-07	0.4	15	1.5	Tbc1d12	Signaling
DMR1:257553001	1	257553001	257555000	2000	1	6.70E-19	0.94	14	0.7	Tbc1d12	Signaling
DMR1:257583001	1	257583001	257584000	1000	1	3.10E-09	0.54	13	1.3	Tbc1d12	Signaling
DMR1:257608001	1	257608001	257609000	1000	1	4.40E-08	0.58	6	0.6	Tbc1d12;LOC100911660;LOC108349465	Signaling
DMR1:257677001	1	257677001	257678000	1000	1	2.90E-11	0.73	5	0.5	LOC685956;Cyp2c11	Metabolism
DMR1:257680001	1	257680001	257681000	1000	1	3.60E-08	0.64	4	0.4	LOC685956;Cyp2c11	Metabolism
DMR1:257687001	1	257687001	257688000	1000	1	9.50E-09	0.54	5	0.5	Cyp2c11;LOC100911914	Metabolism
DMR1:257733001	1	257733001	257735000	2000	1	1.40E-08	-0.44	6	0.3	Cyp2c11;LOC100911914;LOC100911865	Metabolism
DMR1:257736001	1	257736001	257738000	2000	1	1.40E-10	0.72	17	0.85	Cyp2c11;LOC100911914;LOC100911865	Metabolism
DMR1:257840001	1	257840001	257841000	1000	1	5.60E-11	0.72	8	0.8	Cyp2c11;LOC685933	Metabolism
DMR1:257869001	1	257869001	257870000	1000	1	7.00E-07	0.47	12	1.2	Cyp2c11;LOC685933	Metabolism
DMR1:257888001	1	257888001	257889000	1000	1	2.60E-10	-0.58	2	0.2	Cyp2c11;LOC685933	Metabolism
DMR1:257895001	1	257895001	257896000	1000	1	4.80E-08	0.59	7	0.7	Cyp2c11;LOC685933;Hells	Metabolism
DMR1:257918001	1	257918001	257919000	1000	1	2.00E-08	0.61	2	0.2	Cyp2c11;Hells;LOC108349702	Metabolism
DMR1:257943001	1	257943001	257944000	1000	1	1.30E-16	0.99	6	0.6	Cyp2c11;Hells	Metabolism
DMR1:257972001	1	257972001	257973000	1000	1	9.90E-14	0.8	5	0.5	Cyp2c11;LOC108348258	Metabolism
DMR1:257978001	1	257978001	257979000	1000	1	5.10E-07	0.54	3	0.3	Cyp2c11	Metabolism
DMR1:257983001	1	257983001	257985000	2000	1	7.30E-07	-0.43	5	0.25	Cyp2c11	Metabolism
DMR1:257990001	1	257990001	257992000	2000	1	1.70E-11	-0.83	18	0.9	Cyp2c11	Metabolism
DMR1:257993001	1	257993001	257997000	4000	2	3.60E-15	-0.48	37	0.92	Cyp2c11	Metabolism
DMR1:258067001	1	258067001	258073000	6000	2	8.00E-11	0.51	54	0.9	Cyp2c24	Metabolism
DMR1:258091001	1	258091001	258092000	1000	1	3.10E-07	-0.25	21	2.1	Cyp2c24	Metabolism
DMR1:258103001	1	258103001	258104000	1000	1	7.10E-08	-0.61	3	0.3	Cyp2c24	Metabolism
DMR1:258105001	1	258105001	258109000	4000	2	2.80E-12	0.79	16	0.4	Cyp2c24	Metabolism
DMR1:258111001	1	258111001	258112000	1000	1	1.20E-07	-0.56	3	0.3	Cyp2c24	Metabolism
DMR1:258124001	1	258124001	258127000	3000	3	9.00E-10	-0.57	31	1.03	Cyp2c24;LOC108349703	Metabolism
DMR1:258142001	1	258142001	258143000	1000	1	3.00E-07	-0.61	1	0.1	Cyp2c24;LOC108349703	Metabolism
DMR1:258723001	1	258723001	258724000	1000	1	3.20E-13	0.72	7	0.7	Cyp2c12	Metabolism
DMR1:258739001	1	258739001	258743000	4000	2	2.70E-27	0.87	16	0.4	Cyp2c12	Metabolism
DMR1:258772001	1	258772001	258774000	2000	1	5.10E-13	-0.42	19	0.95	Cyp2c12	Metabolism
DMR1:258784001	1	258784001	258789000	5000	2	8.90E-15	-0.62	39	0.78	Cyp2c13	Metabolism
DMR1:258805001	1	258805001	258807000	2000	2	1.50E-08	-0.43	19	0.95	Cyp2c13	Metabolism
DMR1:258821001	1	258821001	258829000	8000	3	3.10E-11	-0.44	72	0.9	Cyp2c13	Metabolism
DMR1:258834001	1	258834001	258838000	4000	1	1.50E-10	-0.48	27	0.68	Cyp2c13	Metabolism
DMR1:258845001	1	258845001	258851000	6000	3	6.10E-16	-0.6	60	1	Cyp2c13	Metabolism
DMR1:258852001	1	258852001	258853000	1000	1	1.10E-18	-0.78	3	0.3	Cyp2c13	Metabolism
DMR1:258854001	1	258854001	258859000	5000	2	1.30E-11	-0.45	51	1.02	Cyp2c13	Metabolism
DMR1:258861001	1	258861001	258865000	4000	1	1.90E-08	0.42	32	0.8	Cyp2c13	Metabolism
DMR1:258867001	1	258867001	258868000	1000	1	1.90E-10	0.61	4	0.4	Cyp2c13	Metabolism
DMR1:258869001	1	258869001	258870000	1000	1	1.20E-07	0.51	3	0.3	Cyp2c13	Metabolism
DMR1:259252001	1	259252001	259253000	1000	1	1.80E-17	0.92	7	0.7	Cyp2c22	

DMR1:259258001	1	259258001	259259000	1000	1	6.40E-15	0.68	4	0.4	Cyp2c22	
DMR1:259268001	1	259268001	259269000	1000	1	3.30E-15	0.55	8	0.8	Cyp2c22	
DMR1:259277001	1	259277001	259283000	6000	1	3.90E-10	0.4	62	1.03	Cyp2c22	
DMR1:259286001	1	259286001	259288000	2000	2	4.20E-07	-0.56	15	0.75	Cyp2c22	
DMR1:259296001	1	259296001	259303000	7000	1	5.80E-13	-0.59	46	0.66	Cyp2c22;Pdlim1	Cytoskeleton
DMR1:259338001	1	259338001	259339000	1000	1	2.60E-27	1.17	5	0.5	Pdlim1	Cytoskeleton
DMR1:259344001	1	259344001	259346000	2000	1	4.00E-11	0.81	12	0.6	Pdlim1	Cytoskeleton
DMR1:259347001	1	259347001	259353000	6000	2	5.40E-18	0.73	71	1.18	Pdlim1	Cytoskeleton
DMR1:259365001	1	259365001	259366000	1000	1	2.20E-10	0.52	3	0.3	Pdlim1;Sorbs1	Cytoskeleton
DMR1:259378001	1	259378001	259385000	7000	2	1.00E-13	0.47	92	1.31	Sorbs1	
DMR1:259393001	1	259393001	259396000	3000	2	3.40E-13	-0.56	54	1.8	Sorbs1	
DMR1:259420001	1	259420001	259424000	4000	3	2.20E-11	-0.62	54	1.35	Sorbs1	
DMR1:259439001	1	259439001	259442000	3000	1	1.80E-07	0.51	27	0.9	Sorbs1	
DMR1:259446001	1	259446001	259449000	3000	1	2.20E-07	0.38	49	1.63	Sorbs1	
DMR1:259462001	1	259462001	259471000	9000	2	3.40E-07	0.33	163	1.81	Sorbs1	
DMR1:259478001	1	259478001	259484000	6000	1	4.10E-08	-0.7	100	1.67	Sorbs1	
DMR1:259490001	1	259490001	259492000	2000	1	9.80E-19	0.6	25	1.25	Sorbs1	
DMR1:259630001	1	259630001	259636000	6000	4	2.90E-14	-0.55	64	1.07	Aldh18a1	Metabolism
DMR1:259637001	1	259637001	259638000	1000	1	2.60E-07	0.49	8	0.8	Aldh18a1	Metabolism
DMR1:259651001	1	259651001	259656000	5000	2	1.30E-11	0.78	99	1.98	Aldh18a1	Metabolism
DMR1:259694001	1	259694001	259698000	4000	2	1.50E-14	0.89	60	1.5	Tctn3;Entpd1;LOC100361643	Signaling
DMR1:259699001	1	259699001	259700000	1000	1	2.10E-07	0.65	8	0.8	Tctn3;Entpd1;LOC100361643	Signaling
DMR1:259703001	1	259703001	259704000	1000	1	2.20E-07	0.49	10	1	Entpd1;LOC100361643	Signaling
DMR1:259725001	1	259725001	259726000	1000	1	2.70E-20	0.93	17	1.7	Entpd1	Signaling
DMR1:259751001	1	259751001	259754000	3000	1	2.00E-08	0.42	32	1.07	Entpd1	Signaling
DMR1:259755001	1	259755001	259758000	3000	1	4.00E-23	0.75	46	1.53	Entpd1	Signaling
DMR1:259773001	1	259773001	259776000	3000	1	1.20E-13	0.65	32	1.07	Entpd1	Signaling
DMR1:259782001	1	259782001	259783000	1000	1	9.50E-08	0.42	16	1.6	Entpd1	Signaling
DMR1:259785001	1	259785001	259786000	1000	1	3.10E-11	0.63	17	1.7	Entpd1	Signaling
DMR1:259812001	1	259812001	259814000	2000	1	4.00E-12	-0.48	26	1.3	Entpd1	Signaling
DMR1:259916001	1	259916001	259917000	1000	1	1.10E-07	0.67	8	0.8	LOC102555476;Ccnj	Signaling
DMR1:259943001	1	259943001	259945000	2000	1	1.40E-07	0.41	21	1.05	Ccnj	Signaling
DMR1:259952001	1	259952001	259953000	1000	1	2.00E-08	0.48	15	1.5	Ccnj;LOC103689954	Signaling;Signaling
DMR1:259954001	1	259954001	259956000	2000	1	2.50E-13	0.7	31	1.55	Ccnj;LOC103689954	Signaling;Signaling
DMR1:260001001	1	260001001	260002000	1000	1	2.30E-15	0.74	4	0.4	Cc2d2b	
DMR1:260049001	1	260049001	260051000	2000	1	8.00E-09	-0.61	28	1.4	Cc2d2b	
DMR1:260070001	1	260070001	260074000	4000	3	6.20E-16	0.96	43	1.07	Cc2d2b	
DMR1:260077001	1	260077001	260083000	6000	3	1.40E-16	0.57	81	1.35	Cc2d2b;Ccnj	Signaling
DMR1:260091001	1	260091001	260092000	1000	1	3.90E-10	0.53	21	2.1	Cc2d2b;Ccnj	Signaling
DMR1:260109001	1	260109001	260112000	3000	1	7.10E-08	0.4	48	1.6	Ccnj	Signaling
DMR1:260116001	1	260116001	260121000	5000	1	1.30E-18	0.72	43	0.86	Ccnj	Signaling
DMR1:260157001	1	260157001	260160000	3000	2	8.60E-10	-0.44	19	0.63	Zfp518a	
DMR1:260211001	1	260211001	260212000	1000	1	8.80E-12	0.44	7	0.7	Blnk	Cytoskeleton
DMR1:260225001	1	260225001	260226000	1000	1	1.50E-24	1.02	8	0.8	Blnk	Cytoskeleton
DMR1:260228001	1	260228001	260231000	3000	2	1.40E-18	0.9	24	0.8	Blnk	Cytoskeleton
DMR1:260252001	1	260252001	260258000	6000	2	3.10E-09	0.33	102	1.7	Blnk	Cytoskeleton
DMR1:260260001	1	260260001	260264000	4000	1	6.20E-09	0.32	48	1.2	Blnk	Cytoskeleton
DMR1:260284001	1	260284001	260291000	7000	1	3.40E-10	0.36	89	1.27	Dntt	Transcription
DMR1:260308001	1	260308001	260313000	5000	2	1.40E-19	0.85	50	1	Dntt	Transcription
DMR1:260319001	1	260319001	260321000	2000	1	5.90E-07	0.56	7	0.35	Dntt;Opalin	Transcription
DMR1:260327001	1	260327001	260331000	4000	1	6.90E-07	-0.62	61	1.52	Dntt;Opalin	Transcription
DMR1:260339001	1	260339001	260341000	2000	1	5.70E-10	0.42	27	1.35	Opalin;Tll2	Protease
DMR1:260349001	1	260349001	260354000	5000	1	7.60E-23	1.03	89	1.78	Opalin;Tll2	Protease
DMR1:260358001	1	260358001	260359000	1000	1	1.30E-17	0.91	5	0.5	Tll2	Protease
DMR1:260376001	1	260376001	260379000	3000	1	2.50E-07	0.28	72	2.4	Tll2	Protease
DMR1:260385001	1	260385001	260386000	1000	1	1.90E-07	0.38	4	0.4	Tll2	Protease
DMR1:260403001	1	260403001	260406000	3000	1	5.30E-08	-0.64	46	1.53	Tll2	Protease
DMR1:260412001	1	260412001	260415000	3000	2	4.40E-18	0.72	54	1.8	Tll2	Protease
DMR1:260456001	1	260456001	260457000	1000	1	8.20E-07	-0.62	19	1.9	Tll2;Tm9sf3	Protease;Transport
DMR1:260485001	1	260485001	260496000	11000	5	1.10E-19	-0.73	127	1.15	Tm9sf3	Transport
DMR1:260503001	1	260503001	260505000	2000	1	6.80E-09	0.51	18	0.9	Tm9sf3	Transport
DMR1:260521001	1	260521001	260523000	2000	2	6.00E-21	0.71	22	1.1	Tm9sf3;Pik3ap1	Transport
DMR1:260530001	1	260530001	260532000	2000	1	3.10E-13	0.61	19	0.95	Pik3ap1	
DMR1:260535001	1	260535001	260538000	3000	1	2.70E-14	0.49	59	1.97	Pik3ap1	
DMR1:260545001	1	260545001	260548000	3000	1	1.00E-07	-0.59	40	1.33	Pik3ap1	

DMR1:260554001	1	260554001	260556000	2000	1	3.10E-15	0.6	24	1.2	Pik3ap1	
DMR1:260569001	1	260569001	260570000	1000	1	3.30E-19	0.66	15	1.5	Pik3ap1	
DMR1:260576001	1	260576001	260582000	6000	1	3.00E-11	-0.65	133	2.22	Pik3ap1	
DMR1:260583001	1	260583001	260586000	3000	1	1.40E-15	0.97	19	0.63	Pik3ap1	
DMR1:260587001	1	260587001	260592000	5000	2	6.40E-14	0.49	97	1.94	Pik3ap1	
DMR1:260599001	1	260599001	260605000	6000	1	2.70E-14	0.61	106	1.77	Pik3ap1	
DMR1:260613001	1	260613001	260614000	1000	1	2.80E-08	0.58	5	0.5	Pik3ap1	
DMR1:260625001	1	260625001	260627000	2000	1	1.60E-08	0.39	22	1.1	Pik3ap1	
DMR1:260645001	1	260645001	260652000	7000	3	5.00E-18	-0.54	94	1.34	Pik3ap1	
DMR1:260656001	1	260656001	260658000	2000	1	9.30E-07	0.39	17	0.85	Pik3ap1	
DMR1:260670001	1	260670001	260677000	7000	1	9.20E-11	0.45	75	1.07	Pik3ap1	
DMR1:260679001	1	260679001	260684000	5000	2	5.10E-08	-0.64	66	1.32	Pik3ap1	
DMR1:260824001	1	260824001	260827000	3000	1	2.40E-07	-0.54	49	1.63	Lcor	
DMR1:260848001	1	260848001	260853000	5000	1	1.30E-10	1.14	59	1.18	Lcor;Slit1	
DMR1:260864001	1	260864001	260869000	5000	1	1.40E-20	0.68	80	1.6	Slit1	
DMR1:260888001	1	260888001	260890000	2000	2	2.30E-10	-0.41	18	0.9	Slit1	
DMR1:260895001	1	260895001	260896000	1000	1	1.40E-15	0.52	29	2.9	Slit1	
DMR1:260897001	1	260897001	260902000	5000	1	3.10E-11	0.64	67	1.34	Slit1	
DMR1:260903001	1	260903001	260905000	2000	1	7.20E-08	0.32	39	1.95	Slit1	
DMR1:260925001	1	260925001	260928000	3000	1	8.30E-11	-0.64	44	1.47	Slit1	
DMR1:260929001	1	260929001	260930000	1000	1	2.20E-08	-0.59	13	1.3	Slit1	
DMR1:260933001	1	260933001	260934000	1000	1	8.80E-07	0.33	9	0.9	Slit1	
DMR1:260940001	1	260940001	260946000	6000	2	1.30E-26	0.8	102	1.7	Slit1;LOC102553433	
DMR1:260947001	1	260947001	260952000	5000	2	5.30E-16	0.63	58	1.16	Slit1;LOC102553433	
DMR1:260964001	1	260964001	260965000	1000	1	6.60E-08	-0.41	28	2.8	Slit1	
DMR1:260982001	1	260982001	260987000	5000	2	4.30E-07	-0.38	65	1.3	Slit1	
DMR1:260997001	1	260997001	260999000	2000	1	4.00E-07	-0.54	46	2.3	Slit1	
DMR1:261007001	1	261007001	261015000	8000	1	1.20E-19	0.58	101	1.26	Arhgap19	Signaling
DMR1:261031001	1	261031001	261032000	1000	1	1.30E-08	-0.38	16	1.6	Arhgap19	Signaling
DMR1:261036001	1	261036001	261038000	2000	2	2.90E-18	0.9	18	0.9	Arhgap19	Signaling
DMR1:261060001	1	261060001	261067000	7000	1	4.10E-11	0.62	99	1.41	Arhgap19;LOC102553733;Frat1	Signaling
DMR1:261069001	1	261069001	261072000	3000	1	3.30E-09	0.54	24	0.8	LOC102553733;Frat1	
DMR1:261092001	1	261092001	261096000	4000	1	5.80E-07	0.36	54	1.35	Frat2	
DMR1:261122001	1	261122001	261125000	3000	1	4.20E-08	-0.54	48	1.6	Rrp12	
DMR1:261128001	1	261128001	261130000	2000	1	3.70E-07	-0.48	23	1.15	Rrp12	
DMR1:261141001	1	261141001	261143000	2000	1	5.30E-12	0.33	25	1.25	Rrp12	
DMR1:261149001	1	261149001	261150000	1000	1	8.40E-21	0.6	15	1.5	Rrp12;Pgam1	Metabolism
DMR1:261185001	1	261185001	261187000	2000	1	3.60E-07	-0.41	38	1.9	Exosc1;Zdhhc16;Mms19	Transcription;Golgi;Cytoskeleton
DMR1:261231001	1	261231001	261232000	1000	1	1.50E-12	0.59	11	1.1	Mms19;Ubtd1	Cytoskeleton;Proteolysis
DMR1:261235001	1	261235001	261237000	2000	1	1.10E-15	0.6	20	1	Mms19;Ubtd1	Cytoskeleton;Proteolysis
DMR1:261252001	1	261252001	261256000	4000	1	1.20E-15	0.65	33	0.82	Ubtd1	Proteolysis
DMR1:261260001	1	261260001	261262000	2000	2	3.00E-19	0.85	22	1.1	Ubtd1	Proteolysis
DMR1:261271001	1	261271001	261280000	9000	1	1.70E-07	-0.48	112	1.24	Ubtd1;Ankrd2	Proteolysis
DMR1:261282001	1	261282001	261284000	2000	1	1.60E-20	0.67	26	1.3	Ubtd1;Ankrd2;Hoga1	Proteolysis;Metabolism
DMR1:261305001	1	261305001	261306000	1000	1	1.60E-09	0.45	5	0.5	Hoga1;MGC93861	Metabolism
DMR1:261307001	1	261307001	261316000	9000	3	4.20E-21	0.84	114	1.27	Hoga1;MGC93861;Morn4	Metabolism
DMR1:261348001	1	261348001	261357000	9000	1	5.10E-08	0.38	93	1.03	Pi4k2a;Avpi1	Signaling
DMR1:261377001	1	261377001	261379000	2000	1	6.20E-08	0.43	16	0.8	Avpi1;LOC102554176	
DMR1:261406001	1	261406001	261408000	2000	2	1.60E-15	0.59	22	1.1	Zfyve27	Transport
DMR1:261410001	1	261410001	261411000	1000	1	6.60E-09	0.41	14	1.4	Zfyve27	Transport
DMR1:261422001	1	261422001	261426000	4000	1	5.60E-07	0.35	73	1.82	Zfyve27	Transport
DMR1:261432001	1	261432001	261434000	2000	1	8.90E-11	-0.71	45	2.25	Zfyve27;Sfrp5	Transport;Receptor
DMR1:261465001	1	261465001	261467000	2000	1	4.30E-20	0.92	13	0.65	RGD1563029	
DMR1:261468001	1	261468001	261471000	3000	1	7.40E-07	-0.5	76	2.53	RGD1563029	
DMR1:261493001	1	261493001	261496000	3000	2	1.10E-24	0.86	37	1.23	LOC102547392;Golga7b	
DMR1:261499001	1	261499001	261500000	1000	1	1.50E-20	0.69	18	1.8	LOC102547392;Golga7b	
DMR1:261501001	1	261501001	261503000	2000	1	5.60E-08	0.51	72	3.6	LOC102547392;Golga7b	
DMR1:261508001	1	261508001	261511000	3000	1	1.10E-10	0.44	60	2	Golga7b	
DMR1:261525001	1	261525001	261529000	4000	1	2.40E-09	0.39	80	2	Mir3085;Crtac1	
DMR1:261532001	1	261532001	261537000	5000	2	2.30E-12	0.47	69	1.38	Mir3085;Crtac1	
DMR1:261539001	1	261539001	261541000	2000	2	4.30E-13	0.46	42	2.1	Crtac1	

DMR1:261546001	1	261546001	261549000	3000	2	6.60E-17	0.68	61	2.03	Crtac1	
DMR1:261550001	1	261550001	261551000	1000	1	1.10E-13	0.44	11	1.1	Crtac1	
DMR1:261558001	1	261558001	261560000	2000	1	4.80E-10	0.45	33	1.65	Crtac1	
DMR1:261566001	1	261566001	261567000	1000	1	7.80E-25	0.7	36	3.6	Crtac1	
DMR1:261576001	1	261576001	261583000	7000	1	2.10E-23	0.82	138	1.97	Crtac1	
DMR1:261611001	1	261611001	261612000	1000	1	1.30E-17	0.62	20	2	Crtac1	
DMR1:261616001	1	261616001	261617000	1000	1	7.10E-08	-0.75	16	1.6	Crtac1	
DMR1:261625001	1	261625001	261629000	4000	1	1.30E-09	0.57	68	1.7	Crtac1	
DMR1:261633001	1	261633001	261637000	4000	1	5.30E-12	0.44	67	1.68	Crtac1	
DMR1:261638001	1	261638001	261649000	11000	3	2.90E-19	0.77	198	1.8	Crtac1	
DMR1:261654001	1	261654001	261656000	2000	2	5.80E-08	0.35	30	1.5	Crtac1	
DMR1:261671001	1	261671001	261676000	5000	1	9.60E-09	0.38	76	1.52	Crtac1	
DMR1:261678001	1	261678001	261679000	1000	1	1.50E-08	0.48	7	0.7	Crtac1	
DMR1:261758001	1	261758001	261759000	1000	1	9.60E-09	0.49	8	0.8	R3hcc1l	
DMR1:261769001	1	261769001	261770000	1000	1	7.90E-09	-0.63	8	0.8	R3hcc1l	
DMR1:261784001	1	261784001	261786000	2000	2	2.70E-08	0.47	15	0.75	R3hcc1l	
DMR1:261790001	1	261790001	261793000	3000	1	4.50E-10	-0.72	29	0.97	R3hcc1l	
DMR1:261826001	1	261826001	261828000	2000	1	4.20E-07	-0.42	18	0.9	R3hcc1l;Loxl4	Metabolism
DMR1:261850001	1	261850001	261851000	1000	1	1.80E-12	0.47	15	1.5	Loxl4	Metabolism
DMR1:261856001	1	261856001	261858000	2000	1	2.00E-11	0.4	37	1.85	Loxl4;LOC102547572	Metabolism
DMR1:261954001	1	261954001	261956000	2000	1	1.40E-12	0.59	29	1.45	Pyroxd2	Metabolism
DMR1:261964001	1	261964001	261969000	5000	1	4.00E-09	0.33	67	1.34	Pyroxd2	Metabolism
DMR1:261971001	1	261971001	261973000	2000	1	3.00E-09	0.47	40	2	Pyroxd2	Metabolism
DMR1:261979001	1	261979001	261981000	2000	1	2.10E-07	0.47	29	1.45	Pyroxd2;Hps1	Metabolism
DMR1:261996001	1	261996001	262003000	7000	1	9.90E-09	0.35	138	1.97	Pyroxd2;Hps1	Metabolism
DMR1:262004001	1	262004001	262014000	10000	2	1.80E-21	0.94	161	1.61	Hps1;LOC108349471;Hpse2	
DMR1:262017001	1	262017001	262018000	1000	1	9.60E-08	-0.58	12	1.2	Hps1;LOC108349471;Hpse2	
DMR1:262079001	1	262079001	262085000	6000	2	1.70E-12	0.51	58	0.97	Hpse2	
DMR1:262100001	1	262100001	262103000	3000	2	2.50E-18	0.96	33	1.1	Hpse2	
DMR1:262118001	1	262118001	262119000	1000	1	3.70E-07	-0.43	12	1.2	Hpse2	
DMR1:262129001	1	262129001	262132000	3000	1	6.70E-08	0.53	22	0.73	Hpse2	
DMR1:262134001	1	262134001	262135000	1000	1	4.00E-08	0.43	5	0.5	Hpse2	
DMR1:262141001	1	262141001	262142000	1000	1	1.20E-21	0.85	11	1.1	Hpse2	
DMR1:262147001	1	262147001	262150000	3000	1	6.50E-08	-0.51	55	1.83	Hpse2	
DMR1:262154001	1	262154001	262155000	1000	1	9.80E-07	-0.62	17	1.7	Hpse2	
DMR1:262158001	1	262158001	262160000	2000	2	1.90E-29	0.89	23	1.15	Hpse2	
DMR1:262166001	1	262166001	262167000	1000	1	2.10E-10	0.54	8	0.8	Hpse2	
DMR1:262170001	1	262170001	262172000	2000	1	3.00E-16	0.68	15	0.75	Hpse2	
DMR1:262183001	1	262183001	262186000	3000	1	4.10E-09	0.42	23	0.77	Hpse2	
DMR1:262195001	1	262195001	262197000	2000	1	2.60E-11	0.54	11	0.55	Hpse2	
DMR1:262198001	1	262198001	262202000	4000	1	7.30E-13	-0.5	28	0.7	Hpse2	
DMR1:262219001	1	262219001	262221000	2000	1	4.00E-09	0.38	19	0.95	Hpse2;LOC102555060	
DMR1:262224001	1	262224001	262228000	4000	2	2.90E-20	0.98	28	0.7	Hpse2;LOC102555060;LOC108349826	
DMR1:262251001	1	262251001	262253000	2000	1	1.90E-12	0.55	24	1.2	Hpse2;LOC108349826	
DMR1:262290001	1	262290001	262293000	3000	1	1.80E-09	-0.51	43	1.43	Hpse2	
DMR1:262327001	1	262327001	262330000	3000	2	8.80E-19	-0.66	22	0.73	Hpse2	
DMR1:262336001	1	262336001	262342000	6000	5	8.70E-24	-0.48	68	1.13	Hpse2	
DMR1:262353001	1	262353001	262354000	1000	1	9.80E-09	0.76	6	0.6	Hpse2	
DMR1:262357001	1	262357001	262362000	5000	3	3.70E-12	0.68	47	0.94	Hpse2	
DMR1:262372001	1	262372001	262374000	2000	1	3.00E-07	-0.36	23	1.15	Hpse2	
DMR1:262388001	1	262388001	262394000	6000	2	3.20E-12	-0.41	86	1.43	Hpse2	
DMR1:262447001	1	262447001	262449000	2000	1	6.00E-15	0.65	24	1.2	Hpse2	
DMR1:262478001	1	262478001	262479000	1000	1	4.00E-08	0.5	14	1.4	Hpse2	
DMR1:262487001	1	262487001	262488000	1000	1	8.80E-07	-0.36	10	1	Hpse2	
DMR1:262512001	1	262512001	262519000	7000	3	4.20E-22	-0.59	78	1.11	Hpse2	
DMR1:262524001	1	262524001	262528000	4000	3	1.20E-17	-0.66	47	1.18	Hpse2	
DMR1:262534001	1	262534001	262540000	6000	4	5.70E-18	-0.52	66	1.1	Hpse2	
DMR1:262546001	1	262546001	262547000	1000	1	7.90E-07	0.55	10	1	Hpse2	
DMR1:262552001	1	262552001	262553000	1000	1	4.00E-09	0.71	9	0.9	Hpse2	
DMR1:262558001	1	262558001	262560000	2000	2	4.90E-17	0.79	23	1.15	Hpse2	
DMR1:262565001	1	262565001	262566000	1000	1	4.90E-07	0.53	9	0.9	Hpse2	
DMR1:262570001	1	262570001	262573000	3000	1	2.50E-11	-0.47	29	0.97	Hpse2	
DMR1:262589001	1	262589001	262593000	4000	3	2.30E-15	-0.46	57	1.43	Hpse2	
DMR1:262615001	1	262615001	262619000	4000	2	9.30E-16	-0.51	39	0.98	Hpse2	

DMR1:262620001	1	262620001	262627000	7000	5	9.60E-18	0.64	73	1.04	Hpse2	
DMR1:262630001	1	262630001	262633000	3000	2	2.60E-13	0.46	24	0.8	Hpse2	
DMR1:262644001	1	262644001	262646000	2000	1	8.30E-07	0.39	33	1.65	Hpse2	
DMR1:262655001	1	262655001	262656000	1000	1	1.10E-23	1.1	3	0.3	Hpse2	
DMR1:262661001	1	262661001	262665000	4000	1	5.10E-27	0.83	51	1.27	Hpse2	
DMR1:262678001	1	262678001	262683000	5000	2	2.90E-12	0.56	43	0.86	Hpse2	
DMR1:262724001	1	262724001	262729000	5000	3	1.40E-20	-0.52	40	0.8	Hpse2	
DMR1:262745001	1	262745001	262748000	3000	1	1.30E-15	0.63	24	0.8	Hpse2	
DMR1:262762001	1	262762001	262768000	6000	2	9.40E-15	-0.56	41	0.68	Hpse2	
DMR1:262773001	1	262773001	262778000	5000	1	1.50E-15	0.71	57	1.14	Hpse2	
DMR1:262791001	1	262791001	262792000	1000	1	5.40E-07	-0.39	5	0.5	Hpse2	
DMR1:262797001	1	262797001	262798000	1000	1	1.20E-07	0.41	9	0.9	Hpse2	
DMR1:262809001	1	262809001	262811000	2000	1	1.20E-10	-0.84	21	1.05	Hpse2	
DMR1:262830001	1	262830001	262832000	2000	1	2.30E-15	0.84	20	1	Hpse2	
DMR1:262884001	1	262884001	262885000	1000	1	7.60E-08	0.61	13	1.3	Hpse2;RGD1564887;LOC100911951	
DMR1:262904001	1	262904001	262906000	2000	1	6.80E-10	0.46	30	1.5	Hpse2;LOC100911951	
DMR1:262914001	1	262914001	262915000	1000	1	1.90E-10	0.39	24	2.4	Hpse2;LOC100911951;LOC108349825	
DMR1:262956001	1	262956001	262958000	2000	1	7.80E-13	0.51	38	1.9	Hpse2	
DMR1:262967001	1	262967001	262968000	1000	1	9.80E-15	0.76	4	0.4	Hpse2	
DMR1:262996001	1	262996001	262997000	1000	1	7.10E-17	0.66	13	1.3	Hpse2;LOC100911700;LOC100911779	
DMR1:263007001	1	263007001	263008000	1000	1	7.90E-07	0.37	16	1.6	Hpse2;LOC100911700;LOC100911779	
DMR1:263025001	1	263025001	263027000	2000	1	6.90E-17	-0.48	14	0.7	Hpse2;LOC100911779;LOC100911855	
DMR1:263028001	1	263028001	263029000	1000	1	1.80E-08	0.46	10	1	Hpse2;LOC100911855	
DMR1:263059001	1	263059001	263062000	3000	2	8.30E-17	-0.5	23	0.77	Hpse2;LOC100911855	
DMR1:263065001	1	263065001	263068000	3000	2	3.70E-09	0.48	19	0.63	Hpse2	
DMR1:263069001	1	263069001	263072000	3000	2	1.30E-12	-0.47	27	0.9	Hpse2	
DMR1:263084001	1	263084001	263088000	4000	2	1.10E-10	0.53	54	1.35	Hpse2	
DMR1:263092001	1	263092001	263094000	2000	1	7.80E-08	0.55	10	0.5	Hpse2	
DMR1:263095001	1	263095001	263098000	3000	1	1.30E-17	0.75	25	0.83	Hpse2	
DMR1:263105001	1	263105001	263115000	10000	4	1.60E-16	0.83	67	0.67	Hpse2	
DMR1:263190001	1	263190001	263196000	6000	1	4.00E-07	0.4	56	0.93	Cnnm1	
DMR1:263211001	1	263211001	263212000	1000	1	1.70E-07	-0.54	15	1.5	Cnnm1	
DMR1:263215001	1	263215001	263216000	1000	1	1.10E-11	0.55	9	0.9	Cnnm1	
DMR1:263228001	1	263228001	263229000	1000	1	1.30E-10	0.42	14	1.4	Cnnm1	
DMR1:263230001	1	263230001	263234000	4000	1	5.70E-07	0.36	66	1.65	Cnnm1	
DMR1:263258001	1	263258001	263259000	1000	1	2.80E-08	0.35	19	1.9	Got1	Metabolism
DMR1:263278001	1	263278001	263281000	3000	1	5.80E-07	-0.56	45	1.5	Got1;LOC102546432	Metabolism
DMR1:263357001	1	263357001	263358000	1000	1	2.90E-09	0.5	12	1.2	Nkx2-3	Development
DMR1:263376001	1	263376001	263377000	1000	1	3.60E-16	0.76	5	0.5	Nkx2-3	Development
DMR1:263418001	1	263418001	263422000	4000	1	3.40E-08	0.38	79	1.98	Slc25a28	
DMR1:263441001	1	263441001	263443000	2000	1	1.20E-18	0.58	38	1.9	Slc25a28;Entpd7	Signaling
DMR1:263444001	1	263444001	263445000	1000	1	1.20E-09	0.47	11	1.1	Entpd7	Signaling
DMR1:263446001	1	263446001	263447000	1000	1	4.80E-17	0.44	6	0.6	Entpd7	Signaling
DMR1:263452001	1	263452001	263459000	7000	2	1.40E-20	1.05	108	1.54	Entpd7	Signaling
DMR1:263464001	1	263464001	263465000	1000	1	1.80E-07	-0.5	18	1.8	Entpd7	Signaling
DMR1:263466001	1	263466001	263467000	1000	1	3.70E-07	-0.55	14	1.4	Entpd7	Signaling
DMR1:263482001	1	263482001	263484000	2000	1	9.70E-07	0.33	64	3.2	Entpd7	Signaling
DMR1:263485001	1	263485001	263486000	1000	1	3.40E-15	0.65	13	1.3	Entpd7;Cox15	Signaling;Transcription
DMR1:263496001	1	263496001	263498000	2000	1	1.80E-09	0.44	22	1.1	Entpd7;Cox15	Signaling;Transcription
DMR1:263529001	1	263529001	263532000	3000	1	7.40E-13	0.52	50	1.67	Cutc	Transport
DMR1:263543001	1	263543001	263547000	4000	3	3.60E-17	-0.55	56	1.4	LOC102546509;Abcc2	Transport
DMR1:263548001	1	263548001	263555000	7000	4	1.00E-09	-0.66	115	1.64	LOC102546509;Abcc2	Transport
DMR1:263556001	1	263556001	263563000	7000	5	2.50E-22	0.58	110	1.57	LOC102546509;Abcc2	Transport
DMR1:263586001	1	263586001	263589000	3000	1	3.80E-07	-0.58	43	1.43	Abcc2	Transport
DMR1:263599001	1	263599001	263600000	1000	1	8.60E-09	-0.52	21	2.1	Abcc2	Transport
DMR1:263601001	1	263601001	263603000	2000	2	9.30E-19	1.03	34	1.7	Abcc2	Transport
DMR1:263608001	1	263608001	263612000	4000	1	2.20E-07	-0.47	78	1.95	Abcc2	Transport
DMR1:263622001	1	263622001	263623000	1000	1	5.20E-07	-0.5	14	1.4	Abcc2;Dnmbp	Transport

DMR1:263633001	1	263633001	263641000	8000	1	2.50E-08	-0.42	134	1.68	Dnmbp	
DMR1:263667001	1	263667001	263670000	3000	1	9.90E-21	0.7	40	1.33	Dnmbp	
DMR1:263674001	1	263674001	263675000	1000	1	8.50E-07	-0.35	17	1.7	Dnmbp	
DMR1:263679001	1	263679001	263682000	3000	2	1.20E-11	0.37	61	2.03	Dnmbp	
DMR1:263684001	1	263684001	263686000	2000	1	7.80E-23	0.83	14	0.7	Dnmbp	
DMR1:263711001	1	263711001	263718000	7000	4	4.50E-15	0.5	122	1.74	Dnmbp	
DMR1:263723001	1	263723001	263726000	3000	1	1.10E-13	0.69	46	1.53	Dnmbp;Cpn1	Protease
DMR1:263728001	1	263728001	263729000	1000	1	1.30E-10	0.51	6	0.6	Dnmbp;Cpn1	Protease
DMR1:263731001	1	263731001	263733000	2000	2	1.10E-18	0.7	22	1.1	Cpn1	Protease
DMR1:263743001	1	263743001	263744000	1000	1	6.10E-11	0.46	12	1.2	Cpn1;LOC102554493	Protease
DMR1:263747001	1	263747001	263749000	2000	1	1.60E-18	0.74	18	0.9	Cpn1;LOC102554493	Protease
DMR1:263750001	1	263750001	263752000	2000	2	1.90E-14	0.44	40	2	Cpn1;LOC102554493	Protease
DMR1:263770001	1	263770001	263775000	5000	2	2.20E-13	0.52	54	1.08	Cpn1;Cyp2c23	Protease;Metabolism
DMR1:263783001	1	263783001	263785000	2000	2	1.00E-10	0.45	42	2.1	Cyp2c23	Metabolism
DMR1:263786001	1	263786001	263787000	1000	1	4.30E-13	-0.84	14	1.4	Cyp2c23	Metabolism
DMR1:263800001	1	263800001	263802000	2000	1	2.40E-09	0.37	32	1.6	Cyp2c23;Erlin1	Metabolism
DMR1:263868001	1	263868001	263869000	1000	1	2.40E-13	0.8	8	0.8	Chuk	Signaling
DMR1:263901001	1	263901001	263904000	3000	1	1.00E-07	-0.51	41	1.37	Cwf19l1;Bloc1s2	
DMR1:263924001	1	263924001	263926000	2000	1	4.90E-08	0.36	45	2.25	Bloc1s2;Pkd2l1	Transport
DMR1:263927001	1	263927001	263929000	2000	1	4.80E-10	0.44	48	2.4	Bloc1s2;Pkd2l1	Transport
DMR1:263947001	1	263947001	263948000	1000	1	1.70E-08	0.31	11	1.1	Pkd2l1	Transport
DMR1:263951001	1	263951001	263956000	5000	1	5.00E-18	0.86	52	1.04	Pkd2l1	Transport
DMR1:263963001	1	263963001	263964000	1000	1	8.80E-07	0.39	12	1.2	Pkd2l1;LOC681458	Transport
DMR1:264081001	1	264081001	264085000	4000	2	2.10E-10	0.62	45	1.12	Scd2;Scd4	
DMR1:264099001	1	264099001	264104000	5000	2	1.30E-16	0.73	64	1.28	Scd4	
DMR1:264109001	1	264109001	264111000	2000	1	2.00E-13	0.58	14	0.7	Scd4	
DMR1:264240001	1	264240001	264242000	2000	1	1.10E-17	0.89	16	0.8	Wnt8b	Signaling
DMR1:264248001	1	264248001	264249000	1000	1	2.20E-17	0.91	5	0.5	Wnt8b	Signaling
DMR1:264254001	1	264254001	264263000	9000	3	5.60E-13	-0.51	125	1.39	Wnt8b;Sec31b	Signaling;Transport
DMR1:264269001	1	264269001	264271000	2000	1	1.90E-08	0.36	34	1.7	Wnt8b;Sec31b	Signaling;Transport
DMR1:264288001	1	264288001	264291000	3000	1	5.50E-12	-0.75	51	1.7	Sec31b;Ndufb8	Transport;Metabolism
DMR1:264311001	1	264311001	264313000	2000	1	5.00E-08	-0.55	21	1.05	Ndufb8;Hif1an	Metabolism
DMR1:264486001	1	264486001	264491000	5000	2	3.20E-23	0.8	124	2.48	LOC102548668;Pax2	
DMR1:264511001	1	264511001	264515000	4000	1	8.90E-09	0.38	78	1.95	Pax2	
DMR1:264517001	1	264517001	264518000	1000	1	8.80E-18	0.54	17	1.7	Pax2	
DMR1:264519001	1	264519001	264528000	9000	3	1.20E-15	0.67	177	1.97	Pax2	
DMR1:264531001	1	264531001	264534000	3000	1	2.20E-17	0.59	53	1.77	Pax2	
DMR1:264535001	1	264535001	264538000	3000	1	9.00E-13	0.45	40	1.33	Pax2	
DMR1:264543001	1	264543001	264545000	2000	1	7.80E-11	0.39	39	1.95	Pax2	
DMR1:264546001	1	264546001	264558000	12000	6	2.20E-20	0.86	176	1.47	Pax2	
DMR1:264559001	1	264559001	264566000	7000	4	1.20E-16	0.61	105	1.5	Pax2	
DMR1:264579001	1	264579001	264582000	3000	2	2.10E-24	0.91	34	1.13	Pax2	
DMR1:264588001	1	264588001	264591000	3000	2	2.00E-21	0.79	41	1.37	Pax2	
DMR1:264594001	1	264594001	264595000	1000	1	8.10E-10	0.39	18	1.8	Pax2	
DMR1:264666001	1	264666001	264668000	2000	1	3.20E-14	0.64	20	1	Slf2	
DMR1:264695001	1	264695001	264697000	2000	2	1.40E-12	0.7	16	0.8	Slf2;LOC108349481	
DMR1:264703001	1	264703001	264709000	6000	1	6.00E-10	0.45	67	1.12	Slf2;LOC108349481	
DMR1:264744001	1	264744001	264747000	3000	2	3.90E-14	0.47	54	1.8	Sema4g;Mrpl43;Peo1	Signaling;Translation
DMR1:264760001	1	264760001	264761000	1000	1	4.20E-07	-0.55	17	1.7	Sema4g;Mrpl43;Peo1;Lzts2	Signaling;Translation
DMR1:264772001	1	264772001	264773000	1000	1	1.40E-11	0.48	15	1.5	Peo1;Lzts2;Pdzd7	Cytoskeleton
DMR1:264784001	1	264784001	264786000	2000	1	9.90E-13	0.54	56	2.8	Lzts2;Pdzd7	Cytoskeleton
DMR1:264802001	1	264802001	264805000	3000	1	2.00E-13	0.57	36	1.2	Pdzd7;Sfxn3	Cytoskeleton;Transport
DMR1:264811001	1	264811001	264812000	1000	1	3.00E-11	0.53	9	0.9	Sfxn3	Transport
DMR1:264833001	1	264833001	264834000	1000	1	1.60E-09	0.48	14	1.4	Kazald1	
DMR1:264836001	1	264836001	264838000	2000	2	8.70E-33	0.79	33	1.65	Kazald1	
DMR1:264888001	1	264888001	264892000	4000	2	1.00E-10	0.44	55	1.38	LOC102548859;Tlx1	
DMR1:264904001	1	264904001	264907000	3000	1	2.50E-07	0.29	26	0.87	Tlx1	
DMR1:264908001	1	264908001	264914000	6000	4	1.00E-26	-0.58	65	1.08	Tlx1	
DMR1:264964001	1	264964001	264965000	1000	1	1.60E-10	0.36	26	2.6	Lbx1	Development
DMR1:264966001	1	264966001	264968000	2000	1	5.30E-08	0.33	32	1.6	Lbx1	Development
DMR1:264984001	1	264984001	264992000	8000	2	1.70E-09	0.46	132	1.65	Lbx1	Development
DMR1:265083001	1	265083001	265099000	16000	7	1.90E-21	0.91	142	0.89	LOC100911827;Btrc	Cytoskeleton

DMR1:265100001	1	265100001	265102000	2000	1	4.80E-10	0.47	63	3.15	Btrc	Cytoskeleton
DMR1:265122001	1	265122001	265124000	2000	1	1.40E-10	0.54	13	0.65	Btrc	Cytoskeleton
DMR1:265143001	1	265143001	265144000	1000	1	6.00E-12	0.74	6	0.6	Btrc	Cytoskeleton
DMR1:265156001	1	265156001	265157000	1000	1	4.40E-07	0.39	2	0.2	Btrc	Cytoskeleton
DMR1:265160001	1	265160001	265162000	2000	1	1.50E-10	0.42	16	0.8	Btrc	Cytoskeleton
DMR1:265223001	1	265223001	265227000	4000	1	4.90E-20	0.82	48	1.2	Btrc	Cytoskeleton
DMR1:265253001	1	265253001	265254000	1000	1	3.70E-09	-0.54	11	1.1	Btrc	Cytoskeleton
DMR1:265258001	1	265258001	265261000	3000	1	6.10E-10	0.4	48	1.6	Btrc	Cytoskeleton
DMR1:265324001	1	265324001	265326000	2000	1	2.60E-11	0.42	23	1.15	Dpcd;Fbxw4	
DMR1:265329001	1	265329001	265330000	1000	1	4.70E-12	-0.76	15	1.5	Fbxw4	
DMR1:265337001	1	265337001	265339000	2000	1	1.70E-08	0.34	26	1.3	Fbxw4	
DMR1:265353001	1	265353001	265358000	5000	2	1.50E-11	0.47	74	1.48	Fbxw4	
DMR1:265361001	1	265361001	265362000	1000	1	3.00E-12	0.56	8	0.8	Fbxw4	
DMR1:265378001	1	265378001	265381000	3000	1	2.60E-12	-0.58	32	1.07	Fbxw4	
DMR1:265388001	1	265388001	265392000	4000	2	8.20E-15	-0.56	63	1.57	Fbxw4	
DMR1:265486001	1	265486001	265487000	1000	1	8.20E-07	0.48	3	0.3	LOC102550220;LOC103691365;Fgf8	Growth Factors
DMR1:265489001	1	265489001	265490000	1000	1	1.20E-09	0.4	10	1	LOC102550220;Fgf8	Growth Factors
DMR1:265514001	1	265514001	265515000	1000	1	6.00E-08	-0.52	9	0.9	Npm3;Mgea5	Transcription
DMR1:265518001	1	265518001	265522000	4000	1	6.30E-07	-0.52	51	1.27	Mgea5	
DMR1:265523001	1	265523001	265525000	2000	1	2.10E-09	-0.51	26	1.3	Mgea5	
DMR1:265546001	1	265546001	265550000	4000	1	8.70E-09	0.41	64	1.6	Mgea5;Kcnip2	
DMR1:265560001	1	265560001	265562000	2000	1	7.10E-09	0.44	29	1.45	Kcnip2	
DMR1:265600001	1	265600001	265601000	1000	1	1.70E-11	0.58	12	1.2	C1H10orf76	
DMR1:265635001	1	265635001	265637000	2000	2	1.60E-14	0.84	22	1.1	C1H10orf76	
DMR1:265646001	1	265646001	265648000	2000	1	1.50E-07	-0.5	31	1.55	C1H10orf76	
DMR1:265654001	1	265654001	265655000	1000	1	7.20E-17	0.67	9	0.9	C1H10orf76	
DMR1:265658001	1	265658001	265661000	3000	1	8.10E-15	0.61	37	1.23	C1H10orf76	
DMR1:265667001	1	265667001	265670000	3000	1	8.80E-11	0.5	29	0.97	C1H10orf76	
DMR1:265684001	1	265684001	265686000	2000	2	2.30E-23	1.09	6	0.3	C1H10orf76	
DMR1:265689001	1	265689001	265695000	6000	2	6.80E-09	-0.48	67	1.12	C1H10orf76	
DMR1:265712001	1	265712001	265714000	2000	1	2.30E-11	0.52	21	1.05	C1H10orf76	
DMR1:265730001	1	265730001	265735000	5000	1	1.10E-08	-0.7	57	1.14	C1H10orf76	
DMR1:265742001	1	265742001	265743000	1000	1	4.00E-08	0.38	7	0.7	C1H10orf76	
DMR1:265748001	1	265748001	265755000	7000	2	1.80E-13	0.47	82	1.17	C1H10orf76;Hps6	
DMR1:265757001	1	265757001	265758000	1000	1	1.70E-08	0.55	33	3.3	C1H10orf76;Hps6	
DMR1:265764001	1	265764001	265766000	2000	1	2.40E-13	0.46	36	1.8	C1H10orf76;Hps6;Ldb1	Transcription
DMR1:265777001	1	265777001	265781000	4000	3	1.30E-17	0.67	65	1.62	Ldb1	Transcription
DMR1:265787001	1	265787001	265788000	1000	1	3.10E-08	0.34	21	2.1	Ldb1	Transcription
DMR1:265789001	1	265789001	265790000	1000	1	4.00E-13	0.43	17	1.7	Ldb1	Transcription
DMR1:265801001	1	265801001	265805000	4000	3	1.30E-11	0.72	43	1.07	Ldb1;Pprc1	Transcription
DMR1:265818001	1	265818001	265819000	1000	1	3.90E-09	0.38	17	1.7	Pprc1	Transcription
DMR1:265843001	1	265843001	265846000	3000	1	9.00E-07	0.37	50	1.67	Nolc1	
DMR1:265877001	1	265877001	265878000	1000	1	4.20E-07	0.55	4	0.4	Elovl3;Pitx3	Metabolism;Development
DMR1:265883001	1	265883001	265887000	4000	1	1.90E-07	0.38	86	2.15	Elovl3;Pitx3	Metabolism;Development
DMR1:265932001	1	265932001	265935000	3000	1	4.30E-08	-0.67	12	0.4	Gbf1	Transcription
DMR1:265941001	1	265941001	265942000	1000	1	7.50E-12	-0.54	10	1	Gbf1	Transcription
DMR1:266008001	1	266008001	266010000	2000	1	5.20E-13	0.49	14	0.7	Gbf1	Transcription
DMR1:266045001	1	266045001	266048000	3000	1	4.30E-07	-0.46	34	1.13	LOC102551485;Nfkb2	Transcription
DMR1:266080001	1	266080001	266084000	4000	1	2.90E-08	0.37	44	1.1	Psd;Fbxl15;Cuedc2;Mir146b	Transcription
DMR1:266090001	1	266090001	266092000	2000	1	4.40E-15	0.58	40	2	Cuedc2;Mir146b;Mfsd13a	
DMR1:266119001	1	266119001	266122000	3000	1	1.40E-10	0.4	58	1.93	Mfsd13a;Actr1a	Cytoskeleton
DMR1:266140001	1	266140001	266141000	1000	1	1.20E-08	0.52	13	1.3	Actr1a;Sufu	Cytoskeleton;Cytoskeleton
DMR1:266151001	1	266151001	266153000	2000	1	9.20E-09	0.36	28	1.4	Actr1a;Sufu	Cytoskeleton;Cytoskeleton
DMR1:266161001	1	266161001	266164000	3000	1	3.40E-13	0.69	30	1	Sufu	Cytoskeleton
DMR1:266167001	1	266167001	266169000	2000	1	3.90E-14	0.71	16	0.8	Sufu	Cytoskeleton
DMR1:266184001	1	266184001	266186000	2000	1	4.50E-09	0.45	28	1.4	Sufu	Cytoskeleton
DMR1:266187001	1	266187001	266189000	2000	1	3.60E-07	-0.61	25	1.25	Sufu	Cytoskeleton
DMR1:266190001	1	266190001	266191000	1000	1	1.70E-07	0.32	18	1.8	Sufu	Cytoskeleton
DMR1:266225001	1	266225001	266226000	1000	1	1.30E-15	0.75	10	1	Sufu	Cytoskeleton
DMR1:266268001	1	266268001	266270000	2000	1	1.60E-10	0.42	54	2.7	Trim8	

DMR1:266277001	1	266277001	266279000	2000	1	1.20E-11	0.51	32	1.6	Trim8;Arl3;LOC108349707	
DMR1:266281001	1	266281001	266284000	3000	2	8.90E-18	0.8	30	1	Arl3;LOC108349707	
DMR1:266294001	1	266294001	266301000	7000	1	1.60E-13	0.53	97	1.39	Arl3;LOC108349707	
DMR1:266302001	1	266302001	266309000	7000	1	7.90E-07	0.37	71	1.01	Arl3;LOC108349707	
DMR1:266311001	1	266311001	266314000	3000	1	4.40E-14	0.69	16	0.53	Arl3	
DMR1:266341001	1	266341001	266344000	3000	2	1.50E-22	0.84	31	1.03	Arl3;Sfxn2	Transport
DMR1:266363001	1	266363001	266365000	2000	1	5.20E-13	0.46	37	1.85	Wbp1l	
DMR1:266382001	1	266382001	266384000	2000	1	4.60E-11	0.44	36	1.8	Wbp1l	
DMR1:266426001	1	266426001	266428000	2000	1	2.00E-08	0.41	35	1.75	Cyp17a1	
DMR1:266434001	1	266434001	266438000	4000	2	2.80E-25	0.84	61	1.52	Cyp17a1	
DMR1:266439001	1	266439001	266444000	5000	1	6.30E-14	0.69	71	1.42	Cyp17a1;Borcs7	
DMR1:266452001	1	266452001	266456000	4000	1	2.20E-10	0.41	57	1.43	Borcs7	
DMR1:266459001	1	266459001	266463000	4000	2	1.40E-20	0.89	47	1.18	Borcs7	
DMR1:266483001	1	266483001	266484000	1000	1	8.90E-07	0.38	28	2.8	As3mt	Epigenetic
DMR1:266519001	1	266519001	266520000	1000	1	1.60E-09	0.5	11	1.1	As3mt;LOC103691369	Epigenetic
DMR1:266521001	1	266521001	266524000	3000	1	8.70E-11	-0.93	22	0.73	As3mt;LOC103691369;Cnnm2	Epigenetic
DMR1:266525001	1	266525001	266526000	1000	1	2.50E-20	0.86	8	0.8	LOC103691369;Cnnm2	
DMR1:266527001	1	266527001	266529000	2000	1	1.30E-08	-0.53	20	1	LOC103691369;Cnnm2	
DMR1:266540001	1	266540001	266543000	3000	1	6.50E-08	0.39	38	1.27	Cnnm2	
DMR1:266544001	1	266544001	266545000	1000	1	5.40E-29	1.08	15	1.5	Cnnm2	
DMR1:266551001	1	266551001	266553000	2000	1	2.40E-07	0.27	18	0.9	Cnnm2	
DMR1:266554001	1	266554001	266556000	2000	1	4.20E-22	0.81	20	1	Cnnm2	
DMR1:266574001	1	266574001	266575000	1000	1	6.60E-09	0.38	14	1.4	Cnnm2	
DMR1:266578001	1	266578001	266579000	1000	1	3.40E-09	0.41	8	0.8	Cnnm2	
DMR1:266597001	1	266597001	266599000	2000	1	1.40E-10	0.36	18	0.9	Cnnm2	
DMR1:266600001	1	266600001	266602000	2000	1	3.30E-07	-0.51	26	1.3	Cnnm2	
DMR1:266605001	1	266605001	266606000	1000	1	8.50E-09	0.56	5	0.5	Cnnm2	
DMR1:266619001	1	266619001	266621000	2000	1	2.90E-07	-0.61	33	1.65	Cnnm2	
DMR1:266625001	1	266625001	266631000	6000	1	3.00E-12	0.49	91	1.52	Cnnm2	
DMR1:266671001	1	266671001	266672000	1000	1	2.60E-08	0.37	15	1.5	Nt5c2	Signaling
DMR1:266673001	1	266673001	266675000	2000	1	5.20E-11	0.67	22	1.1	Nt5c2	Signaling
DMR1:266676001	1	266676001	266678000	2000	1	2.40E-08	0.45	15	0.75	Nt5c2	Signaling
DMR1:266679001	1	266679001	266680000	1000	1	1.70E-07	-0.39	14	1.4	Nt5c2	Signaling
DMR1:266736001	1	266736001	266739000	3000	1	1.70E-07	-0.51	46	1.53	Nt5c2	Signaling
DMR1:266743001	1	266743001	266747000	4000	2	5.80E-09	0.36	72	1.8	Nt5c2	Signaling
DMR1:266772001	1	266772001	266774000	2000	1	1.40E-12	0.72	25	1.25	Nt5c2;Trnar-ucu;Ina	Signaling
DMR1:266775001	1	266775001	266777000	2000	1	4.70E-11	0.54	14	0.7	Nt5c2;Trnar-ucu;Ina	Signaling
DMR1:266787001	1	266787001	266792000	5000	2	6.70E-08	-0.7	74	1.48	Nt5c2;Ina	Signaling
DMR1:266794001	1	266794001	266795000	1000	1	1.40E-12	0.47	11	1.1	Ina	
DMR1:266799001	1	266799001	266806000	7000	2	9.60E-15	-0.58	50	0.71	Ina;Pcgf6	Epigenetic
DMR1:266815001	1	266815001	266817000	2000	1	8.30E-08	-0.4	24	1.2	Pcgf6	Epigenetic
DMR1:266832001	1	266832001	266834000	2000	1	2.90E-07	-0.54	27	1.35	Pcgf6	Epigenetic
DMR1:266836001	1	266836001	266838000	2000	1	6.90E-13	0.75	14	0.7	Pcgf6;Taf5	Epigenetic
DMR1:266847001	1	266847001	266849000	2000	1	8.80E-11	0.47	14	0.7	Taf5	
DMR1:266850001	1	266850001	266852000	2000	1	2.50E-07	-0.58	30	1.5	Taf5;Usmg5	
DMR1:266864001	1	266864001	266866000	2000	1	3.50E-09	0.6	17	0.85	Taf5;Usmg5;Pdcd11	Metabolism
DMR1:266868001	1	266868001	266875000	7000	1	1.60E-10	0.46	105	1.5	Taf5;Usmg5;Pdcd11	Metabolism
DMR1:266876001	1	266876001	266882000	6000	2	3.10E-07	-0.52	91	1.52	Usmg5;Pdcd11	Metabolism
DMR1:266884001	1	266884001	266887000	3000	2	5.30E-23	0.94	29	0.97	Pdcd11	Metabolism
DMR1:266890001	1	266890001	266898000	8000	1	1.00E-07	-0.67	94	1.18	Pdcd11	Metabolism
DMR1:266922001	1	266922001	266924000	2000	2	1.20E-09	-0.73	19	0.95	Calhm2;Calhm1;Calhm3	
DMR1:266935001	1	266935001	266937000	2000	1	2.60E-11	0.53	31	1.55	Calhm3	
DMR1:266938001	1	266938001	266943000	5000	2	1.00E-24	0.85	41	0.82	Calhm3	
DMR1:266956001	1	266956001	266957000	1000	1	2.20E-30	1.02	11	1.1	Neurl1	Proteolysis
DMR1:266958001	1	266958001	266961000	3000	1	4.50E-15	0.57	43	1.43	Neurl1	Proteolysis
DMR1:266973001	1	266973001	266974000	1000	1	1.60E-13	0.46	22	2.2	Neurl1	Proteolysis
DMR1:266976001	1	266976001	266981000	5000	2	4.60E-25	0.86	84	1.68	Neurl1	Proteolysis
DMR1:266982001	1	266982001	266987000	5000	3	9.50E-19	0.55	96	1.92	Neurl1	Proteolysis
DMR1:266992001	1	266992001	266998000	6000	3	2.00E-16	-0.6	79	1.32	Neurl1	Proteolysis
DMR1:267004001	1	267004001	267006000	2000	1	1.50E-07	0.32	27	1.35	Neurl1;LOC108349530	Proteolysis
DMR1:267007001	1	267007001	267008000	1000	1	7.20E-16	0.57	26	2.6	Neurl1;LOC108349530	Proteolysis
DMR1:267018001	1	267018001	267019000	1000	1	8.90E-09	0.32	33	3.3	Neurl1;LOC108349530	Proteolysis
DMR1:267024001	1	267024001	267029000	5000	1	1.30E-08	-0.69	89	1.78	Neurl1	Proteolysis
DMR1:267072001	1	267072001	267074000	2000	1	1.30E-09	0.46	37	1.85	Sh3pxd2a;LOC102552122	
DMR1:267083001	1	267083001	267090000	7000	1	2.20E-07	-0.61	126	1.8	Sh3pxd2a;LOC102552122	

DMR1:267117001	1	267117001	267118000	1000	1	1.10E-24	0.89	34	3.4	Sh3pxd2a	
DMR1:267129001	1	267129001	267134000	5000	2	3.60E-28	1.16	61	1.22	Sh3pxd2a	
DMR1:267156001	1	267156001	267160000	4000	2	3.10E-07	0.44	43	1.07	Sh3pxd2a	
DMR1:267168001	1	267168001	267169000	1000	1	8.40E-17	0.69	7	0.7	Sh3pxd2a	
DMR1:267183001	1	267183001	267185000	2000	2	9.90E-12	0.5	41	2.05	Sh3pxd2a	
DMR1:267208001	1	267208001	267210000	2000	1	1.10E-15	0.58	21	1.05	Sh3pxd2a	
DMR1:267215001	1	267215001	267217000	2000	1	1.30E-15	0.58	26	1.3	Sh3pxd2a	
DMR1:267222001	1	267222001	267228000	6000	1	2.70E-08	0.35	104	1.73	Sh3pxd2a	
DMR1:267233001	1	267233001	267234000	1000	1	8.20E-07	0.32	23	2.3	Sh3pxd2a	
DMR1:267236001	1	267236001	267239000	3000	2	3.40E-21	0.53	50	1.67	Sh3pxd2a	
DMR1:267240001	1	267240001	267243000	3000	1	7.80E-07	0.29	50	1.67	Sh3pxd2a	
DMR1:267279001	1	267279001	267282000	3000	1	8.00E-07	0.31	57	1.9	Obfc1;LOC108349533	
DMR1:267286001	1	267286001	267289000	3000	1	2.90E-11	0.56	44	1.47	Obfc1;LOC108349533	
DMR1:267302001	1	267302001	267305000	3000	1	2.10E-08	-0.58	44	1.47	Obfc1	
DMR1:267318001	1	267318001	267320000	2000	2	5.50E-14	0.49	26	1.3	Obfc1	
DMR1:267322001	1	267322001	267323000	1000	1	1.70E-35	1.44	7	0.7	Obfc1	
DMR1:267365001	1	267365001	267366000	1000	1	1.20E-09	0.47	12	1.2	Slk	
DMR1:267386001	1	267386001	267387000	1000	1	6.50E-07	-0.57	17	1.7	Slk	
DMR1:267394001	1	267394001	267400000	6000	2	2.70E-09	-0.63	66	1.1	Slk	
DMR1:267407001	1	267407001	267413000	6000	1	3.90E-19	0.59	78	1.3	Slk;Col17a1	Extracellular Matrix
DMR1:267415001	1	267415001	267418000	3000	1	9.00E-07	-0.46	48	1.6	Slk;Col17a1	Extracellular Matrix
DMR1:267439001	1	267439001	267441000	2000	1	1.00E-17	0.79	53	2.65	Col17a1;LOC103691371	Extracellular Matrix
DMR1:267450001	1	267450001	267452000	2000	1	1.40E-09	0.29	34	1.7	Col17a1;LOC103691371	Extracellular Matrix
DMR1:267457001	1	267457001	267463000	6000	1	9.00E-09	0.36	102	1.7	Col17a1;LOC103691371	Extracellular Matrix
DMR1:267496001	1	267496001	267499000	3000	1	4.40E-15	0.47	38	1.27	Sfr1	Transcription
DMR1:267505001	1	267505001	267506000	1000	1	4.80E-18	1.12	66	6.6	Sfr1;Cfap43	Transcription
DMR1:267513001	1	267513001	267516000	3000	1	1.00E-14	0.68	43	1.43	Sfr1;Cfap43	Transcription
DMR1:267523001	1	267523001	267527000	4000	1	2.90E-08	0.31	70	1.75	Cfap43	
DMR1:267529001	1	267529001	267532000	3000	2	3.30E-13	0.46	53	1.77	Cfap43	
DMR1:267533001	1	267533001	267534000	1000	1	2.00E-09	0.6	11	1.1	Cfap43	
DMR1:267538001	1	267538001	267539000	1000	1	2.30E-11	0.68	6	0.6	Cfap43	
DMR1:267540001	1	267540001	267542000	2000	2	2.10E-15	0.73	28	1.4	Cfap43	
DMR1:267543001	1	267543001	267544000	1000	1	4.40E-07	0.31	23	2.3	Cfap43	
DMR1:267556001	1	267556001	267562000	6000	3	2.50E-40	1.11	100	1.67	Cfap43	
DMR1:267563001	1	267563001	267565000	2000	1	3.50E-07	0.34	63	3.15	Cfap43	
DMR1:267578001	1	267578001	267581000	3000	1	1.20E-13	0.4	69	2.3	Cfap43	
DMR1:267584001	1	267584001	267586000	2000	1	2.50E-14	0.54	26	1.3	Cfap43	
DMR1:267591001	1	267591001	267592000	1000	1	3.80E-09	0.4	13	1.3	Cfap43	
DMR1:267620001	1	267620001	267624000	4000	2	8.30E-14	0.5	55	1.38	Gsto1;Gsto2	
DMR1:267685001	1	267685001	267687000	2000	1	9.80E-10	-0.62	42	2.1	Cfap58	Development
DMR1:267690001	1	267690001	267691000	1000	1	5.10E-07	0.59	14	1.4	Cfap58	Development
DMR1:267697001	1	267697001	267698000	1000	1	4.00E-11	0.56	10	1	Cfap58	Development
DMR1:267720001	1	267720001	267725000	5000	2	4.10E-15	0.74	104	2.08	Cfap58;LOC108349708	Development
DMR1:267757001	1	267757001	267759000	2000	1	1.20E-07	0.38	24	1.2	Cfap58	Development
DMR1:267764001	1	267764001	267767000	3000	3	2.80E-21	1.08	42	1.4	Cfap58	Development
DMR1:267773001	1	267773001	267775000	2000	1	7.30E-08	-0.47	11	0.55	Cfap58	Development
DMR1:267776001	1	267776001	267777000	1000	1	3.40E-07	0.48	7	0.7	Cfap58	Development
DMR1:267783001	1	267783001	267784000	1000	1	3.40E-07	-0.33	10	1	Cfap58	Development
DMR1:267786001	1	267786001	267787000	1000	1	1.40E-08	0.51	13	1.3	Cfap58	Development
DMR1:267802001	1	267802001	267810000	8000	5	3.70E-21	-0.58	108	1.35	Cfap58	Development
DMR1:267921001	1	267921001	267922000	1000	1	6.70E-11	0.59	15	1.5	Trnak-cuu	
DMR1:267997001	1	267997001	267998000	1000	1	1.90E-08	0.59	3	0.3	Sorcs3	Transport
DMR1:268002001	1	268002001	268009000	7000	1	4.00E-15	0.71	82	1.17	Sorcs3	Transport
DMR1:268011001	1	268011001	268018000	7000	6	9.50E-21	-0.52	88	1.26	Sorcs3	Transport
DMR1:268021001	1	268021001	268025000	4000	1	6.90E-14	0.55	64	1.6	Sorcs3	Transport
DMR1:268028001	1	268028001	268029000	1000	1	1.20E-15	0.66	7	0.7	Sorcs3	Transport
DMR1:268038001	1	268038001	268039000	1000	1	4.00E-10	0.39	21	2.1	Sorcs3	Transport
DMR1:268050001	1	268050001	268051000	1000	1	1.30E-07	0.45	6	0.6	Sorcs3	Transport
DMR1:268055001	1	268055001	268057000	2000	2	4.90E-16	0.62	23	1.15	Sorcs3	Transport
DMR1:268075001	1	268075001	268077000	2000	1	4.20E-07	0.34	31	1.55	Sorcs3	Transport
DMR1:268078001	1	268078001	268080000	2000	1	8.90E-08	0.32	26	1.3	Sorcs3	Transport
DMR1:268089001	1	268089001	268097000	8000	4	1.30E-22	-0.48	120	1.5	Sorcs3	Transport
DMR1:268126001	1	268126001	268127000	1000	1	1.10E-09	0.51	10	1	Sorcs3	Transport
DMR1:268132001	1	268132001	268133000	1000	1	3.40E-09	0.56	14	1.4	Sorcs3	Transport
DMR1:268150001	1	268150001	268153000	3000	3	1.30E-15	0.85	41	1.37	Sorcs3	Transport

DMR1:268177001	1	268177001	268180000	3000	2	2.30E-12	0.77	23	0.77	Sorcs3	Transport
DMR1:268183001	1	268183001	268187000	4000	2	2.10E-08	0.52	44	1.1	Sorcs3	Transport
DMR1:268189001	1	268189001	268190000	1000	1	2.80E-07	0.52	11	1.1	Sorcs3	Transport
DMR1:268223001	1	268223001	268224000	1000	1	5.30E-07	0.51	3	0.3	Sorcs3	Transport
DMR1:268228001	1	268228001	268229000	1000	1	1.30E-08	-0.62	0	0	Sorcs3	Transport
DMR1:268235001	1	268235001	268236000	1000	1	2.40E-07	-0.47	6	0.6	Sorcs3	Transport
DMR1:268242001	1	268242001	268250000	8000	5	5.00E-18	-0.56	109	1.36	Sorcs3	Transport
DMR1:268262001	1	268262001	268264000	2000	1	3.10E-07	-0.58	11	0.55	Sorcs3	Transport
DMR1:268266001	1	268266001	268267000	1000	1	3.90E-19	0.66	19	1.9	Sorcs3	Transport
DMR1:268271001	1	268271001	268272000	1000	1	1.20E-18	0.63	11	1.1	Sorcs3	Transport
DMR1:268273001	1	268273001	268274000	1000	1	4.50E-08	0.44	6	0.6	Sorcs3	Transport
DMR1:268279001	1	268279001	268281000	2000	1	2.60E-11	0.36	24	1.2	Sorcs3	Transport
DMR1:268297001	1	268297001	268303000	6000	1	2.70E-08	-0.63	66	1.1	Sorcs3	Transport
DMR1:268320001	1	268320001	268321000	1000	1	4.90E-12	0.64	5	0.5	Sorcs3	Transport
DMR1:268341001	1	268341001	268343000	2000	1	8.00E-07	-0.46	28	1.4	Sorcs3	Transport
DMR1:268347001	1	268347001	268348000	1000	1	4.90E-15	0.75	10	1	Sorcs3	Transport
DMR1:268373001	1	268373001	268376000	3000	1	9.90E-08	0.39	30	1	Sorcs3	Transport
DMR1:268382001	1	268382001	268384000	2000	2	2.40E-15	0.83	9	0.45	Sorcs3	Transport
DMR1:268387001	1	268387001	268390000	3000	1	5.30E-11	0.53	33	1.1	Sorcs3	Transport
DMR1:268408001	1	268408001	268409000	1000	1	4.10E-10	0.54	9	0.9	Sorcs3	Transport
DMR1:268422001	1	268422001	268423000	1000	1	3.40E-08	0.39	8	0.8	Sorcs3	Transport
DMR1:268429001	1	268429001	268430000	1000	1	1.10E-08	0.38	3	0.3	Sorcs3	Transport
DMR1:268444001	1	268444001	268446000	2000	1	7.60E-07	-0.54	19	0.95	Sorcs3	Transport
DMR1:268458001	1	268458001	268462000	4000	1	5.80E-11	0.55	35	0.88	Sorcs3	Transport
DMR1:268469001	1	268469001	268472000	3000	2	9.00E-15	0.85	21	0.7	Sorcs3	Transport
DMR1:268487001	1	268487001	268489000	2000	2	1.40E-09	-0.52	19	0.95	Sorcs3	Transport
DMR1:268524001	1	268524001	268525000	1000	1	9.50E-18	0.98	11	1.1	Sorcs3	Transport
DMR1:268536001	1	268536001	268538000	2000	1	3.90E-07	0.5	14	0.7	Sorcs3	Transport
DMR1:268546001	1	268546001	268547000	1000	1	2.40E-10	0.52	10	1	Sorcs3	Transport
DMR1:268566001	1	268566001	268568000	2000	2	1.70E-13	0.64	24	1.2	Sorcs3	Transport
DMR1:268576001	1	268576001	268579000	3000	1	1.60E-08	0.41	23	0.77	Sorcs3	Transport
DMR1:268629001	1	268629001	268632000	3000	1	7.50E-15	0.75	22	0.73	Sorcs3	Transport
DMR1:269558001	1	269558001	269560000	2000	1	4.10E-07	-0.52	23	1.15	RGD1561154	
DMR1:270058001	1	270058001	270064000	6000	4	1.20E-19	-0.57	55	0.92	Sorcs1	Transport
DMR1:270069001	1	270069001	270071000	2000	1	2.30E-07	0.36	16	0.8	Sorcs1	Transport
DMR1:270092001	1	270092001	270096000	4000	1	6.90E-08	0.48	22	0.55	Sorcs1	Transport
DMR1:270108001	1	270108001	270109000	1000	1	1.40E-13	0.82	5	0.5	Sorcs1	Transport
DMR1:270180001	1	270180001	270183000	3000	2	2.30E-16	-0.62	11	0.37	Sorcs1	Transport
DMR1:270199001	1	270199001	270200000	1000	1	2.80E-08	0.47	6	0.6	Sorcs1	Transport
DMR1:270211001	1	270211001	270215000	4000	1	1.60E-10	-0.57	64	1.6	Sorcs1	Transport
DMR1:270219001	1	270219001	270221000	2000	1	7.30E-10	0.56	11	0.55	Sorcs1	Transport
DMR1:270228001	1	270228001	270232000	4000	3	9.50E-10	0.62	30	0.75	Sorcs1	Transport
DMR1:270241001	1	270241001	270244000	3000	1	2.20E-07	0.48	31	1.03	Sorcs1	Transport
DMR1:270255001	1	270255001	270257000	2000	1	1.60E-07	0.5	14	0.7	Sorcs1	Transport
DMR1:270258001	1	270258001	270259000	1000	1	9.90E-09	-0.54	20	2	Sorcs1	Transport
DMR1:270270001	1	270270001	270276000	6000	1	4.90E-10	0.46	62	1.03	Sorcs1	Transport
DMR1:270291001	1	270291001	270293000	2000	1	1.10E-18	0.74	17	0.85	Sorcs1	Transport
DMR1:270298001	1	270298001	270300000	2000	2	2.70E-09	0.58	19	0.95	Sorcs1	Transport
DMR1:270322001	1	270322001	270324000	2000	1	1.20E-08	0.55	24	1.2	Sorcs1	Transport
DMR1:270326001	1	270326001	270327000	1000	1	5.30E-11	0.67	10	1	Sorcs1	Transport
DMR1:270333001	1	270333001	270334000	1000	1	1.30E-12	0.48	12	1.2	Sorcs1	Transport
DMR1:270341001	1	270341001	270343000	2000	1	2.20E-17	0.6	24	1.2	Sorcs1	Transport
DMR1:270352001	1	270352001	270355000	3000	1	7.20E-08	0.4	59	1.97	Sorcs1	Transport
DMR1:270379001	1	270379001	270380000	1000	1	4.30E-11	0.75	0	0	Sorcs1	Transport
DMR1:270384001	1	270384001	270387000	3000	1	1.40E-08	0.48	20	0.67	Sorcs1	Transport
DMR1:270397001	1	270397001	270398000	1000	1	7.20E-11	0.59	6	0.6	Sorcs1	Transport
DMR1:270403001	1	270403001	270405000	2000	1	1.70E-16	0.77	16	0.8	Sorcs1	Transport
DMR1:270423001	1	270423001	270426000	3000	1	1.30E-12	0.55	32	1.07	Sorcs1	Transport
DMR1:270429001	1	270429001	270431000	2000	1	1.30E-07	0.41	17	0.85	Sorcs1	Transport
DMR1:271298001	1	271298001	271301000	3000	2	4.40E-09	-0.63	37	1.23	Ccdc147	
DMR1:271309001	1	271309001	271311000	2000	1	9.50E-09	0.64	21	1.05	Ccdc147;LOC108349546	
DMR1:271323001	1	271323001	271328000	5000	2	4.80E-17	0.75	106	2.12	Ccdc147;LOC108349546	
DMR1:271345001	1	271345001	271346000	1000	1	1.30E-08	0.46	12	1.2	Ccdc147	
DMR1:271365001	1	271365001	271370000	5000	3	1.40E-21	1.02	77	1.54	Ccdc147	
DMR1:271388001	1	271388001	271390000	2000	1	4.30E-07	0.44	28	1.4	Ccdc147	

DMR1:271405001	1	271405001	271411000	6000	3	1.30E-14	-0.48	82	1.37	Ccdc147	
DMR1:271643001	1	271643001	271644000	1000	1	3.80E-13	0.78	12	1.2	Trnak-cuu	
DMR1:272795001	1	272795001	272798000	3000	1	2.50E-12	-0.52	30	1	Ins1	Growth Factors
DMR1:272805001	1	272805001	272806000	1000	1	1.80E-14	0.78	7	0.7	Ins1	Growth Factors
DMR1:272852001	1	272852001	272859000	7000	2	4.20E-07	-0.52	67	0.96	Olr385	
DMR1:272896001	1	272896001	272897000	1000	1	3.50E-08	0.58	14	1.4	Olr385	
DMR1:273548001	1	273548001	273555000	7000	4	2.10E-27	-0.58	82	1.17	RGD1561333	
DMR1:273557001	1	273557001	273558000	1000	1	3.40E-07	0.33	7	0.7	RGD1561333	
DMR1:273571001	1	273571001	273572000	1000	1	9.30E-15	0.65	4	0.4	RGD1561333	
DMR1:273701001	1	273701001	273705000	4000	2	7.50E-15	0.69	38	0.95	Xpnpep1	Protease
DMR1:273718001	1	273718001	273726000	8000	1	1.40E-08	0.35	179	2.24	Xpnpep1	Protease
DMR1:273741001	1	273741001	273749000	8000	3	1.10E-22	0.86	118	1.48	Xpnpep1;LOC102550358	Protease
DMR1:273762001	1	273762001	273768000	6000	1	5.20E-11	0.38	103	1.72	Xpnpep1;LOC102550358	Protease
DMR1:273841001	1	273841001	273851000	10000	1	2.30E-07	-0.56	190	1.9	LOC103691374;Add3	Cytoskeleton
DMR1:273869001	1	273869001	273871000	2000	1	1.10E-10	0.58	20	1	Add3	Cytoskeleton
DMR1:273878001	1	273878001	273879000	1000	1	2.70E-08	-0.66	10	1	Add3	Cytoskeleton
DMR1:273881001	1	273881001	273885000	4000	1	6.30E-09	0.49	35	0.88	Add3	Cytoskeleton
DMR1:273887001	1	273887001	273890000	3000	1	1.60E-07	0.44	28	0.93	Add3	Cytoskeleton
DMR1:273901001	1	273901001	273902000	1000	1	2.00E-09	-0.64	11	1.1	Add3	Cytoskeleton
DMR1:273907001	1	273907001	273909000	2000	1	1.60E-10	-0.71	23	1.15	Add3	Cytoskeleton
DMR1:273940001	1	273940001	273941000	1000	1	1.60E-13	0.57	11	1.1	Add3	Cytoskeleton
DMR1:273942001	1	273942001	273943000	1000	1	1.80E-10	0.4	13	1.3	Add3	Cytoskeleton
DMR1:273946001	1	273946001	273948000	2000	1	3.50E-08	-0.55	24	1.2	Add3	Cytoskeleton
DMR1:273950001	1	273950001	273952000	2000	2	3.50E-10	-0.63	29	1.45	Add3	Cytoskeleton
DMR1:273954001	1	273954001	273957000	3000	1	2.10E-10	0.35	44	1.47	Add3	Cytoskeleton
DMR1:273961001	1	273961001	273966000	5000	1	5.80E-12	0.42	93	1.86	Add3	Cytoskeleton
DMR1:273968001	1	273968001	273971000	3000	1	1.00E-06	0.46	42	1.4	Add3	Cytoskeleton
DMR1:274068001	1	274068001	274069000	1000	1	5.40E-07	-0.51	18	1.8	Mxi1;LOC108349609	Transcription
DMR1:274080001	1	274080001	274082000	2000	1	3.50E-21	0.54	28	1.4	Mxi1;LOC108349609	Transcription
DMR1:274092001	1	274092001	274094000	2000	1	2.70E-08	-0.55	16	0.8	Mxi1;LOC108349609;Smndc1	Transcription;Translation
DMR1:274110001	1	274110001	274111000	1000	1	2.70E-09	0.48	21	2.1	Smndc1	Translation
DMR1:274117001	1	274117001	274120000	3000	1	1.10E-07	0.41	40	1.33	Smndc1	Translation
DMR1:274241001	1	274241001	274244000	3000	2	2.10E-09	-0.42	57	1.9	Dusp5	Signaling
DMR1:274247001	1	274247001	274252000	5000	1	2.00E-11	0.75	74	1.48	Dusp5	Signaling
DMR1:274266001	1	274266001	274267000	1000	1	4.30E-12	0.48	15	1.5	Dusp5	Signaling
DMR1:274268001	1	274268001	274271000	3000	1	1.20E-19	1.05	23	0.77	Dusp5	Signaling
DMR1:274294001	1	274294001	274298000	4000	3	1.60E-11	0.61	55	1.38	Rps12l2	
DMR1:274361001	1	274361001	274366000	5000	2	3.70E-13	0.72	65	1.3	Smc3	
DMR1:274383001	1	274383001	274388000	5000	1	6.60E-09	0.39	79	1.58	Rbm20	
DMR1:274433001	1	274433001	274435000	2000	1	8.30E-22	0.67	22	1.1	Rbm20	
DMR1:274437001	1	274437001	274441000	4000	2	5.00E-09	0.41	57	1.43	Rbm20	
DMR1:274446001	1	274446001	274449000	3000	2	4.40E-13	0.5	64	2.13	Rbm20	
DMR1:274460001	1	274460001	274463000	3000	1	2.70E-08	0.39	46	1.53	Rbm20	
DMR1:274470001	1	274470001	274474000	4000	1	4.00E-10	0.54	48	1.2	Rbm20	
DMR1:274479001	1	274479001	274480000	1000	1	6.60E-10	0.56	6	0.6	Rbm20	
DMR1:274481001	1	274481001	274485000	4000	2	7.00E-22	0.65	72	1.8	Rbm20	
DMR1:274486001	1	274486001	274488000	2000	1	7.30E-12	0.44	20	1	Rbm20	
DMR1:274489001	1	274489001	274492000	3000	1	3.30E-09	0.36	43	1.43	Rbm20	
DMR1:274498001	1	274498001	274504000	6000	3	1.20E-15	-0.52	67	1.12	Rbm20	
DMR1:274533001	1	274533001	274542000	9000	1	2.10E-13	0.47	129	1.43	Rbm20	
DMR1:274546001	1	274546001	274549000	3000	1	5.40E-10	0.47	38	1.27	Rbm20	
DMR1:274556001	1	274556001	274558000	2000	1	4.30E-12	0.5	28	1.4	Rbm20	
DMR1:274566001	1	274566001	274569000	3000	1	4.20E-08	-0.45	48	1.6	Rbm20	
DMR1:274582001	1	274582001	274583000	1000	1	1.90E-07	0.35	6	0.6	Rbm20	
DMR1:274593001	1	274593001	274596000	3000	1	1.90E-07	0.56	37	1.23	Rbm20	
DMR1:274597001	1	274597001	274602000	5000	1	1.70E-09	0.53	70	1.4	Rbm20	
DMR1:274605001	1	274605001	274609000	4000	1	2.10E-12	-0.66	50	1.25	Pdcd4	Translation
DMR1:274615001	1	274615001	274618000	3000	2	9.50E-20	0.95	38	1.27	Pdcd4	Translation
DMR1:274624001	1	274624001	274625000	1000	1	5.30E-10	0.5	27	2.7	Pdcd4	Translation
DMR1:274635001	1	274635001	274637000	2000	1	4.70E-07	0.32	22	1.1	Pdcd4	Translation
DMR1:274638001	1	274638001	274640000	2000	2	1.20E-23	0.76	18	0.9	Pdcd4;Bbip1	Translation
DMR1:274643001	1	274643001	274648000	5000	1	2.00E-07	0.38	77	1.54	Pdcd4;Bbip1	Translation
DMR1:274691001	1	274691001	274694000	3000	1	2.60E-20	0.75	27	0.9	Shoc2	Cytoskeleton
DMR1:274708001	1	274708001	274709000	1000	1	3.20E-19	0.85	7	0.7	Shoc2	Cytoskeleton

DMR1:274725001	1	274725001	274727000	2000	1	1.70E-07	-0.49	25	1.25	Shoc2	Cytoskeleton
DMR1:274733001	1	274733001	274740000	7000	6	7.40E-24	-0.46	94	1.34	Shoc2	Cytoskeleton
DMR1:275842001	1	275842001	275852000	10000	4	3.10E-28	0.6	153	1.53	Gpam;LOC102553769	Metabolism
DMR1:275853001	1	275853001	275856000	3000	2	1.20E-15	0.77	38	1.27	Gpam;LOC102553769	Metabolism
DMR1:275859001	1	275859001	275860000	1000	1	1.70E-08	0.36	6	0.6	Gpam	Metabolism
DMR1:275887001	1	275887001	275888000	1000	1	5.90E-16	0.63	12	1.2	Gpam	Metabolism
DMR1:275891001	1	275891001	275893000	2000	1	1.90E-13	0.77	115	5.75	Gpam	Metabolism
DMR1:275894001	1	275894001	275902000	8000	3	1.00E-11	0.49	116	1.45	Gpam	Metabolism
DMR1:275977001	1	275977001	275979000	2000	2	9.10E-15	0.58	25	1.25	Tectb;LOC108348158	Receptor
DMR1:275981001	1	275981001	275982000	1000	1	3.80E-10	0.63	4	0.4	Tectb;LOC108348158	Receptor
DMR1:275983001	1	275983001	275988000	5000	1	6.00E-10	0.59	80	1.6	Tectb;LOC108348158	Receptor
DMR1:276176001	1	276176001	276178000	2000	1	1.60E-10	0.4	32	1.6	Gucy2g	Signaling
DMR1:276189001	1	276189001	276192000	3000	2	6.40E-20	0.9	37	1.23	Gucy2g	Signaling
DMR1:276196001	1	276196001	276200000	4000	2	4.10E-12	0.52	39	0.98	Gucy2g	Signaling
DMR1:276209001	1	276209001	276213000	4000	1	5.00E-11	0.39	78	1.95	Gucy2g	Signaling
DMR1:276261001	1	276261001	276267000	6000	3	2.80E-18	0.64	92	1.53	Acsl5	Metabolism
DMR1:276294001	1	276294001	276297000	3000	1	5.00E-07	0.34	40	1.33	Acsl5;Zdhhc6	Metabolism
DMR1:276322001	1	276322001	276323000	1000	1	4.90E-08	0.31	18	1.8	Vti1a	Transcription
DMR1:276328001	1	276328001	276329000	1000	1	1.70E-12	0.43	8	0.8	Vti1a	Transcription
DMR1:276330001	1	276330001	276335000	5000	1	5.20E-07	0.35	61	1.22	Vti1a	Transcription
DMR1:276336001	1	276336001	276338000	2000	1	1.00E-07	-0.63	29	1.45	Vti1a	Transcription
DMR1:276355001	1	276355001	276357000	2000	1	4.90E-07	-0.52	29	1.45	Vti1a	Transcription
DMR1:276372001	1	276372001	276374000	2000	1	1.10E-07	0.36	36	1.8	Vti1a	Transcription
DMR1:276379001	1	276379001	276382000	3000	1	3.50E-15	-0.69	48	1.6	Vti1a	Transcription
DMR1:276420001	1	276420001	276422000	2000	1	2.20E-08	0.52	15	0.75	Vti1a;LOC103695066	Transcription
DMR1:276428001	1	276428001	276429000	1000	1	4.00E-22	0.78	11	1.1	Vti1a	Transcription
DMR1:276430001	1	276430001	276432000	2000	1	1.30E-08	-0.66	19	0.95	Vti1a	Transcription
DMR1:276439001	1	276439001	276443000	4000	3	5.30E-25	0.79	74	1.85	Vti1a	Transcription
DMR1:276470001	1	276470001	276476000	6000	1	8.10E-09	-0.47	97	1.62	Vti1a;LOC103691376	Transcription
DMR1:276480001	1	276480001	276490000	10000	2	4.10E-09	-0.58	161	1.61	Vti1a	Transcription
DMR1:276491001	1	276491001	276496000	5000	2	2.80E-09	0.39	72	1.44	Vti1a	Transcription
DMR1:276497001	1	276497001	276500000	3000	1	2.50E-09	0.41	57	1.9	Vti1a	Transcription
DMR1:276675001	1	276675001	276685000	10000	3	6.00E-11	-0.77	165	1.65	Tcf7l2	Transcription
DMR1:276706001	1	276706001	276708000	2000	1	2.80E-16	0.52	39	1.95	Tcf7l2	Transcription
DMR1:276712001	1	276712001	276717000	5000	1	7.20E-32	0.77	107	2.14	Tcf7l2	Transcription
DMR1:276723001	1	276723001	276731000	8000	2	2.20E-14	0.55	135	1.69	Tcf7l2	Transcription
DMR1:276733001	1	276733001	276737000	4000	2	3.80E-10	-0.48	57	1.43	Tcf7l2	Transcription
DMR1:277062001	1	277062001	277064000	2000	1	6.20E-32	0.94	22	1.1	LOC102546997;LOC102546765;Habp2	Protease
DMR1:277070001	1	277070001	277072000	2000	1	1.20E-08	0.43	22	1.1	LOC102546997;LOC102546765;Habp2	Protease
DMR1:277087001	1	277087001	277089000	2000	1	2.00E-10	0.53	22	1.1	Habp2	Protease
DMR1:277098001	1	277098001	277103000	5000	1	2.50E-09	0.57	70	1.4	Habp2;Nrap	Protease
DMR1:277119001	1	277119001	277121000	2000	1	2.70E-10	0.41	49	2.45	Nrap	
DMR1:277127001	1	277127001	277130000	3000	2	4.10E-07	0.45	49	1.63	Nrap	
DMR1:277136001	1	277136001	277139000	3000	2	1.30E-13	0.82	39	1.3	Nrap	
DMR1:277142001	1	277142001	277145000	3000	2	3.80E-10	0.59	32	1.07	Nrap	
DMR1:277146001	1	277146001	277151000	5000	4	1.10E-32	0.99	87	1.74	Nrap	
DMR1:277153001	1	277153001	277157000	4000	2	5.00E-19	0.73	35	0.88	Nrap	
DMR1:277173001	1	277173001	277174000	1000	1	4.30E-07	0.37	10	1	Nrap	
DMR1:277192001	1	277192001	277196000	4000	2	6.30E-23	0.97	70	1.75	Casp7	Protease
DMR1:277217001	1	277217001	277218000	1000	1	7.00E-12	0.47	18	1.8	Casp7	Protease
DMR1:277222001	1	277222001	277227000	5000	2	1.00E-29	1.04	77	1.54	Casp7	Protease
DMR1:277229001	1	277229001	277230000	1000	1	1.60E-11	0.45	24	2.4	Casp7	Protease
DMR1:277235001	1	277235001	277236000	1000	1	1.00E-11	0.46	20	2	Casp7	Protease
DMR1:277240001	1	277240001	277242000	2000	1	1.20E-16	0.64	29	1.45	Casp7	Protease
DMR1:277243001	1	277243001	277245000	2000	1	1.50E-14	0.47	53	2.65	Casp7	Protease
DMR1:277250001	1	277250001	277255000	5000	2	3.30E-10	0.42	85	1.7	Casp7;Plekhs1	Protease
DMR1:277256001	1	277256001	277257000	1000	1	2.50E-07	0.44	8	0.8	Plekhs1	
DMR1:277258001	1	277258001	277259000	1000	1	1.20E-13	0.5	14	1.4	Plekhs1	
DMR1:277267001	1	277267001	277271000	4000	1	1.30E-35	1.1	59	1.48	Plekhs1	
DMR1:277275001	1	277275001	277277000	2000	1	2.90E-11	0.44	24	1.2	Plekhs1	
DMR1:277278001	1	277278001	277279000	1000	1	9.80E-07	0.46	14	1.4	Plekhs1	
DMR1:277294001	1	277294001	277296000	2000	1	7.80E-21	0.94	46	2.3	Plekhs1;LOC102547071	
DMR1:277345001	1	277345001	277352000	7000	1	7.00E-14	0.71	116	1.66	Dclre1a;Nhlrc2	Transcription

DMR1:277382001	1	277382001	277383000	1000	1	5.20E-08	0.47	9	0.9	Nhlrc2	
DMR1:277388001	1	277388001	277390000	2000	1	6.30E-19	0.7	23	1.15	Nhlrc2	
DMR1:277401001	1	277401001	277403000	2000	1	9.40E-07	-0.34	33	1.65	Nhlrc2	
DMR1:277404001	1	277404001	277406000	2000	1	2.40E-07	-0.55	36	1.8	Nhlrc2	
DMR1:277425001	1	277425001	277428000	3000	1	5.40E-13	0.6	42	1.4	Nhlrc2	
DMR1:277529001	1	277529001	277532000	3000	1	3.10E-07	-0.41	57	1.9	LOC102547715;Adrb1	Signaling
DMR1:277540001	1	277540001	277542000	2000	1	5.20E-08	-0.5	35	1.75	LOC102547715;Adrb1	Signaling
DMR1:277626001	1	277626001	277627000	1000	1	4.60E-19	0.73	11	1.1	Ccdc186	
DMR1:277628001	1	277628001	277632000	4000	1	5.10E-14	0.6	75	1.88	Ccdc186;Tdrd1	Cytoskeleton
DMR1:277685001	1	277685001	277687000	2000	1	5.50E-10	0.55	24	1.2	Tdrd1;Vwa2	Cytoskeleton;Extracellular Matrix
DMR1:277698001	1	277698001	277701000	3000	1	1.90E-17	0.52	53	1.77	Vwa2	Extracellular Matrix
DMR1:277706001	1	277706001	277710000	4000	2	7.10E-11	-0.5	42	1.05	Vwa2	Extracellular Matrix
DMR1:277734001	1	277734001	277738000	4000	1	5.50E-09	0.46	67	1.68	Vwa2;Afap1l2	Extracellular Matrix
DMR1:277754001	1	277754001	277756000	2000	1	2.00E-09	-0.62	25	1.25	Afap1l2	
DMR1:277762001	1	277762001	277765000	3000	1	6.40E-07	-0.51	47	1.57	Afap1l2	
DMR1:277795001	1	277795001	277799000	4000	4	3.60E-14	0.53	41	1.02	Afap1l2	
DMR1:277806001	1	277806001	277807000	1000	1	6.60E-26	0.91	40	4	Afap1l2	
DMR1:277821001	1	277821001	277822000	1000	1	1.20E-07	0.36	14	1.4	Afap1l2	
DMR1:277834001	1	277834001	277835000	1000	1	1.60E-16	0.92	10	1	Afap1l2	
DMR1:277843001	1	277843001	277844000	1000	1	2.60E-10	0.44	14	1.4	Ablim1	
DMR1:277855001	1	277855001	277858000	3000	1	7.90E-08	-0.53	66	2.2	Ablim1	
DMR1:277859001	1	277859001	277863000	4000	1	2.20E-07	-0.54	64	1.6	Ablim1	
DMR1:277878001	1	277878001	277883000	5000	1	8.60E-11	0.46	94	1.88	Ablim1	
DMR1:277884001	1	277884001	277886000	2000	1	1.30E-09	0.57	21	1.05	Ablim1	
DMR1:277888001	1	277888001	277892000	4000	2	2.60E-14	0.64	61	1.52	Ablim1	
DMR1:277896001	1	277896001	277897000	1000	1	4.70E-07	0.36	23	2.3	Ablim1	
DMR1:277905001	1	277905001	277911000	6000	3	1.10E-13	0.63	63	1.05	Ablim1	
DMR1:277922001	1	277922001	277923000	1000	1	6.90E-14	0.59	9	0.9	Ablim1;LOC103691383	
DMR1:277927001	1	277927001	277928000	1000	1	3.80E-08	-0.66	13	1.3	Ablim1;LOC103691383	
DMR1:277931001	1	277931001	277934000	3000	2	1.20E-24	0.94	68	2.27	Ablim1;LOC103691383	
DMR1:277945001	1	277945001	277947000	2000	1	5.00E-11	0.5	32	1.6	Ablim1	
DMR1:277955001	1	277955001	277956000	1000	1	7.10E-08	0.38	16	1.6	Ablim1	
DMR1:277991001	1	277991001	277993000	2000	1	7.10E-08	0.35	29	1.45	Ablim1	
DMR1:278006001	1	278006001	278012000	6000	1	2.30E-07	-0.43	122	2.03	Ablim1;LOC102550100	
DMR1:278020001	1	278020001	278022000	2000	1	5.30E-07	-0.39	31	1.55	Ablim1;LOC102550100	
DMR1:278030001	1	278030001	278034000	4000	1	5.30E-09	0.4	68	1.7	Ablim1;LOC102550100	
DMR1:278049001	1	278049001	278051000	2000	1	7.90E-08	-0.48	33	1.65	Ablim1	
DMR1:278191001	1	278191001	278195000	4000	1	1.20E-11	0.65	79	1.98	LOC102550567;Fam160b1	
DMR1:278205001	1	278205001	278208000	3000	1	1.40E-07	0.34	47	1.57	Fam160b1	
DMR1:278212001	1	278212001	278216000	4000	1	2.80E-08	0.29	94	2.35	Fam160b1	
DMR1:278217001	1	278217001	278219000	2000	1	1.50E-07	0.45	31	1.55	Fam160b1	
DMR1:278227001	1	278227001	278230000	3000	1	7.50E-11	0.46	59	1.97	Fam160b1	
DMR1:278306001	1	278306001	278311000	5000	1	1.30E-11	-0.64	35	0.7	Mir6316;Trub1	Metabolism
DMR1:278313001	1	278313001	278316000	3000	1	4.80E-10	-0.64	30	1	Trub1	Metabolism
DMR1:278317001	1	278317001	278319000	2000	1	1.10E-07	0.4	35	1.75	Trub1	Metabolism
DMR1:278328001	1	278328001	278334000	6000	1	8.70E-09	0.36	102	1.7	Trub1	Metabolism
DMR1:278342001	1	278342001	278343000	1000	1	1.00E-08	-0.57	14	1.4	Trub1	Metabolism
DMR1:278383001	1	278383001	278384000	1000	1	2.20E-16	0.63	14	1.4	Trub1	Metabolism
DMR1:278389001	1	278389001	278394000	5000	1	4.80E-07	0.3	24	0.48	Trub1	Metabolism
DMR1:278395001	1	278395001	278398000	3000	1	1.90E-20	0.34	16	0.53	Trub1	Metabolism
DMR1:278427001	1	278427001	278429000	2000	1	2.50E-08	0.35	22	1.1	Trub1;LOC108349700	Metabolism
DMR1:278556001	1	278556001	278557000	1000	1	4.00E-11	0.68	14	1.4	Atrnl1	Extracellular Matrix
DMR1:278562001	1	278562001	278564000	2000	1	6.00E-10	-0.47	27	1.35	Atrnl1	Extracellular Matrix
DMR1:278565001	1	278565001	278566000	1000	1	2.10E-12	0.57	18	1.8	Atrnl1	Extracellular Matrix
DMR1:278573001	1	278573001	278576000	3000	3	6.50E-15	0.66	29	0.97	Atrnl1	Extracellular Matrix
DMR1:278581001	1	278581001	278583000	2000	1	3.40E-07	-0.62	30	1.5	Atrnl1	Extracellular Matrix
DMR1:278609001	1	278609001	278612000	3000	3	2.50E-24	0.96	33	1.1	Atrnl1	Extracellular Matrix
DMR1:278613001	1	278613001	278614000	1000	1	6.20E-07	-0.47	18	1.8	Atrnl1	Extracellular Matrix
DMR1:278625001	1	278625001	278635000	10000	1	3.90E-08	-0.51	146	1.46	Atrnl1	Extracellular Matrix
DMR1:278663001	1	278663001	278667000	4000	1	1.70E-08	0.32	58	1.45	Atrnl1	Extracellular Matrix
DMR1:278672001	1	278672001	278673000	1000	1	4.20E-07	0.57	8	0.8	Atrnl1	Extracellular Matrix
DMR1:278682001	1	278682001	278685000	3000	1	2.90E-16	0.52	38	1.27	Atrnl1	Extracellular Matrix
DMR1:278686001	1	278686001	278690000	4000	1	2.50E-09	-0.62	51	1.27	Atrnl1	Extracellular Matrix
DMR1:278706001	1	278706001	278711000	5000	2	2.40E-08	-0.45	39	0.78	Atrnl1	Extracellular Matrix

DMR1:278725001	1	278725001	278728000	3000	2	6.20E-26	1.12	27	0.9	Atrnl1	Extracellular Matrix
DMR1:278729001	1	278729001	278734000	5000	1	7.40E-07	-0.44	59	1.18	Atrnl1	Extracellular Matrix
DMR1:278737001	1	278737001	278740000	3000	1	7.90E-12	-0.6	51	1.7	Atrnl1	Extracellular Matrix
DMR1:278749001	1	278749001	278755000	6000	2	7.40E-10	0.42	87	1.45	Atrnl1	Extracellular Matrix
DMR1:278766001	1	278766001	278771000	5000	3	4.40E-10	-0.57	98	1.96	Atrnl1	Extracellular Matrix
DMR1:278776001	1	278776001	278780000	4000	2	1.40E-12	0.51	59	1.48	Atrnl1	Extracellular Matrix
DMR1:278790001	1	278790001	278791000	1000	1	3.90E-07	-0.58	29	2.9	Atrnl1	Extracellular Matrix
DMR1:278792001	1	278792001	278797000	5000	2	1.80E-20	0.9	67	1.34	Atrnl1	Extracellular Matrix
DMR1:278799001	1	278799001	278802000	3000	1	1.30E-07	-0.45	68	2.27	Atrnl1	Extracellular Matrix
DMR1:278805001	1	278805001	278806000	1000	1	8.70E-08	-0.44	13	1.3	Atrnl1;LOC108349827	Extracellular Matrix
DMR1:278815001	1	278815001	278816000	1000	1	4.50E-09	0.52	16	1.6	Atrnl1;LOC108349827	Extracellular Matrix
DMR1:278822001	1	278822001	278828000	6000	1	4.50E-08	0.4	72	1.2	Atrnl1;LOC108349827	Extracellular Matrix
DMR1:278846001	1	278846001	278847000	1000	1	1.60E-10	0.43	7	0.7	Atrnl1	Extracellular Matrix
DMR1:278848001	1	278848001	278851000	3000	1	4.50E-14	-0.59	11	0.37	Atrnl1	Extracellular Matrix
DMR1:278869001	1	278869001	278871000	2000	1	9.60E-19	0.73	24	1.2	Atrnl1	Extracellular Matrix
DMR1:278876001	1	278876001	278878000	2000	1	2.10E-08	0.49	13	0.65	Atrnl1	Extracellular Matrix
DMR1:278881001	1	278881001	278883000	2000	1	3.00E-20	0.8	26	1.3	Atrnl1	Extracellular Matrix
DMR1:278891001	1	278891001	278893000	2000	1	2.80E-26	0.95	28	1.4	Atrnl1	Extracellular Matrix
DMR1:278894001	1	278894001	278895000	1000	1	7.50E-15	0.65	8	0.8	Atrnl1	Extracellular Matrix
DMR1:278910001	1	278910001	278911000	1000	1	7.50E-14	-0.82	16	1.6	Atrnl1	Extracellular Matrix
DMR1:278920001	1	278920001	278921000	1000	1	6.00E-14	0.72	12	1.2	Atrnl1	Extracellular Matrix
DMR1:278943001	1	278943001	278945000	2000	1	4.50E-29	0.73	25	1.25	Atrnl1	Extracellular Matrix
DMR1:278957001	1	278957001	278959000	2000	1	3.80E-07	-0.48	38	1.9	Atrnl1	Extracellular Matrix
DMR1:278963001	1	278963001	278966000	3000	3	1.00E-34	1.21	33	1.1	Atrnl1	Extracellular Matrix
DMR1:278972001	1	278972001	278974000	2000	1	2.20E-07	0.37	32	1.6	Atrnl1	Extracellular Matrix
DMR1:278988001	1	278988001	278991000	3000	2	5.70E-19	0.84	44	1.47	Atrnl1	Extracellular Matrix
DMR1:279012001	1	279012001	279016000	4000	1	2.70E-11	0.47	55	1.38	Atrnl1	Extracellular Matrix
DMR1:279042001	1	279042001	279048000	6000	1	1.00E-07	0.31	96	1.6	Atrnl1	Extracellular Matrix
DMR1:279079001	1	279079001	279083000	4000	1	1.00E-09	0.42	57	1.43	Atrnl1	Extracellular Matrix
DMR1:279099001	1	279099001	279101000	2000	2	2.50E-08	-0.46	26	1.3	Atrnl1	Extracellular Matrix
DMR1:279108001	1	279108001	279111000	3000	2	4.70E-11	0.44	70	2.33	Atrnl1	Extracellular Matrix
DMR1:279201001	1	279201001	279202000	1000	1	8.00E-16	0.59	17	1.7	Gfra1	Receptor
DMR1:279215001	1	279215001	279218000	3000	1	6.70E-07	-0.39	47	1.57	Gfra1;LOC102556644	Receptor
DMR1:279234001	1	279234001	279243000	9000	7	1.90E-28	-0.65	113	1.26	Gfra1;LOC102556644	Receptor
DMR1:279247001	1	279247001	279249000	2000	2	6.20E-17	0.75	28	1.4	Gfra1;LOC102556644	Receptor
DMR1:279271001	1	279271001	279274000	3000	1	5.60E-07	0.33	55	1.83	Gfra1	Receptor
DMR1:279295001	1	279295001	279296000	1000	1	5.20E-10	-0.6	13	1.3	Gfra1	Receptor
DMR1:279310001	1	279310001	279311000	1000	1	2.10E-07	0.5	28	2.8	Gfra1	Receptor
DMR1:279384001	1	279384001	279386000	2000	1	6.10E-07	0.68	19	0.95	Gfra1	Receptor
DMR1:279415001	1	279415001	279417000	2000	2	2.50E-12	0.57	44	2.2	Gfra1	Receptor
DMR1:279418001	1	279418001	279419000	1000	1	1.10E-07	0.51	16	1.6	Gfra1	Receptor
DMR1:279429001	1	279429001	279431000	2000	1	6.80E-13	0.79	23	1.15	Gfra1	Receptor
DMR1:279472001	1	279472001	279475000	3000	1	8.50E-07	-0.49	9	0.3	Gfra1	Receptor
DMR1:279479001	1	279479001	279481000	2000	1	1.20E-07	0.58	28	1.4	Gfra1	Receptor
DMR1:279520001	1	279520001	279521000	1000	1	1.50E-08	0.69	7	0.7	Gfra1	Receptor
DMR1:279536001	1	279536001	279540000	4000	2	1.50E-16	0.7	64	1.6	Gfra1	Receptor
DMR1:279555001	1	279555001	279556000	1000	1	7.30E-15	0.76	15	1.5	Gfra1	Receptor
DMR1:279647001	1	279647001	279660000	13000	2	5.70E-35	1.05	166	1.28	Ccdc172;LOC108349704	
DMR1:279685001	1	279685001	279688000	3000	1	4.90E-10	0.47	43	1.43	Ccdc172	
DMR1:279689001	1	279689001	279693000	4000	1	1.00E-08	0.51	32	0.8	Ccdc172	
DMR1:279790001	1	279790001	279793000	3000	2	5.90E-10	0.48	36	1.2	LOC108349706;Pnlip	Metabolism
DMR1:279796001	1	279796001	279798000	2000	1	5.50E-13	0.45	40	2	Pnlip	Metabolism
DMR1:279873001	1	279873001	279874000	1000	1	6.80E-11	0.57	12	1.2	Pnliprp1	Metabolism
DMR1:279879001	1	279879001	279880000	1000	1	6.10E-08	-0.54	12	1.2	Pnliprp1	Metabolism
DMR1:279887001	1	279887001	279888000	1000	1	5.40E-16	0.69	10	1	Pnliprp1;Pnliprp2	Metabolism
DMR1:279894001	1	279894001	279895000	1000	1	2.70E-23	1	5	0.5	Pnliprp2	Metabolism
DMR1:279903001	1	279903001	279905000	2000	1	3.70E-11	0.55	28	1.4	Pnliprp2	Metabolism
DMR1:279915001	1	279915001	279918000	3000	1	5.40E-07	0.39	33	1.1	Pnliprp2	Metabolism
DMR1:279920001	1	279920001	279922000	2000	1	2.20E-16	0.78	15	0.75	Pnliprp2	Metabolism
DMR1:279925001	1	279925001	279926000	1000	1	6.40E-09	0.39	15	1.5	Pnliprp2	Metabolism
DMR1:279938001	1	279938001	279945000	7000	3	4.90E-34	1.01	83	1.19	LOC681006;Hspa12a	
DMR1:279951001	1	279951001	279956000	5000	2	3.50E-08	0.52	57	1.14	LOC681006;Hspa12a	
DMR1:279969001	1	279969001	279973000	4000	1	5.30E-10	0.59	65	1.62	Hspa12a	
DMR1:279974001	1	279974001	279978000	4000	3	4.80E-18	0.64	54	1.35	Hspa12a	
DMR1:280018001	1	280018001	280020000	2000	1	2.50E-07	-0.51	20	1	Hspa12a;LOC103691386	

DMR1:280021001	1	280021001	280023000	2000	1	2.40E-07	0.41	24	1.2	Hspa12a;LOC103691386	
DMR1:280029001	1	280029001	280042000	13000	4	1.10E-18	0.95	198	1.52	Hspa12a;LOC100363557	
DMR1:280046001	1	280046001	280050000	4000	2	6.10E-28	0.99	32	0.8	Hspa12a;LOC100363557	
DMR1:280051001	1	280051001	280054000	3000	1	7.60E-09	-0.61	61	2.03	Hspa12a;LOC100363557	
DMR1:280061001	1	280061001	280063000	2000	1	1.20E-17	0.81	37	1.85	Hspa12a	
DMR1:280064001	1	280064001	280066000	2000	1	1.60E-07	0.32	47	2.35	Hspa12a	
DMR1:280072001	1	280072001	280075000	3000	1	6.70E-16	0.6	42	1.4	Hspa12a	
DMR1:280101001	1	280101001	280103000	2000	1	6.90E-17	0.58	37	1.85	Hspa12a;Eno4	Metabolism
DMR1:280119001	1	280119001	280122000	3000	1	1.30E-07	-0.45	55	1.83	Eno4;LOC103691387;Shtn1	Metabolism
DMR1:280137001	1	280137001	280145000	8000	1	1.80E-10	0.39	117	1.46	Eno4;Shtn1	Metabolism
DMR1:280147001	1	280147001	280150000	3000	1	3.30E-08	-0.45	42	1.4	Shtn1	
DMR1:280159001	1	280159001	280161000	2000	1	2.50E-12	0.61	15	0.75	Shtn1	
DMR1:280165001	1	280165001	280168000	3000	1	5.90E-19	0.87	27	0.9	Shtn1	
DMR1:280170001	1	280170001	280176000	6000	2	1.00E-15	0.46	72	1.2	Shtn1	
DMR1:280243001	1	280243001	280247000	4000	1	1.70E-07	-0.5	44	1.1	Shtn1	
DMR1:280325001	1	280325001	280329000	4000	1	7.70E-11	0.52	60	1.5	Vax1	Development
DMR1:280339001	1	280339001	280340000	1000	1	9.00E-09	0.71	35	3.5	Vax1;LOC108349709	Development
DMR1:280380001	1	280380001	280381000	1000	1	1.10E-14	0.65	6	0.6	Kcnk18	Transport
DMR1:280384001	1	280384001	280385000	1000	1	5.70E-07	-0.46	13	1.3	Kcnk18	Transport
DMR1:280386001	1	280386001	280395000	9000	3	6.10E-20	0.84	97	1.08	Kcnk18;Slc18a2	Transport;Transport
DMR1:280409001	1	280409001	280411000	2000	1	1.50E-24	0.76	41	2.05	Slc18a2	Transport
DMR1:280415001	1	280415001	280416000	1000	1	4.30E-12	0.64	6	0.6	Slc18a2	Transport
DMR1:280438001	1	280438001	280440000	2000	1	6.60E-14	0.63	19	0.95	Slc18a2	Transport
DMR1:280443001	1	280443001	280449000	6000	1	3.40E-11	0.46	73	1.22	Slc18a2;Pdzd8	Transport;Cytoskeleton
DMR1:280481001	1	280481001	280484000	3000	1	7.80E-09	0.6	27	0.9	Pdzd8	Cytoskeleton
DMR1:280639001	1	280639001	280641000	2000	1	9.40E-07	0.34	30	1.5	LOC103691392;Emx2	Development
DMR1:281065001	1	281065001	281066000	1000	1	2.40E-15	0.63	20	2	Rab11fip2	
DMR1:281765001	1	281765001	281766000	1000	1	3.90E-10	0.55	4	0.4	Prlhr	Signaling
DMR1:281807001	1	281807001	281809000	2000	1	2.40E-09	0.45	13	0.65	Cacul1	
DMR1:281824001	1	281824001	281827000	3000	1	4.90E-07	-0.46	26	0.87	Cacul1	
DMR1:281829001	1	281829001	281830000	1000	1	2.50E-08	-0.55	5	0.5	Cacul1	
DMR1:281842001	1	281842001	281844000	2000	1	2.30E-09	0.43	19	0.95	Cacul1	
DMR1:281851001	1	281851001	281852000	1000	1	3.20E-13	0.63	4	0.4	Cacul1	
DMR1:281857001	1	281857001	281858000	1000	1	8.10E-09	0.67	2	0.2	Cacul1;LOC100359574	
DMR1:282127001	1	282127001	282128000	1000	1	5.40E-11	0.62	7	0.7	Nanos1	Metabolism
DMR1:282131001	1	282131001	282133000	2000	1	8.30E-11	0.55	18	0.9	Nanos1;Eif3a	Metabolism;Translation
DMR1:282145001	1	282145001	282146000	1000	1	4.70E-12	0.58	21	2.1	Nanos1;Eif3a	Metabolism;Translation
DMR1:282147001	1	282147001	282150000	3000	1	1.00E-11	0.54	30	1	Nanos1;Eif3a	Metabolism;Translation
DMR1:282225001	1	282225001	282226000	1000	1	9.40E-07	0.4	3	0.3	Sfxn4	Transport
DMR1:282243001	1	282243001	282245000	2000	1	3.70E-11	0.61	21	1.05	Sfxn4;Prdx3	Transport;Metabolism
DMR1:282249001	1	282249001	282251000	2000	2	2.60E-11	0.67	34	1.7	Prdx3	Metabolism
DMR1:282261001	1	282261001	282262000	1000	1	4.20E-10	0.6	4	0.4	Prdx3;Grk5	Metabolism;Signaling
DMR1:282279001	1	282279001	282283000	4000	2	2.40E-16	0.82	28	0.7	Grk5	Signaling
DMR1:282284001	1	282284001	282286000	2000	1	4.80E-10	-0.63	11	0.55	Grk5	Signaling
DMR1:282298001	1	282298001	282300000	2000	1	2.30E-10	-0.51	22	1.1	Grk5	Signaling
DMR1:282303001	1	282303001	282304000	1000	1	3.80E-08	0.55	2	0.2	Grk5	Signaling
DMR1:282348001	1	282348001	282350000	2000	1	7.00E-08	0.34	23	1.15	Grk5	Signaling
DMR1:282367001	1	282367001	282369000	2000	1	4.30E-12	0.67	16	0.8	Grk5	Signaling
DMR1:282393001	1	282393001	282394000	1000	1	8.10E-12	0.47	18	1.8	Grk5	Signaling
DMR1:282432001	1	282432001	282436000	4000	2	4.50E-09	-0.54	13	0.32	Grk5	Signaling
DMR1:282439001	1	282439001	282441000	2000	2	7.10E-19	0.99	9	0.45	Grk5	Signaling
DMR1:282477001	1	282477001	282478000	1000	1	6.60E-08	-0.64	8	0.8	Grk5;LOC102550291	Signaling
DMR1:282553001	1	282553001	282557000	4000	1	6.90E-09	0.43	23	0.58	LOC102550729;RGD1563099	
DMR1:282558001	1	282558001	282560000	2000	1	1.90E-14	0.59	40	2	LOC102550729;RGD1563099;Ces2i	Metabolism
DMR1:282688001	1	282688001	282689000	1000	1	1.10E-10	-0.37	9	0.9	LOC108348093;Ces2	
DMR2:1400001	2	1400001	1403000	3000	1	1.70E-11	-0.6	21	0.7	LOC102548635;Erap1	Protease
DMR2:1406001	2	1406001	1407000	1000	1	2.40E-10	0.68	4	0.4	LOC102548635;Erap1	Protease
DMR2:1408001	2	1408001	1409000	1000	1	2.90E-07	0.6	5	0.5	LOC102548635;Erap1	Protease

DMR2:1429001	2	1429001	1430000	1000	1	1.10E-09	0.59	3	0.3	Erap1	Protease
DMR2:1431001	2	1431001	1438000	7000	5	5.60E-18	-0.45	74	1.06	Erap1	Protease
DMR2:1456001	2	1456001	1459000	3000	3	2.40E-14	0.82	13	0.43	Erap1;Cast	Protease;Protease; Proteolysis
DMR2:1468001	2	1468001	1469000	1000	1	7.10E-17	0.92	5	0.5	Cast	Protease; Proteolysis
DMR2:1470001	2	1470001	1474000	4000	1	3.10E-07	-0.58	13	0.32	Cast	Protease; Proteolysis
DMR2:1483001	2	1483001	1484000	1000	1	1.90E-14	0.76	4	0.4	Cast	Protease; Proteolysis
DMR2:1485001	2	1485001	1487000	2000	2	1.10E-22	0.98	17	0.85	Cast	Protease; Proteolysis
DMR2:1490001	2	1490001	1492000	2000	2	2.20E-08	-0.55	10	0.5	Cast	Protease; Proteolysis
DMR2:1498001	2	1498001	1499000	1000	1	8.70E-12	0.73	4	0.4	Cast	Protease; Proteolysis
DMR2:1525001	2	1525001	1529000	4000	1	2.60E-08	-0.38	49	1.23	Cast	Protease; Proteolysis
DMR2:1552001	2	1552001	1553000	1000	1	8.40E-08	0.6	4	0.4	Cast	Protease; Proteolysis
DMR2:2131001	2	2131001	2134000	3000	3	1.80E-22	-0.55	30	1	Rpl17-ps1	
DMR2:2136001	2	2136001	2137000	1000	1	2.60E-15	0.6	6	0.6	Rpl17-ps1	
DMR2:2140001	2	2140001	2142000	2000	1	9.00E-07	-0.59	10	0.5	Rpl17-ps1	
DMR2:2148001	2	2148001	2154000	6000	3	3.40E-12	-0.62	44	0.73	Rpl17-ps1	
DMR2:2465001	2	2465001	2466000	1000	1	1.10E-14	0.77	6	0.6	LOC102548988;Ell2;LOC108349911	Transcription
DMR2:2467001	2	2467001	2468000	1000	1	1.50E-11	0.64	3	0.3	Ell2;LOC108349911	Transcription
DMR2:2497001	2	2497001	2499000	2000	1	1.50E-16	0.71	19	0.95	Ell2;LOC108350152	Transcription
DMR2:2516001	2	2516001	2517000	1000	1	2.00E-20	0.67	9	0.9	Ell2;LOC108350152	Transcription
DMR2:2527001	2	2527001	2528000	1000	1	4.40E-17	0.91	3	0.3	Ell2;LOC102549076	Transcription
DMR2:2623001	2	2623001	2625000	2000	1	3.10E-08	0.58	6	0.3	Glrx;Rhobtb3	Metabolism;Signaling
DMR2:2629001	2	2629001	2630000	1000	1	3.10E-07	-0.51	5	0.5	Rhobtb3	Signaling
DMR2:2668001	2	2668001	2671000	3000	1	3.40E-09	-0.65	32	1.07	Rhobtb3	Signaling
DMR2:2683001	2	2683001	2684000	1000	1	6.30E-09	0.58	57	5.7	Rhobtb3	Signaling
DMR2:2687001	2	2687001	2689000	2000	1	1.30E-11	-0.77	6	0.3	Rhobtb3	Signaling
DMR2:2722001	2	2722001	2726000	4000	3	2.00E-14	0.93	32	0.8	Spata9	
DMR2:2736001	2	2736001	2738000	2000	1	1.90E-07	0.51	4	0.2	Spata9	
DMR2:2740001	2	2740001	2744000	4000	2	6.50E-12	-0.67	16	0.4	Spata9	
DMR2:2752001	2	2752001	2758000	6000	5	7.20E-13	-0.65	54	0.9	Spata9;Rfesd	Metabolism
DMR2:2771001	2	2771001	2779000	8000	5	3.90E-12	-0.51	65	0.81	Spata9;Rfesd;LOC108349912	Metabolism
DMR2:2817001	2	2817001	2824000	7000	2	4.60E-08	-0.51	66	0.94	Gpr150	Signaling
DMR2:2832001	2	2832001	2834000	2000	1	1.80E-08	0.48	8	0.4	Arsk	
DMR2:2850001	2	2850001	2855000	5000	1	1.80E-11	-0.36	30	0.6	Arsk	
DMR2:2860001	2	2860001	2862000	2000	1	3.30E-08	0.61	16	0.8	Arsk	
DMR2:2866001	2	2866001	2869000	3000	1	1.50E-12	-0.53	22	0.73	Arsk	
DMR2:2873001	2	2873001	2875000	2000	1	7.80E-23	0.73	15	0.75	Arsk	
DMR2:2892001	2	2892001	2894000	2000	2	7.30E-22	1.05	16	0.8	Arsk;Ttc37	
DMR2:2899001	2	2899001	2905000	6000	4	1.30E-24	-0.78	62	1.03	Arsk;Ttc37	
DMR2:2906001	2	2906001	2908000	2000	1	4.20E-10	0.52	16	0.8	Ttc37	
DMR2:2913001	2	2913001	2916000	3000	1	1.30E-08	0.47	12	0.4	Ttc37	
DMR2:2919001	2	2919001	2927000	8000	1	2.00E-15	-0.58	69	0.86	Ttc37	
DMR2:2928001	2	2928001	2930000	2000	1	1.60E-07	-0.49	12	0.6	Ttc37	
DMR2:2954001	2	2954001	2956000	2000	1	1.90E-07	-0.65	17	0.85	Ttc37	
DMR2:2972001	2	2972001	2973000	1000	1	5.00E-11	0.57	10	1	Ttc37	
DMR2:2974001	2	2974001	2981000	7000	3	6.40E-15	-0.63	57	0.81	Ttc37	
DMR2:2982001	2	2982001	2987000	5000	4	6.80E-29	-0.57	62	1.24	Ttc37	
DMR2:2989001	2	2989001	2991000	2000	1	2.70E-07	-0.31	10	0.5	Ttc37	
DMR2:3010001	2	3010001	3012000	2000	1	8.20E-07	0.45	15	0.75	Ttc37;Fam81b	
DMR2:3014001	2	3014001	3015000	1000	1	6.40E-08	0.39	10	1	Ttc37;Fam81b	
DMR2:3028001	2	3028001	3031000	3000	1	8.60E-10	-0.43	18	0.6	Ttc37;Fam81b	
DMR2:3032001	2	3032001	3033000	1000	1	1.30E-17	0.63	9	0.9	Ttc37;Fam81b	
DMR2:3065001	2	3065001	3067000	2000	2	6.70E-09	0.63	1	0.05	Fam81b	
DMR2:3071001	2	3071001	3072000	1000	1	3.90E-10	0.61	4	0.4	Fam81b	
DMR2:3075001	2	3075001	3083000	8000	5	4.50E-16	-0.47	85	1.06	Fam81b	

DMR2:3085001	2	3085001	3086000	1000	1	1.20E-16	-0.65	1	0.1	Fam81b	
DMR2:3087001	2	3087001	3088000	1000	1	1.50E-08	-0.66	4	0.4	Fam81b	
DMR2:3094001	2	3094001	3095000	1000	1	2.40E-12	0.61	1	0.1	Fam81b	
DMR2:3101001	2	3101001	3107000	6000	3	7.80E-18	-0.76	21	0.35	Fam81b	
DMR2:3108001	2	3108001	3111000	3000	1	5.90E-08	-0.47	16	0.53	Fam81b	
DMR2:3125001	2	3125001	3127000	2000	1	2.90E-08	0.35	21	1.05	Fam81b	
DMR2:3139001	2	3139001	3141000	2000	1	4.00E-07	-0.36	21	1.05	Fam81b	
DMR2:3142001	2	3142001	3147000	5000	2	1.40E-10	-0.43	42	0.84	Fam81b	
DMR2:3398001	2	3398001	3400000	2000	1	1.10E-09	0.55	10	0.5	Mctp1	
DMR2:3417001	2	3417001	3420000	3000	3	1.20E-13	-0.67	9	0.3	Mctp1	
DMR2:3428001	2	3428001	3429000	1000	1	4.30E-07	0.52	8	0.8	Mctp1	
DMR2:3440001	2	3440001	3443000	3000	2	1.00E-09	-0.53	23	0.77	Mctp1	
DMR2:3459001	2	3459001	3465000	6000	3	1.50E-20	-0.59	44	0.73	Mctp1	
DMR2:3481001	2	3481001	3488000	7000	1	7.30E-08	0.38	47	0.67	Mctp1	
DMR2:3496001	2	3496001	3503000	7000	3	2.60E-17	-0.66	59	0.84	Mctp1	
DMR2:3513001	2	3513001	3515000	2000	1	3.60E-15	0.82	8	0.4	Mctp1	
DMR2:3523001	2	3523001	3524000	1000	1	9.00E-08	-0.61	2	0.2	Mctp1	
DMR2:3540001	2	3540001	3545000	5000	2	2.40E-13	-0.67	22	0.44	Mctp1	
DMR2:3550001	2	3550001	3552000	2000	1	3.20E-08	0.38	45	2.25	Mctp1	
DMR2:3572001	2	3572001	3579000	7000	5	6.70E-20	-0.58	81	1.16	Mctp1	
DMR2:3601001	2	3601001	3606000	5000	3	4.90E-20	-0.51	53	1.06	Mctp1	
DMR2:3607001	2	3607001	3608000	1000	1	1.80E-07	0.45	4	0.4	Mctp1	
DMR2:3630001	2	3630001	3633000	3000	2	2.80E-24	0.97	28	0.93	Mctp1	
DMR2:3649001	2	3649001	3654000	5000	2	8.20E-13	0.49	40	0.8	Mctp1;LOC309927	
DMR2:3661001	2	3661001	3662000	1000	1	2.40E-11	0.65	4	0.4	Mctp1;LOC309927	
DMR2:3667001	2	3667001	3670000	3000	1	1.30E-07	-0.35	12	0.4	Mctp1;LOC309927;LOC680518	
DMR2:3673001	2	3673001	3674000	1000	1	2.90E-10	0.53	9	0.9	Mctp1;LOC680518	
DMR2:3678001	2	3678001	3679000	1000	1	1.10E-08	0.63	3	0.3	Mctp1;LOC680518	
DMR2:3690001	2	3690001	3694000	4000	1	1.00E-16	-0.71	16	0.4	Mctp1	
DMR2:3695001	2	3695001	3701000	6000	2	1.10E-15	-0.57	51	0.85	Mctp1	
DMR2:3709001	2	3709001	3710000	1000	1	9.90E-15	0.78	0	0	Mctp1	
DMR2:3712001	2	3712001	3723000	11000	8	1.00E-17	-0.68	98	0.89	Mctp1	
DMR2:3743001	2	3743001	3746000	3000	1	9.10E-07	-0.36	11	0.37	Mctp1	
DMR2:3778001	2	3778001	3780000	2000	1	9.20E-08	0.55	20	1	Mctp1	
DMR2:3795001	2	3795001	3798000	3000	2	7.60E-10	-0.63	10	0.33	Mctp1	
DMR2:3799001	2	3799001	3806000	7000	3	5.80E-14	-0.54	52	0.74	Mctp1	
DMR2:3811001	2	3811001	3817000	6000	5	1.00E-19	-0.52	66	1.1	Mctp1	
DMR2:3863001	2	3863001	3866000	3000	2	2.30E-20	0.76	23	0.77	Mctp1	
DMR2:3870001	2	3870001	3873000	3000	3	1.20E-13	0.81	19	0.63	Mctp1	
DMR2:3892001	2	3892001	3897000	5000	3	8.50E-18	0.94	16	0.32	Mctp1	
DMR2:3899001	2	3899001	3901000	2000	2	1.60E-17	-0.74	9	0.45	Mctp1	
DMR2:3907001	2	3907001	3913000	6000	4	5.40E-25	-0.63	69	1.15	Mctp1	
DMR2:3925001	2	3925001	3932000	7000	3	5.90E-11	-0.54	33	0.47	Mctp1	
DMR2:3969001	2	3969001	3971000	2000	1	1.60E-12	-0.68	7	0.35	Mctp1	
DMR2:3972001	2	3972001	3973000	1000	1	2.00E-07	-0.46	1	0.1	Mctp1	
DMR2:3982001	2	3982001	3985000	3000	2	3.90E-12	-0.6	10	0.33	Mctp1	
DMR2:3996001	2	3996001	4003000	7000	3	6.20E-11	-0.52	59	0.84	Mctp1	
DMR2:4004001	2	4004001	4006000	2000	1	9.80E-09	0.42	13	0.65	Mctp1	
DMR2:4007001	2	4007001	4011000	4000	4	3.00E-25	0.8	64	1.6	Mctp1	
DMR2:4023001	2	4023001	4025000	2000	1	2.20E-29	0.82	12	0.6	Mctp1	
DMR2:4036001	2	4036001	4038000	2000	2	2.00E-10	-0.5	8	0.4	Mctp1	
DMR2:4040001	2	4040001	4043000	3000	1	4.20E-12	-0.42	17	0.57	Mctp1	
DMR2:4047001	2	4047001	4054000	7000	2	1.70E-13	-0.48	42	0.6	Mctp1	
DMR2:4055001	2	4055001	4059000	4000	2	1.90E-10	-0.45	22	0.55	Mctp1	
DMR2:4063001	2	4063001	4066000	3000	1	2.30E-15	0.89	26	0.87	Mctp1	
DMR2:4104001	2	4104001	4111000	7000	2	3.20E-18	-0.51	70	1	Slf1	
DMR2:4126001	2	4126001	4127000	1000	1	1.30E-11	0.71	5	0.5	Slf1	
DMR2:4130001	2	4130001	4133000	3000	2	3.90E-15	0.6	27	0.9	Slf1	
DMR2:4134001	2	4134001	4142000	8000	5	3.50E-19	0.59	92	1.15	Slf1	
DMR2:4148001	2	4148001	4150000	2000	2	3.80E-09	-0.6	12	0.6	Slf1	
DMR2:4165001	2	4165001	4170000	5000	2	2.30E-09	-0.55	31	0.62	Slf1	
DMR2:4177001	2	4177001	4181000	4000	2	2.20E-09	-0.66	17	0.42	Slf1	
DMR2:4199001	2	4199001	4201000	2000	1	7.00E-09	0.42	14	0.7	Slf1;RGD1560883	
DMR2:4206001	2	4206001	4214000	8000	2	5.50E-11	0.64	45	0.56	RGD1560883	
DMR2:4222001	2	4222001	4224000	2000	2	1.70E-14	0.82	6	0.3	RGD1560883	

DMR2:4241001	2	4241001	4242000	1000	1	1.10E-10	0.82	0	0	RGD1560883;LOC102546510	
DMR2:4254001	2	4254001	4259000	5000	1	6.70E-09	0.61	23	0.46	RGD1560883	
DMR2:4275001	2	4275001	4283000	8000	5	1.10E-16	0.7	76	0.95	RGD1560883	
DMR2:4285001	2	4285001	4286000	1000	1	1.20E-08	0.63	8	0.8	RGD1560883	
DMR2:4287001	2	4287001	4298000	11000	5	2.20E-22	-0.54	74	0.67	RGD1560883	
DMR2:4299001	2	4299001	4302000	3000	1	1.50E-10	0.65	22	0.73	RGD1560883	
DMR2:4357001	2	4357001	4362000	5000	1	8.00E-07	0.48	43	0.86	RGD1560883;LOC680595	
DMR2:4364001	2	4364001	4365000	1000	1	6.20E-12	0.76	1	0.1	RGD1560883;LOC680595	
DMR2:4375001	2	4375001	4377000	2000	1	1.90E-09	0.49	14	0.7	RGD1560883	
DMR2:4385001	2	4385001	4392000	7000	4	1.10E-16	-0.48	51	0.73	RGD1560883	
DMR2:4393001	2	4393001	4397000	4000	1	2.80E-07	-0.63	13	0.32	RGD1560883	
DMR2:4398001	2	4398001	4402000	4000	2	1.40E-13	-0.66	35	0.88	RGD1560883	
DMR2:4413001	2	4413001	4414000	1000	1	1.10E-07	-0.3	10	1	RGD1560883	
DMR2:4415001	2	4415001	4420000	5000	2	1.30E-13	-0.46	64	1.28	RGD1560883	
DMR2:4435001	2	4435001	4436000	1000	1	4.10E-07	-0.5	11	1.1	RGD1560883	
DMR2:4439001	2	4439001	4441000	2000	1	4.70E-24	0.9	19	0.95	RGD1560883	
DMR2:4449001	2	4449001	4451000	2000	1	2.70E-10	-0.43	16	0.8	RGD1560883	
DMR2:4452001	2	4452001	4458000	6000	4	6.10E-21	-0.5	62	1.03	RGD1560883	
DMR2:4461001	2	4461001	4462000	1000	1	1.20E-08	0.62	5	0.5	RGD1560883	
DMR2:4475001	2	4475001	4478000	3000	2	8.50E-08	0.51	15	0.5	RGD1560883	
DMR2:4480001	2	4480001	4484000	4000	1	8.60E-07	-0.41	25	0.62	RGD1560883	
DMR2:4485001	2	4485001	4486000	1000	1	2.10E-07	-0.39	11	1.1	RGD1560883	
DMR2:4495001	2	4495001	4500000	5000	2	5.40E-30	1.09	15	0.3	RGD1560883	
DMR2:4522001	2	4522001	4524000	2000	1	1.70E-07	-0.58	7	0.35	RGD1560883	
DMR2:4525001	2	4525001	4527000	2000	1	1.10E-09	0.52	10	0.5	RGD1560883	
DMR2:4530001	2	4530001	4531000	1000	1	2.30E-07	0.44	3	0.3	RGD1560883	
DMR2:4539001	2	4539001	4542000	3000	2	6.70E-10	-0.73	11	0.37	RGD1560883	
DMR2:4559001	2	4559001	4564000	5000	4	2.00E-24	-0.52	45	0.9	RGD1560883	
DMR2:4565001	2	4565001	4571000	6000	4	3.30E-31	-0.65	70	1.17	RGD1560883	
DMR2:4575001	2	4575001	4577000	2000	1	2.30E-09	0.6	10	0.5	RGD1560883	
DMR2:4602001	2	4602001	4603000	1000	1	1.90E-07	-0.39	4	0.4	RGD1560883	
DMR2:4604001	2	4604001	4605000	1000	1	1.90E-11	0.56	7	0.7	RGD1560883	
DMR2:4612001	2	4612001	4613000	1000	1	2.30E-09	0.58	2	0.2	RGD1560883	
DMR2:4616001	2	4616001	4625000	9000	5	9.80E-22	-0.48	93	1.03	RGD1560883	
DMR2:4636001	2	4636001	4638000	2000	1	2.10E-09	0.61	7	0.35	RGD1560883	
DMR2:4651001	2	4651001	4654000	3000	1	1.50E-09	0.64	10	0.33	RGD1560883	
DMR2:4669001	2	4669001	4671000	2000	1	7.20E-12	0.67	10	0.5	RGD1560883	
DMR2:4673001	2	4673001	4677000	4000	2	7.00E-15	-0.51	28	0.7	RGD1560883	
DMR2:4678001	2	4678001	4683000	5000	5	1.60E-21	-0.7	49	0.98	RGD1560883	
DMR2:4695001	2	4695001	4701000	6000	2	5.80E-14	-0.5	45	0.75	RGD1560883	
DMR2:4703001	2	4703001	4704000	1000	1	1.10E-07	0.42	6	0.6	RGD1560883	
DMR2:4711001	2	4711001	4713000	2000	2	1.90E-10	-0.55	14	0.7	RGD1560883	
DMR2:4720001	2	4720001	4730000	10000	4	3.20E-28	-0.6	86	0.86	RGD1560883	
DMR2:4741001	2	4741001	4749000	8000	6	3.00E-13	-0.41	86	1.07	RGD1560883	
DMR2:4758001	2	4758001	4764000	6000	4	1.90E-16	-0.46	78	1.3	RGD1560883	
DMR2:4786001	2	4786001	4792000	6000	3	7.70E-15	-0.68	53	0.88	Olr206	Receptor
DMR2:4793001	2	4793001	4796000	3000	3	3.50E-09	0.54	28	0.93	Olr206	Receptor
DMR2:4804001	2	4804001	4806000	2000	2	3.10E-22	1.08	6	0.3	Olr206	Receptor
DMR2:4809001	2	4809001	4813000	4000	4	3.10E-21	-0.54	43	1.07	Olr206;Olr205	Receptor
DMR2:4814001	2	4814001	4817000	3000	2	9.30E-14	-0.5	27	0.9	Olr205	Receptor
DMR2:4821001	2	4821001	4823000	2000	1	6.90E-14	0.54	13	0.65	Olr205	Receptor
DMR2:4824001	2	4824001	4825000	1000	1	2.40E-07	0.53	3	0.3	Olr205	Receptor
DMR2:4826001	2	4826001	4827000	1000	1	5.90E-11	0.77	3	0.3	Olr205	Receptor
DMR2:4864001	2	4864001	4866000	2000	2	1.70E-12	0.86	10	0.5	Olr204	Receptor
DMR2:4868001	2	4868001	4869000	1000	1	3.20E-11	0.58	5	0.5	Olr204	Receptor
DMR2:4932001	2	4932001	4937000	5000	1	6.40E-09	-0.44	31	0.62	Fam172a	
DMR2:4948001	2	4948001	4950000	2000	1	8.60E-11	0.51	17	0.85	Fam172a	
DMR2:4951001	2	4951001	4955000	4000	2	2.80E-17	0.76	21	0.52	Fam172a	
DMR2:4956001	2	4956001	4958000	2000	1	9.30E-09	0.43	11	0.55	Fam172a	
DMR2:4983001	2	4983001	4984000	1000	1	3.60E-12	-0.51	13	1.3	Fam172a	
DMR2:4995001	2	4995001	5003000	8000	5	6.10E-12	-0.52	77	0.96	Fam172a	
DMR2:5009001	2	5009001	5014000	5000	1	5.80E-19	0.72	40	0.8	Fam172a	
DMR2:5018001	2	5018001	5021000	3000	1	1.10E-10	-0.48	24	0.8	Fam172a	
DMR2:5033001	2	5033001	5042000	9000	4	2.70E-16	0.91	61	0.68	Fam172a	
DMR2:5043001	2	5043001	5047000	4000	2	9.70E-13	-0.41	56	1.4	Fam172a	

DMR2:5054001	2	5054001	5055000	1000	1	4.60E-17	0.97	7	0.7	Fam172a	
DMR2:5061001	2	5061001	5065000	4000	1	9.50E-09	-0.41	42	1.05	Fam172a	
DMR2:5066001	2	5066001	5067000	1000	1	5.20E-15	0.92	7	0.7	Fam172a	
DMR2:5082001	2	5082001	5083000	1000	1	3.20E-09	0.75	1	0.1	Fam172a	
DMR2:5124001	2	5124001	5129000	5000	2	2.60E-10	-0.55	36	0.72	Fam172a	
DMR2:5154001	2	5154001	5155000	1000	1	8.10E-08	0.43	12	1.2	Fam172a;LOC100911678	
DMR2:5160001	2	5160001	5163000	3000	1	3.20E-10	0.78	13	0.43	Fam172a;LOC100911678	
DMR2:5196001	2	5196001	5197000	1000	1	3.70E-17	0.93	7	0.7	Fam172a	
DMR2:5203001	2	5203001	5207000	4000	2	1.50E-12	-0.47	50	1.25	Fam172a	
DMR2:5208001	2	5208001	5209000	1000	1	6.40E-09	0.66	9	0.9	Fam172a	
DMR2:5223001	2	5223001	5224000	1000	1	1.00E-07	0.53	5	0.5	Fam172a	
DMR2:5265001	2	5265001	5269000	4000	1	2.70E-07	-0.59	29	0.72	Fam172a	
DMR2:5293001	2	5293001	5296000	3000	2	3.20E-08	0.45	24	0.8	Fam172a;LOC680638	
DMR2:5306001	2	5306001	5311000	5000	3	2.30E-22	1.01	20	0.4	Fam172a;LOC680638	
DMR2:5319001	2	5319001	5329000	10000	3	1.70E-28	-0.57	93	0.93	Fam172a	
DMR2:5331001	2	5331001	5334000	3000	2	1.60E-07	-0.45	16	0.53	Fam172a	
DMR2:5345001	2	5345001	5348000	3000	3	5.30E-20	-0.45	27	0.9	Fam172a	
DMR2:5349001	2	5349001	5357000	8000	5	7.60E-12	0.63	53	0.66	Fam172a	
DMR2:5358001	2	5358001	5359000	1000	1	3.10E-08	0.4	8	0.8	Fam172a	
DMR2:5360001	2	5360001	5361000	1000	1	2.80E-07	-0.46	3	0.3	Fam172a	
DMR2:5362001	2	5362001	5363000	1000	1	5.80E-12	0.72	4	0.4	Fam172a	
DMR2:5373001	2	5373001	5376000	3000	2	1.80E-17	0.62	22	0.73	Fam172a	
DMR2:5394001	2	5394001	5402000	8000	4	2.30E-22	-0.59	65	0.81	Fam172a	
DMR2:5405001	2	5405001	5408000	3000	1	3.10E-08	-0.5	23	0.77	Fam172a	
DMR2:5414001	2	5414001	5416000	2000	2	6.90E-26	0.8	19	0.95	Fam172a	
DMR2:5442001	2	5442001	5445000	3000	1	2.10E-08	-0.63	17	0.57	Fam172a;Pou5f2	
DMR2:5452001	2	5452001	5460000	8000	4	1.40E-20	-0.58	91	1.14	Fam172a	
DMR2:5470001	2	5470001	5474000	4000	3	1.90E-11	-0.54	47	1.18	Fam172a	
DMR2:5475001	2	5475001	5478000	3000	1	6.30E-11	-0.4	23	0.77	Fam172a	
DMR2:5488001	2	5488001	5490000	2000	1	3.10E-17	0.55	19	0.95	Fam172a	
DMR2:5497001	2	5497001	5498000	1000	1	5.20E-10	-0.53	4	0.4	Fam172a	
DMR2:5505001	2	5505001	5506000	1000	1	2.40E-12	0.64	7	0.7	Fam172a	
DMR2:5513001	2	5513001	5516000	3000	3	1.30E-12	-0.45	31	1.03	Fam172a	
DMR2:5519001	2	5519001	5520000	1000	1	5.80E-14	0.67	4	0.4	Fam172a	
DMR2:5524001	2	5524001	5525000	1000	1	2.80E-14	0.72	5	0.5	Fam172a;LOC102548021	
DMR2:5536001	2	5536001	5540000	4000	2	1.40E-13	-0.5	41	1.02	Fam172a;LOC102548021	
DMR2:5581001	2	5581001	5583000	2000	1	1.20E-10	0.75	62	3.1	Nr2f1;LOC684998	Transcription
DMR2:8566001	2	8566001	8574000	8000	4	2.10E-13	-0.48	73	0.91	LOC100360354;Rps8-ps1	
DMR2:8658001	2	8658001	8660000	2000	1	9.10E-07	0.44	22	1.1	Tdg-ps1	
DMR2:8668001	2	8668001	8672000	4000	3	2.20E-17	-0.59	46	1.15	Tdg-ps1	
DMR2:8725001	2	8725001	8727000	2000	1	8.60E-11	0.52	11	0.55	Arrdc3	
DMR2:8932001	2	8932001	8934000	2000	1	6.30E-16	0.72	16	0.8	Adgrv1	Signaling
DMR2:8942001	2	8942001	8945000	3000	2	3.80E-37	1.24	17	0.57	Adgrv1	Signaling
DMR2:8948001	2	8948001	8950000	2000	2	1.20E-17	0.62	16	0.8	Adgrv1	Signaling
DMR2:8958001	2	8958001	8960000	2000	1	4.50E-09	0.45	27	1.35	Adgrv1	Signaling
DMR2:8966001	2	8966001	8971000	5000	1	1.20E-07	0.43	30	0.6	Adgrv1	Signaling
DMR2:8977001	2	8977001	8978000	1000	1	5.40E-18	0.77	9	0.9	Adgrv1	Signaling
DMR2:8987001	2	8987001	8989000	2000	1	9.30E-09	0.45	22	1.1	Adgrv1	Signaling
DMR2:8991001	2	8991001	8996000	5000	2	1.70E-11	0.63	37	0.74	Adgrv1	Signaling
DMR2:9007001	2	9007001	9008000	1000	1	1.80E-08	-0.52	10	1	Adgrv1	Signaling
DMR2:9009001	2	9009001	9013000	4000	2	6.80E-11	0.44	33	0.82	Adgrv1	Signaling
DMR2:9026001	2	9026001	9030000	4000	2	3.00E-20	-0.5	40	1	Adgrv1	Signaling
DMR2:9031001	2	9031001	9033000	2000	2	5.70E-16	-0.59	16	0.8	Adgrv1	Signaling
DMR2:9037001	2	9037001	9040000	3000	3	3.10E-11	-0.55	14	0.47	Adgrv1	Signaling
DMR2:9042001	2	9042001	9043000	1000	1	5.00E-07	0.37	7	0.7	Adgrv1	Signaling
DMR2:9044001	2	9044001	9045000	1000	1	2.20E-12	0.71	5	0.5	Adgrv1	Signaling
DMR2:9065001	2	9065001	9066000	1000	1	9.00E-08	0.61	4	0.4	Adgrv1	Signaling
DMR2:9081001	2	9081001	9082000	1000	1	7.50E-11	0.69	3	0.3	Adgrv1	Signaling
DMR2:9086001	2	9086001	9091000	5000	2	1.70E-16	-0.83	26	0.52	Adgrv1	Signaling
DMR2:9097001	2	9097001	9099000	2000	2	8.40E-19	0.95	10	0.5	Adgrv1	Signaling
DMR2:9111001	2	9111001	9112000	1000	1	1.00E-07	0.7	6	0.6	Adgrv1	Signaling
DMR2:9132001	2	9132001	9133000	1000	1	9.70E-08	-0.43	14	1.4	Adgrv1	Signaling
DMR2:9166001	2	9166001	9169000	3000	1	7.60E-10	0.4	45	1.5	Adgrv1	Signaling
DMR2:9184001	2	9184001	9188000	4000	2	1.10E-12	0.68	36	0.9	Adgrv1	Signaling
DMR2:9204001	2	9204001	9207000	3000	3	3.50E-18	-0.5	35	1.17	Adgrv1	Signaling

DMR2:9208001	2	9208001	9212000	4000	2	2.80E-18	-0.54	32	0.8	Adgrv1	Signaling
DMR2:9220001	2	9220001	9221000	1000	1	2.60E-09	0.4	19	1.9	Adgrv1;LOC103691409	Signaling
DMR2:9231001	2	9231001	9238000	7000	4	2.70E-13	0.62	43	0.61	Adgrv1;LOC103691409	Signaling
DMR2:9254001	2	9254001	9255000	1000	1	3.60E-15	0.88	6	0.6	Adgrv1	Signaling
DMR2:9268001	2	9268001	9269000	1000	1	2.40E-10	-0.62	1	0.1	Adgrv1	Signaling
DMR2:9275001	2	9275001	9279000	4000	3	4.20E-17	0.75	29	0.72	Adgrv1;LOC108349913	Signaling
DMR2:9297001	2	9297001	9299000	2000	1	1.40E-08	0.51	29	1.45	Adgrv1	Signaling
DMR2:9325001	2	9325001	9327000	2000	1	1.80E-08	-0.51	25	1.25	Adgrv1	Signaling
DMR2:9346001	2	9346001	9349000	3000	1	3.90E-07	-0.59	35	1.17	Adgrv1	Signaling
DMR2:9360001	2	9360001	9361000	1000	1	7.80E-08	0.45	8	0.8	Adgrv1	Signaling
DMR2:9374001	2	9374001	9376000	2000	1	3.10E-21	0.73	39	1.95	Adgrv1	Signaling
DMR2:9393001	2	9393001	9395000	2000	1	8.90E-16	0.48	30	1.5	Adgrv1	Signaling
DMR2:9400001	2	9400001	9403000	3000	1	2.90E-07	-0.5	57	1.9	Adgrv1	Signaling
DMR2:9426001	2	9426001	9430000	4000	2	1.60E-10	-0.51	21	0.52	Adgrv1	Signaling
DMR2:9435001	2	9435001	9436000	1000	1	6.10E-20	0.61	13	1.3	Adgrv1	Signaling
DMR2:9438001	2	9438001	9443000	5000	2	1.20E-08	-0.62	74	1.48	Adgrv1	Signaling
DMR2:9445001	2	9445001	9452000	7000	1	9.20E-07	-0.52	113	1.61	Adgrv1	Signaling
DMR2:9459001	2	9459001	9464000	5000	1	9.30E-07	-0.51	77	1.54	Adgrv1	Signaling
DMR2:9465001	2	9465001	9466000	1000	1	1.50E-07	-0.35	15	1.5	Adgrv1	Signaling
DMR2:9476001	2	9476001	9477000	1000	1	2.00E-14	0.75	12	1.2	Adgrv1	Signaling
DMR2:9478001	2	9478001	9480000	2000	1	8.50E-16	0.59	28	1.4	Adgrv1	Signaling
DMR2:9485001	2	9485001	9486000	1000	1	1.40E-07	0.48	6	0.6	Adgrv1	Signaling
DMR2:9490001	2	9490001	9495000	5000	1	2.90E-07	0.33	58	1.16	Adgrv1;LOC499494	Signaling
DMR2:9505001	2	9505001	9508000	3000	1	1.80E-08	-0.67	36	1.2	Adgrv1;LOC499494;LOC685441	Signaling
DMR2:9509001	2	9509001	9513000	4000	1	7.40E-09	0.48	62	1.55	Adgrv1;LOC685441	Signaling
DMR2:9522001	2	9522001	9523000	1000	1	8.30E-07	0.45	6	0.6	LOC685441;Lysmd3	
DMR2:9535001	2	9535001	9545000	10000	2	7.20E-18	0.66	132	1.32	Lysmd3;Polr3g	Transcription
DMR2:9546001	2	9546001	9549000	3000	1	1.00E-10	0.42	45	1.5	Polr3g	Transcription
DMR2:9558001	2	9558001	9559000	1000	1	2.20E-07	-0.55	21	2.1	Polr3g	Transcription
DMR2:9565001	2	9565001	9573000	8000	4	1.80E-23	0.75	96	1.2	Polr3g;Mblac2	Transcription;Metabolism
DMR2:9582001	2	9582001	9587000	5000	2	4.90E-15	-0.74	42	0.84	Polr3g;Mblac2	Transcription;Metabolism
DMR2:9589001	2	9589001	9595000	6000	4	2.10E-13	-0.63	34	0.57	Mblac2	Metabolism
DMR2:9613001	2	9613001	9616000	3000	1	2.00E-08	-0.5	10	0.33	Mblac2	Metabolism
DMR2:9619001	2	9619001	9620000	1000	1	2.80E-11	0.47	11	1.1	Mblac2	Metabolism
DMR2:11064001	2	11064001	11067000	3000	1	8.40E-18	0.87	16	0.53	Rps27a-ps29	
DMR2:11075001	2	11075001	11077000	2000	1	4.20E-07	0.53	6	0.3	Rps27a-ps29	
DMR2:11649001	2	11649001	11651000	2000	1	1.80E-12	0.65	10	0.5	LOC102552429;Mef2c	Transcription
DMR2:11656001	2	11656001	11658000	2000	1	2.50E-16	0.81	17	0.85	LOC102552429;Mef2c	Transcription
DMR2:11683001	2	11683001	11684000	1000	1	1.10E-12	0.68	13	1.3	Mef2c	Transcription
DMR2:11728001	2	11728001	11731000	3000	1	2.20E-14	0.54	50	1.67	Mef2c	Transcription
DMR2:11733001	2	11733001	11737000	4000	2	1.30E-08	-0.78	42	1.05	Mef2c	Transcription
DMR2:11739001	2	11739001	11741000	2000	1	2.30E-07	0.52	14	0.7	Mef2c	Transcription
DMR2:11746001	2	11746001	11747000	1000	1	3.00E-08	0.44	15	1.5	Mef2c	Transcription
DMR2:11762001	2	11762001	11764000	2000	2	4.90E-11	0.61	21	1.05	Mef2c	Transcription
DMR2:11765001	2	11765001	11766000	1000	1	2.70E-07	0.53	8	0.8	Mef2c	Transcription
DMR2:11767001	2	11767001	11768000	1000	1	3.10E-10	0.57	7	0.7	Mef2c	Transcription
DMR2:11769001	2	11769001	11771000	2000	1	1.30E-21	0.78	26	1.3	Mef2c	Transcription
DMR2:11877001	2	11877001	11878000	1000	1	7.10E-07	0.48	15	1.5	Mir3597-2;Mir9-2	
DMR2:11889001	2	11889001	11890000	1000	1	2.00E-19	0.8	4	0.4	Mir3597-2;Mir9-2	
DMR2:12242001	2	12242001	12244000	2000	1	8.80E-11	-0.5	8	0.4	Tmem161b	
DMR2:12262001	2	12262001	12265000	3000	1	2.80E-13	-0.81	24	0.8	Tmem161b	
DMR2:12274001	2	12274001	12279000	5000	1	1.20E-07	-0.34	31	0.62	Tmem161b	
DMR2:12288001	2	12288001	12291000	3000	1	9.10E-07	0.39	35	1.17	Tmem161b	
DMR2:12309001	2	12309001	12311000	2000	1	5.50E-07	0.47	18	0.9	Tmem161b	
DMR2:12318001	2	12318001	12319000	1000	1	3.90E-14	0.65	15	1.5	Tmem161b	
DMR2:12344001	2	12344001	12345000	1000	1	2.20E-15	-0.67	2	0.2	Tmem161b	
DMR2:12352001	2	12352001	12355000	3000	1	1.80E-07	-0.53	9	0.3	Tmem161b	
DMR2:12364001	2	12364001	12375000	11000	4	1.60E-22	-0.55	70	0.64	Tmem161b	
DMR2:12392001	2	12392001	12394000	2000	1	5.20E-12	0.57	8	0.4	Tmem161b;LOC100909625	
DMR2:12414001	2	12414001	12416000	2000	1	3.50E-14	0.66	16	0.8	Tmem161b	
DMR2:12417001	2	12417001	12418000	1000	1	8.30E-07	0.35	6	0.6	Tmem161b	
DMR2:12434001	2	12434001	12436000	2000	1	9.00E-16	0.85	18	0.9	Tmem161b;LOC102554178;LOC108349917	

DMR2:12469001	2	12469001	12471000	2000	1	1.00E-10	0.53	23	1.15	Tmem161b;LOC108349917	
DMR2:12498001	2	12498001	12500000	2000	1	6.00E-07	0.49	9	0.45	Tmem161b;LOC102554243	
DMR2:12501001	2	12501001	12502000	1000	1	4.70E-08	0.52	4	0.4	Tmem161b	
DMR2:12533001	2	12533001	12536000	3000	2	2.30E-20	0.67	32	1.07	Tmem161b	
DMR2:12539001	2	12539001	12541000	2000	1	3.20E-11	0.54	15	0.75	Tmem161b	
DMR2:12542001	2	12542001	12544000	2000	2	3.30E-15	0.89	20	1	Tmem161b	
DMR2:12558001	2	12558001	12562000	4000	1	6.20E-11	-0.58	16	0.4	Tmem161b	
DMR2:13591001	2	13591001	13592000	1000	1	1.90E-13	0.65	8	0.8	Ccnh	Signaling
DMR2:13641001	2	13641001	13642000	1000	1	8.70E-11	0.6	6	0.6	Rasa1	Signaling
DMR2:13678001	2	13678001	13679000	1000	1	6.30E-07	0.5	7	0.7	Rasa1	Signaling
DMR2:13686001	2	13686001	13688000	2000	1	2.00E-08	-0.59	13	0.65	Rasa1	Signaling
DMR2:14272001	2	14272001	14274000	2000	2	2.70E-08	-0.6	7	0.35	RGD1559647	
DMR2:14706001	2	14706001	14708000	2000	2	7.70E-19	0.75	16	0.8	Cox7c;LOC252890	Metabolism
DMR2:14711001	2	14711001	14715000	4000	3	1.30E-14	-0.48	34	0.85	Cox7c	Metabolism
DMR2:17608001	2	17608001	17610000	2000	1	4.00E-07	-0.46	16	0.8	Edil3	Metabolism
DMR2:17668001	2	17668001	17670000	2000	1	5.70E-12	0.6	15	0.75	Edil3	Metabolism
DMR2:17701001	2	17701001	17704000	3000	1	9.20E-09	-0.76	26	0.87	Edil3	Metabolism
DMR2:17725001	2	17725001	17727000	2000	1	9.50E-09	0.5	11	0.55	Edil3	Metabolism
DMR2:17761001	2	17761001	17762000	1000	1	5.50E-08	-0.52	5	0.5	Edil3	Metabolism
DMR2:17792001	2	17792001	17793000	1000	1	4.20E-13	0.7	6	0.6	Edil3	Metabolism
DMR2:17794001	2	17794001	17795000	1000	1	2.00E-08	-0.59	8	0.8	Edil3	Metabolism
DMR2:17817001	2	17817001	17818000	1000	1	2.50E-11	0.6	6	0.6	Edil3	Metabolism
DMR2:17871001	2	17871001	17872000	1000	1	1.30E-08	0.51	2	0.2	Edil3	Metabolism
DMR2:17882001	2	17882001	17883000	1000	1	6.00E-08	0.43	11	1.1	Edil3	Metabolism
DMR2:17902001	2	17902001	17904000	2000	1	3.00E-07	0.62	5	0.25	Edil3	Metabolism
DMR2:17935001	2	17935001	17937000	2000	1	1.20E-11	-0.66	7	0.35	Edil3	Metabolism
DMR2:17973001	2	17973001	17974000	1000	1	9.10E-09	0.55	6	0.6	Edil3	Metabolism
DMR2:17995001	2	17995001	17998000	3000	1	1.00E-09	-0.48	20	0.67	Edil3	Metabolism
DMR2:18036001	2	18036001	18041000	5000	4	1.20E-17	-0.63	34	0.68	Edil3	Metabolism
DMR2:18053001	2	18053001	18054000	1000	1	2.00E-15	0.72	8	0.8	Edil3	Metabolism
DMR2:18068001	2	18068001	18069000	1000	1	2.30E-08	0.58	3	0.3	Edil3	Metabolism
DMR2:18075001	2	18075001	18078000	3000	2	6.60E-24	-0.58	26	0.87	Edil3	Metabolism
DMR2:18079001	2	18079001	18082000	3000	1	5.20E-07	-0.4	23	0.77	Edil3	Metabolism
DMR2:18123001	2	18123001	18125000	2000	2	4.30E-12	0.53	22	1.1	Edil3	Metabolism
DMR2:18348001	2	18348001	18349000	1000	1	2.00E-07	-0.57	10	1	LOC688411;Hapln1	Extracellular Matrix
DMR2:18360001	2	18360001	18362000	2000	1	7.60E-25	0.77	20	1	Hapln1	Extracellular Matrix
DMR2:18399001	2	18399001	18400000	1000	1	6.50E-08	0.41	7	0.7	Hapln1	Extracellular Matrix
DMR2:18480001	2	18480001	18482000	2000	1	5.70E-09	-0.43	23	1.15	Vcan	Extracellular Matrix
DMR2:18505001	2	18505001	18506000	1000	1	2.90E-07	0.29	8	0.8	Vcan	Extracellular Matrix
DMR2:18589001	2	18589001	18590000	1000	1	8.40E-07	-0.52	12	1.2	Vcan	Extracellular Matrix
DMR2:18597001	2	18597001	18599000	2000	2	1.80E-18	0.85	11	0.55	Vcan	Extracellular Matrix
DMR2:18665001	2	18665001	18668000	3000	1	5.10E-08	-0.59	31	1.03	Xrcc4	Transcription
DMR2:18669001	2	18669001	18672000	3000	2	1.50E-10	-0.57	12	0.4	Xrcc4	Transcription
DMR2:18733001	2	18733001	18736000	3000	2	1.10E-15	-0.64	16	0.53	Xrcc4	Transcription
DMR2:18738001	2	18738001	18740000	2000	1	8.10E-22	0.78	10	0.5	Xrcc4	Transcription
DMR2:18745001	2	18745001	18755000	10000	8	1.20E-29	0.65	131	1.31	Xrcc4	Transcription
DMR2:18814001	2	18814001	18815000	1000	1	3.70E-08	-0.57	11	1.1	Xrcc4	Transcription
DMR2:18816001	2	18816001	18817000	1000	1	3.10E-07	0.61	10	1	Xrcc4	Transcription
DMR2:18848001	2	18848001	18856000	8000	3	1.90E-18	-0.45	107	1.34	Xrcc4	Transcription
DMR2:18857001	2	18857001	18860000	3000	1	2.50E-08	-0.37	28	0.93	Xrcc4	Transcription
DMR2:18862001	2	18862001	18868000	6000	4	5.40E-30	-0.67	65	1.08	Xrcc4;LOC108350153	Transcription
DMR2:18872001	2	18872001	18874000	2000	1	7.40E-09	0.51	12	0.6	Xrcc4;LOC108350153	Transcription
DMR2:18877001	2	18877001	18878000	1000	1	4.10E-07	0.41	12	1.2	Xrcc4;LOC108350153	Transcription
DMR2:18888001	2	18888001	18896000	8000	2	2.80E-12	-0.49	71	0.89	Xrcc4;LOC108350153	Transcription
DMR2:18897001	2	18897001	18899000	2000	1	2.60E-07	0.65	10	0.5	Xrcc4	Transcription
DMR2:18936001	2	18936001	18937000	1000	1	6.10E-08	-0.59	8	0.8	Xrcc4;Tmem167a	Transcription
DMR2:18949001	2	18949001	18952000	3000	1	8.30E-17	0.71	32	1.07	Tmem167a	
DMR2:19774001	2	19774001	19778000	4000	2	1.30E-11	-0.61	32	0.8	Atp6ap1l	Metabolism
DMR2:19779001	2	19779001	19781000	2000	1	1.00E-08	0.43	19	0.95	Atp6ap1l	Metabolism
DMR2:19789001	2	19789001	19794000	5000	2	7.30E-11	-0.63	21	0.42	Atp6ap1l	Metabolism
DMR2:19814001	2	19814001	19817000	3000	1	2.60E-11	0.43	47	1.57	Atp6ap1l;Rps23	Metabolism;Translation
DMR2:19819001	2	19819001	19821000	2000	1	1.20E-11	0.47	25	1.25	Atp6ap1l;Rps23	Metabolism;Translation

DMR2:19833001	2	19833001	19845000	12000	3	1.90E-11	-0.52	106	0.88	Rps23;Atg10	Translation;Proteolysis
DMR2:19857001	2	19857001	19860000	3000	1	8.80E-08	0.54	14	0.47	Atg10	Proteolysis
DMR2:19885001	2	19885001	19888000	3000	1	2.00E-08	-0.42	23	0.77	Atg10	Proteolysis
DMR2:19897001	2	19897001	19902000	5000	1	1.50E-09	-0.54	75	1.5	Atg10	Proteolysis
DMR2:19915001	2	19915001	19916000	1000	1	4.80E-07	0.42	0	0	Atg10	Proteolysis
DMR2:19921001	2	19921001	19924000	3000	3	3.00E-17	-0.58	26	0.87	Atg10	Proteolysis
DMR2:19937001	2	19937001	19942000	5000	3	5.90E-21	0.72	46	0.92	Atg10	Proteolysis
DMR2:19949001	2	19949001	19955000	6000	2	4.80E-10	0.49	84	1.4	Atg10	Proteolysis
DMR2:19958001	2	19958001	19960000	2000	1	2.10E-08	0.49	17	0.85	Atg10	Proteolysis
DMR2:19965001	2	19965001	19968000	3000	2	6.40E-13	-0.64	37	1.23	Atg10	Proteolysis
DMR2:19992001	2	19992001	19993000	1000	1	6.00E-10	-0.64	3	0.3	Atg10	Proteolysis
DMR2:20005001	2	20005001	20007000	2000	2	3.30E-13	0.49	20	1	Atg10	Proteolysis
DMR2:20008001	2	20008001	20015000	7000	3	1.10E-19	-0.64	86	1.23	Atg10	Proteolysis
DMR2:20016001	2	20016001	20021000	5000	2	2.20E-16	0.65	79	1.58	Atg10	Proteolysis
DMR2:20033001	2	20033001	20036000	3000	2	4.60E-23	0.61	31	1.03	Atg10	Proteolysis
DMR2:20046001	2	20046001	20047000	1000	1	6.70E-13	0.77	4	0.4	Atg10	Proteolysis
DMR2:20072001	2	20072001	20074000	2000	1	2.00E-09	0.44	11	0.55	Atg10	Proteolysis
DMR2:20084001	2	20084001	20090000	6000	2	1.50E-18	0.61	100	1.67	Atg10	Proteolysis
DMR2:20093001	2	20093001	20098000	5000	2	2.30E-12	0.46	58	1.16	Atg10	Proteolysis
DMR2:20106001	2	20106001	20109000	3000	1	1.70E-08	0.3	50	1.67	Atg10	Proteolysis
DMR2:20157001	2	20157001	20159000	2000	1	3.60E-12	0.53	31	1.55	Atg10	Proteolysis
DMR2:20161001	2	20161001	20168000	7000	3	1.10E-20	-0.46	72	1.03	Atg10	Proteolysis
DMR2:20415001	2	20415001	20417000	2000	1	3.20E-09	0.59	43	2.15	Ssbp2	Transcription
DMR2:20424001	2	20424001	20428000	4000	1	7.00E-08	-0.57	51	1.27	Ssbp2	Transcription
DMR2:20431001	2	20431001	20432000	1000	1	2.80E-14	0.56	15	1.5	Ssbp2	Transcription
DMR2:20449001	2	20449001	20454000	5000	2	1.90E-10	-0.41	45	0.9	Ssbp2	Transcription
DMR2:20460001	2	20460001	20464000	4000	1	3.70E-07	0.37	31	0.78	Ssbp2	Transcription
DMR2:20467001	2	20467001	20468000	1000	1	4.80E-15	0.99	1	0.1	Ssbp2	Transcription
DMR2:20472001	2	20472001	20476000	4000	1	1.40E-08	0.59	35	0.88	Ssbp2	Transcription
DMR2:20478001	2	20478001	20488000	10000	6	2.60E-18	-0.83	114	1.14	Ssbp2	Transcription
DMR2:20489001	2	20489001	20493000	4000	2	9.80E-12	-0.58	41	1.02	Ssbp2	Transcription
DMR2:20514001	2	20514001	20516000	2000	1	1.50E-15	0.66	29	1.45	Ssbp2	Transcription
DMR2:20523001	2	20523001	20526000	3000	1	1.50E-17	0.56	26	0.87	Ssbp2	Transcription
DMR2:20535001	2	20535001	20542000	7000	3	1.60E-14	0.57	66	0.94	Ssbp2	Transcription
DMR2:20545001	2	20545001	20546000	1000	1	1.40E-12	0.51	11	1.1	Ssbp2	Transcription
DMR2:20547001	2	20547001	20549000	2000	2	7.00E-09	-0.6	12	0.6	Ssbp2	Transcription
DMR2:20559001	2	20559001	20560000	1000	1	4.80E-09	0.66	6	0.6	Ssbp2	Transcription
DMR2:20561001	2	20561001	20564000	3000	1	1.60E-10	-0.37	27	0.9	Ssbp2	Transcription
DMR2:20574001	2	20574001	20577000	3000	1	9.10E-25	0.87	38	1.27	Ssbp2;LOC103691419	Transcription
DMR2:20578001	2	20578001	20581000	3000	1	5.90E-16	0.82	26	0.87	Ssbp2;LOC103691419	Transcription
DMR2:20591001	2	20591001	20597000	6000	1	3.30E-07	0.47	51	0.85	Ssbp2;LOC103691419	Transcription
DMR2:20601001	2	20601001	20604000	3000	1	2.70E-07	0.51	26	0.87	Ssbp2	Transcription
DMR2:20637001	2	20637001	20641000	4000	2	2.30E-08	0.59	42	1.05	Ssbp2	Transcription
DMR2:20650001	2	20650001	20651000	1000	1	5.80E-10	0.67	4	0.4	Ssbp2	Transcription
DMR2:20657001	2	20657001	20660000	3000	1	7.80E-13	0.58	19	0.63	Ssbp2	Transcription
DMR2:20684001	2	20684001	20685000	1000	1	1.10E-07	0.43	8	0.8	Ssbp2	Transcription
DMR2:20701001	2	20701001	20702000	1000	1	1.80E-12	0.52	23	2.3	Ssbp2	Transcription
DMR2:20716001	2	20716001	20718000	2000	2	1.80E-08	-0.41	20	1	Ssbp2	Transcription
DMR2:20850001	2	20850001	20852000	2000	1	5.30E-14	-0.43	26	1.3	Acot12	Metabolism
DMR2:20853001	2	20853001	20856000	3000	1	2.70E-07	0.41	41	1.37	Acot12	Metabolism
DMR2:20861001	2	20861001	20863000	2000	1	9.00E-08	0.42	32	1.6	Acot12	Metabolism
DMR2:20894001	2	20894001	20896000	2000	1	5.10E-19	0.79	21	1.05	Acot12	Metabolism
DMR2:20900001	2	20900001	20903000	3000	2	1.10E-10	-0.66	40	1.33	Acot12;Zcchc9	Metabolism
DMR2:20905001	2	20905001	20906000	1000	1	3.10E-13	0.45	6	0.6	Acot12;Zcchc9	Metabolism
DMR2:20921001	2	20921001	20922000	1000	1	5.70E-10	0.57	15	1.5	Zcchc9	
DMR2:20926001	2	20926001	20927000	1000	1	2.00E-10	0.39	27	2.7	Zcchc9	
DMR2:21435001	2	21435001	21436000	1000	1	9.30E-18	1.07	10	1	Ckmt2	Signaling
DMR2:21442001	2	21442001	21443000	1000	1	3.10E-10	-0.6	12	1.2	Ckmt2	Signaling
DMR2:21449001	2	21449001	21450000	1000	1	6.40E-20	0.99	5	0.5	Ckmt2	Signaling
DMR2:21460001	2	21460001	21462000	2000	1	6.70E-08	0.5	30	1.5	Ckmt2;Rasgrf2	Signaling;Transcription
DMR2:21465001	2	21465001	21466000	1000	1	2.50E-09	0.47	13	1.3	Rasgrf2	Transcription
DMR2:21479001	2	21479001	21483000	4000	2	8.10E-14	-0.73	11	0.28	Rasgrf2	Transcription
DMR2:21493001	2	21493001	21494000	1000	1	2.90E-19	0.87	8	0.8	Rasgrf2	Transcription

DMR2:21518001	2	21518001	21521000	3000	2	2.00E-18	0.63	46	1.53	Rasgrf2;LOC108349920	Transcription
DMR2:21534001	2	21534001	21538000	4000	3	2.00E-17	0.59	53	1.32	Rasgrf2	Transcription
DMR2:21540001	2	21540001	21542000	2000	2	7.00E-10	-0.76	13	0.65	Rasgrf2	Transcription
DMR2:21546001	2	21546001	21549000	3000	1	9.90E-18	0.86	16	0.53	Rasgrf2	Transcription
DMR2:21574001	2	21574001	21576000	2000	1	8.80E-08	-0.61	32	1.6	Rasgrf2	Transcription
DMR2:21581001	2	21581001	21582000	1000	1	2.60E-10	-0.54	10	1	Rasgrf2	Transcription
DMR2:21592001	2	21592001	21595000	3000	1	8.90E-22	0.79	38	1.27	Rasgrf2	Transcription
DMR2:21634001	2	21634001	21635000	1000	1	1.50E-08	0.38	13	1.3	Rasgrf2	Transcription
DMR2:21637001	2	21637001	21638000	1000	1	2.20E-07	0.37	8	0.8	Rasgrf2	Transcription
DMR2:21682001	2	21682001	21685000	3000	1	2.10E-12	0.57	27	0.9	Rasgrf2	Transcription
DMR2:21786001	2	21786001	21791000	5000	2	8.90E-31	1.29	90	1.8	Msh3	Transcription
DMR2:21796001	2	21796001	21800000	4000	1	3.90E-08	-0.4	66	1.65	Msh3	Transcription
DMR2:21807001	2	21807001	21810000	3000	2	2.40E-18	0.82	30	1	Msh3	Transcription
DMR2:21812001	2	21812001	21814000	2000	1	3.50E-15	0.52	27	1.35	Msh3	Transcription
DMR2:21824001	2	21824001	21827000	3000	2	5.10E-26	1.11	26	0.87	Msh3	Transcription
DMR2:21839001	2	21839001	21844000	5000	2	2.10E-07	-0.47	61	1.22	Msh3	Transcription
DMR2:21848001	2	21848001	21853000	5000	1	4.60E-07	-0.47	66	1.32	Msh3	Transcription
DMR2:21867001	2	21867001	21869000	2000	1	3.00E-10	0.43	16	0.8	Msh3	Transcription
DMR2:21882001	2	21882001	21885000	3000	2	2.40E-15	-0.61	24	0.8	Msh3	Transcription
DMR2:21887001	2	21887001	21888000	1000	1	1.00E-08	0.45	5	0.5	Msh3	Transcription
DMR2:21890001	2	21890001	21895000	5000	3	1.20E-15	-0.71	59	1.18	Msh3	Transcription
DMR2:21898001	2	21898001	21899000	1000	1	1.70E-10	0.62	6	0.6	Msh3	Transcription
DMR2:21900001	2	21900001	21901000	1000	1	8.00E-16	0.65	6	0.6	Msh3	Transcription
DMR2:21906001	2	21906001	21909000	3000	1	1.00E-15	0.54	43	1.43	Msh3	Transcription
DMR2:21910001	2	21910001	21913000	3000	1	6.50E-12	0.52	37	1.23	Msh3	Transcription
DMR2:21916001	2	21916001	21919000	3000	1	4.20E-07	-0.58	48	1.6	Msh3	Transcription
DMR2:21959001	2	21959001	21963000	4000	1	9.30E-07	0.37	49	1.23	Dhfr	Metabolism
DMR2:21990001	2	21990001	21991000	1000	1	1.40E-09	0.65	1	0.1	Ankrd34b	
DMR2:22007001	2	22007001	22008000	1000	1	6.30E-07	-0.61	10	1	Ankrd34b	
DMR2:22009001	2	22009001	22013000	4000	1	1.60E-13	0.57	60	1.5	Ankrd34b	
DMR2:22017001	2	22017001	22020000	3000	3	2.10E-22	0.78	52	1.73	Ankrd34b;Fam151b	
DMR2:22022001	2	22022001	22025000	3000	1	1.80E-11	-0.74	47	1.57	Ankrd34b;Fam151b	
DMR2:22030001	2	22030001	22033000	3000	1	2.30E-08	0.5	45	1.5	Fam151b	
DMR2:22037001	2	22037001	22043000	6000	4	4.60E-27	1.04	128	2.13	Fam151b	
DMR2:22052001	2	22052001	22057000	5000	4	1.40E-11	-0.53	53	1.06	Fam151b;Zfyve16	
DMR2:22064001	2	22064001	22067000	3000	1	6.20E-10	0.4	27	0.9	Fam151b;Zfyve16	
DMR2:22068001	2	22068001	22069000	1000	1	6.80E-11	-0.7	12	1.2	Fam151b;Zfyve16	
DMR2:22110001	2	22110001	22113000	3000	1	2.00E-14	0.7	22	0.73	Zfyve16	
DMR2:22115001	2	22115001	22119000	4000	3	6.90E-11	-0.63	12	0.3	Zfyve16	
DMR2:22133001	2	22133001	22137000	4000	2	6.00E-15	0.64	47	1.18	Spz1	Transcription
DMR2:22142001	2	22142001	22144000	2000	1	2.00E-12	0.48	10	0.5	Spz1	Transcription
DMR2:22154001	2	22154001	22157000	3000	1	3.50E-10	0.41	39	1.3	Spz1	Transcription
DMR2:22198001	2	22198001	22200000	2000	1	1.70E-10	0.54	16	0.8	Serinc5	Signaling
DMR2:22243001	2	22243001	22248000	5000	3	8.80E-19	0.68	77	1.54	Serinc5	Signaling
DMR2:22259001	2	22259001	22260000	1000	1	3.20E-14	0.58	11	1.1	Serinc5	Signaling
DMR2:22261001	2	22261001	22265000	4000	1	1.20E-11	0.47	62	1.55	Serinc5	Signaling
DMR2:22348001	2	22348001	22349000	1000	1	1.10E-12	0.69	8	0.8	Thbs4	
DMR2:22357001	2	22357001	22358000	1000	1	1.50E-07	0.33	18	1.8	Thbs4	
DMR2:22362001	2	22362001	22367000	5000	1	3.00E-09	-0.46	71	1.42	Thbs4	
DMR2:22389001	2	22389001	22391000	2000	1	4.30E-13	0.5	33	1.65	Thbs4	
DMR2:22393001	2	22393001	22397000	4000	1	2.60E-09	0.41	65	1.62	Thbs4	
DMR2:22419001	2	22419001	22421000	2000	1	1.90E-08	-0.47	26	1.3	Mtx3	
DMR2:22422001	2	22422001	22424000	2000	1	2.10E-08	0.44	18	0.9	Mtx3	
DMR2:22431001	2	22431001	22435000	4000	2	2.30E-07	-0.64	60	1.5	Mtx3	
DMR2:22436001	2	22436001	22440000	4000	1	5.50E-13	0.46	45	1.12	Mtx3	
DMR2:22450001	2	22450001	22451000	1000	1	2.30E-07	-0.55	17	1.7	Mtx3;LOC108350222	
DMR2:22634001	2	22634001	22637000	3000	1	4.40E-25	0.88	33	1.1	Cmya5	Proteolysis
DMR2:22638001	2	22638001	22639000	1000	1	7.10E-11	0.45	17	1.7	Cmya5	Proteolysis
DMR2:22643001	2	22643001	22644000	1000	1	4.20E-07	0.4	11	1.1	Cmya5	Proteolysis
DMR2:22655001	2	22655001	22660000	5000	1	7.90E-07	-0.39	71	1.42	Cmya5	Proteolysis
DMR2:22661001	2	22661001	22662000	1000	1	2.10E-09	0.5	15	1.5	Cmya5	Proteolysis
DMR2:22679001	2	22679001	22682000	3000	1	2.70E-16	0.91	57	1.9	Cmya5	Proteolysis
DMR2:22735001	2	22735001	22741000	6000	3	2.70E-21	0.91	69	1.15	Cmya5;Papd4	Proteolysis
DMR2:22766001	2	22766001	22769000	3000	3	8.60E-16	-0.45	26	0.87	Papd4	
DMR2:22770001	2	22770001	22773000	3000	1	6.60E-10	-0.46	25	0.83	Papd4	

DMR2:22912001	2	22912001	22913000	1000	1	8.10E-09	-0.67	14	1.4	Homer1	
DMR2:22931001	2	22931001	22934000	3000	1	4.00E-07	-0.48	28	0.93	Homer1	
DMR2:22936001	2	22936001	22937000	1000	1	2.30E-10	0.4	15	1.5	Homer1	
DMR2:22957001	2	22957001	22961000	4000	1	5.00E-07	0.32	54	1.35	Homer1	
DMR2:22981001	2	22981001	22982000	1000	1	8.60E-08	-0.6	17	1.7	Homer1	
DMR2:22986001	2	22986001	22988000	2000	1	1.00E-06	0.36	23	1.15	Homer1	
DMR2:23003001	2	23003001	23005000	2000	1	2.00E-12	0.49	22	1.1	Homer1	
DMR2:23045001	2	23045001	23047000	2000	1	8.90E-08	-0.54	22	1.1	Jmy	
DMR2:23050001	2	23050001	23056000	6000	1	3.30E-09	-0.69	54	0.9	Jmy	
DMR2:23077001	2	23077001	23078000	1000	1	6.50E-12	-0.66	7	0.7	Jmy	
DMR2:23225001	2	23225001	23227000	2000	1	2.50E-12	0.62	41	2.05	Bhmt	Epigenetic
DMR2:23230001	2	23230001	23232000	2000	1	6.50E-18	0.89	15	0.75	Bhmt	Epigenetic
DMR2:23264001	2	23264001	23265000	1000	1	2.30E-12	0.68	9	0.9	Bhmt;LOC103691423;Bhmt2	Epigenetic
DMR2:23281001	2	23281001	23283000	2000	2	3.70E-16	0.76	16	0.8	Bhmt2;Dmgdh	Epigenetic;Metabolism
DMR2:23303001	2	23303001	23306000	3000	1	2.20E-07	-0.46	36	1.2	Dmgdh	Metabolism
DMR2:23307001	2	23307001	23309000	2000	1	3.40E-07	0.36	24	1.2	Dmgdh	Metabolism
DMR2:23312001	2	23312001	23318000	6000	2	2.10E-15	0.84	64	1.07	Dmgdh	Metabolism
DMR2:23351001	2	23351001	23356000	5000	3	2.90E-21	0.8	82	1.64	Dmgdh	Metabolism
DMR2:23411001	2	23411001	23417000	6000	1	7.30E-08	-0.46	130	2.17	Arsb	
DMR2:23418001	2	23418001	23420000	2000	1	2.40E-08	0.41	32	1.6	Arsb	
DMR2:23427001	2	23427001	23434000	7000	3	1.70E-16	0.76	100	1.43	Arsb	
DMR2:23452001	2	23452001	23456000	4000	1	5.30E-07	0.38	65	1.62	Arsb	
DMR2:23473001	2	23473001	23475000	2000	1	1.90E-11	0.52	30	1.5	Arsb	
DMR2:23476001	2	23476001	23479000	3000	1	6.80E-09	-0.4	47	1.57	Arsb	
DMR2:23485001	2	23485001	23486000	1000	1	6.30E-09	0.37	11	1.1	Arsb	
DMR2:23490001	2	23490001	23491000	1000	1	2.00E-07	-0.49	14	1.4	Arsb	
DMR2:23493001	2	23493001	23499000	6000	1	2.40E-07	-0.43	81	1.35	Arsb	
DMR2:23503001	2	23503001	23506000	3000	1	4.20E-10	0.42	35	1.17	Arsb	
DMR2:23516001	2	23516001	23518000	2000	1	6.50E-07	0.3	23	1.15	Arsb	
DMR2:23523001	2	23523001	23526000	3000	1	7.30E-12	0.53	40	1.33	Arsb	
DMR2:23527001	2	23527001	23534000	7000	4	1.90E-22	0.9	98	1.4	Arsb	
DMR2:23546001	2	23546001	23549000	3000	1	1.60E-08	-0.65	61	2.03	Arsb	
DMR2:23730001	2	23730001	23733000	3000	1	7.10E-10	0.49	37	1.23	Lhfpl2	
DMR2:23734001	2	23734001	23741000	7000	2	7.90E-08	-0.53	136	1.94	Lhfpl2	
DMR2:23743001	2	23743001	23750000	7000	2	9.60E-10	-0.64	119	1.7	Lhfpl2	
DMR2:23755001	2	23755001	23757000	2000	1	3.90E-10	-0.65	46	2.3	Lhfpl2	
DMR2:23758001	2	23758001	23759000	1000	1	2.30E-31	0.92	4	0.4	Lhfpl2	
DMR2:23795001	2	23795001	23797000	2000	1	9.50E-14	0.4	56	2.8	Lhfpl2	
DMR2:23831001	2	23831001	23836000	5000	2	8.40E-15	0.48	89	1.78	Lhfpl2;LOC100360196;LOC685256	
DMR2:23844001	2	23844001	23846000	2000	1	8.90E-07	-0.52	19	0.95	Lhfpl2;LOC100360196;LOC685256	
DMR2:23851001	2	23851001	23852000	1000	1	1.70E-07	-0.56	20	2	Lhfpl2;LOC100360196;LOC685256;Scamp1	Transport
DMR2:23857001	2	23857001	23859000	2000	1	1.70E-07	-0.53	38	1.9	Lhfpl2;Scamp1	Transport
DMR2:23861001	2	23861001	23864000	3000	1	8.80E-07	0.3	45	1.5	Lhfpl2;Scamp1	Transport
DMR2:23872001	2	23872001	23878000	6000	2	4.20E-18	0.59	102	1.7	Scamp1	Transport
DMR2:23880001	2	23880001	23882000	2000	1	2.60E-07	-0.61	29	1.45	Scamp1	Transport
DMR2:23885001	2	23885001	23888000	3000	1	3.60E-09	0.42	46	1.53	Scamp1	Transport
DMR2:23895001	2	23895001	23896000	1000	1	7.00E-11	0.51	11	1.1	Scamp1	Transport
DMR2:23907001	2	23907001	23909000	2000	1	4.20E-08	0.35	23	1.15	Scamp1	Transport
DMR2:23913001	2	23913001	23915000	2000	1	4.10E-11	0.56	31	1.55	Scamp1	Transport
DMR2:23928001	2	23928001	23929000	1000	1	6.80E-10	-0.71	9	0.9	Scamp1	Transport
DMR2:23945001	2	23945001	23948000	3000	1	3.50E-10	0.52	38	1.27	Scamp1	Transport
DMR2:24020001	2	24020001	24021000	1000	1	5.60E-08	0.53	8	0.8	Ap3b1	Transport
DMR2:24032001	2	24032001	24035000	3000	1	1.90E-09	0.41	62	2.07	Ap3b1	Transport
DMR2:24037001	2	24037001	24040000	3000	3	3.40E-22	0.78	23	0.77	Ap3b1	Transport
DMR2:24041001	2	24041001	24045000	4000	1	2.10E-09	0.44	64	1.6	Ap3b1	Transport
DMR2:24053001	2	24053001	24055000	2000	1	7.70E-11	0.59	16	0.8	Ap3b1	Transport
DMR2:24056001	2	24056001	24059000	3000	1	7.40E-13	0.48	42	1.4	Ap3b1	Transport
DMR2:24069001	2	24069001	24072000	3000	1	1.10E-11	-0.58	20	0.67	Ap3b1	Transport
DMR2:24085001	2	24085001	24087000	2000	2	5.90E-08	-0.48	20	1	Ap3b1	Transport
DMR2:24088001	2	24088001	24094000	6000	2	1.10E-08	0.38	81	1.35	Ap3b1	Transport
DMR2:24098001	2	24098001	24101000	3000	1	1.70E-11	0.39	43	1.43	Ap3b1	Transport

DMR2:24102001	2	24102001	24103000	1000	1	1.90E-11	0.47	19	1.9	Ap3b1	Transport
DMR2:24104001	2	24104001	24109000	5000	1	1.40E-09	0.38	71	1.42	Ap3b1	Transport
DMR2:24110001	2	24110001	24111000	1000	1	5.60E-07	0.33	12	1.2	Ap3b1	Transport
DMR2:24118001	2	24118001	24122000	4000	1	1.70E-10	0.43	61	1.52	Ap3b1	Transport
DMR2:24133001	2	24133001	24144000	11000	1	7.10E-07	-0.49	162	1.47	Ap3b1	Transport
DMR2:24148001	2	24148001	24153000	5000	1	2.30E-18	0.66	88	1.76	Ap3b1	Transport
DMR2:24155001	2	24155001	24157000	2000	2	8.30E-14	0.63	28	1.4	Ap3b1	Transport
DMR2:24172001	2	24172001	24174000	2000	1	2.80E-07	-0.52	28	1.4	Ap3b1	Transport
DMR2:24193001	2	24193001	24199000	6000	3	9.40E-16	0.75	97	1.62	Ap3b1	Transport
DMR2:24201001	2	24201001	24209000	8000	2	3.30E-07	-0.48	146	1.82	Ap3b1	Transport
DMR2:24441001	2	24441001	24444000	3000	3	5.50E-11	0.48	48	1.6	Tbca	Translation
DMR2:24479001	2	24479001	24483000	4000	1	8.20E-07	0.36	41	1.02	Tbca	Translation
DMR2:24495001	2	24495001	24496000	1000	1	8.90E-15	-0.66	14	1.4	Tbca	Translation
DMR2:24540001	2	24540001	24541000	1000	1	4.90E-12	0.83	36	3.6	Otp	Development
DMR2:24673001	2	24673001	24675000	2000	1	4.90E-07	-0.53	41	2.05	Wdr41	
DMR2:24677001	2	24677001	24678000	1000	1	3.60E-19	0.88	24	2.4	Wdr41	
DMR2:24681001	2	24681001	24683000	2000	1	8.00E-13	0.54	29	1.45	Wdr41	
DMR2:24691001	2	24691001	24694000	3000	1	3.60E-07	-0.45	10	0.33	Wdr41	
DMR2:24699001	2	24699001	24702000	3000	1	2.20E-07	0.37	45	1.5	Wdr41	
DMR2:24712001	2	24712001	24728000	16000	2	1.60E-10	-0.53	344	2.15	Wdr41;Pde8b	Signaling
DMR2:24734001	2	24734001	24741000	7000	2	4.30E-08	0.38	54	0.77	Pde8b	Signaling
DMR2:24742001	2	24742001	24746000	4000	1	2.70E-08	0.55	70	1.75	Pde8b	Signaling
DMR2:24747001	2	24747001	24748000	1000	1	1.90E-07	0.33	7	0.7	Pde8b	Signaling
DMR2:24753001	2	24753001	24758000	5000	4	8.70E-21	0.87	45	0.9	Pde8b	Signaling
DMR2:24786001	2	24786001	24788000	2000	2	6.70E-08	-0.67	28	1.4	Pde8b	Signaling
DMR2:24796001	2	24796001	24798000	2000	2	9.10E-12	0.7	21	1.05	Pde8b	Signaling
DMR2:24804001	2	24804001	24807000	3000	1	8.60E-11	0.54	37	1.23	Pde8b	Signaling
DMR2:24819001	2	24819001	24820000	1000	1	3.90E-07	-0.56	8	0.8	Pde8b	Signaling
DMR2:24823001	2	24823001	24829000	6000	1	9.00E-07	0.38	65	1.08	Pde8b	Signaling
DMR2:24834001	2	24834001	24835000	1000	1	4.30E-09	0.48	13	1.3	Pde8b	Signaling
DMR2:24838001	2	24838001	24839000	1000	1	1.10E-19	0.67	14	1.4	Pde8b	Signaling
DMR2:24841001	2	24841001	24843000	2000	1	1.60E-07	0.34	15	0.75	Pde8b	Signaling
DMR2:24845001	2	24845001	24846000	1000	1	4.80E-08	0.51	8	0.8	Pde8b	Signaling
DMR2:24848001	2	24848001	24849000	1000	1	7.00E-09	0.39	10	1	Pde8b	Signaling
DMR2:24851001	2	24851001	24852000	1000	1	2.90E-12	0.54	6	0.6	Pde8b	Signaling
DMR2:24853001	2	24853001	24855000	2000	1	1.20E-08	0.4	9	0.45	Pde8b	Signaling
DMR2:24857001	2	24857001	24859000	2000	1	1.80E-20	0.54	30	1.5	Pde8b	Signaling
DMR2:24876001	2	24876001	24878000	2000	2	3.40E-09	-0.46	23	1.15	Pde8b	Signaling
DMR2:24881001	2	24881001	24891000	10000	1	3.10E-08	0.27	132	1.32	Pde8b	Signaling
DMR2:24915001	2	24915001	24916000	1000	1	8.60E-07	-0.58	9	0.9	Pde8b	Signaling
DMR2:24917001	2	24917001	24918000	1000	1	3.30E-12	0.61	7	0.7	Pde8b	Signaling
DMR2:24927001	2	24927001	24929000	2000	1	1.70E-09	0.38	31	1.55	Pde8b	Signaling
DMR2:24941001	2	24941001	24946000	5000	2	1.00E-08	0.44	68	1.36	Pde8b;LOC102548698	Signaling
DMR2:24947001	2	24947001	24948000	1000	1	6.80E-09	-0.49	18	1.8	Pde8b;LOC102548698	Signaling
DMR2:24957001	2	24957001	24959000	2000	1	1.40E-08	0.46	17	0.85	Pde8b;LOC102548698	Signaling
DMR2:24965001	2	24965001	24966000	1000	1	7.40E-21	0.97	9	0.9	Pde8b;LOC102548698	Signaling
DMR2:25042001	2	25042001	25045000	3000	1	3.80E-15	0.51	52	1.73	LOC102548698;Zbed3	
DMR2:25051001	2	25051001	25053000	2000	1	4.00E-15	0.62	43	2.15	Zbed3;LOC679528	
DMR2:25067001	2	25067001	25072000	5000	1	2.60E-11	0.48	90	1.8	Aggf1	
DMR2:25101001	2	25101001	25106000	5000	1	1.90E-09	0.48	77	1.54	Aggf1	
DMR2:25179001	2	25179001	25180000	1000	1	1.40E-08	0.58	51	5.1	S100z	Signaling
DMR2:25189001	2	25189001	25191000	2000	1	2.10E-18	0.45	43	2.15	S100z	Signaling
DMR2:25192001	2	25192001	25194000	2000	1	2.90E-11	0.63	22	1.1	S100z	Signaling
DMR2:25197001	2	25197001	25198000	1000	1	7.50E-07	-0.51	25	2.5	S100z	Signaling
DMR2:25201001	2	25201001	25204000	3000	2	5.30E-11	0.57	56	1.87	S100z	Signaling
DMR2:25205001	2	25205001	25207000	2000	1	3.00E-10	0.45	46	2.3	S100z	Signaling
DMR2:25210001	2	25210001	25211000	1000	1	3.50E-07	-0.43	16	1.6	S100z	Signaling
DMR2:25219001	2	25219001	25225000	6000	1	3.00E-08	0.32	127	2.12	F2rl1	Signaling
DMR2:25240001	2	25240001	25241000	1000	1	1.80E-09	0.41	17	1.7	F2rl1;LOC102550768	Signaling
DMR2:25747001	2	25747001	25748000	1000	1	3.90E-07	0.3	18	1.8	Arhgef28	
DMR2:25749001	2	25749001	25751000	2000	2	2.60E-08	0.45	16	0.8	Arhgef28	
DMR2:25802001	2	25802001	25805000	3000	1	1.10E-07	-0.56	46	1.53	Arhgef28	
DMR2:25808001	2	25808001	25811000	3000	1	4.50E-10	0.39	47	1.57	Arhgef28	
DMR2:25812001	2	25812001	25813000	1000	1	5.00E-07	-0.7	14	1.4	Arhgef28	
DMR2:25829001	2	25829001	25831000	2000	1	5.90E-13	0.55	39	1.95	Arhgef28	

DMR2:25835001	2	25835001	25840000	5000	2	1.50E-16	0.71	62	1.24	Arhgef28	
DMR2:25849001	2	25849001	25852000	3000	2	4.70E-32	0.96	37	1.23	Arhgef28	
DMR2:25861001	2	25861001	25865000	4000	2	1.50E-08	-0.45	47	1.18	Arhgef28	
DMR2:25869001	2	25869001	25877000	8000	1	5.60E-15	0.49	156	1.95	Arhgef28	
DMR2:25893001	2	25893001	25895000	2000	2	8.70E-11	0.5	44	2.2	Arhgef28	
DMR2:25896001	2	25896001	25897000	1000	1	3.70E-11	0.72	7	0.7	Arhgef28	
DMR2:25898001	2	25898001	25899000	1000	1	7.50E-10	0.48	12	1.2	Arhgef28	
DMR2:25905001	2	25905001	25908000	3000	1	3.30E-08	0.41	54	1.8	Arhgef28	
DMR2:25916001	2	25916001	25920000	4000	3	5.60E-10	-0.51	67	1.68	Arhgef28	
DMR2:25921001	2	25921001	25922000	1000	1	7.50E-09	-0.44	16	1.6	Arhgef28	
DMR2:25946001	2	25946001	25947000	1000	1	3.70E-15	0.69	11	1.1	Arhgef28	
DMR2:26010001	2	26010001	26016000	6000	1	6.10E-21	0.73	115	1.92	Arhgef28;RGD1566212	
DMR2:26034001	2	26034001	26035000	1000	1	4.30E-08	0.53	8	0.8	RGD1566212	
DMR2:26040001	2	26040001	26042000	2000	1	7.70E-14	0.77	29	1.45	RGD1566212	
DMR2:26047001	2	26047001	26048000	1000	1	1.80E-08	0.53	3	0.3	RGD1566212	
DMR2:26050001	2	26050001	26055000	5000	2	1.50E-17	-0.65	23	0.46	RGD1566212	
DMR2:26062001	2	26062001	26066000	4000	1	2.40E-11	0.51	73	1.82	RGD1566212	
DMR2:26067001	2	26067001	26071000	4000	1	2.10E-17	0.66	58	1.45	RGD1566212	
DMR2:26085001	2	26085001	26088000	3000	1	3.20E-13	0.56	32	1.07	RGD1566212	
DMR2:26095001	2	26095001	26096000	1000	1	4.30E-20	0.63	10	1	RGD1566212	
DMR2:26108001	2	26108001	26112000	4000	1	2.50E-07	0.27	70	1.75	F2r	Signaling
DMR2:26116001	2	26116001	26117000	1000	1	3.60E-12	0.78	13	1.3	F2r	Signaling
DMR2:26131001	2	26131001	26134000	3000	1	1.40E-11	0.45	55	1.83	F2r	Signaling
DMR2:26151001	2	26151001	26152000	1000	1	3.80E-14	0.73	9	0.9	Iqgap2	Signaling
DMR2:26159001	2	26159001	26160000	1000	1	1.90E-12	0.6	11	1.1	Iqgap2	Signaling
DMR2:26167001	2	26167001	26171000	4000	1	4.70E-12	0.53	58	1.45	Iqgap2	Signaling
DMR2:26187001	2	26187001	26191000	4000	2	1.60E-09	0.52	57	1.43	Iqgap2	Signaling
DMR2:26195001	2	26195001	26199000	4000	1	2.40E-13	0.54	51	1.27	Iqgap2	Signaling
DMR2:26234001	2	26234001	26237000	3000	2	2.40E-12	-0.49	41	1.37	Iqgap2;F2rl2	Signaling;Signaling
DMR2:26244001	2	26244001	26246000	2000	1	5.40E-11	0.48	45	2.25	Iqgap2;F2rl2	Signaling;Signaling
DMR2:26248001	2	26248001	26252000	4000	2	1.60E-08	-0.67	68	1.7	Iqgap2;F2rl2	Signaling;Signaling
DMR2:26253001	2	26253001	26254000	1000	1	2.70E-09	0.53	13	1.3	Iqgap2;F2rl2	Signaling;Signaling
DMR2:26256001	2	26256001	26257000	1000	1	3.50E-14	0.8	2	0.2	Iqgap2	Signaling
DMR2:26258001	2	26258001	26259000	1000	1	2.60E-11	0.46	12	1.2	Iqgap2	Signaling
DMR2:26273001	2	26273001	26279000	6000	1	5.60E-08	-0.41	85	1.42	Iqgap2	Signaling
DMR2:26311001	2	26311001	26313000	2000	1	7.00E-08	0.45	22	1.1	Iqgap2	Signaling
DMR2:26314001	2	26314001	26318000	4000	1	4.60E-08	0.36	66	1.65	Iqgap2	Signaling
DMR2:26322001	2	26322001	26326000	4000	1	3.70E-09	0.41	71	1.77	Iqgap2	Signaling
DMR2:26329001	2	26329001	26334000	5000	1	1.20E-09	-0.59	54	1.08	Iqgap2	Signaling
DMR2:26352001	2	26352001	26354000	2000	1	9.30E-17	0.74	27	1.35	Iqgap2	Signaling
DMR2:26357001	2	26357001	26361000	4000	1	5.30E-08	0.46	58	1.45	Iqgap2	Signaling
DMR2:26364001	2	26364001	26366000	2000	1	1.50E-07	0.3	23	1.15	Iqgap2	Signaling
DMR2:26371001	2	26371001	26373000	2000	1	6.50E-07	0.32	32	1.6	Iqgap2	Signaling
DMR2:26400001	2	26400001	26402000	2000	1	2.70E-07	0.37	20	1	Iqgap2	Signaling
DMR2:26407001	2	26407001	26409000	2000	2	7.80E-09	0.42	22	1.1	Iqgap2	Signaling
DMR2:26410001	2	26410001	26412000	2000	1	5.30E-14	0.75	29	1.45	Iqgap2	Signaling
DMR2:26428001	2	26428001	26429000	1000	1	1.20E-20	0.67	13	1.3	Iqgap2	Signaling
DMR2:26436001	2	26436001	26437000	1000	1	9.00E-11	-1	24	2.4	Iqgap2	Signaling
DMR2:26447001	2	26447001	26450000	3000	1	9.20E-07	0.52	41	1.37	Iqgap2	Signaling
DMR2:26461001	2	26461001	26464000	3000	1	1.20E-08	0.33	59	1.97	Ndufs6	Metabolism
DMR2:26499001	2	26499001	26506000	7000	1	1.20E-16	0.74	115	1.64	Sv2c	
DMR2:26509001	2	26509001	26515000	6000	3	2.10E-11	-0.49	71	1.18	Sv2c	
DMR2:26516001	2	26516001	26521000	5000	2	7.00E-22	-0.47	64	1.28	Sv2c	
DMR2:26523001	2	26523001	26529000	6000	5	3.70E-21	0.76	71	1.18	Sv2c	
DMR2:26533001	2	26533001	26536000	3000	2	1.80E-20	0.86	32	1.07	Sv2c	
DMR2:26543001	2	26543001	26550000	7000	6	1.60E-17	-0.54	101	1.44	Sv2c	
DMR2:26567001	2	26567001	26569000	2000	2	2.80E-18	0.78	30	1.5	Sv2c	
DMR2:26582001	2	26582001	26584000	2000	1	4.50E-08	-0.63	25	1.25	Sv2c	
DMR2:26586001	2	26586001	26588000	2000	1	2.80E-07	0.37	34	1.7	Sv2c	
DMR2:26601001	2	26601001	26607000	6000	4	4.20E-18	0.96	70	1.17	Sv2c	
DMR2:26608001	2	26608001	26610000	2000	2	9.10E-09	-0.57	7	0.35	Sv2c	
DMR2:26619001	2	26619001	26620000	1000	1	1.60E-07	-0.34	7	0.7	Sv2c	
DMR2:26622001	2	26622001	26623000	1000	1	2.70E-09	0.49	11	1.1	Sv2c	
DMR2:26624001	2	26624001	26625000	1000	1	2.30E-12	0.76	3	0.3	Sv2c	
DMR2:26637001	2	26637001	26644000	7000	2	1.40E-11	-0.61	98	1.4	Sv2c	

DMR2:26650001	2	26650001	26653000	3000	1	9.20E-13	0.45	43	1.43	Sv2c	
DMR2:26655001	2	26655001	26661000	6000	2	1.80E-19	-0.51	100	1.67	Sv2c	
DMR2:26669001	2	26669001	26674000	5000	1	8.40E-12	0.49	60	1.2	Sv2c	
DMR2:26691001	2	26691001	26693000	2000	1	2.00E-18	0.71	24	1.2	Sv2c	
DMR2:26992001	2	26992001	26994000	2000	2	2.60E-13	1.03	27	1.35	LOC365650;Poc5	
DMR2:26996001	2	26996001	26998000	2000	2	1.20E-18	0.79	20	1	LOC365650;Poc5	
DMR2:27011001	2	27011001	27012000	1000	1	2.00E-09	0.38	13	1.3	Poc5	
DMR2:27034001	2	27034001	27037000	3000	2	6.60E-33	1.14	36	1.2	Poc5;Ankdd1b	
DMR2:27044001	2	27044001	27045000	1000	1	5.00E-07	-0.59	4	0.4	Ankdd1b	
DMR2:27059001	2	27059001	27060000	1000	1	3.30E-07	0.61	0	0	Ankdd1b	
DMR2:27061001	2	27061001	27064000	3000	3	2.50E-08	-0.54	26	0.87	Ankdd1b	
DMR2:27067001	2	27067001	27068000	1000	1	7.20E-09	0.48	5	0.5	Ankdd1b	
DMR2:27122001	2	27122001	27124000	2000	2	3.70E-13	0.99	17	0.85	Ankdd1b;Arhgef26	Transcription
DMR2:27160001	2	27160001	27162000	2000	1	1.80E-07	-0.62	22	1.1	Ankdd1b;Arhgef26	Transcription
DMR2:27175001	2	27175001	27176000	1000	1	1.50E-16	0.55	14	1.4	Ankdd1b;Arhgef26	Transcription
DMR2:27196001	2	27196001	27198000	2000	2	9.30E-07	0.36	17	0.85	Ankdd1b;Arhgef26	Transcription
DMR2:27209001	2	27209001	27210000	1000	1	4.30E-07	0.45	13	1.3	Ankdd1b;Arhgef26	Transcription
DMR2:27220001	2	27220001	27221000	1000	1	4.00E-07	0.44	15	1.5	Ankdd1b;Arhgef26	Transcription
DMR2:27224001	2	27224001	27228000	4000	1	9.70E-16	0.68	29	0.72	Ankdd1b;Arhgef26	Transcription
DMR2:27248001	2	27248001	27249000	1000	1	1.40E-18	0.62	11	1.1	Ankdd1b	
DMR2:27251001	2	27251001	27254000	3000	2	1.50E-10	0.59	28	0.93	Ankdd1b	
DMR2:27270001	2	27270001	27271000	1000	1	8.40E-07	-0.42	28	2.8	Ankdd1b	
DMR2:27290001	2	27290001	27292000	2000	2	1.70E-08	0.48	29	1.45	Ankdd1b	
DMR2:27298001	2	27298001	27300000	2000	2	2.40E-25	0.83	29	1.45	Ankdd1b;Polk	Transcription
DMR2:27309001	2	27309001	27316000	7000	3	3.10E-14	0.52	121	1.73	Polk	Transcription
DMR2:27319001	2	27319001	27322000	3000	3	6.30E-11	-0.49	33	1.1	Polk	Transcription
DMR2:27323001	2	27323001	27327000	4000	2	3.10E-11	0.56	52	1.3	Polk	Transcription
DMR2:27357001	2	27357001	27360000	3000	2	3.50E-35	1.32	15	0.5	Polk;Col4a3bp	Transcription
DMR2:27367001	2	27367001	27369000	2000	1	3.30E-15	0.73	13	0.65	Polk;Col4a3bp	Transcription
DMR2:27370001	2	27370001	27373000	3000	1	7.40E-07	-0.31	25	0.83	Polk;Col4a3bp	Transcription
DMR2:27384001	2	27384001	27386000	2000	1	2.90E-08	0.59	10	0.5	Col4a3bp	
DMR2:27414001	2	27414001	27416000	2000	1	4.90E-22	0.99	9	0.45	Col4a3bp	
DMR2:27429001	2	27429001	27434000	5000	2	1.90E-11	-0.56	26	0.52	Col4a3bp	
DMR2:27437001	2	27437001	27439000	2000	2	3.00E-21	0.83	18	0.9	Col4a3bp	
DMR2:27463001	2	27463001	27467000	4000	1	9.30E-07	-0.65	49	1.23	Col4a3bp	
DMR2:27470001	2	27470001	27471000	1000	1	2.90E-08	0.35	23	2.3	Col4a3bp;Hmgcr	Metabolism
DMR2:27475001	2	27475001	27480000	5000	1	1.60E-07	0.41	50	1	Col4a3bp;Hmgcr	Metabolism
DMR2:27492001	2	27492001	27497000	5000	1	3.00E-07	-0.55	91	1.82	Hmgcr	Metabolism
DMR2:27507001	2	27507001	27511000	4000	1	2.20E-07	-0.52	84	2.1	Hmgcr	Metabolism
DMR2:27568001	2	27568001	27569000	1000	1	8.90E-07	0.38	7	0.7	LOC103691437;Ankrd31;LOC108350154	
DMR2:27574001	2	27574001	27576000	2000	1	8.00E-07	0.37	19	0.95	Ankrd31;LOC108350154	
DMR2:27577001	2	27577001	27578000	1000	1	9.20E-08	0.42	4	0.4	Ankrd31;LOC108350154	
DMR2:27589001	2	27589001	27590000	1000	1	2.50E-09	-0.66	7	0.7	Ankrd31	
DMR2:27595001	2	27595001	27596000	1000	1	9.20E-07	-0.57	3	0.3	Ankrd31	
DMR2:27599001	2	27599001	27600000	1000	1	5.90E-11	0.66	5	0.5	Ankrd31	
DMR2:27601001	2	27601001	27603000	2000	1	2.40E-08	-0.56	8	0.4	Ankrd31	
DMR2:27646001	2	27646001	27649000	3000	2	1.70E-10	-0.55	17	0.57	Ankrd31	
DMR2:27653001	2	27653001	27661000	8000	4	2.50E-17	0.63	83	1.04	Ankrd31	
DMR2:27670001	2	27670001	27674000	4000	2	4.50E-11	0.64	26	0.65	Ankrd31;LOC102554085	
DMR2:27680001	2	27680001	27682000	2000	1	5.40E-14	0.58	11	0.55	Ankrd31;LOC102554085	
DMR2:27692001	2	27692001	27693000	1000	1	9.50E-12	-0.79	11	1.1	Ankrd31	
DMR2:27696001	2	27696001	27699000	3000	1	2.80E-07	0.4	18	0.6	Ankrd31	
DMR2:27700001	2	27700001	27702000	2000	2	8.60E-08	-0.38	7	0.35	Ankrd31	
DMR2:27705001	2	27705001	27709000	4000	1	5.00E-07	0.44	38	0.95	Ankrd31	
DMR2:27713001	2	27713001	27716000	3000	2	8.70E-08	-0.62	52	1.73	Ankrd31	
DMR2:27727001	2	27727001	27728000	1000	1	8.50E-08	0.46	7	0.7	Ankrd31	
DMR2:27732001	2	27732001	27738000	6000	3	2.60E-13	-0.49	34	0.57	Ankrd31	
DMR2:27872001	2	27872001	27875000	3000	1	6.60E-18	0.5	49	1.63	Fam169a	
DMR2:27894001	2	27894001	27895000	1000	1	1.70E-10	0.42	11	1.1	Fam169a;LOC108349924	
DMR2:27911001	2	27911001	27912000	1000	1	2.00E-33	0.88	16	1.6	Fam169a	
DMR2:27914001	2	27914001	27918000	4000	1	2.40E-13	0.57	55	1.38	Fam169a	
DMR2:27964001	2	27964001	27967000	3000	2	9.90E-13	0.47	43	1.43	Gfm2	Translation
DMR2:27970001	2	27970001	27973000	3000	2	2.20E-07	-0.63	47	1.57	Gfm2	Translation

DMR2:27984001	2	27984001	27986000	2000	1	8.50E-07	-0.36	33	1.65	Gfm2;Hexb	Translation;Metabolism
DMR2:27997001	2	27997001	27998000	1000	1	5.80E-13	0.41	17	1.7	Hexb	Metabolism
DMR2:28011001	2	28011001	28013000	2000	1	1.10E-07	0.41	19	0.95	Hexb	Metabolism
DMR2:28066001	2	28066001	28067000	1000	1	2.90E-10	0.48	14	1.4	Enc1	
DMR2:28353001	2	28353001	28355000	2000	1	3.70E-07	-0.49	35	1.75	LOC108349925;Utp15	Translation
DMR2:28366001	2	28366001	28368000	2000	1	1.50E-08	-0.46	25	1.25	Utp15;LOC100910438	Translation;Signaling
DMR2:28411001	2	28411001	28413000	2000	1	1.20E-09	-0.65	31	1.55	LOC108349926;Btf3	Transcription
DMR2:28434001	2	28434001	28437000	3000	1	3.30E-14	0.56	45	1.5	Btf3	Transcription
DMR2:28451001	2	28451001	28457000	6000	3	1.60E-24	0.95	61	1.02	Foxd1	Transcription
DMR2:28740001	2	28740001	28741000	1000	1	3.10E-12	0.77	1	0.1	Tmem174	
DMR2:28811001	2	28811001	28812000	1000	1	7.20E-07	0.31	14	1.4	Tmem171;LOC102546725	
DMR2:28918001	2	28918001	28920000	2000	2	1.60E-17	0.75	10	0.5	Fcho2	Cytoskeleton
DMR2:28921001	2	28921001	28922000	1000	1	6.70E-17	0.77	13	1.3	Fcho2	Cytoskeleton
DMR2:28936001	2	28936001	28938000	2000	2	7.90E-15	0.64	13	0.65	Fcho2;LOC100364548	Cytoskeleton
DMR2:28940001	2	28940001	28943000	3000	3	1.30E-27	0.8	37	1.23	Fcho2;LOC100364548	Cytoskeleton
DMR2:28979001	2	28979001	28983000	4000	1	1.90E-15	-0.75	36	0.9	Fcho2	Cytoskeleton
DMR2:29035001	2	29035001	29036000	1000	1	1.00E-11	0.51	11	1.1	Fcho2	Cytoskeleton
DMR2:29053001	2	29053001	29057000	4000	1	5.50E-10	0.45	49	1.23	Tnpo1	Transport
DMR2:29062001	2	29062001	29068000	6000	1	1.40E-07	-0.65	69	1.15	Tnpo1	Transport
DMR2:29076001	2	29076001	29078000	2000	1	5.40E-09	-0.73	39	1.95	Tnpo1	Transport
DMR2:29082001	2	29082001	29084000	2000	1	2.50E-08	0.37	18	0.9	Tnpo1	Transport
DMR2:29086001	2	29086001	29088000	2000	2	2.00E-09	-0.36	35	1.75	Tnpo1	Transport
DMR2:29135001	2	29135001	29137000	2000	2	3.80E-07	-0.48	20	1	Tnpo1	Transport
DMR2:29400001	2	29400001	29401000	1000	1	1.70E-14	0.84	9	0.9	Zfp366	Transcription
DMR2:29402001	2	29402001	29405000	3000	2	1.20E-10	-0.54	64	2.13	Zfp366	Transcription
DMR2:29414001	2	29414001	29416000	2000	1	4.10E-07	-0.51	41	2.05	Zfp366	Transcription
DMR2:29424001	2	29424001	29432000	8000	4	9.30E-14	0.7	95	1.19	Zfp366	Transcription
DMR2:29456001	2	29456001	29458000	2000	1	8.70E-08	-0.53	34	1.7	Zfp366;LOC108349928	Transcription
DMR2:29462001	2	29462001	29463000	1000	1	1.10E-09	-0.42	14	1.4	Zfp366;LOC108349928	Transcription
DMR2:29465001	2	29465001	29469000	4000	1	1.10E-18	0.82	22	0.55	Zfp366;LOC108349928	Transcription
DMR2:29506001	2	29506001	29507000	1000	1	4.50E-09	0.45	8	0.8	Zfp366	Transcription
DMR2:29585001	2	29585001	29590000	5000	2	5.90E-10	-0.58	66	1.32	Ptcd2;Mrps27	Translation
DMR2:29591001	2	29591001	29593000	2000	1	4.20E-16	0.54	38	1.9	Ptcd2;Mrps27	Translation
DMR2:29625001	2	29625001	29626000	1000	1	2.70E-10	0.45	16	1.6	Mrps27	Translation
DMR2:29633001	2	29633001	29635000	2000	1	3.40E-22	0.78	23	1.15	Mrps27	Translation
DMR2:29637001	2	29637001	29639000	2000	1	7.10E-14	0.49	26	1.3	Mrps27	Translation
DMR2:29642001	2	29642001	29643000	1000	1	4.40E-08	-0.44	11	1.1	Mrps27	Translation
DMR2:29644001	2	29644001	29648000	4000	2	5.90E-07	0.39	46	1.15	Mrps27	Translation
DMR2:29658001	2	29658001	29661000	3000	1	1.40E-09	-0.7	37	1.23	Mrps27	Translation
DMR2:29667001	2	29667001	29668000	1000	1	3.00E-09	0.38	16	1.6	Mrps27;Map1b	Translation;Cytoskeleton
DMR2:29670001	2	29670001	29673000	3000	1	5.90E-08	0.44	37	1.23	Mrps27;Map1b	Translation;Cytoskeleton
DMR2:29676001	2	29676001	29678000	2000	1	2.70E-08	-0.56	34	1.7	Mrps27;Map1b	Translation;Cytoskeleton
DMR2:29679001	2	29679001	29682000	3000	2	3.20E-16	-0.67	73	2.43	Map1b	Cytoskeleton
DMR2:29685001	2	29685001	29691000	6000	1	1.10E-08	0.45	180	3	Map1b	Cytoskeleton
DMR2:29694001	2	29694001	29700000	6000	1	1.60E-08	-0.63	93	1.55	Map1b;LOC108349930	Cytoskeleton
DMR2:29721001	2	29721001	29726000	5000	1	1.80E-09	0.4	70	1.4	Map1b;LOC108349930	Cytoskeleton
DMR2:29774001	2	29774001	29776000	2000	1	6.80E-12	0.56	26	1.3	Map1b;LOC102553545	Cytoskeleton
DMR2:30132001	2	30132001	30133000	1000	1	6.80E-08	0.41	13	1.3	Cartpt	
DMR2:30162001	2	30162001	30166000	4000	1	1.50E-12	0.43	68	1.7	Mccc2	Metabolism
DMR2:30171001	2	30171001	30176000	5000	3	2.50E-21	0.6	71	1.42	Mccc2	Metabolism
DMR2:30179001	2	30179001	30180000	1000	1	3.70E-09	0.36	15	1.5	Mccc2	Metabolism
DMR2:30188001	2	30188001	30193000	5000	1	8.30E-08	-0.46	71	1.42	Mccc2	Metabolism
DMR2:30194001	2	30194001	30200000	6000	1	1.10E-14	0.86	70	1.17	Mccc2	Metabolism
DMR2:30203001	2	30203001	30204000	1000	1	2.40E-09	0.37	15	1.5	Mccc2	Metabolism
DMR2:30205001	2	30205001	30210000	5000	1	8.70E-08	-0.46	68	1.36	Mccc2	Metabolism
DMR2:30213001	2	30213001	30214000	1000	1	9.20E-10	0.51	6	0.6	Mccc2	Metabolism
DMR2:30215001	2	30215001	30224000	9000	1	1.20E-08	-0.57	119	1.32	Mccc2	Metabolism
DMR2:30228001	2	30228001	30230000	2000	2	2.00E-10	0.42	11	0.55	Mccc2	Metabolism
DMR2:30239001	2	30239001	30243000	4000	1	3.10E-10	0.44	54	1.35	Mccc2;Bdp1	Metabolism;Transcription

DMR2:30254001	2	30254001	30255000	1000	1	7.20E-08	0.4	9	0.9	Mccc2;Bdp1	Metabolism;Transcription
DMR2:30265001	2	30265001	30268000	3000	2	5.00E-09	-0.66	29	0.97	Bdp1	Transcription
DMR2:30292001	2	30292001	30293000	1000	1	2.80E-10	0.41	19	1.9	Bdp1	Transcription
DMR2:30307001	2	30307001	30309000	2000	1	2.40E-11	0.43	25	1.25	Bdp1	Transcription
DMR2:30310001	2	30310001	30314000	4000	1	6.40E-09	-0.54	38	0.95	Bdp1	Transcription
DMR2:30361001	2	30361001	30363000	2000	1	4.00E-08	-0.61	22	1.1	Serf1;Smn1	Translation
DMR2:30386001	2	30386001	30390000	4000	2	6.60E-08	-0.44	63	1.57	Naip6	
DMR2:30409001	2	30409001	30410000	1000	1	1.20E-08	0.46	5	0.5	Naip6	
DMR2:30411001	2	30411001	30412000	1000	1	1.20E-09	0.94	5	0.5	Naip6	
DMR2:30418001	2	30418001	30424000	6000	2	5.50E-10	0.43	48	0.8	Naip6;Naip5	
DMR2:30425001	2	30425001	30428000	3000	1	3.20E-13	0.47	25	0.83	Naip6;Naip5	
DMR2:30431001	2	30431001	30434000	3000	2	1.50E-15	0.57	35	1.17	Naip5	
DMR2:30435001	2	30435001	30436000	1000	1	1.60E-17	0.68	12	1.2	Naip5	
DMR2:30441001	2	30441001	30444000	3000	1	1.60E-10	-0.56	34	1.13	Naip5	
DMR2:30461001	2	30461001	30468000	7000	2	4.00E-14	0.54	170	2.43	Naip5	
DMR2:30495001	2	30495001	30497000	2000	1	1.10E-27	1.21	11	0.55	LOC102554754;Gtf2h2	Transcription
DMR2:30503001	2	30503001	30505000	2000	1	2.20E-10	0.59	13	0.65	Gtf2h2	Transcription
DMR2:30516001	2	30516001	30521000	5000	3	1.30E-21	0.63	35	0.7	Gtf2h2;LOC365655;Ocln	Transcription
DMR2:30540001	2	30540001	30542000	2000	1	8.10E-09	0.43	24	1.2	Ocln	Transcription
DMR2:30556001	2	30556001	30557000	1000	1	3.00E-13	0.54	10	1	Ocln	Transcription
DMR2:30569001	2	30569001	30570000	1000	1	1.10E-07	0.48	14	1.4	Ocln	Transcription
DMR2:30573001	2	30573001	30576000	3000	1	7.20E-07	0.44	48	1.6	Ocln	Transcription
DMR2:30579001	2	30579001	30582000	3000	1	1.00E-11	0.42	41	1.37	Ocln	Transcription
DMR2:30585001	2	30585001	30587000	2000	1	7.20E-11	0.41	26	1.3	Ocln	Transcription
DMR2:30601001	2	30601001	30607000	6000	3	9.60E-13	0.72	61	1.02	LOC100361161;LOC102557393;Marveld2	Transcription
DMR2:30608001	2	30608001	30609000	1000	1	3.60E-09	0.52	10	1	LOC100361161;LOC102557393;Marveld2	Transcription
DMR2:30614001	2	30614001	30615000	1000	1	7.90E-09	0.37	17	1.7	LOC102557393;Marveld2	Transcription
DMR2:30626001	2	30626001	30628000	2000	1	3.00E-17	0.81	39	1.95	LOC102557393;Marveld2;Rad17	Transcription
DMR2:30637001	2	30637001	30638000	1000	1	2.40E-07	-0.63	8	0.8	Marveld2;Rad17	Transcription
DMR2:30662001	2	30662001	30663000	1000	1	2.50E-09	-0.58	12	1.2	Rad17;Ak6;Taf9	Epigenetic
DMR2:30667001	2	30667001	30668000	1000	1	2.40E-07	-0.61	11	1.1	Rad17;Ak6;Taf9	Epigenetic
DMR2:30693001	2	30693001	30697000	4000	2	7.20E-33	1.17	56	1.4	Ccdc125	
DMR2:30699001	2	30699001	30703000	4000	1	1.90E-18	0.56	81	2.02	Ccdc125;Cdk7	Signaling
DMR2:30719001	2	30719001	30720000	1000	1	3.70E-07	0.39	7	0.7	Ccdc125;Cdk7	Signaling
DMR2:30721001	2	30721001	30724000	3000	1	3.40E-09	0.46	47	1.57	Ccdc125;Cdk7	Signaling
DMR2:30733001	2	30733001	30734000	1000	1	6.70E-13	-0.69	10	1	Ccdc125;Cdk7;Mrps36	Signaling;Translation
DMR2:30743001	2	30743001	30746000	3000	1	4.60E-07	-0.42	36	1.2	Cdk7;Mrps36	Signaling;Translation
DMR2:30768001	2	30768001	30772000	4000	1	3.40E-07	-0.46	56	1.4	Cenph	
DMR2:30794001	2	30794001	30796000	2000	1	3.50E-20	0.86	29	1.45	Ccnb1	Signaling
DMR2:30804001	2	30804001	30806000	2000	1	9.80E-07	-0.44	16	0.8	Slc30a5	
DMR2:30819001	2	30819001	30821000	2000	1	7.70E-19	0.83	21	1.05	Slc30a5	
DMR2:30834001	2	30834001	30835000	1000	1	6.90E-12	0.45	13	1.3	Slc30a5	
DMR2:30837001	2	30837001	30840000	3000	1	1.80E-08	-0.62	34	1.13	Slc30a5	
DMR2:30847001	2	30847001	30848000	1000	1	2.60E-07	0.38	15	1.5	Slc30a5	
DMR2:31743001	2	31743001	31744000	1000	1	3.40E-07	0.35	21	2.1	Pik3r1	Signaling
DMR2:31759001	2	31759001	31765000	6000	4	1.60E-17	0.56	100	1.67	Pik3r1	Signaling
DMR2:31770001	2	31770001	31776000	6000	1	1.80E-09	-0.59	114	1.9	Pik3r1	Signaling
DMR2:31778001	2	31778001	31781000	3000	1	5.30E-07	0.36	72	2.4	Pik3r1	Signaling
DMR2:31794001	2	31794001	31795000	1000	1	5.30E-15	0.59	19	1.9	Pik3r1	Signaling
DMR2:31800001	2	31800001	31802000	2000	1	4.00E-20	0.67	36	1.8	Pik3r1	Signaling
DMR2:31803001	2	31803001	31806000	3000	2	4.70E-09	0.44	35	1.17	Pik3r1	Signaling
DMR2:31811001	2	31811001	31816000	5000	1	2.10E-17	0.79	100	2	Pik3r1	Signaling
DMR2:31835001	2	31835001	31836000	1000	1	7.80E-09	0.36	18	1.8	Pik3r1;LOC103691440;LOC103691441;LOC103691442	Signaling
DMR2:32489001	2	32489001	32493000	4000	1	5.30E-09	0.45	91	2.28	Mast4;LOC108349933	Signaling
DMR2:32506001	2	32506001	32508000	2000	1	4.20E-11	0.57	28	1.4	Mast4	Signaling
DMR2:32509001	2	32509001	32512000	3000	1	8.80E-07	-0.58	47	1.57	Mast4	Signaling
DMR2:32525001	2	32525001	32527000	2000	1	3.90E-10	0.6	10	0.5	Mast4;LOC102546726	Signaling
DMR2:32827001	2	32827001	32832000	5000	1	1.90E-07	-0.51	63	1.26	Cd180	Receptor
DMR2:32856001	2	32856001	32858000	2000	1	4.70E-08	0.46	18	0.9	NEWGENE_1310139	

DMR2:32903001	2	32903001	32908000	5000	1	2.00E-09	0.43	102	2.04	NEWGENE_1310139;LOC108350209	
DMR2:32949001	2	32949001	32950000	1000	1	1.10E-09	0.34	24	2.4	NEWGENE_1310139	
DMR2:32958001	2	32958001	32959000	1000	1	2.50E-10	0.57	15	1.5	NEWGENE_1310139	
DMR2:32970001	2	32970001	32972000	2000	1	7.00E-07	-0.51	35	1.75	NEWGENE_1310139	
DMR2:32973001	2	32973001	32977000	4000	1	1.50E-07	0.35	42	1.05	NEWGENE_1310139	
DMR2:32998001	2	32998001	32999000	1000	1	1.80E-10	-0.47	12	1.2	NEWGENE_1310139	
DMR2:33003001	2	33003001	33005000	2000	1	2.00E-13	0.5	36	1.8	NEWGENE_1310139	
DMR2:33008001	2	33008001	33010000	2000	1	8.60E-07	-0.47	35	1.75	NEWGENE_1310139	
DMR2:33017001	2	33017001	33019000	2000	1	1.10E-15	0.65	33	1.65	NEWGENE_1310139	
DMR2:33042001	2	33042001	33043000	1000	1	2.60E-10	-0.53	11	1.1	NEWGENE_1310139	
DMR2:33059001	2	33059001	33066000	7000	1	6.30E-12	0.52	84	1.2	NEWGENE_1310139	
DMR2:33087001	2	33087001	33088000	1000	1	2.50E-12	0.46	9	0.9	NEWGENE_1310139	
DMR2:33103001	2	33103001	33105000	2000	1	4.80E-07	0.3	31	1.55	NEWGENE_1310139	
DMR2:33110001	2	33110001	33112000	2000	1	3.40E-08	0.41	20	1	NEWGENE_1310139	
DMR2:33123001	2	33123001	33128000	5000	3	2.90E-18	0.63	54	1.08	NEWGENE_1310139	
DMR2:33132001	2	33132001	33134000	2000	1	2.90E-08	0.42	19	0.95	NEWGENE_1310139	
DMR2:33136001	2	33136001	33140000	4000	1	8.10E-14	0.52	56	1.4	NEWGENE_1310139	
DMR2:33142001	2	33142001	33145000	3000	1	1.00E-06	-0.45	40	1.33	NEWGENE_1310139	
DMR2:33146001	2	33146001	33149000	3000	1	1.70E-14	0.58	57	1.9	NEWGENE_1310139	
DMR2:33151001	2	33151001	33152000	1000	1	1.00E-07	0.32	18	1.8	NEWGENE_1310139	
DMR2:33159001	2	33159001	33161000	2000	1	6.10E-09	-0.55	36	1.8	NEWGENE_1310139	
DMR2:33166001	2	33166001	33169000	3000	3	9.90E-13	0.47	28	0.93	NEWGENE_1310139	
DMR2:33181001	2	33181001	33184000	3000	1	6.40E-32	1.07	42	1.4	NEWGENE_1310139	
DMR2:33197001	2	33197001	33202000	5000	1	2.60E-08	0.35	61	1.22	NEWGENE_1310139	
DMR2:33212001	2	33212001	33213000	1000	1	2.20E-17	0.68	8	0.8	NEWGENE_1310139	
DMR2:33307001	2	33307001	33308000	1000	1	6.60E-10	0.36	9	0.9	NEWGENE_1310139	
DMR2:33315001	2	33315001	33318000	3000	1	2.60E-11	0.6	28	0.93	NEWGENE_1310139	
DMR2:33322001	2	33322001	33324000	2000	1	6.00E-10	0.46	18	0.9	NEWGENE_1310139	
DMR2:33328001	2	33328001	33334000	6000	3	6.20E-11	0.46	74	1.23	NEWGENE_1310139	
DMR2:33336001	2	33336001	33338000	2000	1	2.80E-07	0.35	29	1.45	NEWGENE_1310139;LOC103695096	
DMR2:33346001	2	33346001	33349000	3000	2	1.40E-10	0.7	40	1.33	NEWGENE_1310139;LOC103695096	
DMR2:33366001	2	33366001	33371000	5000	1	1.20E-09	0.35	60	1.2	NEWGENE_1310139	
DMR2:33381001	2	33381001	33383000	2000	2	1.40E-23	0.9	20	1	NEWGENE_1310139	
DMR2:33389001	2	33389001	33391000	2000	1	6.60E-16	0.51	30	1.5	NEWGENE_1310139	
DMR2:33394001	2	33394001	33395000	1000	1	2.60E-07	-0.44	14	1.4	NEWGENE_1310139	
DMR2:33401001	2	33401001	33404000	3000	1	2.00E-07	-0.46	46	1.53	NEWGENE_1310139	
DMR2:33408001	2	33408001	33409000	1000	1	5.50E-08	0.38	18	1.8	NEWGENE_1310139	
DMR2:33415001	2	33415001	33418000	3000	1	4.60E-08	0.36	27	0.9	NEWGENE_1310139	
DMR2:33426001	2	33426001	33430000	4000	1	9.80E-07	0.31	57	1.43	NEWGENE_1310139	
DMR2:33433001	2	33433001	33436000	3000	1	2.20E-12	0.73	38	1.27	NEWGENE_1310139	
DMR2:33437001	2	33437001	33440000	3000	2	6.90E-12	0.75	28	0.93	NEWGENE_1310139	
DMR2:33446001	2	33446001	33447000	1000	1	6.90E-09	0.31	9	0.9	NEWGENE_1310139	
DMR2:33448001	2	33448001	33450000	2000	1	2.40E-07	-0.54	27	1.35	NEWGENE_1310139	
DMR2:33802001	2	33802001	33806000	4000	2	1.50E-11	-0.57	42	1.05	Srek1	Translation
DMR2:33818001	2	33818001	33819000	1000	1	6.80E-17	0.67	21	2.1	Srek1	Translation
DMR2:33826001	2	33826001	33828000	2000	1	2.50E-07	-0.56	19	0.95	Srek1	Translation
DMR2:33843001	2	33843001	33844000	1000	1	2.10E-12	0.51	11	1.1	Srek1	Translation
DMR2:33848001	2	33848001	33850000	2000	1	1.00E-07	0.4	21	1.05	Srek1	Translation
DMR2:33870001	2	33870001	33871000	1000	1	2.00E-07	-0.56	10	1	Erbin	Cytoskeleton
DMR2:33875001	2	33875001	33880000	5000	4	1.50E-26	-0.57	71	1.42	Erbin	Cytoskeleton
DMR2:33899001	2	33899001	33900000	1000	1	2.20E-09	0.42	13	1.3	Erbin	Cytoskeleton
DMR2:33905001	2	33905001	33906000	1000	1	4.60E-07	-0.52	8	0.8	Erbin	Cytoskeleton
DMR2:33911001	2	33911001	33913000	2000	1	5.20E-09	0.51	20	1	Erbin	Cytoskeleton
DMR2:33925001	2	33925001	33931000	6000	2	7.70E-14	0.66	31	0.52	Erbin	Cytoskeleton
DMR2:34093001	2	34093001	34094000	1000	1	5.60E-17	0.63	9	0.9	Nln	Protease
DMR2:34095001	2	34095001	34101000	6000	1	4.40E-10	0.42	89	1.48	Nln	Protease
DMR2:34103001	2	34103001	34105000	2000	1	6.00E-07	-0.59	25	1.25	Nln	Protease
DMR2:34108001	2	34108001	34112000	4000	2	5.50E-12	-0.49	61	1.52	Nln	Protease
DMR2:34117001	2	34117001	34123000	6000	2	9.40E-09	-0.39	50	0.83	Nln	Protease
DMR2:34124001	2	34124001	34130000	6000	4	3.50E-23	-0.55	89	1.48	Nln	Protease
DMR2:34132001	2	34132001	34133000	1000	1	1.50E-12	0.65	7	0.7	Nln	Protease
DMR2:34135001	2	34135001	34140000	5000	1	1.50E-19	0.92	59	1.18	Nln	Protease

DMR2:34144001	2	34144001	34147000	3000	1	5.50E-08	0.45	37	1.23	Nln	Protease
DMR2:34148001	2	34148001	34150000	2000	1	2.50E-09	0.53	25	1.25	Nln	Protease
DMR2:34154001	2	34154001	34158000	4000	1	6.70E-11	0.38	45	1.12	Nln	Protease
DMR2:34161001	2	34161001	34165000	4000	1	7.80E-12	0.5	46	1.15	Nln	Protease
DMR2:34183001	2	34183001	34184000	1000	1	5.80E-07	0.42	7	0.7	Nln;Sgtb	Protease
DMR2:34188001	2	34188001	34190000	2000	1	7.80E-08	0.36	37	1.85	Nln;Sgtb	Protease
DMR2:34191001	2	34191001	34193000	2000	1	9.40E-08	-0.38	18	0.9	Nln;Sgtb	Protease
DMR2:34194001	2	34194001	34197000	3000	1	6.80E-09	-0.61	59	1.97	Nln;Sgtb	Protease
DMR2:34236001	2	34236001	34242000	6000	3	7.40E-14	0.52	59	0.98	Trappc13	
DMR2:34243001	2	34243001	34244000	1000	1	4.50E-12	0.56	7	0.7	Trappc13	
DMR2:34259001	2	34259001	34260000	1000	1	3.60E-18	0.8	4	0.4	Trappc13;Trim23	Signaling
DMR2:34261001	2	34261001	34265000	4000	1	7.30E-08	0.36	30	0.75	Trappc13;Trim23	Signaling
DMR2:34275001	2	34275001	34278000	3000	1	5.10E-08	0.46	16	0.53	Trim23	Signaling
DMR2:34320001	2	34320001	34322000	2000	1	1.00E-18	0.7	13	0.65	Ppwd1;Cenpk	Transcription
DMR2:34340001	2	34340001	34342000	2000	1	1.30E-07	0.5	10	0.5	Cenpk	
DMR2:34345001	2	34345001	34346000	1000	1	2.20E-11	0.63	9	0.9	Cenpk	
DMR2:34357001	2	34357001	34359000	2000	1	1.30E-07	-0.5	19	0.95	Cenpk	
DMR2:34381001	2	34381001	34385000	4000	1	5.50E-07	0.5	31	0.78	Cenpk;Adamts6	Protease
DMR2:34408001	2	34408001	34413000	5000	1	3.30E-18	0.64	38	0.76	Adamts6;LOC108349936	Protease
DMR2:34441001	2	34441001	34442000	1000	1	6.80E-07	-0.56	8	0.8	Adamts6	Protease
DMR2:34444001	2	34444001	34450000	6000	4	2.30E-24	-0.56	81	1.35	Adamts6	Protease
DMR2:34453001	2	34453001	34455000	2000	1	1.10E-09	-0.42	16	0.8	Adamts6	Protease
DMR2:34457001	2	34457001	34460000	3000	2	2.60E-10	-0.33	44	1.47	Adamts6	Protease
DMR2:34461001	2	34461001	34463000	2000	1	3.40E-08	-0.39	17	0.85	Adamts6	Protease
DMR2:34471001	2	34471001	34474000	3000	2	1.80E-20	-0.63	28	0.93	Adamts6	Protease
DMR2:34481001	2	34481001	34484000	3000	2	9.40E-08	-0.35	37	1.23	Adamts6	Protease
DMR2:34499001	2	34499001	34501000	2000	2	8.80E-18	0.77	8	0.4	Adamts6	Protease
DMR2:34542001	2	34542001	34544000	2000	1	3.30E-08	0.36	15	0.75	Adamts6	Protease
DMR2:34548001	2	34548001	34549000	1000	1	5.00E-07	-0.58	9	0.9	Adamts6	Protease
DMR2:34553001	2	34553001	34554000	1000	1	6.80E-25	0.97	21	2.1	Adamts6	Protease
DMR2:34571001	2	34571001	34572000	1000	1	9.20E-13	0.5	13	1.3	Adamts6;LOC108349937	Protease
DMR2:34579001	2	34579001	34581000	2000	1	1.00E-07	-0.58	21	1.05	Adamts6;LOC108349937	Protease
DMR2:34596001	2	34596001	34598000	2000	1	3.40E-10	0.45	32	1.6	Adamts6	Protease
DMR2:34719001	2	34719001	34720000	1000	1	1.60E-09	0.37	17	1.7	Cwc27	Transcription
DMR2:34752001	2	34752001	34757000	5000	1	4.50E-15	-0.63	41	0.82	Cwc27	Transcription
DMR2:34759001	2	34759001	34760000	1000	1	1.70E-10	0.56	8	0.8	Cwc27	Transcription
DMR2:34783001	2	34783001	34785000	2000	1	1.30E-08	-0.4	13	0.65	Cwc27	Transcription
DMR2:34804001	2	34804001	34806000	2000	1	2.90E-08	-0.36	20	1	Cwc27	Transcription
DMR2:34825001	2	34825001	34826000	1000	1	1.00E-15	0.76	8	0.8	Cwc27	Transcription
DMR2:34884001	2	34884001	34888000	4000	2	9.40E-10	-0.6	29	0.72	Cwc27	Transcription
DMR2:34894001	2	34894001	34899000	5000	1	8.00E-09	0.51	40	0.8	Cwc27	Transcription
DMR2:34928001	2	34928001	34929000	1000	1	7.70E-11	0.66	10	1	Cwc27;Srek1ip1	Transcription
DMR2:34935001	2	34935001	34936000	1000	1	6.00E-22	0.73	9	0.9	Srek1ip1	
DMR2:34951001	2	34951001	34954000	3000	1	1.60E-09	0.54	40	1.33	Fam159b	
DMR2:34974001	2	34974001	34978000	4000	1	1.30E-13	0.54	60	1.5	Fam159b	
DMR2:35061001	2	35061001	35067000	6000	3	3.50E-21	0.81	76	1.27	Rgs7bp	
DMR2:35082001	2	35082001	35086000	4000	1	2.90E-10	0.43	68	1.7	Rgs7bp	
DMR2:35101001	2	35101001	35102000	1000	1	7.20E-07	0.76	10	1	Rgs7bp	
DMR2:35120001	2	35120001	35121000	1000	1	1.90E-07	0.48	4	0.4	Rgs7bp	
DMR2:35126001	2	35126001	35132000	6000	2	2.00E-19	0.72	74	1.23	Rgs7bp	
DMR2:35133001	2	35133001	35134000	1000	1	8.60E-21	1.02	4	0.4	Rgs7bp	
DMR2:35245001	2	35245001	35247000	2000	1	3.30E-09	-0.5	10	0.5	Rgs7bp	
DMR2:35278001	2	35278001	35279000	1000	1	7.60E-09	0.79	6	0.6	Rgs7bp;LOC100910077	Receptor
DMR2:35343001	2	35343001	35345000	2000	1	5.10E-09	-0.39	18	0.9	Rgs7bp;LOC100910217;LOC100910253	
DMR2:35346001	2	35346001	35350000	4000	3	9.70E-16	-0.46	50	1.25	Rgs7bp;LOC100910253;Olr1270-ps	
DMR2:35361001	2	35361001	35362000	1000	1	2.00E-08	0.4	18	1.8	Rgs7bp;LOC100910253;Olr1270-ps	
DMR2:35470001	2	35470001	35471000	1000	1	3.40E-09	0.77	4	0.4	Rgs7bp	
DMR2:35477001	2	35477001	35478000	1000	1	1.40E-07	0.66	3	0.3	Rgs7bp;Olr1276-ps	
DMR2:35481001	2	35481001	35482000	1000	1	3.80E-09	0.82	4	0.4	Rgs7bp;Olr1276-ps	
DMR2:35483001	2	35483001	35485000	2000	1	2.30E-07	-0.35	21	1.05	Rgs7bp;Olr1276-ps	
DMR2:35524001	2	35524001	35525000	1000	1	1.10E-08	-0.47	7	0.7	Rgs7bp;LOC103691445	
DMR2:35534001	2	35534001	35537000	3000	2	1.90E-19	-0.55	47	1.57	Rgs7bp;LOC103691445	

DMR2:35623001	2	35623001	35624000	1000	1	7.10E-08	-0.58	12	1.2	Rgs7bp	
DMR2:35706001	2	35706001	35711000	5000	1	3.50E-14	0.65	62	1.24	LOC502504;Rnf180	Proteolysis
DMR2:35741001	2	35741001	35749000	8000	2	4.90E-09	0.45	117	1.46	Rnf180	Proteolysis
DMR2:35750001	2	35750001	35752000	2000	1	6.30E-09	0.53	14	0.7	Rnf180	Proteolysis
DMR2:35769001	2	35769001	35770000	1000	1	4.30E-07	0.58	6	0.6	Rnf180	Proteolysis
DMR2:35772001	2	35772001	35775000	3000	1	2.20E-09	-0.49	7	0.23	Rnf180	Proteolysis
DMR2:35776001	2	35776001	35778000	2000	1	7.70E-13	0.88	6	0.3	Rnf180	Proteolysis
DMR2:35809001	2	35809001	35815000	6000	1	1.10E-14	0.65	68	1.13	Rnf180	Proteolysis
DMR2:35836001	2	35836001	35838000	2000	2	1.50E-09	-0.72	16	0.8	Rnf180	Proteolysis
DMR2:35842001	2	35842001	35844000	2000	1	5.70E-12	0.66	25	1.25	Rnf180	Proteolysis
DMR2:36240001	2	36240001	36244000	4000	2	1.40E-13	-0.61	50	1.25	Htr1a	Signaling
DMR2:36245001	2	36245001	36247000	2000	1	4.70E-09	0.67	51	2.55	Htr1a	Signaling
DMR2:36798001	2	36798001	36800000	2000	2	1.70E-12	-0.8	11	0.55	RGD1560462	
DMR2:37943001	2	37943001	37948000	5000	1	3.00E-10	0.48	60	1.2	Ipo11	Transport
DMR2:37961001	2	37961001	37962000	1000	1	4.80E-07	0.51	4	0.4	Ipo11	Transport
DMR2:38020001	2	38020001	38023000	3000	3	2.60E-16	-0.67	13	0.43	Ipo11	Transport
DMR2:38087001	2	38087001	38091000	4000	1	7.20E-13	0.51	34	0.85	Ipo11	Transport
DMR2:38093001	2	38093001	38095000	2000	1	4.70E-10	-0.42	23	1.15	Ipo11	Transport
DMR2:38102001	2	38102001	38105000	3000	1	1.80E-07	-0.48	38	1.27	Ipo11	Transport
DMR2:38115001	2	38115001	38116000	1000	1	4.30E-07	0.47	7	0.7	Ipo11;Dimt1	Transport;Epigenetic
DMR2:38129001	2	38129001	38131000	2000	1	1.20E-07	0.31	20	1	Ipo11;Dimt1	Transport;Epigenetic
DMR2:38138001	2	38138001	38139000	1000	1	5.90E-12	-0.59	6	0.6	Dimt1;Kif2a	Epigenetic;Cytoskeleton
DMR2:38212001	2	38212001	38218000	6000	4	1.10E-20	-0.58	75	1.25	Kif2a	Cytoskeleton
DMR2:38219001	2	38219001	38221000	2000	1	7.10E-10	0.33	31	1.55	RGD1564606	Translation
DMR2:38222001	2	38222001	38224000	2000	2	2.10E-09	-0.51	19	0.95	RGD1564606	Translation
DMR2:38225001	2	38225001	38228000	3000	2	1.80E-22	0.71	53	1.77	RGD1564606	Translation
DMR2:38969001	2	38969001	38971000	2000	1	3.20E-16	0.72	18	0.9	Zswim6	
DMR2:38993001	2	38993001	38996000	3000	1	3.40E-10	0.61	31	1.03	Zswim6	
DMR2:39000001	2	39000001	39003000	3000	1	9.70E-07	-0.53	41	1.37	Zswim6	
DMR2:39023001	2	39023001	39027000	4000	2	3.00E-13	-0.49	49	1.23	Zswim6	
DMR2:39045001	2	39045001	39049000	4000	1	3.90E-14	0.56	62	1.55	Zswim6	
DMR2:39331001	2	39331001	39332000	1000	1	4.70E-08	0.43	11	1.1	Ndufaf2	Transcription
DMR2:39363001	2	39363001	39365000	2000	1	2.40E-18	0.84	15	0.75	Ndufaf2	Transcription
DMR2:39366001	2	39366001	39369000	3000	1	6.00E-11	0.61	33	1.1	Ndufaf2	Transcription
DMR2:39376001	2	39376001	39382000	6000	2	3.30E-12	-0.56	42	0.7	Ndufaf2	Transcription
DMR2:39391001	2	39391001	39392000	1000	1	6.00E-14	0.66	13	1.3	Ndufaf2	Transcription
DMR2:39401001	2	39401001	39403000	2000	1	8.30E-18	0.75	15	0.75	Ndufaf2	Transcription
DMR2:39411001	2	39411001	39413000	2000	2	9.40E-19	0.72	22	1.1	Ndufaf2	Transcription
DMR2:39425001	2	39425001	39429000	4000	1	2.40E-17	0.65	55	1.38	Ndufaf2;Ercc8	Transcription;Transcription
DMR2:39435001	2	39435001	39437000	2000	1	1.20E-08	0.44	17	0.85	Ndufaf2;Ercc8	Transcription;Transcription
DMR2:39448001	2	39448001	39451000	3000	1	2.70E-07	0.44	28	0.93	Ercc8	Transcription
DMR2:39475001	2	39475001	39483000	8000	5	1.10E-16	0.52	86	1.07	Ercc8	Transcription
DMR2:39518001	2	39518001	39520000	2000	1	3.80E-07	0.37	2	0.1	RGD1560076	
DMR2:39970001	2	39970001	39972000	2000	1	6.30E-09	0.59	15	0.75	Depdc1b	Cytoskeleton
DMR2:39992001	2	39992001	39995000	3000	1	2.30E-09	0.57	14	0.47	Depdc1b	Cytoskeleton
DMR2:39997001	2	39997001	39998000	1000	1	1.90E-08	0.42	9	0.9	Depdc1b	Cytoskeleton
DMR2:39999001	2	39999001	4.00E+07	1000	1	4.30E-09	0.67	15	1.5	Depdc1b	Cytoskeleton
DMR2:40006001	2	40006001	40008000	2000	2	1.40E-08	-0.7	31	1.55	Depdc1b	Cytoskeleton
DMR2:40011001	2	40011001	40020000	9000	5	3.30E-13	0.72	88	0.98	Depdc1b	Cytoskeleton
DMR2:40047001	2	40047001	40051000	4000	1	6.20E-07	-0.52	35	0.88	Depdc1b	Cytoskeleton
DMR2:40072001	2	40072001	40074000	2000	1	2.10E-21	0.79	21	1.05	Depdc1b	Cytoskeleton
DMR2:40077001	2	40077001	40078000	1000	1	1.30E-07	0.44	12	1.2	Depdc1b	Cytoskeleton
DMR2:40208001	2	40208001	40210000	2000	1	2.60E-18	0.71	13	0.65	Pde4d	Signaling
DMR2:40226001	2	40226001	40228000	2000	1	1.20E-07	-0.55	21	1.05	Pde4d	Signaling
DMR2:40231001	2	40231001	40234000	3000	1	8.90E-08	-0.34	24	0.8	Pde4d	Signaling
DMR2:40236001	2	40236001	40237000	1000	1	4.40E-09	0.52	8	0.8	Pde4d	Signaling
DMR2:40253001	2	40253001	40254000	1000	1	5.10E-08	0.61	9	0.9	Pde4d	Signaling
DMR2:40274001	2	40274001	40275000	1000	1	4.10E-08	0.47	6	0.6	Pde4d	Signaling
DMR2:40278001	2	40278001	40279000	1000	1	3.30E-07	0.49	8	0.8	Pde4d	Signaling
DMR2:40284001	2	40284001	40288000	4000	2	1.70E-10	0.91	18	0.45	Pde4d	Signaling
DMR2:40299001	2	40299001	40300000	1000	1	2.20E-15	-0.76	2	0.2	Pde4d	Signaling
DMR2:40353001	2	40353001	40355000	2000	1	3.10E-22	0.81	13	0.65	Pde4d	Signaling

DMR2:40365001	2	40365001	40371000	6000	2	6.30E-17	0.79	39	0.65	Pde4d;Elovl7;LOC108350211	Signaling;Metabolism
DMR2:40374001	2	40374001	40375000	1000	1	1.50E-13	0.52	11	1.1	Pde4d;Elovl7;LOC108350211	Signaling;Metabolism
DMR2:40380001	2	40380001	40382000	2000	1	2.20E-07	-0.56	25	1.25	Pde4d;Elovl7;LOC108350211	Signaling;Metabolism
DMR2:40394001	2	40394001	40395000	1000	1	1.20E-11	0.75	3	0.3	Pde4d;Elovl7	Signaling;Metabolism
DMR2:40397001	2	40397001	40398000	1000	1	3.80E-07	0.54	4	0.4	Pde4d	Signaling
DMR2:40431001	2	40431001	40433000	2000	1	4.70E-09	0.55	11	0.55	Pde4d	Signaling
DMR2:40441001	2	40441001	40445000	4000	3	6.30E-08	0.7	20	0.5	Pde4d	Signaling
DMR2:40520001	2	40520001	40526000	6000	5	7.50E-16	-0.55	57	0.95	Pde4d	Signaling
DMR2:40546001	2	40546001	40547000	1000	1	1.60E-08	0.65	0	0	Pde4d	Signaling
DMR2:40571001	2	40571001	40572000	1000	1	1.90E-08	0.69	7	0.7	Pde4d	Signaling
DMR2:40592001	2	40592001	40593000	1000	1	4.10E-14	-0.61	10	1	Pde4d	Signaling
DMR2:40656001	2	40656001	40658000	2000	1	2.90E-08	-0.46	27	1.35	Pde4d	Signaling
DMR2:40661001	2	40661001	40663000	2000	1	1.60E-07	0.5	12	0.6	Pde4d	Signaling
DMR2:40668001	2	40668001	40669000	1000	1	6.20E-09	-0.52	12	1.2	Pde4d	Signaling
DMR2:40681001	2	40681001	40684000	3000	1	1.60E-09	-0.53	22	0.73	Pde4d	Signaling
DMR2:40703001	2	40703001	40704000	1000	1	1.00E-08	-0.56	7	0.7	Pde4d	Signaling
DMR2:40709001	2	40709001	40712000	3000	2	4.20E-09	0.4	26	0.87	Pde4d	Signaling
DMR2:40728001	2	40728001	40729000	1000	1	1.40E-08	-0.53	14	1.4	Pde4d	Signaling
DMR2:40753001	2	40753001	40757000	4000	1	2.00E-20	0.66	54	1.35	Pde4d	Signaling
DMR2:40758001	2	40758001	40761000	3000	2	3.40E-16	0.54	36	1.2	Pde4d	Signaling
DMR2:40767001	2	40767001	40769000	2000	2	3.40E-15	0.72	12	0.6	Pde4d	Signaling
DMR2:40774001	2	40774001	40777000	3000	2	4.10E-26	0.82	39	1.3	Pde4d	Signaling
DMR2:40781001	2	40781001	40783000	2000	1	8.60E-07	-0.55	24	1.2	Pde4d	Signaling
DMR2:40811001	2	40811001	40814000	3000	1	1.80E-07	0.45	29	0.97	Pde4d	Signaling
DMR2:40829001	2	40829001	40830000	1000	1	8.30E-09	0.38	12	1.2	Pde4d	Signaling
DMR2:40835001	2	40835001	40837000	2000	1	2.30E-09	0.62	13	0.65	Pde4d	Signaling
DMR2:40849001	2	40849001	40850000	1000	1	4.50E-12	0.89	10	1	Pde4d	Signaling
DMR2:40858001	2	40858001	40859000	1000	1	2.20E-10	0.54	7	0.7	Pde4d;Mir582	Signaling
DMR2:40860001	2	40860001	40861000	1000	1	3.10E-10	-0.77	13	1.3	Pde4d;Mir582	Signaling
DMR2:40871001	2	40871001	40872000	1000	1	1.20E-10	0.53	8	0.8	Pde4d;Mir582	Signaling
DMR2:40909001	2	40909001	40910000	1000	1	3.10E-09	0.59	7	0.7	Pde4d;LOC108350210	Signaling
DMR2:40913001	2	40913001	40914000	1000	1	7.00E-14	0.54	11	1.1	Pde4d;LOC108350210	Signaling
DMR2:40940001	2	40940001	40942000	2000	2	2.20E-13	0.62	19	0.95	Pde4d;LOC108350210	Signaling
DMR2:40943001	2	40943001	40946000	3000	3	6.20E-12	0.56	27	0.9	Pde4d;LOC108350210	Signaling
DMR2:40954001	2	40954001	40956000	2000	1	4.80E-08	0.35	27	1.35	Pde4d;LOC108350210	Signaling
DMR2:40964001	2	40964001	40966000	2000	1	1.00E-07	0.57	5	0.25	Pde4d;LOC108350210	Signaling
DMR2:40997001	2	40997001	40999000	2000	1	4.40E-09	-0.55	26	1.3	Pde4d	Signaling
DMR2:41033001	2	41033001	41035000	2000	1	8.00E-10	0.46	12	0.6	Pde4d	Signaling
DMR2:41036001	2	41036001	41039000	3000	1	3.60E-13	0.54	25	0.83	Pde4d	Signaling
DMR2:41062001	2	41062001	41064000	2000	1	1.40E-10	0.53	16	0.8	Pde4d	Signaling
DMR2:41080001	2	41080001	41082000	2000	1	3.60E-08	-0.57	18	0.9	Pde4d	Signaling
DMR2:41086001	2	41086001	41088000	2000	1	7.90E-10	0.49	15	0.75	Pde4d	Signaling
DMR2:41095001	2	41095001	41097000	2000	2	1.60E-13	-0.6	17	0.85	Pde4d	Signaling
DMR2:41107001	2	41107001	41108000	1000	1	5.30E-07	-0.53	11	1.1	Pde4d	Signaling
DMR2:41109001	2	41109001	41110000	1000	1	1.80E-10	0.65	8	0.8	Pde4d	Signaling
DMR2:41115001	2	41115001	41116000	1000	1	2.40E-11	0.68	3	0.3	Pde4d	Signaling
DMR2:41131001	2	41131001	41135000	4000	2	4.20E-10	0.65	36	0.9	Pde4d	Signaling
DMR2:41150001	2	41150001	41155000	5000	3	6.60E-11	0.46	62	1.24	Pde4d	Signaling
DMR2:41159001	2	41159001	41162000	3000	1	1.70E-07	-0.57	29	0.97	Pde4d	Signaling
DMR2:41175001	2	41175001	41177000	2000	1	4.80E-16	0.82	12	0.6	Pde4d	Signaling
DMR2:41202001	2	41202001	41203000	1000	1	5.70E-07	-0.47	10	1	Pde4d	Signaling
DMR2:41206001	2	41206001	41208000	2000	1	2.00E-07	-0.49	21	1.05	Pde4d	Signaling
DMR2:41217001	2	41217001	41218000	1000	1	6.30E-07	0.37	11	1.1	Pde4d	Signaling
DMR2:41233001	2	41233001	41235000	2000	1	3.10E-07	0.28	28	1.4	Pde4d	Signaling
DMR2:41256001	2	41256001	41257000	1000	1	1.50E-08	0.41	13	1.3	Pde4d	Signaling
DMR2:41263001	2	41263001	41264000	1000	1	3.20E-10	0.49	7	0.7	Pde4d	Signaling
DMR2:41285001	2	41285001	41287000	2000	1	3.30E-08	-0.42	26	1.3	Pde4d	Signaling
DMR2:41309001	2	41309001	41313000	4000	1	6.10E-20	0.66	38	0.95	Pde4d	Signaling
DMR2:41314001	2	41314001	41316000	2000	1	4.40E-08	0.34	17	0.85	Pde4d	Signaling
DMR2:41371001	2	41371001	41372000	1000	1	1.60E-10	0.48	8	0.8	Pde4d	Signaling
DMR2:41378001	2	41378001	41379000	1000	1	1.90E-16	0.72	9	0.9	Pde4d	Signaling

DMR2:41407001	2	41407001	41408000	1000	1	4.10E-08	0.36	6	0.6	Pde4d	Signaling
DMR2:41429001	2	41429001	41434000	5000	1	4.60E-08	-0.46	80	1.6	Pde4d	Signaling
DMR2:41438001	2	41438001	41441000	3000	1	6.70E-19	0.6	55	1.83	Pde4d	Signaling
DMR2:41454001	2	41454001	41455000	1000	1	3.50E-15	0.61	4	0.4	Pde4d	Signaling
DMR2:41476001	2	41476001	41477000	1000	1	1.20E-07	0.44	8	0.8	Pde4d	Signaling
DMR2:41590001	2	41590001	41592000	2000	1	5.00E-12	0.65	19	0.95	Rab3c	
DMR2:41637001	2	41637001	41639000	2000	1	1.70E-09	0.43	22	1.1	Rab3c	
DMR2:41677001	2	41677001	41678000	1000	1	8.70E-10	0.5	6	0.6	Rab3c	
DMR2:41699001	2	41699001	41701000	2000	1	9.00E-13	0.81	8	0.4	Rab3c	
DMR2:41730001	2	41730001	41731000	1000	1	2.50E-07	0.44	10	1	Rab3c	
DMR2:41734001	2	41734001	41737000	3000	1	3.70E-14	0.55	39	1.3	Rab3c	
DMR2:41751001	2	41751001	41753000	2000	1	4.10E-24	0.82	20	1	Rab3c	
DMR2:41761001	2	41761001	41763000	2000	1	3.80E-07	-0.64	7	0.35	Rab3c	
DMR2:41779001	2	41779001	41780000	1000	1	9.00E-16	0.67	5	0.5	Rab3c	
DMR2:41869001	2	41869001	41870000	1000	1	1.00E-10	0.53	7	0.7	Gapt	
DMR2:41903001	2	41903001	41908000	5000	1	5.80E-09	-0.69	26	0.52	Plk2	Signaling
DMR2:42718001	2	42718001	42721000	3000	2	6.80E-07	0.55	18	0.6	RGD1560402	Metabolism
DMR2:42731001	2	42731001	42732000	1000	1	5.30E-08	0.52	4	0.4	RGD1560402	Metabolism
DMR2:42833001	2	42833001	42834000	1000	1	8.40E-07	0.47	6	0.6	LOC103691452;Actbl2	Cytoskeleton
DMR2:43074001	2	43074001	43076000	2000	1	6.20E-19	0.65	26	1.3	Gpbp1	
DMR2:43257001	2	43257001	43260000	3000	1	1.90E-09	0.37	25	0.83	Mier3	Development
DMR2:43288001	2	43288001	43293000	5000	1	5.60E-07	-0.56	68	1.36	Mier3;LOC103691453;LOC100362101	Development
DMR2:43297001	2	43297001	43300000	3000	2	4.50E-08	-0.61	36	1.2	Mier3;LOC100362101	Development
DMR2:43305001	2	43305001	43308000	3000	1	2.50E-23	0.81	34	1.13	Mier3;LOC100362101	Development
DMR2:43323001	2	43323001	43325000	2000	1	4.60E-13	0.67	18	0.9	Map3k1	
DMR2:43344001	2	43344001	43345000	1000	1	1.90E-07	0.45	12	1.2	Map3k1	
DMR2:43352001	2	43352001	43353000	1000	1	2.00E-09	-0.55	17	1.7	Map3k1	
DMR2:43812001	2	43812001	43813000	1000	1	5.00E-09	-0.45	11	1.1	Ankrd55	Cytoskeleton
DMR2:43820001	2	43820001	43821000	1000	1	4.10E-10	0.37	18	1.8	Ankrd55	Cytoskeleton
DMR2:43828001	2	43828001	43829000	1000	1	6.10E-10	0.45	11	1.1	Ankrd55	Cytoskeleton
DMR2:43840001	2	43840001	43843000	3000	1	1.10E-11	0.41	64	2.13	Ankrd55	Cytoskeleton
DMR2:43845001	2	43845001	43846000	1000	1	1.50E-07	-0.38	16	1.6	Ankrd55	Cytoskeleton
DMR2:43849001	2	43849001	43852000	3000	3	9.30E-18	0.88	22	0.73	Ankrd55	Cytoskeleton
DMR2:43869001	2	43869001	43871000	2000	1	3.20E-09	0.57	21	1.05	Ankrd55	Cytoskeleton
DMR2:43921001	2	43921001	43924000	3000	1	2.10E-07	0.48	20	0.67	Ankrd55	Cytoskeleton
DMR2:43927001	2	43927001	43928000	1000	1	1.20E-11	0.62	15	1.5	Ankrd55	Cytoskeleton
DMR2:43951001	2	43951001	43952000	1000	1	5.40E-13	0.54	12	1.2	Ankrd55;LOC100912399	Cytoskeleton;Signaling
DMR2:44014001	2	44014001	44016000	2000	1	5.30E-12	0.61	16	0.8	Ankrd55;LOC108349944	Cytoskeleton
DMR2:44020001	2	44020001	44021000	1000	1	6.50E-09	0.7	2	0.2	Ankrd55;LOC108349944	Cytoskeleton
DMR2:44104001	2	44104001	44105000	1000	1	2.00E-07	-0.45	10	1	Ankrd55;LOC103691456	Cytoskeleton
DMR2:44110001	2	44110001	44112000	2000	2	2.40E-27	0.87	12	0.6	Ankrd55;LOC103691456	Cytoskeleton
DMR2:44122001	2	44122001	44126000	4000	2	4.00E-24	0.77	49	1.23	Ankrd55;LOC103691456	Cytoskeleton
DMR2:44127001	2	44127001	44130000	3000	2	1.10E-07	-0.68	32	1.07	Ankrd55;LOC103691456	Cytoskeleton
DMR2:44141001	2	44141001	44143000	2000	1	1.50E-07	-0.53	24	1.2	Ankrd55;LOC103691456	Cytoskeleton
DMR2:44154001	2	44154001	44155000	1000	1	4.30E-08	0.36	9	0.9	Ankrd55;LOC103691456	Cytoskeleton
DMR2:44170001	2	44170001	44173000	3000	1	4.90E-11	0.37	60	2	Ankrd55;LOC103691456	Cytoskeleton
DMR2:44186001	2	44186001	44189000	3000	1	3.00E-08	0.41	33	1.1	Ankrd55	Cytoskeleton
DMR2:44196001	2	44196001	44201000	5000	1	1.70E-21	0.88	65	1.3	Ankrd55	Cytoskeleton
DMR2:44268001	2	44268001	44270000	2000	1	7.20E-10	0.46	21	1.05	Il6st	Receptor
DMR2:44296001	2	44296001	44299000	3000	1	4.30E-09	-0.65	38	1.27	Il6st	Receptor
DMR2:44324001	2	44324001	44328000	4000	1	3.20E-08	0.4	49	1.23	Il6st;Il31ra	Receptor
DMR2:44329001	2	44329001	44332000	3000	1	3.10E-07	0.42	39	1.3	Il6st;Il31ra	Receptor
DMR2:44353001	2	44353001	44355000	2000	2	7.40E-11	0.59	21	1.05	Il31ra;LOC103691462	Receptor
DMR2:44356001	2	44356001	44358000	2000	2	1.70E-18	0.7	27	1.35	Il31ra	Receptor
DMR2:44362001	2	44362001	44364000	2000	1	1.90E-15	0.76	18	0.9	Il31ra	Receptor
DMR2:44366001	2	44366001	44369000	3000	2	1.80E-09	-0.66	7	0.23	Il31ra	Receptor
DMR2:44370001	2	44370001	44373000	3000	2	9.60E-08	-0.53	38	1.27	Il31ra	Receptor
DMR2:44384001	2	44384001	44388000	4000	2	1.30E-13	-0.56	21	0.52	Il31ra	Receptor
DMR2:44409001	2	44409001	44410000	1000	1	3.90E-07	0.55	3	0.3	Il31ra	Receptor
DMR2:44411001	2	44411001	44420000	9000	5	1.00E-26	0.94	109	1.21	Il31ra	Receptor
DMR2:44446001	2	44446001	44448000	2000	1	2.60E-15	0.72	20	1	Ddx4	
DMR2:44462001	2	44462001	44464000	2000	2	1.20E-10	0.6	15	0.75	Ddx4	
DMR2:44493001	2	44493001	44496000	3000	1	3.60E-10	-0.47	34	1.13	Ddx4	

DMR2:44499001	2	44499001	44500000	1000	1	1.10E-09	0.52	8	0.8	Ddx4	
DMR2:44539001	2	44539001	44543000	4000	1	2.30E-08	0.37	51	1.27	Slc38a9	Transport
DMR2:44550001	2	44550001	44555000	5000	1	4.60E-10	0.54	57	1.14	Slc38a9;LOC102551224	Transport
DMR2:44557001	2	44557001	44559000	2000	1	6.40E-22	0.97	22	1.1	Slc38a9;LOC102551224	Transport
DMR2:44567001	2	44567001	44568000	1000	1	7.10E-07	-0.41	7	0.7	Slc38a9;LOC102551224	Transport
DMR2:44586001	2	44586001	44592000	6000	3	6.90E-20	0.62	85	1.42	Slc38a9	Transport
DMR2:44695001	2	44695001	44697000	2000	1	3.30E-14	0.67	20	1	Plpp1	Signaling
DMR2:44712001	2	44712001	44716000	4000	1	2.90E-10	0.5	39	0.98	Plpp1	Signaling
DMR2:44732001	2	44732001	44733000	1000	1	4.10E-07	-0.51	20	2	Plpp1;Skiv2l2	Signaling
DMR2:44765001	2	44765001	44767000	2000	1	5.50E-08	-0.63	20	1	Skiv2l2	
DMR2:44773001	2	44773001	44777000	4000	1	1.20E-15	-0.78	42	1.05	Skiv2l2	
DMR2:44778001	2	44778001	44779000	1000	1	8.50E-16	0.88	4	0.4	Skiv2l2;Dhx29	Transcription
DMR2:44791001	2	44791001	44794000	3000	1	1.30E-08	-0.5	33	1.1	Skiv2l2;Dhx29	Transcription
DMR2:44814001	2	44814001	44816000	2000	1	8.30E-07	-0.5	32	1.6	Dhx29	Transcription
DMR2:44818001	2	44818001	44820000	2000	1	1.50E-21	0.78	16	0.8	Dhx29	Transcription
DMR2:44821001	2	44821001	44833000	12000	4	1.30E-18	-0.49	150	1.25	Dhx29	Transcription
DMR2:44848001	2	44848001	44849000	1000	1	5.40E-33	1.22	1	0.1	Ccno	Signaling
DMR2:44850001	2	44850001	44853000	3000	1	3.30E-18	0.91	30	1	Ccno	Signaling
DMR2:44858001	2	44858001	44863000	5000	1	1.30E-14	0.74	114	2.28	Ccno;Mcidas	Signaling
DMR2:44866001	2	44866001	44868000	2000	1	6.30E-18	0.9	24	1.2	Ccno;Mcidas	Signaling
DMR2:44876001	2	44876001	44878000	2000	1	2.10E-08	-0.55	23	1.15	Mcidas	
DMR2:44879001	2	44879001	44882000	3000	1	5.60E-08	0.42	47	1.57	Mcidas	
DMR2:44892001	2	44892001	44893000	1000	1	1.60E-10	0.75	30	3	Mir449c;Mir449a	
DMR2:44908001	2	44908001	44909000	1000	1	3.00E-14	0.55	12	1.2	Gpx8;LOC108350166;Cdc20b	Metabolism
DMR2:44919001	2	44919001	44923000	4000	1	1.70E-14	0.48	36	0.9	LOC108350166;Cdc20b	
DMR2:44934001	2	44934001	44936000	2000	2	3.60E-12	0.57	17	0.85	Cdc20b	
DMR2:44938001	2	44938001	44940000	2000	1	5.70E-11	0.44	29	1.45	Cdc20b	
DMR2:44944001	2	44944001	44946000	2000	1	5.90E-08	0.46	25	1.25	Cdc20b	
DMR2:44960001	2	44960001	44962000	2000	1	2.80E-07	0.46	11	0.55	Gzma	Protease
DMR2:44971001	2	44971001	44972000	1000	1	4.00E-17	0.7	10	1	Gzma	Protease
DMR2:44973001	2	44973001	44976000	3000	1	2.40E-10	0.55	21	0.7	Gzma	Protease
DMR2:44985001	2	44985001	44987000	2000	1	4.80E-20	0.73	28	1.4	Gzma	Protease
DMR2:45074001	2	45074001	45078000	4000	1	7.40E-09	0.45	54	1.35	Gzmk	Protease
DMR2:45081001	2	45081001	45084000	3000	2	9.60E-11	0.77	25	0.83	Gzmk	Protease
DMR2:45117001	2	45117001	45119000	2000	1	5.20E-08	-0.53	42	2.1	Esm1	
DMR2:45177001	2	45177001	45181000	4000	1	1.30E-07	0.39	36	0.9	RGD1561161	
DMR2:45199001	2	45199001	45203000	4000	1	9.60E-07	-0.6	41	1.02	RGD1561161	
DMR2:45215001	2	45215001	45221000	6000	2	1.60E-11	-0.47	73	1.22	RGD1561161	
DMR2:45233001	2	45233001	45243000	10000	5	2.80E-12	-0.79	117	1.17	RGD1561161	
DMR2:45448001	2	45448001	45449000	1000	1	2.00E-08	0.33	9	0.9	Snx18	Cytoskeleton
DMR2:45467001	2	45467001	45469000	2000	1	7.30E-22	0.95	30	1.5	Snx18	Cytoskeleton
DMR2:45486001	2	45486001	45488000	2000	1	1.10E-07	-0.47	28	1.4	Snx18;LOC108349945	Cytoskeleton
DMR2:45489001	2	45489001	45493000	4000	1	2.50E-13	0.6	64	1.6	Snx18;LOC108349945	Cytoskeleton
DMR2:45519001	2	45519001	45522000	3000	2	3.10E-08	0.4	50	1.67	Hspb3	
DMR2:45663001	2	45663001	45667000	4000	2	1.30E-21	-0.6	45	1.12	Arl15	
DMR2:45670001	2	45670001	45672000	2000	1	2.20E-18	0.63	44	2.2	Arl15	
DMR2:45684001	2	45684001	45685000	1000	1	9.50E-07	-0.55	14	1.4	Arl15	
DMR2:45702001	2	45702001	45703000	1000	1	1.00E-07	-0.5	13	1.3	Arl15	
DMR2:45705001	2	45705001	45706000	1000	1	5.30E-09	0.33	12	1.2	Arl15	
DMR2:45715001	2	45715001	45717000	2000	1	9.50E-08	-0.48	37	1.85	Arl15	
DMR2:45723001	2	45723001	45726000	3000	1	3.20E-08	0.42	30	1	Arl15	
DMR2:45744001	2	45744001	45745000	1000	1	3.00E-16	0.51	17	1.7	Arl15	
DMR2:45750001	2	45750001	45753000	3000	2	1.70E-08	0.51	58	1.93	Arl15	
DMR2:45754001	2	45754001	45756000	2000	1	2.40E-17	0.51	43	2.15	Arl15	
DMR2:45757001	2	45757001	45765000	8000	1	2.10E-07	-0.49	117	1.46	Arl15;LOC100362624	
DMR2:45768001	2	45768001	45780000	12000	3	1.20E-09	-0.61	184	1.53	Arl15;LOC100362624	
DMR2:45785001	2	45785001	45786000	1000	1	2.80E-12	0.59	12	1.2	Arl15	
DMR2:45791001	2	45791001	45793000	2000	1	2.30E-21	0.61	27	1.35	Arl15	
DMR2:45821001	2	45821001	45822000	1000	1	3.10E-08	-0.5	13	1.3	Arl15	
DMR2:45828001	2	45828001	45830000	2000	1	5.40E-10	-0.49	45	2.25	Arl15	
DMR2:45838001	2	45838001	45840000	2000	1	8.40E-07	-0.38	19	0.95	Arl15	
DMR2:45841001	2	45841001	45844000	3000	2	8.00E-11	0.45	26	0.87	Arl15	
DMR2:45904001	2	45904001	45905000	1000	1	1.00E-09	-0.53	24	2.4	Arl15	
DMR2:45915001	2	45915001	45918000	3000	1	5.20E-14	0.52	36	1.2	Arl15	
DMR2:45919001	2	45919001	45923000	4000	3	1.50E-20	0.9	37	0.92	Arl15	

DMR2:45941001	2	45941001	45944000	3000	1	5.40E-09	0.53	49	1.63	Arl15	
DMR2:45947001	2	45947001	45949000	2000	1	1.20E-08	0.37	26	1.3	Arl15;LOC103691458	
DMR2:45958001	2	45958001	45959000	1000	1	3.40E-11	0.53	19	1.9	Arl15;LOC103691458	
DMR2:45960001	2	45960001	45961000	1000	1	2.80E-07	0.31	28	2.8	Arl15;LOC103691458	
DMR2:45967001	2	45967001	45973000	6000	1	3.50E-08	0.34	78	1.3	Arl15;LOC103691458	
DMR2:45978001	2	45978001	45980000	2000	2	2.00E-12	0.66	25	1.25	Arl15;LOC103691458	
DMR2:45986001	2	45986001	45991000	5000	3	9.00E-08	-0.43	59	1.18	Arl15	
DMR2:46002001	2	46002001	46004000	2000	1	1.30E-17	0.63	26	1.3	Arl15	
DMR2:46008001	2	46008001	46013000	5000	2	1.00E-15	-0.66	79	1.58	Arl15	
DMR2:46021001	2	46021001	46026000	5000	1	2.70E-08	0.36	96	1.92	Arl15	
DMR2:46027001	2	46027001	46031000	4000	1	8.30E-13	0.48	52	1.3	Arl15	
DMR2:46038001	2	46038001	46041000	3000	1	2.20E-07	0.31	48	1.6	Arl15	
DMR2:46043001	2	46043001	46048000	5000	1	4.00E-09	0.41	108	2.16	Arl15	
DMR2:46059001	2	46059001	46064000	5000	1	1.20E-12	0.39	84	1.68	Arl15;LOC108350225	Receptor
DMR2:46404001	2	46404001	46408000	4000	3	2.20E-12	-0.71	49	1.23	Ndufs4	Metabolism
DMR2:46420001	2	46420001	46423000	3000	1	1.80E-30	0.95	15	0.5	Ndufs4	Metabolism
DMR2:46443001	2	46443001	46446000	3000	2	1.60E-20	0.69	23	0.77	Ndufs4	Metabolism
DMR2:46448001	2	46448001	46452000	4000	1	1.20E-08	0.42	31	0.78	Ndufs4	Metabolism
DMR2:46469001	2	46469001	46471000	2000	1	3.80E-18	0.51	68	3.4	Ndufs4	Metabolism
DMR2:46478001	2	46478001	46482000	4000	2	1.70E-14	0.62	26	0.65	Ndufs4	Metabolism
DMR2:46483001	2	46483001	46487000	4000	3	1.60E-12	0.52	48	1.2	Ndufs4	Metabolism
DMR2:46533001	2	46533001	46535000	2000	1	1.10E-15	0.64	22	1.1	Fst	Protease; Proteolysis
DMR2:46538001	2	46538001	46541000	3000	1	7.60E-10	0.42	46	1.53	Fst	Protease; Proteolysis
DMR2:46545001	2	46545001	46547000	2000	1	1.50E-07	0.56	97	4.85	Fst	Protease; Proteolysis
DMR2:47039001	2	47039001	47044000	5000	1	6.00E-09	-0.77	56	1.12	Itga2	Extracellular Matrix
DMR2:47045001	2	47045001	47046000	1000	1	2.20E-07	0.41	14	1.4	Itga2	Extracellular Matrix
DMR2:47090001	2	47090001	47091000	1000	1	4.70E-15	0.54	13	1.3	Itga2	Extracellular Matrix
DMR2:47093001	2	47093001	47095000	2000	1	3.20E-07	0.4	23	1.15	Itga2	Extracellular Matrix
DMR2:47098001	2	47098001	47101000	3000	1	2.20E-07	-0.39	27	0.9	Itga2	Extracellular Matrix
DMR2:47118001	2	47118001	47119000	1000	1	3.30E-12	0.71	4	0.4	Itga1	Extracellular Matrix
DMR2:47129001	2	47129001	47130000	1000	1	2.20E-14	0.57	11	1.1	Itga1	Extracellular Matrix
DMR2:47144001	2	47144001	47145000	1000	1	3.70E-08	0.44	11	1.1	Itga1	Extracellular Matrix
DMR2:47166001	2	47166001	47168000	2000	1	2.50E-08	-0.53	28	1.4	Itga1	Extracellular Matrix
DMR2:47169001	2	47169001	47171000	2000	1	1.10E-12	0.8	23	1.15	Itga1	Extracellular Matrix
DMR2:47185001	2	47185001	47186000	1000	1	1.00E-09	-0.63	17	1.7	Itga1	Extracellular Matrix
DMR2:47215001	2	47215001	47216000	1000	1	1.40E-19	0.73	7	0.7	Itga1	Extracellular Matrix
DMR2:47253001	2	47253001	47254000	1000	1	2.80E-13	0.56	5	0.5	Itga1	Extracellular Matrix
DMR2:47256001	2	47256001	47257000	1000	1	2.10E-15	0.58	12	1.2	Itga1;Pelo	Extracellular Matrix;Translation
DMR2:47290001	2	47290001	47292000	2000	1	1.10E-12	0.64	12	0.6	Itga1	Extracellular Matrix
DMR2:49089001	2	49089001	49090000	1000	1	1.10E-13	0.57	10	1	Parp8	
DMR2:49133001	2	49133001	49138000	5000	1	4.90E-11	-0.81	49	0.98	Parp8	
DMR2:49141001	2	49141001	49146000	5000	1	1.20E-08	0.46	40	0.8	Parp8	
DMR2:49161001	2	49161001	49162000	1000	1	9.60E-07	-0.51	11	1.1	Parp8	
DMR2:49165001	2	49165001	49167000	2000	1	1.10E-07	-0.58	15	0.75	Parp8	
DMR2:49180001	2	49180001	49184000	4000	2	1.30E-14	0.55	32	0.8	Parp8	
DMR2:49197001	2	49197001	49198000	1000	1	2.00E-18	0.78	10	1	Parp8	
DMR2:49218001	2	49218001	49219000	1000	1	8.00E-10	0.48	6	0.6	Parp8	
DMR2:49226001	2	49226001	49227000	1000	1	3.40E-11	0.53	15	1.5	Parp8	
DMR2:49240001	2	49240001	49244000	4000	2	1.50E-07	-0.58	13	0.32	Parp8	
DMR2:49245001	2	49245001	49250000	5000	2	2.60E-09	-0.49	31	0.62	Parp8	
DMR2:49259001	2	49259001	49261000	2000	1	8.10E-11	-0.83	21	1.05	Parp8	
DMR2:49279001	2	49279001	49281000	2000	1	1.50E-13	0.59	18	0.9	Parp8	
DMR2:49316001	2	49316001	49331000	15000	8	4.90E-10	-0.39	433	2.89	Parp8;LOC100910954	
DMR2:49352001	2	49352001	49367000	15000	5	3.60E-08	-0.34	444	2.96	Parp8;LOC100910954	
DMR2:49371001	2	49371001	49372000	1000	1	1.10E-08	0.46	28	2.8	Parp8;LOC100910954	
DMR2:49384001	2	49384001	49388000	4000	2	2.90E-11	-0.47	40	1	Parp8;LOC100910954	
DMR2:49393001	2	49393001	49397000	4000	1	3.80E-19	0.76	110	2.75	Parp8;LOC100910954	
DMR2:49419001	2	49419001	49421000	2000	2	2.00E-13	0.7	18	0.9	Parp8	
DMR2:49440001	2	49440001	49441000	1000	1	8.70E-16	0.85	3	0.3	Parp8	
DMR2:49460001	2	49460001	49464000	4000	1	7.00E-07	-0.52	57	1.43	Parp8	
DMR2:49669001	2	49669001	49674000	5000	3	3.30E-20	0.83	57	1.14	Emb	Cytoskeleton

DMR2:49683001	2	49683001	49686000	3000	1	1.90E-10	0.51	64	2.13	Emb	Cytoskeleton
DMR2:49702001	2	49702001	49703000	1000	1	5.30E-07	-0.45	15	1.5	Emb	Cytoskeleton
DMR2:49712001	2	49712001	49716000	4000	1	3.10E-07	0.31	51	1.27	Emb	Cytoskeleton
DMR2:49729001	2	49729001	49733000	4000	1	8.40E-11	-0.63	51	1.27	Emb	Cytoskeleton
DMR2:49740001	2	49740001	49741000	1000	1	7.30E-17	0.59	13	1.3	Emb	Cytoskeleton
DMR2:50099001	2	50099001	50100000	1000	1	5.60E-11	0.76	50	5	Hcn1	Transport
DMR2:50125001	2	50125001	50127000	2000	1	2.20E-10	0.64	15	0.75	Hcn1	Transport
DMR2:50129001	2	50129001	50130000	1000	1	1.20E-10	0.65	2	0.2	Hcn1	Transport
DMR2:50134001	2	50134001	50135000	1000	1	2.60E-08	0.53	16	1.6	Hcn1	Transport
DMR2:50143001	2	50143001	50150000	7000	5	1.60E-22	-0.51	72	1.03	Hcn1	Transport
DMR2:50185001	2	50185001	50187000	2000	1	1.60E-08	0.45	13	0.65	Hcn1	Transport
DMR2:50189001	2	50189001	50190000	1000	1	4.20E-17	0.81	5	0.5	Hcn1	Transport
DMR2:50191001	2	50191001	50193000	2000	1	4.00E-07	-0.42	10	0.5	Hcn1	Transport
DMR2:50203001	2	50203001	50204000	1000	1	4.90E-16	0.64	6	0.6	Hcn1	Transport
DMR2:50227001	2	50227001	50234000	7000	5	1.60E-19	-0.49	61	0.87	Hcn1	Transport
DMR2:50245001	2	50245001	50246000	1000	1	1.10E-12	0.81	3	0.3	Hcn1	Transport
DMR2:50251001	2	50251001	50252000	1000	1	3.50E-08	0.47	9	0.9	Hcn1	Transport
DMR2:50294001	2	50294001	50297000	3000	3	1.80E-09	-0.62	13	0.43	Hcn1	Transport
DMR2:50320001	2	50320001	50321000	1000	1	5.10E-15	0.9	4	0.4	Hcn1	Transport
DMR2:50332001	2	50332001	50339000	7000	4	4.40E-24	-0.58	74	1.06	Hcn1	Transport
DMR2:50382001	2	50382001	50385000	3000	3	5.30E-14	-0.59	13	0.43	Hcn1	Transport
DMR2:50394001	2	50394001	50395000	1000	1	2.80E-08	0.36	11	1.1	Hcn1	Transport
DMR2:50428001	2	50428001	50429000	1000	1	1.50E-10	0.6	6	0.6	Hcn1	Transport
DMR2:50462001	2	50462001	50463000	1000	1	1.40E-08	0.75	2	0.2	Hcn1	Transport
DMR2:51034001	2	51034001	51036000	2000	1	8.70E-14	0.79	18	0.9	Mrps30	Translation
DMR2:51054001	2	51054001	51056000	2000	2	4.30E-09	0.59	16	0.8	Mrps30	Translation
DMR2:51057001	2	51057001	51059000	2000	1	2.50E-09	0.44	14	0.7	Mrps30	Translation
DMR2:51685001	2	51685001	51687000	2000	1	7.80E-15	-0.66	22	1.1	Fgf10	Growth Factors
DMR2:51689001	2	51689001	51692000	3000	1	1.70E-07	-0.4	38	1.27	Fgf10	Growth Factors
DMR2:51697001	2	51697001	51699000	2000	1	5.60E-07	0.45	22	1.1	Fgf10	Growth Factors
DMR2:52152001	2	52152001	52162000	10000	7	3.30E-19	0.63	67	0.67	RGD1561520	
DMR2:52163001	2	52163001	52165000	2000	2	9.40E-10	0.55	8	0.4	RGD1561520	
DMR2:52166001	2	52166001	52171000	5000	2	8.10E-23	0.84	58	1.16	RGD1561520	
DMR2:52172001	2	52172001	52175000	3000	1	1.80E-08	-0.45	41	1.37	RGD1561520	
DMR2:52204001	2	52204001	52205000	1000	1	1.20E-11	0.57	3	0.3	Nnt	Metabolism
DMR2:52219001	2	52219001	52220000	1000	1	6.00E-08	0.57	3	0.3	Nnt	Metabolism
DMR2:52229001	2	52229001	52232000	3000	1	1.90E-08	0.71	24	0.8	Nnt	Metabolism
DMR2:52244001	2	52244001	52247000	3000	2	9.70E-20	0.86	26	0.87	Nnt	Metabolism
DMR2:52249001	2	52249001	52252000	3000	2	1.40E-13	0.51	48	1.6	Nnt	Metabolism
DMR2:52256001	2	52256001	52258000	2000	1	4.20E-07	0.29	17	0.85	Nnt	Metabolism
DMR2:52275001	2	52275001	52276000	1000	1	5.30E-23	0.99	2	0.2	Nnt;LOC108349949	Metabolism
DMR2:52283001	2	52283001	52285000	2000	1	1.70E-17	0.75	32	1.6	Nnt;LOC108349949	Metabolism
DMR2:52287001	2	52287001	52288000	1000	1	3.40E-08	0.56	9	0.9	Nnt;LOC108349949	Metabolism
DMR2:52307001	2	52307001	52309000	2000	1	2.50E-13	0.75	11	0.55	Paip1	Metabolism
DMR2:52340001	2	52340001	52344000	4000	1	8.80E-07	-0.52	67	1.68	RGD1306227	
DMR2:52371001	2	52371001	52377000	6000	2	5.40E-12	0.65	82	1.37	Tmem267;LOC102556362;Ccl28	Growth Factors
DMR2:52402001	2	52402001	52404000	2000	1	3.90E-07	-0.4	23	1.15	Ccl28;LOC102547360;LOC103691467	Growth Factors
DMR2:52408001	2	52408001	52409000	1000	1	1.70E-08	0.71	3	0.3	Ccl28;LOC102547360;LOC103691467	Growth Factors
DMR2:52440001	2	52440001	52443000	3000	2	3.10E-08	-0.6	48	1.6	Hmgcs1;Nim1k	Signaling
DMR2:52446001	2	52446001	52447000	1000	1	5.30E-11	0.51	11	1.1	Hmgcs1;Nim1k	Signaling
DMR2:52472001	2	52472001	52473000	1000	1	3.10E-10	-0.71	16	1.6	Nim1k	Signaling
DMR2:52486001	2	52486001	52490000	4000	2	6.00E-16	0.69	34	0.85	Nim1k	Signaling
DMR2:53102001	2	53102001	53104000	2000	1	8.70E-12	0.7	18	0.9	LOC102548571;Sepp1	
DMR2:53108001	2	53108001	53109000	1000	1	2.00E-19	0.86	4	0.4	Sepp1;Ccdc152	
DMR2:53117001	2	53117001	53121000	4000	1	1.00E-10	0.46	37	0.92	Sepp1;Ccdc152;LOC103691472	
DMR2:53122001	2	53122001	53123000	1000	1	3.10E-07	-0.54	12	1.2	Sepp1;Ccdc152;LOC103691472	
DMR2:53125001	2	53125001	53128000	3000	2	8.20E-11	0.57	16	0.53	Sepp1;Ccdc152;LOC103691472	
DMR2:53129001	2	53129001	53131000	2000	1	1.90E-12	0.85	3	0.15	Ccdc152;LOC103691472	
DMR2:53137001	2	53137001	53138000	1000	1	9.60E-10	0.52	12	1.2	Ccdc152;LOC103691472	
DMR2:53166001	2	53166001	53167000	1000	1	1.50E-12	0.55	12	1.2	Ghr	Receptor
DMR2:53171001	2	53171001	53178000	7000	4	1.10E-23	0.62	62	0.89	Ghr	Receptor
DMR2:53179001	2	53179001	53180000	1000	1	8.30E-07	-0.52	12	1.2	Ghr	Receptor

DMR2:53201001	2	53201001	53207000	6000	2	6.20E-15	-0.55	60	1	Ghr	Receptor
DMR2:53208001	2	53208001	53211000	3000	3	2.90E-15	0.71	11	0.37	Ghr	Receptor
DMR2:53213001	2	53213001	53214000	1000	1	3.20E-09	-0.73	10	1	Ghr	Receptor
DMR2:53215001	2	53215001	53216000	1000	1	5.30E-09	0.52	9	0.9	Ghr	Receptor
DMR2:53223001	2	53223001	53230000	7000	5	7.10E-19	-0.5	88	1.26	Ghr	Receptor
DMR2:53239001	2	53239001	53240000	1000	1	2.70E-24	1.23	3	0.3	Ghr	Receptor
DMR2:53326001	2	53326001	53327000	1000	1	1.80E-15	0.68	11	1.1	Ghr	Receptor
DMR2:53332001	2	53332001	53334000	2000	1	1.60E-09	-0.42	23	1.15	Ghr	Receptor
DMR2:53357001	2	53357001	53359000	2000	1	1.50E-11	0.64	10	0.5	Ghr	Receptor
DMR2:53397001	2	53397001	53399000	2000	1	5.70E-07	0.35	23	1.15	Ghr	Receptor
DMR2:53406001	2	53406001	53407000	1000	1	3.10E-10	0.44	32	3.2	Ghr;LOC102548637	Receptor
DMR2:53410001	2	53410001	53411000	1000	1	2.10E-11	0.5	11	1.1	Ghr;LOC102548637	Receptor
DMR2:53798001	2	53798001	53800000	2000	1	2.30E-13	0.6	17	0.85	Fbxo4	
DMR2:53833001	2	53833001	53834000	1000	1	2.90E-13	0.71	17	1.7	RGD1305938	
DMR2:53836001	2	53836001	53837000	1000	1	3.60E-07	0.39	8	0.8	RGD1305938	
DMR2:53853001	2	53853001	53854000	1000	1	2.80E-08	-0.43	14	1.4	Oxct1	Transport
DMR2:53861001	2	53861001	53863000	2000	2	2.30E-14	0.86	28	1.4	Oxct1	Transport
DMR2:53864001	2	53864001	53866000	2000	1	3.40E-13	0.51	27	1.35	Oxct1	Transport
DMR2:53886001	2	53886001	53889000	3000	1	1.70E-10	-0.69	41	1.37	Oxct1	Transport
DMR2:53890001	2	53890001	53891000	1000	1	7.00E-11	0.47	13	1.3	Oxct1	Transport
DMR2:53902001	2	53902001	53903000	1000	1	9.10E-07	-0.6	11	1.1	Oxct1	Transport
DMR2:53908001	2	53908001	53909000	1000	1	1.00E-10	0.4	22	2.2	Oxct1	Transport
DMR2:53922001	2	53922001	53924000	2000	1	1.60E-08	-0.4	32	1.6	Oxct1	Transport
DMR2:53930001	2	53930001	53933000	3000	2	3.40E-10	-0.59	44	1.47	Oxct1	Transport
DMR2:53944001	2	53944001	53947000	3000	1	1.30E-09	-0.62	56	1.87	Oxct1	Transport
DMR2:53956001	2	53956001	53957000	1000	1	8.30E-08	-0.5	14	1.4	Oxct1	Transport
DMR2:53981001	2	53981001	53984000	3000	1	1.30E-12	-0.47	44	1.47	Oxct1	Transport
DMR2:54145001	2	54145001	54150000	5000	2	1.40E-10	0.42	32	0.64	RGD1566344	
DMR2:54161001	2	54161001	54163000	2000	1	6.70E-07	0.45	13	0.65	RGD1566344	
DMR2:54200001	2	54200001	54202000	2000	1	1.00E-16	0.89	15	0.75	LOC108349953;Plcxd3	
DMR2:54203001	2	54203001	54204000	1000	1	1.30E-09	0.47	6	0.6	Plcxd3	
DMR2:54227001	2	54227001	54229000	2000	1	2.30E-11	-0.7	18	0.9	Plcxd3	
DMR2:54231001	2	54231001	54233000	2000	2	2.80E-10	0.5	18	0.9	Plcxd3	
DMR2:54242001	2	54242001	54247000	5000	4	1.10E-10	-0.46	46	0.92	Plcxd3	
DMR2:54253001	2	54253001	54254000	1000	1	8.20E-10	0.58	2	0.2	Plcxd3	
DMR2:54263001	2	54263001	54265000	2000	1	4.80E-08	0.41	16	0.8	Plcxd3	
DMR2:54270001	2	54270001	54273000	3000	1	3.10E-07	-0.43	32	1.07	Plcxd3	
DMR2:54274001	2	54274001	54277000	3000	2	2.00E-12	-0.61	12	0.4	Plcxd3	
DMR2:54280001	2	54280001	54282000	2000	2	2.90E-11	0.63	10	0.5	Plcxd3	
DMR2:54287001	2	54287001	54293000	6000	2	2.80E-27	-0.61	71	1.18	Plcxd3	
DMR2:54312001	2	54312001	54313000	1000	1	1.70E-07	-0.52	3	0.3	Plcxd3	
DMR2:54323001	2	54323001	54324000	1000	1	5.50E-08	0.48	7	0.7	Plcxd3	
DMR2:54338001	2	54338001	54339000	1000	1	5.40E-16	0.75	13	1.3	Plcxd3	
DMR2:54347001	2	54347001	54351000	4000	2	3.40E-08	-0.49	15	0.38	Plcxd3	
DMR2:54368001	2	54368001	54369000	1000	1	5.50E-17	0.97	7	0.7	Plcxd3	
DMR2:54455001	2	54455001	54457000	2000	1	8.00E-09	0.59	9	0.45	C6	
DMR2:54459001	2	54459001	54460000	1000	1	3.50E-07	0.45	7	0.7	C6	
DMR2:54463001	2	54463001	54466000	3000	1	1.40E-12	0.49	34	1.13	C6	
DMR2:54519001	2	54519001	54522000	3000	1	6.60E-07	0.42	25	0.83	C6	
DMR2:54530001	2	54530001	54532000	2000	1	1.50E-07	0.45	10	0.5	C6	
DMR2:54620001	2	54620001	54621000	1000	1	2.20E-08	0.61	3	0.3	Mroh2b	
DMR2:54630001	2	54630001	54632000	2000	2	6.40E-14	0.77	13	0.65	Mroh2b	
DMR2:54648001	2	54648001	54649000	1000	1	7.30E-08	-0.5	5	0.5	Mroh2b	
DMR2:54651001	2	54651001	54653000	2000	1	9.40E-11	0.64	13	0.65	Mroh2b	
DMR2:54657001	2	54657001	54658000	1000	1	2.80E-07	0.36	11	1.1	Mroh2b	
DMR2:54661001	2	54661001	54668000	7000	2	1.30E-14	0.48	75	1.07	Mroh2b;LOC102553573	
DMR2:54709001	2	54709001	54714000	5000	2	7.30E-08	-0.45	54	1.08	C7	
DMR2:54726001	2	54726001	54728000	2000	2	2.60E-09	0.6	11	0.55	C7	
DMR2:54746001	2	54746001	54751000	5000	4	9.60E-20	-0.57	45	0.9	C7	
DMR2:54761001	2	54761001	54762000	1000	1	6.60E-18	0.63	3	0.3	C7	
DMR2:54778001	2	54778001	54779000	1000	1	6.70E-09	0.36	14	1.4	C7	
DMR2:54783001	2	54783001	54784000	1000	1	3.20E-09	0.52	16	1.6	C7	
DMR2:54785001	2	54785001	54790000	5000	1	3.80E-10	0.65	53	1.06	C7	
DMR2:54806001	2	54806001	54811000	5000	2	1.90E-16	0.86	35	0.7	Card6	
DMR2:54812001	2	54812001	54816000	4000	1	1.00E-15	0.71	54	1.35	Card6	

DMR2:54821001	2	54821001	54822000	1000	1	2.40E-10	0.47	6	0.6	Card6		
DMR2:54872001	2	54872001	54880000	8000	3	1.40E-09	-0.5	122	1.52	Prkaa1	Signaling	
DMR2:54883001	2	54883001	54886000	3000	2	2.50E-36	1.26	23	0.77	Prkaa1	Signaling	
DMR2:54905001	2	54905001	54908000	3000	2	5.90E-15	0.78	19	0.63	Ttc33;LOC108349955		
DMR2:54909001	2	54909001	54910000	1000	1	2.80E-08	0.47	7	0.7	Ttc33;LOC108349955		
DMR2:54928001	2	54928001	54937000	9000	1	4.00E-09	-0.44	78	0.87	Ttc33;LOC108349955		
DMR2:54945001	2	54945001	54949000	4000	3	1.20E-18	0.7	20	0.5	Ttc33;Ptger4	Signaling	
DMR2:54969001	2	54969001	54970000	1000	1	3.10E-16	0.52	13	1.3	Ptger4	Signaling	
DMR2:55737001	2	55737001	55740000	3000	1	3.00E-07	0.42	24	0.8	Dab2	Cytoskeleton	
DMR2:55744001	2	55744001	55745000	1000	1	4.30E-08	0.47	8	0.8	Dab2	Cytoskeleton	
DMR2:55750001	2	55750001	55753000	3000	1	7.00E-10	0.44	26	0.87	Dab2	Cytoskeleton	
DMR2:55759001	2	55759001	55762000	3000	2	5.80E-10	-0.69	66	2.2	Dab2	Cytoskeleton	
DMR2:55783001	2	55783001	55786000	3000	2	3.00E-09	0.49	28	0.93	C9		
DMR2:55810001	2	55810001	55813000	3000	1	2.60E-07	-0.56	48	1.6	C9		
DMR2:55819001	2	55819001	55820000	1000	1	2.50E-07	0.61	3	0.3	C9		
DMR2:55824001	2	55824001	55825000	1000	1	6.20E-13	0.53	14	1.4	C9;Fyb		
DMR2:55855001	2	55855001	55858000	3000	1	1.90E-08	-0.57	9	0.3	Fyb		
DMR2:55875001	2	55875001	55879000	4000	2	4.40E-21	1.2	27	0.68	Fyb		
DMR2:55921001	2	55921001	55924000	3000	1	2.60E-07	0.56	26	0.87	Fyb		
DMR2:55929001	2	55929001	55933000	4000	3	4.60E-31	1.37	49	1.23	Fyb		
DMR2:55934001	2	55934001	55935000	1000	1	1.60E-22	0.73	14	1.4	Fyb		
DMR2:55942001	2	55942001	55944000	2000	1	4.90E-07	0.48	14	0.7	Fyb		
DMR2:55947001	2	55947001	55950000	3000	2	3.80E-25	1.06	30	1	Fyb		
DMR2:55954001	2	55954001	55957000	3000	1	1.10E-13	0.54	29	0.97	Fyb		
DMR2:55958001	2	55958001	55959000	1000	1	9.70E-08	0.6	4	0.4	Fyb		
DMR2:55968001	2	55968001	55971000	3000	3	1.80E-22	-0.54	27	0.9	Fyb		
DMR2:55972001	2	55972001	55974000	2000	1	1.40E-07	-0.38	20	1	Fyb		
DMR2:55975001	2	55975001	55985000	10000	3	9.60E-19	0.8	131	1.31	Fyb		
DMR2:55992001	2	55992001	55993000	1000	1	5.30E-11	0.61	12	1.2	Fyb		
DMR2:56036001	2	56036001	56038000	2000	1	2.60E-11	0.48	10	0.5	Rictor		
DMR2:56042001	2	56042001	56045000	3000	1	2.00E-09	0.46	29	0.97	Rictor		
DMR2:56124001	2	56124001	56129000	5000	1	9.40E-08	-0.47	67	1.34	Osmr	Receptor	
DMR2:56420001	2	56420001	56422000	2000	1	1.00E-12	0.75	23	1.15	Lifr	Receptor	
DMR2:56437001	2	56437001	56438000	1000	1	7.00E-10	0.37	21	2.1	Lifr	Receptor	
DMR2:56451001	2	56451001	56453000	2000	2	3.70E-12	0.41	27	1.35	Lifr	Receptor	
DMR2:56464001	2	56464001	56466000	2000	1	4.40E-07	0.33	42	2.1	Lifr	Receptor	
DMR2:56473001	2	56473001	56475000	2000	1	1.20E-09	0.46	26	1.3	Lifr	Receptor	
DMR2:56477001	2	56477001	56480000	3000	2	2.30E-13	0.52	42	1.4	Lifr	Receptor	
DMR2:56490001	2	56490001	56493000	3000	1	4.00E-09	-0.47	45	1.5	Lifr	Receptor	
DMR2:56513001	2	56513001	56515000	2000	1	2.70E-13	0.51	23	1.15	Egflam	Extracellular Matrix	
DMR2:56518001	2	56518001	56521000	3000	1	6.00E-07	-0.49	47	1.57	Egflam	Extracellular Matrix	
DMR2:56529001	2	56529001	56530000	1000	1	2.50E-07	-0.53	14	1.4	Egflam;LOC108349957	Extracellular Matrix	
DMR2:56536001	2	56536001	56538000	2000	1	5.00E-10	0.39	30	1.5	Egflam;LOC108349957	Extracellular Matrix	
DMR2:56539001	2	56539001	56542000	3000	1	1.20E-08	0.37	33	1.1	Egflam;LOC108349957	Extracellular Matrix	
DMR2:56547001	2	56547001	56548000	1000	1	6.10E-08	-0.63	13	1.3	Egflam;LOC108349957	Extracellular Matrix	
DMR2:56558001	2	56558001	56564000	6000	1	5.30E-07	-0.51	74	1.23	Egflam;LOC108349957	Extracellular Matrix	
DMR2:56566001	2	56566001	56570000	4000	2	3.40E-08	0.61	35	0.88	Egflam	Extracellular Matrix	
DMR2:56580001	2	56580001	56581000	1000	1	3.00E-08	0.51	8	0.8	Egflam	Extracellular Matrix	
DMR2:56585001	2	56585001	56587000	2000	1	7.10E-07	0.34	23	1.15	Egflam	Extracellular Matrix	
DMR2:56636001	2	56636001	56638000	2000	1	1.00E-13	0.65	13	0.65	Egflam;LOC102550930	Extracellular Matrix	
DMR2:56648001	2	56648001	56650000	2000	1	2.30E-08	0.44	20	1	Egflam	Extracellular Matrix	
DMR2:56884001	2	56884001	56886000	2000	1	1.30E-09	-0.49	29	1.45	Gdnf;LOC103691480	Growth Factors	
DMR2:56896001	2	56896001	56898000	2000	2	9.70E-10	0.52	19	0.95	Gdnf;LOC103691480	Growth Factors	
DMR2:56900001	2	56900001	56901000	1000	1	4.10E-11	0.57	9	0.9	Gdnf;LOC103691480	Growth Factors	
DMR2:56905001	2	56905001	56908000	3000	2	3.70E-23	0.88	27	0.9	Gdnf	Growth Factors	
DMR2:56912001	2	56912001	56915000	3000	1	1.20E-07	0.31	36	1.2	Gdnf	Growth Factors	
DMR2:56919001	2	56919001	56921000	2000	1	7.40E-11	0.52	15	0.75	Gdnf	Growth Factors	
DMR2:56934001	2	56934001	56938000	4000	3	3.80E-34	1.23	33	0.82	Wdr70		
DMR2:56966001	2	56966001	56970000	4000	1	4.50E-07	0.41	61	1.52	Wdr70		
DMR2:56971001	2	56971001	56980000	9000	4	1.90E-24	-0.58	112	1.24	Wdr70		
DMR2:56982001	2	56982001	56985000	3000	3	5.30E-22	0.87	32	1.07	Wdr70		
DMR2:56998001	2	56998001	5.70E+07	2000	1	2.00E-10	0.57	8	0.4	Wdr70		
DMR2:57008001	2	57008001	57010000	2000	1	1.20E-09	0.73	14	0.7	Wdr70		
DMR2:57014001	2	57014001	57017000	3000	2	1.00E-15	0.61	22	0.73	Wdr70;LOC103691492		
DMR2:57038001	2	57038001	57039000	1000	1	3.10E-13	0.84	5	0.5	Wdr70		

DMR2:57047001	2	57047001	57049000	2000	2	6.70E-08	-0.71	21	1.05	Wdr70	
DMR2:57052001	2	57052001	57055000	3000	1	1.50E-07	-0.6	31	1.03	Wdr70	
DMR2:57060001	2	57060001	57066000	6000	5	4.60E-21	-0.54	54	0.9	Wdr70	
DMR2:57079001	2	57079001	57081000	2000	1	8.80E-07	0.33	24	1.2	Wdr70	
DMR2:57110001	2	57110001	57113000	3000	2	2.80E-12	-0.43	50	1.67	Wdr70	
DMR2:57115001	2	57115001	57118000	3000	1	3.30E-18	0.55	29	0.97	Wdr70	
DMR2:57119001	2	57119001	57120000	1000	1	2.10E-11	0.54	6	0.6	Wdr70	
DMR2:57132001	2	57132001	57134000	2000	2	6.10E-09	0.54	17	0.85	Wdr70	
DMR2:57137001	2	57137001	57138000	1000	1	9.10E-09	0.61	4	0.4	Wdr70	
DMR2:57139001	2	57139001	57147000	8000	3	1.90E-12	0.64	71	0.89	Wdr70	
DMR2:57161001	2	57161001	57162000	1000	1	8.80E-08	0.46	11	1.1	Wdr70	
DMR2:57164001	2	57164001	57170000	6000	2	2.10E-22	0.73	99	1.65	Wdr70	
DMR2:57174001	2	57174001	57177000	3000	1	6.60E-07	-0.43	25	0.83	Wdr70	
DMR2:57179001	2	57179001	57181000	2000	1	3.10E-08	0.5	14	0.7	Wdr70	
DMR2:57225001	2	57225001	57228000	3000	1	1.60E-07	-0.47	30	1	Nup155	Transport
DMR2:57233001	2	57233001	57234000	1000	1	3.70E-07	0.41	10	1	Nup155	Transport
DMR2:57276001	2	57276001	57280000	4000	3	1.00E-23	1.16	39	0.98	RGD1310081	
DMR2:57297001	2	57297001	57300000	3000	1	6.20E-09	0.39	39	1.3	RGD1310081;LOC365694	
DMR2:57302001	2	57302001	57303000	1000	1	6.80E-10	0.53	14	1.4	RGD1310081;LOC365694	
DMR2:57313001	2	57313001	57315000	2000	2	1.70E-11	0.47	34	1.7	RGD1310081	
DMR2:57332001	2	57332001	57336000	4000	2	7.10E-07	-0.47	49	1.23	RGD1310081	
DMR2:57340001	2	57340001	57342000	2000	1	3.00E-07	-0.45	7	0.35	RGD1310081;LOC100911004	
DMR2:57350001	2	57350001	57353000	3000	1	7.40E-17	0.65	39	1.3	RGD1310081;LOC100911004	
DMR2:57473001	2	57473001	57478000	5000	2	1.50E-22	0.64	54	1.08	RGD1310081;LOC499538	
DMR2:57479001	2	57479001	57482000	3000	1	4.50E-12	-0.49	12	0.4	RGD1310081;LOC499538	
DMR2:57579001	2	57579001	57582000	3000	1	2.70E-10	0.64	32	1.07	Nipbl	Epigenetic
DMR2:57606001	2	57606001	57608000	2000	1	1.40E-08	-0.54	5	0.25	Nipbl	Epigenetic
DMR2:57609001	2	57609001	57611000	2000	1	1.40E-08	0.38	9	0.45	Nipbl	Epigenetic
DMR2:57615001	2	57615001	57617000	2000	1	1.40E-11	-0.71	17	0.85	Nipbl	Epigenetic
DMR2:57620001	2	57620001	57622000	2000	1	1.50E-08	0.45	8	0.4	Nipbl	Epigenetic
DMR2:57641001	2	57641001	57642000	1000	1	4.20E-09	-0.62	13	1.3	Nipbl;LOC108349959	Epigenetic
DMR2:57864001	2	57864001	57867000	3000	1	1.10E-11	0.5	35	1.17	Slc1a3	Transport
DMR2:57886001	2	57886001	57887000	1000	1	8.90E-22	0.78	9	0.9	Slc1a3	Transport
DMR2:57889001	2	57889001	57891000	2000	1	3.20E-12	0.41	45	2.25	Slc1a3	Transport
DMR2:57894001	2	57894001	57899000	5000	3	2.60E-15	0.72	57	1.14	Slc1a3	Transport
DMR2:57914001	2	57914001	57915000	1000	1	4.00E-07	-0.39	17	1.7	Slc1a3	Transport
DMR2:57916001	2	57916001	57921000	5000	3	2.90E-17	0.84	45	0.9	Slc1a3	Transport
DMR2:57926001	2	57926001	57929000	3000	2	2.40E-19	0.77	58	1.93	Slc1a3	Transport
DMR2:58218001	2	58218001	58219000	1000	1	2.20E-08	-0.53	12	1.2	Ranbp3l	Cytoskeleton
DMR2:58229001	2	58229001	58230000	1000	1	7.30E-18	0.58	16	1.6	Ranbp3l;LOC679871	Cytoskeleton
DMR2:58237001	2	58237001	58238000	1000	1	1.60E-07	0.51	28	2.8	Ranbp3l;LOC679871	Cytoskeleton
DMR2:58246001	2	58246001	58250000	4000	2	9.60E-18	0.73	29	0.72	Ranbp3l;LOC679871	Cytoskeleton
DMR2:58251001	2	58251001	58253000	2000	2	1.10E-15	0.88	6	0.3	Ranbp3l	Cytoskeleton
DMR2:58254001	2	58254001	58260000	6000	3	2.70E-13	-0.46	35	0.58	Ranbp3l;LOC365693	Cytoskeleton
DMR2:58262001	2	58262001	58264000	2000	1	3.00E-07	-0.45	15	0.75	Ranbp3l;LOC365693	Cytoskeleton
DMR2:58269001	2	58269001	58270000	1000	1	6.50E-09	0.64	2	0.2	Ranbp3l;LOC365693	Cytoskeleton
DMR2:58271001	2	58271001	58272000	1000	1	3.00E-14	0.68	8	0.8	Ranbp3l;LOC365693	Cytoskeleton
DMR2:58277001	2	58277001	58281000	4000	3	4.90E-24	-0.58	48	1.2	Ranbp3l;LOC365693	Cytoskeleton
DMR2:58298001	2	58298001	58300000	2000	1	1.50E-19	0.77	27	1.35	Ranbp3l	Cytoskeleton
DMR2:58312001	2	58312001	58315000	3000	1	1.40E-18	0.63	32	1.07	Ranbp3l	Cytoskeleton
DMR2:58318001	2	58318001	58321000	3000	2	1.30E-19	1.01	37	1.23	Ranbp3l	Cytoskeleton
DMR2:58335001	2	58335001	58336000	1000	1	2.00E-17	0.64	15	1.5	Ranbp3l	Cytoskeleton
DMR2:58351001	2	58351001	58357000	6000	5	7.90E-21	-0.51	67	1.12	Ranbp3l;LOC108350155	Cytoskeleton
DMR2:58359001	2	58359001	58363000	4000	2	2.20E-09	0.45	33	0.82	Ranbp3l;LOC108350155	Cytoskeleton
DMR2:58370001	2	58370001	58371000	1000	1	5.20E-09	-0.59	21	2.1	Ranbp3l;LOC108350155	Cytoskeleton
DMR2:58376001	2	58376001	58382000	6000	3	1.00E-09	0.59	66	1.1	Ranbp3l	Cytoskeleton
DMR2:58384001	2	58384001	58386000	2000	2	6.80E-10	0.42	17	0.85	Ranbp3l	Cytoskeleton
DMR2:58389001	2	58389001	58394000	5000	1	4.60E-09	0.5	46	0.92	Ranbp3l	Cytoskeleton
DMR2:58419001	2	58419001	58420000	1000	1	1.30E-08	0.58	2	0.2	Ranbp3l	Cytoskeleton
DMR2:58424001	2	58424001	58425000	1000	1	1.30E-10	0.82	5	0.5	Ranbp3l	Cytoskeleton
DMR2:58475001	2	58475001	58478000	3000	1	6.70E-10	0.4	45	1.5	Nadk2	
DMR2:58482001	2	58482001	58485000	3000	1	4.90E-09	-0.48	54	1.8	Nadk2	
DMR2:58488001	2	58488001	58492000	4000	1	1.10E-07	0.29	58	1.45	Nadk2	
DMR2:58505001	2	58505001	58508000	3000	2	6.80E-08	-0.48	60	2	Nadk2;Skp2	
DMR2:58513001	2	58513001	58514000	1000	1	6.70E-11	0.47	18	1.8	Nadk2;Skp2	

DMR2:58531001	2	58531001	58532000	1000	1	5.60E-07	-0.45	10	1	Skp2;Lmbrd2	
DMR2:58541001	2	58541001	58543000	2000	1	2.10E-07	-0.57	15	0.75	Skp2;Lmbrd2	
DMR2:58553001	2	58553001	58554000	1000	1	3.70E-19	0.75	19	1.9	Lmbrd2	
DMR2:58558001	2	58558001	58559000	1000	1	2.10E-11	0.83	1	0.1	Lmbrd2	
DMR2:58565001	2	58565001	58566000	1000	1	1.70E-13	0.81	5	0.5	Lmbrd2	
DMR2:58596001	2	58596001	58599000	3000	3	2.50E-21	0.72	30	1	Lmbrd2	
DMR2:58679001	2	58679001	58682000	3000	2	2.40E-07	0.42	19	0.63	Ugt3a2	
DMR2:58719001	2	58719001	58720000	1000	1	2.50E-09	0.62	6	0.6	Capsl	Signaling
DMR2:58721001	2	58721001	58724000	3000	1	2.40E-09	0.51	26	0.87	Capsl	Signaling
DMR2:58737001	2	58737001	58740000	3000	1	5.20E-08	0.48	16	0.53	Capsl	Signaling
DMR2:58904001	2	58904001	58906000	2000	1	2.20E-08	0.58	18	0.9	Spef2;LOC108349963	
DMR2:58952001	2	58952001	58959000	7000	5	4.00E-19	-0.48	90	1.29	Spef2	
DMR2:58960001	2	58960001	58964000	4000	2	7.60E-17	-0.52	27	0.68	Spef2	
DMR2:58966001	2	58966001	58968000	2000	1	7.60E-10	0.51	15	0.75	Spef2	
DMR2:58981001	2	58981001	58982000	1000	1	1.90E-09	0.54	3	0.3	Spef2	
DMR2:58989001	2	58989001	58991000	2000	1	4.40E-07	0.47	4	0.2	Spef2	
DMR2:58994001	2	58994001	58997000	3000	1	4.10E-07	-0.4	17	0.57	Spef2	
DMR2:58998001	2	58998001	5.90E+07	2000	1	2.60E-08	0.46	19	0.95	Spef2	
DMR2:59022001	2	59022001	59026000	4000	1	6.80E-08	-0.45	34	0.85	Spef2	
DMR2:59036001	2	59036001	59037000	1000	1	1.40E-07	0.42	7	0.7	Spef2	
DMR2:59059001	2	59059001	59060000	1000	1	4.00E-13	0.56	13	1.3	Spef2	
DMR2:60128001	2	60128001	60130000	2000	1	2.80E-07	0.35	25	1.25	Prlr	Receptor
DMR2:60193001	2	60193001	60201000	8000	4	3.20E-23	-0.57	85	1.06	Prlr	Receptor
DMR2:60224001	2	60224001	60227000	3000	1	6.40E-07	-0.48	36	1.2	Prlr	Receptor
DMR2:60236001	2	60236001	60238000	2000	1	8.50E-07	0.4	17	0.85	Prlr	Receptor
DMR2:60239001	2	60239001	60241000	2000	2	1.10E-11	0.7	8	0.4	Prlr	Receptor
DMR2:60252001	2	60252001	60254000	2000	1	4.30E-13	0.68	17	0.85	Prlr	Receptor
DMR2:60267001	2	60267001	60268000	1000	1	4.10E-07	-0.58	14	1.4	Prlr	Receptor
DMR2:60306001	2	60306001	60307000	1000	1	1.60E-08	0.44	11	1.1	Prlr	Receptor
DMR2:60324001	2	60324001	60326000	2000	2	1.20E-08	0.47	18	0.9	Prlr	Receptor
DMR2:60336001	2	60336001	60338000	2000	2	1.50E-09	0.53	10	0.5	Agxt2	
DMR2:60340001	2	60340001	60347000	7000	2	9.20E-08	0.62	40	0.57	Agxt2	
DMR2:60355001	2	60355001	60357000	2000	1	4.60E-12	0.45	26	1.3	Agxt2	
DMR2:60370001	2	60370001	60371000	1000	1	5.90E-10	0.61	5	0.5	Agxt2	
DMR2:60388001	2	60388001	60394000	6000	2	2.30E-16	-0.4	54	0.9	Agxt2	
DMR2:60412001	2	60412001	60414000	2000	1	2.00E-07	-0.38	16	0.8	Dnajc21	Transcription
DMR2:60415001	2	60415001	60416000	1000	1	4.50E-10	-0.79	13	1.3	Dnajc21	Transcription
DMR2:60421001	2	60421001	60428000	7000	4	8.60E-26	-0.6	85	1.21	Dnajc21	Transcription
DMR2:60436001	2	60436001	60440000	4000	1	4.80E-11	0.47	60	1.5	Dnajc21	Transcription
DMR2:60443001	2	60443001	60446000	3000	2	1.40E-08	0.36	49	1.63	Dnajc21;Brix1	Transcription;Metabolism
DMR2:60455001	2	60455001	60456000	1000	1	5.20E-09	0.45	9	0.9	Dnajc21;Brix1;Rad1	Transcription;Metabolism;DNA Repair
DMR2:60476001	2	60476001	60477000	1000	1	1.80E-07	-0.51	14	1.4	Rad1;LOC102548295;LOC108349965	DNA Repair
DMR2:60478001	2	60478001	60479000	1000	1	1.20E-14	0.59	10	1	Rad1;LOC102548295;LOC108349965	DNA Repair
DMR2:60487001	2	60487001	60489000	2000	2	3.10E-10	0.64	8	0.4	LOC108349965;Ttc23l	
DMR2:60500001	2	60500001	60507000	7000	1	9.20E-08	-0.48	42	0.6	Ttc23l	
DMR2:60528001	2	60528001	60530000	2000	2	3.20E-09	0.66	5	0.25	Ttc23l;LOC108350156	
DMR2:60531001	2	60531001	60534000	3000	2	1.70E-13	0.56	33	1.1	Ttc23l;LOC108350156	
DMR2:60544001	2	60544001	60547000	3000	1	9.60E-08	-0.58	39	1.3	Ttc23l;Rai14	
DMR2:60556001	2	60556001	60560000	4000	1	6.60E-08	-0.49	50	1.25	Rai14	
DMR2:60565001	2	60565001	60570000	5000	1	1.80E-10	0.41	72	1.44	Rai14	
DMR2:60578001	2	60578001	60580000	2000	1	2.00E-07	0.37	27	1.35	Rai14	
DMR2:60581001	2	60581001	60584000	3000	1	5.80E-07	-0.41	59	1.97	Rai14	
DMR2:60590001	2	60590001	60591000	1000	1	4.70E-08	0.61	22	2.2	Rai14	
DMR2:60607001	2	60607001	60614000	7000	2	1.70E-10	0.58	88	1.26	Rai14	
DMR2:60644001	2	60644001	60645000	1000	1	2.20E-14	0.75	6	0.6	Rai14	
DMR2:60646001	2	60646001	60647000	1000	1	1.10E-07	-0.65	9	0.9	Rai14	
DMR2:60648001	2	60648001	60649000	1000	1	7.00E-14	0.44	14	1.4	Rai14	
DMR2:60653001	2	60653001	60654000	1000	1	2.90E-08	0.34	15	1.5	Rai14	
DMR2:60659001	2	60659001	60660000	1000	1	1.30E-07	-0.42	14	1.4	Rai14	
DMR2:60674001	2	60674001	60676000	2000	2	4.60E-19	0.94	30	1.5	Rai14	
DMR2:60679001	2	60679001	60680000	1000	1	3.90E-34	0.97	5	0.5	Rai14	

DMR2:60930001	2	60930001	60934000	4000	2	1.00E-30	1.1	54	1.35	C1qtnf3	
DMR2:60943001	2	60943001	60945000	2000	2	7.90E-11	0.64	20	1	C1qtnf3;Amacr	
DMR2:60950001	2	60950001	60954000	4000	3	3.00E-09	0.55	60	1.5	C1qtnf3;Amacr	
DMR2:60955001	2	60955001	60958000	3000	2	2.80E-19	0.57	54	1.8	Amacr;Slc45a2	Transport
DMR2:60962001	2	60962001	60964000	2000	1	2.00E-13	0.55	24	1.2	Amacr;Slc45a2	Transport
DMR2:60969001	2	60969001	60971000	2000	1	3.10E-11	0.54	27	1.35	Amacr;Slc45a2	Transport
DMR2:60976001	2	60976001	60982000	6000	3	3.00E-17	-0.63	79	1.32	Slc45a2	Transport
DMR2:60996001	2	60996001	60997000	1000	1	1.80E-15	0.58	7	0.7	Slc45a2;Rxfp3	Transport;Signaling
DMR2:61002001	2	61002001	61004000	2000	1	4.90E-11	0.47	21	1.05	Slc45a2;Rxfp3	Transport;Signaling
DMR2:61015001	2	61015001	61020000	5000	2	7.10E-15	0.65	42	0.84	Rxfp3	Signaling
DMR2:61053001	2	61053001	61057000	4000	1	2.80E-07	-0.47	37	0.92	Adamts12	Protease
DMR2:61058001	2	61058001	61059000	1000	1	7.10E-15	0.97	6	0.6	Adamts12	Protease
DMR2:61066001	2	61066001	61068000	2000	1	7.60E-10	0.52	17	0.85	Adamts12	Protease
DMR2:61102001	2	61102001	61103000	1000	1	5.70E-07	0.35	6	0.6	Adamts12	Protease
DMR2:61110001	2	61110001	61112000	2000	1	3.40E-12	0.71	9	0.45	Adamts12	Protease
DMR2:61134001	2	61134001	61135000	1000	1	2.30E-12	0.69	5	0.5	Adamts12	Protease
DMR2:61151001	2	61151001	61154000	3000	2	1.00E-10	0.67	26	0.87	Adamts12	Protease
DMR2:61171001	2	61171001	61172000	1000	1	5.20E-19	1	0	0	Adamts12	Protease
DMR2:61177001	2	61177001	61182000	5000	3	8.00E-15	0.71	38	0.76	Adamts12	Protease
DMR2:61192001	2	61192001	61195000	3000	2	7.20E-18	0.75	27	0.9	Adamts12	Protease
DMR2:61220001	2	61220001	61222000	2000	2	5.70E-11	0.64	12	0.6	Adamts12	Protease
DMR2:61225001	2	61225001	61226000	1000	1	2.30E-17	0.79	9	0.9	Adamts12	Protease
DMR2:61243001	2	61243001	61245000	2000	1	1.10E-08	-0.61	9	0.45	Adamts12	Protease
DMR2:61250001	2	61250001	61251000	1000	1	2.30E-10	0.61	3	0.3	Adamts12	Protease
DMR2:61252001	2	61252001	61254000	2000	1	2.60E-12	0.44	14	0.7	Adamts12	Protease
DMR2:61273001	2	61273001	61276000	3000	1	4.60E-19	0.73	25	0.83	Adamts12	Protease
DMR2:61277001	2	61277001	61278000	1000	1	3.90E-17	0.75	1	0.1	Adamts12	Protease
DMR2:61292001	2	61292001	61295000	3000	1	9.60E-09	0.65	18	0.6	Adamts12	Protease
DMR2:61299001	2	61299001	61301000	2000	1	4.50E-13	0.77	19	0.95	Adamts12	Protease
DMR2:61305001	2	61305001	61306000	1000	1	3.70E-08	-0.54	10	1	Adamts12	Protease
DMR2:61405001	2	61405001	61410000	5000	1	1.30E-08	-0.55	93	1.86	Tars	
DMR2:61419001	2	61419001	61425000	6000	2	4.20E-16	-0.46	58	0.97	Tars	
DMR2:61888001	2	61888001	61890000	2000	2	4.30E-07	-0.55	25	1.25	Npr3	Receptor
DMR2:61927001	2	61927001	61930000	3000	2	4.30E-13	0.51	44	1.47	Npr3	Receptor
DMR2:61958001	2	61958001	61963000	5000	1	2.50E-08	-0.39	59	1.18	Npr3	Receptor
DMR2:62011001	2	62011001	62016000	5000	4	3.50E-09	-0.53	61	1.22	LOC499544;Sub1	Epigenetic;Transcription
DMR2:62017001	2	62017001	62018000	1000	1	1.70E-07	0.42	11	1.1	LOC499544;Sub1	Epigenetic;Transcription
DMR2:62035001	2	62035001	62038000	3000	2	4.30E-10	-0.78	36	1.2	Sub1	Transcription
DMR2:62141001	2	62141001	62145000	4000	1	8.40E-07	0.54	29	0.72	Zfr	Metabolism
DMR2:62191001	2	62191001	62193000	2000	1	4.20E-14	0.75	21	1.05	Zfr	Metabolism
DMR2:62217001	2	62217001	62218000	1000	1	1.60E-07	0.44	10	1	Zfr	Metabolism
DMR2:62230001	2	62230001	62231000	1000	1	1.20E-08	0.35	11	1.1	Mtmr12	Signaling
DMR2:62272001	2	62272001	62274000	2000	1	3.00E-07	-0.46	11	0.55	Mtmr12	Signaling
DMR2:62288001	2	62288001	62289000	1000	1	2.60E-13	0.5	15	1.5	Mtmr12	Signaling
DMR2:62358001	2	62358001	62359000	1000	1	6.60E-10	0.48	8	0.8	Golph3	
DMR2:62413001	2	62413001	62415000	2000	1	6.30E-08	-0.45	39	1.95	Pdzd2	Cytokine
DMR2:62416001	2	62416001	62418000	2000	1	3.10E-07	-0.57	27	1.35	Pdzd2	Cytokine
DMR2:62421001	2	62421001	62422000	1000	1	1.10E-09	0.4	23	2.3	Pdzd2	Cytokine
DMR2:62435001	2	62435001	62439000	4000	1	9.60E-07	-0.44	34	0.85	Pdzd2	Cytokine
DMR2:62446001	2	62446001	62448000	2000	1	5.20E-11	0.37	42	2.1	Pdzd2	Cytokine
DMR2:62456001	2	62456001	62459000	3000	1	4.40E-13	0.56	31	1.03	Pdzd2	Cytokine
DMR2:62493001	2	62493001	62495000	2000	1	1.30E-15	0.65	23	1.15	Pdzd2	Cytokine
DMR2:62500001	2	62500001	62502000	2000	1	9.50E-07	-0.6	31	1.55	Pdzd2	Cytokine
DMR2:62506001	2	62506001	62510000	4000	1	1.30E-10	0.35	87	2.17	Pdzd2	Cytokine
DMR2:62519001	2	62519001	62523000	4000	1	3.90E-13	0.49	59	1.48	Pdzd2	Cytokine
DMR2:62524001	2	62524001	62526000	2000	1	3.00E-11	0.57	19	0.95	Pdzd2	Cytokine
DMR2:62863001	2	62863001	62868000	5000	2	3.20E-08	-0.59	55	1.1	RGD1306502	
DMR2:62894001	2	62894001	62899000	5000	3	4.10E-29	0.87	132	2.64	RGD1306502;Drosha	Translation
DMR2:62932001	2	62932001	62933000	1000	1	1.20E-11	0.56	15	1.5	Drosha	Translation
DMR2:62941001	2	62941001	62945000	4000	1	2.50E-09	0.52	55	1.38	Drosha	Translation
DMR2:62955001	2	62955001	62959000	4000	1	4.10E-07	-0.36	58	1.45	Drosha	Translation
DMR2:62973001	2	62973001	62976000	3000	2	1.10E-11	-0.47	40	1.33	Drosha	Translation
DMR2:62993001	2	62993001	62994000	1000	1	1.30E-13	-0.79	12	1.2	Drosha	Translation

DMR2:63094001	2	63094001	63095000	1000	1	7.00E-09	0.5	6	0.6	LOC103695100;Cdh6	Cytoskeleton
DMR2:63143001	2	63143001	63158000	15000	8	1.40E-29	0.91	169	1.13	Cdh6	Cytoskeleton
DMR2:63160001	2	63160001	63162000	2000	1	3.30E-14	0.66	35	1.75	Cdh6	Cytoskeleton
DMR2:63178001	2	63178001	63182000	4000	1	2.10E-07	-0.38	28	0.7	Cdh6	Cytoskeleton
DMR2:63184001	2	63184001	63185000	1000	1	4.80E-14	0.58	14	1.4	Cdh6	Cytoskeleton
DMR2:63221001	2	63221001	63224000	3000	2	2.70E-21	0.8	13	0.43	Cdh6	Cytoskeleton
DMR2:63236001	2	63236001	63240000	4000	1	7.50E-07	-0.5	44	1.1	Cdh6	Cytoskeleton
DMR2:66593001	2	66593001	66598000	5000	4	3.40E-14	-0.54	32	0.64	RGD1564125	
DMR2:66607001	2	66607001	66608000	1000	1	1.40E-07	0.37	31	3.1	RGD1564125	
DMR2:66613001	2	66613001	66615000	2000	1	5.20E-11	0.72	14	0.7	RGD1564125	
DMR2:66931001	2	66931001	66934000	3000	1	1.30E-11	-0.49	20	0.67	Cdh9	Cytoskeleton
DMR2:66946001	2	66946001	66947000	1000	1	5.40E-09	0.55	0	0	Cdh9	Cytoskeleton
DMR2:66967001	2	66967001	66968000	1000	1	2.20E-09	0.54	4	0.4	Cdh9	Cytoskeleton
DMR2:66995001	2	66995001	66997000	2000	1	1.20E-07	0.57	3	0.15	Cdh9	Cytoskeleton
DMR2:67002001	2	67002001	67003000	1000	1	2.30E-11	0.61	10	1	Cdh9	Cytoskeleton
DMR2:67007001	2	67007001	67009000	2000	1	4.70E-08	0.42	15	0.75	Cdh9	Cytoskeleton
DMR2:67021001	2	67021001	67023000	2000	1	3.20E-10	0.65	5	0.25	Cdh9	Cytoskeleton
DMR2:67027001	2	67027001	67028000	1000	1	7.30E-11	0.55	5	0.5	Cdh9	Cytoskeleton
DMR2:67034001	2	67034001	67036000	2000	1	5.40E-17	-0.45	31	1.55	Cdh9	Cytoskeleton
DMR2:67037001	2	67037001	67039000	2000	1	1.10E-15	-0.57	12	0.6	Cdh9	Cytoskeleton
DMR2:67060001	2	67060001	67061000	1000	1	5.10E-07	0.45	2	0.2	Cdh9	Cytoskeleton
DMR2:67065001	2	67065001	67066000	1000	1	7.70E-09	-0.51	7	0.7	Cdh9	Cytoskeleton
DMR2:68861001	2	68861001	68862000	1000	1	6.40E-11	0.65	12	1.2	Egf	Receptor
DMR2:68866001	2	68866001	68867000	1000	1	9.10E-08	0.56	7	0.7	Egf	Receptor
DMR2:68883001	2	68883001	68885000	2000	1	1.90E-08	0.5	38	1.9	Egf	Receptor
DMR2:68899001	2	68899001	68900000	1000	1	5.90E-11	-0.64	2	0.2	Egf	Receptor
DMR2:69412001	2	69412001	69413000	1000	1	8.60E-07	0.39	11	1.1	Cdh10	Cytoskeleton
DMR2:69470001	2	69470001	69476000	6000	4	1.30E-15	-0.47	62	1.03	Cdh10;LOC103691484	Cytoskeleton
DMR2:69478001	2	69478001	69480000	2000	1	3.90E-12	0.66	16	0.8	Cdh10	Cytoskeleton
DMR2:69550001	2	69550001	69554000	4000	2	2.30E-18	-0.45	48	1.2	Cdh10	Cytoskeleton
DMR2:69559001	2	69559001	69562000	3000	1	1.80E-08	-0.63	32	1.07	Cdh10	Cytoskeleton
DMR2:69572001	2	69572001	69581000	9000	5	3.40E-17	-0.47	91	1.01	Cdh10	Cytoskeleton
DMR2:69594001	2	69594001	69595000	1000	1	2.90E-11	0.52	7	0.7	Cdh10	Cytoskeleton
DMR2:69596001	2	69596001	69597000	1000	1	8.40E-09	0.58	2	0.2	Cdh10	Cytoskeleton
DMR2:71332001	2	71332001	71336000	4000	1	2.70E-09	-0.45	37	0.92	Cdh12	Cytoskeleton
DMR2:71358001	2	71358001	71359000	1000	1	4.50E-08	0.48	6	0.6	Cdh12	Cytoskeleton
DMR2:71366001	2	71366001	71369000	3000	2	7.50E-08	-0.42	24	0.8	Cdh12	Cytoskeleton
DMR2:71404001	2	71404001	71406000	2000	1	6.30E-07	-0.4	20	1	Cdh12	Cytoskeleton
DMR2:71436001	2	71436001	71438000	2000	1	7.50E-08	-0.73	15	0.75	Cdh12	Cytoskeleton
DMR2:71439001	2	71439001	71441000	2000	1	2.80E-09	0.44	19	0.95	Cdh12	Cytoskeleton
DMR2:71450001	2	71450001	71452000	2000	1	3.80E-08	0.59	8	0.4	Cdh12	Cytoskeleton
DMR2:71466001	2	71466001	71468000	2000	1	6.10E-12	0.61	11	0.55	Cdh12	Cytoskeleton
DMR2:71485001	2	71485001	71487000	2000	1	2.40E-11	0.6	13	0.65	Cdh12	Cytoskeleton
DMR2:71510001	2	71510001	71512000	2000	1	7.30E-10	-0.7	6	0.3	Cdh12	Cytoskeleton
DMR2:71513001	2	71513001	71514000	1000	1	6.70E-07	0.5	10	1	Cdh12	Cytoskeleton
DMR2:71520001	2	71520001	71522000	2000	1	3.30E-18	1.1	7	0.35	Cdh12	Cytoskeleton
DMR2:71555001	2	71555001	71556000	1000	1	1.90E-13	0.66	6	0.6	Cdh12	Cytoskeleton
DMR2:71557001	2	71557001	71560000	3000	1	1.30E-08	0.56	15	0.5	Cdh12	Cytoskeleton
DMR2:71626001	2	71626001	71628000	2000	1	1.10E-13	0.6	16	0.8	Cdh12	Cytoskeleton
DMR2:71662001	2	71662001	71663000	1000	1	5.60E-10	0.6	6	0.6	Cdh12;RGD1565422	Cytoskeleton
DMR2:71667001	2	71667001	71668000	1000	1	4.10E-08	0.51	11	1.1	Cdh12;RGD1565422	Cytoskeleton
DMR2:71683001	2	71683001	71685000	2000	2	6.70E-07	-0.4	20	1	Cdh12;RGD1565422	Cytoskeleton
DMR2:71710001	2	71710001	71712000	2000	1	5.80E-09	0.4	14	0.7	Cdh12;RGD1565422	Cytoskeleton
DMR2:71734001	2	71734001	71735000	1000	1	2.40E-19	0.73	5	0.5	Cdh12;RGD1565422	Cytoskeleton
DMR2:71764001	2	71764001	71765000	1000	1	2.50E-10	0.53	8	0.8	Cdh12;RGD1565422	Cytoskeleton
DMR2:71773001	2	71773001	71774000	1000	1	2.20E-10	0.62	0	0	Cdh12	Cytoskeleton
DMR2:71797001	2	71797001	71803000	6000	3	1.10E-18	-0.55	34	0.57	Cdh12	Cytoskeleton
DMR2:71840001	2	71840001	71842000	2000	2	3.70E-11	0.68	8	0.4	Cdh12	Cytoskeleton
DMR2:71850001	2	71850001	71851000	1000	1	6.50E-08	0.54	3	0.3	Cdh12	Cytoskeleton
DMR2:71892001	2	71892001	71894000	2000	1	4.30E-14	0.67	15	0.75	Cdh12	Cytoskeleton
DMR2:71968001	2	71968001	71969000	1000	1	4.40E-07	-0.37	5	0.5	Cdh12	Cytoskeleton
DMR2:71987001	2	71987001	71990000	3000	1	3.20E-08	0.67	24	0.8	Cdh12	Cytoskeleton
DMR2:72026001	2	72026001	72027000	1000	1	1.00E-11	0.8	4	0.4	Cdh12	Cytoskeleton
DMR2:72062001	2	72062001	72064000	2000	1	2.10E-07	0.48	18	0.9	Cdh12	Cytoskeleton
DMR2:72146001	2	72146001	72148000	2000	1	2.90E-08	0.44	11	0.55	Cdh12	Cytoskeleton

DMR2:72190001	2	72190001	72192000	2000	1	2.30E-08	-0.43	14	0.7	Cdh12	Cytoskeleton
DMR2:72193001	2	72193001	72197000	4000	3	1.10E-19	-0.45	57	1.43	Cdh12	Cytoskeleton
DMR2:72201001	2	72201001	72206000	5000	2	1.10E-14	-0.43	49	0.98	Cdh12	Cytoskeleton
DMR2:72212001	2	72212001	72214000	2000	1	7.70E-12	-0.53	8	0.4	Cdh12	Cytoskeleton
DMR2:72218001	2	72218001	72220000	2000	1	3.10E-07	-0.37	19	0.95	Cdh12	Cytoskeleton
DMR2:72232001	2	72232001	72233000	1000	1	7.10E-09	0.45	7	0.7	Cdh12	Cytoskeleton
DMR2:72244001	2	72244001	72245000	1000	1	2.10E-09	0.66	8	0.8	Cdh12	Cytoskeleton
DMR2:72284001	2	72284001	72286000	2000	1	4.30E-12	0.69	16	0.8	Cdh12	Cytoskeleton
DMR2:72298001	2	72298001	72301000	3000	3	3.60E-18	-0.52	30	1	Cdh12	Cytoskeleton
DMR2:72361001	2	72361001	72364000	3000	1	3.90E-09	0.53	20	0.67	Cdh12	Cytoskeleton
DMR2:72365001	2	72365001	72370000	5000	1	1.60E-08	-0.5	46	0.92	Cdh12	Cytoskeleton
DMR2:72402001	2	72402001	72407000	5000	2	2.20E-10	-0.66	29	0.58	Cdh12	Cytoskeleton
DMR2:72412001	2	72412001	72413000	1000	1	3.70E-08	-0.57	3	0.3	Cdh12	Cytoskeleton
DMR2:72426001	2	72426001	72433000	7000	4	8.60E-27	-0.56	75	1.07	Cdh12	Cytoskeleton
DMR2:72446001	2	72446001	72449000	3000	2	4.50E-15	-0.72	13	0.43	Cdh12	Cytoskeleton
DMR2:72460001	2	72460001	72462000	2000	1	1.60E-12	0.57	13	0.65	Cdh12	Cytoskeleton
DMR2:72470001	2	72470001	72476000	6000	2	2.80E-21	-0.72	37	0.62	Cdh12	Cytoskeleton
DMR2:72484001	2	72484001	72486000	2000	2	4.60E-12	0.79	7	0.35	Cdh12	Cytoskeleton
DMR2:72507001	2	72507001	72509000	2000	2	2.10E-08	0.49	13	0.65	Cdh12	Cytoskeleton
DMR2:72518001	2	72518001	72525000	7000	4	1.00E-23	-0.55	100	1.43	Cdh12	Cytoskeleton
DMR2:72528001	2	72528001	72531000	3000	1	1.80E-07	-0.31	35	1.17	Cdh12	Cytoskeleton
DMR2:72544001	2	72544001	72545000	1000	1	5.00E-07	0.54	1	0.1	Cdh12	Cytoskeleton
DMR2:72968001	2	72968001	72970000	2000	2	4.30E-14	-0.79	9	0.45	RGD1562936	
DMR2:72972001	2	72972001	72973000	1000	1	2.20E-09	0.58	5	0.5	RGD1562936	
DMR2:73678001	2	73678001	73679000	1000	1	1.90E-09	0.42	11	1.1	Cdh18	Cytoskeleton
DMR2:73701001	2	73701001	73702000	1000	1	5.50E-14	0.74	2	0.2	Cdh18	Cytoskeleton
DMR2:73704001	2	73704001	73705000	1000	1	1.20E-16	0.68	6	0.6	Cdh18	Cytoskeleton
DMR2:73715001	2	73715001	73716000	1000	1	1.80E-09	0.66	5	0.5	Cdh18	Cytoskeleton
DMR2:73764001	2	73764001	73766000	2000	1	5.90E-27	0.94	11	0.55	Cdh18	Cytoskeleton
DMR2:73781001	2	73781001	73785000	4000	1	2.10E-07	-0.59	20	0.5	Cdh18	Cytoskeleton
DMR2:73787001	2	73787001	73788000	1000	1	9.60E-07	0.54	6	0.6	Cdh18	Cytoskeleton
DMR2:73790001	2	73790001	73792000	2000	1	3.00E-08	-0.51	7	0.35	Cdh18	Cytoskeleton
DMR2:73929001	2	73929001	73931000	2000	1	6.90E-09	0.59	11	0.55	Cdh18	Cytoskeleton
DMR2:73933001	2	73933001	73935000	2000	1	6.40E-10	0.4	17	0.85	Cdh18	Cytoskeleton
DMR2:73969001	2	73969001	73970000	1000	1	2.60E-08	0.56	4	0.4	Cdh18	Cytoskeleton
DMR2:73974001	2	73974001	73977000	3000	1	1.10E-10	-0.42	22	0.73	Cdh18	Cytoskeleton
DMR2:73980001	2	73980001	73987000	7000	4	1.50E-25	-0.65	76	1.09	Cdh18	Cytoskeleton
DMR2:74002001	2	74002001	74003000	1000	1	4.90E-11	0.62	7	0.7	Cdh18	Cytoskeleton
DMR2:74007001	2	74007001	74008000	1000	1	5.50E-10	0.51	7	0.7	Cdh18	Cytoskeleton
DMR2:74013001	2	74013001	74014000	1000	1	9.20E-10	0.51	5	0.5	Cdh18	Cytoskeleton
DMR2:74059001	2	74059001	74060000	1000	1	1.00E-08	-0.56	4	0.4	Cdh18	Cytoskeleton
DMR2:74061001	2	74061001	74064000	3000	1	6.00E-10	-0.42	18	0.6	Cdh18	Cytoskeleton
DMR2:74065001	2	74065001	74067000	2000	1	1.30E-09	0.7	4	0.2	Cdh18	Cytoskeleton
DMR2:74095001	2	74095001	74099000	4000	1	1.80E-08	-0.58	25	0.62	Cdh18;LOC102554526	Cytoskeleton
DMR2:74115001	2	74115001	74116000	1000	1	7.80E-15	-0.48	10	1	Cdh18	Cytoskeleton
DMR2:74130001	2	74130001	74134000	4000	4	2.70E-15	-0.66	22	0.55	Cdh18	Cytoskeleton
DMR2:74174001	2	74174001	74176000	2000	2	2.10E-14	-0.68	8	0.4	Cdh18	Cytoskeleton
DMR2:74178001	2	74178001	74179000	1000	1	3.20E-08	0.55	11	1.1	Cdh18	Cytoskeleton
DMR2:74217001	2	74217001	74219000	2000	1	2.80E-09	0.57	11	0.55	Cdh18	Cytoskeleton
DMR2:74221001	2	74221001	74229000	8000	5	1.40E-16	-0.51	77	0.96	Cdh18	Cytoskeleton
DMR2:74250001	2	74250001	74251000	1000	1	1.40E-09	0.52	5	0.5	Cdh18	Cytoskeleton
DMR2:74272001	2	74272001	74275000	3000	1	8.90E-08	0.43	22	0.73	Cdh18	Cytoskeleton
DMR2:74314001	2	74314001	74317000	3000	2	2.80E-23	-0.48	42	1.4	Cdh18	Cytoskeleton
DMR2:74318001	2	74318001	74320000	2000	2	1.10E-13	-0.53	16	0.8	Cdh18	Cytoskeleton
DMR2:74343001	2	74343001	74349000	6000	4	1.30E-19	-0.51	68	1.13	Cdh18	Cytoskeleton
DMR2:74354001	2	74354001	74359000	5000	2	3.00E-16	-0.45	48	0.96	Cdh18	Cytoskeleton
DMR2:74413001	2	74413001	74415000	2000	1	1.60E-08	-0.39	17	0.85	Cdh18	Cytoskeleton
DMR2:74425001	2	74425001	74427000	2000	1	7.40E-07	0.41	5	0.25	Cdh18	Cytoskeleton
DMR2:74443001	2	74443001	74445000	2000	2	6.80E-10	-0.53	11	0.55	Cdh18	Cytoskeleton
DMR2:74470001	2	74470001	74472000	2000	1	2.70E-08	0.56	13	0.65	Cdh18	Cytoskeleton
DMR2:74474001	2	74474001	74475000	1000	1	1.50E-12	0.69	11	1.1	Cdh18	Cytoskeleton
DMR2:74483001	2	74483001	74486000	3000	1	9.70E-11	0.59	17	0.57	Cdh18	Cytoskeleton
DMR2:74494001	2	74494001	74497000	3000	1	2.30E-08	0.49	11	0.37	Cdh18	Cytoskeleton
DMR2:74498001	2	74498001	74500000	2000	1	9.40E-11	-0.64	25	1.25	Cdh18	Cytoskeleton
DMR2:74514001	2	74514001	74516000	2000	2	5.70E-17	-0.44	26	1.3	Cdh18	Cytoskeleton

DMR2:74549001	2	74549001	74551000	2000	2	1.50E-08	0.5	2	0.1	Cdh18	Cytoskeleton
DMR2:74562001	2	74562001	74565000	3000	2	5.50E-09	0.63	24	0.8	Cdh18	Cytoskeleton
DMR2:74570001	2	74570001	74571000	1000	1	7.90E-10	0.61	7	0.7	Cdh18	Cytoskeleton
DMR2:74660001	2	74660001	74662000	2000	1	7.50E-09	-0.39	25	1.25	Cdh18	Cytoskeleton
DMR2:76685001	2	76685001	76687000	2000	1	1.10E-07	-0.55	58	2.9	Sirpd	Receptor
DMR2:76688001	2	76688001	76689000	1000	1	6.60E-11	0.81	6	0.6	Sirpd	Receptor
DMR2:76904001	2	76904001	76907000	3000	2	1.90E-08	-0.46	19	0.63	Sirpd	Receptor
DMR2:77575001	2	77575001	77577000	2000	2	1.50E-08	0.48	23	1.15	Basp1	
DMR2:77861001	2	77861001	77865000	4000	1	7.10E-11	0.56	42	1.05	Myo10	
DMR2:77874001	2	77874001	77875000	1000	1	1.60E-09	0.52	9	0.9	Myo10	
DMR2:77885001	2	77885001	77892000	7000	1	1.50E-09	0.36	98	1.4	Myo10	
DMR2:77897001	2	77897001	77900000	3000	1	6.50E-07	-0.53	30	1	Myo10	
DMR2:77923001	2	77923001	77924000	1000	1	3.90E-07	0.42	9	0.9	Myo10	
DMR2:77929001	2	77929001	77934000	5000	1	2.40E-07	0.28	77	1.54	Myo10	
DMR2:77944001	2	77944001	77947000	3000	1	9.00E-09	-0.53	43	1.43	Myo10	
DMR2:77948001	2	77948001	77949000	1000	1	3.50E-07	-0.57	13	1.3	Myo10	
DMR2:77952001	2	77952001	77954000	2000	1	1.20E-08	-0.56	31	1.55	Myo10	
DMR2:78006001	2	78006001	78007000	1000	1	3.60E-07	-0.47	10	1	Myo10	
DMR2:78027001	2	78027001	78028000	1000	1	1.30E-08	0.37	22	2.2	Myo10	
DMR2:78065001	2	78065001	78067000	2000	1	3.00E-08	0.35	36	1.8	Myo10	
DMR2:78379001	2	78379001	78382000	3000	1	7.30E-14	-0.39	29	0.97	LOC100911470;Fam134b	
DMR2:78398001	2	78398001	78400000	2000	1	4.40E-19	0.71	31	1.55	Fam134b;Zfp622	
DMR2:78412001	2	78412001	78413000	1000	1	1.20E-24	1.11	7	0.7	Zfp622	
DMR2:78430001	2	78430001	78434000	4000	2	1.20E-16	-0.52	49	1.23	Zfp622;LOC310182	
DMR2:78811001	2	78811001	78818000	7000	3	7.90E-17	-0.55	61	0.87	11-Mar	
DMR2:78820001	2	78820001	78822000	2000	1	8.50E-15	0.84	10	0.5	11-Mar	
DMR2:78823001	2	78823001	78825000	2000	1	1.90E-21	0.87	37	1.85	11-Mar	
DMR2:78841001	2	78841001	78842000	1000	1	1.10E-08	0.66	2	0.2	11-Mar	
DMR2:78879001	2	78879001	78882000	3000	1	1.50E-08	0.54	17	0.57	11-Mar	
DMR2:78889001	2	78889001	78893000	4000	2	7.70E-11	0.66	30	0.75	11-Mar	
DMR2:78894001	2	78894001	78896000	2000	1	2.40E-11	0.58	16	0.8	11-Mar	
DMR2:78916001	2	78916001	78917000	1000	1	4.90E-10	0.74	1	0.1	11-Mar	
DMR2:78920001	2	78920001	78921000	1000	1	5.60E-08	0.44	8	0.8	11-Mar	
DMR2:78929001	2	78929001	78930000	1000	1	1.10E-11	0.71	9	0.9	11-Mar	
DMR2:78932001	2	78932001	78933000	1000	1	1.60E-11	0.64	8	0.8	11-Mar	
DMR2:79055001	2	79055001	79058000	3000	1	3.60E-12	-0.57	18	0.6	Fbxl7	
DMR2:79062001	2	79062001	79063000	1000	1	6.70E-12	0.76	3	0.3	Fbxl7	
DMR2:79127001	2	79127001	79128000	1000	1	2.50E-09	0.42	13	1.3	Fbxl7	
DMR2:79138001	2	79138001	79140000	2000	1	2.90E-15	0.71	9	0.45	Fbxl7	
DMR2:79160001	2	79160001	79164000	4000	4	1.50E-15	0.92	25	0.62	Fbxl7	
DMR2:79172001	2	79172001	79173000	1000	1	1.10E-13	0.78	6	0.6	Fbxl7	
DMR2:79197001	2	79197001	79198000	1000	1	8.80E-08	0.61	8	0.8	Fbxl7	
DMR2:79199001	2	79199001	79200000	1000	1	1.40E-12	0.75	1	0.1	Fbxl7	
DMR2:79201001	2	79201001	79207000	6000	3	7.70E-14	-0.38	57	0.95	Fbxl7	
DMR2:79211001	2	79211001	79213000	2000	1	1.90E-13	0.82	11	0.55	Fbxl7	
DMR2:79223001	2	79223001	79226000	3000	2	5.10E-28	1.09	35	1.17	Fbxl7	
DMR2:79235001	2	79235001	79236000	1000	1	2.40E-10	0.62	7	0.7	Fbxl7	
DMR2:79241001	2	79241001	79243000	2000	1	7.00E-07	0.53	11	0.55	Fbxl7	
DMR2:79244001	2	79244001	79246000	2000	1	2.40E-08	-0.53	16	0.8	Fbxl7	
DMR2:79257001	2	79257001	79258000	1000	1	1.90E-14	0.79	5	0.5	Fbxl7	
DMR2:79278001	2	79278001	79279000	1000	1	1.30E-10	0.67	3	0.3	Fbxl7	
DMR2:79281001	2	79281001	79284000	3000	2	2.90E-22	1.06	19	0.63	Fbxl7	
DMR2:79289001	2	79289001	79291000	2000	1	7.50E-08	0.43	19	0.95	Fbxl7	
DMR2:79310001	2	79310001	79312000	2000	2	1.20E-14	0.92	14	0.7	Fbxl7	
DMR2:79318001	2	79318001	79319000	1000	1	9.50E-07	-0.45	7	0.7	Fbxl7	
DMR2:79321001	2	79321001	79323000	2000	2	5.70E-08	-0.53	23	1.15	Fbxl7	
DMR2:79324001	2	79324001	79325000	1000	1	3.40E-10	0.53	8	0.8	Fbxl7	
DMR2:79347001	2	79347001	79349000	2000	1	7.10E-11	0.75	11	0.55	Fbxl7	
DMR2:79367001	2	79367001	79369000	2000	1	8.30E-07	0.37	30	1.5	Fbxl7	
DMR2:79375001	2	79375001	79377000	2000	1	1.30E-07	0.46	18	0.9	Fbxl7	
DMR2:79382001	2	79382001	79388000	6000	4	1.10E-13	-0.49	60	1	Fbxl7	
DMR2:79406001	2	79406001	79407000	1000	1	7.30E-07	-0.48	11	1.1	Fbxl7	
DMR2:79409001	2	79409001	79410000	1000	1	8.70E-08	0.48	3	0.3	Fbxl7	
DMR2:79446001	2	79446001	79447000	1000	1	8.20E-10	0.56	8	0.8	Fbxl7	
DMR2:80137001	2	80137001	80138000	1000	1	2.90E-11	0.53	12	1.2	Ankh	
DMR2:80156001	2	80156001	80158000	2000	1	2.10E-13	0.47	31	1.55	Ankh	

DMR2:80164001	2	80164001	80166000	2000	2	7.90E-09	0.56	23	1.15	Ankh	
DMR2:80175001	2	80175001	80176000	1000	1	6.90E-07	-0.44	14	1.4	Ankh	
DMR2:80181001	2	80181001	80184000	3000	2	4.80E-15	0.56	53	1.77	Ankh	
DMR2:80185001	2	80185001	80189000	4000	1	8.70E-07	-0.39	58	1.45	Ankh	
DMR2:80194001	2	80194001	80198000	4000	1	9.30E-09	-0.69	55	1.38	Ankh	
DMR2:80200001	2	80200001	80204000	4000	1	9.80E-07	0.33	61	1.52	Ankh	
DMR2:80205001	2	80205001	80212000	7000	1	1.30E-07	0.35	110	1.57	Ankh	
DMR2:80213001	2	80213001	80218000	5000	1	1.20E-11	0.56	68	1.36	Ankh	
DMR2:80271001	2	80271001	80274000	3000	1	1.60E-19	0.79	28	0.93	Otulin	
DMR2:80402001	2	80402001	80405000	3000	1	2.40E-15	0.59	48	1.6	Fam105a	
DMR2:80410001	2	80410001	80414000	4000	2	4.50E-12	0.59	37	0.92	Fam105a	
DMR2:80477001	2	80477001	80479000	2000	1	1.40E-12	0.43	30	1.5	Trio	Transcription
DMR2:80492001	2	80492001	80493000	1000	1	5.60E-09	0.47	17	1.7	Trio	Transcription
DMR2:80494001	2	80494001	80498000	4000	1	3.80E-07	-0.47	59	1.48	Trio	Transcription
DMR2:80500001	2	80500001	80503000	3000	1	8.70E-20	0.68	62	2.07	Trio	Transcription
DMR2:80525001	2	80525001	80529000	4000	1	4.00E-07	0.62	56	1.4	Trio	Transcription
DMR2:80530001	2	80530001	80531000	1000	1	8.90E-07	0.38	13	1.3	Trio	Transcription
DMR2:80537001	2	80537001	80541000	4000	1	1.70E-09	-0.66	48	1.2	Trio	Transcription
DMR2:80558001	2	80558001	80560000	2000	1	1.50E-14	0.51	38	1.9	Trio	Transcription
DMR2:80568001	2	80568001	80572000	4000	1	9.80E-07	-0.38	66	1.65	Trio	Transcription
DMR2:80578001	2	80578001	80579000	1000	1	9.30E-08	0.42	11	1.1	Trio	Transcription
DMR2:80581001	2	80581001	80587000	6000	1	6.90E-07	-0.63	110	1.83	Trio	Transcription
DMR2:80613001	2	80613001	80618000	5000	2	2.50E-12	-0.62	80	1.6	Trio	Transcription
DMR2:80619001	2	80619001	80624000	5000	1	2.40E-12	0.49	94	1.88	Trio	Transcription
DMR2:80635001	2	80635001	80638000	3000	1	3.30E-08	-0.5	48	1.6	Trio	Transcription
DMR2:80643001	2	80643001	80646000	3000	1	4.40E-14	0.74	44	1.47	Trio	Transcription
DMR2:80664001	2	80664001	80673000	9000	3	6.30E-18	0.98	145	1.61	Trio	Transcription
DMR2:80678001	2	80678001	80681000	3000	1	4.60E-07	0.34	42	1.4	Trio	Transcription
DMR2:80720001	2	80720001	80722000	2000	1	5.80E-07	-0.46	27	1.35	Trio;LOC103691490	Transcription
DMR2:80750001	2	80750001	80756000	6000	1	7.80E-15	0.65	86	1.43	Trio	Transcription
DMR2:80757001	2	80757001	80759000	2000	1	4.50E-17	0.63	17	0.85	Trio	Transcription
DMR2:80944001	2	80944001	80946000	2000	1	1.40E-08	-0.69	13	0.65	Dnah5	Cytoskeleton
DMR2:80948001	2	80948001	80951000	3000	2	2.00E-09	-0.64	38	1.27	Dnah5	Cytoskeleton
DMR2:80957001	2	80957001	80958000	1000	1	2.40E-07	-0.59	13	1.3	Dnah5	Cytoskeleton
DMR2:80972001	2	80972001	80975000	3000	1	9.10E-15	0.55	38	1.27	Dnah5	Cytoskeleton
DMR2:80982001	2	80982001	80983000	1000	1	1.00E-14	0.8	7	0.7	Dnah5	Cytoskeleton
DMR2:80987001	2	80987001	80990000	3000	1	4.80E-10	0.48	48	1.6	Dnah5	Cytoskeleton
DMR2:81033001	2	81033001	81035000	2000	2	3.00E-10	-0.47	25	1.25	Dnah5	Cytoskeleton
DMR2:81038001	2	81038001	81044000	6000	3	1.10E-17	-0.5	64	1.07	Dnah5	Cytoskeleton
DMR2:81066001	2	81066001	81067000	1000	1	8.40E-15	0.94	6	0.6	Dnah5	Cytoskeleton
DMR2:81072001	2	81072001	81073000	1000	1	1.70E-07	0.61	8	0.8	Dnah5	Cytoskeleton
DMR2:81157001	2	81157001	81158000	1000	1	5.10E-13	0.77	6	0.6	Dnah5	Cytoskeleton
DMR2:81166001	2	81166001	81167000	1000	1	5.40E-08	-0.38	8	0.8	Dnah5	Cytoskeleton
DMR2:81172001	2	81172001	81173000	1000	1	2.50E-07	-0.59	6	0.6	Dnah5	Cytoskeleton
DMR2:81181001	2	81181001	81182000	1000	1	1.50E-17	1.06	6	0.6	Dnah5	Cytoskeleton
DMR2:81204001	2	81204001	81205000	1000	1	2.70E-10	0.68	7	0.7	Dnah5	Cytoskeleton
DMR2:81232001	2	81232001	81233000	1000	1	7.80E-09	0.76	1	0.1	Dnah5	Cytoskeleton
DMR2:81285001	2	81285001	81291000	6000	3	6.90E-29	-0.63	68	1.13	Dnah5	Cytoskeleton
DMR2:81292001	2	81292001	81300000	8000	2	1.40E-09	-0.46	65	0.81	Dnah5	Cytoskeleton
DMR2:81310001	2	81310001	81311000	1000	1	3.30E-10	-0.69	15	1.5	Dnah5;LOC108349969	Cytoskeleton
DMR2:81320001	2	81320001	81324000	4000	1	1.00E-07	0.48	36	0.9	Dnah5;LOC108349969	Cytoskeleton
DMR2:81328001	2	81328001	81329000	1000	1	5.40E-13	0.7	6	0.6	Dnah5;LOC108349969	Cytoskeleton
DMR2:81339001	2	81339001	81343000	4000	2	4.00E-12	-0.49	27	0.68	Dnah5	Cytoskeleton
DMR2:83216001	2	83216001	83218000	2000	1	4.90E-14	-0.64	6	0.3	Ctnnd2	Cytoskeleton
DMR2:83235001	2	83235001	83239000	4000	1	2.00E-09	-0.59	58	1.45	Ctnnd2	Cytoskeleton
DMR2:83244001	2	83244001	83246000	2000	1	1.40E-08	0.63	16	0.8	Ctnnd2	Cytoskeleton
DMR2:83248001	2	83248001	83253000	5000	1	3.80E-22	0.84	32	0.64	Ctnnd2	Cytoskeleton
DMR2:83260001	2	83260001	83262000	2000	1	5.20E-07	0.39	20	1	Ctnnd2	Cytoskeleton
DMR2:83267001	2	83267001	83268000	1000	1	9.50E-07	0.4	4	0.4	Ctnnd2	Cytoskeleton
DMR2:83309001	2	83309001	83310000	1000	1	7.80E-26	0.95	15	1.5	Ctnnd2	Cytoskeleton
DMR2:83312001	2	83312001	83314000	2000	2	2.90E-17	1	11	0.55	Ctnnd2	Cytoskeleton
DMR2:83328001	2	83328001	83331000	3000	1	6.30E-09	-0.51	28	0.93	Ctnnd2	Cytoskeleton
DMR2:83347001	2	83347001	83350000	3000	1	1.50E-08	-0.59	22	0.73	Ctnnd2	Cytoskeleton
DMR2:83361001	2	83361001	83365000	4000	2	1.10E-08	0.42	50	1.25	Ctnnd2	Cytoskeleton
DMR2:83371001	2	83371001	83375000	4000	1	1.50E-12	0.62	43	1.07	Ctnnd2	Cytoskeleton

DMR2:83376001	2	83376001	83378000	2000	1	3.90E-07	-0.56	28	1.4	Ctnnd2	Cytoskeleton
DMR2:83388001	2	83388001	83389000	1000	1	2.10E-08	0.39	13	1.3	Ctnnd2	Cytoskeleton
DMR2:83396001	2	83396001	83398000	2000	1	8.20E-07	0.4	17	0.85	Ctnnd2	Cytoskeleton
DMR2:83399001	2	83399001	83400000	1000	1	4.50E-08	-0.59	11	1.1	Ctnnd2	Cytoskeleton
DMR2:83403001	2	83403001	83404000	1000	1	1.40E-07	-0.49	18	1.8	Ctnnd2	Cytoskeleton
DMR2:83440001	2	83440001	83444000	4000	1	6.10E-19	1.01	30	0.75	Ctnnd2	Cytoskeleton
DMR2:83450001	2	83450001	83452000	2000	1	3.90E-08	-0.66	12	0.6	Ctnnd2	Cytoskeleton
DMR2:83459001	2	83459001	83461000	2000	1	4.30E-09	-0.43	21	1.05	Ctnnd2	Cytoskeleton
DMR2:83462001	2	83462001	83465000	3000	3	1.10E-22	-0.53	33	1.1	Ctnnd2	Cytoskeleton
DMR2:83470001	2	83470001	83471000	1000	1	2.70E-08	0.57	4	0.4	Ctnnd2	Cytoskeleton
DMR2:83489001	2	83489001	83491000	2000	1	5.40E-07	-0.48	14	0.7	Ctnnd2	Cytoskeleton
DMR2:83509001	2	83509001	83512000	3000	2	3.20E-14	-0.7	13	0.43	Ctnnd2	Cytoskeleton
DMR2:83590001	2	83590001	83592000	2000	2	6.90E-12	0.69	15	0.75	Ctnnd2	Cytoskeleton
DMR2:83593001	2	83593001	83594000	1000	1	3.80E-12	0.7	6	0.6	Ctnnd2	Cytoskeleton
DMR2:83619001	2	83619001	83621000	2000	1	2.10E-09	0.34	24	1.2	Ctnnd2;LOC102553378	Cytoskeleton
DMR2:83639001	2	83639001	83644000	5000	2	9.40E-08	0.52	50	1	Ctnnd2	Cytoskeleton
DMR2:83702001	2	83702001	83704000	2000	1	2.10E-07	0.34	22	1.1	Ctnnd2	Cytoskeleton
DMR2:83750001	2	83750001	83752000	2000	1	3.20E-07	-0.39	33	1.65	Ctnnd2	Cytoskeleton
DMR2:83754001	2	83754001	83758000	4000	1	7.70E-08	-0.6	63	1.57	Ctnnd2	Cytoskeleton
DMR2:83762001	2	83762001	83766000	4000	1	3.40E-11	0.75	26	0.65	Ctnnd2	Cytoskeleton
DMR2:83768001	2	83768001	83770000	2000	1	9.00E-12	0.48	15	0.75	Ctnnd2	Cytoskeleton
DMR2:83771001	2	83771001	83774000	3000	1	1.20E-16	0.78	31	1.03	Ctnnd2	Cytoskeleton
DMR2:83800001	2	83800001	83801000	1000	1	1.60E-17	0.93	3	0.3	Ctnnd2	Cytoskeleton
DMR2:83821001	2	83821001	83826000	5000	1	7.90E-17	0.83	44	0.88	Ctnnd2	Cytoskeleton
DMR2:83836001	2	83836001	83840000	4000	1	5.50E-07	-0.47	51	1.27	Ctnnd2	Cytoskeleton
DMR2:83864001	2	83864001	83867000	3000	2	1.60E-15	0.69	27	0.9	Ctnnd2	Cytoskeleton
DMR2:83868001	2	83868001	83869000	1000	1	8.30E-10	0.44	14	1.4	Ctnnd2	Cytoskeleton
DMR2:83890001	2	83890001	83894000	4000	1	4.10E-08	0.45	30	0.75	Ctnnd2	Cytoskeleton
DMR2:83910001	2	83910001	83914000	4000	1	1.20E-13	-0.5	39	0.98	Ctnnd2	Cytoskeleton
DMR2:83930001	2	83930001	83932000	2000	1	2.20E-07	0.49	18	0.9	Ctnnd2	Cytoskeleton
DMR2:83964001	2	83964001	83966000	2000	1	6.30E-07	0.4	21	1.05	Ctnnd2	Cytoskeleton
DMR2:83971001	2	83971001	83972000	1000	1	3.10E-08	0.44	12	1.2	Ctnnd2	Cytoskeleton
DMR2:84000001	2	84000001	84003000	3000	1	2.50E-07	-0.45	47	1.57	Ctnnd2	Cytoskeleton
DMR2:84012001	2	84012001	84013000	1000	1	1.40E-08	0.37	11	1.1	Ctnnd2	Cytoskeleton
DMR2:84034001	2	84034001	84037000	3000	1	2.50E-13	0.6	20	0.67	Ctnnd2	Cytoskeleton
DMR2:84057001	2	84057001	84059000	2000	2	6.40E-09	-0.54	30	1.5	Ctnnd2	Cytoskeleton
DMR2:84080001	2	84080001	84084000	4000	1	1.20E-20	0.76	70	1.75	Ctnnd2	Cytoskeleton
DMR2:84089001	2	84089001	84091000	2000	1	1.90E-09	0.52	20	1	Ctnnd2	Cytoskeleton
DMR2:84092001	2	84092001	84095000	3000	1	1.80E-08	0.37	51	1.7	Ctnnd2	Cytoskeleton
DMR2:84104001	2	84104001	84108000	4000	1	2.90E-19	0.79	43	1.07	Ctnnd2	Cytoskeleton
DMR2:84261001	2	84261001	84266000	5000	1	5.70E-15	0.56	55	1.1	Dap	
DMR2:84268001	2	84268001	84269000	1000	1	1.10E-08	0.66	5	0.5	Dap	
DMR2:84275001	2	84275001	84276000	1000	1	4.60E-09	0.61	41	4.1	Dap	
DMR2:84295001	2	84295001	84299000	4000	2	6.10E-20	-0.54	62	1.55	Dap	
DMR2:84330001	2	84330001	84331000	1000	1	5.30E-08	-0.59	10	1	Dap	
DMR2:84335001	2	84335001	84336000	1000	1	3.40E-11	0.37	38	3.8	Dap	
DMR2:84351001	2	84351001	84356000	5000	1	5.10E-07	-0.46	61	1.22	Ankrd33b	
DMR2:84380001	2	84380001	84381000	1000	1	1.30E-16	0.58	9	0.9	Ankrd33b	
DMR2:84382001	2	84382001	84384000	2000	1	3.10E-10	0.38	12	0.6	Ankrd33b	
DMR2:84387001	2	84387001	84391000	4000	1	2.90E-19	0.62	44	1.1	Ankrd33b	
DMR2:84398001	2	84398001	84402000	4000	1	1.40E-13	0.45	64	1.6	Ankrd33b	
DMR2:84406001	2	84406001	84408000	2000	1	9.40E-08	-0.54	29	1.45	Ankrd33b	
DMR2:84410001	2	84410001	84413000	3000	1	7.80E-07	0.37	32	1.07	Ankrd33b	
DMR2:84417001	2	84417001	84426000	9000	1	8.90E-11	0.36	116	1.29	Ankrd33b	
DMR2:84549001	2	84549001	84550000	1000	1	3.60E-09	0.54	3	0.3	6-Mar	Proteolysis
DMR2:84553001	2	84553001	84557000	4000	3	3.90E-32	-0.61	59	1.48	6-Mar	Proteolysis
DMR2:84560001	2	84560001	84562000	2000	1	2.30E-11	0.45	14	0.7	6-Mar	Proteolysis
DMR2:84581001	2	84581001	84582000	1000	1	6.80E-08	0.46	12	1.2	6-Mar	Proteolysis
DMR2:84606001	2	84606001	84607000	1000	1	5.50E-07	-0.53	12	1.2	6-Mar	Proteolysis
DMR2:84642001	2	84642001	84646000	4000	2	4.40E-12	0.77	41	1.02	Cmbl	
DMR2:84653001	2	84653001	84658000	5000	1	6.00E-10	-0.48	47	0.94	Cmbl;Cct5	Translation
DMR2:84680001	2	84680001	84683000	3000	1	1.20E-07	-0.51	23	0.77	Cct5;Fam173b	Translation
DMR2:85309001	2	85309001	85310000	1000	1	4.20E-07	0.48	2	0.2	Tas2r119;LOC100362858	Receptor
DMR2:85368001	2	85368001	85369000	1000	1	3.90E-27	0.84	4	0.4	LOC100909539;Sema5a	Signaling
DMR2:85381001	2	85381001	85385000	4000	1	3.50E-09	0.45	42	1.05	LOC100909539;Sema5a	Signaling

DMR2:85404001	2	85404001	85406000	2000	1	1.40E-14	0.71	8	0.4	Sema5a	Signaling
DMR2:85432001	2	85432001	85433000	1000	1	3.40E-10	0.69	9	0.9	Sema5a	Signaling
DMR2:85434001	2	85434001	85435000	1000	1	1.10E-11	-0.73	9	0.9	Sema5a	Signaling
DMR2:85462001	2	85462001	85463000	1000	1	1.10E-08	-0.64	14	1.4	Sema5a	Signaling
DMR2:85475001	2	85475001	85478000	3000	2	7.80E-10	0.45	29	0.97	Sema5a	Signaling
DMR2:85482001	2	85482001	85484000	2000	1	3.70E-14	0.58	29	1.45	Sema5a	Signaling
DMR2:85523001	2	85523001	85526000	3000	2	3.10E-09	0.5	29	0.97	Sema5a	Signaling
DMR2:85533001	2	85533001	85535000	2000	1	8.70E-09	-0.65	13	0.65	Sema5a	Signaling
DMR2:85545001	2	85545001	85548000	3000	1	6.60E-08	0.42	29	0.97	Sema5a	Signaling
DMR2:85550001	2	85550001	85552000	2000	1	1.90E-08	-0.76	19	0.95	Sema5a	Signaling
DMR2:85607001	2	85607001	85609000	2000	1	1.70E-07	0.47	11	0.55	Sema5a	Signaling
DMR2:85688001	2	85688001	85689000	1000	1	1.00E-09	0.67	5	0.5	Sema5a	Signaling
DMR2:85710001	2	85710001	85711000	1000	1	8.50E-12	0.74	9	0.9	Sema5a	Signaling
DMR2:85718001	2	85718001	85720000	2000	1	1.80E-14	0.66	17	0.85	Sema5a	Signaling
DMR2:85740001	2	85740001	85741000	1000	1	2.30E-11	0.7	4	0.4	Sema5a	Signaling
DMR2:85755001	2	85755001	85756000	1000	1	2.80E-10	0.62	1	0.1	Sema5a	Signaling
DMR2:85783001	2	85783001	85784000	1000	1	1.40E-15	0.87	5	0.5	Sema5a	Signaling
DMR2:85801001	2	85801001	85802000	1000	1	1.20E-11	0.59	11	1.1	Sema5a	Signaling
DMR2:86964001	2	86964001	86968000	4000	1	3.00E-12	0.62	26	0.65	LOC108349973;Zfp458	Transcription
DMR2:86970001	2	86970001	86971000	1000	1	1.30E-08	0.75	9	0.9	LOC108349973;Zfp458	Transcription
DMR2:87012001	2	87012001	87014000	2000	2	6.80E-07	0.42	12	0.6	Zfp455;Rsl1	
DMR2:87019001	2	87019001	87023000	4000	1	6.90E-08	-0.38	35	0.88	Zfp455;Rsl1	
DMR2:87540001	2	87540001	87543000	3000	1	5.80E-09	-0.34	30	1	Sirpb2l1	
DMR2:87629001	2	87629001	87630000	1000	1	1.70E-07	0.48	2	0.2	Sirpb2l1	
DMR2:88103001	2	88103001	88104000	1000	1	1.30E-08	0.38	7	0.7	Car2	
DMR2:88118001	2	88118001	88120000	2000	1	5.00E-07	0.4	15	0.75	Car2;Car3	
DMR2:88138001	2	88138001	88140000	2000	1	8.20E-17	0.94	8	0.4	Car3	
DMR2:88272001	2	88272001	88273000	1000	1	9.30E-13	0.42	31	3.1	Car13	
DMR2:88290001	2	88290001	88291000	1000	1	1.20E-12	0.52	7	0.7	Car13	
DMR2:88329001	2	88329001	88338000	9000	5	4.40E-11	0.54	50	0.56	LOC100361118;RGD1565641	
DMR2:88347001	2	88347001	88348000	1000	1	2.30E-07	0.44	8	0.8	RGD1565641;E2f5	Transcription
DMR2:88369001	2	88369001	88372000	3000	2	3.00E-10	-0.43	28	0.93	E2f5	Transcription
DMR2:88373001	2	88373001	88378000	5000	3	4.00E-13	-0.5	38	0.76	E2f5;Lrrcc1	Transcription;Cytoskeleton
DMR2:88392001	2	88392001	88397000	5000	1	9.10E-17	0.7	30	0.6	Lrrcc1	Cytoskeleton
DMR2:88563001	2	88563001	88565000	2000	1	6.50E-07	-0.42	7	0.35	LOC361914;Slc7a12	Transport
DMR2:88611001	2	88611001	88615000	4000	1	1.80E-07	-0.33	39	0.98	Slc7a12	Transport
DMR2:88655001	2	88655001	88657000	2000	1	4.00E-08	-0.46	19	0.95	Slc7a12	Transport
DMR2:88658001	2	88658001	88659000	1000	1	8.50E-15	0.67	2	0.2	Slc7a12;LOC100910566	Transport
DMR2:89029001	2	89029001	89032000	3000	1	6.10E-07	0.45	20	0.67	LOC103691502;Ralyl	
DMR2:89043001	2	89043001	89045000	2000	1	3.10E-07	0.51	18	0.9	LOC103691502;Ralyl	
DMR2:89059001	2	89059001	89062000	3000	2	1.10E-11	-0.57	11	0.37	Ralyl	
DMR2:89070001	2	89070001	89072000	2000	1	7.30E-08	-0.43	5	0.25	Ralyl	
DMR2:89090001	2	89090001	89093000	3000	2	3.10E-10	-0.36	31	1.03	Ralyl	
DMR2:89108001	2	89108001	89109000	1000	1	1.40E-07	0.45	5	0.5	Ralyl	
DMR2:89112001	2	89112001	89114000	2000	1	6.00E-08	0.46	14	0.7	Ralyl	
DMR2:89120001	2	89120001	89122000	2000	1	4.60E-09	0.6	9	0.45	Ralyl	
DMR2:89131001	2	89131001	89138000	7000	5	2.20E-20	-0.52	101	1.44	Ralyl	
DMR2:89191001	2	89191001	89193000	2000	2	8.40E-15	0.94	15	0.75	Ralyl	
DMR2:89207001	2	89207001	89210000	3000	2	8.10E-13	0.67	16	0.53	Ralyl	
DMR2:89228001	2	89228001	89235000	7000	5	4.40E-24	-0.51	73	1.04	Ralyl	
DMR2:89247001	2	89247001	89249000	2000	2	6.20E-09	0.65	11	0.55	Ralyl	
DMR2:89254001	2	89254001	89260000	6000	4	4.00E-16	-0.51	66	1.1	Ralyl	
DMR2:89262001	2	89262001	89264000	2000	1	8.20E-07	-0.48	14	0.7	Ralyl	
DMR2:89320001	2	89320001	89321000	1000	1	1.00E-10	0.7	5	0.5	Ralyl	
DMR2:89331001	2	89331001	89335000	4000	2	1.30E-15	-0.79	9	0.22	Ralyl	
DMR2:89338001	2	89338001	89339000	1000	1	2.90E-07	0.47	7	0.7	Ralyl;LOC108349976	
DMR2:89340001	2	89340001	89349000	9000	5	3.60E-18	0.77	71	0.79	Ralyl;LOC108349976	
DMR2:89354001	2	89354001	89355000	1000	1	1.20E-11	-0.6	5	0.5	Ralyl;LOC108349976	
DMR2:89360001	2	89360001	89362000	2000	2	5.00E-27	0.85	15	0.75	Ralyl	
DMR2:89368001	2	89368001	89370000	2000	1	1.50E-13	0.73	13	0.65	Ralyl	
DMR2:89382001	2	89382001	89383000	1000	1	2.70E-12	0.62	9	0.9	Ralyl	
DMR2:89396001	2	89396001	89397000	1000	1	4.20E-08	-0.46	1	0.1	Ralyl	
DMR2:89408001	2	89408001	89410000	2000	1	5.40E-07	-0.57	14	0.7	Ralyl	
DMR2:89428001	2	89428001	89430000	2000	1	2.40E-08	-0.42	20	1	Ralyl	

DMR2:89458001	2	89458001	89460000	2000	1	8.40E-09	0.51	27	1.35	Ralyl	
DMR2:89475001	2	89475001	89476000	1000	1	1.90E-07	0.54	6	0.6	Ralyl	
DMR2:89495001	2	89495001	89496000	1000	1	2.60E-14	0.79	2	0.2	Ralyl	
DMR2:89503001	2	89503001	89504000	1000	1	3.40E-15	0.75	7	0.7	Ralyl	
DMR2:89531001	2	89531001	89535000	4000	2	2.30E-13	-0.52	24	0.6	Ralyl	
DMR2:89542001	2	89542001	89543000	1000	1	6.50E-20	0.89	2	0.2	Ralyl	
DMR2:89571001	2	89571001	89573000	2000	1	2.80E-07	0.37	7	0.35	Ralyl	
DMR2:89586001	2	89586001	89594000	8000	5	1.70E-24	-0.5	73	0.91	Ralyl	
DMR2:89603001	2	89603001	89611000	8000	4	9.70E-23	0.67	78	0.98	Ralyl	
DMR2:89633001	2	89633001	89634000	1000	1	8.70E-16	0.78	6	0.6	Ralyl	
DMR2:89639001	2	89639001	89642000	3000	2	8.50E-18	0.74	17	0.57	Ralyl	
DMR2:89646001	2	89646001	89647000	1000	1	6.20E-10	0.67	7	0.7	Ralyl	
DMR2:89714001	2	89714001	89715000	1000	1	6.40E-10	0.61	4	0.4	Ralyl	
DMR2:89733001	2	89733001	89735000	2000	1	5.20E-07	0.43	16	0.8	Ralyl	
DMR2:89737001	2	89737001	89738000	1000	1	1.30E-18	0.91	11	1.1	Ralyl	
DMR2:89754001	2	89754001	89756000	2000	2	3.70E-11	0.63	10	0.5	Ralyl	
DMR2:89773001	2	89773001	89775000	2000	2	2.90E-10	-0.61	9	0.45	Ralyl	
DMR2:91486001	2	91486001	91487000	1000	1	3.30E-12	0.42	10	1	Pcsk1;LOC102548697	Protease
DMR2:92542001	2	92542001	92543000	1000	1	2.50E-11	0.39	4	0.4	tGap1	Signaling
DMR2:92544001	2	92544001	92545000	1000	1	9.40E-09	0.44	6	0.6	tGap1	Signaling
DMR2:92552001	2	92552001	92553000	1000	1	2.70E-17	0.89	5	0.5	tGap1;LOC102553273;LOC100363165	Signaling
DMR2:92570001	2	92570001	92571000	1000	1	3.70E-12	0.61	8	0.8	tGap1	Signaling
DMR2:92600001	2	92600001	92601000	1000	1	2.10E-08	0.52	7	0.7	tGap1	Signaling
DMR2:92635001	2	92635001	92637000	2000	1	2.30E-10	-0.68	8	0.4	tGap1	Signaling
DMR2:93543001	2	93543001	93544000	1000	1	9.70E-08	-0.59	1	0.1	Snx16	
DMR2:93568001	2	93568001	93569000	1000	1	4.60E-07	-0.53	15	1.5	Chmp4c	Transport
DMR2:93578001	2	93578001	93579000	1000	1	1.80E-07	-0.39	13	1.3	Chmp4c	Transport
DMR2:93604001	2	93604001	93606000	2000	1	2.10E-09	-0.41	17	0.85	Chmp4c	Transport
DMR2:93610001	2	93610001	93618000	8000	4	1.10E-15	-0.52	81	1.01	Chmp4c	Transport
DMR2:93644001	2	93644001	93646000	2000	1	1.80E-11	0.47	22	1.1	Chmp4c	Transport
DMR2:93648001	2	93648001	93649000	1000	1	4.50E-12	0.47	14	1.4	Chmp4c;Zfand1	Transport;Metabolism
DMR2:93663001	2	93663001	93666000	3000	1	8.20E-11	0.39	46	1.53	Zfand1;Slc10a5;Impa1	Metabolism;Transport;Signaling
DMR2:93687001	2	93687001	93692000	5000	1	4.40E-09	0.43	76	1.52	Impa1	Signaling
DMR2:93706001	2	93706001	93710000	4000	2	1.50E-08	-0.42	25	0.62	Impa1;Fabp12	Signaling
DMR2:93713001	2	93713001	93718000	5000	1	5.20E-07	-0.55	69	1.38	Fabp12	
DMR2:93719001	2	93719001	93720000	1000	1	1.60E-07	-0.63	6	0.6	Fabp12	
DMR2:93721001	2	93721001	93728000	7000	3	3.00E-13	-0.68	96	1.37	Fabp12	
DMR2:93734001	2	93734001	93736000	2000	1	3.00E-17	0.75	11	0.55	Fabp12	
DMR2:93748001	2	93748001	93754000	6000	1	3.60E-13	0.83	58	0.97	Fabp12;LOC108349977	
DMR2:93756001	2	93756001	93757000	1000	1	1.70E-13	0.46	17	1.7	Fabp12;LOC108349977	
DMR2:93762001	2	93762001	93767000	5000	1	1.70E-14	0.54	68	1.36	Fabp12;LOC108349977	
DMR2:93802001	2	93802001	93803000	1000	1	1.30E-17	0.72	8	0.8	Fabp4;Fabp9	
DMR2:93812001	2	93812001	93814000	2000	1	3.40E-09	-0.52	7	0.35	Fabp9	
DMR2:93968001	2	93968001	93973000	5000	2	4.90E-10	0.53	49	0.98	Fabp5	
DMR2:93980001	2	93980001	93984000	4000	1	8.80E-07	-0.54	66	1.65	Fabp5	
DMR2:94274001	2	94274001	94277000	3000	1	8.90E-07	0.33	37	1.23	Pag1	
DMR2:94287001	2	94287001	94288000	1000	1	6.20E-16	0.61	12	1.2	Pag1	
DMR2:94367001	2	94367001	94369000	2000	1	2.10E-09	0.49	22	1.1	LOC102556687;Zfp704	Transcription
DMR2:94370001	2	94370001	94375000	5000	1	1.20E-07	0.3	62	1.24	LOC102556687;Zfp704	Transcription
DMR2:94381001	2	94381001	94389000	8000	2	3.60E-22	-0.58	107	1.34	LOC102556687;Zfp704	Transcription
DMR2:94395001	2	94395001	94397000	2000	1	4.20E-22	0.69	38	1.9	Zfp704	Transcription
DMR2:94402001	2	94402001	94403000	1000	1	1.20E-11	-0.59	4	0.4	Zfp704	Transcription
DMR2:94411001	2	94411001	94412000	1000	1	1.30E-08	0.35	17	1.7	Zfp704	Transcription
DMR2:94420001	2	94420001	94422000	2000	1	2.30E-09	0.39	37	1.85	Zfp704	Transcription
DMR2:94461001	2	94461001	94462000	1000	1	5.80E-08	0.39	12	1.2	Zfp704;LOC103691512	Transcription
DMR2:94468001	2	94468001	94473000	5000	1	8.00E-09	-0.54	76	1.52	Zfp704;LOC103691512	Transcription
DMR2:94525001	2	94525001	94526000	1000	1	7.20E-09	-0.57	8	0.8	Zfp704	Transcription
DMR2:94534001	2	94534001	94541000	7000	1	1.10E-07	0.31	102	1.46	Zfp704	Transcription
DMR2:94546001	2	94546001	94548000	2000	1	9.60E-17	0.54	40	2	Zfp704	Transcription
DMR2:94693001	2	94693001	94696000	3000	1	3.40E-08	-0.58	36	1.2	Zbtb10	Cytoskeleton
DMR2:94703001	2	94703001	94704000	1000	1	1.40E-10	0.4	13	1.3	Zbtb10	Cytoskeleton
DMR2:94956001	2	94956001	94959000	3000	1	1.60E-07	0.47	32	1.07	Tpd52	

DMR2:94963001	2	94963001	94968000	5000	2	5.10E-23	0.86	54	1.08	Tpd52	
DMR2:94975001	2	94975001	94977000	2000	2	1.80E-09	0.38	59	2.95	Tpd52	
DMR2:94996001	2	94996001	9.50E+07	4000	1	3.10E-13	0.54	66	1.65	Tpd52;LOC102546934	
DMR2:95004001	2	95004001	95007000	3000	2	1.70E-11	0.45	41	1.37	Tpd52;LOC102546934	
DMR2:95023001	2	95023001	95024000	1000	1	6.40E-10	-0.52	16	1.6	Tpd52	
DMR2:95029001	2	95029001	95032000	3000	2	2.00E-10	0.42	55	1.83	Tpd52	
DMR2:95078001	2	95078001	95080000	2000	1	5.80E-08	0.6	20	1	Mrps28;LOC499573	Translation
DMR2:95084001	2	95084001	95086000	2000	1	8.10E-11	0.44	19	0.95	Mrps28;LOC499573	Translation
DMR2:95088001	2	95088001	95092000	4000	2	1.10E-12	0.68	29	0.72	Mrps28;LOC499573;LOC103695108	Translation
DMR2:95314001	2	95314001	95315000	1000	1	3.10E-07	0.57	12	1.2	Hey1;LOC108349980	Transcription
DMR2:95328001	2	95328001	95330000	2000	2	1.30E-12	0.69	17	0.85	Hey1;LOC108349980	Transcription
DMR2:95448001	2	95448001	95450000	2000	1	8.30E-09	0.69	6	0.3	Stmn2	
DMR2:95481001	2	95481001	95486000	5000	2	4.60E-14	0.56	24	0.48	Stmn2	
DMR2:96427001	2	96427001	96428000	1000	1	4.80E-15	0.89	5	0.5	Il7	
DMR2:96445001	2	96445001	96448000	3000	1	9.60E-09	0.53	30	1	Il7	
DMR2:96459001	2	96459001	96461000	2000	1	2.00E-08	0.51	11	0.55	Il7	
DMR2:96499001	2	96499001	96501000	2000	1	2.60E-07	-0.68	31	1.55	Zc2hc1a	Transcription
DMR2:96581001	2	96581001	96585000	4000	1	1.70E-07	0.39	26	0.65	Pkia	Signaling
DMR2:96597001	2	96597001	96598000	1000	1	1.50E-14	0.51	21	2.1	Pkia	Signaling
DMR2:96604001	2	96604001	96606000	2000	2	5.60E-13	0.76	16	0.8	Pkia	Signaling
DMR2:96622001	2	96622001	96625000	3000	1	1.70E-09	-0.7	17	0.57	Pkia	Signaling
DMR2:96627001	2	96627001	96630000	3000	1	9.70E-09	0.5	20	0.67	Pkia	Signaling
DMR2:96646001	2	96646001	96648000	2000	1	5.60E-16	0.75	21	1.05	Pkia	Signaling
DMR2:97692001	2	97692001	97694000	2000	1	6.20E-07	-0.43	14	0.7	RGD1564247	
DMR2:98247001	2	98247001	98248000	1000	1	3.50E-11	0.53	5	0.5	Pex2	Transcription
DMR2:98263001	2	98263001	98265000	2000	1	2.60E-07	0.43	10	0.5	Pex2	Transcription
DMR2:98438001	2	98438001	98439000	1000	1	1.30E-11	0.45	14	1.4	Zfhx4	Transcription
DMR2:98486001	2	98486001	98488000	2000	1	3.20E-12	0.61	32	1.6	Zfhx4	Transcription
DMR2:98533001	2	98533001	98534000	1000	1	8.10E-14	0.68	6	0.6	Zfhx4	Transcription
DMR2:100219001	2	100219001	100221000	2000	1	6.20E-15	0.53	11	0.55	Hnf4g	Transcription
DMR2:100230001	2	100230001	100231000	1000	1	2.20E-10	0.51	7	0.7	Hnf4g	Transcription
DMR2:100266001	2	100266001	100267000	1000	1	2.30E-13	0.68	1	0.1	Hnf4g	Transcription
DMR2:100291001	2	100291001	100292000	1000	1	5.30E-09	-0.47	5	0.5	Hnf4g	Transcription
DMR2:100294001	2	100294001	100295000	1000	1	8.70E-09	0.56	4	0.4	Hnf4g	Transcription
DMR2:100317001	2	100317001	100318000	1000	1	5.10E-07	0.39	6	0.6	Hnf4g	Transcription
DMR2:100337001	2	100337001	100339000	2000	2	2.10E-17	-0.61	16	0.8	Hnf4g	Transcription
DMR2:100344001	2	100344001	100346000	2000	1	7.80E-11	-0.63	8	0.4	Hnf4g	Transcription
DMR2:100350001	2	100350001	100352000	2000	1	1.00E-10	0.47	11	0.55	Hnf4g	Transcription
DMR2:100377001	2	100377001	100378000	1000	1	1.00E-08	0.63	5	0.5	Hnf4g	Transcription
DMR2:100381001	2	100381001	100384000	3000	3	1.70E-28	0.91	19	0.63	Hnf4g	Transcription
DMR2:100703001	2	100703001	100704000	1000	1	8.00E-07	0.51	1	0.1	Ythdf3	
DMR2:100719001	2	100719001	100720000	1000	1	8.60E-08	0.51	5	0.5	Ythdf3	
DMR2:102446001	2	102446001	102447000	1000	1	2.70E-11	0.64	10	1	Pramel3	
DMR2:102448001	2	102448001	102449000	1000	1	4.10E-09	0.58	6	0.6	Pramel3	
DMR2:102676001	2	102676001	102678000	2000	1	1.40E-28	0.91	18	0.9	Bhlhe22	Transcription
DMR2:102679001	2	102679001	102680000	1000	1	7.10E-18	0.83	8	0.8	Bhlhe22	Transcription
DMR2:102733001	2	102733001	102734000	1000	1	9.40E-16	0.66	9	0.9	Cyp7b1	Metabolism
DMR2:102757001	2	102757001	102758000	1000	1	6.20E-07	-0.46	4	0.4	Cyp7b1	Metabolism
DMR2:102768001	2	102768001	102770000	2000	1	8.10E-08	0.42	8	0.4	Cyp7b1	Metabolism
DMR2:102775001	2	102775001	102776000	1000	1	6.60E-08	0.5	3	0.3	Cyp7b1	Metabolism
DMR2:103921001	2	103921001	103923000	2000	1	4.80E-13	0.6	9	0.45	Armc1	
DMR2:103945001	2	103945001	103951000	6000	2	1.90E-08	-0.43	68	1.13	Armc1	
DMR2:103954001	2	103954001	103956000	2000	1	2.80E-23	0.84	7	0.35	Armc1	
DMR2:103963001	2	103963001	103965000	2000	1	1.20E-10	-0.46	13	0.65	Armc1	
DMR2:103988001	2	103988001	103989000	1000	1	6.90E-10	0.66	5	0.5	Armc1	
DMR2:103998001	2	103998001	1.04E+08	2000	1	2.80E-11	0.47	14	0.7	Mtfr1	
DMR2:104004001	2	104004001	104005000	1000	1	1.70E-07	-0.67	9	0.9	Mtfr1	
DMR2:104018001	2	104018001	104019000	1000	1	4.90E-08	0.43	8	0.8	Mtfr1	
DMR2:104059001	2	104059001	104061000	2000	2	2.20E-17	1.06	12	0.6	Pde7a	Signaling
DMR2:104073001	2	104073001	104074000	1000	1	6.30E-11	0.6	9	0.9	Pde7a	Signaling
DMR2:104081001	2	104081001	104083000	2000	2	6.60E-09	0.45	20	1	Pde7a	Signaling
DMR2:104090001	2	104090001	104092000	2000	1	8.10E-21	0.95	10	0.5	Pde7a	Signaling
DMR2:104101001	2	104101001	104102000	1000	1	1.50E-17	0.55	9	0.9	Pde7a	Signaling
DMR2:104112001	2	104112001	104113000	1000	1	2.60E-12	0.47	4	0.4	Pde7a	Signaling

DMR2:104115001	2	104115001	104119000	4000	1	8.10E-11	0.53	28	0.7	Pde7a	Signaling
DMR2:104296001	2	104296001	104297000	1000	1	7.20E-11	0.53	5	0.5	Dnajc5b	Transcription
DMR2:104298001	2	104298001	104300000	2000	1	4.60E-09	0.5	10	0.5	Dnajc5b	Transcription
DMR2:104303001	2	104303001	104305000	2000	1	7.30E-09	0.38	23	1.15	Dnajc5b	Transcription
DMR2:104307001	2	104307001	104309000	2000	1	9.90E-12	0.42	15	0.75	Dnajc5b	Transcription
DMR2:104310001	2	104310001	104311000	1000	1	3.30E-12	0.49	15	1.5	Dnajc5b	Transcription
DMR2:104313001	2	104313001	104314000	1000	1	1.40E-09	-0.8	12	1.2	Dnajc5b	Transcription
DMR2:104323001	2	104323001	104324000	1000	1	1.20E-07	0.44	6	0.6	Dnajc5b	Transcription
DMR2:104344001	2	104344001	104345000	1000	1	3.30E-07	0.54	17	1.7	Dnajc5b	Transcription
DMR2:104351001	2	104351001	104353000	2000	2	9.30E-14	0.46	14	0.7	Dnajc5b	Transcription
DMR2:104355001	2	104355001	104357000	2000	1	6.70E-09	-0.67	12	0.6	Dnajc5b	Transcription
DMR2:104385001	2	104385001	104386000	1000	1	2.50E-08	-0.63	10	1	Dnajc5b	Transcription
DMR2:104417001	2	104417001	104420000	3000	1	3.10E-20	0.78	38	1.27	Trim55	Proteolysis
DMR2:104435001	2	104435001	104437000	2000	1	1.30E-18	0.89	7	0.35	Trim55	Proteolysis
DMR2:104765001	2	104765001	104771000	6000	3	1.70E-18	-0.53	66	1.1	Cp	Metabolism
DMR2:104786001	2	104786001	104787000	1000	1	6.40E-07	0.53	4	0.4	Cp;Hps3	Metabolism
DMR2:104828001	2	104828001	104829000	1000	1	8.90E-08	0.59	5	0.5	Hps3	
DMR2:104833001	2	104833001	104834000	1000	1	5.40E-12	0.78	4	0.4	Hps3	
DMR2:104835001	2	104835001	104840000	5000	4	5.90E-26	0.86	21	0.42	Hps3	
DMR2:104842001	2	104842001	104843000	1000	1	7.60E-08	0.42	24	2.4	Hps3	
DMR2:104844001	2	104844001	104846000	2000	1	3.20E-13	0.63	10	0.5	Hltf	
DMR2:104847001	2	104847001	104852000	5000	2	3.70E-14	-0.58	26	0.52	Hltf	
DMR2:104857001	2	104857001	104860000	3000	1	2.30E-20	0.75	26	0.87	Hltf	
DMR2:104879001	2	104879001	104880000	1000	1	4.60E-07	-0.47	21	2.1	Hltf	
DMR2:104888001	2	104888001	104892000	4000	1	1.20E-09	0.46	30	0.75	Hltf	
DMR2:104893001	2	104893001	104896000	3000	2	1.50E-20	0.8	23	0.77	Hltf	
DMR2:104898001	2	104898001	104899000	1000	1	1.70E-07	0.51	6	0.6	Hltf	
DMR2:104910001	2	104910001	104916000	6000	3	3.60E-12	0.55	50	0.83	Hltf;Gyg1	Golgi
DMR2:104938001	2	104938001	104940000	2000	2	7.20E-12	0.63	11	0.55	Gyg1	Golgi
DMR2:104956001	2	104956001	104957000	1000	1	1.60E-10	0.45	11	1.1	Gyg1	Golgi
DMR2:105002001	2	105002001	105007000	5000	4	2.40E-10	-0.41	49	0.98	LOC682402;Cpa3	Protease
DMR2:105020001	2	105020001	105027000	7000	4	2.60E-15	-0.54	68	0.97	Cpa3	Protease
DMR2:105048001	2	105048001	105049000	1000	1	7.20E-15	0.82	3	0.3	Cpa3	Protease
DMR2:105050001	2	105050001	105059000	9000	4	1.50E-30	-0.57	88	0.98	Cpa3;Cpb1	Protease
DMR2:105064001	2	105064001	105067000	3000	1	4.90E-21	0.92	17	0.57	Cpb1	Protease
DMR2:105085001	2	105085001	105086000	1000	1	2.90E-08	-0.56	7	0.7	Cpb1	Protease
DMR2:105135001	2	105135001	105140000	5000	2	5.70E-17	0.68	41	0.82	Agtr1b	Signaling
DMR2:105143001	2	105143001	105145000	2000	1	1.30E-11	0.63	13	0.65	Agtr1b	Signaling
DMR2:105154001	2	105154001	105156000	2000	2	3.60E-17	0.75	11	0.55	Agtr1b	Signaling
DMR2:105195001	2	105195001	105198000	3000	1	5.70E-12	-0.48	31	1.03	Agtr1b	Signaling
DMR2:105212001	2	105212001	105216000	4000	2	5.00E-08	-0.49	13	0.32	Agtr1b	Signaling
DMR2:105605001	2	105605001	105608000	3000	1	1.20E-13	-0.52	38	1.27	Med14-ps1	
DMR2:107230001	2	107230001	107232000	2000	1	1.10E-11	0.54	16	0.8	Tbl1xr1	
DMR2:107247001	2	107247001	107248000	1000	1	9.40E-09	0.42	10	1	Tbl1xr1	
DMR2:107262001	2	107262001	107264000	2000	1	2.60E-12	0.57	24	1.2	Tbl1xr1	
DMR2:107300001	2	107300001	107305000	5000	2	5.00E-08	-0.5	31	0.62	Tbl1xr1	
DMR2:107310001	2	107310001	107311000	1000	1	3.80E-09	0.47	9	0.9	Tbl1xr1	
DMR2:107337001	2	107337001	107338000	1000	1	5.10E-09	-0.6	18	1.8	Tbl1xr1	
DMR2:109114001	2	109114001	109117000	3000	1	3.90E-07	-0.46	11	0.37	Naaladl2	
DMR2:109120001	2	109120001	109121000	1000	1	4.90E-11	-0.77	5	0.5	Naaladl2	
DMR2:109197001	2	109197001	109199000	2000	2	1.20E-10	-0.61	11	0.55	Naaladl2	
DMR2:109214001	2	109214001	109216000	2000	1	2.60E-09	0.59	8	0.4	Naaladl2	
DMR2:109229001	2	109229001	109231000	2000	1	2.20E-18	0.89	11	0.55	Naaladl2	
DMR2:109247001	2	109247001	109249000	2000	2	3.20E-10	-0.63	20	1	Naaladl2	
DMR2:109268001	2	109268001	109269000	1000	1	3.70E-08	0.56	3	0.3	Naaladl2	
DMR2:109300001	2	109300001	109306000	6000	1	8.30E-10	-0.47	52	0.87	Naaladl2	
DMR2:109320001	2	109320001	109321000	1000	1	9.40E-07	-0.43	19	1.9	Naaladl2;LOC682571	
DMR2:109323001	2	109323001	109324000	1000	1	1.80E-08	0.53	6	0.6	Naaladl2;LOC682571	
DMR2:109340001	2	109340001	109341000	1000	1	9.40E-07	-0.6	13	1.3	Naaladl2	
DMR2:109342001	2	109342001	109343000	1000	1	2.00E-09	0.48	4	0.4	Naaladl2	
DMR2:109347001	2	109347001	109348000	1000	1	1.70E-07	-0.5	3	0.3	Naaladl2	
DMR2:109351001	2	109351001	109352000	1000	1	4.00E-07	0.54	6	0.6	Naaladl2	
DMR2:109360001	2	109360001	109361000	1000	1	7.20E-12	-0.69	9	0.9	Naaladl2	
DMR2:109370001	2	109370001	109371000	1000	1	7.80E-07	0.43	11	1.1	Naaladl2	
DMR2:109395001	2	109395001	109396000	1000	1	4.30E-12	0.43	10	1	Naaladl2	

DMR2:109399001	2	109399001	109403000	4000	1	1.50E-10	0.58	35	0.88	Naaladl2		
DMR2:109424001	2	109424001	109428000	4000	2	2.20E-20	0.82	30	0.75	Naaladl2		
DMR2:109440001	2	109440001	109441000	1000	1	3.70E-13	0.74	3	0.3	Naaladl2		
DMR2:109445001	2	109445001	109446000	1000	1	6.00E-08	-0.72	5	0.5	Naaladl2		
DMR2:109472001	2	109472001	109473000	1000	1	1.00E-11	0.51	6	0.6	Naaladl2		
DMR2:109477001	2	109477001	109479000	2000	1	1.40E-08	-0.61	8	0.4	Naaladl2		
DMR2:109485001	2	109485001	109486000	1000	1	9.10E-11	0.42	12	1.2	Naaladl2		
DMR2:109488001	2	109488001	109493000	5000	4	1.80E-09	-0.45	46	0.92	Naaladl2		
DMR2:109504001	2	109504001	109507000	3000	2	1.60E-07	-0.46	25	0.83	Naaladl2		
DMR2:109531001	2	109531001	109537000	6000	2	3.30E-10	0.74	44	0.73	Naaladl2		
DMR2:109550001	2	109550001	109557000	7000	3	5.80E-25	-0.55	94	1.34	Naaladl2		
DMR2:109587001	2	109587001	109589000	2000	1	9.60E-07	-0.54	10	0.5	Naaladl2		
DMR2:109647001	2	109647001	109648000	1000	1	5.40E-12	0.72	3	0.3	Naaladl2		
DMR2:109667001	2	109667001	109669000	2000	1	2.60E-15	0.72	9	0.45	Naaladl2		
DMR2:109673001	2	109673001	109675000	2000	1	1.70E-15	0.77	8	0.4	Naaladl2		
DMR2:109676001	2	109676001	109678000	2000	1	3.50E-27	1	2	0.1	Naaladl2		
DMR2:109711001	2	109711001	109715000	4000	2	7.50E-11	0.54	32	0.8	Naaladl2		
DMR2:109721001	2	109721001	109723000	2000	1	9.60E-09	-0.38	15	0.75	Naaladl2		
DMR2:109725001	2	109725001	109726000	1000	1	4.70E-13	0.79	4	0.4	Naaladl2		
DMR2:109755001	2	109755001	109757000	2000	2	1.40E-13	0.64	14	0.7	Naaladl2		
DMR2:109803001	2	109803001	109806000	3000	3	6.60E-17	-0.57	29	0.97	Naaladl2		
DMR2:109828001	2	109828001	109829000	1000	1	2.40E-17	-0.69	4	0.4	Naaladl2		
DMR2:109851001	2	109851001	109852000	1000	1	8.80E-07	0.49	5	0.5	Naaladl2		
DMR2:109859001	2	109859001	109860000	1000	1	3.40E-10	0.94	1	0.1	Naaladl2		
DMR2:109880001	2	109880001	109882000	2000	1	1.00E-12	0.63	8	0.4	Naaladl2		
DMR2:109903001	2	109903001	109904000	1000	1	5.50E-07	0.48	5	0.5	Naaladl2		
DMR2:109906001	2	109906001	109909000	3000	1	4.80E-14	0.78	24	0.8	Naaladl2		
DMR2:109928001	2	109928001	109929000	1000	1	3.90E-09	0.5	6	0.6	Naaladl2		
DMR2:109978001	2	109978001	109979000	1000	1	6.70E-11	0.65	6	0.6	Naaladl2		
DMR2:109995001	2	109995001	109996000	1000	1	5.80E-08	0.44	7	0.7	Naaladl2		
DMR2:109998001	2	109998001	109999000	1000	1	7.90E-07	0.48	3	0.3	Naaladl2		
DMR2:110000001	2	110000001	110006000	6000	2	2.40E-20	-0.56	43	0.72	Naaladl2		
DMR2:110015001	2	110015001	110018000	3000	1	2.20E-13	0.62	19	0.63	Naaladl2		
DMR2:110019001	2	110019001	110021000	2000	1	1.90E-21	0.97	18	0.9	Naaladl2		
DMR2:110043001	2	110043001	110045000	2000	1	1.50E-14	-0.65	10	0.5	Naaladl2		
DMR2:110073001	2	110073001	110074000	1000	1	1.70E-15	0.83	2	0.2	Naaladl2		
DMR2:110109001	2	110109001	110111000	2000	1	7.50E-10	0.62	8	0.4	Naaladl2		
DMR2:110146001	2	110146001	110148000	2000	2	2.20E-15	0.83	18	0.9	Naaladl2		
DMR2:110176001	2	110176001	110179000	3000	1	3.20E-07	-0.53	56	1.87	Naaladl2		
DMR2:110192001	2	110192001	110193000	1000	1	1.10E-11	0.6	4	0.4	Naaladl2		
DMR2:110211001	2	110211001	110212000	1000	1	2.80E-07	0.43	3	0.3	Naaladl2		
DMR2:110218001	2	110218001	110219000	1000	1	7.00E-12	0.55	5	0.5	Naaladl2		
DMR2:110263001	2	110263001	110264000	1000	1	1.60E-15	0.7	9	0.9	Naaladl2		
DMR2:110287001	2	110287001	110289000	2000	1	8.00E-09	-0.5	18	0.9	Naaladl2		
DMR2:110307001	2	110307001	110308000	1000	1	1.50E-14	0.93	6	0.6	Naaladl2;LOC499584		
DMR2:110316001	2	110316001	110317000	1000	1	3.70E-09	0.58	5	0.5	Naaladl2;LOC499584		
DMR2:110323001	2	110323001	110324000	1000	1	5.20E-16	0.87	4	0.4	Naaladl2;LOC499584		
DMR2:110351001	2	110351001	110355000	4000	1	5.50E-08	0.39	30	0.75	Naaladl2		
DMR2:110380001	2	110380001	110383000	3000	1	2.40E-07	-0.39	25	0.83	Naaladl2		
DMR2:110393001	2	110393001	110395000	2000	1	5.40E-09	0.41	13	0.65	Naaladl2		
DMR2:110398001	2	110398001	110399000	1000	1	4.50E-07	0.44	2	0.2	Naaladl2		
DMR2:110420001	2	110420001	110421000	1000	1	5.60E-07	0.4	3	0.3	Naaladl2		
DMR2:111047001	2	111047001	111050000	3000	2	7.80E-10	0.5	24	0.8	Nlgn1	Cytoskeleton	
DMR2:111066001	2	111066001	111067000	1000	1	5.70E-20	0.84	6	0.6	Nlgn1	Cytoskeleton	
DMR2:111082001	2	111082001	111083000	1000	1	6.60E-09	-0.69	5	0.5	Nlgn1	Cytoskeleton	
DMR2:111097001	2	111097001	111104000	7000	6	2.70E-28	-0.6	93	1.33	Nlgn1	Cytoskeleton	
DMR2:111132001	2	111132001	111133000	1000	1	2.60E-11	0.77	1	0.1	Nlgn1	Cytoskeleton	
DMR2:111180001	2	111180001	111181000	1000	1	8.60E-08	0.45	6	0.6	Nlgn1	Cytoskeleton	
DMR2:111185001	2	111185001	111186000	1000	1	1.30E-09	-0.59	11	1.1	Nlgn1	Cytoskeleton	
DMR2:111229001	2	111229001	111233000	4000	1	9.30E-10	0.73	22	0.55	Nlgn1	Cytoskeleton	
DMR2:111235001	2	111235001	111236000	1000	1	1.60E-12	0.7	5	0.5	Nlgn1	Cytoskeleton	
DMR2:111245001	2	111245001	111248000	3000	3	8.80E-25	-0.44	37	1.23	Nlgn1	Cytoskeleton	
DMR2:111270001	2	111270001	111271000	1000	1	1.60E-11	0.57	11	1.1	Nlgn1	Cytoskeleton	
DMR2:111312001	2	111312001	111313000	1000	1	4.90E-18	0.92	8	0.8	Nlgn1	Cytoskeleton	
DMR2:111320001	2	111320001	111321000	1000	1	8.10E-09	0.49	17	1.7	Nlgn1	Cytoskeleton	

DMR2:111355001	2	111355001	111361000	6000	3	2.60E-38	-0.58	73	1.22	Nlgn1	Cytoskeleton
DMR2:111422001	2	111422001	111423000	1000	1	2.70E-07	-0.58	15	1.5	Nlgn1	Cytoskeleton
DMR2:111428001	2	111428001	111429000	1000	1	1.20E-20	1.05	7	0.7	Nlgn1	Cytoskeleton
DMR2:111446001	2	111446001	111447000	1000	1	5.20E-07	-0.32	10	1	Nlgn1	Cytoskeleton
DMR2:111450001	2	111450001	111453000	3000	1	3.00E-08	0.39	25	0.83	Nlgn1	Cytoskeleton
DMR2:111455001	2	111455001	111456000	1000	1	9.90E-09	0.56	7	0.7	Nlgn1	Cytoskeleton
DMR2:111460001	2	111460001	111462000	2000	1	1.10E-14	0.63	21	1.05	Nlgn1	Cytoskeleton
DMR2:111470001	2	111470001	111473000	3000	2	3.60E-11	0.53	12	0.4	Nlgn1	Cytoskeleton
DMR2:111478001	2	111478001	111480000	2000	1	5.00E-14	0.79	9	0.45	Nlgn1	Cytoskeleton
DMR2:111483001	2	111483001	111484000	1000	1	2.30E-08	0.52	8	0.8	Nlgn1	Cytoskeleton
DMR2:111505001	2	111505001	111506000	1000	1	2.30E-13	0.76	4	0.4	Nlgn1;LOC108350212	Cytoskeleton
DMR2:111507001	2	111507001	111509000	2000	1	4.00E-07	0.38	12	0.6	Nlgn1;LOC108350212	Cytoskeleton
DMR2:111515001	2	111515001	111518000	3000	2	7.70E-13	0.84	33	1.1	Nlgn1;LOC108350212	Cytoskeleton
DMR2:111581001	2	111581001	111583000	2000	1	5.60E-10	0.49	12	0.6	Nlgn1	Cytoskeleton
DMR2:111585001	2	111585001	111587000	2000	1	4.10E-07	0.38	20	1	Nlgn1	Cytoskeleton
DMR2:111599001	2	111599001	111600000	1000	1	6.30E-08	0.51	11	1.1	Nlgn1	Cytoskeleton
DMR2:111614001	2	111614001	111615000	1000	1	2.10E-13	0.79	5	0.5	Nlgn1	Cytoskeleton
DMR2:111618001	2	111618001	111622000	4000	1	5.10E-09	0.63	25	0.62	Nlgn1	Cytoskeleton
DMR2:111623001	2	111623001	111626000	3000	1	3.20E-09	-0.39	23	0.77	Nlgn1	Cytoskeleton
DMR2:111662001	2	111662001	111665000	3000	2	2.00E-14	-0.55	37	1.23	Nlgn1	Cytoskeleton
DMR2:111673001	2	111673001	111675000	2000	1	8.20E-08	0.59	15	0.75	Nlgn1	Cytoskeleton
DMR2:111684001	2	111684001	111689000	5000	2	1.90E-10	-0.41	48	0.96	Nlgn1	Cytoskeleton
DMR2:111739001	2	111739001	111740000	1000	1	1.00E-07	0.35	6	0.6	Nlgn1	Cytoskeleton
DMR2:111763001	2	111763001	111765000	2000	2	9.50E-14	0.84	13	0.65	Nlgn1	Cytoskeleton
DMR2:111770001	2	111770001	111772000	2000	1	2.00E-07	-0.5	21	1.05	Nlgn1	Cytoskeleton
DMR2:111807001	2	111807001	111809000	2000	2	1.60E-17	1.04	12	0.6	Nlgn1	Cytoskeleton
DMR2:111818001	2	111818001	111820000	2000	1	1.50E-08	0.47	6	0.3	Nlgn1	Cytoskeleton
DMR2:111836001	2	111836001	111837000	1000	1	9.20E-10	0.48	7	0.7	Nlgn1	Cytoskeleton
DMR2:111889001	2	111889001	111897000	8000	2	3.80E-12	0.54	80	1	Nlgn1	Cytoskeleton
DMR2:111899001	2	111899001	111901000	2000	1	4.10E-15	0.59	22	1.1	Nlgn1	Cytoskeleton
DMR2:111910001	2	111910001	111911000	1000	1	1.30E-13	0.71	4	0.4	Nlgn1	Cytoskeleton
DMR2:111914001	2	111914001	111915000	1000	1	1.20E-08	0.5	9	0.9	Nlgn1	Cytoskeleton
DMR2:111936001	2	111936001	111937000	1000	1	4.40E-09	0.39	13	1.3	Nlgn1	Cytoskeleton
DMR2:111944001	2	111944001	111945000	1000	1	8.70E-07	0.48	6	0.6	Nlgn1	Cytoskeleton
DMR2:112283001	2	112283001	112284000	1000	1	6.40E-07	0.42	18	1.8	Spata16	
DMR2:112304001	2	112304001	112306000	2000	1	2.60E-07	-0.62	7	0.35	Spata16	
DMR2:112320001	2	112320001	112321000	1000	1	5.00E-15	0.74	9	0.9	Spata16	
DMR2:112324001	2	112324001	112331000	7000	3	5.70E-21	-0.45	83	1.19	Spata16	
DMR2:112339001	2	112339001	112341000	2000	2	1.70E-28	0.93	11	0.55	Spata16	
DMR2:112354001	2	112354001	112364000	10000	7	3.40E-20	-0.61	92	0.92	Spata16	
DMR2:112367001	2	112367001	112375000	8000	5	1.90E-23	-0.59	100	1.25	Spata16	
DMR2:112389001	2	112389001	112390000	1000	1	4.80E-27	0.81	12	1.2	Spata16	
DMR2:112415001	2	112415001	112418000	3000	2	5.30E-07	-0.55	7	0.23	Spata16	
DMR2:112423001	2	112423001	112429000	6000	4	1.80E-16	-0.53	62	1.03	Spata16	
DMR2:112434001	2	112434001	112435000	1000	1	5.60E-08	-0.37	19	1.9	Spata16	
DMR2:112449001	2	112449001	112451000	2000	1	1.40E-10	0.46	16	0.8	Spata16	
DMR2:112480001	2	112480001	112481000	1000	1	6.70E-07	0.42	5	0.5	Spata16	
DMR2:112488001	2	112488001	112490000	2000	2	1.80E-16	0.93	14	0.7	Spata16	
DMR2:112504001	2	112504001	112506000	2000	2	1.60E-16	0.88	14	0.7	Spata16	
DMR2:112528001	2	112528001	112530000	2000	1	1.80E-07	0.48	30	1.5	Spata16	
DMR2:112532001	2	112532001	112534000	2000	1	4.10E-08	0.42	34	1.7	Spata16	
DMR2:112536001	2	112536001	112538000	2000	1	2.10E-07	-0.4	22	1.1	Spata16	
DMR2:112560001	2	112560001	112561000	1000	1	3.00E-08	0.59	11	1.1	Spata16	
DMR2:112589001	2	112589001	112594000	5000	2	6.90E-15	0.49	58	1.16	Spata16	
DMR2:112604001	2	112604001	112607000	3000	2	9.00E-11	0.59	17	0.57	Spata16	
DMR2:112624001	2	112624001	112625000	1000	1	2.40E-12	0.54	6	0.6	Spata16	
DMR2:112637001	2	112637001	112640000	3000	1	1.00E-06	0.41	20	0.67	Spata16	
DMR2:112642001	2	112642001	112648000	6000	4	6.80E-18	-0.47	60	1	Spata16	
DMR2:112650001	2	112650001	112653000	3000	1	3.10E-07	-0.49	5	0.17	Spata16	
DMR2:112657001	2	112657001	112660000	3000	1	2.50E-09	0.41	26	0.87	Spata16	
DMR2:112661001	2	112661001	112662000	1000	1	3.60E-07	0.4	3	0.3	Spata16	
DMR2:112665001	2	112665001	112668000	3000	1	2.90E-10	0.56	23	0.77	Spata16	
DMR2:112756001	2	112756001	112761000	5000	1	1.50E-08	0.39	41	0.82	LOC102555205;Ect2	Transcription
DMR2:112779001	2	112779001	112780000	1000	1	6.30E-14	-0.52	8	0.8	Ect2	Transcription
DMR2:112784001	2	112784001	112785000	1000	1	7.60E-09	0.36	13	1.3	Ect2	Transcription

DMR2:112836001	2	112836001	112840000	4000	2	4.50E-08	-0.56	88	2.2	Ect2	Transcription
DMR2:112893001	2	112893001	112896000	3000	1	8.10E-12	0.51	31	1.03	Nceh1	Metabolism
DMR2:112912001	2	112912001	112914000	2000	1	1.30E-25	1.14	29	1.45	Nceh1	Metabolism
DMR2:112921001	2	112921001	112923000	2000	1	1.50E-18	0.7	21	1.05	Nceh1	Metabolism
DMR2:113001001	2	113001001	113003000	2000	1	7.50E-07	0.34	34	1.7	Tnfsf10	
DMR2:113008001	2	113008001	113011000	3000	2	5.10E-17	0.79	28	0.93	Tnfsf10	
DMR2:113015001	2	113015001	113016000	1000	1	4.40E-07	-0.6	18	1.8	Tnfsf10	
DMR2:113022001	2	113022001	113024000	2000	1	2.90E-21	0.81	31	1.55	Tnfsf10	
DMR2:113032001	2	113032001	113035000	3000	2	8.20E-17	0.72	33	1.1	Tnfsf10	
DMR2:113057001	2	113057001	113062000	5000	2	1.40E-09	-0.73	25	0.5	LOC103691520;Ghsr	Signaling
DMR2:113080001	2	113080001	113088000	8000	5	2.80E-13	-0.54	49	0.61	Ghsr	Signaling
DMR2:113118001	2	113118001	113120000	2000	1	1.00E-24	0.77	24	1.2	Fndc3b	Proteolysis
DMR2:113168001	2	113168001	113171000	3000	1	2.90E-08	0.53	35	1.17	Fndc3b	Proteolysis
DMR2:113181001	2	113181001	113190000	9000	2	1.70E-07	0.39	123	1.37	Fndc3b	Proteolysis
DMR2:113196001	2	113196001	113197000	1000	1	6.20E-11	0.46	13	1.3	Fndc3b	Proteolysis
DMR2:113198001	2	113198001	113202000	4000	2	1.70E-22	0.97	48	1.2	Fndc3b	Proteolysis
DMR2:113203001	2	113203001	113206000	3000	1	1.70E-07	-0.54	39	1.3	Fndc3b	Proteolysis
DMR2:113216001	2	113216001	113218000	2000	2	1.40E-09	0.44	22	1.1	Fndc3b	Proteolysis
DMR2:113222001	2	113222001	113223000	1000	1	4.40E-19	0.89	11	1.1	Fndc3b	Proteolysis
DMR2:113227001	2	113227001	113228000	1000	1	7.60E-08	0.41	7	0.7	Fndc3b	Proteolysis
DMR2:113269001	2	113269001	113270000	1000	1	1.20E-12	0.66	11	1.1	Fndc3b	Proteolysis
DMR2:113287001	2	113287001	113291000	4000	2	1.10E-20	-0.65	46	1.15	Fndc3b	Proteolysis
DMR2:113296001	2	113296001	113299000	3000	1	1.10E-07	-0.58	38	1.27	Fndc3b	Proteolysis
DMR2:113308001	2	113308001	113311000	3000	1	5.10E-12	0.51	36	1.2	Fndc3b	Proteolysis
DMR2:113318001	2	113318001	113319000	1000	1	9.10E-11	0.41	13	1.3	Fndc3b	Proteolysis
DMR2:113330001	2	113330001	113332000	2000	1	9.00E-07	0.29	24	1.2	Fndc3b	Proteolysis
DMR2:113349001	2	113349001	113350000	1000	1	1.70E-13	0.47	15	1.5	Fndc3b	Proteolysis
DMR2:113363001	2	113363001	113365000	2000	1	8.20E-18	0.69	28	1.4	Fndc3b	Proteolysis
DMR2:113372001	2	113372001	113373000	1000	1	7.70E-10	0.4	8	0.8	Fndc3b	Proteolysis
DMR2:113581001	2	113581001	113584000	3000	3	2.50E-17	0.73	39	1.3	Tmem212;LOC102555566	Transport
DMR2:113587001	2	113587001	113588000	1000	1	6.20E-11	-0.59	10	1	Tmem212;LOC102555566	Transport
DMR2:113593001	2	113593001	113595000	2000	2	7.30E-09	-0.47	32	1.6	Tmem212;LOC102555566	Transport
DMR2:113600001	2	113600001	113601000	1000	1	2.00E-08	0.41	15	1.5	Tmem212;LOC102555566	Transport
DMR2:113604001	2	113604001	113606000	2000	2	9.00E-09	0.4	26	1.3	Tmem212;LOC102555566	Transport
DMR2:113622001	2	113622001	113624000	2000	2	7.90E-15	0.77	22	1.1	Tmem212	Transport
DMR2:113625001	2	113625001	113628000	3000	1	1.30E-16	0.97	37	1.23	Tmem212	Transport
DMR2:113640001	2	113640001	113644000	4000	1	8.00E-07	-0.56	55	1.38	Pld1	Metabolism
DMR2:113691001	2	113691001	113693000	2000	1	4.20E-19	0.79	18	0.9	Pld1	Metabolism
DMR2:113699001	2	113699001	113701000	2000	1	5.80E-16	0.77	19	0.95	Pld1	Metabolism
DMR2:113703001	2	113703001	113704000	1000	1	5.70E-08	-0.62	6	0.6	Pld1	Metabolism
DMR2:113724001	2	113724001	113728000	4000	1	2.60E-07	-0.43	67	1.68	Pld1	Metabolism
DMR2:113733001	2	113733001	113737000	4000	1	6.50E-11	-0.61	38	0.95	Pld1	Metabolism
DMR2:113755001	2	113755001	113757000	2000	1	2.50E-13	0.45	20	1	Pld1	Metabolism
DMR2:113777001	2	113777001	113778000	1000	1	5.20E-10	0.52	4	0.4	Pld1	Metabolism
DMR2:113815001	2	113815001	113816000	1000	1	1.60E-07	0.34	16	1.6	Pld1	Metabolism
DMR2:113972001	2	113972001	113977000	5000	1	3.90E-09	0.39	84	1.68	Tnik	Signaling
DMR2:113980001	2	113980001	113981000	1000	1	6.90E-17	0.81	12	1.2	Tnik	Signaling
DMR2:114002001	2	114002001	114004000	2000	1	1.60E-09	0.4	11	0.55	Tnik	Signaling
DMR2:114008001	2	114008001	114010000	2000	1	2.00E-11	0.44	33	1.65	Tnik	Signaling
DMR2:114032001	2	114032001	114033000	1000	1	1.40E-08	0.47	7	0.7	Tnik	Signaling
DMR2:114037001	2	114037001	114038000	1000	1	1.20E-10	-0.58	15	1.5	Tnik	Signaling
DMR2:114070001	2	114070001	114071000	1000	1	1.30E-08	0.39	16	1.6	Tnik	Signaling
DMR2:114072001	2	114072001	114076000	4000	1	1.20E-14	0.68	42	1.05	Tnik	Signaling
DMR2:114103001	2	114103001	114104000	1000	1	7.50E-07	-0.44	13	1.3	Tnik	Signaling
DMR2:114113001	2	114113001	114114000	1000	1	1.20E-07	0.36	8	0.8	Tnik	Signaling
DMR2:114115001	2	114115001	114116000	1000	1	3.90E-11	0.54	1	0.1	Tnik	Signaling
DMR2:114122001	2	114122001	114125000	3000	1	7.80E-08	-0.53	35	1.17	Tnik	Signaling
DMR2:114200001	2	114200001	114201000	1000	1	3.30E-07	-0.45	5	0.5	Tnik	Signaling
DMR2:114207001	2	114207001	114210000	3000	2	1.40E-12	0.59	30	1	Tnik	Signaling
DMR2:114217001	2	114217001	114218000	1000	1	9.60E-13	0.61	13	1.3	Tnik	Signaling
DMR2:114256001	2	114256001	114257000	1000	1	6.00E-07	0.47	10	1	Tnik	Signaling
DMR2:114262001	2	114262001	114263000	1000	1	5.50E-10	0.6	8	0.8	Tnik	Signaling
DMR2:114270001	2	114270001	114271000	1000	1	6.10E-12	0.56	6	0.6	Tnik	Signaling
DMR2:114288001	2	114288001	114291000	3000	2	7.50E-15	0.53	50	1.67	Tnik	Signaling
DMR2:114308001	2	114308001	114311000	3000	2	1.00E-08	0.58	22	0.73	Tnik	Signaling

DMR2:114313001	2	114313001	114318000	5000	1	6.50E-19	0.52	73	1.46	Tnik	Signaling
DMR2:114342001	2	114342001	114344000	2000	2	2.90E-16	0.8	7	0.35	Tnik	Signaling
DMR2:114369001	2	114369001	114372000	3000	1	3.40E-08	-0.6	37	1.23	Tnik	Signaling
DMR2:114373001	2	114373001	114376000	3000	1	6.20E-10	-0.52	32	1.07	Tnik	Signaling
DMR2:114401001	2	114401001	114404000	3000	1	1.40E-14	0.72	19	0.63	Tnik;Slc2a2	Signaling
DMR2:114412001	2	114412001	114414000	2000	1	5.50E-07	0.32	21	1.05	Slc2a2	
DMR2:114415001	2	114415001	114416000	1000	1	6.60E-08	-0.59	14	1.4	Slc2a2	
DMR2:114448001	2	114448001	114449000	1000	1	1.70E-08	0.54	3	0.3	Slc2a2	
DMR2:114450001	2	114450001	114454000	4000	1	6.20E-15	0.5	67	1.68	Slc2a2	
DMR2:115087001	2	115087001	115090000	3000	1	5.40E-15	-0.5	31	1.03	Olr1279	
DMR2:115329001	2	115329001	115331000	2000	1	1.10E-11	0.63	32	1.6	Eif5a2	Translation
DMR2:115364001	2	115364001	115367000	3000	1	4.10E-08	-0.41	30	1	Eif5a2;Rpl22l1	Translation;Translation
DMR2:115374001	2	115374001	115376000	2000	1	1.80E-12	0.46	18	0.9	Rpl22l1	Translation
DMR2:115690001	2	115690001	115692000	2000	1	7.80E-07	0.33	26	1.3	Slc7a14	Transport
DMR2:115717001	2	115717001	115722000	5000	1	9.00E-09	0.36	46	0.92	Slc7a14	Transport
DMR2:115760001	2	115760001	115764000	4000	1	7.00E-08	-0.51	67	1.68	Slc7a14	Transport
DMR2:115766001	2	115766001	115767000	1000	1	3.90E-18	0.76	12	1.2	Slc7a14	Transport
DMR2:115776001	2	115776001	115777000	1000	1	1.00E-07	0.59	12	1.2	Slc7a14	Transport
DMR2:115782001	2	115782001	115784000	2000	1	1.10E-08	0.42	26	1.3	Slc7a14	Transport
DMR2:115794001	2	115794001	115797000	3000	1	9.70E-09	-0.58	46	1.53	Slc7a14	Transport
DMR2:115812001	2	115812001	115814000	2000	1	7.30E-25	0.67	35	1.75	Cldn11	Cell Junction
DMR2:115815001	2	115815001	115816000	1000	1	8.50E-13	0.57	11	1.1	Cldn11	Cell Junction
DMR2:115825001	2	115825001	115827000	2000	1	3.80E-07	-0.43	22	1.1	Cldn11	Cell Junction
DMR2:115842001	2	115842001	115847000	5000	2	1.30E-19	-0.72	26	0.52	Cldn11	Cell Junction
DMR2:115855001	2	115855001	115857000	2000	1	8.10E-09	0.38	41	2.05	Skil	
DMR2:115858001	2	115858001	115860000	2000	2	2.50E-13	0.4	29	1.45	Skil	
DMR2:115932001	2	115932001	115936000	4000	1	5.80E-08	0.31	69	1.73	LOC102547647;LOC102547718;Prkci	Signaling
DMR2:115952001	2	115952001	115956000	4000	2	1.30E-09	-0.52	47	1.18	Prkci	Signaling
DMR2:115966001	2	115966001	115972000	6000	1	1.00E-11	0.4	69	1.15	Prkci	Signaling
DMR2:115977001	2	115977001	115980000	3000	2	5.50E-14	-0.63	35	1.17	Prkci	Signaling
DMR2:115985001	2	115985001	115988000	3000	2	2.20E-07	0.53	21	0.7	Prkci	Signaling
DMR2:115994001	2	115994001	115996000	2000	1	5.20E-08	0.44	24	1.2	Prkci	Signaling
DMR2:116010001	2	116010001	116011000	1000	1	8.60E-07	0.36	10	1	Prkci;LOC100910976	Signaling
DMR2:116041001	2	116041001	116042000	1000	1	5.80E-10	-0.54	13	1.3	Phc3	Epigenetic
DMR2:116058001	2	116058001	116059000	1000	1	8.20E-07	0.42	8	0.8	Phc3	Epigenetic
DMR2:116063001	2	116063001	116068000	5000	1	3.60E-13	0.48	45	0.9	Phc3	Epigenetic
DMR2:116078001	2	116078001	116080000	2000	1	1.60E-09	-0.46	9	0.45	Phc3	Epigenetic
DMR2:116083001	2	116083001	116085000	2000	2	4.00E-19	0.56	19	0.95	Phc3	Epigenetic
DMR2:116119001	2	116119001	116127000	8000	7	1.60E-17	-0.68	43	0.54	Gpr160	Signaling
DMR2:116158001	2	116158001	116159000	1000	1	4.90E-15	0.69	8	0.8	Gpr160;LOC102547815	Signaling
DMR2:116164001	2	116164001	116169000	5000	3	3.10E-12	0.51	82	1.64	Gpr160;LOC102547815	Signaling
DMR2:116200001	2	116200001	116201000	1000	1	1.40E-07	0.34	18	1.8	Sec62	
DMR2:116202001	2	116202001	116203000	1000	1	9.40E-10	0.51	5	0.5	Sec62	
DMR2:116217001	2	116217001	116219000	2000	2	3.00E-14	0.77	9	0.45	Sec62	
DMR2:116221001	2	116221001	116222000	1000	1	1.20E-07	0.33	6	0.6	Sec62	
DMR2:116231001	2	116231001	116234000	3000	1	1.40E-07	0.35	30	1	Sec62;Samd7	Epigenetic
DMR2:116269001	2	116269001	116272000	3000	1	4.00E-19	0.84	32	1.07	Samd7;LOC685033	Epigenetic
DMR2:116309001	2	116309001	116312000	3000	1	1.80E-08	0.39	36	1.2	LOC685065;Lrrc31	
DMR2:116325001	2	116325001	116328000	3000	1	9.00E-13	0.5	60	2	Lrrc31	
DMR2:116337001	2	116337001	116339000	2000	1	1.50E-07	0.33	44	2.2	Lrrc31;Lrriq4	Cytoskeleton
DMR2:116348001	2	116348001	116352000	4000	1	3.10E-07	-0.47	36	0.9	Lrrc31;Lrriq4	Cytoskeleton
DMR2:116355001	2	116355001	116362000	7000	3	1.00E-25	1.19	96	1.37	Lrriq4;Lrrc34	Cytoskeleton
DMR2:116375001	2	116375001	116377000	2000	1	3.00E-08	0.38	38	1.9	Lrriq4;Lrrc34	Cytoskeleton
DMR2:116418001	2	116418001	116420000	2000	1	7.90E-11	0.6	27	1.35	Mynn;Trnav-aac;Actrt3	Transcription;Cytoskeleton
DMR2:116436001	2	116436001	116441000	5000	1	1.00E-08	0.41	46	0.92	Terc;LOC108350239	
DMR2:116443001	2	116443001	116445000	2000	1	2.50E-09	-0.52	18	0.9	Terc;LOC108350239	
DMR2:116512001	2	116512001	116517000	5000	1	1.10E-12	-0.69	32	0.64	Egfem1	
DMR2:116538001	2	116538001	116539000	1000	1	6.90E-21	0.68	10	1	Egfem1	
DMR2:116554001	2	116554001	116557000	3000	1	8.90E-16	0.54	35	1.17	Egfem1	
DMR2:116583001	2	116583001	116584000	1000	1	4.30E-22	0.82	5	0.5	Egfem1	
DMR2:116594001	2	116594001	116595000	1000	1	2.60E-07	-0.54	9	0.9	Egfem1	
DMR2:116600001	2	116600001	116601000	1000	1	2.30E-08	0.44	8	0.8	Egfem1	

DMR2:116615001	2	116615001	116616000	1000	1	7.30E-13	0.54	8	0.8	Egfem1	
DMR2:116626001	2	116626001	116627000	1000	1	4.50E-10	0.7	7	0.7	Egfem1	
DMR2:116635001	2	116635001	116637000	2000	1	5.60E-11	0.75	17	0.85	Egfem1	
DMR2:116638001	2	116638001	116645000	7000	5	5.10E-20	-0.5	88	1.26	Egfem1	
DMR2:116646001	2	116646001	116648000	2000	1	8.20E-14	-0.4	43	2.15	Egfem1	
DMR2:116695001	2	116695001	116697000	2000	1	1.10E-07	-0.7	5	0.25	Egfem1	
DMR2:116702001	2	116702001	116704000	2000	1	4.50E-09	0.5	13	0.65	Egfem1	
DMR2:116730001	2	116730001	116731000	1000	1	8.20E-07	0.29	13	1.3	Egfem1	
DMR2:116762001	2	116762001	116765000	3000	1	1.80E-13	0.46	29	0.97	Egfem1	
DMR2:116775001	2	116775001	116781000	6000	3	3.20E-13	-0.42	46	0.77	Egfem1	
DMR2:116786001	2	116786001	116787000	1000	1	1.40E-08	0.59	8	0.8	Egfem1	
DMR2:116792001	2	116792001	116797000	5000	2	3.10E-10	-0.65	26	0.52	Egfem1	
DMR2:116808001	2	116808001	116813000	5000	1	2.90E-08	-0.37	45	0.9	Egfem1	
DMR2:116874001	2	116874001	116875000	1000	1	8.30E-09	0.44	13	1.3	Egfem1;Mir551b	
DMR2:116907001	2	116907001	116909000	2000	2	1.00E-13	0.63	11	0.55	Egfem1	
DMR2:116911001	2	116911001	116912000	1000	1	7.40E-07	0.45	3	0.3	Egfem1	
DMR2:116913001	2	116913001	116919000	6000	4	6.00E-14	-0.43	73	1.22	Egfem1	
DMR2:116920001	2	116920001	116921000	1000	1	2.20E-20	1.06	5	0.5	Egfem1	
DMR2:116922001	2	116922001	116923000	1000	1	2.10E-13	0.78	3	0.3	Egfem1	
DMR2:116930001	2	116930001	116934000	4000	2	9.30E-16	-0.63	21	0.52	Egfem1;LOC103691523	
DMR2:116948001	2	116948001	116956000	8000	4	2.60E-23	-0.46	78	0.98	Egfem1;LOC103691523	
DMR2:116978001	2	116978001	116979000	1000	1	3.20E-08	0.51	6	0.6	Egfem1	
DMR2:116980001	2	116980001	116984000	4000	3	1.70E-14	-0.53	37	0.92	Egfem1	
DMR2:116992001	2	116992001	116993000	1000	1	9.70E-19	0.88	5	0.5	Egfem1	
DMR2:117006001	2	117006001	117008000	2000	1	2.50E-11	0.77	8	0.4	Egfem1	
DMR2:117011001	2	117011001	117013000	2000	2	3.20E-22	0.74	27	1.35	Egfem1	
DMR2:117040001	2	117040001	117042000	2000	1	3.50E-12	0.55	16	0.8	Egfem1	
DMR2:117045001	2	117045001	117050000	5000	1	1.60E-09	-0.38	52	1.04	Egfem1	
DMR2:117063001	2	117063001	117064000	1000	1	3.50E-09	0.37	8	0.8	Egfem1	
DMR2:117089001	2	117089001	117091000	2000	1	1.70E-10	-0.49	6	0.3	Egfem1	
DMR2:117113001	2	117113001	117116000	3000	3	3.80E-16	0.83	16	0.53	Egfem1	
DMR2:117145001	2	117145001	117148000	3000	1	2.50E-10	0.57	19	0.63	Egfem1	
DMR2:117157001	2	117157001	117158000	1000	1	1.30E-07	-0.46	15	1.5	Egfem1	
DMR2:117399001	2	117399001	117400000	1000	1	9.60E-12	0.59	4	0.4	Mecom	Transcription
DMR2:117425001	2	117425001	117426000	1000	1	1.20E-07	-0.42	22	2.2	Mecom	Transcription
DMR2:117438001	2	117438001	117439000	1000	1	2.70E-08	0.36	10	1	Mecom	Transcription
DMR2:117440001	2	117440001	117441000	1000	1	5.90E-12	0.52	7	0.7	Mecom	Transcription
DMR2:117463001	2	117463001	117466000	3000	1	5.40E-07	-0.42	66	2.2	Mecom	Transcription
DMR2:117492001	2	117492001	117497000	5000	1	2.60E-10	0.45	70	1.4	Mecom	Transcription
DMR2:117500001	2	117500001	117508000	8000	3	5.40E-24	0.86	104	1.3	Mecom	Transcription
DMR2:117514001	2	117514001	117516000	2000	1	1.70E-10	0.43	32	1.6	Mecom	Transcription
DMR2:117569001	2	117569001	117571000	2000	1	8.60E-07	-0.49	20	1	Mecom	Transcription
DMR2:117625001	2	117625001	117626000	1000	1	1.30E-09	-0.63	17	1.7	Mecom	Transcription
DMR2:117647001	2	117647001	117651000	4000	1	1.60E-08	-0.45	82	2.05	Mecom	Transcription
DMR2:117669001	2	117669001	117671000	2000	1	1.20E-15	-0.74	24	1.2	Mecom;LOC103691544	Transcription
DMR2:117739001	2	117739001	117741000	2000	1	4.90E-07	-0.56	47	2.35	Mecom;LOC103691544	Transcription
DMR2:117743001	2	117743001	117744000	1000	1	9.30E-08	0.6	5	0.5	Mecom;LOC103691544	Transcription
DMR2:117764001	2	117764001	117766000	2000	1	1.20E-13	0.45	35	1.75	Mecom;LOC103691544	Transcription
DMR2:117775001	2	117775001	117779000	4000	1	2.00E-11	0.56	33	0.82	Mecom;LOC103691544	Transcription
DMR2:117781001	2	117781001	117784000	3000	1	3.00E-08	-0.55	37	1.23	Mecom;LOC103691544	Transcription
DMR2:117788001	2	117788001	117794000	6000	1	2.30E-08	0.54	68	1.13	Mecom;LOC103691544	Transcription
DMR2:117806001	2	117806001	117808000	2000	1	3.40E-07	0.35	15	0.75	Mecom;LOC103691544	Transcription
DMR2:117809001	2	117809001	117814000	5000	2	5.50E-14	-0.73	73	1.46	Mecom;LOC103691544	Transcription
DMR2:117828001	2	117828001	117830000	2000	1	1.40E-07	0.48	14	0.7	Mecom;LOC103691544	Transcription
DMR2:117840001	2	117840001	117849000	9000	2	1.30E-15	0.48	129	1.43	Mecom;LOC103691544	Transcription
DMR2:117851001	2	117851001	117855000	4000	1	3.00E-09	-0.59	66	1.65	Mecom;LOC103691544	Transcription
DMR2:117878001	2	117878001	117884000	6000	4	4.90E-08	-0.57	79	1.32	Mecom;LOC103691544	Transcription
DMR2:117885001	2	117885001	117887000	2000	1	2.10E-16	0.93	8	0.4	Mecom;LOC103691544	Transcription
DMR2:117895001	2	117895001	117898000	3000	2	1.70E-09	0.44	18	0.6	Mecom;LOC103691544	Transcription
DMR2:117919001	2	117919001	117920000	1000	1	1.50E-11	0.64	9	0.9	Mecom;LOC103691544	Transcription
DMR2:117938001	2	117938001	117942000	4000	1	5.40E-22	0.78	53	1.32	Mecom;LOC103691544	Transcription
DMR2:117943001	2	117943001	117945000	2000	1	1.80E-07	0.32	26	1.3	Mecom;LOC103691544	Transcription
DMR2:117976001	2	117976001	117977000	1000	1	6.80E-22	1.03	6	0.6	Mecom	Transcription
DMR2:117980001	2	117980001	117981000	1000	1	5.10E-11	0.7	16	1.6	Mecom	Transcription
DMR2:117987001	2	117987001	117989000	2000	1	3.20E-08	0.39	31	1.55	Mecom	Transcription

DMR2:118266001	2	118266001	118267000	1000	1	3.40E-08	0.48	6	0.6	LOC108349990;Kcnmb2	Transport
DMR2:118276001	2	118276001	118279000	3000	1	6.00E-16	0.68	32	1.07	Kcnmb2;LOC102549015	Transport
DMR2:118288001	2	118288001	118292000	4000	2	2.70E-18	0.72	34	0.85	Kcnmb2;LOC102549015	Transport
DMR2:118307001	2	118307001	118308000	1000	1	1.60E-13	0.66	11	1.1	Kcnmb2	Transport
DMR2:118310001	2	118310001	118313000	3000	1	4.50E-09	-0.4	27	0.9	Kcnmb2	Transport
DMR2:118324001	2	118324001	118325000	1000	1	3.60E-11	0.73	5	0.5	Kcnmb2	Transport
DMR2:118336001	2	118336001	118339000	3000	2	8.60E-14	0.56	29	0.97	Kcnmb2	Transport
DMR2:118343001	2	118343001	118345000	2000	1	5.40E-11	-0.7	9	0.45	Kcnmb2	Transport
DMR2:118346001	2	118346001	118347000	1000	1	2.20E-08	0.37	7	0.7	Kcnmb2	Transport
DMR2:118349001	2	118349001	118351000	2000	1	1.20E-10	0.5	20	1	Kcnmb2	Transport
DMR2:118352001	2	118352001	118353000	1000	1	2.00E-14	0.62	15	1.5	Kcnmb2	Transport
DMR2:118360001	2	118360001	118361000	1000	1	1.50E-10	0.82	3	0.3	Kcnmb2	Transport
DMR2:118368001	2	118368001	118369000	1000	1	2.60E-12	0.45	13	1.3	Kcnmb2;LOC102548736	Transport
DMR2:118370001	2	118370001	118374000	4000	2	1.60E-07	-0.55	16	0.4	Kcnmb2;LOC102548736	Transport
DMR2:118400001	2	118400001	118402000	2000	2	3.10E-17	0.79	5	0.25	Kcnmb2;LOC102548736	Transport
DMR2:118426001	2	118426001	118427000	1000	1	2.50E-11	0.47	13	1.3	Kcnmb2	Transport
DMR2:118435001	2	118435001	118436000	1000	1	9.80E-11	0.51	6	0.6	Kcnmb2	Transport
DMR2:118437001	2	118437001	118438000	1000	1	7.40E-11	0.51	15	1.5	Kcnmb2	Transport
DMR2:118449001	2	118449001	118451000	2000	1	1.40E-07	0.54	12	0.6	Kcnmb2	Transport
DMR2:118475001	2	118475001	118476000	1000	1	1.40E-21	0.69	10	1	Kcnmb2	Transport
DMR2:118491001	2	118491001	118495000	4000	4	1.50E-29	-0.58	69	1.73	Kcnmb2	Transport
DMR2:118497001	2	118497001	118503000	6000	4	1.50E-19	0.89	55	0.92	Kcnmb2	Transport
DMR2:118510001	2	118510001	118511000	1000	1	1.20E-12	0.62	10	1	Kcnmb2	Transport
DMR2:118512001	2	118512001	118513000	1000	1	1.90E-11	0.54	9	0.9	Kcnmb2	Transport
DMR2:118533001	2	118533001	118534000	1000	1	3.00E-07	0.5	10	1	Kcnmb2	Transport
DMR2:118546001	2	118546001	118547000	1000	1	2.20E-08	0.45	12	1.2	Kcnmb2	Transport
DMR2:118559001	2	118559001	118564000	5000	2	2.90E-08	-0.65	55	1.1	Kcnmb2	Transport
DMR2:118570001	2	118570001	118575000	5000	3	7.30E-11	0.63	47	0.94	Kcnmb2	Transport
DMR2:118589001	2	118589001	118592000	3000	1	3.20E-07	0.69	13	0.43	Kcnmb2	Transport
DMR2:118725001	2	118725001	118729000	4000	1	9.40E-07	0.29	71	1.77	Zmat3	
DMR2:118732001	2	118732001	118733000	1000	1	3.80E-12	0.49	20	2	Zmat3	
DMR2:118857001	2	118857001	118859000	2000	1	9.00E-10	-0.7	30	1.5	Pik3ca;Kcnmb3	Signaling;Transport
DMR2:118866001	2	118866001	118871000	5000	1	2.50E-08	-0.61	73	1.46	Pik3ca;Kcnmb3	Signaling;Transport
DMR2:118879001	2	118879001	118884000	5000	1	2.60E-07	-0.56	53	1.06	Kcnmb3;LOC103691525	Transport
DMR2:118887001	2	118887001	118889000	2000	1	1.40E-09	0.61	21	1.05	Kcnmb3;LOC103691525	Transport
DMR2:118900001	2	118900001	118904000	4000	2	1.90E-07	-0.7	58	1.45	Zfp639	
DMR2:118931001	2	118931001	118933000	2000	1	2.80E-20	1	36	1.8	Mfn1	
DMR2:118935001	2	118935001	118937000	2000	1	7.00E-09	-0.51	19	0.95	Mfn1	
DMR2:118957001	2	118957001	118962000	5000	3	1.10E-08	-0.66	60	1.2	Mfn1	
DMR2:118971001	2	118971001	118974000	3000	1	2.50E-07	-0.59	35	1.17	Mfn1;Gnb4	Signaling
DMR2:118990001	2	118990001	118992000	2000	1	5.80E-11	0.49	53	2.65	Gnb4;LOC103691541	Signaling
DMR2:119004001	2	119004001	119005000	1000	1	8.90E-09	0.34	28	2.8	Gnb4;LOC103691541	Signaling
DMR2:119135001	2	119135001	119137000	2000	1	2.90E-07	-0.59	17	0.85	Actl6a;Mrpl47;Ndufb5	Cytoskeleton;Translation;Metabolism
DMR2:119203001	2	119203001	119205000	2000	1	4.30E-07	0.31	18	0.9	Usp13	Protease
DMR2:119208001	2	119208001	119209000	1000	1	1.50E-15	0.56	6	0.6	Usp13	Protease
DMR2:119217001	2	119217001	119220000	3000	1	4.60E-08	0.3	46	1.53	Usp13	Protease
DMR2:119223001	2	119223001	119228000	5000	1	3.50E-07	0.39	62	1.24	Usp13	Protease
DMR2:119229001	2	119229001	119233000	4000	1	3.00E-10	0.4	40	1	Usp13	Protease
DMR2:119234001	2	119234001	119237000	3000	1	5.20E-07	0.37	24	0.8	Usp13	Protease
DMR2:119239001	2	119239001	119240000	1000	1	4.90E-07	0.34	13	1.3	Usp13	Protease
DMR2:119267001	2	119267001	119270000	3000	1	1.80E-09	0.4	34	1.13	Usp13	Protease
DMR2:119298001	2	119298001	119303000	5000	1	2.20E-16	0.92	111	2.22	Usp13	Protease
DMR2:119309001	2	119309001	119313000	4000	1	1.20E-11	0.46	43	1.07	Usp13	Protease
DMR2:119318001	2	119318001	119319000	1000	1	1.50E-10	0.51	9	0.9	Usp13	Protease
DMR2:119322001	2	119322001	119324000	2000	2	9.10E-14	0.5	36	1.8	Usp13;Pex5l	Protease;Transport
DMR2:119347001	2	119347001	119348000	1000	1	3.10E-11	0.55	10	1	Pex5l	Transport
DMR2:119350001	2	119350001	119351000	1000	1	1.60E-10	0.62	4	0.4	Pex5l	Transport
DMR2:119368001	2	119368001	119369000	1000	1	4.60E-07	-0.56	10	1	Pex5l	Transport
DMR2:119410001	2	119410001	119411000	1000	1	8.10E-09	0.65	9	0.9	Pex5l	Transport
DMR2:119412001	2	119412001	119414000	2000	1	1.50E-10	0.42	10	0.5	Pex5l	Transport
DMR2:120256001	2	120256001	120259000	3000	1	1.20E-11	-0.54	74	2.47	Ttc14	
DMR2:120287001	2	120287001	120294000	7000	3	8.30E-10	0.66	46	0.66	Ttc14;Ccdc39	
DMR2:120302001	2	120302001	120306000	4000	1	2.30E-07	0.48	34	0.85	Ccdc39	
DMR2:120314001	2	120314001	120316000	2000	1	3.20E-11	0.72	19	0.95	Ccdc39	

DMR2:120335001	2	120335001	120337000	2000	1	2.30E-24	0.83	20	1	Ccdc39	
DMR2:120349001	2	120349001	120350000	1000	1	2.10E-07	0.38	12	1.2	Ccdc39	
DMR2:120360001	2	120360001	120362000	2000	1	1.00E-19	0.88	33	1.65	Ccdc39	
DMR2:120368001	2	120368001	120370000	2000	1	1.20E-11	0.79	14	0.7	Ccdc39	
DMR2:120494001	2	120494001	120497000	3000	2	1.40E-15	-0.86	30	1	Dnajc19	
DMR2:120559001	2	120559001	120560000	1000	1	1.00E-08	-0.54	14	1.4	Fxr1	Translation
DMR2:120573001	2	120573001	120575000	2000	2	2.20E-07	-0.6	4	0.2	Fxr1	Translation
DMR2:120580001	2	120580001	120581000	1000	1	6.90E-12	0.49	13	1.3	Fxr1	Translation
DMR2:122334001	2	122334001	122335000	1000	1	4.20E-11	0.43	8	0.8	Atp11b	Transport
DMR2:122368001	2	122368001	122369000	1000	1	1.60E-11	0.67	3	0.3	Atp11b	Transport
DMR2:122370001	2	122370001	122373000	3000	1	3.80E-07	0.33	25	0.83	Atp11b	Transport
DMR2:122417001	2	122417001	122418000	1000	1	6.10E-12	0.65	6	0.6	Atp11b	Transport
DMR2:122449001	2	122449001	122455000	6000	2	1.70E-13	-0.44	54	0.9	Atp11b	Transport
DMR2:122466001	2	122466001	122467000	1000	1	4.20E-13	0.43	13	1.3	Dcun1d1	
DMR2:122468001	2	122468001	122470000	2000	1	2.30E-07	0.32	27	1.35	Dcun1d1	
DMR2:122483001	2	122483001	122489000	6000	2	2.20E-09	-0.52	61	1.02	Dcun1d1	
DMR2:122522001	2	122522001	122524000	2000	1	3.00E-07	0.65	14	0.7	Dcun1d1;LOC102552069	
DMR2:122539001	2	122539001	122541000	2000	1	4.80E-09	0.53	16	0.8	LOC102552069;Mccc1	Metabolism
DMR2:122551001	2	122551001	122553000	2000	1	6.20E-09	0.56	36	1.8	Mccc1	Metabolism
DMR2:122555001	2	122555001	122556000	1000	1	7.00E-09	-0.59	14	1.4	Mccc1	Metabolism
DMR2:122559001	2	122559001	122560000	1000	1	4.30E-09	0.34	18	1.8	Mccc1	Metabolism
DMR2:122561001	2	122561001	122565000	4000	1	9.50E-07	-0.52	67	1.68	Mccc1	Metabolism
DMR2:122584001	2	122584001	122586000	2000	1	2.60E-08	0.43	20	1	Mccc1	Metabolism
DMR2:122606001	2	122606001	122607000	1000	1	2.40E-08	0.67	8	0.8	Mccc1;LOC102553014	Metabolism
DMR2:122610001	2	122610001	122611000	1000	1	1.80E-14	0.8	11	1.1	Mccc1;LOC102553014	Metabolism
DMR2:122613001	2	122613001	122614000	1000	1	9.70E-07	0.4	19	1.9	Mccc1;LOC102553014	Metabolism
DMR2:122629001	2	122629001	122630000	1000	1	3.40E-10	0.53	10	1	Mccc1;LOC102553014	Metabolism
DMR2:122669001	2	122669001	122671000	2000	1	1.20E-08	-0.37	21	1.05	Mccc1	Metabolism
DMR2:122684001	2	122684001	122689000	5000	2	1.30E-16	-0.62	83	1.66	Mccc1	Metabolism
DMR2:122705001	2	122705001	122710000	5000	2	2.60E-17	0.5	60	1.2	Ccdc144b	
DMR2:122711001	2	122711001	122714000	3000	1	3.10E-07	-0.37	30	1	Ccdc144b	
DMR2:122743001	2	122743001	122745000	2000	1	5.00E-08	-0.5	14	0.7	Ccdc144b	
DMR2:122799001	2	122799001	122801000	2000	1	3.30E-08	0.32	22	1.1	Acad9	Metabolism
DMR2:122806001	2	122806001	122808000	2000	2	4.10E-14	0.64	13	0.65	Acad9	Metabolism
DMR2:122845001	2	122845001	122846000	1000	1	4.70E-08	0.4	12	1.2	Rslcan18	
DMR2:122858001	2	122858001	122860000	2000	1	7.20E-09	0.32	27	1.35	Rslcan18;LOC102556166;LOC108349996	
DMR2:122868001	2	122868001	122869000	1000	1	1.10E-08	-0.7	15	1.5	Rslcan18;LOC102556166;LOC108349996;Arse	Metabolism
DMR2:122881001	2	122881001	122883000	2000	1	3.50E-10	0.56	61	3.05	Rslcan18;Arse;Qrfpr	Metabolism;Signaling
DMR2:122894001	2	122894001	122895000	1000	1	9.50E-08	-0.5	15	1.5	Arse;Qrfpr	Metabolism;Signaling
DMR2:122913001	2	122913001	122914000	1000	1	8.80E-14	0.61	8	0.8	Qrfpr	Signaling
DMR2:122915001	2	122915001	122918000	3000	2	1.00E-17	0.92	33	1.1	Qrfpr	Signaling
DMR2:122939001	2	122939001	122940000	1000	1	2.00E-19	0.73	12	1.2	Qrfpr	Signaling
DMR2:122941001	2	122941001	122943000	2000	2	2.20E-07	0.39	39	1.95	Qrfpr	Signaling
DMR2:122945001	2	122945001	122947000	2000	1	1.40E-16	0.91	9	0.45	Qrfpr	Signaling
DMR2:122950001	2	122950001	122952000	2000	1	2.80E-08	0.37	21	1.05	Qrfpr	Signaling
DMR2:123159001	2	123159001	123167000	8000	1	9.00E-08	0.34	159	1.99	Anxa5	Signaling
DMR2:123171001	2	123171001	123172000	1000	1	8.10E-09	0.37	11	1.1	Anxa5	Signaling
DMR2:123176001	2	123176001	123179000	3000	1	6.70E-16	0.84	36	1.2	Anxa5	Signaling
DMR2:123191001	2	123191001	123193000	2000	1	4.70E-18	0.79	42	2.1	Anxa5	Signaling
DMR2:123297001	2	123297001	123300000	3000	1	1.00E-09	-0.59	6	0.2	Bbs7	
DMR2:123311001	2	123311001	123317000	6000	2	4.30E-12	0.52	73	1.22	Bbs7	
DMR2:123318001	2	123318001	123321000	3000	2	2.50E-07	-0.55	27	0.9	Bbs7;Trpc3	Transport
DMR2:123340001	2	123340001	123342000	2000	1	2.00E-16	0.75	19	0.95	Trpc3	Transport
DMR2:123352001	2	123352001	123353000	1000	1	3.50E-08	-0.47	22	2.2	Trpc3	Transport
DMR2:123359001	2	123359001	123360000	1000	1	1.20E-12	0.55	13	1.3	Trpc3	Transport
DMR2:123361001	2	123361001	123365000	4000	1	1.50E-23	0.78	65	1.62	Trpc3	Transport
DMR2:123389001	2	123389001	123393000	4000	4	3.30E-12	-0.63	37	0.92	Trpc3	Transport
DMR2:123394001	2	123394001	123395000	1000	1	2.50E-07	0.42	15	1.5	Trpc3	Transport
DMR2:123419001	2	123419001	123421000	2000	1	3.20E-09	0.4	32	1.6	Trpc3;LOC365768	Transport
DMR2:123432001	2	123432001	123433000	1000	1	7.00E-07	0.41	8	0.8	Trpc3;LOC365768	Transport
DMR2:123436001	2	123436001	123441000	5000	2	4.30E-15	-0.47	60	1.2	Trpc3;LOC365768	Transport

DMR2:123463001	2	123463001	123467000	4000	4	1.10E-18	0.54	31	0.78	Trpc3	Transport
DMR2:123545001	2	123545001	123551000	6000	2	2.20E-18	-0.75	44	0.73	RGD1307100	
DMR2:123554001	2	123554001	123555000	1000	1	1.20E-07	-0.58	10	1	RGD1307100	
DMR2:123563001	2	123563001	123567000	4000	2	2.40E-12	0.7	28	0.7	RGD1307100	
DMR2:123570001	2	123570001	123574000	4000	1	1.90E-13	0.73	24	0.6	RGD1307100	
DMR2:123577001	2	123577001	123581000	4000	3	2.90E-15	0.75	22	0.55	RGD1307100	
DMR2:123590001	2	123590001	123597000	7000	5	4.00E-27	-0.57	66	0.94	RGD1307100	
DMR2:123601001	2	123601001	123612000	11000	2	4.80E-11	-0.62	45	0.41	RGD1307100	
DMR2:123620001	2	123620001	123628000	8000	4	1.20E-13	0.62	83	1.04	RGD1307100	
DMR2:123633001	2	123633001	123635000	2000	2	2.20E-25	0.95	13	0.65	RGD1307100	
DMR2:123637001	2	123637001	123638000	1000	1	7.10E-10	-0.6	12	1.2	RGD1307100	
DMR2:123640001	2	123640001	123643000	3000	1	2.90E-09	-0.5	36	1.2	RGD1307100	
DMR2:123646001	2	123646001	123651000	5000	3	1.30E-25	-0.51	73	1.46	RGD1307100	
DMR2:123654001	2	123654001	123656000	2000	1	3.10E-22	0.9	15	0.75	RGD1307100	
DMR2:123666001	2	123666001	123673000	7000	3	7.20E-20	-0.55	82	1.17	RGD1307100	
DMR2:123701001	2	123701001	123703000	2000	2	7.50E-12	0.6	12	0.6	RGD1307100	
DMR2:123704001	2	123704001	123705000	1000	1	1.50E-14	0.88	1	0.1	RGD1307100	
DMR2:123726001	2	123726001	123729000	3000	2	1.10E-11	-0.47	29	0.97	RGD1307100	
DMR2:123754001	2	123754001	123757000	3000	1	7.00E-13	0.5	37	1.23	RGD1307100	
DMR2:123776001	2	123776001	123779000	3000	1	2.00E-09	0.5	8	0.27	RGD1307100	
DMR2:123802001	2	123802001	123804000	2000	1	2.50E-11	0.55	11	0.55	Adad1	Metabolism
DMR2:123813001	2	123813001	123814000	1000	1	2.00E-07	-0.47	7	0.7	Adad1	Metabolism
DMR2:123815001	2	123815001	123819000	4000	1	4.80E-07	-0.41	31	0.78	Adad1	Metabolism
DMR2:123835001	2	123835001	123839000	4000	1	1.80E-07	-0.38	25	0.62	Adad1;Il2	Metabolism
DMR2:123842001	2	123842001	123845000	3000	2	5.10E-25	0.85	10	0.33	Adad1;Il2	Metabolism
DMR2:123856001	2	123856001	123857000	1000	1	1.90E-12	0.64	8	0.8	Adad1;Il2	Metabolism
DMR2:123860001	2	123860001	123861000	1000	1	1.60E-09	0.59	5	0.5	Il2	
DMR2:123980001	2	123980001	123982000	2000	1	1.10E-13	0.51	20	1	Il21	Cytokine
DMR2:124028001	2	124028001	124037000	9000	3	2.80E-16	-0.44	107	1.19	Cetn4	Signaling
DMR2:124058001	2	124058001	124059000	1000	1	2.90E-07	0.51	8	0.8	Cetn4;Bbs12	Signaling
DMR2:124075001	2	124075001	124079000	4000	1	8.20E-17	0.67	47	1.18	Fgf2	Growth Factors
DMR2:124104001	2	124104001	124106000	2000	1	5.70E-07	0.34	19	0.95	Fgf2	Growth Factors
DMR2:124117001	2	124117001	124118000	1000	1	9.90E-09	0.38	13	1.3	Fgf2	Growth Factors
DMR2:124122001	2	124122001	124128000	6000	1	5.40E-11	0.57	62	1.03	Fgf2;Nudt6	Growth Factors;Signaling
DMR2:124164001	2	124164001	124166000	2000	2	4.60E-12	-0.5	19	0.95	Spata5	
DMR2:124178001	2	124178001	124179000	1000	1	3.00E-13	0.75	4	0.4	Spata5;LOC108349998	
DMR2:124184001	2	124184001	124185000	1000	1	6.30E-25	0.75	11	1.1	Spata5;LOC108349998	
DMR2:124191001	2	124191001	124199000	8000	6	7.90E-21	-0.58	79	0.99	Spata5;LOC108349998	
DMR2:124212001	2	124212001	124215000	3000	3	5.80E-09	-0.58	12	0.4	Spata5	
DMR2:124216001	2	124216001	124217000	1000	1	5.00E-07	0.55	4	0.4	Spata5	
DMR2:124270001	2	124270001	124271000	1000	1	3.90E-08	0.4	4	0.4	Spata5	
DMR2:124289001	2	124289001	124294000	5000	1	4.20E-10	-0.52	41	0.82	Spata5;LOC102546767	
DMR2:124307001	2	124307001	124309000	2000	1	3.90E-09	0.51	8	0.4	Spata5	
DMR2:124320001	2	124320001	124322000	2000	2	4.70E-15	0.84	11	0.55	Spata5	
DMR2:124349001	2	124349001	124351000	2000	1	5.30E-07	-0.48	24	1.2	Spata5;LOC108350242	
DMR2:125243001	2	125243001	125245000	2000	1	2.30E-11	0.44	17	0.85	RGD1564378	
DMR2:125249001	2	125249001	125251000	2000	1	3.10E-18	0.94	9	0.45	RGD1564378	
DMR2:125256001	2	125256001	125259000	3000	1	1.60E-17	0.88	21	0.7	RGD1564378	
DMR2:125296001	2	125296001	125299000	3000	1	9.30E-09	-0.61	49	1.63	Ankrd50	Cytoskeleton
DMR2:125302001	2	125302001	125304000	2000	1	4.70E-11	0.6	18	0.9	Ankrd50	Cytoskeleton
DMR2:125311001	2	125311001	125312000	1000	1	1.10E-17	0.56	16	1.6	Ankrd50	Cytoskeleton
DMR2:125317001	2	125317001	125319000	2000	1	4.70E-11	0.65	12	0.6	Ankrd50	Cytoskeleton
DMR2:125742001	2	125742001	125744000	2000	1	8.80E-08	0.49	12	0.6	Fat4	Cytoskeleton
DMR2:125745001	2	125745001	125748000	3000	1	2.10E-08	-0.43	37	1.23	Fat4	Cytoskeleton
DMR2:125764001	2	125764001	125765000	1000	1	3.20E-20	0.66	7	0.7	Fat4	Cytoskeleton
DMR2:125796001	2	125796001	125797000	1000	1	1.10E-07	-0.53	7	0.7	Fat4	Cytoskeleton
DMR2:125803001	2	125803001	125804000	1000	1	1.00E-09	0.41	13	1.3	Fat4	Cytoskeleton
DMR2:125817001	2	125817001	125819000	2000	1	9.60E-12	0.65	10	0.5	Fat4	Cytoskeleton
DMR2:125827001	2	125827001	125829000	2000	1	9.50E-12	0.61	14	0.7	Fat4	Cytoskeleton
DMR2:125857001	2	125857001	125858000	1000	1	5.50E-11	-0.62	10	1	Fat4	Cytoskeleton
DMR2:125889001	2	125889001	125890000	1000	1	3.80E-10	0.55	9	0.9	Fat4	Cytoskeleton
DMR2:127479001	2	127479001	127480000	1000	1	9.50E-09	0.57	14	1.4	Intu	
DMR2:127490001	2	127490001	127491000	1000	1	3.30E-09	0.42	6	0.6	Intu	
DMR2:127499001	2	127499001	127501000	2000	1	5.20E-18	0.8	19	0.95	Intu	

DMR2:127505001	2	127505001	127507000	2000	1	5.90E-09	-0.4	20	1	Intu	
DMR2:127513001	2	127513001	127517000	4000	2	5.00E-16	0.98	44	1.1	Intu	
DMR2:127532001	2	127532001	127534000	2000	1	8.70E-08	-0.47	8	0.4	LOC100360560;Slc25a31	Transport
DMR2:127602001	2	127602001	127604000	2000	1	6.50E-09	0.45	13	0.65	Intu	
DMR2:127611001	2	127611001	127614000	3000	1	3.60E-13	0.68	33	1.1	Intu	
DMR2:127615001	2	127615001	127620000	5000	1	7.00E-07	-0.35	34	0.68	Intu;Hspa4l	
DMR2:127621001	2	127621001	127623000	2000	1	1.00E-10	0.47	10	0.5	Intu;Hspa4l	
DMR2:127641001	2	127641001	127643000	2000	1	7.70E-09	-0.67	14	0.7	Hspa4l	
DMR2:127651001	2	127651001	127652000	1000	1	1.50E-25	0.9	7	0.7	Hspa4l	
DMR2:127660001	2	127660001	127661000	1000	1	1.50E-08	0.46	4	0.4	Hspa4l	
DMR2:127696001	2	127696001	127697000	1000	1	5.10E-10	0.53	10	1	Plk4;Mfsd8	Signaling
DMR2:127758001	2	127758001	127765000	7000	3	1.30E-11	-0.51	63	0.9	Mfsd8;Abhd18	
DMR2:127779001	2	127779001	127782000	3000	1	9.90E-10	0.38	31	1.03	Mfsd8;Abhd18;LOC365778	
DMR2:127805001	2	127805001	127811000	6000	4	6.30E-12	0.6	44	0.73	Abhd18	
DMR2:127823001	2	127823001	127824000	1000	1	2.80E-13	0.68	1	0.1	Abhd18	
DMR2:127840001	2	127840001	127841000	1000	1	3.80E-07	0.73	6	0.6	Larp1b	Metabolism
DMR2:127853001	2	127853001	127854000	1000	1	1.10E-09	0.44	4	0.4	Larp1b	Metabolism
DMR2:127865001	2	127865001	127866000	1000	1	8.00E-08	0.43	10	1	Larp1b	Metabolism
DMR2:127882001	2	127882001	127883000	1000	1	2.00E-13	0.66	4	0.4	Larp1b	Metabolism
DMR2:127932001	2	127932001	127938000	6000	3	1.40E-19	-0.55	57	0.95	RGD1565989	
DMR2:127945001	2	127945001	127949000	4000	1	1.00E-11	0.61	33	0.82	RGD1565989	
DMR2:128493001	2	128493001	128497000	4000	1	1.20E-08	0.32	46	1.15	Jade1	Transcription
DMR2:128536001	2	128536001	128538000	2000	1	2.60E-07	-0.49	10	0.5	Sclt1;LOC102553838	
DMR2:128612001	2	128612001	128613000	1000	1	7.00E-09	0.4	11	1.1	Sclt1	
DMR2:128639001	2	128639001	128640000	1000	1	3.80E-07	0.42	4	0.4	Sclt1	
DMR2:128650001	2	128650001	128651000	1000	1	1.30E-24	0.81	5	0.5	Sclt1	
DMR2:128657001	2	128657001	128665000	8000	4	4.70E-24	-0.58	93	1.16	Sclt1	
DMR2:128672001	2	128672001	128673000	1000	1	6.00E-22	1.27	4	0.4	Sclt1;RGD1359508	
DMR2:128684001	2	128684001	128685000	1000	1	3.80E-11	0.66	6	0.6	Sclt1;RGD1359508	
DMR2:128686001	2	128686001	128690000	4000	2	5.90E-13	-0.41	48	1.2	RGD1359508	
DMR2:128712001	2	128712001	128716000	4000	2	2.00E-10	-0.66	14	0.35	RGD1359508	
DMR2:128729001	2	128729001	128730000	1000	1	4.70E-07	0.46	7	0.7	RGD1359508	
DMR2:128747001	2	128747001	128750000	3000	1	3.90E-09	0.35	64	2.13	RGD1359508	
DMR2:130650001	2	130650001	130651000	1000	1	4.50E-08	0.53	5	0.5	Pabpc4l	
DMR2:130662001	2	130662001	130665000	3000	2	1.50E-11	-0.47	20	0.67	Pabpc4l	
DMR2:130667001	2	130667001	130670000	3000	2	2.20E-11	-0.45	27	0.9	Pabpc4l	
DMR2:132238001	2	132238001	132239000	1000	1	8.70E-28	1.22	69	6.9	RGD1563562	Signaling
DMR2:132283001	2	132283001	132285000	2000	1	1.90E-07	0.44	8	0.4	RGD1563562;LOC102555656	Signaling
DMR2:136993001	2	136993001	136994000	1000	1	6.10E-10	0.68	38	3.8	LOC102549579;Pcdh10	Cytoskeleton
DMR2:138188001	2	138188001	138189000	1000	1	7.70E-07	0.48	2	0.2	RGD1307595	Transcription
DMR2:138203001	2	138203001	138204000	1000	1	8.60E-08	0.65	4	0.4	RGD1307595	Transcription
DMR2:139500001	2	139500001	139502000	2000	2	4.30E-17	0.87	15	0.75	Slc7a11	Transport
DMR2:139532001	2	139532001	139534000	2000	1	2.10E-13	0.78	12	0.6	Slc7a11	Transport
DMR2:140275001	2	140275001	140277000	2000	1	2.90E-07	0.36	23	1.15	Noct	Translation
DMR2:140318001	2	140318001	140320000	2000	1	5.80E-18	0.86	15	0.75	Elf2	Transcription
DMR2:140321001	2	140321001	140322000	1000	1	1.70E-07	0.37	6	0.6	Elf2	Transcription
DMR2:140340001	2	140340001	140343000	3000	1	1.50E-16	0.62	58	1.93	Elf2	Transcription
DMR2:140344001	2	140344001	140347000	3000	1	9.30E-12	0.42	51	1.7	Elf2	Transcription
DMR2:140369001	2	140369001	140371000	2000	2	8.80E-24	1.02	25	1.25	Elf2	Transcription
DMR2:140375001	2	140375001	140382000	7000	1	8.40E-09	-0.66	104	1.49	Elf2	Transcription
DMR2:140385001	2	140385001	140386000	1000	1	6.90E-07	0.53	7	0.7	Elf2	Transcription
DMR2:140440001	2	140440001	140442000	2000	2	1.20E-13	0.46	21	1.05	Mgarp	
DMR2:140451001	2	140451001	140452000	1000	1	3.00E-07	0.49	8	0.8	Mgarp;Ndufc1	Metabolism
DMR2:140476001	2	140476001	140477000	1000	1	2.50E-08	0.63	1	0.1	Naa15	Metabolism
DMR2:140482001	2	140482001	140484000	2000	2	8.20E-14	-0.79	11	0.55	Naa15	Metabolism
DMR2:140520001	2	140520001	140521000	1000	1	3.90E-08	0.53	6	0.6	Naa15	Metabolism
DMR2:140557001	2	140557001	140560000	3000	1	5.70E-13	0.58	40	1.33	Rab33b	
DMR2:140561001	2	140561001	140565000	4000	1	6.40E-10	-0.47	60	1.5	Rab33b	
DMR2:140567001	2	140567001	140571000	4000	1	9.30E-08	-0.58	51	1.27	Setd7	Epigenetic
DMR2:140612001	2	140612001	140613000	1000	1	4.40E-15	0.52	17	1.7	Setd7;LOC103691564	Epigenetic
DMR2:140704001	2	140704001	140707000	3000	1	1.10E-08	0.56	40	1.33	Mgst2	Transport
DMR2:140713001	2	140713001	140715000	2000	2	7.20E-14	0.57	27	1.35	Mgst2	Transport
DMR2:140740001	2	140740001	140742000	2000	1	3.10E-07	-0.44	16	0.8	Maml3	
DMR2:140747001	2	140747001	140749000	2000	1	2.20E-07	-0.6	28	1.4	Maml3	
DMR2:140777001	2	140777001	140778000	1000	1	1.70E-10	0.54	16	1.6	Maml3	

DMR2:140812001	2	140812001	140815000	3000	2	6.00E-14	0.73	37	1.23	Maml3;LOC679491	
DMR2:140820001	2	140820001	140826000	6000	4	4.60E-15	-0.55	52	0.87	Maml3;Dusp14l1	
DMR2:140828001	2	140828001	140833000	5000	1	5.10E-10	0.41	65	1.3	Maml3;Dusp14l1	
DMR2:140834001	2	140834001	140837000	3000	1	4.60E-11	0.51	65	2.17	Maml3;Dusp14l1	
DMR2:140839001	2	140839001	140843000	4000	1	6.50E-08	0.65	30	0.75	Maml3;Dusp14l1	
DMR2:140844001	2	140844001	140845000	1000	1	5.90E-07	0.4	11	1.1	Maml3;Dusp14l1	
DMR2:140852001	2	140852001	140855000	3000	1	1.30E-12	0.69	27	0.9	Maml3	
DMR2:140861001	2	140861001	140862000	1000	1	9.80E-08	0.52	20	2	Maml3	
DMR2:140873001	2	140873001	140876000	3000	2	2.80E-25	1.06	36	1.2	Maml3	
DMR2:140882001	2	140882001	140886000	4000	1	3.10E-12	0.56	47	1.18	Maml3	
DMR2:140901001	2	140901001	140907000	6000	1	2.10E-08	-0.66	90	1.5	Maml3	
DMR2:140917001	2	140917001	140918000	1000	1	7.20E-07	-0.6	19	1.9	Maml3	
DMR2:140940001	2	140940001	140941000	1000	1	1.10E-09	0.51	16	1.6	Maml3	
DMR2:140959001	2	140959001	140964000	5000	1	1.00E-10	0.42	103	2.06	Maml3	
DMR2:140979001	2	140979001	140984000	5000	1	1.90E-08	-0.59	79	1.58	Maml3	
DMR2:140985001	2	140985001	140986000	1000	1	3.10E-15	0.49	13	1.3	Maml3	
DMR2:140987001	2	140987001	140992000	5000	1	5.10E-08	-0.5	87	1.74	Maml3	
DMR2:141000001	2	141000001	141001000	1000	1	2.00E-20	0.68	18	1.8	Maml3	
DMR2:141005001	2	141005001	141008000	3000	1	6.50E-10	-0.76	39	1.3	Maml3	
DMR2:141021001	2	141021001	141022000	1000	1	2.50E-14	0.57	13	1.3	Maml3	
DMR2:141025001	2	141025001	141031000	6000	1	7.60E-07	0.36	116	1.93	Maml3	
DMR2:141042001	2	141042001	141051000	9000	1	2.40E-07	-0.47	177	1.97	Maml3	
DMR2:141059001	2	141059001	141060000	1000	1	7.10E-07	0.31	13	1.3	Maml3	
DMR2:141061001	2	141061001	141067000	6000	1	1.80E-27	0.86	98	1.63	Maml3	
DMR2:141084001	2	141084001	141087000	3000	1	1.40E-08	0.43	41	1.37	Maml3	
DMR2:141090001	2	141090001	141092000	2000	1	1.20E-09	0.47	13	0.65	Maml3	
DMR2:141094001	2	141094001	141095000	1000	1	1.10E-10	0.44	12	1.2	Maml3	
DMR2:141101001	2	141101001	141108000	7000	2	1.80E-14	0.56	116	1.66	Maml3	
DMR2:141138001	2	141138001	141141000	3000	1	5.70E-13	0.53	37	1.23	Maml3	
DMR2:141146001	2	141146001	141148000	2000	1	6.60E-07	-0.35	32	1.6	Maml3	
DMR2:141156001	2	141156001	141159000	3000	1	4.60E-15	0.6	35	1.17	Maml3	
DMR2:141163001	2	141163001	141164000	1000	1	1.80E-07	-0.63	9	0.9	Maml3	
DMR2:141192001	2	141192001	141196000	4000	1	6.60E-08	-0.45	63	1.57	Maml3	
DMR2:141199001	2	141199001	141200000	1000	1	8.70E-13	0.51	9	0.9	Maml3	
DMR2:141203001	2	141203001	141213000	10000	2	2.50E-12	0.41	134	1.34	Maml3	
DMR2:141219001	2	141219001	141221000	2000	1	3.60E-11	0.39	38	1.9	Maml3	
DMR2:141231001	2	141231001	141236000	5000	1	6.60E-10	-0.61	86	1.72	Maml3	
DMR2:141239001	2	141239001	141244000	5000	1	2.20E-10	-0.73	74	1.48	Maml3	
DMR2:141282001	2	141282001	141284000	2000	1	6.50E-19	0.87	25	1.25	Maml3	
DMR2:141443001	2	141443001	141445000	2000	1	2.70E-07	-0.53	25	1.25	LOC103691555;Foxo1	
DMR2:141459001	2	141459001	141461000	2000	1	2.30E-11	-0.77	18	0.9	LOC103691555;Foxo1	
DMR2:141471001	2	141471001	141473000	2000	1	7.20E-07	-0.5	16	0.8	Foxo1	
DMR2:141481001	2	141481001	141482000	1000	1	4.60E-12	0.48	15	1.5	Foxo1	
DMR2:141486001	2	141486001	141487000	1000	1	3.40E-10	0.42	27	2.7	Foxo1	
DMR2:141526001	2	141526001	141529000	3000	1	4.20E-11	0.56	40	1.33	Foxo1	
DMR2:141875001	2	141875001	141879000	4000	2	1.10E-08	-0.57	23	0.58	Trnak-cuu	
DMR2:141889001	2	141889001	141891000	2000	2	1.50E-13	0.51	23	1.15	Trnak-cuu	
DMR2:142188001	2	142188001	142191000	3000	1	9.70E-14	0.52	14	0.47	Cog6	
DMR2:142217001	2	142217001	142219000	2000	1	1.50E-26	1.05	18	0.9	Cog6	
DMR2:142241001	2	142241001	142243000	2000	1	7.20E-07	0.45	16	0.8	Cog6	
DMR2:142253001	2	142253001	142255000	2000	2	8.00E-16	0.9	15	0.75	Lhfp	
DMR2:142258001	2	142258001	142261000	3000	2	2.50E-09	-0.52	48	1.6	Lhfp	
DMR2:142269001	2	142269001	142273000	4000	1	4.80E-07	-0.39	56	1.4	Lhfp	
DMR2:142292001	2	142292001	142294000	2000	1	3.20E-11	0.48	24	1.2	Lhfp	
DMR2:142315001	2	142315001	142318000	3000	1	1.20E-12	0.57	24	0.8	Lhfp	
DMR2:142336001	2	142336001	142339000	3000	1	1.60E-16	0.95	26	0.87	Lhfp	
DMR2:142373001	2	142373001	142376000	3000	3	1.90E-10	-0.64	35	1.17	Lhfp	
DMR2:142378001	2	142378001	142380000	2000	2	1.70E-10	-0.65	31	1.55	Lhfp	
DMR2:142401001	2	142401001	142404000	3000	1	3.90E-11	0.42	29	0.97	Lhfp	
DMR2:142463001	2	142463001	142465000	2000	1	4.10E-07	-0.45	39	1.95	Lhfp	
DMR2:142674001	2	142674001	142677000	3000	1	7.80E-09	0.41	46	1.53	Nhlrc3;LOC102549317;Proser1	Proteolysis
DMR2:142699001	2	142699001	142700000	1000	1	8.90E-07	0.33	18	1.8	Proser1	
DMR2:142715001	2	142715001	142718000	3000	1	3.30E-07	0.35	48	1.6	Proser1;Stoml3	Cytoskeleton
DMR2:142721001	2	142721001	142724000	3000	1	7.30E-16	0.61	30	1	Stoml3	Cytoskeleton
DMR2:142732001	2	142732001	142740000	8000	1	1.70E-07	0.58	123	1.54	Stoml3;Frem2	Cytoskeleton

DMR2:142745001	2	142745001	142749000	4000	1	2.90E-12	0.47	87	2.17	Frem2	
DMR2:142762001	2	142762001	142765000	3000	2	3.20E-09	-0.48	37	1.23	Frem2	
DMR2:142771001	2	142771001	142775000	4000	1	5.50E-10	0.37	48	1.2	Frem2	
DMR2:142776001	2	142776001	142777000	1000	1	2.40E-07	-0.63	14	1.4	Frem2	
DMR2:142779001	2	142779001	142786000	7000	2	1.50E-10	-0.54	100	1.43	Frem2	
DMR2:142791001	2	142791001	142793000	2000	1	1.50E-13	0.65	31	1.55	Frem2	
DMR2:142806001	2	142806001	142811000	5000	1	1.50E-07	0.44	85	1.7	Frem2	
DMR2:142823001	2	142823001	142828000	5000	3	1.70E-15	0.59	68	1.36	Frem2	
DMR2:142837001	2	142837001	142850000	13000	4	1.00E-19	0.66	188	1.45	Frem2	
DMR2:142894001	2	142894001	142900000	6000	4	6.00E-29	-0.58	68	1.13	Frem2	
DMR2:143462001	2	143462001	143464000	2000	1	5.50E-07	-0.45	19	0.95	Trpc4	Transport
DMR2:143481001	2	143481001	143489000	8000	6	2.50E-35	-0.61	109	1.36	Trpc4	Transport
DMR2:143528001	2	143528001	143531000	3000	1	5.60E-07	0.45	31	1.03	Trpc4	Transport
DMR2:143543001	2	143543001	143546000	3000	1	6.70E-07	0.42	40	1.33	Trpc4	Transport
DMR2:143570001	2	143570001	143571000	1000	1	3.60E-14	0.62	7	0.7	Trpc4	Transport
DMR2:143575001	2	143575001	143576000	1000	1	4.60E-10	0.38	12	1.2	Trpc4	Transport
DMR2:143598001	2	143598001	143599000	1000	1	5.60E-15	0.84	5	0.5	Trpc4	Transport
DMR2:143611001	2	143611001	143617000	6000	2	9.00E-17	-0.55	63	1.05	Trpc4	Transport
DMR2:143670001	2	143670001	143673000	3000	1	3.10E-10	-0.69	37	1.23	Postn	Cytoskeleton
DMR2:143675001	2	143675001	143676000	1000	1	4.70E-18	0.59	12	1.2	Postn	Cytoskeleton
DMR2:143689001	2	143689001	143694000	5000	1	7.90E-09	-0.49	60	1.2	Postn	Cytoskeleton
DMR2:143695001	2	143695001	143697000	2000	2	9.50E-14	0.69	21	1.05	Postn	Cytoskeleton
DMR2:143824001	2	143824001	143825000	1000	1	5.40E-16	0.6	10	1	Rexo1l1-ps1	Transcription
DMR2:143828001	2	143828001	143829000	1000	1	1.90E-07	0.42	12	1.2	Rexo1l1-ps1	Transcription
DMR2:143834001	2	143834001	143837000	3000	1	8.50E-08	-0.4	29	0.97	Rexo1l1-ps1	Transcription
DMR2:143839001	2	143839001	143840000	1000	1	5.30E-10	0.49	10	1	Rexo1l1-ps1	Transcription
DMR2:143884001	2	143884001	143887000	3000	1	3.00E-10	0.42	24	0.8	Supt20h	Epigenetic
DMR2:143901001	2	143901001	143903000	2000	1	6.20E-10	0.56	14	0.7	Supt20h	Epigenetic
DMR2:143913001	2	143913001	143914000	1000	1	2.80E-08	-0.51	6	0.6	Supt20h	Epigenetic
DMR2:143937001	2	143937001	143940000	3000	1	1.00E-07	-0.44	57	1.9	Exosc8;Alg5	Transcription;Golgi
DMR2:143943001	2	143943001	143944000	1000	1	4.40E-12	0.44	23	2.3	Alg5;Smad9	Golgi;Transcription
DMR2:143949001	2	143949001	143950000	1000	1	7.40E-09	-0.61	20	2	Alg5;Smad9	Golgi;Transcription
DMR2:143968001	2	143968001	143970000	2000	1	4.40E-12	0.48	23	1.15	Smad9	Transcription
DMR2:143974001	2	143974001	143976000	2000	1	2.00E-07	0.23	12	0.6	Smad9	Transcription
DMR2:143988001	2	143988001	143990000	2000	1	9.70E-07	0.37	30	1.5	Smad9	Transcription
DMR2:144221001	2	144221001	144222000	1000	1	3.10E-13	0.58	3	0.3	LOC103691556;Rfxapl1	
DMR2:144227001	2	144227001	144236000	9000	4	4.80E-23	0.68	97	1.08	Rfxapl1	
DMR2:144297001	2	144297001	144298000	1000	1	1.80E-13	0.45	13	1.3	Sertm1	
DMR2:144300001	2	144300001	144303000	3000	1	6.10E-18	0.72	27	0.9	Sertm1	
DMR2:144327001	2	144327001	144328000	1000	1	1.60E-12	0.74	8	0.8	Sertm1	
DMR2:144330001	2	144330001	144332000	2000	1	7.10E-08	0.43	22	1.1	Sertm1	
DMR2:144461001	2	144461001	144467000	6000	1	1.80E-13	0.66	79	1.32	Ccna1	Signaling
DMR2:144505001	2	144505001	144509000	4000	1	1.60E-08	0.55	54	1.35	Ccna1	Signaling
DMR2:144566001	2	144566001	144567000	1000	1	9.00E-08	0.42	12	1.2	Ccdc169	
DMR2:144568001	2	144568001	144569000	1000	1	6.70E-08	0.44	14	1.4	Ccdc169	
DMR2:144575001	2	144575001	144577000	2000	1	4.60E-09	-0.59	22	1.1	Ccdc169	
DMR2:144621001	2	144621001	144624000	3000	1	3.70E-07	0.38	28	0.93	Sohlh2	Transcription
DMR2:144641001	2	144641001	144643000	2000	1	4.40E-07	-0.54	33	1.65	Dclk1	Signaling
DMR2:144650001	2	144650001	144653000	3000	1	2.60E-09	0.41	49	1.63	Dclk1	Signaling
DMR2:144667001	2	144667001	144669000	2000	1	7.40E-11	0.67	23	1.15	Dclk1;LOC108350012	Signaling
DMR2:144694001	2	144694001	144696000	2000	1	1.20E-07	0.36	21	1.05	Dclk1	Signaling
DMR2:144708001	2	144708001	144714000	6000	1	3.40E-12	0.62	70	1.17	Dclk1	Signaling
DMR2:144730001	2	144730001	144731000	1000	1	4.50E-21	0.91	10	1	Dclk1	Signaling
DMR2:144751001	2	144751001	144753000	2000	1	5.90E-13	0.67	27	1.35	Dclk1	Signaling
DMR2:144762001	2	144762001	144766000	4000	2	1.50E-08	-0.39	61	1.52	Dclk1	Signaling
DMR2:144767001	2	144767001	144769000	2000	1	3.60E-16	0.79	18	0.9	Dclk1	Signaling
DMR2:144775001	2	144775001	144779000	4000	1	4.50E-09	-0.55	55	1.38	Dclk1	Signaling
DMR2:144804001	2	144804001	144806000	2000	1	4.40E-07	-0.68	24	1.2	Dclk1;LOC102552324	Signaling
DMR2:144837001	2	144837001	144842000	5000	1	2.60E-16	0.77	64	1.28	Dclk1	Signaling
DMR2:144855001	2	144855001	144857000	2000	1	4.40E-22	0.66	31	1.55	Dclk1	Signaling
DMR2:144871001	2	144871001	144876000	5000	1	6.40E-09	-0.4	83	1.66	Dclk1	Signaling
DMR2:144885001	2	144885001	144886000	1000	1	1.80E-07	-0.54	12	1.2	Dclk1	Signaling
DMR2:144895001	2	144895001	144898000	3000	1	2.10E-11	0.53	37	1.23	Dclk1	Signaling
DMR2:144899001	2	144899001	144901000	2000	1	7.80E-09	0.43	25	1.25	Dclk1	Signaling
DMR2:144902001	2	144902001	144905000	3000	1	7.50E-19	0.63	43	1.43	Dclk1	Signaling

DMR2:144907001	2	144907001	144910000	3000	1	1.90E-09	-0.45	30	1	Dclk1	Signaling
DMR2:144924001	2	144924001	144929000	5000	1	8.00E-10	-0.52	77	1.54	Dclk1	Signaling
DMR2:144931001	2	144931001	144934000	3000	1	6.40E-09	0.37	50	1.67	Dclk1	Signaling
DMR2:144935001	2	144935001	144939000	4000	3	2.70E-15	-0.59	65	1.62	Dclk1	Signaling
DMR2:144941001	2	144941001	144949000	8000	4	1.10E-08	0.44	101	1.26	Dclk1	Signaling
DMR2:145007001	2	145007001	145009000	2000	1	9.50E-07	-0.36	19	0.95	Nbea	
DMR2:145027001	2	145027001	145028000	1000	1	6.20E-10	0.63	8	0.8	Nbea	
DMR2:145061001	2	145061001	145063000	2000	1	3.50E-12	0.59	19	0.95	Nbea	
DMR2:145071001	2	145071001	145074000	3000	2	1.30E-13	0.5	27	0.9	Nbea	
DMR2:145085001	2	145085001	145088000	3000	1	1.20E-08	-0.39	30	1	Nbea	
DMR2:145098001	2	145098001	145106000	8000	4	1.40E-28	-0.59	91	1.14	Nbea	
DMR2:145111001	2	145111001	145113000	2000	1	3.70E-09	0.48	16	0.8	Nbea	
DMR2:145124001	2	145124001	145126000	2000	2	1.30E-13	0.58	111	5.55	Nbea	
DMR2:145137001	2	145137001	145140000	3000	1	1.80E-08	-0.55	33	1.1	Nbea;LOC102552834	
DMR2:145205001	2	145205001	145207000	2000	1	1.40E-08	0.55	15	0.75	Nbea	
DMR2:145211001	2	145211001	145212000	1000	1	1.80E-09	0.61	6	0.6	Nbea;LOC102552778	
DMR2:145245001	2	145245001	145246000	1000	1	4.00E-11	0.53	6	0.6	Nbea	
DMR2:145253001	2	145253001	145254000	1000	1	2.10E-17	0.94	5	0.5	Nbea	
DMR2:145256001	2	145256001	145265000	9000	4	3.20E-20	-0.58	102	1.13	Nbea	
DMR2:145286001	2	145286001	145287000	1000	1	4.30E-11	0.58	3	0.3	Nbea	
DMR2:145290001	2	145290001	145294000	4000	1	2.40E-08	0.53	37	0.92	Nbea	
DMR2:145300001	2	145300001	145301000	1000	1	1.50E-15	0.6	13	1.3	Nbea	
DMR2:145308001	2	145308001	145316000	8000	6	4.50E-16	0.61	62	0.78	Nbea	
DMR2:145351001	2	145351001	145352000	1000	1	9.30E-12	0.66	5	0.5	Nbea	
DMR2:145375001	2	145375001	145377000	2000	2	8.40E-14	-0.77	5	0.25	Nbea	
DMR2:145433001	2	145433001	145438000	5000	2	3.70E-10	0.59	39	0.78	Nbea	
DMR2:145445001	2	145445001	145446000	1000	1	2.70E-08	0.4	9	0.9	Nbea	
DMR2:145453001	2	145453001	145456000	3000	1	4.60E-08	0.37	29	0.97	Nbea	
DMR2:145465001	2	145465001	145466000	1000	1	9.40E-09	0.5	8	0.8	Nbea	
DMR2:145472001	2	145472001	145475000	3000	1	9.40E-09	-0.53	16	0.53	Nbea	
DMR2:145519001	2	145519001	145522000	3000	2	2.10E-12	0.97	25	0.83	Nbea	
DMR2:145933001	2	145933001	145936000	3000	1	4.80E-18	0.88	24	0.8	Eif3i-ps1	
DMR2:146946001	2	146946001	146950000	4000	1	3.90E-08	-0.47	9	0.22	RGD1563340	
DMR2:147007001	2	147007001	147008000	1000	1	1.90E-10	-0.58	8	0.8	RGD1306704	
DMR2:147012001	2	147012001	147015000	3000	1	2.20E-16	0.6	30	1	RGD1306704	
DMR2:147017001	2	147017001	147019000	2000	1	2.50E-08	0.41	8	0.4	RGD1306704	
DMR2:147026001	2	147026001	147029000	3000	1	4.30E-08	0.49	13	0.43	RGD1306704	
DMR2:147470001	2	147470001	147473000	3000	2	3.70E-07	-0.6	12	0.4	RGD1563943	
DMR2:147483001	2	147483001	147484000	1000	1	4.30E-25	-0.88	5	0.5	RGD1563943	
DMR2:147567001	2	147567001	147570000	3000	1	9.50E-09	0.44	25	0.83	Wwtr1	Transcription
DMR2:147600001	2	147600001	147602000	2000	2	2.00E-26	1.03	13	0.65	Wwtr1	Transcription
DMR2:147615001	2	147615001	147617000	2000	1	4.30E-08	-0.57	18	0.9	Wwtr1	Transcription
DMR2:147618001	2	147618001	147620000	2000	1	2.50E-11	0.66	22	1.1	Wwtr1	Transcription
DMR2:147622001	2	147622001	147624000	2000	2	4.00E-11	-0.54	21	1.05	Wwtr1	Transcription
DMR2:147634001	2	147634001	147640000	6000	1	3.50E-11	0.57	79	1.32	Wwtr1	Transcription
DMR2:147645001	2	147645001	147646000	1000	1	3.30E-11	0.56	6	0.6	Wwtr1	Transcription
DMR2:147654001	2	147654001	147657000	3000	1	1.20E-09	0.55	30	1	Wwtr1	Transcription
DMR2:147663001	2	147663001	147665000	2000	1	7.60E-07	-0.49	24	1.2	Wwtr1	Transcription
DMR2:147680001	2	147680001	147682000	2000	1	1.30E-07	0.41	21	1.05	Wwtr1	Transcription
DMR2:147689001	2	147689001	147691000	2000	1	3.70E-09	-0.44	32	1.6	Wwtr1	Transcription
DMR2:147698001	2	147698001	147699000	1000	1	6.30E-10	0.5	10	1	Wwtr1	Transcription
DMR2:147700001	2	147700001	147702000	2000	1	2.00E-15	0.61	24	1.2	Wwtr1;LOC100909433	Transcription
DMR2:147784001	2	147784001	147786000	2000	1	9.10E-08	0.37	24	1.2	Commd2;LOC108350015;Ankub1	
DMR2:147793001	2	147793001	147798000	5000	4	4.20E-09	-0.53	46	0.92	LOC108350015;Ankub1	
DMR2:147820001	2	147820001	147822000	2000	2	1.10E-17	0.74	13	0.65	Ankub1;Rpl36a-ps1	
DMR2:147826001	2	147826001	147829000	3000	2	3.90E-15	0.63	42	1.4	Ankub1;Rpl36a-ps1	
DMR2:147843001	2	147843001	147846000	3000	2	1.00E-16	0.9	23	0.77	Rnf13	
DMR2:147863001	2	147863001	147865000	2000	1	2.50E-23	1.22	13	0.65	Rnf13	
DMR2:147868001	2	147868001	147870000	2000	1	8.50E-25	0.88	14	0.7	Rnf13	
DMR2:147873001	2	147873001	147874000	1000	1	3.10E-07	-0.39	10	1	Rnf13	
DMR2:147889001	2	147889001	147890000	1000	1	1.80E-07	0.49	6	0.6	Rnf13	
DMR2:147910001	2	147910001	147913000	3000	1	8.70E-08	-0.59	14	0.47	Rnf13	
DMR2:147922001	2	147922001	147923000	1000	1	7.30E-17	1.01	3	0.3	Rnf13	
DMR2:147933001	2	147933001	147934000	1000	1	4.20E-07	-0.35	10	1	Rnf13	

DMR2:147966001	2	147966001	147982000	16000	13	5.90E-29	-0.67	156	0.98	Pfn2	Cytoskeleton
DMR2:148664001	2	148664001	148665000	1000	1	3.00E-08	0.61	4	0.4	Tsc22d2	
DMR2:148666001	2	148666001	148670000	4000	2	4.20E-08	-0.52	16	0.4	Tsc22d2	
DMR2:148710001	2	148710001	148711000	1000	1	7.00E-12	0.55	14	1.4	Serp1	Transcription
DMR2:148749001	2	148749001	148751000	2000	1	2.10E-10	0.66	8	0.4	Eif2a	Translation
DMR2:148810001	2	148810001	148811000	1000	1	2.50E-21	1.04	7	0.7	Erich6	
DMR2:148817001	2	148817001	148820000	3000	1	2.90E-08	-0.68	32	1.07	Erich6	
DMR2:148826001	2	148826001	148828000	2000	2	3.30E-23	0.84	15	0.75	Erich6	
DMR2:148830001	2	148830001	148831000	1000	1	6.30E-07	-0.44	11	1.1	Erich6	
DMR2:148838001	2	148838001	148840000	2000	1	3.90E-15	0.93	35	1.75	Erich6	
DMR2:148843001	2	148843001	148846000	3000	3	7.20E-20	-0.47	46	1.53	Erich6	
DMR2:148847001	2	148847001	148849000	2000	1	2.60E-07	0.31	14	0.7	Erich6	
DMR2:148856001	2	148856001	148858000	2000	1	1.10E-08	-0.54	14	0.7	Erich6	
DMR2:148866001	2	148866001	148874000	8000	1	1.40E-14	0.72	103	1.29	Erich6;Siah2	Proteolysis
DMR2:148896001	2	148896001	148899000	3000	3	4.00E-11	-0.78	21	0.7	Siah2	Proteolysis
DMR2:149005001	2	149005001	149007000	2000	1	3.10E-15	0.69	30	1.5	Fam188b2	
DMR2:149016001	2	149016001	149017000	1000	1	3.00E-07	-0.4	15	1.5	Fam188b2	
DMR2:149070001	2	149070001	149071000	1000	1	4.40E-18	0.86	7	0.7	Clrn1	
DMR2:149089001	2	149089001	149091000	2000	1	9.50E-09	0.43	27	1.35	Clrn1;LOC108350018	
DMR2:149095001	2	149095001	149098000	3000	1	6.70E-10	0.49	34	1.13	Clrn1;LOC108350018	
DMR2:149221001	2	149221001	149223000	2000	1	1.00E-21	0.8	31	1.55	Med12l	Transcription
DMR2:149226001	2	149226001	149228000	2000	1	2.40E-07	-0.51	30	1.5	Med12l	Transcription
DMR2:149229001	2	149229001	149233000	4000	2	1.90E-12	0.41	64	1.6	Med12l	Transcription
DMR2:149242001	2	149242001	149246000	4000	2	4.40E-12	0.46	61	1.52	Med12l	Transcription
DMR2:149248001	2	149248001	149252000	4000	1	1.50E-08	-0.65	73	1.82	Med12l	Transcription
DMR2:149255001	2	149255001	149257000	2000	1	2.40E-07	0.34	24	1.2	Med12l	Transcription
DMR2:149271001	2	149271001	149273000	2000	1	9.00E-10	0.4	37	1.85	Med12l	Transcription
DMR2:149279001	2	149279001	149280000	1000	1	2.50E-07	-0.39	13	1.3	Med12l	Transcription
DMR2:149284001	2	149284001	149285000	1000	1	1.80E-10	0.52	12	1.2	Med12l	Transcription
DMR2:149289001	2	149289001	149290000	1000	1	5.90E-08	-0.58	14	1.4	Med12l	Transcription
DMR2:149294001	2	149294001	149300000	6000	4	7.20E-12	-0.44	55	0.92	Med12l	Transcription
DMR2:149311001	2	149311001	149315000	4000	1	1.40E-08	-0.45	39	0.98	Med12l;Gpr171	Transcription;Signaling
DMR2:149319001	2	149319001	149323000	4000	1	5.80E-07	0.57	47	1.18	Med12l;Gpr171;P2ry14	Transcription;Signaling
DMR2:149335001	2	149335001	149337000	2000	1	2.30E-07	0.39	31	1.55	Gpr171;P2ry14	Signaling
DMR2:149339001	2	149339001	149342000	3000	1	3.80E-07	0.37	40	1.33	P2ry14	Signaling
DMR2:149382001	2	149382001	149383000	1000	1	3.60E-09	-0.58	6	0.6	P2ry14	Signaling
DMR2:149386001	2	149386001	149392000	6000	3	2.70E-12	0.48	38	0.63	P2ry14;Gpr87	Signaling
DMR2:149393001	2	149393001	149395000	2000	1	4.10E-07	-0.49	35	1.75	Gpr87	Signaling
DMR2:149399001	2	149399001	149406000	7000	2	2.30E-10	-0.57	114	1.63	Gpr87	Signaling
DMR2:149424001	2	149424001	149425000	1000	1	7.10E-16	0.56	7	0.7	Gpr87;P2ry13	Signaling
DMR2:149435001	2	149435001	149436000	1000	1	2.00E-12	-0.6	12	1.2	P2ry13;P2ry12	Signaling
DMR2:149453001	2	149453001	149455000	2000	1	1.90E-08	0.46	42	2.1	P2ry12	Signaling
DMR2:149471001	2	149471001	149475000	4000	1	1.10E-09	-0.56	38	0.95	P2ry12	Signaling
DMR2:149479001	2	149479001	149482000	3000	1	7.90E-10	0.39	38	1.27	P2ry12	Signaling
DMR2:149535001	2	149535001	149538000	3000	1	8.70E-10	-0.47	61	2.03	Igsf10	
DMR2:149543001	2	149543001	149545000	2000	1	7.20E-08	0.56	14	0.7	Igsf10	
DMR2:149558001	2	149558001	149559000	1000	1	2.40E-08	0.48	7	0.7	Igsf10	
DMR2:149570001	2	149570001	149574000	4000	3	2.50E-11	0.69	23	0.58	Igsf10	
DMR2:149739001	2	149739001	149741000	2000	2	2.80E-09	0.52	12	0.6	RGD1559622;LOC103691561	Metabolism
DMR2:149744001	2	149744001	149749000	5000	2	2.00E-11	-0.59	30	0.6	RGD1559622;LOC103691561	Metabolism
DMR2:149754001	2	149754001	149760000	6000	1	2.80E-12	0.68	34	0.57	RGD1559622	Metabolism
DMR2:149833001	2	149833001	149837000	4000	2	3.40E-15	-0.55	29	0.72	RGD1561998	Metabolism
DMR2:149842001	2	149842001	149843000	1000	1	1.30E-07	0.4	5	0.5	RGD1561998	Metabolism
DMR2:149846001	2	149846001	149851000	5000	2	1.70E-10	-0.71	21	0.42	RGD1561998	Metabolism
DMR2:149861001	2	149861001	149862000	1000	1	1.30E-09	-0.69	5	0.5	RGD1561998	Metabolism
DMR2:149892001	2	149892001	149893000	1000	1	7.40E-07	-0.5	3	0.3	RGD1560324	Metabolism
DMR2:149911001	2	149911001	149912000	1000	1	4.70E-07	0.51	9	0.9	RGD1560324	Metabolism
DMR2:149914001	2	149914001	149918000	4000	2	2.70E-18	0.88	26	0.65	RGD1560324	Metabolism
DMR2:149919001	2	149919001	149920000	1000	1	8.00E-10	-0.47	11	1.1	RGD1560324	Metabolism
DMR2:149928001	2	149928001	149929000	1000	1	1.70E-19	0.9	11	1.1	RGD1560324	Metabolism
DMR2:149996001	2	149996001	149999000	3000	1	8.20E-07	0.47	21	0.7	RGD1560324	Metabolism
DMR2:150000001	2	150000001	150002000	2000	1	3.90E-22	0.88	14	0.7	RGD1560324	Metabolism
DMR2:150047001	2	150047001	150055000	8000	5	3.60E-20	-0.45	73	0.91	RGD1560162	

DMR2:150063001	2	150063001	150065000	2000	1	8.90E-09	-0.44	60	3	RGD1560162	
DMR2:150072001	2	150072001	150073000	1000	1	3.70E-08	-0.43	17	1.7	RGD1560162	
DMR2:150092001	2	150092001	150098000	6000	3	3.50E-13	-0.47	36	0.6	RGD1560162;LOC108350019;LOC100910567	
DMR2:150161001	2	150161001	150162000	1000	1	1.60E-13	0.72	3	0.3	Aadac	Metabolism
DMR2:150357001	2	150357001	150358000	1000	1	3.70E-16	1	6	0.6	Aadacl2	Metabolism
DMR2:150359001	2	150359001	150362000	3000	1	5.60E-09	-0.54	18	0.6	Aadacl2	Metabolism
DMR2:150701001	2	150701001	150703000	2000	1	4.00E-08	-0.67	29	1.45	Mbnl1	Translation
DMR2:150704001	2	150704001	150706000	2000	1	1.30E-09	0.48	14	0.7	Mbnl1	Translation
DMR2:150715001	2	150715001	150722000	7000	4	4.50E-24	-0.5	80	1.14	Mbnl1	Translation
DMR2:150735001	2	150735001	150736000	1000	1	1.10E-11	0.7	7	0.7	Mbnl1	Translation
DMR2:150794001	2	150794001	150796000	2000	1	1.80E-08	-0.46	18	0.9	Mbnl1	Translation
DMR2:150845001	2	150845001	150848000	3000	1	3.30E-08	-0.81	36	1.2	Mbnl1	Translation
DMR2:150862001	2	150862001	150865000	3000	2	7.30E-09	-0.57	42	1.4	Mbnl1;LOC100910956	Translation
DMR2:151307001	2	151307001	151308000	1000	1	5.00E-07	0.49	8	0.8	P2ry1	Signaling
DMR2:151311001	2	151311001	151312000	1000	1	1.30E-08	0.53	7	0.7	P2ry1	Signaling
DMR2:152795001	2	152795001	152796000	1000	1	1.10E-12	0.72	8	0.8	Dhx36	Transcription
DMR2:152799001	2	152799001	152800000	1000	1	2.40E-18	0.89	1	0.1	Dhx36	Transcription
DMR2:152816001	2	152816001	152820000	4000	1	2.00E-17	0.64	24	0.6	Dhx36	Transcription
DMR2:152853001	2	152853001	152860000	7000	4	3.60E-26	-0.5	76	1.09	Gpr149	Signaling
DMR2:152873001	2	152873001	152876000	3000	1	1.80E-18	-0.46	36	1.2	Gpr149	Signaling
DMR2:152890001	2	152890001	152891000	1000	1	1.80E-19	0.93	3	0.3	Gpr149	Signaling
DMR2:152906001	2	152906001	152908000	2000	2	2.30E-14	0.92	16	0.8	Gpr149	Signaling
DMR2:153825001	2	153825001	153826000	1000	1	1.10E-08	0.51	5	0.5	Mme	Protease
DMR2:153828001	2	153828001	153830000	2000	2	4.50E-14	0.62	16	0.8	Mme	Protease
DMR2:153839001	2	153839001	153840000	1000	1	4.60E-07	0.46	5	0.5	Mme	Protease
DMR2:153849001	2	153849001	153853000	4000	2	1.10E-10	0.59	41	1.02	Mme	Protease
DMR2:153864001	2	153864001	153865000	1000	1	2.30E-11	0.51	7	0.7	Mme	Protease
DMR2:153907001	2	153907001	153912000	5000	2	5.70E-08	-0.64	38	0.76	LOC108350023;RGD1560703	
DMR2:153913001	2	153913001	153914000	1000	1	4.10E-07	-0.65	6	0.6	RGD1560703	
DMR2:154255001	2	154255001	154256000	1000	1	9.40E-09	0.57	6	0.6	Plch1	Metabolism
DMR2:154271001	2	154271001	154272000	1000	1	3.50E-08	-0.62	14	1.4	Plch1	Metabolism
DMR2:154292001	2	154292001	154294000	2000	1	1.70E-19	0.66	18	0.9	Plch1	Metabolism
DMR2:154306001	2	154306001	154307000	1000	1	3.20E-11	0.49	5	0.5	Plch1	Metabolism
DMR2:154318001	2	154318001	154319000	1000	1	3.70E-16	0.62	9	0.9	Plch1	Metabolism
DMR2:154358001	2	154358001	154359000	1000	1	6.50E-07	0.45	6	0.6	Plch1	Metabolism
DMR2:154361001	2	154361001	154363000	2000	1	1.90E-07	-0.33	13	0.65	Plch1	Metabolism
DMR2:154386001	2	154386001	154393000	7000	3	3.50E-14	-0.5	71	1.01	Plch1	Metabolism
DMR2:154394001	2	154394001	154398000	4000	1	1.50E-08	-0.52	31	0.78	Plch1	Metabolism
DMR2:154403001	2	154403001	154406000	3000	1	8.90E-08	-0.53	30	1	Plch1	Metabolism
DMR2:154412001	2	154412001	154414000	2000	2	4.40E-20	0.69	19	0.95	Plch1	Metabolism
DMR2:154426001	2	154426001	154428000	2000	1	1.00E-15	0.79	12	0.6	Plch1	Metabolism
DMR2:154435001	2	154435001	154436000	1000	1	1.20E-08	-0.67	11	1.1	Plch1	Metabolism
DMR2:154446001	2	154446001	154449000	3000	1	1.40E-14	0.59	23	0.77	Plch1	Metabolism
DMR2:154450001	2	154450001	154451000	1000	1	2.70E-17	0.69	9	0.9	Plch1	Metabolism
DMR2:154471001	2	154471001	154478000	7000	6	2.50E-24	-0.51	60	0.86	Plch1;RGD1565059	Metabolism
DMR2:154496001	2	154496001	154502000	6000	3	3.80E-19	-0.4	75	1.25	RGD1565059	
DMR2:154515001	2	154515001	154516000	1000	1	5.20E-10	0.46	7	0.7	RGD1565059;Slc33a1	Transport
DMR2:154524001	2	154524001	154526000	2000	1	8.80E-09	0.63	18	0.9	Slc33a1	Transport
DMR2:154563001	2	154563001	154565000	2000	2	1.20E-12	0.59	10	0.5	Gmps	Metabolism
DMR2:154629001	2	154629001	154630000	1000	1	4.70E-13	0.52	8	0.8	Vom2r44	Signaling
DMR2:154638001	2	154638001	154639000	1000	1	3.90E-14	0.77	2	0.2	Vom2r44	Signaling
DMR2:154641001	2	154641001	154644000	3000	1	1.70E-08	0.55	14	0.47	Vom2r44	Signaling
DMR2:154669001	2	154669001	154670000	1000	1	7.50E-07	0.49	9	0.9	Vom2r45	
DMR2:154684001	2	154684001	154685000	1000	1	1.50E-14	0.65	6	0.6	Vom2r45	
DMR2:154687001	2	154687001	154688000	1000	1	2.30E-08	0.55	7	0.7	Vom2r45	
DMR2:154690001	2	154690001	154694000	4000	2	5.70E-15	-0.43	71	1.77	Vom2r45	
DMR2:154695001	2	154695001	154696000	1000	1	1.00E-11	0.83	6	0.6	Vom2r45	
DMR2:154699001	2	154699001	154700000	1000	1	6.20E-12	-0.84	10	1	Vom2r45	
DMR2:154701001	2	154701001	154702000	1000	1	1.70E-12	0.61	7	0.7	Vom2r45	
DMR2:154709001	2	154709001	154714000	5000	2	3.30E-19	0.93	28	0.56	Vom2r45	
DMR2:154719001	2	154719001	154721000	2000	1	4.00E-08	-0.5	9	0.45	Vom2r46	Signaling
DMR2:154724001	2	154724001	154725000	1000	1	8.70E-11	0.7	4	0.4	Vom2r46	Signaling
DMR2:154727001	2	154727001	154728000	1000	1	5.40E-12	0.47	14	1.4	Vom2r46	Signaling
DMR2:154743001	2	154743001	154747000	4000	2	3.00E-11	-0.6	19	0.48	Vom2r46	Signaling

DMR2:155358001	2	155358001	155360000	2000	2	7.30E-12	-0.78	5	0.25	Vom2r47	Signaling
DMR2:155380001	2	155380001	155382000	2000	1	9.20E-23	1.08	13	0.65	Vom2r47	Signaling
DMR2:155384001	2	155384001	155385000	1000	1	1.30E-15	0.71	8	0.8	Vom2r47	Signaling
DMR2:155482001	2	155482001	155484000	2000	1	9.00E-07	0.45	4	0.2	Vom2r-ps74	
DMR2:155546001	2	155546001	155547000	1000	1	1.40E-09	-0.6	7	0.7	Kcnab1	
DMR2:155552001	2	155552001	155553000	1000	1	9.10E-07	0.4	5	0.5	Kcnab1	
DMR2:155560001	2	155560001	155562000	2000	2	5.10E-18	0.84	14	0.7	Kcnab1	
DMR2:155564001	2	155564001	155565000	1000	1	4.80E-08	0.38	9	0.9	Kcnab1	
DMR2:155571001	2	155571001	155575000	4000	2	5.00E-11	-0.56	18	0.45	Kcnab1	
DMR2:155615001	2	155615001	155617000	2000	1	3.70E-15	0.55	19	0.95	Kcnab1	
DMR2:155639001	2	155639001	155641000	2000	1	2.00E-15	-0.8	4	0.2	Kcnab1	
DMR2:155651001	2	155651001	155655000	4000	1	2.40E-11	0.48	31	0.78	Kcnab1	
DMR2:155667001	2	155667001	155668000	1000	1	7.40E-17	0.8	5	0.5	Kcnab1	
DMR2:155669001	2	155669001	155673000	4000	2	1.90E-28	-0.56	46	1.15	Kcnab1	
DMR2:155703001	2	155703001	155704000	1000	1	4.60E-10	-0.56	4	0.4	Kcnab1	
DMR2:155705001	2	155705001	155706000	1000	1	1.20E-07	0.36	10	1	Kcnab1	
DMR2:155710001	2	155710001	155712000	2000	2	7.10E-10	0.44	13	0.65	Kcnab1	
DMR2:155757001	2	155757001	155758000	1000	1	6.50E-08	0.63	4	0.4	Kcnab1	
DMR2:155762001	2	155762001	155769000	7000	5	3.20E-16	-0.51	78	1.11	Kcnab1	
DMR2:155773001	2	155773001	155776000	3000	1	5.30E-13	0.46	30	1	Kcnab1	
DMR2:155783001	2	155783001	155786000	3000	2	4.30E-10	0.57	16	0.53	Kcnab1	
DMR2:155794001	2	155794001	155795000	1000	1	5.00E-07	0.62	7	0.7	Kcnab1	
DMR2:155834001	2	155834001	155836000	2000	2	5.50E-10	0.65	10	0.5	Kcnab1	
DMR2:155845001	2	155845001	155849000	4000	1	1.20E-17	0.7	29	0.72	Kcnab1	
DMR2:155851001	2	155851001	155854000	3000	1	5.80E-14	0.52	29	0.97	Kcnab1	
DMR2:155870001	2	155870001	155872000	2000	1	6.40E-17	0.99	5	0.25	Kcnab1	
DMR2:155875001	2	155875001	155877000	2000	1	5.60E-08	0.51	11	0.55	Kcnab1	
DMR2:155878001	2	155878001	155881000	3000	1	3.10E-07	-0.54	13	0.43	Kcnab1	
DMR2:155883001	2	155883001	155889000	6000	4	2.40E-20	-0.5	56	0.93	Kcnab1	
DMR2:155895001	2	155895001	155897000	2000	2	7.40E-29	0.75	23	1.15	Kcnab1	
DMR2:155899001	2	155899001	155901000	2000	1	2.20E-07	0.39	8	0.4	Kcnab1	
DMR2:155944001	2	155944001	155949000	5000	3	1.50E-14	-0.52	55	1.1	Kcnab1	
DMR2:155950001	2	155950001	155954000	4000	3	2.10E-21	-0.59	47	1.18	Kcnab1	
DMR2:155956001	2	155956001	155957000	1000	1	2.30E-11	0.68	7	0.7	Kcnab1	
DMR2:155983001	2	155983001	155984000	1000	1	4.30E-15	0.81	6	0.6	Kcnab1	
DMR2:155988001	2	155988001	155994000	6000	1	4.50E-07	-0.43	133	2.22	Kcnab1;LOC108350024	
DMR2:156028001	2	156028001	156029000	1000	1	1.30E-11	0.51	6	0.6	Ssr3	
DMR2:156030001	2	156030001	156031000	1000	1	3.90E-07	-0.33	7	0.7	Ssr3	
DMR2:157318001	2	157318001	157319000	1000	1	2.10E-09	0.51	8	0.8	LOC102554278;Tiparp	
DMR2:157326001	2	157326001	157328000	2000	1	6.90E-09	0.5	20	1	LOC102554278;Tiparp	
DMR2:157331001	2	157331001	157333000	2000	1	1.20E-09	0.5	5	0.25	Tiparp	
DMR2:157335001	2	157335001	157336000	1000	1	2.50E-12	-0.62	9	0.9	Tiparp;LOC108350031	
DMR2:157464001	2	157464001	157465000	1000	1	5.50E-10	-0.64	3	0.3	Lekr1	
DMR2:157484001	2	157484001	157486000	2000	1	3.20E-07	-0.43	8	0.4	Lekr1	
DMR2:157525001	2	157525001	157527000	2000	1	3.10E-07	-0.36	19	0.95	Lekr1	
DMR2:157540001	2	157540001	157546000	6000	2	3.30E-19	0.58	52	0.87	Lekr1	
DMR2:157566001	2	157566001	157567000	1000	1	3.30E-10	0.64	3	0.3	Lekr1	
DMR2:157859001	2	157859001	157862000	3000	2	1.20E-18	0.48	24	0.8	LOC108350032;Veph1	
DMR2:157869001	2	157869001	157870000	1000	1	4.60E-17	0.66	18	1.8	Veph1	
DMR2:157874001	2	157874001	157876000	2000	1	9.60E-11	0.63	14	0.7	Veph1	
DMR2:157880001	2	157880001	157882000	2000	2	7.50E-14	-0.47	15	0.75	Veph1	
DMR2:157883001	2	157883001	157884000	1000	1	9.30E-07	-0.46	11	1.1	Veph1	
DMR2:157896001	2	157896001	157899000	3000	1	3.20E-07	-0.52	49	1.63	Veph1	
DMR2:157900001	2	157900001	157901000	1000	1	9.70E-07	-0.54	9	0.9	Veph1	
DMR2:157922001	2	157922001	157923000	1000	1	1.20E-23	0.79	21	2.1	Veph1	
DMR2:157941001	2	157941001	157949000	8000	5	6.30E-20	0.94	61	0.76	Veph1	
DMR2:157951001	2	157951001	157956000	5000	3	1.00E-31	-0.64	63	1.26	Veph1	
DMR2:157960001	2	157960001	157962000	2000	1	6.50E-20	1.01	16	0.8	Veph1	
DMR2:157970001	2	157970001	157971000	1000	1	3.30E-07	-0.68	13	1.3	Veph1	
DMR2:157985001	2	157985001	157987000	2000	1	9.30E-10	0.47	22	1.1	Veph1	
DMR2:158006001	2	158006001	158007000	1000	1	5.40E-10	0.52	9	0.9	Veph1	
DMR2:158029001	2	158029001	158031000	2000	1	5.50E-12	0.81	19	0.95	Veph1	
DMR2:158045001	2	158045001	158047000	2000	1	7.40E-11	-0.66	10	0.5	Veph1	
DMR2:158069001	2	158069001	158071000	2000	1	1.10E-12	0.51	19	0.95	Veph1	
DMR2:158082001	2	158082001	158083000	1000	1	2.60E-13	0.86	9	0.9	Veph1	

DMR2:158089001	2	158089001	158092000	3000	1	9.00E-09	0.33	32	1.07	Veph1;Ptx3	
DMR2:158094001	2	158094001	158096000	2000	1	1.30E-09	-0.55	14	0.7	Veph1;Ptx3	
DMR2:158108001	2	158108001	158113000	5000	3	2.50E-13	0.68	48	0.96	Veph1;Ptx3	
DMR2:158115001	2	158115001	158116000	1000	1	2.80E-19	0.61	9	0.9	Veph1	
DMR2:158154001	2	158154001	158155000	1000	1	2.60E-11	-0.79	9	0.9	Veph1	
DMR2:158160001	2	158160001	158162000	2000	1	1.00E-23	0.87	15	0.75	Veph1	
DMR2:164133001	2	164133001	164135000	2000	1	5.70E-09	-0.74	14	0.7	Shox2;Rsrc1	Development
DMR2:164136001	2	164136001	164137000	1000	1	3.30E-13	0.86	3	0.3	Shox2;Rsrc1	Development
DMR2:164138001	2	164138001	164140000	2000	1	5.00E-18	0.68	19	0.95	Rsrc1	
DMR2:164142001	2	164142001	164145000	3000	1	1.90E-08	0.36	19	0.63	Rsrc1	
DMR2:164152001	2	164152001	164154000	2000	1	6.10E-10	-0.8	19	0.95	Rsrc1	
DMR2:164158001	2	164158001	164163000	5000	2	5.40E-08	0.56	35	0.7	Rsrc1	
DMR2:164172001	2	164172001	164175000	3000	3	1.60E-16	0.54	85	2.83	Rsrc1	
DMR2:164185001	2	164185001	164187000	2000	1	5.80E-15	0.71	14	0.7	Rsrc1	
DMR2:164188001	2	164188001	164189000	1000	1	2.30E-14	1.02	6	0.6	Rsrc1	
DMR2:164210001	2	164210001	164213000	3000	1	6.00E-17	0.74	13	0.43	Rsrc1	
DMR2:164217001	2	164217001	164218000	1000	1	2.70E-14	0.68	11	1.1	Rsrc1	
DMR2:164219001	2	164219001	164221000	2000	2	2.80E-23	0.96	12	0.6	Rsrc1	
DMR2:164240001	2	164240001	164244000	4000	1	6.50E-08	0.47	26	0.65	Rsrc1	
DMR2:164245001	2	164245001	164248000	3000	1	2.60E-07	-0.5	16	0.53	Rsrc1	
DMR2:164275001	2	164275001	164277000	2000	1	1.80E-14	-0.76	8	0.4	Rsrc1	
DMR2:164279001	2	164279001	164280000	1000	1	1.50E-16	0.65	10	1	Rsrc1	
DMR2:164286001	2	164286001	164287000	1000	1	6.50E-17	0.91	5	0.5	Rsrc1	
DMR2:164289001	2	164289001	164294000	5000	1	1.30E-21	0.59	40	0.8	Rsrc1	
DMR2:164344001	2	164344001	164351000	7000	3	1.90E-19	0.85	56	0.8	Rsrc1	
DMR2:164352001	2	164352001	164356000	4000	1	4.30E-17	0.7	20	0.5	Rsrc1	
DMR2:164358001	2	164358001	164363000	5000	1	9.70E-10	0.36	34	0.68	Rsrc1	
DMR2:164375001	2	164375001	164376000	1000	1	7.70E-08	0.54	6	0.6	Rsrc1	
DMR2:164378001	2	164378001	164385000	7000	3	5.10E-27	-0.55	84	1.2	Rsrc1	
DMR2:164424001	2	164424001	164425000	1000	1	1.80E-11	0.52	5	0.5	Rsrc1	
DMR2:164468001	2	164468001	164473000	5000	3	2.40E-09	-0.45	32	0.64	Rsrc1	
DMR2:164474001	2	164474001	164476000	2000	2	7.70E-20	0.91	10	0.5	Rsrc1	
DMR2:164485001	2	164485001	164487000	2000	1	4.10E-07	0.43	12	0.6	Rsrc1	
DMR2:164493001	2	164493001	164494000	1000	1	2.00E-10	0.71	5	0.5	Rsrc1	
DMR2:164508001	2	164508001	164509000	1000	1	5.40E-15	0.67	6	0.6	Rsrc1	
DMR2:164529001	2	164529001	164531000	2000	1	1.80E-09	0.41	30	1.5	Rsrc1	
DMR2:164532001	2	164532001	164533000	1000	1	7.10E-07	-0.5	11	1.1	Rsrc1	
DMR2:164549001	2	164549001	164550000	1000	1	4.50E-12	0.85	49	4.9	Mlf1	Signaling
DMR2:164564001	2	164564001	164566000	2000	1	2.50E-15	0.78	8	0.4	Mlf1	Signaling
DMR2:164574001	2	164574001	164576000	2000	1	2.00E-10	-0.46	10	0.5	Mlf1	Signaling
DMR2:164578001	2	164578001	164580000	2000	2	1.40E-20	0.8	11	0.55	Mlf1	Signaling
DMR2:164585001	2	164585001	164594000	9000	6	5.00E-24	-0.55	117	1.3	Mlf1;Gfm1	Signaling
DMR2:164617001	2	164617001	164619000	2000	1	1.90E-12	0.61	30	1.5	Gfm1;Lxn	Protease; Proteolysis
DMR2:164621001	2	164621001	164623000	2000	1	1.30E-08	-0.67	28	1.4	Gfm1;Lxn	Protease; Proteolysis
DMR2:164653001	2	164653001	164657000	4000	1	1.70E-07	0.36	54	1.35	Gfm1;Rarres1	Protease; Proteolysis
DMR2:164679001	2	164679001	164682000	3000	1	5.10E-07	-0.41	65	2.17	Rarres1	Protease; Proteolysis
DMR2:164687001	2	164687001	164688000	1000	1	5.00E-07	-0.58	13	1.3	Rarres1	Protease; Proteolysis
DMR2:164750001	2	164750001	164752000	2000	2	8.30E-10	-0.63	21	1.05	Mfsd1	
DMR2:164769001	2	164769001	164770000	1000	1	1.50E-09	0.44	10	1	Mfsd1;LOC310487	
DMR2:164771001	2	164771001	164774000	3000	2	1.30E-11	-0.55	23	0.77	Mfsd1;LOC310487	
DMR2:165085001	2	165085001	165086000	1000	1	1.60E-08	0.26	17	1.7	Il12a	
DMR2:165089001	2	165089001	165090000	1000	1	1.10E-19	0.61	13	1.3	Il12a	
DMR2:165521001	2	165521001	165522000	1000	1	1.90E-17	0.7	7	0.7	Ift80	
DMR2:165544001	2	165544001	165545000	1000	1	9.30E-09	-0.65	8	0.8	Ift80	
DMR2:165562001	2	165562001	165563000	1000	1	4.20E-07	-0.47	11	1.1	Ift80	
DMR2:165576001	2	165576001	165577000	1000	1	6.80E-10	0.46	5	0.5	Ift80	
DMR2:165598001	2	165598001	165599000	1000	1	5.90E-07	0.51	1	0.1	Ift80;Smc4;Mir15b;Mir16	
DMR2:165650001	2	165650001	165659000	9000	3	6.80E-18	0.6	75	0.83	Trim59	
DMR2:165684001	2	165684001	165685000	1000	1	1.10E-17	0.45	14	1.4	Kpna4	Transport
DMR2:165696001	2	165696001	165697000	1000	1	3.90E-10	0.55	8	0.8	Kpna4	Transport

DMR2:165752001	2	165752001	165754000	2000	1	5.90E-07	-0.37	38	1.9	Arl14	Signaling
DMR2:165755001	2	165755001	165756000	1000	1	1.60E-11	0.62	12	1.2	Arl14	Signaling
DMR2:165760001	2	165760001	165761000	1000	1	7.50E-07	0.31	17	1.7	Arl14	Signaling
DMR2:165763001	2	165763001	165767000	4000	1	2.60E-22	0.73	57	1.43	Arl14	Signaling
DMR2:165772001	2	165772001	165775000	3000	1	1.10E-08	0.51	30	1	Arl14	Signaling
DMR2:165820001	2	165820001	165821000	1000	1	7.60E-07	-0.46	9	0.9	RGD1563962	
DMR2:165833001	2	165833001	165835000	2000	1	9.50E-12	0.51	24	1.2	NEWGENE_1585613	
DMR2:165837001	2	165837001	165841000	4000	3	4.30E-16	0.65	47	1.18	NEWGENE_1585613	
DMR2:165842001	2	165842001	165844000	2000	1	7.10E-08	0.39	16	0.8	NEWGENE_1585613	
DMR2:165845001	2	165845001	165848000	3000	1	8.80E-14	0.44	45	1.5	NEWGENE_1585613	
DMR2:165924001	2	165924001	165925000	1000	1	1.30E-17	0.63	6	0.6	Ppm1l	Signaling
DMR2:165938001	2	165938001	165939000	1000	1	1.00E-07	0.4	19	1.9	Ppm1l	Signaling
DMR2:165952001	2	165952001	165954000	2000	1	1.20E-07	0.44	23	1.15	Ppm1l	Signaling
DMR2:165987001	2	165987001	165990000	3000	1	1.70E-14	0.69	45	1.5	Ppm1l	Signaling
DMR2:165993001	2	165993001	165995000	2000	1	3.20E-15	0.47	27	1.35	Ppm1l	Signaling
DMR2:166008001	2	166008001	166012000	4000	1	2.70E-09	0.43	29	0.72	Ppm1l	Signaling
DMR2:166013001	2	166013001	166020000	7000	1	1.50E-07	0.37	102	1.46	Ppm1l	Signaling
DMR2:166070001	2	166070001	166071000	1000	1	1.70E-17	0.83	11	1.1	Ppm1l;LOC102548099	Signaling
DMR2:166124001	2	166124001	166126000	2000	1	9.10E-08	0.47	29	1.45	Ppm1l	Signaling
DMR2:166132001	2	166132001	166135000	3000	2	1.70E-10	0.5	45	1.5	Ppm1l	Signaling
DMR2:166142001	2	166142001	166146000	4000	2	2.10E-28	0.79	75	1.88	Ppm1l	Signaling
DMR2:166155001	2	166155001	166156000	1000	1	5.30E-18	0.68	13	1.3	Ppm1l	Signaling
DMR2:166169001	2	166169001	166177000	8000	2	5.80E-10	-0.59	76	0.95	Ppm1l	Signaling
DMR2:166181001	2	166181001	166183000	2000	1	1.70E-08	0.4	13	0.65	Ppm1l	Signaling
DMR2:166189001	2	166189001	166190000	1000	1	1.70E-22	0.65	20	2	Ppm1l	Signaling
DMR2:166191001	2	166191001	166194000	3000	1	4.40E-09	-0.44	35	1.17	Ppm1l	Signaling
DMR2:166204001	2	166204001	166205000	1000	1	8.70E-08	-0.57	6	0.6	Ppm1l	Signaling
DMR2:166344001	2	166344001	166346000	2000	1	1.20E-07	-0.4	10	0.5	RGD1563620	
DMR2:166351001	2	166351001	166353000	2000	1	1.10E-15	0.71	32	1.6	RGD1563620	
DMR2:166394001	2	166394001	166395000	1000	1	5.40E-15	0.72	7	0.7	Nmd3	
DMR2:166396001	2	166396001	166397000	1000	1	6.70E-08	0.36	9	0.9	Nmd3	
DMR2:166658001	2	166658001	166659000	1000	1	8.10E-09	0.49	8	0.8	Sptssb	Golgi
DMR2:166675001	2	166675001	166677000	2000	1	1.30E-08	0.53	13	0.65	Sptssb	Golgi
DMR2:166689001	2	166689001	166694000	5000	3	3.60E-20	0.82	41	0.82	Sptssb	Golgi
DMR2:166834001	2	166834001	166837000	3000	1	1.60E-16	0.68	31	1.03	Otol1	Extracellular Matrix
DMR2:166875001	2	166875001	166876000	1000	1	4.10E-07	0.37	13	1.3	Otol1	Extracellular Matrix
DMR2:168236001	2	168236001	168240000	4000	1	2.70E-10	0.4	25	0.62	Vom1r56	
DMR2:168250001	2	168250001	168251000	1000	1	1.20E-09	0.7	3	0.3	Vom1r55	Receptor
DMR2:168258001	2	168258001	168260000	2000	1	1.20E-10	0.54	11	0.55	Vom1r55	Receptor
DMR2:170219001	2	170219001	170220000	1000	1	1.00E-09	0.51	4	0.4	Si	Metabolism
DMR2:170232001	2	170232001	170233000	1000	1	5.70E-07	-0.53	7	0.7	Si	Metabolism
DMR2:170276001	2	170276001	170277000	1000	1	7.30E-09	0.73	7	0.7	Si	Metabolism
DMR2:170310001	2	170310001	170312000	2000	2	1.10E-27	1.01	10	0.5	Si	Metabolism
DMR2:170436001	2	170436001	170437000	1000	1	2.10E-12	0.64	7	0.7	Slitrk3	
DMR2:170446001	2	170446001	170447000	1000	1	3.70E-10	0.82	4	0.4	Slitrk3	
DMR2:171143001	2	171143001	171144000	1000	1	2.90E-08	0.59	4	0.4	Bche	Metabolism
DMR2:171165001	2	171165001	171166000	1000	1	1.00E-07	0.58	20	2	Bche	Metabolism
DMR2:171203001	2	171203001	171205000	2000	1	2.80E-08	0.52	24	1.2	Bche	Metabolism
DMR2:172325001	2	172325001	172326000	1000	1	2.20E-09	0.51	6	0.6	Schip1	
DMR2:172329001	2	172329001	172338000	9000	6	1.90E-25	-0.53	112	1.24	Schip1	
DMR2:172339001	2	172339001	172345000	6000	2	1.30E-08	0.37	79	1.32	Schip1	
DMR2:172353001	2	172353001	172354000	1000	1	9.60E-07	-0.47	20	2	Schip1	
DMR2:172356001	2	172356001	172359000	3000	1	1.60E-08	0.38	47	1.57	Schip1	
DMR2:172367001	2	172367001	172369000	2000	1	4.40E-10	0.39	24	1.2	Schip1	
DMR2:172388001	2	172388001	172393000	5000	1	1.30E-15	0.54	61	1.22	Schip1	
DMR2:172435001	2	172435001	172440000	5000	2	1.30E-12	0.59	86	1.72	Schip1	
DMR2:172443001	2	172443001	172444000	1000	1	3.90E-07	-0.42	15	1.5	Schip1	
DMR2:172447001	2	172447001	172451000	4000	1	2.00E-19	0.59	50	1.25	Schip1	
DMR2:172534001	2	172534001	172536000	2000	1	1.60E-08	-0.52	10	0.5	Schip1	
DMR2:172539001	2	172539001	172540000	1000	1	9.00E-07	0.34	12	1.2	Schip1	
DMR2:172566001	2	172566001	172568000	2000	1	3.20E-08	0.43	17	0.85	Schip1	
DMR2:172599001	2	172599001	172601000	2000	1	2.40E-07	0.44	27	1.35	Schip1	
DMR2:172605001	2	172605001	172606000	1000	1	1.30E-08	0.54	6	0.6	Schip1	
DMR2:172618001	2	172618001	172619000	1000	1	4.20E-07	0.31	7	0.7	Schip1	
DMR2:172650001	2	172650001	172659000	9000	4	4.40E-20	0.71	97	1.08	Schip1;LOC100910449	

DMR2:172669001	2	172669001	172671000	2000	1	1.40E-10	0.49	17	0.85	Schip1;LOC100910449	
DMR2:172683001	2	172683001	172684000	1000	1	1.40E-12	0.56	7	0.7	Schip1;LOC100910449	
DMR2:172692001	2	172692001	172693000	1000	1	3.30E-23	0.66	3	0.3	Schip1	
DMR2:172696001	2	172696001	172699000	3000	1	7.60E-09	0.57	11	0.37	Schip1	
DMR2:172703001	2	172703001	172704000	1000	1	6.60E-09	0.48	5	0.5	Schip1	
DMR2:172705001	2	172705001	172707000	2000	1	2.00E-17	0.83	18	0.9	Schip1	
DMR2:172739001	2	172739001	172740000	1000	1	5.20E-07	0.31	12	1.2	Schip1	
DMR2:172774001	2	172774001	172775000	1000	1	6.50E-17	0.61	7	0.7	Schip1	
DMR2:172824001	2	172824001	172825000	1000	1	1.10E-14	0.65	6	0.6	Schip1	
DMR2:172845001	2	172845001	172849000	4000	2	1.10E-15	-0.59	46	1.15	Schip1	
DMR2:172858001	2	172858001	172860000	2000	1	7.00E-07	0.43	14	0.7	Schip1	
DMR2:172882001	2	172882001	172883000	1000	1	5.90E-08	0.42	5	0.5	Schip1	
DMR2:172891001	2	172891001	172897000	6000	3	2.50E-17	-0.49	63	1.05	Schip1	
DMR2:172899001	2	172899001	172900000	1000	1	4.60E-08	-0.47	3	0.3	Schip1	
DMR2:172939001	2	172939001	172942000	3000	1	2.30E-08	0.44	28	0.93	Schip1	
DMR2:172946001	2	172946001	172947000	1000	1	4.60E-07	-0.58	10	1	Schip1	
DMR2:172953001	2	172953001	172954000	1000	1	1.80E-08	0.34	4	0.4	Schip1	
DMR2:172961001	2	172961001	172962000	1000	1	5.30E-09	0.49	7	0.7	Schip1	
DMR2:172971001	2	172971001	172972000	1000	1	1.60E-17	0.65	7	0.7	Schip1	
DMR2:173023001	2	173023001	173027000	4000	1	3.60E-24	0.81	33	0.82	Schip1	
DMR2:173035001	2	173035001	173036000	1000	1	4.50E-10	0.57	3	0.3	Schip1	
DMR2:173040001	2	173040001	173041000	1000	1	1.10E-11	0.62	5	0.5	Schip1	
DMR2:173054001	2	173054001	173056000	2000	1	3.10E-09	0.55	8	0.4	Schip1	
DMR2:173057001	2	173057001	173058000	1000	1	9.80E-12	0.77	1	0.1	Schip1	
DMR2:173061001	2	173061001	173064000	3000	1	3.40E-08	-0.66	30	1	Schip1	
DMR2:173070001	2	173070001	173071000	1000	1	1.60E-14	0.72	6	0.6	Schip1	
DMR2:173082001	2	173082001	173083000	1000	1	3.90E-10	-0.71	7	0.7	Schip1	
DMR2:173097001	2	173097001	173104000	7000	4	2.70E-18	-0.59	64	0.91	Schip1	
DMR2:173443001	2	173443001	173447000	4000	2	4.90E-10	-0.59	22	0.55	LOC365821;Zbbx	
DMR2:173448001	2	173448001	173451000	3000	1	6.10E-11	0.59	21	0.7	Zbbx	
DMR2:173477001	2	173477001	173481000	4000	2	3.40E-09	-0.65	15	0.38	Zbbx	
DMR2:173532001	2	173532001	173533000	1000	1	9.60E-16	0.88	5	0.5	Zbbx	
DMR2:173570001	2	173570001	173571000	1000	1	2.60E-13	0.65	5	0.5	Zbbx	
DMR2:173572001	2	173572001	173574000	2000	1	2.30E-15	0.89	3	0.15	Zbbx	
DMR2:173666001	2	173666001	173667000	1000	1	2.50E-12	0.66	3	0.3	Serpini2;Wdr49	Protease; Proteolysis
DMR2:173697001	2	173697001	173699000	2000	1	1.10E-17	0.8	7	0.35	Wdr49	
DMR2:173702001	2	173702001	173704000	2000	1	3.10E-07	0.51	9	0.45	Wdr49	
DMR2:173714001	2	173714001	173719000	5000	2	3.10E-07	0.5	32	0.64	Wdr49	
DMR2:173722001	2	173722001	173724000	2000	1	4.30E-08	-0.56	13	0.65	Wdr49	
DMR2:173741001	2	173741001	173742000	1000	1	6.40E-07	0.47	5	0.5	Wdr49	
DMR2:173758001	2	173758001	173759000	1000	1	2.30E-09	0.56	5	0.5	Wdr49	
DMR2:173766001	2	173766001	173771000	5000	1	5.90E-12	-0.37	47	0.94	Wdr49;LOC102556029	
DMR2:173781001	2	173781001	173784000	3000	1	2.90E-09	0.47	20	0.67	Wdr49;LOC102556029	
DMR2:173787001	2	173787001	173791000	4000	1	1.50E-12	-0.39	34	0.85	Wdr49	
DMR2:173796001	2	173796001	173798000	2000	1	2.80E-07	0.42	17	0.85	Wdr49	
DMR2:173806001	2	173806001	173814000	8000	4	3.30E-11	0.62	62	0.78	Wdr49	
DMR2:174027001	2	174027001	174029000	2000	1	1.30E-11	0.49	15	0.75	Serpini1	Protease; Proteolysis
DMR2:174053001	2	174053001	174056000	3000	1	3.40E-10	0.53	14	0.47	Serpini1	Protease; Proteolysis
DMR2:174066001	2	174066001	174069000	3000	1	7.10E-21	-0.51	23	0.77	Serpini1	Protease; Proteolysis
DMR2:174078001	2	174078001	174082000	4000	2	3.30E-08	0.44	40	1	Serpini1	Protease; Proteolysis
DMR2:174087001	2	174087001	174088000	1000	1	1.20E-10	0.46	6	0.6	Serpini1	Protease; Proteolysis
DMR2:174089001	2	174089001	174093000	4000	2	2.20E-15	1.01	23	0.58	Serpini1	Protease; Proteolysis
DMR2:174100001	2	174100001	174101000	1000	1	1.00E-13	0.84	3	0.3	Serpini1	Protease; Proteolysis
DMR2:174118001	2	174118001	174120000	2000	1	1.20E-07	0.43	22	1.1	Serpini1	Protease; Proteolysis
DMR2:174313001	2	174313001	174316000	3000	1	7.60E-14	0.66	35	1.17	Golim4	
DMR2:174328001	2	174328001	174329000	1000	1	7.00E-07	0.4	7	0.7	Golim4	

DMR2:174331001	2	174331001	174332000	1000	1	1.40E-08	0.44	17	1.7	Golim4	
DMR2:174349001	2	174349001	174350000	1000	1	3.30E-07	-0.39	10	1	Golim4	
DMR2:174362001	2	174362001	174364000	2000	1	9.00E-07	0.42	12	0.6	Golim4	
DMR2:174373001	2	174373001	174375000	2000	2	5.30E-08	-0.34	24	1.2	Golim4	
DMR2:174376001	2	174376001	174380000	4000	3	2.30E-21	-0.62	54	1.35	Golim4	
DMR2:174387001	2	174387001	174394000	7000	4	6.10E-22	-0.53	74	1.06	Golim4	
DMR2:174420001	2	174420001	174421000	1000	1	6.80E-09	0.57	1	0.1	Golim4;LOC102547227	
DMR2:174422001	2	174422001	174423000	1000	1	6.20E-07	-0.6	2	0.2	Golim4;LOC102547227	
DMR2:174528001	2	174528001	174533000	5000	2	4.00E-08	-0.43	35	0.7	Fstl5	Protease; Proteolysis
DMR2:174537001	2	174537001	174538000	1000	1	1.50E-11	0.66	9	0.9	Fstl5	Protease; Proteolysis
DMR2:174565001	2	174565001	174567000	2000	1	1.50E-14	0.69	16	0.8	Fstl5	Protease; Proteolysis
DMR2:174572001	2	174572001	174573000	1000	1	6.50E-09	0.5	13	1.3	Fstl5	Protease; Proteolysis
DMR2:174575001	2	174575001	174576000	1000	1	4.40E-07	-0.51	4	0.4	Fstl5	Protease; Proteolysis
DMR2:174602001	2	174602001	174604000	2000	1	1.50E-10	0.62	14	0.7	Fstl5	Protease; Proteolysis
DMR2:174608001	2	174608001	174609000	1000	1	2.60E-09	0.62	2	0.2	Fstl5	Protease; Proteolysis
DMR2:174622001	2	174622001	174623000	1000	1	1.30E-14	0.69	2	0.2	Fstl5	Protease; Proteolysis
DMR2:174628001	2	174628001	174629000	1000	1	6.80E-10	0.45	8	0.8	Fstl5	Protease; Proteolysis
DMR2:174634001	2	174634001	174642000	8000	5	2.30E-20	0.54	63	0.79	Fstl5	Protease; Proteolysis
DMR2:174669001	2	174669001	174671000	2000	1	4.30E-08	0.59	6	0.3	Fstl5	Protease; Proteolysis
DMR2:174696001	2	174696001	174697000	1000	1	2.80E-11	-0.5	6	0.6	Fstl5	Protease; Proteolysis
DMR2:174708001	2	174708001	174711000	3000	1	4.10E-08	0.6	23	0.77	Fstl5	Protease; Proteolysis
DMR2:174755001	2	174755001	174757000	2000	2	5.60E-10	-0.64	7	0.35	Fstl5	Protease; Proteolysis
DMR2:174768001	2	174768001	174769000	1000	1	9.70E-09	0.5	8	0.8	Fstl5	Protease; Proteolysis
DMR2:174776001	2	174776001	174779000	3000	1	6.70E-08	-0.35	16	0.53	Fstl5	Protease; Proteolysis
DMR2:174808001	2	174808001	174810000	2000	1	1.10E-08	0.62	13	0.65	Fstl5	Protease; Proteolysis
DMR2:174830001	2	174830001	174832000	2000	2	1.80E-19	1	8	0.4	Fstl5	Protease; Proteolysis
DMR2:174858001	2	174858001	174860000	2000	1	3.00E-09	-0.69	7	0.35	Fstl5	Protease; Proteolysis
DMR2:174898001	2	174898001	174904000	6000	2	9.70E-09	-0.36	63	1.05	Fstl5	Protease; Proteolysis
DMR2:174908001	2	174908001	174909000	1000	1	5.80E-18	1.03	9	0.9	Fstl5	Protease; Proteolysis
DMR2:174932001	2	174932001	174934000	2000	1	4.20E-09	-0.38	17	0.85	Fstl5	Protease; Proteolysis
DMR2:174967001	2	174967001	174969000	2000	2	4.10E-10	0.66	18	0.9	Fstl5	Protease; Proteolysis
DMR2:174981001	2	174981001	174983000	2000	2	2.30E-19	1	5	0.25	Fstl5	Protease; Proteolysis
DMR2:175026001	2	175026001	175028000	2000	1	1.90E-10	0.52	13	0.65	Fstl5	Protease; Proteolysis
DMR2:175034001	2	175034001	175037000	3000	2	6.70E-09	0.51	18	0.6	Fstl5;LOC108350037	Protease; Proteolysis
DMR2:175058001	2	175058001	175060000	2000	1	1.60E-07	0.58	14	0.7	Fstl5;LOC108350037	Protease; Proteolysis
DMR2:175078001	2	175078001	175079000	1000	1	3.40E-20	1.03	3	0.3	Fstl5	Protease; Proteolysis

DMR2:175117001	2	175117001	175118000	1000	1	1.40E-12	0.75	1	0.1	Fstl5	Protease; Proteolysis
DMR2:175131001	2	175131001	175132000	1000	1	1.20E-15	0.78	3	0.3	Fstl5	Protease; Proteolysis
DMR2:175162001	2	175162001	175163000	1000	1	1.50E-09	-0.55	4	0.4	Fstl5	Protease; Proteolysis
DMR2:175177001	2	175177001	175179000	2000	1	9.70E-13	0.74	15	0.75	Fstl5	Protease; Proteolysis
DMR2:175184001	2	175184001	175185000	1000	1	1.80E-08	0.47	9	0.9	Fstl5	Protease; Proteolysis
DMR2:175196001	2	175196001	175197000	1000	1	3.60E-07	0.45	4	0.4	Fstl5	Protease; Proteolysis
DMR2:175241001	2	175241001	175243000	2000	2	3.80E-08	0.52	10	0.5	Fstl5	Protease; Proteolysis
DMR2:177642001	2	177642001	177649000	7000	4	3.20E-19	-0.5	72	1.03	LOC361966;Hsc70-ps1;LOC100363048	
DMR2:177656001	2	177656001	177657000	1000	1	1.80E-11	0.73	6	0.6	LOC361966;Hsc70-ps1;LOC100363048	
DMR2:177845001	2	177845001	177847000	2000	1	3.10E-08	0.37	12	0.6	Rapgef2	Transcription
DMR2:177897001	2	177897001	177899000	2000	1	1.70E-07	0.42	16	0.8	Rapgef2	Transcription
DMR2:177901001	2	177901001	177902000	1000	1	6.60E-13	0.57	12	1.2	Rapgef2	Transcription
DMR2:177913001	2	177913001	177917000	4000	2	9.80E-23	-0.65	38	0.95	Rapgef2	Transcription
DMR2:177936001	2	177936001	177943000	7000	2	4.70E-08	-0.46	44	0.63	Rapgef2	Transcription
DMR2:177947001	2	177947001	177958000	11000	1	2.30E-08	-0.57	145	1.32	Rapgef2	Transcription
DMR2:177973001	2	177973001	177974000	1000	1	6.40E-09	-0.5	13	1.3	Rapgef2	Transcription
DMR2:177979001	2	177979001	177985000	6000	1	1.60E-07	0.41	86	1.43	Rapgef2;LOC103691592	Transcription
DMR2:178009001	2	178009001	178013000	4000	1	1.80E-09	0.43	53	1.32	Rapgef2	Transcription
DMR2:178019001	2	178019001	178020000	1000	1	3.30E-08	-0.66	10	1	Rapgef2	Transcription
DMR2:178023001	2	178023001	178031000	8000	1	2.10E-08	-0.63	81	1.01	Rapgef2	Transcription
DMR2:178041001	2	178041001	178045000	4000	1	2.50E-07	-0.45	62	1.55	Rapgef2	Transcription
DMR2:178058001	2	178058001	178060000	2000	1	2.10E-09	0.54	41	2.05	Rapgef2	Transcription
DMR2:178064001	2	178064001	178065000	1000	1	1.00E-07	0.46	6	0.6	Rapgef2	Transcription
DMR2:178067001	2	178067001	178070000	3000	1	4.50E-17	0.56	27	0.9	Rapgef2	Transcription
DMR2:178238001	2	178238001	178241000	3000	1	4.40E-09	0.51	33	1.1	LOC499643;Fnip2	
DMR2:178246001	2	178246001	178247000	1000	1	1.70E-09	0.47	17	1.7	Fnip2	
DMR2:178249001	2	178249001	178250000	1000	1	5.40E-07	-0.46	9	0.9	Fnip2	
DMR2:178258001	2	178258001	178262000	4000	1	7.10E-09	0.54	49	1.23	Fnip2	
DMR2:178292001	2	178292001	178293000	1000	1	2.00E-21	0.83	9	0.9	Fnip2	
DMR2:178300001	2	178300001	178303000	3000	1	4.50E-17	0.57	22	0.73	Fnip2	
DMR2:178312001	2	178312001	178313000	1000	1	1.70E-14	0.69	9	0.9	Fnip2	
DMR2:178321001	2	178321001	178324000	3000	1	5.40E-07	0.27	32	1.07	Fnip2	
DMR2:178325001	2	178325001	178326000	1000	1	4.60E-18	0.59	13	1.3	Fnip2	
DMR2:178335001	2	178335001	178336000	1000	1	6.90E-09	0.78	44	4.4	Fnip2	
DMR2:178338001	2	178338001	178342000	4000	2	1.70E-18	0.82	32	0.8	Fnip2	
DMR2:178351001	2	178351001	178352000	1000	1	1.20E-12	0.68	14	1.4	Ppid	Transcription
DMR2:178357001	2	178357001	178358000	1000	1	1.40E-07	-0.62	7	0.7	Ppid;Etfdh	Transcription;Metabolism
DMR2:178395001	2	178395001	178400000	5000	1	1.80E-13	0.46	54	1.08	Etfdh;RGD1560010;Rxfp1	Metabolism;Signaling
DMR2:178431001	2	178431001	178434000	3000	2	7.00E-11	-0.58	27	0.9	Rxfp1	Signaling
DMR2:178436001	2	178436001	178437000	1000	1	5.00E-26	0.89	18	1.8	Rxfp1	Signaling
DMR2:178445001	2	178445001	178448000	3000	1	1.60E-14	0.48	33	1.1	Rxfp1	Signaling
DMR2:178449001	2	178449001	178456000	7000	2	1.80E-14	0.97	72	1.03	Rxfp1	Signaling
DMR2:178473001	2	178473001	178478000	5000	2	6.30E-12	-0.5	30	0.6	Rxfp1	Signaling
DMR2:178482001	2	178482001	178485000	3000	1	3.30E-12	0.47	33	1.1	Rxfp1	Signaling
DMR2:178490001	2	178490001	178493000	3000	1	1.10E-07	-0.41	29	0.97	Rxfp1	Signaling
DMR2:178512001	2	178512001	178517000	5000	1	2.90E-10	-0.75	64	1.28	Rxfp1;LOC108350043	Signaling
DMR2:178520001	2	178520001	178522000	2000	1	5.70E-08	0.54	16	0.8	Rxfp1;LOC108350043	Signaling
DMR2:178597001	2	178597001	178603000	6000	1	6.60E-19	0.66	86	1.43	Tmem144	
DMR2:178620001	2	178620001	178621000	1000	1	4.50E-09	0.45	9	0.9	Tmem144;LOC102556854	
DMR2:178622001	2	178622001	178623000	1000	1	2.00E-07	0.41	12	1.2	Tmem144;LOC102556854	
DMR2:178673001	2	178673001	178674000	1000	1	7.20E-10	0.57	7	0.7	Fam198b	
DMR2:178689001	2	178689001	178690000	1000	1	4.70E-07	0.56	4	0.4	Fam198b	
DMR2:178691001	2	178691001	178695000	4000	1	1.80E-07	0.41	28	0.7	Fam198b	
DMR2:178699001	2	178699001	178701000	2000	1	4.40E-10	-0.64	24	1.2	Fam198b	

DMR2:178723001	2	178723001	178726000	3000	2	1.20E-17	-0.45	51	1.7	Fam198b	
DMR2:178727001	2	178727001	178729000	2000	1	3.60E-09	-0.56	30	1.5	Fam198b	
DMR2:179590001	2	179590001	179592000	2000	1	2.30E-11	0.64	32	1.6	Gria2;Mir2985	Receptor
DMR2:179604001	2	179604001	179606000	2000	2	8.30E-23	0.79	5	0.25	Gria2	Receptor
DMR2:179611001	2	179611001	179614000	3000	1	7.30E-07	-0.46	33	1.1	Gria2	Receptor
DMR2:179634001	2	179634001	179639000	5000	3	1.50E-12	-0.47	54	1.08	Gria2	Receptor
DMR2:179676001	2	179676001	179677000	1000	1	2.00E-07	-0.67	10	1	Gria2	Receptor
DMR2:179682001	2	179682001	179684000	2000	1	1.20E-08	0.42	18	0.9	Gria2	Receptor
DMR2:179685001	2	179685001	179687000	2000	1	4.50E-08	0.45	14	0.7	Gria2	Receptor
DMR2:179691001	2	179691001	179692000	1000	1	4.40E-26	0.89	7	0.7	Gria2	Receptor
DMR2:179694001	2	179694001	179695000	1000	1	3.40E-13	0.75	4	0.4	Gria2	Receptor
DMR2:179706001	2	179706001	179707000	1000	1	1.70E-08	-0.7	11	1.1	Gria2	Receptor
DMR2:179713001	2	179713001	179719000	6000	3	3.30E-28	-0.63	73	1.22	Gria2	Receptor
DMR2:179812001	2	179812001	179815000	3000	1	1.10E-15	0.54	20	0.67	Glrb	Ion Channel
DMR2:179816001	2	179816001	179817000	1000	1	1.00E-10	-0.7	9	0.9	Glrb	Ion Channel
DMR2:179820001	2	179820001	179823000	3000	1	2.70E-07	0.51	19	0.63	Glrb	Ion Channel
DMR2:179824001	2	179824001	179829000	5000	3	1.70E-11	-0.71	43	0.86	Glrb	Ion Channel
DMR2:179837001	2	179837001	179838000	1000	1	4.90E-17	0.66	6	0.6	Glrb	Ion Channel
DMR2:179845001	2	179845001	179846000	1000	1	8.10E-10	0.43	8	0.8	Glrb	Ion Channel
DMR2:179963001	2	179963001	179966000	3000	1	9.90E-22	0.93	16	0.53	Pdgfc	Growth Factors
DMR2:179970001	2	179970001	179975000	5000	2	5.30E-08	0.58	37	0.74	Pdgfc	Growth Factors
DMR2:180004001	2	180004001	180005000	1000	1	2.40E-07	0.45	5	0.5	Pdgfc	Growth Factors
DMR2:180007001	2	180007001	180010000	3000	2	2.40E-13	-0.37	48	1.6	Pdgfc	Growth Factors
DMR2:180023001	2	180023001	180024000	1000	1	2.30E-18	0.72	9	0.9	Pdgfc	Growth Factors
DMR2:180037001	2	180037001	180038000	1000	1	1.90E-17	0.65	20	2	Pdgfc	Growth Factors
DMR2:180046001	2	180046001	180048000	2000	1	3.10E-08	-0.57	26	1.3	Pdgfc	Growth Factors
DMR2:180075001	2	180075001	180078000	3000	3	1.60E-12	-0.7	14	0.47	Pdgfc	Growth Factors
DMR2:180101001	2	180101001	180102000	1000	1	2.00E-07	0.48	7	0.7	Pdgfc	Growth Factors
DMR2:180132001	2	180132001	180135000	3000	2	2.00E-09	-0.55	26	0.87	Pdgfc	Growth Factors
DMR2:180860001	2	180860001	180861000	1000	1	5.00E-09	-0.59	17	1.7	Ctso	
DMR2:180921001	2	180921001	180923000	2000	1	6.40E-09	-0.54	26	1.3	Tdo2	Metabolism
DMR2:180938001	2	180938001	180940000	2000	2	2.30E-22	0.77	23	1.15	Asic5	Transport
DMR2:180953001	2	180953001	180955000	2000	2	1.50E-09	0.51	8	0.4	Asic5	Transport
DMR2:180961001	2	180961001	180965000	4000	2	4.30E-16	0.82	20	0.5	Asic5	Transport
DMR2:180969001	2	180969001	180970000	1000	1	1.00E-14	0.62	20	2	Asic5;Gucy1b3	Transport
DMR2:181008001	2	181008001	181009000	1000	1	1.70E-08	0.34	15	1.5	Gucy1b3	
DMR2:181018001	2	181018001	181020000	2000	1	4.30E-09	0.46	27	1.35	Gucy1b3	
DMR2:181026001	2	181026001	181028000	2000	1	3.60E-07	0.48	17	0.85	Gucy1b3	
DMR2:181031001	2	181031001	181034000	3000	3	3.80E-13	0.57	29	0.97	Gucy1b3	
DMR2:181041001	2	181041001	181042000	1000	1	2.20E-10	0.5	5	0.5	Gucy1a3	
DMR2:181063001	2	181063001	181064000	1000	1	6.40E-07	0.49	6	0.6	Gucy1a3	
DMR2:181074001	2	181074001	181076000	2000	1	5.30E-07	0.42	12	0.6	Gucy1a3	
DMR2:181091001	2	181091001	181094000	3000	1	2.10E-25	0.81	21	0.7	Gucy1a3	
DMR2:181325001	2	181325001	181326000	1000	1	7.00E-11	0.43	44	4.4	Map9	
DMR2:181339001	2	181339001	181340000	1000	1	1.30E-13	0.47	19	1.9	Map9	
DMR2:181341001	2	181341001	181347000	6000	1	6.50E-19	0.58	109	1.82	Map9	
DMR2:181359001	2	181359001	181363000	4000	2	1.40E-19	0.53	48	1.2	Map9	
DMR2:181521001	2	181521001	181525000	4000	1	2.30E-11	0.4	23	0.58	Npy2r	Signaling
DMR2:181881001	2	181881001	181884000	3000	1	4.20E-11	0.58	21	0.7	Rbm46;LOC102555909	Metabolism
DMR2:181894001	2	181894001	181895000	1000	1	5.10E-09	-0.59	2	0.2	LOC102555909;Lrat	Metabolism
DMR2:181980001	2	181980001	181983000	3000	1	3.70E-07	-0.43	49	1.63	LOC102556113;Fgg	Signaling
DMR2:181995001	2	181995001	181996000	1000	1	3.70E-10	0.49	13	1.3	Fgg;Fga	Signaling
DMR2:181998001	2	181998001	181999000	1000	1	3.10E-11	0.66	7	0.7	Fgg;Fga	Signaling
DMR2:182003001	2	182003001	182006000	3000	2	4.90E-14	0.85	19	0.63	Fgg;Fga	Signaling
DMR2:182019001	2	182019001	182023000	4000	3	1.00E-16	-0.78	22	0.55	Fga;Fgb	Signaling
DMR2:182024001	2	182024001	182028000	4000	1	9.60E-07	0.26	53	1.32	Fgb	Signaling
DMR2:182038001	2	182038001	182039000	1000	1	3.90E-10	-0.66	14	1.4	Fgb;Plrg1	Signaling
DMR2:182044001	2	182044001	182054000	10000	2	1.50E-16	0.62	118	1.18	Fgb;Plrg1	Signaling
DMR2:182097001	2	182097001	182105000	8000	4	1.80E-21	-0.51	100	1.25	Dchs2	
DMR2:182125001	2	182125001	182126000	1000	1	1.10E-08	0.6	11	1.1	Dchs2	
DMR2:182127001	2	182127001	182130000	3000	1	1.40E-13	0.83	12	0.4	Dchs2	
DMR2:182142001	2	182142001	182145000	3000	2	3.60E-09	-0.47	38	1.27	Dchs2	
DMR2:182149001	2	182149001	182150000	1000	1	2.20E-09	-0.5	14	1.4	Dchs2	
DMR2:182151001	2	182151001	182155000	4000	1	2.10E-08	-0.67	51	1.27	Dchs2	
DMR2:182168001	2	182168001	182169000	1000	1	6.60E-10	-0.68	11	1.1	Dchs2	

DMR2:182189001	2	182189001	182192000	3000	2	8.70E-08	0.42	26	0.87	Dchs2	
DMR2:182193001	2	182193001	182194000	1000	1	1.30E-09	0.44	9	0.9	Dchs2	
DMR2:182195001	2	182195001	182196000	1000	1	8.20E-29	0.81	6	0.6	Dchs2	
DMR2:182215001	2	182215001	182221000	6000	1	1.90E-08	0.39	70	1.17	Dchs2	
DMR2:182224001	2	182224001	182225000	1000	1	7.60E-09	0.56	1	0.1	Dchs2	
DMR2:182265001	2	182265001	182268000	3000	1	1.00E-07	0.38	37	1.23	Dchs2	
DMR2:182291001	2	182291001	182292000	1000	1	5.10E-07	-0.55	9	0.9	Dchs2	
DMR2:182294001	2	182294001	182295000	1000	1	2.20E-13	0.63	6	0.6	Dchs2	
DMR2:182299001	2	182299001	182302000	3000	2	3.90E-08	-0.64	47	1.57	Dchs2	
DMR2:182306001	2	182306001	182307000	1000	1	9.60E-08	0.45	13	1.3	Dchs2	
DMR2:182322001	2	182322001	182324000	2000	1	1.00E-07	0.39	25	1.25	Dchs2;LOC100360775	
DMR2:182716001	2	182716001	182720000	4000	1	1.30E-08	-0.59	59	1.48	LOC108350198;Sfrp2	Receptor
DMR2:182827001	2	182827001	182837000	10000	4	1.20E-20	-0.52	124	1.24	LOC102551394;Tlr2	
DMR2:182849001	2	182849001	182850000	1000	1	1.40E-07	0.47	8	0.8	Tlr2	
DMR2:183063001	2	183063001	183066000	3000	1	5.60E-10	0.55	29	0.97	LOC103691596;Mnd1	
DMR2:183078001	2	183078001	183080000	2000	1	3.00E-09	-0.72	20	1	LOC103691596;Mnd1	
DMR2:183113001	2	183113001	183115000	2000	1	3.50E-14	0.52	24	1.2	Mnd1	
DMR2:183117001	2	183117001	183119000	2000	2	7.50E-15	0.79	15	0.75	Mnd1	
DMR2:183131001	2	183131001	183133000	2000	1	6.30E-08	0.32	11	0.55	Mnd1	
DMR2:183165001	2	183165001	183166000	1000	1	1.30E-09	0.43	25	2.5	Trim2	Proteolysis
DMR2:183206001	2	183206001	183209000	3000	1	1.70E-07	-0.46	34	1.13	Trim2	Proteolysis
DMR2:183221001	2	183221001	183223000	2000	1	1.60E-12	0.65	20	1	Trim2;LOC102550635	Proteolysis
DMR2:183230001	2	183230001	183231000	1000	1	6.80E-09	0.53	13	1.3	Trim2;LOC102550635	Proteolysis
DMR2:183438001	2	183438001	183445000	7000	2	9.00E-29	-0.53	80	1.14	Fhdc1	
DMR2:183447001	2	183447001	183449000	2000	1	2.50E-08	0.45	24	1.2	Fhdc1	
DMR2:183461001	2	183461001	183465000	4000	1	3.20E-08	0.34	80	2	Fhdc1	
DMR2:183507001	2	183507001	183509000	2000	1	8.70E-10	-0.49	21	1.05	LOC102550864;Arfip1	Transport
DMR2:183513001	2	183513001	183514000	1000	1	1.80E-08	0.44	9	0.9	Arfip1	Transport
DMR2:183527001	2	183527001	183529000	2000	1	6.20E-08	-0.43	16	0.8	Arfip1	Transport
DMR2:183530001	2	183530001	183535000	5000	2	2.60E-13	0.53	53	1.06	Arfip1	Transport
DMR2:183567001	2	183567001	183571000	4000	1	3.40E-07	0.38	35	0.88	Arfip1	Transport
DMR2:183585001	2	183585001	183595000	10000	8	3.30E-22	0.88	105	1.05	Arfip1;Tigd4	Transport;Epigenetic
DMR2:183669001	2	183669001	183670000	1000	1	8.90E-18	0.77	7	0.7	Tmem154	
DMR2:183679001	2	183679001	183683000	4000	1	6.20E-13	0.56	46	1.15	Tmem154;LOC108350047	
DMR2:183684001	2	183684001	183691000	7000	3	6.10E-12	-0.62	97	1.39	Tmem154;LOC108350047	
DMR2:183692001	2	183692001	183696000	4000	2	1.90E-08	0.43	72	1.8	Tmem154;LOC108350047	
DMR2:183703001	2	183703001	183706000	3000	1	2.10E-17	0.54	38	1.27	Tmem154	
DMR2:183707001	2	183707001	183708000	1000	1	3.50E-07	-0.55	13	1.3	Tmem154	
DMR2:183709001	2	183709001	183712000	3000	1	3.50E-08	0.36	34	1.13	Tmem154	
DMR2:183715001	2	183715001	183719000	4000	1	3.00E-08	0.42	47	1.18	Tmem154	
DMR2:183720001	2	183720001	183723000	3000	2	1.50E-17	0.91	35	1.17	Tmem154	
DMR2:184229001	2	184229001	184231000	2000	1	4.90E-09	-0.58	29	1.45	Fbxw7	Proteolysis
DMR2:184232001	2	184232001	184233000	1000	1	2.40E-08	-0.37	21	2.1	Fbxw7	Proteolysis
DMR2:184308001	2	184308001	184309000	1000	1	1.20E-20	0.7	11	1.1	Fbxw7	Proteolysis
DMR2:184316001	2	184316001	184318000	2000	1	7.90E-09	0.36	28	1.4	Fbxw7	Proteolysis
DMR2:184616001	2	184616001	184618000	2000	1	1.50E-07	-0.6	26	1.3	Gatb	Metabolism
DMR2:184622001	2	184622001	184624000	2000	1	4.90E-09	0.42	11	0.55	Gatb	Metabolism
DMR2:184662001	2	184662001	184665000	3000	1	5.10E-07	0.59	35	1.17	Gatb	Metabolism
DMR2:184673001	2	184673001	184675000	2000	1	5.30E-07	-0.29	19	0.95	Gatb;Fam160a1	Metabolism
DMR2:184678001	2	184678001	184685000	7000	1	7.00E-07	-0.5	98	1.4	Gatb;Fam160a1	Metabolism
DMR2:184690001	2	184690001	184694000	4000	2	1.90E-14	-0.58	84	2.1	Fam160a1	
DMR2:184732001	2	184732001	184733000	1000	1	3.40E-23	0.88	5	0.5	Fam160a1	
DMR2:184744001	2	184744001	184747000	3000	1	9.20E-19	0.9	12	0.4	Fam160a1	
DMR2:184752001	2	184752001	184756000	4000	2	2.50E-07	0.48	18	0.45	Fam160a1	
DMR2:184765001	2	184765001	184767000	2000	1	3.40E-07	0.4	5	0.25	Fam160a1	
DMR2:184840001	2	184840001	184841000	1000	1	1.10E-10	0.93	6	0.6	Fam160a1;LOC680510	
DMR2:184865001	2	184865001	184868000	3000	1	1.10E-08	0.46	33	1.1	Fam160a1	
DMR2:184896001	2	184896001	184897000	1000	1	5.30E-07	0.46	10	1	Fam160a1;LOC108348208;LOC100910255	
DMR2:184927001	2	184927001	184929000	2000	2	5.00E-13	0.77	15	0.75	Fam160a1;LOC100910284	
DMR2:184998001	2	184998001	185003000	5000	1	5.70E-10	0.47	81	1.62	Fam160a1	
DMR2:185274001	2	185274001	185279000	5000	1	5.30E-07	-0.49	64	1.28	Sh3d19;Prss48	Protease
DMR2:185282001	2	185282001	185283000	1000	1	2.60E-07	0.41	11	1.1	Sh3d19;Prss48	Protease
DMR2:185309001	2	185309001	185313000	4000	1	8.40E-13	0.46	54	1.35	Sh3d19;Prss48	Protease
DMR2:185319001	2	185319001	185322000	3000	1	3.40E-08	0.32	46	1.53	Sh3d19	

DMR2:185327001	2	185327001	185329000	2000	1	2.90E-28	1.05	19	0.95	Sh3d19	
DMR2:185351001	2	185351001	185352000	1000	1	4.50E-14	0.47	25	2.5	Sh3d19	
DMR2:185358001	2	185358001	185360000	2000	1	7.70E-11	0.52	29	1.45	Sh3d19	
DMR2:185361001	2	185361001	185365000	4000	1	6.80E-08	0.38	61	1.52	Sh3d19	
DMR2:185380001	2	185380001	185381000	1000	1	1.70E-12	0.59	8	0.8	Sh3d19	
DMR2:185382001	2	185382001	185383000	1000	1	4.20E-09	0.44	10	1	Sh3d19	
DMR2:185387001	2	185387001	185390000	3000	1	3.50E-16	0.6	32	1.07	Sh3d19	
DMR2:185401001	2	185401001	185404000	3000	1	8.90E-10	0.42	40	1.33	Sh3d19	
DMR2:185440001	2	185440001	185443000	3000	3	8.80E-12	-0.59	32	1.07	Sh3d19;Rps3a	Translation
DMR2:185447001	2	185447001	185449000	2000	1	5.50E-07	0.35	42	2.1	Rps3a	Translation
DMR2:185587001	2	185587001	185589000	2000	1	2.10E-07	-0.56	22	1.1	Lrba	
DMR2:185597001	2	185597001	185600000	3000	1	1.10E-15	0.57	44	1.47	Lrba	
DMR2:185637001	2	185637001	185639000	2000	1	1.40E-10	0.51	19	0.95	Lrba	
DMR2:185651001	2	185651001	185655000	4000	2	1.70E-09	-0.58	56	1.4	Lrba	
DMR2:185658001	2	185658001	185661000	3000	1	1.10E-08	0.39	36	1.2	Lrba	
DMR2:185700001	2	185700001	185704000	4000	1	4.70E-14	0.67	33	0.82	Lrba	
DMR2:185711001	2	185711001	185713000	2000	2	9.40E-15	0.58	20	1	Lrba	
DMR2:185732001	2	185732001	185734000	2000	1	2.20E-16	0.72	12	0.6	Lrba	
DMR2:185740001	2	185740001	185746000	6000	2	1.10E-13	-0.51	50	0.83	Lrba	
DMR2:185756001	2	185756001	185758000	2000	1	2.20E-07	0.48	8	0.4	Lrba	
DMR2:185760001	2	185760001	185764000	4000	1	4.40E-09	0.4	33	0.82	Lrba	
DMR2:185766001	2	185766001	185767000	1000	1	3.30E-08	0.46	11	1.1	Lrba	
DMR2:185771001	2	185771001	185773000	2000	1	5.10E-09	0.53	14	0.7	Lrba	
DMR2:185812001	2	185812001	185815000	3000	1	8.00E-07	0.36	33	1.1	Lrba;LOC103691614	
DMR2:185821001	2	185821001	185825000	4000	3	8.30E-14	0.71	28	0.7	Lrba	
DMR2:185867001	2	185867001	185868000	1000	1	1.20E-17	0.95	4	0.4	Lrba	
DMR2:185875001	2	185875001	185876000	1000	1	1.20E-07	0.47	6	0.6	Lrba	
DMR2:185887001	2	185887001	185891000	4000	1	9.60E-10	0.48	41	1.02	Lrba	
DMR2:185913001	2	185913001	185914000	1000	1	1.10E-13	0.53	8	0.8	Lrba	
DMR2:185919001	2	185919001	185921000	2000	1	5.00E-15	-0.51	21	1.05	Lrba	
DMR2:185928001	2	185928001	185929000	1000	1	1.00E-07	-0.47	4	0.4	Lrba	
DMR2:185940001	2	185940001	185941000	1000	1	8.20E-09	0.6	6	0.6	Lrba	
DMR2:185989001	2	185989001	185992000	3000	3	7.00E-12	0.55	26	0.87	Lrba	
DMR2:185993001	2	185993001	185999000	6000	3	5.50E-20	-0.6	63	1.05	Lrba	
DMR2:186001001	2	186001001	186004000	3000	3	3.60E-16	0.69	40	1.33	Lrba	
DMR2:186014001	2	186014001	186019000	5000	2	1.80E-12	-0.39	57	1.14	Lrba	
DMR2:186047001	2	186047001	186048000	1000	1	1.00E-07	0.53	10	1	Lrba	
DMR2:186078001	2	186078001	186079000	1000	1	6.40E-16	0.59	4	0.4	Lrba	
DMR2:186110001	2	186110001	186112000	2000	2	2.50E-08	-0.55	23	1.15	Lrba;Dclk2	Signaling
DMR2:186135001	2	186135001	186136000	1000	1	7.40E-16	0.57	18	1.8	Dclk2	Signaling
DMR2:186141001	2	186141001	186142000	1000	1	7.00E-07	-0.64	10	1	Dclk2	Signaling
DMR2:186145001	2	186145001	186146000	1000	1	7.90E-07	-0.47	23	2.3	Dclk2	Signaling
DMR2:186148001	2	186148001	186152000	4000	1	6.80E-17	0.6	55	1.38	Dclk2	Signaling
DMR2:186172001	2	186172001	186175000	3000	1	9.10E-13	0.73	24	0.8	Dclk2	Signaling
DMR2:186198001	2	186198001	186202000	4000	1	1.80E-07	-0.38	36	0.9	Dclk2	Signaling
DMR2:186206001	2	186206001	186210000	4000	2	7.00E-09	-0.54	49	1.23	Dclk2	Signaling
DMR2:186252001	2	186252001	186253000	1000	1	1.80E-10	0.53	13	1.3	Dclk2	Signaling
DMR2:186326001	2	186326001	186327000	1000	1	1.30E-24	0.91	9	0.9	Cd1d1	
DMR2:186334001	2	186334001	186336000	2000	1	1.90E-07	0.46	23	1.15	Cd1d1	
DMR2:186337001	2	186337001	186339000	2000	1	3.10E-11	-0.44	27	1.35	Cd1d1	
DMR2:186343001	2	186343001	186344000	1000	1	1.70E-09	0.43	9	0.9	Cd1d1	
DMR2:186425001	2	186425001	186432000	7000	5	3.90E-24	0.68	146	2.09	Kirrel	
DMR2:186437001	2	186437001	186440000	3000	1	5.00E-12	0.43	68	2.27	Kirrel	
DMR2:186447001	2	186447001	186451000	4000	1	3.90E-18	0.58	56	1.4	Kirrel	
DMR2:186453001	2	186453001	186456000	3000	1	1.30E-15	0.54	38	1.27	Kirrel	
DMR2:186464001	2	186464001	186466000	2000	1	6.60E-15	0.47	25	1.25	Kirrel	
DMR2:186467001	2	186467001	186471000	4000	1	2.00E-11	-0.66	52	1.3	Kirrel	
DMR2:186473001	2	186473001	186477000	4000	1	8.40E-07	-0.53	47	1.18	Kirrel	
DMR2:186482001	2	186482001	186485000	3000	1	2.10E-10	0.64	48	1.6	Kirrel	
DMR2:186491001	2	186491001	186495000	4000	4	9.00E-11	0.57	36	0.9	Kirrel	
DMR2:186496001	2	186496001	186499000	3000	2	7.10E-09	0.48	32	1.07	Kirrel	
DMR2:186503001	2	186503001	186505000	2000	1	2.50E-26	1.13	11	0.55	Kirrel	
DMR2:186512001	2	186512001	186514000	2000	2	8.80E-09	0.36	20	1	Kirrel	
DMR2:186590001	2	186590001	186591000	1000	1	1.10E-15	0.66	15	1.5	Fcrl2	Immune
DMR2:186605001	2	186605001	186606000	1000	1	1.80E-15	0.76	10	1	Fcrl2	Immune

DMR2:186613001	2	186613001	186616000	3000	3	3.50E-25	0.75	34	1.13	Fcrl2	Immune
DMR2:186678001	2	186678001	186679000	1000	1	4.10E-09	0.39	5	0.5	Cd5l	
DMR2:186690001	2	186690001	186692000	2000	2	6.90E-11	0.43	29	1.45	Cd5l	
DMR2:186704001	2	186704001	186706000	2000	2	4.80E-12	0.51	33	1.65	Cd5l;Fcrl1	Immune
DMR2:186717001	2	186717001	186719000	2000	1	4.60E-11	0.5	16	0.8	Fcrl1	Immune
DMR2:186727001	2	186727001	186728000	1000	1	4.40E-08	0.4	10	1	Fcrl1	Immune
DMR2:186729001	2	186729001	186731000	2000	1	2.80E-07	0.45	7	0.35	Fcrl1	Immune
DMR2:186778001	2	186778001	186779000	1000	1	3.40E-10	0.54	11	1.1	Fcrl3	
DMR2:186780001	2	186780001	186781000	1000	1	2.10E-10	0.5	11	1.1	Fcrl3	
DMR2:186783001	2	186783001	186785000	2000	1	8.90E-10	0.4	21	1.05	Fcrl3	
DMR2:186794001	2	186794001	186797000	3000	3	3.40E-15	0.85	9	0.3	Fcrl3;LOC103691616	
DMR2:186798001	2	186798001	186799000	1000	1	1.10E-13	0.5	20	2	Fcrl3;LOC103691616	
DMR2:186804001	2	186804001	186807000	3000	2	1.50E-10	0.5	42	1.4	Fcrl3;LOC103691616	
DMR2:186891001	2	186891001	186893000	2000	1	1.20E-20	0.75	33	1.65	Etv3;Etv3l	Transcription
DMR2:186901001	2	186901001	186904000	3000	1	9.60E-08	0.45	24	0.8	Etv3l;LOC108350054	Transcription
DMR2:186905001	2	186905001	186909000	4000	2	1.40E-09	0.51	71	1.77	Etv3l;LOC108350054	Transcription
DMR2:186919001	2	186919001	186922000	3000	1	2.80E-10	0.51	33	1.1	Etv3l;LOC108350054;LOC100361532	Transcription
DMR2:186923001	2	186923001	186924000	1000	1	9.00E-07	-0.43	12	1.2	LOC108350054;LOC100361532;RGD1563314	
DMR2:186925001	2	186925001	186927000	2000	1	5.20E-07	0.39	22	1.1	LOC108350054;LOC100361532;RGD1563314;LOC680772	
DMR2:186973001	2	186973001	186975000	2000	1	1.00E-11	0.67	23	1.15	LOC102546356;Arhgef11	Transcription
DMR2:186976001	2	186976001	186978000	2000	1	2.30E-09	0.51	10	0.5	LOC102546356;Arhgef11	Transcription
DMR2:186996001	2	186996001	186998000	2000	1	1.20E-07	0.54	23	1.15	Arhgef11	Transcription
DMR2:187002001	2	187002001	187005000	3000	1	4.50E-08	-0.47	36	1.2	Arhgef11	Transcription
DMR2:187021001	2	187021001	187023000	2000	1	7.20E-13	0.52	25	1.25	Arhgef11	Transcription
DMR2:187028001	2	187028001	187031000	3000	1	2.80E-07	0.46	15	0.5	Arhgef11	Transcription
DMR2:187041001	2	187041001	187043000	2000	2	1.90E-08	0.51	10	0.5	Arhgef11	Transcription
DMR2:187049001	2	187049001	187051000	2000	2	7.30E-10	0.44	25	1.25	Arhgef11	Transcription
DMR2:187053001	2	187053001	187056000	3000	1	1.10E-07	0.58	26	0.87	Arhgef11	Transcription
DMR2:187078001	2	187078001	187083000	5000	2	7.00E-09	0.49	45	0.9	Arhgef11	Transcription
DMR2:187100001	2	187100001	187102000	2000	1	6.90E-08	-0.53	21	1.05	Arhgef11;Lrrc71	Transcription
DMR2:187112001	2	187112001	187113000	1000	1	3.40E-11	0.46	17	1.7	Arhgef11;Lrrc71;Pear1	Transcription;Signaling
DMR2:187115001	2	187115001	187116000	1000	1	4.70E-10	0.63	15	1.5	Lrrc71;Pear1	Signaling
DMR2:187119001	2	187119001	187121000	2000	1	1.10E-12	0.43	37	1.85	Lrrc71;Pear1	Signaling
DMR2:187125001	2	187125001	187127000	2000	1	1.20E-16	0.56	24	1.2	Pear1	Signaling
DMR2:187129001	2	187129001	187130000	1000	1	6.80E-07	0.33	18	1.8	Pear1	Signaling
DMR2:187133001	2	187133001	187134000	1000	1	4.10E-08	0.51	31	3.1	Pear1;Ntrk1	Signaling;Receptor
DMR2:187139001	2	187139001	187143000	4000	1	2.50E-20	0.58	54	1.35	Pear1;Ntrk1	Signaling;Receptor
DMR2:187158001	2	187158001	187159000	1000	1	1.50E-22	0.87	18	1.8	Ntrk1;Insrr	Receptor
DMR2:187179001	2	187179001	187181000	2000	2	3.60E-10	0.57	26	1.3	Insrr	Receptor
DMR2:187206001	2	187206001	187210000	4000	4	1.10E-19	-0.62	58	1.45	Sh2d2a	Immune
DMR2:187211001	2	187211001	187213000	2000	1	1.60E-08	0.41	26	1.3	Sh2d2a	Immune
DMR2:187214001	2	187214001	187216000	2000	1	4.70E-08	0.39	16	0.8	Sh2d2a	Immune
DMR2:187222001	2	187222001	187224000	2000	2	6.10E-15	0.54	28	1.4	Sh2d2a;Prcc	Immune
DMR2:187280001	2	187280001	187283000	3000	3	3.00E-18	0.76	44	1.47	Hdgf;mrpl24	Transcription
DMR2:187340001	2	187340001	187341000	1000	1	2.10E-09	0.51	9	0.9	Nes	
DMR2:187356001	2	187356001	187357000	1000	1	2.60E-09	0.5	8	0.8	Nes;Bcan	Extracellular Matrix
DMR2:187364001	2	187364001	187366000	2000	1	1.10E-21	0.84	36	1.8	Bcan	Extracellular Matrix
DMR2:187407001	2	187407001	187411000	4000	1	2.20E-09	-0.48	45	1.12	Hapln2;Gpatch4	Extracellular Matrix;Metabolism
DMR2:187413001	2	187413001	187414000	1000	1	3.70E-11	0.48	13	1.3	Gpatch4	Metabolism
DMR2:187423001	2	187423001	187424000	1000	1	7.80E-11	0.54	8	0.8	Gpatch4;Naxe;Ttc24	Metabolism;Metabolism
DMR2:187439001	2	187439001	187443000	4000	1	1.10E-12	-0.83	43	1.07	Ttc24;Iqgap3	Signaling
DMR2:187458001	2	187458001	187462000	4000	2	9.00E-11	0.41	49	1.23	Iqgap3	Signaling
DMR2:187472001	2	187472001	187475000	3000	1	8.00E-07	-0.43	61	2.03	Iqgap3	Signaling
DMR2:187476001	2	187476001	187478000	2000	1	5.40E-26	0.97	31	1.55	Iqgap3	Signaling
DMR2:187490001	2	187490001	187491000	1000	1	6.30E-10	0.38	10	1	Iqgap3;LOC108350055	Signaling
DMR2:187495001	2	187495001	187497000	2000	1	8.70E-09	-0.53	30	1.5	Iqgap3;LOC108350055	Signaling
DMR2:187498001	2	187498001	187499000	1000	1	1.10E-08	0.43	9	0.9	Iqgap3;LOC108350055	Signaling
DMR2:187503001	2	187503001	187505000	2000	2	1.80E-18	0.92	6	0.3	LOC108350055;Mef2d	Transcription
DMR2:187537001	2	187537001	187538000	1000	1	1.50E-09	0.43	17	1.7	Mef2d	Transcription

DMR2:187573001	2	187573001	187576000	3000	2	3.60E-20	0.67	41	1.37	LOC102547113;LOC100911992;Mir3597-1;Mir9-1	
DMR2:187589001	2	187589001	187591000	2000	1	7.60E-09	0.44	27	1.35	LOC100911992;Mir3597-1;Mir9-1	
DMR2:187630001	2	187630001	187633000	3000	1	2.00E-12	0.42	37	1.23	Rhbg	Transport
DMR2:187661001	2	187661001	187666000	5000	1	1.40E-12	0.53	45	0.9	LOC103691617;Tsacc;Cct3	Transcription;Translation
DMR2:187681001	2	187681001	187682000	1000	1	4.60E-13	0.55	13	1.3	Cct3	Translation
DMR2:187716001	2	187716001	187717000	1000	1	1.70E-10	0.4	10	1	Tmem79;Smg5	Metabolism
DMR2:187731001	2	187731001	187732000	1000	1	4.30E-10	0.42	12	1.2	Smg5;Paqr6;Bglap	Metabolism;Signaling
DMR2:187751001	2	187751001	187755000	4000	1	5.30E-11	0.63	55	1.38	Paqr6;Bglap;Pmf1	Signaling
DMR2:187760001	2	187760001	187761000	1000	1	4.60E-23	0.62	14	1.4	Pmf1	
DMR2:187775001	2	187775001	187776000	1000	1	4.50E-09	0.37	13	1.3	Pmf1;Slc25a44	Transport
DMR2:187791001	2	187791001	187796000	5000	1	5.80E-10	-0.55	73	1.46	Slc25a44;Sema4a	Transport;Signaling
DMR2:187798001	2	187798001	187800000	2000	1	1.10E-12	0.55	25	1.25	Sema4a	Signaling
DMR2:187820001	2	187820001	187822000	2000	1	9.50E-25	0.69	28	1.4	Sema4a	Signaling
DMR2:187830001	2	187830001	187832000	2000	1	3.20E-11	0.6	20	1	Sema4a	Signaling
DMR2:187850001	2	187850001	187851000	1000	1	1.60E-10	0.44	5	0.5	Lmna	
DMR2:187871001	2	187871001	187879000	8000	2	1.60E-12	0.48	92	1.15	Lmna	
DMR2:187896001	2	187896001	187898000	2000	1	5.20E-11	0.41	70	3.5	Mex3a;Rab25	Metabolism
DMR2:187900001	2	187900001	187901000	1000	1	6.80E-14	0.54	10	1	Mex3a;Rab25	Metabolism
DMR2:187926001	2	187926001	187928000	2000	1	6.30E-09	0.42	27	1.35	Ubqln4	
DMR2:187935001	2	187935001	187936000	1000	1	7.60E-07	-0.4	4	0.4	Ubqln4	
DMR2:187956001	2	187956001	187958000	2000	1	4.40E-10	0.47	30	1.5	Ssr2;Arhgef2	Transport
DMR2:187966001	2	187966001	187967000	1000	1	1.30E-07	0.4	18	1.8	Ssr2;Arhgef2	Transport
DMR2:187968001	2	187968001	187972000	4000	1	4.10E-10	-0.53	51	1.27	Ssr2;Arhgef2	Transport
DMR2:187973001	2	187973001	187975000	2000	1	8.10E-13	0.73	19	0.95	Arhgef2	
DMR2:187979001	2	187979001	187983000	4000	1	8.10E-07	0.36	46	1.15	Arhgef2	
DMR2:187995001	2	187995001	187998000	3000	1	2.60E-09	0.4	49	1.63	Arhgef2	
DMR2:188013001	2	188013001	188014000	1000	1	8.70E-07	-0.42	21	2.1	Arhgef2	
DMR2:188022001	2	188022001	188023000	1000	1	1.30E-15	0.59	18	1.8	Arhgef2;Rxfp4-ps1	
DMR2:188050001	2	188050001	188051000	1000	1	2.90E-07	0.58	6	0.6	RGD1565775	
DMR2:188107001	2	188107001	188110000	3000	1	1.50E-07	0.56	29	0.97	Rit1;Syt11	Signaling;Transport
DMR2:188122001	2	188122001	188123000	1000	1	3.60E-21	0.57	12	1.2	Syt11	Transport
DMR2:188147001	2	188147001	188148000	1000	1	2.00E-08	-0.61	8	0.8	Syt11;LOC108350057;Gon4l	Transport;Transcription
DMR2:188157001	2	188157001	188162000	5000	1	1.70E-09	0.53	59	1.18	Gon4l	Transcription
DMR2:188164001	2	188164001	188166000	2000	1	4.00E-10	0.42	24	1.2	Gon4l	Transcription
DMR2:188171001	2	188171001	188172000	1000	1	3.90E-10	0.48	11	1.1	Gon4l	Transcription
DMR2:188173001	2	188173001	188175000	2000	1	1.10E-10	0.51	20	1	Gon4l	Transcription
DMR2:188184001	2	188184001	188186000	2000	1	1.00E-08	-0.47	33	1.65	Gon4l	Transcription
DMR2:188192001	2	188192001	188199000	7000	1	5.50E-07	-0.45	113	1.61	Gon4l	Transcription
DMR2:188227001	2	188227001	188228000	1000	1	2.70E-07	-0.52	17	1.7	Dap3	Translation
DMR2:188229001	2	188229001	188232000	3000	2	6.50E-14	0.43	46	1.53	Dap3	Translation
DMR2:188238001	2	188238001	188240000	2000	1	1.70E-07	-0.49	25	1.25	Dap3	Translation
DMR2:188252001	2	188252001	188253000	1000	1	5.50E-28	1.07	75	7.5	Dap3;Ash1l	Translation;Epigenetic
DMR2:188258001	2	188258001	188259000	1000	1	1.20E-10	0.52	7	0.7	Dap3;Ash1l	Translation;Epigenetic
DMR2:188280001	2	188280001	188282000	2000	1	2.90E-07	0.41	22	1.1	Ash1l	Epigenetic
DMR2:188315001	2	188315001	188318000	3000	1	1.20E-12	0.68	33	1.1	Ash1l	Epigenetic
DMR2:188321001	2	188321001	188322000	1000	1	5.30E-17	0.73	5	0.5	Ash1l	Epigenetic
DMR2:188338001	2	188338001	188339000	1000	1	2.00E-09	-0.56	6	0.6	Ash1l	Epigenetic
DMR2:188341001	2	188341001	188348000	7000	4	2.50E-20	0.84	57	0.81	Ash1l	Epigenetic
DMR2:188358001	2	188358001	188365000	7000	1	2.40E-09	0.4	85	1.21	Ash1l;LOC108350157	Epigenetic
DMR2:188372001	2	188372001	188373000	1000	1	1.40E-14	0.69	14	1.4	Ash1l;LOC108350157	Epigenetic
DMR2:188380001	2	188380001	188382000	2000	2	4.80E-16	0.61	23	1.15	Ash1l;LOC108350157	Epigenetic
DMR2:188383001	2	188383001	188384000	1000	1	5.40E-07	0.46	9	0.9	Ash1l;LOC108350157	Epigenetic
DMR2:188442001	2	188442001	188445000	3000	2	4.80E-16	0.81	39	1.3	Pklr	Signaling
DMR2:188446001	2	188446001	188448000	2000	1	2.90E-10	0.42	14	0.7	Pklr	Signaling
DMR2:188462001	2	188462001	188464000	2000	1	2.10E-08	0.5	32	1.6	Pklr;Hcn3	Signaling;Transport
DMR2:188465001	2	188465001	188467000	2000	1	1.10E-10	-0.45	24	1.2	Pklr;Hcn3;Clk2	Signaling;Transport;Signaling
DMR2:188479001	2	188479001	188480000	1000	1	3.00E-14	0.52	15	1.5	Hcn3;Clk2;Mir3541;Scamp3	Transport;Signaling

DMR2:188481001	2	188481001	188482000	1000	1	4.60E-19	0.55	44	4.4	Hcn3;Clk2;Mir3541;Scamp3	Transport;Signaling
DMR2:188518001	2	188518001	188519000	1000	1	3.80E-07	0.32	16	1.6	LOC102550011;Gba;Mtx1;Thbs3	
DMR2:188525001	2	188525001	188527000	2000	1	2.00E-14	0.57	18	0.9	Gba;Mtx1;Thbs3	
DMR2:188533001	2	188533001	188534000	1000	1	1.30E-14	0.51	25	2.5	Mtx1;Thbs3;Mir92b;Muc1	Cytoskeleton
DMR2:188549001	2	188549001	188555000	6000	4	8.10E-30	0.84	136	2.27	Thbs3;Mir92b;Muc1;Trim46;Krtcap2	Cytoskeleton;Proteolysis
DMR2:188581001	2	188581001	188583000	2000	2	6.60E-23	0.93	8	0.4	Dpm3;Slc50a1;Efna1	Transport;Signaling
DMR2:188593001	2	188593001	188594000	1000	1	8.90E-17	0.56	19	1.9	Dpm3;Slc50a1;Efna1;LOC102550325	Transport;Signaling
DMR2:188600001	2	188600001	188605000	5000	1	1.70E-20	0.96	51	1.02	Efna1;LOC102550325	Signaling
DMR2:188662001	2	188662001	188665000	3000	1	2.10E-11	0.42	47	1.57	Efna4;Adam15;Dcst1	Signaling;Protease
DMR2:188673001	2	188673001	188674000	1000	1	4.40E-11	0.42	12	1.2	Adam15;Dcst1	Protease
DMR2:188678001	2	188678001	188682000	4000	2	3.00E-17	0.6	90	2.25	Adam15;Dcst1;Dcst2	Protease
DMR2:188683001	2	188683001	188684000	1000	1	3.00E-10	0.38	23	2.3	Dcst1;Dcst2	
DMR2:188688001	2	188688001	188690000	2000	1	4.70E-07	0.37	35	1.75	Dcst1;Dcst2	
DMR2:188691001	2	188691001	188694000	3000	2	1.20E-12	0.49	30	1	Dcst1;Dcst2	
DMR2:188702001	2	188702001	188704000	2000	1	2.10E-08	0.37	24	1.2	Dcst2;Zbtb7b	
DMR2:188707001	2	188707001	188708000	1000	1	1.90E-09	0.45	19	1.9	Dcst2;Zbtb7b	
DMR2:188711001	2	188711001	188715000	4000	2	2.40E-15	0.51	61	1.52	Dcst2;Zbtb7b	
DMR2:188718001	2	188718001	188719000	1000	1	3.60E-20	1.17	42	4.2	Zbtb7b;Flad1;Lenep	Transport
DMR2:188733001	2	188733001	188734000	1000	1	3.60E-10	0.44	25	2.5	Flad1;Lenep;Cks1b	Transport;Cytoskeleton
DMR2:188754001	2	188754001	188755000	1000	1	1.30E-16	0.64	16	1.6	Cks1b;Shc1;Pygo2;Pbxip1	Cytoskeleton
DMR2:188766001	2	188766001	188767000	1000	1	8.60E-17	0.79	14	1.4	Shc1;Pygo2;Pbxip1	Cytoskeleton
DMR2:188768001	2	188768001	188769000	1000	1	1.90E-08	0.42	9	0.9	Pygo2;Pbxip1	
DMR2:188771001	2	188771001	188775000	4000	3	1.20E-16	0.62	70	1.75	Pygo2;Pbxip1;Pmvk	Signaling
DMR2:188821001	2	188821001	188828000	7000	5	5.00E-13	0.64	79	1.13	Kcnn3	Transport
DMR2:188880001	2	188880001	188886000	6000	2	2.80E-16	0.71	78	1.3	Kcnn3	Transport
DMR2:188891001	2	188891001	188894000	3000	2	6.50E-12	0.49	43	1.43	Kcnn3	Transport
DMR2:188900001	2	188900001	188904000	4000	1	7.20E-09	0.34	61	1.52	Kcnn3	Transport
DMR2:188920001	2	188920001	188923000	3000	1	2.40E-08	-0.55	29	0.97	Kcnn3	Transport
DMR2:188950001	2	188950001	188951000	1000	1	2.10E-08	-0.51	9	0.9	Kcnn3	Transport
DMR2:188954001	2	188954001	188959000	5000	1	3.30E-10	0.44	57	1.14	Kcnn3	Transport
DMR2:188961001	2	188961001	188963000	2000	1	7.40E-08	0.38	23	1.15	Kcnn3	Transport
DMR2:188977001	2	188977001	188980000	3000	1	6.50E-08	0.36	44	1.47	Kcnn3	Transport
DMR2:188984001	2	188984001	188987000	3000	2	1.90E-22	0.9	63	2.1	Kcnn3	Transport
DMR2:189036001	2	189036001	189040000	4000	2	2.20E-18	0.67	20	0.5	Adar	Metabolism
DMR2:189041001	2	189041001	189043000	2000	1	1.70E-07	-0.55	16	0.8	Adar	Metabolism
DMR2:189047001	2	189047001	189049000	2000	1	4.80E-07	0.36	9	0.45	Adar	Metabolism
DMR2:189052001	2	189052001	189053000	1000	1	7.90E-17	0.78	3	0.3	Adar	Metabolism
DMR2:189075001	2	189075001	189077000	2000	1	7.10E-11	0.48	27	1.35	Adar	Metabolism
DMR2:189089001	2	189089001	189092000	3000	1	1.00E-08	0.33	47	1.57	Adar;Chrnb2	Metabolism;Ion Channel
DMR2:189102001	2	189102001	189103000	1000	1	3.30E-07	0.44	6	0.6	Chrnb2;Ube2q1	Ion Channel;Proteolysis
DMR2:189119001	2	189119001	189125000	6000	1	1.10E-07	-0.62	73	1.22	Ube2q1	Proteolysis
DMR2:189159001	2	189159001	189162000	3000	1	1.10E-14	0.62	36	1.2	She	
DMR2:189165001	2	189165001	189169000	4000	1	8.40E-16	0.54	44	1.1	She	
DMR2:189174001	2	189174001	189176000	2000	1	1.70E-10	0.56	24	1.2	She	
DMR2:189177001	2	189177001	189179000	2000	1	1.80E-09	0.5	26	1.3	She	
DMR2:189199001	2	189199001	189201000	2000	2	1.00E-13	0.83	14	0.7	She;Il6r	Receptor
DMR2:189202001	2	189202001	189203000	1000	1	1.10E-27	0.7	15	1.5	She;Il6r	Receptor
DMR2:189221001	2	189221001	189224000	3000	1	9.40E-12	0.53	38	1.27	Il6r	Receptor
DMR2:189227001	2	189227001	189233000	6000	1	1.30E-29	1.1	75	1.25	Il6r	Receptor
DMR2:189240001	2	189240001	189242000	2000	1	4.90E-07	0.3	19	0.95	Il6r	Receptor
DMR2:189260001	2	189260001	189263000	3000	1	7.30E-07	-0.59	49	1.63	Il6r;LOC108350271	Receptor
DMR2:189330001	2	189330001	189331000	1000	1	9.30E-07	0.46	12	1.2	Hax1;Atp8b2;LOC103691621	Transport
DMR2:189335001	2	189335001	189338000	3000	1	1.90E-07	0.44	21	0.7	Hax1;Atp8b2;LOC103691621	Transport
DMR2:189341001	2	189341001	189342000	1000	1	9.90E-12	-0.61	2	0.2	Hax1;Atp8b2;LOC103691621	Transport
DMR2:189375001	2	189375001	189376000	1000	1	4.80E-12	0.77	4	0.4	Atp8b2	Transport
DMR2:189387001	2	189387001	189389000	2000	1	1.60E-13	0.42	21	1.05	Atp8b2	Transport
DMR2:189421001	2	189421001	189423000	2000	1	1.60E-07	0.55	8	0.4	LOC361985;RGD1564171;LOC108350058;Mir190b;Tpm3	Cytoskeleton
DMR2:189470001	2	189470001	189472000	2000	1	1.80E-08	0.45	22	1.1	Nup210l	Transport

DMR2:189481001	2	189481001	189486000	5000	1	2.60E-07	-0.52	71	1.42	Nup210l	Transport
DMR2:189487001	2	189487001	189490000	3000	1	1.40E-15	0.68	30	1	Nup210l	Transport
DMR2:189503001	2	189503001	189505000	2000	2	3.20E-14	-0.49	9	0.45	Nup210l	Transport
DMR2:189507001	2	189507001	189509000	2000	1	9.90E-09	0.47	18	0.9	Nup210l	Transport
DMR2:189514001	2	189514001	189517000	3000	1	2.40E-09	0.32	104	3.47	Nup210l	Transport
DMR2:189521001	2	189521001	189522000	1000	1	7.50E-07	0.38	13	1.3	Nup210l	Transport
DMR2:189523001	2	189523001	189525000	2000	1	5.50E-10	0.52	12	0.6	Nup210l	Transport
DMR2:189541001	2	189541001	189546000	5000	1	2.50E-07	0.44	51	1.02	Nup210l	Transport
DMR2:189579001	2	189579001	189580000	1000	1	3.80E-14	0.72	12	1.2	Nup210l;Rps27;Rab13	Transport;Translation
DMR2:189585001	2	189585001	189589000	4000	1	3.50E-21	0.72	49	1.23	Rab13;Jtb;Creb3l4	
DMR2:189594001	2	189594001	189595000	1000	1	9.20E-10	0.41	2	0.2	Rab13;Jtb;Creb3l4	
DMR2:189604001	2	189604001	189607000	3000	1	3.60E-07	0.43	34	1.13	Jtb;Creb3l4;Slc39a1;Crtc2	Transport;Transcription
DMR2:189617001	2	189617001	189624000	7000	2	4.80E-20	0.64	75	1.07	Slc39a1;Crtc2;Dennd4b	Transport;Transcription
DMR2:189628001	2	189628001	189631000	3000	2	7.90E-15	0.51	49	1.63	Crtc2;Dennd4b;LOC102551157	Transcription
DMR2:189704001	2	189704001	189706000	2000	2	7.70E-11	0.6	13	0.65	Gatad2b	Transcription
DMR2:189731001	2	189731001	189733000	2000	1	7.30E-14	0.41	33	1.65	Gatad2b	Transcription
DMR2:189745001	2	189745001	189746000	1000	1	1.10E-09	0.57	10	1	Gatad2b	Transcription
DMR2:189782001	2	189782001	189785000	3000	2	2.80E-10	-0.77	29	0.97	Ints3	
DMR2:189799001	2	189799001	189801000	2000	1	2.30E-07	0.39	15	0.75	Ints3	
DMR2:189824001	2	189824001	189827000	3000	1	5.80E-07	0.45	30	1	Ints3;LOC108350272;LOC100362048	
DMR2:189844001	2	189844001	189846000	2000	1	1.60E-11	0.51	26	1.3	LOC100362048;Npr1	Signaling
DMR2:189854001	2	189854001	189855000	1000	1	4.90E-10	0.56	27	2.7	Npr1;Ilf2	Signaling;Transcription
DMR2:189880001	2	189880001	189882000	2000	2	1.50E-17	0.88	59	2.95	Ilf2;Snapin;LOC361990	Transcription
DMR2:189908001	2	189908001	189910000	2000	1	6.70E-13	0.68	10	0.5	LOC361990;S100a1;S100a13	Signaling
DMR2:189917001	2	189917001	189918000	1000	1	4.90E-07	0.37	7	0.7	S100a13;S100a16	Signaling
DMR2:189922001	2	189922001	189923000	1000	1	2.00E-15	0.66	9	0.9	S100a13;S100a16	Signaling
DMR2:189924001	2	189924001	189925000	1000	1	2.20E-19	0.57	10	1	S100a16	Signaling
DMR2:189930001	2	189930001	189936000	6000	1	9.90E-08	-0.62	32	0.53	S100a16	Signaling
DMR2:189937001	2	189937001	189941000	4000	2	5.10E-10	0.5	29	0.72	S100a16	Signaling
DMR2:189970001	2	189970001	189972000	2000	2	3.90E-19	0.76	16	0.8	S100a3;LOC685396;LOC100910139	Signaling
DMR2:189997001	2	189997001	189998000	1000	1	1.20E-09	0.38	11	1.1	S100a3;S100a4;S100a5;S100a6	Signaling
DMR2:190011001	2	190011001	190013000	2000	1	7.00E-11	0.51	21	1.05	S100a5;S100a6	Signaling
DMR2:190045001	2	190045001	190049000	4000	2	4.80E-11	-0.46	29	0.72	S100a7a	Signaling
DMR2:190063001	2	190063001	190064000	1000	1	4.70E-07	0.36	4	0.4	S100a7a;S100a8	Signaling
DMR2:190068001	2	190068001	190069000	1000	1	3.90E-10	0.59	12	1.2	S100a7a;S100a8	Signaling
DMR2:190077001	2	190077001	190078000	1000	1	2.90E-07	-0.62	7	0.7	S100a8	Signaling
DMR2:190101001	2	190101001	190102000	1000	1	1.20E-12	0.61	10	1	S100a9	Signaling
DMR2:190122001	2	190122001	190124000	2000	1	2.60E-09	-0.56	1	0.05	LOC685528;Pglyrp4	
DMR2:190131001	2	190131001	190142000	11000	6	3.00E-14	0.81	87	0.79	Pglyrp4	
DMR2:190145001	2	190145001	190147000	2000	2	1.30E-11	0.53	12	0.6	Pglyrp4	
DMR2:190150001	2	190150001	190153000	3000	1	6.60E-10	-0.45	15	0.5	Pglyrp4	
DMR2:190160001	2	190160001	190161000	1000	1	1.50E-12	0.55	2	0.2	Pglyrp4	
DMR2:190192001	2	190192001	190199000	7000	4	1.50E-16	0.8	37	0.53	Pglyrp4	
DMR2:190200001	2	190200001	190202000	2000	1	5.40E-10	0.48	12	0.6	Pglyrp4	
DMR2:190205001	2	190205001	190211000	6000	1	5.40E-08	-0.37	63	1.05	Pglyrp4	
DMR2:190271001	2	190271001	190273000	2000	1	2.60E-08	-0.33	41	2.05	RGD1562917	
DMR2:190614001	2	190614001	190621000	7000	2	8.60E-12	-0.45	50	0.71	S100vp	Signaling
DMR2:190632001	2	190632001	190633000	1000	1	2.90E-13	0.52	9	0.9	S100vp	Signaling
DMR2:190639001	2	190639001	190642000	3000	1	3.70E-07	-0.43	9	0.3	S100vp	Signaling
DMR2:191890001	2	191890001	191891000	1000	1	7.50E-11	0.48	7	0.7	LOC108350200;Pglyrp3b	
DMR2:191930001	2	191930001	191931000	1000	1	1.70E-09	0.66	8	0.8	Pglyrp3;LOC365846	
DMR2:191934001	2	191934001	191936000	2000	1	4.70E-13	0.58	13	0.65	Pglyrp3;LOC365846	
DMR2:191939001	2	191939001	191940000	1000	1	1.90E-11	0.42	12	1.2	Pglyrp3;LOC365846;LOC100911387	
DMR2:191943001	2	191943001	191946000	3000	1	2.80E-14	-0.56	18	0.6	Pglyrp3;LOC365846;LOC100911387	
DMR2:191948001	2	191948001	191950000	2000	1	2.70E-24	0.85	9	0.45	Pglyrp3;LOC100911387	
DMR2:192002001	2	192002001	192003000	1000	1	3.30E-15	0.69	6	0.6	Pglyrp3	
DMR2:192027001	2	192027001	192028000	1000	1	1.00E-14	0.69	8	0.8	Pglyrp3;Prr9	

DMR2:192039001	2	192039001	192041000	2000	2	8.90E-15	0.81	17	0.85	Pglyrp3;Prr9	
DMR2:192052001	2	192052001	192053000	1000	1	1.90E-18	0.63	10	1	Pglyrp3	
DMR2:192060001	2	192060001	192061000	1000	1	2.00E-15	0.76	7	0.7	Pglyrp3	
DMR2:192075001	2	192075001	192076000	1000	1	2.60E-12	0.58	7	0.7	Pglyrp3	
DMR2:192090001	2	192090001	192091000	1000	1	3.10E-07	0.44	11	1.1	Pglyrp3	
DMR2:192101001	2	192101001	192102000	1000	1	5.20E-07	0.36	6	0.6	Pglyrp3	
DMR2:192118001	2	192118001	192120000	2000	2	3.10E-15	0.58	19	0.95	Pglyrp3	
DMR2:192124001	2	192124001	192125000	1000	1	5.20E-13	0.7	4	0.4	Pglyrp3	
DMR2:192126001	2	192126001	192129000	3000	3	3.10E-13	-0.6	15	0.5	Pglyrp3	
DMR2:192150001	2	192150001	192152000	2000	1	1.40E-07	0.61	4	0.2	Pglyrp3;LOC108350059	
DMR2:192157001	2	192157001	192159000	2000	1	3.60E-09	0.43	15	0.75	Pglyrp3;LOC108350059	
DMR2:192165001	2	192165001	192167000	2000	1	1.50E-08	0.57	9	0.45	Pglyrp3	
DMR2:192488001	2	192488001	192492000	4000	2	2.10E-13	0.81	20	0.5	Sprr2d	
DMR2:192616001	2	192616001	192617000	1000	1	8.30E-08	0.48	4	0.4	LOC102550257;Sprr1b	
DMR2:192624001	2	192624001	192626000	2000	1	7.50E-14	0.56	15	0.75	LOC102550257;Sprr1b;LOC108350274	
DMR2:192680001	2	192680001	192681000	1000	1	1.20E-08	0.59	2	0.2	Sprr1a	
DMR2:192704001	2	192704001	192705000	1000	1	7.70E-07	0.52	3	0.3	Sprr4	
DMR2:192769001	2	192769001	192773000	4000	1	7.70E-07	0.51	18	0.45	Ivl;Smcp	
DMR2:192778001	2	192778001	192780000	2000	2	1.10E-18	0.96	7	0.35	Ivl;Smcp	
DMR2:192999001	2	192999001	1.93E+08	1000	1	3.30E-09	0.49	8	0.8	Lce1f;LOC102552128	
DMR2:193146001	2	193146001	193149000	3000	1	7.40E-09	-0.49	15	0.5	Kprp	
DMR2:193304001	2	193304001	193305000	1000	1	4.40E-14	0.84	6	0.6	RGD1564972	
DMR2:193315001	2	193315001	193317000	2000	2	2.30E-15	0.97	31	1.55	RGD1564972	
DMR2:193353001	2	193353001	193356000	3000	2	4.00E-23	-0.7	17	0.57	RGD1561111	
DMR2:193427001	2	193427001	193429000	2000	2	3.00E-11	0.72	13	0.65	Crnn;LOC108350061	Signaling
DMR2:193441001	2	193441001	193443000	2000	2	2.30E-07	-0.5	15	0.75	Crnn;LOC108350061	Signaling
DMR2:193484001	2	193484001	193486000	2000	1	3.30E-08	0.47	12	0.6	Flg2	Cytoskeleton
DMR2:193494001	2	193494001	193496000	2000	1	8.30E-09	0.52	13	0.65	Flg2	Cytoskeleton
DMR2:193571001	2	193571001	193576000	5000	2	6.60E-12	-0.98	111	2.22	LOC108350062;Flg	
DMR2:193630001	2	193630001	193638000	8000	6	3.50E-10	-0.85	115	1.44	Hrnr	Cytoskeleton
DMR2:193643001	2	193643001	193650000	7000	3	1.60E-10	-0.53	53	0.76	Hrnr	Cytoskeleton
DMR2:193712001	2	193712001	193713000	1000	1	5.80E-15	0.85	2	0.2	Rptn;RGD1560208	
DMR2:193739001	2	193739001	193740000	1000	1	9.90E-12	0.58	9	0.9	Rptn	
DMR2:193791001	2	193791001	193792000	1000	1	9.60E-07	0.56	3	0.3	Tchh;LOC502545	
DMR2:193857001	2	193857001	193861000	4000	1	8.30E-11	0.51	37	0.92	S100a11	Signaling
DMR2:193867001	2	193867001	193869000	2000	1	5.20E-14	0.69	30	1.5	S100a11	Signaling
DMR2:193870001	2	193870001	193871000	1000	1	2.50E-09	0.45	5	0.5	S100a11	Signaling
DMR2:193872001	2	193872001	193873000	1000	1	1.20E-07	0.44	8	0.8	S100a11	Signaling
DMR2:193874001	2	193874001	193876000	2000	1	1.50E-11	0.65	16	0.8	S100a11	Signaling
DMR2:194003001	2	194003001	194006000	3000	2	2.50E-17	0.66	25	0.83	LOC688743;RGD1564513	
DMR2:194009001	2	194009001	194011000	2000	1	3.10E-14	0.56	14	0.7	LOC688743;RGD1564513	
DMR2:194311001	2	194311001	194315000	4000	1	1.60E-09	0.55	18	0.45	RGD1560096;LOC100911153	
DMR2:194320001	2	194320001	194321000	1000	1	1.60E-09	-0.44	5	0.5	RGD1560096;LOC100911153	
DMR2:194369001	2	194369001	194371000	2000	2	4.10E-18	0.52	6	0.3	LOC102548071;RGD1563451	Proteolysis
DMR2:194372001	2	194372001	194374000	2000	1	1.90E-11	0.48	6	0.3	LOC102548071;RGD1563451	Proteolysis
DMR2:194380001	2	194380001	194383000	3000	2	2.00E-10	-0.37	32	1.07	RGD1563451;RGD1564915	
DMR2:194387001	2	194387001	194389000	2000	1	4.40E-07	0.47	6	0.3	RGD1564915	
DMR2:194419001	2	194419001	194428000	9000	3	7.30E-16	0.65	87	0.97	RGD1564313	Proteolysis
DMR2:194429001	2	194429001	194432000	3000	2	1.60E-15	0.65	8	0.27	RGD1564313	Proteolysis
DMR2:194505001	2	194505001	194507000	2000	1	5.40E-10	0.64	14	0.7	LOC365852;LOC100363449;RGD1560510	Proteolysis
DMR2:194511001	2	194511001	194513000	2000	1	5.80E-09	0.39	5	0.25	LOC365852;LOC100363449;RGD1560510	Proteolysis
DMR2:194515001	2	194515001	194516000	1000	1	3.80E-17	0.57	1	0.1	LOC365852;LOC100363449;RGD1560510	Proteolysis
DMR2:194517001	2	194517001	194520000	3000	2	5.30E-14	0.52	13	0.43	RGD1560510	Proteolysis
DMR2:194549001	2	194549001	194550000	1000	1	1.00E-08	0.36	4	0.4	RGD1565369	
DMR2:194551001	2	194551001	194552000	1000	1	2.60E-13	0.65	3	0.3	RGD1565369	
DMR2:194554001	2	194554001	194555000	1000	1	1.60E-15	0.86	3	0.3	RGD1565369	
DMR2:194556001	2	194556001	194558000	2000	1	4.00E-09	0.59	6	0.3	RGD1565369	
DMR2:194566001	2	194566001	194568000	2000	2	6.60E-18	0.46	6	0.3	RGD1565369	
DMR2:194604001	2	194604001	194617000	13000	4	5.00E-23	0.73	110	0.85	LOC689026;Tdpoz1	
DMR2:194753001	2	194753001	194757000	4000	3	5.10E-23	0.64	19	0.48	RGD1560661;LOC295200	
DMR2:194758001	2	194758001	194765000	7000	2	4.80E-11	-0.58	56	0.8	RGD1560661;LOC295200	

DMR2:194872001	2	194872001	194873000	1000	1	8.60E-11	0.67	0	0	RGD1566337	Proteolysis
DMR2:194876001	2	194876001	194878000	2000	2	6.00E-09	0.5	5	0.25	RGD1566337	Proteolysis
DMR2:194881001	2	194881001	194883000	2000	1	1.20E-20	1.01	6	0.3	RGD1566337	Proteolysis
DMR2:194886001	2	194886001	194890000	4000	1	3.30E-17	0.42	29	0.72	RGD1566337	Proteolysis
DMR2:194924001	2	194924001	194930000	6000	1	3.60E-07	-0.39	40	0.67	RGD1562104;RGD1564386	
DMR2:194939001	2	194939001	194941000	2000	2	5.10E-11	0.46	7	0.35	RGD1562104;RGD1564386	
DMR2:194942001	2	194942001	194945000	3000	2	2.00E-16	0.61	17	0.57	RGD1564386	
DMR2:194946001	2	194946001	194947000	1000	1	3.80E-21	0.59	2	0.2	RGD1564386	
DMR2:195029001	2	195029001	195032000	3000	2	3.40E-21	0.6	8	0.27	RGD1563392	
DMR2:195035001	2	195035001	195040000	5000	3	3.10E-13	0.69	13	0.26	RGD1563392	
DMR2:195042001	2	195042001	195045000	3000	2	2.00E-08	-0.39	36	1.2	RGD1563392	
DMR2:195046001	2	195046001	195053000	7000	2	2.80E-15	-0.52	63	0.9	RGD1563392	
DMR2:195148001	2	195148001	195149000	1000	1	4.40E-08	0.69	0	0	RGD1563667	Proteolysis
DMR2:195155001	2	195155001	195156000	1000	1	4.30E-22	0.7	5	0.5	RGD1563667	Proteolysis
DMR2:195280001	2	195280001	195281000	1000	1	8.70E-07	0.45	1	0.1	RGD1563667	Proteolysis
DMR2:195283001	2	195283001	195284000	1000	1	2.40E-15	0.61	6	0.6	RGD1563667	Proteolysis
DMR2:195305001	2	195305001	195306000	1000	1	2.20E-18	0.72	1	0.1	RGD1563667	Proteolysis
DMR2:195316001	2	195316001	195321000	5000	3	1.80E-14	-0.46	50	1	RGD1563667	Proteolysis
DMR2:195333001	2	195333001	195335000	2000	1	5.10E-15	0.48	10	0.5	RGD1563667	Proteolysis
DMR2:195336001	2	195336001	195340000	4000	1	3.00E-10	0.49	23	0.58	RGD1563667	Proteolysis
DMR2:195378001	2	195378001	195379000	1000	1	5.50E-07	0.38	12	1.2	RGD1563667	Proteolysis
DMR2:195477001	2	195477001	195478000	1000	1	3.60E-18	0.72	7	0.7	RGD1563667;RGD1560554	Proteolysis
DMR2:195487001	2	195487001	195490000	3000	3	2.50E-19	0.7	12	0.4	RGD1563667;RGD1560554	Proteolysis
DMR2:195491001	2	195491001	195493000	2000	2	1.50E-12	0.58	6	0.3	RGD1563667;RGD1560554;LOC310649	Proteolysis
DMR2:195496001	2	195496001	195498000	2000	2	2.00E-10	-0.55	10	0.5	RGD1563667;RGD1560554;LOC310649	Proteolysis
DMR2:195506001	2	195506001	195510000	4000	2	6.30E-12	0.56	32	0.8	RGD1563667;RGD1560554;LOC310649	Proteolysis
DMR2:195513001	2	195513001	195514000	1000	1	8.10E-10	-0.49	1	0.1	RGD1563667;RGD1560554;LOC310649	Proteolysis
DMR2:195515001	2	195515001	195519000	4000	2	1.80E-08	-0.59	50	1.25	RGD1563667;RGD1560554	Proteolysis
DMR2:195521001	2	195521001	195525000	4000	1	8.70E-14	0.57	27	0.68	RGD1563667;RGD1560554	Proteolysis
DMR2:195528001	2	195528001	195531000	3000	1	3.30E-09	-0.75	13	0.43	RGD1563667;RGD1560554	Proteolysis
DMR2:195545001	2	195545001	195547000	2000	1	6.30E-20	0.69	18	0.9	RGD1563667;RGD1560554;Them4	Proteolysis;Metabolism
DMR2:195562001	2	195562001	195564000	2000	2	8.10E-24	0.77	21	1.05	RGD1563667;RGD1560554;Them4	Proteolysis;Metabolism
DMR2:195571001	2	195571001	195573000	2000	1	7.80E-25	0.7	32	1.6	Them4;Them5	Metabolism
DMR2:195588001	2	195588001	195593000	5000	2	1.50E-12	0.58	53	1.06	Them5;C2cd4d	Metabolism
DMR2:195594001	2	195594001	195595000	1000	1	7.50E-09	0.5	1	0.1	Them5;C2cd4d	Metabolism
DMR2:195612001	2	195612001	195613000	1000	1	6.70E-08	0.57	31	3.1	C2cd4d;Rorc;Lingo4	Transcription;Receptor
DMR2:195658001	2	195658001	195660000	2000	1	1.20E-26	0.99	10	0.5	Tdrkh;LOC103691639	Cytoskeleton
DMR2:195661001	2	195661001	195666000	5000	2	2.70E-14	0.79	30	0.6	Tdrkh;LOC103691639;Oaz3	Cytoskeleton;Signaling
DMR2:195695001	2	195695001	195698000	3000	1	8.00E-09	0.66	39	1.3	mrpl9;LOC102556390;Riiad1	
DMR2:195713001	2	195713001	195721000	8000	4	4.00E-26	0.64	223	2.79	Riiad1;Celf3	
DMR2:195733001	2	195733001	195737000	4000	1	7.70E-07	-0.53	44	1.1	Celf3;Snx27	Cytoskeleton
DMR2:195743001	2	195743001	195746000	3000	1	1.10E-10	-0.48	39	1.3	Snx27	Cytoskeleton
DMR2:195772001	2	195772001	195773000	1000	1	4.10E-09	0.41	9	0.9	Snx27	Cytoskeleton
DMR2:195829001	2	195829001	195830000	1000	1	1.00E-08	-0.44	17	1.7	Snx27	Cytoskeleton
DMR2:195842001	2	195842001	195847000	5000	3	1.40E-07	-0.42	50	1	RGD1560263	
DMR2:195848001	2	195848001	195851000	3000	2	2.80E-14	0.48	34	1.13	RGD1560263	
DMR2:195861001	2	195861001	195862000	1000	1	2.60E-08	-0.51	8	0.8	RGD1560263;Tuft1	
DMR2:195896001	2	195896001	195898000	2000	1	1.20E-25	0.76	19	0.95	Tuft1	
DMR2:195901001	2	195901001	195902000	1000	1	1.90E-08	0.49	3	0.3	Tuft1;Cgn	
DMR2:195929001	2	195929001	195931000	2000	1	1.30E-15	0.56	23	1.15	Cgn;LOC100363268	
DMR2:195933001	2	195933001	195935000	2000	1	2.30E-14	0.68	23	1.15	Cgn;LOC100363268	
DMR2:196011001	2	196011001	196017000	6000	2	3.50E-25	1.05	65	1.08	Pogz;LOC108350064	Transcription
DMR2:196026001	2	196026001	196027000	1000	1	2.70E-10	0.45	4	0.4	Pogz	Transcription
DMR2:196034001	2	196034001	196035000	1000	1	7.70E-11	0.54	12	1.2	Pogz;Psmb4	Transcription;Protease
DMR2:196047001	2	196047001	196049000	2000	2	1.40E-14	0.63	12	0.6	Pogz;Psmb4;LOC108350065	Transcription;Protease

DMR2:196131001	2	196131001	196134000	3000	1	1.30E-08	0.43	45	1.5	Selenbp1;Rfx5;LOC108350066;Pi4kb	Immune;Transcription;Signaling
DMR2:196140001	2	196140001	196141000	1000	1	6.80E-11	0.58	35	3.5	Selenbp1;LOC108350066;Pi4kb	Immune;Signaling
DMR2:196156001	2	196156001	196157000	1000	1	1.60E-09	0.49	6	0.6	Selenbp1;Pi4kb;LOC102547008	Immune;Signaling
DMR2:196170001	2	196170001	196171000	1000	1	1.50E-14	0.76	13	1.3	Pi4kb;LOC102547008;LOC100363361	Signaling
DMR2:196190001	2	196190001	196194000	4000	1	2.70E-11	0.4	47	1.18	LOC100363361;Psmd4	Protease
DMR2:196213001	2	196213001	196214000	1000	1	1.30E-07	0.32	16	1.6	Psmd4	Protease
DMR2:196222001	2	196222001	196225000	3000	1	8.00E-10	-0.46	90	3	Pip5k1a	Signaling
DMR2:196269001	2	196269001	196271000	2000	1	9.20E-19	0.77	59	2.95	Pip5k1a;Vps72	Signaling;Transcription
DMR2:196287001	2	196287001	196290000	3000	2	1.90E-19	0.61	44	1.47	Vps72;Tmod4	Transcription;Cytoskeleton
DMR2:196297001	2	196297001	196300000	3000	1	6.60E-10	0.38	62	2.07	Vps72;Tmod4;Scnm1;Lysmd1;Tnfaip8l2	Transcription;Cytoskeleton;Transport
DMR2:196312001	2	196312001	196313000	1000	1	1.80E-09	0.4	20	2	Scnm1;Lysmd1;Tnfaip8l2	Transport
DMR2:196321001	2	196321001	196322000	1000	1	7.50E-27	1.11	10	1	Lysmd1;Tnfaip8l2;Sema6c	Signaling
DMR2:196336001	2	196336001	196340000	4000	2	2.20E-10	0.6	56	1.4	Tnfaip8l2;Sema6c	Signaling
DMR2:196348001	2	196348001	196349000	1000	1	4.80E-14	0.66	9	0.9	Sema6c;Gabpb2	Signaling
DMR2:196397001	2	196397001	196399000	2000	1	7.30E-07	-0.63	27	1.35	Gabpb2;Mllt11;Cdc42se1	
DMR2:196404001	2	196404001	196406000	2000	1	2.40E-09	0.44	32	1.6	Mllt11;Cdc42se1;RGD1359334;Bnipl	
DMR2:196417001	2	196417001	196421000	4000	3	4.20E-13	0.51	66	1.65	Cdc42se1;RGD1359334;Bnipl;Prune	
DMR2:196463001	2	196463001	196466000	3000	1	1.90E-07	-0.53	34	1.13	Prune;Fam63a;Anxa9	Signaling
DMR2:196475001	2	196475001	196477000	2000	1	1.20E-09	0.47	19	0.95	Fam63a;Anxa9	Signaling
DMR2:196511001	2	196511001	196513000	2000	1	8.00E-08	-0.46	17	0.85	Setdb1	Epigenetic
DMR2:196527001	2	196527001	196528000	1000	1	1.70E-13	0.67	27	2.7	Setdb1	Epigenetic
DMR2:196530001	2	196530001	196532000	2000	2	1.10E-17	0.7	23	1.15	Setdb1	Epigenetic
DMR2:196624001	2	196624001	196627000	3000	1	2.50E-07	0.38	42	1.4	Arnt	Transcription
DMR2:196634001	2	196634001	196635000	1000	1	8.00E-07	0.38	9	0.9	Arnt	Transcription
DMR2:196650001	2	196650001	196655000	5000	1	4.20E-10	0.55	49	0.98	Arnt;Ctsk;LOC102547324	Transcription;Protease
DMR2:196663001	2	196663001	196666000	3000	2	1.70E-13	0.49	41	1.37	Ctsk;LOC102547324	Protease
DMR2:196669001	2	196669001	196673000	4000	1	7.20E-08	0.37	45	1.12	Ctsk;LOC102547324	Protease
DMR2:196808001	2	196808001	196809000	1000	1	1.90E-21	0.96	10	1	Olr1864-ps	
DMR2:196956001	2	196956001	196959000	3000	1	2.80E-07	-0.52	28	0.93	RGD1566258	
DMR2:196960001	2	196960001	196962000	2000	2	8.40E-08	-0.67	20	1	RGD1566258	
DMR2:196964001	2	196964001	196970000	6000	1	5.90E-16	0.53	94	1.57	RGD1566258	
DMR2:197643001	2	197643001	197647000	4000	2	8.40E-16	0.81	48	1.2	Ctss	Protease
DMR2:197663001	2	197663001	197671000	8000	1	1.10E-21	0.67	122	1.52	Ctss	Protease
DMR2:197700001	2	197700001	197705000	5000	2	2.10E-11	-0.46	38	0.76	Hormad1;LOC102547423	
DMR2:197726001	2	197726001	197727000	1000	1	1.20E-08	0.44	11	1.1	Hormad1;Golph3l	
DMR2:197799001	2	197799001	197800000	1000	1	1.40E-10	-0.59	14	1.4	Adamtsl4	Protease
DMR2:197850001	2	197850001	197851000	1000	1	1.80E-07	-0.52	10	1	Ecm1	
DMR2:197856001	2	197856001	197858000	2000	1	5.70E-07	0.38	65	3.25	Ecm1;Tars2	Translation
DMR2:197861001	2	197861001	197867000	6000	1	1.80E-08	0.36	112	1.87	Ecm1;Tars2	Translation
DMR2:197922001	2	197922001	197924000	2000	1	4.80E-20	0.62	20	1	Rprd2	Signaling
DMR2:197958001	2	197958001	197961000	3000	1	1.50E-07	-0.54	29	0.97	Prpf3	Translation
DMR2:197989001	2	197989001	197991000	2000	1	2.90E-08	0.42	43	2.15	Mrps21;Ciart;LOC108348218	Translation;Transcription
DMR2:198025001	2	198025001	198026000	1000	1	2.60E-09	0.52	9	0.9	Car14;LOC103691643	
DMR2:198027001	2	198027001	198029000	2000	1	2.80E-07	-0.42	26	1.3	Car14;LOC103691643	
DMR2:198035001	2	198035001	198038000	3000	2	3.40E-16	0.7	24	0.8	LOC103691643;Anp32e	Epigenetic
DMR2:198063001	2	198063001	198064000	1000	1	3.40E-07	-0.57	10	1	Anp32e	Epigenetic
DMR2:198082001	2	198082001	198085000	3000	2	1.40E-14	0.52	44	1.47	RGD1562196;LOC108350069	
DMR2:198112001	2	198112001	198114000	2000	1	2.80E-07	-0.4	30	1.5	Plekho1;Vps45	Transport
DMR2:198117001	2	198117001	198118000	1000	1	2.70E-13	0.82	69	6.9	Plekho1;Vps45	Transport
DMR2:198175001	2	198175001	198177000	2000	2	6.40E-12	0.59	19	0.95	Vps45	Transport
DMR2:198253001	2	198253001	198254000	1000	1	6.10E-07	0.35	14	1.4	Otud7b;LOC102548788	Protease
DMR2:198262001	2	198262001	198263000	1000	1	3.50E-07	-0.53	15	1.5	Otud7b;LOC102548788	Protease
DMR2:198266001	2	198266001	198269000	3000	1	4.10E-15	0.46	37	1.23	Otud7b;LOC102548788	Protease
DMR2:198270001	2	198270001	198271000	1000	1	8.90E-07	0.36	9	0.9	Otud7b;LOC102548788	Protease
DMR2:198273001	2	198273001	198275000	2000	2	1.60E-09	-0.65	20	1	Otud7b;LOC102548788	Protease
DMR2:198296001	2	198296001	198298000	2000	1	2.50E-07	0.31	34	1.7	Otud7b;Mtmr11	Protease;Signaling

DMR2:198307001	2	198307001	198311000	4000	1	1.40E-08	0.43	61	1.52	Otud7b;Mtmr11;Sf3b4	Protease;Signaling;Translation
DMR2:198315001	2	198315001	198319000	4000	2	3.00E-13	0.51	66	1.65	Mtmr11;Sf3b4;Sv2a	Signaling;Translation
DMR2:198325001	2	198325001	198330000	5000	1	7.80E-11	0.36	105	2.1	Sf3b4;Sv2a;Bola1	Translation
DMR2:198331001	2	198331001	198338000	7000	3	2.40E-16	0.56	95	1.36	Sv2a;Bola1	
DMR2:198340001	2	198340001	198341000	1000	1	2.40E-14	0.73	12	1.2	Sv2a;Bola1	
DMR2:198342001	2	198342001	198345000	3000	2	5.30E-25	0.83	36	1.2	Sv2a;Bola1	
DMR2:198366001	2	198366001	198367000	1000	1	5.50E-13	0.45	9	0.9	Hist2h2ab;Hist2h2ac;Hist2h2be	Epigenetic
DMR2:198397001	2	198397001	198398000	1000	1	9.50E-08	0.44	5	0.5	Hist2h2aa3;Hist2h3c2	Epigenetic
DMR2:198405001	2	198405001	198407000	2000	1	6.10E-07	-0.54	28	1.4	Hist2h3c2;Hist2h4	Epigenetic
DMR2:198408001	2	198408001	198410000	2000	2	2.30E-07	-0.59	36	1.8	Hist2h3c2;Hist2h4;LOC100912338	Epigenetic
DMR2:198443001	2	198443001	198446000	3000	1	5.50E-08	0.45	37	1.23	Fcgr1a;LOC100359862	Immune
DMR2:198457001	2	198457001	198460000	3000	1	5.30E-09	0.38	35	1.17	LOC100359862;Trnag-ccc	
DMR2:198474001	2	198474001	198479000	5000	1	5.80E-07	-0.59	56	1.12	Trnan-guu	
DMR2:198496001	2	198496001	198499000	3000	2	6.70E-14	0.5	33	1.1	Trnav-cac;Trnan-guu;LOC690204;Trnaq-cug	
DMR2:198512001	2	198512001	198513000	1000	1	3.40E-13	0.7	21	2.1	Trnaq-cug;Trnae-uuc;Trnag-ccc	
DMR2:198535001	2	198535001	198537000	2000	1	2.10E-08	0.39	38	1.9	Trnaq-cug;Trnan-guu;Trnae-uuc;Trnah-gug	
DMR2:198548001	2	198548001	198551000	3000	1	9.40E-11	0.58	35	1.17	Trnan-guu;Trnae-uuc;Trnah-gug	
DMR2:198556001	2	198556001	198559000	3000	1	3.20E-11	0.42	44	1.47	Trnah-gug;Trnag-ccc;Trnak-cuu	
DMR2:198572001	2	198572001	198582000	10000	1	2.30E-10	0.46	150	1.5	Trnag-ccc;Trnak-cuu;Trnah-gug;LOC103691644;LOC100360229	
DMR2:198609001	2	198609001	198610000	1000	1	6.10E-15	0.68	4	0.4	LOC103691644;Trnaq-cug	
DMR2:198617001	2	198617001	198620000	3000	1	7.00E-08	0.37	36	1.2	Trnaq-cug	
DMR2:198644001	2	198644001	198646000	2000	1	1.80E-09	-0.46	31	1.55	Trnan-guu;Trnak-cuu;Trnah-gug;Trnag-ucc;Trnae-cuc;Hfe2	
DMR2:198676001	2	198676001	198677000	1000	1	6.50E-19	0.6	14	1.4	LOC103691645;Txnip	
DMR2:198690001	2	198690001	198697000	7000	1	4.60E-08	-0.7	85	1.21	LOC103691645;Txnip;Polr3gl	Transcription
DMR2:198724001	2	198724001	198725000	1000	1	2.30E-07	0.54	22	2.2	Polr3gl;LOC102552578;Ankrd34a;Lix1l	Transcription
DMR2:198728001	2	198728001	198729000	1000	1	1.40E-14	0.54	22	2.2	Polr3gl;Ankrd34a;Lix1l	Transcription
DMR2:198735001	2	198735001	198736000	1000	1	5.00E-07	-0.44	13	1.3	Ankrd34a;Lix1l	
DMR2:198744001	2	198744001	198747000	3000	2	1.50E-16	0.63	42	1.4	Lix1l;Rbm8a	
DMR2:198768001	2	198768001	198773000	5000	2	8.70E-12	0.47	70	1.4	Rbm8a;Pex11b;Itga10	Extracellular Matrix
DMR2:198774001	2	198774001	198776000	2000	1	9.90E-07	-0.47	30	1.5	Pex11b;Itga10	Extracellular Matrix
DMR2:198777001	2	198777001	198778000	1000	1	3.50E-15	0.49	26	2.6	Pex11b;Itga10	Extracellular Matrix
DMR2:198786001	2	198786001	198790000	4000	1	1.00E-08	0.43	43	1.07	Itga10;Ankrd35	Extracellular Matrix
DMR2:198796001	2	198796001	198801000	5000	2	1.50E-15	0.72	90	1.8	Itga10;Ankrd35	Extracellular Matrix
DMR2:198804001	2	198804001	198806000	2000	1	3.40E-07	-0.55	31	1.55	Ankrd35	
DMR2:198842001	2	198842001	198845000	3000	1	3.80E-10	0.48	40	1.33	Nudt17;Polr3c;Rnf115	Metabolism;Transcription;Proteolysis
DMR2:198868001	2	198868001	198874000	6000	3	4.30E-11	-0.39	60	1	Rnf115	Proteolysis
DMR2:198895001	2	198895001	198898000	3000	1	1.50E-09	-0.7	12	0.4	Rnf115	Proteolysis
DMR2:198902001	2	198902001	198903000	1000	1	2.00E-09	-0.61	5	0.5	Rnf115	Proteolysis
DMR2:198904001	2	198904001	198905000	1000	1	2.90E-10	0.53	3	0.3	Rnf115	Proteolysis
DMR2:198914001	2	198914001	198918000	4000	2	3.70E-10	0.44	54	1.35	Rnf115	Proteolysis
DMR2:198924001	2	198924001	198929000	5000	4	1.50E-16	-0.46	55	1.1	Rnf115;Cd160	Proteolysis
DMR2:198942001	2	198942001	198945000	3000	1	2.10E-11	0.46	36	1.2	Cd160	
DMR2:198950001	2	198950001	198954000	4000	1	2.10E-08	0.45	49	1.23	Cd160	
DMR2:198967001	2	198967001	198968000	1000	1	3.40E-07	-0.43	20	2	Cd160;Pdzk1	
DMR2:198970001	2	198970001	198971000	1000	1	3.60E-22	1.06	24	2.4	Cd160;Pdzk1	
DMR2:198980001	2	198980001	198982000	2000	1	1.60E-25	0.83	26	1.3	Pdzk1	
DMR2:198983001	2	198983001	198989000	6000	1	2.90E-08	0.41	70	1.17	Pdzk1	
DMR2:199012001	2	199012001	199014000	2000	1	8.20E-07	-0.49	21	1.05	Gpr89b	Signaling
DMR2:199020001	2	199020001	199021000	1000	1	6.30E-12	0.58	12	1.2	Gpr89b	Signaling
DMR2:199044001	2	199044001	199046000	2000	1	3.50E-07	0.38	7	0.35	Gpr89b;Gja8	Signaling;Cytoskeleton
DMR2:199150001	2	199150001	199153000	3000	3	3.20E-11	-0.82	49	1.63	LOC690353;Gja5	Cytoskeleton
DMR2:199160001	2	199160001	199161000	1000	1	2.50E-09	0.44	14	1.4	Gja5	Cytoskeleton
DMR2:199162001	2	199162001	199163000	1000	1	2.70E-23	0.73	12	1.2	Gja5	Cytoskeleton

DMR2:199191001	2	199191001	199192000	1000	1	1.60E-09	0.5	12	1.2	Gja5;LOC100909441	Cytoskeleton;Cytoskeleton
DMR2:199279001	2	199279001	199281000	2000	1	2.50E-08	0.39	22	1.1	Acp6	Signaling
DMR2:199295001	2	199295001	199297000	2000	1	3.50E-09	0.37	46	2.3	Acp6	Signaling
DMR2:199306001	2	199306001	199312000	6000	3	3.50E-11	-0.53	64	1.07	Acp6	Signaling
DMR2:199327001	2	199327001	199330000	3000	2	1.20E-09	0.49	52	1.73	Bcl9	
DMR2:199333001	2	199333001	199336000	3000	2	1.40E-15	0.47	69	2.3	Bcl9	
DMR2:199338001	2	199338001	199339000	1000	1	9.30E-11	0.4	17	1.7	Bcl9	
DMR2:199353001	2	199353001	199356000	3000	1	5.70E-07	-0.54	43	1.43	Bcl9	
DMR2:199360001	2	199360001	199363000	3000	2	2.40E-08	-0.55	44	1.47	Bcl9	
DMR2:199374001	2	199374001	199379000	5000	1	5.70E-17	0.89	55	1.1	Bcl9	
DMR2:199390001	2	199390001	199393000	3000	1	5.80E-07	0.34	35	1.17	Bcl9	
DMR2:199395001	2	199395001	199399000	4000	1	5.60E-09	0.39	69	1.73	Bcl9	
DMR2:199409001	2	199409001	199411000	2000	1	8.30E-11	0.6	29	1.45	Bcl9;LOC102549654	
DMR2:199420001	2	199420001	199423000	3000	2	5.00E-19	0.89	47	1.57	Bcl9;LOC102549654	
DMR2:199429001	2	199429001	199430000	1000	1	2.10E-09	0.39	3	0.3	Bcl9;LOC102549654;LOC102550046	
DMR2:199523001	2	199523001	199525000	2000	1	1.80E-10	0.64	12	0.6	Olr390	Signaling
DMR2:199551001	2	199551001	199553000	2000	2	4.40E-12	-0.45	12	0.6	Olr391-ps	
DMR2:199555001	2	199555001	199556000	1000	1	1.50E-11	0.46	19	1.9	Olr391-ps	
DMR2:199559001	2	199559001	199560000	1000	1	8.80E-09	0.56	5	0.5	Olr391-ps	
DMR2:199704001	2	199704001	199712000	8000	6	4.50E-14	-0.58	81	1.01	Chd1l	
DMR2:199713001	2	199713001	199714000	1000	1	1.50E-08	-0.66	8	0.8	Chd1l	
DMR2:199718001	2	199718001	199719000	1000	1	4.40E-07	0.39	20	2	Chd1l	
DMR2:199726001	2	199726001	199728000	2000	1	2.10E-07	-0.54	39	1.95	Chd1l	
DMR2:199732001	2	199732001	199733000	1000	1	2.80E-09	0.38	8	0.8	Chd1l	
DMR2:199743001	2	199743001	199746000	3000	1	1.40E-11	-0.46	13	0.43	Chd1l	
DMR2:199747001	2	199747001	199752000	5000	1	1.40E-07	-0.4	77	1.54	Chd1l	
DMR2:199753001	2	199753001	199762000	9000	2	3.10E-08	0.4	143	1.59	Chd1l	
DMR2:199774001	2	199774001	199775000	1000	1	2.00E-07	0.35	12	1.2	Chd1l	
DMR2:199781001	2	199781001	199785000	4000	3	1.50E-11	-0.49	31	0.78	Chd1l	
DMR2:199802001	2	199802001	199803000	1000	1	2.10E-08	0.42	13	1.3	Chd1l;Fmo5	Metabolism
DMR2:199810001	2	199810001	199815000	5000	1	7.20E-11	0.43	62	1.24	Fmo5	Metabolism
DMR2:199816001	2	199816001	199817000	1000	1	1.60E-07	0.38	10	1	Fmo5	Metabolism
DMR2:199825001	2	199825001	199826000	1000	1	1.80E-07	-0.46	7	0.7	Fmo5;Prkab2	Metabolism;Signaling
DMR2:199850001	2	199850001	199852000	2000	2	5.60E-10	0.41	16	0.8	Prkab2;Trnaq-cug;LOC102550575	Signaling
DMR2:199854001	2	199854001	199856000	2000	1	2.90E-08	-0.33	20	1	Prkab2;Trnaq-cug;LOC102550575	Signaling
DMR2:199865001	2	199865001	199867000	2000	1	1.40E-26	0.86	9	0.45	Trnaq-cug;LOC102550575;Pde4dip	
DMR2:199873001	2	199873001	199875000	2000	1	1.40E-15	0.7	29	1.45	LOC102550575;Pde4dip	
DMR2:199876001	2	199876001	199880000	4000	2	7.00E-17	0.52	42	1.05	LOC102550575;Pde4dip	
DMR2:199897001	2	199897001	199898000	1000	1	5.90E-07	0.37	13	1.3	Pde4dip	
DMR2:199899001	2	199899001	199900000	1000	1	5.00E-09	0.44	10	1	Pde4dip	
DMR2:199905001	2	199905001	199906000	1000	1	6.10E-07	0.42	12	1.2	Pde4dip	
DMR2:199909001	2	199909001	199911000	2000	1	5.40E-18	0.65	27	1.35	Pde4dip	
DMR2:199932001	2	199932001	199934000	2000	1	1.80E-30	0.88	37	1.85	Pde4dip	
DMR2:199958001	2	199958001	199961000	3000	3	5.20E-16	0.77	30	1	Pde4dip	
DMR2:199965001	2	199965001	199967000	2000	1	2.20E-07	0.38	21	1.05	Pde4dip	
DMR2:199990001	2	199990001	199993000	3000	1	3.00E-12	0.52	28	0.93	Pde4dip	
DMR2:200023001	2	200023001	200027000	4000	1	5.40E-12	0.53	46	1.15	Pde4dip	
DMR2:200078001	2	200078001	200079000	1000	1	2.20E-09	0.45	15	1.5	Sec22b	
DMR2:200088001	2	200088001	200090000	2000	1	1.80E-08	-0.66	23	1.15	Sec22b;LOC102550637	
DMR2:200094001	2	200094001	200100000	6000	1	3.20E-07	-0.5	107	1.78	Sec22b;LOC102550637	
DMR2:200196001	2	200196001	200201000	5000	1	9.00E-07	-0.51	79	1.58	Notch2	Extracellular Matrix
DMR2:200207001	2	200207001	200209000	2000	1	5.80E-07	-0.46	23	1.15	Notch2	Extracellular Matrix
DMR2:200236001	2	200236001	200239000	3000	2	6.30E-09	0.4	42	1.4	Notch2	Extracellular Matrix
DMR2:200248001	2	200248001	200250000	2000	1	2.10E-09	-0.49	24	1.2	Notch2	Extracellular Matrix
DMR2:200252001	2	200252001	200255000	3000	1	1.50E-09	0.47	38	1.27	Notch2	Extracellular Matrix
DMR2:200257001	2	200257001	200265000	8000	3	3.10E-14	0.58	108	1.35	Notch2	Extracellular Matrix
DMR2:200266001	2	200266001	200271000	5000	2	4.30E-30	1.05	80	1.6	Notch2	Extracellular Matrix
DMR2:200274001	2	200274001	200283000	9000	1	1.50E-10	-0.66	161	1.79	Notch2	Extracellular Matrix
DMR2:200289001	2	200289001	200292000	3000	2	6.30E-19	0.76	18	0.6	Notch2	Extracellular Matrix

DMR2:200299001	2	200299001	200302000	3000	1	1.60E-20	0.8	52	1.73	Notch2	Extracellular Matrix
DMR2:200332001	2	200332001	200333000	1000	1	8.20E-08	-0.64	9	0.9	Adam30;LOC100361630	Protease
DMR2:200381001	2	200381001	200386000	5000	3	1.10E-34	0.89	71	1.42	Reg4	
DMR2:200387001	2	200387001	200389000	2000	1	9.90E-10	0.61	21	1.05	Reg4	
DMR2:200390001	2	200390001	200392000	2000	1	1.90E-15	0.55	20	1	Reg4	
DMR2:200401001	2	200401001	200402000	1000	1	5.60E-13	0.59	18	1.8	Reg4	
DMR2:200407001	2	200407001	200409000	2000	1	5.90E-16	0.55	62	3.1	Reg4	
DMR2:200413001	2	200413001	200417000	4000	1	1.00E-14	0.71	41	1.02	Reg4	
DMR2:200449001	2	200449001	200451000	2000	1	4.30E-09	0.49	24	1.2	Hmgcs2	
DMR2:200456001	2	200456001	200462000	6000	1	8.90E-09	-0.54	78	1.3	Hmgcs2	
DMR2:200470001	2	200470001	200472000	2000	1	6.40E-08	0.39	23	1.15	Hmgcs2	
DMR2:200475001	2	200475001	200479000	4000	2	2.80E-08	0.33	59	1.48	Hmgcs2;Phgdh	Metabolism
DMR2:200493001	2	200493001	200494000	1000	1	1.70E-11	0.48	13	1.3	Phgdh	Metabolism
DMR2:200500001	2	200500001	200502000	2000	1	1.70E-11	-0.49	23	1.15	Phgdh	Metabolism
DMR2:200509001	2	200509001	200510000	1000	1	6.40E-09	0.36	8	0.8	Phgdh	Metabolism
DMR2:200587001	2	200587001	200589000	2000	1	3.30E-10	0.56	36	1.8	Zfp697;LOC108348209	Transcription
DMR2:200617001	2	200617001	200620000	3000	2	1.60E-07	0.43	49	1.63	LOC102550892;Hsd3b5	Metabolism
DMR2:200626001	2	200626001	200629000	3000	1	9.80E-07	-0.54	25	0.83	Hsd3b5	Metabolism
DMR2:200635001	2	200635001	200636000	1000	1	6.50E-07	0.53	13	1.3	Hsd3b5	Metabolism
DMR2:200640001	2	200640001	200644000	4000	2	1.00E-15	0.63	54	1.35	Hsd3b5	Metabolism
DMR2:200701001	2	200701001	200707000	6000	3	2.60E-14	0.52	31	0.52	LOC502588;Hsd3b6	Metabolism
DMR2:200730001	2	200730001	200732000	2000	1	9.70E-18	0.79	23	1.15	Hsd3b6	Metabolism
DMR2:200747001	2	200747001	200749000	2000	2	8.80E-10	-0.45	5	0.25	Hsd3b3	
DMR2:200771001	2	200771001	200772000	1000	1	7.30E-12	0.77	7	0.7	Hsd3b3	
DMR2:200779001	2	200779001	200780000	1000	1	8.10E-15	0.57	13	1.3	Hao2	Metabolism
DMR2:200784001	2	200784001	200787000	3000	1	1.40E-08	0.43	23	0.77	Hao2	Metabolism
DMR2:200805001	2	200805001	200808000	3000	1	8.50E-09	0.52	17	0.57	Hao2	Metabolism
DMR2:201107001	2	201107001	201109000	2000	1	1.70E-08	0.33	47	2.35	Snrpgl2	
DMR2:201111001	2	201111001	201112000	1000	1	4.30E-11	-0.63	12	1.2	Snrpgl2	
DMR2:201161001	2	201161001	201165000	4000	2	2.60E-12	0.53	37	0.92	LOC690649;Wars2	Translation
DMR2:201169001	2	201169001	201171000	2000	1	1.10E-09	0.46	16	0.8	LOC690649;Wars2	Translation
DMR2:201181001	2	201181001	201182000	1000	1	2.60E-10	0.43	10	1	Wars2	Translation
DMR2:201183001	2	201183001	201184000	1000	1	1.20E-16	0.62	12	1.2	Wars2	Translation
DMR2:201193001	2	201193001	201196000	3000	1	7.20E-11	0.48	26	0.87	Wars2	Translation
DMR2:201197001	2	201197001	201199000	2000	1	8.40E-08	0.41	24	1.2	Wars2	Translation
DMR2:201214001	2	201214001	201216000	2000	2	2.90E-12	0.54	27	1.35	Wars2	Translation
DMR2:201225001	2	201225001	201227000	2000	1	2.30E-07	-0.3	38	1.9	Wars2	Translation
DMR2:201242001	2	201242001	201245000	3000	1	1.50E-08	-0.47	31	1.03	Wars2	Translation
DMR2:201248001	2	201248001	201252000	4000	3	9.90E-21	-0.69	64	1.6	Wars2	Translation
DMR2:201287001	2	201287001	201288000	1000	1	7.80E-17	0.72	13	1.3	Tbx15	Transcription
DMR2:201326001	2	201326001	201327000	1000	1	4.80E-09	-0.58	14	1.4	Tbx15	Transcription
DMR2:201332001	2	201332001	201333000	1000	1	7.80E-17	0.86	7	0.7	Tbx15	Transcription
DMR2:201343001	2	201343001	201349000	6000	1	7.80E-07	-0.55	66	1.1	Tbx15	Transcription
DMR2:201352001	2	201352001	201354000	2000	1	2.30E-07	0.34	27	1.35	Tbx15	Transcription
DMR2:201415001	2	201415001	201416000	1000	1	7.80E-10	0.6	6	0.6	LOC500213;RGD1561508	
DMR2:201417001	2	201417001	201425000	8000	4	8.90E-24	-0.58	95	1.19	RGD1561508	
DMR2:202177001	2	202177001	202181000	4000	3	2.80E-23	-0.56	67	1.68	Spag17	
DMR2:202217001	2	202217001	202220000	3000	1	2.70E-10	0.41	30	1	Spag17	
DMR2:202222001	2	202222001	202224000	2000	1	8.40E-13	0.53	17	0.85	Spag17	
DMR2:202248001	2	202248001	202252000	4000	3	1.00E-18	0.76	23	0.58	Spag17	
DMR2:202259001	2	202259001	202263000	4000	1	5.30E-16	0.9	34	0.85	Spag17	
DMR2:202274001	2	202274001	202277000	3000	1	1.30E-07	0.36	32	1.07	Spag17	
DMR2:202278001	2	202278001	202280000	2000	1	6.40E-10	0.58	15	0.75	Spag17	
DMR2:202308001	2	202308001	202309000	1000	1	5.30E-07	-0.45	13	1.3	Spag17	
DMR2:202313001	2	202313001	202314000	1000	1	8.50E-14	0.82	9	0.9	Spag17	
DMR2:202329001	2	202329001	202331000	2000	2	1.00E-09	-0.57	10	0.5	Spag17	
DMR2:202338001	2	202338001	202340000	2000	1	6.50E-10	-0.47	13	0.65	Spag17;LOC108348086	
DMR2:202359001	2	202359001	202360000	1000	1	3.30E-30	0.87	16	1.6	Spag17;LOC108348086	
DMR2:202371001	2	202371001	202373000	2000	1	1.10E-07	-0.36	13	0.65	Spag17	
DMR2:202387001	2	202387001	202389000	2000	1	1.40E-10	0.48	21	1.05	Spag17	
DMR2:202394001	2	202394001	202396000	2000	1	8.20E-07	0.48	9	0.45	Spag17	
DMR2:202417001	2	202417001	202419000	2000	1	1.20E-10	-0.36	14	0.7	Spag17	
DMR2:202429001	2	202429001	202430000	1000	1	1.10E-19	0.9	5	0.5	Spag17	
DMR2:202432001	2	202432001	202436000	4000	1	1.70E-07	-0.63	53	1.32	Spag17	
DMR2:202443001	2	202443001	202447000	4000	1	5.30E-09	0.47	31	0.78	Spag17;Wdr3	

DMR2:202465001	2	202465001	202468000	3000	1	1.10E-13	0.54	40	1.33	Wdr3;Gdap2	
DMR2:202478001	2	202478001	202479000	1000	1	1.70E-11	0.68	7	0.7	Wdr3;Gdap2	
DMR2:202488001	2	202488001	202491000	3000	1	1.20E-08	0.59	38	1.27	Gdap2	
DMR2:202503001	2	202503001	202508000	5000	2	8.60E-12	-0.69	48	0.96	Gdap2	
DMR2:202512001	2	202512001	202513000	1000	1	5.80E-08	0.41	17	1.7	Gdap2	
DMR2:202517001	2	202517001	202522000	5000	1	1.70E-12	0.42	72	1.44	Gdap2	
DMR2:202532001	2	202532001	202533000	1000	1	1.80E-10	0.54	7	0.7	Gdap2	
DMR2:202534001	2	202534001	202535000	1000	1	2.00E-08	-0.54	1	0.1	Gdap2	
DMR2:202811001	2	202811001	202814000	3000	1	4.60E-25	0.77	36	1.2	Fam46c	
DMR2:202914001	2	202914001	202917000	3000	1	2.50E-07	-0.53	42	1.4	Man1a2	Golgi
DMR2:202927001	2	202927001	202929000	2000	1	2.80E-13	0.63	16	0.8	Man1a2	Golgi
DMR2:202948001	2	202948001	202955000	7000	4	6.30E-22	-0.56	82	1.17	Man1a2	Golgi
DMR2:202956001	2	202956001	202958000	2000	1	1.00E-07	0.32	18	0.9	Man1a2	Golgi
DMR2:202965001	2	202965001	202966000	1000	1	6.30E-09	-0.57	9	0.9	Man1a2	Golgi
DMR2:202970001	2	202970001	202974000	4000	2	6.10E-11	0.54	44	1.1	Man1a2	Golgi
DMR2:202990001	2	202990001	202993000	3000	1	1.40E-12	0.67	15	0.5	Man1a2	Golgi
DMR2:202996001	2	202996001	2.03E+08	4000	2	7.40E-11	0.47	43	1.07	Man1a2	Golgi
DMR2:203001001	2	203001001	203003000	2000	1	4.40E-08	-0.46	13	0.65	Man1a2	Golgi
DMR2:203018001	2	203018001	203024000	6000	2	1.60E-10	0.45	61	1.02	Man1a2;LOC683192	Golgi
DMR2:203050001	2	203050001	203054000	4000	1	2.90E-07	-0.63	35	0.88	Man1a2	Golgi
DMR2:203199001	2	203199001	203200000	1000	1	6.70E-10	0.72	3	0.3	Vtcn1	Immune
DMR2:203203001	2	203203001	203205000	2000	1	4.10E-09	0.39	36	1.8	Vtcn1	Immune
DMR2:203208001	2	203208001	203210000	2000	1	9.70E-08	0.34	27	1.35	Vtcn1	Immune
DMR2:203217001	2	203217001	203218000	1000	1	9.30E-07	0.31	16	1.6	Vtcn1	Immune
DMR2:203221001	2	203221001	203222000	1000	1	3.90E-13	0.49	6	0.6	Vtcn1	Immune
DMR2:203240001	2	203240001	203242000	2000	1	6.00E-19	0.57	32	1.6	Vtcn1	Immune
DMR2:203244001	2	203244001	203248000	4000	2	1.30E-16	-0.69	46	1.15	Vtcn1	Immune
DMR2:203250001	2	203250001	203252000	2000	1	1.50E-07	0.42	6	0.3	Vtcn1	Immune
DMR2:203258001	2	203258001	203262000	4000	3	3.20E-11	0.57	47	1.18	Vtcn1	Immune
DMR2:203263001	2	203263001	203265000	2000	1	3.90E-09	0.49	24	1.2	Vtcn1	Immune
DMR2:203299001	2	203299001	203300000	1000	1	3.20E-07	0.4	8	0.8	Trim45	
DMR2:203303001	2	203303001	203304000	1000	1	6.00E-08	-0.59	16	1.6	Trim45	
DMR2:203324001	2	203324001	203325000	1000	1	1.30E-20	0.66	15	1.5	Ttf2	
DMR2:203326001	2	203326001	203332000	6000	2	3.60E-21	0.75	102	1.7	Ttf2	
DMR2:203333001	2	203333001	203334000	1000	1	3.60E-12	-0.77	15	1.5	Ttf2	
DMR2:203338001	2	203338001	203342000	4000	1	4.90E-11	0.49	56	1.4	Ttf2	
DMR2:203368001	2	203368001	203370000	2000	1	8.70E-09	0.33	27	1.35	Cd101	Immune
DMR2:203377001	2	203377001	203384000	7000	1	5.60E-11	0.45	109	1.56	Cd101	Immune
DMR2:203407001	2	203407001	203415000	8000	2	5.00E-09	-0.43	96	1.2	Cd101;Ptgfrn	Immune
DMR2:203418001	2	203418001	203420000	2000	1	4.80E-08	0.45	22	1.1	Cd101;Ptgfrn	Immune
DMR2:203441001	2	203441001	203445000	4000	2	1.50E-11	0.44	70	1.75	Ptgfrn	Immune
DMR2:203446001	2	203446001	203451000	5000	2	5.70E-13	0.45	73	1.46	Ptgfrn;LOC108350075	Immune
DMR2:203452001	2	203452001	203458000	6000	1	5.40E-09	0.33	100	1.67	Ptgfrn;LOC108350075	Immune
DMR2:203464001	2	203464001	203470000	6000	1	8.70E-10	0.54	82	1.37	Ptgfrn;LOC108350075	Immune
DMR2:203485001	2	203485001	203493000	8000	3	5.70E-11	0.43	134	1.68	Ptgfrn	Immune
DMR2:203499001	2	203499001	203501000	2000	2	4.60E-12	0.5	31	1.55	Ptgfrn	Immune
DMR2:203680001	2	203680001	203681000	1000	1	5.30E-11	0.69	8	0.8	Cd2	Immune
DMR2:203764001	2	203764001	203766000	2000	2	9.90E-20	0.6	25	1.25	LOC103691649;Igsf3	Immune
DMR2:203784001	2	203784001	203785000	1000	1	1.90E-11	0.52	12	1.2	Igsf3	Immune
DMR2:203790001	2	203790001	203792000	2000	1	3.90E-07	0.37	23	1.15	Igsf3	Immune
DMR2:203797001	2	203797001	203799000	2000	1	1.10E-11	0.41	31	1.55	Igsf3	Immune
DMR2:203804001	2	203804001	203805000	1000	1	3.20E-14	0.75	7	0.7	Igsf3	Immune
DMR2:203838001	2	203838001	203841000	3000	1	2.10E-07	0.36	42	1.4	Igsf3	Immune
DMR2:203842001	2	203842001	203846000	4000	1	1.70E-15	0.56	71	1.77	Igsf3	Immune
DMR2:203849001	2	203849001	203850000	1000	1	1.30E-09	0.42	13	1.3	Igsf3	Immune
DMR2:203855001	2	203855001	203856000	1000	1	1.80E-18	0.86	12	1.2	Igsf3	Immune
DMR2:203996001	2	203996001	203998000	2000	1	6.10E-21	0.62	36	1.8	Atp1a1	Transport
DMR2:204226001	2	204226001	204227000	1000	1	5.70E-08	0.4	10	1	Mab21l3	
DMR2:204234001	2	204234001	204237000	3000	1	1.20E-07	0.36	50	1.67	Mab21l3	
DMR2:204239001	2	204239001	204240000	1000	1	8.00E-09	0.72	2	0.2	Mab21l3	
DMR2:204247001	2	204247001	204248000	1000	1	2.10E-11	0.5	10	1	Mab21l3	
DMR2:204254001	2	204254001	204258000	4000	2	9.90E-17	0.59	68	1.7	Mab21l3	
DMR2:204264001	2	204264001	204267000	3000	1	2.10E-07	-0.38	37	1.23	Mab21l3	
DMR2:204278001	2	204278001	204280000	2000	2	1.40E-13	0.84	12	0.6	Mab21l3;Slc22a15	Transport
DMR2:204287001	2	204287001	204288000	1000	1	1.40E-09	0.39	11	1.1	Slc22a15	Transport

DMR2:204292001	2	204292001	204294000	2000	1	8.10E-09	0.44	19	0.95	Slc22a15	Transport
DMR2:204295001	2	204295001	204297000	2000	1	1.10E-07	-0.42	30	1.5	Slc22a15	Transport
DMR2:204318001	2	204318001	204320000	2000	1	3.70E-17	0.59	23	1.15	Slc22a15	Transport
DMR2:204345001	2	204345001	204346000	1000	1	7.10E-10	0.66	4	0.4	Slc22a15	Transport
DMR2:204350001	2	204350001	204351000	1000	1	4.60E-11	0.51	5	0.5	Slc22a15	Transport
DMR2:204438001	2	204438001	204440000	2000	1	4.80E-07	-0.49	24	1.2	Nhlh2	Transcription
DMR2:204442001	2	204442001	204443000	1000	1	4.00E-10	0.44	7	0.7	Nhlh2	Transcription
DMR2:204510001	2	204510001	204511000	1000	1	6.30E-12	0.5	15	1.5	Casq2	Signaling
DMR2:204525001	2	204525001	204533000	8000	1	6.10E-08	0.47	95	1.19	Casq2	Signaling
DMR2:204582001	2	204582001	204590000	8000	1	9.20E-07	-0.42	127	1.59	Vangl1	
DMR2:204626001	2	204626001	204627000	1000	1	4.90E-07	0.42	12	1.2	Vangl1	
DMR2:204632001	2	204632001	204634000	2000	1	2.50E-09	0.44	11	0.55	Vangl1	
DMR2:204635001	2	204635001	204638000	3000	1	1.70E-12	0.81	26	0.87	Vangl1	
DMR2:204881001	2	204881001	204882000	1000	1	9.60E-15	0.57	8	0.8	LOC102549693;Ngf	Growth Factors
DMR2:204895001	2	204895001	204897000	2000	1	7.90E-12	0.72	13	0.65	Ngf	Growth Factors
DMR2:204917001	2	204917001	204919000	2000	1	1.80E-08	0.63	11	0.55	Ngf	Growth Factors
DMR2:204924001	2	204924001	204925000	1000	1	2.70E-18	0.85	14	1.4	Ngf	Growth Factors
DMR2:204947001	2	204947001	204950000	3000	2	2.80E-15	0.88	26	0.87	Ngf	Growth Factors
DMR2:205150001	2	205150001	205158000	8000	3	1.50E-24	0.84	65	0.81	LOC108350076;Tspan2	
DMR2:205191001	2	205191001	205193000	2000	1	1.10E-08	-0.58	26	1.3	Tspan2	
DMR2:205195001	2	205195001	205200000	5000	2	1.70E-17	-0.6	67	1.34	Tspan2;Tshb	Hormone
DMR2:205215001	2	205215001	205216000	1000	1	4.10E-11	0.6	3	0.3	Tshb	Hormone
DMR2:205274001	2	205274001	205276000	2000	1	3.50E-11	0.47	20	1	Sycp1	
DMR2:205281001	2	205281001	205286000	5000	2	8.80E-13	-0.46	38	0.76	Sycp1	
DMR2:205289001	2	205289001	205291000	2000	2	3.60E-09	0.49	15	0.75	Sycp1	
DMR2:205293001	2	205293001	205294000	1000	1	1.40E-08	0.51	6	0.6	Sycp1	
DMR2:205296001	2	205296001	205297000	1000	1	1.40E-12	-0.46	6	0.6	Sycp1	
DMR2:205304001	2	205304001	205306000	2000	2	8.20E-16	0.91	14	0.7	Sycp1	
DMR2:205313001	2	205313001	205315000	2000	1	1.60E-14	0.58	12	0.6	Sycp1	
DMR2:205319001	2	205319001	205321000	2000	1	7.20E-07	0.44	12	0.6	Sycp1	
DMR2:205322001	2	205322001	205323000	1000	1	2.80E-07	0.43	6	0.6	Sycp1	
DMR2:205342001	2	205342001	205344000	2000	1	6.90E-17	0.76	16	0.8	Sycp1	
DMR2:205345001	2	205345001	205353000	8000	5	2.30E-16	-0.49	100	1.25	Sycp1	
DMR2:205354001	2	205354001	205361000	7000	3	2.20E-15	-0.44	74	1.06	Sycp1	
DMR2:205369001	2	205369001	205374000	5000	4	3.20E-17	-0.58	52	1.04	Sycp1	
DMR2:205375001	2	205375001	205378000	3000	3	1.00E-16	-0.5	37	1.23	Sycp1	
DMR2:205383001	2	205383001	205387000	4000	1	1.70E-09	-0.47	27	0.68	Sycp1	
DMR2:205393001	2	205393001	205398000	5000	3	3.40E-17	-0.54	42	0.84	Sycp1	
DMR2:205401001	2	205401001	205402000	1000	1	8.20E-11	0.68	2	0.2	Sycp1	
DMR2:205403001	2	205403001	205405000	2000	2	1.10E-16	0.82	8	0.4	Sycp1	
DMR2:205407001	2	205407001	205412000	5000	1	6.50E-09	-0.41	35	0.7	Sycp1	
DMR2:205414001	2	205414001	205418000	4000	2	1.30E-17	0.75	22	0.55	Sycp1	
DMR2:205433001	2	205433001	205435000	2000	1	1.20E-10	0.52	9	0.45	Sycp1;Nr1h5	Transcription
DMR2:205440001	2	205440001	205445000	5000	2	9.50E-17	0.96	65	1.3	Nr1h5	Transcription
DMR2:205446001	2	205446001	205451000	5000	1	3.60E-10	0.42	112	2.24	Nr1h5	Transcription
DMR2:205452001	2	205452001	205455000	3000	1	5.00E-13	0.69	19	0.63	Nr1h5	Transcription
DMR2:205458001	2	205458001	205459000	1000	1	7.40E-08	0.38	12	1.2	Nr1h5	Transcription
DMR2:205460001	2	205460001	205461000	1000	1	1.20E-11	0.57	9	0.9	Nr1h5	Transcription
DMR2:205481001	2	205481001	205483000	2000	1	1.90E-19	0.54	13	0.65	LOC108350203;Sike1	
DMR2:205524001	2	205524001	205525000	1000	1	7.50E-07	-0.51	6	0.6	Csde1	
DMR2:205559001	2	205559001	205561000	2000	1	5.20E-08	-0.66	22	1.1	Csde1;Nras;Ampd1	Signaling;Metabolism
DMR2:205578001	2	205578001	205580000	2000	1	1.90E-09	0.41	19	0.95	Ampd1	Metabolism
DMR2:205581001	2	205581001	205584000	3000	1	1.60E-27	0.76	46	1.53	Ampd1;Dennd2c	Metabolism
DMR2:205585001	2	205585001	205591000	6000	3	1.00E-22	0.75	89	1.48	Ampd1;Dennd2c	Metabolism
DMR2:205597001	2	205597001	205599000	2000	2	1.10E-15	0.76	18	0.9	Ampd1;Dennd2c	Metabolism
DMR2:205613001	2	205613001	205616000	3000	1	1.50E-17	0.8	24	0.8	Dennd2c	
DMR2:205618001	2	205618001	205627000	9000	2	1.70E-08	-0.69	128	1.42	Dennd2c	
DMR2:205628001	2	205628001	205633000	5000	1	5.70E-08	0.36	83	1.66	Dennd2c	
DMR2:205636001	2	205636001	205641000	5000	1	7.60E-17	-0.88	79	1.58	Dennd2c	
DMR2:205776001	2	205776001	205778000	2000	1	7.00E-07	0.47	6	0.3	Trim33	Epigenetic
DMR2:205807001	2	205807001	205811000	4000	1	1.00E-10	0.46	36	0.9	Trim33	Epigenetic
DMR2:205822001	2	205822001	205823000	1000	1	6.00E-16	0.8	4	0.4	Trim33	Epigenetic
DMR2:205853001	2	205853001	205854000	1000	1	8.70E-10	0.52	6	0.6	Trim33	Epigenetic
DMR2:205869001	2	205869001	205875000	6000	2	2.00E-16	0.62	62	1.03	Trim33	Epigenetic

DMR2:206086001	2	206086001	206091000	5000	2	7.70E-16	0.83	58	1.16	Syt6	Transport
DMR2:206098001	2	206098001	206101000	3000	2	1.50E-14	0.59	23	0.77	Syt6	Transport
DMR2:206102001	2	206102001	206104000	2000	1	1.30E-10	0.44	28	1.4	Syt6	Transport
DMR2:206114001	2	206114001	206117000	3000	2	7.60E-08	-0.51	35	1.17	Syt6	Transport
DMR2:206209001	2	206209001	206212000	3000	1	2.80E-09	-0.51	42	1.4	LOC102547265;Olfml3	Development
DMR2:206225001	2	206225001	206230000	5000	2	7.10E-10	0.44	67	1.34	LOC102547265;Olfml3;Hipk1	Development
DMR2:206256001	2	206256001	206260000	4000	1	1.60E-07	-0.4	46	1.15	Hipk1;LOC108350077	
DMR2:206264001	2	206264001	206265000	1000	1	6.50E-26	0.93	9	0.9	Hipk1;LOC108350077	
DMR2:206276001	2	206276001	206278000	2000	1	1.00E-09	0.47	11	0.55	Hipk1;LOC108350077;Dclre1b	Transcription
DMR2:206319001	2	206319001	206322000	3000	2	3.80E-14	0.83	21	0.7	Bcl2l15	
DMR2:206328001	2	206328001	206329000	1000	1	6.00E-09	0.57	9	0.9	Bcl2l15	
DMR2:206360001	2	206360001	206363000	3000	1	1.10E-16	0.93	29	0.97	Ptpn22	
DMR2:206369001	2	206369001	206375000	6000	1	1.90E-09	0.41	93	1.55	Ptpn22	
DMR2:206383001	2	206383001	206384000	1000	1	3.60E-07	-0.63	11	1.1	Ptpn22;Rsbn1	
DMR2:206413001	2	206413001	206416000	3000	1	3.40E-12	0.49	24	0.8	Rsbn1	
DMR2:206418001	2	206418001	206419000	1000	1	5.70E-07	0.45	8	0.8	Rsbn1	
DMR2:206425001	2	206425001	206427000	2000	1	1.20E-07	0.46	60	3	Rsbn1	
DMR2:206430001	2	206430001	206432000	2000	2	1.00E-09	0.48	84	4.2	Rsbn1	
DMR2:206463001	2	206463001	206464000	1000	1	4.30E-15	0.71	6	0.6	Phtf1	Development
DMR2:206483001	2	206483001	206486000	3000	1	5.40E-08	-0.58	38	1.27	Phtf1	Development
DMR2:206518001	2	206518001	206519000	1000	1	6.00E-10	0.59	6	0.6	Phtf1;Magi3	Development
DMR2:206534001	2	206534001	206535000	1000	1	7.30E-08	0.35	7	0.7	Magi3	
DMR2:206537001	2	206537001	206538000	1000	1	1.60E-13	0.54	10	1	Magi3	
DMR2:206556001	2	206556001	206558000	2000	1	9.60E-13	0.62	22	1.1	Magi3	
DMR2:206562001	2	206562001	206563000	1000	1	1.60E-19	0.84	4	0.4	Magi3	
DMR2:206589001	2	206589001	206591000	2000	2	2.50E-16	0.62	10	0.5	Magi3	
DMR2:206593001	2	206593001	206596000	3000	2	1.80E-14	-0.56	19	0.63	Magi3	
DMR2:206626001	2	206626001	206629000	3000	2	2.10E-19	-0.57	45	1.5	Magi3	
DMR2:206635001	2	206635001	206644000	9000	5	7.00E-20	-0.52	145	1.61	Magi3	
DMR2:206660001	2	206660001	206662000	2000	2	9.30E-12	0.55	13	0.65	Magi3	
DMR2:206665001	2	206665001	206666000	1000	1	7.00E-13	0.48	11	1.1	Magi3	
DMR2:206672001	2	206672001	206675000	3000	2	3.50E-12	0.63	11	0.37	Magi3	
DMR2:206678001	2	206678001	206680000	2000	1	2.70E-11	-0.52	4	0.2	Magi3	
DMR2:206900001	2	206900001	206902000	2000	2	1.90E-20	0.9	13	0.65	LOC103691658;RGD1564469	
DMR2:206907001	2	206907001	206909000	2000	1	3.30E-07	0.46	23	1.15	LOC103691658;RGD1564469	
DMR2:206918001	2	206918001	206920000	2000	1	1.40E-07	-0.52	35	1.75	Lrig2	Receptor
DMR2:206935001	2	206935001	206936000	1000	1	7.40E-09	-0.54	19	1.9	Lrig2	Receptor
DMR2:207126001	2	207126001	207130000	4000	1	3.60E-13	0.46	48	1.2	Slc16a1	Transport
DMR2:207135001	2	207135001	207136000	1000	1	3.10E-07	0.42	21	2.1	Slc16a1;LOC102547750	Transport
DMR2:207247001	2	207247001	207249000	2000	1	1.20E-08	-0.41	34	1.7	Fam19a3	
DMR2:207265001	2	207265001	207269000	4000	1	1.20E-09	0.36	49	1.23	Fam19a3;Ppm1j;Rhoc;Mov10	Signaling;Signaling
DMR2:207276001	2	207276001	207283000	7000	1	2.90E-14	0.45	126	1.8	Ppm1j;Rhoc;Mov10	Signaling;Signaling
DMR2:207288001	2	207288001	207292000	4000	1	6.50E-09	0.37	63	1.57	Rhoc;Mov10	Signaling
DMR2:207295001	2	207295001	207296000	1000	1	4.80E-08	0.42	6	0.6	Mov10;Capza1	Cytoskeleton
DMR2:207297001	2	207297001	207298000	1000	1	1.10E-11	0.63	0	0	Mov10;Capza1	Cytoskeleton
DMR2:207349001	2	207349001	207350000	1000	1	9.50E-09	0.62	11	1.1	Capza1;St7l	Cytoskeleton
DMR2:207369001	2	207369001	207370000	1000	1	1.00E-23	0.93	7	0.7	St7l	
DMR2:207384001	2	207384001	207385000	1000	1	8.30E-09	0.45	8	0.8	St7l	
DMR2:207386001	2	207386001	207395000	9000	5	4.90E-23	-0.56	132	1.47	St7l	
DMR2:207404001	2	207404001	207406000	2000	1	2.60E-11	0.56	18	0.9	St7l	
DMR2:207421001	2	207421001	207422000	1000	1	1.20E-14	0.78	6	0.6	St7l	
DMR2:207438001	2	207438001	207440000	2000	1	7.20E-08	0.45	17	0.85	Wnt2b	Signaling
DMR2:207443001	2	207443001	207448000	5000	2	3.00E-12	-0.46	73	1.46	Wnt2b	Signaling
DMR2:207509001	2	207509001	207510000	1000	1	5.80E-08	-0.58	8	0.8	Cttnbp2nl;LOC103691661	
DMR2:207532001	2	207532001	207534000	2000	1	3.20E-08	-0.65	20	1	Cttnbp2nl	
DMR2:207537001	2	207537001	207539000	2000	1	1.40E-09	0.34	23	1.15	Cttnbp2nl	
DMR2:207565001	2	207565001	207567000	2000	1	1.80E-08	-0.56	36	1.8	RGD1564583	
DMR2:207581001	2	207581001	207583000	2000	1	6.10E-08	-0.41	25	1.25	RGD1564583;LOC102548357;LOC102548297	
DMR2:207917001	2	207917001	207920000	3000	2	5.60E-16	0.82	27	0.9	Kcnd3	Transport
DMR2:207928001	2	207928001	207931000	3000	1	3.70E-22	0.53	52	1.73	Kcnd3	Transport
DMR2:207933001	2	207933001	207934000	1000	1	3.00E-07	0.39	5	0.5	Kcnd3	Transport
DMR2:207946001	2	207946001	207949000	3000	1	3.50E-07	-0.53	27	0.9	Kcnd3	Transport
DMR2:207960001	2	207960001	207963000	3000	1	6.70E-09	0.63	26	0.87	Kcnd3	Transport
DMR2:207969001	2	207969001	207970000	1000	1	1.90E-08	0.42	16	1.6	Kcnd3	Transport

DMR2:207981001	2	207981001	207983000	2000	1	1.10E-09	0.41	22	1.1	Kcnd3	Transport
DMR2:207986001	2	207986001	207987000	1000	1	2.00E-09	0.71	4	0.4	Kcnd3	Transport
DMR2:208015001	2	208015001	208017000	2000	2	1.70E-14	0.75	18	0.9	Kcnd3	Transport
DMR2:208020001	2	208020001	208021000	1000	1	3.00E-16	0.68	10	1	Kcnd3	Transport
DMR2:208024001	2	208024001	208028000	4000	1	2.30E-11	0.8	40	1	Kcnd3	Transport
DMR2:208037001	2	208037001	208043000	6000	2	6.50E-25	0.95	75	1.25	Kcnd3	Transport
DMR2:208046001	2	208046001	208048000	2000	1	9.00E-19	0.68	19	0.95	Kcnd3	Transport
DMR2:208071001	2	208071001	208072000	1000	1	2.90E-20	0.92	7	0.7	Kcnd3	Transport
DMR2:208076001	2	208076001	208077000	1000	1	1.50E-10	0.39	16	1.6	Kcnd3	Transport
DMR2:208091001	2	208091001	208092000	1000	1	2.80E-13	0.48	41	4.1	Kcnd3	Transport
DMR2:208093001	2	208093001	208095000	2000	1	5.30E-08	0.37	23	1.15	Kcnd3	Transport
DMR2:208101001	2	208101001	208104000	3000	2	9.00E-13	0.65	27	0.9	Kcnd3	Transport
DMR2:208119001	2	208119001	208123000	4000	1	1.20E-07	0.38	58	1.45	Kcnd3	Transport
DMR2:208133001	2	208133001	208135000	2000	2	1.90E-10	0.53	11	0.55	Kcnd3;Ddx20	Transport
DMR2:208179001	2	208179001	208182000	3000	1	2.20E-12	0.69	37	1.23	Fam212b	
DMR2:208190001	2	208190001	208192000	2000	2	4.20E-23	0.77	22	1.1	Fam212b;Rap1a	Signaling
DMR2:208210001	2	208210001	208214000	4000	2	7.80E-17	0.54	41	1.02	Rap1a;LOC108350082	Signaling
DMR2:208223001	2	208223001	208225000	2000	1	6.30E-07	0.3	13	0.65	Rap1a;LOC108350082	Signaling
DMR2:208334001	2	208334001	208335000	1000	1	2.70E-07	0.53	4	0.4	LOC100911347;Adora3	Signaling
DMR2:208344001	2	208344001	208345000	1000	1	1.30E-11	0.77	8	0.8	LOC100911347;Adora3	Signaling
DMR2:208385001	2	208385001	208386000	1000	1	5.30E-08	0.42	19	1.9	LOC100911347;Adora3	Signaling
DMR2:208492001	2	208492001	208494000	2000	1	2.00E-07	-0.62	26	1.3	Tmigd3;LOC100911796	Immune
DMR2:208499001	2	208499001	208500000	1000	1	4.00E-10	0.68	8	0.8	Tmigd3;LOC100911796	Immune
DMR2:208534001	2	208534001	208535000	1000	1	1.90E-09	0.63	13	1.3	Tmigd3;LOC100911796	Immune
DMR2:208549001	2	208549001	208550000	1000	1	1.80E-13	0.68	9	0.9	Tmigd3;LOC100363228	Immune
DMR2:208565001	2	208565001	208566000	1000	1	2.80E-12	0.67	6	0.6	Tmigd3;LOC100363228;Atp5f1	Immune
DMR2:208596001	2	208596001	208600000	4000	3	4.10E-14	-0.52	41	1.02	Wdr77;LOC102553341	
DMR2:208608001	2	208608001	208611000	3000	2	1.40E-08	-0.48	16	0.53	Pifo	
DMR2:208620001	2	208620001	208621000	1000	1	8.50E-09	0.48	7	0.7	Pifo	
DMR2:208632001	2	208632001	208633000	1000	1	5.10E-32	1.18	9	0.9	Pifo;LOC103691665;LOC108350084	
DMR2:208642001	2	208642001	208646000	4000	2	2.30E-19	0.75	29	0.72	Pifo;LOC103691665;LOC108350084	
DMR2:208729001	2	208729001	208734000	5000	2	5.90E-17	0.8	23	0.46	Prdx1l1	
DMR2:208735001	2	208735001	208737000	2000	1	2.10E-12	0.64	17	0.85	Prdx1l1	
DMR2:208743001	2	208743001	208745000	2000	2	4.30E-17	0.8	11	0.55	Prdx1l1;Chia	Metabolism
DMR2:208760001	2	208760001	208765000	5000	3	7.50E-23	-0.55	43	0.86	Chia	Metabolism
DMR2:208767001	2	208767001	208768000	1000	1	7.30E-07	0.48	0	0	Chia	Metabolism
DMR2:208782001	2	208782001	208783000	1000	1	2.50E-09	0.6	2	0.2	Chia	Metabolism
DMR2:208788001	2	208788001	208796000	8000	5	3.40E-16	-0.42	103	1.29	Chia	Metabolism
DMR2:208890001	2	208890001	208895000	5000	1	1.10E-12	-0.43	38	0.76	Chi3l3	Metabolism
DMR2:208973001	2	208973001	208975000	2000	2	2.40E-11	-0.68	6	0.3	RGD1309110	Metabolism
DMR2:208976001	2	208976001	208979000	3000	1	2.80E-08	-0.43	13	0.43	RGD1309110	Metabolism
DMR2:208980001	2	208980001	208981000	1000	1	1.80E-09	0.64	5	0.5	RGD1309110	Metabolism
DMR2:208987001	2	208987001	208990000	3000	1	5.40E-08	-0.53	13	0.43	RGD1309110	Metabolism
DMR2:209112001	2	209112001	209114000	2000	1	4.40E-09	0.6	7	0.35	Dennd2d;Cept1	Transport
DMR2:209169001	2	209169001	209170000	1000	1	1.90E-14	0.57	12	1.2	Cept1;Dram2;LOC103691666	Transport
DMR2:209179001	2	209179001	209181000	2000	1	1.30E-08	0.47	14	0.7	Dram2;LOC103691666	
DMR2:209191001	2	209191001	209192000	1000	1	2.90E-15	0.63	11	1.1	Dram2	
DMR2:209197001	2	209197001	209202000	5000	3	6.70E-11	-0.55	69	1.38	Dram2	
DMR2:209363001	2	209363001	209365000	2000	2	4.20E-16	0.83	12	0.6	Lrif1	
DMR2:209371001	2	209371001	209372000	1000	1	5.70E-08	-0.6	3	0.3	Lrif1	
DMR2:209377001	2	209377001	209381000	4000	1	6.30E-07	-0.37	23	0.58	Lrif1	
DMR2:209389001	2	209389001	209392000	3000	1	6.30E-07	0.4	8	0.27	Lrif1	
DMR2:209394001	2	209394001	209396000	2000	1	1.60E-12	-0.63	8	0.4	Lrif1	
DMR2:209405001	2	209405001	209409000	4000	2	5.30E-13	-0.67	15	0.38	Lrif1	
DMR2:209416001	2	209416001	209417000	1000	1	1.10E-08	0.44	6	0.6	Lrif1	
DMR2:209420001	2	209420001	209422000	2000	1	3.90E-18	0.72	13	0.65	Lrif1	
DMR2:209423001	2	209423001	209430000	7000	3	1.20E-11	-0.44	46	0.66	Lrif1	
DMR2:209438001	2	209438001	209440000	2000	1	8.20E-07	-0.56	15	0.75	Lrif1	
DMR2:209478001	2	209478001	209481000	3000	1	1.10E-11	0.6	23	0.77	Cd53	
DMR2:209501001	2	209501001	209502000	1000	1	7.80E-09	0.63	5	0.5	Cd53	
DMR2:209508001	2	209508001	209512000	4000	2	1.90E-07	0.41	29	0.72	Cd53	
DMR2:209514001	2	209514001	209517000	3000	1	7.00E-11	-0.6	21	0.7	Cd53	
DMR2:209520001	2	209520001	209526000	6000	4	2.60E-12	-0.68	15	0.25	Cd53	

DMR2:209529001	2	209529001	209530000	1000	1	3.30E-10	0.47	4	0.4	Cd53	
DMR2:209538001	2	209538001	209540000	2000	1	4.90E-10	0.51	7	0.35	Cd53	
DMR2:209546001	2	209546001	209549000	3000	1	8.70E-13	0.53	17	0.57	Cd53	
DMR2:209759001	2	209759001	209760000	1000	1	2.80E-08	0.56	4	0.4	LOC108350087;Kcna3;LOC103691668	Transport
DMR2:209762001	2	209762001	209763000	1000	1	3.60E-19	0.69	6	0.6	LOC108350087;Kcna3;LOC103691668	Transport
DMR2:209776001	2	209776001	209777000	1000	1	1.80E-10	0.42	12	1.2	LOC108350087;Kcna3;LOC103691668	Transport
DMR2:209851001	2	209851001	209855000	4000	1	7.50E-11	0.42	43	1.07	Kcna2	Transport
DMR2:209919001	2	209919001	209920000	1000	1	3.10E-08	0.51	3	0.3	Kcna10	Transport
DMR2:209950001	2	209950001	209957000	7000	5	1.50E-20	-0.49	81	1.16	LOC102557194;Cym	
DMR2:209958001	2	209958001	209961000	3000	1	8.40E-08	0.29	31	1.03	LOC102557194;Cym	
DMR2:209966001	2	209966001	209967000	1000	1	9.00E-26	0.87	9	0.9	LOC102557194;Cym;LOC103691675	
DMR2:209982001	2	209982001	209985000	3000	1	2.10E-11	0.48	49	1.63	Cym;LOC103691675;LOC108350205;Prok1	Hormone
DMR2:209996001	2	209996001	2.10E+08	4000	1	1.60E-10	0.55	38	0.95	Prok1;LOC103691669	Hormone
DMR2:210026001	2	210026001	210030000	4000	1	3.30E-12	0.64	51	1.27	LOC103691669;Lamtor5	
DMR2:210056001	2	210056001	210057000	1000	1	9.30E-09	0.55	6	0.6	Slc16a4	Transport
DMR2:210122001	2	210122001	210123000	1000	1	8.00E-08	0.42	5	0.5	Slc16a4;LOC684509;LOC108350089	Transport;Metabolism
DMR2:210235001	2	210235001	210236000	1000	1	5.20E-15	0.47	14	1.4	Kcnc4	Transport
DMR2:210249001	2	210249001	210253000	4000	1	5.80E-07	0.37	50	1.25	Kcnc4;Slc6a17	Transport;Transport
DMR2:210259001	2	210259001	210263000	4000	1	2.10E-20	0.76	31	0.78	Slc6a17	Transport
DMR2:210265001	2	210265001	210268000	3000	2	3.80E-11	0.47	40	1.33	Slc6a17	Transport
DMR2:210274001	2	210274001	210275000	1000	1	5.80E-09	-0.82	10	1	Slc6a17	Transport
DMR2:210302001	2	210302001	210304000	2000	2	4.30E-16	0.58	17	0.85	Slc6a17	Transport
DMR2:210378001	2	210378001	210379000	1000	1	3.60E-08	0.77	54	5.4	Alx3	Development
DMR2:210407001	2	210407001	210409000	2000	1	9.20E-10	0.39	38	1.9	Strip1	
DMR2:210415001	2	210415001	210418000	3000	1	3.20E-14	0.65	41	1.37	Strip1	
DMR2:210429001	2	210429001	210430000	1000	1	7.40E-08	-0.38	12	1.2	Ahcyl1	Metabolism
DMR2:210459001	2	210459001	210460000	1000	1	2.00E-09	-0.65	9	0.9	Ahcyl1	Metabolism
DMR2:210528001	2	210528001	210529000	1000	1	3.30E-15	0.75	7	0.7	Csf1	
DMR2:210534001	2	210534001	210535000	1000	1	1.20E-07	0.35	8	0.8	Csf1	
DMR2:210549001	2	210549001	210550000	1000	1	1.80E-22	0.82	11	1.1	Csf1	
DMR2:210661001	2	210661001	210668000	7000	1	2.50E-16	0.6	71	1.01	Eps8l3	Cytoskeleton
DMR2:210681001	2	210681001	210685000	4000	1	1.90E-08	-0.51	40	1	Eps8l3;Gstm3	Cytoskeleton
DMR2:210688001	2	210688001	210693000	5000	2	1.60E-13	-0.66	81	1.62	Eps8l3;Gstm3;Gstm6l	Cytoskeleton;Transport
DMR2:210694001	2	210694001	210697000	3000	1	1.10E-10	0.51	15	0.5	Gstm3;Gstm6l	Transport
DMR2:210714001	2	210714001	210715000	1000	1	8.70E-13	0.55	6	0.6	Gstm7	Transport
DMR2:210724001	2	210724001	210725000	1000	1	7.90E-07	0.3	22	2.2	Gstm7;Gstm6	Transport
DMR2:210729001	2	210729001	210732000	3000	1	5.20E-10	0.52	32	1.07	Gstm7;Gstm6	Transport
DMR2:210754001	2	210754001	210756000	2000	1	2.20E-08	0.37	26	1.3	LOC102550297;LOC102550192;Gstm3	
DMR2:210759001	2	210759001	210760000	1000	1	4.50E-12	0.75	5	0.5	LOC102550297;LOC102550192;Gstm3	
DMR2:210778001	2	210778001	210779000	1000	1	5.80E-12	0.43	16	1.6	Gstm2	Transport
DMR2:210790001	2	210790001	210793000	3000	1	5.90E-07	-0.39	30	1	Gstm2	Transport
DMR2:210804001	2	210804001	210805000	1000	1	1.60E-07	0.42	7	0.7	Gstm1	Transport
DMR2:210830001	2	210830001	210833000	3000	1	1.70E-08	0.31	44	1.47	Gstm4	Transport
DMR2:210841001	2	210841001	210843000	2000	1	8.30E-07	-0.46	21	1.05	Gstm4	Transport
DMR2:210853001	2	210853001	210855000	2000	1	7.90E-14	0.47	38	1.9	Ampd2	Metabolism
DMR2:210856001	2	210856001	210857000	1000	1	4.60E-10	0.49	10	1	Ampd2	Metabolism
DMR2:210867001	2	210867001	210869000	2000	1	1.70E-16	0.45	37	1.85	Ampd2	Metabolism
DMR2:210877001	2	210877001	210879000	2000	1	8.90E-16	0.48	20	1	Ampd2;Gnat2	Metabolism;Signaling
DMR2:210881001	2	210881001	210890000	9000	3	6.20E-13	0.64	157	1.74	Ampd2;Gnat2;Gnai3	Metabolism;Signaling
DMR2:210892001	2	210892001	210894000	2000	1	5.80E-14	0.66	21	1.05	Gnat2;Gnai3	Signaling
DMR2:210901001	2	210901001	210904000	3000	1	3.00E-09	-0.58	38	1.27	Gnai3	Signaling
DMR2:210913001	2	210913001	210915000	2000	2	1.80E-12	0.53	21	1.05	Gnai3	Signaling
DMR2:210931001	2	210931001	210932000	1000	1	1.50E-07	-0.69	11	1.1	Gnai3;LOC108350090;Gpr61	Signaling;Signaling
DMR2:210941001	2	210941001	210942000	1000	1	9.30E-07	-0.61	15	1.5	Gnai3;LOC108350090;Gpr61	Signaling;Signaling

DMR2:210951001	2	210951001	210958000	7000	3	2.50E-21	0.84	90	1.29	Gpr61	Signaling
DMR2:210999001	2	210999001	211001000	2000	1	2.10E-10	0.72	34	1.7	Cyb561d1;Atxn7l2;Sypl2	Transport
DMR2:211011001	2	211011001	211012000	1000	1	3.40E-14	0.45	15	1.5	Atxn7l2;Sypl2	Transport
DMR2:211027001	2	211027001	211030000	3000	2	6.50E-09	0.46	37	1.23	Sypl2	Transport
DMR2:211044001	2	211044001	211045000	1000	1	2.10E-07	-0.42	12	1.2	Psma5	Protease
DMR2:211064001	2	211064001	211065000	1000	1	1.40E-12	0.82	5	0.5	Psma5	Protease
DMR2:211067001	2	211067001	211069000	2000	1	5.80E-10	0.55	11	0.55	Psma5;Sort1	Protease;Transport
DMR2:211072001	2	211072001	211073000	1000	1	1.20E-07	0.37	12	1.2	Psma5;Sort1	Protease;Transport
DMR2:211084001	2	211084001	211087000	3000	1	8.90E-07	0.32	35	1.17	Sort1	Transport
DMR2:211088001	2	211088001	211091000	3000	1	5.30E-07	0.45	42	1.4	Sort1	Transport
DMR2:211096001	2	211096001	211097000	1000	1	1.60E-13	0.59	10	1	Sort1	Transport
DMR2:211115001	2	211115001	211116000	1000	1	2.90E-13	0.62	10	1	Sort1	Transport
DMR2:211119001	2	211119001	211122000	3000	2	3.40E-12	-0.45	52	1.73	Sort1	Transport
DMR2:211129001	2	211129001	211130000	1000	1	1.10E-09	0.41	15	1.5	Sort1	Transport
DMR2:211134001	2	211134001	211138000	4000	1	1.50E-31	1.02	42	1.05	Sort1	Transport
DMR2:211144001	2	211144001	211153000	9000	1	1.10E-07	0.32	140	1.56	Sort1;Mybphl	Transport
DMR2:211162001	2	211162001	211168000	6000	1	9.40E-13	0.52	79	1.32	Sort1;Mybphl;Psrc1	Transport
DMR2:211169001	2	211169001	211170000	1000	1	2.40E-12	-0.74	22	2.2	Mybphl;Psrc1	
DMR2:211172001	2	211172001	211173000	1000	1	2.80E-07	-0.52	13	1.3	Mybphl;Psrc1	
DMR2:211174001	2	211174001	211176000	2000	1	3.30E-08	0.57	32	1.6	Mybphl;Psrc1;Celsr2	Cytoskeleton
DMR2:211177001	2	211177001	211180000	3000	1	9.80E-11	0.46	60	2	Mybphl;Psrc1;Celsr2	Cytoskeleton
DMR2:211199001	2	211199001	211204000	5000	3	5.10E-11	0.46	95	1.9	Celsr2;LOC108350091	Cytoskeleton
DMR2:211210001	2	211210001	211214000	4000	1	7.70E-07	-0.39	66	1.65	Celsr2;LOC108350091;Sars	Cytoskeleton
DMR2:211228001	2	211228001	211232000	4000	1	1.50E-08	-0.53	41	1.02	LOC108350091;Sars	
DMR2:211260001	2	211260001	211264000	4000	2	1.10E-12	0.48	73	1.82	RGD1310209	
DMR2:211276001	2	211276001	211280000	4000	4	6.90E-20	0.86	14	0.35	RGD1310209	
DMR2:211281001	2	211281001	211286000	5000	2	5.80E-08	-0.39	56	1.12	RGD1310209	
DMR2:211298001	2	211298001	211299000	1000	1	2.30E-07	-0.38	20	2	RGD1310209	
DMR2:211331001	2	211331001	211332000	1000	1	2.20E-14	0.58	12	1.2	RGD1310209;RGD1309139;Cox6b1	Metabolism
DMR2:211362001	2	211362001	211365000	3000	1	1.40E-08	-0.41	39	1.3	Tmem167b;Taf13	Transcription
DMR2:211375001	2	211375001	211376000	1000	1	4.80E-16	0.77	7	0.7	Taf13;Wdr47	Transcription
DMR2:211377001	2	211377001	211380000	3000	2	1.50E-23	0.97	24	0.8	Taf13;Wdr47	Transcription
DMR2:211395001	2	211395001	211396000	1000	1	8.10E-09	0.62	4	0.4	Wdr47	
DMR2:211401001	2	211401001	211405000	4000	2	5.20E-15	-0.49	46	1.15	Wdr47	
DMR2:211458001	2	211458001	211459000	1000	1	6.00E-12	0.55	17	1.7	Clcc1	Transport
DMR2:211482001	2	211482001	211493000	11000	4	2.90E-18	-0.49	159	1.45	Clcc1;Gpsm2	Transport
DMR2:211497001	2	211497001	211502000	5000	3	1.60E-12	-0.76	73	1.46	Gpsm2	
DMR2:211505001	2	211505001	211508000	3000	2	2.00E-07	-0.45	51	1.7	Gpsm2	
DMR2:211509001	2	211509001	211513000	4000	2	5.50E-18	0.59	114	2.85	Gpsm2	
DMR2:211514001	2	211514001	211515000	1000	1	3.60E-07	0.26	17	1.7	Gpsm2	
DMR2:211523001	2	211523001	211524000	1000	1	7.40E-08	-0.49	11	1.1	Gpsm2	
DMR2:211546001	2	211546001	211549000	3000	1	5.30E-24	1.01	37	1.23	Aknad1	
DMR2:211550001	2	211550001	211553000	3000	1	5.30E-07	0.46	41	1.37	Aknad1	
DMR2:211575001	2	211575001	211578000	3000	1	6.90E-12	0.41	44	1.47	Aknad1;LOC102547393	
DMR2:211630001	2	211630001	211632000	2000	1	2.20E-09	-0.52	26	1.3	Stxbp3	Transport
DMR2:211635001	2	211635001	211637000	2000	1	1.80E-07	0.37	23	1.15	Stxbp3	Transport
DMR2:211666001	2	211666001	211668000	2000	1	2.40E-23	0.7	23	1.15	Fndc7	
DMR2:211671001	2	211671001	211673000	2000	1	3.50E-07	0.33	36	1.8	Fndc7;Mir6314	
DMR2:211695001	2	211695001	211696000	1000	1	1.50E-10	0.54	5	0.5	Fndc7	
DMR2:211697001	2	211697001	211701000	4000	3	1.90E-22	0.7	37	0.92	Fndc7;Prpf38b	
DMR2:211707001	2	211707001	211709000	2000	1	1.20E-20	0.69	44	2.2	Prpf38b	
DMR2:211722001	2	211722001	211724000	2000	1	3.20E-13	0.49	22	1.1	Prpf38b	
DMR2:211725001	2	211725001	211727000	2000	1	4.80E-09	0.37	26	1.3	Prpf38b	
DMR2:211743001	2	211743001	211744000	1000	1	2.90E-11	0.7	7	0.7	Henmt1	
DMR2:211751001	2	211751001	211754000	3000	1	3.40E-07	0.37	43	1.43	Henmt1;Fam102b	
DMR2:211758001	2	211758001	211760000	2000	1	5.50E-07	0.29	23	1.15	Henmt1;Fam102b	
DMR2:211771001	2	211771001	211773000	2000	2	6.50E-20	0.78	15	0.75	Fam102b	
DMR2:211794001	2	211794001	211798000	4000	3	1.30E-12	-0.55	47	1.18	Fam102b	
DMR2:211804001	2	211804001	211806000	2000	1	4.00E-09	0.37	19	0.95	Fam102b	
DMR2:211836001	2	211836001	211837000	1000	1	2.20E-07	0.32	10	1	Fam102b	
DMR2:211869001	2	211869001	211871000	2000	1	4.60E-08	-0.48	25	1.25	LOC108350282;Slc25a54	Transport
DMR2:211872001	2	211872001	211874000	2000	1	3.60E-11	0.62	15	0.75	LOC108350282;Slc25a54	Transport
DMR2:211878001	2	211878001	211880000	2000	1	2.10E-07	0.38	25	1.25	LOC108350282;Slc25a54	Transport
DMR2:211890001	2	211890001	211891000	1000	1	1.20E-15	0.61	15	1.5	Slc25a54	Transport

DMR2:211897001	2	211897001	211898000	1000	1	9.30E-08	-0.54	3	0.3	Slc25a54	Transport
DMR2:211914001	2	211914001	211917000	3000	1	1.50E-12	0.62	34	1.13	Slc25a54	Transport
DMR2:211948001	2	211948001	211950000	2000	2	4.50E-17	0.62	18	0.9	Slc25a24	Transport
DMR2:211958001	2	211958001	211965000	7000	4	1.20E-17	0.62	92	1.31	Slc25a24	Transport
DMR2:212244001	2	212244001	212245000	1000	1	3.70E-09	0.45	3	0.3	Vav3	
DMR2:212252001	2	212252001	212255000	3000	2	3.10E-10	0.49	29	0.97	Vav3	
DMR2:212264001	2	212264001	212268000	4000	2	1.30E-16	0.58	42	1.05	Vav3	
DMR2:212280001	2	212280001	212284000	4000	1	3.70E-07	-0.44	45	1.12	Vav3	
DMR2:212285001	2	212285001	212286000	1000	1	4.70E-09	0.36	16	1.6	Vav3	
DMR2:212300001	2	212300001	212303000	3000	1	8.50E-11	0.57	24	0.8	Vav3	
DMR2:212319001	2	212319001	212321000	2000	1	6.30E-08	0.4	22	1.1	Vav3	
DMR2:212327001	2	212327001	212328000	1000	1	7.50E-07	-0.54	10	1	Vav3	
DMR2:212332001	2	212332001	212333000	1000	1	2.30E-11	0.55	10	1	Vav3	
DMR2:212343001	2	212343001	212344000	1000	1	4.80E-10	0.44	12	1.2	Vav3	
DMR2:212359001	2	212359001	212360000	1000	1	4.60E-10	0.4	14	1.4	Vav3	
DMR2:212361001	2	212361001	212367000	6000	5	4.70E-16	0.7	56	0.93	Vav3	
DMR2:212370001	2	212370001	212372000	2000	1	5.90E-12	0.67	17	0.85	Vav3	
DMR2:212388001	2	212388001	212391000	3000	1	5.30E-14	0.52	38	1.27	Vav3	
DMR2:212403001	2	212403001	212406000	3000	1	8.70E-09	0.37	43	1.43	Vav3	
DMR2:212417001	2	212417001	212418000	1000	1	1.80E-07	-0.45	16	1.6	Vav3	
DMR2:212439001	2	212439001	212443000	4000	3	1.30E-11	-0.41	33	0.82	Vav3;Mrps17-ps1	
DMR2:212451001	2	212451001	212452000	1000	1	7.30E-09	0.42	10	1	Vav3	
DMR2:212687001	2	212687001	212688000	1000	1	5.10E-16	0.56	11	1.1	Ntng1	Extracellular Matrix
DMR2:212730001	2	212730001	212731000	1000	1	1.40E-08	0.55	4	0.4	Ntng1	Extracellular Matrix
DMR2:212732001	2	212732001	212735000	3000	2	5.30E-20	0.84	25	0.83	Ntng1	Extracellular Matrix
DMR2:212736001	2	212736001	212737000	1000	1	6.50E-08	0.4	7	0.7	Ntng1	Extracellular Matrix
DMR2:212746001	2	212746001	212747000	1000	1	4.40E-08	-0.6	13	1.3	Ntng1	Extracellular Matrix
DMR2:212759001	2	212759001	212761000	2000	2	4.60E-22	1	27	1.35	Ntng1	Extracellular Matrix
DMR2:212817001	2	212817001	212819000	2000	1	3.00E-13	0.66	15	0.75	Ntng1	Extracellular Matrix
DMR2:212820001	2	212820001	212823000	3000	1	2.00E-20	1.02	13	0.43	Ntng1	Extracellular Matrix
DMR2:212828001	2	212828001	212829000	1000	1	4.40E-07	-0.55	16	1.6	Ntng1	Extracellular Matrix
DMR2:212864001	2	212864001	212865000	1000	1	2.60E-07	0.46	7	0.7	Ntng1	Extracellular Matrix
DMR2:212868001	2	212868001	212869000	1000	1	2.00E-07	0.45	10	1	Ntng1	Extracellular Matrix
DMR2:212895001	2	212895001	212896000	1000	1	1.30E-07	0.48	7	0.7	Ntng1	Extracellular Matrix
DMR2:212897001	2	212897001	212898000	1000	1	3.30E-08	0.5	9	0.9	Ntng1	Extracellular Matrix
DMR2:212918001	2	212918001	212919000	1000	1	6.60E-08	0.4	5	0.5	Ntng1	Extracellular Matrix
DMR2:212929001	2	212929001	212931000	2000	1	3.50E-08	0.51	8	0.4	Ntng1	Extracellular Matrix
DMR2:213004001	2	213004001	213005000	1000	1	2.40E-07	0.3	13	1.3	Ntng1	Extracellular Matrix
DMR2:213027001	2	213027001	213036000	9000	4	2.10E-20	-0.49	93	1.03	Ntng1	Extracellular Matrix
DMR2:214101001	2	214101001	214102000	1000	1	3.20E-11	0.8	17	1.7	RGD1562088	
DMR2:215371001	2	215371001	215377000	6000	3	2.40E-20	-0.42	63	1.05	Hmox2-ps1	
DMR2:215380001	2	215380001	215381000	1000	1	1.20E-07	0.61	6	0.6	Hmox2-ps1	
DMR2:215920001	2	215920001	215922000	2000	1	1.40E-08	-0.68	20	1	Amd1-ps1	
DMR2:216226001	2	216226001	216228000	2000	1	2.40E-07	-0.4	30	1.5	Amy2-ps2	
DMR2:216237001	2	216237001	216239000	2000	2	1.40E-10	0.57	10	0.5	Amy2-ps2	
DMR2:216244001	2	216244001	216247000	3000	1	4.50E-14	-0.48	44	1.47	Amy2-ps2	
DMR2:216363001	2	216363001	216372000	9000	4	5.10E-10	0.49	44	0.49	Amy2a3	
DMR2:216447001	2	216447001	216449000	2000	1	1.90E-11	0.56	10	0.5	Amy1a	Metabolism
DMR2:216481001	2	216481001	216482000	1000	1	1.30E-16	0.83	7	0.7	Rnpc3	
DMR2:216883001	2	216883001	216885000	2000	1	5.60E-19	0.78	13	0.65	Col11a1	Extracellular Matrix
DMR2:216889001	2	216889001	216890000	1000	1	7.70E-07	0.43	9	0.9	Col11a1	Extracellular Matrix
DMR2:216968001	2	216968001	216969000	1000	1	5.30E-12	0.79	6	0.6	Col11a1	Extracellular Matrix
DMR2:216985001	2	216985001	216986000	1000	1	3.60E-11	0.75	5	0.5	Col11a1	Extracellular Matrix
DMR2:217013001	2	217013001	217015000	2000	1	4.50E-20	0.74	28	1.4	Col11a1	Extracellular Matrix
DMR2:217772001	2	217772001	217773000	1000	1	2.10E-08	0.42	2	0.2	Olfm3	Development
DMR2:217778001	2	217778001	217779000	1000	1	1.10E-13	0.78	4	0.4	Olfm3	Development
DMR2:217801001	2	217801001	217802000	1000	1	3.50E-10	0.59	1	0.1	Olfm3	Development
DMR2:217811001	2	217811001	217812000	1000	1	5.30E-10	0.55	6	0.6	Olfm3	Development
DMR2:217820001	2	217820001	217821000	1000	1	2.10E-18	0.76	9	0.9	Olfm3	Development
DMR2:217824001	2	217824001	217825000	1000	1	1.00E-12	0.63	7	0.7	Olfm3	Development
DMR2:217829001	2	217829001	217832000	3000	2	6.00E-07	0.4	30	1	Olfm3	Development
DMR2:217849001	2	217849001	217850000	1000	1	2.60E-10	-0.41	13	1.3	Olfm3	Development
DMR2:217852001	2	217852001	217853000	1000	1	5.70E-07	0.39	1	0.1	Olfm3	Development
DMR2:217877001	2	217877001	217879000	2000	1	9.50E-15	0.53	24	1.2	Olfm3	Development
DMR2:217880001	2	217880001	217881000	1000	1	4.40E-08	-0.58	10	1	Olfm3	Development

DMR2:217888001	2	217888001	217890000	2000	2	5.80E-15	0.86	8	0.4	Olfm3	Development
DMR2:217911001	2	217911001	217916000	5000	2	1.80E-26	-0.57	37	0.74	Olfm3	Development
DMR2:218826001	2	218826001	218828000	2000	1	1.90E-07	-0.47	20	1	Dph5	Epigenetic
DMR2:218835001	2	218835001	218837000	2000	1	1.00E-15	0.8	19	0.95	Dph5	Epigenetic
DMR2:218841001	2	218841001	218843000	2000	1	5.00E-09	0.44	18	0.9	Dph5	Epigenetic
DMR2:218858001	2	218858001	218859000	1000	1	1.80E-09	0.48	14	1.4	Dph5	Epigenetic
DMR2:218871001	2	218871001	218876000	5000	2	4.60E-09	-0.48	29	0.58	Dph5	Epigenetic
DMR2:218915001	2	218915001	218921000	6000	1	1.50E-11	0.54	68	1.13	Slc30a7	
DMR2:218936001	2	218936001	218937000	1000	1	2.20E-08	0.55	9	0.9	Slc30a7	
DMR2:218943001	2	218943001	218945000	2000	1	4.50E-13	0.57	16	0.8	Slc30a7;Extl2	Golgi
DMR2:218969001	2	218969001	218972000	3000	2	7.60E-07	0.53	34	1.13	Extl2	Golgi
DMR2:219032001	2	219032001	219034000	2000	1	3.30E-07	-0.34	19	0.95	RGD1559607	
DMR2:219040001	2	219040001	219044000	4000	2	2.50E-21	0.85	39	0.98	RGD1559607	
DMR2:219054001	2	219054001	219055000	1000	1	1.30E-10	0.68	4	0.4	RGD1559607;Rps27a-ps24	
DMR2:219057001	2	219057001	219058000	1000	1	2.30E-07	0.49	11	1.1	RGD1559607;Rps27a-ps24	
DMR2:219069001	2	219069001	219073000	4000	1	1.50E-08	0.39	40	1	Rps27a-ps24;Vcam1	
DMR2:219078001	2	219078001	219082000	4000	1	2.50E-13	0.77	53	1.32	Vcam1;LOC100912479	
DMR2:219314001	2	219314001	219316000	2000	1	2.50E-08	0.39	24	1.2	Cdc14a	Signaling
DMR2:219318001	2	219318001	219320000	2000	1	2.50E-20	0.69	21	1.05	Cdc14a	Signaling
DMR2:219325001	2	219325001	219326000	1000	1	6.30E-10	-0.47	12	1.2	Cdc14a	Signaling
DMR2:219344001	2	219344001	219346000	2000	1	5.20E-07	0.38	39	1.95	Cdc14a	Signaling
DMR2:219347001	2	219347001	219349000	2000	2	3.50E-11	0.66	13	0.65	Cdc14a	Signaling
DMR2:219353001	2	219353001	219354000	1000	1	7.30E-15	0.57	13	1.3	Cdc14a	Signaling
DMR2:219361001	2	219361001	219363000	2000	2	5.60E-23	0.81	27	1.35	Cdc14a	Signaling
DMR2:219368001	2	219368001	219372000	4000	2	3.10E-12	0.62	69	1.73	Cdc14a	Signaling
DMR2:219377001	2	219377001	219380000	3000	2	1.20E-11	0.7	35	1.17	Cdc14a	Signaling
DMR2:219390001	2	219390001	219393000	3000	1	1.30E-10	0.36	45	1.5	Cdc14a	Signaling
DMR2:219414001	2	219414001	219416000	2000	1	1.90E-09	0.34	24	1.2	Cdc14a	Signaling
DMR2:219420001	2	219420001	219431000	11000	3	2.60E-15	0.54	164	1.49	Cdc14a	Signaling
DMR2:219443001	2	219443001	219444000	1000	1	1.30E-13	0.59	15	1.5	Cdc14a	Signaling
DMR2:219445001	2	219445001	219452000	7000	3	2.30E-20	0.85	70	1	Cdc14a	Signaling
DMR2:219522001	2	219522001	219528000	6000	5	7.00E-20	-0.49	70	1.17	Rtcd1	
DMR2:219564001	2	219564001	219569000	5000	2	9.20E-14	0.57	74	1.48	Rtcd1;Dbt	Metabolism
DMR2:219571001	2	219571001	219575000	4000	1	1.00E-07	0.38	70	1.75	Dbt	Metabolism
DMR2:219591001	2	219591001	219596000	5000	1	4.20E-14	0.54	77	1.54	Dbt;Lrrc39	Metabolism;Cytoskeleton
DMR2:219598001	2	219598001	219600000	2000	1	6.20E-09	0.58	18	0.9	Dbt;Lrrc39	Metabolism;Cytoskeleton
DMR2:219609001	2	219609001	219610000	1000	1	3.20E-10	-0.52	16	1.6	Lrrc39;Trmt13	Cytoskeleton
DMR2:219619001	2	219619001	219621000	2000	1	1.90E-11	0.54	17	0.85	Lrrc39;Trmt13;Sass6	Cytoskeleton
DMR2:219631001	2	219631001	219632000	1000	1	5.90E-07	0.41	15	1.5	Trmt13;Sass6	
DMR2:219668001	2	219668001	219670000	2000	1	2.40E-07	0.48	12	0.6	Sass6;NEWGENE_1305281	
DMR2:219673001	2	219673001	219674000	1000	1	8.90E-17	0.69	9	0.9	NEWGENE_1305281	
DMR2:219699001	2	219699001	219702000	3000	1	4.50E-08	-0.42	29	0.97	NEWGENE_1305281;LOC102548510;Slc35a3	Transport
DMR2:219749001	2	219749001	219751000	2000	2	7.80E-11	0.47	8	0.4	Slc35a3	Transport
DMR2:219779001	2	219779001	219780000	1000	1	3.00E-10	0.54	6	0.6	Agl	
DMR2:219782001	2	219782001	219783000	1000	1	1.10E-07	0.42	19	1.9	Agl	
DMR2:219787001	2	219787001	219789000	2000	1	7.50E-15	0.64	22	1.1	Agl	
DMR2:219793001	2	219793001	219794000	1000	1	2.90E-07	-0.52	12	1.2	Agl	
DMR2:219799001	2	219799001	219802000	3000	2	1.20E-09	-0.72	36	1.2	Agl	
DMR2:219806001	2	219806001	219811000	5000	1	7.50E-09	0.47	66	1.32	Agl	
DMR2:219882001	2	219882001	219889000	7000	4	1.40E-13	0.57	58	0.83	Plppr5	Signaling
DMR2:219909001	2	219909001	219911000	2000	1	5.80E-12	0.74	16	0.8	Plppr5	Signaling
DMR2:219970001	2	219970001	219971000	1000	1	1.50E-14	-0.57	8	0.8	Plppr5	Signaling
DMR2:219980001	2	219980001	219981000	1000	1	1.10E-07	0.49	5	0.5	Plppr5	Signaling
DMR2:219985001	2	219985001	219986000	1000	1	6.00E-14	0.84	6	0.6	Plppr5	Signaling
DMR2:220003001	2	220003001	220004000	1000	1	2.50E-07	0.54	8	0.8	Plppr5;LOC108348210	Signaling
DMR2:220010001	2	220010001	220011000	1000	1	2.00E-21	1.16	1	0.1	Plppr5;LOC108348210	Signaling
DMR2:220030001	2	220030001	220034000	4000	3	3.90E-19	-0.77	40	1	Plppr5;LOC108348210	Signaling
DMR2:220066001	2	220066001	220068000	2000	1	3.60E-11	0.55	16	0.8	Plppr5	Signaling
DMR2:220087001	2	220087001	220088000	1000	1	2.10E-07	0.59	5	0.5	Plppr5	Signaling
DMR2:220323001	2	220323001	220324000	1000	1	7.20E-07	0.35	12	1.2	Plppr4	Signaling
DMR2:220343001	2	220343001	220347000	4000	2	7.40E-12	-0.38	52	1.3	Plppr4	Signaling
DMR2:220350001	2	220350001	220357000	7000	4	8.50E-18	-0.47	78	1.11	Plppr4	Signaling

DMR2:220420001	2	220420001	220422000	2000	2	4.30E-23	0.8	16	0.8	LOC102548573;Frrs1	Metabolism
DMR2:220423001	2	220423001	220425000	2000	2	9.40E-18	0.77	17	0.85	LOC102548573;Frrs1	Metabolism
DMR2:220430001	2	220430001	220431000	1000	1	3.90E-07	0.38	16	1.6	Frrs1	Metabolism
DMR2:220464001	2	220464001	220466000	2000	1	3.90E-08	0.36	43	2.15	Frrs1	Metabolism
DMR2:220476001	2	220476001	220481000	5000	2	1.40E-09	0.45	60	1.2	Frrs1;Palmd	Metabolism
DMR2:220493001	2	220493001	220496000	3000	1	2.10E-07	0.43	35	1.17	Palmd	
DMR2:220497001	2	220497001	220499000	2000	1	4.90E-11	0.56	18	0.9	Palmd	
DMR2:220504001	2	220504001	220506000	2000	1	7.50E-18	0.77	12	0.6	Palmd	
DMR2:220511001	2	220511001	220513000	2000	1	8.30E-10	0.53	25	1.25	Palmd	
DMR2:220524001	2	220524001	220528000	4000	1	1.90E-08	0.44	46	1.15	Palmd;LOC102548639	
DMR2:220535001	2	220535001	220537000	2000	1	5.20E-07	0.39	35	1.75	Palmd;LOC102548639	
DMR2:220542001	2	220542001	220546000	4000	1	3.40E-08	0.57	22	0.55	Palmd;LOC102548639	
DMR2:221010001	2	221010001	221013000	3000	3	4.20E-12	-0.87	14	0.47	Snx7	Cytoskeleton
DMR2:221032001	2	221032001	221033000	1000	1	3.90E-35	1.51	0	0	Snx7	Cytoskeleton
DMR2:221048001	2	221048001	221049000	1000	1	3.10E-08	0.57	4	0.4	Snx7	Cytoskeleton
DMR2:221058001	2	221058001	221060000	2000	1	8.70E-21	1.08	17	0.85	Snx7	Cytoskeleton
DMR2:221065001	2	221065001	221067000	2000	1	5.10E-07	-0.54	21	1.05	Snx7	Cytoskeleton
DMR2:221820001	2	221820001	221823000	3000	2	4.30E-10	0.65	21	0.7	LOC108350213;Dpyd	Metabolism
DMR2:221831001	2	221831001	221833000	2000	1	6.90E-07	0.37	18	0.9	LOC108350213;Dpyd	Metabolism
DMR2:221836001	2	221836001	221840000	4000	1	7.20E-09	0.44	44	1.1	Dpyd	Metabolism
DMR2:221864001	2	221864001	221866000	2000	2	9.10E-10	0.6	20	1	Dpyd	Metabolism
DMR2:221870001	2	221870001	221874000	4000	3	1.10E-32	-0.68	59	1.48	Dpyd	Metabolism
DMR2:221876001	2	221876001	221877000	1000	1	6.20E-07	-0.59	11	1.1	Dpyd	Metabolism
DMR2:221888001	2	221888001	221889000	1000	1	5.90E-08	0.42	8	0.8	Dpyd	Metabolism
DMR2:221929001	2	221929001	221930000	1000	1	1.40E-14	-0.9	14	1.4	Dpyd	Metabolism
DMR2:221948001	2	221948001	221950000	2000	2	6.30E-10	0.57	8	0.4	Dpyd	Metabolism
DMR2:221970001	2	221970001	221971000	1000	1	4.50E-08	0.51	14	1.4	Dpyd	Metabolism
DMR2:221987001	2	221987001	221988000	1000	1	2.50E-08	0.58	2	0.2	Dpyd	Metabolism
DMR2:222017001	2	222017001	222020000	3000	2	1.70E-19	0.92	34	1.13	Dpyd	Metabolism
DMR2:222029001	2	222029001	222030000	1000	1	5.90E-10	0.64	5	0.5	Dpyd	Metabolism
DMR2:222035001	2	222035001	222037000	2000	1	8.30E-07	0.38	14	0.7	Dpyd	Metabolism
DMR2:222043001	2	222043001	222044000	1000	1	1.40E-08	-0.56	3	0.3	Dpyd	Metabolism
DMR2:222053001	2	222053001	222057000	4000	1	1.60E-08	-0.52	23	0.58	Dpyd	Metabolism
DMR2:222062001	2	222062001	222066000	4000	3	2.10E-16	0.86	39	0.98	Dpyd	Metabolism
DMR2:222131001	2	222131001	222135000	4000	2	8.50E-08	-0.6	42	1.05	Dpyd	Metabolism
DMR2:222139001	2	222139001	222143000	4000	2	4.60E-16	0.84	38	0.95	Dpyd	Metabolism
DMR2:222162001	2	222162001	222165000	3000	1	1.90E-08	0.5	45	1.5	Dpyd	Metabolism
DMR2:222193001	2	222193001	222201000	8000	5	4.40E-26	-0.68	94	1.18	Dpyd	Metabolism
DMR2:222210001	2	222210001	222213000	3000	1	1.30E-10	0.53	26	0.87	Dpyd	Metabolism
DMR2:222219001	2	222219001	222220000	1000	1	6.50E-08	-0.35	7	0.7	Dpyd	Metabolism
DMR2:222226001	2	222226001	222227000	1000	1	3.80E-09	0.45	10	1	Dpyd	Metabolism
DMR2:222230001	2	222230001	222232000	2000	1	1.10E-13	0.57	11	0.55	Dpyd	Metabolism
DMR2:222249001	2	222249001	222251000	2000	1	3.30E-07	0.45	13	0.65	Dpyd	Metabolism
DMR2:222273001	2	222273001	222277000	4000	2	2.90E-14	-0.86	21	0.52	Dpyd	Metabolism
DMR2:222279001	2	222279001	222280000	1000	1	1.40E-07	0.42	6	0.6	Dpyd	Metabolism
DMR2:222323001	2	222323001	222325000	2000	1	2.70E-07	0.58	8	0.4	Dpyd	Metabolism
DMR2:222330001	2	222330001	222335000	5000	2	5.80E-09	-0.62	21	0.42	Dpyd	Metabolism
DMR2:222339001	2	222339001	222344000	5000	3	5.10E-17	0.79	36	0.72	Dpyd	Metabolism
DMR2:222382001	2	222382001	222391000	9000	5	2.30E-24	-0.55	102	1.13	Dpyd	Metabolism
DMR2:222397001	2	222397001	222399000	2000	2	1.50E-11	0.63	10	0.5	Dpyd	Metabolism
DMR2:222405001	2	222405001	222413000	8000	4	2.90E-11	0.54	85	1.06	Dpyd	Metabolism
DMR2:222429001	2	222429001	222433000	4000	2	7.80E-14	0.74	31	0.78	Dpyd	Metabolism
DMR2:222434001	2	222434001	222438000	4000	3	3.10E-17	-0.49	53	1.32	Dpyd	Metabolism
DMR2:222440001	2	222440001	222442000	2000	1	8.70E-07	0.55	13	0.65	Dpyd	Metabolism
DMR2:222530001	2	222530001	222532000	2000	2	4.50E-21	0.94	14	0.7	Dpyd	Metabolism
DMR2:222533001	2	222533001	222537000	4000	3	2.70E-09	0.54	38	0.95	Dpyd	Metabolism
DMR2:222539001	2	222539001	222540000	1000	1	8.60E-12	-0.76	3	0.3	Dpyd	Metabolism
DMR2:222552001	2	222552001	222553000	1000	1	1.20E-09	-0.57	2	0.2	Dpyd	Metabolism
DMR2:222569001	2	222569001	222576000	7000	2	1.70E-10	0.4	51	0.73	Dpyd	Metabolism
DMR2:222599001	2	222599001	222601000	2000	1	3.20E-07	0.35	18	0.9	Dpyd	Metabolism
DMR2:222608001	2	222608001	222609000	1000	1	2.60E-07	0.56	7	0.7	Dpyd	Metabolism
DMR2:222615001	2	222615001	222618000	3000	1	8.50E-13	0.7	29	0.97	Dpyd	Metabolism
DMR2:222667001	2	222667001	222678000	11000	6	1.10E-17	0.62	101	0.92	Dpyd	Metabolism
DMR2:222681001	2	222681001	222683000	2000	1	1.20E-09	-0.69	11	0.55	Dpyd	Metabolism
DMR2:222684001	2	222684001	222686000	2000	1	8.70E-12	0.51	22	1.1	Dpyd	Metabolism

DMR2:222691001	2	222691001	222693000	2000	1	2.20E-09	0.67	19	0.95	Dpyd	Metabolism
DMR2:223253001	2	223253001	223257000	4000	3	2.50E-15	-0.61	49	1.23	LOC103691683;Ptbp2	
DMR2:223266001	2	223266001	223269000	3000	3	5.00E-16	0.6	31	1.03	Ptbp2	
DMR2:223276001	2	223276001	223280000	4000	1	9.60E-08	-0.57	47	1.18	Ptbp2	
DMR2:223289001	2	223289001	223292000	3000	1	4.30E-07	0.47	15	0.5	Ptbp2	
DMR2:223330001	2	223330001	223333000	3000	1	3.50E-08	-0.56	30	1	Ptbp2	
DMR2:224724001	2	224724001	224731000	7000	2	1.00E-07	-0.51	79	1.13	Rwdd3;LOC691737	
DMR2:224733001	2	224733001	224736000	3000	3	5.40E-11	-0.7	5	0.17	Rwdd3;LOC691737	
DMR2:224782001	2	224782001	224786000	4000	1	9.90E-07	-0.43	50	1.25	Tmem56	
DMR2:224789001	2	224789001	224790000	1000	1	1.20E-07	0.47	11	1.1	Tmem56	
DMR2:224809001	2	224809001	224810000	1000	1	3.20E-07	0.4	9	0.9	Tmem56	
DMR2:224815001	2	224815001	224816000	1000	1	7.50E-08	-0.45	14	1.4	Tmem56	
DMR2:224830001	2	224830001	224835000	5000	2	1.40E-11	0.53	64	1.28	Tmem56	
DMR2:224836001	2	224836001	224838000	2000	1	7.70E-07	-0.45	29	1.45	Tmem56	
DMR2:224844001	2	224844001	224847000	3000	1	1.70E-11	0.45	42	1.4	Tmem56;Alg14	Transport
DMR2:224860001	2	224860001	224861000	1000	1	1.00E-10	-0.62	6	0.6	Alg14	Transport
DMR2:224865001	2	224865001	224866000	1000	1	3.30E-26	1.03	10	1	Alg14	Transport
DMR2:224869001	2	224869001	224870000	1000	1	9.60E-26	0.91	5	0.5	Alg14;LOC108350097	Transport
DMR2:224877001	2	224877001	224878000	1000	1	2.30E-08	0.4	9	0.9	Alg14;LOC108350097	Transport
DMR2:224899001	2	224899001	224903000	4000	1	1.20E-14	0.55	54	1.35	Alg14	Transport
DMR2:224911001	2	224911001	224916000	5000	1	3.20E-13	0.6	73	1.46	Alg14	Transport
DMR2:224917001	2	224917001	224920000	3000	2	1.20E-08	-0.56	35	1.17	Alg14	Transport
DMR2:224925001	2	224925001	224929000	4000	1	1.20E-12	0.57	45	1.12	Alg14	Transport
DMR2:224999001	2	224999001	2.25E+08	1000	1	3.50E-09	0.41	11	1.1	Cnn3	Cytoskeleton
DMR2:225015001	2	225015001	225018000	3000	1	2.50E-07	0.36	46	1.53	Cnn3	Cytoskeleton
DMR2:225032001	2	225032001	225036000	4000	1	1.10E-08	-0.69	71	1.77	Cnn3;Slc44a3	Cytoskeleton;Transpo rt
DMR2:225040001	2	225040001	225042000	2000	1	2.80E-07	0.44	35	1.75	Cnn3;Slc44a3	Cytoskeleton;Transpo rt
DMR2:225056001	2	225056001	225063000	7000	3	3.50E-15	-0.64	84	1.2	Slc44a3	Transport
DMR2:225064001	2	225064001	225065000	1000	1	3.40E-18	0.66	15	1.5	Slc44a3	Transport
DMR2:225070001	2	225070001	225074000	4000	1	9.40E-08	-0.66	57	1.43	Slc44a3	Transport
DMR2:225075001	2	225075001	225077000	2000	1	2.20E-07	-0.6	23	1.15	Slc44a3	Transport
DMR2:225078001	2	225078001	225082000	4000	1	3.70E-11	0.46	39	0.98	Slc44a3	Transport
DMR2:225109001	2	225109001	225112000	3000	1	4.10E-07	0.45	41	1.37	Slc44a3	Transport
DMR2:225113001	2	225113001	225114000	1000	1	1.60E-09	0.42	35	3.5	Slc44a3	Transport
DMR2:225317001	2	225317001	225320000	3000	1	9.30E-07	-0.49	52	1.73	LOC102555482;F3	Receptor
DMR2:225337001	2	225337001	225338000	1000	1	4.20E-17	0.91	18	1.8	Abcd3	Transport
DMR2:225342001	2	225342001	225345000	3000	1	4.90E-21	0.66	43	1.43	Abcd3	Transport
DMR2:225380001	2	225380001	225382000	2000	1	3.10E-17	-0.73	32	1.6	Abcd3;LOC108350099	Transport
DMR2:225545001	2	225545001	225546000	1000	1	1.70E-15	0.69	4	0.4	LOC108350286;LOC103695137; Arhgap29	
DMR2:225567001	2	225567001	225569000	2000	1	1.10E-15	0.77	12	0.6	Arhgap29	
DMR2:225583001	2	225583001	225584000	1000	1	8.50E-11	-0.67	10	1	Arhgap29	
DMR2:225588001	2	225588001	225592000	4000	1	7.20E-12	0.5	53	1.32	Arhgap29	
DMR2:225636001	2	225636001	225637000	1000	1	1.30E-08	0.44	13	1.3	Abca4	Transport
DMR2:225646001	2	225646001	225648000	2000	2	4.70E-14	0.51	28	1.4	Abca4	Transport
DMR2:225652001	2	225652001	225658000	6000	4	3.20E-14	0.83	66	1.1	Abca4	Transport
DMR2:225659001	2	225659001	225660000	1000	1	1.70E-09	0.51	7	0.7	Abca4	Transport
DMR2:225671001	2	225671001	225674000	3000	2	1.30E-14	0.66	23	0.77	Abca4	Transport
DMR2:225682001	2	225682001	225683000	1000	1	1.60E-07	-0.48	14	1.4	Abca4	Transport
DMR2:225698001	2	225698001	225700000	2000	1	2.80E-12	0.71	10	0.5	Abca4	Transport
DMR2:225701001	2	225701001	225705000	4000	2	2.00E-13	0.69	53	1.32	Abca4	Transport
DMR2:225709001	2	225709001	225712000	3000	1	4.10E-11	0.51	44	1.47	Abca4	Transport
DMR2:225713001	2	225713001	225714000	1000	1	2.60E-11	0.5	22	2.2	Abca4	Transport
DMR2:225736001	2	225736001	225737000	1000	1	6.00E-13	0.66	7	0.7	Abca4	Transport
DMR2:225743001	2	225743001	225747000	4000	1	2.90E-09	0.35	52	1.3	Abca4	Transport
DMR2:225771001	2	225771001	225774000	3000	1	3.10E-09	0.54	26	0.87	Abca4	Transport
DMR2:225793001	2	225793001	225797000	4000	1	1.30E-12	0.67	42	1.05	Abca4;LOC102556705	Transport
DMR2:225812001	2	225812001	225820000	8000	3	1.40E-24	-0.67	97	1.21	LOC102556705;LOC102555028; Gclm	Metabolism
DMR2:225845001	2	225845001	225846000	1000	1	5.70E-14	0.55	9	0.9	Gclm;Dnttip2	Metabolism
DMR2:225847001	2	225847001	225850000	3000	1	4.40E-09	-0.59	31	1.03	Gclm;Dnttip2	Metabolism
DMR2:225880001	2	225880001	225881000	1000	1	4.10E-14	0.49	15	1.5	Trnar-ucu;Mir760	
DMR2:225883001	2	225883001	225885000	2000	1	1.60E-13	0.42	19	0.95	Mir760	

DMR2:226556001	2	226556001	226557000	1000	1	1.40E-07	0.47	11	1.1	Bcar3	
DMR2:226559001	2	226559001	226560000	1000	1	2.70E-07	-0.56	18	1.8	Bcar3	
DMR2:226570001	2	226570001	226571000	1000	1	6.00E-10	0.44	15	1.5	Bcar3	
DMR2:226604001	2	226604001	226605000	1000	1	2.00E-27	0.81	24	2.4	Bcar3	
DMR2:226609001	2	226609001	226615000	6000	2	3.80E-21	0.72	91	1.52	Bcar3	
DMR2:226634001	2	226634001	226642000	8000	2	5.00E-09	0.36	112	1.4	Bcar3	
DMR2:226652001	2	226652001	226654000	2000	1	1.30E-09	-0.53	28	1.4	Bcar3;LOC108350102	
DMR2:226685001	2	226685001	226687000	2000	2	2.40E-17	0.62	19	0.95	Bcar3;Fnbp1l	
DMR2:226697001	2	226697001	226698000	1000	1	4.80E-07	0.34	7	0.7	Fnbp1l	
DMR2:226727001	2	226727001	226730000	3000	2	1.10E-07	0.48	17	0.57	Fnbp1l	
DMR2:226736001	2	226736001	226737000	1000	1	1.50E-14	0.71	7	0.7	Fnbp1l	
DMR2:226748001	2	226748001	226753000	5000	1	8.70E-07	-0.31	39	0.78	Fnbp1l	
DMR2:226909001	2	226909001	226915000	6000	5	3.10E-16	-0.48	67	1.12	Pde5a	Signaling
DMR2:226944001	2	226944001	226945000	1000	1	5.80E-11	0.5	8	0.8	Pde5a	Signaling
DMR2:226954001	2	226954001	226956000	2000	1	1.10E-07	0.33	39	1.95	Pde5a	Signaling
DMR2:226980001	2	226980001	226986000	6000	1	4.10E-08	-0.53	97	1.62	Pde5a	Signaling
DMR2:226993001	2	226993001	226998000	5000	1	3.30E-08	-0.44	40	0.8	Pde5a	Signaling
DMR2:227005001	2	227005001	227010000	5000	1	9.70E-07	-0.37	79	1.58	Pde5a	Signaling
DMR2:227017001	2	227017001	227019000	2000	1	3.40E-12	0.58	23	1.15	Pde5a	Signaling
DMR2:227029001	2	227029001	227031000	2000	1	1.40E-07	-0.49	16	0.8	Pde5a	Signaling
DMR2:227034001	2	227034001	227037000	3000	1	8.30E-10	-0.54	10	0.33	Pde5a	Signaling
DMR2:227071001	2	227071001	227072000	1000	1	1.30E-07	0.48	2	0.2	LOC102550474;Fabp2	
DMR2:227076001	2	227076001	227077000	1000	1	1.70E-08	0.42	13	1.3	LOC102550474;Fabp2	
DMR2:227090001	2	227090001	227091000	1000	1	3.70E-07	0.41	7	0.7	LOC102550474;Fabp2;LOC691807;Usp53	Protease
DMR2:227105001	2	227105001	227111000	6000	4	6.80E-18	0.68	44	0.73	LOC102550474;LOC691807;Usp53	Protease
DMR2:227113001	2	227113001	227117000	4000	1	3.80E-08	0.47	23	0.58	Usp53	Protease
DMR2:227129001	2	227129001	227132000	3000	1	3.60E-12	0.51	33	1.1	Usp53	Protease
DMR2:227136001	2	227136001	227138000	2000	1	4.90E-08	0.37	25	1.25	Usp53	Protease
DMR2:227153001	2	227153001	227154000	1000	1	8.70E-08	-0.6	6	0.6	Usp53	Protease
DMR2:227155001	2	227155001	227157000	2000	1	2.30E-08	0.53	15	0.75	Usp53	Protease
DMR2:227181001	2	227181001	227184000	3000	1	6.40E-12	0.58	36	1.2	Myoz2	Cytoskeleton
DMR2:227199001	2	227199001	227200000	1000	1	1.50E-07	-0.52	18	1.8	Myoz2	Cytoskeleton
DMR2:227204001	2	227204001	227207000	3000	1	7.80E-07	-0.55	45	1.5	Myoz2	Cytoskeleton
DMR2:227208001	2	227208001	227209000	1000	1	1.60E-11	0.7	3	0.3	Myoz2;LOC365926	Cytoskeleton
DMR2:227240001	2	227240001	227247000	7000	1	1.80E-15	0.59	94	1.34	Synpo2	Cytoskeleton
DMR2:227254001	2	227254001	227258000	4000	2	6.00E-23	1	48	1.2	Synpo2	Cytoskeleton
DMR2:227272001	2	227272001	227281000	9000	3	7.00E-26	-0.59	103	1.14	Synpo2	Cytoskeleton
DMR2:227282001	2	227282001	227283000	1000	1	1.90E-08	0.62	8	0.8	Synpo2	Cytoskeleton
DMR2:227285001	2	227285001	227286000	1000	1	1.50E-09	-0.66	24	2.4	Synpo2	Cytoskeleton
DMR2:227313001	2	227313001	227316000	3000	1	5.70E-07	0.33	33	1.1	Synpo2	Cytoskeleton
DMR2:227320001	2	227320001	227323000	3000	1	2.70E-13	0.56	25	0.83	Synpo2	Cytoskeleton
DMR2:227325001	2	227325001	227329000	4000	2	9.40E-12	0.41	50	1.25	Synpo2	Cytoskeleton
DMR2:227330001	2	227330001	227331000	1000	1	6.50E-13	0.68	6	0.6	Synpo2	Cytoskeleton
DMR2:227332001	2	227332001	227333000	1000	1	4.70E-10	0.49	71	7.1	Synpo2	Cytoskeleton
DMR2:227341001	2	227341001	227343000	2000	1	8.00E-07	0.35	15	0.75	Synpo2	Cytoskeleton
DMR2:227357001	2	227357001	227358000	1000	1	9.30E-07	0.34	14	1.4	Synpo2	Cytoskeleton
DMR2:227388001	2	227388001	227391000	3000	1	2.20E-07	0.36	39	1.3	Synpo2	Cytoskeleton
DMR2:227414001	2	227414001	227415000	1000	1	1.10E-12	0.51	10	1	Synpo2	Cytoskeleton
DMR2:227417001	2	227417001	227419000	2000	1	2.50E-12	0.64	10	0.5	Synpo2	Cytoskeleton
DMR2:227471001	2	227471001	227473000	2000	1	4.90E-08	0.37	31	1.55	Sec24d	Transport
DMR2:227475001	2	227475001	227477000	2000	2	7.00E-25	0.67	20	1	Sec24d	Transport
DMR2:227495001	2	227495001	227496000	1000	1	1.70E-10	0.4	11	1.1	Sec24d	Transport
DMR2:227499001	2	227499001	227501000	2000	2	2.60E-20	0.78	20	1	Sec24d	Transport
DMR2:227553001	2	227553001	227555000	2000	1	7.70E-26	0.63	57	2.85	Sec24d	Transport
DMR2:227557001	2	227557001	227559000	2000	1	4.50E-07	-0.48	21	1.05	Sec24d;Mettl14	Transport;Translation
DMR2:227573001	2	227573001	227574000	1000	1	5.10E-07	-0.63	11	1.1	Mettl14	Translation
DMR2:227649001	2	227649001	227650000	1000	1	2.50E-08	0.41	13	1.3	LOC108350103;Prss12	
DMR2:227661001	2	227661001	227662000	1000	1	1.20E-07	-0.53	14	1.4	LOC108350103;Prss12	
DMR2:227669001	2	227669001	227670000	1000	1	1.20E-10	0.39	11	1.1	Prss12	
DMR2:227684001	2	227684001	227685000	1000	1	5.70E-16	0.82	2	0.2	Prss12	
DMR2:227697001	2	227697001	227699000	2000	1	1.60E-07	-0.61	26	1.3	Prss12	
DMR2:227703001	2	227703001	227709000	6000	1	1.60E-12	0.6	83	1.38	Prss12;LOC103691688	

DMR2:227722001	2	227722001	227725000	3000	2	1.20E-09	0.47	46	1.53	Prss12;LOC103691688	
DMR2:227727001	2	227727001	227732000	5000	2	1.20E-14	-0.63	84	1.68	Prss12;LOC103691688;Ndst3	Transport
DMR2:227733001	2	227733001	227739000	6000	1	1.50E-08	0.51	94	1.57	Ndst3	Transport
DMR2:227753001	2	227753001	227755000	2000	1	2.30E-17	0.64	30	1.5	Ndst3	Transport
DMR2:227757001	2	227757001	227759000	2000	1	4.20E-08	-0.52	29	1.45	Ndst3	Transport
DMR2:227771001	2	227771001	227774000	3000	1	2.20E-18	0.64	35	1.17	Ndst3	Transport
DMR2:227787001	2	227787001	227789000	2000	1	3.50E-08	0.51	11	0.55	Ndst3	Transport
DMR2:227792001	2	227792001	227793000	1000	1	1.80E-23	0.98	12	1.2	Ndst3	Transport
DMR2:227794001	2	227794001	227797000	3000	1	8.30E-08	-0.52	33	1.1	Ndst3	Transport
DMR2:227809001	2	227809001	227810000	1000	1	1.10E-11	0.49	35	3.5	Ndst3	Transport
DMR2:227814001	2	227814001	227815000	1000	1	6.80E-09	0.46	15	1.5	Ndst3	Transport
DMR2:227841001	2	227841001	227846000	5000	1	3.80E-07	-0.39	40	0.8	Ndst3	Transport
DMR2:227847001	2	227847001	227848000	1000	1	1.80E-10	0.47	24	2.4	Ndst3	Transport
DMR2:227849001	2	227849001	227851000	2000	1	3.60E-09	0.48	24	1.2	Ndst3	Transport
DMR2:227894001	2	227894001	227898000	4000	1	1.30E-20	0.77	50	1.25	Ndst3	Transport
DMR2:228033001	2	228033001	228035000	2000	2	3.60E-09	0.56	28	1.4	Hspd1-ps12	
DMR2:228045001	2	228045001	228053000	8000	2	1.50E-07	-0.54	36	0.45	Hspd1-ps12	
DMR2:228533001	2	228533001	228537000	4000	1	2.30E-15	0.62	39	0.98	LOC103691690;Tram1l1	
DMR2:228538001	2	228538001	228539000	1000	1	2.70E-17	0.6	9	0.9	LOC103691690;Tram1l1	
DMR2:228645001	2	228645001	228646000	1000	1	6.00E-10	0.54	9	0.9	RGD1306519	Signaling
DMR2:228662001	2	228662001	228665000	3000	1	2.80E-21	0.82	34	1.13	RGD1306519	Signaling
DMR2:228672001	2	228672001	228673000	1000	1	2.60E-12	0.59	7	0.7	RGD1306519;LOC691855	Signaling
DMR2:228679001	2	228679001	228681000	2000	2	6.90E-16	0.59	19	0.95	RGD1306519;LOC691855	Signaling
DMR2:229179001	2	229179001	229180000	1000	1	8.80E-07	0.39	7	0.7	Ndst4	Transport
DMR2:229222001	2	229222001	229223000	1000	1	3.40E-12	0.65	8	0.8	Ndst4	Transport
DMR2:229244001	2	229244001	229246000	2000	1	9.80E-10	-0.58	6	0.3	Ndst4	Transport
DMR2:229258001	2	229258001	229259000	1000	1	3.70E-08	0.56	8	0.8	Ndst4	Transport
DMR2:229266001	2	229266001	229268000	2000	1	1.10E-14	0.83	7	0.35	Ndst4;LOC108350104	Transport
DMR2:229295001	2	229295001	229296000	1000	1	2.30E-10	0.57	6	0.6	Ndst4	Transport
DMR2:229297001	2	229297001	229298000	1000	1	4.90E-11	-0.64	3	0.3	Ndst4	Transport
DMR2:229316001	2	229316001	229320000	4000	1	4.90E-11	0.53	33	0.82	Ndst4	Transport
DMR2:229323001	2	229323001	229325000	2000	1	2.70E-07	-0.59	13	0.65	Ndst4	Transport
DMR2:229361001	2	229361001	229367000	6000	4	5.80E-19	-0.45	62	1.03	Ndst4	Transport
DMR2:229368001	2	229368001	229369000	1000	1	5.10E-11	0.68	6	0.6	Ndst4	Transport
DMR2:229373001	2	229373001	229374000	1000	1	9.60E-09	-0.65	12	1.2	Ndst4	Transport
DMR2:229391001	2	229391001	229392000	1000	1	6.80E-21	0.94	7	0.7	Ndst4	Transport
DMR2:229402001	2	229402001	229404000	2000	1	5.50E-17	0.7	11	0.55	Ndst4	Transport
DMR2:229421001	2	229421001	229427000	6000	2	3.90E-15	-0.47	53	0.88	Ndst4	Transport
DMR2:229428001	2	229428001	229429000	1000	1	8.60E-14	0.68	5	0.5	Ndst4	Transport
DMR2:229430001	2	229430001	229431000	1000	1	2.30E-11	0.75	18	1.8	Ndst4	Transport
DMR2:229438001	2	229438001	229442000	4000	2	1.20E-07	0.35	59	1.48	Ndst4	Transport
DMR2:229462001	2	229462001	229463000	1000	1	6.60E-07	-0.49	8	0.8	Ndst4	Transport
DMR2:229483001	2	229483001	229484000	1000	1	1.30E-12	0.66	6	0.6	Ndst4	Transport
DMR2:229506001	2	229506001	229507000	1000	1	3.70E-07	0.43	10	1	Ndst4	Transport
DMR2:229665001	2	229665001	229668000	3000	1	3.10E-07	0.37	26	0.87	Ugt8	
DMR2:229693001	2	229693001	229695000	2000	2	7.00E-16	0.86	10	0.5	Ugt8	
DMR2:230191001	2	230191001	230193000	2000	1	1.20E-07	0.37	43	2.15	Arsj;LOC103689977;Mcub	
DMR2:230223001	2	230223001	230226000	3000	3	5.60E-11	0.59	49	1.63	Arsj;Mcub	
DMR2:230261001	2	230261001	230265000	4000	2	6.50E-16	0.95	33	0.82	Arsj;Mcub	
DMR2:230266001	2	230266001	230268000	2000	2	2.00E-23	0.92	19	0.95	Arsj;Mcub	
DMR2:230281001	2	230281001	230289000	8000	1	3.60E-13	0.73	105	1.31	Arsj;Mcub;Sec24b	Transport
DMR2:230292001	2	230292001	230297000	5000	1	3.50E-08	0.38	87	1.74	Arsj;Sec24b	Transport
DMR2:230342001	2	230342001	230343000	1000	1	4.10E-09	0.41	22	2.2	Arsj;Sec24b	Transport
DMR2:230354001	2	230354001	230355000	1000	1	4.10E-16	0.58	11	1.1	Arsj;Sec24b	Transport
DMR2:230358001	2	230358001	230360000	2000	1	1.30E-13	0.68	17	0.85	Arsj;Sec24b	Transport
DMR2:230395001	2	230395001	230396000	1000	1	1.90E-07	0.39	13	1.3	Arsj;LOC103691692	
DMR2:230402001	2	230402001	230403000	1000	1	1.50E-07	-0.74	10	1	Arsj	
DMR2:230441001	2	230441001	230445000	4000	1	6.30E-09	0.5	37	0.92	Arsj	
DMR2:230452001	2	230452001	230456000	4000	1	4.60E-20	0.86	56	1.4	Arsj	
DMR2:230457001	2	230457001	230459000	2000	1	1.20E-08	0.36	25	1.25	Arsj	
DMR2:230460001	2	230460001	230466000	6000	2	4.20E-13	-0.55	87	1.45	Arsj;LOC102553088	
DMR2:230489001	2	230489001	230490000	1000	1	1.20E-16	0.46	18	1.8	Arsj;LOC102553088	
DMR2:230500001	2	230500001	230501000	1000	1	7.40E-07	-0.5	11	1.1	Arsj	
DMR2:230525001	2	230525001	230527000	2000	1	2.00E-08	-0.57	15	0.75	Arsj	
DMR2:230535001	2	230535001	230539000	4000	1	2.60E-11	0.57	67	1.68	Arsj	

DMR2:230544001	2	230544001	230546000	2000	1	3.90E-09	0.45	22	1.1	Arsj	
DMR2:230558001	2	230558001	230560000	2000	1	3.20E-09	0.48	25	1.25	Arsj	
DMR2:230593001	2	230593001	230598000	5000	2	1.80E-07	0.57	46	0.92	Arsj;LOC108350105	
DMR2:230620001	2	230620001	230621000	1000	1	1.90E-07	0.54	7	0.7	Arsj;LOC108350105	
DMR2:230623001	2	230623001	230625000	2000	1	6.90E-09	0.44	20	1	Arsj;LOC108350105	
DMR2:230648001	2	230648001	230649000	1000	1	1.90E-10	0.53	2	0.2	Arsj	
DMR2:230894001	2	230894001	230898000	4000	2	1.40E-09	-0.69	44	1.1	Camk2d	Signaling
DMR2:230918001	2	230918001	230921000	3000	1	1.60E-07	0.39	40	1.33	Camk2d	Signaling
DMR2:230942001	2	230942001	230943000	1000	1	8.40E-09	0.53	8	0.8	Camk2d	Signaling
DMR2:230945001	2	230945001	230946000	1000	1	5.10E-13	0.64	10	1	Camk2d	Signaling
DMR2:230956001	2	230956001	230957000	1000	1	1.50E-12	0.69	6	0.6	Camk2d	Signaling
DMR2:230966001	2	230966001	230967000	1000	1	1.60E-10	0.64	7	0.7	Camk2d	Signaling
DMR2:230985001	2	230985001	230987000	2000	2	1.30E-09	-0.66	30	1.5	Camk2d	Signaling
DMR2:231005001	2	231005001	231011000	6000	1	1.50E-08	-0.67	94	1.57	Camk2d	Signaling
DMR2:231023001	2	231023001	231025000	2000	1	2.20E-11	-0.49	24	1.2	Camk2d	Signaling
DMR2:231026001	2	231026001	231029000	3000	2	3.90E-08	-0.42	30	1	Camk2d	Signaling
DMR2:231044001	2	231044001	231045000	1000	1	8.20E-08	-0.62	10	1	Camk2d;LOC108350106	Signaling
DMR2:231086001	2	231086001	231090000	4000	1	6.20E-07	-0.32	39	0.98	Camk2d	Signaling
DMR2:231097001	2	231097001	231098000	1000	1	5.20E-09	0.44	17	1.7	Camk2d	Signaling
DMR2:231104001	2	231104001	231110000	6000	1	7.80E-09	0.45	58	0.97	Camk2d	Signaling
DMR2:231112001	2	231112001	231113000	1000	1	2.50E-10	0.54	11	1.1	Camk2d	Signaling
DMR2:231121001	2	231121001	231122000	1000	1	7.80E-10	-0.65	2	0.2	Camk2d	Signaling
DMR2:231126001	2	231126001	231130000	4000	1	6.40E-07	-0.51	53	1.32	Camk2d	Signaling
DMR2:231215001	2	231215001	231218000	3000	2	1.20E-13	-0.62	10	0.33	Ank2	
DMR2:231224001	2	231224001	231227000	3000	1	2.10E-09	0.44	31	1.03	Ank2	
DMR2:231229001	2	231229001	231231000	2000	1	8.10E-24	0.94	22	1.1	Ank2	
DMR2:231243001	2	231243001	231247000	4000	2	2.50E-19	0.91	53	1.32	Ank2	
DMR2:231270001	2	231270001	231273000	3000	1	3.50E-09	-0.48	44	1.47	Ank2	
DMR2:231291001	2	231291001	231292000	1000	1	3.30E-07	-0.44	18	1.8	Ank2	
DMR2:231297001	2	231297001	231299000	2000	1	8.20E-07	-0.51	36	1.8	Ank2	
DMR2:231302001	2	231302001	231307000	5000	2	1.80E-11	-0.69	79	1.58	Ank2	
DMR2:231314001	2	231314001	231315000	1000	1	8.20E-09	-0.5	11	1.1	Ank2	
DMR2:231327001	2	231327001	231328000	1000	1	2.30E-08	-0.49	21	2.1	Ank2	
DMR2:231335001	2	231335001	231340000	5000	2	4.00E-10	0.51	68	1.36	Ank2	
DMR2:231353001	2	231353001	231354000	1000	1	2.10E-15	0.86	2	0.2	Ank2	
DMR2:231355001	2	231355001	231358000	3000	1	3.50E-09	-0.65	44	1.47	Ank2	
DMR2:231366001	2	231366001	231368000	2000	1	3.30E-18	0.91	13	0.65	Ank2	
DMR2:231373001	2	231373001	231376000	3000	2	4.10E-11	-0.69	15	0.5	Ank2	
DMR2:231383001	2	231383001	231386000	3000	1	3.10E-09	0.42	50	1.67	Ank2	
DMR2:231394001	2	231394001	231400000	6000	2	1.50E-19	0.69	65	1.08	Ank2	
DMR2:231405001	2	231405001	231406000	1000	1	1.60E-12	0.59	11	1.1	Ank2	
DMR2:231425001	2	231425001	231427000	2000	2	3.00E-12	-0.73	26	1.3	Ank2	
DMR2:231428001	2	231428001	231431000	3000	1	8.60E-07	0.34	42	1.4	Ank2	
DMR2:231436001	2	231436001	231437000	1000	1	1.30E-11	0.5	4	0.4	Ank2	
DMR2:231443001	2	231443001	231450000	7000	1	4.70E-07	0.34	84	1.2	Ank2	
DMR2:231482001	2	231482001	231485000	3000	1	5.20E-08	-0.54	31	1.03	Ank2	
DMR2:231488001	2	231488001	231489000	1000	1	9.90E-11	0.53	8	0.8	Ank2	
DMR2:231495001	2	231495001	231501000	6000	2	9.10E-09	0.42	103	1.72	Ank2	
DMR2:231503001	2	231503001	231506000	3000	2	6.10E-08	0.43	38	1.27	Ank2	
DMR2:231508001	2	231508001	231511000	3000	1	6.80E-19	0.57	59	1.97	Ank2	
DMR2:231522001	2	231522001	231523000	1000	1	5.80E-07	0.31	16	1.6	Ank2	
DMR2:231854001	2	231854001	231857000	3000	1	1.30E-10	0.51	38	1.27	Larp7	Metabolism
DMR2:231867001	2	231867001	231868000	1000	1	1.70E-07	0.35	13	1.3	Larp7	Metabolism
DMR2:231926001	2	231926001	231930000	4000	1	4.70E-10	0.54	30	0.75	Zgrf1	
DMR2:231936001	2	231936001	231937000	1000	1	3.40E-08	0.38	39	3.9	Zgrf1	
DMR2:231948001	2	231948001	231954000	6000	1	4.20E-09	-0.63	58	0.97	Zgrf1;Neurog2	Transcription
DMR2:231971001	2	231971001	231974000	3000	3	4.90E-13	0.64	31	1.03	Neurog2	Transcription
DMR2:231999001	2	231999001	232003000	4000	1	6.30E-09	-0.8	42	1.05	Alpk1	Signaling
DMR2:232008001	2	232008001	232009000	1000	1	1.90E-07	0.37	20	2	Alpk1	Signaling
DMR2:232031001	2	232031001	232032000	1000	1	5.10E-10	0.41	19	1.9	Alpk1;LOC103691697;LOC103691696	Signaling
DMR2:232041001	2	232041001	232043000	2000	2	4.70E-12	0.51	29	1.45	Alpk1;LOC103691697;LOC103691696	Signaling
DMR2:232049001	2	232049001	232053000	4000	1	4.00E-09	0.42	74	1.85	Alpk1	Signaling
DMR2:232056001	2	232056001	232059000	3000	2	7.80E-30	1.06	78	2.6	Alpk1	Signaling

DMR2:232062001	2	232062001	232065000	3000	2	2.30E-10	0.58	29	0.97	Alpk1	Signaling
DMR2:232077001	2	232077001	232079000	2000	1	9.80E-08	0.47	13	0.65	Alpk1	Signaling
DMR2:232094001	2	232094001	232095000	1000	1	7.90E-09	0.4	10	1	Alpk1;Tifa	Signaling
DMR2:232096001	2	232096001	232100000	4000	2	4.90E-21	0.68	30	0.75	Alpk1;Tifa	Signaling
DMR2:232103001	2	232103001	232104000	1000	1	9.50E-08	0.39	11	1.1	Alpk1;Tifa	Signaling
DMR2:232111001	2	232111001	232114000	3000	2	1.40E-09	0.43	33	1.1	Alpk1;Tifa	Signaling
DMR2:232118001	2	232118001	232120000	2000	1	4.10E-15	0.93	12	0.6	Alpk1;Tifa	Signaling
DMR2:232121001	2	232121001	232123000	2000	1	2.20E-09	0.48	24	1.2	Alpk1;Tifa	Signaling
DMR2:232124001	2	232124001	232125000	1000	1	5.90E-25	1.01	2	0.2	Alpk1;Tifa	Signaling
DMR2:232145001	2	232145001	232150000	5000	1	2.70E-16	0.56	102	2.04	Tifa;Ap1ar	
DMR2:232154001	2	232154001	232158000	4000	1	2.00E-09	0.36	53	1.32	Ap1ar	
DMR2:232167001	2	232167001	232169000	2000	1	6.20E-09	-0.55	29	1.45	Ap1ar	
DMR2:232306001	2	232306001	232307000	1000	1	1.00E-07	0.46	15	1.5	RGD1565088	
DMR2:232314001	2	232314001	232318000	4000	2	8.40E-20	0.68	74	1.85	RGD1565088	
DMR2:233601001	2	233601001	233602000	1000	1	4.00E-10	0.69	26	2.6	Pitx2	Development
DMR2:233611001	2	233611001	233612000	1000	1	7.60E-07	0.32	6	0.6	Pitx2	Development
DMR2:233630001	2	233630001	233631000	1000	1	4.30E-07	0.39	13	1.3	Pitx2;LOC102546772	Development
DMR2:233659001	2	233659001	233660000	1000	1	9.60E-14	0.53	10	1	Enpep	Protease
DMR2:233669001	2	233669001	233670000	1000	1	2.10E-07	0.37	4	0.4	Enpep	Protease
DMR2:233671001	2	233671001	233672000	1000	1	3.80E-12	0.55	20	2	Enpep	Protease
DMR2:233677001	2	233677001	233678000	1000	1	2.60E-07	-0.64	19	1.9	Enpep;LOC108350158	Protease
DMR2:233699001	2	233699001	233702000	3000	1	2.70E-22	0.87	51	1.7	Enpep;LOC108350158	Protease
DMR2:233734001	2	233734001	233735000	1000	1	1.10E-16	0.98	6	0.6	Enpep	Protease
DMR2:233740001	2	233740001	233741000	1000	1	7.70E-07	0.45	15	1.5	Enpep	Protease
DMR2:233746001	2	233746001	233747000	1000	1	1.30E-08	0.46	15	1.5	Enpep	Protease
DMR2:233906001	2	233906001	233908000	2000	1	3.00E-08	0.47	22	1.1	Dcm5	
DMR2:234175001	2	234175001	234180000	5000	1	7.20E-09	0.56	77	1.54	Elovl6	Metabolism
DMR2:234186001	2	234186001	234190000	4000	2	1.10E-10	0.5	79	1.98	Elovl6	Metabolism
DMR2:234223001	2	234223001	234224000	1000	1	1.00E-17	0.78	15	1.5	Elovl6	Metabolism
DMR2:234240001	2	234240001	234241000	1000	1	1.10E-07	0.47	15	1.5	Elovl6	Metabolism
DMR2:234285001	2	234285001	234287000	2000	2	3.50E-10	0.57	25	1.25	Elovl6	Metabolism
DMR2:234981001	2	234981001	234982000	1000	1	4.30E-07	0.42	8	0.8	RGD1560484	
DMR2:235077001	2	235077001	235078000	1000	1	2.40E-12	0.48	22	2.2	Egf	Receptor
DMR2:235080001	2	235080001	235081000	1000	1	2.00E-07	-0.61	20	2	Egf	Receptor
DMR2:235103001	2	235103001	235105000	2000	1	9.00E-07	0.46	33	1.65	Egf	Receptor
DMR2:235162001	2	235162001	235163000	1000	1	8.50E-07	0.38	21	2.1	Egf	Receptor
DMR2:235173001	2	235173001	235176000	3000	1	4.60E-08	0.31	65	2.17	Egf	Receptor
DMR2:235198001	2	235198001	235210000	12000	3	9.30E-22	1.1	153	1.27	Lrit3	
DMR2:235222001	2	235222001	235223000	1000	1	1.40E-09	0.46	8	0.8	Lrit3	
DMR2:235224001	2	235224001	235226000	2000	1	3.80E-11	0.59	16	0.8	Lrit3	
DMR2:235234001	2	235234001	235237000	3000	2	1.40E-25	0.85	40	1.33	Lrit3;Rrh	Signaling
DMR2:235251001	2	235251001	235253000	2000	1	1.20E-09	0.55	29	1.45	Rrh;Gar1	Signaling;Metabolism
DMR2:235278001	2	235278001	235285000	7000	4	7.90E-31	-0.65	83	1.19	Cfi	Protease
DMR2:235301001	2	235301001	235304000	3000	1	1.30E-10	-0.62	41	1.37	Cfi;Pla2g12a	Protease;Metabolism
DMR2:235310001	2	235310001	235311000	1000	1	2.50E-13	0.56	16	1.6	Cfi;Pla2g12a	Protease;Metabolism
DMR2:235312001	2	235312001	235315000	3000	1	3.80E-24	1.05	72	2.4	Cfi;Pla2g12a	Protease;Metabolism
DMR2:235346001	2	235346001	235350000	4000	1	1.40E-08	0.42	79	1.98	Casp6;Mcub	Protease
DMR2:235366001	2	235366001	235367000	1000	1	1.50E-07	0.37	11	1.1	Mcub	
DMR2:235516001	2	235516001	235519000	3000	2	1.40E-21	0.81	43	1.43	Col25a1;LOC103691705	Extracellular Matrix
DMR2:235534001	2	235534001	235538000	4000	1	2.60E-11	0.45	46	1.15	Col25a1;LOC103691705	Extracellular Matrix
DMR2:235558001	2	235558001	235560000	2000	1	5.70E-12	0.37	30	1.5	Col25a1	Extracellular Matrix
DMR2:235561001	2	235561001	235563000	2000	1	9.10E-08	0.42	28	1.4	Col25a1	Extracellular Matrix
DMR2:235566001	2	235566001	235569000	3000	1	1.50E-16	0.68	65	2.17	Col25a1	Extracellular Matrix
DMR2:235572001	2	235572001	235577000	5000	3	3.10E-08	-0.49	76	1.52	Col25a1	Extracellular Matrix
DMR2:235591001	2	235591001	235593000	2000	1	1.90E-19	0.69	24	1.2	Col25a1	Extracellular Matrix
DMR2:235609001	2	235609001	235612000	3000	1	1.50E-19	0.7	37	1.23	Col25a1	Extracellular Matrix
DMR2:235631001	2	235631001	235636000	5000	1	2.20E-07	-0.42	71	1.42	Col25a1	Extracellular Matrix
DMR2:235643001	2	235643001	235646000	3000	1	4.00E-09	-0.58	56	1.87	Col25a1	Extracellular Matrix
DMR2:235663001	2	235663001	235667000	4000	1	1.80E-08	-0.45	57	1.43	Col25a1	Extracellular Matrix
DMR2:235692001	2	235692001	235694000	2000	2	2.50E-16	0.52	31	1.55	Col25a1	Extracellular Matrix
DMR2:235736001	2	235736001	235740000	4000	1	2.60E-15	0.59	62	1.55	Etnppl	

DMR2:235742001	2	235742001	235744000	2000	1	2.10E-07	-0.51	48	2.4	Etnppl	
DMR2:235745001	2	235745001	235746000	1000	1	1.20E-15	0.57	10	1	Etnppl	
DMR2:235769001	2	235769001	235772000	3000	1	1.20E-17	0.67	44	1.47	Etnppl	
DMR2:235805001	2	235805001	235807000	2000	2	1.80E-24	0.78	19	0.95	Ostc	Transport
DMR2:235815001	2	235815001	235818000	3000	1	7.60E-07	0.47	34	1.13	Ostc	Transport
DMR2:235821001	2	235821001	235823000	2000	1	6.00E-10	0.45	29	1.45	Ostc	Transport
DMR2:235850001	2	235850001	235852000	2000	1	1.10E-07	0.35	28	1.4	Rpl34	Translation
DMR2:236259001	2	236259001	236261000	2000	1	3.50E-08	0.31	46	2.3	Lef1;LOC103691708	Transcription
DMR2:236263001	2	236263001	236266000	3000	2	5.80E-20	1.23	38	1.27	Lef1;LOC103691708	Transcription
DMR2:236292001	2	236292001	236295000	3000	2	2.00E-15	0.51	46	1.53	Lef1	Transcription
DMR2:236316001	2	236316001	236317000	1000	1	1.10E-09	0.36	12	1.2	Lef1	Transcription
DMR2:236331001	2	236331001	236333000	2000	1	9.00E-08	0.35	28	1.4	Lef1	Transcription
DMR2:236343001	2	236343001	236347000	4000	1	3.90E-07	-0.46	100	2.5	Lef1;Hadh	Transcription
DMR2:236348001	2	236348001	236349000	1000	1	6.50E-10	0.39	20	2	Lef1;Hadh	Transcription
DMR2:236362001	2	236362001	236366000	4000	1	2.60E-08	0.3	69	1.73	Hadh	
DMR2:236367001	2	236367001	236377000	10000	2	4.10E-11	-0.54	155	1.55	Hadh	
DMR2:236390001	2	236390001	236391000	1000	1	2.00E-12	0.85	1	0.1	Hadh	
DMR2:236399001	2	236399001	236403000	4000	1	2.40E-07	-0.53	64	1.6	Hadh	
DMR2:236405001	2	236405001	236410000	5000	2	4.10E-10	-0.69	23	0.46	Hadh;Cyp2u1	Metabolism
DMR2:236411001	2	236411001	236414000	3000	1	7.60E-24	0.84	43	1.43	Cyp2u1	Metabolism
DMR2:236436001	2	236436001	236440000	4000	1	1.60E-11	0.48	64	1.6	Cyp2u1;LOC102552472	Metabolism
DMR2:236457001	2	236457001	236460000	3000	1	3.40E-07	0.28	44	1.47	Sgms2	
DMR2:236500001	2	236500001	236504000	4000	1	4.70E-07	-0.57	71	1.77	Sgms2;LOC108350108;LOC108350109	
DMR2:236510001	2	236510001	236511000	1000	1	4.10E-21	0.83	10	1	Sgms2;LOC108350108;LOC108350109	
DMR2:236517001	2	236517001	236520000	3000	2	3.60E-22	0.6	61	2.03	Sgms2;LOC108350109	
DMR2:236534001	2	236534001	236535000	1000	1	2.00E-14	0.83	5	0.5	Sgms2;LOC108350110	
DMR2:236610001	2	236610001	236611000	1000	1	1.70E-09	0.35	15	1.5	Papss1	
DMR2:236612001	2	236612001	236614000	2000	2	1.70E-13	0.45	20	1	Papss1	
DMR2:236620001	2	236620001	236623000	3000	1	5.70E-23	0.78	27	0.9	Papss1	
DMR2:236635001	2	236635001	236642000	7000	1	1.80E-07	-0.44	113	1.61	Papss1	
DMR2:236644001	2	236644001	236652000	8000	1	1.70E-09	0.36	121	1.51	Papss1	
DMR2:236656001	2	236656001	236660000	4000	2	5.60E-10	0.4	72	1.8	Papss1	
DMR2:236661001	2	236661001	236665000	4000	1	6.30E-13	0.51	65	1.62	Papss1	
DMR2:237139001	2	237139001	237140000	1000	1	1.80E-10	0.48	8	0.8	LOC680243;Dkk2	
DMR2:237146001	2	237146001	237147000	1000	1	6.20E-07	0.36	6	0.6	Dkk2	
DMR2:237157001	2	237157001	237159000	2000	1	9.90E-07	-0.41	24	1.2	Dkk2	
DMR2:237166001	2	237166001	237169000	3000	1	9.00E-08	0.5	35	1.17	Dkk2	
DMR2:237179001	2	237179001	237182000	3000	1	6.80E-12	0.75	22	0.73	Dkk2	
DMR2:237230001	2	237230001	237232000	2000	2	9.50E-34	1.08	14	0.7	Dkk2	
DMR2:237245001	2	237245001	237246000	1000	1	2.70E-07	0.62	3	0.3	Dkk2	
DMR2:237247001	2	237247001	237249000	2000	1	6.00E-08	0.4	14	0.7	Dkk2	
DMR2:237680001	2	237680001	237682000	2000	1	4.70E-10	-0.56	19	0.95	LOC102547683;Gimd1	Signaling
DMR2:237691001	2	237691001	237692000	1000	1	2.50E-09	0.53	5	0.5	Gimd1	Signaling
DMR2:237696001	2	237696001	237697000	1000	1	4.20E-08	0.46	6	0.6	Gimd1	Signaling
DMR2:237700001	2	237700001	237704000	4000	1	3.00E-07	-0.52	33	0.82	Gimd1	Signaling
DMR2:237727001	2	237727001	237728000	1000	1	6.50E-08	-0.58	12	1.2	Aimp1	Translation
DMR2:237730001	2	237730001	237731000	1000	1	2.80E-12	0.55	9	0.9	Aimp1	Translation
DMR2:237754001	2	237754001	237756000	2000	1	1.30E-07	0.33	20	1	Aimp1;Tbck	Translation;Signaling
DMR2:237766001	2	237766001	237768000	2000	1	4.60E-09	-0.51	9	0.45	Tbck	Signaling
DMR2:237770001	2	237770001	237771000	1000	1	3.60E-09	0.41	16	1.6	Tbck	Signaling
DMR2:237787001	2	237787001	237789000	2000	1	2.30E-07	0.39	13	0.65	Tbck	Signaling
DMR2:237797001	2	237797001	237800000	3000	1	1.50E-09	0.5	37	1.23	Tbck	Signaling
DMR2:237805001	2	237805001	237806000	1000	1	2.20E-08	0.37	14	1.4	Tbck	Signaling
DMR2:237817001	2	237817001	237825000	8000	7	8.90E-19	0.5	97	1.21	Tbck	Signaling
DMR2:237857001	2	237857001	237867000	10000	6	5.20E-25	0.59	108	1.08	Tbck	Signaling
DMR2:237882001	2	237882001	237887000	5000	4	2.00E-29	-0.51	51	1.02	Tbck	Signaling
DMR2:237888001	2	237888001	237889000	1000	1	3.60E-07	-0.41	10	1	Tbck	Signaling
DMR2:237908001	2	237908001	237910000	2000	2	2.90E-13	0.77	22	1.1	Tbck;LOC108350113	Signaling
DMR2:237933001	2	237933001	237936000	3000	1	1.10E-07	0.36	25	0.83	Tbck;LOC102553582	Signaling
DMR2:237938001	2	237938001	237941000	3000	2	7.40E-25	0.9	27	0.9	Tbck	Signaling
DMR2:237958001	2	237958001	237960000	2000	1	6.30E-08	0.48	12	0.6	Tbck;LOC680335;LOC102556143	Signaling
DMR2:238013001	2	238013001	238018000	5000	2	5.30E-08	-0.52	100	2	Npnt	

DMR2:238020001	2	238020001	238021000	1000	1	6.00E-07	0.45	12	1.2	Npnt	
DMR2:238026001	2	238026001	238028000	2000	1	2.40E-08	0.33	48	2.4	Npnt	
DMR2:238029001	2	238029001	238031000	2000	1	1.20E-09	0.37	39	1.95	Npnt	
DMR2:238040001	2	238040001	238049000	9000	1	9.90E-15	0.51	127	1.41	Npnt	
DMR2:238053001	2	238053001	238054000	1000	1	4.10E-07	0.39	15	1.5	Npnt	
DMR2:238077001	2	238077001	238079000	2000	1	3.40E-09	0.47	20	1	Npnt;LOC365942	
DMR2:238109001	2	238109001	238114000	5000	1	3.60E-07	0.38	65	1.3	LOC102548236;Gstcd	
DMR2:238115001	2	238115001	238116000	1000	1	2.00E-09	0.62	6	0.6	LOC102548236;Gstcd	
DMR2:238127001	2	238127001	238129000	2000	1	1.20E-11	0.37	23	1.15	Gstcd	
DMR2:238144001	2	238144001	238147000	3000	1	2.90E-16	0.59	47	1.57	Gstcd	
DMR2:238179001	2	238179001	238180000	1000	1	1.00E-07	0.56	3	0.3	Gstcd	
DMR2:238182001	2	238182001	238186000	4000	1	5.70E-18	0.64	66	1.65	Gstcd	
DMR2:238193001	2	238193001	238194000	1000	1	3.70E-16	0.54	19	1.9	Gstcd	
DMR2:238201001	2	238201001	238208000	7000	3	3.10E-18	-0.52	84	1.2	Gstcd	
DMR2:238217001	2	238217001	238218000	1000	1	3.30E-15	0.74	11	1.1	Gstcd	
DMR2:238257001	2	238257001	238258000	1000	1	5.90E-08	-0.69	15	1.5	Gstcd;Ints12	
DMR2:238285001	2	238285001	238287000	2000	2	1.80E-13	0.62	24	1.2	Arhgef38	
DMR2:238302001	2	238302001	238306000	4000	1	4.70E-07	0.48	55	1.38	Arhgef38	
DMR2:238329001	2	238329001	238336000	7000	6	2.10E-21	-0.54	74	1.06	Arhgef38	
DMR2:238345001	2	238345001	238348000	3000	2	1.30E-11	-0.45	29	0.97	Arhgef38	
DMR2:238358001	2	238358001	238359000	1000	1	8.20E-07	0.38	13	1.3	Arhgef38	
DMR2:238518001	2	238518001	238520000	2000	1	2.50E-11	-0.65	18	0.9	LOC102546359;Ppa2	Signaling
DMR2:238534001	2	238534001	238535000	1000	1	2.00E-10	-0.5	29	2.9	LOC102546359;Ppa2	Signaling
DMR2:238536001	2	238536001	238540000	4000	2	2.90E-13	0.57	76	1.9	LOC102546359;Ppa2	Signaling
DMR2:238554001	2	238554001	238556000	2000	1	1.80E-07	-0.44	15	0.75	Ppa2	Signaling
DMR2:238563001	2	238563001	238566000	3000	2	1.20E-10	0.47	35	1.17	Ppa2	Signaling
DMR2:238567001	2	238567001	238569000	2000	1	1.70E-10	0.42	48	2.4	Ppa2	Signaling
DMR2:238570001	2	238570001	238572000	2000	1	4.20E-09	0.5	33	1.65	Ppa2	Signaling
DMR2:238574001	2	238574001	238576000	2000	1	2.90E-18	0.71	28	1.4	Ppa2	Signaling
DMR2:238577001	2	238577001	238579000	2000	1	6.40E-22	0.6	42	2.1	Ppa2	Signaling
DMR2:238587001	2	238587001	238588000	1000	1	1.10E-08	0.38	16	1.6	Ppa2	Signaling
DMR2:238590001	2	238590001	238594000	4000	1	1.30E-07	0.35	58	1.45	Ppa2	Signaling
DMR2:238688001	2	238688001	238689000	1000	1	9.00E-11	-0.47	4	0.4	Gpbp1l2	
DMR2:238693001	2	238693001	238697000	4000	1	2.20E-10	-0.58	63	1.57	Gpbp1l2	
DMR2:238711001	2	238711001	238716000	5000	1	1.10E-08	0.55	71	1.42	Tet2	
DMR2:238740001	2	238740001	238743000	3000	2	2.70E-07	0.55	21	0.7	Tet2	
DMR2:238758001	2	238758001	238759000	1000	1	4.90E-11	-0.79	8	0.8	Tet2	
DMR2:238769001	2	238769001	238772000	3000	2	5.20E-11	0.44	40	1.33	Tet2	
DMR2:238782001	2	238782001	238784000	2000	2	1.70E-11	-0.53	33	1.65	Tet2	
DMR2:238789001	2	238789001	238793000	4000	2	1.10E-09	-0.66	47	1.18	Tet2	
DMR2:239444001	2	239444001	239451000	7000	4	2.10E-14	-0.45	53	0.76	Cxxc4	
DMR2:240054001	2	240054001	240057000	3000	2	1.80E-13	0.88	25	0.83	Tacr3	Signaling
DMR2:240077001	2	240077001	240078000	1000	1	1.10E-08	0.36	3	0.3	Tacr3	Signaling
DMR2:240108001	2	240108001	240110000	2000	1	7.40E-08	0.46	10	0.5	Tacr3	Signaling
DMR2:240400001	2	240400001	240404000	4000	2	7.90E-08	0.38	39	0.98	Cenpe	
DMR2:240428001	2	240428001	240429000	1000	1	1.00E-24	0.63	16	1.6	Cenpe	
DMR2:240452001	2	240452001	240455000	3000	1	2.40E-08	-0.48	51	1.7	Cenpe;Bdh2	Metabolism
DMR2:240475001	2	240475001	240476000	1000	1	1.00E-09	0.42	22	2.2	Bdh2;Slc9b2	Metabolism
DMR2:240487001	2	240487001	240496000	9000	2	4.00E-13	0.53	124	1.38	Bdh2;Slc9b2	Metabolism
DMR2:240520001	2	240520001	240523000	3000	2	1.70E-23	1	37	1.23	Slc9b2;Slc9b1	
DMR2:240549001	2	240549001	240551000	2000	2	6.30E-19	0.7	12	0.6	Slc9b1	
DMR2:240556001	2	240556001	240563000	7000	3	1.80E-24	-0.6	88	1.26	Slc9b1	
DMR2:240570001	2	240570001	240571000	1000	1	1.30E-24	0.79	10	1	Slc9b1	
DMR2:240586001	2	240586001	240590000	4000	1	2.20E-07	-0.5	48	1.2	Slc9b1;Cisd2	
DMR2:240591001	2	240591001	240599000	8000	4	8.30E-23	-0.56	101	1.26	Slc9b1;Cisd2	
DMR2:240670001	2	240670001	240671000	1000	1	4.20E-09	0.42	8	0.8	Manba	Golgi
DMR2:240673001	2	240673001	240675000	2000	1	2.00E-07	0.36	40	2	Manba	Golgi
DMR2:240676001	2	240676001	240681000	5000	3	1.00E-21	0.85	78	1.56	Manba	Golgi
DMR2:240698001	2	240698001	240699000	1000	1	2.80E-13	0.69	11	1.1	Manba	Golgi
DMR2:240700001	2	240700001	240704000	4000	1	1.50E-09	0.49	61	1.52	Manba	Golgi
DMR2:240705001	2	240705001	240708000	3000	1	5.00E-08	-0.52	59	1.97	Manba	Golgi
DMR2:240711001	2	240711001	240721000	10000	6	2.70E-23	-0.57	112	1.12	Manba	Golgi
DMR2:240731001	2	240731001	240734000	3000	2	6.20E-22	0.88	36	1.2	Manba	Golgi
DMR2:240766001	2	240766001	240767000	1000	1	2.50E-16	0.72	8	0.8	Manba;Nfkb1	Golgi;Transcription
DMR2:240771001	2	240771001	240773000	2000	1	5.20E-12	0.51	29	1.45	Nfkb1	Transcription

DMR2:240775001	2	240775001	240777000	2000	1	2.20E-25	0.73	36	1.8	Nfkb1	Transcription
DMR2:240787001	2	240787001	240793000	6000	2	4.50E-21	0.78	101	1.68	Nfkb1;LOC103691715	Transcription
DMR2:240796001	2	240796001	240800000	4000	1	7.30E-12	-0.88	79	1.98	Nfkb1;LOC103691715	Transcription
DMR2:240805001	2	240805001	240808000	3000	1	2.20E-13	0.44	66	2.2	Nfkb1;LOC103691715	Transcription
DMR2:240813001	2	240813001	240816000	3000	2	6.60E-13	0.54	50	1.67	Nfkb1;LOC103691715	Transcription
DMR2:240827001	2	240827001	240832000	5000	1	9.80E-15	0.49	86	1.72	Nfkb1	Transcription
DMR2:240833001	2	240833001	240835000	2000	1	8.80E-08	0.29	19	0.95	Nfkb1	Transcription
DMR2:240852001	2	240852001	240853000	1000	1	4.90E-10	0.37	17	1.7	Nfkb1	Transcription
DMR2:240856001	2	240856001	240858000	2000	1	5.40E-07	0.34	26	1.3	Nfkb1	Transcription
DMR2:240865001	2	240865001	240866000	1000	1	1.80E-07	-0.51	20	2	Nfkb1	Transcription
DMR2:240872001	2	240872001	240875000	3000	1	2.50E-07	0.41	42	1.4	Nfkb1	Transcription
DMR2:240880001	2	240880001	240885000	5000	2	2.30E-13	0.52	75	1.5	Nfkb1	Transcription
DMR2:240894001	2	240894001	240896000	2000	1	8.30E-07	-0.52	25	1.25	Nfkb1	Transcription
DMR2:241036001	2	241036001	241037000	1000	1	2.80E-15	1.01	6	0.6	Slc39a8	Transport
DMR2:241043001	2	241043001	241044000	1000	1	2.20E-07	0.4	6	0.6	Slc39a8	Transport
DMR2:241048001	2	241048001	241050000	2000	2	5.20E-12	0.6	26	1.3	Slc39a8	Transport
DMR2:241073001	2	241073001	241074000	1000	1	7.60E-10	0.61	8	0.8	Slc39a8	Transport
DMR2:241083001	2	241083001	241086000	3000	1	1.60E-15	0.74	19	0.63	Slc39a8	Transport
DMR2:241279001	2	241279001	241280000	1000	1	2.80E-08	0.65	5	0.5	Bank1	
DMR2:241296001	2	241296001	241298000	2000	1	3.00E-13	0.59	13	0.65	Bank1	
DMR2:241301001	2	241301001	241302000	1000	1	2.40E-09	0.53	6	0.6	Bank1	
DMR2:241306001	2	241306001	241311000	5000	1	1.70E-07	-0.59	61	1.22	Bank1	
DMR2:241328001	2	241328001	241330000	2000	1	3.00E-07	0.48	13	0.65	Bank1	
DMR2:241335001	2	241335001	241338000	3000	2	4.70E-09	-0.57	11	0.37	Bank1	
DMR2:241348001	2	241348001	241349000	1000	1	2.60E-07	-0.4	7	0.7	Bank1	
DMR2:241354001	2	241354001	241355000	1000	1	2.70E-09	0.39	10	1	Bank1	
DMR2:241379001	2	241379001	241382000	3000	1	2.30E-12	0.62	24	0.8	Bank1	
DMR2:241393001	2	241393001	241395000	2000	1	1.70E-09	0.47	14	0.7	Bank1	
DMR2:241396001	2	241396001	241398000	2000	1	4.60E-07	-0.54	15	0.75	Bank1	
DMR2:241404001	2	241404001	241407000	3000	1	6.80E-07	0.51	25	0.83	Bank1	
DMR2:241467001	2	241467001	241469000	2000	1	9.10E-23	0.62	21	1.05	Bank1	
DMR2:241484001	2	241484001	241485000	1000	1	1.30E-10	0.47	14	1.4	Bank1	
DMR2:241506001	2	241506001	241508000	2000	2	4.20E-22	0.82	10	0.5	Bank1	
DMR2:241540001	2	241540001	241541000	1000	1	1.00E-08	0.48	8	0.8	Bank1	
DMR2:241913001	2	241913001	241922000	9000	1	4.60E-09	0.35	152	1.69	Ppp3ca	Signaling
DMR2:241923001	2	241923001	241927000	4000	1	3.30E-08	-0.55	47	1.18	Ppp3ca	Signaling
DMR2:241944001	2	241944001	241949000	5000	1	4.70E-09	0.61	51	1.02	Ppp3ca	Signaling
DMR2:241953001	2	241953001	241955000	2000	1	4.90E-13	0.65	10	0.5	Ppp3ca	Signaling
DMR2:241958001	2	241958001	241961000	3000	2	2.60E-14	-0.54	46	1.53	Ppp3ca	Signaling
DMR2:241963001	2	241963001	241966000	3000	1	7.10E-10	0.52	33	1.1	Ppp3ca	Signaling
DMR2:241974001	2	241974001	241983000	9000	2	5.90E-10	0.46	145	1.61	Ppp3ca	Signaling
DMR2:241984001	2	241984001	241986000	2000	1	8.10E-09	-0.53	29	1.45	Ppp3ca	Signaling
DMR2:242018001	2	242018001	242022000	4000	1	1.90E-14	0.61	41	1.02	Ppp3ca;LOC103691732	Signaling
DMR2:242055001	2	242055001	242059000	4000	1	3.00E-08	0.41	31	0.78	Ppp3ca	Signaling
DMR2:242062001	2	242062001	242064000	2000	1	2.60E-10	0.5	25	1.25	Ppp3ca	Signaling
DMR2:242075001	2	242075001	242083000	8000	1	7.10E-07	-0.41	99	1.24	Ppp3ca	Signaling
DMR2:242085001	2	242085001	242088000	3000	1	2.60E-07	-0.47	30	1	Ppp3ca	Signaling
DMR2:242102001	2	242102001	242103000	1000	1	1.50E-12	0.82	12	1.2	Ppp3ca	Signaling
DMR2:242141001	2	242141001	242142000	1000	1	2.60E-07	0.46	13	1.3	Ppp3ca	Signaling
DMR2:242144001	2	242144001	242145000	1000	1	8.20E-07	-0.55	8	0.8	Ppp3ca	Signaling
DMR2:242148001	2	242148001	242153000	5000	1	4.40E-10	-0.52	79	1.58	Ppp3ca	Signaling
DMR2:242170001	2	242170001	242177000	7000	2	1.90E-13	0.59	96	1.37	Ppp3ca	Signaling
DMR2:242179001	2	242179001	242187000	8000	1	2.60E-07	-0.42	143	1.79	Ppp3ca	Signaling
DMR2:242622001	2	242622001	242625000	3000	2	7.80E-22	0.97	15	0.5	Emcn	
DMR2:242627001	2	242627001	242628000	1000	1	2.60E-07	-0.42	18	1.8	Emcn	
DMR2:242636001	2	242636001	242637000	1000	1	7.70E-10	0.61	10	1	Emcn	
DMR2:242647001	2	242647001	242648000	1000	1	2.80E-09	-0.66	13	1.3	Emcn;LOC108350120	
DMR2:242653001	2	242653001	242656000	3000	2	5.60E-11	-0.71	33	1.1	Emcn;LOC108350120	
DMR2:242657001	2	242657001	242659000	2000	2	5.30E-16	0.63	31	1.55	Emcn;LOC108350120	
DMR2:242705001	2	242705001	242707000	2000	1	1.00E-16	0.65	26	1.3	Emcn;LOC108350120	
DMR2:242722001	2	242722001	242725000	3000	1	2.70E-10	0.45	34	1.13	Emcn;LOC108350120	
DMR2:242876001	2	242876001	242877000	1000	1	2.30E-08	0.43	13	1.3	Ddit4l	
DMR2:242890001	2	242890001	242893000	3000	1	1.20E-07	0.36	44	1.47	Ddit4l	
DMR2:243128001	2	243128001	243130000	2000	1	8.90E-10	-0.5	33	1.65	Dnajb14	
DMR2:243135001	2	243135001	243140000	5000	2	2.30E-07	-0.58	72	1.44	Dnajb14	

DMR2:243186001	2	243186001	243188000	2000	1	6.10E-07	0.37	22	1.1	Dapp1	
DMR2:243192001	2	243192001	243193000	1000	1	4.10E-09	0.47	10	1	Dapp1	
DMR2:243209001	2	243209001	243214000	5000	1	6.20E-13	0.67	88	1.76	Dapp1	
DMR2:243220001	2	243220001	243221000	1000	1	1.60E-12	0.62	11	1.1	Dapp1;LOC102555357	
DMR2:243225001	2	243225001	243227000	2000	1	2.60E-22	0.9	15	0.75	Dapp1;LOC102555357	
DMR2:243356001	2	243356001	243358000	2000	2	3.50E-32	0.76	24	1.2	LOC102555814;Mttp	Transport
DMR2:243361001	2	243361001	243363000	2000	1	4.20E-14	0.45	40	2	LOC102555814;Mttp	Transport
DMR2:243377001	2	243377001	243386000	9000	3	4.00E-10	0.63	128	1.42	Mttp	Transport
DMR2:243394001	2	243394001	243396000	2000	1	2.00E-07	-0.47	30	1.5	Mttp	Transport
DMR2:243404001	2	243404001	243410000	6000	2	1.70E-12	0.47	85	1.42	Mttp	Transport
DMR2:243415001	2	243415001	243416000	1000	1	5.40E-10	0.43	24	2.4	Mttp;Trmt10a	Transport;Epigenetic
DMR2:243435001	2	243435001	243439000	4000	1	1.50E-07	-0.6	46	1.15	Trmt10a;RGD1309170	Epigenetic
DMR2:243441001	2	243441001	243443000	2000	1	7.80E-10	0.39	21	1.05	Trmt10a;RGD1309170	Epigenetic
DMR2:243448001	2	243448001	243452000	4000	2	1.80E-09	0.57	44	1.1	RGD1309170	
DMR2:243466001	2	243466001	243467000	1000	1	4.10E-09	0.32	12	1.2	RGD1309170	
DMR2:243490001	2	243490001	243494000	4000	1	1.50E-09	0.41	69	1.73	LOC681022;Adh7	Metabolism
DMR2:243497001	2	243497001	243506000	9000	2	7.40E-10	-0.51	138	1.53	LOC681022;Adh7	Metabolism
DMR2:243508001	2	243508001	243511000	3000	1	7.50E-19	0.88	60	2	Adh7	Metabolism
DMR2:243525001	2	243525001	243527000	2000	1	1.60E-08	0.5	17	0.85	Adh7;LOC102556058	Metabolism
DMR2:243533001	2	243533001	243542000	9000	1	5.10E-12	0.45	165	1.83	LOC102556058;Adh1	Metabolism
DMR2:243547001	2	243547001	243549000	2000	1	5.60E-07	-0.4	27	1.35	Adh1	Metabolism
DMR2:243555001	2	243555001	243557000	2000	1	3.20E-07	0.26	34	1.7	Adh1	Metabolism
DMR2:243573001	2	243573001	243575000	2000	2	7.80E-22	0.79	20	1	LOC102556144;Adh6a	Metabolism
DMR2:243582001	2	243582001	243590000	8000	3	5.70E-24	-0.57	95	1.19	LOC102556144;Adh6a	Metabolism
DMR2:243671001	2	243671001	243675000	4000	2	1.30E-12	-0.68	16	0.4	Adh6	Metabolism
DMR2:243676001	2	243676001	243681000	5000	2	3.00E-17	0.86	45	0.9	Adh6	Metabolism
DMR2:243698001	2	243698001	243700000	2000	1	1.40E-12	0.69	26	1.3	Adh4;LOC102556299	Metabolism
DMR2:243703001	2	243703001	243704000	1000	1	2.50E-14	0.6	8	0.8	Adh4;LOC102556299	Metabolism
DMR2:243712001	2	243712001	243716000	4000	3	2.20E-18	-0.66	50	1.25	Adh4;LOC102556299	Metabolism
DMR2:243737001	2	243737001	243742000	5000	1	1.20E-11	0.36	83	1.66	Adh5;Metap1	Metabolism;Protease
DMR2:243753001	2	243753001	243759000	6000	2	2.80E-15	0.58	78	1.3	Metap1	Protease
DMR2:243762001	2	243762001	243764000	2000	1	1.30E-15	0.61	17	0.85	Metap1	Protease
DMR2:243779001	2	243779001	243780000	1000	1	4.90E-13	0.65	13	1.3	Metap1	Protease
DMR2:243784001	2	243784001	243786000	2000	1	1.60E-07	-0.55	42	2.1	Metap1	Protease
DMR2:243787001	2	243787001	243788000	1000	1	2.10E-07	0.4	12	1.2	Metap1	Protease
DMR2:243810001	2	243810001	243811000	1000	1	4.20E-07	-0.48	14	1.4	LOC102556448;Eif4e;Mir1956	Translation
DMR2:243828001	2	243828001	243830000	2000	1	1.60E-08	0.37	18	0.9	Eif4e;Mir1956	Translation
DMR2:244072001	2	244072001	244073000	1000	1	8.80E-07	-0.52	9	0.9	Tspan5	
DMR2:244082001	2	244082001	244083000	1000	1	1.20E-12	0.59	13	1.3	Tspan5	
DMR2:244084001	2	244084001	244086000	2000	1	8.20E-09	0.39	41	2.05	Tspan5	
DMR2:244095001	2	244095001	244097000	2000	2	3.60E-24	0.82	25	1.25	Tspan5	
DMR2:244098001	2	244098001	244103000	5000	1	7.50E-07	0.3	108	2.16	Tspan5	
DMR2:244105001	2	244105001	244109000	4000	2	2.80E-17	0.63	49	1.23	Tspan5	
DMR2:244122001	2	244122001	244123000	1000	1	3.50E-09	0.72	1	0.1	Tspan5	
DMR2:244135001	2	244135001	244138000	3000	2	2.20E-13	0.58	51	1.7	Tspan5	
DMR2:244160001	2	244160001	244164000	4000	1	1.80E-35	0.94	59	1.48	Tspan5	
DMR2:244170001	2	244170001	244172000	2000	1	2.60E-10	-0.36	33	1.65	Tspan5	
DMR2:244178001	2	244178001	244184000	6000	1	5.10E-07	0.26	110	1.83	Tspan5	
DMR2:244202001	2	244202001	244203000	1000	1	5.50E-10	0.41	12	1.2	Tspan5	
DMR2:244227001	2	244227001	244228000	1000	1	2.80E-07	-0.68	15	1.5	Tspan5	
DMR2:244229001	2	244229001	244232000	3000	2	1.10E-10	-0.58	29	0.97	Tspan5	
DMR2:244233001	2	244233001	244236000	3000	1	2.60E-21	0.9	36	1.2	Tspan5	
DMR2:244240001	2	244240001	244243000	3000	1	1.40E-07	-0.39	21	0.7	RGD1560590	
DMR2:244248001	2	244248001	244252000	4000	2	1.50E-07	-0.58	28	0.7	RGD1560590;Rap1gds1	
DMR2:244264001	2	244264001	244268000	4000	1	1.20E-07	-0.34	54	1.35	Rap1gds1	
DMR2:244269001	2	244269001	244274000	5000	3	1.90E-08	-0.53	73	1.46	Rap1gds1	
DMR2:244286001	2	244286001	244292000	6000	1	4.60E-09	-0.56	101	1.68	Rap1gds1	
DMR2:244293001	2	244293001	244294000	1000	1	6.90E-09	-0.57	7	0.7	Rap1gds1	
DMR2:244317001	2	244317001	244319000	2000	1	1.90E-08	0.48	16	0.8	Rap1gds1	
DMR2:244323001	2	244323001	244325000	2000	1	1.70E-10	0.45	27	1.35	Rap1gds1	
DMR2:244345001	2	244345001	244348000	3000	2	1.60E-08	0.49	44	1.47	Rap1gds1	
DMR2:244359001	2	244359001	244363000	4000	1	2.90E-08	0.29	42	1.05	Rap1gds1	
DMR2:244364001	2	244364001	244366000	2000	1	5.10E-08	0.38	29	1.45	Rap1gds1	
DMR2:244367001	2	244367001	244368000	1000	1	3.60E-14	0.76	5	0.5	Rap1gds1	

DMR2:244374001	2	244374001	244375000	1000	1	2.30E-14	0.7	3	0.3	Rap1gds1	
DMR2:244376001	2	244376001	244381000	5000	3	3.70E-14	-0.6	31	0.62	Rap1gds1	
DMR2:244513001	2	244513001	244515000	2000	1	4.10E-07	-0.45	10	0.5	Stpg2	Development
DMR2:244516001	2	244516001	244518000	2000	2	4.10E-11	0.58	15	0.75	Stpg2	Development
DMR2:244541001	2	244541001	244556000	15000	8	2.50E-27	-0.58	165	1.1	Stpg2	Development
DMR2:244567001	2	244567001	244571000	4000	2	4.00E-10	-0.71	19	0.48	Stpg2	Development
DMR2:244572001	2	244572001	244574000	2000	1	9.10E-07	0.35	19	0.95	Stpg2	Development
DMR2:244579001	2	244579001	244580000	1000	1	5.30E-07	0.55	4	0.4	Stpg2	Development
DMR2:244581001	2	244581001	244583000	2000	1	4.10E-13	0.74	10	0.5	Stpg2	Development
DMR2:244585001	2	244585001	244592000	7000	7	7.20E-21	0.73	73	1.04	Stpg2	Development
DMR2:244612001	2	244612001	244613000	1000	1	1.40E-25	0.93	10	1	Stpg2	Development
DMR2:244624001	2	244624001	244628000	4000	2	1.00E-14	-0.77	12	0.3	Stpg2	Development
DMR2:244629001	2	244629001	244631000	2000	1	4.70E-11	-0.56	9	0.45	Stpg2	Development
DMR2:244633001	2	244633001	244635000	2000	1	2.80E-13	0.76	18	0.9	Stpg2	Development
DMR2:244650001	2	244650001	244655000	5000	4	3.60E-11	-0.38	54	1.08	Stpg2	Development
DMR2:244659001	2	244659001	244666000	7000	4	9.30E-13	-0.61	45	0.64	Stpg2	Development
DMR2:244680001	2	244680001	244684000	4000	3	1.30E-18	-0.5	31	0.78	Stpg2	Development
DMR2:244687001	2	244687001	244691000	4000	1	2.10E-08	-0.33	56	1.4	Stpg2	Development
DMR2:244696001	2	244696001	244698000	2000	1	5.40E-22	0.72	11	0.55	Stpg2	Development
DMR2:244700001	2	244700001	244702000	2000	1	1.40E-13	0.65	21	1.05	Stpg2	Development
DMR2:244707001	2	244707001	244710000	3000	1	9.00E-12	0.61	20	0.67	Stpg2	Development
DMR2:244716001	2	244716001	244717000	1000	1	7.20E-07	0.5	2	0.2	Stpg2	Development
DMR2:244737001	2	244737001	244741000	4000	1	2.80E-13	-0.39	24	0.6	Stpg2	Development
DMR2:244749001	2	244749001	244750000	1000	1	3.00E-08	-0.54	8	0.8	Stpg2	Development
DMR2:244755001	2	244755001	244758000	3000	1	7.80E-09	-0.57	22	0.73	Stpg2	Development
DMR2:244773001	2	244773001	244775000	2000	2	5.00E-12	-0.35	27	1.35	Stpg2	Development
DMR2:244779001	2	244779001	244782000	3000	1	4.90E-09	0.51	19	0.63	Stpg2	Development
DMR2:244784001	2	244784001	244785000	1000	1	1.10E-09	0.44	6	0.6	Stpg2	Development
DMR2:244801001	2	244801001	244802000	1000	1	3.40E-13	0.57	9	0.9	Stpg2	Development
DMR2:244816001	2	244816001	244817000	1000	1	2.30E-14	0.69	6	0.6	Stpg2;LOC100910816	Development
DMR2:244824001	2	244824001	244830000	6000	3	5.20E-28	-0.58	61	1.02	Stpg2;LOC100910816	Development
DMR2:244831001	2	244831001	244833000	2000	2	8.20E-13	0.45	26	1.3	Stpg2;LOC100910816	Development
DMR2:244835001	2	244835001	244837000	2000	2	1.10E-21	0.91	10	0.5	Stpg2	Development
DMR2:244848001	2	244848001	244856000	8000	5	4.40E-21	-0.45	90	1.12	Stpg2	Development
DMR2:244857001	2	244857001	244860000	3000	1	2.30E-11	-0.63	24	0.8	Stpg2	Development
DMR2:244861001	2	244861001	244863000	2000	1	5.00E-08	0.43	19	0.95	Stpg2	Development
DMR2:244882001	2	244882001	244883000	1000	1	2.40E-07	0.46	2	0.2	Stpg2	Development
DMR2:244909001	2	244909001	244911000	2000	1	1.80E-14	0.91	8	0.4	Stpg2	Development
DMR2:244912001	2	244912001	244915000	3000	1	1.40E-14	-0.49	24	0.8	Stpg2	Development
DMR2:244916001	2	244916001	244917000	1000	1	1.70E-08	0.63	3	0.3	Stpg2	Development
DMR2:244926001	2	244926001	244932000	6000	5	4.80E-23	-0.52	66	1.1	Stpg2	Development
DMR2:244998001	2	244998001	245005000	7000	4	1.30E-20	-0.56	78	1.11	Stpg2	Development
DMR2:245008001	2	245008001	245009000	1000	1	1.40E-13	0.65	11	1.1	Stpg2	Development
DMR2:245016001	2	245016001	245021000	5000	2	4.80E-10	-0.77	59	1.18	Stpg2	Development
DMR2:245022001	2	245022001	245023000	1000	1	5.80E-12	-0.6	16	1.6	Stpg2	Development
DMR2:245042001	2	245042001	245043000	1000	1	1.20E-09	0.49	11	1.1	Stpg2	Development
DMR2:245045001	2	245045001	245046000	1000	1	2.60E-07	-0.41	13	1.3	Stpg2	Development
DMR2:246732001	2	246732001	246734000	2000	2	5.10E-18	0.84	10	0.5	LOC103691720;Pdha2	Metabolism
DMR2:246740001	2	246740001	246741000	1000	1	2.00E-08	0.56	7	0.7	LOC103691720;Pdha2	Metabolism
DMR2:247060001	2	247060001	247061000	1000	1	1.20E-07	0.43	14	1.4	Unc5c	Receptor
DMR2:247064001	2	247064001	247065000	1000	1	6.20E-07	0.48	9	0.9	Unc5c	Receptor
DMR2:247070001	2	247070001	247075000	5000	1	6.20E-10	-0.53	55	1.1	Unc5c	Receptor
DMR2:247157001	2	247157001	247159000	2000	1	4.60E-12	0.44	22	1.1	Unc5c	Receptor
DMR2:247198001	2	247198001	247199000	1000	1	1.20E-08	0.38	18	1.8	Unc5c	Receptor
DMR2:247213001	2	247213001	247216000	3000	2	4.90E-08	-0.62	25	0.83	Unc5c	Receptor
DMR2:247220001	2	247220001	247221000	1000	1	8.20E-09	0.56	6	0.6	Unc5c	Receptor
DMR2:247237001	2	247237001	247239000	2000	2	1.60E-16	0.63	12	0.6	Unc5c	Receptor
DMR2:247307001	2	247307001	247311000	4000	1	4.10E-12	0.49	40	1	Unc5c	Receptor
DMR2:247312001	2	247312001	247314000	2000	2	1.40E-14	0.63	12	0.6	Unc5c	Receptor
DMR2:247357001	2	247357001	247359000	2000	1	4.40E-10	0.71	18	0.9	Unc5c	Receptor
DMR2:247360001	2	247360001	247361000	1000	1	1.80E-15	0.6	14	1.4	Unc5c	Receptor
DMR2:247383001	2	247383001	247385000	2000	1	3.70E-08	0.55	10	0.5	Unc5c;Bmpr1b	Receptor;Signaling
DMR2:247401001	2	247401001	247405000	4000	1	6.90E-08	-0.66	49	1.23	Unc5c;Bmpr1b	Receptor;Signaling
DMR2:247437001	2	247437001	247438000	1000	1	4.80E-07	-0.54	11	1.1	Bmpr1b	Signaling
DMR2:247483001	2	247483001	247486000	3000	1	1.20E-07	-0.49	26	0.87	Bmpr1b	Signaling

DMR2:247495001	2	247495001	247501000	6000	1	9.50E-13	0.6	75	1.25	Bmpr1b	Signaling
DMR2:247511001	2	247511001	247514000	3000	1	3.40E-07	0.47	23	0.77	Bmpr1b;LOC108350217	Signaling
DMR2:247531001	2	247531001	247533000	2000	1	4.90E-07	-0.56	15	0.75	Bmpr1b;LOC108350217	Signaling
DMR2:247545001	2	247545001	247546000	1000	1	2.30E-14	0.88	3	0.3	Bmpr1b	Signaling
DMR2:247576001	2	247576001	247578000	2000	1	2.30E-11	-0.78	27	1.35	Bmpr1b	Signaling
DMR2:247586001	2	247586001	247587000	1000	1	1.90E-08	-0.72	16	1.6	Bmpr1b	Signaling
DMR2:247607001	2	247607001	247608000	1000	1	1.90E-09	0.45	8	0.8	Bmpr1b	Signaling
DMR2:247627001	2	247627001	247632000	5000	2	8.30E-12	-0.6	58	1.16	Bmpr1b	Signaling
DMR2:247645001	2	247645001	247648000	3000	2	1.60E-09	0.54	39	1.3	Bmpr1b	Signaling
DMR2:247658001	2	247658001	247660000	2000	1	1.90E-14	0.57	33	1.65	Bmpr1b	Signaling
DMR2:247839001	2	247839001	247840000	1000	1	3.40E-07	0.47	9	0.9	Pdlim5	Cytoskeleton
DMR2:247848001	2	247848001	247852000	4000	1	6.60E-12	0.56	48	1.2	Pdlim5	Cytoskeleton
DMR2:247861001	2	247861001	247864000	3000	1	3.40E-09	0.64	37	1.23	Pdlim5	Cytoskeleton
DMR2:247867001	2	247867001	247870000	3000	2	4.80E-15	0.91	18	0.6	Pdlim5	Cytoskeleton
DMR2:247886001	2	247886001	247887000	1000	1	1.10E-13	0.94	5	0.5	Pdlim5	Cytoskeleton
DMR2:247892001	2	247892001	247894000	2000	1	1.30E-11	0.47	20	1	Pdlim5	Cytoskeleton
DMR2:247899001	2	247899001	247900000	1000	1	5.60E-13	0.71	7	0.7	Pdlim5	Cytoskeleton
DMR2:247903001	2	247903001	247904000	1000	1	1.80E-11	0.88	5	0.5	Pdlim5	Cytoskeleton
DMR2:247905001	2	247905001	247906000	1000	1	7.70E-12	0.65	14	1.4	Pdlim5	Cytoskeleton
DMR2:247909001	2	247909001	247911000	2000	1	8.40E-07	-0.45	42	2.1	Pdlim5	Cytoskeleton
DMR2:247913001	2	247913001	247914000	1000	1	3.70E-10	-0.84	11	1.1	Pdlim5	Cytoskeleton
DMR2:247933001	2	247933001	247936000	3000	1	7.70E-20	0.88	26	0.87	Pdlim5	Cytoskeleton
DMR2:247957001	2	247957001	247959000	2000	1	1.00E-07	-0.55	23	1.15	Pdlim5	Cytoskeleton
DMR2:247969001	2	247969001	247972000	3000	2	6.40E-24	1	35	1.17	Pdlim5	Cytoskeleton
DMR2:247999001	2	247999001	248004000	5000	2	2.30E-15	-0.6	28	0.56	Pdlim5	Cytoskeleton
DMR2:248197001	2	248197001	248199000	2000	1	9.40E-09	0.4	16	0.8	Gbp5	Signaling
DMR2:248200001	2	248200001	248204000	4000	3	2.40E-11	-0.47	13	0.32	Gbp5	Signaling
DMR2:248242001	2	248242001	248246000	4000	1	4.30E-11	0.7	31	0.78	LOC685067;Gbp4	Signaling
DMR2:248282001	2	248282001	248284000	2000	1	2.00E-07	0.4	15	0.75	Gbp2	Signaling
DMR2:248344001	2	248344001	248345000	1000	1	4.70E-17	0.85	9	0.9	Gbp1	Signaling
DMR2:248385001	2	248385001	248387000	2000	1	2.50E-10	0.59	9	0.45	Gbp1	Signaling
DMR2:248389001	2	248389001	248390000	1000	1	5.50E-15	0.69	5	0.5	Gbp1	Signaling
DMR2:248396001	2	248396001	248398000	2000	1	7.00E-09	-0.64	14	0.7	Gbp1	Signaling
DMR2:248414001	2	248414001	248418000	4000	1	1.70E-07	-0.34	27	0.68	Gbp1	Signaling
DMR2:248428001	2	248428001	248431000	3000	2	1.40E-12	-1.11	20	0.67	Gbp1	Signaling
DMR2:248468001	2	248468001	248471000	3000	1	1.70E-08	-0.56	9	0.3	Gbp3;LOC102553780	Signaling
DMR2:248475001	2	248475001	248477000	2000	1	6.20E-07	0.43	15	0.75	Gbp3;LOC102553780	Signaling
DMR2:248499001	2	248499001	248500000	1000	1	1.20E-15	0.99	6	0.6	Gbp3	Signaling
DMR2:248528001	2	248528001	248529000	1000	1	9.20E-14	-0.54	7	0.7	RGD1560801	
DMR2:248547001	2	248547001	248548000	1000	1	9.30E-20	0.67	12	1.2	RGD1560801	
DMR2:248555001	2	248555001	248561000	6000	3	4.30E-13	-0.62	44	0.73	RGD1560801	
DMR2:248569001	2	248569001	248570000	1000	1	5.80E-09	-0.45	6	0.6	RGD1560801	
DMR2:248650001	2	248650001	248653000	3000	1	4.40E-22	0.64	36	1.2	Kyat3	Metabolism
DMR2:248670001	2	248670001	248671000	1000	1	3.50E-12	0.59	10	1	Kyat3	Metabolism
DMR2:248680001	2	248680001	248681000	1000	1	9.80E-11	-0.58	19	1.9	Kyat3	Metabolism
DMR2:248684001	2	248684001	248687000	3000	2	5.20E-14	0.59	51	1.7	Kyat3	Metabolism
DMR2:248688001	2	248688001	248692000	4000	1	2.40E-13	0.75	69	1.73	Kyat3	Metabolism
DMR2:248701001	2	248701001	248704000	3000	1	1.50E-16	0.63	42	1.4	LOC108350125;Gtf2b	Transcription
DMR2:248705001	2	248705001	248708000	3000	1	3.20E-09	0.52	31	1.03	LOC108350125;Gtf2b	Transcription
DMR2:248714001	2	248714001	248718000	4000	2	2.90E-08	0.46	52	1.3	LOC108350125;Gtf2b	Transcription
DMR2:248751001	2	248751001	248754000	3000	1	1.40E-10	0.41	35	1.17	Pkn2	Signaling
DMR2:248768001	2	248768001	248769000	1000	1	2.10E-08	0.47	7	0.7	Pkn2	Signaling
DMR2:248775001	2	248775001	248778000	3000	1	4.70E-11	0.77	24	0.8	Pkn2	Signaling
DMR2:248780001	2	248780001	248782000	2000	1	1.70E-07	-0.53	19	0.95	Pkn2	Signaling
DMR2:248804001	2	248804001	248806000	2000	2	2.10E-17	-0.55	30	1.5	Pkn2	Signaling
DMR2:248845001	2	248845001	248846000	1000	1	6.40E-07	0.38	8	0.8	Pkn2	Signaling
DMR2:250206001	2	250206001	250212000	6000	2	3.10E-15	0.89	74	1.23	Lmo4	
DMR2:250214001	2	250214001	250217000	3000	1	8.20E-07	-0.44	54	1.8	Lmo4	
DMR2:250462001	2	250462001	250466000	4000	1	1.10E-09	-0.57	77	1.93	LOC102549483;Hs2st1	Transport
DMR2:250467001	2	250467001	250471000	4000	1	6.60E-07	-0.45	81	2.02	LOC102549483;Hs2st1	Transport
DMR2:250483001	2	250483001	250485000	2000	1	2.10E-07	0.32	31	1.55	Hs2st1	Transport
DMR2:250491001	2	250491001	250495000	4000	1	3.20E-08	0.3	72	1.8	Hs2st1	Transport
DMR2:250496001	2	250496001	250500000	4000	1	4.30E-10	-0.67	79	1.98	Hs2st1	Transport
DMR2:250502001	2	250502001	250505000	3000	1	1.90E-07	-0.57	27	0.9	Hs2st1	Transport
DMR2:250524001	2	250524001	250529000	5000	2	2.00E-15	0.47	66	1.32	Hs2st1	Transport

DMR2:250568001	2	250568001	250573000	5000	1	6.30E-12	0.46	67	1.34	Hs2st1	Transport
DMR2:250574001	2	250574001	250576000	2000	1	1.40E-07	0.34	24	1.2	Hs2st1	Transport
DMR2:250626001	2	250626001	250627000	1000	1	1.20E-13	-0.61	2	0.2	15-Sep	
DMR2:250628001	2	250628001	250630000	2000	1	2.60E-07	-0.51	9	0.45	15-Sep	
DMR2:250642001	2	250642001	250645000	3000	2	3.50E-08	0.39	34	1.13	15-Sep	
DMR2:250711001	2	250711001	250714000	3000	1	4.70E-15	0.44	39	1.3	Sh3glb1	
DMR2:250746001	2	250746001	250747000	1000	1	4.30E-20	0.65	17	1.7	Sh3glb1;Clca2	Transport
DMR2:250751001	2	250751001	250755000	4000	2	9.30E-08	-0.43	21	0.52	Sh3glb1;Clca2	Transport
DMR2:250768001	2	250768001	250769000	1000	1	8.80E-09	0.46	8	0.8	Clca2	Transport
DMR2:250793001	2	250793001	250801000	8000	4	1.90E-18	0.81	125	1.56	Clca4l;LOC108350129;LOC102549543	Transport
DMR2:250810001	2	250810001	250812000	2000	1	6.30E-07	0.35	21	1.05	Clca4l;LOC102549543	Transport
DMR2:250813001	2	250813001	250817000	4000	1	3.60E-07	-0.36	20	0.5	Clca4l;LOC102549543	Transport
DMR2:250835001	2	250835001	250842000	7000	4	8.50E-18	-0.61	53	0.76	Clca4	Transport
DMR2:250870001	2	250870001	250872000	2000	2	5.10E-17	0.59	26	1.3	Clca4	Transport
DMR2:250874001	2	250874001	250881000	7000	2	3.60E-09	-0.54	87	1.24	Clca4	Transport
DMR2:250890001	2	250890001	250895000	5000	1	4.50E-25	0.72	50	1	Clca1	Transport
DMR2:250920001	2	250920001	250922000	2000	1	8.60E-07	0.38	14	0.7	Clca1;LOC108350130	Transport
DMR2:250972001	2	250972001	250977000	5000	1	3.30E-07	-0.55	75	1.5	Clca5;LOC103691724	Transport
DMR2:251013001	2	251013001	251016000	3000	3	2.30E-11	0.43	24	0.8	Odf2l	
DMR2:251023001	2	251023001	251025000	2000	1	4.60E-07	0.32	37	1.85	Odf2l	
DMR2:251041001	2	251041001	251045000	4000	1	3.50E-07	-0.35	20	0.5	Odf2l	
DMR2:251188001	2	251188001	251193000	5000	1	7.40E-07	0.31	69	1.38	LOC102549633;Col24a1	Extracellular Matrix
DMR2:251202001	2	251202001	251206000	4000	1	8.90E-07	-0.46	38	0.95	Col24a1	Extracellular Matrix
DMR2:251215001	2	251215001	251216000	1000	1	2.70E-10	0.42	12	1.2	Col24a1	Extracellular Matrix
DMR2:251226001	2	251226001	251229000	3000	1	8.50E-08	-0.6	43	1.43	Col24a1	Extracellular Matrix
DMR2:251232001	2	251232001	251233000	1000	1	4.30E-14	0.77	9	0.9	Col24a1	Extracellular Matrix
DMR2:251235001	2	251235001	251237000	2000	1	3.00E-07	-0.49	29	1.45	Col24a1	Extracellular Matrix
DMR2:251255001	2	251255001	251257000	2000	1	1.80E-07	-0.46	23	1.15	Col24a1	Extracellular Matrix
DMR2:251258001	2	251258001	251261000	3000	2	1.60E-16	0.67	47	1.57	Col24a1	Extracellular Matrix
DMR2:251274001	2	251274001	251275000	1000	1	5.80E-11	0.59	1	0.1	Col24a1	Extracellular Matrix
DMR2:251282001	2	251282001	251286000	4000	3	3.20E-14	-0.65	57	1.43	Col24a1	Extracellular Matrix
DMR2:251292001	2	251292001	251295000	3000	2	2.50E-15	0.8	30	1	Col24a1	Extracellular Matrix
DMR2:251311001	2	251311001	251313000	2000	1	7.90E-08	0.31	17	0.85	Col24a1	Extracellular Matrix
DMR2:251314001	2	251314001	251315000	1000	1	2.90E-07	0.38	7	0.7	Col24a1	Extracellular Matrix
DMR2:251327001	2	251327001	251335000	8000	1	9.10E-09	-0.64	136	1.7	Col24a1	Extracellular Matrix
DMR2:251349001	2	251349001	251353000	4000	1	3.30E-11	0.64	29	0.72	Col24a1;LOC108350131	Extracellular Matrix
DMR2:251361001	2	251361001	251362000	1000	1	3.40E-08	0.33	10	1	Col24a1	Extracellular Matrix
DMR2:251373001	2	251373001	251374000	1000	1	1.20E-07	-0.63	14	1.4	Col24a1;LOC103691726	Extracellular Matrix
DMR2:251397001	2	251397001	251399000	2000	2	1.60E-09	0.5	23	1.15	Col24a1	Extracellular Matrix
DMR2:251400001	2	251400001	251406000	6000	1	4.00E-10	0.42	100	1.67	Col24a1	Extracellular Matrix
DMR2:251407001	2	251407001	251412000	5000	3	8.10E-11	-0.64	24	0.48	Col24a1	Extracellular Matrix
DMR2:251428001	2	251428001	251433000	5000	1	7.60E-09	0.43	72	1.44	Col24a1	Extracellular Matrix
DMR2:251434001	2	251434001	251435000	1000	1	6.20E-15	0.69	9	0.9	Col24a1	Extracellular Matrix
DMR2:251448001	2	251448001	251452000	4000	1	5.60E-15	0.52	39	0.98	Znhit6	
DMR2:251453001	2	251453001	251455000	2000	2	1.20E-14	0.69	71	3.55	Znhit6	
DMR2:251456001	2	251456001	251457000	1000	1	1.50E-08	0.37	18	1.8	Znhit6	
DMR2:251461001	2	251461001	251462000	1000	1	9.20E-15	0.63	10	1	Znhit6	
DMR2:251489001	2	251489001	251490000	1000	1	2.30E-09	0.56	7	0.7	Znhit6	
DMR2:251494001	2	251494001	251498000	4000	1	5.90E-09	-0.57	64	1.6	Znhit6	
DMR2:251537001	2	251537001	251539000	2000	1	4.20E-07	-0.54	27	1.35	Cyr61;LOC102553018	
DMR2:251541001	2	251541001	251548000	7000	2	9.30E-19	0.6	86	1.23	Cyr61;LOC102553018	
DMR2:251624001	2	251624001	251625000	1000	1	9.90E-13	0.54	19	1.9	Ddah1	Metabolism
DMR2:251628001	2	251628001	251631000	3000	1	1.20E-08	0.34	35	1.17	Ddah1	Metabolism
DMR2:251638001	2	251638001	251643000	5000	2	7.20E-13	-0.52	92	1.84	Ddah1	Metabolism
DMR2:251648001	2	251648001	251650000	2000	1	2.40E-11	-0.72	25	1.25	Ddah1;LOC108350132	Metabolism
DMR2:251652001	2	251652001	251654000	2000	2	2.90E-15	0.52	40	2	Ddah1;LOC108350132	Metabolism
DMR2:251657001	2	251657001	251660000	3000	1	8.40E-09	0.35	59	1.97	Ddah1;LOC108350132	Metabolism
DMR2:251676001	2	251676001	251677000	1000	1	1.90E-09	0.44	21	2.1	Ddah1	Metabolism
DMR2:251684001	2	251684001	251688000	4000	1	7.80E-17	0.59	42	1.05	Ddah1	Metabolism
DMR2:251689001	2	251689001	251693000	4000	1	7.00E-07	-0.48	72	1.8	Ddah1	Metabolism
DMR2:251708001	2	251708001	251709000	1000	1	2.90E-11	0.47	22	2.2	Ddah1	Metabolism
DMR2:251710001	2	251710001	251711000	1000	1	3.40E-12	-0.64	23	2.3	Ddah1	Metabolism
DMR2:251720001	2	251720001	251723000	3000	1	1.30E-11	0.39	35	1.17	Ddah1;LOC103691727	Metabolism
DMR2:251727001	2	251727001	251730000	3000	1	3.20E-11	0.51	44	1.47	Ddah1;LOC103691727	Metabolism

DMR2:251731001	2	251731001	251736000	5000	1	2.40E-09	-0.57	67	1.34	Ddah1;LOC103691727	Metabolism
DMR2:251738001	2	251738001	251741000	3000	1	4.50E-11	0.57	38	1.27	Ddah1;LOC103691727	Metabolism
DMR2:251742001	2	251742001	251744000	2000	1	1.40E-16	0.51	28	1.4	Ddah1	Metabolism
DMR2:251750001	2	251750001	251756000	6000	1	3.90E-07	0.48	97	1.62	Ddah1	Metabolism
DMR2:251819001	2	251819001	251825000	6000	1	1.60E-09	-0.49	92	1.53	Bcl10;RGD1560065;LOC102553118	
DMR2:251826001	2	251826001	251828000	2000	1	8.10E-19	0.77	18	0.9	RGD1560065;LOC102553118	
DMR2:251854001	2	251854001	251857000	3000	1	3.10E-09	-0.44	31	1.03	Syde2	Signaling
DMR2:251879001	2	251879001	251880000	1000	1	3.50E-07	0.21	17	1.7	Syde2	Signaling
DMR2:251907001	2	251907001	251910000	3000	1	4.60E-13	0.64	48	1.6	Wdr63	Cytoskeleton
DMR2:251914001	2	251914001	251919000	5000	4	8.00E-19	0.57	87	1.74	Wdr63	Cytoskeleton
DMR2:251930001	2	251930001	251932000	2000	1	9.10E-07	-0.62	29	1.45	Wdr63;Mcoln3	Cytoskeleton;Transport
DMR2:251934001	2	251934001	251935000	1000	1	4.20E-10	-0.56	17	1.7	Wdr63;Mcoln3	Cytoskeleton;Transport
DMR2:251936001	2	251936001	251938000	2000	1	2.40E-14	0.52	25	1.25	Wdr63;Mcoln3	Cytoskeleton;Transport
DMR2:251944001	2	251944001	251945000	1000	1	2.80E-07	-0.35	27	2.7	Wdr63;Mcoln3	Cytoskeleton;Transport
DMR2:251955001	2	251955001	251956000	1000	1	1.00E-10	0.44	15	1.5	Wdr63;Mcoln3	Cytoskeleton;Transport
DMR2:251972001	2	251972001	251974000	2000	1	8.40E-08	0.35	22	1.1	Wdr63;Mcoln3	Cytoskeleton;Transport
DMR2:251991001	2	251991001	251992000	1000	1	1.30E-14	0.65	7	0.7	Mcoln3	Transport
DMR2:251993001	2	251993001	252001000	8000	4	1.20E-14	0.62	115	1.44	Mcoln3	Transport
DMR2:252003001	2	252003001	252009000	6000	3	7.80E-19	0.82	97	1.62	Mcoln3;Mcoln2	Transport
DMR2:252027001	2	252027001	252031000	4000	1	7.30E-22	1.08	44	1.1	Mcoln2	Transport
DMR2:252056001	2	252056001	252057000	1000	1	8.90E-07	0.5	38	3.8	Mcoln2	Transport
DMR2:252074001	2	252074001	252078000	4000	1	8.20E-09	0.46	36	0.9	Mcoln2	Transport
DMR2:252081001	2	252081001	252084000	3000	1	4.80E-07	0.47	26	0.87	Lpar3	Signaling
DMR2:252109001	2	252109001	252110000	1000	1	2.10E-08	0.32	17	1.7	Lpar3	Signaling
DMR2:252129001	2	252129001	252136000	7000	1	4.10E-09	0.35	102	1.46	Lpar3	Signaling
DMR2:252154001	2	252154001	252157000	3000	2	3.40E-13	0.48	39	1.3	Lpar3	Signaling
DMR2:252159001	2	252159001	252165000	6000	1	2.50E-08	-0.52	82	1.37	Lpar3	Signaling
DMR2:252167001	2	252167001	252169000	2000	2	2.90E-17	1.02	46	2.3	Lpar3	Signaling
DMR2:252254001	2	252254001	252257000	3000	1	6.10E-09	0.51	47	1.57	Ssx2ip	
DMR2:252281001	2	252281001	252285000	4000	2	6.90E-32	0.97	79	1.98	Ssx2ip	
DMR2:252340001	2	252340001	252342000	2000	1	2.90E-07	0.33	20	1	Spata1	
DMR2:252343001	2	252343001	252350000	7000	4	9.50E-21	-0.59	58	0.83	Spata1;Gng5	Signaling
DMR2:252352001	2	252352001	252353000	1000	1	3.60E-14	0.61	12	1.2	Spata1;Gng5	Signaling
DMR2:252361001	2	252361001	252362000	1000	1	7.70E-16	0.55	9	0.9	Spata1;Gng5;Rpf1	Signaling;Translation
DMR2:252369001	2	252369001	252370000	1000	1	1.20E-07	-0.57	14	1.4	Spata1;Gng5;Rpf1	Signaling;Translation
DMR2:252378001	2	252378001	252381000	3000	1	3.70E-07	-0.56	48	1.6	Gng5;Rpf1	Signaling;Translation
DMR2:252389001	2	252389001	252390000	1000	1	9.60E-09	-0.73	16	1.6	Rpf1	Translation
DMR2:252434001	2	252434001	252435000	1000	1	1.10E-12	0.73	5	0.5	Dnase2b	Transcription
DMR2:252436001	2	252436001	252440000	4000	3	2.90E-19	0.65	65	1.62	Dnase2b	Transcription
DMR2:252455001	2	252455001	252459000	4000	3	8.30E-25	1.05	78	1.95	Dnase2b;Uox	Transcription
DMR2:252471001	2	252471001	252474000	3000	1	9.20E-15	0.63	41	1.37	Dnase2b;Uox	Transcription
DMR2:252488001	2	252488001	252491000	3000	1	4.30E-10	0.41	34	1.13	Uox;LOC56764	
DMR2:252609001	2	252609001	252610000	1000	1	2.90E-09	0.52	24	2.4	Prkacb	Signaling
DMR2:252624001	2	252624001	252627000	3000	3	5.00E-11	-0.68	49	1.63	Prkacb	Signaling
DMR2:252629001	2	252629001	252631000	2000	1	2.60E-15	0.81	19	0.95	Prkacb	Signaling
DMR2:252644001	2	252644001	252645000	1000	1	9.00E-17	0.5	13	1.3	Prkacb	Signaling
DMR2:252660001	2	252660001	252661000	1000	1	5.90E-25	0.72	13	1.3	Prkacb	Signaling
DMR2:252664001	2	252664001	252669000	5000	2	1.60E-20	0.85	42	0.84	Prkacb	Signaling
DMR2:252698001	2	252698001	252700000	2000	1	6.20E-14	0.84	15	0.75	Prkacb	Signaling
DMR2:252780001	2	252780001	252785000	5000	1	4.10E-15	0.46	61	1.22	LOC108350133;Ttll7	Cytoskeleton
DMR2:252786001	2	252786001	252787000	1000	1	3.20E-11	0.47	21	2.1	Ttll7	Cytoskeleton
DMR2:252788001	2	252788001	252791000	3000	1	4.30E-08	0.31	78	2.6	Ttll7	Cytoskeleton
DMR2:252797001	2	252797001	252799000	2000	2	6.50E-14	0.62	30	1.5	Ttll7	Cytoskeleton
DMR2:252802001	2	252802001	252808000	6000	2	1.40E-09	0.33	105	1.75	Ttll7	Cytoskeleton
DMR2:252810001	2	252810001	252813000	3000	1	4.90E-08	-0.59	56	1.87	Ttll7	Cytoskeleton
DMR2:252814001	2	252814001	252816000	2000	1	3.20E-15	0.78	25	1.25	Ttll7	Cytoskeleton
DMR2:252824001	2	252824001	252827000	3000	2	2.80E-13	0.56	43	1.43	Ttll7	Cytoskeleton
DMR2:252830001	2	252830001	252841000	11000	6	3.80E-20	0.71	99	0.9	Ttll7	Cytoskeleton

DMR2:252843001	2	252843001	252849000	6000	1	1.40E-11	-0.56	124	2.07	Ttll7	Cytoskeleton
DMR2:255929001	2	255929001	255932000	3000	1	2.60E-09	-0.54	7	0.23	RGD1566247	
DMR2:255934001	2	255934001	255936000	2000	2	5.70E-25	1.04	4	0.2	RGD1566247	
DMR2:256156001	2	256156001	256157000	1000	1	2.00E-07	0.55	5	0.5	Calm-ps1	
DMR2:256645001	2	256645001	256649000	4000	1	2.10E-08	0.39	36	0.9	Adgrl4	Signaling
DMR2:256688001	2	256688001	256695000	7000	2	1.10E-11	0.43	85	1.21	Adgrl4	Signaling
DMR2:256705001	2	256705001	256706000	1000	1	3.50E-20	1.08	4	0.4	Adgrl4	Signaling
DMR2:256709001	2	256709001	256710000	1000	1	7.80E-07	-0.48	14	1.4	Adgrl4	Signaling
DMR2:256890001	2	256890001	256895000	5000	1	1.60E-17	-0.65	29	0.58	Ifi44	
DMR2:256902001	2	256902001	256904000	2000	2	2.20E-11	0.51	14	0.7	Ifi44	
DMR2:256909001	2	256909001	256911000	2000	1	5.80E-07	0.49	18	0.9	Ifi44	
DMR2:256949001	2	256949001	256950000	1000	1	3.40E-10	0.41	16	1.6	Ifi44l	
DMR2:256968001	2	256968001	256975000	7000	1	2.60E-08	0.43	43	0.61	Ifi44l	
DMR2:256982001	2	256982001	256985000	3000	1	5.40E-19	0.9	42	1.4	Ifi44l	
DMR2:257005001	2	257005001	257007000	2000	1	8.40E-17	0.79	20	1	Ptgfr	Signaling
DMR2:257288001	2	257288001	257289000	1000	1	1.30E-08	-0.58	28	2.8	Gipc2	Cytoskeleton
DMR2:257297001	2	257297001	257300000	3000	1	1.20E-12	0.48	41	1.37	Gipc2;LOC108350142	Cytoskeleton
DMR2:257312001	2	257312001	257315000	3000	1	1.80E-07	-0.69	41	1.37	Gipc2;LOC108350142	Cytoskeleton
DMR2:257318001	2	257318001	257323000	5000	2	1.20E-10	-0.52	69	1.38	Gipc2;LOC108350142	Cytoskeleton
DMR2:257324001	2	257324001	257330000	6000	3	2.90E-14	0.6	49	0.82	Gipc2	Cytoskeleton
DMR2:257343001	2	257343001	257348000	5000	1	1.10E-07	0.3	64	1.28	Gipc2	Cytoskeleton
DMR2:257362001	2	257362001	257368000	6000	1	9.90E-15	0.69	84	1.4	Gipc2	Cytoskeleton
DMR2:257371001	2	257371001	257376000	5000	2	6.30E-20	0.71	80	1.6	Gipc2	Cytoskeleton
DMR2:257388001	2	257388001	257389000	1000	1	1.10E-07	0.51	19	1.9	LOC108350143;Dnajb4	Transcription
DMR2:257418001	2	257418001	257419000	1000	1	1.80E-13	0.6	12	1.2	Dnajb4;Fubp1	Transcription;Metabolism
DMR2:257464001	2	257464001	257466000	2000	1	4.50E-11	0.55	18	0.9	Nexn;LOC102548862	Cytoskeleton
DMR2:257467001	2	257467001	257469000	2000	1	6.50E-07	-0.49	27	1.35	Nexn;LOC102548862	Cytoskeleton
DMR2:257471001	2	257471001	257473000	2000	1	5.60E-10	0.44	29	1.45	Nexn;LOC102548862	Cytoskeleton
DMR2:257486001	2	257486001	257489000	3000	1	5.20E-07	-0.52	64	2.13	Nexn;Miga1	Cytoskeleton
DMR2:257505001	2	257505001	257506000	1000	1	1.80E-08	-0.7	13	1.3	Miga1	
DMR2:257525001	2	257525001	257526000	1000	1	6.70E-07	-0.36	19	1.9	Miga1	
DMR2:257544001	2	257544001	257547000	3000	1	8.60E-07	-0.57	39	1.3	Miga1;LOC108350144	
DMR2:257562001	2	257562001	257563000	1000	1	5.00E-18	0.73	7	0.7	LOC108350144;Usp33	Protease
DMR2:257573001	2	257573001	257574000	1000	1	1.50E-08	-0.62	9	0.9	Usp33	Protease
DMR2:257670001	2	257670001	257673000	3000	1	2.40E-07	-0.49	32	1.07	Zzz3;LOC365985	Epigenetic
DMR2:257674001	2	257674001	257678000	4000	1	4.50E-10	-0.5	70	1.75	Zzz3;LOC365985	Epigenetic
DMR2:257919001	2	257919001	257921000	2000	1	2.50E-07	-0.53	22	1.1	Pigk	
DMR2:257966001	2	257966001	257968000	2000	1	1.40E-07	0.46	21	1.05	Pigk	
DMR2:257992001	2	257992001	2.58E+08	8000	2	1.40E-08	-0.49	106	1.32	Pigk	
DMR2:258028001	2	258028001	258029000	1000	1	7.80E-07	-0.52	10	1	St6galnac5	
DMR2:258052001	2	258052001	258057000	5000	1	4.90E-10	-0.55	87	1.74	St6galnac5	
DMR2:258120001	2	258120001	258128000	8000	4	1.40E-13	0.62	92	1.15	St6galnac5	
DMR2:258129001	2	258129001	258131000	2000	1	2.30E-08	0.5	26	1.3	St6galnac5	
DMR2:258136001	2	258136001	258138000	2000	1	3.20E-16	0.7	32	1.6	St6galnac5	
DMR2:258149001	2	258149001	258151000	2000	1	3.00E-17	0.63	15	0.75	St6galnac5;LOC100361005	
DMR2:258167001	2	258167001	258168000	1000	1	7.90E-18	0.87	4	0.4	St6galnac5;LOC100361005	
DMR2:258176001	2	258176001	258177000	1000	1	8.70E-07	-0.44	9	0.9	St6galnac5	
DMR2:258185001	2	258185001	258190000	5000	1	2.30E-11	0.52	70	1.4	St6galnac5	
DMR2:258191001	2	258191001	258195000	4000	2	8.90E-14	0.48	48	1.2	St6galnac5	
DMR2:258204001	2	258204001	258205000	1000	1	2.00E-07	-0.48	11	1.1	St6galnac5	
DMR2:258217001	2	258217001	258218000	1000	1	9.80E-16	0.93	6	0.6	St6galnac5	
DMR2:258233001	2	258233001	258235000	2000	1	2.00E-07	-0.54	20	1	St6galnac5;LOC108350145	
DMR2:258281001	2	258281001	258282000	1000	1	3.20E-15	0.86	4	0.4	St6galnac5	
DMR2:258297001	2	258297001	258300000	3000	1	2.60E-12	-0.79	40	1.33	St6galnac5	
DMR2:258320001	2	258320001	258326000	6000	1	7.30E-09	-0.55	71	1.18	St6galnac5	
DMR2:258337001	2	258337001	258340000	3000	1	1.00E-08	0.34	35	1.17	St6galnac5	
DMR2:258369001	2	258369001	258371000	2000	1	4.50E-07	0.34	12	0.6	St6galnac5	
DMR2:258439001	2	258439001	258440000	1000	1	4.50E-16	0.65	7	0.7	St6galnac5	
DMR2:258460001	2	258460001	258463000	3000	1	1.40E-07	-0.38	40	1.33	St6galnac5	
DMR2:258464001	2	258464001	258465000	1000	1	3.90E-07	0.55	5	0.5	St6galnac5	
DMR2:258474001	2	258474001	258480000	6000	1	1.00E-13	0.64	73	1.22	St6galnac5	
DMR2:258499001	2	258499001	258503000	4000	2	7.00E-21	0.97	27	0.68	St6galnac5	
DMR2:258514001	2	258514001	258515000	1000	1	2.40E-12	0.6	8	0.8	St6galnac5	
DMR2:258527001	2	258527001	258529000	2000	1	1.60E-07	-0.51	22	1.1	St6galnac5	

DMR2:258534001	2	258534001	258537000	3000	1	1.00E-07	0.37	22	0.73	St6galnac5	
DMR2:258540001	2	258540001	258544000	4000	2	1.60E-18	-0.58	51	1.27	St6galnac5	
DMR2:258560001	2	258560001	258562000	2000	1	6.50E-15	0.46	21	1.05	St6galnac5	
DMR2:258591001	2	258591001	258592000	1000	1	4.30E-10	0.67	5	0.5	St6galnac5	
DMR2:258599001	2	258599001	258600000	1000	1	4.90E-11	0.57	5	0.5	St6galnac5	
DMR2:258602001	2	258602001	258604000	2000	2	1.20E-15	0.57	25	1.25	St6galnac5	
DMR2:258614001	2	258614001	258615000	1000	1	1.60E-08	-0.68	12	1.2	St6galnac5	
DMR2:258623001	2	258623001	258625000	2000	1	6.70E-08	-0.53	26	1.3	St6galnac5	
DMR2:258634001	2	258634001	258637000	3000	3	2.40E-17	-0.47	47	1.57	St6galnac5	
DMR2:258640001	2	258640001	258641000	1000	1	1.30E-12	0.59	19	1.9	St6galnac5	
DMR2:258642001	2	258642001	258644000	2000	1	2.90E-08	-0.42	36	1.8	St6galnac5	
DMR2:258659001	2	258659001	258661000	2000	1	3.30E-08	0.42	27	1.35	St6galnac5	
DMR2:258671001	2	258671001	258674000	3000	1	4.90E-10	0.39	50	1.67	St6galnac5	
DMR2:258675001	2	258675001	258676000	1000	1	2.50E-08	-0.7	15	1.5	St6galnac5	
DMR2:258677001	2	258677001	258684000	7000	2	1.10E-09	-0.56	90	1.29	St6galnac5	
DMR2:258685001	2	258685001	258687000	2000	1	7.60E-15	0.56	40	2	St6galnac5	
DMR2:258691001	2	258691001	258696000	5000	4	5.50E-15	0.87	75	1.5	St6galnac5	
DMR2:258698001	2	258698001	258700000	2000	1	4.30E-13	0.82	27	1.35	St6galnac5	
DMR2:258705001	2	258705001	258706000	1000	1	5.30E-15	0.71	9	0.9	St6galnac5	
DMR2:258709001	2	258709001	258710000	1000	1	1.50E-20	0.72	5	0.5	St6galnac5	
DMR2:258715001	2	258715001	258717000	2000	2	6.90E-22	0.99	18	0.9	St6galnac5	
DMR2:258735001	2	258735001	258738000	3000	2	1.50E-12	-0.79	28	0.93	St6galnac5	
DMR2:258739001	2	258739001	258740000	1000	1	8.70E-18	0.78	6	0.6	St6galnac5	
DMR2:258742001	2	258742001	258745000	3000	1	6.30E-17	0.76	28	0.93	St6galnac5	
DMR2:258749001	2	258749001	258754000	5000	1	1.30E-11	0.53	64	1.28	St6galnac5	
DMR2:258755001	2	258755001	258756000	1000	1	4.20E-07	-0.53	9	0.9	St6galnac5	
DMR2:258767001	2	258767001	258773000	6000	2	3.10E-12	0.51	90	1.5	St6galnac5	
DMR2:258775001	2	258775001	258778000	3000	1	8.50E-07	0.4	39	1.3	St6galnac5	
DMR2:258782001	2	258782001	258784000	2000	1	4.10E-07	-0.56	30	1.5	St6galnac5;Adgrl2	Signaling
DMR2:258787001	2	258787001	258789000	2000	2	8.80E-14	0.54	26	1.3	St6galnac5;Adgrl2	Signaling
DMR2:258803001	2	258803001	258804000	1000	1	2.20E-07	-0.37	24	2.4	St6galnac5;Adgrl2	Signaling
DMR2:258811001	2	258811001	258814000	3000	1	1.60E-07	-0.49	60	2	St6galnac5;Adgrl2	Signaling
DMR2:258817001	2	258817001	258819000	2000	1	2.10E-09	0.36	41	2.05	St6galnac5;Adgrl2	Signaling
DMR2:258824001	2	258824001	258833000	9000	2	3.90E-13	-0.55	172	1.91	St6galnac5;Adgrl2	Signaling
DMR2:258865001	2	258865001	258871000	6000	1	4.10E-08	-0.56	118	1.97	St6galnac5;Adgrl2	Signaling
DMR2:258906001	2	258906001	258911000	5000	1	1.80E-29	0.93	65	1.3	St6galnac5;Adgrl2	Signaling
DMR2:258915001	2	258915001	258918000	3000	1	1.30E-15	0.54	31	1.03	St6galnac5;Adgrl2	Signaling
DMR2:258929001	2	258929001	258932000	3000	1	3.40E-10	-0.55	59	1.97	St6galnac5;Adgrl2	Signaling
DMR2:258933001	2	258933001	258934000	1000	1	3.50E-08	-0.45	15	1.5	St6galnac5;Adgrl2	Signaling
DMR2:258944001	2	258944001	258945000	1000	1	7.00E-07	0.29	17	1.7	St6galnac5;Adgrl2	Signaling
DMR2:258946001	2	258946001	258949000	3000	1	4.20E-15	0.66	59	1.97	St6galnac5;Adgrl2	Signaling
DMR2:258960001	2	258960001	258962000	2000	1	3.00E-07	-0.66	25	1.25	St6galnac5;Adgrl2;LOC103690134	Signaling
DMR2:258980001	2	258980001	258986000	6000	3	4.10E-20	0.74	95	1.58	St6galnac5;Adgrl2;LOC103690134	Signaling
DMR2:258999001	2	258999001	259007000	8000	2	1.10E-22	0.73	149	1.86	St6galnac5;Adgrl2	Signaling
DMR2:259008001	2	259008001	259009000	1000	1	3.20E-10	0.55	14	1.4	St6galnac5	
DMR2:259012001	2	259012001	259015000	3000	1	2.00E-07	0.43	34	1.13	St6galnac5	
DMR2:259021001	2	259021001	259027000	6000	2	6.70E-19	0.88	104	1.73	St6galnac5	
DMR2:259040001	2	259040001	259042000	2000	1	5.60E-08	0.43	20	1	St6galnac5	
DMR2:259046001	2	259046001	259048000	2000	2	7.70E-15	0.6	35	1.75	St6galnac5	
DMR2:259051001	2	259051001	259057000	6000	2	7.80E-09	0.41	128	2.13	St6galnac5	
DMR2:259060001	2	259060001	259062000	2000	1	3.10E-08	0.55	14	0.7	St6galnac5	
DMR2:259066001	2	259066001	259067000	1000	1	3.00E-09	0.45	18	1.8	St6galnac5	
DMR2:259081001	2	259081001	259088000	7000	1	1.00E-14	0.58	117	1.67	St6galnac5	
DMR2:259092001	2	259092001	259093000	1000	1	7.30E-10	0.48	13	1.3	St6galnac5	
DMR2:259099001	2	259099001	259106000	7000	1	9.40E-19	0.74	97	1.39	St6galnac5	
DMR2:259118001	2	259118001	259122000	4000	2	1.40E-08	0.36	60	1.5	St6galnac5	
DMR2:259139001	2	259139001	259142000	3000	2	4.60E-08	0.4	28	0.93	St6galnac5	
DMR2:259144001	2	259144001	259149000	5000	3	2.60E-16	0.85	66	1.32	St6galnac5	
DMR2:259156001	2	259156001	259160000	4000	2	2.30E-14	-0.55	61	1.52	St6galnac5	
DMR2:259381001	2	259381001	259385000	4000	1	2.80E-10	0.62	55	1.38	St6galnac3	
DMR2:259389001	2	259389001	259393000	4000	3	6.50E-12	-0.53	25	0.62	St6galnac3	
DMR2:259407001	2	259407001	259408000	1000	1	5.50E-12	0.49	12	1.2	St6galnac3	
DMR2:259411001	2	259411001	259413000	2000	2	1.50E-25	1.04	17	0.85	St6galnac3	

DMR2:259416001	2	259416001	259422000	6000	1	3.50E-10	-0.72	121	2.02	St6galnac3	
DMR2:259424001	2	259424001	259426000	2000	1	1.80E-07	-0.38	36	1.8	St6galnac3	
DMR2:259448001	2	259448001	259454000	6000	2	5.10E-21	0.51	114	1.9	St6galnac3	
DMR2:259485001	2	259485001	259490000	5000	1	2.70E-10	-0.6	82	1.64	St6galnac3	
DMR2:259497001	2	259497001	259499000	2000	1	2.80E-08	0.51	22	1.1	St6galnac3	
DMR2:259501001	2	259501001	259505000	4000	3	3.80E-12	0.45	52	1.3	St6galnac3	
DMR2:259522001	2	259522001	259523000	1000	1	9.60E-09	0.47	2	0.2	St6galnac3	
DMR2:259549001	2	259549001	259552000	3000	1	9.20E-07	-0.32	61	2.03	St6galnac3	
DMR2:259556001	2	259556001	259563000	7000	1	7.40E-08	0.41	97	1.39	St6galnac3	
DMR2:259564001	2	259564001	259567000	3000	1	7.80E-10	0.39	48	1.6	St6galnac3	
DMR2:259568001	2	259568001	259572000	4000	3	3.20E-10	0.38	66	1.65	St6galnac3	
DMR2:259600001	2	259600001	259606000	6000	1	2.80E-09	0.58	98	1.63	St6galnac3	
DMR2:259623001	2	259623001	259624000	1000	1	2.40E-13	0.56	13	1.3	St6galnac3	
DMR2:259625001	2	259625001	259626000	1000	1	5.10E-15	0.94	4	0.4	St6galnac3	
DMR2:259628001	2	259628001	259629000	1000	1	2.50E-17	0.66	8	0.8	St6galnac3	
DMR2:259634001	2	259634001	259635000	1000	1	3.50E-13	0.62	8	0.8	St6galnac3	
DMR2:259640001	2	259640001	259643000	3000	1	5.00E-07	0.38	23	0.77	St6galnac3	
DMR2:259662001	2	259662001	259663000	1000	1	5.30E-07	0.47	10	1	St6galnac3	
DMR2:259665001	2	259665001	259667000	2000	1	1.50E-07	-0.38	19	0.95	St6galnac3	
DMR2:259680001	2	259680001	259682000	2000	1	3.10E-08	-0.57	34	1.7	St6galnac3	
DMR2:259690001	2	259690001	259692000	2000	1	9.10E-14	0.46	30	1.5	St6galnac3	
DMR2:259713001	2	259713001	259720000	7000	2	1.80E-11	0.65	84	1.2	St6galnac3;LOC108350218	
DMR2:259721001	2	259721001	259732000	11000	6	1.30E-21	0.66	128	1.16	St6galnac3;LOC108350218;LOC108350219	
DMR2:259736001	2	259736001	259737000	1000	1	1.20E-10	0.41	15	1.5	St6galnac3;LOC108350218;LOC108350219	
DMR2:259751001	2	259751001	259758000	7000	2	2.10E-14	0.89	68	0.97	St6galnac3;LOC108350219	
DMR2:259759001	2	259759001	259763000	4000	1	9.30E-10	0.45	63	1.57	St6galnac3;LOC108350219	
DMR2:259764001	2	259764001	259767000	3000	1	7.40E-13	0.56	47	1.57	St6galnac3;LOC108350219	
DMR2:259789001	2	259789001	259791000	2000	1	1.20E-21	0.65	34	1.7	St6galnac3;LOC108350219	
DMR2:259815001	2	259815001	259817000	2000	1	3.60E-09	-0.69	37	1.85	St6galnac3	
DMR2:259834001	2	259834001	259838000	4000	2	6.30E-15	0.61	51	1.27	St6galnac3	
DMR2:259845001	2	259845001	259856000	11000	2	3.10E-11	0.43	191	1.74	St6galnac3	
DMR2:259870001	2	259870001	259871000	1000	1	2.40E-07	0.52	15	1.5	St6galnac3	
DMR2:259875001	2	259875001	259879000	4000	1	2.00E-09	0.42	53	1.32	St6galnac3	
DMR2:259882001	2	259882001	259883000	1000	1	4.60E-08	0.33	19	1.9	St6galnac3	
DMR2:259900001	2	259900001	259903000	3000	2	9.60E-12	0.48	36	1.2	St6galnac3	
DMR2:260059001	2	260059001	260063000	4000	2	7.70E-15	0.6	45	1.12	Asb17;Msh4	Transcription
DMR2:260106001	2	260106001	260107000	1000	1	4.10E-20	0.68	18	1.8	Msh4;Rabggtb	Transcription;Metabolism
DMR2:260128001	2	260128001	260130000	2000	1	1.40E-08	-0.47	27	1.35	Acadm	Metabolism
DMR2:260140001	2	260140001	260141000	1000	1	3.10E-09	-0.66	18	1.8	Acadm	Metabolism
DMR2:260152001	2	260152001	260153000	1000	1	6.10E-07	0.47	4	0.4	Acadm;LOC108350296	Metabolism
DMR2:260184001	2	260184001	260188000	4000	1	3.50E-12	0.84	56	1.4	Slc44a5	Transport
DMR2:260189001	2	260189001	260191000	2000	1	8.80E-09	-0.61	27	1.35	Slc44a5	Transport
DMR2:260199001	2	260199001	260201000	2000	2	2.50E-07	-0.51	29	1.45	Slc44a5	Transport
DMR2:260202001	2	260202001	260204000	2000	1	1.50E-10	0.48	24	1.2	Slc44a5	Transport
DMR2:260205001	2	260205001	260206000	1000	1	1.10E-10	0.67	3	0.3	Slc44a5	Transport
DMR2:260211001	2	260211001	260215000	4000	1	6.80E-12	0.51	52	1.3	Slc44a5	Transport
DMR2:260217001	2	260217001	260219000	2000	2	1.30E-16	0.84	15	0.75	Slc44a5	Transport
DMR2:260245001	2	260245001	260248000	3000	1	5.70E-13	0.78	20	0.67	Slc44a5	Transport
DMR2:260252001	2	260252001	260257000	5000	2	7.00E-10	-0.52	72	1.44	Slc44a5	Transport
DMR2:260261001	2	260261001	260264000	3000	2	4.80E-08	0.45	54	1.8	Slc44a5	Transport
DMR2:260265001	2	260265001	260266000	1000	1	5.70E-18	0.64	11	1.1	Slc44a5	Transport
DMR2:260272001	2	260272001	260273000	1000	1	3.50E-16	0.52	25	2.5	Slc44a5	Transport
DMR2:260289001	2	260289001	260291000	2000	1	5.10E-09	-0.57	38	1.9	Slc44a5	Transport
DMR2:260307001	2	260307001	260309000	2000	1	2.00E-18	0.89	39	1.95	Slc44a5;LOC102553583	Transport
DMR2:260311001	2	260311001	260312000	1000	1	4.10E-07	-0.58	17	1.7	Slc44a5;LOC102553583	Transport
DMR2:260340001	2	260340001	260342000	2000	1	2.80E-13	0.59	26	1.3	Slc44a5;LOC102553583	Transport
DMR2:260346001	2	260346001	260351000	5000	2	1.80E-17	0.56	56	1.12	Slc44a5;LOC102553583	Transport
DMR2:260352001	2	260352001	260355000	3000	2	6.70E-22	-0.54	46	1.53	Slc44a5	Transport
DMR2:260367001	2	260367001	260374000	7000	1	4.70E-10	-0.68	89	1.27	Slc44a5	Transport
DMR2:260375001	2	260375001	260384000	9000	6	2.70E-21	-0.75	132	1.47	Slc44a5	Transport
DMR2:260387001	2	260387001	260390000	3000	1	3.40E-07	0.53	26	0.87	Slc44a5	Transport
DMR2:260392001	2	260392001	260396000	4000	1	1.40E-11	0.53	10	0.25	Slc44a5	Transport

DMR2:260406001	2	260406001	260411000	5000	2	1.00E-12	0.51	72	1.44	Slc44a5	Transport
DMR2:260421001	2	260421001	260427000	6000	2	1.20E-21	0.71	110	1.83	Slc44a5	Transport
DMR2:260506001	2	260506001	260507000	1000	1	1.50E-07	0.39	10	1	Slc44a5	Transport
DMR2:260510001	2	260510001	260513000	3000	1	3.60E-13	0.66	36	1.2	Slc44a5	Transport
DMR2:260523001	2	260523001	260524000	1000	1	6.90E-08	0.35	11	1.1	Slc44a5	Transport
DMR2:260530001	2	260530001	260532000	2000	1	1.50E-11	0.46	27	1.35	Slc44a5	Transport
DMR2:260601001	2	260601001	260602000	1000	1	1.70E-08	0.57	32	3.2	Lhx8	Development
DMR2:260603001	2	260603001	260608000	5000	3	1.90E-13	0.8	61	1.22	Lhx8	Development
DMR2:260850001	2	260850001	260852000	2000	1	1.60E-20	0.63	32	1.6	Tyw3	Epigenetic
DMR2:260855001	2	260855001	260857000	2000	1	1.20E-07	0.32	16	0.8	Tyw3	Epigenetic
DMR2:260868001	2	260868001	260877000	9000	4	2.70E-13	-0.7	81	0.9	Tyw3;Cryz	Epigenetic;Metabolism
DMR2:260889001	2	260889001	260891000	2000	1	8.00E-10	-0.67	27	1.35	Tyw3;Cryz;LOC108350160	Epigenetic;Metabolism
DMR2:260904001	2	260904001	260907000	3000	1	2.20E-07	-0.69	43	1.43	Cryz;LOC108350160	Metabolism
DMR2:260909001	2	260909001	260910000	1000	1	9.40E-08	0.33	18	1.8	Cryz	Metabolism
DMR2:260914001	2	260914001	260915000	1000	1	1.60E-08	0.51	11	1.1	Cryz	Metabolism
DMR2:260919001	2	260919001	260922000	3000	1	1.30E-11	0.39	29	0.97	Cryz	Metabolism
DMR2:260941001	2	260941001	260945000	4000	2	7.20E-14	-0.79	38	0.95	Erich3	
DMR2:260946001	2	260946001	260947000	1000	1	2.50E-08	0.67	4	0.4	Erich3;LOC102555516	
DMR2:260966001	2	260966001	260974000	8000	4	6.20E-19	-0.46	80	1	Erich3;LOC102555516	
DMR2:260989001	2	260989001	260990000	1000	1	2.30E-07	0.34	16	1.6	Erich3;LOC102555516;LOC108350146	
DMR2:261011001	2	261011001	261012000	1000	1	1.70E-07	0.37	10	1	Erich3	
DMR2:261013001	2	261013001	261015000	2000	1	7.20E-15	0.6	7	0.35	Erich3	
DMR2:261017001	2	261017001	261020000	3000	1	3.60E-09	0.41	46	1.53	Erich3	
DMR2:261026001	2	261026001	261028000	2000	1	3.60E-09	-0.64	27	1.35	Erich3	
DMR2:261059001	2	261059001	261061000	2000	1	8.20E-10	0.4	34	1.7	Erich3;LOC102555624;Tnni3k	
DMR2:261062001	2	261062001	261066000	4000	1	2.10E-07	0.37	60	1.5	LOC102555624;Tnni3k	
DMR2:261069001	2	261069001	261070000	1000	1	1.20E-07	-0.55	18	1.8	LOC102555624;Tnni3k	
DMR2:261072001	2	261072001	261073000	1000	1	3.40E-07	-0.54	9	0.9	LOC102555624;Tnni3k	
DMR2:261074001	2	261074001	261076000	2000	2	4.10E-12	0.42	37	1.85	LOC102555624;Tnni3k	
DMR2:261079001	2	261079001	261082000	3000	1	3.20E-17	0.72	29	0.97	LOC102555624;Tnni3k	
DMR2:261083001	2	261083001	261085000	2000	1	8.00E-11	0.56	25	1.25	LOC102555624;Tnni3k	
DMR2:261108001	2	261108001	261109000	1000	1	8.10E-13	0.56	12	1.2	LOC102555624;Tnni3k	
DMR2:261118001	2	261118001	261122000	4000	1	2.20E-07	-0.62	54	1.35	LOC102555624;Tnni3k	
DMR2:261137001	2	261137001	261139000	2000	1	1.40E-15	0.65	27	1.35	LOC102555624;Tnni3k	
DMR2:261151001	2	261151001	261153000	2000	1	2.60E-07	0.37	13	0.65	Tnni3k	
DMR2:261158001	2	261158001	261165000	7000	2	3.30E-08	0.44	64	0.91	Tnni3k	
DMR2:261174001	2	261174001	261177000	3000	1	2.20E-16	-0.76	32	1.07	Tnni3k	
DMR2:261185001	2	261185001	261187000	2000	1	4.00E-07	0.37	18	0.9	Tnni3k	
DMR2:261196001	2	261196001	261200000	4000	1	2.50E-13	0.58	51	1.27	Tnni3k	
DMR2:261201001	2	261201001	261202000	1000	1	4.90E-08	0.44	14	1.4	Tnni3k	
DMR2:261237001	2	261237001	261238000	1000	1	1.10E-08	0.6	5	0.5	Tnni3k	
DMR2:261239001	2	261239001	261241000	2000	1	3.70E-09	-0.68	9	0.45	Tnni3k	
DMR2:261244001	2	261244001	261246000	2000	1	7.80E-11	-0.5	22	1.1	Tnni3k	
DMR2:261268001	2	261268001	261269000	1000	1	3.60E-09	0.44	17	1.7	Tnni3k	
DMR2:261289001	2	261289001	261290000	1000	1	5.50E-09	-0.51	5	0.5	Tnni3k	
DMR2:261323001	2	261323001	261324000	1000	1	1.50E-07	0.4	11	1.1	Tnni3k	
DMR2:261329001	2	261329001	261330000	1000	1	1.20E-07	0.55	8	0.8	Tnni3k	
DMR2:261339001	2	261339001	261344000	5000	2	1.20E-14	0.77	52	1.04	Tnni3k	
DMR2:261383001	2	261383001	261384000	1000	1	2.40E-07	0.59	3	0.3	Fpgt	Transport
DMR2:261386001	2	261386001	261387000	1000	1	4.00E-07	0.53	3	0.3	Fpgt	Transport
DMR2:261428001	2	261428001	261429000	1000	1	2.40E-07	-0.58	13	1.3	Lrriq3	
DMR2:261431001	2	261431001	261432000	1000	1	6.20E-07	0.44	0	0	Lrriq3	
DMR2:261440001	2	261440001	261444000	4000	1	4.20E-07	-0.45	55	1.38	Lrriq3	
DMR2:261474001	2	261474001	261477000	3000	1	9.50E-07	-0.45	16	0.53	Lrriq3	
DMR2:261480001	2	261480001	261482000	2000	1	2.50E-11	0.57	27	1.35	Lrriq3	
DMR2:262914001	2	262914001	262916000	2000	2	2.00E-16	0.95	64	3.2	Negr1	Immune
DMR2:262976001	2	262976001	262978000	2000	2	3.20E-10	-0.54	9	0.45	Negr1	Immune
DMR2:262983001	2	262983001	262984000	1000	1	1.20E-07	0.5	9	0.9	Negr1	Immune
DMR2:263023001	2	263023001	263024000	1000	1	4.00E-07	-0.51	17	1.7	Negr1	Immune
DMR2:263026001	2	263026001	263027000	1000	1	6.30E-11	0.55	7	0.7	Negr1	Immune
DMR2:263029001	2	263029001	263030000	1000	1	1.80E-10	0.61	9	0.9	Negr1	Immune
DMR2:263047001	2	263047001	263048000	1000	1	1.20E-13	0.76	14	1.4	Negr1	Immune

DMR2:263071001	2	263071001	263072000	1000	1	5.20E-09	0.42	4	0.4	Negr1	Immune
DMR2:263088001	2	263088001	263090000	2000	2	2.50E-23	0.88	12	0.6	Negr1	Immune
DMR2:263097001	2	263097001	263102000	5000	1	1.80E-09	-0.49	66	1.32	Negr1	Immune
DMR2:263111001	2	263111001	263113000	2000	1	4.20E-10	0.66	11	0.55	Negr1	Immune
DMR2:263120001	2	263120001	263126000	6000	1	4.00E-22	1.1	80	1.33	Negr1	Immune
DMR2:263135001	2	263135001	263136000	1000	1	7.60E-07	0.44	3	0.3	Negr1	Immune
DMR2:263166001	2	263166001	263170000	4000	1	7.30E-07	0.52	44	1.1	Negr1	Immune
DMR2:263241001	2	263241001	263242000	1000	1	2.30E-11	0.62	8	0.8	Negr1	Immune
DMR2:263262001	2	263262001	263266000	4000	1	5.50E-14	0.71	25	0.62	Negr1	Immune
DMR2:263272001	2	263272001	263274000	2000	2	8.20E-12	0.86	12	0.6	Negr1	Immune
DMR2:263281001	2	263281001	263284000	3000	1	4.40E-07	0.5	23	0.77	Negr1	Immune
DMR2:263291001	2	263291001	263293000	2000	1	6.70E-17	0.84	10	0.5	Negr1	Immune
DMR2:263304001	2	263304001	263305000	1000	1	9.60E-07	0.37	14	1.4	Negr1	Immune
DMR2:263358001	2	263358001	263359000	1000	1	2.40E-10	0.52	6	0.6	Negr1	Immune
DMR2:263427001	2	263427001	263428000	1000	1	2.20E-08	-0.61	10	1	Negr1	Immune
DMR2:263435001	2	263435001	263437000	2000	2	8.00E-08	-0.65	14	0.7	Negr1	Immune
DMR2:263453001	2	263453001	263458000	5000	1	9.00E-22	1.03	34	0.68	Negr1	Immune
DMR2:263536001	2	263536001	263537000	1000	1	1.10E-07	0.55	9	0.9	Negr1	Immune
DMR2:263567001	2	263567001	263569000	2000	1	2.20E-16	0.64	25	1.25	Negr1	Immune
DMR2:263588001	2	263588001	263591000	3000	1	1.00E-09	-0.58	18	0.6	Negr1	Immune
DMR2:263600001	2	263600001	263602000	2000	1	2.00E-08	0.53	19	0.95	Negr1	Immune
DMR2:263625001	2	263625001	263627000	2000	1	7.20E-10	-0.71	19	0.95	Negr1	Immune
DMR2:263628001	2	263628001	263635000	7000	5	8.30E-28	-0.58	85	1.21	Negr1	Immune
DMR2:263646001	2	263646001	263647000	1000	1	3.50E-09	0.55	9	0.9	Negr1	Immune
DMR2:263651001	2	263651001	263653000	2000	1	6.80E-07	0.46	17	0.85	Negr1	Immune
DMR2:263897001	2	263897001	263898000	1000	1	5.20E-10	0.7	24	2.4	Ptger3	Signaling
DMR2:263914001	2	263914001	263916000	2000	1	3.90E-09	0.45	20	1	Ptger3	Signaling
DMR2:263935001	2	263935001	263936000	1000	1	6.10E-07	0.45	7	0.7	Ptger3;LOC108350148	Signaling
DMR2:263965001	2	263965001	263966000	1000	1	9.80E-13	0.44	10	1	Ptger3	Signaling
DMR2:263989001	2	263989001	263992000	3000	1	3.40E-07	-0.58	20	0.67	Ptger3	Signaling
DMR2:264046001	2	264046001	264047000	1000	1	3.40E-18	0.68	9	0.9	RGD1564920	
DMR2:264049001	2	264049001	264052000	3000	1	1.40E-18	0.77	33	1.1	RGD1564920	
DMR2:264260001	2	264260001	264261000	1000	1	5.80E-07	0.45	6	0.6	Cth	Metabolism
DMR2:264262001	2	264262001	264263000	1000	1	6.90E-13	0.77	6	0.6	Cth	Metabolism
DMR2:264268001	2	264268001	264269000	1000	1	4.30E-07	0.55	3	0.3	Cth	Metabolism
DMR2:264270001	2	264270001	264271000	1000	1	1.50E-07	0.34	17	1.7	Cth	Metabolism
DMR2:264691001	2	264691001	264695000	4000	1	2.00E-11	-0.75	22	0.55	Depdc1	Cytoskeleton
DMR2:264696001	2	264696001	264697000	1000	1	3.90E-12	0.57	10	1	Depdc1	Cytoskeleton
DMR2:264699001	2	264699001	264700000	1000	1	2.50E-20	-0.68	6	0.6	Depdc1	Cytoskeleton
DMR2:264701001	2	264701001	264705000	4000	4	4.50E-17	1.11	41	1.02	Depdc1	Cytoskeleton
DMR2:264712001	2	264712001	264714000	2000	2	3.80E-17	0.76	12	0.6	Depdc1	Cytoskeleton
DMR2:264726001	2	264726001	264727000	1000	1	1.50E-08	0.62	2	0.2	Depdc1	Cytoskeleton
DMR2:264729001	2	264729001	264732000	3000	2	1.30E-07	0.48	16	0.53	Depdc1	Cytoskeleton
DMR2:264740001	2	264740001	264745000	5000	1	5.70E-16	-0.56	43	0.86	Depdc1;LOC103691744	Cytoskeleton
DMR2:264776001	2	264776001	264777000	1000	1	8.20E-10	0.53	5	0.5	Ankrd13c	
DMR2:264817001	2	264817001	264818000	1000	1	1.30E-07	-0.6	8	0.8	Ankrd13c	
DMR2:264849001	2	264849001	264850000	1000	1	8.80E-11	0.64	8	0.8	Srsf11	Translation
DMR2:264871001	2	264871001	264872000	1000	1	2.60E-08	0.43	10	1	Srsf11;Lrrc40	Translation;Cytoskeleton
DMR2:264880001	2	264880001	264883000	3000	1	6.70E-12	0.48	25	0.83	Lrrc40	Cytoskeleton
DMR2:264919001	2	264919001	264920000	1000	1	1.20E-09	0.67	5	0.5	Lrrc7	Cytoskeleton
DMR2:264924001	2	264924001	264926000	2000	1	5.60E-07	-0.43	20	1	Lrrc7	Cytoskeleton
DMR2:264981001	2	264981001	264983000	2000	1	8.10E-10	0.51	33	1.65	Lrrc7	Cytoskeleton
DMR2:264994001	2	264994001	264995000	1000	1	2.40E-08	0.53	5	0.5	Lrrc7	Cytoskeleton
DMR2:265002001	2	265002001	265003000	1000	1	2.00E-07	0.45	7	0.7	Lrrc7	Cytoskeleton
DMR2:265004001	2	265004001	265005000	1000	1	2.80E-19	0.65	7	0.7	Lrrc7	Cytoskeleton
DMR2:265018001	2	265018001	265019000	1000	1	2.20E-08	-0.6	7	0.7	Lrrc7	Cytoskeleton
DMR2:265054001	2	265054001	265055000	1000	1	8.30E-08	0.54	8	0.8	Lrrc7	Cytoskeleton
DMR2:265063001	2	265063001	265064000	1000	1	4.10E-11	0.45	9	0.9	Lrrc7	Cytoskeleton
DMR2:265078001	2	265078001	265080000	2000	2	1.70E-08	0.67	16	0.8	Lrrc7	Cytoskeleton
DMR2:265097001	2	265097001	265098000	1000	1	3.50E-19	0.88	11	1.1	Lrrc7	Cytoskeleton
DMR2:265118001	2	265118001	265120000	2000	1	7.60E-08	-0.54	15	0.75	Lrrc7	Cytoskeleton
DMR2:265135001	2	265135001	265138000	3000	1	5.70E-07	0.34	33	1.1	Lrrc7	Cytoskeleton
DMR2:265152001	2	265152001	265153000	1000	1	2.60E-13	-0.76	7	0.7	Lrrc7	Cytoskeleton
DMR2:265199001	2	265199001	265205000	6000	4	1.90E-17	-0.52	48	0.8	Lrrc7	Cytoskeleton

DMR2:265225001	2	265225001	265226000	1000	1	6.40E-20	0.92	2	0.2	Lrrc7	Cytoskeleton
DMR2:265230001	2	265230001	265231000	1000	1	2.50E-20	0.9	4	0.4	Lrrc7	Cytoskeleton
DMR2:265249001	2	265249001	265252000	3000	1	2.60E-09	0.63	19	0.63	Lrrc7	Cytoskeleton
DMR2:265259001	2	265259001	265263000	4000	1	1.80E-07	-0.5	17	0.42	Lrrc7	Cytoskeleton
DMR2:265290001	2	265290001	265291000	1000	1	3.00E-11	0.63	5	0.5	Lrrc7	Cytoskeleton
DMR2:265306001	2	265306001	265308000	2000	2	1.30E-09	-0.58	7	0.35	Lrrc7	Cytoskeleton
DMR2:266158001	2	266158001	266159000	1000	1	5.70E-11	0.56	2	0.2	Rpe65	Metabolism
DMR2:266170001	2	266170001	266171000	1000	1	1.70E-13	0.63	3	0.3	Rpe65	Metabolism
DMR2:266175001	2	266175001	266177000	2000	1	5.40E-13	0.72	7	0.35	Rpe65	Metabolism
DMR2:266178001	2	266178001	266179000	1000	1	1.60E-15	0.94	6	0.6	Rpe65	Metabolism
DMR2:266312001	2	266312001	266314000	2000	1	6.40E-07	0.44	10	0.5	Trnai-aau;Wls;LOC108350151	
DMR2:266337001	2	266337001	266339000	2000	1	2.00E-08	-0.53	13	0.65	Wls	
DMR2:266344001	2	266344001	266345000	1000	1	7.70E-18	0.85	12	1.2	Wls	
DMR2:266348001	2	266348001	266349000	1000	1	2.30E-07	0.54	3	0.3	Wls	
DMR2:266362001	2	266362001	266365000	3000	2	1.10E-11	-0.41	31	1.03	Wls	
DMR2:266383001	2	266383001	266385000	2000	1	4.80E-19	0.75	9	0.45	Wls	
DMR2:266393001	2	266393001	266394000	1000	1	3.00E-07	0.44	7	0.7	Wls	
DMR2:266405001	2	266405001	266410000	5000	3	7.00E-20	-0.56	53	1.06	Wls	
DMR2:266418001	2	266418001	266419000	1000	1	2.40E-17	-0.71	9	0.9	Wls	
DMR2:266429001	2	266429001	266434000	5000	1	9.40E-16	0.56	42	0.84	Wls	
DMR3:1	3	1	1000	1000	1	6.40E-15	0.67	6	0.6	RGD1562465	
DMR3:15001	3	15001	17000	2000	1	3.60E-07	-0.56	44	2.2	RGD1562465	
DMR3:70001	3	70001	71000	1000	1	2.50E-20	0.95	3	0.3	Nxph2	Signaling
DMR3:80001	3	80001	82000	2000	2	2.30E-20	0.81	11	0.55	Nxph2	Signaling
DMR3:83001	3	83001	86000	3000	2	2.60E-10	-0.61	14	0.47	Nxph2	Signaling
DMR3:94001	3	94001	95000	1000	1	3.40E-10	0.65	9	0.9	Nxph2	Signaling
DMR3:106001	3	106001	107000	1000	1	6.30E-22	0.89	11	1.1	Nxph2	Signaling
DMR3:108001	3	108001	109000	1000	1	4.30E-07	0.57	9	0.9	Nxph2	Signaling
DMR3:123001	3	123001	124000	1000	1	1.50E-07	0.36	15	1.5	Nxph2	Signaling
DMR3:143001	3	143001	145000	2000	1	1.20E-15	0.59	13	0.65	Nxph2;LOC499742	Signaling
DMR3:146001	3	146001	150000	4000	2	1.10E-19	-0.54	33	0.82	Nxph2;LOC499742	Signaling
DMR3:155001	3	155001	157000	2000	1	8.50E-10	-0.57	7	0.35	Nxph2;LOC499742	Signaling
DMR3:181001	3	181001	185000	4000	1	6.70E-07	-0.32	40	1	Nxph2	Signaling
DMR3:195001	3	195001	198000	3000	1	6.00E-12	-0.5	23	0.77	Nxph2	Signaling
DMR3:201001	3	201001	204000	3000	1	1.30E-15	-0.57	20	0.67	Nxph2	Signaling
DMR3:225001	3	225001	226000	1000	1	8.20E-09	0.63	3	0.3	Nxph2	Signaling
DMR3:236001	3	236001	245000	9000	6	1.70E-14	-0.64	64	0.71	Nxph2	Signaling
DMR3:247001	3	247001	251000	4000	2	9.20E-11	0.55	17	0.42	Nxph2;LOC679914	Signaling
DMR3:268001	3	268001	271000	3000	1	5.90E-07	-0.45	8	0.27	Nxph2;LOC679914	Signaling
DMR3:282001	3	282001	289000	7000	1	1.90E-08	-0.4	43	0.61	Nxph2	Signaling
DMR3:290001	3	290001	291000	1000	1	8.20E-25	0.89	5	0.5	Nxph2	Signaling
DMR3:292001	3	292001	296000	4000	2	2.70E-08	-0.6	13	0.32	Nxph2	Signaling
DMR3:301001	3	301001	306000	5000	1	4.20E-10	0.61	28	0.56	Nxph2	Signaling
DMR3:309001	3	309001	312000	3000	1	1.50E-07	0.5	14	0.47	Nxph2	Signaling
DMR3:390001	3	390001	394000	4000	1	1.10E-07	0.62	25	0.62	Spopl	Proteolysis
DMR3:411001	3	411001	412000	1000	1	9.10E-09	-0.64	3	0.3	Spopl	Proteolysis
DMR3:417001	3	417001	422000	5000	1	2.10E-11	-0.54	23	0.46	Spopl	Proteolysis
DMR3:434001	3	434001	435000	1000	1	1.80E-08	0.69	3	0.3	Spopl	Proteolysis
DMR3:442001	3	442001	446000	4000	2	2.10E-11	-0.53	12	0.3	Spopl	Proteolysis
DMR3:453001	3	453001	461000	8000	1	7.40E-21	0.84	40	0.5	Spopl	Proteolysis
DMR3:475001	3	475001	476000	1000	1	2.60E-17	-0.52	6	0.6	Spopl	Proteolysis
DMR3:563001	3	563001	569000	6000	2	5.70E-17	-0.74	94	1.57	LOC679995;Psx1	
DMR3:570001	3	570001	573000	3000	1	3.50E-07	-0.42	10	0.33	Psx1	
DMR3:576001	3	576001	577000	1000	1	3.40E-11	0.53	7	0.7	Psx1	
DMR3:578001	3	578001	583000	5000	3	2.70E-08	-0.52	24	0.48	Psx1	
DMR3:681001	3	681001	685000	4000	1	3.90E-08	-0.48	15	0.38	RGD1564974	
DMR3:896001	3	896001	898000	2000	1	4.60E-09	-0.41	16	0.8	Hnmt	
DMR3:903001	3	903001	905000	2000	1	5.90E-09	0.8	5	0.25	Hnmt	
DMR3:911001	3	911001	913000	2000	1	1.90E-09	0.47	14	0.7	Hnmt	
DMR3:926001	3	926001	928000	2000	1	3.60E-07	0.36	15	0.75	Hnmt	
DMR3:929001	3	929001	934000	5000	2	3.60E-10	-0.52	43	0.86	Hnmt	
DMR3:938001	3	938001	939000	1000	1	2.10E-08	-0.49	6	0.6	Hnmt	
DMR3:1091001	3	1091001	1092000	1000	1	1.30E-14	0.62	4	0.4	Tbpl2	Transcription
DMR3:1100001	3	1100001	1102000	2000	1	1.80E-12	0.65	11	0.55	Tbpl2	Transcription
DMR3:1104001	3	1104001	1105000	1000	1	2.90E-07	-0.57	2	0.2	Tbpl2	Transcription

DMR3:1283001	3	1283001	1285000	2000	1	1.10E-07	0.43	15	0.75	Il36g	Cytokine
DMR3:1296001	3	1296001	1302000	6000	2	2.00E-17	0.76	43	0.72	Il36g	Cytokine
DMR3:1318001	3	1318001	1321000	3000	2	4.00E-09	-0.51	17	0.57	Il36a	Cytokine
DMR3:1325001	3	1325001	1326000	1000	1	1.10E-07	0.49	6	0.6	Il36a	Cytokine
DMR3:1348001	3	1348001	1352000	4000	2	9.80E-11	-0.42	31	0.78	RGD1559890	
DMR3:1358001	3	1358001	1359000	1000	1	7.70E-10	0.47	12	1.2	Il36b	Cytokine
DMR3:1365001	3	1365001	1366000	1000	1	1.60E-11	0.54	12	1.2	Il36b	Cytokine
DMR3:1381001	3	1381001	1383000	2000	2	1.10E-07	-0.6	3	0.15	Il36b;Il36rn	Cytokine
DMR3:1394001	3	1394001	1408000	14000	9	1.50E-14	-0.58	142	1.01	Il36b;Il36rn;Il1f10	Cytokine
DMR3:1417001	3	1417001	1426000	9000	2	7.50E-12	0.59	87	0.97	Il36b;Il1f10	Cytokine
DMR3:1433001	3	1433001	1437000	4000	1	3.10E-13	0.63	34	0.85	Il36b	Cytokine
DMR3:1446001	3	1446001	1448000	2000	1	1.30E-16	0.96	12	0.6	Il36b;Il1rn	Cytokine
DMR3:1469001	3	1469001	1470000	1000	1	4.30E-14	0.72	7	0.7	Il36b;Il1rn;Psd4	Cytokine;Transcriptio n
DMR3:1472001	3	1472001	1473000	1000	1	2.60E-07	0.48	5	0.5	Il36b;Il1rn;Psd4	Cytokine;Transcriptio n
DMR3:1474001	3	1474001	1477000	3000	1	1.00E-09	0.46	29	0.97	Il36b;Il1rn;Psd4	Cytokine;Transcriptio n
DMR3:1492001	3	1492001	1493000	1000	1	6.30E-15	0.58	19	1.9	Psd4	Transcription
DMR3:1523001	3	1523001	1524000	1000	1	3.90E-14	0.65	17	1.7	Psd4;Pax8;LOC102549321	Transcription
DMR3:1529001	3	1529001	1534000	5000	2	1.50E-16	0.87	56	1.12	Pax8;LOC102549321	
DMR3:1537001	3	1537001	1540000	3000	1	8.20E-07	0.38	25	0.83	Pax8;LOC102549321	
DMR3:1554001	3	1554001	1557000	3000	1	6.10E-07	0.46	27	0.9	Pax8	
DMR3:1561001	3	1561001	1562000	1000	1	1.20E-15	0.67	21	2.1	Pax8	
DMR3:1566001	3	1566001	1569000	3000	2	8.00E-20	0.87	25	0.83	Pax8	
DMR3:1591001	3	1591001	1594000	3000	1	1.70E-09	0.4	35	1.17	Pax8	
DMR3:1725001	3	1725001	1736000	11000	7	2.30E-20	0.75	126	1.15	Cacna1b	Transport
DMR3:1746001	3	1746001	1749000	3000	2	5.10E-15	0.52	33	1.1	Cacna1b	Transport
DMR3:1778001	3	1778001	1779000	1000	1	2.10E-07	0.29	19	1.9	Cacna1b	Transport
DMR3:1807001	3	1807001	1811000	4000	2	3.10E-19	0.88	37	0.92	Cacna1b	Transport
DMR3:1812001	3	1812001	1817000	5000	1	1.10E-10	0.48	70	1.4	Cacna1b	Transport
DMR3:1822001	3	1822001	1824000	2000	1	3.20E-18	0.88	11	0.55	Cacna1b	Transport
DMR3:1826001	3	1826001	1828000	2000	2	1.10E-15	0.77	11	0.55	Cacna1b	Transport
DMR3:1834001	3	1834001	1836000	2000	2	3.10E-12	0.8	46	2.3	Cacna1b	Transport
DMR3:1850001	3	1850001	1851000	1000	1	2.00E-08	0.33	15	1.5	Cacna1b	Transport
DMR3:1865001	3	1865001	1868000	3000	1	2.20E-07	-0.52	47	1.57	Cacna1b	Transport
DMR3:1884001	3	1884001	1885000	1000	1	3.70E-21	0.83	18	1.8	Cacna1b	Transport
DMR3:1886001	3	1886001	1888000	2000	1	1.30E-16	0.68	24	1.2	Cacna1b	Transport
DMR3:1892001	3	1892001	1896000	4000	2	1.90E-12	0.63	62	1.55	Cacna1b	Transport
DMR3:1898001	3	1898001	1903000	5000	1	3.60E-10	-0.62	57	1.14	Cacna1b	Transport
DMR3:1915001	3	1915001	1920000	5000	2	1.00E-21	1.09	70	1.4	Cacna1b	Transport
DMR3:1979001	3	1979001	1984000	5000	1	9.00E-08	-0.58	59	1.18	Ehmt1	
DMR3:1986001	3	1986001	1987000	1000	1	6.50E-07	-0.61	7	0.7	Ehmt1	
DMR3:1994001	3	1994001	1996000	2000	1	1.70E-09	-0.64	18	0.9	Ehmt1	
DMR3:2002001	3	2002001	2003000	1000	1	3.80E-10	0.56	8	0.8	Ehmt1	
DMR3:2055001	3	2055001	2058000	3000	1	4.10E-08	-0.52	33	1.1	Ehmt1	
DMR3:2072001	3	2072001	2073000	1000	1	2.60E-11	0.79	8	0.8	Ehmt1	
DMR3:2076001	3	2076001	2078000	2000	1	6.00E-10	-0.63	20	1	Ehmt1	
DMR3:2079001	3	2079001	2081000	2000	1	1.00E-09	0.47	20	1	Ehmt1	
DMR3:2107001	3	2107001	2109000	2000	1	2.60E-07	0.35	19	0.95	Ehmt1	
DMR3:2141001	3	2141001	2142000	1000	1	1.10E-07	0.48	9	0.9	Arrdc1	
DMR3:2182001	3	2182001	2183000	1000	1	6.40E-09	0.73	55	5.5	Dph7;Mrpl41;Pnpla7	Epigenetic;Translatio n;Metabolism
DMR3:2195001	3	2195001	2196000	1000	1	1.80E-08	0.58	12	1.2	Pnpla7	Metabolism
DMR3:2203001	3	2203001	2204000	1000	1	1.90E-22	1.07	6	0.6	Pnpla7	Metabolism
DMR3:2226001	3	2226001	2227000	1000	1	9.20E-14	0.57	13	1.3	Pnpla7;LOC681261	Metabolism
DMR3:2231001	3	2231001	2232000	1000	1	1.60E-08	-0.49	20	2	Pnpla7;LOC681261	Metabolism
DMR3:2235001	3	2235001	2241000	6000	1	1.00E-12	0.48	80	1.33	Pnpla7;LOC681261	Metabolism
DMR3:2242001	3	2242001	2245000	3000	2	3.50E-18	0.89	29	0.97	Pnpla7;LOC681261	Metabolism
DMR3:2264001	3	2264001	2270000	6000	1	7.90E-15	0.58	125	2.08	Pnpla7;Nsmf	Metabolism
DMR3:2284001	3	2284001	2286000	2000	1	4.10E-11	0.58	22	1.1	Entpd8	Signaling
DMR3:2291001	3	2291001	2294000	3000	1	3.40E-26	0.89	42	1.4	Entpd8;Noxa1	Signaling;Signaling
DMR3:2298001	3	2298001	2301000	3000	1	2.30E-19	0.89	40	1.33	Entpd8;Noxa1	Signaling;Signaling
DMR3:2305001	3	2305001	2308000	3000	1	3.50E-21	0.74	46	1.53	Entpd8;Noxa1	Signaling;Signaling
DMR3:2418001	3	2418001	2420000	2000	2	1.80E-15	0.92	13	0.65	Tor4a;Nelfb	Transcription

DMR3:2446001	3	2446001	2448000	2000	1	2.80E-14	0.45	14	0.7	Fam166a;Tubb4b;Slc34a3;Rnf224	Cytoskeleton;Transport
DMR3:2449001	3	2449001	2450000	1000	1	2.00E-11	0.5	15	1.5	Fam166a;Tubb4b;Slc34a3;Rnf224;Cysrt1	Cytoskeleton;Transport
DMR3:2452001	3	2452001	2454000	2000	1	9.30E-07	0.36	29	1.45	Tubb4b;Slc34a3;Rnf224;Cysrt1;Rnf208	Cytoskeleton;Transport
DMR3:2510001	3	2510001	2511000	1000	1	1.10E-19	0.59	24	2.4	Anapc2;Tmem210;Lrrc26;Grin1	Receptor
DMR3:2530001	3	2530001	2532000	2000	1	3.40E-12	0.58	15	0.75	Grin1	Receptor
DMR3:2552001	3	2552001	2554000	2000	1	3.10E-08	-0.65	26	1.3	Man1b1;LOC103691747	Golgi
DMR3:2555001	3	2555001	2557000	2000	1	4.80E-07	0.39	18	0.9	Man1b1;LOC103691747	Golgi
DMR3:2559001	3	2559001	2561000	2000	2	8.00E-23	0.78	55	2.75	Man1b1;LOC103691747;Dpp7	Golgi;Protease
DMR3:2562001	3	2562001	2563000	1000	1	2.70E-13	0.53	8	0.8	Man1b1;LOC103691747;Dpp7	Golgi;Protease
DMR3:2586001	3	2586001	2591000	5000	2	1.90E-16	0.62	93	1.86	Uap1l1;LOC108350589;Sapcd2	
DMR3:2594001	3	2594001	2602000	8000	2	9.90E-17	0.82	122	1.52	Uap1l1;LOC108350589;Sapcd2	
DMR3:2614001	3	2614001	2616000	2000	2	1.20E-10	0.6	22	1.1	Entpd2;Npdc1	Signaling
DMR3:2628001	3	2628001	2630000	2000	1	1.90E-14	0.58	32	1.6	Entpd2;Npdc1;LOC366006;Fut7	Signaling;Golgi
DMR3:2646001	3	2646001	2647000	1000	1	2.00E-07	0.42	8	0.8	Fut7;Abca2	Golgi;Transport
DMR3:2655001	3	2655001	2658000	3000	1	1.00E-07	0.37	63	2.1	Fut7;Abca2	Golgi;Transport
DMR3:2664001	3	2664001	2670000	6000	2	5.20E-10	0.39	186	3.1	Abca2;Clic3;RGD1306215	Transport;Transport
DMR3:2695001	3	2695001	2697000	2000	1	5.80E-09	-0.6	33	1.65	Ptgds	Transport
DMR3:2734001	3	2734001	2737000	3000	2	6.90E-09	0.39	50	1.67	C8g;Fbxw5;Traf2	Cytoskeleton
DMR3:2738001	3	2738001	2742000	4000	1	1.40E-12	0.55	63	1.57	Fbxw5;Traf2	Cytoskeleton
DMR3:2760001	3	2760001	2763000	3000	1	2.40E-08	-0.55	39	1.3	Traf2	Cytoskeleton
DMR3:2765001	3	2765001	2769000	4000	3	1.30E-08	0.44	57	1.43	Traf2	Cytoskeleton
DMR3:2777001	3	2777001	2779000	2000	1	1.70E-11	-0.6	34	1.7	Traf2;Edf1;Mamdc4	Cytoskeleton
DMR3:2847001	3	2847001	2849000	2000	1	1.30E-07	0.37	23	1.15	Ccdc183;Tmem141;Fcna	
DMR3:2853001	3	2853001	2854000	1000	1	8.30E-08	0.36	19	1.9	Ccdc183;Tmem141;Fcna	
DMR3:2861001	3	2861001	2865000	4000	1	2.00E-07	0.44	46	1.15	Fcna;Lcn8	Transport
DMR3:2866001	3	2866001	2868000	2000	1	7.60E-08	0.34	20	1	Lcn8;Lcn5	Transport
DMR3:2875001	3	2875001	2876000	1000	1	8.00E-08	0.41	14	1.4	Lcn8;Lcn5;LOC108350548	Transport
DMR3:2883001	3	2883001	2885000	2000	2	3.90E-12	0.49	32	1.6	Lcn8;Lcn5;LOC108350548;Lcn6;Lcn10	Transport
DMR3:2895001	3	2895001	2898000	3000	1	3.10E-20	0.67	42	1.4	LOC108350548;Lcn6;Lcn10	Transport
DMR3:2901001	3	2901001	2904000	3000	1	2.20E-13	0.75	31	1.03	Lcn6;Lcn10;Obp2a	Transport
DMR3:2924001	3	2924001	2926000	2000	1	1.20E-09	-0.47	29	1.45	Bmyc;LOC100360027;Lcn11	Transport
DMR3:2928001	3	2928001	2930000	2000	2	9.50E-17	0.77	13	0.65	LOC100360027;Lcn11	Transport
DMR3:2941001	3	2941001	2943000	2000	2	8.90E-29	0.96	18	0.9	LOC100360027;Lcn11;Lcn3	Transport
DMR3:2944001	3	2944001	2948000	4000	1	1.70E-12	0.45	60	1.5	LOC100360027;Lcn11;Lcn3	Transport
DMR3:3245001	3	3245001	3247000	2000	1	1.20E-14	0.55	29	1.45	LOC108348105;LOC100359901;LOC108350339;Glt6d1	Golgi
DMR3:3248001	3	3248001	3260000	12000	3	3.70E-12	-0.52	202	1.68	LOC108348105;LOC100359901;LOC108350339;Glt6d1	Golgi
DMR3:3264001	3	3264001	3265000	1000	1	5.10E-12	0.44	18	1.8	Glt6d1;Lcn9	Golgi;Transport
DMR3:3286001	3	3286001	3288000	2000	1	4.20E-10	0.35	33	1.65	Sohlh1	Transcription
DMR3:3307001	3	3307001	3308000	1000	1	4.80E-11	0.46	10	1	Kcnt1	Transport
DMR3:3321001	3	3321001	3325000	4000	1	1.20E-09	0.39	81	2.02	Kcnt1	Transport
DMR3:3335001	3	3335001	3341000	6000	2	4.70E-19	0.67	88	1.47	Kcnt1	Transport
DMR3:3378001	3	3378001	3382000	4000	1	1.20E-11	0.52	51	1.27	Camsap1;LOC100360117	
DMR3:3406001	3	3406001	3409000	3000	1	1.50E-07	-0.4	27	0.9	Camsap1	
DMR3:3422001	3	3422001	3424000	2000	2	6.20E-18	0.75	18	0.9	Camsap1	
DMR3:3433001	3	3433001	3434000	1000	1	5.00E-09	0.76	111	11.1	Camsap1;LOC102550543;LOC102552327	
DMR3:3441001	3	3441001	3443000	2000	1	8.90E-09	-0.64	27	1.35	Camsap1;LOC102550543;LOC102552327	
DMR3:3457001	3	3457001	3464000	7000	1	8.20E-07	-0.55	94	1.34	LOC102552327;Ubac1	
DMR3:3478001	3	3478001	3479000	1000	1	7.10E-19	0.88	10	1	Ubac1	
DMR3:3504001	3	3504001	3505000	1000	1	1.50E-08	-0.56	13	1.3	Nacc2	
DMR3:3518001	3	3518001	3520000	2000	1	2.10E-13	0.49	33	1.65	Nacc2	
DMR3:3524001	3	3524001	3526000	2000	1	1.80E-08	0.46	30	1.5	Nacc2	
DMR3:3529001	3	3529001	3531000	2000	1	3.50E-11	0.42	32	1.6	Nacc2	
DMR3:3550001	3	3550001	3553000	3000	1	8.40E-14	0.45	46	1.53	Nacc2	
DMR3:3582001	3	3582001	3586000	4000	1	1.30E-18	0.68	61	1.52	Nacc2;LOC103691752;RGD1564379	
DMR3:3598001	3	3598001	3605000	7000	3	1.10E-32	0.9	132	1.89	LOC103691752;RGD1564379	
DMR3:3639001	3	3639001	3645000	6000	2	3.80E-11	0.53	99	1.65	Lhx3	Development

DMR3:3671001	3	3671001	3672000	1000	1	2.70E-18	0.64	13	1.3	Lhx3;Qsox2	Development;Metabolism
DMR3:3679001	3	3679001	3682000	3000	1	1.60E-18	0.63	39	1.3	Qsox2;LOC103691753	Metabolism
DMR3:3696001	3	3696001	3697000	1000	1	3.10E-11	0.49	21	2.1	Qsox2;LOC103691753	Metabolism
DMR3:3712001	3	3712001	3722000	10000	1	5.40E-07	0.33	142	1.42	Ccdc187	Cytoskeleton
DMR3:3762001	3	3762001	3766000	4000	2	1.20E-16	0.94	52	1.3	Gpsm1	
DMR3:3774001	3	3774001	3776000	2000	1	3.00E-10	0.39	29	1.45	Gpsm1	
DMR3:3859001	3	3859001	3864000	5000	1	8.50E-14	0.46	71	1.42	Inpp5e;Sec16a	Signaling
DMR3:3869001	3	3869001	3872000	3000	2	1.20E-18	0.59	47	1.57	Sec16a	
DMR3:3876001	3	3876001	3877000	1000	1	3.60E-07	-0.67	18	1.8	Sec16a	
DMR3:3883001	3	3883001	3884000	1000	1	2.90E-19	0.54	9	0.9	Sec16a	
DMR3:3885001	3	3885001	3889000	4000	1	7.80E-08	-0.52	46	1.15	Sec16a	
DMR3:3890001	3	3890001	3891000	1000	1	3.90E-12	0.91	65	6.5	Sec16a	
DMR3:3906001	3	3906001	3908000	2000	1	3.40E-07	0.34	37	1.85	Notch1	Extracellular Matrix
DMR3:3940001	3	3940001	3942000	2000	1	1.40E-07	0.38	21	1.05	Notch1	Extracellular Matrix
DMR3:4024001	3	4024001	4028000	4000	2	9.60E-16	0.51	84	2.1	LOC108350341;Egfl7	Signaling
DMR3:4047001	3	4047001	4049000	2000	2	4.70E-17	0.57	34	1.7	Egfl7;Mir126a;Mir126;Agpat2	Signaling;Metabolism
DMR3:4052001	3	4052001	4054000	2000	1	9.90E-09	0.56	14	0.7	Egfl7;Mir126a;Mir126;Agpat2	Signaling;Metabolism
DMR3:4058001	3	4058001	4059000	1000	1	1.50E-17	0.88	6	0.6	Agpat2	Metabolism
DMR3:4064001	3	4064001	4065000	1000	1	8.60E-23	0.71	6	0.6	Agpat2	Metabolism
DMR3:4098001	3	4098001	4099000	1000	1	5.10E-08	-0.56	17	1.7	Fam69b;LOC100911266;LOC681186;LOC102547612	
DMR3:4367001	3	4367001	4369000	2000	1	4.80E-11	0.44	32	1.6	LOC100911704;Abo	Golgi
DMR3:4373001	3	4373001	4376000	3000	1	3.60E-08	0.43	46	1.53	LOC100911704;Abo	Golgi
DMR3:4384001	3	4384001	4386000	2000	2	2.20E-11	-0.43	20	1	LOC100911704;Abo	Golgi
DMR3:4391001	3	4391001	4392000	1000	1	7.80E-08	0.55	9	0.9	LOC100911704;Abo	Golgi
DMR3:4397001	3	4397001	4400000	3000	1	7.00E-15	0.56	34	1.13	LOC100911704;Abo	Golgi
DMR3:5102001	3	5102001	5103000	1000	1	2.80E-09	0.53	7	0.7	Gbgt1	
DMR3:5105001	3	5105001	5106000	1000	1	1.00E-19	0.98	3	0.3	Gbgt1	
DMR3:5109001	3	5109001	5110000	1000	1	3.20E-09	0.62	10	1	Gbgt1	
DMR3:5114001	3	5114001	5115000	1000	1	9.20E-09	0.52	5	0.5	Gbgt1	
DMR3:5133001	3	5133001	5134000	1000	1	1.20E-08	0.54	3	0.3	Gbgt1	
DMR3:5137001	3	5137001	5140000	3000	1	2.50E-11	-0.48	21	0.7	Gbgt1	
DMR3:5376001	3	5376001	5380000	4000	2	2.10E-13	-0.41	42	1.05	Abo2	Golgi
DMR3:5381001	3	5381001	5383000	2000	1	2.70E-07	-0.35	19	0.95	Abo2	Golgi
DMR3:5406001	3	5406001	5407000	1000	1	8.00E-09	0.35	9	0.9	Abo2	Golgi
DMR3:5410001	3	5410001	5412000	2000	1	5.90E-22	0.9	14	0.7	Abo2	Golgi
DMR3:5437001	3	5437001	5439000	2000	1	2.10E-13	0.49	33	1.65	LOC102548384;Surf6	Metabolism
DMR3:5442001	3	5442001	5450000	8000	1	3.00E-07	-0.49	155	1.94	LOC102548384;Surf6;Med22;Rpl7a	Metabolism;Transcription;Translation
DMR3:5457001	3	5457001	5458000	1000	1	1.40E-08	0.47	16	1.6	Surf6;Med22;Rpl7a;Surf1;Surf2	Metabolism;Transcription;Translation
DMR3:5484001	3	5484001	5487000	3000	2	3.30E-26	0.77	32	1.07	Surf4;Stkld1	Transcription;Signaling
DMR3:5491001	3	5491001	5494000	3000	1	1.70E-07	0.4	34	1.13	Surf4;Stkld1;Rexo4	Transcription;Signaling;Transcription
DMR3:5522001	3	5522001	5524000	2000	1	9.80E-18	0.51	26	1.3	Adamts13	Protease
DMR3:5525001	3	5525001	5534000	9000	5	4.90E-20	0.86	61	0.68	Adamts13	Protease
DMR3:5535001	3	5535001	5536000	1000	1	1.50E-14	0.65	16	1.6	Adamts13	Protease
DMR3:5602001	3	5602001	5606000	4000	2	2.60E-10	-0.64	53	1.32	Tmem8c	
DMR3:5622001	3	5622001	5624000	2000	1	1.40E-09	0.36	30	1.5	Tmem8c;Adamtsl2	Protease
DMR3:5640001	3	5640001	5643000	3000	2	1.10E-15	1	40	1.33	Adamtsl2	Protease
DMR3:5666001	3	5666001	5670000	4000	1	1.20E-08	0.35	58	1.45	Fam163b	
DMR3:5680001	3	5680001	5684000	4000	1	4.70E-11	0.41	47	1.18	Fam163b	
DMR3:5697001	3	5697001	5699000	2000	1	5.60E-11	0.56	33	1.65	Fam163b	
DMR3:5720001	3	5720001	5722000	2000	1	6.80E-07	0.32	38	1.9	Dbh	Metabolism
DMR3:5727001	3	5727001	5728000	1000	1	3.80E-07	0.4	17	1.7	Dbh;Sardh	Metabolism;Metabolism
DMR3:5730001	3	5730001	5732000	2000	1	1.10E-07	-0.48	34	1.7	Dbh;Sardh	Metabolism;Metabolism
DMR3:5736001	3	5736001	5738000	2000	1	9.60E-10	0.51	25	1.25	Dbh;Sardh	Metabolism;Metabolism

DMR3:5739001	3	5739001	5741000	2000	1	6.50E-12	0.78	55	2.75	Dbh;Sardh	Metabolism;Metabolism
DMR3:5754001	3	5754001	5756000	2000	2	7.30E-29	0.73	36	1.8	Sardh;LOC102548789	Metabolism
DMR3:5763001	3	5763001	5765000	2000	1	1.90E-09	0.47	22	1.1	Sardh;LOC102548789	Metabolism
DMR3:5766001	3	5766001	5768000	2000	1	3.20E-11	0.5	24	1.2	Sardh;LOC102548789	Metabolism
DMR3:5769001	3	5769001	5773000	4000	1	1.00E-13	0.47	78	1.95	Sardh;LOC102548789	Metabolism
DMR3:5790001	3	5790001	5791000	1000	1	8.30E-18	0.74	18	1.8	Sardh	Metabolism
DMR3:5798001	3	5798001	5800000	2000	1	1.30E-20	1.03	16	0.8	Sardh;LOC103691757	Metabolism
DMR3:5850001	3	5850001	5853000	3000	1	7.10E-07	-0.54	57	1.9	Vav2	
DMR3:5865001	3	5865001	5866000	1000	1	1.90E-11	0.56	10	1	Vav2	
DMR3:5885001	3	5885001	5893000	8000	2	1.90E-20	0.79	106	1.32	Vav2	
DMR3:5906001	3	5906001	5910000	4000	1	1.10E-20	0.94	66	1.65	Vav2	
DMR3:5919001	3	5919001	5926000	7000	3	1.50E-25	1	104	1.49	Vav2	
DMR3:5928001	3	5928001	5929000	1000	1	2.10E-08	0.36	21	2.1	Vav2	
DMR3:5931001	3	5931001	5938000	7000	5	7.60E-19	0.85	88	1.26	Vav2	
DMR3:5944001	3	5944001	5950000	6000	2	7.80E-08	0.36	99	1.65	Vav2	
DMR3:5960001	3	5960001	5962000	2000	2	3.80E-24	0.76	29	1.45	Vav2	
DMR3:5971001	3	5971001	5974000	3000	1	1.90E-18	0.6	43	1.43	Vav2	
DMR3:5978001	3	5978001	5980000	2000	1	1.80E-25	0.72	34	1.7	Vav2	
DMR3:5981001	3	5981001	5982000	1000	1	2.90E-25	0.71	15	1.5	Vav2;LOC100911432	
DMR3:5983001	3	5983001	5986000	3000	2	1.30E-15	0.58	38	1.27	Vav2;LOC100911432;Brd3	
DMR3:5987001	3	5987001	5988000	1000	1	6.20E-11	0.52	11	1.1	LOC100911432;Brd3	
DMR3:5990001	3	5990001	5993000	3000	2	3.40E-14	0.82	70	2.33	LOC100911432;Brd3;Mir7578	
DMR3:6005001	3	6005001	6009000	4000	1	9.00E-07	-0.55	58	1.45	Brd3;Mir7578	
DMR3:6011001	3	6011001	6015000	4000	1	2.50E-13	0.49	76	1.9	Brd3	
DMR3:6033001	3	6033001	6038000	5000	1	3.60E-23	0.76	78	1.56	Brd3	
DMR3:6049001	3	6049001	6050000	1000	1	3.10E-08	0.51	13	1.3	Brd3	
DMR3:6054001	3	6054001	6058000	4000	3	6.90E-12	0.75	50	1.25	Brd3;Wdr5	
DMR3:6087001	3	6087001	6089000	2000	1	5.60E-08	-0.49	15	0.75	Wdr5	
DMR3:6265001	3	6265001	6267000	2000	2	1.30E-14	0.61	26	1.3	Rxra	Transcription
DMR3:6270001	3	6270001	6272000	2000	1	1.10E-12	-0.57	39	1.95	Rxra	Transcription
DMR3:6273001	3	6273001	6278000	5000	1	1.40E-13	0.66	94	1.88	Rxra	Transcription
DMR3:6288001	3	6288001	6292000	4000	2	2.20E-15	-0.59	100	2.5	Rxra	Transcription
DMR3:6301001	3	6301001	6303000	2000	1	1.70E-24	1	30	1.5	Rxra	Transcription
DMR3:6304001	3	6304001	6308000	4000	3	1.10E-25	1.01	62	1.55	Rxra	Transcription
DMR3:6451001	3	6451001	6456000	5000	2	3.90E-12	0.48	67	1.34	Col5a1	Extracellular Matrix
DMR3:6457001	3	6457001	6459000	2000	1	5.80E-17	0.87	10	0.5	Col5a1	Extracellular Matrix
DMR3:6465001	3	6465001	6467000	2000	1	9.90E-09	-0.73	32	1.6	Col5a1	Extracellular Matrix
DMR3:6472001	3	6472001	6474000	2000	1	5.10E-07	0.41	55	2.75	Col5a1	Extracellular Matrix
DMR3:6481001	3	6481001	6484000	3000	2	1.80E-23	0.83	38	1.27	Col5a1	Extracellular Matrix
DMR3:6485001	3	6485001	6487000	2000	1	9.40E-14	0.49	27	1.35	Col5a1	Extracellular Matrix
DMR3:6488001	3	6488001	6496000	8000	2	1.40E-16	0.82	111	1.39	Col5a1	Extracellular Matrix
DMR3:6515001	3	6515001	6517000	2000	1	2.20E-09	0.42	26	1.3	Col5a1	Extracellular Matrix
DMR3:6535001	3	6535001	6537000	2000	1	1.70E-09	0.32	37	1.85	Col5a1	Extracellular Matrix
DMR3:6540001	3	6540001	6541000	1000	1	4.90E-09	0.64	13	1.3	Col5a1	Extracellular Matrix
DMR3:6560001	3	6560001	6565000	5000	2	2.40E-20	0.64	57	1.14	Col5a1	Extracellular Matrix
DMR3:6604001	3	6604001	6608000	4000	2	4.40E-19	0.85	48	1.2	Fcnb	
DMR3:6615001	3	6615001	6616000	1000	1	6.40E-15	0.44	17	1.7	Fcnb	
DMR3:6629001	3	6629001	6632000	3000	1	1.90E-08	0.45	43	1.43	Fcnb	
DMR3:6636001	3	6636001	6638000	2000	1	4.60E-07	0.3	48	2.4	Fcnb	
DMR3:6769001	3	6769001	6772000	3000	3	1.50E-19	0.72	26	0.87	Olfm1	Development
DMR3:6785001	3	6785001	6786000	1000	1	2.00E-22	0.76	11	1.1	Olfm1	Development
DMR3:6808001	3	6808001	6809000	1000	1	9.50E-11	0.45	10	1	Olfm1;LOC102549127	Development
DMR3:6970001	3	6970001	6972000	2000	1	3.20E-07	-0.56	14	0.7	RGD1564492	
DMR3:6980001	3	6980001	6984000	4000	3	8.90E-17	0.76	37	0.92	RGD1564492	
DMR3:7027001	3	7027001	7028000	1000	1	2.30E-09	0.53	13	1.3	Ppp1r26	
DMR3:7129001	3	7129001	7135000	6000	1	9.30E-13	0.48	111	1.85	Ralgds;Cel;Gtf3c5	Transcription;Cytoskeleton;Transcription
DMR3:7170001	3	7170001	7174000	4000	4	5.00E-23	0.86	55	1.38	Gtf3c5	Transcription
DMR3:7211001	3	7211001	7218000	7000	2	2.50E-14	0.64	84	1.2	Gfi1b;Tsc1	Transcription
DMR3:7226001	3	7226001	7228000	2000	1	9.40E-13	0.44	23	1.15	Tsc1	
DMR3:7232001	3	7232001	7234000	2000	2	1.60E-12	0.64	14	0.7	Tsc1	
DMR3:7257001	3	7257001	7259000	2000	1	3.10E-20	0.85	25	1.25	Tsc1	
DMR3:7260001	3	7260001	7263000	3000	1	9.10E-07	-0.58	54	1.8	Tsc1;Spaca9	
DMR3:7280001	3	7280001	7281000	1000	1	3.70E-10	0.45	13	1.3	Spaca9;Ak8	Signaling

DMR3:7292001	3	7292001	7293000	1000	1	1.80E-11	0.48	5	0.5	Ak8	Signaling
DMR3:7299001	3	7299001	7307000	8000	3	3.30E-19	0.65	131	1.64	Ak8	Signaling
DMR3:7309001	3	7309001	7311000	2000	1	7.90E-12	0.68	24	1.2	Ak8	Signaling
DMR3:7321001	3	7321001	7326000	5000	1	1.40E-17	0.62	86	1.72	Ak8	Signaling
DMR3:7337001	3	7337001	7339000	2000	1	1.00E-46	1.22	27	1.35	Ak8	Signaling
DMR3:7348001	3	7348001	7350000	2000	1	3.90E-19	0.73	38	1.9	Ak8	Signaling
DMR3:7354001	3	7354001	7355000	1000	1	5.30E-09	0.52	11	1.1	Ak8	Signaling
DMR3:7382001	3	7382001	7384000	2000	1	5.10E-07	0.32	27	1.35	Ak8	Signaling
DMR3:7390001	3	7390001	7391000	1000	1	6.70E-13	0.51	15	1.5	Ak8	Signaling
DMR3:7396001	3	7396001	7400000	4000	2	3.30E-14	0.58	48	1.2	Ak8;Gtf3c4	Signaling;Transcription
DMR3:7401001	3	7401001	7402000	1000	1	1.10E-35	1.18	4	0.4	Ak8;Gtf3c4	Signaling;Transcription
DMR3:7412001	3	7412001	7413000	1000	1	3.60E-12	0.63	13	1.3	Gtf3c4;Ddx31	Transcription
DMR3:7448001	3	7448001	7451000	3000	1	5.30E-10	-0.57	35	1.17	Ddx31	
DMR3:7452001	3	7452001	7455000	3000	1	2.90E-07	-0.43	44	1.47	Ddx31	
DMR3:7457001	3	7457001	7459000	2000	1	8.20E-11	-0.61	34	1.7	Ddx31	
DMR3:7471001	3	7471001	7474000	3000	2	7.40E-22	0.77	32	1.07	Ddx31	
DMR3:7476001	3	7476001	7478000	2000	1	3.90E-07	-0.51	22	1.1	Ddx31	
DMR3:7484001	3	7484001	7485000	1000	1	5.20E-16	0.61	16	1.6	Ddx31;Barhl1	Development
DMR3:7506001	3	7506001	7510000	4000	2	2.80E-07	0.35	55	1.38	Barhl1;Cfap77	Development;Development
DMR3:7518001	3	7518001	7519000	1000	1	6.30E-12	0.43	23	2.3	Cfap77	Development
DMR3:7522001	3	7522001	7527000	5000	2	2.90E-20	0.94	93	1.86	Cfap77	Development
DMR3:7531001	3	7531001	7533000	2000	2	5.50E-14	0.86	11	0.55	Cfap77	Development
DMR3:7576001	3	7576001	7579000	3000	1	1.00E-23	0.84	48	1.6	Cfap77	Development
DMR3:7587001	3	7587001	7591000	4000	1	2.80E-07	0.42	80	2	Cfap77	Development
DMR3:7618001	3	7618001	7620000	2000	2	9.20E-09	0.49	38	1.9	Cfap77	Development
DMR3:7652001	3	7652001	7656000	4000	1	2.90E-08	0.6	35	0.88	Ttf1;LOC108350345	
DMR3:7673001	3	7673001	7674000	1000	1	5.70E-08	0.41	15	1.5	LOC108350345;Setx	
DMR3:7675001	3	7675001	7676000	1000	1	7.40E-11	0.44	9	0.9	LOC108350345;Setx	
DMR3:7682001	3	7682001	7683000	1000	1	1.90E-08	0.36	11	1.1	LOC108350345;Setx	
DMR3:7710001	3	7710001	7711000	1000	1	2.00E-07	-0.53	14	1.4	Setx	
DMR3:7735001	3	7735001	7736000	1000	1	1.50E-07	0.37	17	1.7	Setx;Ntng2	Extracellular Matrix
DMR3:7741001	3	7741001	7743000	2000	1	3.20E-13	0.87	67	3.35	Setx;Ntng2	Extracellular Matrix
DMR3:7763001	3	7763001	7765000	2000	1	4.60E-08	0.67	9	0.45	Ntng2	Extracellular Matrix
DMR3:7766001	3	7766001	7770000	4000	2	4.00E-23	0.85	40	1	Ntng2	Extracellular Matrix
DMR3:7774001	3	7774001	7775000	1000	1	9.80E-18	0.76	11	1.1	Ntng2	Extracellular Matrix
DMR3:7792001	3	7792001	7794000	2000	1	1.90E-19	0.61	34	1.7	Ntng2	Extracellular Matrix
DMR3:7880001	3	7880001	7882000	2000	1	9.10E-07	-0.36	14	0.7	Med27	Transcription
DMR3:7901001	3	7901001	7902000	1000	1	6.00E-07	-0.46	15	1.5	Med27	Transcription
DMR3:7922001	3	7922001	7925000	3000	1	8.80E-07	-0.53	44	1.47	Med27	Transcription
DMR3:7935001	3	7935001	7938000	3000	1	3.60E-07	-0.53	44	1.47	Med27	Transcription
DMR3:7939001	3	7939001	7944000	5000	1	8.30E-11	-0.77	57	1.14	Med27	Transcription
DMR3:7956001	3	7956001	7957000	1000	1	9.40E-09	-0.58	12	1.2	Med27	Transcription
DMR3:8015001	3	8015001	8017000	2000	1	7.60E-10	0.41	28	1.4	Med27	Transcription
DMR3:8035001	3	8035001	8036000	1000	1	1.30E-09	0.54	12	1.2	Med27	Transcription
DMR3:8039001	3	8039001	8042000	3000	1	4.80E-09	-0.44	36	1.2	Med27	Transcription
DMR3:8196001	3	8196001	8197000	1000	1	3.80E-07	-0.55	12	1.2	Rapgef1	Transcription
DMR3:8198001	3	8198001	8204000	6000	3	2.10E-10	0.57	71	1.18	Rapgef1	Transcription
DMR3:8214001	3	8214001	8218000	4000	1	4.70E-21	0.67	56	1.4	Rapgef1	Transcription
DMR3:8230001	3	8230001	8232000	2000	1	8.90E-09	-0.43	15	0.75	Rapgef1	Transcription
DMR3:8234001	3	8234001	8237000	3000	1	2.40E-08	-0.63	32	1.07	Rapgef1	Transcription
DMR3:8254001	3	8254001	8256000	2000	2	2.80E-07	-0.55	27	1.35	Rapgef1	Transcription
DMR3:8264001	3	8264001	8267000	3000	1	5.60E-10	0.49	30	1	Rapgef1	Transcription
DMR3:8272001	3	8272001	8273000	1000	1	2.10E-15	0.7	8	0.8	Rapgef1	Transcription
DMR3:8285001	3	8285001	8287000	2000	1	1.70E-18	0.52	40	2	Rapgef1	Transcription
DMR3:8314001	3	8314001	8315000	1000	1	5.10E-12	0.63	12	1.2	Rapgef1;LOC102546892;LOC102547011	Transcription
DMR3:8402001	3	8402001	8403000	1000	1	9.40E-09	-0.75	13	1.3	Urm1;Mir2964;Mir219-2	
DMR3:8411001	3	8411001	8412000	1000	1	7.80E-10	0.66	13	1.3	Urm1;Mir2964;Mir219-2;Cercam	Golgi
DMR3:8427001	3	8427001	8429000	2000	1	7.00E-14	0.39	30	1.5	Cercam	Golgi
DMR3:8446001	3	8446001	8449000	3000	2	1.70E-12	0.59	30	1	Cercam;LOC108350590;Odf2	Golgi
DMR3:8454001	3	8454001	8458000	4000	2	2.50E-24	0.96	60	1.5	Cercam;LOC108350590;Odf2	Golgi

DMR3:8463001	3	8463001	8467000	4000	1	9.30E-10	-0.78	57	1.43	Odf2	
DMR3:8495001	3	8495001	8496000	1000	1	3.10E-08	-0.59	12	1.2	Odf2;LOC108350347;Gle1	Translation
DMR3:8530001	3	8530001	8531000	1000	1	3.20E-11	0.38	17	1.7	Gle1;Sptan1	Translation
DMR3:8551001	3	8551001	8552000	1000	1	1.90E-07	-0.55	8	0.8	Sptan1	
DMR3:8616001	3	8616001	8623000	7000	1	2.50E-07	-0.5	91	1.3	Wdr34;Pkn3	Cytoskeleton
DMR3:8636001	3	8636001	8642000	6000	1	6.80E-12	0.44	90	1.5	Pkn3	
DMR3:8649001	3	8649001	8654000	5000	1	2.40E-09	0.56	83	1.66	Pkn3;Zdhhc12;Zer1	
DMR3:8697001	3	8697001	8701000	4000	2	1.00E-20	1.15	39	0.98	Zer1;Tbc1d13	Signaling
DMR3:8734001	3	8734001	8740000	6000	3	2.50E-18	-0.51	61	1.02	Endog;LOC499770	
DMR3:8765001	3	8765001	8769000	4000	2	1.60E-12	0.63	73	1.82	Kyat1	Metabolism
DMR3:8776001	3	8776001	8777000	1000	1	1.10E-09	0.51	13	1.3	Kyat1	Metabolism
DMR3:8792001	3	8792001	8796000	4000	1	2.60E-12	0.5	53	1.32	Kyat1;LOC108350591;Lrrc8a	Metabolism;Cytoskeleton
DMR3:8811001	3	8811001	8815000	4000	1	1.30E-07	0.57	57	1.43	Lrrc8a	Cytoskeleton
DMR3:8820001	3	8820001	8823000	3000	1	4.00E-11	0.45	102	3.4	Lrrc8a;Phyhd1	Cytoskeleton;Metabolism
DMR3:8824001	3	8824001	8830000	6000	3	7.90E-14	-0.52	94	1.57	Lrrc8a;Phyhd1	Cytoskeleton;Metabolism
DMR3:8832001	3	8832001	8836000	4000	1	2.70E-15	0.5	57	1.43	Lrrc8a;Phyhd1	Cytoskeleton;Metabolism
DMR3:8866001	3	8866001	8871000	5000	1	7.70E-12	0.46	83	1.66	Nup188	
DMR3:8877001	3	8877001	8879000	2000	1	2.80E-07	-0.47	17	0.85	Nup188	
DMR3:8884001	3	8884001	8889000	5000	1	1.50E-08	-0.64	75	1.5	Nup188	
DMR3:8893001	3	8893001	8896000	3000	2	4.30E-14	0.56	60	2	Nup188	
DMR3:8917001	3	8917001	8919000	2000	1	6.00E-11	0.43	41	2.05	Nup188;Sh3glb2;Miga2	
DMR3:8937001	3	8937001	8939000	2000	1	2.20E-07	0.34	31	1.55	Miga2	
DMR3:8978001	3	8978001	8981000	3000	1	4.90E-17	0.62	45	1.5	Crat;Ptpa	Metabolism;Signaling
DMR3:9006001	3	9006001	9007000	1000	1	3.20E-10	0.46	14	1.4	Ptpa	Signaling
DMR3:9233001	3	9233001	9234000	1000	1	1.70E-07	0.38	9	0.9	Nup214;Aif1l	Transport;Signaling
DMR3:9263001	3	9263001	9266000	3000	1	5.00E-20	0.57	52	1.73	Aif1l;Lamc3	Signaling;Extracellular Matrix
DMR3:9303001	3	9303001	9304000	1000	1	1.30E-24	0.97	9	0.9	Lamc3	Extracellular Matrix
DMR3:9305001	3	9305001	9307000	2000	2	1.80E-38	1.19	25	1.25	Lamc3	Extracellular Matrix
DMR3:9308001	3	9308001	9310000	2000	1	6.70E-07	0.39	29	1.45	Lamc3	Extracellular Matrix
DMR3:9313001	3	9313001	9314000	1000	1	1.10E-09	0.44	12	1.2	Lamc3	Extracellular Matrix
DMR3:9321001	3	9321001	9322000	1000	1	4.80E-07	0.38	23	2.3	Lamc3	Extracellular Matrix
DMR3:9331001	3	9331001	9333000	2000	1	6.00E-13	0.47	32	1.6	Lamc3;LOC102554154	Extracellular Matrix
DMR3:9336001	3	9336001	9338000	2000	1	2.60E-07	0.46	25	1.25	Lamc3;LOC102554154	Extracellular Matrix
DMR3:9363001	3	9363001	9364000	1000	1	2.40E-19	1.01	6	0.6	Fibcd1	Signaling
DMR3:9390001	3	9390001	9391000	1000	1	8.30E-28	1.34	10	1	Fibcd1	Signaling
DMR3:9621001	3	9621001	9625000	4000	1	4.30E-09	0.52	57	1.43	RGD1311084	
DMR3:9668001	3	9668001	9669000	1000	1	4.30E-10	0.37	9	0.9	Ntmt1;Asb6	Epigenetic;Cytoskeleton
DMR3:9684001	3	9684001	9685000	1000	1	1.70E-07	0.48	9	0.9	Prrx2	
DMR3:9698001	3	9698001	9701000	3000	1	3.60E-18	0.65	38	1.27	Prrx2	
DMR3:9710001	3	9710001	9711000	1000	1	5.10E-07	-0.53	23	2.3	Prrx2	
DMR3:9714001	3	9714001	9717000	3000	1	6.80E-30	1.08	62	2.07	Prrx2	
DMR3:9719001	3	9719001	9724000	5000	4	4.30E-16	0.71	64	1.28	Prrx2;Ptges	Transport
DMR3:9784001	3	9784001	9786000	2000	1	2.10E-14	0.58	27	1.35	Tor1b	Transcription
DMR3:9809001	3	9809001	9814000	5000	2	3.30E-12	0.48	65	1.3	Tor1a;RGD1305178;Usp20	Transcription;Protease
DMR3:9861001	3	9861001	9866000	5000	3	1.20E-14	0.5	122	2.44	Usp20;Fnbp1	Protease
DMR3:9867001	3	9867001	9868000	1000	1	1.60E-15	0.51	12	1.2	Fnbp1	
DMR3:9872001	3	9872001	9873000	1000	1	1.50E-17	0.45	22	2.2	Fnbp1	
DMR3:9884001	3	9884001	9889000	5000	3	6.30E-23	0.62	118	2.36	Fnbp1	
DMR3:9918001	3	9918001	9920000	2000	1	1.40E-32	0.9	35	1.75	Fnbp1	
DMR3:9931001	3	9931001	9935000	4000	1	7.20E-08	0.44	79	1.98	Fnbp1;LOC102554659	
DMR3:9938001	3	9938001	9939000	1000	1	1.80E-36	1.18	9	0.9	Fnbp1;LOC102554659	
DMR3:9941001	3	9941001	9942000	1000	1	5.60E-07	0.38	12	1.2	Fnbp1;LOC102554659	
DMR3:9945001	3	9945001	9947000	2000	1	6.40E-08	0.41	36	1.8	Fnbp1;LOC102554659	
DMR3:9994001	3	9994001	9997000	3000	2	1.70E-16	0.95	45	1.5	Fibcd1l1	
DMR3:10022001	3	10022001	10024000	2000	2	2.30E-21	0.99	38	1.9	Fibcd1l1	
DMR3:10060001	3	10060001	10063000	3000	1	2.60E-09	0.41	47	1.57	Abl1	
DMR3:10136001	3	10136001	10140000	4000	2	2.50E-11	-0.42	62	1.55	Abl1	

DMR3:10167001	3	10167001	10172000	5000	4	5.20E-19	-0.7	63	1.26	Exosc2;Prdm12	Translation;Transcription
DMR3:10175001	3	10175001	10177000	2000	1	4.50E-09	0.36	31	1.55	Prdm12	Transcription
DMR3:10184001	3	10184001	10191000	7000	1	6.90E-08	0.46	114	1.63	Prdm12;LOC108350349	Transcription
DMR3:10204001	3	10204001	10205000	1000	1	3.20E-10	0.45	2	0.2	Prdm12;LOC108350349	Transcription
DMR3:10208001	3	10208001	10209000	1000	1	7.10E-23	0.8	9	0.9	Prdm12;LOC108350349	Transcription
DMR3:10210001	3	10210001	10212000	2000	2	5.90E-22	0.67	35	1.75	LOC108350349;Fubp3	Metabolism
DMR3:10264001	3	10264001	10265000	1000	1	9.50E-10	-0.71	14	1.4	Fubp3	Metabolism
DMR3:10320001	3	10320001	10324000	4000	3	6.20E-13	0.76	49	1.23	Ass1	Metabolism
DMR3:10342001	3	10342001	10344000	2000	1	1.50E-08	0.37	36	1.8	Ass1	Metabolism
DMR3:10347001	3	10347001	10348000	1000	1	2.50E-09	0.39	28	2.8	Ass1	Metabolism
DMR3:10356001	3	10356001	10361000	5000	3	2.80E-20	0.83	81	1.62	Ass1	Metabolism
DMR3:10377001	3	10377001	10379000	2000	1	2.30E-19	1.08	30	1.5	Ass1;Hmcn2	Metabolism
DMR3:10401001	3	10401001	10406000	5000	1	4.60E-09	-0.5	57	1.14	Hmcn2	
DMR3:10443001	3	10443001	10444000	1000	1	2.00E-19	0.78	9	0.9	Hmcn2	
DMR3:10450001	3	10450001	10453000	3000	1	1.50E-15	0.8	47	1.57	Hmcn2	
DMR3:10455001	3	10455001	10456000	1000	1	3.00E-25	0.86	15	1.5	Hmcn2	
DMR3:10458001	3	10458001	10459000	1000	1	2.60E-13	0.75	4	0.4	Hmcn2	
DMR3:10488001	3	10488001	10491000	3000	1	4.00E-08	0.39	55	1.83	Hmcn2	
DMR3:10500001	3	10500001	10501000	1000	1	2.10E-10	0.45	24	2.4	Hmcn2	
DMR3:10502001	3	10502001	10507000	5000	4	6.80E-23	1	75	1.5	Hmcn2	
DMR3:10538001	3	10538001	10539000	1000	1	5.20E-12	0.62	11	1.1	Hmcn2;LOC108350350	
DMR3:10546001	3	10546001	10548000	2000	1	6.50E-08	-0.44	33	1.65	Hmcn2;LOC108350350;Ncs1	
DMR3:10555001	3	10555001	10559000	4000	3	1.40E-13	-0.61	90	2.25	LOC108350350;Ncs1	
DMR3:10576001	3	10576001	10577000	1000	1	4.80E-10	-0.57	19	1.9	Ncs1	
DMR3:10582001	3	10582001	10586000	4000	2	6.30E-13	0.49	76	1.9	Ncs1;LOC103691761	
DMR3:10589001	3	10589001	10591000	2000	1	1.50E-13	0.46	34	1.7	Ncs1;LOC103691761	
DMR3:10608001	3	10608001	10609000	1000	1	6.70E-14	0.46	12	1.2	Ncs1	
DMR3:10610001	3	10610001	10613000	3000	1	1.20E-17	0.7	50	1.67	Ncs1	
DMR3:10651001	3	10651001	10655000	4000	1	2.80E-07	-0.5	71	1.77	Gpr107	Signaling
DMR3:10665001	3	10665001	10667000	2000	1	4.50E-17	0.51	29	1.45	Gpr107	Signaling
DMR3:10679001	3	10679001	10680000	1000	1	5.80E-07	-0.57	15	1.5	Gpr107	Signaling
DMR3:10990001	3	10990001	10993000	3000	2	9.70E-08	0.49	63	2.1	Nup214	Transport
DMR3:10994001	3	10994001	10996000	2000	2	5.20E-13	0.54	35	1.75	Nup214	Transport
DMR3:11004001	3	11004001	11009000	5000	3	2.30E-12	0.51	62	1.24	Nup214	Transport
DMR3:11090001	3	11090001	11094000	4000	1	1.90E-08	-0.56	71	1.77	LOC102555629;Fam78a	
DMR3:11130001	3	11130001	11135000	5000	3	6.80E-11	0.47	68	1.36	Plpp7	Signaling
DMR3:11156001	3	11156001	11161000	5000	1	1.50E-09	0.49	90	1.8	Prrc2b	Metabolism
DMR3:11169001	3	11169001	11170000	1000	1	1.40E-08	-0.52	21	2.1	Prrc2b	Metabolism
DMR3:11174001	3	11174001	11175000	1000	1	6.10E-09	0.4	32	3.2	Prrc2b	Metabolism
DMR3:11180001	3	11180001	11181000	1000	1	8.50E-14	1.16	33	3.3	Prrc2b	Metabolism
DMR3:11183001	3	11183001	11184000	1000	1	8.20E-07	-0.44	18	1.8	Prrc2b	Metabolism
DMR3:11194001	3	11194001	11196000	2000	1	6.10E-15	0.46	46	2.3	Prrc2b	Metabolism
DMR3:11217001	3	11217001	11222000	5000	1	6.70E-07	-0.37	105	2.1	Prrc2b	Metabolism
DMR3:11242001	3	11242001	11244000	2000	1	4.30E-20	0.77	27	1.35	Prrc2b;LOC103691765;Pomt1	Metabolism;Transport
DMR3:11280001	3	11280001	11281000	1000	1	4.60E-09	0.43	12	1.2	Pomt1;Uck1	Transport;Signaling
DMR3:11301001	3	11301001	11304000	3000	2	1.80E-08	-0.44	23	0.77	LOC103691766;Swi5	Transcription
DMR3:11322001	3	11322001	11324000	2000	1	6.50E-11	0.51	32	1.6	Swi5;Golga2	Transcription;Transport
DMR3:11338001	3	11338001	11339000	1000	1	2.90E-09	0.41	20	2	Golga2;Dnm1	Transport
DMR3:11346001	3	11346001	11347000	1000	1	2.40E-14	0.68	8	0.8	Golga2;Dnm1	Transport
DMR3:11348001	3	11348001	11354000	6000	1	1.80E-11	0.54	122	2.03	Dnm1	Transport
DMR3:11355001	3	11355001	11357000	2000	1	5.40E-20	0.58	35	1.75	Dnm1	Transport
DMR3:11362001	3	11362001	11364000	2000	2	3.00E-16	0.58	42	2.1	Dnm1	Transport
DMR3:11366001	3	11366001	11368000	2000	2	1.20E-07	-0.5	49	2.45	Dnm1	Transport
DMR3:11405001	3	11405001	11406000	1000	1	1.60E-16	0.59	15	1.5	Ciz1;RGD1561113;Lcn2	Transport
DMR3:11419001	3	11419001	11421000	2000	2	3.40E-15	0.9	21	1.05	Ciz1;RGD1561113;Lcn2;Ptges2	Transport;Metabolism
DMR3:11432001	3	11432001	11433000	1000	1	1.30E-09	0.39	23	2.3	Ptges2;Slc25a25	Metabolism;Transport
DMR3:11572001	3	11572001	11577000	5000	2	6.20E-13	0.47	80	1.6	Fam102a	
DMR3:11608001	3	11608001	11612000	4000	2	2.70E-11	0.52	61	1.52	St6galnac4;St6galnac6	
DMR3:11634001	3	11634001	11636000	2000	2	1.40E-11	0.44	29	1.45	St6galnac6	
DMR3:11637001	3	11637001	11641000	4000	3	9.00E-09	-0.46	67	1.68	St6galnac6;LOC108350550	

DMR3:11657001	3	11657001	11660000	3000	1	1.50E-12	0.75	40	1.33	LOC108350550;Ak1	Signaling
DMR3:11682001	3	11682001	11683000	1000	1	2.20E-18	0.61	12	1.2	Eng	Receptor
DMR3:11713001	3	11713001	11715000	2000	1	2.40E-20	0.79	30	1.5	Eng;Fpgs	Receptor;Metabolism
DMR3:11736001	3	11736001	11739000	3000	1	1.40E-09	0.36	47	1.57	Fpgs;LOC103691767;Cdk9	Metabolism;Signaling
DMR3:11756001	3	11756001	11760000	4000	1	1.80E-13	0.54	34	0.85	Cdk9;Sh2d3c	Signaling
DMR3:11771001	3	11771001	11777000	6000	3	6.70E-12	0.6	61	1.02	Sh2d3c	
DMR3:11801001	3	11801001	11805000	4000	1	6.90E-07	0.32	52	1.3	Sh2d3c;Tor2a;Ttc16;Ptrh1;Cfap157	Transcription;Metabolism;Development
DMR3:11807001	3	11807001	11808000	1000	1	2.40E-07	0.4	12	1.2	Tor2a;Ttc16;Ptrh1;Cfap157	Transcription;Metabolism;Development
DMR3:11815001	3	11815001	11817000	2000	1	1.20E-08	0.38	48	2.4	Ttc16;Ptrh1;Cfap157;Stxbp1	Metabolism;Development;Transport
DMR3:11848001	3	11848001	11850000	2000	2	7.40E-21	0.66	41	2.05	Stxbp1	Transport
DMR3:11851001	3	11851001	11853000	2000	2	1.50E-22	0.84	40	2	Stxbp1	Transport
DMR3:11876001	3	11876001	11878000	2000	2	2.90E-11	0.41	30	1.5	Stxbp1	Transport
DMR3:11879001	3	11879001	11880000	1000	1	7.70E-15	0.63	7	0.7	Stxbp1	Transport
DMR3:11884001	3	11884001	11889000	5000	2	6.20E-18	1.02	154	3.08	Stxbp1	Transport
DMR3:11913001	3	11913001	11915000	2000	1	4.90E-11	0.45	35	1.75	Fam129b	
DMR3:11916001	3	11916001	11919000	3000	2	3.00E-10	0.43	32	1.07	Fam129b	
DMR3:11924001	3	11924001	11925000	1000	1	2.00E-12	0.57	19	1.9	Fam129b	
DMR3:11926001	3	11926001	11927000	1000	1	7.70E-14	0.63	17	1.7	Fam129b	
DMR3:11930001	3	11930001	11934000	4000	1	2.20E-07	-0.49	57	1.43	Fam129b	
DMR3:11949001	3	11949001	11951000	2000	1	6.50E-09	0.39	41	2.05	Fam129b	
DMR3:11955001	3	11955001	11958000	3000	1	5.00E-13	0.48	54	1.8	Fam129b	
DMR3:11960001	3	11960001	11964000	4000	1	2.10E-08	-0.58	67	1.68	Fam129b;Lrsam1	
DMR3:11967001	3	11967001	11971000	4000	1	7.10E-11	0.43	104	2.6	Fam129b;Lrsam1	
DMR3:11972001	3	11972001	11973000	1000	1	7.90E-10	0.47	22	2.2	Fam129b;Lrsam1	
DMR3:11981001	3	11981001	11985000	4000	1	5.30E-07	-0.42	88	2.2	Fam129b;Lrsam1	
DMR3:12124001	3	12124001	12127000	3000	1	2.80E-07	0.36	28	0.93	Garnl3;LOC102557449	Signaling
DMR3:12130001	3	12130001	12134000	4000	2	1.50E-17	0.57	54	1.35	Garnl3;LOC102557449	Signaling
DMR3:12144001	3	12144001	12147000	3000	1	5.80E-10	0.41	22	0.73	Garnl3	Signaling
DMR3:12164001	3	12164001	12165000	1000	1	1.50E-08	-0.73	11	1.1	Garnl3	Signaling
DMR3:12176001	3	12176001	12179000	3000	1	4.50E-11	0.63	129	4.3	Garnl3;Ralgps1	Signaling;Transcription
DMR3:12223001	3	12223001	12224000	1000	1	6.10E-07	-0.45	14	1.4	Ralgps1	Transcription
DMR3:12227001	3	12227001	12230000	3000	1	4.20E-10	0.51	28	0.93	Ralgps1	Transcription
DMR3:12240001	3	12240001	12243000	3000	2	2.90E-11	0.45	31	1.03	Ralgps1	Transcription
DMR3:12257001	3	12257001	12259000	2000	1	4.50E-09	0.49	25	1.25	Ralgps1;Angptl2	Transcription;Signaling
DMR3:12316001	3	12316001	12317000	1000	1	1.70E-08	0.52	9	0.9	Ralgps1	Transcription
DMR3:12335001	3	12335001	12336000	1000	1	2.00E-08	0.46	9	0.9	Ralgps1	Transcription
DMR3:12370001	3	12370001	12371000	1000	1	5.00E-16	0.53	10	1	Ralgps1	Transcription
DMR3:12390001	3	12390001	12395000	5000	1	8.80E-10	-0.41	52	1.04	Ralgps1	Transcription
DMR3:12396001	3	12396001	12398000	2000	1	3.50E-11	0.55	16	0.8	Ralgps1	Transcription
DMR3:12463001	3	12463001	12466000	3000	1	7.80E-09	-0.7	36	1.2	Zbtb34	Transcription
DMR3:12480001	3	12480001	12487000	7000	1	2.70E-10	0.35	113	1.61	Zbtb43	Cytoskeleton
DMR3:12499001	3	12499001	12502000	3000	1	1.00E-09	0.35	41	1.37	Zbtb43	Cytoskeleton
DMR3:12608001	3	12608001	12612000	4000	1	1.50E-20	0.71	84	2.1	Lmx1b	Development
DMR3:12614001	3	12614001	12615000	1000	1	3.80E-07	0.31	21	2.1	Lmx1b	Development
DMR3:12620001	3	12620001	12623000	3000	1	5.80E-10	0.39	52	1.73	Lmx1b	Development
DMR3:12624001	3	12624001	12625000	1000	1	4.50E-22	0.89	17	1.7	Lmx1b	Development
DMR3:12636001	3	12636001	12638000	2000	1	1.90E-15	0.48	36	1.8	Lmx1b	Development
DMR3:12642001	3	12642001	12643000	1000	1	2.60E-07	0.36	3	0.3	Lmx1b	Development
DMR3:12648001	3	12648001	12649000	1000	1	4.60E-15	0.63	12	1.2	Lmx1b	Development
DMR3:12652001	3	12652001	12653000	1000	1	5.50E-09	0.44	10	1	Lmx1b	Development
DMR3:12654001	3	12654001	12657000	3000	1	1.70E-16	0.74	34	1.13	Lmx1b	Development
DMR3:12659001	3	12659001	12661000	2000	1	9.50E-12	0.44	21	1.05	Lmx1b	Development
DMR3:12674001	3	12674001	12675000	1000	1	7.20E-25	0.93	2	0.2	Lmx1b	Development
DMR3:12777001	3	12777001	12782000	5000	1	1.00E-07	0.32	96	1.92	Mvb12b	
DMR3:12784001	3	12784001	12786000	2000	2	8.70E-09	0.43	25	1.25	Mvb12b	
DMR3:12794001	3	12794001	12798000	4000	1	3.80E-11	0.4	75	1.88	Mvb12b	
DMR3:12802001	3	12802001	12804000	2000	1	1.90E-12	0.5	27	1.35	Mvb12b	
DMR3:12831001	3	12831001	12835000	4000	1	1.20E-07	0.4	69	1.73	Mvb12b	

DMR3:12842001	3	12842001	12843000	1000	1	1.30E-12	0.49	10	1	Mvb12b	
DMR3:12853001	3	12853001	12855000	2000	1	3.00E-10	0.43	42	2.1	Mvb12b	
DMR3:12864001	3	12864001	12866000	2000	1	5.60E-08	-0.43	34	1.7	Mvb12b	
DMR3:12897001	3	12897001	12900000	3000	1	4.90E-19	0.83	53	1.77	Mvb12b	
DMR3:12901001	3	12901001	12902000	1000	1	3.30E-09	0.75	8	0.8	Mvb12b	
DMR3:12904001	3	12904001	12905000	1000	1	1.60E-07	-0.58	17	1.7	Mvb12b	
DMR3:12932001	3	12932001	12936000	4000	1	5.60E-14	0.5	82	2.05	Mvb12b	
DMR3:13229001	3	13229001	13232000	3000	1	2.20E-10	0.43	33	1.1	Pbx3	Development
DMR3:13292001	3	13292001	13296000	4000	2	7.60E-14	-0.72	51	1.27	Pbx3	Development
DMR3:13328001	3	13328001	13329000	1000	1	8.40E-10	0.57	7	0.7	Pbx3	Development
DMR3:13349001	3	13349001	13351000	2000	1	6.00E-11	-0.97	20	1	Pbx3	Development
DMR3:13355001	3	13355001	13356000	1000	1	3.20E-07	0.4	8	0.8	Pbx3	Development
DMR3:13366001	3	13366001	13371000	5000	1	9.30E-08	-0.51	48	0.96	Pbx3	Development
DMR3:13401001	3	13401001	13403000	2000	1	8.80E-13	0.59	24	1.2	Pbx3;LOC103691768	Development
DMR3:13495001	3	13495001	13498000	3000	1	2.30E-07	0.49	33	1.1	Mapkap1	Cytoskeleton
DMR3:13501001	3	13501001	13503000	2000	1	6.70E-08	-0.49	26	1.3	Mapkap1	Cytoskeleton
DMR3:13510001	3	13510001	13511000	1000	1	1.30E-13	0.49	12	1.2	Mapkap1;LOC100911753	Cytoskeleton
DMR3:13514001	3	13514001	13515000	1000	1	2.90E-23	1	4	0.4	Mapkap1;LOC100911753	Cytoskeleton
DMR3:13517001	3	13517001	13520000	3000	1	1.60E-07	0.35	25	0.83	Mapkap1;LOC100911753	Cytoskeleton
DMR3:13537001	3	13537001	13539000	2000	2	3.50E-33	1.09	16	0.8	Mapkap1	Cytoskeleton
DMR3:13604001	3	13604001	13609000	5000	1	5.10E-08	-0.57	64	1.28	Mapkap1	Cytoskeleton
DMR3:13610001	3	13610001	13612000	2000	1	2.30E-09	0.44	23	1.15	Mapkap1	Cytoskeleton
DMR3:13653001	3	13653001	13654000	1000	1	3.60E-07	0.37	15	1.5	Mapkap1	Cytoskeleton
DMR3:13655001	3	13655001	13659000	4000	1	7.40E-17	0.56	55	1.38	Mapkap1	Cytoskeleton
DMR3:13660001	3	13660001	13663000	3000	2	2.30E-13	0.44	37	1.23	Mapkap1	Cytoskeleton
DMR3:13664001	3	13664001	13667000	3000	1	3.90E-07	-0.36	39	1.3	Mapkap1	Cytoskeleton
DMR3:13694001	3	13694001	13695000	1000	1	4.70E-09	0.42	11	1.1	Mapkap1	Cytoskeleton
DMR3:13742001	3	13742001	13745000	3000	2	1.90E-12	0.48	40	1.33	Gapvd1	Transcription
DMR3:13790001	3	13790001	13792000	2000	1	4.00E-07	-0.45	22	1.1	Gapvd1	Transcription
DMR3:13793001	3	13793001	13794000	1000	1	5.50E-17	0.57	9	0.9	Gapvd1	Transcription
DMR3:13850001	3	13850001	13864000	14000	2	7.40E-25	0.69	206	1.47	Hspa5;Rabepk;Fbxw2	
DMR3:13922001	3	13922001	13932000	10000	1	9.20E-07	0.31	156	1.56	Psmd5;RGD1564854	Protease;Transport
DMR3:14002001	3	14002001	14005000	3000	2	8.80E-22	0.87	50	1.67	LOC102550940;Traf1	Cytoskeleton
DMR3:14030001	3	14030001	14031000	1000	1	1.70E-07	-0.53	18	1.8	Traf1	Cytoskeleton
DMR3:14054001	3	14054001	14055000	1000	1	1.80E-08	0.35	15	1.5	C5	
DMR3:14061001	3	14061001	14064000	3000	2	5.70E-21	0.93	36	1.2	C5	
DMR3:14079001	3	14079001	14080000	1000	1	3.10E-08	0.37	10	1	C5	
DMR3:14091001	3	14091001	14094000	3000	1	5.20E-19	0.63	44	1.47	C5	
DMR3:14097001	3	14097001	14098000	1000	1	7.10E-15	-0.75	5	0.5	C5	
DMR3:14099001	3	14099001	14100000	1000	1	3.20E-08	-0.43	6	0.6	C5	
DMR3:14116001	3	14116001	14118000	2000	1	4.40E-08	-0.38	16	0.8	C5	
DMR3:14205001	3	14205001	14206000	1000	1	3.20E-11	0.62	13	1.3	LOC100912221;C5	
DMR3:14207001	3	14207001	14209000	2000	2	4.50E-15	0.66	29	1.45	LOC100912221;C5	
DMR3:14216001	3	14216001	14218000	2000	1	6.10E-07	0.32	24	1.2	C5	
DMR3:14229001	3	14229001	14234000	5000	4	2.10E-27	-0.84	55	1.1	C5	
DMR3:14262001	3	14262001	14265000	3000	3	3.20E-16	0.7	30	1	LOC103691771;RGD1308742	
DMR3:14267001	3	14267001	14269000	2000	1	2.70E-08	0.3	19	0.95	LOC103691771;RGD1308742	
DMR3:14272001	3	14272001	14275000	3000	1	7.10E-07	-0.45	48	1.6	RGD1308742	
DMR3:14293001	3	14293001	14296000	3000	1	4.80E-07	0.34	61	2.03	RGD1308742;Cntrl	
DMR3:14301001	3	14301001	14302000	1000	1	2.70E-07	0.48	8	0.8	RGD1308742;Cntrl	
DMR3:14313001	3	14313001	14314000	1000	1	6.80E-08	0.4	6	0.6	Cntrl	
DMR3:14328001	3	14328001	14330000	2000	1	4.00E-07	-0.42	27	1.35	Cntrl	
DMR3:14336001	3	14336001	14342000	6000	1	1.50E-08	0.39	76	1.27	Cntrl	
DMR3:14372001	3	14372001	14374000	2000	1	5.40E-11	-0.68	26	1.3	Cntrl;Rab14	
DMR3:14444001	3	14444001	14450000	6000	1	5.70E-20	0.64	77	1.28	LOC102554040;Gsn	Cytoskeleton
DMR3:14451001	3	14451001	14453000	2000	2	4.80E-12	-0.79	32	1.6	LOC102554040;Gsn	Cytoskeleton
DMR3:14473001	3	14473001	14474000	1000	1	2.60E-11	0.48	13	1.3	Gsn	Cytoskeleton
DMR3:14486001	3	14486001	14488000	2000	2	1.20E-08	-0.68	33	1.65	Gsn	Cytoskeleton
DMR3:14489001	3	14489001	14492000	3000	1	2.20E-09	0.36	50	1.67	Gsn	Cytoskeleton
DMR3:14498001	3	14498001	14500000	2000	1	4.60E-18	0.47	43	2.15	Gsn	Cytoskeleton
DMR3:14501001	3	14501001	14504000	3000	1	1.60E-10	0.55	37	1.23	Gsn	Cytoskeleton
DMR3:14508001	3	14508001	14511000	3000	1	1.60E-07	-0.36	59	1.97	Gsn;Stom	Cytoskeleton;Cytoskeleton
DMR3:14520001	3	14520001	14521000	1000	1	3.60E-13	0.48	19	1.9	Stom;LOC108350355	Cytoskeleton
DMR3:14542001	3	14542001	14543000	1000	1	3.00E-10	0.48	19	1.9	Stom	Cytoskeleton

DMR3:14549001	3	14549001	14550000	1000	1	4.10E-09	0.39	16	1.6	Ggta1l1	Golgi
DMR3:14559001	3	14559001	14560000	1000	1	1.80E-18	0.62	9	0.9	Ggta1l1	Golgi
DMR3:14564001	3	14564001	14566000	2000	1	7.20E-17	0.87	24	1.2	Ggta1l1;LOC102548933	Golgi
DMR3:14569001	3	14569001	14570000	1000	1	6.00E-15	0.53	12	1.2	Ggta1l1;LOC102548933	Golgi
DMR3:14572001	3	14572001	14575000	3000	1	3.10E-08	0.47	34	1.13	Ggta1l1;LOC102548933	Golgi
DMR3:14623001	3	14623001	14628000	5000	3	2.70E-13	0.58	88	1.76	LOC502618;Ggta1	Golgi
DMR3:14638001	3	14638001	14640000	2000	1	1.10E-08	-0.54	47	2.35	Ggta1	Golgi
DMR3:14672001	3	14672001	14676000	4000	1	2.70E-07	-0.43	78	1.95	Ggta1	Golgi
DMR3:14678001	3	14678001	14683000	5000	2	2.10E-16	0.56	73	1.46	Ggta1	Golgi
DMR3:14690001	3	14690001	14694000	4000	2	3.90E-16	0.59	65	1.62	Ggta1	Golgi
DMR3:14865001	3	14865001	14869000	4000	1	8.70E-07	0.49	67	1.68	RGD1564343	
DMR3:14876001	3	14876001	14879000	3000	2	2.30E-11	0.46	66	2.2	RGD1564343	
DMR3:14899001	3	14899001	14902000	3000	2	5.00E-13	0.48	44	1.47	Dab2ip	Signaling
DMR3:14908001	3	14908001	14911000	3000	1	1.00E-12	0.45	40	1.33	Dab2ip;LOC108350356	Signaling
DMR3:14931001	3	14931001	14934000	3000	1	1.30E-13	0.76	34	1.13	Dab2ip;LOC108350356	Signaling
DMR3:14941001	3	14941001	14943000	2000	2	4.90E-12	0.44	35	1.75	Dab2ip	Signaling
DMR3:14945001	3	14945001	14947000	2000	1	1.00E-19	0.74	20	1	Dab2ip	Signaling
DMR3:14967001	3	14967001	14968000	1000	1	2.80E-07	0.3	21	2.1	Dab2ip	Signaling
DMR3:14972001	3	14972001	14975000	3000	2	2.60E-10	0.4	83	2.77	Dab2ip	Signaling
DMR3:14980001	3	14980001	14981000	1000	1	2.30E-07	0.37	12	1.2	Dab2ip	Signaling
DMR3:15027001	3	15027001	15032000	5000	1	4.30E-08	0.49	46	0.92	Dab2ip	Signaling
DMR3:15063001	3	15063001	15069000	6000	2	2.00E-09	-0.62	92	1.53	Dab2ip	Signaling
DMR3:15070001	3	15070001	15072000	2000	1	1.30E-21	0.65	41	2.05	Dab2ip	Signaling
DMR3:15087001	3	15087001	15092000	5000	2	3.10E-23	0.92	54	1.08	Ttll11	Cytoskeleton
DMR3:15102001	3	15102001	15103000	1000	1	3.20E-22	0.66	20	2	Ttll11	Cytoskeleton
DMR3:15104001	3	15104001	15115000	11000	4	4.20E-15	0.62	161	1.46	Ttll11	Cytoskeleton
DMR3:15120001	3	15120001	15122000	2000	1	1.20E-10	0.5	28	1.4	Ttll11	Cytoskeleton
DMR3:15138001	3	15138001	15145000	7000	1	8.90E-09	-0.47	122	1.74	Ttll11	Cytoskeleton
DMR3:15146001	3	15146001	15149000	3000	1	2.00E-07	0.38	58	1.93	Ttll11	Cytoskeleton
DMR3:15160001	3	15160001	15162000	2000	1	9.70E-07	-0.61	21	1.05	Ttll11	Cytoskeleton
DMR3:15174001	3	15174001	15182000	8000	4	2.20E-24	0.7	155	1.94	Ttll11	Cytoskeleton
DMR3:15208001	3	15208001	15211000	3000	1	3.40E-09	-0.65	50	1.67	Ttll11;LOC108350357	Cytoskeleton
DMR3:15230001	3	15230001	15233000	3000	1	1.40E-14	0.42	28	0.93	Ttll11	Cytoskeleton
DMR3:15251001	3	15251001	15253000	2000	1	7.60E-07	-0.59	26	1.3	Ttll11	Cytoskeleton
DMR3:15261001	3	15261001	15263000	2000	1	1.80E-07	0.36	31	1.55	Ttll11	Cytoskeleton
DMR3:15264001	3	15264001	15266000	2000	1	1.30E-14	0.85	26	1.3	Ttll11	Cytoskeleton
DMR3:15278001	3	15278001	15279000	1000	1	6.20E-14	0.55	28	2.8	Ttll11	Cytoskeleton
DMR3:15322001	3	15322001	15323000	1000	1	4.40E-23	0.7	13	1.3	Ttll11	Cytoskeleton
DMR3:15361001	3	15361001	15363000	2000	1	1.60E-25	0.75	28	1.4	Ndufa8	Metabolism
DMR3:15389001	3	15389001	15391000	2000	2	1.60E-08	0.43	38	1.9	Ndufa8;Morn5	Metabolism
DMR3:15409001	3	15409001	15413000	4000	1	7.00E-12	0.47	76	1.9	Morn5;Lhx6	Development
DMR3:15442001	3	15442001	15446000	4000	1	6.90E-33	0.93	47	1.18	Lhx6;Rbm18	Development;Metabolism
DMR3:15484001	3	15484001	15491000	7000	3	1.00E-14	-0.58	77	1.1	Mrrf	Translation
DMR3:15498001	3	15498001	15499000	1000	1	3.40E-07	-0.59	15	1.5	Mrrf	Translation
DMR3:15509001	3	15509001	15512000	3000	1	4.90E-11	0.54	22	0.73	Mrrf;LOC108350359	Translation
DMR3:15525001	3	15525001	15527000	2000	1	1.30E-12	0.45	24	1.2	Mrrf;LOC108350359	Translation
DMR3:15549001	3	15549001	15553000	4000	2	1.30E-11	0.6	29	0.72	Ptgs1	Metabolism
DMR3:15562001	3	15562001	15567000	5000	1	4.60E-08	-0.51	67	1.34	Ptgs1	Metabolism
DMR3:15575001	3	15575001	15577000	2000	1	3.10E-08	0.34	23	1.15	Ptgs1	Metabolism
DMR3:15583001	3	15583001	15586000	3000	2	5.20E-08	0.44	26	0.87	Ptgs1	Metabolism
DMR3:15605001	3	15605001	15606000	1000	1	1.50E-07	0.48	7	0.7	RGD1561766	
DMR3:15610001	3	15610001	15611000	1000	1	6.60E-16	0.63	10	1	Olr393-ps	
DMR3:15621001	3	15621001	15626000	5000	1	5.10E-09	-0.62	29	0.58	Olr393-ps	
DMR3:15628001	3	15628001	15639000	11000	7	7.70E-28	-0.51	110	1	Olr393-ps;Olr394-ps	
DMR3:15646001	3	15646001	15651000	5000	2	4.20E-11	-0.34	37	0.74	Olr394-ps	
DMR3:15688001	3	15688001	15690000	2000	2	1.10E-10	0.72	12	0.6	Olr395	Receptor
DMR3:15693001	3	15693001	15701000	8000	3	2.50E-18	0.59	89	1.11	Olr395	Receptor
DMR3:15739001	3	15739001	15742000	3000	1	8.70E-12	-0.53	9	0.3	Olr396	Receptor
DMR3:15752001	3	15752001	15756000	4000	1	9.00E-12	-0.52	26	0.65	Olr396	Receptor
DMR3:15763001	3	15763001	15770000	7000	3	1.30E-11	-0.59	31	0.44	Klf5-ps1	
DMR3:15780001	3	15780001	15781000	1000	1	7.60E-09	0.47	12	1.2	Klf5-ps1;Olr397	Receptor
DMR3:15816001	3	15816001	15818000	2000	1	4.90E-13	0.64	13	0.65	Olr398	Receptor
DMR3:15920001	3	15920001	15921000	1000	1	2.10E-08	-0.55	3	0.3	Olr401	Receptor
DMR3:15922001	3	15922001	15923000	1000	1	1.30E-08	0.7	3	0.3	Olr401	Receptor

DMR3:15924001	3	15924001	15926000	2000	1	8.30E-12	0.58	15	0.75	Olr401	Receptor
DMR3:15936001	3	15936001	15938000	2000	1	3.40E-09	0.52	10	0.5	Olr401	Receptor
DMR3:16011001	3	16011001	16013000	2000	1	3.30E-08	-0.34	17	0.85	Olr402	Receptor
DMR3:16042001	3	16042001	16048000	6000	4	3.90E-22	-0.6	66	1.1	Olr403	Receptor
DMR3:16107001	3	16107001	16112000	5000	1	5.60E-07	-0.48	33	0.66	Olr404	Receptor
DMR3:16175001	3	16175001	16176000	1000	1	3.50E-10	0.66	4	0.4	Olr406	Receptor
DMR3:16187001	3	16187001	16189000	2000	1	1.90E-08	-0.42	19	0.95	Olr406	Receptor
DMR3:16230001	3	16230001	16238000	8000	5	1.90E-23	-0.46	83	1.04	Olr407	Receptor
DMR3:16248001	3	16248001	16254000	6000	3	6.60E-14	-0.49	60	1	Olr407	Receptor
DMR3:16293001	3	16293001	16294000	1000	1	1.30E-08	-0.52	1	0.1	Olr408	Receptor
DMR3:17174001	3	17174001	17175000	1000	1	2.00E-09	0.64	6	0.6	RGD1565463	
DMR3:17660001	3	17660001	17661000	1000	1	9.80E-07	0.55	10	1	LOC690737;RGD1563602	
DMR3:17666001	3	17666001	17667000	1000	1	1.00E-08	0.5	5	0.5	LOC690737;RGD1563602	
DMR3:17675001	3	17675001	17676000	1000	1	2.90E-13	0.65	8	0.8	RGD1563602	
DMR3:17865001	3	17865001	17867000	2000	1	3.60E-14	0.51	12	0.6	Igkv28	
DMR3:17868001	3	17868001	17870000	2000	1	2.30E-19	0.69	17	0.85	Igkv28	
DMR3:17876001	3	17876001	17879000	3000	1	5.10E-07	0.38	23	0.77	Igkv28	
DMR3:18307001	3	18307001	18310000	3000	1	6.10E-09	-0.51	16	0.53	RGD1565546	
DMR3:19122001	3	19122001	19123000	1000	1	5.60E-10	0.71	8	0.8	RGD1564476	
DMR3:19184001	3	19184001	19187000	3000	1	1.50E-09	0.58	18	0.6	LOC690580;RGD1563500	
DMR3:20010001	3	20010001	20014000	4000	2	6.20E-07	-0.47	20	0.5	RGD1563433;LOC102551399	
DMR3:20347001	3	20347001	20349000	2000	1	3.80E-15	-0.58	9	0.45	RGD1565269	
DMR3:20839001	3	20839001	20843000	4000	2	1.70E-15	0.75	12	0.3	Olr411	Receptor
DMR3:20844001	3	20844001	20845000	1000	1	8.00E-12	0.68	4	0.4	Olr411	Receptor
DMR3:20870001	3	20870001	20871000	1000	1	1.70E-14	0.86	4	0.4	Olr412-ps;Olr413	Receptor
DMR3:20875001	3	20875001	20876000	1000	1	2.50E-11	-0.72	7	0.7	Olr412-ps;Olr413;Olr414	Receptor
DMR3:20901001	3	20901001	20904000	3000	1	5.00E-09	-0.58	13	0.43	Olr415;Olr416	Receptor
DMR3:20966001	3	20966001	20968000	2000	1	2.30E-07	0.51	14	0.7	Olr417	Receptor
DMR3:20972001	3	20972001	20973000	1000	1	6.60E-07	0.57	1	0.1	Olr417;Olr418	Receptor
DMR3:21055001	3	21055001	21056000	1000	1	1.30E-07	-0.47	2	0.2	Olr422	Receptor
DMR3:21071001	3	21071001	21074000	3000	1	3.10E-19	0.62	23	0.77	Olr422	Receptor
DMR3:21098001	3	21098001	21105000	7000	1	8.90E-22	0.82	31	0.44	Olr423	Receptor
DMR3:21123001	3	21123001	21125000	2000	1	4.70E-10	-0.53	5	0.25	Olr423;Olr424	Receptor
DMR3:21148001	3	21148001	21151000	3000	1	5.00E-13	0.7	24	0.8	Olr425	Receptor
DMR3:21211001	3	21211001	21213000	2000	1	3.70E-08	-0.52	4	0.2	Olr427	Receptor
DMR3:21214001	3	21214001	21222000	8000	3	3.80E-25	-0.55	104	1.3	Olr427	Receptor
DMR3:21280001	3	21280001	21281000	1000	1	8.00E-07	0.55	6	0.6	Olr428	Receptor
DMR3:21354001	3	21354001	21358000	4000	1	9.90E-12	-0.59	9	0.22	Olr434	Signaling
DMR3:21370001	3	21370001	21372000	2000	1	3.40E-09	0.47	10	0.5	Olr434;LOC690155	Signaling
DMR3:21568001	3	21568001	21571000	3000	2	1.70E-09	0.43	18	0.6	LOC100911251;Klf5-ps2	
DMR3:21572001	3	21572001	21580000	8000	3	3.50E-10	0.47	57	0.71	LOC100911251;Klf5-ps2;LOC690273;LOC108350364	
DMR3:21597001	3	21597001	21599000	2000	2	4.60E-12	0.57	16	0.8	LOC108350364;Pdcl	
DMR3:21613001	3	21613001	21614000	1000	1	2.60E-07	0.43	11	1.1	Pdcl;LOC102556236;Rc3h2	
DMR3:21615001	3	21615001	21617000	2000	1	3.00E-07	0.4	12	0.6	Pdcl;LOC102556236;Rc3h2	
DMR3:21632001	3	21632001	21633000	1000	1	2.40E-07	0.33	5	0.5	Rc3h2	
DMR3:21640001	3	21640001	21641000	1000	1	8.60E-09	0.61	5	0.5	Rc3h2	
DMR3:21663001	3	21663001	21665000	2000	1	2.10E-12	0.47	16	0.8	Rc3h2	
DMR3:21737001	3	21737001	21739000	2000	1	3.60E-08	0.49	8	0.4	Rabgap1	Signaling
DMR3:21742001	3	21742001	21744000	2000	1	1.20E-07	0.34	16	0.8	Rabgap1	Signaling
DMR3:21756001	3	21756001	21758000	2000	1	4.00E-18	0.68	19	0.95	Rabgap1;Gpr21	Signaling;Signaling
DMR3:21801001	3	21801001	21802000	1000	1	5.30E-10	0.61	8	0.8	Rabgap1	Signaling
DMR3:21835001	3	21835001	21838000	3000	1	3.20E-08	0.38	20	0.67	Strbp	Metabolism
DMR3:21853001	3	21853001	21854000	1000	1	5.30E-13	0.86	6	0.6	Strbp	Metabolism
DMR3:21866001	3	21866001	21871000	5000	1	1.30E-18	1.04	19	0.38	Strbp	Metabolism
DMR3:21910001	3	21910001	21914000	4000	2	5.40E-09	0.44	15	0.38	Strbp	Metabolism
DMR3:21935001	3	21935001	21936000	1000	1	1.90E-10	0.6	6	0.6	Strbp	Metabolism
DMR3:21965001	3	21965001	21967000	2000	1	1.30E-11	0.6	26	1.3	Strbp	Metabolism
DMR3:22049001	3	22049001	22051000	2000	1	3.70E-10	0.38	58	2.9	Crb2	Cytoskeleton
DMR3:22079001	3	22079001	22081000	2000	2	1.00E-10	0.46	31	1.55	Dennd1a	
DMR3:22097001	3	22097001	22098000	1000	1	4.90E-17	0.79	6	0.6	Dennd1a	
DMR3:22105001	3	22105001	22106000	1000	1	7.50E-20	0.73	7	0.7	Dennd1a	
DMR3:22112001	3	22112001	22113000	1000	1	8.90E-08	0.42	11	1.1	Dennd1a	
DMR3:22124001	3	22124001	22125000	1000	1	1.70E-10	0.49	9	0.9	Dennd1a	
DMR3:22165001	3	22165001	22166000	1000	1	3.50E-07	0.41	9	0.9	Dennd1a	

DMR3:22183001	3	22183001	22184000	1000	1	7.40E-09	-0.57	6	0.6	Dennd1a	
DMR3:22287001	3	22287001	22290000	3000	1	6.50E-07	0.42	29	0.97	Dennd1a	
DMR3:22299001	3	22299001	22300000	1000	1	3.70E-07	0.34	8	0.8	Dennd1a	
DMR3:22316001	3	22316001	22317000	1000	1	3.60E-15	0.66	7	0.7	Dennd1a	
DMR3:22399001	3	22399001	22401000	2000	1	8.40E-07	0.37	19	0.95	Dennd1a	
DMR3:22406001	3	22406001	22407000	1000	1	1.30E-15	0.78	2	0.2	Dennd1a	
DMR3:22426001	3	22426001	22430000	4000	2	4.60E-11	-0.6	32	0.8	Dennd1a	
DMR3:22443001	3	22443001	22448000	5000	3	1.90E-10	0.61	39	0.78	Dennd1a	
DMR3:22451001	3	22451001	22452000	1000	1	2.00E-08	0.58	2	0.2	Dennd1a	
DMR3:22467001	3	22467001	22468000	1000	1	2.00E-08	-0.51	6	0.6	Dennd1a	
DMR3:22486001	3	22486001	22493000	7000	3	8.30E-17	-0.72	59	0.84	Dennd1a	
DMR3:22661001	3	22661001	22663000	2000	1	1.70E-16	0.64	25	1.25	Lhx2	Development
DMR3:22830001	3	22830001	22831000	1000	1	4.40E-08	-0.34	25	2.5	Nek6	
DMR3:22853001	3	22853001	22855000	2000	1	7.60E-09	-0.54	40	2	Nek6	
DMR3:22856001	3	22856001	22859000	3000	1	1.10E-08	0.44	49	1.63	Nek6	
DMR3:22896001	3	22896001	22897000	1000	1	9.60E-07	0.45	4	0.4	Nek6;Psmb7	Protease
DMR3:22928001	3	22928001	22929000	1000	1	7.00E-07	-0.58	9	0.9	Psmb7	Protease
DMR3:22939001	3	22939001	22940000	1000	1	5.80E-09	0.49	5	0.5	Psmb7	Protease
DMR3:22957001	3	22957001	22958000	1000	1	5.20E-11	0.51	10	1	Psmb7;LOC690401;LOC100362149	Protease
DMR3:22992001	3	22992001	22993000	1000	1	3.50E-08	0.4	16	1.6	Nr5a1	Transcription
DMR3:23024001	3	23024001	23025000	1000	1	2.20E-15	0.74	10	1	Nr5a1;LOC102554503;Nr6a1	Transcription
DMR3:23064001	3	23064001	23066000	2000	1	1.40E-07	-0.46	23	1.15	Nr6a1	
DMR3:23068001	3	23068001	23069000	1000	1	1.30E-09	0.58	3	0.3	Nr6a1	
DMR3:23072001	3	23072001	23073000	1000	1	6.80E-07	0.38	5	0.5	Nr6a1	
DMR3:23097001	3	23097001	23099000	2000	2	4.00E-18	1.09	10	0.5	Nr6a1	
DMR3:23107001	3	23107001	23108000	1000	1	1.10E-17	1.06	2	0.2	Nr6a1	
DMR3:23113001	3	23113001	23114000	1000	1	1.10E-13	0.49	8	0.8	Nr6a1	
DMR3:23148001	3	23148001	23150000	2000	1	5.10E-10	0.5	16	0.8	Nr6a1;LOC102554311;Mir181a2;Mir181b2	
DMR3:23161001	3	23161001	23162000	1000	1	6.10E-10	0.66	7	0.7	Nr6a1;Mir181b2	
DMR3:23168001	3	23168001	23169000	1000	1	8.40E-12	0.58	6	0.6	Nr6a1	
DMR3:23174001	3	23174001	23178000	4000	3	1.30E-09	0.63	9	0.22	Nr6a1	
DMR3:23225001	3	23225001	23227000	2000	1	3.10E-08	-0.48	23	1.15	Nr6a1;Olfml2a	Development
DMR3:23250001	3	23250001	23253000	3000	1	1.00E-07	0.58	29	0.97	Olfml2a;LOC102554600	Development
DMR3:23261001	3	23261001	23265000	4000	2	1.60E-13	0.73	34	0.85	Olfml2a;LOC102554600	Development
DMR3:23365001	3	23365001	23366000	1000	1	3.30E-08	0.62	2	0.2	LOC102546322;Scai	
DMR3:23368001	3	23368001	23369000	1000	1	1.00E-13	0.56	8	0.8	LOC102546322;Scai	
DMR3:23372001	3	23372001	23373000	1000	1	4.40E-13	0.62	4	0.4	Scai	
DMR3:23394001	3	23394001	23395000	1000	1	2.00E-13	0.6	10	1	Scai	
DMR3:23403001	3	23403001	23406000	3000	1	5.00E-08	-0.42	33	1.1	Scai	
DMR3:23424001	3	23424001	23425000	1000	1	8.10E-16	0.67	4	0.4	Scai	
DMR3:23433001	3	23433001	23440000	7000	4	1.00E-21	-0.44	73	1.04	Scai	
DMR3:23458001	3	23458001	23460000	2000	1	1.10E-10	0.43	9	0.45	Scai	
DMR3:23481001	3	23481001	23482000	1000	1	8.60E-08	0.38	17	1.7	Scai;Ppp6c	Signaling
DMR3:23502001	3	23502001	23503000	1000	1	1.30E-07	0.36	14	1.4	Ppp6c	Signaling
DMR3:23518001	3	23518001	23519000	1000	1	8.60E-10	0.76	3	0.3	Ppp6c	Signaling
DMR3:25154001	3	25154001	25156000	2000	1	1.30E-11	0.52	12	0.6	RGD1561736	
DMR3:25188001	3	25188001	25193000	5000	1	4.60E-07	-0.41	44	0.88	Lrp1b	
DMR3:25276001	3	25276001	25279000	3000	2	1.50E-08	0.51	17	0.57	Lrp1b	
DMR3:25330001	3	25330001	25331000	1000	1	2.30E-18	0.85	5	0.5	Lrp1b	
DMR3:25336001	3	25336001	25337000	1000	1	8.80E-09	-0.63	7	0.7	Lrp1b	
DMR3:25338001	3	25338001	25339000	1000	1	6.10E-08	0.53	5	0.5	Lrp1b	
DMR3:25360001	3	25360001	25361000	1000	1	6.50E-19	0.9	3	0.3	Lrp1b	
DMR3:25383001	3	25383001	25384000	1000	1	2.00E-10	0.63	4	0.4	Lrp1b	
DMR3:25438001	3	25438001	25443000	5000	1	1.20E-08	0.59	18	0.36	Lrp1b	
DMR3:25533001	3	25533001	25534000	1000	1	3.80E-08	0.63	4	0.4	Lrp1b	
DMR3:25541001	3	25541001	25543000	2000	1	1.00E-12	0.53	8	0.4	Lrp1b	
DMR3:25550001	3	25550001	25551000	1000	1	2.60E-25	0.9	3	0.3	Lrp1b	
DMR3:25555001	3	25555001	25556000	1000	1	9.60E-07	0.41	5	0.5	Lrp1b	
DMR3:25564001	3	25564001	25568000	4000	1	3.20E-10	0.59	19	0.48	Lrp1b	
DMR3:25589001	3	25589001	25596000	7000	5	1.00E-19	-0.49	85	1.21	Lrp1b	
DMR3:25612001	3	25612001	25614000	2000	1	1.60E-11	0.55	10	0.5	Lrp1b	
DMR3:25620001	3	25620001	25622000	2000	1	4.20E-13	0.61	12	0.6	Lrp1b	
DMR3:25627001	3	25627001	25630000	3000	1	6.20E-10	-0.37	26	0.87	Lrp1b	

DMR3:25644001	3	25644001	25645000	1000	1	2.20E-07	-0.41	9	0.9	Lrp1b	
DMR3:25649001	3	25649001	25650000	1000	1	8.10E-22	1.04	10	1	Lrp1b	
DMR3:25653001	3	25653001	25654000	1000	1	4.60E-09	0.66	4	0.4	Lrp1b	
DMR3:25658001	3	25658001	25660000	2000	1	4.50E-08	0.59	9	0.45	Lrp1b	
DMR3:25666001	3	25666001	25670000	4000	1	5.70E-09	-0.69	13	0.32	Lrp1b	
DMR3:25711001	3	25711001	25713000	2000	1	1.20E-07	-0.5	13	0.65	Lrp1b	
DMR3:25728001	3	25728001	25734000	6000	3	6.50E-16	-0.64	33	0.55	Lrp1b	
DMR3:25761001	3	25761001	25762000	1000	1	8.10E-09	0.6	6	0.6	Lrp1b	
DMR3:25783001	3	25783001	25785000	2000	1	4.50E-08	-0.5	9	0.45	Lrp1b	
DMR3:25794001	3	25794001	25795000	1000	1	1.80E-07	0.5	5	0.5	Lrp1b	
DMR3:25838001	3	25838001	25841000	3000	1	4.80E-10	-0.51	15	0.5	Lrp1b	
DMR3:25866001	3	25866001	25869000	3000	3	4.60E-12	0.55	21	0.7	Lrp1b	
DMR3:25884001	3	25884001	25886000	2000	1	7.70E-07	-0.5	5	0.25	Lrp1b	
DMR3:25890001	3	25890001	25893000	3000	2	3.10E-19	0.83	19	0.63	Lrp1b	
DMR3:25912001	3	25912001	25913000	1000	1	4.10E-12	0.58	9	0.9	Lrp1b;LOC108350579	
DMR3:25943001	3	25943001	25945000	2000	1	3.10E-14	0.8	9	0.45	Lrp1b	
DMR3:25946001	3	25946001	25949000	3000	1	5.80E-10	-0.51	8	0.27	Lrp1b	
DMR3:25979001	3	25979001	25982000	3000	1	2.20E-07	-0.53	19	0.63	Lrp1b	
DMR3:25984001	3	25984001	25986000	2000	1	7.50E-07	-0.39	13	0.65	Lrp1b	
DMR3:25990001	3	25990001	25991000	1000	1	6.20E-08	0.64	4	0.4	Lrp1b	
DMR3:26043001	3	26043001	26045000	2000	1	4.40E-13	0.8	8	0.4	Lrp1b	
DMR3:26086001	3	26086001	26087000	1000	1	8.60E-10	0.59	4	0.4	Lrp1b	
DMR3:26132001	3	26132001	26133000	1000	1	6.50E-16	0.6	11	1.1	Lrp1b	
DMR3:26156001	3	26156001	26157000	1000	1	2.00E-11	0.62	7	0.7	Lrp1b	
DMR3:26171001	3	26171001	26175000	4000	1	6.00E-11	0.55	22	0.55	Lrp1b	
DMR3:26201001	3	26201001	26203000	2000	1	1.80E-17	0.67	13	0.65	Lrp1b	
DMR3:26215001	3	26215001	26218000	3000	2	1.50E-19	-0.52	33	1.1	Lrp1b	
DMR3:26240001	3	26240001	26242000	2000	2	2.90E-07	0.51	14	0.7	Lrp1b	
DMR3:26256001	3	26256001	26257000	1000	1	5.10E-07	0.33	21	2.1	Lrp1b	
DMR3:26261001	3	26261001	26262000	1000	1	6.60E-14	0.49	19	1.9	Lrp1b	
DMR3:26285001	3	26285001	26287000	2000	1	9.50E-07	-0.33	10	0.5	Lrp1b	
DMR3:26289001	3	26289001	26290000	1000	1	3.00E-19	0.93	6	0.6	Lrp1b	
DMR3:26292001	3	26292001	26293000	1000	1	6.40E-15	0.82	4	0.4	Lrp1b	
DMR3:26294001	3	26294001	26295000	1000	1	2.00E-08	0.4	7	0.7	Lrp1b	
DMR3:26331001	3	26331001	26332000	1000	1	5.20E-08	0.48	4	0.4	Lrp1b	
DMR3:26351001	3	26351001	26354000	3000	1	1.40E-10	0.48	78	2.6	Lrp1b	
DMR3:26368001	3	26368001	26370000	2000	1	8.60E-08	-0.68	8	0.4	Lrp1b	
DMR3:26402001	3	26402001	26404000	2000	1	3.80E-08	0.43	20	1	Lrp1b	
DMR3:26405001	3	26405001	26412000	7000	5	6.80E-20	-0.51	62	0.89	Lrp1b	
DMR3:26431001	3	26431001	26432000	1000	1	1.80E-13	0.54	6	0.6	Lrp1b	
DMR3:26445001	3	26445001	26446000	1000	1	6.20E-17	0.85	2	0.2	Lrp1b	
DMR3:26450001	3	26450001	26454000	4000	2	2.70E-08	-0.68	16	0.4	Lrp1b	
DMR3:26462001	3	26462001	26464000	2000	2	1.30E-22	0.86	13	0.65	Lrp1b	
DMR3:26511001	3	26511001	26513000	2000	1	3.80E-07	0.55	32	1.6	Lrp1b	
DMR3:26551001	3	26551001	26553000	2000	2	1.20E-16	0.95	7	0.35	Lrp1b	
DMR3:26565001	3	26565001	26570000	5000	3	6.00E-20	-0.56	55	1.1	Lrp1b	
DMR3:26583001	3	26583001	26584000	1000	1	8.00E-12	0.7	6	0.6	Lrp1b	
DMR3:26604001	3	26604001	26606000	2000	1	5.20E-14	0.46	46	2.3	Lrp1b	
DMR3:26613001	3	26613001	26615000	2000	1	3.30E-11	0.69	17	0.85	Lrp1b	
DMR3:26620001	3	26620001	26628000	8000	5	6.00E-20	-0.61	91	1.14	Lrp1b	
DMR3:26655001	3	26655001	26656000	1000	1	5.00E-09	-0.48	4	0.4	Lrp1b	
DMR3:26682001	3	26682001	26683000	1000	1	8.20E-08	-0.35	15	1.5	Lrp1b	
DMR3:26684001	3	26684001	26687000	3000	1	2.70E-07	-0.6	33	1.1	Lrp1b	
DMR3:26688001	3	26688001	26690000	2000	2	9.60E-17	0.65	20	1	Lrp1b	
DMR3:26725001	3	26725001	26727000	2000	1	9.00E-09	0.4	11	0.55	Lrp1b	
DMR3:26744001	3	26744001	26745000	1000	1	1.30E-13	0.94	3	0.3	Lrp1b	
DMR3:26756001	3	26756001	26761000	5000	2	3.10E-09	-0.56	28	0.56	Lrp1b	
DMR3:26762001	3	26762001	26764000	2000	1	2.60E-07	0.46	11	0.55	Lrp1b	
DMR3:26815001	3	26815001	26822000	7000	6	1.00E-23	-0.54	84	1.2	Lrp1b	
DMR3:26834001	3	26834001	26836000	2000	2	2.00E-10	0.49	12	0.6	Lrp1b	
DMR3:26850001	3	26850001	26851000	1000	1	8.40E-16	0.72	12	1.2	Lrp1b	
DMR3:26871001	3	26871001	26873000	2000	1	9.90E-07	-0.48	23	1.15	Lrp1b	
DMR3:26885001	3	26885001	26887000	2000	2	2.30E-21	0.68	10	0.5	Lrp1b	
DMR3:26911001	3	26911001	26915000	4000	1	4.90E-13	-0.56	43	1.07	Lrp1b	
DMR3:26918001	3	26918001	26922000	4000	1	6.40E-14	0.68	35	0.88	Lrp1b	

DMR3:26970001	3	26970001	26971000	1000	1	6.60E-14	0.72	10	1	Lrp1b	
DMR3:26991001	3	26991001	26993000	2000	1	2.20E-07	-0.33	27	1.35	Lrp1b	
DMR3:27015001	3	27015001	27016000	1000	1	2.30E-11	0.64	8	0.8	Lrp1b	
DMR3:27019001	3	27019001	27020000	1000	1	2.70E-09	0.44	7	0.7	Lrp1b	
DMR3:27041001	3	27041001	27042000	1000	1	3.80E-25	1.05	0	0	Lrp1b	
DMR3:27049001	3	27049001	27051000	2000	1	3.20E-12	0.72	10	0.5	Lrp1b	
DMR3:27087001	3	27087001	27088000	1000	1	4.40E-21	0.88	5	0.5	Lrp1b	
DMR3:27122001	3	27122001	27124000	2000	1	4.60E-09	0.54	5	0.25	Lrp1b	
DMR3:27197001	3	27197001	27198000	1000	1	6.70E-17	0.74	7	0.7	Lrp1b	
DMR3:27237001	3	27237001	27238000	1000	1	9.90E-07	-0.39	16	1.6	Lrp1b	
DMR3:27256001	3	27256001	27257000	1000	1	3.40E-07	0.43	9	0.9	Lrp1b	
DMR3:27281001	3	27281001	27282000	1000	1	1.10E-14	0.57	9	0.9	Lrp1b	
DMR3:27297001	3	27297001	27299000	2000	2	9.10E-14	-0.59	17	0.85	Lrp1b	
DMR3:27300001	3	27300001	27302000	2000	1	2.40E-15	0.66	14	0.7	Lrp1b	
DMR3:27307001	3	27307001	27309000	2000	2	1.30E-21	0.84	16	0.8	Lrp1b	
DMR3:27310001	3	27310001	27311000	1000	1	6.00E-11	0.72	3	0.3	Lrp1b	
DMR3:27327001	3	27327001	27330000	3000	1	9.90E-09	-0.46	32	1.07	Lrp1b	
DMR3:27353001	3	27353001	27357000	4000	2	5.60E-14	0.7	73	1.82	Lrp1b	
DMR3:28411001	3	28411001	28412000	1000	1	8.80E-11	0.45	13	1.3	Kynu	Metabolism
DMR3:28434001	3	28434001	28441000	7000	5	8.50E-10	-0.61	54	0.77	Kynu;LOC102547849	Metabolism
DMR3:28472001	3	28472001	28473000	1000	1	7.00E-07	0.33	14	1.4	Kynu	Metabolism
DMR3:28487001	3	28487001	28488000	1000	1	2.60E-13	0.77	7	0.7	Kynu	Metabolism
DMR3:28508001	3	28508001	28511000	3000	2	5.70E-12	0.67	21	0.7	Kynu	Metabolism
DMR3:28512001	3	28512001	28513000	1000	1	4.00E-07	-0.49	12	1.2	Kynu	Metabolism
DMR3:28516001	3	28516001	28517000	1000	1	4.40E-12	0.63	4	0.4	Kynu	Metabolism
DMR3:28527001	3	28527001	28530000	3000	1	9.30E-18	0.71	23	0.77	Kynu	Metabolism
DMR3:28534001	3	28534001	28538000	4000	1	6.70E-08	0.45	26	0.65	Kynu	Metabolism
DMR3:28547001	3	28547001	28555000	8000	4	2.70E-15	-0.52	91	1.14	Kynu	Metabolism
DMR3:28568001	3	28568001	28570000	2000	1	1.30E-09	0.57	8	0.4	Kynu	Metabolism
DMR3:28616001	3	28616001	28618000	2000	1	9.00E-07	-0.6	36	1.8	Arhgap15	Signaling
DMR3:28640001	3	28640001	28644000	4000	2	4.00E-11	-0.55	22	0.55	Arhgap15	Signaling
DMR3:28646001	3	28646001	28649000	3000	1	1.30E-08	0.56	23	0.77	Arhgap15	Signaling
DMR3:28651001	3	28651001	28653000	2000	1	1.40E-09	-0.42	17	0.85	Arhgap15	Signaling
DMR3:28660001	3	28660001	28662000	2000	1	5.80E-12	0.72	13	0.65	Arhgap15	Signaling
DMR3:28673001	3	28673001	28676000	3000	2	2.10E-11	-0.68	50	1.67	Arhgap15	Signaling
DMR3:28688001	3	28688001	28689000	1000	1	1.40E-14	0.79	5	0.5	Arhgap15	Signaling
DMR3:28695001	3	28695001	28696000	1000	1	2.10E-09	0.47	9	0.9	Arhgap15	Signaling
DMR3:28697001	3	28697001	28698000	1000	1	8.20E-07	-0.57	15	1.5	Arhgap15	Signaling
DMR3:28709001	3	28709001	28711000	2000	1	9.10E-07	0.39	16	0.8	Arhgap15	Signaling
DMR3:28717001	3	28717001	28718000	1000	1	6.60E-07	-0.51	9	0.9	Arhgap15	Signaling
DMR3:28720001	3	28720001	28721000	1000	1	4.40E-21	0.74	11	1.1	Arhgap15	Signaling
DMR3:28726001	3	28726001	28727000	1000	1	2.50E-10	0.67	7	0.7	Arhgap15	Signaling
DMR3:28775001	3	28775001	28777000	2000	1	5.80E-07	-0.41	31	1.55	Arhgap15	Signaling
DMR3:28784001	3	28784001	28787000	3000	1	1.50E-09	0.54	33	1.1	Arhgap15	Signaling
DMR3:28788001	3	28788001	28790000	2000	1	2.50E-07	-0.47	27	1.35	Arhgap15	Signaling
DMR3:28811001	3	28811001	28812000	1000	1	4.50E-13	-0.88	13	1.3	Arhgap15	Signaling
DMR3:28885001	3	28885001	28887000	2000	1	7.50E-08	-0.51	32	1.6	Arhgap15	Signaling
DMR3:28908001	3	28908001	28909000	1000	1	8.40E-12	0.55	11	1.1	Arhgap15	Signaling
DMR3:28916001	3	28916001	28918000	2000	1	9.80E-09	0.43	38	1.9	Arhgap15	Signaling
DMR3:28926001	3	28926001	28927000	1000	1	2.20E-07	-0.44	4	0.4	Arhgap15	Signaling
DMR3:28934001	3	28934001	28935000	1000	1	2.90E-07	-0.62	15	1.5	Arhgap15	Signaling
DMR3:28944001	3	28944001	28945000	1000	1	1.20E-08	-0.5	13	1.3	Arhgap15	Signaling
DMR3:28970001	3	28970001	28972000	2000	1	1.60E-07	0.38	20	1	Arhgap15	Signaling
DMR3:28973001	3	28973001	28975000	2000	1	5.10E-08	-0.55	28	1.4	Arhgap15	Signaling
DMR3:28976001	3	28976001	28977000	1000	1	1.00E-08	-0.4	20	2	Arhgap15	Signaling
DMR3:28984001	3	28984001	28986000	2000	1	2.40E-14	0.46	35	1.75	Arhgap15	Signaling
DMR3:28989001	3	28989001	28990000	1000	1	3.00E-18	0.66	13	1.3	Arhgap15	Signaling
DMR3:29013001	3	29013001	29015000	2000	1	9.70E-15	-0.59	15	0.75	Arhgap15	Signaling
DMR3:29016001	3	29016001	29019000	3000	1	4.80E-08	0.44	30	1	Arhgap15	Signaling
DMR3:29030001	3	29030001	29033000	3000	2	3.00E-07	-0.57	42	1.4	Arhgap15	Signaling
DMR3:29058001	3	29058001	29061000	3000	2	3.70E-11	-0.5	37	1.23	Arhgap15	Signaling
DMR3:29063001	3	29063001	29067000	4000	2	9.70E-17	0.68	35	0.88	Arhgap15	Signaling
DMR3:29075001	3	29075001	29078000	3000	3	2.80E-12	0.55	26	0.87	Arhgap15	Signaling
DMR3:29084001	3	29084001	29086000	2000	1	2.40E-07	-0.47	23	1.15	Arhgap15	Signaling
DMR3:29100001	3	29100001	29107000	7000	1	3.90E-09	0.46	112	1.6	Arhgap15	Signaling

DMR3:29109001	3	29109001	29113000	4000	3	3.80E-14	0.78	37	0.92	Arhgap15	Signaling
DMR3:29123001	3	29123001	29125000	2000	1	2.90E-07	-0.46	18	0.9	Arhgap15	Signaling
DMR3:29128001	3	29128001	29136000	8000	1	3.20E-07	-0.6	117	1.46	Arhgap15	Signaling
DMR3:29182001	3	29182001	29183000	1000	1	4.10E-12	0.57	13	1.3	Arhgap15	Signaling
DMR3:29187001	3	29187001	29191000	4000	1	7.30E-14	0.53	48	1.2	Arhgap15	Signaling
DMR3:29405001	3	29405001	29406000	1000	1	9.10E-14	0.56	15	1.5	LOC108350368;Gtdc1	Golgi
DMR3:29411001	3	29411001	29413000	2000	1	1.90E-07	0.33	27	1.35	LOC108350368;Gtdc1	Golgi
DMR3:29425001	3	29425001	29427000	2000	1	1.10E-07	0.39	22	1.1	Gtdc1	Golgi
DMR3:29456001	3	29456001	29457000	1000	1	7.10E-10	0.5	4	0.4	Gtdc1	Golgi
DMR3:29479001	3	29479001	29482000	3000	1	4.10E-07	-0.54	17	0.57	Gtdc1	Golgi
DMR3:29505001	3	29505001	29506000	1000	1	6.60E-09	0.57	6	0.6	Gtdc1	Golgi
DMR3:29509001	3	29509001	29512000	3000	1	7.80E-08	0.39	15	0.5	Gtdc1;LOC108350367	Golgi
DMR3:29515001	3	29515001	29517000	2000	2	5.40E-21	1.16	21	1.05	Gtdc1;LOC108350367	Golgi
DMR3:29575001	3	29575001	29579000	4000	1	2.00E-11	0.7	22	0.55	Gtdc1	Golgi
DMR3:29583001	3	29583001	29585000	2000	1	4.90E-10	0.53	14	0.7	Gtdc1	Golgi
DMR3:29586001	3	29586001	29590000	4000	1	7.80E-12	0.63	26	0.65	Gtdc1	Golgi
DMR3:29601001	3	29601001	29605000	4000	1	7.80E-08	0.41	27	0.68	Gtdc1	Golgi
DMR3:29638001	3	29638001	29639000	1000	1	8.70E-11	0.62	8	0.8	Gtdc1	Golgi
DMR3:29640001	3	29640001	29642000	2000	2	2.20E-10	-0.48	24	1.2	Gtdc1	Golgi
DMR3:29646001	3	29646001	29647000	1000	1	8.60E-12	0.47	8	0.8	Gtdc1	Golgi
DMR3:29655001	3	29655001	29658000	3000	2	6.80E-25	1.15	15	0.5	Gtdc1	Golgi
DMR3:29668001	3	29668001	29671000	3000	2	2.20E-08	0.5	29	0.97	Gtdc1	Golgi
DMR3:29681001	3	29681001	29682000	1000	1	7.50E-15	0.91	13	1.3	Gtdc1	Golgi
DMR3:29685001	3	29685001	29688000	3000	1	4.40E-08	0.42	23	0.77	Gtdc1	Golgi
DMR3:29690001	3	29690001	29691000	1000	1	2.00E-09	0.45	4	0.4	Gtdc1	Golgi
DMR3:29711001	3	29711001	29718000	7000	5	1.10E-20	-0.9	68	0.97	Gtdc1	Golgi
DMR3:29878001	3	29878001	29879000	1000	1	4.80E-11	0.57	7	0.7	Zeb2	Transcription
DMR3:29904001	3	29904001	29906000	2000	1	2.90E-10	-0.73	19	0.95	Zeb2	Transcription
DMR3:29917001	3	29917001	29918000	1000	1	4.70E-07	-0.42	24	2.4	Zeb2	Transcription
DMR3:29929001	3	29929001	29930000	1000	1	5.00E-08	0.48	5	0.5	Zeb2	Transcription
DMR3:29941001	3	29941001	29942000	1000	1	1.20E-14	0.49	12	1.2	Zeb2	Transcription
DMR3:29957001	3	29957001	29960000	3000	2	3.10E-08	-0.47	53	1.77	Zeb2	Transcription
DMR3:29963001	3	29963001	29965000	2000	1	3.10E-11	0.38	27	1.35	Zeb2	Transcription
DMR3:29966001	3	29966001	29970000	4000	2	2.30E-10	0.43	71	1.77	Zeb2	Transcription
DMR3:29971001	3	29971001	29972000	1000	1	8.70E-07	-0.43	17	1.7	Zeb2	Transcription
DMR3:29990001	3	29990001	29993000	3000	1	4.40E-12	0.47	47	1.57	Zeb2;LOC100912577;Zeb2os	Transcription
DMR3:31813001	3	31813001	31814000	1000	1	3.80E-10	0.72	7	0.7	LOC690840;Olr435-ps	
DMR3:31820001	3	31820001	31823000	3000	2	1.10E-14	-0.61	9	0.3	Olr435-ps	
DMR3:32939001	3	32939001	32940000	1000	1	1.20E-07	0.43	6	0.6	LOC102553549;Acvr2a	Signaling
DMR3:32976001	3	32976001	32979000	3000	1	7.00E-08	0.55	20	0.67	Acvr2a	Signaling
DMR3:32981001	3	32981001	32982000	1000	1	2.90E-07	0.48	5	0.5	Acvr2a	Signaling
DMR3:33000001	3	33000001	33001000	1000	1	3.20E-09	0.69	12	1.2	Acvr2a	Signaling
DMR3:33031001	3	33031001	33032000	1000	1	2.70E-08	-0.57	11	1.1	Acvr2a;Orc4	Signaling;Cell Cycle
DMR3:33044001	3	33044001	33045000	1000	1	3.60E-08	0.47	10	1	Acvr2a;Orc4	Signaling;Cell Cycle
DMR3:33059001	3	33059001	33061000	2000	2	2.10E-09	-0.69	13	0.65	Orc4	Cell Cycle
DMR3:33355001	3	33355001	33357000	2000	1	1.10E-10	0.42	20	1	Mbd5	
DMR3:33373001	3	33373001	33375000	2000	1	7.80E-09	0.42	11	0.55	Mbd5	
DMR3:33377001	3	33377001	33380000	3000	3	1.80E-18	-0.53	30	1	Mbd5	
DMR3:33389001	3	33389001	33390000	1000	1	1.60E-10	0.52	16	1.6	Mbd5	
DMR3:33401001	3	33401001	33402000	1000	1	1.50E-16	0.84	10	1	Mbd5	
DMR3:33409001	3	33409001	33410000	1000	1	4.80E-10	0.47	11	1.1	Mbd5	
DMR3:33411001	3	33411001	33419000	8000	3	1.90E-20	-0.54	61	0.76	Mbd5	
DMR3:33479001	3	33479001	33482000	3000	1	2.70E-12	0.45	24	0.8	Mbd5	
DMR3:33485001	3	33485001	33486000	1000	1	2.10E-15	0.68	10	1	Mbd5	
DMR3:33491001	3	33491001	33492000	1000	1	1.70E-07	-0.53	15	1.5	Mbd5	
DMR3:33665001	3	33665001	33667000	2000	1	1.90E-16	0.69	17	0.85	Epc2	Epigenetic
DMR3:33677001	3	33677001	33682000	5000	4	1.90E-22	-0.51	49	0.98	Epc2	Epigenetic
DMR3:33691001	3	33691001	33699000	8000	4	4.60E-25	-0.5	89	1.11	Epc2	Epigenetic
DMR3:33743001	3	33743001	33744000	1000	1	3.20E-19	0.65	12	1.2	Epc2	Epigenetic
DMR3:33750001	3	33750001	33755000	5000	2	6.50E-22	0.74	58	1.16	Epc2	Epigenetic
DMR3:35010001	3	35010001	35013000	3000	1	2.60E-18	0.49	47	1.57	Kif5c	
DMR3:35020001	3	35020001	35026000	6000	3	1.20E-24	0.7	120	2	Kif5c	
DMR3:35031001	3	35031001	35032000	1000	1	2.40E-17	0.99	11	1.1	Kif5c	
DMR3:35068001	3	35068001	35069000	1000	1	7.40E-17	0.68	3	0.3	Kif5c	
DMR3:35128001	3	35128001	35132000	4000	1	1.50E-19	0.64	46	1.15	Kif5c	

DMR3:35137001	3	35137001	35140000	3000	1	7.80E-10	-0.59	29	0.97	Kif5c	
DMR3:35193001	3	35193001	35194000	1000	1	2.30E-14	0.56	6	0.6	Kif5c	
DMR3:35225001	3	35225001	35226000	1000	1	1.50E-20	0.72	10	1	Kif5c	
DMR3:35228001	3	35228001	35232000	4000	1	7.10E-13	0.55	63	1.57	Kif5c	
DMR3:35233001	3	35233001	35237000	4000	1	4.20E-07	-0.44	38	0.95	Kif5c	
DMR3:35245001	3	35245001	35247000	2000	1	9.10E-07	0.39	13	0.65	Kif5c	
DMR3:35273001	3	35273001	35279000	6000	3	9.30E-11	0.4	90	1.5	Lypd6b	
DMR3:35300001	3	35300001	35301000	1000	1	1.50E-10	0.46	22	2.2	Lypd6b	
DMR3:35328001	3	35328001	35329000	1000	1	1.60E-11	-0.78	9	0.9	Lypd6b	
DMR3:35355001	3	35355001	35358000	3000	1	3.90E-07	-0.58	31	1.03	Lypd6b	
DMR3:35360001	3	35360001	35363000	3000	1	7.20E-07	0.33	34	1.13	Lypd6b	
DMR3:35396001	3	35396001	35397000	1000	1	2.40E-07	-0.39	14	1.4	Lypd6b	
DMR3:35408001	3	35408001	35411000	3000	1	2.70E-09	-0.66	43	1.43	Lypd6b	
DMR3:35540001	3	35540001	35542000	2000	1	1.10E-09	0.42	38	1.9	Ns5atp4	
DMR3:35543001	3	35543001	35544000	1000	1	4.40E-10	-0.78	7	0.7	Ns5atp4	
DMR3:35557001	3	35557001	35560000	3000	2	3.40E-15	0.69	27	0.9	Lypd6	
DMR3:35576001	3	35576001	35577000	1000	1	2.40E-08	-0.57	11	1.1	Lypd6	
DMR3:35578001	3	35578001	35580000	2000	1	3.00E-09	0.49	20	1	Lypd6	
DMR3:35587001	3	35587001	35591000	4000	1	4.70E-11	0.42	66	1.65	Lypd6	
DMR3:35599001	3	35599001	35600000	1000	1	7.10E-14	0.46	16	1.6	Lypd6	
DMR3:35601001	3	35601001	35603000	2000	1	2.10E-11	0.73	17	0.85	Lypd6	
DMR3:35604001	3	35604001	35605000	1000	1	2.30E-14	0.51	21	2.1	Lypd6	
DMR3:35635001	3	35635001	35641000	6000	2	1.30E-09	-0.44	67	1.12	Lypd6;Trnav-aac	
DMR3:35653001	3	35653001	35655000	2000	1	2.00E-07	-0.47	21	1.05	Lypd6;LOC102555146	
DMR3:35679001	3	35679001	35681000	2000	2	2.60E-09	-0.64	35	1.75	Lypd6	
DMR3:35682001	3	35682001	35683000	1000	1	1.80E-17	0.66	12	1.2	Lypd6	
DMR3:35692001	3	35692001	35695000	3000	1	3.60E-07	-0.42	38	1.27	Lypd6	
DMR3:35710001	3	35710001	35711000	1000	1	2.90E-07	-0.6	5	0.5	Lypd6	
DMR3:35782001	3	35782001	35783000	1000	1	2.80E-07	-0.61	11	1.1	Mmadhc	
DMR3:35806001	3	35806001	35811000	5000	3	8.40E-23	0.85	46	0.92	Mmadhc;LOC102555425	
DMR3:36645001	3	36645001	36646000	1000	1	1.40E-07	-0.52	12	1.2	Rnd3	Signaling
DMR3:36663001	3	36663001	36664000	1000	1	1.70E-21	1	4	0.4	Rnd3	Signaling
DMR3:37144001	3	37144001	37145000	1000	1	8.80E-07	0.31	13	1.3	Tas2r134	Receptor
DMR3:37438001	3	37438001	37439000	1000	1	4.10E-15	0.58	11	1.1	Rbm43	Transcription
DMR3:37475001	3	37475001	37478000	3000	3	3.10E-16	0.67	41	1.37	Nmi	Transcription
DMR3:37535001	3	37535001	37537000	2000	2	3.70E-09	0.57	15	0.75	Tnfaip6	
DMR3:37538001	3	37538001	37540000	2000	1	9.80E-09	0.55	21	1.05	Tnfaip6	
DMR3:37554001	3	37554001	37557000	3000	1	7.50E-10	0.48	34	1.13	Tnfaip6	
DMR3:37558001	3	37558001	37560000	2000	1	1.30E-21	0.69	23	1.15	Tnfaip6	
DMR3:37569001	3	37569001	37572000	3000	1	6.70E-07	0.42	33	1.1	Tnfaip6	
DMR3:37595001	3	37595001	37597000	2000	1	7.80E-15	0.49	16	0.8	Rif1	Transcription
DMR3:37602001	3	37602001	37607000	5000	1	1.30E-13	0.48	43	0.86	Rif1	Transcription
DMR3:37627001	3	37627001	37628000	1000	1	1.10E-07	0.58	2	0.2	Rif1	Transcription
DMR3:37636001	3	37636001	37638000	2000	1	4.50E-07	-0.43	18	0.9	Rif1	Transcription
DMR3:37662001	3	37662001	37669000	7000	1	1.30E-12	0.7	76	1.09	Neb	
DMR3:37675001	3	37675001	37677000	2000	1	1.20E-10	0.4	26	1.3	Neb	
DMR3:37685001	3	37685001	37687000	2000	1	1.30E-08	-0.6	22	1.1	Neb	
DMR3:37689001	3	37689001	37690000	1000	1	5.70E-11	0.51	9	0.9	Neb	
DMR3:37691001	3	37691001	37694000	3000	1	3.30E-07	-0.47	60	2	Neb	
DMR3:37722001	3	37722001	37723000	1000	1	2.60E-10	-0.63	18	1.8	Neb	
DMR3:37740001	3	37740001	37743000	3000	1	4.30E-12	0.51	32	1.07	Neb	
DMR3:37769001	3	37769001	37775000	6000	3	2.10E-14	-0.41	93	1.55	Neb	
DMR3:37776001	3	37776001	37780000	4000	2	6.10E-14	0.62	50	1.25	Neb	
DMR3:37804001	3	37804001	37807000	3000	1	7.10E-07	-0.53	53	1.77	Neb	
DMR3:37812001	3	37812001	37815000	3000	2	5.70E-09	-0.46	45	1.5	Neb	
DMR3:37823001	3	37823001	37825000	2000	1	4.60E-14	0.48	37	1.85	Neb	
DMR3:37845001	3	37845001	37847000	2000	1	1.70E-08	-0.46	36	1.8	Neb	
DMR3:37856001	3	37856001	37858000	2000	1	7.20E-09	-0.68	19	0.95	Neb	
DMR3:37860001	3	37860001	37861000	1000	1	3.00E-07	0.34	12	1.2	Neb	
DMR3:37863001	3	37863001	37865000	2000	1	3.10E-10	0.42	13	0.65	Neb	
DMR3:37870001	3	37870001	37872000	2000	2	5.50E-09	0.49	12	0.6	RGD1563584	
DMR3:37873001	3	37873001	37875000	2000	2	2.10E-08	-0.54	17	0.85	RGD1563584	
DMR3:37876001	3	37876001	37878000	2000	1	2.30E-08	0.48	21	1.05	RGD1563584	
DMR3:37918001	3	37918001	37921000	3000	1	4.00E-07	0.39	46	1.53	Arl5a	Signaling
DMR3:37942001	3	37942001	37943000	1000	1	4.40E-19	0.59	14	1.4	Arl5a;Cacnb4	Signaling;Transport

DMR3:37944001	3	37944001	37946000	2000	1	1.10E-07	-0.6	26	1.3	Arl5a;Cacnb4	Signaling;Transport
DMR3:37956001	3	37956001	37957000	1000	1	2.30E-08	-0.68	10	1	Arl5a;Cacnb4	Signaling;Transport
DMR3:37960001	3	37960001	37961000	1000	1	5.00E-08	-0.49	8	0.8	Cacnb4	Transport
DMR3:37962001	3	37962001	37968000	6000	2	7.70E-37	1.01	63	1.05	Cacnb4	Transport
DMR3:37978001	3	37978001	37981000	3000	1	2.30E-14	0.62	38	1.27	Cacnb4	Transport
DMR3:37988001	3	37988001	37994000	6000	1	1.10E-14	0.72	77	1.28	Cacnb4	Transport
DMR3:38000001	3	38000001	38002000	2000	1	5.40E-07	-0.47	29	1.45	Cacnb4	Transport
DMR3:38003001	3	38003001	38004000	1000	1	2.00E-15	0.5	22	2.2	Cacnb4	Transport
DMR3:38014001	3	38014001	38022000	8000	5	1.30E-30	-0.6	119	1.49	Cacnb4	Transport
DMR3:38040001	3	38040001	38050000	10000	3	5.20E-24	0.86	139	1.39	Cacnb4	Transport
DMR3:38057001	3	38057001	38059000	2000	1	1.00E-07	-0.57	38	1.9	Cacnb4	Transport
DMR3:38081001	3	38081001	38084000	3000	1	1.30E-13	0.61	34	1.13	Cacnb4	Transport
DMR3:38085001	3	38085001	38090000	5000	2	4.90E-16	-0.58	62	1.24	Cacnb4	Transport
DMR3:38092001	3	38092001	38094000	2000	1	2.50E-08	0.47	14	0.7	Cacnb4	Transport
DMR3:38097001	3	38097001	38099000	2000	2	1.50E-09	-0.61	23	1.15	Cacnb4	Transport
DMR3:38130001	3	38130001	38132000	2000	1	3.10E-08	0.3	53	2.65	Cacnb4	Transport
DMR3:38173001	3	38173001	38176000	3000	1	1.10E-07	0.31	43	1.43	Cacnb4	Transport
DMR3:38187001	3	38187001	38189000	2000	1	1.20E-14	0.57	33	1.65	Cacnb4	Transport
DMR3:38194001	3	38194001	38198000	4000	2	3.90E-09	-0.69	37	0.92	Cacnb4	Transport
DMR3:38213001	3	38213001	38217000	4000	1	1.10E-10	-0.57	44	1.1	Cacnb4	Transport
DMR3:38220001	3	38220001	38223000	3000	1	2.30E-07	-0.48	36	1.2	Cacnb4;Stam2	Transport;Cytoskeleto n
DMR3:38236001	3	38236001	38238000	2000	1	3.50E-07	-0.53	27	1.35	Stam2	Cytoskeleton
DMR3:38241001	3	38241001	38245000	4000	1	8.60E-07	0.28	52	1.3	Stam2	Cytoskeleton
DMR3:38249001	3	38249001	38252000	3000	1	5.60E-11	0.35	48	1.6	Stam2	Cytoskeleton
DMR3:38256001	3	38256001	38260000	4000	2	2.60E-08	-0.63	51	1.27	Stam2	Cytoskeleton
DMR3:38352001	3	38352001	38359000	7000	3	1.10E-16	0.56	71	1.01	RGD1564306	
DMR3:38368001	3	38368001	38370000	2000	1	3.70E-15	0.57	20	1	RGD1564306	
DMR3:38385001	3	38385001	38391000	6000	1	7.60E-13	0.64	73	1.22	RGD1560248	
DMR3:38395001	3	38395001	38396000	1000	1	1.30E-09	-0.56	10	1	RGD1560248	
DMR3:38419001	3	38419001	38425000	6000	2	1.40E-11	-0.7	81	1.35	RGD1560248	
DMR3:38435001	3	38435001	38439000	4000	1	1.30E-07	0.36	72	1.8	RGD1560248	
DMR3:38460001	3	38460001	38463000	3000	1	2.80E-07	-0.65	33	1.1	RGD1560248	
DMR3:38498001	3	38498001	38500000	2000	1	7.00E-17	0.74	13	0.65	RGD1560248	
DMR3:38501001	3	38501001	38503000	2000	1	4.90E-07	0.32	38	1.9	RGD1560248	
DMR3:38507001	3	38507001	38509000	2000	1	1.00E-08	0.41	16	0.8	RGD1560248	
DMR3:38516001	3	38516001	38517000	1000	1	2.10E-09	0.52	4	0.4	RGD1560248	
DMR3:38555001	3	38555001	38558000	3000	2	5.20E-14	0.6	60	2	RGD1560248	
DMR3:38562001	3	38562001	38564000	2000	1	2.10E-07	-0.52	20	1	RGD1560248	
DMR3:38567001	3	38567001	38570000	3000	1	2.80E-11	0.62	47	1.57	RGD1560248	
DMR3:38571001	3	38571001	38575000	4000	1	2.10E-16	0.54	59	1.48	RGD1560248	
DMR3:38581001	3	38581001	38584000	3000	2	1.70E-13	0.44	48	1.6	RGD1560248	
DMR3:38600001	3	38600001	38601000	1000	1	2.30E-11	0.64	6	0.6	RGD1560248	
DMR3:38609001	3	38609001	38613000	4000	2	4.70E-10	0.43	31	0.78	RGD1560248	
DMR3:38669001	3	38669001	38671000	2000	1	2.90E-07	-0.5	25	1.25	RGD1560248;Prpf40a	
DMR3:38692001	3	38692001	38693000	1000	1	1.50E-13	0.48	14	1.4	Prpf40a	
DMR3:38706001	3	38706001	38708000	2000	1	3.30E-11	-0.37	27	1.35	Prpf40a	
DMR3:38721001	3	38721001	38722000	1000	1	3.80E-09	-0.65	5	0.5	Prpf40a	
DMR3:38864001	3	38864001	38866000	2000	1	1.80E-23	0.87	19	0.95	Rps27a-ps7;LOC105650978	
DMR3:39964001	3	39964001	39966000	2000	1	3.60E-11	0.53	19	0.95	Galnt13	Golgi
DMR3:39973001	3	39973001	39975000	2000	1	1.00E-08	0.43	28	1.4	Galnt13	Golgi
DMR3:39981001	3	39981001	39983000	2000	1	2.80E-12	0.77	7	0.35	Galnt13	Golgi
DMR3:39992001	3	39992001	39993000	1000	1	8.60E-09	0.52	5	0.5	Galnt13	Golgi
DMR3:40010001	3	40010001	40012000	2000	2	5.70E-16	0.79	12	0.6	Galnt13	Golgi
DMR3:40016001	3	40016001	40019000	3000	1	1.80E-07	-0.47	25	0.83	Galnt13	Golgi
DMR3:40021001	3	40021001	40023000	2000	2	4.90E-15	0.96	7	0.35	Galnt13	Golgi
DMR3:40040001	3	40040001	40042000	2000	2	2.90E-10	-0.45	16	0.8	Galnt13	Golgi
DMR3:40045001	3	40045001	40048000	3000	1	3.20E-08	0.51	19	0.63	Galnt13	Golgi
DMR3:40053001	3	40053001	40056000	3000	1	6.70E-09	0.52	17	0.57	Galnt13	Golgi
DMR3:40076001	3	40076001	40078000	2000	1	2.30E-08	0.38	13	0.65	Galnt13	Golgi
DMR3:40092001	3	40092001	40093000	1000	1	4.10E-08	0.5	3	0.3	Galnt13	Golgi
DMR3:40096001	3	40096001	40098000	2000	1	1.80E-16	0.82	12	0.6	Galnt13	Golgi
DMR3:40120001	3	40120001	40121000	1000	1	5.30E-09	0.68	4	0.4	Galnt13	Golgi
DMR3:40127001	3	40127001	40132000	5000	2	1.80E-12	0.75	18	0.36	Galnt13	Golgi
DMR3:40152001	3	40152001	40153000	1000	1	4.60E-14	-0.57	2	0.2	Galnt13	Golgi

DMR3:40157001	3	40157001	40159000	2000	1	3.40E-07	0.47	17	0.85	Galnt13	Golgi
DMR3:40172001	3	40172001	40174000	2000	1	1.70E-17	-0.62	13	0.65	Galnt13	Golgi
DMR3:40209001	3	40209001	40210000	1000	1	4.80E-07	0.51	5	0.5	Galnt13	Golgi
DMR3:40218001	3	40218001	40219000	1000	1	4.10E-08	0.63	4	0.4	Galnt13	Golgi
DMR3:40234001	3	40234001	40235000	1000	1	2.70E-09	0.51	8	0.8	Galnt13	Golgi
DMR3:40255001	3	40255001	40256000	1000	1	4.70E-15	0.82	10	1	Galnt13	Golgi
DMR3:40258001	3	40258001	40260000	2000	2	3.40E-11	0.63	19	0.95	Galnt13	Golgi
DMR3:40261001	3	40261001	40262000	1000	1	2.50E-10	0.7	2	0.2	Galnt13	Golgi
DMR3:40270001	3	40270001	40273000	3000	2	5.70E-08	-0.68	12	0.4	Galnt13	Golgi
DMR3:40279001	3	40279001	40280000	1000	1	6.00E-10	-0.65	7	0.7	Galnt13	Golgi
DMR3:40283001	3	40283001	40286000	3000	2	3.80E-10	0.64	19	0.63	Galnt13	Golgi
DMR3:40298001	3	40298001	40299000	1000	1	6.00E-11	0.59	7	0.7	Galnt13	Golgi
DMR3:40310001	3	40310001	40311000	1000	1	3.00E-12	0.79	11	1.1	Galnt13	Golgi
DMR3:40342001	3	40342001	40348000	6000	4	2.90E-17	-0.55	56	0.93	Galnt13	Golgi
DMR3:40377001	3	40377001	40379000	2000	2	9.00E-17	1	6	0.3	Galnt13	Golgi
DMR3:40388001	3	40388001	40389000	1000	1	1.10E-09	0.61	2	0.2	Galnt13	Golgi
DMR3:40403001	3	40403001	40404000	1000	1	1.10E-13	0.79	7	0.7	Galnt13	Golgi
DMR3:40406001	3	40406001	40408000	2000	1	9.70E-11	0.63	17	0.85	Galnt13	Golgi
DMR3:40483001	3	40483001	40484000	1000	1	1.40E-10	-0.75	4	0.4	Galnt13	Golgi
DMR3:40485001	3	40485001	40487000	2000	1	3.40E-07	-0.48	7	0.35	Galnt13	Golgi
DMR3:40512001	3	40512001	40514000	2000	1	7.60E-07	-0.64	7	0.35	Galnt13	Golgi
DMR3:40542001	3	40542001	40544000	2000	1	4.00E-15	0.81	14	0.7	Galnt13	Golgi
DMR3:40551001	3	40551001	40552000	1000	1	5.00E-07	-0.64	8	0.8	Galnt13	Golgi
DMR3:40559001	3	40559001	40560000	1000	1	2.50E-10	0.56	6	0.6	Galnt13	Golgi
DMR3:40563001	3	40563001	40565000	2000	2	5.50E-14	0.83	19	0.95	Galnt13	Golgi
DMR3:40581001	3	40581001	40584000	3000	1	4.10E-13	0.52	17	0.57	Galnt13	Golgi
DMR3:40586001	3	40586001	40588000	2000	1	3.70E-12	0.64	7	0.35	Galnt13	Golgi
DMR3:40595001	3	40595001	40596000	1000	1	1.00E-07	0.72	4	0.4	Galnt13;LOC679905	Golgi
DMR3:40612001	3	40612001	40614000	2000	1	4.20E-07	0.55	10	0.5	Galnt13;LOC679905	Golgi
DMR3:41021001	3	41021001	41022000	1000	1	5.60E-13	0.71	7	0.7	LOC108350374;Kcnj3	Transport
DMR3:41035001	3	41035001	41037000	2000	2	2.00E-08	0.5	20	1	Kcnj3	Transport
DMR3:41049001	3	41049001	41050000	1000	1	4.90E-09	0.58	6	0.6	Kcnj3	Transport
DMR3:41068001	3	41068001	41069000	1000	1	1.00E-08	0.56	7	0.7	Kcnj3	Transport
DMR3:41127001	3	41127001	41128000	1000	1	4.80E-10	0.65	4	0.4	Kcnj3	Transport
DMR3:41146001	3	41146001	41147000	1000	1	3.70E-12	0.61	4	0.4	Kcnj3	Transport
DMR3:41185001	3	41185001	41186000	1000	1	8.50E-09	0.49	8	0.8	Kcnj3	Transport
DMR3:43131001	3	43131001	43133000	2000	1	7.90E-07	0.3	37	1.85	Nr4a2	Transcription
DMR3:43176001	3	43176001	43177000	1000	1	5.90E-15	0.77	6	0.6	LOC108350375;Trnaa-cgc;Trnag-gcc	
DMR3:43178001	3	43178001	43180000	2000	1	6.80E-12	0.59	20	1	LOC108350375;Trnaa-cgc;Trnag-gcc	
DMR3:43181001	3	43181001	43182000	1000	1	2.60E-12	0.58	10	1	LOC108350375;Trnaa-cgc;Trnag-gcc	
DMR3:43184001	3	43184001	43187000	3000	1	3.00E-09	0.5	46	1.53	LOC108350375;Trnaa-cgc;Trnag-gcc	
DMR3:43211001	3	43211001	43214000	3000	2	2.40E-07	-0.42	36	1.2	Gpd2	Metabolism
DMR3:43215001	3	43215001	43217000	2000	1	3.40E-08	0.48	26	1.3	Gpd2	Metabolism
DMR3:43225001	3	43225001	43227000	2000	2	8.90E-08	0.51	22	1.1	Gpd2	Metabolism
DMR3:43228001	3	43228001	43231000	3000	1	1.30E-30	0.91	26	0.87	Gpd2	Metabolism
DMR3:43243001	3	43243001	43245000	2000	1	2.00E-16	0.6	35	1.75	Gpd2	Metabolism
DMR3:43250001	3	43250001	43251000	1000	1	1.70E-08	0.55	6	0.6	Gpd2	Metabolism
DMR3:43256001	3	43256001	43257000	1000	1	5.30E-08	0.49	7	0.7	Gpd2	Metabolism
DMR3:43265001	3	43265001	43268000	3000	3	2.30E-16	0.97	35	1.17	Gpd2	Metabolism
DMR3:43269001	3	43269001	43270000	1000	1	6.80E-08	0.34	12	1.2	Gpd2	Metabolism
DMR3:43281001	3	43281001	43287000	6000	5	6.10E-19	-0.51	57	0.95	Gpd2	Metabolism
DMR3:43310001	3	43310001	43314000	4000	2	1.90E-10	-0.42	54	1.35	Gpd2	Metabolism
DMR3:43324001	3	43324001	43325000	1000	1	2.20E-16	0.81	9	0.9	Gpd2	Metabolism
DMR3:43347001	3	43347001	43349000	2000	1	1.20E-09	0.47	49	2.45	Gpd2	Metabolism
DMR3:43357001	3	43357001	43362000	5000	1	4.00E-12	0.45	81	1.62	Gpd2;LOC366056	Metabolism
DMR3:43366001	3	43366001	43367000	1000	1	7.70E-07	-0.49	20	2	Gpd2;LOC366056	Metabolism
DMR3:44033001	3	44033001	44035000	2000	1	1.30E-10	0.58	15	0.75	Galnt5	Golgi
DMR3:44050001	3	44050001	44056000	6000	4	1.30E-30	-0.6	47	0.78	Galnt5	Golgi
DMR3:44074001	3	44074001	44076000	2000	1	6.60E-09	-0.5	22	1.1	Galnt5;Ermn	Golgi
DMR3:44096001	3	44096001	44097000	1000	1	7.30E-07	0.61	5	0.5	Ermn	
DMR3:44153001	3	44153001	44154000	1000	1	7.40E-12	0.57	5	0.5	Cytip	

DMR3:44189001	3	44189001	44190000	1000	1	9.40E-08	0.43	8	0.8	Cytip;LOC102555754	
DMR3:44191001	3	44191001	44193000	2000	1	3.80E-09	0.43	40	2	Cytip;LOC102555754	
DMR3:44207001	3	44207001	44211000	4000	4	1.70E-11	-0.53	22	0.55	Cytip;LOC103691810	
DMR3:44267001	3	44267001	44268000	1000	1	4.60E-08	-0.56	11	1.1	Acvr1c	Signaling
DMR3:44292001	3	44292001	44295000	3000	1	2.80E-07	0.49	33	1.1	Acvr1c	Signaling
DMR3:44296001	3	44296001	44297000	1000	1	2.20E-25	1.04	8	0.8	Acvr1c	Signaling
DMR3:44322001	3	44322001	44324000	2000	1	4.20E-09	0.53	18	0.9	Acvr1c	Signaling
DMR3:44349001	3	44349001	44350000	1000	1	6.30E-20	0.77	6	0.6	Acvr1c	Signaling
DMR3:44424001	3	44424001	44427000	3000	2	2.10E-25	0.86	24	0.8	Acvr1	Signaling
DMR3:44429001	3	44429001	44430000	1000	1	2.80E-07	-0.48	12	1.2	Acvr1	Signaling
DMR3:44433001	3	44433001	44436000	3000	2	2.30E-08	-0.65	52	1.73	Acvr1	Signaling
DMR3:44438001	3	44438001	44440000	2000	1	2.20E-20	0.89	28	1.4	Acvr1	Signaling
DMR3:44476001	3	44476001	44482000	6000	2	2.20E-16	0.84	74	1.23	Acvr1	Signaling
DMR3:44497001	3	44497001	44499000	2000	1	1.50E-16	0.59	28	1.4	Acvr1	Signaling
DMR3:44522001	3	44522001	44525000	3000	2	6.00E-13	-0.67	31	1.03	Acvr1	Signaling
DMR3:44531001	3	44531001	44533000	2000	1	8.30E-12	0.46	29	1.45	Acvr1	Signaling
DMR3:44818001	3	44818001	44819000	1000	1	1.40E-11	0.42	10	1	Upp2	Signaling
DMR3:44825001	3	44825001	44826000	1000	1	3.60E-09	0.52	5	0.5	Upp2	Signaling
DMR3:44830001	3	44830001	44835000	5000	1	1.90E-23	0.68	95	1.9	Upp2	Signaling
DMR3:44886001	3	44886001	44888000	2000	1	2.10E-10	0.46	30	1.5	Ccdc148;LOC102556209	
DMR3:44889001	3	44889001	44890000	1000	1	1.90E-08	-0.5	9	0.9	Ccdc148;LOC102556209	
DMR3:44899001	3	44899001	44903000	4000	3	1.70E-23	0.74	53	1.32	Ccdc148;LOC102556209	
DMR3:44937001	3	44937001	44939000	2000	1	1.00E-11	-0.66	10	0.5	Ccdc148;LOC102556209	
DMR3:44948001	3	44948001	44949000	1000	1	8.30E-07	-0.52	20	2	Ccdc148	
DMR3:44950001	3	44950001	44952000	2000	2	1.00E-10	0.61	17	0.85	Ccdc148	
DMR3:44993001	3	44993001	44998000	5000	2	2.40E-12	-0.63	44	0.88	Ccdc148	
DMR3:45009001	3	45009001	45015000	6000	2	5.10E-08	-0.58	82	1.37	Ccdc148	
DMR3:45017001	3	45017001	45020000	3000	2	5.30E-10	0.51	33	1.1	Ccdc148	
DMR3:45049001	3	45049001	45053000	4000	2	3.00E-09	0.48	35	0.88	Ccdc148	
DMR3:45073001	3	45073001	45075000	2000	1	3.50E-08	0.45	27	1.35	Ccdc148	
DMR3:45077001	3	45077001	45078000	1000	1	3.50E-09	-0.6	13	1.3	Ccdc148	
DMR3:45083001	3	45083001	45087000	4000	1	1.90E-08	-0.55	53	1.32	Ccdc148	
DMR3:45100001	3	45100001	45105000	5000	2	7.60E-14	-0.66	23	0.46	Ccdc148	
DMR3:45124001	3	45124001	45130000	6000	3	5.00E-16	-0.44	65	1.08	Ccdc148	
DMR3:45137001	3	45137001	45139000	2000	1	2.20E-13	0.73	19	0.95	Ccdc148	
DMR3:45152001	3	45152001	45153000	1000	1	8.30E-11	0.46	12	1.2	Ccdc148	
DMR3:45174001	3	45174001	45179000	5000	2	5.10E-16	-0.77	50	1	Ccdc148	
DMR3:45257001	3	45257001	45258000	1000	1	1.40E-08	0.49	8	0.8	Pkp4	Cytoskeleton
DMR3:45259001	3	45259001	45261000	2000	1	8.20E-26	0.82	24	1.2	Pkp4	Cytoskeleton
DMR3:45265001	3	45265001	45266000	1000	1	8.30E-10	0.4	17	1.7	Pkp4	Cytoskeleton
DMR3:45289001	3	45289001	45292000	3000	1	6.50E-09	0.48	30	1	Pkp4	Cytoskeleton
DMR3:45297001	3	45297001	45298000	1000	1	1.30E-07	0.42	8	0.8	Pkp4	Cytoskeleton
DMR3:45314001	3	45314001	45316000	2000	1	3.30E-09	0.67	17	0.85	Pkp4	Cytoskeleton
DMR3:45372001	3	45372001	45375000	3000	2	1.10E-19	0.78	52	1.73	Pkp4	Cytoskeleton
DMR3:45379001	3	45379001	45387000	8000	1	7.10E-07	-0.64	132	1.65	Pkp4	Cytoskeleton
DMR3:45389001	3	45389001	45393000	4000	1	6.40E-09	-0.64	58	1.45	Pkp4	Cytoskeleton
DMR3:45408001	3	45408001	45409000	1000	1	8.20E-10	0.38	17	1.7	Pkp4	Cytoskeleton
DMR3:45416001	3	45416001	45419000	3000	1	1.40E-07	0.37	48	1.6	Pkp4	Cytoskeleton
DMR3:45525001	3	45525001	45529000	4000	1	5.00E-19	0.69	78	1.95	LOC108350378;Dapl1	
DMR3:45541001	3	45541001	45544000	3000	1	4.10E-07	0.3	40	1.33	LOC108350378;Dapl1	
DMR3:45551001	3	45551001	45559000	8000	2	1.70E-07	0.37	97	1.21	LOC108350378;Dapl1	
DMR3:45562001	3	45562001	45564000	2000	1	1.40E-09	0.4	37	1.85	LOC108350378;Dapl1	
DMR3:45678001	3	45678001	45679000	1000	1	1.50E-07	0.47	10	1	Tanc1	
DMR3:45681001	3	45681001	45682000	1000	1	6.90E-10	0.36	13	1.3	Tanc1	
DMR3:45687001	3	45687001	45693000	6000	2	6.00E-08	-0.52	95	1.58	Tanc1	
DMR3:45717001	3	45717001	45719000	2000	1	9.40E-09	0.38	31	1.55	Tanc1	
DMR3:45723001	3	45723001	45725000	2000	1	5.90E-07	0.38	30	1.5	Tanc1	
DMR3:45726001	3	45726001	45733000	7000	1	1.90E-08	-0.64	84	1.2	Tanc1	
DMR3:45746001	3	45746001	45755000	9000	1	1.40E-09	0.38	139	1.54	Tanc1	
DMR3:45756001	3	45756001	45757000	1000	1	5.30E-13	0.49	27	2.7	Tanc1	
DMR3:45758001	3	45758001	45763000	5000	1	3.10E-14	-0.79	81	1.62	Tanc1	
DMR3:45792001	3	45792001	45794000	2000	1	8.00E-07	-0.49	36	1.8	Tanc1	
DMR3:45812001	3	45812001	45819000	7000	2	3.90E-11	0.46	130	1.86	Tanc1	
DMR3:45825001	3	45825001	45838000	13000	3	8.10E-13	0.43	240	1.85	Tanc1	
DMR3:45875001	3	45875001	45878000	3000	1	4.40E-09	0.4	39	1.3	Tanc1	

DMR3:45919001	3	45919001	45922000	3000	1	7.80E-09	0.42	39	1.3	Tanc1;Wdsub1	
DMR3:45953001	3	45953001	45955000	2000	2	1.20E-10	0.48	29	1.45	Wdsub1;LOC108350580	
DMR3:45967001	3	45967001	45969000	2000	1	1.80E-11	0.53	21	1.05	LOC108350580;Baz2b	Epigenetic
DMR3:46000001	3	46000001	46004000	4000	2	2.00E-12	-0.58	39	0.98	Baz2b	Epigenetic
DMR3:46036001	3	46036001	46040000	4000	1	7.60E-17	0.55	69	1.73	Baz2b	Epigenetic
DMR3:46073001	3	46073001	46074000	1000	1	9.90E-11	0.66	23	2.3	Baz2b	Epigenetic
DMR3:46083001	3	46083001	46086000	3000	2	7.90E-13	-0.68	25	0.83	Baz2b	Epigenetic
DMR3:46139001	3	46139001	46146000	7000	3	2.70E-19	0.67	68	0.97	Baz2b	Epigenetic
DMR3:46166001	3	46166001	46171000	5000	1	5.90E-08	0.35	54	1.08	Baz2b	Epigenetic
DMR3:46187001	3	46187001	46188000	1000	1	7.70E-09	-0.43	19	1.9	Baz2b;LOC100361645	Epigenetic;Transcription
DMR3:46209001	3	46209001	46214000	5000	1	2.70E-14	0.61	60	1.2	Baz2b;LOC100361645	Epigenetic;Transcription
DMR3:46219001	3	46219001	46226000	7000	3	1.40E-07	-0.65	40	0.57	Baz2b;LOC100361645;LOC102551618	Epigenetic;Transcription
DMR3:46234001	3	46234001	46235000	1000	1	5.10E-10	0.42	16	1.6	Baz2b;LOC100361645;LOC102551618	Epigenetic;Transcription
DMR3:46239001	3	46239001	46240000	1000	1	6.30E-07	0.5	5	0.5	Baz2b;LOC100361645;LOC102551618	Epigenetic;Transcription
DMR3:46263001	3	46263001	46266000	3000	1	7.90E-20	0.7	44	1.47	Baz2b	Epigenetic
DMR3:46346001	3	46346001	46347000	1000	1	1.90E-10	0.43	17	1.7	Cd302	
DMR3:46355001	3	46355001	46360000	5000	2	1.40E-18	1.05	77	1.54	Cd302;Mir6216;Ly75	
DMR3:46379001	3	46379001	46383000	4000	1	1.10E-17	0.65	62	1.55	Ly75	
DMR3:46410001	3	46410001	46414000	4000	1	9.10E-09	0.36	58	1.45	Ly75	
DMR3:46435001	3	46435001	46437000	2000	1	1.60E-09	0.37	37	1.85	Ly75	
DMR3:46442001	3	46442001	46445000	3000	1	1.80E-07	0.3	51	1.7	Ly75	
DMR3:46446001	3	46446001	46448000	2000	1	9.20E-14	0.48	16	0.8	Ly75;LOC108350581	
DMR3:46459001	3	46459001	46468000	9000	2	3.40E-09	0.43	118	1.31	Ly75;LOC108350581	
DMR3:46496001	3	46496001	46498000	2000	1	1.10E-07	-0.55	26	1.3	LOC108350581;Pla2r1	
DMR3:46524001	3	46524001	46527000	3000	1	2.30E-27	0.88	30	1	Pla2r1	
DMR3:46535001	3	46535001	46541000	6000	3	6.60E-09	0.47	56	0.93	Pla2r1	
DMR3:46548001	3	46548001	46550000	2000	1	3.60E-18	0.89	16	0.8	Pla2r1	
DMR3:46574001	3	46574001	46578000	4000	3	5.90E-17	0.75	73	1.82	Pla2r1	
DMR3:46606001	3	46606001	46608000	2000	2	5.00E-15	0.75	21	1.05	Pla2r1	
DMR3:46654001	3	46654001	46657000	3000	2	1.80E-08	0.48	31	1.03	Itgb6	Extracellular Matrix
DMR3:46658001	3	46658001	46662000	4000	1	3.80E-12	0.6	53	1.32	Itgb6	Extracellular Matrix
DMR3:46671001	3	46671001	46672000	1000	1	1.50E-19	0.83	4	0.4	Itgb6	Extracellular Matrix
DMR3:46673001	3	46673001	46674000	1000	1	8.90E-08	-0.55	14	1.4	Itgb6	Extracellular Matrix
DMR3:46677001	3	46677001	46678000	1000	1	6.70E-10	0.48	14	1.4	Itgb6	Extracellular Matrix
DMR3:46729001	3	46729001	46733000	4000	2	2.60E-12	0.43	65	1.62	Itgb6;LOC108350379	Extracellular Matrix
DMR3:46741001	3	46741001	46742000	1000	1	6.70E-08	0.48	7	0.7	Itgb6;LOC108350379	Extracellular Matrix
DMR3:46754001	3	46754001	46757000	3000	3	2.10E-21	0.73	20	0.67	Itgb6;LOC108350379	Extracellular Matrix
DMR3:46758001	3	46758001	46760000	2000	1	2.00E-07	0.36	26	1.3	Itgb6;LOC108350379	Extracellular Matrix
DMR3:46767001	3	46767001	46769000	2000	1	4.40E-10	0.46	37	1.85	Itgb6;LOC102551550	Extracellular Matrix
DMR3:46780001	3	46780001	46785000	5000	1	1.30E-07	-0.59	45	0.9	Itgb6;LOC102551550	Extracellular Matrix
DMR3:46791001	3	46791001	46794000	3000	1	4.70E-08	0.34	49	1.63	LOC102551550;Rbms1	
DMR3:46801001	3	46801001	46802000	1000	1	9.80E-07	-0.44	12	1.2	Rbms1	
DMR3:46836001	3	46836001	46838000	2000	1	4.20E-13	-0.58	31	1.55	Rbms1	
DMR3:46857001	3	46857001	46859000	2000	1	3.90E-07	-0.55	26	1.3	Rbms1	
DMR3:46866001	3	46866001	46867000	1000	1	1.10E-07	0.42	15	1.5	Rbms1	
DMR3:46871001	3	46871001	46874000	3000	1	1.20E-08	0.38	40	1.33	Rbms1	
DMR3:46937001	3	46937001	46938000	1000	1	5.10E-21	0.87	4	0.4	Rbms1	
DMR3:46948001	3	46948001	46949000	1000	1	1.50E-08	-0.49	9	0.9	Rbms1	
DMR3:46952001	3	46952001	46954000	2000	2	4.70E-09	0.6	16	0.8	Rbms1	
DMR3:46978001	3	46978001	46986000	8000	4	1.80E-26	-0.56	95	1.19	Rbms1	
DMR3:46994001	3	46994001	46995000	1000	1	2.10E-10	0.67	8	0.8	Rbms1	
DMR3:47002001	3	47002001	47005000	3000	2	1.70E-17	0.59	45	1.5	Rbms1	
DMR3:47018001	3	47018001	47021000	3000	1	1.00E-07	-0.44	33	1.1	Rbms1	
DMR3:47456001	3	47456001	47457000	1000	1	7.20E-07	0.42	11	1.1	Tank	
DMR3:47482001	3	47482001	47484000	2000	2	9.90E-09	0.47	17	0.85	Tank	
DMR3:47492001	3	47492001	47493000	1000	1	4.90E-07	0.42	6	0.6	Tank	
DMR3:47499001	3	47499001	47500000	1000	1	3.30E-10	0.58	4	0.4	Tank	
DMR3:47525001	3	47525001	47526000	1000	1	2.30E-15	0.54	19	1.9	Tank	
DMR3:47573001	3	47573001	47576000	3000	3	7.90E-18	0.96	19	0.63	Psmd14	Translation
DMR3:47624001	3	47624001	47627000	3000	1	1.50E-10	0.56	24	0.8	Psmd14;LOC103691831	Translation

DMR3:47634001	3	47634001	47635000	1000	1	8.60E-07	0.3	10	1	Psmd14	Translation
DMR3:47646001	3	47646001	47648000	2000	1	1.90E-14	0.92	13	0.65	Psmd14	Translation
DMR3:47660001	3	47660001	47661000	1000	1	3.40E-10	0.49	5	0.5	Psmd14	Translation
DMR3:47809001	3	47809001	47813000	4000	1	3.40E-09	-0.53	31	0.78	RGD1566198	
DMR3:47818001	3	47818001	47820000	2000	1	4.40E-10	-0.53	16	0.8	RGD1566198	
DMR3:47821001	3	47821001	47823000	2000	1	4.60E-09	-0.7	12	0.6	RGD1566198	
DMR3:47987001	3	47987001	47988000	1000	1	4.90E-10	-0.63	3	0.3	Slc4a10	Transport
DMR3:48015001	3	48015001	48017000	2000	1	2.10E-14	0.79	11	0.55	Slc4a10	Transport
DMR3:48043001	3	48043001	48044000	1000	1	2.60E-15	0.69	2	0.2	Slc4a10	Transport
DMR3:48054001	3	48054001	48057000	3000	2	2.20E-12	-0.61	29	0.97	Slc4a10	Transport
DMR3:48060001	3	48060001	48062000	2000	1	5.80E-09	0.46	12	0.6	Slc4a10	Transport
DMR3:48070001	3	48070001	48071000	1000	1	3.70E-07	0.52	7	0.7	Slc4a10	Transport
DMR3:48080001	3	48080001	48082000	2000	1	1.20E-07	-0.52	15	0.75	Slc4a10	Transport
DMR3:48083001	3	48083001	48085000	2000	1	2.80E-09	0.66	11	0.55	Slc4a10	Transport
DMR3:48088001	3	48088001	48090000	2000	1	7.50E-07	0.39	17	0.85	Slc4a10	Transport
DMR3:48111001	3	48111001	48112000	1000	1	1.10E-12	0.6	7	0.7	Slc4a10	Transport
DMR3:48118001	3	48118001	48121000	3000	1	1.20E-07	0.54	14	0.47	Slc4a10	Transport
DMR3:48143001	3	48143001	48147000	4000	1	5.90E-10	-0.42	32	0.8	Slc4a10	Transport
DMR3:48162001	3	48162001	48163000	1000	1	1.40E-10	-0.54	5	0.5	Slc4a10	Transport
DMR3:48164001	3	48164001	48166000	2000	2	1.00E-16	-0.62	8	0.4	Slc4a10	Transport
DMR3:48184001	3	48184001	48190000	6000	5	6.00E-14	-0.47	50	0.83	Slc4a10	Transport
DMR3:48211001	3	48211001	48212000	1000	1	3.20E-13	-0.73	12	1.2	Slc4a10	Transport
DMR3:48251001	3	48251001	48252000	1000	1	1.90E-10	0.53	6	0.6	Slc4a10	Transport
DMR3:48269001	3	48269001	48272000	3000	1	5.60E-07	0.31	44	1.47	Slc4a10	Transport
DMR3:48278001	3	48278001	48279000	1000	1	5.20E-09	0.49	12	1.2	Slc4a10	Transport
DMR3:48300001	3	48300001	48301000	1000	1	3.40E-07	0.41	7	0.7	Dpp4	Protease
DMR3:48305001	3	48305001	48311000	6000	1	2.00E-08	-0.67	79	1.32	Dpp4	Protease
DMR3:48318001	3	48318001	48325000	7000	2	4.80E-09	-0.5	105	1.5	Dpp4	Protease
DMR3:48346001	3	48346001	48349000	3000	1	1.60E-07	0.38	51	1.7	Dpp4	Protease
DMR3:48353001	3	48353001	48354000	1000	1	4.20E-14	0.82	8	0.8	Dpp4	Protease
DMR3:48360001	3	48360001	48365000	5000	1	4.80E-07	0.37	53	1.06	Dpp4	Protease
DMR3:48377001	3	48377001	48378000	1000	1	5.00E-11	0.47	10	1	Dpp4	Protease
DMR3:48380001	3	48380001	48385000	5000	3	1.80E-12	0.49	60	1.2	Dpp4	Protease
DMR3:48440001	3	48440001	48443000	3000	2	2.10E-16	0.75	32	1.07	Gcg	
DMR3:48457001	3	48457001	48458000	1000	1	2.60E-07	-0.58	12	1.2	Gcg;Fap	Protease
DMR3:48481001	3	48481001	48482000	1000	1	7.30E-07	-0.49	16	1.6	Fap	Protease
DMR3:48496001	3	48496001	48498000	2000	1	3.00E-12	-0.41	20	1	Fap	Protease
DMR3:48518001	3	48518001	48519000	1000	1	8.70E-09	0.56	8	0.8	Fap	Protease
DMR3:48533001	3	48533001	48536000	3000	1	5.10E-09	-0.6	40	1.33	Fap	Protease
DMR3:48543001	3	48543001	48547000	4000	1	2.60E-15	0.59	65	1.62	Fap	Protease
DMR3:48560001	3	48560001	48568000	8000	1	1.00E-10	-0.85	128	1.6	Ifih1	
DMR3:48573001	3	48573001	48575000	2000	1	7.20E-10	-0.68	23	1.15	Ifih1	
DMR3:48576001	3	48576001	48584000	8000	1	6.70E-07	-0.53	120	1.5	Ifih1	
DMR3:48587001	3	48587001	48588000	1000	1	1.50E-08	-0.47	22	2.2	Ifih1	
DMR3:48597001	3	48597001	48598000	1000	1	4.10E-08	0.43	9	0.9	Ifih1	
DMR3:48601001	3	48601001	48603000	2000	1	2.90E-12	0.4	32	1.6	Ifih1	
DMR3:48606001	3	48606001	48611000	5000	2	6.60E-14	0.73	39	0.78	Ifih1	
DMR3:48616001	3	48616001	48619000	3000	1	3.30E-07	0.45	26	0.87	Gca	
DMR3:48630001	3	48630001	48632000	2000	2	2.40E-12	-0.58	26	1.3	Gca;LOC102548992	
DMR3:48633001	3	48633001	48637000	4000	2	8.20E-12	-0.57	38	0.95	Gca;LOC102548992	
DMR3:48653001	3	48653001	48654000	1000	1	4.10E-18	0.68	7	0.7	Gca;Kcnh7	Transport
DMR3:48657001	3	48657001	48662000	5000	1	1.10E-07	0.58	40	0.8	Gca;Kcnh7	Transport
DMR3:48663001	3	48663001	48667000	4000	2	2.80E-08	-0.56	79	1.98	Gca;Kcnh7	Transport
DMR3:48692001	3	48692001	48694000	2000	1	1.00E-07	-0.65	16	0.8	Kcnh7	Transport
DMR3:48703001	3	48703001	48705000	2000	1	7.50E-16	0.98	55	2.75	Kcnh7	Transport
DMR3:48743001	3	48743001	48745000	2000	2	1.30E-10	0.51	24	1.2	Kcnh7	Transport
DMR3:48767001	3	48767001	48768000	1000	1	1.80E-12	-0.62	3	0.3	Kcnh7	Transport
DMR3:48816001	3	48816001	48817000	1000	1	1.70E-08	0.63	4	0.4	Kcnh7	Transport
DMR3:48819001	3	48819001	48821000	2000	1	7.60E-07	0.45	9	0.45	Kcnh7	Transport
DMR3:48851001	3	48851001	48854000	3000	2	7.60E-09	-0.5	15	0.5	Kcnh7	Transport
DMR3:48875001	3	48875001	48876000	1000	1	3.10E-07	0.41	10	1	Kcnh7	Transport
DMR3:48904001	3	48904001	48905000	1000	1	5.50E-07	0.38	11	1.1	Kcnh7	Transport
DMR3:48909001	3	48909001	48912000	3000	2	1.10E-14	0.69	17	0.57	Kcnh7	Transport
DMR3:48914001	3	48914001	48915000	1000	1	4.80E-07	0.42	11	1.1	Kcnh7	Transport
DMR3:48919001	3	48919001	48920000	1000	1	3.10E-08	0.52	5	0.5	Kcnh7	Transport

DMR3:48923001	3	48923001	48925000	2000	1	1.40E-09	0.53	15	0.75	Kcnh7	Transport
DMR3:48930001	3	48930001	48932000	2000	1	3.80E-10	0.52	26	1.3	Kcnh7	Transport
DMR3:48940001	3	48940001	48943000	3000	1	5.50E-09	0.42	15	0.5	Kcnh7	Transport
DMR3:48964001	3	48964001	48966000	2000	2	1.50E-08	-0.58	8	0.4	Kcnh7	Transport
DMR3:48980001	3	48980001	48981000	1000	1	4.90E-10	0.57	11	1.1	Kcnh7	Transport
DMR3:48997001	3	48997001	48998000	1000	1	1.70E-08	-0.66	9	0.9	Kcnh7	Transport
DMR3:48999001	3	48999001	49001000	2000	2	4.50E-18	0.72	14	0.7	Kcnh7	Transport
DMR3:49009001	3	49009001	49010000	1000	1	5.20E-07	0.51	5	0.5	Kcnh7	Transport
DMR3:49034001	3	49034001	49038000	4000	1	5.00E-09	-0.48	53	1.32	Kcnh7	Transport
DMR3:49047001	3	49047001	49049000	2000	1	1.70E-11	0.68	11	0.55	Kcnh7	Transport
DMR3:49052001	3	49052001	49059000	7000	4	1.70E-19	-0.54	69	0.99	Kcnh7	Transport
DMR3:49060001	3	49060001	49061000	1000	1	2.40E-12	0.61	5	0.5	Kcnh7	Transport
DMR3:49083001	3	49083001	49084000	1000	1	9.50E-07	0.51	3	0.3	Kcnh7	Transport
DMR3:49097001	3	49097001	49099000	2000	1	1.10E-14	0.6	19	0.95	Kcnh7	Transport
DMR3:49111001	3	49111001	49112000	1000	1	2.00E-08	0.47	4	0.4	Kcnh7	Transport
DMR3:49134001	3	49134001	49136000	2000	1	3.00E-07	0.44	18	0.9	Kcnh7	Transport
DMR3:49177001	3	49177001	49178000	1000	1	1.30E-13	0.66	12	1.2	Kcnh7;LOC108350383	Transport
DMR3:49990001	3	49990001	49992000	2000	1	7.90E-07	0.47	26	1.3	LOC108350385;Fign	Cytoskeleton
DMR3:50002001	3	50002001	50005000	3000	1	3.10E-10	-0.62	44	1.47	Fign	Cytoskeleton
DMR3:50041001	3	50041001	50045000	4000	1	7.00E-11	0.5	48	1.2	Fign	Cytoskeleton
DMR3:50056001	3	50056001	50057000	1000	1	2.00E-09	0.44	19	1.9	Fign	Cytoskeleton
DMR3:50085001	3	50085001	50089000	4000	1	7.80E-07	-0.62	56	1.4	Fign	Cytoskeleton
DMR3:50095001	3	50095001	50101000	6000	1	6.20E-08	0.53	92	1.53	Fign	Cytoskeleton
DMR3:50109001	3	50109001	50110000	1000	1	1.00E-07	-0.5	8	0.8	Fign	Cytoskeleton
DMR3:50111001	3	50111001	50116000	5000	1	3.50E-10	-0.75	68	1.36	Fign	Cytoskeleton
DMR3:50130001	3	50130001	50131000	1000	1	4.20E-10	0.42	12	1.2	Fign	Cytoskeleton
DMR3:50933001	3	50933001	50935000	2000	1	1.10E-13	0.58	18	0.9	Grb14	Cytoskeleton
DMR3:50953001	3	50953001	50955000	2000	1	8.30E-23	1.11	22	1.1	Grb14	Cytoskeleton
DMR3:50960001	3	50960001	50963000	3000	1	1.70E-08	-0.8	36	1.2	Grb14	Cytoskeleton
DMR3:50979001	3	50979001	50982000	3000	2	5.30E-13	-0.43	36	1.2	Grb14	Cytoskeleton
DMR3:50984001	3	50984001	50985000	1000	1	1.40E-11	-0.51	8	0.8	Grb14	Cytoskeleton
DMR3:50986001	3	50986001	50987000	1000	1	1.60E-13	0.52	9	0.9	Grb14	Cytoskeleton
DMR3:51001001	3	51001001	51005000	4000	1	1.10E-14	-0.48	60	1.5	Grb14;LOC102552585	Cytoskeleton
DMR3:51006001	3	51006001	51008000	2000	2	4.30E-07	-0.51	36	1.8	Grb14;LOC102552585	Cytoskeleton
DMR3:51023001	3	51023001	51028000	5000	3	2.00E-21	1.01	44	0.88	Grb14;LOC102552585	Cytoskeleton
DMR3:51030001	3	51030001	51038000	8000	2	2.00E-15	0.67	87	1.09	Grb14	Cytoskeleton
DMR3:51136001	3	51136001	51137000	1000	1	3.40E-17	0.96	18	1.8	LOC100911508;Cobll1	
DMR3:51140001	3	51140001	51142000	2000	2	2.90E-16	0.92	13	0.65	Cobll1	
DMR3:51143001	3	51143001	51145000	2000	1	1.50E-12	0.6	15	0.75	Cobll1	
DMR3:51150001	3	51150001	51157000	7000	6	2.30E-21	-0.46	82	1.17	Cobll1	
DMR3:51161001	3	51161001	51164000	3000	1	1.40E-08	0.38	35	1.17	Cobll1	
DMR3:51169001	3	51169001	51170000	1000	1	7.60E-13	0.53	10	1	Cobll1	
DMR3:51175001	3	51175001	51178000	3000	1	5.40E-12	0.59	34	1.13	Cobll1	
DMR3:51183001	3	51183001	51185000	2000	2	4.20E-26	0.92	26	1.3	Cobll1	
DMR3:51186001	3	51186001	51190000	4000	1	7.10E-10	-0.55	70	1.75	Cobll1	
DMR3:51193001	3	51193001	51194000	1000	1	2.70E-08	0.45	7	0.7	Cobll1	
DMR3:51208001	3	51208001	51212000	4000	1	1.20E-12	0.63	49	1.23	Cobll1	
DMR3:51230001	3	51230001	51231000	1000	1	1.10E-08	-0.57	16	1.6	Cobll1	
DMR3:51232001	3	51232001	51239000	7000	1	1.00E-09	0.52	103	1.47	Cobll1	
DMR3:51243001	3	51243001	51245000	2000	1	1.00E-07	0.62	36	1.8	Cobll1	
DMR3:51267001	3	51267001	51269000	2000	1	1.70E-07	-0.46	21	1.05	Cobll1	
DMR3:51283001	3	51283001	51284000	1000	1	1.10E-11	-0.8	12	1.2	Cobll1;LOC108350543	
DMR3:51360001	3	51360001	51363000	3000	1	1.40E-14	0.63	43	1.43	Slc38a11	Transport
DMR3:51374001	3	51374001	51378000	4000	1	1.60E-09	0.46	42	1.05	Slc38a11	Transport
DMR3:51385001	3	51385001	51386000	1000	1	1.20E-09	0.59	4	0.4	Slc38a11	Transport
DMR3:51402001	3	51402001	51403000	1000	1	3.00E-07	-0.37	12	1.2	Slc38a11	Transport
DMR3:51522001	3	51522001	51524000	2000	2	4.30E-10	0.71	13	0.65	LOC102553383;Scn3a	Transport
DMR3:51537001	3	51537001	51540000	3000	3	3.30E-22	0.65	40	1.33	LOC102553383;Scn3a	Transport
DMR3:51587001	3	51587001	51588000	1000	1	3.00E-08	-0.61	14	1.4	LOC102553383;Scn3a	Transport
DMR3:51679001	3	51679001	51682000	3000	1	5.40E-11	0.43	30	1	Scn2a	Transport
DMR3:51699001	3	51699001	51705000	6000	1	2.80E-09	0.5	39	0.65	Scn2a	Transport
DMR3:51706001	3	51706001	51707000	1000	1	1.80E-09	-0.66	7	0.7	Scn2a	Transport
DMR3:51713001	3	51713001	51714000	1000	1	4.20E-14	0.53	20	2	Scn2a	Transport
DMR3:51720001	3	51720001	51721000	1000	1	2.30E-13	0.72	4	0.4	Scn2a	Transport
DMR3:51724001	3	51724001	51725000	1000	1	3.10E-16	0.6	15	1.5	Scn2a	Transport

DMR3:51732001	3	51732001	51733000	1000	1	3.90E-08	0.67	6	0.6	Scn2a	Transport
DMR3:51754001	3	51754001	51755000	1000	1	1.20E-08	-0.72	15	1.5	Scn2a	Transport
DMR3:51761001	3	51761001	51762000	1000	1	2.50E-07	-0.37	7	0.7	Scn2a	Transport
DMR3:51785001	3	51785001	51788000	3000	1	1.90E-10	0.52	17	0.57	Scn2a	Transport
DMR3:51789001	3	51789001	51793000	4000	1	7.60E-08	0.37	60	1.5	Scn2a	Transport
DMR3:51808001	3	51808001	51810000	2000	1	5.00E-07	-0.52	20	1	Scn2a	Transport
DMR3:51827001	3	51827001	51829000	2000	1	1.00E-07	-0.46	21	1.05	Scn2a	Transport
DMR3:51874001	3	51874001	51875000	1000	1	1.90E-13	0.61	20	2	Csrnp3	
DMR3:51889001	3	51889001	51891000	2000	1	3.70E-19	0.8	26	1.3	Csrnp3	
DMR3:51900001	3	51900001	51903000	3000	1	2.80E-08	-0.51	53	1.77	Csrnp3	
DMR3:51913001	3	51913001	51914000	1000	1	5.00E-09	0.53	6	0.6	Csrnp3	
DMR3:51918001	3	51918001	51922000	4000	1	2.90E-08	-0.59	46	1.15	Csrnp3	
DMR3:51923001	3	51923001	51927000	4000	1	3.40E-07	0.35	40	1	Csrnp3	
DMR3:51938001	3	51938001	51939000	1000	1	1.90E-09	0.56	7	0.7	Csrnp3	
DMR3:51949001	3	51949001	51958000	9000	3	5.90E-14	0.61	154	1.71	Csrnp3	
DMR3:51963001	3	51963001	51965000	2000	1	2.60E-09	-0.55	30	1.5	Csrnp3	
DMR3:51972001	3	51972001	51975000	3000	1	9.30E-07	0.4	34	1.13	Csrnp3	
DMR3:51996001	3	51996001	51998000	2000	1	1.40E-09	-0.42	33	1.65	Csrnp3	
DMR3:52020001	3	52020001	52021000	1000	1	1.10E-09	0.43	14	1.4	Csrnp3	
DMR3:52037001	3	52037001	52038000	1000	1	4.70E-07	-0.54	10	1	Csrnp3	
DMR3:52052001	3	52052001	52064000	12000	10	2.80E-23	0.64	91	0.76	Csrnp3	
DMR3:52069001	3	52069001	52077000	8000	3	2.90E-09	-0.49	82	1.02	Csrnp3	
DMR3:52092001	3	52092001	52094000	2000	1	8.00E-09	0.64	30	1.5	Csrnp3	
DMR3:52165001	3	52165001	52169000	4000	1	1.10E-08	0.42	61	1.52	Galnt3	Golgi
DMR3:52180001	3	52180001	52181000	1000	1	3.60E-07	0.34	9	0.9	Galnt3	Golgi
DMR3:52182001	3	52182001	52184000	2000	1	6.40E-13	0.51	31	1.55	Galnt3	Golgi
DMR3:52185001	3	52185001	52186000	1000	1	5.20E-09	-0.46	15	1.5	Galnt3	Golgi
DMR3:52187001	3	52187001	52188000	1000	1	3.10E-07	0.31	18	1.8	Galnt3	Golgi
DMR3:52204001	3	52204001	52207000	3000	2	3.80E-08	0.49	32	1.07	Galnt3	Golgi
DMR3:52222001	3	52222001	52224000	2000	1	4.10E-07	-0.32	23	1.15	Galnt3	Golgi
DMR3:52279001	3	52279001	52282000	3000	1	5.60E-10	0.37	33	1.1	Ttc21b	
DMR3:52292001	3	52292001	52295000	3000	1	6.80E-12	-0.64	33	1.1	Ttc21b	
DMR3:52327001	3	52327001	52329000	2000	1	7.40E-14	0.54	36	1.8	Ttc21b;LOC108350386	
DMR3:52341001	3	52341001	52343000	2000	2	1.40E-12	0.55	23	1.15	Ttc21b	
DMR3:52368001	3	52368001	52369000	1000	1	1.30E-08	0.43	10	1	Ttc21b	
DMR3:52370001	3	52370001	52371000	1000	1	3.90E-08	0.58	4	0.4	Ttc21b	
DMR3:52425001	3	52425001	52428000	3000	2	9.40E-18	0.84	16	0.53	Scn1a	Transport
DMR3:52442001	3	52442001	52443000	1000	1	7.90E-08	0.5	19	1.9	Scn1a	Transport
DMR3:52444001	3	52444001	52446000	2000	2	4.40E-14	0.57	33	1.65	Scn1a	Transport
DMR3:52459001	3	52459001	52460000	1000	1	1.50E-16	0.67	9	0.9	Scn1a	Transport
DMR3:52471001	3	52471001	52477000	6000	3	4.50E-21	0.93	37	0.62	Scn1a	Transport
DMR3:52480001	3	52480001	52486000	6000	3	1.50E-10	0.53	53	0.88	Scn1a	Transport
DMR3:52487001	3	52487001	52488000	1000	1	4.10E-08	0.39	8	0.8	Scn1a	Transport
DMR3:52519001	3	52519001	52523000	4000	2	4.10E-09	-0.55	29	0.72	Scn1a;LOC108350388	Transport
DMR3:52535001	3	52535001	52536000	1000	1	1.90E-08	-0.39	13	1.3	Scn1a;LOC108350387	Transport
DMR3:52570001	3	52570001	52575000	5000	4	1.80E-12	-0.46	36	0.72	LOC108350387;Scn9a	Transport
DMR3:52577001	3	52577001	52579000	2000	2	4.20E-17	0.83	9	0.45	LOC108350387;Scn9a	Transport
DMR3:52588001	3	52588001	52590000	2000	1	1.00E-07	0.54	26	1.3	LOC108350387;Scn9a	Transport
DMR3:52614001	3	52614001	52616000	2000	1	1.30E-07	-0.5	33	1.65	LOC108350387;Scn9a	Transport
DMR3:52624001	3	52624001	52626000	2000	1	1.60E-12	0.51	21	1.05	LOC108350387;Scn9a	Transport
DMR3:52646001	3	52646001	52647000	1000	1	3.10E-07	0.41	9	0.9	LOC108350387;Scn9a	Transport
DMR3:52765001	3	52765001	52766000	1000	1	1.20E-11	0.57	9	0.9	Scn7a	Transport
DMR3:52769001	3	52769001	52770000	1000	1	3.80E-12	0.75	6	0.6	Scn7a	Transport
DMR3:52791001	3	52791001	52792000	1000	1	9.70E-17	0.6	12	1.2	Scn7a	Transport
DMR3:52848001	3	52848001	52849000	1000	1	8.50E-09	0.52	5	0.5	Scn7a	Transport
DMR3:52995001	3	52995001	52997000	2000	1	1.90E-23	0.81	5	0.25	RGD1560831	
DMR3:53002001	3	53002001	53005000	3000	1	2.50E-12	0.72	23	0.77	RGD1560831	
DMR3:53557001	3	53557001	53559000	2000	1	6.10E-09	-0.55	4	0.2	Xirp2	Cytoskeleton
DMR3:53583001	3	53583001	53584000	1000	1	7.20E-13	0.65	7	0.7	Xirp2	Cytoskeleton
DMR3:53611001	3	53611001	53612000	1000	1	7.10E-11	0.55	9	0.9	Xirp2	Cytoskeleton
DMR3:53620001	3	53620001	53621000	1000	1	1.70E-08	0.6	11	1.1	Xirp2	Cytoskeleton
DMR3:53638001	3	53638001	53641000	3000	1	3.50E-07	-0.73	46	1.53	Xirp2	Cytoskeleton
DMR3:53660001	3	53660001	53662000	2000	2	2.50E-08	-0.71	25	1.25	Xirp2	Cytoskeleton
DMR3:53737001	3	53737001	53740000	3000	1	2.90E-11	0.38	43	1.43	B3galt1	Golgi
DMR3:53741001	3	53741001	53742000	1000	1	1.60E-10	-0.52	10	1	B3galt1	Golgi

DMR3:53757001	3	53757001	53758000	1000	1	7.50E-14	0.61	9	0.9	B3galt1	Golgi
DMR3:53764001	3	53764001	53765000	1000	1	2.10E-09	0.65	3	0.3	B3galt1	Golgi
DMR3:53770001	3	53770001	53771000	1000	1	2.00E-07	0.43	9	0.9	B3galt1	Golgi
DMR3:53789001	3	53789001	53790000	1000	1	4.80E-07	0.45	5	0.5	B3galt1	Golgi
DMR3:53856001	3	53856001	53857000	1000	1	7.70E-09	-0.65	9	0.9	B3galt1	Golgi
DMR3:53890001	3	53890001	53891000	1000	1	4.50E-08	0.47	9	0.9	B3galt1	Golgi
DMR3:53909001	3	53909001	53913000	4000	2	4.00E-20	-0.47	51	1.27	B3galt1	Golgi
DMR3:53949001	3	53949001	53951000	2000	1	4.10E-08	0.55	22	1.1	B3galt1	Golgi
DMR3:53966001	3	53966001	53968000	2000	1	1.90E-08	0.49	9	0.45	B3galt1	Golgi
DMR3:54039001	3	54039001	54040000	1000	1	1.00E-07	0.53	3	0.3	B3galt1	Golgi
DMR3:54053001	3	54053001	54060000	7000	5	4.70E-16	-0.48	62	0.89	B3galt1	Golgi
DMR3:54081001	3	54081001	54083000	2000	1	7.30E-08	0.56	19	0.95	B3galt1	Golgi
DMR3:54085001	3	54085001	54090000	5000	2	1.00E-19	0.75	39	0.78	B3galt1	Golgi
DMR3:54095001	3	54095001	54097000	2000	1	9.10E-07	0.32	19	0.95	B3galt1	Golgi
DMR3:54101001	3	54101001	54102000	1000	1	8.00E-10	0.59	6	0.6	B3galt1	Golgi
DMR3:54118001	3	54118001	54119000	1000	1	8.80E-09	0.46	9	0.9	B3galt1	Golgi
DMR3:54121001	3	54121001	54123000	2000	2	1.90E-10	0.68	15	0.75	B3galt1	Golgi
DMR3:54154001	3	54154001	54158000	4000	1	7.60E-07	-0.67	55	1.38	B3galt1	Golgi
DMR3:54185001	3	54185001	54187000	2000	1	2.60E-11	0.49	13	0.65	B3galt1	Golgi
DMR3:54197001	3	54197001	54200000	3000	1	1.00E-07	0.28	33	1.1	B3galt1	Golgi
DMR3:54203001	3	54203001	54206000	3000	1	1.10E-09	0.52	26	0.87	B3galt1	Golgi
DMR3:54207001	3	54207001	54210000	3000	1	1.10E-07	-0.52	11	0.37	B3galt1	Golgi
DMR3:54222001	3	54222001	54223000	1000	1	1.00E-08	0.59	2	0.2	B3galt1	Golgi
DMR3:54224001	3	54224001	54228000	4000	1	1.10E-09	-0.4	30	0.75	B3galt1	Golgi
DMR3:54230001	3	54230001	54231000	1000	1	6.20E-18	0.85	8	0.8	B3galt1	Golgi
DMR3:54273001	3	54273001	54280000	7000	1	2.40E-07	0.53	63	0.9	B3galt1	Golgi
DMR3:54362001	3	54362001	54368000	6000	1	9.90E-17	0.58	107	1.78	Stk39;LOC100911723	
DMR3:54378001	3	54378001	54381000	3000	1	2.80E-13	0.74	39	1.3	Stk39;LOC100911723	
DMR3:54385001	3	54385001	54389000	4000	1	4.60E-07	0.27	43	1.07	Stk39;LOC100911723	
DMR3:54392001	3	54392001	54393000	1000	1	2.60E-09	0.4	35	3.5	Stk39	
DMR3:54394001	3	54394001	54396000	2000	1	5.80E-20	0.96	16	0.8	Stk39	
DMR3:54407001	3	54407001	54408000	1000	1	8.10E-18	0.76	11	1.1	Stk39	
DMR3:54415001	3	54415001	54417000	2000	1	1.40E-09	0.37	20	1	Stk39	
DMR3:54418001	3	54418001	54419000	1000	1	3.80E-08	0.34	22	2.2	Stk39	
DMR3:54442001	3	54442001	54444000	2000	1	3.40E-09	0.44	17	0.85	Stk39	
DMR3:54452001	3	54452001	54453000	1000	1	1.80E-07	0.62	6	0.6	Stk39	
DMR3:54457001	3	54457001	54461000	4000	2	1.20E-08	-0.54	50	1.25	Stk39	
DMR3:54483001	3	54483001	54487000	4000	2	3.10E-13	0.56	83	2.08	Stk39	
DMR3:54495001	3	54495001	54504000	9000	1	3.50E-13	-0.41	121	1.34	Stk39	
DMR3:54505001	3	54505001	54507000	2000	1	1.10E-07	0.33	26	1.3	Stk39	
DMR3:54508001	3	54508001	54514000	6000	1	5.30E-16	0.5	104	1.73	Stk39	
DMR3:54527001	3	54527001	54538000	11000	3	2.30E-17	-0.5	154	1.4	Stk39	
DMR3:54556001	3	54556001	54559000	3000	2	3.90E-10	-0.43	49	1.63	Stk39	
DMR3:54560001	3	54560001	54562000	2000	2	3.60E-21	0.61	38	1.9	Stk39	
DMR3:54564001	3	54564001	54566000	2000	1	7.70E-07	-0.46	44	2.2	Stk39	
DMR3:54573001	3	54573001	54574000	1000	1	9.10E-15	-0.6	16	1.6	Stk39	
DMR3:54597001	3	54597001	54603000	6000	1	1.50E-08	-0.4	98	1.63	Stk39	
DMR3:54606001	3	54606001	54609000	3000	1	1.90E-17	0.84	40	1.33	Stk39	
DMR3:54611001	3	54611001	54612000	1000	1	8.60E-16	0.75	8	0.8	Stk39	
DMR3:54618001	3	54618001	54619000	1000	1	2.90E-14	0.56	15	1.5	Stk39	
DMR3:54631001	3	54631001	54635000	4000	1	1.60E-21	0.93	35	0.88	Stk39	
DMR3:55009001	3	55009001	55013000	4000	1	7.00E-12	0.73	46	1.15	RGD1564400	
DMR3:55017001	3	55017001	55018000	1000	1	2.10E-12	0.88	4	0.4	RGD1564400	
DMR3:55026001	3	55026001	55027000	1000	1	4.60E-16	0.59	9	0.9	RGD1564400	
DMR3:55028001	3	55028001	55036000	8000	2	2.40E-14	0.59	77	0.96	RGD1564400	
DMR3:55086001	3	55086001	55087000	1000	1	8.60E-08	0.52	20	2	Cers6	
DMR3:55092001	3	55092001	55094000	2000	1	6.30E-16	0.66	9	0.45	Cers6	
DMR3:55099001	3	55099001	55103000	4000	1	1.50E-14	0.52	44	1.1	Cers6	
DMR3:55126001	3	55126001	55131000	5000	1	4.10E-08	-0.6	74	1.48	Cers6	
DMR3:55139001	3	55139001	55141000	2000	1	5.30E-13	0.53	15	0.75	Cers6	
DMR3:55151001	3	55151001	55152000	1000	1	3.70E-07	0.68	4	0.4	Cers6	
DMR3:55154001	3	55154001	55157000	3000	1	7.80E-07	0.37	28	0.93	Cers6	
DMR3:55165001	3	55165001	55168000	3000	1	1.10E-18	0.63	49	1.63	Cers6	
DMR3:55179001	3	55179001	55180000	1000	1	5.30E-09	0.4	12	1.2	Cers6	
DMR3:55183001	3	55183001	55187000	4000	1	9.40E-08	0.37	40	1	Cers6	

DMR3:55197001	3	55197001	55200000	3000	1	9.60E-08	0.35	42	1.4	Cers6	
DMR3:55205001	3	55205001	55206000	1000	1	3.00E-10	0.49	8	0.8	Cers6	
DMR3:55214001	3	55214001	55215000	1000	1	3.40E-14	0.61	11	1.1	Cers6	
DMR3:55216001	3	55216001	55219000	3000	1	1.60E-11	0.54	32	1.07	Cers6	
DMR3:55222001	3	55222001	55224000	2000	1	8.60E-22	0.8	15	0.75	Cers6	
DMR3:55237001	3	55237001	55239000	2000	1	8.20E-09	-0.51	36	1.8	Cers6	
DMR3:55246001	3	55246001	55247000	1000	1	2.80E-07	-0.47	10	1	Cers6	
DMR3:55249001	3	55249001	55252000	3000	1	3.80E-15	0.56	45	1.5	Cers6	
DMR3:55257001	3	55257001	55259000	2000	1	1.80E-12	0.44	24	1.2	Cers6	
DMR3:55263001	3	55263001	55266000	3000	1	4.00E-07	-0.58	33	1.1	Cers6	
DMR3:55267001	3	55267001	55270000	3000	1	1.30E-10	0.35	40	1.33	Cers6	
DMR3:55278001	3	55278001	55280000	2000	1	8.80E-13	0.7	15	0.75	Cers6	
DMR3:55298001	3	55298001	55302000	4000	1	5.80E-11	-0.36	58	1.45	Cers6	
DMR3:55303001	3	55303001	55305000	2000	2	2.70E-08	0.45	9	0.45	Cers6	
DMR3:55347001	3	55347001	55351000	4000	2	2.30E-15	0.6	59	1.48	Cers6;LOC366068	
DMR3:55356001	3	55356001	55360000	4000	1	8.80E-24	0.98	50	1.25	LOC366068;Nostrin	
DMR3:55377001	3	55377001	55378000	1000	1	9.90E-09	0.4	13	1.3	Nostrin	
DMR3:55390001	3	55390001	55393000	3000	1	2.40E-08	-0.7	35	1.17	Nostrin	
DMR3:55417001	3	55417001	55419000	2000	1	1.40E-10	0.53	20	1	Nostrin	
DMR3:55420001	3	55420001	55434000	14000	3	3.90E-15	0.59	207	1.48	Nostrin;Spc25	
DMR3:55443001	3	55443001	55446000	3000	1	5.30E-07	0.39	36	1.2	Nostrin;Spc25	
DMR3:55456001	3	55456001	55457000	1000	1	4.20E-10	0.48	14	1.4	Spc25;LOC108350389;G6pc2	
DMR3:55550001	3	55550001	55554000	4000	1	1.50E-07	0.36	45	1.12	Abcb11	Transport
DMR3:55558001	3	55558001	55562000	4000	2	1.00E-07	-0.57	32	0.8	Abcb11	Transport
DMR3:55580001	3	55580001	55581000	1000	1	3.50E-10	0.4	15	1.5	Abcb11	Transport
DMR3:55584001	3	55584001	55586000	2000	2	1.10E-17	0.63	20	1	Abcb11	Transport
DMR3:55635001	3	55635001	55640000	5000	1	1.10E-08	0.4	69	1.38	Dhrs9	Metabolism
DMR3:55645001	3	55645001	55648000	3000	2	1.40E-07	-0.39	53	1.77	Dhrs9	Metabolism
DMR3:55652001	3	55652001	55657000	5000	1	1.60E-10	0.72	51	1.02	Dhrs9;Lrp2	Metabolism
DMR3:55693001	3	55693001	55694000	1000	1	6.60E-09	0.33	27	2.7	Lrp2	
DMR3:55698001	3	55698001	55702000	4000	1	5.20E-20	0.55	86	2.15	Lrp2	
DMR3:55709001	3	55709001	55711000	2000	1	4.80E-10	0.51	25	1.25	Lrp2	
DMR3:55717001	3	55717001	55719000	2000	1	7.40E-08	-0.4	44	2.2	Lrp2	
DMR3:55723001	3	55723001	55725000	2000	1	3.10E-08	0.46	24	1.2	Lrp2	
DMR3:55741001	3	55741001	55745000	4000	1	4.90E-07	-0.55	87	2.17	Lrp2	
DMR3:55765001	3	55765001	55768000	3000	1	7.00E-08	-0.54	49	1.63	Lrp2	
DMR3:55769001	3	55769001	55770000	1000	1	7.00E-09	0.46	13	1.3	Lrp2	
DMR3:55815001	3	55815001	55817000	2000	1	4.30E-12	0.6	20	1	Lrp2	
DMR3:55875001	3	55875001	55879000	4000	1	1.80E-09	0.41	53	1.32	LOC108350390;Bbs5	
DMR3:55880001	3	55880001	55882000	2000	1	9.00E-15	0.49	28	1.4	LOC108350390;Bbs5	
DMR3:55894001	3	55894001	55895000	1000	1	2.10E-08	-0.42	18	1.8	Bbs5	
DMR3:55897001	3	55897001	55898000	1000	1	7.40E-15	0.76	16	1.6	Bbs5	
DMR3:55901001	3	55901001	55903000	2000	1	2.20E-14	0.59	18	0.9	Bbs5;Klhl41	Cytoskeleton
DMR3:55904001	3	55904001	55905000	1000	1	6.20E-08	-0.68	11	1.1	Bbs5;Klhl41	Cytoskeleton
DMR3:55923001	3	55923001	55925000	2000	1	6.00E-08	-0.55	29	1.45	Klhl41;Fastkd1	Cytoskeleton
DMR3:55930001	3	55930001	55932000	2000	1	1.70E-12	-0.72	22	1.1	Klhl41;Fastkd1	Cytoskeleton
DMR3:55937001	3	55937001	55938000	1000	1	3.00E-08	0.34	20	2	Fastkd1	
DMR3:55940001	3	55940001	55942000	2000	1	1.50E-07	-0.34	32	1.6	Fastkd1	
DMR3:55943001	3	55943001	55944000	1000	1	5.70E-11	0.53	13	1.3	Fastkd1	
DMR3:55969001	3	55969001	55971000	2000	1	6.00E-07	-0.45	20	1	Ppig;Ddit4l2	Transcription
DMR3:55986001	3	55986001	55988000	2000	2	3.60E-11	0.47	33	1.65	Ppig;Ccdc173	Transcription
DMR3:56000001	3	56000001	56004000	4000	2	1.40E-12	-0.6	42	1.05	Ppig;Ccdc173	Transcription
DMR3:56006001	3	56006001	56007000	1000	1	1.00E-12	-0.62	6	0.6	Ccdc173	
DMR3:56008001	3	56008001	56009000	1000	1	3.80E-11	0.48	9	0.9	Ccdc173	
DMR3:56016001	3	56016001	56019000	3000	2	2.50E-19	0.6	48	1.6	Ccdc173	
DMR3:56070001	3	56070001	56074000	4000	1	1.20E-20	0.94	58	1.45	Klhl23	Cytoskeleton
DMR3:56079001	3	56079001	56081000	2000	1	5.90E-07	-0.34	21	1.05	Klhl23	Cytoskeleton
DMR3:56082001	3	56082001	56084000	2000	1	2.60E-13	0.65	22	1.1	Ssb	Metabolism
DMR3:56143001	3	56143001	56145000	2000	1	8.70E-10	-0.62	9	0.45	Ubr3	Proteolysis
DMR3:56163001	3	56163001	56165000	2000	1	2.40E-07	0.45	10	0.5	Ubr3	Proteolysis
DMR3:56168001	3	56168001	56169000	1000	1	1.30E-09	0.55	11	1.1	Ubr3	Proteolysis
DMR3:56171001	3	56171001	56173000	2000	1	2.20E-09	0.62	7	0.35	Ubr3	Proteolysis
DMR3:56178001	3	56178001	56179000	1000	1	5.80E-09	0.48	8	0.8	Ubr3	Proteolysis
DMR3:56191001	3	56191001	56192000	1000	1	7.30E-12	0.74	4	0.4	Ubr3	Proteolysis
DMR3:56197001	3	56197001	56198000	1000	1	3.10E-20	1.05	2	0.2	Ubr3	Proteolysis

DMR3:56201001	3	56201001	56203000	2000	1	9.60E-12	0.52	21	1.05	Ubr3	Proteolysis
DMR3:56228001	3	56228001	56230000	2000	1	5.50E-10	0.47	13	0.65	Ubr3	Proteolysis
DMR3:56231001	3	56231001	56232000	1000	1	5.80E-09	-0.64	11	1.1	Ubr3	Proteolysis
DMR3:56268001	3	56268001	56270000	2000	1	4.10E-07	0.43	17	0.85	Ubr3	Proteolysis
DMR3:56287001	3	56287001	56288000	1000	1	8.00E-07	-0.62	7	0.7	Ubr3	Proteolysis
DMR3:56292001	3	56292001	56298000	6000	2	4.70E-15	0.82	96	1.6	Ubr3	Proteolysis
DMR3:56361001	3	56361001	56366000	5000	1	2.20E-09	0.45	60	1.2	Myo3b	
DMR3:56377001	3	56377001	56378000	1000	1	3.20E-21	0.55	13	1.3	Myo3b	
DMR3:56381001	3	56381001	56389000	8000	2	8.20E-09	-0.62	126	1.57	Myo3b	
DMR3:56395001	3	56395001	56400000	5000	1	5.70E-11	-0.58	72	1.44	Myo3b	
DMR3:56438001	3	56438001	56441000	3000	3	1.40E-25	-0.84	15	0.5	Myo3b	
DMR3:56460001	3	56460001	56461000	1000	1	2.00E-13	0.68	8	0.8	Myo3b	
DMR3:56471001	3	56471001	56474000	3000	2	3.80E-09	0.4	64	2.13	Myo3b	
DMR3:56488001	3	56488001	56496000	8000	5	3.30E-14	0.9	79	0.99	Myo3b	
DMR3:56500001	3	56500001	56506000	6000	2	1.90E-13	0.57	62	1.03	Myo3b	
DMR3:56507001	3	56507001	56510000	3000	1	5.30E-11	-0.79	47	1.57	Myo3b	
DMR3:56521001	3	56521001	56525000	4000	1	3.10E-07	-0.59	59	1.48	Myo3b	
DMR3:56556001	3	56556001	56560000	4000	1	6.90E-12	0.72	25	0.62	Myo3b	
DMR3:56567001	3	56567001	56569000	2000	1	1.60E-08	0.42	25	1.25	Myo3b	
DMR3:56581001	3	56581001	56586000	5000	2	4.50E-10	0.37	78	1.56	Myo3b	
DMR3:56588001	3	56588001	56590000	2000	1	4.40E-14	0.65	22	1.1	Myo3b	
DMR3:56591001	3	56591001	56594000	3000	1	1.00E-08	0.39	44	1.47	Myo3b	
DMR3:56620001	3	56620001	56621000	1000	1	1.90E-08	0.52	9	0.9	Myo3b	
DMR3:56641001	3	56641001	56643000	2000	1	2.00E-16	0.92	22	1.1	Myo3b	
DMR3:56646001	3	56646001	56647000	1000	1	4.90E-07	0.49	20	2	Myo3b	
DMR3:56664001	3	56664001	56670000	6000	1	4.00E-13	1	62	1.03	Myo3b	
DMR3:56726001	3	56726001	56729000	3000	1	2.00E-18	0.87	24	0.8	Myo3b	
DMR3:56813001	3	56813001	56817000	4000	1	1.70E-07	0.46	51	1.27	Erich2;LOC103691815	
DMR3:56830001	3	56830001	56831000	1000	1	1.40E-16	0.96	7	0.7	Erich2	
DMR3:56841001	3	56841001	56843000	2000	1	6.20E-12	0.53	11	0.55	Erich2	
DMR3:56845001	3	56845001	56846000	1000	1	1.30E-10	0.52	14	1.4	Erich2	
DMR3:56869001	3	56869001	56870000	1000	1	2.30E-18	0.93	4	0.4	Gad1	Metabolism
DMR3:56874001	3	56874001	56875000	1000	1	9.10E-08	0.4	7	0.7	Gad1	Metabolism
DMR3:56888001	3	56888001	56889000	1000	1	3.20E-07	0.36	7	0.7	Gad1	Metabolism
DMR3:56897001	3	56897001	56899000	2000	1	2.20E-19	0.76	22	1.1	Gad1	Metabolism
DMR3:56904001	3	56904001	56908000	4000	1	2.50E-07	0.47	29	0.72	Gad1	Metabolism
DMR3:57096001	3	57096001	57097000	1000	1	2.20E-07	0.59	6	0.6	Tlk1	Signaling
DMR3:57104001	3	57104001	57106000	2000	1	2.40E-07	0.41	12	0.6	Tlk1	Signaling
DMR3:57146001	3	57146001	57148000	2000	1	7.40E-08	0.4	35	1.75	Tlk1	Signaling
DMR3:57153001	3	57153001	57156000	3000	1	1.50E-15	0.52	32	1.07	Tlk1	Signaling
DMR3:57157001	3	57157001	57159000	2000	1	6.90E-07	-0.48	28	1.4	Tlk1	Signaling
DMR3:57168001	3	57168001	57171000	3000	1	1.50E-08	0.38	56	1.87	Tlk1	Signaling
DMR3:57174001	3	57174001	57182000	8000	2	2.50E-12	-0.64	81	1.01	Tlk1;RGD1565767	Signaling;Translation
DMR3:57208001	3	57208001	57214000	6000	2	3.20E-08	0.39	69	1.15	Tlk1	Signaling
DMR3:57215001	3	57215001	57216000	1000	1	2.90E-09	0.39	18	1.8	Tlk1	Signaling
DMR3:57217001	3	57217001	57219000	2000	1	7.40E-25	0.78	28	1.4	Tlk1	Signaling
DMR3:57225001	3	57225001	57227000	2000	1	3.80E-07	0.43	33	1.65	Tlk1	Signaling
DMR3:57246001	3	57246001	57249000	3000	1	2.00E-16	0.67	42	1.4	Tlk1;Mettl8	Signaling
DMR3:57391001	3	57391001	57392000	1000	1	1.60E-09	0.64	8	0.8	Mettl8;LOC103690017	Signaling
DMR3:57400001	3	57400001	57403000	3000	1	2.30E-19	0.7	29	0.97	Mettl8;LOC103690017	Signaling
DMR3:57574001	3	57574001	57577000	3000	2	4.20E-19	0.77	24	0.8	Mettl8;LOC102548511	
DMR3:57601001	3	57601001	57602000	1000	1	1.40E-07	0.33	7	0.7	Mettl8;LOC102548511	
DMR3:57603001	3	57603001	57604000	1000	1	3.00E-07	-0.41	14	1.4	Mettl8;LOC102548511	
DMR3:57617001	3	57617001	57621000	4000	1	2.80E-13	0.44	49	1.23	Mettl8;LOC102548511	
DMR3:57628001	3	57628001	57635000	7000	2	9.80E-16	0.55	121	1.73	Mettl8	
DMR3:57677001	3	57677001	57685000	8000	3	5.90E-14	-0.54	109	1.36	Dcaf17	
DMR3:57707001	3	57707001	57711000	4000	1	2.70E-13	0.57	48	1.2	Cybrd1	Metabolism
DMR3:57720001	3	57720001	57726000	6000	1	6.10E-08	0.35	76	1.27	Cybrd1	Metabolism
DMR3:57733001	3	57733001	57736000	3000	2	1.10E-08	-0.4	38	1.27	Cybrd1	Metabolism
DMR3:57737001	3	57737001	57742000	5000	3	2.20E-12	0.53	58	1.16	Cybrd1	Metabolism
DMR3:57743001	3	57743001	57745000	2000	1	1.70E-11	0.4	50	2.5	Cybrd1	Metabolism
DMR3:57806001	3	57806001	57808000	2000	1	2.50E-11	0.49	27	1.35	Dync1i2	Cytoskeleton
DMR3:57810001	3	57810001	57812000	2000	1	1.80E-07	0.34	15	0.75	Dync1i2	Cytoskeleton
DMR3:57816001	3	57816001	57817000	1000	1	1.00E-23	1.02	5	0.5	Dync1i2	Cytoskeleton
DMR3:57819001	3	57819001	57820000	1000	1	2.60E-08	-0.55	12	1.2	Dync1i2	Cytoskeleton

DMR3:57839001	3	57839001	57841000	2000	1	7.50E-07	-0.48	15	0.75	Dync1i2	Cytoskeleton
DMR3:57853001	3	57853001	57855000	2000	1	1.70E-17	0.71	18	0.9	Dync1i2	Cytoskeleton
DMR3:57859001	3	57859001	57861000	2000	2	4.00E-09	0.42	20	1	Dync1i2	Cytoskeleton
DMR3:57876001	3	57876001	57878000	2000	1	3.50E-07	0.35	21	1.05	Dync1i2;Slc25a12	Cytoskeleton;Transport
DMR3:57915001	3	57915001	57917000	2000	1	6.90E-09	0.52	10	0.5	Slc25a12	Transport
DMR3:57943001	3	57943001	57944000	1000	1	1.60E-08	0.38	19	1.9	Slc25a12	Transport
DMR3:57963001	3	57963001	57964000	1000	1	6.70E-10	0.42	11	1.1	Slc25a12	Transport
DMR3:57974001	3	57974001	57976000	2000	2	2.40E-32	1.15	18	0.9	Slc25a12	Transport
DMR3:58001001	3	58001001	58002000	1000	1	1.20E-08	0.58	7	0.7	Slc25a12	Transport
DMR3:58005001	3	58005001	58008000	3000	1	7.70E-07	-0.51	30	1	Slc25a12	Transport
DMR3:58089001	3	58089001	58093000	4000	1	5.40E-08	0.39	51	1.27	LOC102549016;Metap1d	Protease
DMR3:58098001	3	58098001	58101000	3000	3	9.80E-24	0.58	42	1.4	Metap1d	Protease
DMR3:58102001	3	58102001	58112000	10000	2	2.90E-12	0.72	113	1.13	Metap1d	Protease
DMR3:58116001	3	58116001	58118000	2000	2	2.50E-13	0.44	21	1.05	Metap1d	Protease
DMR3:58145001	3	58145001	58149000	4000	2	6.50E-33	0.86	75	1.88	Metap1d;LOC108350393	Protease
DMR3:58155001	3	58155001	58161000	6000	2	8.40E-12	0.47	100	1.67	Metap1d;LOC108350393;Dlx1;LOC100910518	Protease
DMR3:58452001	3	58452001	58456000	4000	2	9.90E-18	0.57	59	1.48	Itga6	Extracellular Matrix
DMR3:58476001	3	58476001	58483000	7000	1	3.20E-13	-0.93	104	1.49	Itga6	Extracellular Matrix
DMR3:58484001	3	58484001	58496000	12000	2	1.90E-11	-0.64	197	1.64	Itga6	Extracellular Matrix
DMR3:58499001	3	58499001	58500000	1000	1	4.10E-08	-0.44	23	2.3	Itga6	Extracellular Matrix
DMR3:58510001	3	58510001	58511000	1000	1	3.20E-10	-0.62	19	1.9	Itga6	Extracellular Matrix
DMR3:58532001	3	58532001	58534000	2000	2	2.10E-10	-0.61	16	0.8	Pdk1	Signaling
DMR3:58543001	3	58543001	58549000	6000	1	3.50E-15	-0.67	97	1.62	Pdk1	Signaling
DMR3:58567001	3	58567001	58569000	2000	1	9.60E-20	0.77	21	1.05	Pdk1	Signaling
DMR3:58570001	3	58570001	58571000	1000	1	2.40E-12	0.48	16	1.6	Pdk1	Signaling
DMR3:58649001	3	58649001	58651000	2000	1	7.10E-12	0.62	13	0.65	Rapgef4	Transcription
DMR3:58653001	3	58653001	58656000	3000	1	9.40E-08	0.4	39	1.3	Rapgef4	Transcription
DMR3:58658001	3	58658001	58659000	1000	1	3.90E-15	0.52	19	1.9	Rapgef4	Transcription
DMR3:58667001	3	58667001	58669000	2000	1	1.20E-15	0.54	26	1.3	Rapgef4	Transcription
DMR3:58674001	3	58674001	58679000	5000	4	7.40E-17	0.54	57	1.14	Rapgef4	Transcription
DMR3:58684001	3	58684001	58685000	1000	1	6.30E-15	0.68	11	1.1	Rapgef4	Transcription
DMR3:58699001	3	58699001	58705000	6000	2	7.50E-12	0.61	100	1.67	Rapgef4	Transcription
DMR3:58706001	3	58706001	58712000	6000	1	5.90E-10	0.43	109	1.82	Rapgef4	Transcription
DMR3:58721001	3	58721001	58722000	1000	1	2.80E-07	0.38	15	1.5	Rapgef4	Transcription
DMR3:58723001	3	58723001	58726000	3000	3	3.60E-12	0.65	36	1.2	Rapgef4	Transcription
DMR3:58757001	3	58757001	58759000	2000	1	3.50E-19	0.51	27	1.35	Rapgef4	Transcription
DMR3:58790001	3	58790001	58796000	6000	1	6.20E-18	0.75	73	1.22	Rapgef4	Transcription
DMR3:58819001	3	58819001	58823000	4000	1	6.20E-07	0.37	59	1.48	Rapgef4	Transcription
DMR3:58828001	3	58828001	58829000	1000	1	8.10E-10	0.5	12	1.2	Rapgef4	Transcription
DMR3:58860001	3	58860001	58868000	8000	1	7.00E-09	0.52	106	1.32	Rapgef4;LOC103695171	Transcription
DMR3:58878001	3	58878001	58880000	2000	2	3.10E-16	0.75	18	0.9	Rapgef4;LOC103695171	Transcription
DMR3:58883001	3	58883001	58890000	7000	2	2.60E-07	-0.57	97	1.39	Rapgef4;LOC103695171	Transcription
DMR3:58891001	3	58891001	58896000	5000	1	3.20E-11	0.46	82	1.64	Rapgef4;LOC103695171	Transcription
DMR3:58899001	3	58899001	58908000	9000	1	3.20E-08	-0.51	163	1.81	Rapgef4;LOC103695171	Transcription
DMR3:58909001	3	58909001	58911000	2000	1	3.60E-08	-0.52	41	2.05	Rapgef4	Transcription
DMR3:58914001	3	58914001	58916000	2000	1	1.90E-15	-0.54	28	1.4	Rapgef4	Transcription
DMR3:58933001	3	58933001	58934000	1000	1	2.30E-12	0.6	11	1.1	Rapgef4	Transcription
DMR3:58935001	3	58935001	58941000	6000	4	6.90E-15	0.81	74	1.23	Rapgef4	Transcription
DMR3:58961001	3	58961001	58963000	2000	1	4.90E-12	0.52	26	1.3	Zak	
DMR3:58974001	3	58974001	58978000	4000	2	1.00E-16	0.77	60	1.5	Zak	
DMR3:58982001	3	58982001	58984000	2000	1	2.50E-09	0.43	40	2	Zak	
DMR3:59011001	3	59011001	59013000	2000	1	1.00E-12	0.51	26	1.3	Zak	
DMR3:59019001	3	59019001	59027000	8000	1	7.60E-07	-0.6	98	1.23	Zak	
DMR3:59034001	3	59034001	59036000	2000	2	5.50E-08	0.37	26	1.3	Zak;LOC102555296	
DMR3:59050001	3	59050001	59055000	5000	1	1.90E-10	0.43	85	1.7	Zak;LOC102555296	
DMR3:59073001	3	59073001	59074000	1000	1	3.90E-08	0.34	12	1.2	Zak	
DMR3:59086001	3	59086001	59088000	2000	1	4.80E-09	0.42	31	1.55	Zak	
DMR3:59093001	3	59093001	59094000	1000	1	6.10E-09	0.49	13	1.3	Zak	
DMR3:59101001	3	59101001	59104000	3000	1	7.90E-07	0.36	49	1.63	Zak	
DMR3:59113001	3	59113001	59117000	4000	3	2.00E-13	0.53	70	1.75	Zak;LOC108350557	
DMR3:59147001	3	59147001	59150000	3000	2	1.90E-11	0.47	34	1.13	LOC108350557;Cdca7	
DMR3:59155001	3	59155001	59160000	5000	1	9.80E-07	-0.62	81	1.62	Cdca7;LOC108350395	
DMR3:59163001	3	59163001	59164000	1000	1	9.60E-07	-0.55	15	1.5	Cdca7;LOC108350395	

DMR3:59165001	3	59165001	59168000	3000	1	3.70E-07	-0.63	47	1.57	Cdca7;LOC108350395	
DMR3:59410001	3	59410001	59412000	2000	1	3.10E-22	0.82	19	0.95	Eno1-ps1	
DMR3:59576001	3	59576001	59579000	3000	1	8.10E-07	-0.5	23	0.77	LOC108350397;RGD1563716	
DMR3:59635001	3	59635001	59636000	1000	1	1.40E-07	0.41	13	1.3	Sp3	Transcription
DMR3:59641001	3	59641001	59642000	1000	1	3.20E-14	0.71	5	0.5	Sp3	Transcription
DMR3:59658001	3	59658001	59661000	3000	2	2.10E-07	0.51	17	0.57	Sp3	Transcription
DMR3:59662001	3	59662001	59665000	3000	1	1.80E-08	0.44	25	0.83	Sp3	Transcription
DMR3:59810001	3	59810001	59812000	2000	1	7.90E-07	-0.46	27	1.35	LOC102546819;Ola1;LOC103695172	Signaling
DMR3:59828001	3	59828001	59830000	2000	2	6.70E-20	0.58	24	1.2	LOC102546819;Ola1;LOC103695172	Signaling
DMR3:59843001	3	59843001	59844000	1000	1	8.50E-08	-0.63	12	1.2	Ola1;LOC103695172	Signaling
DMR3:59851001	3	59851001	59854000	3000	1	1.40E-12	0.57	36	1.2	Ola1	Signaling
DMR3:59900001	3	59900001	59901000	1000	1	9.40E-14	0.62	9	0.9	Ola1	Signaling
DMR3:59979001	3	59979001	59980000	1000	1	6.60E-07	0.64	24	2.4	Sp9	Transcription
DMR3:60028001	3	60028001	60030000	2000	1	1.60E-07	-0.46	24	1.2	Cir1;LOC108350582;Scrn3	
DMR3:60036001	3	60036001	60038000	2000	1	3.10E-07	-0.56	19	0.95	Scrn3	
DMR3:60050001	3	60050001	60052000	2000	1	1.90E-17	0.61	27	1.35	Scrn3;Gpr155	Transcription
DMR3:60067001	3	60067001	60076000	9000	5	2.70E-26	-0.51	103	1.14	Gpr155	Transcription
DMR3:60079001	3	60079001	60082000	3000	1	1.60E-07	0.43	38	1.27	Gpr155	Transcription
DMR3:60100001	3	60100001	60101000	1000	1	1.90E-07	0.38	12	1.2	Gpr155	Transcription
DMR3:60112001	3	60112001	60117000	5000	1	3.20E-13	0.72	32	0.64	Gpr155	Transcription
DMR3:60161001	3	60161001	60165000	4000	1	8.60E-10	0.48	69	1.73	Wipf1	Cytoskeleton
DMR3:60198001	3	60198001	60201000	3000	1	2.70E-07	-0.56	56	1.87	Wipf1	Cytoskeleton
DMR3:60202001	3	60202001	60203000	1000	1	7.80E-10	0.39	14	1.4	Wipf1	Cytoskeleton
DMR3:60204001	3	60204001	60205000	1000	1	3.40E-08	0.29	18	1.8	Wipf1	Cytoskeleton
DMR3:60207001	3	60207001	60211000	4000	1	6.90E-07	-0.4	70	1.75	Wipf1	Cytoskeleton
DMR3:60441001	3	60441001	60442000	1000	1	2.70E-07	0.33	26	2.6	LOC108350398;Chrna1	Ion Channel
DMR3:60464001	3	60464001	60467000	3000	2	4.70E-12	0.58	57	1.9	Chrna1;LOC102552170	Ion Channel
DMR3:60468001	3	60468001	60470000	2000	1	2.30E-07	0.41	30	1.5	Chrna1;LOC102552170	Ion Channel
DMR3:60504001	3	60504001	60505000	1000	1	5.60E-08	0.44	13	1.3	LOC102552170;Chn1	
DMR3:60512001	3	60512001	60518000	6000	1	1.20E-08	0.32	88	1.47	LOC102552170;Chn1;LOC102552328	
DMR3:60562001	3	60562001	60563000	1000	1	1.10E-07	-0.39	8	0.8	Chn1	
DMR3:60570001	3	60570001	60571000	1000	1	5.00E-09	0.47	9	0.9	Chn1	
DMR3:60602001	3	60602001	60605000	3000	1	1.30E-14	0.67	24	0.8	Chn1	
DMR3:60606001	3	60606001	60611000	5000	3	9.50E-09	-0.45	65	1.3	Chn1	
DMR3:60625001	3	60625001	60626000	1000	1	4.70E-13	0.54	12	1.2	Chn1	
DMR3:60635001	3	60635001	60639000	4000	2	6.50E-09	-0.61	16	0.4	Chn1	
DMR3:60651001	3	60651001	60657000	6000	2	3.20E-08	0.45	105	1.75	Chn1	
DMR3:60731001	3	60731001	60735000	4000	1	2.20E-07	-0.49	31	0.78	Atf2;LOC108350399	Transcription
DMR3:60741001	3	60741001	60743000	2000	1	4.90E-11	-0.61	9	0.45	Atf2	Transcription
DMR3:60748001	3	60748001	60750000	2000	1	4.10E-07	0.4	22	1.1	Atf2	Transcription
DMR3:60816001	3	60816001	60819000	3000	1	6.20E-18	0.88	20	0.67	Atp5g3	
DMR3:61472001	3	61472001	61473000	1000	1	4.00E-09	-0.74	9	0.9	Lnp	
DMR3:61479001	3	61479001	61484000	5000	2	6.20E-25	1.15	28	0.56	Lnp	
DMR3:61496001	3	61496001	61500000	4000	1	6.40E-07	-0.38	40	1	Lnp;LOC100364636	
DMR3:61554001	3	61554001	61555000	1000	1	8.40E-08	0.37	26	2.6	LOC100364636;LOC502637;Evx2	
DMR3:61561001	3	61561001	61563000	2000	2	2.50E-09	0.42	32	1.6	LOC502637;Evx2	
DMR3:61679001	3	61679001	61682000	3000	2	1.50E-23	0.71	33	1.1	Hoxd3;Hoxd1	Development
DMR3:61693001	3	61693001	61695000	2000	1	2.70E-13	0.68	15	0.75	Hoxd1	Development
DMR3:61696001	3	61696001	61697000	1000	1	1.60E-07	-0.59	9	0.9	Hoxd1	Development
DMR3:61738001	3	61738001	61748000	10000	2	2.70E-10	0.38	115	1.15	Mtx2	
DMR3:61765001	3	61765001	61770000	5000	2	1.60E-12	-0.47	30	0.6	Mtx2	
DMR3:61782001	3	61782001	61783000	1000	1	7.20E-08	-0.65	8	0.8	Mtx2	
DMR3:62484001	3	62484001	62488000	4000	2	3.40E-17	0.61	24	0.6	Hnrnpa3;Nfe2l2	Transcription
DMR3:62531001	3	62531001	62534000	3000	1	8.90E-07	0.52	27	0.9	Nfe2l2;LOC102555850	Transcription
DMR3:62731001	3	62731001	62732000	1000	1	4.00E-12	0.66	3	0.3	Agps	
DMR3:62741001	3	62741001	62742000	1000	1	1.50E-09	0.54	9	0.9	Agps	
DMR3:62764001	3	62764001	62768000	4000	1	9.60E-21	0.83	53	1.32	Ttc30b	
DMR3:62770001	3	62770001	62774000	4000	2	7.10E-12	-0.71	57	1.43	Ttc30b	
DMR3:62792001	3	62792001	62793000	1000	1	3.90E-07	0.38	14	1.4	Ttc30a1	
DMR3:62800001	3	62800001	62803000	3000	1	5.90E-07	-0.54	42	1.4	Ttc30a1;Ttc30a;LOC102551299	
DMR3:62807001	3	62807001	62808000	1000	1	1.30E-18	0.91	4	0.4	Ttc30a1;Ttc30a;LOC102551299	

DMR3:62824001	3	62824001	62833000	9000	2	2.10E-07	-0.43	153	1.7	LOC102551299;Pde11a	Signaling
DMR3:62834001	3	62834001	62838000	4000	1	4.80E-13	0.53	89	2.22	Pde11a	Signaling
DMR3:62863001	3	62863001	62865000	2000	1	9.10E-07	-0.45	33	1.65	Pde11a	Signaling
DMR3:62871001	3	62871001	62872000	1000	1	7.00E-13	0.49	13	1.3	Pde11a	Signaling
DMR3:62884001	3	62884001	62886000	2000	1	9.30E-08	0.31	36	1.8	Pde11a	Signaling
DMR3:62896001	3	62896001	62899000	3000	1	1.90E-11	0.75	41	1.37	Pde11a	Signaling
DMR3:62924001	3	62924001	62927000	3000	1	4.50E-08	-0.79	54	1.8	Pde11a	Signaling
DMR3:62928001	3	62928001	62929000	1000	1	5.70E-07	-0.58	6	0.6	Pde11a	Signaling
DMR3:62935001	3	62935001	62936000	1000	1	1.20E-08	0.34	16	1.6	Pde11a	Signaling
DMR3:62948001	3	62948001	62949000	1000	1	3.00E-11	0.43	25	2.5	Pde11a	Signaling
DMR3:62959001	3	62959001	62962000	3000	2	1.70E-14	1.02	44	1.47	Pde11a	Signaling
DMR3:62966001	3	62966001	62967000	1000	1	6.50E-07	0.33	15	1.5	Pde11a	Signaling
DMR3:62972001	3	62972001	62973000	1000	1	1.90E-12	0.58	9	0.9	Pde11a	Signaling
DMR3:62992001	3	62992001	62993000	1000	1	3.90E-07	0.32	15	1.5	Pde11a	Signaling
DMR3:62998001	3	62998001	62999000	1000	1	6.00E-08	0.43	9	0.9	Pde11a	Signaling
DMR3:63009001	3	63009001	63011000	2000	1	1.00E-07	-0.62	31	1.55	Pde11a	Signaling
DMR3:63024001	3	63024001	63027000	3000	1	2.40E-22	0.77	32	1.07	Pde11a	Signaling
DMR3:63050001	3	63050001	63051000	1000	1	1.40E-16	0.66	14	1.4	Pde11a	Signaling
DMR3:63056001	3	63056001	63061000	5000	2	5.90E-13	0.59	59	1.18	Pde11a	Signaling
DMR3:63064001	3	63064001	63065000	1000	1	1.90E-08	0.37	7	0.7	Pde11a	Signaling
DMR3:63074001	3	63074001	63076000	2000	1	1.10E-08	0.46	31	1.55	Pde11a	Signaling
DMR3:63083001	3	63083001	63085000	2000	1	6.30E-10	0.46	18	0.9	Pde11a	Signaling
DMR3:63098001	3	63098001	63102000	4000	1	1.20E-14	0.77	47	1.18	Pde11a	Signaling
DMR3:63103001	3	63103001	63107000	4000	1	7.90E-16	0.86	38	0.95	Pde11a	Signaling
DMR3:63109001	3	63109001	63121000	12000	11	1.30E-21	-0.5	137	1.14	Pde11a	Signaling
DMR3:63130001	3	63130001	63134000	4000	1	1.50E-13	0.71	62	1.55	Pde11a	Signaling
DMR3:63170001	3	63170001	63180000	10000	5	4.70E-21	-0.49	134	1.34	Pde11a	Signaling
DMR3:63184001	3	63184001	63186000	2000	1	8.90E-07	0.34	24	1.2	Pde11a	Signaling
DMR3:63190001	3	63190001	63191000	1000	1	5.70E-08	0.33	16	1.6	Pde11a	Signaling
DMR3:63195001	3	63195001	63199000	4000	1	1.40E-07	0.47	42	1.05	Pde11a;Cyct;LOC678730	Signaling
DMR3:63205001	3	63205001	63206000	1000	1	5.80E-09	0.47	3	0.3	Pde11a;Cyct;LOC678730	Signaling
DMR3:63207001	3	63207001	63209000	2000	1	1.40E-10	0.51	22	1.1	Pde11a;Cyct;LOC678730	Signaling
DMR3:63227001	3	63227001	63229000	2000	1	5.50E-07	-0.43	33	1.65	Rbm45	
DMR3:63255001	3	63255001	63256000	1000	1	3.60E-08	-0.41	17	1.7	Osbpl6	
DMR3:63266001	3	63266001	63270000	4000	1	9.80E-13	0.41	61	1.52	Osbpl6	
DMR3:63271001	3	63271001	63274000	3000	2	4.20E-10	0.43	49	1.63	Osbpl6	
DMR3:63287001	3	63287001	63296000	9000	4	4.50E-17	0.59	122	1.36	Osbpl6	
DMR3:63299001	3	63299001	63302000	3000	2	4.00E-14	-0.45	26	0.87	Osbpl6	
DMR3:63303001	3	63303001	63308000	5000	2	6.40E-13	0.58	57	1.14	Osbpl6	
DMR3:63312001	3	63312001	63313000	1000	1	5.30E-14	0.75	7	0.7	Osbpl6	
DMR3:63314001	3	63314001	63317000	3000	1	2.20E-12	0.46	56	1.87	Osbpl6	
DMR3:63318001	3	63318001	63321000	3000	2	5.10E-16	0.55	62	2.07	Osbpl6	
DMR3:63334001	3	63334001	63345000	11000	5	3.60E-19	-0.48	155	1.41	Osbpl6	
DMR3:63354001	3	63354001	63364000	10000	2	1.50E-10	0.55	126	1.26	Osbpl6	
DMR3:63371001	3	63371001	63373000	2000	1	1.70E-08	0.43	26	1.3	Osbpl6	
DMR3:63378001	3	63378001	63384000	6000	2	1.20E-09	-0.45	95	1.58	Osbpl6	
DMR3:63385001	3	63385001	63386000	1000	1	5.40E-09	-0.65	14	1.4	Osbpl6	
DMR3:63395001	3	63395001	63397000	2000	2	2.20E-09	0.42	21	1.05	Osbpl6	
DMR3:63401001	3	63401001	63407000	6000	2	6.70E-13	0.53	101	1.68	Osbpl6	
DMR3:63421001	3	63421001	63424000	3000	1	5.70E-10	0.37	73	2.43	Osbpl6	
DMR3:63432001	3	63432001	63440000	8000	1	5.10E-15	0.53	147	1.84	Osbpl6	
DMR3:63463001	3	63463001	63465000	2000	1	9.00E-19	0.61	45	2.25	Osbpl6	
DMR3:63479001	3	63479001	63481000	2000	1	1.80E-07	0.35	72	3.6	Prkra	
DMR3:63485001	3	63485001	63488000	3000	1	5.80E-07	-0.47	26	0.87	Prkra	
DMR3:63493001	3	63493001	63495000	2000	2	1.70E-09	0.47	17	0.85	Prkra	
DMR3:63496001	3	63496001	63498000	2000	1	1.50E-09	0.4	27	1.35	Prkra	
DMR3:63513001	3	63513001	63515000	2000	2	2.70E-14	0.69	38	1.9	Prkra;Dfnb59;Fkbp7	Transcription
DMR3:63526001	3	63526001	63527000	1000	1	4.20E-15	-0.67	21	2.1	Dfnb59;Fkbp7;Plekha3	Transcription
DMR3:63546001	3	63546001	63549000	3000	1	1.60E-16	0.52	29	0.97	Plekha3	
DMR3:63580001	3	63580001	63590000	10000	1	5.80E-08	-0.47	268	2.68	Ttn	
DMR3:63612001	3	63612001	63616000	4000	2	4.60E-08	-0.66	90	2.25	Ttn	
DMR3:63617001	3	63617001	63619000	2000	1	1.70E-07	-0.42	40	2	Ttn	
DMR3:63630001	3	63630001	63634000	4000	1	4.30E-15	-0.76	65	1.62	Ttn	
DMR3:63640001	3	63640001	63641000	1000	1	4.90E-08	-0.62	15	1.5	Ttn	
DMR3:63683001	3	63683001	63687000	4000	1	5.10E-08	-0.48	67	1.68	Ttn	

DMR3:63726001	3	63726001	63727000	1000	1	3.80E-08	0.49	8	0.8	Ttn	
DMR3:63728001	3	63728001	63733000	5000	2	1.10E-10	-0.54	106	2.12	Ttn	
DMR3:63734001	3	63734001	63737000	3000	1	1.10E-07	0.32	58	1.93	Ttn	
DMR3:63738001	3	63738001	63740000	2000	1	3.30E-07	0.43	21	1.05	Ttn	
DMR3:63758001	3	63758001	63760000	2000	2	4.00E-10	-0.63	40	2	Ttn	
DMR3:63828001	3	63828001	63831000	3000	1	2.00E-07	0.4	35	1.17	Ttn	
DMR3:63842001	3	63842001	63843000	1000	1	3.80E-13	0.69	7	0.7	Ttn	
DMR3:63888001	3	63888001	63889000	1000	1	5.20E-08	0.4	8	0.8	Ccdc141	
DMR3:63904001	3	63904001	63905000	1000	1	3.20E-14	0.61	16	1.6	Ccdc141	
DMR3:63963001	3	63963001	63966000	3000	1	2.50E-12	0.67	18	0.6	Ccdc141	
DMR3:63973001	3	63973001	63974000	1000	1	3.60E-10	0.49	11	1.1	Ccdc141	
DMR3:63976001	3	63976001	63979000	3000	2	4.70E-13	0.65	16	0.53	Ccdc141	
DMR3:63990001	3	63990001	63994000	4000	2	1.40E-12	0.76	41	1.02	Ccdc141	
DMR3:63995001	3	63995001	63999000	4000	3	1.50E-09	0.46	36	0.9	Ccdc141	
DMR3:64008001	3	64008001	64012000	4000	2	2.60E-21	0.73	46	1.15	Ccdc141	
DMR3:64029001	3	64029001	64033000	4000	1	5.10E-14	0.52	43	1.07	Ccdc141;Sestd1	
DMR3:64043001	3	64043001	64045000	2000	1	5.50E-12	0.5	23	1.15	Sestd1	
DMR3:64054001	3	64054001	64059000	5000	2	1.70E-13	0.48	75	1.5	Sestd1	
DMR3:64075001	3	64075001	64079000	4000	1	1.40E-07	-0.44	45	1.12	Sestd1	
DMR3:64084001	3	64084001	64085000	1000	1	1.80E-09	-0.55	21	2.1	Sestd1	
DMR3:64092001	3	64092001	64095000	3000	2	2.70E-15	0.71	42	1.4	Sestd1	
DMR3:64108001	3	64108001	64109000	1000	1	5.20E-08	0.37	18	1.8	Sestd1	
DMR3:64113001	3	64113001	64115000	2000	1	1.50E-09	0.44	22	1.1	Sestd1	
DMR3:64125001	3	64125001	64128000	3000	1	1.70E-15	0.52	42	1.4	Sestd1	
DMR3:64264001	3	64264001	64265000	1000	1	1.30E-09	0.45	9	0.9	Zfp385b	
DMR3:64271001	3	64271001	64275000	4000	1	1.50E-09	-0.73	68	1.7	Zfp385b	
DMR3:64276001	3	64276001	64279000	3000	2	1.60E-12	0.47	40	1.33	Zfp385b	
DMR3:64286001	3	64286001	64290000	4000	2	5.60E-09	-0.56	27	0.68	Zfp385b	
DMR3:64306001	3	64306001	64311000	5000	1	5.70E-08	-0.45	83	1.66	Zfp385b	
DMR3:64318001	3	64318001	64324000	6000	1	4.10E-09	-0.67	111	1.85	Zfp385b	
DMR3:64366001	3	64366001	64369000	3000	1	4.60E-07	-0.52	50	1.67	Zfp385b	
DMR3:64379001	3	64379001	64381000	2000	1	3.00E-21	0.67	33	1.65	Zfp385b	
DMR3:64387001	3	64387001	64392000	5000	1	3.10E-07	-0.66	70	1.4	Zfp385b	
DMR3:64401001	3	64401001	64403000	2000	1	2.90E-11	0.68	21	1.05	Zfp385b	
DMR3:64405001	3	64405001	64411000	6000	2	4.80E-13	0.48	100	1.67	Zfp385b	
DMR3:64412001	3	64412001	64417000	5000	1	1.10E-12	-0.63	63	1.26	Zfp385b	
DMR3:64443001	3	64443001	64446000	3000	1	2.30E-07	0.34	42	1.4	Zfp385b	
DMR3:64457001	3	64457001	64459000	2000	1	4.10E-08	0.33	37	1.85	Zfp385b	
DMR3:64462001	3	64462001	64464000	2000	1	8.80E-08	-0.56	26	1.3	Zfp385b	
DMR3:64466001	3	64466001	64470000	4000	1	7.00E-10	-0.6	92	2.3	Zfp385b	
DMR3:64523001	3	64523001	64526000	3000	1	6.60E-07	-0.47	46	1.53	Zfp385b	
DMR3:64552001	3	64552001	64554000	2000	1	9.70E-09	-0.53	47	2.35	Zfp385b;LOC102553814	
DMR3:64565001	3	64565001	64570000	5000	3	8.40E-20	0.77	48	0.96	Zfp385b;LOC102553814;LOC103691828	
DMR3:64731001	3	64731001	64733000	2000	1	5.40E-08	0.39	30	1.5	Cwc22	Translation
DMR3:64737001	3	64737001	64739000	2000	1	2.50E-19	0.76	34	1.7	Cwc22	Translation
DMR3:64774001	3	64774001	64776000	2000	2	5.90E-24	0.93	17	0.85	Cwc22	Translation
DMR3:64788001	3	64788001	64793000	5000	5	6.20E-13	0.53	35	0.7	Cwc22;LOC295681	Translation
DMR3:65667001	3	65667001	65668000	1000	1	3.10E-10	0.43	18	1.8	RGD1564319	Signaling
DMR3:65811001	3	65811001	65812000	1000	1	4.00E-07	0.33	14	1.4	LOC108350407;Ube2e3	Proteolysis
DMR3:65821001	3	65821001	65822000	1000	1	1.70E-09	-0.58	10	1	LOC108350407;Ube2e3	Proteolysis
DMR3:65832001	3	65832001	65833000	1000	1	7.60E-07	-0.36	10	1	Ube2e3	Proteolysis
DMR3:65836001	3	65836001	65838000	2000	1	3.50E-09	0.44	10	0.5	Ube2e3	Proteolysis
DMR3:65839001	3	65839001	65841000	2000	2	4.30E-16	-0.62	13	0.65	Ube2e3	Proteolysis
DMR3:65865001	3	65865001	65867000	2000	1	2.00E-15	0.55	11	0.55	Ube2e3	Proteolysis
DMR3:65876001	3	65876001	65878000	2000	1	5.20E-08	0.37	18	0.9	Ube2e3	Proteolysis
DMR3:66187001	3	66187001	66188000	1000	1	6.20E-10	0.58	6	0.6	Itga4	Extracellular Matrix
DMR3:66189001	3	66189001	66191000	2000	1	7.60E-13	0.6	23	1.15	Itga4	Extracellular Matrix
DMR3:66231001	3	66231001	66232000	1000	1	3.80E-07	0.39	5	0.5	Itga4	Extracellular Matrix
DMR3:66242001	3	66242001	66245000	3000	2	1.20E-08	0.59	28	0.93	Itga4	Extracellular Matrix
DMR3:66266001	3	66266001	66267000	1000	1	1.10E-15	0.78	16	1.6	Itga4;Cerkl	Extracellular Matrix;Signaling
DMR3:66272001	3	66272001	66275000	3000	1	1.20E-09	0.64	17	0.57	Itga4;Cerkl	Extracellular Matrix;Signaling
DMR3:66282001	3	66282001	66284000	2000	2	1.70E-11	0.55	16	0.8	Cerkl	Signaling

DMR3:66312001	3	66312001	66314000	2000	1	3.20E-07	-0.63	26	1.3	Cerkl	Signaling
DMR3:66316001	3	66316001	66323000	7000	5	3.80E-14	-0.48	67	0.96	Cerkl	Signaling
DMR3:66403001	3	66403001	66404000	1000	1	1.60E-07	-0.53	8	0.8	Cerkl;LOC102553021	Signaling
DMR3:66585001	3	66585001	66586000	1000	1	4.00E-08	-0.63	14	1.4	LOC103691830;Ssfa2	
DMR3:66596001	3	66596001	66599000	3000	1	6.80E-12	-0.57	38	1.27	LOC103691830;Ssfa2	
DMR3:66618001	3	66618001	66621000	3000	1	1.70E-07	-0.43	51	1.7	Ssfa2	
DMR3:66698001	3	66698001	66700000	2000	1	8.30E-13	0.57	28	1.4	Ppp1r1c	Signaling
DMR3:66717001	3	66717001	66718000	1000	1	4.30E-08	-0.48	4	0.4	Ppp1r1c	Signaling
DMR3:66729001	3	66729001	66730000	1000	1	4.20E-10	-0.57	17	1.7	Ppp1r1c	Signaling
DMR3:66739001	3	66739001	66741000	2000	2	7.00E-16	-0.52	11	0.55	Ppp1r1c	Signaling
DMR3:66742001	3	66742001	66744000	2000	2	2.40E-13	-0.46	19	0.95	Ppp1r1c	Signaling
DMR3:66756001	3	66756001	66757000	1000	1	1.40E-23	0.97	10	1	Ppp1r1c	Signaling
DMR3:66766001	3	66766001	66768000	2000	1	4.30E-15	0.63	15	0.75	Ppp1r1c	Signaling
DMR3:66800001	3	66800001	66801000	1000	1	5.50E-09	0.59	5	0.5	Pde1a	Signaling
DMR3:66832001	3	66832001	66833000	1000	1	9.50E-19	0.62	13	1.3	Pde1a	Signaling
DMR3:66878001	3	66878001	66879000	1000	1	1.20E-07	-0.39	12	1.2	Pde1a	Signaling
DMR3:66913001	3	66913001	66917000	4000	2	3.90E-14	-0.48	45	1.12	Pde1a	Signaling
DMR3:66943001	3	66943001	66944000	1000	1	7.10E-07	0.55	9	0.9	Pde1a	Signaling
DMR3:66948001	3	66948001	66951000	3000	1	2.50E-14	0.94	18	0.6	Pde1a	Signaling
DMR3:66958001	3	66958001	66959000	1000	1	1.80E-08	0.49	13	1.3	Pde1a	Signaling
DMR3:66964001	3	66964001	66966000	2000	2	5.00E-09	0.72	10	0.5	Pde1a	Signaling
DMR3:67009001	3	67009001	67010000	1000	1	3.90E-20	1.08	4	0.4	Pde1a	Signaling
DMR3:67072001	3	67072001	67076000	4000	2	1.80E-15	-0.58	46	1.15	Pde1a	Signaling
DMR3:67112001	3	67112001	67113000	1000	1	7.40E-08	0.49	14	1.4	Pde1a	Signaling
DMR3:67118001	3	67118001	67120000	2000	2	7.60E-12	0.82	9	0.45	Pde1a	Signaling
DMR3:67165001	3	67165001	67167000	2000	2	2.60E-19	1.07	5	0.25	Pde1a	Signaling
DMR3:67212001	3	67212001	67218000	6000	2	9.30E-10	-0.42	41	0.68	Pde1a	Signaling
DMR3:67228001	3	67228001	67235000	7000	3	3.60E-13	-0.46	82	1.17	Pde1a	Signaling
DMR3:67248001	3	67248001	67249000	1000	1	4.50E-13	0.87	4	0.4	Pde1a	Signaling
DMR3:67522001	3	67522001	67529000	7000	3	1.60E-15	0.71	41	0.59	Dnajc10	Transcription
DMR3:67530001	3	67530001	67531000	1000	1	5.40E-19	0.72	14	1.4	Dnajc10	Transcription
DMR3:67571001	3	67571001	67576000	5000	1	4.70E-12	0.51	59	1.18	Dnajc10	Transcription
DMR3:67640001	3	67640001	67642000	2000	1	2.40E-14	0.52	33	1.65	Frzb	Receptor
DMR3:67648001	3	67648001	67653000	5000	1	7.40E-15	0.55	54	1.08	Frzb	Receptor
DMR3:67664001	3	67664001	67665000	1000	1	3.30E-12	0.56	7	0.7	Frzb	Receptor
DMR3:67676001	3	67676001	67678000	2000	1	1.20E-07	0.38	37	1.85	Frzb	Receptor
DMR3:67729001	3	67729001	67731000	2000	2	1.00E-09	-0.66	30	1.5	Nckap1	
DMR3:67780001	3	67780001	67782000	2000	1	4.50E-12	0.46	13	0.65	Nckap1	
DMR3:67793001	3	67793001	67794000	1000	1	2.10E-11	0.44	20	2	Nckap1	
DMR3:67798001	3	67798001	67799000	1000	1	4.10E-09	0.44	8	0.8	Nckap1	
DMR3:67854001	3	67854001	67855000	1000	1	2.60E-11	0.45	16	1.6	Dusp19	Signaling
DMR3:67863001	3	67863001	67865000	2000	1	1.50E-09	0.45	20	1	Dusp19;Nup35	Signaling
DMR3:67866001	3	67866001	67868000	2000	2	6.50E-09	-0.64	19	0.95	Dusp19;Nup35	Signaling
DMR3:67876001	3	67876001	67878000	2000	1	2.20E-12	-0.71	9	0.45	Nup35	
DMR3:69288001	3	69288001	69290000	2000	1	1.00E-19	0.97	10	0.5	Zfp804a	
DMR3:69316001	3	69316001	69317000	1000	1	5.00E-07	0.44	7	0.7	Zfp804a	
DMR3:69318001	3	69318001	69319000	1000	1	3.80E-08	-0.54	3	0.3	Zfp804a	
DMR3:69345001	3	69345001	69346000	1000	1	1.80E-13	0.78	4	0.4	Zfp804a	
DMR3:69347001	3	69347001	69348000	1000	1	2.70E-12	0.68	2	0.2	Zfp804a	
DMR3:69380001	3	69380001	69381000	1000	1	1.90E-10	0.59	5	0.5	Zfp804a	
DMR3:69443001	3	69443001	69445000	2000	1	2.50E-07	0.56	7	0.35	Zfp804a	
DMR3:69450001	3	69450001	69451000	1000	1	7.30E-09	0.59	10	1	Zfp804a	
DMR3:69457001	3	69457001	69458000	1000	1	1.30E-07	0.57	11	1.1	Zfp804a	
DMR3:69462001	3	69462001	69464000	2000	1	1.60E-07	0.54	8	0.4	Zfp804a	
DMR3:69466001	3	69466001	69467000	1000	1	2.30E-12	-0.77	4	0.4	Zfp804a	
DMR3:69477001	3	69477001	69483000	6000	1	3.90E-09	-0.53	29	0.48	Zfp804a	
DMR3:69555001	3	69555001	69557000	2000	1	8.30E-09	0.36	8	0.4	Zfp804a	
DMR3:69558001	3	69558001	69560000	2000	1	4.90E-11	0.62	11	0.55	Zfp804a	
DMR3:70276001	3	70276001	70279000	3000	2	5.00E-11	-0.6	13	0.43	Fsip2	
DMR3:70313001	3	70313001	70319000	6000	2	1.70E-09	-0.54	47	0.78	Fsip2	
DMR3:70324001	3	70324001	70333000	9000	1	1.60E-07	-0.62	124	1.38	Fsip2	
DMR3:70342001	3	70342001	70343000	1000	1	1.10E-07	0.44	13	1.3	Fsip2	
DMR3:70362001	3	70362001	70365000	3000	1	1.70E-10	0.46	25	0.83	Fsip2	
DMR3:71024001	3	71024001	71025000	1000	1	6.80E-07	0.49	9	0.9	Zc3h15	Transcription
DMR3:71033001	3	71033001	71037000	4000	1	4.00E-09	0.43	35	0.88	Zc3h15	Transcription

DMR3:71038001	3	71038001	71040000	2000	1	7.20E-20	0.75	21	1.05	Zc3h15	Transcription
DMR3:71103001	3	71103001	71105000	2000	2	5.30E-12	0.58	11	0.55	LOC102548277;Itgav	Extracellular Matrix
DMR3:71114001	3	71114001	71115000	1000	1	3.90E-17	0.98	61	6.1	LOC102548277;Itgav	Extracellular Matrix
DMR3:71157001	3	71157001	71160000	3000	2	9.40E-13	0.72	37	1.23	Itgav	Extracellular Matrix
DMR3:71191001	3	71191001	71194000	3000	2	3.20E-12	-0.66	55	1.83	Itgav	Extracellular Matrix
DMR3:71195001	3	71195001	71197000	2000	2	2.90E-11	0.49	33	1.65	Itgav	Extracellular Matrix
DMR3:71199001	3	71199001	71200000	1000	1	1.50E-07	-0.37	16	1.6	Itgav	Extracellular Matrix
DMR3:71201001	3	71201001	71203000	2000	1	8.00E-12	0.42	43	2.15	Itgav;Fam171b	Extracellular Matrix
DMR3:71230001	3	71230001	71232000	2000	2	9.10E-19	0.69	24	1.2	Fam171b;LOC102548485	
DMR3:71263001	3	71263001	71265000	2000	1	2.40E-09	-0.52	26	1.3	Fam171b;LOC108350410;Zswim2	Proteolysis
DMR3:71273001	3	71273001	71276000	3000	1	2.30E-07	0.43	36	1.2	Fam171b;LOC108350410;Zswim2	Proteolysis
DMR3:71278001	3	71278001	71279000	1000	1	3.40E-19	0.85	12	1.2	LOC108350410;Zswim2	Proteolysis
DMR3:71280001	3	71280001	71281000	1000	1	4.20E-07	-0.66	10	1	Zswim2	Proteolysis
DMR3:71289001	3	71289001	71290000	1000	1	1.20E-11	0.58	13	1.3	Zswim2	Proteolysis
DMR3:71308001	3	71308001	71309000	1000	1	5.30E-11	0.62	4	0.4	Trnap-agg	
DMR3:71742001	3	71742001	71747000	5000	2	5.50E-26	0.82	64	1.28	Calcrl	Receptor
DMR3:71764001	3	71764001	71765000	1000	1	6.10E-16	0.74	9	0.9	Calcrl	Receptor
DMR3:71770001	3	71770001	71777000	7000	3	3.30E-09	0.43	79	1.13	Calcrl	Receptor
DMR3:71781001	3	71781001	71786000	5000	3	1.30E-12	-0.78	73	1.46	Calcrl	Receptor
DMR3:71789001	3	71789001	71791000	2000	1	4.00E-18	0.86	29	1.45	Calcrl	Receptor
DMR3:71794001	3	71794001	71797000	3000	2	5.10E-16	-0.45	31	1.03	Calcrl	Receptor
DMR3:71821001	3	71821001	71825000	4000	2	3.70E-13	-0.53	38	0.95	Calcrl;LOC108350411	Receptor
DMR3:71830001	3	71830001	71835000	5000	1	4.30E-07	0.44	33	0.66	Calcrl;LOC108350411	Receptor
DMR3:71855001	3	71855001	71856000	1000	1	4.40E-09	0.43	29	2.9	Calcrl;Tfpi	Receptor;Protease; Proteolysis
DMR3:71894001	3	71894001	71899000	5000	2	7.80E-15	0.53	65	1.3	Tfpi	Protease; Proteolysis
DMR3:71906001	3	71906001	71907000	1000	1	1.00E-08	0.39	8	0.8	Tfpi	Protease; Proteolysis
DMR3:71995001	3	71995001	71996000	1000	1	7.10E-11	0.71	3	0.3	Ctnnd1	Cytoskeleton
DMR3:72001001	3	72001001	72005000	4000	1	2.00E-12	0.58	45	1.12	Ctnnd1	Cytoskeleton
DMR3:72027001	3	72027001	72028000	1000	1	1.00E-09	0.53	5	0.5	Ctnnd1	Cytoskeleton
DMR3:72111001	3	72111001	72112000	1000	1	9.40E-07	-0.56	8	0.8	Zdhhc5	
DMR3:72132001	3	72132001	72136000	4000	1	5.80E-15	0.69	48	1.2	Clp1;Ypel4;Mir130a;Mir3590	Translation
DMR3:72137001	3	72137001	72139000	2000	1	3.50E-07	0.36	35	1.75	Clp1;Ypel4;Mir130a;Mir3590	Translation
DMR3:72143001	3	72143001	72144000	1000	1	6.60E-10	0.68	28	2.8	Clp1;Ypel4;Mir130a;Mir3590	Translation
DMR3:72154001	3	72154001	72156000	2000	2	7.80E-10	0.43	17	0.85	Serping1	Protease; Proteolysis
DMR3:72157001	3	72157001	72160000	3000	2	1.30E-19	0.98	21	0.7	Serping1	Protease; Proteolysis
DMR3:72174001	3	72174001	72176000	2000	2	1.60E-09	0.61	18	0.9	Serping1	Protease; Proteolysis
DMR3:72183001	3	72183001	72187000	4000	1	3.30E-13	0.64	45	1.12	Ube2l6	Proteolysis
DMR3:72234001	3	72234001	72236000	2000	1	8.80E-07	0.33	19	0.95	Timm10;Slc43a1	Transport
DMR3:72270001	3	72270001	72272000	2000	2	6.60E-11	0.41	25	1.25	Slc43a1;LOC108350413;Rtn4rl2; LOC102554396	Receptor
DMR3:72277001	3	72277001	72279000	2000	1	9.60E-18	0.56	35	1.75	LOC108350413;Rtn4rl2;LOC102554396	Receptor
DMR3:72282001	3	72282001	72284000	2000	2	6.10E-18	0.69	29	1.45	Rtn4rl2;LOC102554396	Receptor
DMR3:72288001	3	72288001	72289000	1000	1	1.00E-23	0.99	27	2.7	Rtn4rl2	Receptor
DMR3:72295001	3	72295001	72299000	4000	1	5.50E-11	-0.8	53	1.32	Rtn4rl2	Receptor
DMR3:72355001	3	72355001	72362000	7000	4	2.90E-23	-0.54	88	1.26	Slc43a3;LOC366097	Transport
DMR3:72379001	3	72379001	72380000	1000	1	2.20E-07	0.56	0	0	LOC366097;Prg2	Extracellular Matrix
DMR3:72387001	3	72387001	72389000	2000	1	2.20E-10	0.4	29	1.45	Prg2;Prg3	Extracellular Matrix
DMR3:72411001	3	72411001	72412000	1000	1	7.60E-08	0.47	10	1	Prg3;P2rx3	Extracellular Matrix;Ion Channel
DMR3:72429001	3	72429001	72431000	2000	1	1.00E-09	0.58	26	1.3	P2rx3	Ion Channel
DMR3:72435001	3	72435001	72439000	4000	2	6.20E-19	0.64	41	1.02	P2rx3;Ssrp1	Ion Channel
DMR3:72440001	3	72440001	72443000	3000	1	4.60E-10	0.37	30	1	P2rx3;Ssrp1	Ion Channel
DMR3:72444001	3	72444001	72447000	3000	1	3.40E-10	0.52	32	1.07	P2rx3;Ssrp1	Ion Channel
DMR3:72456001	3	72456001	72458000	2000	1	1.00E-08	0.34	25	1.25	P2rx3;Ssrp1;Tnks1bp1	Ion Channel
DMR3:72460001	3	72460001	72462000	2000	2	7.00E-15	0.53	31	1.55	Ssrp1;Tnks1bp1	
DMR3:72465001	3	72465001	72469000	4000	2	1.30E-19	0.65	53	1.32	Ssrp1;Tnks1bp1	

DMR3:72475001	3	72475001	72477000	2000	1	1.60E-12	0.59	18	0.9	Tnks1bp1	
DMR3:72481001	3	72481001	72482000	1000	1	1.80E-07	0.33	17	1.7	Tnks1bp1	
DMR3:72542001	3	72542001	72544000	2000	1	1.50E-10	0.59	19	0.95	Aplnr	Signaling
DMR3:72545001	3	72545001	72546000	1000	1	1.30E-15	0.66	6	0.6	Aplnr	Signaling
DMR3:72554001	3	72554001	72557000	3000	2	1.20E-15	0.71	28	0.93	Aplnr	Signaling
DMR3:72580001	3	72580001	72581000	1000	1	5.30E-07	0.42	3	0.3	LOC102554972;Lrrc55	
DMR3:72602001	3	72602001	72607000	5000	3	5.50E-09	0.49	58	1.16	Lrrc55	
DMR3:72608001	3	72608001	72611000	3000	1	1.80E-13	0.82	25	0.83	Lrrc55	
DMR3:72735001	3	72735001	72736000	1000	1	8.70E-21	0.84	5	0.5	Olr436	Receptor
DMR3:72769001	3	72769001	72771000	2000	2	9.40E-09	0.56	16	0.8	Olr438-ps;Olr439	Receptor
DMR3:72779001	3	72779001	72780000	1000	1	1.50E-28	1.17	5	0.5	Olr439;Olr440	Receptor
DMR3:72790001	3	72790001	72791000	1000	1	8.00E-08	0.59	5	0.5	Olr440	
DMR3:72798001	3	72798001	72801000	3000	2	2.70E-13	-0.67	23	0.77	Olr441	Receptor
DMR3:72808001	3	72808001	72810000	2000	1	2.70E-18	0.88	17	0.85	Olr441;Olr442	Receptor
DMR3:72847001	3	72847001	72852000	5000	2	6.60E-14	0.75	16	0.32	LOC680873;Fads2l1	
DMR3:72864001	3	72864001	72865000	1000	1	1.10E-13	-0.47	12	1.2	Fads2l1	
DMR3:72878001	3	72878001	72879000	1000	1	1.80E-27	0.85	10	1	Fads2l1	
DMR3:72894001	3	72894001	72896000	2000	1	4.90E-10	0.65	17	0.85	Fads2l1	
DMR3:72897001	3	72897001	72901000	4000	2	5.10E-19	0.81	28	0.7	Fads2l1	
DMR3:72905001	3	72905001	72907000	2000	2	5.40E-21	0.81	13	0.65	Fads2l1	
DMR3:72967001	3	72967001	72972000	5000	2	1.30E-13	-0.75	26	0.52	Olr443	Signaling
DMR3:72981001	3	72981001	72988000	7000	4	9.20E-16	0.76	44	0.63	Olr443;Olr444;Olr445	Signaling
DMR3:72994001	3	72994001	72996000	2000	1	2.30E-12	0.55	14	0.7	Olr444;Olr445;Olr446	Signaling
DMR3:73027001	3	73027001	73034000	7000	4	1.60E-26	-0.57	77	1.1	Olr448	Signaling
DMR3:73043001	3	73043001	73044000	1000	1	9.50E-08	0.48	7	0.7	Olr448;Olr449-ps;Olr450	Signaling;Receptor
DMR3:73068001	3	73068001	73072000	4000	2	2.60E-11	-0.79	12	0.3	LOC684539;Olr452-ps	Receptor
DMR3:73073001	3	73073001	73074000	1000	1	3.60E-13	0.62	7	0.7	LOC684539;Olr452-ps	Receptor
DMR3:73091001	3	73091001	73092000	1000	1	2.00E-13	0.81	2	0.2	Olr453	Signaling
DMR3:73100001	3	73100001	73101000	1000	1	9.60E-08	0.61	7	0.7	Olr453	Signaling
DMR3:73130001	3	73130001	73134000	4000	1	1.80E-07	-0.63	18	0.45	Olr455;Olr456	Receptor
DMR3:73164001	3	73164001	73167000	3000	1	1.90E-18	1.03	10	0.33	Olr458	Receptor
DMR3:73182001	3	73182001	73183000	1000	1	4.30E-09	-0.4	11	1.1	Olr459;Olr460-ps	Signaling
DMR3:73195001	3	73195001	73197000	2000	2	5.40E-20	0.88	6	0.3	Olr460-ps;Olr461	Receptor
DMR3:73210001	3	73210001	73213000	3000	1	3.30E-11	-0.63	9	0.3	Olr461	Receptor
DMR3:73216001	3	73216001	73219000	3000	2	6.40E-09	0.68	23	0.77	Olr462	Signaling
DMR3:73238001	3	73238001	73239000	1000	1	5.10E-08	0.48	7	0.7	Olr463	Receptor
DMR3:73241001	3	73241001	73245000	4000	2	2.20E-08	-0.61	15	0.38	Olr463;Olr464;Olr465	Receptor
DMR3:73301001	3	73301001	73303000	2000	1	2.50E-11	0.63	15	0.75	Olr468;Olr469	Receptor
DMR3:73308001	3	73308001	73309000	1000	1	9.00E-07	-0.52	9	0.9	Olr468;Olr469	Receptor
DMR3:73310001	3	73310001	73311000	1000	1	5.70E-09	0.55	8	0.8	Olr468;Olr469	Receptor
DMR3:73332001	3	73332001	73333000	1000	1	3.10E-09	0.55	9	0.9	LOC684683;Olr470	Receptor
DMR3:73357001	3	73357001	73358000	1000	1	1.90E-07	0.56	0	0	Olr472;Olr473	Receptor
DMR3:73368001	3	73368001	73373000	5000	3	3.00E-13	0.63	16	0.32	Olr473	Receptor
DMR3:73393001	3	73393001	73394000	1000	1	4.60E-07	0.42	9	0.9	LOC103691839;Olr475;Olr476	Receptor
DMR3:73409001	3	73409001	73410000	1000	1	4.10E-08	0.45	4	0.4	Olr476	Receptor
DMR3:73417001	3	73417001	73420000	3000	2	3.40E-11	0.63	11	0.37	Olr477	Receptor
DMR3:73440001	3	73440001	73447000	7000	3	2.20E-27	-0.59	66	0.94	Olr478-ps;Olr479	Receptor
DMR3:73448001	3	73448001	73449000	1000	1	6.60E-20	1.11	4	0.4	Olr478-ps;Olr479	Receptor
DMR3:73463001	3	73463001	73464000	1000	1	1.90E-08	0.46	5	0.5	Olr479	Receptor
DMR3:73468001	3	73468001	73470000	2000	2	7.70E-10	-0.45	15	0.75	Olr480	Receptor
DMR3:73502001	3	73502001	73503000	1000	1	2.30E-07	-0.51	4	0.4	Olr482;Olr483	Receptor
DMR3:73514001	3	73514001	73516000	2000	2	1.50E-08	-0.59	7	0.35	Olr483;Olr484	Receptor
DMR3:73521001	3	73521001	73522000	1000	1	1.20E-08	0.79	0	0	Olr484	Receptor
DMR3:73536001	3	73536001	73539000	3000	1	5.20E-09	0.54	24	0.8	Olr485	Receptor
DMR3:73547001	3	73547001	73554000	7000	5	1.10E-19	-0.5	78	1.11	Olr485;Olr486	Receptor
DMR3:73555001	3	73555001	73556000	1000	1	6.50E-09	0.59	5	0.5	Olr486	Receptor
DMR3:73557001	3	73557001	73559000	2000	1	2.30E-18	1.01	11	0.55	Olr486;Olr487-ps	Receptor
DMR3:73587001	3	73587001	73591000	4000	1	1.90E-19	-0.5	34	0.85	Olr488;Olr489-ps	Receptor
DMR3:73592001	3	73592001	73605000	13000	5	1.00E-30	-0.62	119	0.92	Olr489-ps	
DMR3:73652001	3	73652001	73653000	1000	1	8.20E-10	0.52	8	0.8	Olr492-ps;Olr493	
DMR3:73666001	3	73666001	73667000	1000	1	1.20E-07	0.55	3	0.3	Olr493;Olr494-ps;Olr495;LOC100912505	Receptor
DMR3:73685001	3	73685001	73688000	3000	2	7.90E-16	-0.78	11	0.37	LOC100912505;Olr497-ps	Receptor
DMR3:73691001	3	73691001	73698000	7000	4	1.70E-20	0.63	67	0.96	Olr497-ps;Olr498-ps	
DMR3:73708001	3	73708001	73712000	4000	1	4.30E-09	-0.41	30	0.75	Olr498-ps;LOC100912540	

DMR3:73805001	3	73805001	73806000	1000	1	2.20E-11	0.39	3	0.3	Olr507	Receptor
DMR3:73834001	3	73834001	73838000	4000	1	1.80E-16	-0.55	44	1.1	Olr508	Receptor
DMR3:73848001	3	73848001	73851000	3000	3	2.00E-17	-0.52	28	0.93	Olr508;Olr509-ps	Receptor
DMR3:73862001	3	73862001	73863000	1000	1	2.20E-07	-0.35	4	0.4	Olr509-ps	
DMR3:73867001	3	73867001	73870000	3000	1	4.10E-09	-0.53	17	0.57	Olr510	Signaling
DMR3:73871001	3	73871001	73873000	2000	1	1.50E-07	0.57	12	0.6	Olr510	Signaling
DMR3:73884001	3	73884001	73886000	2000	2	1.10E-14	0.84	7	0.35	Olr510	Signaling
DMR3:73889001	3	73889001	73890000	1000	1	4.00E-13	0.81	3	0.3	Olr510;Olr511-ps	Signaling
DMR3:73907001	3	73907001	73909000	2000	1	1.00E-09	0.49	17	0.85	Olr511-ps;Olr512-ps	
DMR3:73918001	3	73918001	73923000	5000	1	6.30E-15	0.65	24	0.48	Olr512-ps	
DMR3:73927001	3	73927001	73930000	3000	3	2.10E-13	0.7	12	0.4	Olr513	Receptor
DMR3:73940001	3	73940001	73941000	1000	1	2.50E-14	0.65	2	0.2	Olr513;Olr514	Receptor
DMR3:73944001	3	73944001	73945000	1000	1	5.70E-11	-0.67	4	0.4	Olr513;Olr514	Receptor
DMR3:73953001	3	73953001	73960000	7000	4	2.00E-22	-0.57	83	1.19	Olr514	Receptor
DMR3:74011001	3	74011001	74013000	2000	1	7.00E-09	0.58	3	0.15	Olr516	Signaling
DMR3:74022001	3	74022001	74031000	9000	5	1.40E-15	-0.62	95	1.06	Olr516	Signaling
DMR3:74046001	3	74046001	74048000	2000	1	9.50E-07	-0.56	6	0.3	Olr517	Signaling
DMR3:74049001	3	74049001	74052000	3000	3	2.80E-12	0.62	11	0.37	Olr517	Signaling
DMR3:74289001	3	74289001	74290000	1000	1	5.60E-10	-0.6	22	2.2	LOC100359810;Olr523-ps	
DMR3:74291001	3	74291001	74293000	2000	1	8.70E-09	0.48	15	0.75	LOC100359810;Olr523-ps	
DMR3:74294001	3	74294001	74295000	1000	1	3.70E-07	0.45	5	0.5	Olr523-ps	
DMR3:74305001	3	74305001	74309000	4000	3	1.10E-12	-0.65	17	0.42	Olr523-ps;Olr524-ps	
DMR3:74326001	3	74326001	74328000	2000	1	1.00E-10	-0.62	6	0.3	Olr524-ps	
DMR3:74343001	3	74343001	74344000	1000	1	1.00E-08	0.59	4	0.4	Olr519	Signaling
DMR3:74361001	3	74361001	74365000	4000	3	5.50E-16	-0.4	40	1	Olr518	Signaling
DMR3:74366001	3	74366001	74369000	3000	2	4.40E-08	0.44	21	0.7	Olr518	Signaling
DMR3:74388001	3	74388001	74389000	1000	1	1.40E-09	0.44	8	0.8	Olr518	Signaling
DMR3:74397001	3	74397001	74400000	3000	1	1.60E-07	-0.56	14	0.47	Olr525-ps	
DMR3:74414001	3	74414001	74417000	3000	1	2.00E-09	-0.49	22	0.73	Olr525-ps;Olr526	Receptor
DMR3:74419001	3	74419001	74422000	3000	1	3.40E-17	0.73	27	0.9	Olr526	Receptor
DMR3:74440001	3	74440001	74441000	1000	1	2.20E-17	0.77	8	0.8	Olr527	Receptor
DMR3:74469001	3	74469001	74471000	2000	2	5.20E-08	0.62	5	0.25	Olr528	Receptor
DMR3:74484001	3	74484001	74485000	1000	1	9.30E-09	0.53	3	0.3	Olr528	Receptor
DMR3:74504001	3	74504001	74509000	5000	2	8.60E-10	-0.43	34	0.68	Olr529	Receptor
DMR3:74518001	3	74518001	74519000	1000	1	4.10E-25	0.9	7	0.7	Olr530	Receptor
DMR3:74529001	3	74529001	74530000	1000	1	6.90E-08	0.66	3	0.3	Olr530	Receptor
DMR3:74587001	3	74587001	74589000	2000	1	8.20E-07	0.57	12	0.6	Olr532;Olr534-ps;Olr535;Olr536	Receptor
DMR3:74614001	3	74614001	74618000	4000	2	4.40E-08	-0.52	29	0.72	Olr537;Olr538-ps	Signaling
DMR3:74635001	3	74635001	74636000	1000	1	1.70E-07	-0.51	4	0.4	Olr538-ps;Olr539	Signaling
DMR3:74706001	3	74706001	74708000	2000	2	8.60E-16	-0.67	16	0.8	Olr541	Signaling
DMR3:74765001	3	74765001	74766000	1000	1	1.00E-08	0.55	8	0.8	Olfr1055;LOC100909900	Receptor
DMR3:74803001	3	74803001	74809000	6000	2	1.10E-14	-0.55	44	0.73	LOC102546972;Olr499	Receptor
DMR3:74867001	3	74867001	74868000	1000	1	2.20E-08	0.56	6	0.6	Olr544	Signaling
DMR3:74897001	3	74897001	74898000	1000	1	1.00E-09	0.65	3	0.3	Olr545	Receptor
DMR3:74940001	3	74940001	74945000	5000	4	1.00E-16	-0.67	71	1.42	Olr549-ps	
DMR3:75011001	3	75011001	75013000	2000	1	3.40E-12	-0.62	12	0.6	Olr547-ps	
DMR3:75029001	3	75029001	75034000	5000	2	5.40E-17	0.55	39	0.78	Olr547-ps	
DMR3:75079001	3	75079001	75084000	5000	3	6.60E-27	-0.51	47	0.94	Olr542	Signaling
DMR3:75090001	3	75090001	75091000	1000	1	3.00E-07	0.47	3	0.3	Olr542	Signaling
DMR3:75174001	3	75174001	75175000	1000	1	6.10E-08	0.31	17	1.7	LOC103691842;Olr546	Signaling
DMR3:75246001	3	75246001	75248000	2000	2	1.30E-09	-0.56	11	0.55	Olr553-ps	
DMR3:75301001	3	75301001	75302000	1000	1	1.20E-15	0.93	2	0.2	Olr555	Receptor
DMR3:75305001	3	75305001	75311000	6000	3	4.40E-32	-0.61	62	1.03	Olr555	Receptor
DMR3:75319001	3	75319001	75320000	1000	1	2.40E-08	0.49	6	0.6	Olr556	Receptor
DMR3:75323001	3	75323001	75324000	1000	1	1.70E-07	0.54	2	0.2	Olr556	Receptor
DMR3:75328001	3	75328001	75333000	5000	2	1.70E-09	-0.42	35	0.7	Olr556	Receptor
DMR3:75350001	3	75350001	75351000	1000	1	8.60E-13	0.7	6	0.6	Olr557	Receptor
DMR3:75360001	3	75360001	75367000	7000	3	8.70E-15	-0.47	64	0.91	Olr557	Receptor
DMR3:75368001	3	75368001	75374000	6000	3	3.70E-14	-0.47	53	0.88	Olr558	Receptor
DMR3:75375001	3	75375001	75376000	1000	1	6.40E-12	-0.38	5	0.5	Olr558	Receptor
DMR3:75386001	3	75386001	75388000	2000	1	1.30E-08	-0.37	19	0.95	Olr558;Olr559	Receptor
DMR3:75392001	3	75392001	75400000	8000	5	4.50E-24	-0.52	76	0.95	Olr559;LOC685743	Receptor
DMR3:75417001	3	75417001	75419000	2000	1	1.70E-10	0.64	3	0.15	Olr560;LOC685772	Receptor
DMR3:75420001	3	75420001	75429000	9000	7	2.70E-18	-0.49	107	1.19	Olr560;LOC685772;Olr561	Receptor

DMR3:75440001	3	75440001	75442000	2000	1	6.50E-14	0.7	10	0.5	Olr561	Receptor
DMR3:75446001	3	75446001	75452000	6000	4	2.40E-18	-0.71	54	0.9	Olr561	Receptor
DMR3:75457001	3	75457001	75458000	1000	1	1.00E-09	0.5	7	0.7	Olr562	Receptor
DMR3:75491001	3	75491001	75492000	1000	1	3.20E-07	0.45	4	0.4	Olr564-ps;LOC108350559	
DMR3:75495001	3	75495001	75499000	4000	1	4.70E-20	-0.5	49	1.23	Olr564-ps;LOC108350559;Olr565-ps	
DMR3:75506001	3	75506001	75509000	3000	1	1.10E-07	0.5	13	0.43	Olr565-ps;Olr566	Receptor
DMR3:75525001	3	75525001	75528000	3000	1	1.40E-07	-0.54	9	0.3	Olr566	Receptor
DMR3:75537001	3	75537001	75539000	2000	2	4.40E-09	0.55	11	0.55	Pramel7	
DMR3:75546001	3	75546001	75548000	2000	1	8.20E-09	0.46	18	0.9	Pramel7	
DMR3:75566001	3	75566001	75567000	1000	1	6.60E-10	0.58	8	0.8	Pramel6;Olr567	Signaling
DMR3:75590001	3	75590001	75593000	3000	1	2.40E-10	-0.68	6	0.2	Olr569;Olr570-ps	Signaling
DMR3:75595001	3	75595001	75598000	3000	2	9.80E-16	0.88	9	0.3	Olr570-ps;Olr571-ps	
DMR3:75601001	3	75601001	75603000	2000	1	4.30E-11	0.63	9	0.45	Olr571-ps	
DMR3:75605001	3	75605001	75613000	8000	4	3.00E-27	-0.6	85	1.06	Olr571-ps	
DMR3:75633001	3	75633001	75634000	1000	1	4.10E-10	0.6	2	0.2	Olr572-ps;LOC100361457	
DMR3:75642001	3	75642001	75643000	1000	1	1.90E-10	-0.31	21	2.1	Olr572-ps;LOC100361457	
DMR3:75645001	3	75645001	75650000	5000	3	9.50E-17	-0.45	51	1.02	Olr572-ps;LOC100361457;LOC100360176;Olr573-ps	
DMR3:75660001	3	75660001	75664000	4000	2	1.80E-09	-0.43	43	1.07	LOC100360176;Olr573-ps	
DMR3:75692001	3	75692001	75701000	9000	8	1.30E-25	-0.59	89	0.99	Olr574-ps	
DMR3:75707001	3	75707001	75708000	1000	1	1.10E-10	0.67	7	0.7	Olr575	Signaling
DMR3:75727001	3	75727001	75730000	3000	1	5.00E-08	-0.45	31	1.03	Olr576;Olr577	Signaling;Receptor
DMR3:75736001	3	75736001	75744000	8000	3	2.10E-09	0.5	35	0.44	Olr577;Olr578	Receptor;Signaling
DMR3:75753001	3	75753001	75754000	1000	1	4.40E-18	0.75	9	0.9	Olr578	Signaling
DMR3:75798001	3	75798001	75800000	2000	1	1.20E-17	0.7	13	0.65	Olr579-ps;Olr580-ps;Olr581	Signaling
DMR3:75802001	3	75802001	75805000	3000	1	1.20E-08	-0.39	36	1.2	Olr579-ps;Olr580-ps;Olr581	Signaling
DMR3:75806001	3	75806001	75808000	2000	1	8.80E-07	-0.5	7	0.35	Olr580-ps;Olr581	Signaling
DMR3:75819001	3	75819001	75824000	5000	2	1.60E-14	-0.69	33	0.66	Olr581;Olr582	Signaling
DMR3:75827001	3	75827001	75833000	6000	5	3.90E-28	-0.6	76	1.27	Olr582	Signaling
DMR3:75837001	3	75837001	75841000	4000	2	9.30E-18	-0.49	37	0.92	Olr582;Olr583	Signaling
DMR3:75856001	3	75856001	75859000	3000	1	8.60E-10	0.51	17	0.57	Olr583	Signaling
DMR3:75869001	3	75869001	75871000	2000	2	2.80E-12	0.6	10	0.5	Olr584	Signaling
DMR3:75882001	3	75882001	75885000	3000	1	1.00E-11	-0.49	27	0.9	Olr584	Signaling
DMR3:75886001	3	75886001	75889000	3000	3	2.60E-13	-0.38	36	1.2	Olr584	Signaling
DMR3:75897001	3	75897001	75904000	7000	5	2.80E-23	-0.52	73	1.04	Olr585-ps;Olr586	Signaling
DMR3:75911001	3	75911001	75912000	1000	1	9.10E-12	0.69	5	0.5	Olr585-ps;Olr586;LOC502650	Signaling
DMR3:75927001	3	75927001	75930000	3000	2	7.60E-09	-0.6	10	0.33	Olr587;Olr588	Signaling;Receptor
DMR3:75940001	3	75940001	75942000	2000	1	1.30E-08	-0.44	13	0.65	Olr588;Olr589-ps	Receptor
DMR3:75946001	3	75946001	75947000	1000	1	2.20E-07	0.59	3	0.3	Olr588;Olr589-ps	Receptor
DMR3:75962001	3	75962001	75963000	1000	1	2.20E-16	0.8	11	1.1	Olr590;Olr591-ps;Olr592	Signaling;Receptor
DMR3:75967001	3	75967001	75968000	1000	1	4.00E-09	-0.7	4	0.4	Olr590;Olr591-ps;Olr592	Signaling;Receptor
DMR3:75974001	3	75974001	75975000	1000	1	1.10E-08	0.5	11	1.1	Olr591-ps;Olr592;Olr593-ps	Receptor
DMR3:76002001	3	76002001	76014000	12000	6	6.70E-24	0.79	90	0.75	Olr595	Receptor
DMR3:76022001	3	76022001	76028000	6000	5	7.20E-22	-0.57	51	0.85	Olr596	Signaling
DMR3:76031001	3	76031001	76033000	2000	1	5.30E-07	-0.47	14	0.7	Olr596	Signaling
DMR3:76071001	3	76071001	76072000	1000	1	4.00E-08	0.65	10	1	LOC686641;Olr600-ps	
DMR3:76121001	3	76121001	76130000	9000	4	5.10E-19	-0.64	83	0.92	LOC102555599;Olr603-ps;Olr604	Receptor
DMR3:76227001	3	76227001	76229000	2000	1	2.70E-09	0.53	18	0.9	Olr606	Receptor
DMR3:76292001	3	76292001	76293000	1000	1	1.60E-08	0.6	2	0.2	Olr609	Receptor
DMR3:76306001	3	76306001	76311000	5000	1	1.10E-09	-0.4	46	0.92	Olr610	
DMR3:76335001	3	76335001	76336000	1000	1	5.90E-08	0.56	5	0.5	LOC108348034;Olr612-ps;Olr613	Signaling
DMR3:76469001	3	76469001	76470000	1000	1	1.60E-07	0.55	8	0.8	Olr619	Receptor
DMR3:76478001	3	76478001	76484000	6000	4	1.90E-13	-0.69	53	0.88	Olr619;Olr620-ps	Receptor
DMR3:76522001	3	76522001	76524000	2000	1	4.70E-12	-0.68	20	1	Olr623;Olr622-ps	Receptor
DMR3:76527001	3	76527001	76529000	2000	1	6.30E-15	0.87	15	0.75	Olr623;Olr622-ps	Receptor
DMR3:76545001	3	76545001	76547000	2000	2	1.10E-18	0.84	5	0.25	Olr621	Receptor
DMR3:76586001	3	76586001	76588000	2000	1	2.50E-09	0.54	18	0.9	Olr624	
DMR3:76591001	3	76591001	76592000	1000	1	8.90E-07	0.53	5	0.5	Olr624	
DMR3:76596001	3	76596001	76599000	3000	1	5.20E-08	-0.76	20	0.67	Olr624	
DMR3:76632001	3	76632001	76636000	4000	3	1.70E-11	-0.67	16	0.4	Olr625	Receptor
DMR3:76641001	3	76641001	76643000	2000	1	1.10E-07	0.39	12	0.6	Olr626-ps	

DMR3:76667001	3	76667001	76674000	7000	3	1.10E-20	-0.48	61	0.87	Olr627-ps;Olr628-ps	
DMR3:76678001	3	76678001	76679000	1000	1	5.00E-07	-0.4	6	0.6	Olr628-ps	
DMR3:76680001	3	76680001	76681000	1000	1	3.40E-13	0.66	7	0.7	Olr628-ps	
DMR3:76684001	3	76684001	76690000	6000	3	6.50E-15	-0.44	46	0.77	Olr628-ps;Olr629	Receptor
DMR3:76713001	3	76713001	76715000	2000	1	2.30E-13	0.86	8	0.4	Olr630;Olr631	Receptor
DMR3:76724001	3	76724001	76725000	1000	1	9.50E-10	0.56	6	0.6	Olr631;Olr632-ps	Receptor
DMR3:76729001	3	76729001	76731000	2000	1	3.10E-08	0.41	6	0.3	Olr632-ps	
DMR3:76735001	3	76735001	76736000	1000	1	2.80E-09	0.7	2	0.2	Olr632-ps	
DMR3:76755001	3	76755001	76756000	1000	1	3.60E-07	0.46	7	0.7	Olr633	Receptor
DMR3:76764001	3	76764001	76765000	1000	1	7.80E-08	-0.55	3	0.3	Olr634	Receptor
DMR3:76774001	3	76774001	76775000	1000	1	1.10E-12	0.74	2	0.2	Olr634	Receptor
DMR3:76831001	3	76831001	76834000	3000	1	1.70E-07	-0.56	20	0.67	Olr636	Receptor
DMR3:76838001	3	76838001	76839000	1000	1	4.00E-12	0.65	12	1.2	Olr636	Receptor
DMR3:76909001	3	76909001	76915000	6000	4	3.80E-18	-0.52	65	1.08	Olr640	Receptor
DMR3:76929001	3	76929001	76930000	1000	1	1.10E-07	0.45	5	0.5	Olr641	Receptor
DMR3:76931001	3	76931001	76932000	1000	1	7.50E-12	0.82	2	0.2	Olr641	Receptor
DMR3:76936001	3	76936001	76937000	1000	1	4.30E-10	-0.53	2	0.2	Olr641	Receptor
DMR3:76960001	3	76960001	76961000	1000	1	2.60E-17	0.74	8	0.8	Olr642-ps;Olr643-ps	
DMR3:76972001	3	76972001	76973000	1000	1	4.80E-09	0.65	3	0.3	Olr643-ps;Olr644-ps	
DMR3:76987001	3	76987001	76991000	4000	2	7.00E-11	-0.5	16	0.4	Olr644-ps;Olr645-ps	
DMR3:77019001	3	77019001	77021000	2000	1	2.10E-09	0.56	11	0.55	Olr646	Receptor
DMR3:77063001	3	77063001	77065000	2000	2	1.50E-12	-0.61	7	0.35	Olr649	Receptor
DMR3:77069001	3	77069001	77070000	1000	1	5.10E-09	0.64	2	0.2	Olr649	Receptor
DMR3:77077001	3	77077001	77091000	14000	5	1.10E-25	-0.53	135	0.96	Olr649;Olr650	Receptor
DMR3:77101001	3	77101001	77107000	6000	1	1.30E-08	-0.58	156	2.6	Olr650;Olr651	Receptor
DMR3:77142001	3	77142001	77143000	1000	1	8.00E-07	-0.54	6	0.6	Olr653	Receptor
DMR3:77146001	3	77146001	77148000	2000	2	2.60E-08	0.57	9	0.45	Olr653	Receptor
DMR3:77153001	3	77153001	77155000	2000	1	3.70E-09	0.51	14	0.7	Olr653	Receptor
DMR3:77156001	3	77156001	77158000	2000	1	5.00E-08	-0.52	10	0.5	Olr653	Receptor
DMR3:77185001	3	77185001	77187000	2000	1	1.80E-09	-0.72	11	0.55	Olr655	
DMR3:77188001	3	77188001	77191000	3000	1	1.60E-12	0.79	10	0.33	Olr655	
DMR3:77201001	3	77201001	77206000	5000	2	4.40E-11	-0.44	39	0.78	Olr655	
DMR3:77372001	3	77372001	77380000	8000	4	3.50E-11	-0.45	84	1.05	LOC686900;Olr657	Receptor
DMR3:77383001	3	77383001	77384000	1000	1	1.70E-07	0.43	6	0.6	Olr657	Receptor
DMR3:77402001	3	77402001	77404000	2000	2	2.40E-11	0.66	10	0.5	Olr658	Receptor
DMR3:77409001	3	77409001	77410000	1000	1	1.00E-14	0.79	4	0.4	Olr658;Olr659	Receptor
DMR3:77419001	3	77419001	77421000	2000	1	1.40E-11	0.52	9	0.45	Olr659	Receptor
DMR3:77439001	3	77439001	77446000	7000	6	1.80E-18	-0.46	89	1.27	Olr660	Receptor
DMR3:77465001	3	77465001	77473000	8000	3	4.10E-26	-0.6	114	1.43	Olr661	Receptor
DMR3:77475001	3	77475001	77476000	1000	1	1.90E-08	0.53	4	0.4	Olr661	Receptor
DMR3:77496001	3	77496001	77497000	1000	1	2.40E-18	0.99	0	0	Olr662	Receptor
DMR3:77516001	3	77516001	77519000	3000	1	4.10E-10	0.68	18	0.6	Olr663	Receptor
DMR3:77523001	3	77523001	77524000	1000	1	1.60E-15	0.78	6	0.6	Olr663	Receptor
DMR3:77527001	3	77527001	77528000	1000	1	5.90E-10	0.5	5	0.5	Olr663	Receptor
DMR3:77574001	3	77574001	77575000	1000	1	1.20E-20	0.87	8	0.8	Olr664	Receptor
DMR3:77588001	3	77588001	77590000	2000	1	3.50E-07	0.35	13	0.65	Olr664;Bex4-ps1	Receptor
DMR3:77610001	3	77610001	77611000	1000	1	2.70E-07	0.38	4	0.4	Olr665;Olr666	Receptor
DMR3:77649001	3	77649001	77650000	1000	1	2.10E-14	0.7	5	0.5	Olr667-ps	
DMR3:77651001	3	77651001	77653000	2000	1	5.70E-10	-0.41	15	0.75	Olr667-ps;Olr668	Receptor
DMR3:77670001	3	77670001	77671000	1000	1	2.60E-09	0.68	6	0.6	Olr668;Olr669	Receptor
DMR3:77683001	3	77683001	77686000	3000	1	2.00E-08	-0.62	16	0.53	Olr669;Olr670	Receptor
DMR3:77688001	3	77688001	77689000	1000	1	4.60E-08	-0.36	15	1.5	Olr670;LOC689219	Receptor
DMR3:77718001	3	77718001	77722000	4000	2	6.90E-17	-0.59	18	0.45	Olr671;Olr672	Receptor
DMR3:77725001	3	77725001	77726000	1000	1	5.00E-10	0.53	7	0.7	Olr672	
DMR3:77731001	3	77731001	77734000	3000	2	1.30E-11	-0.61	20	0.67	Olr672	
DMR3:77781001	3	77781001	77783000	2000	1	1.00E-09	-0.45	24	1.2	Olr673	
DMR3:77796001	3	77796001	77798000	2000	1	1.90E-13	0.71	7	0.35	Olr673;Olr674	
DMR3:77823001	3	77823001	77825000	2000	2	2.70E-18	0.97	6	0.3	Olr675	Receptor
DMR3:77842001	3	77842001	77843000	1000	1	7.90E-16	0.73	2	0.2	Olr676-ps	
DMR3:77845001	3	77845001	77847000	2000	1	7.00E-16	0.72	16	0.8	Olr676-ps	
DMR3:77857001	3	77857001	77858000	1000	1	2.40E-11	-0.61	10	1	Olr676-ps	
DMR3:77860001	3	77860001	77874000	14000	5	3.40E-12	-0.62	117	0.84	Olr677	Receptor
DMR3:77905001	3	77905001	77909000	4000	2	3.60E-10	0.51	29	0.72	Olr679	Receptor
DMR3:77990001	3	77990001	77997000	7000	3	1.70E-15	-0.54	57	0.81	Olr684;Olr685-ps	Receptor
DMR3:78004001	3	78004001	78006000	2000	2	6.60E-10	0.7	7	0.35	Olr685-ps;Olr686;Olr687	Receptor

DMR3:78175001	3	78175001	78183000	8000	5	8.20E-19	-0.52	92	1.15	Olr696	Receptor
DMR3:78185001	3	78185001	78187000	2000	1	2.70E-08	0.36	17	0.85	Olr696	Receptor
DMR3:78219001	3	78219001	78225000	6000	2	1.40E-09	-0.66	33	0.55	Olr697;Olr698	Receptor
DMR3:78227001	3	78227001	78230000	3000	1	9.30E-11	0.5	9	0.3	Olr698	Receptor
DMR3:78253001	3	78253001	78255000	2000	1	8.80E-07	0.52	7	0.35	Olr699-ps;Olr700-ps	
DMR3:78274001	3	78274001	78277000	3000	2	2.70E-09	-0.51	27	0.9	Olr701;Olr702	Receptor
DMR3:78317001	3	78317001	78318000	1000	1	1.10E-07	0.64	4	0.4	Olr695	
DMR3:78417001	3	78417001	78423000	6000	3	3.60E-20	-0.48	68	1.13	Olr704;Olr705	Receptor
DMR3:78491001	3	78491001	78492000	1000	1	8.30E-20	0.94	1	0.1	Olr707	
DMR3:78521001	3	78521001	78523000	2000	2	3.70E-12	0.74	7	0.35	Olr709	Receptor
DMR3:78534001	3	78534001	78537000	3000	2	2.40E-12	0.54	20	0.67	Olr709;Olr710	Receptor
DMR3:78552001	3	78552001	78554000	2000	1	2.30E-08	0.51	12	0.6	Olr710;Olr711	Receptor
DMR3:78588001	3	78588001	78590000	2000	1	6.80E-08	-0.7	6	0.3	Olr715	Receptor
DMR3:78604001	3	78604001	78605000	1000	1	6.10E-08	-0.63	1	0.1	Olr715	Receptor
DMR3:78612001	3	78612001	78618000	6000	1	1.70E-08	-0.42	37	0.62	Olr716	Receptor
DMR3:78619001	3	78619001	78620000	1000	1	7.80E-12	0.69	1	0.1	Olr716	Receptor
DMR3:78626001	3	78626001	78629000	3000	1	7.20E-09	-0.63	16	0.53	Olr716	Receptor
DMR3:78653001	3	78653001	78654000	1000	1	9.10E-08	-0.49	3	0.3	Olr718	Receptor
DMR3:78673001	3	78673001	78678000	5000	2	4.70E-17	-0.59	34	0.68	Olr718	Receptor
DMR3:78704001	3	78704001	78705000	1000	1	1.30E-10	-0.41	5	0.5	Olr719-ps	
DMR3:78732001	3	78732001	78734000	2000	1	3.90E-14	-0.75	13	0.65	Olr721	Receptor
DMR3:78739001	3	78739001	78740000	1000	1	1.10E-11	0.53	8	0.8	Olr722	Receptor
DMR3:78747001	3	78747001	78748000	1000	1	6.90E-15	0.6	7	0.7	Olr722	Receptor
DMR3:78754001	3	78754001	78759000	5000	1	1.40E-07	-0.31	30	0.6	Olr722	Receptor
DMR3:78763001	3	78763001	78769000	6000	3	1.60E-26	-0.62	67	1.12	Olr1258	
DMR3:78776001	3	78776001	78778000	2000	1	1.80E-07	-0.41	22	1.1	Olr1258	
DMR3:78782001	3	78782001	78784000	2000	1	1.00E-11	0.64	10	0.5	Olr1258;Olr724	Receptor
DMR3:78796001	3	78796001	78797000	1000	1	2.80E-08	0.39	27	2.7	Olr724	Receptor
DMR3:78838001	3	78838001	78839000	1000	1	6.50E-10	0.62	7	0.7	Olr725	Receptor
DMR3:78843001	3	78843001	78844000	1000	1	3.10E-07	-0.39	5	0.5	Olr725;Olr726	Receptor
DMR3:78845001	3	78845001	78846000	1000	1	3.70E-10	0.54	5	0.5	Olr725;Olr726	Receptor
DMR3:78856001	3	78856001	78859000	3000	1	3.40E-07	0.52	10	0.33	Olr726;Olr727	Receptor
DMR3:78867001	3	78867001	78869000	2000	1	4.20E-10	-0.4	18	0.9	Olr727;Olr728	Receptor
DMR3:78875001	3	78875001	78877000	2000	2	1.30E-14	0.92	8	0.4	Olr728;Olr729	Receptor
DMR3:78884001	3	78884001	78885000	1000	1	5.90E-19	0.74	6	0.6	Olr728;Olr729	Receptor
DMR3:78886001	3	78886001	78888000	2000	1	1.70E-24	0.89	13	0.65	Olr728;Olr729	Receptor
DMR3:78893001	3	78893001	78894000	1000	1	3.50E-11	0.63	9	0.9	Olr729	Receptor
DMR3:78925001	3	78925001	78931000	6000	5	2.50E-23	-0.5	58	0.97	Olr730-ps	
DMR3:78956001	3	78956001	78958000	2000	1	1.20E-12	-0.61	9	0.45	Olr733;Olr732-ps	Receptor
DMR3:78960001	3	78960001	78961000	1000	1	8.50E-07	0.43	5	0.5	Olr733;Olr732-ps	Receptor
DMR3:78962001	3	78962001	78971000	9000	3	6.70E-15	0.48	87	0.97	Olr733;Olr732-ps	Receptor
DMR3:78977001	3	78977001	78978000	1000	1	9.00E-10	0.63	2	0.2	Olr733;Olr732-ps;Olr734	Receptor
DMR3:78988001	3	78988001	78992000	4000	3	9.30E-14	0.64	21	0.52	Olr734	Receptor
DMR3:78999001	3	78999001	79008000	9000	5	9.20E-31	-0.51	81	0.9	Olr735;Olr736	Receptor
DMR3:79024001	3	79024001	79027000	3000	1	3.00E-23	-0.79	12	0.4	Olr736	Receptor
DMR3:79028001	3	79028001	79036000	8000	5	3.40E-12	-0.59	60	0.75	Olr736	Receptor
DMR3:79053001	3	79053001	79054000	1000	1	9.20E-07	0.35	10	1	Olr737;Olr738-ps	Receptor
DMR3:79056001	3	79056001	79057000	1000	1	4.70E-09	-0.75	6	0.6	Olr737;Olr738-ps	Receptor
DMR3:79058001	3	79058001	79059000	1000	1	6.60E-09	-0.57	5	0.5	Olr737;Olr738-ps;Olr739-ps;LOC100911542	Receptor
DMR3:79062001	3	79062001	79068000	6000	3	1.00E-14	-0.42	62	1.03	Olr738-ps;Olr739-ps;LOC100911542	
DMR3:79113001	3	79113001	79117000	4000	2	2.20E-22	1	38	0.95	Olr741	Receptor
DMR3:79118001	3	79118001	79119000	1000	1	2.80E-19	0.87	6	0.6	Olr741	Receptor
DMR3:79122001	3	79122001	79125000	3000	1	3.50E-09	-0.5	26	0.87	Olr741;Olr742	Receptor
DMR3:79143001	3	79143001	79150000	7000	2	3.00E-12	-0.42	48	0.69	Olr742;Olr743-ps	Receptor
DMR3:79168001	3	79168001	79173000	5000	1	8.80E-07	-0.49	13	0.26	Olr744	Receptor
DMR3:79174001	3	79174001	79182000	8000	3	1.10E-23	-0.52	86	1.07	Olr744;Olr745	Receptor
DMR3:79186001	3	79186001	79187000	1000	1	1.80E-12	0.59	7	0.7	Olr745	Receptor
DMR3:79251001	3	79251001	79257000	6000	2	7.60E-13	0.56	97	1.62	Ptprj	Signaling
DMR3:79261001	3	79261001	79264000	3000	2	7.70E-08	-0.56	38	1.27	Ptprj	Signaling
DMR3:79265001	3	79265001	79269000	4000	1	9.00E-07	-0.43	57	1.43	Ptprj	Signaling
DMR3:79277001	3	79277001	79281000	4000	1	1.80E-07	-0.42	70	1.75	Ptprj	Signaling
DMR3:79297001	3	79297001	79298000	1000	1	4.80E-24	0.78	12	1.2	Ptprj	Signaling
DMR3:79323001	3	79323001	79324000	1000	1	1.50E-08	0.37	21	2.1	Ptprj	Signaling

DMR3:79327001	3	79327001	79331000	4000	1	3.20E-15	0.55	55	1.38	Ptprj	Signaling
DMR3:79342001	3	79342001	79344000	2000	1	6.40E-10	0.45	25	1.25	Ptprj	Signaling
DMR3:79395001	3	79395001	79398000	3000	2	1.40E-13	0.54	38	1.27	Ptprj;LOC102555454	Signaling
DMR3:79484001	3	79484001	79494000	10000	5	1.60E-19	-0.45	123	1.23	LOC102555600;LOC103691844;Nup160	Transport
DMR3:79499001	3	79499001	79501000	2000	1	1.60E-13	0.57	19	0.95	LOC102555600;Nup160	Transport
DMR3:79520001	3	79520001	79521000	1000	1	2.80E-13	0.69	7	0.7	Nup160	Transport
DMR3:79554001	3	79554001	79555000	1000	1	1.70E-16	0.69	4	0.4	Nup160	Transport
DMR3:79556001	3	79556001	79558000	2000	1	1.00E-07	0.45	16	0.8	Nup160	Transport
DMR3:79565001	3	79565001	79566000	1000	1	2.90E-07	0.41	5	0.5	Nup160;Fnbp4	Transport
DMR3:79590001	3	79590001	79594000	4000	1	3.70E-07	-0.62	39	0.98	Fnbp4	
DMR3:79629001	3	79629001	79636000	7000	1	5.10E-08	0.44	126	1.8	LOC108350562;Agbl2	Protease
DMR3:79687001	3	79687001	79688000	1000	1	1.90E-07	-0.4	11	1.1	Mtch2	Transport
DMR3:79707001	3	79707001	79710000	3000	2	6.00E-21	0.71	30	1	C1qtnf4;Fam180b	
DMR3:79711001	3	79711001	79713000	2000	1	9.00E-09	0.51	21	1.05	C1qtnf4;Fam180b;Ndufs3	Metabolism
DMR3:79718001	3	79718001	79719000	1000	1	4.50E-07	-0.65	12	1.2	C1qtnf4;Fam180b;Ndufs3;Kbtbd4	Metabolism
DMR3:79762001	3	79762001	79764000	2000	1	3.10E-07	-0.56	18	0.9	LOC690017;Celf1	
DMR3:79820001	3	79820001	79821000	1000	1	2.20E-18	0.82	6	0.6	Celf1	
DMR3:79826001	3	79826001	79827000	1000	1	1.40E-08	-0.57	19	1.9	Celf1	
DMR3:79858001	3	79858001	79863000	5000	1	1.40E-08	0.43	65	1.3	Rapsn	
DMR3:79878001	3	79878001	79879000	1000	1	3.10E-08	-0.52	14	1.4	Rapsn;Psmc3;Slc39a13	Protease;Transport
DMR3:79910001	3	79910001	79912000	2000	1	1.00E-22	0.74	30	1.5	LOC103691845;LOC102555995;Spi1	Transcription
DMR3:79915001	3	79915001	79922000	7000	3	2.60E-24	0.86	97	1.39	LOC103691845;LOC102555995;Spi1	Transcription
DMR3:79924001	3	79924001	79927000	3000	2	9.00E-25	0.95	26	0.87	Spi1	Transcription
DMR3:79931001	3	79931001	79932000	1000	1	5.60E-13	0.49	14	1.4	Spi1;Mybpc3	Transcription
DMR3:79933001	3	79933001	79936000	3000	1	3.30E-09	0.41	44	1.47	Spi1;Mybpc3	Transcription
DMR3:79946001	3	79946001	79950000	4000	2	3.20E-17	0.54	61	1.52	Spi1;Mybpc3	Transcription
DMR3:79951001	3	79951001	79957000	6000	1	8.90E-07	-0.59	107	1.78	Mybpc3;Madd	
DMR3:79964001	3	79964001	79966000	2000	1	4.30E-08	0.29	50	2.5	Mybpc3;Madd	
DMR3:79979001	3	79979001	79980000	1000	1	4.60E-08	0.49	10	1	Madd	
DMR3:79981001	3	79981001	79983000	2000	1	9.80E-10	-0.63	23	1.15	Madd	
DMR3:79991001	3	79991001	79992000	1000	1	2.80E-17	0.5	17	1.7	Madd	
DMR3:79996001	3	79996001	80001000	5000	1	5.40E-09	0.36	97	1.94	Madd;Nr1h3	Transcription
DMR3:80004001	3	80004001	80005000	1000	1	2.70E-08	0.38	21	2.1	Madd;Nr1h3	Transcription
DMR3:80015001	3	80015001	80017000	2000	1	4.10E-13	0.57	30	1.5	Nr1h3;Acp2	Transcription;Signaling
DMR3:80045001	3	80045001	80046000	1000	1	1.30E-10	0.47	12	1.2	Ddb2	DNA Repair
DMR3:80054001	3	80054001	80055000	1000	1	2.30E-08	-0.6	11	1.1	Ddb2	DNA Repair
DMR3:80099001	3	80099001	80101000	2000	2	1.80E-24	1.03	12	0.6	Arfgap2;RGD1309540	Signaling
DMR3:80121001	3	80121001	80124000	3000	2	7.00E-33	1.05	30	1	RGD1309540	
DMR3:80129001	3	80129001	80132000	3000	1	2.80E-08	0.37	41	1.37	RGD1309540	
DMR3:80168001	3	80168001	80169000	1000	1	3.00E-08	0.57	8	0.8	RGD1309540	
DMR3:80170001	3	80170001	80172000	2000	1	3.50E-07	0.55	26	1.3	RGD1309540	
DMR3:80180001	3	80180001	80181000	1000	1	1.90E-07	0.57	7	0.7	RGD1309540	
DMR3:80188001	3	80188001	80191000	3000	1	2.40E-21	0.9	35	1.17	RGD1309540	
DMR3:80218001	3	80218001	80221000	3000	3	3.20E-10	-0.47	26	0.87	RGD1309540	
DMR3:80244001	3	80244001	80248000	4000	3	4.50E-24	1.25	29	0.72	RGD1309540	
DMR3:80259001	3	80259001	80260000	1000	1	4.40E-12	0.61	1	0.1	RGD1309540	
DMR3:80276001	3	80276001	80278000	2000	1	2.10E-08	0.4	26	1.3	RGD1309540	
DMR3:80296001	3	80296001	80299000	3000	1	3.30E-07	-0.47	42	1.4	RGD1309540	
DMR3:80302001	3	80302001	80305000	3000	2	6.40E-16	0.83	24	0.8	RGD1309540	
DMR3:80316001	3	80316001	80317000	1000	1	5.90E-07	0.49	2	0.2	RGD1309540	
DMR3:80322001	3	80322001	80324000	2000	1	4.40E-08	0.49	8	0.4	RGD1309540	
DMR3:80360001	3	80360001	80361000	1000	1	4.50E-07	0.54	23	2.3	Lrp4	Binding Proteins
DMR3:80371001	3	80371001	80373000	2000	1	5.90E-10	0.52	31	1.55	Lrp4	Binding Proteins
DMR3:80380001	3	80380001	80382000	2000	1	2.90E-07	0.31	50	2.5	Lrp4	Binding Proteins
DMR3:80389001	3	80389001	80390000	1000	1	1.40E-10	0.54	5	0.5	Lrp4	Binding Proteins
DMR3:80402001	3	80402001	80404000	2000	1	8.60E-08	0.35	37	1.85	Lrp4	Binding Proteins
DMR3:80448001	3	80448001	80449000	1000	1	8.80E-07	-0.53	8	0.8	Ckap5	Cytoskeleton
DMR3:80532001	3	80532001	80534000	2000	1	1.50E-14	0.5	21	1.05	Ckap5;F2;LOC103691846	Cytoskeleton;Protease
DMR3:80545001	3	80545001	80546000	1000	1	1.20E-12	0.5	8	0.8	F2;Znf408;Arhgap1	Protease;Signaling

DMR3:80569001	3	80569001	80570000	1000	1	8.00E-10	0.48	5	0.5	Arhgap1	Signaling
DMR3:80572001	3	80572001	80575000	3000	1	7.30E-14	0.7	44	1.47	Arhgap1;Atg13	Signaling
DMR3:80586001	3	80586001	80587000	1000	1	2.00E-19	0.7	14	1.4	Arhgap1;Atg13	Signaling
DMR3:80615001	3	80615001	80618000	3000	2	1.50E-09	0.76	43	1.43	Atg13;Harbi1	
DMR3:80629001	3	80629001	80632000	3000	1	4.30E-15	0.62	31	1.03	Harbi1;Ambra1	
DMR3:80652001	3	80652001	80653000	1000	1	1.50E-18	0.93	9	0.9	Ambra1	
DMR3:80655001	3	80655001	80659000	4000	1	1.80E-08	-0.42	34	0.85	Ambra1	
DMR3:80662001	3	80662001	80663000	1000	1	1.30E-11	0.68	4	0.4	Ambra1	
DMR3:80675001	3	80675001	80677000	2000	2	9.10E-15	0.56	32	1.6	Ambra1	
DMR3:80702001	3	80702001	80704000	2000	1	1.40E-13	0.87	10	0.5	Ambra1	
DMR3:80711001	3	80711001	80712000	1000	1	2.10E-08	0.52	6	0.6	Ambra1	
DMR3:80720001	3	80720001	80721000	1000	1	8.70E-09	0.56	5	0.5	Ambra1	
DMR3:80733001	3	80733001	80734000	1000	1	2.30E-08	0.45	11	1.1	Ambra1	
DMR3:80768001	3	80768001	80769000	1000	1	9.90E-11	0.52	12	1.2	Ambra1	
DMR3:80800001	3	80800001	80802000	2000	1	7.80E-17	0.69	17	0.85	Ambra1	
DMR3:80809001	3	80809001	80810000	1000	1	3.50E-07	0.45	8	0.8	Ambra1	
DMR3:80819001	3	80819001	80821000	2000	1	1.90E-07	-0.4	19	0.95	Ambra1	
DMR3:80846001	3	80846001	80848000	2000	1	2.60E-11	0.45	39	1.95	Chrm4;Mdk;Dgkz	Signaling;Growth Factors;Signaling
DMR3:80857001	3	80857001	80859000	2000	1	1.90E-09	0.45	22	1.1	Dgkz;LOC102557176	Signaling
DMR3:80883001	3	80883001	80884000	1000	1	1.20E-08	0.38	11	1.1	Dgkz;LOC103691847;Creb3l1	Signaling
DMR3:80890001	3	80890001	80896000	6000	3	1.40E-32	0.98	73	1.22	Dgkz;LOC103691847;Creb3l1	Signaling
DMR3:80898001	3	80898001	80902000	4000	2	3.90E-11	-0.71	76	1.9	LOC103691847;Creb3l1	
DMR3:80903001	3	80903001	80904000	1000	1	1.30E-13	0.57	13	1.3	LOC103691847;Creb3l1	
DMR3:80906001	3	80906001	80908000	2000	2	1.40E-11	-0.41	32	1.6	LOC103691847;Creb3l1	
DMR3:80924001	3	80924001	80926000	2000	1	7.30E-10	0.55	17	0.85	LOC103691847;Creb3l1	
DMR3:81146001	3	81146001	81149000	3000	2	2.60E-17	0.65	24	0.8	Phf21a	
DMR3:81197001	3	81197001	81202000	5000	1	1.80E-07	0.5	46	0.92	Phf21a	
DMR3:81211001	3	81211001	81215000	4000	2	4.10E-12	-0.53	41	1.02	Phf21a	
DMR3:81224001	3	81224001	81225000	1000	1	4.10E-16	0.9	6	0.6	Phf21a	
DMR3:81251001	3	81251001	81253000	2000	1	7.70E-07	-0.59	19	0.95	Phf21a	
DMR3:81255001	3	81255001	81258000	3000	1	2.10E-07	-0.6	30	1	Phf21a	
DMR3:81301001	3	81301001	81302000	1000	1	7.20E-10	0.63	13	1.3	Pex16;RGD1563263;Mapk8ip1	Cytoskeleton
DMR3:81349001	3	81349001	81350000	1000	1	7.90E-07	-0.65	12	1.2	Cry2;LOC102546693	DNA Repair
DMR3:81366001	3	81366001	81367000	1000	1	7.90E-08	-0.65	19	1.9	Slc35c1	Transport
DMR3:81431001	3	81431001	81437000	6000	1	3.00E-15	0.57	51	0.85	LOC108350417;Chst1	Transport
DMR3:81443001	3	81443001	81446000	3000	1	4.90E-09	0.36	39	1.3	LOC108350417;Chst1	Transport
DMR3:81447001	3	81447001	81450000	3000	1	3.50E-10	0.57	23	0.77	Chst1	Transport
DMR3:81466001	3	81466001	81467000	1000	1	1.50E-09	0.48	19	1.9	Chst1	Transport
DMR3:81471001	3	81471001	81473000	2000	1	2.70E-14	0.45	23	1.15	Chst1	Transport
DMR3:81490001	3	81490001	81492000	2000	2	3.70E-20	0.95	10	0.5	Chst1	Transport
DMR3:81805001	3	81805001	81806000	1000	1	2.50E-12	0.59	9	0.9	Syt13	Transport
DMR3:81812001	3	81812001	81817000	5000	2	1.20E-10	0.69	87	1.74	Syt13	Transport
DMR3:81868001	3	81868001	81870000	2000	1	3.60E-10	0.33	47	2.35	Prdm11	Transcription
DMR3:81885001	3	81885001	81887000	2000	1	1.20E-11	0.42	41	2.05	Prdm11;LOC102547078	Transcription
DMR3:81918001	3	81918001	81921000	3000	1	1.70E-08	0.39	38	1.27	Prdm11	Transcription
DMR3:81946001	3	81946001	81948000	2000	1	7.50E-09	-0.67	35	1.75	Prdm11	Transcription
DMR3:82074001	3	82074001	82076000	2000	1	2.20E-10	0.74	45	2.25	Tp53i11	
DMR3:82110001	3	82110001	82111000	1000	1	5.10E-18	0.59	14	1.4	Tspan18	
DMR3:82116001	3	82116001	82121000	5000	2	8.70E-21	0.74	52	1.04	Tspan18	
DMR3:82125001	3	82125001	82129000	4000	2	3.00E-07	-0.38	30	0.75	Tspan18	
DMR3:82136001	3	82136001	82137000	1000	1	7.20E-15	0.86	3	0.3	Tspan18	
DMR3:82146001	3	82146001	82147000	1000	1	6.10E-09	0.59	4	0.4	Tspan18	
DMR3:82160001	3	82160001	82162000	2000	2	2.90E-08	0.51	31	1.55	Tspan18	
DMR3:82176001	3	82176001	82177000	1000	1	8.70E-17	0.75	7	0.7	Tspan18	
DMR3:82178001	3	82178001	82179000	1000	1	5.90E-07	-0.42	17	1.7	Tspan18	
DMR3:82206001	3	82206001	82207000	1000	1	1.10E-10	0.46	9	0.9	Tspan18	
DMR3:82214001	3	82214001	82217000	3000	2	4.00E-25	0.73	33	1.1	Tspan18	
DMR3:82242001	3	82242001	82243000	1000	1	6.40E-08	0.7	5	0.5	Tspan18	
DMR3:82338001	3	82338001	82340000	2000	1	2.80E-08	0.35	11	0.55	Cd82	
DMR3:82539001	3	82539001	82540000	1000	1	3.40E-15	0.68	2	0.2	Alx4	Development
DMR3:82567001	3	82567001	82569000	2000	1	4.60E-15	0.53	32	1.6	Alx4	Development
DMR3:82570001	3	82570001	82571000	1000	1	1.30E-09	0.47	8	0.8	Alx4	Development
DMR3:82576001	3	82576001	82578000	2000	1	4.80E-07	0.35	26	1.3	Alx4	Development
DMR3:82579001	3	82579001	82581000	2000	1	4.20E-11	0.45	30	1.5	Alx4	Development

DMR3:82596001	3	82596001	82597000	1000	1	2.40E-17	1	6	0.6	Ext2	Golgi
DMR3:82599001	3	82599001	82601000	2000	1	3.70E-13	0.57	30	1.5	Ext2	Golgi
DMR3:82626001	3	82626001	82627000	1000	1	2.90E-11	0.62	7	0.7	Ext2	Golgi
DMR3:82632001	3	82632001	82633000	1000	1	8.90E-11	0.66	5	0.5	Ext2	Golgi
DMR3:82635001	3	82635001	82638000	3000	1	7.20E-12	0.61	29	0.97	Ext2	Golgi
DMR3:82681001	3	82681001	82684000	3000	1	1.60E-10	0.71	32	1.07	Ext2	Golgi
DMR3:82688001	3	82688001	82689000	1000	1	2.20E-08	-0.61	15	1.5	Ext2	Golgi
DMR3:82690001	3	82690001	82693000	3000	1	9.90E-07	-0.46	33	1.1	Ext2	Golgi
DMR3:82704001	3	82704001	82705000	1000	1	2.00E-07	0.41	11	1.1	Ext2	Golgi
DMR3:82720001	3	82720001	82724000	4000	1	5.70E-14	0.44	70	1.75	Ext2	Golgi
DMR3:82738001	3	82738001	82741000	3000	1	7.00E-13	-0.4	34	1.13	Ext2;Accs	Golgi;Metabolism
DMR3:82745001	3	82745001	82746000	1000	1	4.10E-08	-0.54	8	0.8	Accs	Metabolism
DMR3:82753001	3	82753001	82754000	1000	1	6.00E-09	0.41	14	1.4	Accs;Accsl	Metabolism
DMR3:82772001	3	82772001	82774000	2000	1	4.20E-09	-0.69	33	1.65	Accsl	Metabolism
DMR3:82775001	3	82775001	82778000	3000	2	1.30E-27	0.82	32	1.07	Accsl	Metabolism
DMR3:82844001	3	82844001	82845000	1000	1	1.40E-14	0.6	17	1.7	LOC100910012;RGD1564664	
DMR3:82877001	3	82877001	82879000	2000	1	8.10E-07	-0.37	26	1.3	Alkbh3	Metabolism
DMR3:82966001	3	82966001	82967000	1000	1	8.80E-08	0.38	8	0.8	Hsd17b12;LOC100362432	
DMR3:82975001	3	82975001	82979000	4000	1	8.90E-24	0.98	38	0.95	Hsd17b12;LOC100362432	
DMR3:83011001	3	83011001	83013000	2000	1	6.70E-08	0.4	19	0.95	Hsd17b12	
DMR3:83036001	3	83036001	83037000	1000	1	1.40E-11	0.55	9	0.9	Hsd17b12	
DMR3:83052001	3	83052001	83053000	1000	1	9.80E-15	0.57	10	1	Hsd17b12	
DMR3:83058001	3	83058001	83063000	5000	4	2.40E-11	-0.53	73	1.46	Hsd17b12	
DMR3:83145001	3	83145001	83147000	2000	1	1.30E-07	-0.48	19	0.95	Mir129-2	
DMR3:83190001	3	83190001	83194000	4000	2	9.20E-11	0.45	40	1	Ttc17	
DMR3:83203001	3	83203001	83206000	3000	2	9.20E-17	0.65	43	1.43	Ttc17	
DMR3:83211001	3	83211001	83213000	2000	1	4.60E-07	-0.71	32	1.6	Ttc17	
DMR3:83214001	3	83214001	83218000	4000	4	2.80E-21	-0.45	60	1.5	Ttc17	
DMR3:83249001	3	83249001	83251000	2000	1	6.80E-09	-0.64	27	1.35	Ttc17	
DMR3:83253001	3	83253001	83257000	4000	2	4.70E-13	0.59	21	0.52	Ttc17	
DMR3:83322001	3	83322001	83326000	4000	3	3.70E-10	-0.71	39	0.98	Api5	
DMR3:85443001	3	85443001	85444000	1000	1	6.70E-08	-0.62	7	0.7	Lrrc4c	
DMR3:85457001	3	85457001	85464000	7000	4	3.00E-12	0.58	43	0.61	Lrrc4c	
DMR3:85512001	3	85512001	85513000	1000	1	7.00E-16	0.74	9	0.9	Lrrc4c	
DMR3:85530001	3	85530001	85532000	2000	1	6.40E-07	0.51	12	0.6	Lrrc4c	
DMR3:85572001	3	85572001	85575000	3000	2	7.50E-09	-0.48	13	0.43	Lrrc4c	
DMR3:85580001	3	85580001	85588000	8000	5	6.60E-23	0.63	68	0.85	Lrrc4c	
DMR3:85595001	3	85595001	85596000	1000	1	7.10E-12	-0.76	9	0.9	Lrrc4c	
DMR3:85602001	3	85602001	85604000	2000	1	1.70E-08	0.59	8	0.4	Lrrc4c	
DMR3:85607001	3	85607001	85608000	1000	1	2.40E-13	0.62	9	0.9	Lrrc4c	
DMR3:85637001	3	85637001	85638000	1000	1	1.30E-08	0.68	1	0.1	Lrrc4c	
DMR3:85649001	3	85649001	85651000	2000	1	7.10E-07	-0.35	9	0.45	Lrrc4c	
DMR3:85700001	3	85700001	85701000	1000	1	2.80E-14	0.71	8	0.8	Lrrc4c	
DMR3:85704001	3	85704001	85705000	1000	1	5.20E-16	0.85	3	0.3	Lrrc4c	
DMR3:85713001	3	85713001	85714000	1000	1	5.50E-07	0.42	10	1	Lrrc4c	
DMR3:85789001	3	85789001	85790000	1000	1	7.20E-12	0.43	7	0.7	Lrrc4c;LOC100361740	
DMR3:85791001	3	85791001	85792000	1000	1	7.10E-09	0.63	6	0.6	Lrrc4c;LOC100361740	
DMR3:85805001	3	85805001	85806000	1000	1	1.20E-07	0.51	5	0.5	Lrrc4c;LOC366130	
DMR3:85828001	3	85828001	85829000	1000	1	1.20E-11	0.53	5	0.5	Lrrc4c;LOC366130	
DMR3:85830001	3	85830001	85842000	12000	4	3.90E-14	-0.61	95	0.79	Lrrc4c;LOC366130	
DMR3:85849001	3	85849001	85850000	1000	1	5.20E-08	0.57	5	0.5	Lrrc4c;LOC366130	
DMR3:85868001	3	85868001	85869000	1000	1	1.20E-15	0.85	6	0.6	Lrrc4c	
DMR3:85875001	3	85875001	85878000	3000	1	8.00E-15	0.85	21	0.7	Lrrc4c	
DMR3:85881001	3	85881001	85882000	1000	1	1.90E-08	0.5	8	0.8	Lrrc4c	
DMR3:85887001	3	85887001	85889000	2000	1	2.60E-10	0.63	13	0.65	Lrrc4c	
DMR3:85897001	3	85897001	85899000	2000	1	5.80E-07	0.56	10	0.5	Lrrc4c	
DMR3:85921001	3	85921001	85923000	2000	1	6.30E-07	0.52	10	0.5	Lrrc4c	
DMR3:85927001	3	85927001	85930000	3000	1	4.70E-08	-0.58	12	0.4	Lrrc4c	
DMR3:85954001	3	85954001	85957000	3000	1	8.50E-13	0.65	21	0.7	Lrrc4c	
DMR3:85970001	3	85970001	85972000	2000	1	1.30E-09	0.56	10	0.5	Lrrc4c	
DMR3:85978001	3	85978001	85981000	3000	1	3.70E-08	0.45	24	0.8	Lrrc4c	
DMR3:86043001	3	86043001	86044000	1000	1	8.30E-10	0.69	3	0.3	Lrrc4c	
DMR3:86069001	3	86069001	86071000	2000	2	1.20E-12	0.77	16	0.8	Lrrc4c	
DMR3:86075001	3	86075001	86077000	2000	1	2.90E-08	0.49	19	0.95	Lrrc4c	
DMR3:86099001	3	86099001	86101000	2000	1	7.90E-07	0.54	17	0.85	Lrrc4c	

DMR3:86136001	3	86136001	86138000	2000	1	2.70E-08	0.59	14	0.7	Lrrc4c	
DMR3:86150001	3	86150001	86151000	1000	1	3.40E-08	0.53	10	1	Lrrc4c	
DMR3:86156001	3	86156001	86157000	1000	1	1.50E-12	0.64	7	0.7	Lrrc4c	
DMR3:86158001	3	86158001	86159000	1000	1	1.50E-09	0.58	2	0.2	Lrrc4c	
DMR3:86189001	3	86189001	86190000	1000	1	2.90E-16	-0.81	6	0.6	Lrrc4c	
DMR3:86192001	3	86192001	86194000	2000	1	1.40E-11	0.64	19	0.95	Lrrc4c	
DMR3:86214001	3	86214001	86215000	1000	1	8.30E-09	0.53	6	0.6	Lrrc4c	
DMR3:86232001	3	86232001	86235000	3000	1	7.00E-08	-0.57	9	0.3	Lrrc4c	
DMR3:86239001	3	86239001	86241000	2000	2	7.50E-27	0.83	15	0.75	Lrrc4c	
DMR3:86248001	3	86248001	86249000	1000	1	3.60E-15	0.83	3	0.3	Lrrc4c	
DMR3:86258001	3	86258001	86259000	1000	1	1.70E-08	0.53	5	0.5	Lrrc4c	
DMR3:86273001	3	86273001	86274000	1000	1	7.40E-10	0.55	4	0.4	Lrrc4c	
DMR3:86277001	3	86277001	86278000	1000	1	2.10E-07	0.54	4	0.4	Lrrc4c	
DMR3:86303001	3	86303001	86304000	1000	1	6.10E-15	0.61	10	1	Lrrc4c	
DMR3:86318001	3	86318001	86319000	1000	1	2.00E-07	0.51	6	0.6	Lrrc4c	
DMR3:86323001	3	86323001	86325000	2000	2	1.60E-07	-0.6	5	0.25	Lrrc4c	
DMR3:86328001	3	86328001	86329000	1000	1	5.80E-13	0.64	5	0.5	Lrrc4c	
DMR3:86434001	3	86434001	86435000	1000	1	8.80E-08	0.48	8	0.8	Lrrc4c	
DMR3:86442001	3	86442001	86444000	2000	1	1.20E-09	0.66	7	0.35	Lrrc4c	
DMR3:86453001	3	86453001	86456000	3000	1	5.50E-13	0.56	20	0.67	Lrrc4c	
DMR3:86463001	3	86463001	86464000	1000	1	2.30E-17	0.92	3	0.3	Lrrc4c	
DMR3:86472001	3	86472001	86473000	1000	1	8.50E-07	0.43	5	0.5	Lrrc4c	
DMR3:86475001	3	86475001	86476000	1000	1	3.70E-08	0.59	5	0.5	Lrrc4c	
DMR3:86514001	3	86514001	86515000	1000	1	5.20E-11	0.62	4	0.4	Lrrc4c	
DMR3:86518001	3	86518001	86519000	1000	1	2.50E-07	-0.5	6	0.6	Lrrc4c	
DMR3:86527001	3	86527001	86531000	4000	3	6.70E-15	0.74	21	0.52	Lrrc4c	
DMR3:86555001	3	86555001	86556000	1000	1	3.30E-07	0.52	3	0.3	Lrrc4c	
DMR3:86565001	3	86565001	86566000	1000	1	2.20E-07	0.54	19	1.9	Lrrc4c	
DMR3:86577001	3	86577001	86578000	1000	1	2.70E-08	0.57	6	0.6	Lrrc4c	
DMR3:86590001	3	86590001	86593000	3000	2	8.70E-11	-0.65	12	0.4	Lrrc4c	
DMR3:86608001	3	86608001	86609000	1000	1	1.80E-13	0.62	5	0.5	Lrrc4c;LOC100362626	
DMR3:86617001	3	86617001	86618000	1000	1	8.30E-07	0.56	2	0.2	Lrrc4c;LOC100362626	
DMR3:86657001	3	86657001	86659000	2000	1	3.90E-08	0.45	12	0.6	Lrrc4c	
DMR3:86686001	3	86686001	86688000	2000	1	4.30E-07	0.48	9	0.45	Lrrc4c	
DMR3:86745001	3	86745001	86746000	1000	1	6.90E-08	0.5	7	0.7	Lrrc4c	
DMR3:86764001	3	86764001	86766000	2000	1	3.60E-07	0.56	3	0.15	Lrrc4c	
DMR3:86778001	3	86778001	86779000	1000	1	2.50E-20	1.11	11	1.1	Lrrc4c	
DMR3:86792001	3	86792001	86795000	3000	1	9.90E-10	0.4	20	0.67	Lrrc4c	
DMR3:86813001	3	86813001	86814000	1000	1	5.10E-08	0.48	5	0.5	Lrrc4c	
DMR3:89563001	3	89563001	89565000	2000	2	1.30E-08	0.63	7	0.35	RGD1561224;LOC108350603	
DMR3:89740001	3	89740001	89742000	2000	1	4.20E-09	0.49	20	1	RGD1560961	
DMR3:91092001	3	91092001	91098000	6000	2	9.70E-17	0.54	120	2	RGD1309730	
DMR3:91115001	3	91115001	91116000	1000	1	1.30E-11	1.06	8	0.8	RGD1309730	
DMR3:91132001	3	91132001	91133000	1000	1	3.80E-07	-0.44	8	0.8	RGD1309730	
DMR3:91138001	3	91138001	91140000	2000	1	3.20E-08	0.55	14	0.7	RGD1309730	
DMR3:91170001	3	91170001	91171000	1000	1	2.70E-09	-0.78	12	1.2	RGD1309730	
DMR3:91178001	3	91178001	91180000	2000	2	2.50E-09	-0.54	3	0.15	RGD1309730	
DMR3:91280001	3	91280001	91285000	5000	1	1.70E-22	0.61	75	1.5	Traf6;Prr5l	Cytoskeleton
DMR3:91295001	3	91295001	91298000	3000	1	5.10E-07	0.33	30	1	Prr5l	
DMR3:91299001	3	91299001	91300000	1000	1	2.70E-10	0.51	7	0.7	Prr5l	
DMR3:91313001	3	91313001	91315000	2000	1	3.60E-09	0.54	18	0.9	Prr5l	
DMR3:91356001	3	91356001	91359000	3000	1	4.80E-09	0.5	24	0.8	Prr5l	
DMR3:91374001	3	91374001	91375000	1000	1	7.10E-07	0.38	8	0.8	Prr5l	
DMR3:91385001	3	91385001	91389000	4000	2	6.50E-10	-0.57	51	1.27	Prr5l	
DMR3:91394001	3	91394001	91395000	1000	1	2.70E-09	0.37	9	0.9	Prr5l	
DMR3:91420001	3	91420001	91421000	1000	1	6.00E-07	0.44	8	0.8	Prr5l	
DMR3:91449001	3	91449001	91451000	2000	1	3.40E-14	0.69	12	0.6	Prr5l	
DMR3:91482001	3	91482001	91483000	1000	1	1.20E-07	0.45	8	0.8	Commd9	
DMR3:91484001	3	91484001	91485000	1000	1	1.50E-07	0.38	16	1.6	Commd9	
DMR3:91644001	3	91644001	91649000	5000	1	3.50E-07	-0.5	31	0.62	Ldlrad3	Binding Proteins
DMR3:91660001	3	91660001	91661000	1000	1	1.20E-32	0.97	1	0.1	Ldlrad3	Binding Proteins
DMR3:91673001	3	91673001	91674000	1000	1	4.90E-14	0.48	13	1.3	Ldlrad3	Binding Proteins
DMR3:91675001	3	91675001	91676000	1000	1	6.70E-14	0.82	17	1.7	Ldlrad3	Binding Proteins
DMR3:91685001	3	91685001	91690000	5000	1	1.60E-09	-0.55	83	1.66	Ldlrad3	Binding Proteins
DMR3:91744001	3	91744001	91746000	2000	1	9.10E-13	0.4	20	1	Ldlrad3	Binding Proteins

DMR3:91772001	3	91772001	91783000	11000	2	1.70E-10	0.65	153	1.39	Ldlrad3	Binding Proteins
DMR3:91793001	3	91793001	91798000	5000	2	9.70E-24	0.83	63	1.26	Ldlrad3	Binding Proteins
DMR3:91811001	3	91811001	91817000	6000	1	1.10E-08	0.37	81	1.35	Ldlrad3	Binding Proteins
DMR3:91823001	3	91823001	91827000	4000	1	2.40E-21	0.9	38	0.95	Ldlrad3	Binding Proteins
DMR3:91840001	3	91840001	91846000	6000	1	3.30E-20	0.66	57	0.95	Ldlrad3	Binding Proteins
DMR3:91958001	3	91958001	91960000	2000	1	1.40E-07	-0.39	20	1	Trim44	Proteolysis
DMR3:92037001	3	92037001	92039000	2000	2	1.30E-24	1.07	11	0.55	Trim44	Proteolysis
DMR3:92040001	3	92040001	92042000	2000	2	4.00E-08	0.71	7	0.35	Trim44	Proteolysis
DMR3:92045001	3	92045001	92049000	4000	3	8.00E-10	-0.64	19	0.48	Trim44	Proteolysis
DMR3:92054001	3	92054001	92057000	3000	2	2.50E-14	0.77	24	0.8	Trim44	Proteolysis
DMR3:92063001	3	92063001	92066000	3000	3	2.40E-24	0.62	28	0.93	Trim44	Proteolysis
DMR3:92069001	3	92069001	92070000	1000	1	6.70E-20	0.87	11	1.1	Trim44	Proteolysis
DMR3:92074001	3	92074001	92075000	1000	1	1.20E-12	0.92	4	0.4	Trim44	Proteolysis
DMR3:92092001	3	92092001	92095000	3000	1	6.80E-17	0.56	28	0.93	Trim44;LOC102551100	Proteolysis
DMR3:92119001	3	92119001	92121000	2000	2	2.10E-12	0.73	14	0.7	Trim44;LOC102551100	Proteolysis
DMR3:92142001	3	92142001	92148000	6000	1	9.30E-16	0.48	60	1	Trim44;LOC102551100	Proteolysis
DMR3:92152001	3	92152001	92153000	1000	1	9.00E-07	0.29	48	4.8	Trim44	Proteolysis
DMR3:92156001	3	92156001	92160000	4000	1	8.80E-09	-0.46	72	1.8	Trim44	Proteolysis
DMR3:92189001	3	92189001	92191000	2000	1	3.30E-07	-0.39	20	1	Trim44	Proteolysis
DMR3:92194001	3	92194001	92196000	2000	1	9.90E-07	-0.57	27	1.35	Trim44	Proteolysis
DMR3:92197001	3	92197001	92198000	1000	1	4.80E-08	0.44	5	0.5	Trim44	Proteolysis
DMR3:92201001	3	92201001	92204000	3000	3	3.80E-21	-0.6	33	1.1	Trim44	Proteolysis
DMR3:92205001	3	92205001	92208000	3000	1	1.40E-14	0.55	24	0.8	Trim44	Proteolysis
DMR3:92233001	3	92233001	92234000	1000	1	4.80E-16	0.93	30	3	Trim44;LOC108350422	Proteolysis
DMR3:92237001	3	92237001	92238000	1000	1	2.90E-09	0.43	9	0.9	Trim44;LOC108350422	Proteolysis
DMR3:92294001	3	92294001	92297000	3000	1	5.30E-07	-0.54	40	1.33	Fjx1	
DMR3:92391001	3	92391001	92395000	4000	1	2.40E-11	0.49	33	0.82	Pamr1	Protease
DMR3:92404001	3	92404001	92406000	2000	1	5.90E-09	0.46	25	1.25	Pamr1	Protease
DMR3:92423001	3	92423001	92427000	4000	2	1.40E-08	0.46	49	1.23	Pamr1	Protease
DMR3:92429001	3	92429001	92430000	1000	1	6.20E-08	-0.53	12	1.2	Pamr1	Protease
DMR3:92431001	3	92431001	92436000	5000	4	8.10E-19	-0.53	41	0.82	Pamr1	Protease
DMR3:92437001	3	92437001	92440000	3000	2	9.70E-12	-0.55	70	2.33	Pamr1	Protease
DMR3:92444001	3	92444001	92446000	2000	2	2.40E-11	0.46	22	1.1	Pamr1	Protease
DMR3:92452001	3	92452001	92454000	2000	1	1.30E-11	0.57	27	1.35	Pamr1	Protease
DMR3:92457001	3	92457001	92461000	4000	2	7.50E-20	0.87	24	0.6	Pamr1	Protease
DMR3:92506001	3	92506001	92507000	1000	1	7.50E-10	0.44	10	1	Pamr1	Protease
DMR3:92532001	3	92532001	92533000	1000	1	9.50E-12	0.53	7	0.7	Slc1a2	Transport
DMR3:92566001	3	92566001	92567000	1000	1	3.70E-11	0.54	6	0.6	Slc1a2	Transport
DMR3:92639001	3	92639001	92640000	1000	1	6.70E-10	0.7	6	0.6	Slc1a2	Transport
DMR3:92719001	3	92719001	92720000	1000	1	2.10E-11	0.75	7	0.7	Cd44	Receptor
DMR3:92747001	3	92747001	92748000	1000	1	8.40E-07	-0.37	11	1.1	Cd44	Receptor
DMR3:92784001	3	92784001	92785000	1000	1	8.40E-10	0.56	14	1.4	Cd44;LOC366141	Receptor
DMR3:92793001	3	92793001	92794000	1000	1	2.90E-17	0.72	3	0.3	Cd44;LOC366141	Receptor
DMR3:92915001	3	92915001	92916000	1000	1	1.30E-07	0.49	7	0.7	Pdhx	Metabolism
DMR3:92974001	3	92974001	92978000	4000	1	1.10E-12	-0.58	15	0.38	Apip	Metabolism
DMR3:93001001	3	93001001	93006000	5000	3	2.20E-09	-0.41	44	0.88	Apip	Metabolism
DMR3:93162001	3	93162001	93167000	5000	1	2.30E-07	0.44	43	0.86	Ehf	Transcription
DMR3:93173001	3	93173001	93174000	1000	1	3.50E-12	0.73	8	0.8	Ehf	Transcription
DMR3:93214001	3	93214001	93217000	3000	1	4.20E-08	-0.68	43	1.43	Ehf	Transcription
DMR3:93222001	3	93222001	93226000	4000	3	1.00E-15	0.7	28	0.7	Ehf	Transcription
DMR3:93326001	3	93326001	93328000	2000	1	1.30E-09	0.57	15	0.75	Elf5	Transcription
DMR3:93334001	3	93334001	93336000	2000	1	2.40E-15	0.53	34	1.7	Elf5	Transcription
DMR3:93338001	3	93338001	93340000	2000	1	8.30E-14	0.71	20	1	Elf5	Transcription
DMR3:93352001	3	93352001	93355000	3000	1	5.10E-07	-0.65	31	1.03	Elf5;LOC102548892	Transcription
DMR3:93375001	3	93375001	93379000	4000	1	1.80E-10	0.4	50	1.25	Elf5;Cat	Transcription;Metabolism
DMR3:93387001	3	93387001	93391000	4000	1	5.50E-12	0.44	66	1.65	Cat	Metabolism
DMR3:93401001	3	93401001	93404000	3000	1	4.20E-10	-0.54	48	1.6	Cat;LOC108350544	Metabolism
DMR3:93645001	3	93645001	93648000	3000	1	8.30E-07	0.37	45	1.5	Abtb2;LOC108350604	Cytoskeleton
DMR3:93667001	3	93667001	93669000	2000	1	3.40E-07	-0.48	39	1.95	Nat10	Translation
DMR3:93681001	3	93681001	93682000	1000	1	1.00E-14	0.5	10	1	Nat10	Translation
DMR3:93758001	3	93758001	93761000	3000	3	1.60E-21	0.7	24	0.8	Caprin1;LOC102549184;LOC108350425	Metabolism
DMR3:93774001	3	93774001	93776000	2000	1	2.10E-10	0.51	16	0.8	Caprin1;LOC108350425	Metabolism
DMR3:93783001	3	93783001	93785000	2000	1	1.20E-07	0.33	30	1.5	Caprin1;LOC108350425	Metabolism

DMR3:93790001	3	93790001	93792000	2000	2	1.50E-15	0.69	16	0.8	Caprin1;LOC108350425	Metabolism
DMR3:93935001	3	93935001	93938000	3000	1	1.50E-12	0.52	25	0.83	Lmo2	
DMR3:93961001	3	93961001	93962000	1000	1	2.30E-08	0.5	16	1.6	LOC108350426;Fbxo3	
DMR3:93972001	3	93972001	93974000	2000	1	5.30E-08	0.48	21	1.05	LOC108350426;Fbxo3	
DMR3:93997001	3	93997001	93999000	2000	2	6.60E-11	0.65	19	0.95	Fbxo3	
DMR3:94026001	3	94026001	94027000	1000	1	1.10E-07	0.57	2	0.2	Cd59;RGD1563222	
DMR3:94039001	3	94039001	94043000	4000	1	2.30E-07	0.3	55	1.38	RGD1563222	
DMR3:94044001	3	94044001	94046000	2000	1	2.30E-07	0.37	25	1.25	RGD1563222	
DMR3:94353001	3	94353001	94355000	2000	1	1.20E-14	0.48	37	1.85	Hipk3	
DMR3:94360001	3	94360001	94363000	3000	1	5.10E-07	-0.48	41	1.37	Hipk3	
DMR3:94412001	3	94412001	94413000	1000	1	1.40E-12	0.79	7	0.7	Hipk3	
DMR3:94425001	3	94425001	94427000	2000	2	6.70E-18	0.77	35	1.75	Hipk3	
DMR3:94520001	3	94520001	94521000	1000	1	2.40E-14	0.45	13	1.3	Cstf3	Translation
DMR3:94525001	3	94525001	94527000	2000	1	8.50E-15	0.81	7	0.35	Cstf3	Translation
DMR3:94556001	3	94556001	94559000	3000	1	4.30E-09	0.63	16	0.53	Cstf3	Translation
DMR3:94562001	3	94562001	94563000	1000	1	3.30E-07	-0.58	3	0.3	Cstf3	Translation
DMR3:94614001	3	94614001	94617000	3000	1	5.10E-07	0.38	23	0.77	Tcp11l1	Cytoskeleton
DMR3:94637001	3	94637001	94641000	4000	1	1.30E-07	-0.62	33	0.82	Tcp11l1	Cytoskeleton
DMR3:94648001	3	94648001	94649000	1000	1	5.20E-17	0.96	3	0.3	Tcp11l1	Cytoskeleton
DMR3:94661001	3	94661001	94663000	2000	1	2.00E-11	-0.53	19	0.95	Tcp11l1;Depdc7	Cytoskeleton;Cytoskeleton
DMR3:94677001	3	94677001	94679000	2000	1	7.20E-15	0.56	26	1.3	Depdc7	Cytoskeleton
DMR3:94698001	3	94698001	94699000	1000	1	3.90E-20	0.67	12	1.2	Qser1	
DMR3:94728001	3	94728001	94729000	1000	1	3.60E-19	0.74	6	0.6	Qser1	
DMR3:94751001	3	94751001	94754000	3000	3	8.00E-17	-0.73	22	0.73	Qser1	
DMR3:94774001	3	94774001	94779000	5000	2	2.40E-11	-0.49	41	0.82	Qser1;Prrg4	Protease
DMR3:94780001	3	94780001	94781000	1000	1	5.10E-11	0.62	3	0.3	Prrg4	Protease
DMR3:94797001	3	94797001	94798000	1000	1	5.60E-09	0.31	10	1	Prrg4	Protease
DMR3:94830001	3	94830001	94832000	2000	1	1.40E-08	-0.43	24	1.2	RGD1560017	
DMR3:94851001	3	94851001	94856000	5000	1	4.60E-11	-0.7	35	0.7	Ccdc73	
DMR3:94870001	3	94870001	94871000	1000	1	3.20E-11	0.58	4	0.4	Ccdc73	
DMR3:94873001	3	94873001	94875000	2000	1	3.80E-14	0.74	12	0.6	Ccdc73	
DMR3:94881001	3	94881001	94889000	8000	7	5.00E-26	0.6	92	1.15	Ccdc73	
DMR3:94902001	3	94902001	94903000	1000	1	3.00E-14	0.67	7	0.7	Ccdc73	
DMR3:94909001	3	94909001	94912000	3000	2	1.10E-16	-0.52	23	0.77	Ccdc73	
DMR3:94921001	3	94921001	94927000	6000	4	2.10E-20	-0.55	59	0.98	Ccdc73	
DMR3:94931001	3	94931001	94933000	2000	1	6.20E-09	0.57	7	0.35	Ccdc73	
DMR3:94935001	3	94935001	94941000	6000	4	1.40E-11	0.48	71	1.18	Ccdc73	
DMR3:94955001	3	94955001	94958000	3000	2	1.50E-11	-0.54	19	0.63	Ccdc73	
DMR3:94959001	3	94959001	94960000	1000	1	8.80E-10	0.63	8	0.8	Ccdc73	
DMR3:94969001	3	94969001	94970000	1000	1	9.00E-07	-0.58	11	1.1	Ccdc73	
DMR3:94976001	3	94976001	94980000	4000	2	3.30E-20	0.72	28	0.7	Ccdc73	
DMR3:94982001	3	94982001	94983000	1000	1	8.90E-10	-0.63	14	1.4	Ccdc73;Eif3m	
DMR3:95000001	3	95000001	95003000	3000	2	5.70E-17	0.59	18	0.6	Eif3m;LOC100912353	
DMR3:95006001	3	95006001	95007000	1000	1	1.50E-18	0.94	10	1	Eif3m;LOC100912353	
DMR3:95016001	3	95016001	95019000	3000	1	5.50E-07	0.46	24	0.8	Eif3m;LOC100912353	
DMR3:95119001	3	95119001	95125000	6000	1	1.20E-08	-0.51	105	1.75	Wt1	Transcription
DMR3:95126001	3	95126001	95129000	3000	1	9.10E-10	0.52	32	1.07	Wt1	Transcription
DMR3:95153001	3	95153001	95155000	2000	1	3.60E-10	0.53	23	1.15	Wt1	Transcription
DMR3:95156001	3	95156001	95157000	1000	1	9.50E-18	0.79	7	0.7	Wt1	Transcription
DMR3:95170001	3	95170001	95172000	2000	2	1.10E-11	0.67	20	1	Wt1	Transcription
DMR3:95185001	3	95185001	95186000	1000	1	1.00E-07	0.52	7	0.7	Wt1	Transcription
DMR3:95407001	3	95407001	95408000	1000	1	9.10E-07	-0.54	17	1.7	LOC691083;Rcn1	Metabolism;Signaling
DMR3:95413001	3	95413001	95414000	1000	1	4.00E-08	0.36	12	1.2	Rcn1	Signaling
DMR3:95416001	3	95416001	95417000	1000	1	1.70E-11	0.54	11	1.1	Rcn1	Signaling
DMR3:95424001	3	95424001	95427000	3000	1	4.30E-12	0.63	26	0.87	Rcn1	Signaling
DMR3:95714001	3	95714001	95717000	3000	2	2.10E-32	0.88	48	1.6	Pax6	
DMR3:95721001	3	95721001	95722000	1000	1	6.50E-13	0.96	30	3	Pax6	
DMR3:95743001	3	95743001	95746000	3000	2	1.90E-18	-0.57	41	1.37	Elp4	
DMR3:95748001	3	95748001	95750000	2000	1	5.20E-10	0.77	20	1	Elp4	
DMR3:95778001	3	95778001	95779000	1000	1	9.50E-18	0.77	13	1.3	Elp4	
DMR3:95815001	3	95815001	95816000	1000	1	5.30E-08	0.43	10	1	Elp4	
DMR3:95817001	3	95817001	95818000	1000	1	2.60E-25	0.84	6	0.6	Elp4	
DMR3:95864001	3	95864001	95865000	1000	1	6.70E-09	0.55	3	0.3	Elp4	

DMR3:95899001	3	95899001	95901000	2000	1	9.60E-07	-0.43	8	0.4	Elp4	
DMR3:95906001	3	95906001	95912000	6000	4	2.10E-22	-0.56	51	0.85	Elp4	
DMR3:95916001	3	95916001	95917000	1000	1	7.40E-10	-0.67	2	0.2	Elp4	
DMR3:95941001	3	95941001	95942000	1000	1	4.10E-07	0.34	11	1.1	Elp4;LOC108350427	
DMR3:95983001	3	95983001	95984000	1000	1	7.80E-11	0.6	10	1	Immp1l	Protease
DMR3:95988001	3	95988001	95989000	1000	1	5.90E-07	0.47	7	0.7	Immp1l	Protease
DMR3:95993001	3	95993001	95994000	1000	1	9.50E-14	0.56	8	0.8	Immp1l	Protease
DMR3:95995001	3	95995001	95996000	1000	1	2.60E-28	0.66	24	2.4	Immp1l	Protease
DMR3:96012001	3	96012001	96013000	1000	1	8.50E-07	0.31	10	1	Immp1l;LOC108350429	Protease
DMR3:96053001	3	96053001	96055000	2000	1	1.00E-08	0.5	12	0.6	Dnajc24	Transcription
DMR3:97205001	3	97205001	97206000	1000	1	1.20E-12	0.47	7	0.7	Dcdc5	
DMR3:97218001	3	97218001	97221000	3000	1	2.50E-14	0.85	34	1.13	Dcdc5	
DMR3:97228001	3	97228001	97229000	1000	1	3.80E-10	0.63	6	0.6	Dcdc5	
DMR3:97230001	3	97230001	97231000	1000	1	4.60E-08	0.43	10	1	Dcdc5	
DMR3:97251001	3	97251001	97255000	4000	1	5.80E-10	0.57	49	1.23	Dcdc5	
DMR3:97256001	3	97256001	97258000	2000	1	1.80E-08	-0.69	36	1.8	Dcdc5	
DMR3:97268001	3	97268001	97271000	3000	2	7.30E-13	0.51	39	1.3	Dcdc5	
DMR3:97272001	3	97272001	97273000	1000	1	7.20E-11	0.52	12	1.2	Dcdc5	
DMR3:97302001	3	97302001	97304000	2000	1	4.20E-18	0.81	7	0.35	Dcdc5	
DMR3:97310001	3	97310001	97311000	1000	1	2.00E-09	0.71	11	1.1	Dcdc5	
DMR3:97335001	3	97335001	97337000	2000	2	3.20E-11	-0.53	16	0.8	Dcdc5	
DMR3:97355001	3	97355001	97358000	3000	3	3.40E-13	-0.67	8	0.27	Dcdc5	
DMR3:97363001	3	97363001	97371000	8000	5	2.60E-28	-0.54	105	1.31	Dcdc5	
DMR3:97372001	3	97372001	97374000	2000	2	3.10E-19	0.73	15	0.75	Dcdc5	
DMR3:97375001	3	97375001	97379000	4000	2	9.70E-13	-0.44	26	0.65	Dcdc5	
DMR3:97402001	3	97402001	97403000	1000	1	2.60E-15	0.54	17	1.7	Dcdc5	
DMR3:97414001	3	97414001	97415000	1000	1	5.40E-11	0.54	1	0.1	Dcdc5	
DMR3:97429001	3	97429001	97431000	2000	2	5.40E-18	0.77	10	0.5	Dcdc5	
DMR3:97735001	3	97735001	97736000	1000	1	7.00E-07	0.36	10	1	Mpped2	Metabolism
DMR3:97738001	3	97738001	97739000	1000	1	1.70E-31	1.21	6	0.6	Mpped2	Metabolism
DMR3:97762001	3	97762001	97764000	2000	1	6.80E-08	-0.55	26	1.3	Mpped2	Metabolism
DMR3:97780001	3	97780001	97781000	1000	1	1.50E-11	0.5	19	1.9	Mpped2	Metabolism
DMR3:97786001	3	97786001	97787000	1000	1	4.60E-07	0.32	14	1.4	Mpped2	Metabolism
DMR3:97792001	3	97792001	97795000	3000	2	4.20E-11	-0.53	32	1.07	Mpped2	Metabolism
DMR3:97809001	3	97809001	97815000	6000	2	2.20E-07	-0.61	118	1.97	Mpped2	Metabolism
DMR3:97839001	3	97839001	97840000	1000	1	6.70E-09	-0.57	23	2.3	Mpped2	Metabolism
DMR3:97841001	3	97841001	97844000	3000	1	1.20E-07	0.35	50	1.67	Mpped2	Metabolism
DMR3:97845001	3	97845001	97849000	4000	1	1.90E-18	0.51	48	1.2	Mpped2	Metabolism
DMR3:97862001	3	97862001	97867000	5000	2	8.80E-08	0.33	76	1.52	Mpped2	Metabolism
DMR3:97883001	3	97883001	97885000	2000	1	5.90E-07	-0.44	24	1.2	Mpped2	Metabolism
DMR3:97886001	3	97886001	97895000	9000	1	9.30E-07	-0.52	131	1.46	Mpped2	Metabolism
DMR3:97976001	3	97976001	97979000	3000	1	1.60E-07	-0.51	49	1.63	LOC103691854;Arl14ep	
DMR3:97983001	3	97983001	97988000	5000	2	2.90E-09	-0.52	70	1.4	LOC103691854;Arl14ep	
DMR3:97989001	3	97989001	97991000	2000	1	2.80E-07	-0.49	26	1.3	Arl14ep	
DMR3:98081001	3	98081001	98083000	2000	1	1.20E-14	0.77	20	1	Fshb	Hormone
DMR3:98084001	3	98084001	98086000	2000	1	8.50E-09	0.56	13	0.65	Fshb	Hormone
DMR3:98094001	3	98094001	98095000	1000	1	3.00E-10	0.55	4	0.4	Fshb	Hormone
DMR3:98101001	3	98101001	98103000	2000	1	9.60E-07	0.36	22	1.1	Fshb	Hormone
DMR3:98286001	3	98286001	98287000	1000	1	6.40E-08	0.5	6	0.6	Kcna4	Transport
DMR3:98294001	3	98294001	98297000	3000	2	2.20E-10	0.71	27	0.9	Kcna4	Transport
DMR3:99444001	3	99444001	99445000	1000	1	2.70E-10	0.38	9	0.9	Olr746-ps	
DMR3:99461001	3	99461001	99463000	2000	2	7.10E-11	0.67	11	0.55	Olr746-ps	
DMR3:100186001	3	100186001	100187000	1000	1	1.80E-12	0.67	6	0.6	Mettl15	Epigenetic
DMR3:100197001	3	100197001	100198000	1000	1	6.10E-07	0.25	4	0.4	Mettl15	Epigenetic
DMR3:100201001	3	100201001	100202000	1000	1	1.30E-16	0.68	6	0.6	Mettl15	Epigenetic
DMR3:100211001	3	100211001	100214000	3000	2	6.90E-08	-0.54	14	0.47	Mettl15	Epigenetic
DMR3:100226001	3	100226001	100227000	1000	1	1.70E-08	0.56	6	0.6	Mettl15	Epigenetic
DMR3:100240001	3	100240001	100241000	1000	1	9.70E-15	0.74	2	0.2	Mettl15	Epigenetic
DMR3:100251001	3	100251001	100254000	3000	2	3.40E-24	1.06	23	0.77	Mettl15	Epigenetic
DMR3:100263001	3	100263001	100265000	2000	2	1.30E-10	0.61	12	0.6	Mettl15;LOC108350435	Epigenetic
DMR3:100270001	3	100270001	100271000	1000	1	1.20E-07	-0.48	13	1.3	Mettl15;LOC108350435	Epigenetic
DMR3:100279001	3	100279001	100280000	1000	1	1.00E-12	0.53	6	0.6	Mettl15;LOC108350435	Epigenetic
DMR3:100290001	3	100290001	100293000	3000	1	3.40E-12	0.69	16	0.53	Mettl15	Epigenetic
DMR3:100298001	3	100298001	100299000	1000	1	5.20E-13	0.62	8	0.8	Mettl15	Epigenetic
DMR3:100303001	3	100303001	100305000	2000	2	3.30E-07	0.46	11	0.55	Mettl15	Epigenetic

DMR3:100322001	3	100322001	100323000	1000	1	7.70E-09	0.46	6	0.6	Mettl15	Epigenetic
DMR3:100333001	3	100333001	100334000	1000	1	4.90E-11	-0.72	11	1.1	Mettl15	Epigenetic
DMR3:100381001	3	100381001	100382000	1000	1	6.30E-10	-0.56	12	1.2	Kif18a	Cytoskeleton
DMR3:100402001	3	100402001	100403000	1000	1	6.90E-07	-0.61	9	0.9	Kif18a	Cytoskeleton
DMR3:100761001	3	100761001	100762000	1000	1	3.10E-08	0.51	5	0.5	Bdnf	Growth Factors
DMR3:100796001	3	100796001	100797000	1000	1	1.60E-08	0.53	4	0.4	Bdnf	Growth Factors
DMR3:100814001	3	100814001	100815000	1000	1	1.20E-07	-0.6	17	1.7	Bdnf	Growth Factors
DMR3:101004001	3	101004001	101005000	1000	1	1.70E-14	0.8	6	0.6	Lin7c	Cytoskeleton
DMR3:101024001	3	101024001	101028000	4000	2	2.70E-16	-0.49	43	1.07	Lin7c	Cytoskeleton
DMR3:101047001	3	101047001	101048000	1000	1	9.30E-07	0.29	11	1.1	Lgr4	
DMR3:101049001	3	101049001	101050000	1000	1	4.00E-12	0.42	5	0.5	Lgr4	
DMR3:101052001	3	101052001	101053000	1000	1	3.10E-07	0.59	52	5.2	Lgr4	
DMR3:101057001	3	101057001	101059000	2000	1	3.90E-07	-0.58	7	0.35	Lgr4	
DMR3:101084001	3	101084001	101088000	4000	1	1.00E-12	0.5	61	1.52	Lgr4	
DMR3:101098001	3	101098001	101101000	3000	1	8.40E-07	-0.62	26	0.87	Lgr4	
DMR3:101162001	3	101162001	101164000	2000	1	1.10E-10	0.59	11	0.55	Lgr4;Ccdc34	
DMR3:101166001	3	101166001	101171000	5000	2	6.30E-12	-0.55	26	0.52	Ccdc34	
DMR3:101195001	3	101195001	101202000	7000	3	1.10E-17	-0.54	54	0.77	Ccdc34	
DMR3:101418001	3	101418001	101420000	2000	1	7.70E-08	0.37	8	0.4	Bbox1	Metabolism
DMR3:101452001	3	101452001	101455000	3000	1	1.00E-10	0.41	28	0.93	Bbox1	Metabolism
DMR3:101484001	3	101484001	101486000	2000	2	5.10E-23	-0.79	10	0.5	Bbox1	Metabolism
DMR3:101780001	3	101780001	101782000	2000	1	7.70E-08	0.41	16	0.8	Slc5a12	Transport
DMR3:101828001	3	101828001	101830000	2000	1	2.10E-11	0.66	12	0.6	Slc5a12	Transport
DMR3:101840001	3	101840001	101841000	1000	1	9.50E-17	0.59	11	1.1	Slc5a12;Ano3	Transport
DMR3:101859001	3	101859001	101863000	4000	2	2.40E-10	-0.72	25	0.62	Ano3	
DMR3:101866001	3	101866001	101867000	1000	1	2.20E-14	0.63	0	0	Ano3	
DMR3:101901001	3	101901001	101902000	1000	1	1.20E-17	0.84	5	0.5	Ano3;Muc15	
DMR3:101976001	3	101976001	101982000	6000	3	6.00E-24	-0.51	49	0.82	Ano3	
DMR3:101984001	3	101984001	101985000	1000	1	4.60E-08	-0.59	8	0.8	Ano3	
DMR3:102015001	3	102015001	102017000	2000	2	2.00E-12	-0.44	23	1.15	Ano3	
DMR3:102058001	3	102058001	102064000	6000	5	9.80E-20	-0.61	55	0.92	Ano3	
DMR3:102070001	3	102070001	102073000	3000	1	2.70E-08	0.44	24	0.8	Ano3	
DMR3:102079001	3	102079001	102080000	1000	1	2.00E-20	0.78	5	0.5	Ano3	
DMR3:102081001	3	102081001	102082000	1000	1	1.80E-09	0.54	15	1.5	Ano3	
DMR3:102086001	3	102086001	102087000	1000	1	8.40E-19	0.78	7	0.7	Ano3	
DMR3:102104001	3	102104001	102105000	1000	1	2.10E-07	0.49	4	0.4	Ano3	
DMR3:102112001	3	102112001	102114000	2000	2	2.80E-08	-0.51	10	0.5	Ano3	
DMR3:102131001	3	102131001	102133000	2000	2	3.50E-23	0.91	9	0.45	Ano3	
DMR3:102162001	3	102162001	102165000	3000	1	3.10E-14	0.68	17	0.57	Ano3	
DMR3:102173001	3	102173001	102175000	2000	1	4.10E-08	0.45	6	0.3	Ano3	
DMR3:102206001	3	102206001	102212000	6000	1	5.30E-08	0.59	28	0.47	Ano3	
DMR3:102310001	3	102310001	102312000	2000	1	8.50E-12	-0.6	6	0.3	Ankrd30a	
DMR3:102342001	3	102342001	102344000	2000	1	1.90E-07	0.53	9	0.45	Ankrd30a	
DMR3:102362001	3	102362001	102364000	2000	1	7.10E-25	0.75	4	0.2	Ankrd30a	
DMR3:102387001	3	102387001	102388000	1000	1	4.40E-10	0.46	16	1.6	Ankrd30a	
DMR3:102389001	3	102389001	102395000	6000	6	9.50E-21	-0.46	66	1.1	Ankrd30a	
DMR3:102409001	3	102409001	102410000	1000	1	1.40E-11	0.69	6	0.6	Ankrd30a	
DMR3:102439001	3	102439001	102449000	10000	5	8.40E-11	-0.6	66	0.66	Ankrd30a;Olr747;Olr748	Receptor
DMR3:102461001	3	102461001	102466000	5000	4	2.10E-09	-0.61	25	0.5	Olr748;Olr749	Receptor
DMR3:102491001	3	102491001	102496000	5000	1	1.90E-07	0.4	33	0.66	Olr750	Receptor
DMR3:102534001	3	102534001	102537000	3000	1	1.70E-12	-0.58	13	0.43	Olr752	Receptor
DMR3:102538001	3	102538001	102544000	6000	6	1.20E-24	-0.58	55	0.92	Olr752;Olr753	Receptor
DMR3:102561001	3	102561001	102562000	1000	1	8.60E-11	-0.56	8	0.8	Olr753	Receptor
DMR3:102568001	3	102568001	102571000	3000	3	3.10E-16	0.74	18	0.6	Olr754	Receptor
DMR3:102584001	3	102584001	102586000	2000	2	8.80E-12	0.76	4	0.2	Olr754;Olr755-ps	Receptor
DMR3:102608001	3	102608001	102609000	1000	1	2.90E-18	0.73	4	0.4	RGD1562507	
DMR3:102668001	3	102668001	102672000	4000	1	1.80E-07	0.48	23	0.58	Olr758;RGD1561276;Olr760;RGD1566059	Receptor
DMR3:102677001	3	102677001	102679000	2000	1	1.50E-10	0.57	12	0.6	Olr760;RGD1566059	Receptor
DMR3:102698001	3	102698001	102699000	1000	1	2.30E-07	0.54	5	0.5	Olr762-ps;Olr763;Olr764-ps	Receptor
DMR3:102700001	3	102700001	102701000	1000	1	2.50E-11	0.61	8	0.8	Olr762-ps;Olr763;Olr764-ps	Receptor
DMR3:102711001	3	102711001	102713000	2000	1	8.80E-07	-0.55	14	0.7	Olr763;Olr764-ps;Olr765	Receptor
DMR3:102793001	3	102793001	102794000	1000	1	3.40E-10	0.58	4	0.4	Olr768	Receptor
DMR3:102799001	3	102799001	102801000	2000	1	4.70E-08	0.54	14	0.7	Olr768	Receptor
DMR3:102803001	3	102803001	102805000	2000	2	9.20E-10	0.66	7	0.35	Olr768	Receptor

DMR3:102832001	3	102832001	102834000	2000	2	1.50E-12	0.73	6	0.3	Olr769;Olr770	Receptor
DMR3:102867001	3	102867001	102869000	2000	1	3.10E-08	0.46	10	0.5	Olr771;LOC108350605	Receptor
DMR3:102914001	3	102914001	102918000	4000	1	2.00E-09	-0.47	22	0.55	Olr772	Receptor
DMR3:102933001	3	102933001	102935000	2000	1	1.90E-12	0.63	9	0.45	Olr772	Receptor
DMR3:102943001	3	102943001	102944000	1000	1	1.90E-15	0.69	4	0.4	Olr773;LOC296027	Receptor
DMR3:102952001	3	102952001	102953000	1000	1	4.50E-07	-0.42	15	1.5	Olr773;LOC296027	Receptor
DMR3:102987001	3	102987001	102989000	2000	1	1.10E-07	0.37	9	0.45	Olr774	Receptor
DMR3:103006001	3	103006001	103007000	1000	1	8.90E-10	0.48	5	0.5	Olr774	Receptor
DMR3:103009001	3	103009001	103012000	3000	2	8.30E-11	-0.47	18	0.6	Olr774	Receptor
DMR3:103029001	3	103029001	103033000	4000	1	2.90E-16	-0.58	31	0.78	LOC108350606;Olr775	Receptor
DMR3:103052001	3	103052001	103056000	4000	1	3.60E-08	0.43	27	0.68	Olr775;Olr776	Receptor
DMR3:103063001	3	103063001	103065000	2000	1	4.70E-16	0.87	8	0.4	Olr776	Receptor
DMR3:103103001	3	103103001	103104000	1000	1	6.30E-12	0.77	0	0	LOC100363400;Olr778	Receptor
DMR3:103116001	3	103116001	103119000	3000	3	1.10E-14	0.58	42	1.4	LOC100363400;Olr778;Olr779	Receptor
DMR3:103134001	3	103134001	103136000	2000	2	7.60E-15	0.84	8	0.4	Olr779;Olr780	Receptor
DMR3:103146001	3	103146001	103147000	1000	1	5.50E-07	0.46	5	0.5	Olr780;Olr781	Receptor
DMR3:103149001	3	103149001	103150000	1000	1	8.50E-09	0.49	8	0.8	Olr780;Olr781	Receptor
DMR3:103160001	3	103160001	103163000	3000	2	1.20E-14	-0.6	19	0.63	Olr781;Olr782	Receptor
DMR3:103193001	3	103193001	103199000	6000	2	1.40E-08	0.5	41	0.68	LOC100363452;Olr783	Translation;Receptor
DMR3:103220001	3	103220001	103222000	2000	1	4.00E-17	0.79	11	0.55	Olr784;Olr785	Receptor
DMR3:103225001	3	103225001	103227000	2000	2	5.80E-19	0.86	7	0.35	Olr784;Olr785	Receptor
DMR3:103232001	3	103232001	103240000	8000	4	7.00E-12	-0.58	53	0.66	Olr785;Olr786	Receptor
DMR3:103242001	3	103242001	103243000	1000	1	5.40E-07	0.55	4	0.4	Olr786	
DMR3:103250001	3	103250001	103252000	2000	1	1.60E-09	-0.46	18	0.9	Olr786	
DMR3:103253001	3	103253001	103256000	3000	2	7.20E-13	-0.73	13	0.43	Olr786;Olr787-ps	
DMR3:103265001	3	103265001	103267000	2000	1	2.30E-11	0.55	10	0.5	Olr787-ps	
DMR3:103272001	3	103272001	103273000	1000	1	1.30E-08	0.54	9	0.9	Olr787-ps	
DMR3:103274001	3	103274001	103282000	8000	2	5.10E-20	-0.54	61	0.76	Olr787-ps	
DMR3:103293001	3	103293001	103296000	3000	2	4.70E-10	-0.41	22	0.73	Olr788	Receptor
DMR3:103305001	3	103305001	103306000	1000	1	4.30E-08	0.57	9	0.9	Olr788	Receptor
DMR3:103310001	3	103310001	103311000	1000	1	1.30E-07	0.5	8	0.8	Olr788	Receptor
DMR3:103337001	3	103337001	103339000	2000	1	2.00E-07	-0.49	7	0.35	Olr789	
DMR3:103345001	3	103345001	103347000	2000	1	9.80E-14	0.94	13	0.65	Olr789	
DMR3:103354001	3	103354001	103358000	4000	1	5.60E-10	0.48	43	1.07	Olr789	
DMR3:103390001	3	103390001	103393000	3000	2	8.90E-09	-0.44	20	0.67	Olr790	Receptor
DMR3:103400001	3	103400001	103402000	2000	1	3.20E-15	0.95	7	0.35	Olr790	Receptor
DMR3:103405001	3	103405001	103406000	1000	1	2.80E-08	0.59	9	0.9	Olr790	Receptor
DMR3:103407001	3	103407001	103410000	3000	1	1.20E-07	-0.38	37	1.23	Olr790	Receptor
DMR3:103438001	3	103438001	103440000	2000	2	3.60E-10	-0.47	13	0.65	Olr791	Receptor
DMR3:103441001	3	103441001	103444000	3000	1	1.70E-07	-0.47	36	1.2	Olr791	Receptor
DMR3:103523001	3	103523001	103524000	1000	1	6.90E-07	0.47	15	1.5	Katnbl1	
DMR3:103544001	3	103544001	103550000	6000	2	4.10E-12	-0.48	53	0.88	Olr792	Receptor
DMR3:103552001	3	103552001	103555000	3000	1	4.80E-08	0.42	17	0.57	Olr792	Receptor
DMR3:103615001	3	103615001	103616000	1000	1	9.70E-13	0.63	5	0.5	Olr794-ps	
DMR3:103633001	3	103633001	103635000	2000	1	3.80E-09	0.59	8	0.4	Olr795	Receptor
DMR3:103639001	3	103639001	103640000	1000	1	3.80E-07	0.53	3	0.3	Olr795	Receptor
DMR3:103643001	3	103643001	103652000	9000	5	3.50E-19	-0.46	84	0.93	Olr795;Olr796	Receptor
DMR3:103664001	3	103664001	103669000	5000	2	1.90E-14	0.57	36	0.72	Olr796	Receptor
DMR3:103720001	3	103720001	103721000	1000	1	4.10E-08	0.37	16	1.6	Lpcat4	Metabolism
DMR3:103729001	3	103729001	103731000	2000	1	1.10E-17	0.5	43	2.15	Lpcat4;Nutm1	Metabolism
DMR3:103733001	3	103733001	103736000	3000	1	4.10E-12	0.41	44	1.47	Lpcat4;Nutm1	Metabolism
DMR3:103738001	3	103738001	103741000	3000	2	4.30E-09	0.44	39	1.3	Lpcat4;Nutm1;Nop10	Metabolism;Metabolism
DMR3:103750001	3	103750001	103752000	2000	2	1.60E-15	0.94	35	1.75	Nutm1;Nop10;Slc12a6	Metabolism;Transport
DMR3:103781001	3	103781001	103782000	1000	1	2.10E-10	0.46	20	2	Slc12a6	Transport
DMR3:103791001	3	103791001	103794000	3000	3	1.80E-21	0.9	16	0.53	Slc12a6	Transport
DMR3:103795001	3	103795001	103801000	6000	3	1.30E-11	0.68	44	0.73	Slc12a6	Transport
DMR3:103874001	3	103874001	103875000	1000	1	2.20E-08	0.45	9	0.9	Katnbl1;LOC102551197	
DMR3:103879001	3	103879001	103882000	3000	1	2.10E-13	0.62	37	1.23	Katnbl1;LOC102551197	
DMR3:103917001	3	103917001	103919000	2000	1	4.40E-10	0.56	19	0.95	Katnbl1	
DMR3:103969001	3	103969001	103971000	2000	1	1.20E-24	0.79	24	1.2	Chrm5;Aven	Signaling
DMR3:103972001	3	103972001	103973000	1000	1	7.20E-10	0.51	10	1	Chrm5;Aven	Signaling
DMR3:103983001	3	103983001	103984000	1000	1	4.30E-09	-0.48	9	0.9	Chrm5;Aven	Signaling
DMR3:103990001	3	103990001	103991000	1000	1	7.30E-07	-0.46	14	1.4	Chrm5;Aven	Signaling

DMR3:104026001	3	104026001	104029000	3000	1	3.50E-08	0.4	21	0.7	Chrm5;Aven	Signaling
DMR3:104030001	3	104030001	104032000	2000	1	1.70E-29	0.8	14	0.7	Aven	
DMR3:104034001	3	104034001	104035000	1000	1	1.40E-15	0.55	16	1.6	Aven	
DMR3:104038001	3	104038001	104039000	1000	1	4.30E-16	0.51	9	0.9	Aven	
DMR3:104041001	3	104041001	104043000	2000	1	3.80E-11	0.47	25	1.25	Aven	
DMR3:104067001	3	104067001	104068000	1000	1	1.40E-10	0.61	5	0.5	Aven;LOC108350586;LOC102551301	
DMR3:104083001	3	104083001	104084000	1000	1	1.80E-07	0.4	12	1.2	Aven;LOC108350584	
DMR3:104088001	3	104088001	104090000	2000	2	7.80E-11	0.43	27	1.35	Aven;LOC108350584	
DMR3:104095001	3	104095001	104096000	1000	1	1.80E-07	0.45	7	0.7	Aven;LOC108350584	
DMR3:104098001	3	104098001	104100000	2000	2	1.30E-10	0.64	13	0.65	Aven;LOC108350584	
DMR3:104107001	3	104107001	104108000	1000	1	5.10E-09	0.71	1	0.1	Aven;Ryr3	Ion Channel
DMR3:104118001	3	104118001	104123000	5000	1	3.30E-16	0.78	41	0.82	Aven;Ryr3	Ion Channel
DMR3:104138001	3	104138001	104140000	2000	1	7.20E-10	0.59	16	0.8	Ryr3	Ion Channel
DMR3:104142001	3	104142001	104148000	6000	2	1.40E-15	0.75	48	0.8	Ryr3	Ion Channel
DMR3:104154001	3	104154001	104156000	2000	1	7.60E-10	0.67	6	0.3	Ryr3	Ion Channel
DMR3:104160001	3	104160001	104161000	1000	1	8.80E-12	0.51	9	0.9	Ryr3	Ion Channel
DMR3:104162001	3	104162001	104163000	1000	1	1.30E-08	-0.51	18	1.8	Ryr3	Ion Channel
DMR3:104173001	3	104173001	104177000	4000	1	6.70E-24	0.85	57	1.43	Ryr3	Ion Channel
DMR3:104202001	3	104202001	104204000	2000	1	1.70E-07	0.49	18	0.9	Ryr3	Ion Channel
DMR3:104218001	3	104218001	104220000	2000	1	1.20E-12	0.64	17	0.85	Ryr3;LOC103691859	Ion Channel
DMR3:104243001	3	104243001	104245000	2000	1	3.00E-12	0.45	28	1.4	Ryr3	Ion Channel
DMR3:104246001	3	104246001	104247000	1000	1	5.40E-07	0.36	11	1.1	Ryr3	Ion Channel
DMR3:104248001	3	104248001	104251000	3000	1	2.20E-14	0.51	34	1.13	Ryr3	Ion Channel
DMR3:104273001	3	104273001	104275000	2000	2	9.80E-21	0.77	12	0.6	Ryr3	Ion Channel
DMR3:104306001	3	104306001	104309000	3000	2	2.20E-14	-0.54	31	1.03	Ryr3	Ion Channel
DMR3:104341001	3	104341001	104342000	1000	1	1.70E-09	0.48	6	0.6	Ryr3	Ion Channel
DMR3:104353001	3	104353001	104355000	2000	1	1.30E-07	-0.56	17	0.85	Ryr3	Ion Channel
DMR3:104367001	3	104367001	104368000	1000	1	2.60E-07	-0.48	11	1.1	Ryr3;LOC108350585	Ion Channel
DMR3:104384001	3	104384001	104385000	1000	1	7.20E-10	0.63	7	0.7	Ryr3;LOC108350585	Ion Channel
DMR3:104392001	3	104392001	104394000	2000	1	5.30E-09	-0.6	16	0.8	Ryr3;LOC108350585	Ion Channel
DMR3:104399001	3	104399001	104400000	1000	1	6.20E-08	0.49	3	0.3	Ryr3	Ion Channel
DMR3:104402001	3	104402001	104404000	2000	1	6.20E-16	0.61	15	0.75	Ryr3	Ion Channel
DMR3:104409001	3	104409001	104411000	2000	2	7.10E-09	-0.68	18	0.9	Ryr3	Ion Channel
DMR3:104419001	3	104419001	104421000	2000	1	4.50E-07	-0.44	27	1.35	Ryr3	Ion Channel
DMR3:104429001	3	104429001	104431000	2000	1	9.90E-07	-0.44	27	1.35	Ryr3	Ion Channel
DMR3:104439001	3	104439001	104440000	1000	1	9.90E-08	-0.64	11	1.1	Ryr3	Ion Channel
DMR3:104477001	3	104477001	104478000	1000	1	2.30E-13	0.66	10	1	Ryr3	Ion Channel
DMR3:104479001	3	104479001	104481000	2000	1	6.80E-13	0.66	15	0.75	Ryr3	Ion Channel
DMR3:104537001	3	104537001	104539000	2000	1	1.20E-20	0.65	20	1	Ryr3	Ion Channel
DMR3:104563001	3	104563001	104564000	1000	1	1.60E-10	0.54	9	0.9	Ryr3	Ion Channel
DMR3:104578001	3	104578001	104579000	1000	1	2.90E-23	0.89	5	0.5	Ryr3	Ion Channel
DMR3:104580001	3	104580001	104582000	2000	1	3.60E-07	0.36	20	1	Ryr3	Ion Channel
DMR3:104591001	3	104591001	104593000	2000	1	1.40E-08	-0.53	20	1	Ryr3	Ion Channel
DMR3:104596001	3	104596001	104597000	1000	1	5.30E-11	0.48	21	2.1	Ryr3	Ion Channel
DMR3:104644001	3	104644001	104646000	2000	1	1.80E-09	-0.65	17	0.85	Ryr3	Ion Channel
DMR3:104674001	3	104674001	104683000	9000	4	1.70E-31	-0.56	111	1.23	Ryr3;Hmgn4	Ion Channel
DMR3:104710001	3	104710001	104715000	5000	4	2.00E-08	-0.72	24	0.48	Tmco5b	
DMR3:104732001	3	104732001	104735000	3000	2	5.20E-17	0.78	14	0.47	Tmco5b	
DMR3:104737001	3	104737001	104739000	2000	2	9.40E-08	-0.61	12	0.6	Tmco5b	
DMR3:104743001	3	104743001	104749000	6000	4	2.70E-28	-0.64	46	0.77	Tmco5b;LOC102553315	
DMR3:104758001	3	104758001	104763000	5000	3	3.70E-21	-0.49	64	1.28	Tmco5b;LOC102553315	
DMR3:104788001	3	104788001	104790000	2000	1	1.90E-07	0.54	10	0.5	Fmn1	
DMR3:104799001	3	104799001	104802000	3000	1	1.70E-09	0.48	32	1.07	Fmn1	
DMR3:104804001	3	104804001	104805000	1000	1	1.60E-11	0.78	4	0.4	Fmn1	
DMR3:104812001	3	104812001	104815000	3000	1	8.70E-07	-0.55	34	1.13	Fmn1	
DMR3:104857001	3	104857001	104858000	1000	1	2.20E-09	0.59	18	1.8	Fmn1	
DMR3:104914001	3	104914001	104916000	2000	1	2.20E-10	0.43	19	0.95	Fmn1	
DMR3:104932001	3	104932001	104935000	3000	1	6.90E-15	0.47	39	1.3	Fmn1	
DMR3:104967001	3	104967001	104968000	1000	1	9.30E-07	-0.49	6	0.6	Fmn1	
DMR3:104971001	3	104971001	104974000	3000	1	3.40E-08	-0.53	33	1.1	Fmn1	
DMR3:104987001	3	104987001	104988000	1000	1	8.30E-08	-0.67	16	1.6	Fmn1	
DMR3:105011001	3	105011001	105012000	1000	1	1.90E-11	0.5	15	1.5	Fmn1	
DMR3:105020001	3	105020001	105023000	3000	1	2.00E-11	0.63	26	0.87	Fmn1	
DMR3:105025001	3	105025001	105029000	4000	3	1.20E-09	-0.58	52	1.3	Fmn1	

DMR3:105046001	3	105046001	105047000	1000	1	1.50E-07	-0.35	16	1.6	Fmn1	
DMR3:105070001	3	105070001	105073000	3000	1	3.20E-15	0.84	36	1.2	Fmn1	
DMR3:105091001	3	105091001	105094000	3000	1	3.50E-08	0.45	28	0.93	Fmn1	
DMR3:105102001	3	105102001	105105000	3000	1	5.80E-13	0.63	32	1.07	Fmn1	
DMR3:105109001	3	105109001	105110000	1000	1	3.60E-07	0.32	8	0.8	Fmn1	
DMR3:105155001	3	105155001	105157000	2000	1	3.40E-07	0.32	27	1.35	Fmn1;LOC102551877;LOC102551792	
DMR3:105169001	3	105169001	105170000	1000	1	1.50E-08	0.48	13	1.3	Fmn1;LOC102551877;LOC102551792;LOC364803	
DMR3:105200001	3	105200001	105204000	4000	1	4.70E-10	0.57	32	0.8	Grem1	
DMR3:105220001	3	105220001	105223000	3000	1	3.20E-17	0.73	29	0.97	Grem1	
DMR3:105244001	3	105244001	105246000	2000	1	7.90E-15	0.61	12	0.6	Scg5	Transcription
DMR3:105273001	3	105273001	105275000	2000	1	1.70E-09	0.42	19	0.95	Scg5;Arhgap11a	Transcription;Signaling
DMR3:105297001	3	105297001	105299000	2000	1	9.30E-07	0.53	65	3.25	Arhgap11a;LOC108350437;LOC108350436	Signaling
DMR3:105504001	3	105504001	105511000	7000	3	9.30E-16	0.66	119	1.7	LOC100910676;LOC102552135;Actc1	Cytoskeleton
DMR3:105518001	3	105518001	105519000	1000	1	1.00E-08	-0.69	8	0.8	LOC100910676;Actc1	Cytoskeleton
DMR3:105594001	3	105594001	105602000	8000	2	1.10E-09	0.46	100	1.25	Aqr	
DMR3:105608001	3	105608001	105613000	5000	1	2.40E-17	0.65	78	1.56	Aqr	
DMR3:105615001	3	105615001	105617000	2000	1	8.40E-07	-0.45	25	1.25	Aqr	
DMR3:105620001	3	105620001	105622000	2000	1	4.40E-15	0.61	23	1.15	Aqr	
DMR3:105637001	3	105637001	105639000	2000	1	2.10E-08	0.49	21	1.05	Aqr	
DMR3:105644001	3	105644001	105647000	3000	1	3.20E-13	-0.73	13	0.43	Aqr	
DMR3:105648001	3	105648001	105653000	5000	3	2.20E-12	-0.66	31	0.62	Aqr;Zfp770	Transcription
DMR3:105655001	3	105655001	105656000	1000	1	9.60E-07	0.4	13	1.3	Zfp770	Transcription
DMR3:105673001	3	105673001	105674000	1000	1	1.30E-09	0.58	2	0.2	Zfp770	Transcription
DMR3:105994001	3	105994001	105998000	4000	1	3.70E-15	0.81	21	0.52	Dph6	
DMR3:106000001	3	106000001	106003000	3000	1	3.20E-08	-0.38	27	0.9	Dph6	
DMR3:106007001	3	106007001	106011000	4000	1	4.30E-12	0.52	24	0.6	Dph6	
DMR3:106025001	3	106025001	106028000	3000	1	5.70E-13	0.68	26	0.87	Dph6	
DMR3:106054001	3	106054001	106055000	1000	1	4.50E-07	0.48	3	0.3	Dph6;LOC108350438	
DMR3:106058001	3	106058001	106059000	1000	1	1.60E-07	0.4	7	0.7	Dph6;LOC108350438	
DMR3:106116001	3	106116001	106120000	4000	2	3.50E-11	-0.52	20	0.5	Dph6	
DMR3:106122001	3	106122001	106123000	1000	1	1.80E-15	0.93	6	0.6	Dph6	
DMR3:107138001	3	107138001	107139000	1000	1	1.00E-07	-0.5	16	1.6	RGD1563680	
DMR3:107154001	3	107154001	107156000	2000	1	5.10E-07	0.5	17	0.85	RGD1563680	
DMR3:107159001	3	107159001	107161000	2000	1	7.70E-14	0.58	25	1.25	RGD1563680	
DMR3:107223001	3	107223001	107224000	1000	1	3.50E-07	0.44	5	0.5	RGD1563680;LOC103691863	
DMR3:107247001	3	107247001	107249000	2000	1	3.30E-08	0.41	20	1	RGD1563680	
DMR3:107255001	3	107255001	107257000	2000	1	8.40E-08	-0.45	21	1.05	RGD1563680	
DMR3:107258001	3	107258001	107263000	5000	2	1.00E-11	0.57	30	0.6	RGD1563680	
DMR3:107312001	3	107312001	107314000	2000	1	2.70E-08	0.4	18	0.9	RGD1563680	
DMR3:107320001	3	107320001	107321000	1000	1	1.10E-07	-0.49	12	1.2	RGD1563680	
DMR3:107330001	3	107330001	107332000	2000	1	2.50E-09	0.49	20	1	RGD1563680	
DMR3:107341001	3	107341001	107348000	7000	2	3.10E-18	0.97	82	1.17	RGD1563680	
DMR3:107598001	3	107598001	107601000	3000	2	7.60E-11	0.57	31	1.03	Meis2	Development
DMR3:107608001	3	107608001	107613000	5000	1	1.60E-07	0.35	74	1.48	Meis2	Development
DMR3:107616001	3	107616001	107620000	4000	1	3.80E-08	0.45	49	1.23	Meis2	Development
DMR3:107650001	3	107650001	107651000	1000	1	3.20E-08	0.5	2	0.2	Meis2	Development
DMR3:107665001	3	107665001	107666000	1000	1	2.40E-07	0.46	4	0.4	Meis2	Development
DMR3:107684001	3	107684001	107686000	2000	2	1.70E-08	0.46	10	0.5	Meis2	Development
DMR3:107694001	3	107694001	107695000	1000	1	8.70E-11	0.48	8	0.8	Meis2	Development
DMR3:107702001	3	107702001	107703000	1000	1	1.10E-08	-0.6	9	0.9	Meis2	Development
DMR3:107730001	3	107730001	107733000	3000	2	1.70E-10	0.45	39	1.3	Meis2;LOC103691866	Development
DMR3:107749001	3	107749001	107752000	3000	1	4.80E-17	0.46	59	1.97	Meis2	Development
DMR3:107755001	3	107755001	107756000	1000	1	1.80E-14	0.69	15	1.5	Meis2	Development
DMR3:107758001	3	107758001	107762000	4000	2	9.90E-11	0.59	180	4.5	Meis2;LOC103691864	Development
DMR3:107767001	3	107767001	107769000	2000	1	2.90E-09	0.66	34	1.7	Meis2;LOC103691864;LOC102555456	Development
DMR3:108536001	3	108536001	108540000	4000	3	4.70E-21	-0.51	56	1.4	Tmco5a	
DMR3:108565001	3	108565001	108566000	1000	1	1.80E-15	0.83	4	0.4	Tmco5a	
DMR3:108569001	3	108569001	108575000	6000	1	4.00E-08	0.62	66	1.1	Tmco5a	
DMR3:108789001	3	108789001	108790000	1000	1	8.20E-13	0.53	12	1.2	LOC108350441;Spred1	Cytoskeleton

DMR3:108841001	3	108841001	108843000	2000	1	5.10E-07	-0.54	16	0.8	Spred1;LOC102555996	Cytoskeleton
DMR3:108937001	3	108937001	108943000	6000	4	9.40E-25	-0.57	58	0.97	Fam98b	
DMR3:108946001	3	108946001	108948000	2000	1	1.20E-12	0.54	15	0.75	Fam98b	
DMR3:108949001	3	108949001	108956000	7000	5	1.00E-21	-0.52	85	1.21	Fam98b	
DMR3:108960001	3	108960001	108961000	1000	1	1.20E-12	0.58	9	0.9	Fam98b	
DMR3:108977001	3	108977001	108979000	2000	1	1.30E-21	1.08	20	1	Fam98b;Rasgrp1	Transcription
DMR3:108994001	3	108994001	108995000	1000	1	4.50E-15	0.56	14	1.4	Rasgrp1	Transcription
DMR3:109008001	3	109008001	109011000	3000	1	2.60E-08	0.55	33	1.1	Rasgrp1	Transcription
DMR3:109026001	3	109026001	109027000	1000	1	3.80E-10	0.5	10	1	Rasgrp1	Transcription
DMR3:109028001	3	109028001	109031000	3000	1	4.20E-13	0.83	29	0.97	Rasgrp1	Transcription
DMR3:109049001	3	109049001	109051000	2000	1	1.30E-15	0.66	17	0.85	Rasgrp1	Transcription
DMR3:109881001	3	109881001	109883000	2000	1	5.10E-22	0.87	20	1	Thbs1;Fsip1	
DMR3:109914001	3	109914001	109915000	1000	1	1.80E-08	0.51	5	0.5	Fsip1	
DMR3:109931001	3	109931001	109932000	1000	1	3.80E-07	0.47	10	1	Fsip1	
DMR3:109971001	3	109971001	109974000	3000	2	6.00E-09	0.46	38	1.27	Fsip1	
DMR3:109975001	3	109975001	109976000	1000	1	2.00E-11	0.58	24	2.4	Fsip1	
DMR3:109985001	3	109985001	109987000	2000	1	1.00E-11	0.64	20	1	Fsip1	
DMR3:110002001	3	110002001	110006000	4000	1	1.10E-09	-0.33	48	1.2	Fsip1	
DMR3:110014001	3	110014001	110018000	4000	2	7.60E-08	-0.61	61	1.52	Fsip1	
DMR3:110027001	3	110027001	110028000	1000	1	6.80E-11	0.48	14	1.4	Fsip1	
DMR3:110032001	3	110032001	110035000	3000	1	5.90E-15	0.61	81	2.7	Gpr176	Signaling
DMR3:110039001	3	110039001	110041000	2000	1	3.70E-09	-0.49	22	1.1	Gpr176	Signaling
DMR3:110055001	3	110055001	110058000	3000	1	7.50E-08	0.47	28	0.93	Gpr176	Signaling
DMR3:110062001	3	110062001	110065000	3000	1	5.50E-16	0.83	26	0.87	Gpr176	Signaling
DMR3:110078001	3	110078001	110079000	1000	1	4.90E-12	0.67	9	0.9	Gpr176	Signaling
DMR3:110092001	3	110092001	110096000	4000	1	7.90E-10	-0.7	47	1.18	Gpr176	Signaling
DMR3:110120001	3	110120001	110125000	5000	1	5.20E-20	0.63	53	1.06	Gpr176	Signaling
DMR3:110129001	3	110129001	110131000	2000	1	2.60E-15	0.69	26	1.3	Gpr176	Signaling
DMR3:110145001	3	110145001	110150000	5000	2	6.40E-15	-0.6	21	0.42	Gpr176;Eif2ak4	Signaling;Signaling
DMR3:110165001	3	110165001	110166000	1000	1	1.20E-11	0.77	5	0.5	Eif2ak4	Signaling
DMR3:110179001	3	110179001	110181000	2000	1	1.80E-07	0.37	16	0.8	Eif2ak4	Signaling
DMR3:110186001	3	110186001	110187000	1000	1	7.60E-14	0.58	14	1.4	Eif2ak4	Signaling
DMR3:110194001	3	110194001	110195000	1000	1	5.00E-08	0.35	9	0.9	Eif2ak4	Signaling
DMR3:110228001	3	110228001	110229000	1000	1	3.10E-07	-0.62	11	1.1	Eif2ak4	Signaling
DMR3:110252001	3	110252001	110254000	2000	1	1.50E-09	0.44	22	1.1	Eif2ak4;Srp14	Signaling;Metabolism
DMR3:110255001	3	110255001	110257000	2000	1	2.20E-07	0.45	21	1.05	Eif2ak4;Srp14	Signaling;Metabolism
DMR3:110297001	3	110297001	110301000	4000	1	9.40E-09	0.42	38	0.95	Bmf	
DMR3:110316001	3	110316001	110318000	2000	2	3.10E-15	0.92	11	0.55	Bmf	
DMR3:110388001	3	110388001	110392000	4000	1	1.40E-08	0.37	51	1.27	Bub1b	Signaling
DMR3:110409001	3	110409001	110414000	5000	2	7.40E-08	0.37	57	1.14	Bub1b	Signaling
DMR3:110473001	3	110473001	110475000	2000	1	4.80E-11	0.72	22	1.1	Pak6	Signaling
DMR3:110482001	3	110482001	110486000	4000	2	3.60E-11	0.42	59	1.48	Pak6;Ankrd63	Signaling
DMR3:110505001	3	110505001	110511000	6000	1	6.90E-15	0.48	72	1.2	Ankrd63;Plcb2;LOC102548045	Metabolism
DMR3:110525001	3	110525001	110526000	1000	1	7.90E-08	0.39	17	1.7	Plcb2;LOC102548045	Metabolism
DMR3:110532001	3	110532001	110534000	2000	1	9.20E-16	0.74	26	1.3	LOC102548045;Inafm2	
DMR3:110544001	3	110544001	110546000	2000	1	1.50E-07	-0.55	36	1.8	Inafm2;LOC102546973;RGD1565536	
DMR3:110557001	3	110557001	110558000	1000	1	1.00E-20	0.68	11	1.1	LOC102546973;RGD1565536;Phgr1	
DMR3:110616001	3	110616001	110618000	2000	1	1.10E-17	0.76	12	0.6	Knstrn	
DMR3:110629001	3	110629001	110632000	3000	1	1.70E-08	0.46	31	1.03	Knstrn	
DMR3:110694001	3	110694001	110696000	2000	1	5.70E-07	0.3	31	1.55	Ivd	Metabolism
DMR3:110742001	3	110742001	110745000	3000	2	3.00E-14	0.92	15	0.5	Bahd1;Chst14	Transport
DMR3:110809001	3	110809001	110810000	1000	1	8.90E-07	0.34	25	2.5	Ccdc32	
DMR3:110813001	3	110813001	110815000	2000	1	2.40E-18	0.94	22	1.1	Ccdc32	
DMR3:110821001	3	110821001	110826000	5000	2	1.70E-12	0.5	56	1.12	Ccdc32;Rpusd2	
DMR3:110900001	3	110900001	110903000	3000	2	1.40E-15	0.7	20	0.67	Knl1	
DMR3:110934001	3	110934001	110938000	4000	1	1.30E-09	0.46	46	1.15	Rad51;Rmdn3	Transcription
DMR3:110977001	3	110977001	110978000	1000	1	8.20E-08	-0.52	14	1.4	LOC108350564;Gchfr;Dnajc17;LOC691418	Signaling;Transcription
DMR3:111003001	3	111003001	111004000	1000	1	1.50E-07	0.38	11	1.1	Dnajc17;Zfyve19	Transcription
DMR3:111007001	3	111007001	111009000	2000	1	8.90E-14	0.68	19	0.95	Dnajc17;Zfyve19	Transcription

DMR3:111038001	3	111038001	111041000	3000	1	6.80E-14	0.72	26	0.87	Ppp1r14d;Spint1	Signaling;Protease; Proteolysis
DMR3:111070001	3	111070001	111073000	3000	2	2.10E-10	0.52	21	0.7	Spint1;Rhov	Protease; Proteolysis;Signaling
DMR3:111077001	3	111077001	111079000	2000	1	5.30E-15	0.65	19	0.95	Rhov	Signaling
DMR3:111089001	3	111089001	111092000	3000	1	1.40E-09	0.47	37	1.23	Rhov;Vps18	Signaling;Transport
DMR3:111093001	3	111093001	111097000	4000	1	2.90E-11	0.56	40	1	Rhov;Vps18	Signaling;Transport
DMR3:111136001	3	111136001	111140000	4000	1	6.70E-18	0.66	87	2.17	LOC103691871;Dll4	
DMR3:111152001	3	111152001	111155000	3000	2	4.20E-24	0.88	28	0.93	Dll4;Chac1	
DMR3:111160001	3	111160001	111164000	4000	1	1.40E-10	0.56	83	2.08	Chac1	
DMR3:111171001	3	111171001	111172000	1000	1	2.30E-11	0.61	6	0.6	Chac1	
DMR3:111179001	3	111179001	111181000	2000	2	1.60E-27	0.78	16	0.8	Ino80	
DMR3:111294001	3	111294001	111296000	2000	1	8.80E-08	-0.49	21	1.05	Dppa3-ps1	
DMR3:111356001	3	111356001	111358000	2000	1	1.30E-13	0.61	13	0.65	Exd1;Chp1	
DMR3:111373001	3	111373001	111374000	1000	1	9.60E-07	-0.34	9	0.9	Chp1	
DMR3:111385001	3	111385001	111387000	2000	1	2.80E-09	-0.52	26	1.3	Chp1	
DMR3:111492001	3	111492001	111494000	2000	1	5.10E-24	1.17	8	0.4	Rtf1	
DMR3:111495001	3	111495001	111497000	2000	1	1.50E-08	-0.46	19	0.95	Rtf1	
DMR3:111573001	3	111573001	111576000	3000	1	7.00E-07	-0.61	48	1.6	Rpap1	
DMR3:111603001	3	111603001	111604000	1000	1	2.40E-07	0.38	12	1.2	Tyro3	Receptor
DMR3:111613001	3	111613001	111615000	2000	1	9.10E-11	0.42	28	1.4	Tyro3	Receptor
DMR3:111621001	3	111621001	111624000	3000	1	5.70E-10	0.4	33	1.1	Tyro3	Receptor
DMR3:111626001	3	111626001	111627000	1000	1	8.30E-09	0.5	5	0.5	Tyro3	Receptor
DMR3:111666001	3	111666001	111668000	2000	1	6.60E-07	0.48	13	0.65	LOC691881;Mga	Transcription
DMR3:111722001	3	111722001	111724000	2000	1	2.10E-07	0.33	16	0.8	Mga	Transcription
DMR3:111779001	3	111779001	111781000	2000	1	1.60E-07	0.36	20	1	Mapkbp1	
DMR3:111790001	3	111790001	111791000	1000	1	5.00E-09	0.55	1	0.1	Mapkbp1	
DMR3:111828001	3	111828001	111833000	5000	1	6.10E-07	0.33	100	2	Jmjd7;Pla2g4b;Sptbn5	Metabolism
DMR3:111843001	3	111843001	111844000	1000	1	1.70E-09	0.45	16	1.6	Pla2g4b;Sptbn5	Metabolism
DMR3:111854001	3	111854001	111856000	2000	1	8.70E-14	0.59	33	1.65	Sptbn5	
DMR3:111867001	3	111867001	111870000	3000	1	3.00E-13	0.52	61	2.03	Sptbn5;Ehd4	Transport
DMR3:111885001	3	111885001	111886000	1000	1	5.20E-09	0.49	13	1.3	Sptbn5;Ehd4	Transport
DMR3:111935001	3	111935001	111936000	1000	1	2.10E-07	0.36	16	1.6	Ehd4	Transport
DMR3:111969001	3	111969001	111971000	2000	1	6.20E-09	0.52	23	1.15	Pla2g4e	Metabolism
DMR3:111979001	3	111979001	111981000	2000	1	1.80E-09	0.53	21	1.05	Pla2g4e	Metabolism
DMR3:111993001	3	111993001	111994000	1000	1	5.00E-10	0.47	13	1.3	Pla2g4e	Metabolism
DMR3:112005001	3	112005001	112006000	1000	1	5.80E-09	0.46	10	1	Pla2g4e	Metabolism
DMR3:112046001	3	112046001	112048000	2000	1	1.30E-09	-0.43	29	1.45	Pla2g4d	Metabolism
DMR3:112050001	3	112050001	112053000	3000	2	3.60E-22	0.87	37	1.23	Pla2g4d	Metabolism
DMR3:112062001	3	112062001	112063000	1000	1	4.00E-08	0.46	7	0.7	Pla2g4d;Pla2g4f	Metabolism
DMR3:112071001	3	112071001	112073000	2000	1	4.10E-07	-0.57	22	1.1	Pla2g4d;Pla2g4f	Metabolism
DMR3:112089001	3	112089001	112092000	3000	1	4.20E-09	-0.61	47	1.57	Pla2g4f;Vps39;LOC691532	Metabolism
DMR3:112145001	3	112145001	112146000	1000	1	5.40E-10	0.37	4	0.4	Tmem87a	Signaling
DMR3:112216001	3	112216001	112218000	2000	1	2.50E-07	-0.4	22	1.1	Ganc;LOC108350445;Capn3	Metabolism;Protease
DMR3:112248001	3	112248001	112250000	2000	1	2.90E-12	0.45	21	1.05	Capn3	Protease
DMR3:112266001	3	112266001	112269000	3000	1	3.30E-08	-0.39	46	1.53	Capn3;LOC108350446	Protease
DMR3:112270001	3	112270001	112271000	1000	1	2.80E-07	0.3	13	1.3	Capn3;LOC108350446;Zfp106	Protease
DMR3:112286001	3	112286001	112291000	5000	1	1.60E-09	0.47	65	1.3	Capn3;Zfp106	Protease
DMR3:112295001	3	112295001	112297000	2000	1	4.60E-08	-0.53	28	1.4	Zfp106	
DMR3:112320001	3	112320001	112321000	1000	1	4.20E-13	0.44	16	1.6	Zfp106	
DMR3:112344001	3	112344001	112345000	1000	1	3.60E-10	0.55	8	0.8	Snap23	Transcription
DMR3:112357001	3	112357001	112358000	1000	1	4.90E-07	0.57	8	0.8	Snap23;Lrrc57	Transcription
DMR3:112361001	3	112361001	112362000	1000	1	2.00E-07	-0.5	16	1.6	Snap23;Lrrc57;LOC691918	Transcription
DMR3:112364001	3	112364001	112366000	2000	1	4.00E-09	-0.5	16	0.8	Snap23;Lrrc57;LOC691918	Transcription
DMR3:112417001	3	112417001	112421000	4000	1	2.60E-12	0.46	68	1.7	Stard9	Cytoskeleton
DMR3:112439001	3	112439001	112442000	3000	2	3.10E-09	-0.5	7	0.23	Stard9	Cytoskeleton
DMR3:112459001	3	112459001	112460000	1000	1	3.30E-08	0.41	9	0.9	Stard9	Cytoskeleton
DMR3:112480001	3	112480001	112482000	2000	1	1.80E-07	-0.61	22	1.1	Stard9	Cytoskeleton
DMR3:112507001	3	112507001	112510000	3000	1	3.70E-17	0.76	20	0.67	Stard9;LOC100911166	Cytoskeleton;Translat ion
DMR3:112514001	3	112514001	112518000	4000	1	9.20E-11	0.67	27	0.68	Stard9;LOC100911166	Cytoskeleton;Translat ion
DMR3:112648001	3	112648001	112652000	4000	2	1.00E-13	-0.55	42	1.05	Stard9;Cdan1	Cytoskeleton
DMR3:112737001	3	112737001	112738000	1000	1	2.70E-09	0.44	5	0.5	Ttbk2	Signaling

DMR3:112758001	3	112758001	112759000	1000	1	8.60E-08	0.4	6	0.6	Ttbk2	Signaling
DMR3:112775001	3	112775001	112781000	6000	1	1.10E-07	-0.38	29	0.48	Ttbk2;LOC108350447	Signaling
DMR3:112786001	3	112786001	112787000	1000	1	1.10E-10	0.66	3	0.3	Ttbk2;LOC108350447	Signaling
DMR3:112793001	3	112793001	112796000	3000	1	7.80E-07	0.48	23	0.77	Ttbk2;LOC108350447;Ubr1	Signaling;Proteolysis
DMR3:112851001	3	112851001	112853000	2000	1	5.00E-07	0.36	23	1.15	Ubr1	Proteolysis
DMR3:112867001	3	112867001	112869000	2000	1	3.50E-07	0.37	14	0.7	Ubr1	Proteolysis
DMR3:112885001	3	112885001	112889000	4000	1	4.10E-08	0.55	24	0.6	Ubr1	Proteolysis
DMR3:112898001	3	112898001	112899000	1000	1	1.60E-07	0.38	11	1.1	Ubr1	Proteolysis
DMR3:112902001	3	112902001	112905000	3000	1	8.50E-12	0.46	25	0.83	Ubr1	Proteolysis
DMR3:112925001	3	112925001	112926000	1000	1	4.00E-15	0.73	11	1.1	Tmem62	Transcription
DMR3:112927001	3	112927001	112931000	4000	1	3.80E-16	0.62	49	1.23	Tmem62	Transcription
DMR3:112955001	3	112955001	112958000	3000	1	1.40E-07	0.59	58	1.93	Tmem62;Ccndbp1;Epb42	Transcription;Transport
DMR3:112975001	3	112975001	112976000	1000	1	4.80E-07	0.39	13	1.3	Epb42	Transport
DMR3:112982001	3	112982001	112985000	3000	1	1.50E-07	-0.42	42	1.4	Epb42	Transport
DMR3:112994001	3	112994001	112995000	1000	1	6.70E-08	0.41	9	0.9	Epb42;Tgm7l1	Transport
DMR3:113004001	3	113004001	113006000	2000	1	1.70E-17	0.81	13	0.65	Tgm7l1	
DMR3:113016001	3	113016001	113018000	2000	1	2.00E-09	-0.63	19	0.95	Tgm7l1	
DMR3:113019001	3	113019001	113020000	1000	1	5.80E-08	0.46	5	0.5	Tgm7l1	
DMR3:113030001	3	113030001	113032000	2000	2	1.10E-15	0.9	4	0.2	Tgm7l1;Tgm7	Transport
DMR3:113034001	3	113034001	113042000	8000	7	3.30E-22	0.93	74	0.92	Tgm7l1;Tgm7	Transport
DMR3:113045001	3	113045001	113049000	4000	1	2.40E-20	0.75	49	1.23	Tgm7	Transport
DMR3:113053001	3	113053001	113058000	5000	1	1.20E-07	-0.5	70	1.4	Tgm7	Transport
DMR3:113060001	3	113060001	113063000	3000	1	7.80E-24	0.86	33	1.1	Tgm7	Transport
DMR3:113139001	3	113139001	113140000	1000	1	9.80E-07	-0.54	4	0.4	Tgm7;Tubgcp4	Transport;Cytoskeleton
DMR3:113144001	3	113144001	113147000	3000	2	2.30E-35	1.19	12	0.4	Tubgcp4	Cytoskeleton
DMR3:113172001	3	113172001	113179000	7000	4	3.70E-19	0.56	64	0.91	Tp53bp1	Transcription
DMR3:113193001	3	113193001	113198000	5000	1	9.00E-14	0.58	57	1.14	Tp53bp1	Transcription
DMR3:113214001	3	113214001	113221000	7000	1	2.10E-15	0.57	54	0.77	Tp53bp1	Transcription
DMR3:113222001	3	113222001	113229000	7000	2	2.00E-19	0.75	50	0.71	Tp53bp1	Transcription
DMR3:113234001	3	113234001	113239000	5000	1	2.60E-07	-0.56	42	0.84	Tp53bp1	Transcription
DMR3:113262001	3	113262001	113266000	4000	1	2.20E-15	0.58	99	2.48	Tp53bp1;Map1a;Ppip5k1	Transcription;Cytoskeleton;Signaling
DMR3:113277001	3	113277001	113280000	3000	3	5.00E-17	0.66	24	0.8	Map1a;Ppip5k1	Cytoskeleton;Signaling
DMR3:113283001	3	113283001	113285000	2000	1	6.00E-14	0.5	28	1.4	Ppip5k1	Signaling
DMR3:113287001	3	113287001	113288000	1000	1	2.00E-09	0.38	17	1.7	Ppip5k1	Signaling
DMR3:113299001	3	113299001	113301000	2000	2	1.10E-23	1.02	13	0.65	Ppip5k1	Signaling
DMR3:113303001	3	113303001	113311000	8000	3	9.80E-15	0.59	74	0.92	Ppip5k1;Ckmt1b	Signaling
DMR3:113314001	3	113314001	113317000	3000	2	5.10E-14	0.59	80	2.67	Ppip5k1;Ckmt1b;Strc	Signaling;Cytoskeleton
DMR3:113326001	3	113326001	113328000	2000	1	1.90E-07	0.33	32	1.6	Ppip5k1;Ckmt1b;Strc	Signaling;Cytoskeleton
DMR3:113337001	3	113337001	113342000	5000	1	4.60E-18	0.7	87	1.74	Strc	Cytoskeleton
DMR3:113358001	3	113358001	113363000	5000	3	1.10E-18	0.72	41	0.82	Catsper2	Transport
DMR3:113379001	3	113379001	113381000	2000	1	1.40E-07	0.49	14	0.7	Catsper2;Pdia3	Transport;Transcription
DMR3:113428001	3	113428001	113430000	2000	1	6.60E-08	0.44	21	1.05	Serf2;Serinc4;Hypk;Mfap1a	Signaling
DMR3:113431001	3	113431001	113433000	2000	2	5.80E-33	0.99	11	0.55	Serinc4;Hypk;Mfap1a	Signaling
DMR3:113438001	3	113438001	113441000	3000	1	2.60E-10	0.45	32	1.07	Mfap1a;Wdr76	
DMR3:113452001	3	113452001	113454000	2000	1	2.20E-11	-0.49	18	0.9	Mfap1a;Wdr76	
DMR3:113470001	3	113470001	113474000	4000	1	9.70E-29	1.09	50	1.25	Wdr76;Frmd5	
DMR3:113475001	3	113475001	113478000	3000	1	8.40E-08	-0.47	38	1.27	Wdr76;Frmd5	
DMR3:113480001	3	113480001	113482000	2000	1	5.60E-09	-0.69	37	1.85	Wdr76;Frmd5	
DMR3:113494001	3	113494001	113497000	3000	1	6.30E-10	0.44	26	0.87	Frmd5	
DMR3:113503001	3	113503001	113509000	6000	1	7.00E-09	0.39	89	1.48	Frmd5	
DMR3:113512001	3	113512001	113514000	2000	1	9.80E-08	-0.46	27	1.35	Frmd5	
DMR3:113524001	3	113524001	113530000	6000	2	2.40E-16	0.64	75	1.25	Frmd5	
DMR3:113554001	3	113554001	113557000	3000	2	2.00E-07	-0.63	44	1.47	Frmd5	
DMR3:113578001	3	113578001	113580000	2000	1	5.80E-10	-0.53	30	1.5	Frmd5	
DMR3:113581001	3	113581001	113589000	8000	5	2.00E-21	-0.55	113	1.41	Frmd5	
DMR3:113597001	3	113597001	113598000	1000	1	5.40E-14	0.64	6	0.6	Frmd5	
DMR3:113599001	3	113599001	113601000	2000	1	5.70E-10	0.51	15	0.75	Frmd5	
DMR3:113603001	3	113603001	113604000	1000	1	3.00E-07	0.45	14	1.4	Frmd5	

DMR3:113612001	3	113612001	113613000	1000	1	8.30E-09	0.46	8	0.8	Frmd5	
DMR3:113614001	3	113614001	113615000	1000	1	1.10E-07	0.33	10	1	Frmd5	
DMR3:113625001	3	113625001	113626000	1000	1	2.00E-07	0.32	10	1	Frmd5	
DMR3:113649001	3	113649001	113651000	2000	1	3.80E-07	0.32	32	1.6	Frmd5;LOC108350449;Rps4x-ps2	
DMR3:113653001	3	113653001	113654000	1000	1	4.90E-10	0.36	12	1.2	Frmd5;LOC108350449;Rps4x-ps2	
DMR3:113655001	3	113655001	113658000	3000	1	9.40E-09	-0.59	52	1.73	Frmd5;LOC108350449;Rps4x-ps2	
DMR3:113672001	3	113672001	113673000	1000	1	3.80E-17	0.6	13	1.3	Frmd5	
DMR3:113678001	3	113678001	113679000	1000	1	8.40E-12	0.59	9	0.9	Frmd5;LOC102552883	
DMR3:113689001	3	113689001	113692000	3000	1	4.70E-08	0.28	23	0.77	Frmd5;LOC102552883	
DMR3:113704001	3	113704001	113706000	2000	1	7.30E-07	0.34	25	1.25	Frmd5	
DMR3:113710001	3	113710001	113714000	4000	2	2.20E-07	-0.38	43	1.07	Frmd5	
DMR3:113716001	3	113716001	113719000	3000	1	5.00E-10	-0.61	40	1.33	Frmd5	
DMR3:113725001	3	113725001	113728000	3000	1	2.70E-19	0.96	44	1.47	Frmd5	
DMR3:113731001	3	113731001	113732000	1000	1	2.20E-12	0.47	14	1.4	Frmd5	
DMR3:113738001	3	113738001	113741000	3000	2	2.70E-20	0.66	32	1.07	Frmd5	
DMR3:113749001	3	113749001	113750000	1000	1	8.80E-07	-0.43	18	1.8	Frmd5	
DMR3:113763001	3	113763001	113766000	3000	1	7.10E-07	0.38	22	0.73	Frmd5	
DMR3:113832001	3	113832001	113833000	1000	1	1.00E-07	-0.6	8	0.8	Casc4;LOC102554762	
DMR3:113870001	3	113870001	113872000	2000	1	8.70E-07	0.36	18	0.9	Casc4	
DMR3:113888001	3	113888001	113895000	7000	4	2.80E-22	-0.51	82	1.17	Casc4	
DMR3:113902001	3	113902001	113903000	1000	1	7.50E-08	0.56	6	0.6	Ctdspl2	
DMR3:114025001	3	114025001	114028000	3000	3	1.10E-09	-0.88	40	1.33	Spg11	
DMR3:114039001	3	114039001	114043000	4000	2	5.90E-21	0.6	50	1.25	Spg11	
DMR3:114055001	3	114055001	114057000	2000	1	4.10E-09	-0.53	35	1.75	Spg11;Patl2	Translation
DMR3:114080001	3	114080001	114081000	1000	1	5.30E-15	0.53	12	1.2	Patl2;B2m	Translation;Immune
DMR3:114088001	3	114088001	114090000	2000	1	8.40E-07	0.29	29	1.45	B2m	Immune
DMR3:114131001	3	114131001	114134000	3000	2	1.40E-22	1.01	28	0.93	Trim69;Terb2	Proteolysis
DMR3:114150001	3	114150001	114153000	3000	2	7.50E-19	0.87	22	0.73	Terb2	
DMR3:114157001	3	114157001	114158000	1000	1	1.20E-20	0.66	8	0.8	Terb2	
DMR3:114166001	3	114166001	114173000	7000	2	1.20E-08	0.57	56	0.8	Sord;LOC108350450	Metabolism
DMR3:114216001	3	114216001	114222000	6000	1	6.90E-10	0.43	80	1.33	Sord;Duox2	Metabolism;Metabolism
DMR3:114232001	3	114232001	114233000	1000	1	1.20E-20	0.67	7	0.7	Duox2;Duoxa2;Duoxa1	Metabolism
DMR3:114241001	3	114241001	114242000	1000	1	1.50E-07	0.38	13	1.3	Duox2;Duoxa2;Duoxa1;Duox1	Metabolism
DMR3:114245001	3	114245001	114249000	4000	1	8.10E-12	0.45	44	1.1	Duox2;Duoxa2;Duoxa1;Duox1	Metabolism
DMR3:114254001	3	114254001	114257000	3000	1	2.10E-10	0.37	45	1.5	Duoxa1;Duox1	Metabolism
DMR3:114295001	3	114295001	114298000	3000	2	1.30E-11	0.42	40	1.33	Duox1;Shf	Metabolism
DMR3:114299001	3	114299001	114300000	1000	1	1.50E-09	0.46	17	1.7	Shf	
DMR3:114345001	3	114345001	114346000	1000	1	8.50E-21	0.85	6	0.6	LOC102557338;Slc28a2	Transport
DMR3:114355001	3	114355001	114360000	5000	3	8.70E-10	-0.54	48	0.96	LOC102557338;Slc28a2	Transport
DMR3:114384001	3	114384001	114388000	4000	2	4.70E-11	-0.56	19	0.48	Slc28a2	Transport
DMR3:114394001	3	114394001	114395000	1000	1	2.70E-11	0.39	25	2.5	Slc28a2	Transport
DMR3:114397001	3	114397001	114398000	1000	1	4.30E-12	0.6	4	0.4	Slc28a2	Transport
DMR3:114399001	3	114399001	114400000	1000	1	3.80E-07	-0.49	9	0.9	Slc28a2	Transport
DMR3:114401001	3	114401001	114403000	2000	1	8.90E-08	-0.34	11	0.55	Slc28a2	Transport
DMR3:114413001	3	114413001	114415000	2000	1	1.00E-07	0.36	30	1.5	Slc28a2	Transport
DMR3:114433001	3	114433001	114435000	2000	1	5.10E-07	0.53	11	0.55	Slc28a2	Transport
DMR3:114438001	3	114438001	114440000	2000	1	9.60E-13	0.67	12	0.6	Slc28a2	Transport
DMR3:114465001	3	114465001	114466000	1000	1	1.40E-07	-0.36	7	0.7	Slc28a2	Transport
DMR3:114479001	3	114479001	114482000	3000	2	4.90E-15	-0.58	18	0.6	Slc28a2	Transport
DMR3:114491001	3	114491001	114494000	3000	1	6.90E-08	-0.35	19	0.63	Slc28a2	Transport
DMR3:114523001	3	114523001	114527000	4000	1	4.00E-07	0.37	31	0.78	Slc28a2	Transport
DMR3:114532001	3	114532001	114534000	2000	2	4.20E-17	-0.54	12	0.6	Slc28a2	Transport
DMR3:114546001	3	114546001	114548000	2000	1	3.50E-09	0.51	10	0.5	Slc28a2	Transport
DMR3:114552001	3	114552001	114553000	1000	1	6.90E-16	0.66	9	0.9	Slc28a2	Transport
DMR3:114601001	3	114601001	114603000	2000	1	3.80E-08	-0.42	12	0.6	Slc28a2	Transport
DMR3:114619001	3	114619001	114621000	2000	2	4.50E-12	-0.71	9	0.45	Slc28a2	Transport
DMR3:114712001	3	114712001	114715000	3000	2	2.40E-07	-0.5	49	1.63	Gatm	Transport
DMR3:114731001	3	114731001	114734000	3000	1	2.80E-12	0.62	34	1.13	Gatm	Transport
DMR3:114743001	3	114743001	114745000	2000	1	1.40E-11	-0.67	22	1.1	Spata5l1	
DMR3:114760001	3	114760001	114762000	2000	1	1.50E-11	0.49	27	1.35	Spata5l1	
DMR3:114770001	3	114770001	114771000	1000	1	4.20E-12	0.45	23	2.3	Spata5l1;MGC105649;Mir147	

DMR3:114836001	3	114836001	114838000	2000	1	2.60E-07	-0.47	16	0.8	Slc30a4;LOC108350451	Transport
DMR3:114860001	3	114860001	114862000	2000	1	9.50E-15	0.8	12	0.6	Bloc1s6	
DMR3:114899001	3	114899001	114900000	1000	1	2.70E-09	-0.67	7	0.7	Sqrdl	
DMR3:114906001	3	114906001	114910000	4000	3	5.00E-13	-0.63	51	1.27	Sqrdl	
DMR3:114925001	3	114925001	114928000	3000	2	3.30E-15	-0.84	36	1.2	Sqrdl	
DMR3:114930001	3	114930001	114931000	1000	1	3.80E-08	0.47	8	0.8	Sqrdl	
DMR3:114942001	3	114942001	114950000	8000	4	5.40E-20	-0.47	86	1.07	Sqrdl	
DMR3:115201001	3	115201001	115202000	1000	1	1.90E-08	0.39	11	1.1	RGD1563036	
DMR3:116893001	3	116893001	116897000	4000	1	1.60E-10	-0.67	58	1.45	Sema6d	Signaling
DMR3:116913001	3	116913001	116916000	3000	1	7.90E-08	-0.57	42	1.4	Sema6d	Signaling
DMR3:116951001	3	116951001	116952000	1000	1	1.20E-07	0.31	14	1.4	Sema6d	Signaling
DMR3:116957001	3	116957001	116959000	2000	1	5.20E-09	-0.5	31	1.55	Sema6d	Signaling
DMR3:116960001	3	116960001	116961000	1000	1	3.70E-09	0.65	37	3.7	Sema6d	Signaling
DMR3:117331001	3	117331001	117334000	3000	1	6.50E-12	-0.67	36	1.2	Slc24a5	Transport
DMR3:117335001	3	117335001	117337000	2000	1	1.10E-07	-0.64	33	1.65	Slc24a5	Transport
DMR3:117360001	3	117360001	117362000	2000	1	1.90E-07	0.39	20	1	Slc24a5;Myef2;LOC103691879	Transport;Translation
DMR3:117363001	3	117363001	117365000	2000	1	1.50E-08	0.32	28	1.4	Slc24a5;Myef2;LOC103691879	Transport;Translation
DMR3:117419001	3	117419001	117423000	4000	1	1.20E-10	0.47	73	1.82	Ctxn2;Slc12a1	Transport
DMR3:117441001	3	117441001	117443000	2000	2	9.30E-19	0.67	19	0.95	Slc12a1	Transport
DMR3:117452001	3	117452001	117453000	1000	1	1.20E-12	0.48	21	2.1	Slc12a1	Transport
DMR3:117460001	3	117460001	117462000	2000	1	5.90E-10	-0.62	40	2	Slc12a1	Transport
DMR3:117486001	3	117486001	117488000	2000	2	7.30E-20	0.83	20	1	Slc12a1;LOC102555270	Transport
DMR3:117501001	3	117501001	117502000	1000	1	2.80E-13	0.65	5	0.5	Slc12a1;LOC102555270;Rpl32-ps1	Transport
DMR3:117505001	3	117505001	117506000	1000	1	1.70E-07	0.35	5	0.5	Slc12a1;LOC102555270;Rpl32-ps1;Dut	Transport;Signaling
DMR3:117508001	3	117508001	117509000	1000	1	7.10E-12	0.49	12	1.2	Slc12a1;LOC102555270;Rpl32-ps1;Dut	Transport;Signaling
DMR3:117516001	3	117516001	117517000	1000	1	4.30E-13	0.62	11	1.1	LOC102555270;Rpl32-ps1;Dut	Signaling
DMR3:117569001	3	117569001	117572000	3000	2	8.00E-11	-0.59	57	1.9	Fbn1	Extracellular Matrix
DMR3:117587001	3	117587001	117590000	3000	1	6.60E-07	-0.62	33	1.1	Fbn1	Extracellular Matrix
DMR3:117598001	3	117598001	117599000	1000	1	5.30E-12	0.55	16	1.6	Fbn1	Extracellular Matrix
DMR3:117645001	3	117645001	117648000	3000	1	4.80E-07	0.39	41	1.37	Fbn1	Extracellular Matrix
DMR3:117669001	3	117669001	117672000	3000	1	3.00E-07	-0.67	37	1.23	Fbn1	Extracellular Matrix
[truncated: 673,150 more chars]
